# Supplementary material for: Spatial snapshots of amyloid precursor protein intramembrane processing via early endosome proteomics
Source: Nat Commun. 2022 Oct 16;13:6112. doi: 10.1038/s41467-022-33881-x (PMC9573879; doi:10.1038/s41467-022-33881-x)
Supplement: Supplementary file 1 — Supplementary Information [file 41467_2022_33881_MOESM1_ESM.pdf]

SUPPLEMENTAL INFORMATION

Spatial Snapshots of Amyloid Precursor Protein Intramembrane Processing via Early Endosome Proteomics

Hankum Park[1,2,^], Frances V. Hundley[1,2*], Qing Yu[1*], Katherine A. Overmyer[3,4], Dain R. Brademan[3,4], Lia Serrano[3], Joao A. Paulo[1], Julia C. Paoli[1,2], Sharan Swarup[1,2,#], Joshua J. Coon[3,4,5], Steven P. Gygi[1], and J. Wade Harper[1,2]

[1]Department of Cell Biology, Harvard Medical School, Boston, MA 02115, USA
[2]Aligning Science Across Parkinson's (ASAP) Collaborative Research Network, Chevy Chase, MD 20815, USA
[3]Department of Biomolecular Chemistry, University of Wisconsin–Madison, Madison, WI 53706, USA
[4]Morgridge Institute for Research, Madison, WI 53715, USA
[5]Department of Chemistry, University of Wisconsin–Madison, Madison, WI 53706, USA
[^]Current address: Department of Dental Science, School of Dentistry and Dental Research Institute, Seoul National University, Seoul 03080, Republic of Korea
[#]Current Address: Casma Therapeutics, Cambridge MA 02139
*, equal contribution

Corresponding author: wade_harper@hms.harvard.edu

Table of Contents

Supplementary Fig 1……….page 2
Supplementary Fig 2……….page 4
Supplementary Fig 3……….page 6
Supplementary Fig 4……….page 8
Supplementary Fig 5……….page 9
Supplementary Fig 6……….page 11
Supplementary Fig 7……….page 13
Supplementary Fig 8……….page 14

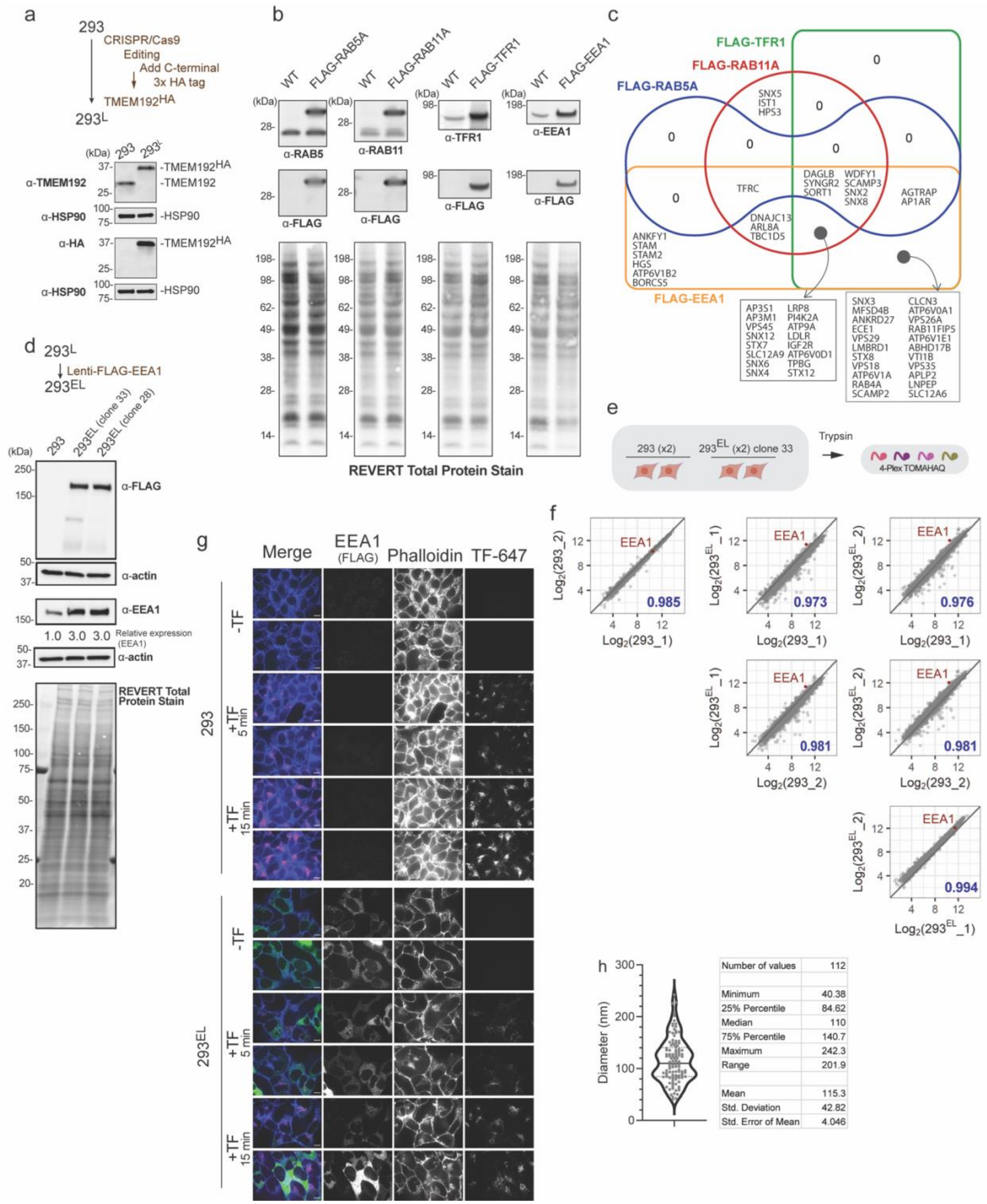

Supplementary Fig. 1. Identification of EEA1 as a candidate affinity reagent for early endosome purification.

a, Scheme summarizing how 293 cells were gene edited to place a C-terminal 3xHA tag on the endogenous TMEM192 gene as described[31] to create 293^{L} cells. Cell extracts from 293 or 293^{L} cells were subjected to immunoblotting with α-TMEM192 and α-HA to demonstrate homozygous targeting of the TMEM192 gene to

introduce the 3xHA tag, with α-HSP90 used as a loading control. α-TMEM192 and α-HA blots were on separate gels with separate α-HSP90 loading control blots.
b, The indicated FLAG-tagged proteins were stably expressed in 293^L cells using a lentiviral construct. Cell extracts were subjected to immunoblotting with the indicated antibodies. Total proteins were stained with REVERT.
c, Overlap in endosomal proteins found in FLAG-EEA1, FLAG-TFR1, FLAG-RAB11, and FLAG-RAB5A immune complexes from non-detergent extracts.
d, 293^L cells were transduced with a lentivirus expressing FLAG-EEA1 to create 293^{EL} cells. Extracts from parental 293 or 293^{EL} cells were subjected to immunoblotting with α-EEA1 or α-FLAG antibodies. Quantification of blots revealed ~3-fold higher levels of FLAG-EEA1 than the endogenous protein. Total proteins were stained with REVERT.
e,f, Total proteome analysis of duplicate cultures of 293 and 293^{EL} cells. The relative abundance of individual proteins based on TMT reporter ion intensities are shown for all pair-wise combinations of biological duplicate cells (n=2) employed in the 8-plex TMT experiment. Pearson's coefficients (R) for pairwise comparisons are shown. The position of EEA1 is indicated by the red circle.
g, 293 or 293^{EL} cells (clone 28) were serum starved for 1h prior to addition of serum-free media with or without Alexa-647-labeled TF (25 μg/mL). Cells were fixed at the indicated times and subjected to imaging to visualize Alexa-647-labeled TF, immunofluorescence of FLAG-EEA1, and phalloidin. Images are representative of 5 fields from two replicate experiments. Scale bar, 10 μm.
h, Size distribution of vesicles identified in FLAG-EEA1 immune complexes by transmission electron microscopy. The diameter for a total of 112 vesicles was measured.

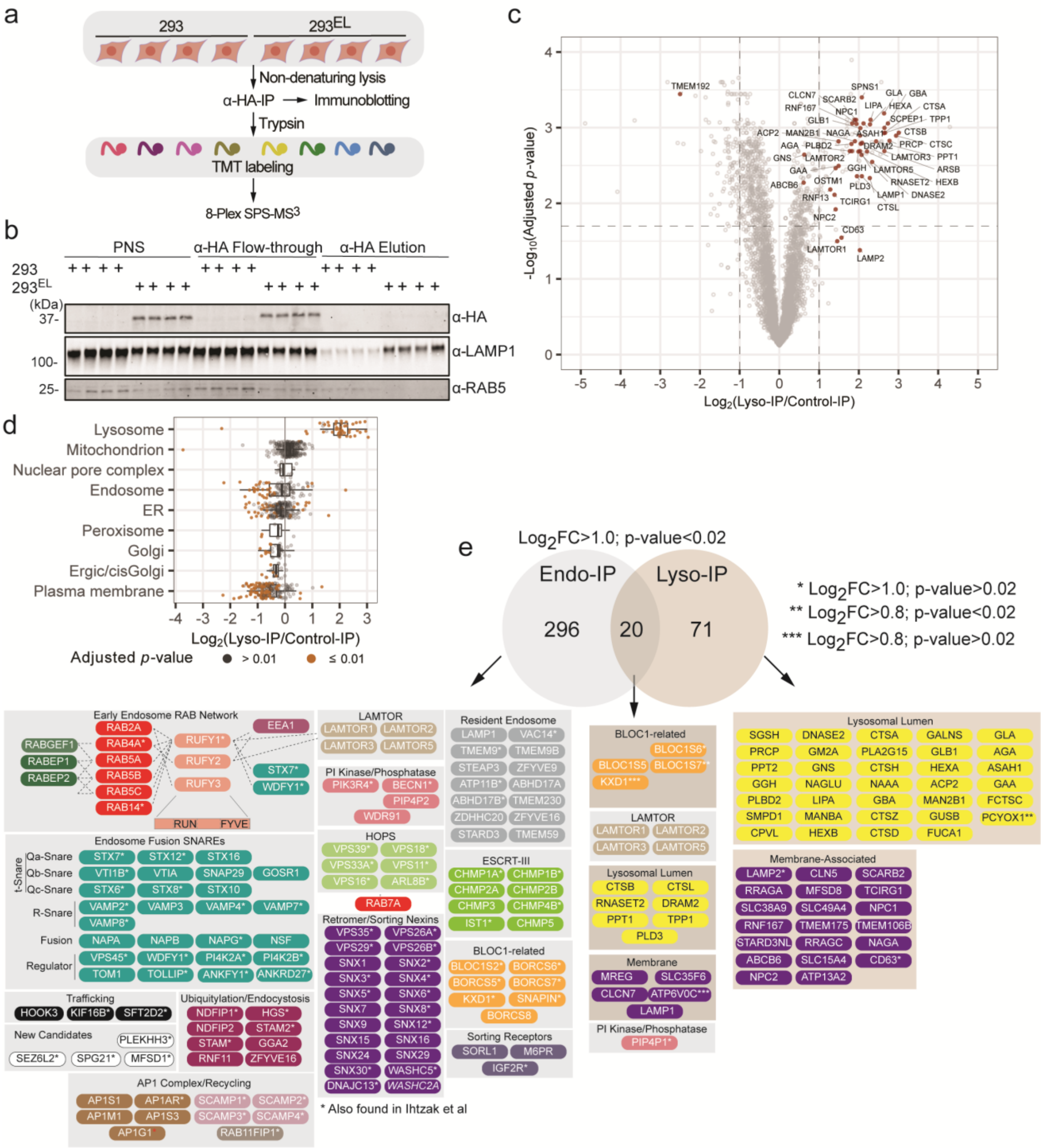

Supplementary Fig. 2. Landscape of the lysosomal proteome and endosomal lipidome.

a, Scheme depicting quantitative proteomics of the Lyso-IP proteome. 293 control cells or 293^{EL} cells (clone 33) in biological quadruplicate (n=4) were subjected to the Lyso-IP protocol (α-HA IP) and either subjected to immunoblotting or trypsinized prior to labeling with TMT and analysis by mass spectrometry.

b, Immunoblot of proteins from the experiment outlined in panel a. Note, TMEM192^{HA} is not released from the α-HA antibody magnetic beads under the elution conditions employed. For input and flow through, 0.05% of total was subjected to immunoblotting, whereas 6% of the elution was analyzed.

c, Proteomic analysis of lysosomes purified by Lyso-IP. Volcano plot for quadruple Lyso-IP associated proteins relative to control cells lacking TMEM192^{HA} is shown. Selected proteins linked with lysosomes are

shown in red. Two-sided Student's t-test was performed and adjusted for multiple comparisons by two-stage Benjamini & Hochberg step-up procedure.
d, Box plots depicting the enrichment of various classes of proteins based on the annotation of Itzhak et al,[32] demonstrating that proteins assigned to lysosomes are the most highly enriched, while endosomal, ER, mitochondrial, and PM proteins are depleted. Orange dots correspond to proteins with an adjusted p-value $<$ 0.01. Left border, interior line, and right border in the box plot represent the 1st quartile, median, and 3rd quartile, respectively.
e, Comparison of proteins associated with Endo- and Lyso-IPs. Proteins with $Log_2FC>1.0$ (p-value<0.02) present in either Endo- or Lyso-IPs were compared in a Venn diagram. Two-sided Student's t-test was performed and adjusted for multiple comparisons by two-stage Benjamini & Hochberg step-up procedure. Proteins that are shared or exclusive to each organelle enrichment method are shown.

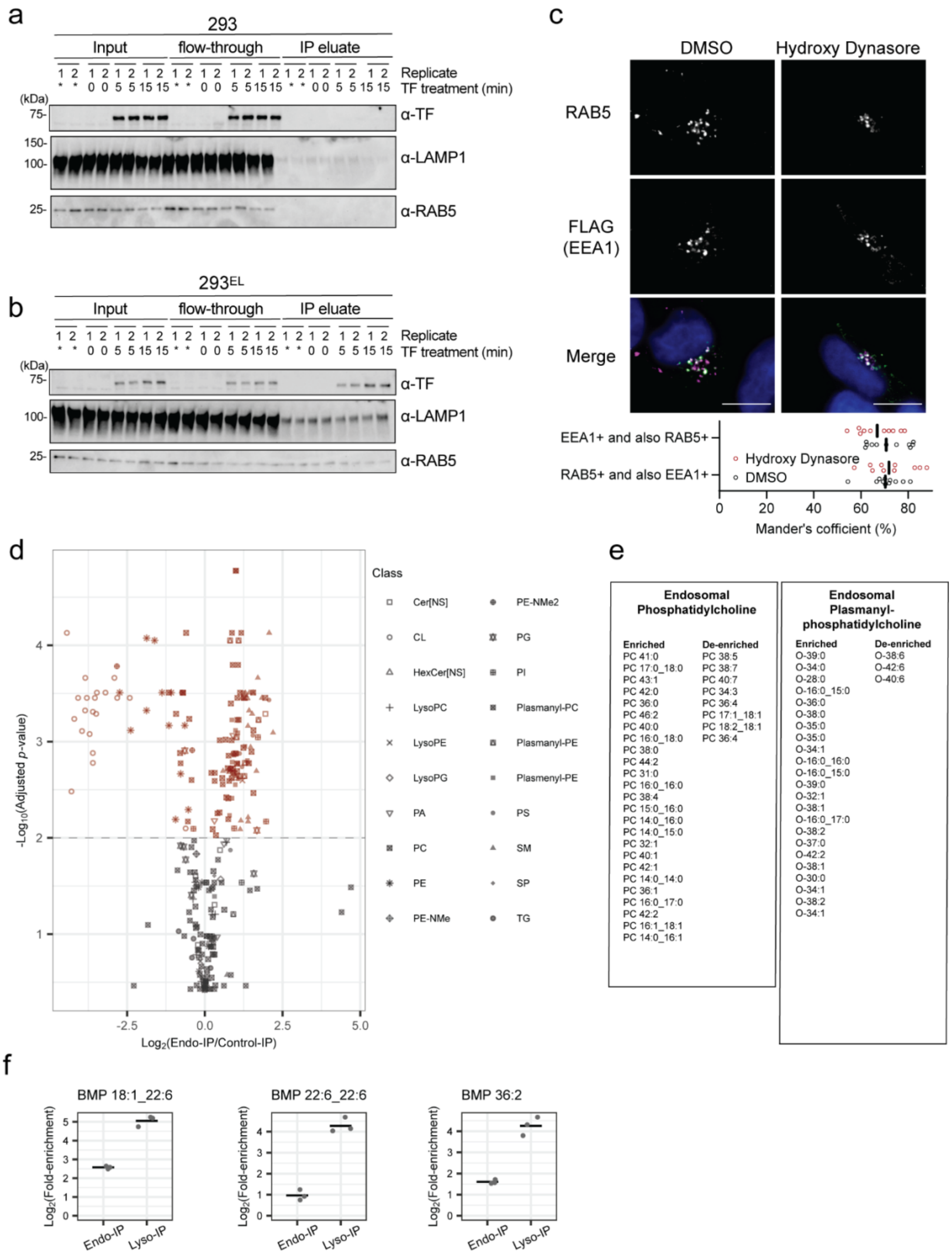

Supplementary Fig. 3. Analysis of Endo-IP for capture of dynamic cargo and lipidome analysis.

a-b, Immunoblots of Endo-IPs from the experiment outlined in Fig. 3a. 293 or 293^{EL} cells (clone 33) were serum starved for 1h prior to addition of serum-free media supplemented with 25 μg/ml holo-transferrin (TF) in duplicate (n=2). At the indicated times, cells were harvested and extracts subjected to Endo-IP followed by immunoblotting with the indicated antibodies. Control cells not serum starved (*). The input and flow-through after the α-FLAG IP are also shown. For input and flow through, 0.5% of total was subjected to immunoblotting, whereas 6% of the elution was analyzed. Proteomic analysis of these samples is shown in **Fig. 3c,d**.
c, 293^{EL} cells (clone 33) were treated with DMSO (as control) or Hydroxy Dynasore (20 μM, 3h) prior to immunofluorescence using α-FLAG (green, to detect FLAG-EEA1) and α-RAB5 (magenta) antibodies, and nuclei were stained with Hoechst 33342 (blue). Image analysis indicates that the Mander's coefficient is ~0.7 for both the overlap of FLAG puncta with RAB5 and RAB5 puncta with FLAG over 10 cells.
d, Volcano plot depicting the enrichment of various lipid classes within Endo-IP as compared with control immune complexes (data from **Supplementary Data 6**) (n=3). Two-sided Student's t-test was performed and adjusted for multiple comparisons by two-stage Benjamini & Hochberg step-up procedure.
e, Individual phosphatidylcholine species found to be enriched or de-enriched in Endo-IPs based on lipidomics (data from **Supplementary Data 6**).
f, Log_2FC for the indicated BMP species identified by lipidomics in either Endo-IP or Lyso-IP samples (n=3) (data from **Supplementary Data 6**).

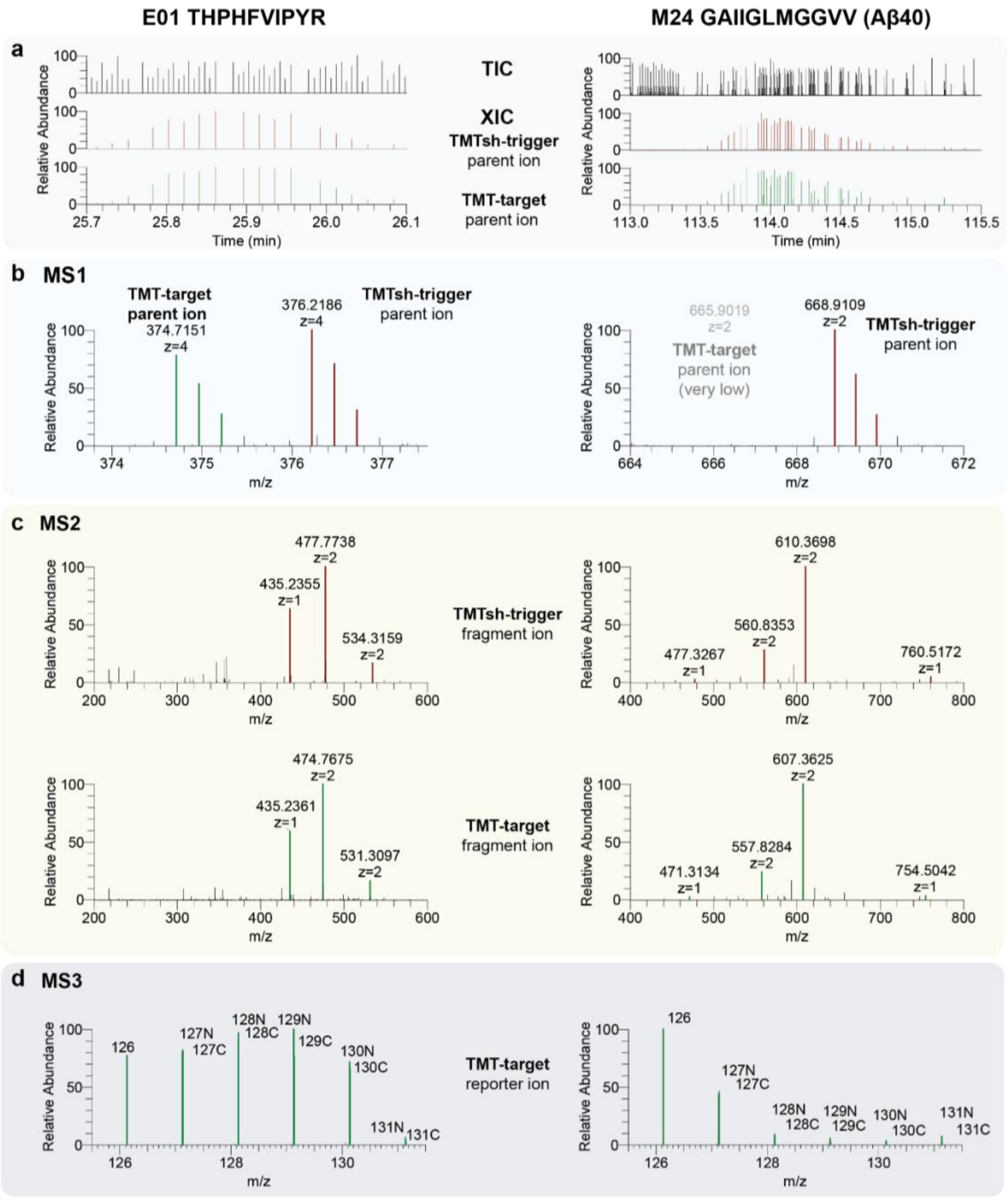

Supplementary Fig. 4. Half-tryptic peptides for quantification of Aβ peptides.
a-d, MS^2 fragmentation patterns for selected APP peptides, indicating fragment ions employed as triggers for TOMAHAQ proteomics.

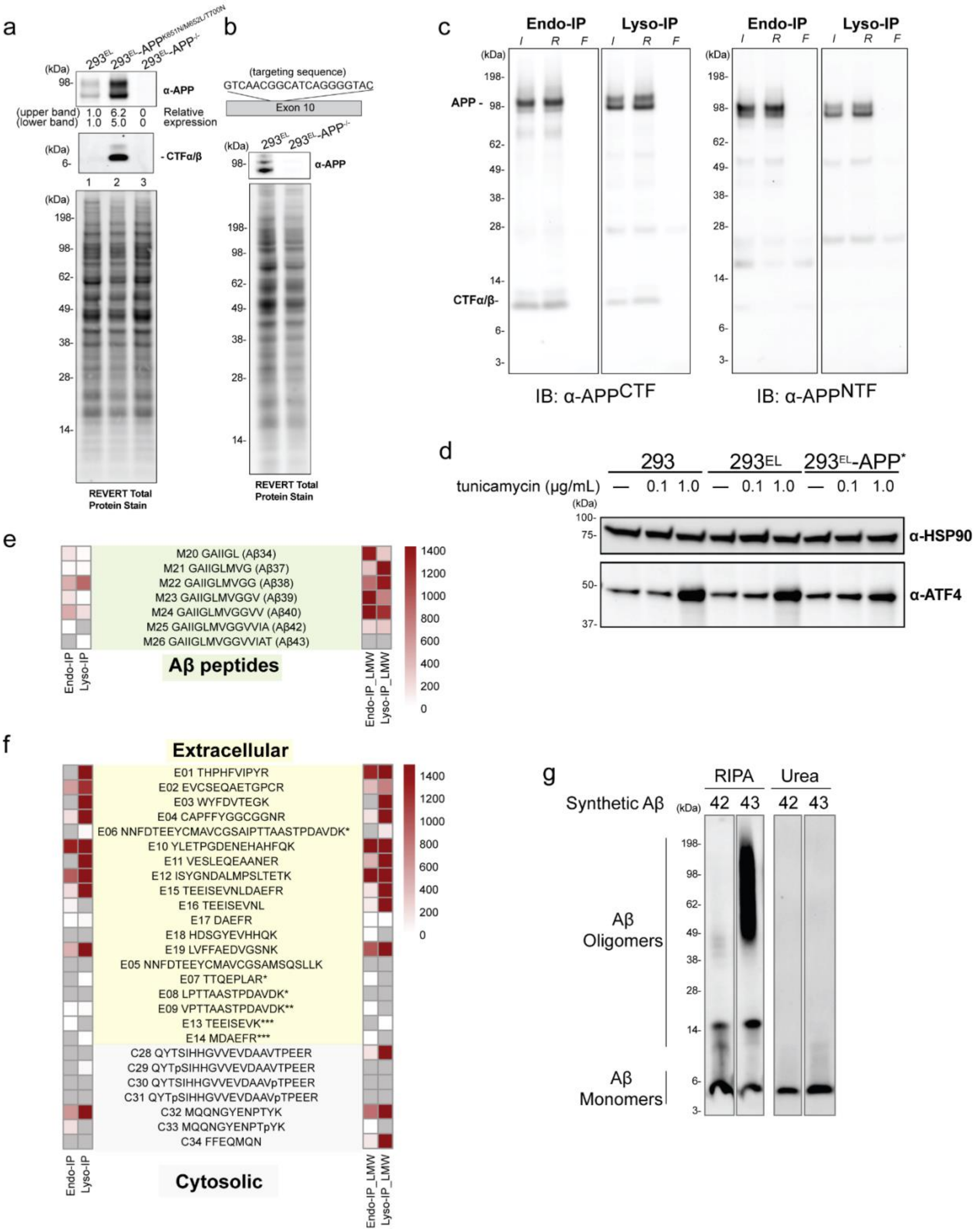

Supplementary Fig. 5. Application of spatial endolysosomal proteomics to analysis of APP processing. a, 293^{EL}-$APP^{-/-}$ cells (see panel b) were transduced with a lentiviral vector expressing $APP^{Sw;T700N}$ to generate 293^{EL}-APP* and extracts from these cells immunoblotted with the indicated antibodies. APP was expressed at

6-fold higher levels than endogenous APP in parental cells. CTFα/β were not appreciably detected in 293^{EL} cells.

b, Creation of 293^{EL} cells lacking APP. The indicated CRISPR gRNA was used to create a frameshift in exon 14 of APP. Extracts from parental and $APP^{-/-}$ cells were subjected to immunoblotting to demonstrate loss of APP, using total amount of loaded proteins as a loading control.

c, Full immunoblots of data shown in **Fig. 5f** probed with α-C-terminal APP (α-APP^{CTF}) and α-N-terminal APP (α-APP^{NTF}) antibodies.

d, 293^{EL} or 293^{EL}-APP* cells were either left untreated or treated with Tunicamycin (1h) as a positive control for induction of the integrated stress response, and cell extracts probed with α-ATF4.

e,f, Heat maps showing the signal/noise (S/N) ratios for APP peptides in the context of biological duplicate Endo-IP or Lyso-IP, with or without LMW enrichment (n=2). Gray squares indicate cases where the endogenous peptide was below the detection limit, whereas white squares indicate peptide detection but with extremely low S/N.

g, SDS-PAGE analysis of synthetic Aβ42/43 solubilized in either RIPA buffer or 8 M urea. Peptides were detected by α-Aβ (6E10). Samples solubilized in RIPA buffer have detectable oligomers and are observed as monomers when solubilized in urea.

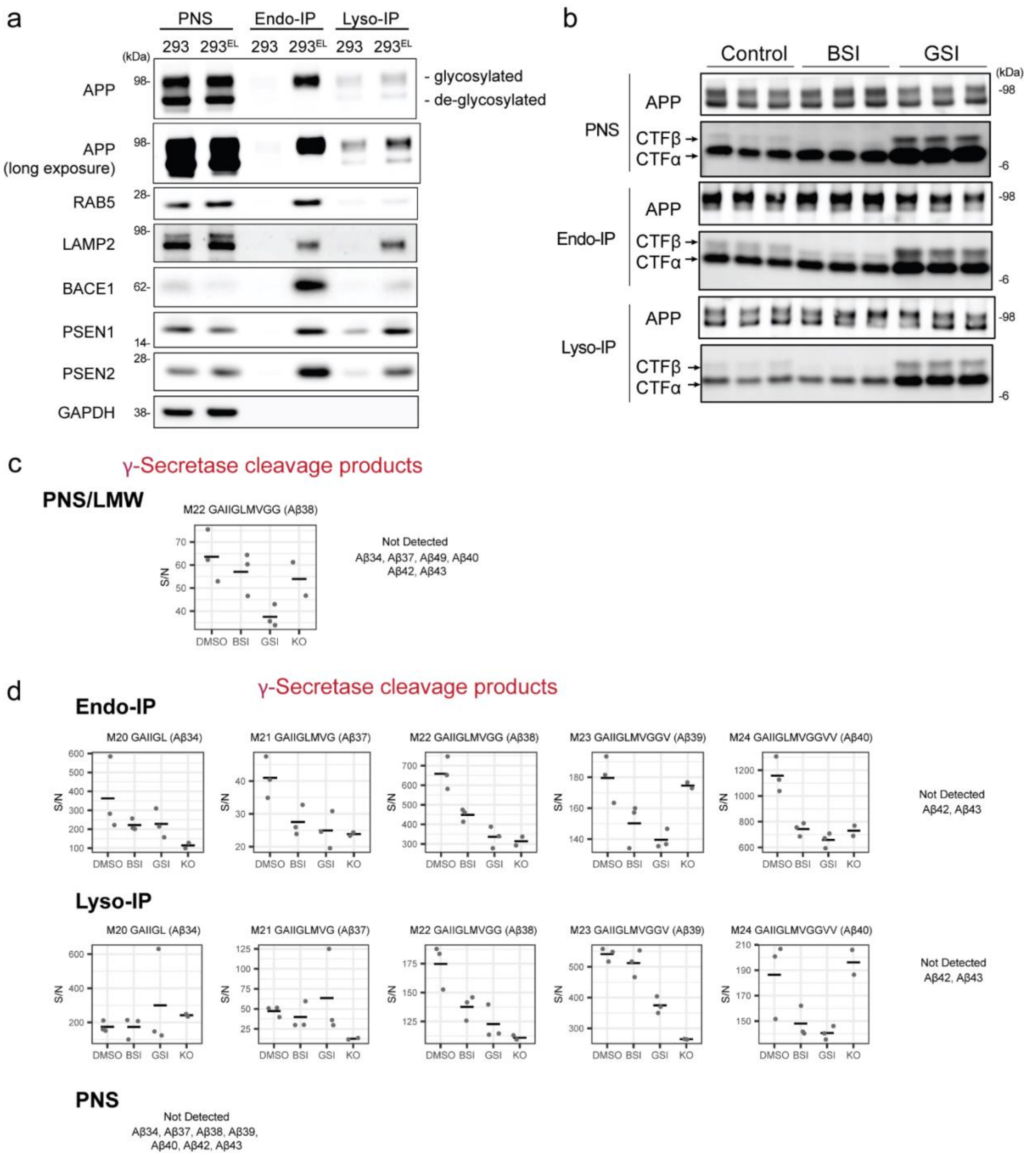

Supplementary Fig. 6. Digital snapshots of APP/Aβ processing in early endosome and lysosomal compartments.

a, 293 or 293^{EL} cells were subjected to Endo-IP (α-FLAG) or Lyso-IP (α-HA) and immune complexes lysed in RIPA buffer prior to immunoblotting with the indicated antibodies. PNS fractions were analyzed in parallel. The positions of glycosylated and de-glycosylated APP are indicated.

b, Immunoblots of full-length APP and the CTFα/β fragments in the PNS, Endo-IP or Lyso-IP corresponding to the experiments shown in **Fig. 6b,c** (biological triplicates, n=3). The images for Endo-IP and Lyso-IP are the same images as in **Fig. 6c** but are shown here to allow comparison with the PNS sample.

c, Signal/noise plots for γ-Secretase cleavage products identified in the PNS sample with LMW enrichment corresponding to **Fig. 6a,b** (biological triplicates, n=3).
d, Signal/noise plots for γ-Secretase cleavage products from PNS, Endo-IP, and Lyso-IP without LMW enrichment. Peptides below the detection limit are indicated (biological triplicates, n=3).

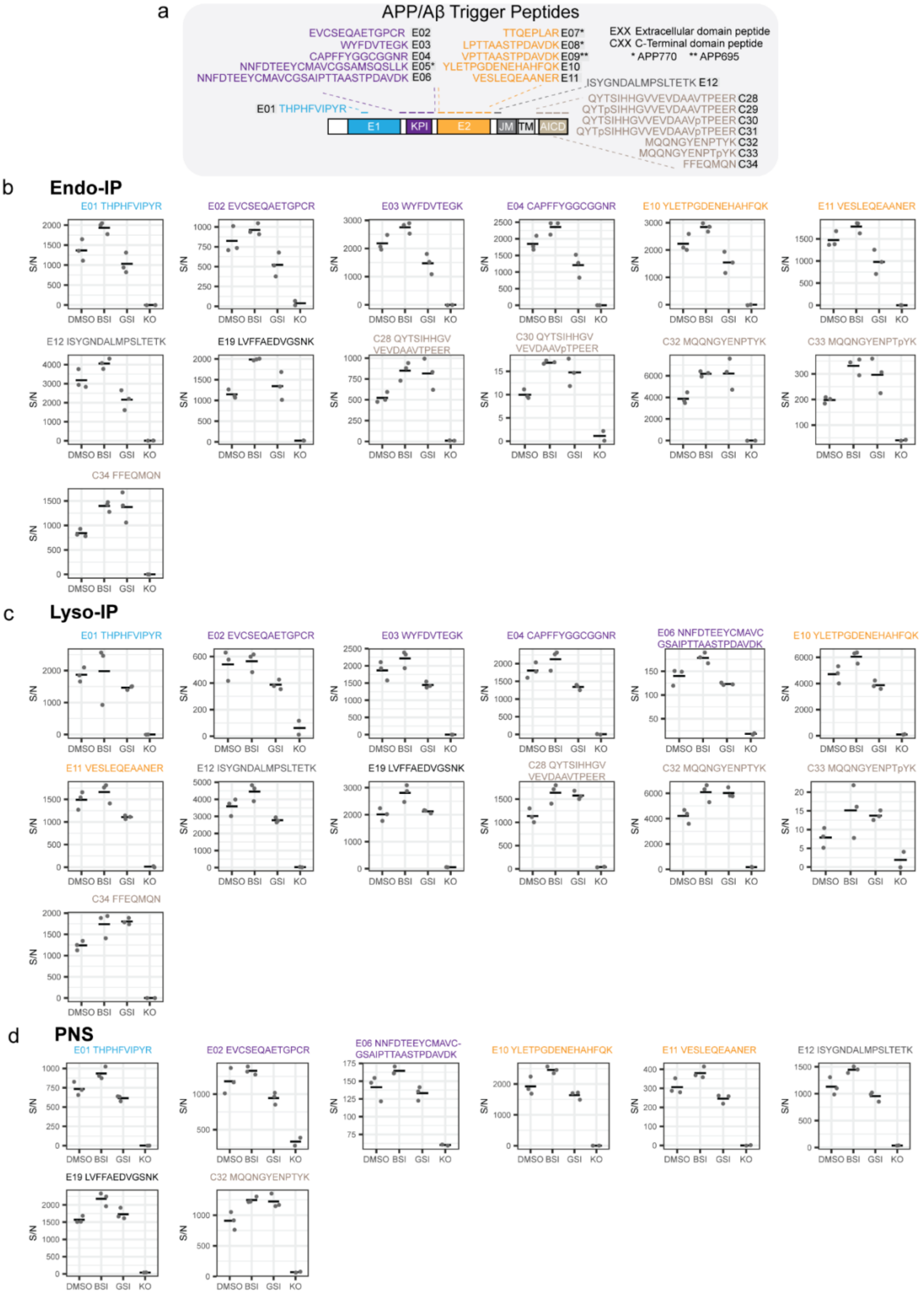

Supplementary Fig. 7. Analysis of peptides derived from extracellular and C-terminal domains in APP present in PNS, Endo-IP and Lyso-IP.

a, Domain schematic of APP indicating the sequences and locations of APP trigger peptides in these regions of the protein.
b-d, Signal/noise plots for peptides derived from the extracellular and C-terminal domains of APP present in the Endo-IP (panel B), Lyso-IP (panel C), and PNS (panel D) without LMW enrichment (corresponding to **Fig. 6a,b**) (biological triplicates, n=3). Color of peptide sequences corresponds to the domain localization in panel A. Peptides not shown were not detected above the signal seen in samples from 293^{EL}-$APP^{-/-}$ cells.

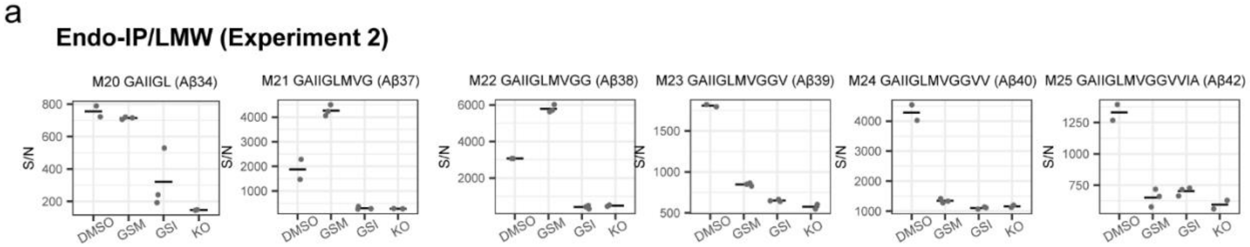

Supplementary Fig. 8. Quantitative assessment of γ-secretase modulator action on APP in early endosomes.
a, Quantitative analysis of Aβ peptides in biological triplicate (n=3) Endo-IP samples after LMW filtration from a fully independent replication of the Endo-IP experiment in **Fig. 7a**. Signal-to-noise for MS^3 intensities (relative to $APP^{-/-}$ cells, n=2) is shown. Asterisks refer to *t*-test of DMSO treated samples versus compound treatment: *n.s.*, not significant; *, $p \leq 0.05$; **, $p \leq 0.01$; ***, $p \leq 0.001$.
