# Supplementary material for: Spatial snapshots of amyloid precursor protein intramembrane processing via early endosome proteomics
Source: Nat Commun. 2022 Oct 16;13:6112. doi: 10.1038/s41467-022-33881-x (PMC9573879; doi:10.1038/s41467-022-33881-x)
Supplement: Supplementary file 3 — Description of Additional Supplementary Files [file 41467_2022_33881_MOESM3_ESM.pdf]

Description of Additional Supplementary Files and Source Data:

Supplementary Data 1. Quantitative proteomic analysis for screening bait proteins to develop Endo-IP (related to **Fig. 1a, Supplementary Fig. 1c**).

Supplementary Data 2. Quantitative total proteomes of cells expressing FLAG-EEA1 (Related to **Supplementary Fig. 1e,f**).

Supplementary Data 3. Quantitative proteomics of FLAG-EEA1-purified endosomes versus control immunoprecipitates (related to **Fig. 2a-e**).

Supplementary Data 4. Quantitative proteomics of TMEM192-HA-purified lysosomes versus control immunoprecipitates (related to **Supplementary Fig. 2a-e**).

Supplementary Data 5. Quantitative proteomics of FLAG-EEA1-purified endosomes in the presence and absence of dynamin inhibitor or TF (related to **Fig. 3a-e; Supplementary Fig. 3a-c**).

Supplementary Data 6. Lipidomic analysis of FLAG-EEA1 immunoprecipitates relative to control cells lacking FLAG-EEA1 (related for **Fig. 3f, Supplementary Fig. 3d-f**).

Supplementary Data 7. APP/Aβ reference peptides employed in TOMAHAQ experiments (related to **Fig. 4e**).

Supplementary Data 8. Aβ40/42/43 in vitro TOMAHAQ data. (related to **Fig. 4c,d**).

Supplementary Data 9. TOMAHAQ analysis of Endo- and Lyso-IP samples with and without filtration (related to **Fig. 5c-g, Supplementary Fig. 5e,f)**).

Supplementary Data 10. TOMAHAQ analysis of Endo- and Lyso-IP samples from cells treated with BSI or GSI (relevant to **Fig. 6a,e,f; Supplementary Fig. 6c,d, Supplementary Fig. 7**).

Supplementary Data 11. Absolute quantification of Endo- and Lyso-IP samples from cells treated with BSI or GSI. (relevant to **Fig. 6a,e,f**).

Supplementary Data 12. TOMAHAQ analysis of Endo- and Lyso-IP samples from cells treated with GSM or GSI (relevant to **Fig. 7c,d**).

Supplementary Data 13. Absolute quantification of Endo- and Lyso-IP samples from cells treated with GSM or GSI. (relevant to **Fig. 7c,d**).

Supplementary Data 14. Details of reagents and materials used in this study.

Supplementary Data 15. Annotated lipid spectra (relevant to **Fig. 3f**).

Source Data 1. Uncropped images from all SDS-PAGE gels in this study.

Source Data 2. Source data for plots.
