# Supplementary material for: Spatial snapshots of amyloid precursor protein intramembrane processing via early endosome proteomics
Source: Nat Commun. 2022 Oct 16;13:6112. doi: 10.1038/s41467-022-33881-x (PMC9573879; doi:10.1038/s41467-022-33881-x)
Supplement: Supplementary file 17 — Supplementary Data 14 [file 41467_2022_33881_MOESM17_ESM.docx]

**Supplementary Data 14. Reagents and materials used in this study.**

| **REAGENT or RESOURCE** | **SOURCE** | **IDENTIFIER** |
| --- | --- | --- |
| **Antibodies [dilution factor]** | | |
| α-EEA1 (C45B10) rabbit mAb [1:1000] | Cell Signaling Technology | 3288; RRID:AB_2096811 |
| α-RAB5 (C8B1) rabbit mAb [1:1000] | Cell Signaling Technology | 3547; RRID:AB_2300649 |
| α-PSEN1 (D39D1) rabbit mAb [1:1000] | Cell Signaling Technology | 5643; RRID:AB10706356_ |
| α-PSEN2/AD5 (EP1515Y) rabbit mAb [1:1000] | Abcam | ab51249; RRID:AB_882202 |
| α-LAMP1 (D2D11) rabbit mAb [1:1000] | Cell Signaling Technology | 9091; RRID:AB_2687579 |
| α-LAMP2 (D5C2P) rabbit mAb [1:1000] | Cell Signaling Technology | 49067; RRID:AB_2799349 |
| α-TMEM192 rabbit pAb [1:1000] | Proteintech | 28263-1-AP; RRID:AB_2881099 |
| α-HA [1:1000] | Biolegend | 901513; RRID:AB_2565335 |
| α-HA (6E2) mouse mAb [1:1000] | Cell Signaling Technology | 2367; RRID:AB_10691311 |
| α-FLAG M2 mouse mAb [1:1000] | Sigma-Aldrich | F1804; RRID:AB_262044 |
| α-ZO-1 rabbit pAb [1:1000] | Proteintech | 21773-1-AP; RRID:AB_10733242 |
| α-Golga1 rabbit pAb [1:1000] | Proteintech | 12640-1-AP; RRID:AB_2115315 |
| α-Calreticulin rabbit pAb [1:1000] | Proteintech | 10292-1-AP; RRID;AB_513777 |
| α-RAB11 (D4F5) rabbit mAb [1:1000] | Cell Signaling Technology | 5589; RRID:AB_10693925 |
| α-Lamin A/C (4C11) mouse mAb [1:1000] | Cell Signaling Technology | 4777; RRID:AB_10545756 |
| α-VDAC1/Porin rabbit pAb [1:1000] | Proteintech | 55259-1-AP; RRID:AB_10837225 |
| α-RAB7 (D95F2) rabbit mAb [1:1000] | Proteintech | 9367 |
| α-DYKDDDDK tag, mouse mAb (FG4R) [1:1000] | Thermo Fisher Scientific | MA1-91878; RRID:AB_1957945 |
| α-GAPDH (D16H11) XP rabbit mAb [1:1000] | Cell Signaling Technology | 5174; RRID: AB_10622025 |
| α-APP CTF (C1/6.1) mouse mAb [1:1000] | BioLegend | 802801; RRID:AB_2564648 |
| α-APP A4 (22C11) mouse mAb [1:1000] | Sigma | MAB348 |
| α-TFRC/CD71 (D7G9X) [1:1000] | Cell Signaling Technology | 13113; RRID:AB_2715594 |
| α-Transferrin [1:1000] | Abcam | Ab82411; RRID:AB_1659060 |
| α-ATF4/CREB (B-3) [1:1000] | Santa Cruz Biotechnology | SC-390063; RRID:AB_2810998 |
| α-PEX19 rabbit pAb [1:1000] | Proteintech | 14713-1-AP; RRID:AB_2162265 |
| α-CD71/TFR1 (D7G9X) rabbit mAb [1:1000] | Cell Signaling Technology | 13113; RRID:AB_2715594 |
| α-HSP90 (3F11C1) mouse mAb [1:1000] | Proteintech | 60318-1-Ig; RRID:AB_2881429 |
| α-BACE1 (D10E5) rabbit mAb [1:1000] | Cell Signaling Technology | 5606; RRID:AB_1903900 |
| IRDye 680RD Goat α-Rabbit IgG secondary antibody [1:10,000] | Li-Cor | 926-68071; RRID:AB_10956166 |
| IRDye 680RD Goat α-Mouse IgG secondary antibody [1:10,000] | Li-Cor | 926-68070; RRID:AB_10956588 |
| IRDye 800CW Goat α-Rabbit IgG secondary antibody [1:10,000] | Li-Cor | 926-32211; RRID:AB_621843 |
| IRDye 800CW Goat α-Mouse IgG secondary antibody [1:10,000] | Li-Cor | 926-32210; RRID:AB_621842 |
| Goat α-Rabbit IgG, HRP-linked antibody [1:5000] | Cell Signaling Technology | 7474P2 |
| Goat α-Rabbit IgG HRP conjugate [1:5000] | Bio-Rad | 1706515; RRID:AB_11125142 |
| Goat α-Mouse IgG HRP conjugate [1:5000] | Bio-Rad | 1706516; RRID:AB_11125547 |
| Alexa Fluor 594 Goat α-Rabbit IgG (H+L) cross-adsorbed secondary antibody [1:400] | Thermo Fisher Scientific | A-11012; RRID:AB_2534079 |
| Alexa Fluor 488 Goat α-Mouse IgG (H+L) highly cross-adsorbed secondary antibody [1:400] | Thermo Fisher Scientific | A-11029; RRID:AB_2534088 |
| **Bacterial and virus strains** |  |  |
| DH5 alpha E. coli cells | ThermoFisher | 18265017 |
| T1R E. coli cells | ThermoFisher | C854003 |
| **Chemicals, peptides, and recombinant proteins** | | |
| Q5 Hot Start High-Fidelity DNA Polymerase | New England BioLabs | M0493 |
| QuikChange II Site-Directed Mutagenesis Kit | Agilent | 200523 |
| α-FLAG M2 magnetic beads | Sigma-Aldrich | M8823 |
| Pierce α-HA magnetic beads | Thermo Fisher Scientific | 88837: RRID:AB_2861399 |
| TMT10plex Isobaric Label Reagent Set plus TMT11-131C Label Reagent | Thermo Fisher Scientific | A34808 |
| TMTPro^TM^ 16Plex Label Reagent set | Thermo Fisher Scientific | A44520 |
| Super Heavy TMT Label Reagent | Thermo Fisher Scientific | A43073 |
| Lipofectamine 3000 | Invitrogen | L3000008 |
| Pierce™ High pH Reversed-Phase Peptide Fractionation Kit | Thermo Fisher Scientific | 84868 |
| HyClone Fetal bovine serum | GE Healthcare | SB30910 |
| Puromycin | Sigma-Aldrich | P9620 |
| G418 (Geneticin) | Invivogen | ant-gn-2 |
| Dulbecco’s MEM (DMEM), high glucose, pyruvate | GIBCO / Invitrogen | 11995 |
| holo-Transferrin, human | Sigma-Aldrich | T0665 |
| Transferrin from human serum, Alexa Fluor 647 conjugate | Thermo Fisher Scientific | T23366 |
| PhosSTOP | Roche | 04906845001 |
| Hoechst 33342, Trihydrochloride, Trihydrate | Thermo Fisher Scientific | H3570 |
| Complete EDTA-free protease inhibitor cocktail | Sigma-Aldrich | 11873580001 |
| Tris(2-carboxyethyl)phosphine hydrochloride solution | Sigma-Aldrich | 646547 |
| Iodoacetamide | Sigma-Aldrich | I1149 |
| Trichloroacetic acid solution 6.1 N | Sigma-Aldrich | T0699 |
| Trifluoroacetic acid | fisher scientific | A11650 |
| Hydroxylamine solution 50 wt. % | Sigma-Aldrich | 438227 |
| Formic Acid | Sigma-Aldrich | 5330020050 |
| Pierce Trypsin Protease, MS grade | Thermo Fisher Scientific | 90305 |
| Lysyl endopeptidaseR (Lys-C) | Wako | 129-02541 |
| Poly-l-lysine solution, 0.01% | Sigma-Aldrich | P4832 |
| Paraformaldehyde, 16% solution | Electron Microscopy Services | 15710 |
| ProLong Glass Antifade Mountant | Thermo Fisher Scientific | P36982 |
| REVERT 700 total protein stain kit | Li-Cor | 926-11016 |
| NuPAGE LDS sample buffer (4X) | Thermo Fisher Scientific | NP0007 |
| NuPAGE sample reducing agent (10X) | Thermo Fisher Scientific | NP0009 |
| Bio-Rad Protein Assay Dye Reagent Concentrate | Bio-Rad | 5000006 |
| NuPAGE MES SDS Running Buffer (20X) | Thermo Fisher Scientific | NP0002 |
| Immobilon-FL PVDF Membrane | Millipore | IPFL00010 |
| Hydrogen peroxide solution, 30% (w/w) in H2O | Sigma-Aldrich | H1009 |
| WHEATON Dounce Tissue Grinder, 7 mL | DWK Life Sciences | 357542 |
| KIMBLE KONTES Dounce Tissue Grinder, 2 mL | DWK Life Sciences | 885300-0002 |
| Hydroxylamine solution | Sigma-Aldrich | 438337 |
| Nonidet P40 substitute | Sigma-Aldrich | 74385 |
| Urea | Sigma-Aldrich | U5378 |
| EPPS 0.2M buffer solution, pH 8.5 | Alfa Aesar | J61476.AE |
| Empore C18 47 mm Extraction Disc, Model 2215 | 3M | 98060402173 |
| Sep-Pak C18 1 cc Vac Cartridge | Waters | WAT054955 |
| Pierce Quantitative Fluorometric Peptide Assay | Thermo Fisher Scientific | 23290 |
| Hydroxy Dynasore (Dyngo^TM^4a) | Cayman Chemical | 29479 |
| Lanabecestat (AZD3293) | Selleckchem | S8193 |
| Semagacestat | Cayman Chemical | 16713 |
| BPN-15606 | MedChemExpress | HY-117482 |
| Beta-Amyloid (1-38) | rPeptide | A-1078-1 |
| Beta-Amyloid (1-40) | rPeptide | A-1175-025 |
| Beta-Amyloid (1-42) | rPeptide | A-1167-025 |
| Beta-Amyloid (1-43) | rPeptide | A-1005-1 |
| Amicon Ultra-0.5 centrifugal filter, 10 kDa NMWL | Millipore | UFC501096 |
| Amicon Ultra-0.5 centrifugal filter, 30 kDa NMWL | Millipore | UFC503024 |
| Amicon Ultra-0.5 centrifugal filter, 50 kDa NMWL | Millipore | UFC505024 |
| RIPA lysis and extraction buffer | Thermo Fisher | 89900 |
| High precision glass cover slip, No 1.5, 12mm diameter | Bioscience Tools | CSHP-No1.5-12 |
| Reference peptides for APP/Aβ (see Supplementary Data Table S7) | Biomatik  Thermo Fisher Scientific | Custom order |
| **Experimental models: Cell lines** |  |  |
| 293T cells | ATCC | CRL-3216; RRID:CVCL_0063 |
| 293 cells | ATCC | CRL-1573; RRID: CVCL_0045 |
| 293^L^: TMEM192-3xHA | This study |  |
| 293^L^-APP^-/-^: TMEM192-3xHA; APP^-/-^ | This study |  |
| 293^EL^-APP^-/-^: TMEM192-3xHA; APP-/-; FLAG-EEA1 | This study |  |
| 293^EL^-APP*: TMEM192-3xHA; APP^-/-^; FLAG-EEA1; APP^Sw;T700N^ | This study |  |
| **Recombinant DNA** |  |  |
| pSMART TMEM192-3xHA (targeting vector for genomic tagging) | Eapen et al., 2021 ^36^ | Addgene #175777; RRID:Addgene_175777 |
| pHAGE-FLAG-EEA1 | This study | Addgene#176491; RRID:Addgene_176491 |
| pHAGE-FLAG-RAB11A | This study | Addgene#176489; RRID:Addgene_176489 |
| pPHAGE-FLAG-TFR1 | This study | Addgene#176490; RRID:Addgene_176490 |
| pPHAGE-FLAG-RAB5A | This study | Addgene#176488; RRID:Addgene_176488 |
| pX459-gRNA-APP (for making APP deletion by CRISPR/Cas9) | This study | Addgene#176487; RRID:Addgene_176488 |
| pENTR221-APP751 | DNA Resource Core, Harvard Medical School | HsCD00431993 |
| pHAGE-APP^Sw;T700N^ | This study | Addgene#177094; RRID:Addgene_177094 |
| **Software and algorithms** |  |  |
| Prism v9 | GraphPad | https://www.graphpad.com/scientificsoftware/prism/ |
| Comet 2019.01 | Eng et al 2013^61^ | http://comet-ms.sourceforge.net/ |
| Fiji | ImageJ and SciJava projects ^78^ | https://imagej.net/software/fiji/ |
| ImageLab v6.0.1 | Biorad | https://www.bio-rad.com/en-us/product/image-lab-software?ID=KRE6P5E8Z&source_wt=imagelabsoftware_surl |
| MetaMorph v7.10 | Molecular Devices | https://www.moleculardevices.com/products/cellular-imaging-systems/acquisition-and-analysis-software/metamorph-microscopy#gref |
| Tomahto v1 API (This API is freely available, although an API license from Thermo is necessary for installation on instrument.) | Yu et al., 2020^35^ | https://gygi.hms.harvard.edu/software.html |
| R 3.6.3 | R Core Team | https://www.r-project.org/ |
| LipiDex v1.1 | Hutchins et al., 2018^50^ | https://github.com/coongroup/LipiDex |
| Compound Discoverer 2.1 | Thermo Fisher Scientific | https://www.thermofisher.com/us/en/home/industrial/mass-spectrometry/liquid-chromatography-mass-spectrometry-lc-ms/lc-ms-software/multi-omics-data-analysis/compound-discoverer-software.html |
| LipiDex Spectrum Annotator | This study | https://github.com/coongroup/LipiDexSpectrumAnnotator |
| Skyline v20.2 | MacLean et al., 2010 ^79^ | https://skyline.ms/project/home/begin.view? |
| **Instrument** |  |  |
| Orbitrap Fusion Lumos Tribrid Mass Spectrometer | Thermo Fisher Scientific | IQLAAEGAAPFADBMBHQ |
| Orbitrap Eclipse Tribrid Mass Spectrometer | Thermo Fisher Scientific | FSN04-10000 |
| Agilent 1260 Infinity HPLC | Agilent | G1311B, G1316A, G1329B, G1315D |
| Aeris 2.6 μm PEPTIDE XB-C18 100 Å, LC column | Phenomenex | 00G-4505-E0 |
