# Supplementary material for: Spatial snapshots of amyloid precursor protein intramembrane processing via early endosome proteomics
Source: Nat Commun. 2022 Oct 16;13:6112. doi: 10.1038/s41467-022-33881-x (PMC9573879; doi:10.1038/s41467-022-33881-x)
Supplement: Supplementary file 19 — Reporting Summary [file 41467_2022_33881_MOESM19_ESM.pdf]

nature portfolio

Corresponding author(s): Harper

Last updated by author(s): Sep 14, 2022

Reporting Summary

Nature Portfolio wishes to improve the reproducibility of the work that we publish. This form provides structure for consistency and transparency in reporting. For further information on Nature Portfolio policies, see our Editorial Policies and the Editorial Policy Checklist.

Statistics

For all statistical analyses, confirm that the following items are present in the figure legend, table legend, main text, or Methods section.

n/a	Confirmed	
☐	☒	The exact sample size (*n*) for each experimental group/condition, given as a discrete number and unit of measurement
☐	☒	A statement on whether measurements were taken from distinct samples or whether the same sample was measured repeatedly
☐	☒	The statistical test(s) used AND whether they are one- or two-sided<br>*Only common tests should be described solely by name; describe more complex techniques in the Methods section.*
☒	☐	A description of all covariates tested
☒	☐	A description of any assumptions or corrections, such as tests of normality and adjustment for multiple comparisons
☐	☒	A full description of the statistical parameters including central tendency (e.g. means) or other basic estimates (e.g. regression coefficient) AND variation (e.g. standard deviation) or associated estimates of uncertainty (e.g. confidence intervals)
☐	☒	For null hypothesis testing, the test statistic (e.g. *F*, *t*, *r*) with confidence intervals, effect sizes, degrees of freedom and *P* value noted<br>*Give P values as exact values whenever suitable.*
☒	☐	For Bayesian analysis, information on the choice of priors and Markov chain Monte Carlo settings
☒	☐	For hierarchical and complex designs, identification of the appropriate level for tests and full reporting of outcomes
☐	☒	Estimates of effect sizes (e.g. Cohen's *d*, Pearson's *r*), indicating how they were calculated

Our web collection on statistics for biologists contains articles on many of the points above.

Software and code

Policy information about availability of computer code

Data collection

Tomahto API v1 (This API is freely available, although an API license from Thermo is necessary for installation on instrument.) Yu et al., 2020 Ref.35; https://gygi.hms.harvard.edu/software.html
Orbitrap Eclipse Tribrid Mass Spectrometer (Cat#FSN04-10000) with FAIMS Pro Interface (#FMS02-10001) - Thermo Fisher Scientific
Orbitrap Fusion Lumos Tribrid MS (Cat#IQLAAEGAAPFADBMBHQ) with or without FAIMS Pro Interface (#FMS02-10001) - Thermo Fisher Scientific
Orbitrap Fusion Tribrid MS (Cat#IQLAAEGAAPFADBMBCX) with FAIMS Pro Interface (#FMS02-10001) - Thermo Fisher Scientific

Data analysis

Quantification of targeted proteomics data: Skyline v20.2 MacLean et al., 2010 Ref. 79 ; https://skyline.ms/project/home/begin.view?
Annotation of lipidomics sprectra: LipiDex Spectrum Annotator This study https://github.com/coongroup/LipiDexSpectrumAnnotator
Lipidomics identification software: Compound Discoverer 2.1 Thermo Fisher Scientific https://www.thermofisher.com/us/en/home/industrial/mass-spectrometry/liquid-chromatography-mass-spectrometry-lc-ms/lc-ms-software/multi-omics-data-analysis/compound-discoverer-software.html
Lipidomics analysis: LipiDex v1.1 Hutchins et al., 2018 Ref. 50 https://github.com/coongroup/LipiDex
Programming language: R 3.6.3 R Core Team https://www.r-project.org/
Analysis of cells visualized by light microscopy: MetaMorph v7.10 Molecular Devices https://www.moleculardevices.com/products/cellular-imaging-systems/acquisition-and-analysis-software/metamorph-microscopy#
Visualization of immunoblots: ImageLab v6.0.1 Biorad https://www.bio-rad.com/en-us/product/image-lab-software?ID=KRE6P5E8Z&source_wt=imagelabsoftware_surl
Quantification of images: Fiji ImageJ and SciJava projects Ref.78 https://imagej.net/software/fiji/
Peptide identification from mass spectrometry data: Comet release 2019.01; Eng et al 2013 Ref. 61; http://comet-ms.sourceforge.net/
Statistical analysis and graphing of data: Prism v9 GraphPad https://www.graphpad.com/scientificsoftware/prism/

For manuscripts utilizing custom algorithms or software that are central to the research but not yet described in published literature, software must be made available to editors and reviewers. We strongly encourage code deposition in a community repository (e.g. GitHub). See the Nature Portfolio guidelines for submitting code & software for further information.

Data

Policy information about availability of data

All manuscripts must include a data availability statement. This statement should provide the following information, where applicable:

- Accession codes, unique identifiers, or web links for publicly available datasets
- A description of any restrictions on data availability
- For clinical datasets or third party data, please ensure that the statement adheres to our policy

All MS raw files have been deposited in MassIVE with the identifiers MSV000088132 (proteomics) [https://doi.org/doi:10.25345/C5RN99] and MSV000088048 (lipidomics) [https://doi.org/doi:10.25345/C5MC31]. Annotated lipid spectra are in Supplementary Data File 15. Uncropped images can be found in Source Data 1. Source data for individual plots can be found in Source Data 2. The searching database for proteomics was constructed from Swiss-Prot human database (released on Jun 17, 2020 at UniProt, [https://ftp.uniprot.org/pub/databases/uniprot/previous_major_releases/release-2020_06/])

Human research participants

Policy information about studies involving human research participants and Sex and Gender in Research.

Reporting on sex and gender	not applicable
Population characteristics	not applicable
Recruitment	not applicable
Ethics oversight	not applicable

Note that full information on the approval of the study protocol must also be provided in the manuscript.

Field-specific reporting

Please select the one below that is the best fit for your research. If you are not sure, read the appropriate sections before making your selection.

☒ Life sciences ☐ Behavioural & social sciences ☐ Ecological, evolutionary & environmental sciences

For a reference copy of the document with all sections, see nature.com/documents/nr-reporting-summary-flat.pdf

Life sciences study design

All studies must disclose on these points even when the disclosure is negative.

Sample size	For proteomics experiments, we chose n=3 or n=4 (biological) given the limitation of the available TMT channels, but because these are multiplexed, the actual "n" for determination of relative abundance is the same of all samples in the plex . Lipidomics were performed in biological triplicate. No sample-size calculation was performed. Sample size was determined to be adequate based on the magnitude and consistency of measurable differences between groups and low observed variability between samples.
Data exclusions	No data were excluded from the analyses.
Replication	We confirm that all attempts at replication were successful and consistent at least two independent experiments.
Randomization	Proteomics samples with TMT and TMTpro reagents were randomly allocated in the TMT/TMTpro group while replicates were in adjacent channels. For other experiments, no randomization was done.
Blinding	Blinding was not performed as the data analysis was not subjective but definitely quantitative.

Reporting for specific materials, systems and methods

We require information from authors about some types of materials, experimental systems and methods used in many studies. Here, indicate whether each material, system or method listed is relevant to your study. If you are not sure if a list item applies to your research, read the appropriate section before selecting a response.

Materials & experimental systems

n/a	Involved in the study
☐	☒ Antibodies
☐	☒ Eukaryotic cell lines
☒	☐ Palaeontology and archaeology
☒	☐ Animals and other organisms
☒	☐ Clinical data
☒	☐ Dual use research of concern

Methods

n/a	Involved in the study
☒	☐ ChIP-seq
☒	☐ Flow cytometry
☒	☐ MRI-based neuroimaging

Antibodies

Antibodies used

anti-EEA1 (C45B10) rabbit mAb Cell Signaling Technology 3288; RRID:AB_2096811
anti-RAB5 (C8B1) rabbit mAb Cell Signaling Technology 3547; RRID:AB_2300649
anti-PSEN1 (D39D1) rabbit mAb Cell Signaling Technology 5643; RRID:AB10706356_
anti-PSEN2/AD5 (EP1515Y) rabbit mAb Abcam ab51249; RRID:AB_882202
anti-LAMP1 (D2D11) rabbit mAb Cell Signaling Technology 9091; RRID:AB_2687579
anti-LAMP2 (D5C2P) rabbit mAb Cell Signaling Technology 49067; RRID:AB_2799349
anti-TMEM192 rabbit pAb Proteintech 28263-1-AP; RRID:AB_2881099
anti-HA Biolegend 901513; RRID:AB_2565335
anti-HA (6E2) mouse mAb Cell Signaling Technology 2367; RRID:AB_10691311
anti-FLAG M2 mouse mAb Sigma-Aldrich F1804; RRID:AB_262044
anti-ZO-1 rabbit pAb Proteintech 21773-1-AP; RRID:AB_10733242
anti-Golga1 rabbit pAb Proteintech 12640-1-AP; RRID:AB_2115315
anti-Calreticulin rabbit pAb Proteintech 10292-1-AP; RRID;AB_513777
anti-RAB11 (D4F5) rabbit mAb Cell Signaling Technology 5589; RRID:AB_10693925
anti-Lamin A/C (4C11) mouse mAb Cell Signaling Technology 4777; RRID:AB_10545756
anti-VDAC1/Porin rabbit pAb Proteintech 55259-1-AP; RRID:AB_10837225
anti-RAB7 (D95F2) rabbit mAb Proteintech 9367
anti-DYKDDDDK tag, mouse mAb (FG4R) Thermo Fisher Scientific MA1-91878; RRID:AB_1957945
anti-GAPDH (D16H11) XP rabbit mAb Cell Signaling Technology 5174; RRID: AB_10622025
anti-APP CTF (C1/6.1) mouse mAb BioLegend 802801; RRID:AB_2564648
anti-APP A4 (22C11) mouse mAb Sigma MAB348
anti-TFRC/CD71 (D7G9X) Cell Signaling Technology 13113; RRID:AB_2715594
anti-Transferrin Abcam Ab82411; RRID:AB_1659060
anti-ATF4/CREB (B-3) Santa Cruz Biotechnology SC-390063; RRID:AB_2810998
anti-PEX19 rabbit pAb Proteintech 14713-1-AP; RRID:AB_2162265
anti-CD71/TFR1 (D7G9X) rabbit mAb Cell Signaling Technology 13113; RRID:AB_2715594
anti-HSP90 (3F11C1) mouse mAb Proteintech 60318-1-Ig; RRID:AB_2881429
anti-BACE1 (D10E5) rabbit mAb Cell Signaling Technology 5606; RRID:AB_1903900
IRDye 680RD Goat anti-Rabbit IgG secondary antibody Li-Cor 926-68071; RRID:AB_10956166
IRDye 680RD Goat anti-Mouse IgG secondary antibody Li-Cor 926-68070; RRID:AB_10956588
IRDye 800CW Goat anti-Rabbit IgG secondary antibody Li-Cor 926-32211; RRID:AB_621843
IRDye 800CW Goat anti-Mouse IgG secondary antibody Li-Cor 926-32210; RRID:AB_621842
Goat anti-Rabbit IgG, HRP-linked antibody Cell Signaling Technology 7474P2
Goat anti-Rabbit IgG HRP conjugate Bio-Rad 1706515; RRID:AB_11125142
Goat anti-Mouse IgG HRP conjugate Bio-Rad 1706516; RRID:AB_11125547
Alexa Fluor 594 Goat anti-Rabbit IgG (H+L) cross-adsorbed secondary antibody Thermo Fisher Scientific A-11012; RRID:AB_2534079
Alexa Fluor 488 Goat anti-Mouse IgG (H+L) highly cross-adsorbed secondary antibody Thermo Fisher Scientific A-11029; RRID:AB_2534088

* Dilution factor for each antibody is described in the Supplementary Table 14.

Validation

Specificity (human) for antibodies against APP, CTF, and A4 were validated using cells that lack APP (created by CRISPR-Cas9 gene deletion). Similarly, PSEN1, PSN2 and BACE1 antibodies were validated using cells lacking the respective genes. All antibodies were commercially obtained, and validation was based on the datasheets from the manufacturer as well as the extensive usage in the field.

Eukaryotic cell lines

Policy information about cell lines and Sex and Gender in Research

Cell line source(s)

Human: HEK293 ATCC CRL-1573; RRID:CVCL_0045.
293_L and 293_EL cells were derived from HEK293 using CRISPR-Cas9 gene editing and lentiviral transduction.

Authentication

Karyotyping (GTG-banded karyotype) of the original HEK293 was performed by Brigham and Women's Hospital Cytogenomics Core Laboratory.

Mycoplasma contamination

All cell lines were found to be free of mycoplasma using Mycoplasma Plus PCR assay kit (Agilent).

Commonly misidentified lines
(See ICLAC register)

none
