# Supplementary material for: Spatial snapshots of amyloid precursor protein intramembrane processing via early endosome proteomics
Source: Nat Commun. 2022 Oct 16;13:6112. doi: 10.1038/s41467-022-33881-x (PMC9573879; doi:10.1038/s41467-022-33881-x)

Fig. 1d

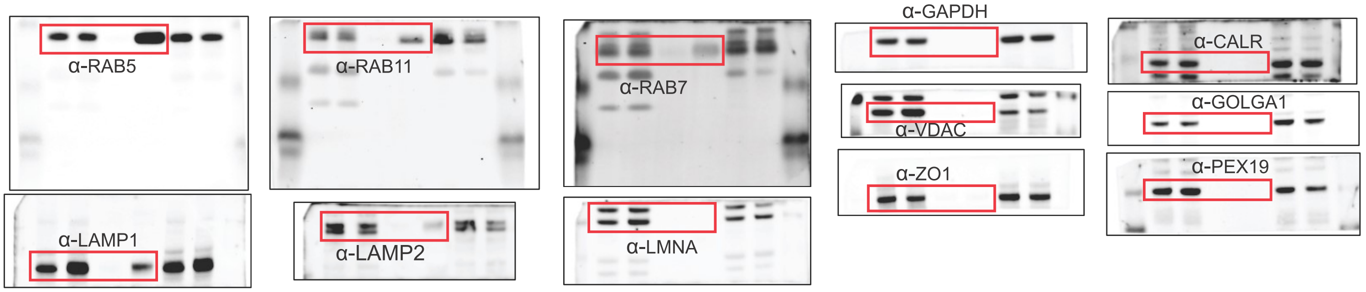

Fig. 3b

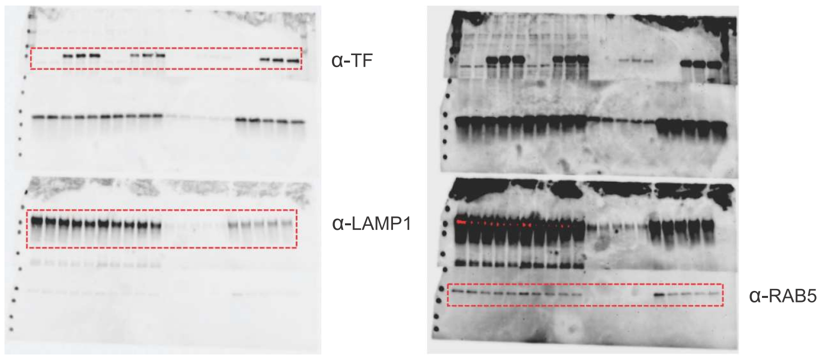

Fig. 5e

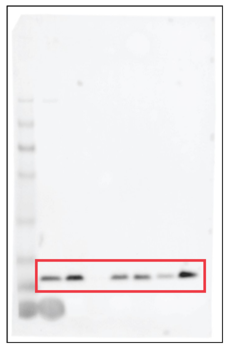

Fig. 5f

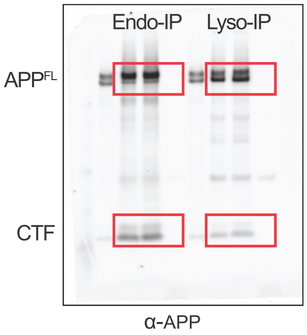

Fig. 5c

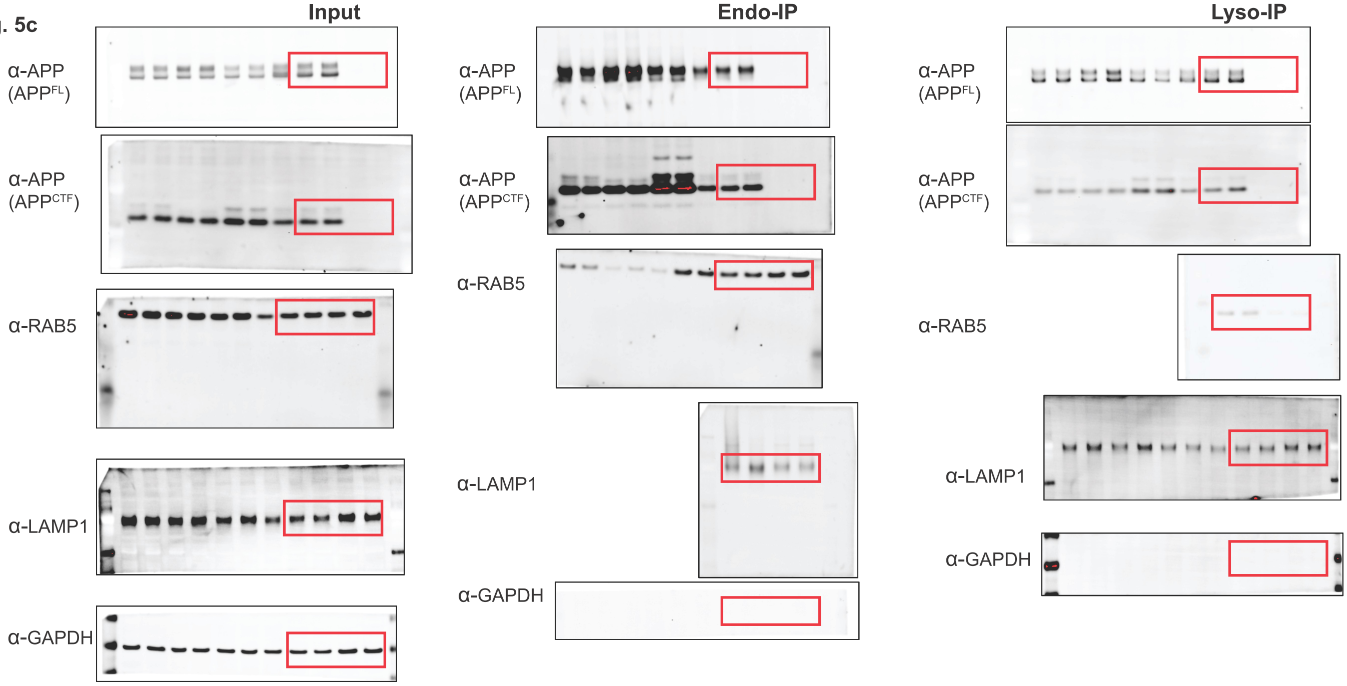

Fig. 6c

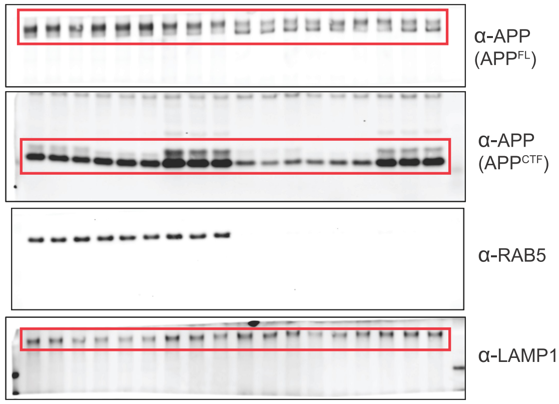

Fig. 7b

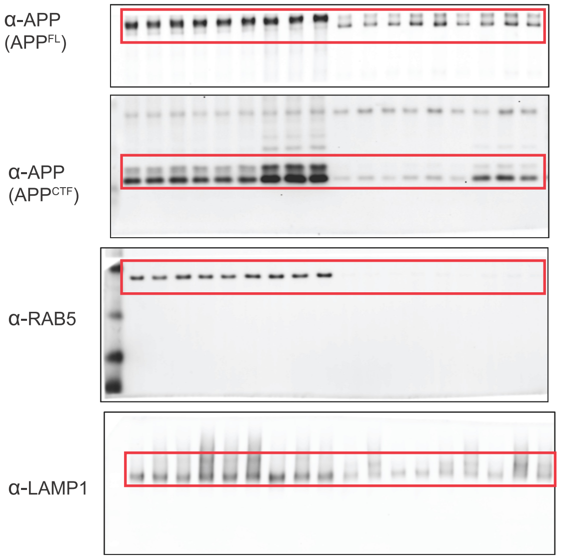

Extended Data Fig. 1a

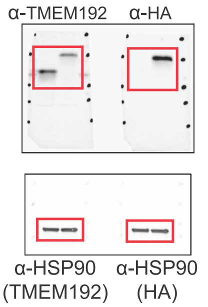

Extended Data Fig. 1b

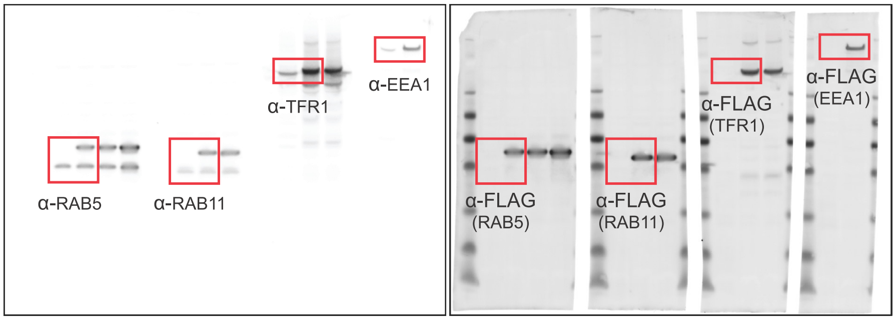

Extendend Data Fig. 1d

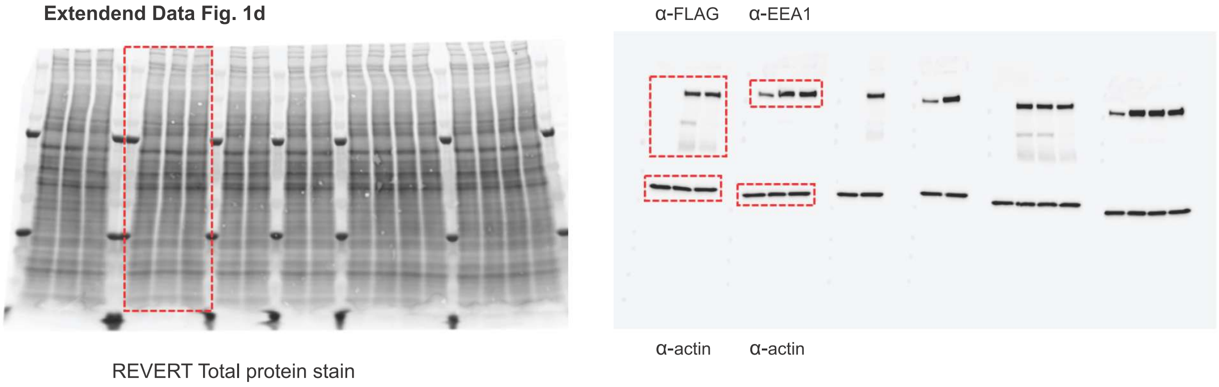

Extended Data Fig. 2b

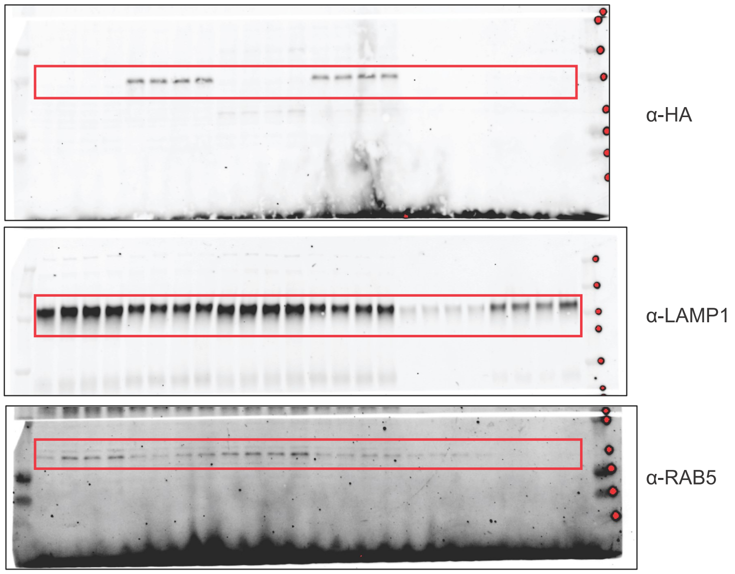

Extended Data Fig. 3a

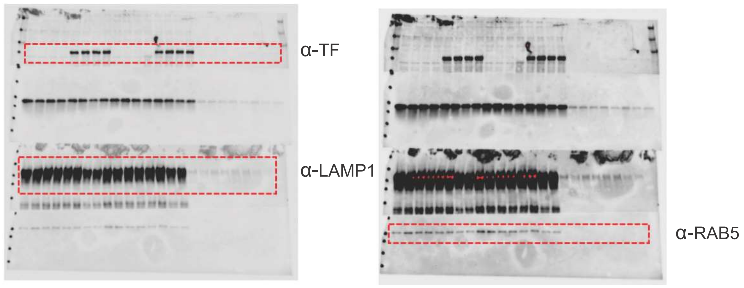

Extended Data Fig. 3b

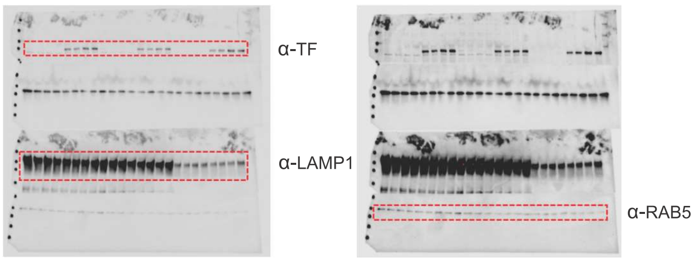

Extended Data Fig. 5a

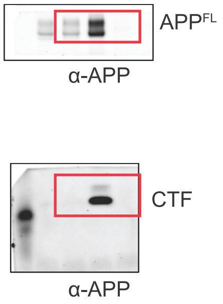

Extended Data Fig. 5b

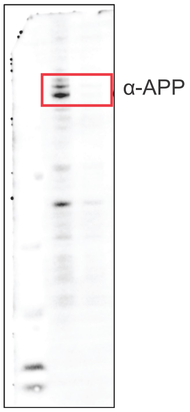

Extended Data Fig. 5c

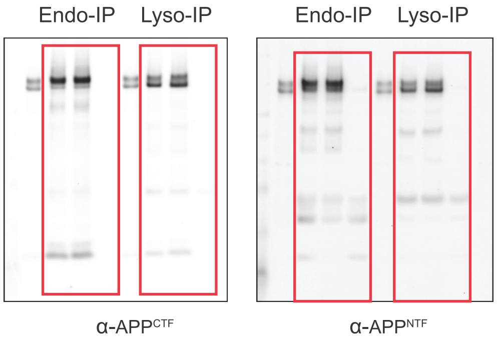

Extendend Data Fig. 5d

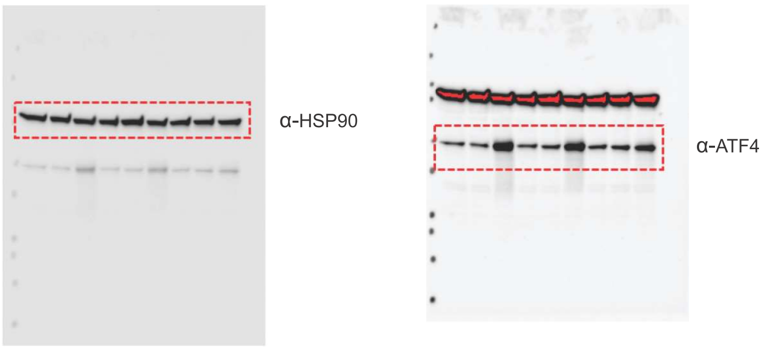

Extended Data Fig. 5g

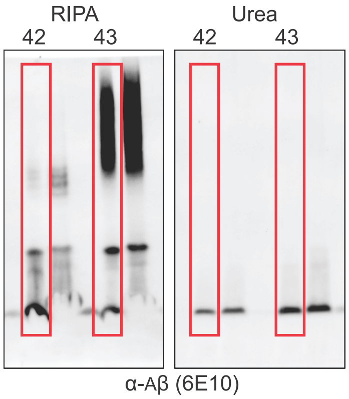

Extended Data Fig. 6a

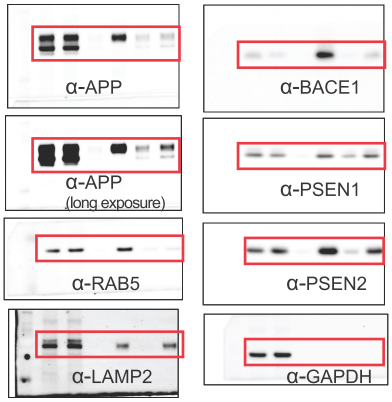

Extended Data Fig. 6b

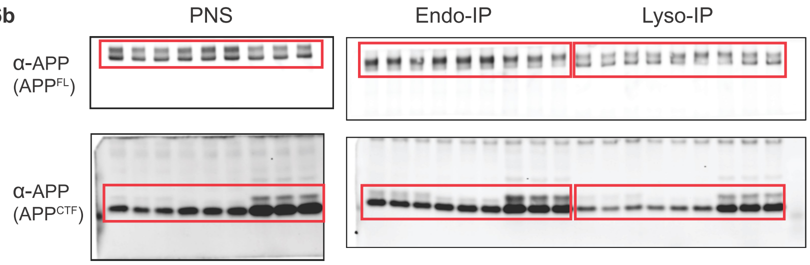

Supplement: Supplementary file 20 — Source Data [file 41467_2022_33881_MOESM20_ESM.zip › Source Data 1_Uncropped WB.pdf]
