# Supplementary material for: Spatial snapshots of amyloid precursor protein intramembrane processing via early endosome proteomics
Source: Nat Commun. 2022 Oct 16;13:6112. doi: 10.1038/s41467-022-33881-x (PMC9573879; doi:10.1038/s41467-022-33881-x)

Alkenyl-DG P-16:0_20:4 [M+H]+ | Ret. Time (min): 8.512 | m/z: 601.519

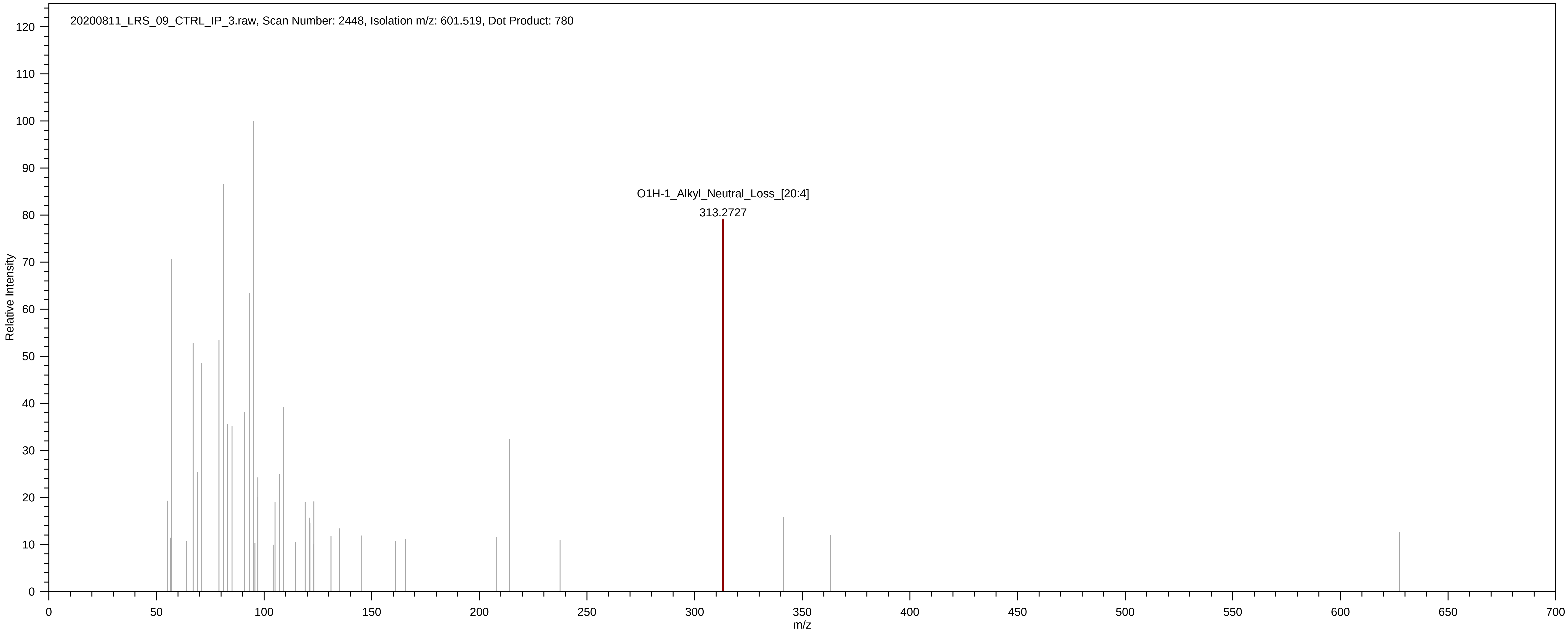

Alkenyl-DG P-16:0_20:4 [M+H]+ | Ret. Time (min): 8.517 | m/z: 601.519

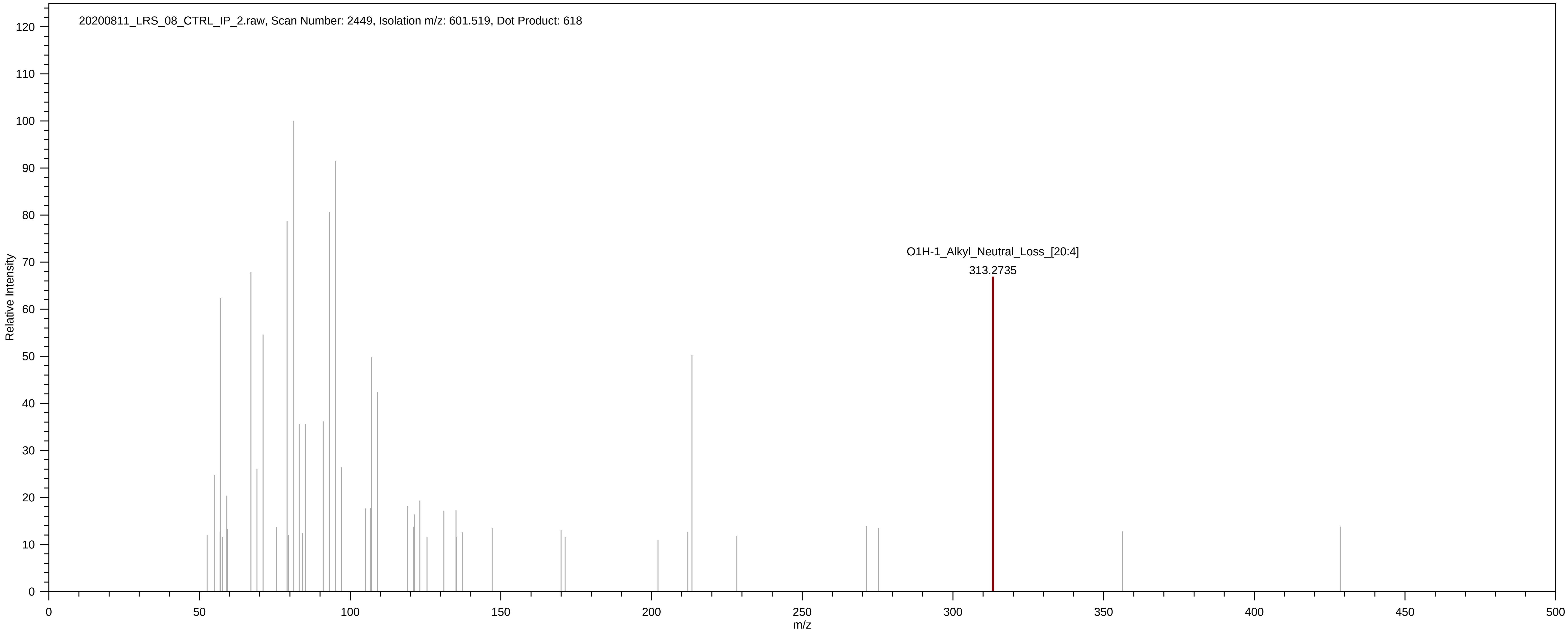

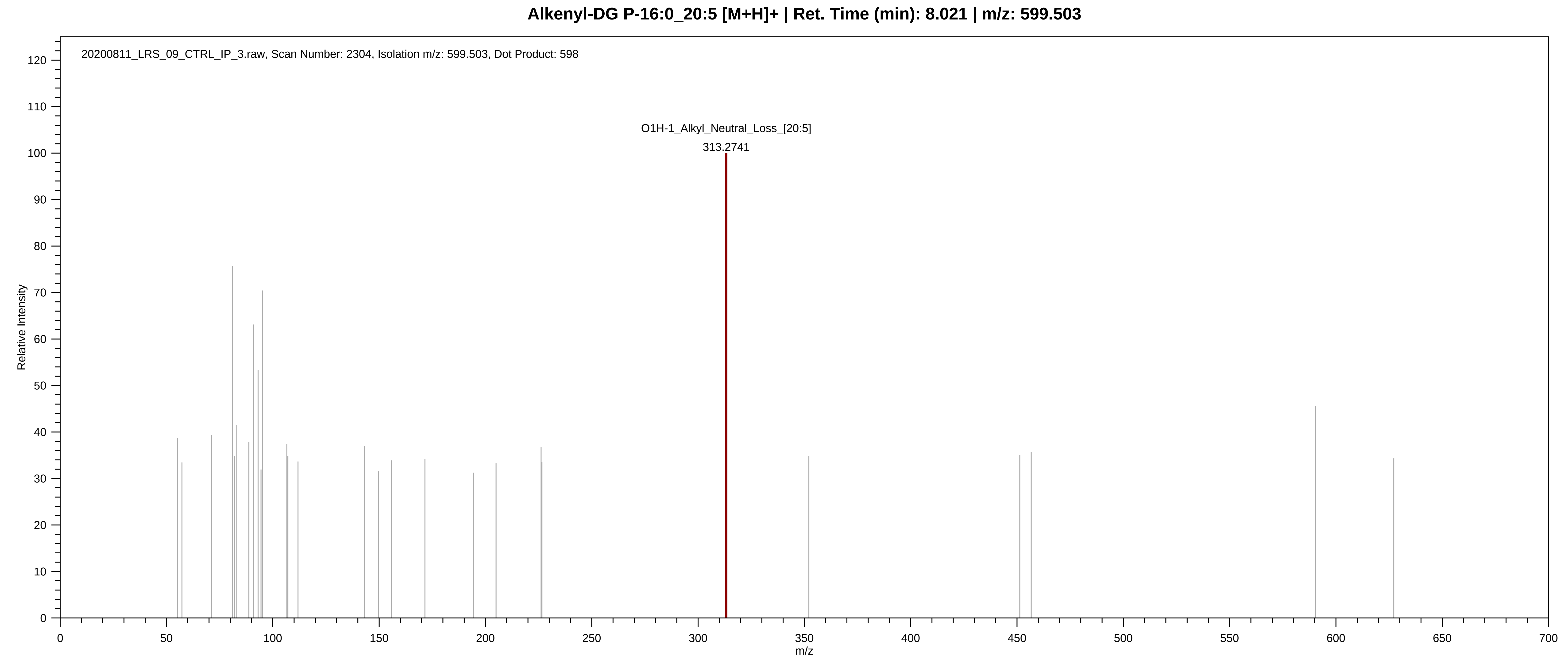

Alkenyl-DG P-16:0_20:5 [M+H]+ | Ret. Time (min): 8.021 | m/z: 599.503
20200811_LRS_09_CTRL_IP_3.raw, Scan Number: 2304, Isolation m/z: 599.503, Dot Product: 598
O1H-1_Alkyl_Neutral_Loss_[20:5]
313.2741
Relative Intensity
m/z

Alkenyl-DG P-16:0_20:5 [M+H]+ | Ret. Time (min): 8.036 | m/z: 599.503

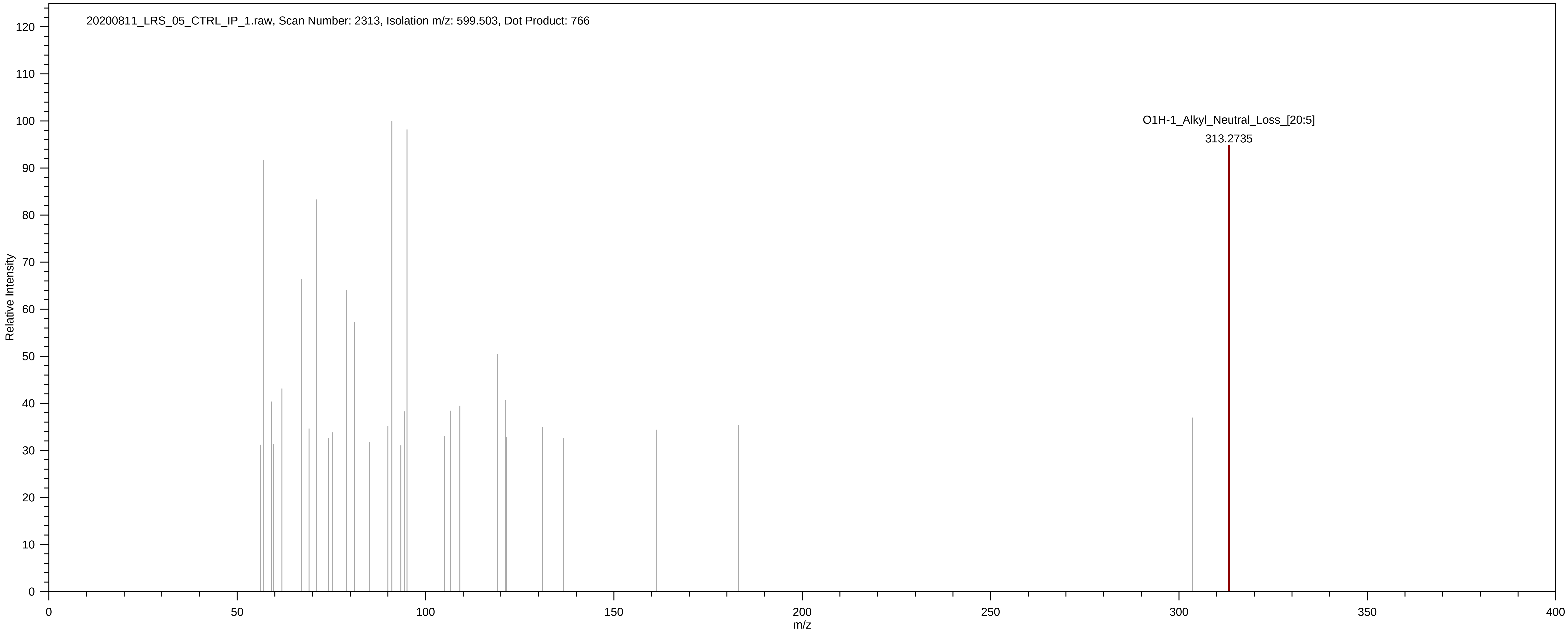

Alkenyl-DG P-18:0_20:4 [M+H]+ | Ret. Time (min): 9.309 | m/z: 629.55

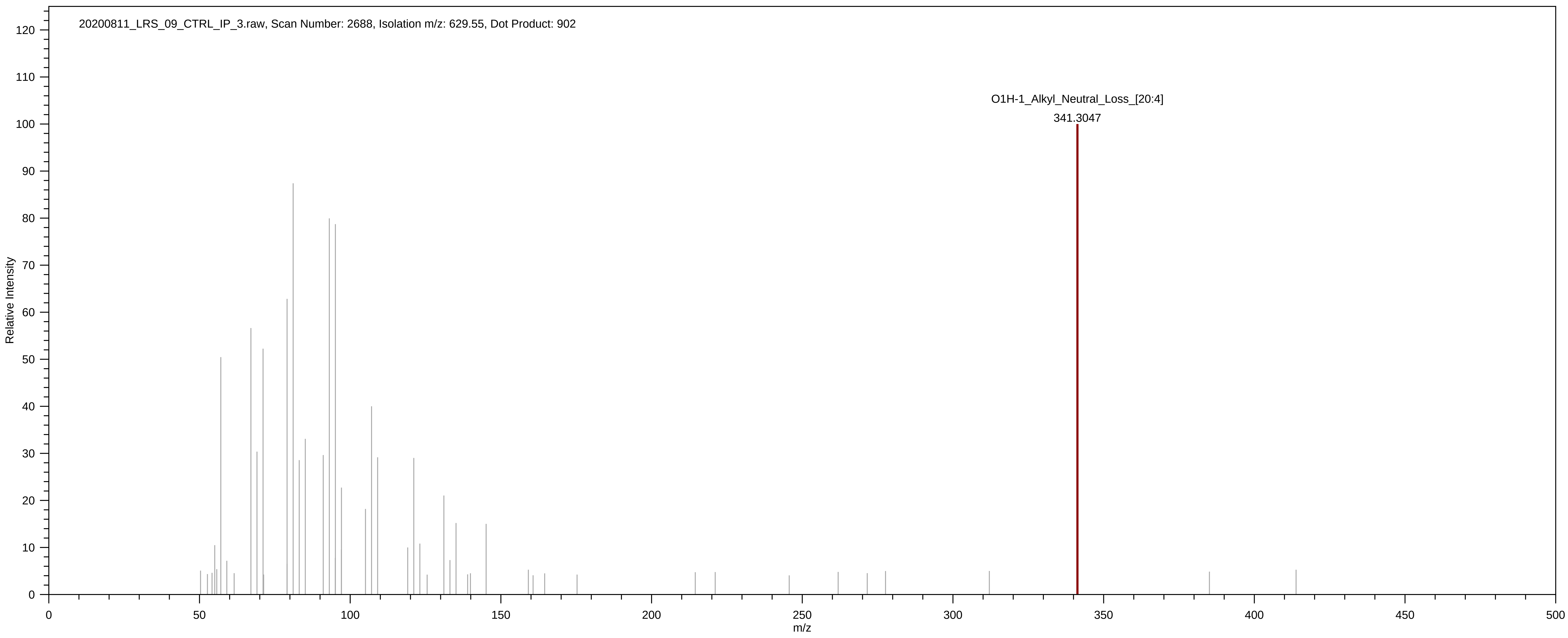

Alkenyl-DG P-18:0_20:4 [M+H]+ | Ret. Time (min): 9.3 | m/z: 629.551

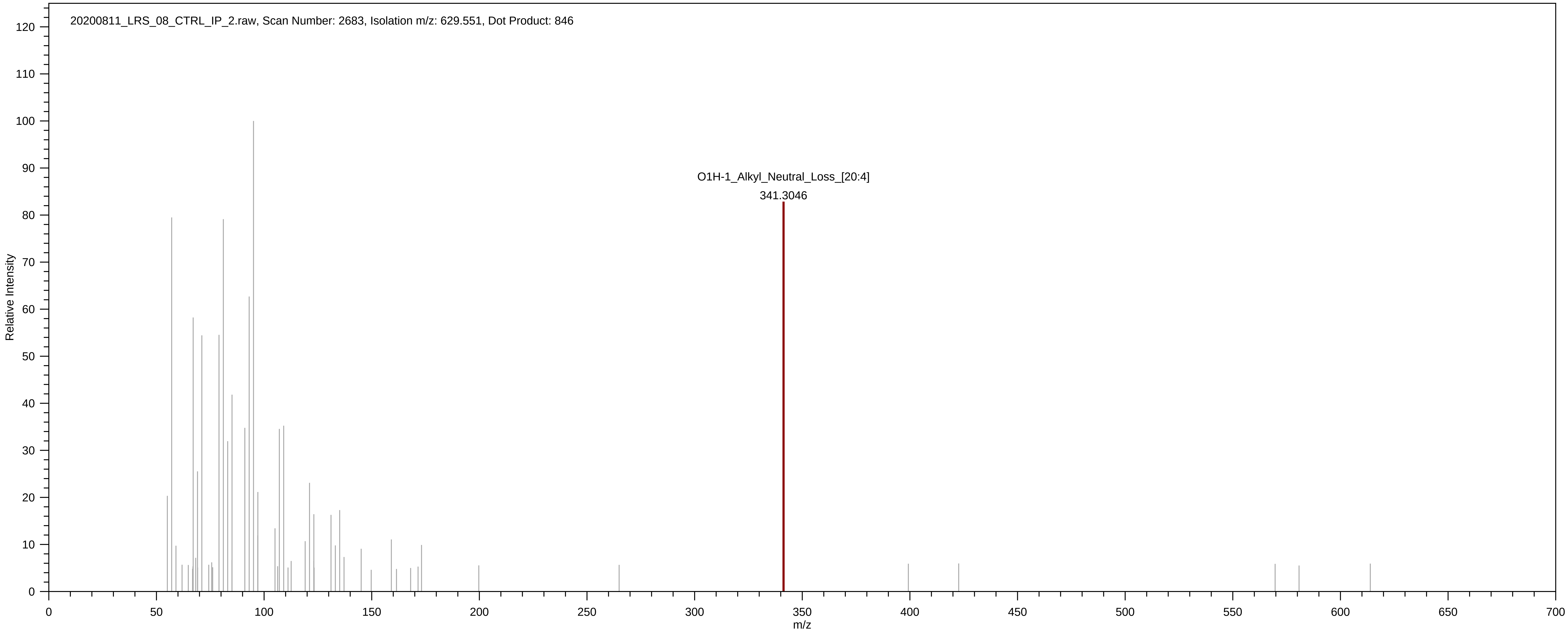

Alkenyl-DG P-18:0_20:4 [M+H]+ | Ret. Time (min): 9.328 | m/z: 629.551

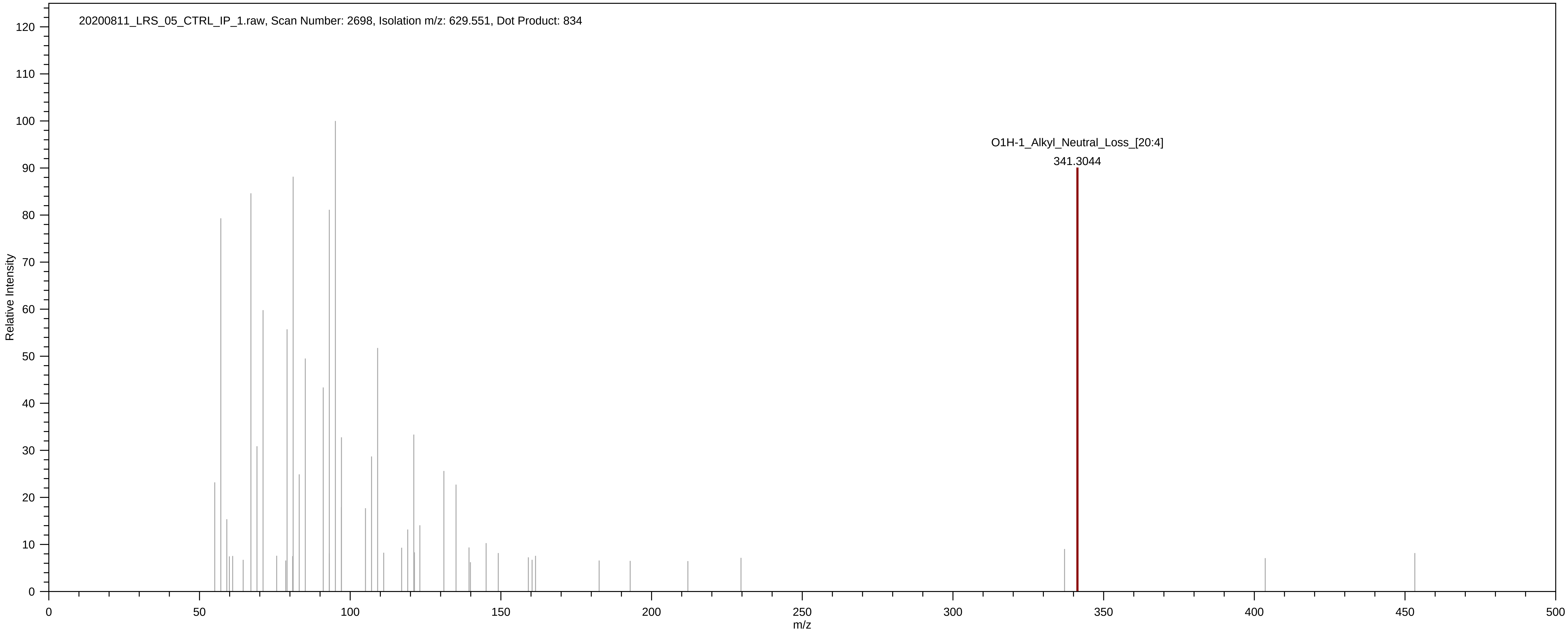

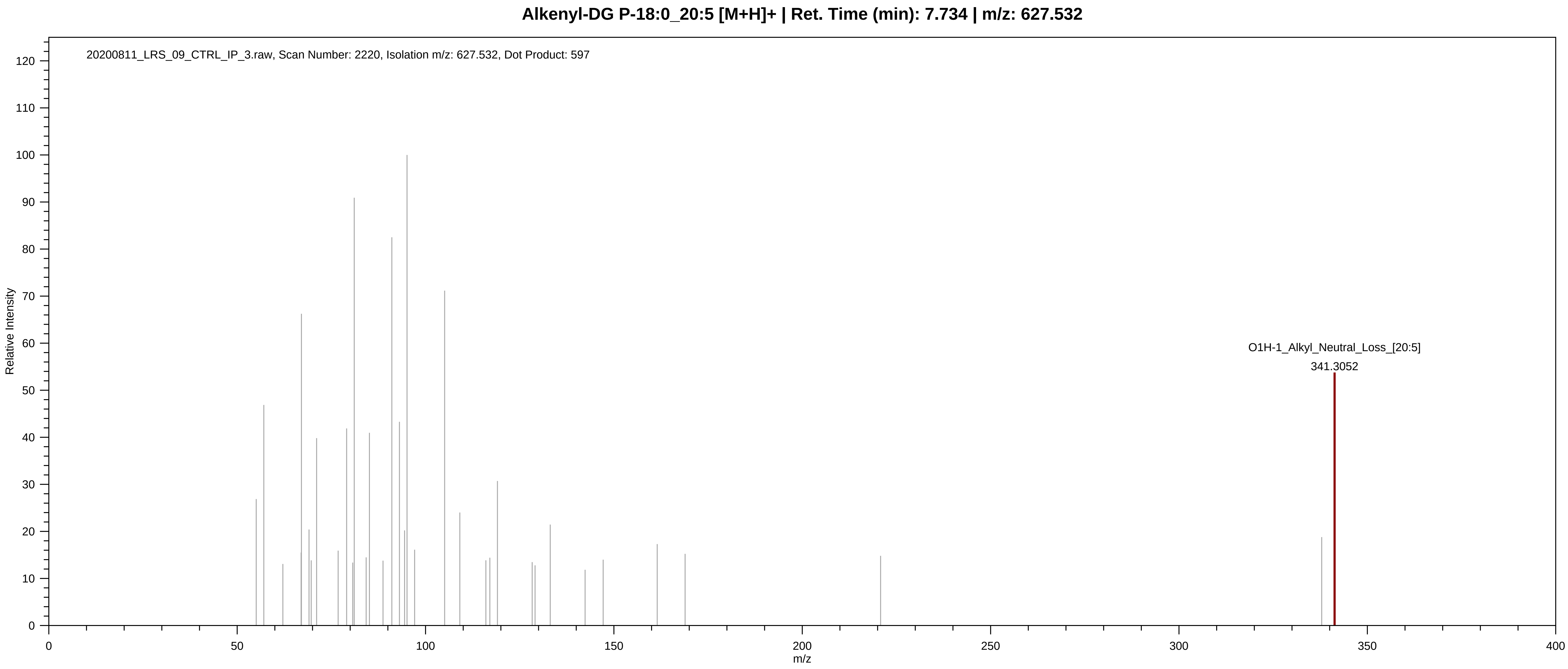

Alkenyl-DG P-18:0_20:5 [M+H]+ | Ret. Time (min): 7.734 | m/z: 627.532
20200811_LRS_09_CTRL_IP_3.raw, Scan Number: 2220, Isolation m/z: 627.532, Dot Product: 597
O1H-1_Alkyl_Neutral_Loss_[20:5]
341.3052
Relative Intensity
m/z

Alkenyl-DG P-18:0_20:5 [M+H]+ | Ret. Time (min): 7.767 | m/z: 627.533

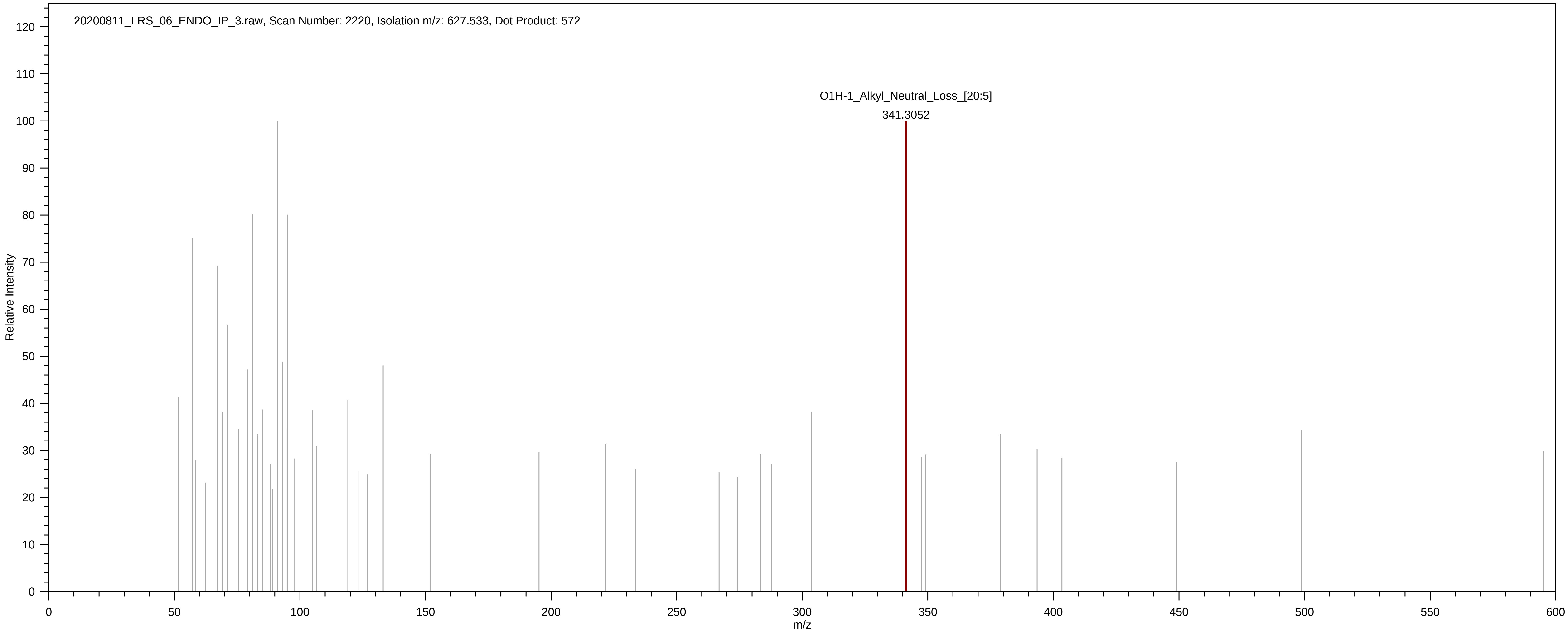

Alkenyl-DG P-18:0_20:5 [M+H]+ | Ret. Time (min): 7.765 | m/z: 627.534

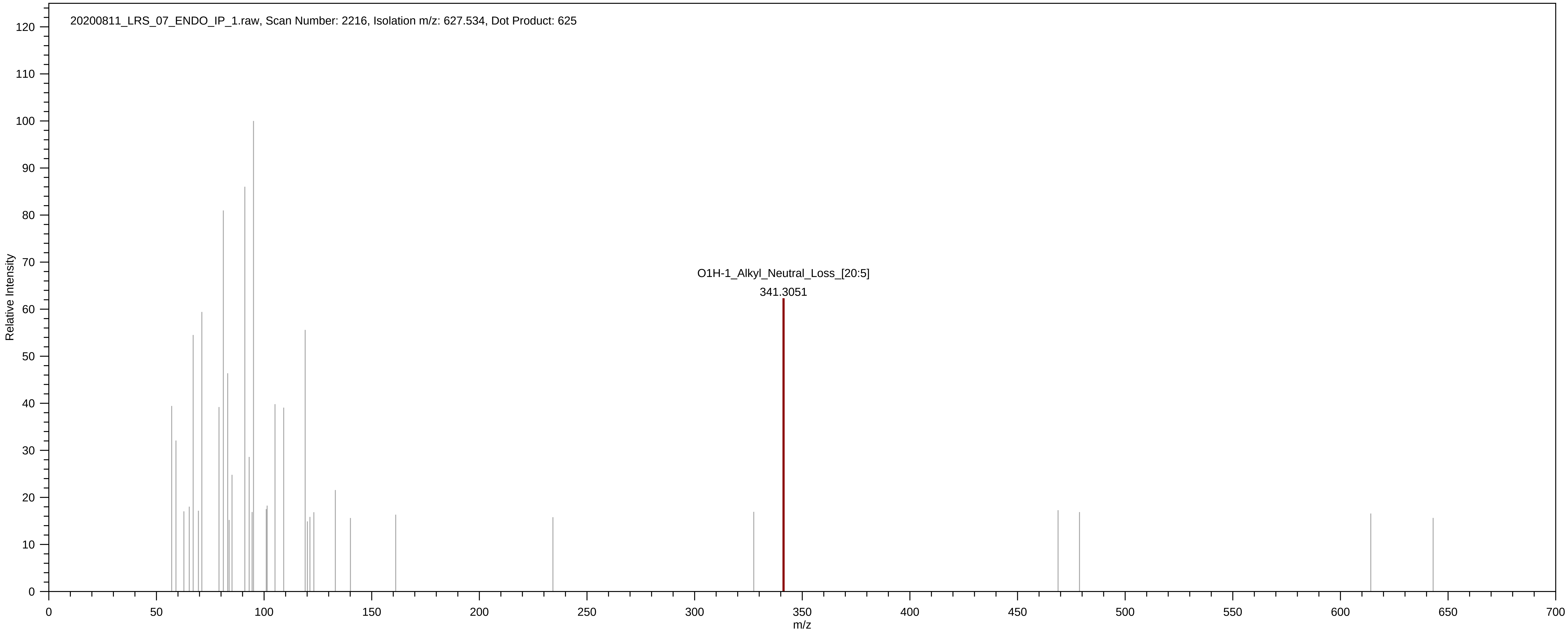

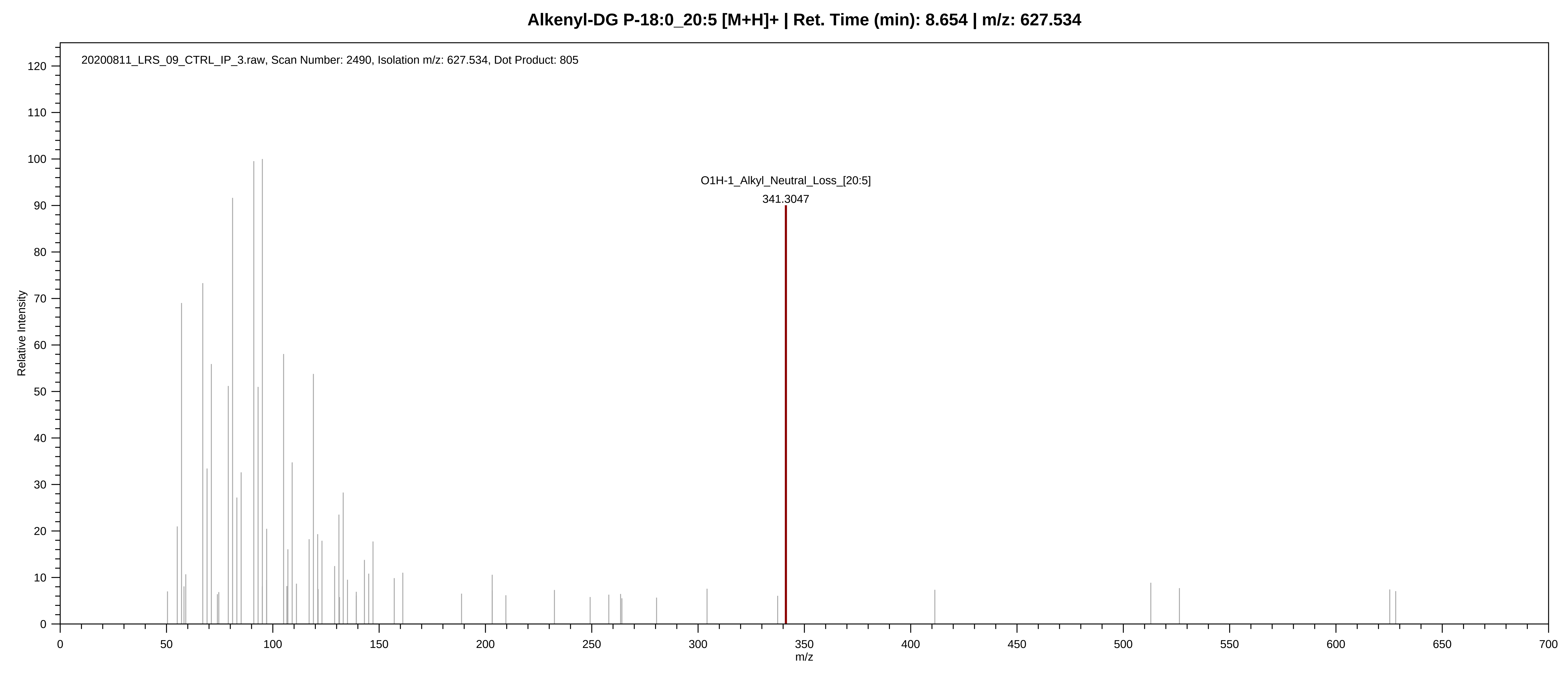
Alkenyl-DG P-18:0_20:5 [M+H]+ | Ret. Time (min): 8.654 | m/z: 627.534
20200811_LRS_09_CTRL_IP_3.raw, Scan Number: 2490, Isolation m/z: 627.534, Dot Product: 805
O1H-1_Alkyl_Neutral_Loss_[20:5]
341.3047
Relative Intensity
m/z

Alkenyl-DG P-18:0_20:5 [M+H]+ | Ret. Time (min): 8.706 | m/z: 627.534

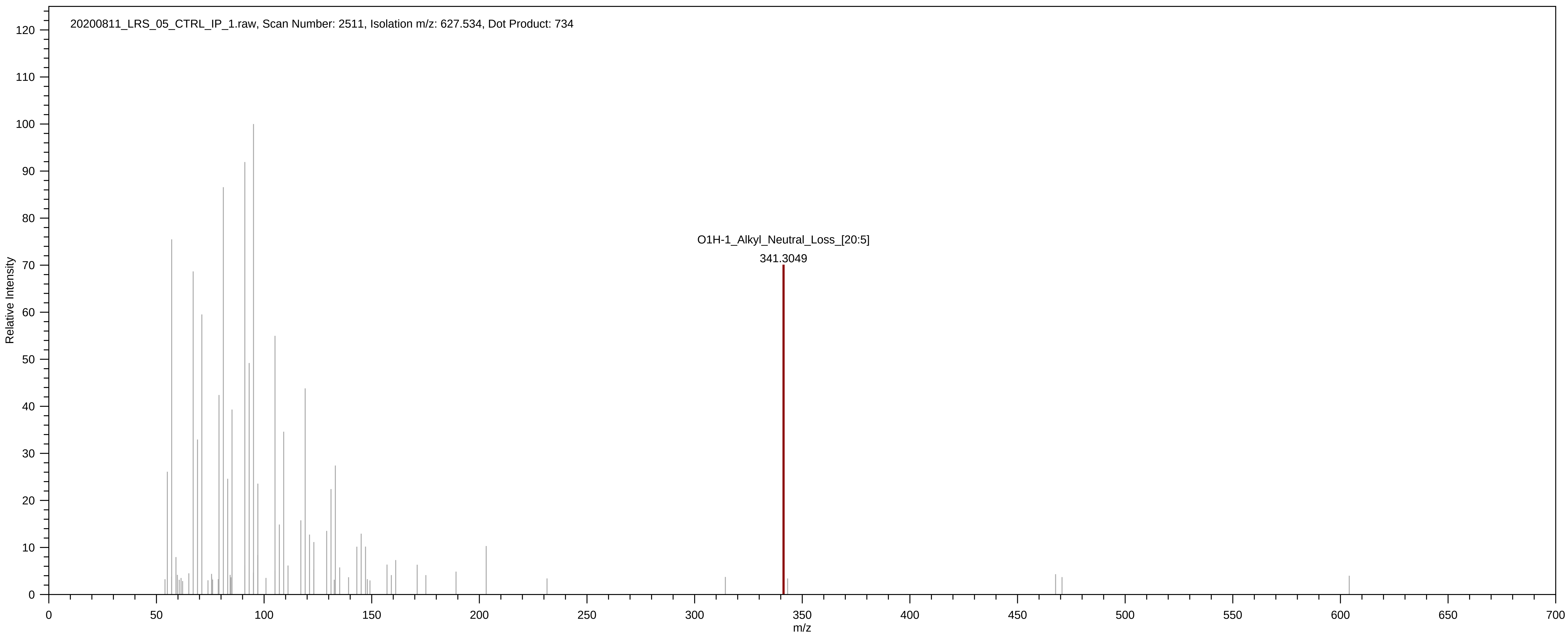

Alkenyl-DG P-18:0_20:5 [M+H]+ | Ret. Time (min): 8.759 | m/z: 627.534

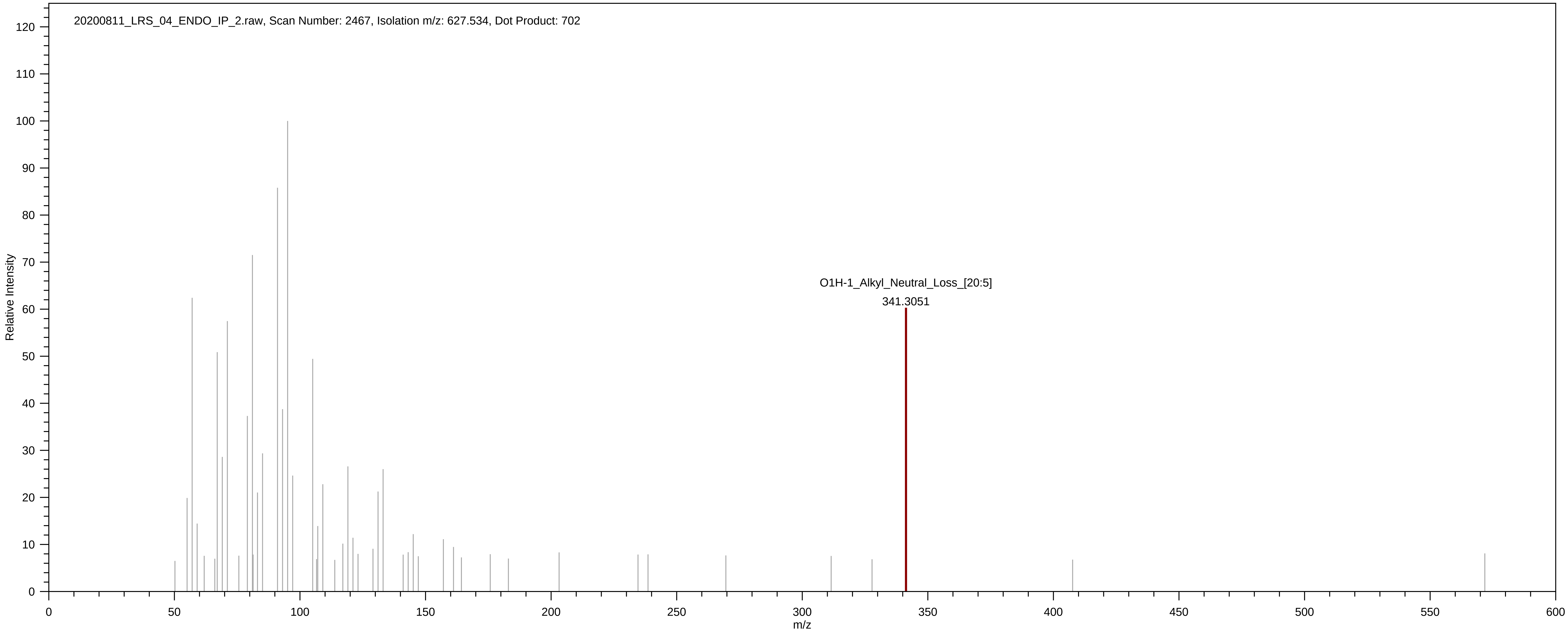

Alkenyl-DG P-18:0_20:5 [M+H]+ | Ret. Time (min): 8.759 | m/z: 627.534

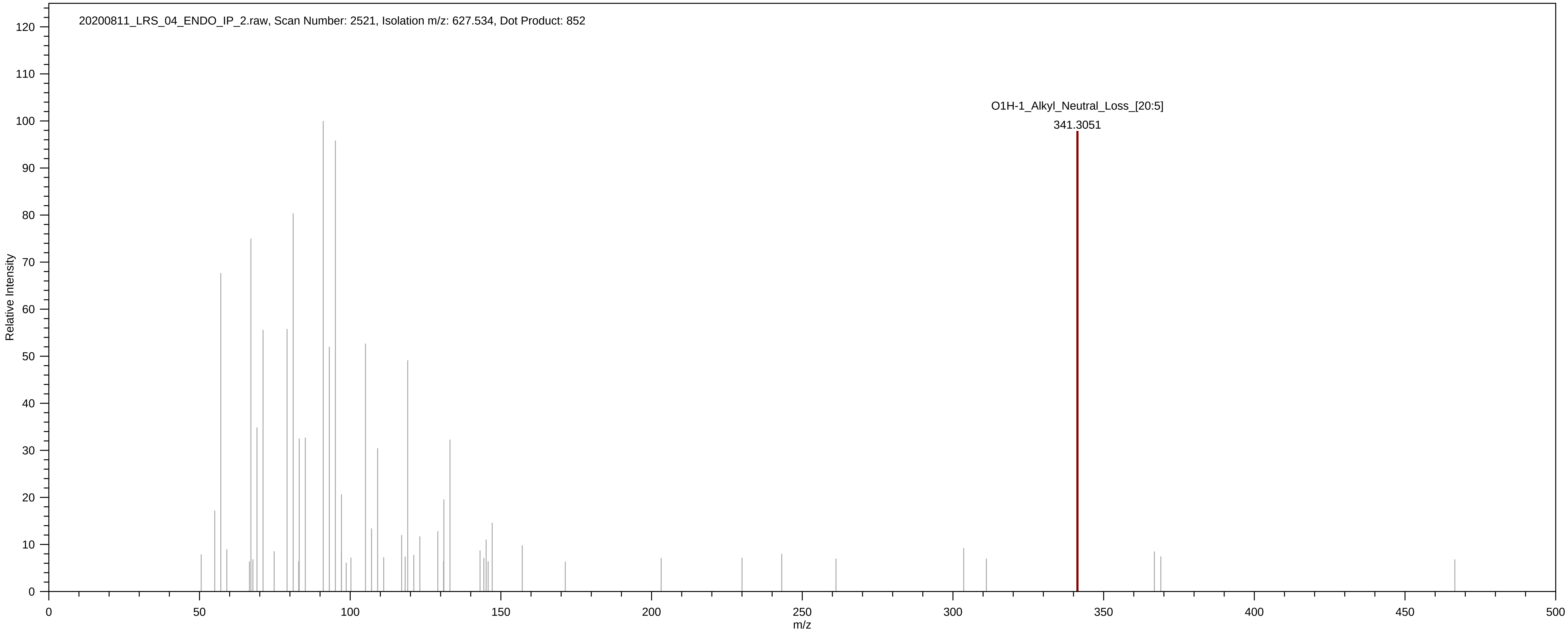

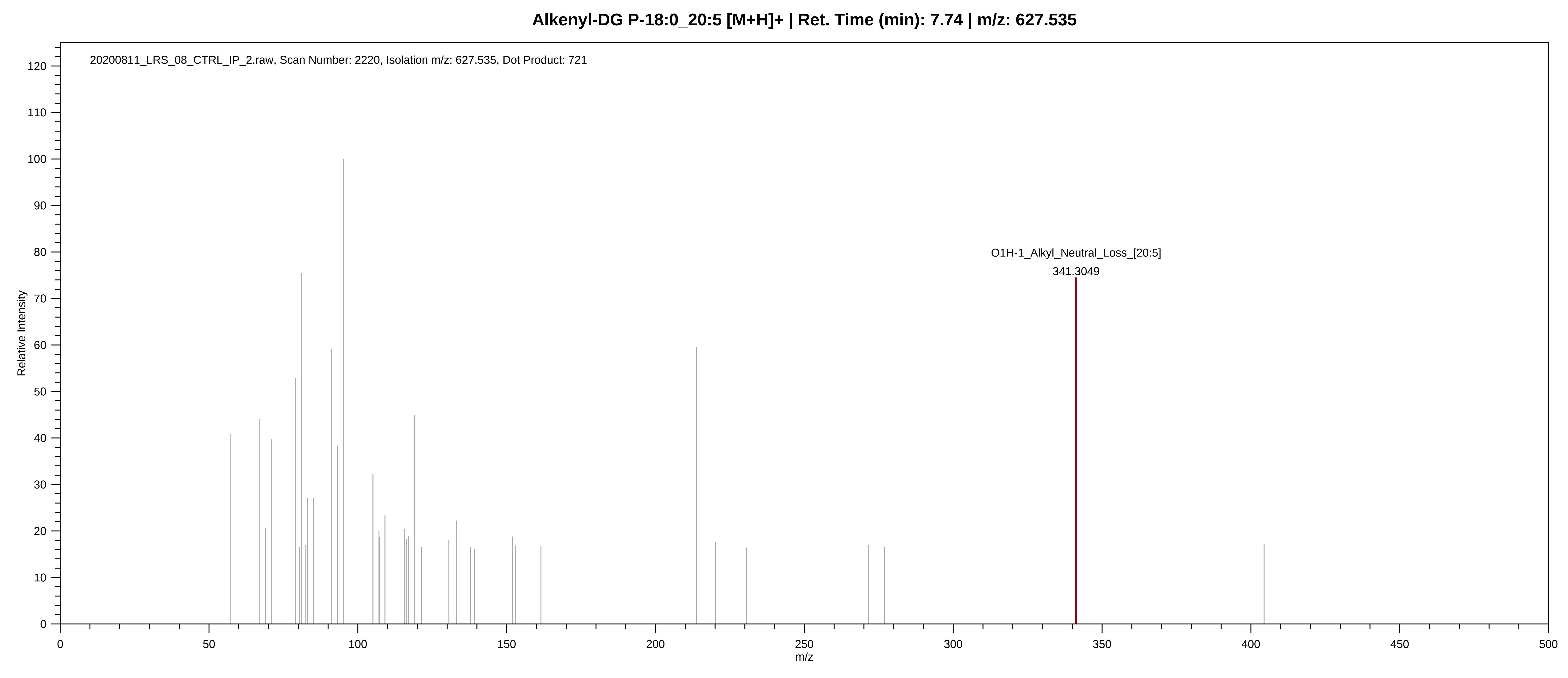

Alkenyl-DG P-18:0_20:5 [M+H]+ | Ret. Time (min): 7.74 | m/z: 627.535
20200811_LRS_08_CTRL_IP_2.raw, Scan Number: 2220, Isolation m/z: 627.535, Dot Product: 721
O1H-1_Alkyl_Neutral_Loss_[20:5]
341.3049
Relative Intensity
m/z

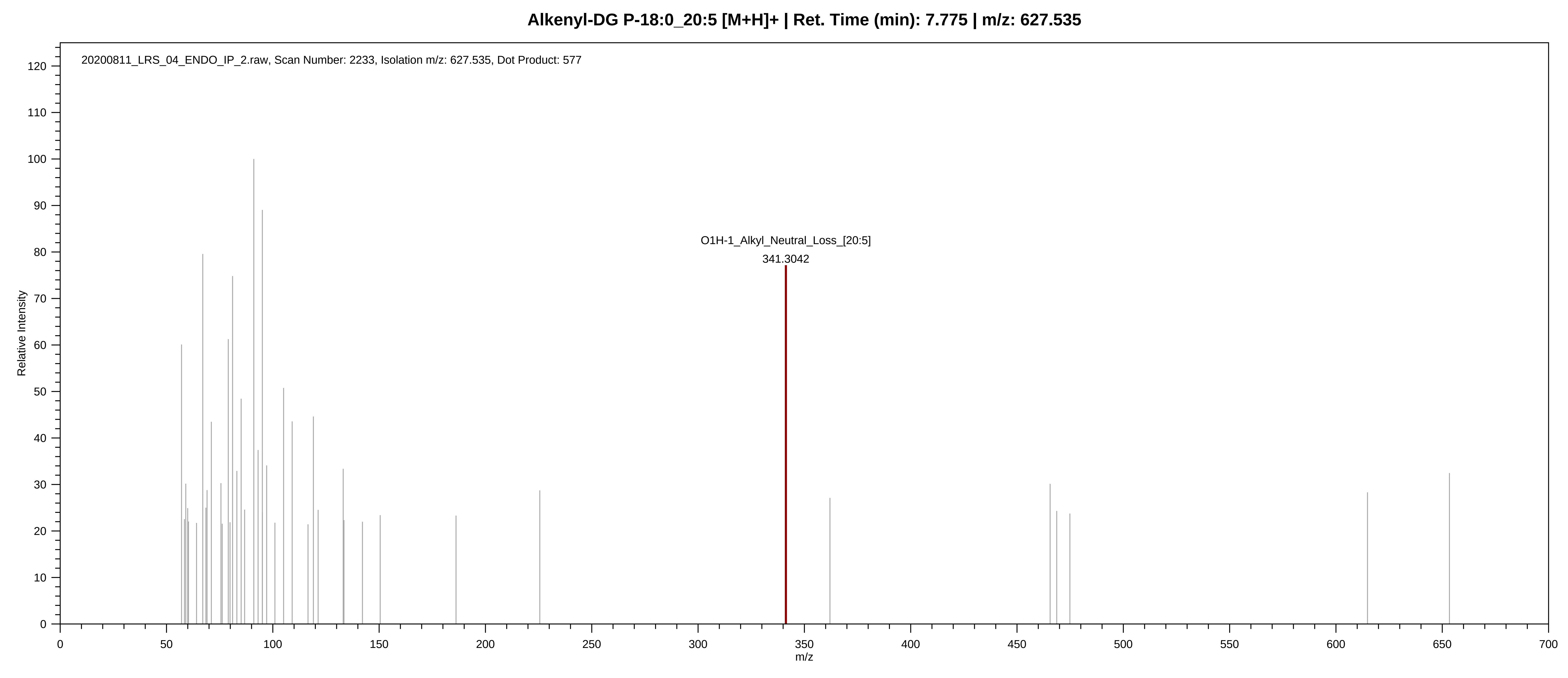

Alkenyl-DG P-18:0_20:5 [M+H]+ | Ret. Time (min): 7.775 | m/z: 627.535
20200811_LRS_04_ENDO_IP_2.raw, Scan Number: 2233, Isolation m/z: 627.535, Dot Product: 577
O1H-1_Alkyl_Neutral_Loss_[20:5]
341.3042
Relative Intensity
m/z

Alkenyl-DG P-18:0_20:5 [M+H]+ | Ret. Time (min): 8.523 | m/z: 627.535

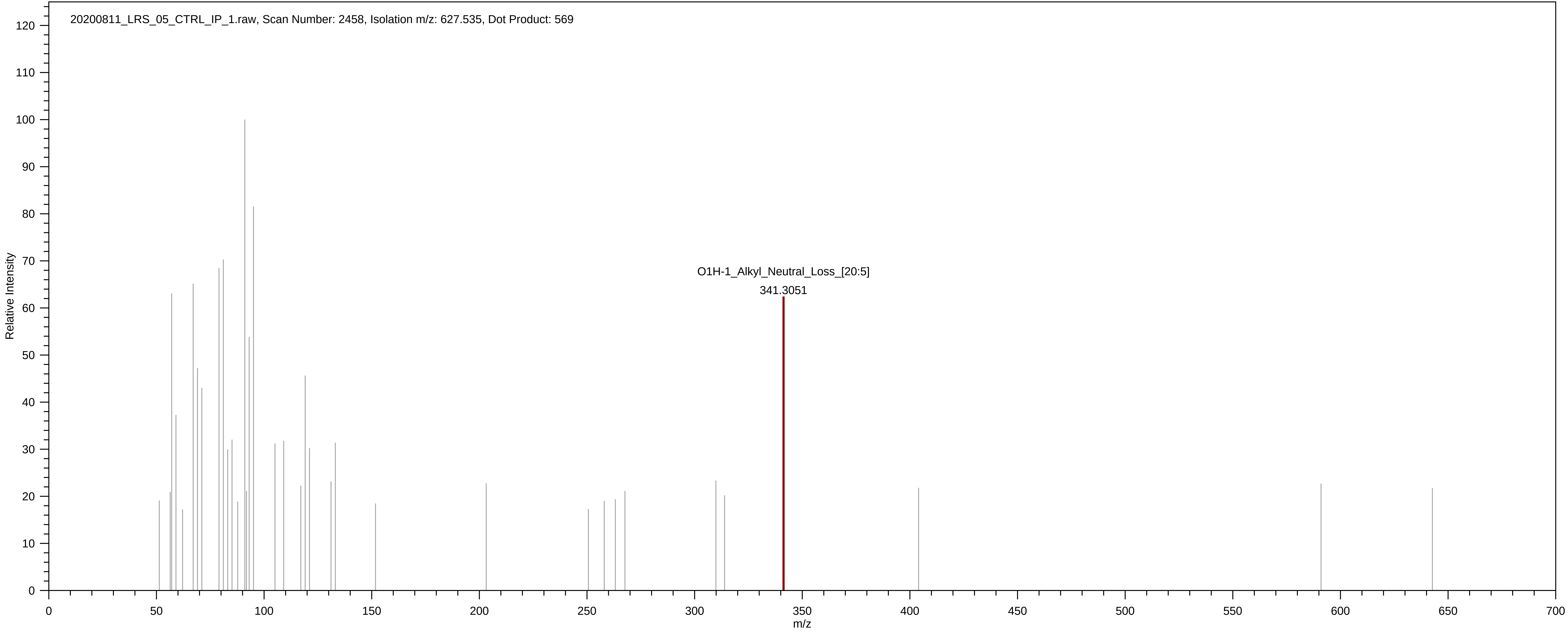

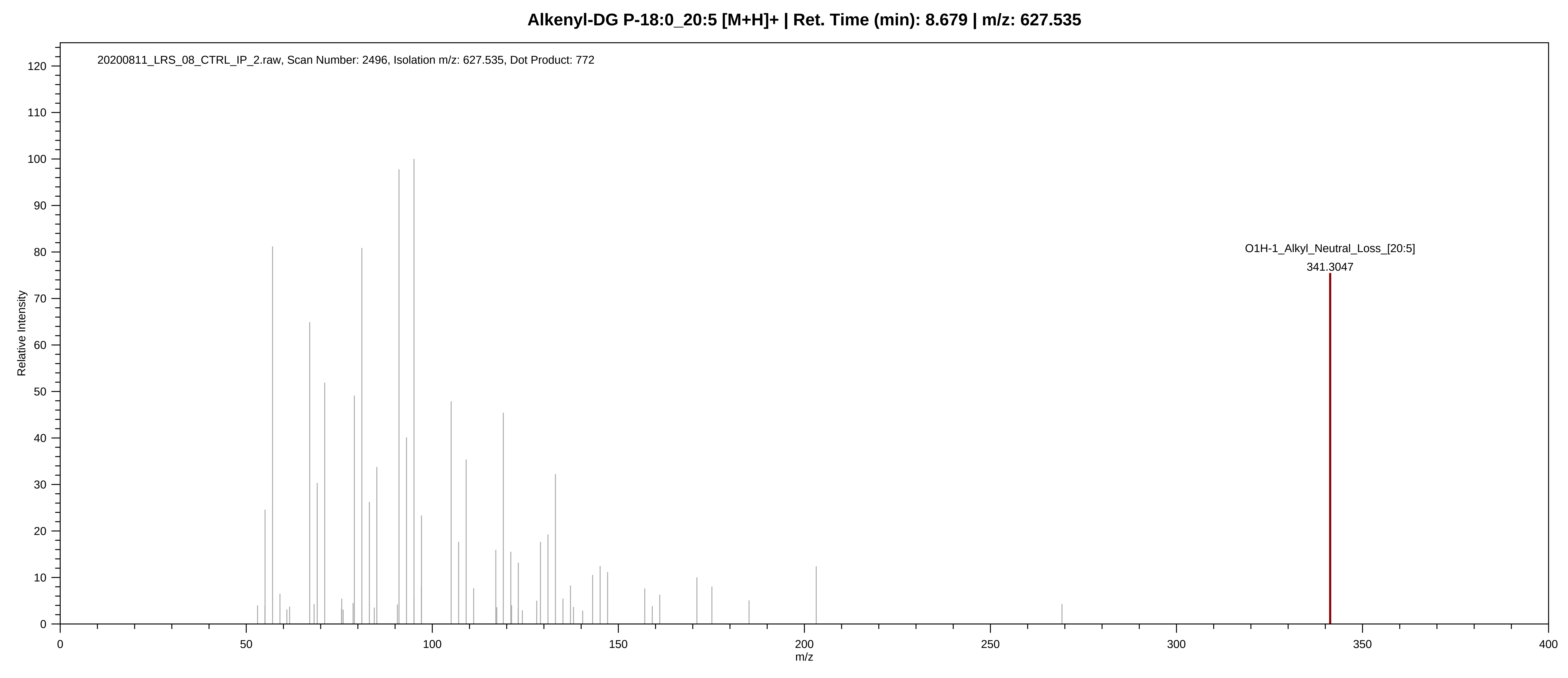

Alkenyl-DG P-18:0_20:5 [M+H]+ | Ret. Time (min): 8.679 | m/z: 627.535
20200811_LRS_08_CTRL_IP_2.raw, Scan Number: 2496, Isolation m/z: 627.535, Dot Product: 772
O1H-1_Alkyl_Neutral_Loss_[20:5]
341.3047
Relative Intensity
m/z

Alkenyl-DG P-18:0_20:5 [M+H]+ | Ret. Time (min): 8.725 | m/z: 627.536

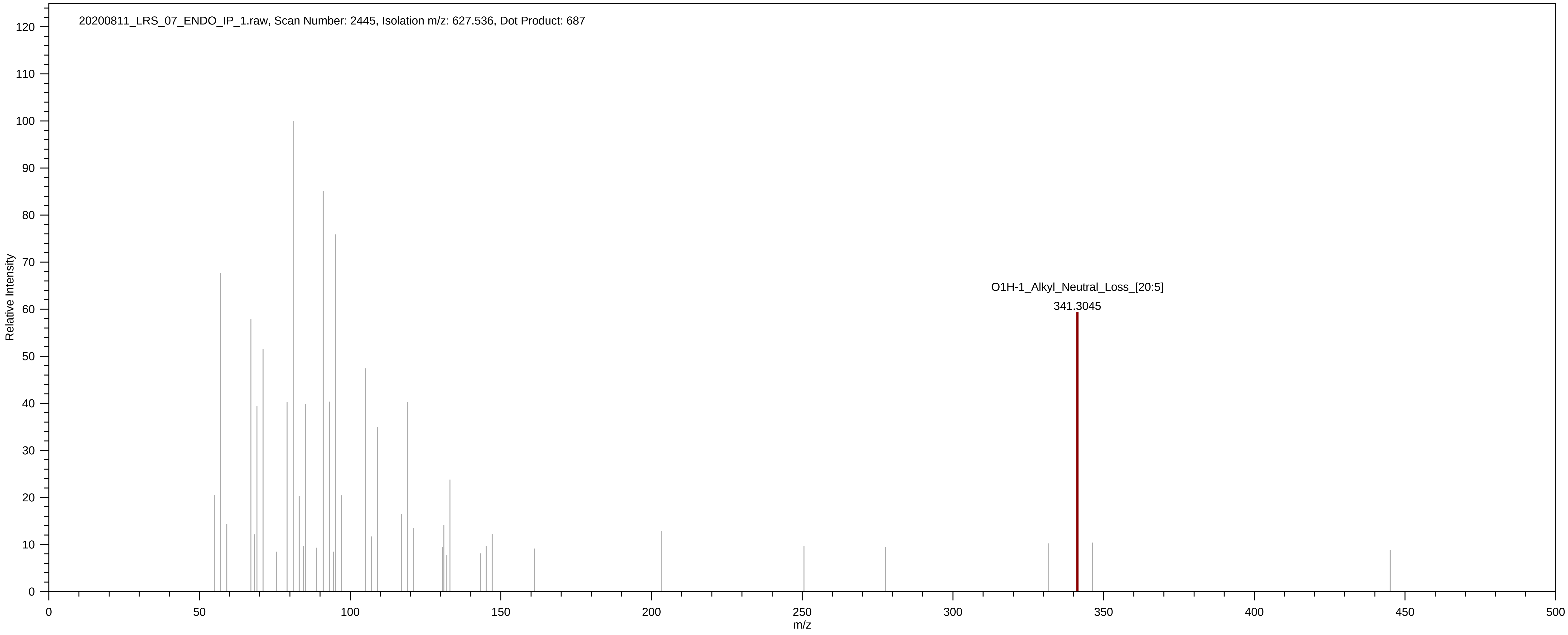

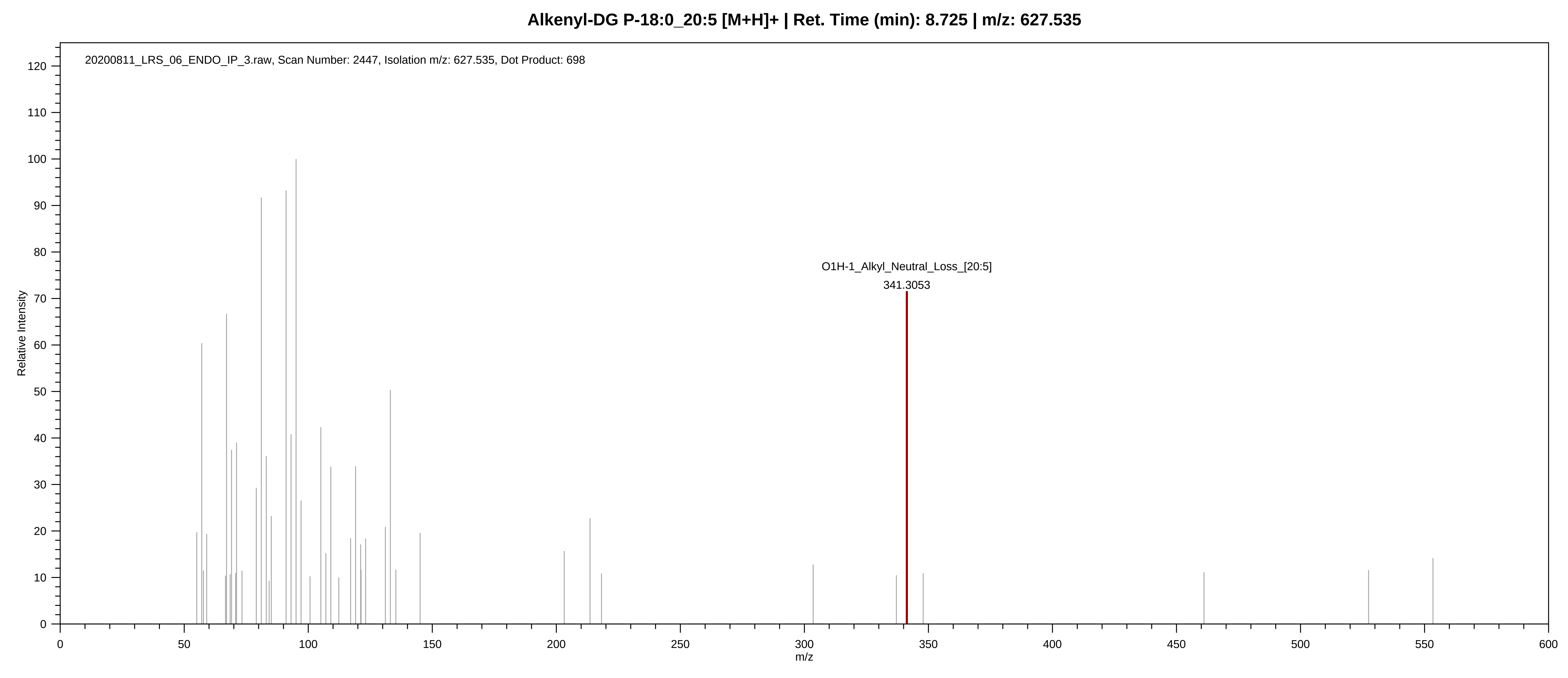

Alkenyl-DG P-18:0_20:5 [M+H]+ | Ret. Time (min): 8.725 | m/z: 627.535
20200811_LRS_06_ENDO_IP_3.raw, Scan Number: 2447, Isolation m/z: 627.535, Dot Product: 698
O1H-1_Alkyl_Neutral_Loss_[20:5]
341.3053
Relative Intensity
m/z

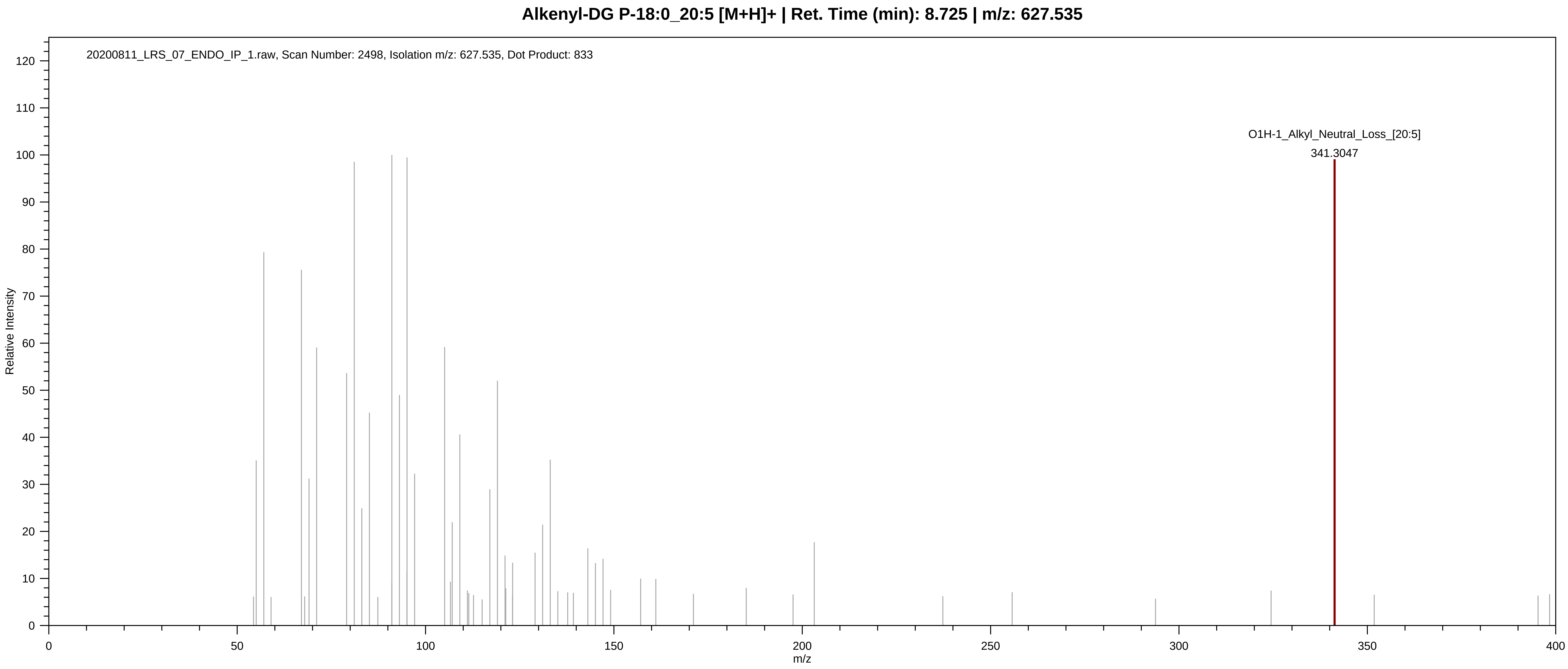

Alkenyl-DG P-18:0_20:5 [M+H]+ | Ret. Time (min): 8.725 | m/z: 627.535
20200811_LRS_07_ENDO_IP_1.raw, Scan Number: 2498, Isolation m/z: 627.535, Dot Product: 833
O1H-1_Alkyl_Neutral_Loss_[20:5]
341.3047
Relative Intensity
m/z

Alkenyl-DG P-18:0_20:5 [M+H]+ | Ret. Time (min): 8.725 | m/z: 627.535

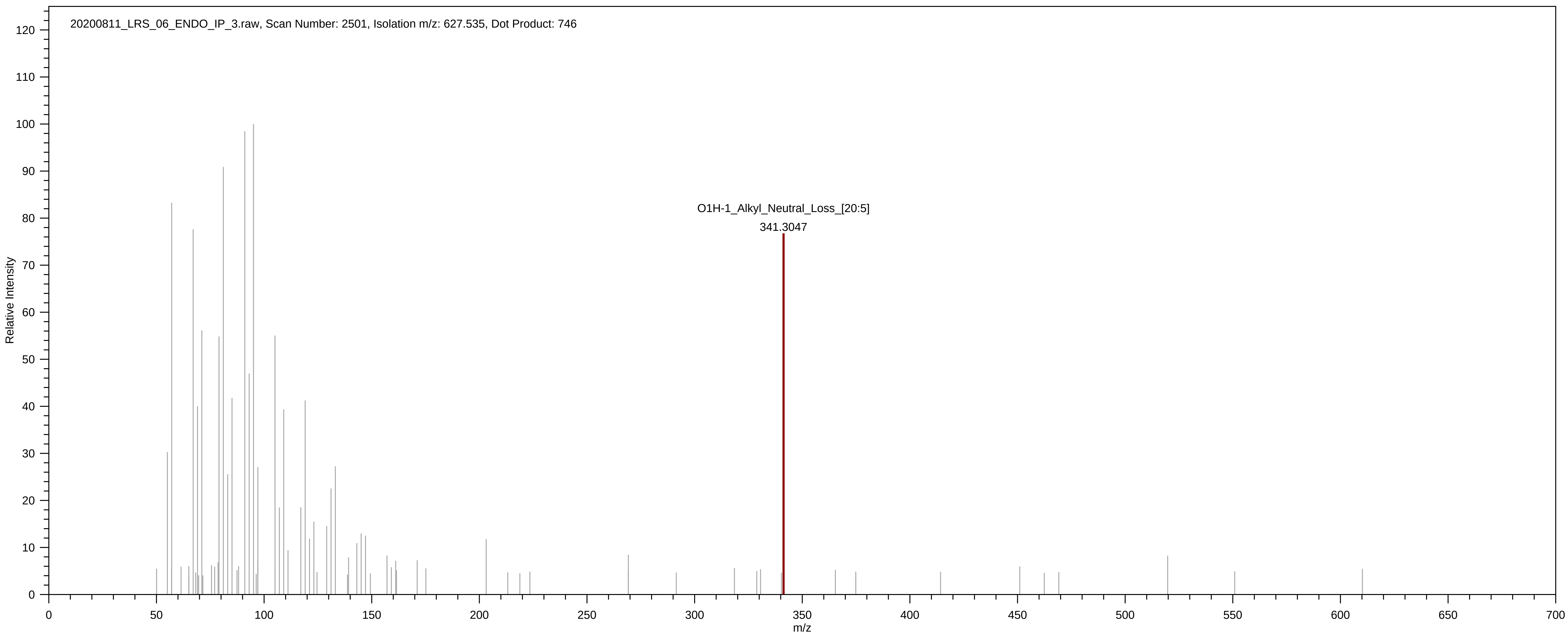

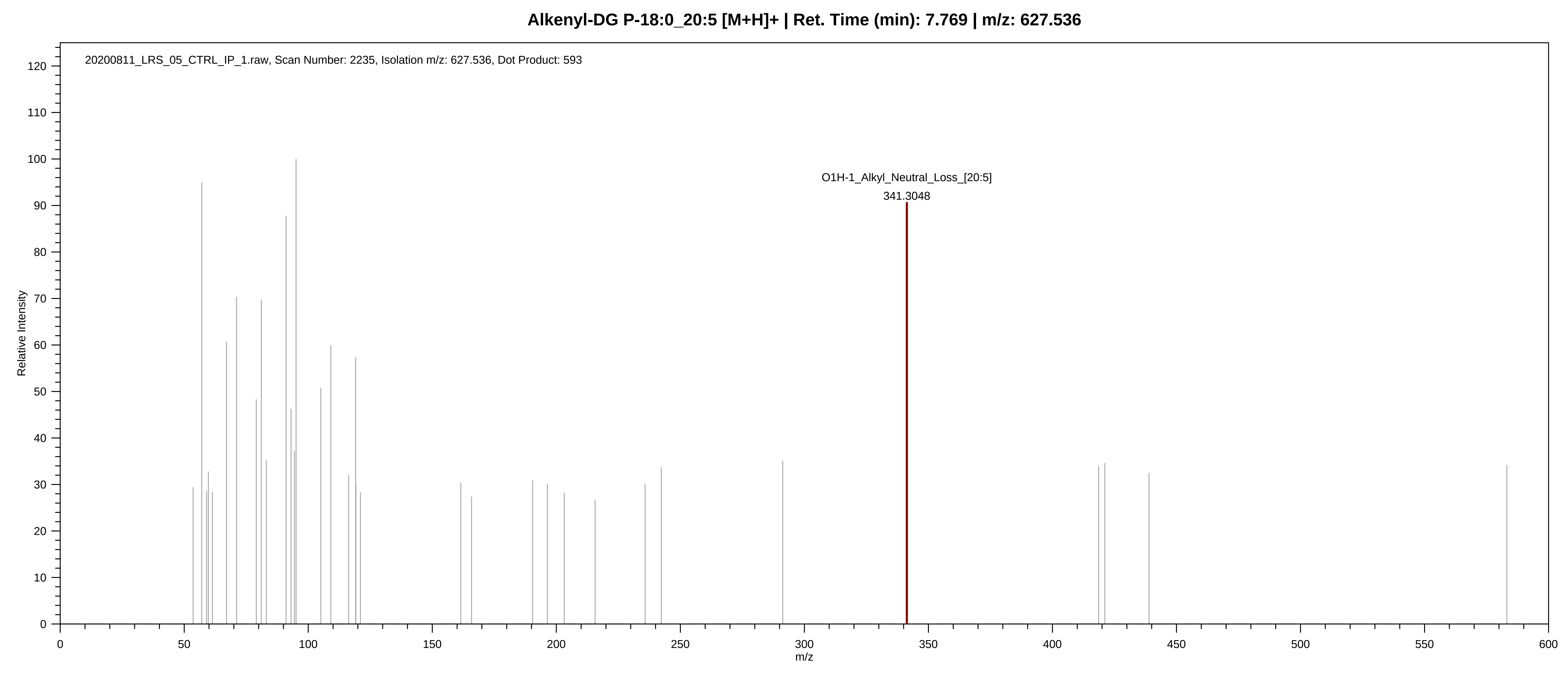

Alkenyl-DG P-18:0_20:5 [M+H]+ | Ret. Time (min): 7.769 | m/z: 627.536
20200811_LRS_05_CTRL_IP_1.raw, Scan Number: 2235, Isolation m/z: 627.536, Dot Product: 593
O1H-1_Alkyl_Neutral_Loss_[20:5]
341.3048
Relative Intensity
m/z

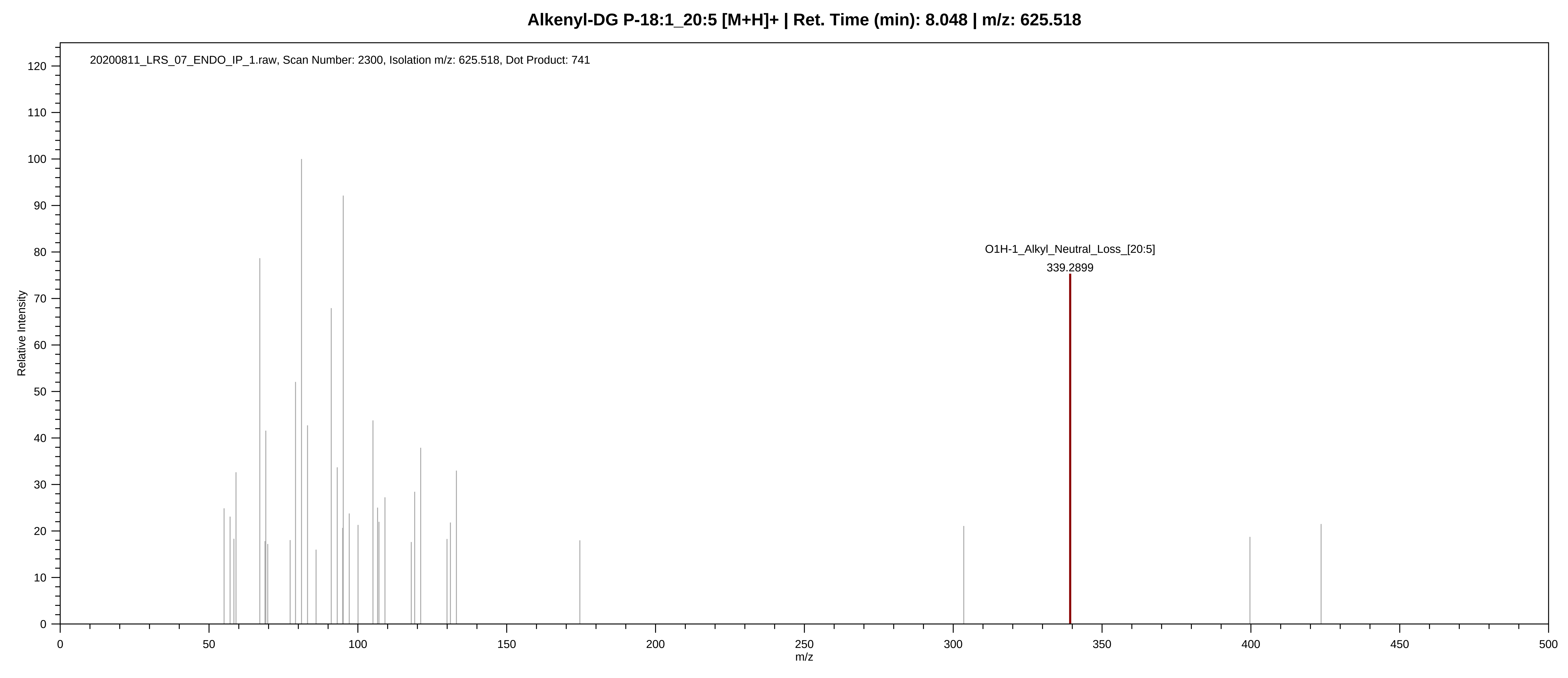
Alkenyl-DG P-18:1_20:5 [M+H]+ | Ret. Time (min): 8.048 | m/z: 625.518
20200811_LRS_07_ENDO_IP_1.raw, Scan Number: 2300, Isolation m/z: 625.518, Dot Product: 741
O1H-1_Alkyl_Neutral_Loss_[20:5]
339.2899
Relative Intensity
m/z
0
10
20
30
40
50
60
70
80
90
100
110
120
0
50
100
150
200
250
300
350
400
450
500

Alkenyl-DG P-18:1_20:5 [M+H]+ | Ret. Time (min): 8.058 | m/z: 625.518

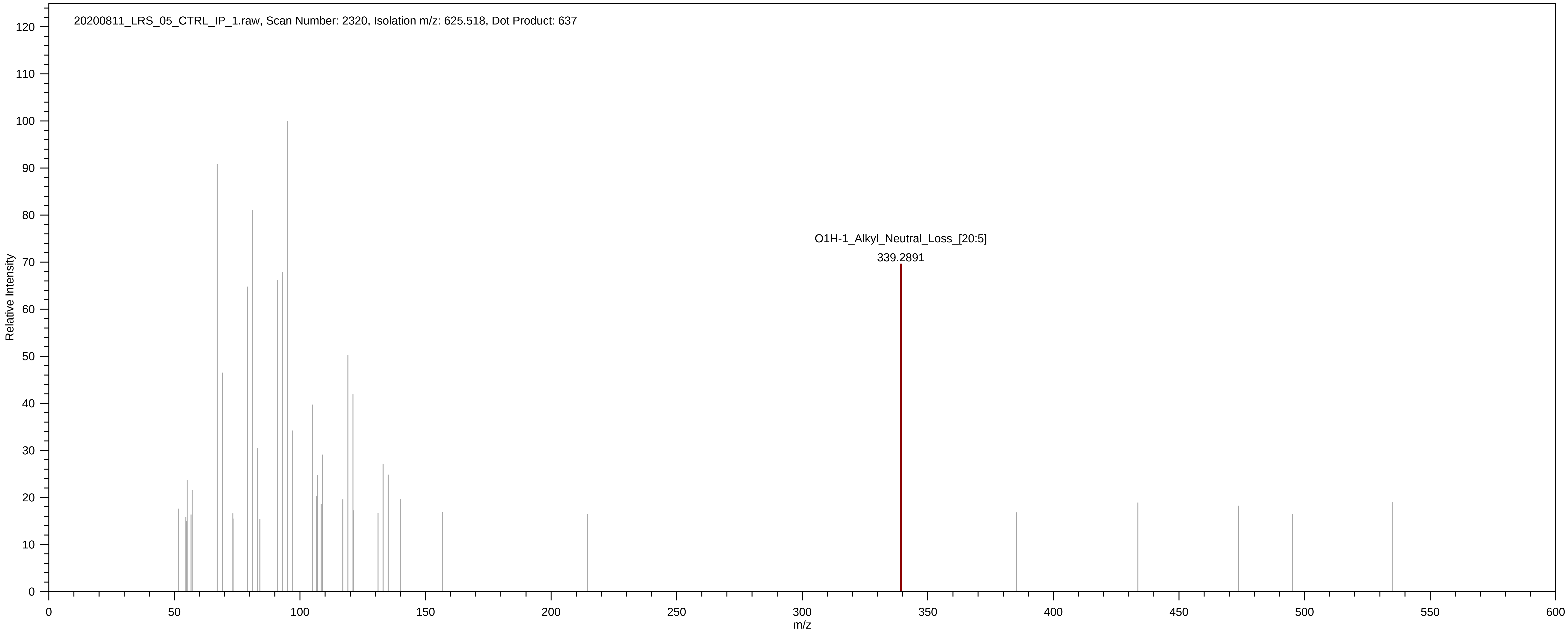

Alkenyl-DG P-18:1_20:5 [M+H]+ | Ret. Time (min): 8.025 | m/z: 625.519

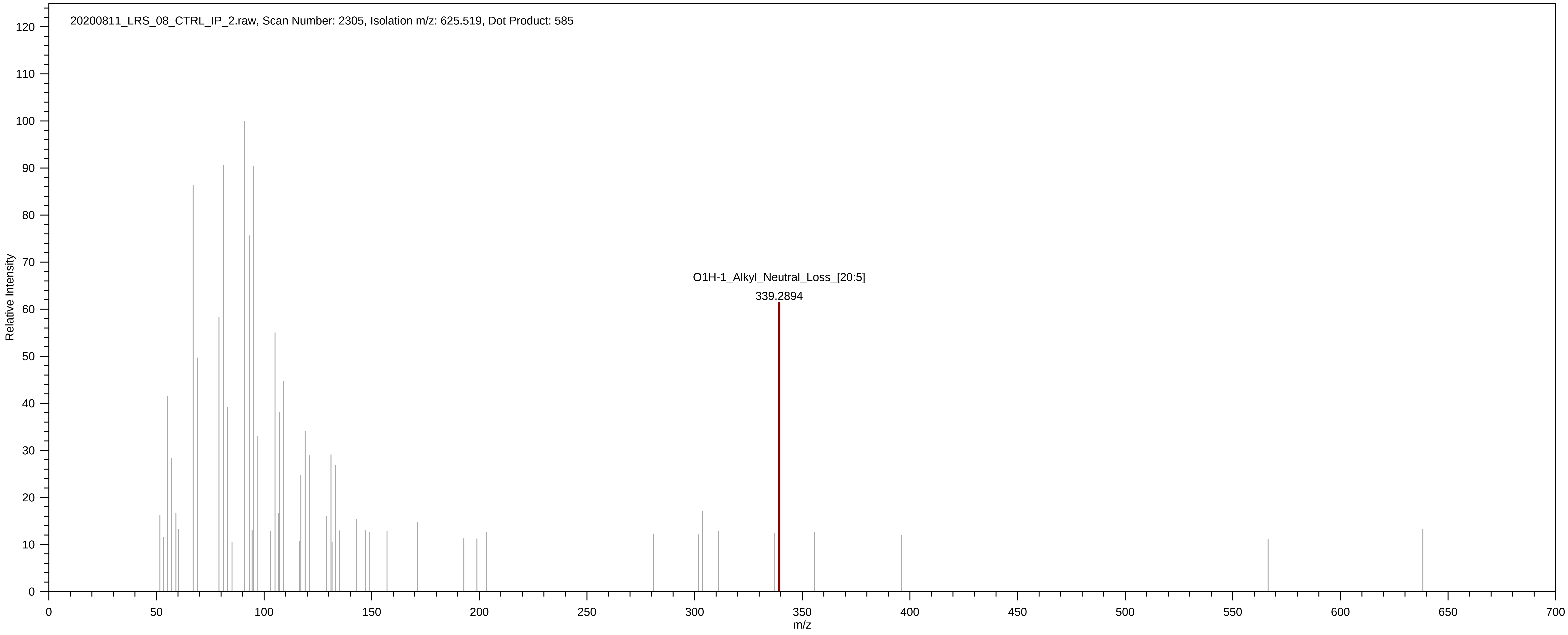

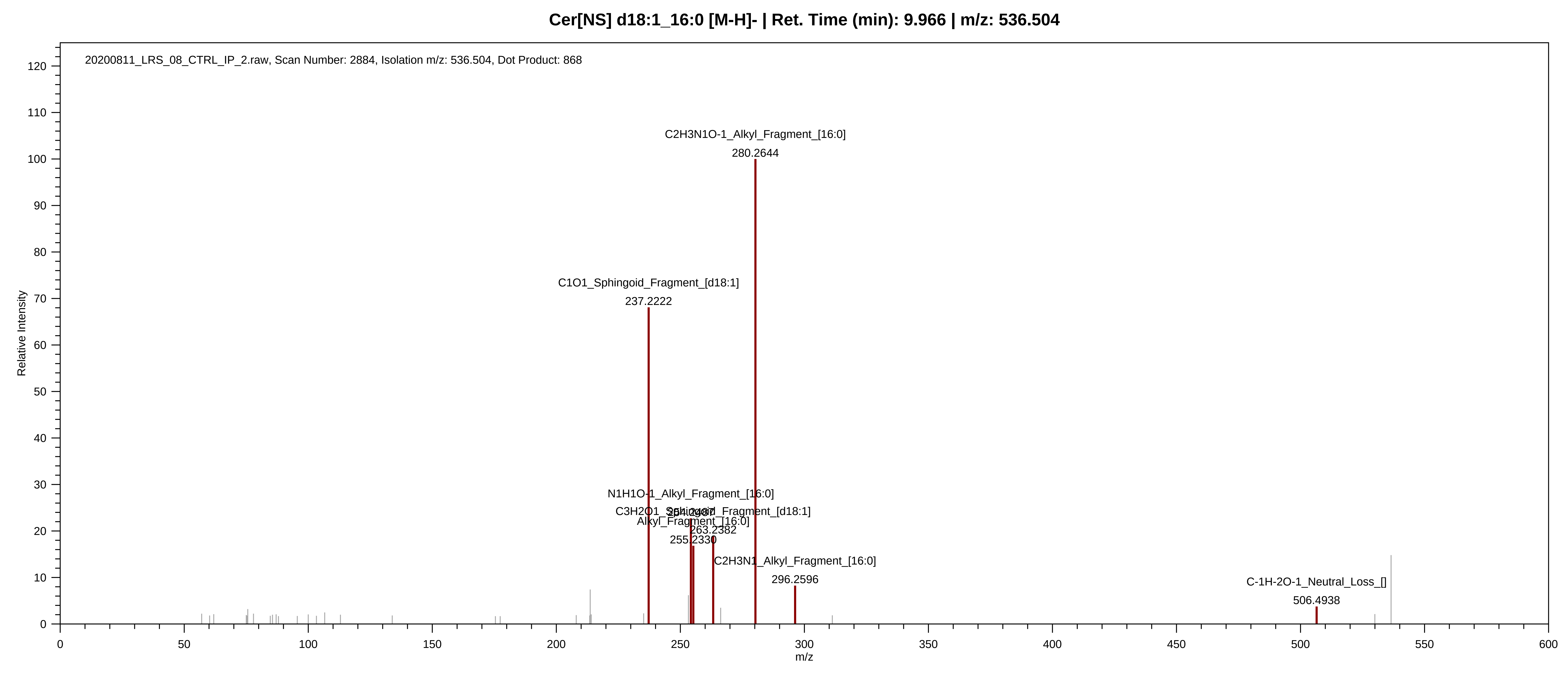
Cer[NS] d18:1_16:0 [M-H]- | Ret. Time (min): 9.966 | m/z: 536.504
20200811_LRS_08_CTRL_IP_2.raw, Scan Number: 2884, Isolation m/z: 536.504, Dot Product: 868
C2H3N1O-1_Alkyl_Fragment_[16:0]
280.2644
C1O1_Sphingoid_Fragment_[d18:1]
237.2222
N1H1O-1_Alkyl_Fragment_[16:0]
C3H2O1_Sphingoid_Fragment_[d18:1]
Alkyl_Fragment_[16:0]
263.2382
255.2330
C2H3N1_Alkyl_Fragment_[16:0]
296.2596
C-1H-2O-1_Neutral_Loss_[]
506.4938
Relative Intensity
m/z

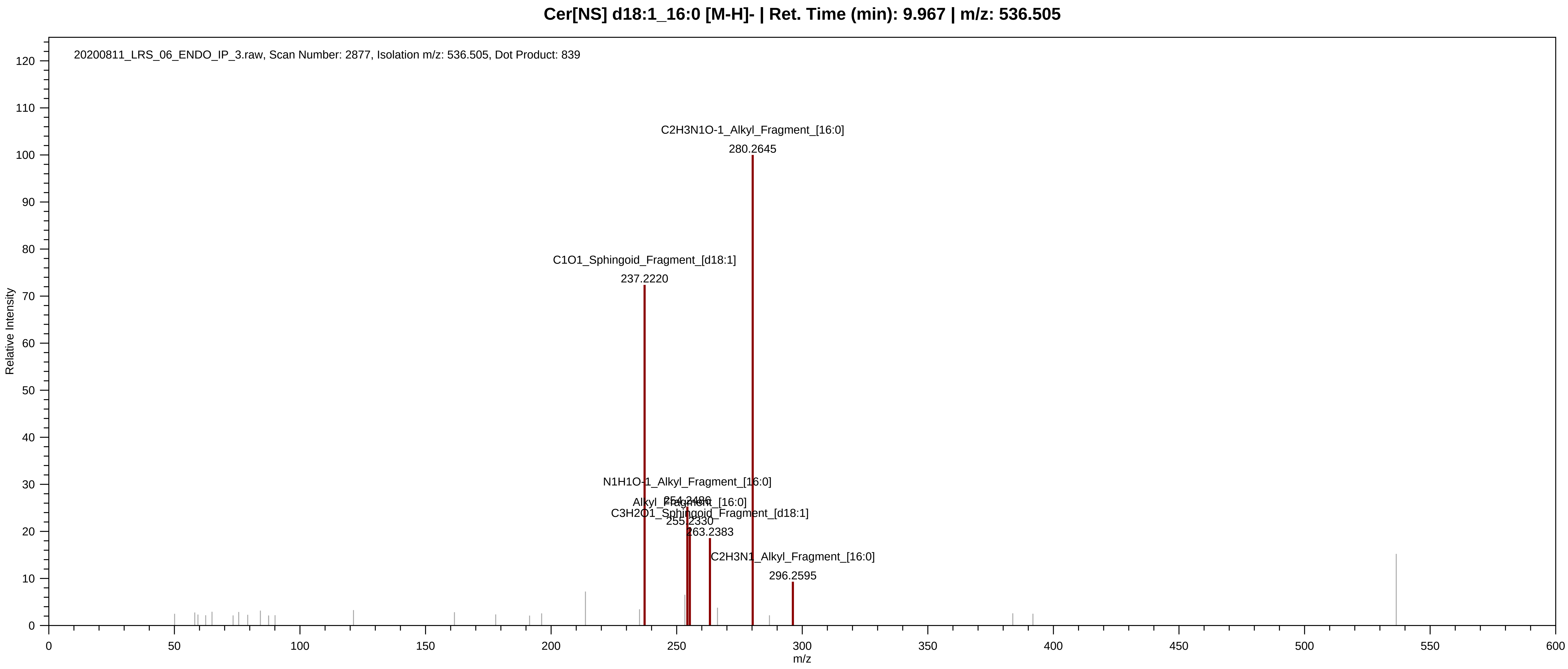

Cer[NS] d18:1_16:0 [M-H]- | Ret. Time (min): 9.967 | m/z: 536.505
20200811_LRS_06_ENDO_IP_3.raw, Scan Number: 2877, Isolation m/z: 536.505, Dot Product: 839
C2H3N1O-1_Alkyl_Fragment_[16:0]
280.2645
C1O1_Sphingoid_Fragment_[d18:1]
237.2220
N1H1O-1_Alkyl_Fragment_[16:0]
254.2486
Alkyl_Fragment_[16:0]
C3H2O1_Sphingoid_Fragment_[d18:1]
255.2330
263.2383
C2H3N1_Alkyl_Fragment_[16:0]
296.2595
Relative Intensity
m/z

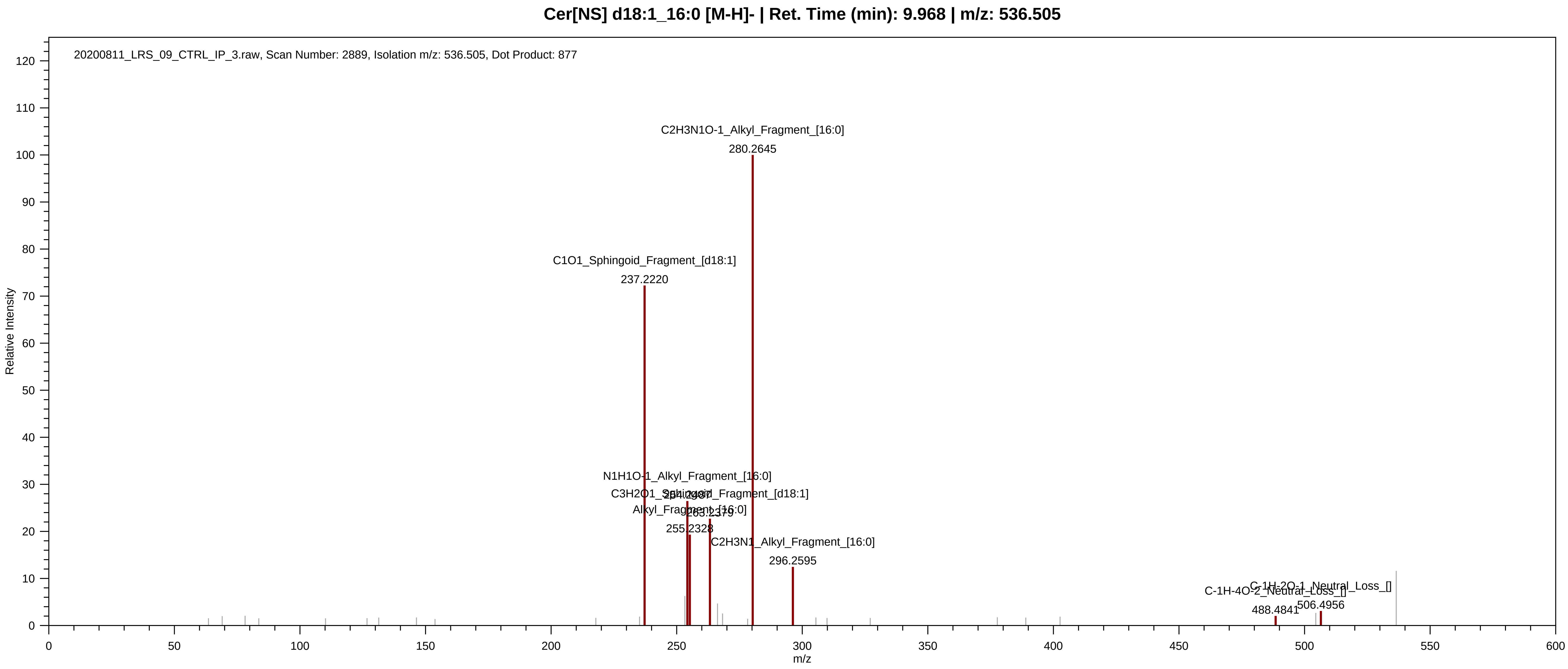

Cer[NS] d18:1_16:0 [M-H]- | Ret. Time (min): 9.968 | m/z: 536.505
20200811_LRS_09_CTRL_IP_3.raw, Scan Number: 2889, Isolation m/z: 536.505, Dot Product: 877
C2H3N1O-1_Alkyl_Fragment_[16:0]
280.2645
C1O1_Sphingoid_Fragment_[d18:1]
237.2220
N1H1O-1_Alkyl_Fragment_[16:0]
C3H2O1_Sphingoid_Fragment_[d18:1]
Alkyl_Fragment_[16:0]
255.2328
C2H3N1_Alkyl_Fragment_[16:0]
296.2595
C-1H-2O-1_Neutral_Loss_[]
C-1H-4O-2_Neutral_Loss_[]
488.4841
506.4956
Relative Intensity
m/z

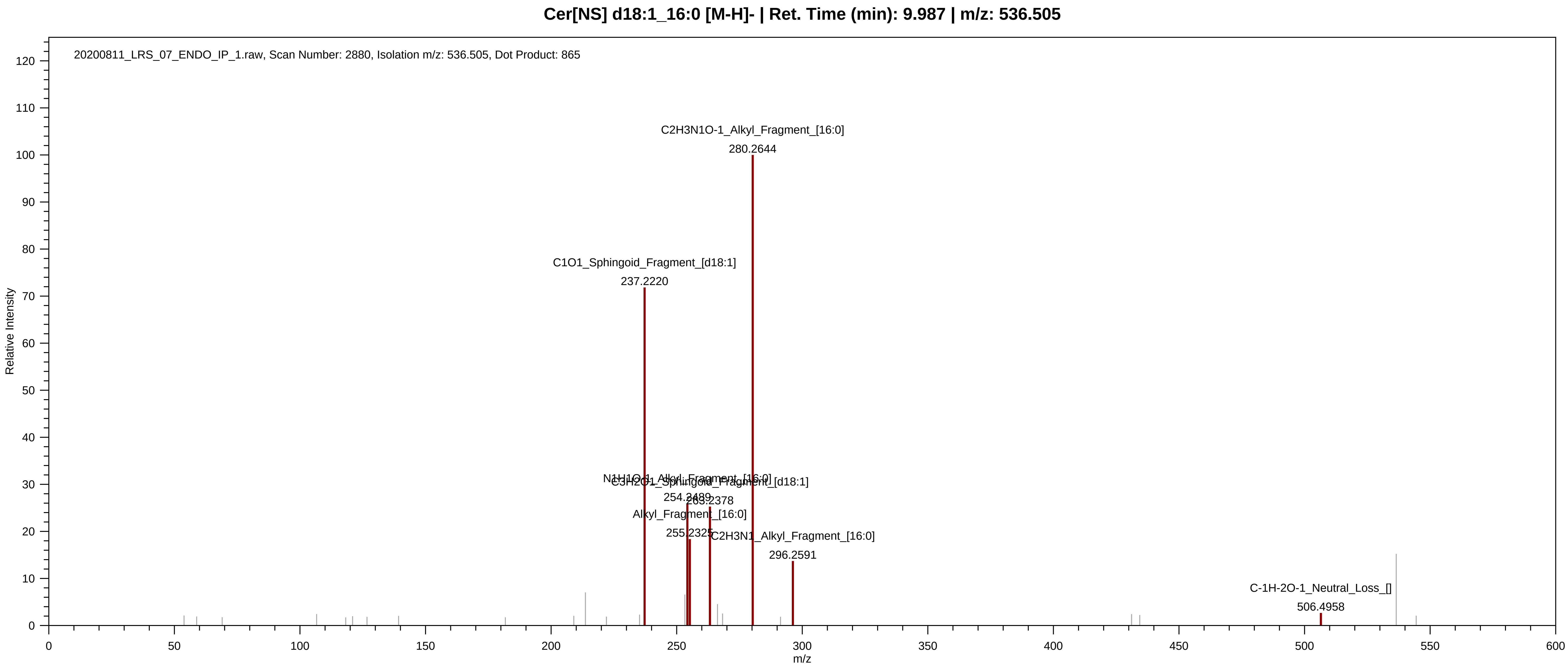
Cer[NS] d18:1_16:0 [M-H]- | Ret. Time (min): 9.987 | m/z: 536.505
20200811_LRS_07_ENDO_IP_1.raw, Scan Number: 2880, Isolation m/z: 536.505, Dot Product: 865
C2H3N1O-1_Alkyl_Fragment_[16:0]
280.2644
C1O1_Sphingoid_Fragment_[d18:1]
237.2220
N1H1O-1_Alkyl_Fragment_[16:0]
C3H2O1_Sphingoid_Fragment_[d18:1]
254.2489
263.2378
Alkyl_Fragment_[16:0]
255.2325
C2H3N1_Alkyl_Fragment_[16:0]
296.2591
C-1H-2O-1_Neutral_Loss_[]
506.4958
Relative Intensity
m/z

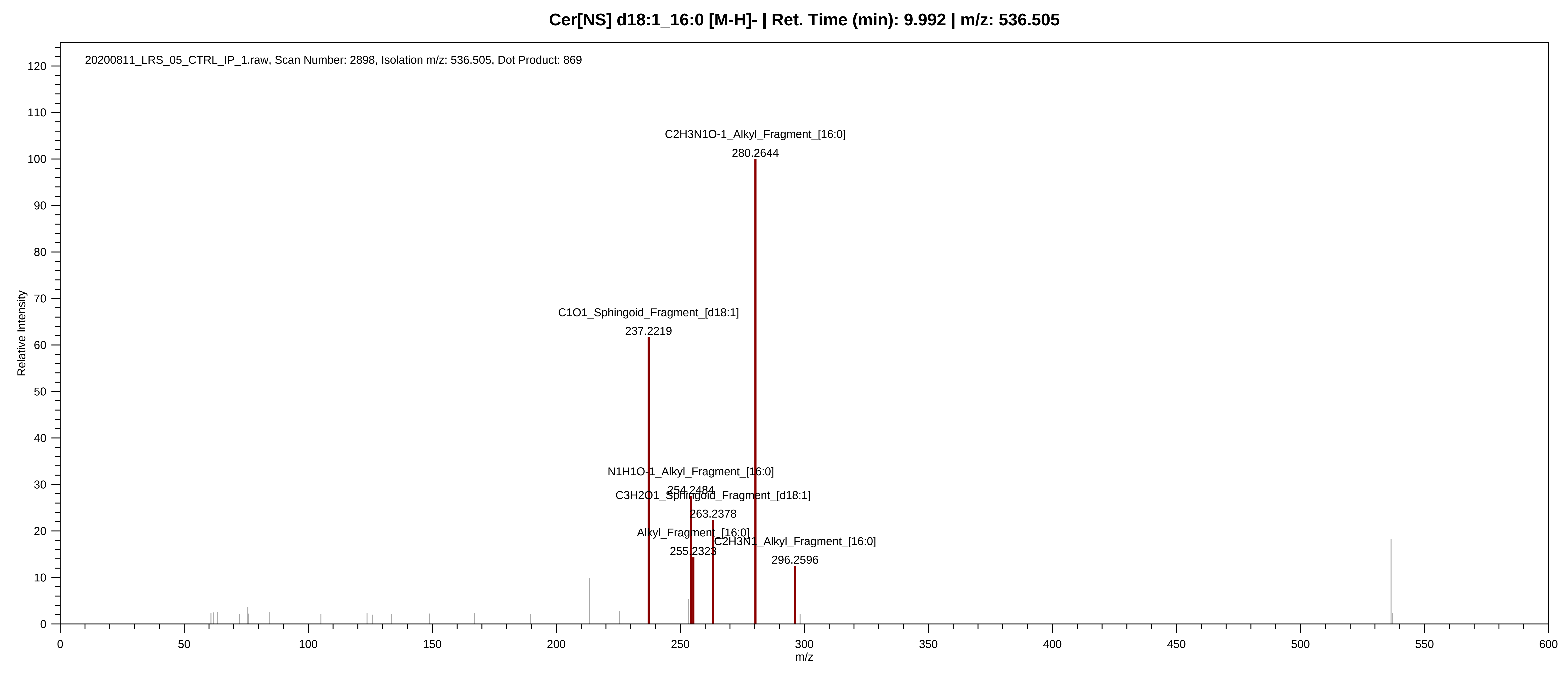

Cer[NS] d18:1_16:0 [M-H]- | Ret. Time (min): 9.992 | m/z: 536.505
20200811_LRS_05_CTRL_IP_1.raw, Scan Number: 2898, Isolation m/z: 536.505, Dot Product: 869
C2H3N1O-1_Alkyl_Fragment_[16:0]
280.2644
C1O1_Sphingoid_Fragment_[d18:1]
237.2219
N1H1O-1_Alkyl_Fragment_[16:0]
254.2484
C3H2O1_Sphingoid_Fragment_[d18:1]
263.2378
Alkyl_Fragment_[16:0]
255.2323
C2H3N1_Alkyl_Fragment_[16:0]
296.2596
Relative Intensity
m/z

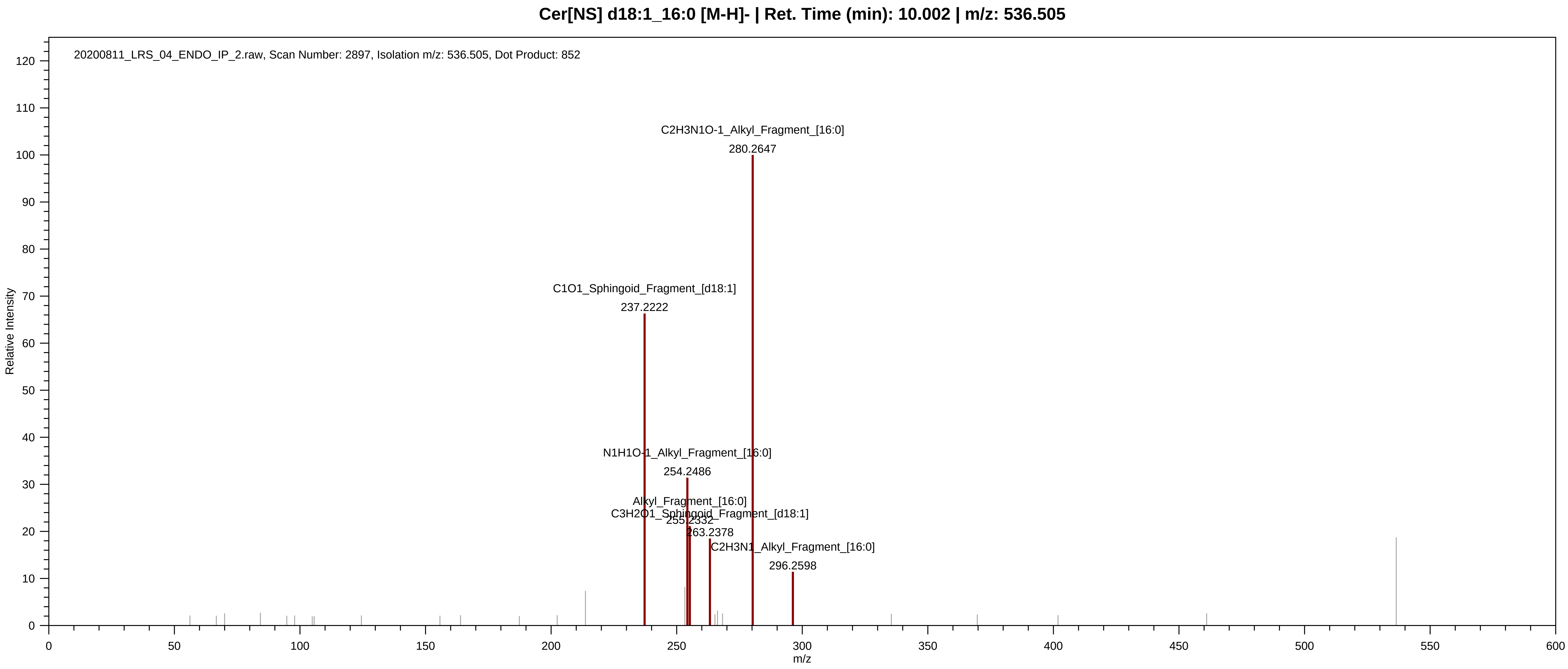

Cer[NS] d18:1_16:0 [M-H]- | Ret. Time (min): 10.002 | m/z: 536.505
20200811_LRS_04_ENDO_IP_2.raw, Scan Number: 2897, Isolation m/z: 536.505, Dot Product: 852
C2H3N1O-1_Alkyl_Fragment_[16:0]
280.2647
C1O1_Sphingoid_Fragment_[d18:1]
237.2222
N1H1O-1_Alkyl_Fragment_[16:0]
254.2486
Alkyl_Fragment_[16:0]
255.2332
C3H2O1_Sphingoid_Fragment_[d18:1]
263.2378
C2H3N1_Alkyl_Fragment_[16:0]
296.2598
Relative Intensity
m/z

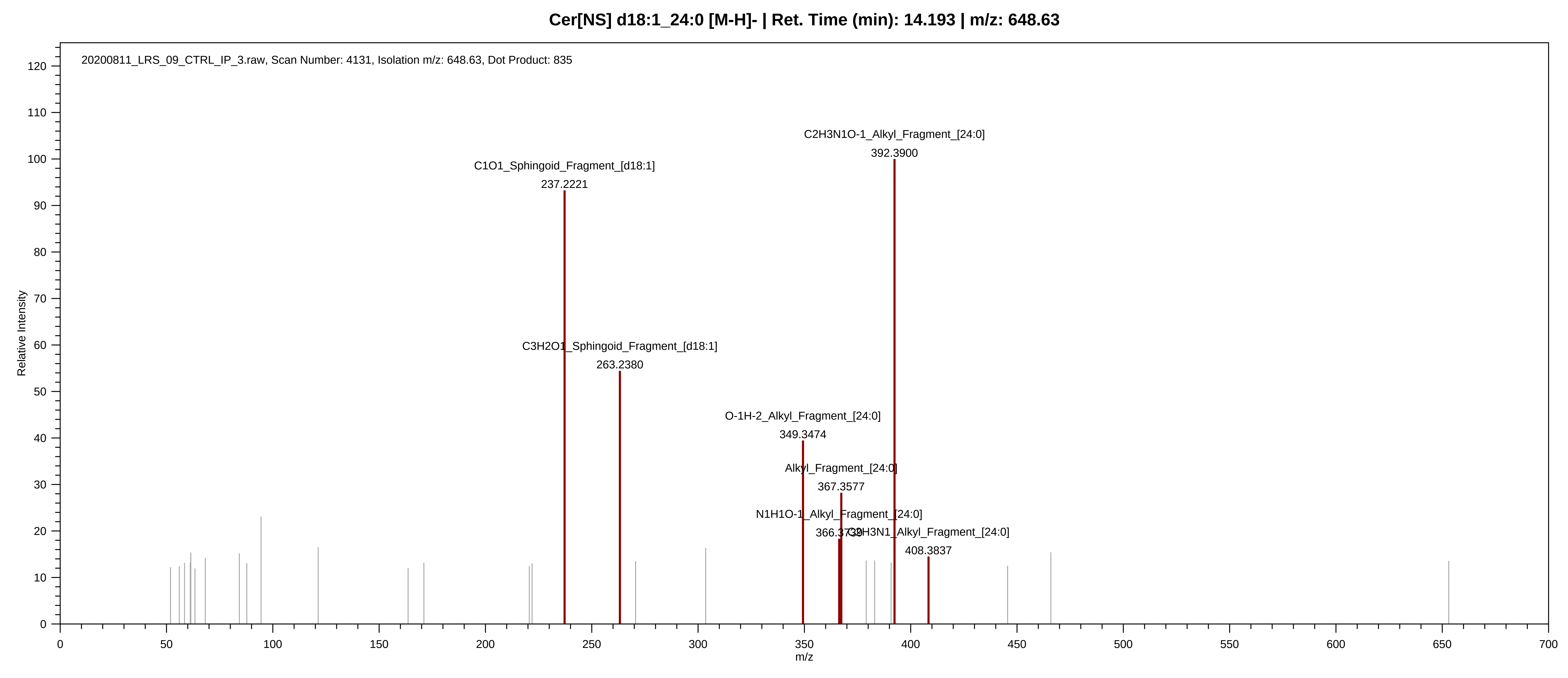

Cer[NS] d18:1_24:0 [M-H]- | Ret. Time (min): 14.193 | m/z: 648.63
20200811_LRS_09_CTRL_IP_3.raw, Scan Number: 4131, Isolation m/z: 648.63, Dot Product: 835
C2H3N1O-1_Alkyl_Fragment_[24:0]
392.3900
C1O1_Sphingoid_Fragment_[d18:1]
237.2221
C3H2O1_Sphingoid_Fragment_[d18:1]
263.2380
O-1H-2_Alkyl_Fragment_[24:0]
349.3474
Alkyl_Fragment_[24:0]
367.3577
N1H1O-1_Alkyl_Fragment_[24:0]
366.3739
C2H3N1_Alkyl_Fragment_[24:0]
408.3837
Relative Intensity
m/z

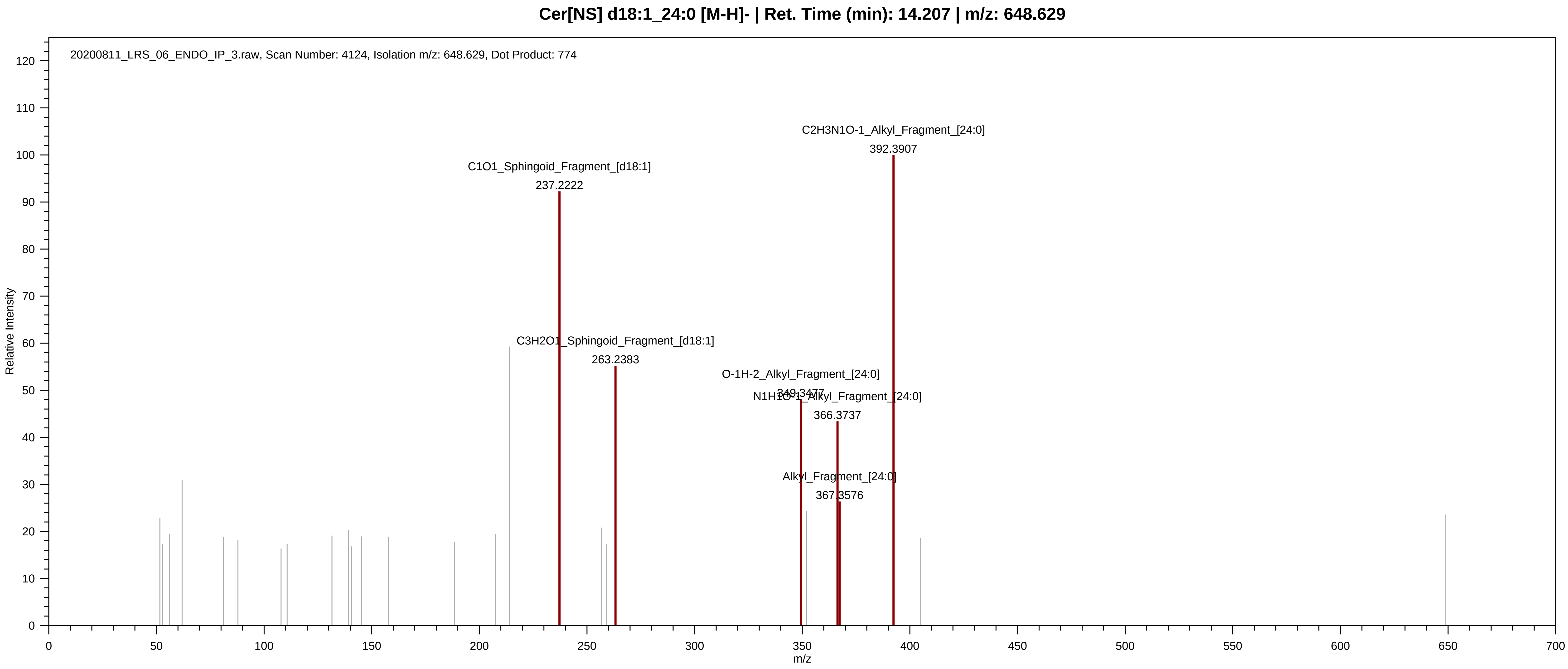

Cer[NS] d18:1_24:0 [M-H]- | Ret. Time (min): 14.207 | m/z: 648.629
20200811_LRS_06_ENDO_IP_3.raw, Scan Number: 4124, Isolation m/z: 648.629, Dot Product: 774
C2H3N1O-1_Alkyl_Fragment_[24:0]
392.3907
C1O1_Sphingoid_Fragment_[d18:1]
237.2222
C3H2O1_Sphingoid_Fragment_[d18:1]
263.2383
O-1H-2_Alkyl_Fragment_[24:0]
349.3477
N1H1O-1_Alkyl_Fragment_[24:0]
366.3737
Alkyl_Fragment_[24:0]
367.3576
Relative Intensity
m/z

Cer[NS] d18:1_24:0 [M-H]- | Ret. Time (min): 14.191 | m/z: 648.631

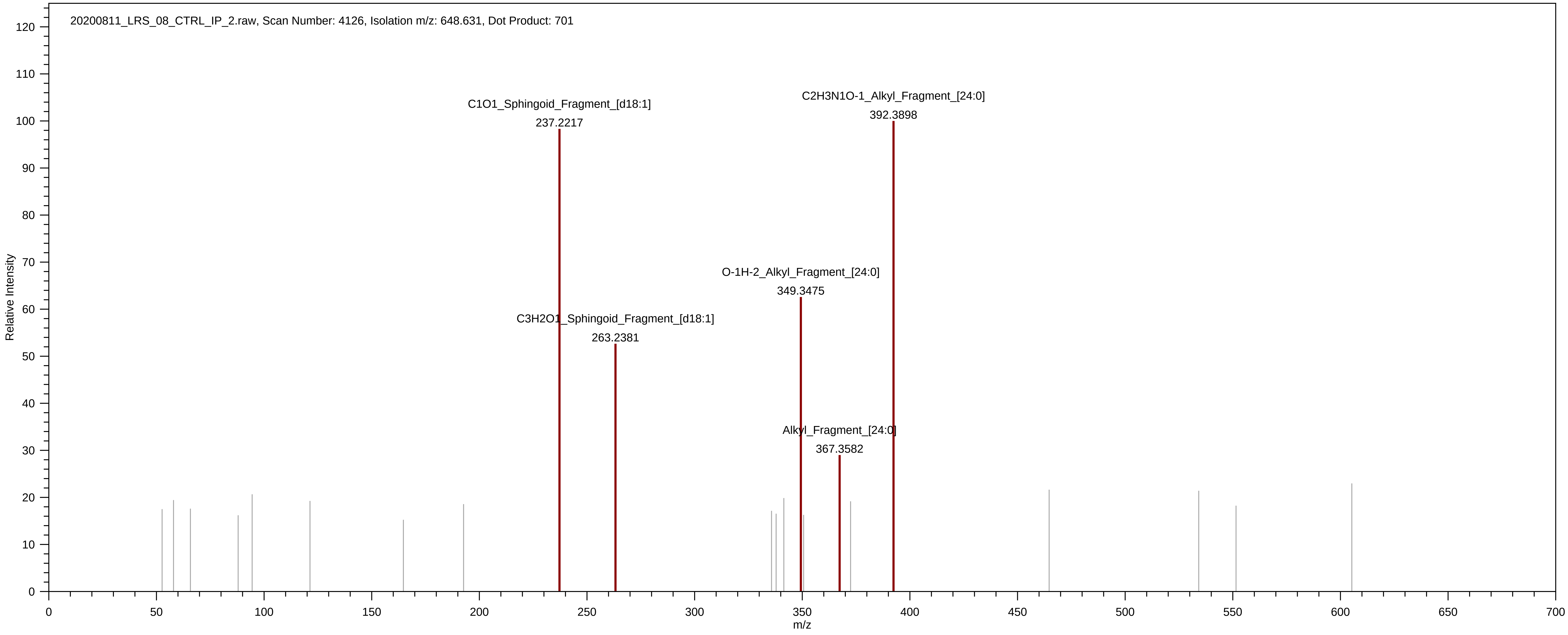

Cer[NS] d18:1_24:0 [M-H]- | Ret. Time (min): 14.203 | m/z: 648.631

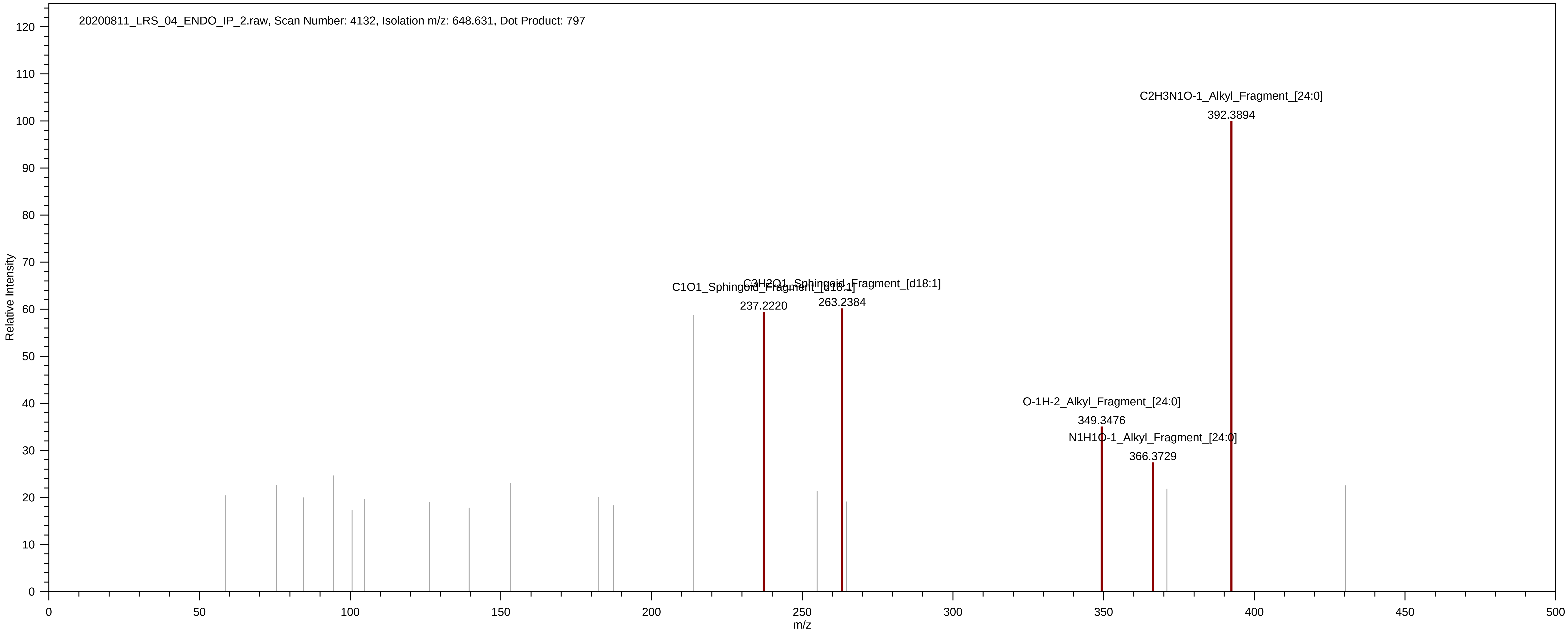

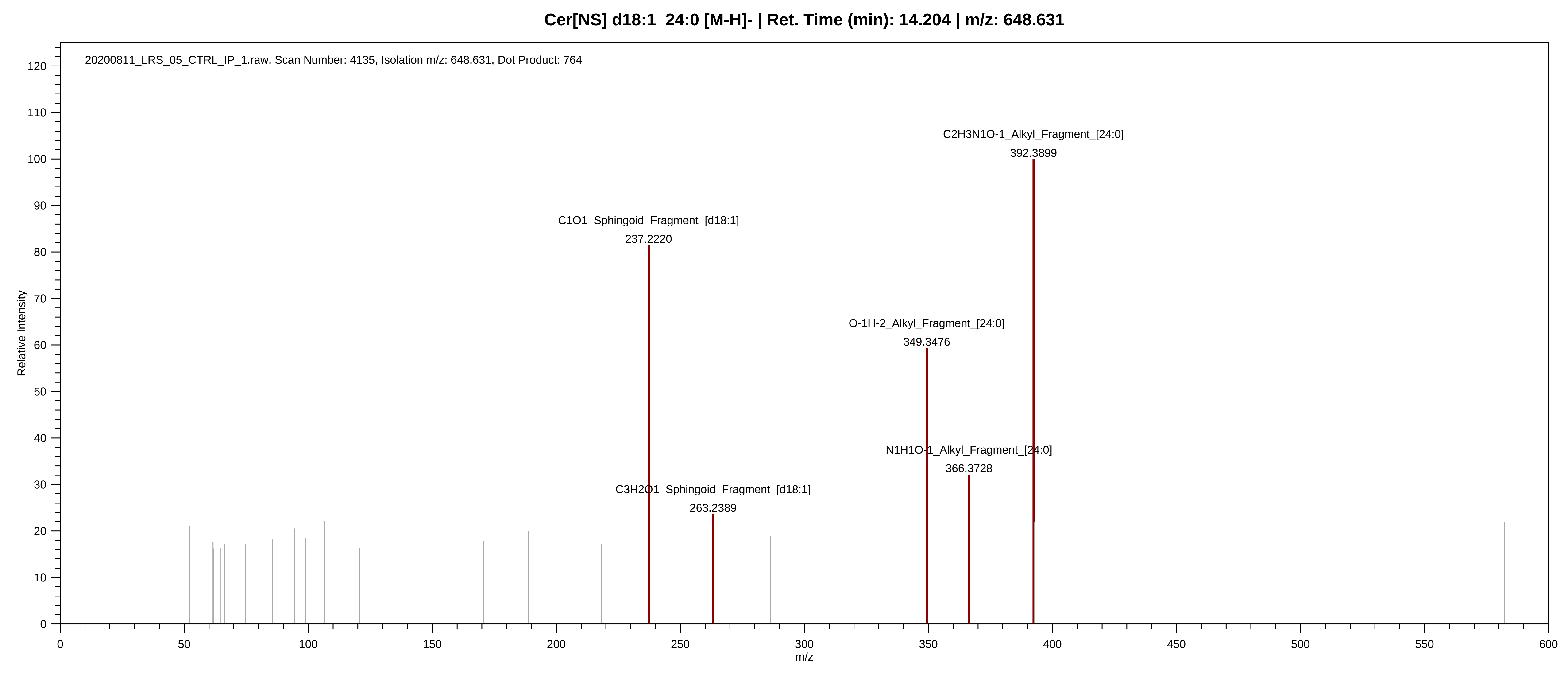
Cer[NS] d18:1_24:0 [M-H]- | Ret. Time (min): 14.204 | m/z: 648.631
20200811_LRS_05_CTRL_IP_1.raw, Scan Number: 4135, Isolation m/z: 648.631, Dot Product: 764
C2H3N1O-1_Alkyl_Fragment_[24:0]
392.3899
C1O1_Sphingoid_Fragment_[d18:1]
237.2220
O-1H-2_Alkyl_Fragment_[24:0]
349.3476
N1H1O-1_Alkyl_Fragment_[24:0]
366.3728
C3H2O1_Sphingoid_Fragment_[d18:1]
263.2389
Relative Intensity
m/z

Cer[NS] d18:1_24:0 [M-H]- | Ret. Time (min): 14.207 | m/z: 648.631

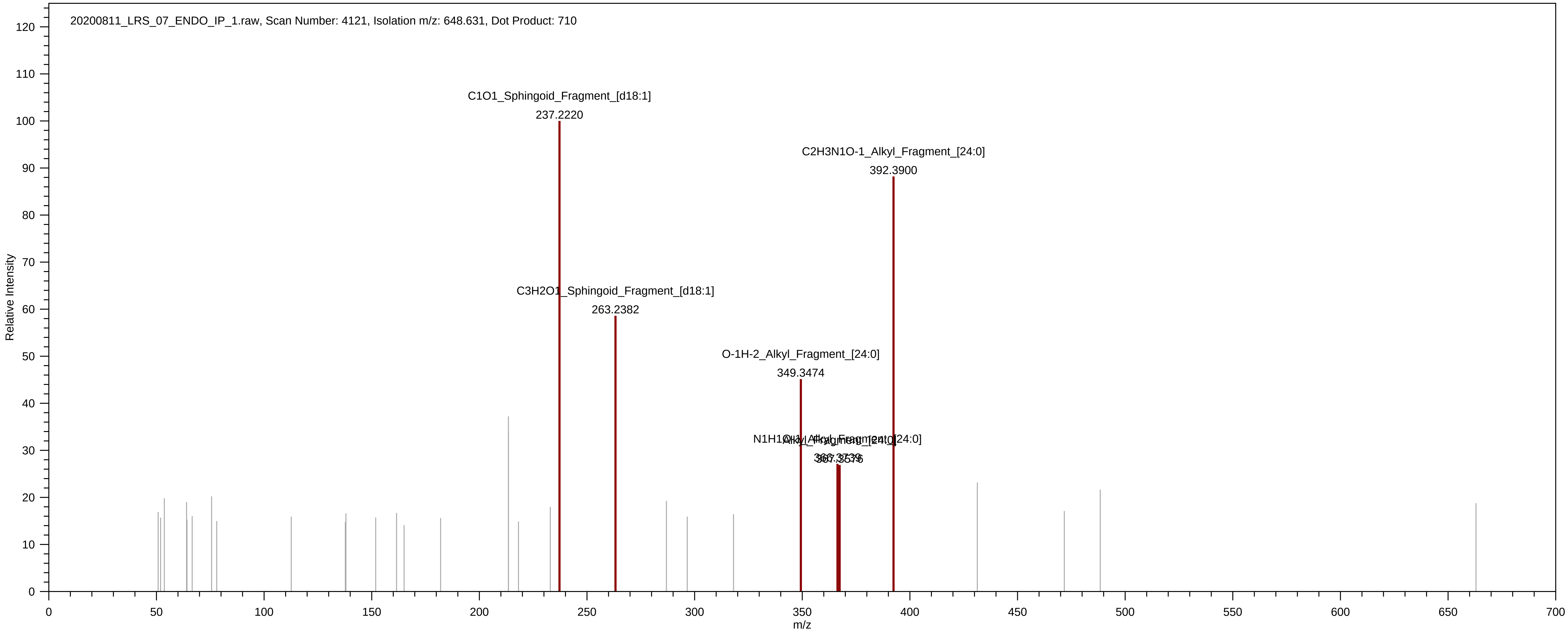

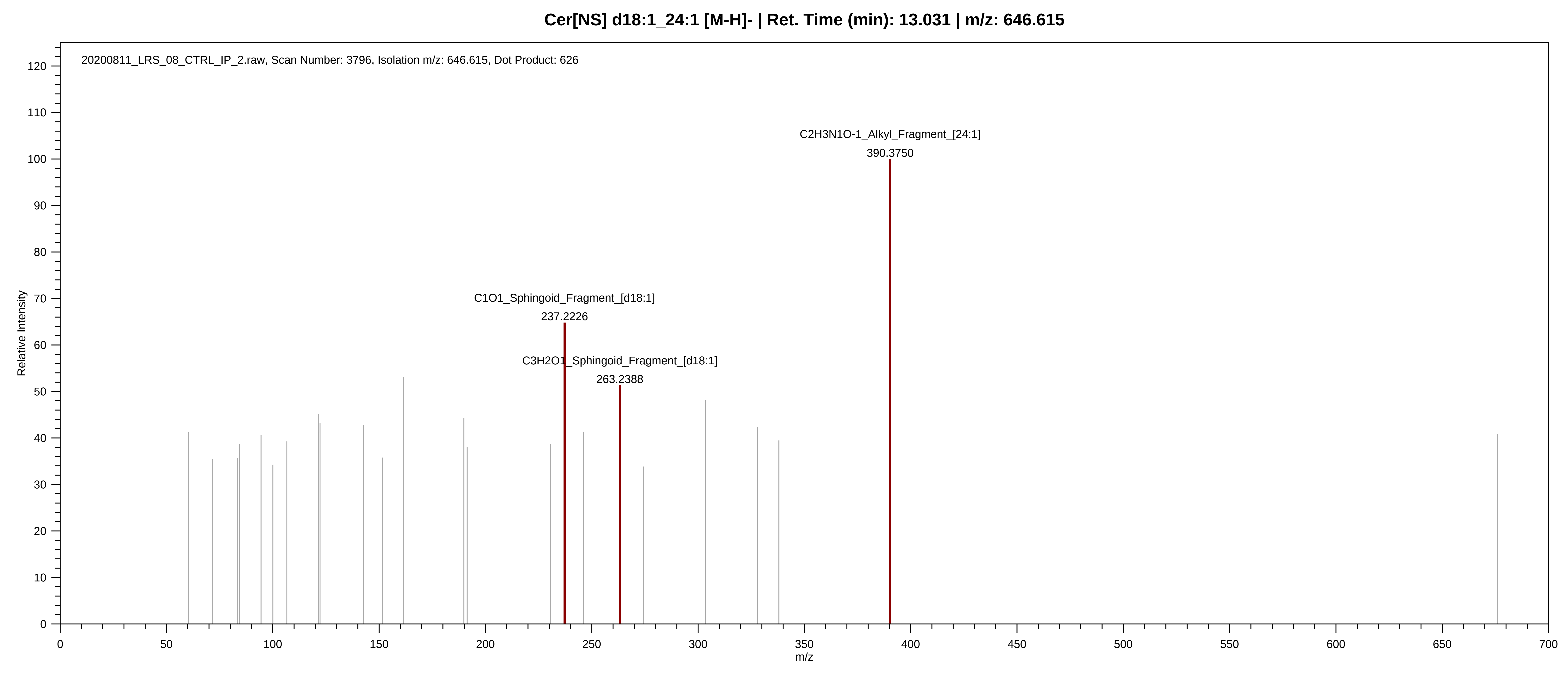
Cer[NS] d18:1_24:1 [M-H]- | Ret. Time (min): 13.031 | m/z: 646.615
20200811_LRS_08_CTRL_IP_2.raw, Scan Number: 3796, Isolation m/z: 646.615, Dot Product: 626
C2H3N1O-1_Alkyl_Fragment_[24:1]
390.3750
C1O1_Sphingoid_Fragment_[d18:1]
237.2226
C3H2O1_Sphingoid_Fragment_[d18:1]
263.2388
Relative Intensity
m/z

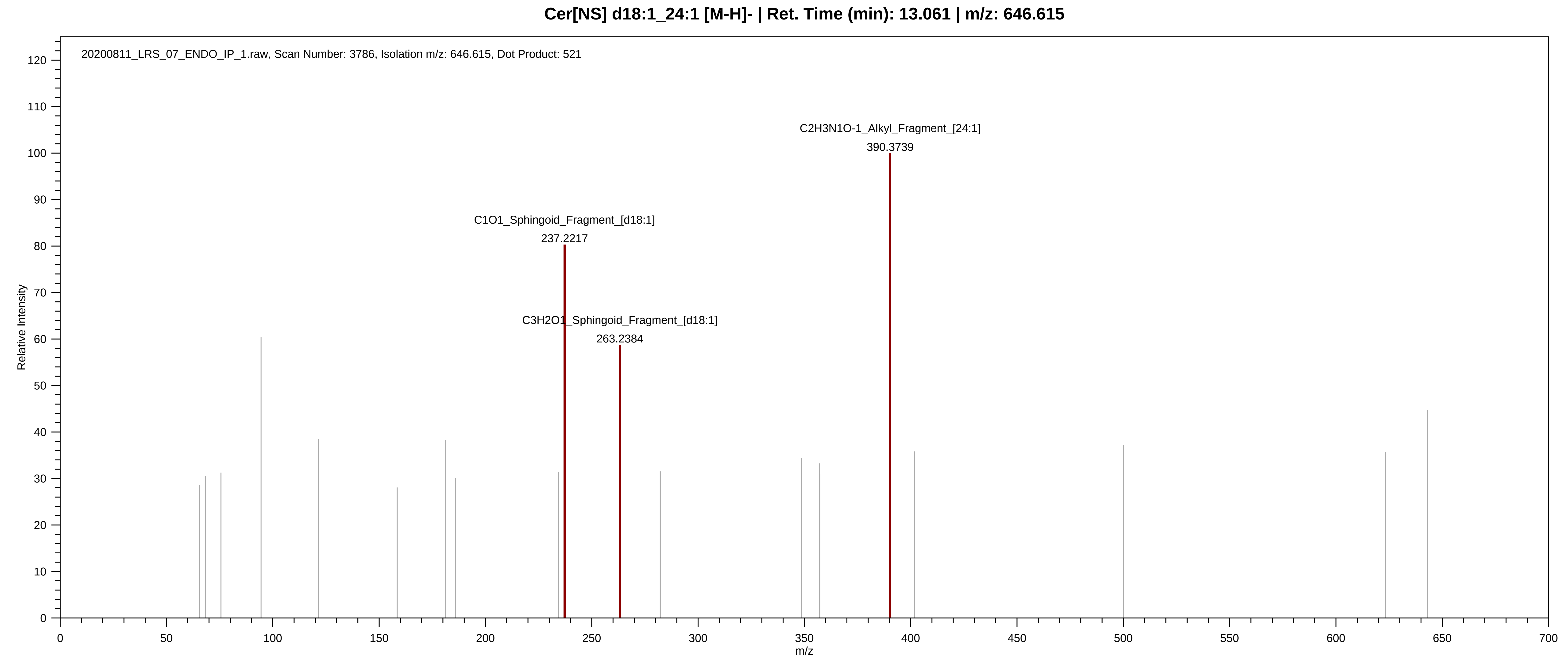
Cer[NS] d18:1_24:1 [M-H]- | Ret. Time (min): 13.061 | m/z: 646.615
20200811_LRS_07_ENDO_IP_1.raw, Scan Number: 3786, Isolation m/z: 646.615, Dot Product: 521
C2H3N1O-1_Alkyl_Fragment_[24:1]
390.3739
C1O1_Sphingoid_Fragment_[d18:1]
237.2217
C3H2O1_Sphingoid_Fragment_[d18:1]
263.2384
Relative Intensity
m/z

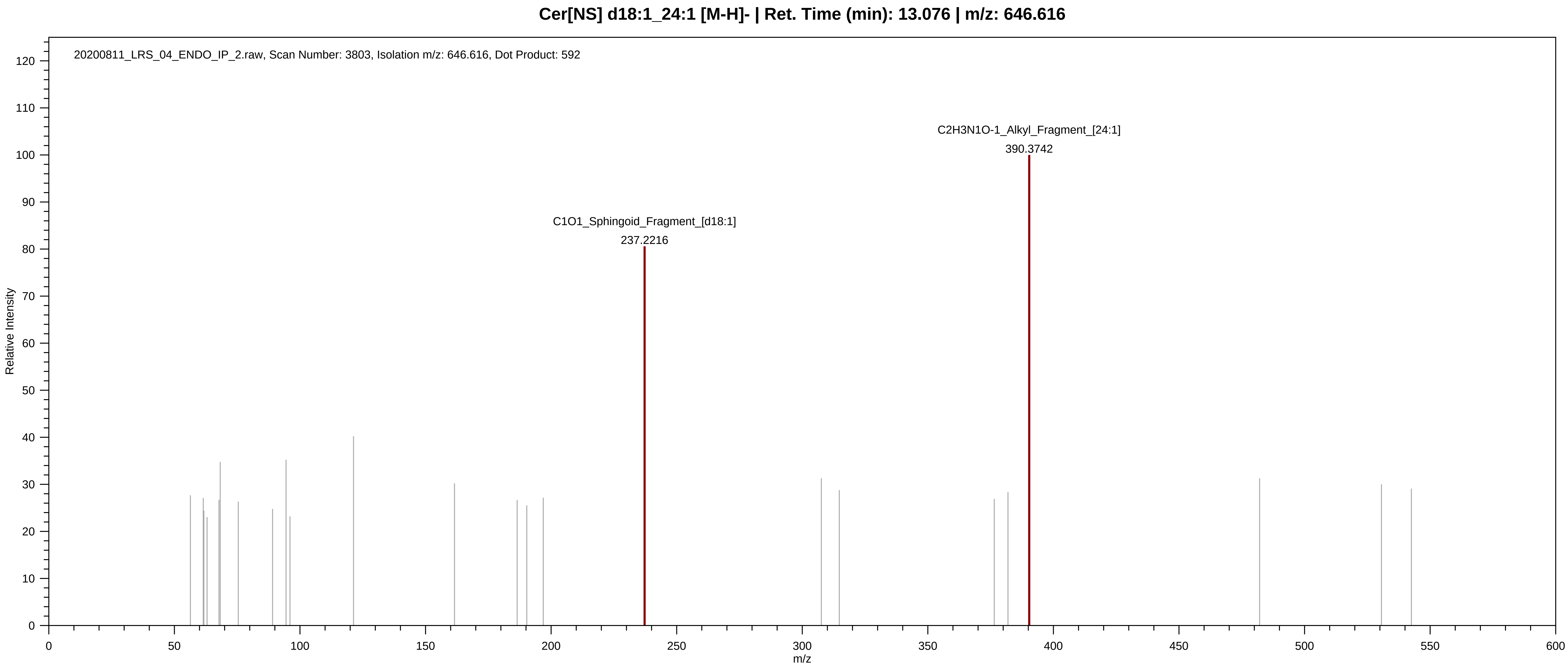

Cer[NS] d18:1_24:1 [M-H]- | Ret. Time (min): 13.076 | m/z: 646.616
20200811_LRS_04_ENDO_IP_2.raw, Scan Number: 3803, Isolation m/z: 646.616, Dot Product: 592
C2H3N1O-1_Alkyl_Fragment_[24:1]
390.3742
C1O1_Sphingoid_Fragment_[d18:1]
237.2216
Relative Intensity
m/z

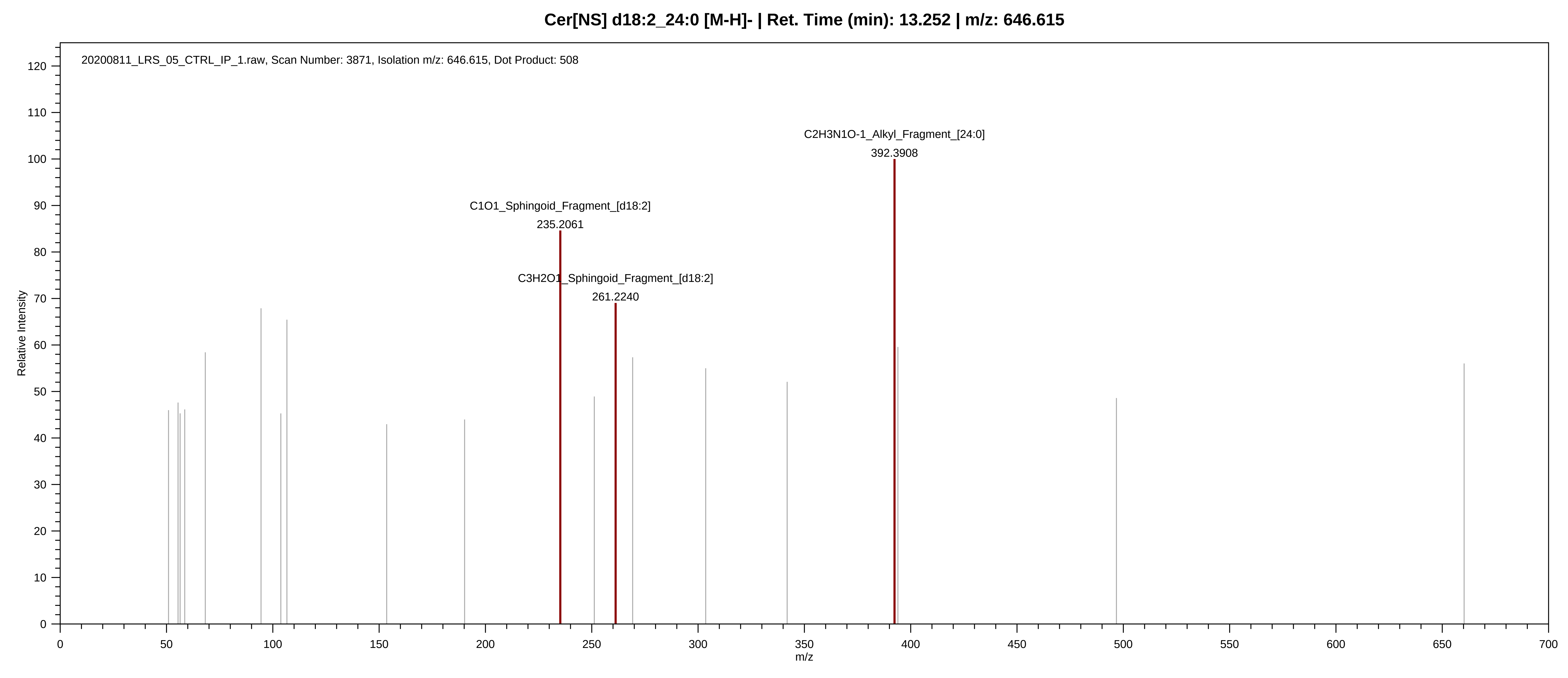

Cer[NS] d18:2_24:0 [M-H]- | Ret. Time (min): 13.252 | m/z: 646.615
20200811_LRS_05_CTRL_IP_1.raw, Scan Number: 3871, Isolation m/z: 646.615, Dot Product: 508
C2H3N1O-1_Alkyl_Fragment_[24:0]
392.3908
C1O1_Sphingoid_Fragment_[d18:2]
235.2061
C3H2O1_Sphingoid_Fragment_[d18:2]
261.2240
Relative Intensity
m/z

Cer[NS] d18:2_24:0 [M-H]- | Ret. Time (min): 13.267 | m/z: 646.615

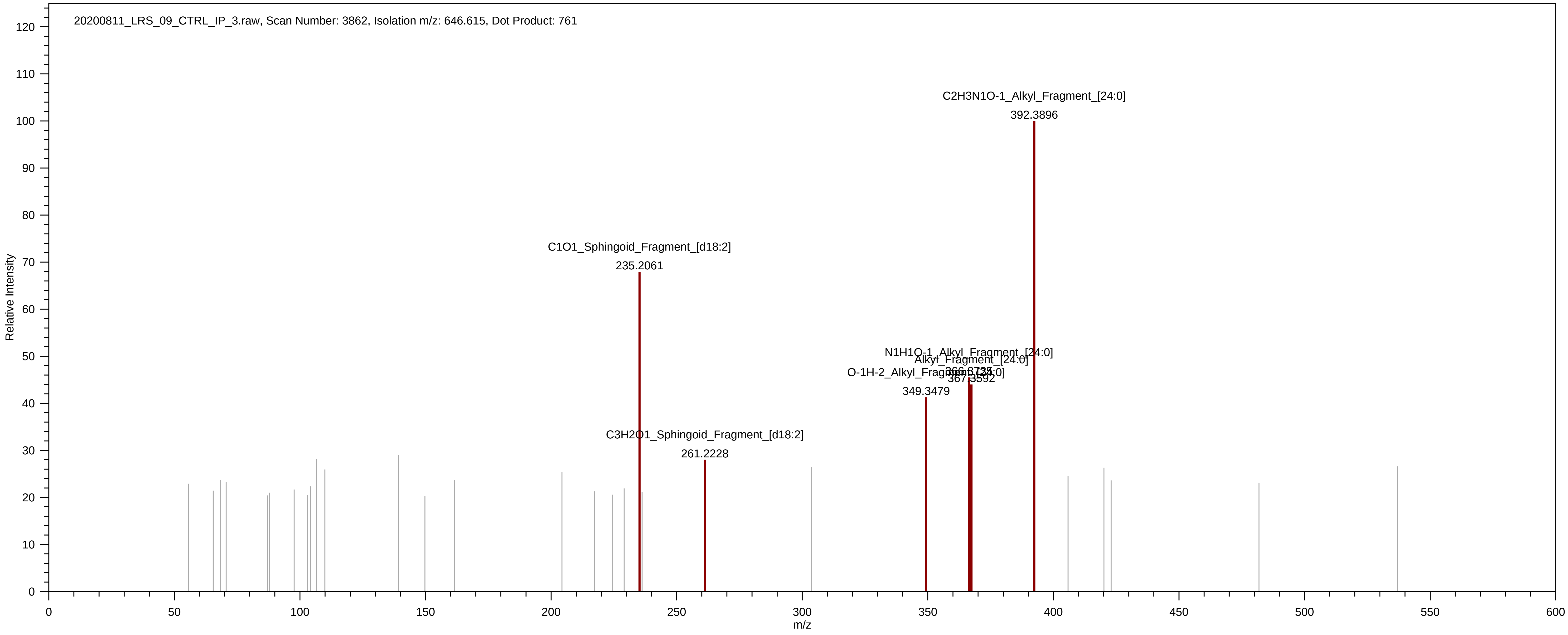

Cer[NS] d18:2_24:0 [M-H]- | Ret. Time (min): 13.287 | m/z: 646.615

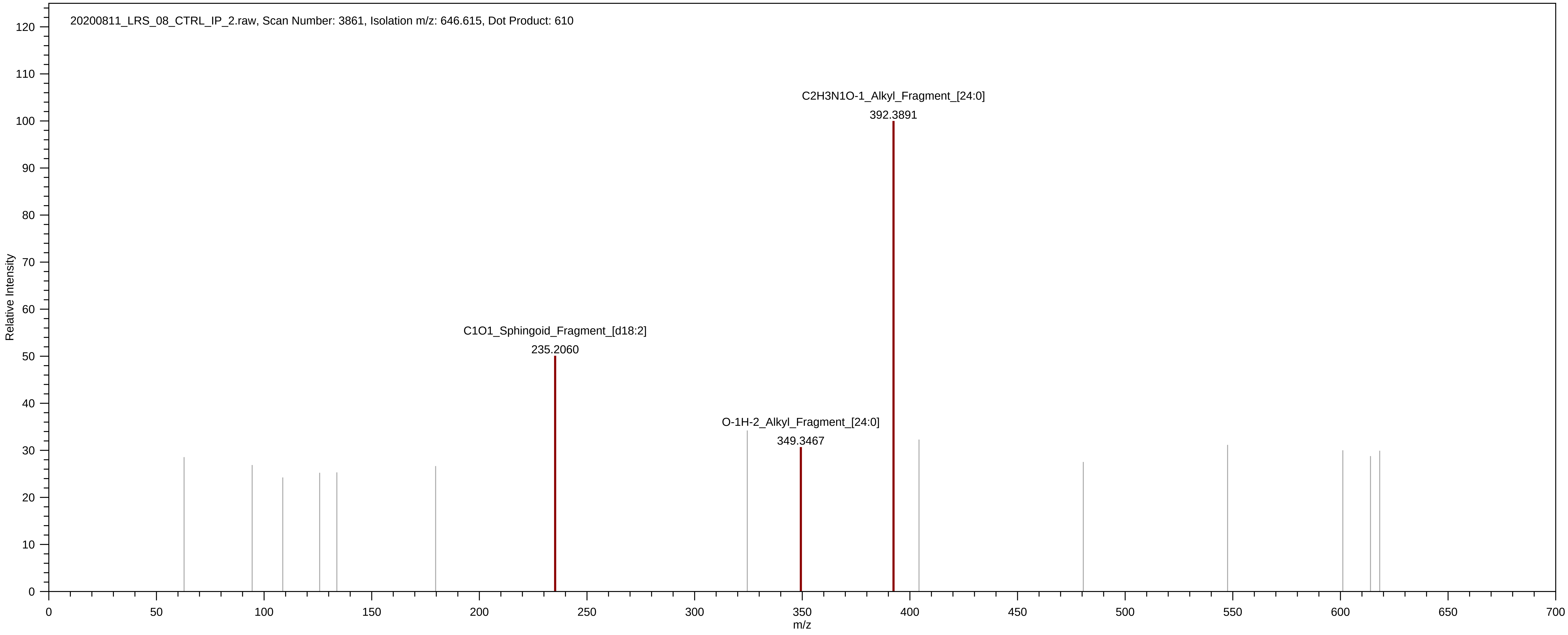

Cer[NS] d34:1 [M+H-H2O]+ | Ret. Time (min): 9.995 | m/z: 520.508

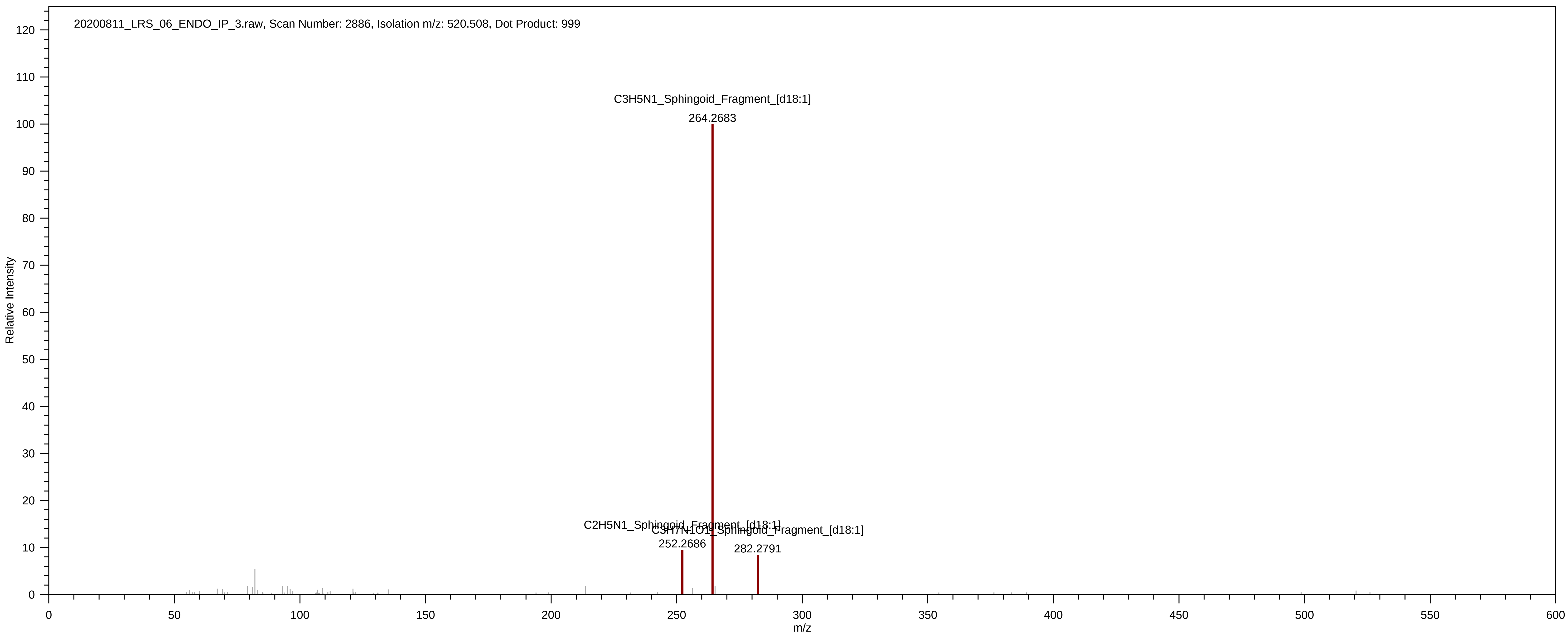

Cer[NS] d34:1 [M+H-H2O]+ | Ret. Time (min): 10.002 | m/z: 520.508

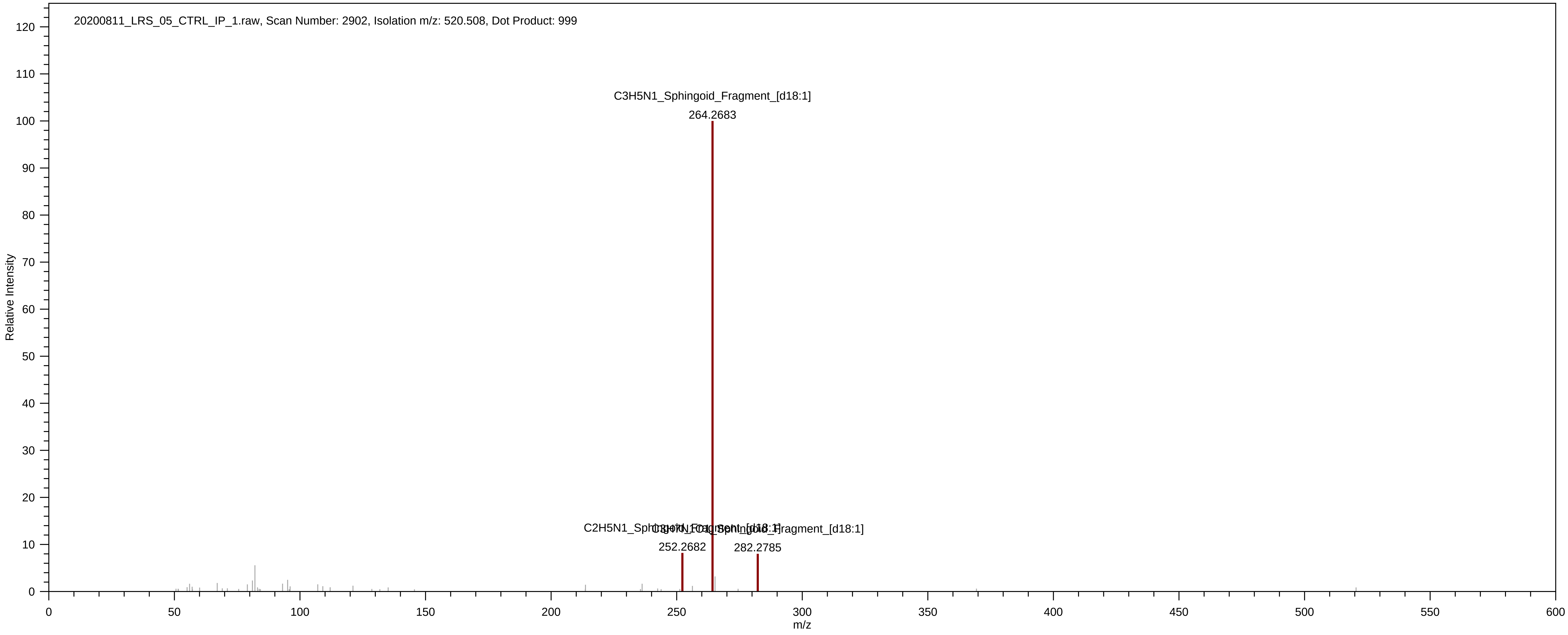

Cer[NS] d34:1 [M+H-H2O]+ | Ret. Time (min): 10.013 | m/z: 520.508

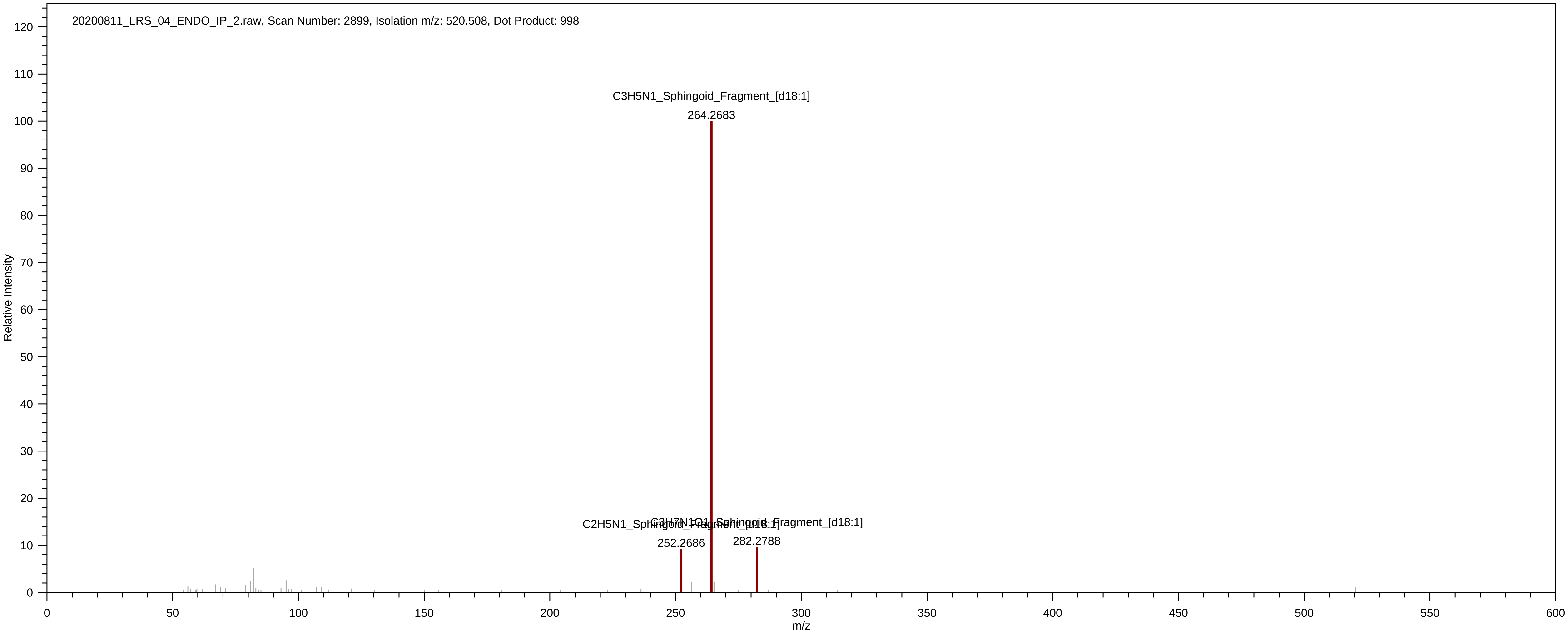

Cer[NS] d34:1 [M+H-H2O]+ | Ret. Time (min): 9.14 | m/z: 520.509

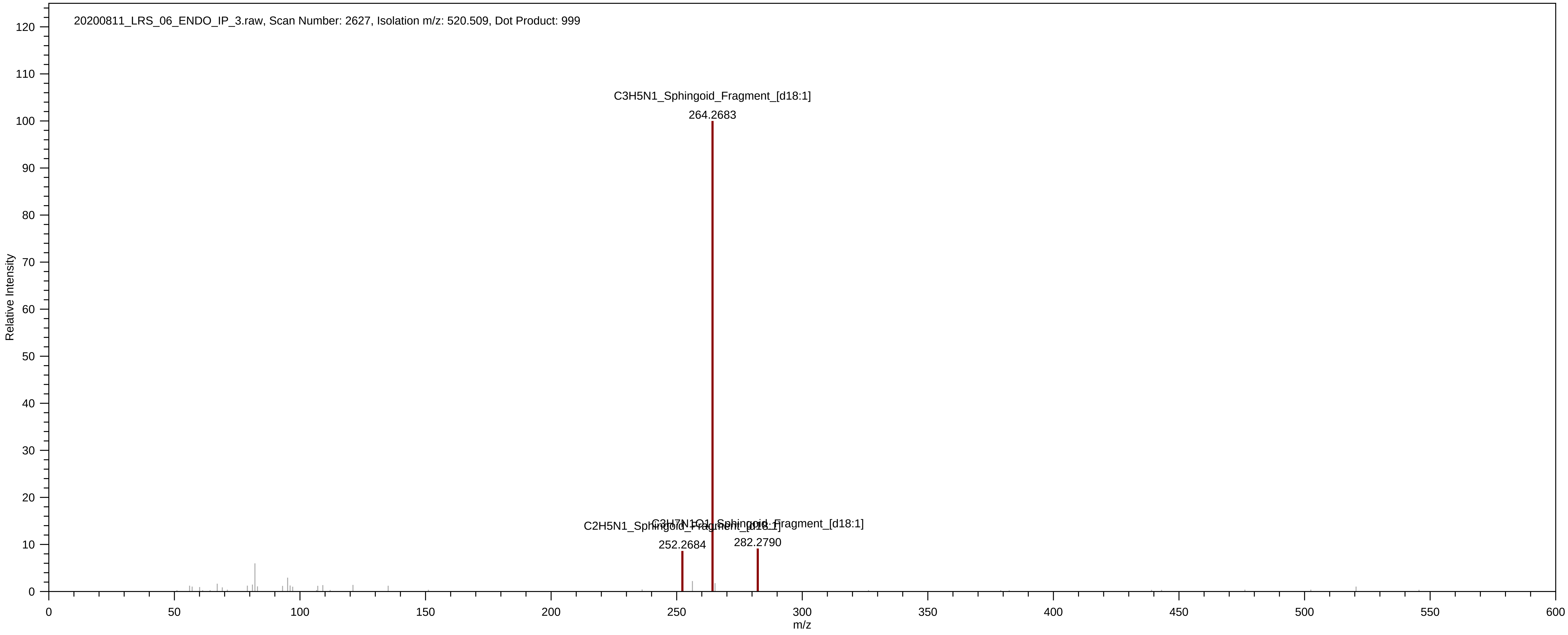

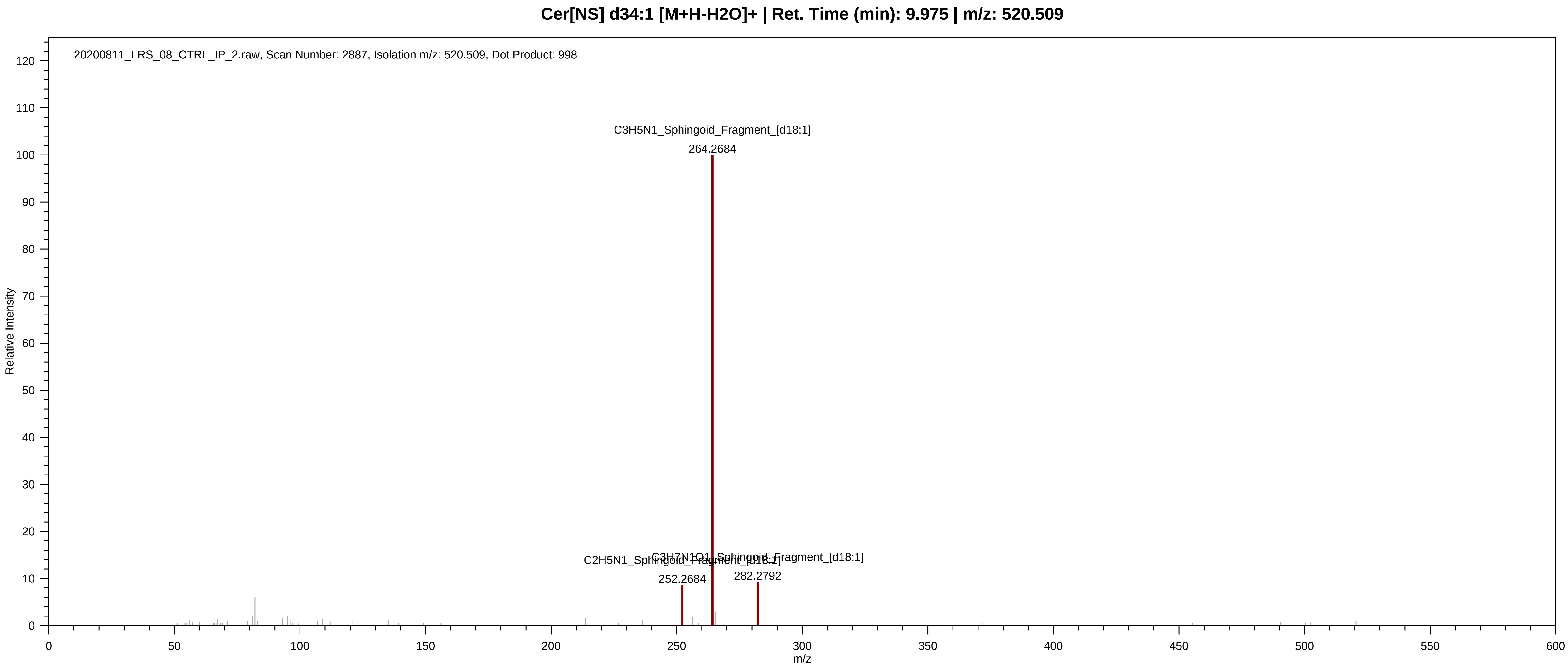
Cer[NS] d34:1 [M+H-H2O]+ | Ret. Time (min): 9.975 | m/z: 520.509
20200811_LRS_08_CTRL_IP_2.raw, Scan Number: 2887, Isolation m/z: 520.509, Dot Product: 998
C3H5N1_Sphingoid_Fragment_[d18:1]
264.2684
C2H5N1_Sphingoid_Fragment_[d18:1]
252.2684
C3H7N1O1_Sphingoid_Fragment_[d18:1]
282.2792
Relative Intensity
m/z

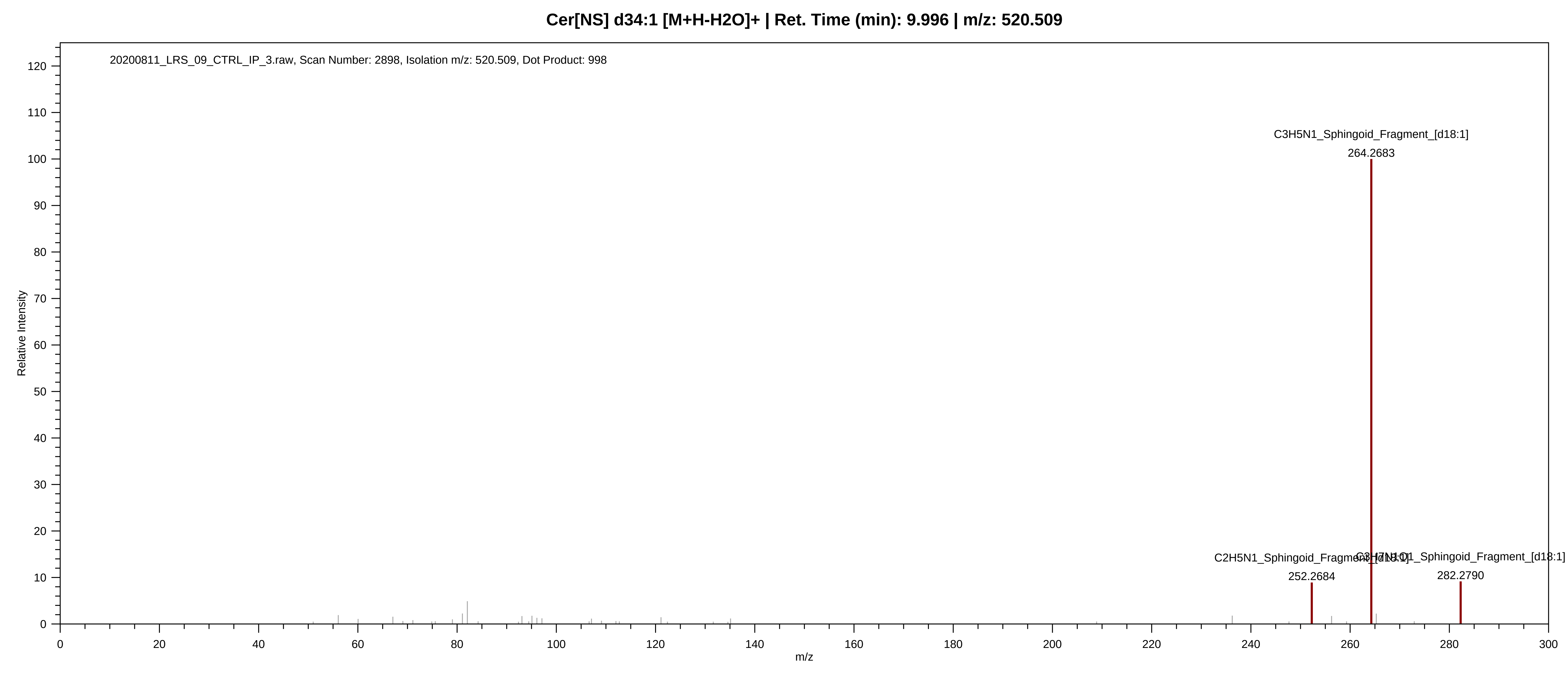

Cer[NS] d34:1 [M+H-H2O]+ | Ret. Time (min): 9.996 | m/z: 520.509
20200811_LRS_09_CTRL_IP_3.raw, Scan Number: 2898, Isolation m/z: 520.509, Dot Product: 998
C3H5N1_Sphingoid_Fragment_[d18:1]
264.2683
C2H5N1_Sphingoid_Fragment_[d18:1]
252.2684
C3H7N1O1_Sphingoid_Fragment_[d18:1]
282.2790
Relative Intensity
m/z

Cer[NS] d34:1 [M+H-H2O]+ | Ret. Time (min): 9.997 | m/z: 520.509

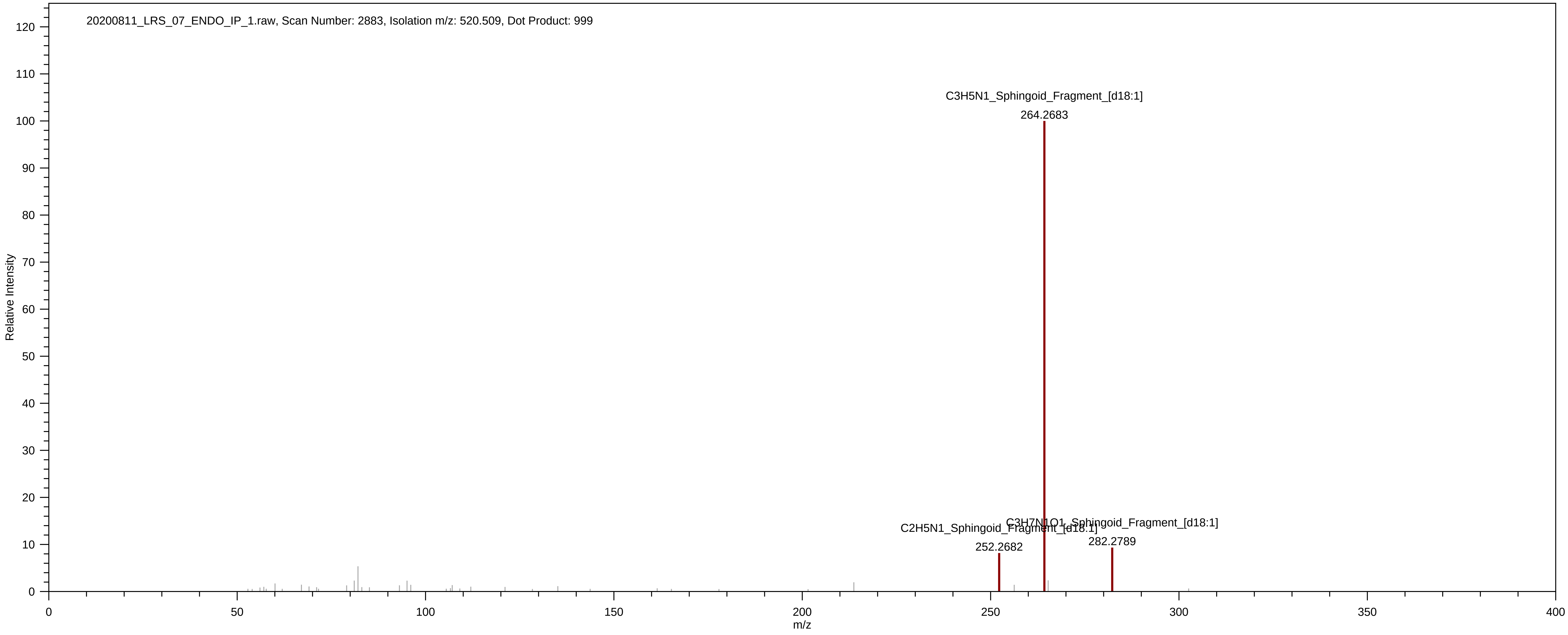

Cer[NS] d40:1 [M+H-H2O]+ | Ret. Time (min): 12.201 | m/z: 604.603

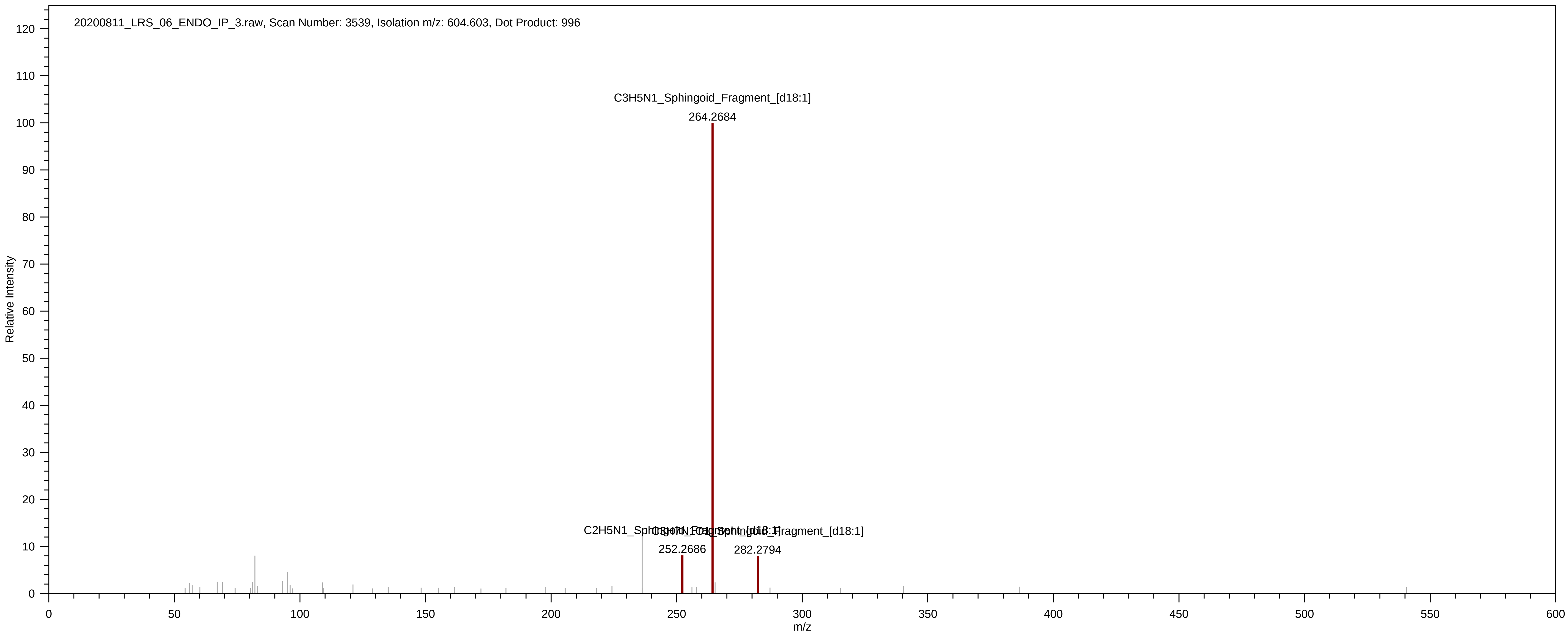

Cer[NS] d40:1 [M+H-H2O]+ | Ret. Time (min): 12.201 | m/z: 604.603

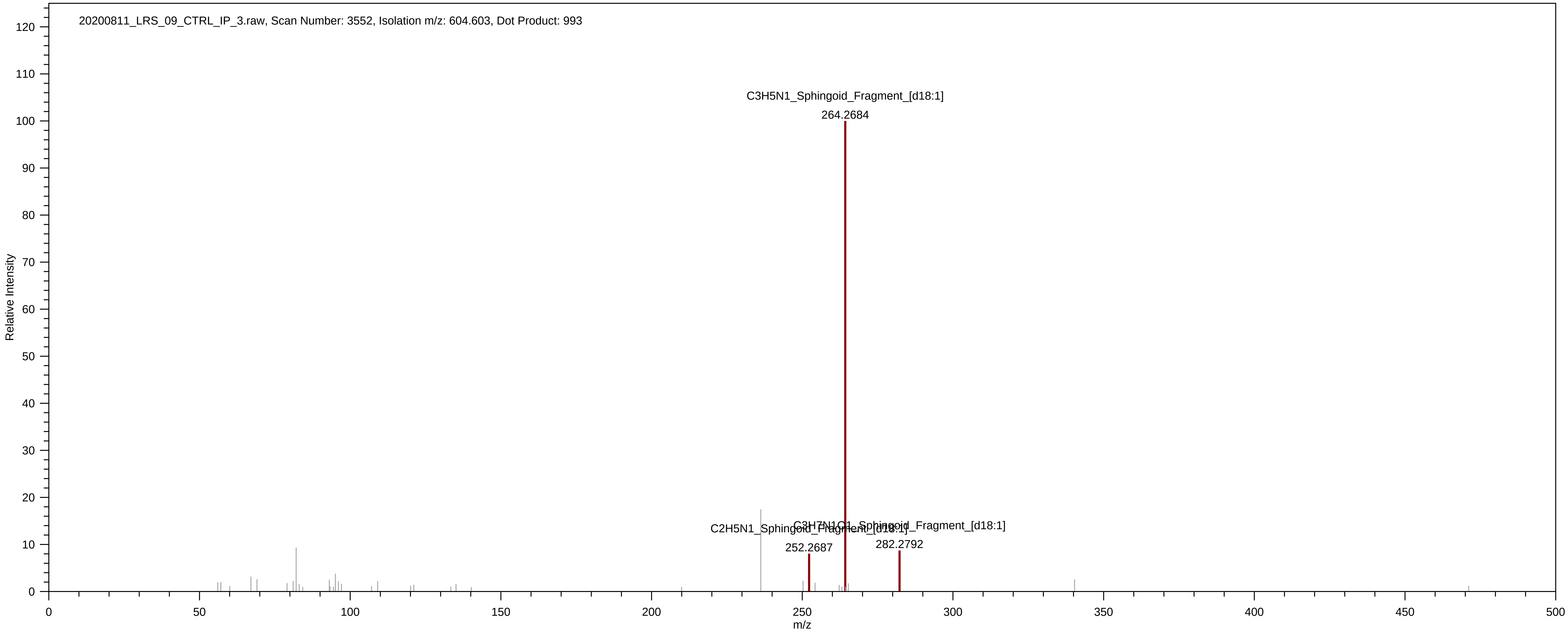

Cer[NS] d40:1 [M+H-H2O]+ | Ret. Time (min): 12.202 | m/z: 604.603

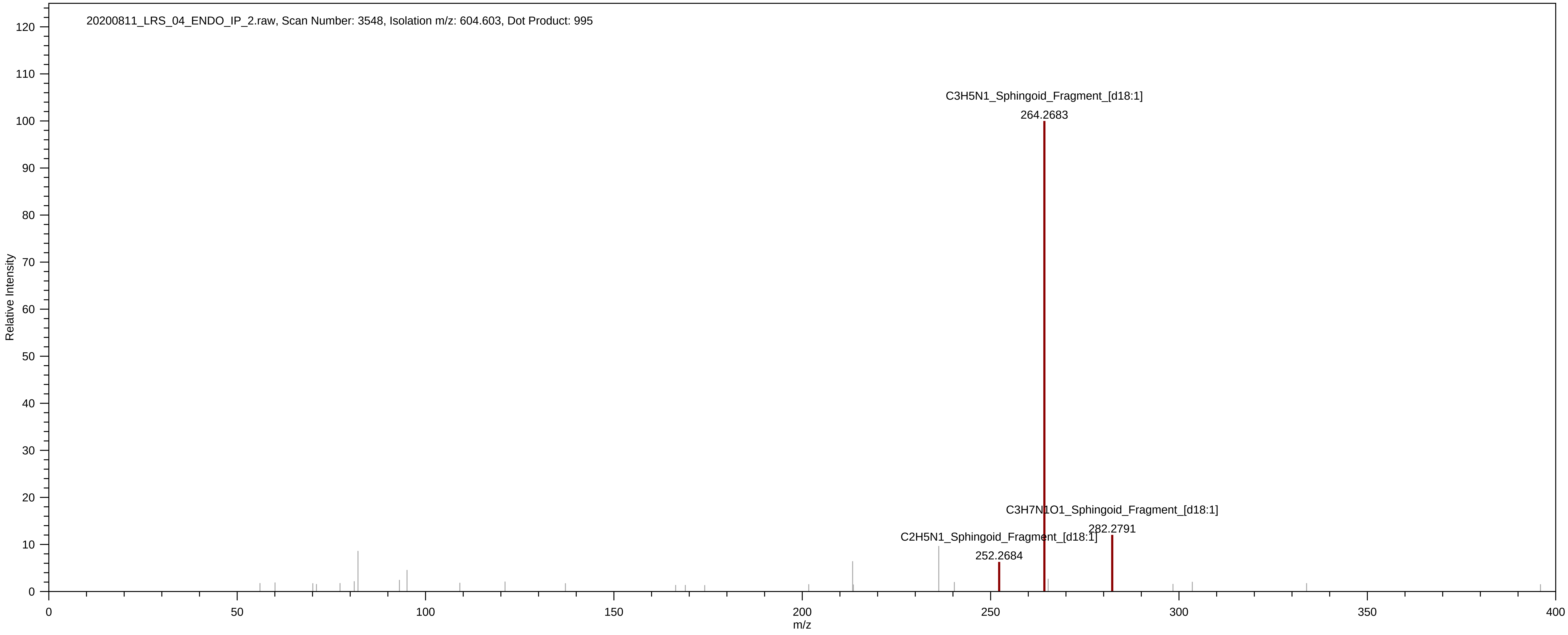

Cer[NS] d40:1 [M+H-H2O]+ | Ret. Time (min): 13.165 | m/z: 604.603

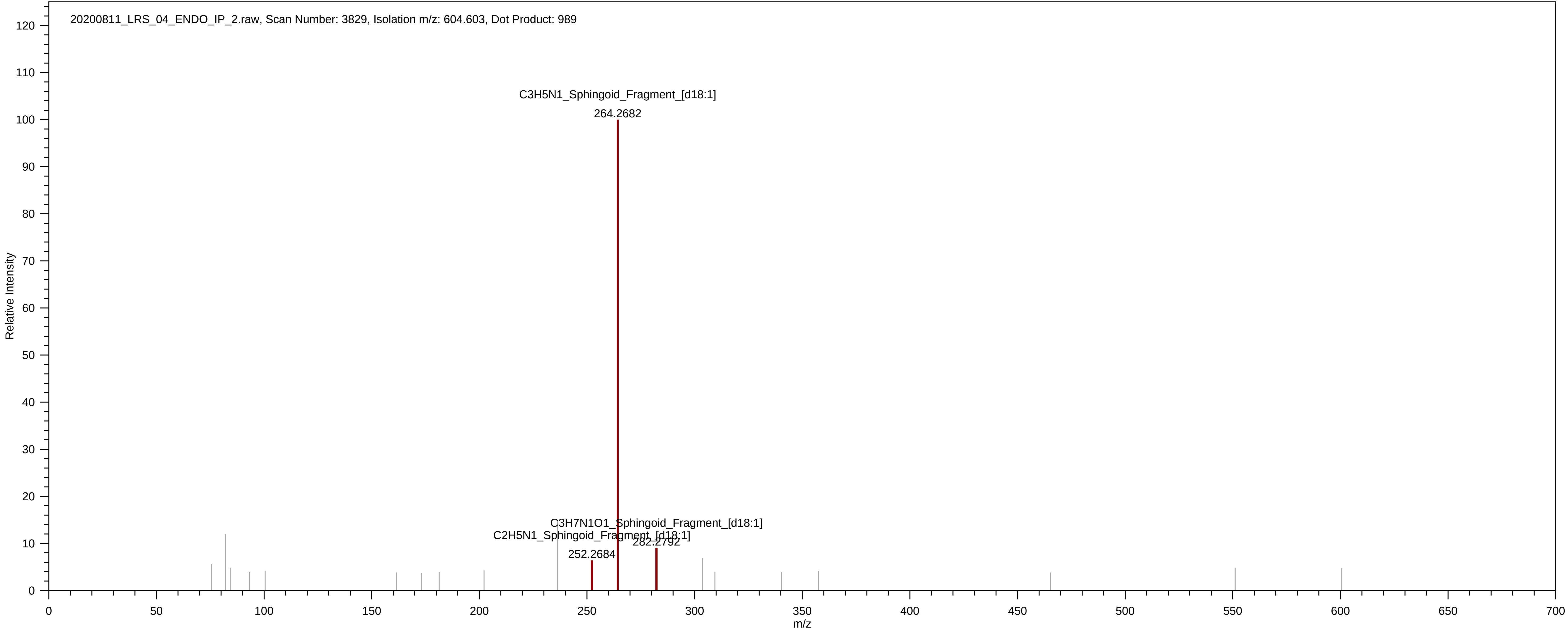

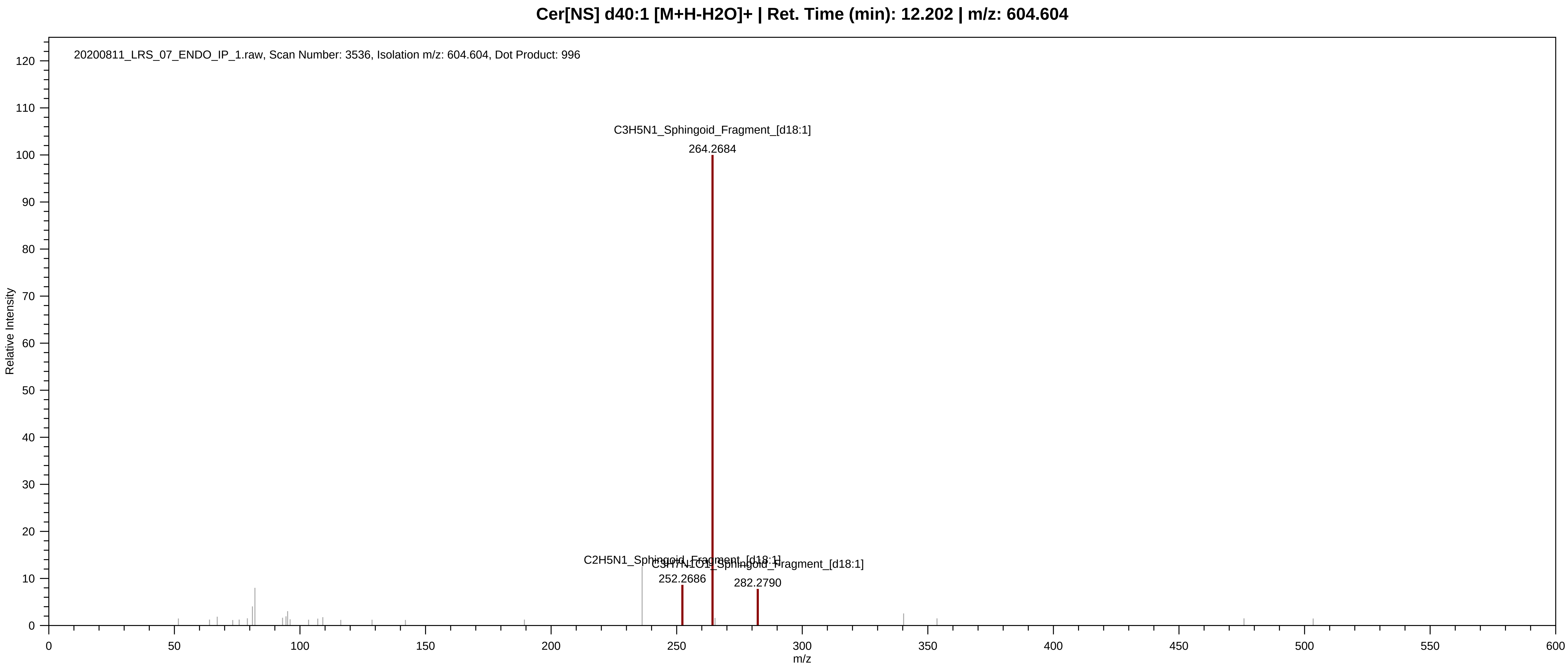
Cer[NS] d40:1 [M+H-H2O]+ | Ret. Time (min): 12.202 | m/z: 604.604
20200811_LRS_07_ENDO_IP_1.raw, Scan Number: 3536, Isolation m/z: 604.604, Dot Product: 996
C3H5N1_Sphingoid_Fragment_[d18:1]
264.2684
C2H5N1_Sphingoid_Fragment_[d18:1]
252.2686
C3H7N1O1_Sphingoid_Fragment_[d18:1]
282.2790
Relative Intensity
m/z

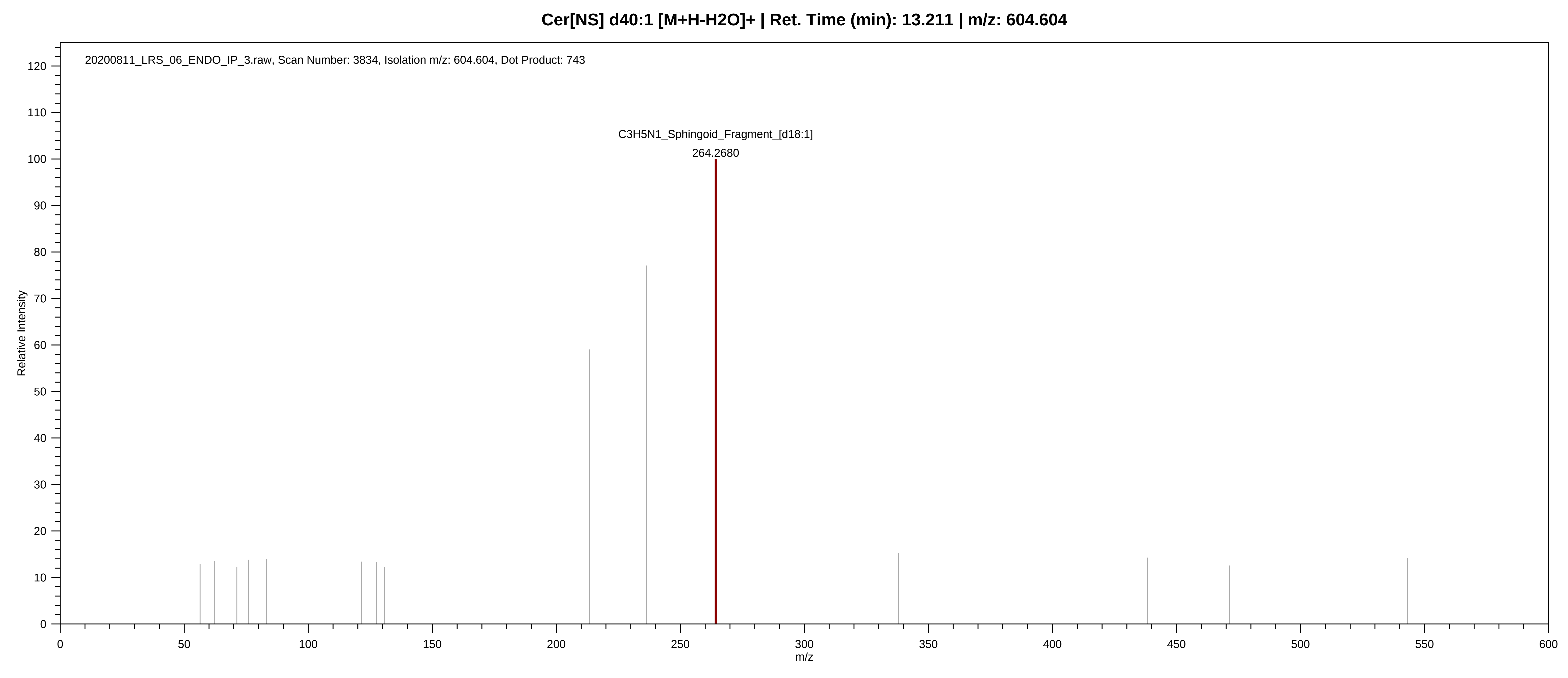

Cer[NS] d40:1 [M+H-H2O]+ | Ret. Time (min): 13.211 | m/z: 604.604
20200811_LRS_06_ENDO_IP_3.raw, Scan Number: 3834, Isolation m/z: 604.604, Dot Product: 743
C3H5N1_Sphingoid_Fragment_[d18:1]
264.2680
Relative Intensity
m/z

Cer[NS] d40:2 [M+H-H2O]+ | Ret. Time (min): 12.223 | m/z: 602.588

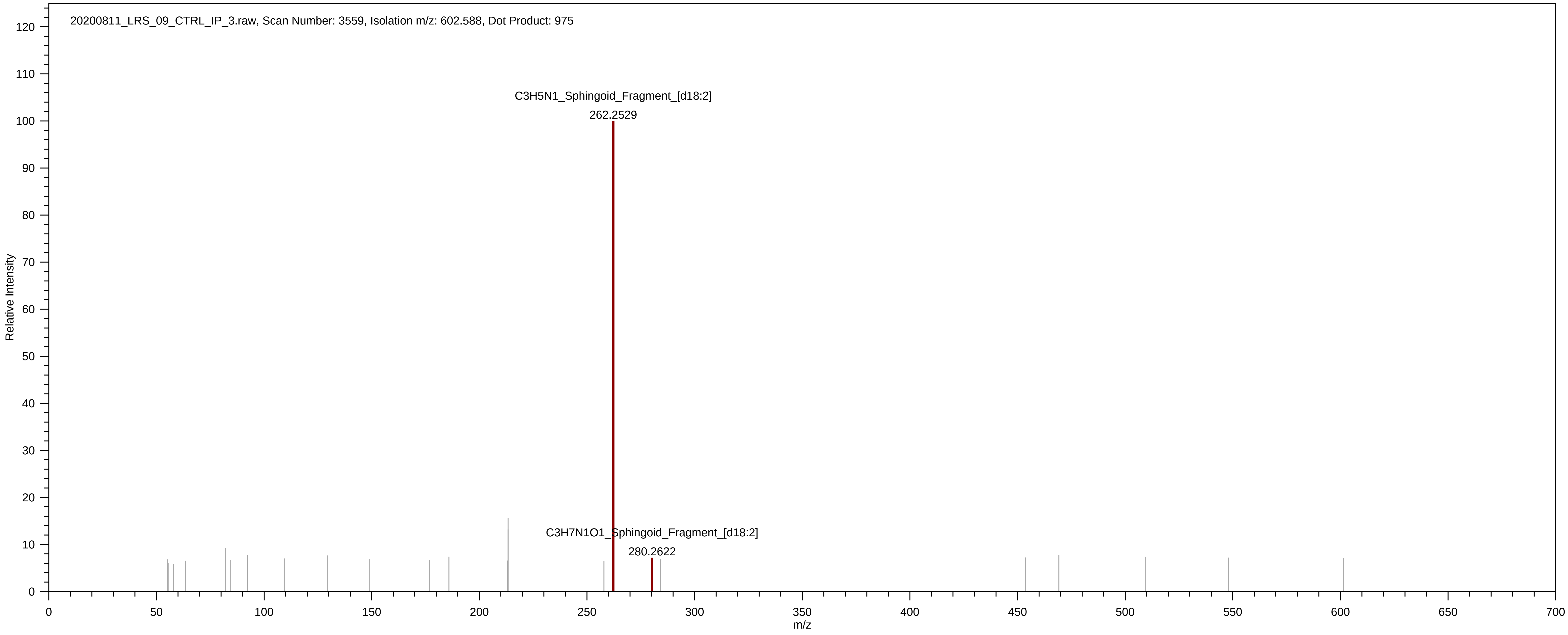

Cer[NS] d41:1 [M+H-H2O]+ | Ret. Time (min): 13.688 | m/z: 618.619

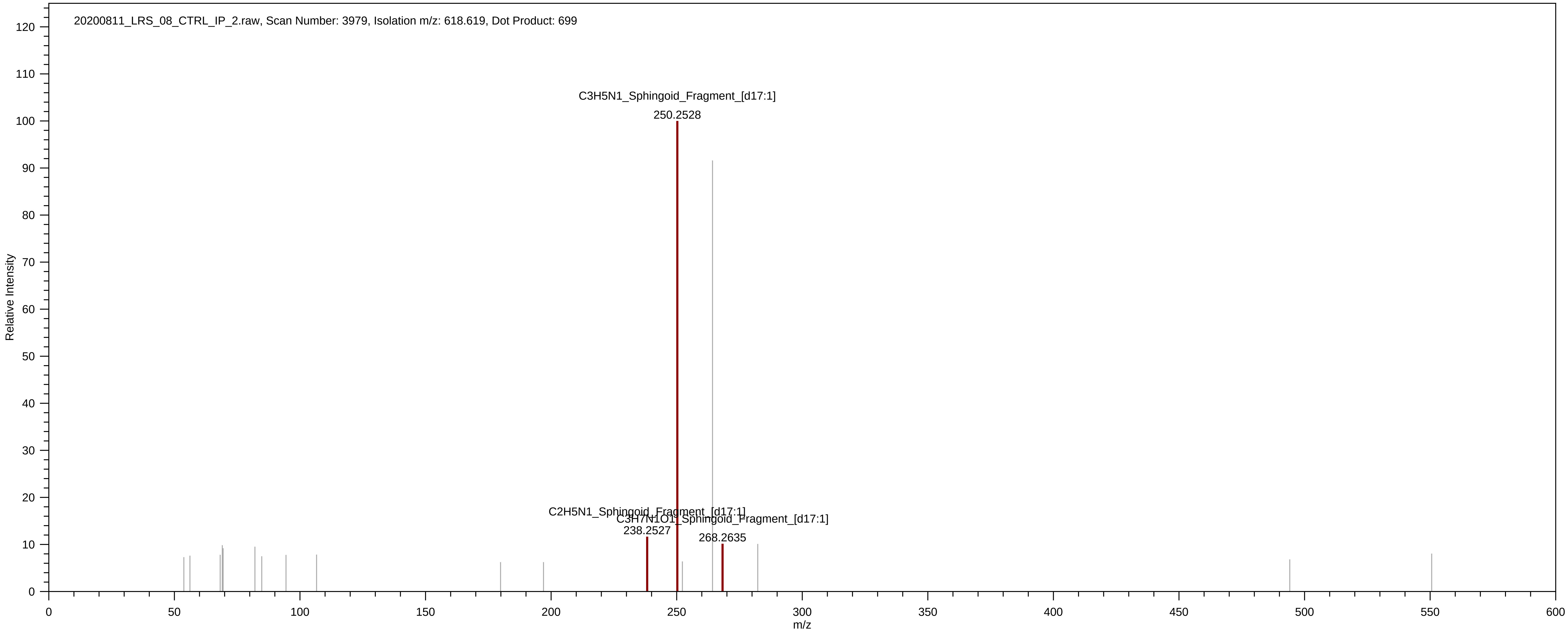

Cer[NS] d41:1 [M+H-H2O]+ | Ret. Time (min): 13.667 | m/z: 618.621

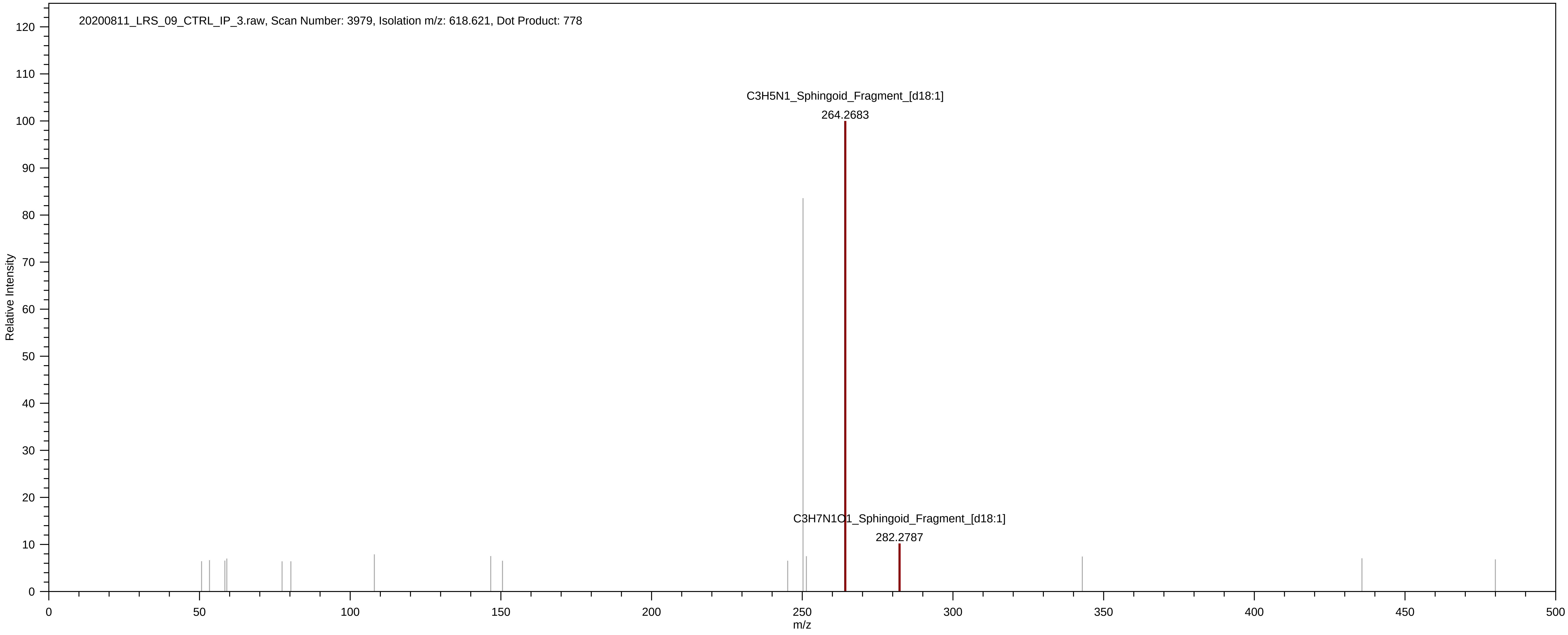

Cer[NS] d42:1 [M+H-H2O]+ | Ret. Time (min): 13.23 | m/z: 632.634

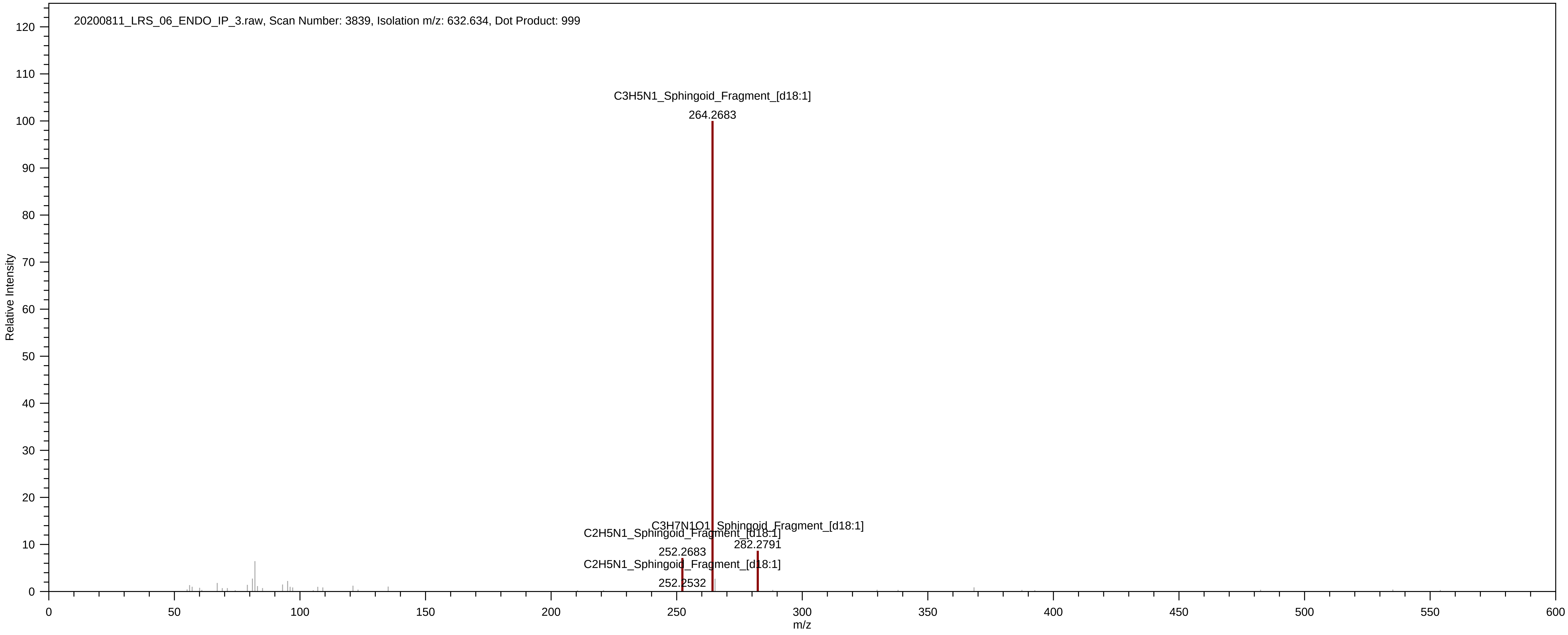

Cer[NS] d42:1 [M+H-H2O]+ | Ret. Time (min): 13.23 | m/z: 632.634

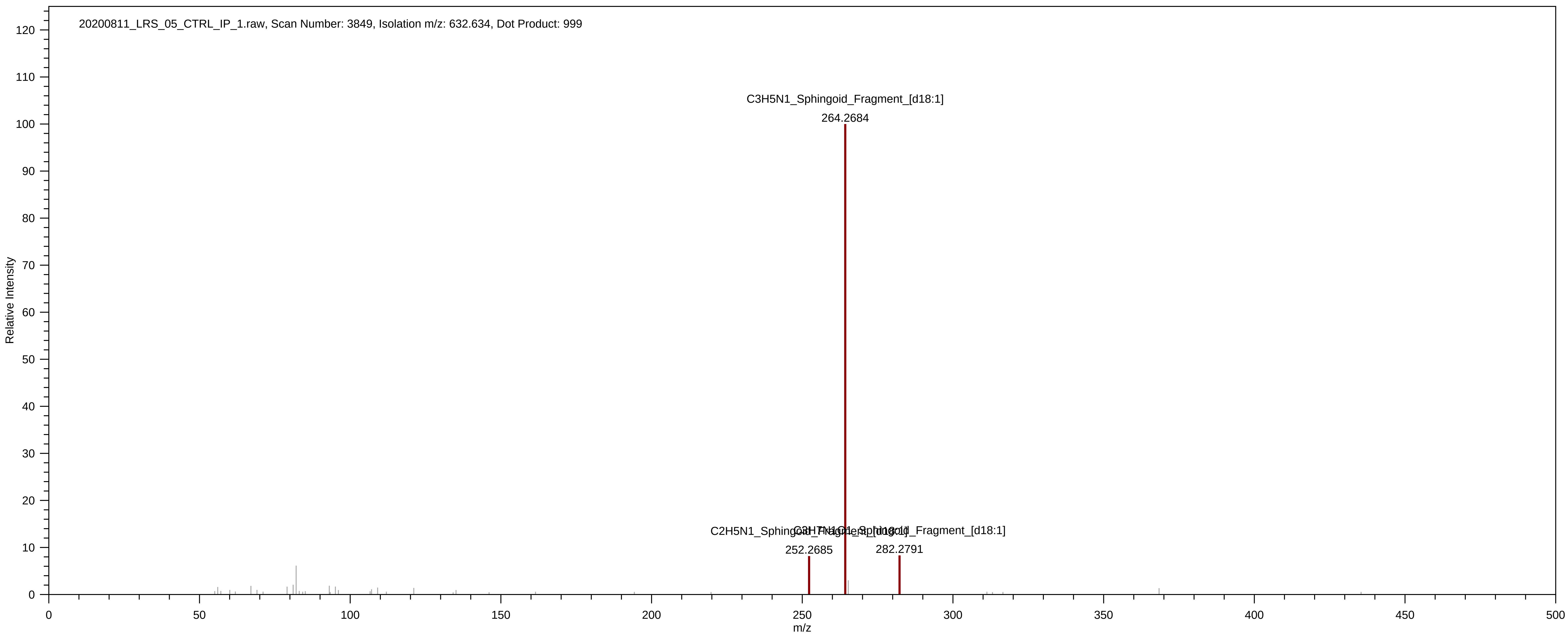

Cer[NS] d42:1 [M+H-H2O]+ | Ret. Time (min): 13.214 | m/z: 632.634

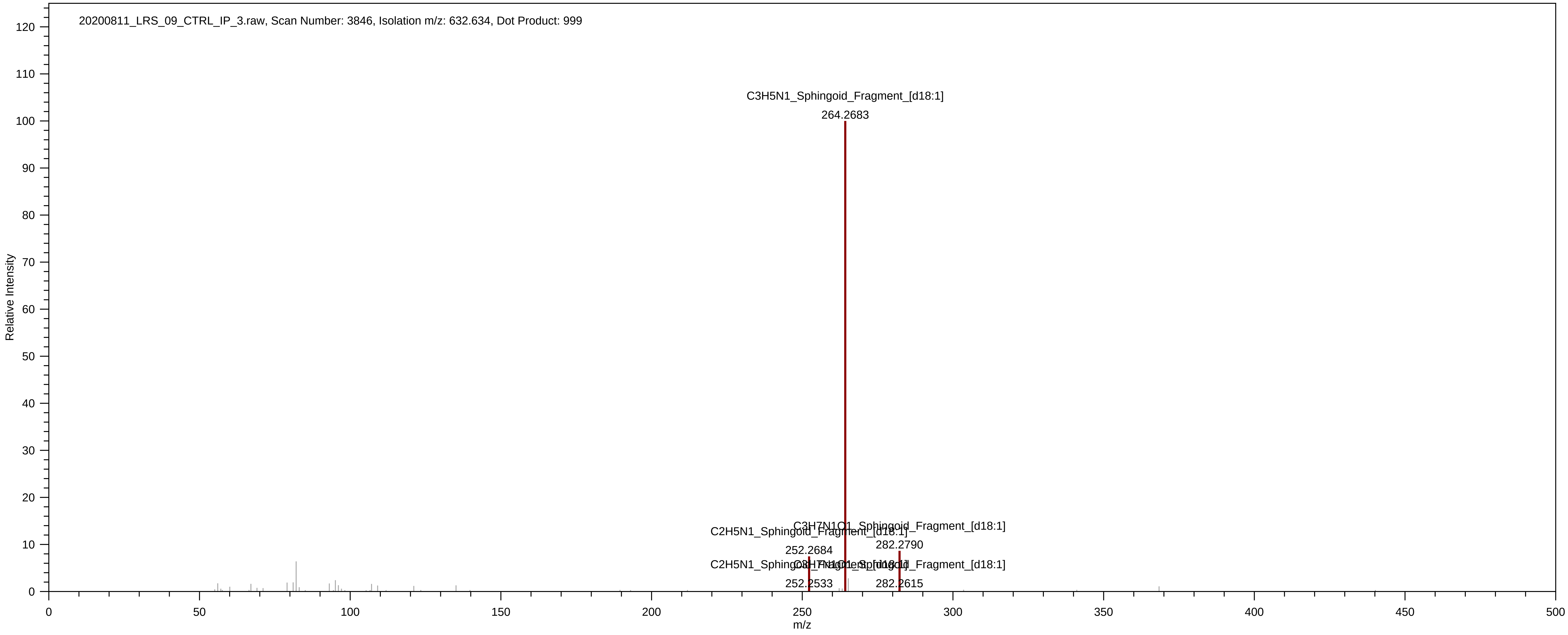

Cer[NS] d42:1 [M+H-H2O]+ | Ret. Time (min): 13.216 | m/z: 632.634

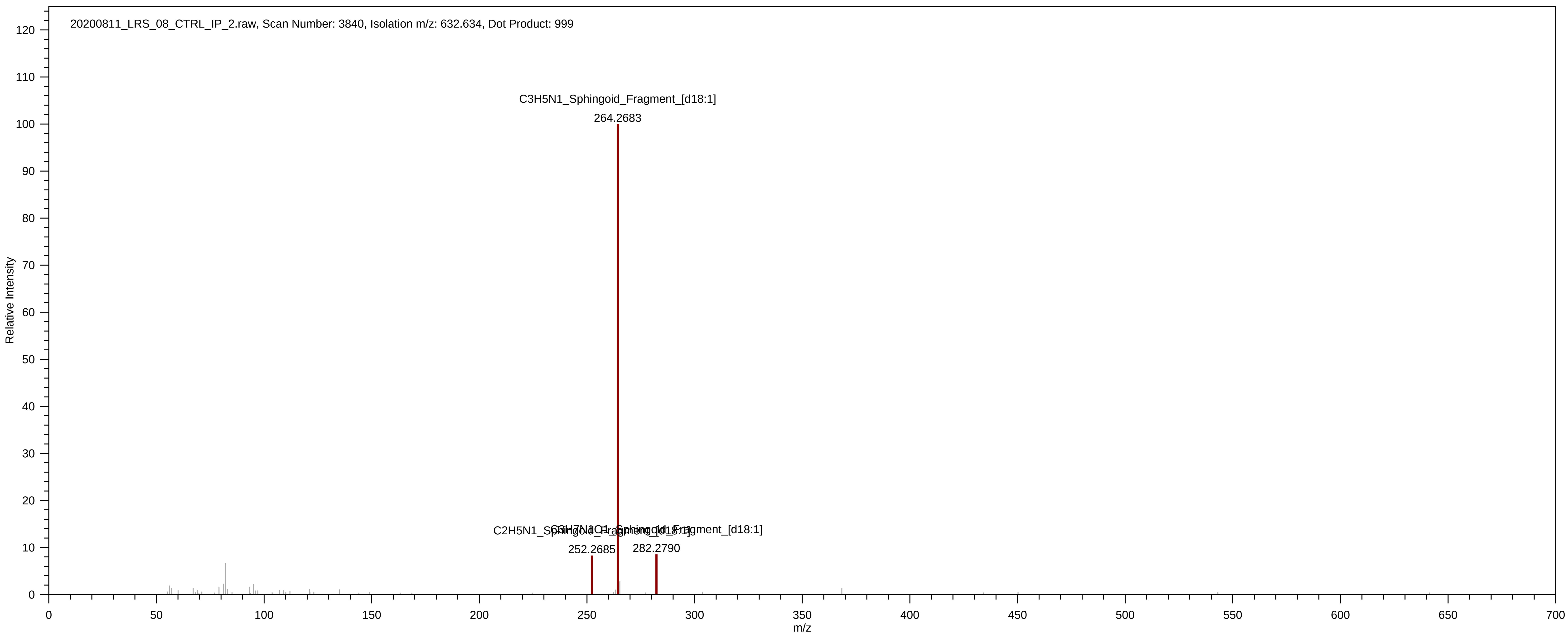

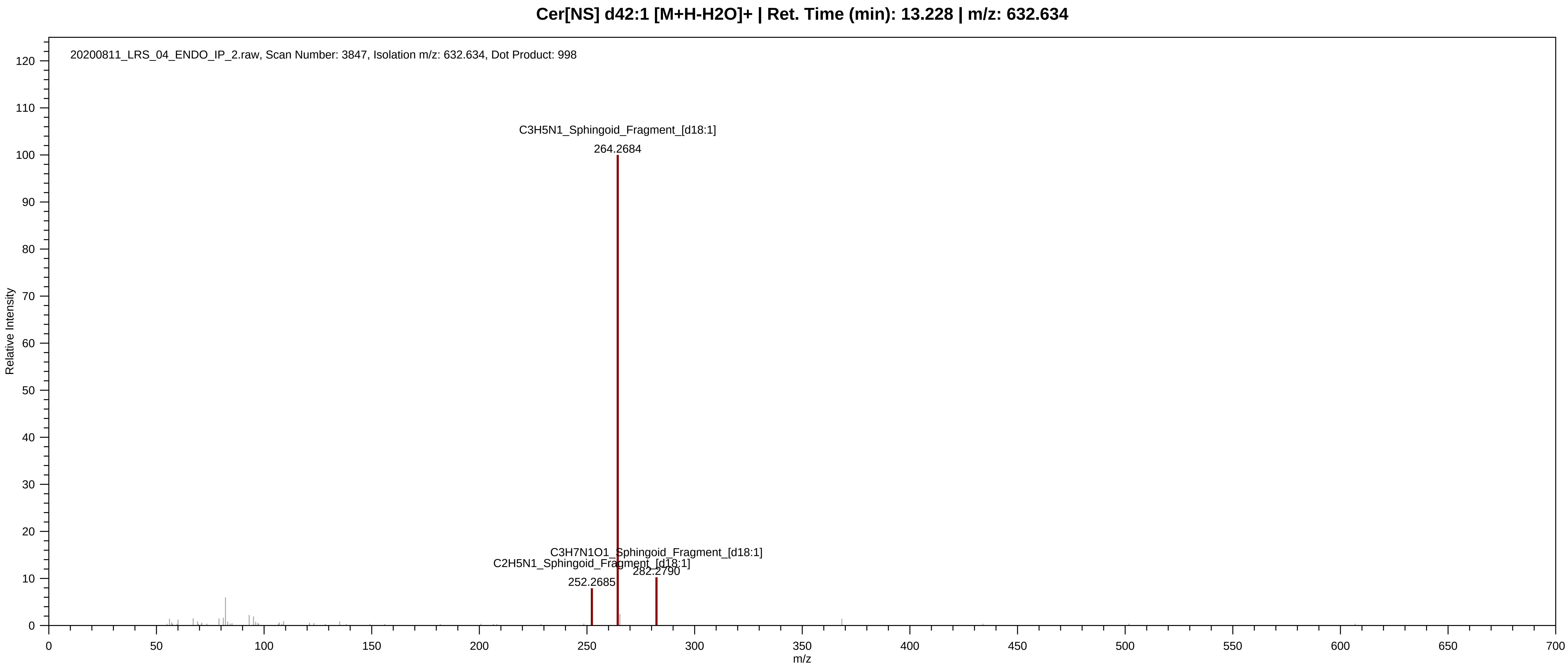
Cer[NS] d42:1 [M+H-H2O]+ | Ret. Time (min): 13.228 | m/z: 632.634
20200811_LRS_04_ENDO_IP_2.raw, Scan Number: 3847, Isolation m/z: 632.634, Dot Product: 998
C3H5N1_Sphingoid_Fragment_[d18:1]
264.2684
C3H7N1O1_Sphingoid_Fragment_[d18:1]
C2H5N1_Sphingoid_Fragment_[d18:1]
282.2790
252.2685
Relative Intensity
m/z

Cer[NS] d42:1 [M+H-H2O]+ | Ret. Time (min): 12.823 | m/z: 632.635

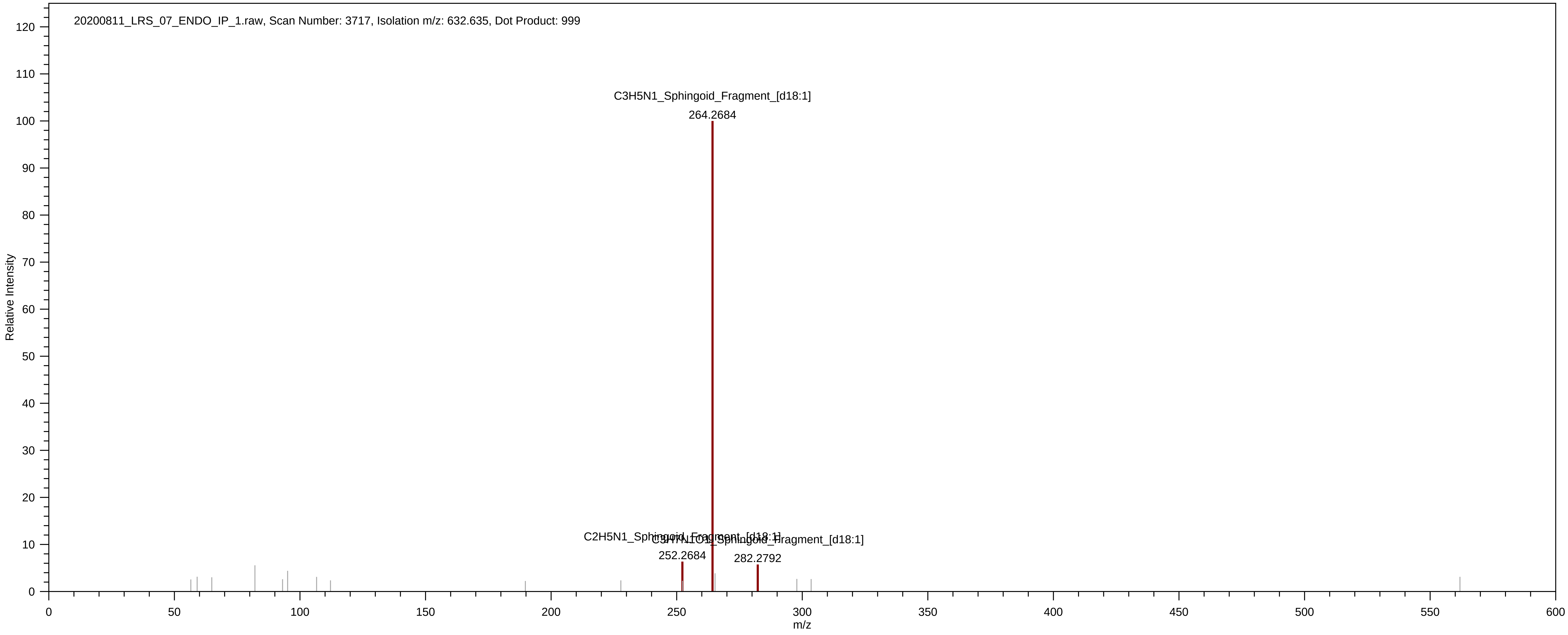

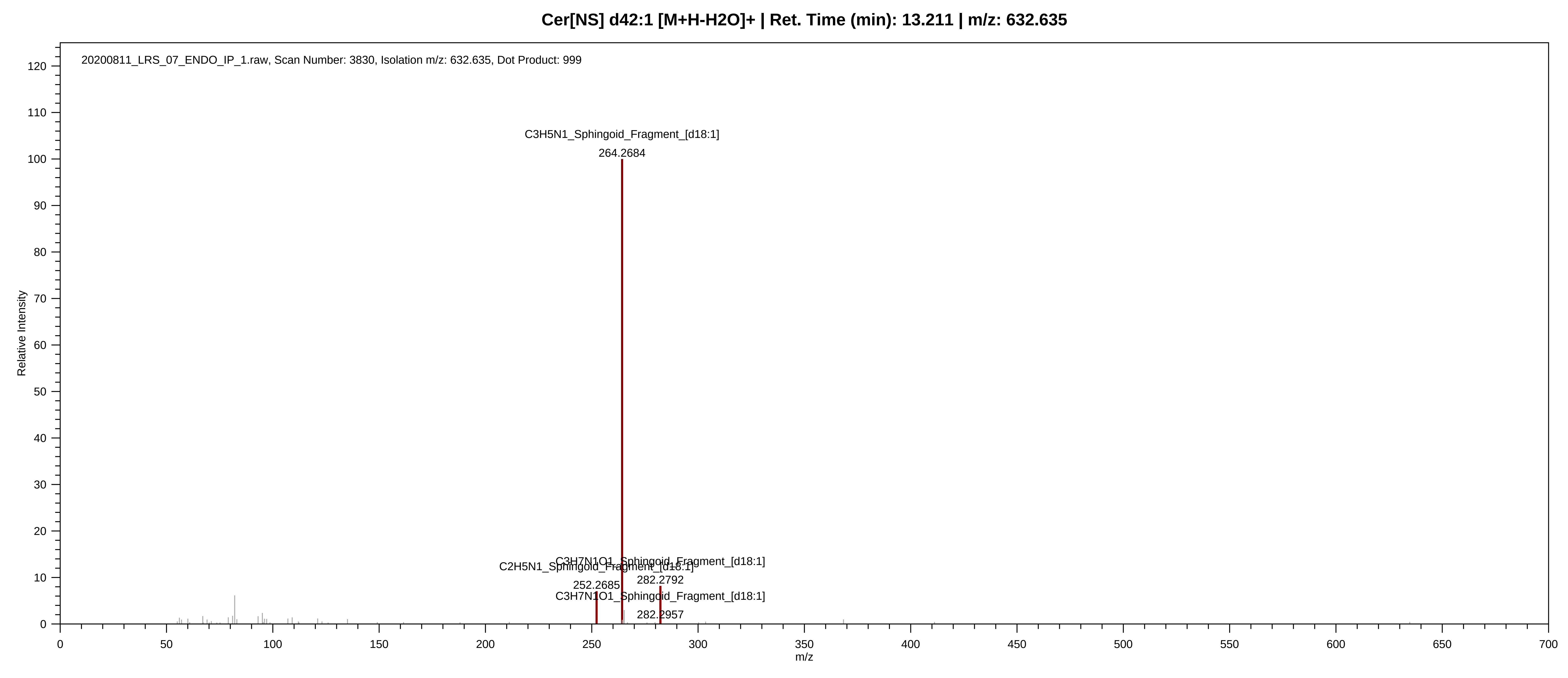

Cer[NS] d42:1 [M+H-H2O]+ | Ret. Time (min): 13.211 | m/z: 632.635
20200811_LRS_07_ENDO_IP_1.raw, Scan Number: 3830, Isolation m/z: 632.635, Dot Product: 999
C3H5N1_Sphingoid_Fragment_[d18:1]
264.2684
C3H7N1O1_Sphingoid_Fragment_[d18:1]
C2H5N1_Sphingoid_Fragment_[d18:1]
282.2792
252.2685
C3H7N1O1_Sphingoid_Fragment_[d18:1]
282.2957
Relative Intensity
m/z

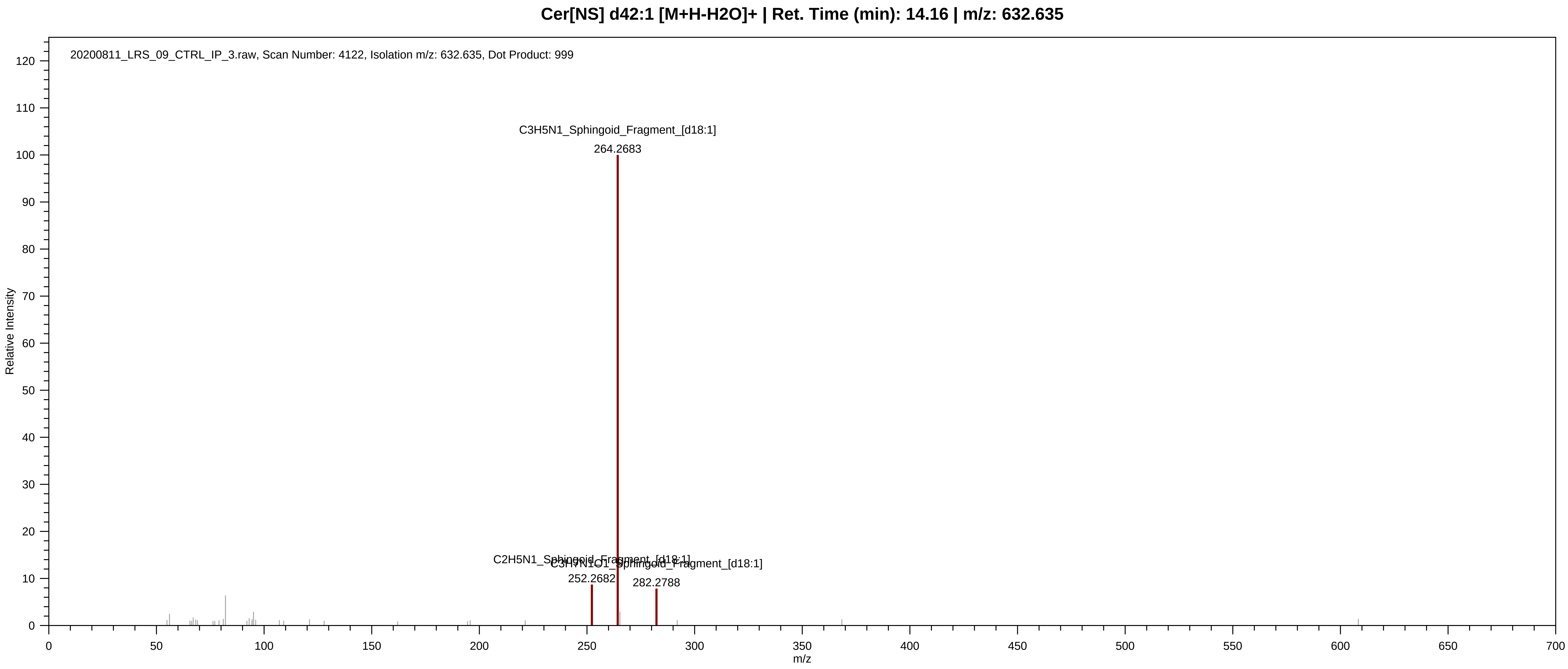

Cer[NS] d42:1 [M+H-H2O]+ | Ret. Time (min): 14.16 | m/z: 632.635
20200811_LRS_09_CTRL_IP_3.raw, Scan Number: 4122, Isolation m/z: 632.635, Dot Product: 999
C3H5N1_Sphingoid_Fragment_[d18:1]
264.2683
C2H5N1_Sphingoid_Fragment_[d18:1]
252.2682
C3H7N1O1_Sphingoid_Fragment_[d18:1]
282.2788
Relative Intensity
m/z

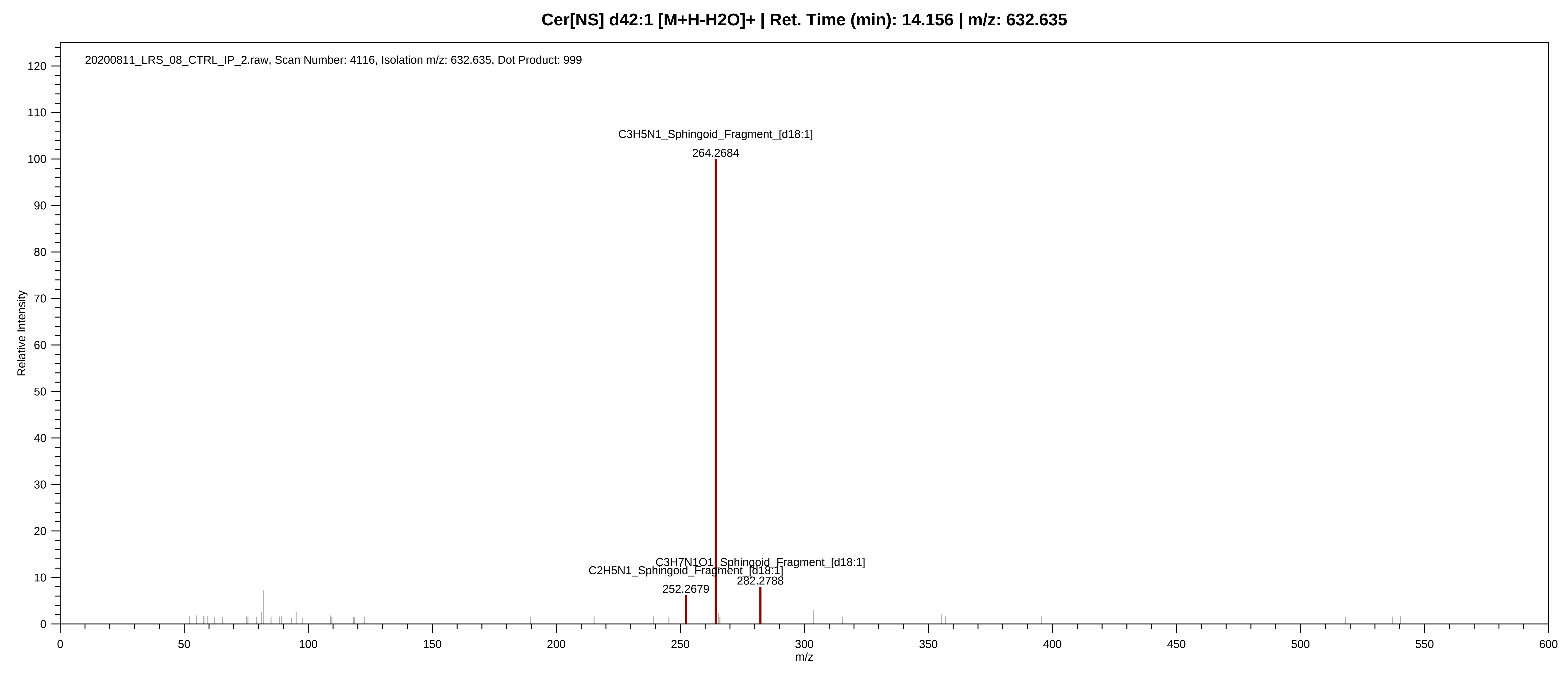
Cer[NS] d42:1 [M+H-H2O]+ | Ret. Time (min): 14.156 | m/z: 632.635
20200811_LRS_08_CTRL_IP_2.raw, Scan Number: 4116, Isolation m/z: 632.635, Dot Product: 999
C3H5N1_Sphingoid_Fragment_[d18:1]
264.2684
C3H7N1O1_Sphingoid_Fragment_[d18:1]
C2H5N1_Sphingoid_Fragment_[d18:1]
252.2679
282.2788
Relative Intensity
m/z

Cer[NS] d42:1 [M+H-H2O]+ | Ret. Time (min): 14.176 | m/z: 632.635

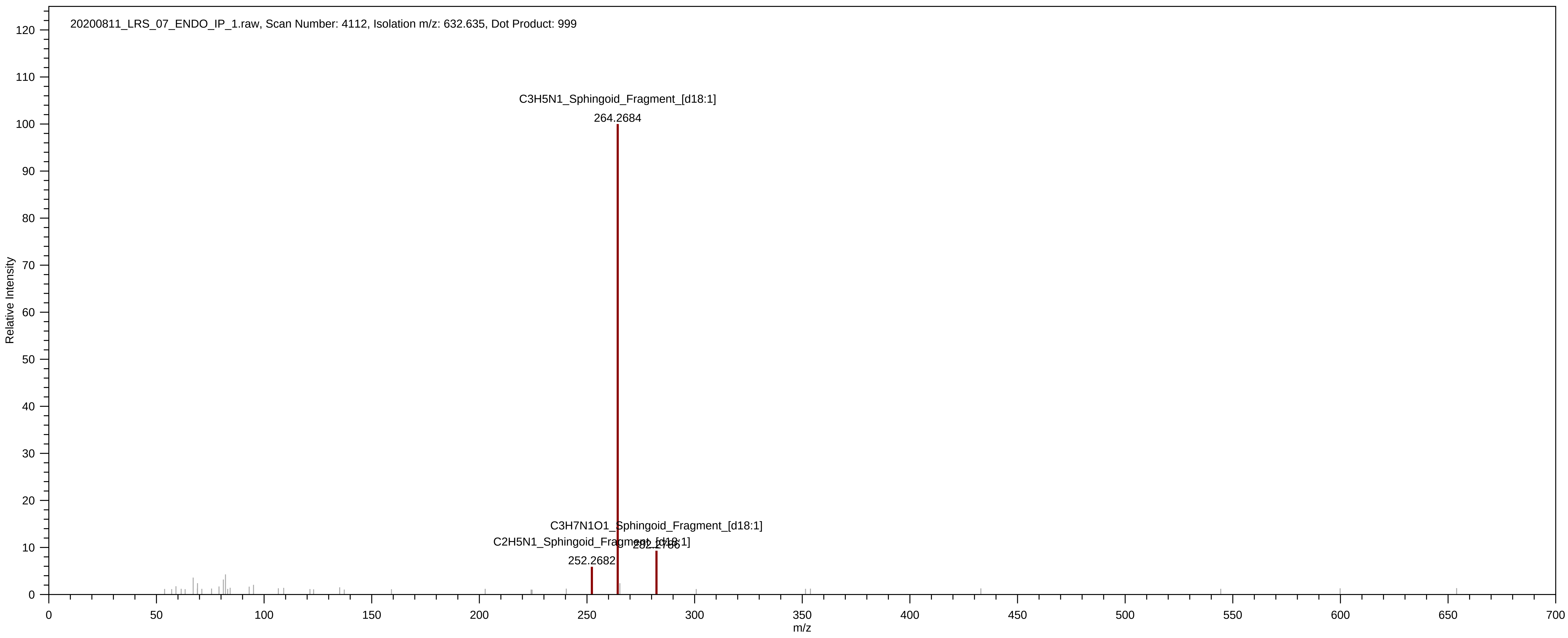

Cer[NS] d42:1 [M+H-H2O]+ | Ret. Time (min): 14.192 | m/z: 632.635

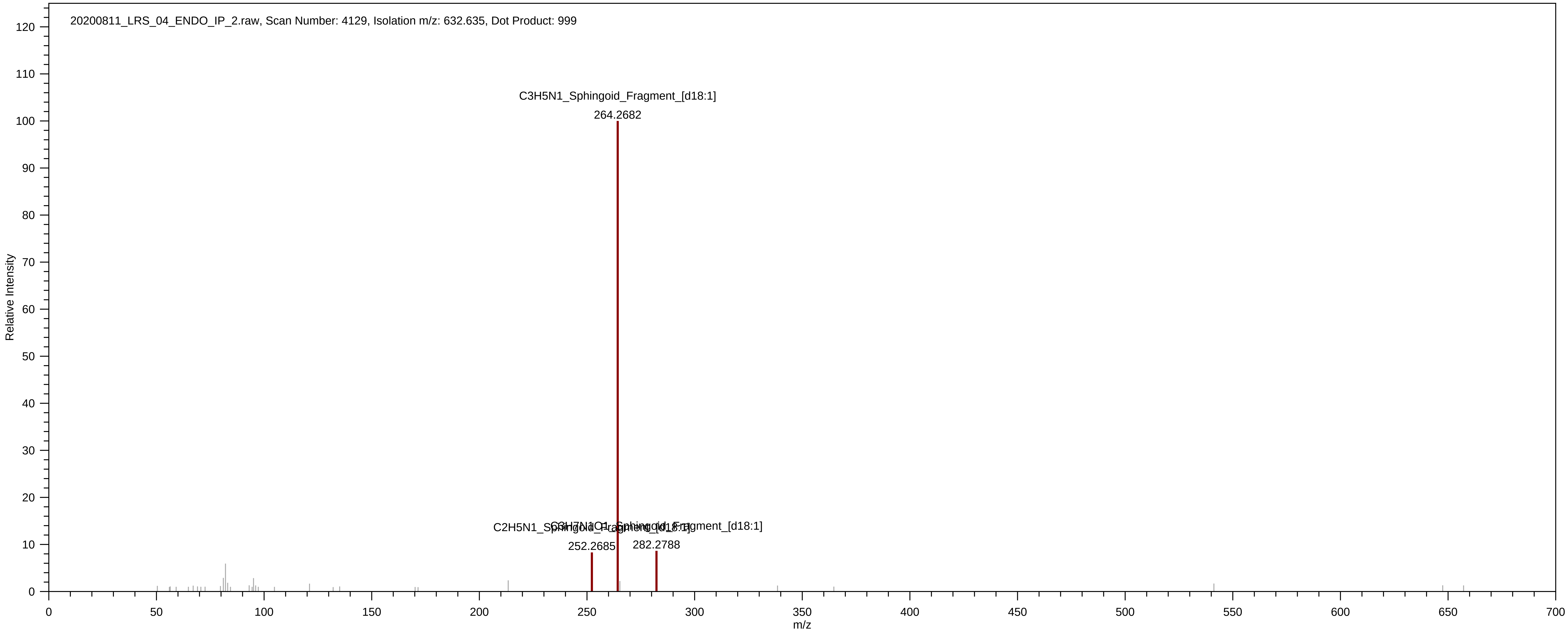

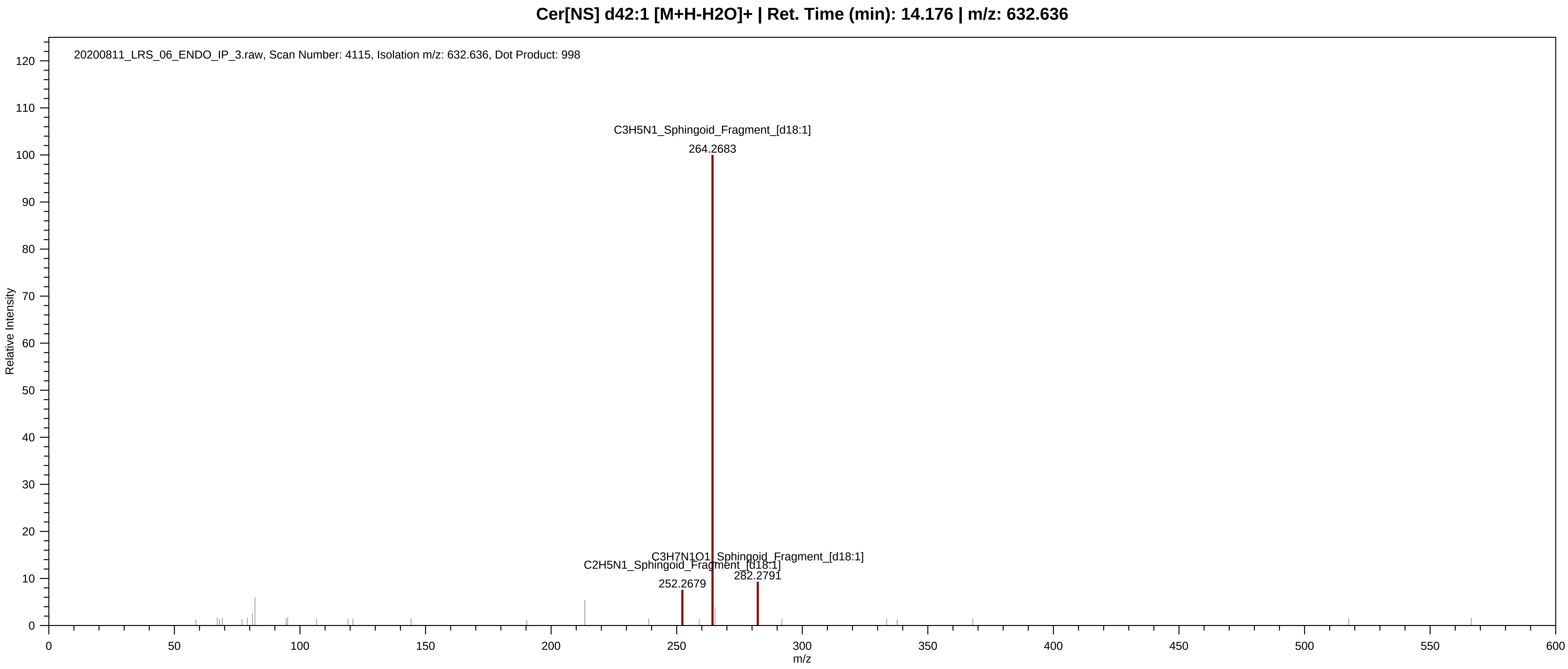

Cer[NS] d42:1 [M+H-H2O]+ | Ret. Time (min): 14.176 | m/z: 632.636
20200811_LRS_06_ENDO_IP_3.raw, Scan Number: 4115, Isolation m/z: 632.636, Dot Product: 998
C3H5N1_Sphingoid_Fragment_[d18:1]
264.2683
C3H7N1O1_Sphingoid_Fragment_[d18:1]
C2H5N1_Sphingoid_Fragment_[d18:1]
282.2791
252.2679
Relative Intensity
m/z

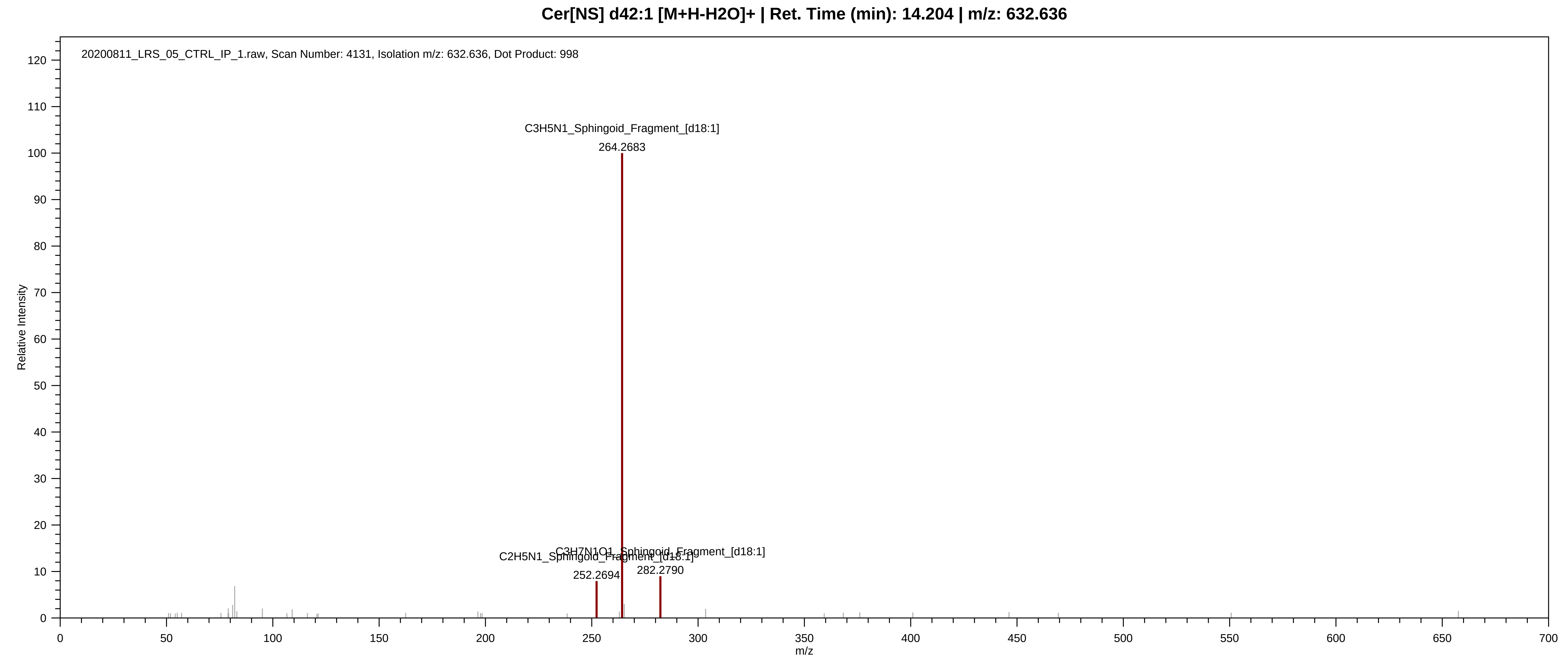

Cer[NS] d42:1 [M+H-H2O]+ | Ret. Time (min): 14.204 | m/z: 632.636
20200811_LRS_05_CTRL_IP_1.raw, Scan Number: 4131, Isolation m/z: 632.636, Dot Product: 998
C3H5N1_Sphingoid_Fragment_[d18:1]
264.2683
C3H7N1O1_Sphingoid_Fragment_[d18:1]
C2H5N1_Sphingoid_Fragment_[d18:1]
252.2694
282.2790
Relative Intensity
m/z

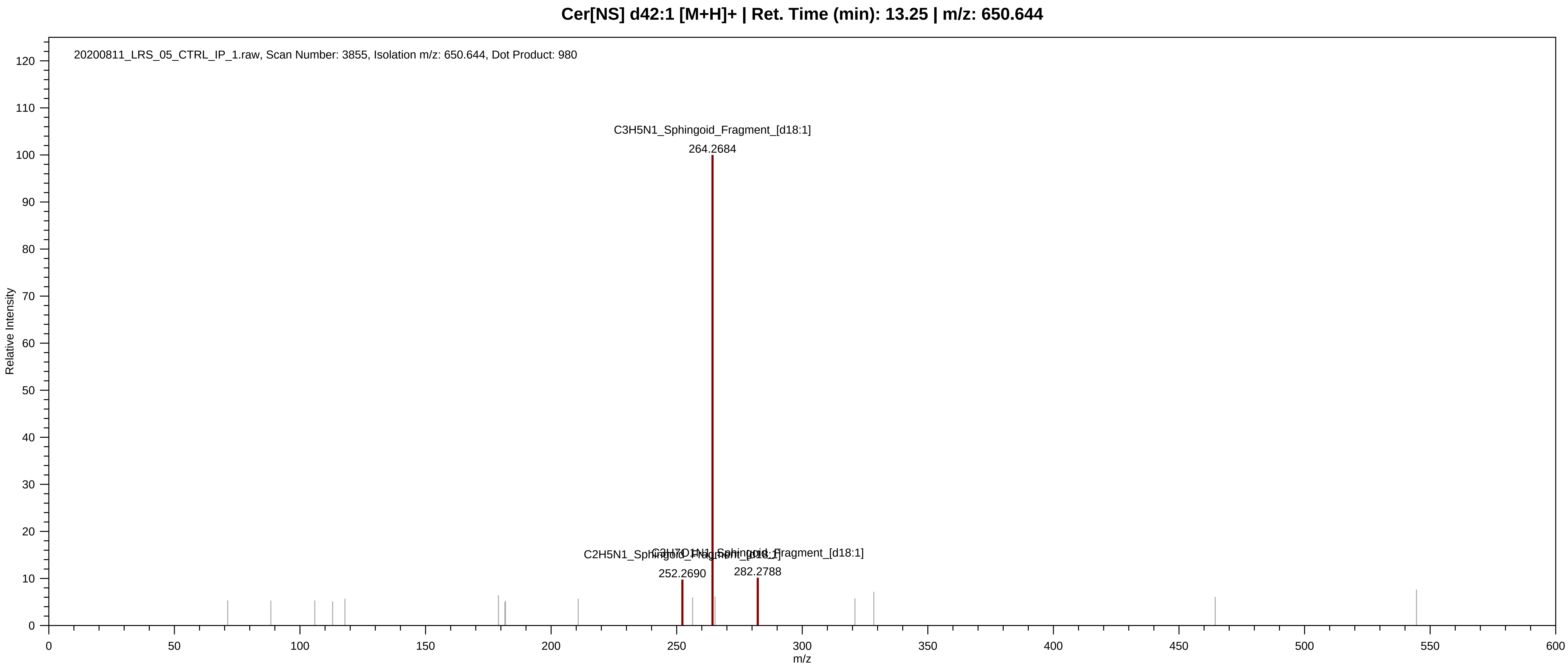

Cer[NS] d42:1 [M+H]+ | Ret. Time (min): 13.25 | m/z: 650.644
20200811_LRS_05_CTRL_IP_1.raw, Scan Number: 3855, Isolation m/z: 650.644, Dot Product: 980
C3H5N1_Sphingoid_Fragment_[d18:1]
264.2684
C2H5N1_Sphingoid_Fragment_[d18:1]
252.2690
C3H7O1N1_Sphingoid_Fragment_[d18:1]
282.2788
Relative Intensity
m/z

Cer[NS] d42:1 [M+H]+ | Ret. Time (min): 13.234 | m/z: 650.644

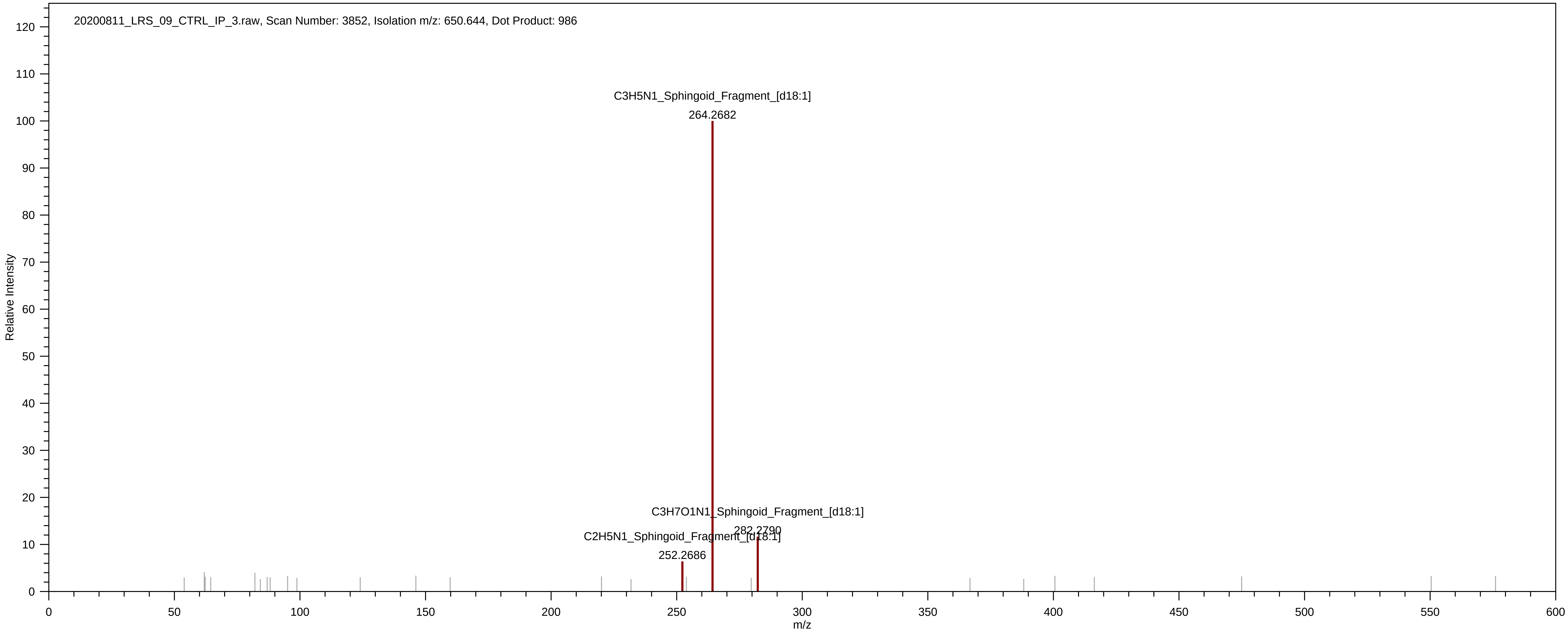

Cer[NS] d42:1 [M+H]+ | Ret. Time (min): 13.213 | m/z: 650.645

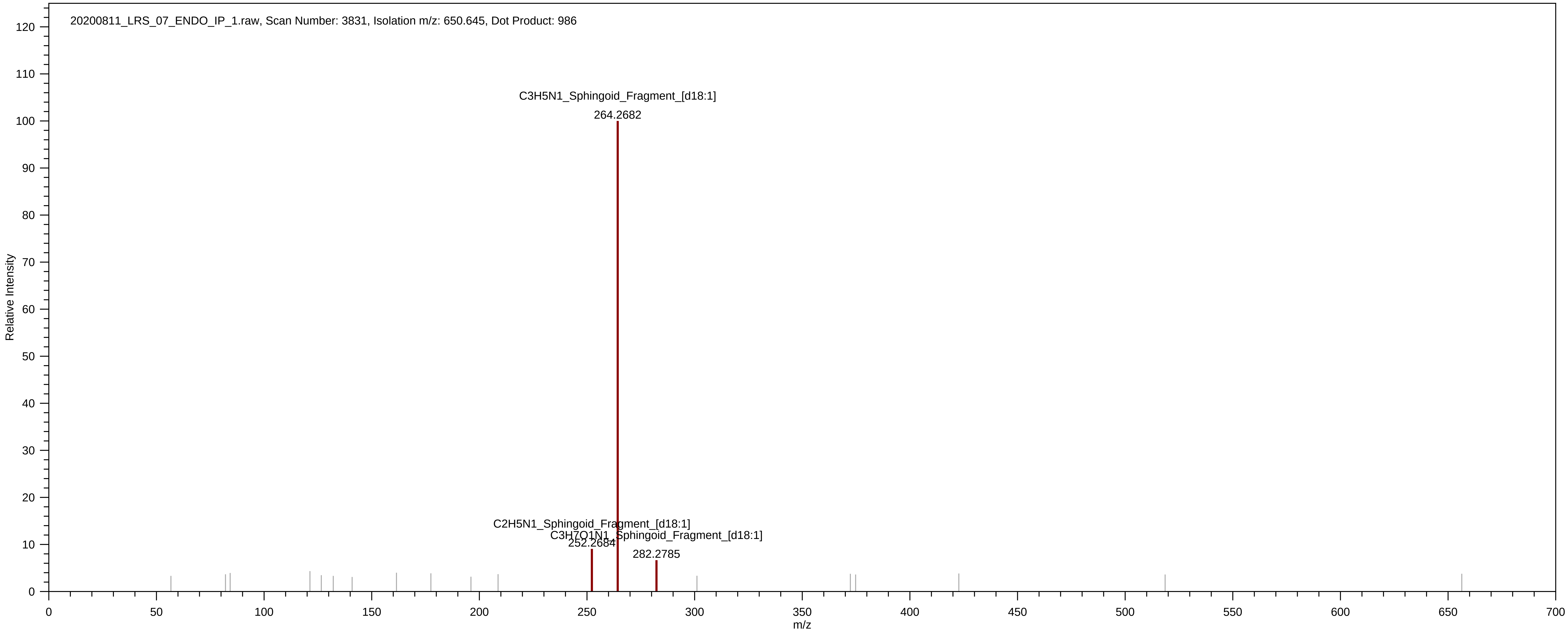

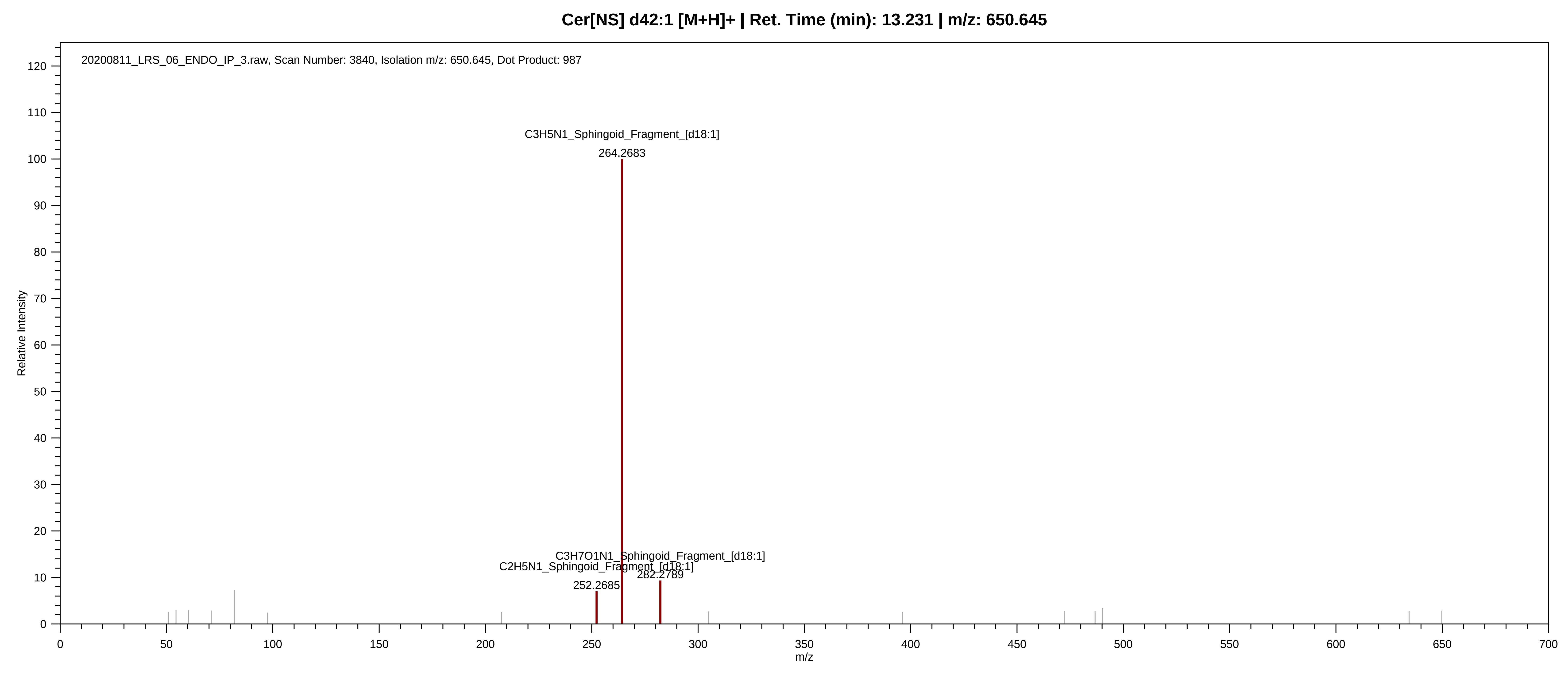

Cer[NS] d42:1 [M+H]+ | Ret. Time (min): 13.231 | m/z: 650.645
20200811_LRS_06_ENDO_IP_3.raw, Scan Number: 3840, Isolation m/z: 650.645, Dot Product: 987
C3H5N1_Sphingoid_Fragment_[d18:1]
264.2683
C3H7O1N1_Sphingoid_Fragment_[d18:1]
C2H5N1_Sphingoid_Fragment_[d18:1]
282.2789
252.2685
Relative Intensity
m/z

Cer[NS] d42:1 [M+H]+ | Ret. Time (min): 13.27 | m/z: 650.646

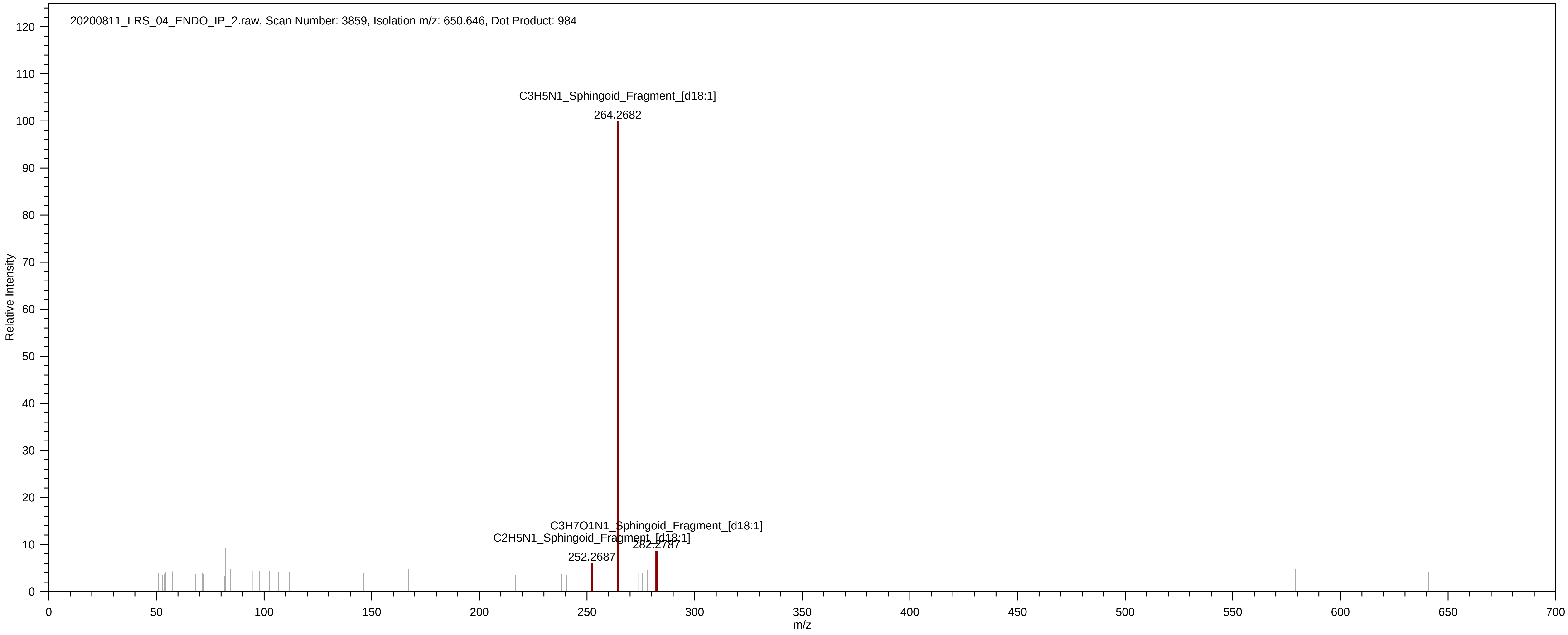

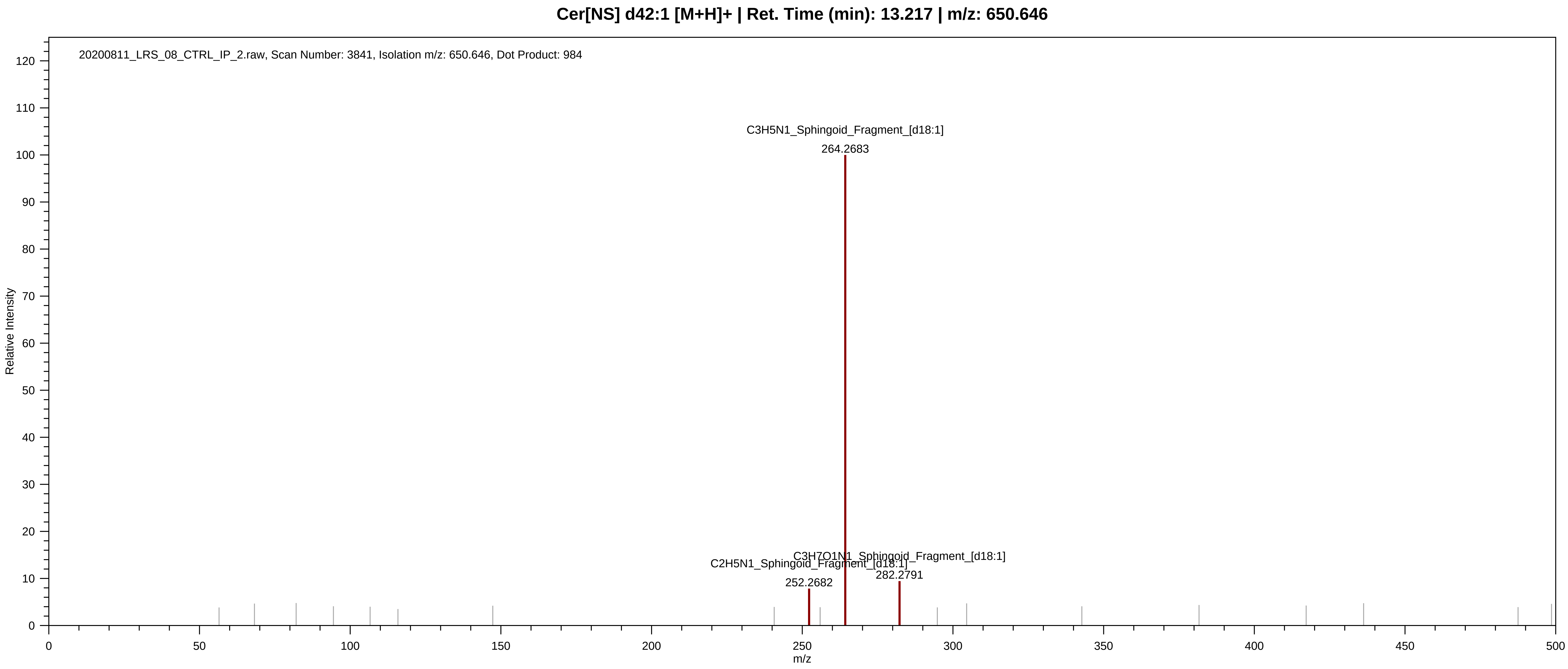
Cer[NS] d42:1 [M+H]+ | Ret. Time (min): 13.217 | m/z: 650.646
20200811_LRS_08_CTRL_IP_2.raw, Scan Number: 3841, Isolation m/z: 650.646, Dot Product: 984
C3H5N1_Sphingoid_Fragment_[d18:1]
264.2683
C3H7O1N1_Sphingoid_Fragment_[d18:1]
282.2791
C2H5N1_Sphingoid_Fragment_[d18:1]
252.2682
Relative Intensity
m/z

Cer[NS] d42:2 [M+H-H2O]+ | Ret. Time (min): 11.713 | m/z: 630.62

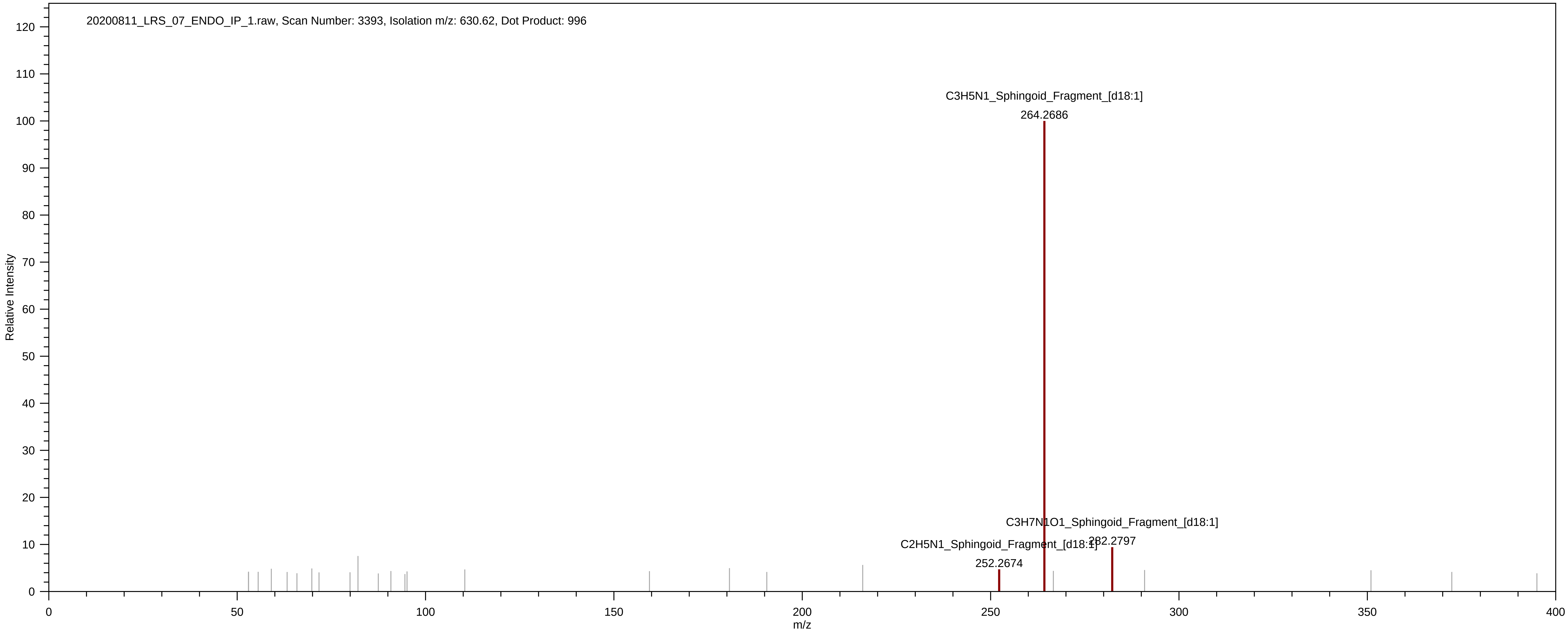

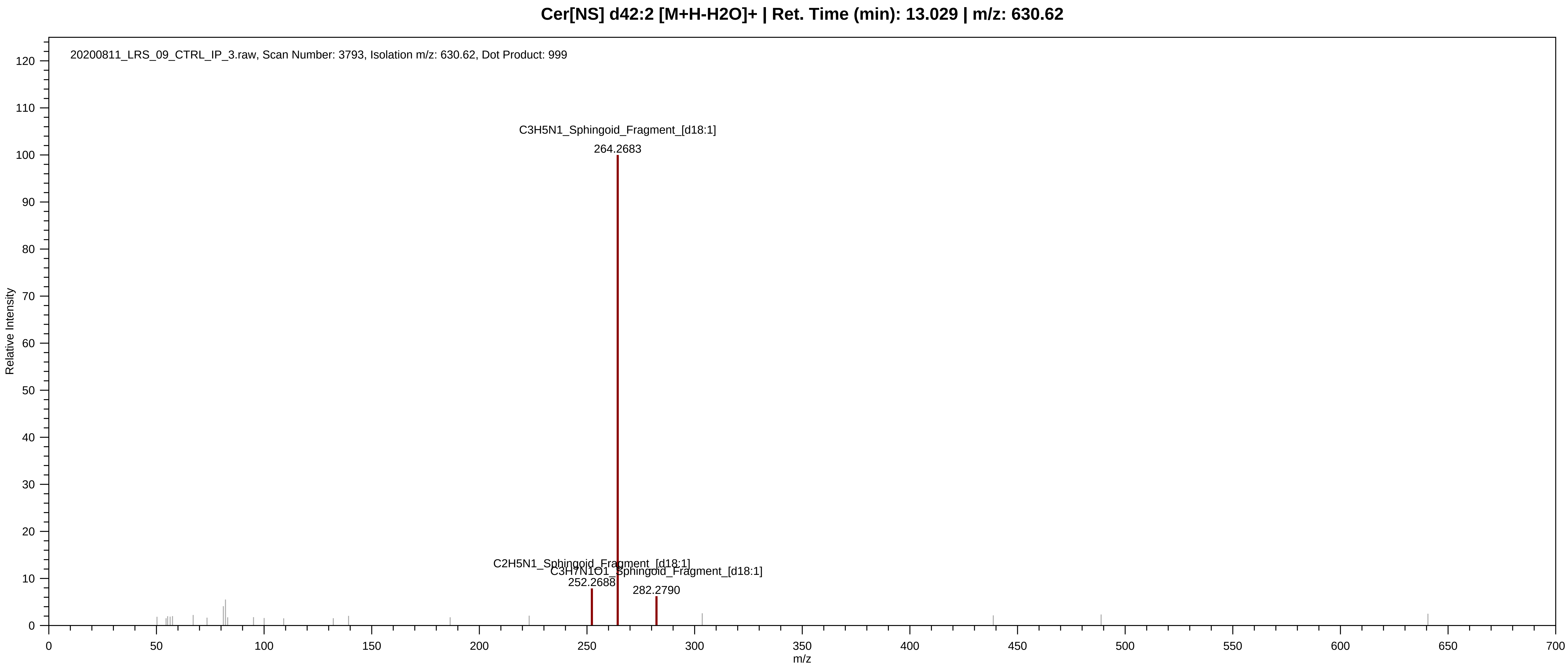
Cer[NS] d42:2 [M+H-H2O]+ | Ret. Time (min): 13.029 | m/z: 630.62
20200811_LRS_09_CTRL_IP_3.raw, Scan Number: 3793, Isolation m/z: 630.62, Dot Product: 999
C3H5N1_Sphingoid_Fragment_[d18:1]
264.2683
C2H5N1_Sphingoid_Fragment_[d18:1]
252.2688
C3H7N1O1_Sphingoid_Fragment_[d18:1]
282.2790
Relative Intensity
m/z

Cer[NS] d42:2 [M+H-H2O]+ | Ret. Time (min): 13.031 | m/z: 630.62

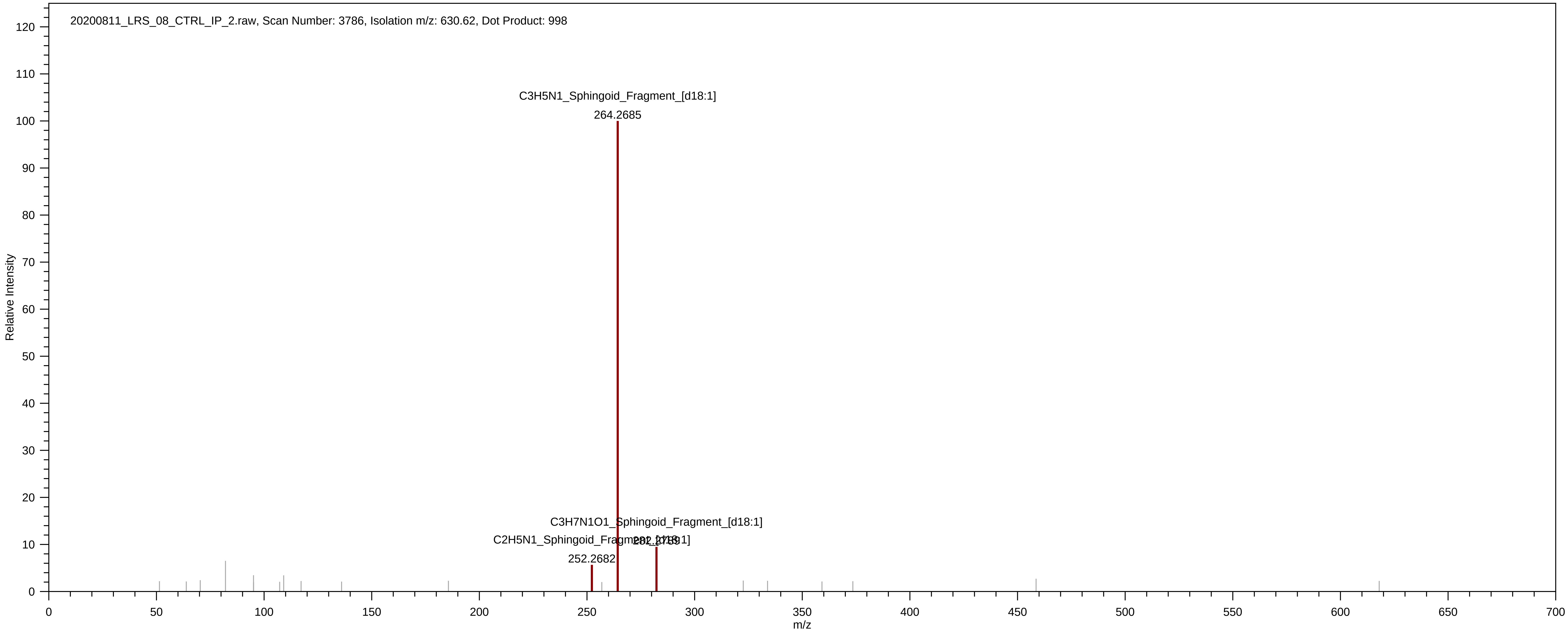

Cer[NS] d42:2 [M+H-H2O]+ | Ret. Time (min): 13.252 | m/z: 630.62

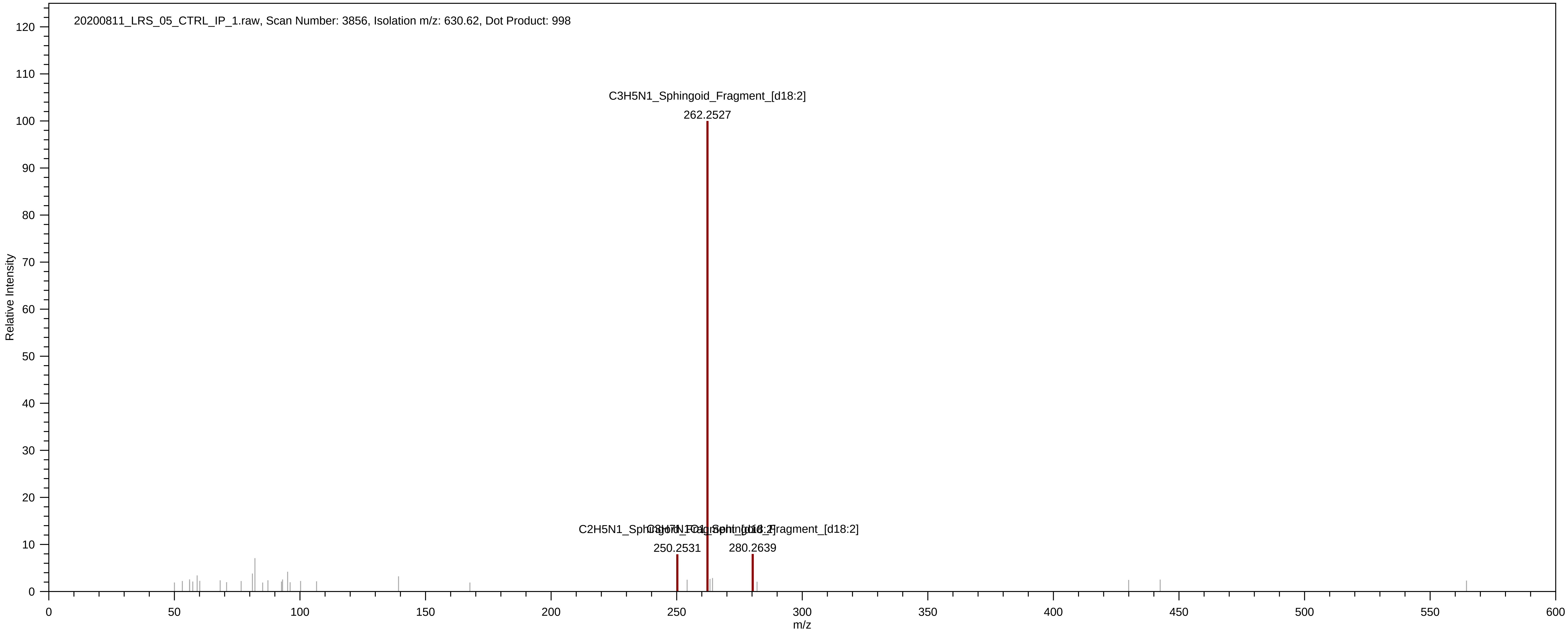

Cer[NS] d42:2 [M+H-H2O]+ | Ret. Time (min): 13.046 | m/z: 630.618

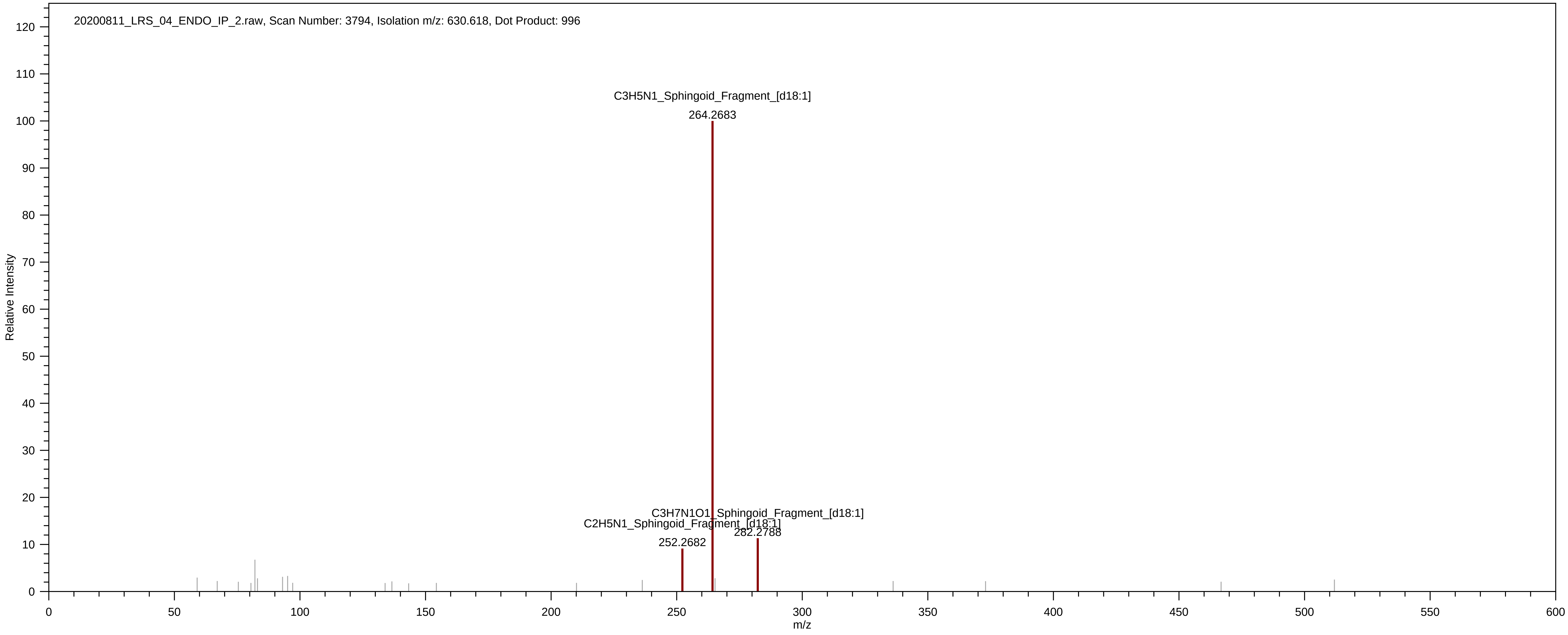

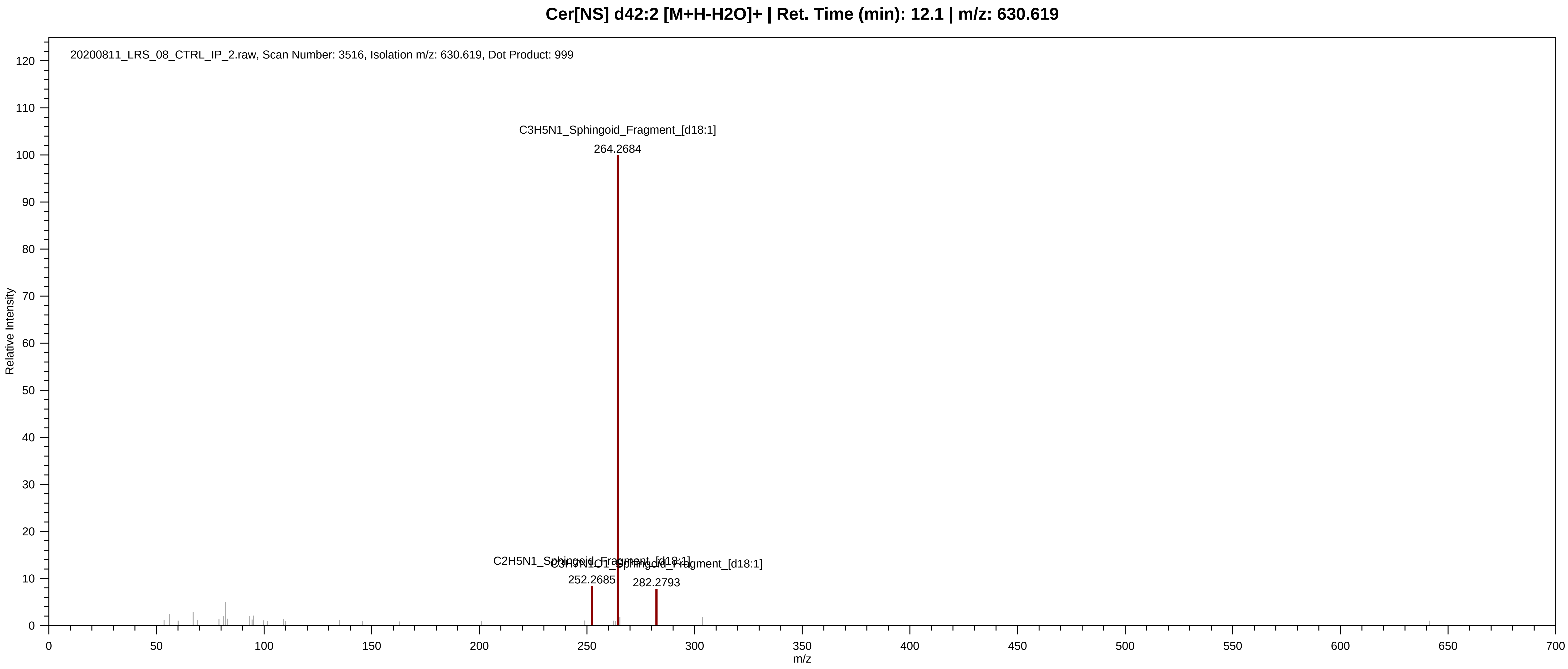

Cer[NS] d42:2 [M+H-H2O]+ | Ret. Time (min): 12.1 | m/z: 630.619
20200811_LRS_08_CTRL_IP_2.raw, Scan Number: 3516, Isolation m/z: 630.619, Dot Product: 999
C3H5N1_Sphingoid_Fragment_[d18:1]
264.2684
C2H5N1_Sphingoid_Fragment_[d18:1]
252.2685
C3H7N1O1_Sphingoid_Fragment_[d18:1]
282.2793
Relative Intensity
m/z

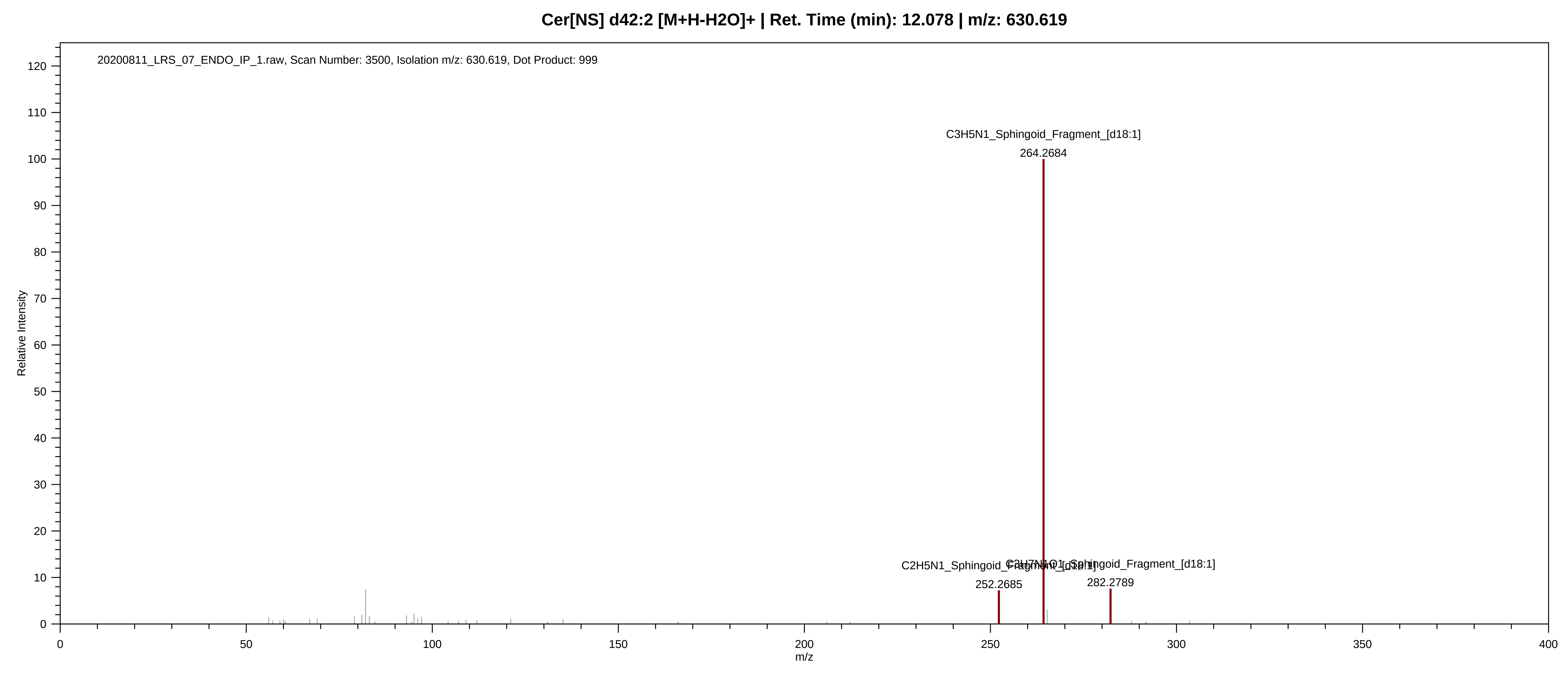

Cer[NS] d42:2 [M+H-H2O]+ | Ret. Time (min): 12.078 | m/z: 630.619
20200811_LRS_07_ENDO_IP_1.raw, Scan Number: 3500, Isolation m/z: 630.619, Dot Product: 999
C3H5N1_Sphingoid_Fragment_[d18:1]
264.2684
C2H5N1_Sphingoid_Fragment_[d18:1]
252.2685
C2H7N1O1_Sphingoid_Fragment_[d18:1]
282.2789
Relative Intensity
m/z

Cer[NS] d42:2 [M+H-H2O]+ | Ret. Time (min): 12.098 | m/z: 630.619

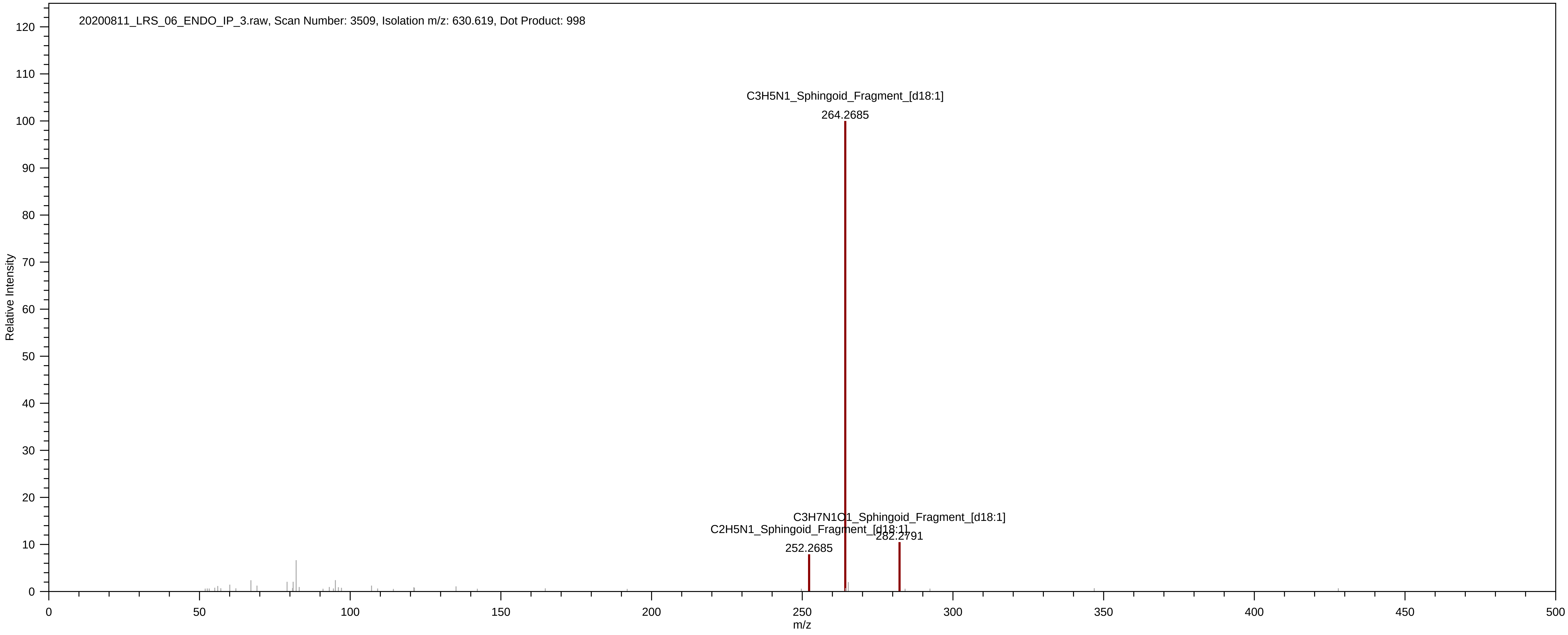

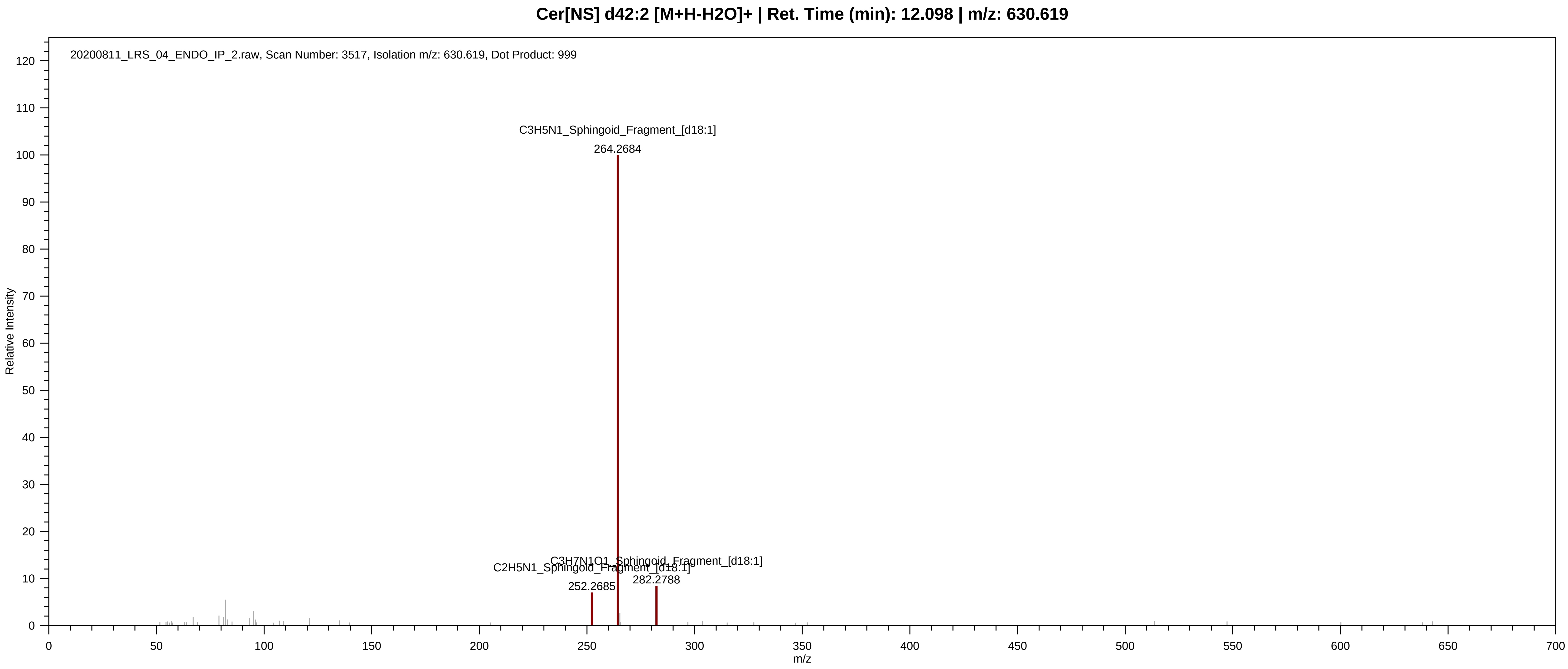

Cer[NS] d42:2 [M+H-H2O]+ | Ret. Time (min): 12.098 | m/z: 630.619
20200811_LRS_04_ENDO_IP_2.raw, Scan Number: 3517, Isolation m/z: 630.619, Dot Product: 999
C3H5N1_Sphingoid_Fragment_[d18:1]
264.2684
C3H7N1O1_Sphingoid_Fragment_[d18:1]
C2H5N1_Sphingoid_Fragment_[d18:1]
252.2685
282.2788
Relative Intensity
m/z

Cer[NS] d42:2 [M+H-H2O]+ | Ret. Time (min): 12.099 | m/z: 630.619

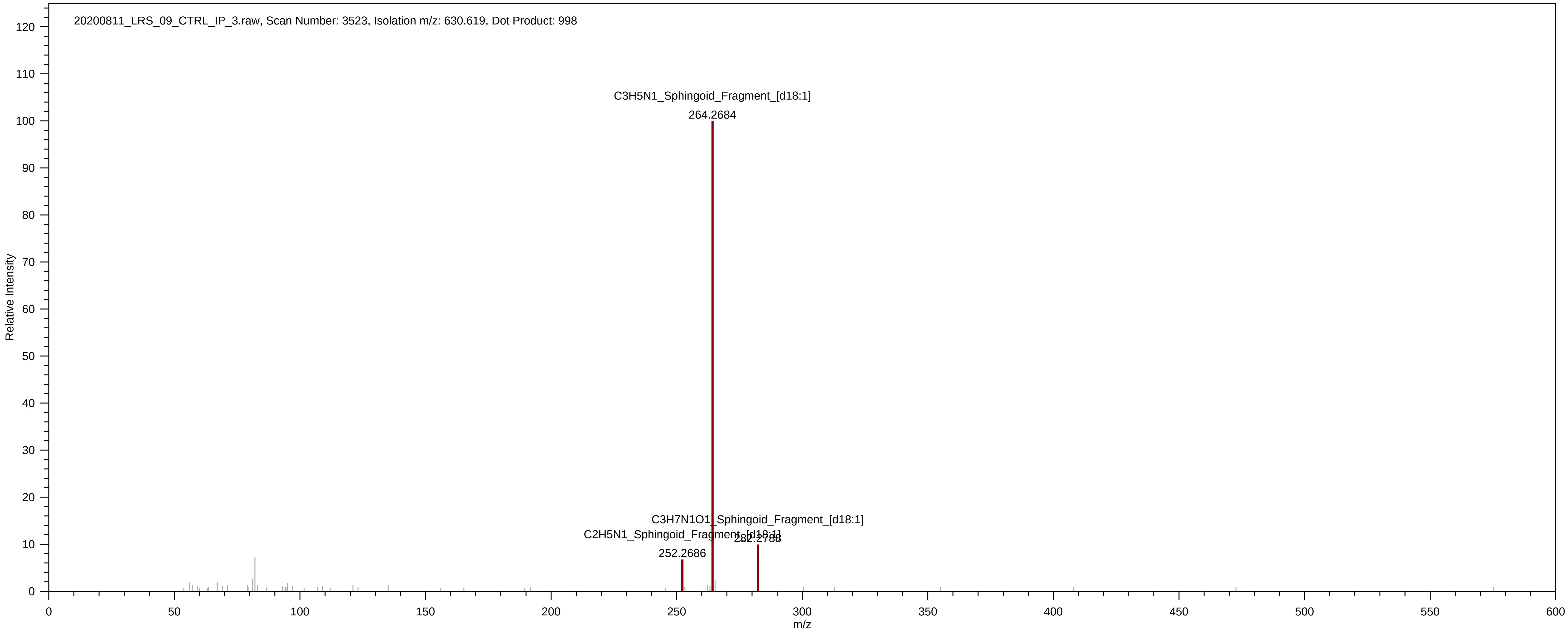

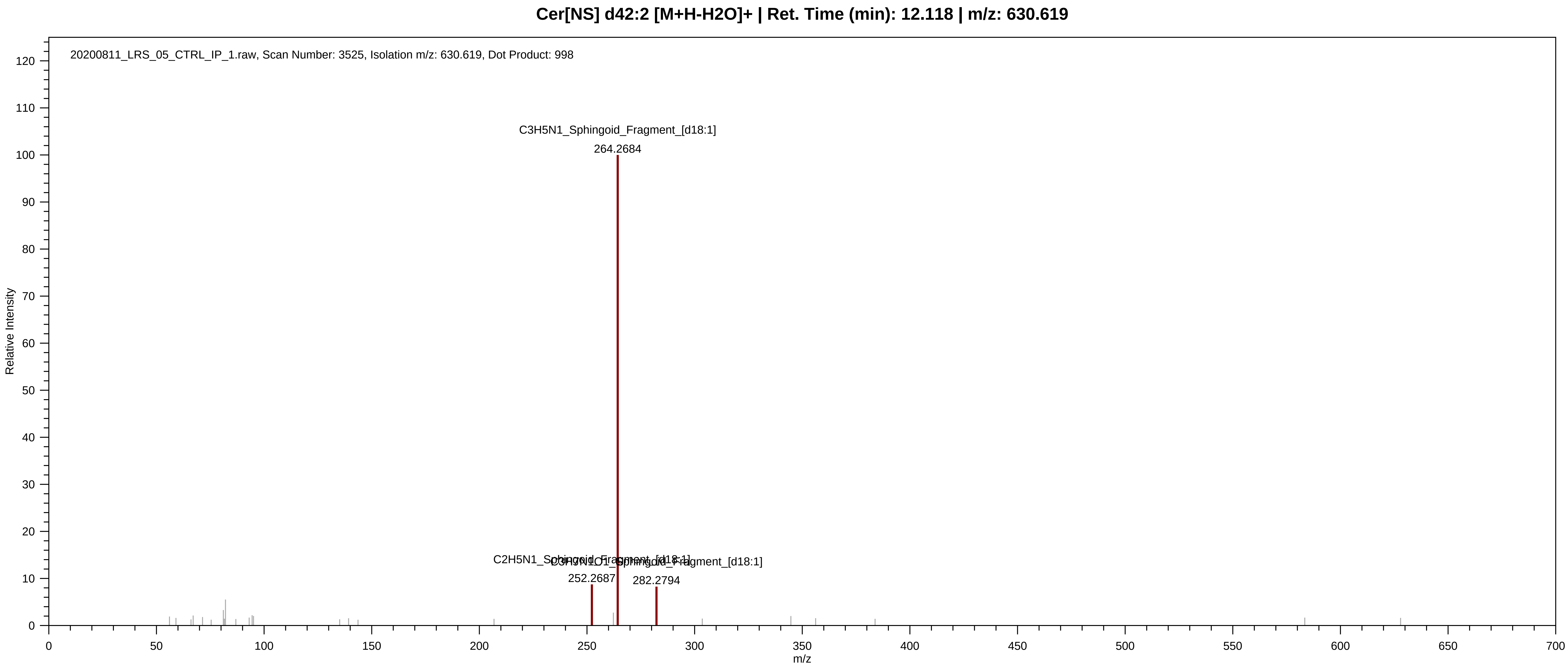

Cer[NS] d42:2 [M+H-H2O]+ | Ret. Time (min): 12.118 | m/z: 630.619
20200811_LRS_05_CTRL_IP_1.raw, Scan Number: 3525, Isolation m/z: 630.619, Dot Product: 998
C3H5N1_Sphingoid_Fragment_[d18:1]
264.2684
C2H5N1_Sphingoid_Fragment_[d18:1]
252.2687
C3H9N1O1_Sphingoid_Fragment_[d18:1]
282.2794
Relative Intensity
m/z

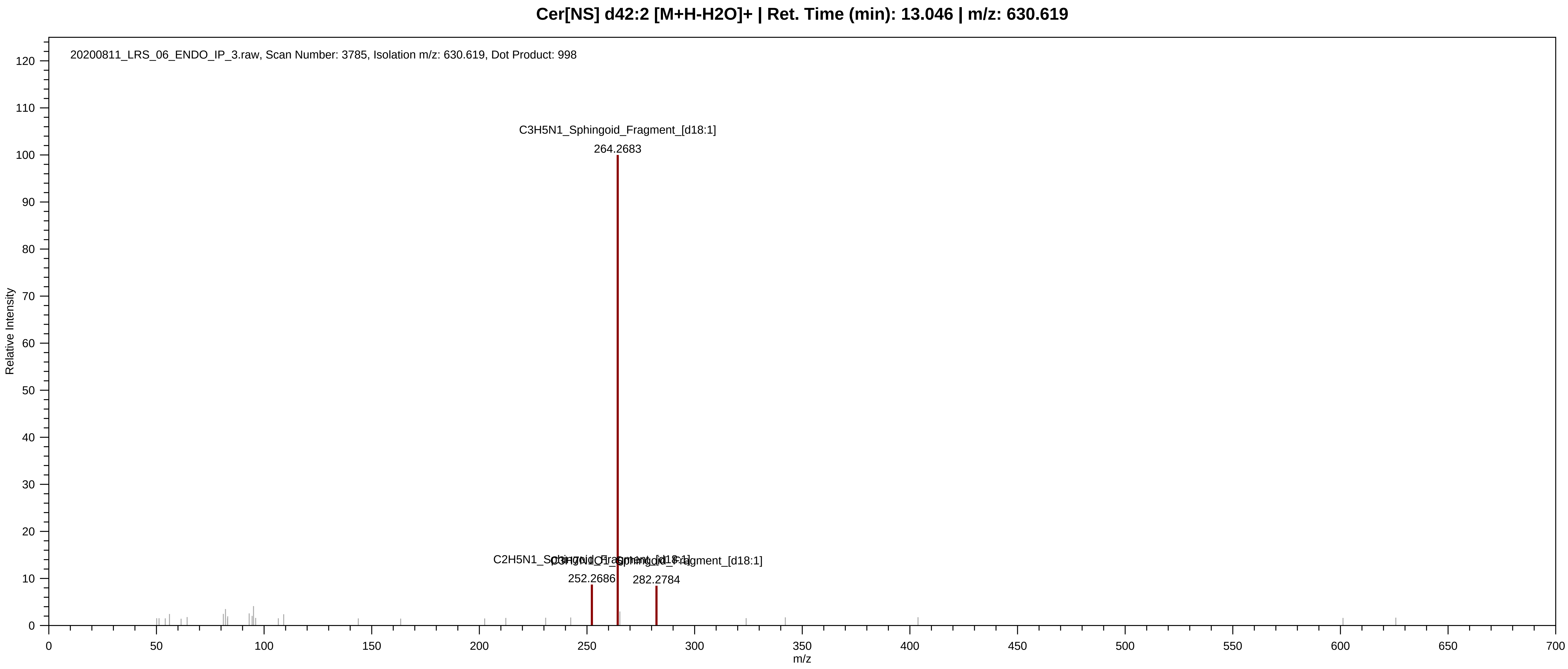

Cer[NS] d42:2 [M+H-H2O]+ | Ret. Time (min): 13.046 | m/z: 630.619
20200811_LRS_06_ENDO_IP_3.raw, Scan Number: 3785, Isolation m/z: 630.619, Dot Product: 998
C3H5N1_Sphingoid_Fragment_[d18:1]
264.2683
C2H5N1_Sphingoid_Fragment_[d18:1]
252.2686
C3H9N1O1_Sphingoid_Fragment_[d18:1]
282.2784
Relative Intensity
m/z

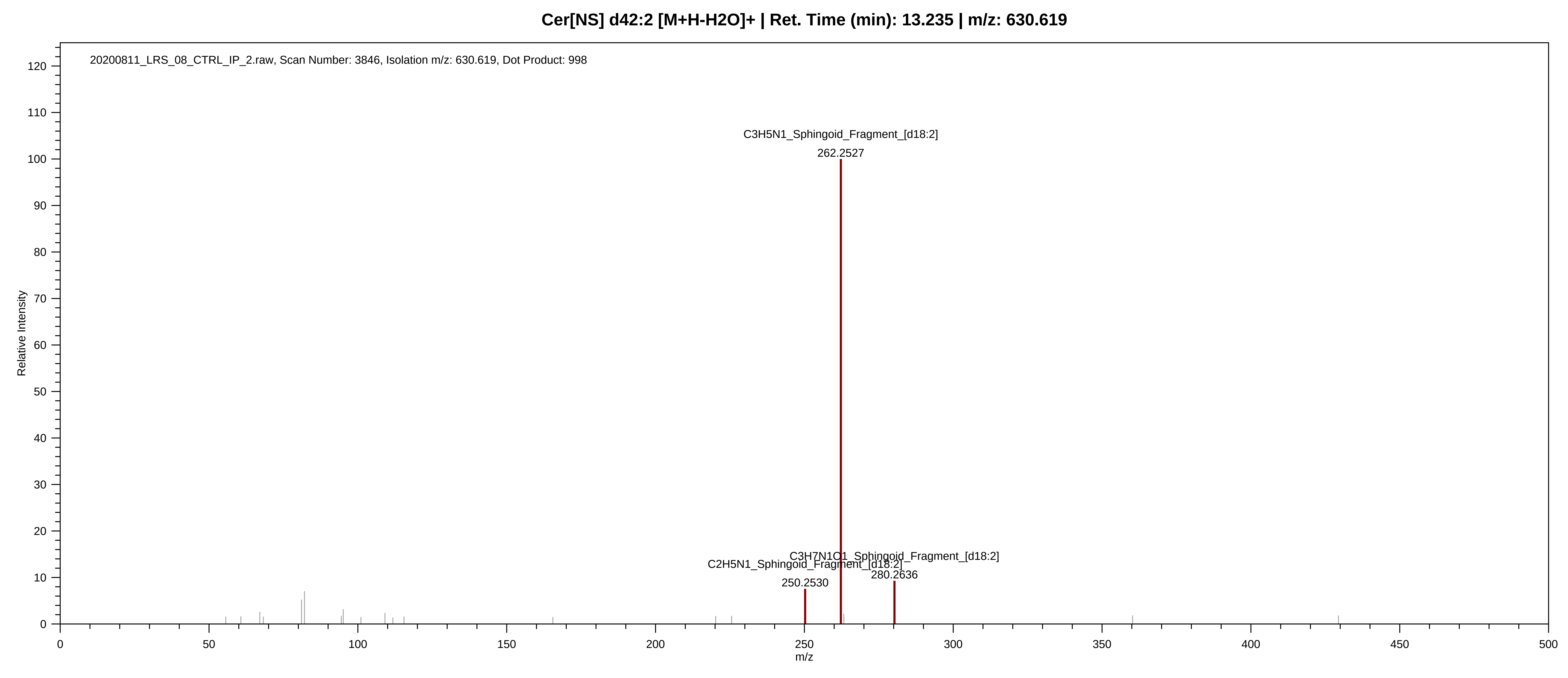
Cer[NS] d42:2 [M+H-H2O]+ | Ret. Time (min): 13.235 | m/z: 630.619
20200811_LRS_08_CTRL_IP_2.raw, Scan Number: 3846, Isolation m/z: 630.619, Dot Product: 998
C3H5N1_Sphingoid_Fragment_[d18:2]
262.2527
C3H7N1O1_Sphingoid_Fragment_[d18:2]
C2H5N1_Sphingoid_Fragment_[d18:2]
280.2636
250.2530
Relative Intensity
m/z
0
10
20
30
40
50
60
70
80
90
100
110
120
0
50
100
150
200
250
300
350
400
450
500

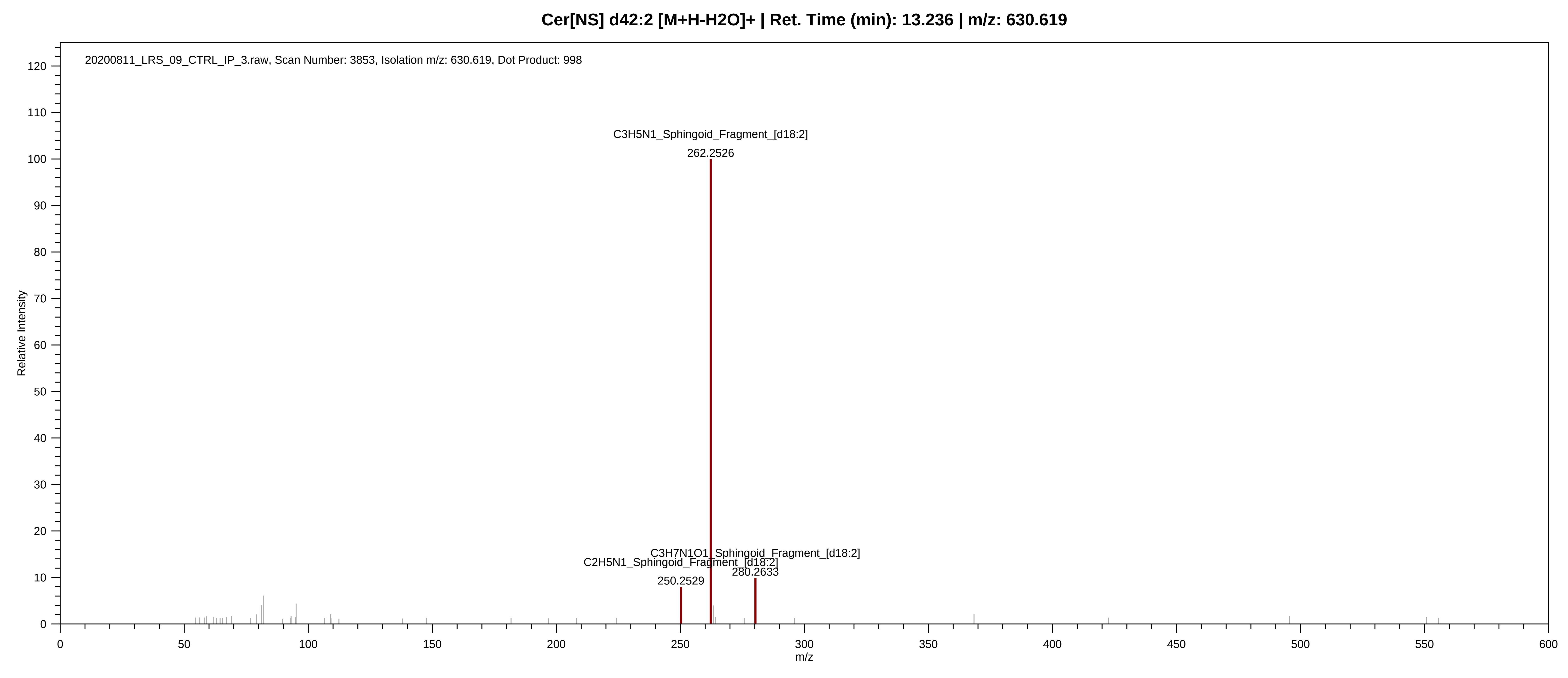
Cer[NS] d42:2 [M+H-H2O]+ | Ret. Time (min): 13.236 | m/z: 630.619
20200811_LRS_09_CTRL_IP_3.raw, Scan Number: 3853, Isolation m/z: 630.619, Dot Product: 998
C3H5N1_Sphingoid_Fragment_[d18:2]
262.2526
C3H7N1O1_Sphingoid_Fragment_[d18:2]
C2H5N1_Sphingoid_Fragment_[d18:2]
280.2633
250.2529
Relative Intensity
m/z

Cer[NS] d42:2 [M+H-H2O]+ | Ret. Time (min): 13.031 | m/z: 630.621

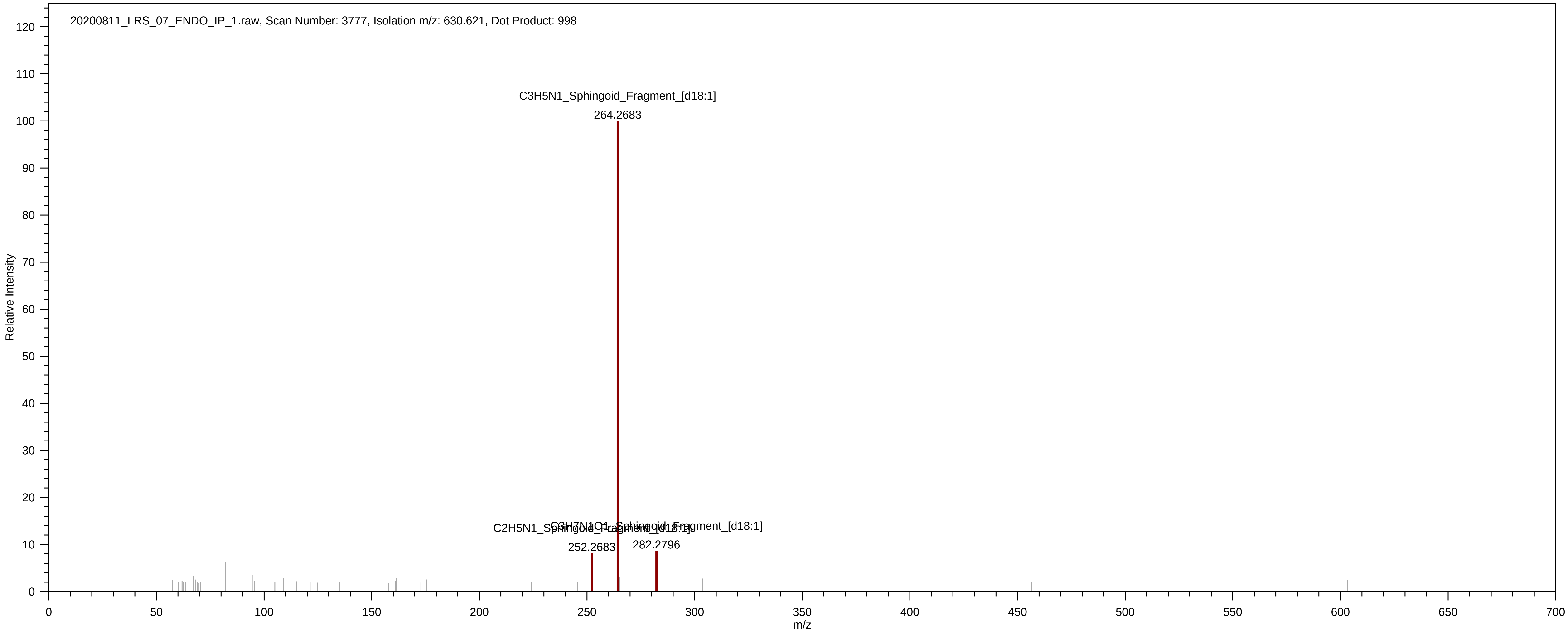

Cer[NS] d42:2 [M+H]+ | Ret. Time (min): 12.1 | m/z: 648.63

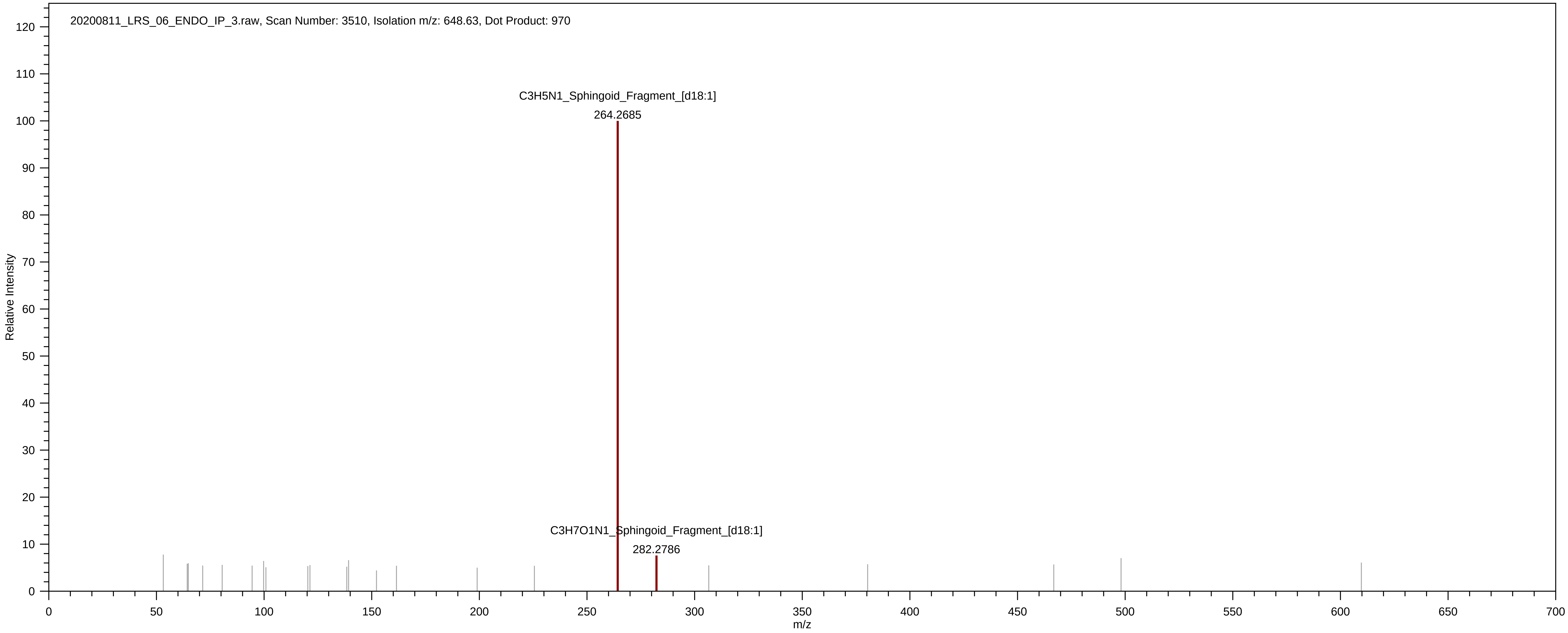

Cer[NS] d42:2 [M+H]+ | Ret. Time (min): 12.101 | m/z: 648.629

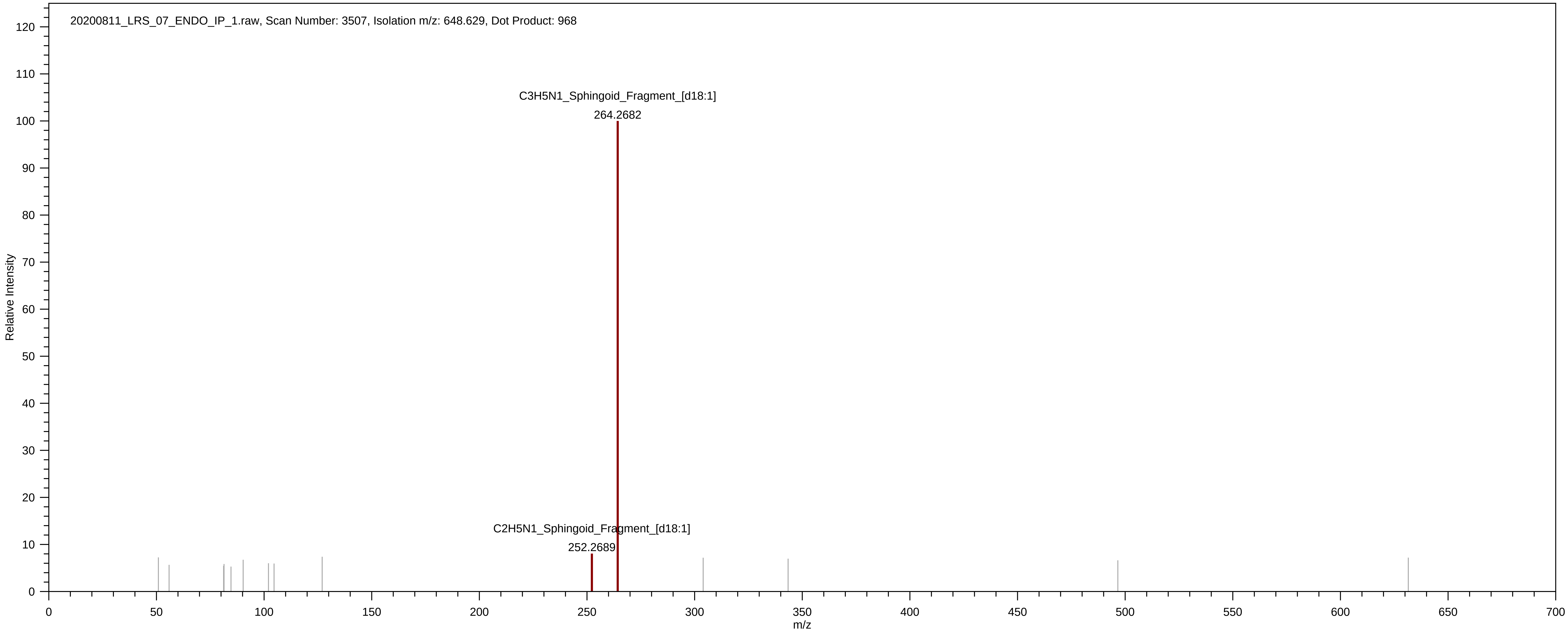

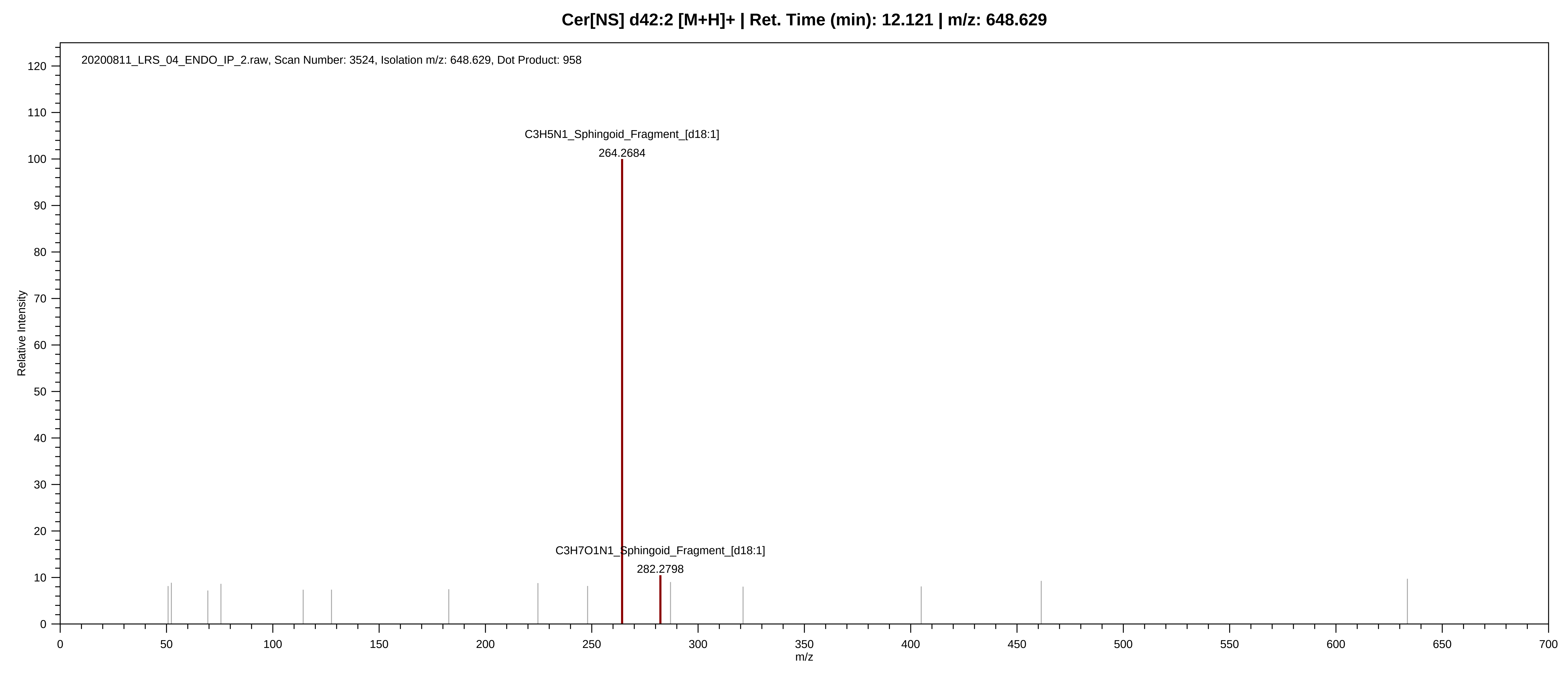

Cer[NS] d42:2 [M+H]+ | Ret. Time (min): 12.121 | m/z: 648.629
20200811_LRS_04_ENDO_IP_2.raw, Scan Number: 3524, Isolation m/z: 648.629, Dot Product: 958
C3H5N1_Sphingoid_Fragment_[d18:1]
264.2684
C3H7O1N1_Sphingoid_Fragment_[d18:1]
282.2798
Relative Intensity
m/z

Cer[NS] d42:3 [M+H-H2O]+ | Ret. Time (min): 12.12 | m/z: 628.603

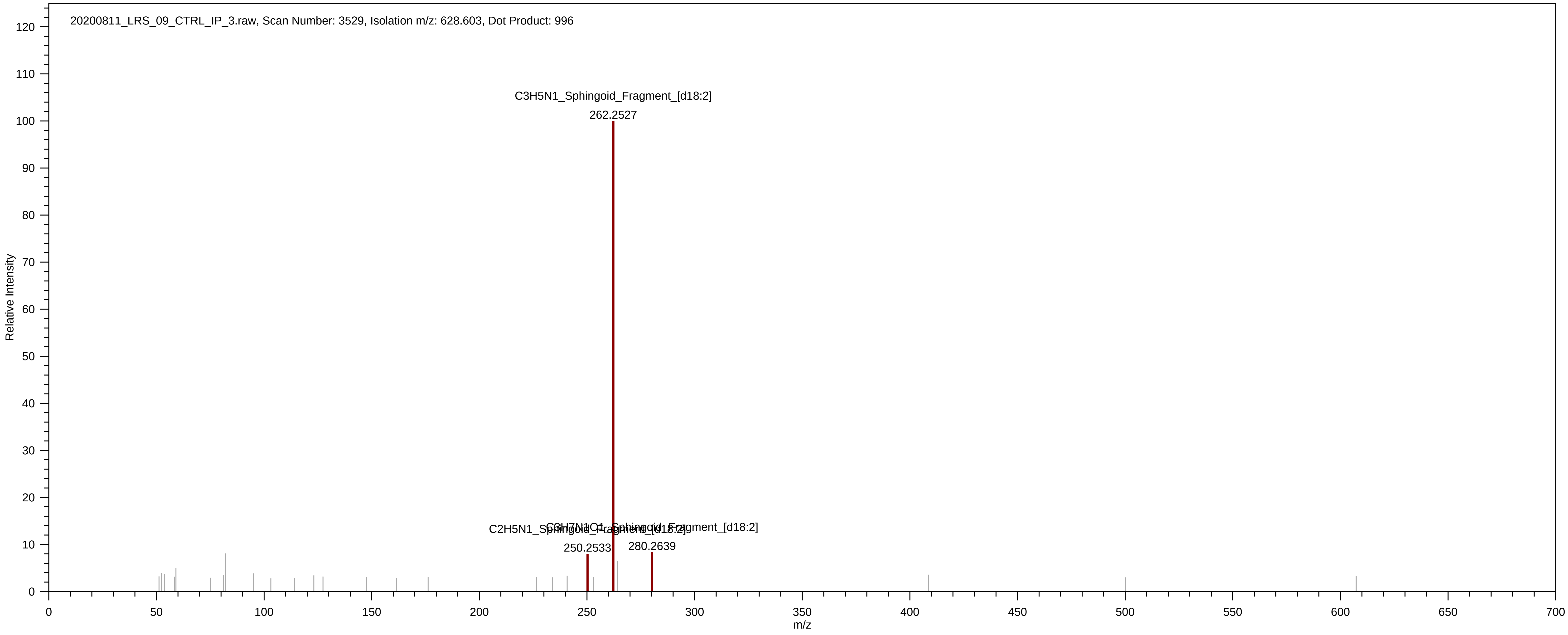

Cer[NS] d42:3 [M+H-H2O]+ | Ret. Time (min): 12.123 | m/z: 628.603

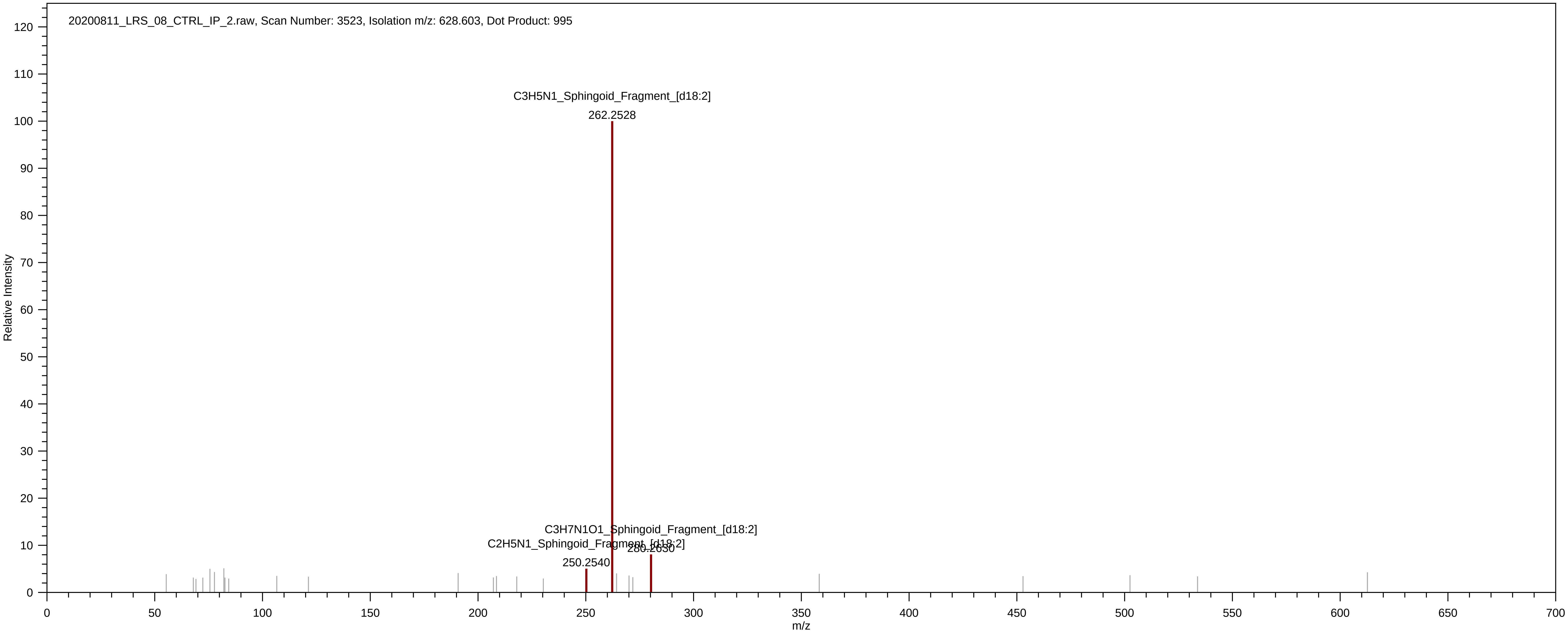

Cer[NS] d42:3 [M+H-H2O]+ | Ret. Time (min): 12.14 | m/z: 628.604

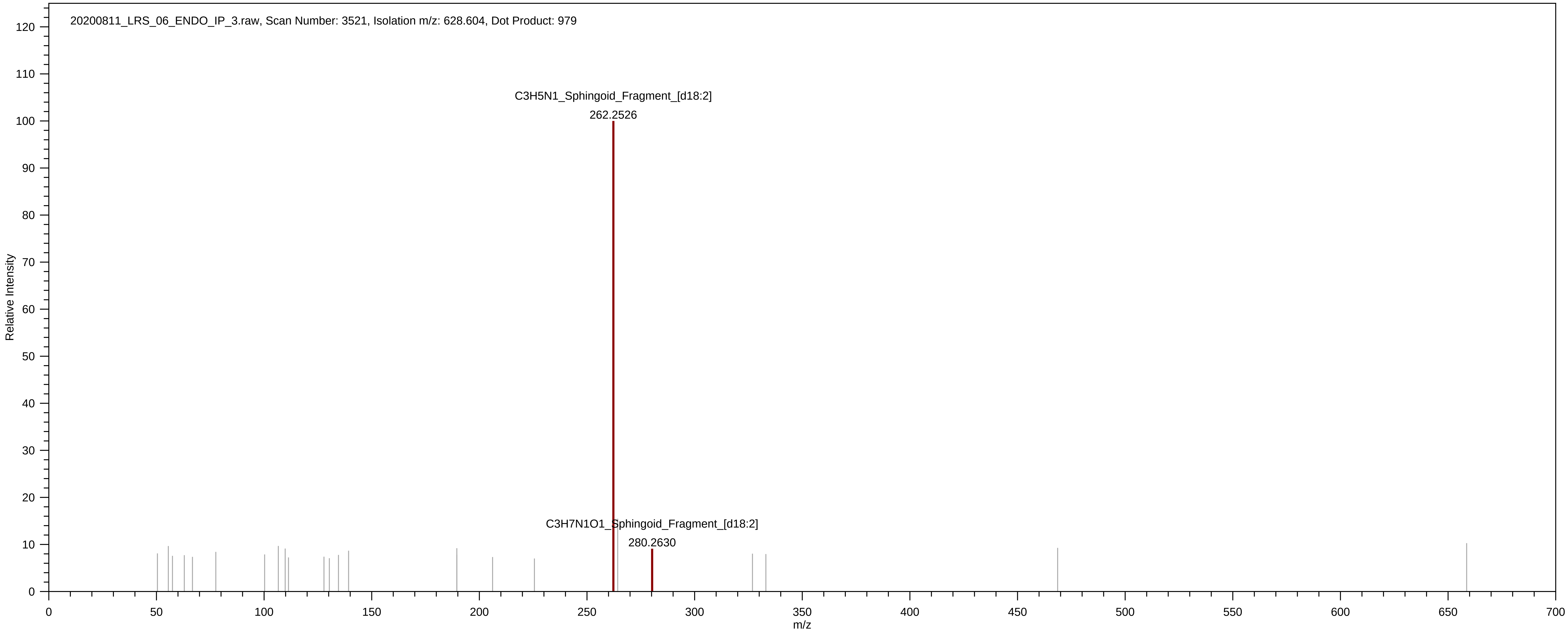

Cer[NS] d42:3 [M+H-H2O]+ | Ret. Time (min): 12.139 | m/z: 628.604

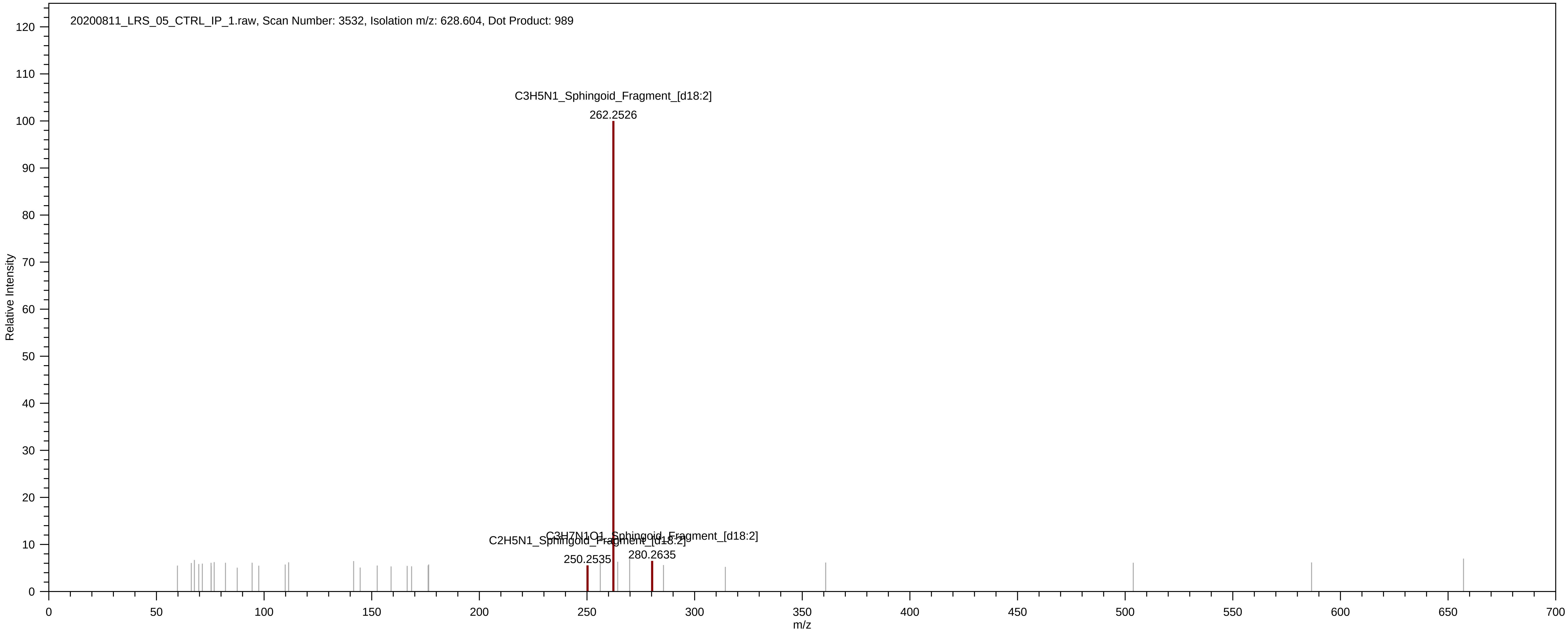

CL 16:0_18:1_16:1_18:1 [M-H]- | Ret. Time (min): 16.217 | m/z: 1401.977

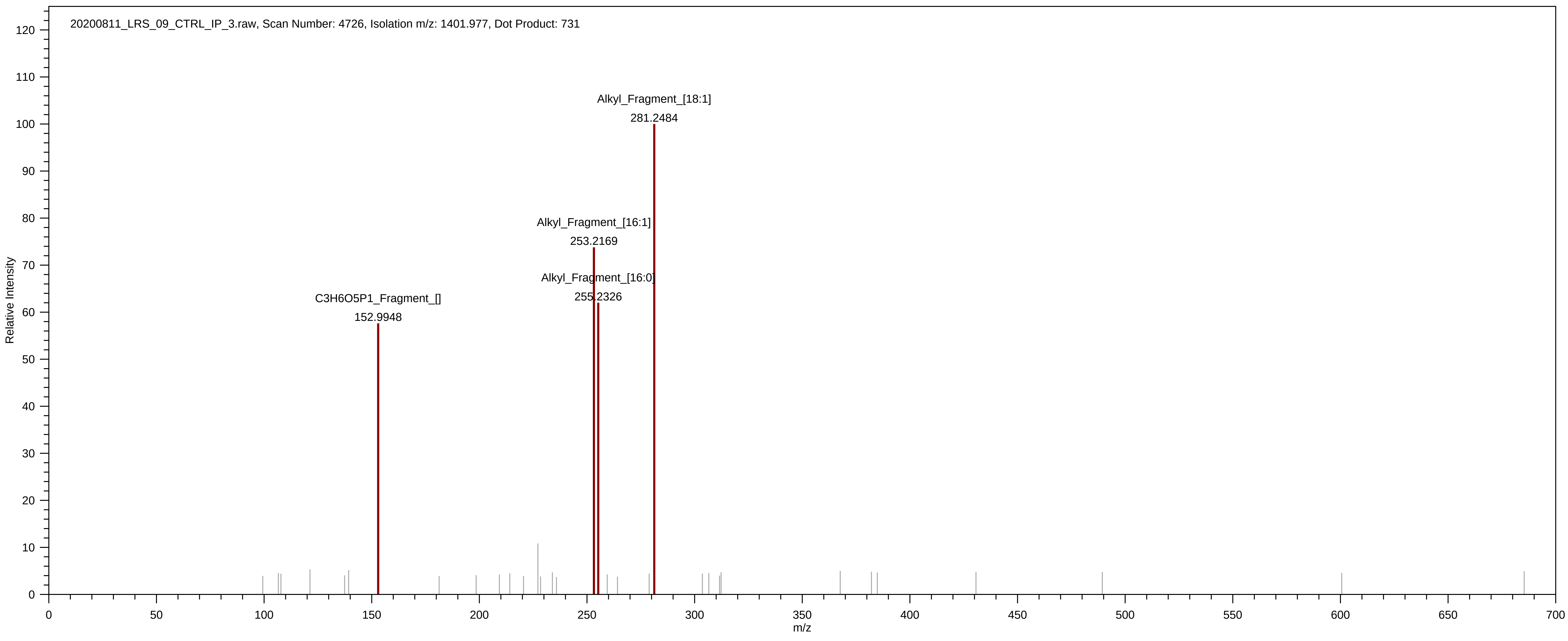

CL 16:0_18:1_16:1_18:1 [M-H]- | Ret. Time (min): 16.295 | m/z: 1401.981

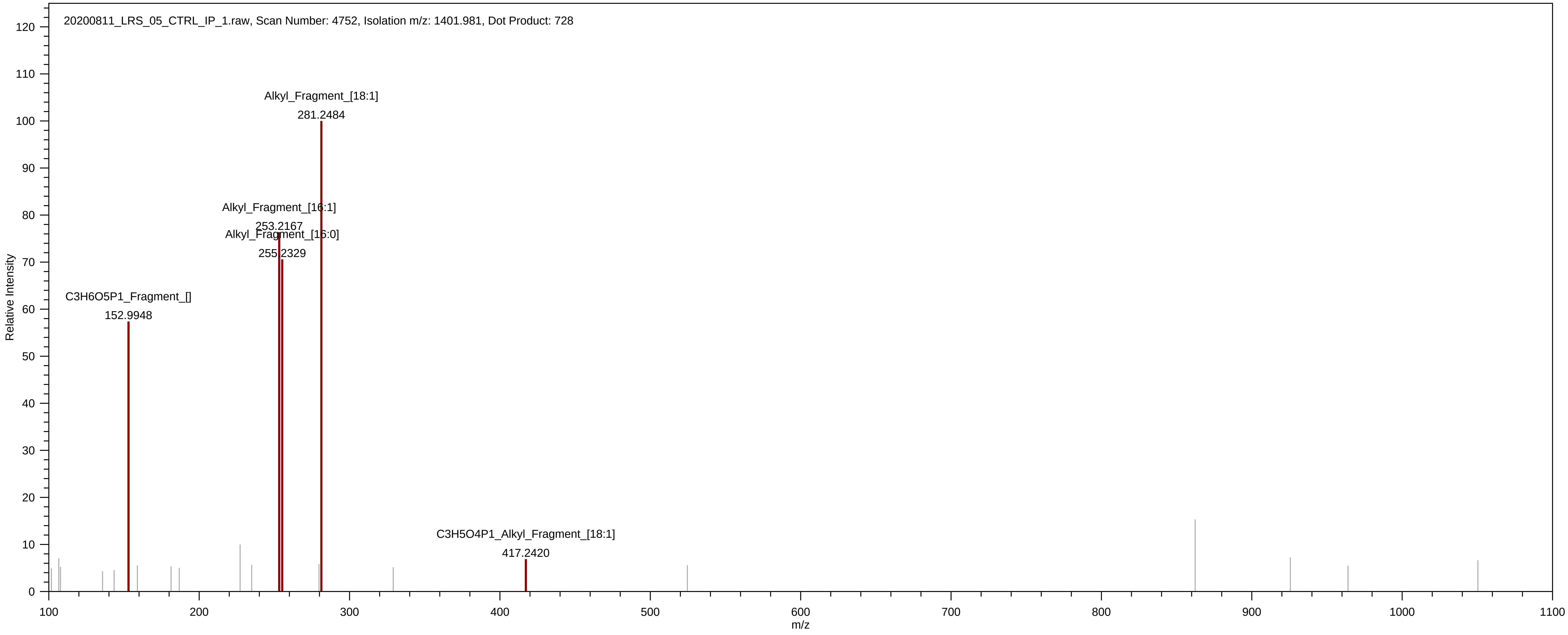

CL 16:0_18:1_16:1_18:1 [M-H]- | Ret. Time (min): 16.232 | m/z: 1401.983

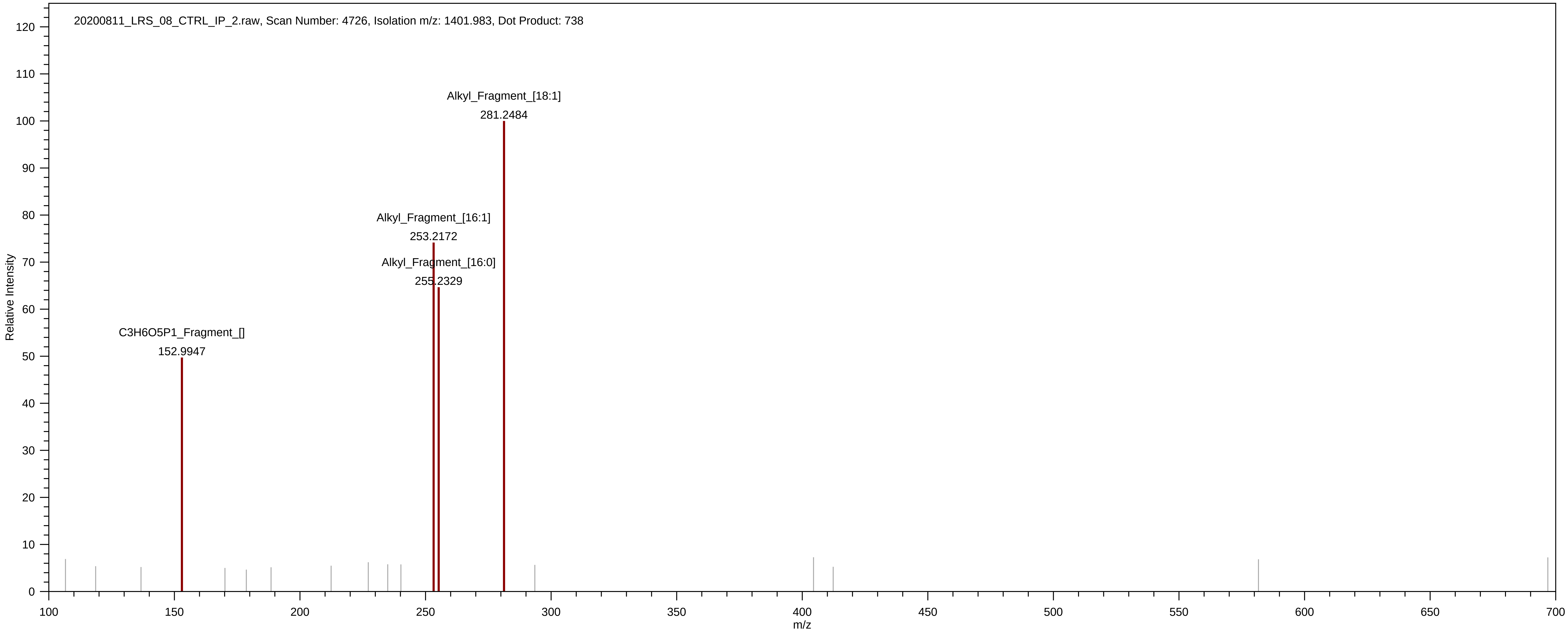

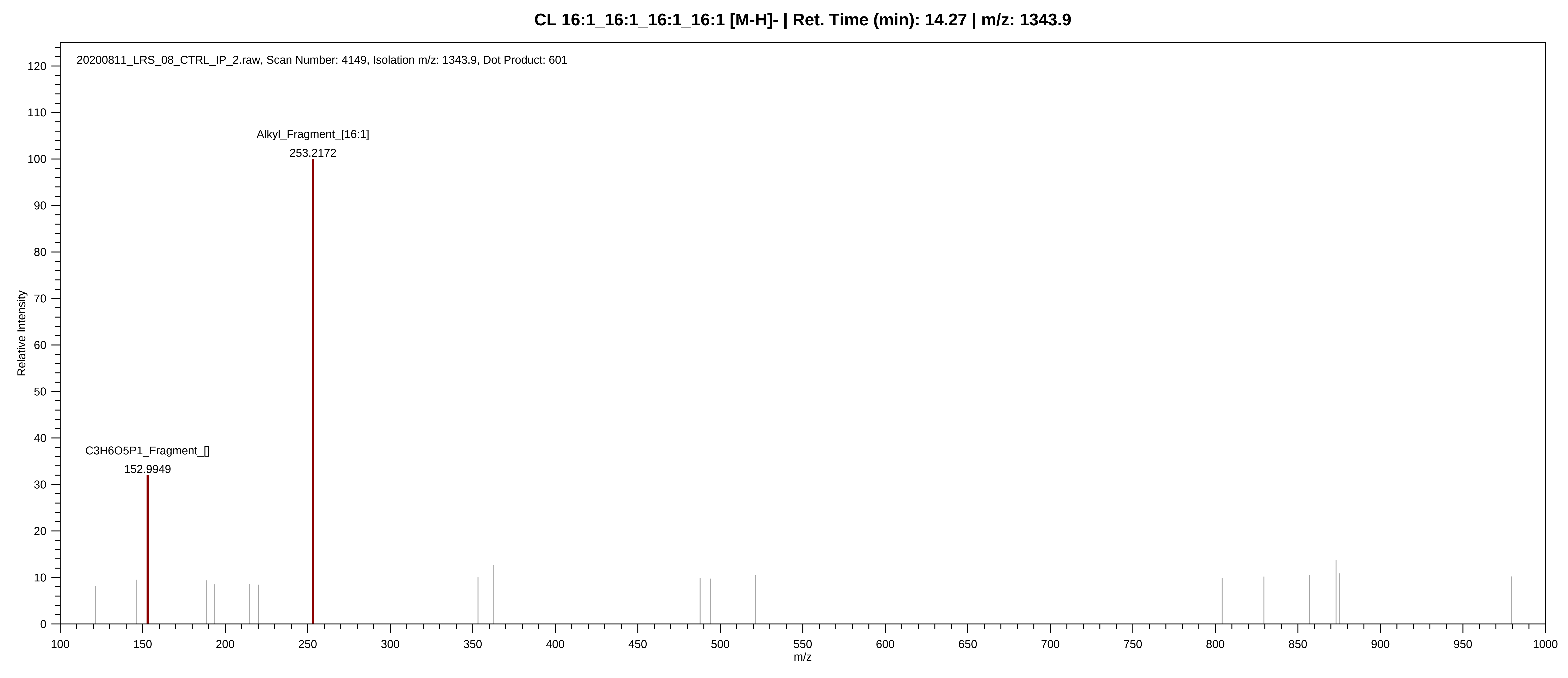
CL 16:1_16:1_16:1_16:1 [M-H]- | Ret. Time (min): 14.27 | m/z: 1343.9
20200811_LRS_08_CTRL_IP_2.raw, Scan Number: 4149, Isolation m/z: 1343.9, Dot Product: 601
Alkyl_Fragment_[16:1]
253.2172
C3H6O5P1_Fragment_[]
152.9949
Relative Intensity
m/z

CL 16:1_16:1_16:1_16:1 [M-H]- | Ret. Time (min): 14.252 | m/z: 1343.899

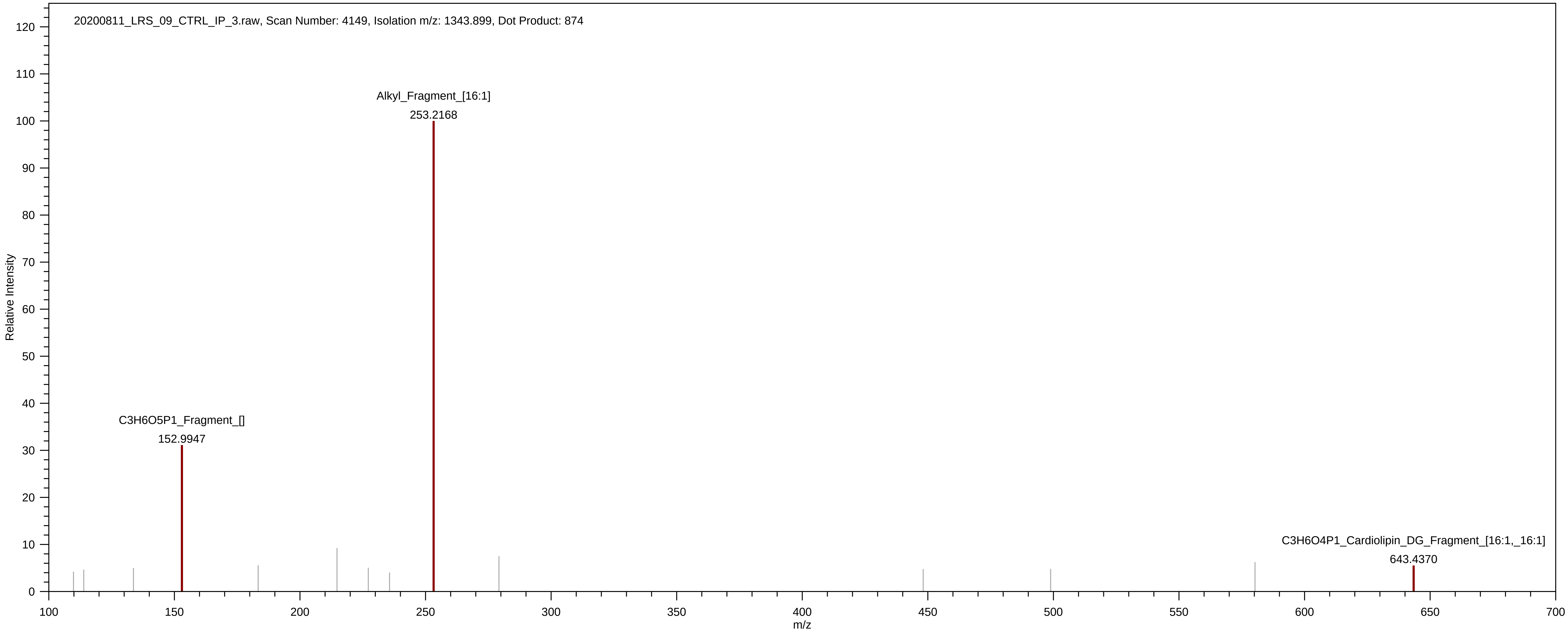

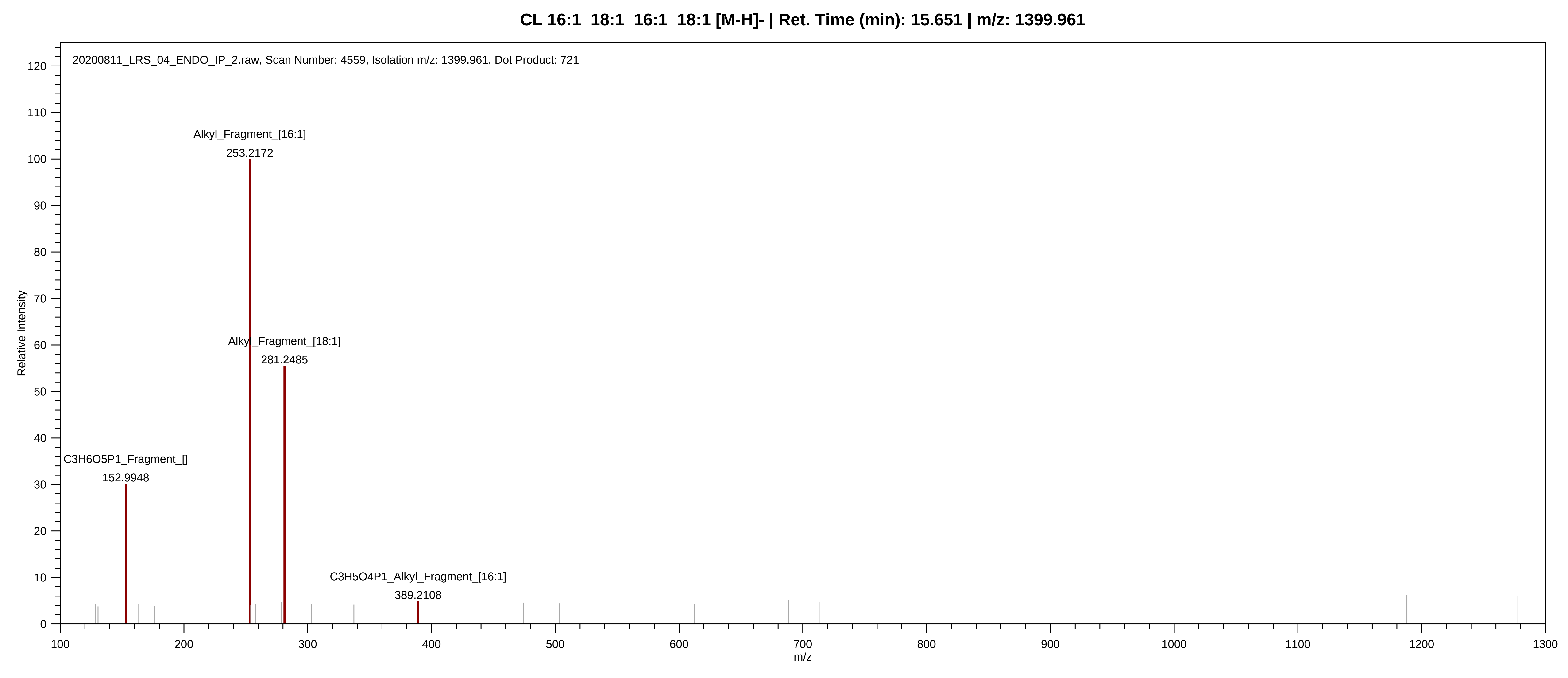

CL 16:1_18:1_16:1_18:1 [M-H]- | Ret. Time (min): 15.651 | m/z: 1399.961
20200811_LRS_04_ENDO_IP_2.raw, Scan Number: 4559, Isolation m/z: 1399.961, Dot Product: 721
Alkyl_Fragment_[16:1]
253.2172
Alkyl_Fragment_[18:1]
281.2485
C3H6O5P1_Fragment_[]
152.9948
C3H5O4P1_Alkyl_Fragment_[16:1]
389.2108
Relative Intensity
m/z

CL 16:1_18:1_16:1_18:1 [M-H]- | Ret. Time (min): 15.523 | m/z: 1399.968

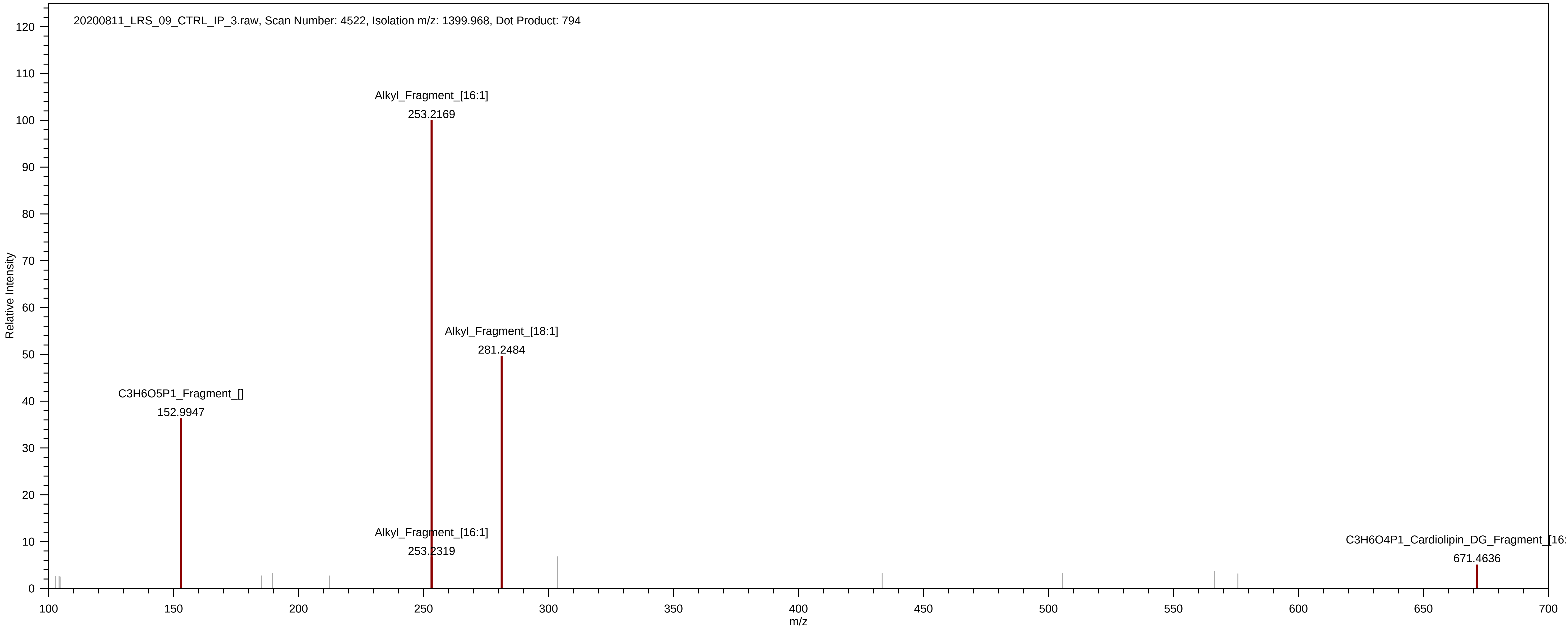

CL 16:1_18:1_16:1_18:2 [M-H]- | Ret. Time (min): 15.038 | m/z: 1397.95

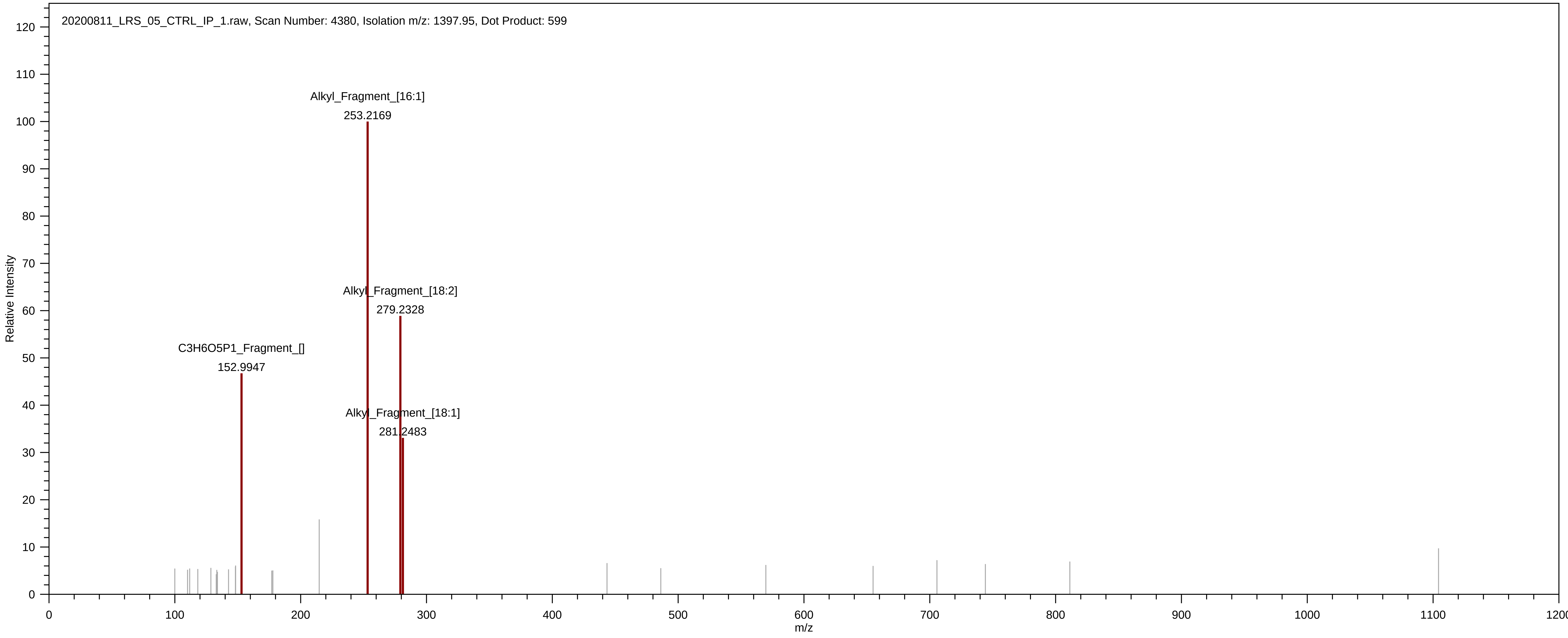

CL 16:1_18:2_16:1_16:1 [M-H]- | Ret. Time (min): 14.335 | m/z: 1369.921

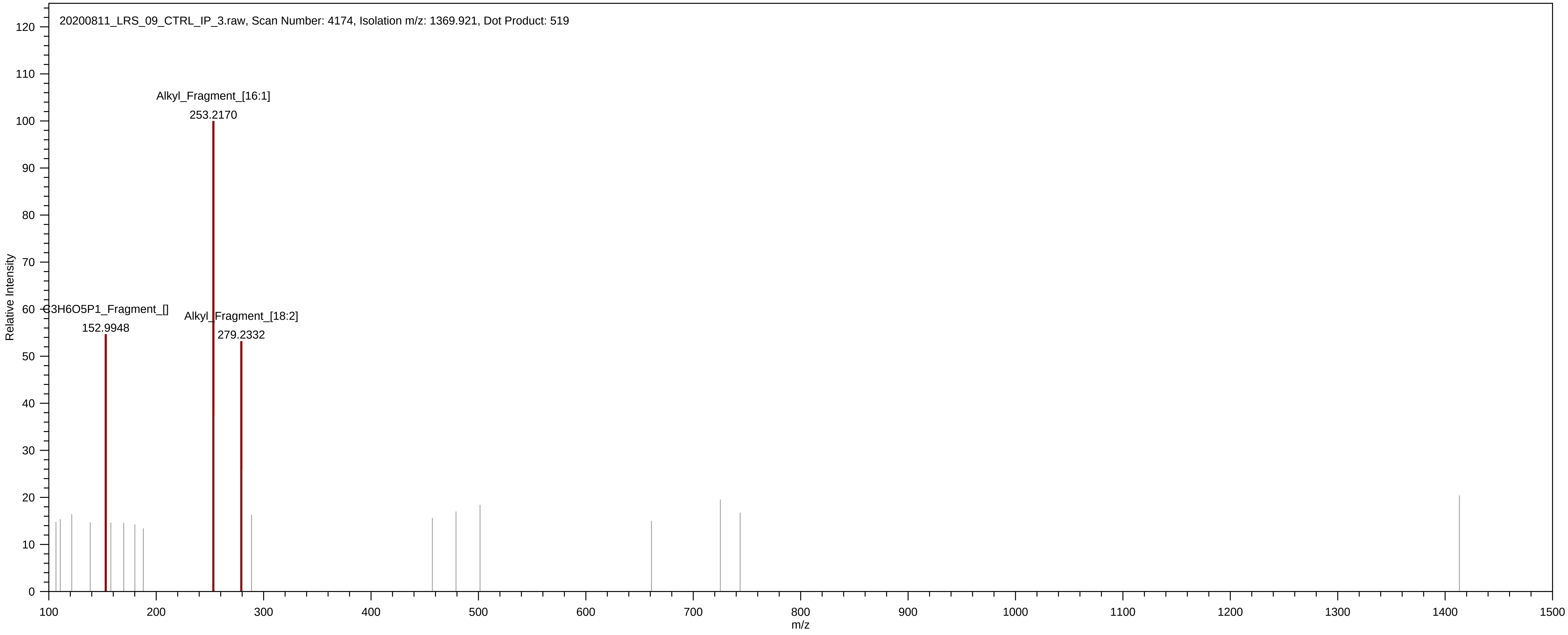

CL 68:2 [M-H]- | Ret. Time (min): 16.941 | m/z: 1403.995

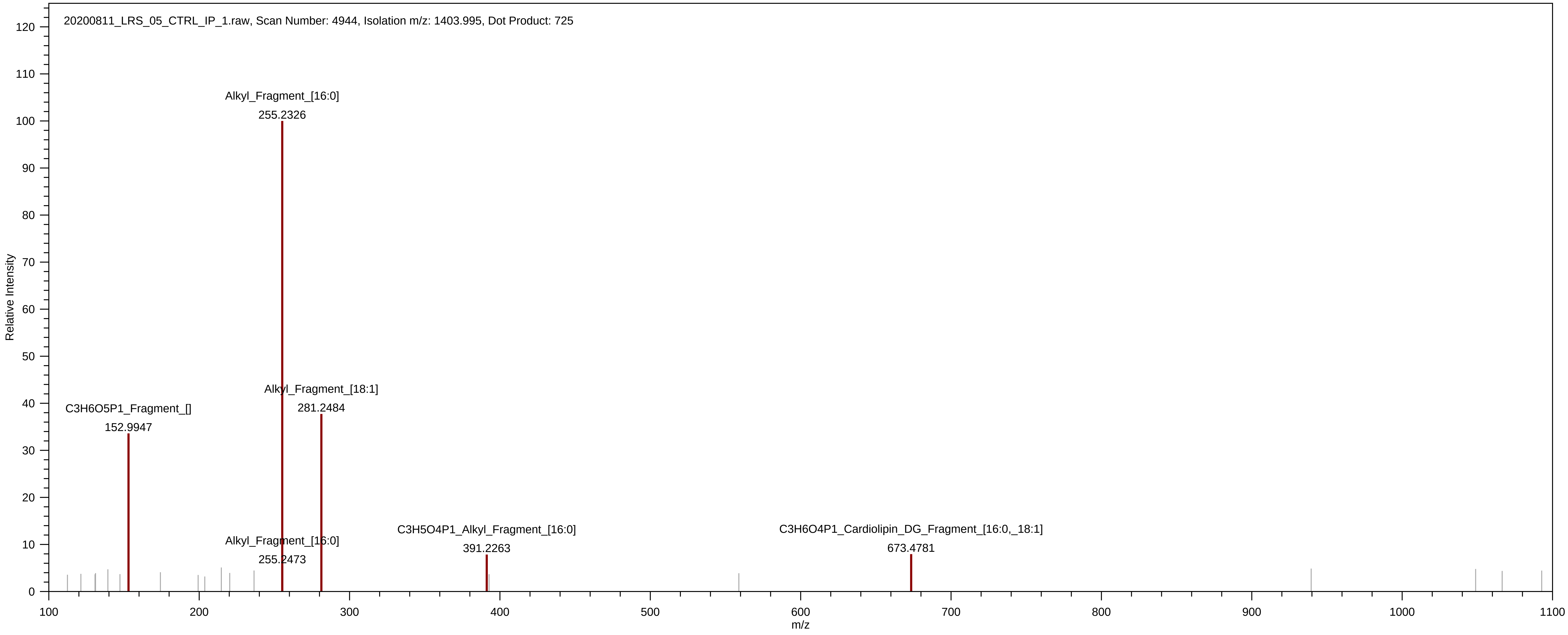

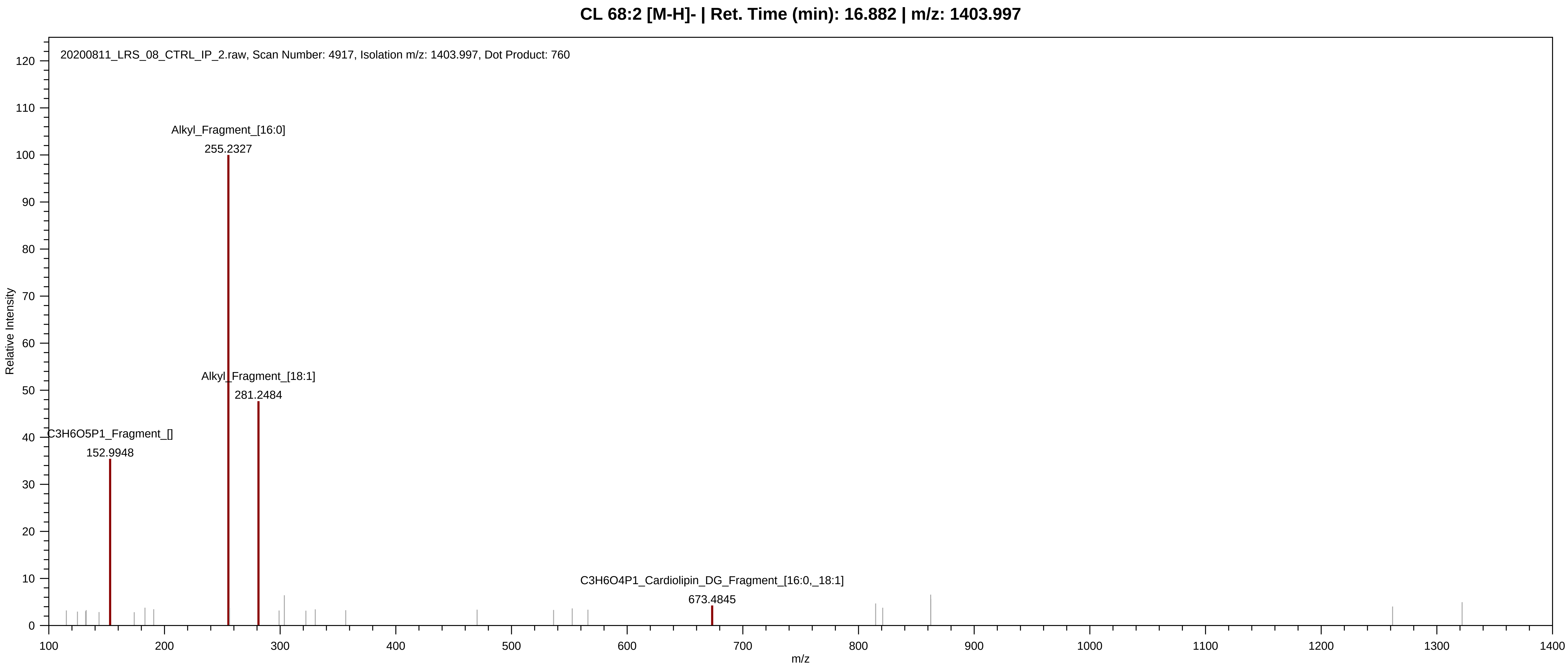

CL 68:2 [M-H]- | Ret. Time (min): 16.882 | m/z: 1403.997
20200811_LRS_08_CTRL_IP_2.raw, Scan Number: 4917, Isolation m/z: 1403.997, Dot Product: 760
Alkyl_Fragment_[16:0]
255.2327
Alkyl_Fragment_[18:1]
281.2484
C3H6O5P1_Fragment_[]
152.9948
C3H6O4P1_Cardiolipin_DG_Fragment_[16:0,_18:1]
673.4845
Relative Intensity
m/z

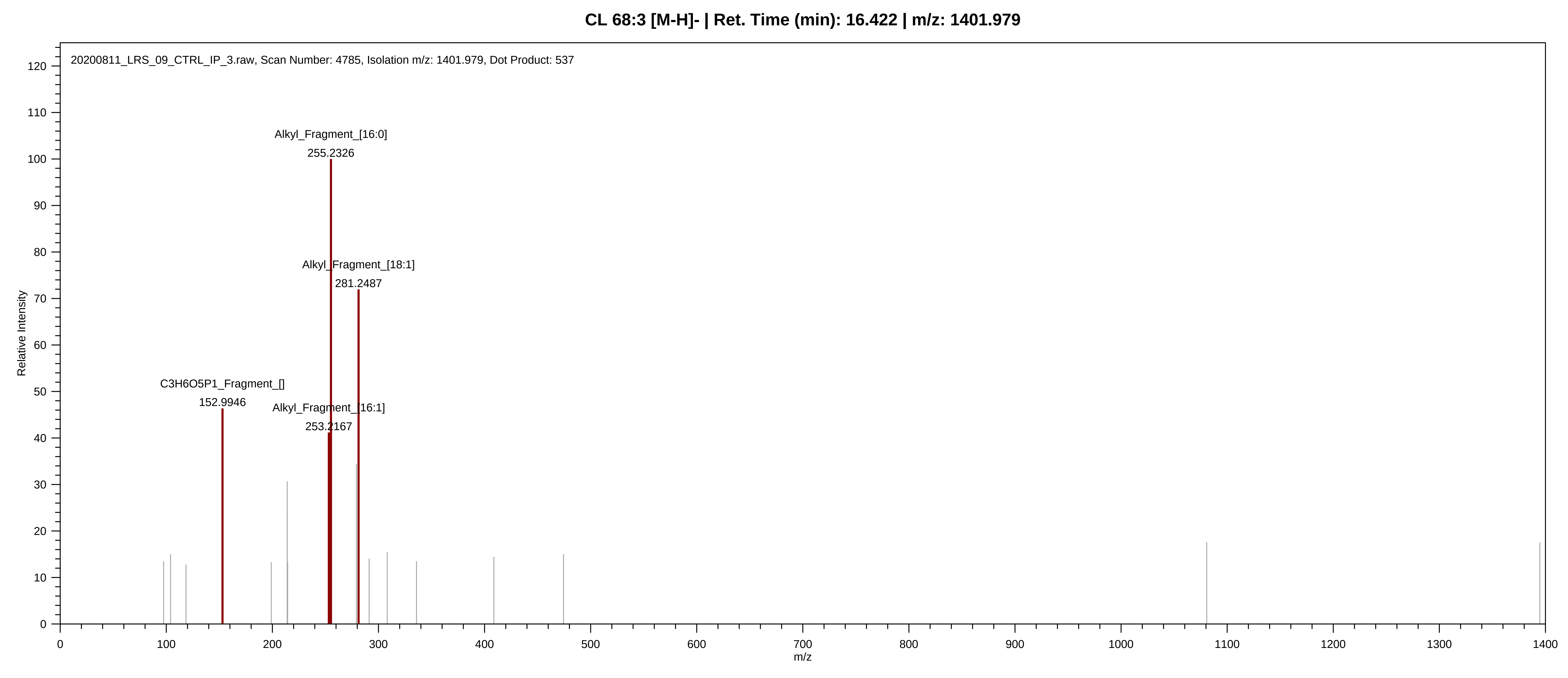

CL 68:3 [M-H]- | Ret. Time (min): 16.422 | m/z: 1401.979
20200811_LRS_09_CTRL_IP_3.raw, Scan Number: 4785, Isolation m/z: 1401.979, Dot Product: 537
Alkyl_Fragment_[16:0]
255.2326
Alkyl_Fragment_[18:1]
281.2487
C3H6O5P1_Fragment_[]
152.9946
Alkyl_Fragment_[16:1]
253.2167
Relative Intensity
m/z

CL 68:4 [M-H]- | Ret. Time (min): 15.596 | m/z: 1399.961

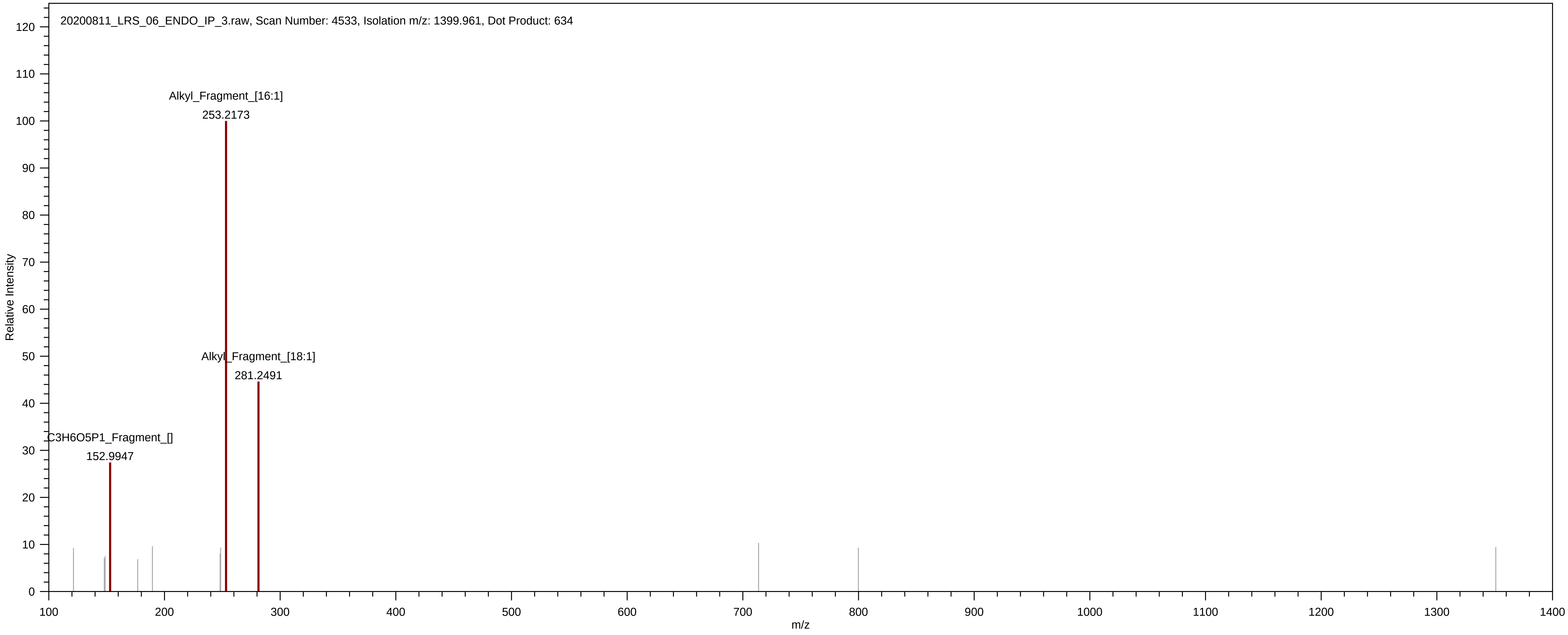

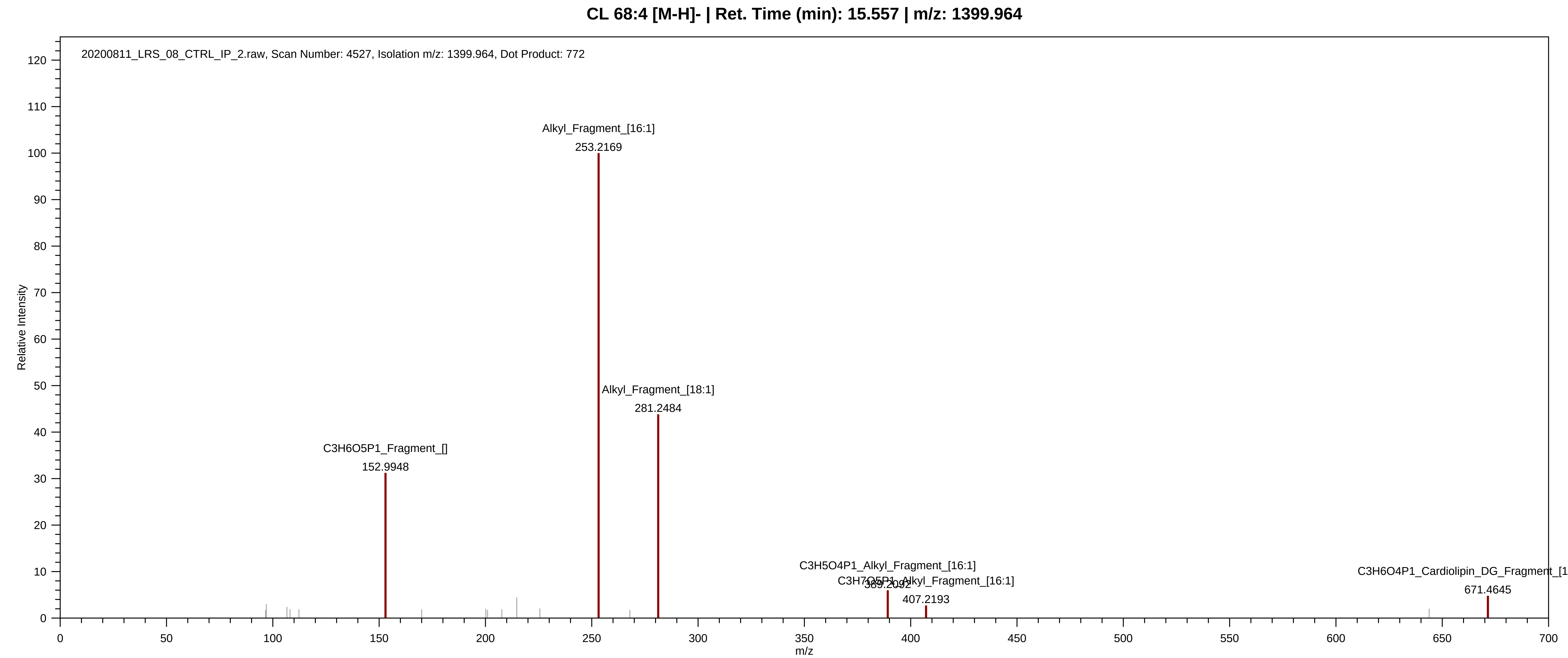

CL 68:4 [M-H]- | Ret. Time (min): 15.557 | m/z: 1399.964
20200811_LRS_08_CTRL_IP_2.raw, Scan Number: 4527, Isolation m/z: 1399.964, Dot Product: 772
Alkyl_Fragment_[16:1]
253.2169
Alkyl_Fragment_[18:1]
281.2484
C3H6O5P1_Fragment_[]
152.9948
C3H5O4P1_Alkyl_Fragment_[16:1]
389.2092
C3H7O5P1_Alkyl_Fragment_[16:1]
407.2193
C3H6O4P1_Cardiolipin_DG_Fragment_[1
671.4645
Relative Intensity
m/z

CL 68:4 [M-H]- | Ret. Time (min): 15.725 | m/z: 1399.964

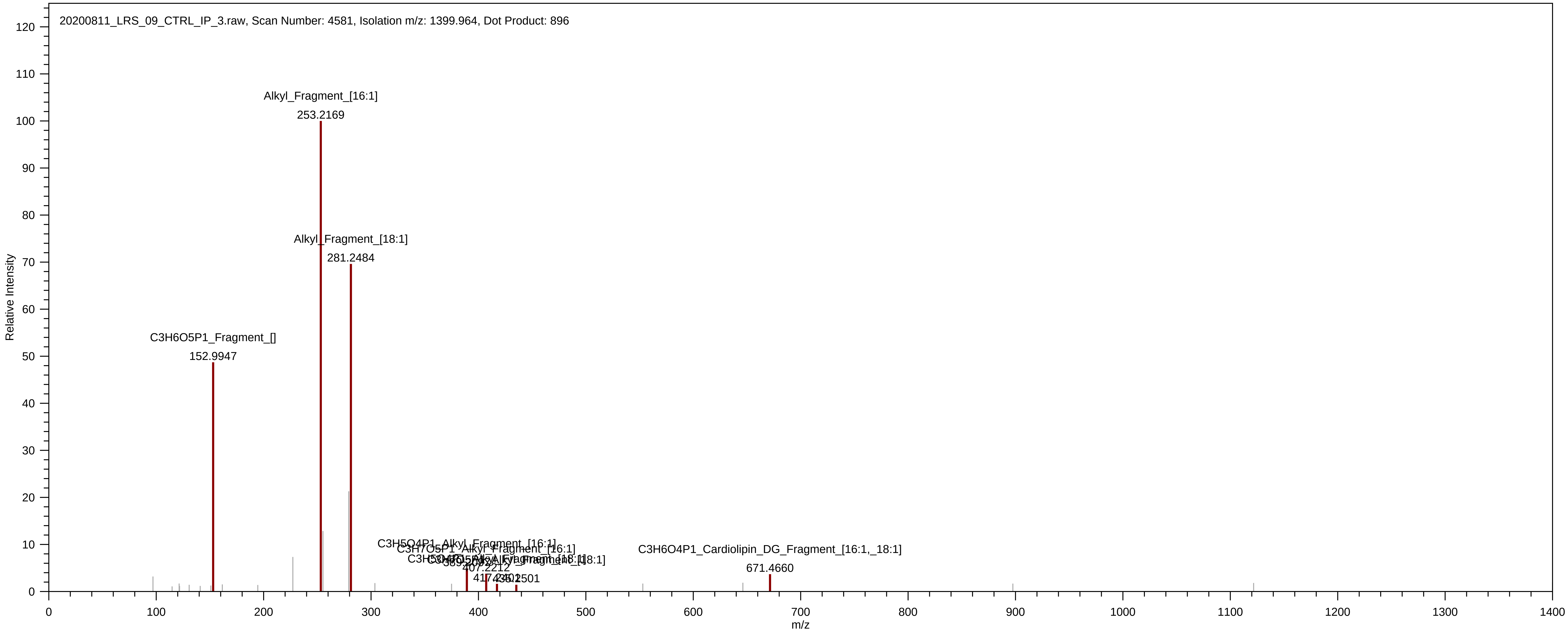

CL 68:4 [M-H]- | Ret. Time (min): 15.742 | m/z: 1399.964

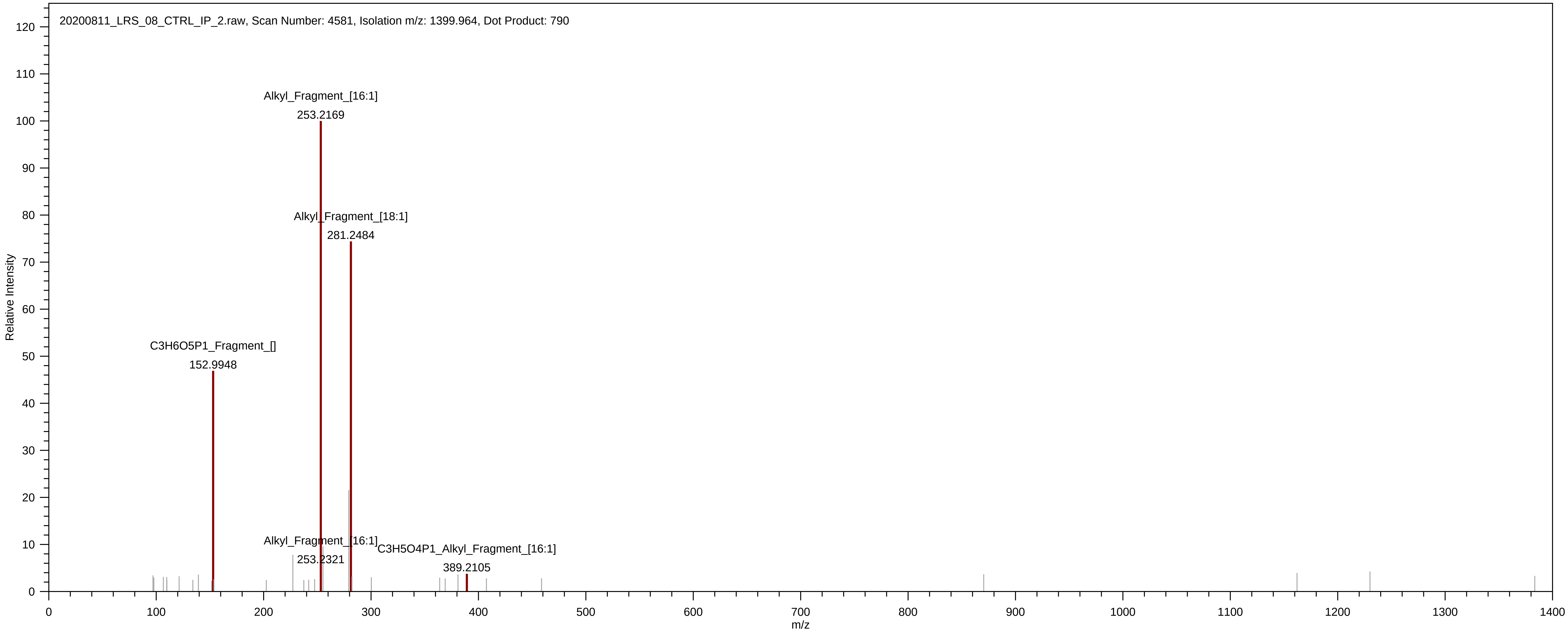

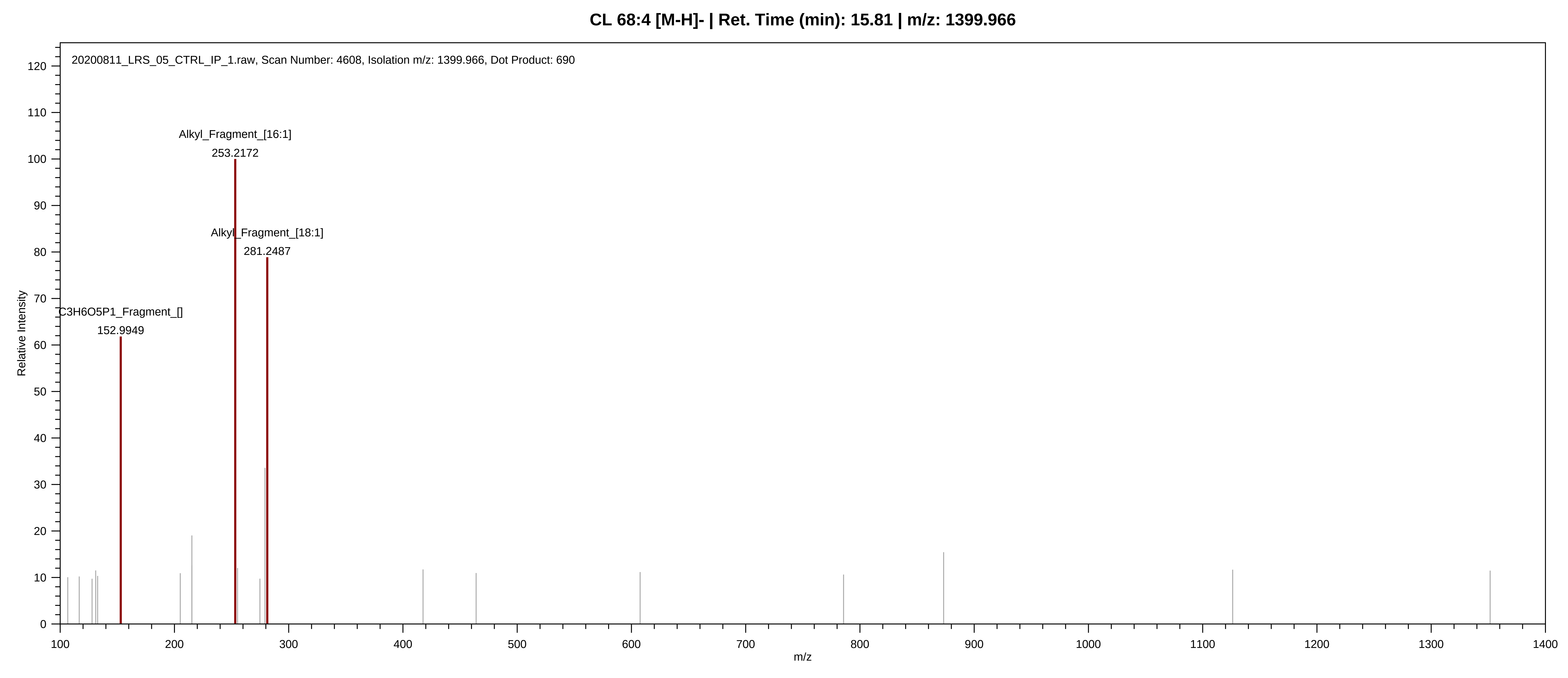

CL 68:4 [M-H]- | Ret. Time (min): 15.81 | m/z: 1399.966
20200811_LRS_05_CTRL_IP_1.raw, Scan Number: 4608, Isolation m/z: 1399.966, Dot Product: 690
Alkyl_Fragment_[16:1]
253.2172
Alkyl_Fragment_[18:1]
281.2487
C3H6O5P1_Fragment_[]
152.9949
Relative Intensity
m/z

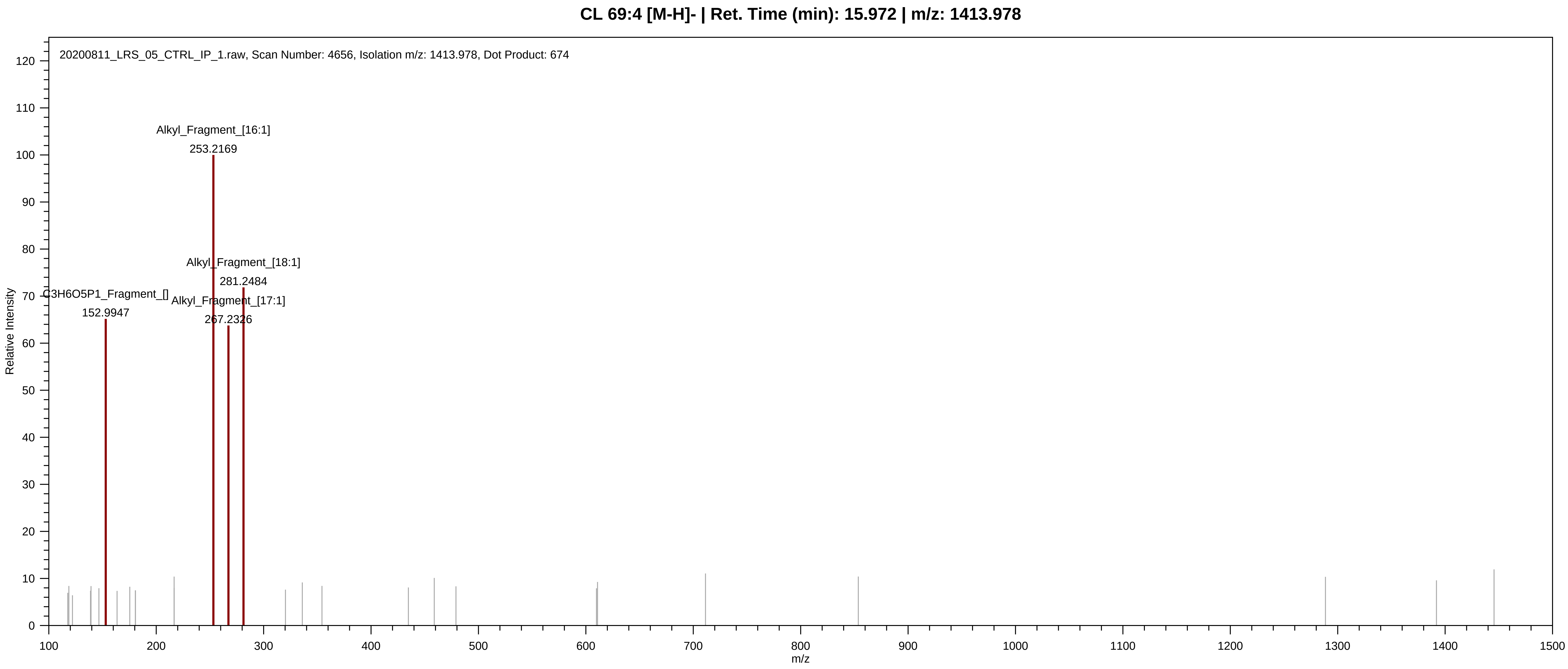
CL 69:4 [M-H]- | Ret. Time (min): 15.972 | m/z: 1413.978
20200811_LRS_05_CTRL_IP_1.raw, Scan Number: 4656, Isolation m/z: 1413.978, Dot Product: 674
Alkyl_Fragment_[16:1]
253.2169
Alkyl_Fragment_[18:1]
281.2484
C3H6O5P1_Fragment_[]
152.9947
Alkyl_Fragment_[17:1]
267.2326
Relative Intensity
m/z

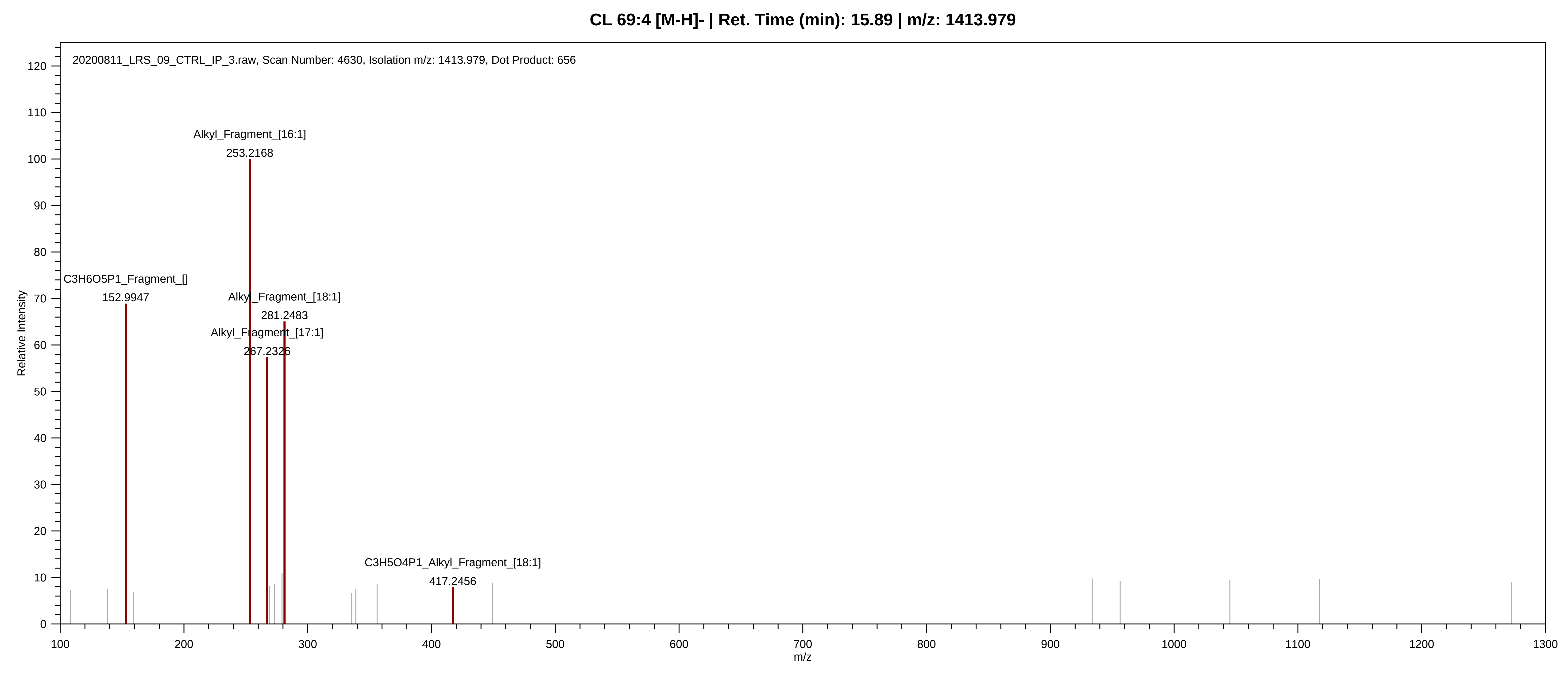
CL 69:4 [M-H]- | Ret. Time (min): 15.89 | m/z: 1413.979
20200811_LRS_09_CTRL_IP_3.raw, Scan Number: 4630, Isolation m/z: 1413.979, Dot Product: 656
Alkyl_Fragment_[16:1]
253.2168
C3H6O5P1_Fragment_[]
152.9947
Alkyl_Fragment_[18:1]
281.2483
Alkyl_Fragment_[17:1]
267.2326
C3H5O4P1_Alkyl_Fragment_[18:1]
417.2456
Relative Intensity
m/z

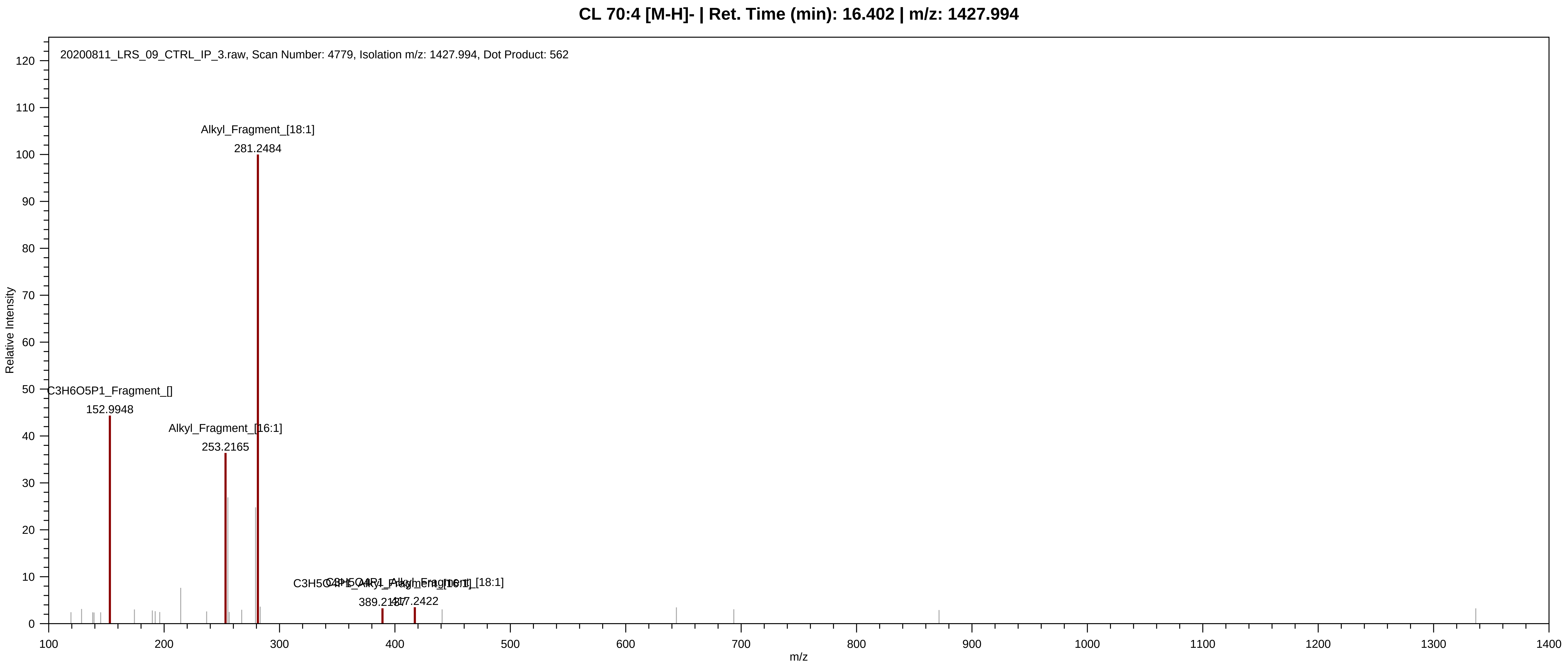

CL 70:4 [M-H]- | Ret. Time (min): 16.402 | m/z: 1427.994
20200811_LRS_09_CTRL_IP_3.raw, Scan Number: 4779, Isolation m/z: 1427.994, Dot Product: 562
Alkyl_Fragment_[18:1]
281.2484
C3H6O5P1_Fragment_[]
152.9948
Alkyl_Fragment_[16:1]
253.2165
389.2137
417.2422
Relative Intensity
m/z

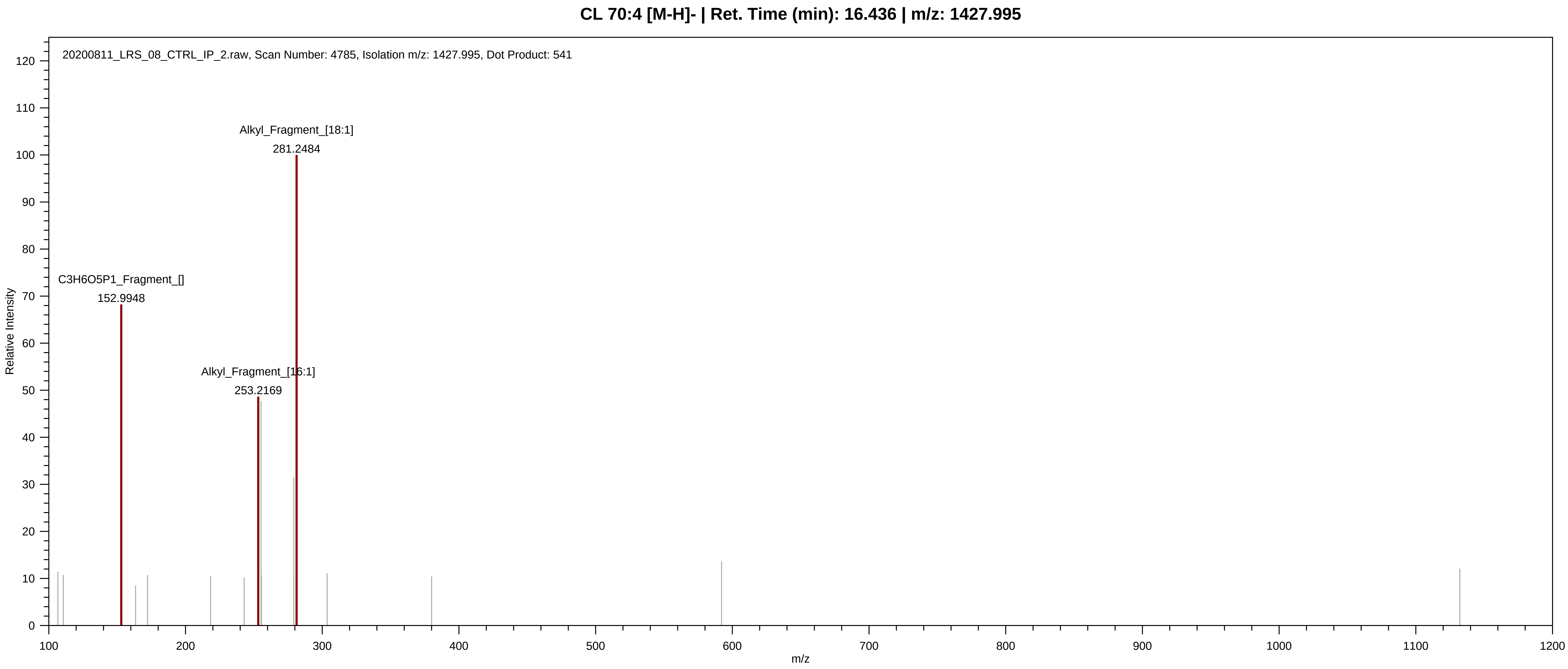
CL 70:4 [M-H]- | Ret. Time (min): 16.436 | m/z: 1427.995
20200811_LRS_08_CTRL_IP_2.raw, Scan Number: 4785, Isolation m/z: 1427.995, Dot Product: 541
Alkyl_Fragment_[18:1]
281.2484
C3H6O5P1_Fragment_[]
152.9948
Alkyl_Fragment_[16:1]
253.2169
Relative Intensity
m/z

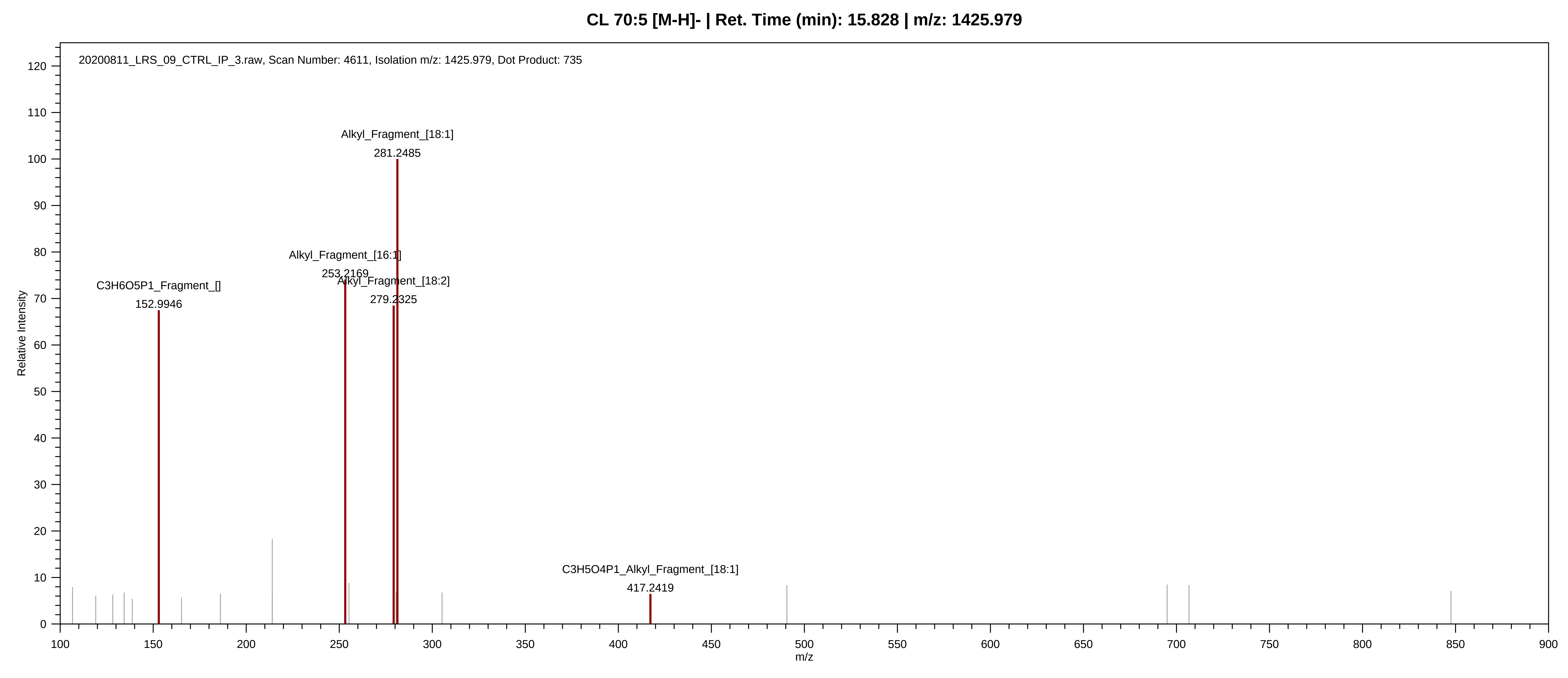

CL 70:5 [M-H]- | Ret. Time (min): 15.828 | m/z: 1425.979
20200811_LRS_09_CTRL_IP_3.raw, Scan Number: 4611, Isolation m/z: 1425.979, Dot Product: 735
Alkyl_Fragment_[18:1]
281.2485
Alkyl_Fragment_[16:1]
253.2169
Alkyl_Fragment_[18:2]
279.2325
C3H6O5P1_Fragment_[]
152.9946
C3H5O4P1_Alkyl_Fragment_[18:1]
417.2419
Relative Intensity
m/z

CL 70:6 [M-H]- | Ret. Time (min): 15.233 | m/z: 1423.964

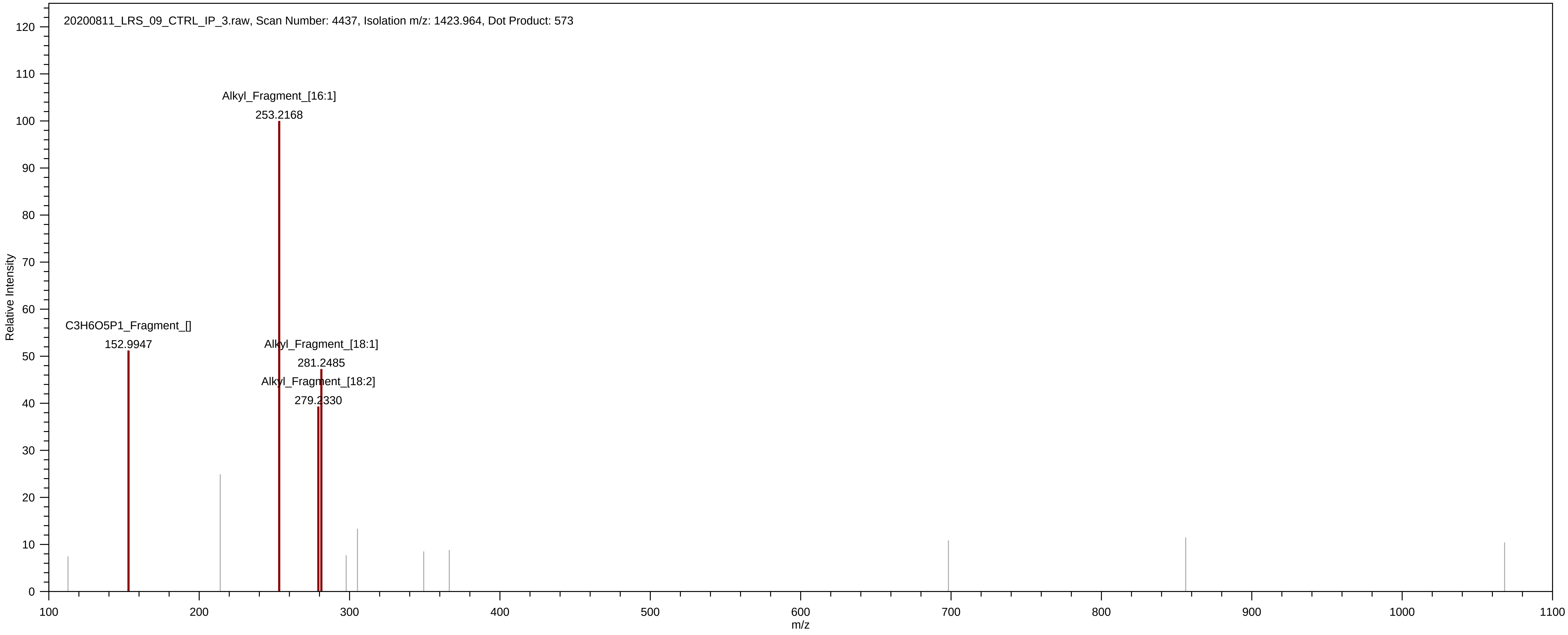

CL 72:6 [M-H]- | Ret. Time (min): 15.767 | m/z: 1451.992

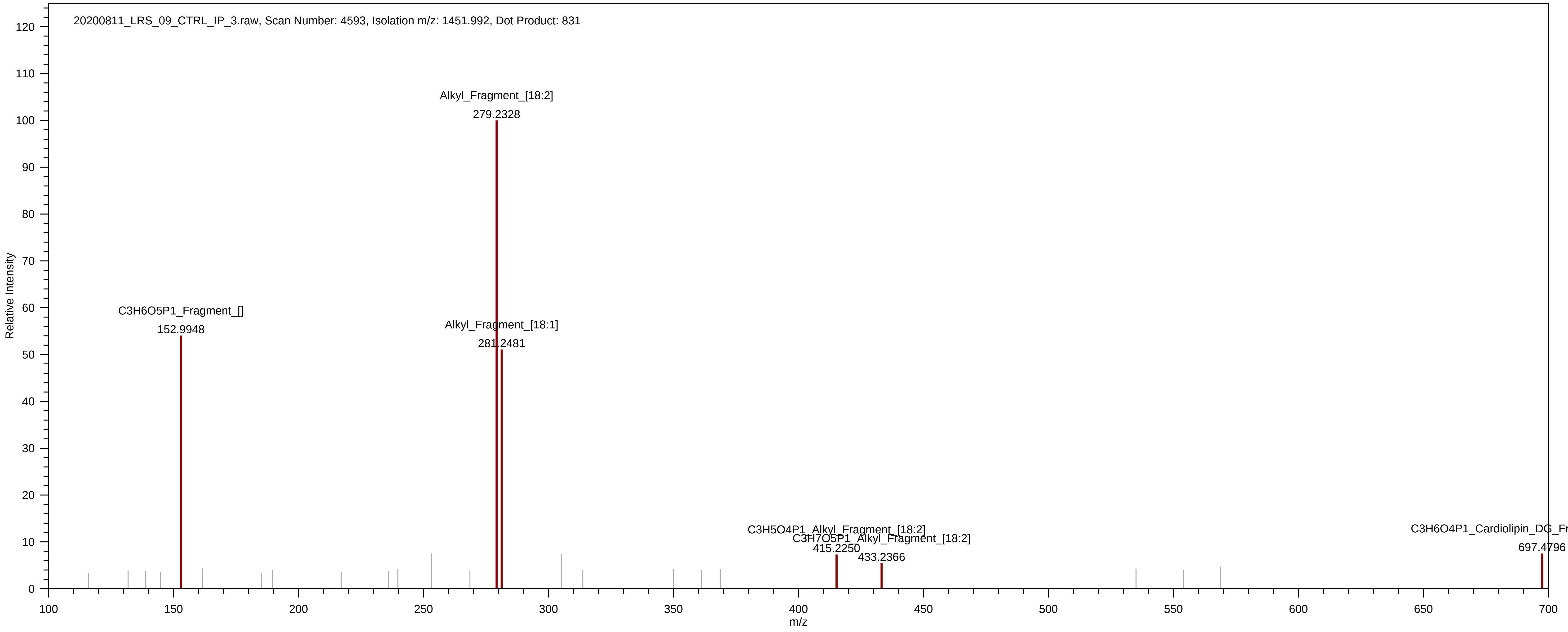

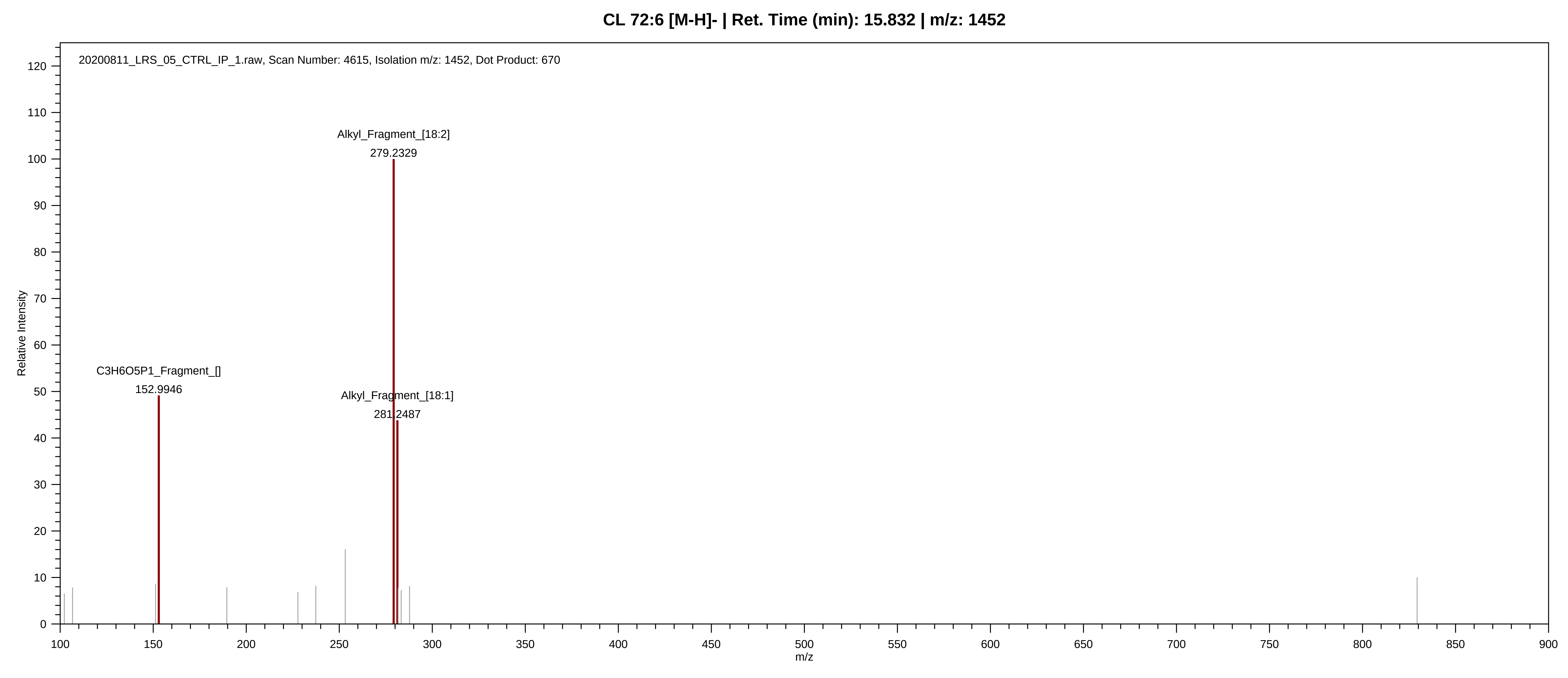
CL 72:6 [M-H]- | Ret. Time (min): 15.832 | m/z: 1452
20200811_LRS_05_CTRL_IP_1.raw, Scan Number: 4615, Isolation m/z: 1452, Dot Product: 670
Alkyl_Fragment_[18:2]
279.2329
C3H6O5P1_Fragment_[]
152.9946
Alkyl_Fragment_[18:1]
281.2487
Relative Intensity
m/z

HexCer[NS] d18:1_16:0 [M-H]- | Ret. Time (min): 9.072 | m/z: 698.558

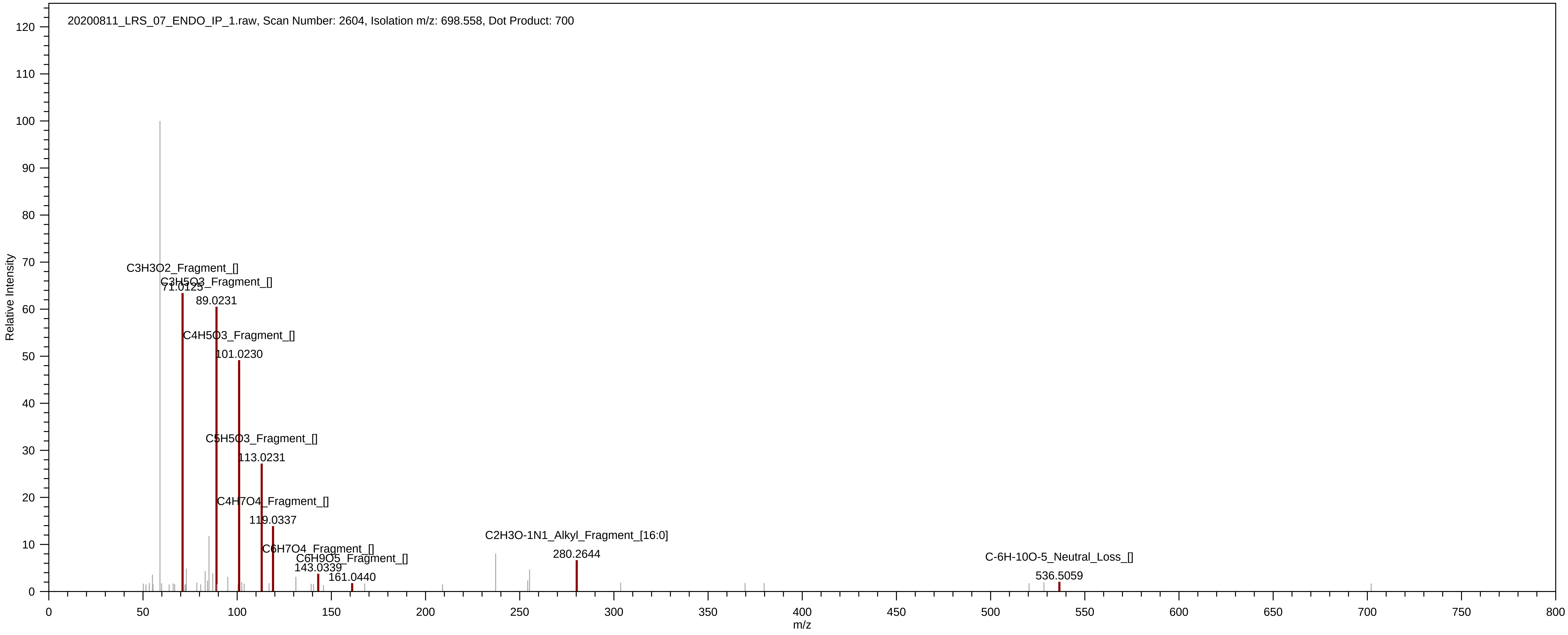

HexCer[NS] d18:1_16:0 [M-H]- | Ret. Time (min): 9.106 | m/z: 698.558

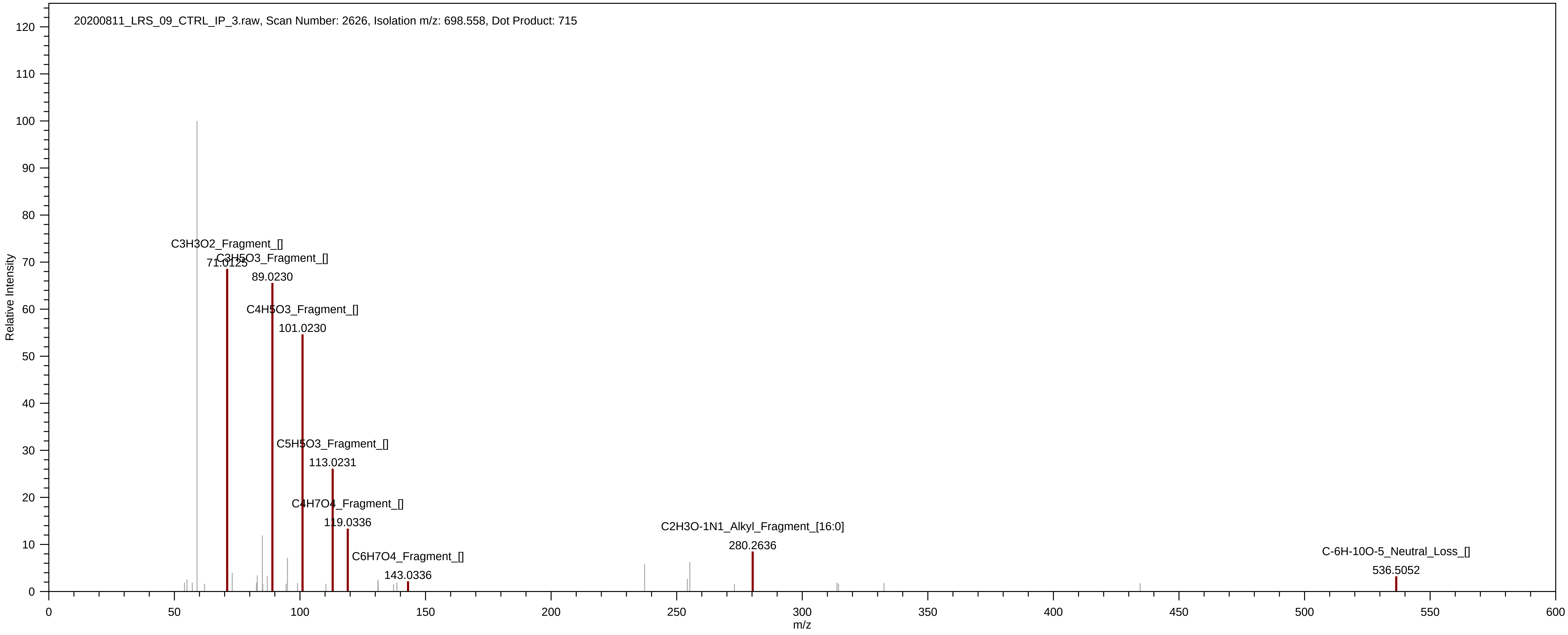

HexCer[NS] d18:1_16:0 [M-H]- | Ret. Time (min): 9.111 | m/z: 698.558

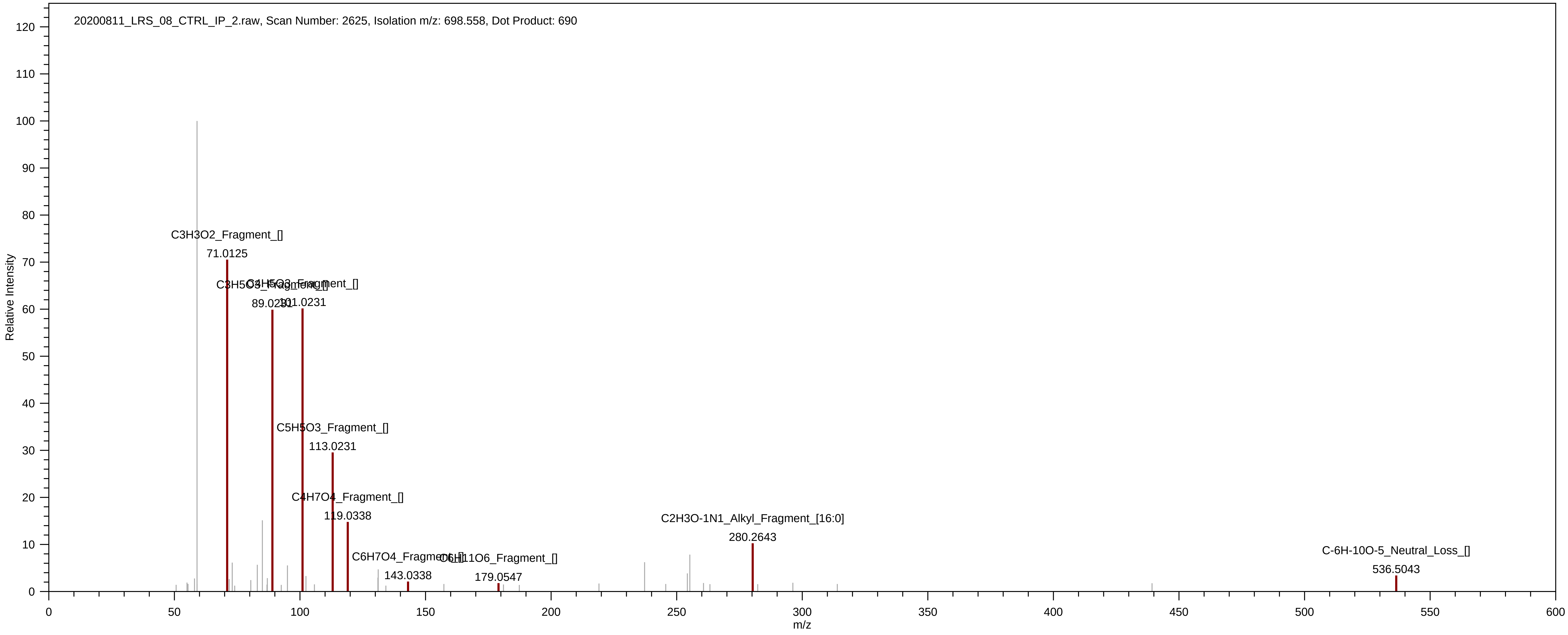

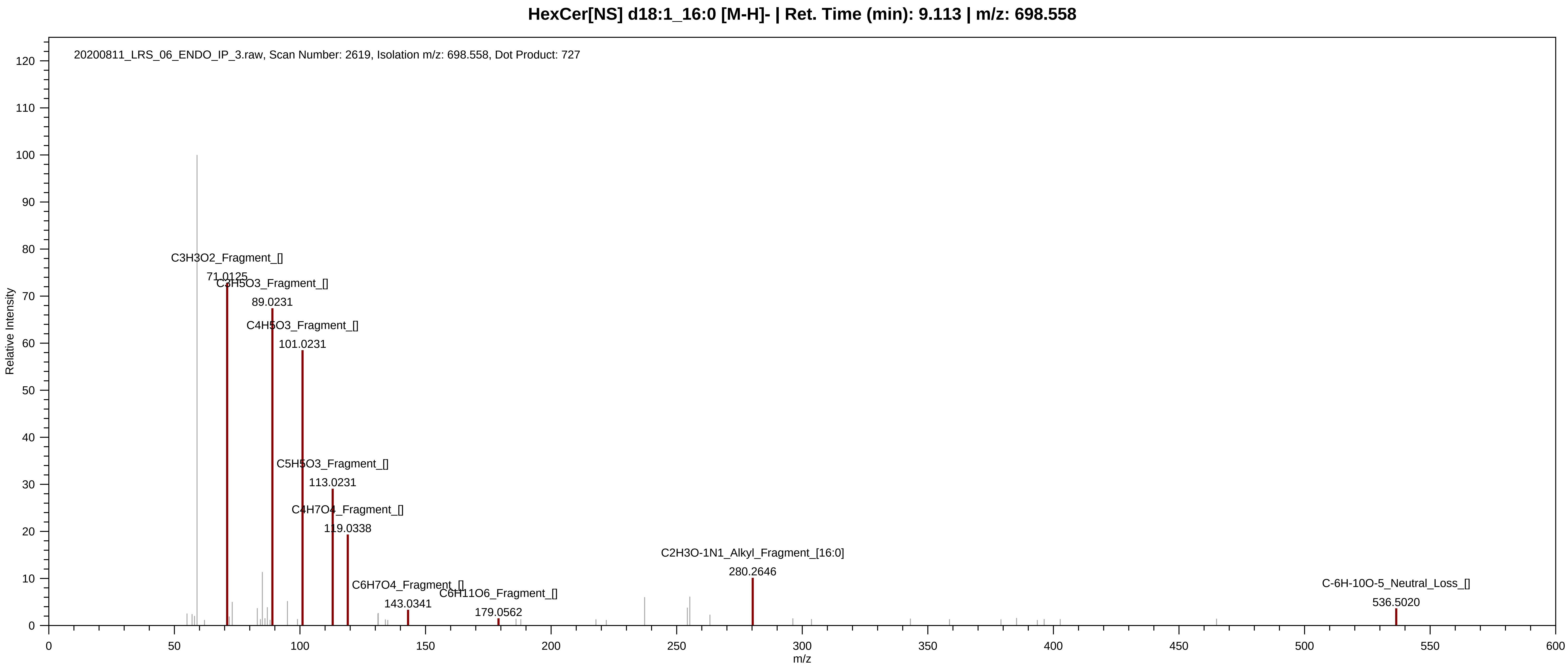
HexCer[NS] d18:1_16:0 [M-H]- | Ret. Time (min): 9.113 | m/z: 698.558
20200811_LRS_06_ENDO_IP_3.raw, Scan Number: 2619, Isolation m/z: 698.558, Dot Product: 727
Relative Intensity
m/z
C3H3O2_Fragment_[]
71.0125
C3H5O3_Fragment_[]
89.0231
C4H5O3_Fragment_[]
101.0231
C5H5O3_Fragment_[]
113.0231
C4H7O4_Fragment_[]
119.0338
C6H7O4_Fragment_[]
143.0341
C6H11O6_Fragment_[]
179.0562
C2H3O-1N1_Alkyl_Fragment_[16:0]
280.2646
C-6H-10O-5_Neutral_Loss_[]
536.5020

HexCer[NS] d34:1 [M+H]+ | Ret. Time (min): 9.072 | m/z: 700.572

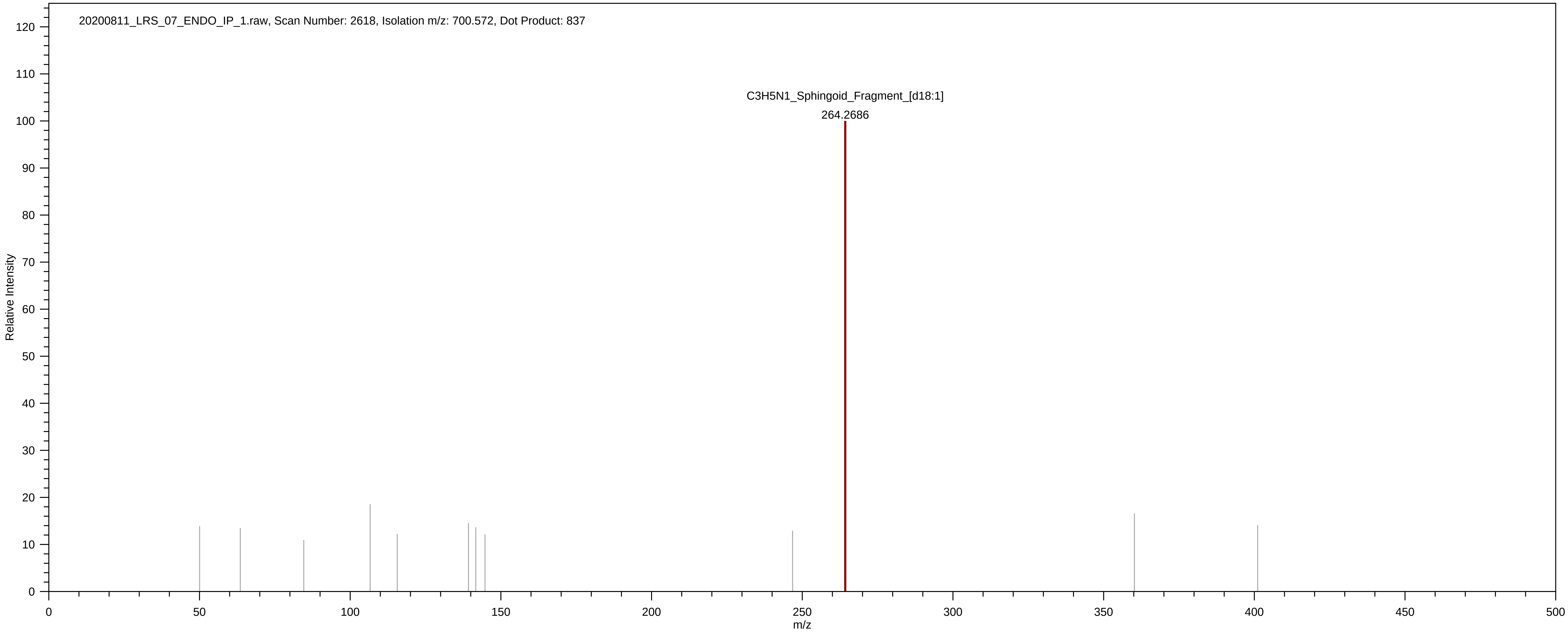

HexCer[NS] d34:1 [M+H]+ | Ret. Time (min): 9.106 | m/z: 700.572

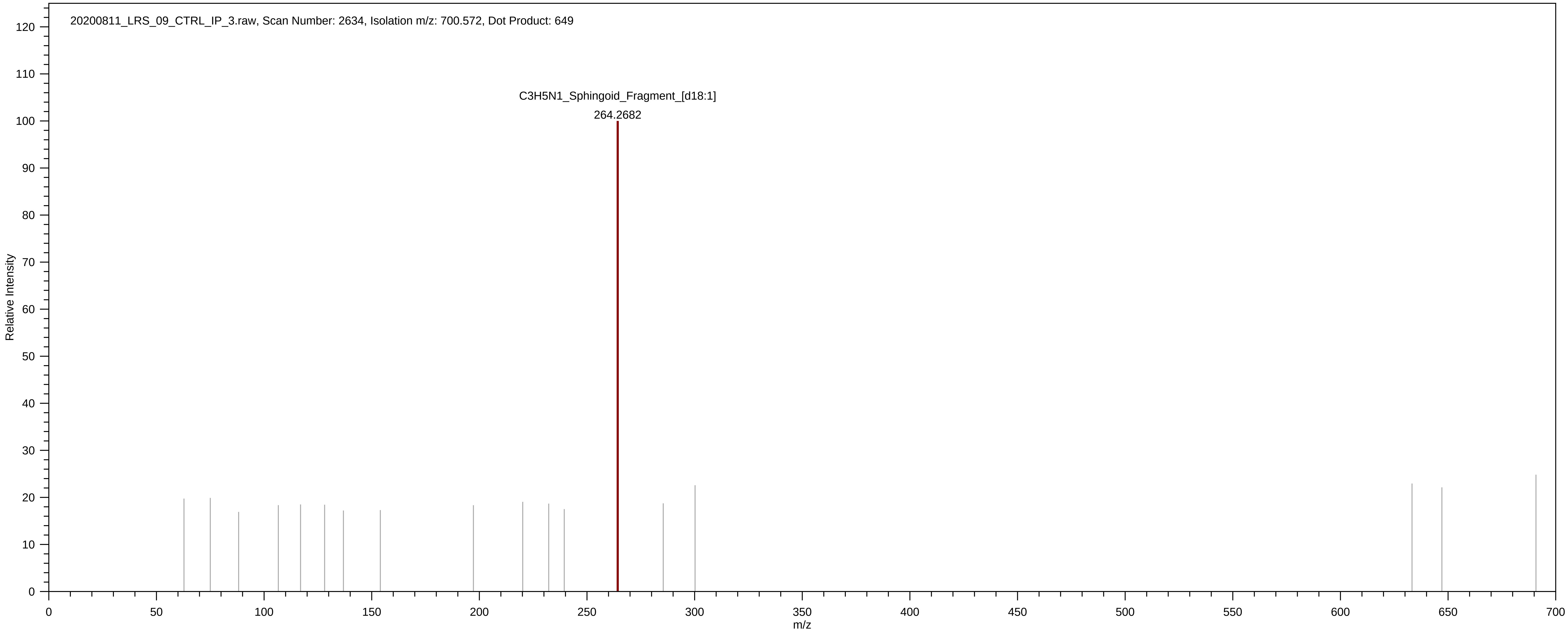

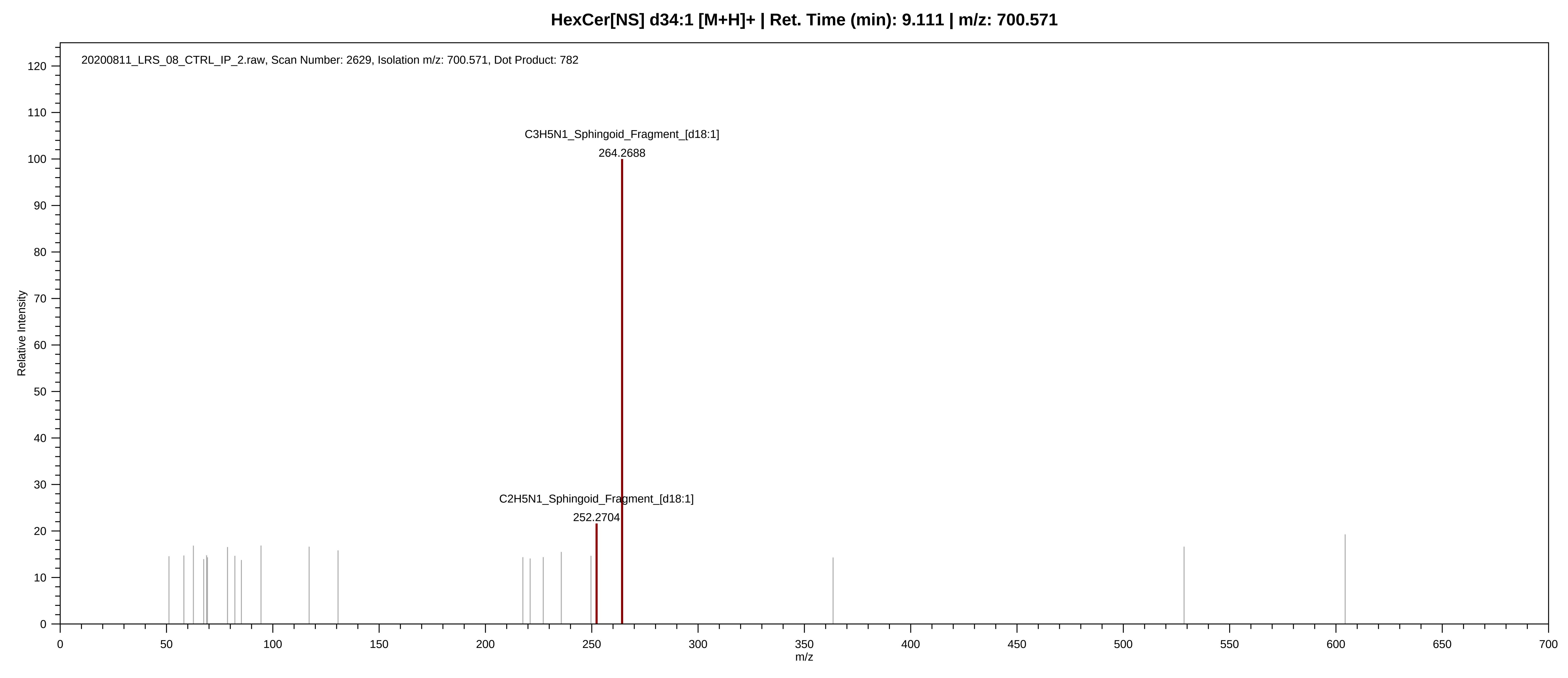
HexCer[NS] d34:1 [M+H]+ | Ret. Time (min): 9.111 | m/z: 700.571
20200811_LRS_08_CTRL_IP_2.raw, Scan Number: 2629, Isolation m/z: 700.571, Dot Product: 782
C3H5N1_Sphingoid_Fragment_[d18:1]
264.2688
C2H5N1_Sphingoid_Fragment_[d18:1]
252.2704
Relative Intensity
m/z

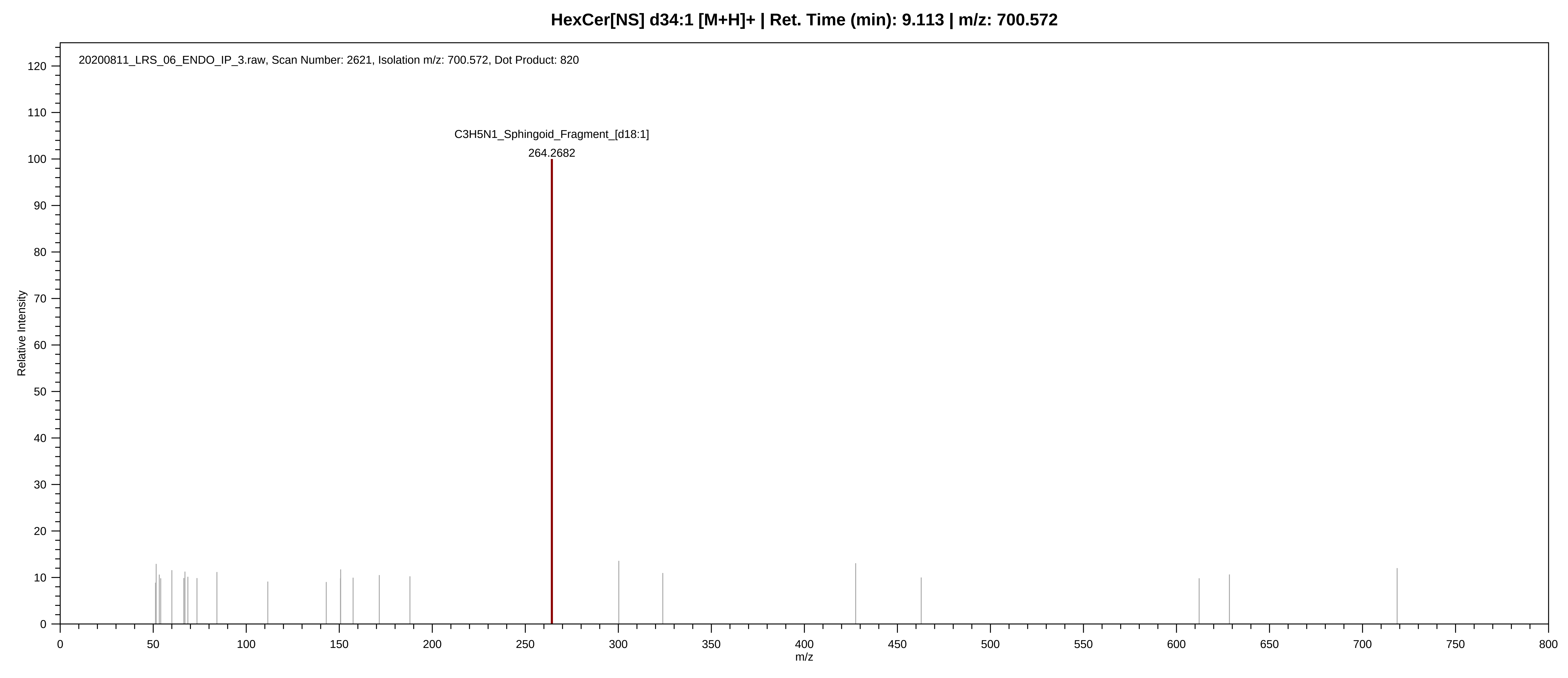
HexCer[NS] d34:1 [M+H]+ | Ret. Time (min): 9.113 | m/z: 700.572
20200811_LRS_06_ENDO_IP_3.raw, Scan Number: 2621, Isolation m/z: 700.572, Dot Product: 820
C3H5N1_Sphingoid_Fragment_[d18:1]
264.2682
Relative Intensity
m/z

HexCer[NS] d42:2 [M+H]+ | Ret. Time (min): 12.926 | m/z: 810.687

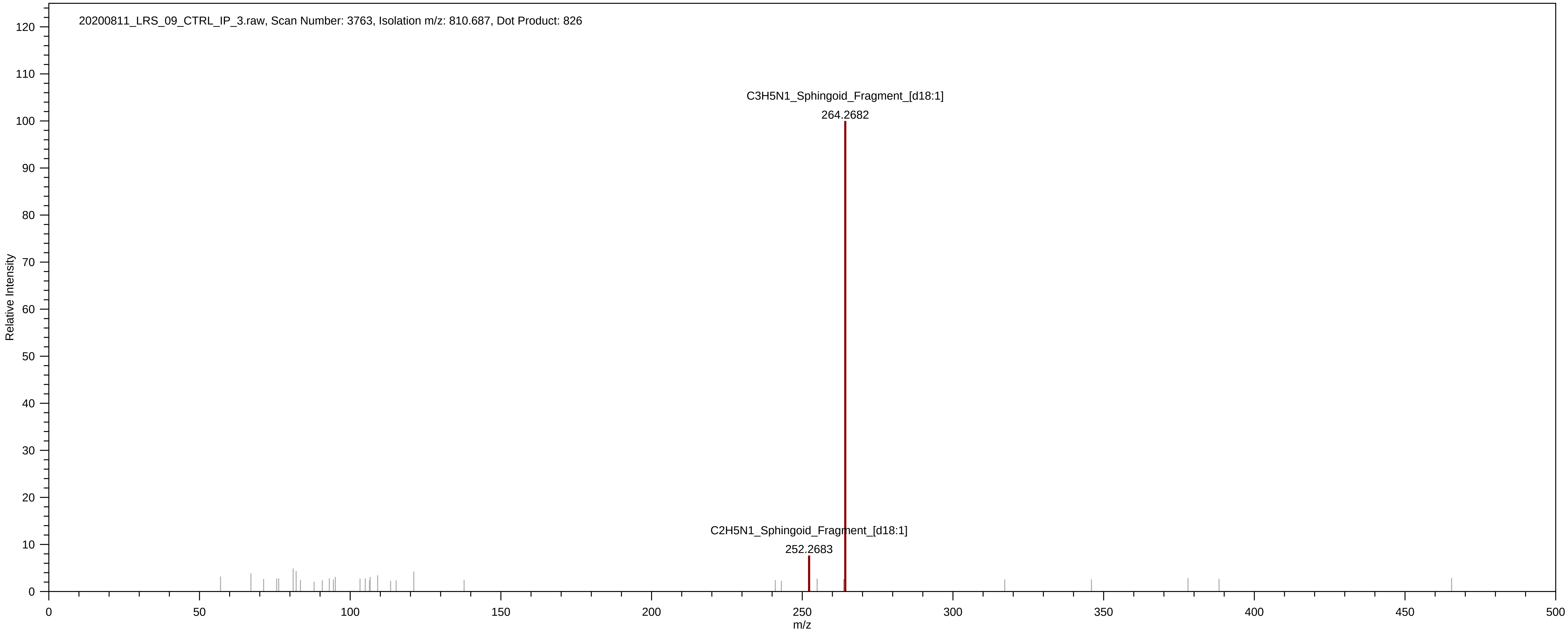

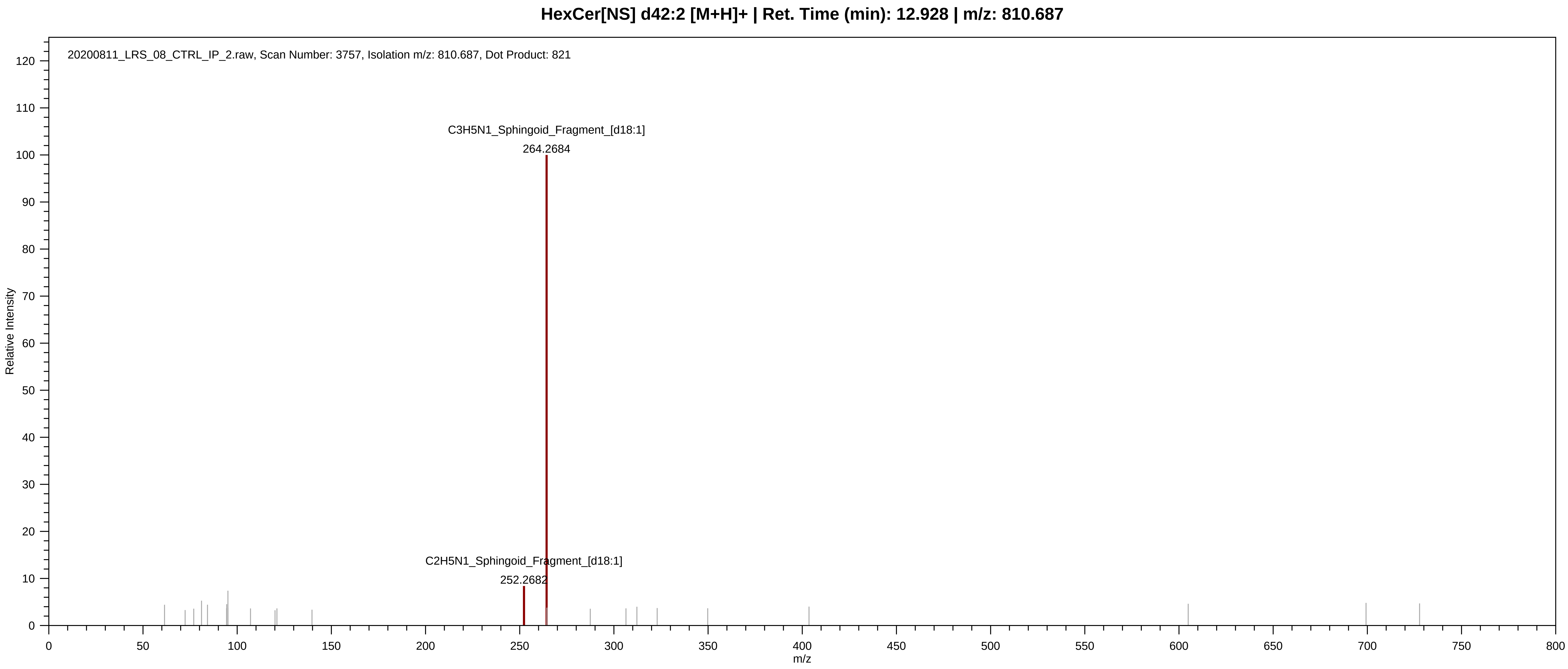
HexCer[NS] d42:2 [M+H]+ | Ret. Time (min): 12.928 | m/z: 810.687
20200811_LRS_08_CTRL_IP_2.raw, Scan Number: 3757, Isolation m/z: 810.687, Dot Product: 821
C3H5N1_Sphingoid_Fragment_[d18:1]
264.2684
C2H5N1_Sphingoid_Fragment_[d18:1]
252.2682
Relative Intensity
m/z

HexCer[NS] d42:2 [M+H]+ | Ret. Time (min): 12.943 | m/z: 810.687

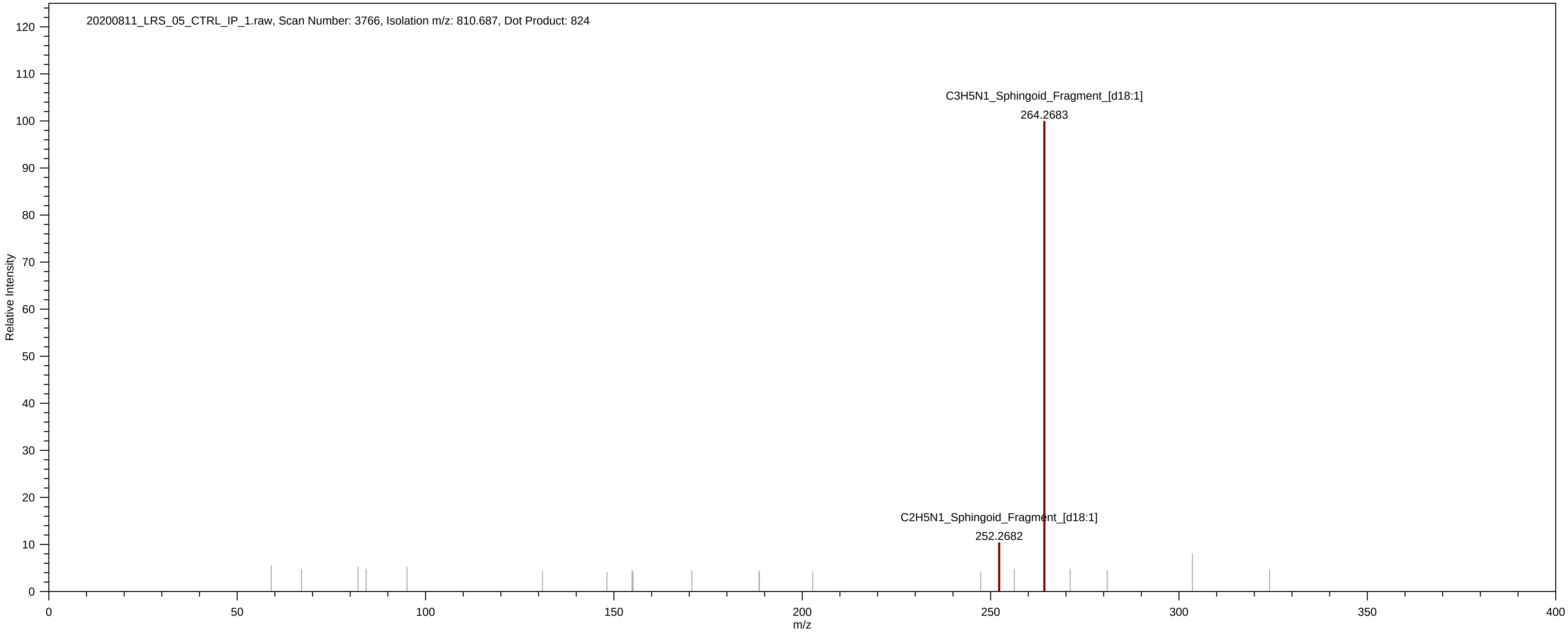

LysoPC 14:0 [M+H]+ | Ret. Time (min): 1.111 | m/z: 468.308

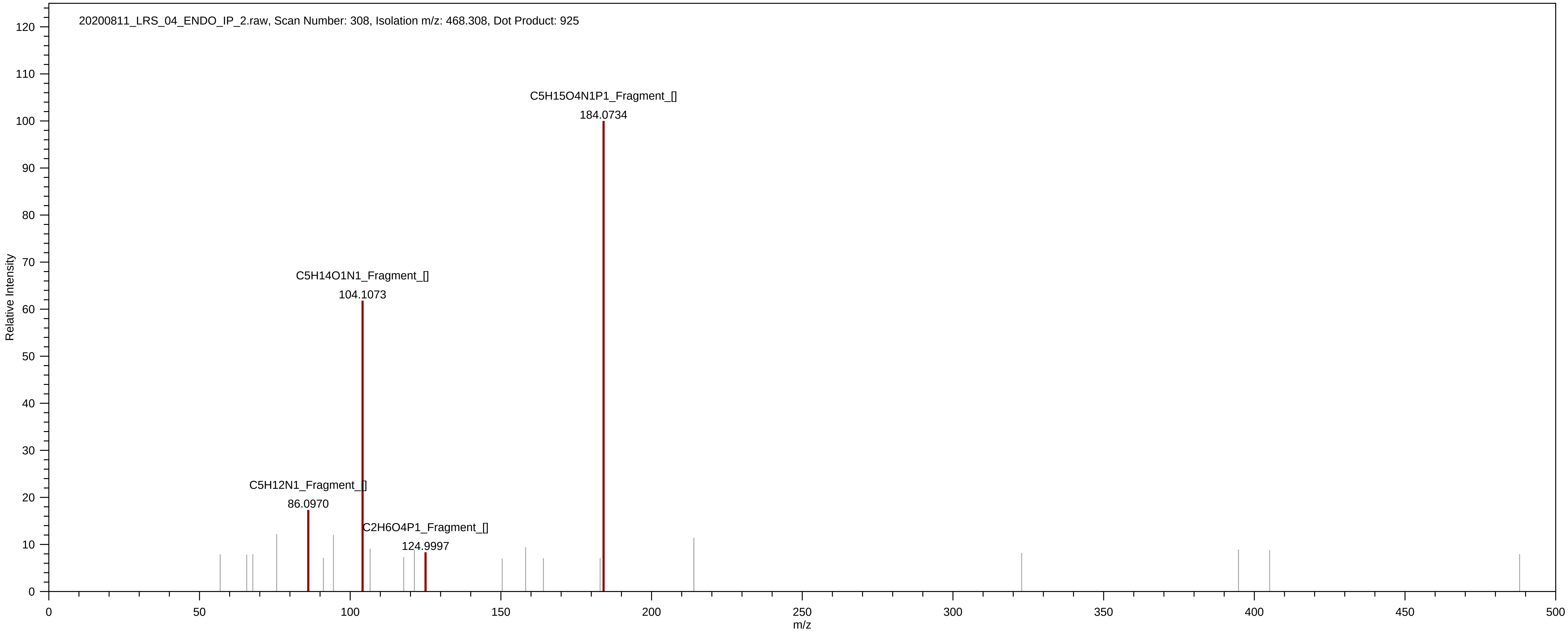

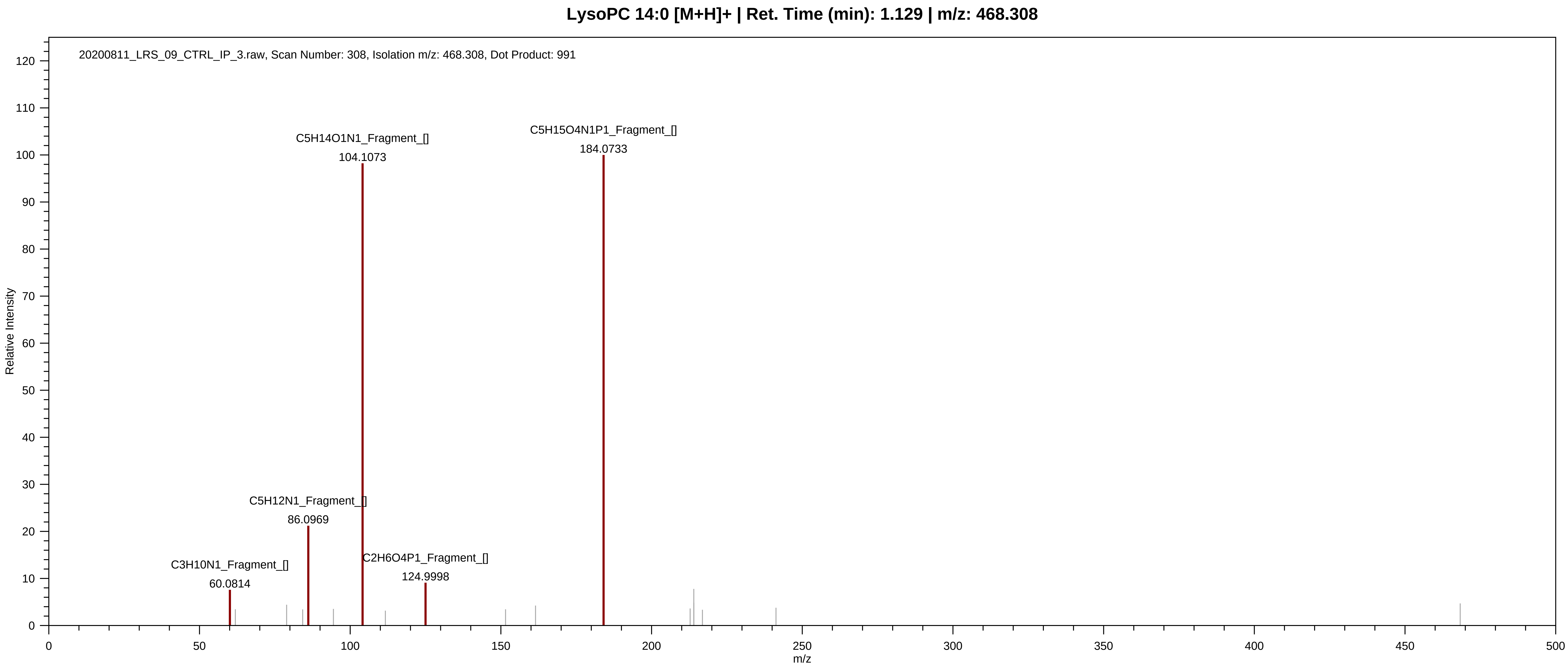
LysoPC 14:0 [M+H]+ | Ret. Time (min): 1.129 | m/z: 468.308
20200811_LRS_09_CTRL_IP_3.raw, Scan Number: 308, Isolation m/z: 468.308, Dot Product: 991
C5H15O4N1P1_Fragment_[]
184.0733
C5H14O1N1_Fragment_[]
104.1073
C5H12N1_Fragment_[]
86.0969
C2H6O4P1_Fragment_[]
124.9998
C3H10N1_Fragment_[]
60.0814
Relative Intensity
m/z

LysoPC 14:0 [M+H]+ | Ret. Time (min): 1.131 | m/z: 468.308

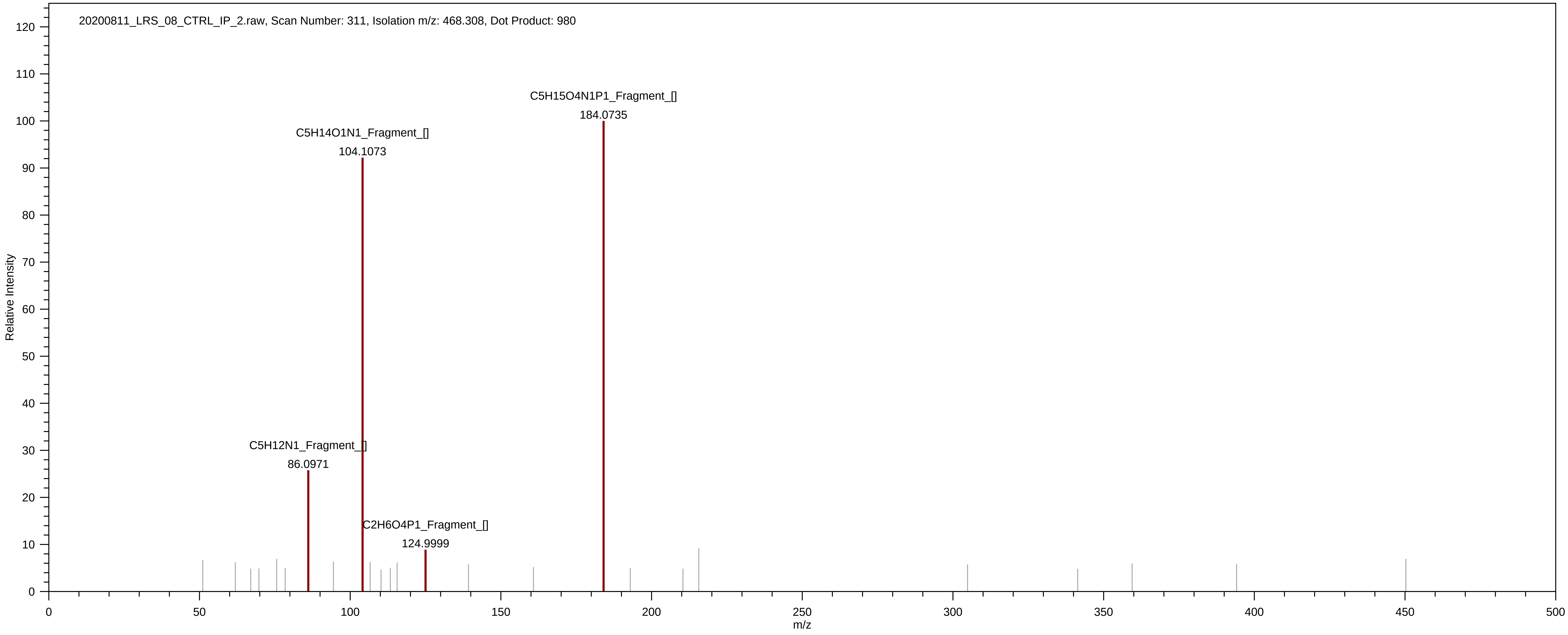

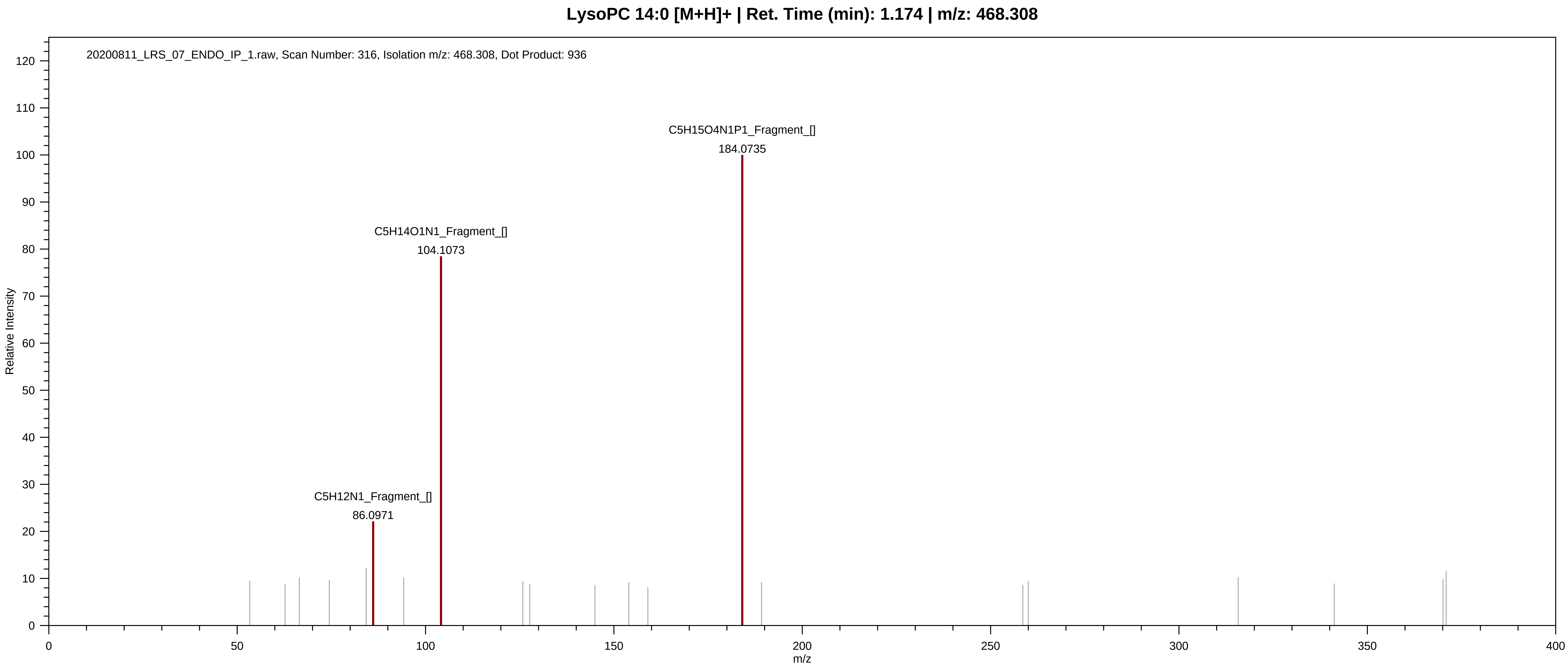

LysoPC 14:0 [M+H]+ | Ret. Time (min): 1.174 | m/z: 468.308
20200811_LRS_07_ENDO_IP_1.raw, Scan Number: 316, Isolation m/z: 468.308, Dot Product: 936
C5H15O4N1P1_Fragment_[]
184.0735
C5H14O1N1_Fragment_[]
104.1073
C5H12N1_Fragment_[]
86.0971
Relative Intensity
m/z

LysoPC 14:0 [M+H]+ | Ret. Time (min): 1.175 | m/z: 468.308

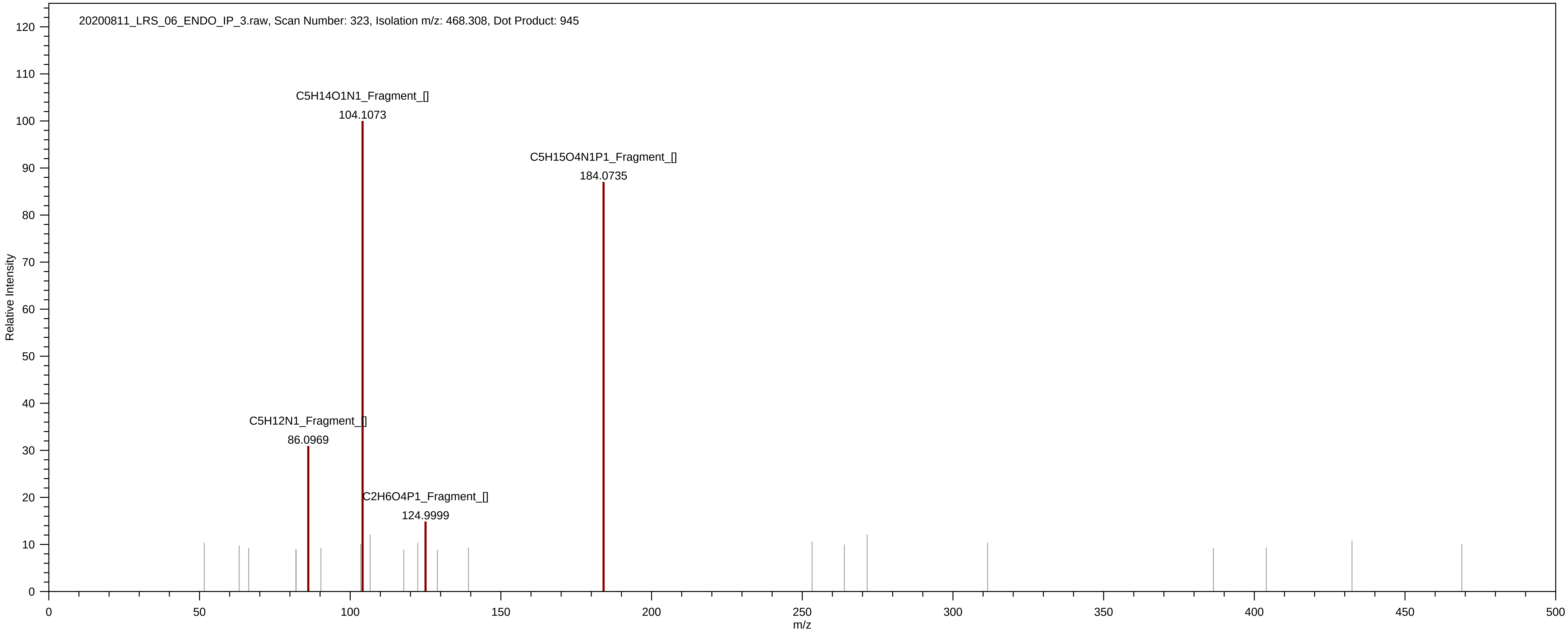

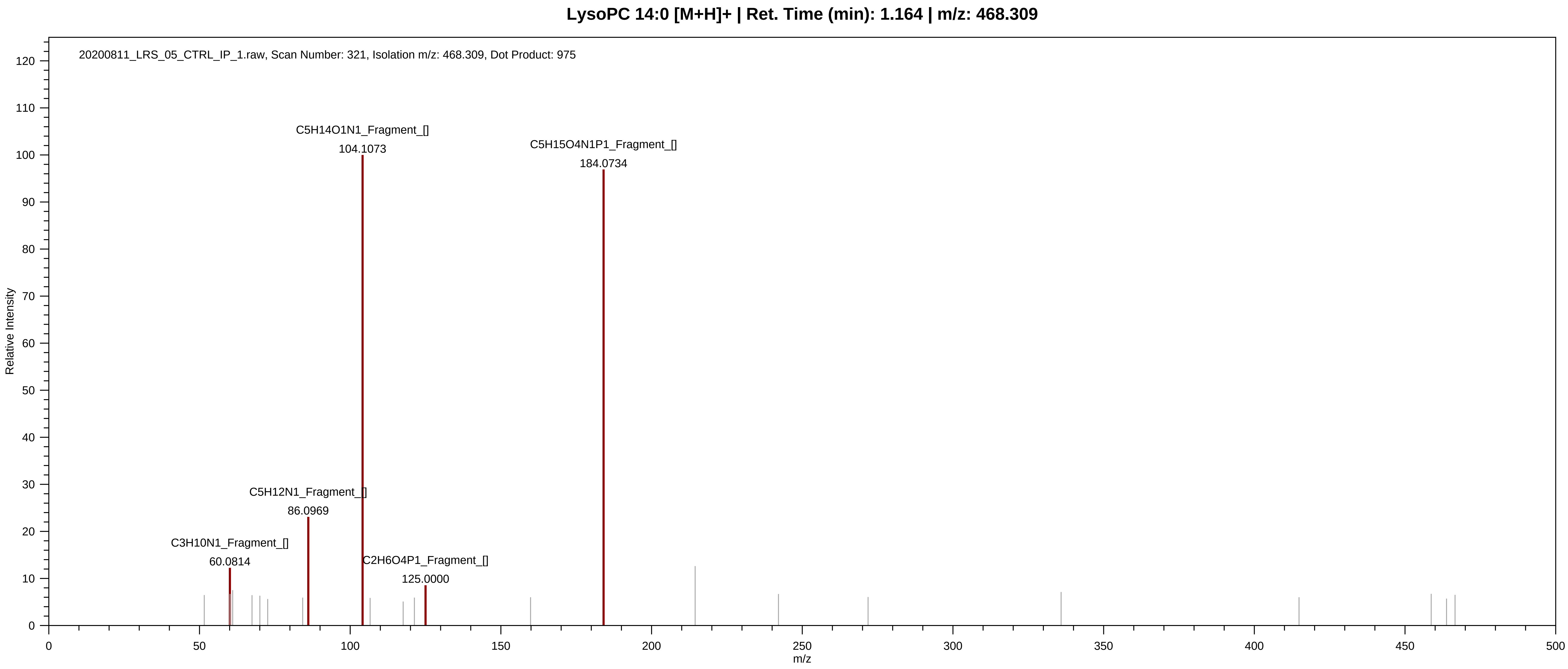
LysoPC 14:0 [M+H]+ | Ret. Time (min): 1.164 | m/z: 468.309
20200811_LRS_05_CTRL_IP_1.raw, Scan Number: 321, Isolation m/z: 468.309, Dot Product: 975
C5H14O1N1_Fragment_[]
104.1073
C5H15O4N1P1_Fragment_[]
184.0734
C5H12N1_Fragment_[]
86.0969
C3H10N1_Fragment_[]
60.0814
C2H6O4P1_Fragment_[]
125.0000
Relative Intensity
m/z

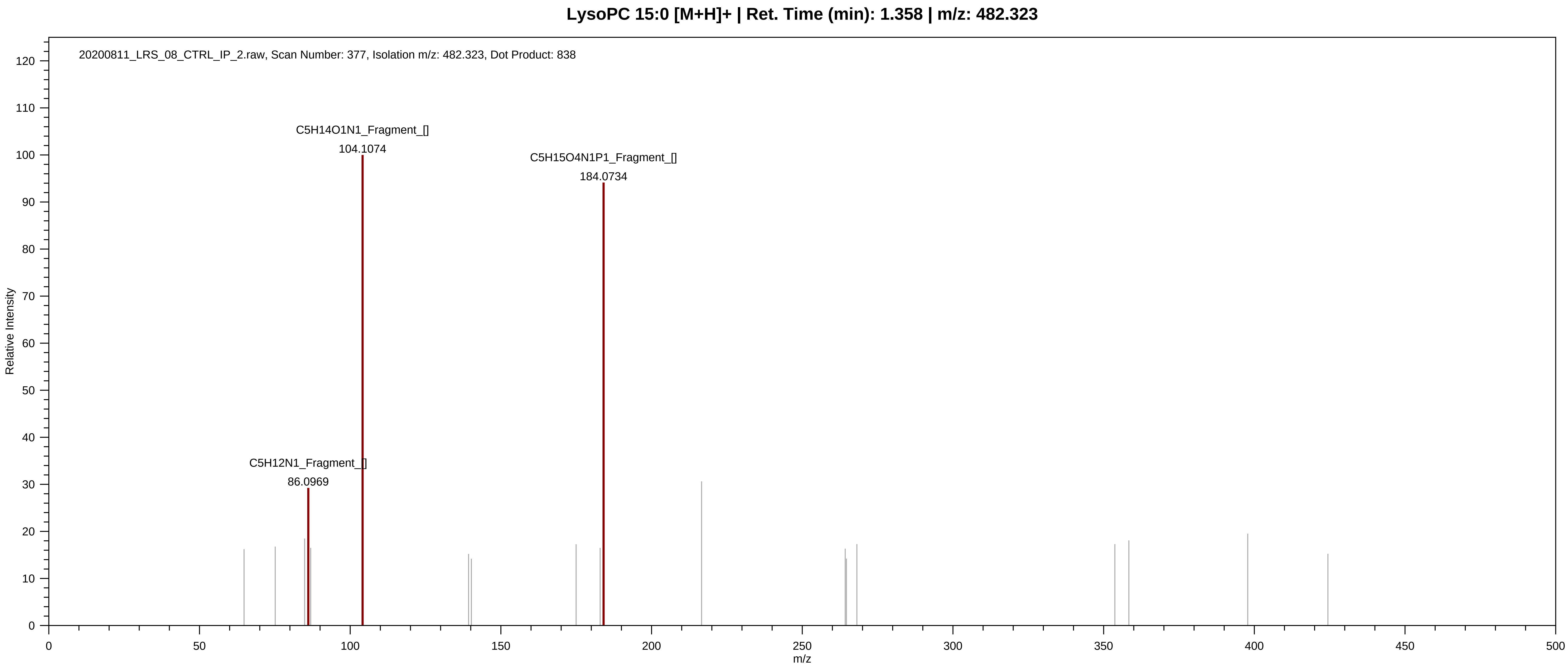

LysoPC 15:0 [M+H]+ | Ret. Time (min): 1.358 | m/z: 482.323
20200811_LRS_08_CTRL_IP_2.raw, Scan Number: 377, Isolation m/z: 482.323, Dot Product: 838
C5H14O1N1_Fragment_[]
104.1074
C5H15O4N1P1_Fragment_[]
184.0734
C5H12N1_Fragment_[]
86.0969
Relative Intensity
m/z

LysoPC 16:0 [M+Ac-H]- | Ret. Time (min): 1.74 | m/z: 554.346

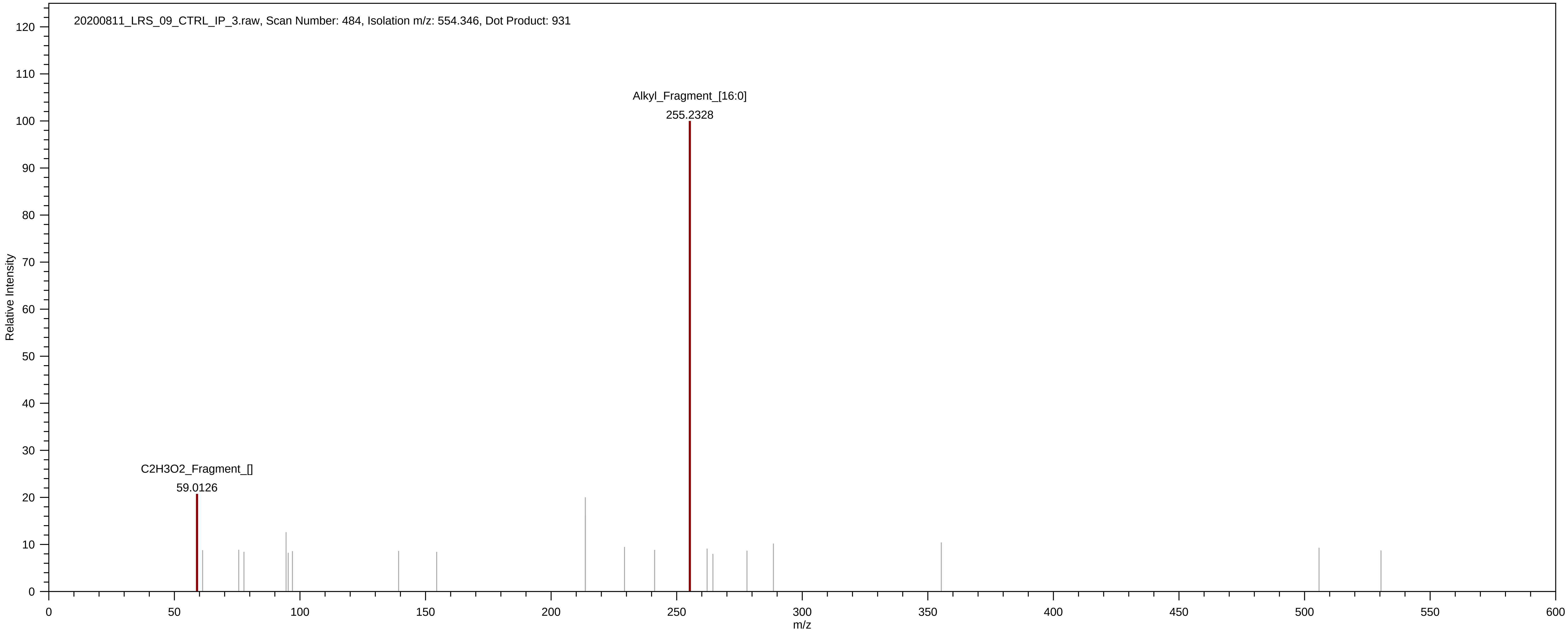

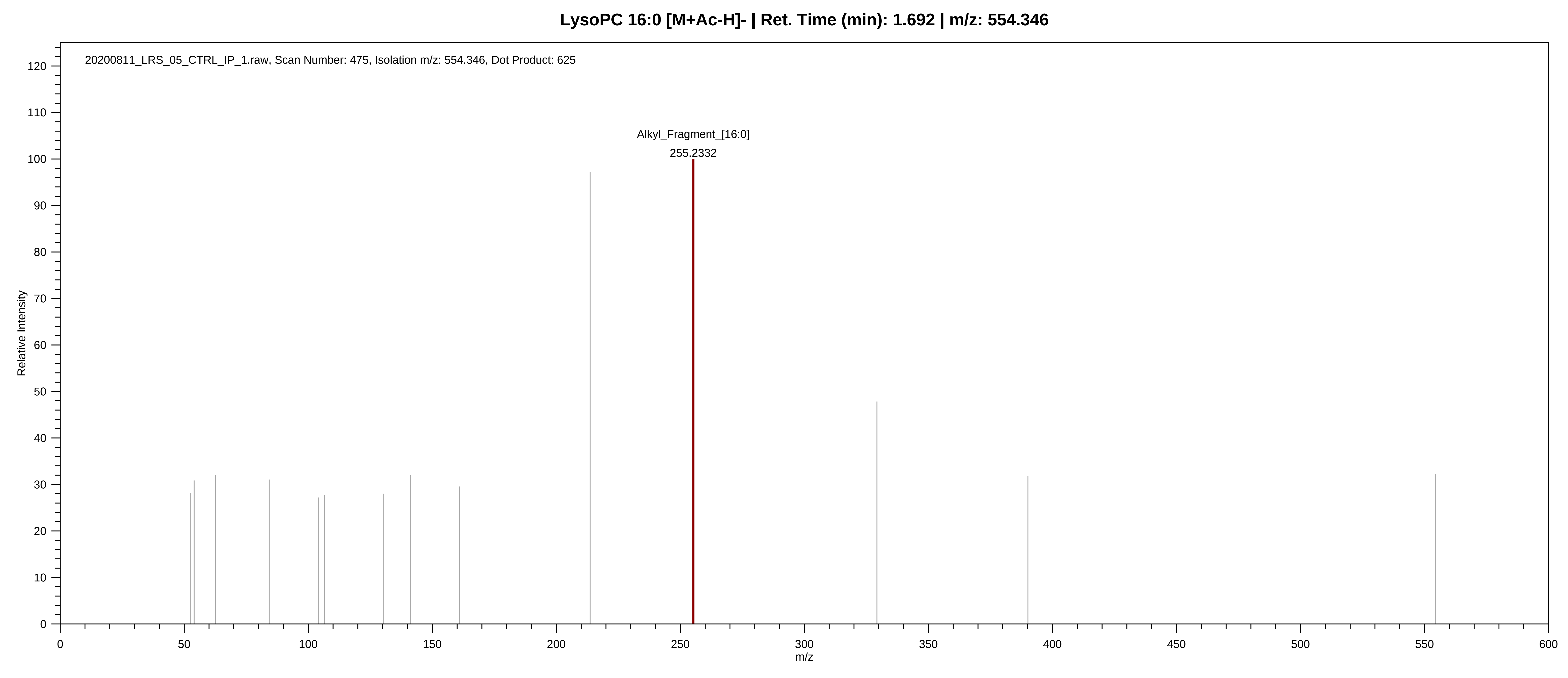
LysoPC 16:0 [M+Ac-H]- | Ret. Time (min): 1.692 | m/z: 554.346
20200811_LRS_05_CTRL_IP_1.raw, Scan Number: 475, Isolation m/z: 554.346, Dot Product: 625
Alkyl_Fragment_[16:0]
255.2332
Relative Intensity
m/z

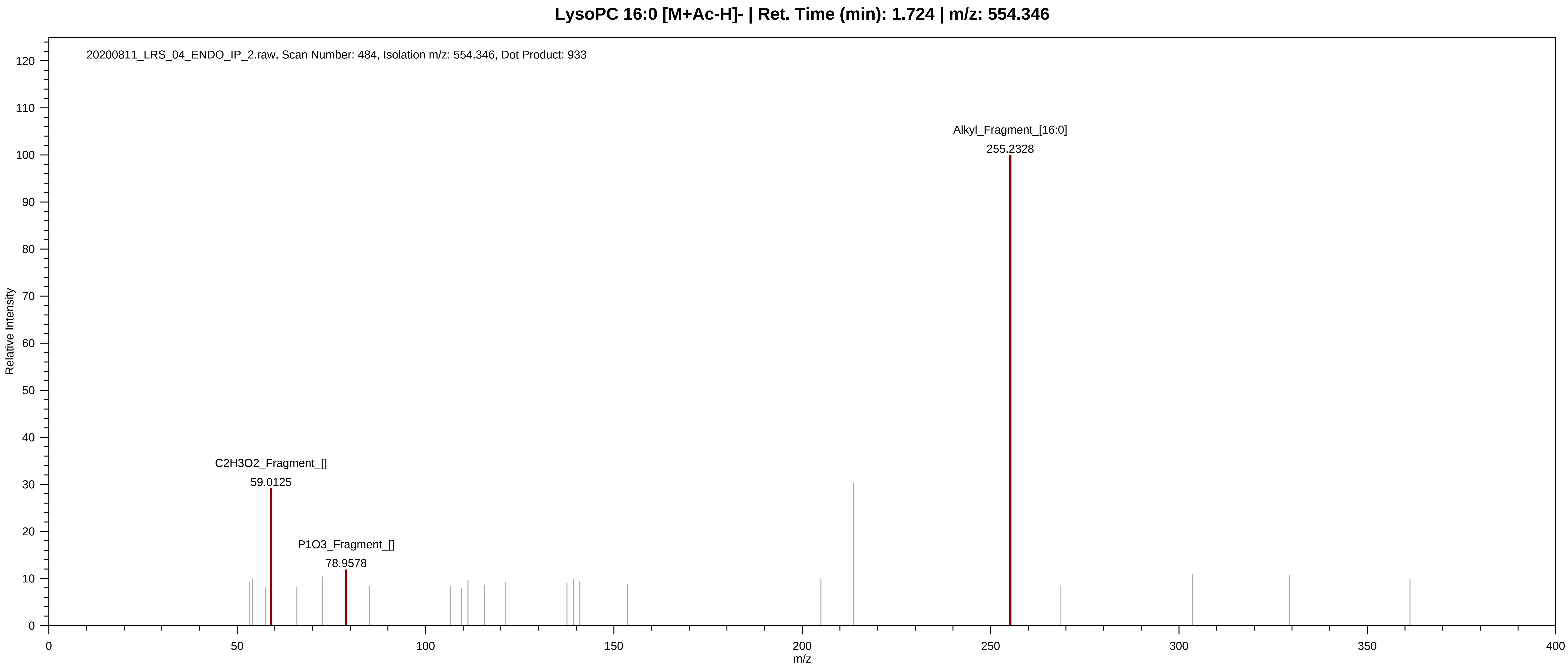
LysoPC 16:0 [M+Ac-H]- | Ret. Time (min): 1.724 | m/z: 554.346
20200811_LRS_04_ENDO_IP_2.raw, Scan Number: 484, Isolation m/z: 554.346, Dot Product: 933
Alkyl_Fragment_[16:0]
255.2328
C2H3O2_Fragment_[]
59.0125
P1O3_Fragment_[]
78.9578
Relative Intensity
m/z

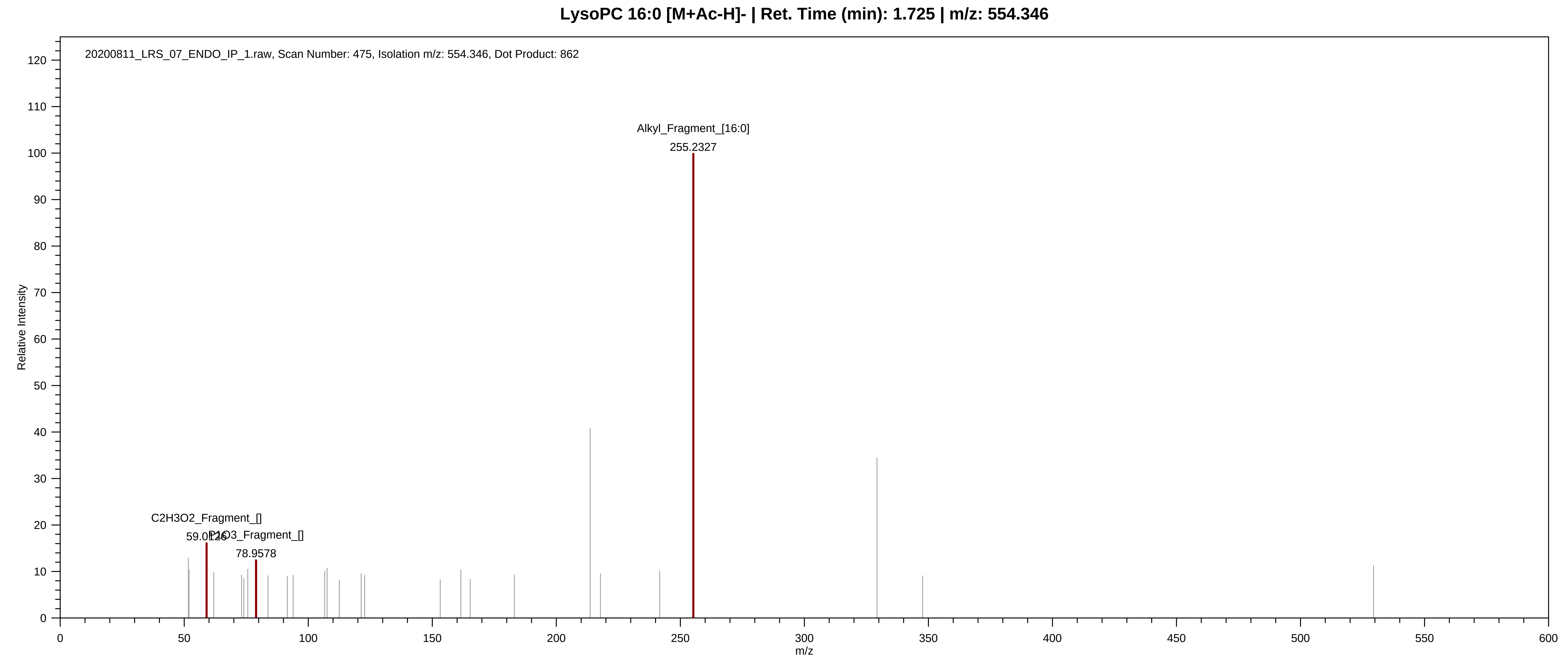
LysoPC 16:0 [M+Ac-H]- | Ret. Time (min): 1.725 | m/z: 554.346
20200811_LRS_07_ENDO_IP_1.raw, Scan Number: 475, Isolation m/z: 554.346, Dot Product: 862
Alkyl_Fragment_[16:0]
255.2327
C2H3O2_Fragment_[]
59.0126
P1O3_Fragment_[]
78.9578
Relative Intensity
m/z

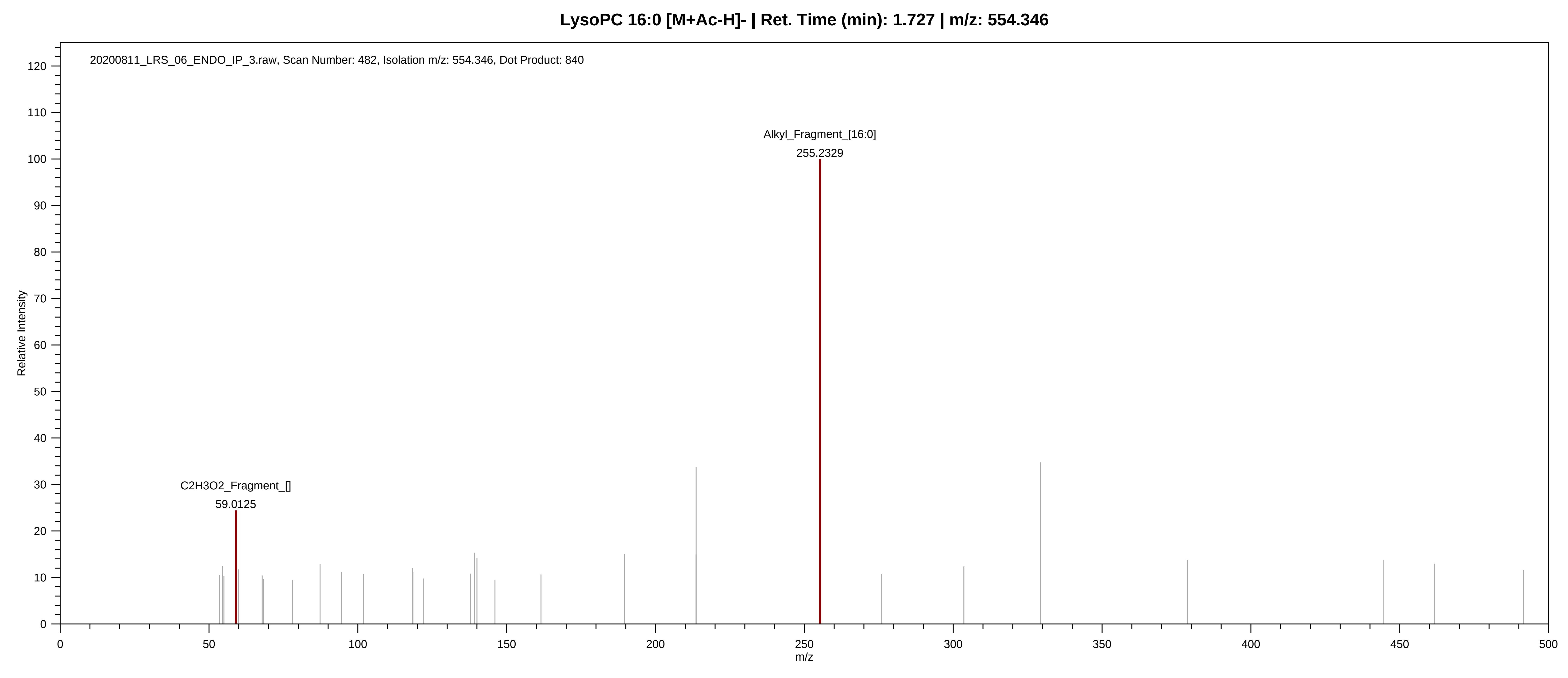
LysoPC 16:0 [M+Ac-H]- | Ret. Time (min): 1.727 | m/z: 554.346
20200811_LRS_06_ENDO_IP_3.raw, Scan Number: 482, Isolation m/z: 554.346, Dot Product: 840
Alkyl_Fragment_[16:0]
255.2329
C2H3O2_Fragment_[]
59.0125
Relative Intensity
m/z

LysoPC 16:0 [M+Ac-H]- | Ret. Time (min): 1.763 | m/z: 554.346

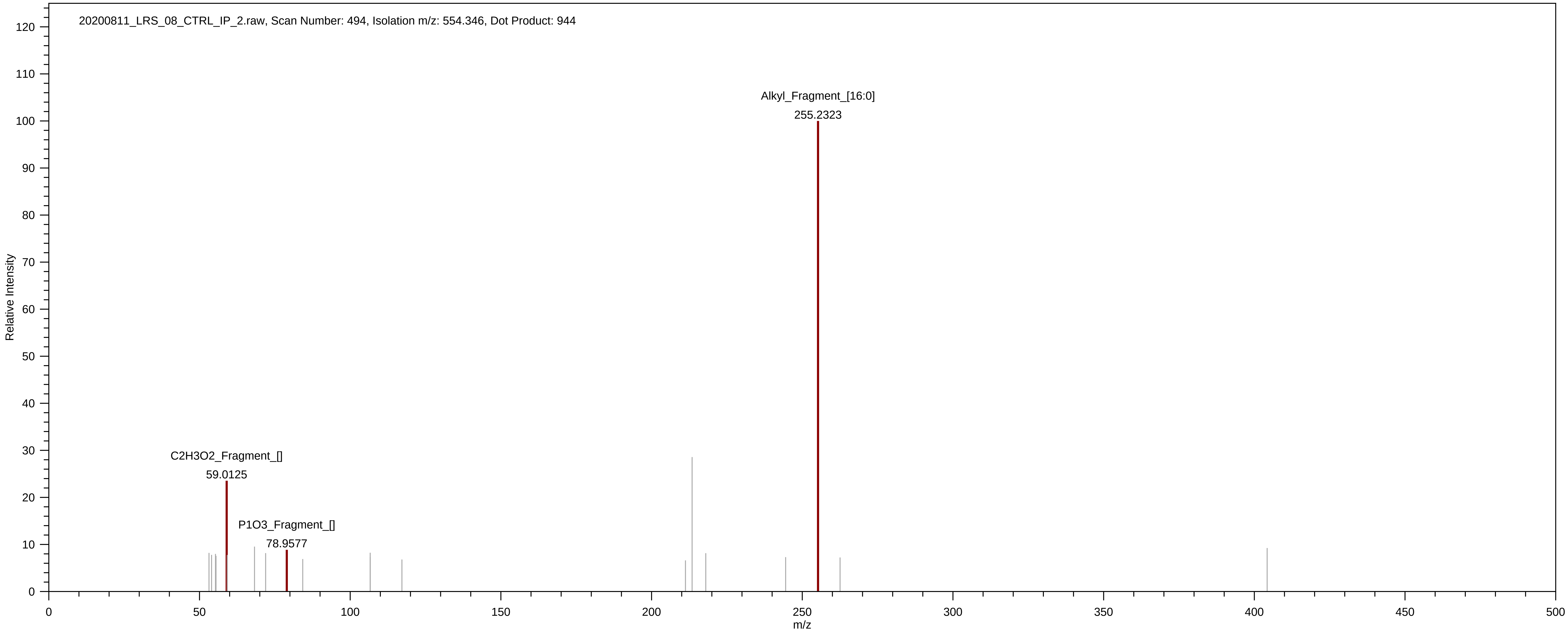

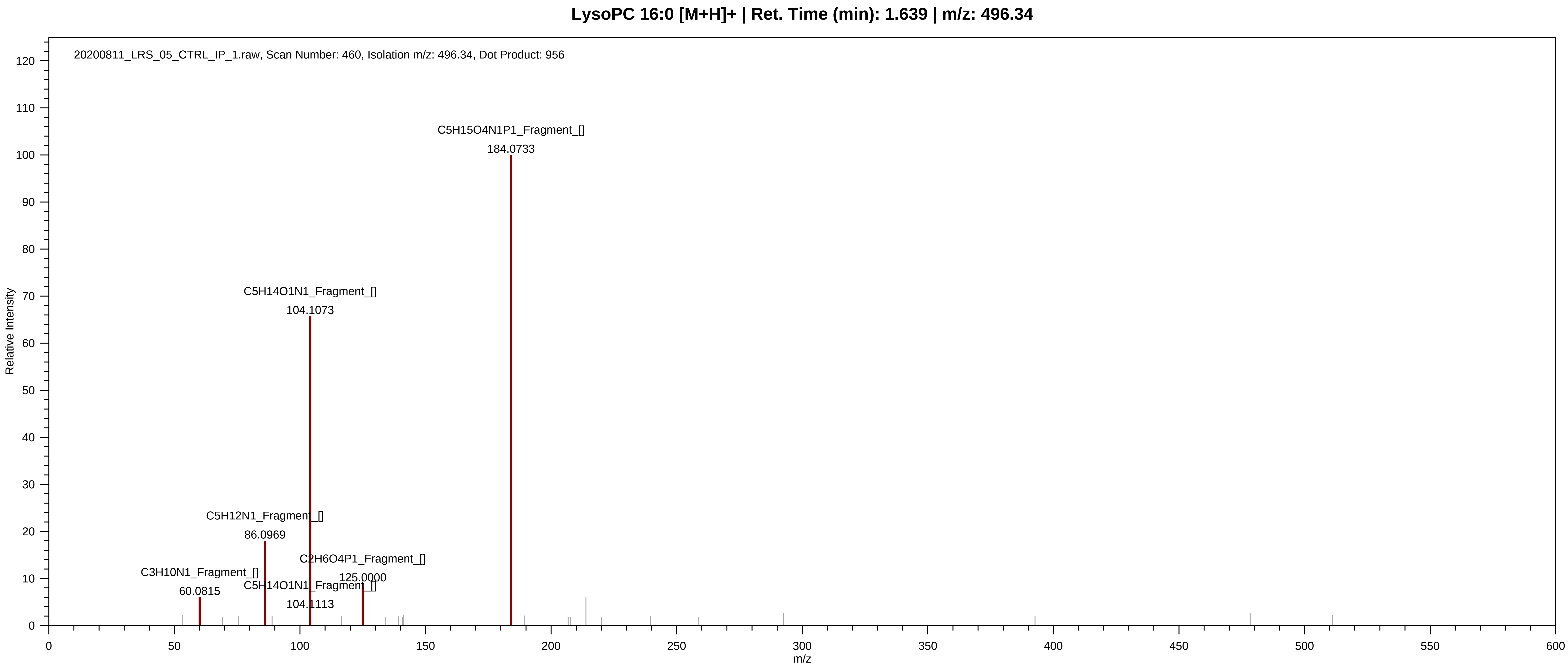
LysoPC 16:0 [M+H]+ | Ret. Time (min): 1.639 | m/z: 496.34
20200811_LRS_05_CTRL_IP_1.raw, Scan Number: 460, Isolation m/z: 496.34, Dot Product: 956
C5H15O4N1P1_Fragment_[]
184.0733
C5H14O1N1_Fragment_[]
104.1073
C5H12N1_Fragment_[]
86.0969
C2H6O4P1_Fragment_[]
125.0000
C3H10N1_Fragment_[]
60.0815
C5H14O1N1_Fragment_[]
104.1113
Relative Intensity
m/z

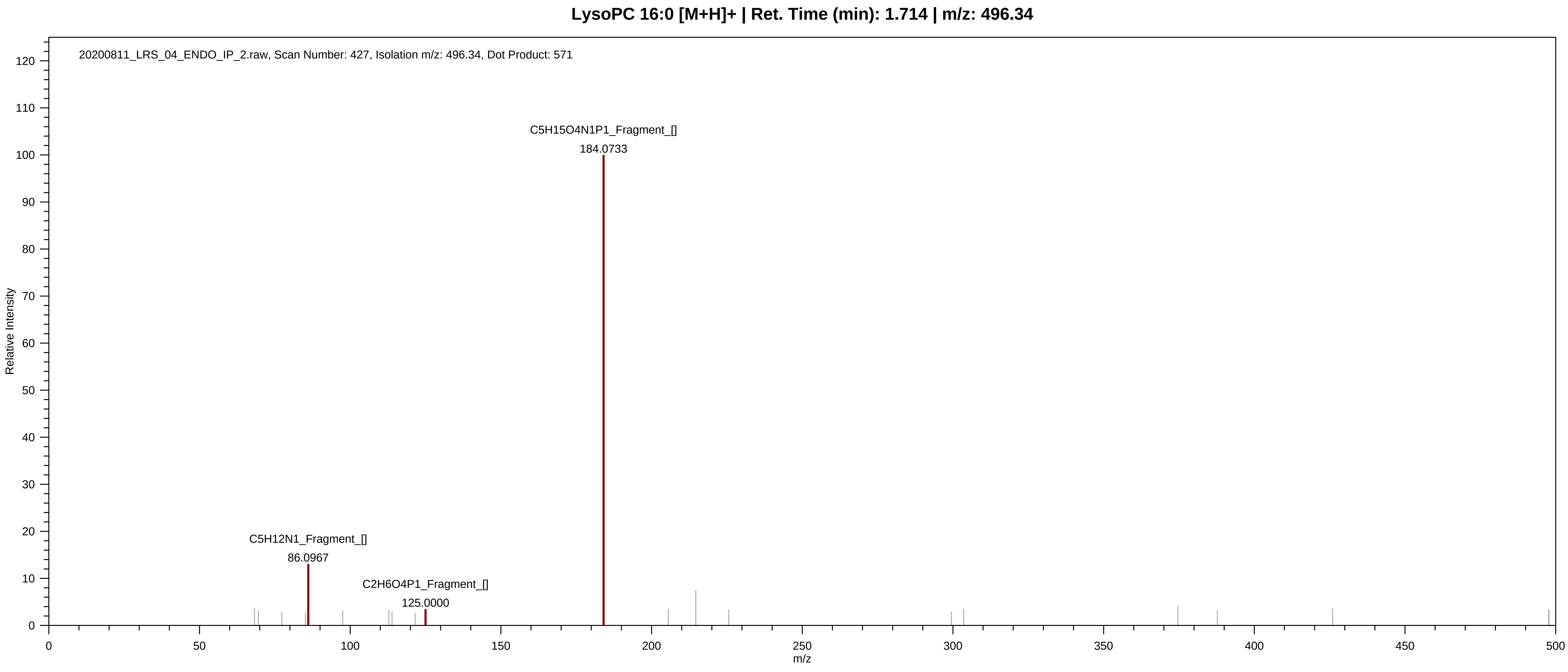
LysoPC 16:0 [M+H]+ | Ret. Time (min): 1.714 | m/z: 496.34
20200811_LRS_04_ENDO_IP_2.raw, Scan Number: 427, Isolation m/z: 496.34, Dot Product: 571
C5H15O4N1P1_Fragment_[]
184.0733
C5H12N1_Fragment_[]
86.0967
C2H6O4P1_Fragment_[]
125.0000
Relative Intensity
m/z
0
10
20
30
40
50
60
70
80
90
100
110
120
0
50
100
150
200
250
300
350
400
450
500

LysoPC 16:0 [M+H]+ | Ret. Time (min): 1.714 | m/z: 496.34

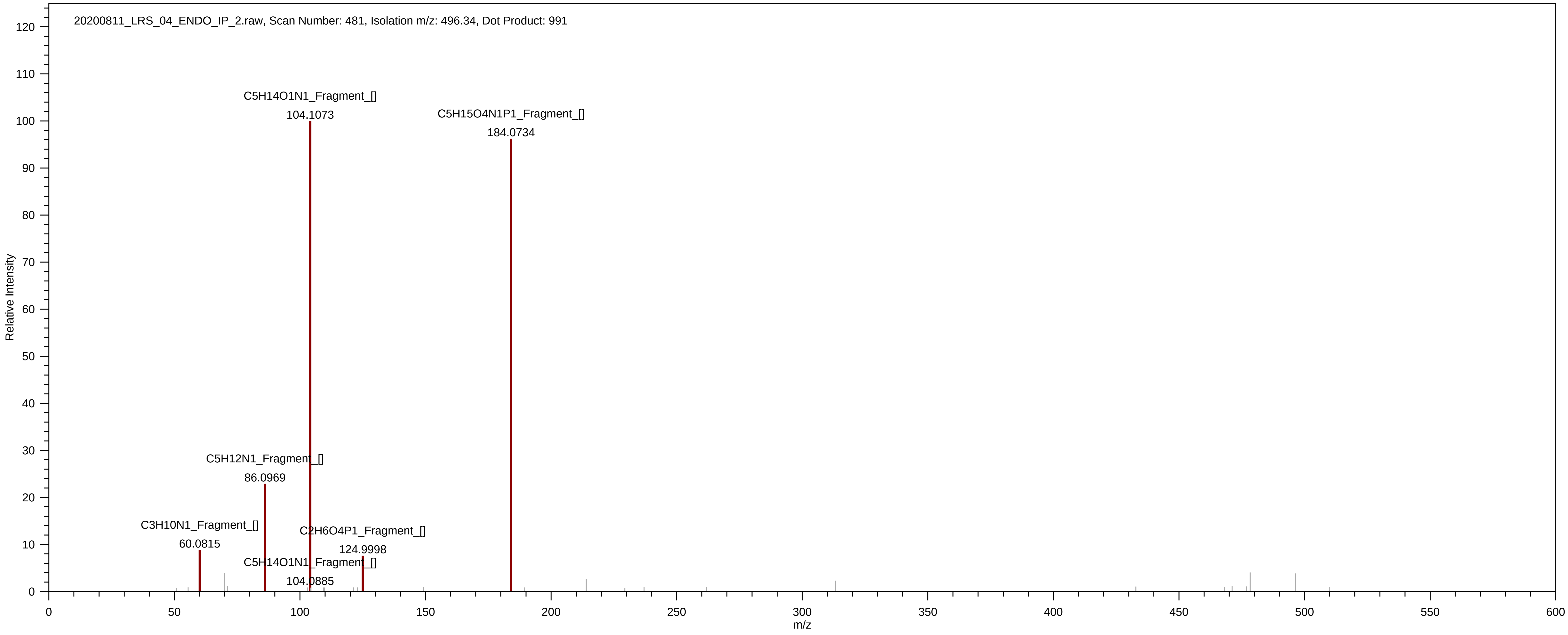

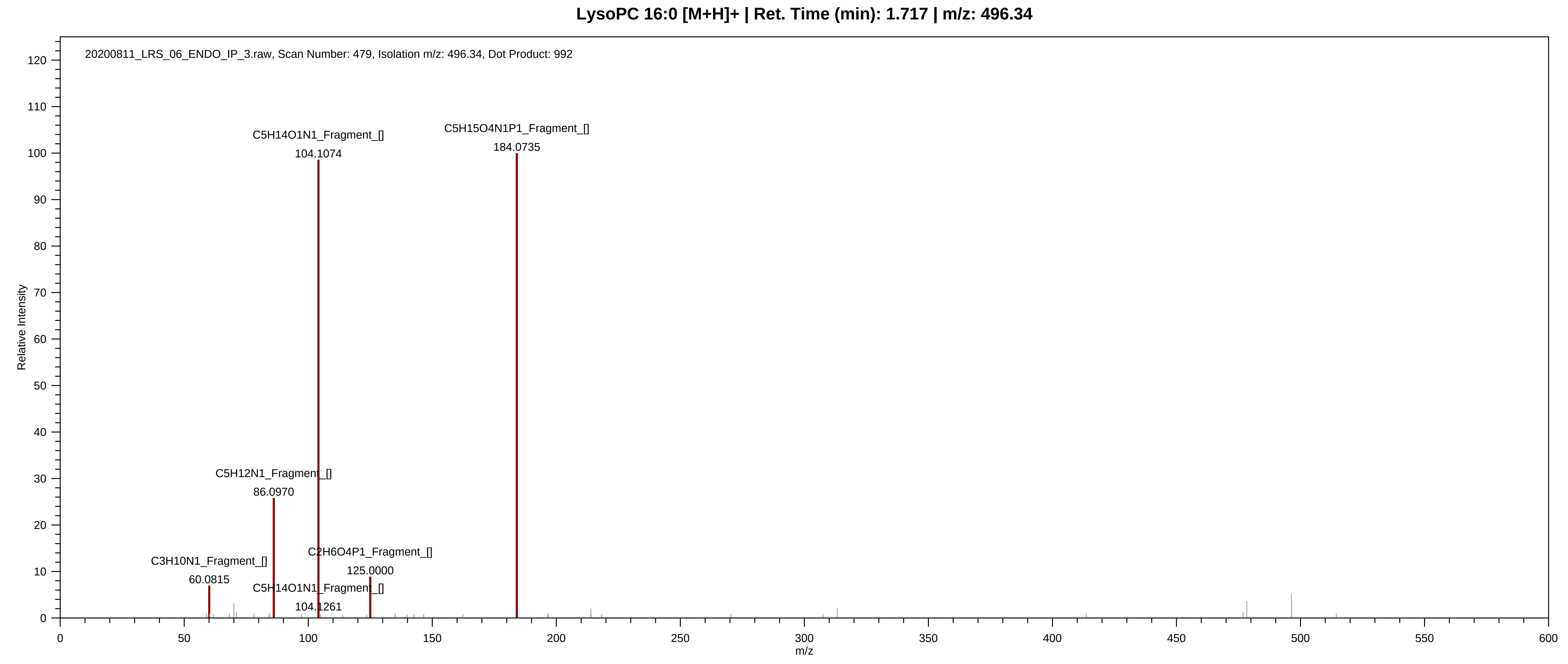

LysoPC 16:0 [M+H]+ | Ret. Time (min): 1.717 | m/z: 496.34
20200811_LRS_06_ENDO_IP_3.raw, Scan Number: 479, Isolation m/z: 496.34, Dot Product: 992
C5H14O1N1_Fragment_[]
104.1074
C5H15O4N1P1_Fragment_[]
184.0735
C5H12N1_Fragment_[]
86.0970
C2H6O4P1_Fragment_[]
125.0000
C3H10N1_Fragment_[]
60.0815
C5H14O1N1_Fragment_[]
104.1261
Relative Intensity
m/z

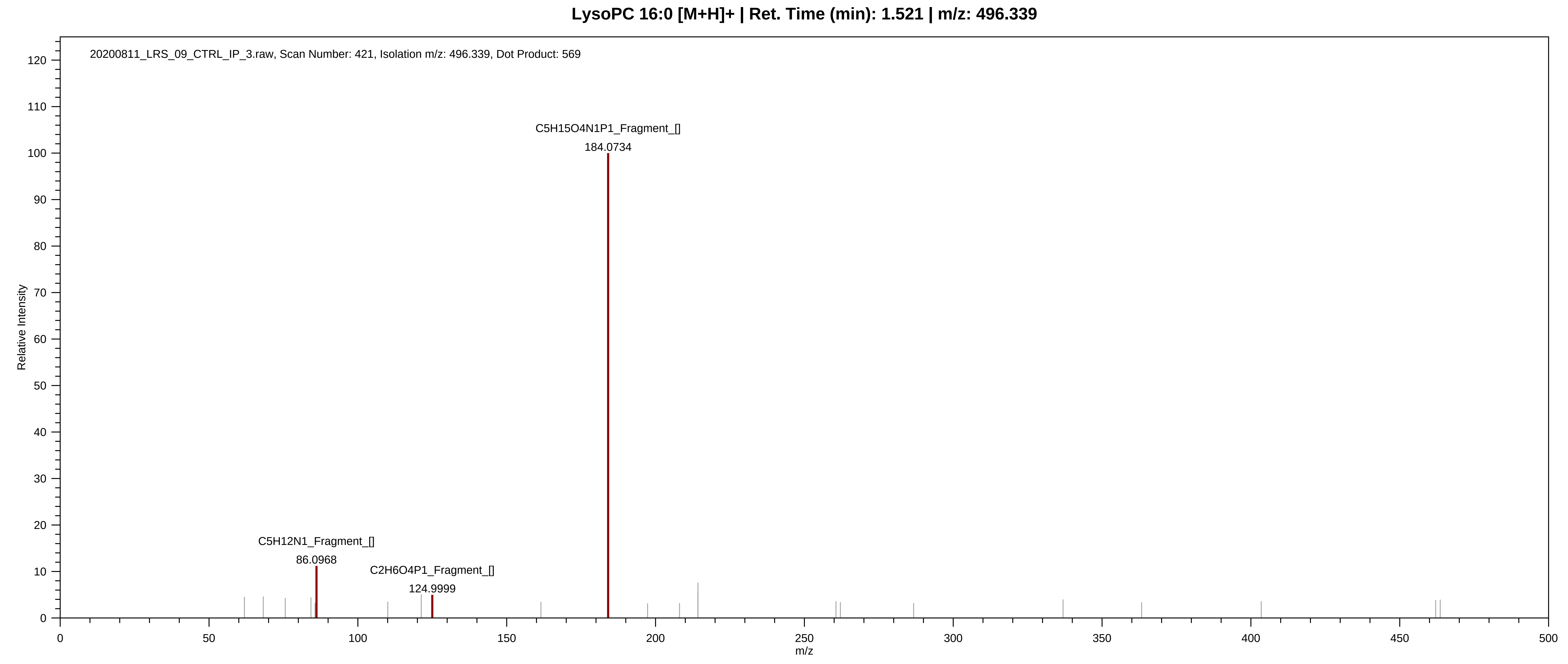

LysoPC 16:0 [M+H]+ | Ret. Time (min): 1.521 | m/z: 496.339
20200811_LRS_09_CTRL_IP_3.raw, Scan Number: 421, Isolation m/z: 496.339, Dot Product: 569
C5H15O4N1P1_Fragment_[]
184.0734
C5H12N1_Fragment_[]
86.0968
C2H6O4P1_Fragment_[]
124.9999
Relative Intensity
m/z

LysoPC 16:1 [M+H]+ | Ret. Time (min): 1.152 | m/z: 494.324

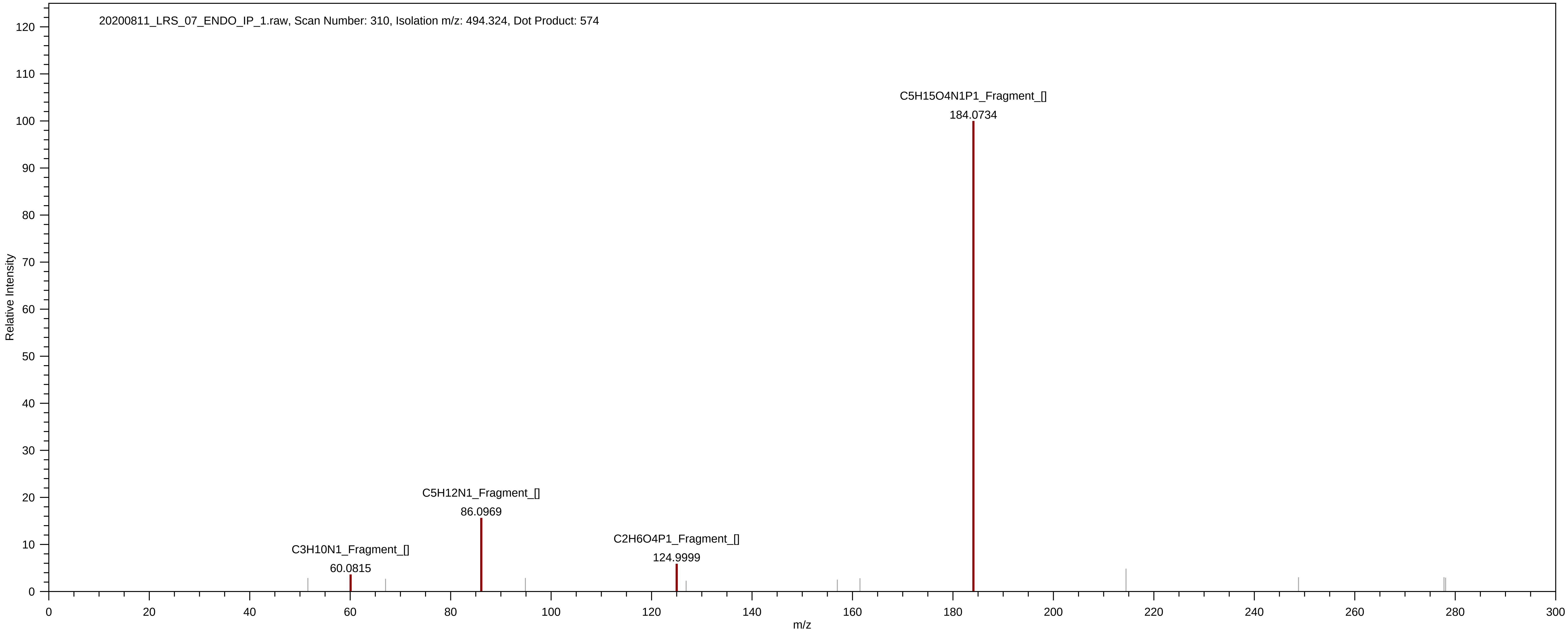

LysoPC 16:1 [M+H]+ | Ret. Time (min): 1.153 | m/z: 494.324

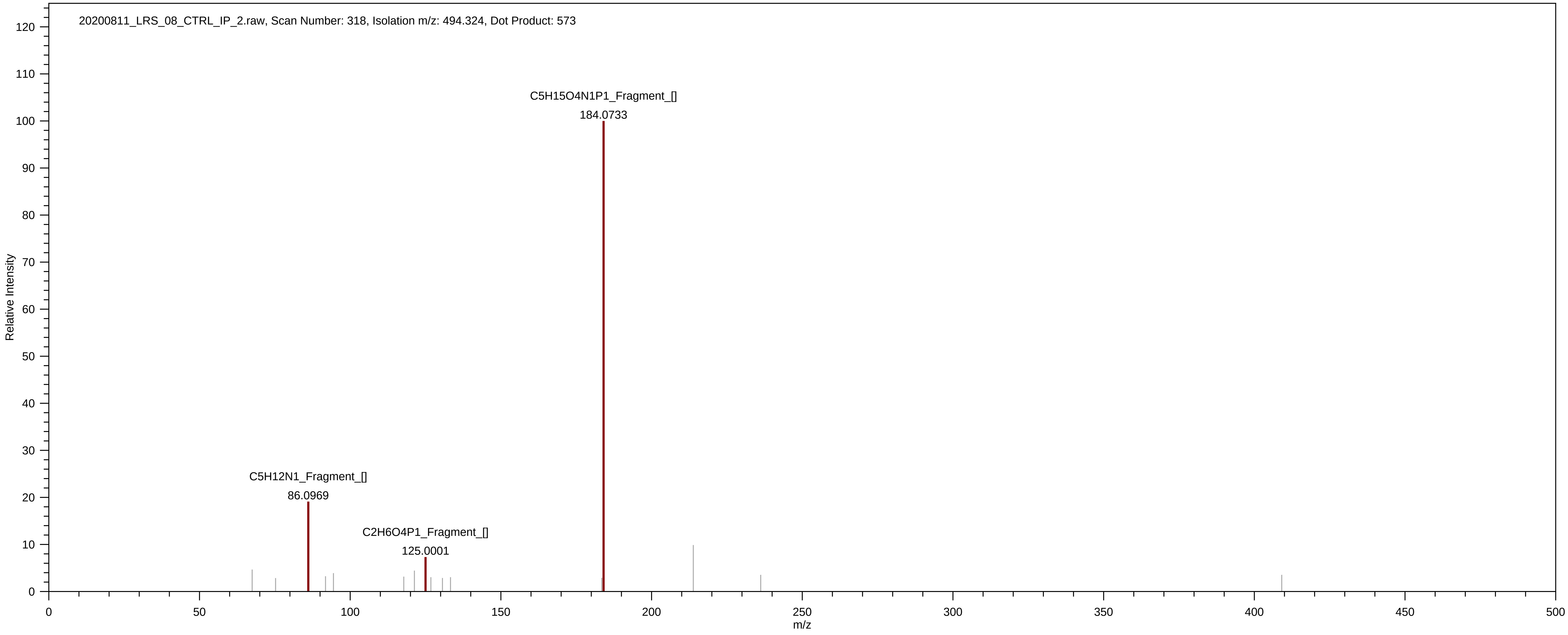

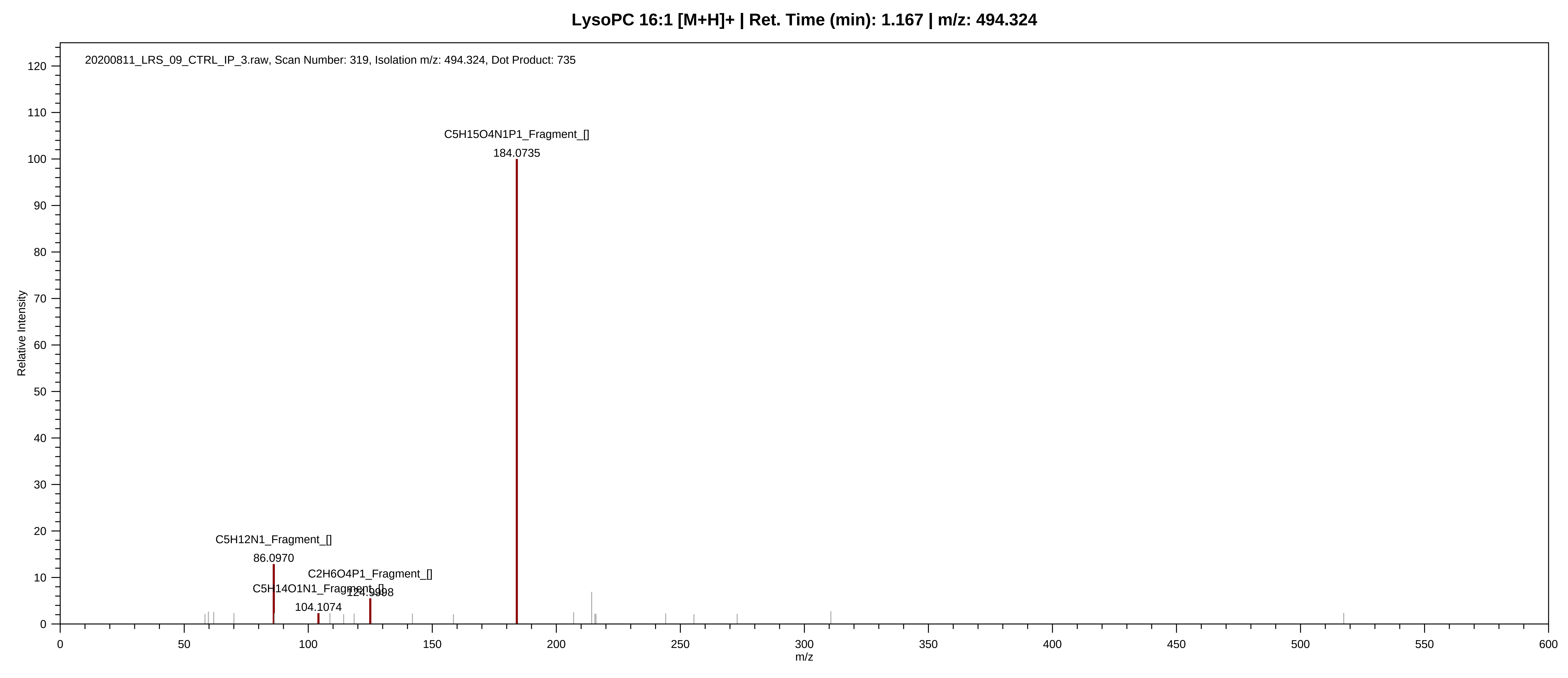

LysoPC 16:1 [M+H]+ | Ret. Time (min): 1.167 | m/z: 494.324
20200811_LRS_09_CTRL_IP_3.raw, Scan Number: 319, Isolation m/z: 494.324, Dot Product: 735
C5H15O4N1P1_Fragment_[]
184.0735
C5H12N1_Fragment_[]
86.0970
C2H6O4P1_Fragment_[]
C5H14O1N1_Fragment_[]
124.9998
104.1074
Relative Intensity
m/z

LysoPC 16:1 [M+H]+ | Ret. Time (min): 1.187 | m/z: 494.324

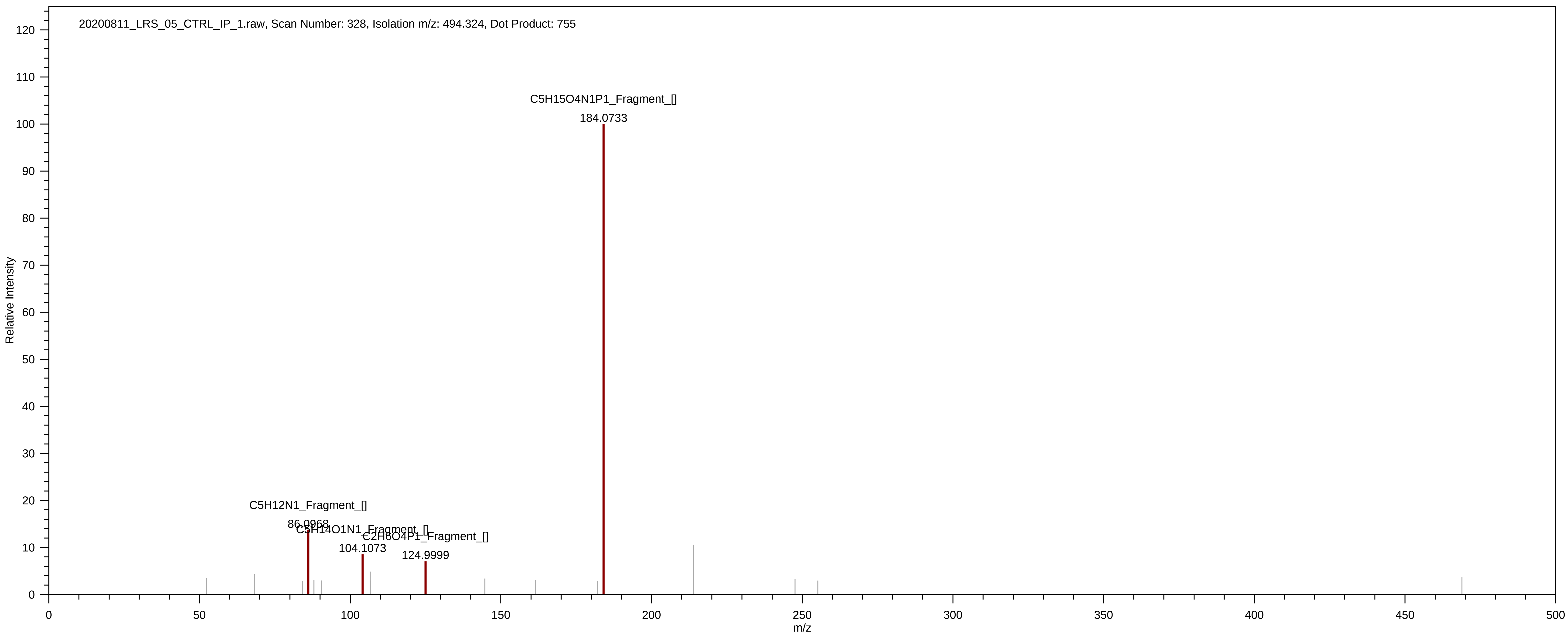

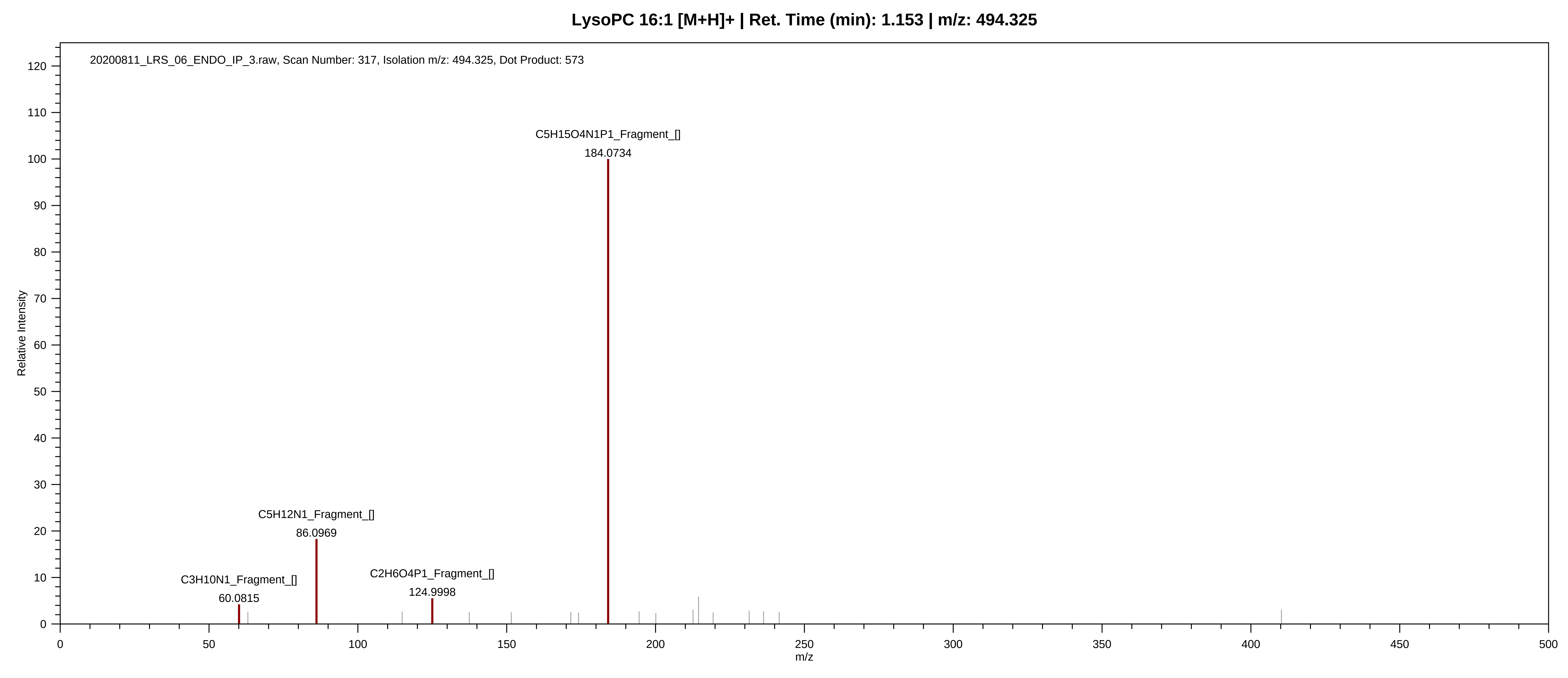
LysoPC 16:1 [M+H]+ | Ret. Time (min): 1.153 | m/z: 494.325
20200811_LRS_06_ENDO_IP_3.raw, Scan Number: 317, Isolation m/z: 494.325, Dot Product: 573
C5H15O4N1P1_Fragment_[]
184.0734
C5H12N1_Fragment_[]
86.0969
C2H6O4P1_Fragment_[]
124.9998
C3H10N1_Fragment_[]
60.0815
Relative Intensity
m/z

LysoPC 17:1 [M+H]+ | Ret. Time (min): 1.481 | m/z: 508.341

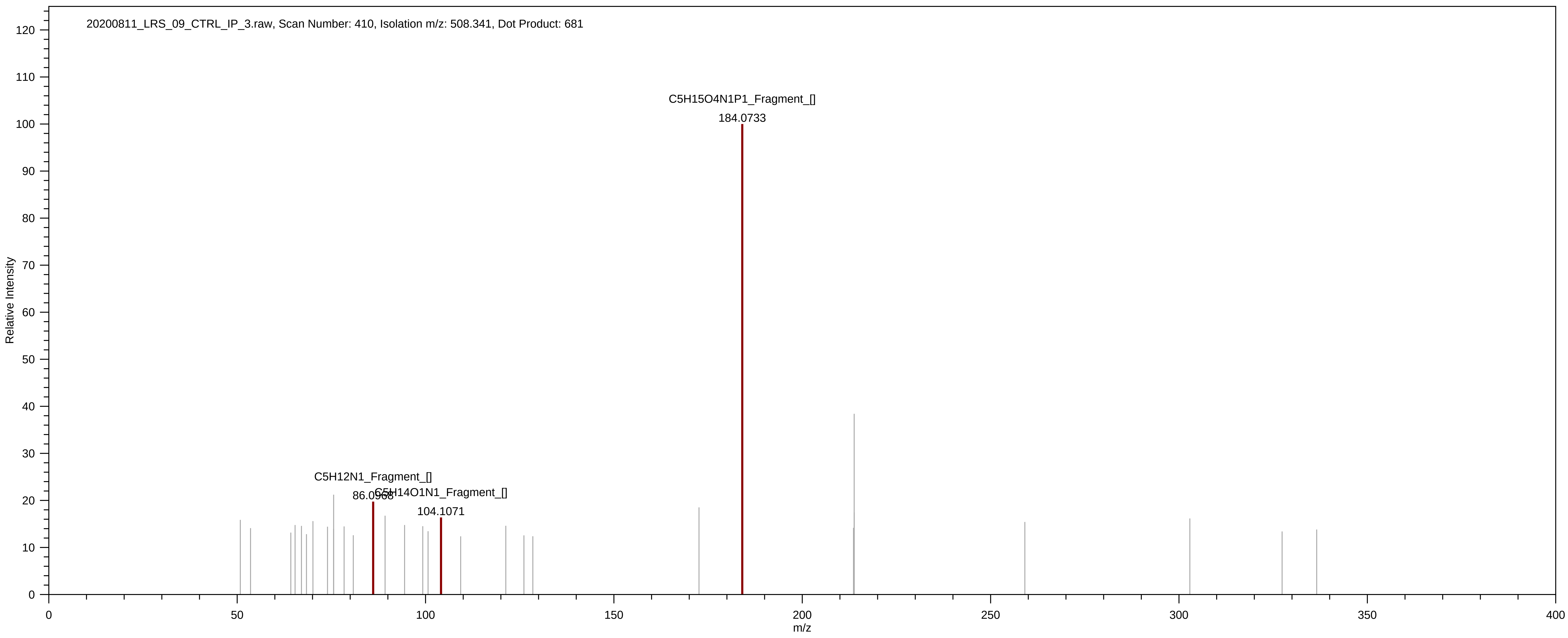

LysoPC 18:0 [M+Ac-H]- | Ret. Time (min): 2.973 | m/z: 582.378

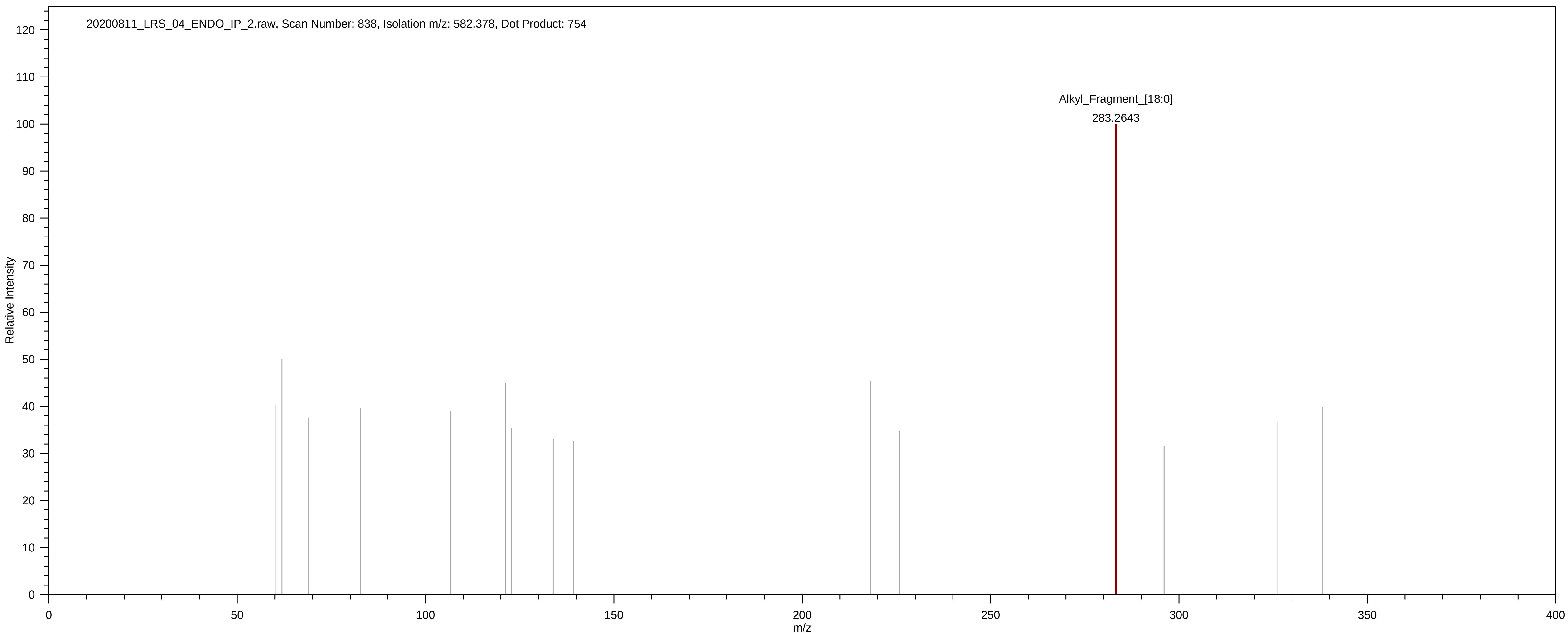

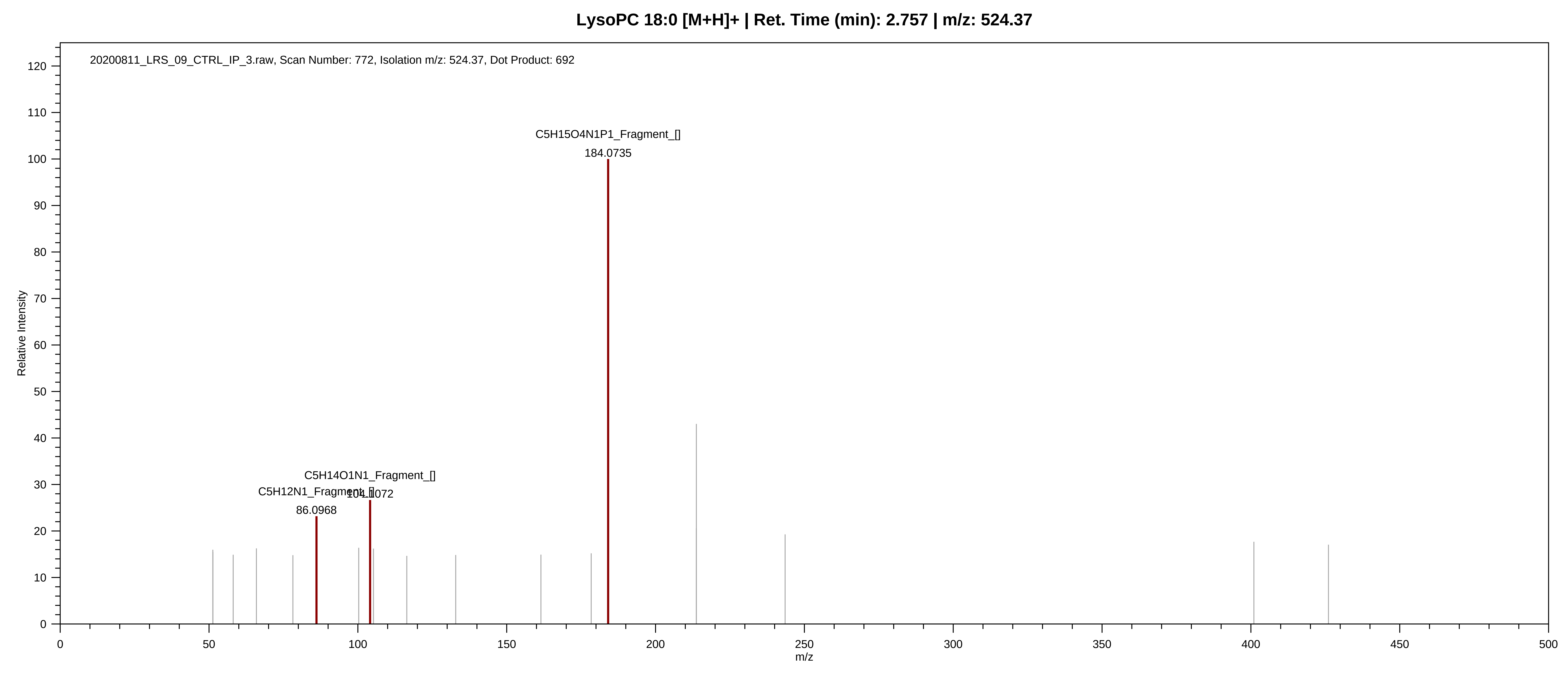

LysoPC 18:0 [M+H]+ | Ret. Time (min): 2.757 | m/z: 524.37
20200811_LRS_09_CTRL_IP_3.raw, Scan Number: 772, Isolation m/z: 524.37, Dot Product: 692
C5H15O4N1P1_Fragment_[]
184.0735
C5H14O1N1_Fragment_[]
104.1072
C5H12N1_Fragment_[]
86.0968
Relative Intensity
m/z

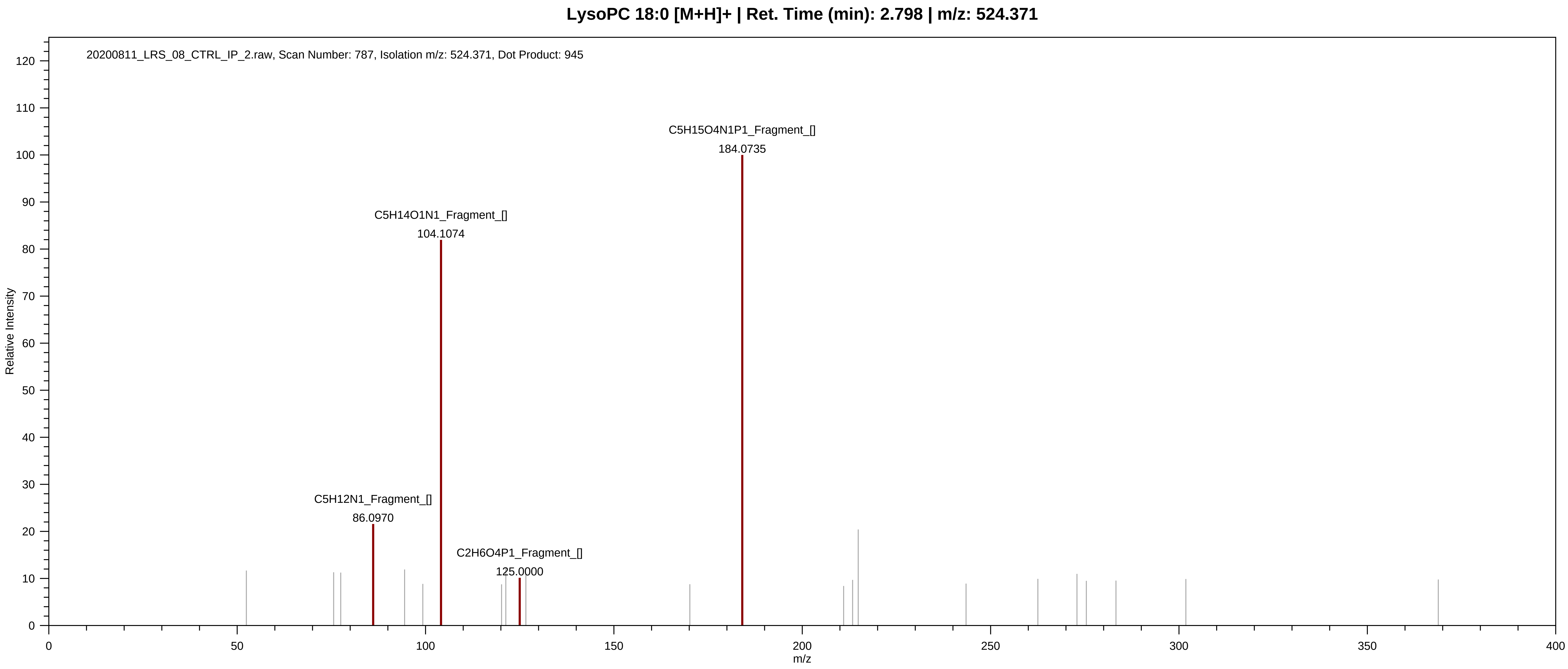

LysoPC 18:0 [M+H]+ | Ret. Time (min): 2.798 | m/z: 524.371
20200811_LRS_08_CTRL_IP_2.raw, Scan Number: 787, Isolation m/z: 524.371, Dot Product: 945
C5H15O4N1P1_Fragment_[]
184.0735
C5H14O1N1_Fragment_[]
104.1074
C5H12N1_Fragment_[]
86.0970
C2H6O4P1_Fragment_[]
125.0000
Relative Intensity
m/z

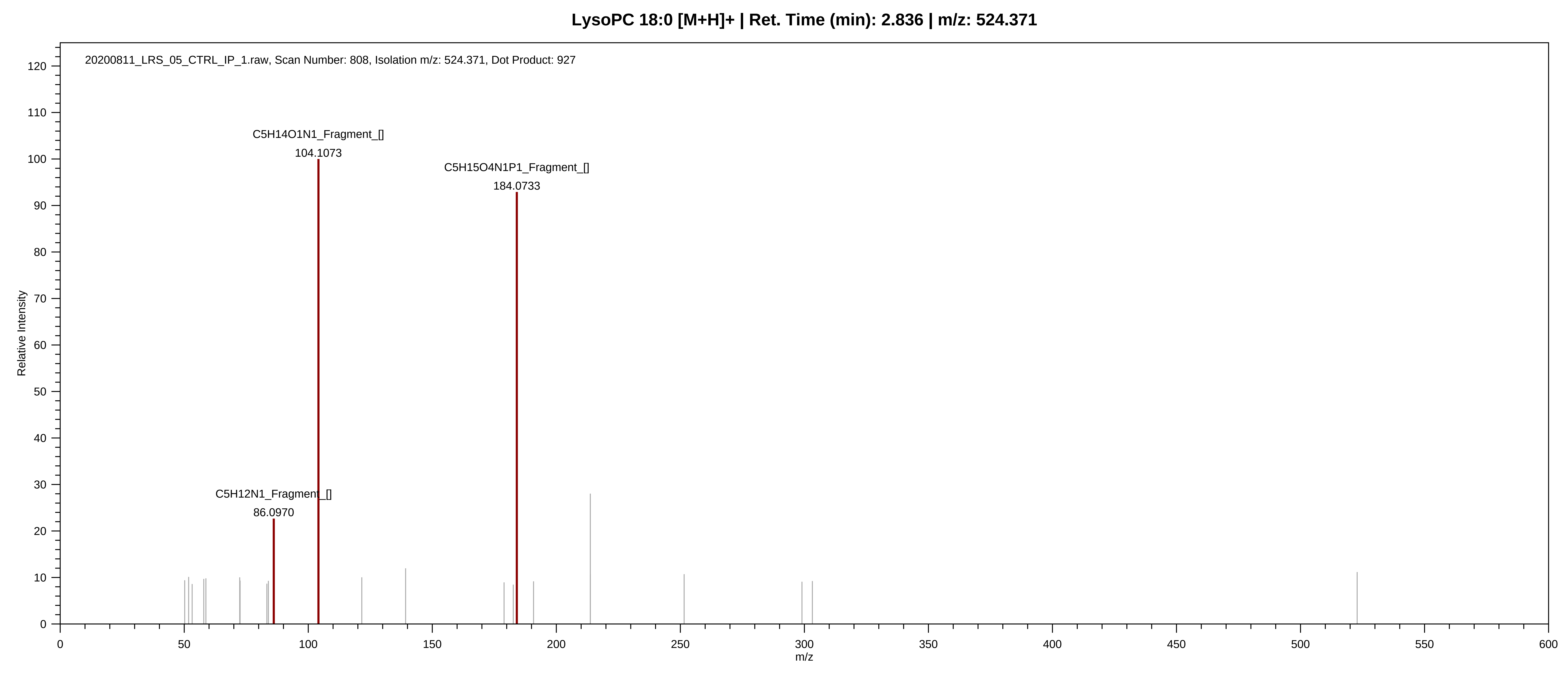
LysoPC 18:0 [M+H]+ | Ret. Time (min): 2.836 | m/z: 524.371
20200811_LRS_05_CTRL_IP_1.raw, Scan Number: 808, Isolation m/z: 524.371, Dot Product: 927
C5H14O1N1_Fragment_[]
104.1073
C5H15O4N1P1_Fragment_[]
184.0733
C5H12N1_Fragment_[]
86.0970
Relative Intensity
m/z

LysoPC 18:0 [M+H]+ | Ret. Time (min): 2.857 | m/z: 524.371

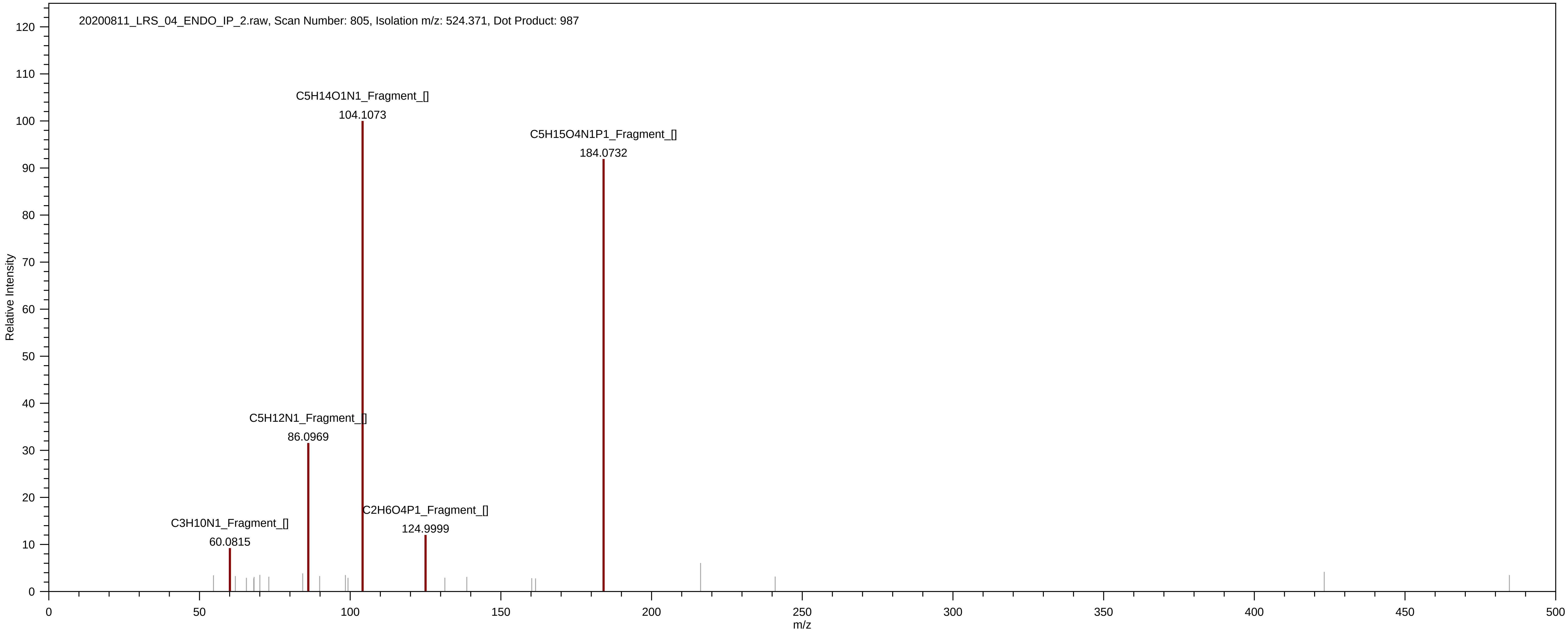

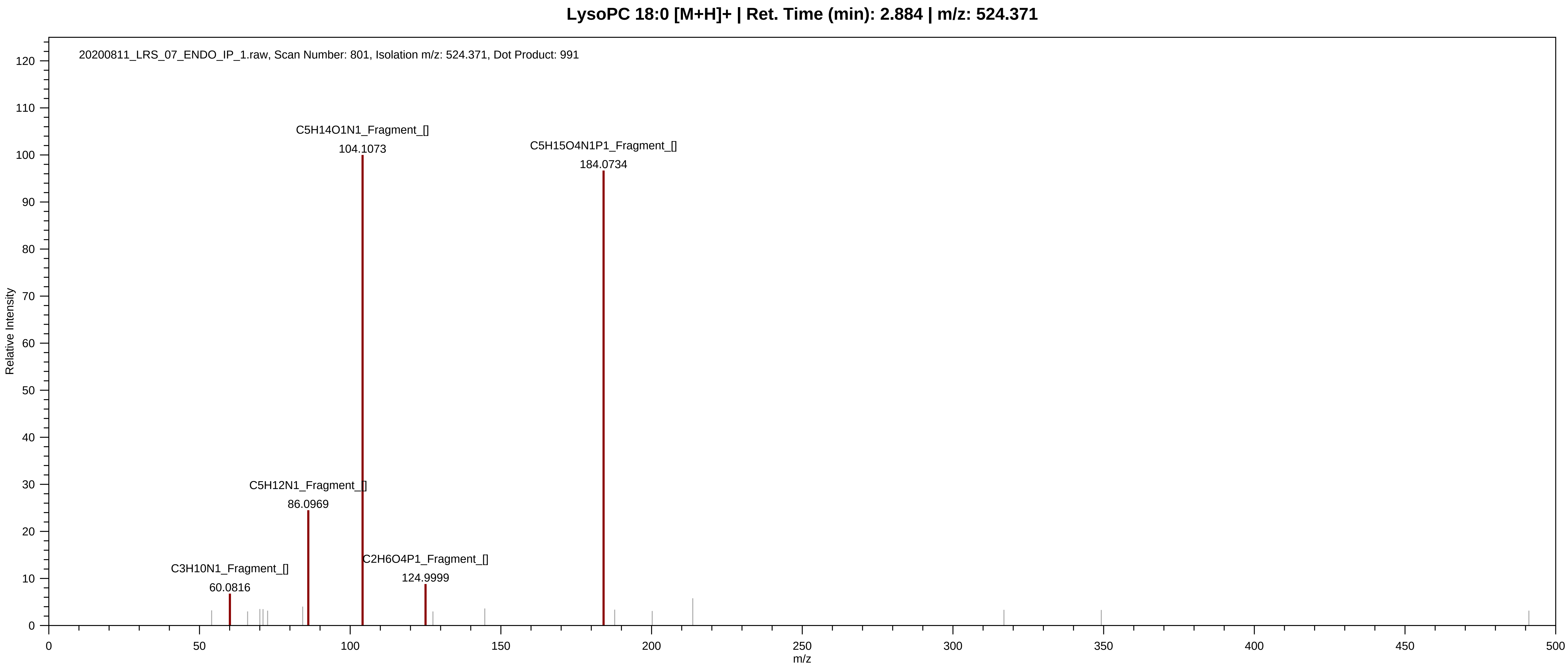
LysoPC 18:0 [M+H]+ | Ret. Time (min): 2.884 | m/z: 524.371
20200811_LRS_07_ENDO_IP_1.raw, Scan Number: 801, Isolation m/z: 524.371, Dot Product: 991
C5H14O1N1_Fragment_[]
104.1073
C5H15O4N1P1_Fragment_[]
184.0734
C5H12N1_Fragment_[]
86.0969
C2H6O4P1_Fragment_[]
124.9999
C3H10N1_Fragment_[]
60.0816
Relative Intensity
m/z

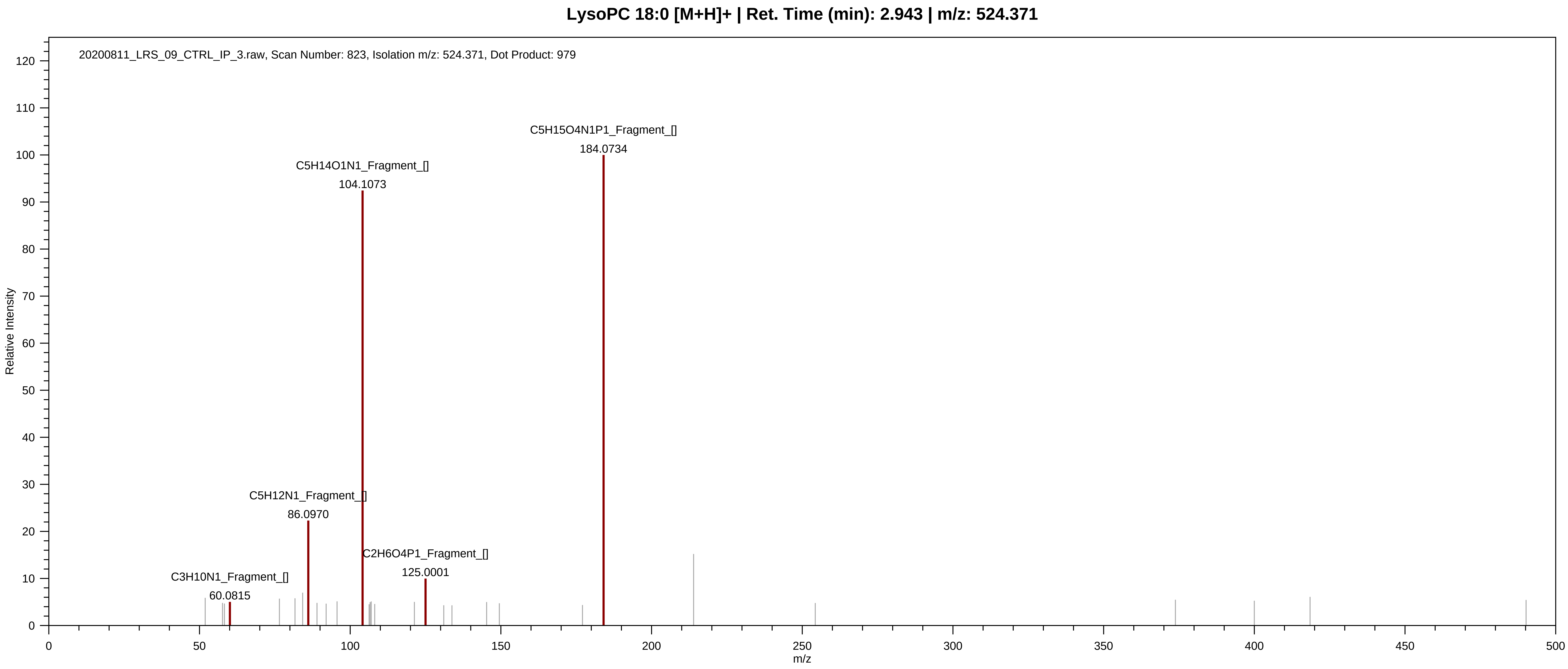

LysoPC 18:0 [M+H]+ | Ret. Time (min): 2.943 | m/z: 524.371
20200811_LRS_09_CTRL_IP_3.raw, Scan Number: 823, Isolation m/z: 524.371, Dot Product: 979
C5H15O4N1P1_Fragment_[]
184.0734
C5H14O1N1_Fragment_[]
104.1073
C5H12N1_Fragment_[]
86.0970
C2H6O4P1_Fragment_[]
125.0001
C3H10N1_Fragment_[]
60.0815
Relative Intensity
m/z

LysoPC 18:0 [M+H]+ | Ret. Time (min): 2.989 | m/z: 524.371

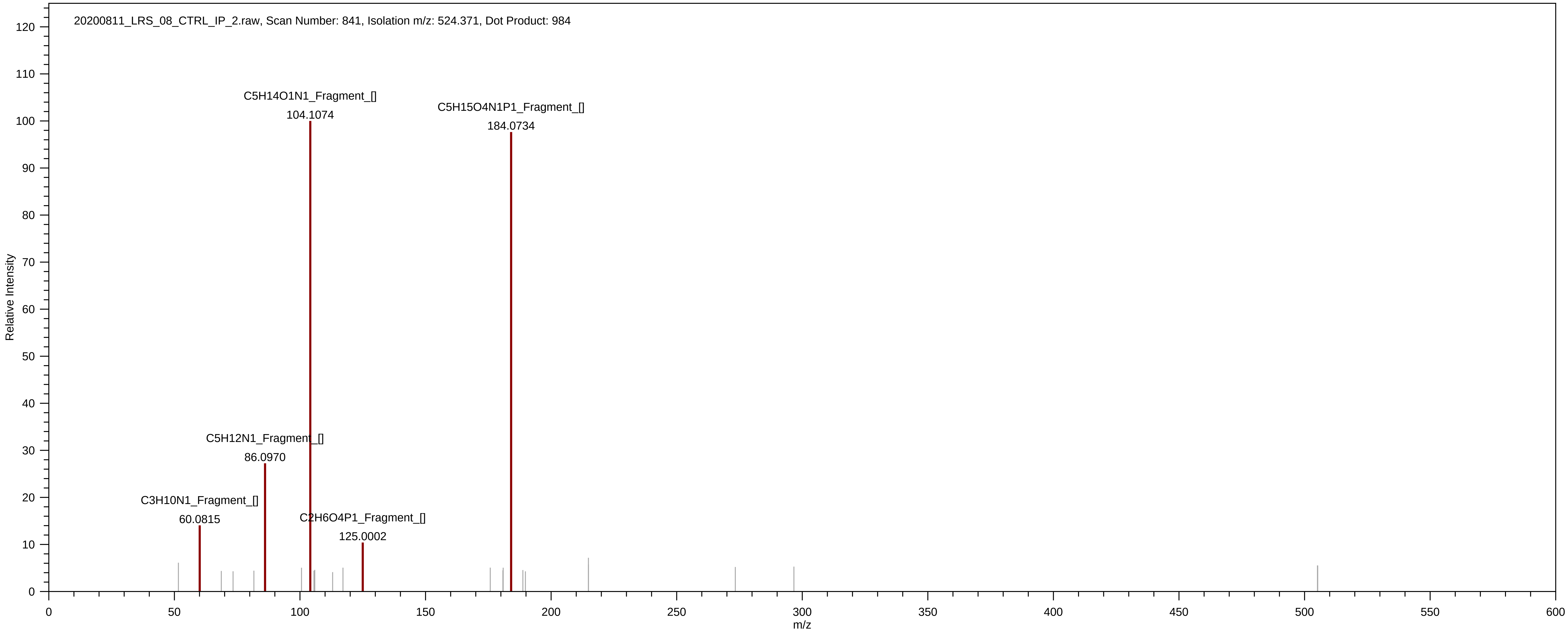

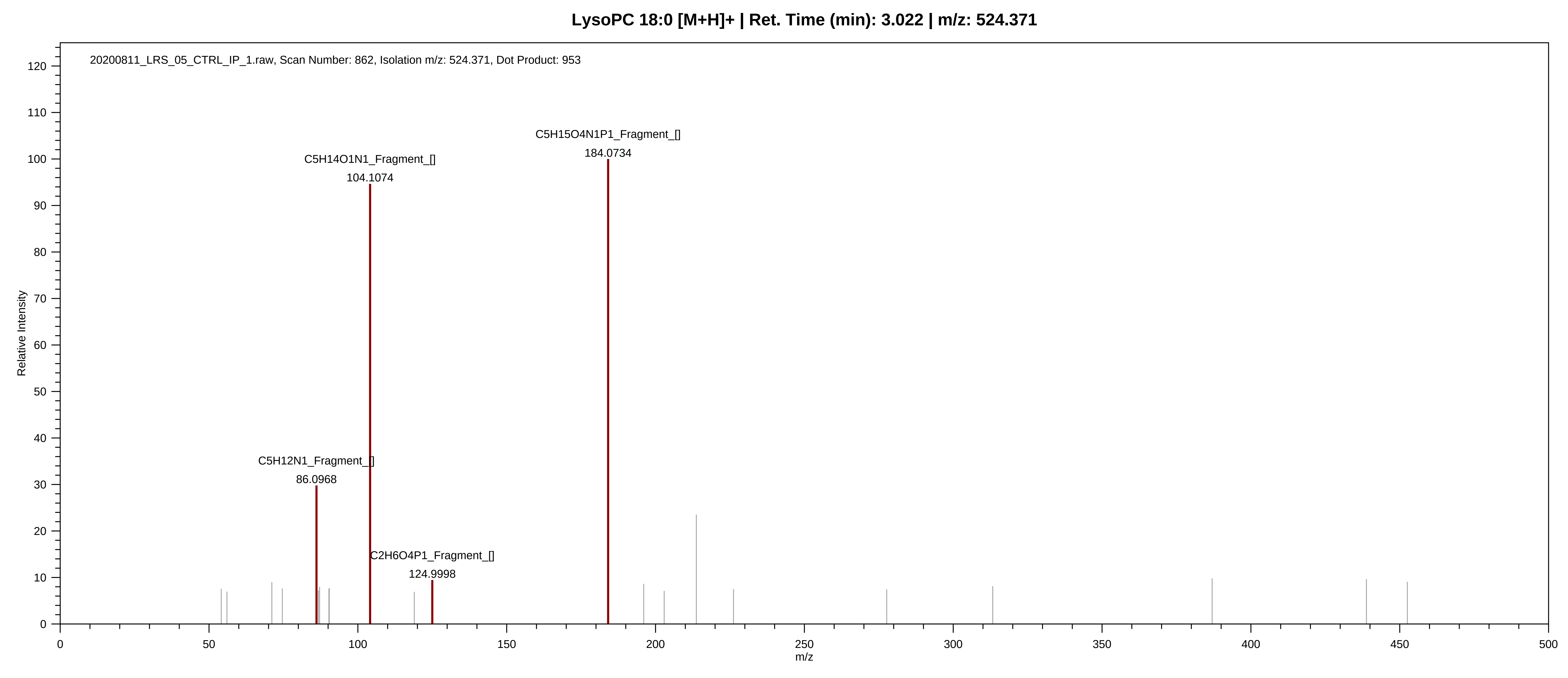
LysoPC 18:0 [M+H]+ | Ret. Time (min): 3.022 | m/z: 524.371
20200811_LRS_05_CTRL_IP_1.raw, Scan Number: 862, Isolation m/z: 524.371, Dot Product: 953
C5H15O4N1P1_Fragment_[]
184.0734
C5H14O1N1_Fragment_[]
104.1074
C5H12N1_Fragment_[]
86.0968
C2H6O4P1_Fragment_[]
124.9998
Relative Intensity
m/z

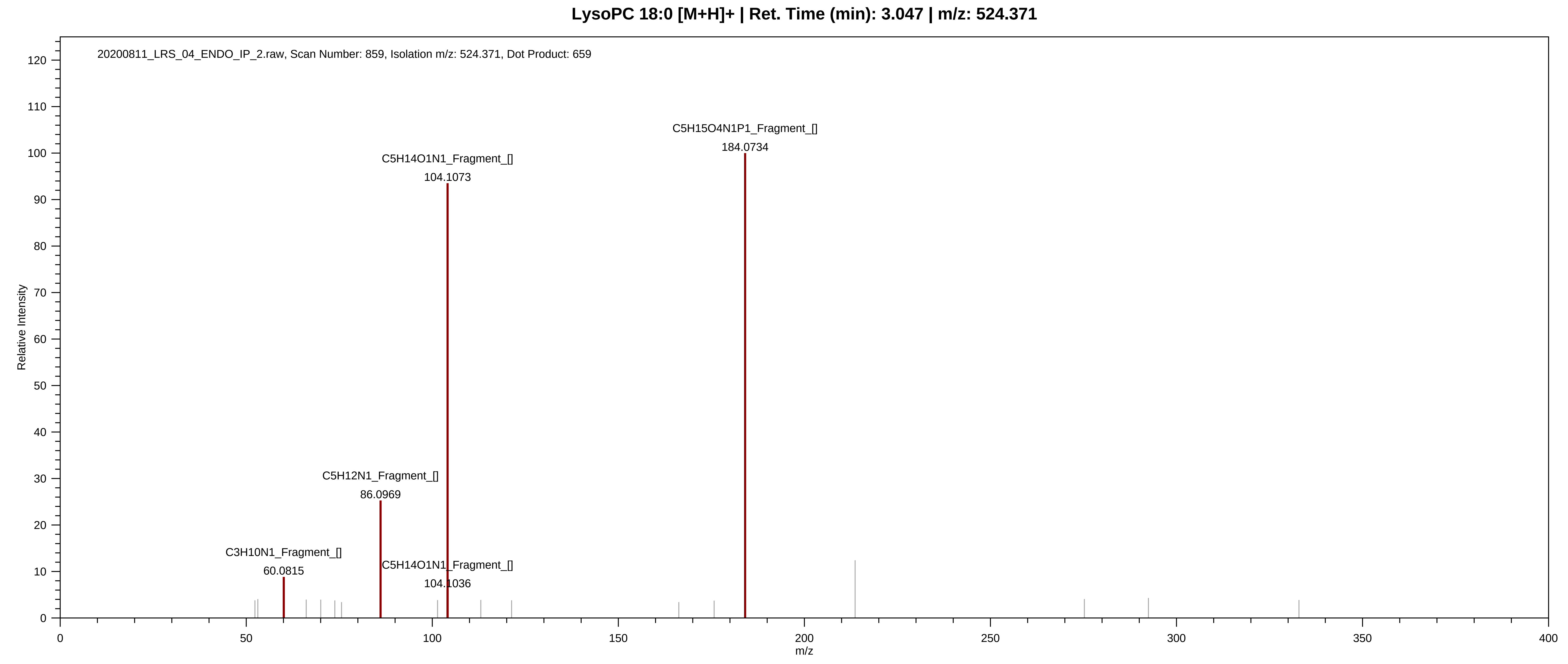
LysoPC 18:0 [M+H]+ | Ret. Time (min): 3.047 | m/z: 524.371
20200811_LRS_04_ENDO_IP_2.raw, Scan Number: 859, Isolation m/z: 524.371, Dot Product: 659
C5H15O4N1P1_Fragment_[]
184.0734
C5H14O1N1_Fragment_[]
104.1073
C5H12N1_Fragment_[]
86.0969
C3H10N1_Fragment_[]
60.0815
C5H14O1N1_Fragment_[]
104.1036
Relative Intensity
m/z

LysoPC 18:0 [M+H]+ | Ret. Time (min): 3.074 | m/z: 524.371

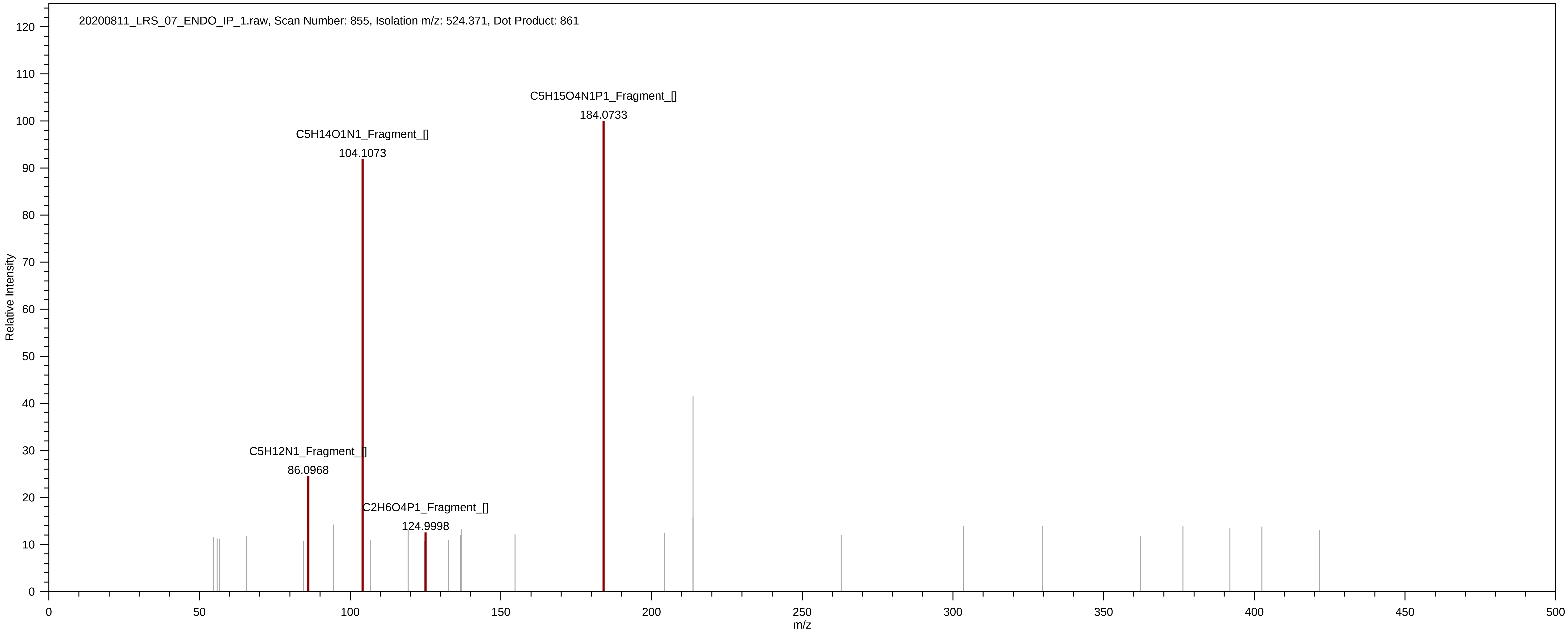

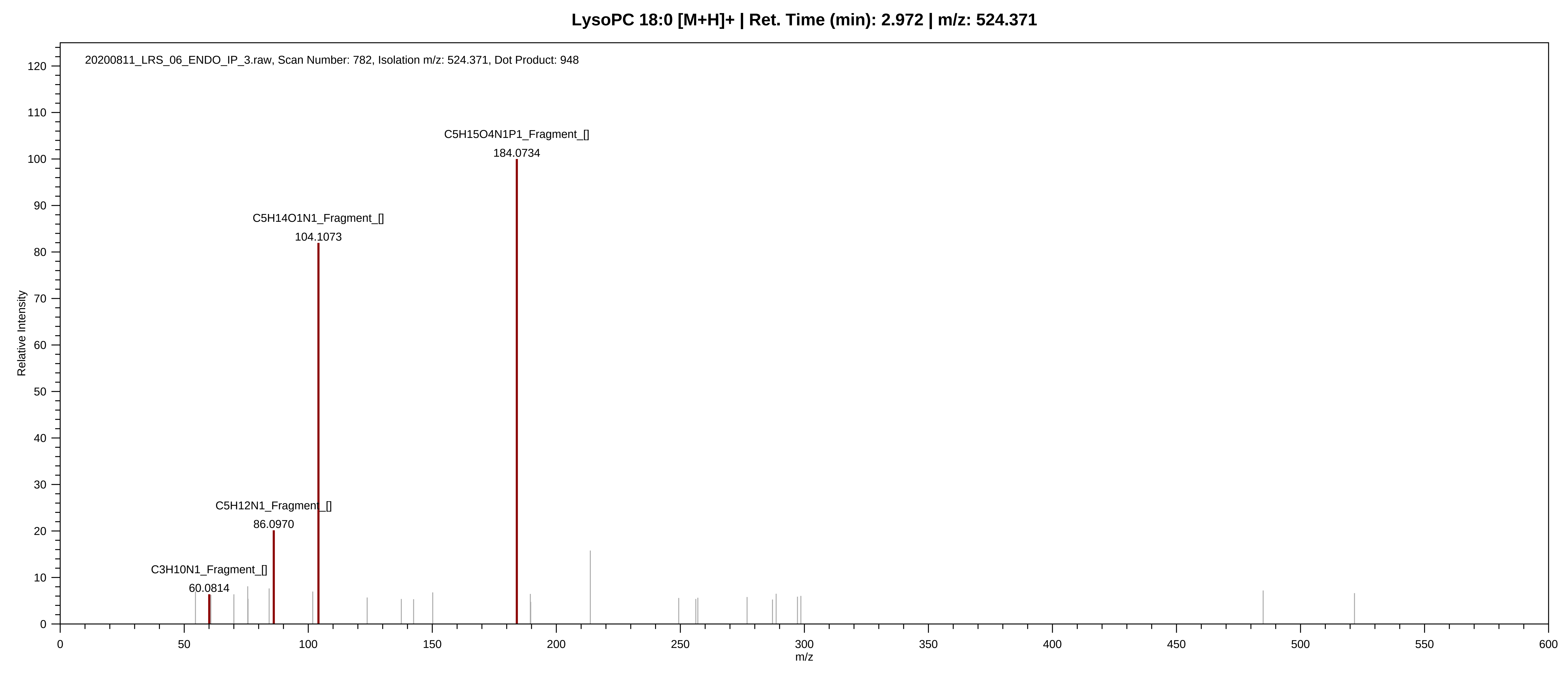

LysoPC 18:0 [M+H]+ | Ret. Time (min): 2.972 | m/z: 524.371
20200811_LRS_06_ENDO_IP_3.raw, Scan Number: 782, Isolation m/z: 524.371, Dot Product: 948
C5H15O4N1P1_Fragment_[]
184.0734
C5H14O1N1_Fragment_[]
104.1073
C5H12N1_Fragment_[]
86.0970
C3H10N1_Fragment_[]
60.0814
Relative Intensity
m/z

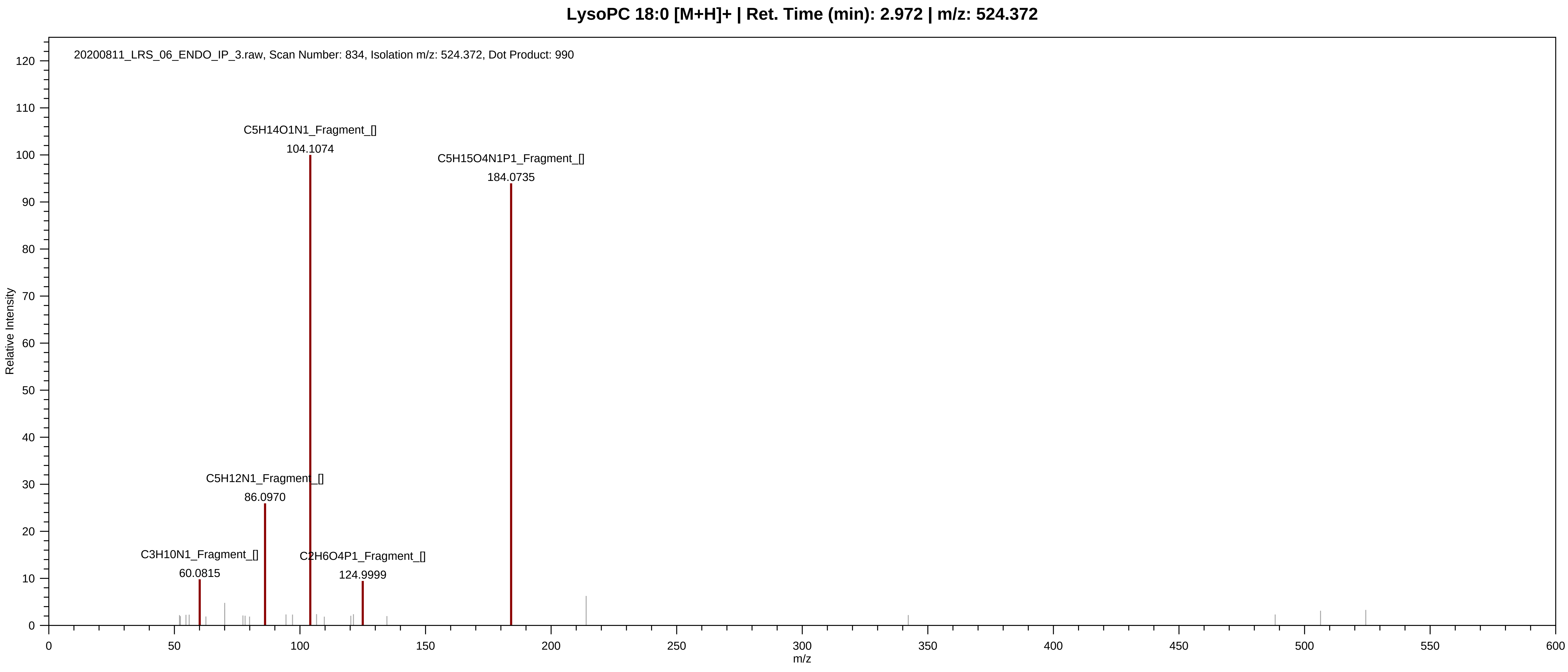

LysoPC 18:0 [M+H]+ | Ret. Time (min): 2.972 | m/z: 524.372
20200811_LRS_06_ENDO_IP_3.raw, Scan Number: 834, Isolation m/z: 524.372, Dot Product: 990
C5H14O1N1_Fragment_[]
104.1074
C5H15O4N1P1_Fragment_[]
184.0735
C5H12N1_Fragment_[]
86.0970
C3H10N1_Fragment_[]
60.0815
C2H6O4P1_Fragment_[]
124.9999
Relative Intensity
m/z

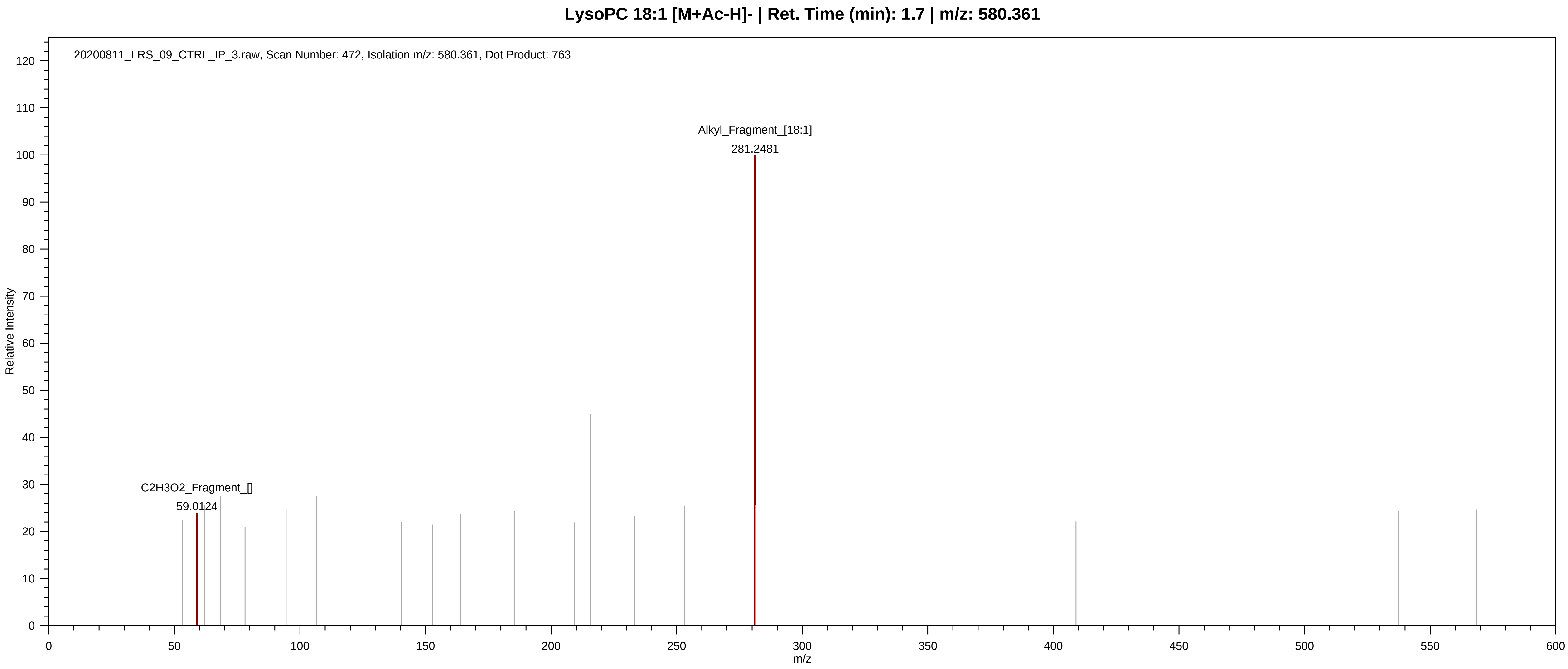

LysoPC 18:1 [M+Ac-H]- | Ret. Time (min): 1.7 | m/z: 580.361
20200811_LRS_09_CTRL_IP_3.raw, Scan Number: 472, Isolation m/z: 580.361, Dot Product: 763
Alkyl_Fragment_[18:1]
281.2481
C2H3O2_Fragment_[]
59.0124
Relative Intensity
m/z

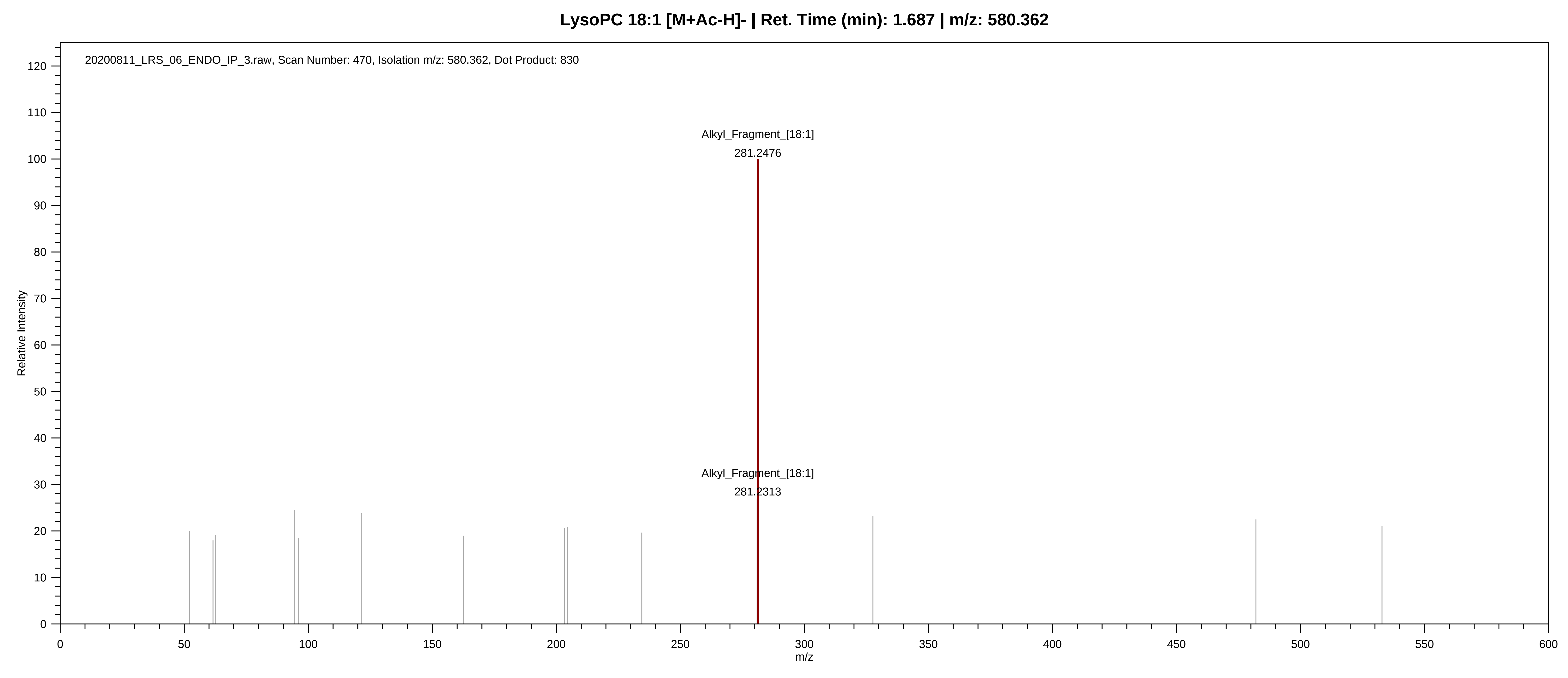

LysoPC 18:1 [M+Ac-H]- | Ret. Time (min): 1.687 | m/z: 580.362
20200811_LRS_06_ENDO_IP_3.raw, Scan Number: 470, Isolation m/z: 580.362, Dot Product: 830
Alkyl_Fragment_[18:1]
281.2476
Alkyl_Fragment_[18:1]
281.2313
Relative Intensity
m/z

LysoPC 18:1 [M+Ac-H]- | Ret. Time (min): 1.704 | m/z: 580.363

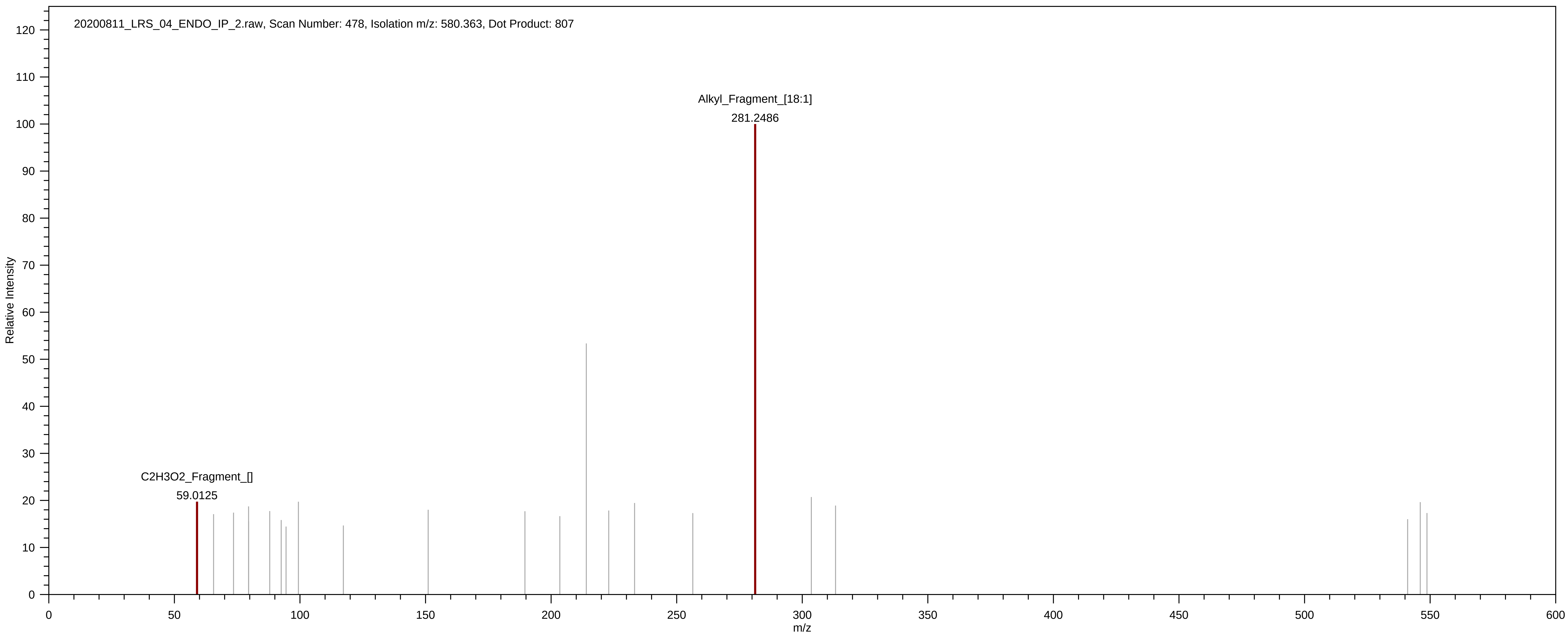

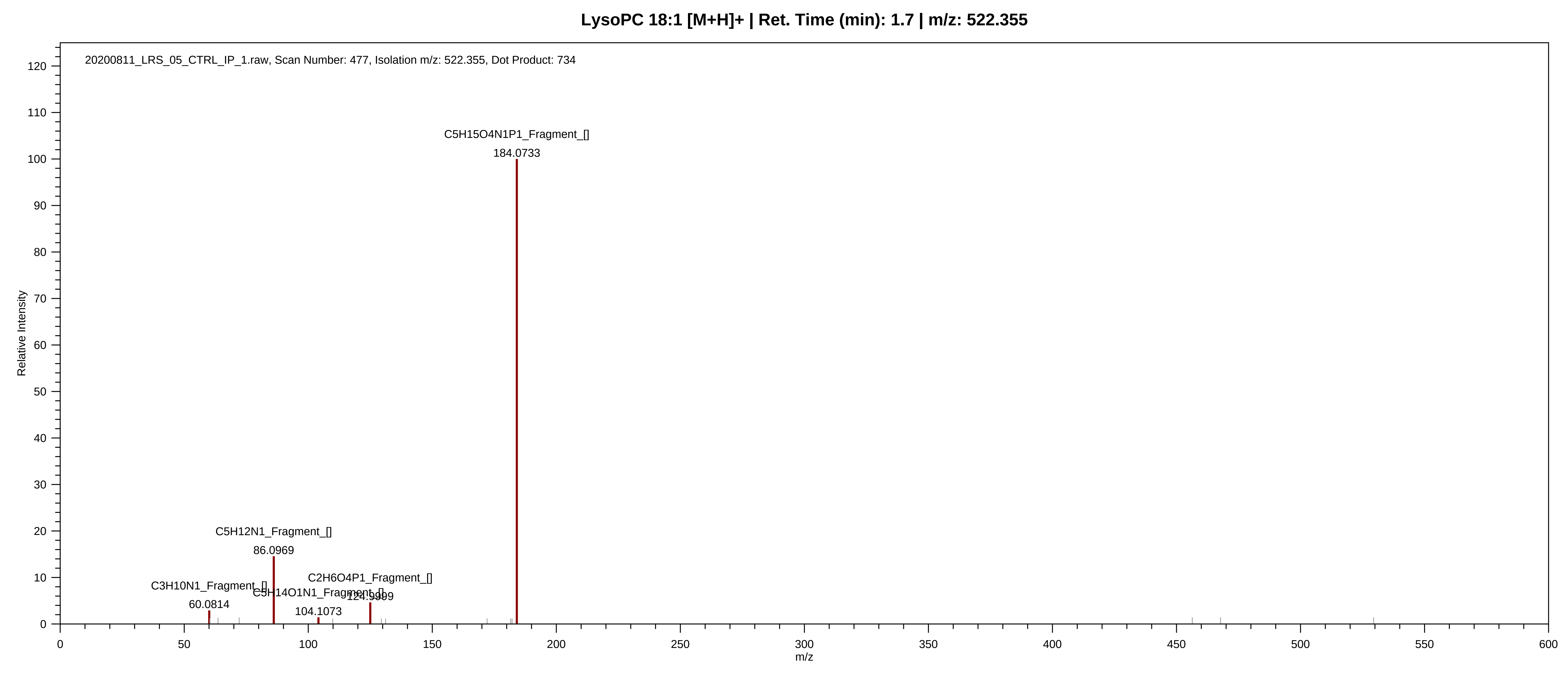
LysoPC 18:1 [M+H]+ | Ret. Time (min): 1.7 | m/z: 522.355
20200811_LRS_05_CTRL_IP_1.raw, Scan Number: 477, Isolation m/z: 522.355, Dot Product: 734
C5H15O4N1P1_Fragment_[]
184.0733
C5H12N1_Fragment_[]
86.0969
C2H6O4P1_Fragment_[]
C3H10N1_Fragment_[]
60.0814
104.1073
Relative Intensity
m/z

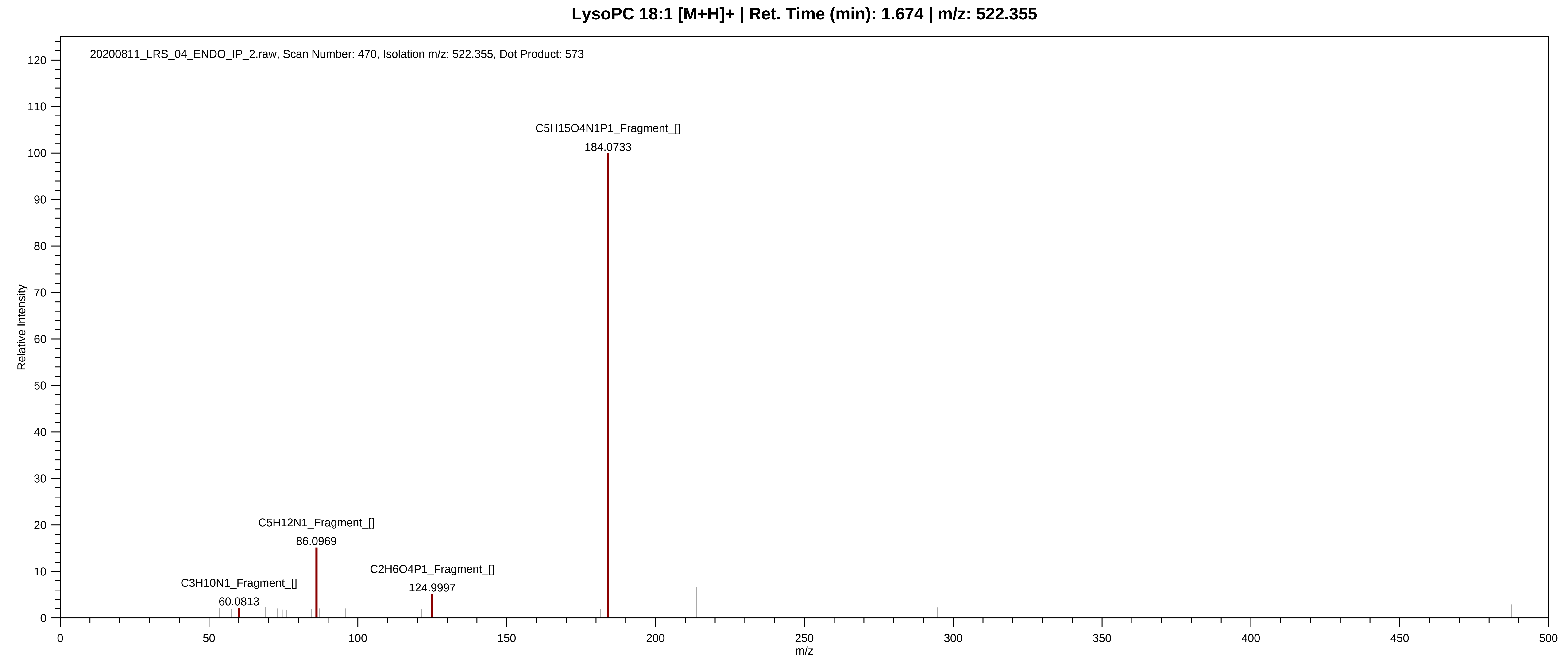
LysoPC 18:1 [M+H]+ | Ret. Time (min): 1.674 | m/z: 522.355
20200811_LRS_04_ENDO_IP_2.raw, Scan Number: 470, Isolation m/z: 522.355, Dot Product: 573
C5H15O4N1P1_Fragment_[]
184.0733
C5H12N1_Fragment_[]
86.0969
C2H6O4P1_Fragment_[]
124.9997
C3H10N1_Fragment_[]
60.0813
Relative Intensity
m/z

LysoPC 18:1 [M+H]+ | Ret. Time (min): 2.066 | m/z: 522.355

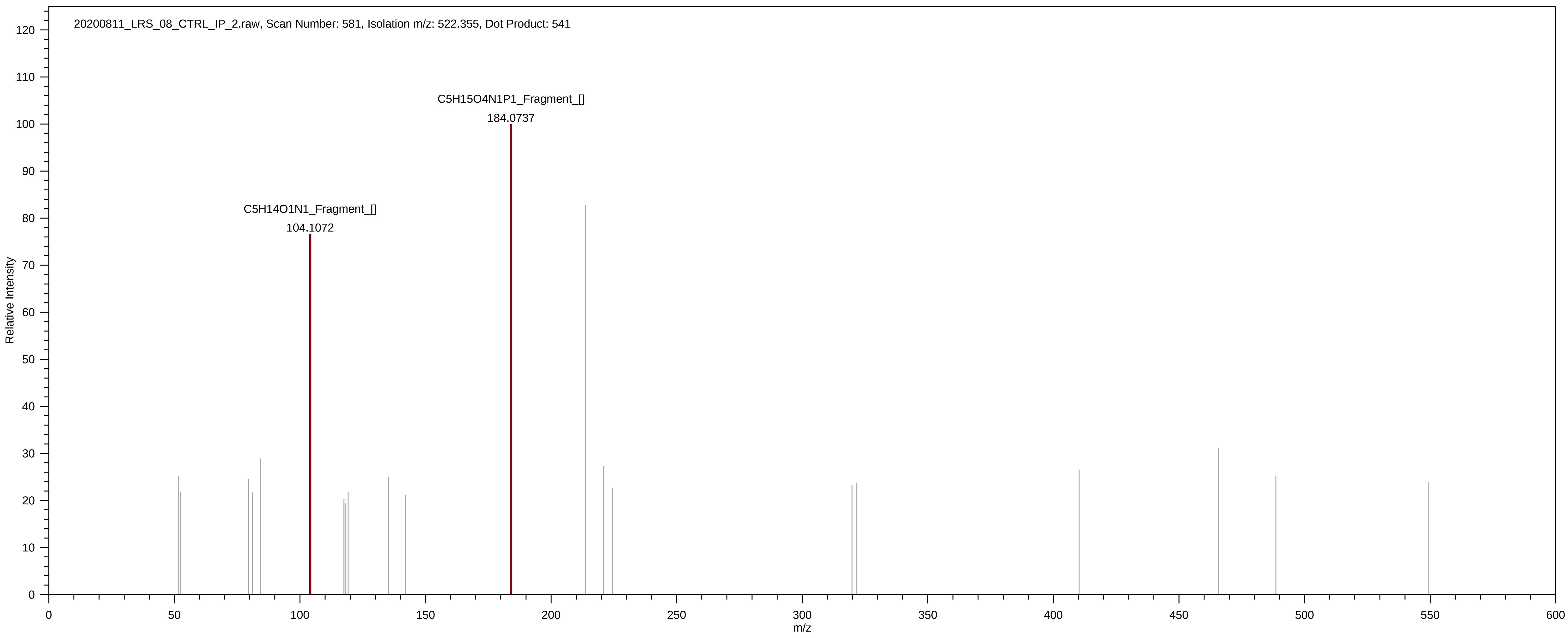

LysoPC 18:1 [M+H]+ | Ret. Time (min): 2.068 | m/z: 522.355

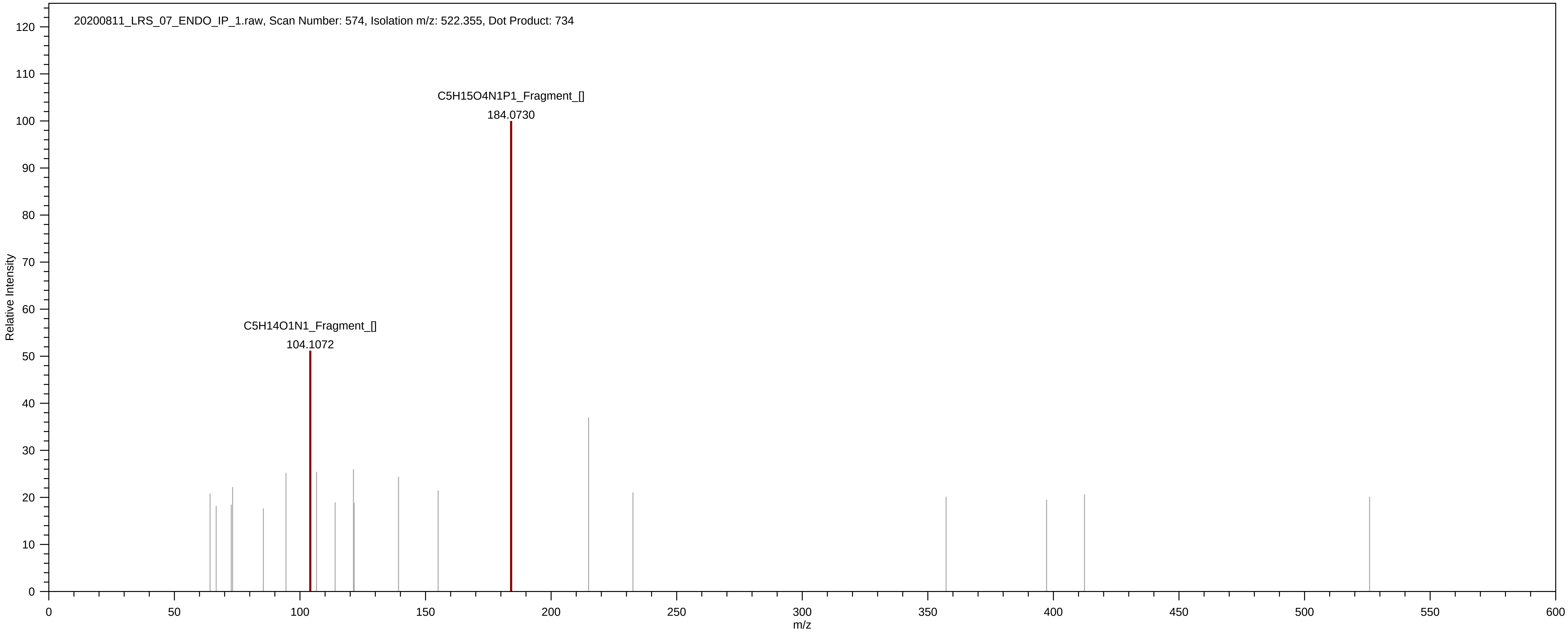

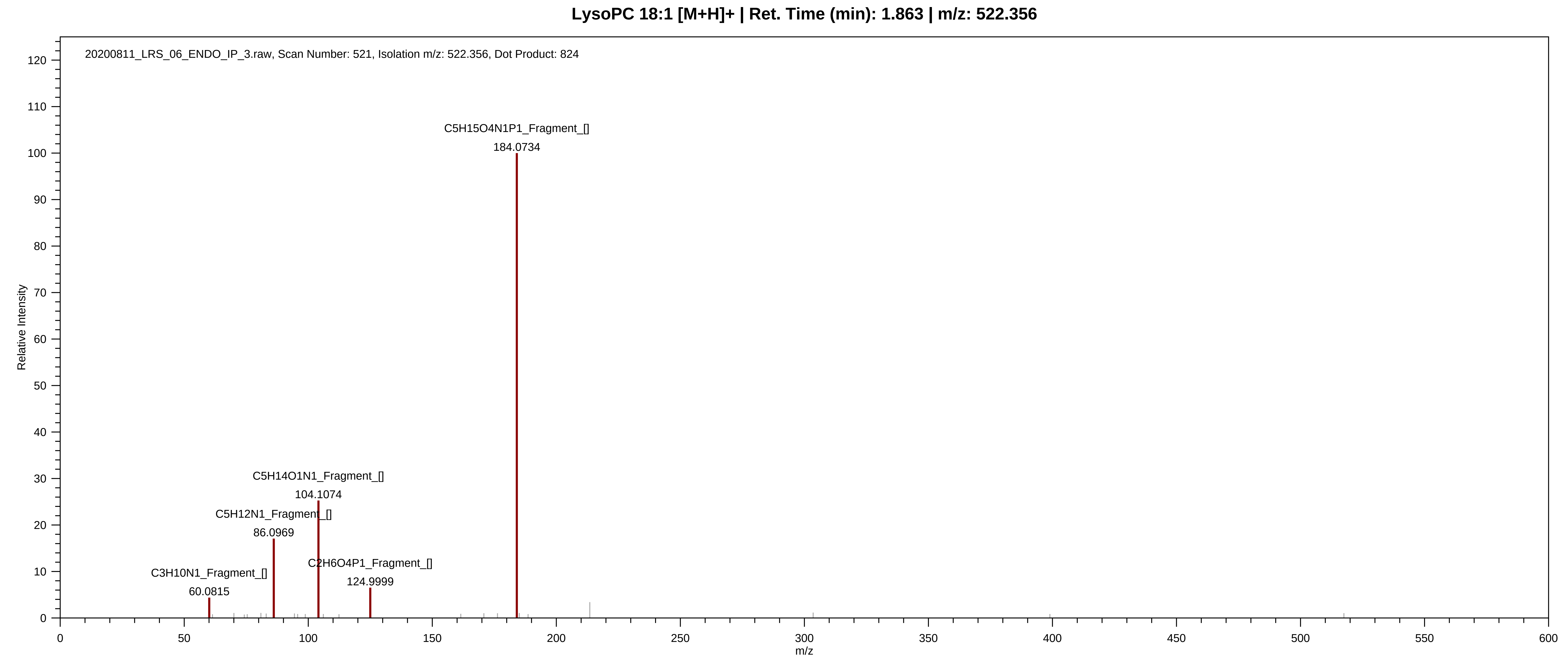

LysoPC 18:1 [M+H]+ | Ret. Time (min): 1.863 | m/z: 522.356
20200811_LRS_06_ENDO_IP_3.raw, Scan Number: 521, Isolation m/z: 522.356, Dot Product: 824
C5H15O4N1P1_Fragment_[]
184.0734
C5H14O1N1_Fragment_[]
104.1074
C5H12N1_Fragment_[]
86.0969
C2H6O4P1_Fragment_[]
124.9999
C3H10N1_Fragment_[]
60.0815
Relative Intensity
m/z

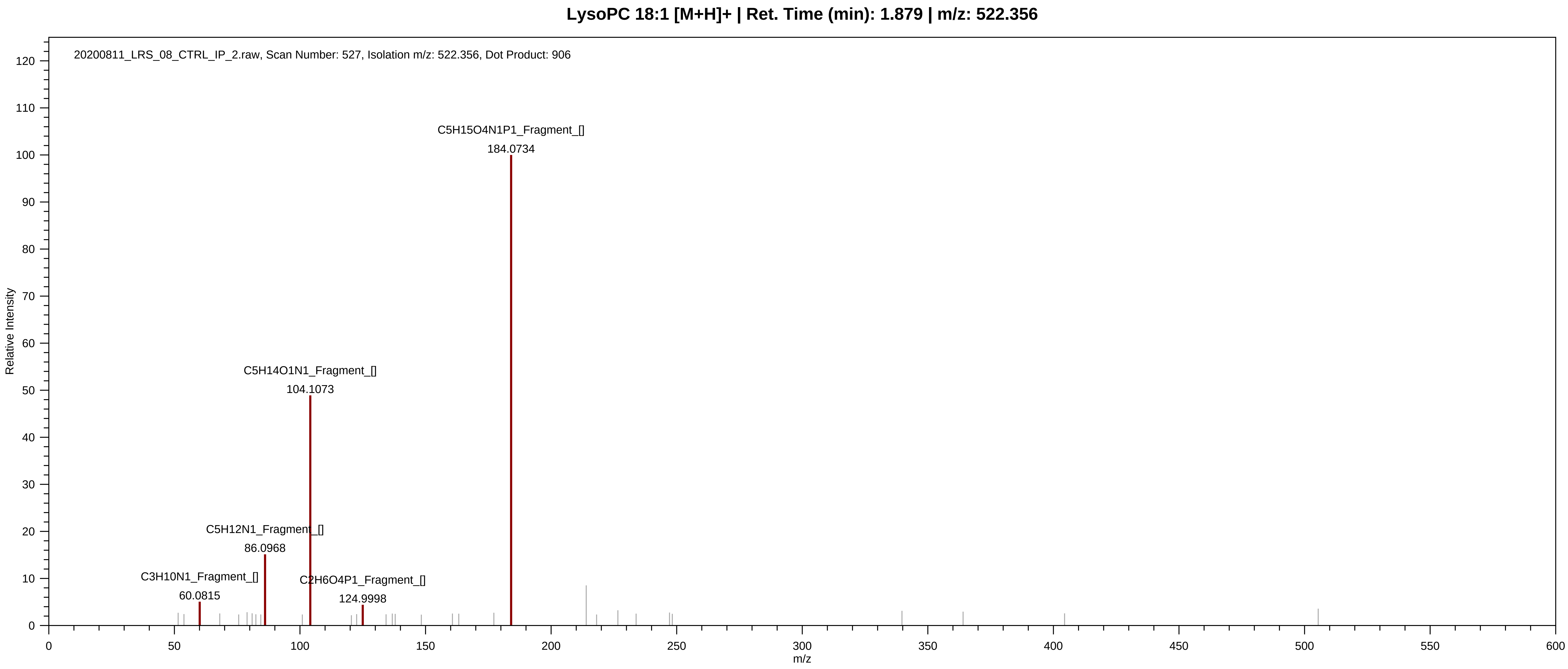

LysoPC 18:1 [M+H]+ | Ret. Time (min): 1.879 | m/z: 522.356
20200811_LRS_08_CTRL_IP_2.raw, Scan Number: 527, Isolation m/z: 522.356, Dot Product: 906
C5H15O4N1P1_Fragment_[]
184.0734
C5H14O1N1_Fragment_[]
104.1073
C5H12N1_Fragment_[]
86.0968
C3H10N1_Fragment_[]
60.0815
C2H6O4P1_Fragment_[]
124.9998
Relative Intensity
m/z

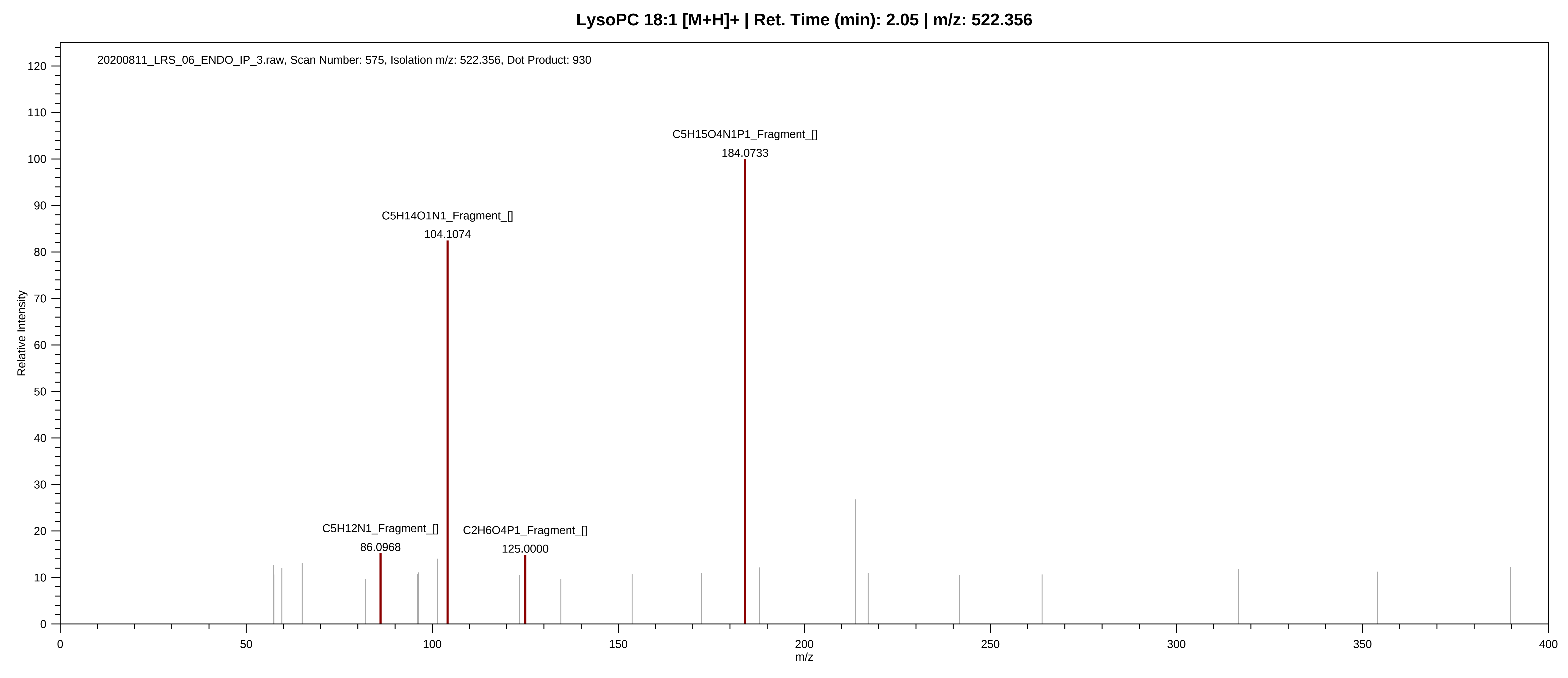

LysoPC 18:1 [M+H]+ | Ret. Time (min): 2.05 | m/z: 522.356
20200811_LRS_06_ENDO_IP_3.raw, Scan Number: 575, Isolation m/z: 522.356, Dot Product: 930
C5H15O4N1P1_Fragment_[]
184.0733
C5H14O1N1_Fragment_[]
104.1074
C5H12N1_Fragment_[]
86.0968
C2H6O4P1_Fragment_[]
125.0000
Relative Intensity
m/z

LysoPC 18:1 [M+H]+ | Ret. Time (min): 2.063 | m/z: 522.356

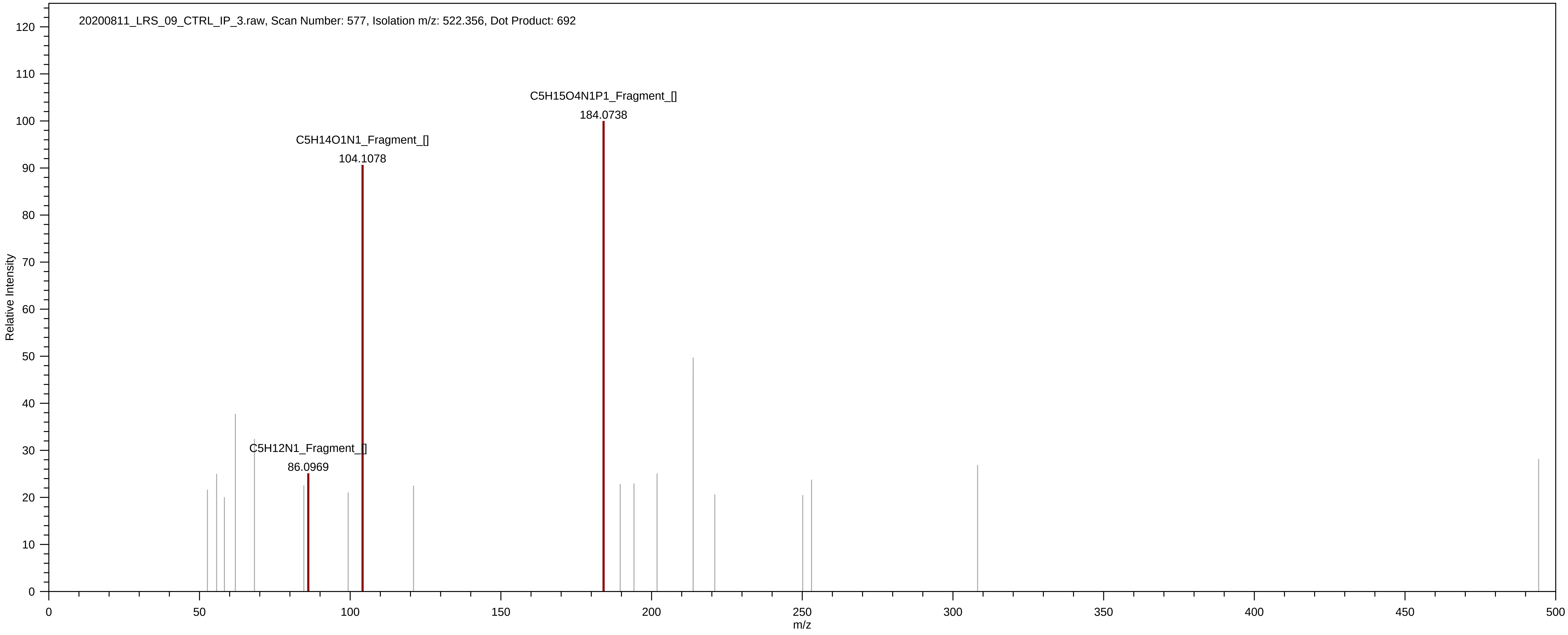

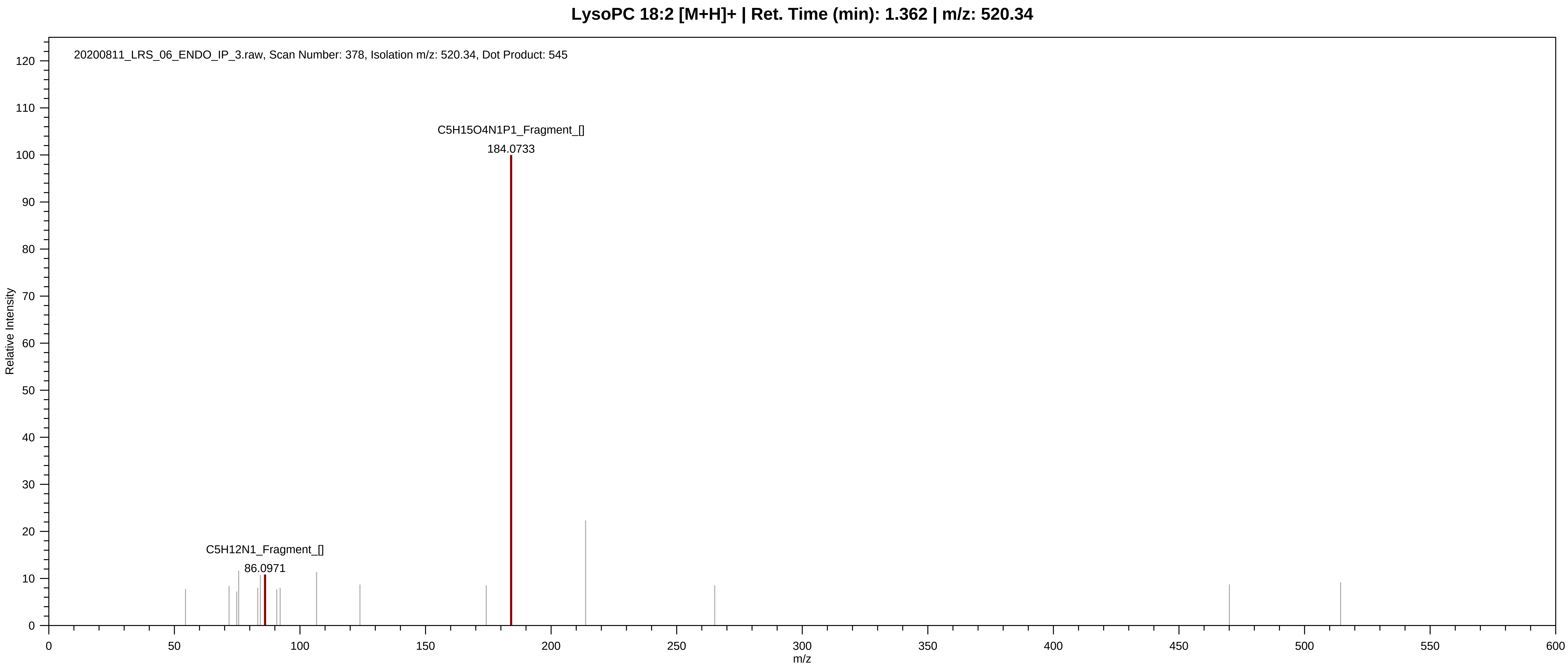
LysoPC 18:2 [M+H]+ | Ret. Time (min): 1.362 | m/z: 520.34
20200811_LRS_06_ENDO_IP_3.raw, Scan Number: 378, Isolation m/z: 520.34, Dot Product: 545
C5H15O4N1P1_Fragment_[]
184.0733
C5H12N1_Fragment_[]
86.0971
Relative Intensity
m/z
0
10
20
30
40
50
60
70
80
90
100
110
120
0
50
100
150
200
250
300
350
400
450
500
550
600

LysoPC 18:2 [M+H]+ | Ret. Time (min): 1.401 | m/z: 520.339

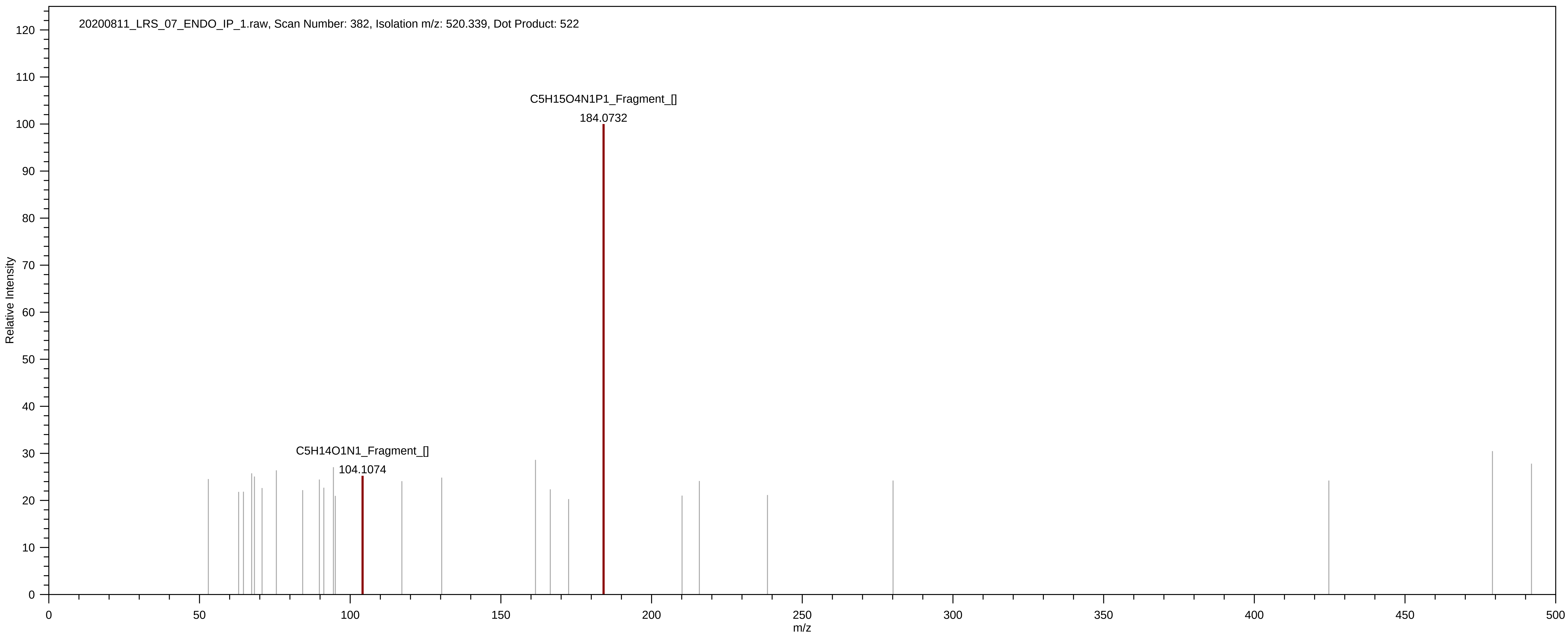

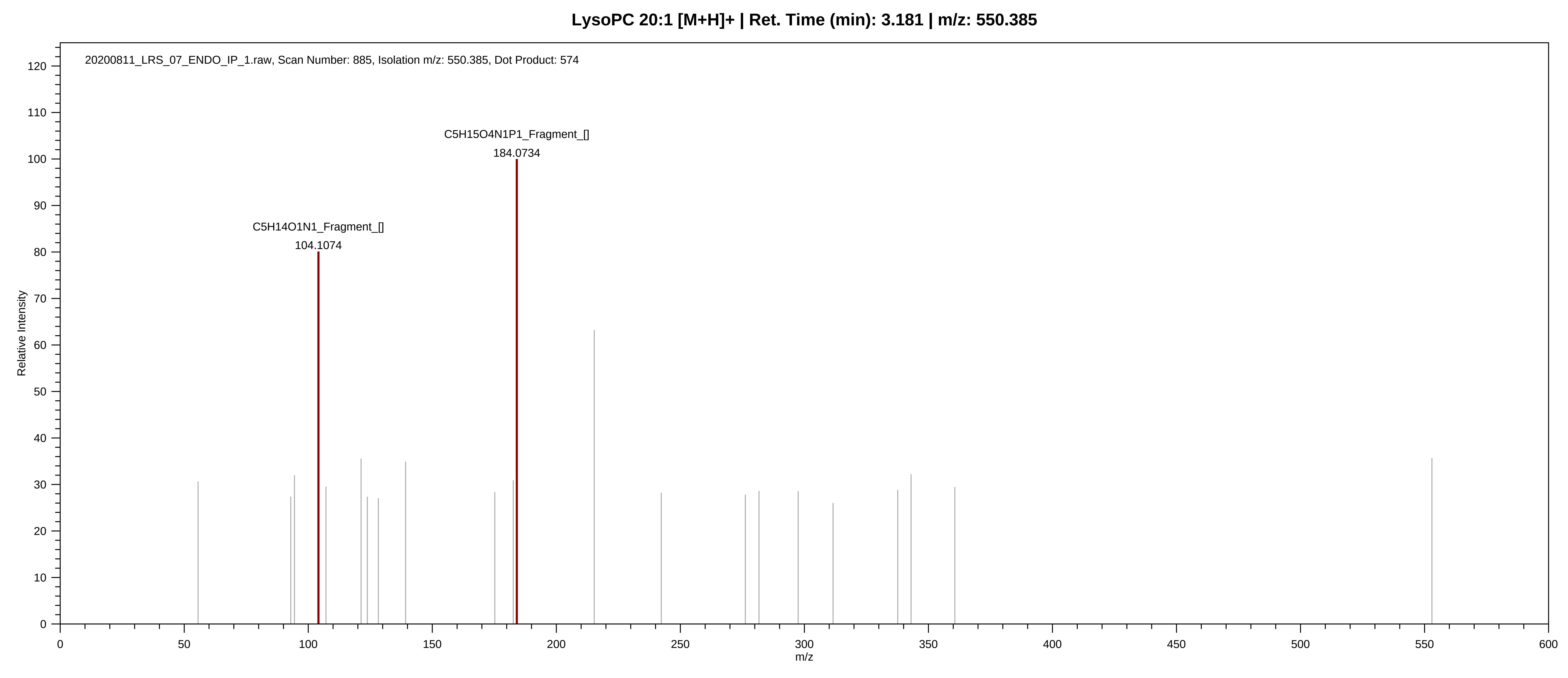
LysoPC 20:1 [M+H]+ | Ret. Time (min): 3.181 | m/z: 550.385
20200811_LRS_07_ENDO_IP_1.raw, Scan Number: 885, Isolation m/z: 550.385, Dot Product: 574
C5H15O4N1P1_Fragment_[]
184.0734
C5H14O1N1_Fragment_[]
104.1074
Relative Intensity
m/z
0
10
20
30
40
50
60
70
80
90
100
110
120
0
50
100
150
200
250
300
350
400
450
500
550
600

LysoPC 20:1 [M+H]+ | Ret. Time (min): 3.141 | m/z: 550.386

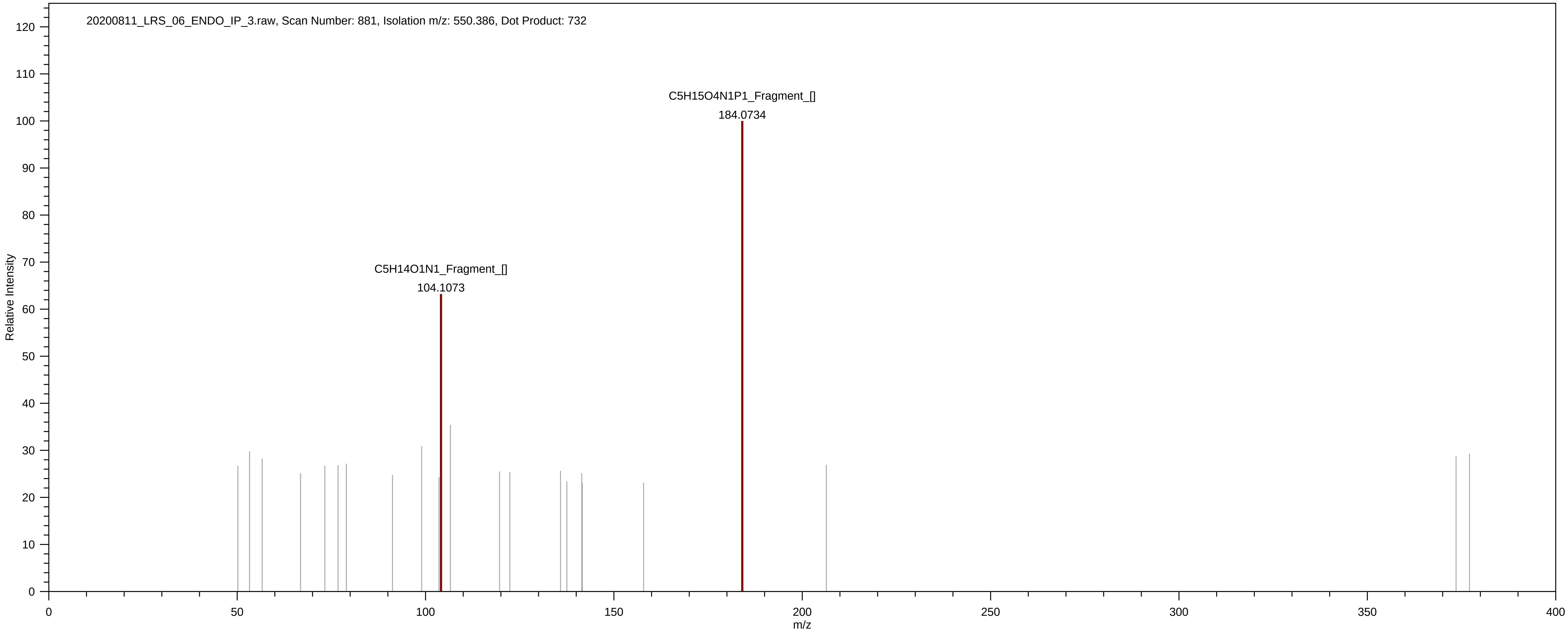

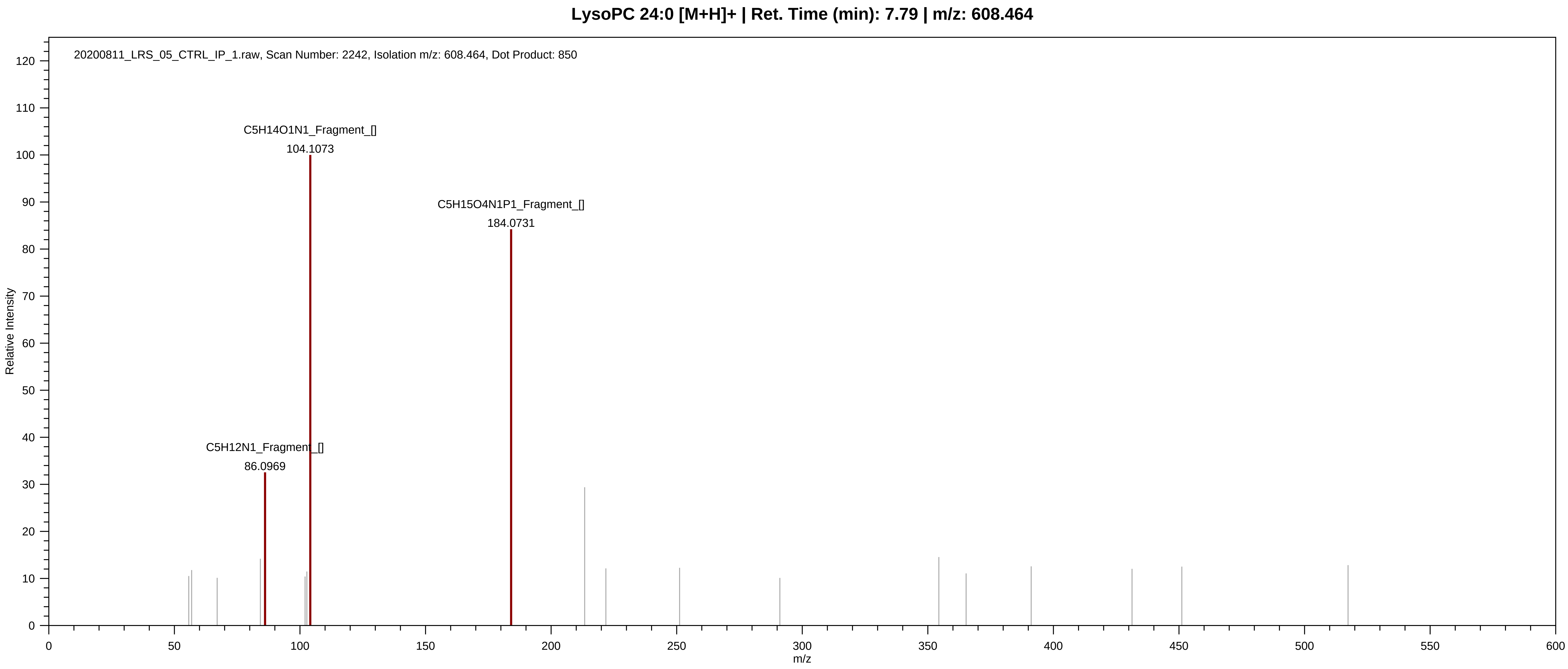
LysoPC 24:0 [M+H]+ | Ret. Time (min): 7.79 | m/z: 608.464
20200811_LRS_05_CTRL_IP_1.raw, Scan Number: 2242, Isolation m/z: 608.464, Dot Product: 850
C5H14O1N1_Fragment_[]
104.1073
C5H15O4N1P1_Fragment_[]
184.0731
C5H12N1_Fragment_[]
86.0969
Relative Intensity
m/z
0
10
20
30
40
50
60
70
80
90
100
110
120
0
50
100
150
200
250
300
350
400
450
500
550
600

LysoPC 24:0 [M+H]+ | Ret. Time (min): 7.758 | m/z: 608.464

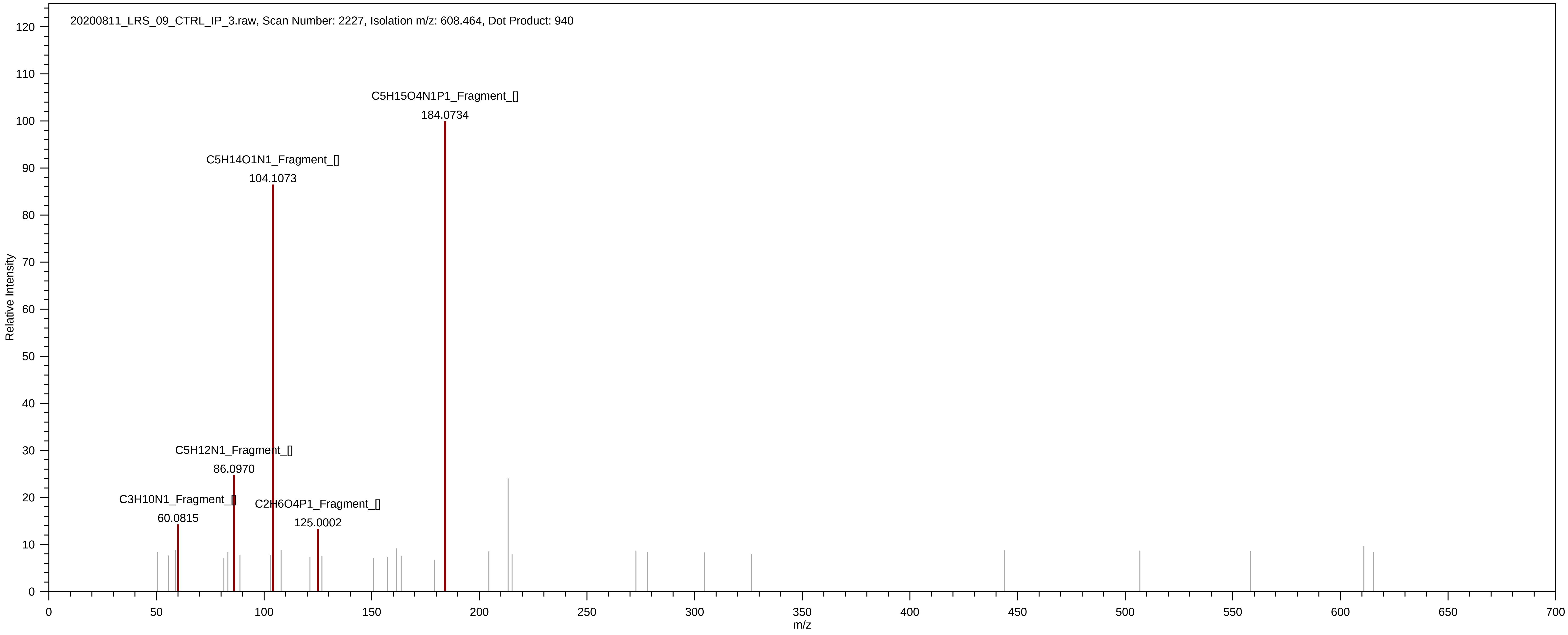

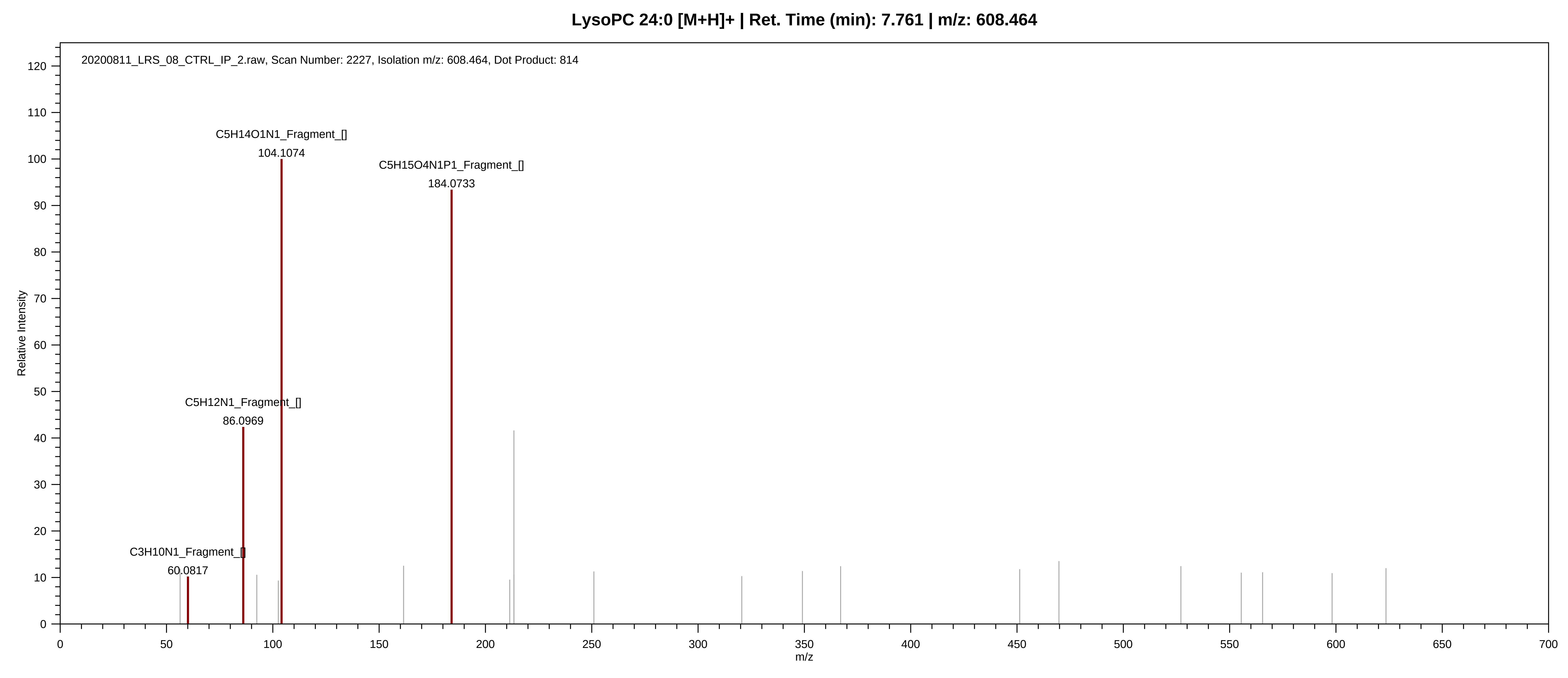

LysoPC 24:0 [M+H]+ | Ret. Time (min): 7.761 | m/z: 608.464
20200811_LRS_08_CTRL_IP_2.raw, Scan Number: 2227, Isolation m/z: 608.464, Dot Product: 814
C5H14O1N1_Fragment_[]
104.1074
C5H15O4N1P1_Fragment_[]
184.0733
C5H12N1_Fragment_[]
86.0969
C3H10N1_Fragment_[]
60.0817
Relative Intensity
m/z

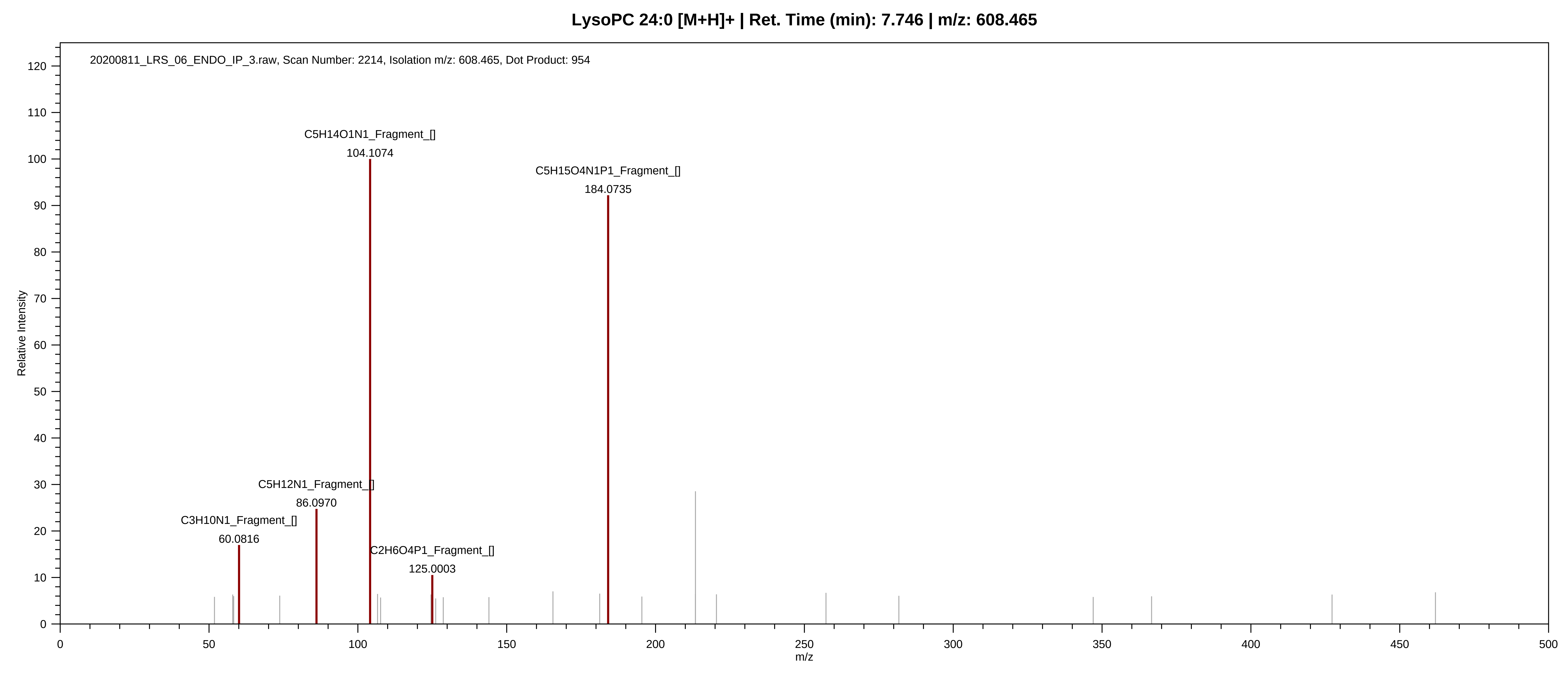
LysoPC 24:0 [M+H]+ | Ret. Time (min): 7.746 | m/z: 608.465
20200811_LRS_06_ENDO_IP_3.raw, Scan Number: 2214, Isolation m/z: 608.465, Dot Product: 954
C5H14O1N1_Fragment_[]
104.1074
C5H15O4N1P1_Fragment_[]
184.0735
C5H12N1_Fragment_[]
86.0970
C3H10N1_Fragment_[]
60.0816
C2H6O4P1_Fragment_[]
125.0003
Relative Intensity
m/z
0
10
20
30
40
50
60
70
80
90
100
110
120
0
50
100
150
200
250
300
350
400
450
500

LysoPC 24:0 [M+H]+ | Ret. Time (min): 7.753 | m/z: 608.465

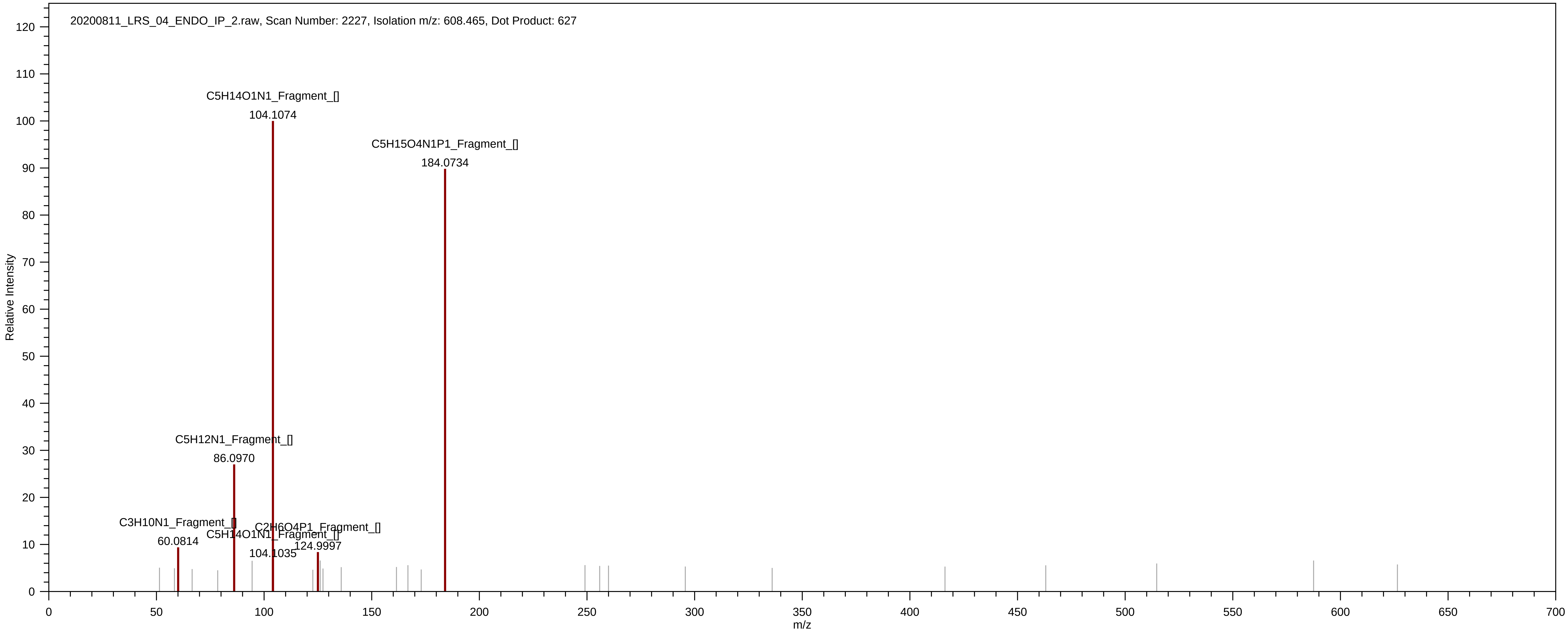

LysoPC 24:1 [M+H]+ | Ret. Time (min): 6.972 | m/z: 606.45

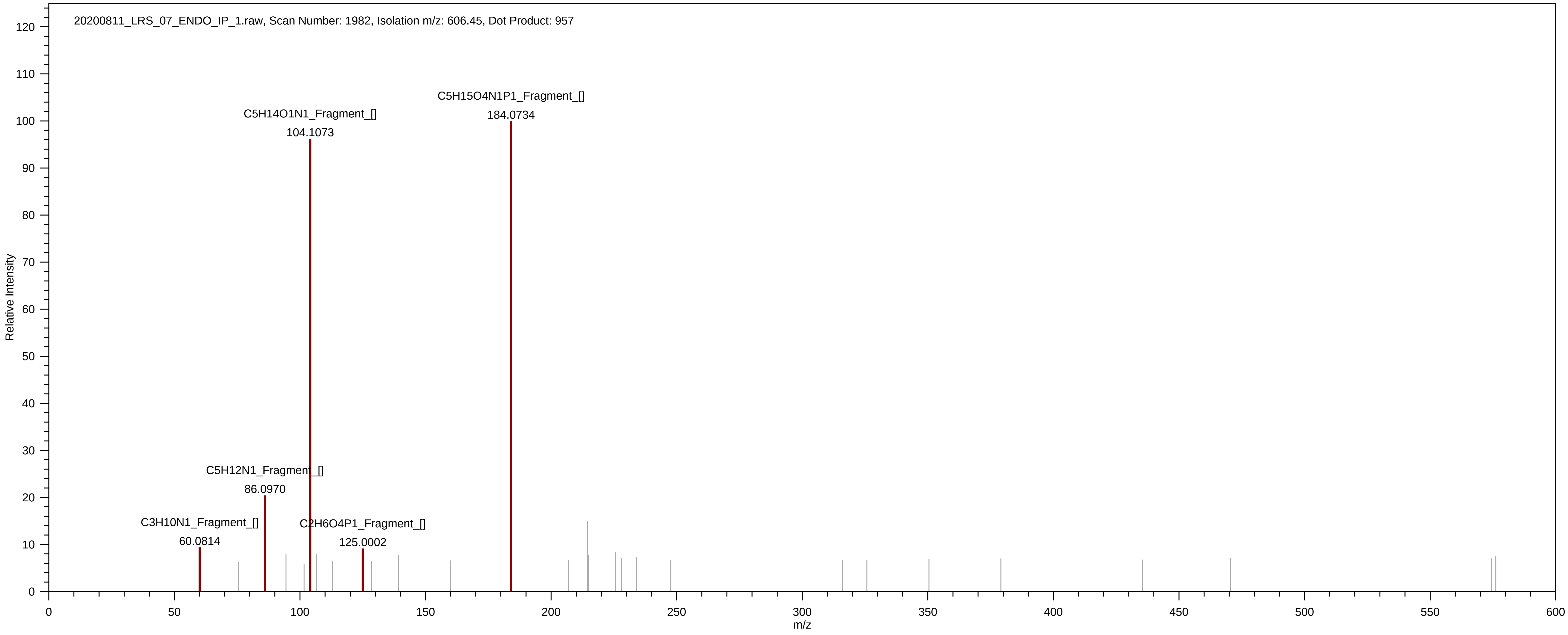

LysoPC 24:1 [M+H]+ | Ret. Time (min): 6.972 | m/z: 606.451

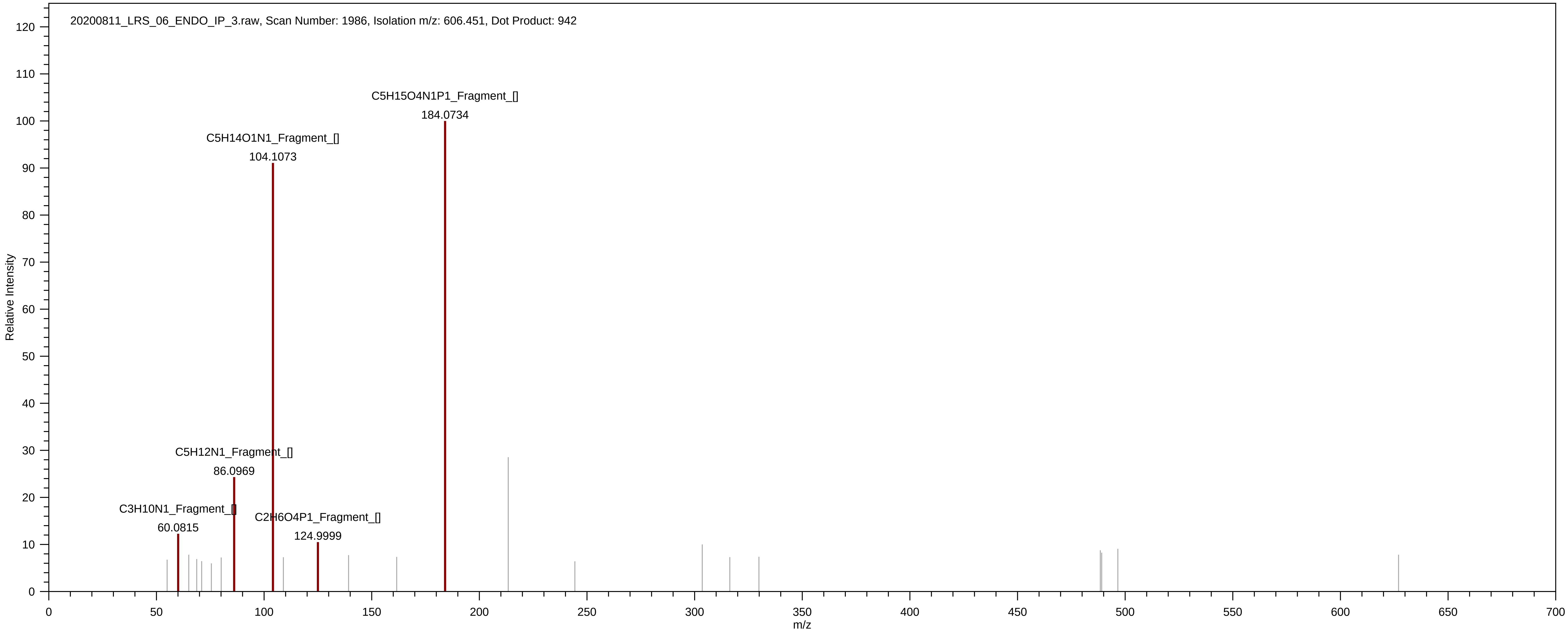

LysoPC 26:0 [M+H]+ | Ret. Time (min): 8.698 | m/z: 636.495

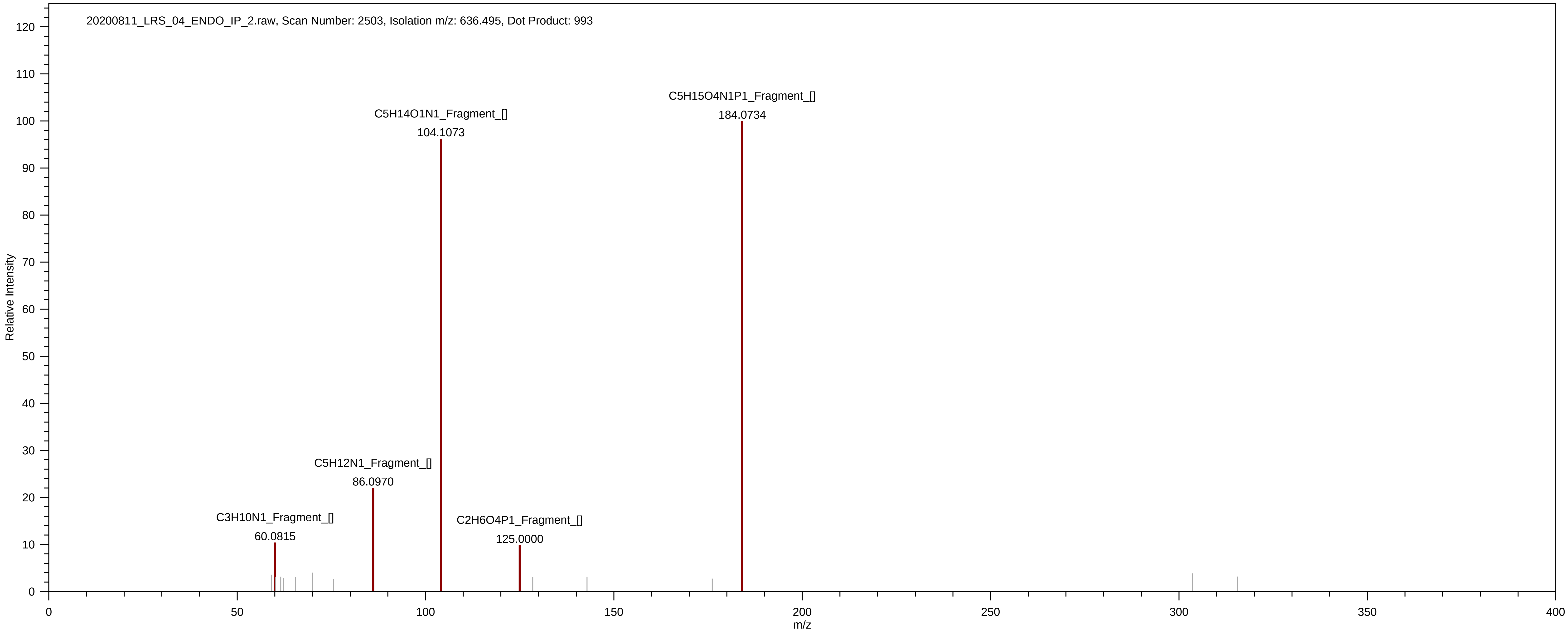

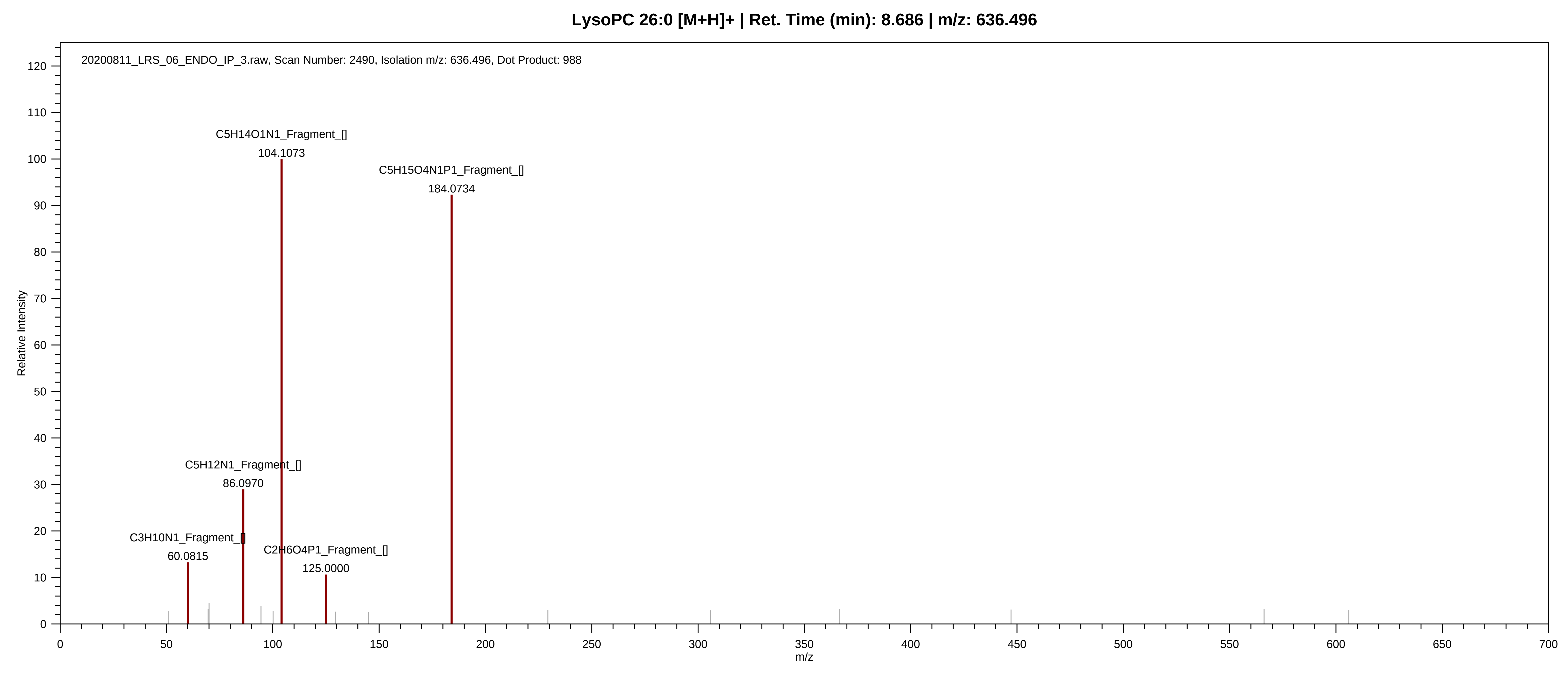
LysoPC 26:0 [M+H]+ | Ret. Time (min): 8.686 | m/z: 636.496
20200811_LRS_06_ENDO_IP_3.raw, Scan Number: 2490, Isolation m/z: 636.496, Dot Product: 988
C5H14O1N1_Fragment_[]
104.1073
C5H15O4N1P1_Fragment_[]
184.0734
C5H12N1_Fragment_[]
86.0970
C3H10N1_Fragment_[]
60.0815
C2H6O4P1_Fragment_[]
125.0000
Relative Intensity
m/z

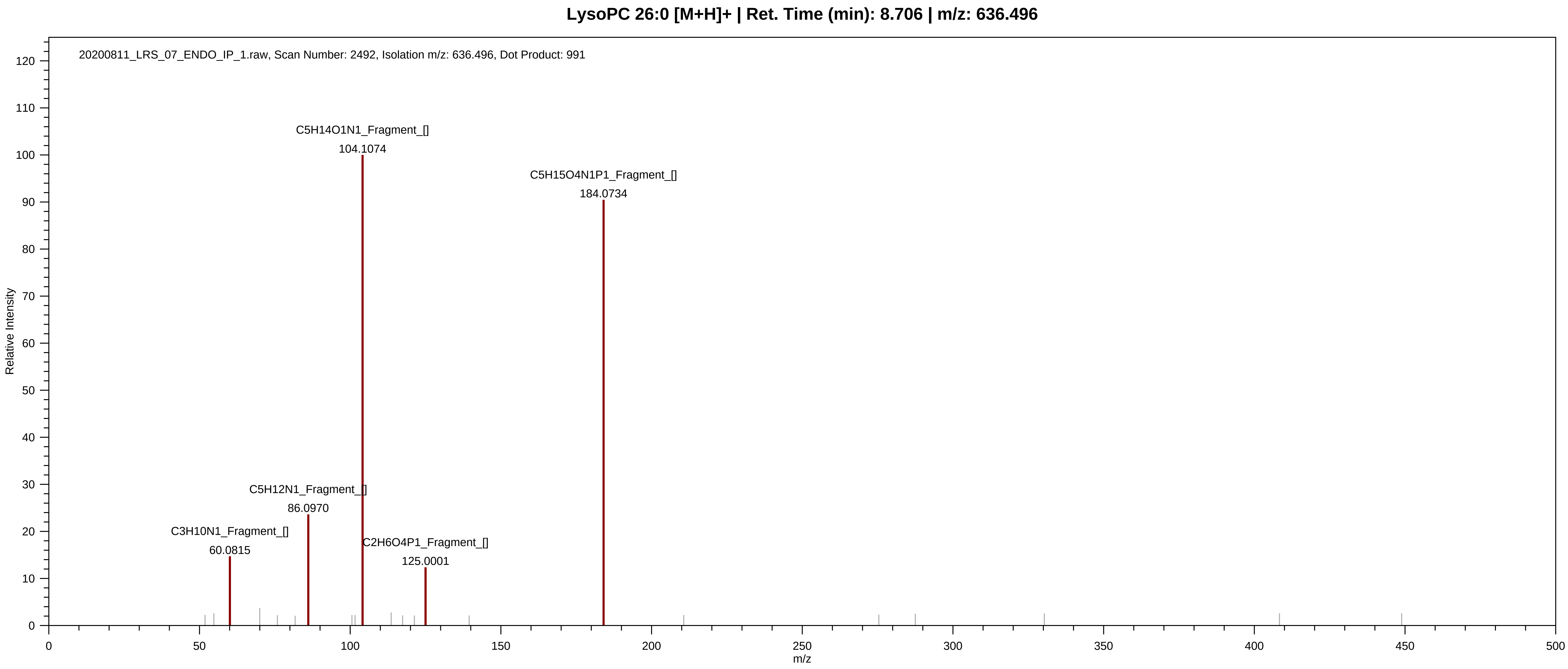

LysoPC 26:0 [M+H]+ | Ret. Time (min): 8.706 | m/z: 636.496
20200811_LRS_07_ENDO_IP_1.raw, Scan Number: 2492, Isolation m/z: 636.496, Dot Product: 991
C5H14O1N1_Fragment_[]
104.1074
C5H15O4N1P1_Fragment_[]
184.0734
C5H12N1_Fragment_[]
86.0970
C3H10N1_Fragment_[]
60.0815
C2H6O4P1_Fragment_[]
125.0001
Relative Intensity
m/z

LysoPC 26:1 [M+H]+ | Ret. Time (min): 7.755 | m/z: 634.481

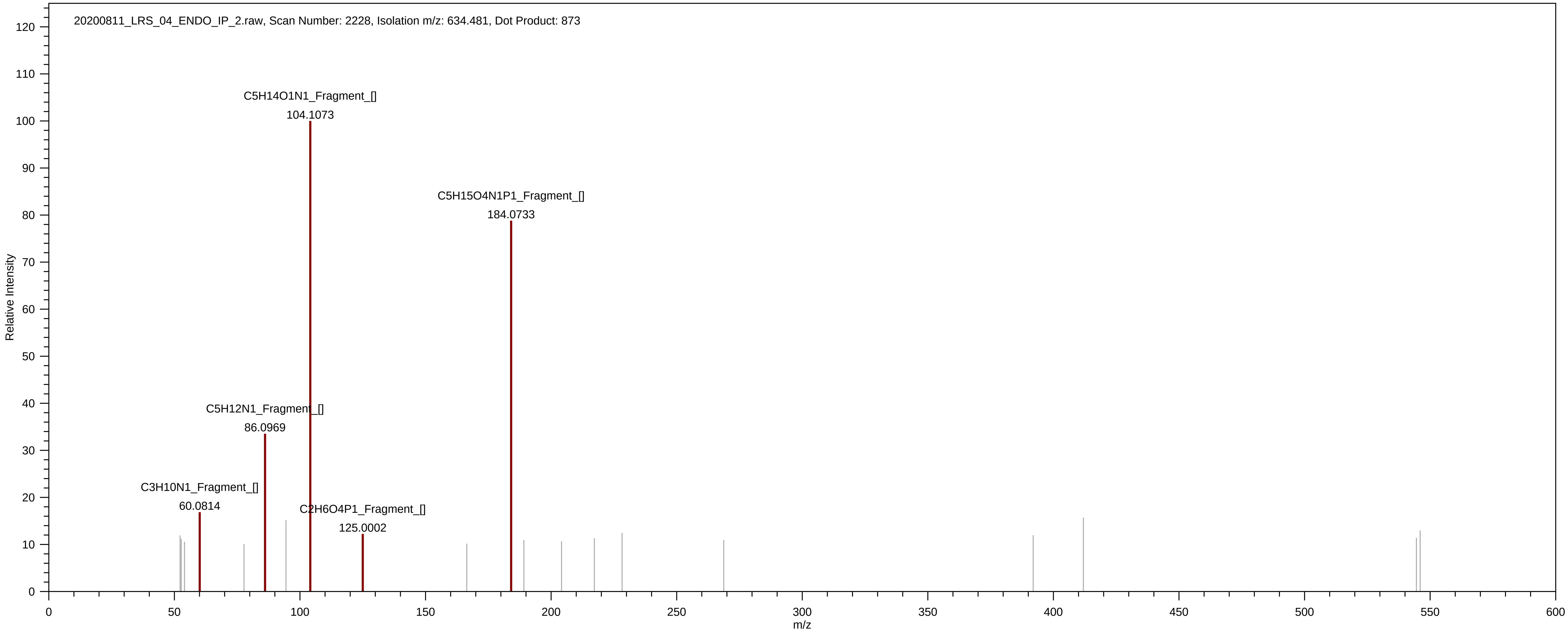

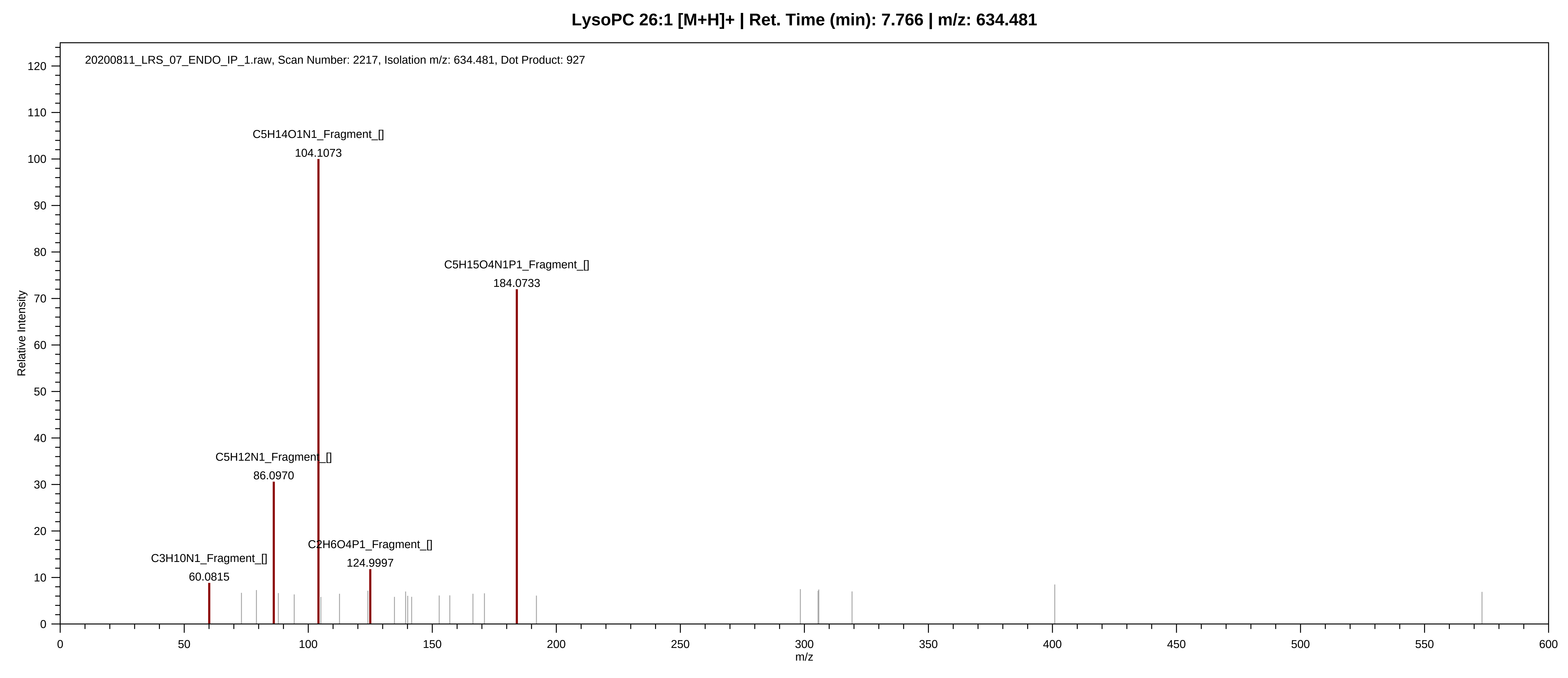

LysoPC 26:1 [M+H]+ | Ret. Time (min): 7.766 | m/z: 634.481
20200811_LRS_07_ENDO_IP_1.raw, Scan Number: 2217, Isolation m/z: 634.481, Dot Product: 927
C5H14O1N1_Fragment_[]
104.1073
C5H15O4N1P1_Fragment_[]
184.0733
C5H12N1_Fragment_[]
86.0970
C2H6O4P1_Fragment_[]
124.9997
C3H10N1_Fragment_[]
60.0815
Relative Intensity
m/z

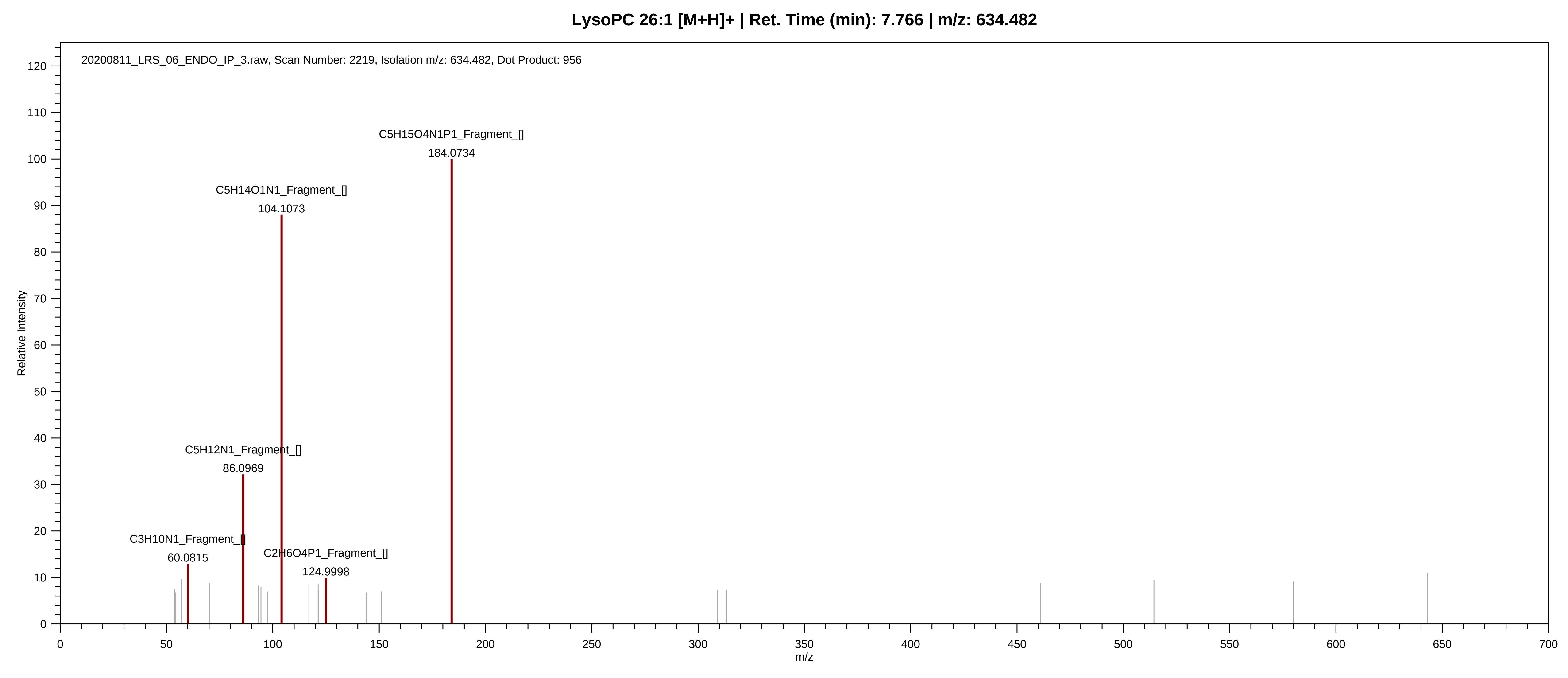

LysoPC 26:1 [M+H]+ | Ret. Time (min): 7.766 | m/z: 634.482
20200811_LRS_06_ENDO_IP_3.raw, Scan Number: 2219, Isolation m/z: 634.482, Dot Product: 956
C5H15O4N1P1_Fragment_[]
184.0734
C5H14O1N1_Fragment_[]
104.1073
C5H12N1_Fragment_[]
86.0969
C3H10N1_Fragment_[]
60.0815
C2H6O4P1_Fragment_[]
124.9998
Relative Intensity
m/z
0
10
20
30
40
50
60
70
80
90
100
110
120
0
50
100
150
200
250
300
350
400
450
500
550
600
650
700

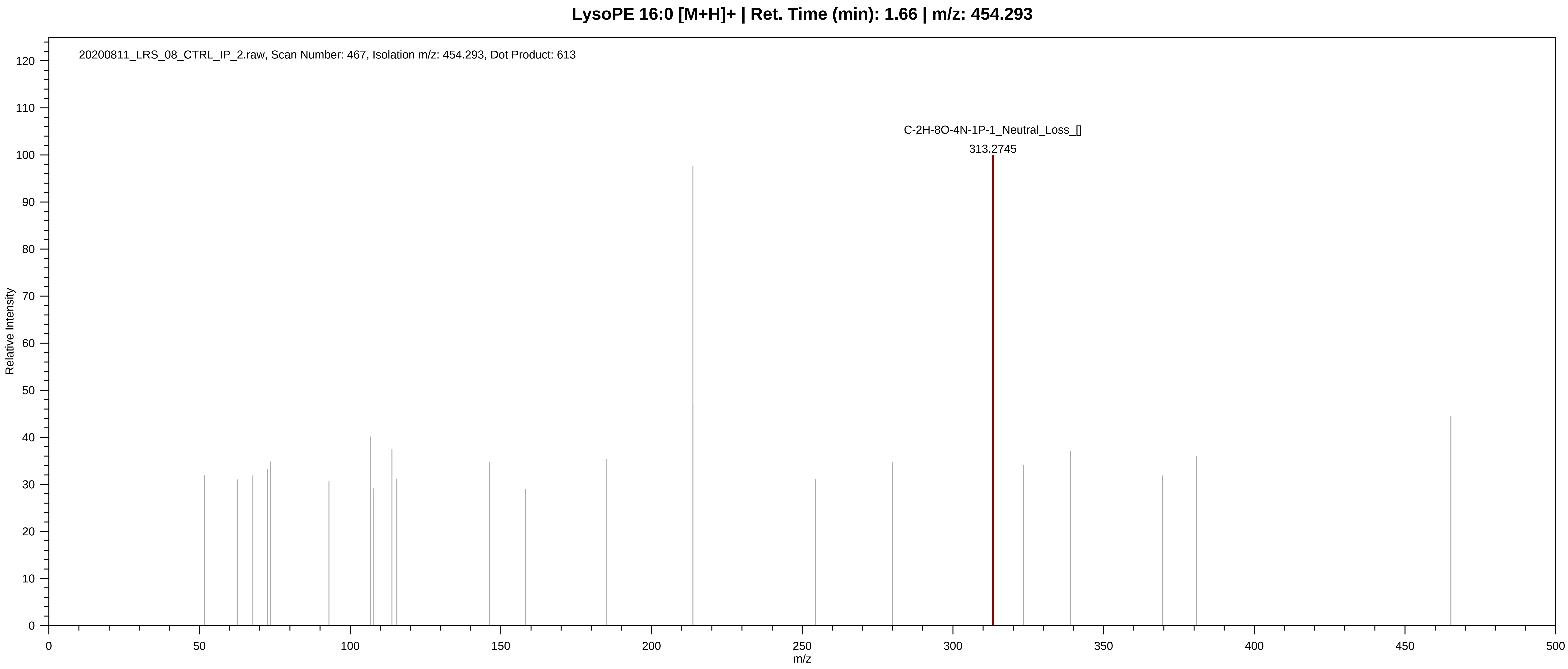

LysoPE 16:0 [M+H]+ | Ret. Time (min): 1.66 | m/z: 454.293
20200811_LRS_08_CTRL_IP_2.raw, Scan Number: 467, Isolation m/z: 454.293, Dot Product: 613
C-2H-8O-4N-1P-1_Neutral_Loss_[]
313.2745
Relative Intensity
m/z

LysoPE 16:0 [M+H]+ | Ret. Time (min): 1.661 | m/z: 454.293

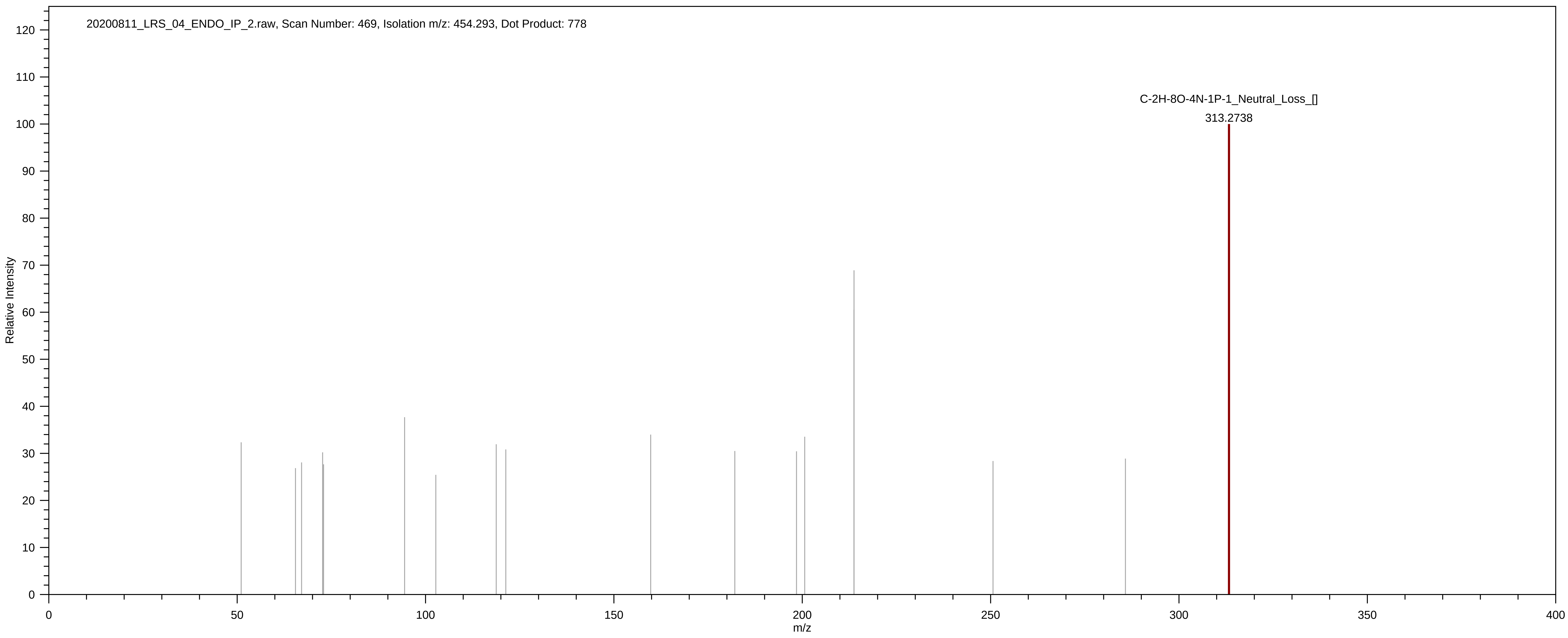

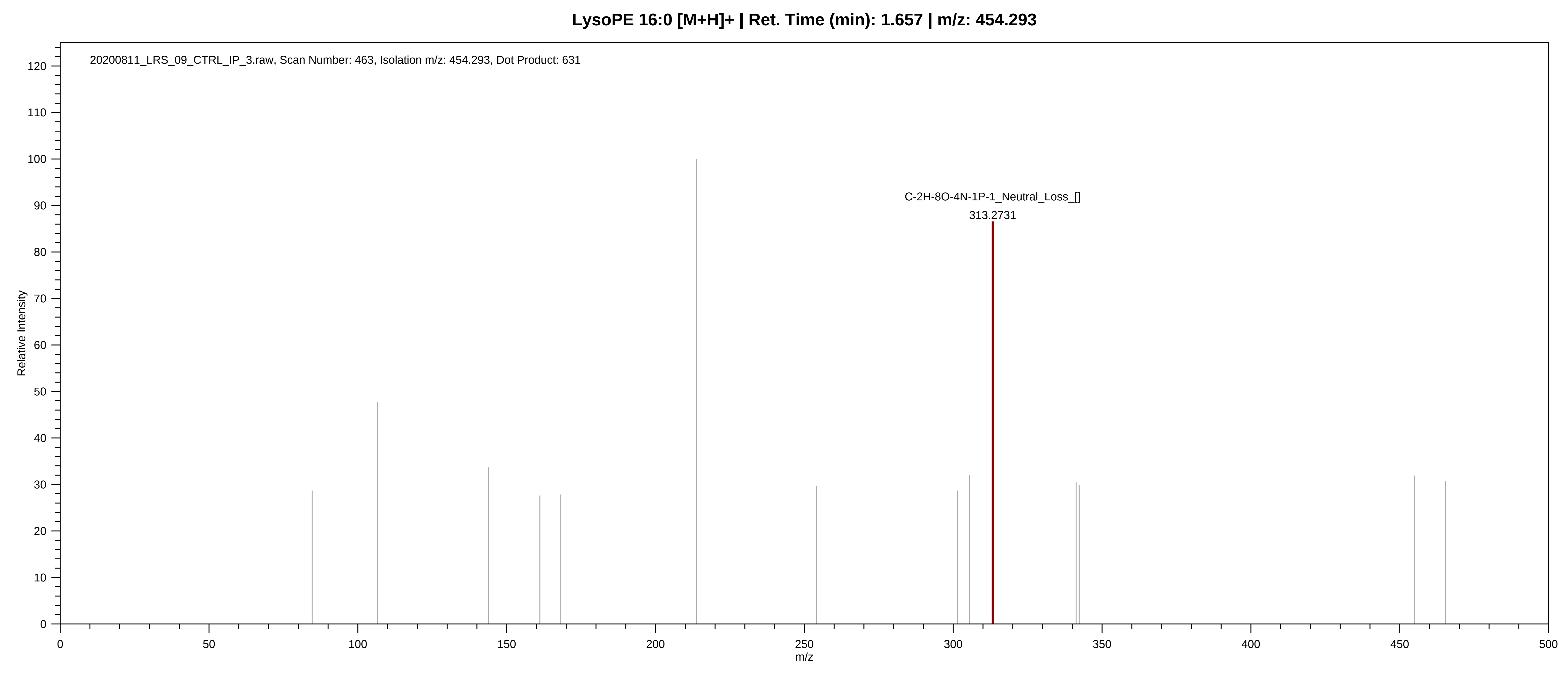

LysoPE 16:0 [M+H]+ | Ret. Time (min): 1.657 | m/z: 454.293
20200811_LRS_09_CTRL_IP_3.raw, Scan Number: 463, Isolation m/z: 454.293, Dot Product: 631
C-2H-8O-4N-1P-1_Neutral_Loss_[]
313.2731
Relative Intensity
m/z

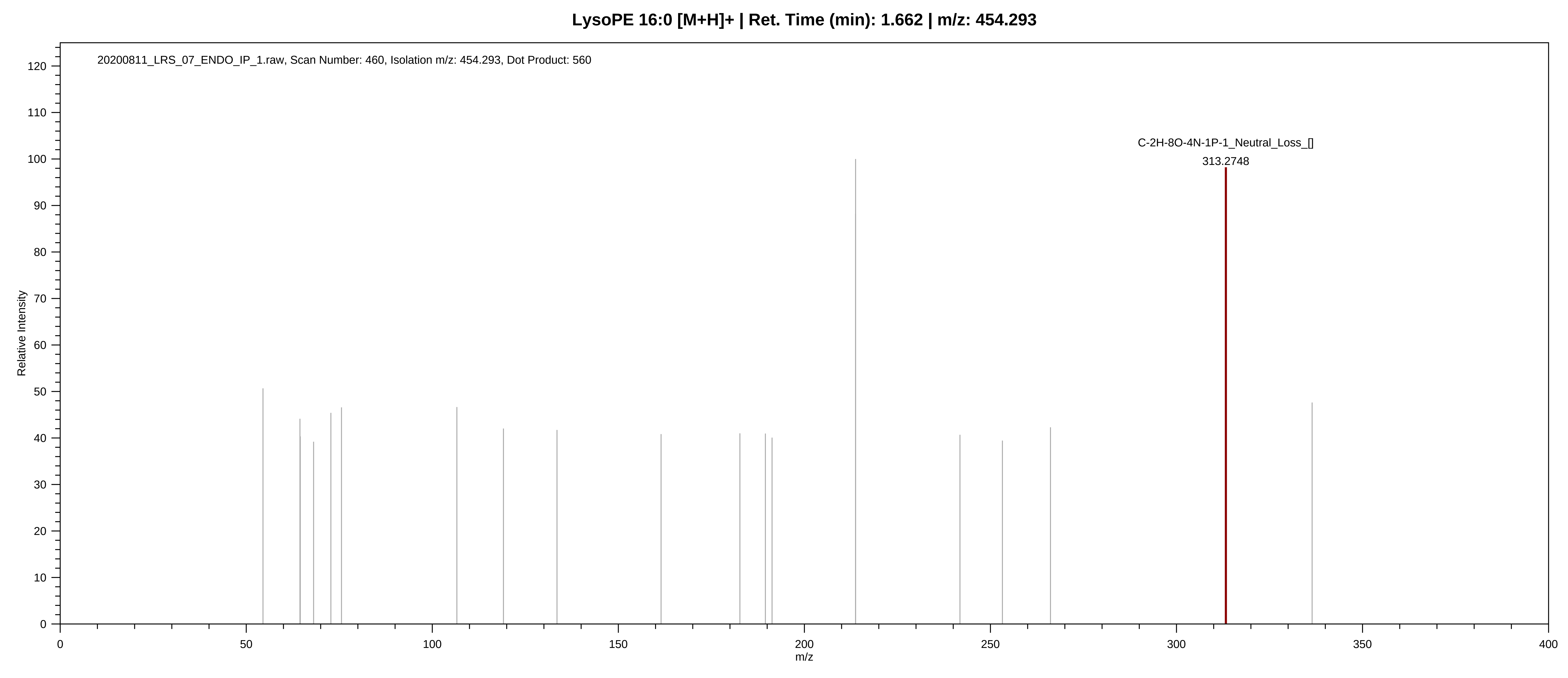

LysoPE 16:0 [M+H]+ | Ret. Time (min): 1.662 | m/z: 454.293
20200811_LRS_07_ENDO_IP_1.raw, Scan Number: 460, Isolation m/z: 454.293, Dot Product: 560
C-2H-8O-4N-1P-1_Neutral_Loss_[]
313.2748
Relative Intensity
m/z

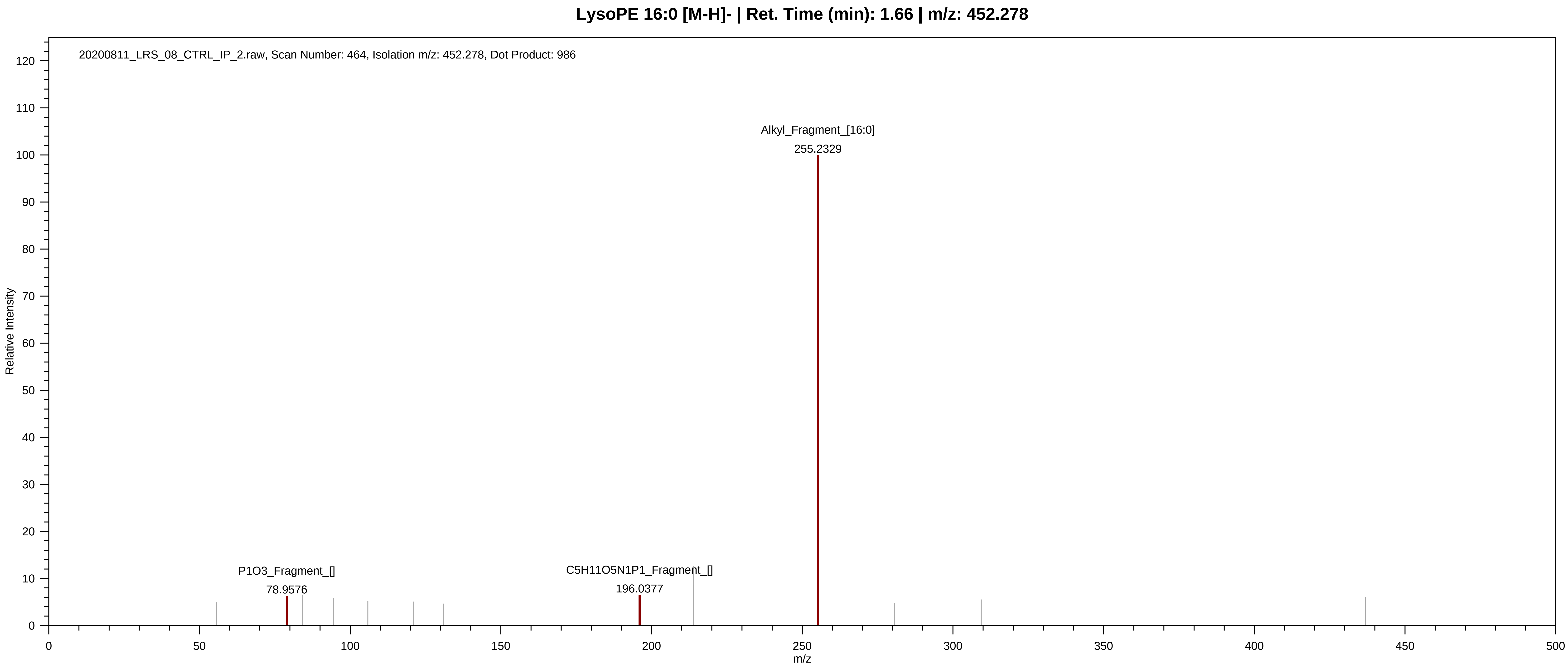
LysoPE 16:0 [M-H]- | Ret. Time (min): 1.66 | m/z: 452.278
20200811_LRS_08_CTRL_IP_2.raw, Scan Number: 464, Isolation m/z: 452.278, Dot Product: 986
Alkyl_Fragment_[16:0]
255.2329
P1O3_Fragment_[]
78.9576
C5H11O5N1P1_Fragment_[]
196.0377
Relative Intensity
m/z
0
10
20
30
40
50
60
70
80
90
100
110
120
0
50
100
150
200
250
300
350
400
450
500

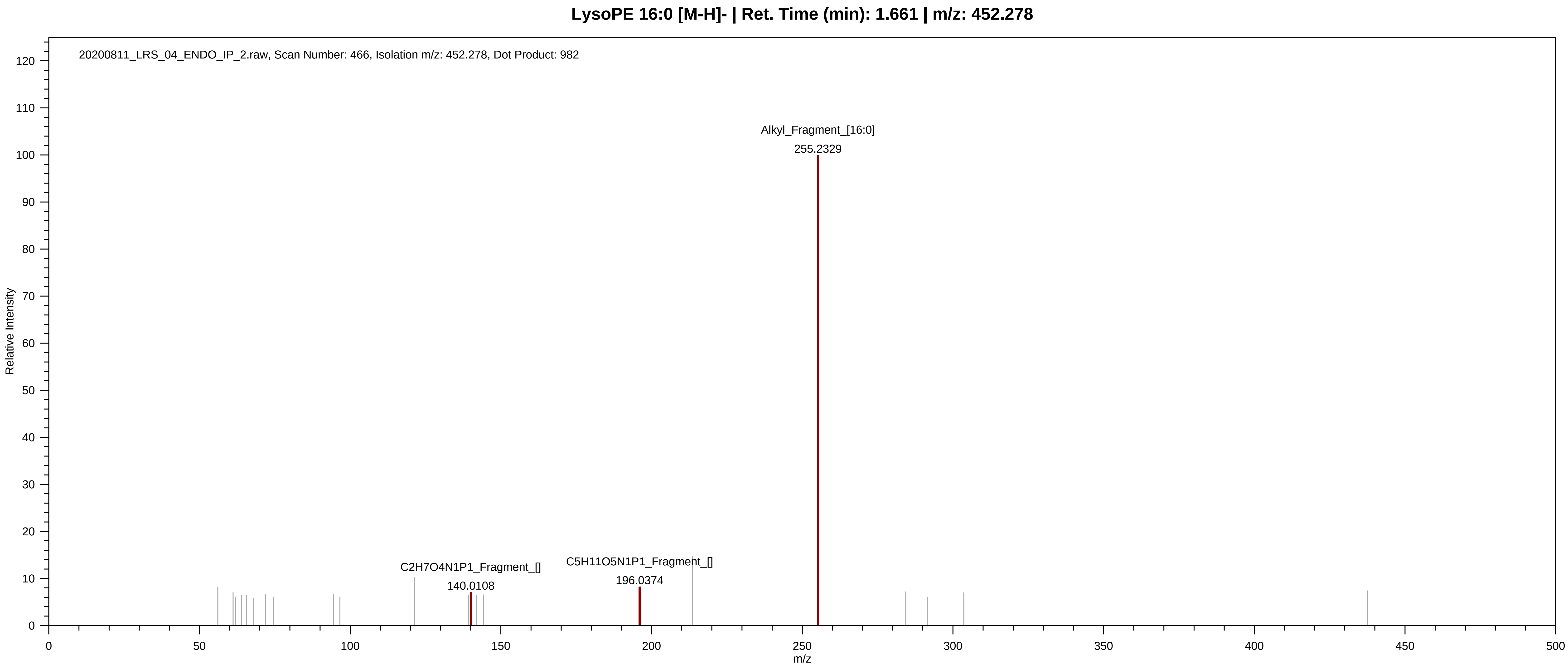
LysoPE 16:0 [M-H]- | Ret. Time (min): 1.661 | m/z: 452.278
20200811_LRS_04_ENDO_IP_2.raw, Scan Number: 466, Isolation m/z: 452.278, Dot Product: 982
Alkyl_Fragment_[16:0]
255.2329
C2H7O4N1P1_Fragment_[]
140.0108
C5H11O5N1P1_Fragment_[]
196.0374
Relative Intensity
m/z

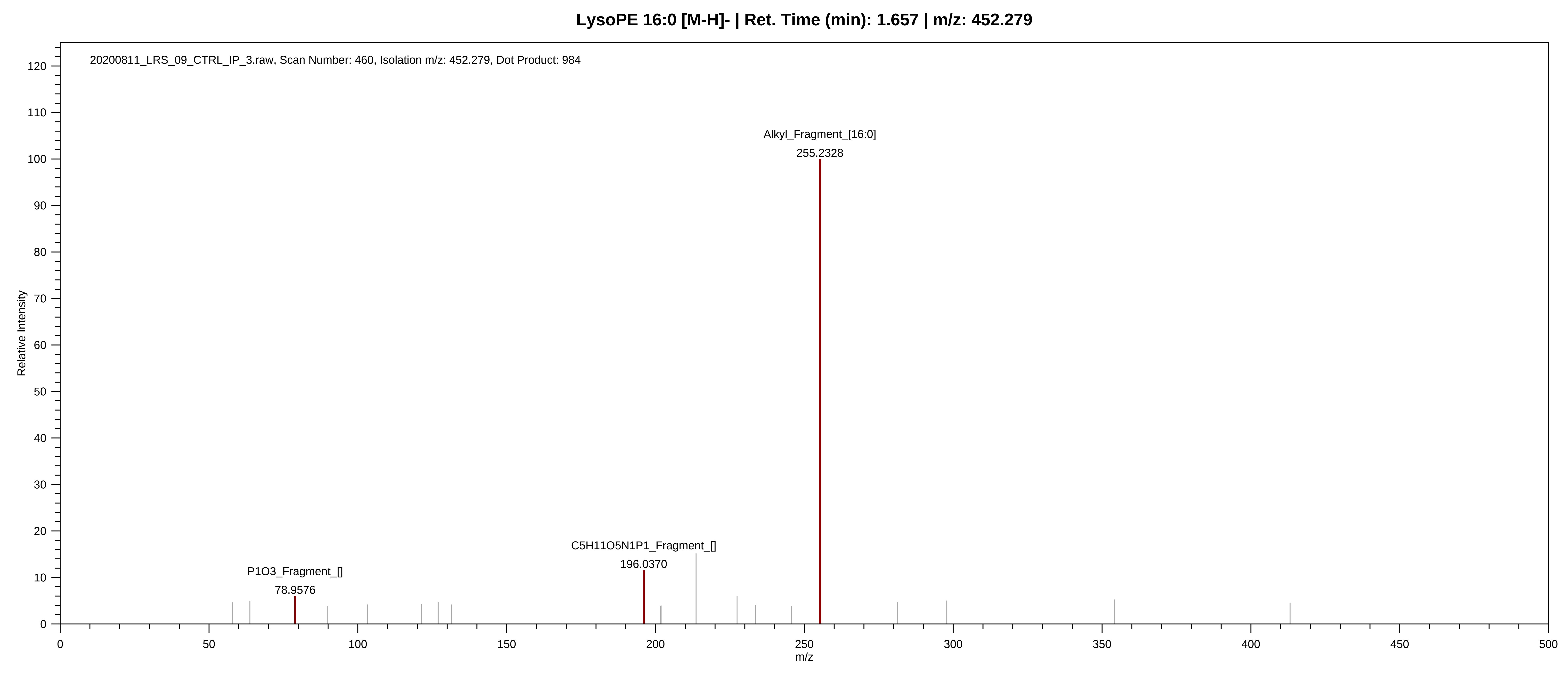
LysoPE 16:0 [M-H]- | Ret. Time (min): 1.657 | m/z: 452.279
20200811_LRS_09_CTRL_IP_3.raw, Scan Number: 460, Isolation m/z: 452.279, Dot Product: 984
Alkyl_Fragment_[16:0]
255.2328
C5H11O5N1P1_Fragment_[]
196.0370
P1O3_Fragment_[]
78.9576
Relative Intensity
m/z

LysoPE 16:0 [M-H]- | Ret. Time (min): 1.662 | m/z: 452.279

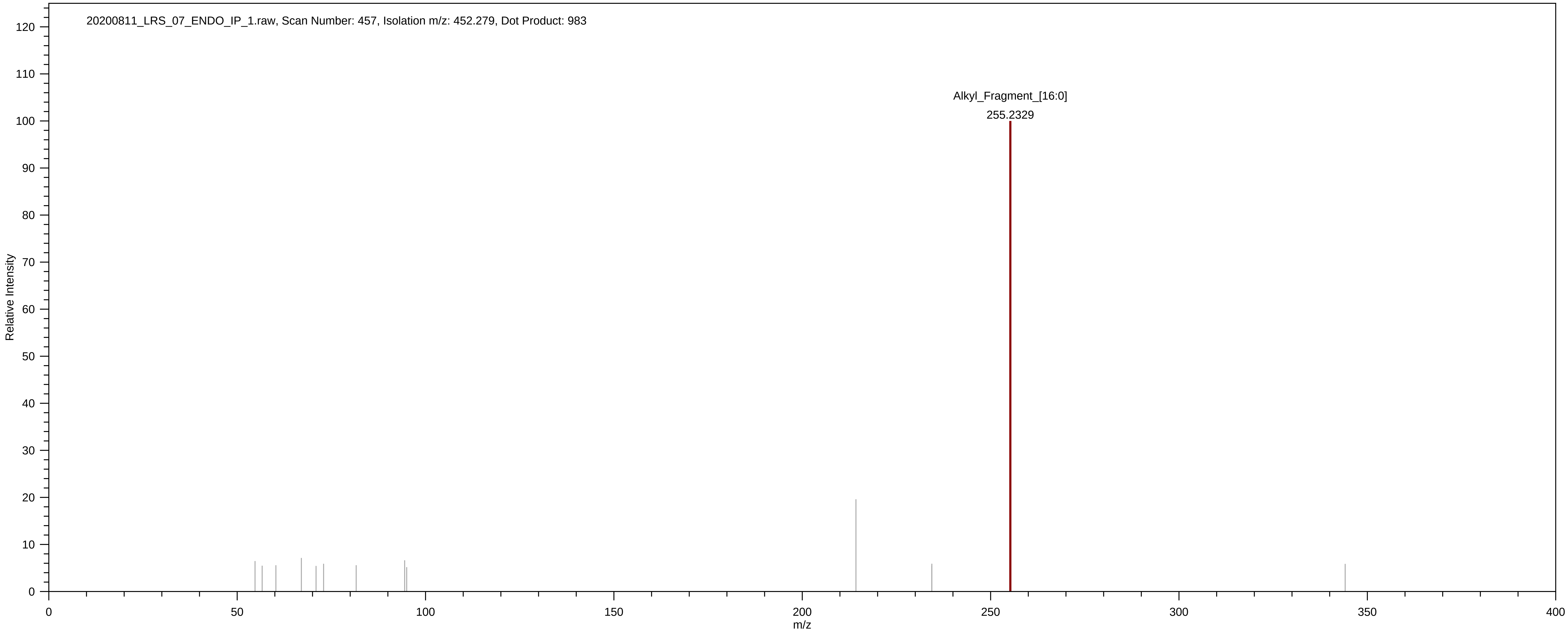

LysoPE 16:0 [M-H]- | Ret. Time (min): 1.664 | m/z: 452.279

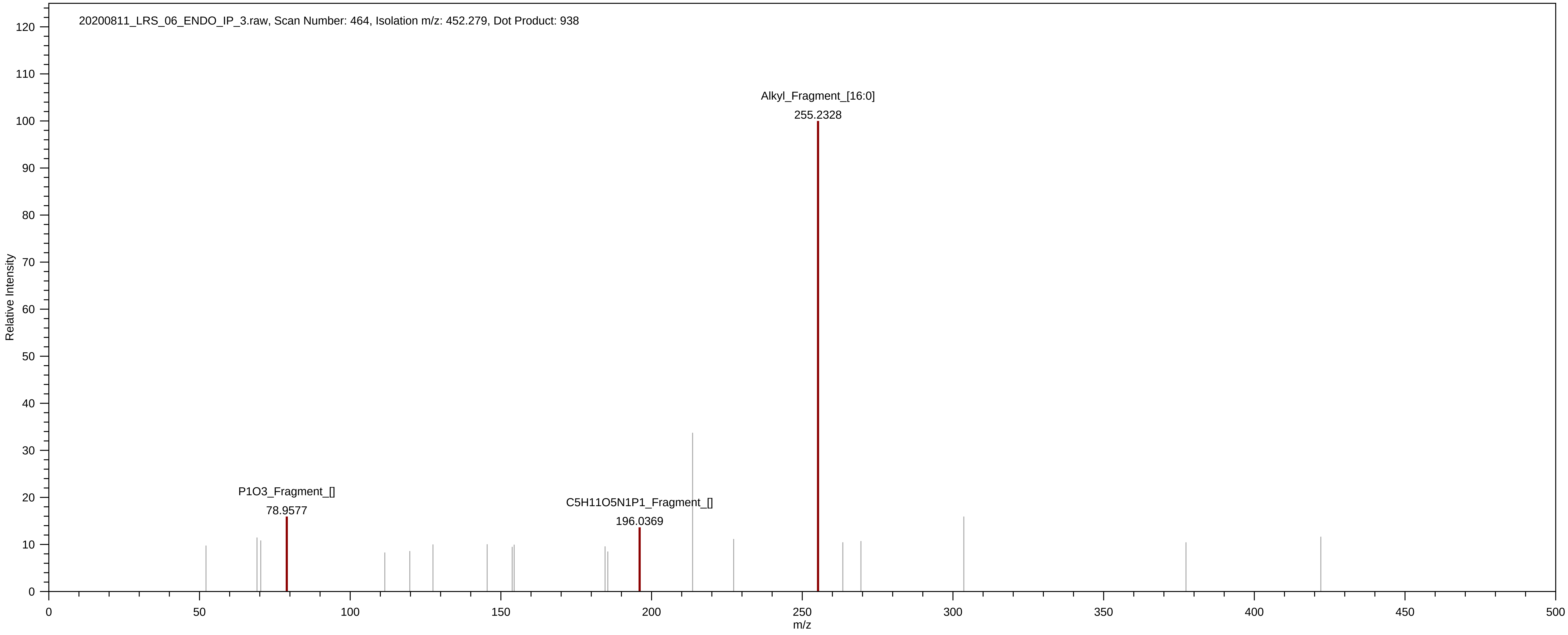

LysoPE 18:0 [M+H]+ | Ret. Time (min): 2.762 | m/z: 482.324

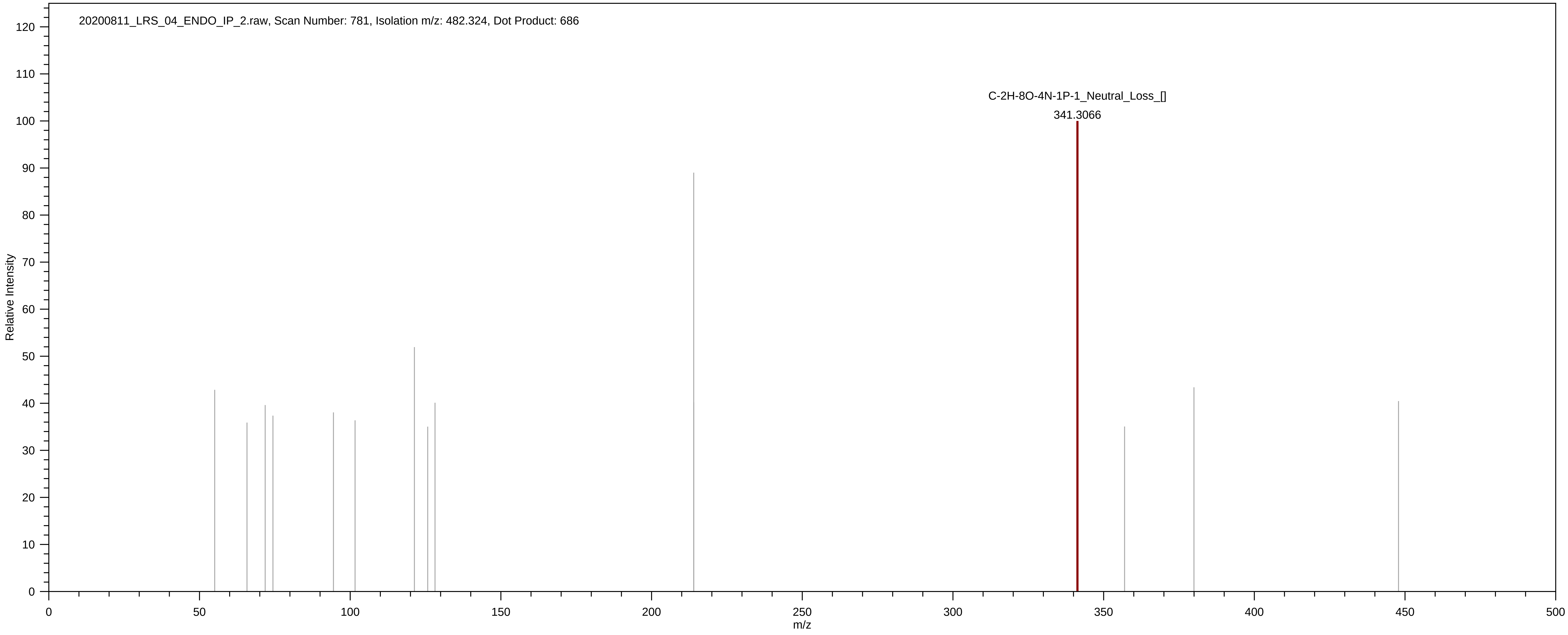

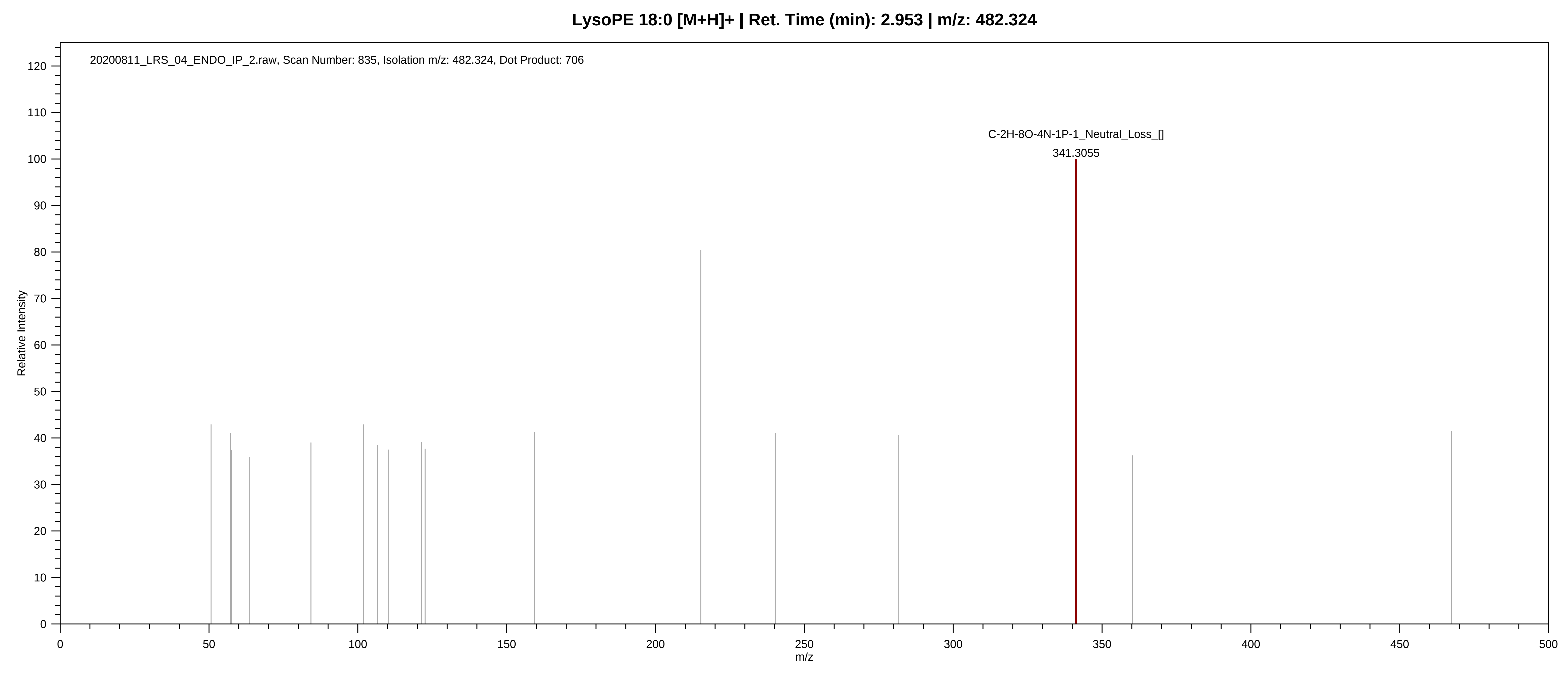

LysoPE 18:0 [M+H]+ | Ret. Time (min): 2.953 | m/z: 482.324
20200811_LRS_04_ENDO_IP_2.raw, Scan Number: 835, Isolation m/z: 482.324, Dot Product: 706
C-2H-8O-4N-1P-1_Neutral_Loss_[]
341.3055
Relative Intensity
m/z

LysoPE 18:0 [M+H]+ | Ret. Time (min): 2.746 | m/z: 482.324

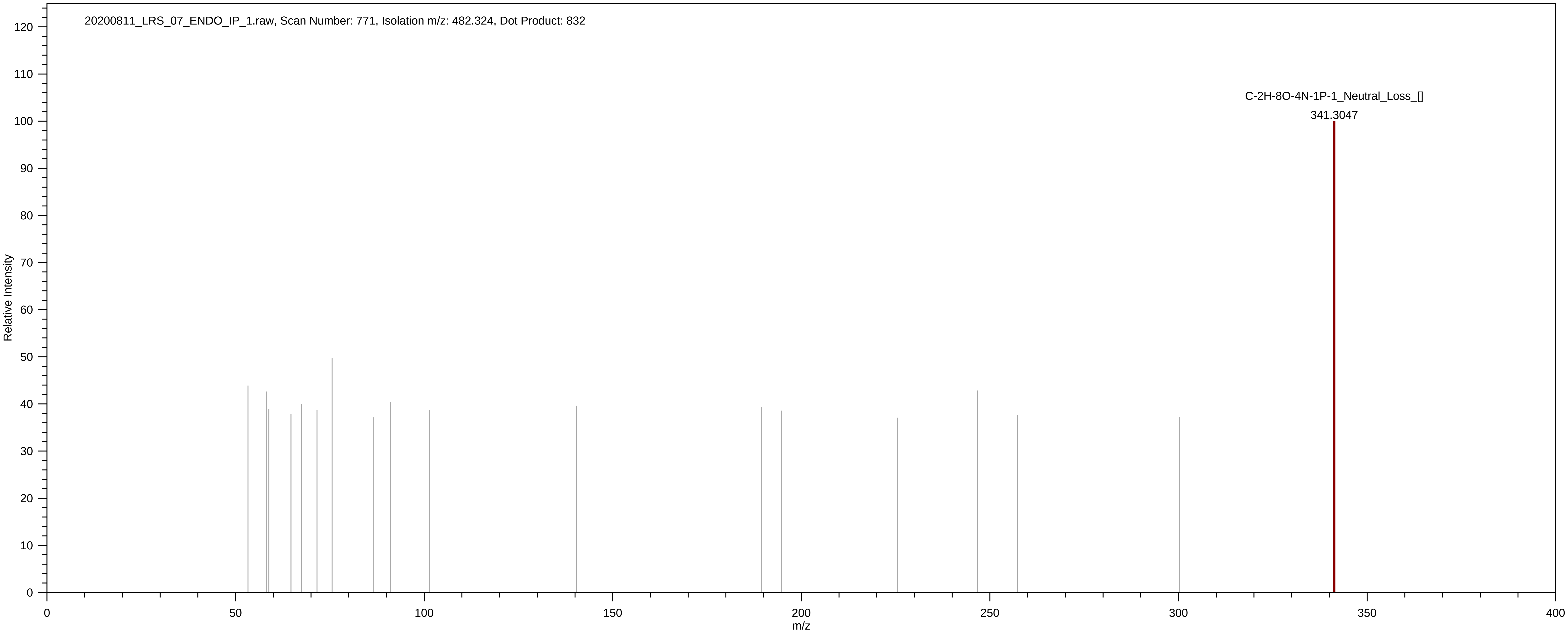

LysoPE 18:0 [M-H]- | Ret. Time (min): 2.75 | m/z: 480.31

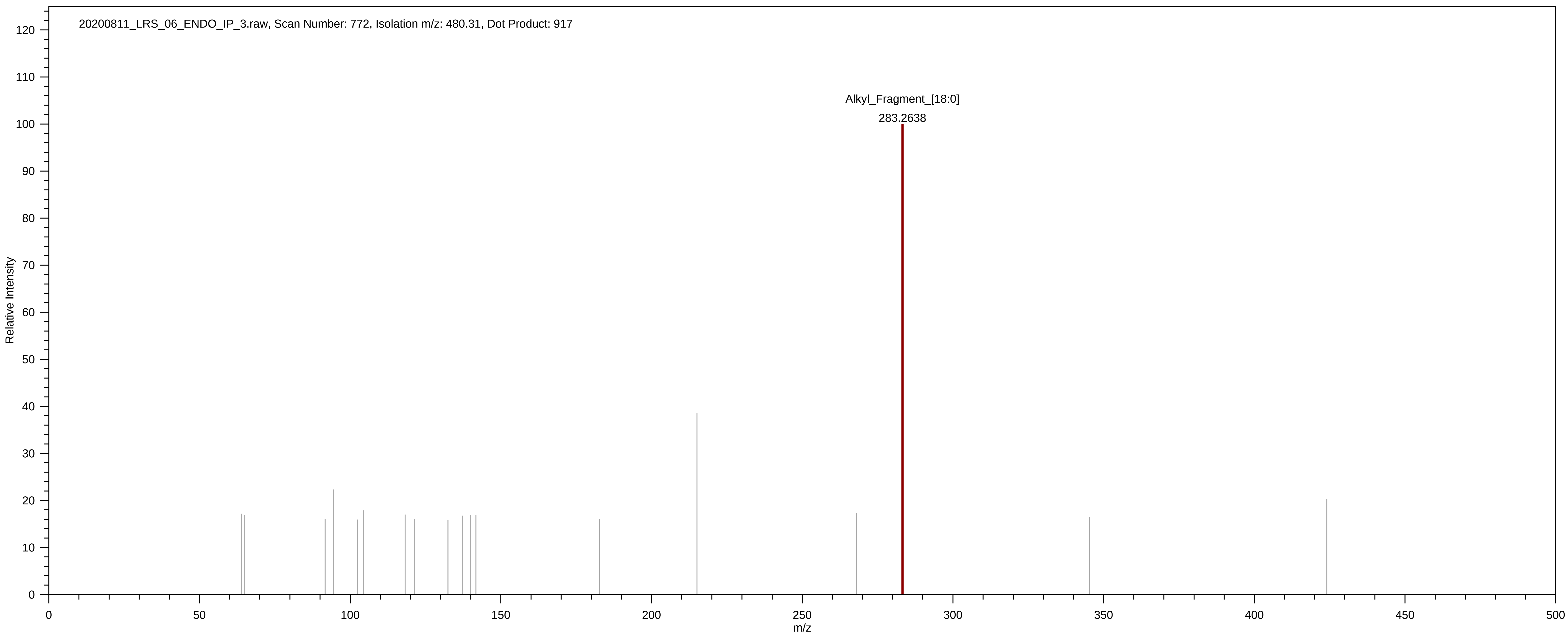

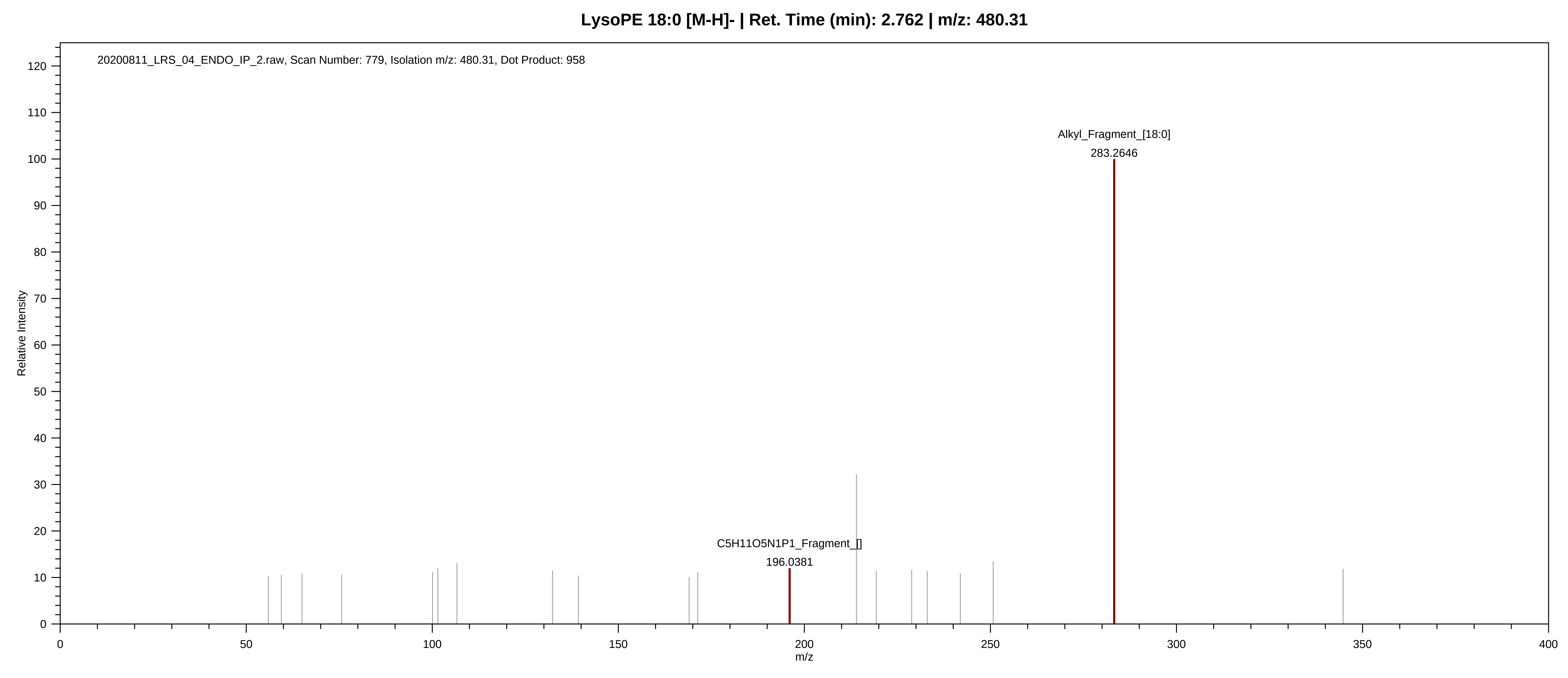
LysoPE 18:0 [M-H]- | Ret. Time (min): 2.762 | m/z: 480.31
20200811_LRS_04_ENDO_IP_2.raw, Scan Number: 779, Isolation m/z: 480.31, Dot Product: 958
Alkyl_Fragment_[18:0]
283.2646
C5H11O5N1P1_Fragment_[]
196.0381
Relative Intensity
m/z

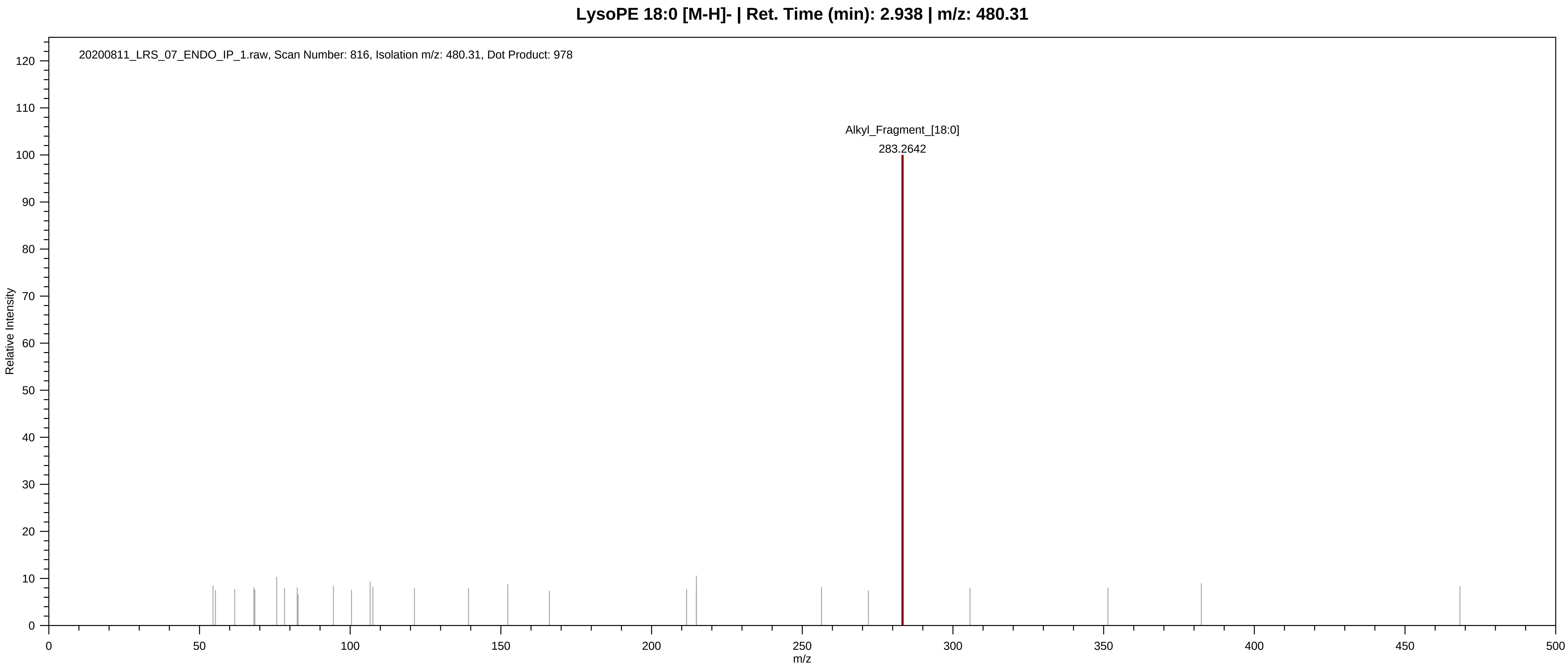

LysoPE 18:0 [M-H]- | Ret. Time (min): 2.938 | m/z: 480.31
20200811_LRS_07_ENDO_IP_1.raw, Scan Number: 816, Isolation m/z: 480.31, Dot Product: 978
Alkyl_Fragment_[18:0]
283.2642
Relative Intensity
m/z

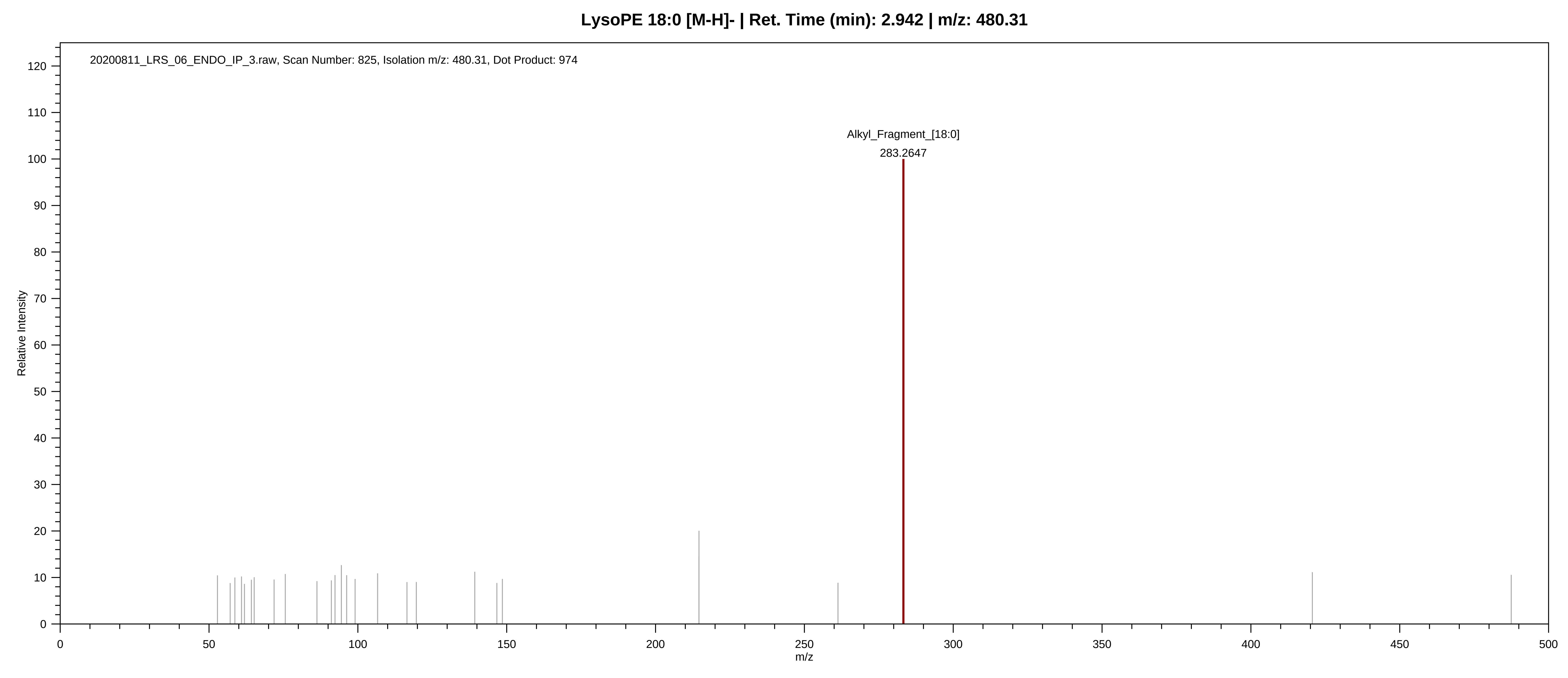
LysoPE 18:0 [M-H]- | Ret. Time (min): 2.942 | m/z: 480.31
20200811_LRS_06_ENDO_IP_3.raw, Scan Number: 825, Isolation m/z: 480.31, Dot Product: 974
Alkyl_Fragment_[18:0]
283.2647
Relative Intensity
m/z
0
10
20
30
40
50
60
70
80
90
100
110
120
0
50
100
150
200
250
300
350
400
450
500

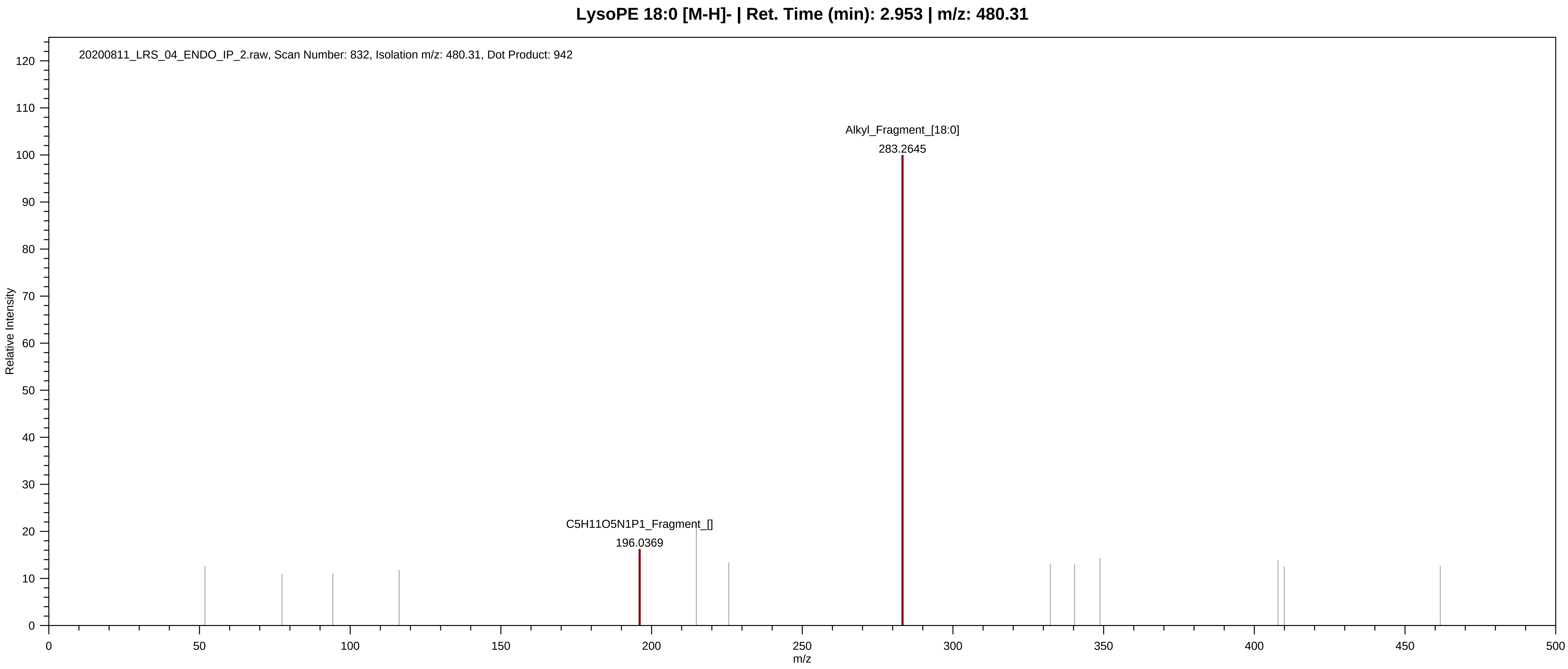

LysoPE 18:0 [M-H]- | Ret. Time (min): 2.953 | m/z: 480.31
20200811_LRS_04_ENDO_IP_2.raw, Scan Number: 832, Isolation m/z: 480.31, Dot Product: 942
Alkyl_Fragment_[18:0]
283.2645
C5H11O5N1P1_Fragment_[]
196.0369
Relative Intensity
m/z

LysoPE 18:0 [M-H]- | Ret. Time (min): 2.746 | m/z: 480.308

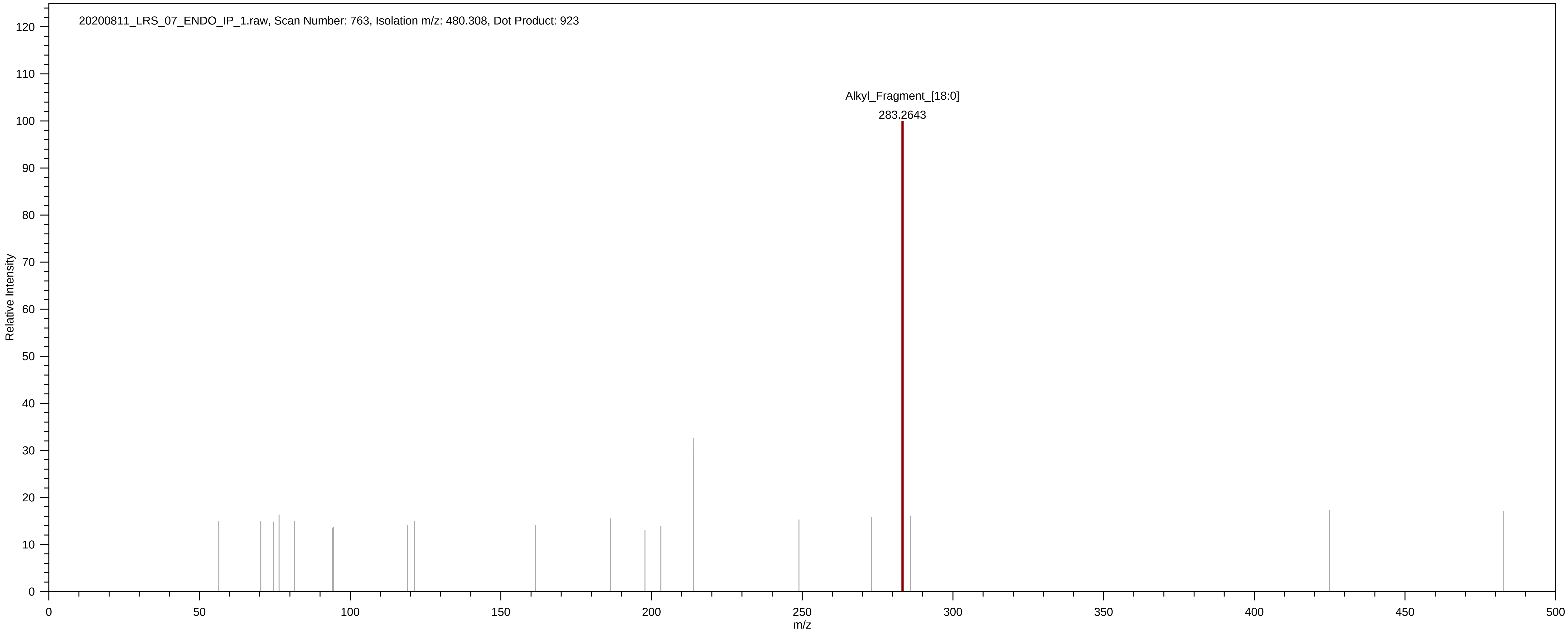

LysoPE 18:0 [M-H]- | Ret. Time (min): 2.75 | m/z: 480.309

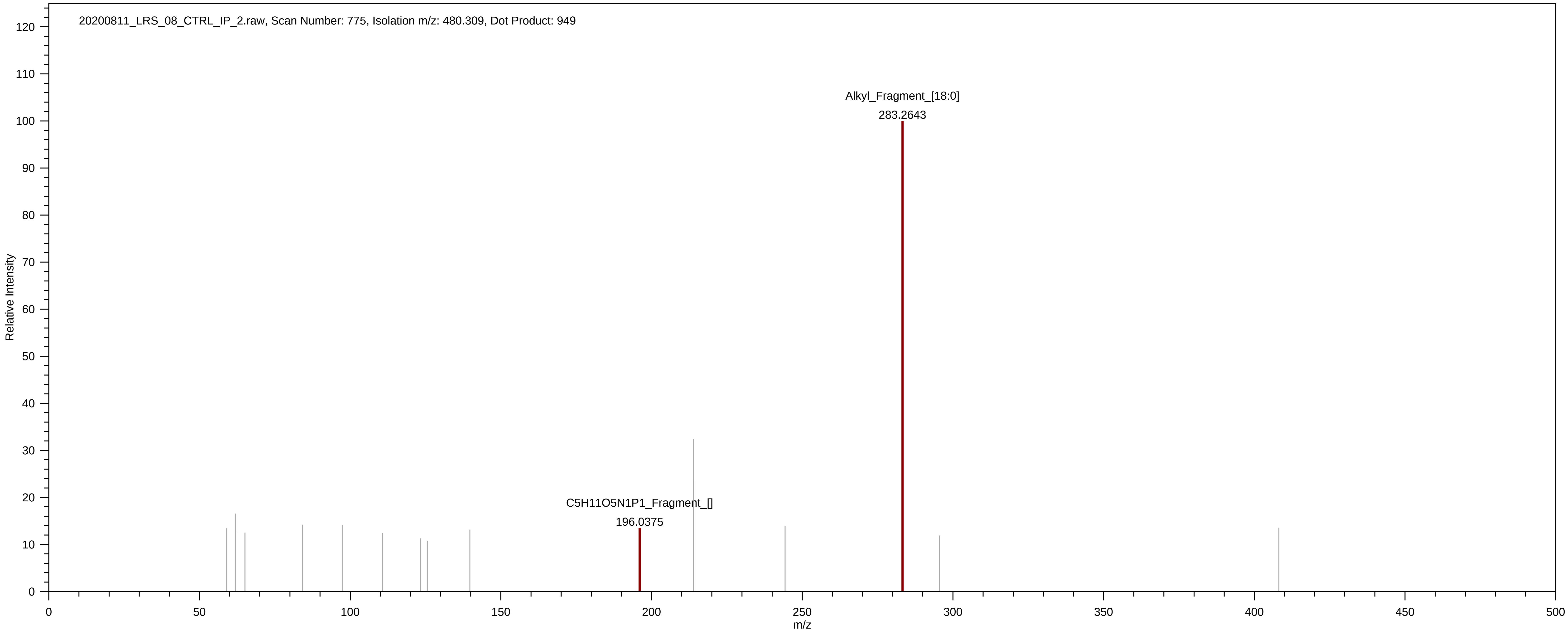

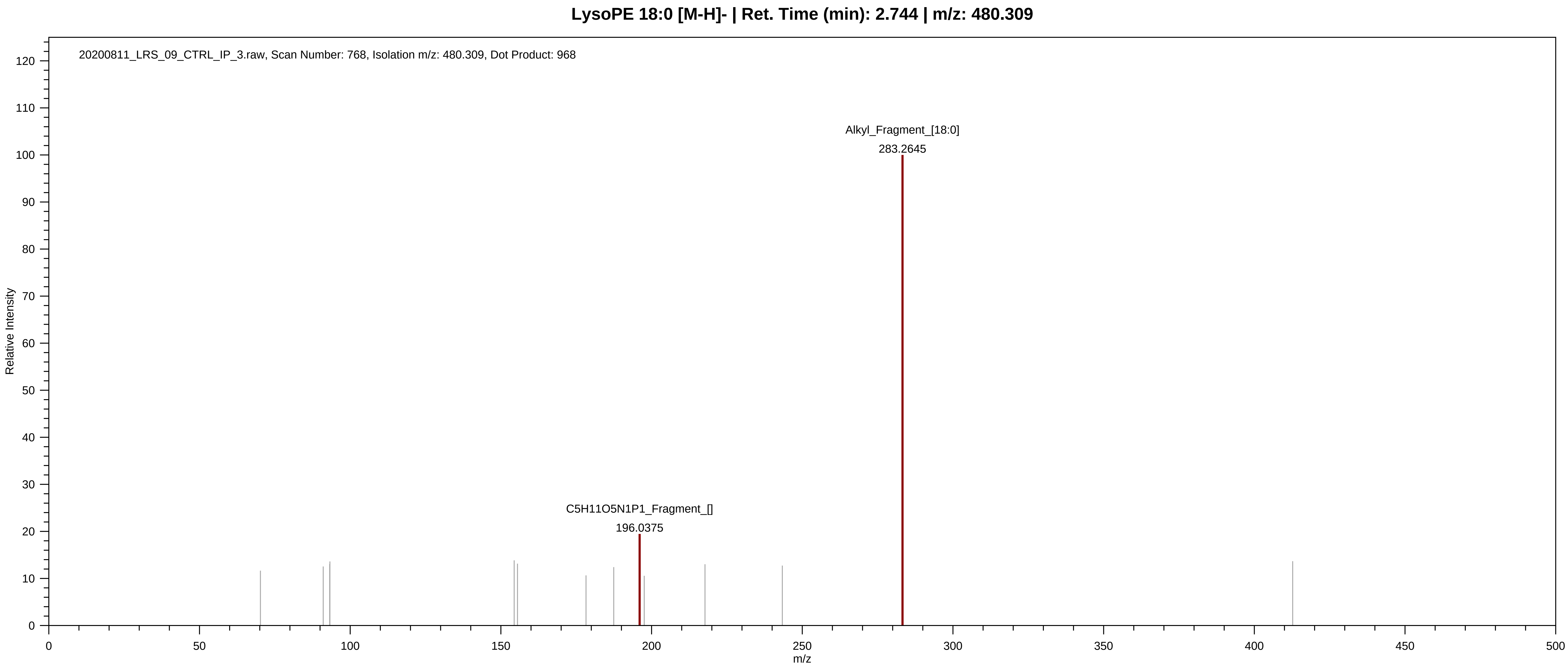
LysoPE 18:0 [M-H]- | Ret. Time (min): 2.744 | m/z: 480.309
20200811_LRS_09_CTRL_IP_3.raw, Scan Number: 768, Isolation m/z: 480.309, Dot Product: 968
Alkyl_Fragment_[18:0]
283.2645
C5H11O5N1P1_Fragment_[]
196.0375
Relative Intensity
m/z

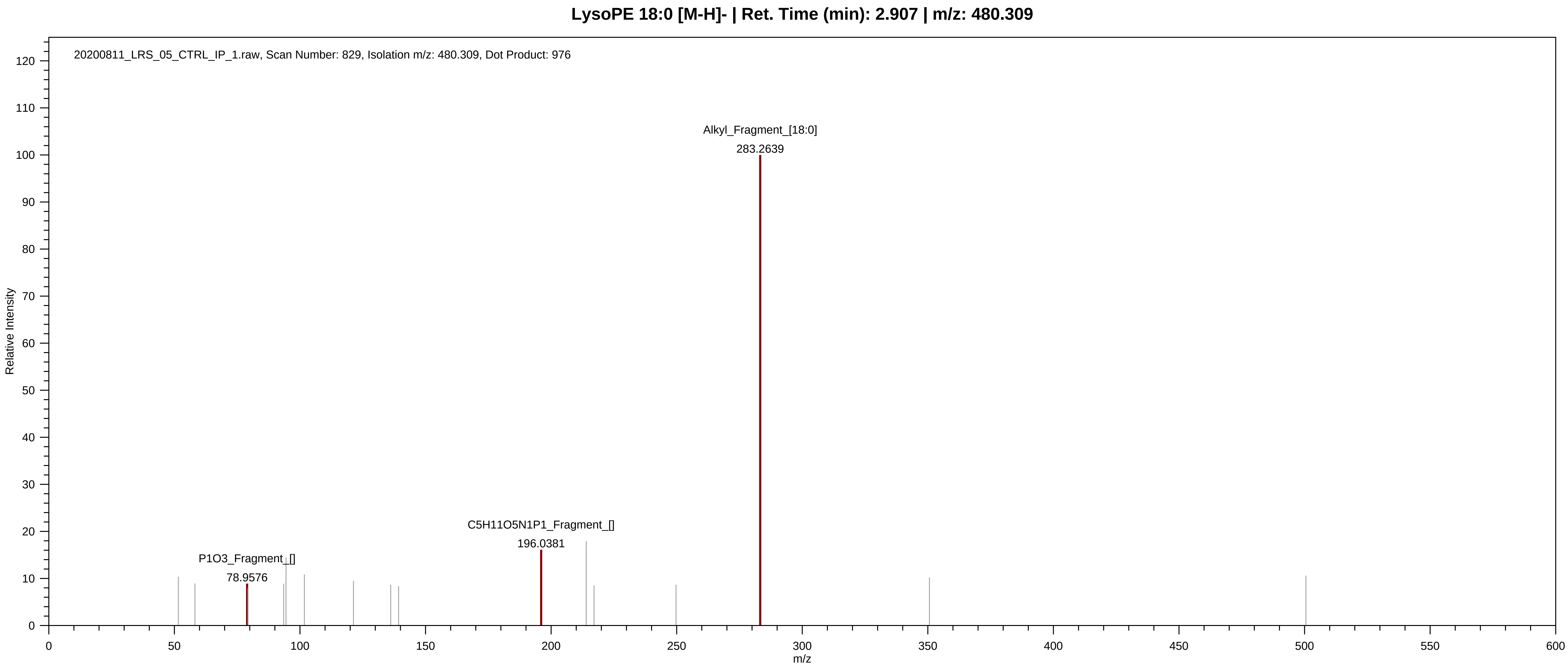

LysoPE 18:0 [M-H]- | Ret. Time (min): 2.907 | m/z: 480.309
20200811_LRS_05_CTRL_IP_1.raw, Scan Number: 829, Isolation m/z: 480.309, Dot Product: 976
Alkyl_Fragment_[18:0]
283.2639
C5H11O5N1P1_Fragment_[]
196.0381
P1O3_Fragment_[]
78.9576
Relative Intensity
m/z

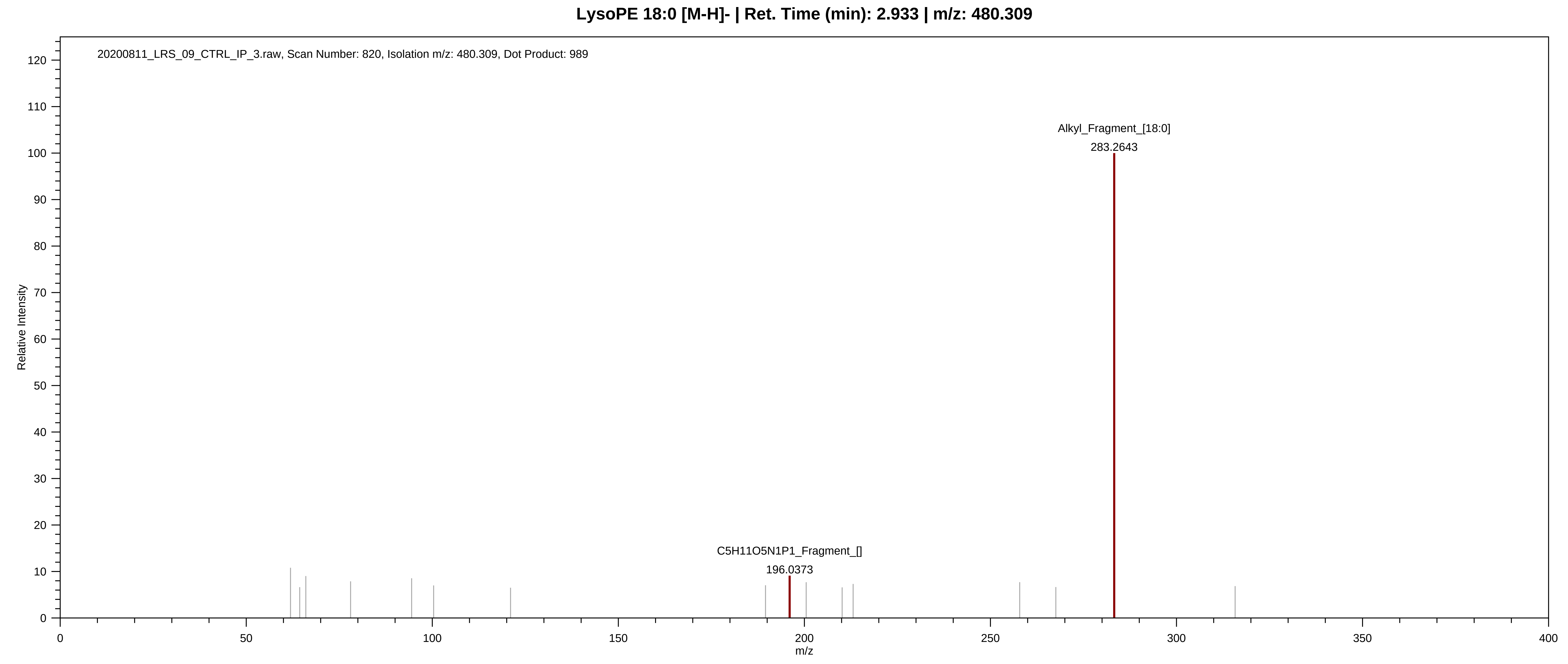
LysoPE 18:0 [M-H]- | Ret. Time (min): 2.933 | m/z: 480.309
20200811_LRS_09_CTRL_IP_3.raw, Scan Number: 820, Isolation m/z: 480.309, Dot Product: 989
Alkyl_Fragment_[18:0]
283.2643
C5H11O5N1P1_Fragment_[]
196.0373
Relative Intensity
m/z

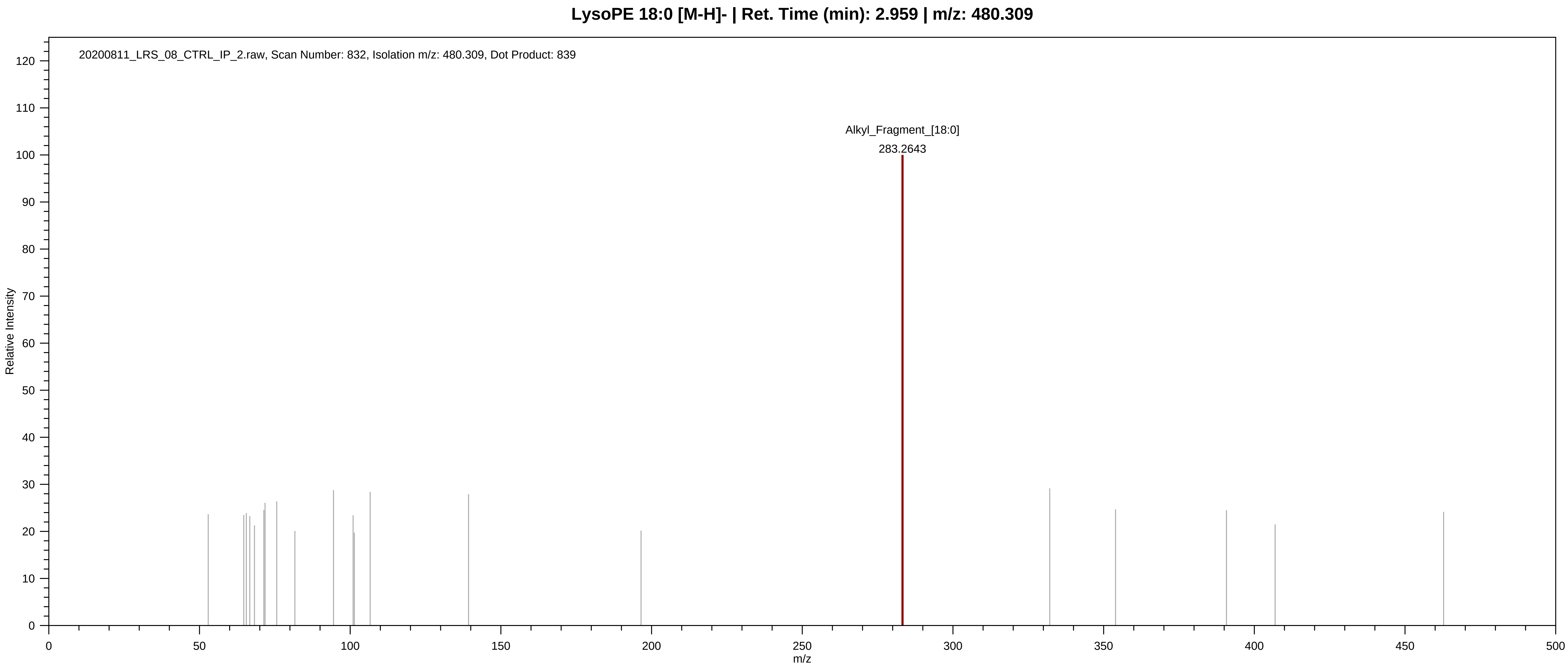
LysoPE 18:0 [M-H]- | Ret. Time (min): 2.959 | m/z: 480.309
20200811_LRS_08_CTRL_IP_2.raw, Scan Number: 832, Isolation m/z: 480.309, Dot Product: 839
Alkyl_Fragment_[18:0]
283.2643
Relative Intensity
m/z
0
10
20
30
40
50
60
70
80
90
100
110
120
0
50
100
150
200
250
300
350
400
450
500

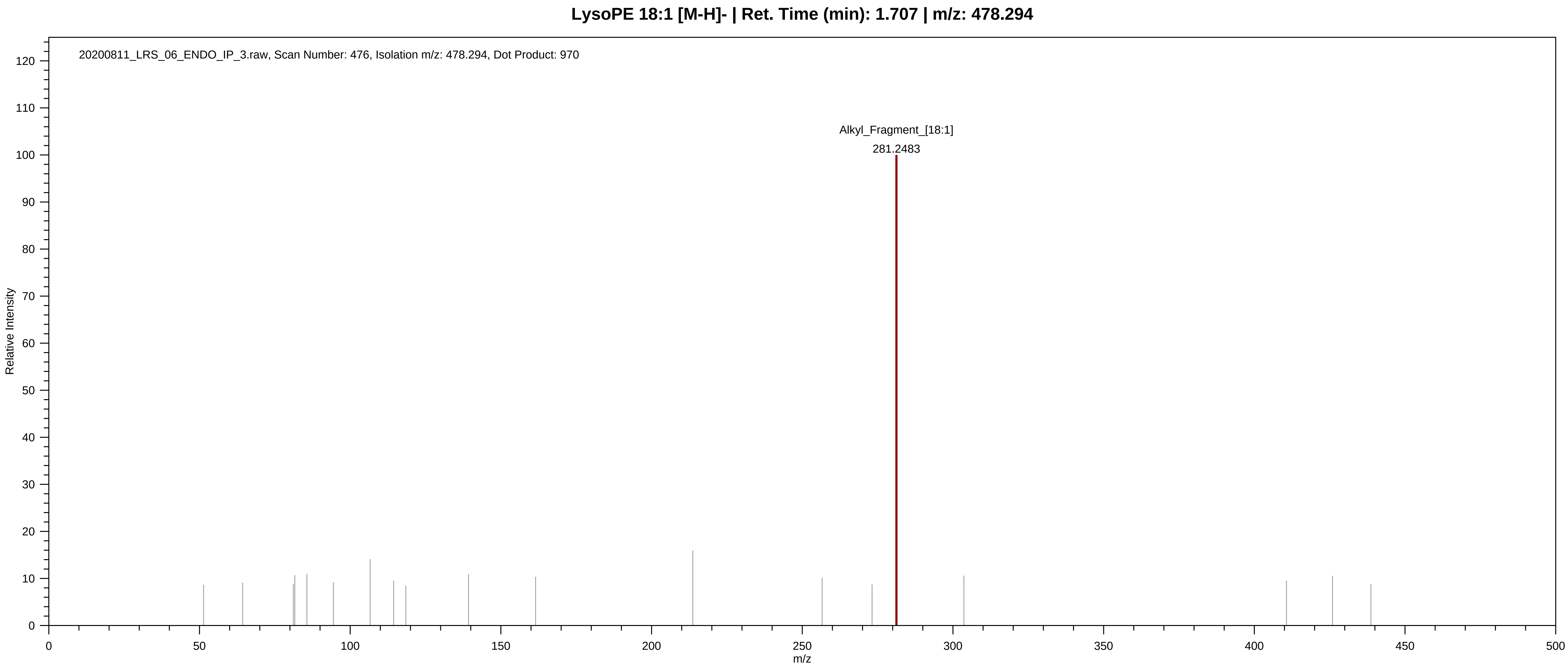
LysoPE 18:1 [M-H]- | Ret. Time (min): 1.707 | m/z: 478.294
20200811_LRS_06_ENDO_IP_3.raw, Scan Number: 476, Isolation m/z: 478.294, Dot Product: 970
Alkyl_Fragment_[18:1]
281.2483
Relative Intensity
m/z
0
10
20
30
40
50
60
70
80
90
100
110
120
0
50
100
150
200
250
300
350
400
450
500

LysoPE 18:1 [M-H]- | Ret. Time (min): 1.723 | m/z: 478.294

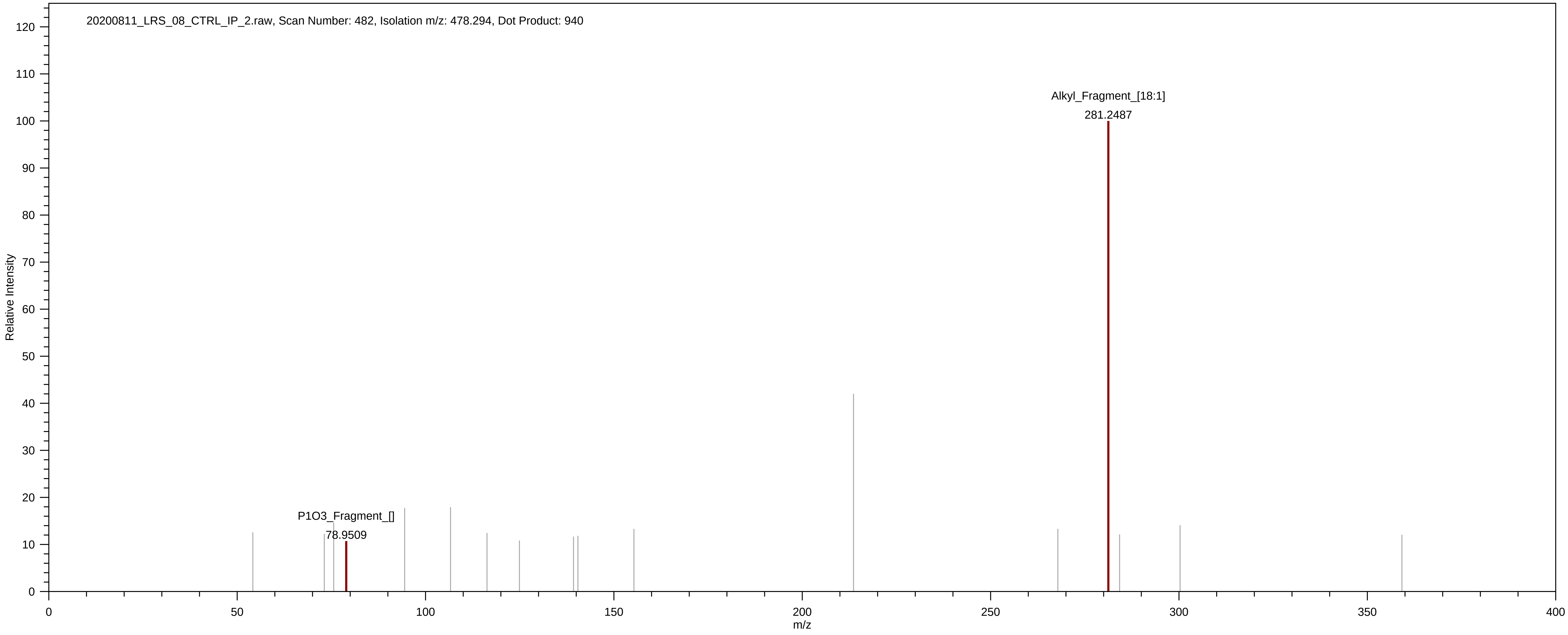

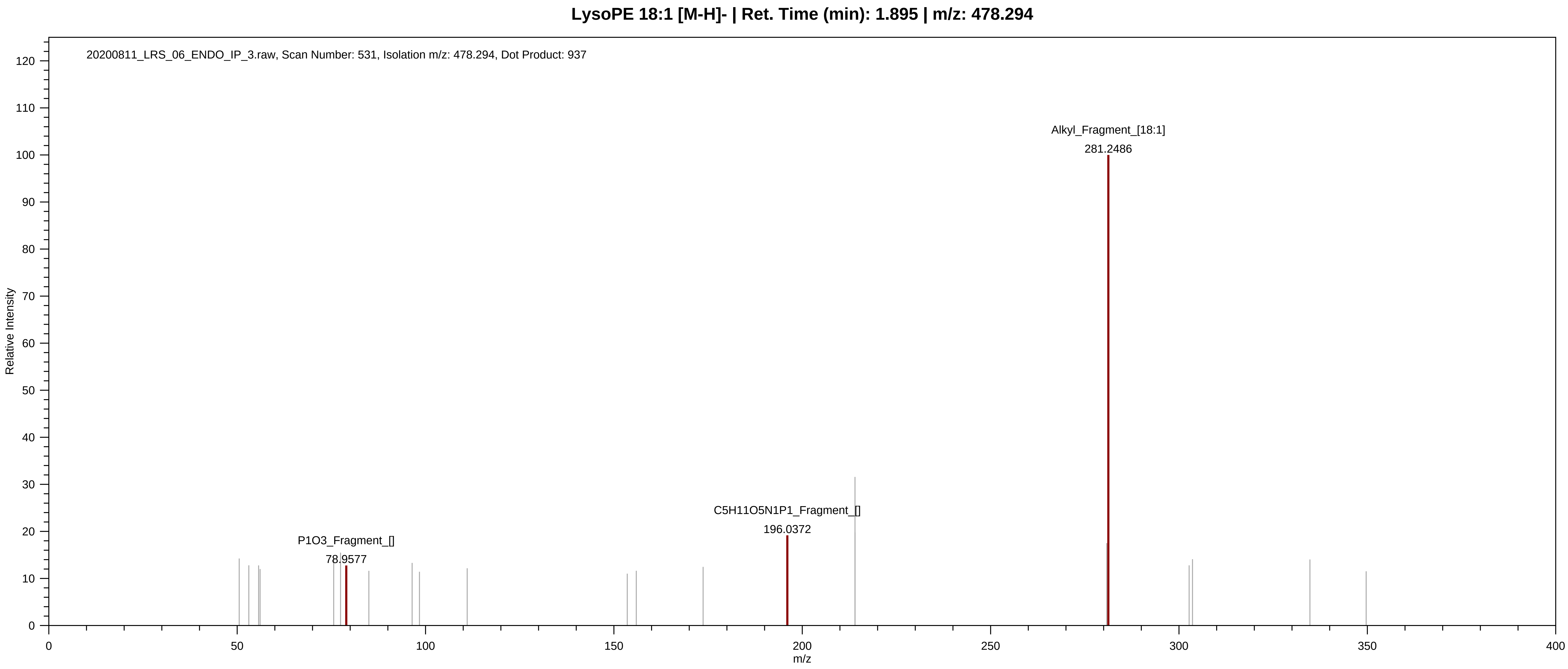
LysoPE 18:1 [M-H]- | Ret. Time (min): 1.895 | m/z: 478.294
20200811_LRS_06_ENDO_IP_3.raw, Scan Number: 531, Isolation m/z: 478.294, Dot Product: 937
Alkyl_Fragment_[18:1]
281.2486
C5H11O5N1P1_Fragment_[]
196.0372
P1O3_Fragment_[]
78.9577
Relative Intensity
m/z

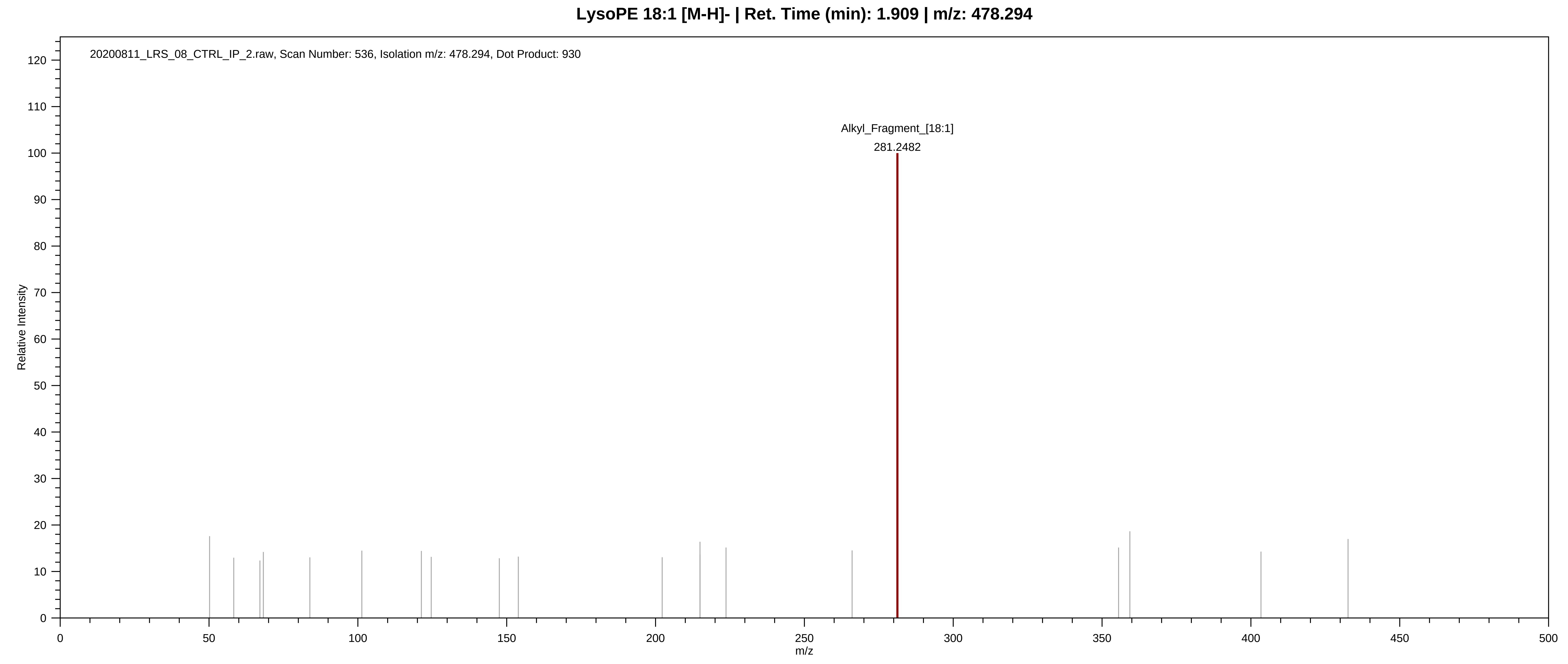
LysoPE 18:1 [M-H]- | Ret. Time (min): 1.909 | m/z: 478.294
20200811_LRS_08_CTRL_IP_2.raw, Scan Number: 536, Isolation m/z: 478.294, Dot Product: 930
Alkyl_Fragment_[18:1]
281.2482
Relative Intensity
m/z
0
10
20
30
40
50
60
70
80
90
100
110
120
0
50
100
150
200
250
300
350
400
450
500

LysoPE 24:0 [M+H]+ | Ret. Time (min): 7.837 | m/z: 566.419

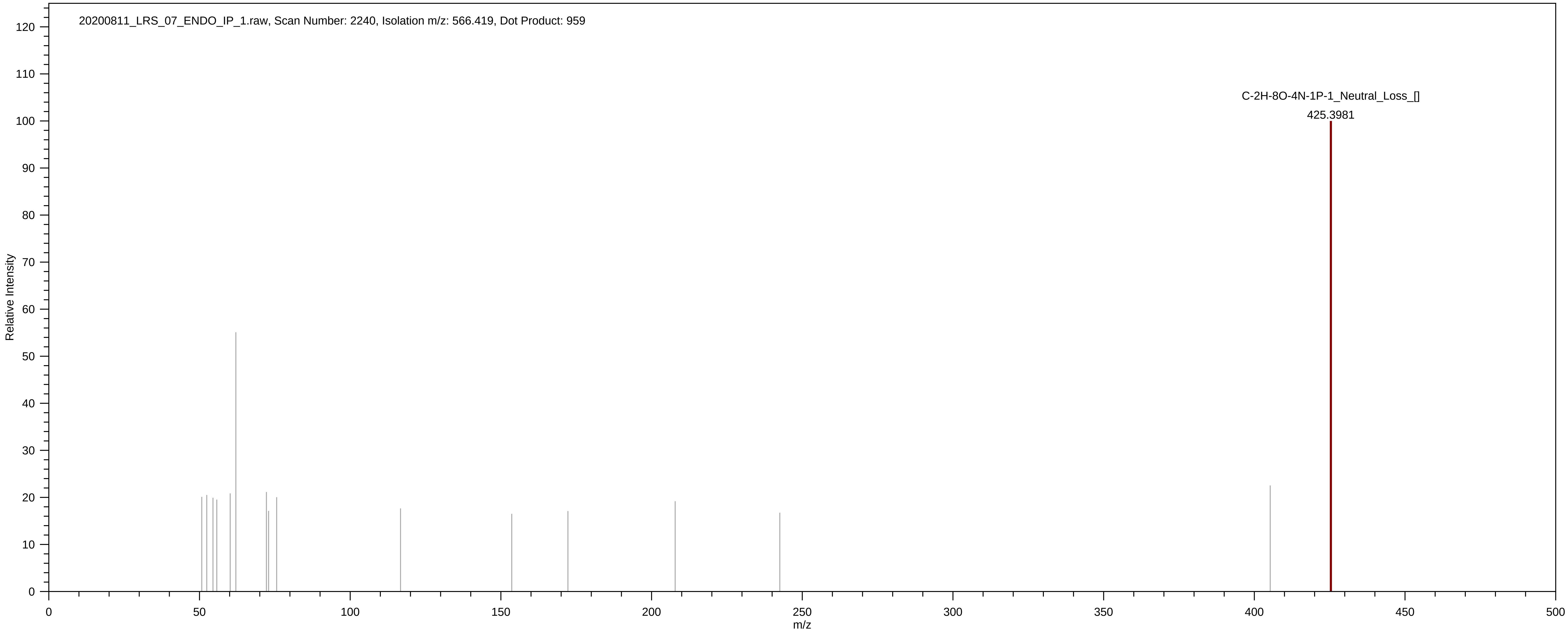

LysoPE 24:0 [M+H]+ | Ret. Time (min): 7.847 | m/z: 566.417

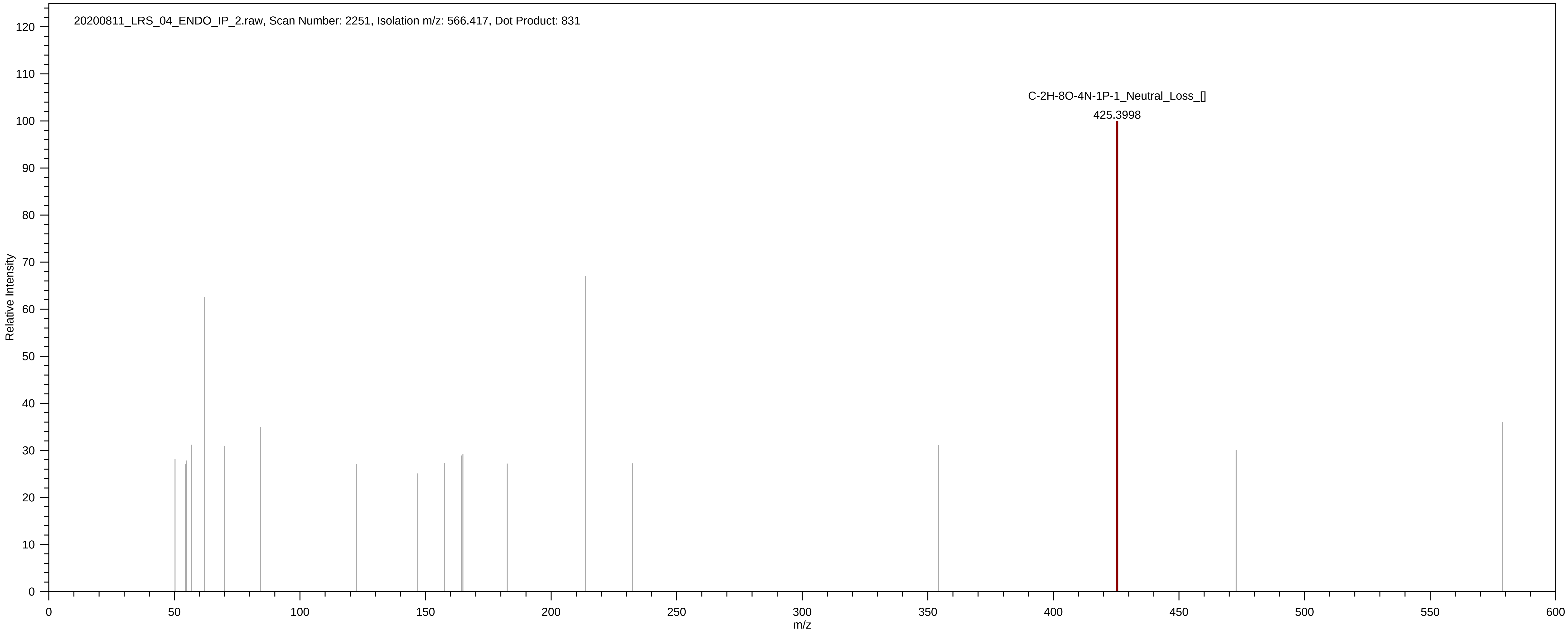

LysoPE 24:0 [M+H]+ | Ret. Time (min): 7.856 | m/z: 566.416

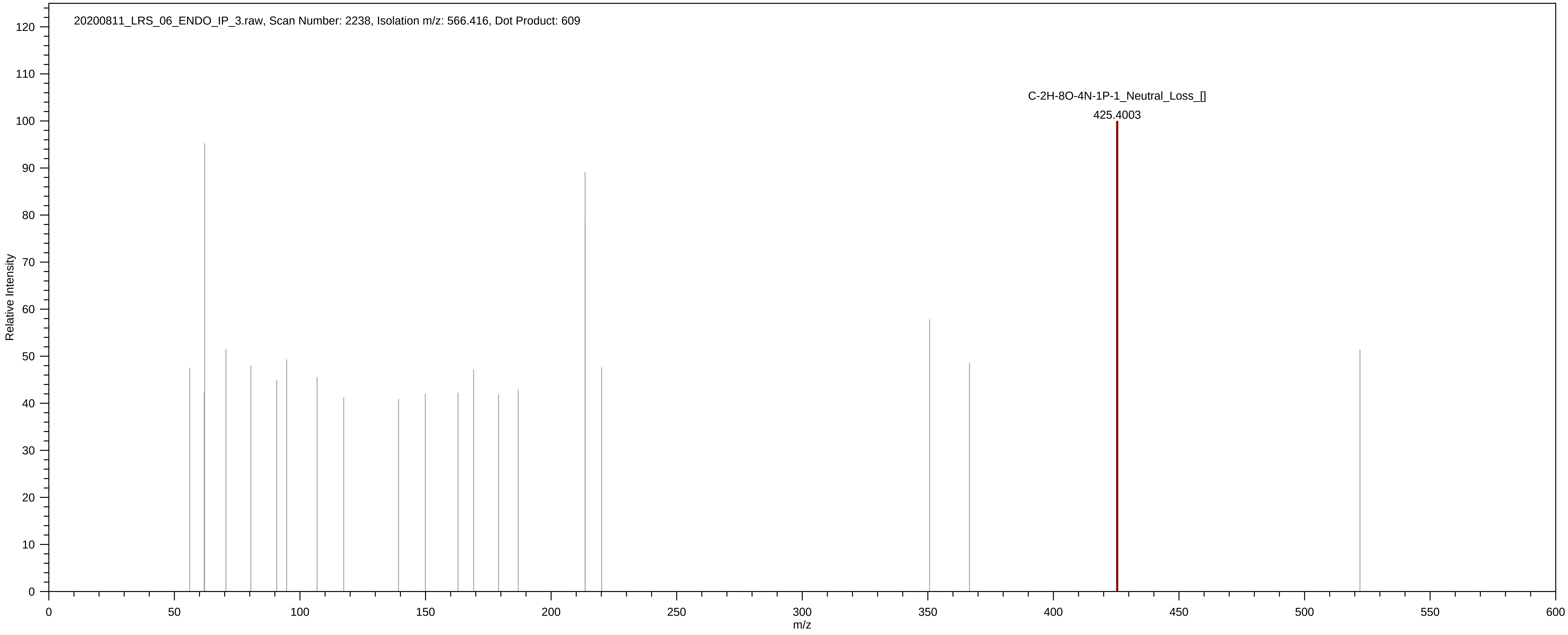

LysoPE 24:0 [M-H]- | Ret. Time (min): 7.837 | m/z: 564.402

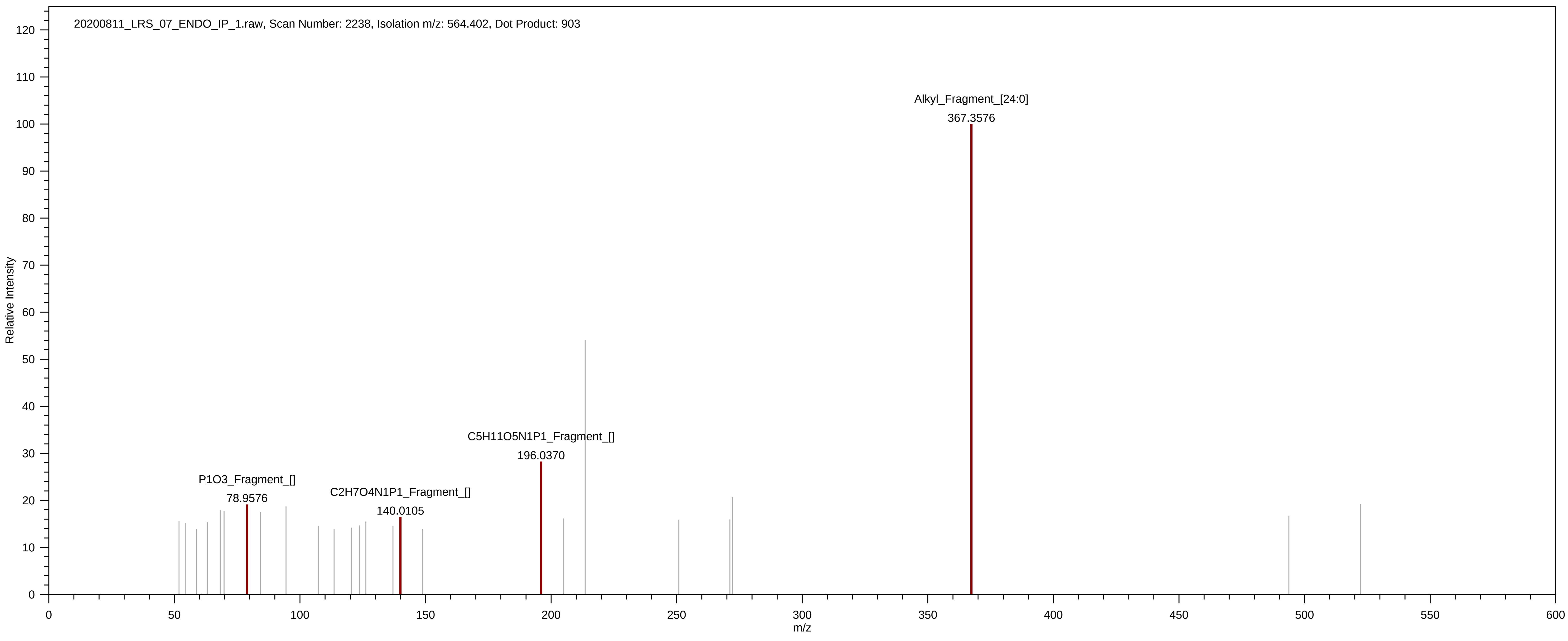

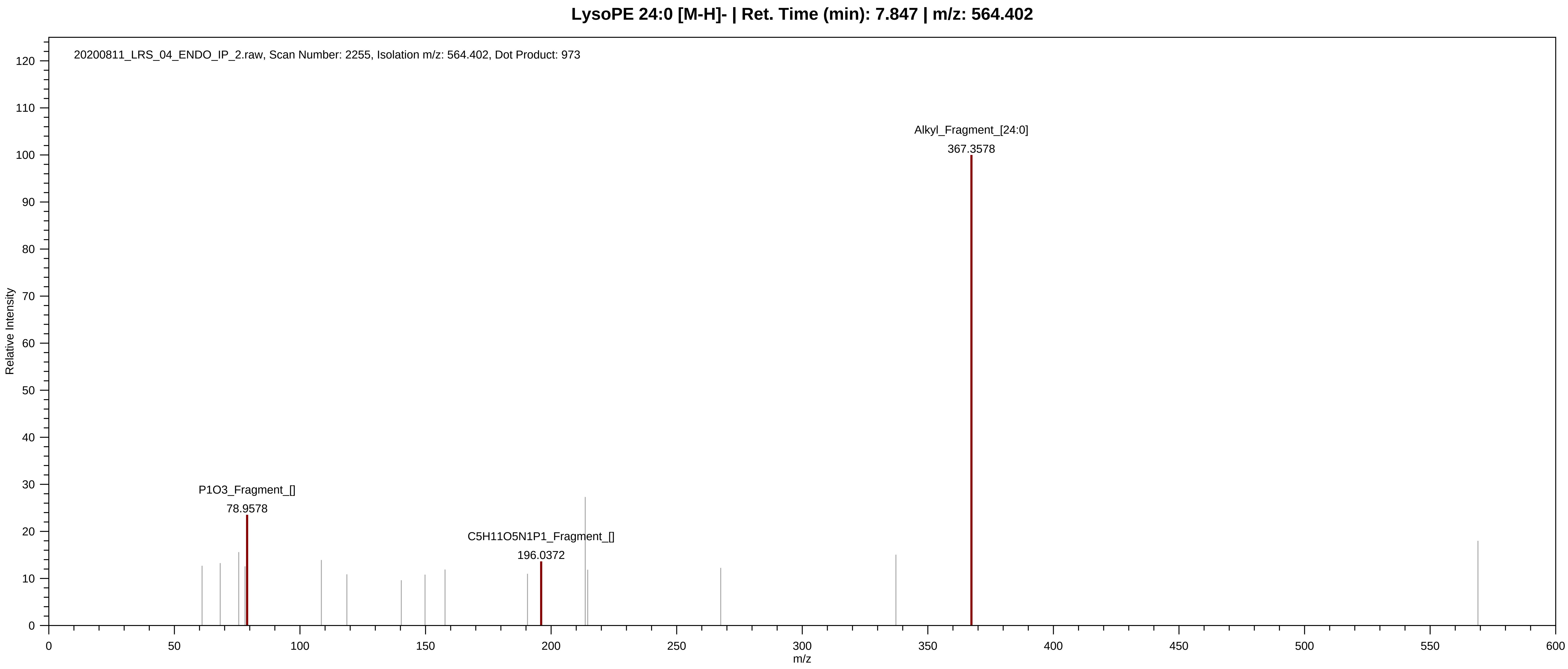

LysoPE 24:0 [M-H]- | Ret. Time (min): 7.847 | m/z: 564.402
20200811_LRS_04_ENDO_IP_2.raw, Scan Number: 2255, Isolation m/z: 564.402, Dot Product: 973
Alkyl_Fragment_[24:0]
367.3578
P1O3_Fragment_[]
78.9578
C5H11O5N1P1_Fragment_[]
196.0372
Relative Intensity
m/z

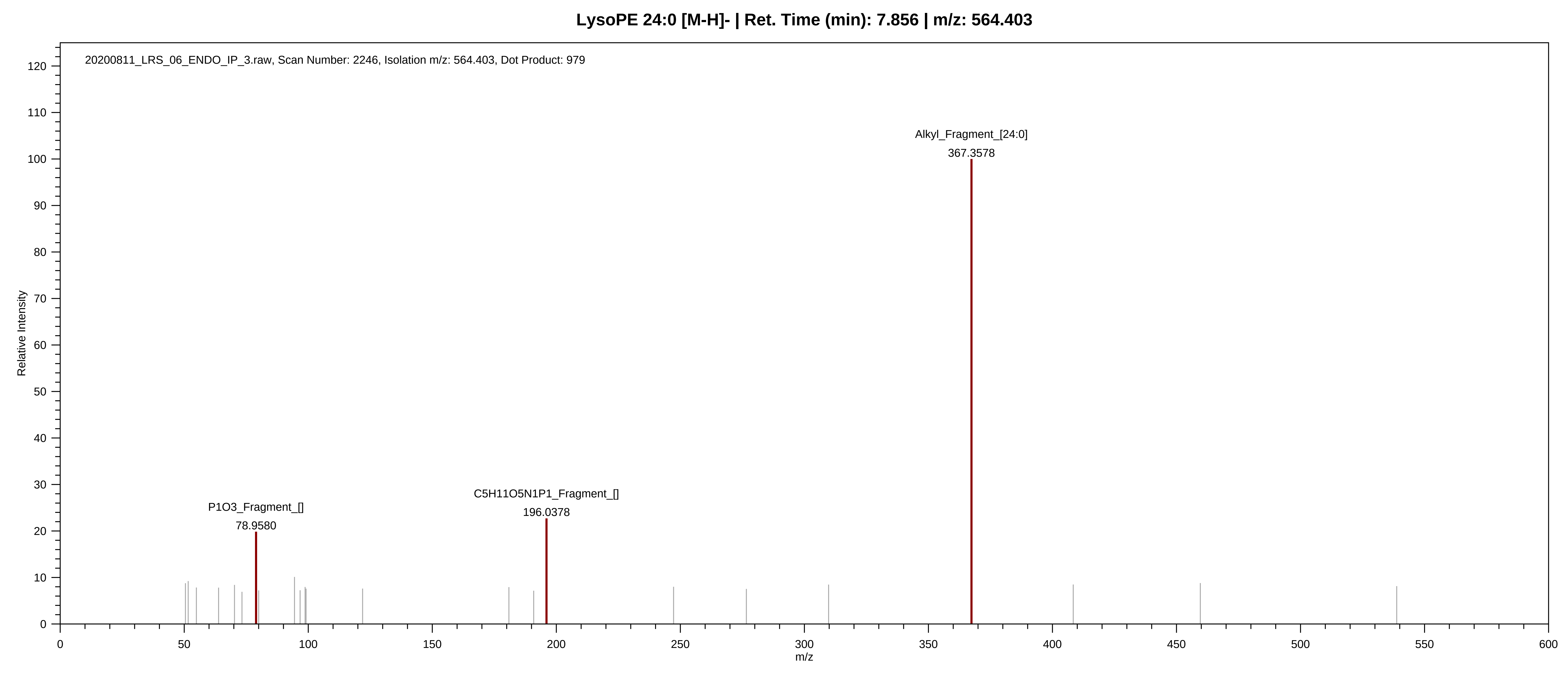

LysoPE 24:0 [M-H]- | Ret. Time (min): 7.856 | m/z: 564.403
20200811_LRS_06_ENDO_IP_3.raw, Scan Number: 2246, Isolation m/z: 564.403, Dot Product: 979
Alkyl_Fragment_[24:0]
367.3578
C5H11O5N1P1_Fragment_[]
196.0378
P1O3_Fragment_[]
78.9580
Relative Intensity
m/z

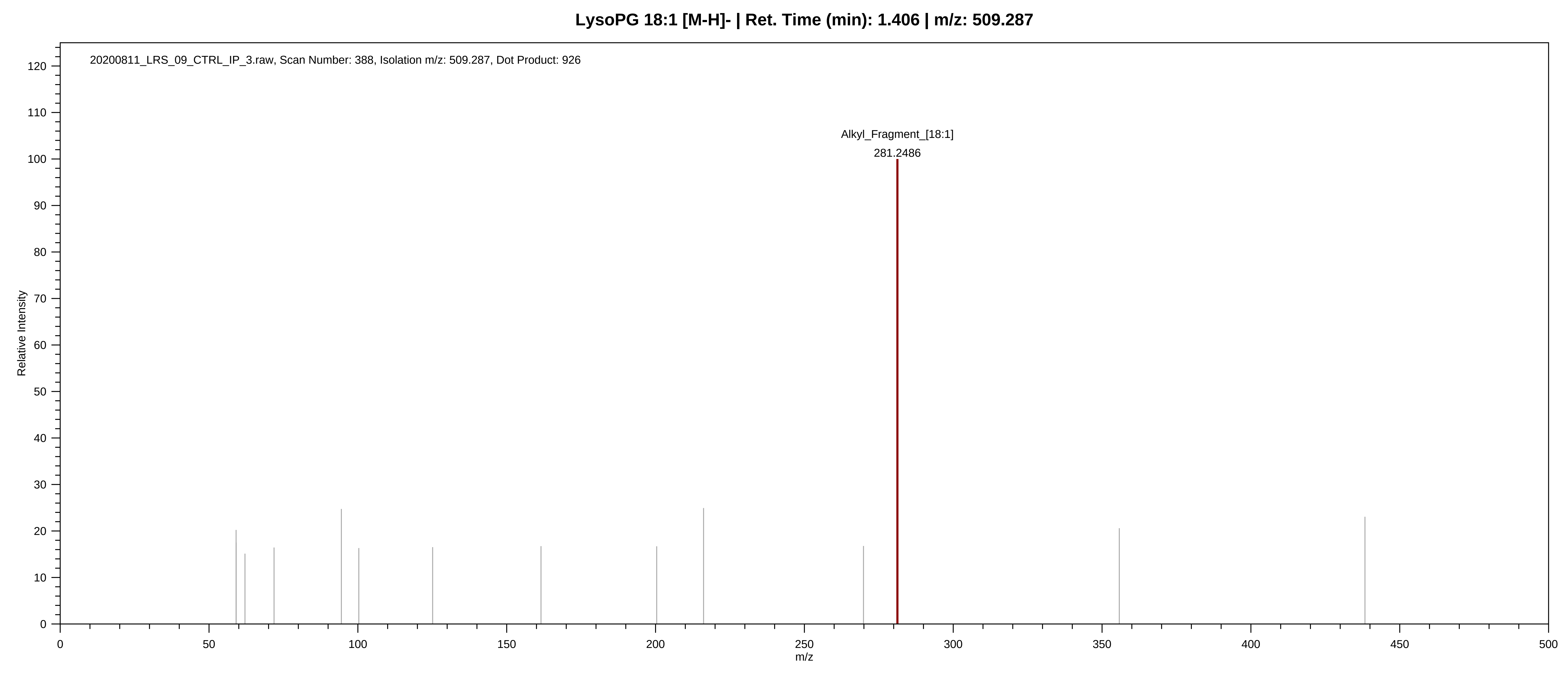

LysoPG 18:1 [M-H]- | Ret. Time (min): 1.406 | m/z: 509.287
20200811_LRS_09_CTRL_IP_3.raw, Scan Number: 388, Isolation m/z: 509.287, Dot Product: 926
Alkyl_Fragment_[18:1]
281.2486
Relative Intensity
m/z

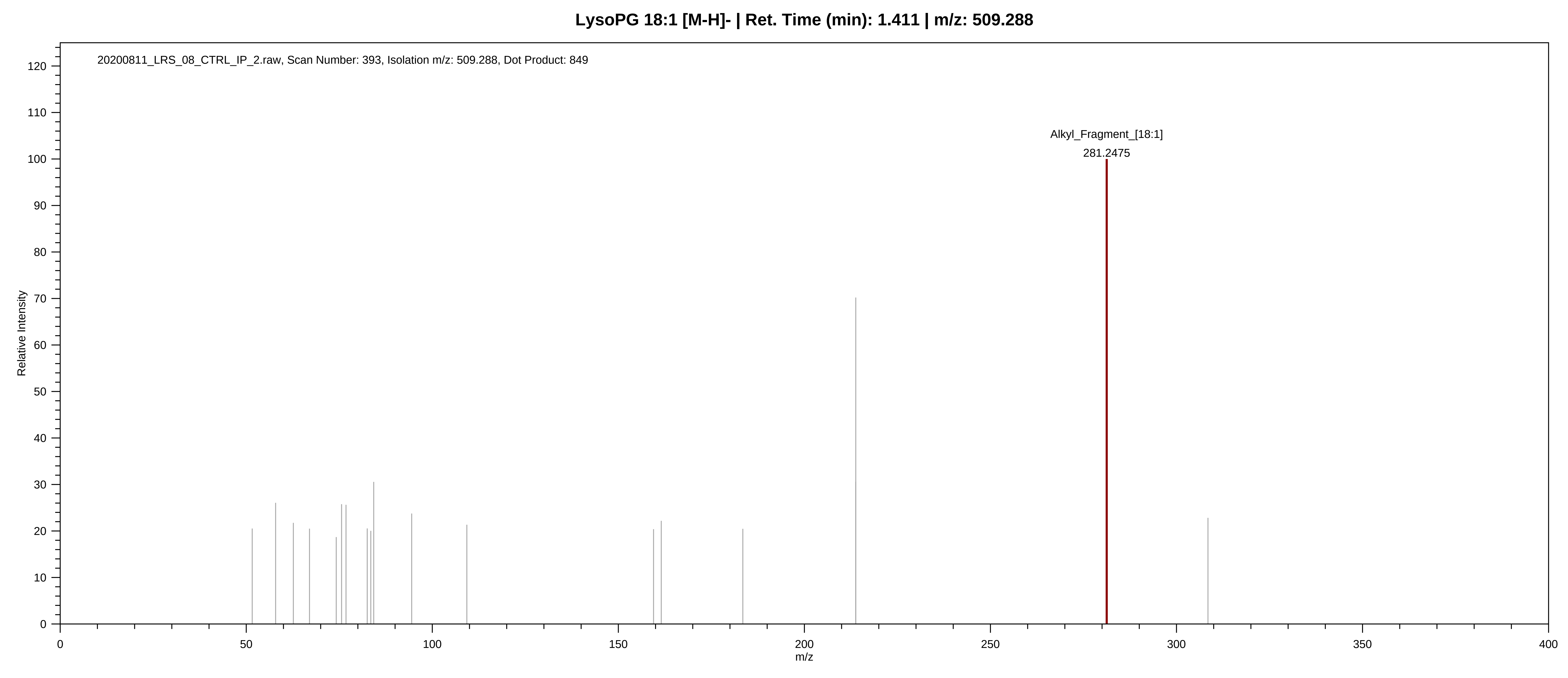

LysoPG 18:1 [M-H]- | Ret. Time (min): 1.411 | m/z: 509.288
20200811_LRS_08_CTRL_IP_2.raw, Scan Number: 393, Isolation m/z: 509.288, Dot Product: 849
Alkyl_Fragment_[18:1]
281.2475
Relative Intensity
m/z

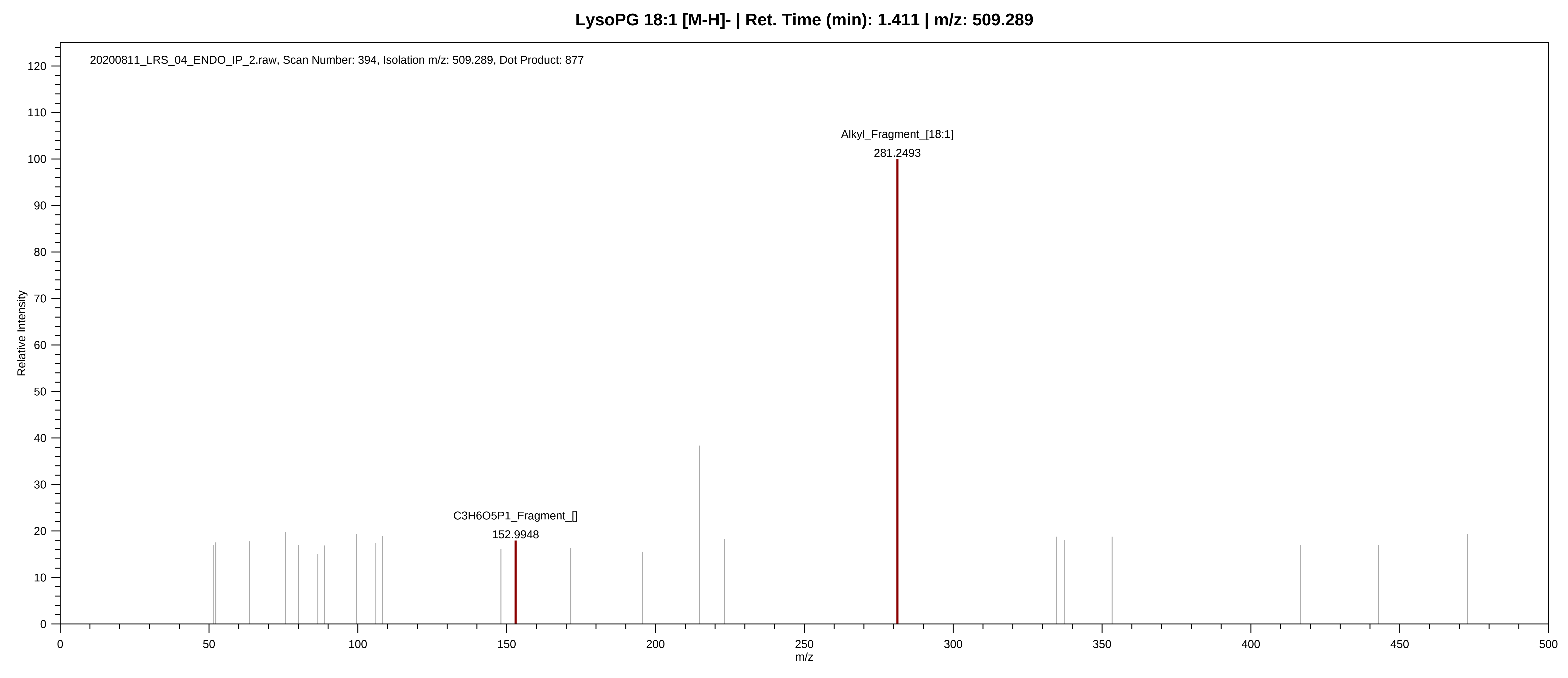
LysoPG 18:1 [M-H]- | Ret. Time (min): 1.411 | m/z: 509.289
20200811_LRS_04_ENDO_IP_2.raw, Scan Number: 394, Isolation m/z: 509.289, Dot Product: 877
Alkyl_Fragment_[18:1]
281.2493
C3H6O5P1_Fragment_[]
152.9948
Relative Intensity
m/z

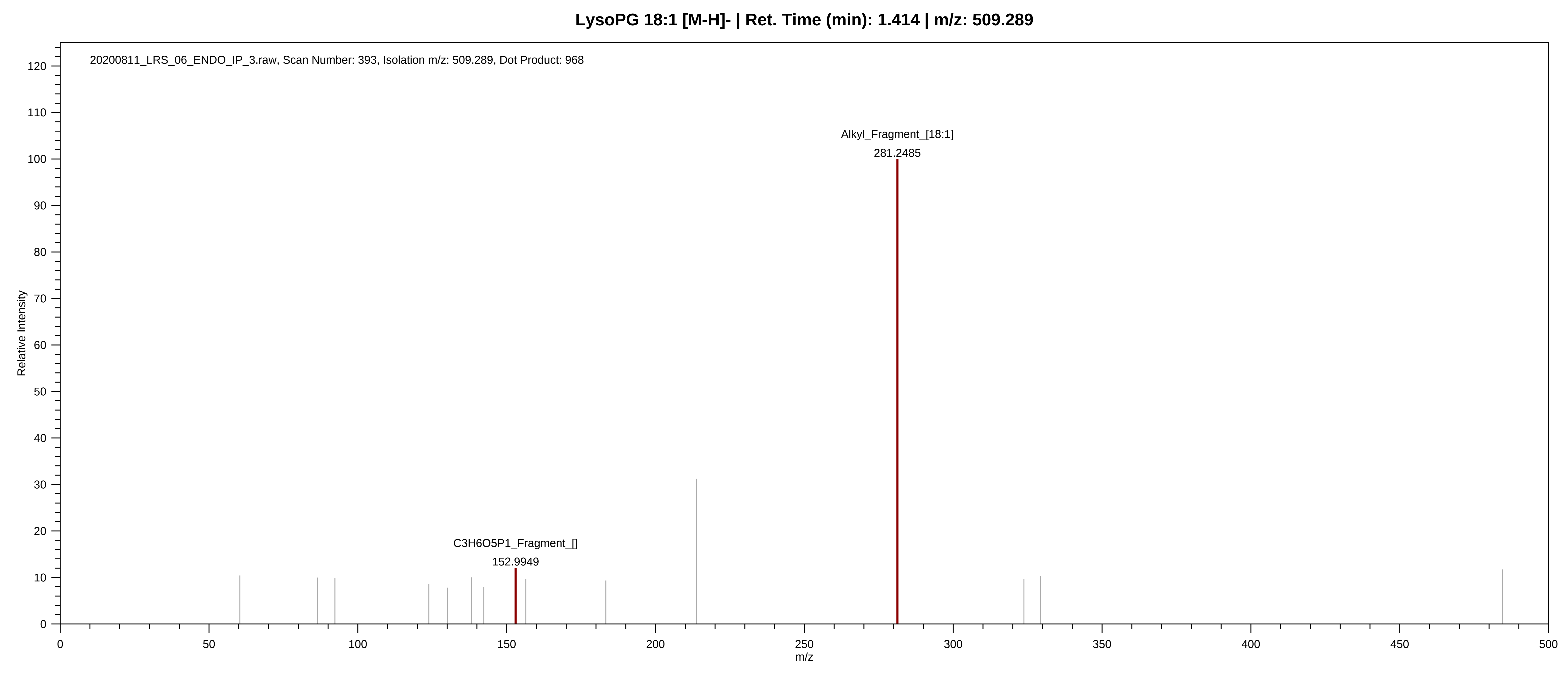
LysoPG 18:1 [M-H]- | Ret. Time (min): 1.414 | m/z: 509.289
20200811_LRS_06_ENDO_IP_3.raw, Scan Number: 393, Isolation m/z: 509.289, Dot Product: 968
Alkyl_Fragment_[18:1]
281.2485
C3H6O5P1_Fragment_[]
152.9949
Relative Intensity
m/z

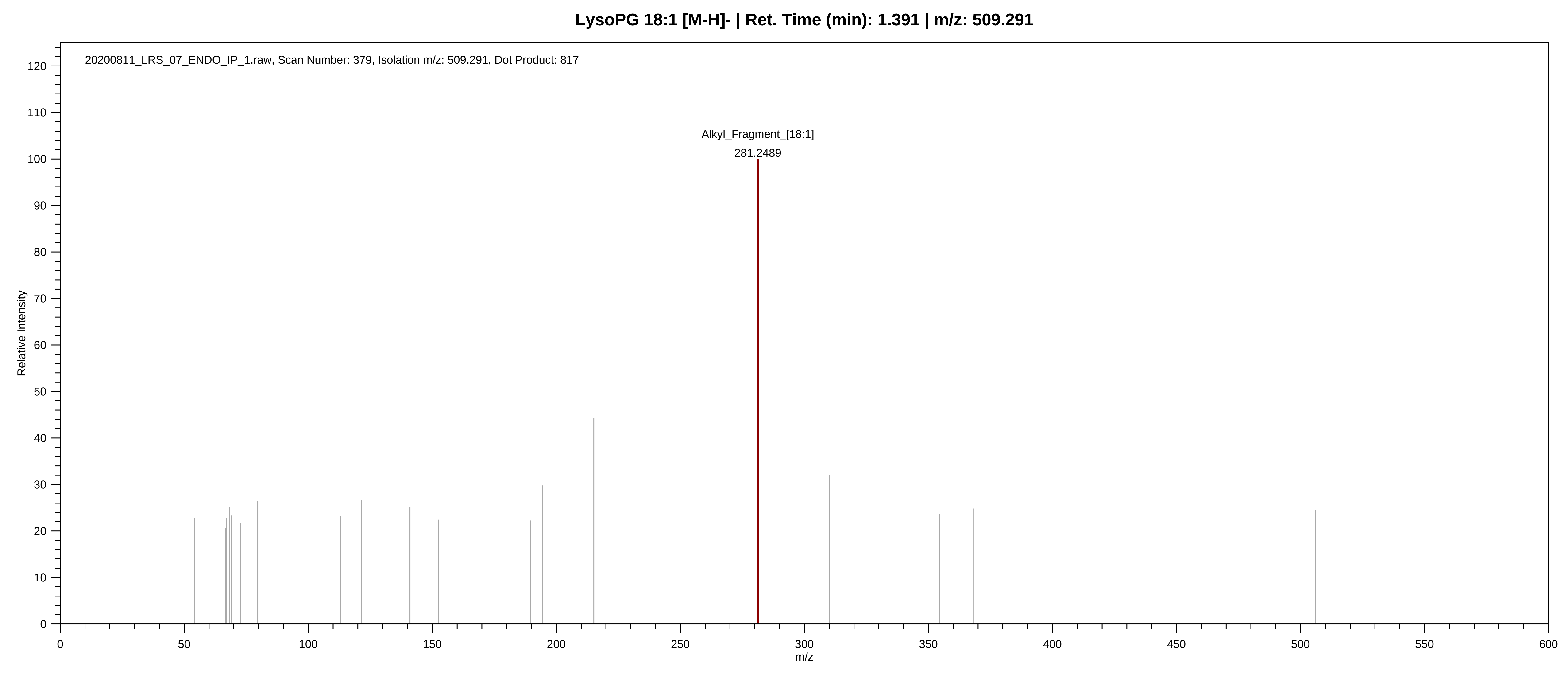
LysoPG 18:1 [M-H]- | Ret. Time (min): 1.391 | m/z: 509.291
20200811_LRS_07_ENDO_IP_1.raw, Scan Number: 379, Isolation m/z: 509.291, Dot Product: 817
Alkyl_Fragment_[18:1]
281.2489
Relative Intensity
m/z

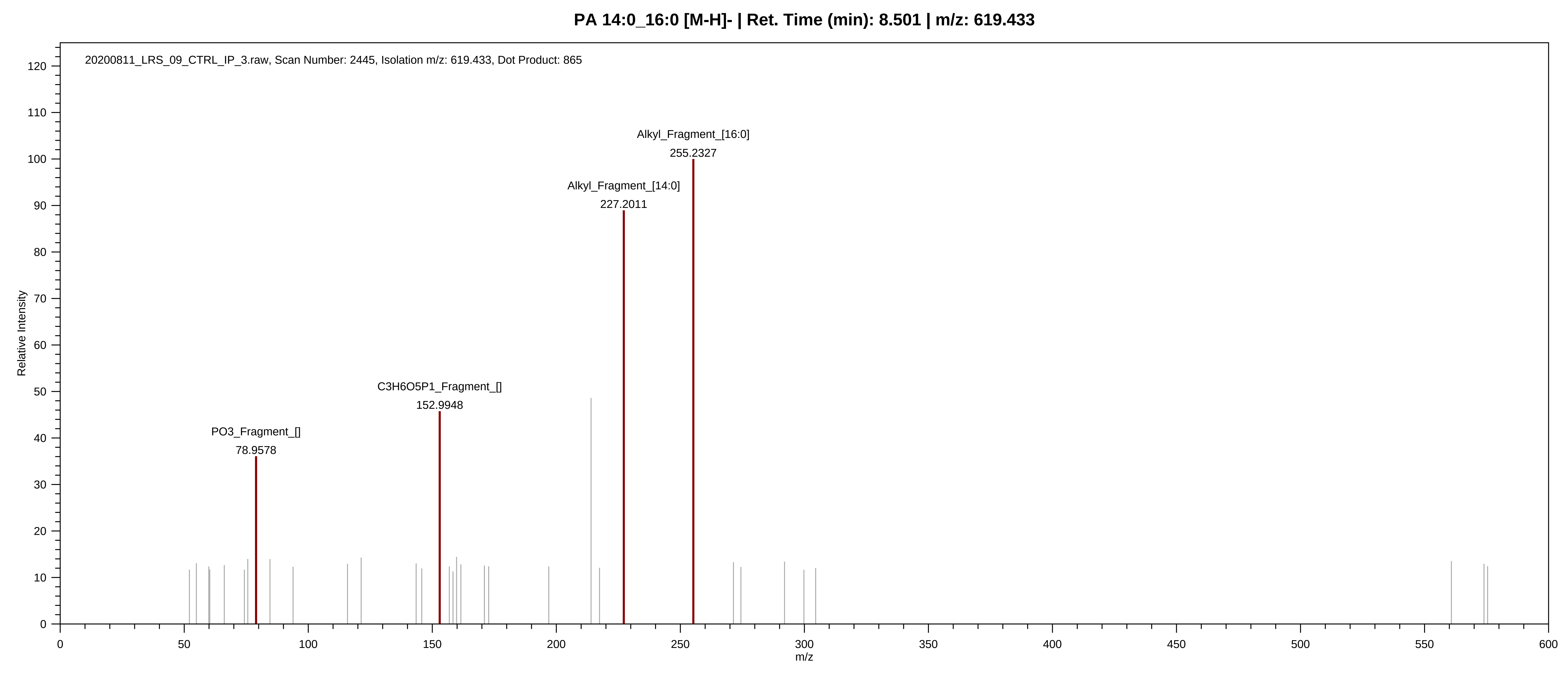

PA 14:0_16:0 [M-H]- | Ret. Time (min): 8.501 | m/z: 619.433
20200811_LRS_09_CTRL_IP_3.raw, Scan Number: 2445, Isolation m/z: 619.433, Dot Product: 865
Alkyl_Fragment_[16:0]
255.2327
Alkyl_Fragment_[14:0]
227.2011
C3H6O5P1_Fragment_[]
152.9948
PO3_Fragment_[]
78.9578
Relative Intensity
m/z

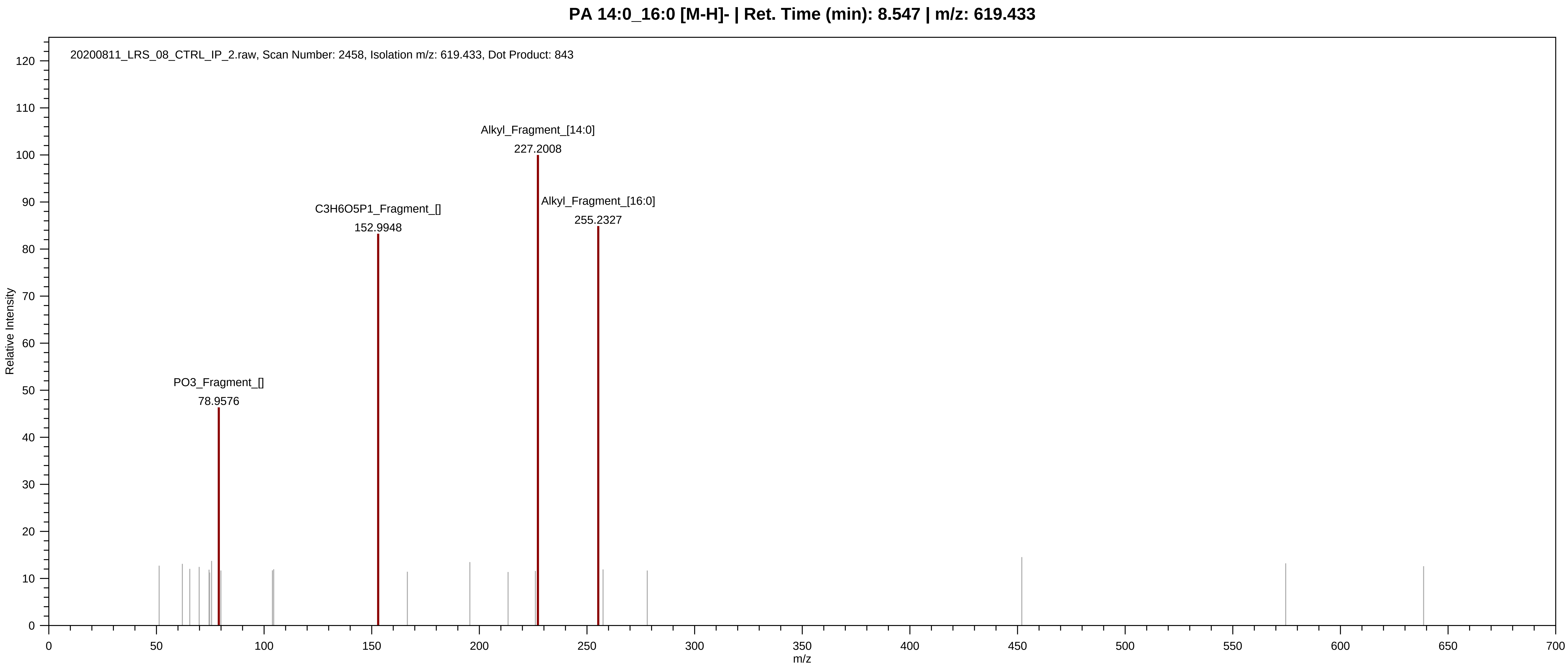
PA 14:0_16:0 [M-H]- | Ret. Time (min): 8.547 | m/z: 619.433
20200811_LRS_08_CTRL_IP_2.raw, Scan Number: 2458, Isolation m/z: 619.433, Dot Product: 843
Alkyl_Fragment_[14:0]
227.2008
Alkyl_Fragment_[16:0]
255.2327
C3H6O5P1_Fragment_[]
152.9948
PO3_Fragment_[]
78.9576
Relative Intensity
m/z

PA 14:0_16:0 [M-H]- | Ret. Time (min): 8.566 | m/z: 619.433

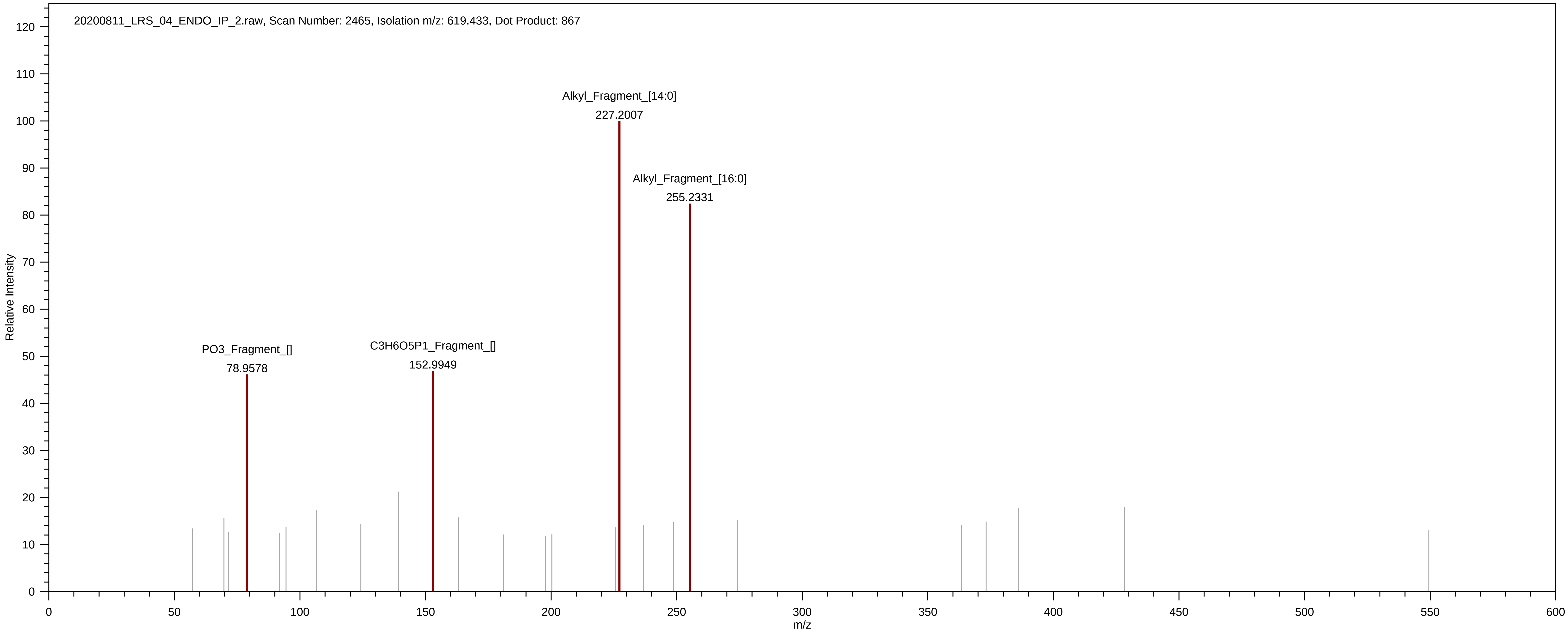

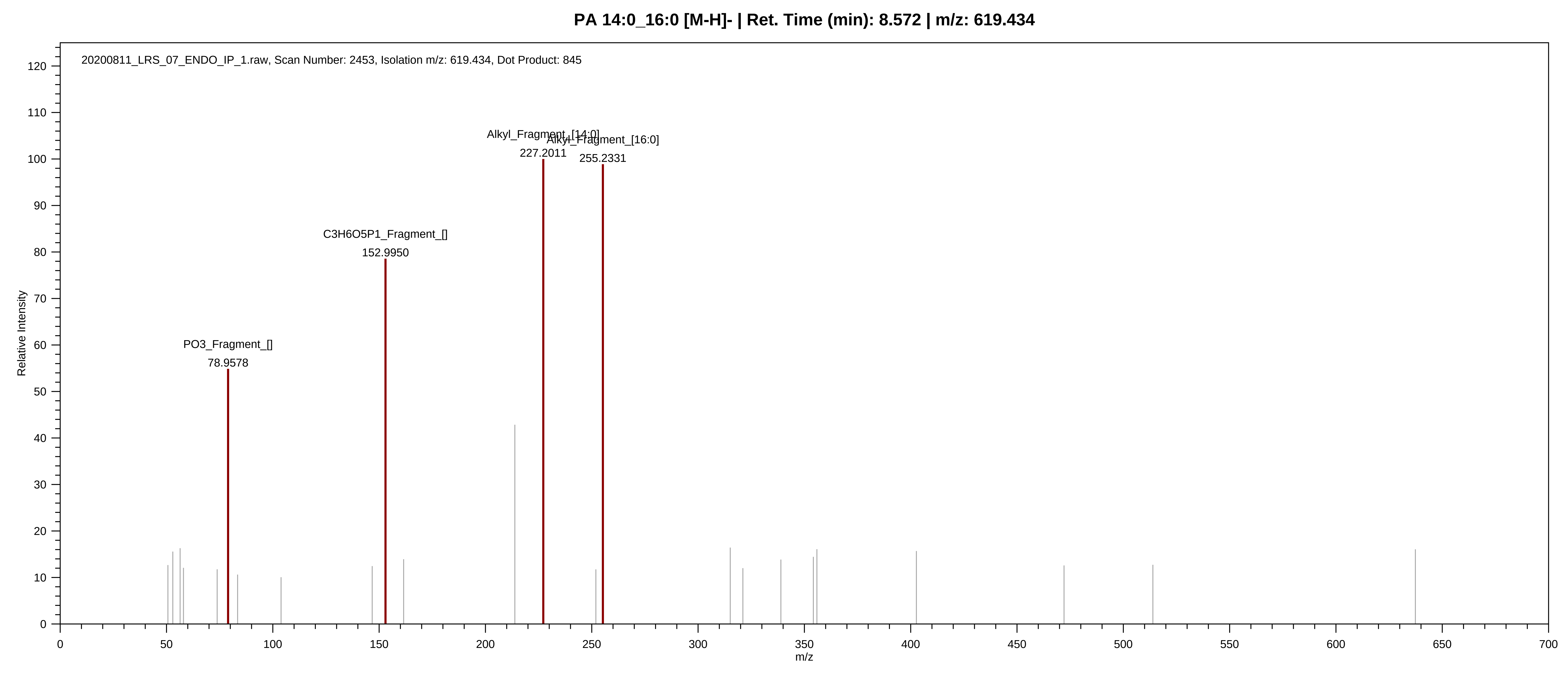
PA 14:0_16:0 [M-H]- | Ret. Time (min): 8.572 | m/z: 619.434
20200811_LRS_07_ENDO_IP_1.raw, Scan Number: 2453, Isolation m/z: 619.434, Dot Product: 845
PO3_Fragment_[]
78.9578
C3H6O5P1_Fragment_[]
152.9950
Alkyl_Fragment_[14:0]
227.2011
Alkyl_Fragment_[16:0]
255.2331
Relative Intensity
m/z

PA 14:0_16:0 [M-H]- | Ret. Time (min): 8.592 | m/z: 619.434

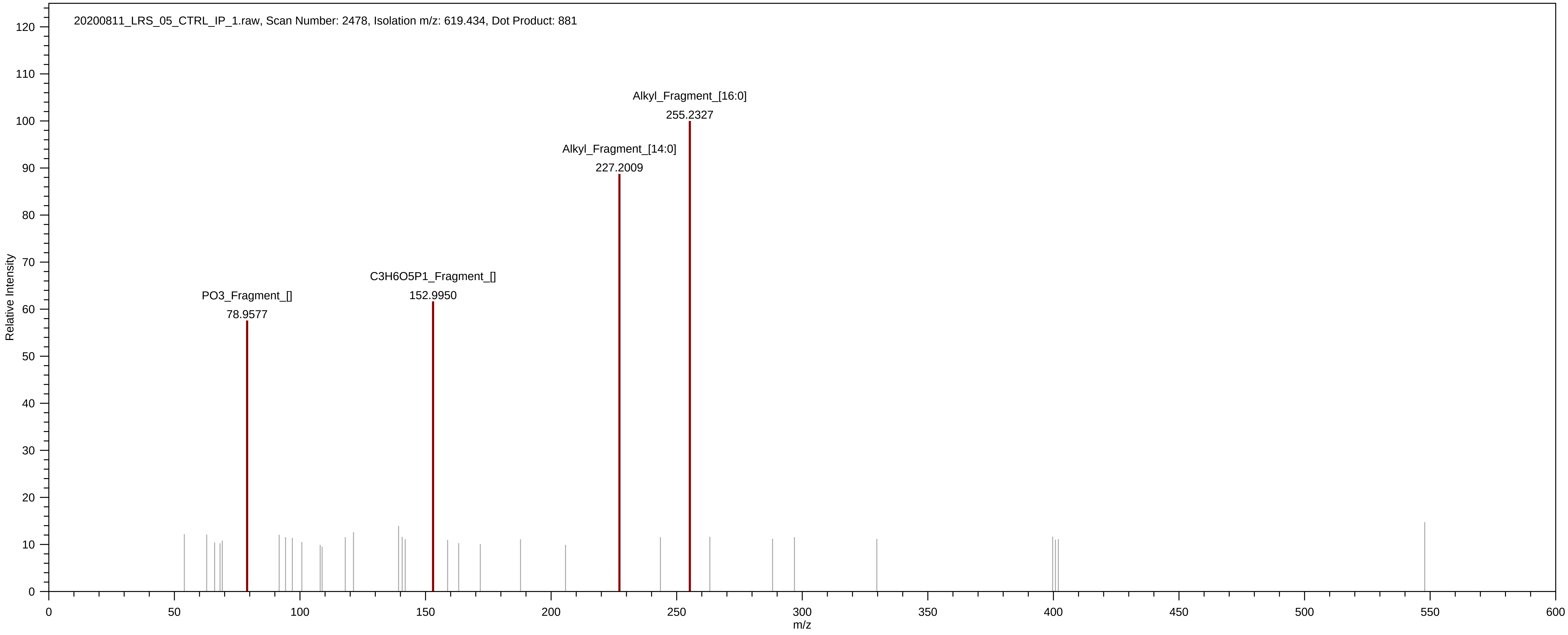

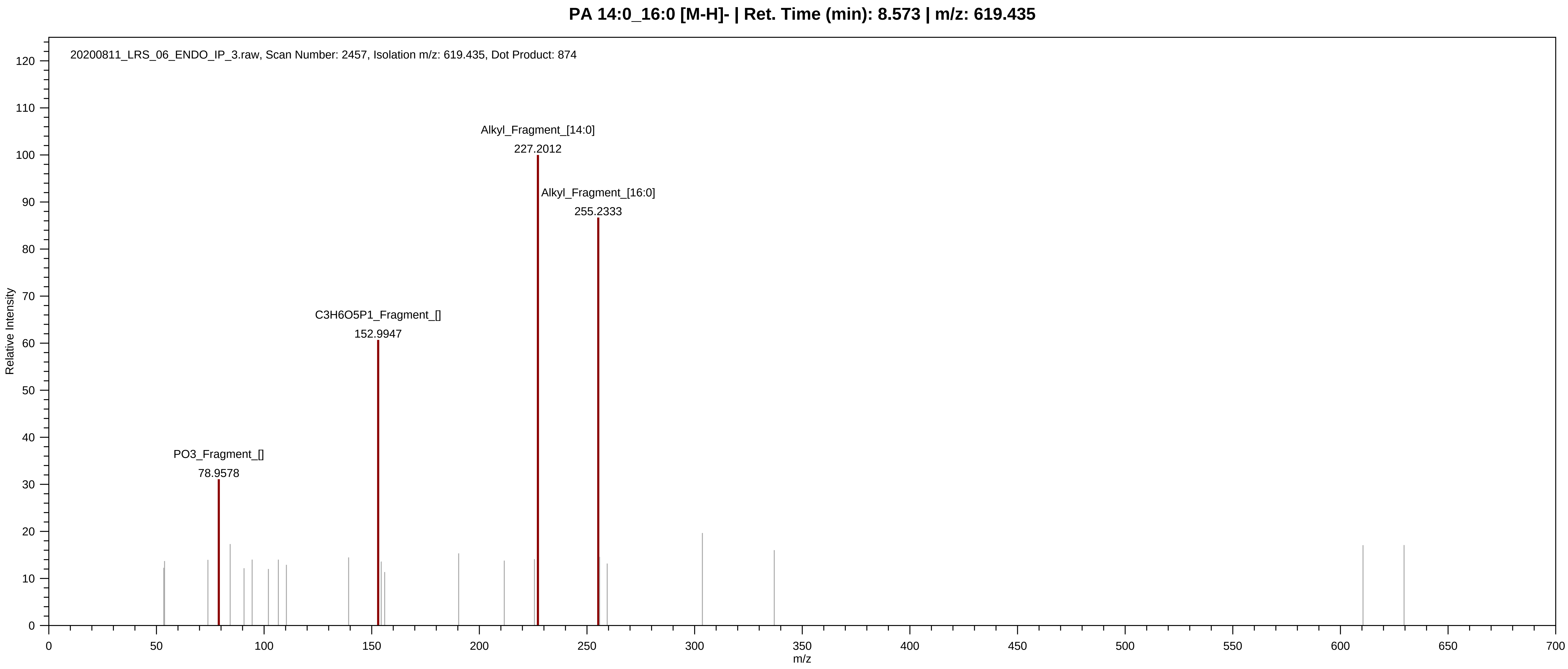
PA 14:0_16:0 [M-H]- | Ret. Time (min): 8.573 | m/z: 619.435
20200811_LRS_06_ENDO_IP_3.raw, Scan Number: 2457, Isolation m/z: 619.435, Dot Product: 874
Alkyl_Fragment_[14:0]
227.2012
Alkyl_Fragment_[16:0]
255.2333
C3H6O5P1_Fragment_[]
152.9947
PO3_Fragment_[]
78.9578
Relative Intensity
m/z

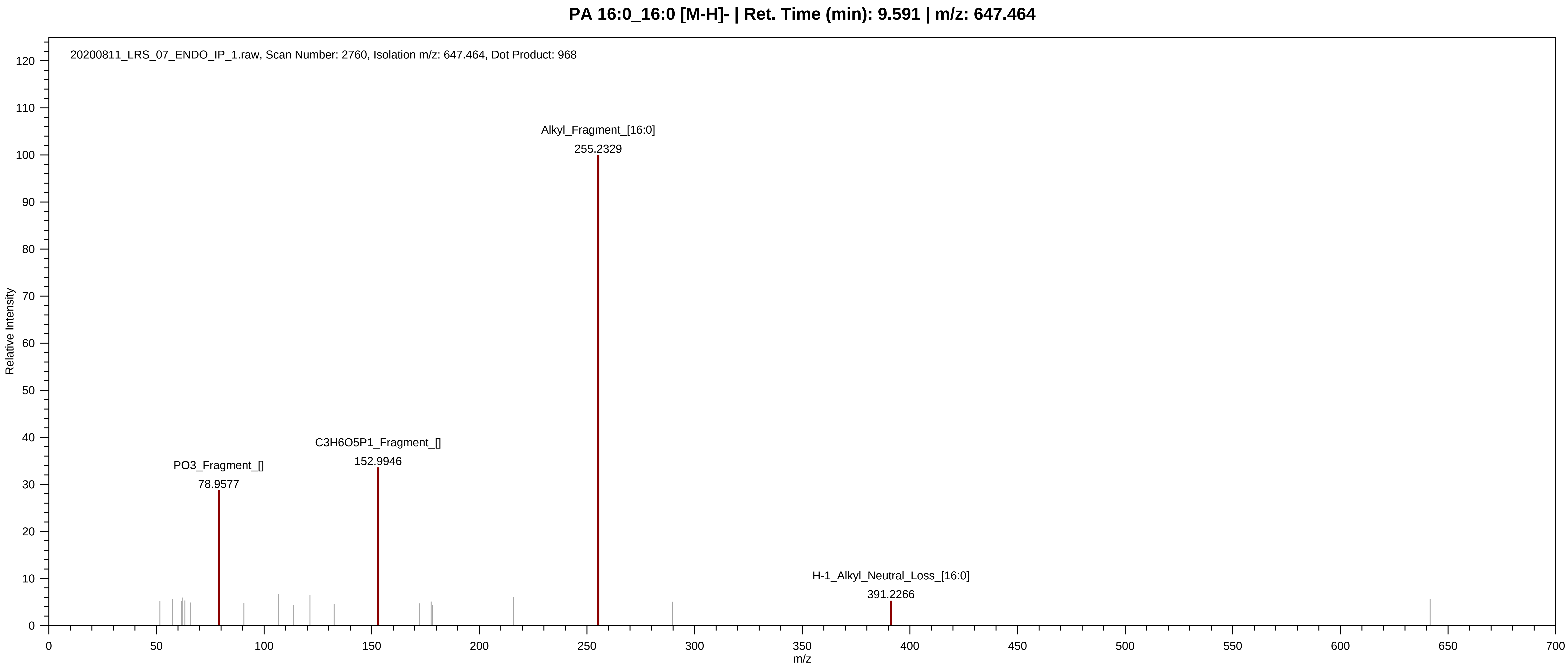

PA 16:0_16:0 [M-H]- | Ret. Time (min): 9.591 | m/z: 647.464
20200811_LRS_07_ENDO_IP_1.raw, Scan Number: 2760, Isolation m/z: 647.464, Dot Product: 968
Alkyl_Fragment_[16:0]
255.2329
C3H6O5P1_Fragment_[]
152.9946
PO3_Fragment_[]
78.9577
H-1_Alkyl_Neutral_Loss_[16:0]
391.2266
Relative Intensity
m/z

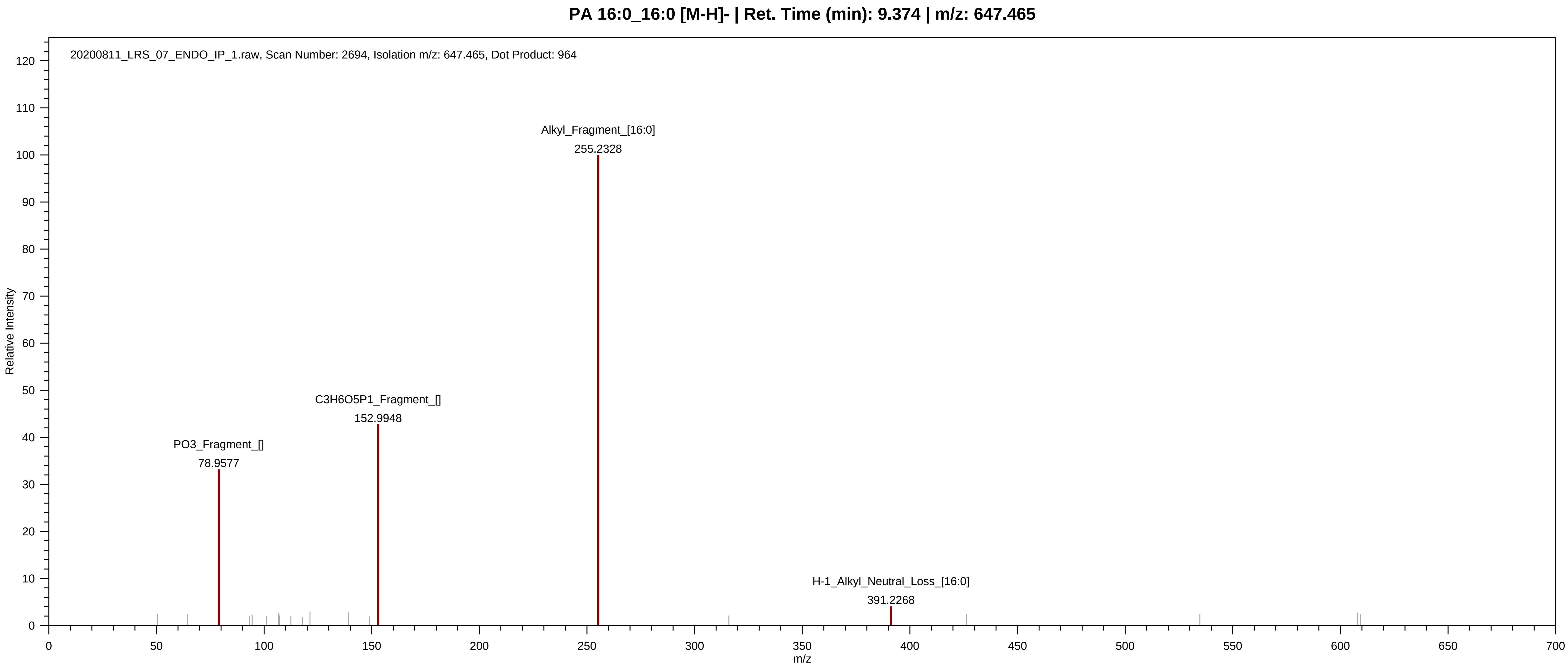

PA 16:0_16:0 [M-H]- | Ret. Time (min): 9.374 | m/z: 647.465
20200811_LRS_07_ENDO_IP_1.raw, Scan Number: 2694, Isolation m/z: 647.465, Dot Product: 964
Alkyl_Fragment_[16:0]
255.2328
C3H6O5P1_Fragment_[]
152.9948
PO3_Fragment_[]
78.9577
H-1_Alkyl_Neutral_Loss_[16:0]
391.2268
Relative Intensity
m/z

PA 16:0_16:0 [M-H]- | Ret. Time (min): 9.628 | m/z: 647.465

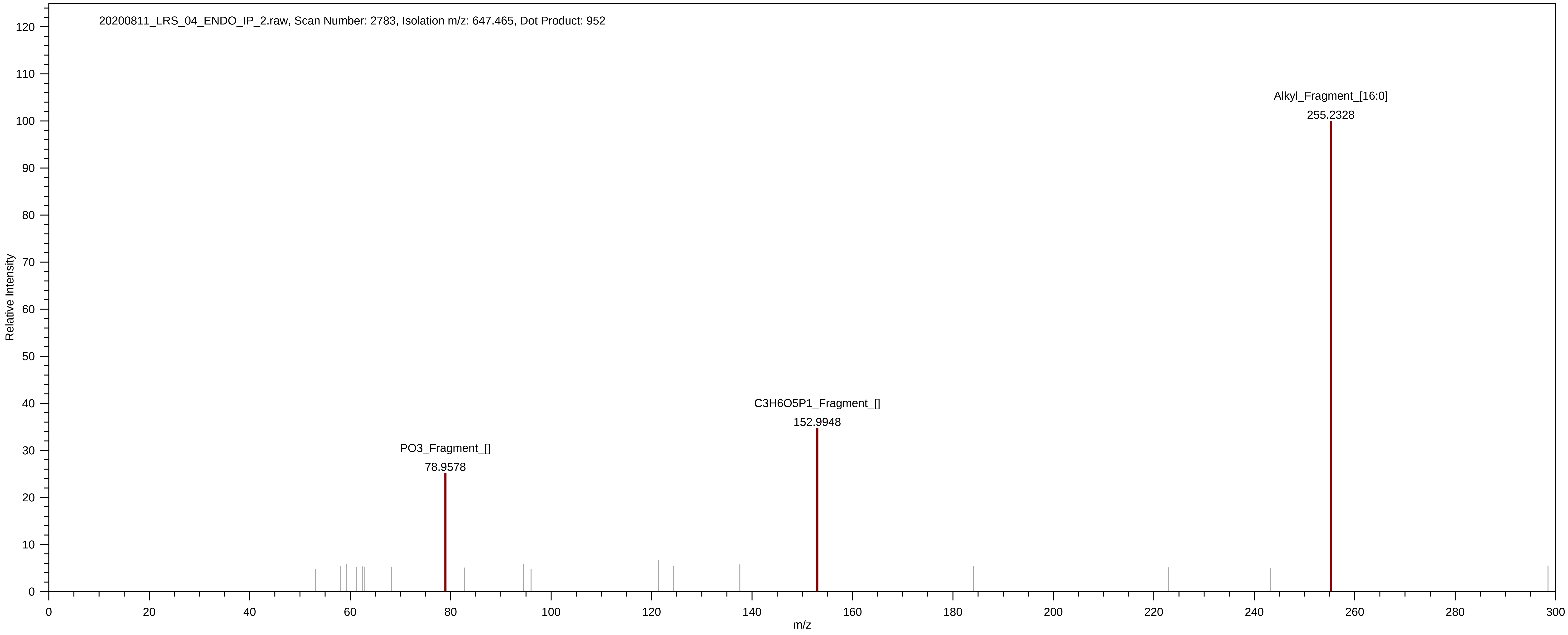

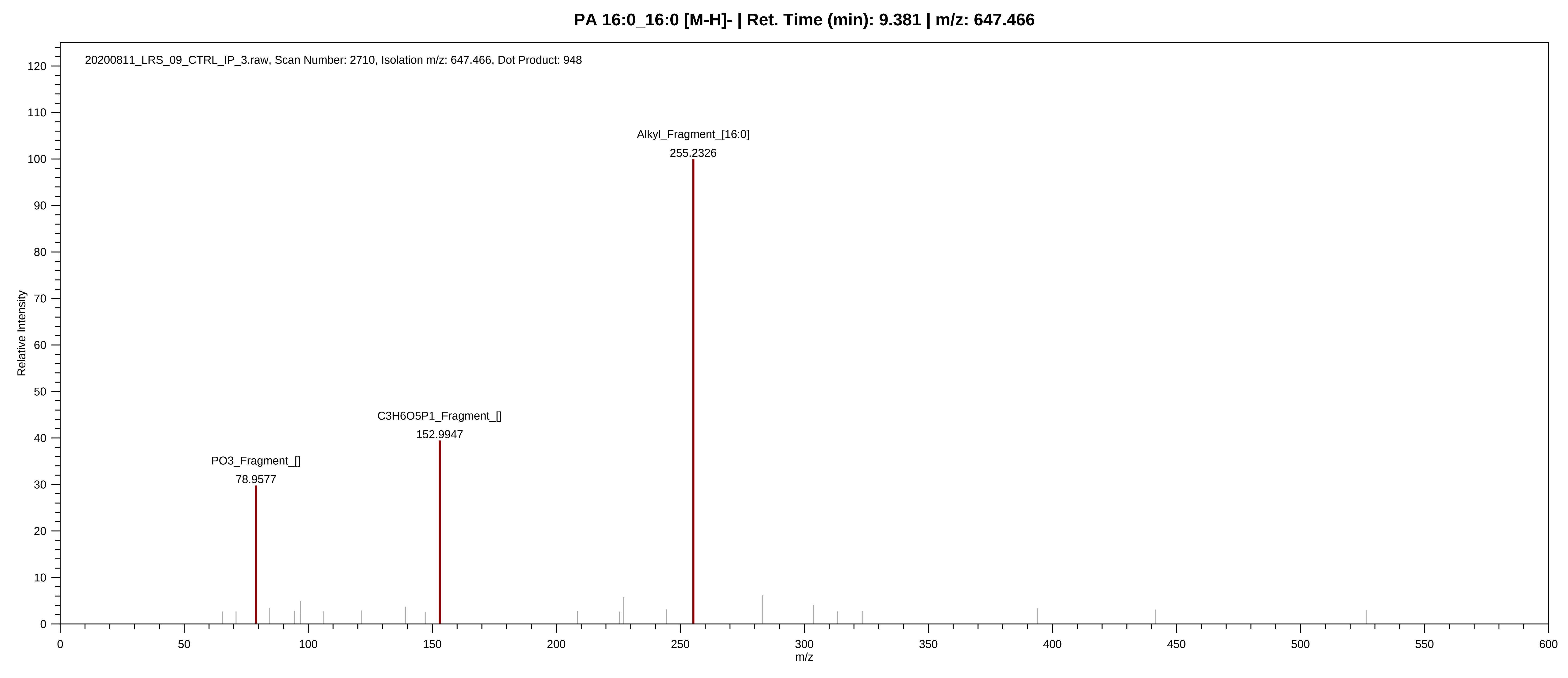

PA 16:0_16:0 [M-H]- | Ret. Time (min): 9.381 | m/z: 647.466
20200811_LRS_09_CTRL_IP_3.raw, Scan Number: 2710, Isolation m/z: 647.466, Dot Product: 948
Alkyl_Fragment_[16:0]
255.2326
C3H6O5P1_Fragment_[]
152.9947
PO3_Fragment_[]
78.9577
Relative Intensity
m/z

PA 16:0_16:0 [M-H]- | Ret. Time (min): 9.394 | m/z: 647.466

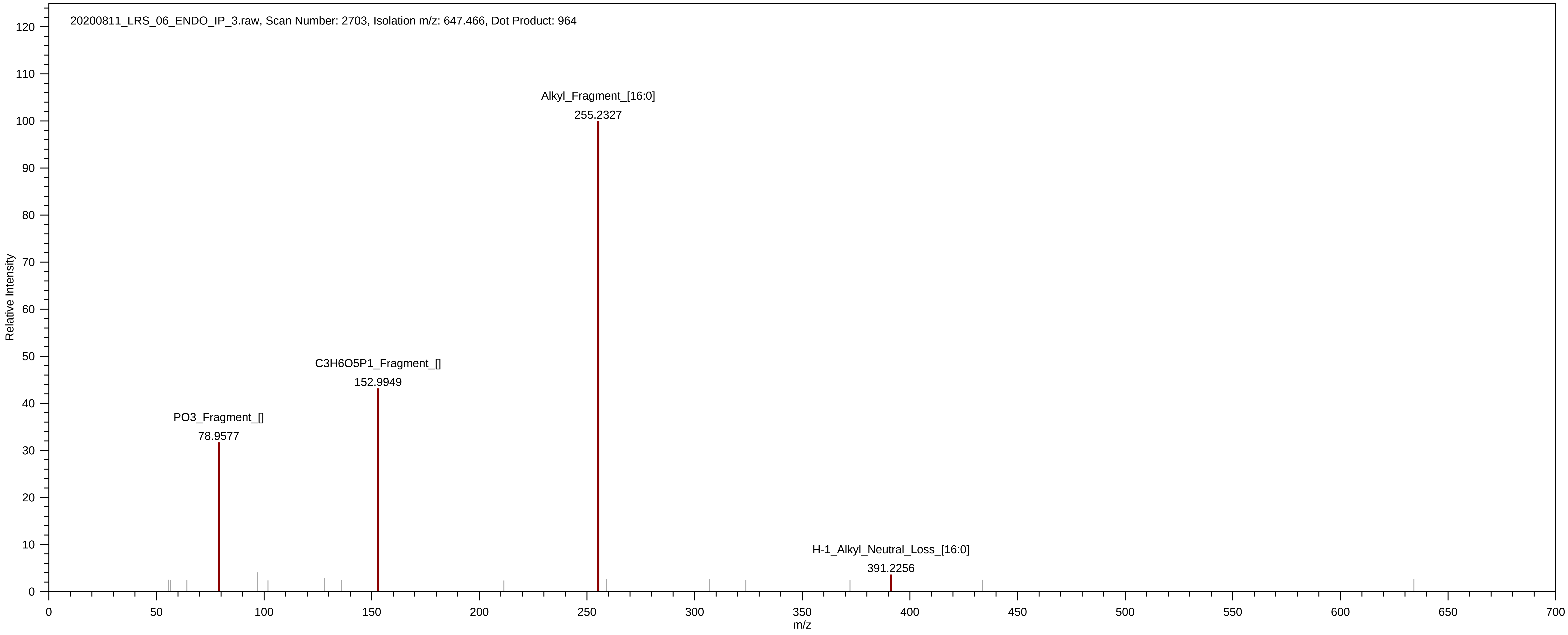

PA 16:0_16:0 [M-H]- | Ret. Time (min): 9.408 | m/z: 647.466

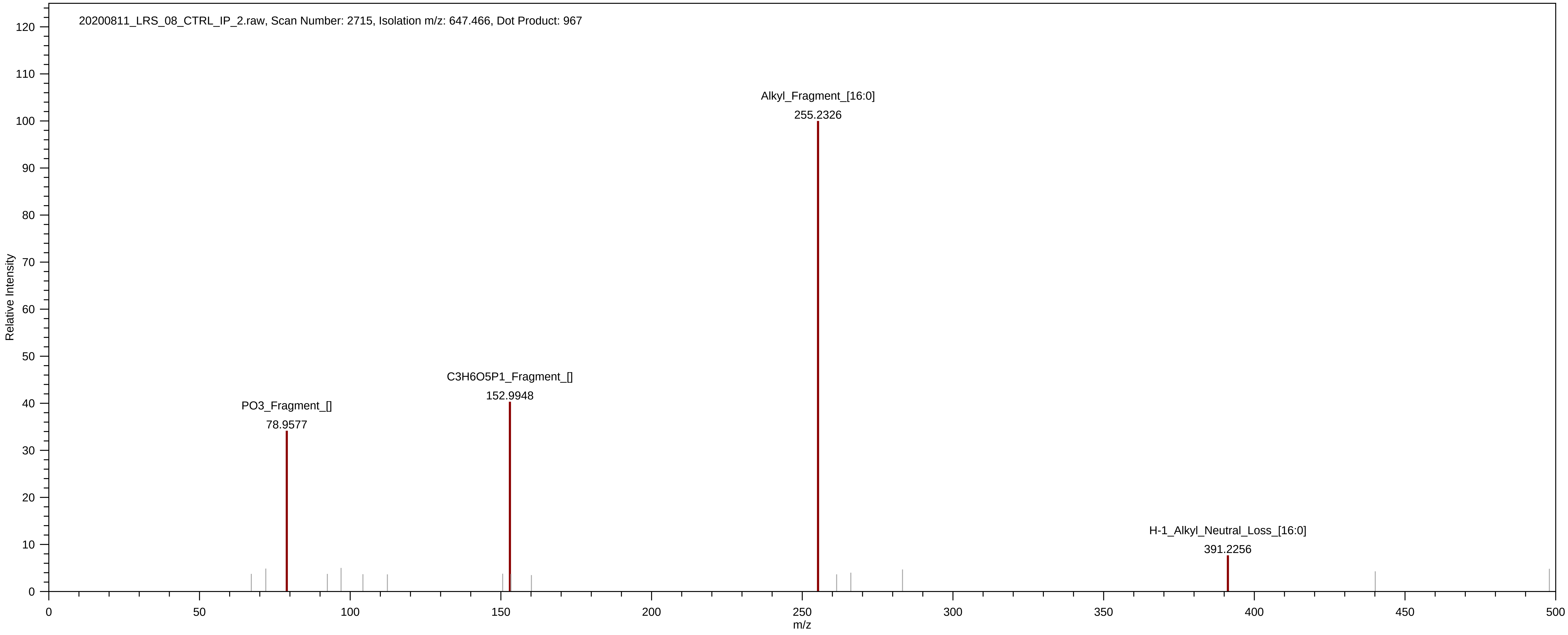

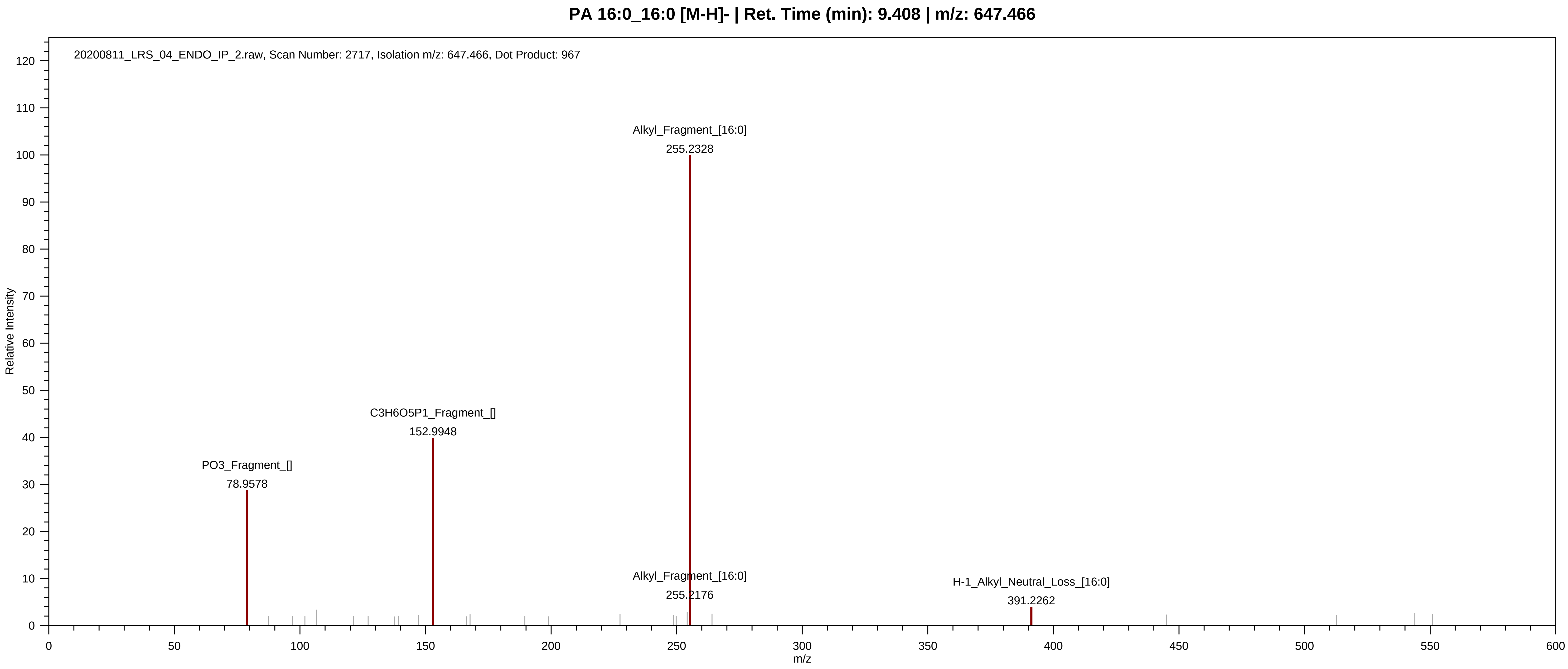

PA 16:0_16:0 [M-H]- | Ret. Time (min): 9.408 | m/z: 647.466
20200811_LRS_04_ENDO_IP_2.raw, Scan Number: 2717, Isolation m/z: 647.466, Dot Product: 967
Alkyl_Fragment_[16:0]
255.2328
C3H6O5P1_Fragment_[]
152.9948
PO3_Fragment_[]
78.9578
Alkyl_Fragment_[16:0]
255.2176
H-1_Alkyl_Neutral_Loss_[16:0]
391.2262
Relative Intensity
m/z

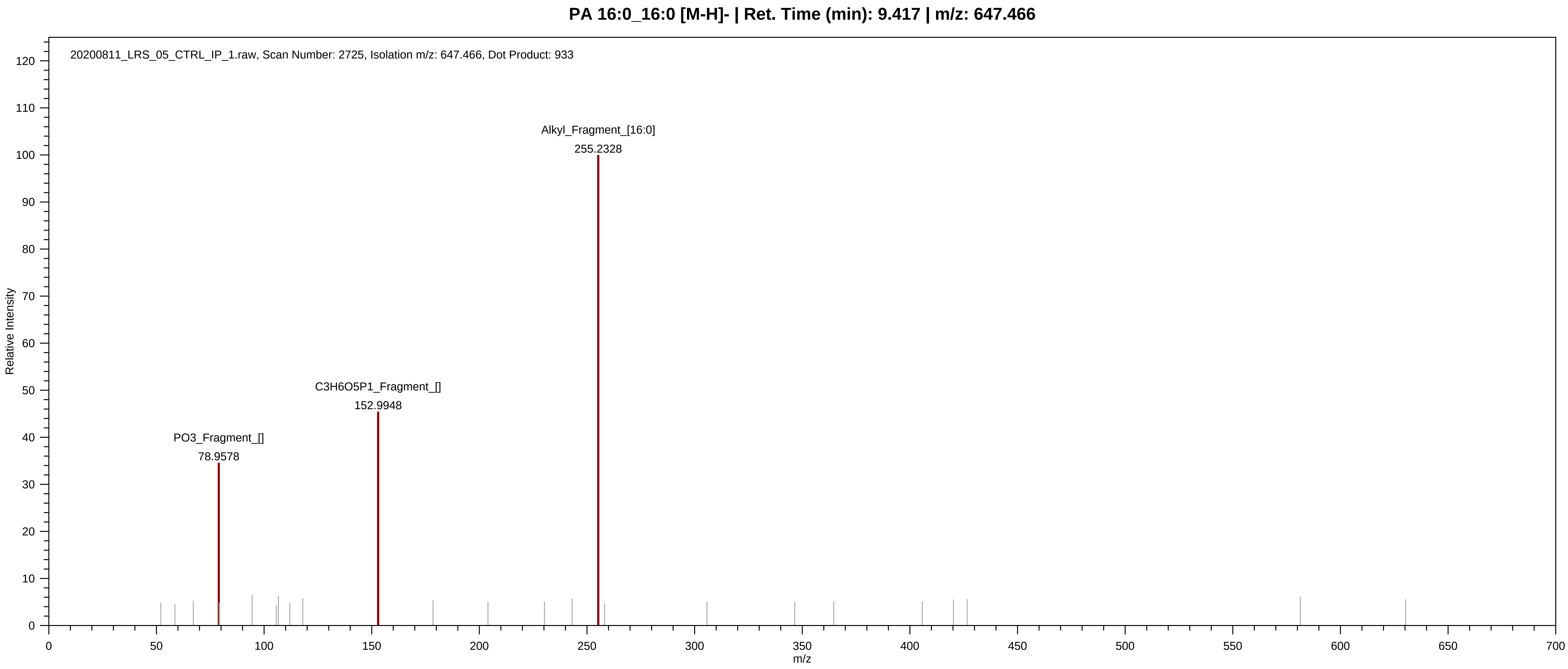

PA 16:0_16:0 [M-H]- | Ret. Time (min): 9.417 | m/z: 647.466
20200811_LRS_05_CTRL_IP_1.raw, Scan Number: 2725, Isolation m/z: 647.466, Dot Product: 933
Alkyl_Fragment_[16:0]
255.2328
C3H6O5P1_Fragment_[]
152.9948
PO3_Fragment_[]
78.9578
Relative Intensity
m/z

PA 16:0_16:0 [M-H]- | Ret. Time (min): 9.651 | m/z: 647.466

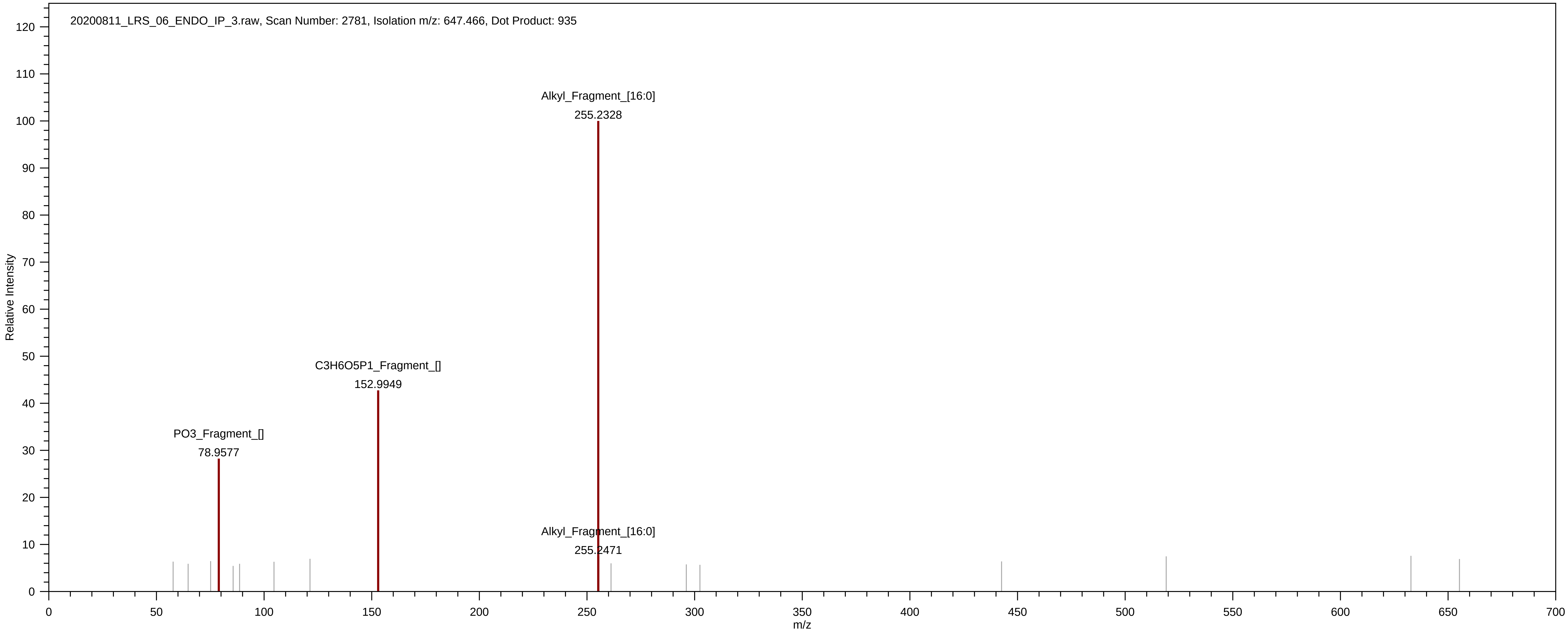

PA 16:0_18:0 [M-H]- | Ret. Time (min): 10.709 | m/z: 675.497

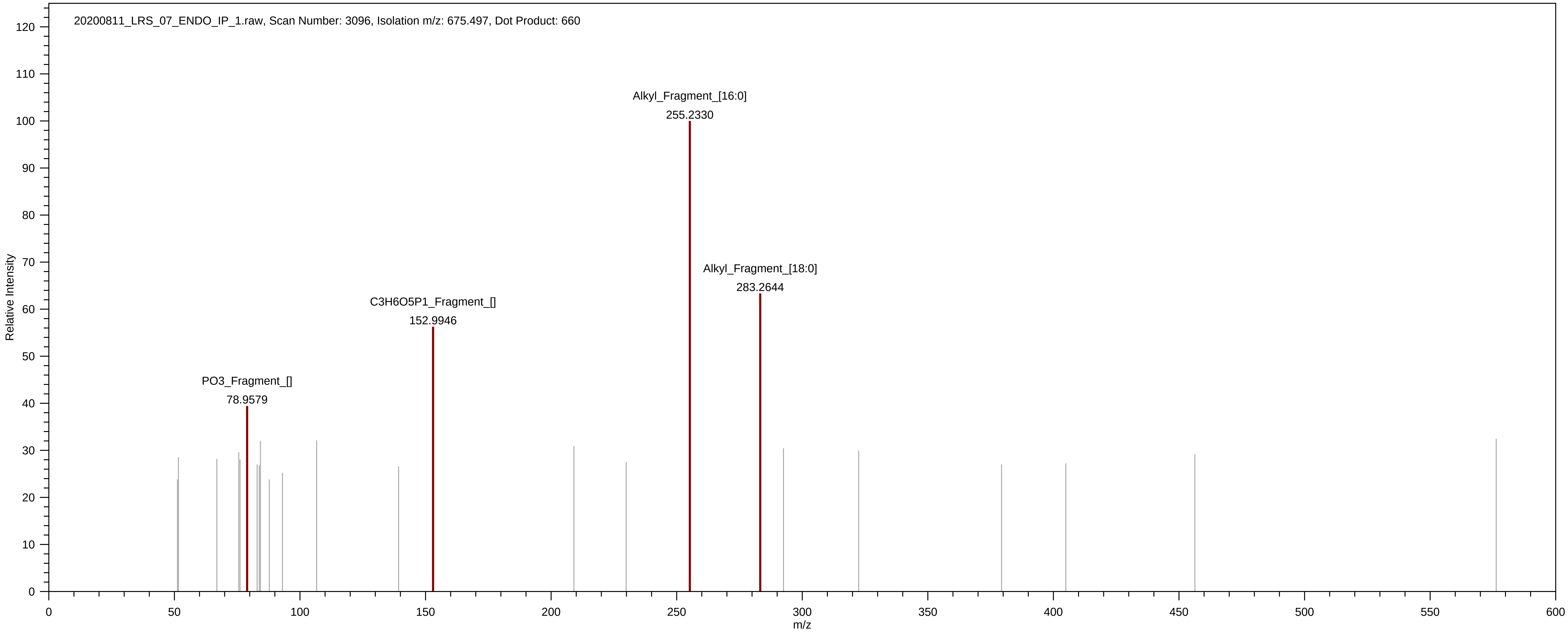

PA 16:0_18:1 [M-H]- | Ret. Time (min): 9.49 | m/z: 673.481

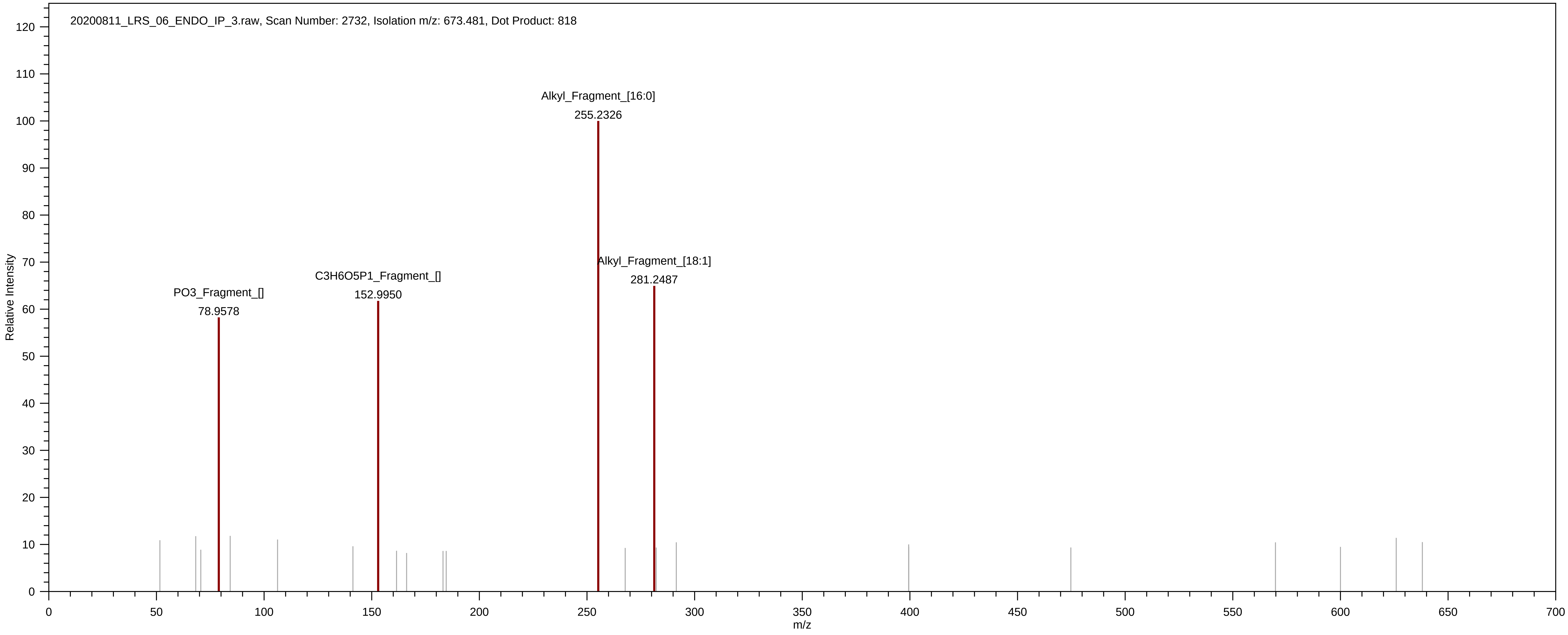

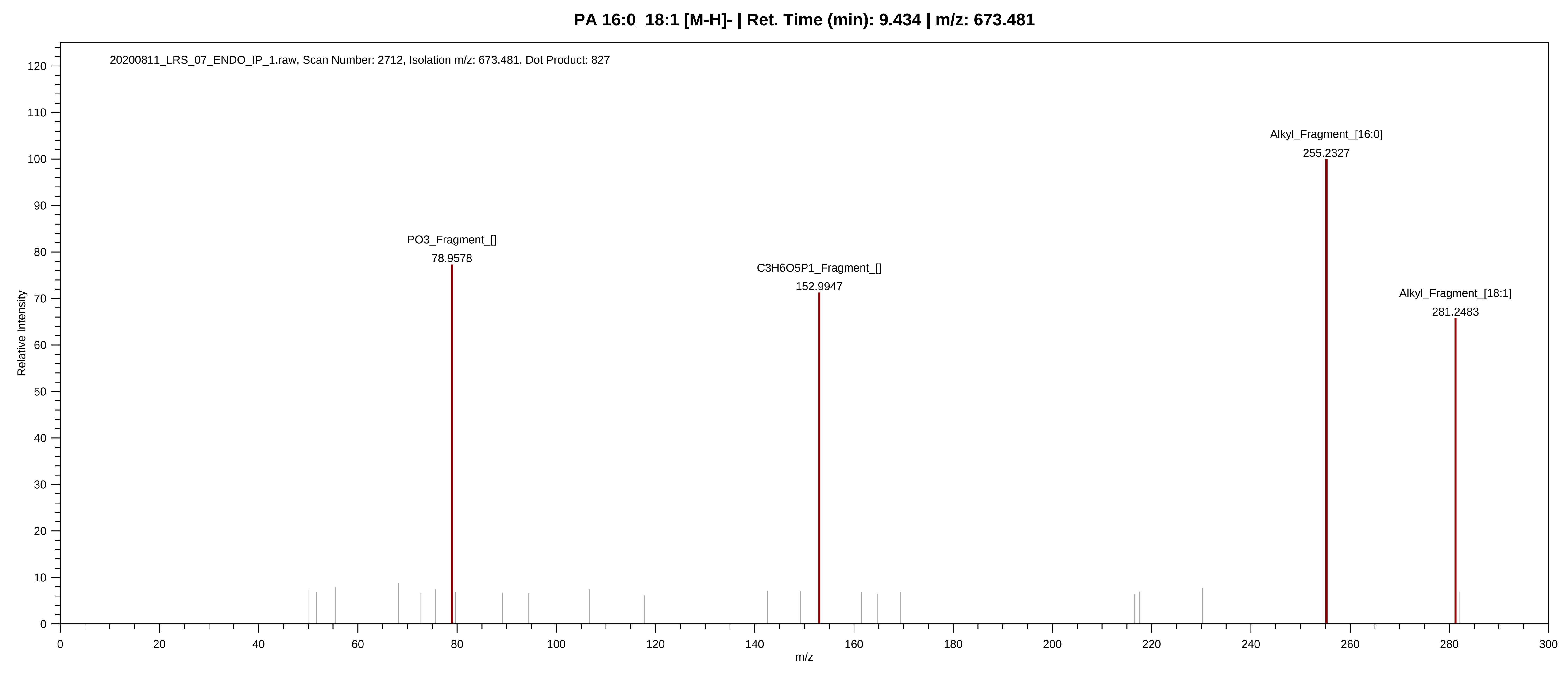
PA 16:0_18:1 [M-H]- | Ret. Time (min): 9.434 | m/z: 673.481
20200811_LRS_07_ENDO_IP_1.raw, Scan Number: 2712, Isolation m/z: 673.481, Dot Product: 827
PO3_Fragment_[]
78.9578
C3H6O5P1_Fragment_[]
152.9947
Alkyl_Fragment_[16:0]
255.2327
Alkyl_Fragment_[18:1]
281.2483
Relative Intensity
m/z

PA 16:0_18:1 [M-H]- | Ret. Time (min): 9.459 | m/z: 673.481

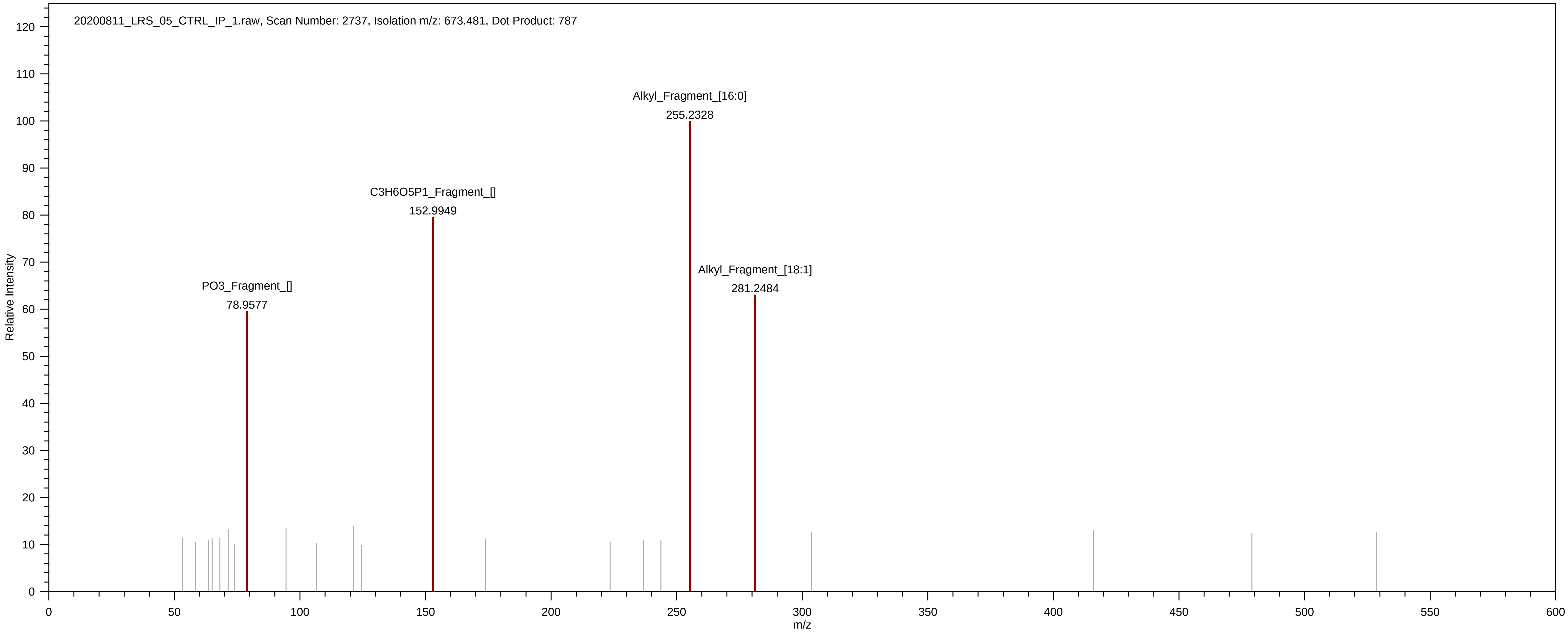

PA 16:0_18:1 [M-H]- | Ret. Time (min): 9.468 | m/z: 673.481

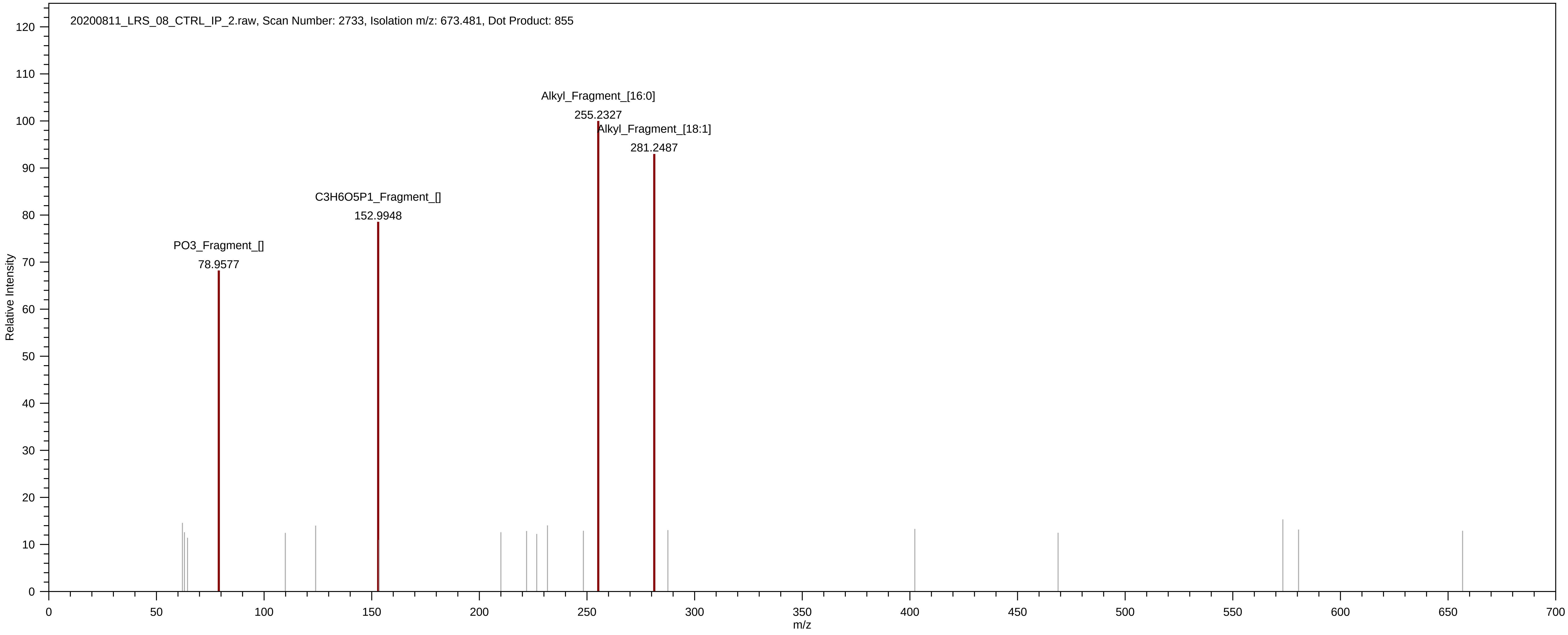

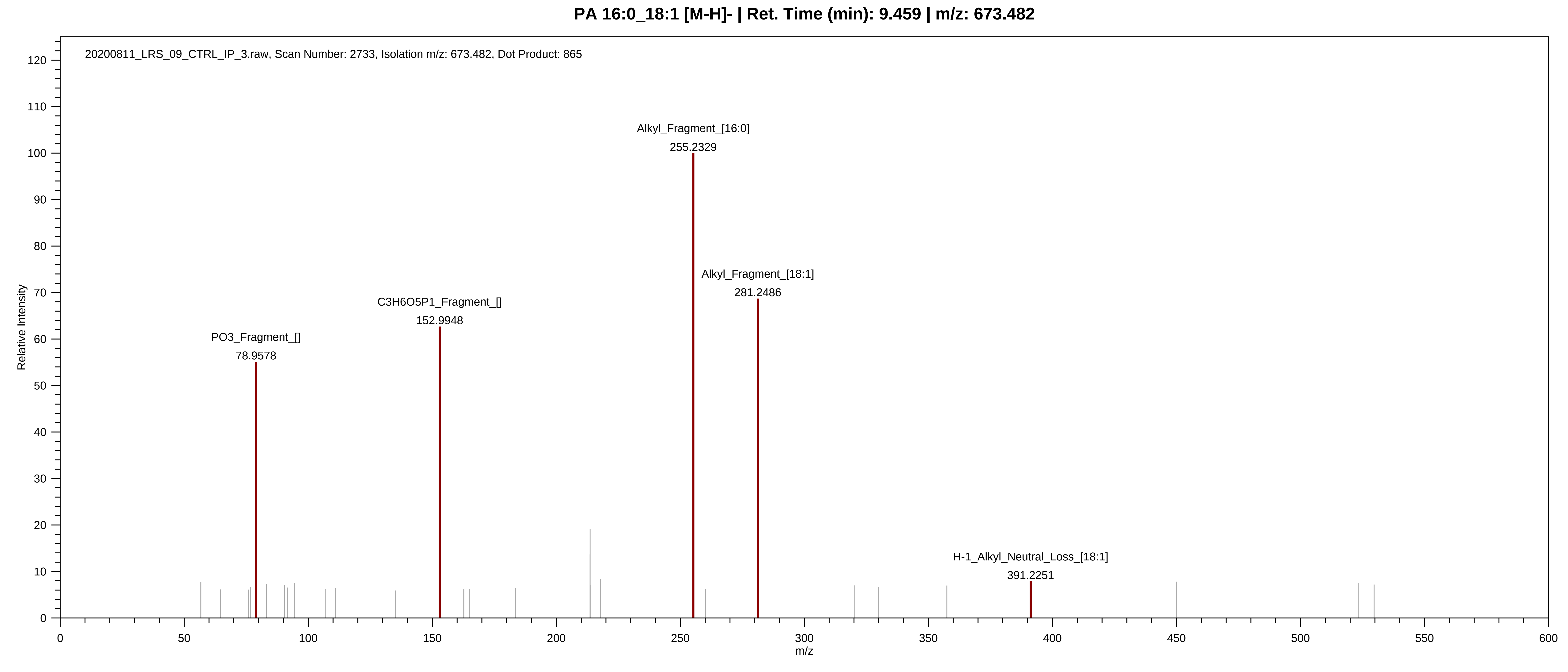

PA 16:0_18:1 [M-H]- | Ret. Time (min): 9.459 | m/z: 673.482
20200811_LRS_09_CTRL_IP_3.raw, Scan Number: 2733, Isolation m/z: 673.482, Dot Product: 865
Alkyl_Fragment_[16:0]
255.2329
Alkyl_Fragment_[18:1]
281.2486
C3H6O5P1_Fragment_[]
152.9948
PO3_Fragment_[]
78.9578
H-1_Alkyl_Neutral_Loss_[18:1]
391.2251
Relative Intensity
m/z

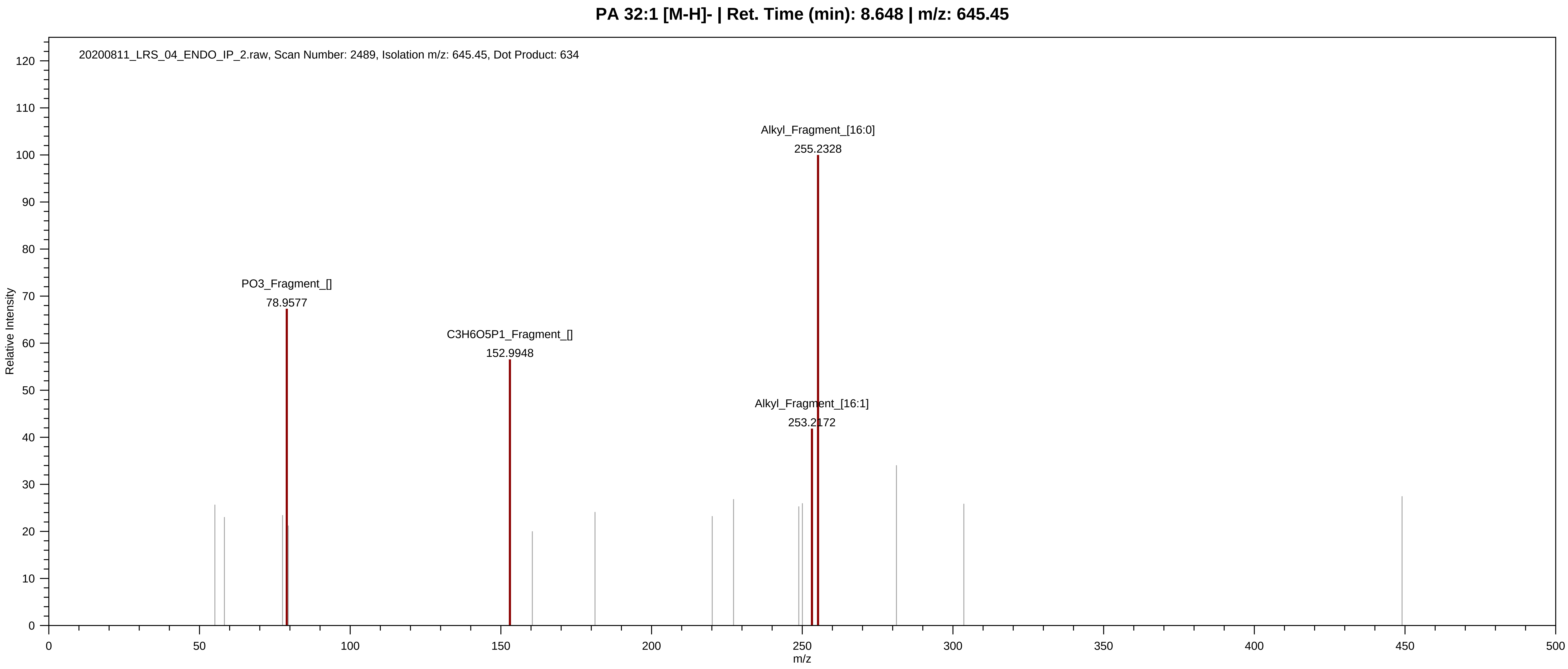

PA 32:1 [M-H]- | Ret. Time (min): 8.648 | m/z: 645.45
20200811_LRS_04_ENDO_IP_2.raw, Scan Number: 2489, Isolation m/z: 645.45, Dot Product: 634
Alkyl_Fragment_[16:0]
255.2328
PO3_Fragment_[]
78.9577
C3H6O5P1_Fragment_[]
152.9948
Alkyl_Fragment_[16:1]
253.2172
Relative Intensity
m/z
0
10
20
30
40
50
60
70
80
90
100
110
120
0
50
100
150
200
250
300
350
400
450
500

PC 14:0_14:0 [M+Ac-H]- | Ret. Time (min): 8.013 | m/z: 736.514

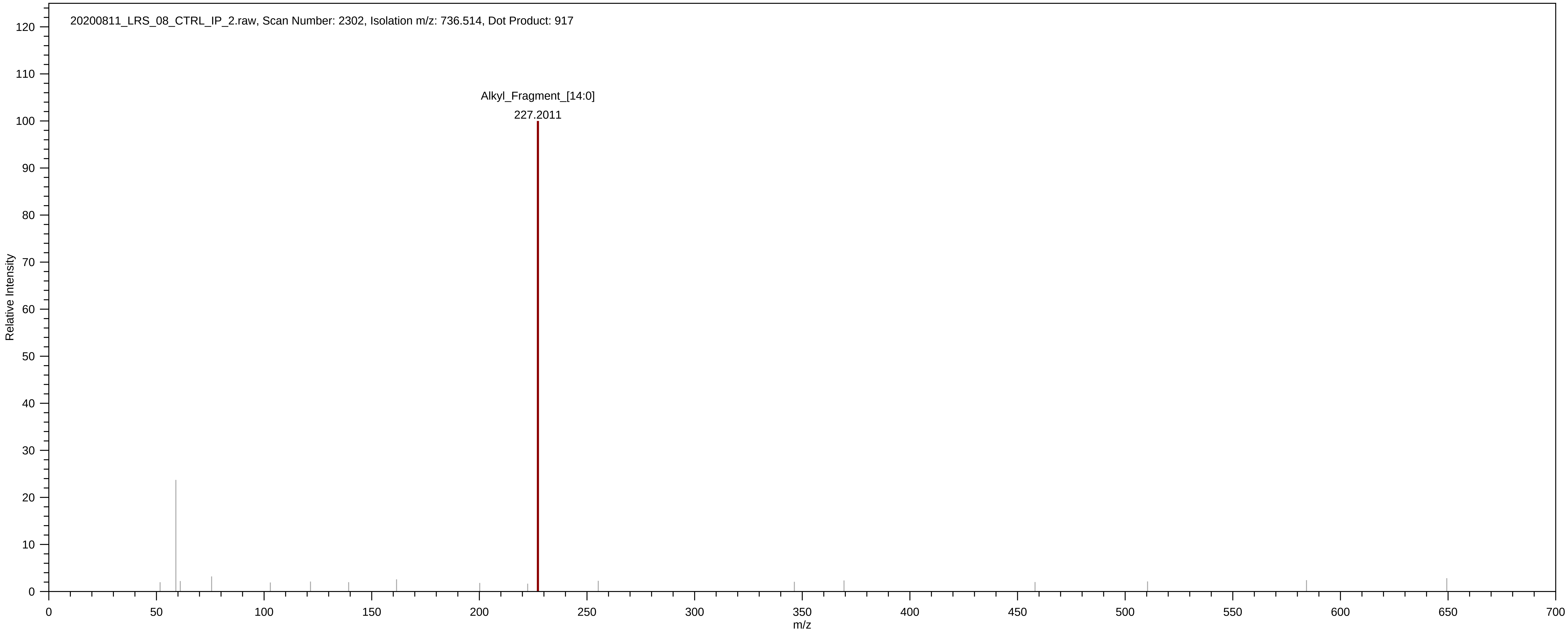

PC 14:0_14:0 [M+Ac-H]- | Ret. Time (min): 8.005 | m/z: 736.515

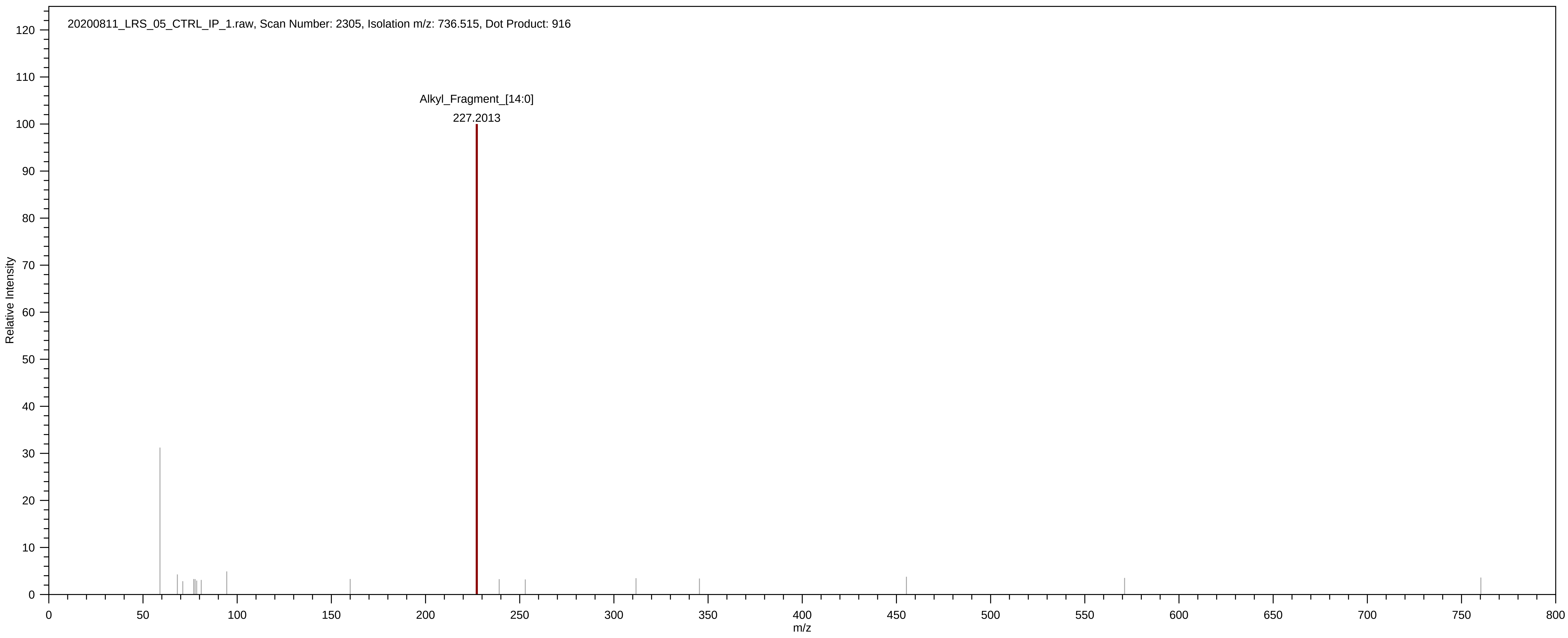

PC 14:0_14:0 [M+Ac-H]- | Ret. Time (min): 8.009 | m/z: 736.515

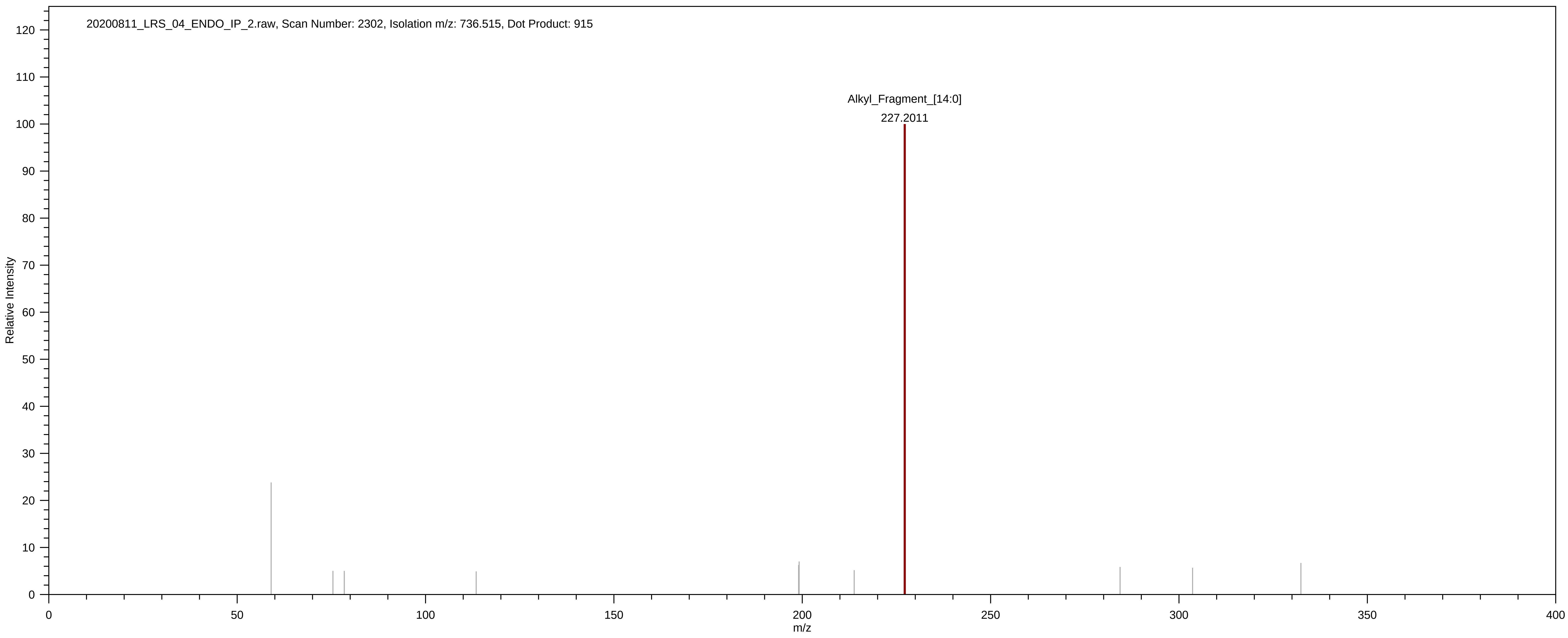

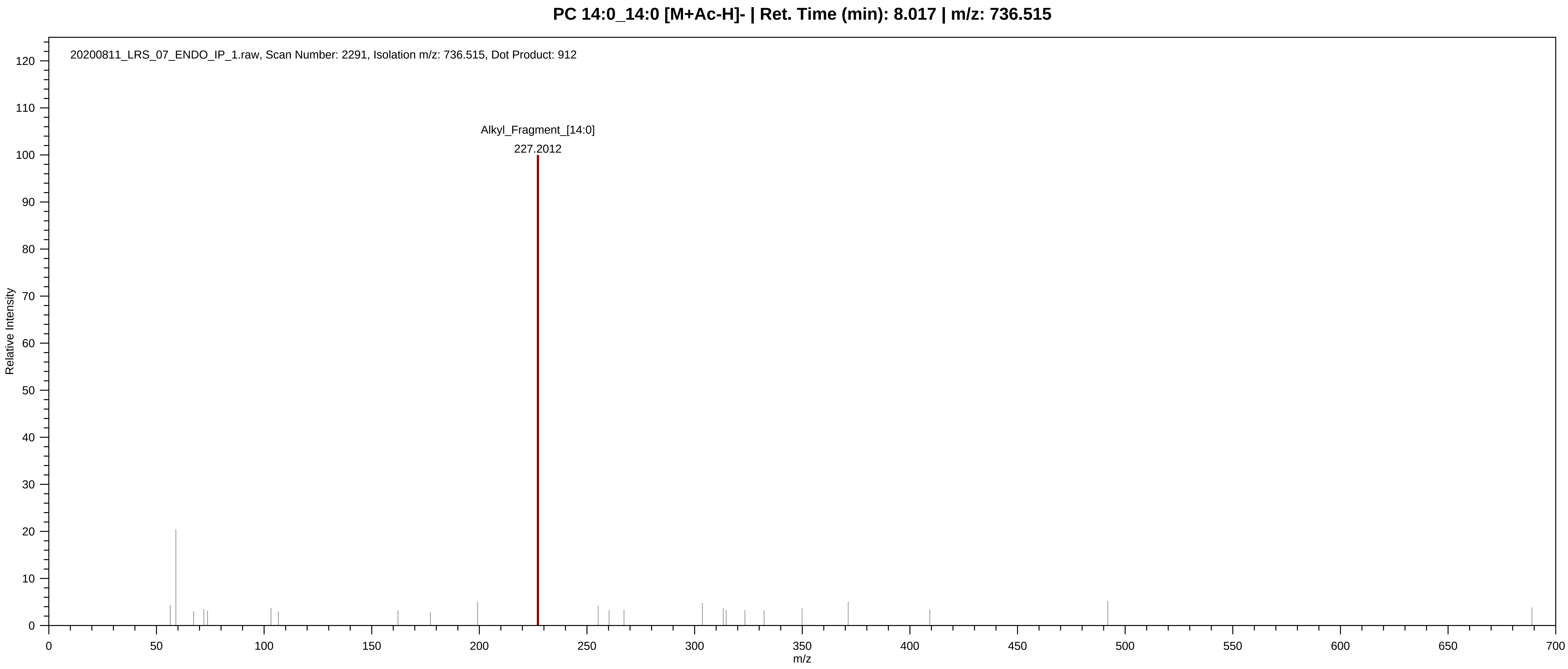
PC 14:0_14:0 [M+Ac-H]- | Ret. Time (min): 8.017 | m/z: 736.515
20200811_LRS_07_ENDO_IP_1.raw, Scan Number: 2291, Isolation m/z: 736.515, Dot Product: 912
Alkyl_Fragment_[14:0]
227.2012
Relative Intensity
m/z

PC 14:0_14:0 [M+Ac-H]- | Ret. Time (min): 8.019 | m/z: 736.515

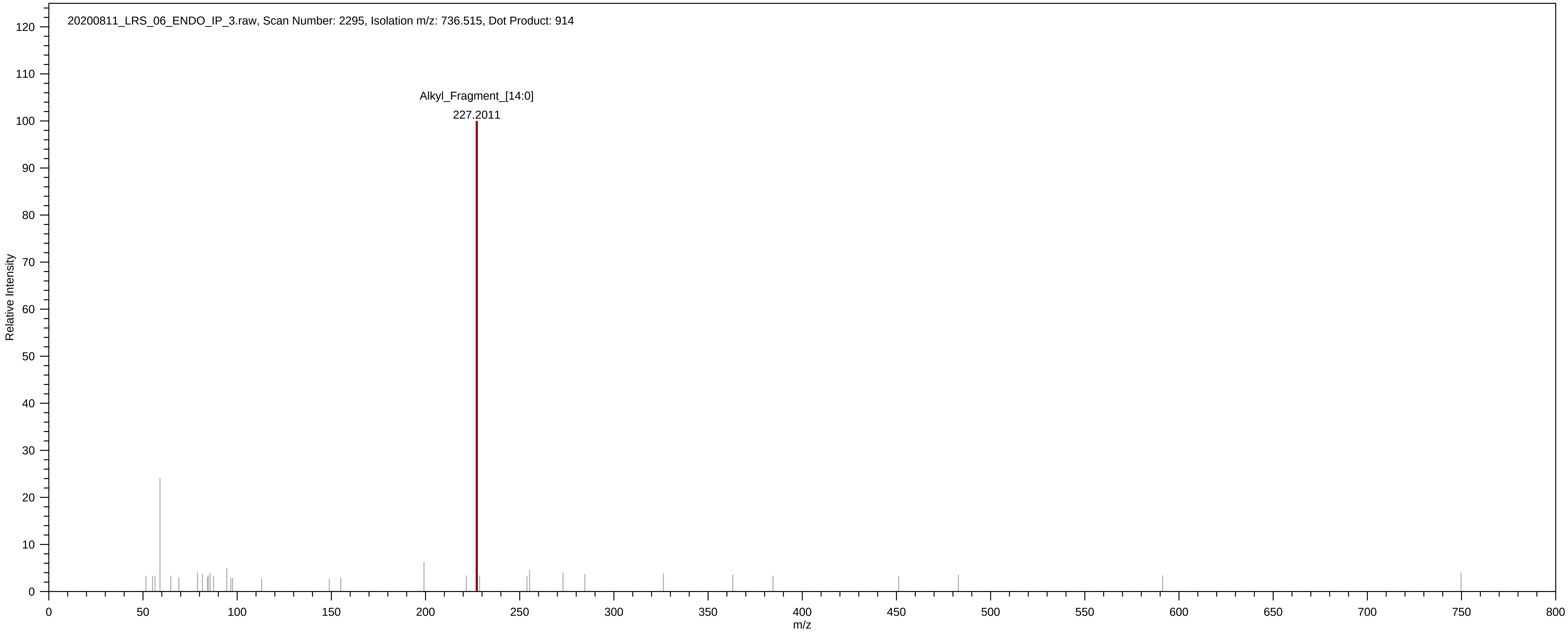

PC 14:0_15:0 [M+Ac-H]- | Ret. Time (min): 8.444 | m/z: 750.53

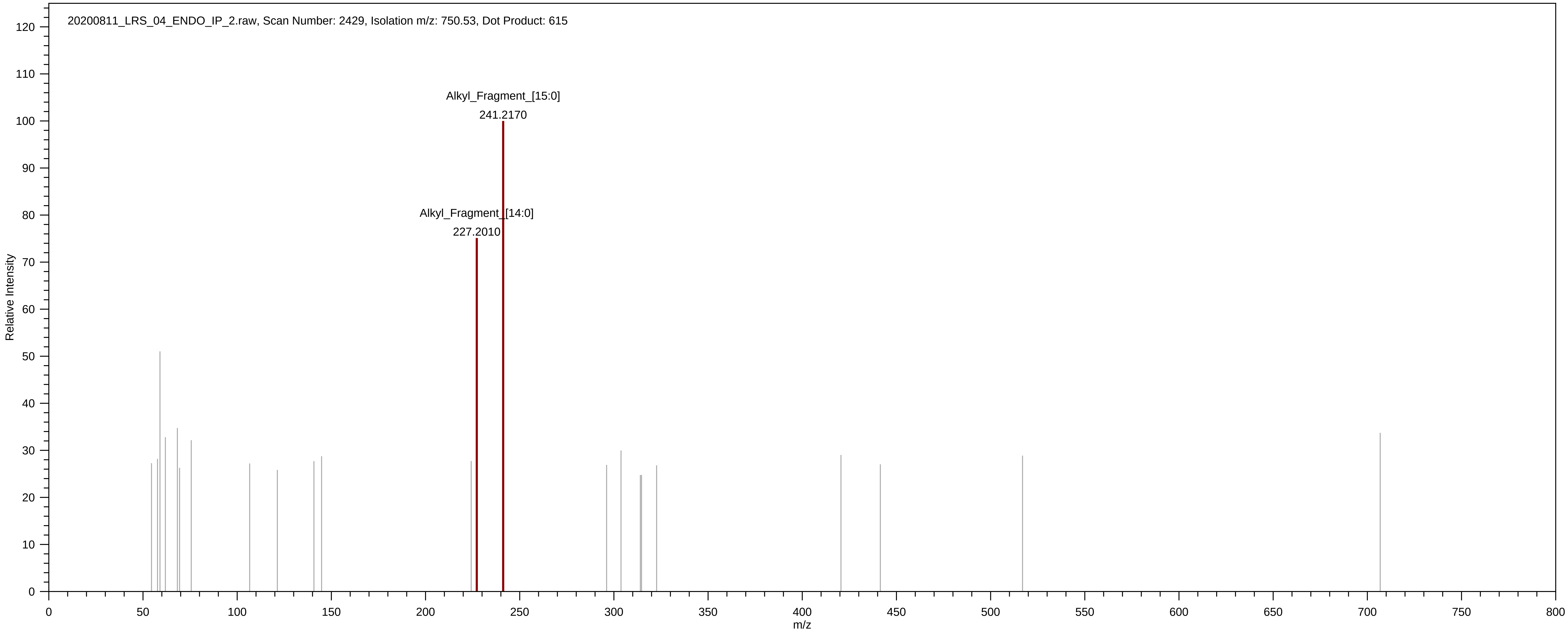

PC 14:0_15:0 [M+Ac-H]- | Ret. Time (min): 8.451 | m/z: 750.529

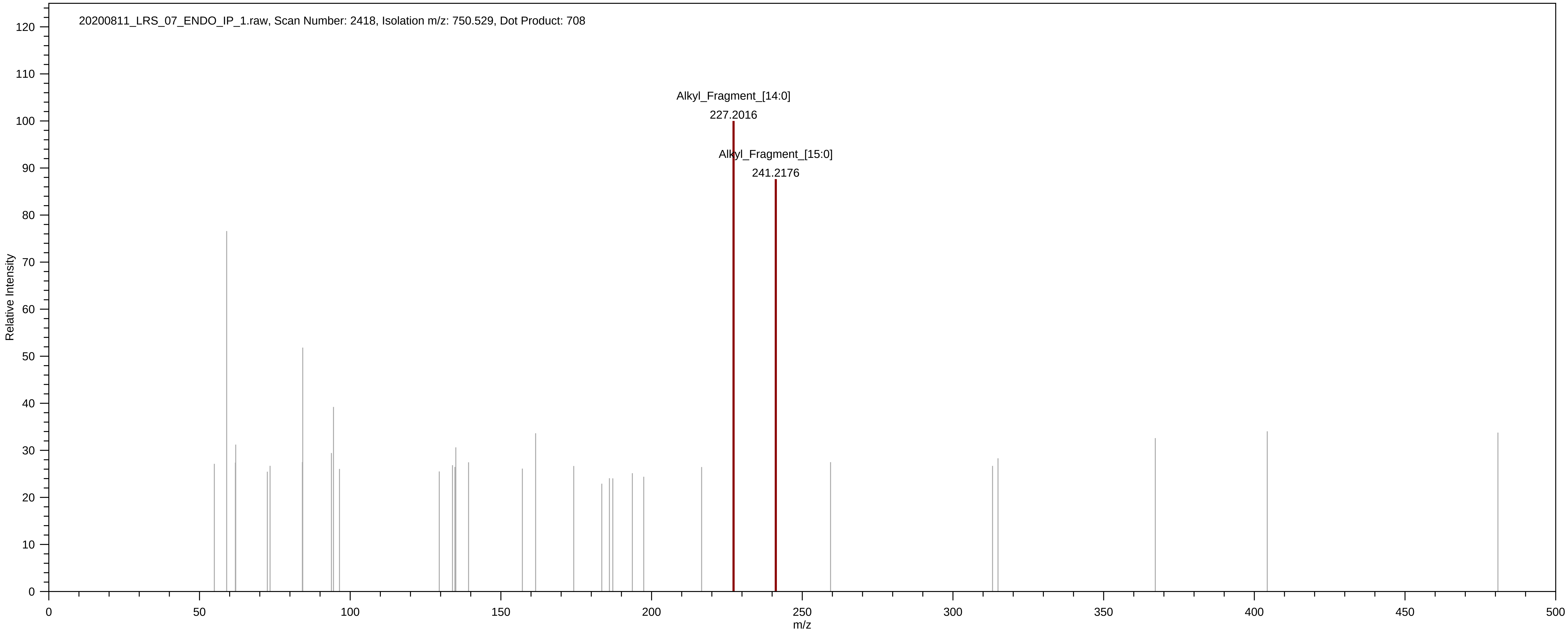

PC 14:0_15:0 [M+Ac-H]- | Ret. Time (min): 8.464 | m/z: 750.529

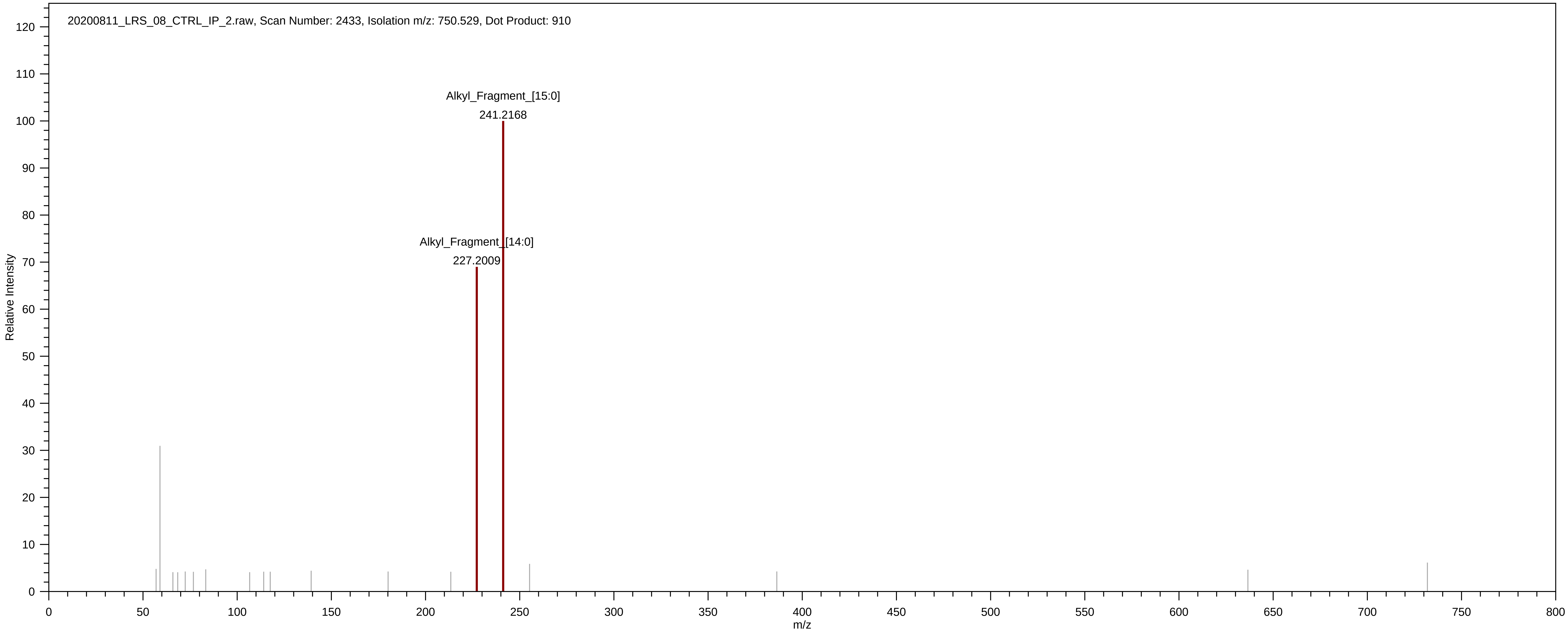

PC 14:0_15:0 [M+Ac-H]- | Ret. Time (min): 8.512 | m/z: 750.529

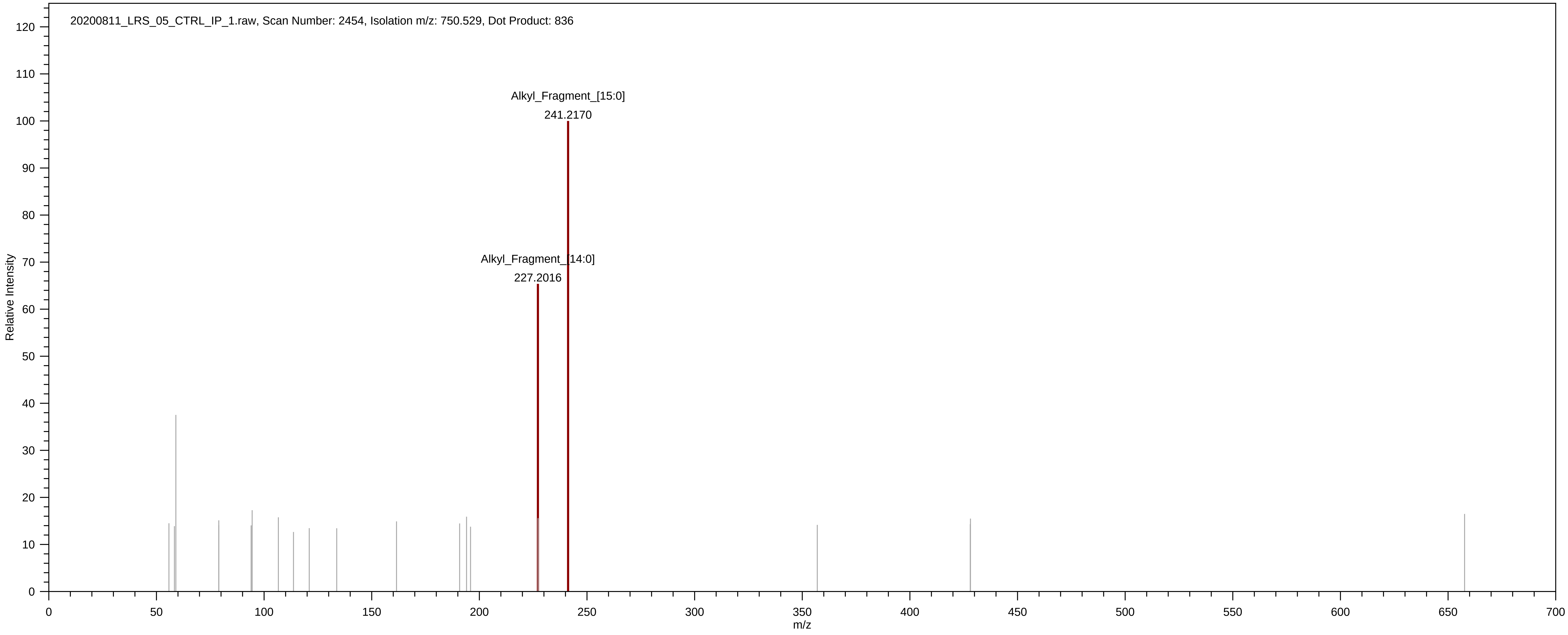

PC 14:0_16:0 [M+Ac-H]- | Ret. Time (min): 8.858 | m/z: 764.544

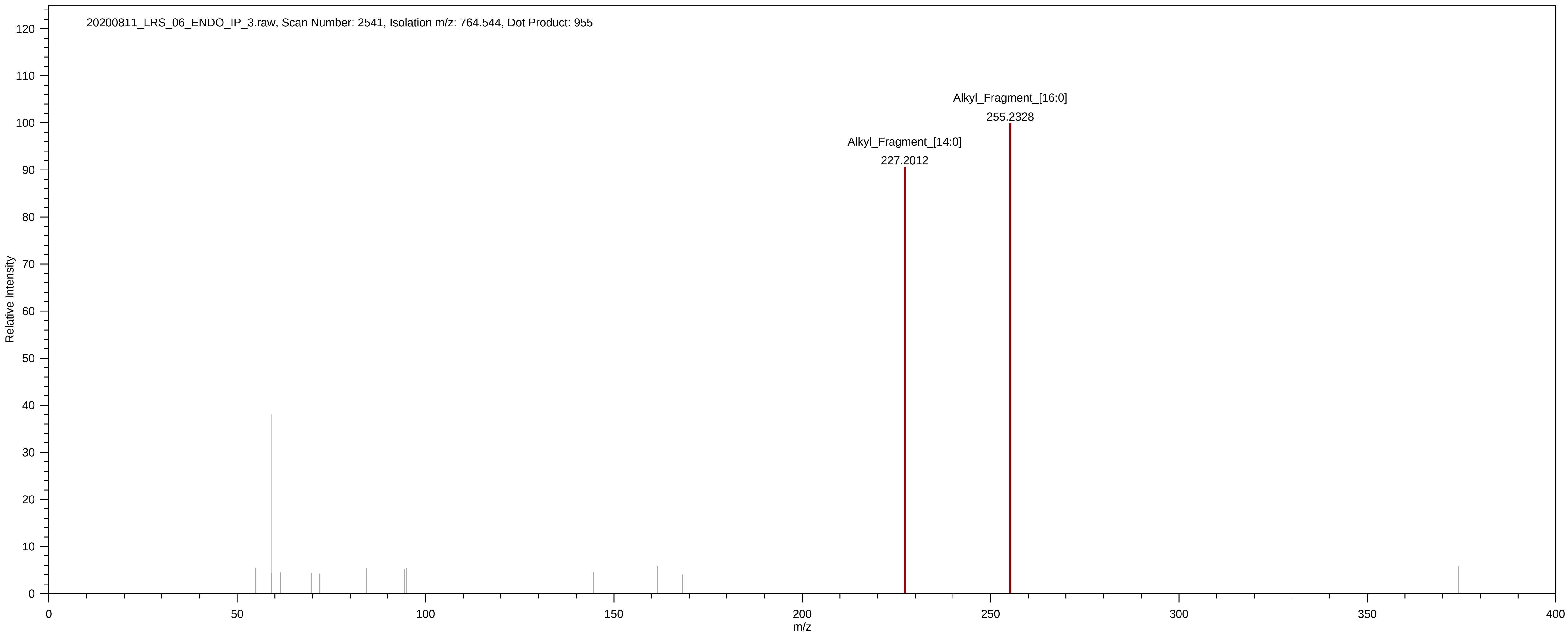

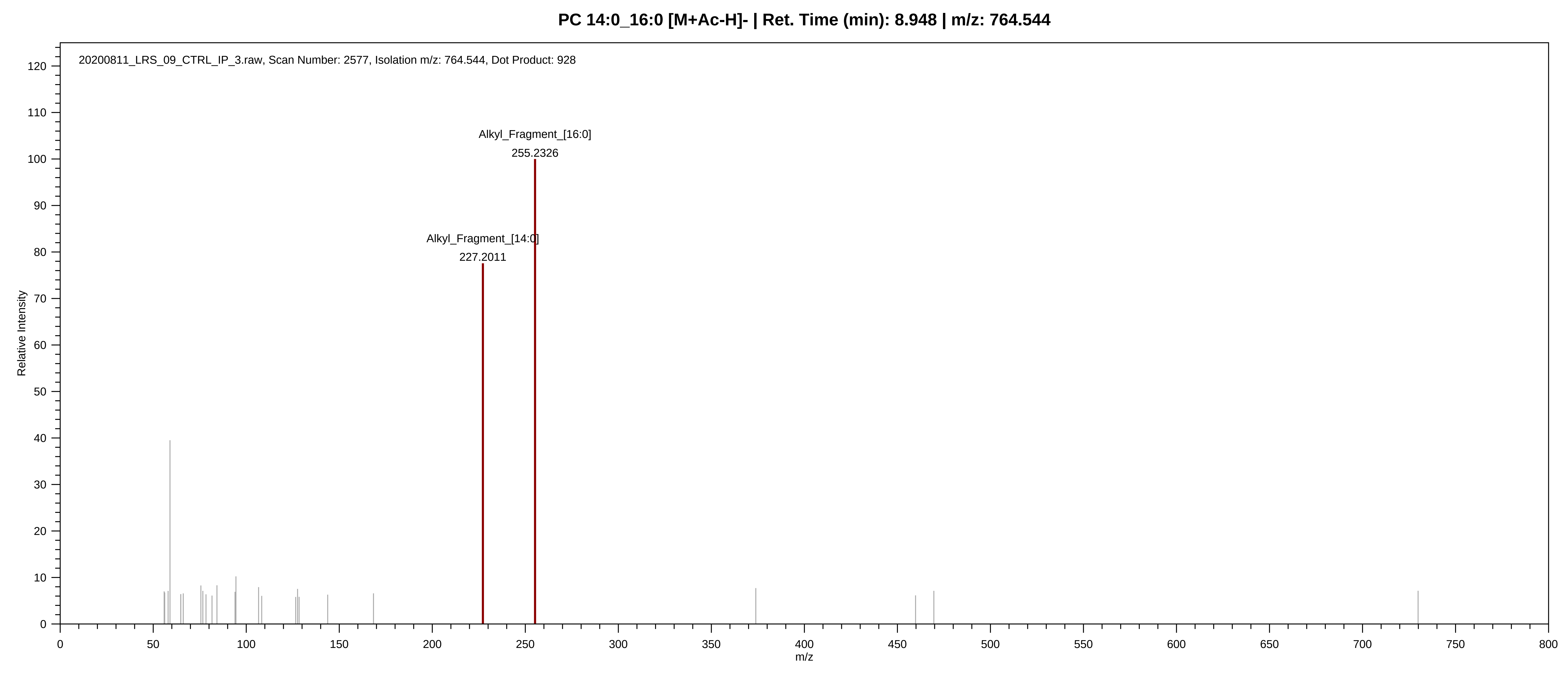
PC 14:0_16:0 [M+Ac-H]- | Ret. Time (min): 8.948 | m/z: 764.544
20200811_LRS_09_CTRL_IP_3.raw, Scan Number: 2577, Isolation m/z: 764.544, Dot Product: 928
Alkyl_Fragment_[16:0]
255.2326
Alkyl_Fragment_[14:0]
227.2011
Relative Intensity
m/z

PC 14:0_16:0 [M+Ac-H]- | Ret. Time (min): 8.952 | m/z: 764.545

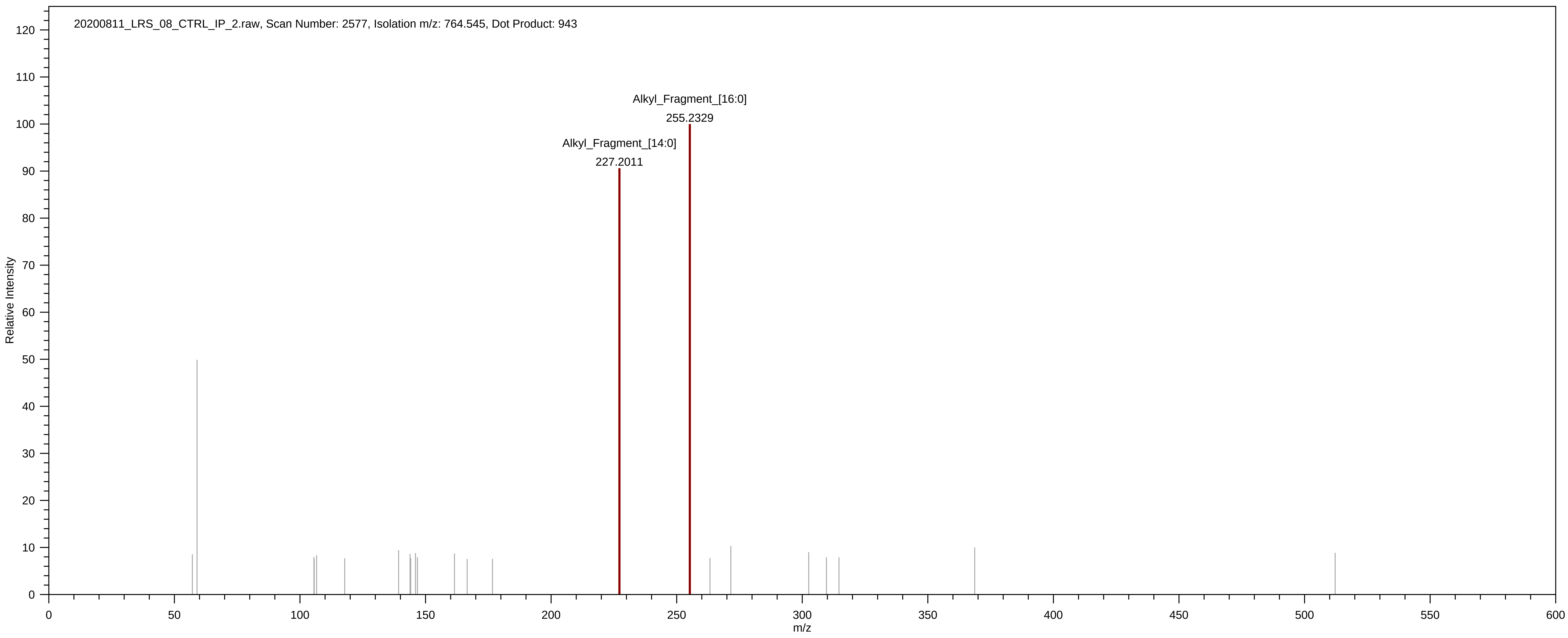

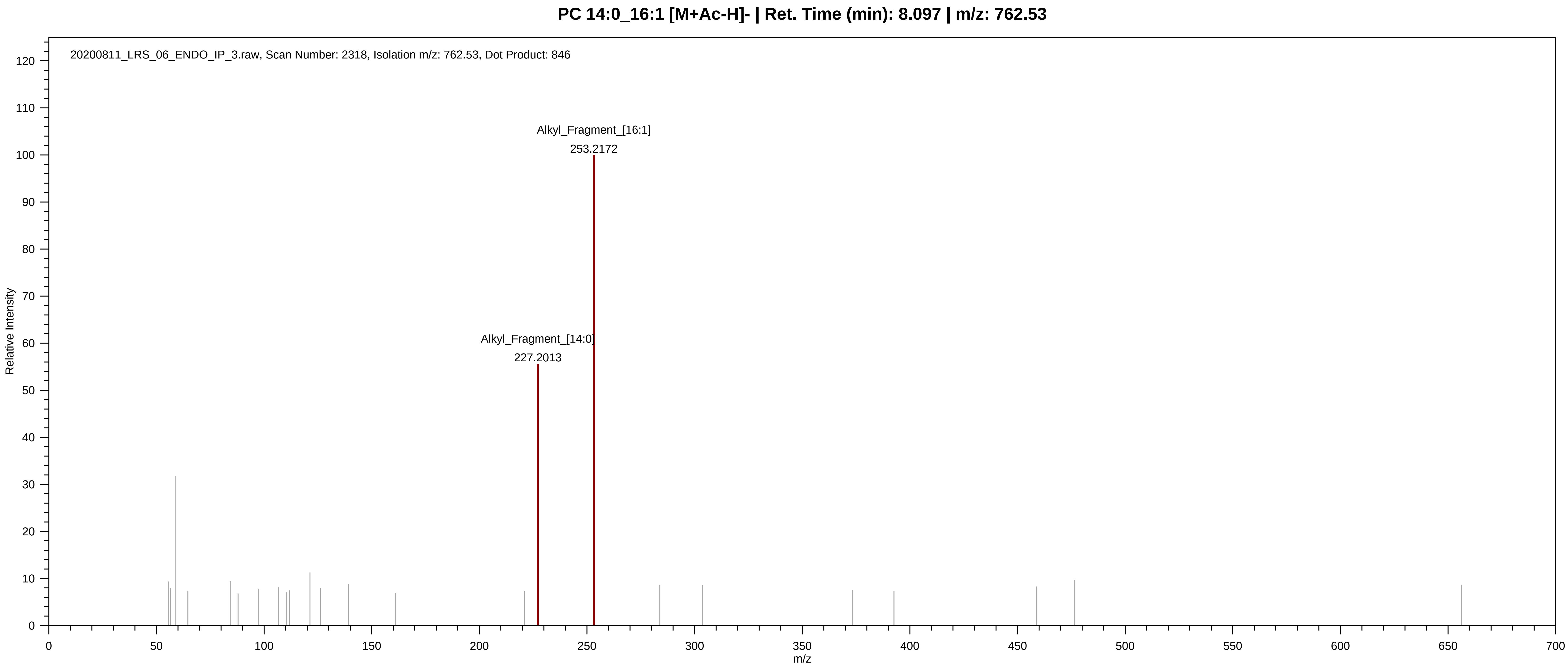

PC 14:0_16:1 [M+Ac-H]- | Ret. Time (min): 8.097 | m/z: 762.53
20200811_LRS_06_ENDO_IP_3.raw, Scan Number: 2318, Isolation m/z: 762.53, Dot Product: 846
Alkyl_Fragment_[16:1]
253.2172
Alkyl_Fragment_[14:0]
227.2013
Relative Intensity
m/z

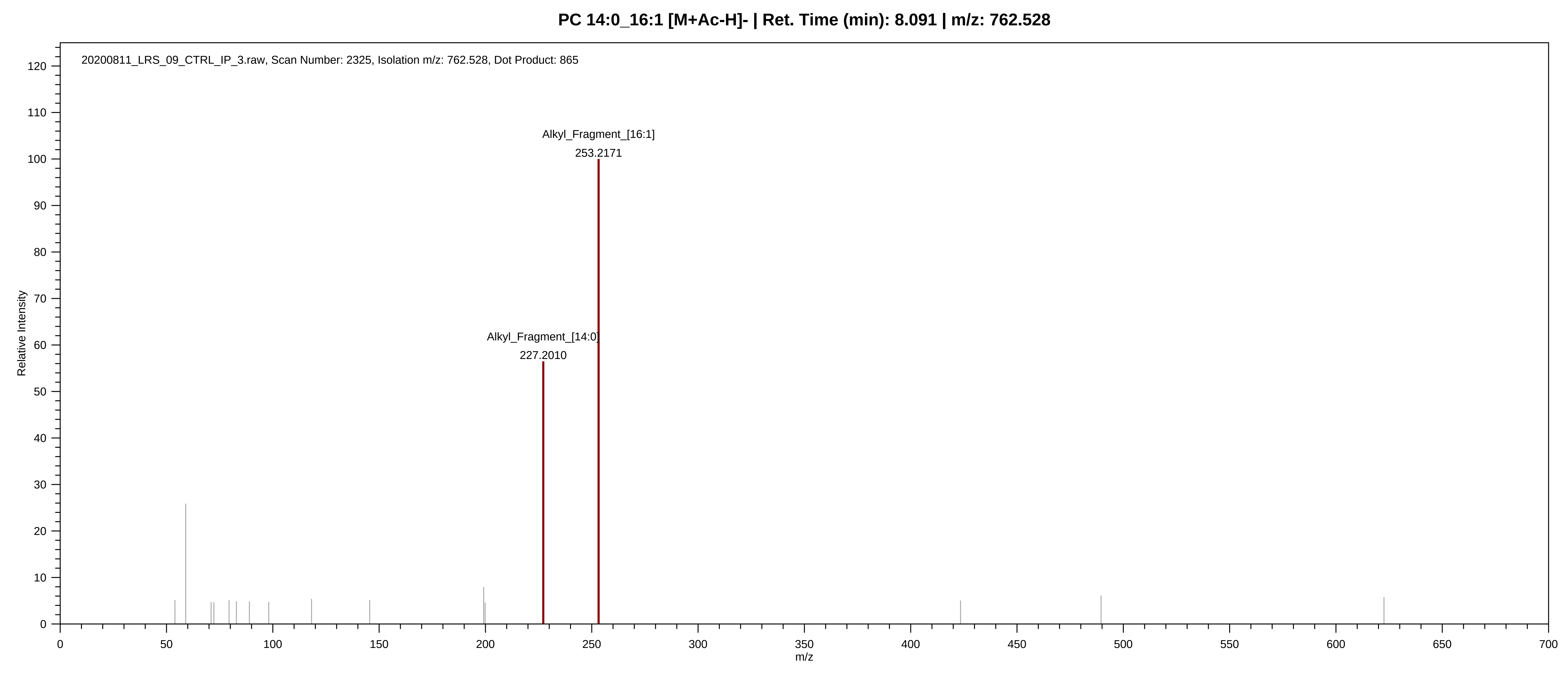

PC 14:0_16:1 [M+Ac-H]- | Ret. Time (min): 8.091 | m/z: 762.528
20200811_LRS_09_CTRL_IP_3.raw, Scan Number: 2325, Isolation m/z: 762.528, Dot Product: 865
Alkyl_Fragment_[16:1]
253.2171
Alkyl_Fragment_[14:0]
227.2010
Relative Intensity
m/z

PC 14:0_16:1 [M+Ac-H]- | Ret. Time (min): 8.112 | m/z: 762.528

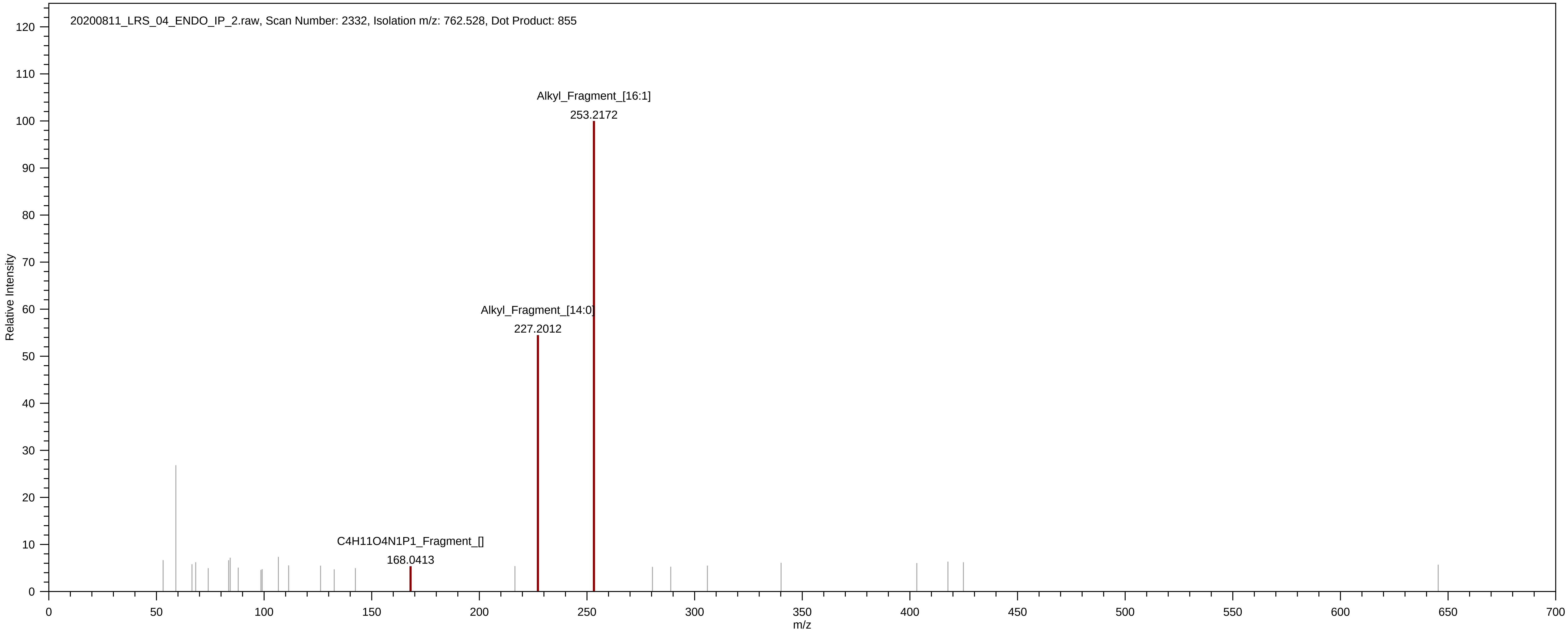

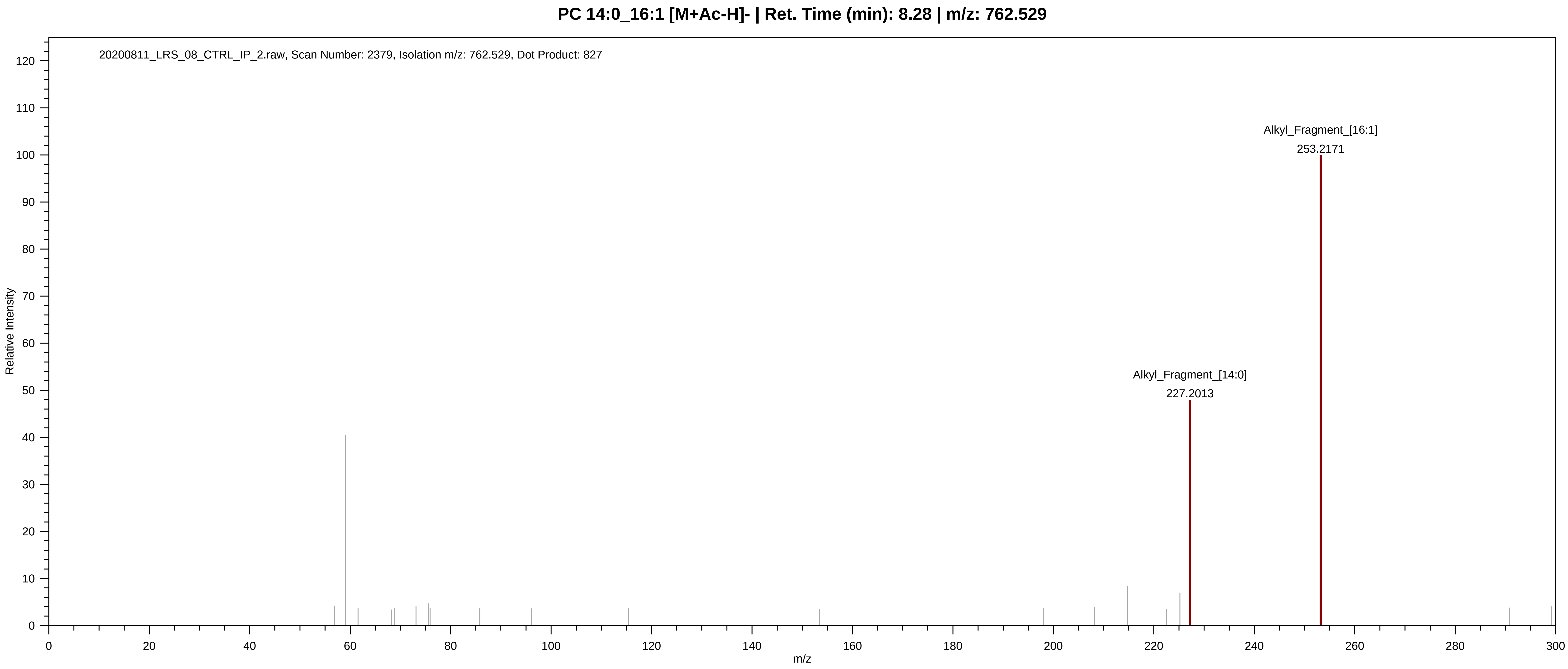
PC 14:0_16:1 [M+Ac-H]- | Ret. Time (min): 8.28 | m/z: 762.529
20200811_LRS_08_CTRL_IP_2.raw, Scan Number: 2379, Isolation m/z: 762.529, Dot Product: 827
Alkyl_Fragment_[16:1]
253.2171
Alkyl_Fragment_[14:0]
227.2013
Relative Intensity
m/z

PC 14:0_16:1 [M+Ac-H]- | Ret. Time (min): 8.099 | m/z: 762.529

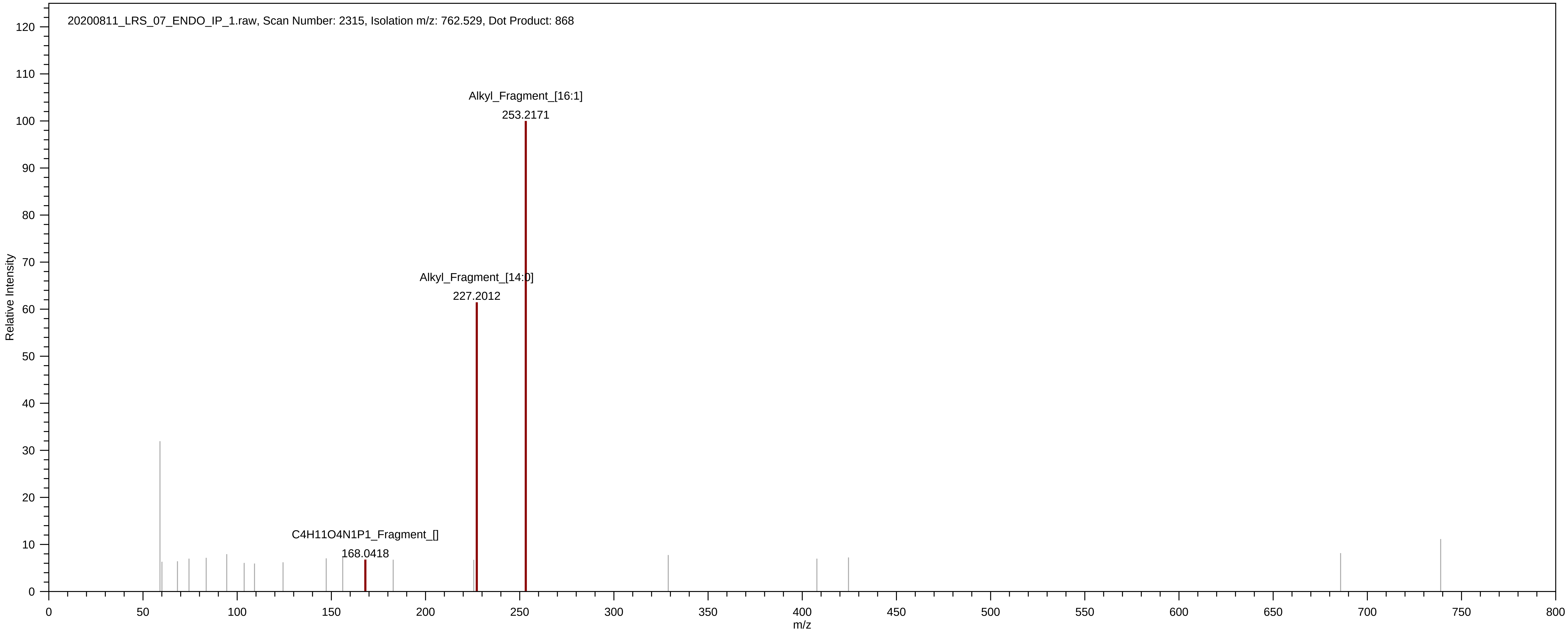

PC 14:0_16:1 [M+Ac-H]- | Ret. Time (min): 8.107 | m/z: 762.529

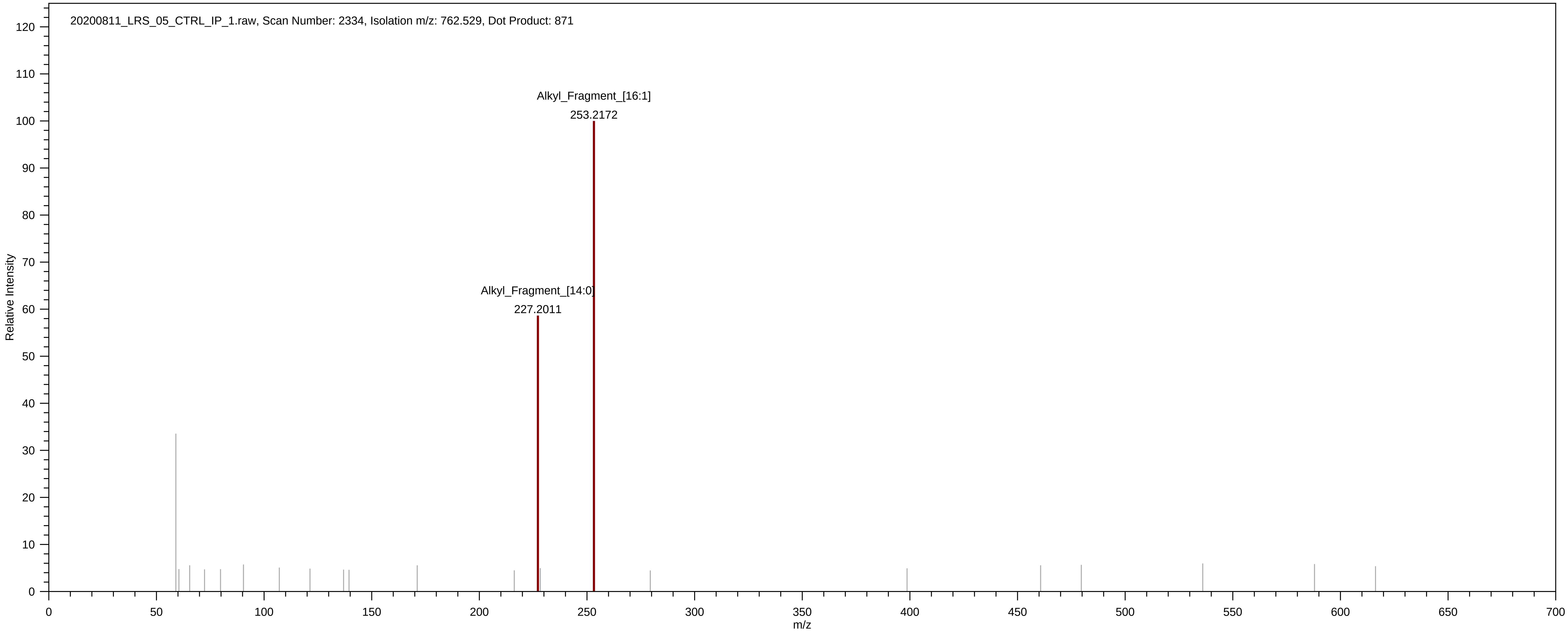

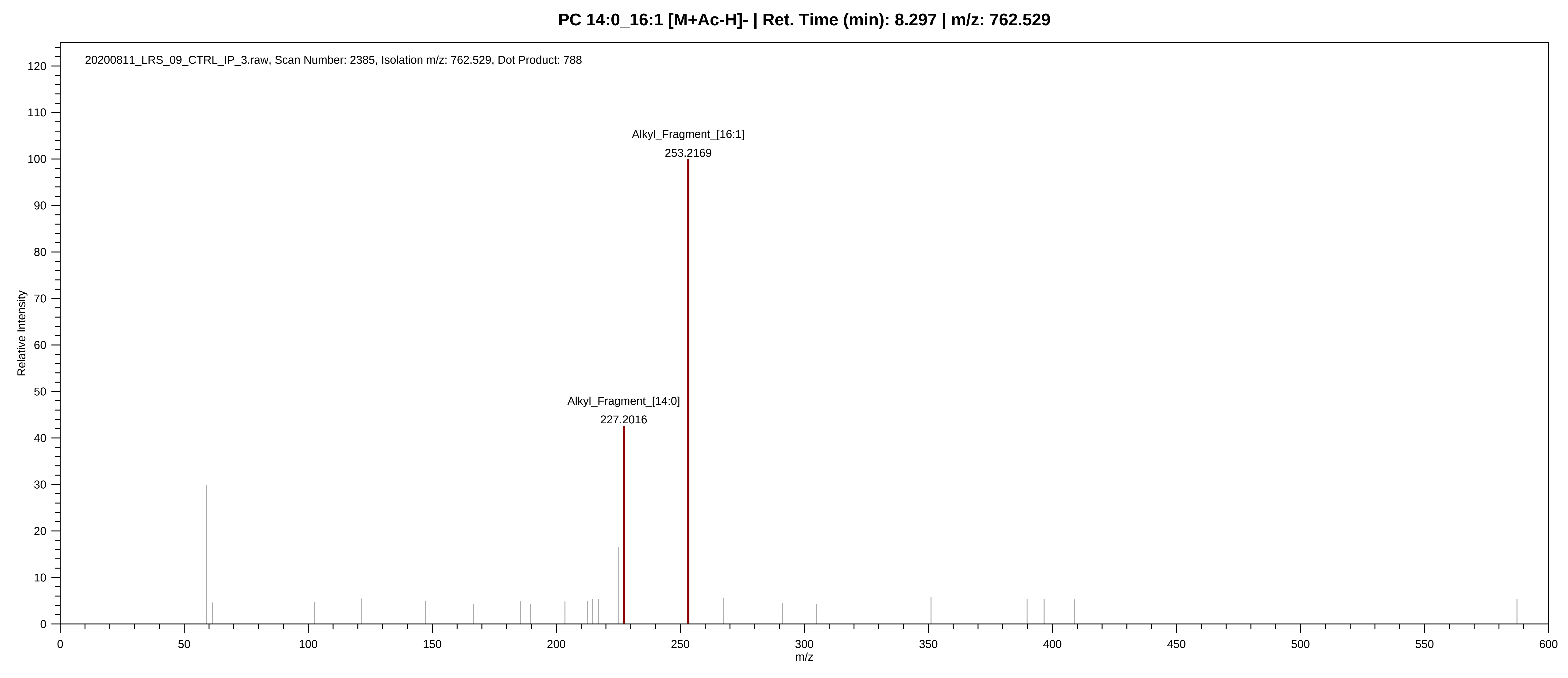

PC 14:0_16:1 [M+Ac-H]- | Ret. Time (min): 8.297 | m/z: 762.529
20200811_LRS_09_CTRL_IP_3.raw, Scan Number: 2385, Isolation m/z: 762.529, Dot Product: 788
Alkyl_Fragment_[16:1]
253.2169
Alkyl_Fragment_[14:0]
227.2016
Relative Intensity
m/z

PC 14:0_16:1 [M+Ac-H]- | Ret. Time (min): 8.309 | m/z: 762.529

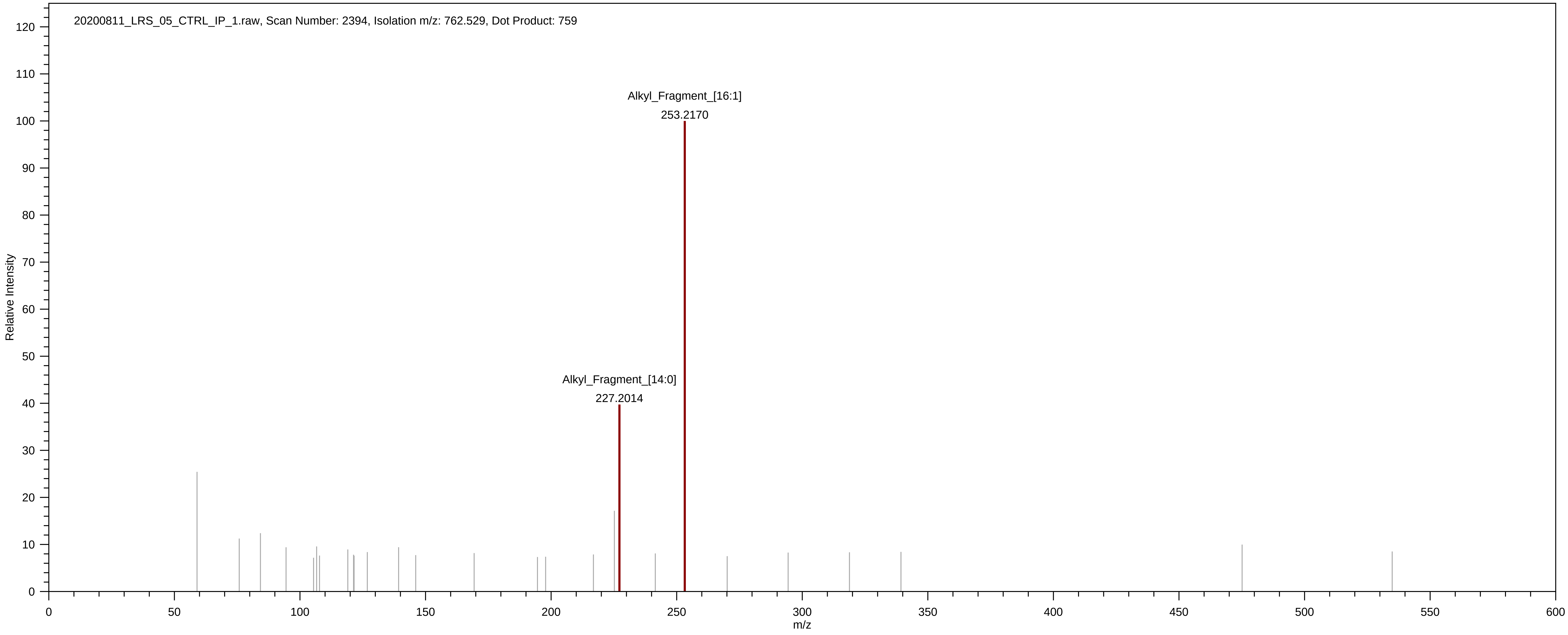

PC 15:0_16:0 [M+Ac-H]- | Ret. Time (min): 9.13 | m/z: 778.56

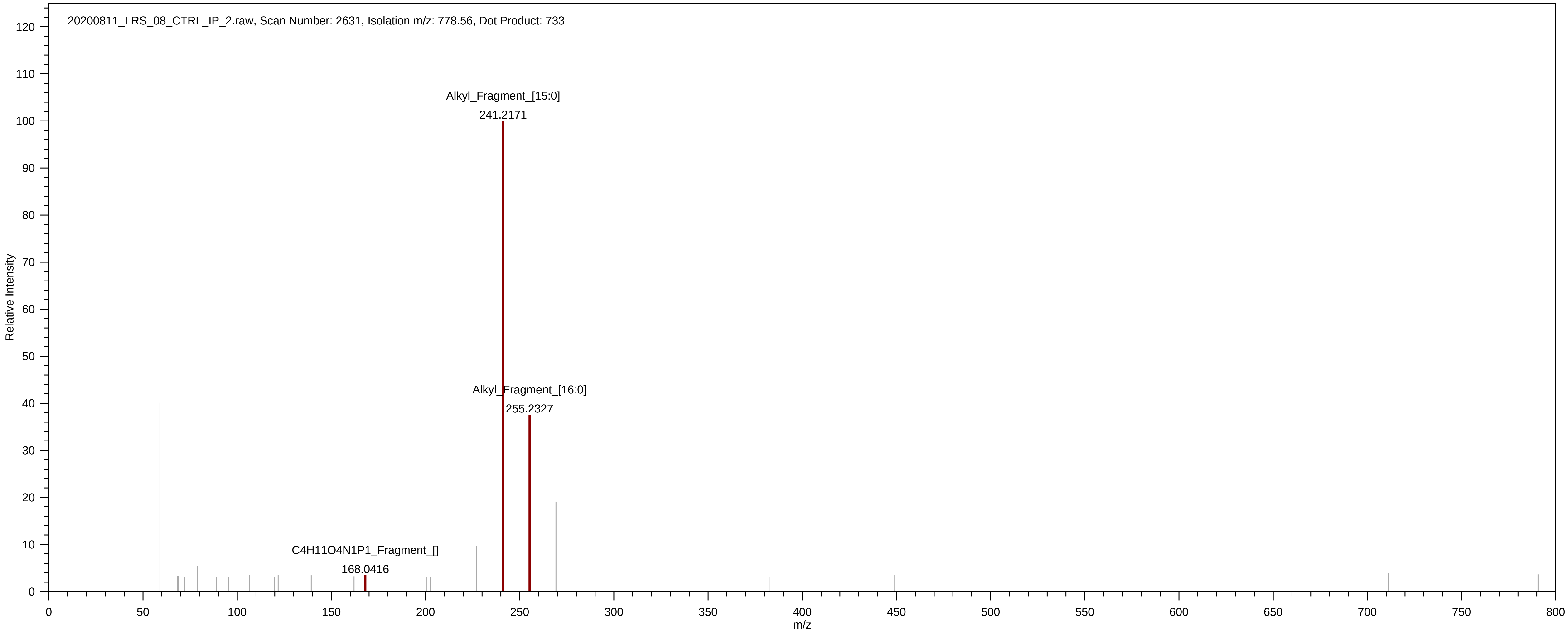

PC 15:0_16:0 [M+Ac-H]- | Ret. Time (min): 9.135 | m/z: 778.56

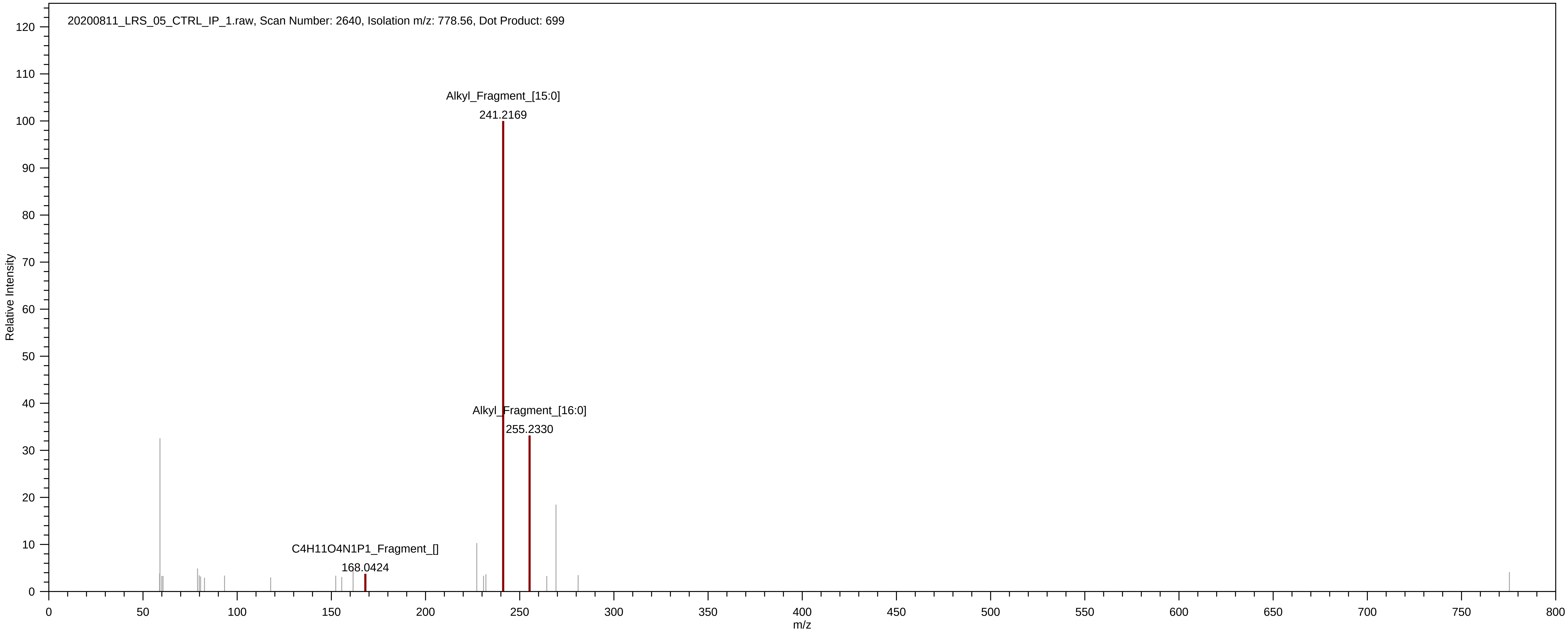

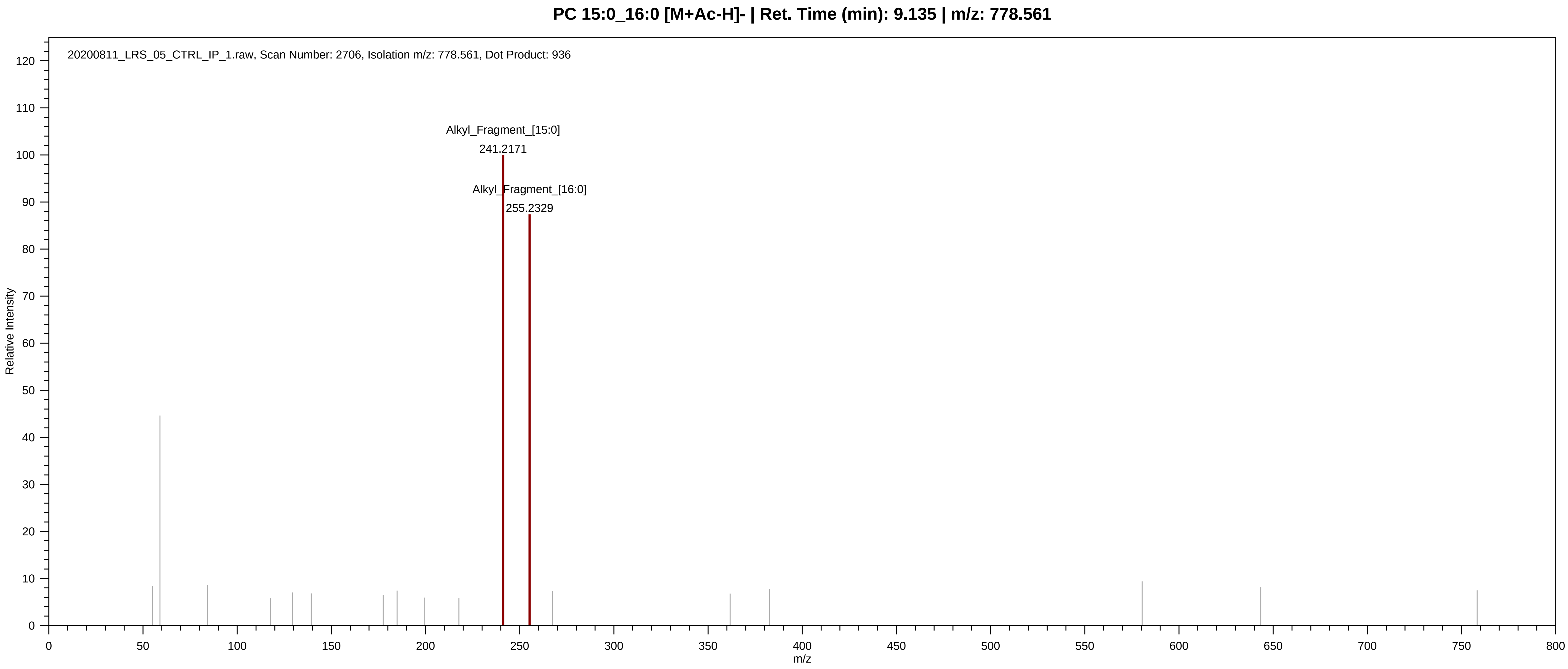
PC 15:0_16:0 [M+Ac-H]- | Ret. Time (min): 9.135 | m/z: 778.561
20200811_LRS_05_CTRL_IP_1.raw, Scan Number: 2706, Isolation m/z: 778.561, Dot Product: 936
Alkyl_Fragment_[15:0]
241.2171
Alkyl_Fragment_[16:0]
255.2329
Relative Intensity
m/z

PC 15:0_16:0 [M+Ac-H]- | Ret. Time (min): 9.226 | m/z: 778.56

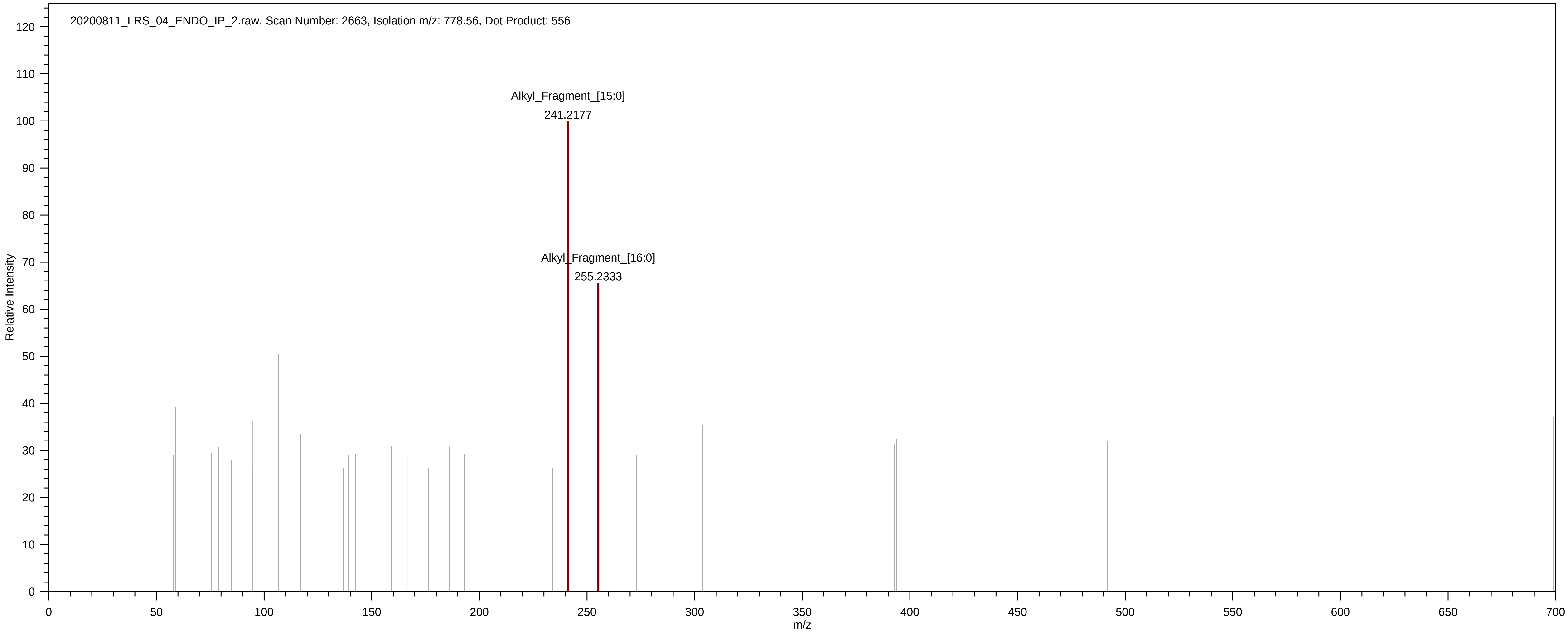

PC 15:0_16:0 [M+Ac-H]- | Ret. Time (min): 9.281 | m/z: 778.56

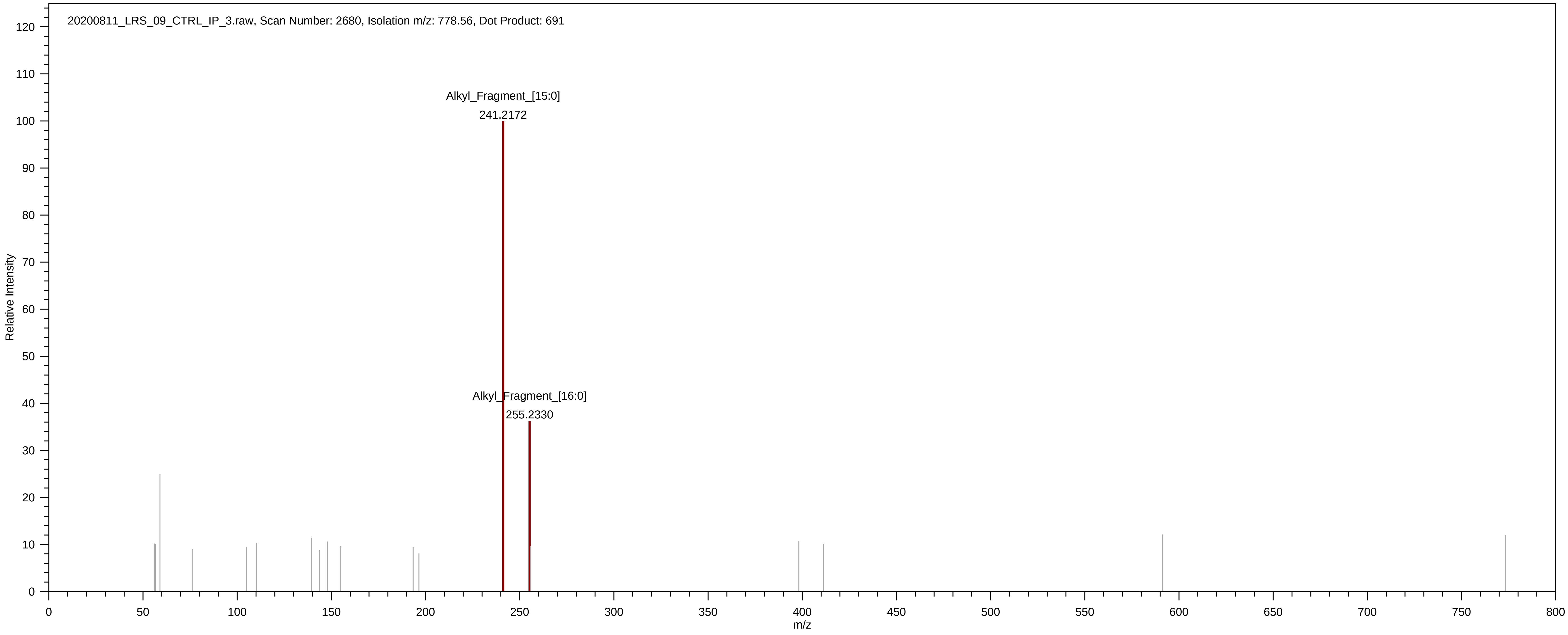

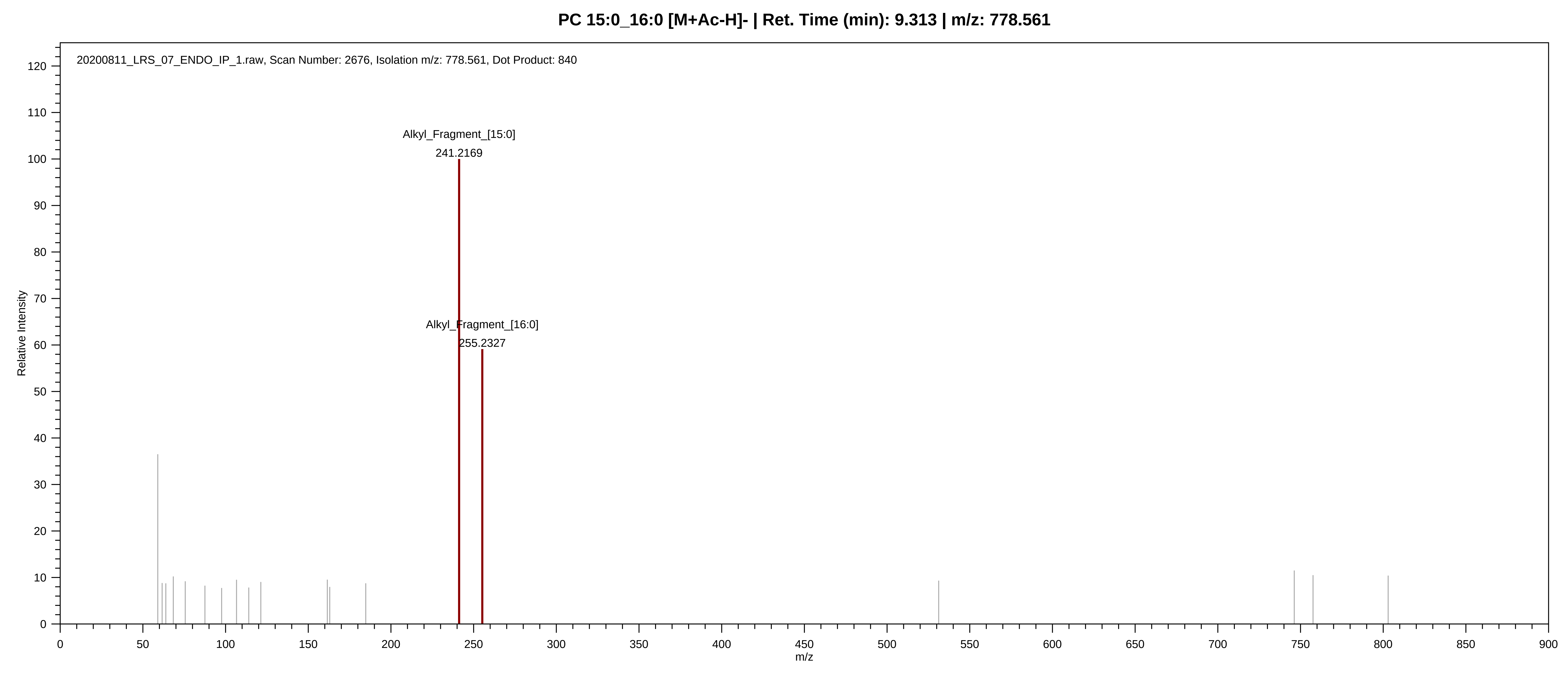
PC 15:0_16:0 [M+Ac-H]- | Ret. Time (min): 9.313 | m/z: 778.561
20200811_LRS_07_ENDO_IP_1.raw, Scan Number: 2676, Isolation m/z: 778.561, Dot Product: 840
Alkyl_Fragment_[15:0]
241.2169
Alkyl_Fragment_[16:0]
255.2327
Relative Intensity
m/z

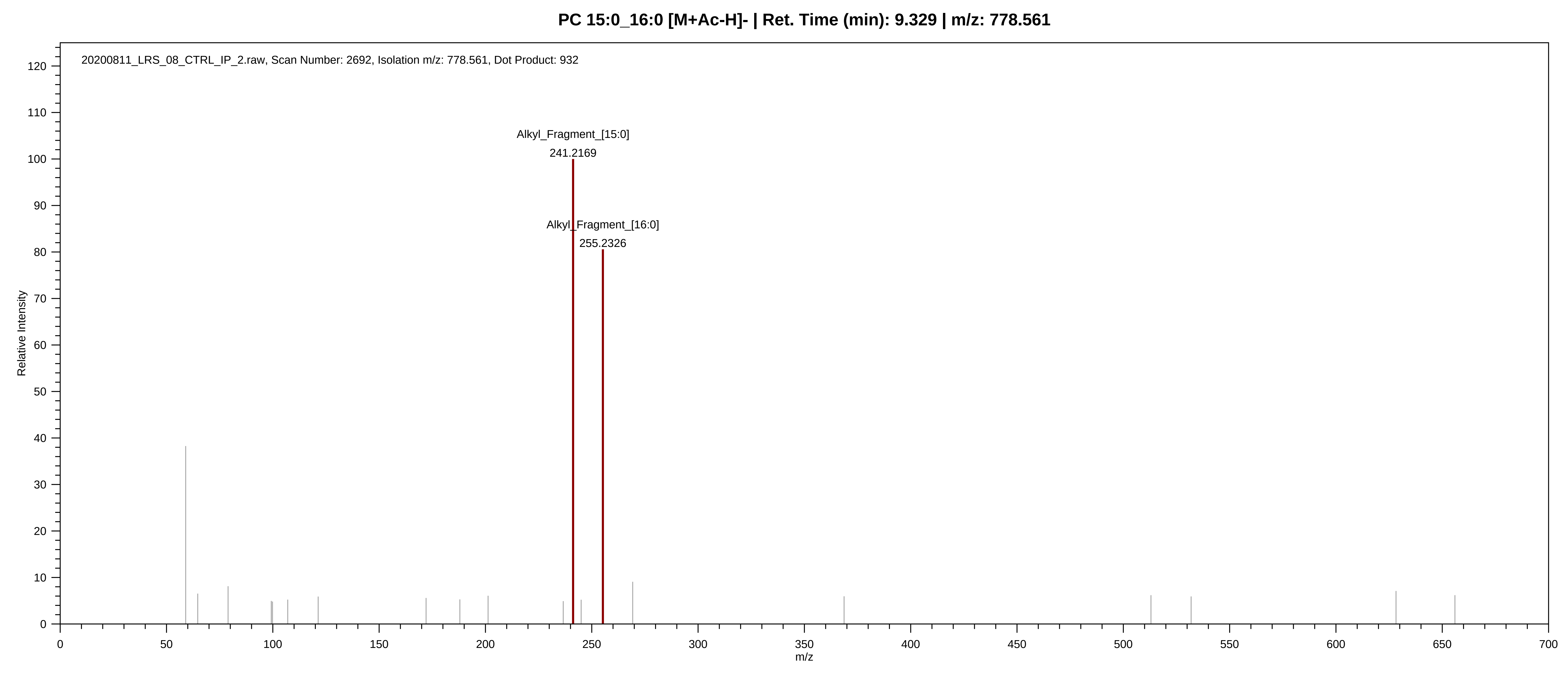

PC 15:0_16:0 [M+Ac-H]- | Ret. Time (min): 9.329 | m/z: 778.561
20200811_LRS_08_CTRL_IP_2.raw, Scan Number: 2692, Isolation m/z: 778.561, Dot Product: 932
Alkyl_Fragment_[15:0]
241.2169
Alkyl_Fragment_[16:0]
255.2326
Relative Intensity
m/z

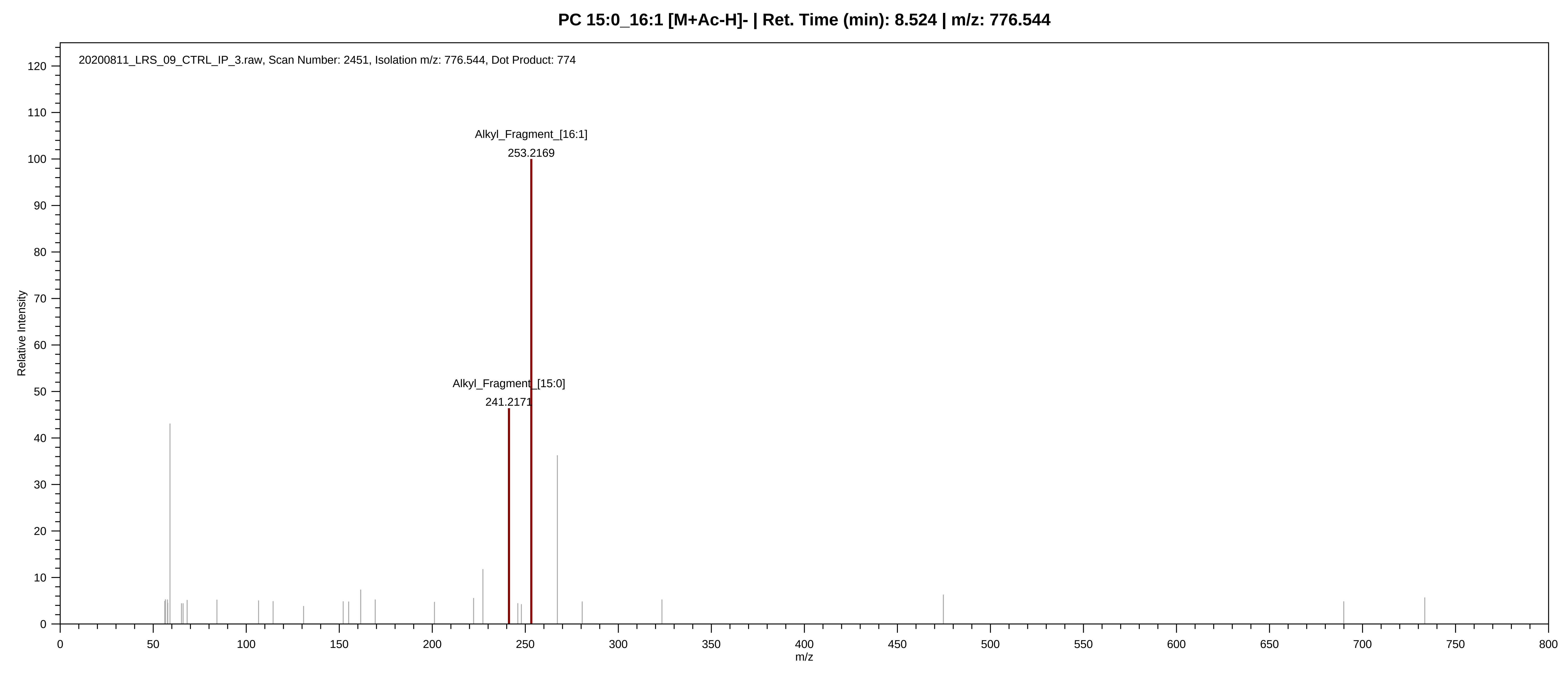
PC 15:0_16:1 [M+Ac-H]- | Ret. Time (min): 8.524 | m/z: 776.544
20200811_LRS_09_CTRL_IP_3.raw, Scan Number: 2451, Isolation m/z: 776.544, Dot Product: 774
Alkyl_Fragment_[16:1]
253.2169
Alkyl_Fragment_[15:0]
241.2171
Relative Intensity
m/z

PC 15:0_16:1 [M+Ac-H]- | Ret. Time (min): 8.524 | m/z: 776.544

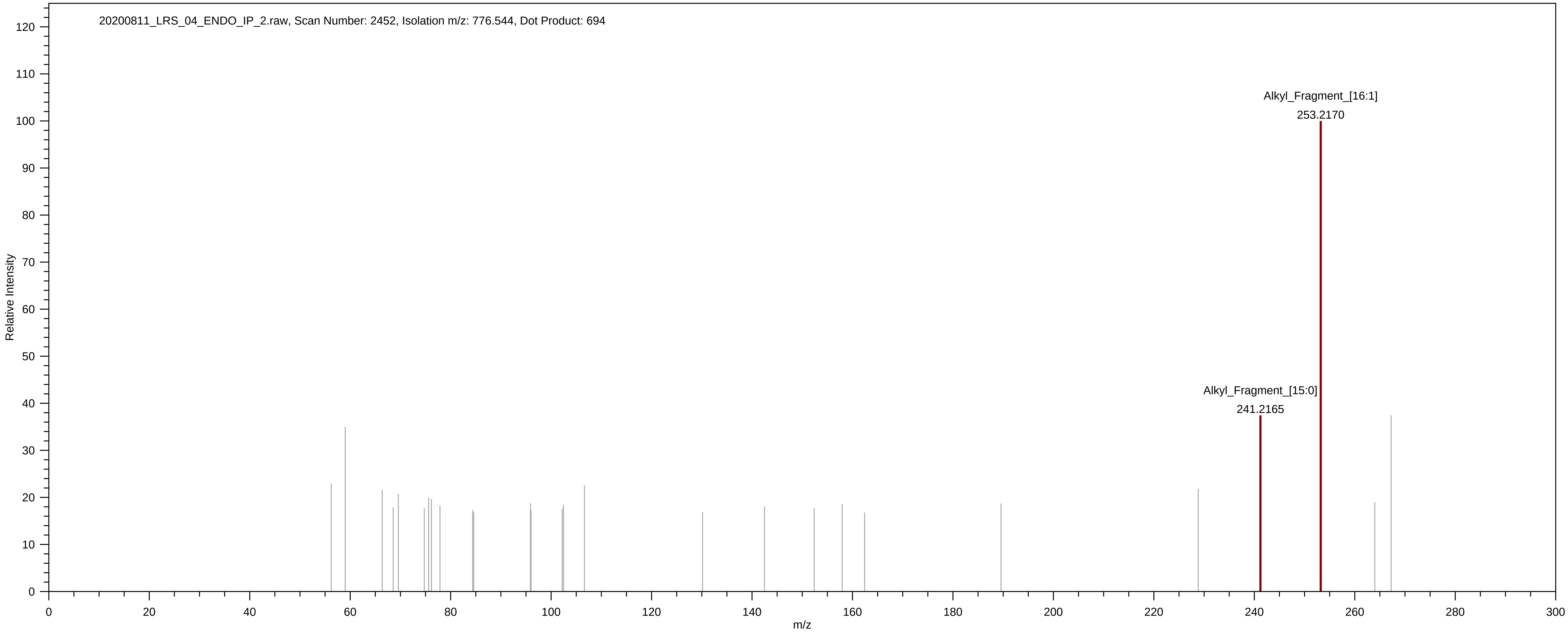

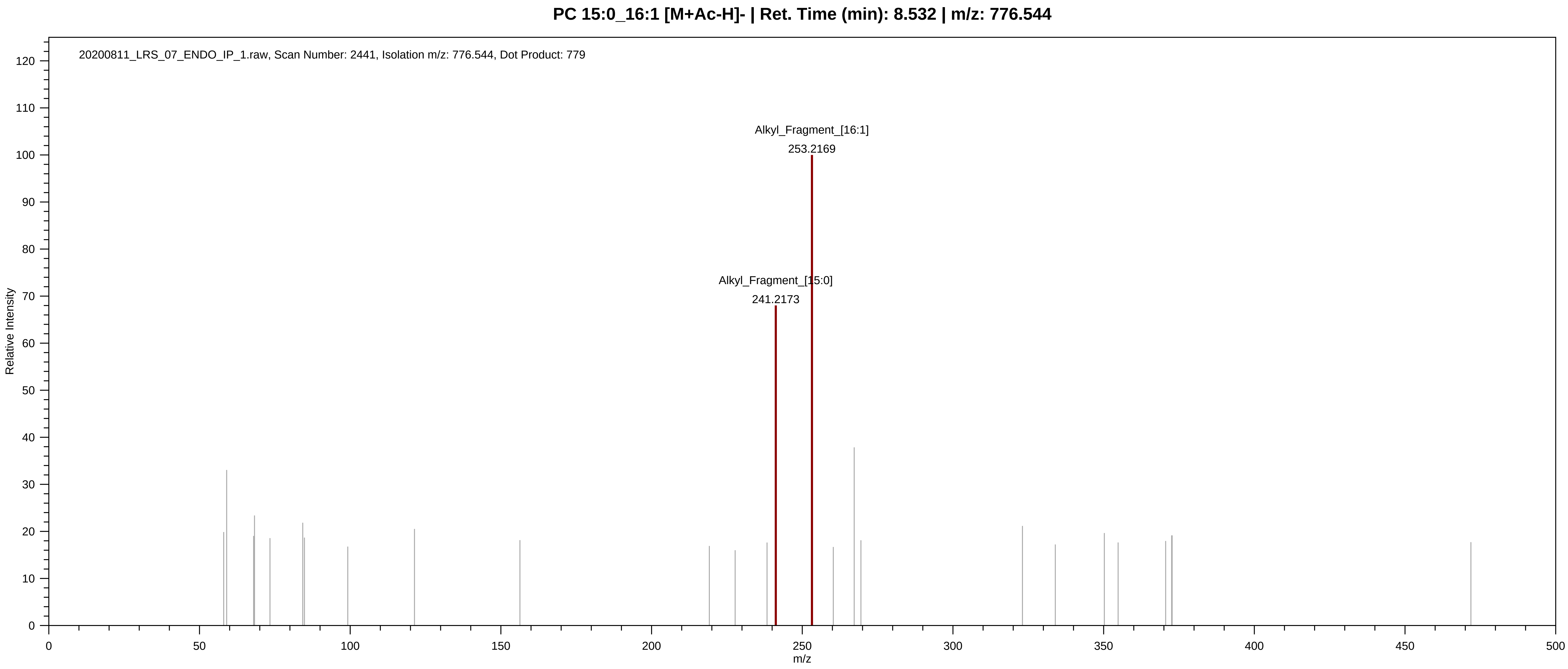
PC 15:0_16:1 [M+Ac-H]- | Ret. Time (min): 8.532 | m/z: 776.544
20200811_LRS_07_ENDO_IP_1.raw, Scan Number: 2441, Isolation m/z: 776.544, Dot Product: 779
Alkyl_Fragment_[16:1]
253.2169
Alkyl_Fragment_[15:0]
241.2173
Relative Intensity
m/z

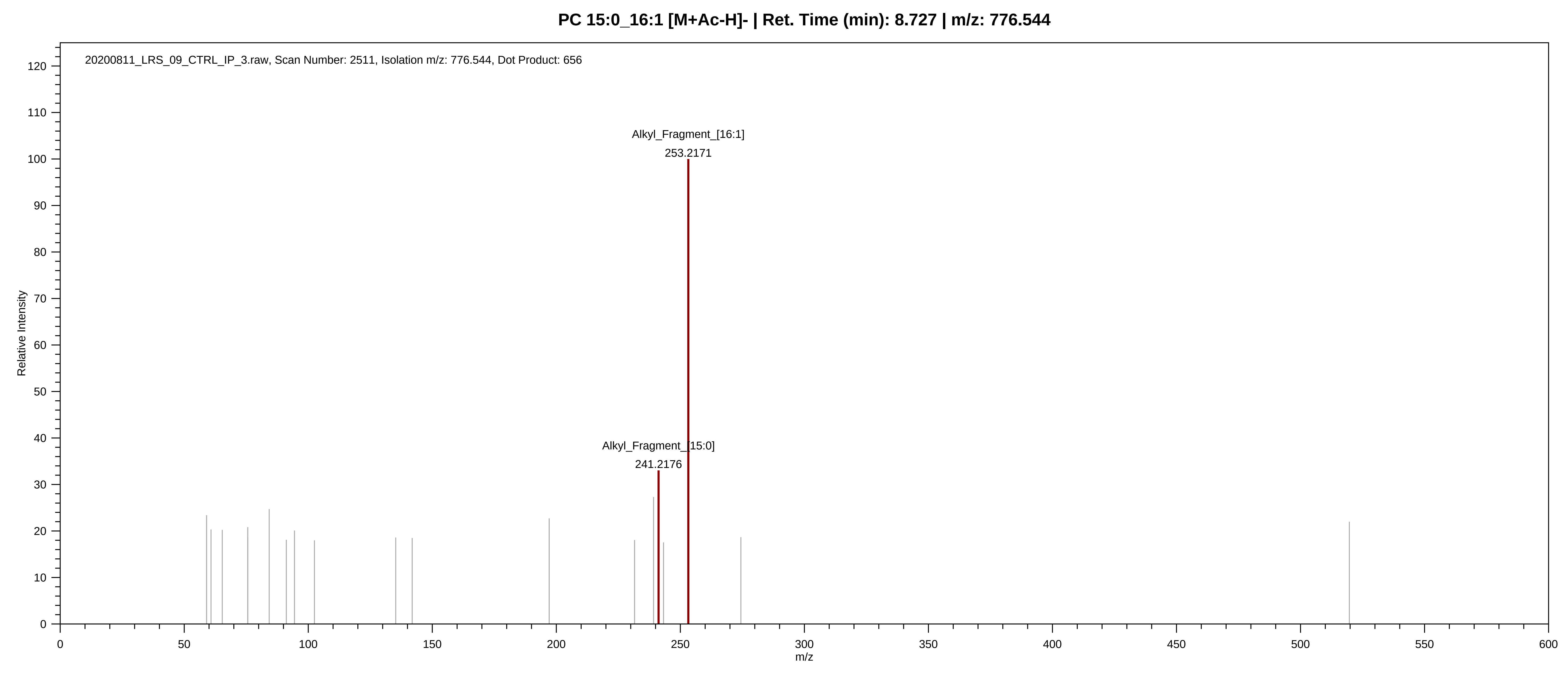
PC 15:0_16:1 [M+Ac-H]- | Ret. Time (min): 8.727 | m/z: 776.544
20200811_LRS_09_CTRL_IP_3.raw, Scan Number: 2511, Isolation m/z: 776.544, Dot Product: 656
Alkyl_Fragment_[16:1]
253.2171
Alkyl_Fragment_[15:0]
241.2176
Relative Intensity
m/z

PC 15:0_16:1 [M+Ac-H]- | Ret. Time (min): 8.528 | m/z: 776.545

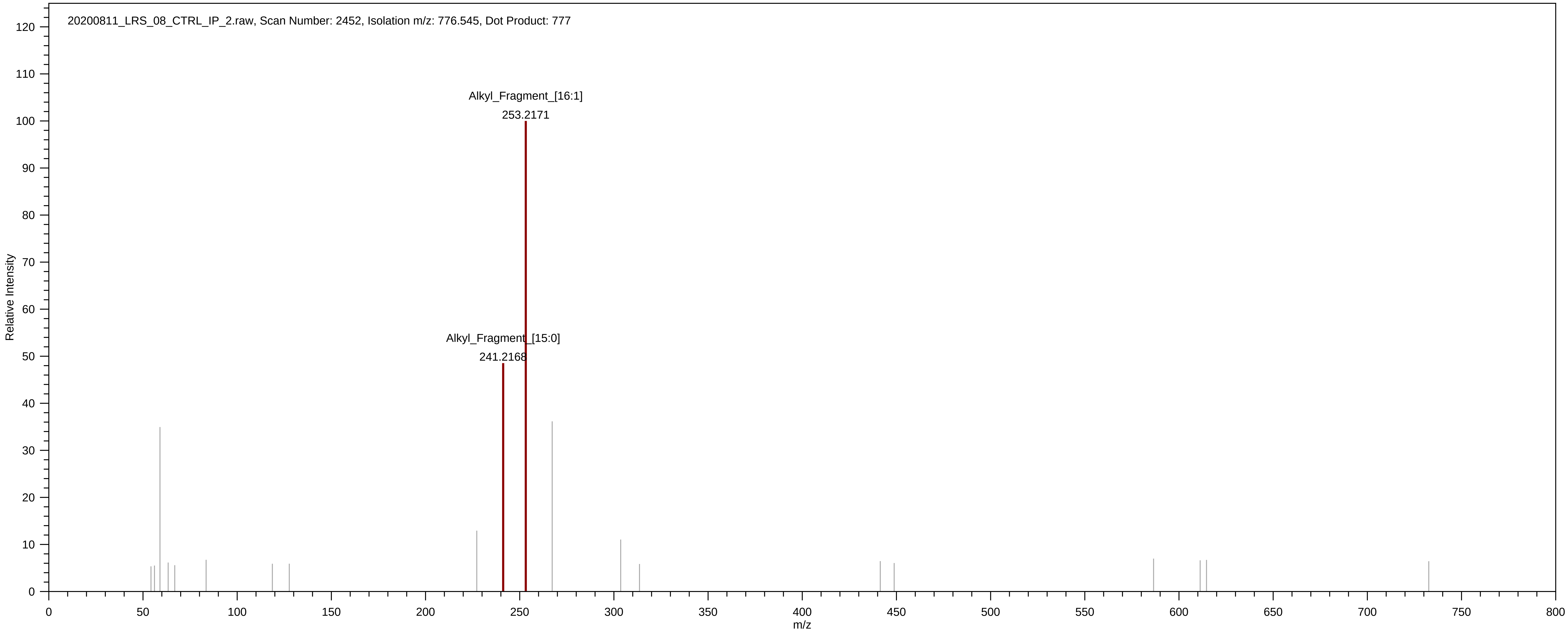

PC 15:0_16:1 [M+Ac-H]- | Ret. Time (min): 8.572 | m/z: 776.545

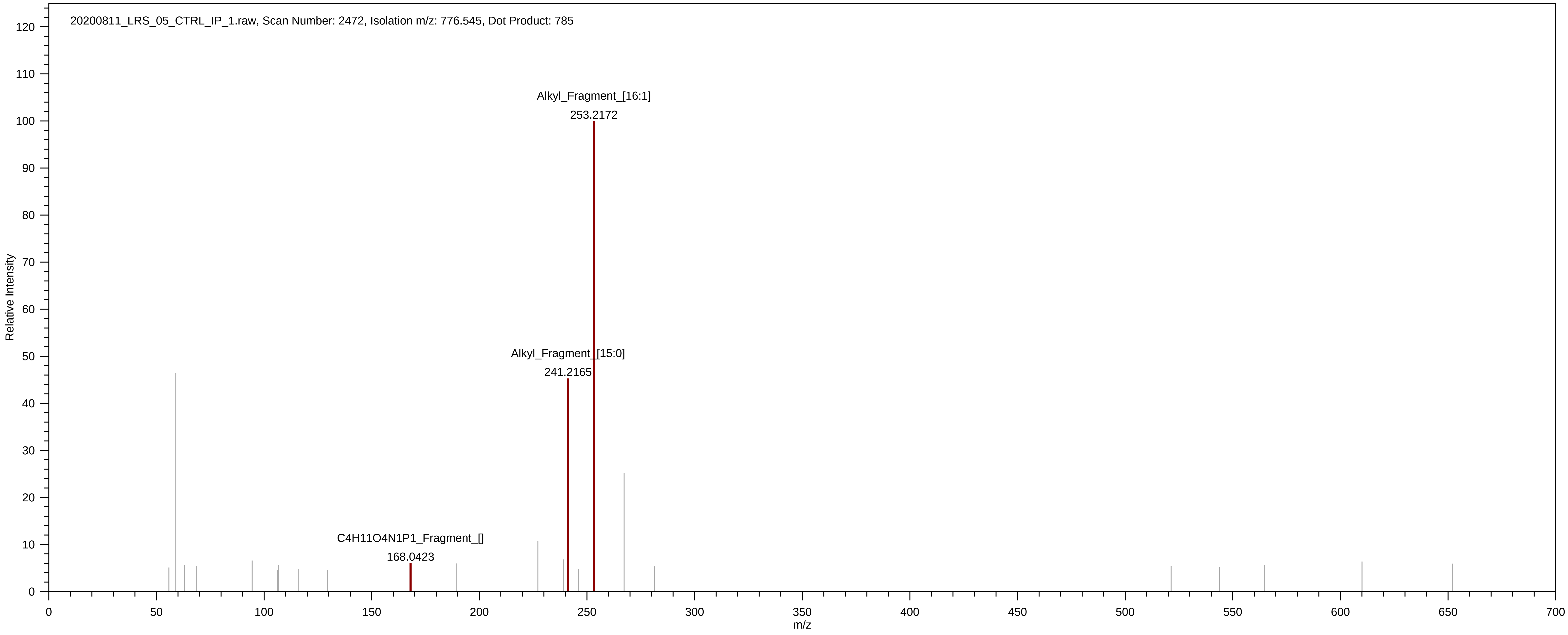

PC 16:0_16:0 [M+Ac-H]- | Ret. Time (min): 9.913 | m/z: 792.574

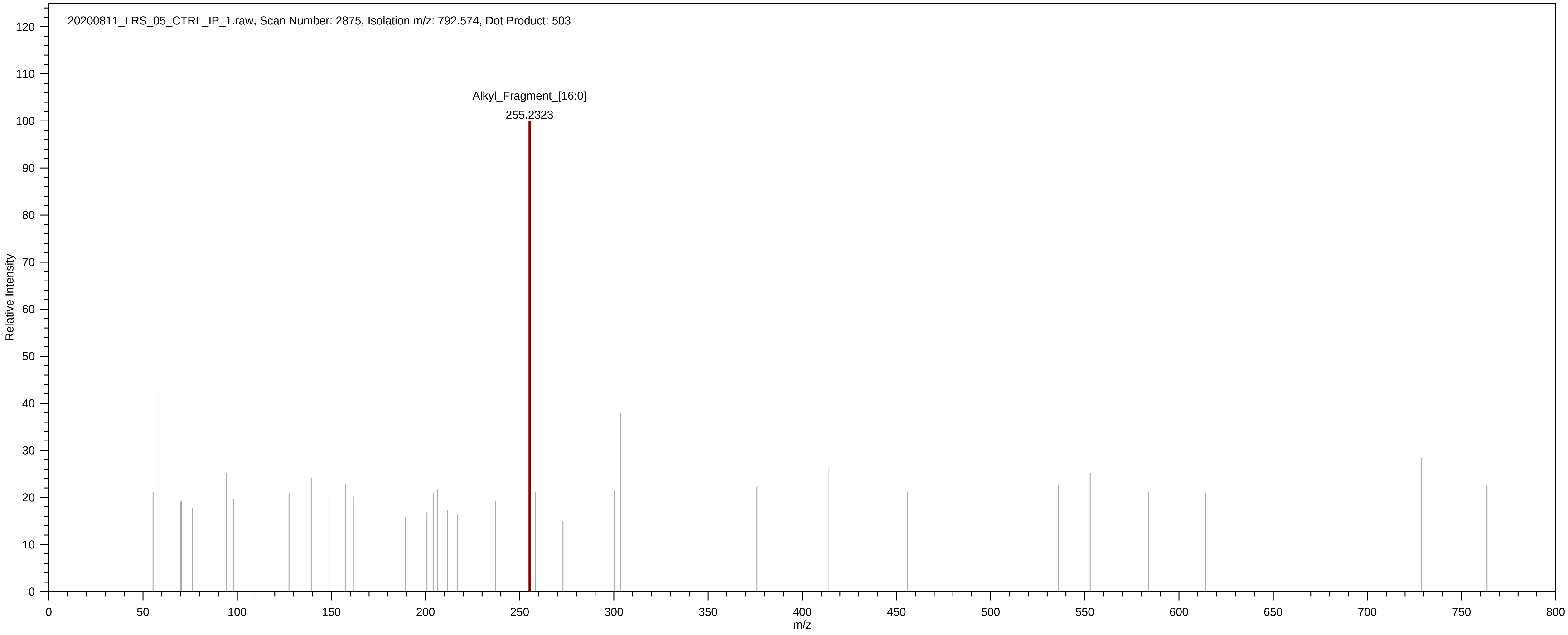

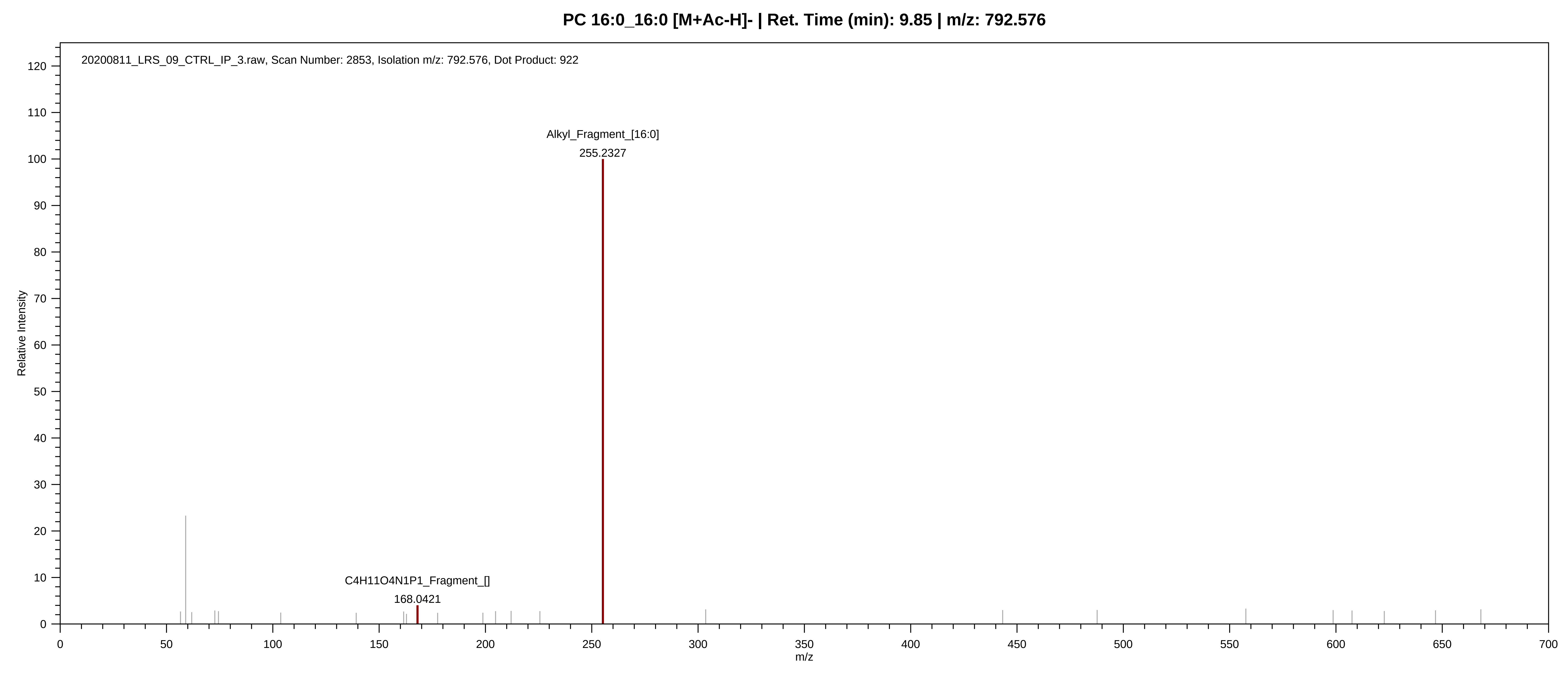
PC 16:0_16:0 [M+Ac-H]- | Ret. Time (min): 9.85 | m/z: 792.576
20200811_LRS_09_CTRL_IP_3.raw, Scan Number: 2853, Isolation m/z: 792.576, Dot Product: 922
Alkyl_Fragment_[16:0]
255.2327
C4H11O4N1P1_Fragment_[]
168.0421
Relative Intensity
m/z

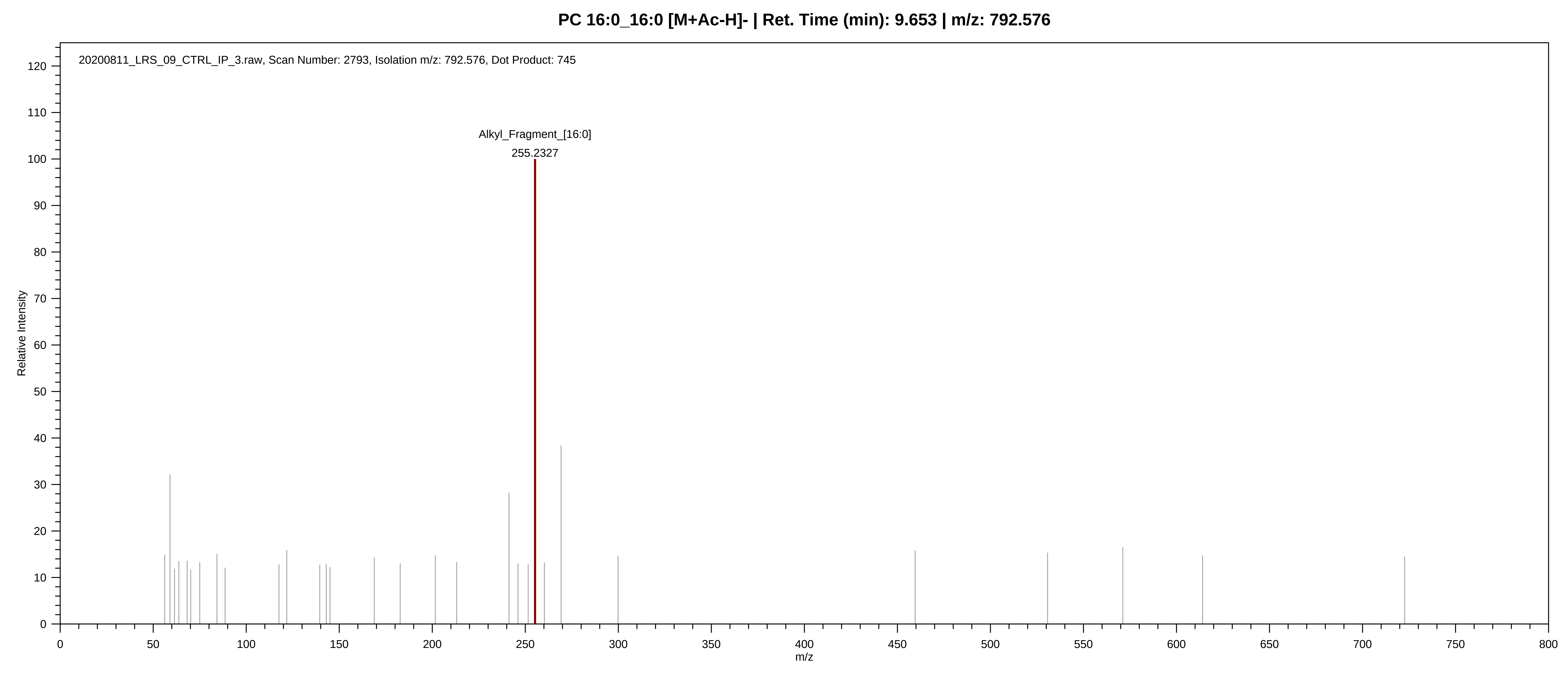

PC 16:0_16:0 [M+Ac-H]- | Ret. Time (min): 9.653 | m/z: 792.576
20200811_LRS_09_CTRL_IP_3.raw, Scan Number: 2793, Isolation m/z: 792.576, Dot Product: 745
Alkyl_Fragment_[16:0]
255.2327
Relative Intensity
m/z

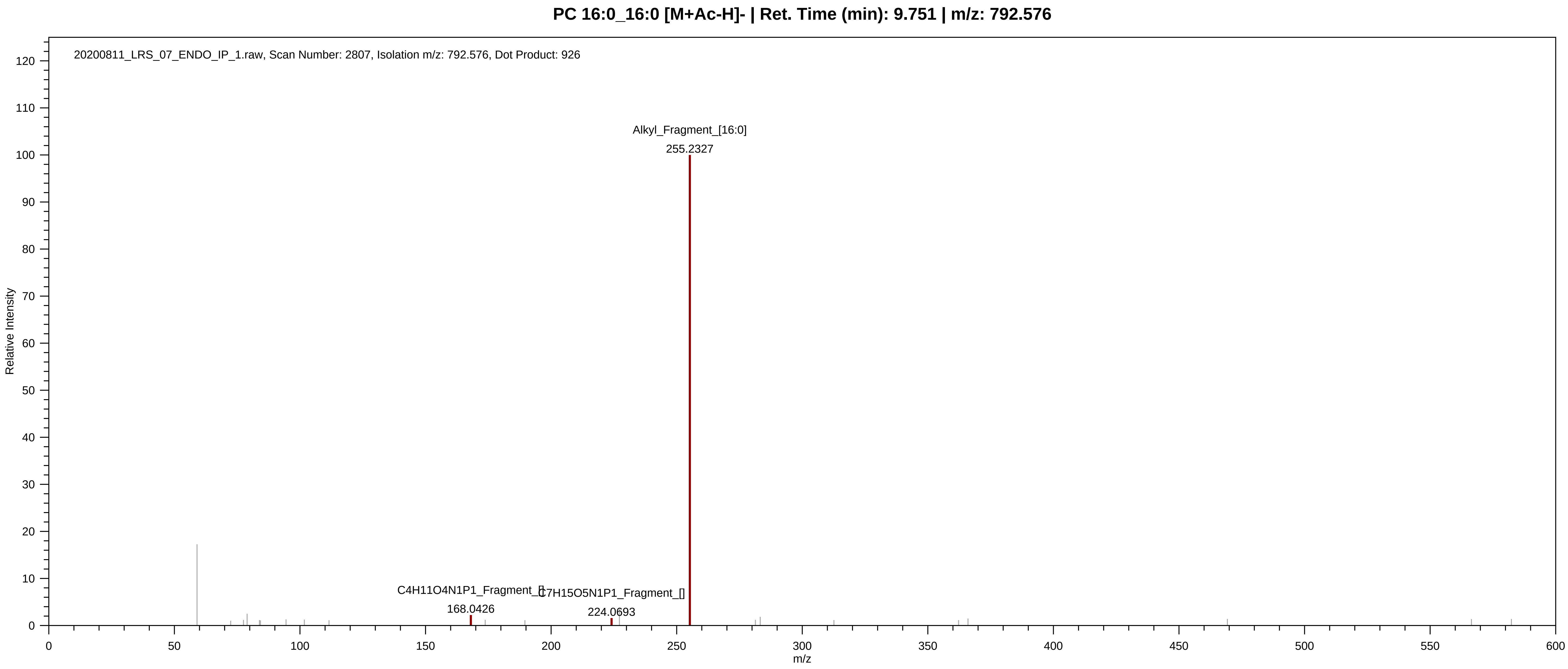
PC 16:0_16:0 [M+Ac-H]- | Ret. Time (min): 9.751 | m/z: 792.576
20200811_LRS_07_ENDO_IP_1.raw, Scan Number: 2807, Isolation m/z: 792.576, Dot Product: 926
Alkyl_Fragment_[16:0]
255.2327
C4H11O4N1P1_Fragment_[]
168.0426
C7H15O5N1P1_Fragment_[]
224.0693
Relative Intensity
m/z
0
10
20
30
40
50
60
70
80
90
100
110
120
0
50
100
150
200
250
300
350
400
450
500
550
600

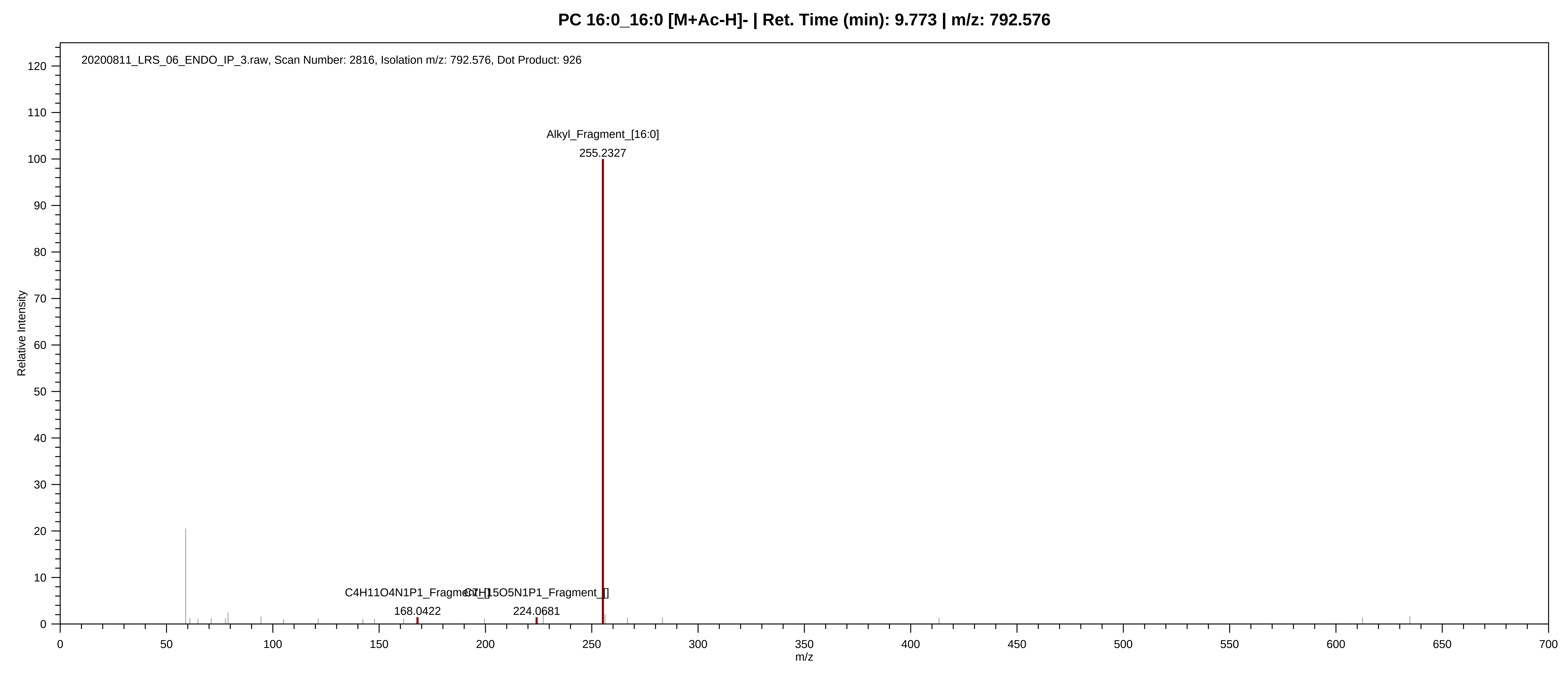

PC 16:0_16:0 [M+Ac-H]- | Ret. Time (min): 9.773 | m/z: 792.576
20200811_LRS_06_ENDO_IP_3.raw, Scan Number: 2816, Isolation m/z: 792.576, Dot Product: 926
Alkyl_Fragment_[16:0]
255.2327
C4H11O4N1P1_Fragment_[]
168.0422
C7H15O5N1P1_Fragment_[]
224.0681
Relative Intensity
m/z

PC 16:0_16:0 [M+Ac-H]- | Ret. Time (min): 9.825 | m/z: 792.576

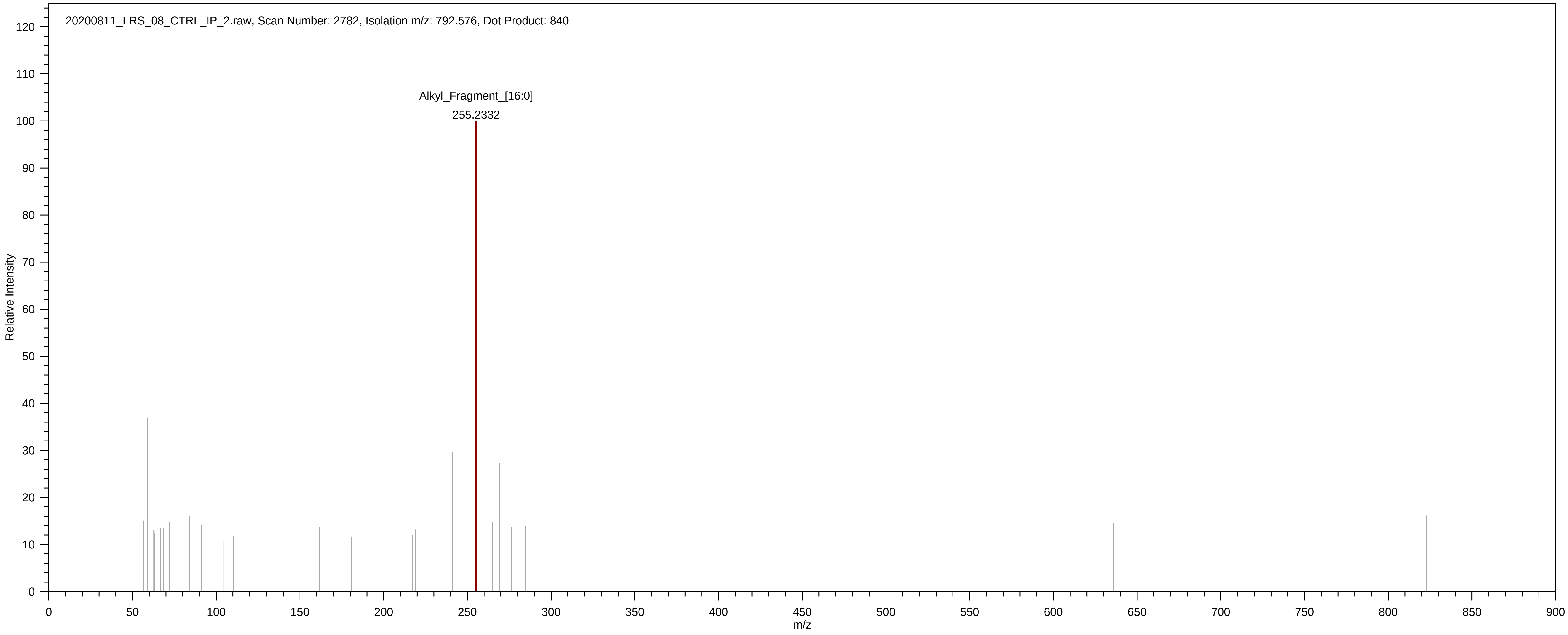

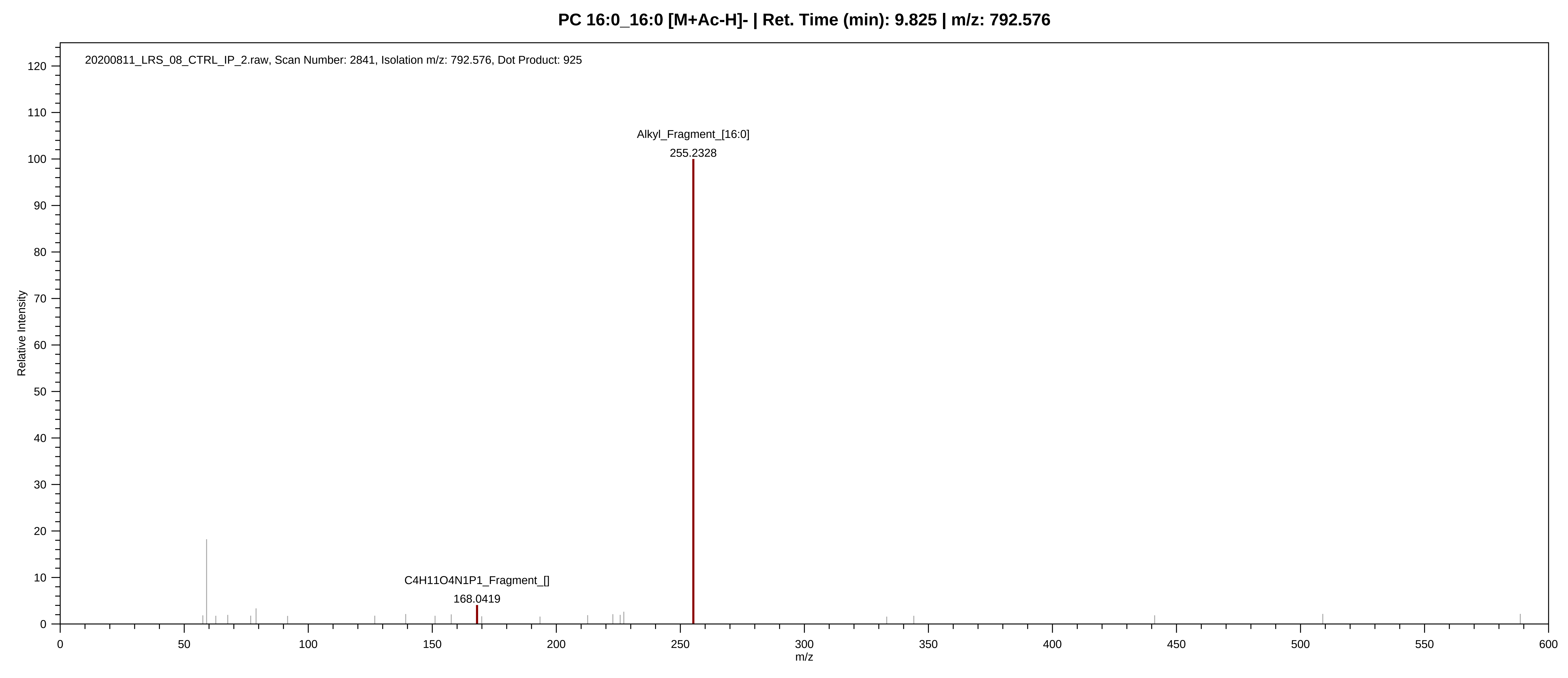
PC 16:0_16:0 [M+Ac-H]- | Ret. Time (min): 9.825 | m/z: 792.576
20200811_LRS_08_CTRL_IP_2.raw, Scan Number: 2841, Isolation m/z: 792.576, Dot Product: 925
Alkyl_Fragment_[16:0]
255.2328
C4H11O4N1P1_Fragment_[]
168.0419
Relative Intensity
m/z

PC 16:0_16:0 [M+Ac-H]- | Ret. Time (min): 9.769 | m/z: 792.577

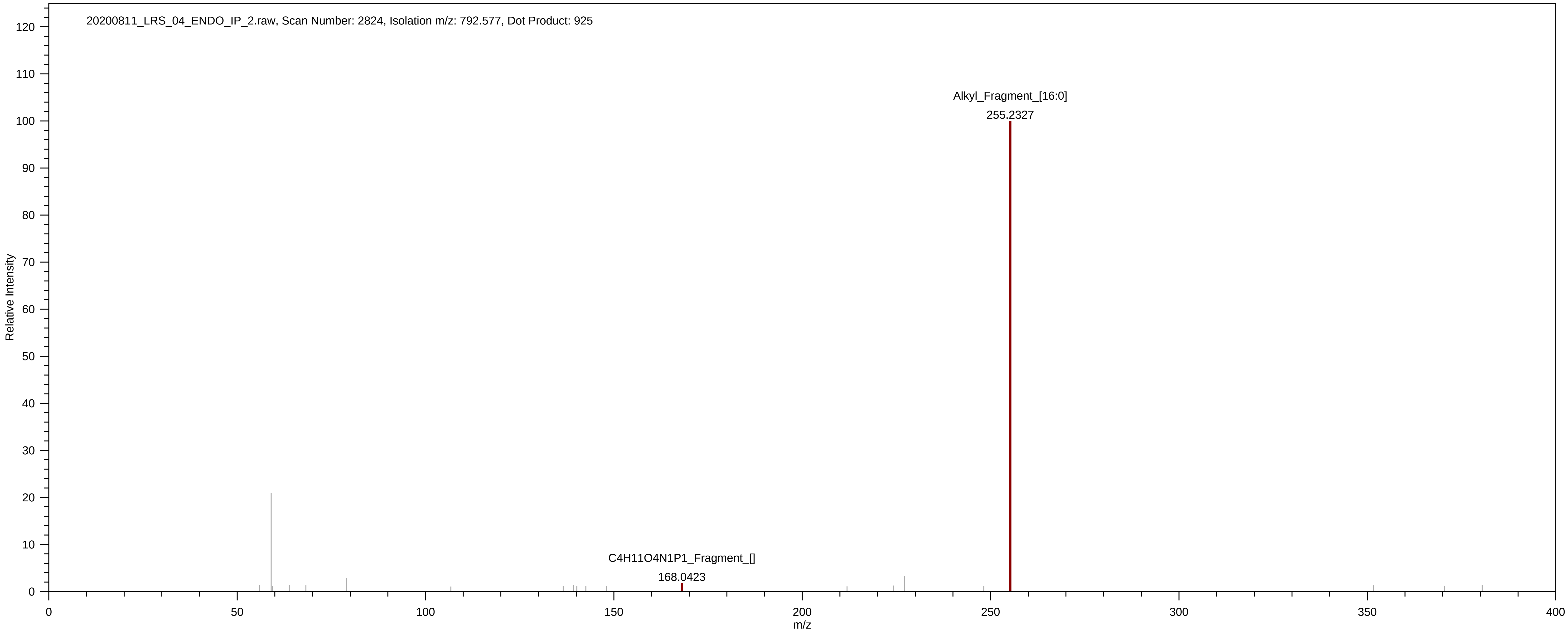

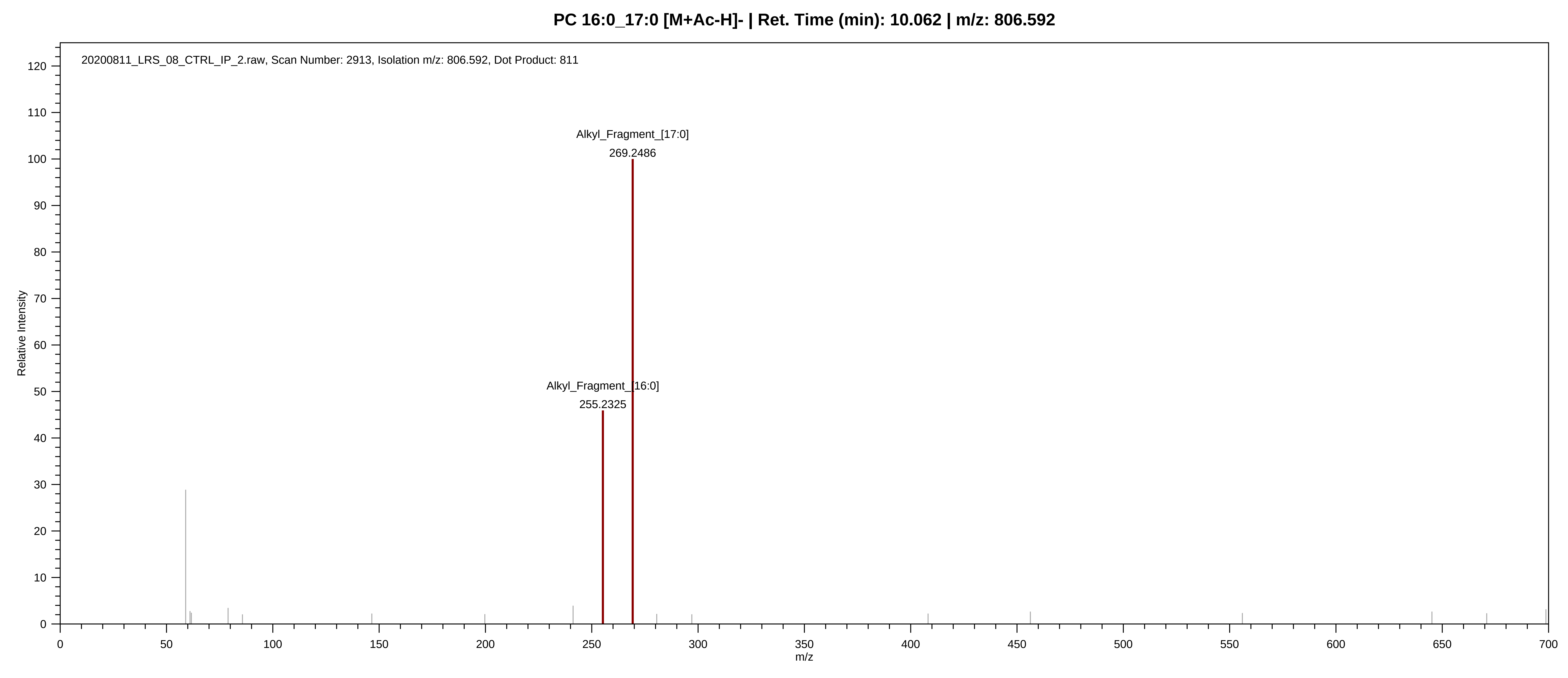

PC 16:0_17:0 [M+Ac-H]- | Ret. Time (min): 10.062 | m/z: 806.592
20200811_LRS_08_CTRL_IP_2.raw, Scan Number: 2913, Isolation m/z: 806.592, Dot Product: 811
Alkyl_Fragment_[17:0]
269.2486
Alkyl_Fragment_[16:0]
255.2325
Relative Intensity
m/z

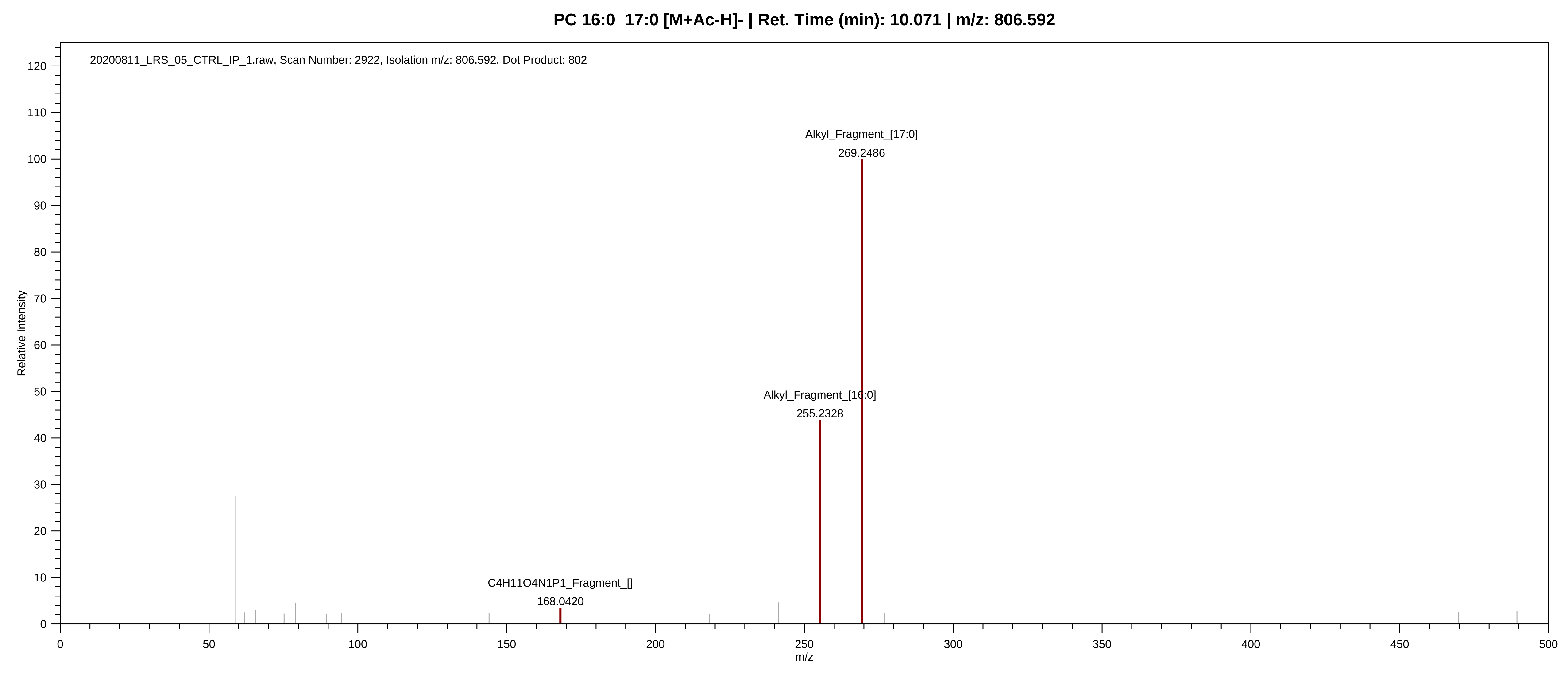
PC 16:0_17:0 [M+Ac-H]- | Ret. Time (min): 10.071 | m/z: 806.592
20200811_LRS_05_CTRL_IP_1.raw, Scan Number: 2922, Isolation m/z: 806.592, Dot Product: 802
Alkyl_Fragment_[17:0]
269.2486
Alkyl_Fragment_[16:0]
255.2328
C4H11O4N1P1_Fragment_[]
168.0420
Relative Intensity
m/z

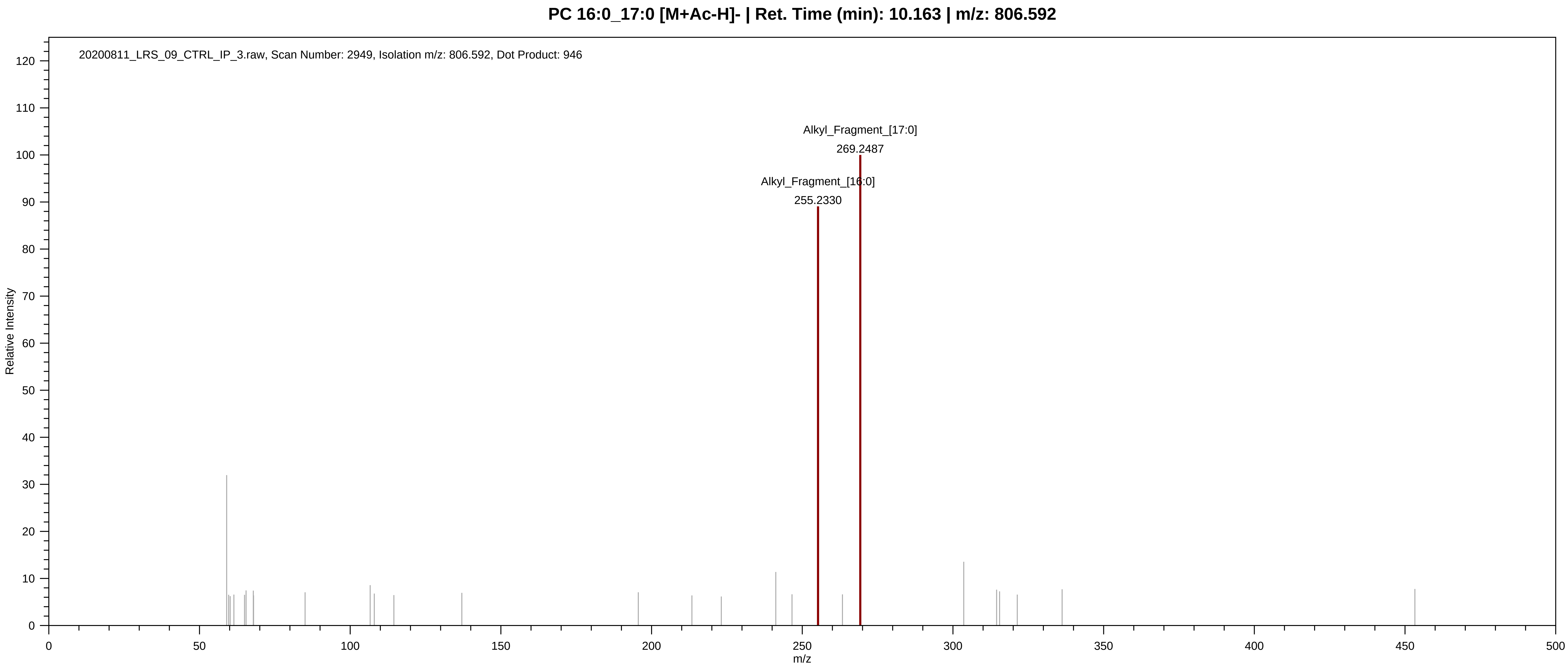

PC 16:0_17:0 [M+Ac-H]- | Ret. Time (min): 10.163 | m/z: 806.592
20200811_LRS_09_CTRL_IP_3.raw, Scan Number: 2949, Isolation m/z: 806.592, Dot Product: 946
Alkyl_Fragment_[17:0]
269.2487
Alkyl_Fragment_[16:0]
255.2330
Relative Intensity
m/z

PC 16:0_17:0 [M+Ac-H]- | Ret. Time (min): 10.068 | m/z: 806.593

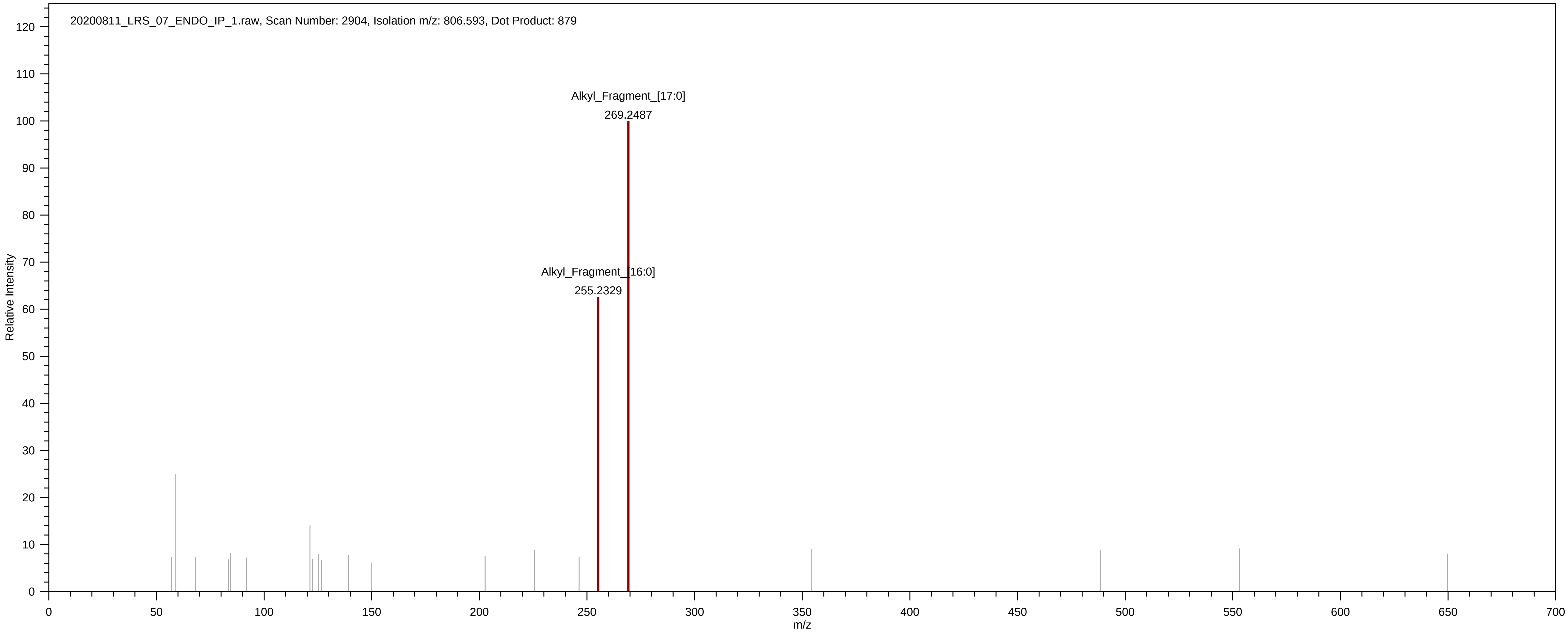

PC 16:0_17:0 [M+Ac-H]- | Ret. Time (min): 10.083 | m/z: 806.593

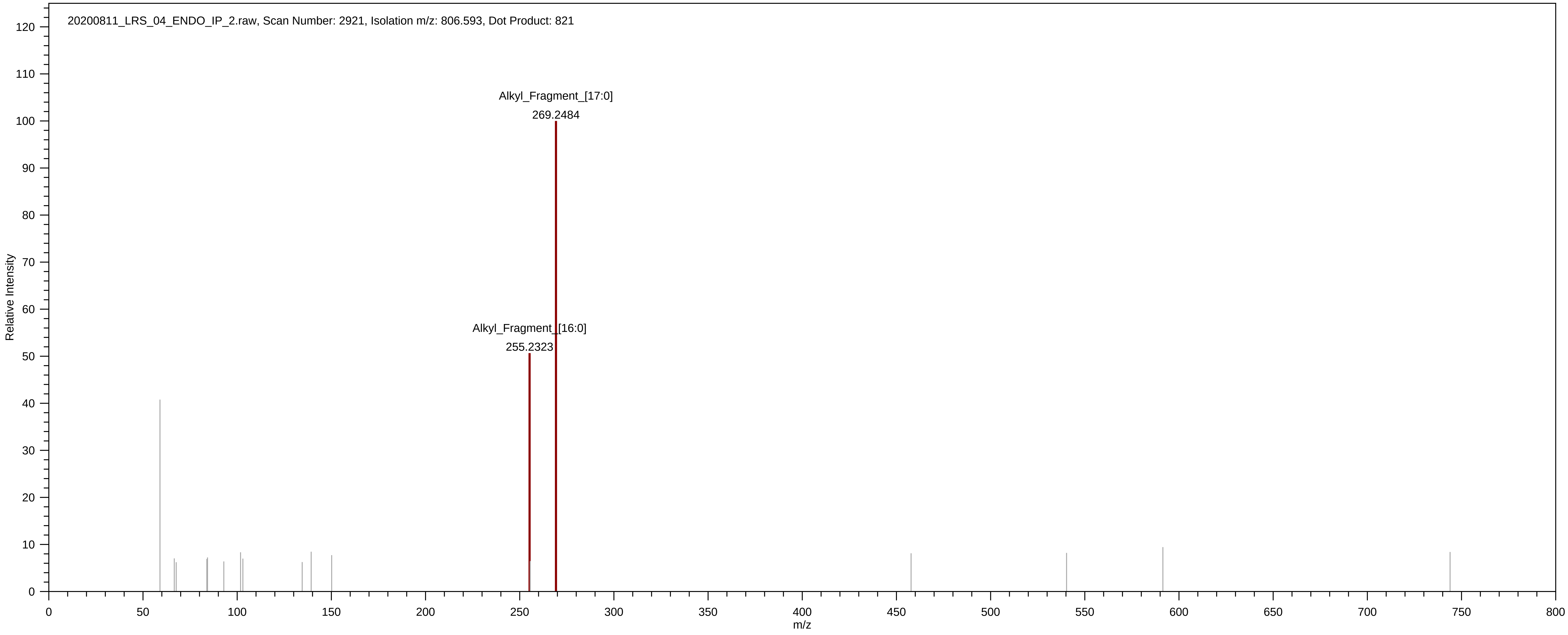

PC 16:0_17:0 [M+Ac-H]- | Ret. Time (min): 10.084 | m/z: 806.593

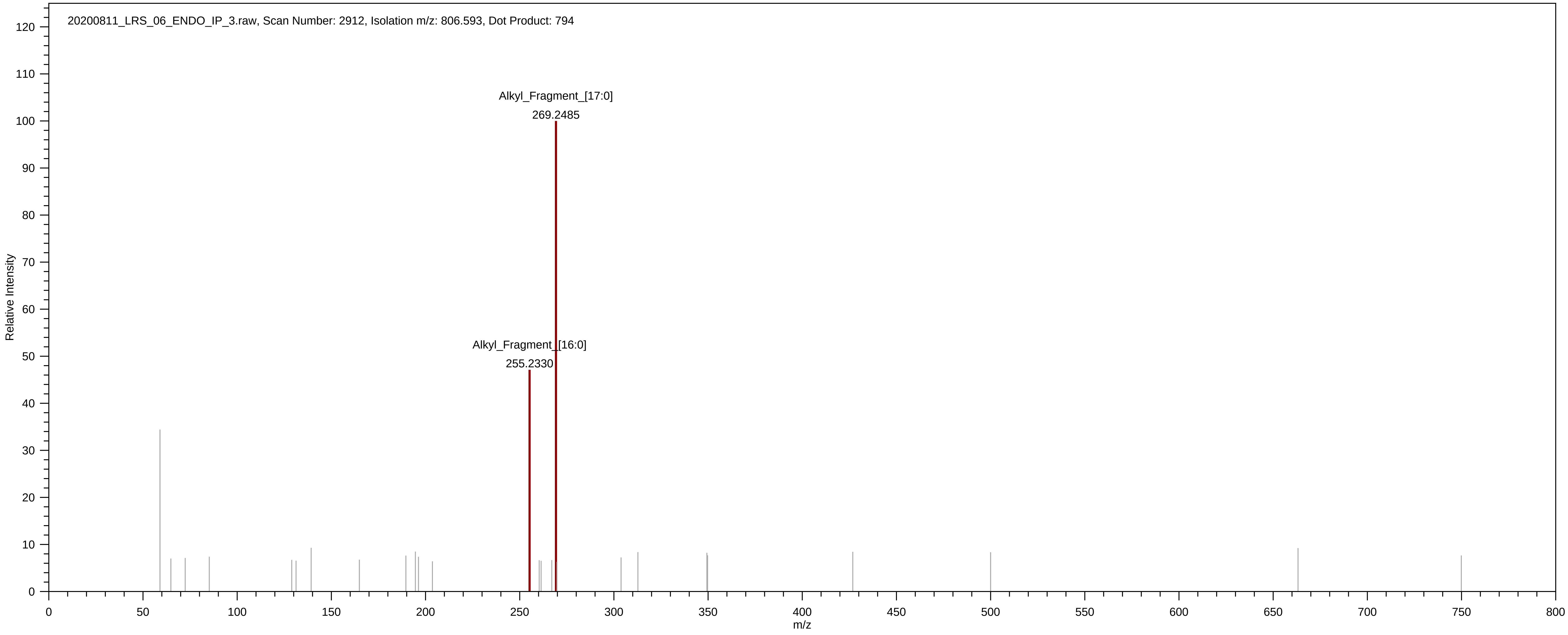

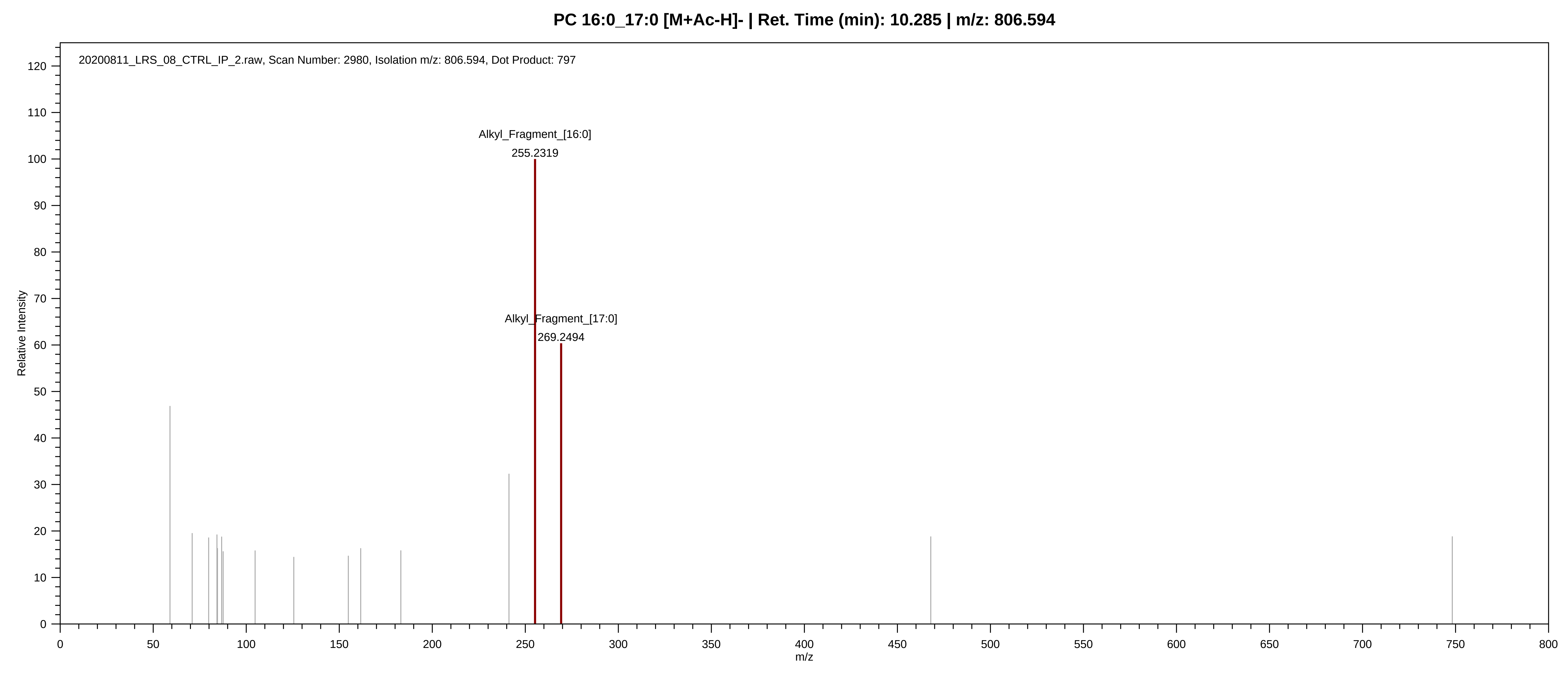
PC 16:0_17:0 [M+Ac-H]- | Ret. Time (min): 10.285 | m/z: 806.594
20200811_LRS_08_CTRL_IP_2.raw, Scan Number: 2980, Isolation m/z: 806.594, Dot Product: 797
Alkyl_Fragment_[16:0]
255.2319
Alkyl_Fragment_[17:0]
269.2494
Relative Intensity
m/z

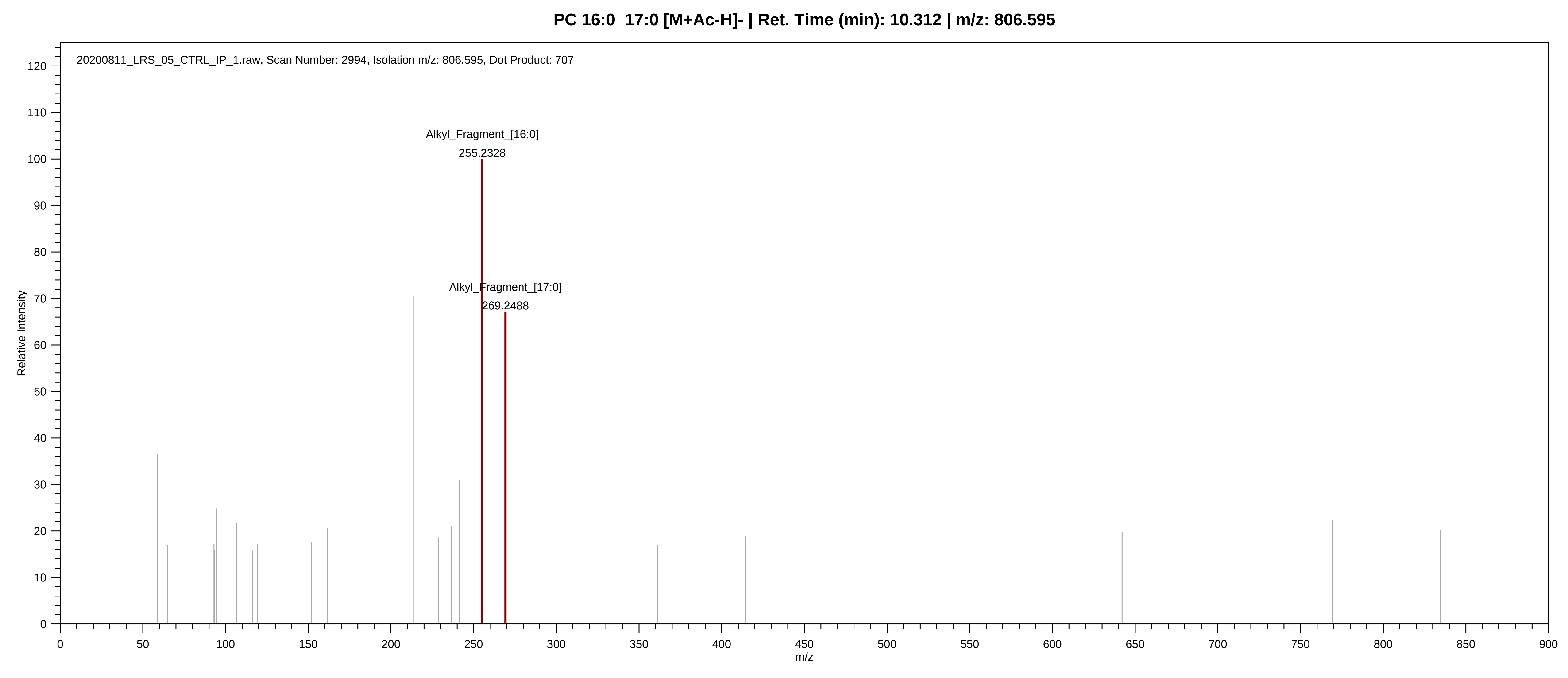
PC 16:0_17:0 [M+Ac-H]- | Ret. Time (min): 10.312 | m/z: 806.595
20200811_LRS_05_CTRL_IP_1.raw, Scan Number: 2994, Isolation m/z: 806.595, Dot Product: 707
Alkyl_Fragment_[16:0]
255.2328
Alkyl_Fragment_[17:0]
269.2488
Relative Intensity
m/z

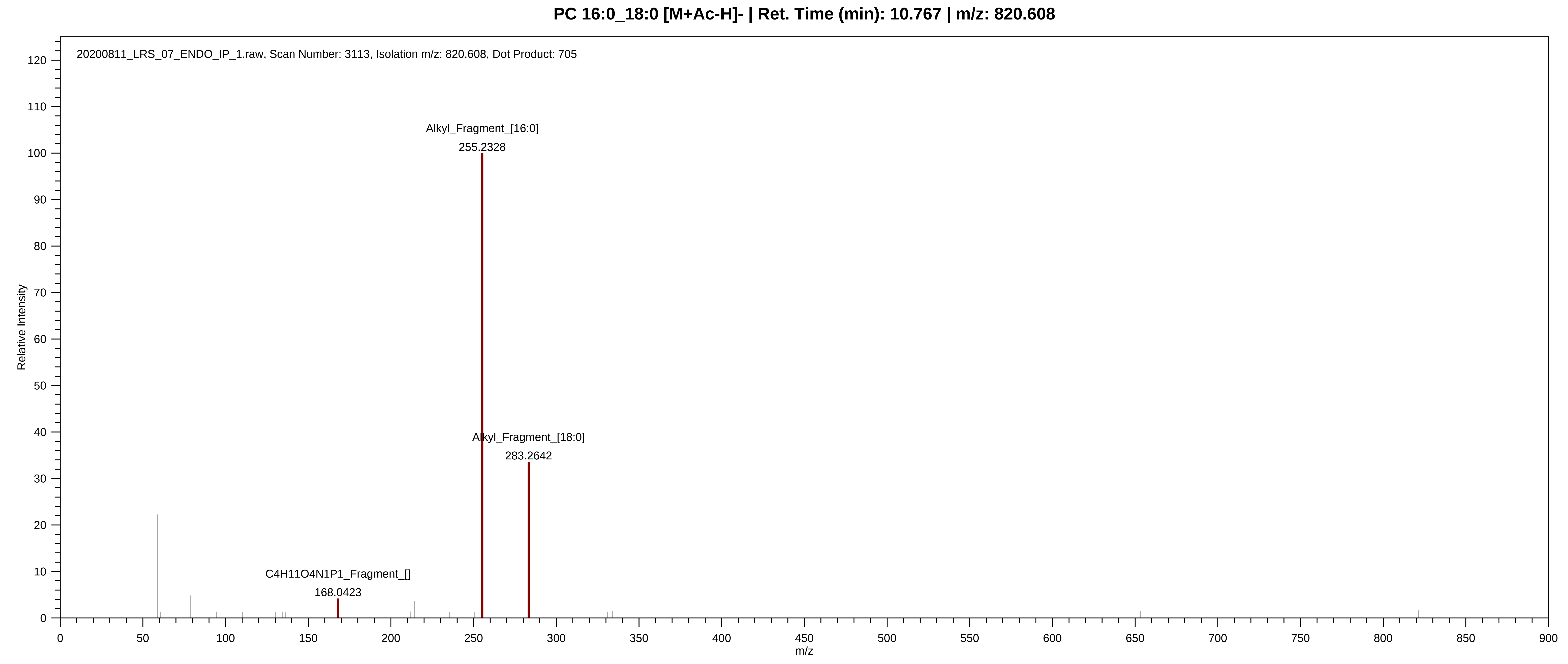
PC 16:0_18:0 [M+Ac-H]- | Ret. Time (min): 10.767 | m/z: 820.608
20200811_LRS_07_ENDO_IP_1.raw, Scan Number: 3113, Isolation m/z: 820.608, Dot Product: 705
Alkyl_Fragment_[16:0]
255.2328
Alkyl_Fragment_[18:0]
283.2642
C4H11O4N1P1_Fragment_[]
168.0423
Relative Intensity
m/z

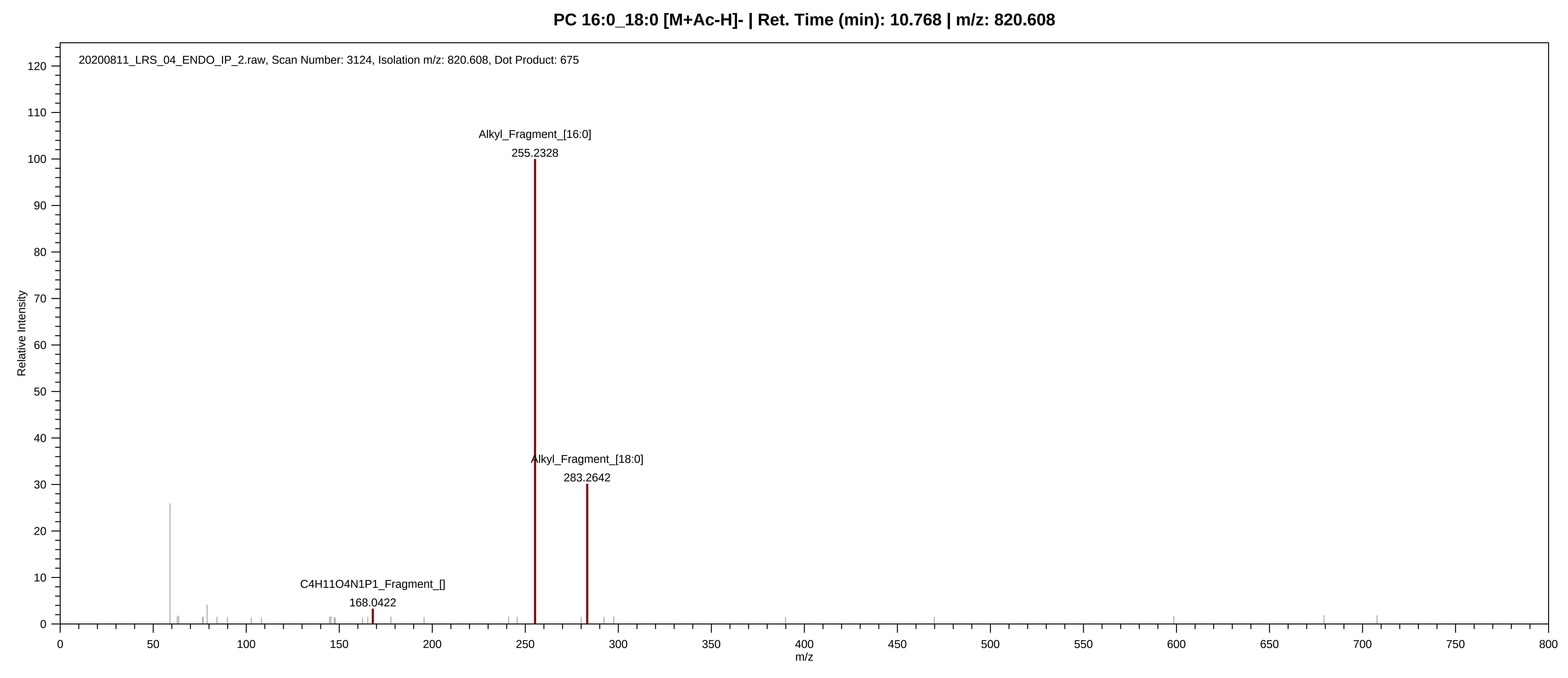
PC 16:0_18:0 [M+Ac-H]- | Ret. Time (min): 10.768 | m/z: 820.608
20200811_LRS_04_ENDO_IP_2.raw, Scan Number: 3124, Isolation m/z: 820.608, Dot Product: 675
Alkyl_Fragment_[16:0]
255.2328
Alkyl_Fragment_[18:0]
283.2642
C4H11O4N1P1_Fragment_[]
168.0422
Relative Intensity
m/z

PC 16:0_18:1 [M+Ac-H]- | Ret. Time (min): 9.754 | m/z: 818.592

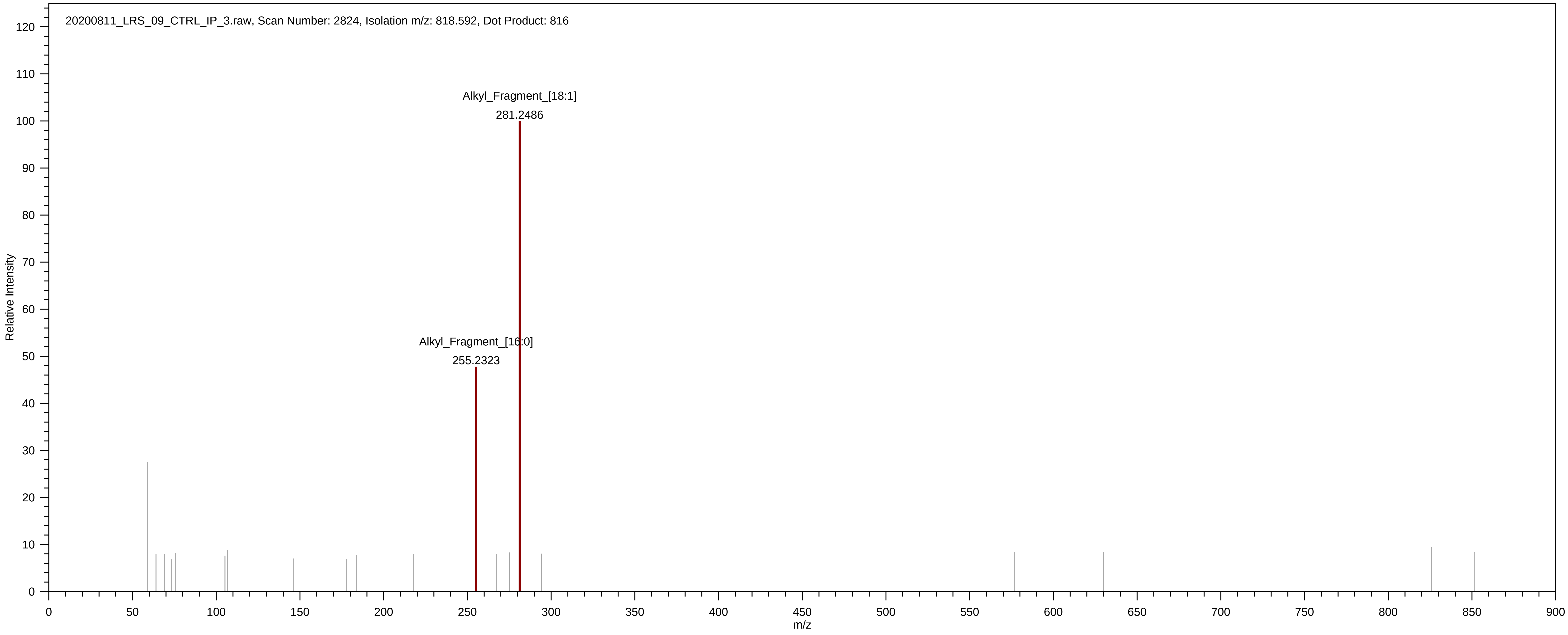

PC 16:0_18:1 [M+Ac-H]- | Ret. Time (min): 9.767 | m/z: 818.592

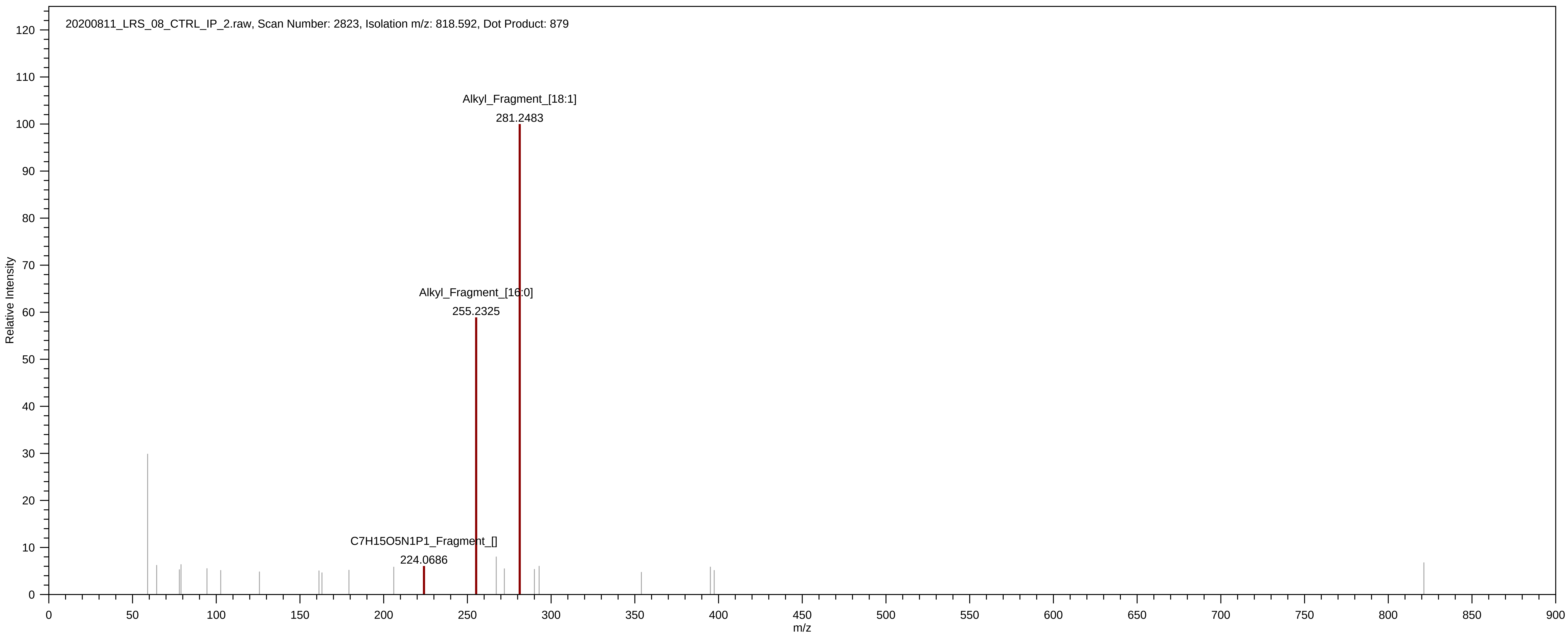

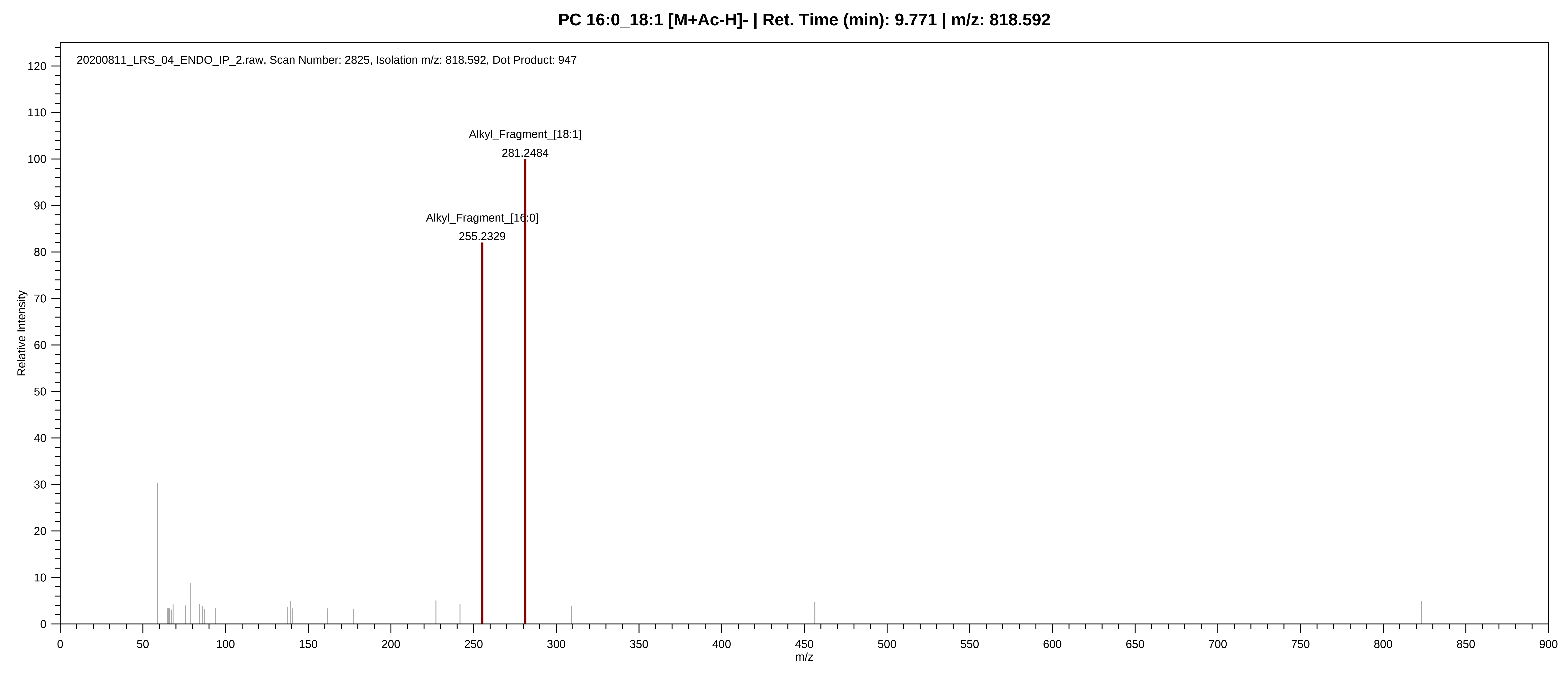
PC 16:0_18:1 [M+Ac-H]- | Ret. Time (min): 9.771 | m/z: 818.592
20200811_LRS_04_ENDO_IP_2.raw, Scan Number: 2825, Isolation m/z: 818.592, Dot Product: 947
Alkyl_Fragment_[18:1]
281.2484
Alkyl_Fragment_[16:0]
255.2329
Relative Intensity
m/z

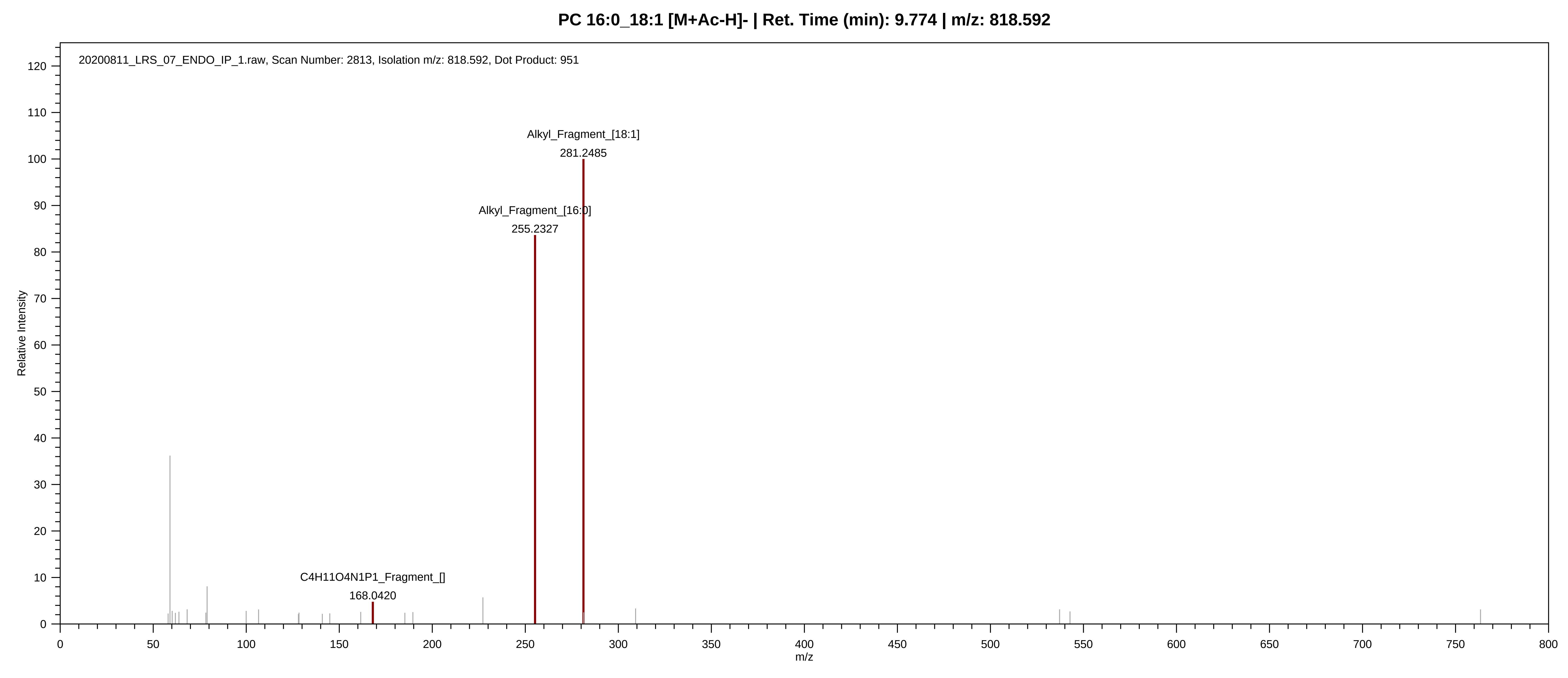
PC 16:0_18:1 [M+Ac-H]- | Ret. Time (min): 9.774 | m/z: 818.592
20200811_LRS_07_ENDO_IP_1.raw, Scan Number: 2813, Isolation m/z: 818.592, Dot Product: 951
Alkyl_Fragment_[18:1]
281.2485
Alkyl_Fragment_[16:0]
255.2327
C4H11O4N1P1_Fragment_[]
168.0420
Relative Intensity
m/z

PC 16:0_18:1 [M+Ac-H]- | Ret. Time (min): 9.774 | m/z: 818.592

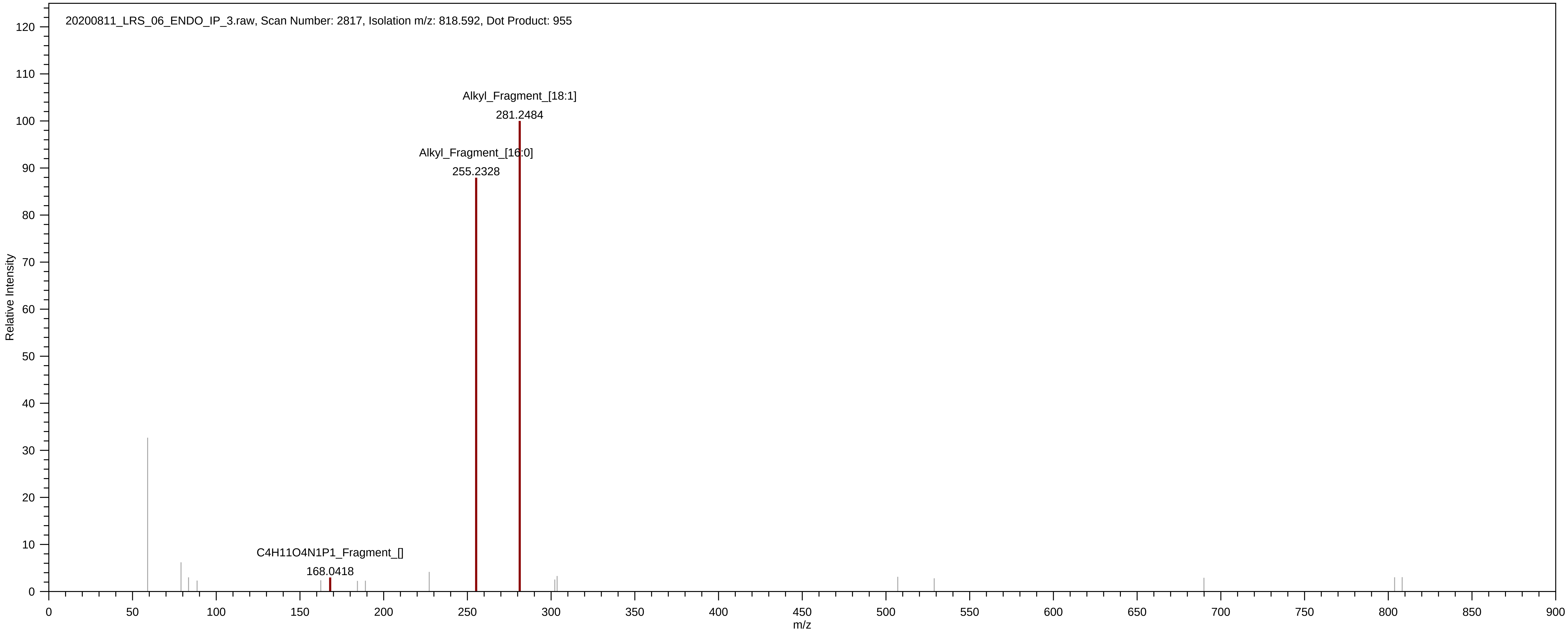

PC 16:0_18:1 [M+Ac-H]- | Ret. Time (min): 9.778 | m/z: 818.592

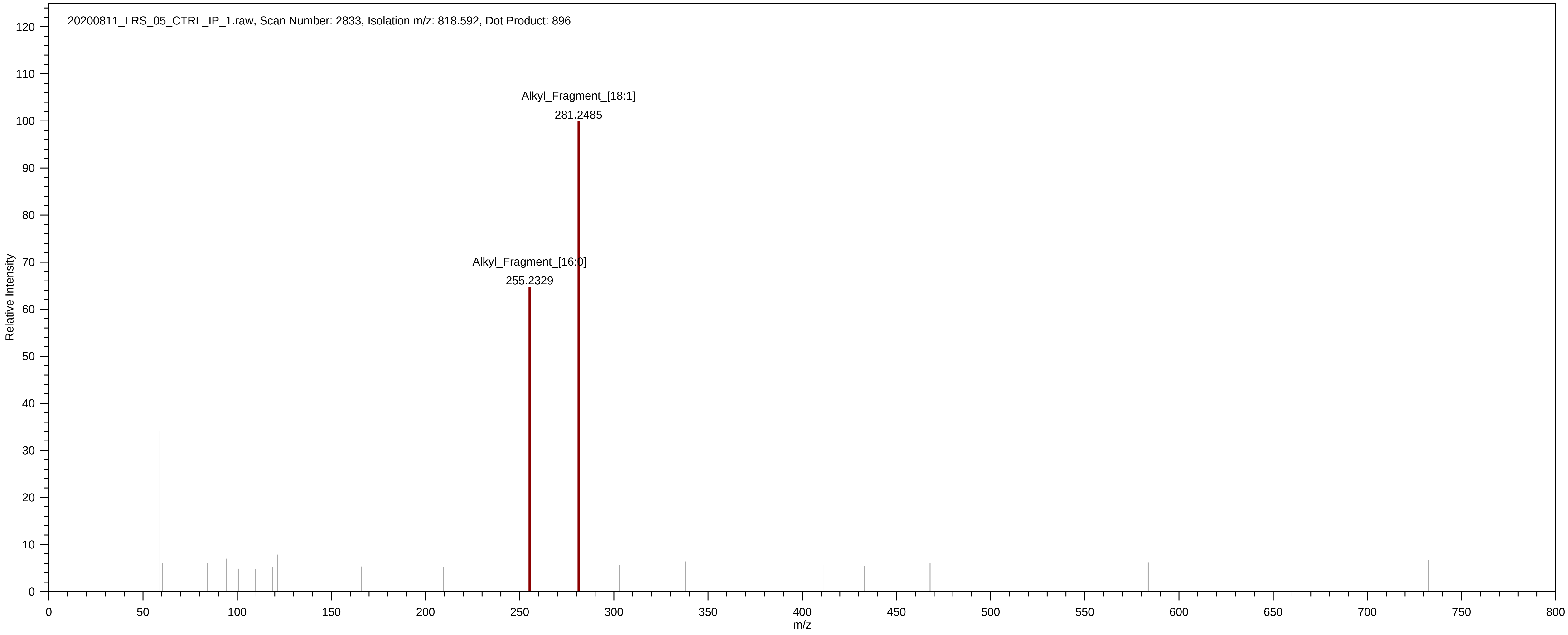

PC 16:0_18:1 [M+Ac-H]- | Ret. Time (min): 9.949 | m/z: 818.592

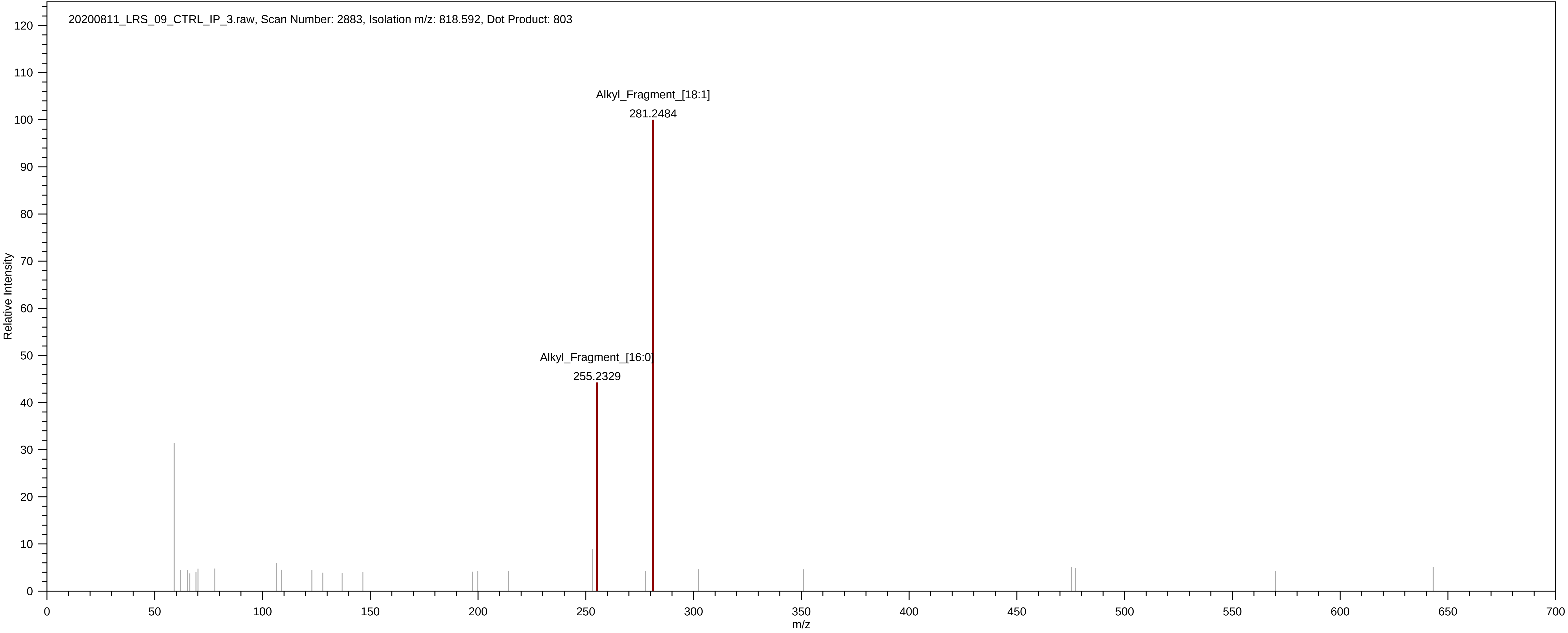

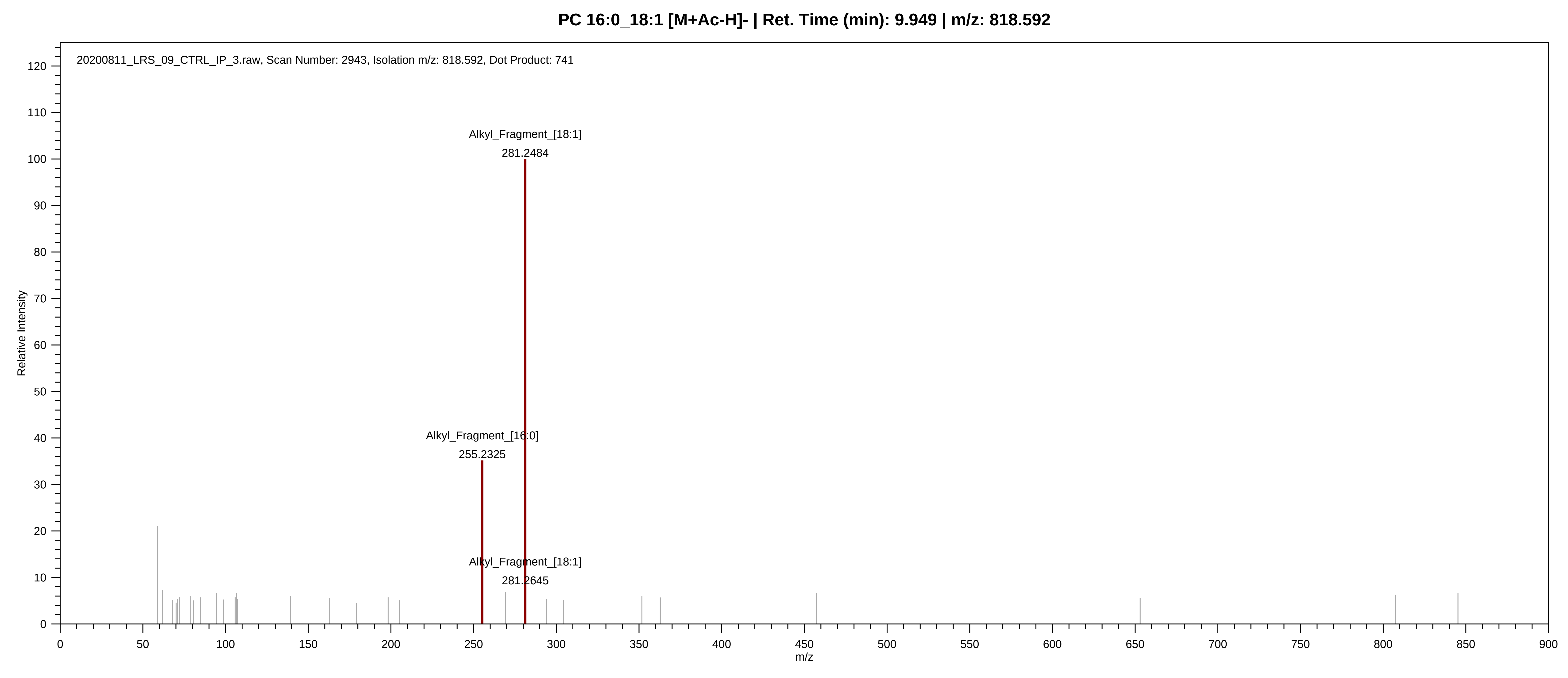

PC 16:0_18:1 [M+Ac-H]- | Ret. Time (min): 9.949 | m/z: 818.592
20200811_LRS_09_CTRL_IP_3.raw, Scan Number: 2943, Isolation m/z: 818.592, Dot Product: 741
Alkyl_Fragment_[18:1]
281.2484
Alkyl_Fragment_[16:0]
255.2325
Alkyl_Fragment_[18:1]
281.2645
Relative Intensity
m/z

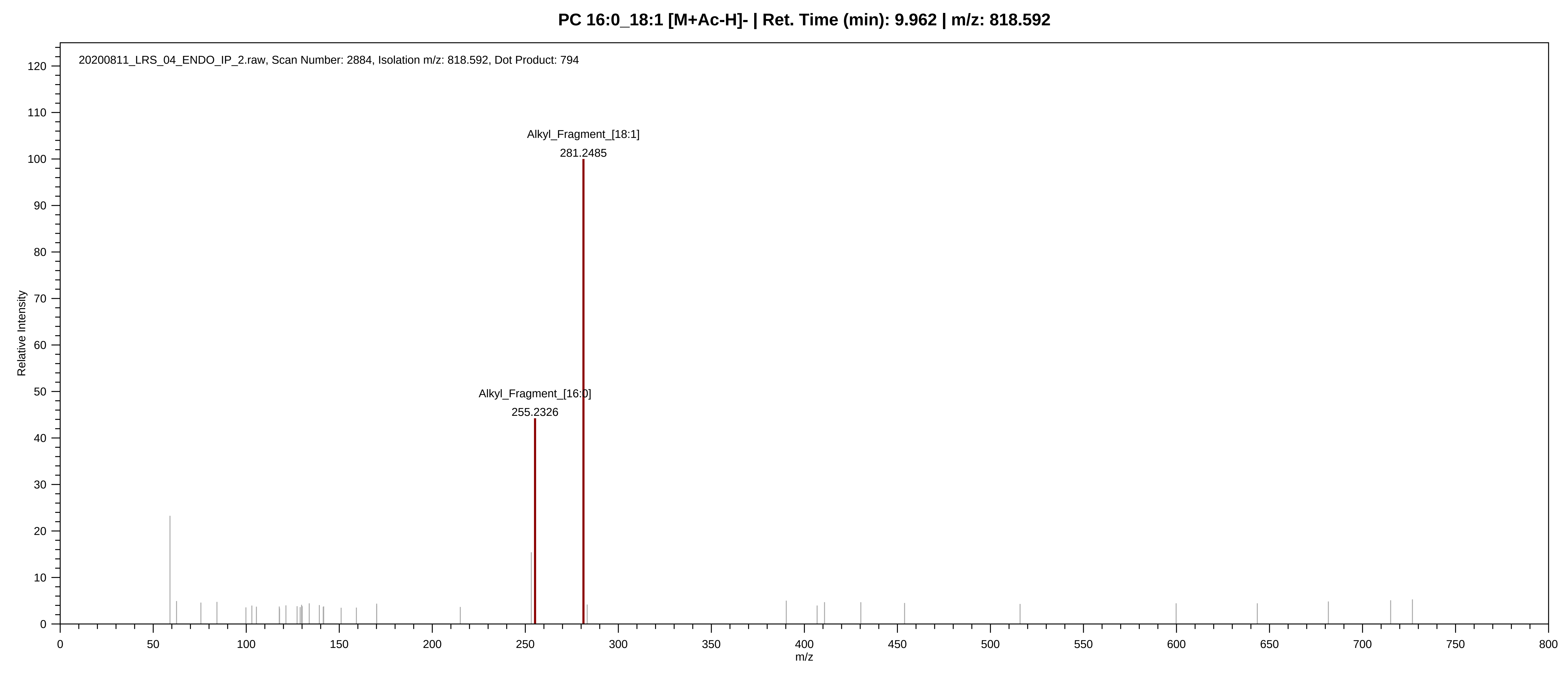
PC 16:0_18:1 [M+Ac-H]- | Ret. Time (min): 9.962 | m/z: 818.592
20200811_LRS_04_ENDO_IP_2.raw, Scan Number: 2884, Isolation m/z: 818.592, Dot Product: 794
Alkyl_Fragment_[18:1]
281.2485
Alkyl_Fragment_[16:0]
255.2326
Relative Intensity
m/z

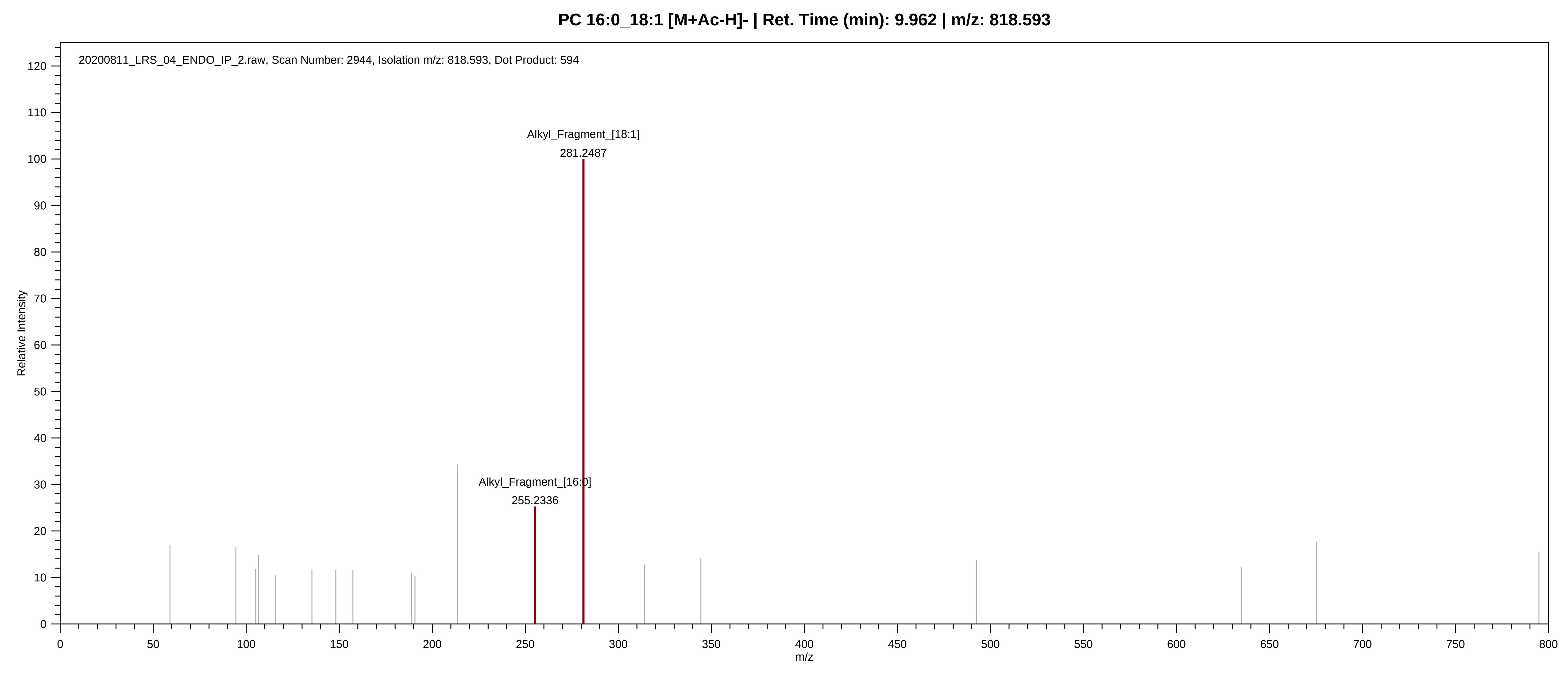

PC 16:0_18:1 [M+Ac-H]- | Ret. Time (min): 9.962 | m/z: 818.593
20200811_LRS_04_ENDO_IP_2.raw, Scan Number: 2944, Isolation m/z: 818.593, Dot Product: 594
Alkyl_Fragment_[18:1]
281.2487
Alkyl_Fragment_[16:0]
255.2336
Relative Intensity
m/z

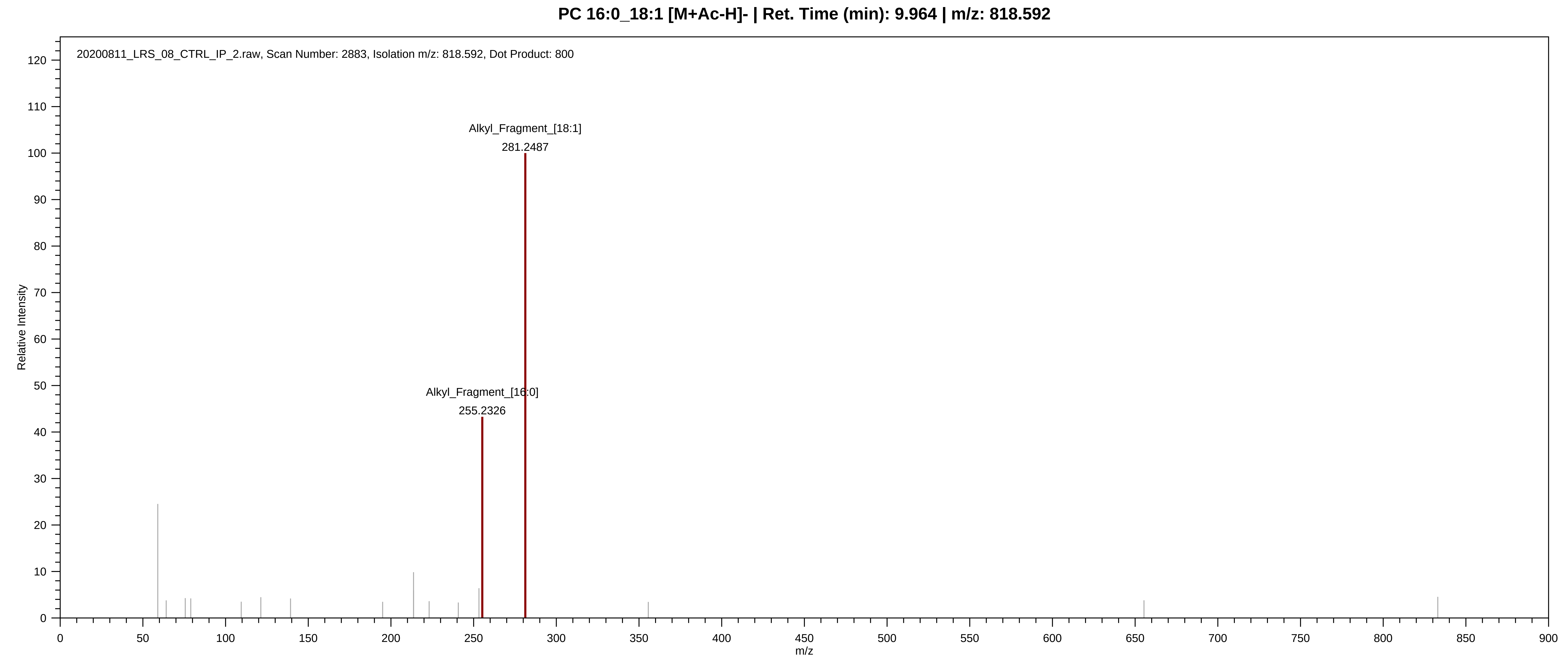

PC 16:0_18:1 [M+Ac-H]- | Ret. Time (min): 9.964 | m/z: 818.592
20200811_LRS_08_CTRL_IP_2.raw, Scan Number: 2883, Isolation m/z: 818.592, Dot Product: 800
Alkyl_Fragment_[18:1]
281.2487
Alkyl_Fragment_[16:0]
255.2326
Relative Intensity
m/z

PC 16:0_18:1 [M+Ac-H]- | Ret. Time (min): 9.964 | m/z: 818.592

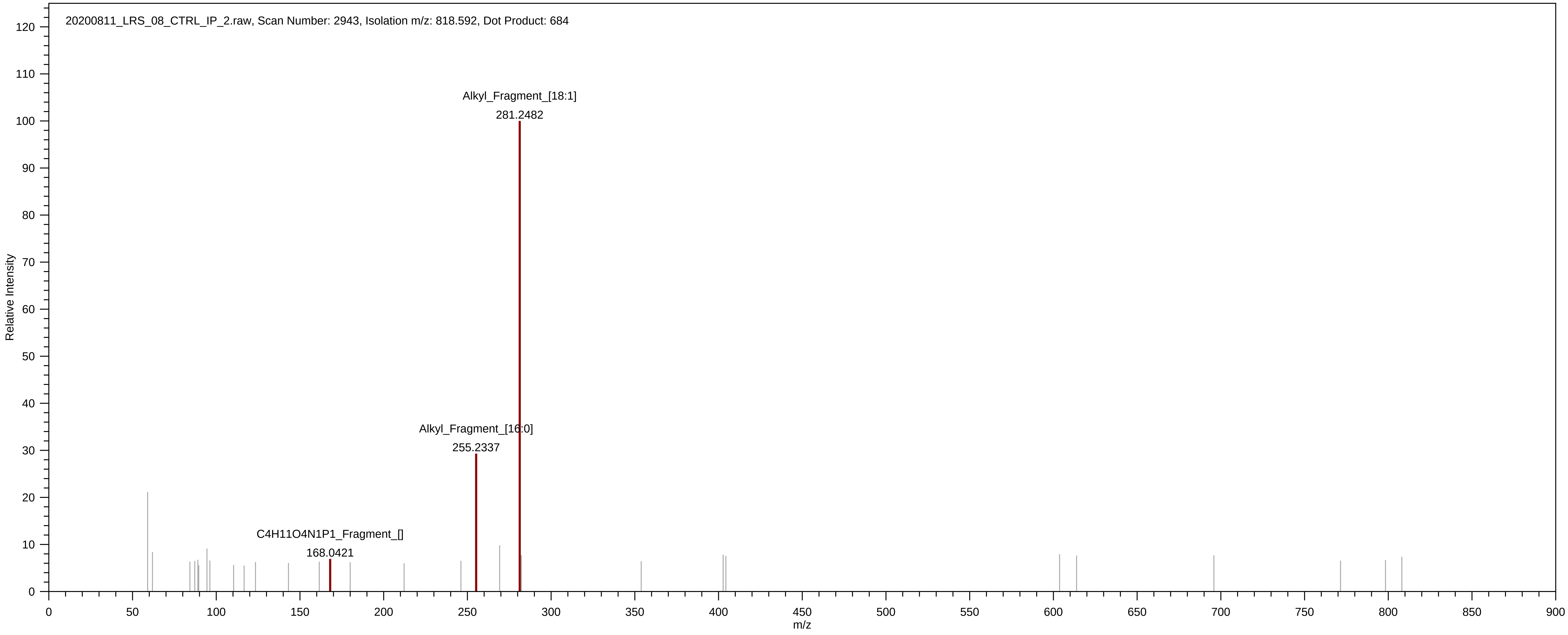

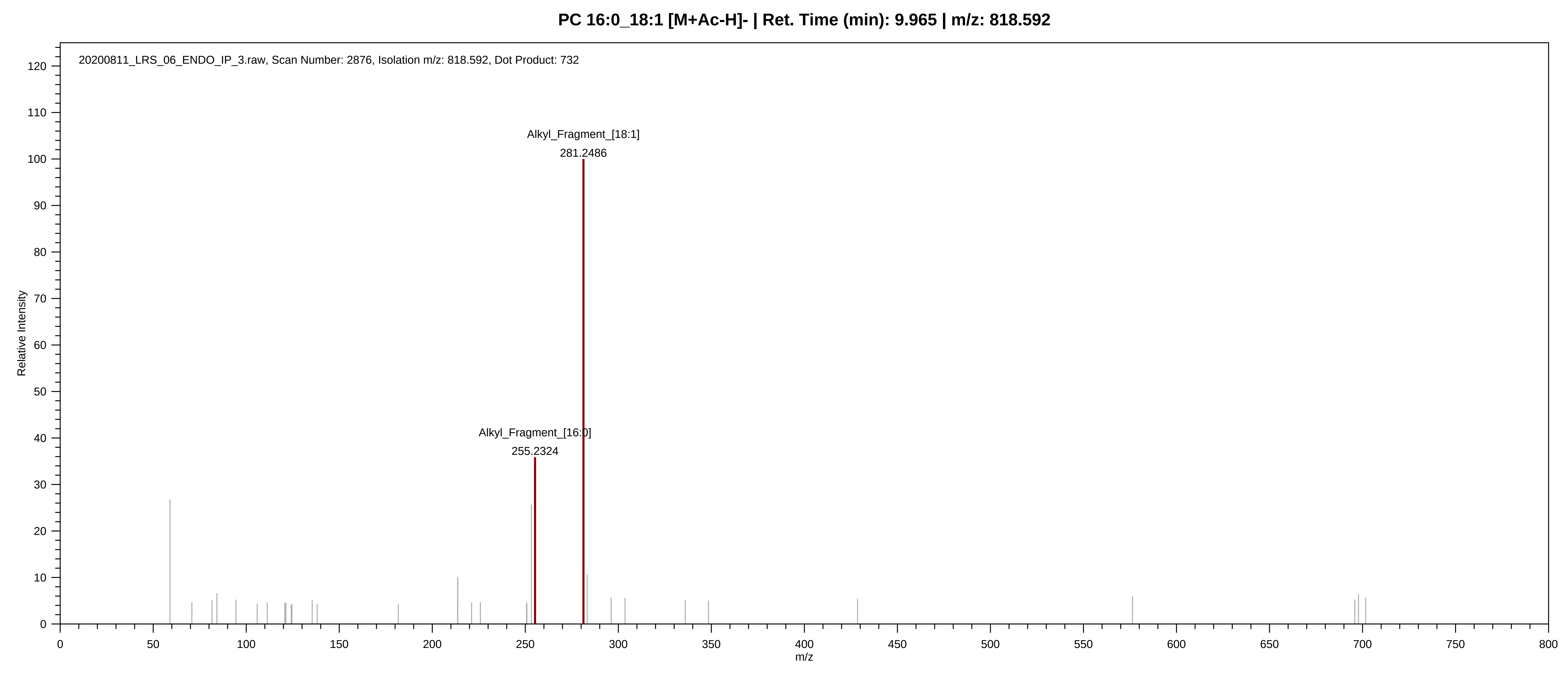
PC 16:0_18:1 [M+Ac-H]- | Ret. Time (min): 9.965 | m/z: 818.592
20200811_LRS_06_ENDO_IP_3.raw, Scan Number: 2876, Isolation m/z: 818.592, Dot Product: 732
Alkyl_Fragment_[18:1]
281.2486
Alkyl_Fragment_[16:0]
255.2324
Relative Intensity
m/z

PC 16:0_18:1 [M+Ac-H]- | Ret. Time (min): 9.965 | m/z: 818.592

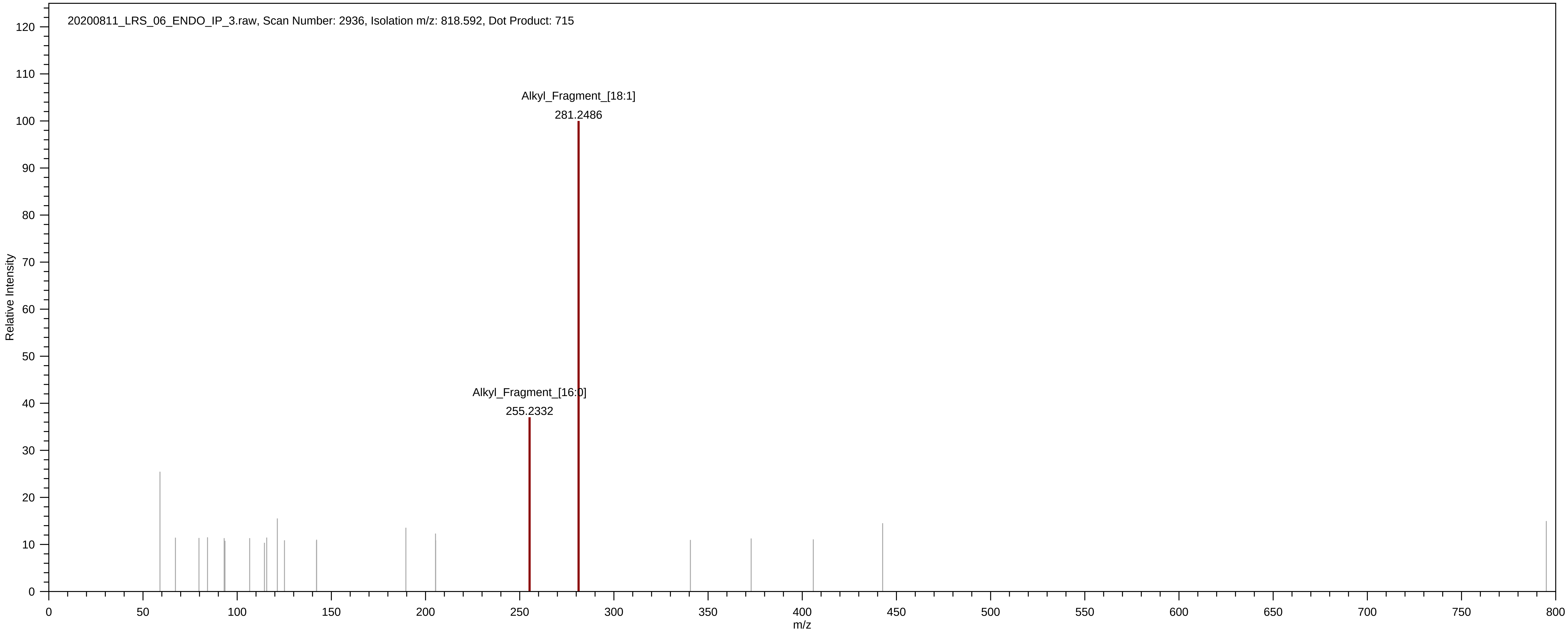

PC 16:0_18:1 [M+Ac-H]- | Ret. Time (min): 9.967 | m/z: 818.592

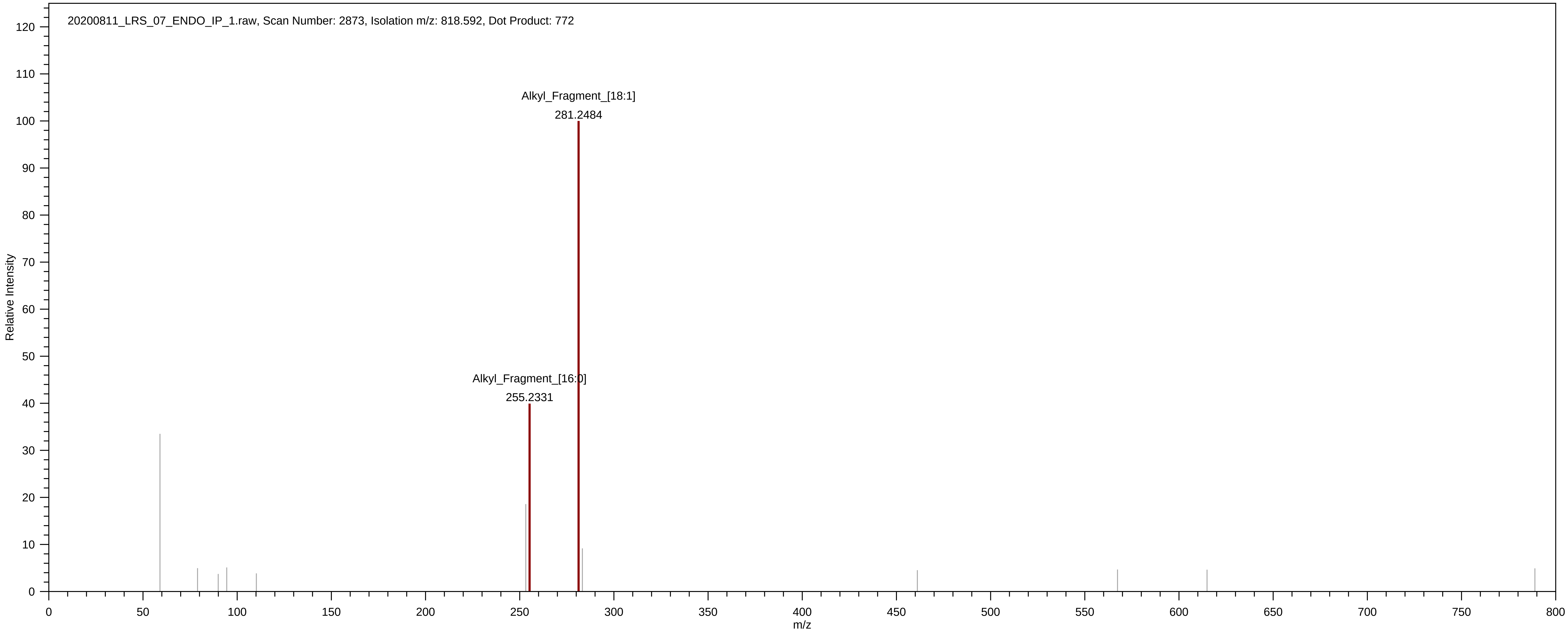

PC 16:0_18:1 [M+Ac-H]- | Ret. Time (min): 9.967 | m/z: 818.593

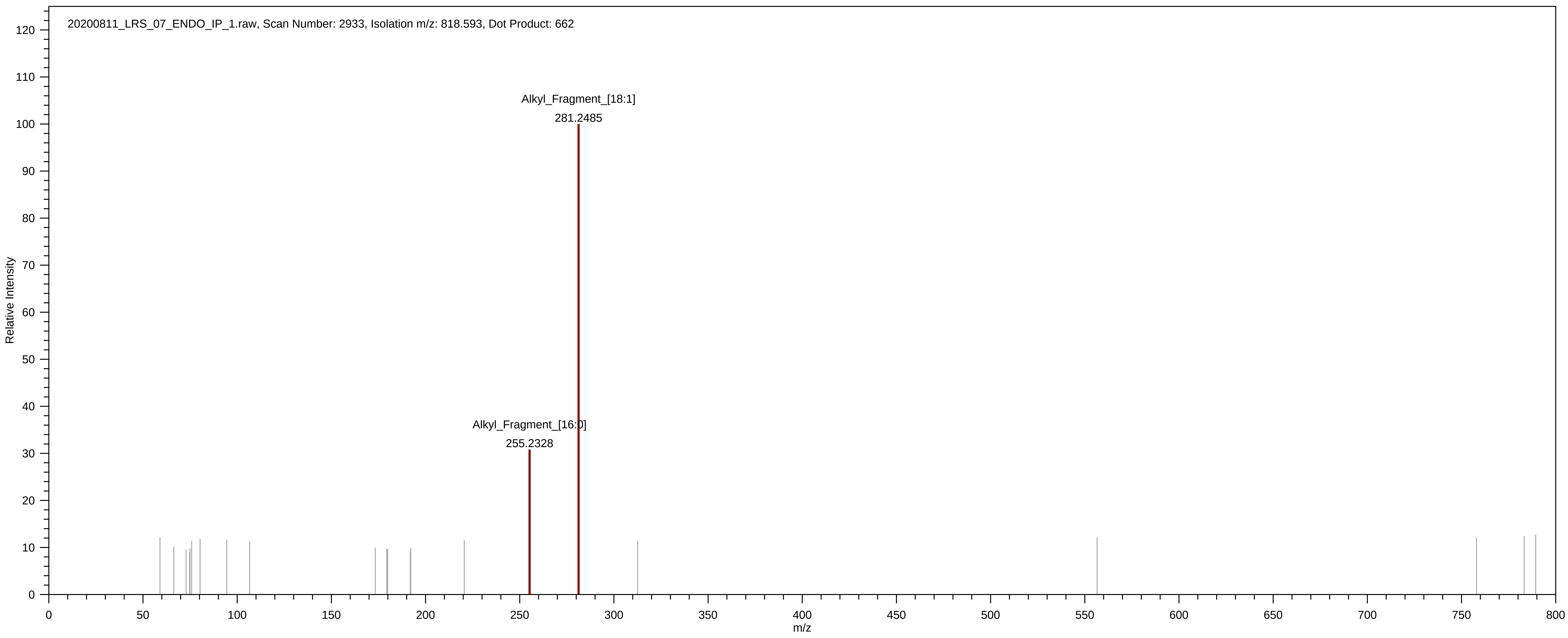

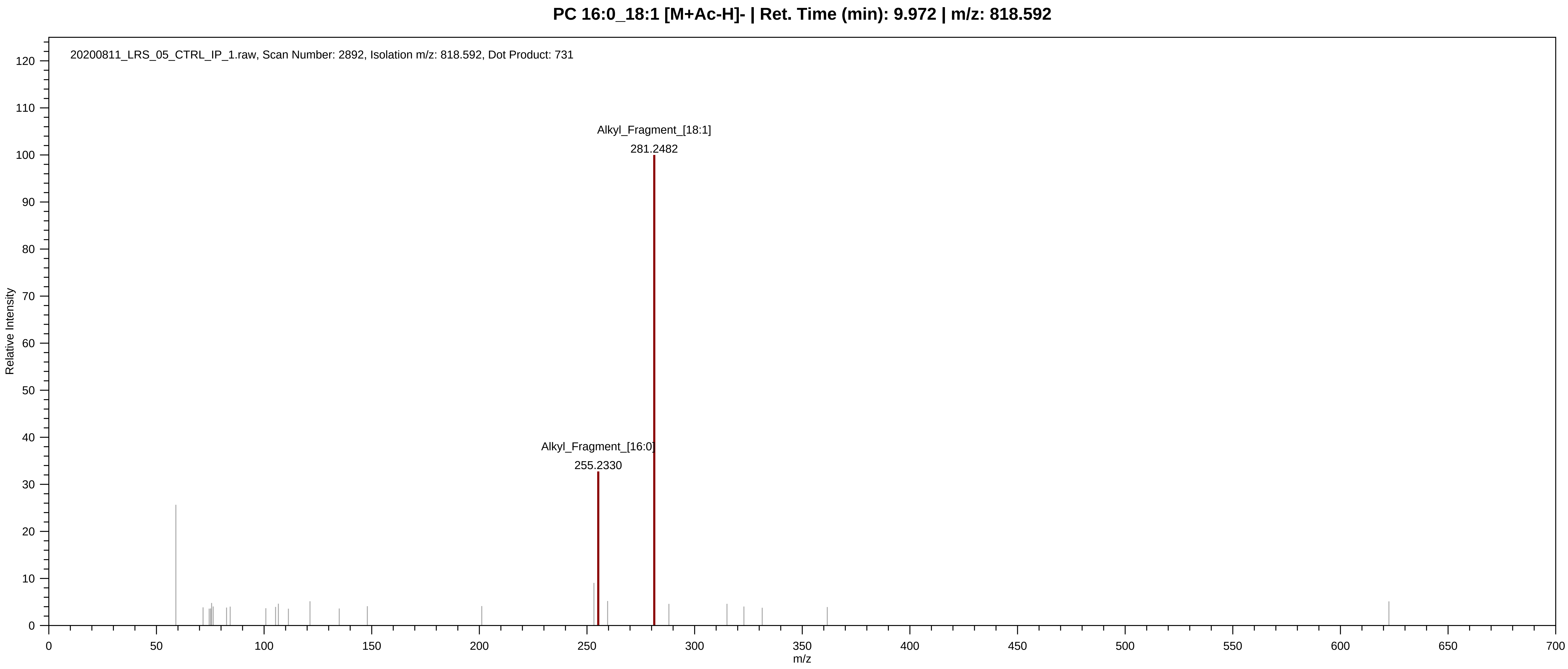
PC 16:0_18:1 [M+Ac-H]- | Ret. Time (min): 9.972 | m/z: 818.592
20200811_LRS_05_CTRL_IP_1.raw, Scan Number: 2892, Isolation m/z: 818.592, Dot Product: 731
Alkyl_Fragment_[18:1]
281.2482
Alkyl_Fragment_[16:0]
255.2330
Relative Intensity
m/z

PC 16:0_18:1 [M+Ac-H]- | Ret. Time (min): 9.972 | m/z: 818.592

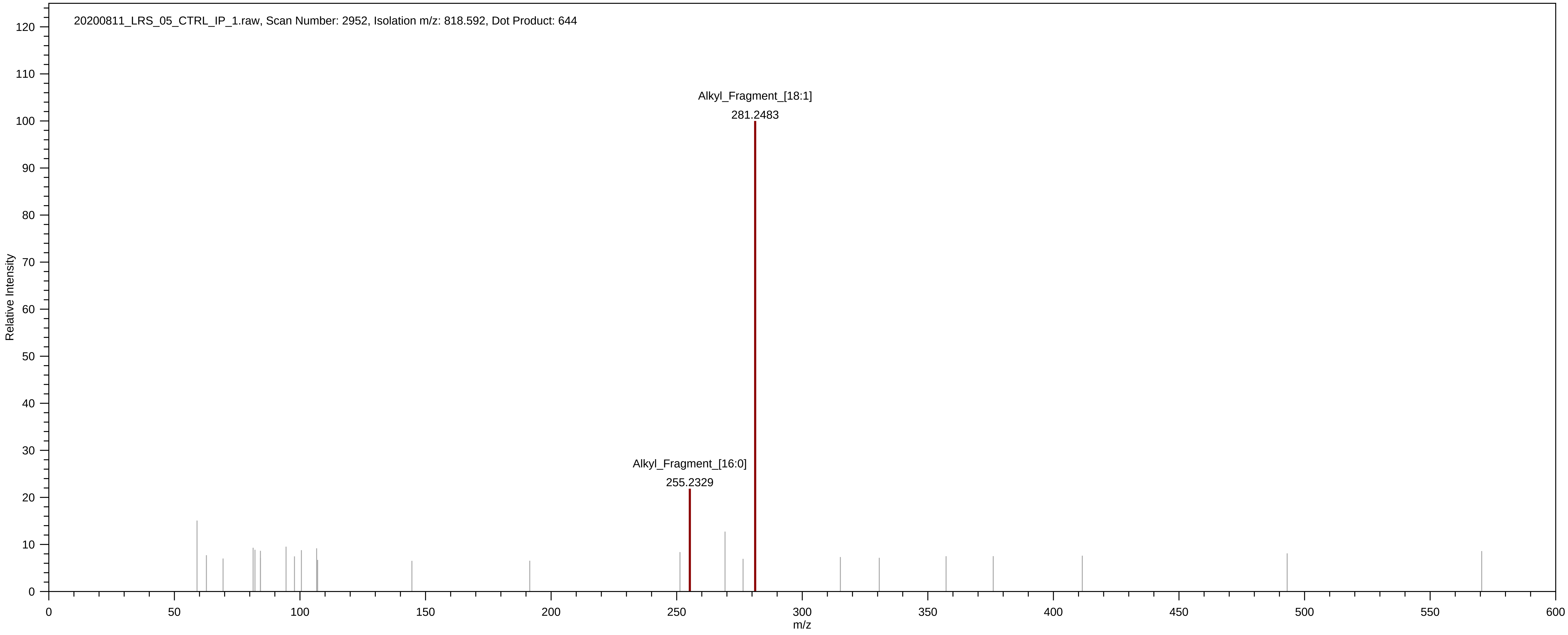

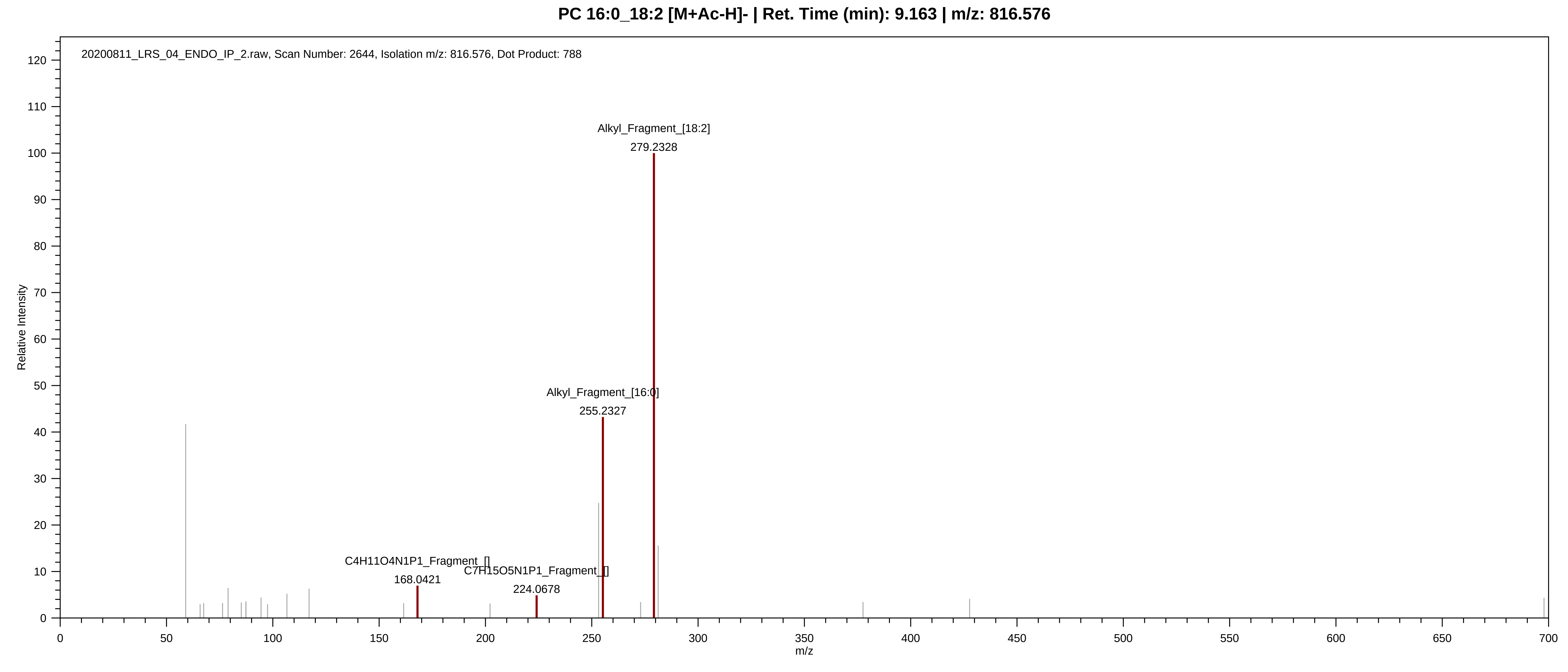
PC 16:0_18:2 [M+Ac-H]- | Ret. Time (min): 9.163 | m/z: 816.576
20200811_LRS_04_ENDO_IP_2.raw, Scan Number: 2644, Isolation m/z: 816.576, Dot Product: 788
Alkyl_Fragment_[18:2]
279.2328
Alkyl_Fragment_[16:0]
255.2327
C4H11O4N1P1_Fragment_[]
168.0421
C7H15O5N1P1_Fragment_[]
224.0678
Relative Intensity
m/z

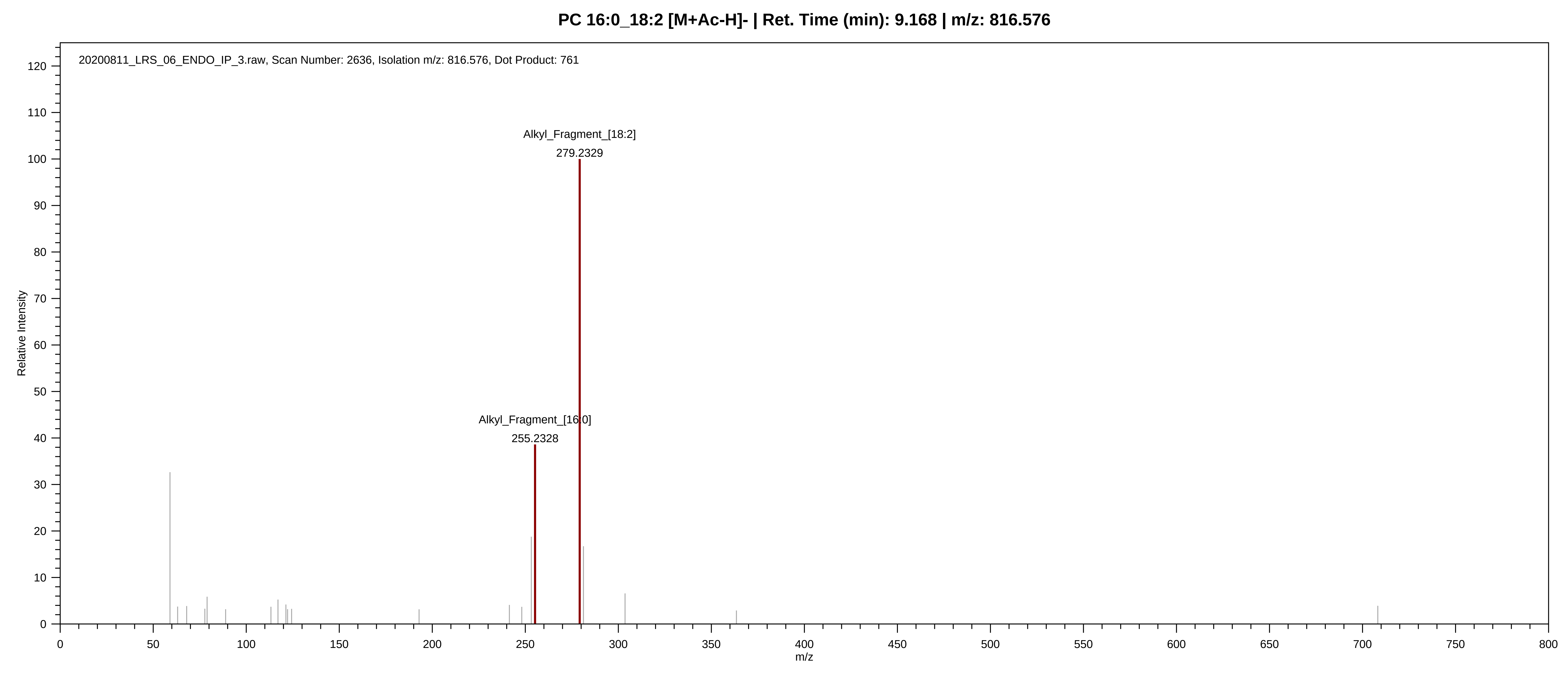

PC 16:0_18:2 [M+Ac-H]- | Ret. Time (min): 9.168 | m/z: 816.576
20200811_LRS_06_ENDO_IP_3.raw, Scan Number: 2636, Isolation m/z: 816.576, Dot Product: 761
Alkyl_Fragment_[18:2]
279.2329
Alkyl_Fragment_[16:0]
255.2328
Relative Intensity
m/z

PC 16:0_18:2 [M+Ac-H]- | Ret. Time (min): 9.169 | m/z: 816.576

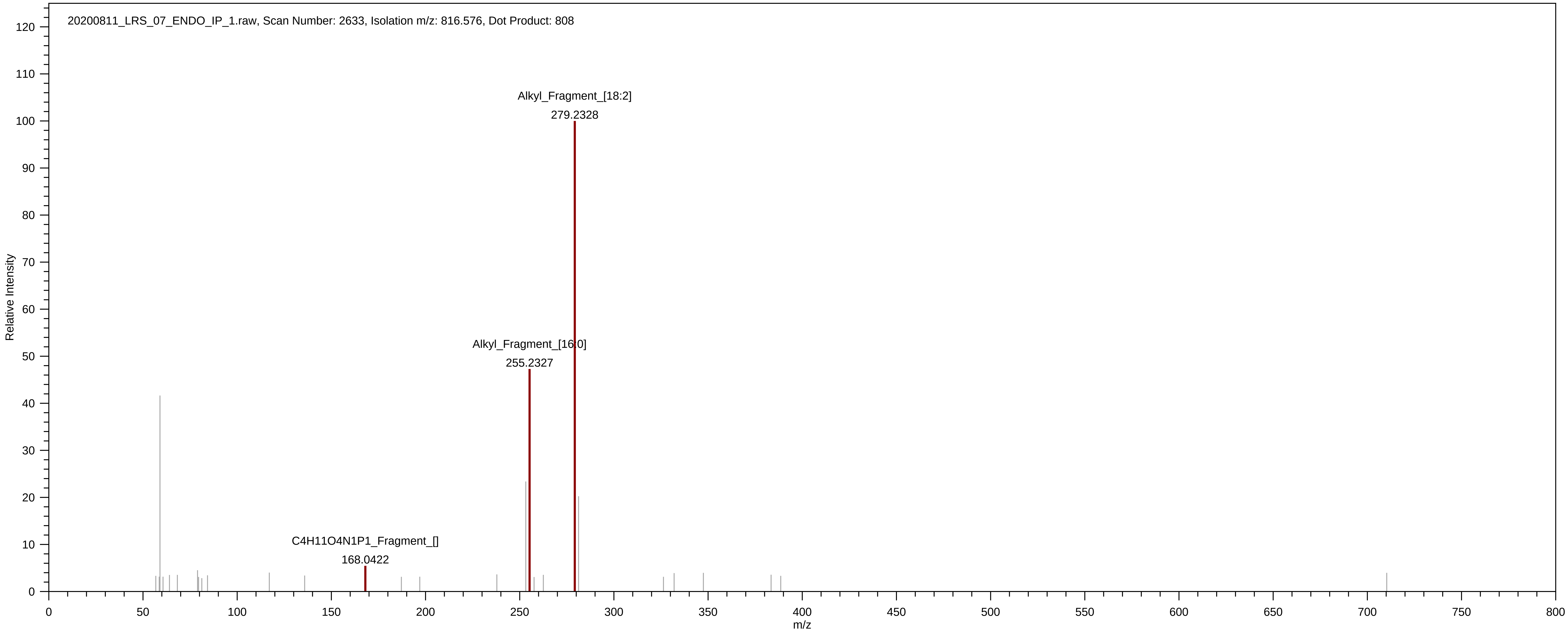

PC 16:0_20:0 [M+Ac-H]- | Ret. Time (min): 11.837 | m/z: 848.641

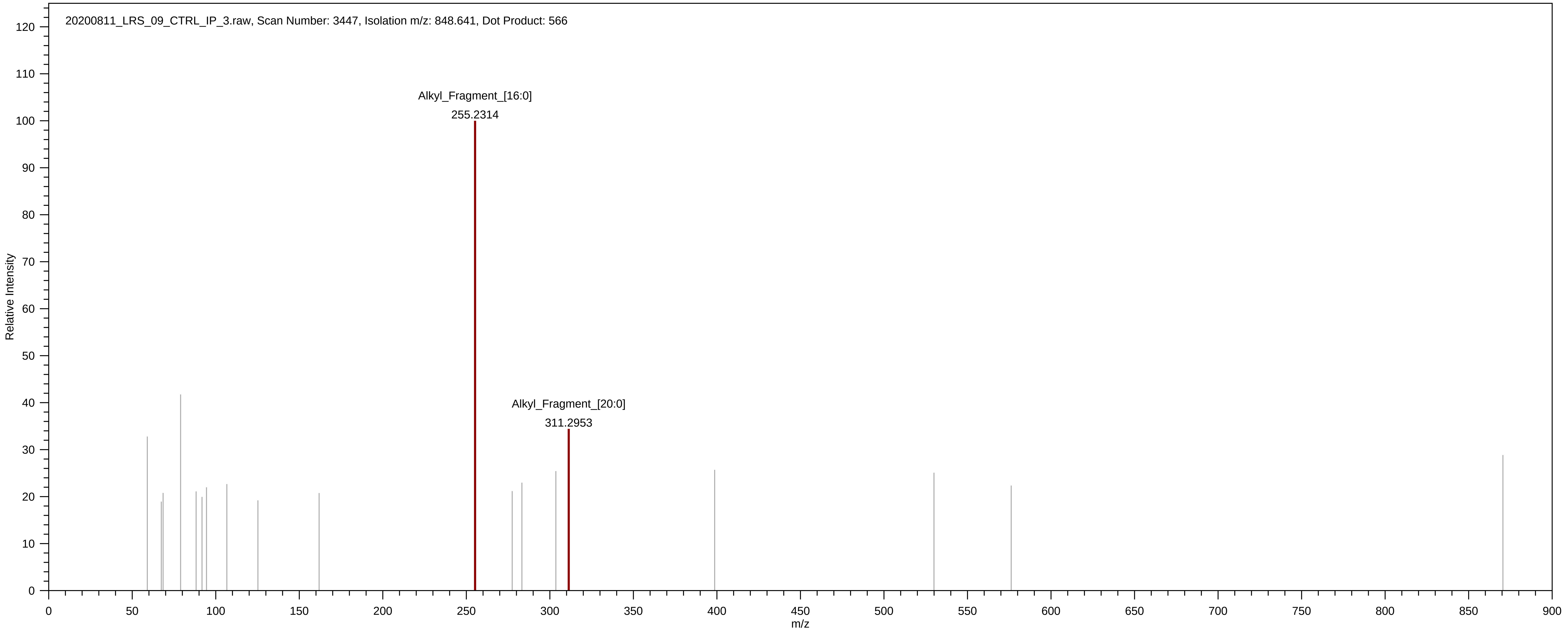

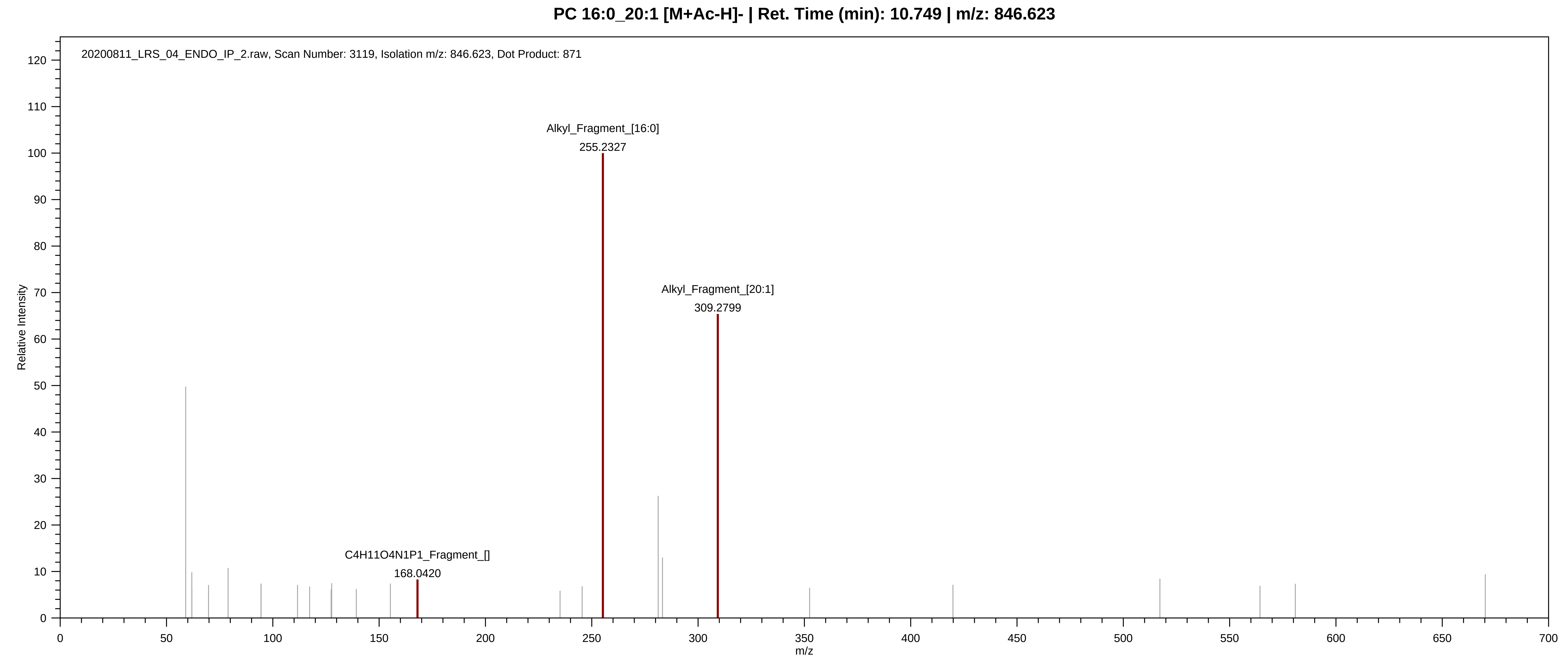

PC 16:0_20:1 [M+Ac-H]- | Ret. Time (min): 10.749 | m/z: 846.623
20200811_LRS_04_ENDO_IP_2.raw, Scan Number: 3119, Isolation m/z: 846.623, Dot Product: 871
Alkyl_Fragment_[16:0]
255.2327
Alkyl_Fragment_[20:1]
309.2799
C4H11O4N1P1_Fragment_[]
168.0420
Relative Intensity
m/z

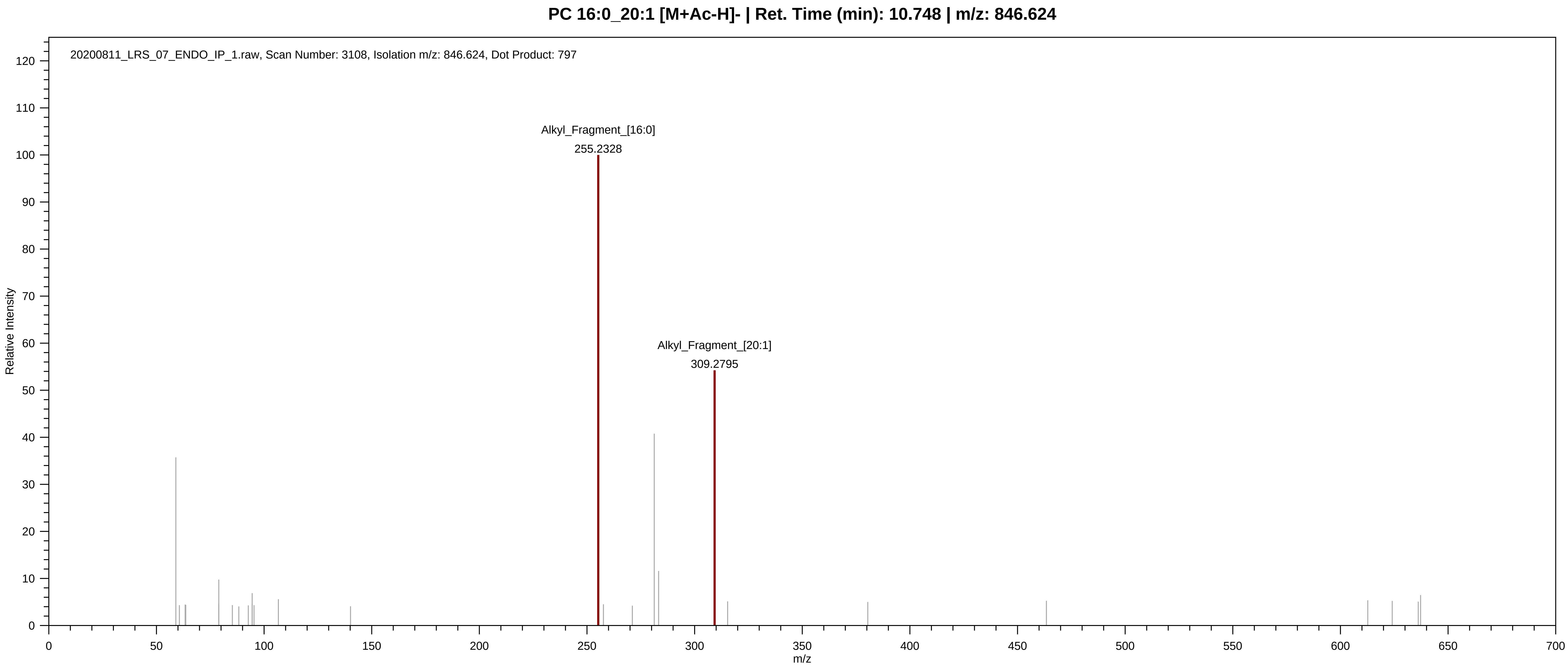

PC 16:0_20:1 [M+Ac-H]- | Ret. Time (min): 10.748 | m/z: 846.624
20200811_LRS_07_ENDO_IP_1.raw, Scan Number: 3108, Isolation m/z: 846.624, Dot Product: 797
Alkyl_Fragment_[16:0]
255.2328
Alkyl_Fragment_[20:1]
309.2795
Relative Intensity
m/z

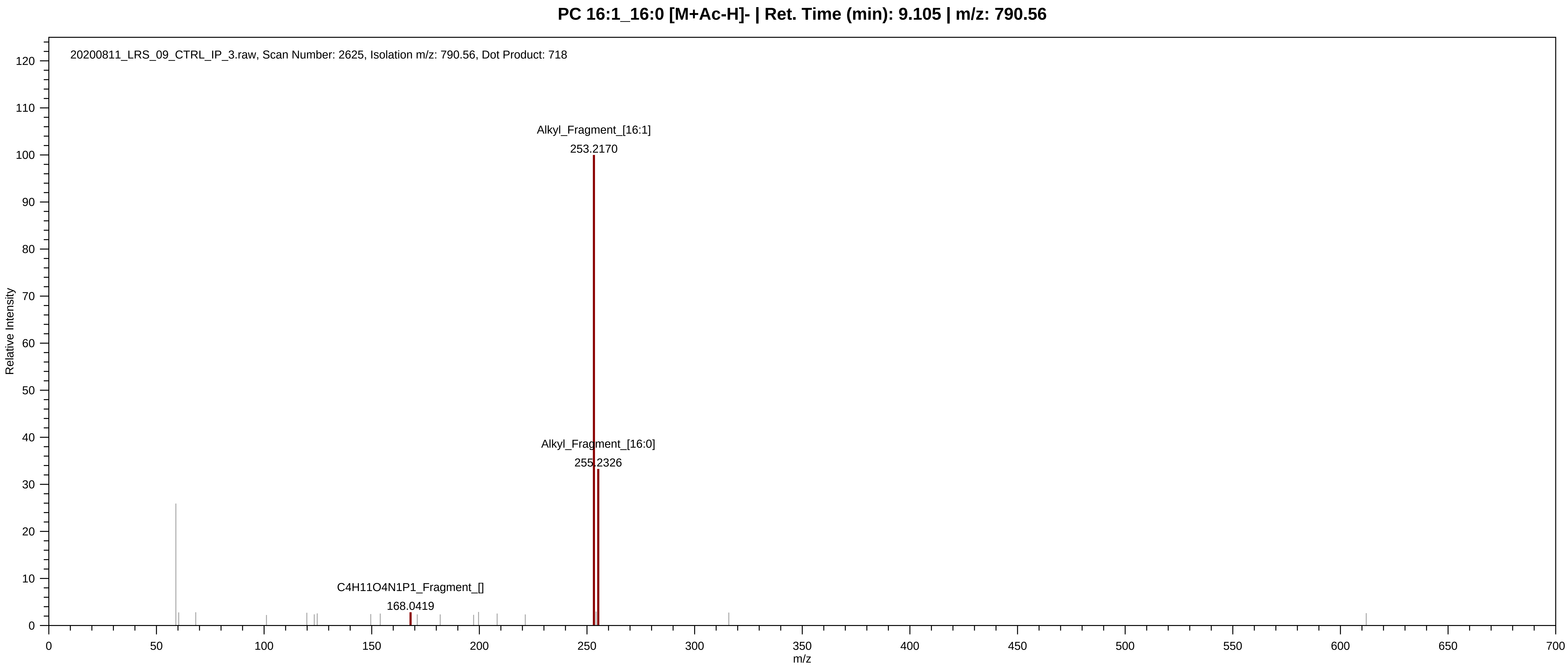
PC 16:1_16:0 [M+Ac-H]- | Ret. Time (min): 9.105 | m/z: 790.56
20200811_LRS_09_CTRL_IP_3.raw, Scan Number: 2625, Isolation m/z: 790.56, Dot Product: 718
Alkyl_Fragment_[16:1]
253.2170
Alkyl_Fragment_[16:0]
255.2326
C4H11O4N1P1_Fragment_[]
168.0419
Relative Intensity
m/z

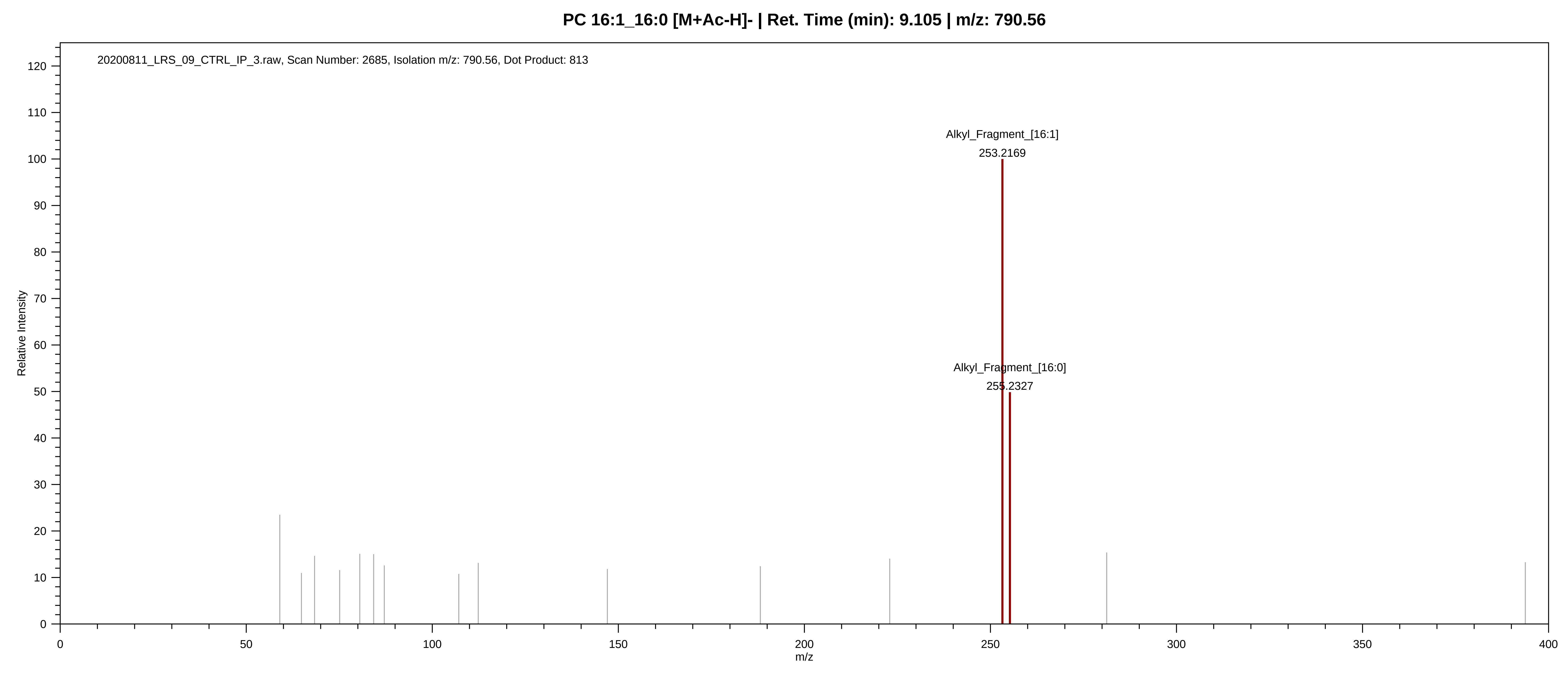

PC 16:1_16:0 [M+Ac-H]- | Ret. Time (min): 9.105 | m/z: 790.56
20200811_LRS_09_CTRL_IP_3.raw, Scan Number: 2685, Isolation m/z: 790.56, Dot Product: 813
Alkyl_Fragment_[16:1]
253.2169
Alkyl_Fragment_[16:0]
255.2327
Relative Intensity
m/z

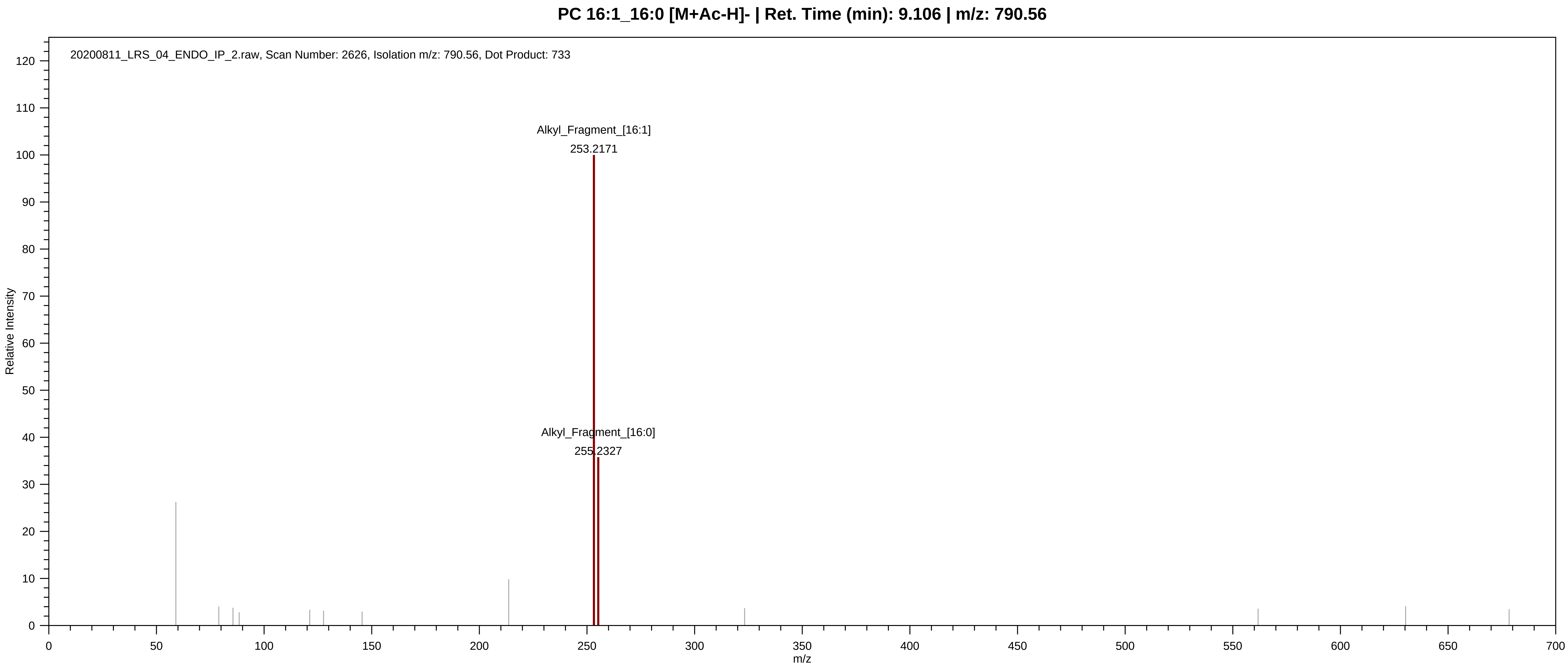

PC 16:1_16:0 [M+Ac-H]- | Ret. Time (min): 9.106 | m/z: 790.56
20200811_LRS_04_ENDO_IP_2.raw, Scan Number: 2626, Isolation m/z: 790.56, Dot Product: 733
Alkyl_Fragment_[16:1]
253.2171
Alkyl_Fragment_[16:0]
255.2327
Relative Intensity
m/z

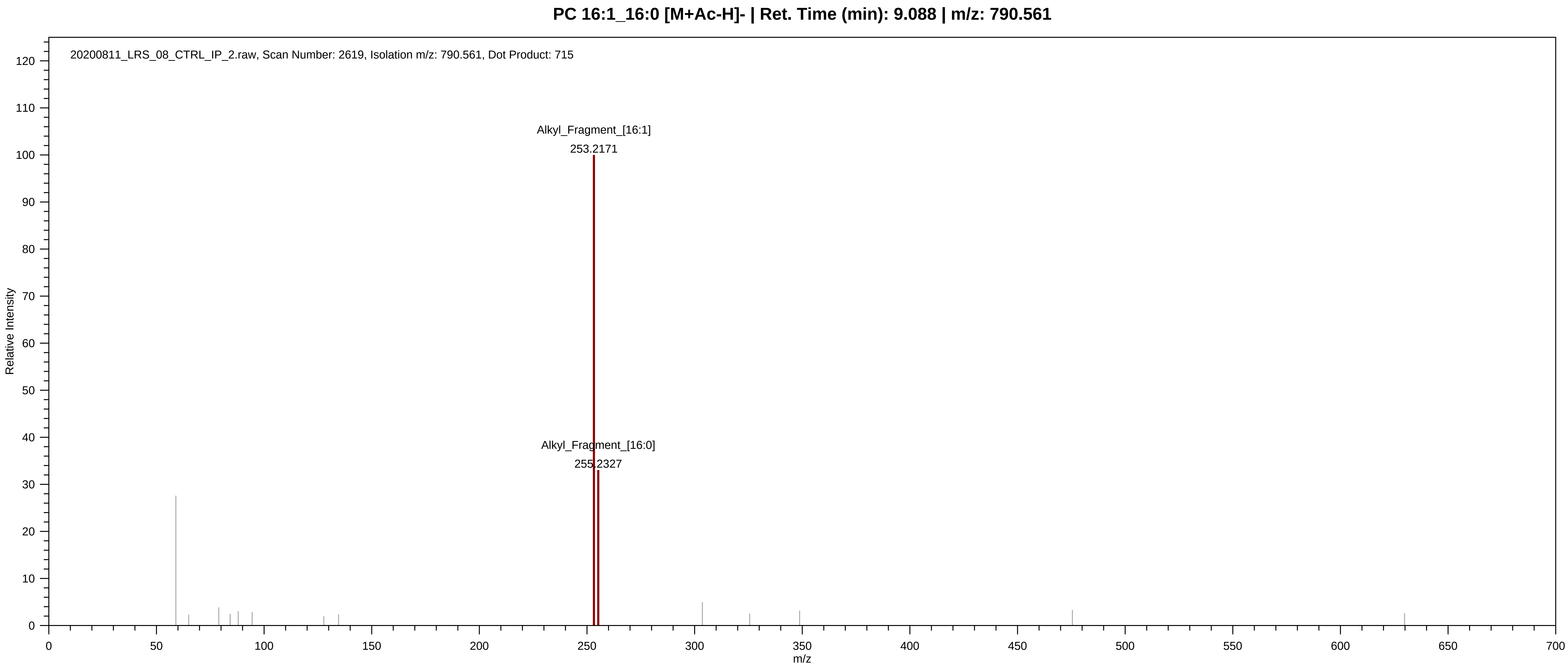

PC 16:1_16:0 [M+Ac-H]- | Ret. Time (min): 9.088 | m/z: 790.561
20200811_LRS_08_CTRL_IP_2.raw, Scan Number: 2619, Isolation m/z: 790.561, Dot Product: 715
Alkyl_Fragment_[16:1]
253.2171
Alkyl_Fragment_[16:0]
255.2327
Relative Intensity
m/z

PC 16:1_16:0 [M+Ac-H]- | Ret. Time (min): 9.088 | m/z: 790.56

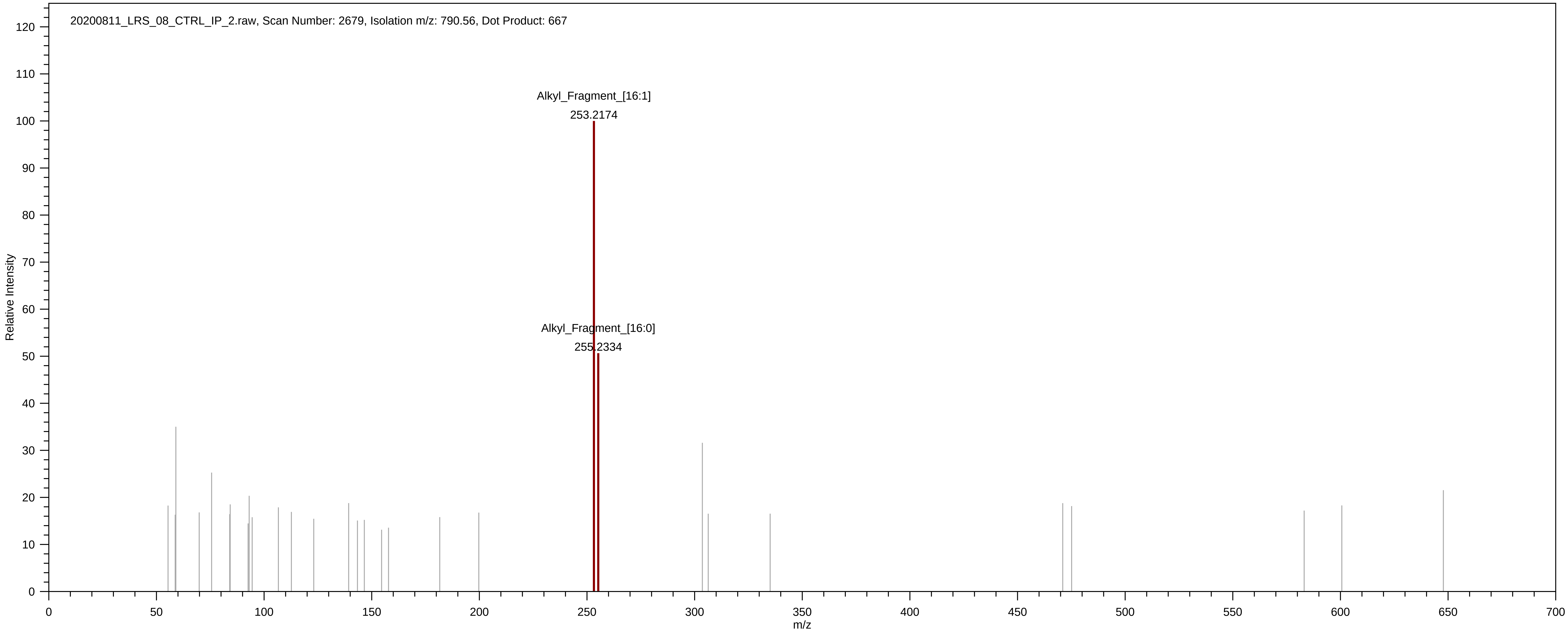

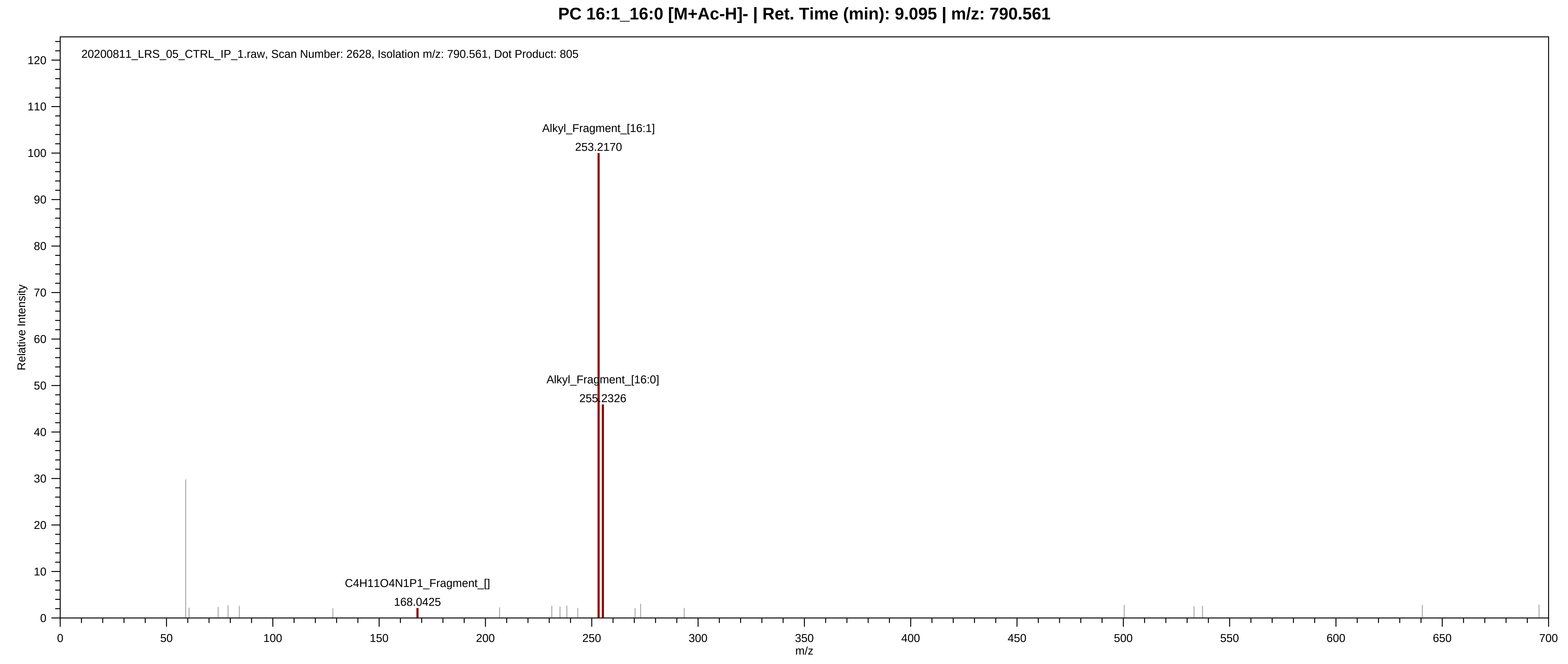
PC 16:1_16:0 [M+Ac-H]- | Ret. Time (min): 9.095 | m/z: 790.561
20200811_LRS_05_CTRL_IP_1.raw, Scan Number: 2628, Isolation m/z: 790.561, Dot Product: 805
Alkyl_Fragment_[16:1]
253.2170
Alkyl_Fragment_[16:0]
255.2326
C4H11O4N1P1_Fragment_[]
168.0425
Relative Intensity
m/z

PC 16:1_16:0 [M+Ac-H]- | Ret. Time (min): 9.095 | m/z: 790.56

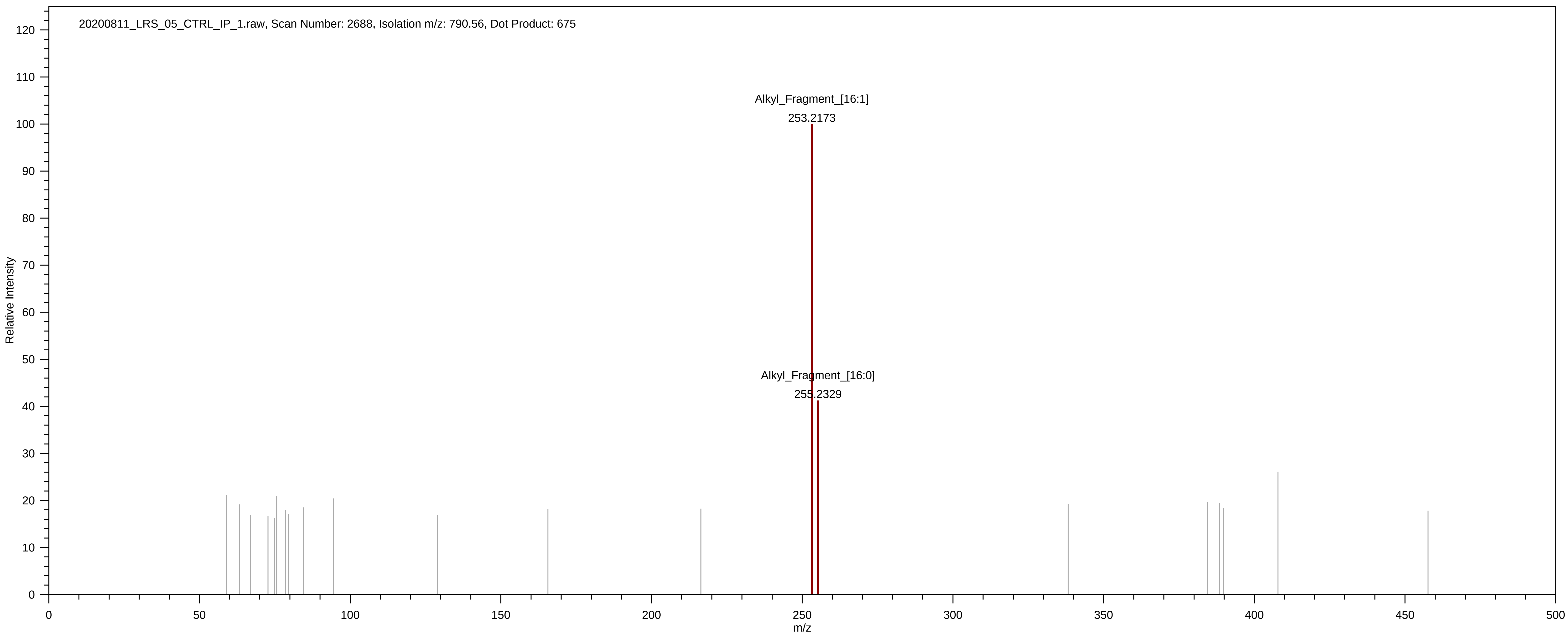

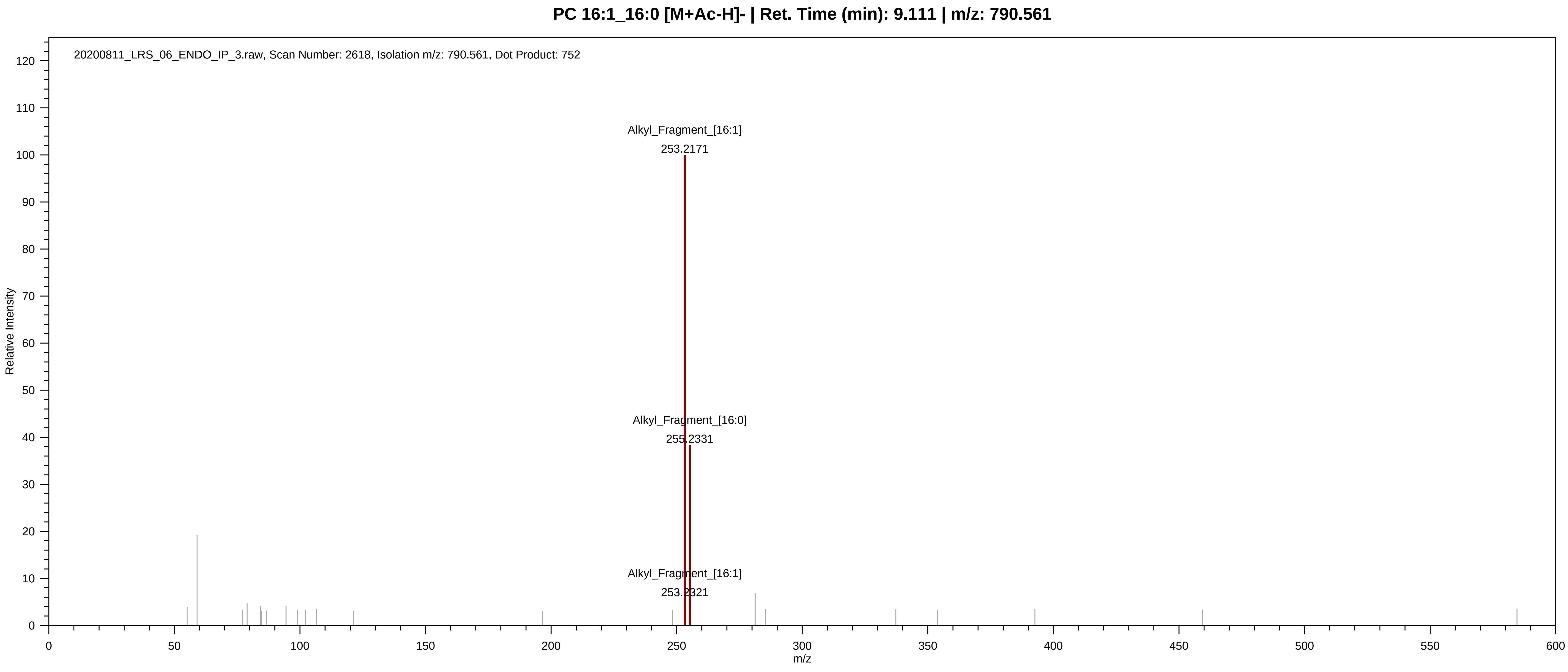

PC 16:1_16:0 [M+Ac-H]- | Ret. Time (min): 9.111 | m/z: 790.561
20200811_LRS_06_ENDO_IP_3.raw, Scan Number: 2618, Isolation m/z: 790.561, Dot Product: 752
Alkyl_Fragment_[16:1]
253.2171
Alkyl_Fragment_[16:0]
255.2331
Alkyl_Fragment_[16:1]
253.2321
Relative Intensity
m/z
0
10
20
30
40
50
60
70
80
90
100
110
120
0
50
100
150
200
250
300
350
400
450
500
550
600

PC 16:1_16:0 [M+Ac-H]- | Ret. Time (min): 9.112 | m/z: 790.561

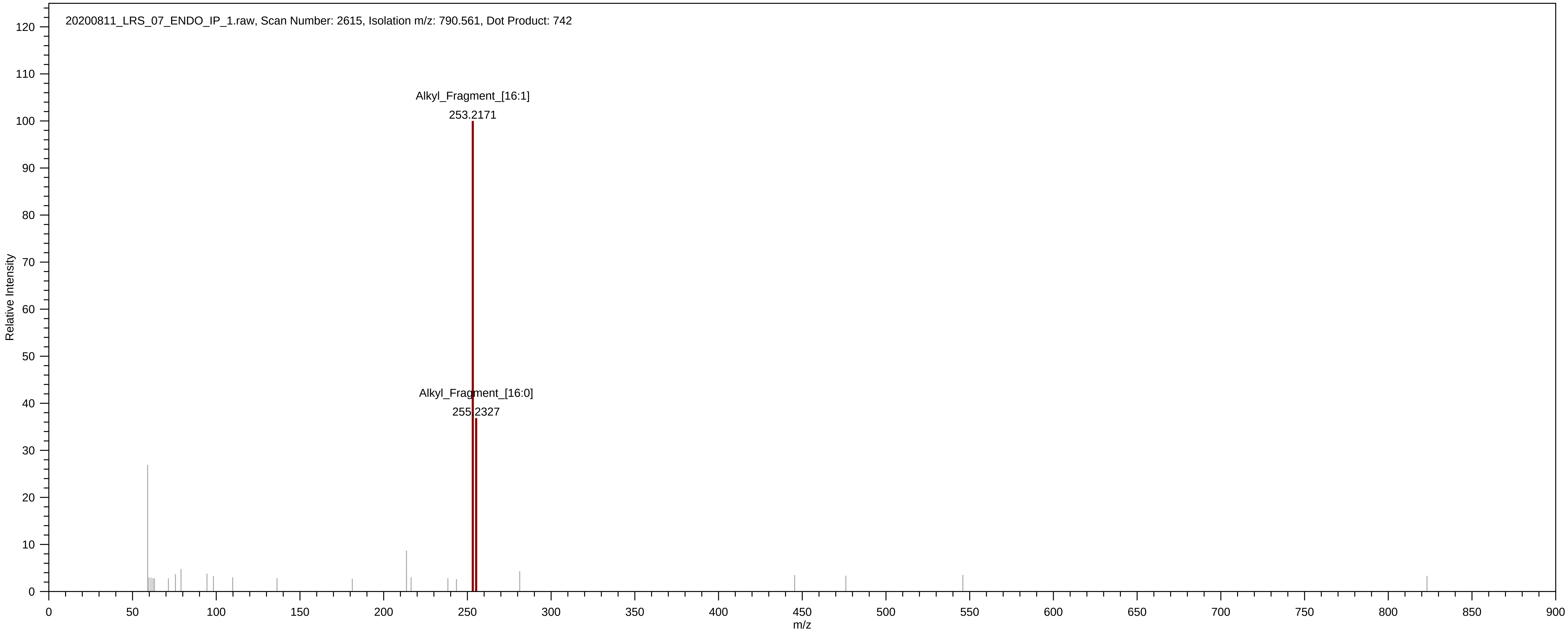

PC 16:1_16:1 [M+Ac-H]- | Ret. Time (min): 8.202 | m/z: 788.544

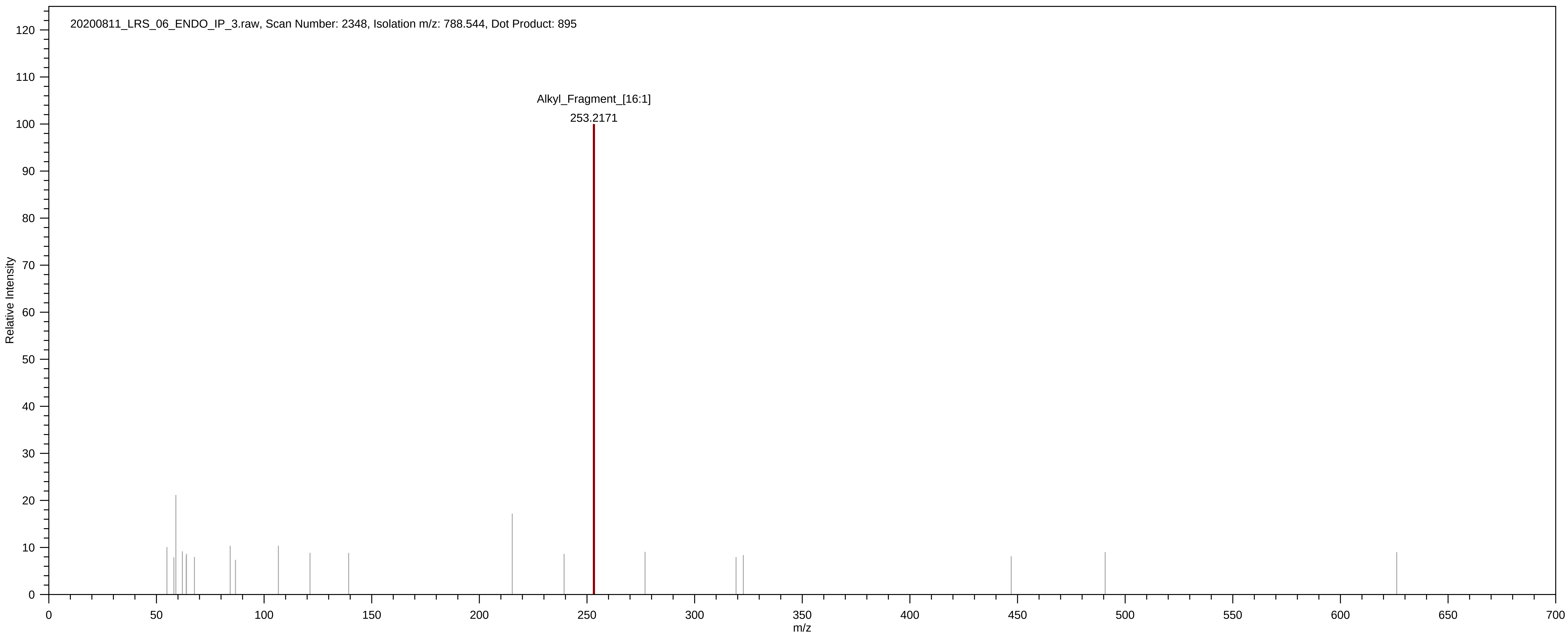

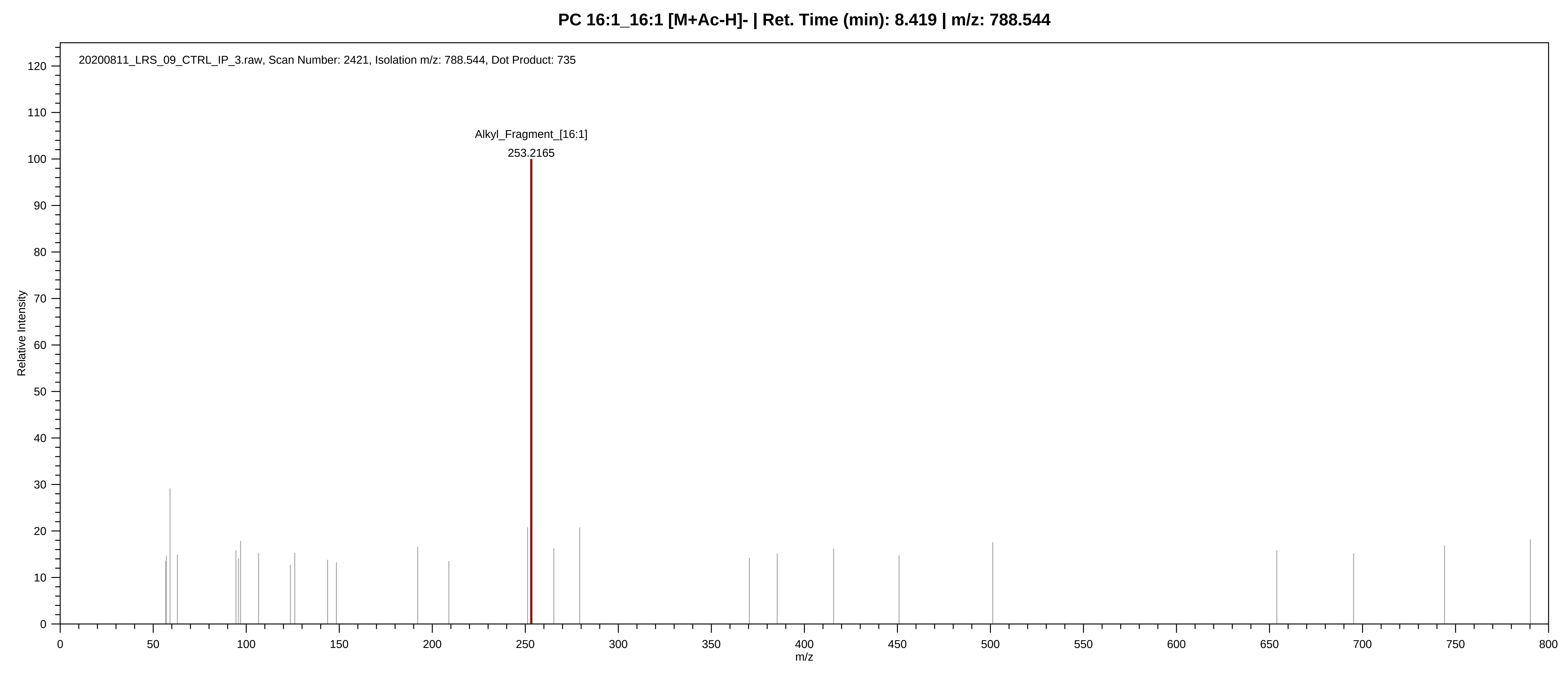

PC 16:1_16:1 [M+Ac-H]- | Ret. Time (min): 8.419 | m/z: 788.544
20200811_LRS_09_CTRL_IP_3.raw, Scan Number: 2421, Isolation m/z: 788.544, Dot Product: 735
Alkyl_Fragment_[16:1]
253.2165
Relative Intensity
m/z

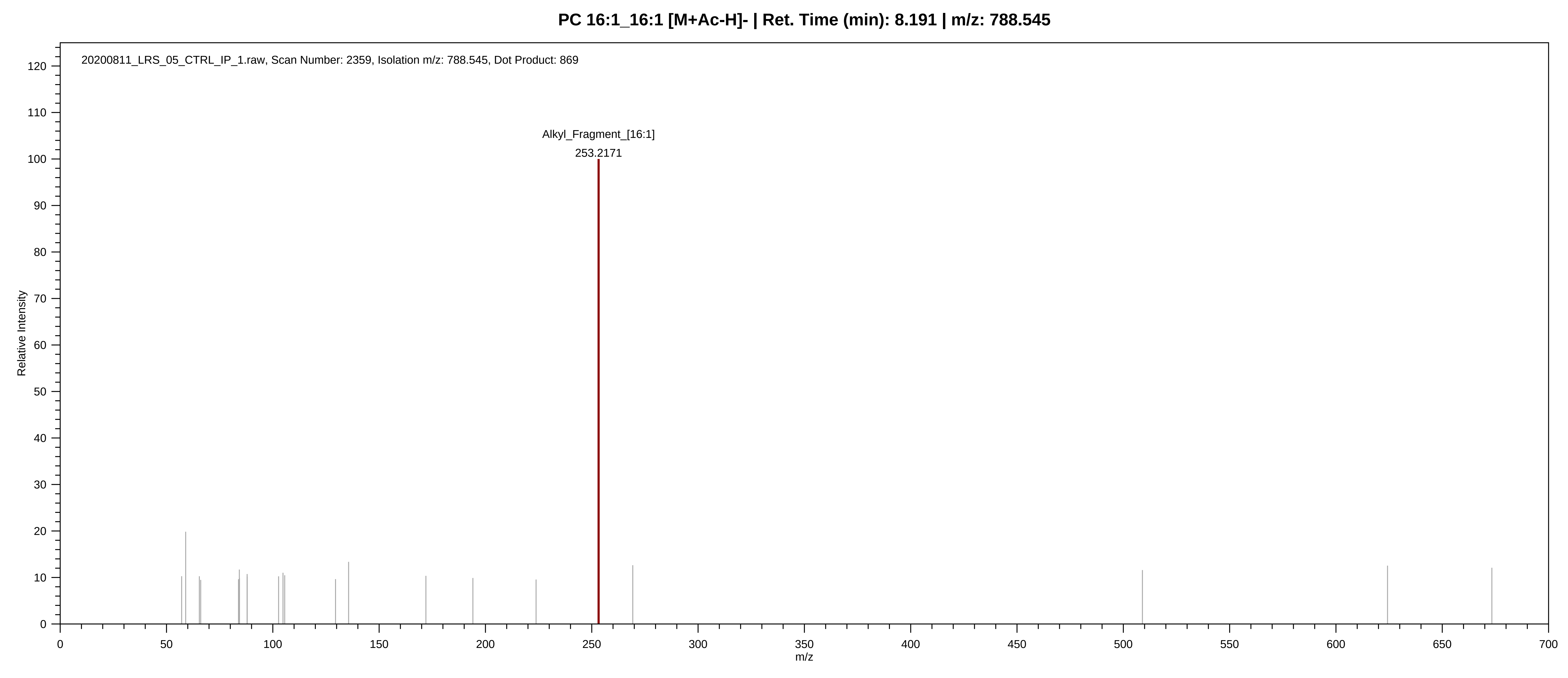

PC 16:1_16:1 [M+Ac-H]- | Ret. Time (min): 8.191 | m/z: 788.545
20200811_LRS_05_CTRL_IP_1.raw, Scan Number: 2359, Isolation m/z: 788.545, Dot Product: 869
Alkyl_Fragment_[16:1]
253.2171
Relative Intensity
m/z

PC 16:1_16:1 [M+Ac-H]- | Ret. Time (min): 8.197 | m/z: 788.545

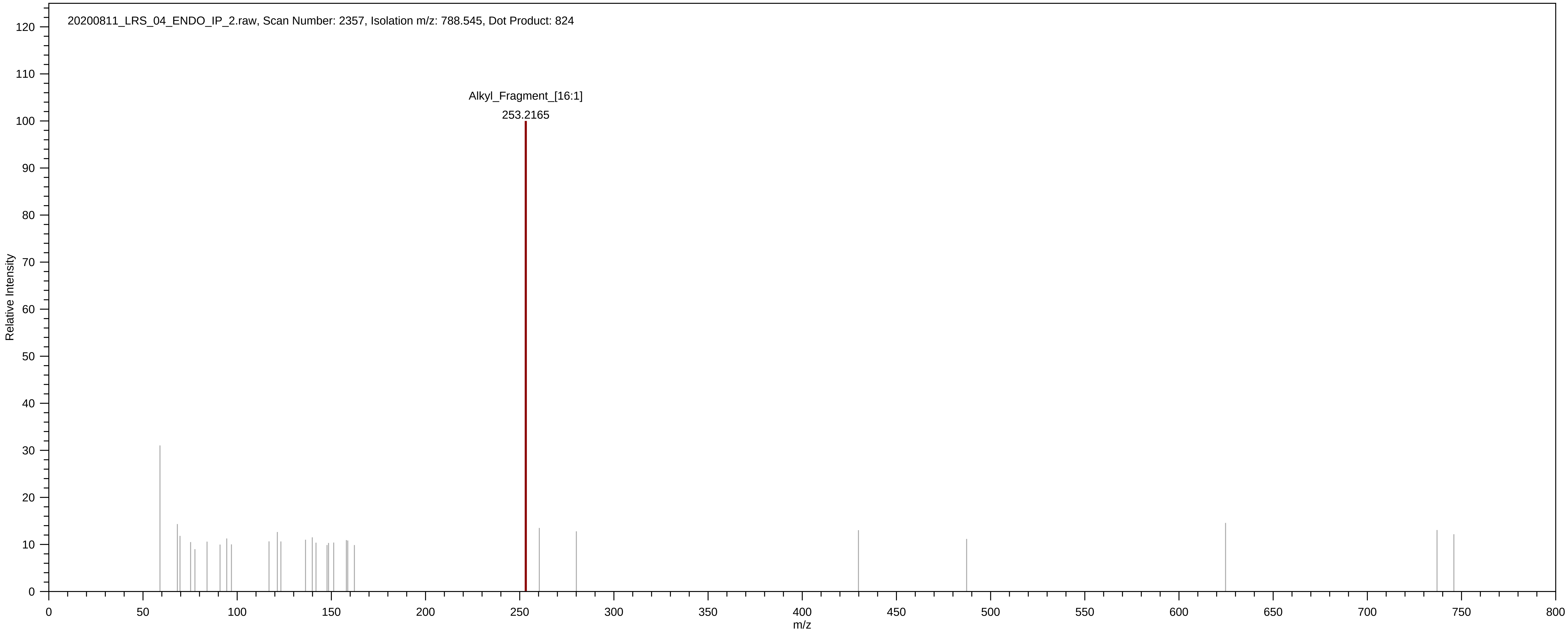

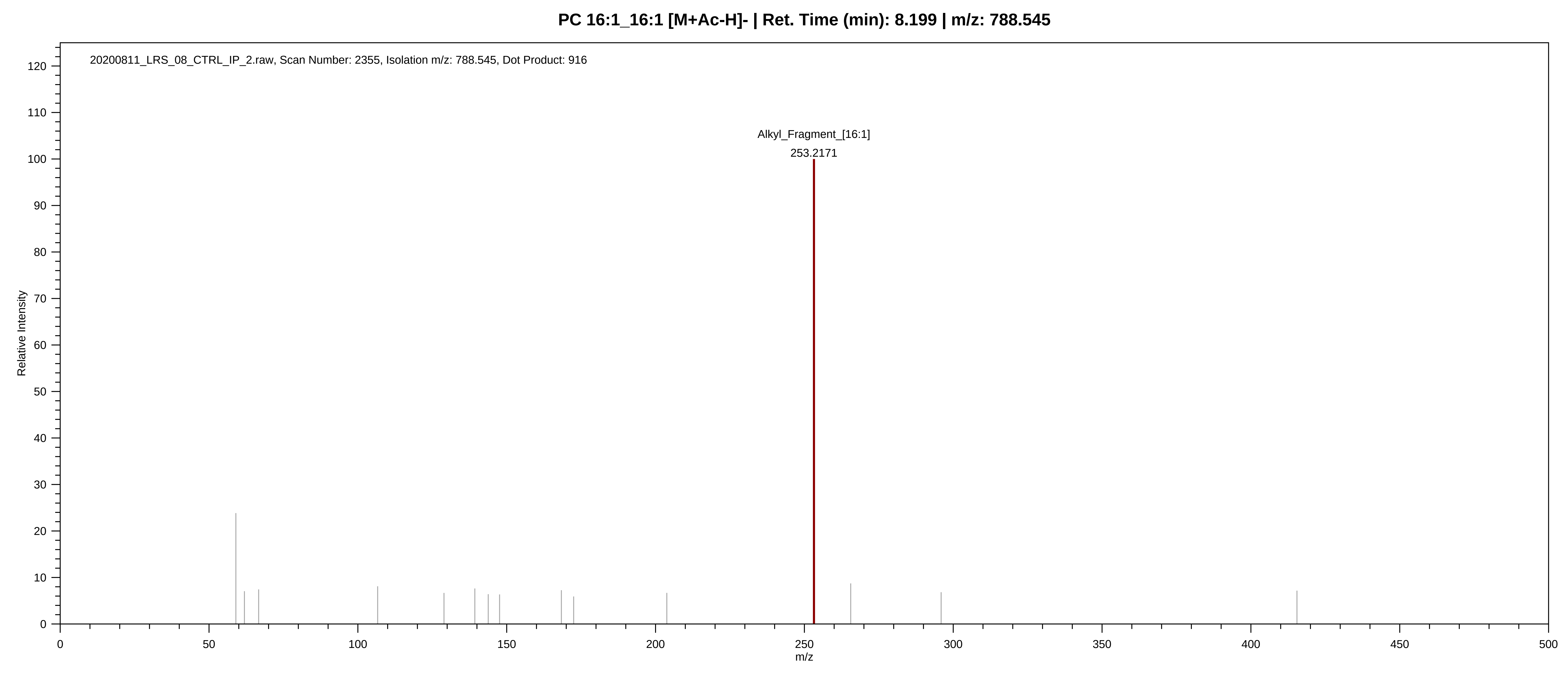

PC 16:1_16:1 [M+Ac-H]- | Ret. Time (min): 8.199 | m/z: 788.545
20200811_LRS_08_CTRL_IP_2.raw, Scan Number: 2355, Isolation m/z: 788.545, Dot Product: 916
Alkyl_Fragment_[16:1]
253.2171
Relative Intensity
m/z

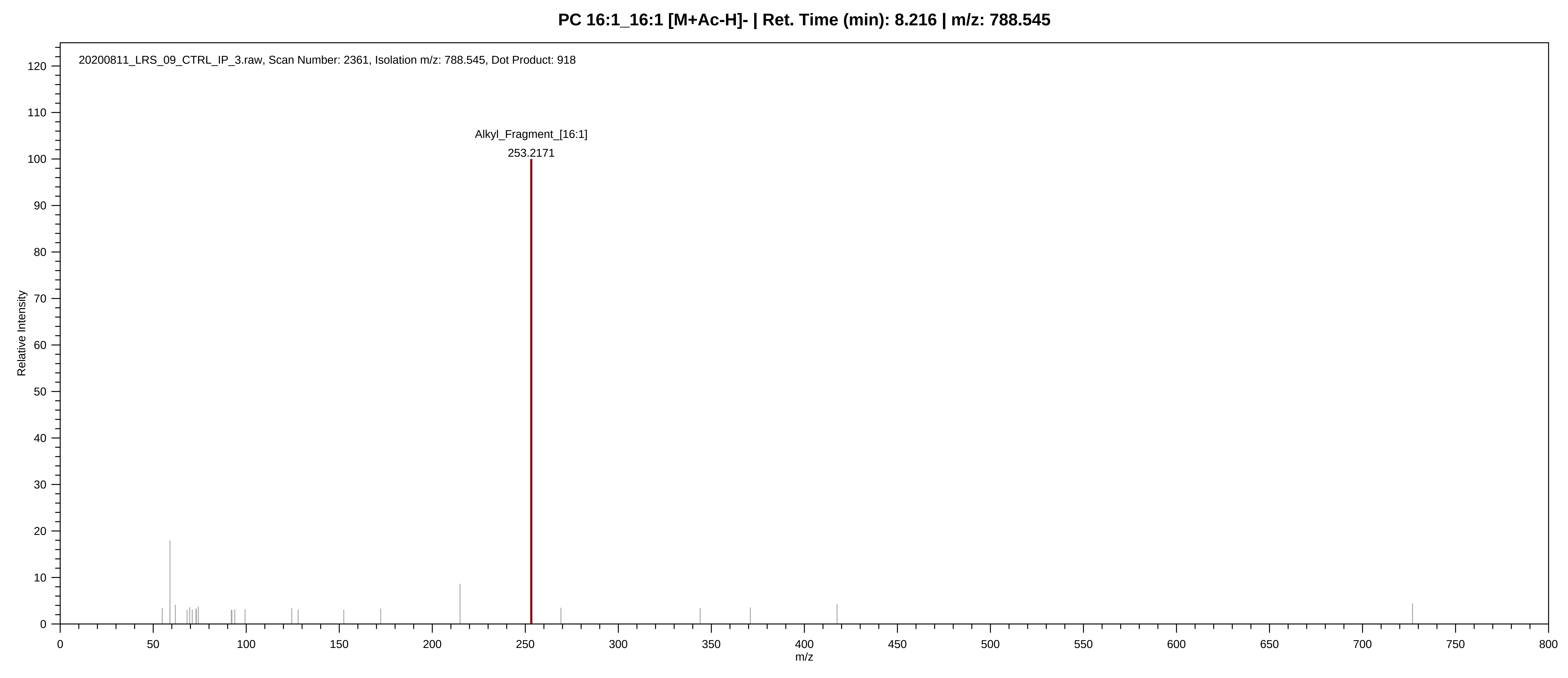
PC 16:1_16:1 [M+Ac-H]- | Ret. Time (min): 8.216 | m/z: 788.545
20200811_LRS_09_CTRL_IP_3.raw, Scan Number: 2361, Isolation m/z: 788.545, Dot Product: 918
Alkyl_Fragment_[16:1]
253.2171
Relative Intensity
m/z

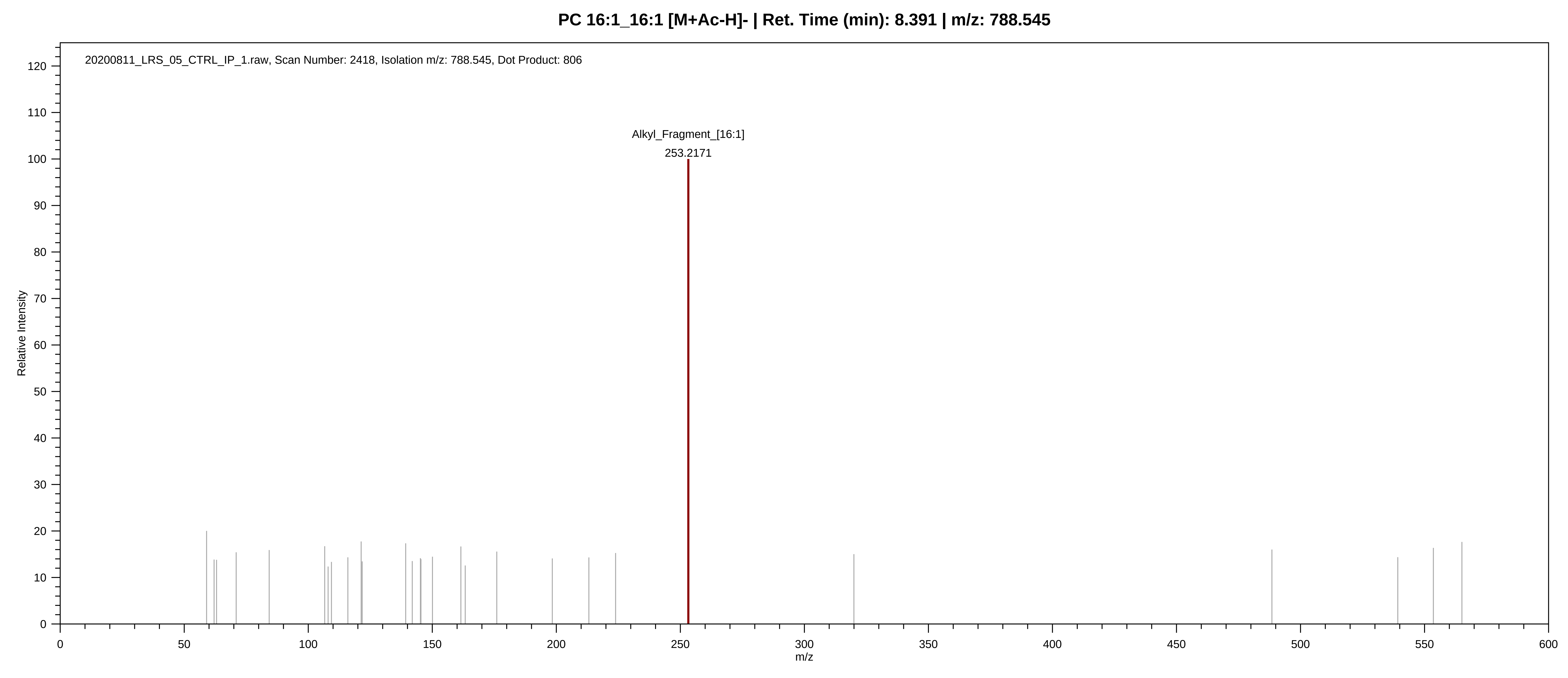

PC 16:1_16:1 [M+Ac-H]- | Ret. Time (min): 8.391 | m/z: 788.545
20200811_LRS_05_CTRL_IP_1.raw, Scan Number: 2418, Isolation m/z: 788.545, Dot Product: 806
Alkyl_Fragment_[16:1]
253.2171
Relative Intensity
m/z

PC 16:1_16:1 [M+Ac-H]- | Ret. Time (min): 8.203 | m/z: 788.546

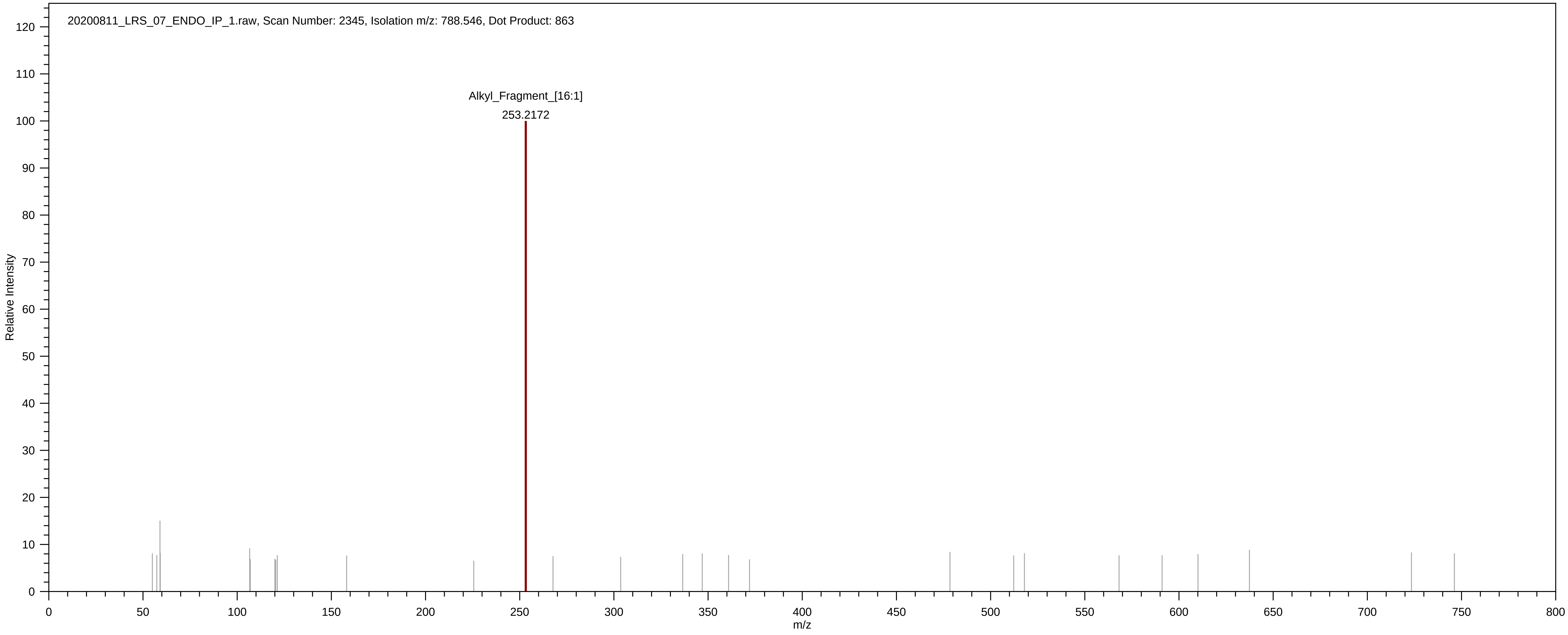

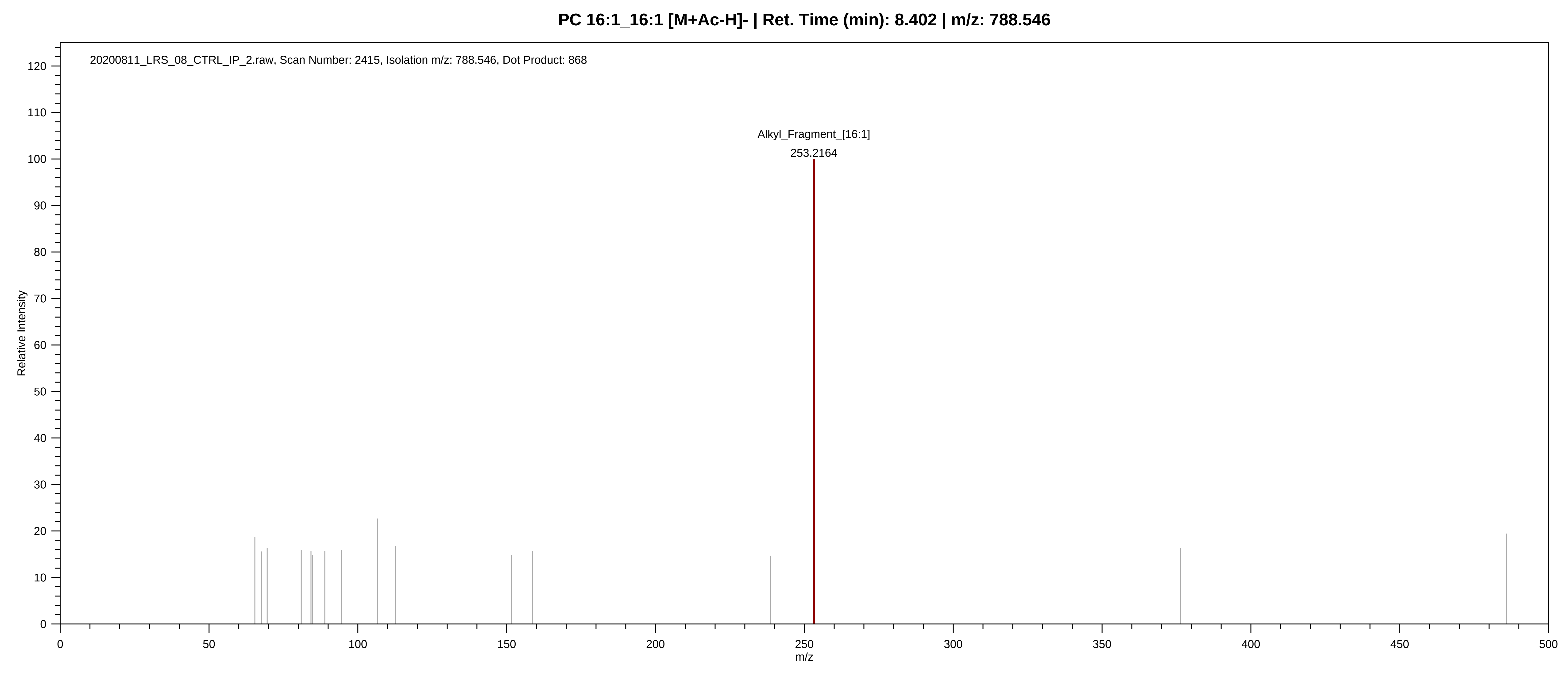

PC 16:1_16:1 [M+Ac-H]- | Ret. Time (min): 8.402 | m/z: 788.546
20200811_LRS_08_CTRL_IP_2.raw, Scan Number: 2415, Isolation m/z: 788.546, Dot Product: 868
Alkyl_Fragment_[16:1]
253.2164
Relative Intensity
m/z

PC 16:1_17:1 [M+Ac-H]- | Ret. Time (min): 8.607 | m/z: 802.56

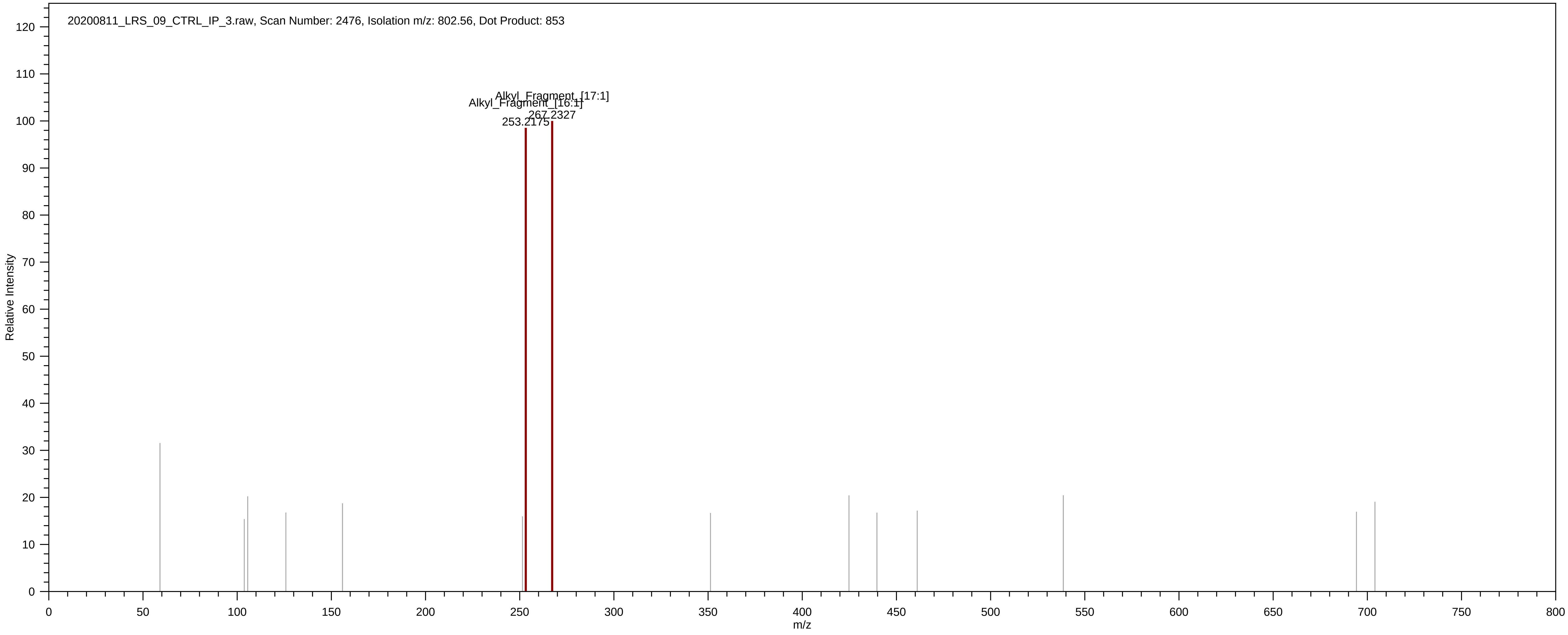

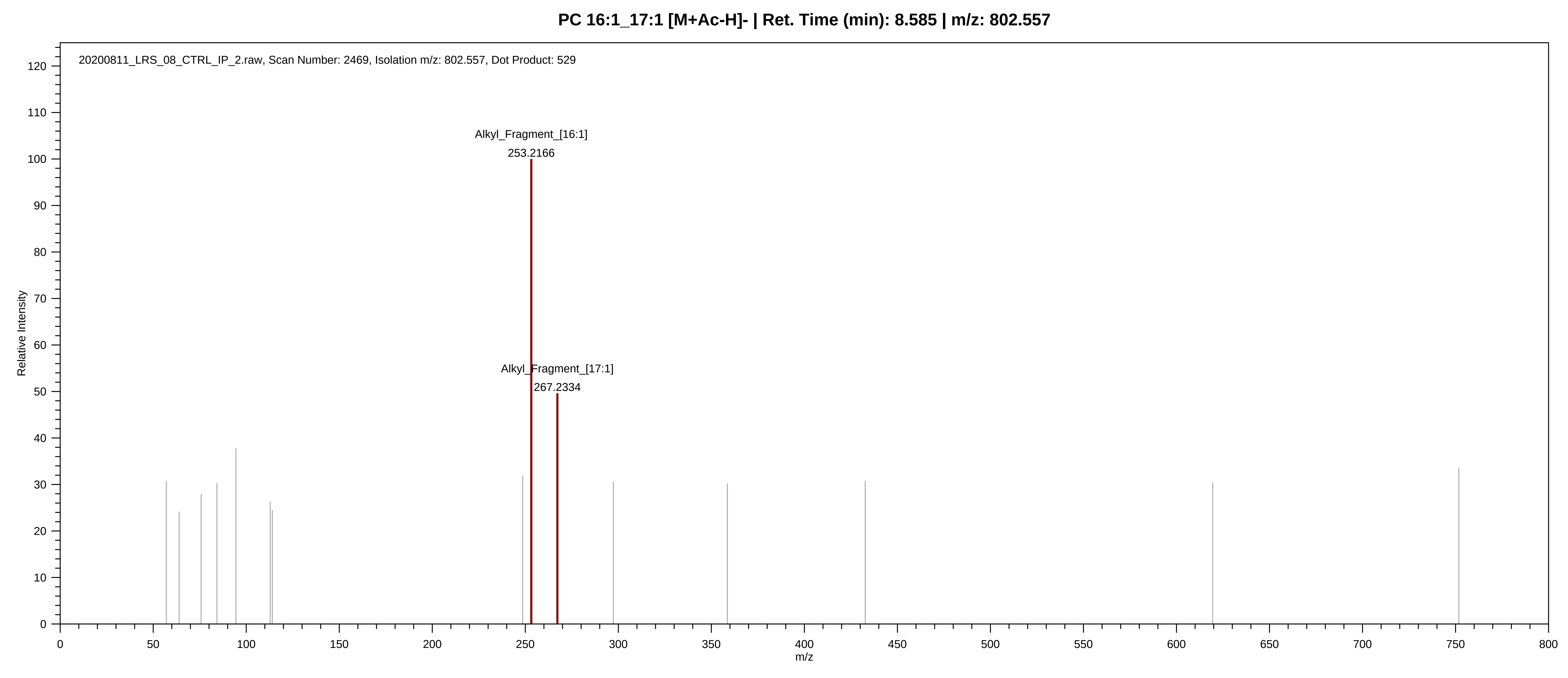
PC 16:1_17:1 [M+Ac-H]- | Ret. Time (min): 8.585 | m/z: 802.557
20200811_LRS_08_CTRL_IP_2.raw, Scan Number: 2469, Isolation m/z: 802.557, Dot Product: 529
Alkyl_Fragment_[16:1]
253.2166
Alkyl_Fragment_[17:1]
267.2334
Relative Intensity
m/z

PC 16:1_17:1 [M+Ac-H]- | Ret. Time (min): 8.635 | m/z: 802.561

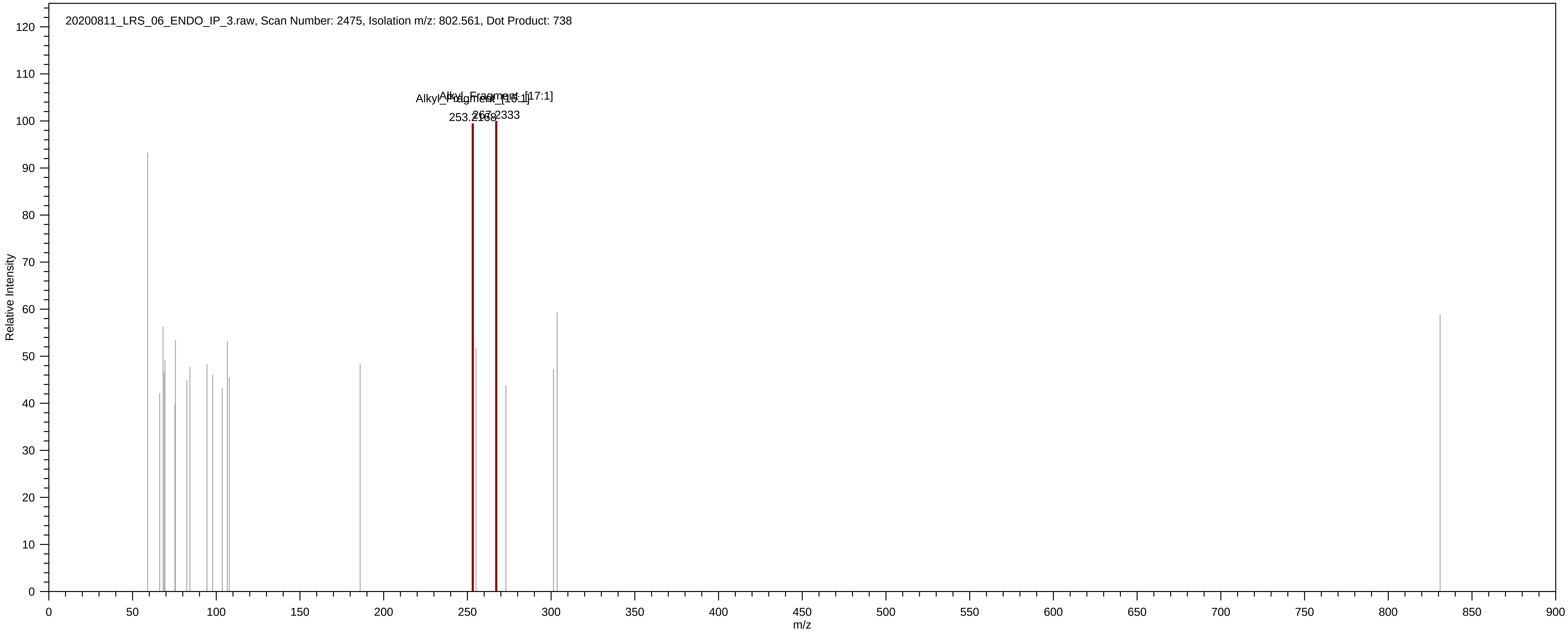

PC 16:1_17:1 [M+Ac-H]- | Ret. Time (min): 8.614 | m/z: 802.562

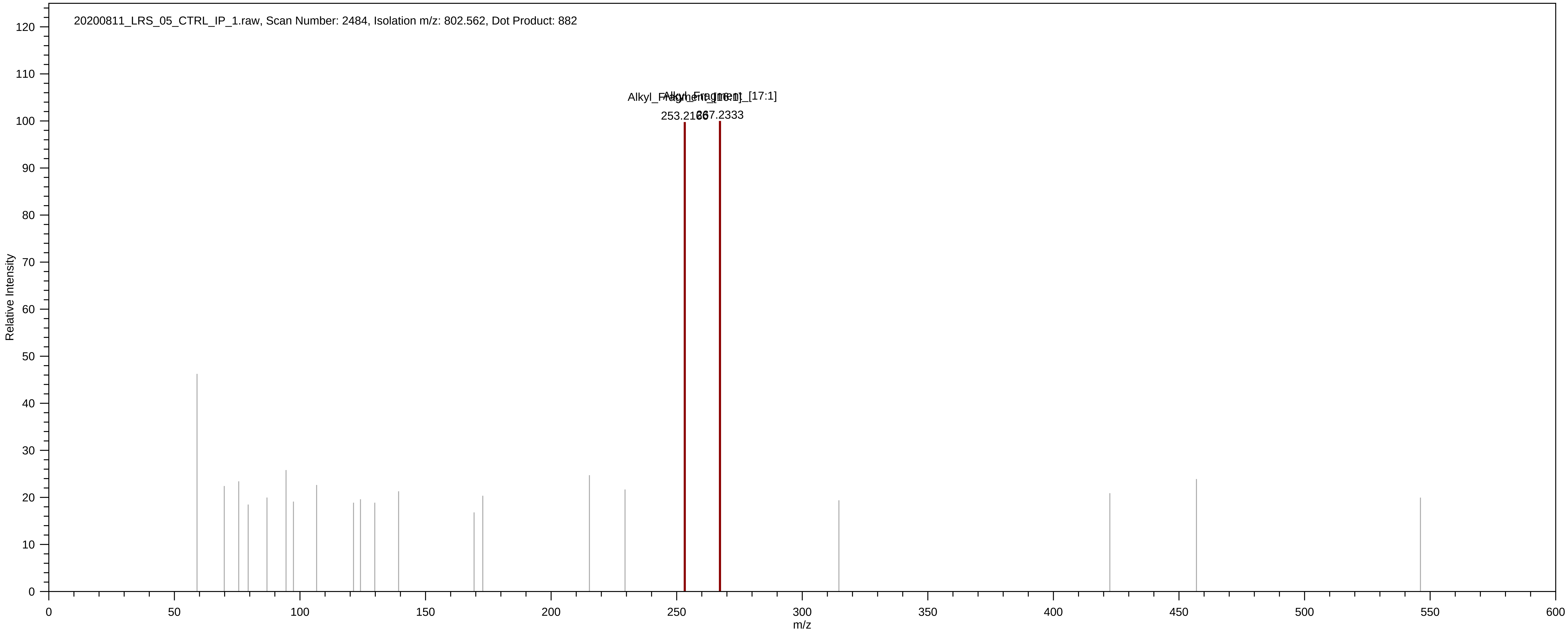

PC 16:1_18:1 [M+Ac-H]- | Ret. Time (min): 8.968 | m/z: 816.58

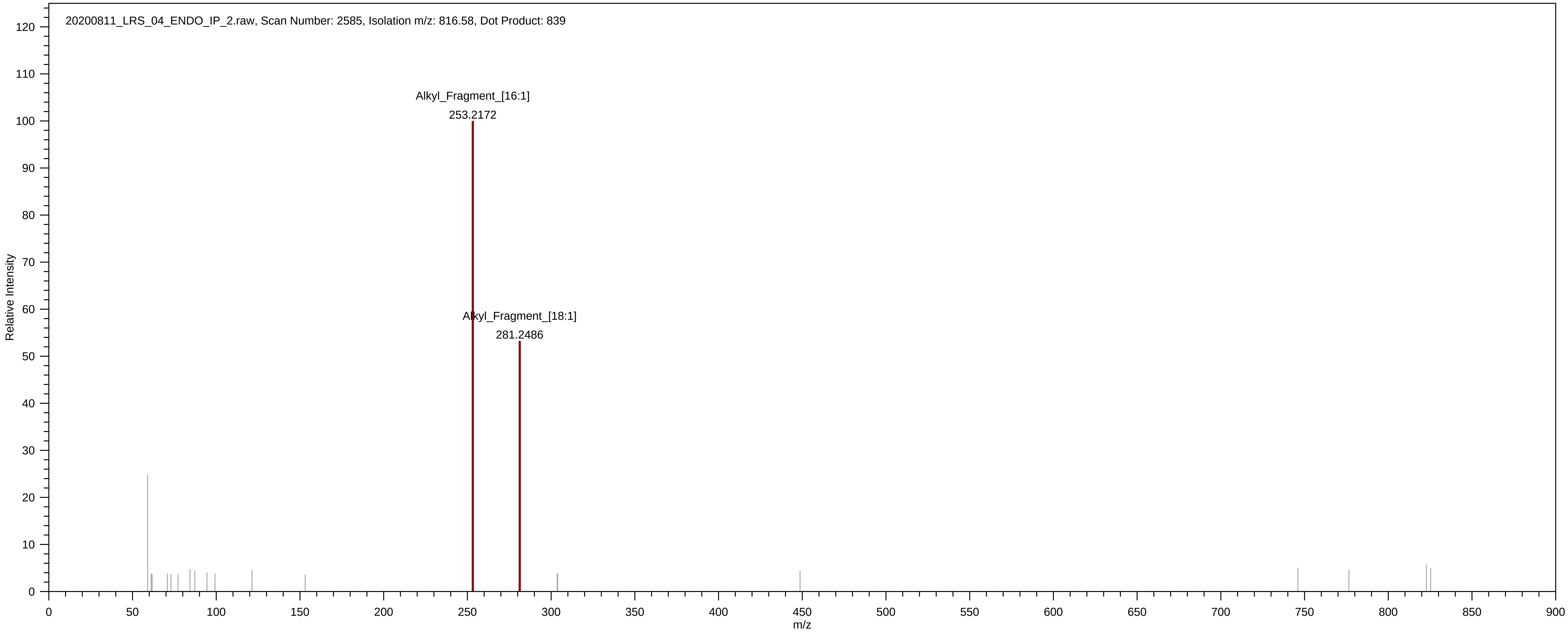

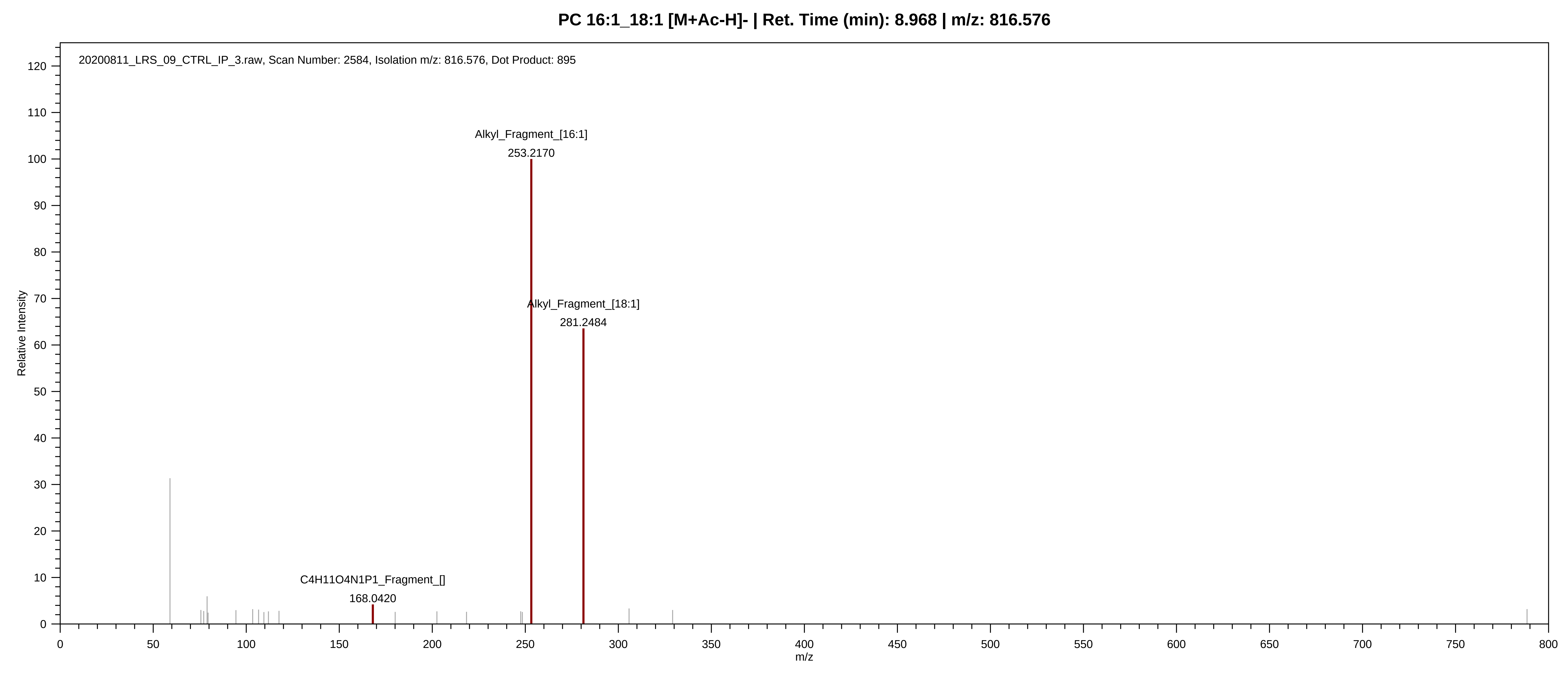
PC 16:1_18:1 [M+Ac-H]- | Ret. Time (min): 8.968 | m/z: 816.576
20200811_LRS_09_CTRL_IP_3.raw, Scan Number: 2584, Isolation m/z: 816.576, Dot Product: 895
Alkyl_Fragment_[16:1]
253.2170
Alkyl_Fragment_[18:1]
281.2484
C4H11O4N1P1_Fragment_[]
168.0420
Relative Intensity
m/z

PC 16:1_18:1 [M+Ac-H]- | Ret. Time (min): 8.953 | m/z: 816.577

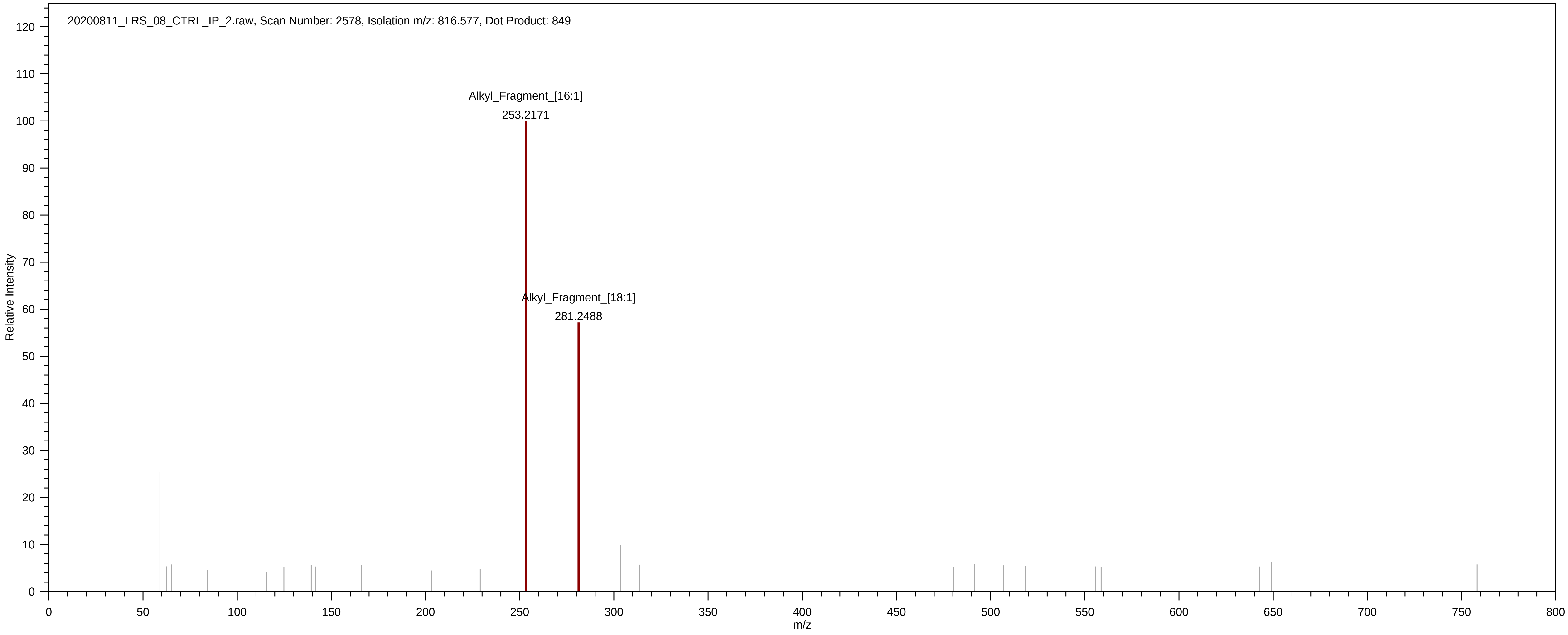

PC 16:1_18:1 [M+Ac-H]- | Ret. Time (min): 8.972 | m/z: 816.577

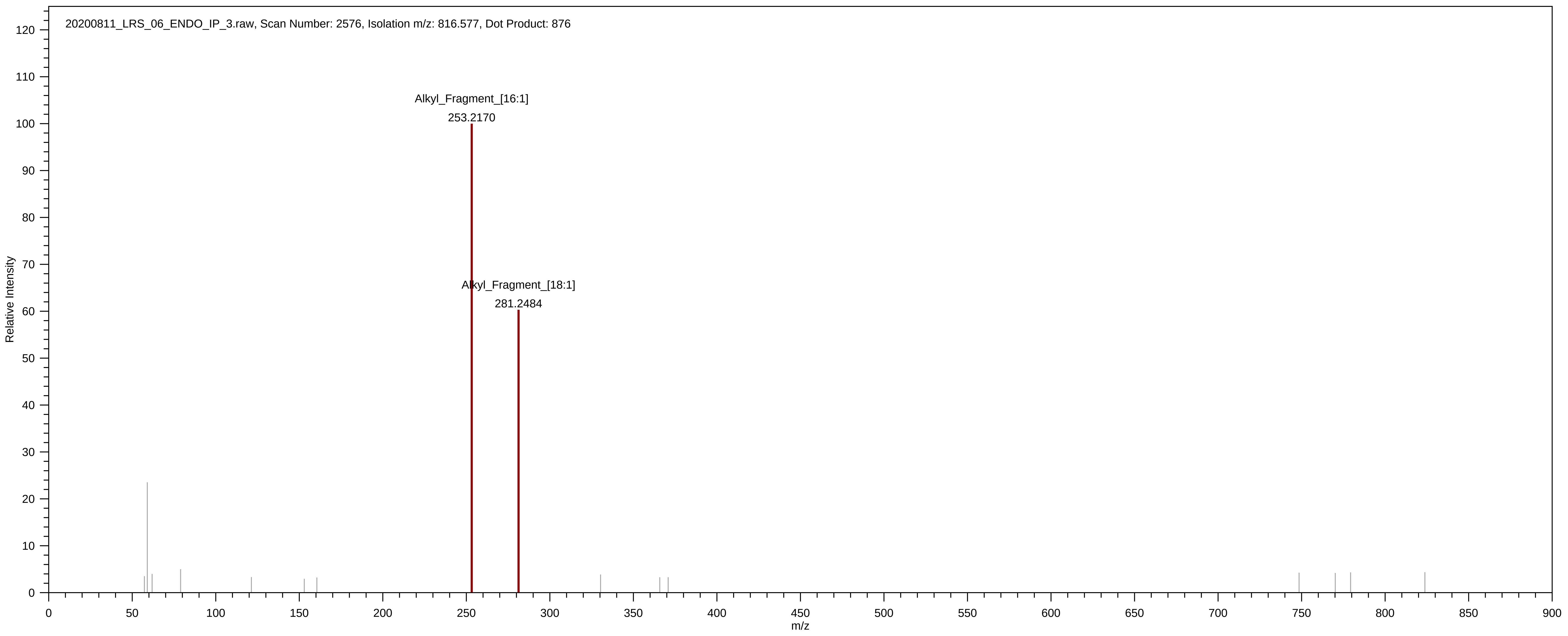

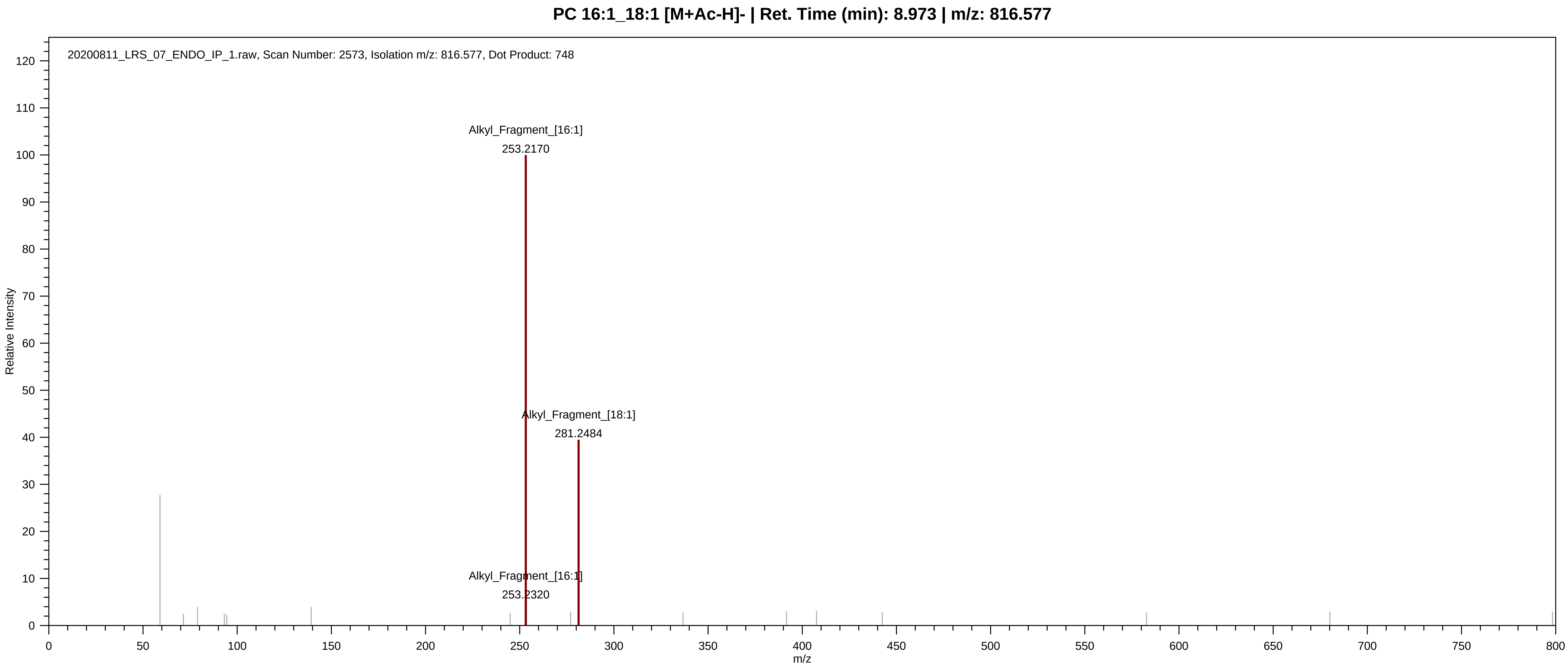

PC 16:1_18:1 [M+Ac-H]- | Ret. Time (min): 8.973 | m/z: 816.577
20200811_LRS_07_ENDO_IP_1.raw, Scan Number: 2573, Isolation m/z: 816.577, Dot Product: 748
Alkyl_Fragment_[16:1]
253.2170
Alkyl_Fragment_[18:1]
281.2484
Alkyl_Fragment_[16:1]
253.2320
Relative Intensity
m/z

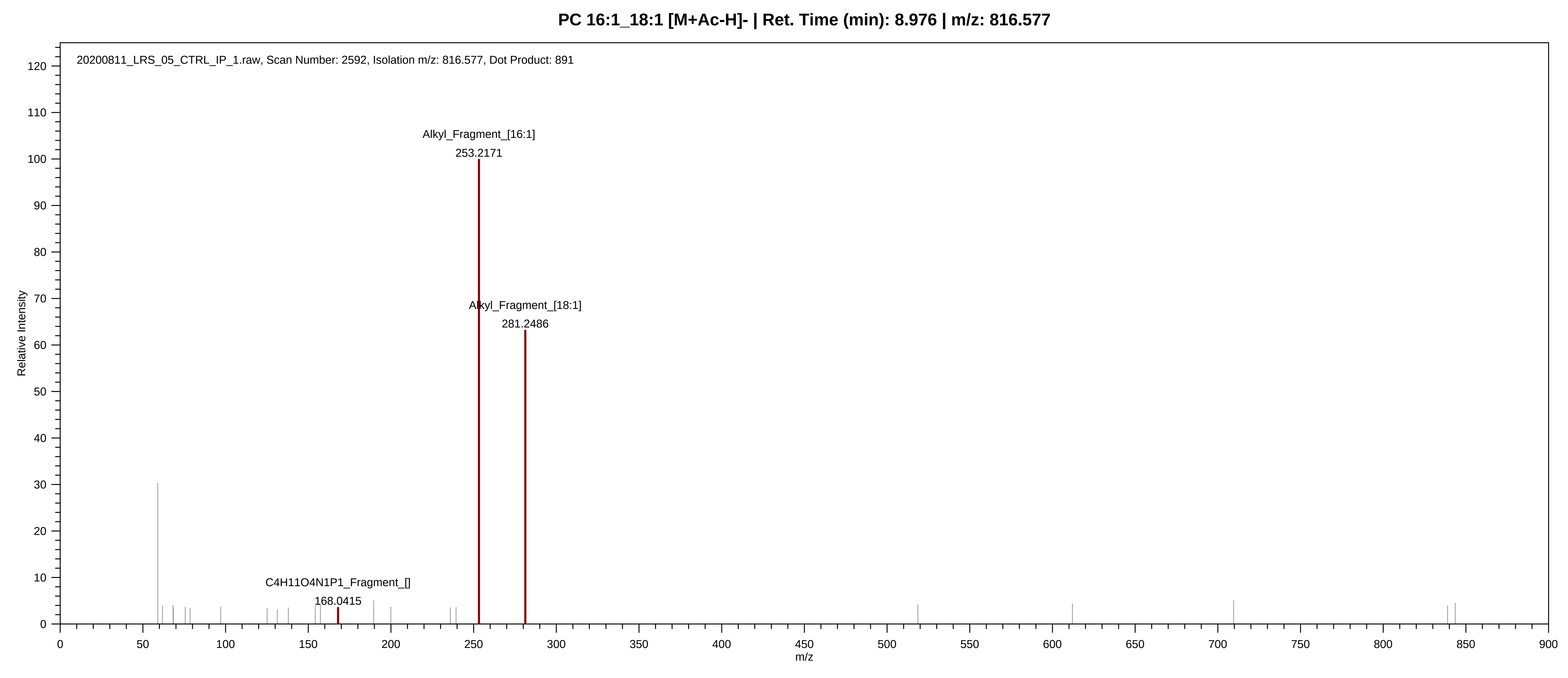

PC 16:1_18:1 [M+Ac-H]- | Ret. Time (min): 8.976 | m/z: 816.577
20200811_LRS_05_CTRL_IP_1.raw, Scan Number: 2592, Isolation m/z: 816.577, Dot Product: 891
Alkyl_Fragment_[16:1]
253.2171
Alkyl_Fragment_[18:1]
281.2486
C4H11O4N1P1_Fragment_[]
168.0415
Relative Intensity
m/z

PC 17:0_18:0 [M+Ac-H]- | Ret. Time (min): 11.085 | m/z: 834.627

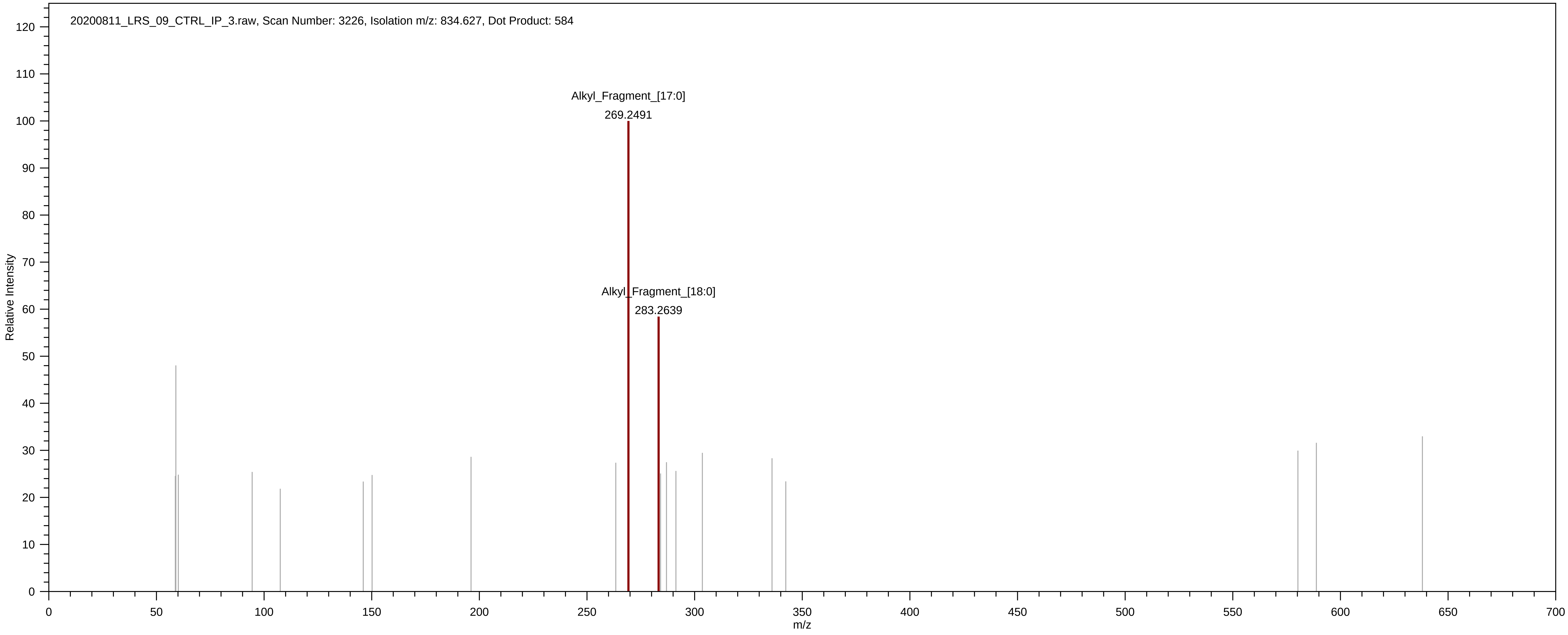

PC 17:0_18:1 [M+Ac-H]- | Ret. Time (min): 10.131 | m/z: 832.607

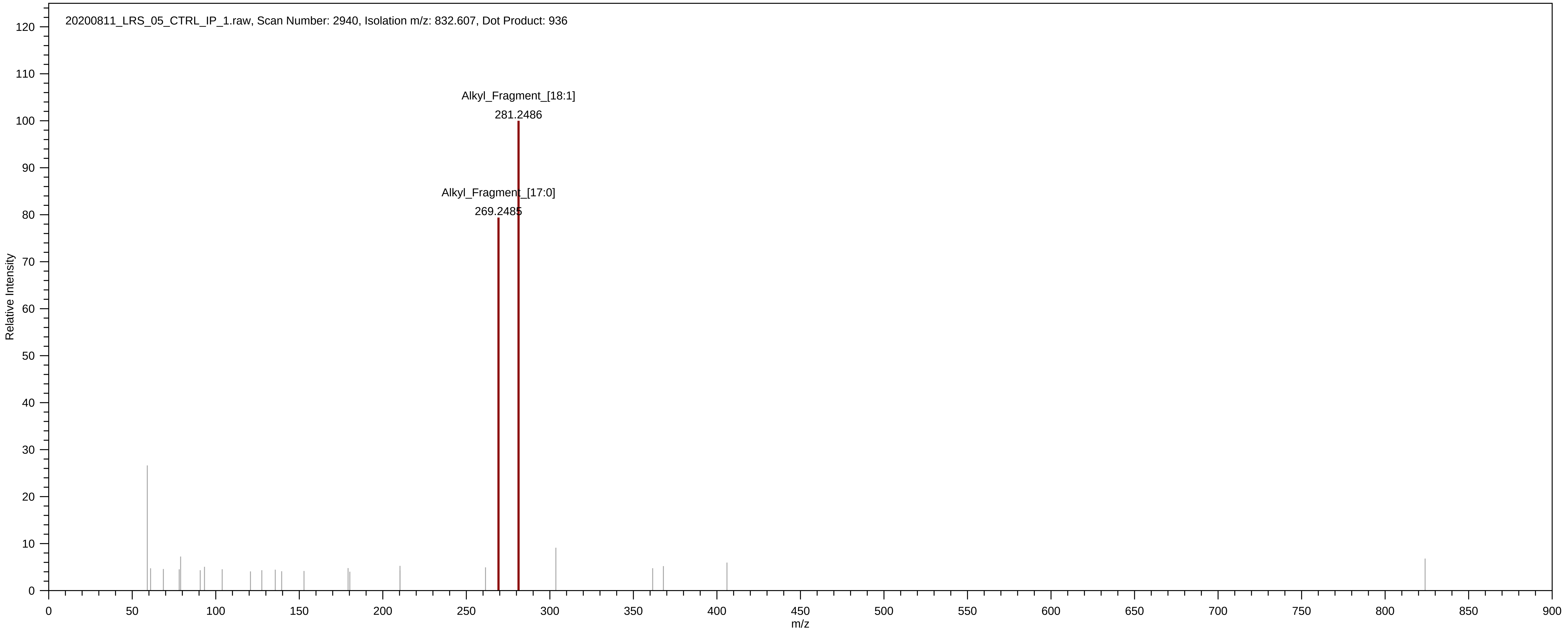

PC 17:0_18:1 [M+Ac-H]- | Ret. Time (min): 10.084 | m/z: 832.608

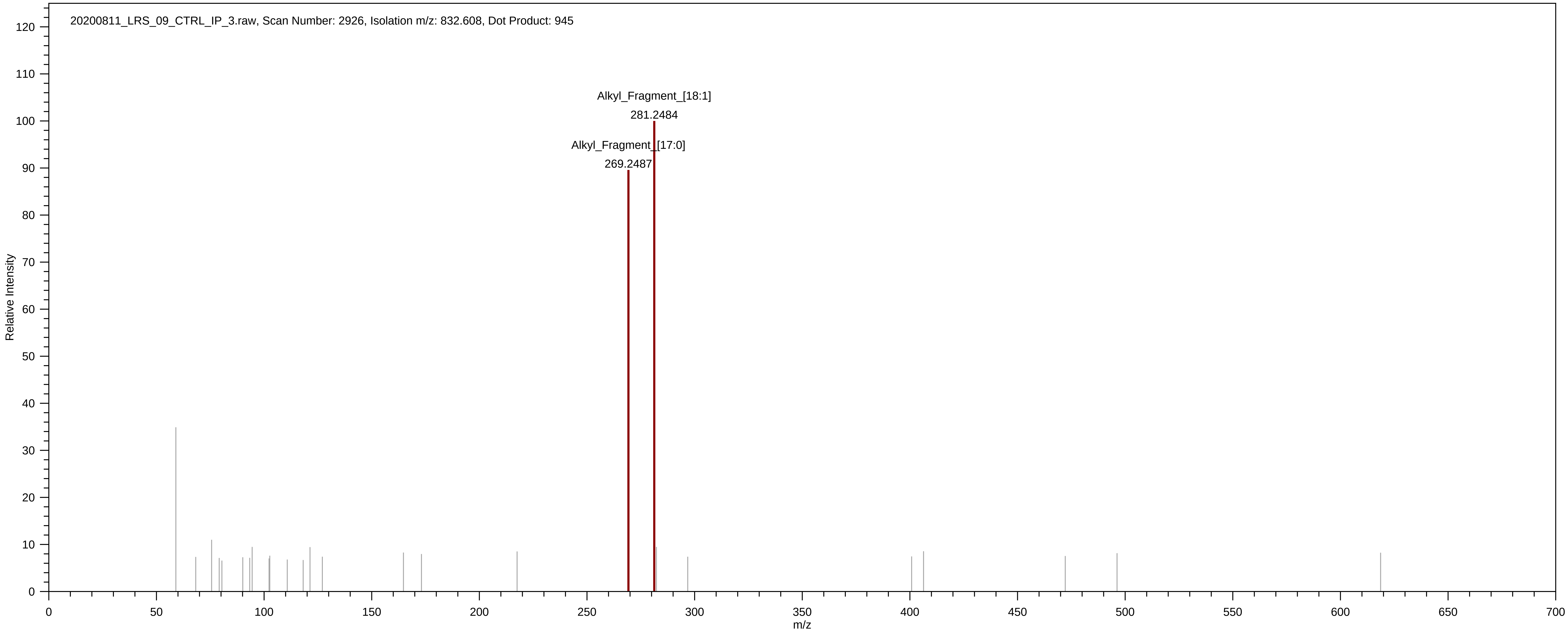

PC 17:0_18:1 [M+Ac-H]- | Ret. Time (min): 10.123 | m/z: 832.608

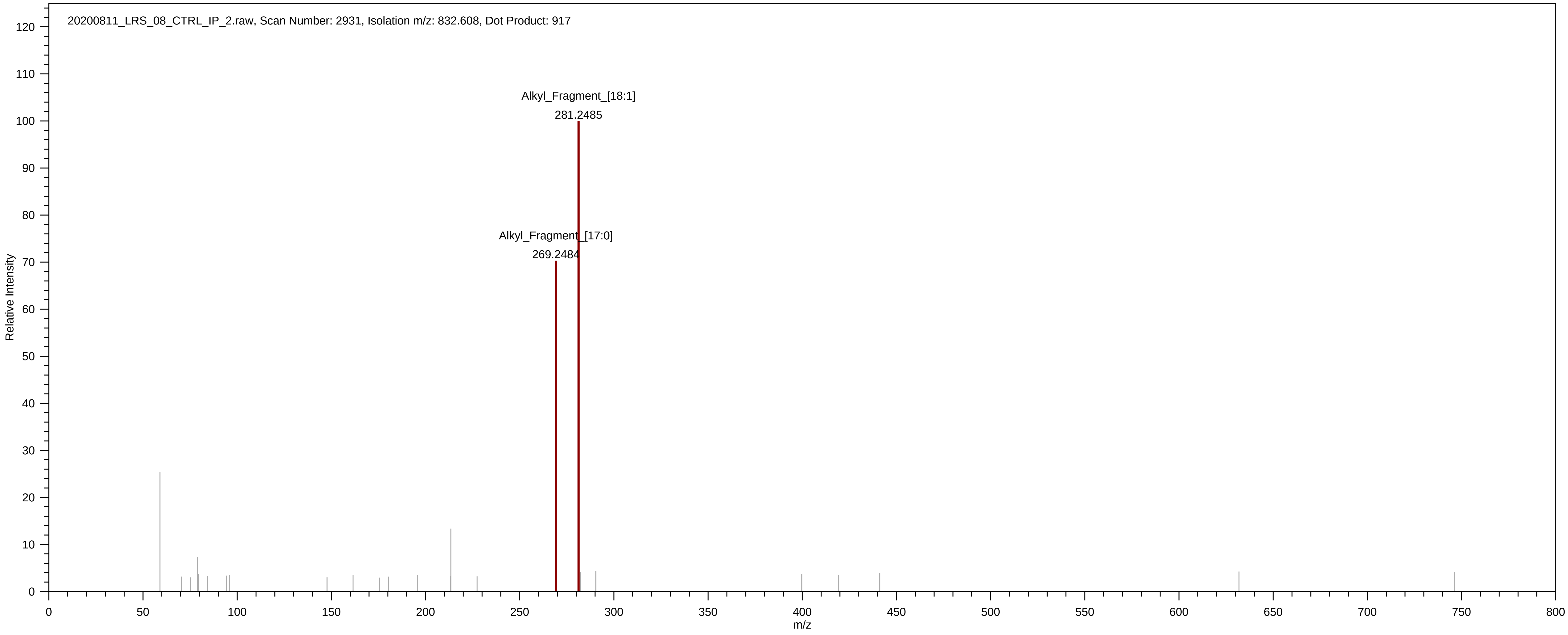

PC 17:0_18:1 [M+Ac-H]- | Ret. Time (min): 10.166 | m/z: 832.608

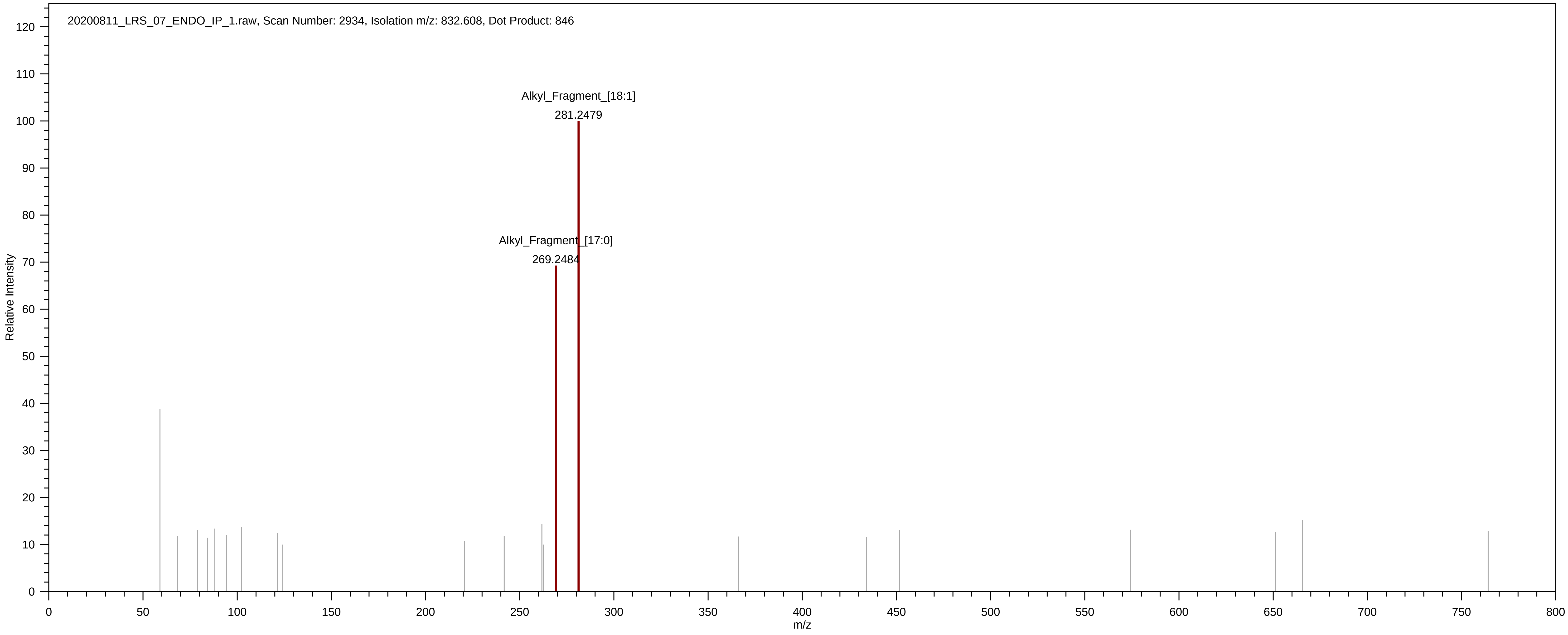

PC 17:0_18:1 [M+Ac-H]- | Ret. Time (min): 10.186 | m/z: 832.608

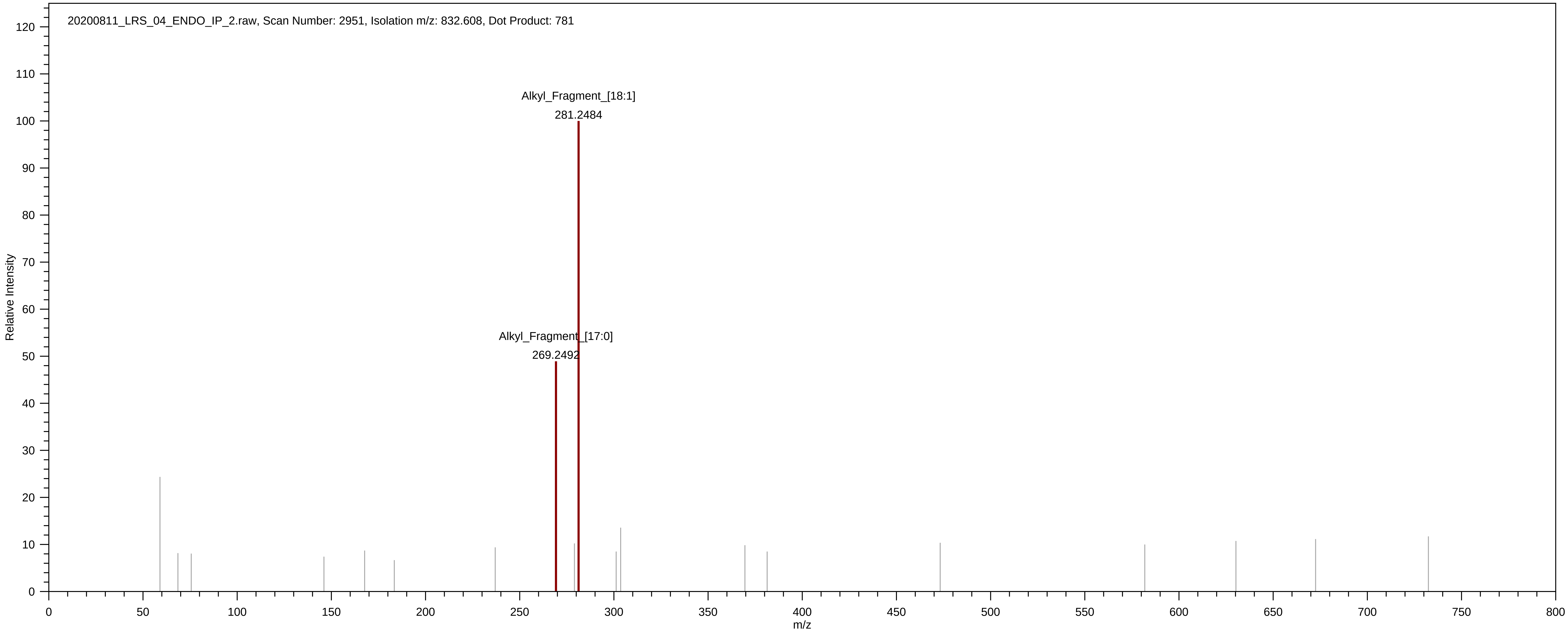

PC 17:0_18:1 [M+Ac-H]- | Ret. Time (min): 10.332 | m/z: 832.608

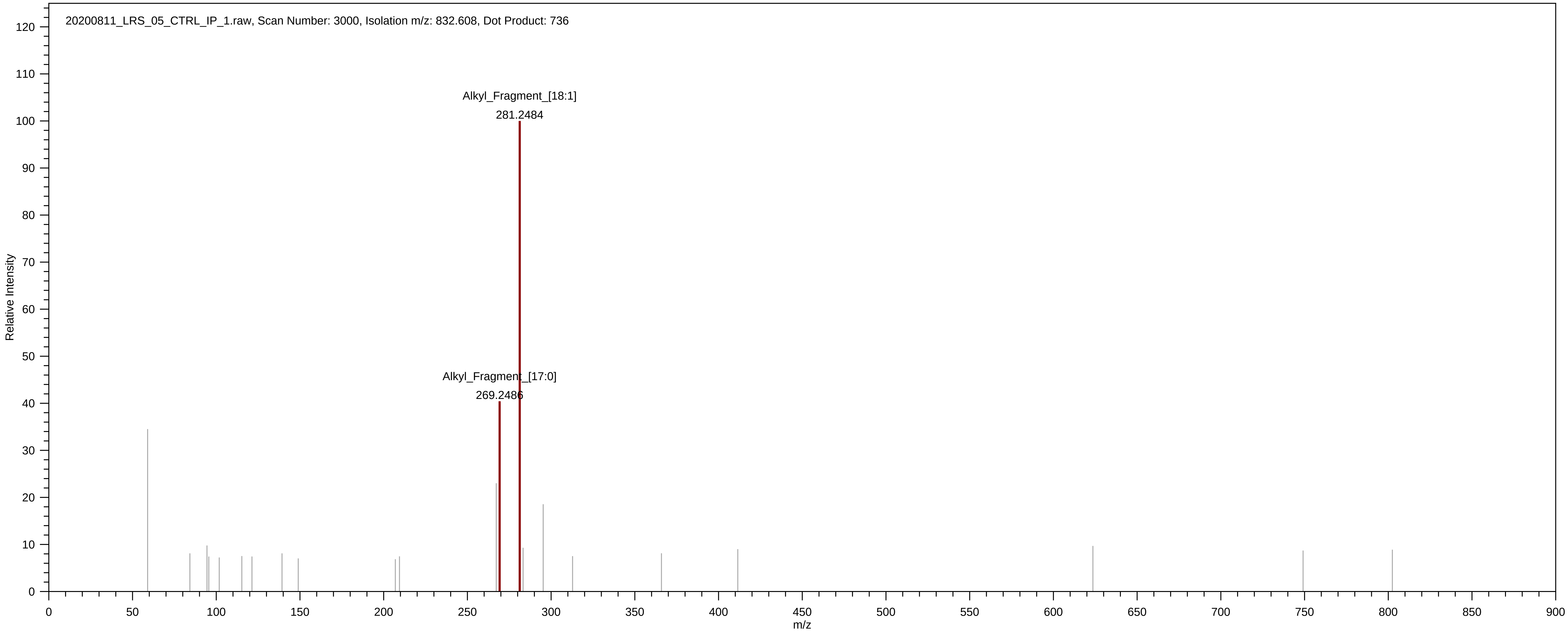

PC 17:0_18:1 [M+Ac-H]- | Ret. Time (min): 10.165 | m/z: 832.609

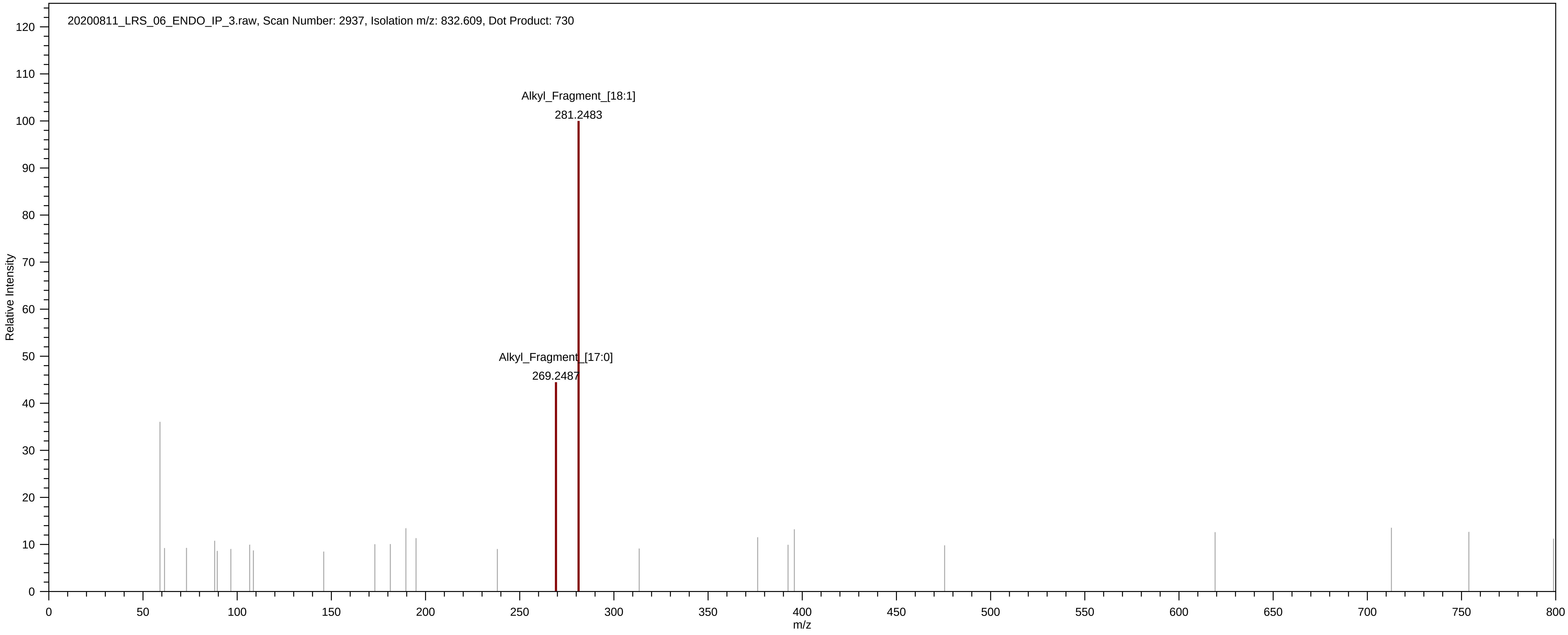

PC 17:0_18:1 [M+Ac-H]- | Ret. Time (min): 10.323 | m/z: 832.609

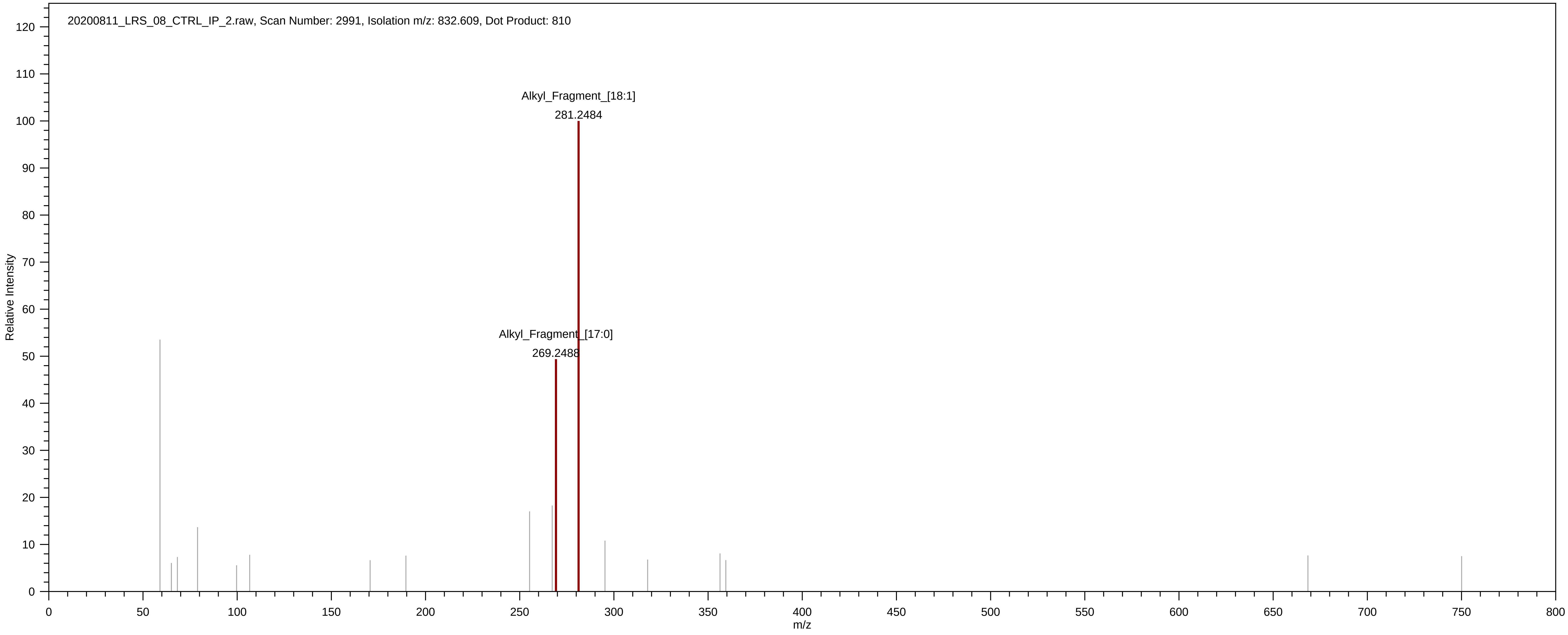

PC 17:1_18:1 [M+Ac-H]- | Ret. Time (min): 9.429 | m/z: 830.592

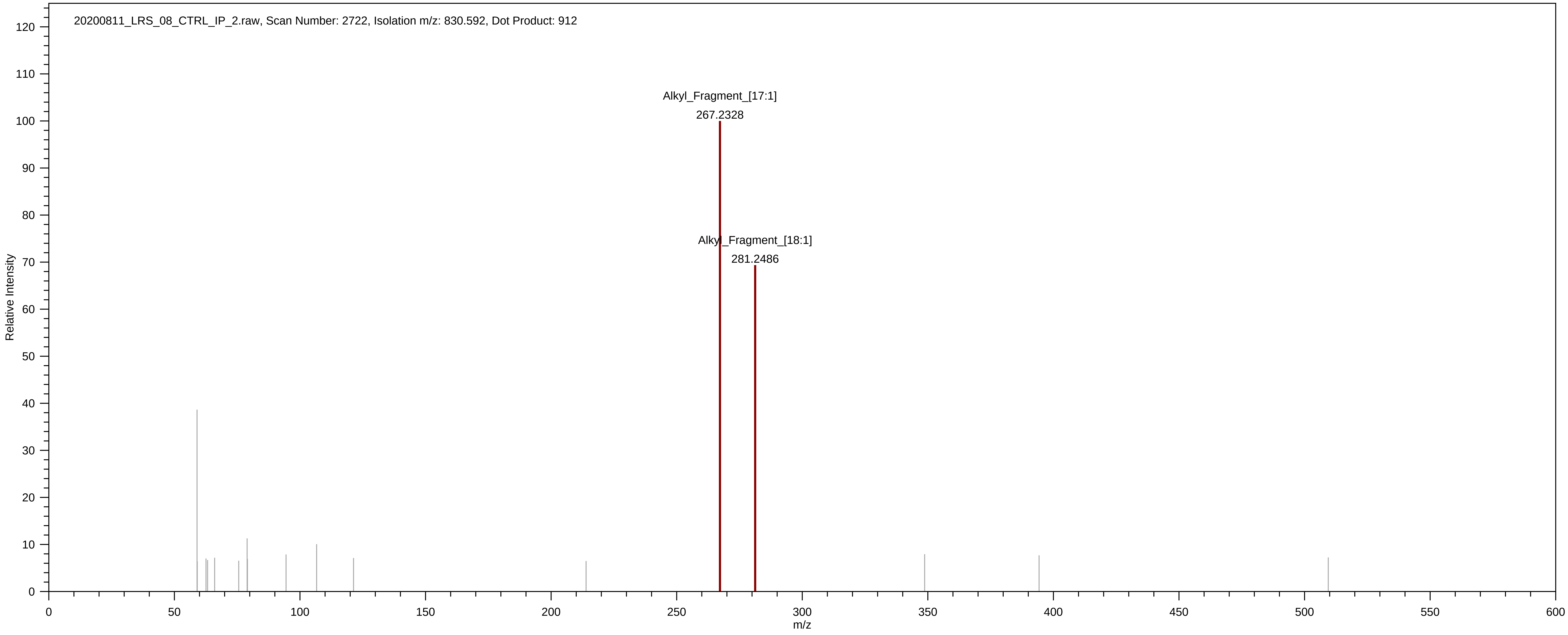

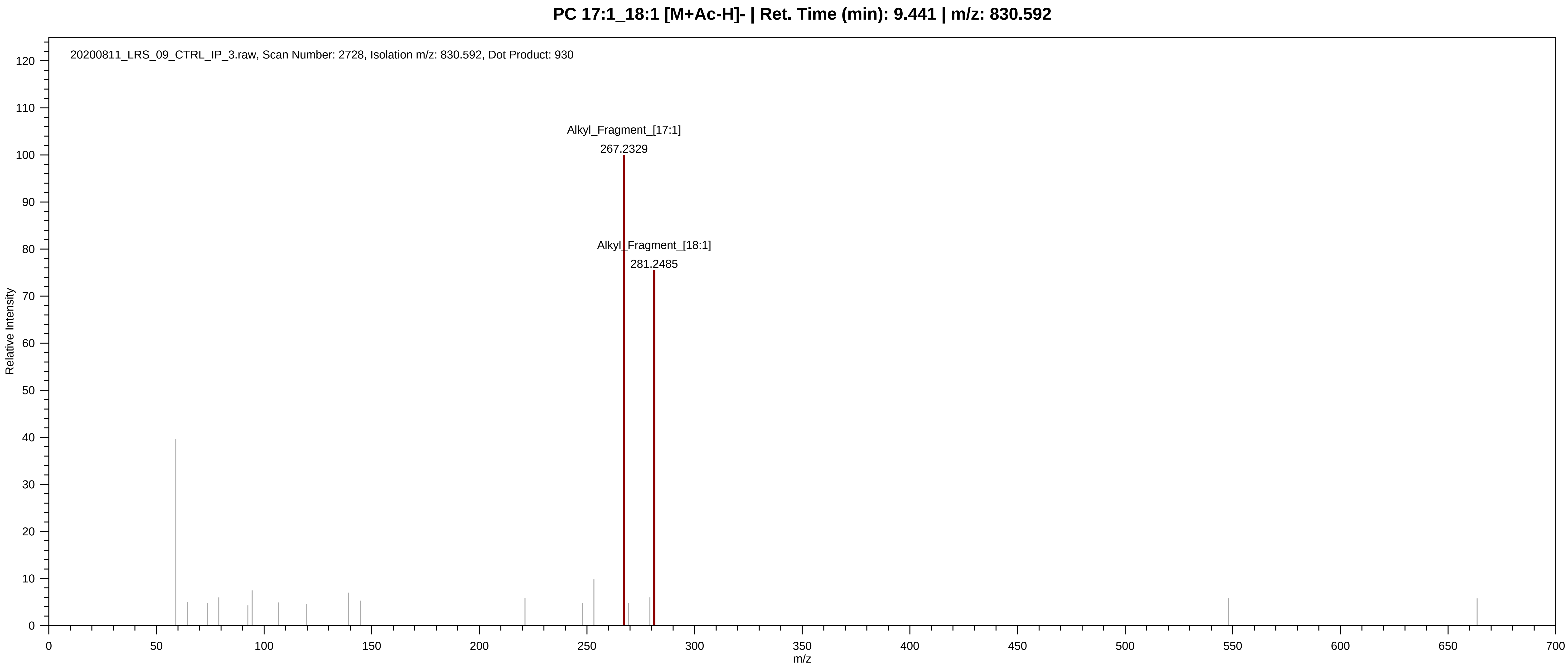

PC 17:1_18:1 [M+Ac-H]- | Ret. Time (min): 9.441 | m/z: 830.592
20200811_LRS_09_CTRL_IP_3.raw, Scan Number: 2728, Isolation m/z: 830.592, Dot Product: 930
Alkyl_Fragment_[17:1]
267.2329
Alkyl_Fragment_[18:1]
281.2485
Relative Intensity
m/z

PC 17:1_18:1 [M+Ac-H]- | Ret. Time (min): 9.45 | m/z: 830.593

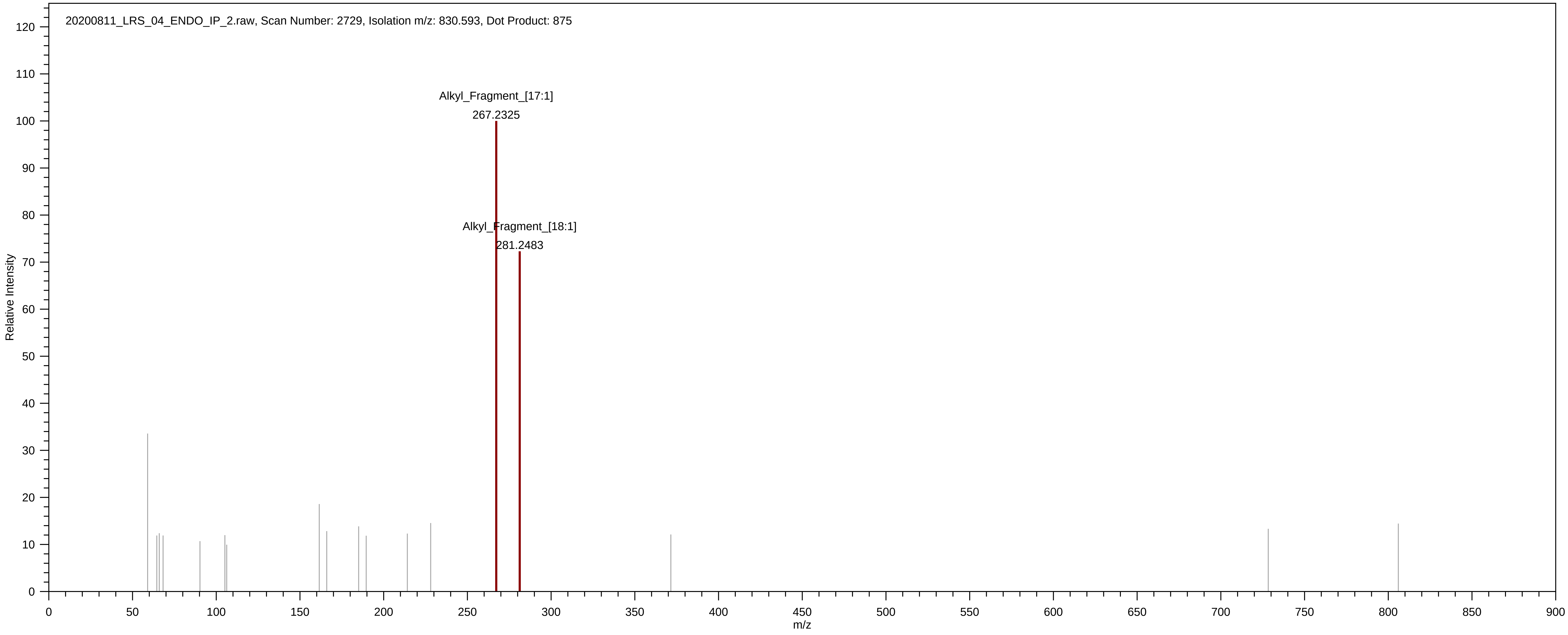

PC 17:1_18:1 [M+Ac-H]- | Ret. Time (min): 9.455 | m/z: 830.593

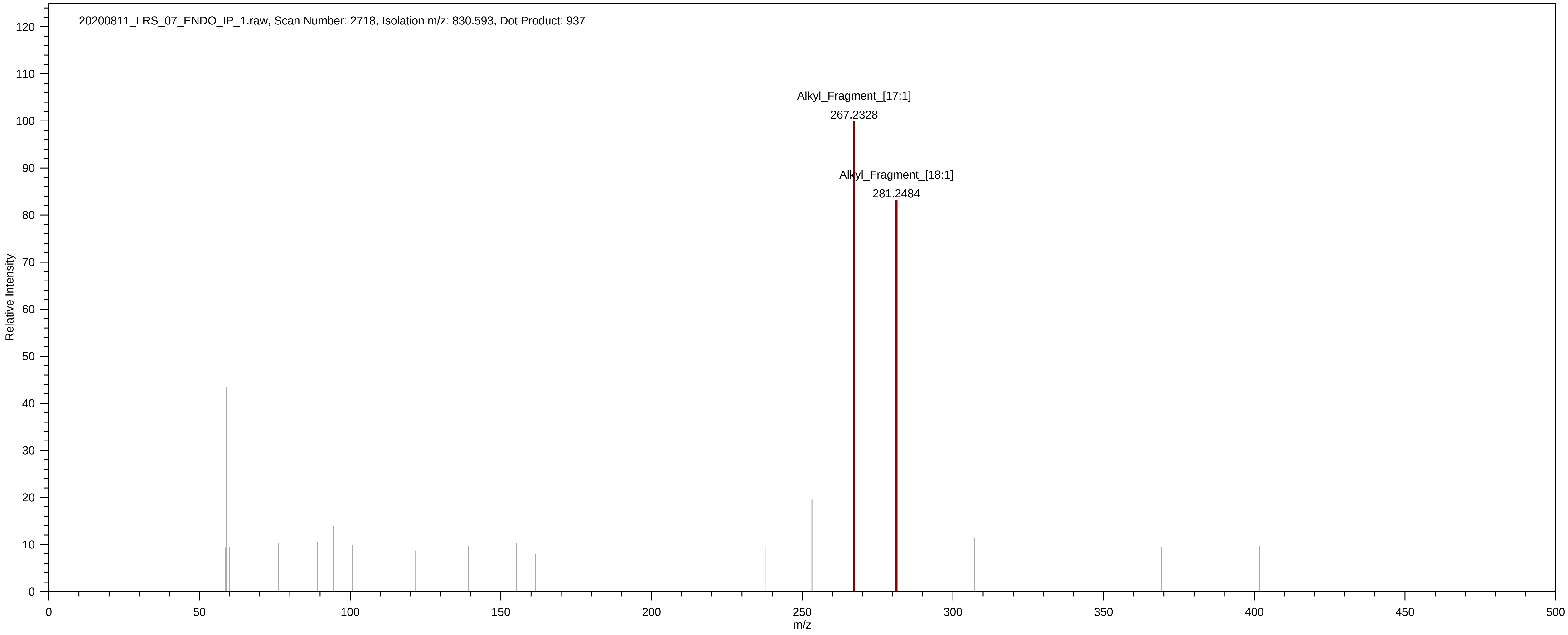

PC 17:1_18:1 [M+Ac-H]- | Ret. Time (min): 9.473 | m/z: 830.593

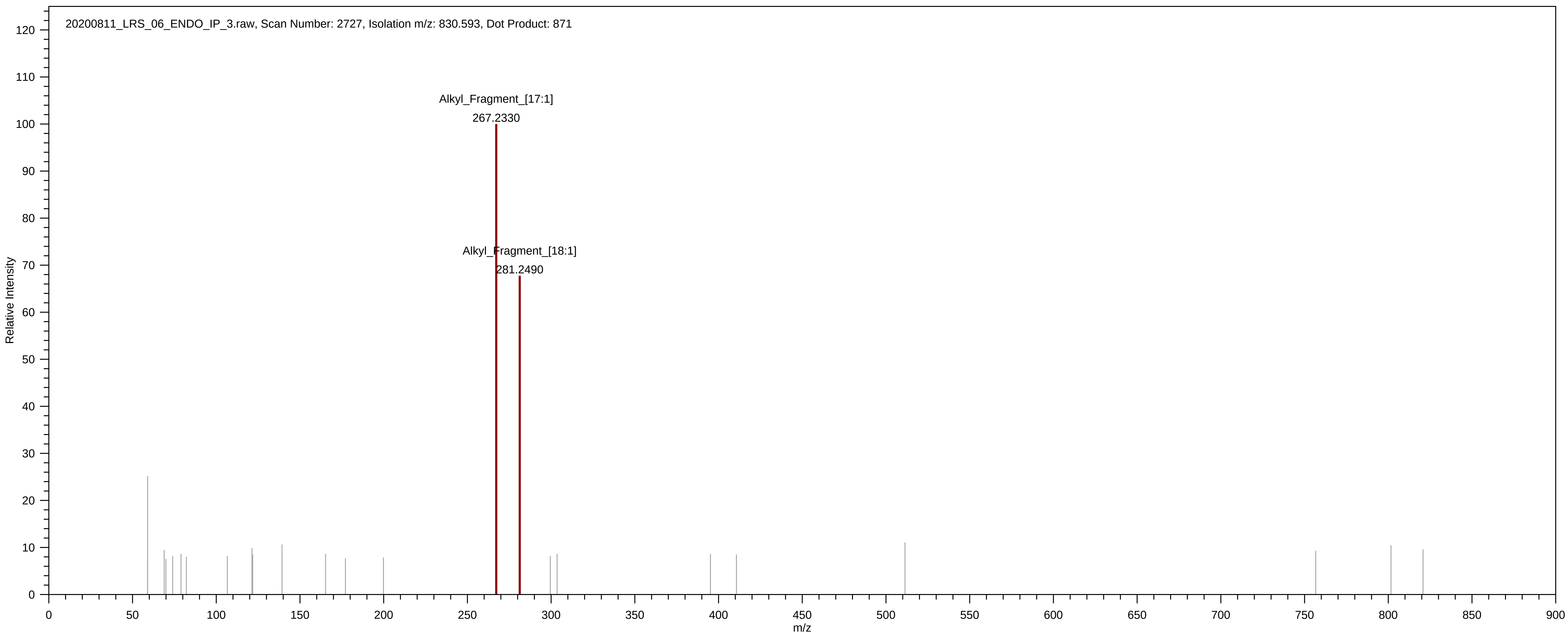

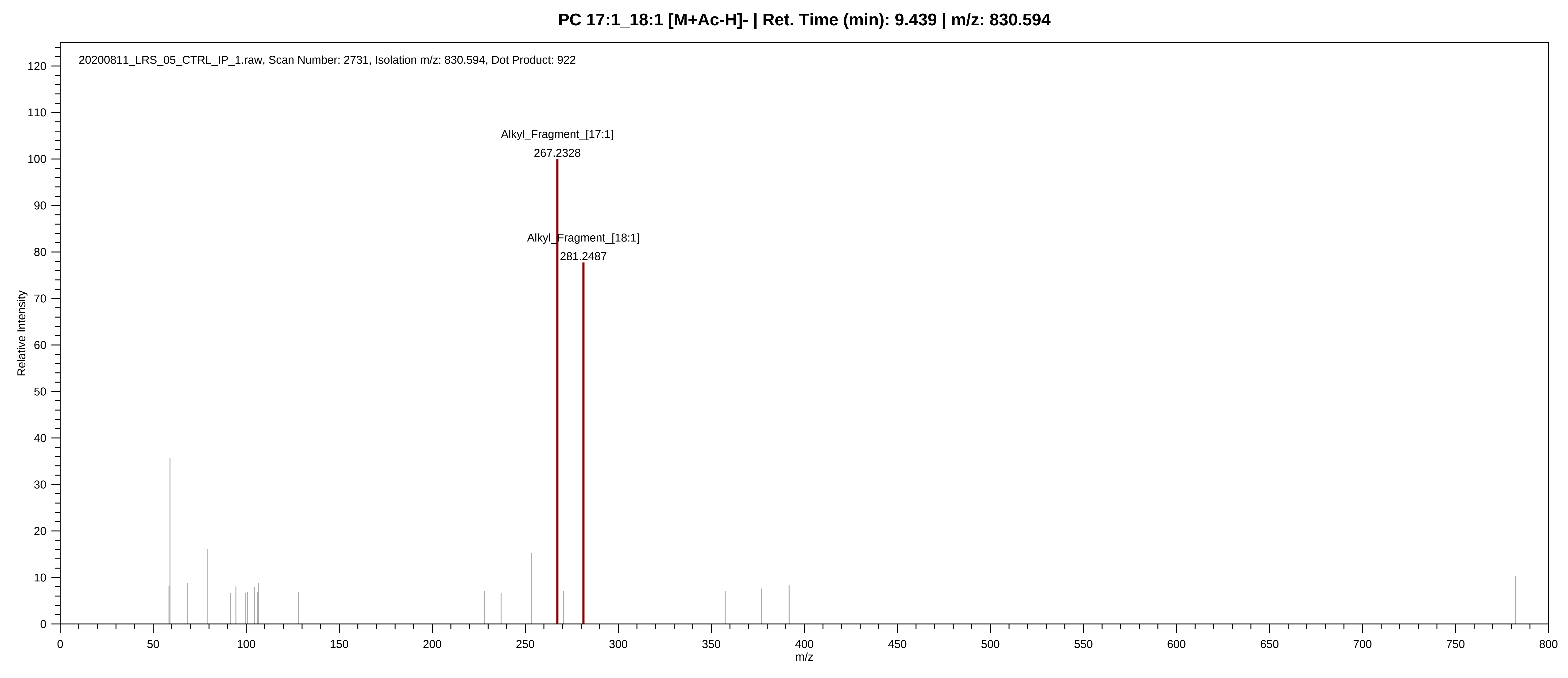
PC 17:1_18:1 [M+Ac-H]- | Ret. Time (min): 9.439 | m/z: 830.594
20200811_LRS_05_CTRL_IP_1.raw, Scan Number: 2731, Isolation m/z: 830.594, Dot Product: 922
Alkyl_Fragment_[17:1]
267.2328
Alkyl_Fragment_[18:1]
281.2487
Relative Intensity
m/z

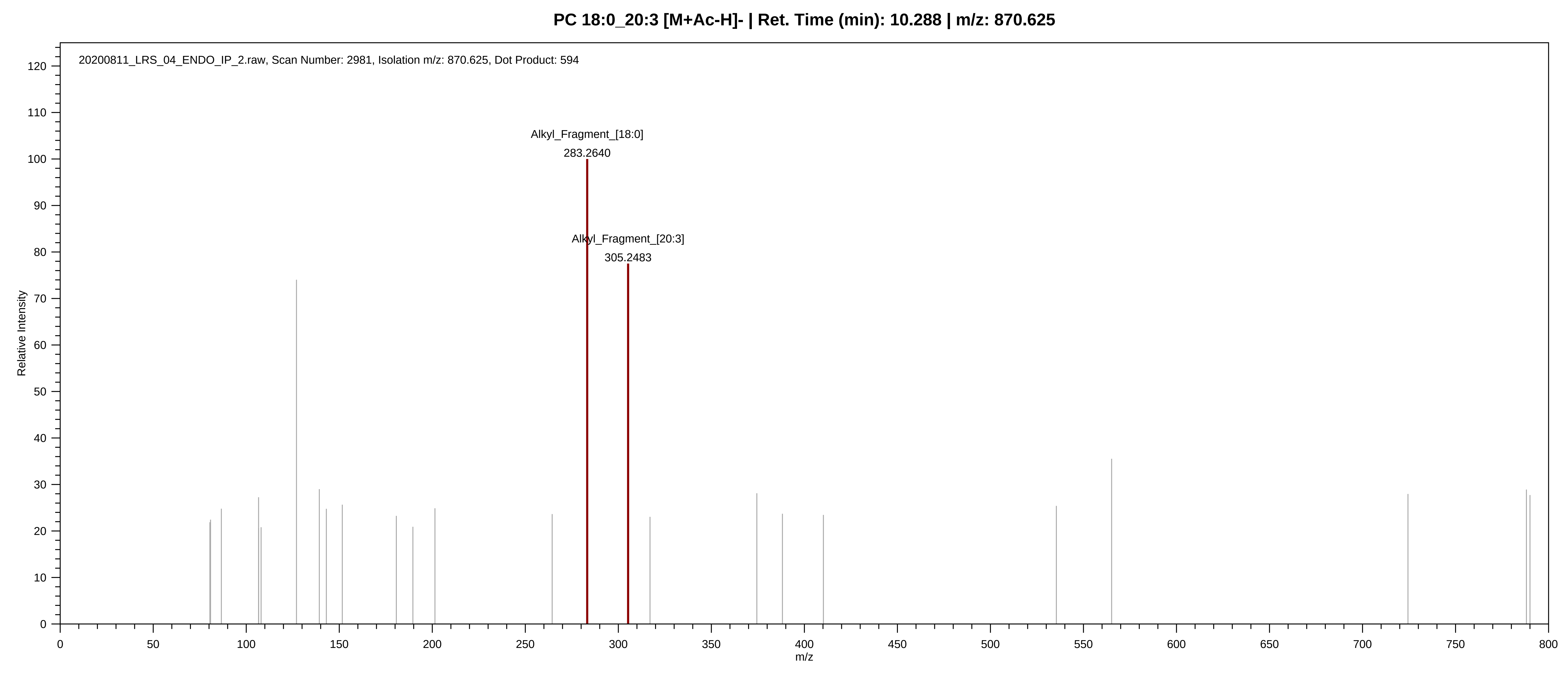
PC 18:0_20:3 [M+Ac-H]- | Ret. Time (min): 10.288 | m/z: 870.625
20200811_LRS_04_ENDO_IP_2.raw, Scan Number: 2981, Isolation m/z: 870.625, Dot Product: 594
Alkyl_Fragment_[18:0]
283.2640
Alkyl_Fragment_[20:3]
305.2483
Relative Intensity
m/z

PC 18:1_18:0 [M+Ac-H]- | Ret. Time (min): 10.998 | m/z: 846.622

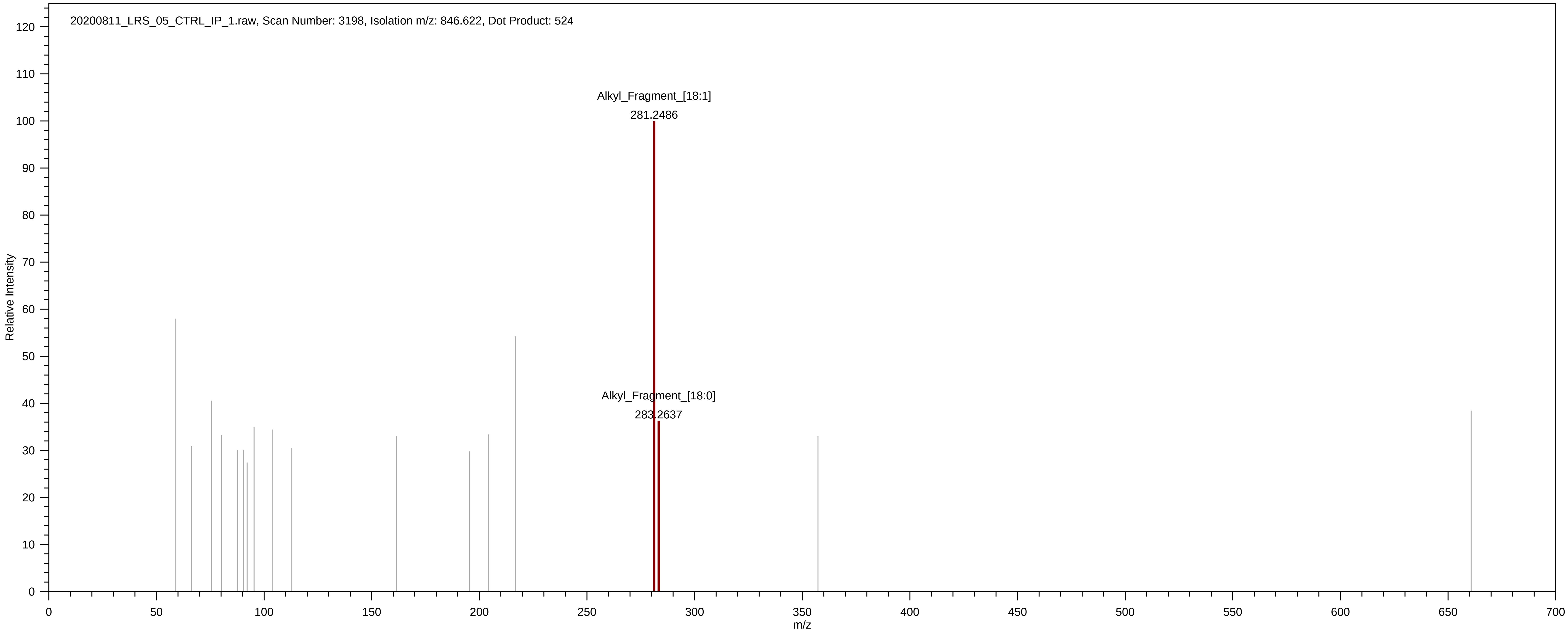

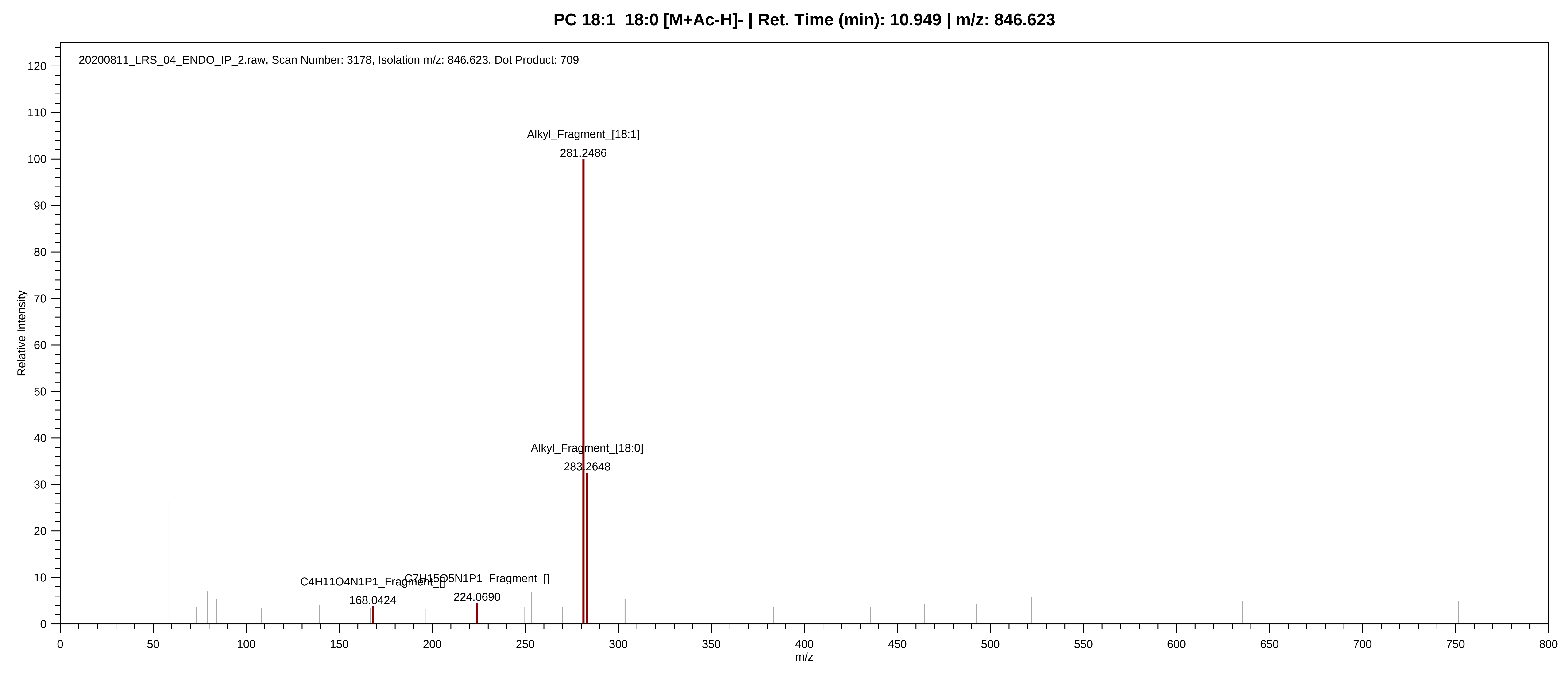
PC 18:1_18:0 [M+Ac-H]- | Ret. Time (min): 10.949 | m/z: 846.623
20200811_LRS_04_ENDO_IP_2.raw, Scan Number: 3178, Isolation m/z: 846.623, Dot Product: 709
Alkyl_Fragment_[18:1]
281.2486
Alkyl_Fragment_[18:0]
283.2648
C4H11O4N1P1_Fragment_[]
168.0424
C7H15O5N1P1_Fragment_[]
224.0690
Relative Intensity
m/z

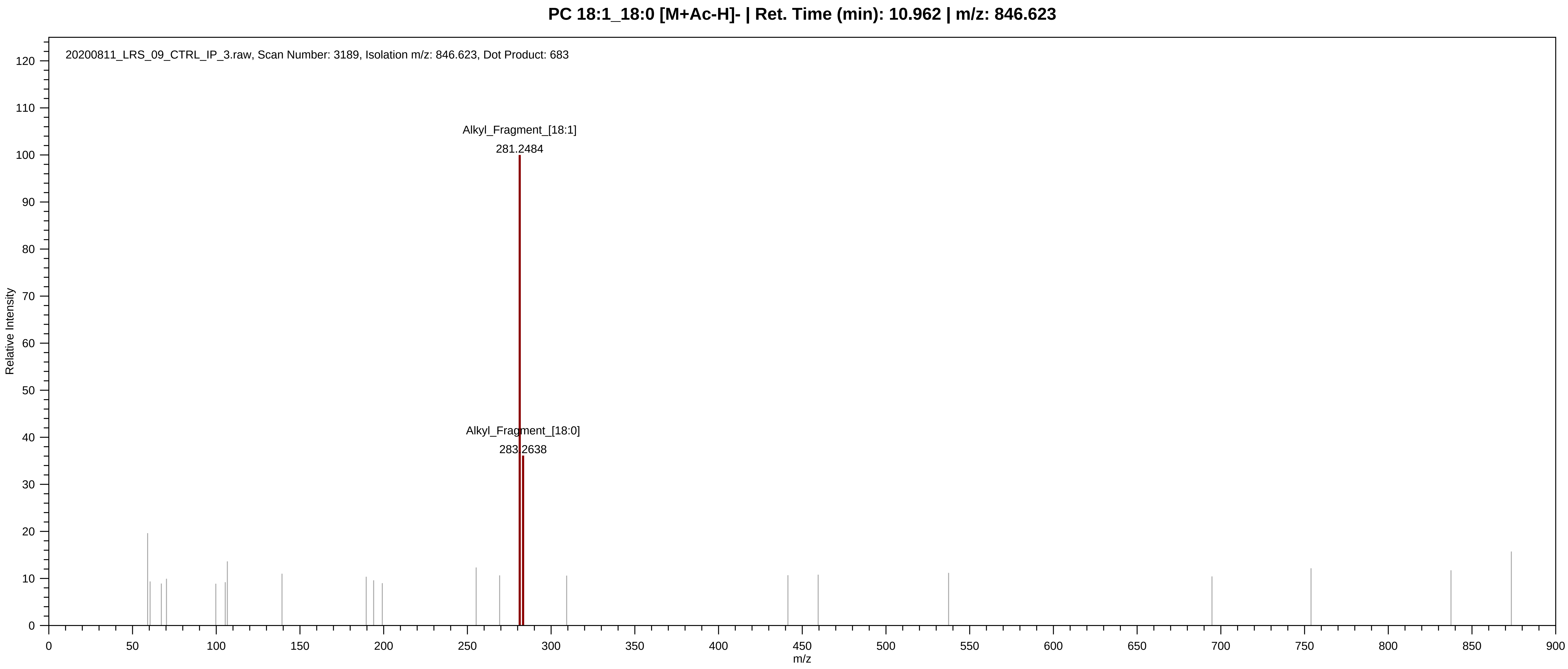

PC 18:1_18:0 [M+Ac-H]- | Ret. Time (min): 10.962 | m/z: 846.623
20200811_LRS_09_CTRL_IP_3.raw, Scan Number: 3189, Isolation m/z: 846.623, Dot Product: 683
Alkyl_Fragment_[18:1]
281.2484
Alkyl_Fragment_[18:0]
283.2638
Relative Intensity
m/z

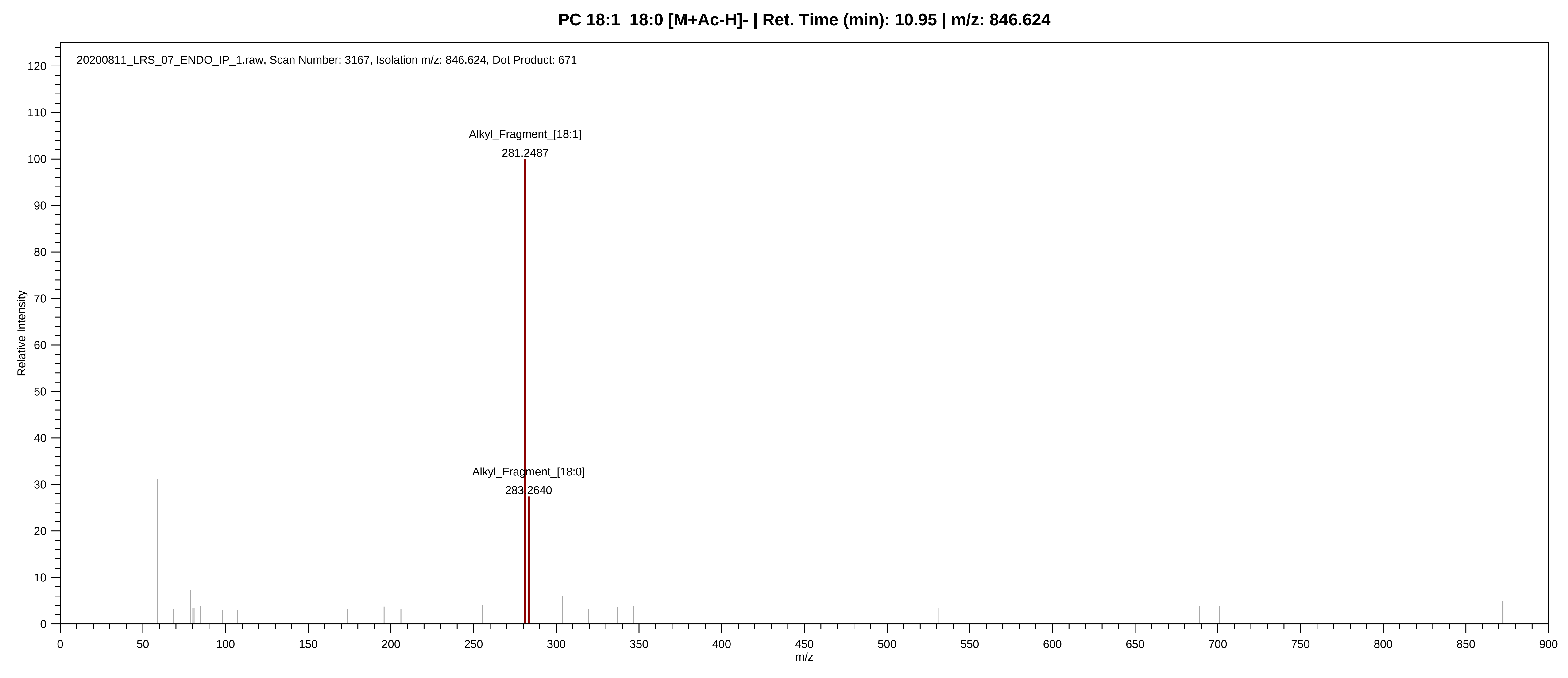
PC 18:1_18:0 [M+Ac-H]- | Ret. Time (min): 10.95 | m/z: 846.624
20200811_LRS_07_ENDO_IP_1.raw, Scan Number: 3167, Isolation m/z: 846.624, Dot Product: 671
Alkyl_Fragment_[18:1]
281.2487
Alkyl_Fragment_[18:0]
283.2640
Relative Intensity
m/z

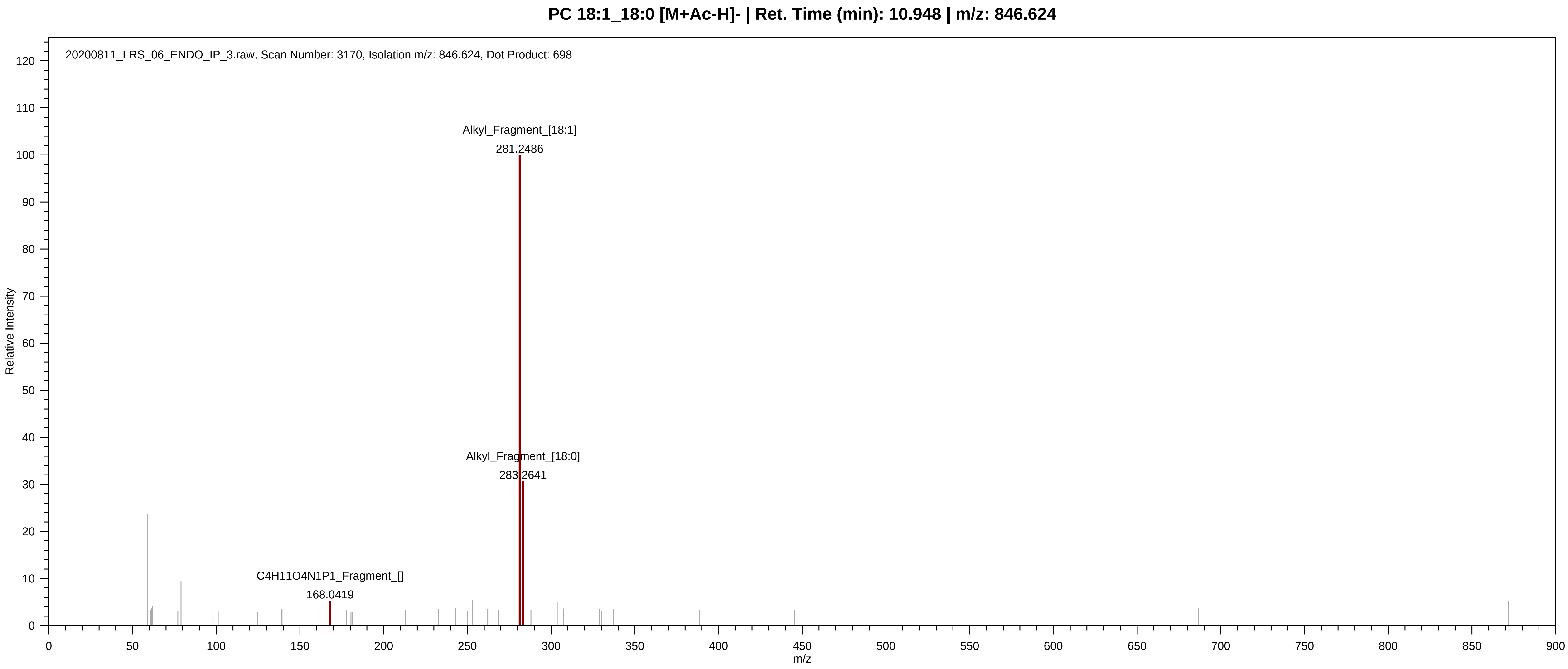
PC 18:1_18:0 [M+Ac-H]- | Ret. Time (min): 10.948 | m/z: 846.624
20200811_LRS_06_ENDO_IP_3.raw, Scan Number: 3170, Isolation m/z: 846.624, Dot Product: 698
Alkyl_Fragment_[18:1]
281.2486
Alkyl_Fragment_[18:0]
283.2641
C4H11O4N1P1_Fragment_[]
168.0419
Relative Intensity
m/z

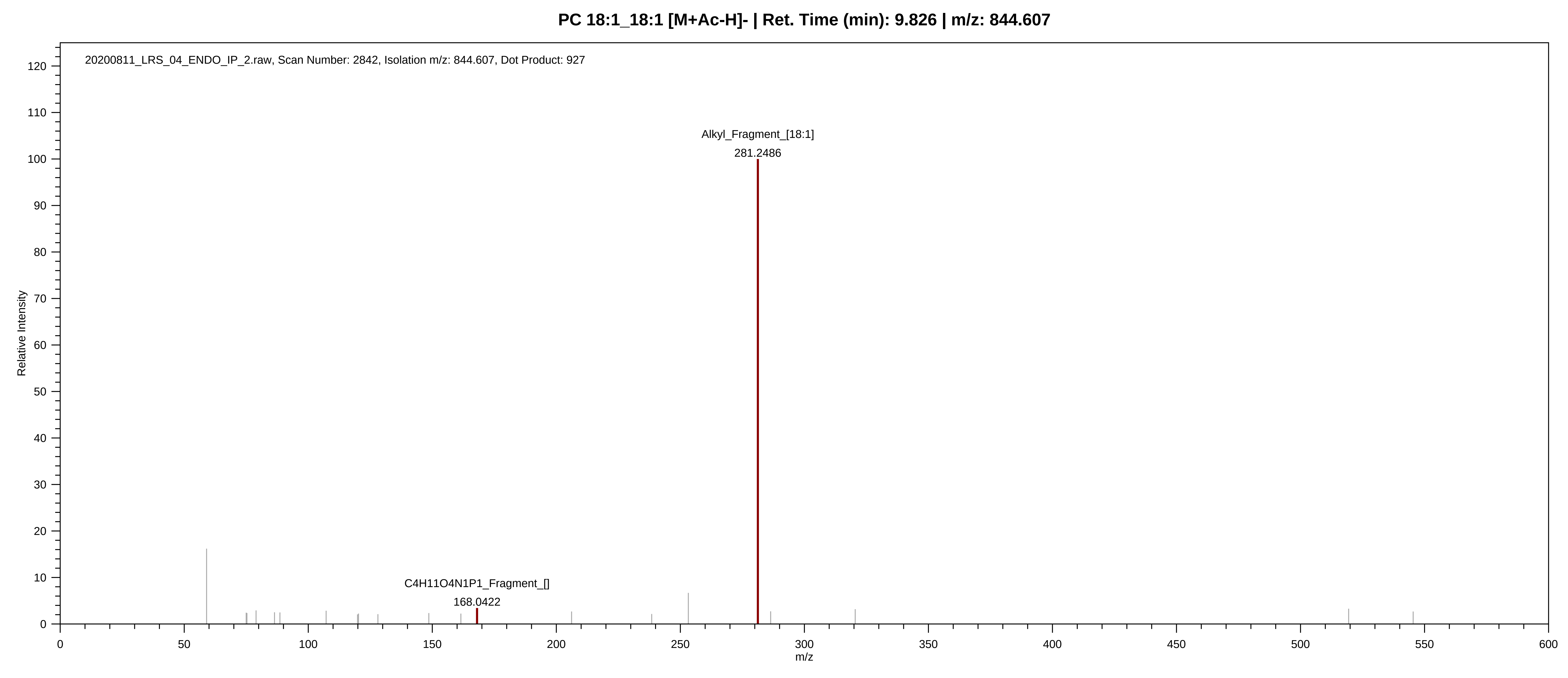

PC 18:1_18:1 [M+Ac-H]- | Ret. Time (min): 9.826 | m/z: 844.607
20200811_LRS_04_ENDO_IP_2.raw, Scan Number: 2842, Isolation m/z: 844.607, Dot Product: 927
Alkyl_Fragment_[18:1]
281.2486
C4H11O4N1P1_Fragment_[]
168.0422
Relative Intensity
m/z

PC 18:1_18:1 [M+Ac-H]- | Ret. Time (min): 9.828 | m/z: 844.607

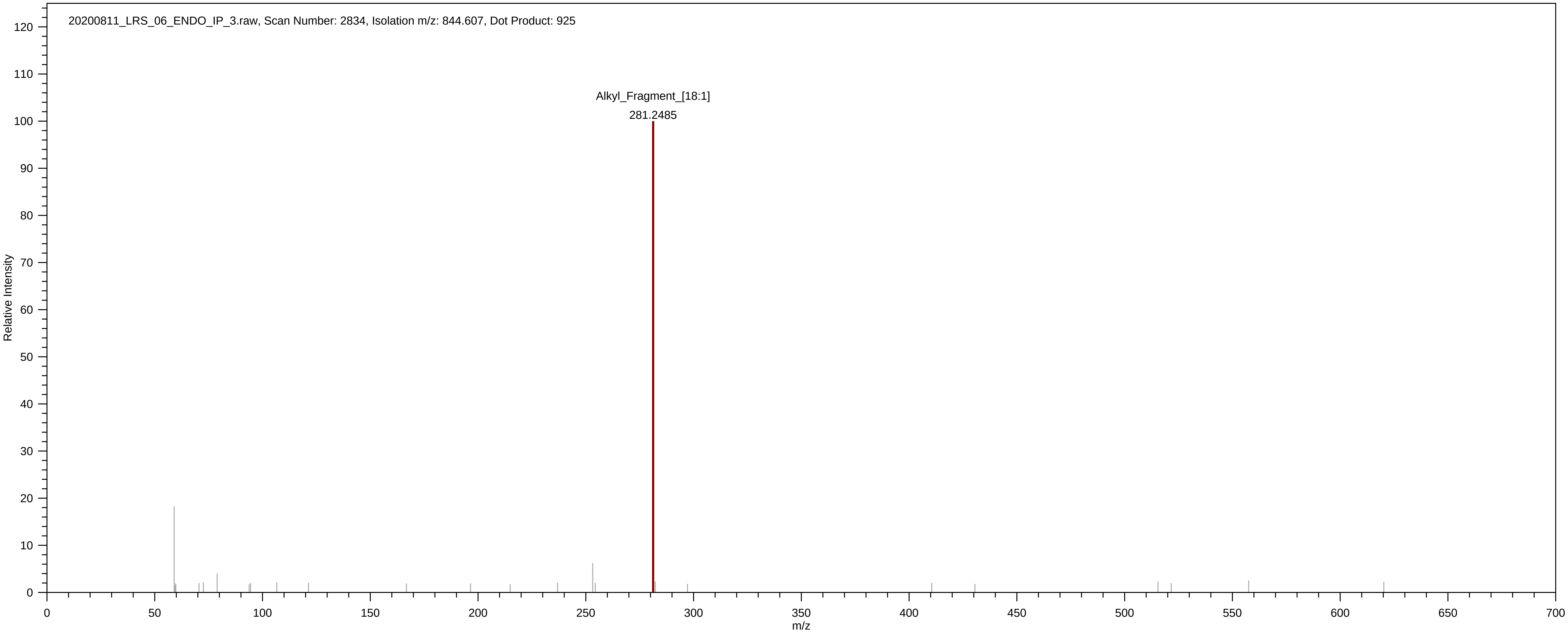

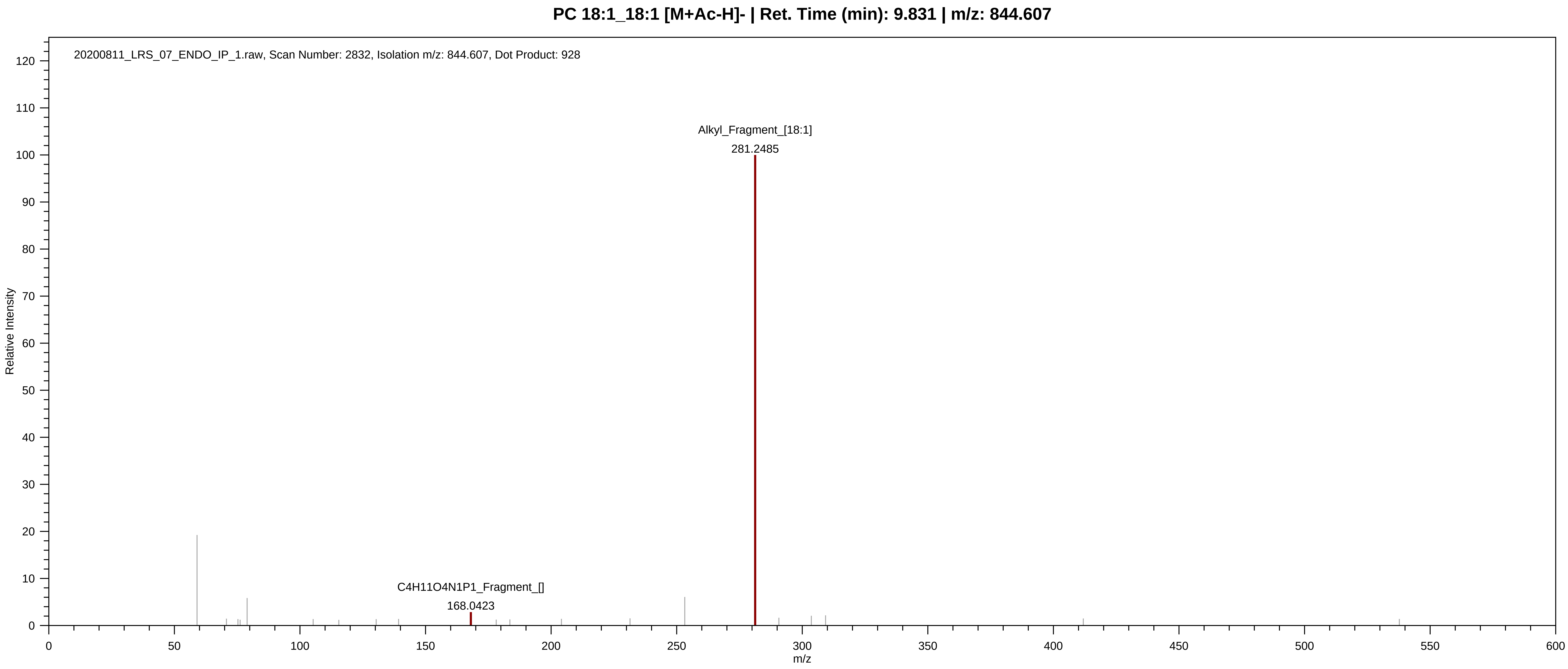
PC 18:1_18:1 [M+Ac-H]- | Ret. Time (min): 9.831 | m/z: 844.607
20200811_LRS_07_ENDO_IP_1.raw, Scan Number: 2832, Isolation m/z: 844.607, Dot Product: 928
Alkyl_Fragment_[18:1]
281.2485
C4H11O4N1P1_Fragment_[]
168.0423
Relative Intensity
m/z

PC 18:1_18:1 [M+Ac-H]- | Ret. Time (min): 9.844 | m/z: 844.607

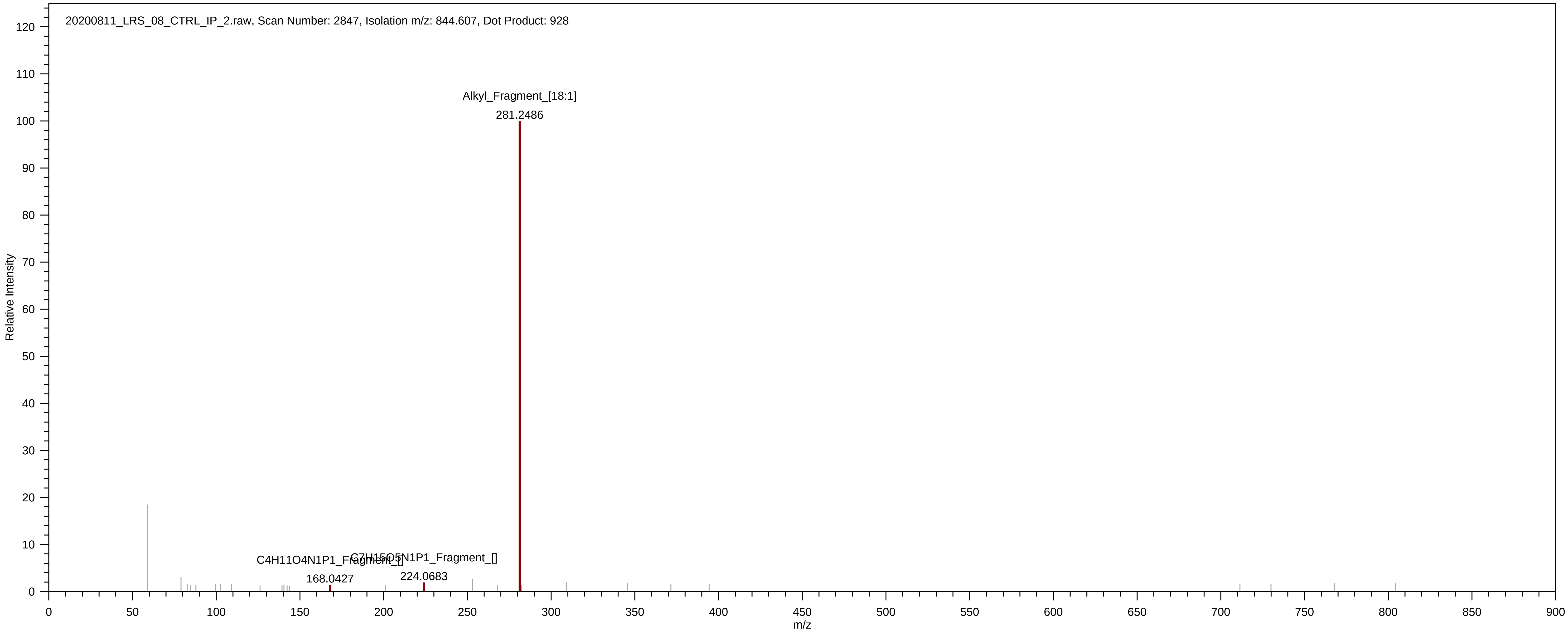

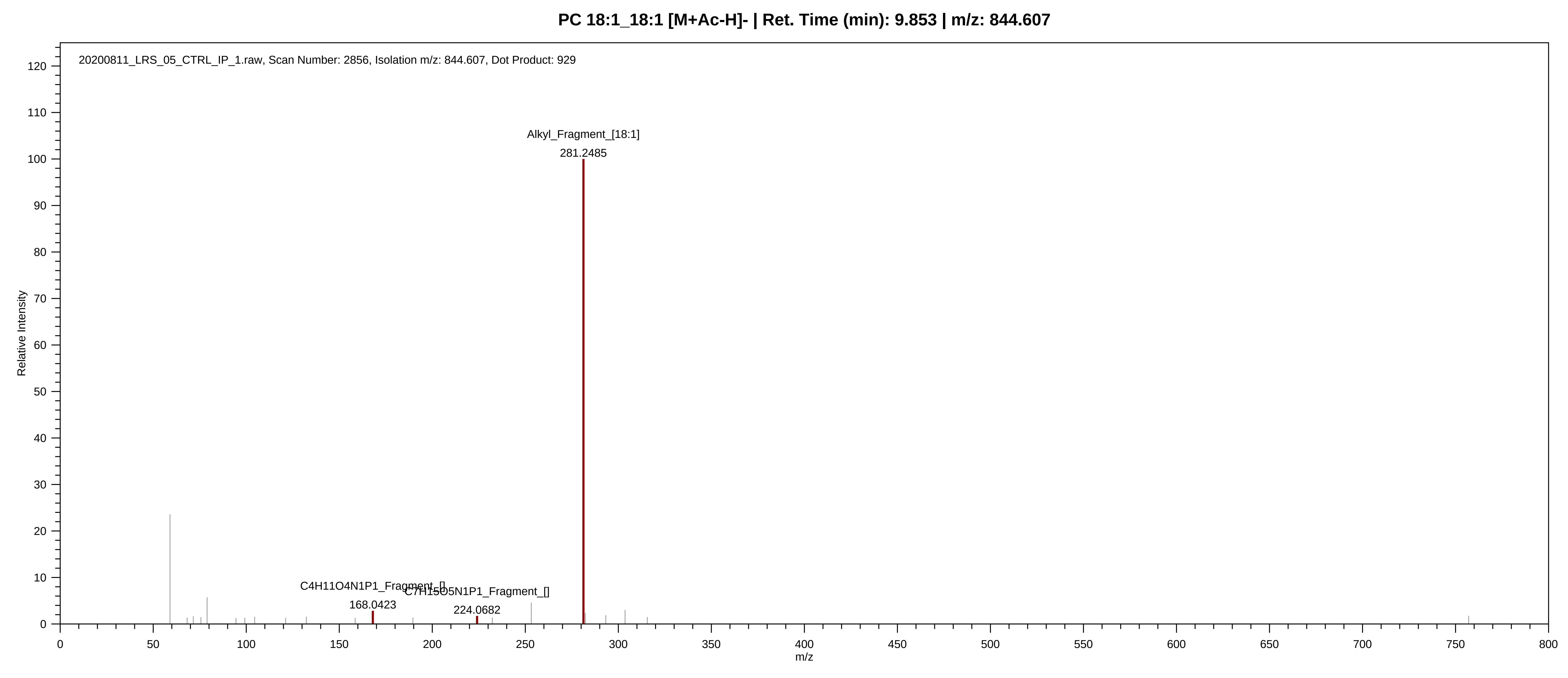

PC 18:1_18:1 [M+Ac-H]- | Ret. Time (min): 9.853 | m/z: 844.607
20200811_LRS_05_CTRL_IP_1.raw, Scan Number: 2856, Isolation m/z: 844.607, Dot Product: 929
Alkyl_Fragment_[18:1]
281.2485
C4H11O4N1P1_Fragment_[]
168.0423
C7H15O5N1P1_Fragment_[]
224.0682
Relative Intensity
m/z

PC 18:1_18:1 [M+Ac-H]- | Ret. Time (min): 9.832 | m/z: 844.608

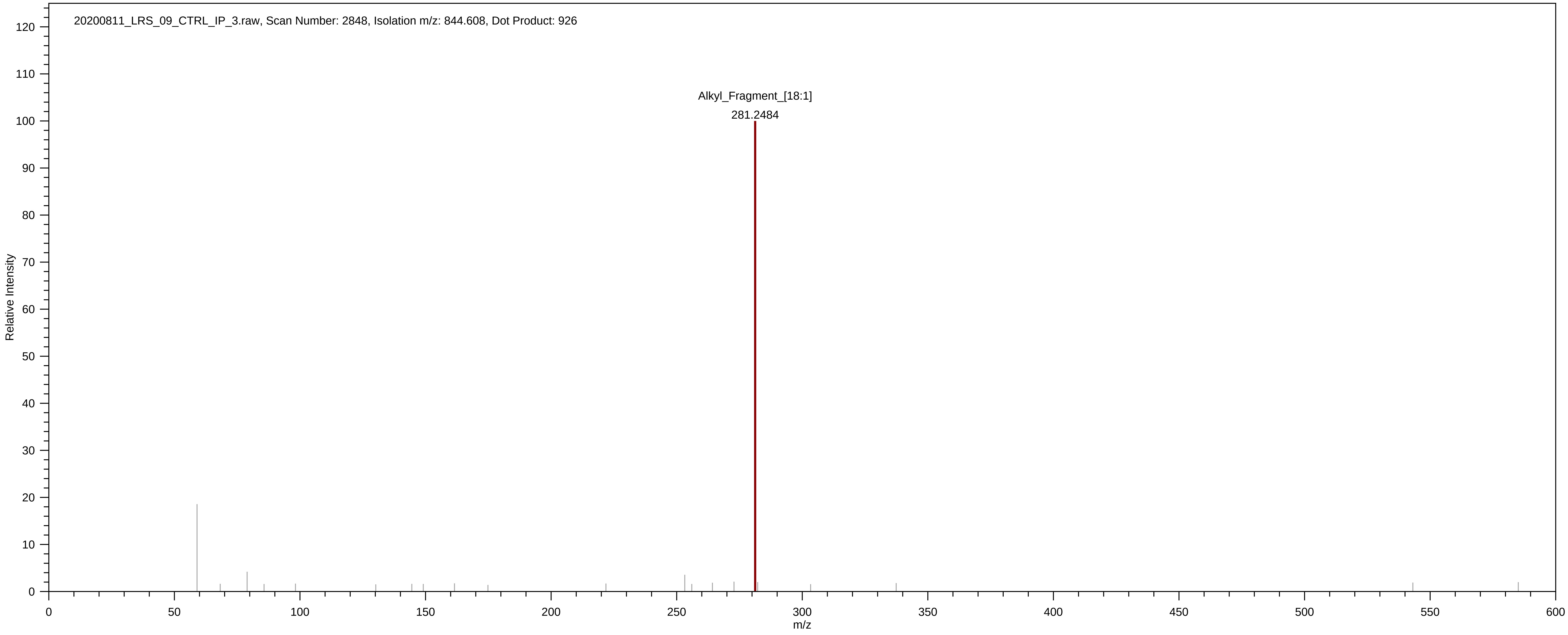

PC 18:1_18:1 [M+Ac-H]- | Ret. Time (min): 10.024 | m/z: 844.608

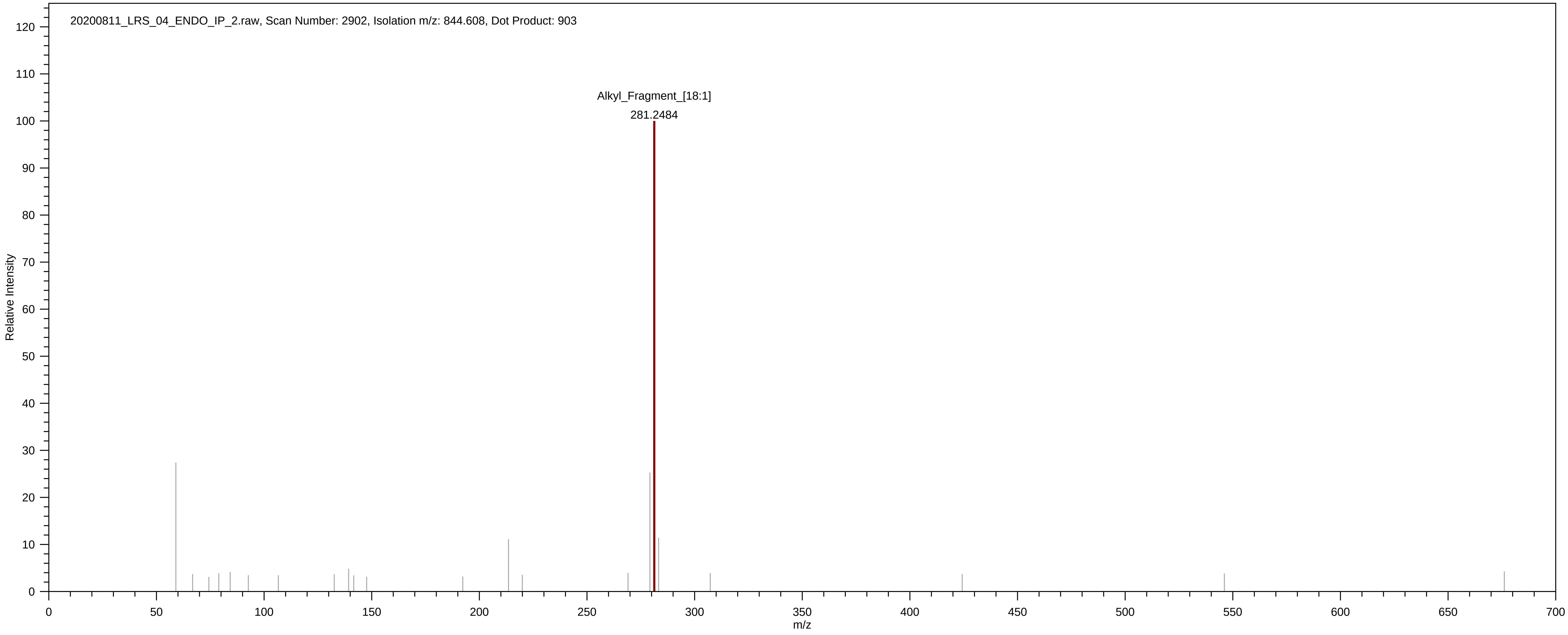

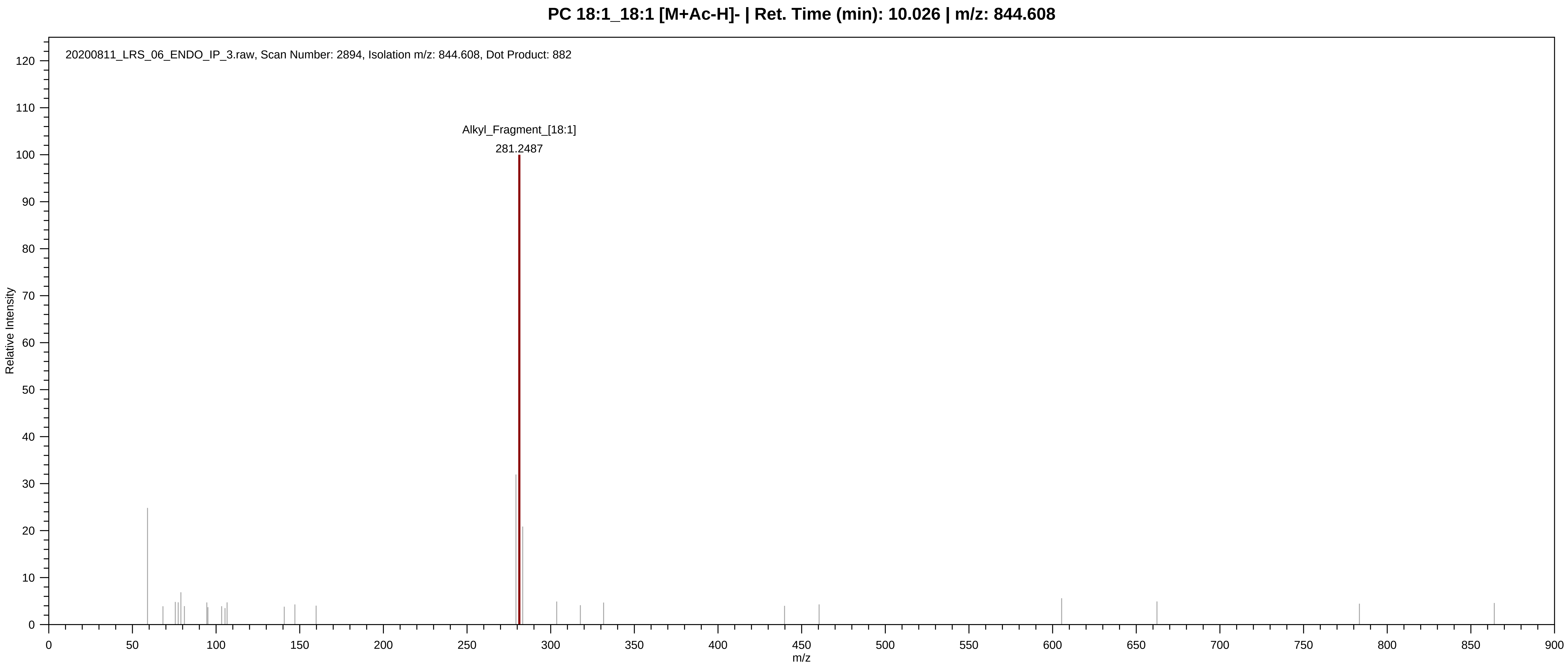

PC 18:1_18:1 [M+Ac-H]- | Ret. Time (min): 10.026 | m/z: 844.608
20200811_LRS_06_ENDO_IP_3.raw, Scan Number: 2894, Isolation m/z: 844.608, Dot Product: 882
Alkyl_Fragment_[18:1]
281.2487
Relative Intensity
m/z

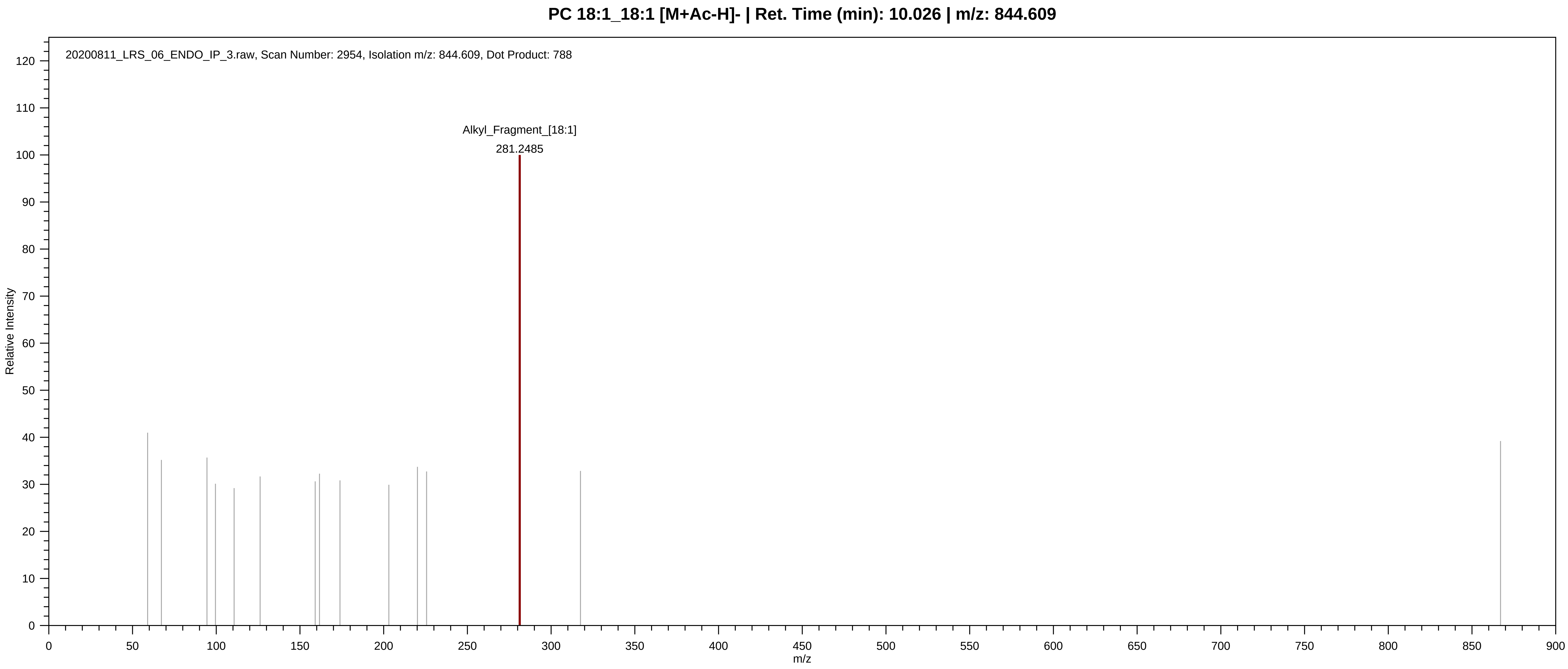
PC 18:1_18:1 [M+Ac-H]- | Ret. Time (min): 10.026 | m/z: 844.609
20200811_LRS_06_ENDO_IP_3.raw, Scan Number: 2954, Isolation m/z: 844.609, Dot Product: 788
Alkyl_Fragment_[18:1]
281.2485
Relative Intensity
m/z

PC 18:1_18:1 [M+Ac-H]- | Ret. Time (min): 10.027 | m/z: 844.608

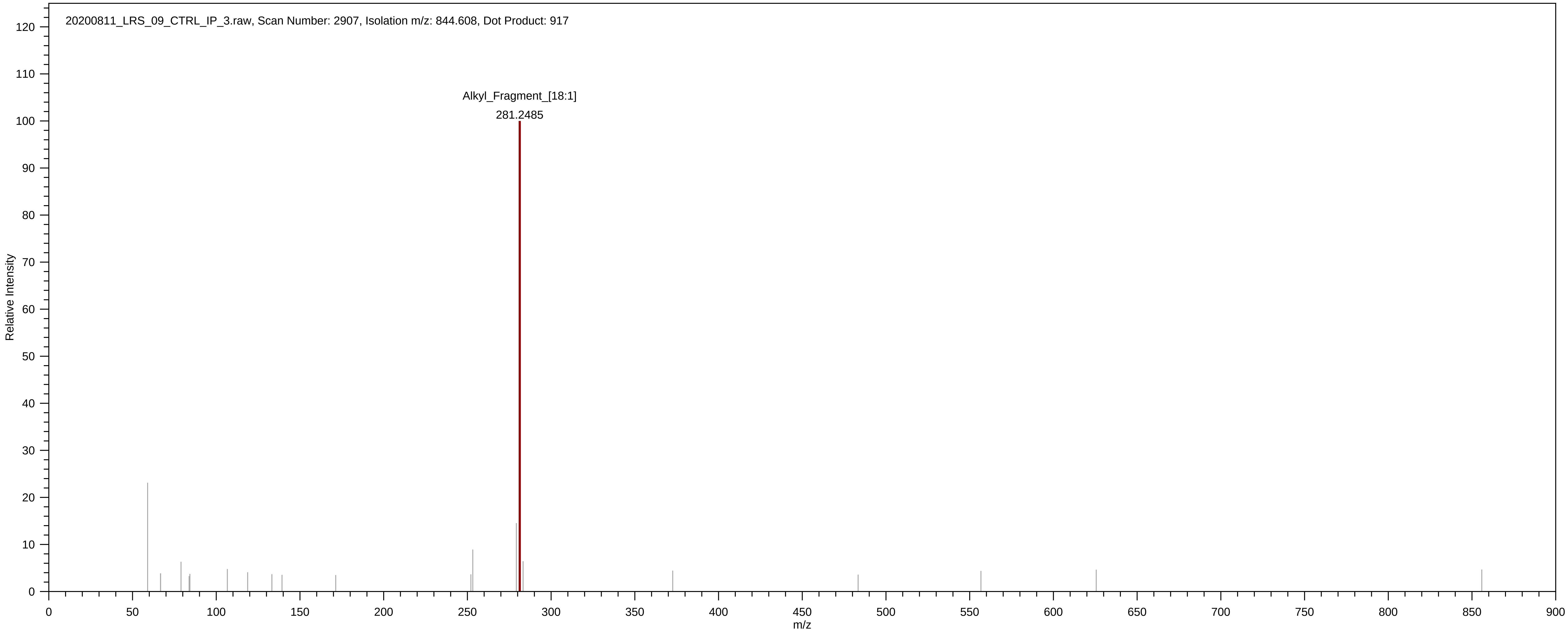

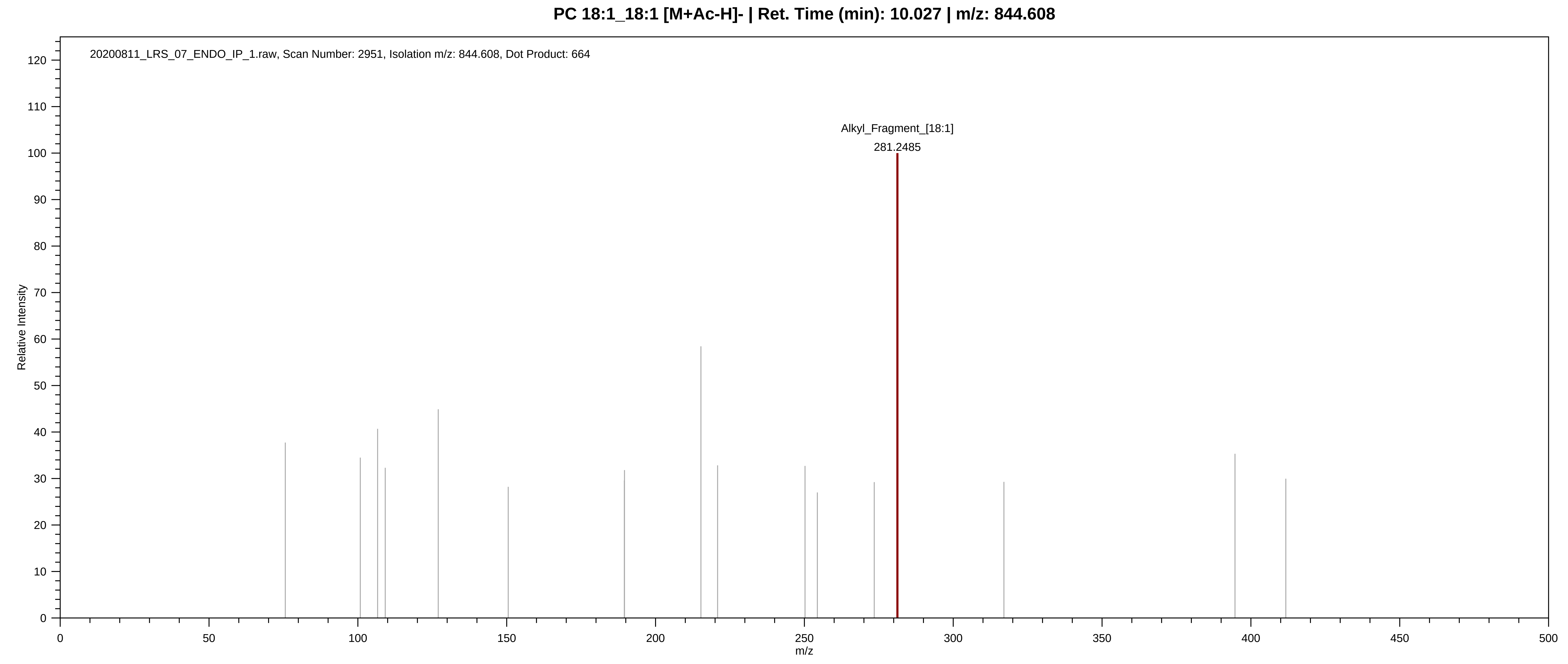

PC 18:1_18:1 [M+Ac-H]- | Ret. Time (min): 10.027 | m/z: 844.608
20200811_LRS_07_ENDO_IP_1.raw, Scan Number: 2951, Isolation m/z: 844.608, Dot Product: 664
Alkyl_Fragment_[18:1]
281.2485
Relative Intensity
m/z
0
10
20
30
40
50
60
70
80
90
100
110
120
0
50
100
150
200
250
300
350
400
450
500

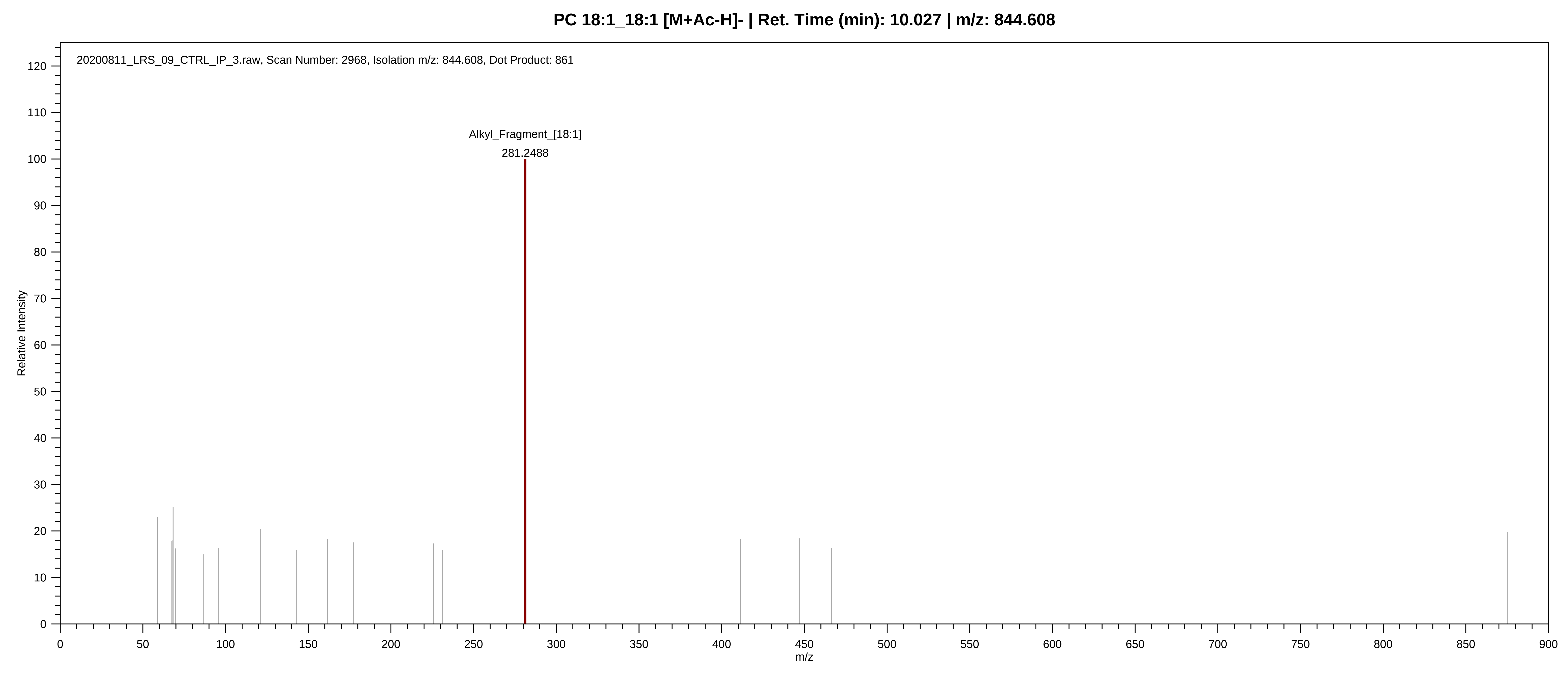
PC 18:1_18:1 [M+Ac-H]- | Ret. Time (min): 10.027 | m/z: 844.608
20200811_LRS_09_CTRL_IP_3.raw, Scan Number: 2968, Isolation m/z: 844.608, Dot Product: 861
Alkyl_Fragment_[18:1]
281.2488
Relative Intensity
m/z

PC 18:1_18:1 [M+Ac-H]- | Ret. Time (min): 10.043 | m/z: 844.608

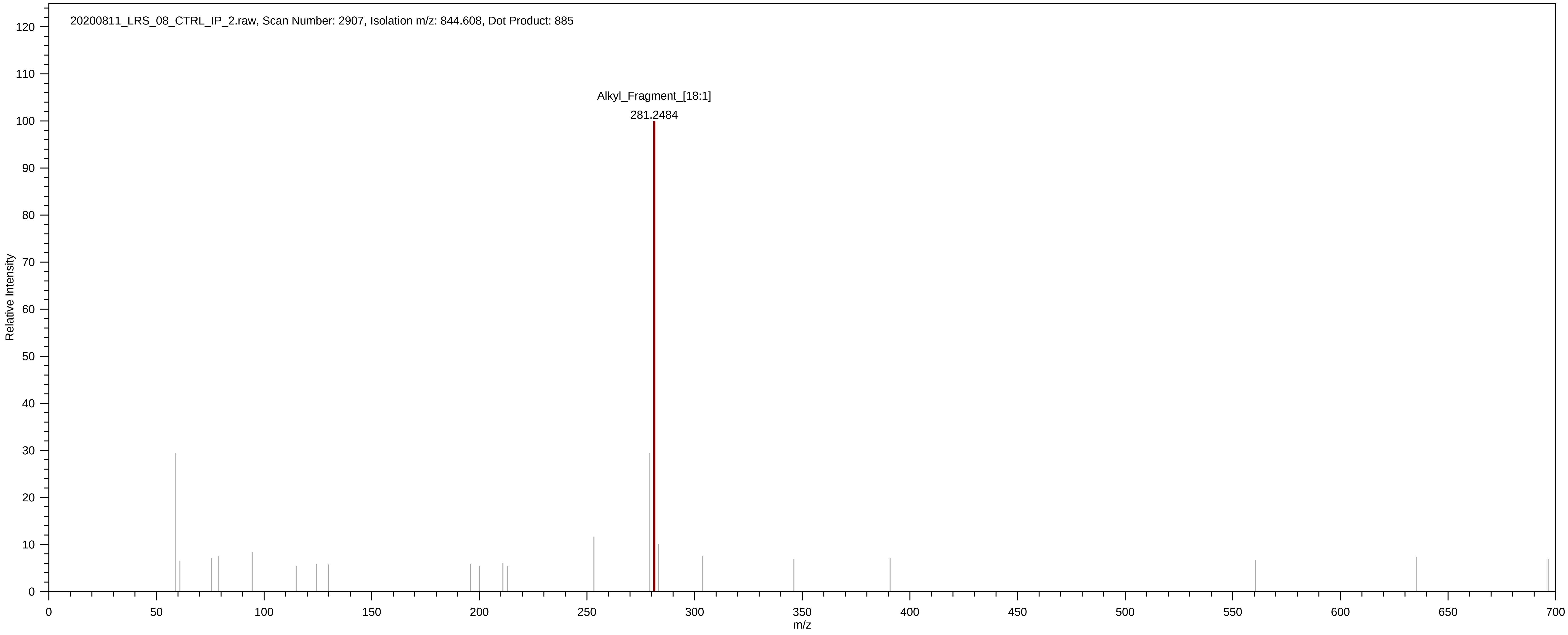

PC 18:1_18:1 [M+Ac-H]- | Ret. Time (min): 10.043 | m/z: 844.609

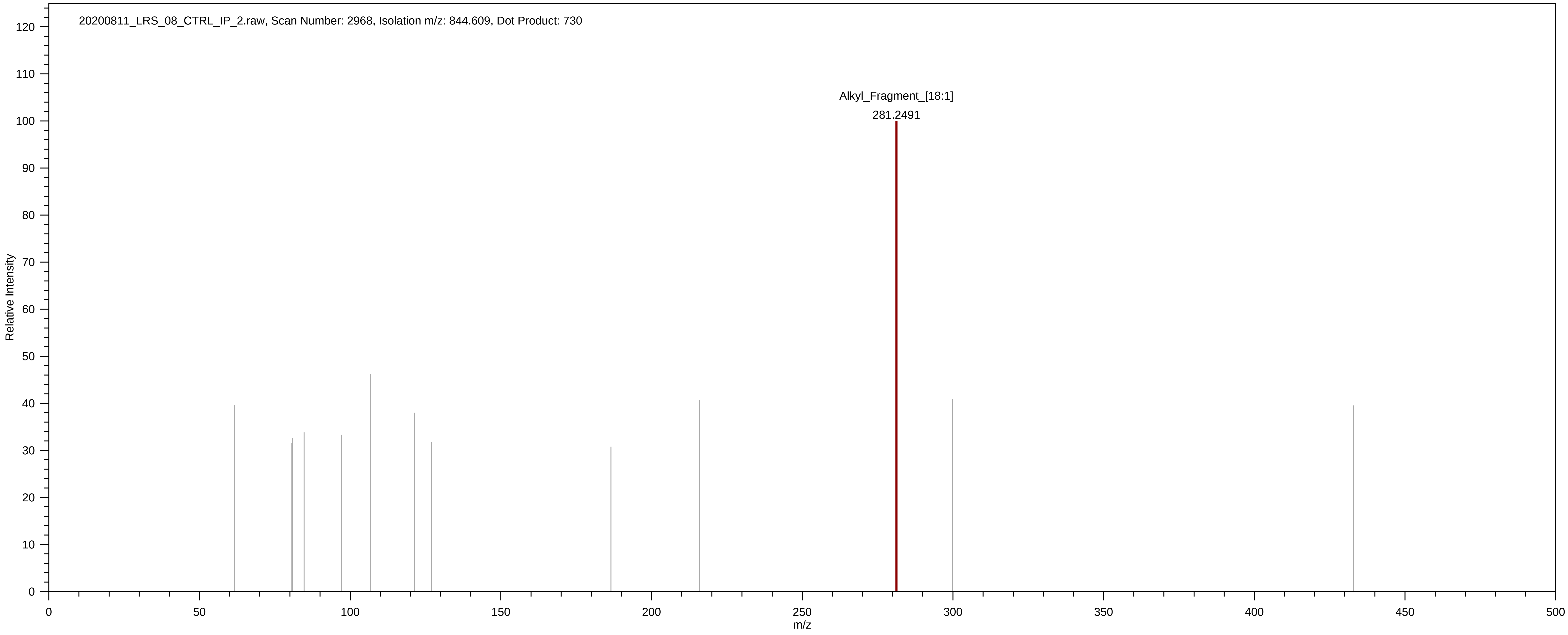

PC 18:1_20:1 [M+Ac-H]- | Ret. Time (min): 10.786 | m/z: 872.64

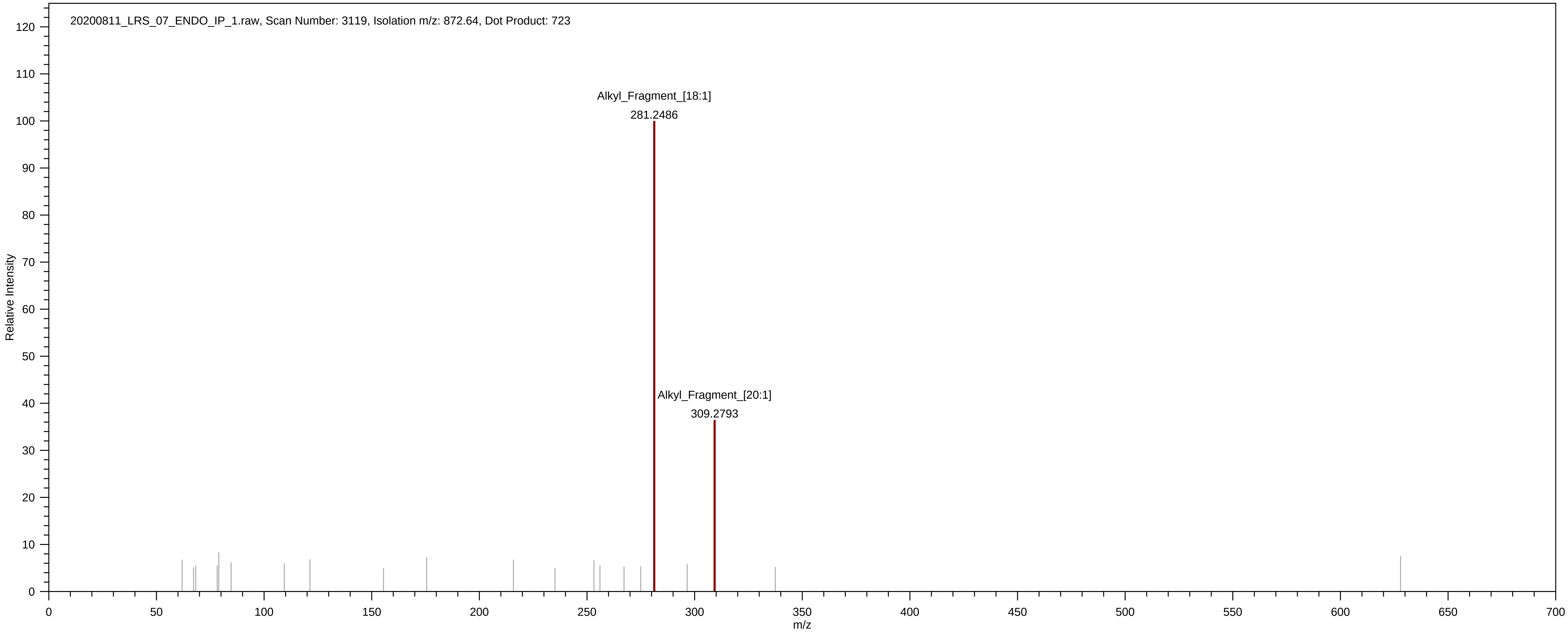

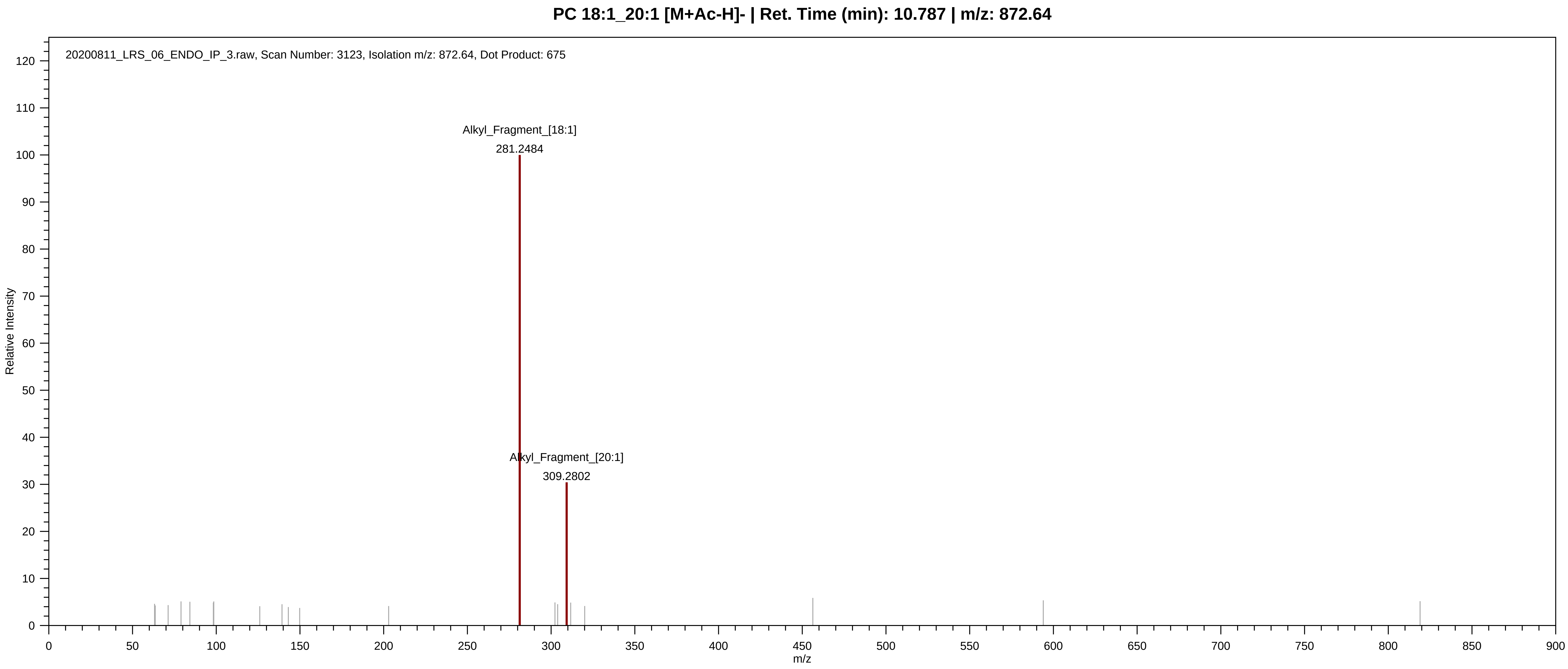
PC 18:1_20:1 [M+Ac-H]- | Ret. Time (min): 10.787 | m/z: 872.64
20200811_LRS_06_ENDO_IP_3.raw, Scan Number: 3123, Isolation m/z: 872.64, Dot Product: 675
Alkyl_Fragment_[18:1]
281.2484
Alkyl_Fragment_[20:1]
309.2802
Relative Intensity
m/z

PC 18:1_20:1 [M+Ac-H]- | Ret. Time (min): 10.788 | m/z: 872.638

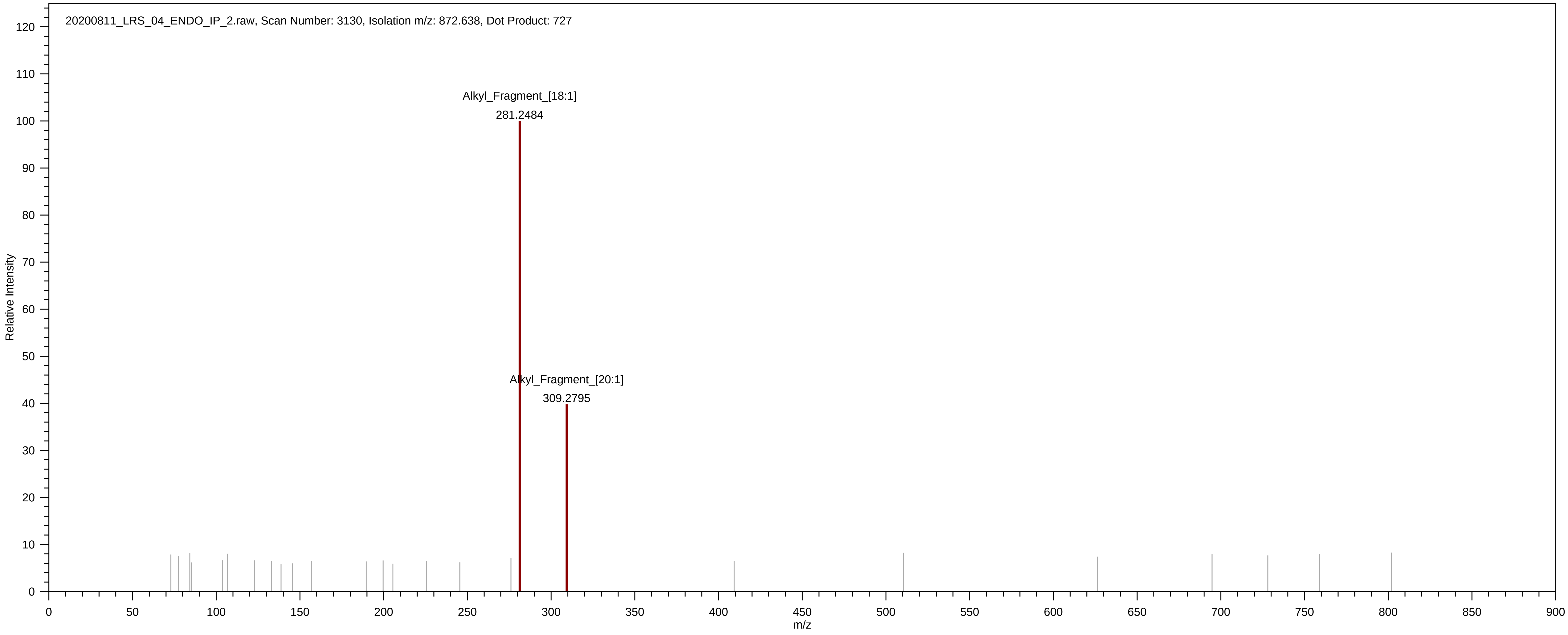

PC 18:1_20:1 [M+Ac-H]- | Ret. Time (min): 10.803 | m/z: 872.639

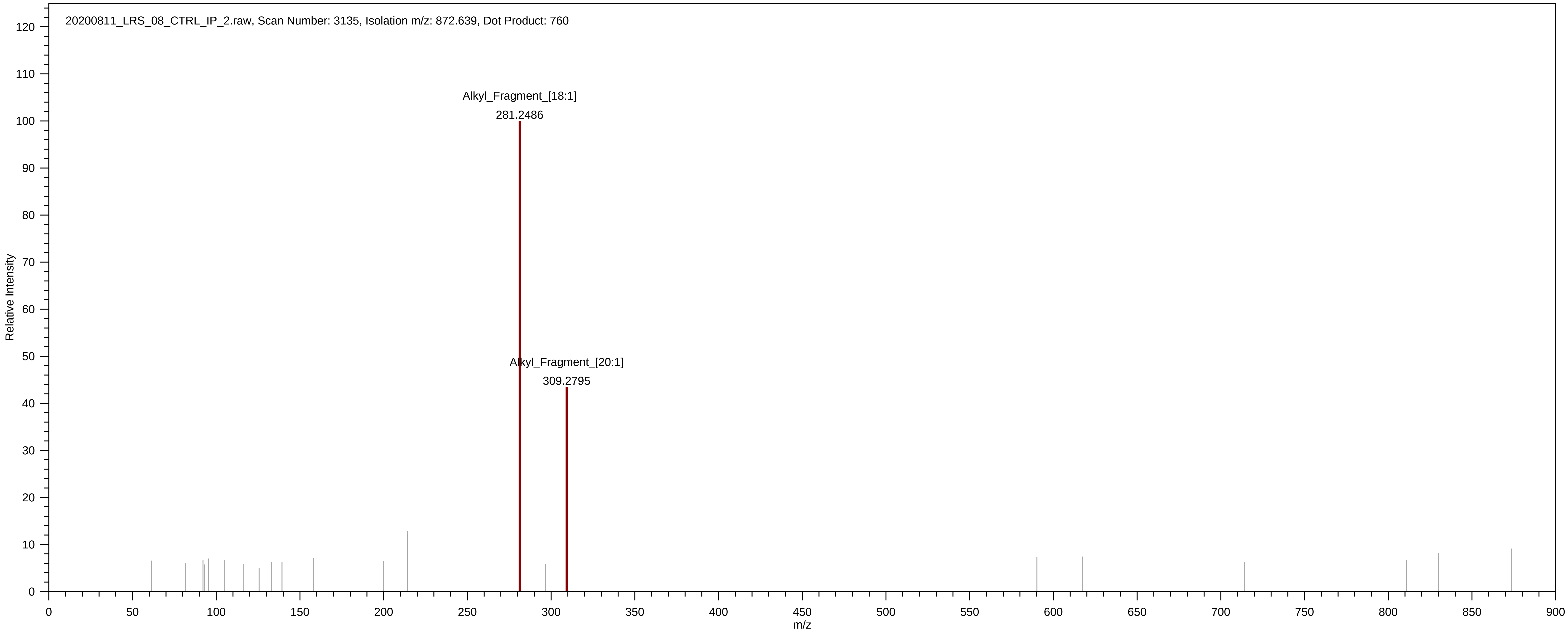

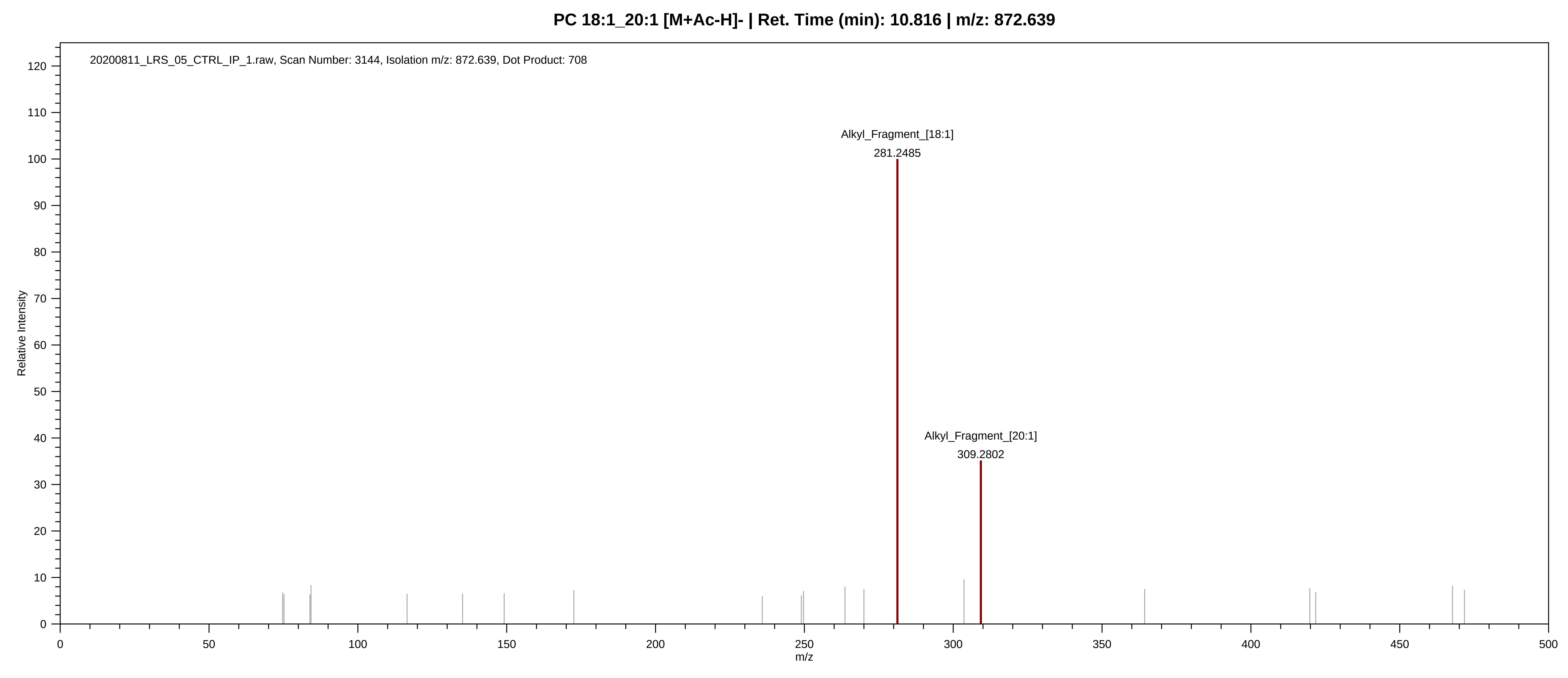
PC 18:1_20:1 [M+Ac-H]- | Ret. Time (min): 10.816 | m/z: 872.639
20200811_LRS_05_CTRL_IP_1.raw, Scan Number: 3144, Isolation m/z: 872.639, Dot Product: 708
Alkyl_Fragment_[18:1]
281.2485
Alkyl_Fragment_[20:1]
309.2802
Relative Intensity
m/z

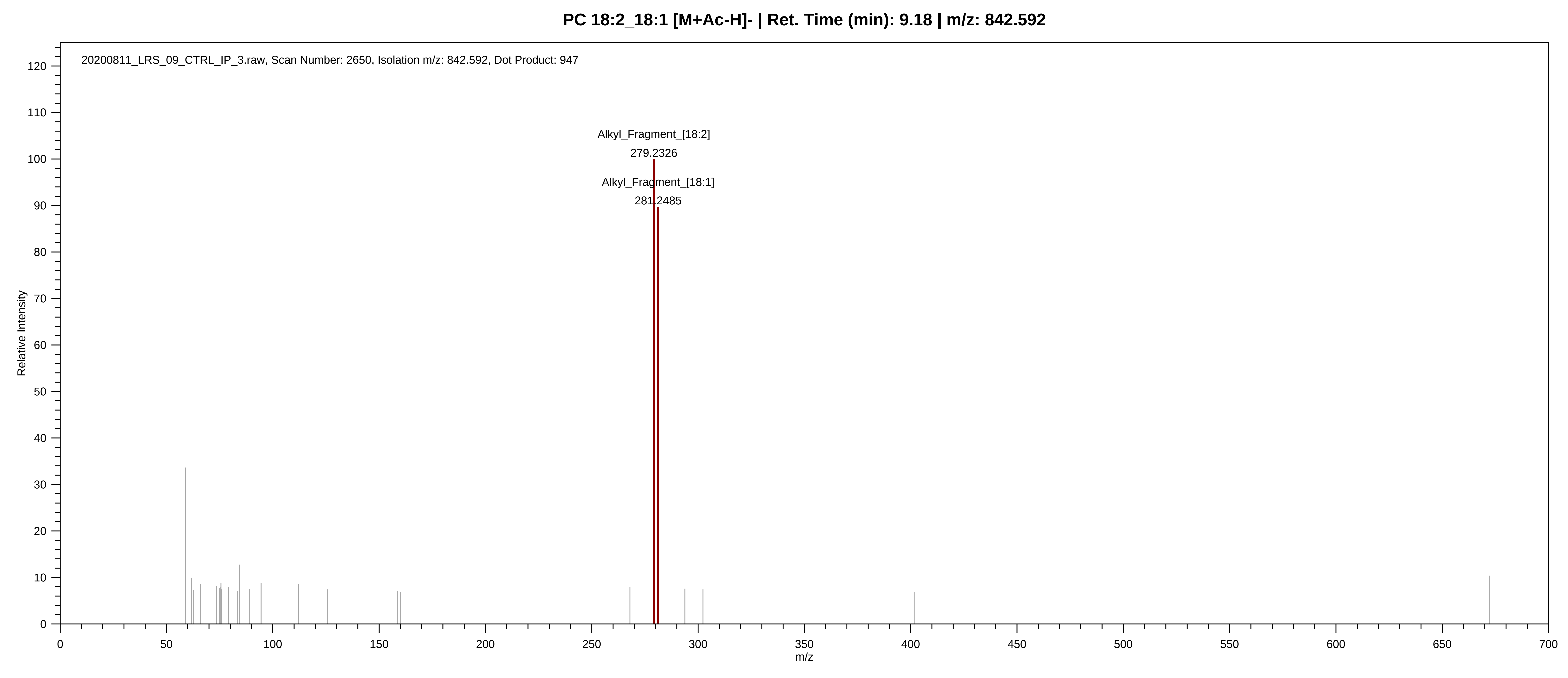
PC 18:2_18:1 [M+Ac-H]- | Ret. Time (min): 9.18 | m/z: 842.592
20200811_LRS_09_CTRL_IP_3.raw, Scan Number: 2650, Isolation m/z: 842.592, Dot Product: 947
Alkyl_Fragment_[18:2]
279.2326
Alkyl_Fragment_[18:1]
281.2485
Relative Intensity
m/z

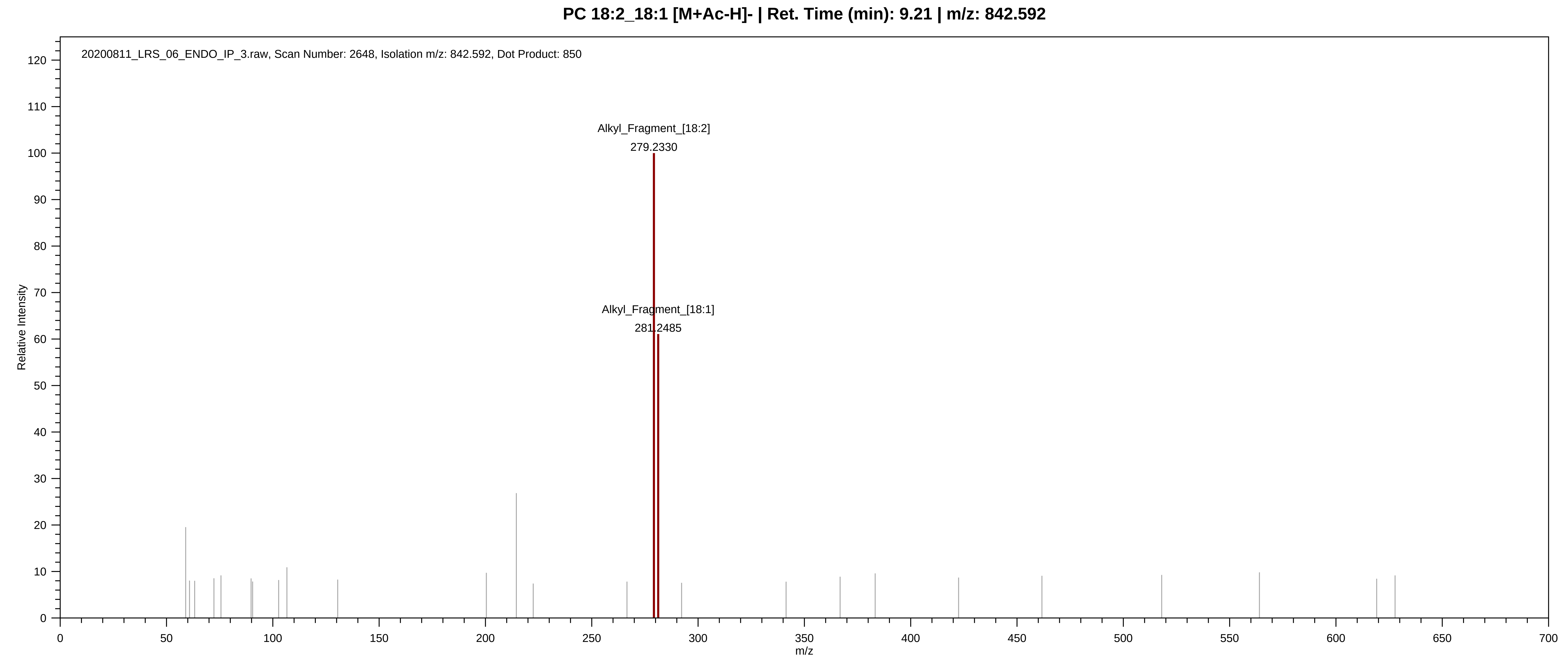
PC 18:2_18:1 [M+Ac-H]- | Ret. Time (min): 9.21 | m/z: 842.592
20200811_LRS_06_ENDO_IP_3.raw, Scan Number: 2648, Isolation m/z: 842.592, Dot Product: 850
Alkyl_Fragment_[18:2]
279.2330
Alkyl_Fragment_[18:1]
281.2485
Relative Intensity
m/z

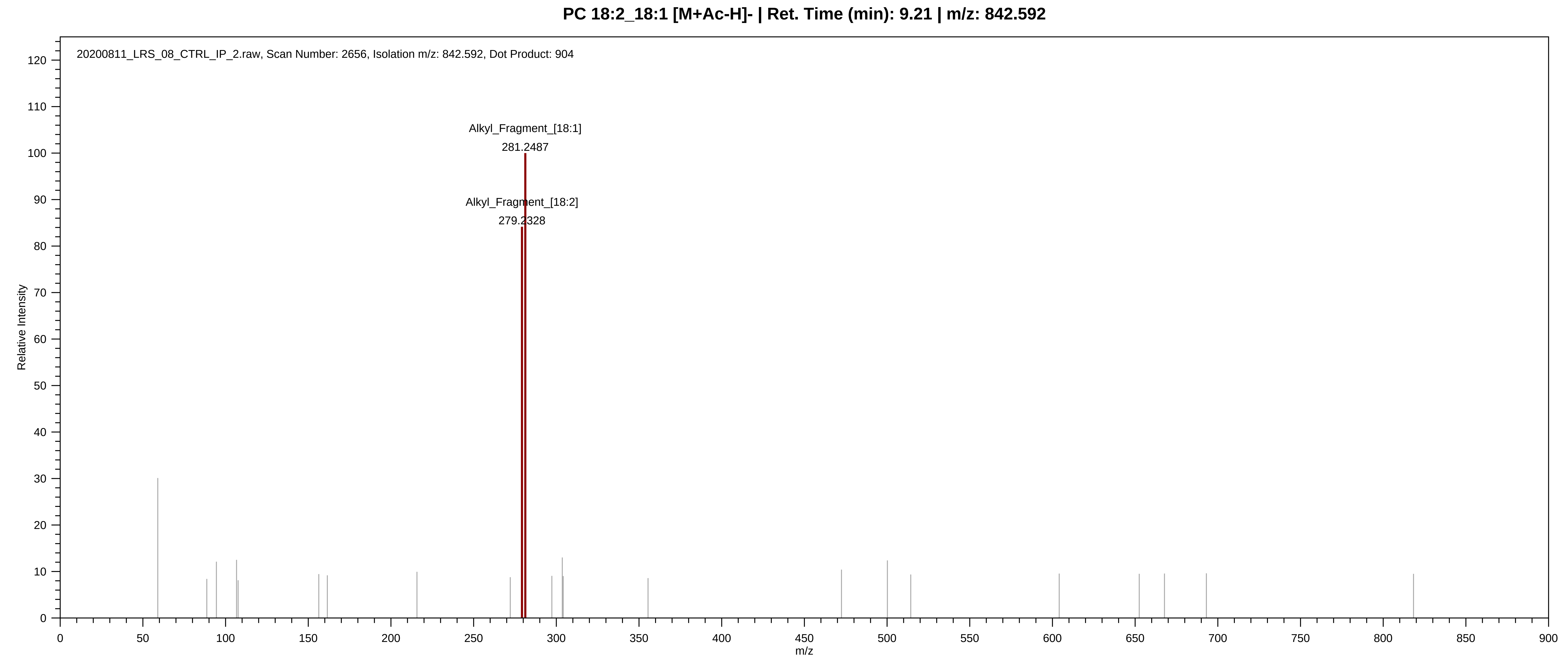

PC 18:2_18:1 [M+Ac-H]- | Ret. Time (min): 9.21 | m/z: 842.592
20200811_LRS_08_CTRL_IP_2.raw, Scan Number: 2656, Isolation m/z: 842.592, Dot Product: 904
Alkyl_Fragment_[18:1]
281.2487
Alkyl_Fragment_[18:2]
279.2328
Relative Intensity
m/z

PC 18:2_18:1 [M+Ac-H]- | Ret. Time (min): 9.217 | m/z: 842.592

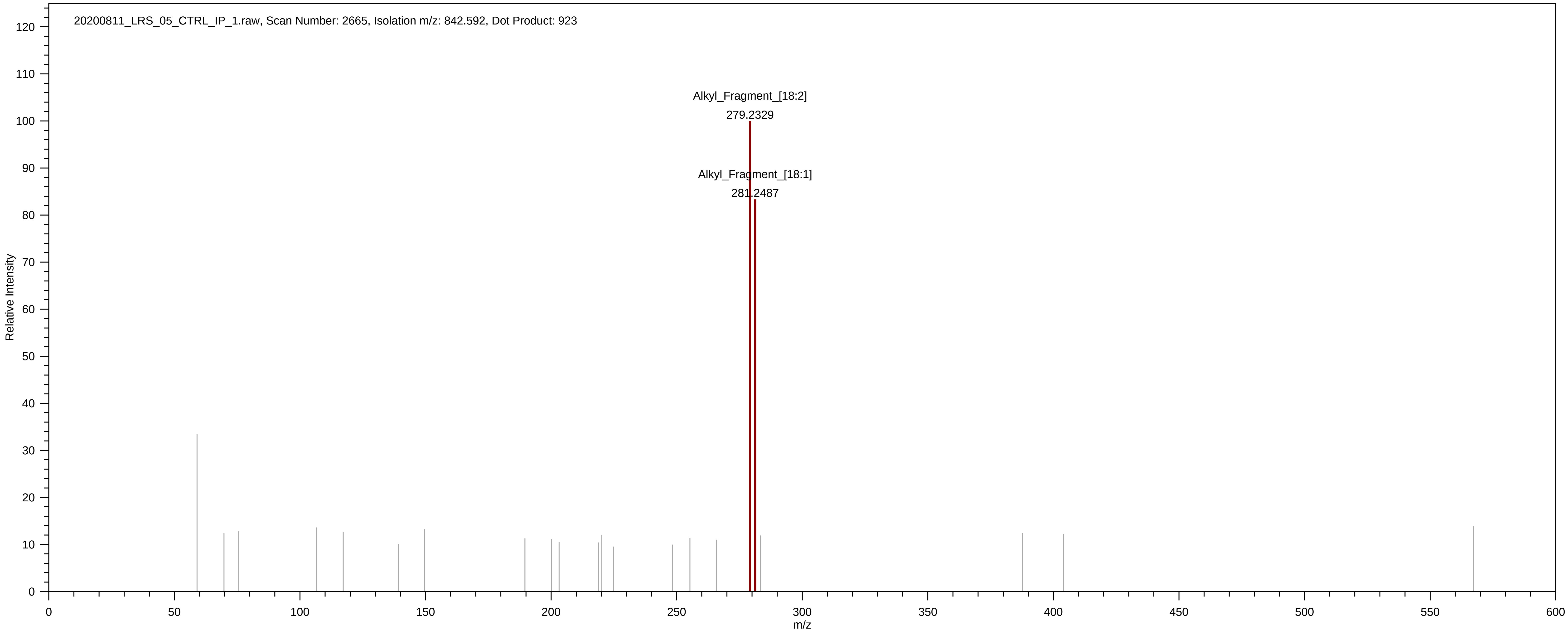

PC 27:0 [M+H]+ | Ret. Time (min): 7.551 | m/z: 664.496

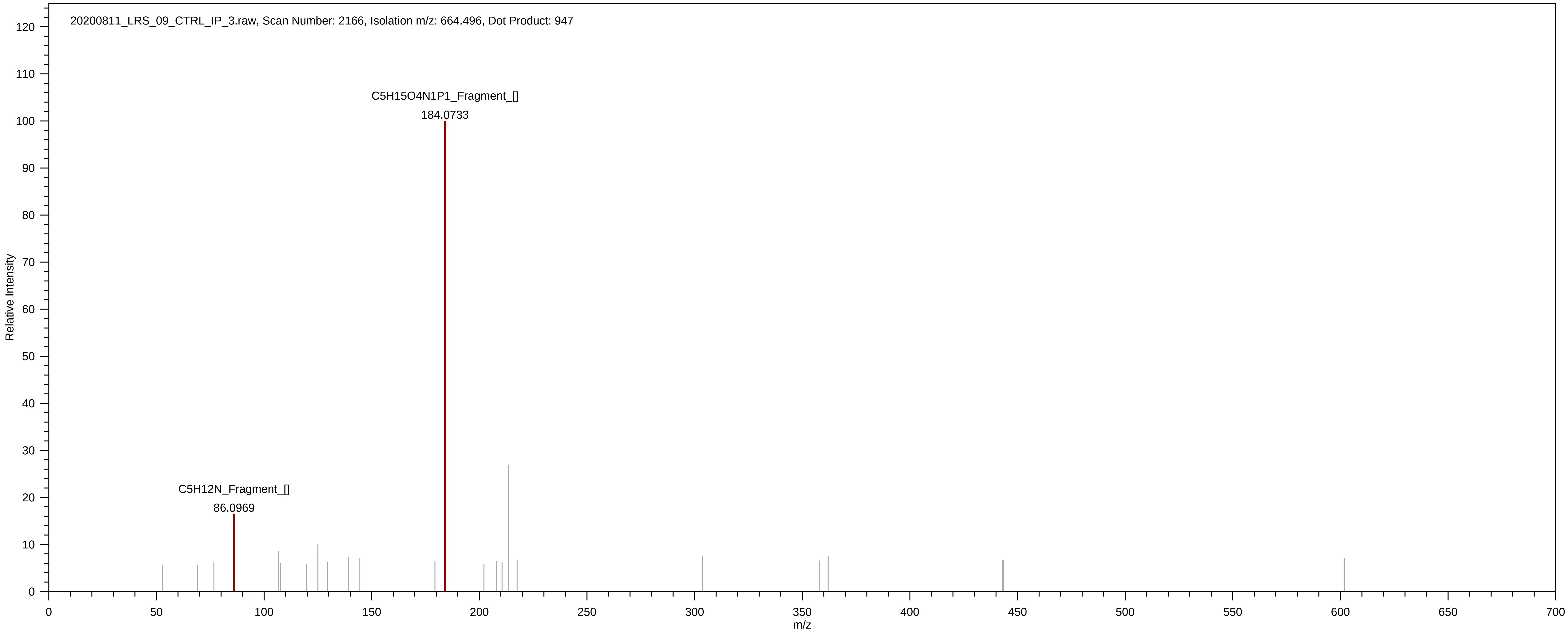

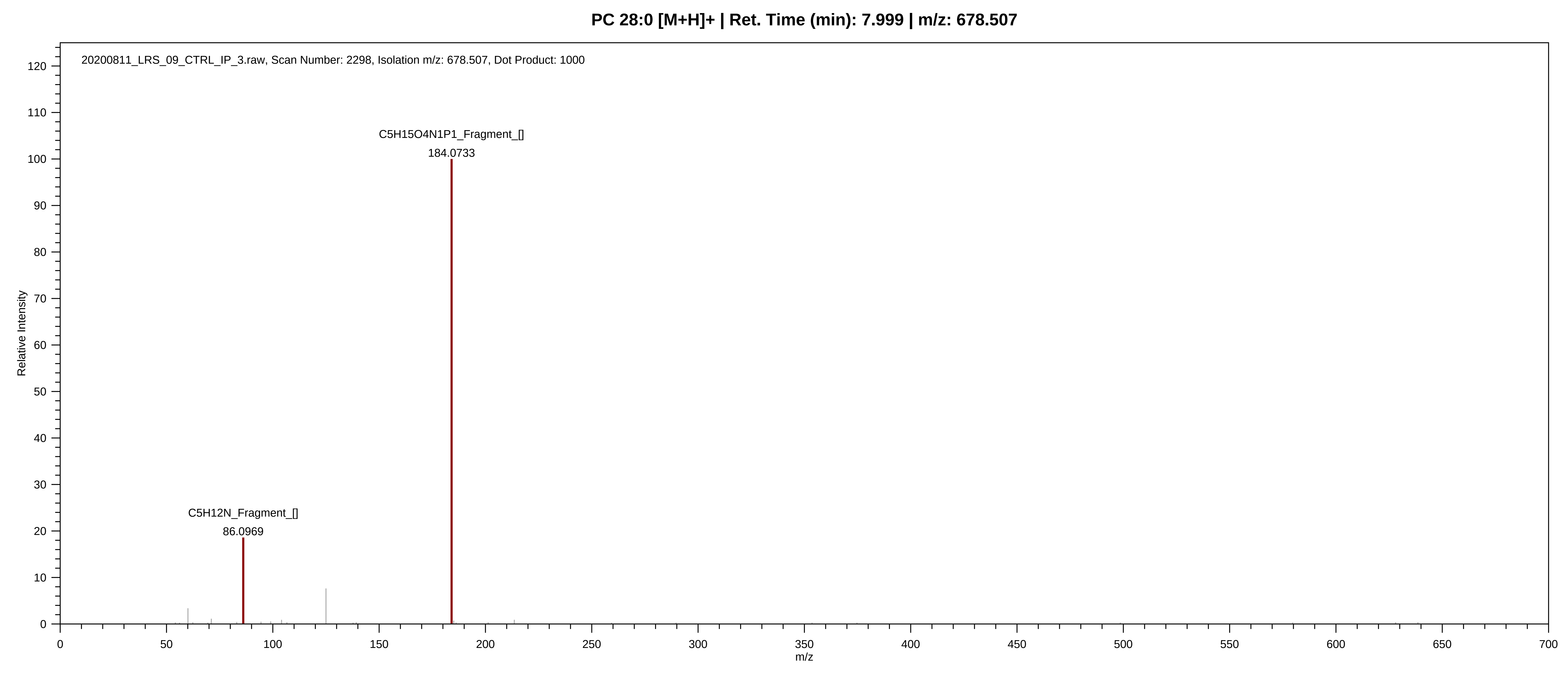

PC 28:0 [M+H]+ | Ret. Time (min): 7.999 | m/z: 678.507
20200811_LRS_09_CTRL_IP_3.raw, Scan Number: 2298, Isolation m/z: 678.507, Dot Product: 1000
C5H15O4N1P1_Fragment_[]
184.0733
C5H12N_Fragment_[]
86.0969
Relative Intensity
m/z

PC 28:0 [M+H]+ | Ret. Time (min): 8.001 | m/z: 678.507

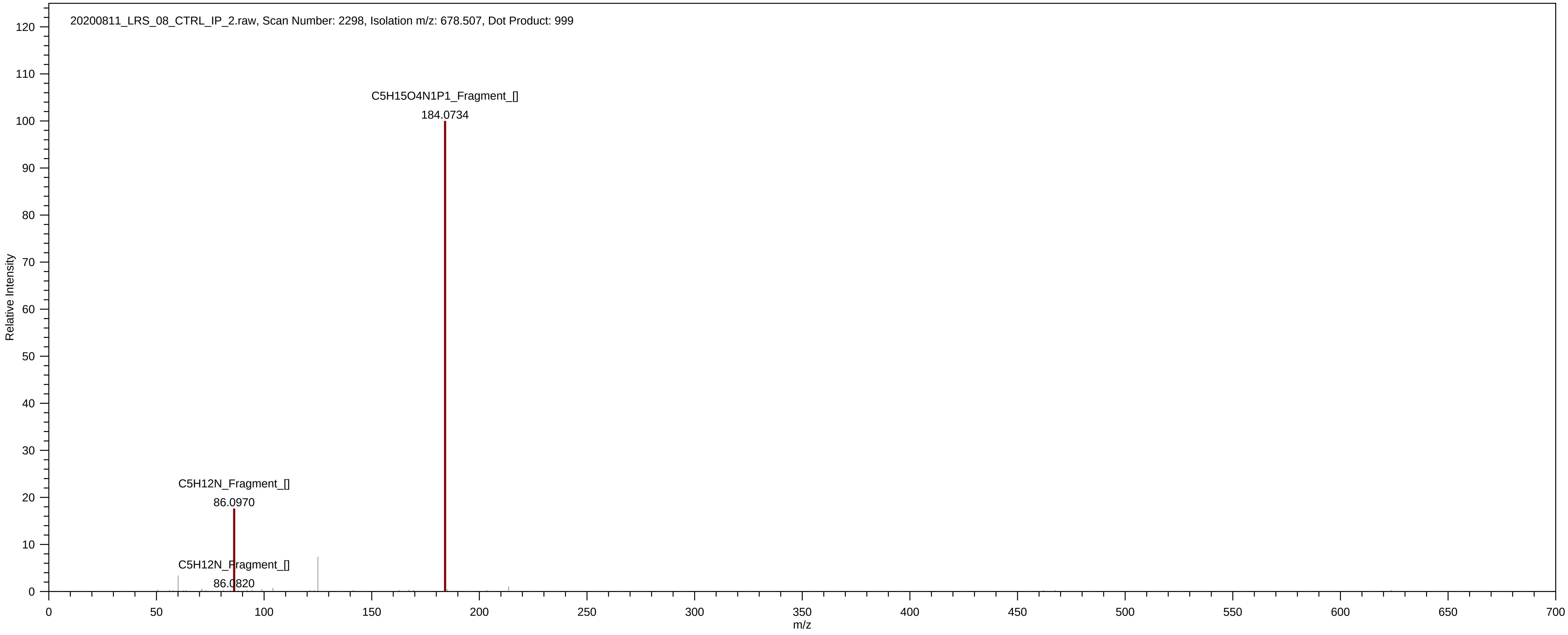

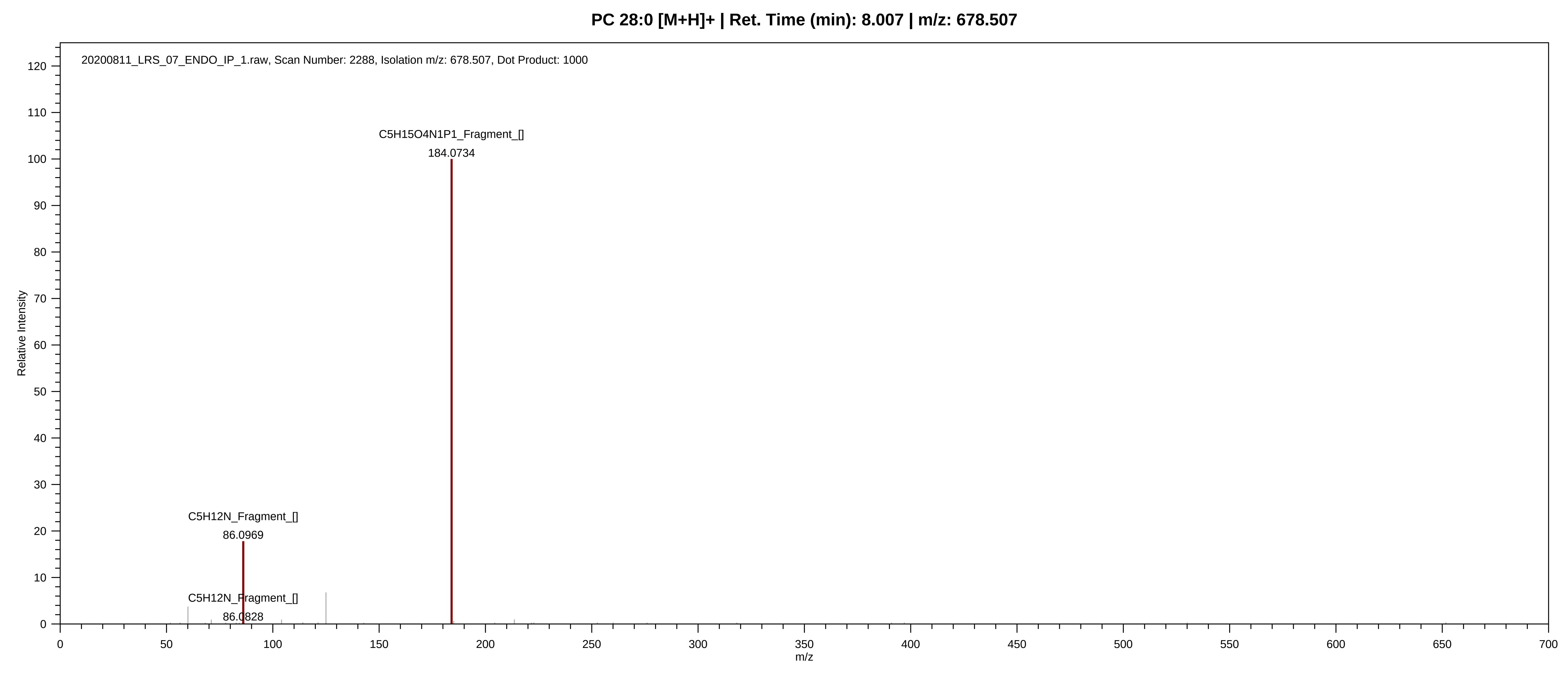
PC 28:0 [M+H]+ | Ret. Time (min): 8.007 | m/z: 678.507
20200811_LRS_07_ENDO_IP_1.raw, Scan Number: 2288, Isolation m/z: 678.507, Dot Product: 1000
C5H15O4N1P1_Fragment_[]
184.0734
C5H12N_Fragment_[]
86.0969
C5H12N_Fragment_[]
86.0828
Relative Intensity
m/z

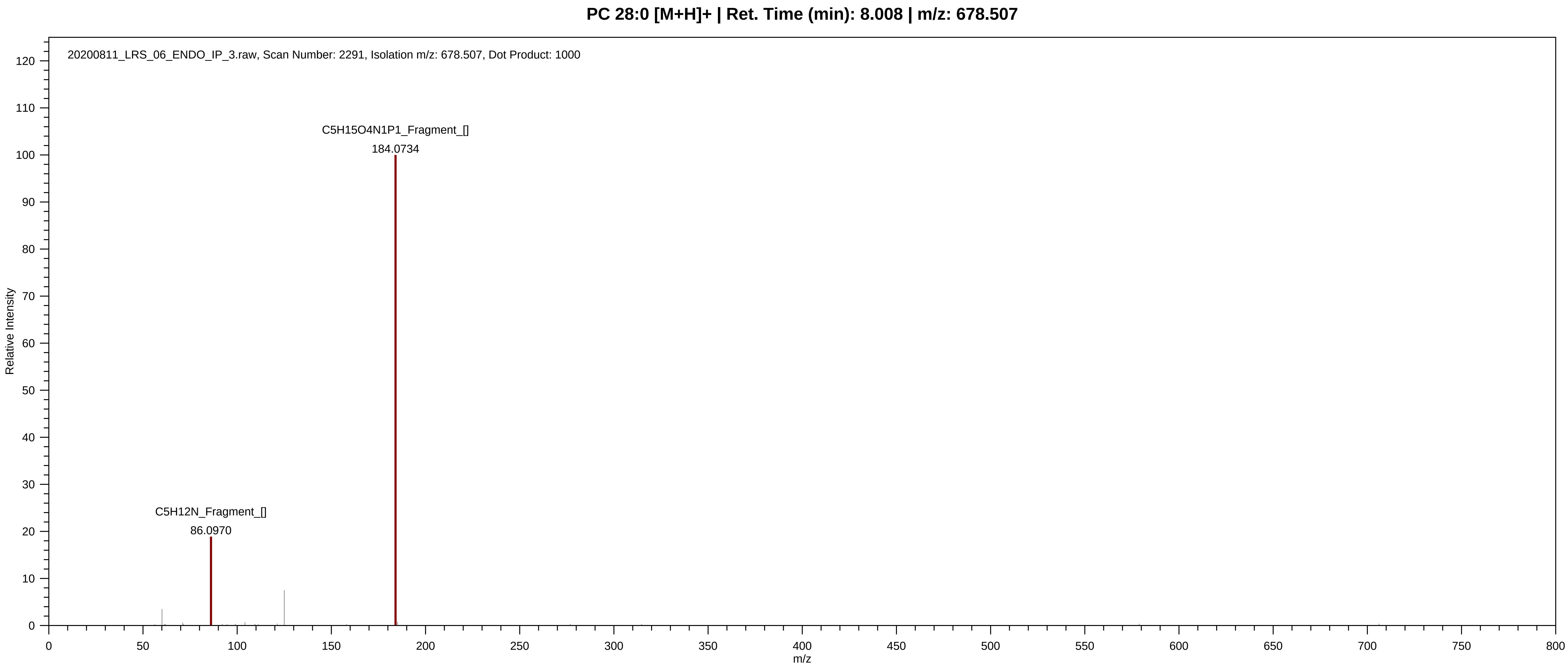
PC 28:0 [M+H]+ | Ret. Time (min): 8.008 | m/z: 678.507
20200811_LRS_06_ENDO_IP_3.raw, Scan Number: 2291, Isolation m/z: 678.507, Dot Product: 1000
C5H15O4N1P1_Fragment_[]
184.0734
C5H12N_Fragment_[]
86.0970
Relative Intensity
m/z

PC 28:0 [M+H]+ | Ret. Time (min): 8.208 | m/z: 678.507

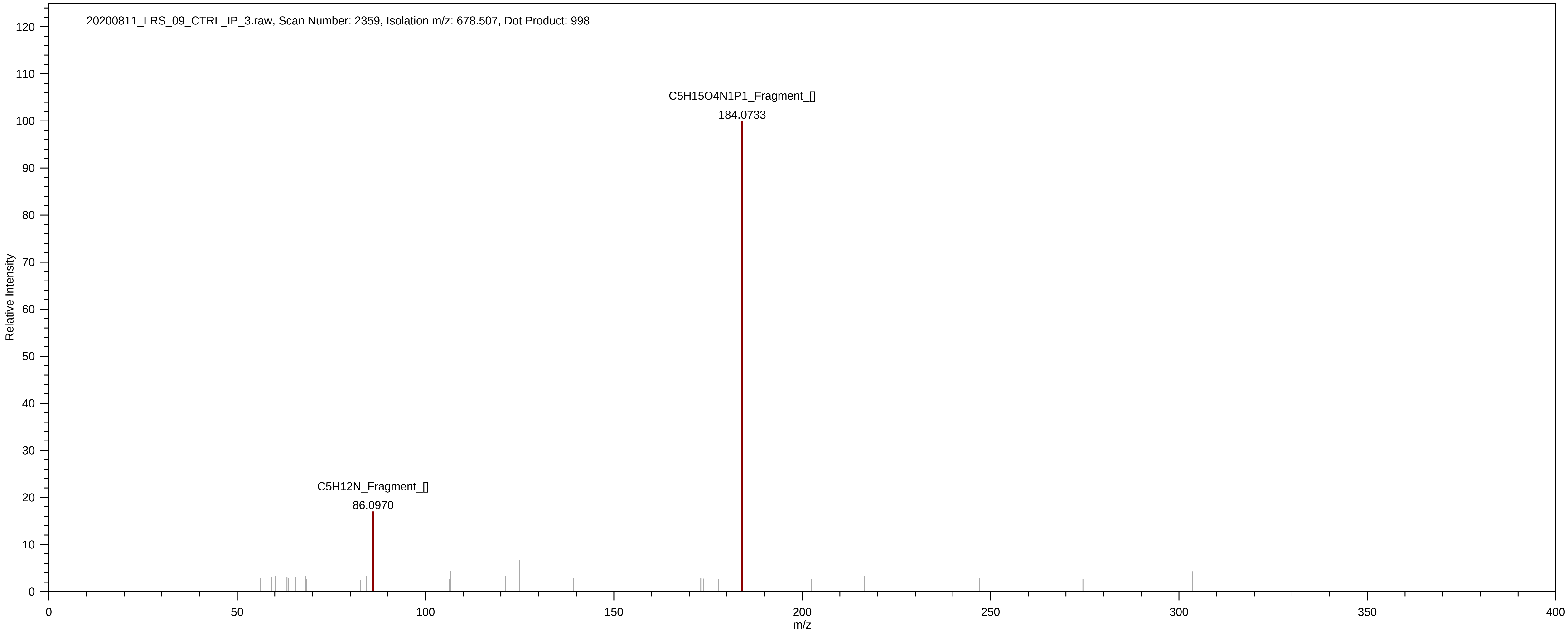

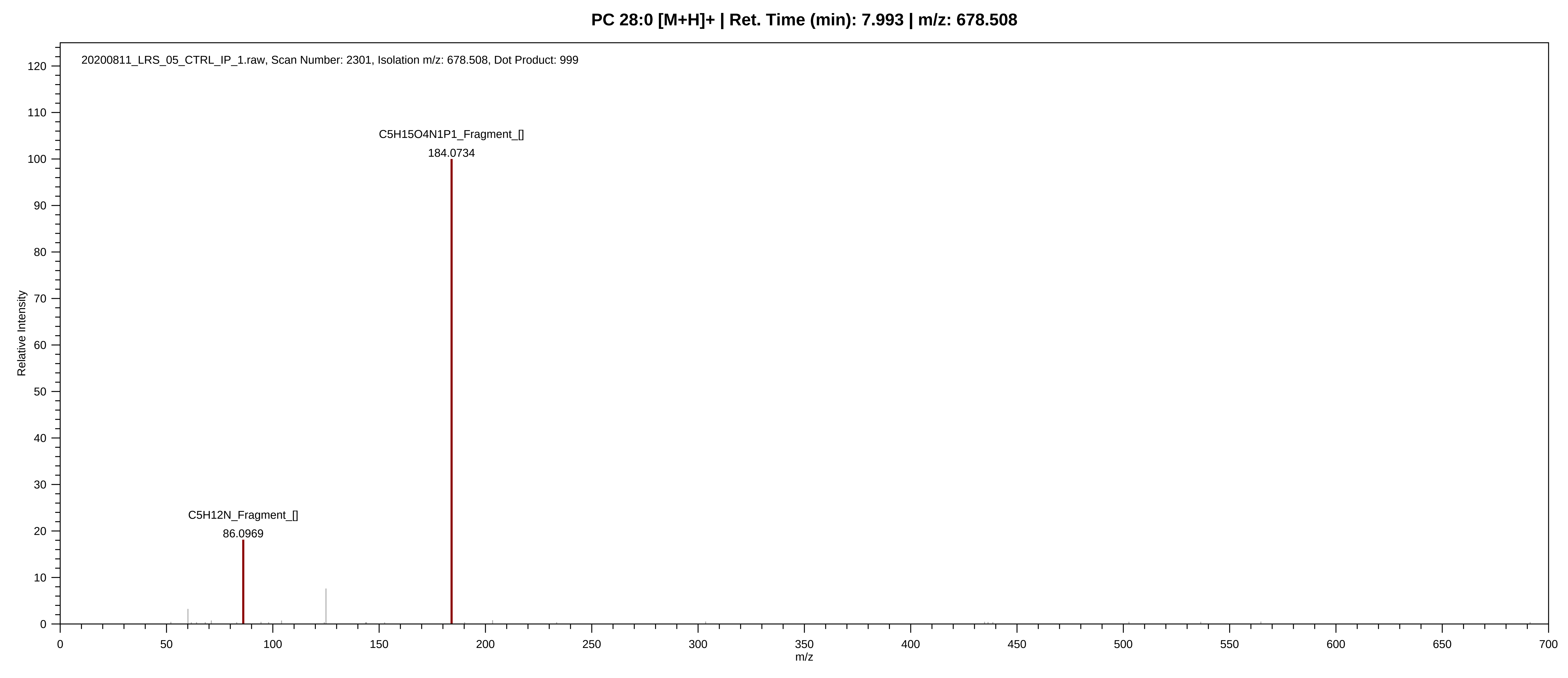
PC 28:0 [M+H]+ | Ret. Time (min): 7.993 | m/z: 678.508
20200811_LRS_05_CTRL_IP_1.raw, Scan Number: 2301, Isolation m/z: 678.508, Dot Product: 999
C5H15O4N1P1_Fragment_[]
184.0734
C5H12N_Fragment_[]
86.0969
Relative Intensity
m/z

PC 28:0 [M+H]+ | Ret. Time (min): 7.999 | m/z: 678.508

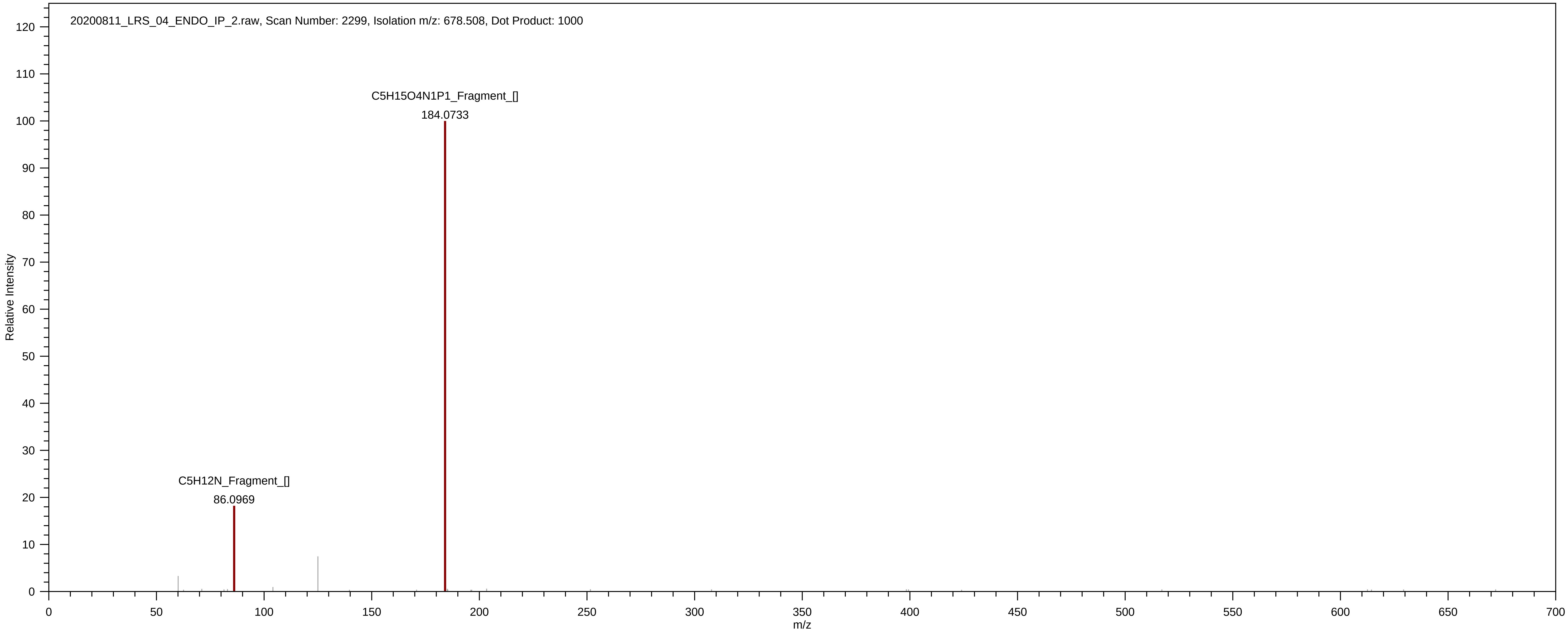

PC 28:0 [M+H]+ | Ret. Time (min): 8.178 | m/z: 678.508

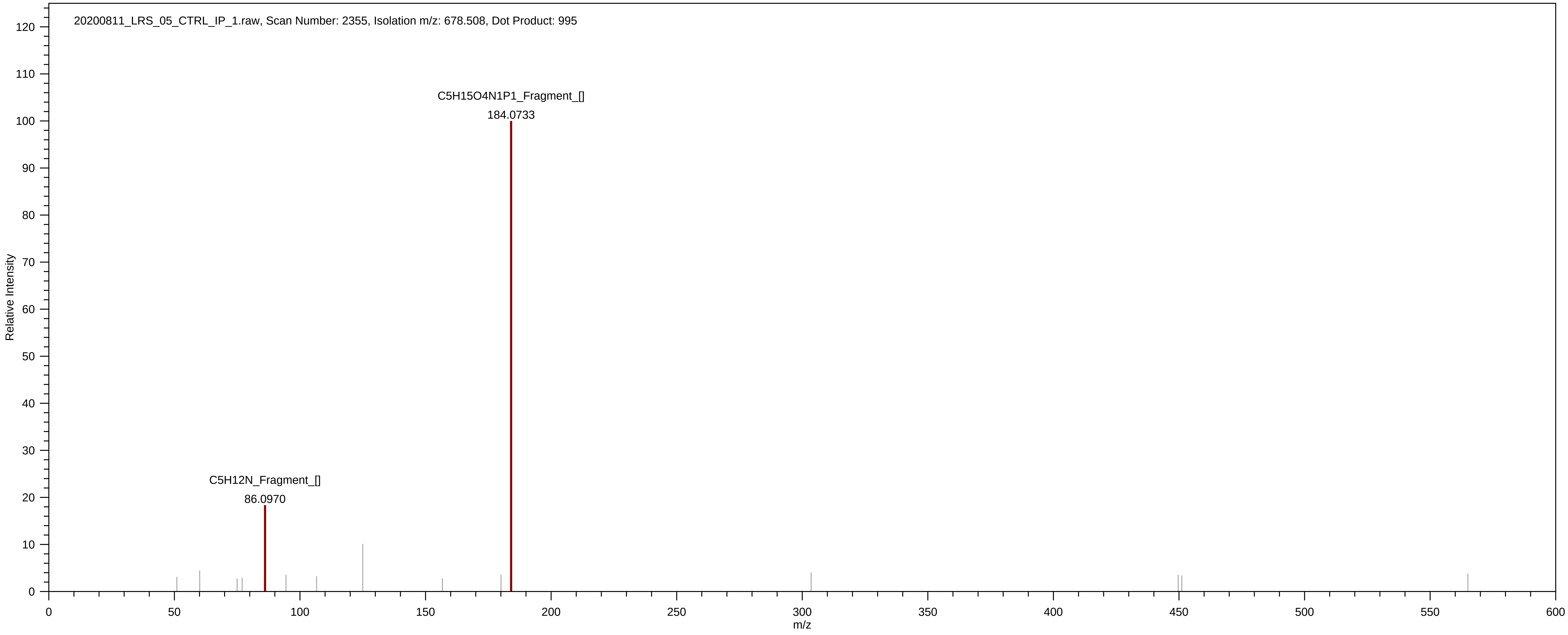

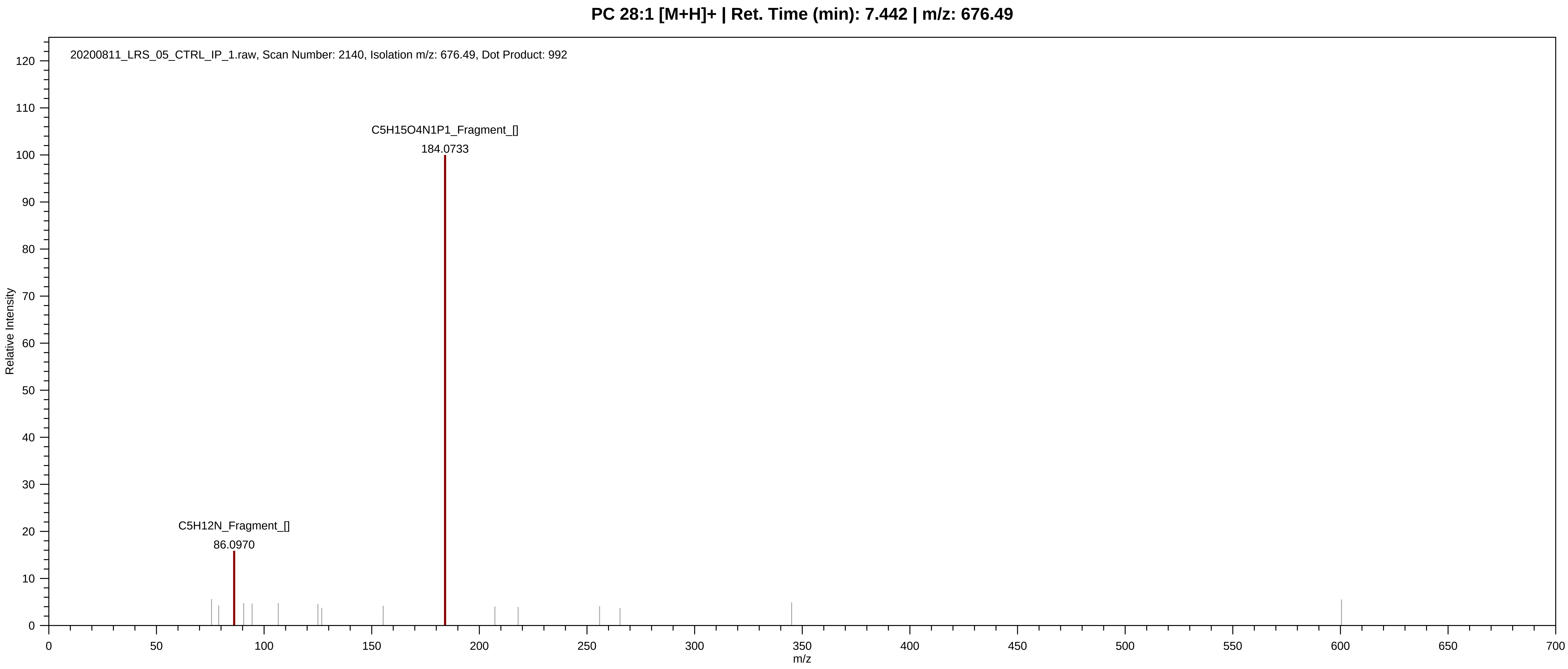

PC 28:1 [M+H]+ | Ret. Time (min): 7.442 | m/z: 676.49
20200811_LRS_05_CTRL_IP_1.raw, Scan Number: 2140, Isolation m/z: 676.49, Dot Product: 992
C5H15O4N1P1_Fragment_[]
184.0733
C5H12N_Fragment_[]
86.0970
Relative Intensity
m/z

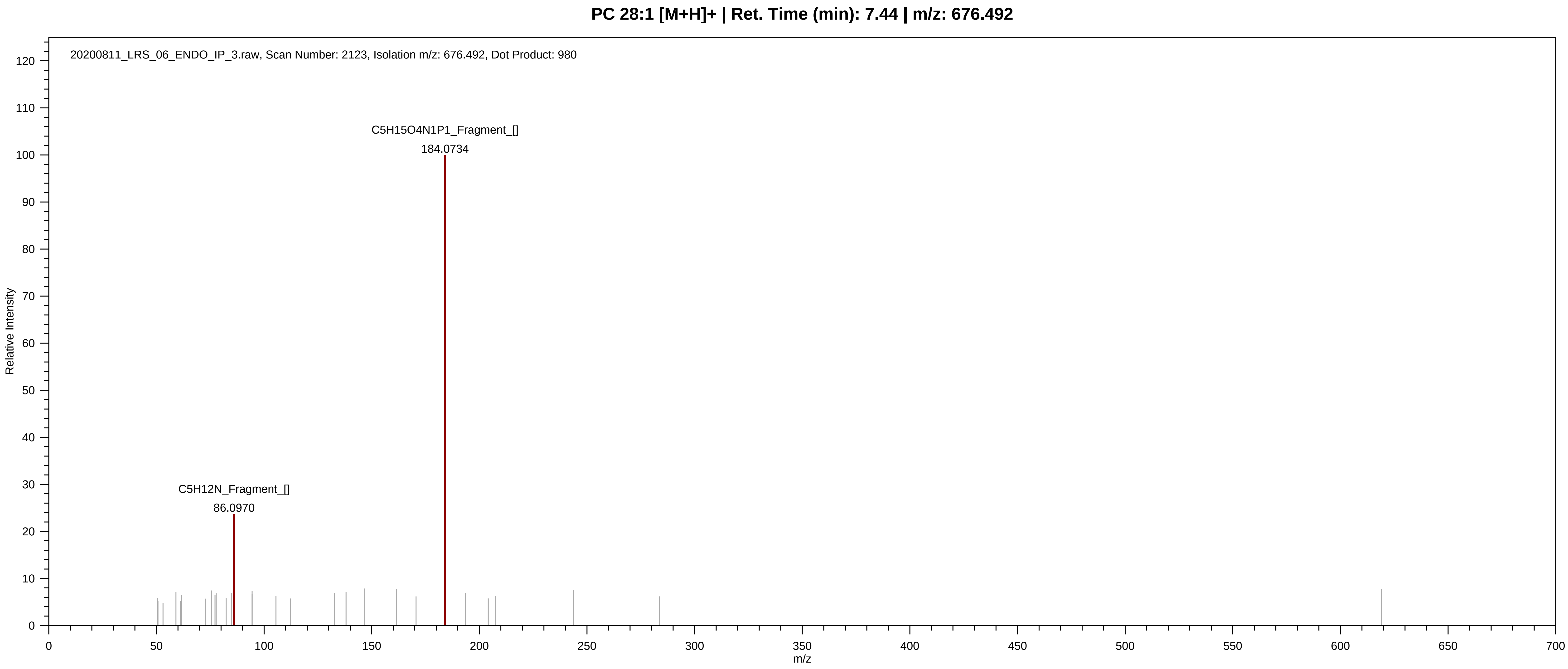
PC 28:1 [M+H]+ | Ret. Time (min): 7.44 | m/z: 676.492
20200811_LRS_06_ENDO_IP_3.raw, Scan Number: 2123, Isolation m/z: 676.492, Dot Product: 980
C5H15O4N1P1_Fragment_[]
184.0734
C5H12N_Fragment_[]
86.0970
Relative Intensity
m/z

PC 28:1 [M+H]+ | Ret. Time (min): 7.408 | m/z: 676.492

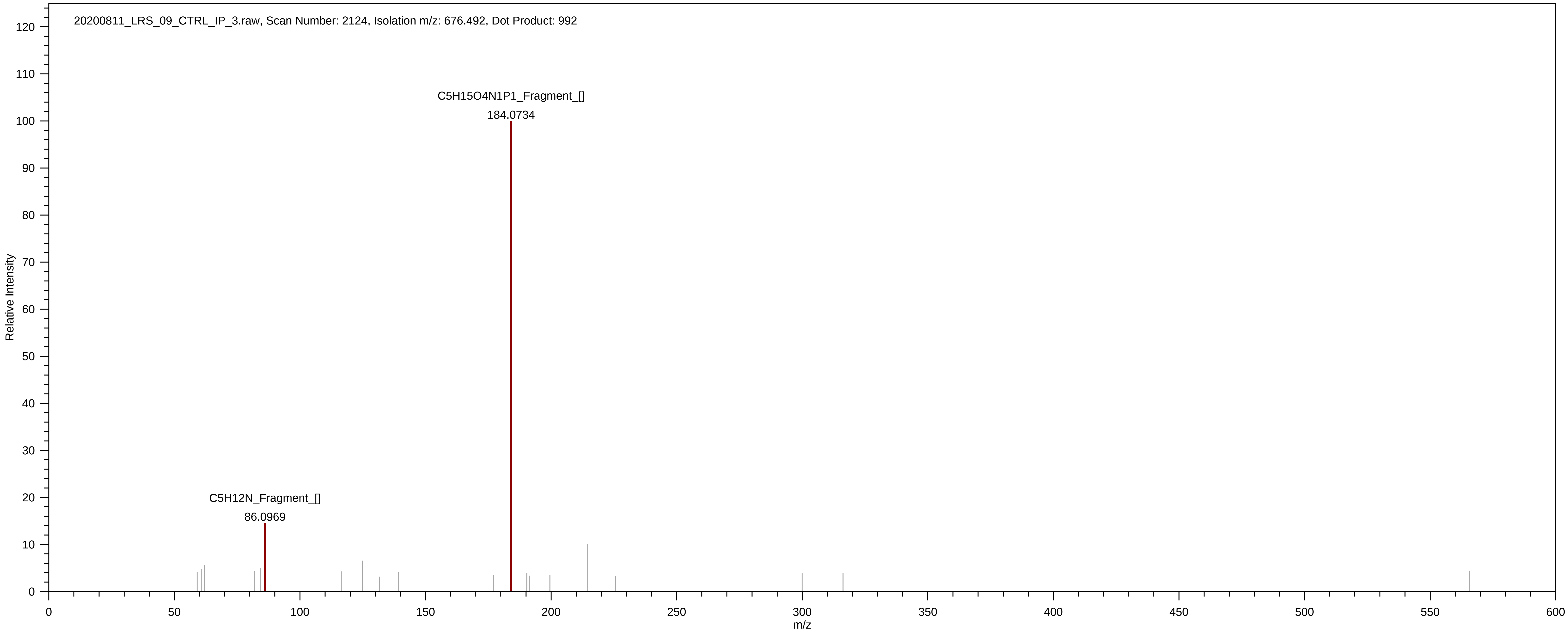

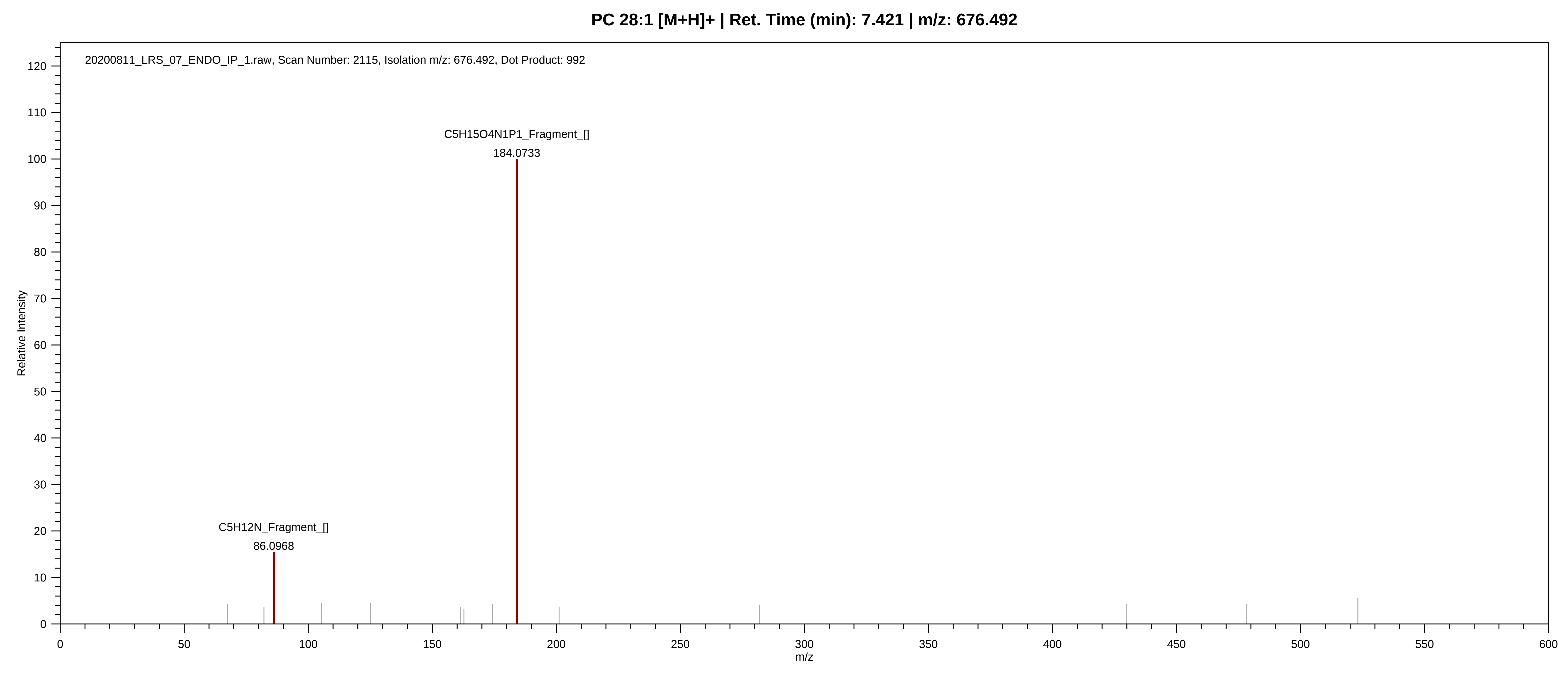

PC 28:1 [M+H]+ | Ret. Time (min): 7.421 | m/z: 676.492
20200811_LRS_07_ENDO_IP_1.raw, Scan Number: 2115, Isolation m/z: 676.492, Dot Product: 992
C5H15O4N1P1_Fragment_[]
184.0733
C5H12N_Fragment_[]
86.0968
Relative Intensity
m/z

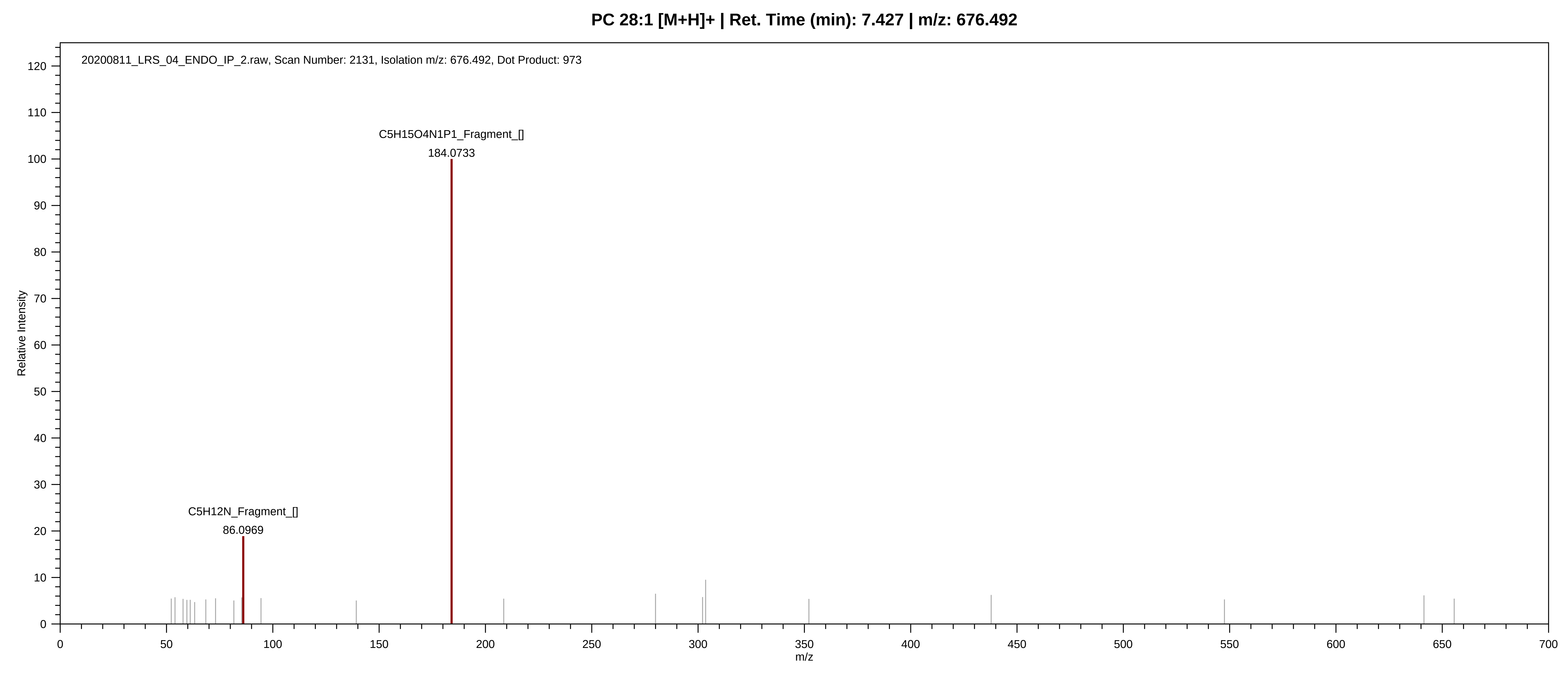
PC 28:1 [M+H]+ | Ret. Time (min): 7.427 | m/z: 676.492
20200811_LRS_04_ENDO_IP_2.raw, Scan Number: 2131, Isolation m/z: 676.492, Dot Product: 973
C5H15O4N1P1_Fragment_[]
184.0733
C5H12N_Fragment_[]
86.0969
Relative Intensity
m/z

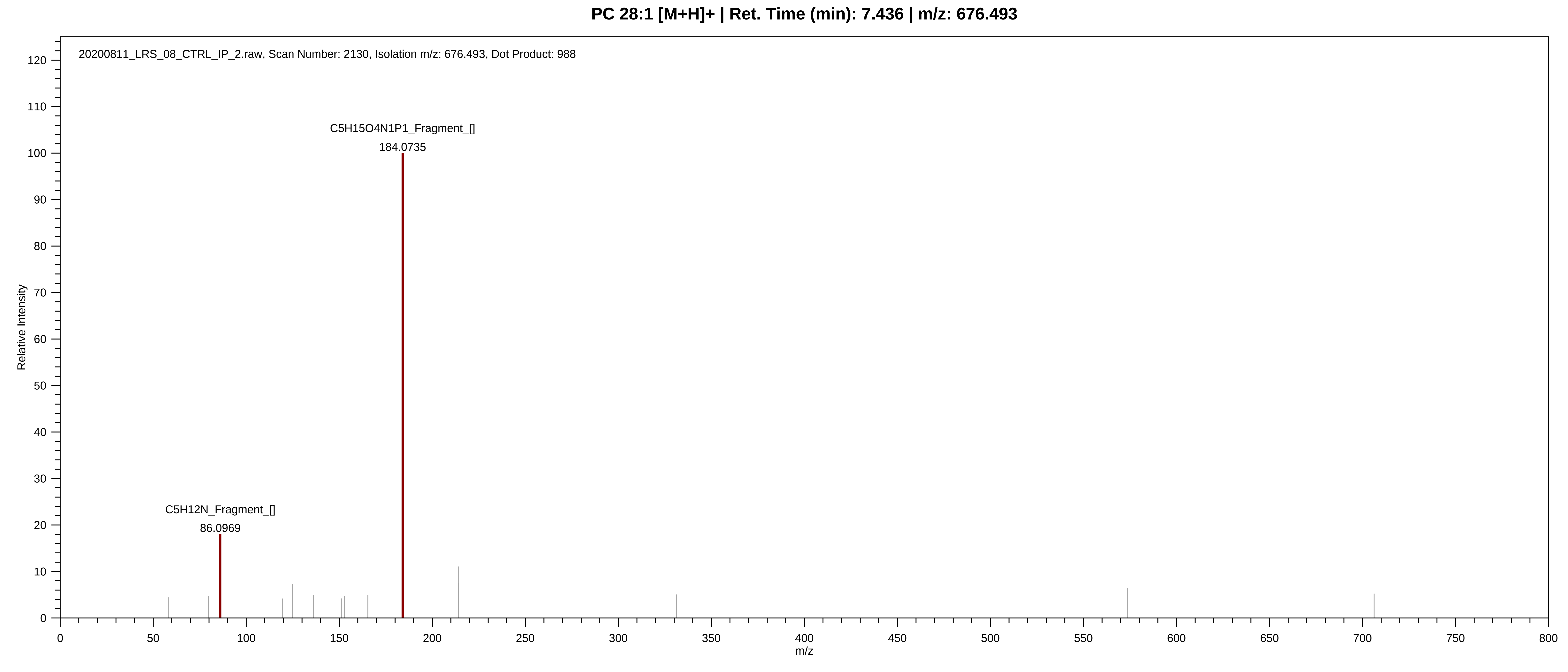
PC 28:1 [M+H]+ | Ret. Time (min): 7.436 | m/z: 676.493
20200811_LRS_08_CTRL_IP_2.raw, Scan Number: 2130, Isolation m/z: 676.493, Dot Product: 988
C5H15O4N1P1_Fragment_[]
184.0735
C5H12N_Fragment_[]
86.0969
Relative Intensity
m/z

PC 29:0 [M+H]+ | Ret. Time (min): 8.308 | m/z: 692.52

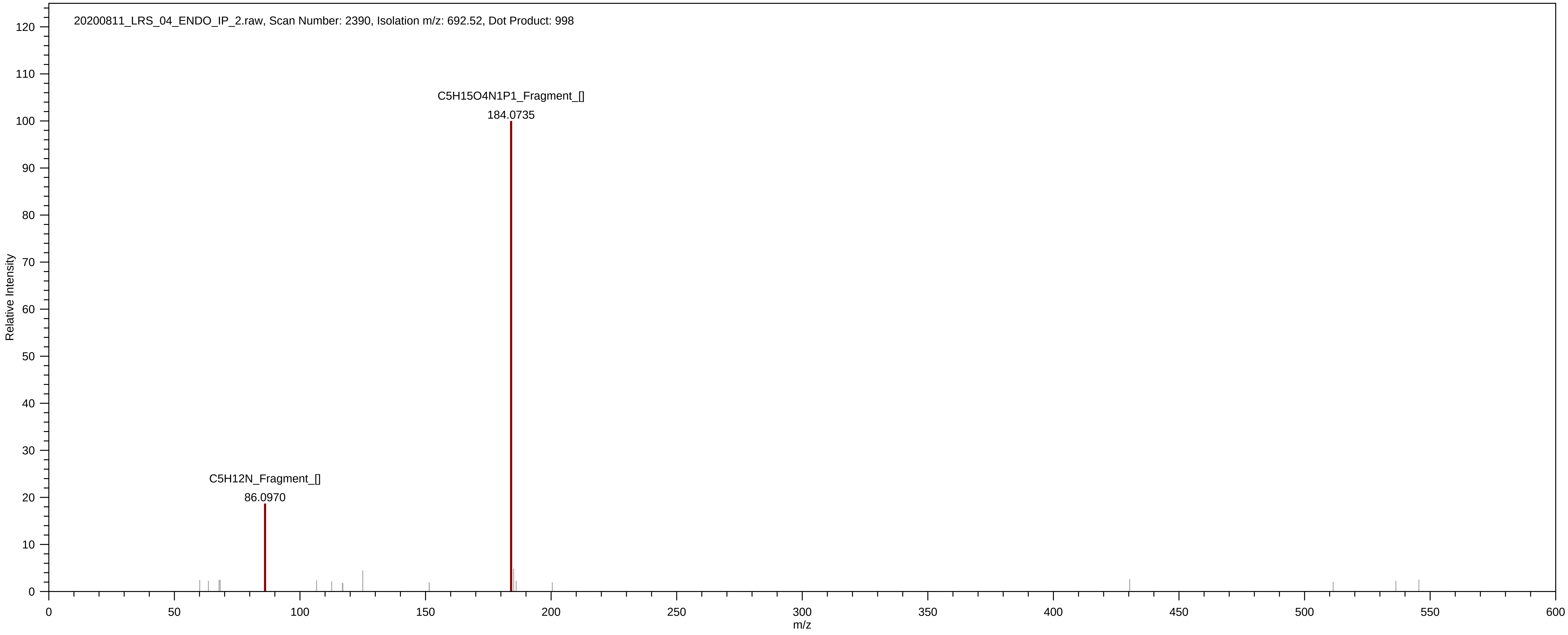

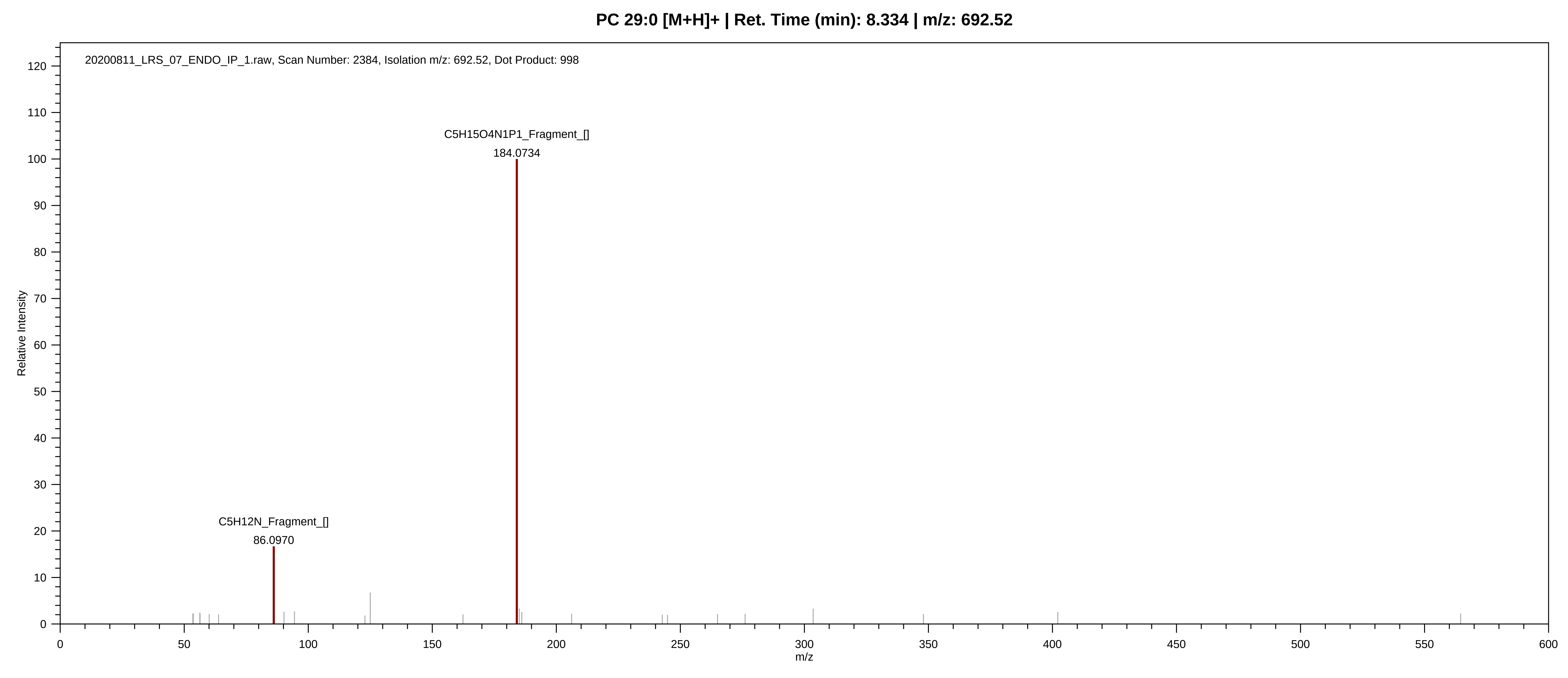

PC 29:0 [M+H]+ | Ret. Time (min): 8.334 | m/z: 692.52
20200811_LRS_07_ENDO_IP_1.raw, Scan Number: 2384, Isolation m/z: 692.52, Dot Product: 998
C5H15O4N1P1_Fragment_[]
184.0734
C5H12N_Fragment_[]
86.0970
Relative Intensity
m/z

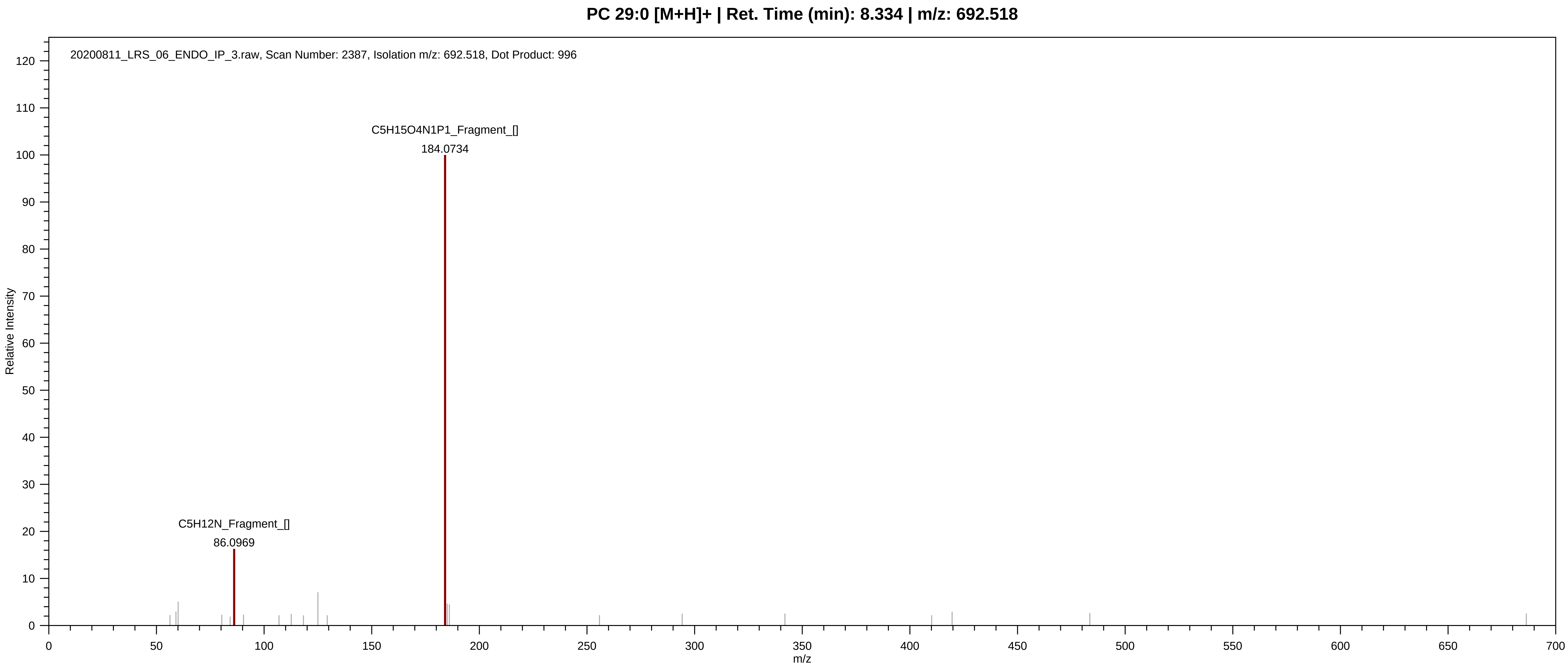

PC 29:0 [M+H]+ | Ret. Time (min): 8.334 | m/z: 692.518
20200811_LRS_06_ENDO_IP_3.raw, Scan Number: 2387, Isolation m/z: 692.518, Dot Product: 996
C5H15O4N1P1_Fragment_[]
184.0734
C5H12N_Fragment_[]
86.0969
Relative Intensity
m/z

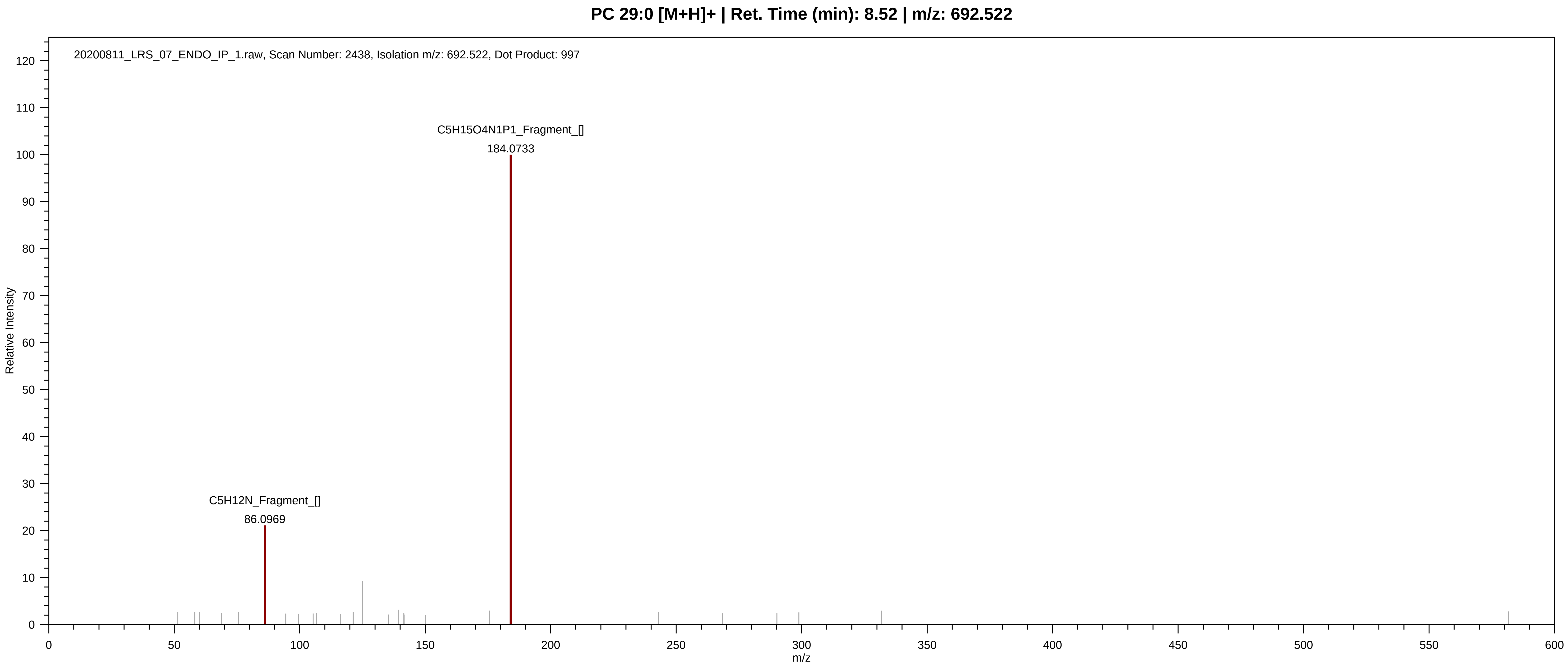
PC 29:0 [M+H]+ | Ret. Time (min): 8.52 | m/z: 692.522
20200811_LRS_07_ENDO_IP_1.raw, Scan Number: 2438, Isolation m/z: 692.522, Dot Product: 997
C5H15O4N1P1_Fragment_[]
184.0733
C5H12N_Fragment_[]
86.0969
Relative Intensity
m/z

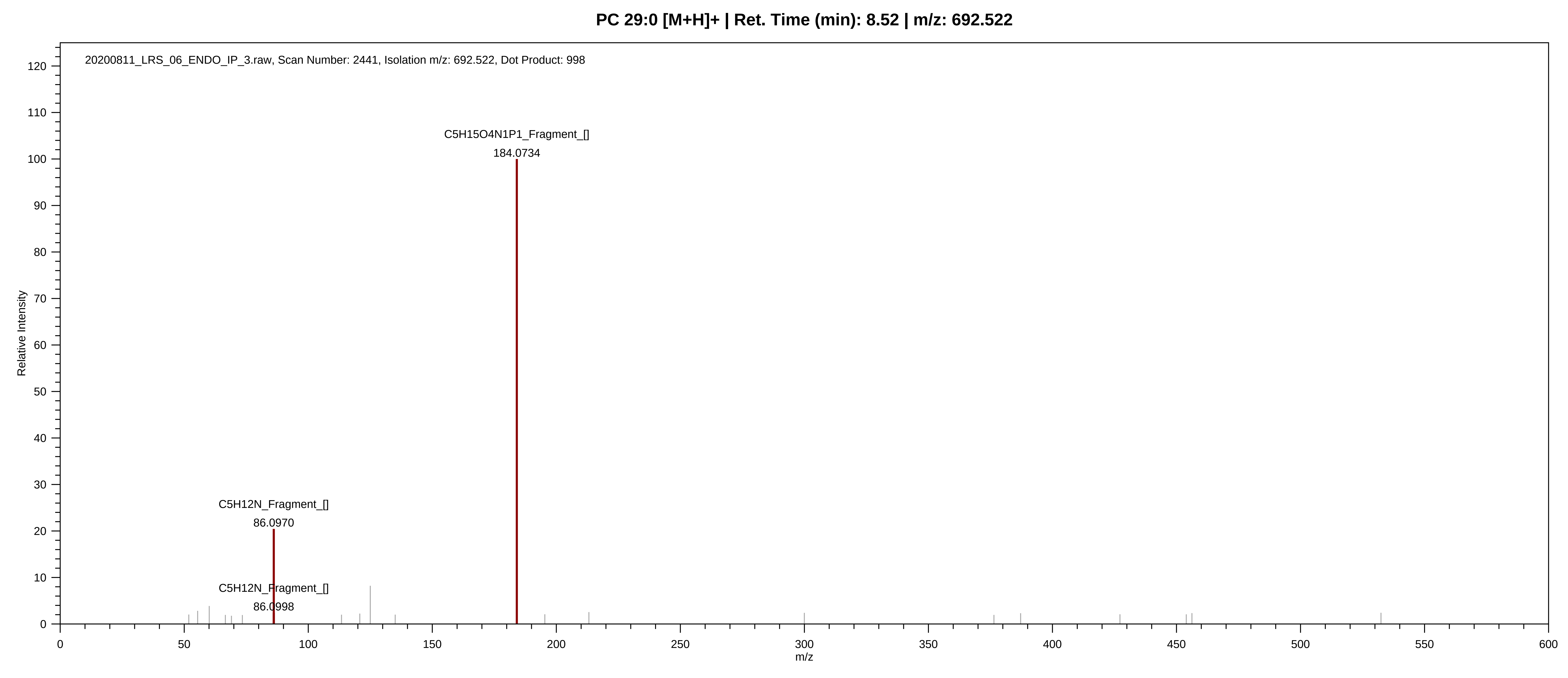

PC 29:0 [M+H]+ | Ret. Time (min): 8.52 | m/z: 692.522
20200811_LRS_06_ENDO_IP_3.raw, Scan Number: 2441, Isolation m/z: 692.522, Dot Product: 998
C5H15O4N1P1_Fragment_[]
184.0734
C5H12N_Fragment_[]
86.0970
C5H12N_Fragment_[]
86.0998
Relative Intensity
m/z

PC 29:0 [M+H]+ | Ret. Time (min): 8.433 | m/z: 692.522

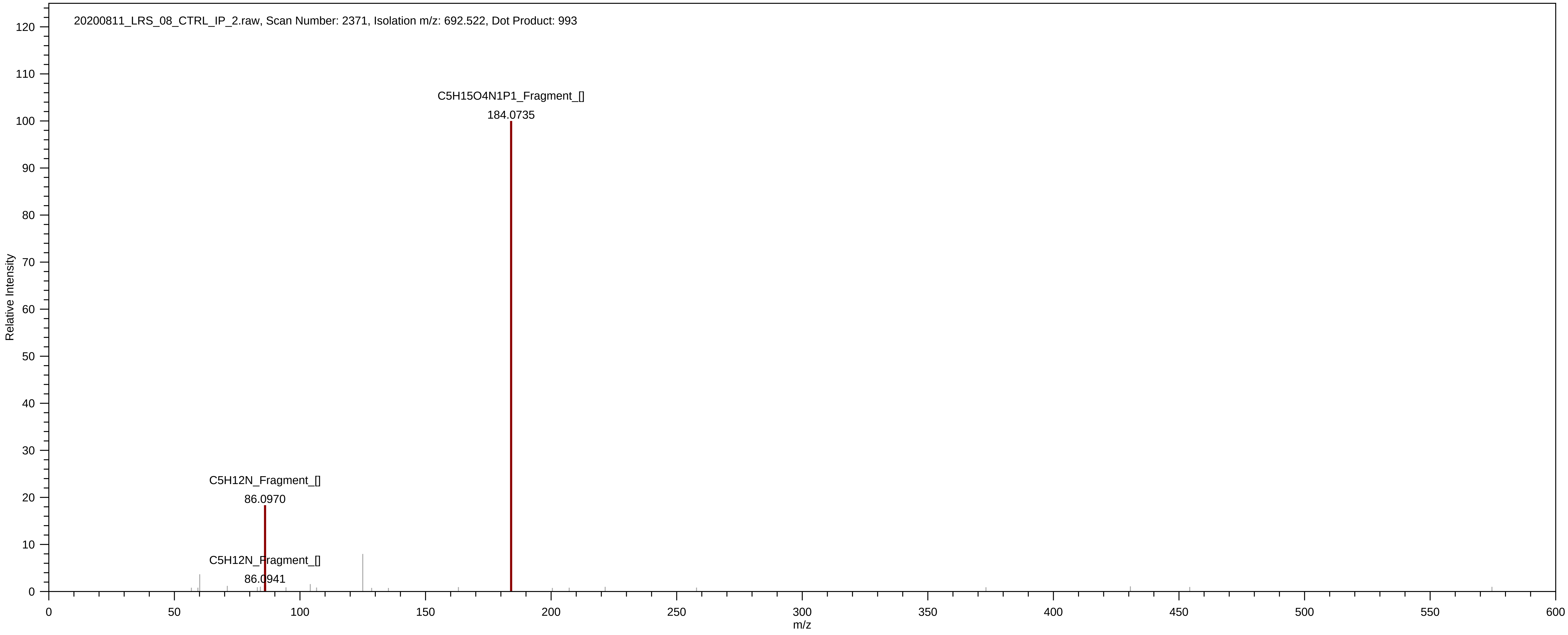

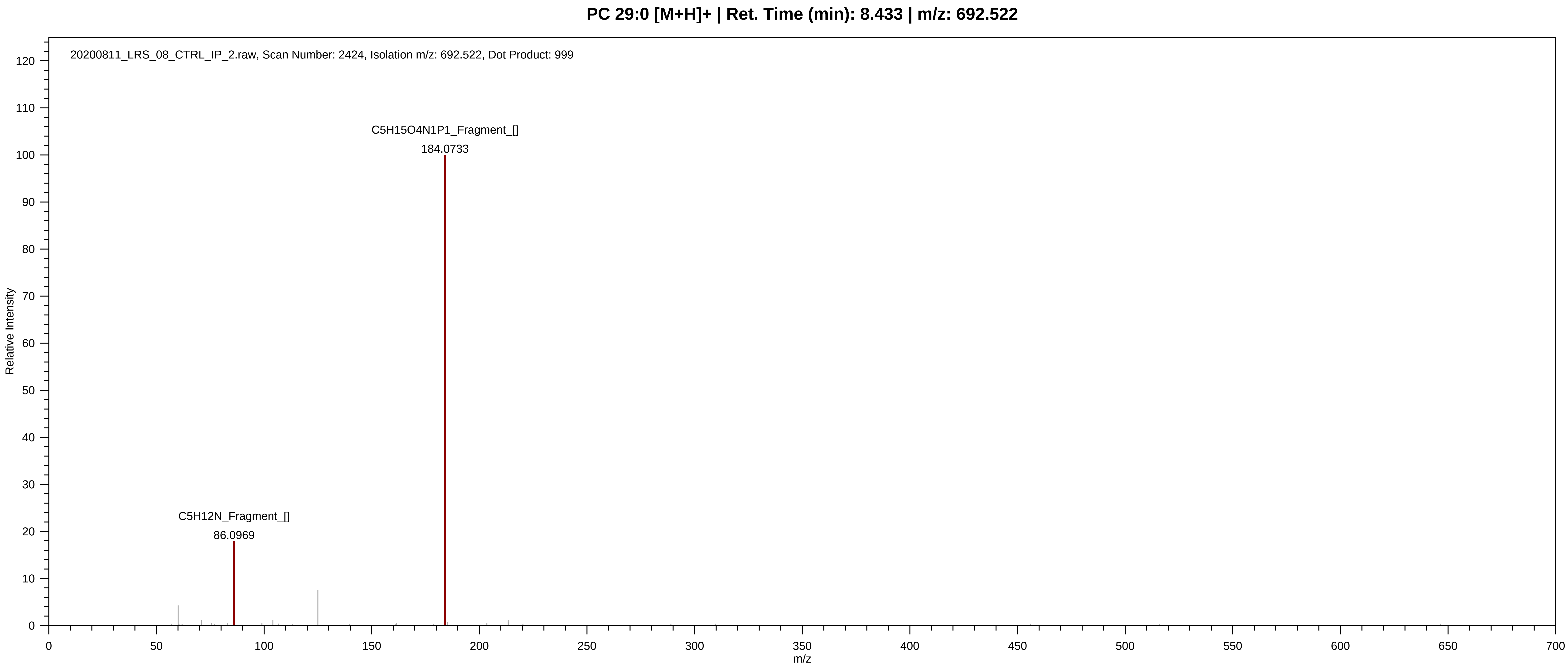
PC 29:0 [M+H]+ | Ret. Time (min): 8.433 | m/z: 692.522
20200811_LRS_08_CTRL_IP_2.raw, Scan Number: 2424, Isolation m/z: 692.522, Dot Product: 999
C5H15O4N1P1_Fragment_[]
184.0733
C5H12N_Fragment_[]
86.0969
Relative Intensity
m/z

PC 29:0 [M+H]+ | Ret. Time (min): 8.451 | m/z: 692.522

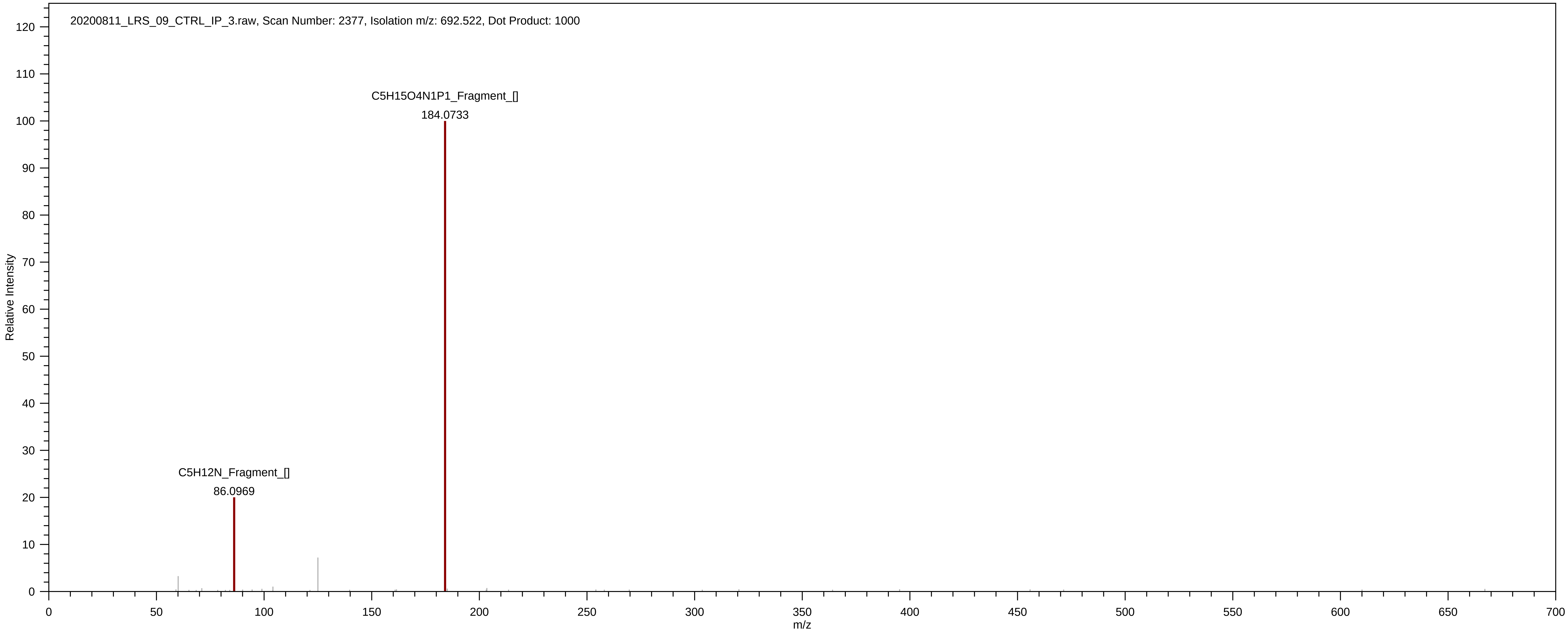

PC 29:0 [M+H]+ | Ret. Time (min): 8.451 | m/z: 692.522

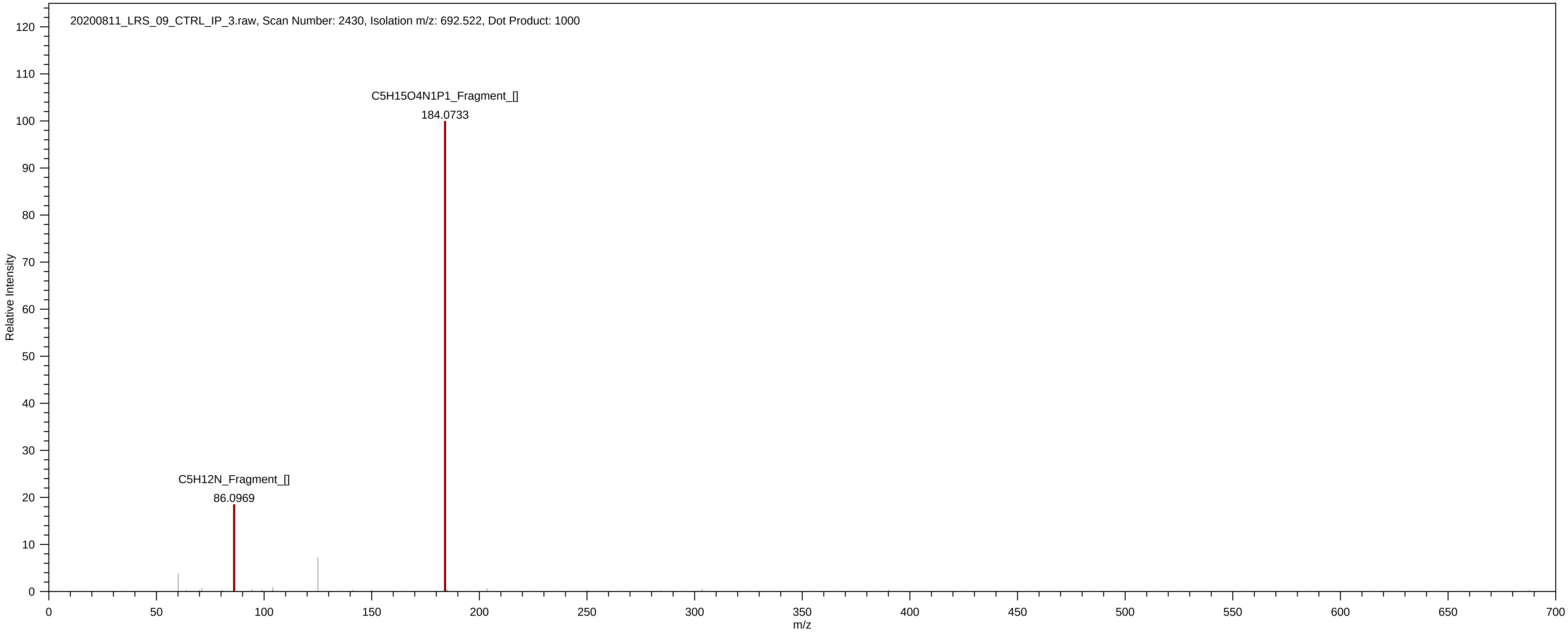

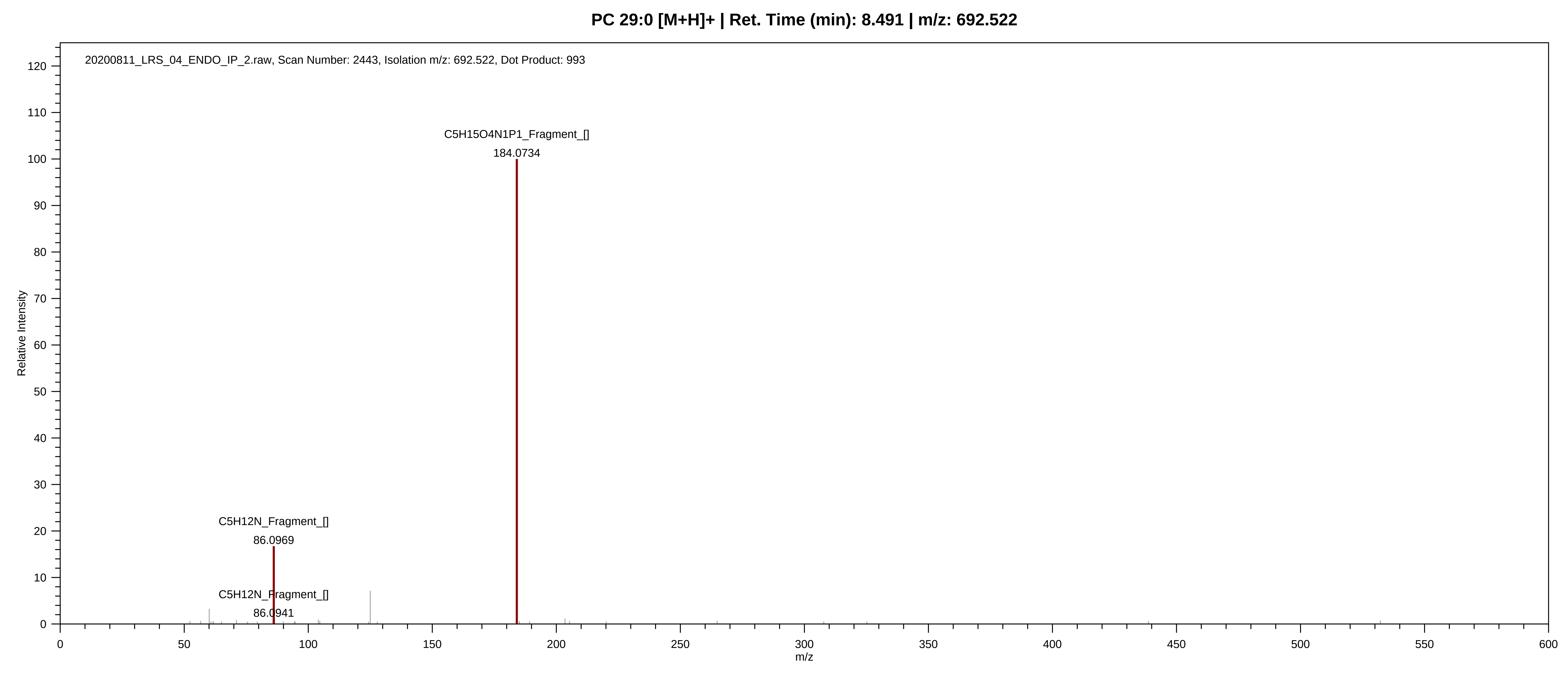

PC 29:0 [M+H]+ | Ret. Time (min): 8.491 | m/z: 692.522
20200811_LRS_04_ENDO_IP_2.raw, Scan Number: 2443, Isolation m/z: 692.522, Dot Product: 993
C5H15O4N1P1_Fragment_[]
184.0734
C5H12N_Fragment_[]
86.0969
C5H12N_Fragment_[]
86.0941
Relative Intensity
m/z

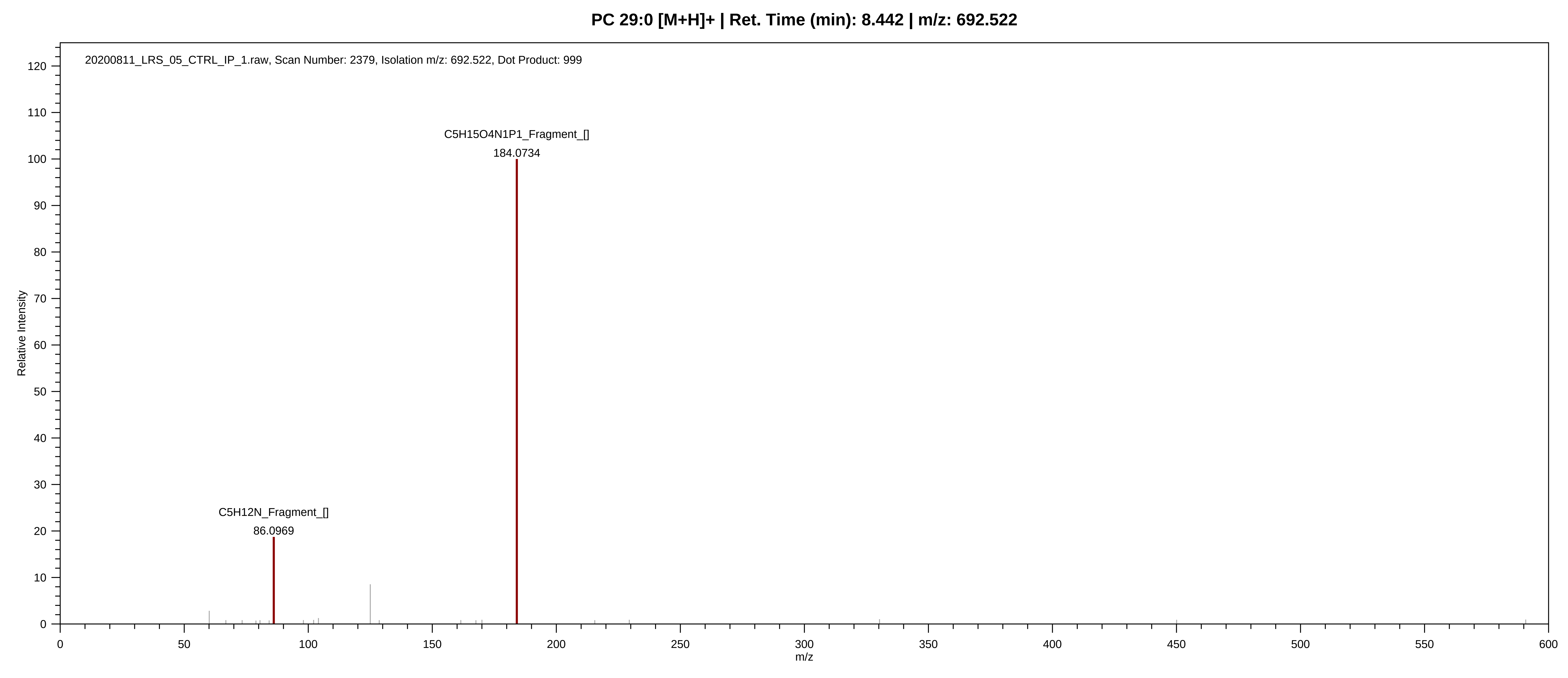
PC 29:0 [M+H]+ | Ret. Time (min): 8.442 | m/z: 692.522
20200811_LRS_05_CTRL_IP_1.raw, Scan Number: 2379, Isolation m/z: 692.522, Dot Product: 999
C5H15O4N1P1_Fragment_[]
184.0734
C5H12N_Fragment_[]
86.0969
Relative Intensity
m/z

PC 29:0 [M+H]+ | Ret. Time (min): 8.442 | m/z: 692.523

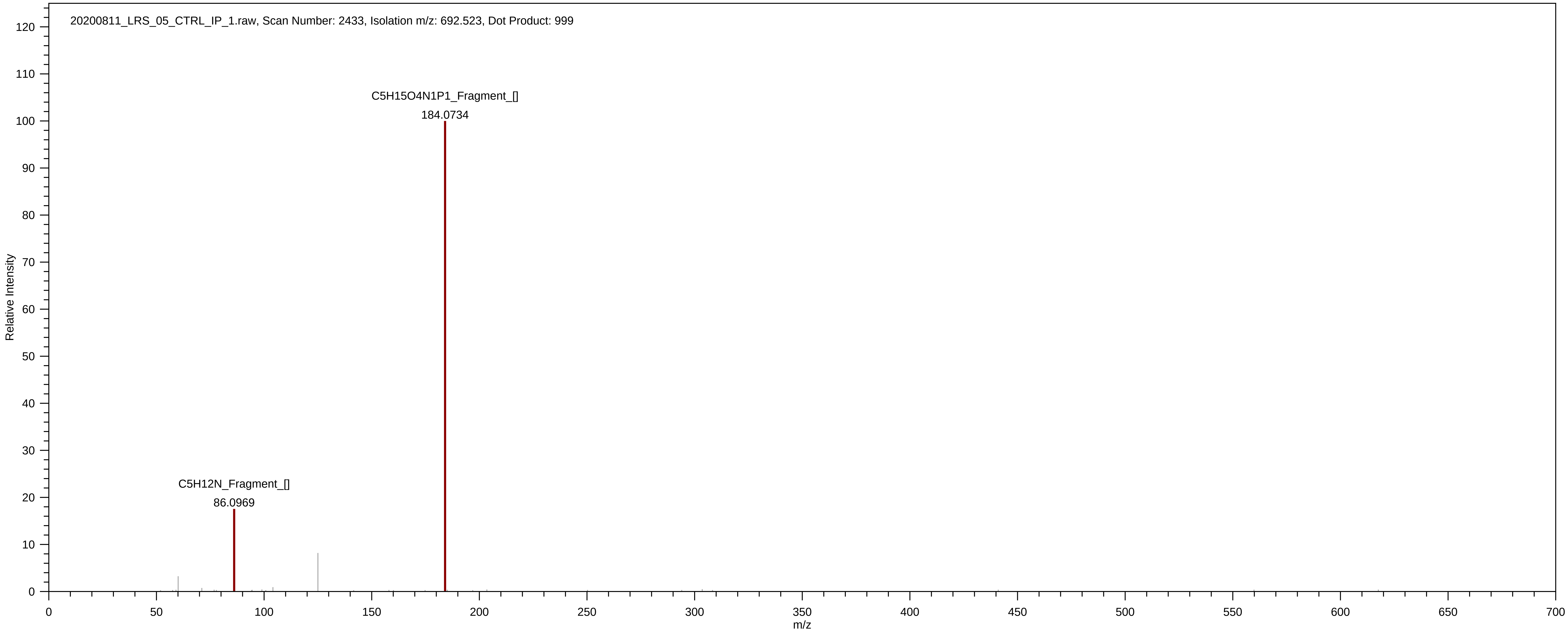

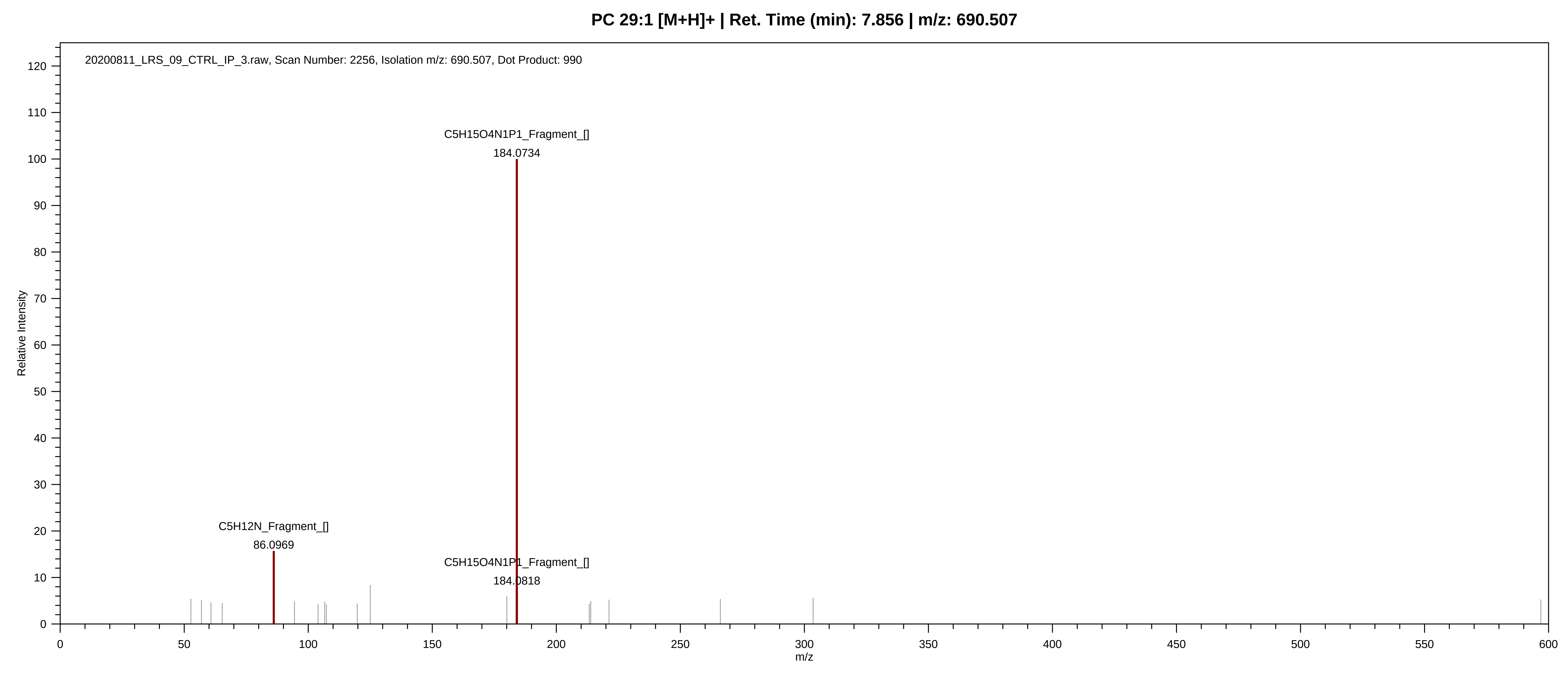
PC 29:1 [M+H]+ | Ret. Time (min): 7.856 | m/z: 690.507
20200811_LRS_09_CTRL_IP_3.raw, Scan Number: 2256, Isolation m/z: 690.507, Dot Product: 990
C5H15O4N1P1_Fragment_[]
184.0734
C5H12N_Fragment_[]
86.0969
C5H15O4N1P1_Fragment_[]
184.0818
Relative Intensity
m/z

PC 30:0 [M+H]+ | Ret. Time (min): 8.827 | m/z: 706.536

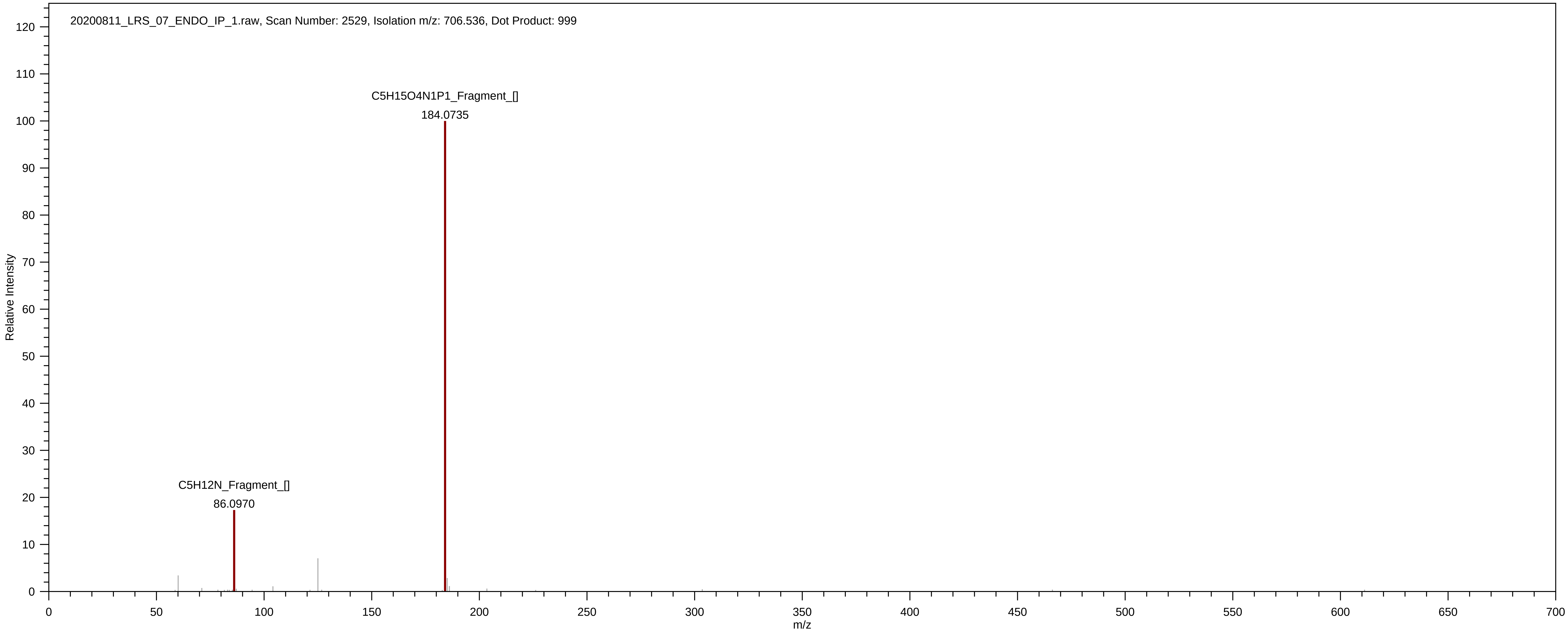

PC 30:0 [M+H]+ | Ret. Time (min): 8.827 | m/z: 706.536

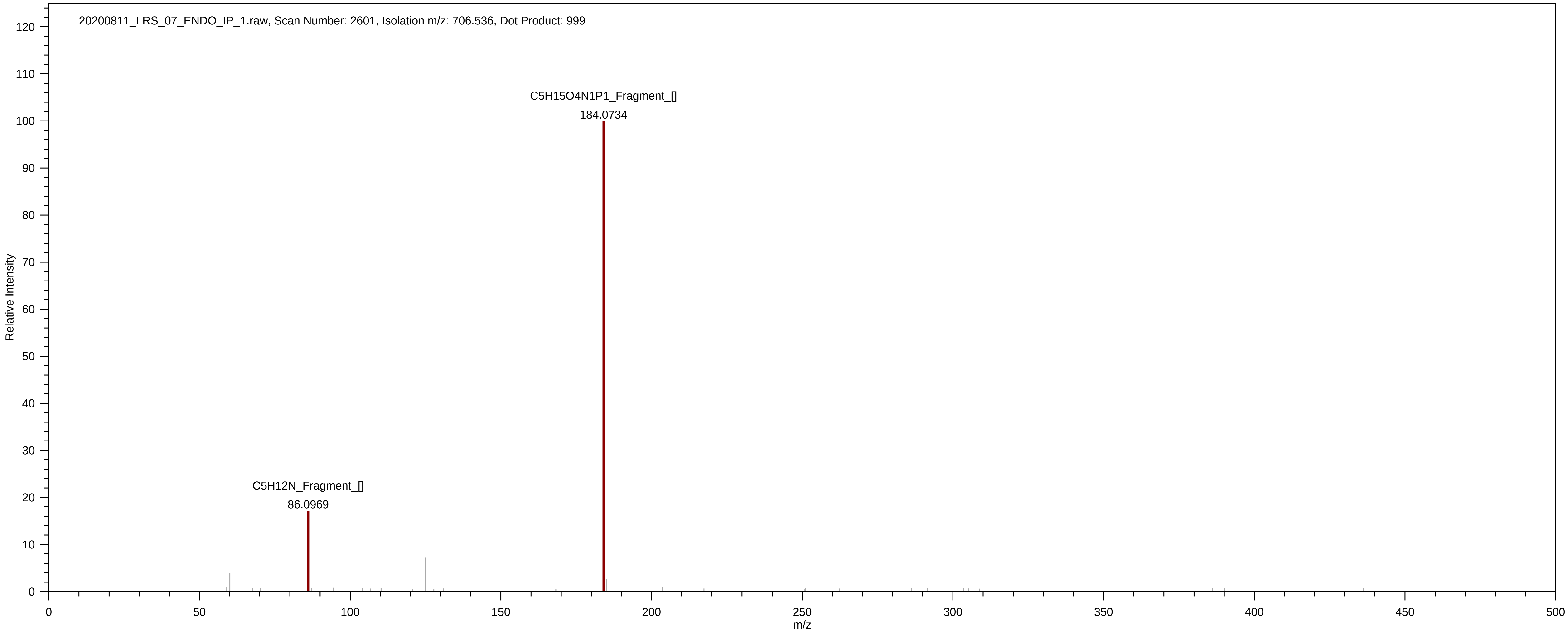

PC 30:0 [M+H]+ | Ret. Time (min): 8.898 | m/z: 706.538

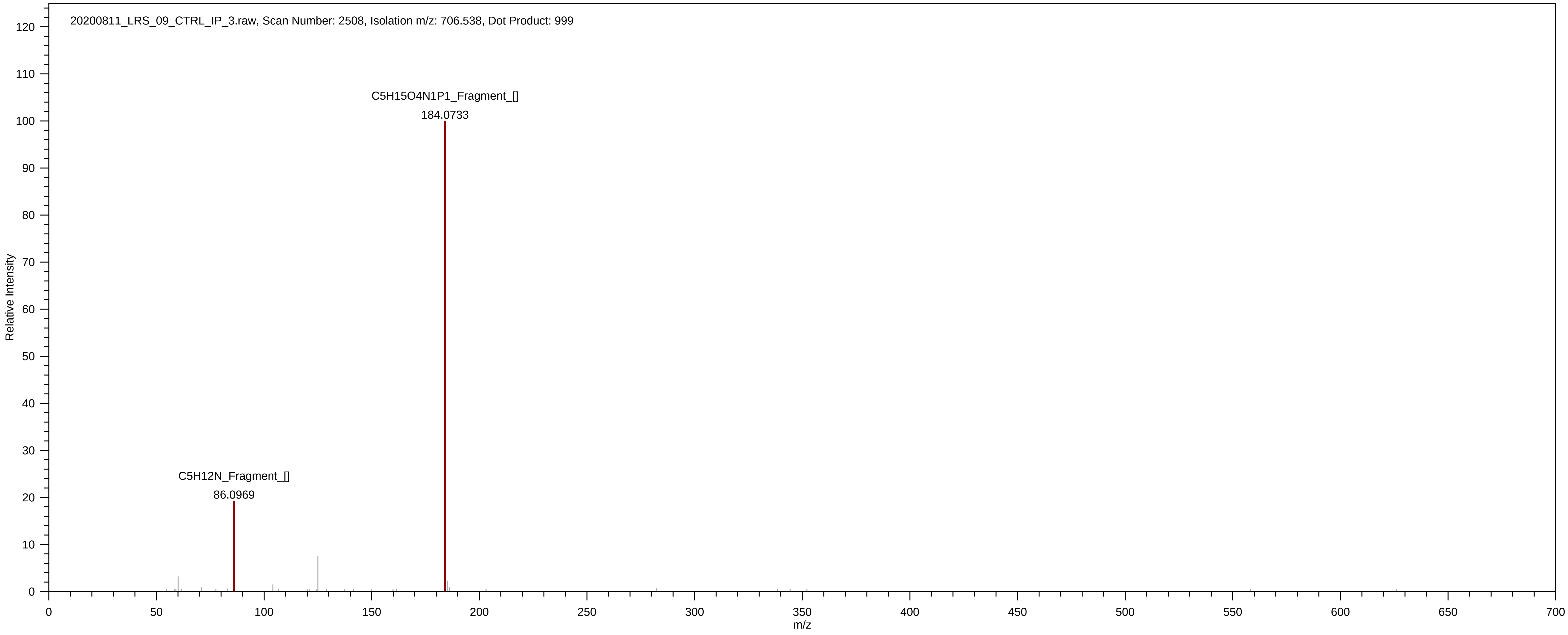

PC 30:0 [M+H]+ | Ret. Time (min): 8.898 | m/z: 706.537

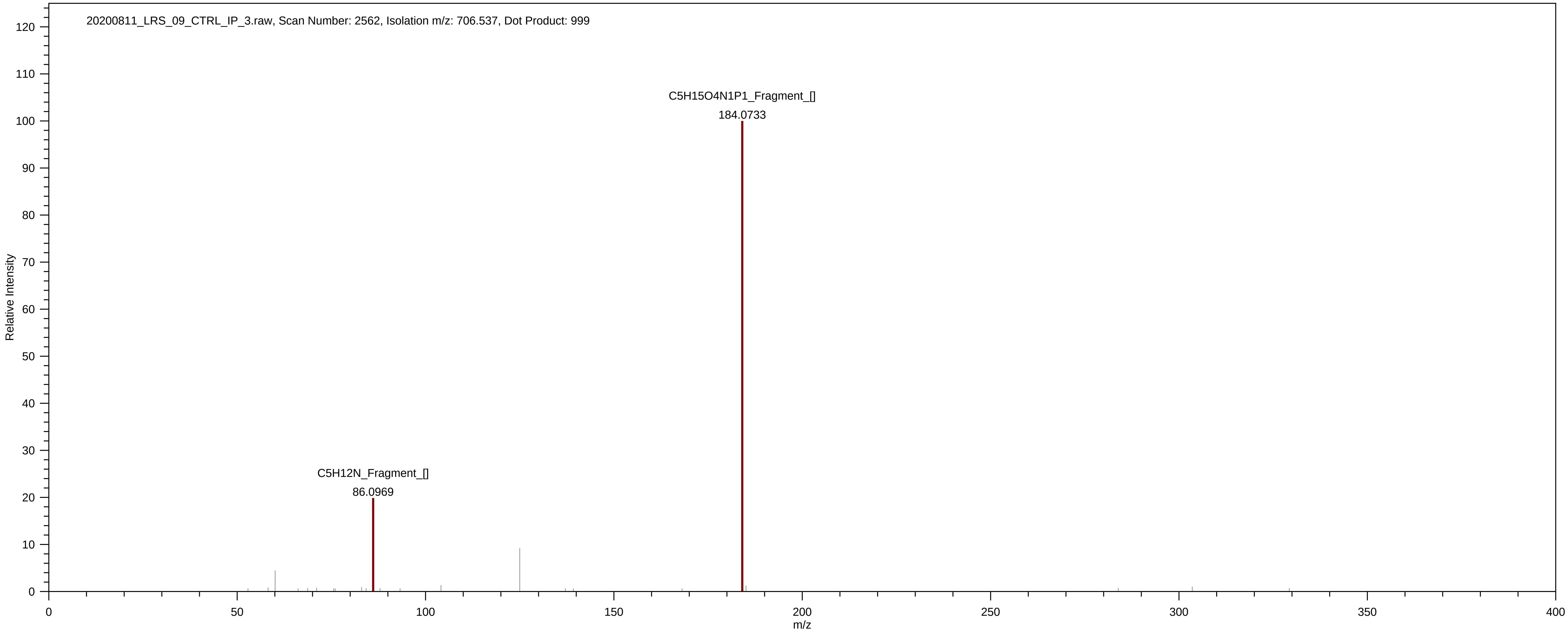

PC 30:0 [M+H]+ | Ret. Time (min): 8.882 | m/z: 706.537

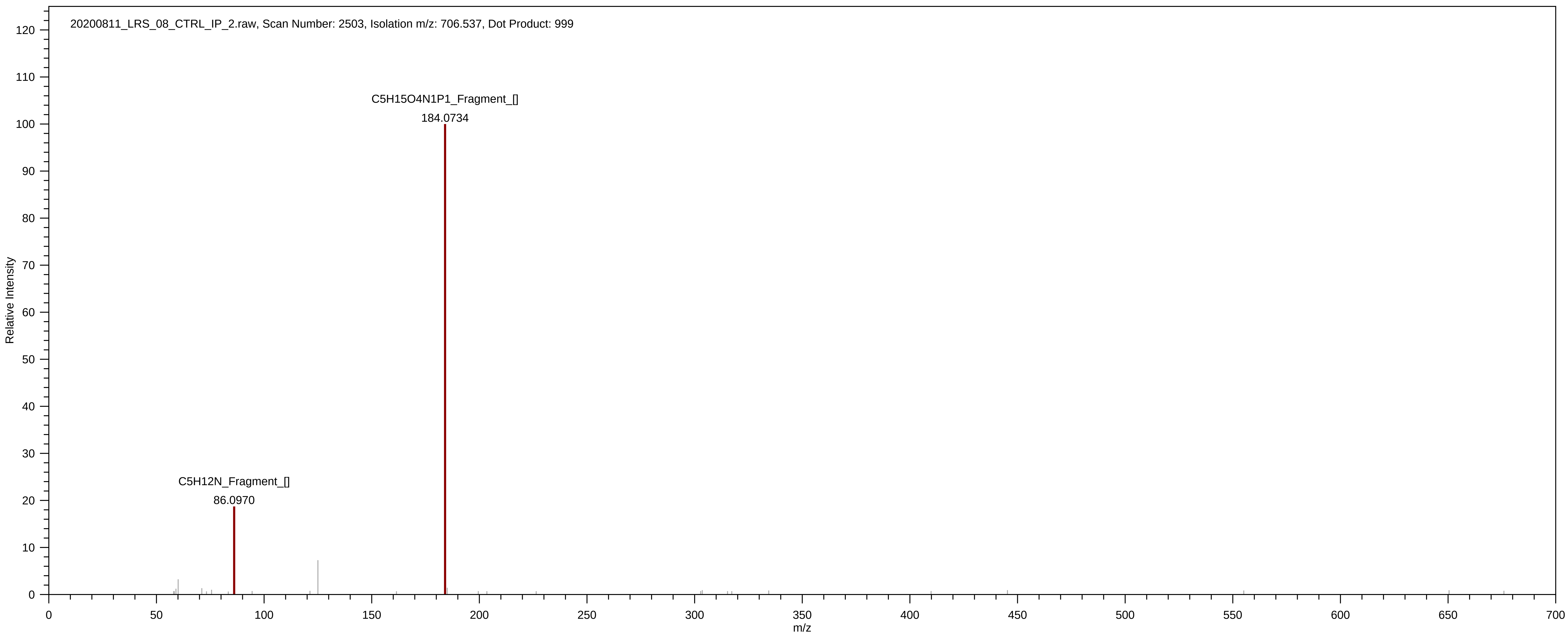

PC 30:0 [M+H]+ | Ret. Time (min): 8.882 | m/z: 706.538

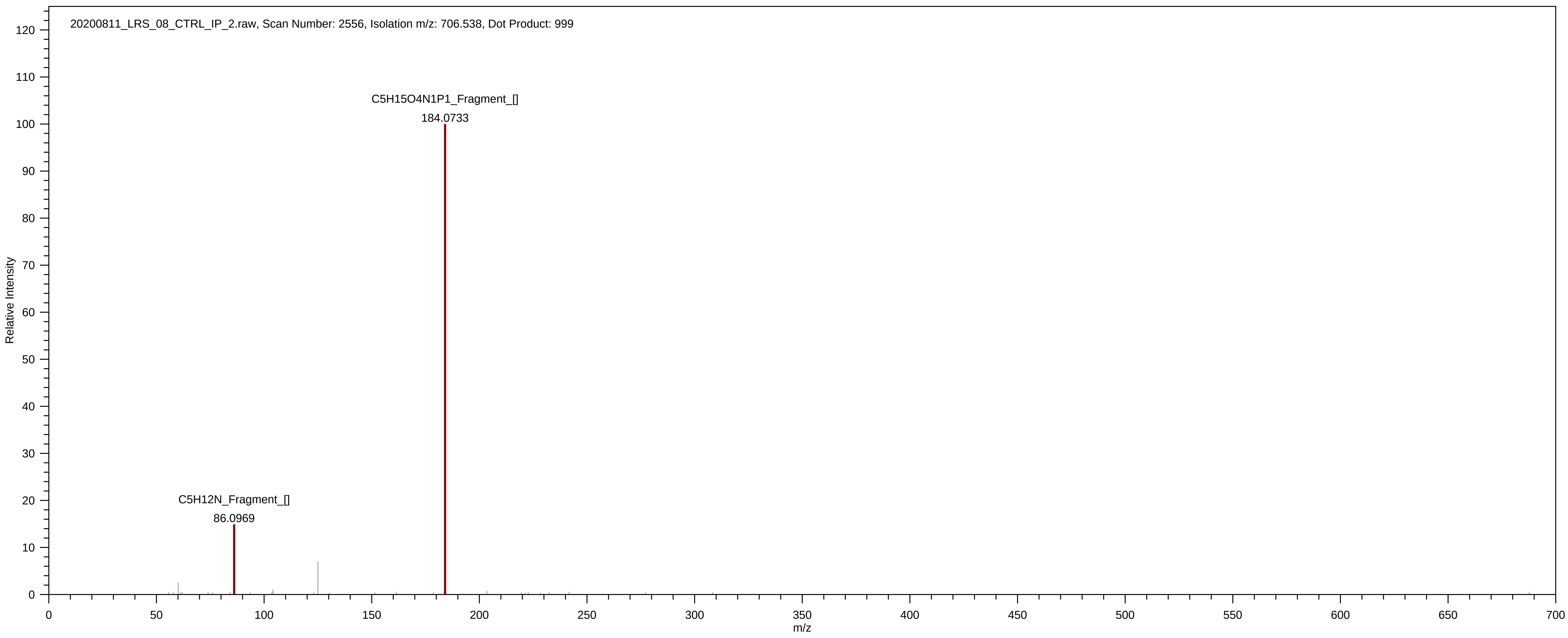

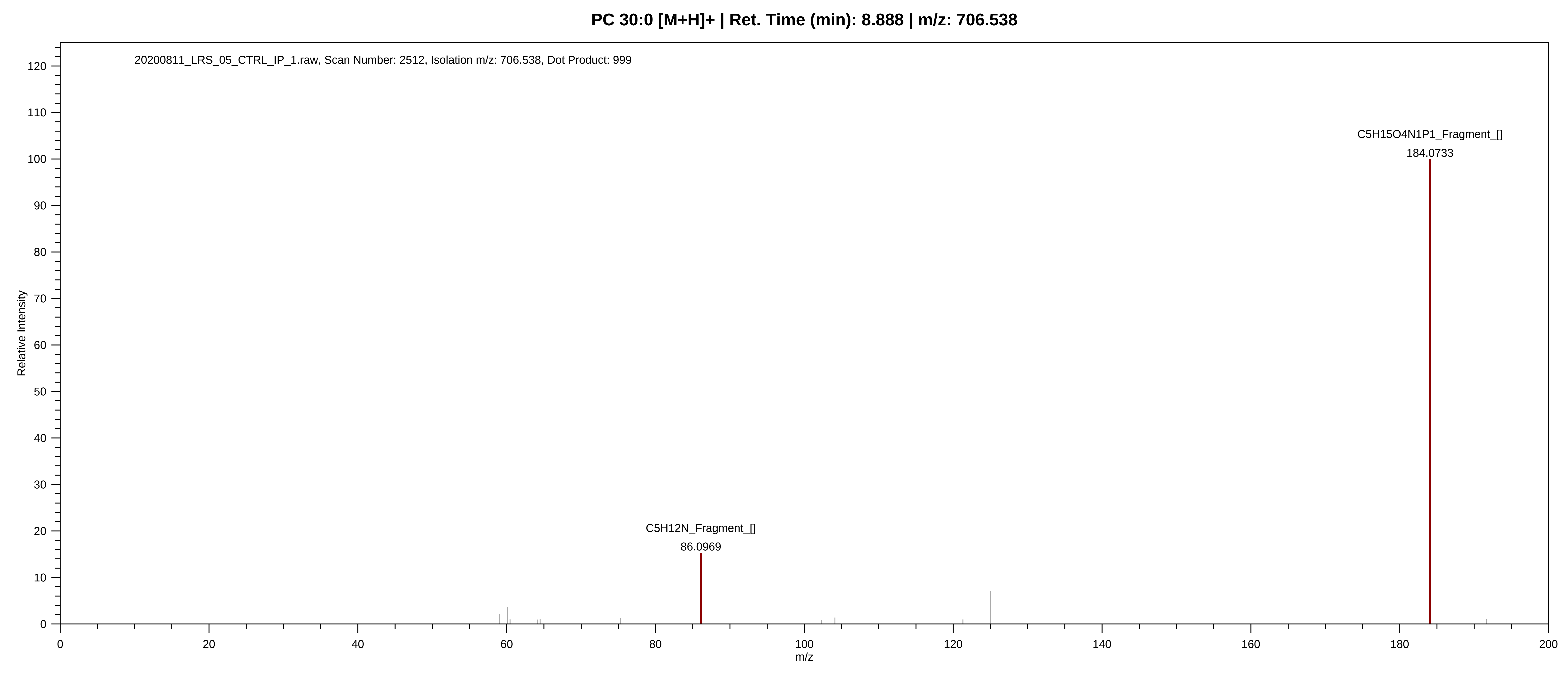
PC 30:0 [M+H]+ | Ret. Time (min): 8.888 | m/z: 706.538
20200811_LRS_05_CTRL_IP_1.raw, Scan Number: 2512, Isolation m/z: 706.538, Dot Product: 999
C5H15O4N1P1_Fragment_[]
184.0733
C5H12N_Fragment_[]
86.0969
Relative Intensity
m/z

PC 30:0 [M+H]+ | Ret. Time (min): 8.888 | m/z: 706.538

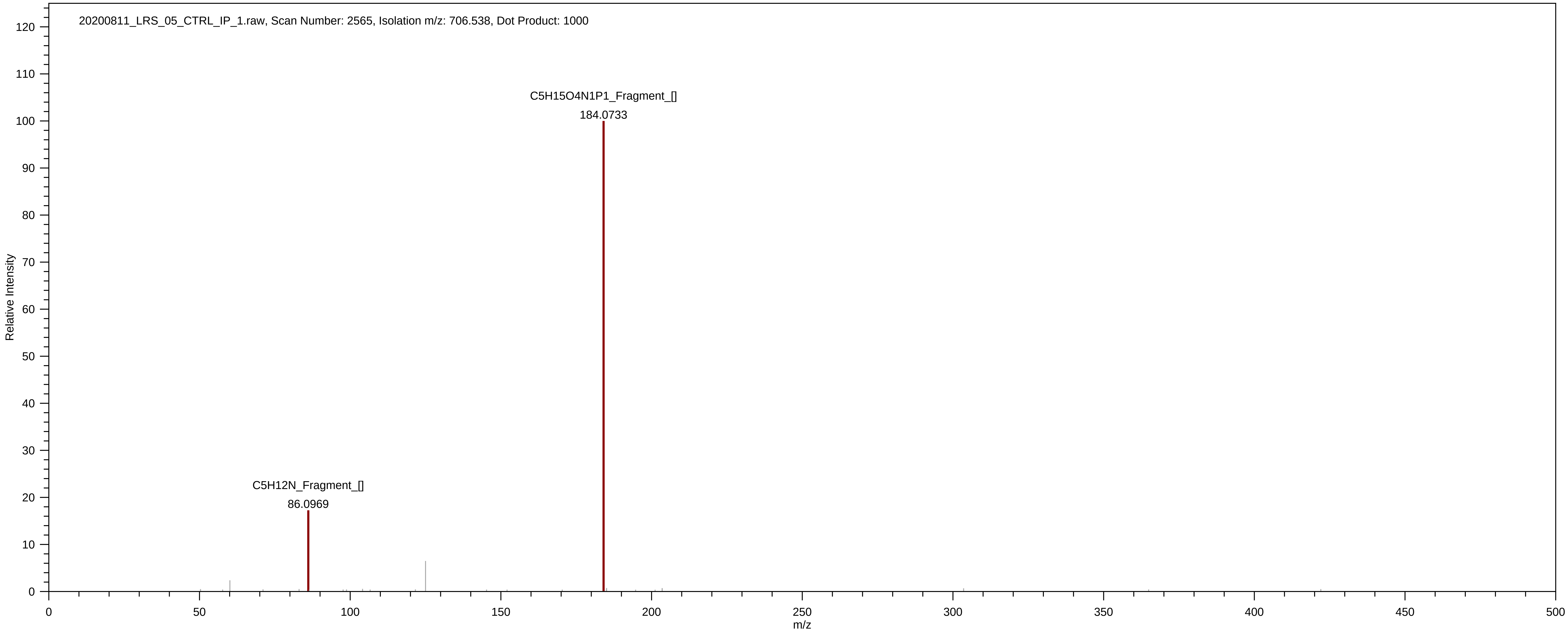

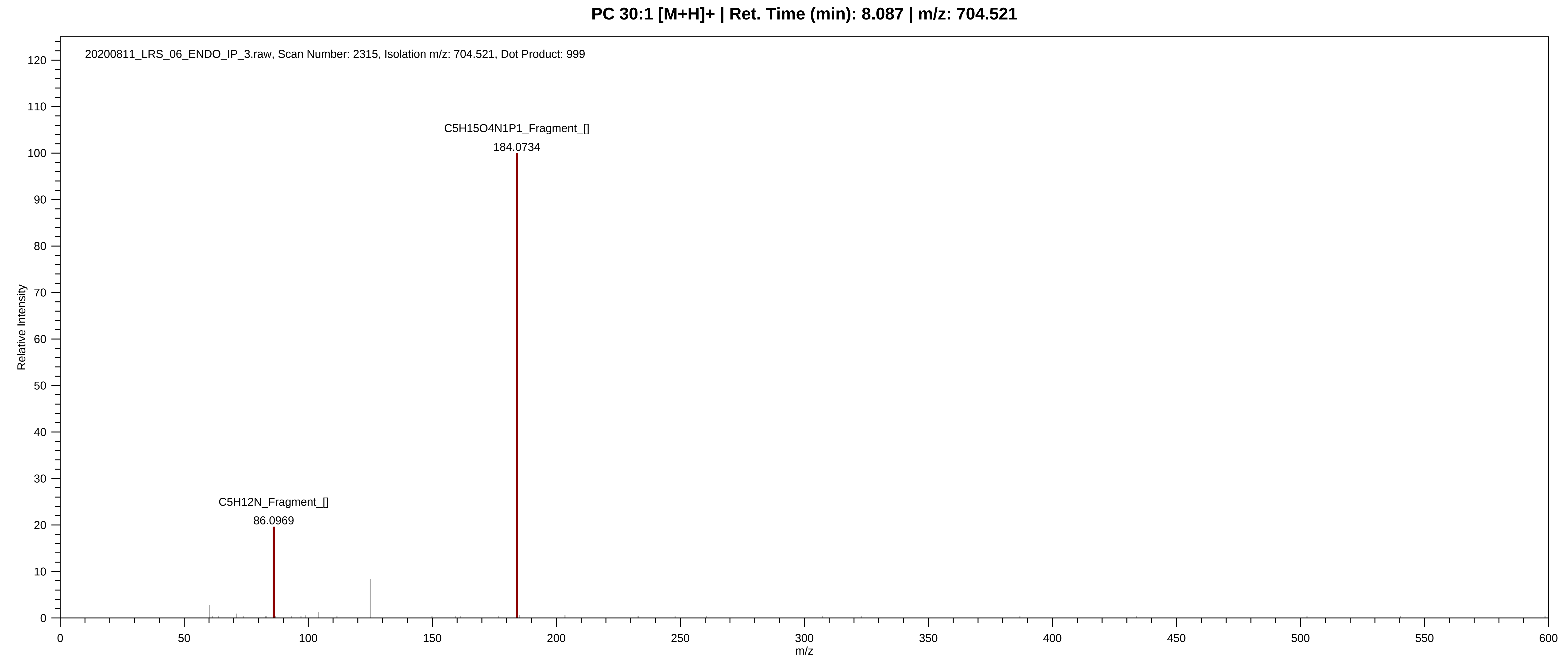
PC 30:1 [M+H]+ | Ret. Time (min): 8.087 | m/z: 704.521
20200811_LRS_06_ENDO_IP_3.raw, Scan Number: 2315, Isolation m/z: 704.521, Dot Product: 999
C5H15O4N1P1_Fragment_[]
184.0734
C5H12N_Fragment_[]
86.0969
Relative Intensity
m/z
0
10
20
30
40
50
60
70
80
90
100
110
120
0
50
100
150
200
250
300
350
400
450
500
550
600

PC 30:1 [M+H]+ | Ret. Time (min): 8.083 | m/z: 704.522

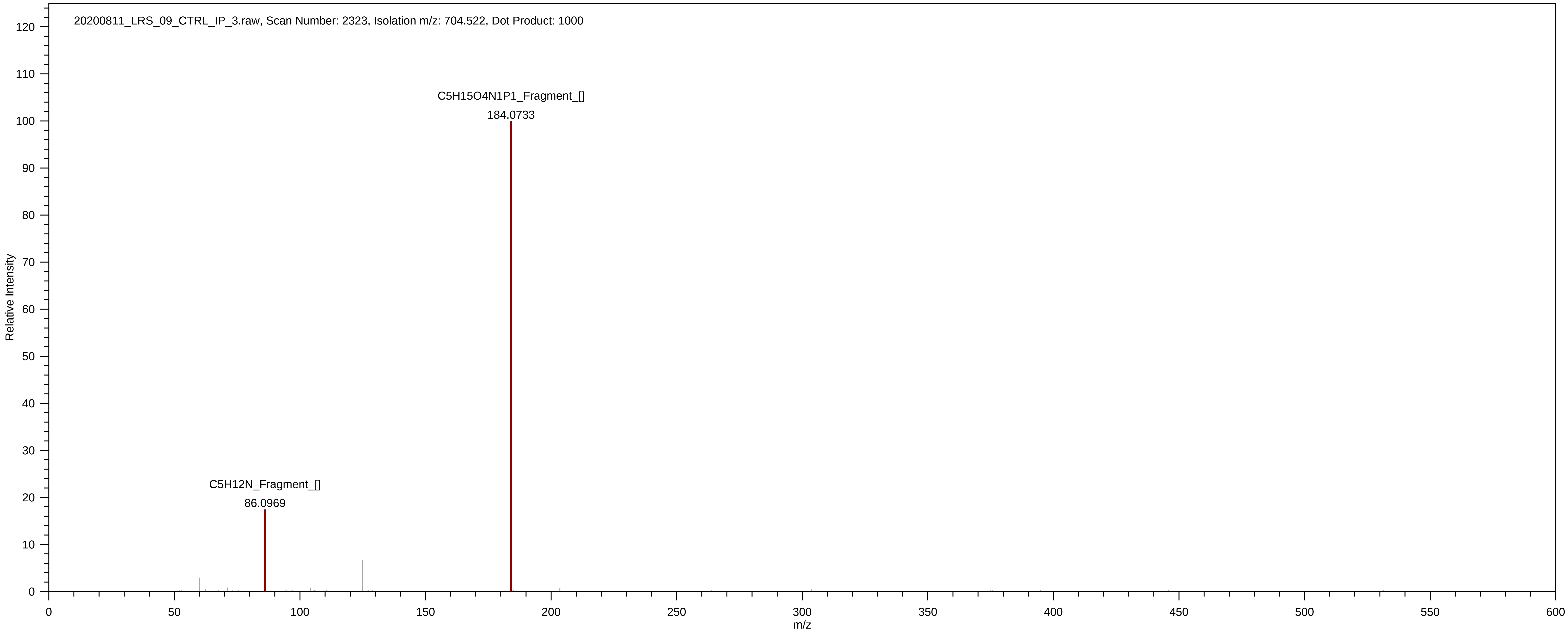

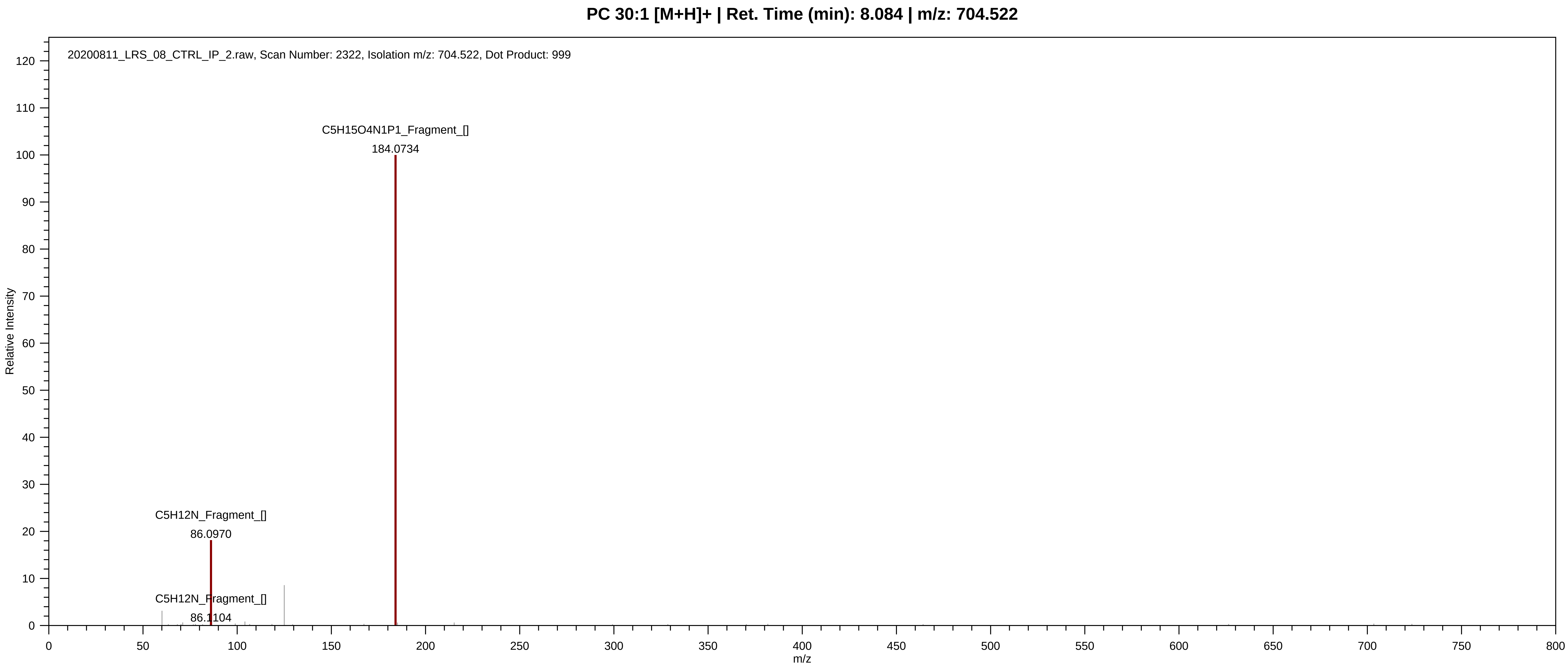
PC 30:1 [M+H]+ | Ret. Time (min): 8.084 | m/z: 704.522
20200811_LRS_08_CTRL_IP_2.raw, Scan Number: 2322, Isolation m/z: 704.522, Dot Product: 999
C5H15O4N1P1_Fragment_[]
184.0734
C5H12N_Fragment_[]
86.0970
C5H12N_Fragment_[]
86.1104
Relative Intensity
m/z

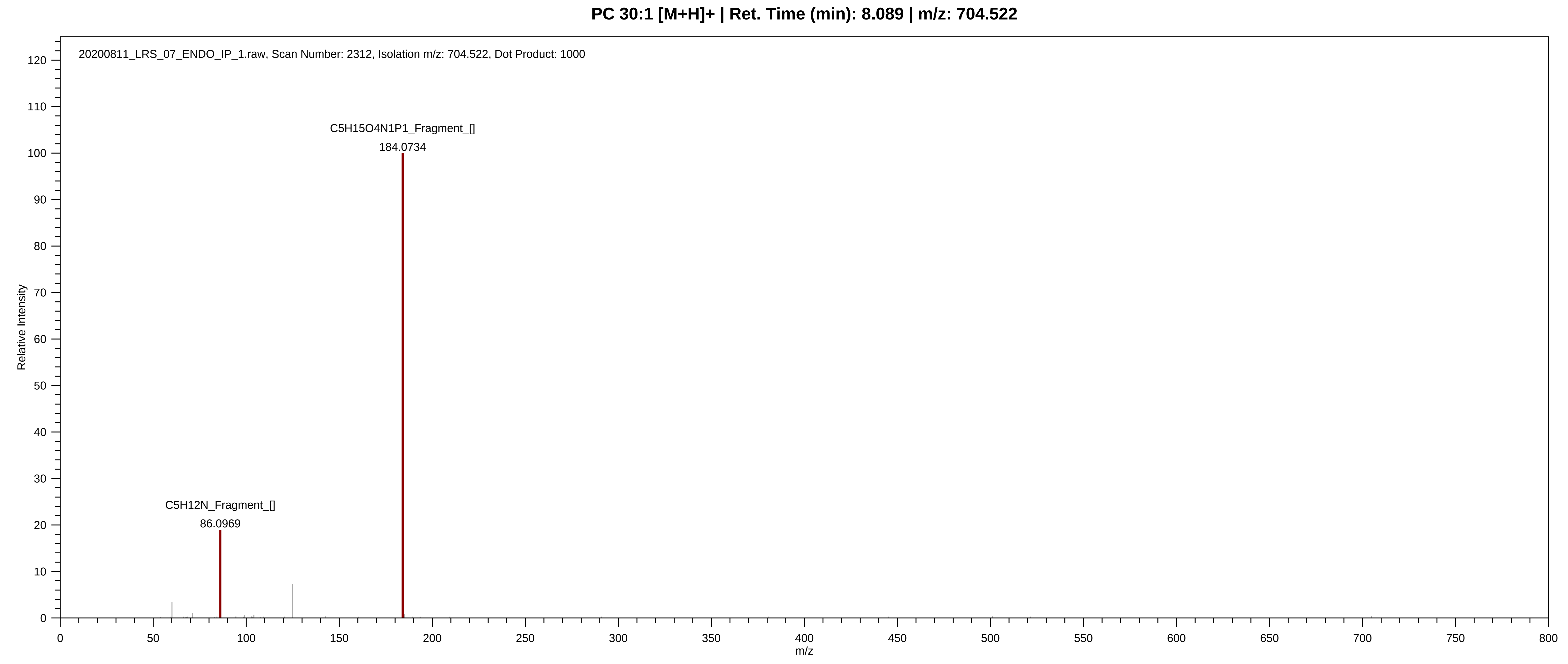
PC 30:1 [M+H]+ | Ret. Time (min): 8.089 | m/z: 704.522
20200811_LRS_07_ENDO_IP_1.raw, Scan Number: 2312, Isolation m/z: 704.522, Dot Product: 1000
C5H15O4N1P1_Fragment_[]
184.0734
C5H12N_Fragment_[]
86.0969
Relative Intensity
m/z
0
10
20
30
40
50
60
70
80
90
100
110
120
0
50
100
150
200
250
300
350
400
450
500
550
600
650
700
750
800

PC 30:1 [M+H]+ | Ret. Time (min): 8.101 | m/z: 704.522

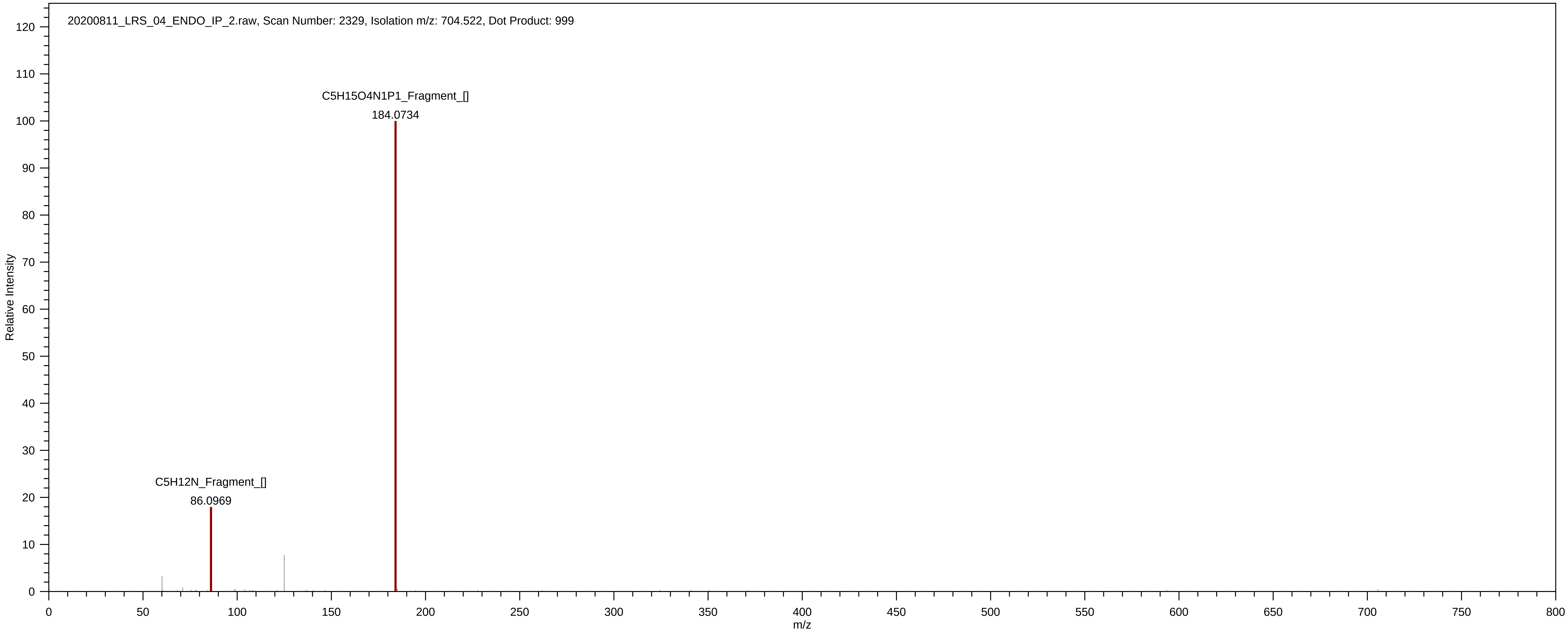

PC 30:1 [M+H]+ | Ret. Time (min): 8.266 | m/z: 704.522

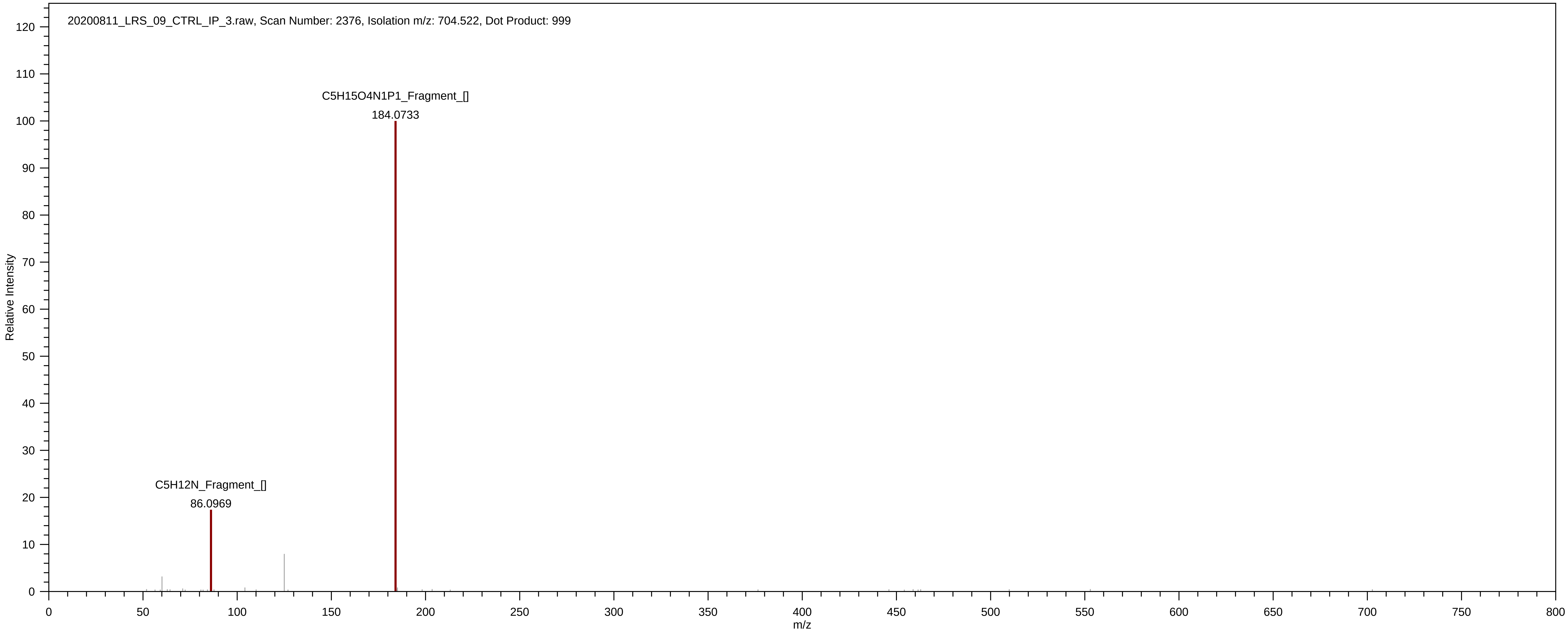

PC 30:1 [M+H]+ | Ret. Time (min): 8.266 | m/z: 704.522

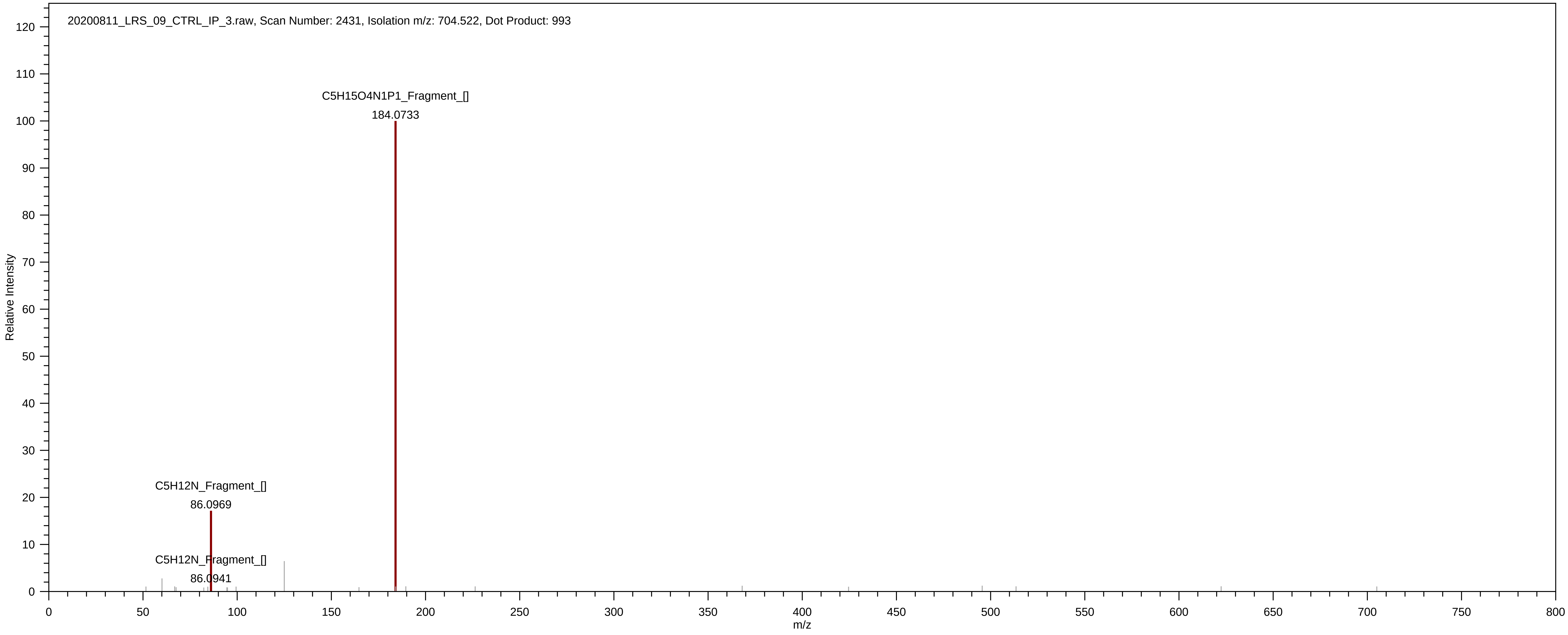

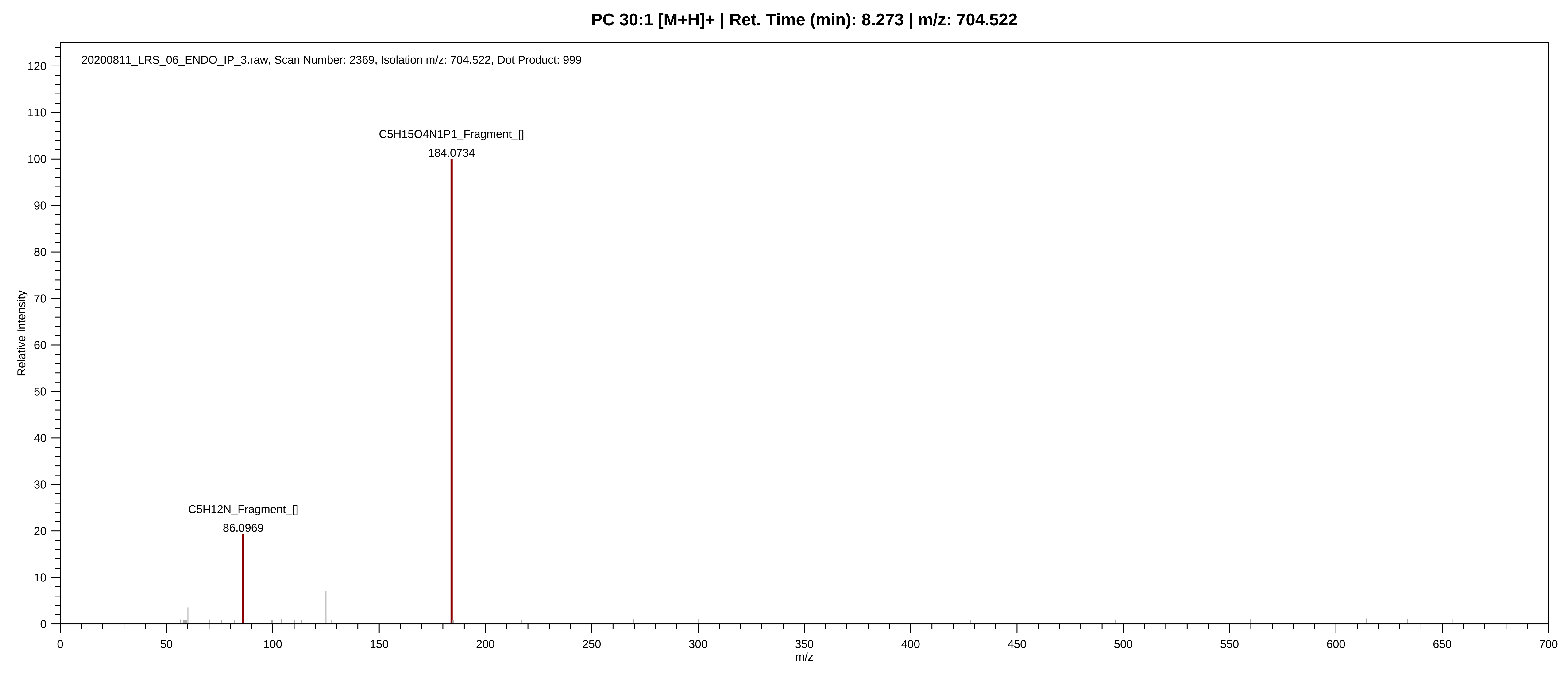
PC 30:1 [M+H]+ | Ret. Time (min): 8.273 | m/z: 704.522
20200811_LRS_06_ENDO_IP_3.raw, Scan Number: 2369, Isolation m/z: 704.522, Dot Product: 999
C5H15O4N1P1_Fragment_[]
184.0734
C5H12N_Fragment_[]
86.0969
Relative Intensity
m/z

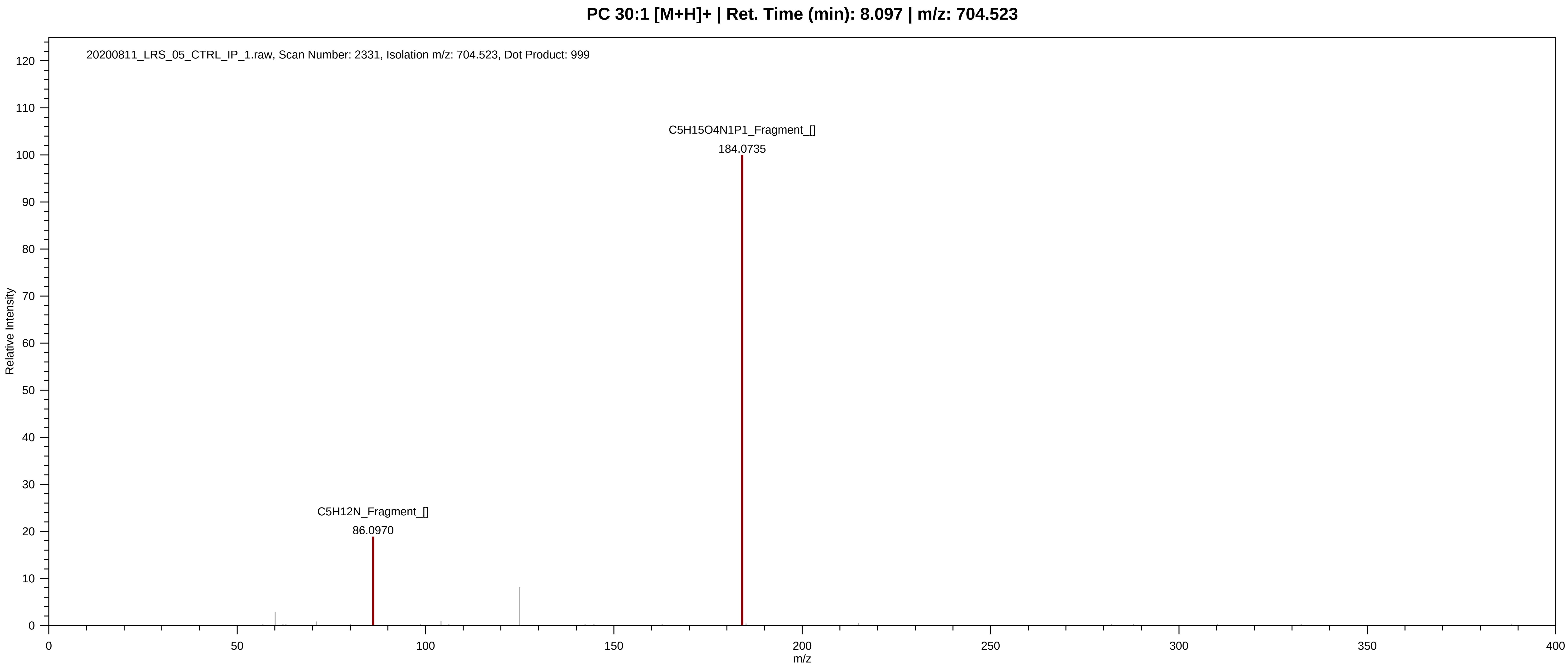
PC 30:1 [M+H]+ | Ret. Time (min): 8.097 | m/z: 704.523
20200811_LRS_05_CTRL_IP_1.raw, Scan Number: 2331, Isolation m/z: 704.523, Dot Product: 999
C5H15O4N1P1_Fragment_[]
184.0735
C5H12N_Fragment_[]
86.0970
Relative Intensity
m/z
0
10
20
30
40
50
60
70
80
90
100
110
120
0
50
100
150
200
250
300
350
400

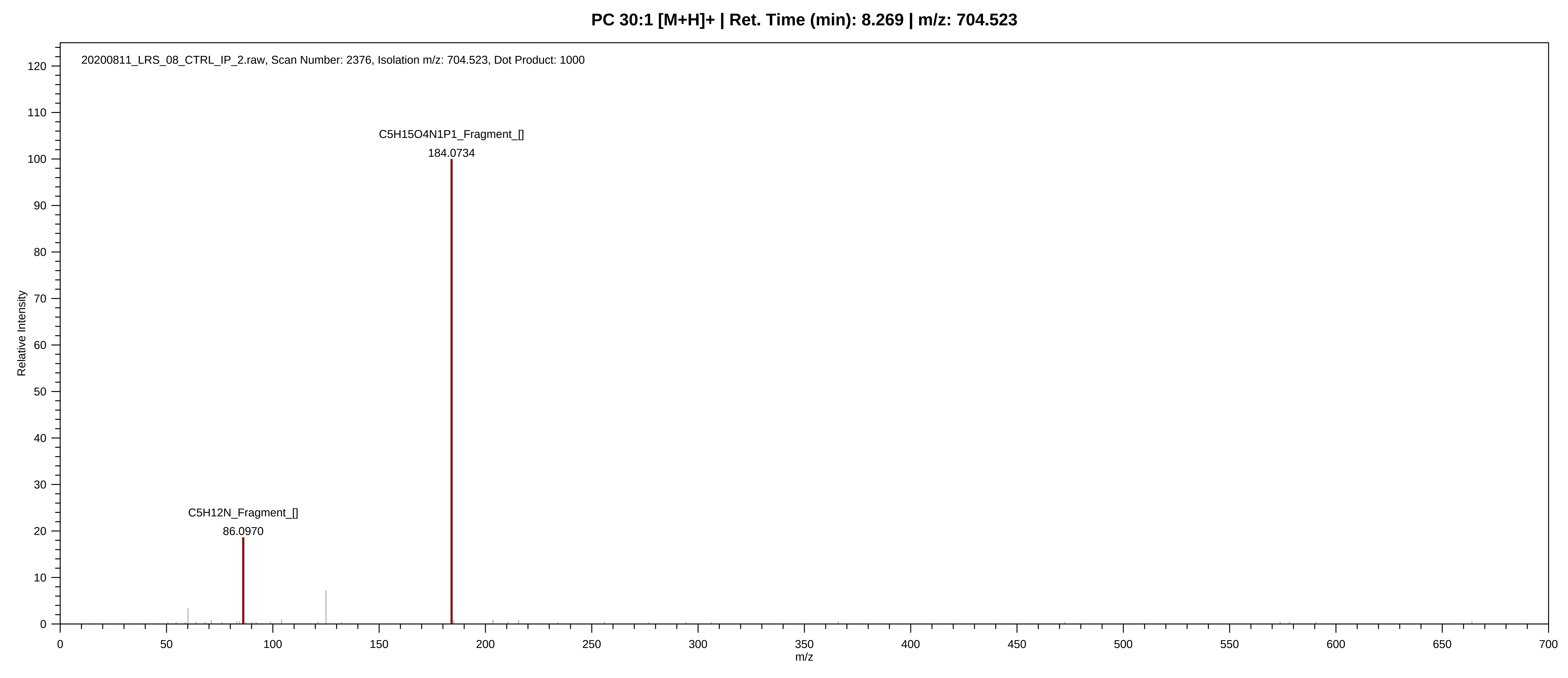
PC 30:1 [M+H]+ | Ret. Time (min): 8.269 | m/z: 704.523
20200811_LRS_08_CTRL_IP_2.raw, Scan Number: 2376, Isolation m/z: 704.523, Dot Product: 1000
C5H15O4N1P1_Fragment_[]
184.0734
C5H12N_Fragment_[]
86.0970
Relative Intensity
m/z

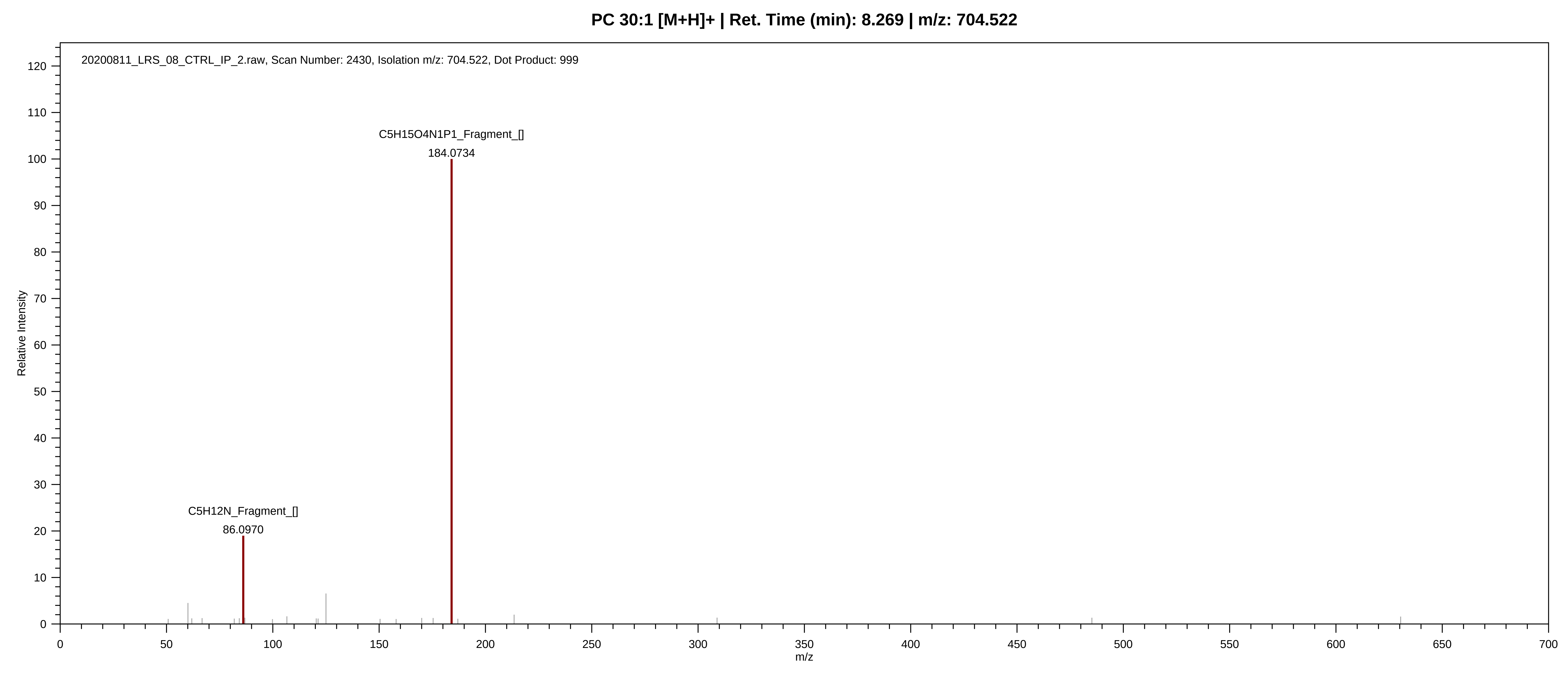

PC 30:1 [M+H]+ | Ret. Time (min): 8.269 | m/z: 704.522
20200811_LRS_08_CTRL_IP_2.raw, Scan Number: 2430, Isolation m/z: 704.522, Dot Product: 999
C5H15O4N1P1_Fragment_[]
184.0734
C5H12N_Fragment_[]
86.0970
Relative Intensity
m/z

PC 30:1 [M+H]+ | Ret. Time (min): 8.273 | m/z: 704.523

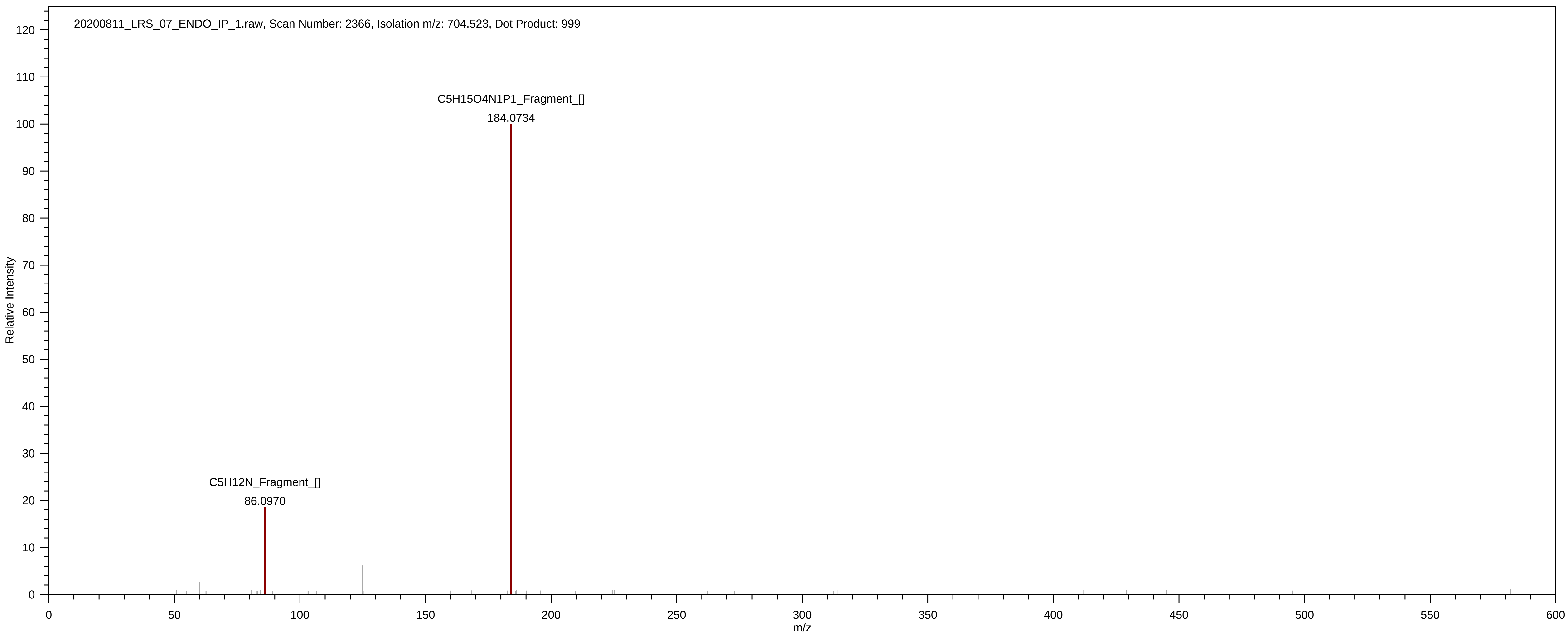

PC 30:1 [M+H]+ | Ret. Time (min): 8.279 | m/z: 704.523

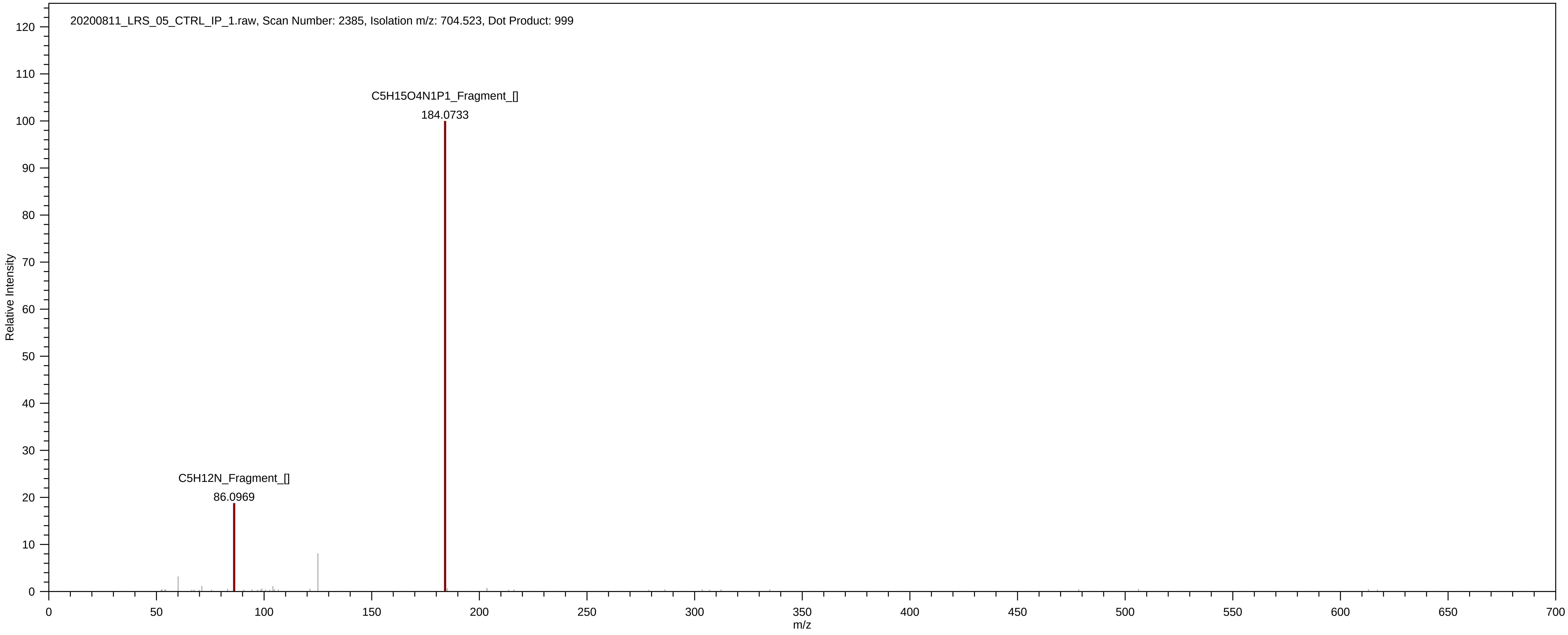

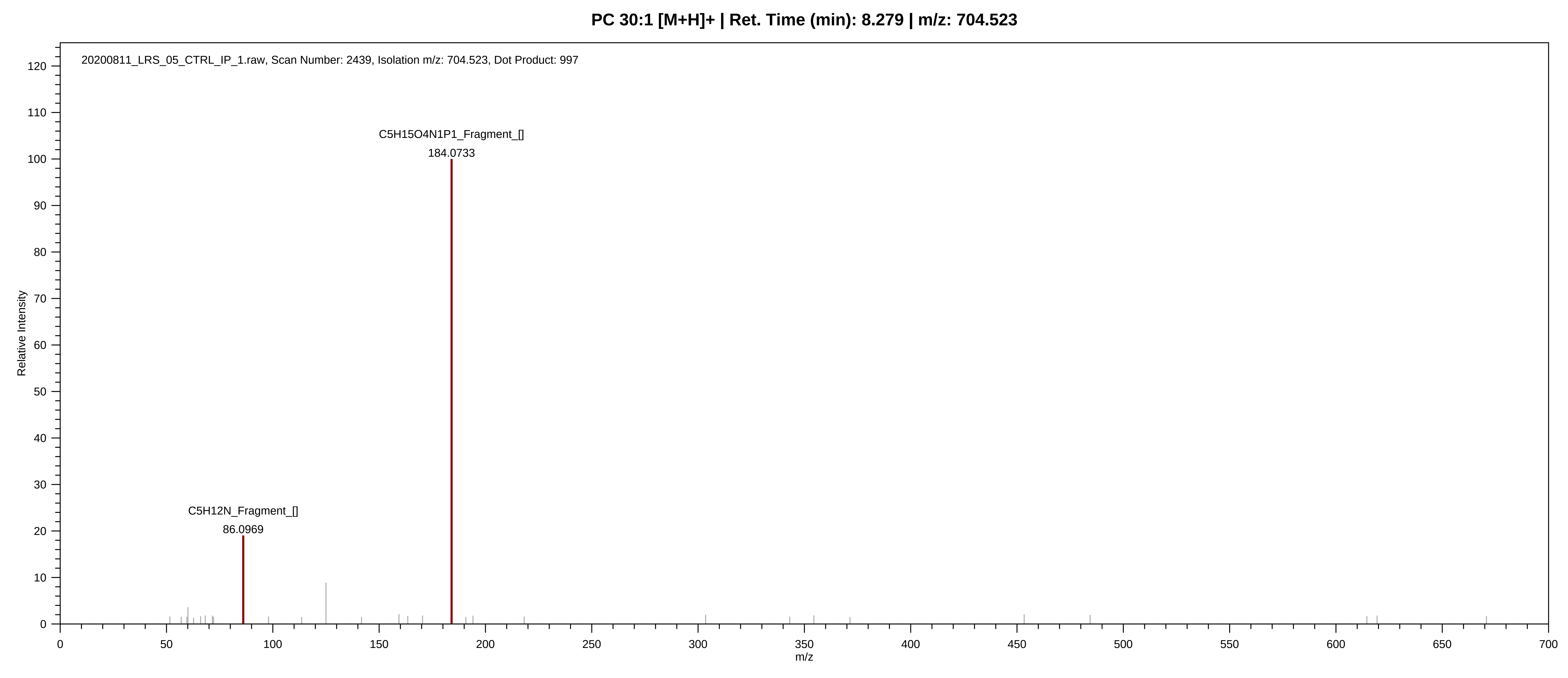
PC 30:1 [M+H]+ | Ret. Time (min): 8.279 | m/z: 704.523
20200811_LRS_05_CTRL_IP_1.raw, Scan Number: 2439, Isolation m/z: 704.523, Dot Product: 997
C5H15O4N1P1_Fragment_[]
184.0733
C5H12N_Fragment_[]
86.0969
Relative Intensity
m/z

PC 30:1 [M+H]+ | Ret. Time (min): 8.287 | m/z: 704.523

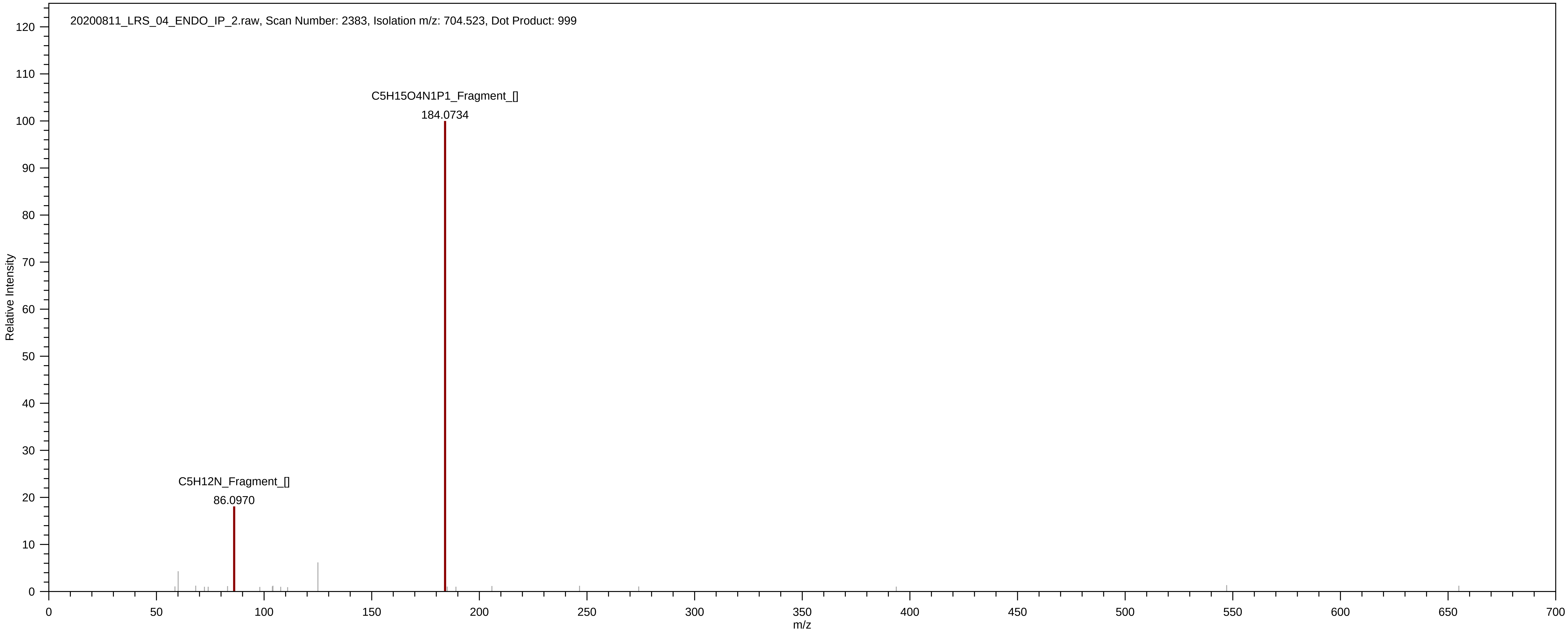

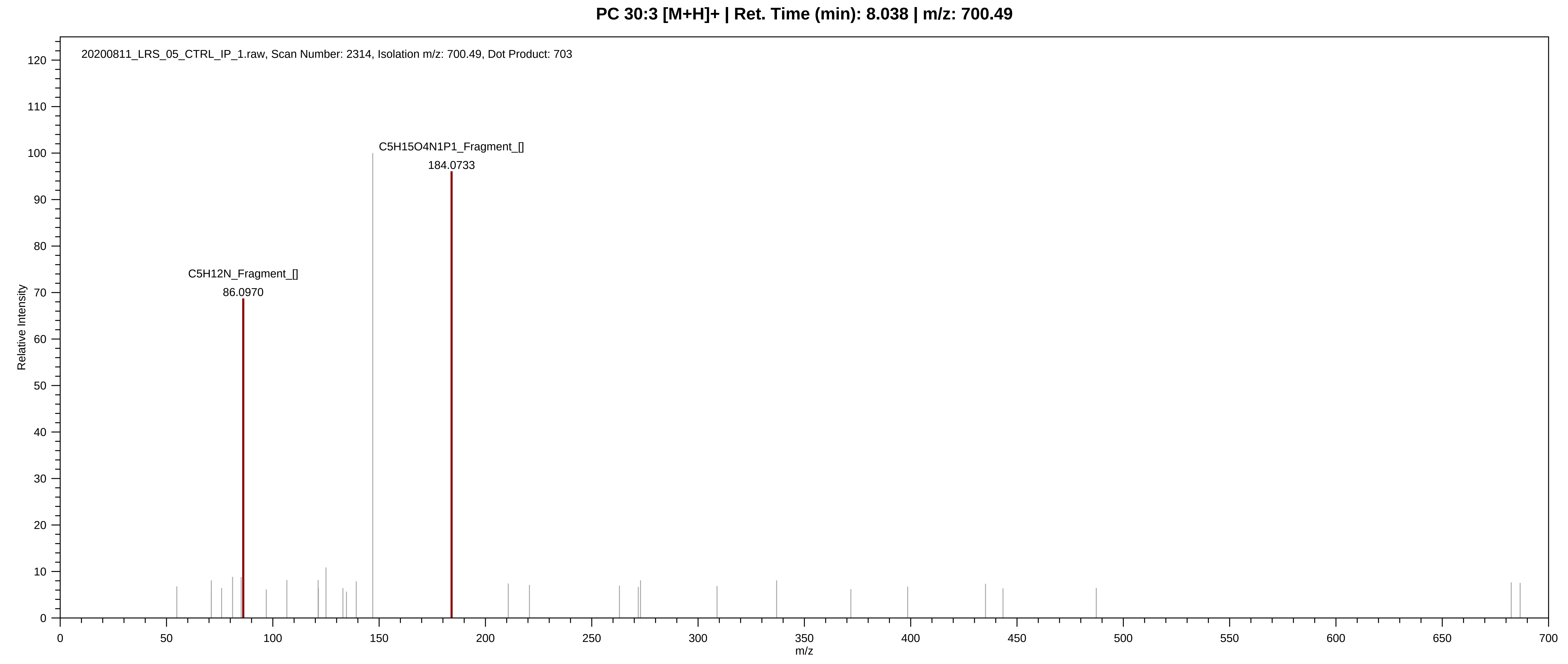
PC 30:3 [M+H]+ | Ret. Time (min): 8.038 | m/z: 700.49
20200811_LRS_05_CTRL_IP_1.raw, Scan Number: 2314, Isolation m/z: 700.49, Dot Product: 703
C5H15O4N1P1_Fragment_[]
184.0733
C5H12N_Fragment_[]
86.0970
Relative Intensity
m/z
0
10
20
30
40
50
60
70
80
90
100
110
120
0
50
100
150
200
250
300
350
400
450
500
550
600
650
700

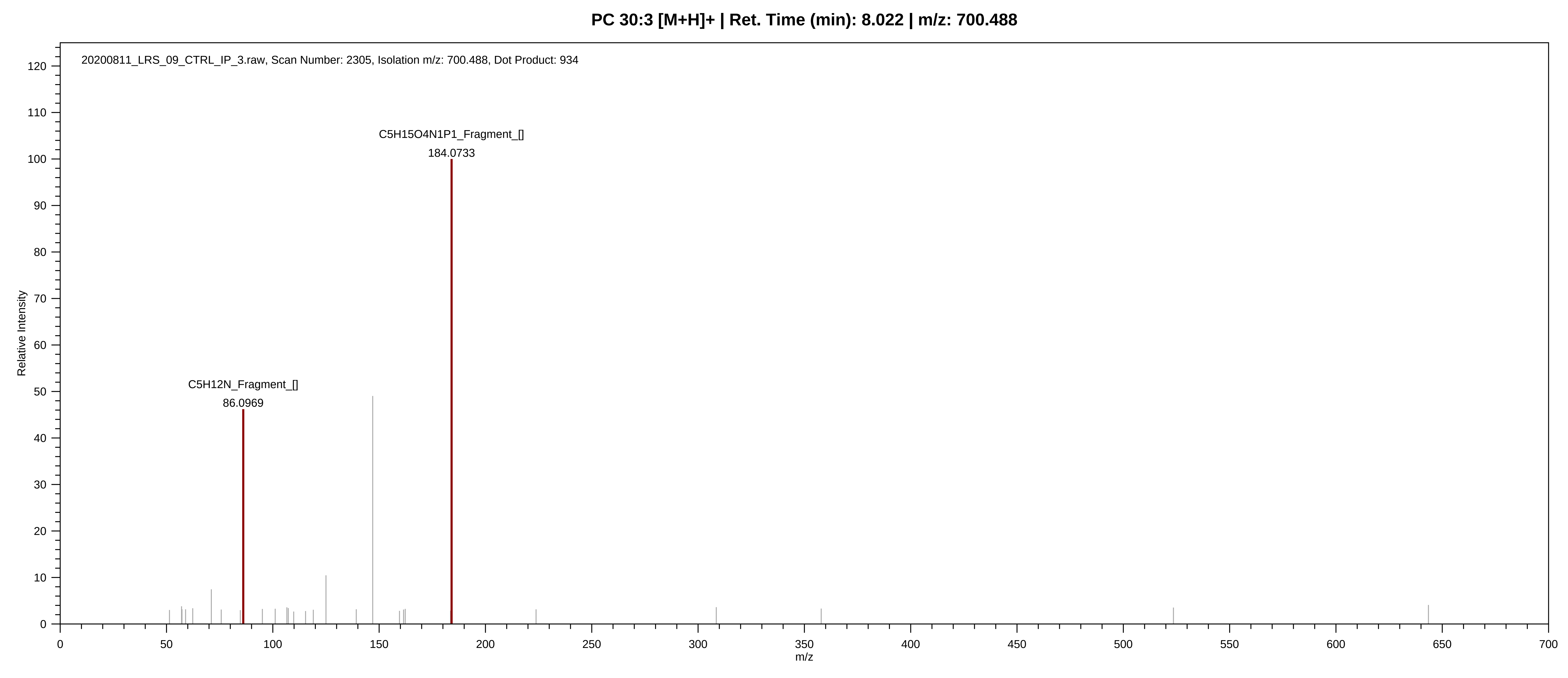

PC 30:3 [M+H]+ | Ret. Time (min): 8.022 | m/z: 700.488
20200811_LRS_09_CTRL_IP_3.raw, Scan Number: 2305, Isolation m/z: 700.488, Dot Product: 934
C5H15O4N1P1_Fragment_[]
184.0733
C5H12N_Fragment_[]
86.0969
Relative Intensity
m/z

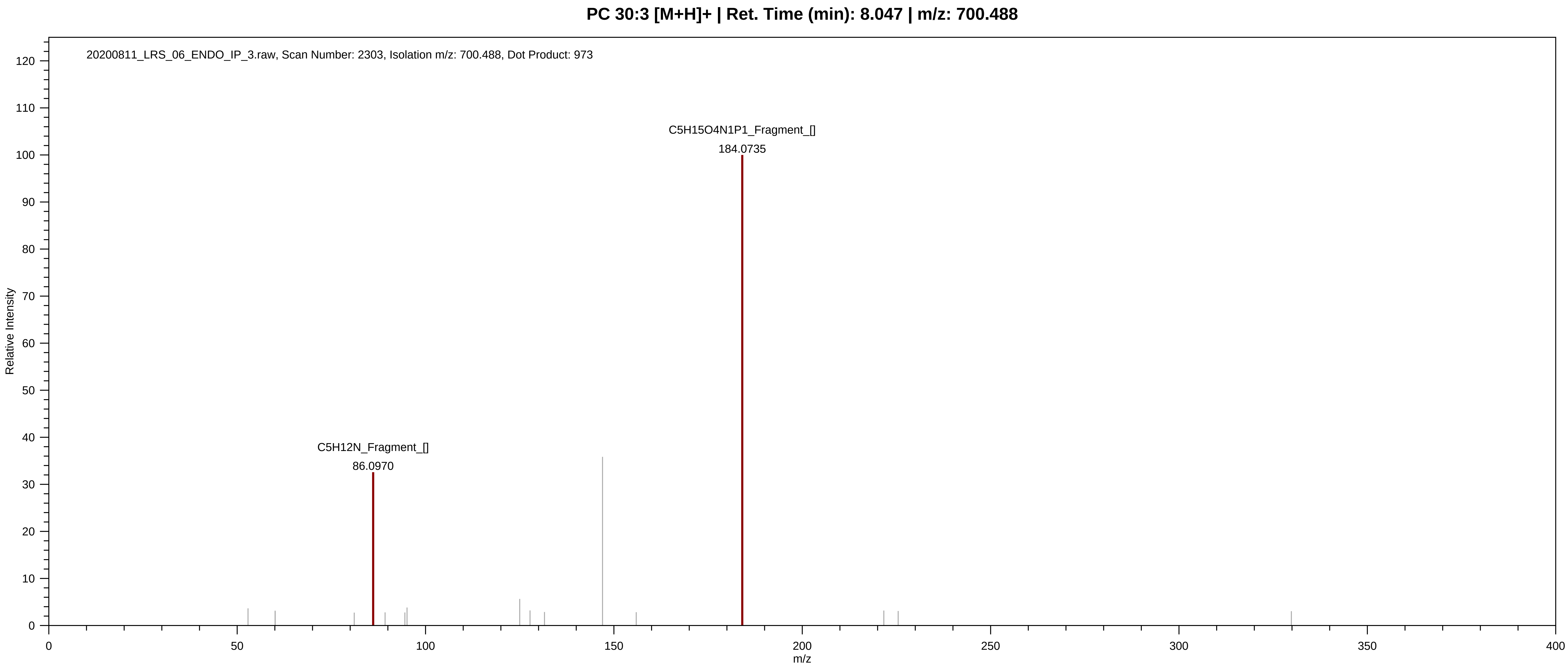
PC 30:3 [M+H]+ | Ret. Time (min): 8.047 | m/z: 700.488
20200811_LRS_06_ENDO_IP_3.raw, Scan Number: 2303, Isolation m/z: 700.488, Dot Product: 973
C5H15O4N1P1_Fragment_[]
184.0735
C5H12N_Fragment_[]
86.0970
Relative Intensity
m/z
0
10
20
30
40
50
60
70
80
90
100
110
120
0
50
100
150
200
250
300
350
400

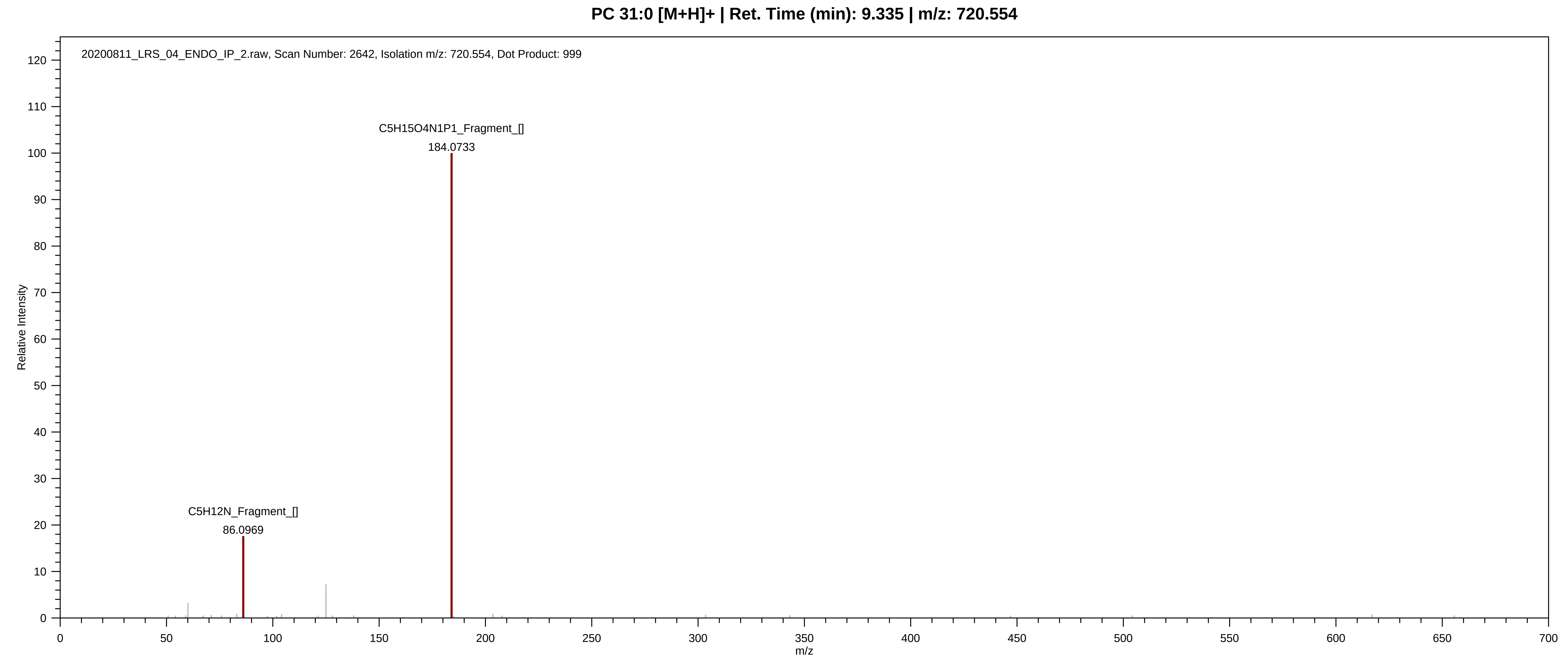
PC 31:0 [M+H]+ | Ret. Time (min): 9.335 | m/z: 720.554
20200811_LRS_04_ENDO_IP_2.raw, Scan Number: 2642, Isolation m/z: 720.554, Dot Product: 999
C5H15O4N1P1_Fragment_[]
184.0733
C5H12N_Fragment_[]
86.0969
Relative Intensity
m/z
0
10
20
30
40
50
60
70
80
90
100
110
120
0
50
100
150
200
250
300
350
400
450
500
550
600
650
700

PC 31:0 [M+H]+ | Ret. Time (min): 9.335 | m/z: 720.553

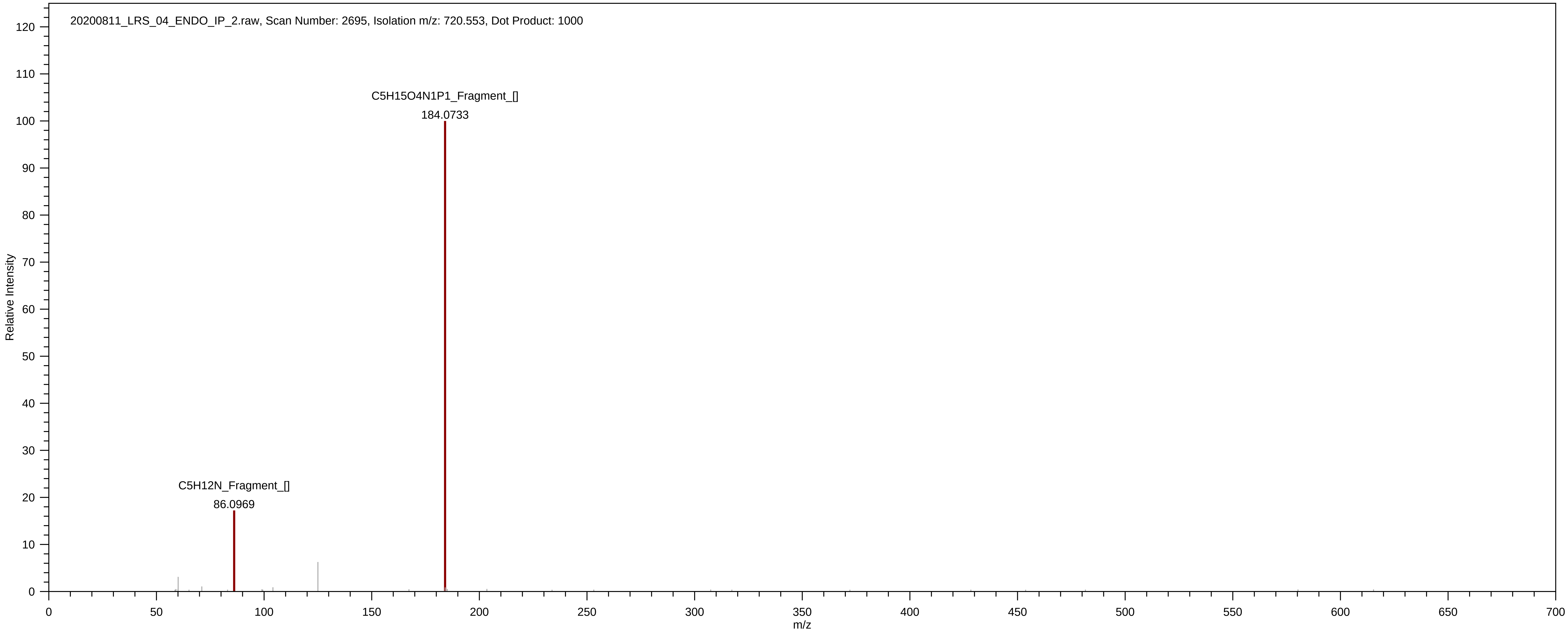

PC 31:0 [M+H]+ | Ret. Time (min): 9.32 | m/z: 720.554

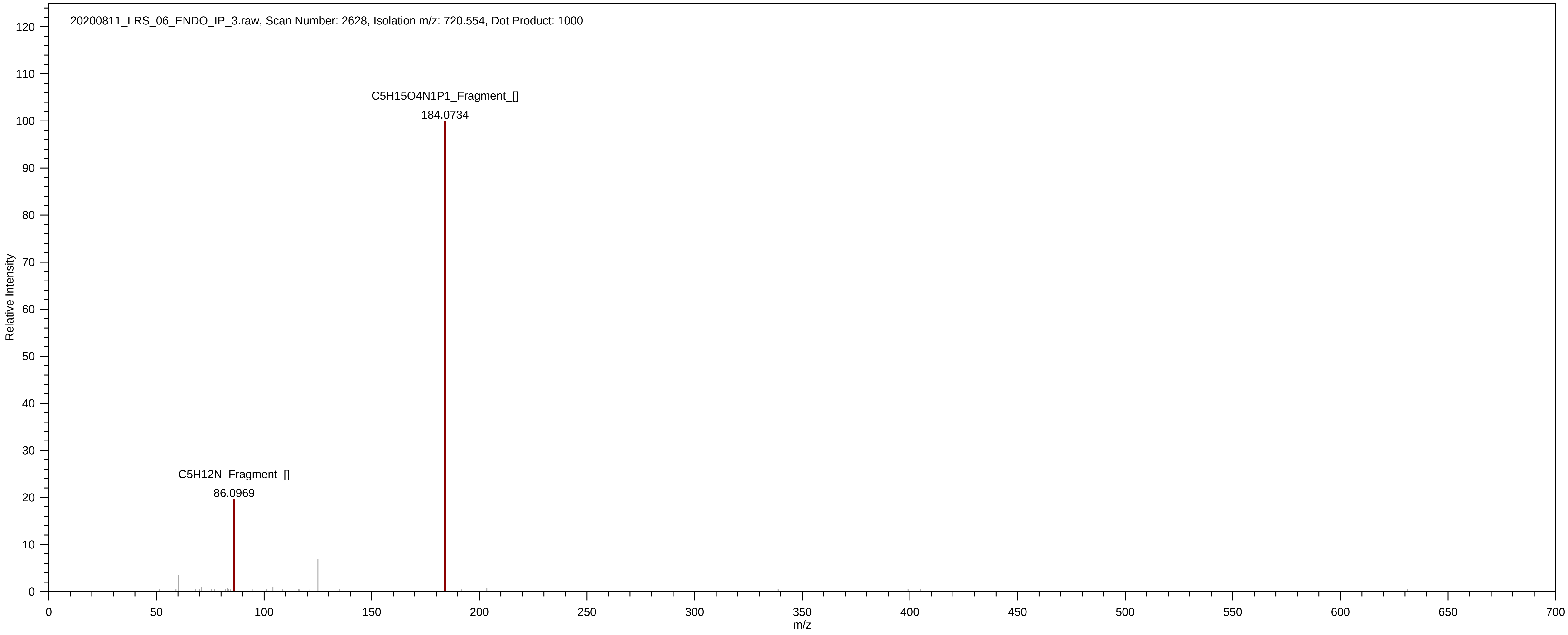

PC 31:0 [M+H]+ | Ret. Time (min): 9.32 | m/z: 720.554

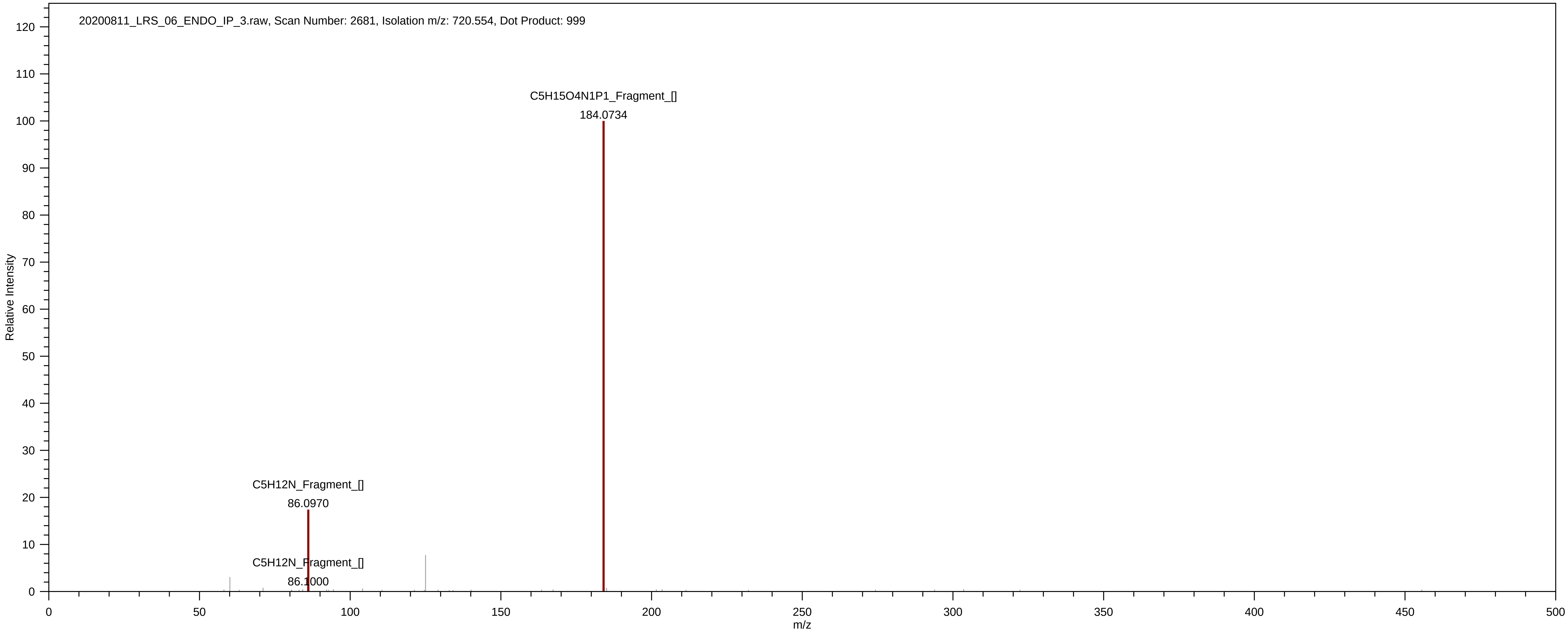

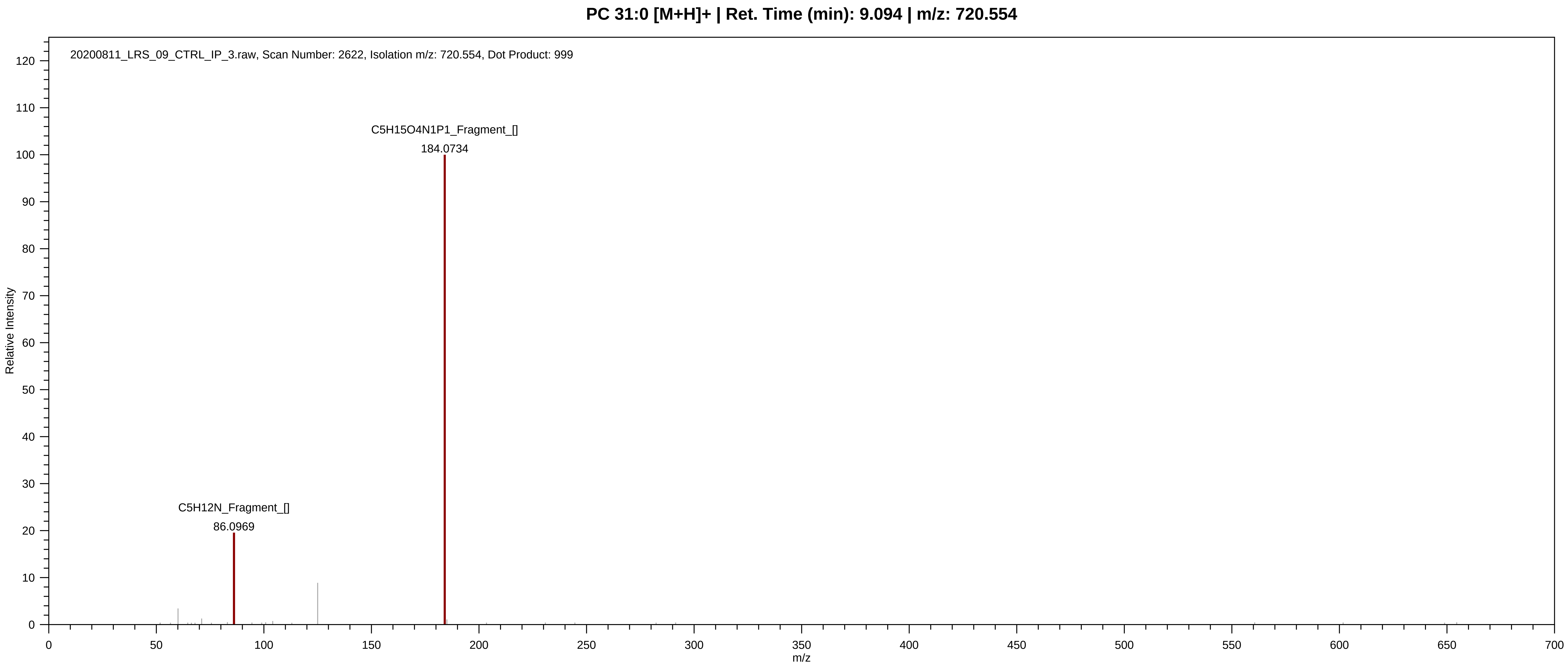
PC 31:0 [M+H]+ | Ret. Time (min): 9.094 | m/z: 720.554
20200811_LRS_09_CTRL_IP_3.raw, Scan Number: 2622, Isolation m/z: 720.554, Dot Product: 999
C5H15O4N1P1_Fragment_[]
184.0734
C5H12N_Fragment_[]
86.0969
Relative Intensity
m/z

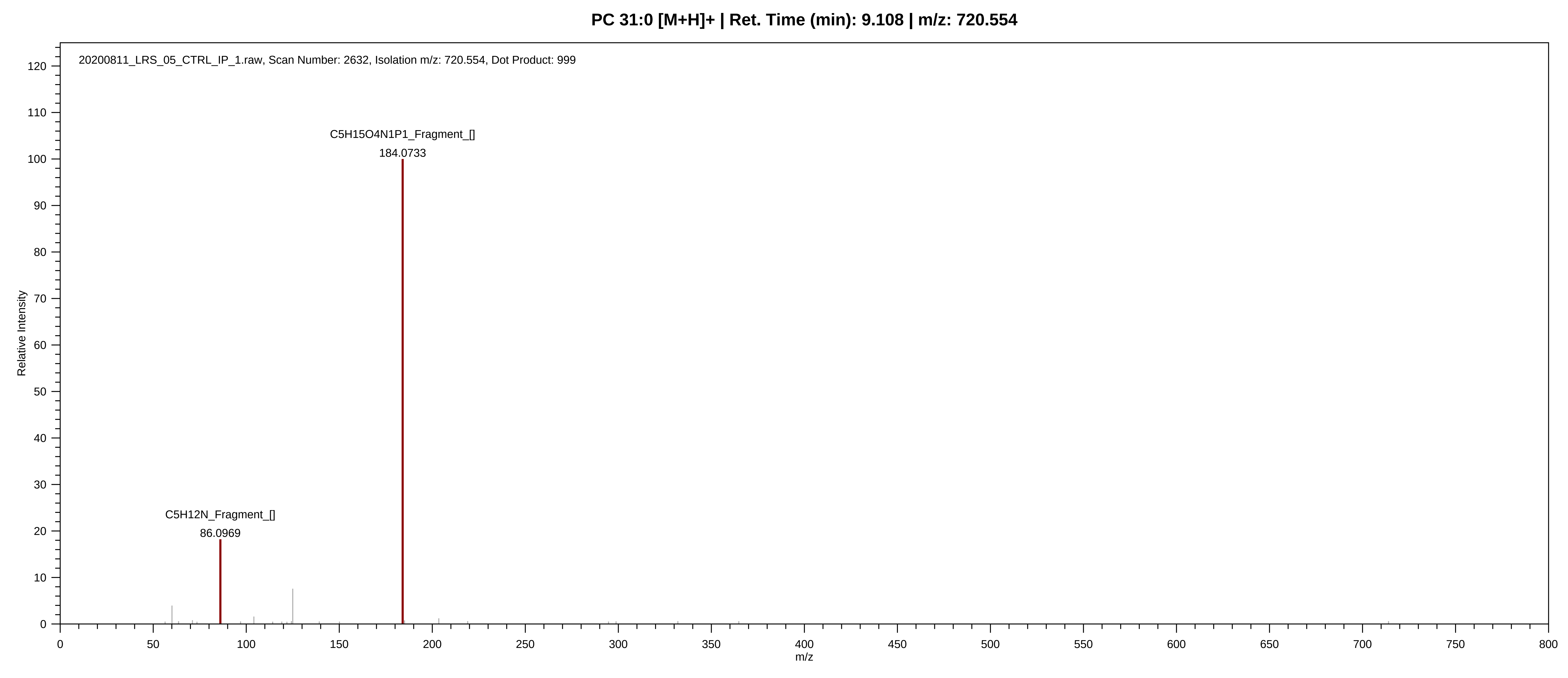
PC 31:0 [M+H]+ | Ret. Time (min): 9.108 | m/z: 720.554
20200811_LRS_05_CTRL_IP_1.raw, Scan Number: 2632, Isolation m/z: 720.554, Dot Product: 999
C5H15O4N1P1_Fragment_[]
184.0733
C5H12N_Fragment_[]
86.0969
Relative Intensity
m/z

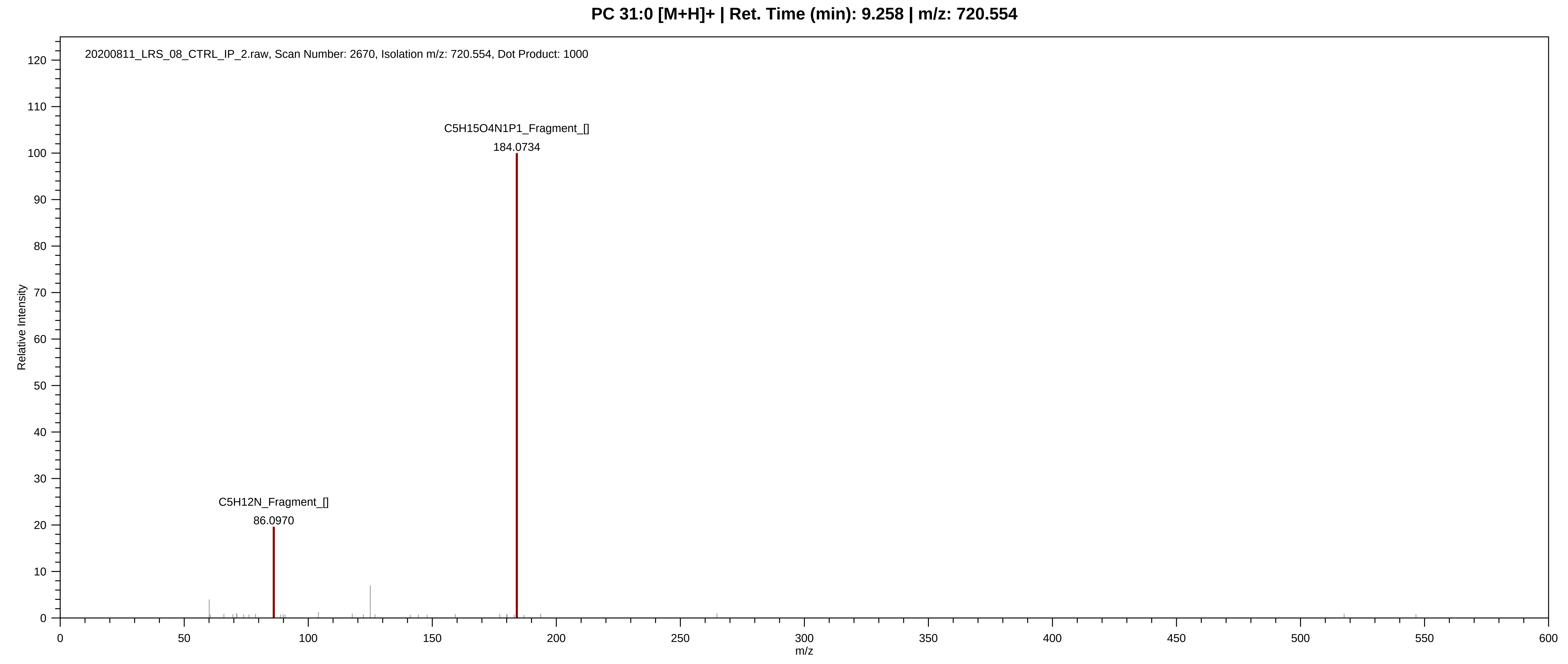
PC 31:0 [M+H]+ | Ret. Time (min): 9.258 | m/z: 720.554
20200811_LRS_08_CTRL_IP_2.raw, Scan Number: 2670, Isolation m/z: 720.554, Dot Product: 1000
C5H15O4N1P1_Fragment_[]
184.0734
C5H12N_Fragment_[]
86.0970
Relative Intensity
m/z
0
10
20
30
40
50
60
70
80
90
100
110
120
0
50
100
150
200
250
300
350
400
450
500
550
600

PC 31:0 [M+H]+ | Ret. Time (min): 9.286 | m/z: 720.554

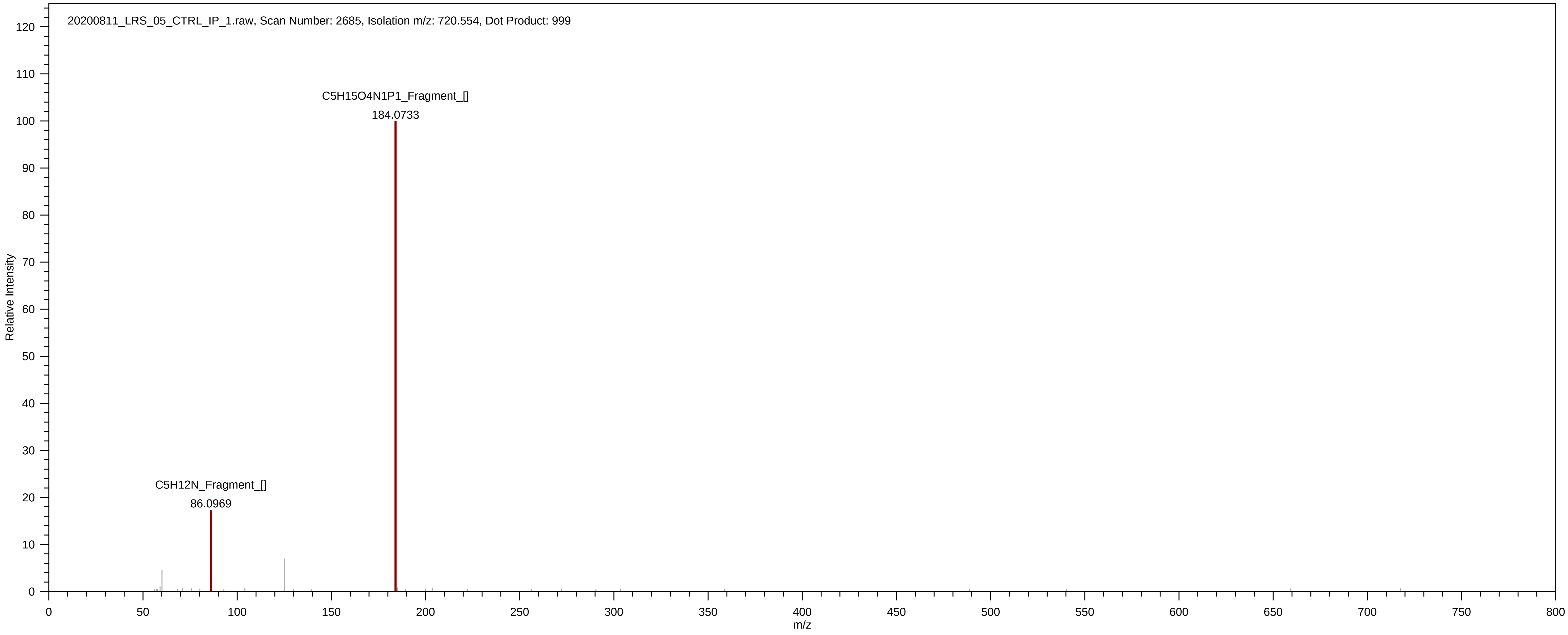

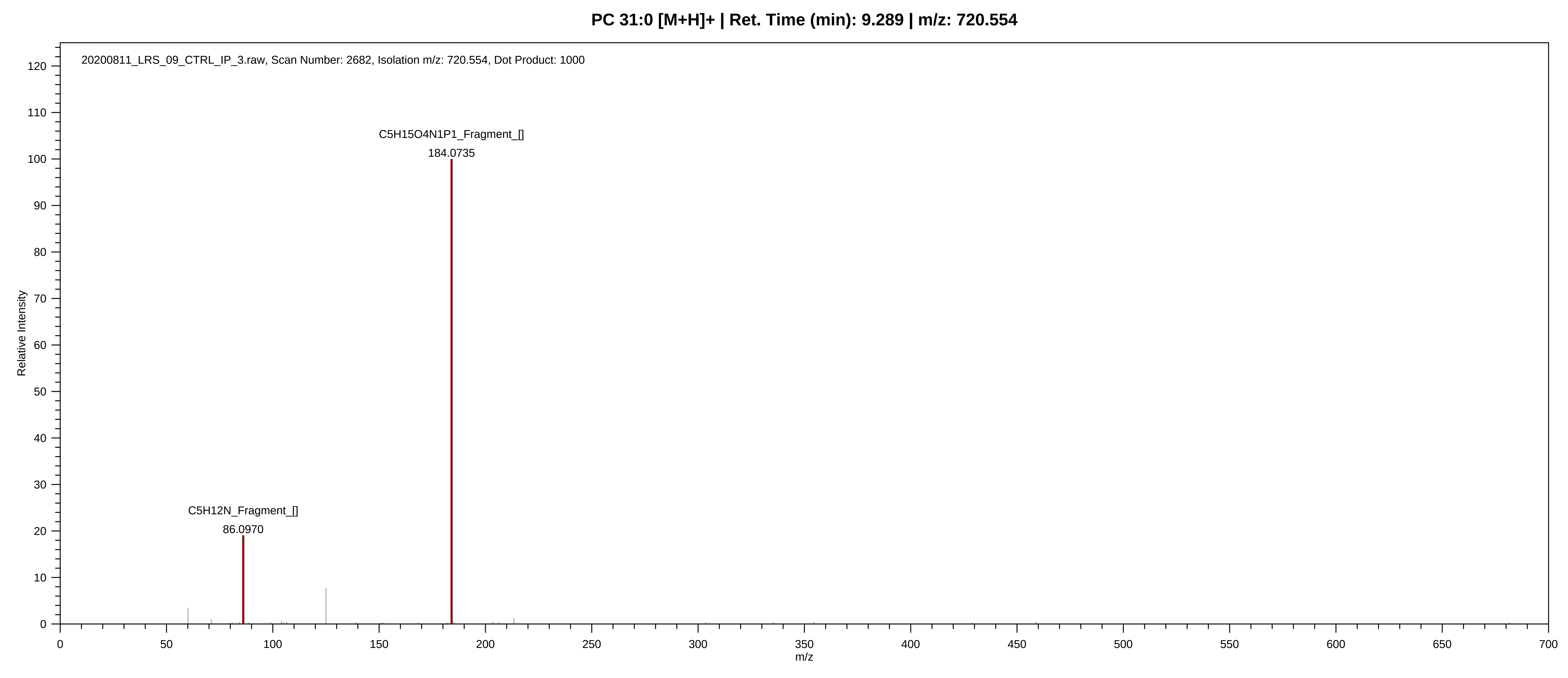

PC 31:0 [M+H]+ | Ret. Time (min): 9.289 | m/z: 720.554
20200811_LRS_09_CTRL_IP_3.raw, Scan Number: 2682, Isolation m/z: 720.554, Dot Product: 1000
C5H15O4N1P1_Fragment_[]
184.0735
C5H12N_Fragment_[]
86.0970
Relative Intensity
m/z

PC 31:0 [M+H]+ | Ret. Time (min): 9.321 | m/z: 720.553

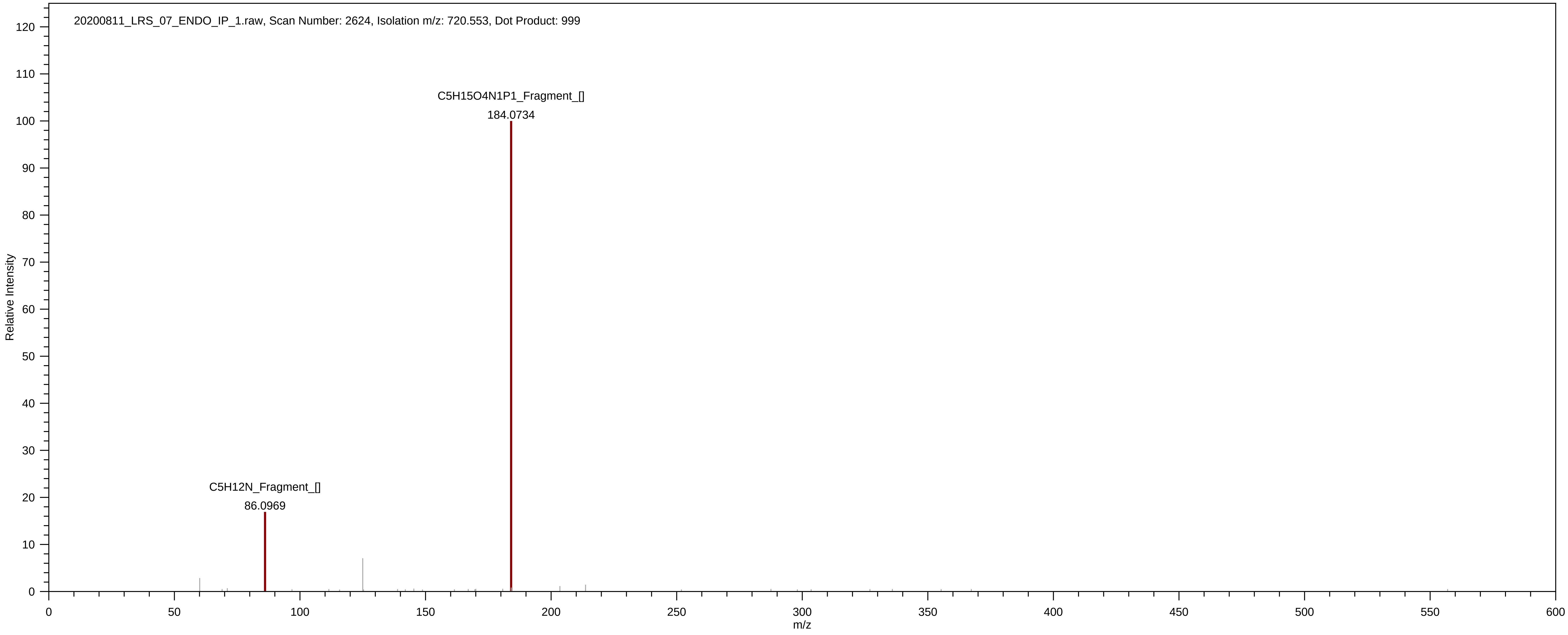

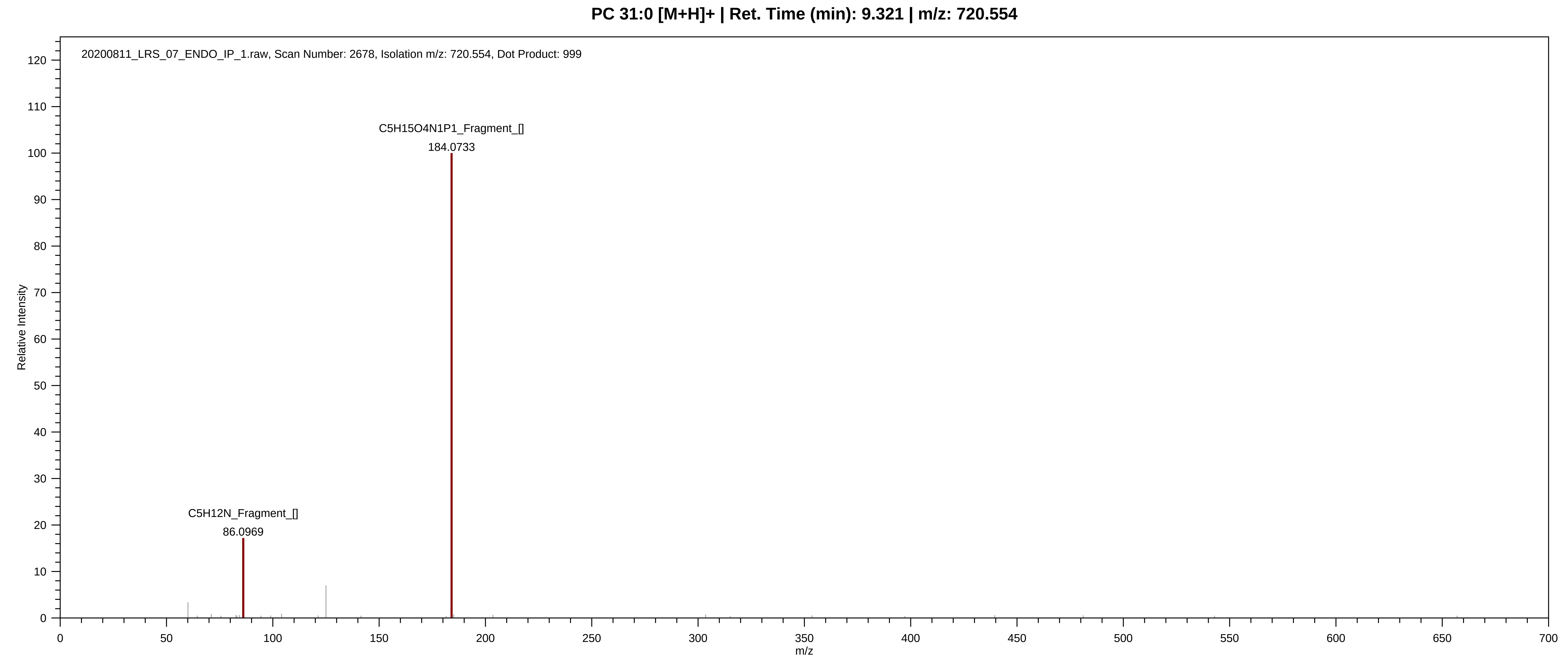
PC 31:0 [M+H]+ | Ret. Time (min): 9.321 | m/z: 720.554
20200811_LRS_07_ENDO_IP_1.raw, Scan Number: 2678, Isolation m/z: 720.554, Dot Product: 999
C5H15O4N1P1_Fragment_[]
184.0733
C5H12N_Fragment_[]
86.0969
Relative Intensity
m/z

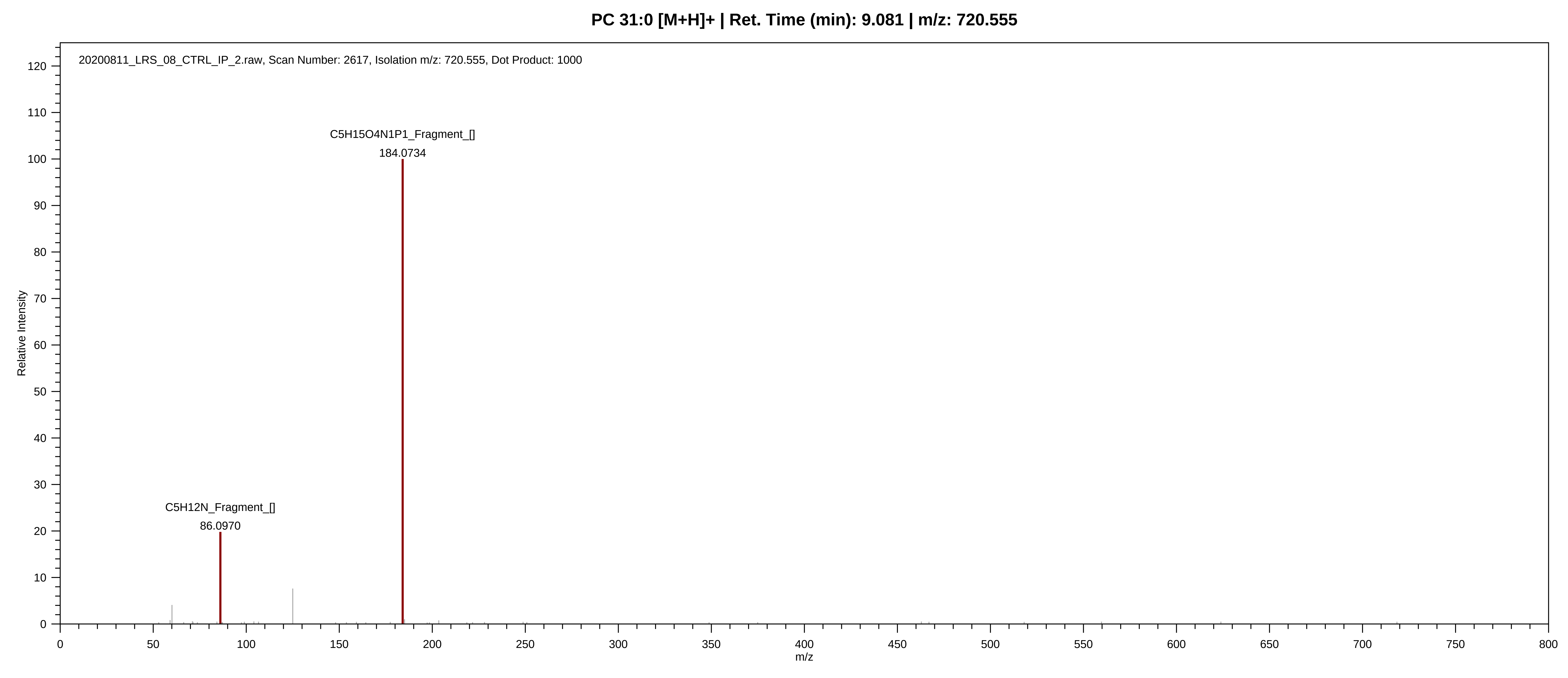
PC 31:0 [M+H]+ | Ret. Time (min): 9.081 | m/z: 720.555
20200811_LRS_08_CTRL_IP_2.raw, Scan Number: 2617, Isolation m/z: 720.555, Dot Product: 1000
C5H15O4N1P1_Fragment_[]
184.0734
C5H12N_Fragment_[]
86.0970
Relative Intensity
m/z

PC 31:1 [M+H]+ | Ret. Time (min): 8.371 | m/z: 718.537

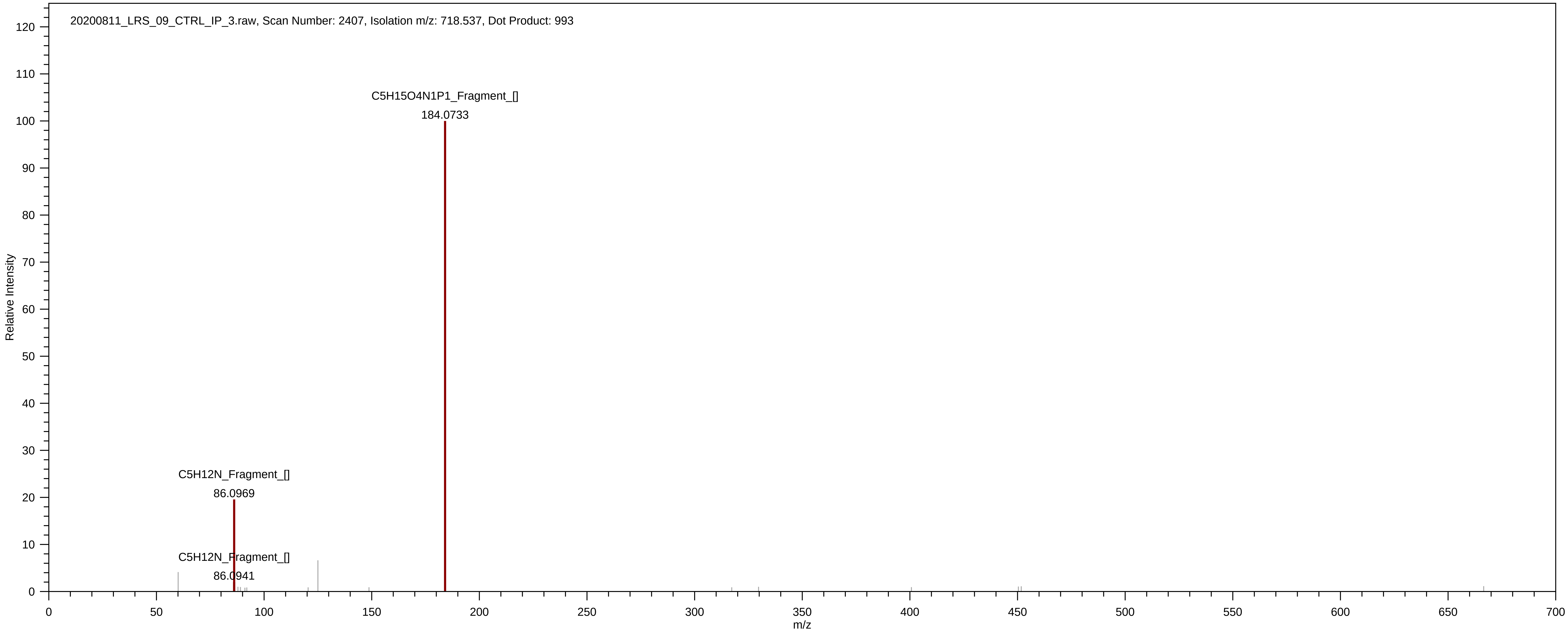

PC 31:1 [M+H]+ | Ret. Time (min): 8.738 | m/z: 718.537

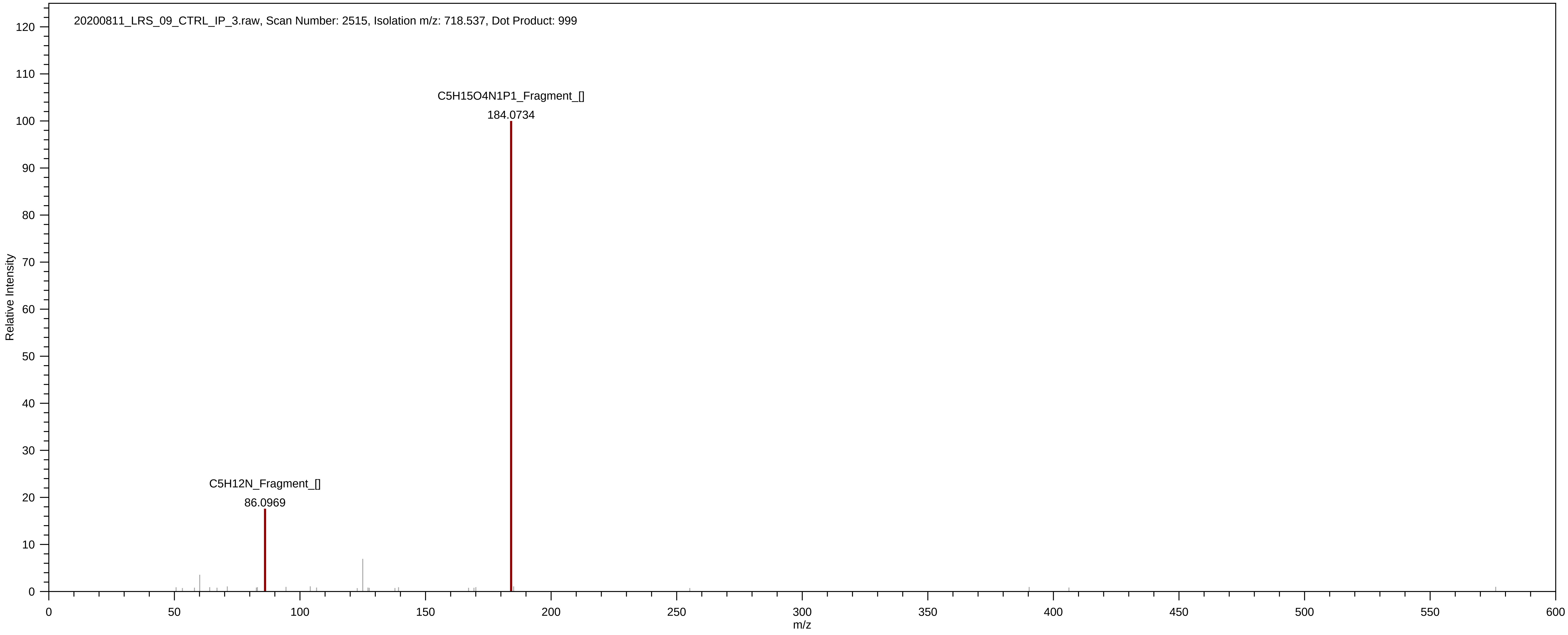

PC 31:1 [M+H]+ | Ret. Time (min): 8.72 | m/z: 718.538

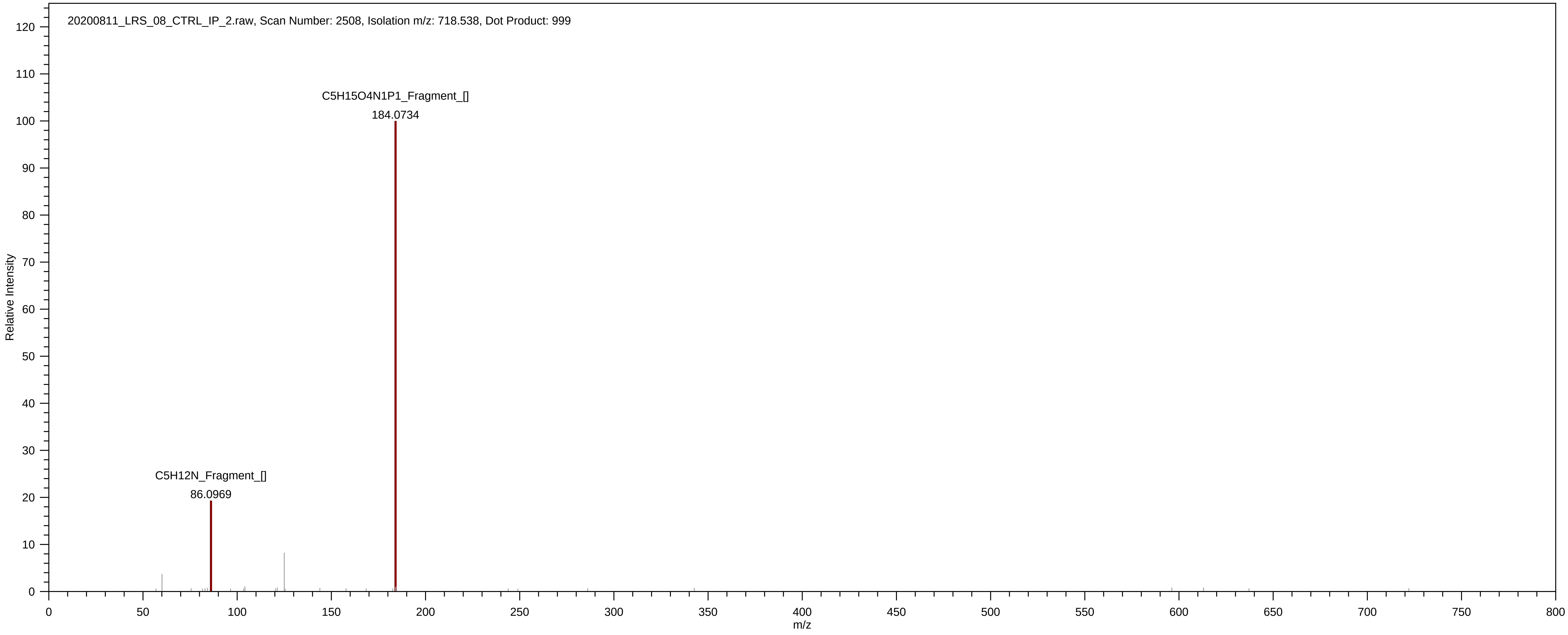

PC 31:1 [M+H]+ | Ret. Time (min): 8.501 | m/z: 718.538

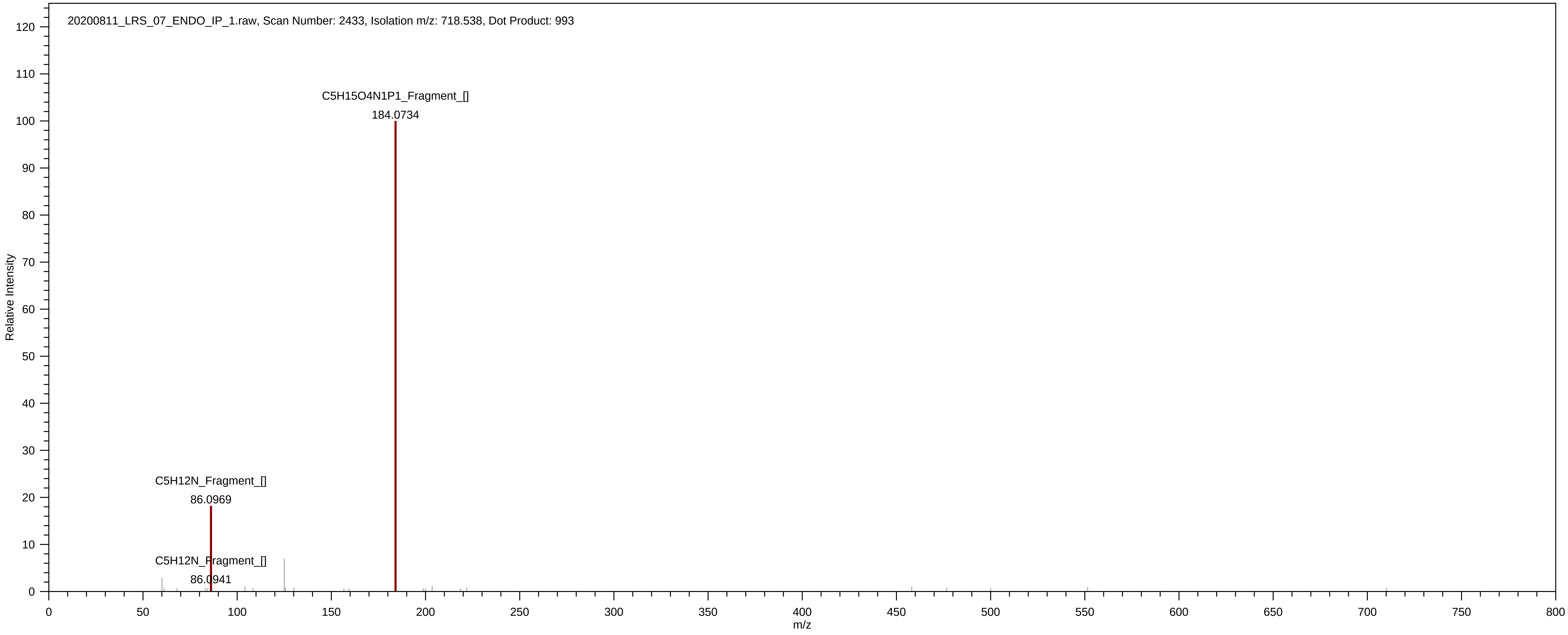

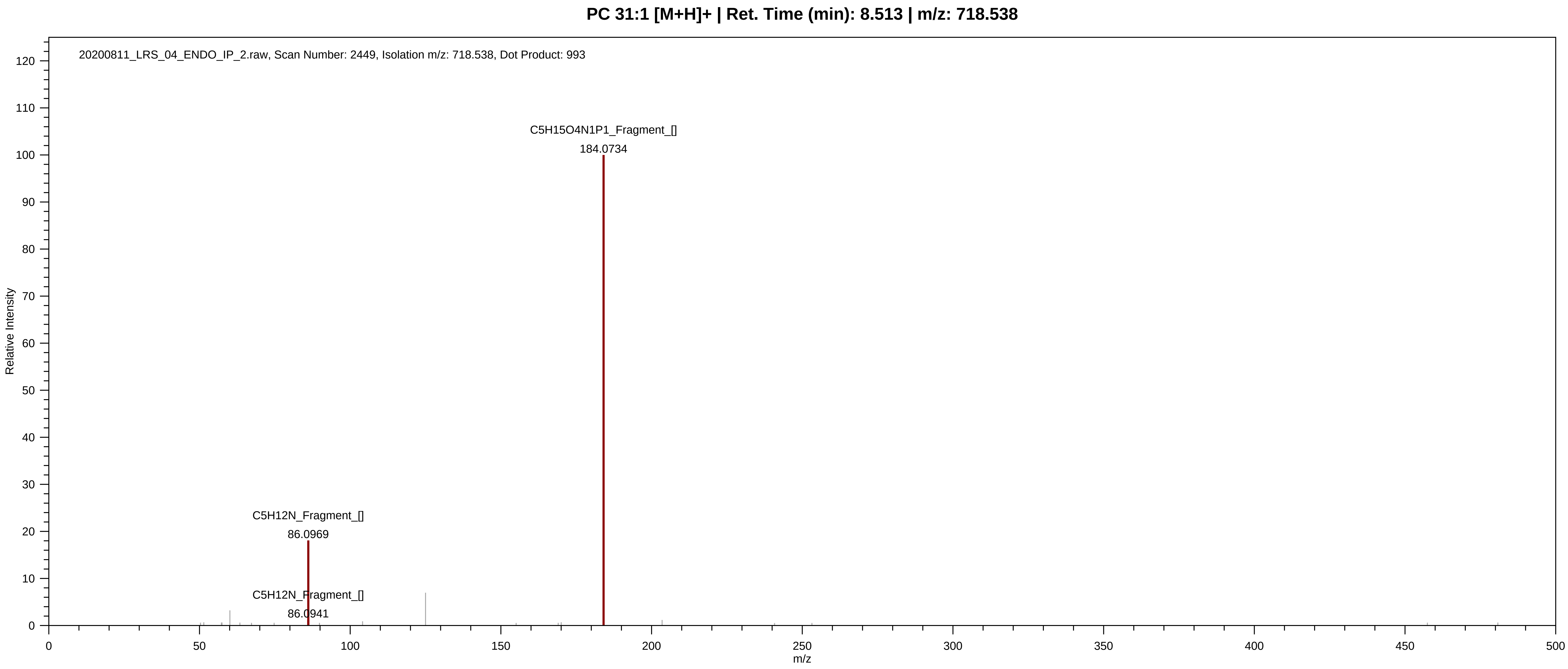

PC 31:1 [M+H]+ | Ret. Time (min): 8.513 | m/z: 718.538
20200811_LRS_04_ENDO_IP_2.raw, Scan Number: 2449, Isolation m/z: 718.538, Dot Product: 993
C5H15O4N1P1_Fragment_[]
184.0734
C5H12N_Fragment_[]
86.0969
C5H12N_Fragment_[]
86.0941
Relative Intensity
m/z

PC 31:1 [M+H]+ | Ret. Time (min): 8.522 | m/z: 718.538

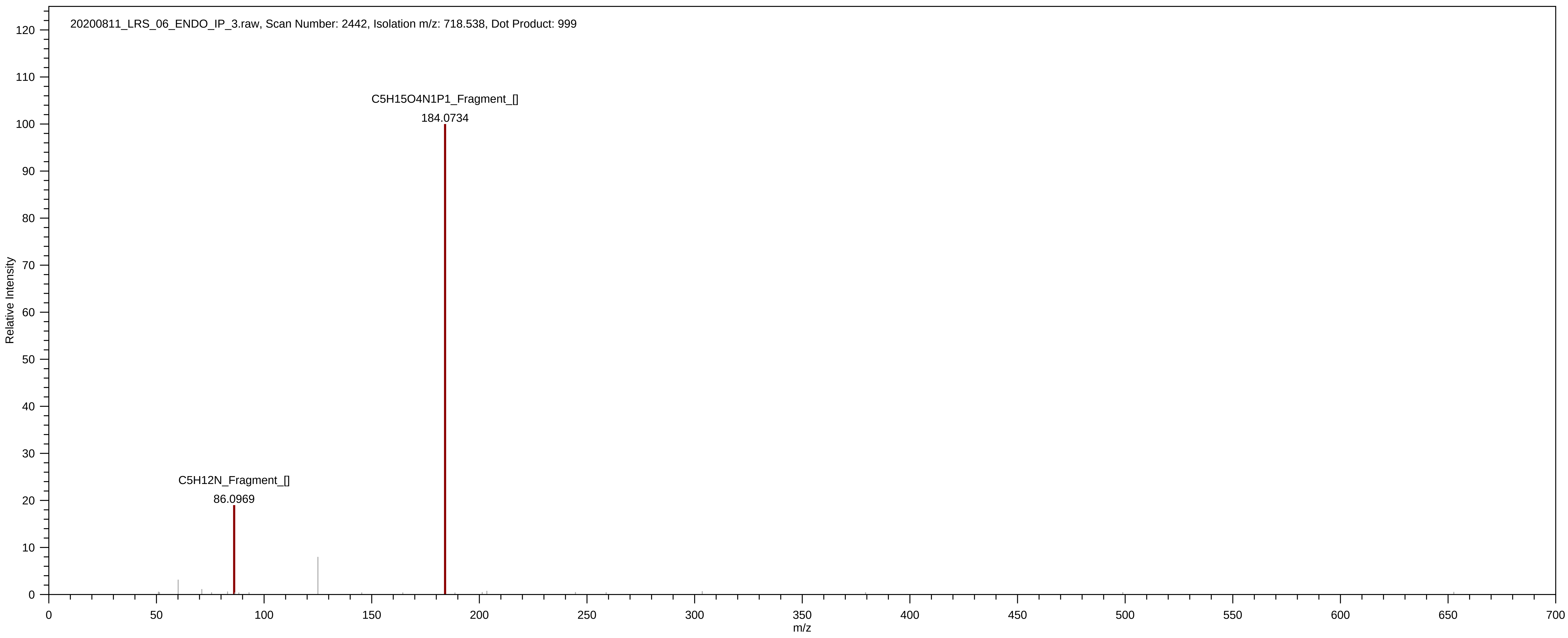

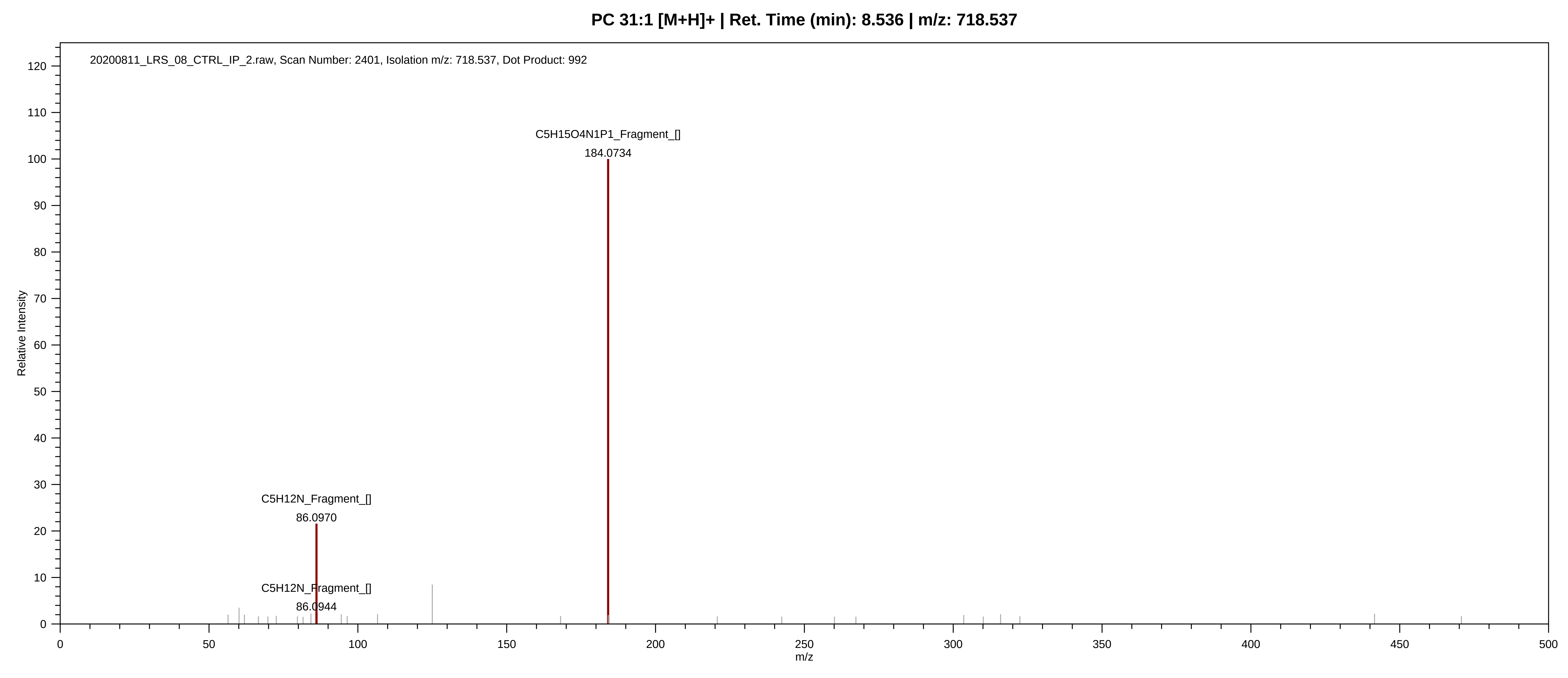
PC 31:1 [M+H]+ | Ret. Time (min): 8.536 | m/z: 718.537
20200811_LRS_08_CTRL_IP_2.raw, Scan Number: 2401, Isolation m/z: 718.537, Dot Product: 992
C5H15O4N1P1_Fragment_[]
184.0734
C5H12N_Fragment_[]
86.0970
C5H12N_Fragment_[]
86.0944
Relative Intensity
m/z

PC 31:1 [M+H]+ | Ret. Time (min): 8.536 | m/z: 718.538

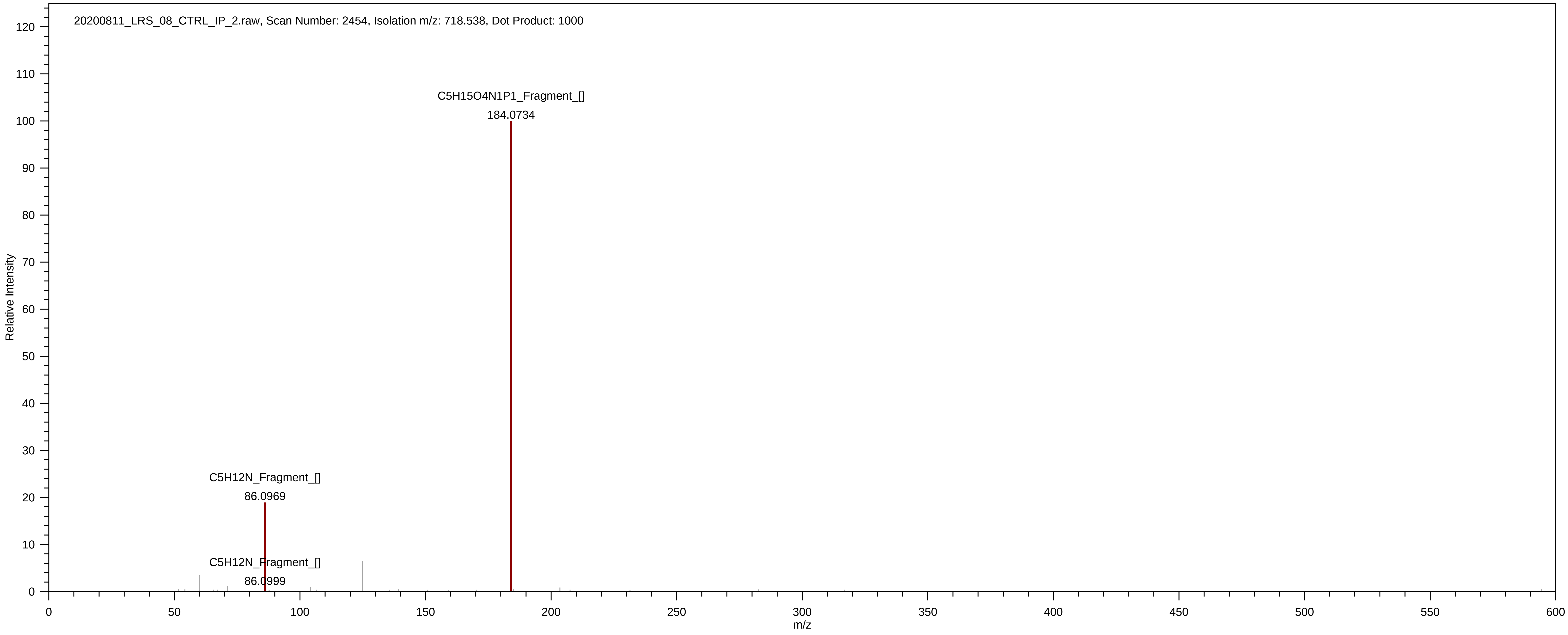

PC 31:1 [M+H]+ | Ret. Time (min): 8.543 | m/z: 718.538

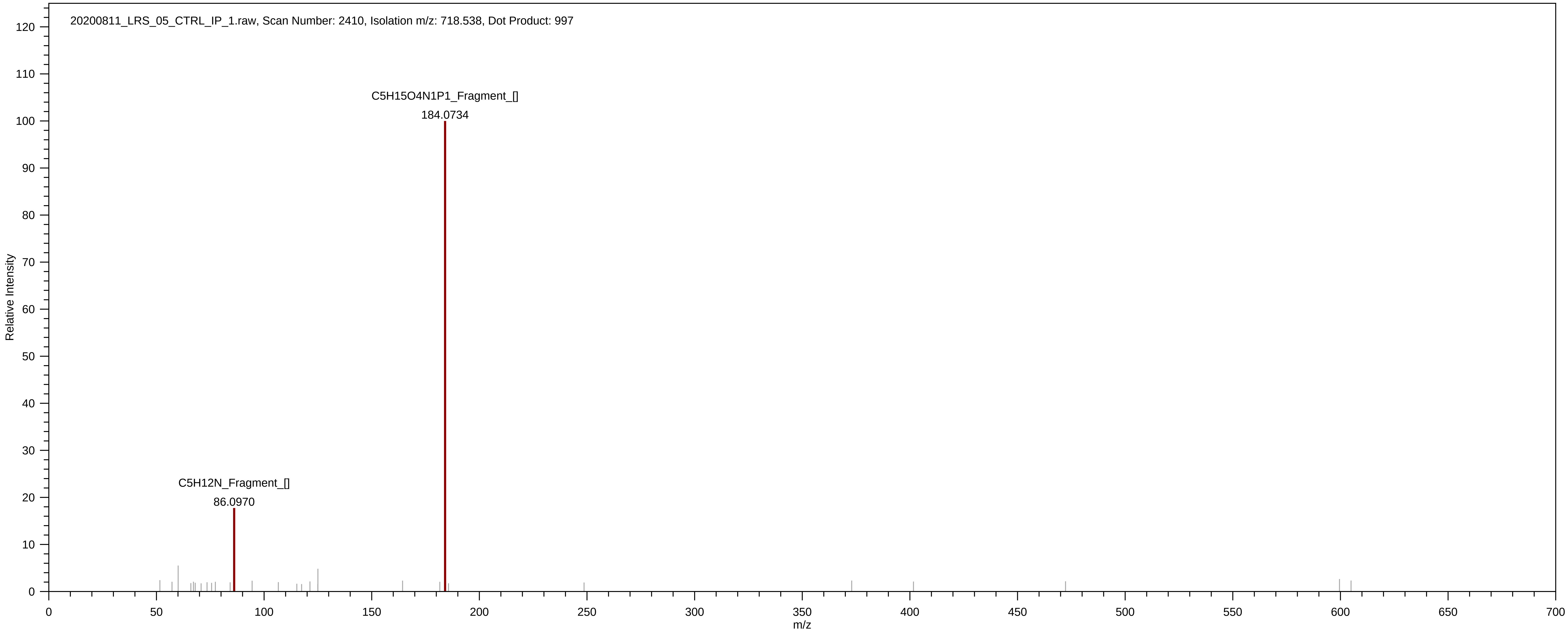

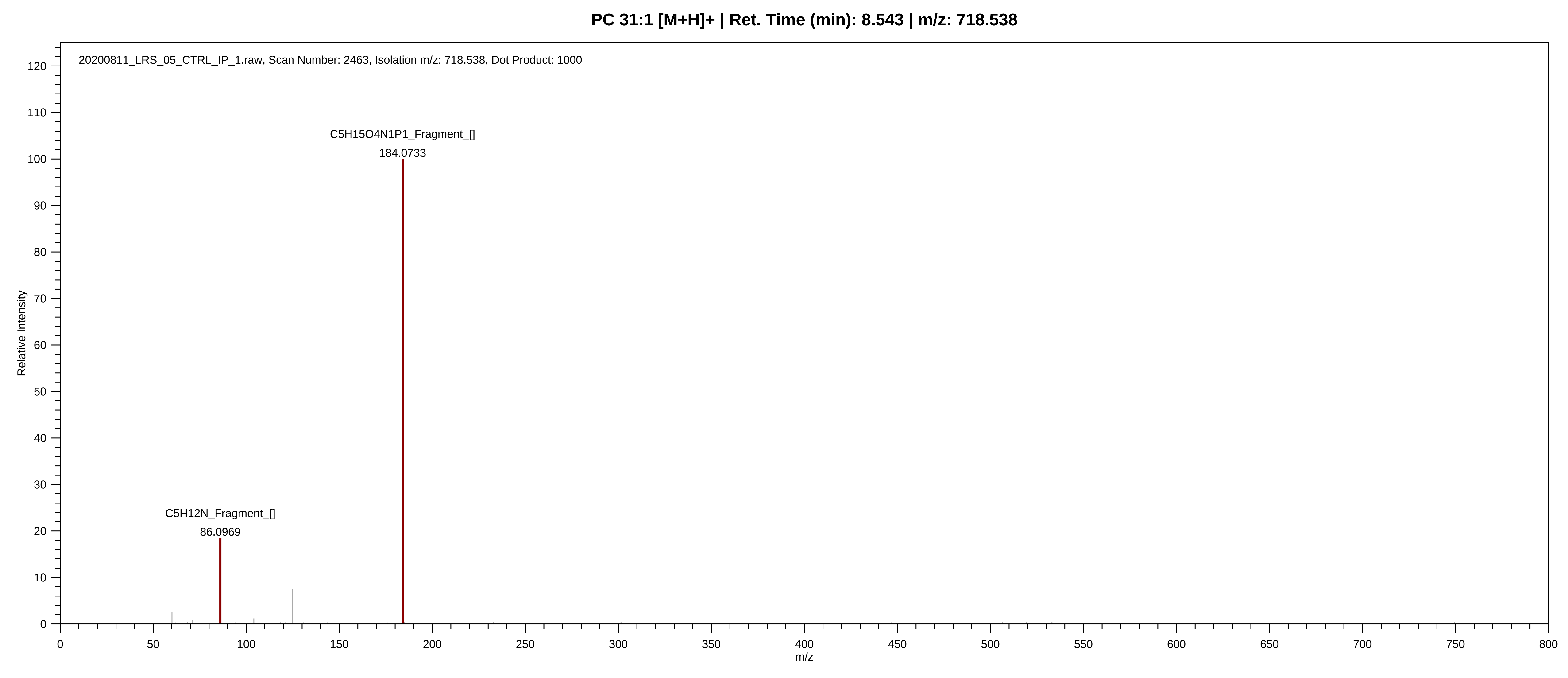
PC 31:1 [M+H]+ | Ret. Time (min): 8.543 | m/z: 718.538
20200811_LRS_05_CTRL_IP_1.raw, Scan Number: 2463, Isolation m/z: 718.538, Dot Product: 1000
C5H15O4N1P1_Fragment_[]
184.0733
C5H12N_Fragment_[]
86.0969
Relative Intensity
m/z

PC 31:1 [M+H]+ | Ret. Time (min): 8.543 | m/z: 718.538

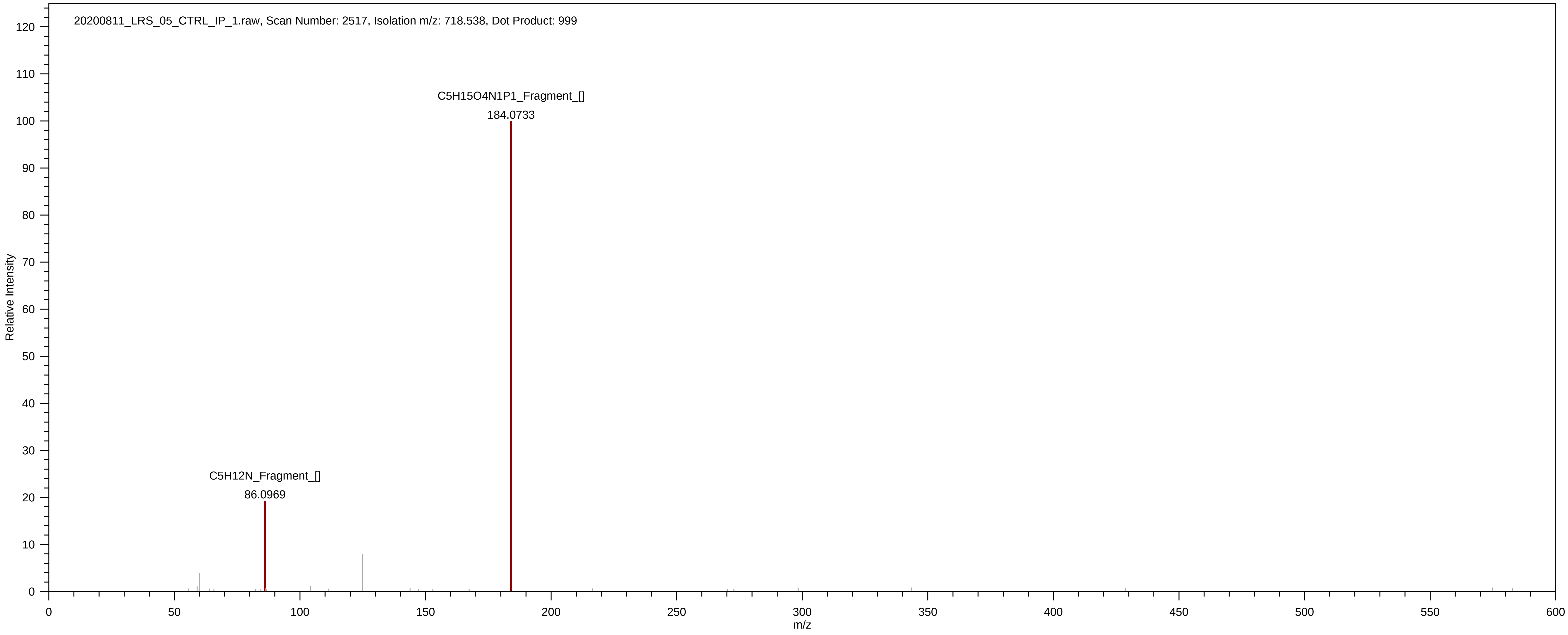

PC 31:1 [M+H]+ | Ret. Time (min): 8.553 | m/z: 718.538

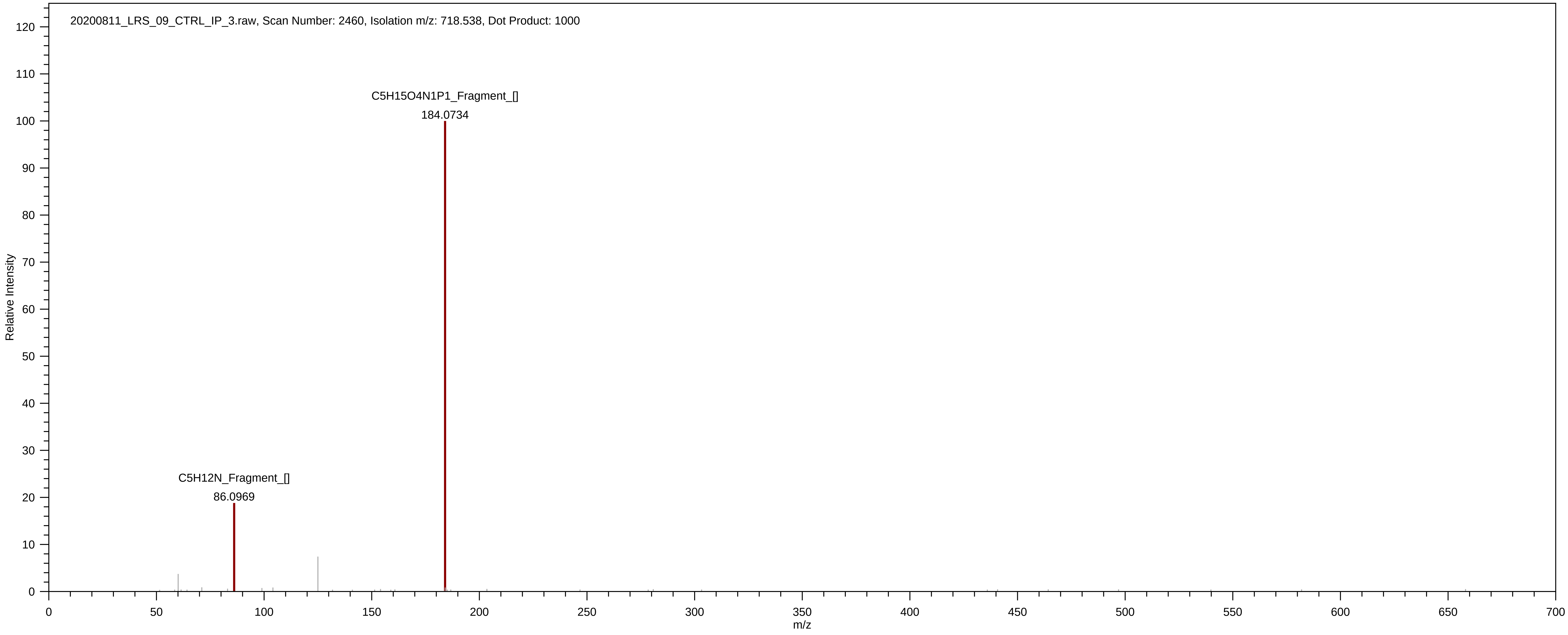

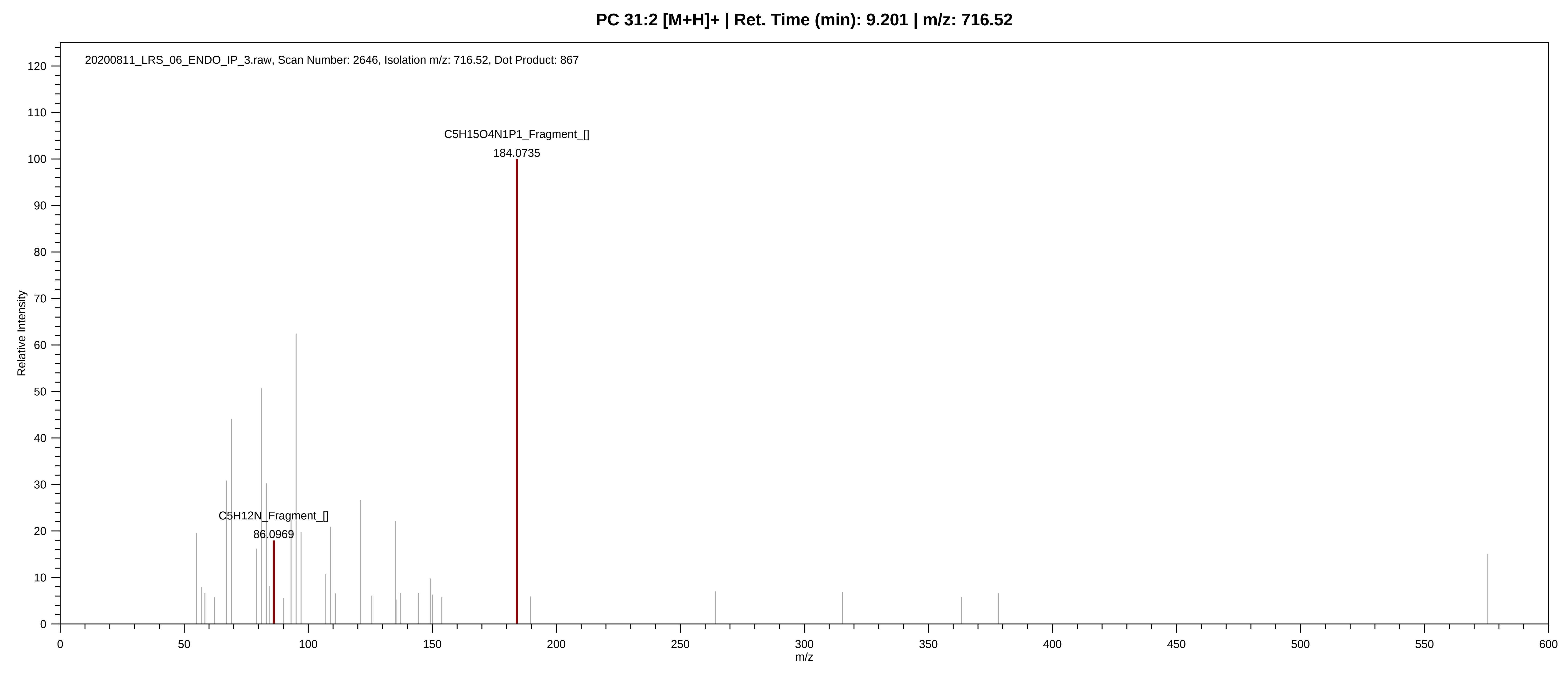

PC 31:2 [M+H]+ | Ret. Time (min): 9.201 | m/z: 716.52
20200811_LRS_06_ENDO_IP_3.raw, Scan Number: 2646, Isolation m/z: 716.52, Dot Product: 867
C5H15O4N1P1_Fragment_[]
184.0735
C5H12N_Fragment_[]
86.0969
Relative Intensity
m/z

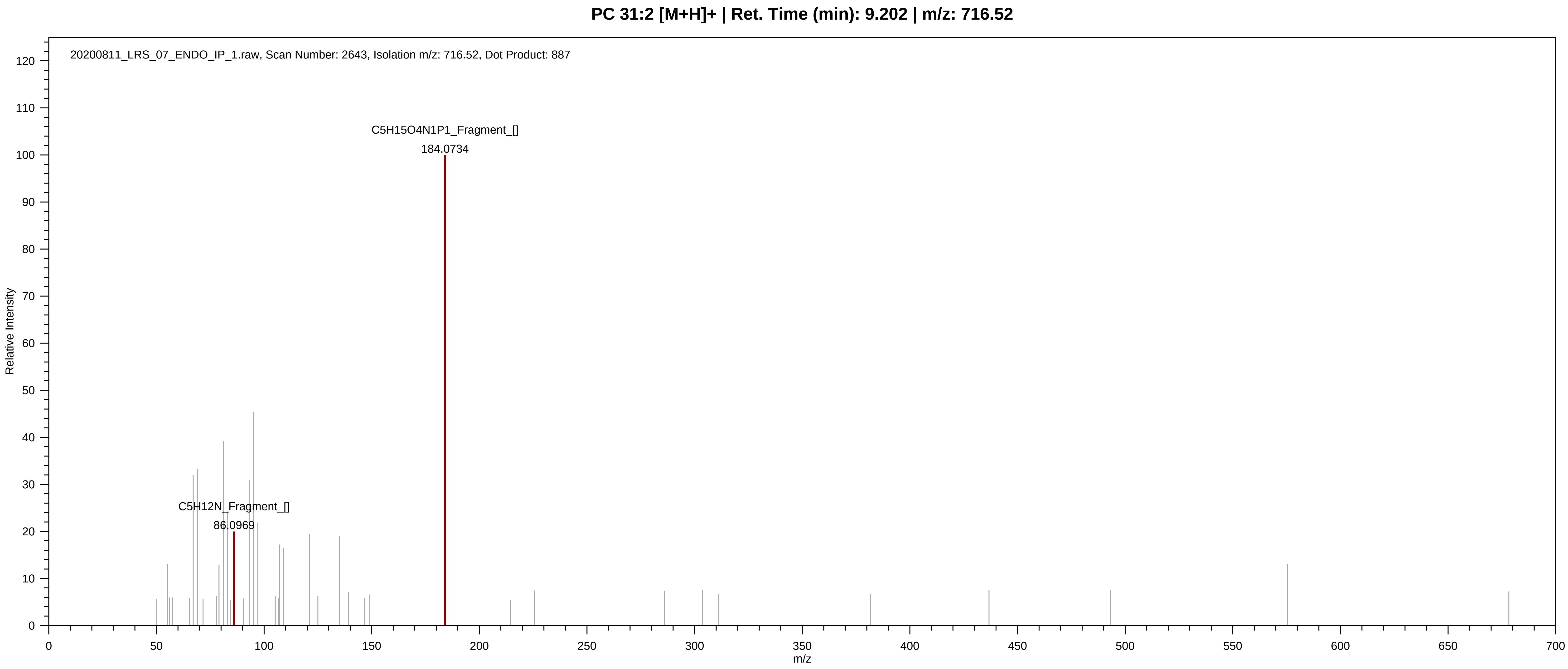
PC 31:2 [M+H]+ | Ret. Time (min): 9.202 | m/z: 716.52
20200811_LRS_07_ENDO_IP_1.raw, Scan Number: 2643, Isolation m/z: 716.52, Dot Product: 887
C5H15O4N1P1_Fragment_[]
184.0734
C5H12N_Fragment_[]
86.0969
Relative Intensity
m/z
0
10
20
30
40
50
60
70
80
90
100
110
120
0
50
100
150
200
250
300
350
400
450
500
550
600
650
700

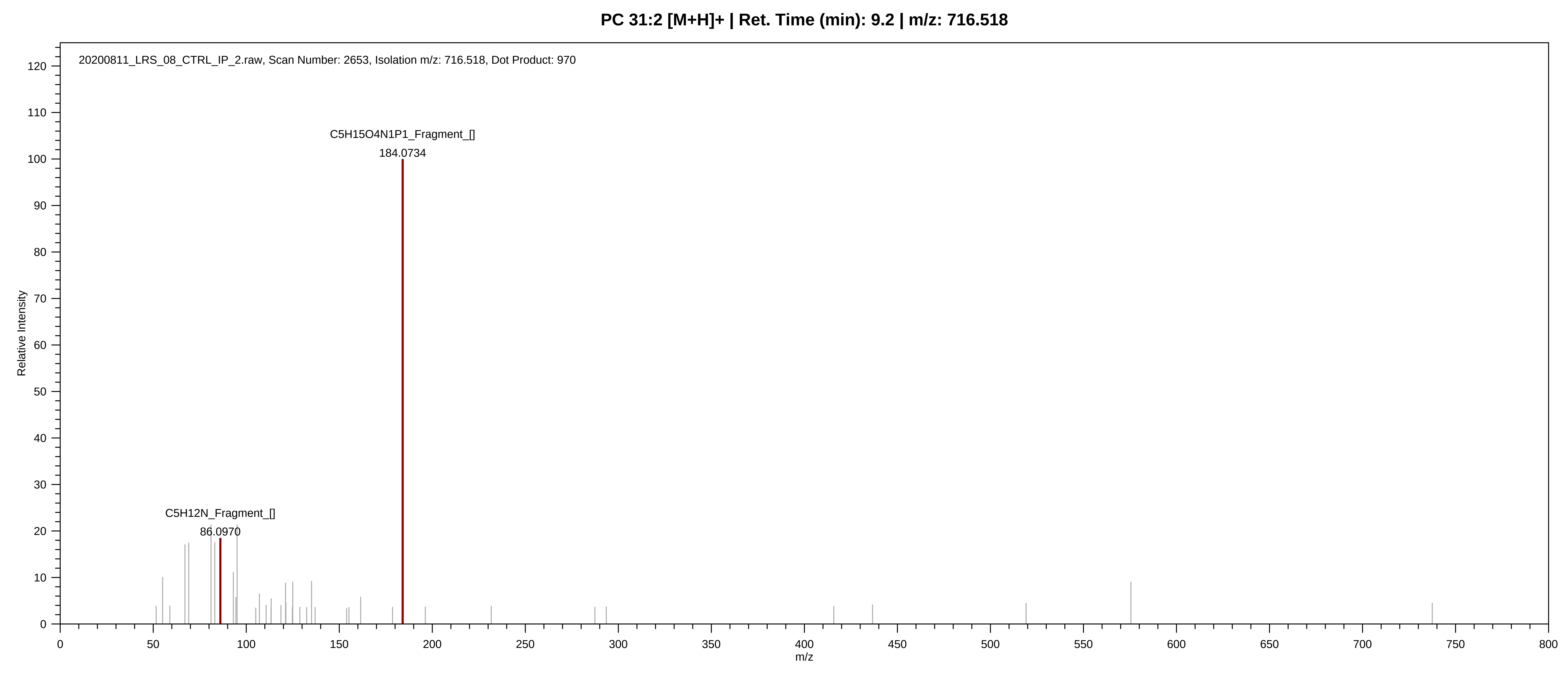
PC 31:2 [M+H]+ | Ret. Time (min): 9.2 | m/z: 716.518
20200811_LRS_08_CTRL_IP_2.raw, Scan Number: 2653, Isolation m/z: 716.518, Dot Product: 970
C5H15O4N1P1_Fragment_[]
184.0734
C5H12N_Fragment_[]
86.0970
Relative Intensity
m/z

PC 31:2 [M+H]+ | Ret. Time (min): 9.226 | m/z: 716.518

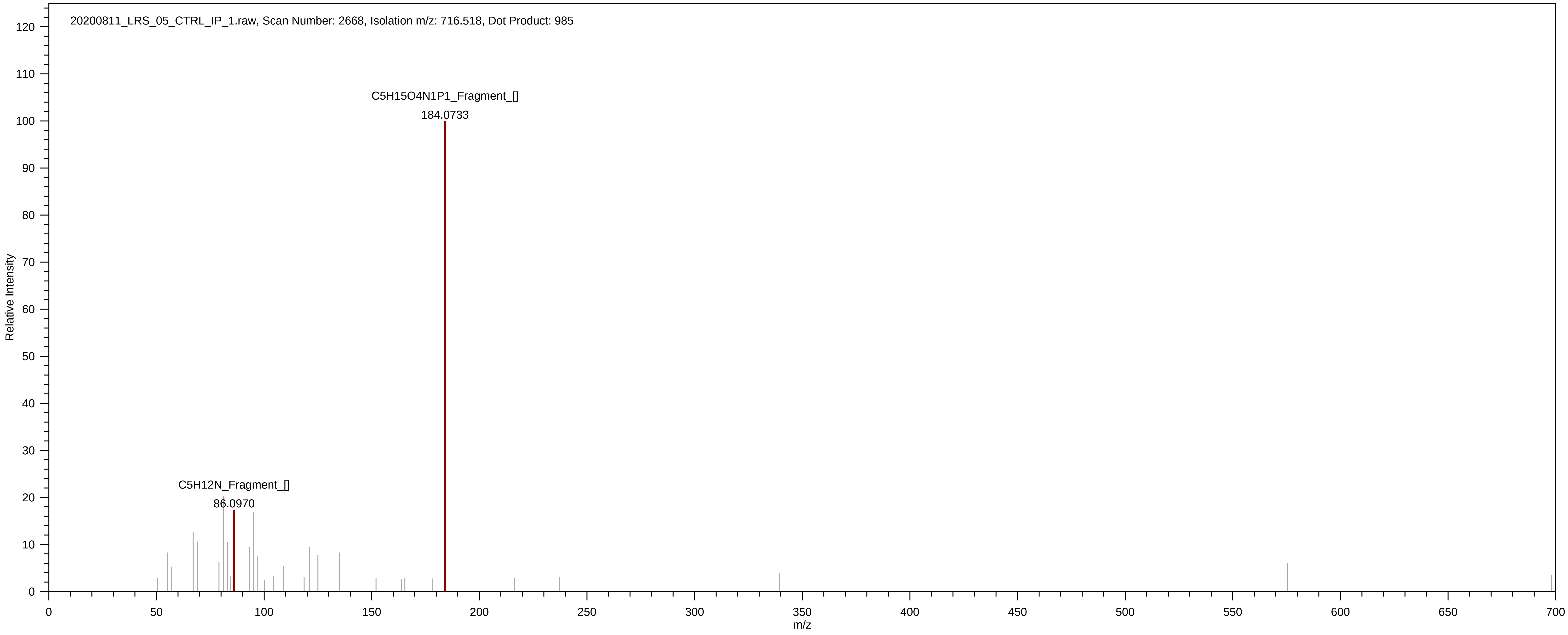

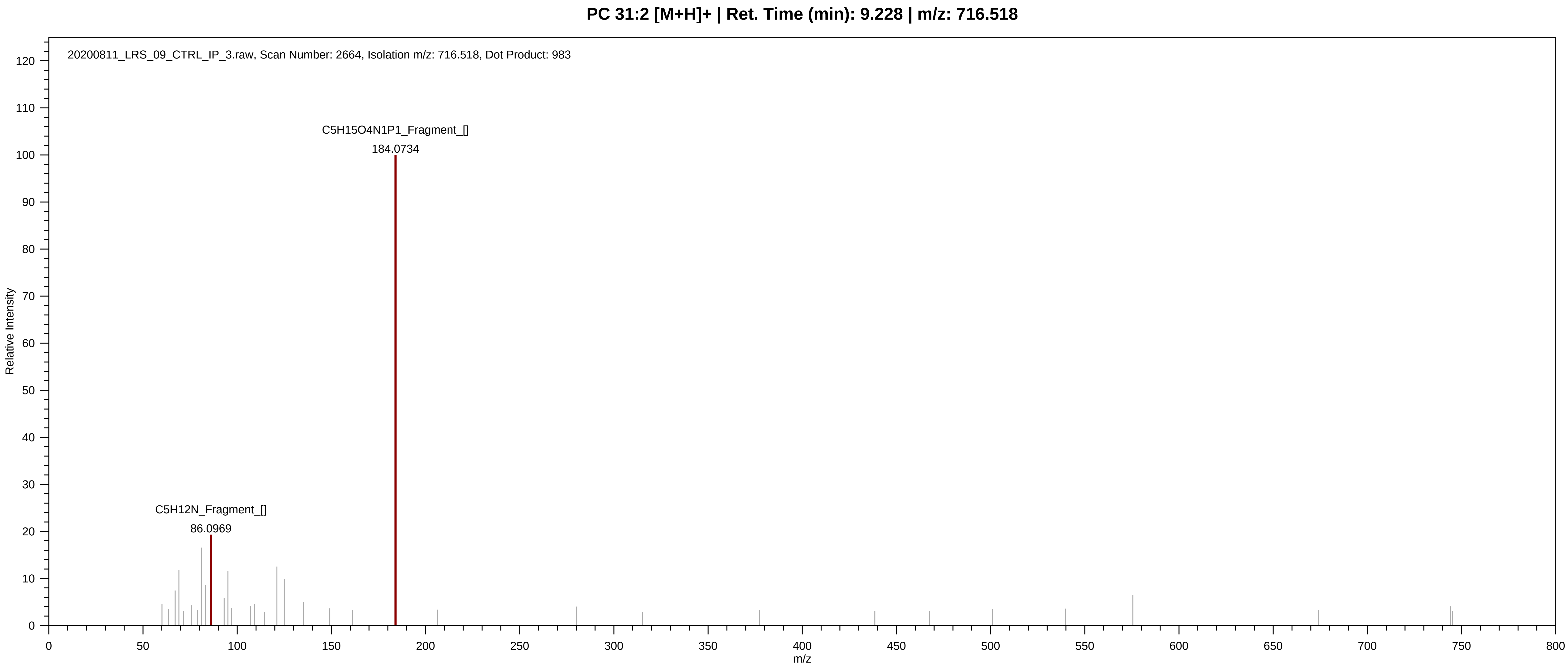
PC 31:2 [M+H]+ | Ret. Time (min): 9.228 | m/z: 716.518
20200811_LRS_09_CTRL_IP_3.raw, Scan Number: 2664, Isolation m/z: 716.518, Dot Product: 983
C5H15O4N1P1_Fragment_[]
184.0734
C5H12N_Fragment_[]
86.0969
Relative Intensity
m/z

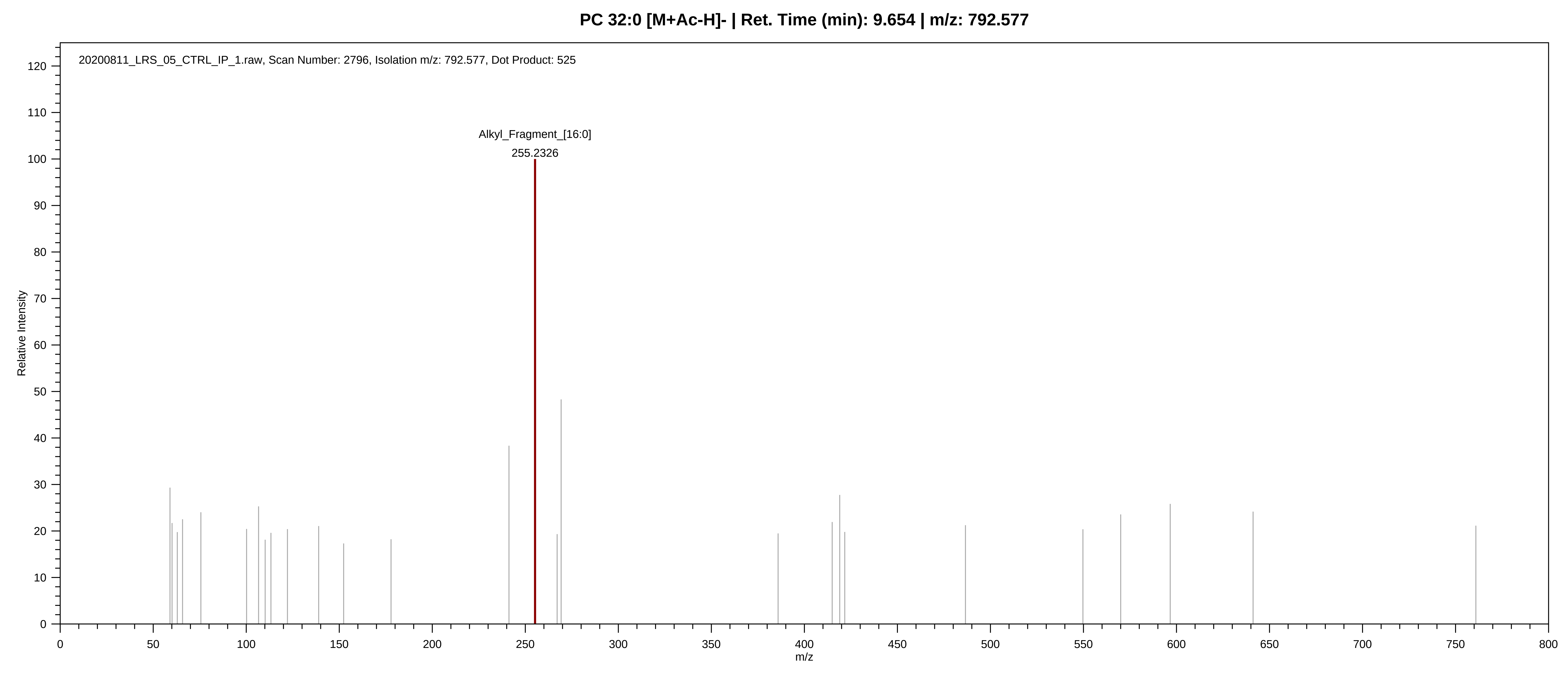
PC 32:0 [M+Ac-H]- | Ret. Time (min): 9.654 | m/z: 792.577
20200811_LRS_05_CTRL_IP_1.raw, Scan Number: 2796, Isolation m/z: 792.577, Dot Product: 525
Alkyl_Fragment_[16:0]
255.2326
Relative Intensity
m/z

PC 32:0 [M+H]+ | Ret. Time (min): 9.661 | m/z: 734.568

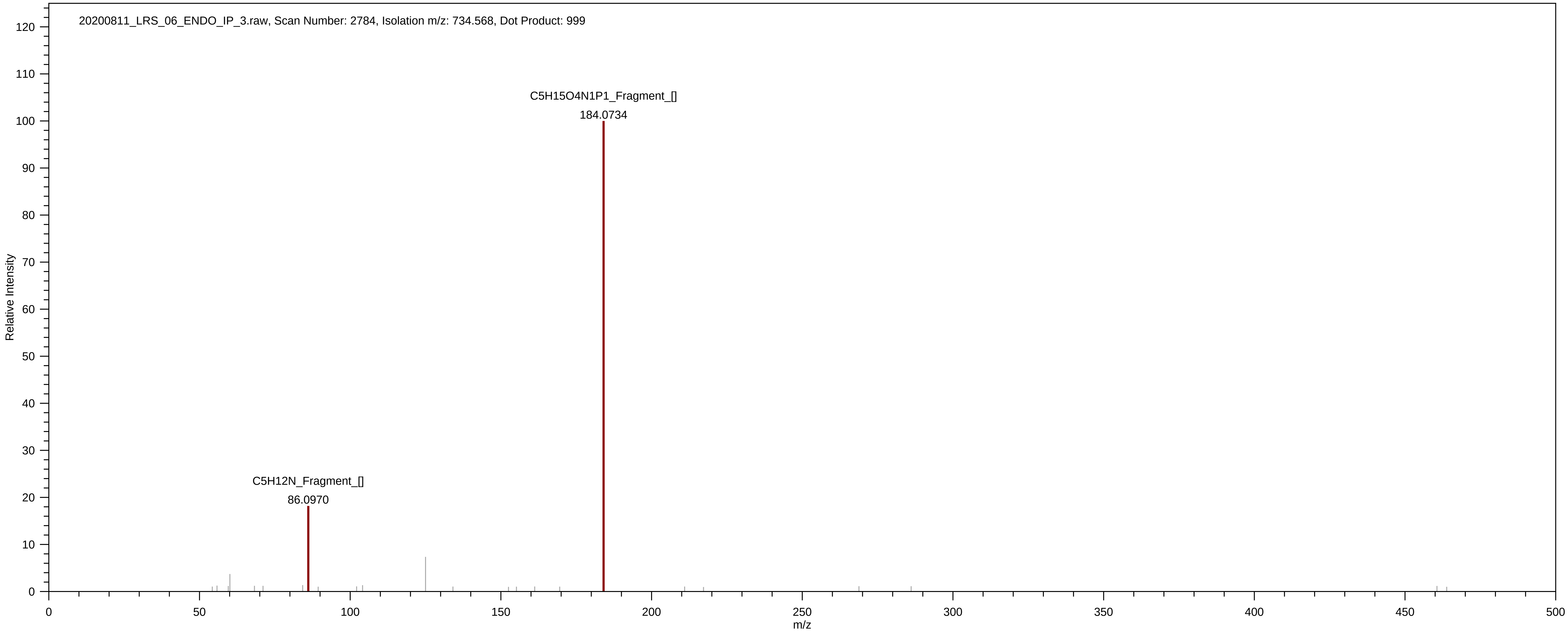

PC 32:0 [M+H]+ | Ret. Time (min): 9.763 | m/z: 734.57

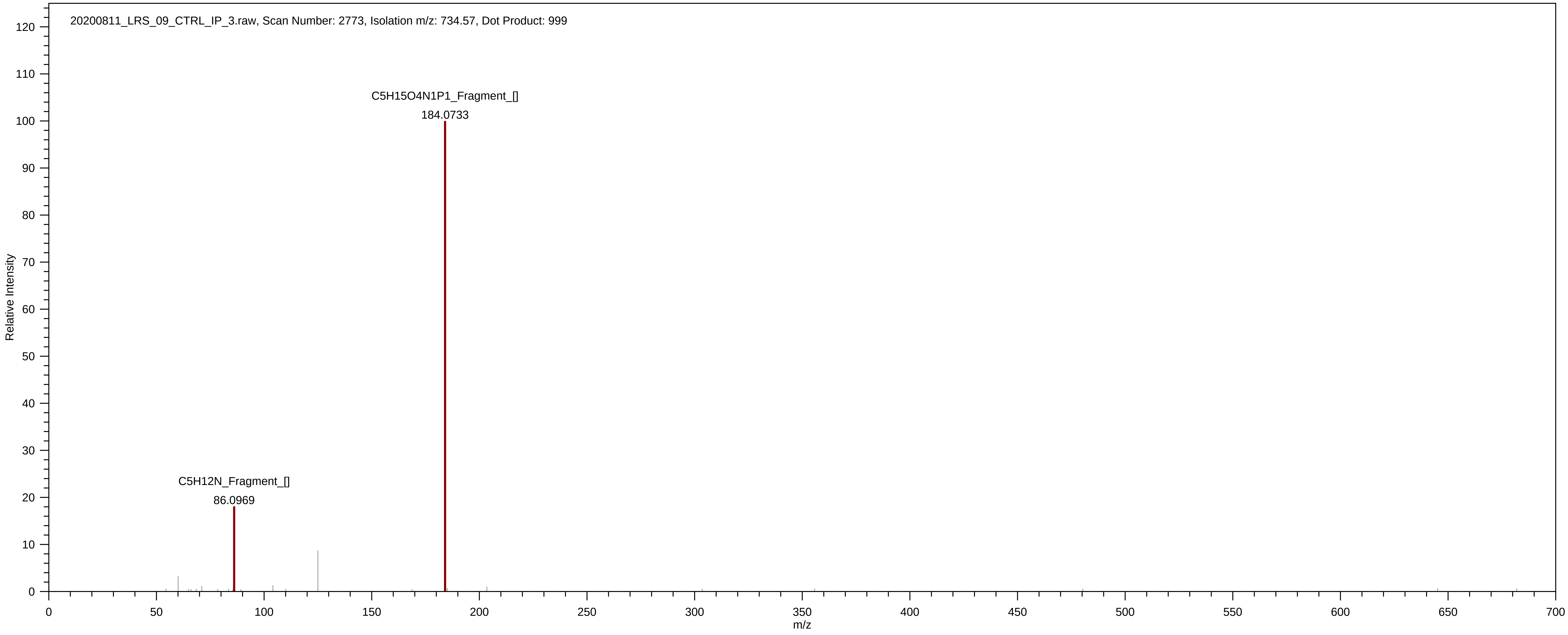

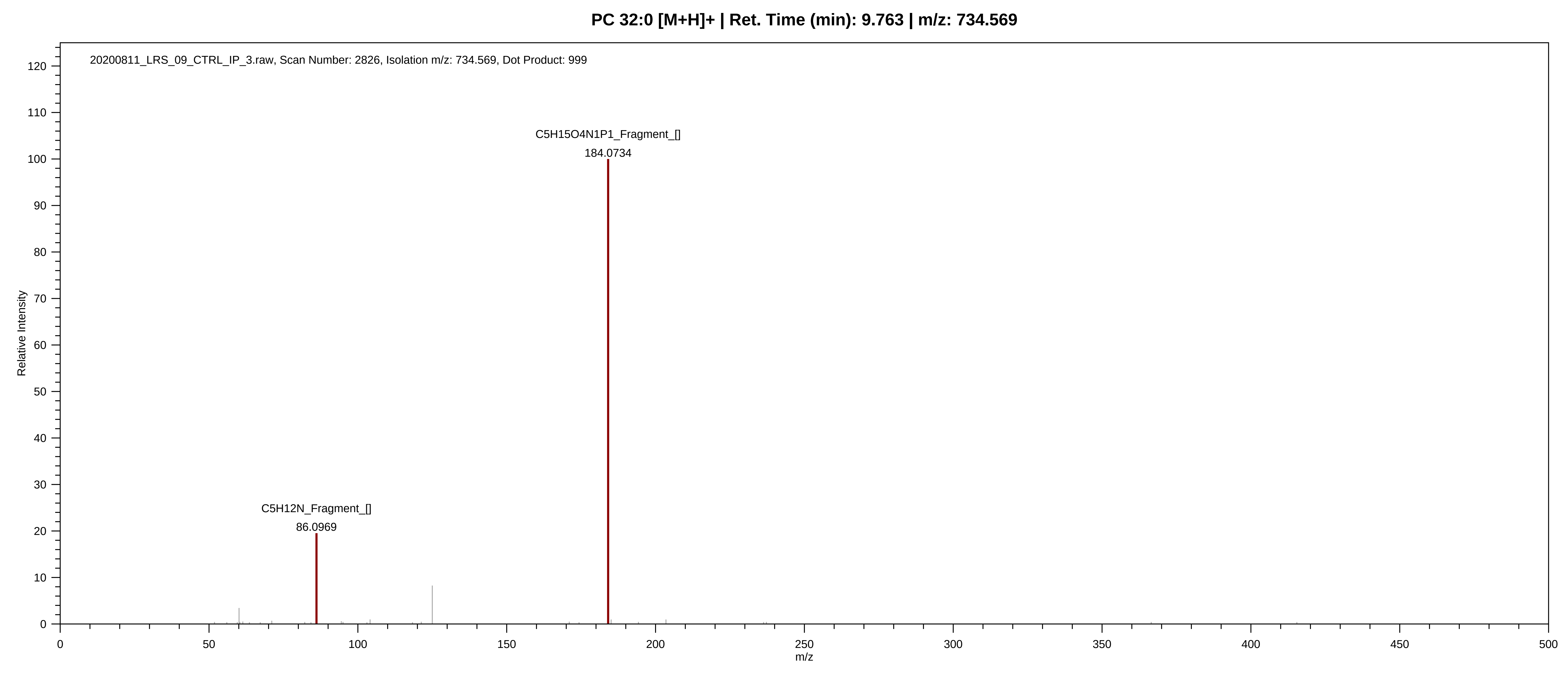
PC 32:0 [M+H]+ | Ret. Time (min): 9.763 | m/z: 734.569
20200811_LRS_09_CTRL_IP_3.raw, Scan Number: 2826, Isolation m/z: 734.569, Dot Product: 999
C5H15O4N1P1_Fragment_[]
184.0734
C5H12N_Fragment_[]
86.0969
Relative Intensity
m/z

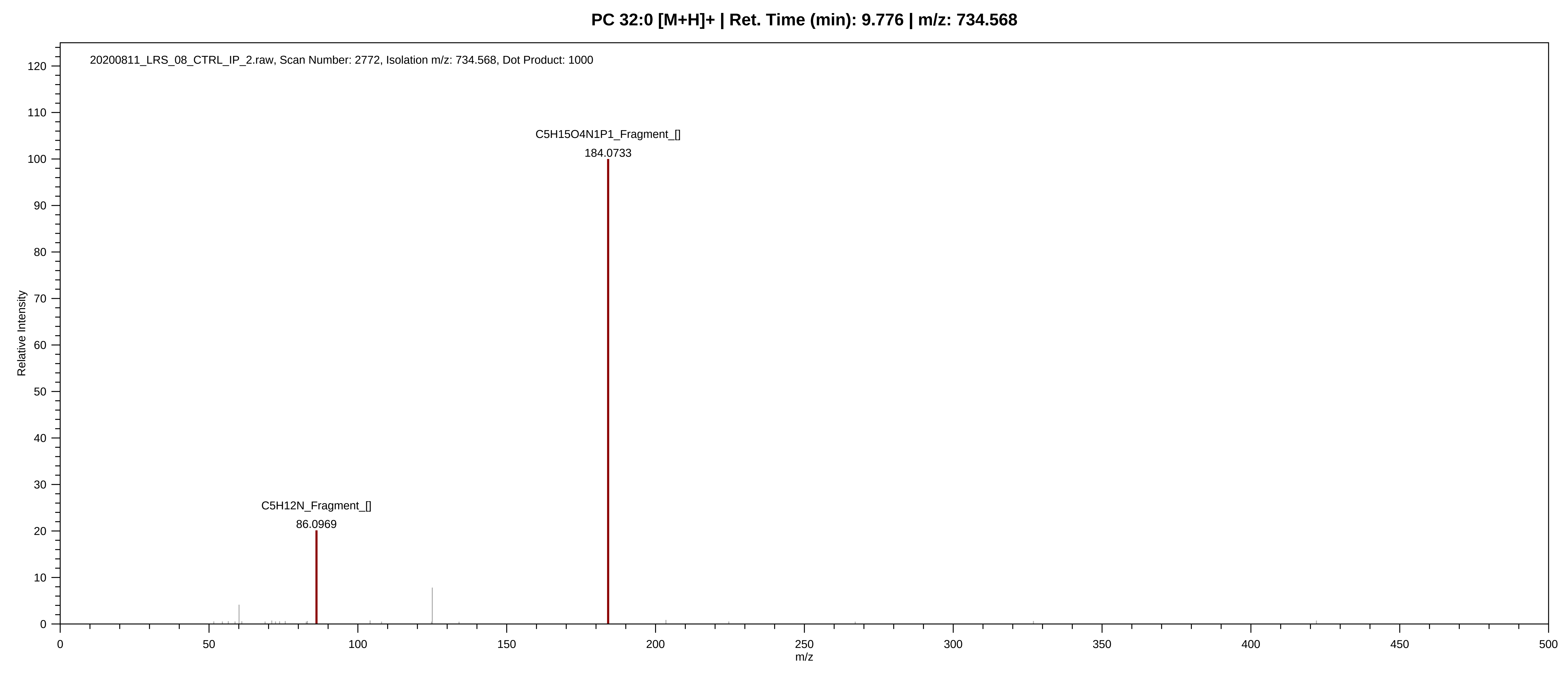

PC 32:0 [M+H]+ | Ret. Time (min): 9.776 | m/z: 734.568
20200811_LRS_08_CTRL_IP_2.raw, Scan Number: 2772, Isolation m/z: 734.568, Dot Product: 1000
C5H15O4N1P1_Fragment_[]
184.0733
C5H12N_Fragment_[]
86.0969
Relative Intensity
m/z

PC 32:0 [M+H]+ | Ret. Time (min): 9.776 | m/z: 734.569

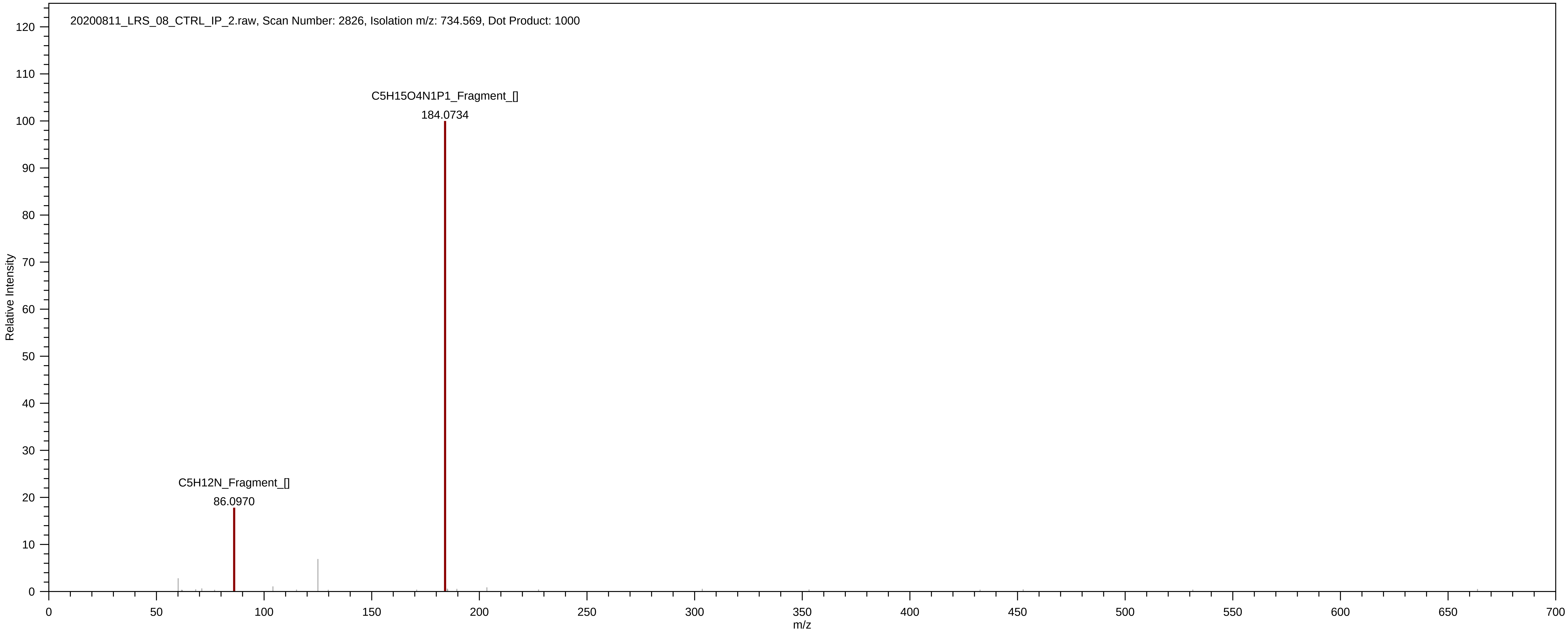

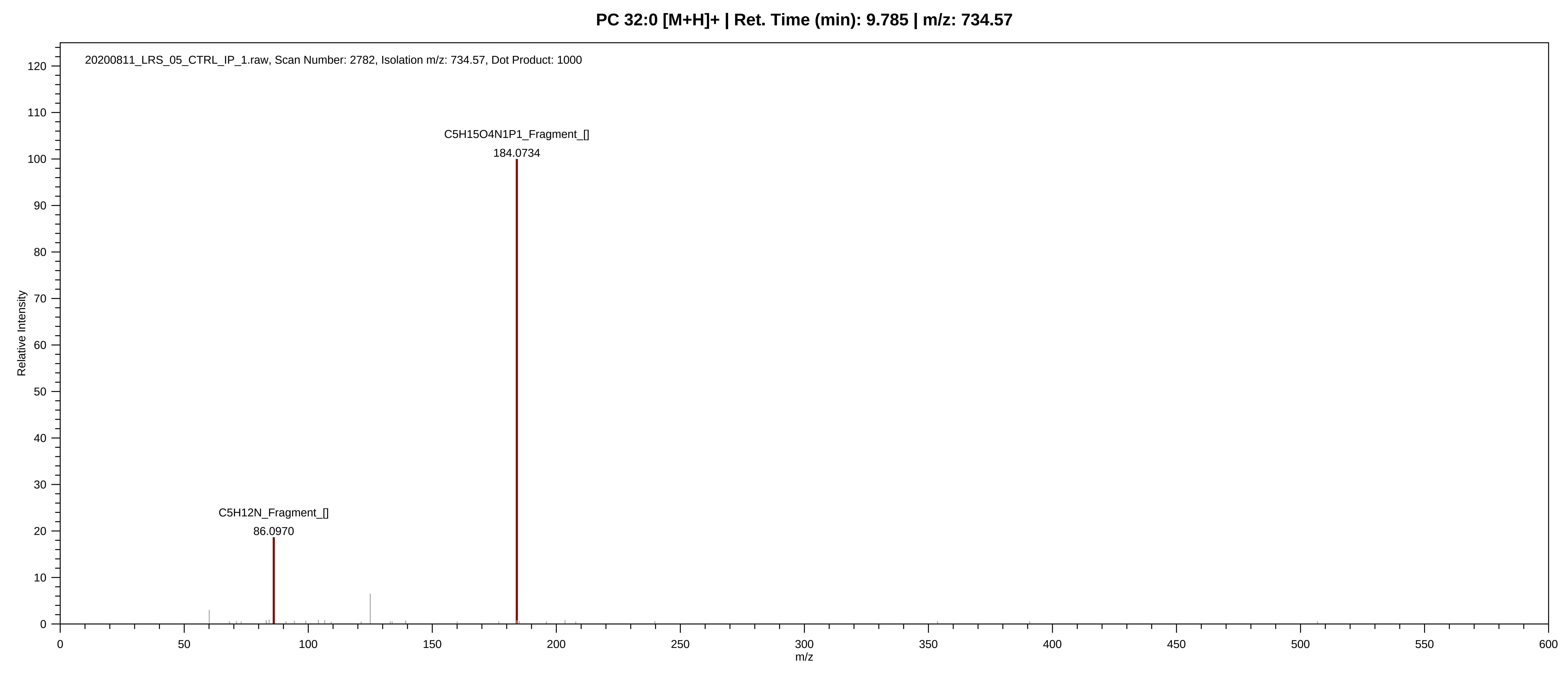
PC 32:0 [M+H]+ | Ret. Time (min): 9.785 | m/z: 734.57
20200811_LRS_05_CTRL_IP_1.raw, Scan Number: 2782, Isolation m/z: 734.57, Dot Product: 1000
C5H15O4N1P1_Fragment_[]
184.0734
C5H12N_Fragment_[]
86.0970
Relative Intensity
m/z

PC 32:0 [M+H]+ | Ret. Time (min): 9.785 | m/z: 734.569

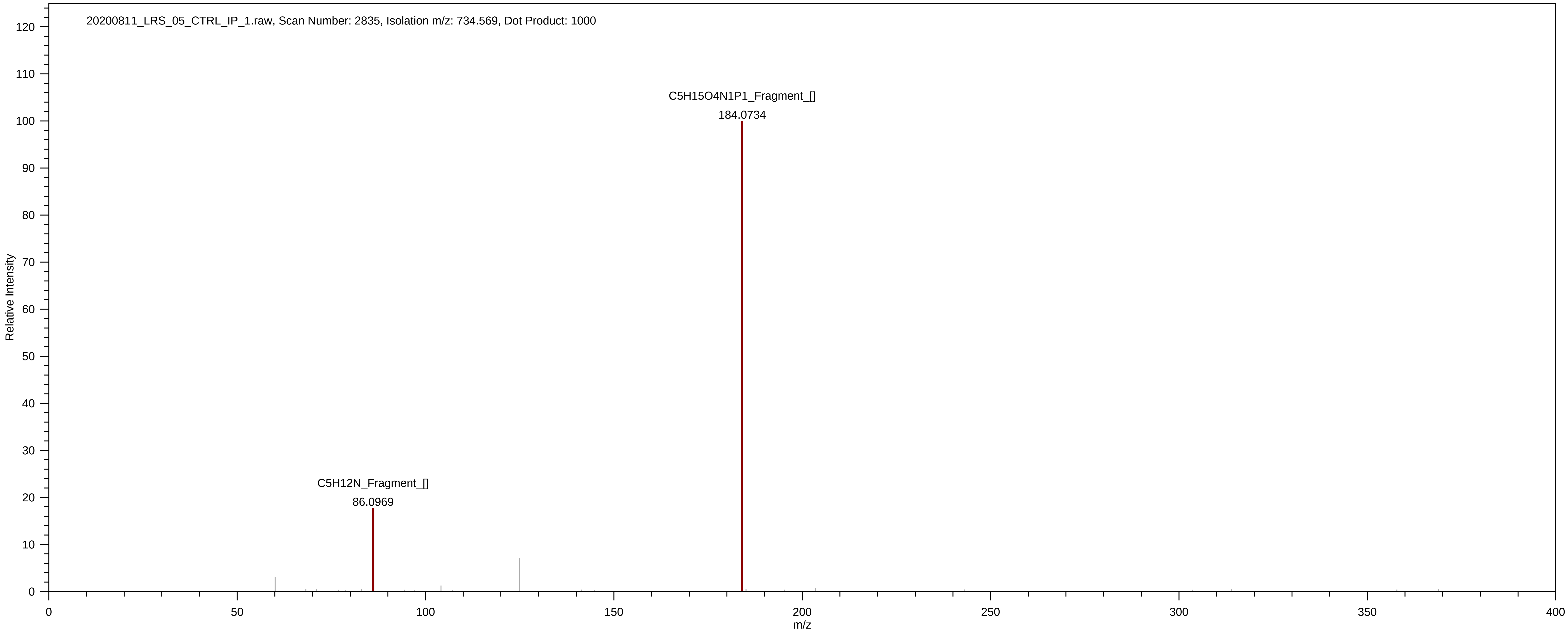

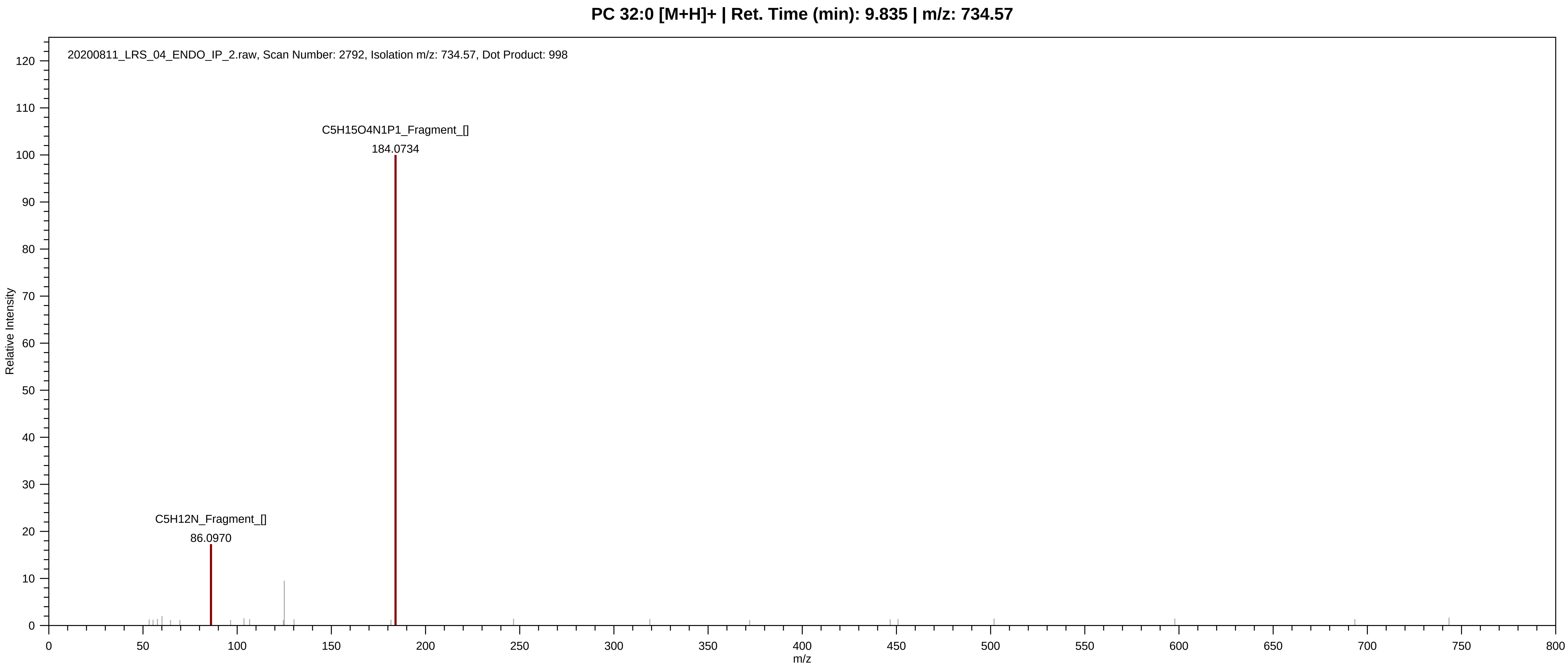
PC 32:0 [M+H]+ | Ret. Time (min): 9.835 | m/z: 734.57
20200811_LRS_04_ENDO_IP_2.raw, Scan Number: 2792, Isolation m/z: 734.57, Dot Product: 998
C5H15O4N1P1_Fragment_[]
184.0734
C5H12N_Fragment_[]
86.0970
Relative Intensity
m/z

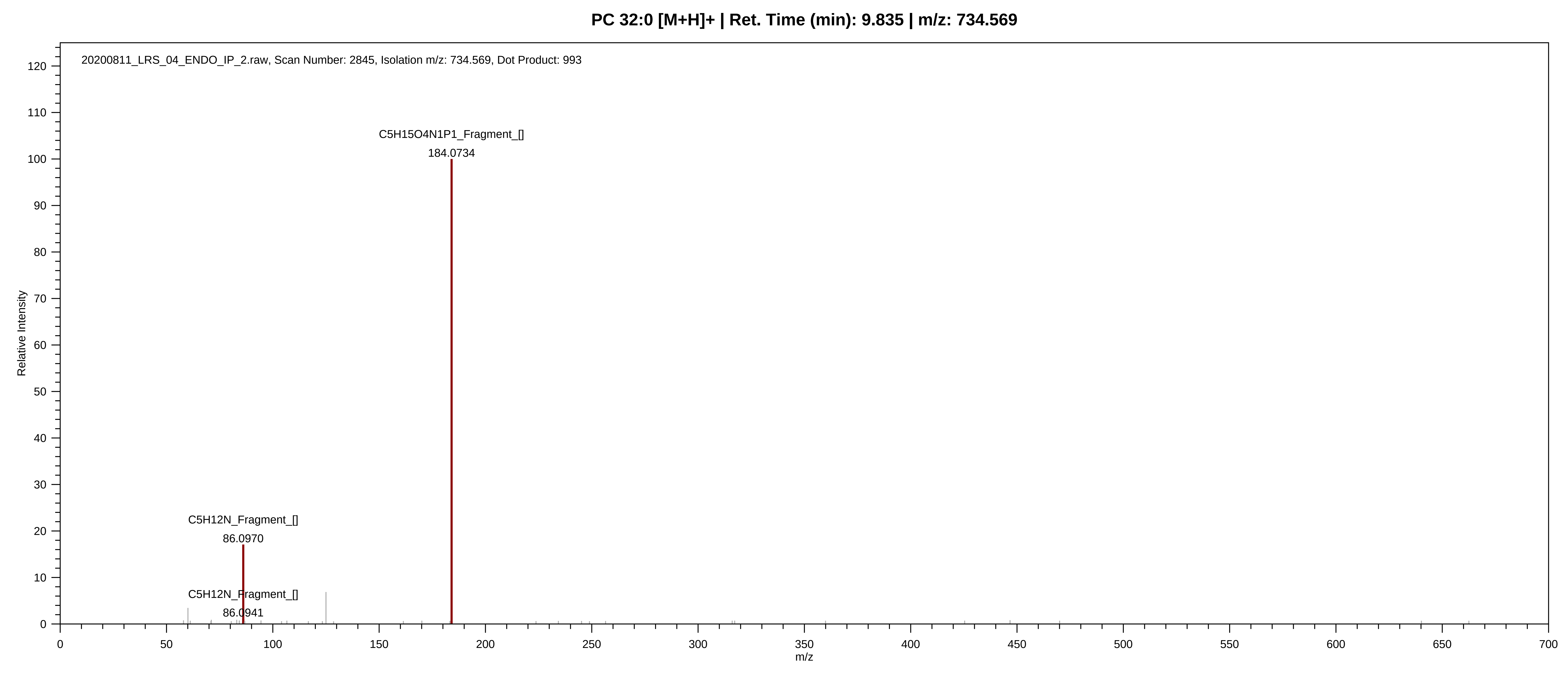

PC 32:0 [M+H]+ | Ret. Time (min): 9.835 | m/z: 734.569
20200811_LRS_04_ENDO_IP_2.raw, Scan Number: 2845, Isolation m/z: 734.569, Dot Product: 993
C5H15O4N1P1_Fragment_[]
184.0734
C5H12N_Fragment_[]
86.0970
C5H12N_Fragment_[]
86.0941
Relative Intensity
m/z

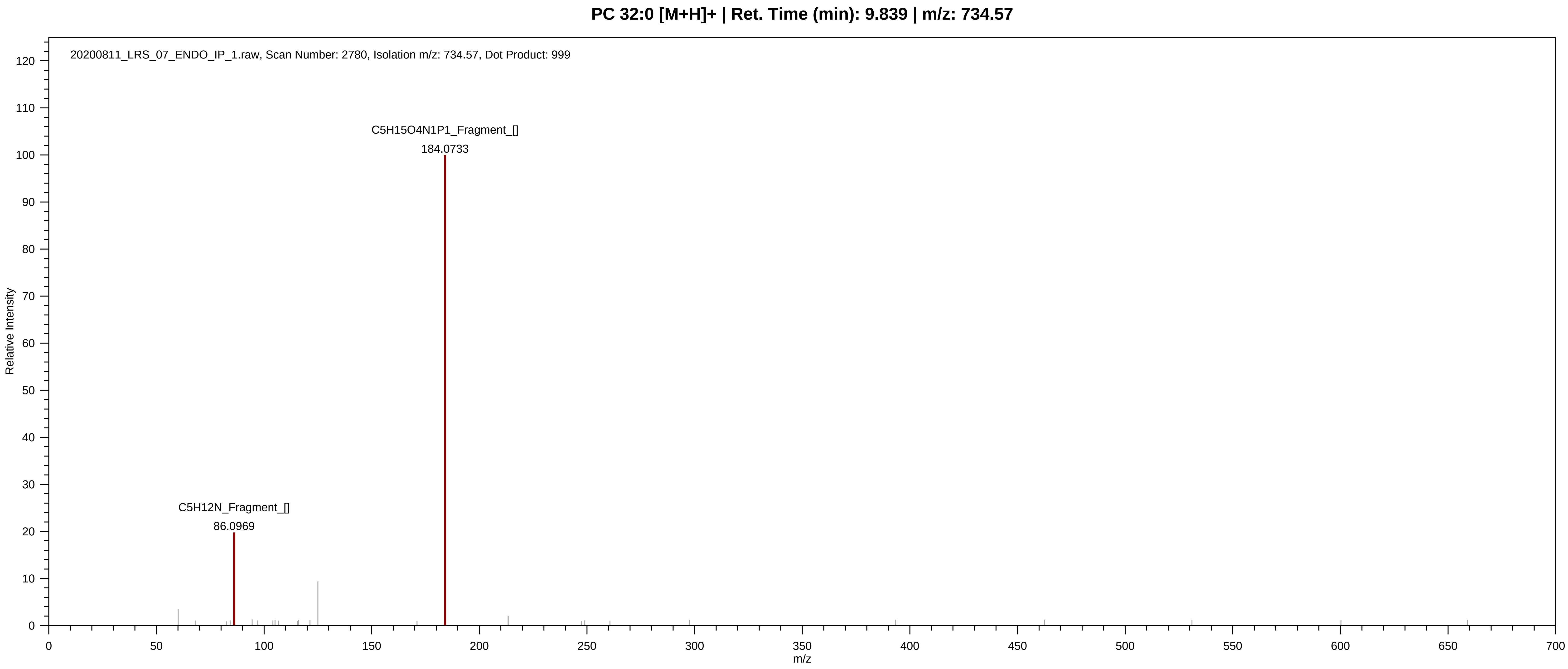

PC 32:0 [M+H]+ | Ret. Time (min): 9.839 | m/z: 734.57
20200811_LRS_07_ENDO_IP_1.raw, Scan Number: 2780, Isolation m/z: 734.57, Dot Product: 999
C5H15O4N1P1_Fragment_[]
184.0733
C5H12N_Fragment_[]
86.0969
Relative Intensity
m/z

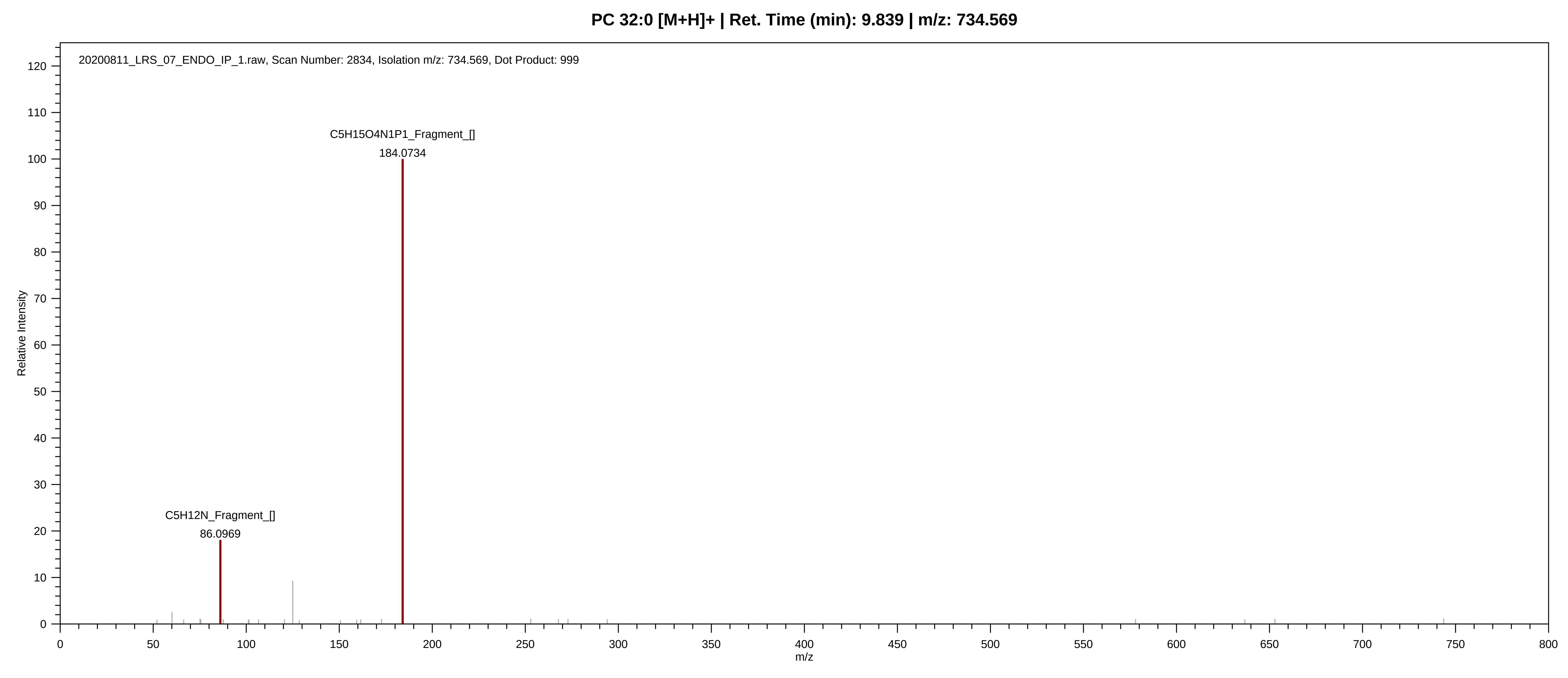
PC 32:0 [M+H]+ | Ret. Time (min): 9.839 | m/z: 734.569
20200811_LRS_07_ENDO_IP_1.raw, Scan Number: 2834, Isolation m/z: 734.569, Dot Product: 999
C5H15O4N1P1_Fragment_[]
184.0734
C5H12N_Fragment_[]
86.0969
Relative Intensity
m/z

PC 32:0 [M+H]+ | Ret. Time (min): 9.839 | m/z: 734.569

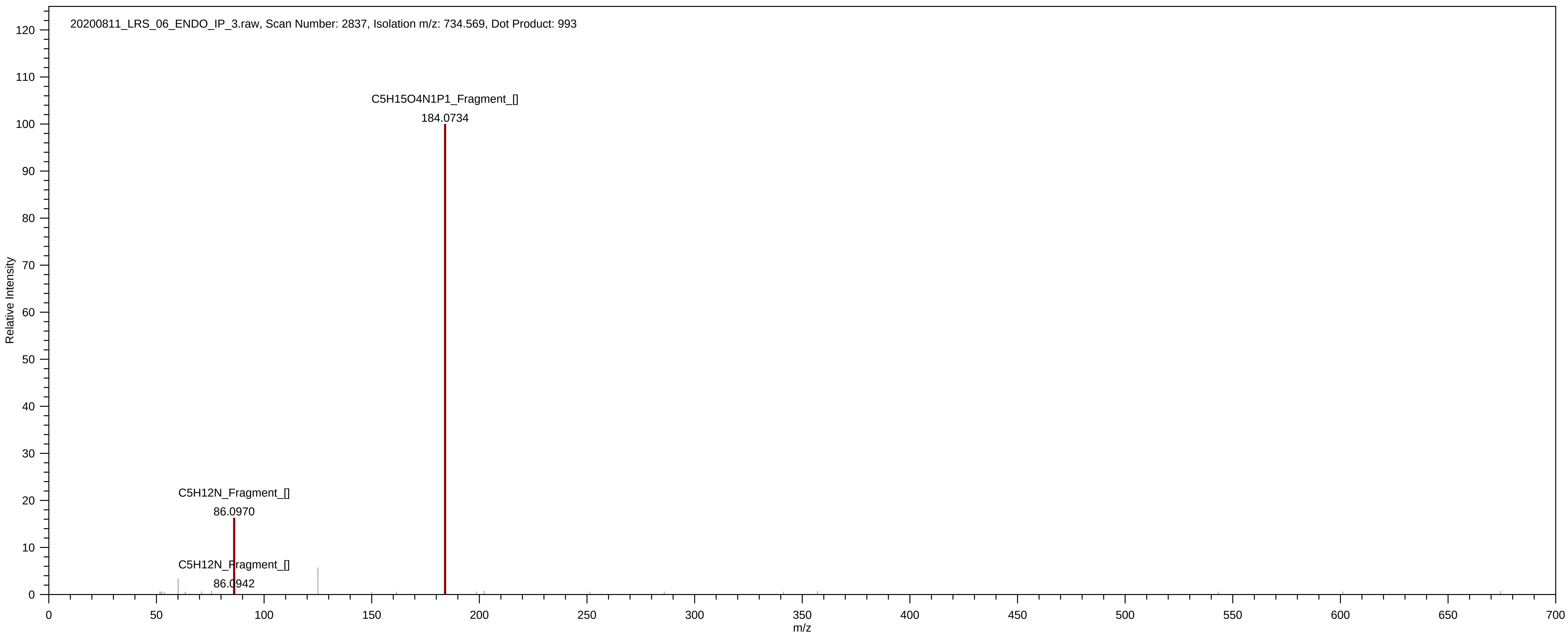

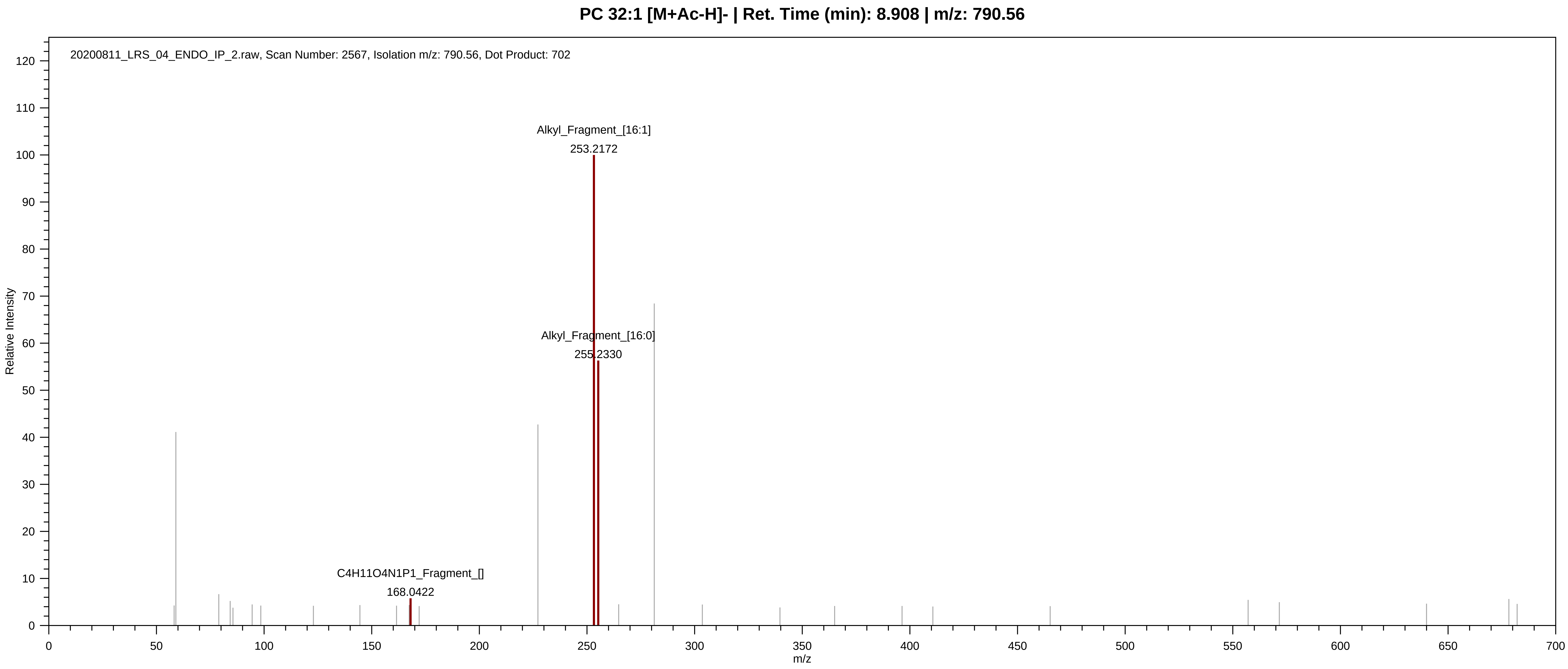

PC 32:1 [M+Ac-H]- | Ret. Time (min): 8.908 | m/z: 790.56
20200811_LRS_04_ENDO_IP_2.raw, Scan Number: 2567, Isolation m/z: 790.56, Dot Product: 702
Alkyl_Fragment_[16:1]
253.2172
Alkyl_Fragment_[16:0]
255.2330
C4H11O4N1P1_Fragment_[]
168.0422
Relative Intensity
m/z

PC 32:1 [M+Ac-H]- | Ret. Time (min): 8.914 | m/z: 790.56

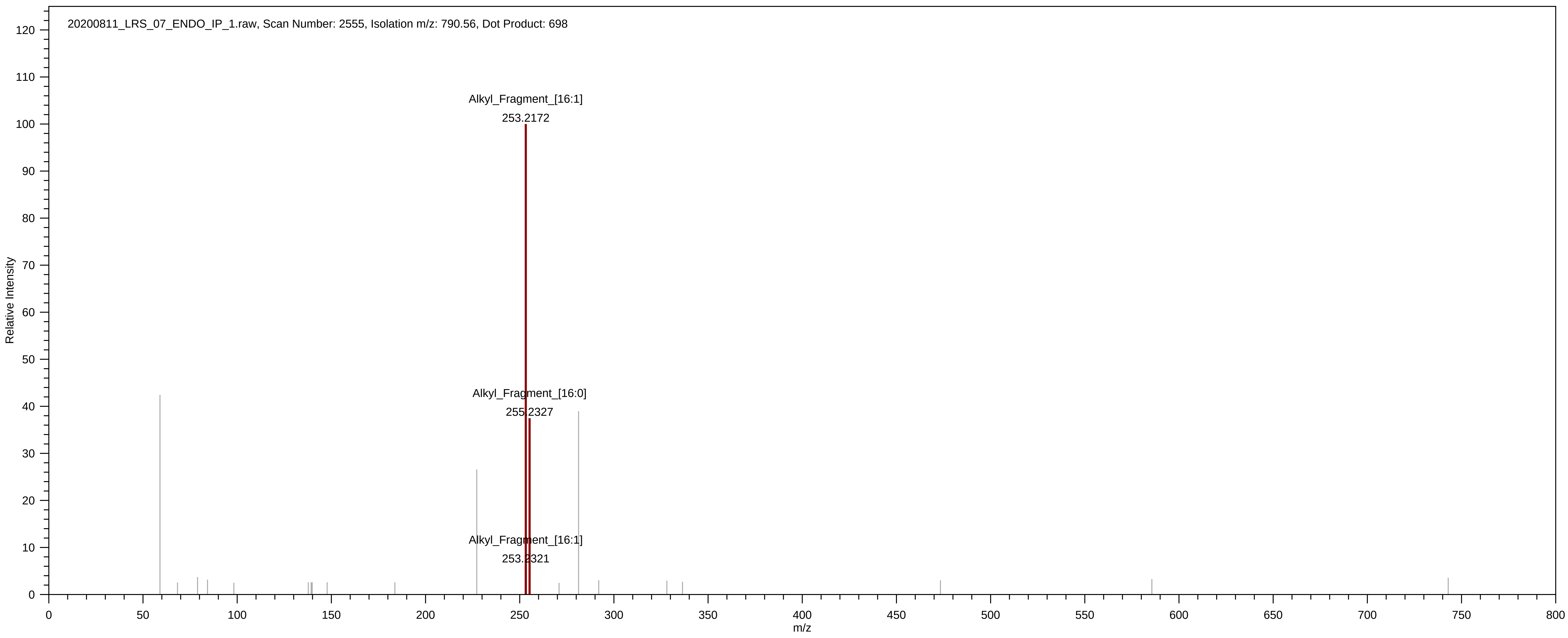

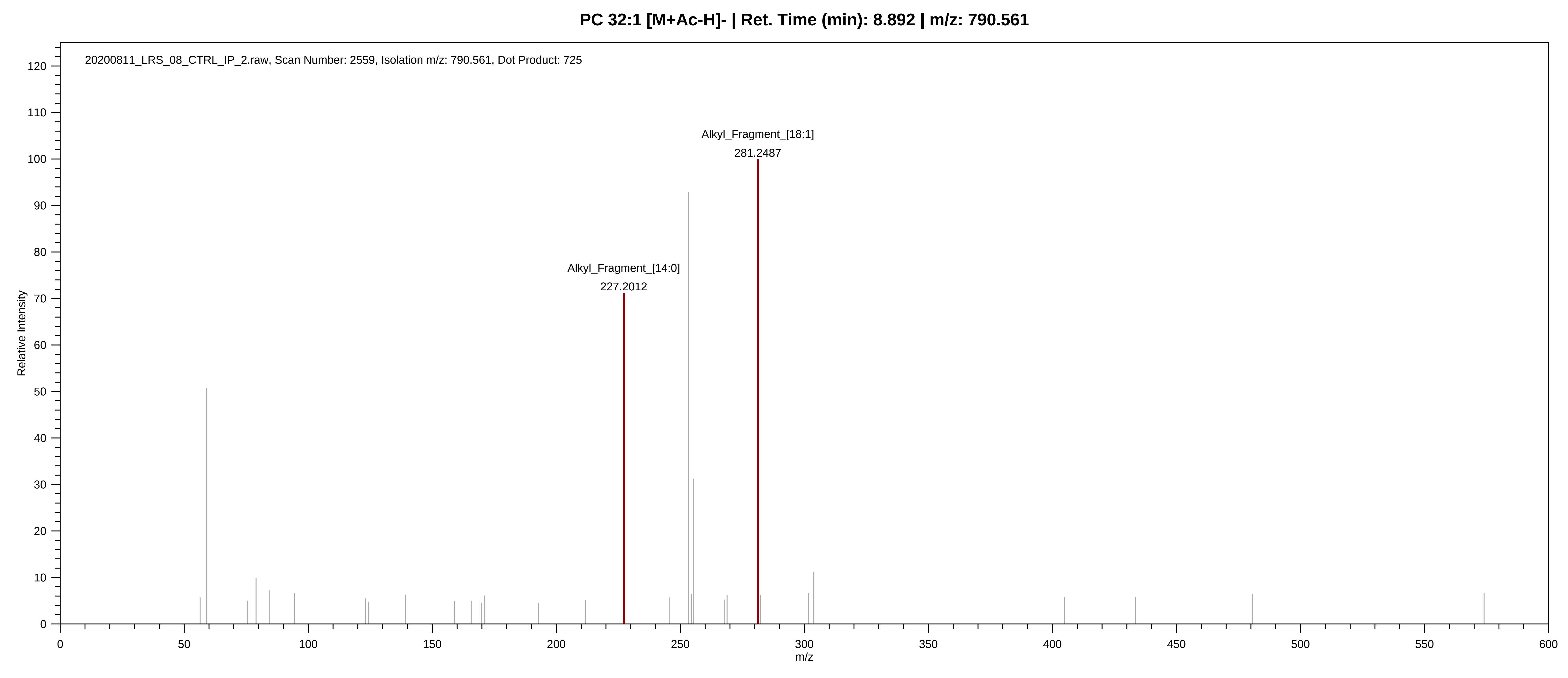
PC 32:1 [M+Ac-H]- | Ret. Time (min): 8.892 | m/z: 790.561
20200811_LRS_08_CTRL_IP_2.raw, Scan Number: 2559, Isolation m/z: 790.561, Dot Product: 725
Alkyl_Fragment_[18:1]
281.2487
Alkyl_Fragment_[14:0]
227.2012
Relative Intensity
m/z

PC 32:1 [M+Ac-H]- | Ret. Time (min): 8.907 | m/z: 790.561

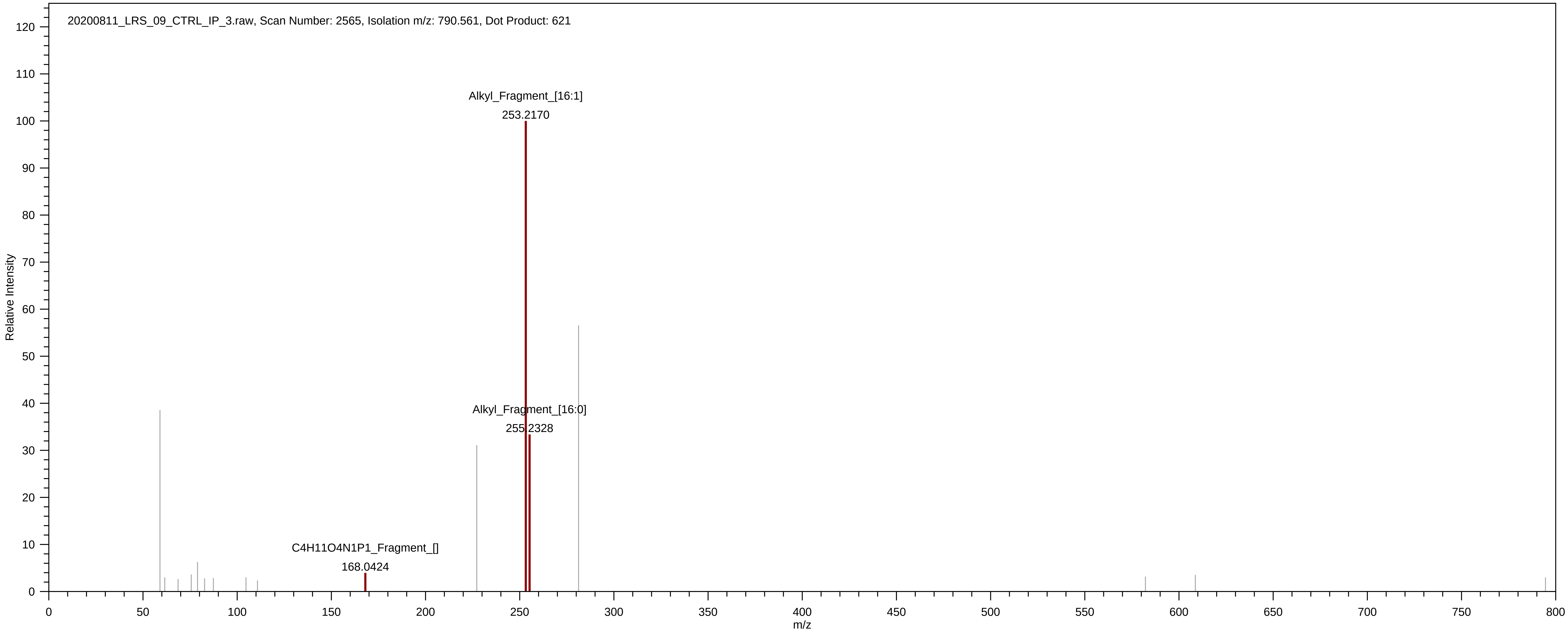

PC 32:1 [M+Ac-H]- | Ret. Time (min): 8.915 | m/z: 790.561

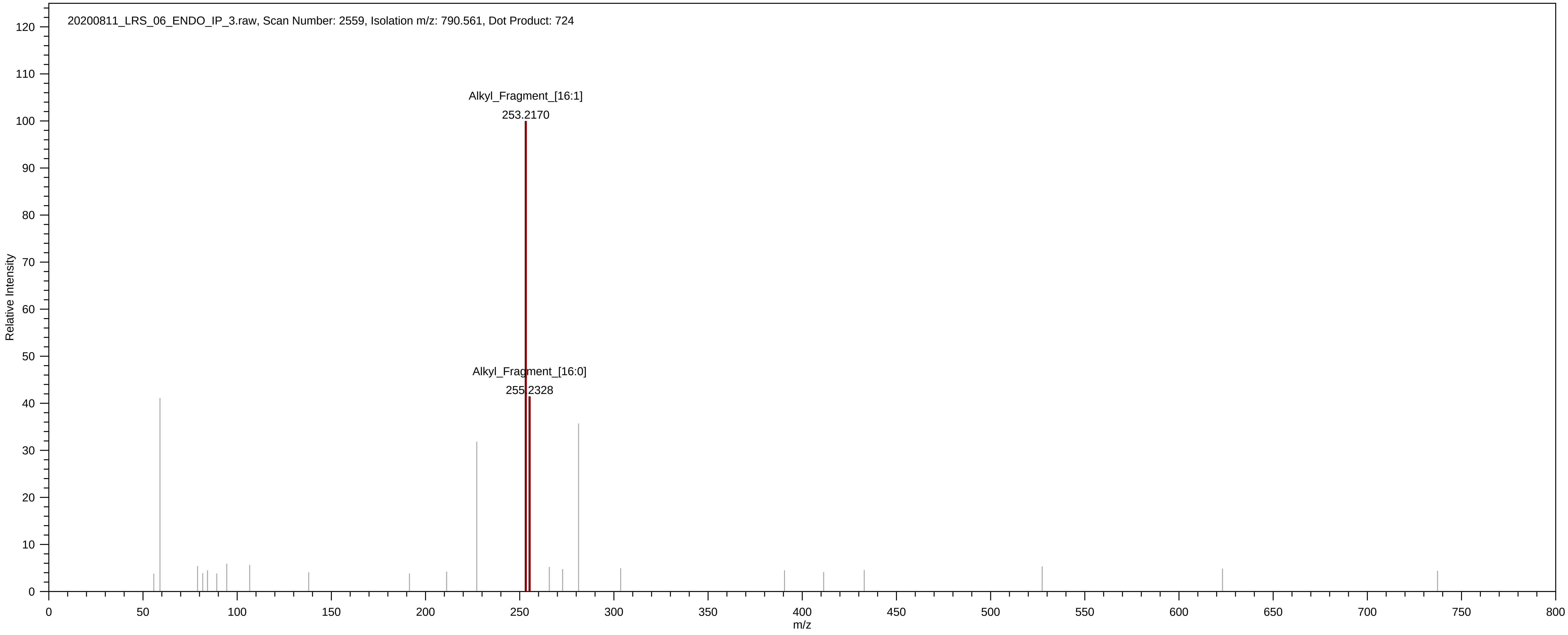

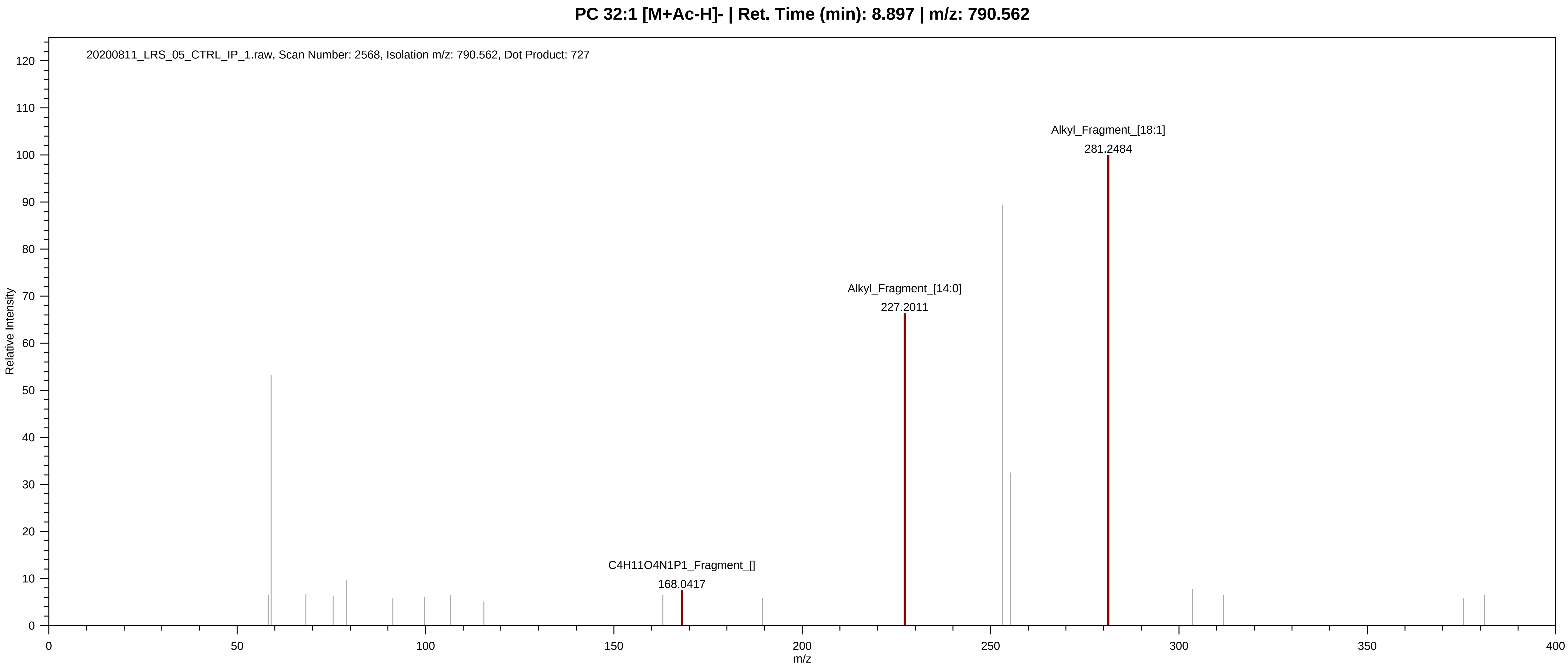

PC 32:1 [M+Ac-H]- | Ret. Time (min): 8.897 | m/z: 790.562
20200811_LRS_05_CTRL_IP_1.raw, Scan Number: 2568, Isolation m/z: 790.562, Dot Product: 727
Alkyl_Fragment_[18:1]
281.2484
Alkyl_Fragment_[14:0]
227.2011
C4H11O4N1P1_Fragment_[]
168.0417
Relative Intensity
m/z

PC 32:1 [M+H]+ | Ret. Time (min): 8.838 | m/z: 732.552

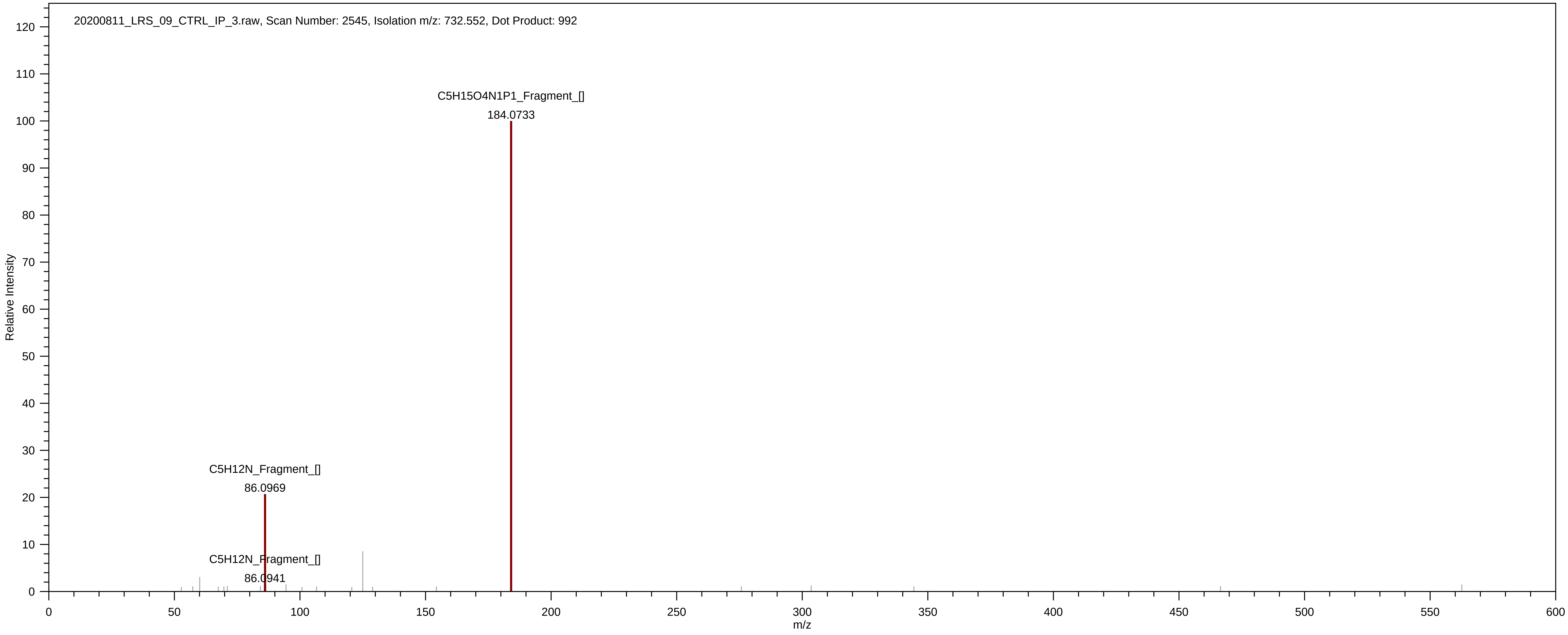

PC 32:1 [M+H]+ | Ret. Time (min): 8.879 | m/z: 732.553

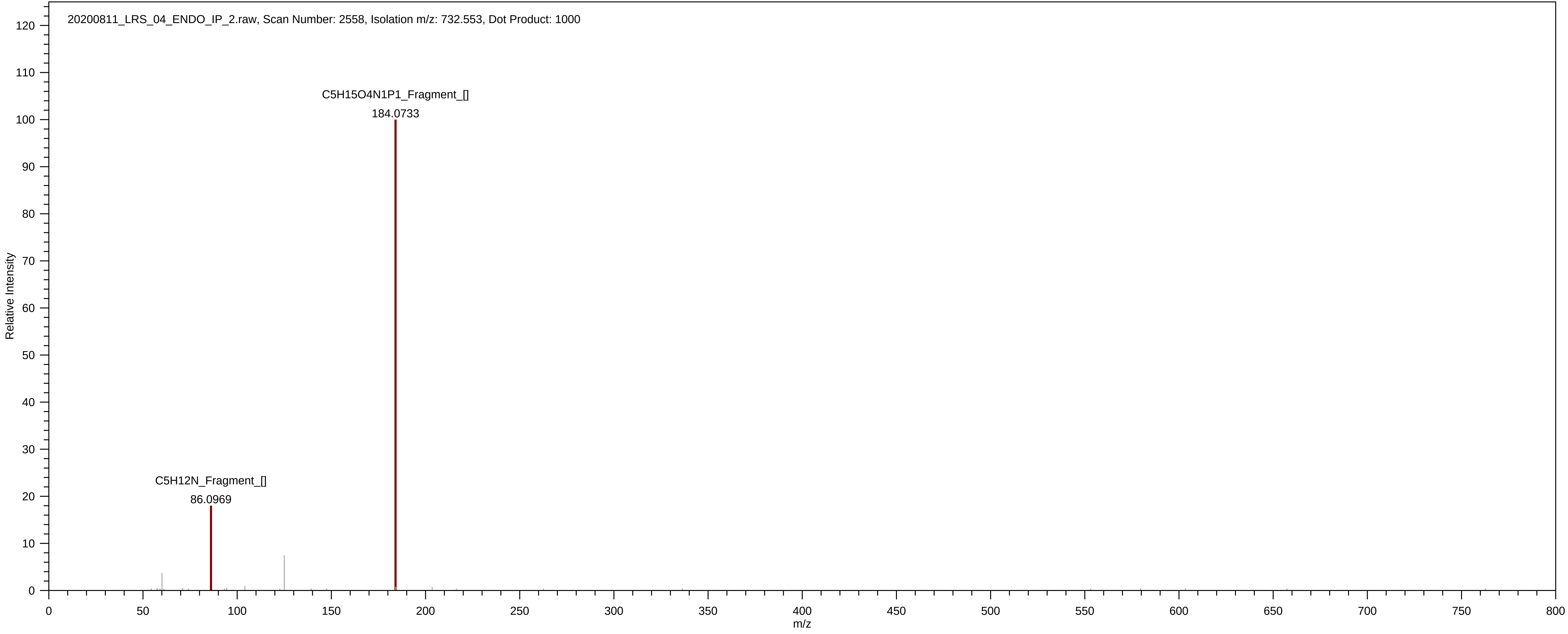

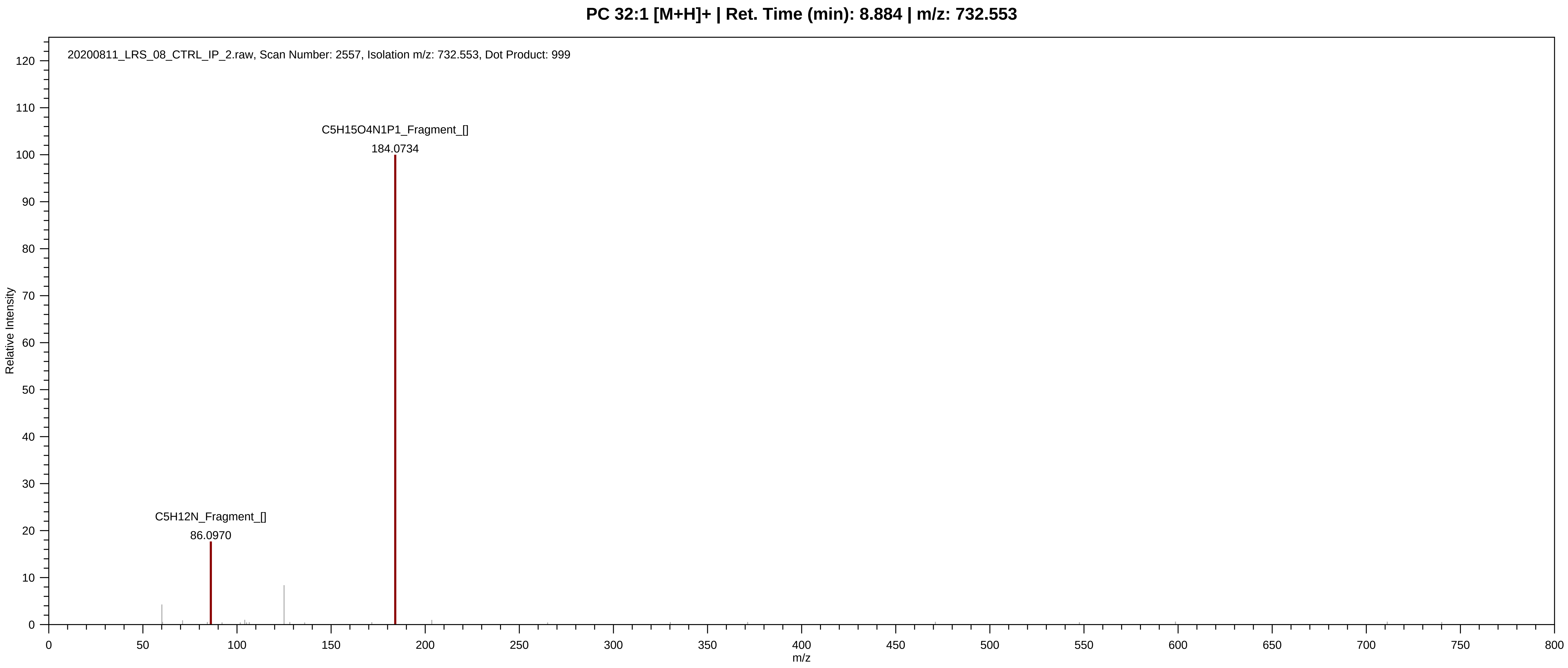

PC 32:1 [M+H]+ | Ret. Time (min): 8.884 | m/z: 732.553
20200811_LRS_08_CTRL_IP_2.raw, Scan Number: 2557, Isolation m/z: 732.553, Dot Product: 999
C5H15O4N1P1_Fragment_[]
184.0734
C5H12N_Fragment_[]
86.0970
Relative Intensity
m/z

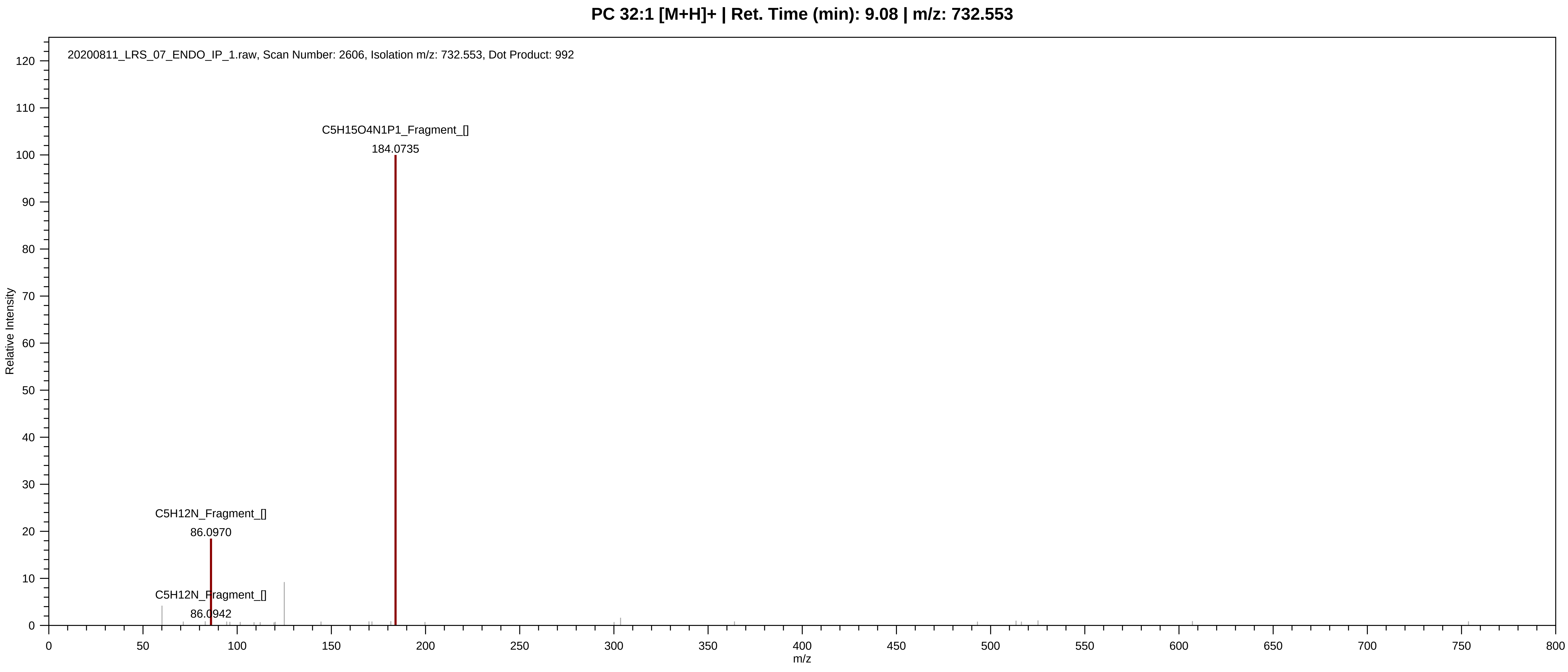
PC 32:1 [M+H]+ | Ret. Time (min): 9.08 | m/z: 732.553
20200811_LRS_07_ENDO_IP_1.raw, Scan Number: 2606, Isolation m/z: 732.553, Dot Product: 992
C5H15O4N1P1_Fragment_[]
184.0735
C5H12N_Fragment_[]
86.0970
C5H12N_Fragment_[]
86.0942
Relative Intensity
m/z

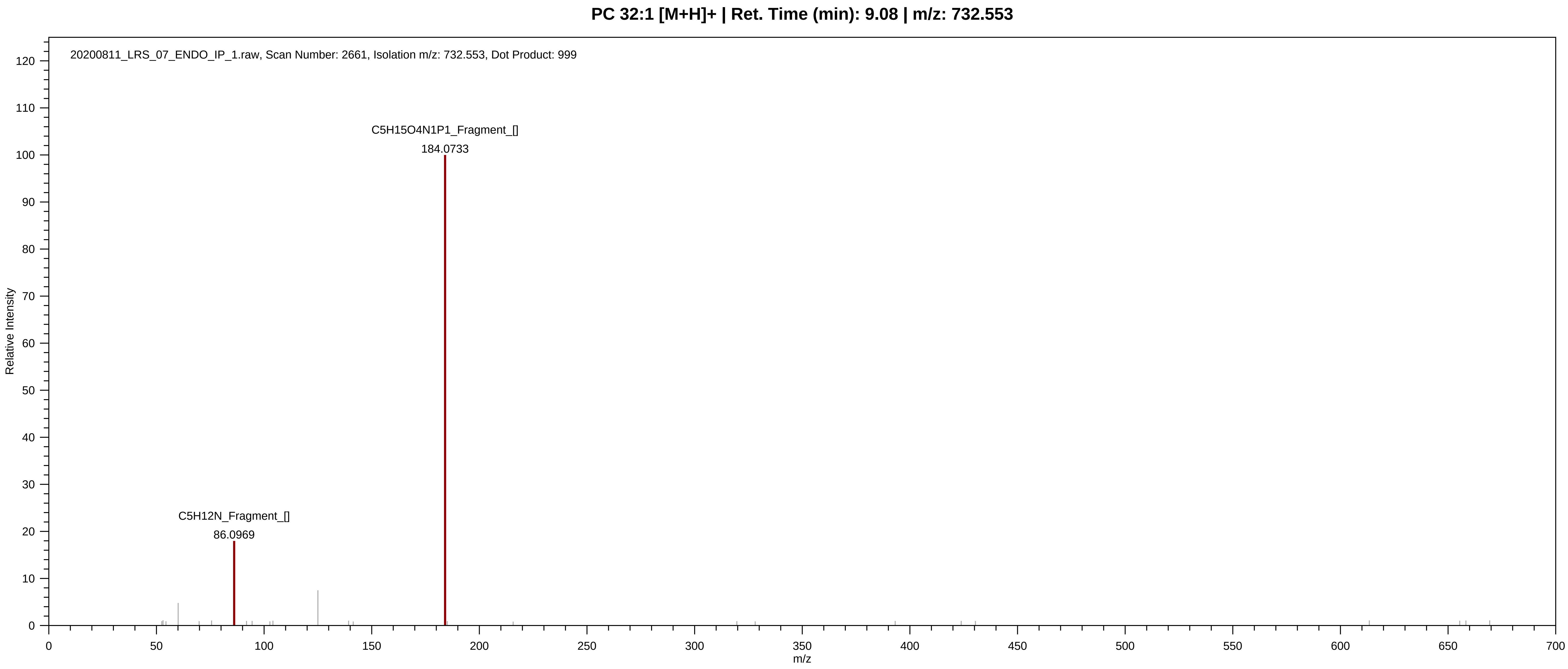
PC 32:1 [M+H]+ | Ret. Time (min): 9.08 | m/z: 732.553
20200811_LRS_07_ENDO_IP_1.raw, Scan Number: 2661, Isolation m/z: 732.553, Dot Product: 999
C5H15O4N1P1_Fragment_[]
184.0733
C5H12N_Fragment_[]
86.0969
Relative Intensity
m/z

PC 32:1 [M+H]+ | Ret. Time (min): 9.015 | m/z: 732.553

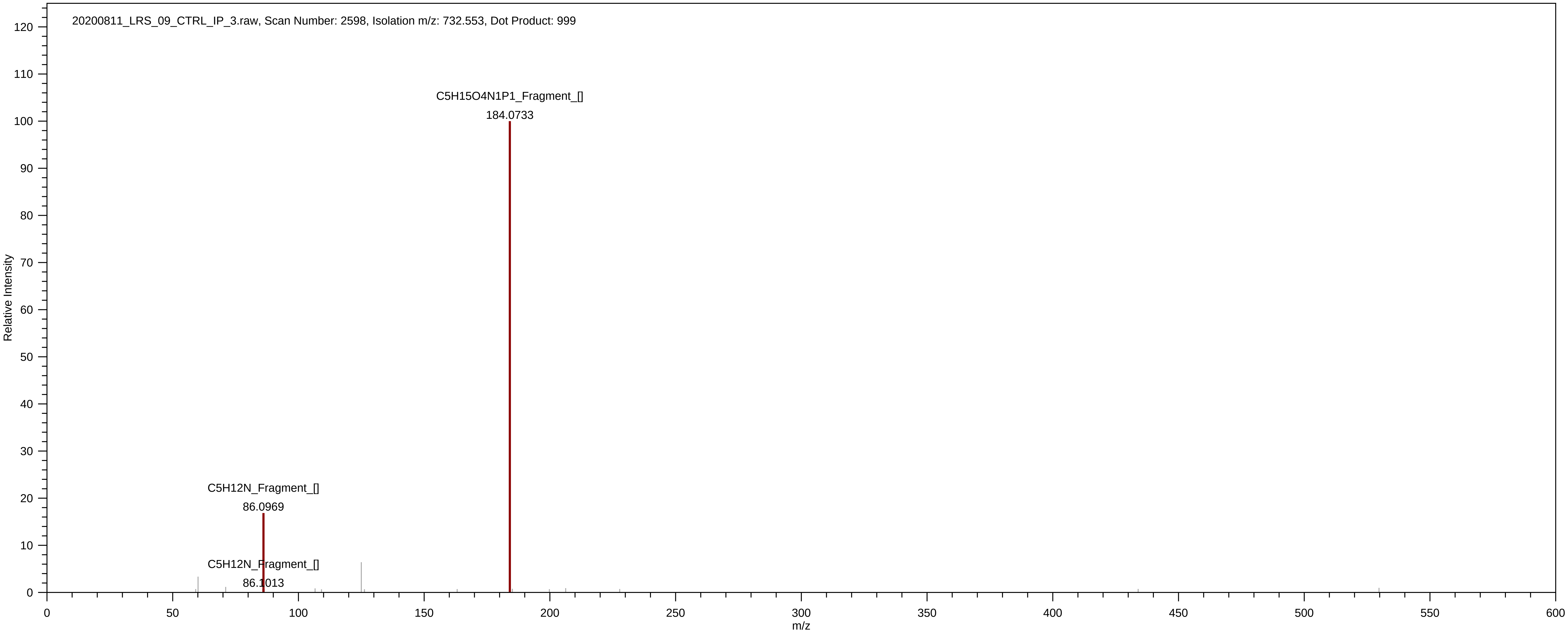

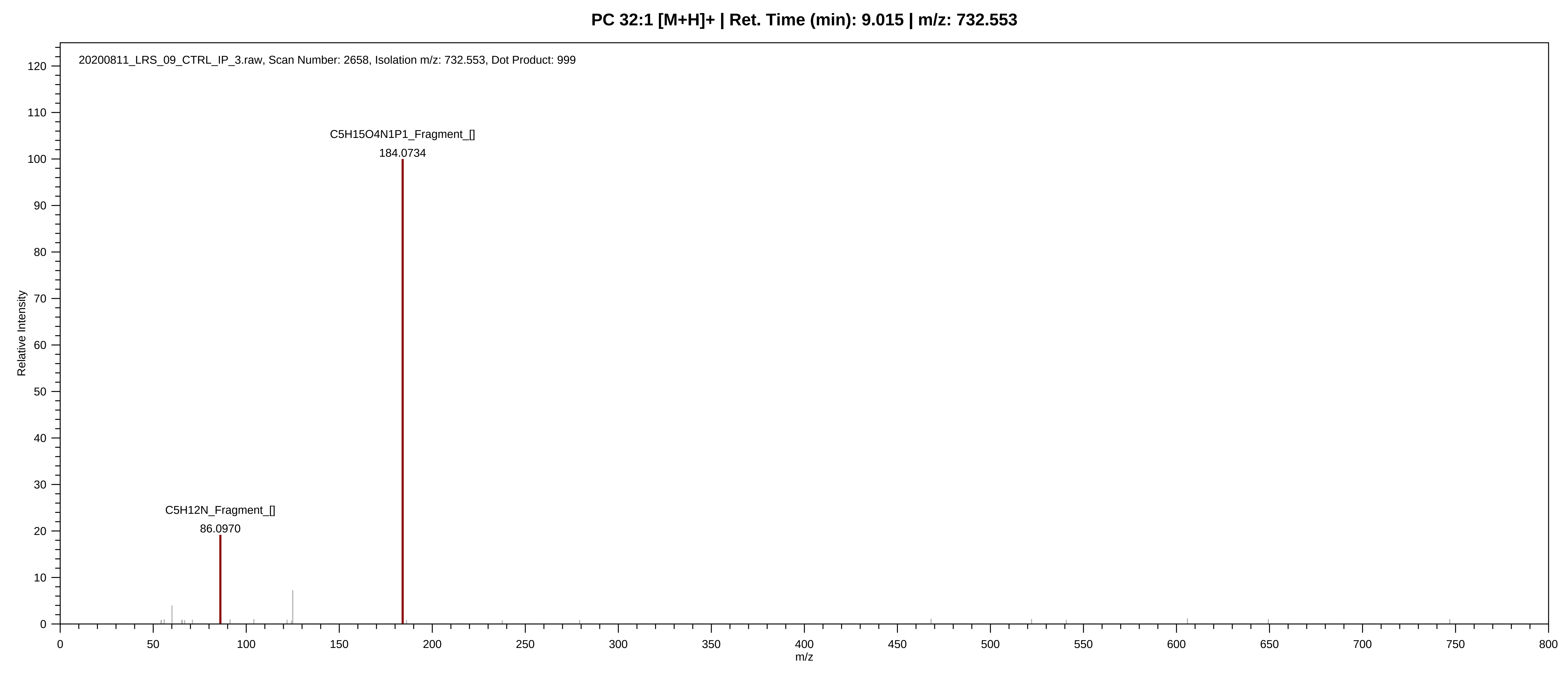

PC 32:1 [M+H]+ | Ret. Time (min): 9.015 | m/z: 732.553
20200811_LRS_09_CTRL_IP_3.raw, Scan Number: 2658, Isolation m/z: 732.553, Dot Product: 999
C5H15O4N1P1_Fragment_[]
184.0734
C5H12N_Fragment_[]
86.0970
Relative Intensity
m/z

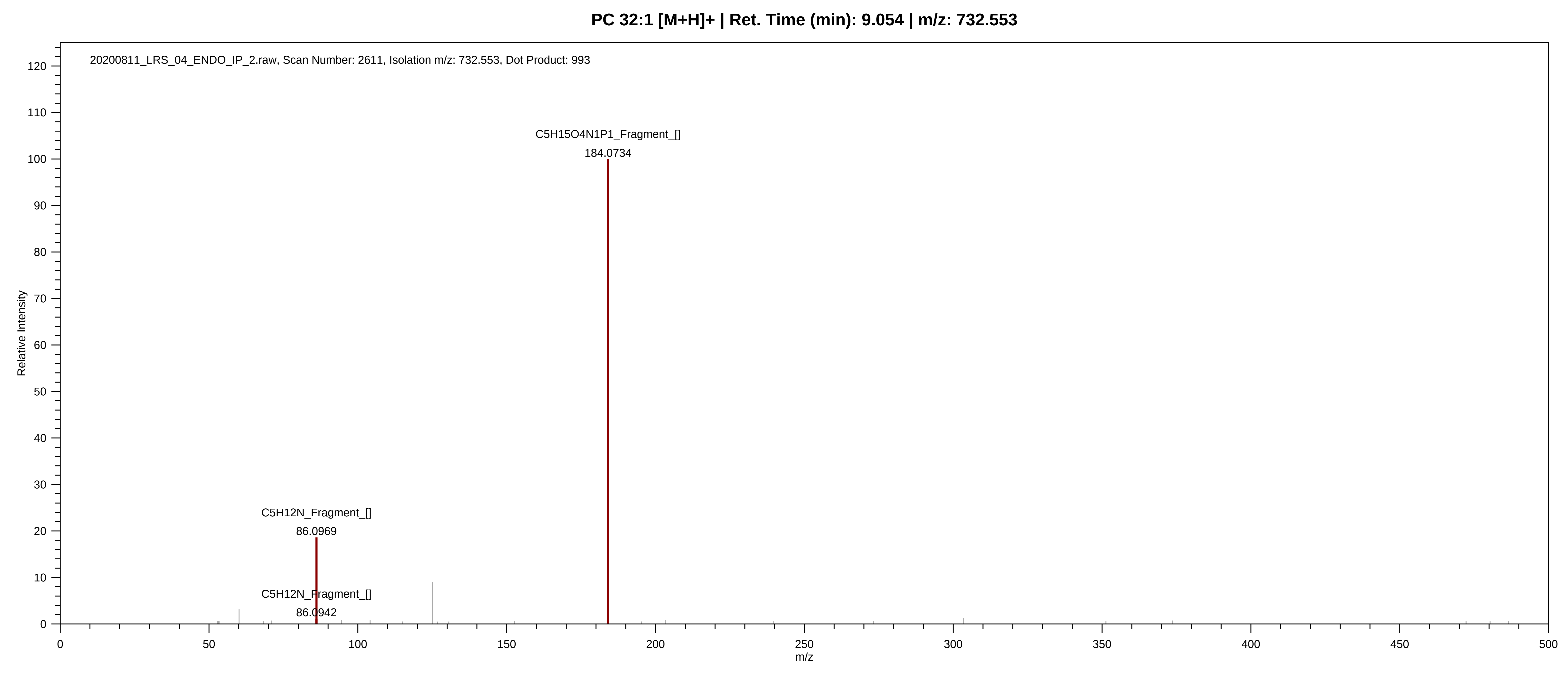
PC 32:1 [M+H]+ | Ret. Time (min): 9.054 | m/z: 732.553
20200811_LRS_04_ENDO_IP_2.raw, Scan Number: 2611, Isolation m/z: 732.553, Dot Product: 993
C5H15O4N1P1_Fragment_[]
184.0734
C5H12N_Fragment_[]
86.0969
C5H12N_Fragment_[]
86.0942
Relative Intensity
m/z
0
10
20
30
40
50
60
70
80
90
100
110
120
0
50
100
150
200
250
300
350
400
450
500

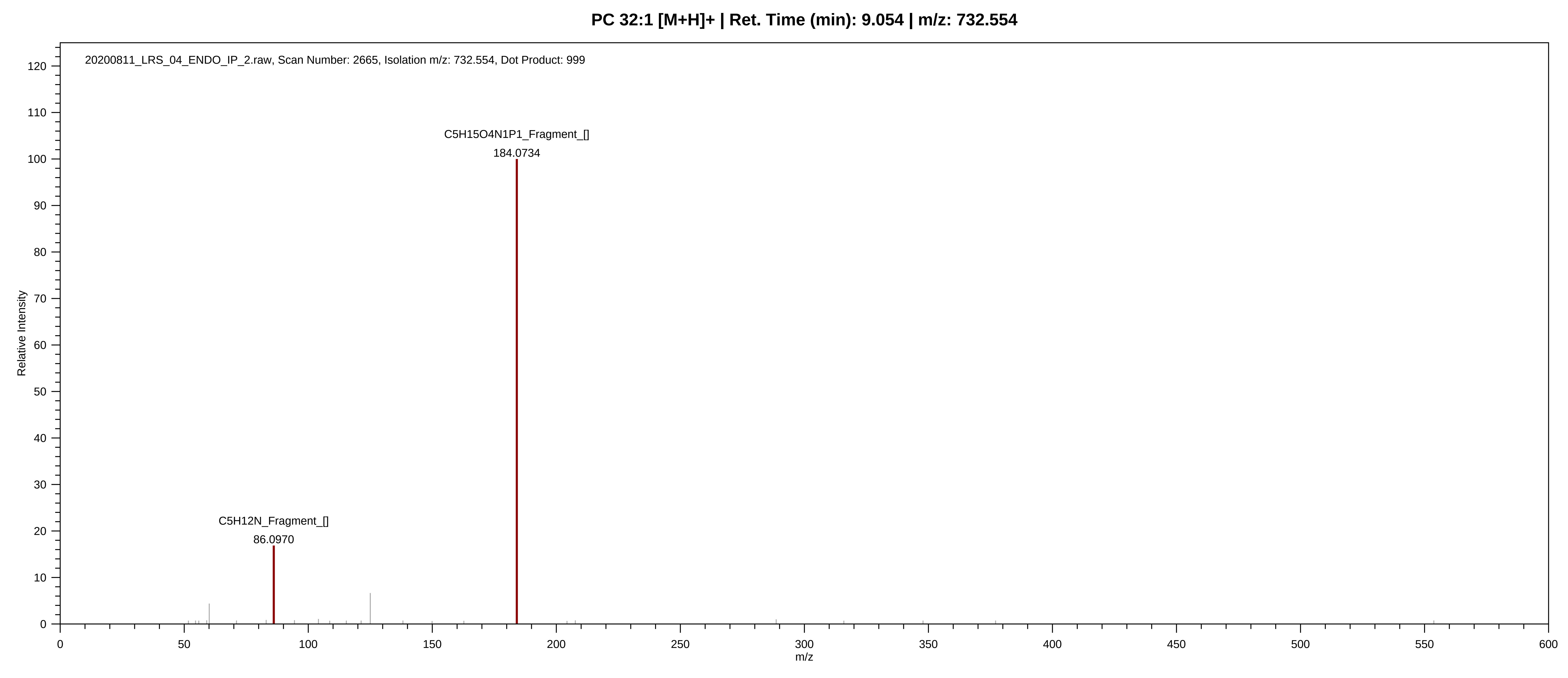
PC 32:1 [M+H]+ | Ret. Time (min): 9.054 | m/z: 732.554
20200811_LRS_04_ENDO_IP_2.raw, Scan Number: 2665, Isolation m/z: 732.554, Dot Product: 999
C5H15O4N1P1_Fragment_[]
184.0734
C5H12N_Fragment_[]
86.0970
Relative Intensity
m/z

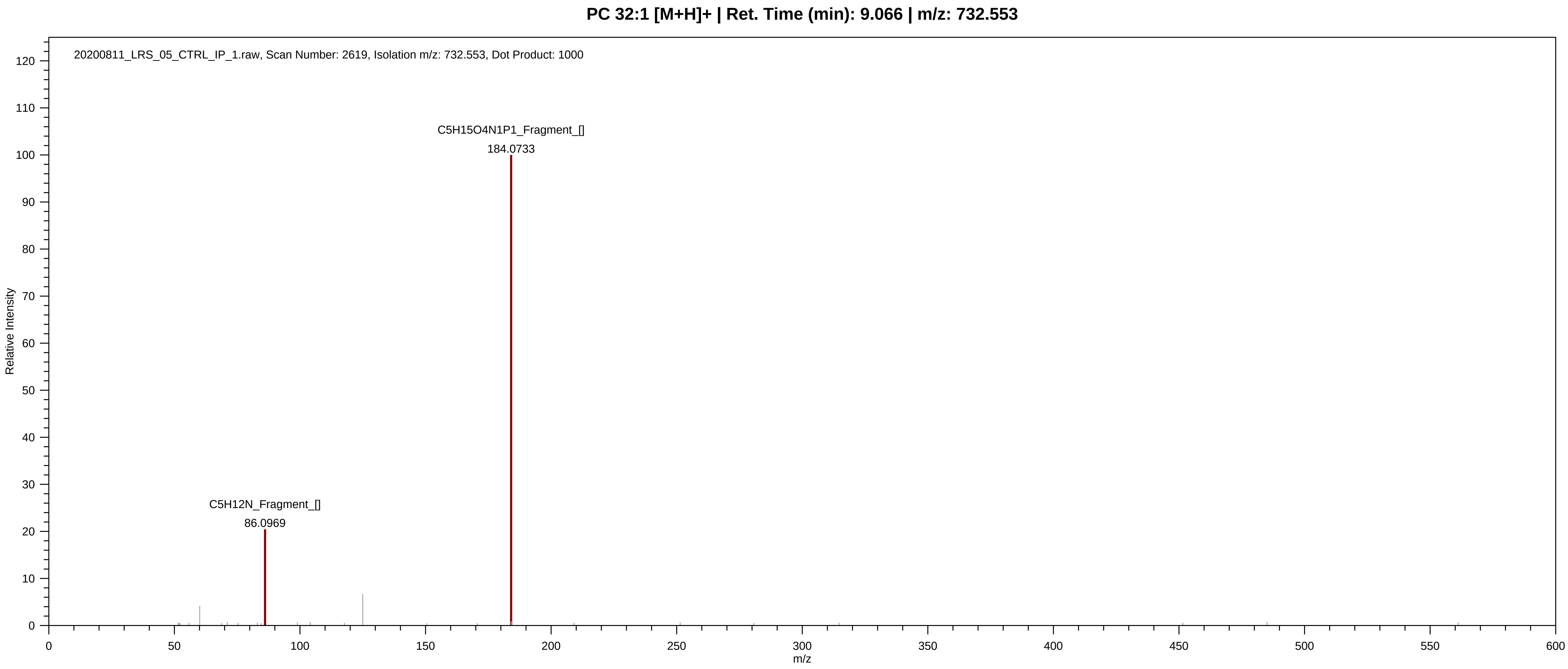

PC 32:1 [M+H]+ | Ret. Time (min): 9.066 | m/z: 732.553
20200811_LRS_05_CTRL_IP_1.raw, Scan Number: 2619, Isolation m/z: 732.553, Dot Product: 1000
C5H15O4N1P1_Fragment_[]
184.0733
C5H12N_Fragment_[]
86.0969
Relative Intensity
m/z

PC 32:1 [M+H]+ | Ret. Time (min): 9.066 | m/z: 732.554

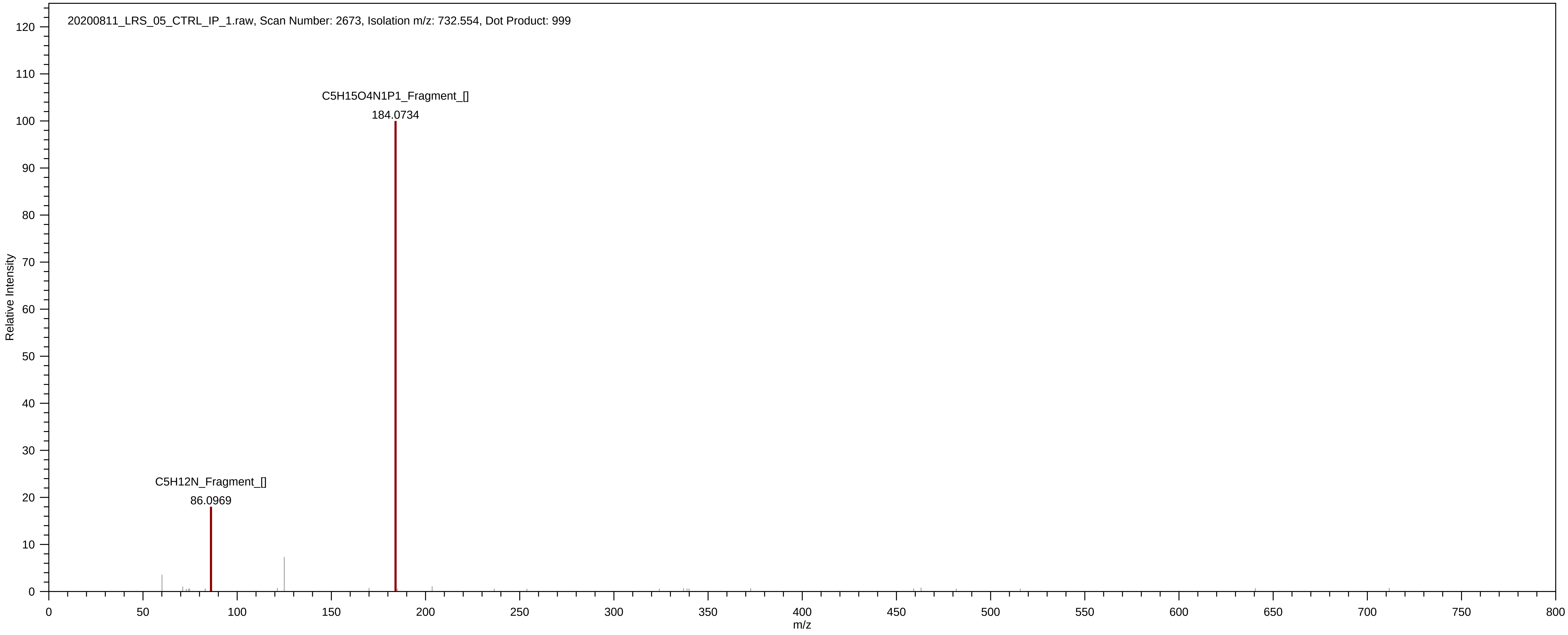

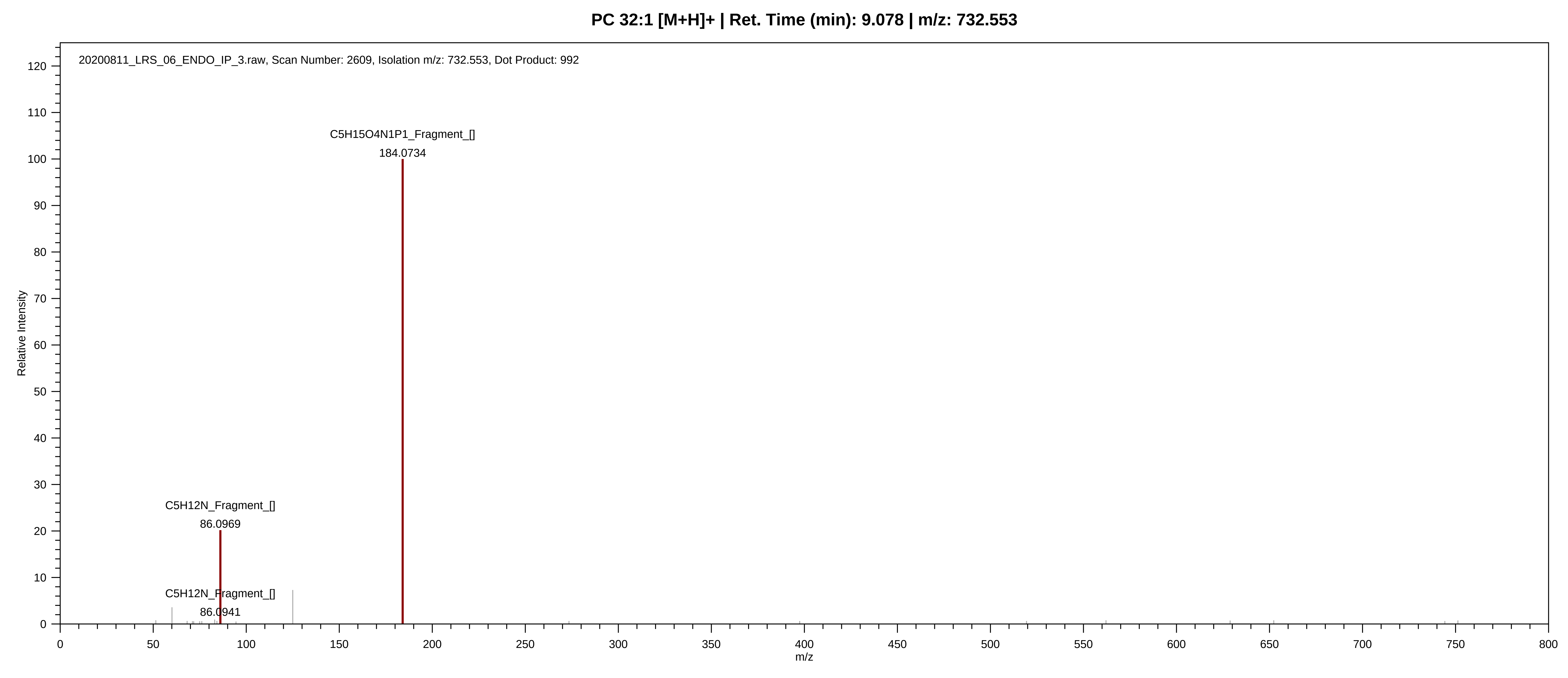

PC 32:1 [M+H]+ | Ret. Time (min): 9.078 | m/z: 732.553
20200811_LRS_06_ENDO_IP_3.raw, Scan Number: 2609, Isolation m/z: 732.553, Dot Product: 992
C5H15O4N1P1_Fragment_[]
184.0734
C5H12N_Fragment_[]
86.0969
C5H12N_Fragment_[]
86.0941
Relative Intensity
m/z

PC 32:1 [M+H]+ | Ret. Time (min): 9.078 | m/z: 732.554

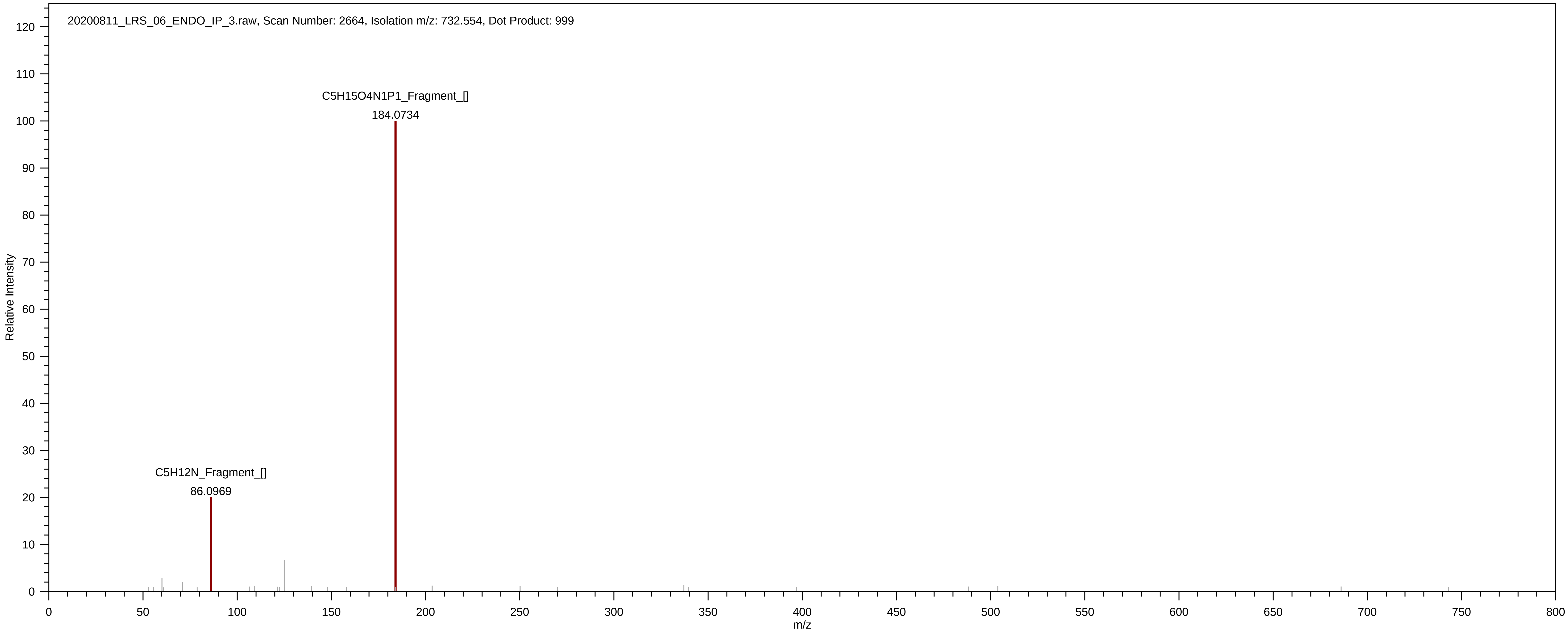

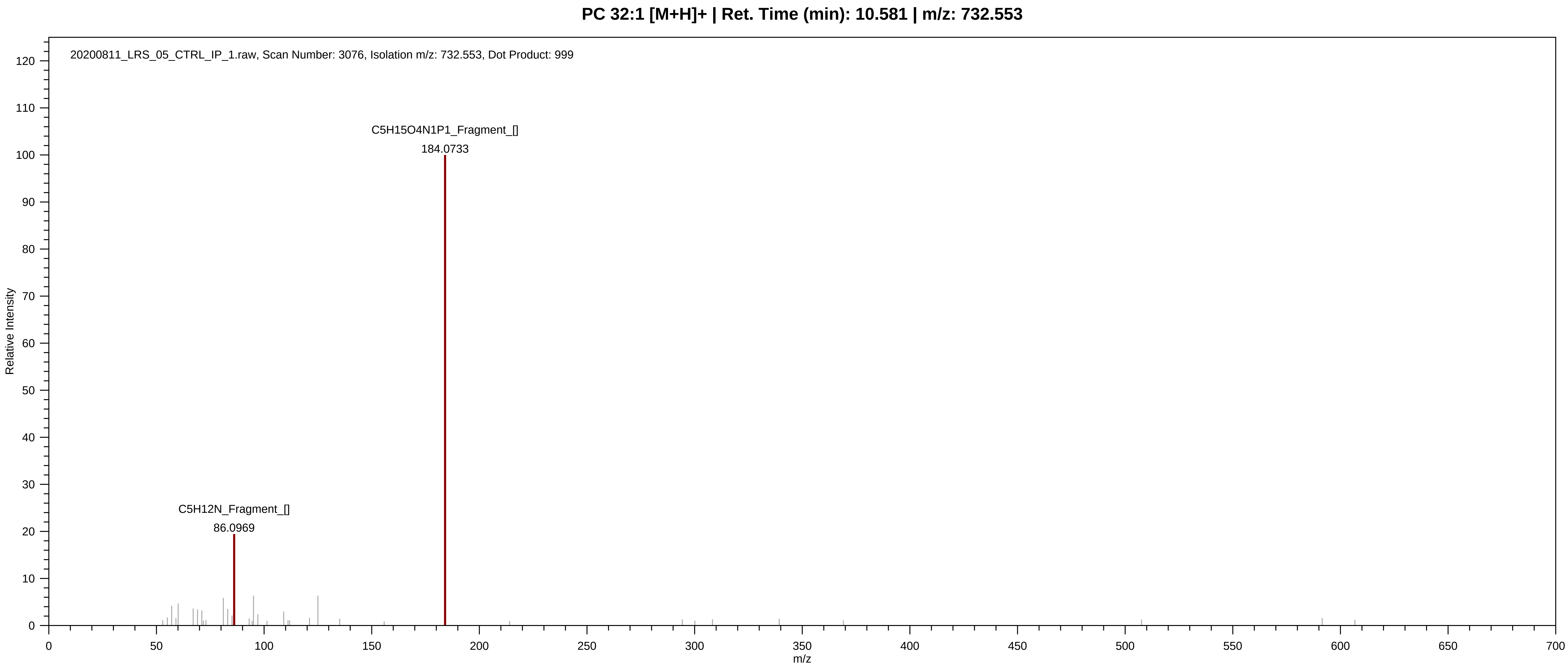
PC 32:1 [M+H]+ | Ret. Time (min): 10.581 | m/z: 732.553
20200811_LRS_05_CTRL_IP_1.raw, Scan Number: 3076, Isolation m/z: 732.553, Dot Product: 999
C5H15O4N1P1_Fragment_[]
184.0733
C5H12N_Fragment_[]
86.0969
Relative Intensity
m/z

PC 32:1 [M+H]+ | Ret. Time (min): 8.885 | m/z: 732.554

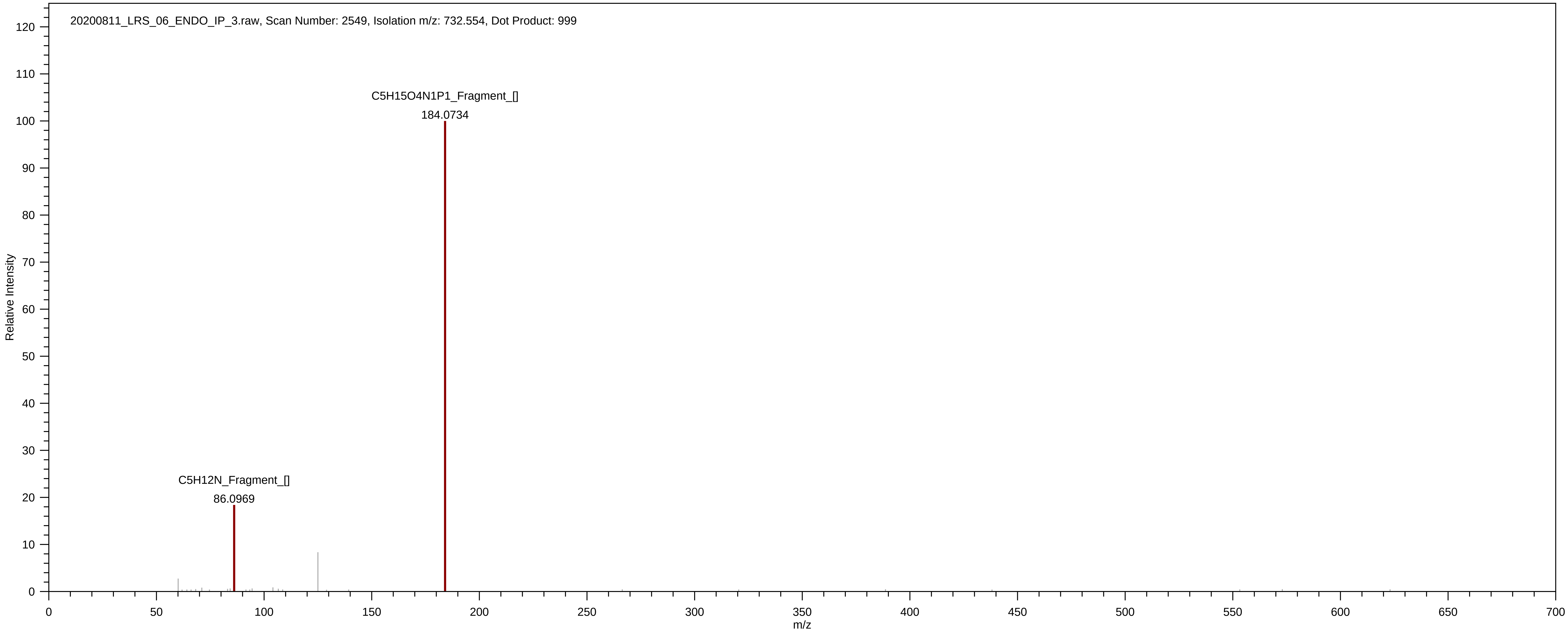

PC 32:1 [M+H]+ | Ret. Time (min): 8.886 | m/z: 732.554

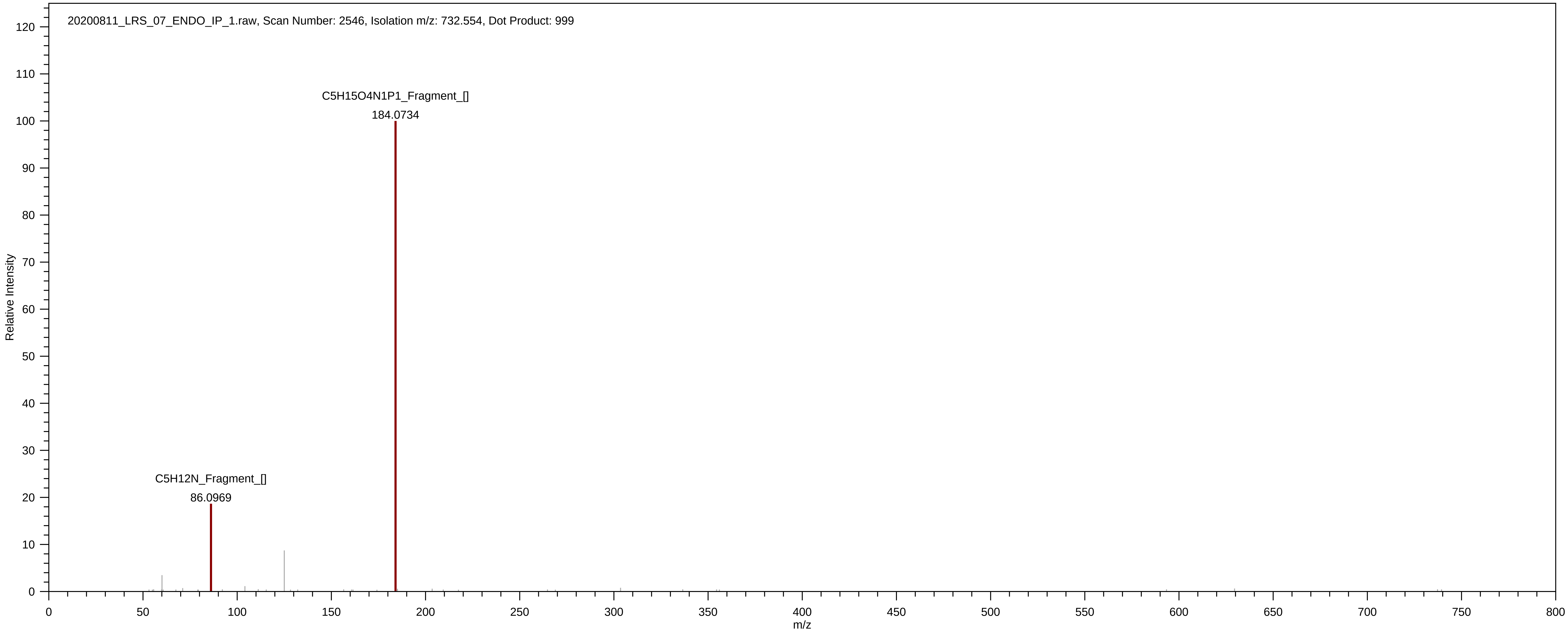

PC 32:1 [M+H]+ | Ret. Time (min): 9.059 | m/z: 732.554

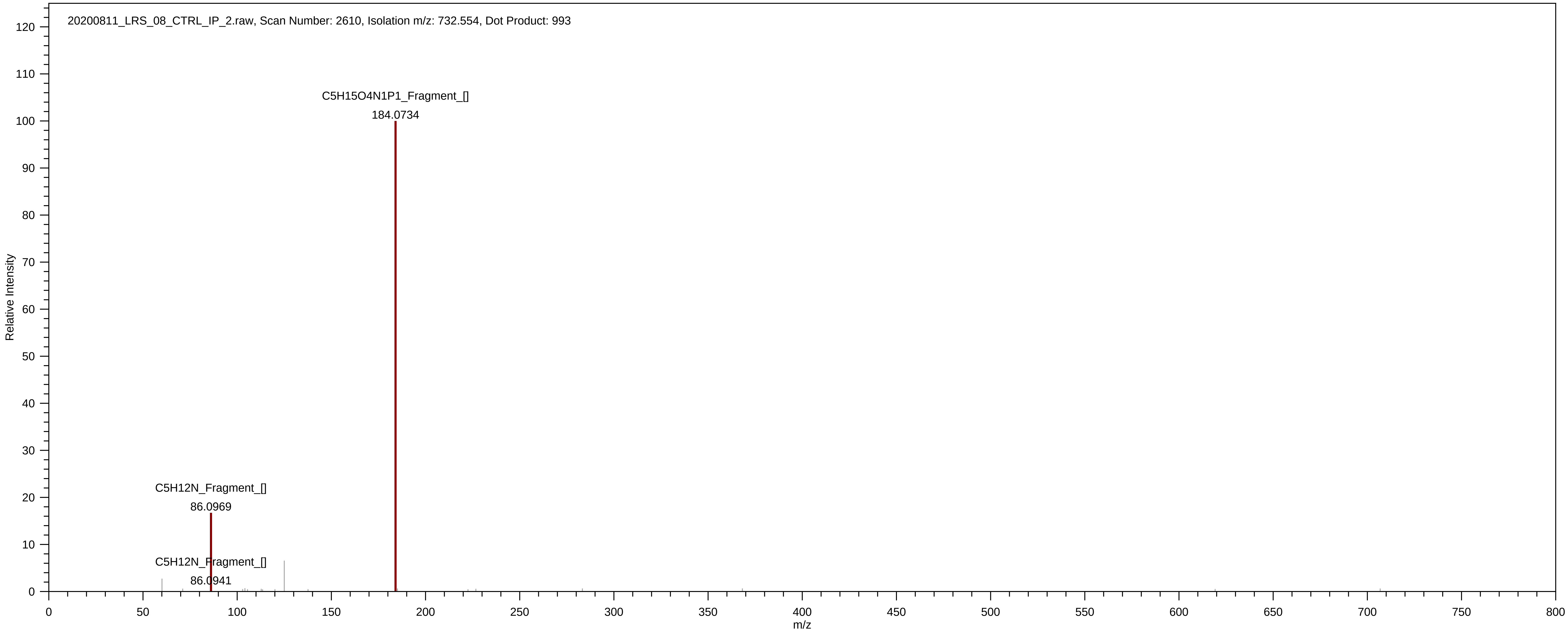

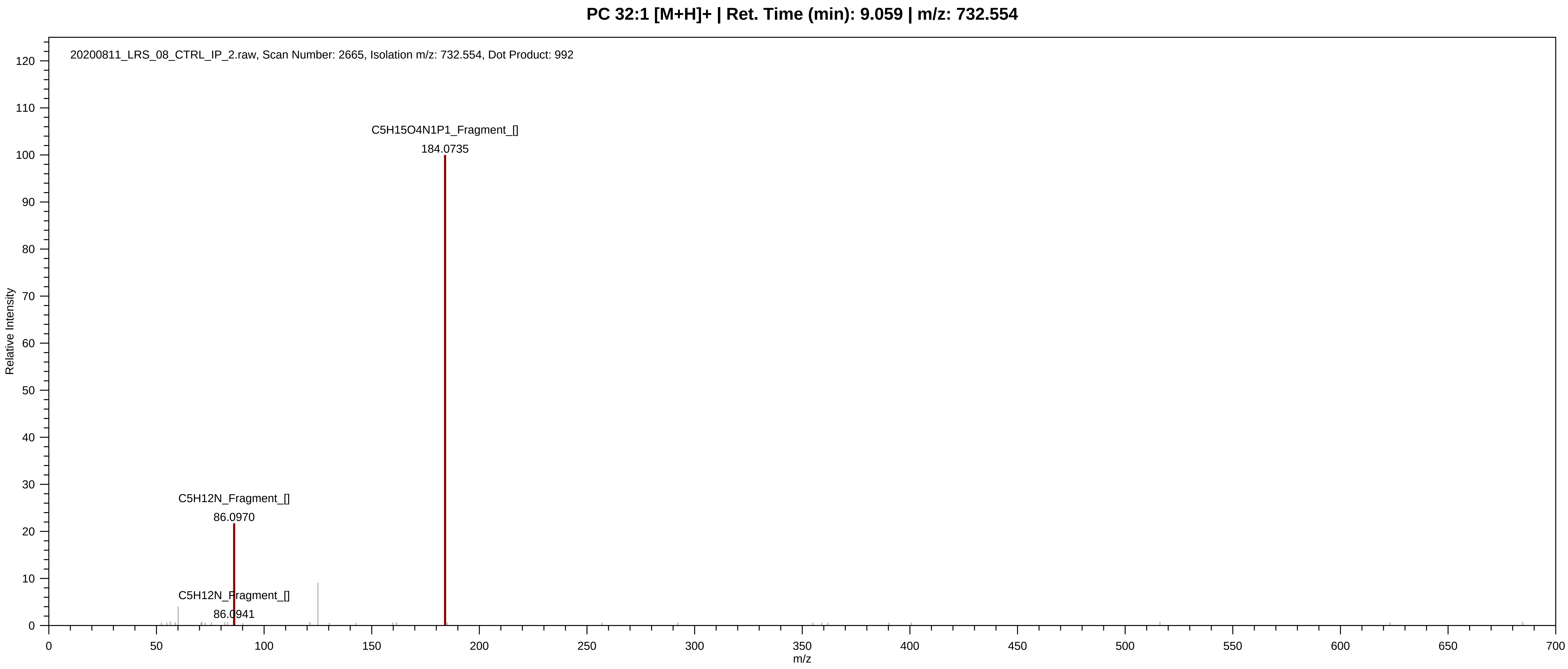
PC 32:1 [M+H]+ | Ret. Time (min): 9.059 | m/z: 732.554
20200811_LRS_08_CTRL_IP_2.raw, Scan Number: 2665, Isolation m/z: 732.554, Dot Product: 992
C5H15O4N1P1_Fragment_[]
184.0735
C5H12N_Fragment_[]
86.0970
C5H12N_Fragment_[]
86.0941
Relative Intensity
m/z

PC 32:1 [M+H]+ | Ret. Time (min): 8.889 | m/z: 732.555

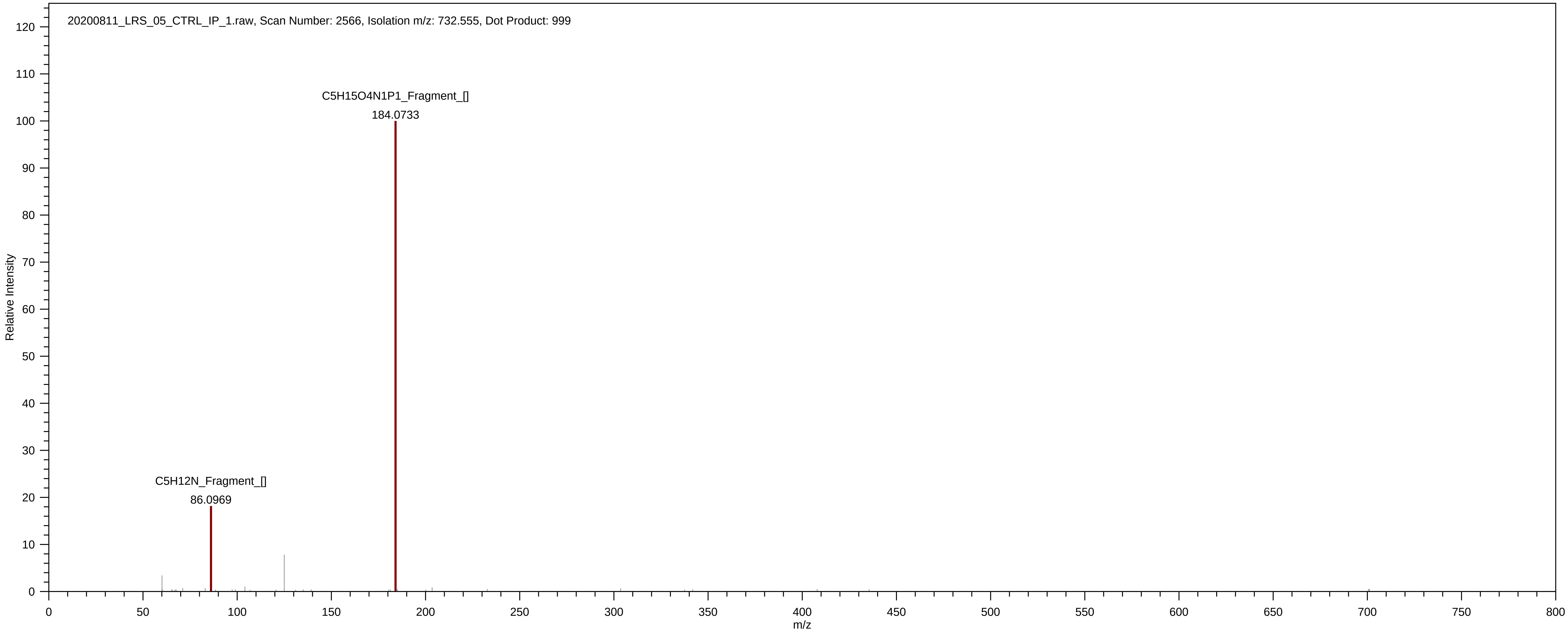

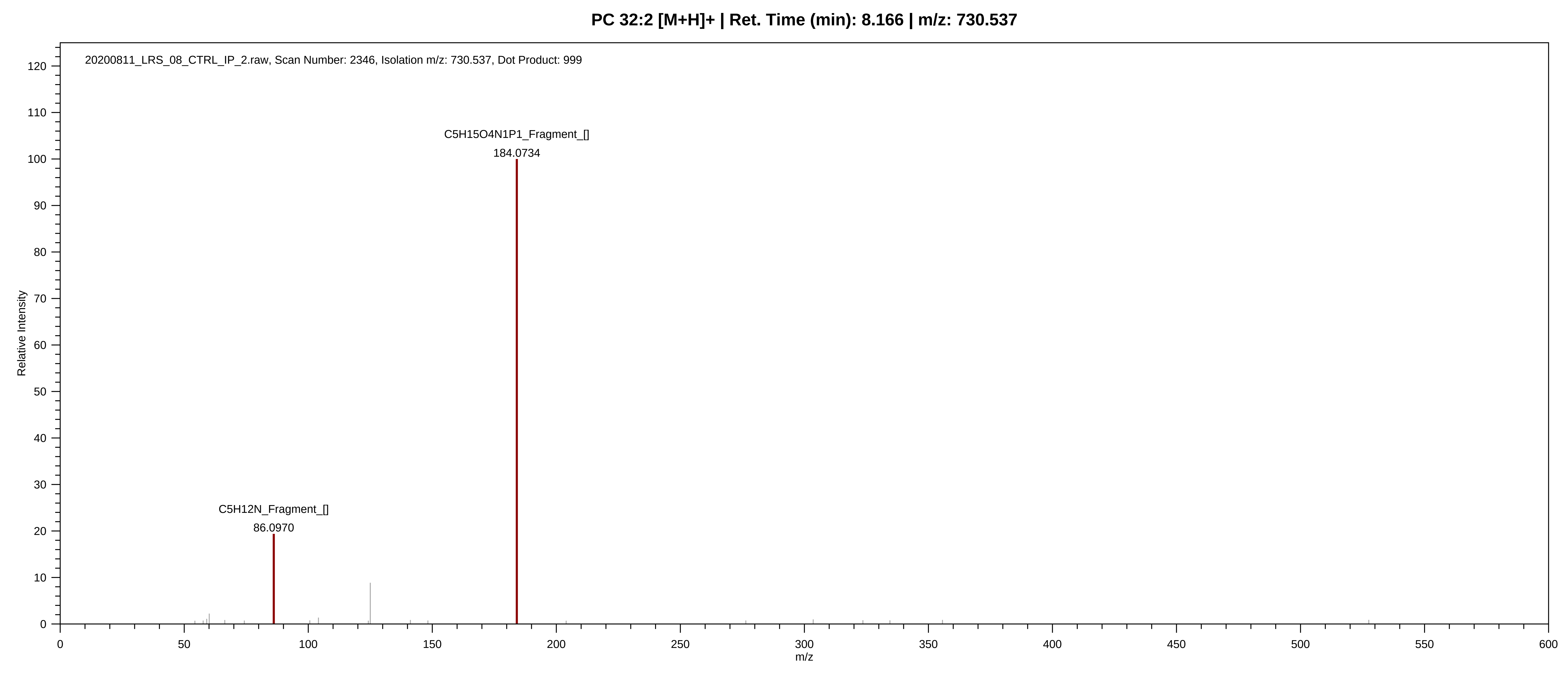
PC 32:2 [M+H]+ | Ret. Time (min): 8.166 | m/z: 730.537
20200811_LRS_08_CTRL_IP_2.raw, Scan Number: 2346, Isolation m/z: 730.537, Dot Product: 999
C5H15O4N1P1_Fragment_[]
184.0734
C5H12N_Fragment_[]
86.0970
Relative Intensity
m/z

PC 32:2 [M+H]+ | Ret. Time (min): 8.184 | m/z: 730.537

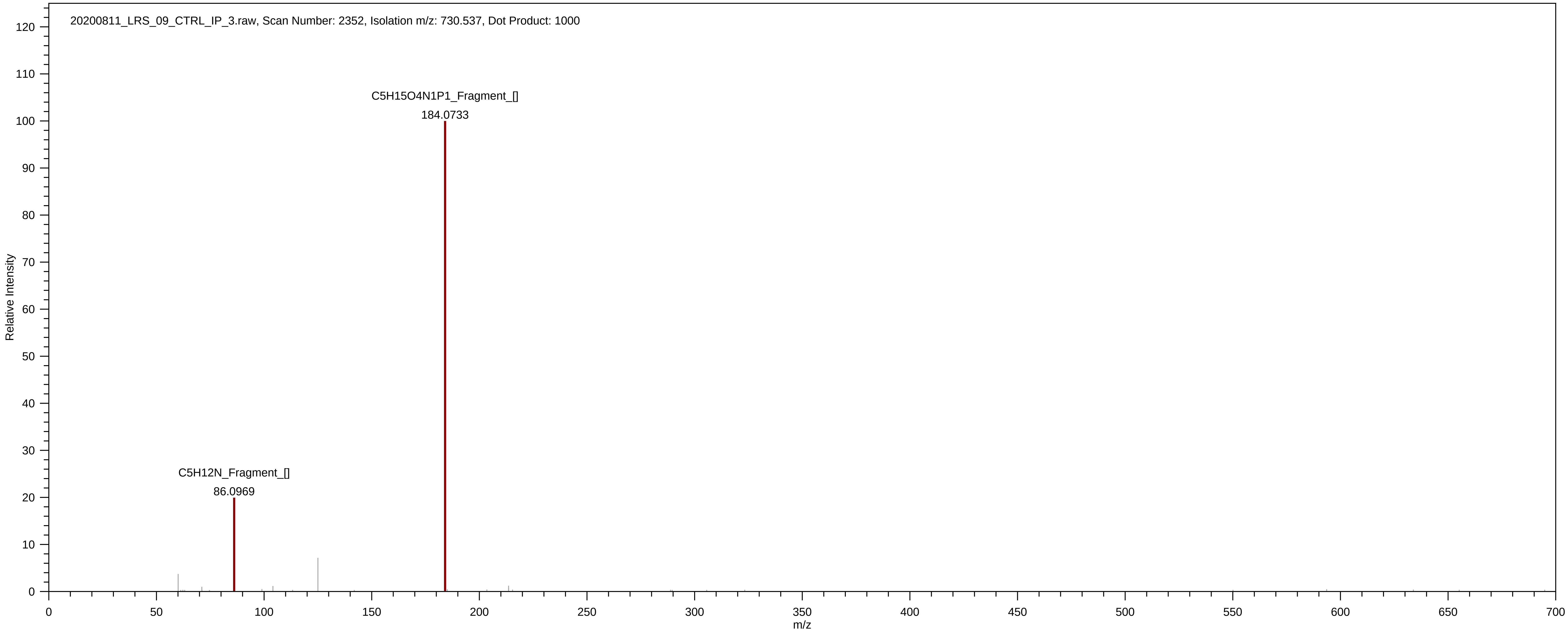

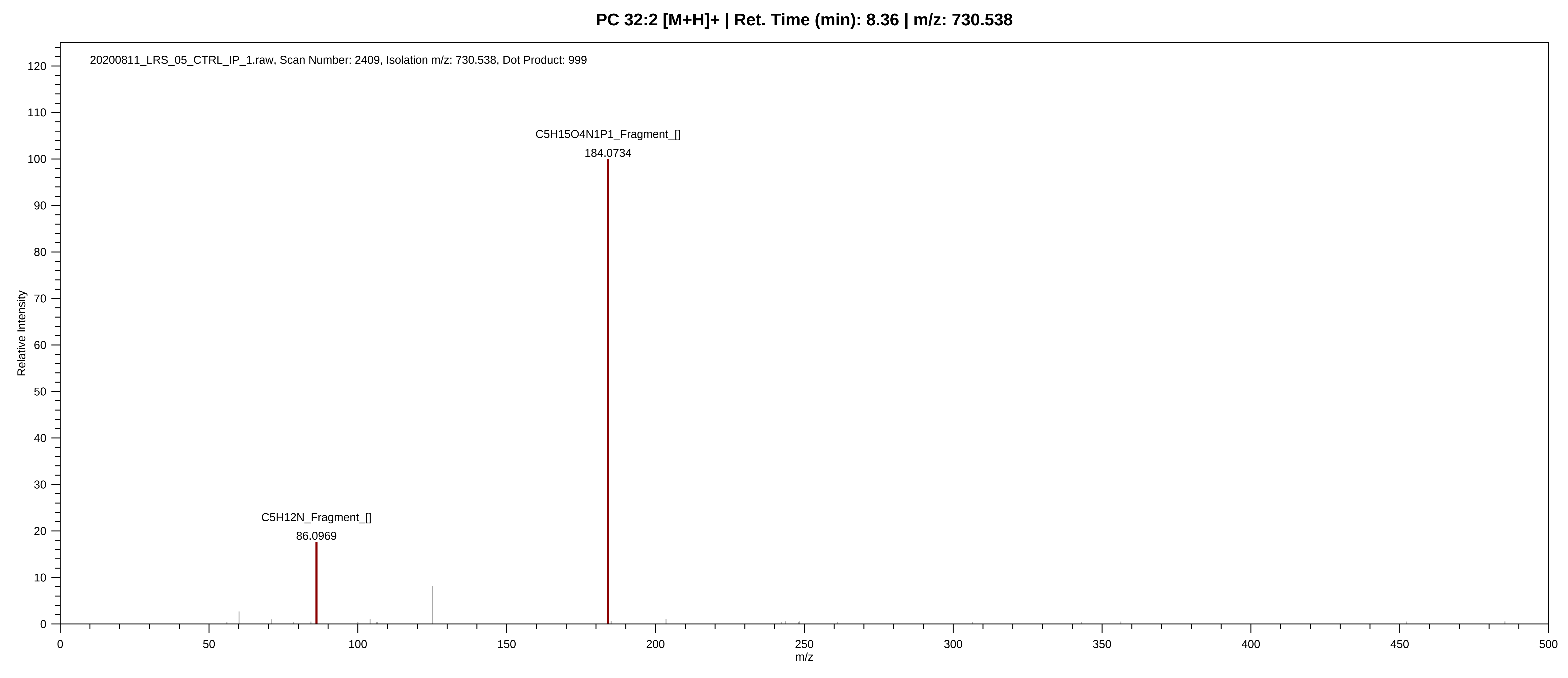

PC 32:2 [M+H]+ | Ret. Time (min): 8.36 | m/z: 730.538
20200811_LRS_05_CTRL_IP_1.raw, Scan Number: 2409, Isolation m/z: 730.538, Dot Product: 999
C5H15O4N1P1_Fragment_[]
184.0734
C5H12N_Fragment_[]
86.0969
Relative Intensity
m/z

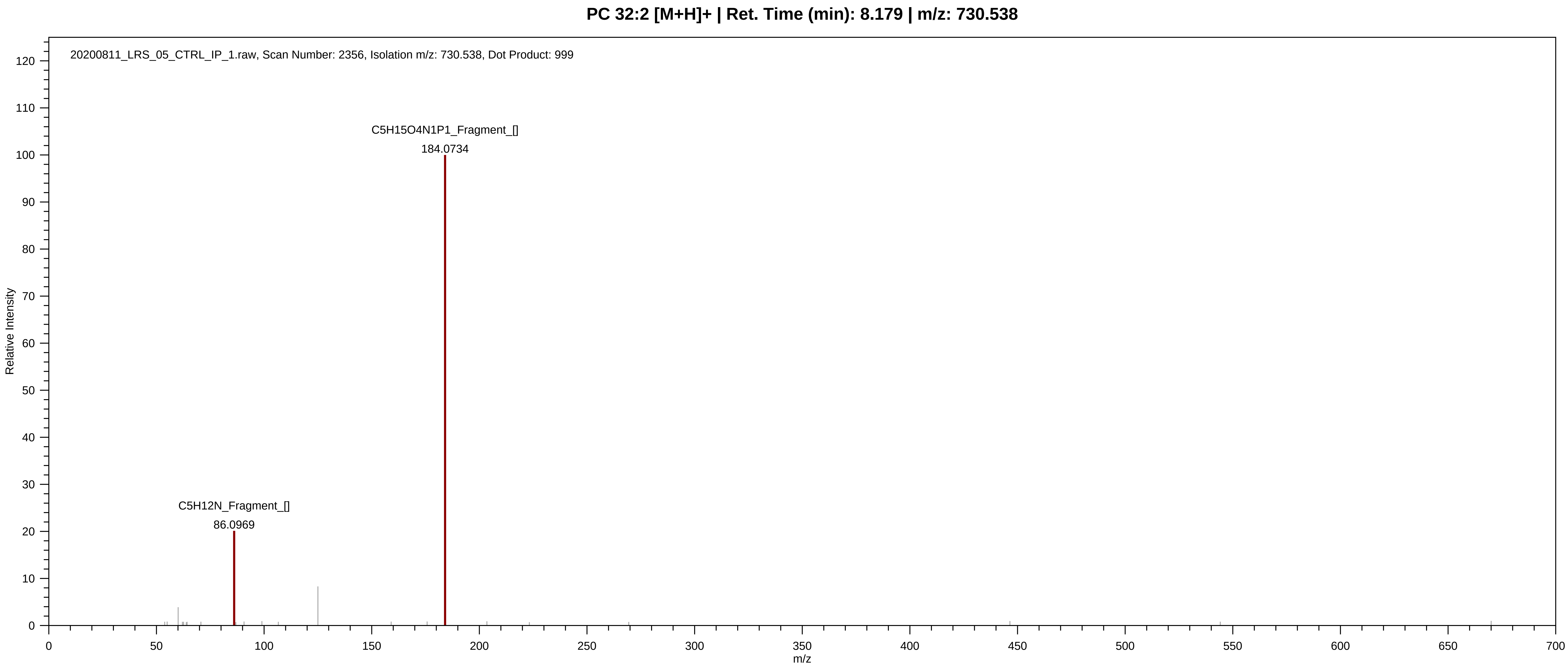
PC 32:2 [M+H]+ | Ret. Time (min): 8.179 | m/z: 730.538
20200811_LRS_05_CTRL_IP_1.raw, Scan Number: 2356, Isolation m/z: 730.538, Dot Product: 999
C5H15O4N1P1_Fragment_[]
184.0734
C5H12N_Fragment_[]
86.0969
Relative Intensity
m/z

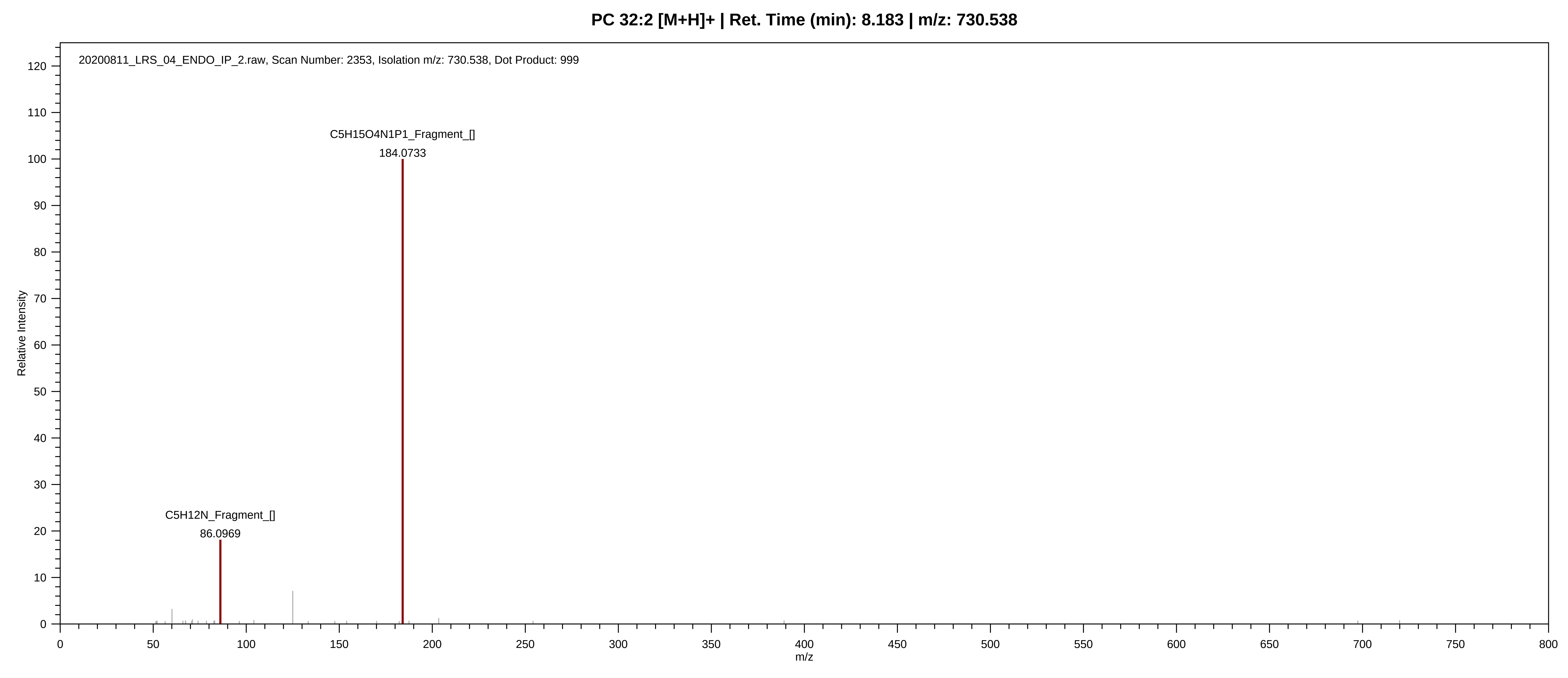
PC 32:2 [M+H]+ | Ret. Time (min): 8.183 | m/z: 730.538
20200811_LRS_04_ENDO_IP_2.raw, Scan Number: 2353, Isolation m/z: 730.538, Dot Product: 999
C5H15O4N1P1_Fragment_[]
184.0733
C5H12N_Fragment_[]
86.0969
Relative Intensity
m/z
0
10
20
30
40
50
60
70
80
90
100
110
120
0
50
100
150
200
250
300
350
400
450
500
550
600
650
700
750
800

PC 32:2 [M+H]+ | Ret. Time (min): 8.191 | m/z: 730.538

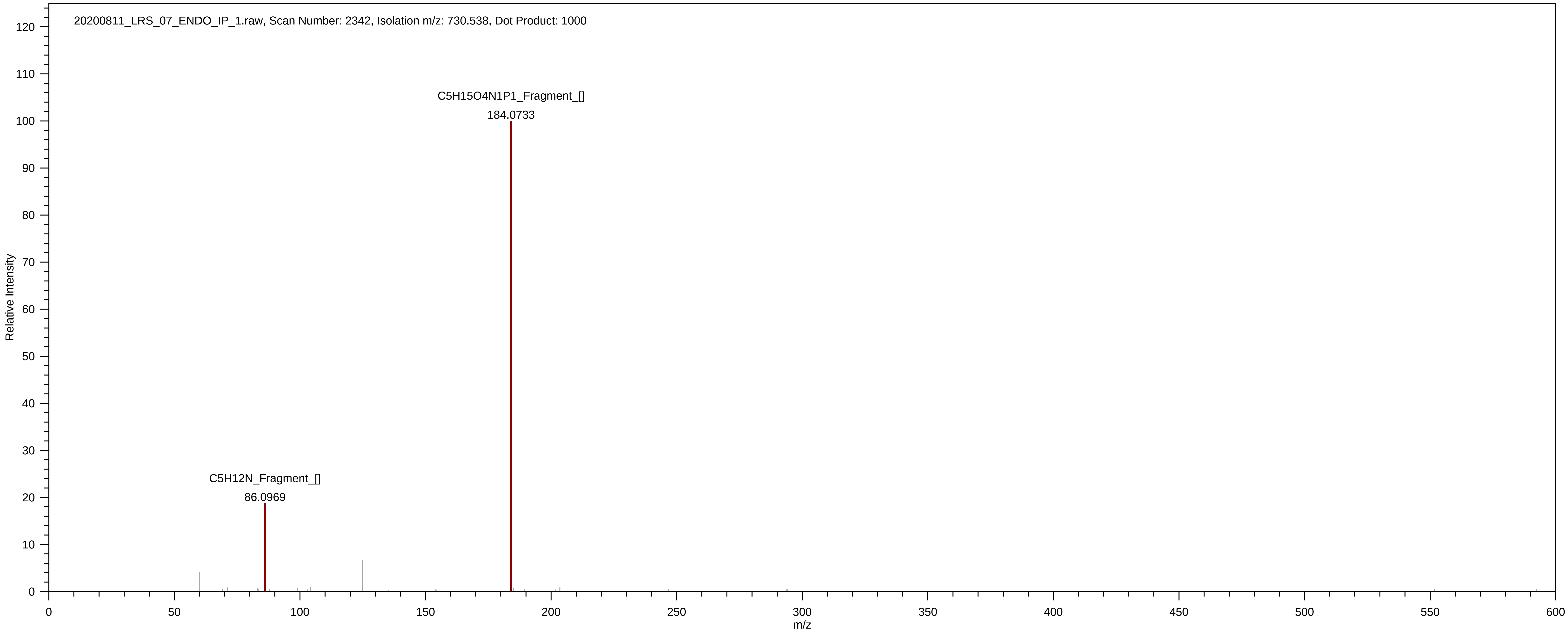

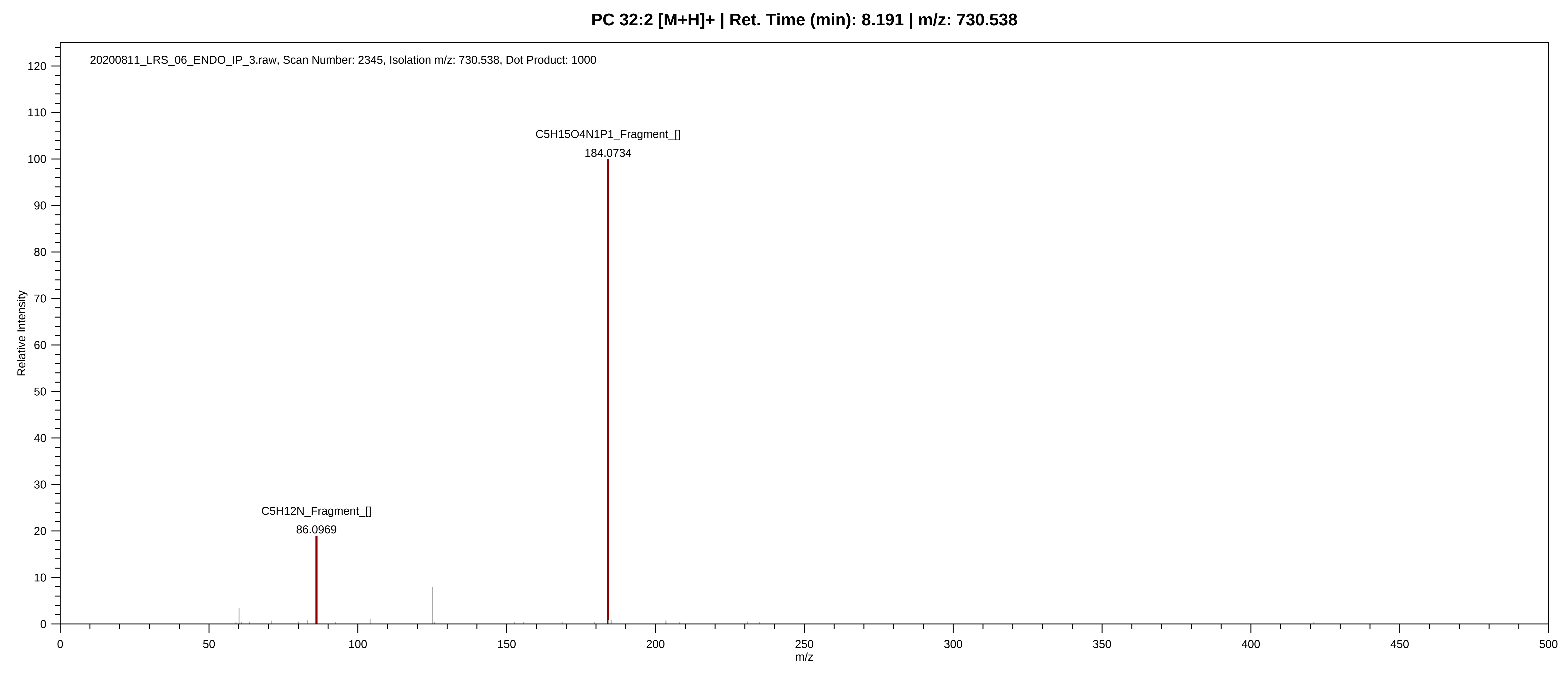

PC 32:2 [M+H]+ | Ret. Time (min): 8.191 | m/z: 730.538
20200811_LRS_06_ENDO_IP_3.raw, Scan Number: 2345, Isolation m/z: 730.538, Dot Product: 1000
C5H15O4N1P1_Fragment_[]
184.0734
C5H12N_Fragment_[]
86.0969
Relative Intensity
m/z

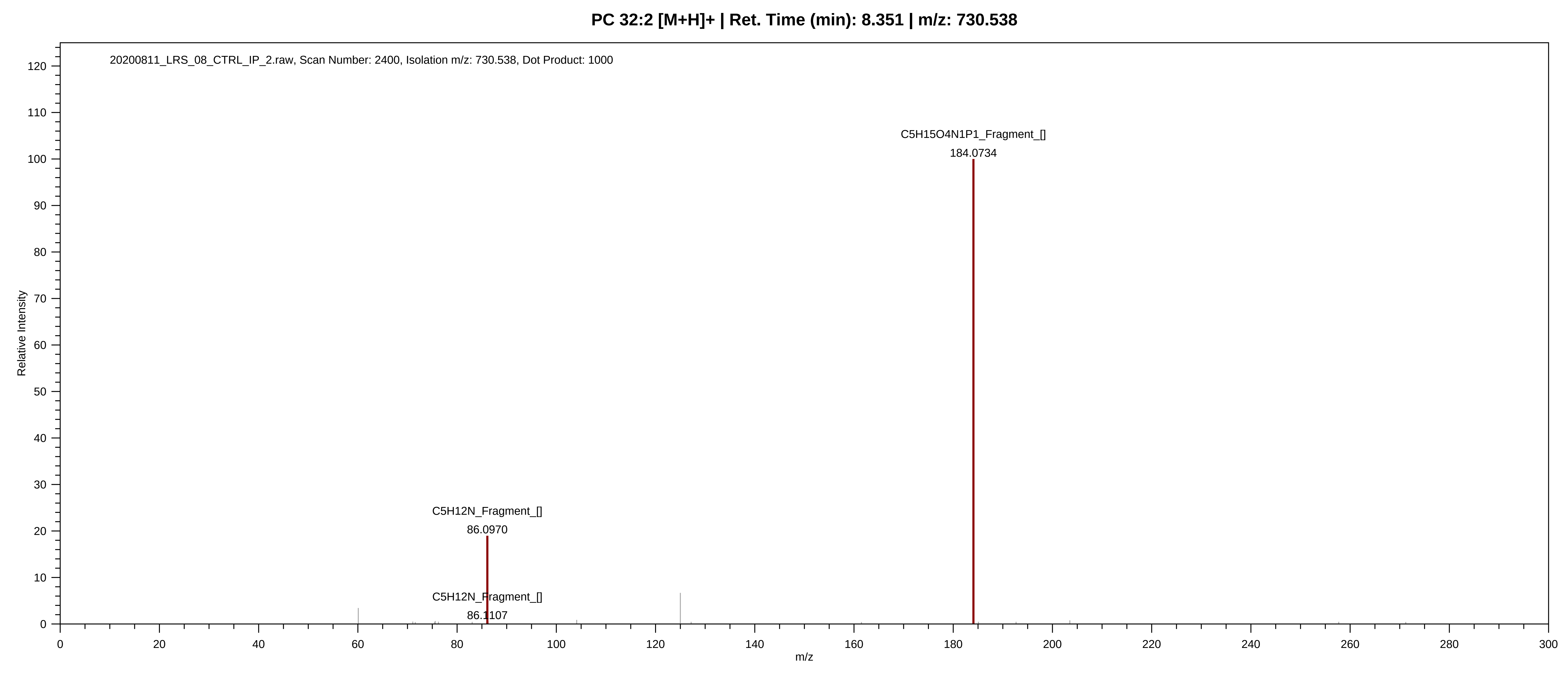

PC 32:2 [M+H]+ | Ret. Time (min): 8.351 | m/z: 730.538
20200811_LRS_08_CTRL_IP_2.raw, Scan Number: 2400, Isolation m/z: 730.538, Dot Product: 1000
C5H15O4N1P1_Fragment_[]
184.0734
C5H12N_Fragment_[]
86.0970
C5H12N_Fragment_[]
86.1107
Relative Intensity
m/z

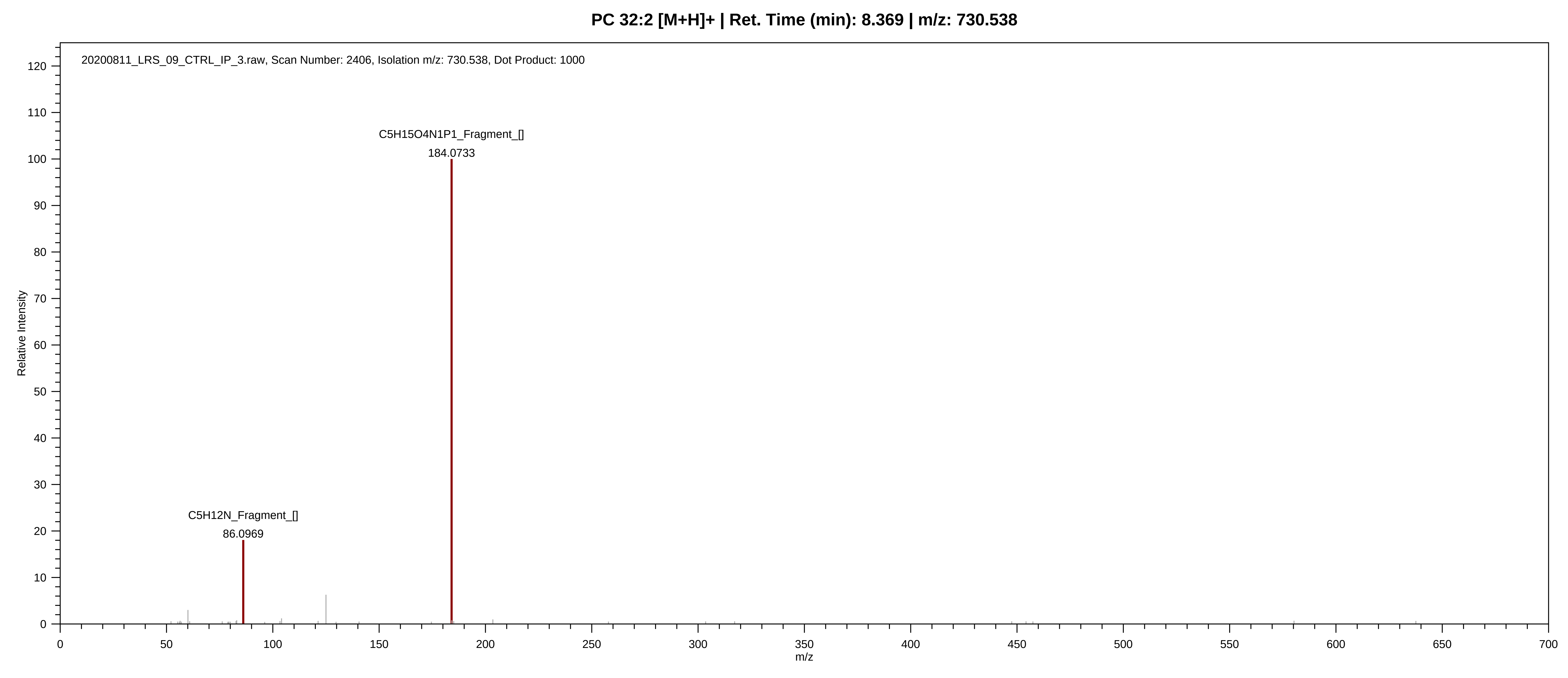
PC 32:2 [M+H]+ | Ret. Time (min): 8.369 | m/z: 730.538
20200811_LRS_09_CTRL_IP_3.raw, Scan Number: 2406, Isolation m/z: 730.538, Dot Product: 1000
C5H15O4N1P1_Fragment_[]
184.0733
C5H12N_Fragment_[]
86.0969
Relative Intensity
m/z

PC 32:2 [M+H]+ | Ret. Time (min): 8.369 | m/z: 730.538

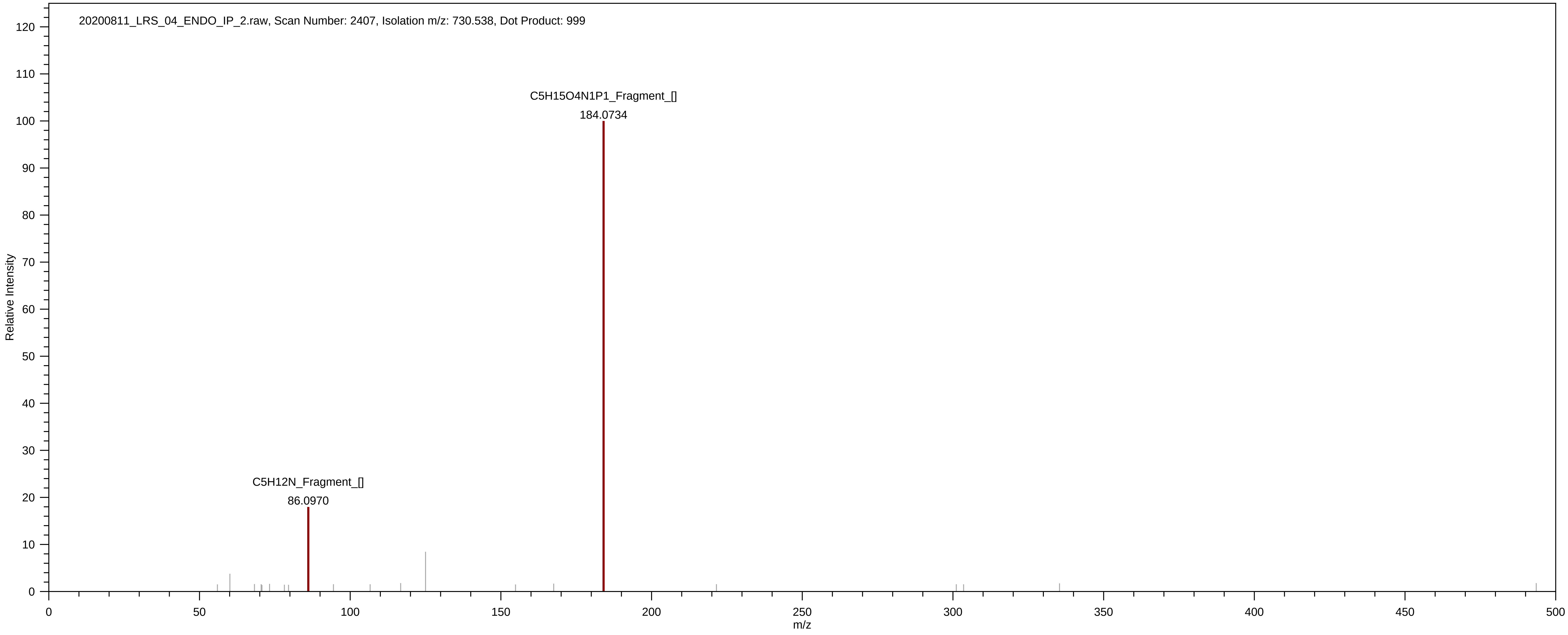

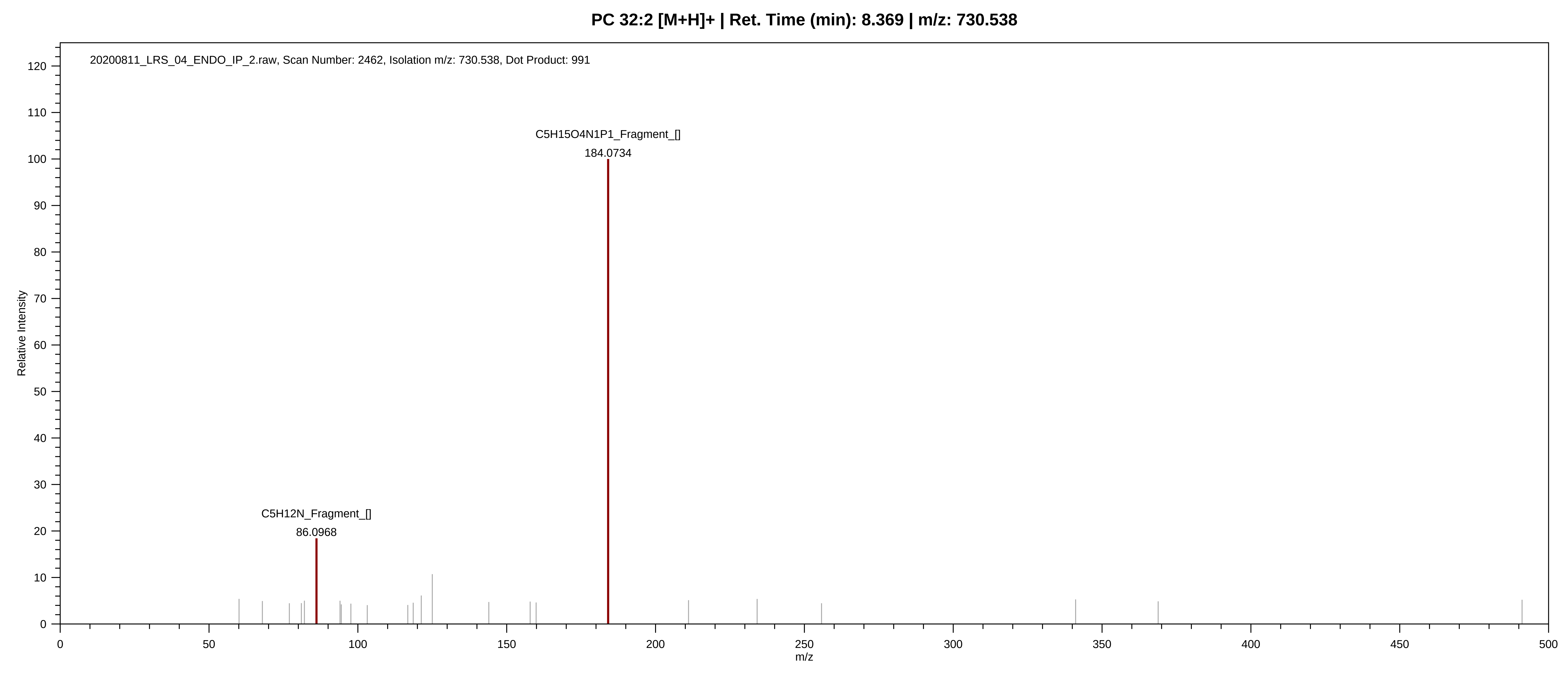
PC 32:2 [M+H]+ | Ret. Time (min): 8.369 | m/z: 730.538
20200811_LRS_04_ENDO_IP_2.raw, Scan Number: 2462, Isolation m/z: 730.538, Dot Product: 991
C5H15O4N1P1_Fragment_[]
184.0734
C5H12N_Fragment_[]
86.0968
Relative Intensity
m/z

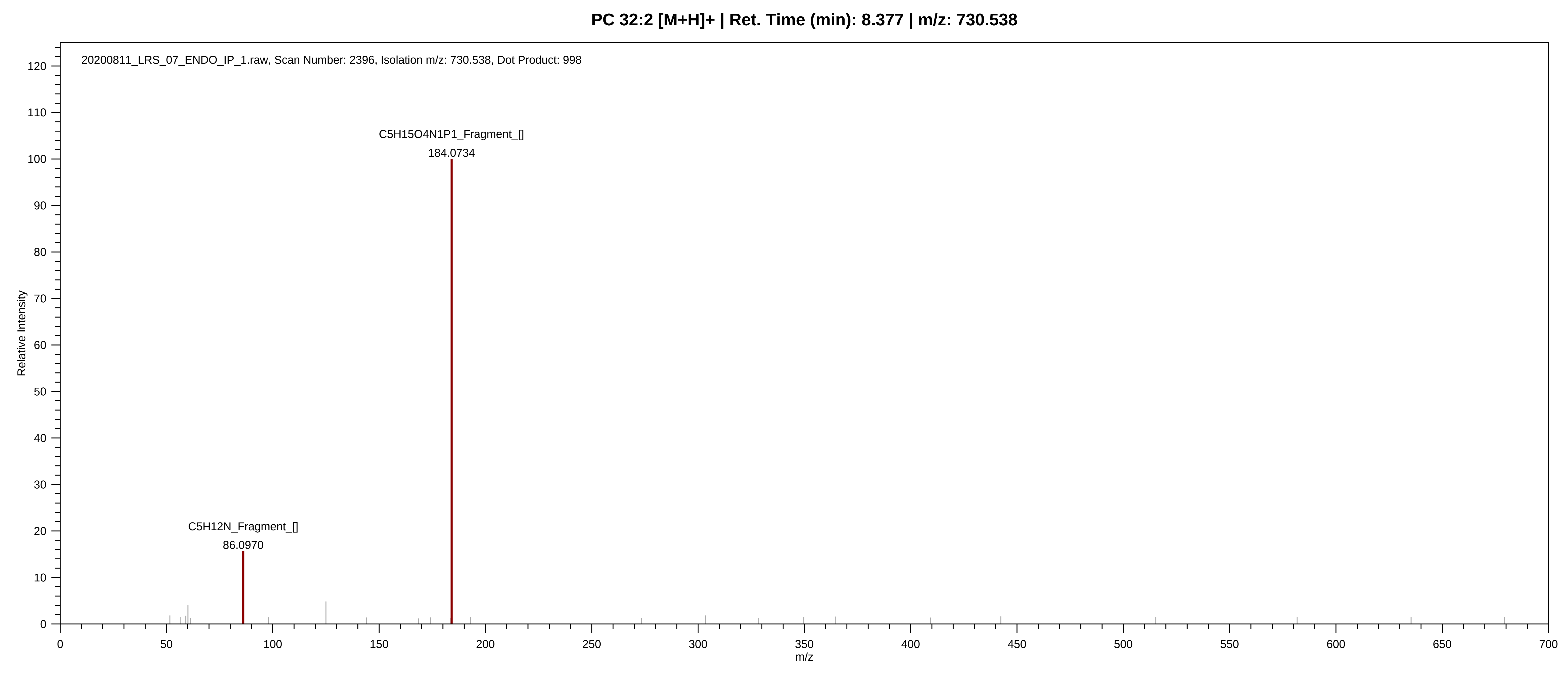
PC 32:2 [M+H]+ | Ret. Time (min): 8.377 | m/z: 730.538
20200811_LRS_07_ENDO_IP_1.raw, Scan Number: 2396, Isolation m/z: 730.538, Dot Product: 998
C5H15O4N1P1_Fragment_[]
184.0734
C5H12N_Fragment_[]
86.0970
Relative Intensity
m/z

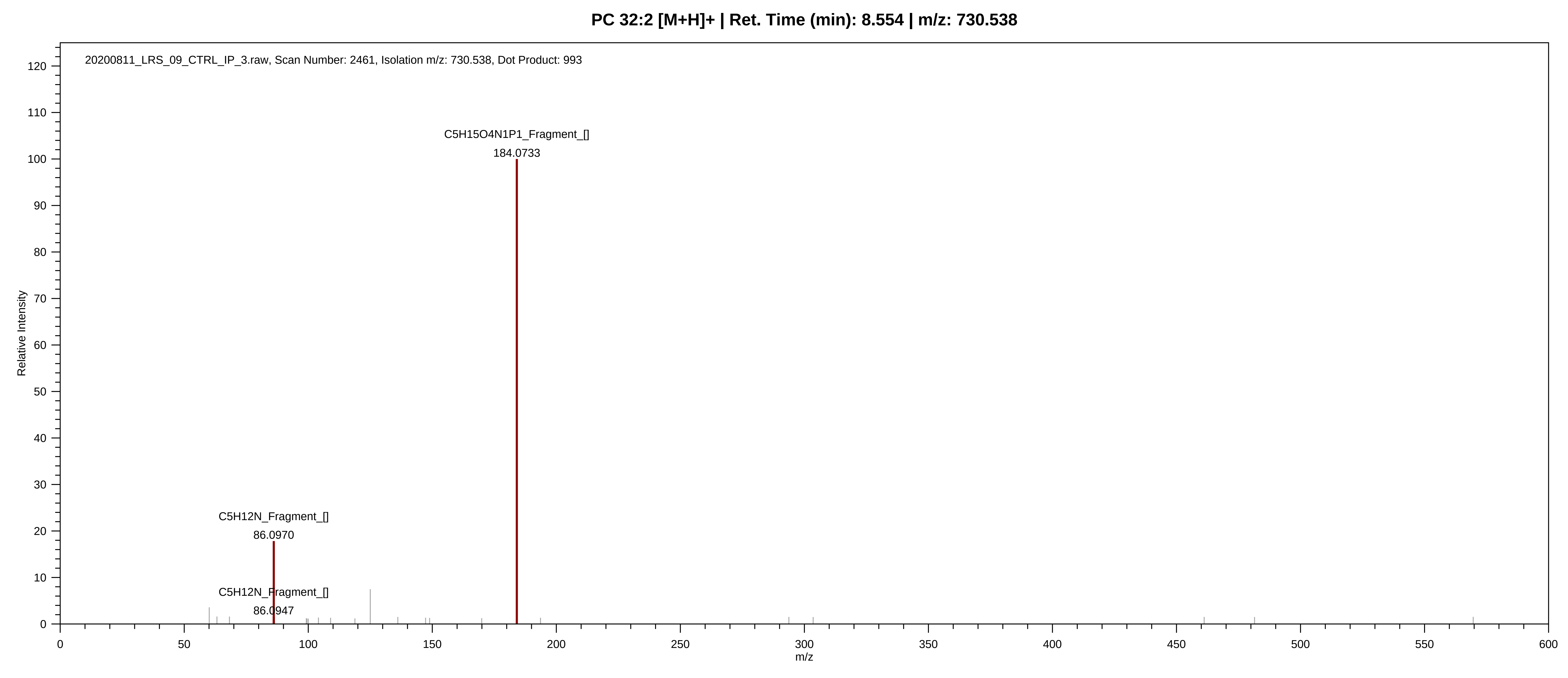

PC 32:2 [M+H]+ | Ret. Time (min): 8.554 | m/z: 730.538
20200811_LRS_09_CTRL_IP_3.raw, Scan Number: 2461, Isolation m/z: 730.538, Dot Product: 993
C5H15O4N1P1_Fragment_[]
184.0733
C5H12N_Fragment_[]
86.0970
C5H12N_Fragment_[]
86.0947
Relative Intensity
m/z

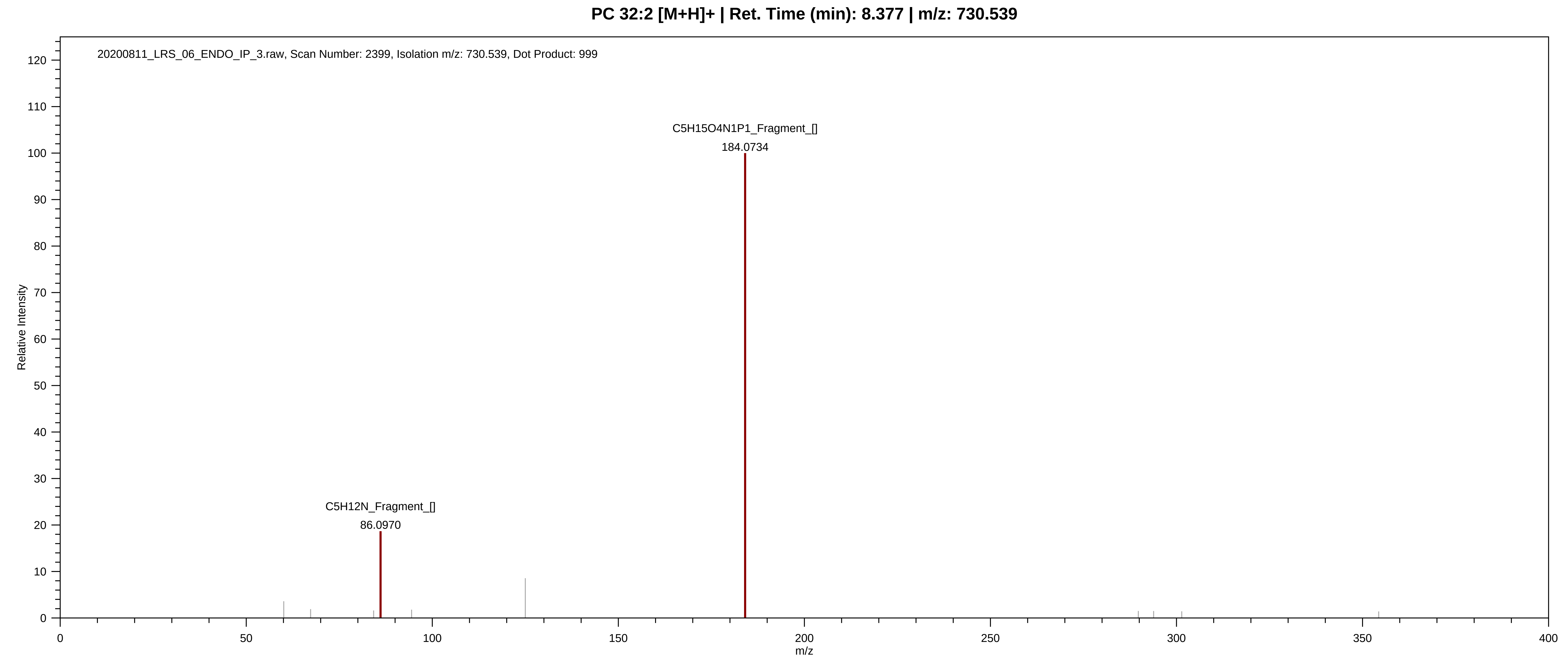

PC 32:2 [M+H]+ | Ret. Time (min): 8.377 | m/z: 730.539
20200811_LRS_06_ENDO_IP_3.raw, Scan Number: 2399, Isolation m/z: 730.539, Dot Product: 999
C5H15O4N1P1_Fragment_[]
184.0734
C5H12N_Fragment_[]
86.0970
Relative Intensity
m/z

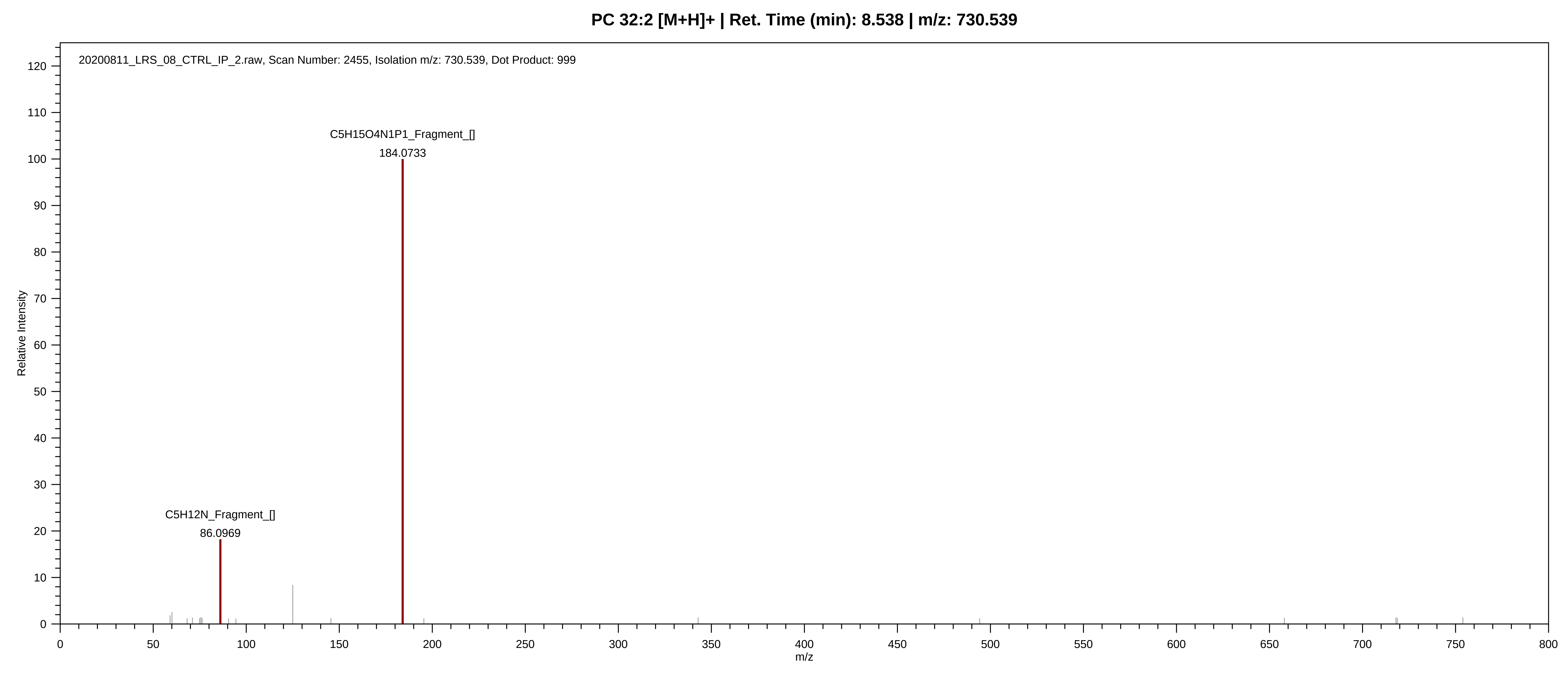
PC 32:2 [M+H]+ | Ret. Time (min): 8.538 | m/z: 730.539
20200811_LRS_08_CTRL_IP_2.raw, Scan Number: 2455, Isolation m/z: 730.539, Dot Product: 999
C5H15O4N1P1_Fragment_[]
184.0733
C5H12N_Fragment_[]
86.0969
Relative Intensity
m/z

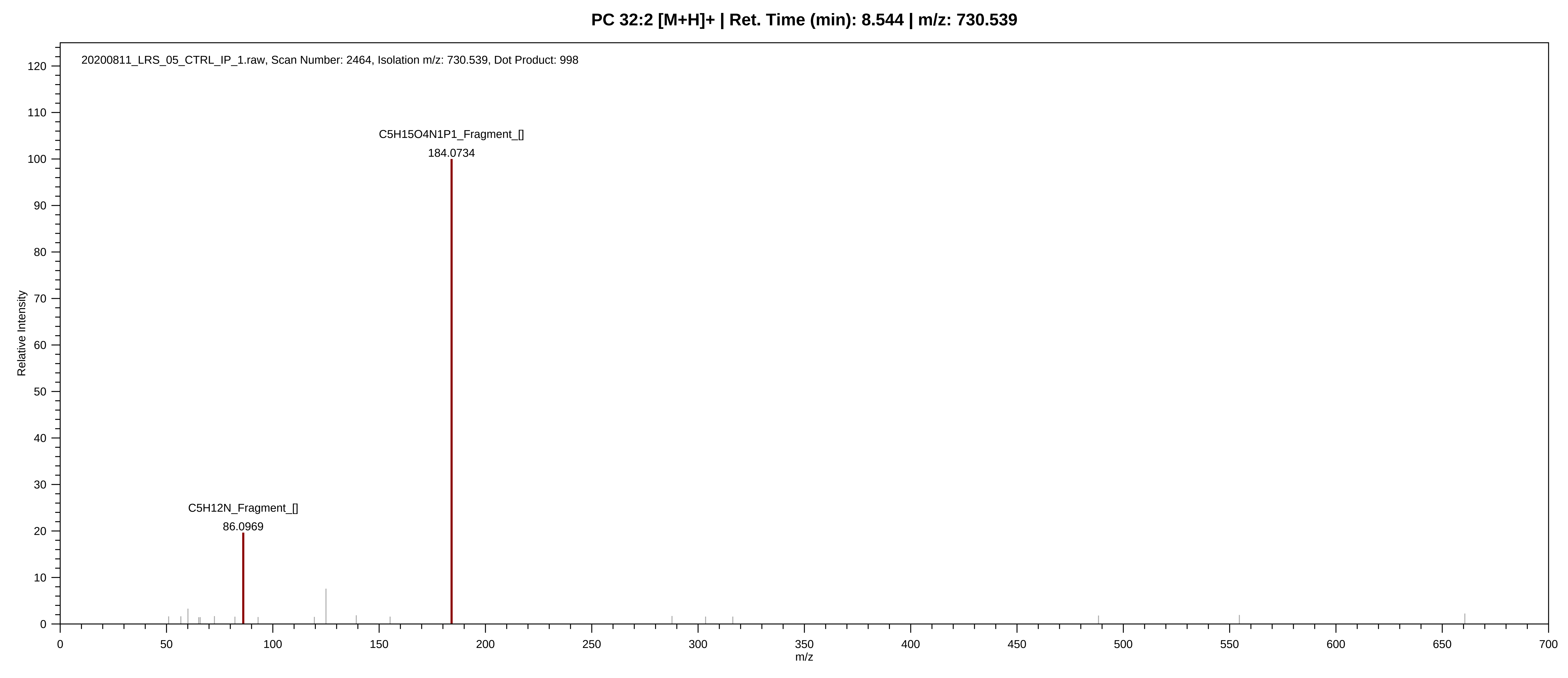

PC 32:2 [M+H]+ | Ret. Time (min): 8.544 | m/z: 730.539
20200811_LRS_05_CTRL_IP_1.raw, Scan Number: 2464, Isolation m/z: 730.539, Dot Product: 998
C5H15O4N1P1_Fragment_[]
184.0734
C5H12N_Fragment_[]
86.0969
Relative Intensity
m/z

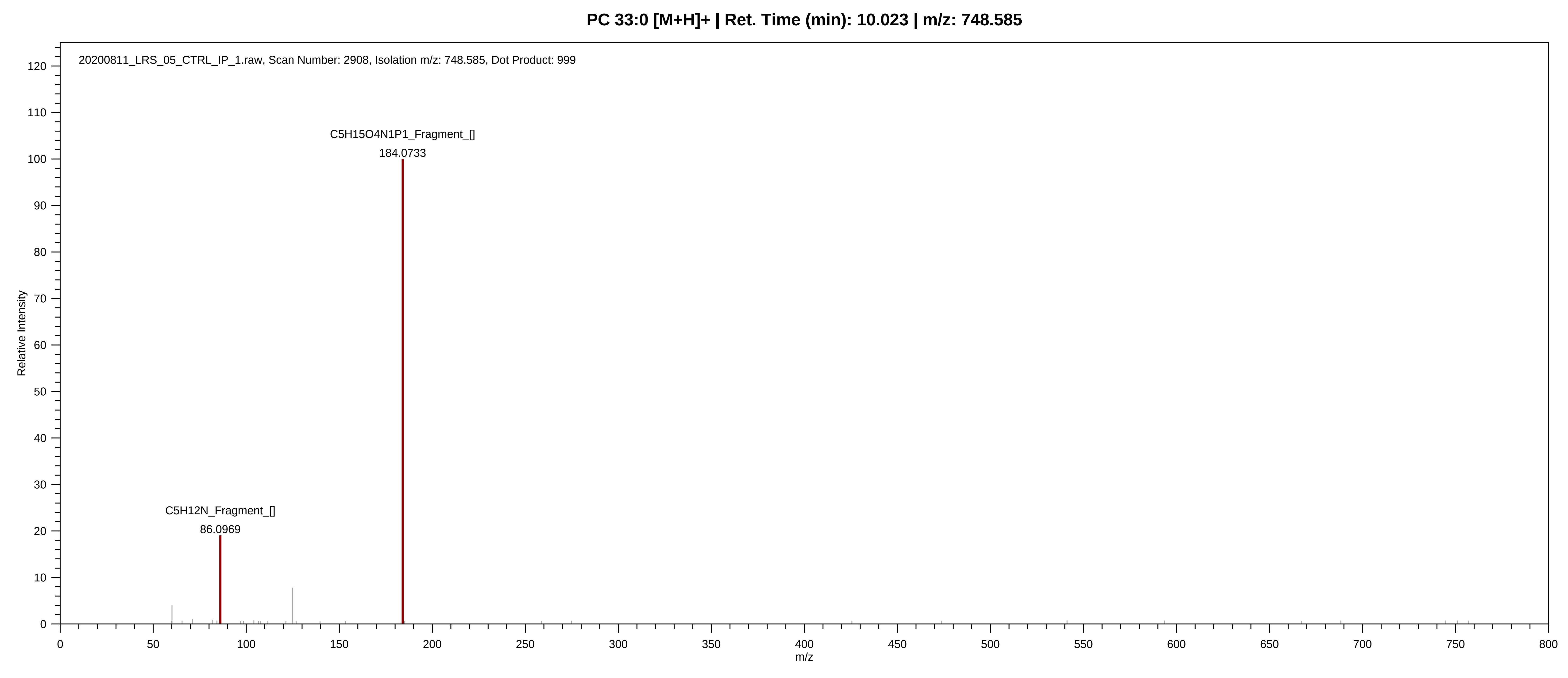
PC 33:0 [M+H]+ | Ret. Time (min): 10.023 | m/z: 748.585
20200811_LRS_05_CTRL_IP_1.raw, Scan Number: 2908, Isolation m/z: 748.585, Dot Product: 999
C5H15O4N1P1_Fragment_[]
184.0733
C5H12N_Fragment_[]
86.0969
Relative Intensity
m/z
0
10
20
30
40
50
60
70
80
90
100
110
120
0
50
100
150
200
250
300
350
400
450
500
550
600
650
700
750
800

PC 33:0 [M+H]+ | Ret. Time (min): 10.033 | m/z: 748.585

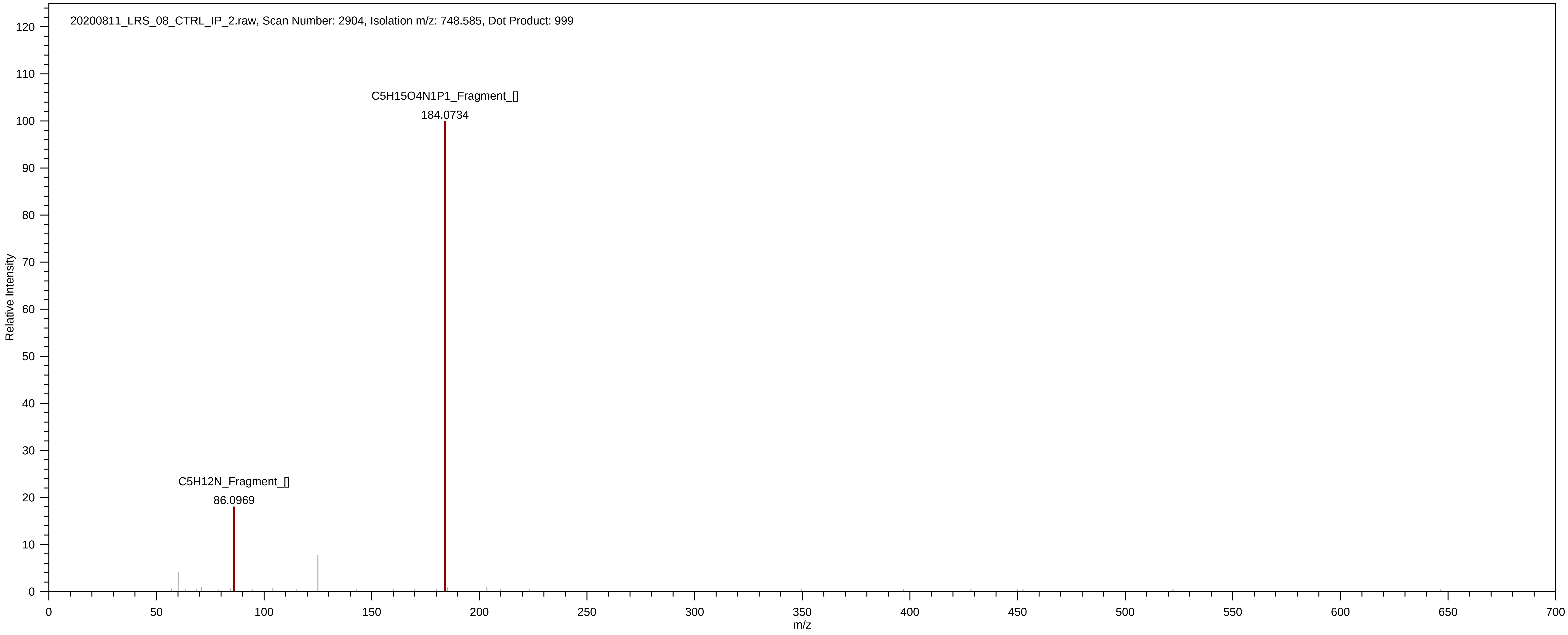

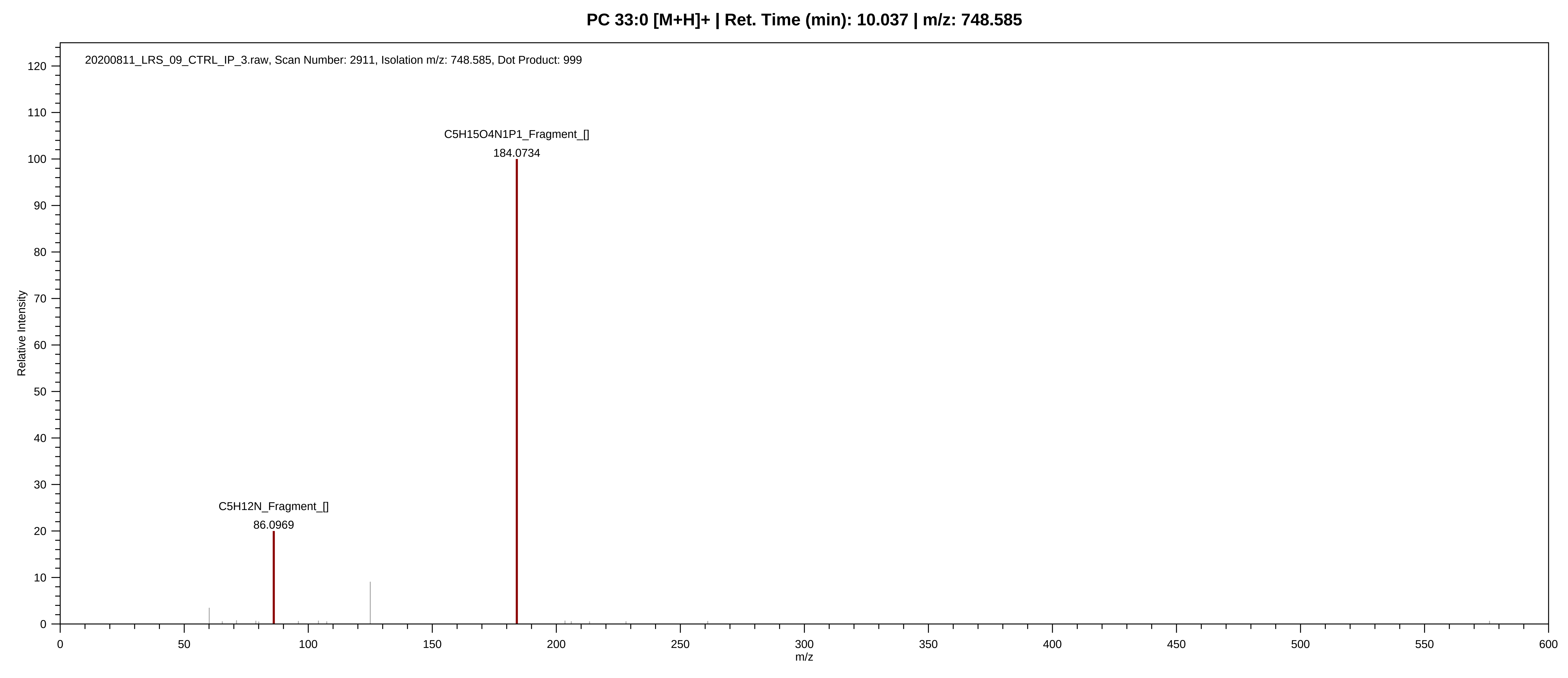

PC 33:0 [M+H]+ | Ret. Time (min): 10.037 | m/z: 748.585
20200811_LRS_09_CTRL_IP_3.raw, Scan Number: 2911, Isolation m/z: 748.585, Dot Product: 999
C5H15O4N1P1_Fragment_[]
184.0734
C5H12N_Fragment_[]
86.0969
Relative Intensity
m/z

PC 33:0 [M+H]+ | Ret. Time (min): 10.203 | m/z: 748.585

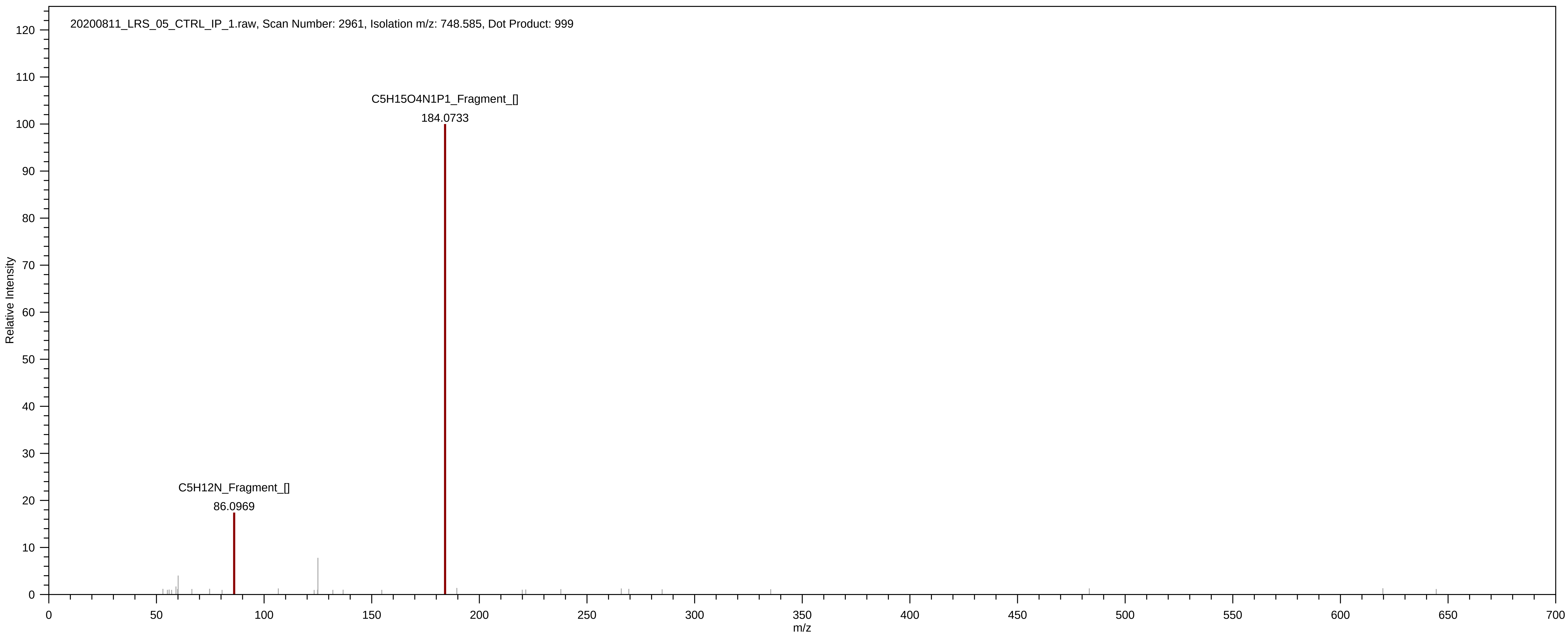

PC 33:0 [M+H]+ | Ret. Time (min): 10.217 | m/z: 748.585

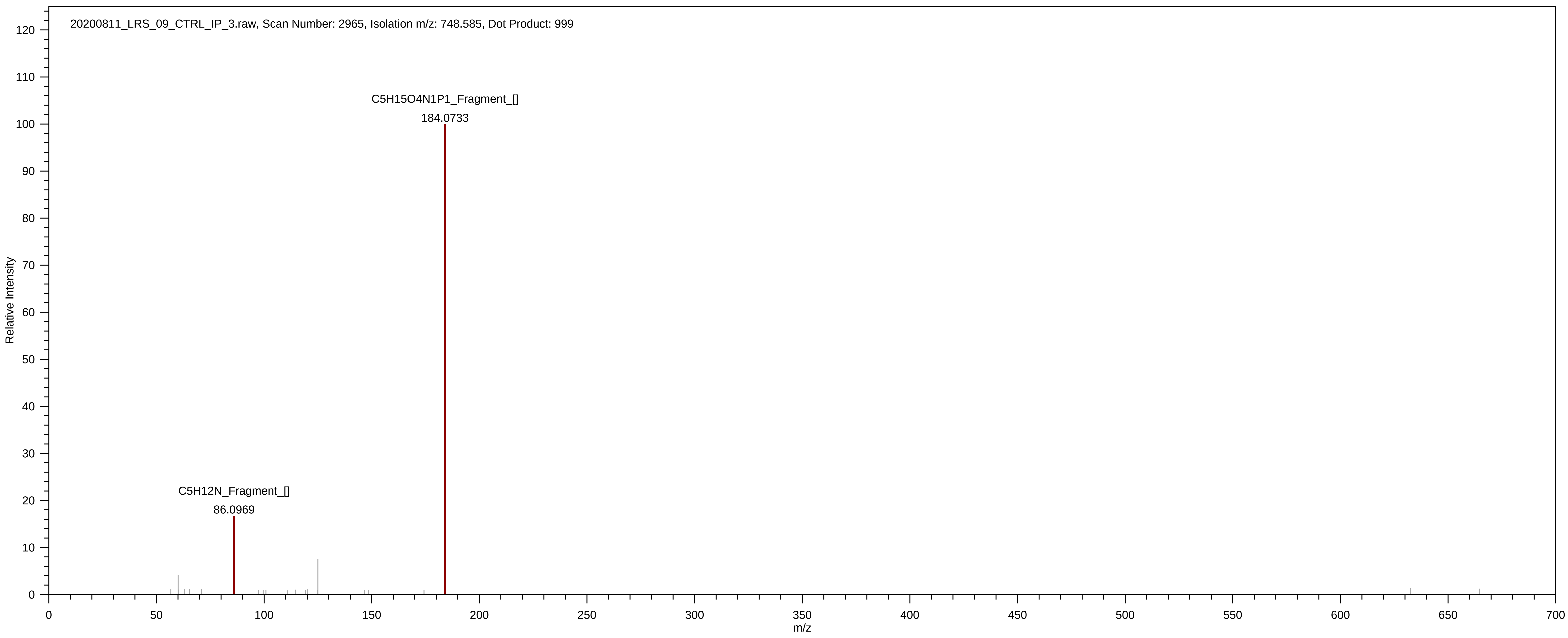

PC 33:0 [M+H]+ | Ret. Time (min): 10.054 | m/z: 748.586

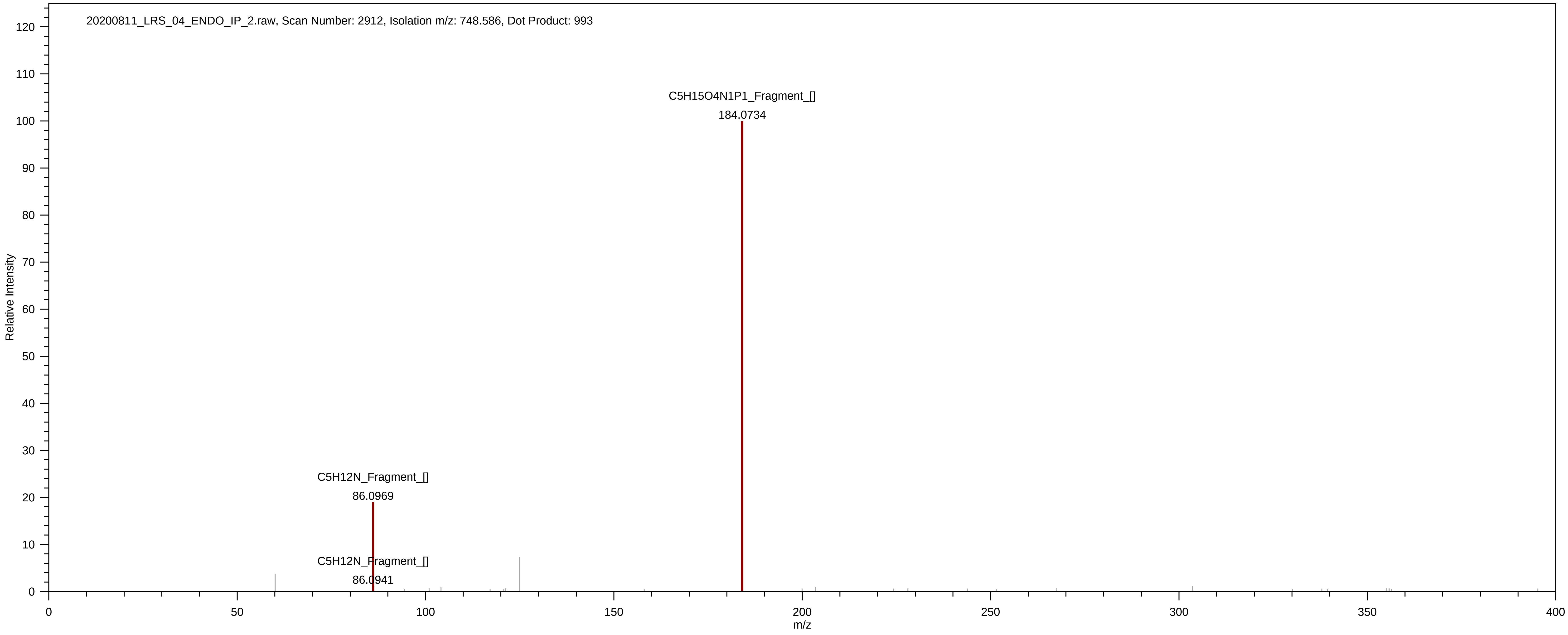

PC 33:0 [M+H]+ | Ret. Time (min): 10.074 | m/z: 748.586

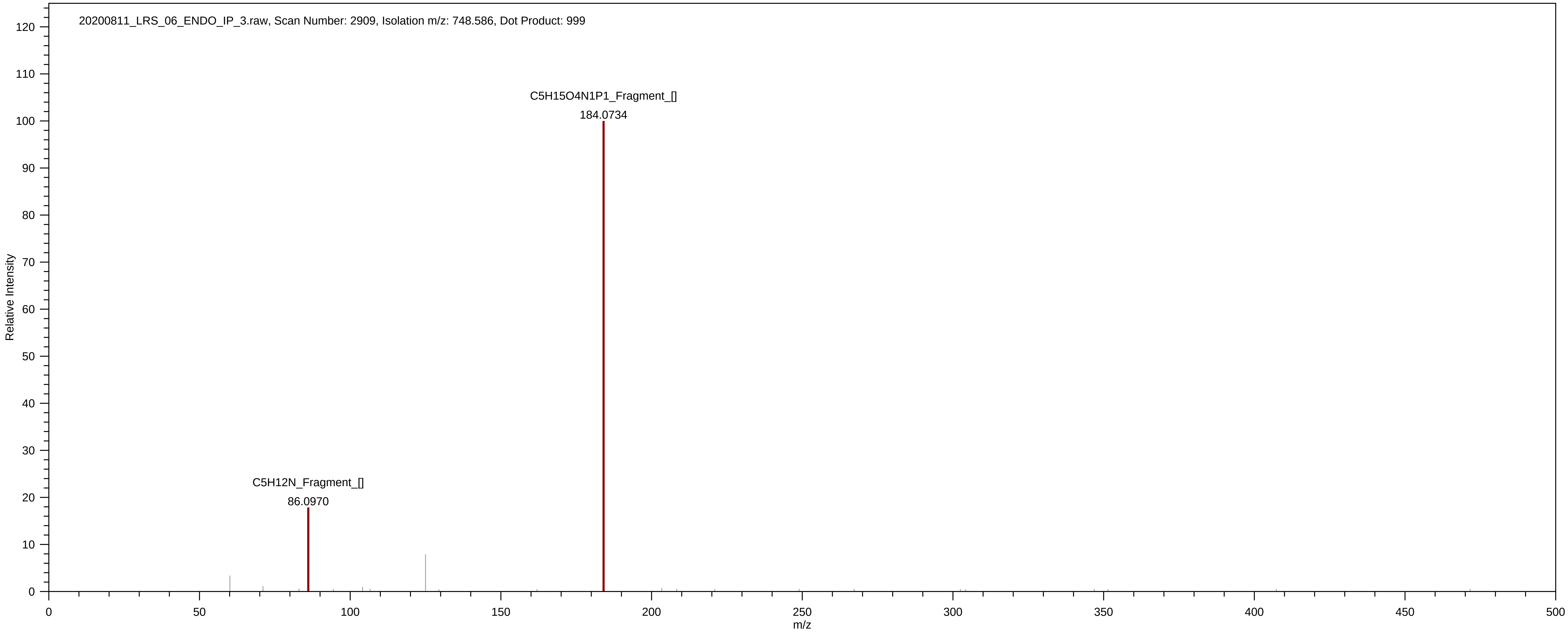

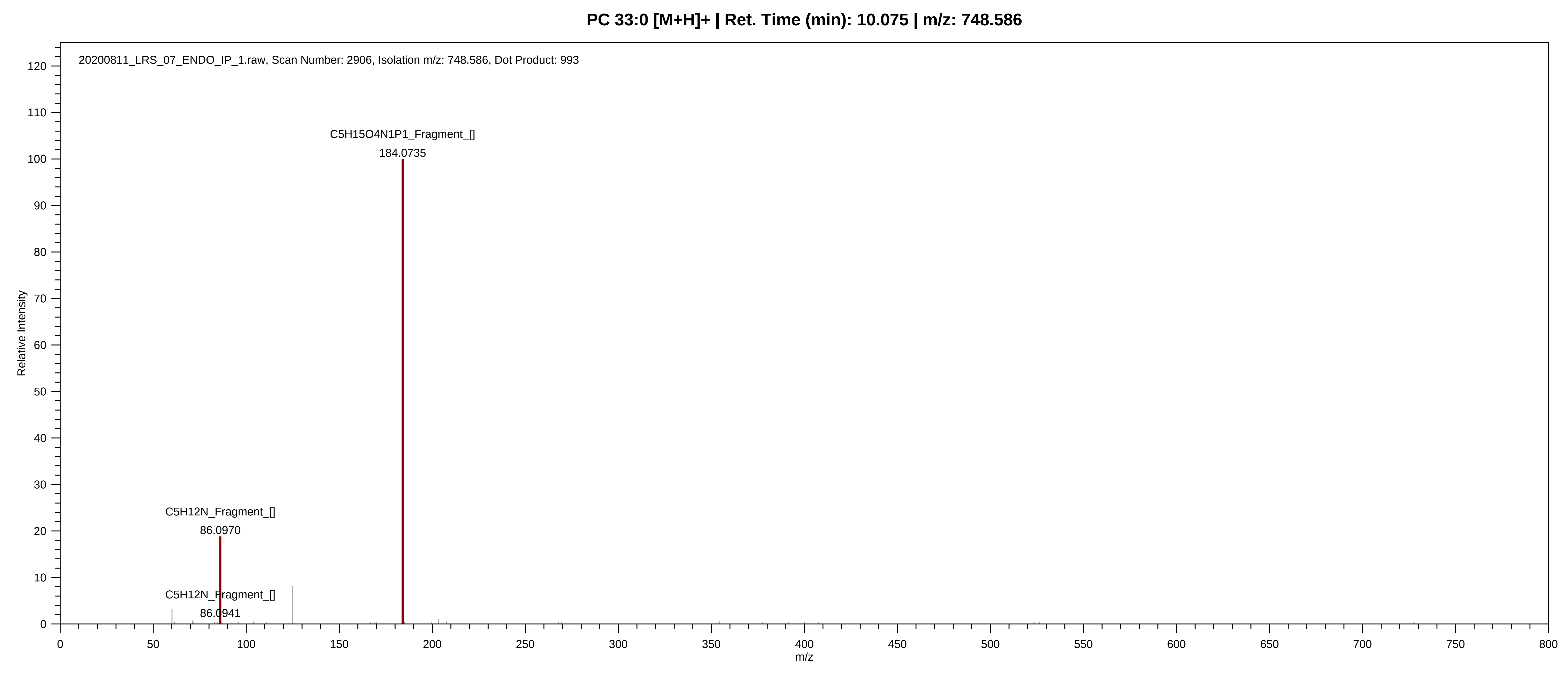
PC 33:0 [M+H]+ | Ret. Time (min): 10.075 | m/z: 748.586
20200811_LRS_07_ENDO_IP_1.raw, Scan Number: 2906, Isolation m/z: 748.586, Dot Product: 993
C5H15O4N1P1_Fragment_[]
184.0735
C5H12N_Fragment_[]
86.0970
C5H12N_Fragment_[]
86.0941
Relative Intensity
m/z

PC 33:0 [M+H]+ | Ret. Time (min): 10.215 | m/z: 748.586

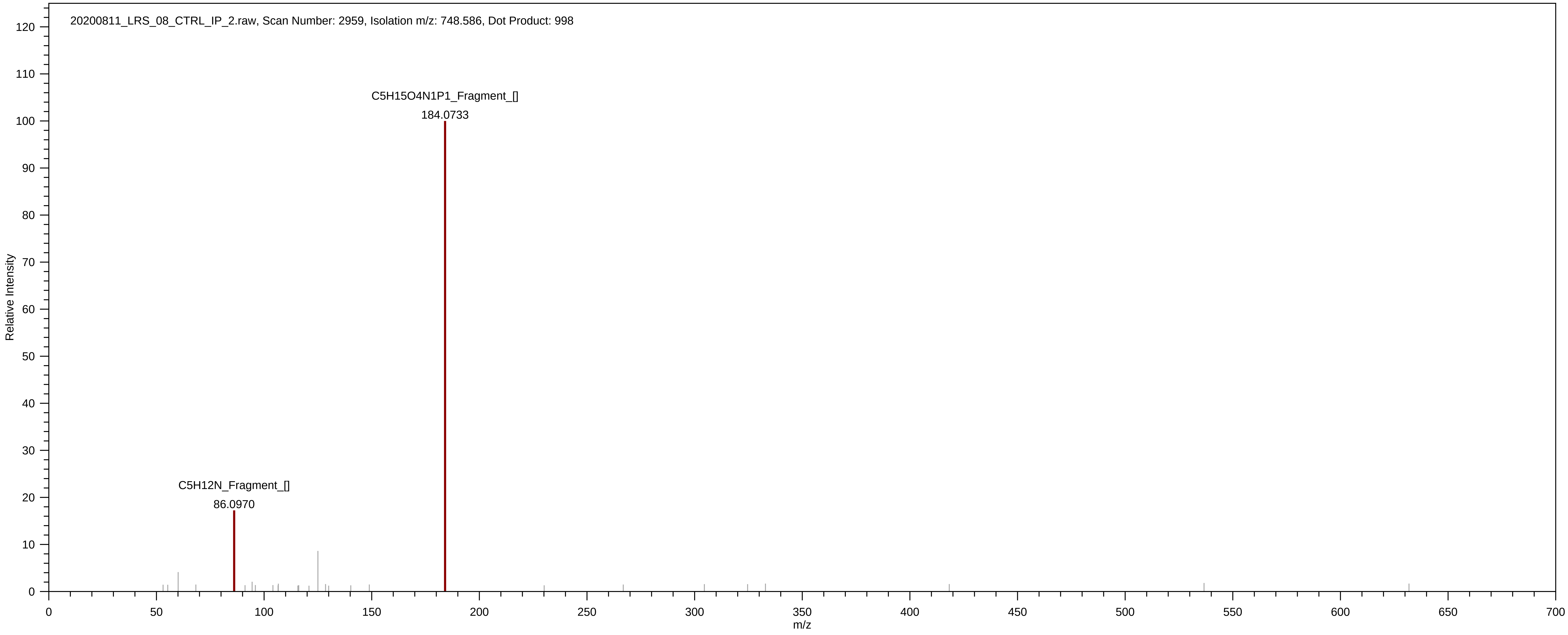

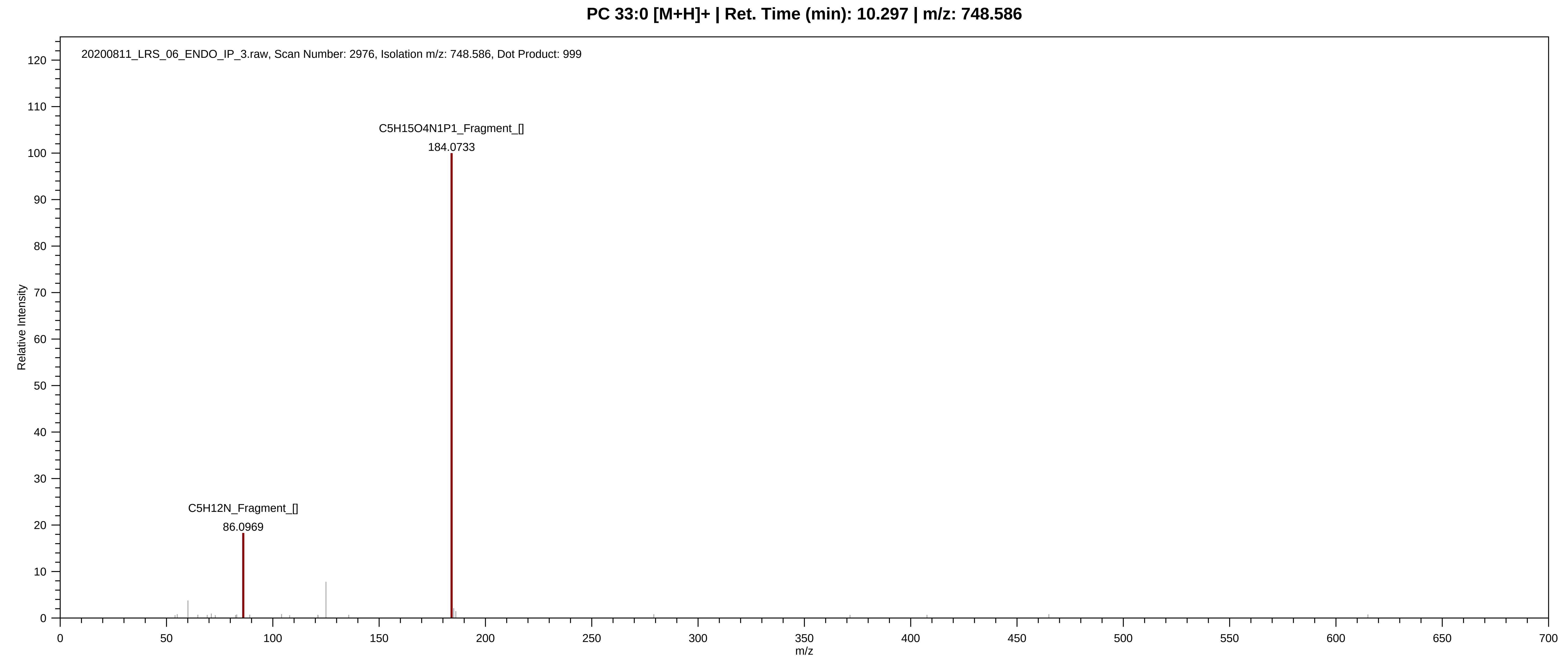

PC 33:0 [M+H]+ | Ret. Time (min): 10.297 | m/z: 748.586
20200811_LRS_06_ENDO_IP_3.raw, Scan Number: 2976, Isolation m/z: 748.586, Dot Product: 999
C5H15O4N1P1_Fragment_[]
184.0733
C5H12N_Fragment_[]
86.0969
Relative Intensity
m/z

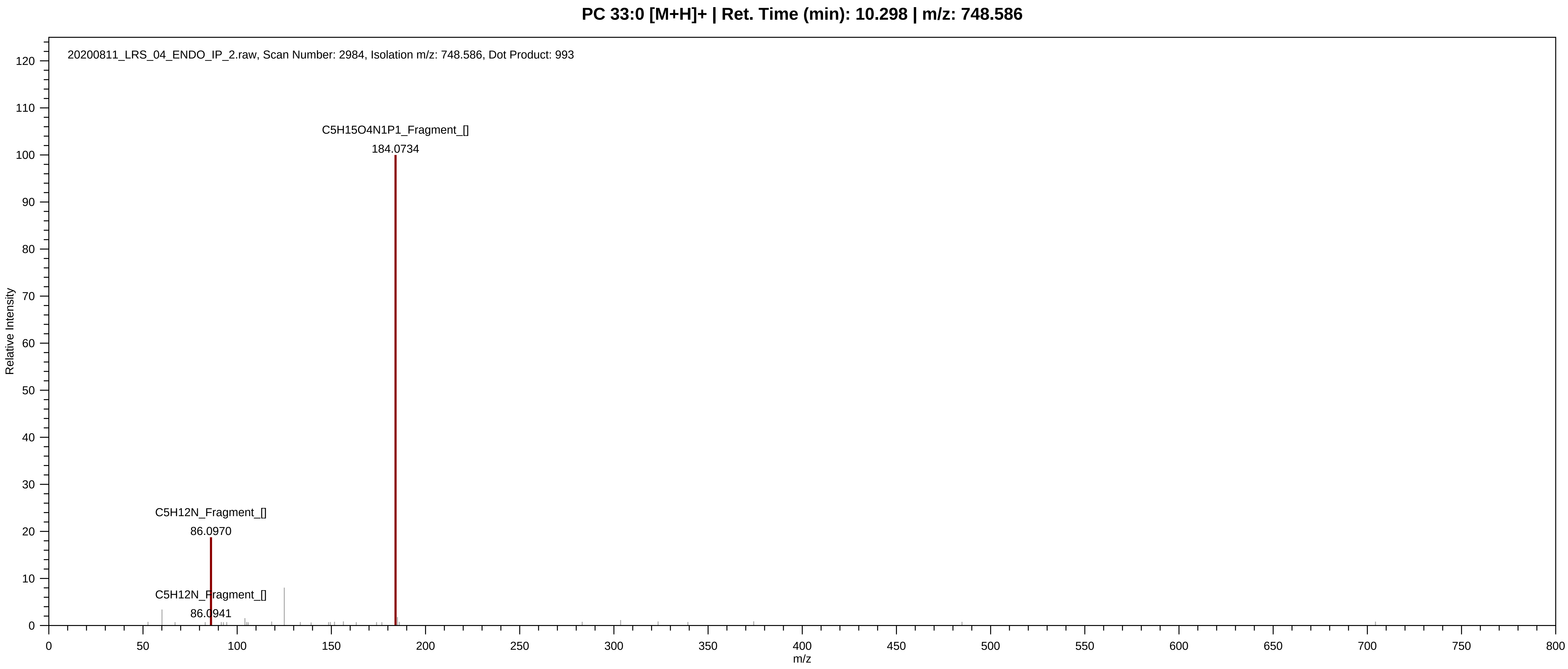

PC 33:0 [M+H]+ | Ret. Time (min): 10.298 | m/z: 748.586
20200811_LRS_04_ENDO_IP_2.raw, Scan Number: 2984, Isolation m/z: 748.586, Dot Product: 993
C5H15O4N1P1_Fragment_[]
184.0734
C5H12N_Fragment_[]
86.0970
C5H12N_Fragment_[]
86.0941
Relative Intensity
m/z

PC 33:1 [M+Ac-H]- | Ret. Time (min): 9.369 | m/z: 804.576

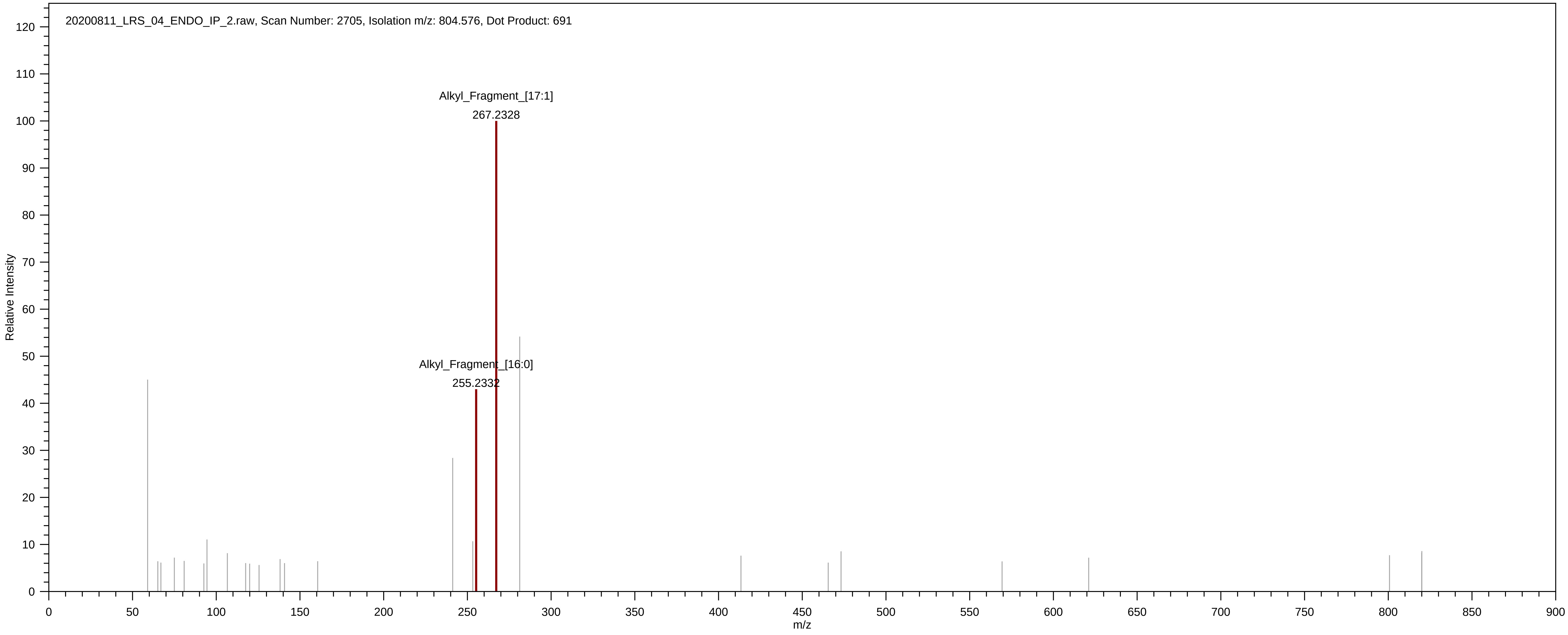

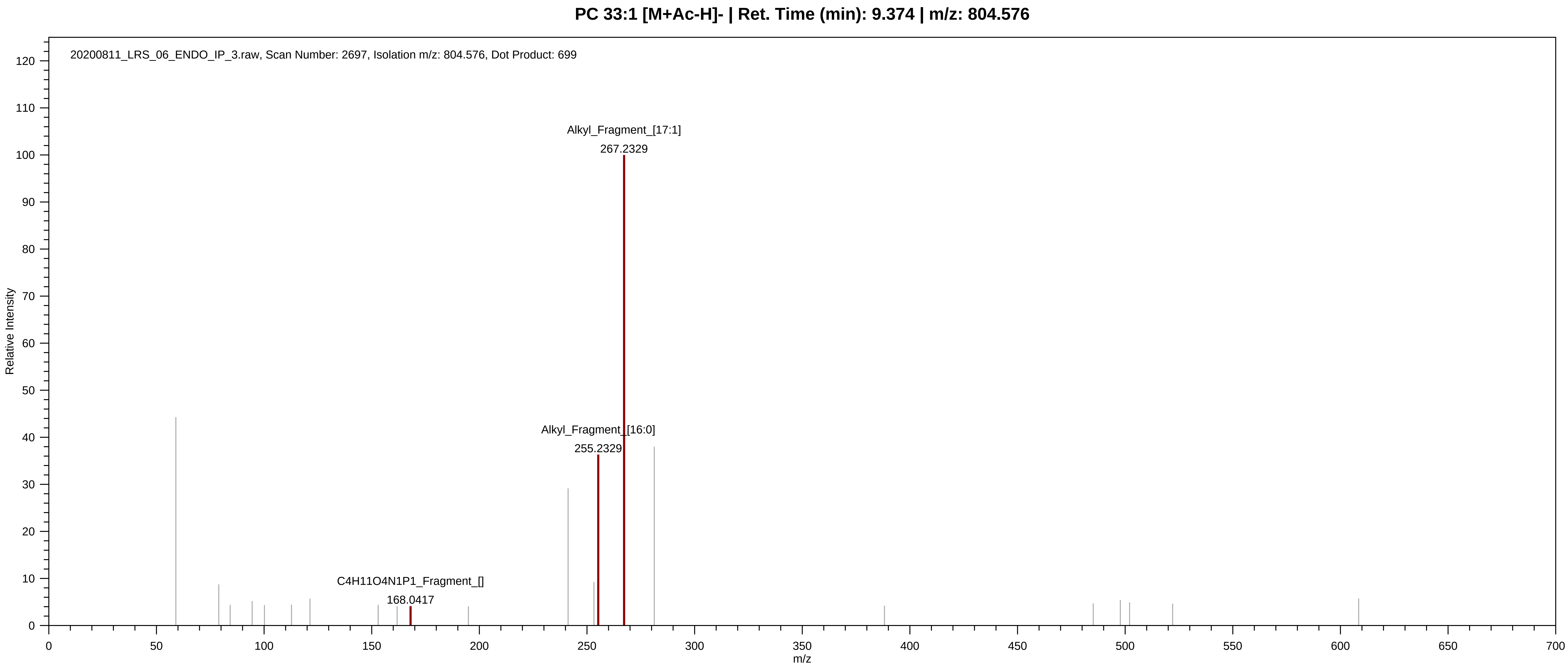
PC 33:1 [M+Ac-H]- | Ret. Time (min): 9.374 | m/z: 804.576
20200811_LRS_06_ENDO_IP_3.raw, Scan Number: 2697, Isolation m/z: 804.576, Dot Product: 699
Alkyl_Fragment_[17:1]
267.2329
Alkyl_Fragment_[16:0]
255.2329
C4H11O4N1P1_Fragment_[]
168.0417
Relative Intensity
m/z

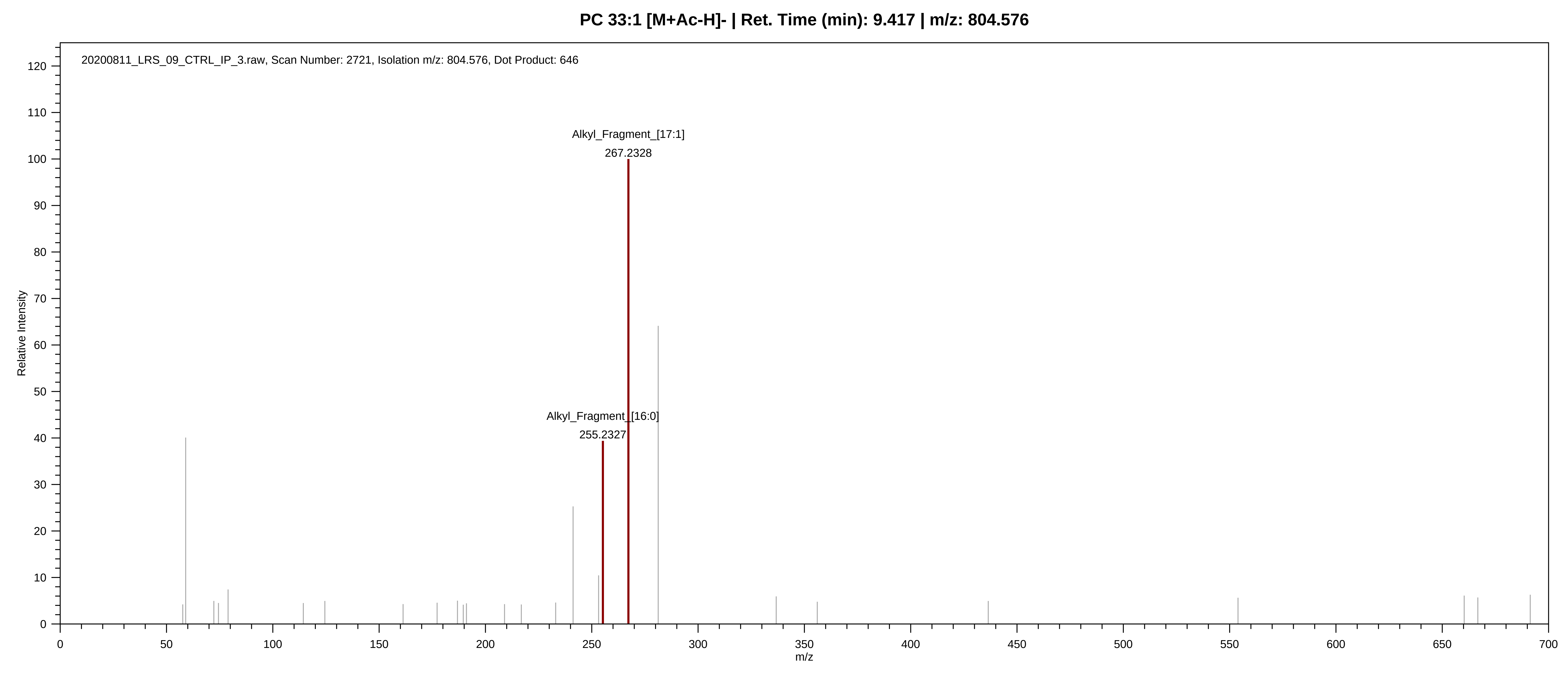
PC 33:1 [M+Ac-H]- | Ret. Time (min): 9.417 | m/z: 804.576
20200811_LRS_09_CTRL_IP_3.raw, Scan Number: 2721, Isolation m/z: 804.576, Dot Product: 646
Alkyl_Fragment_[17:1]
267.2328
Alkyl_Fragment_[16:0]
255.2327
Relative Intensity
m/z

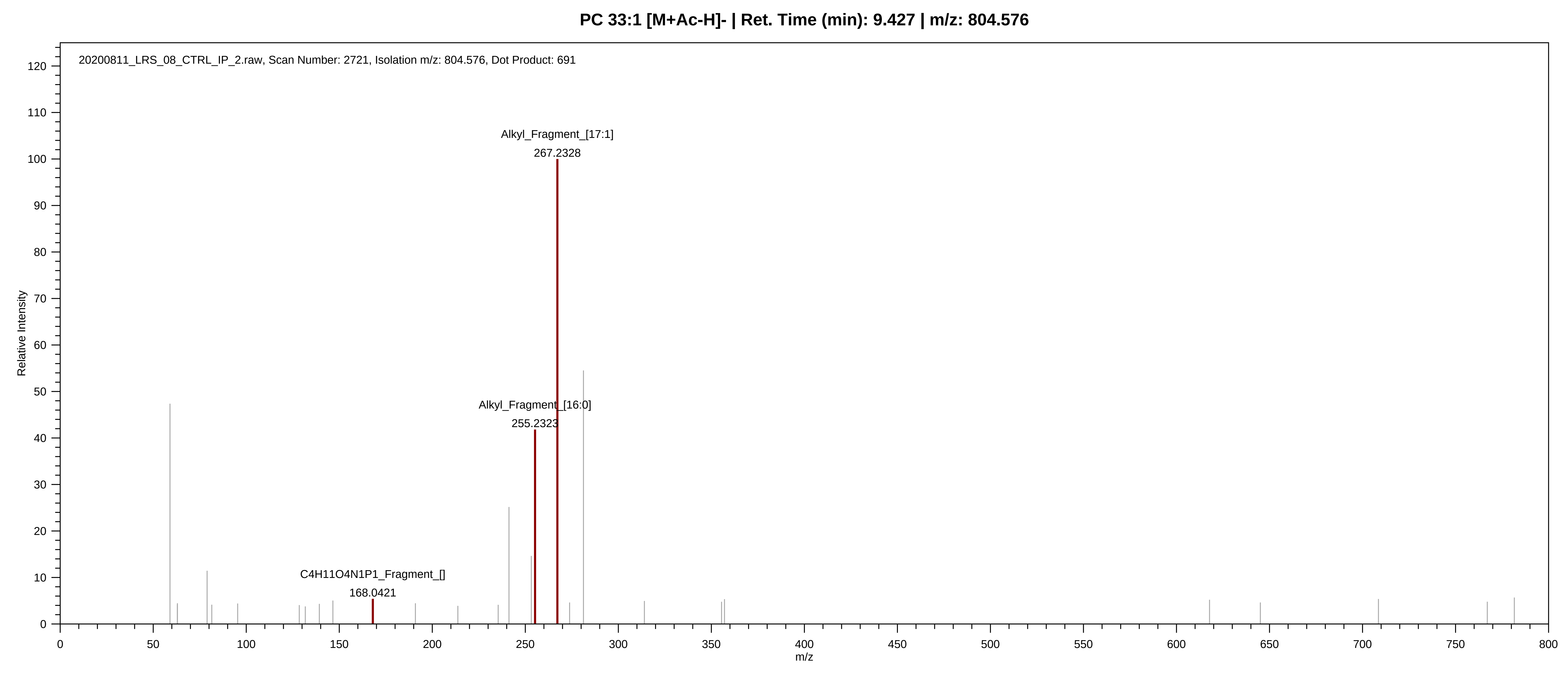
PC 33:1 [M+Ac-H]- | Ret. Time (min): 9.427 | m/z: 804.576
20200811_LRS_08_CTRL_IP_2.raw, Scan Number: 2721, Isolation m/z: 804.576, Dot Product: 691
Alkyl_Fragment_[17:1]
267.2328
Alkyl_Fragment_[16:0]
255.2323
C4H11O4N1P1_Fragment_[]
168.0421
Relative Intensity
m/z

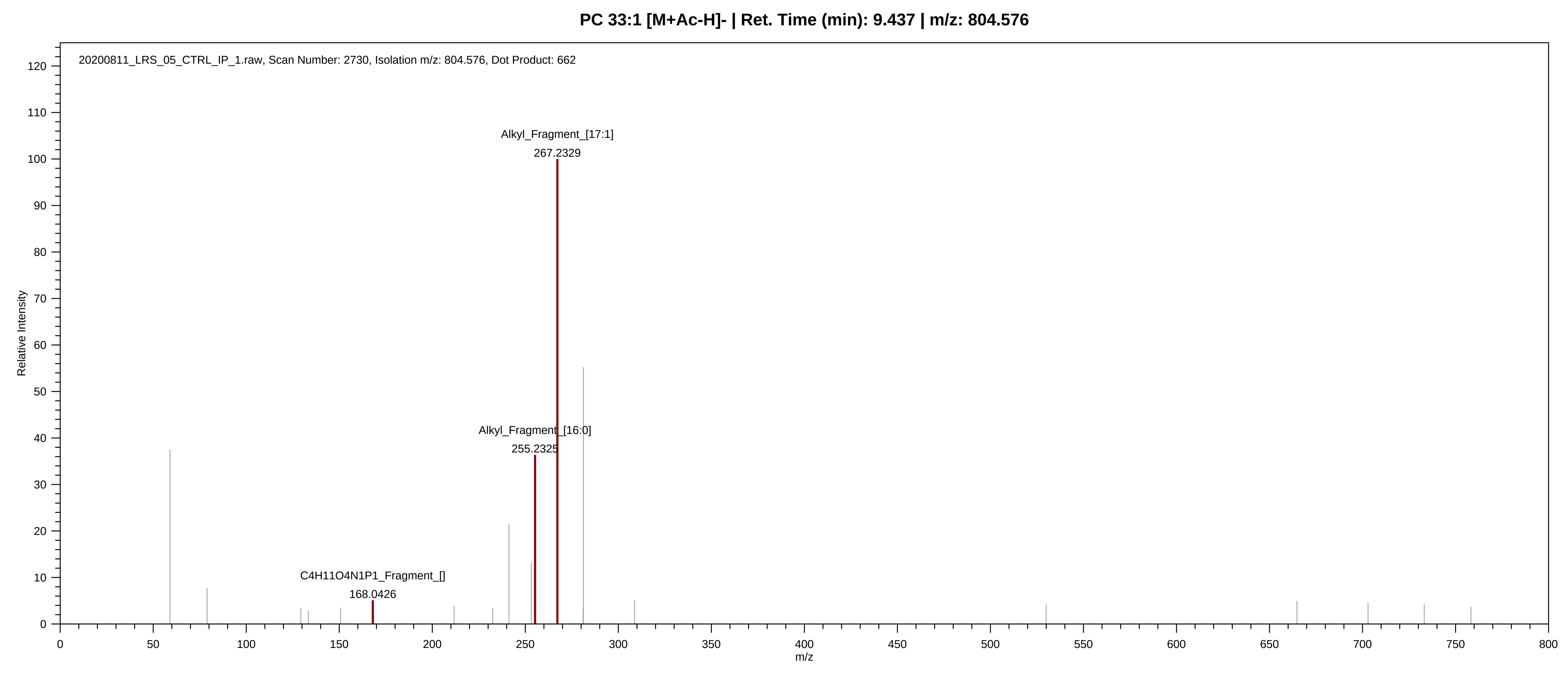

PC 33:1 [M+Ac-H]- | Ret. Time (min): 9.437 | m/z: 804.576
20200811_LRS_05_CTRL_IP_1.raw, Scan Number: 2730, Isolation m/z: 804.576, Dot Product: 662
Alkyl_Fragment_[17:1]
267.2329
Alkyl_Fragment_[16:0]
255.2325
C4H11O4N1P1_Fragment_[]
168.0426
Relative Intensity
m/z

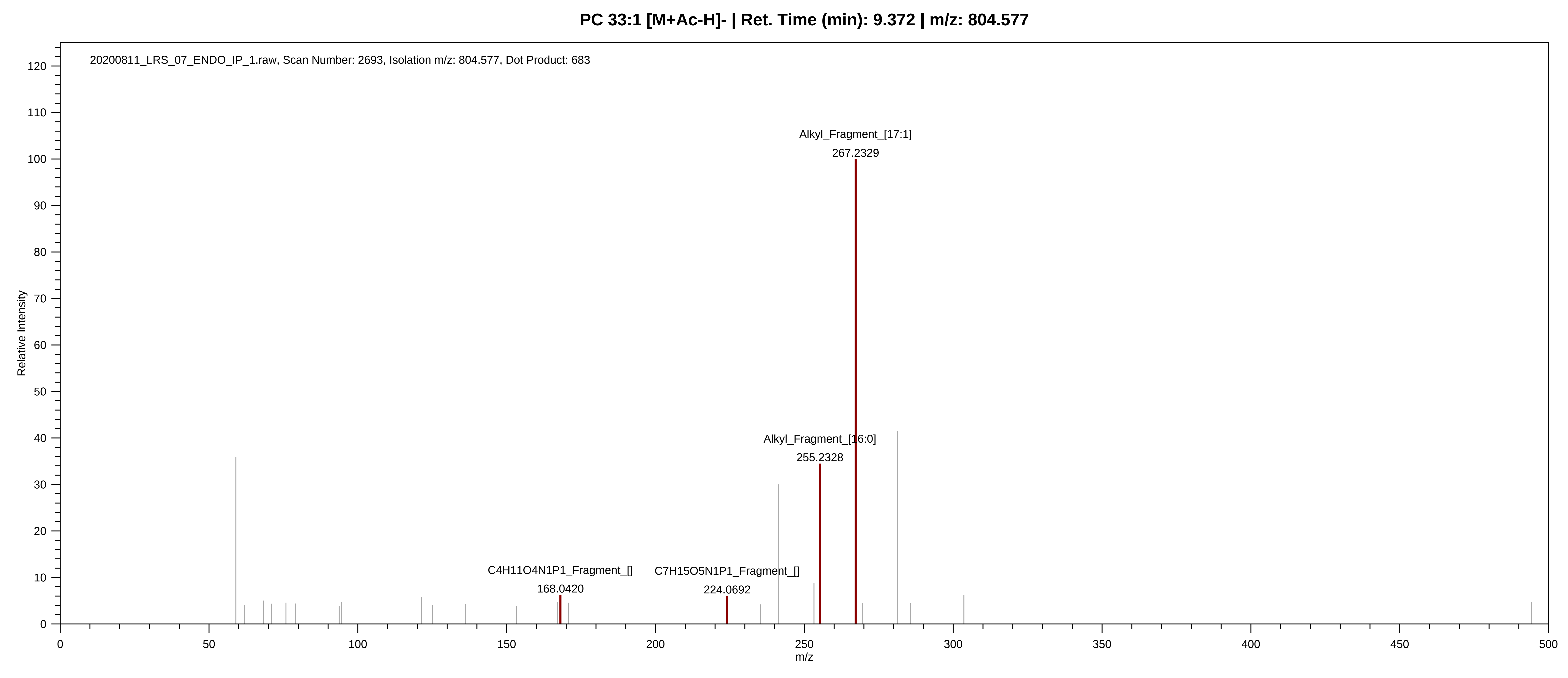

PC 33:1 [M+Ac-H]- | Ret. Time (min): 9.372 | m/z: 804.577
20200811_LRS_07_ENDO_IP_1.raw, Scan Number: 2693, Isolation m/z: 804.577, Dot Product: 683
Alkyl_Fragment_[17:1]
267.2329
Alkyl_Fragment_[16:0]
255.2328
C4H11O4N1P1_Fragment_[]
168.0420
C7H15O5N1P1_Fragment_[]
224.0692
Relative Intensity
m/z

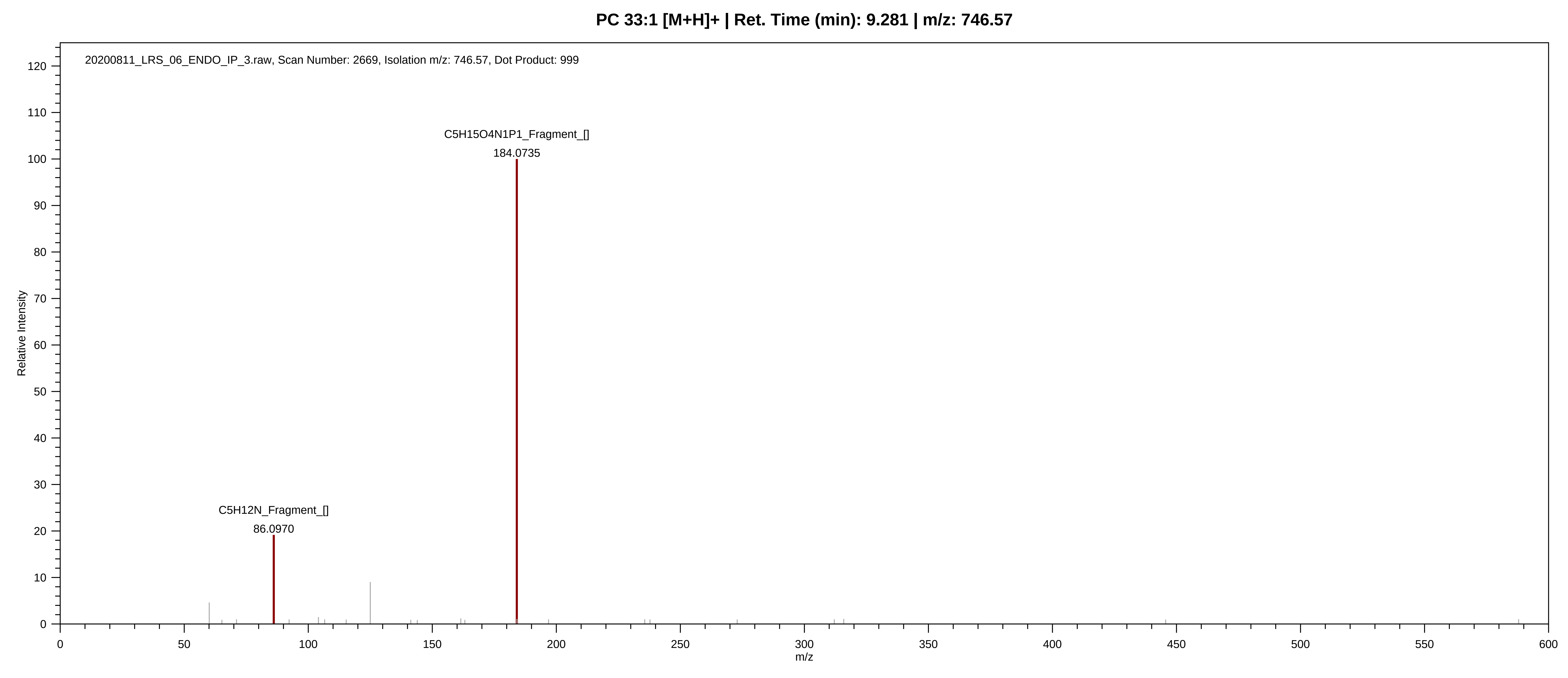

PC 33:1 [M+H]+ | Ret. Time (min): 9.281 | m/z: 746.57
20200811_LRS_06_ENDO_IP_3.raw, Scan Number: 2669, Isolation m/z: 746.57, Dot Product: 999
C5H15O4N1P1_Fragment_[]
184.0735
C5H12N_Fragment_[]
86.0970
Relative Intensity
m/z

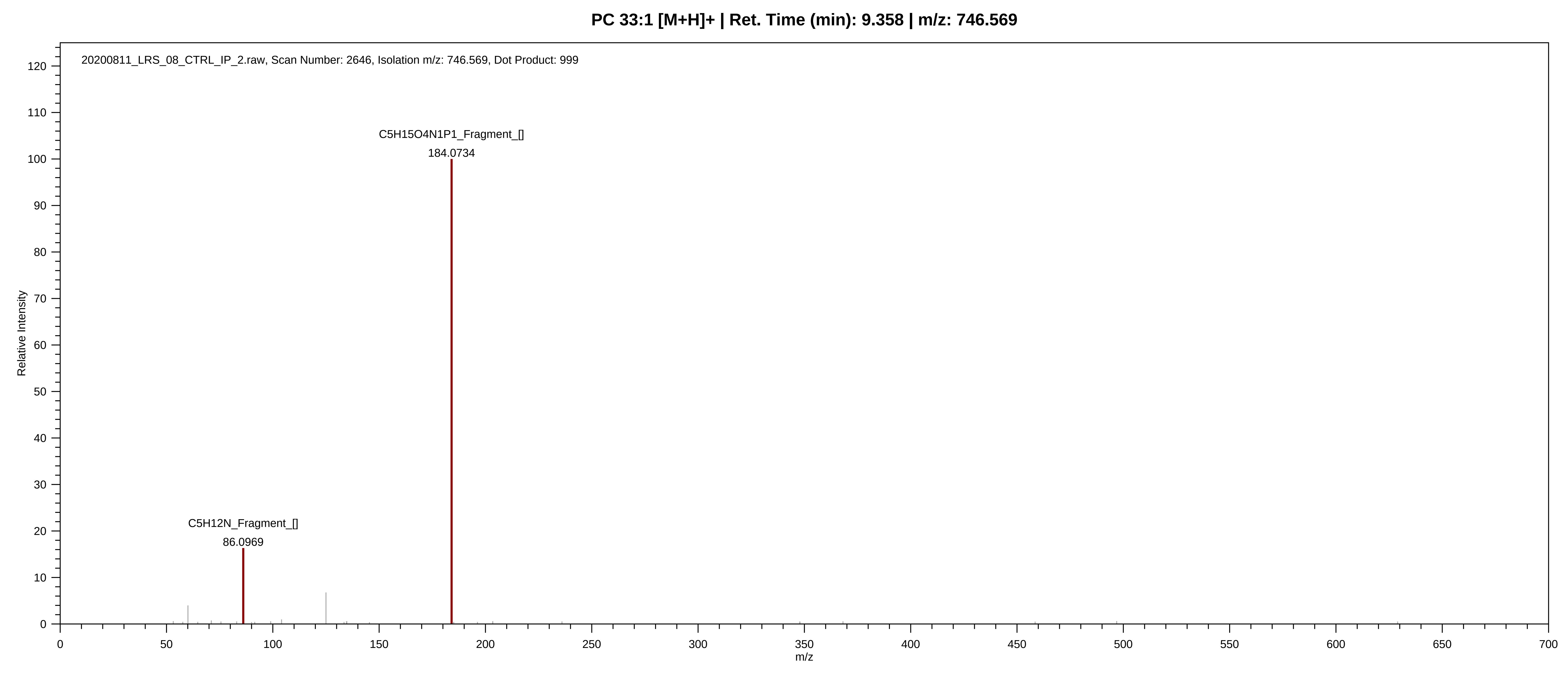
PC 33:1 [M+H]+ | Ret. Time (min): 9.358 | m/z: 746.569
20200811_LRS_08_CTRL_IP_2.raw, Scan Number: 2646, Isolation m/z: 746.569, Dot Product: 999
C5H15O4N1P1_Fragment_[]
184.0734
C5H12N_Fragment_[]
86.0969
Relative Intensity
m/z

PC 33:1 [M+H]+ | Ret. Time (min): 9.358 | m/z: 746.57

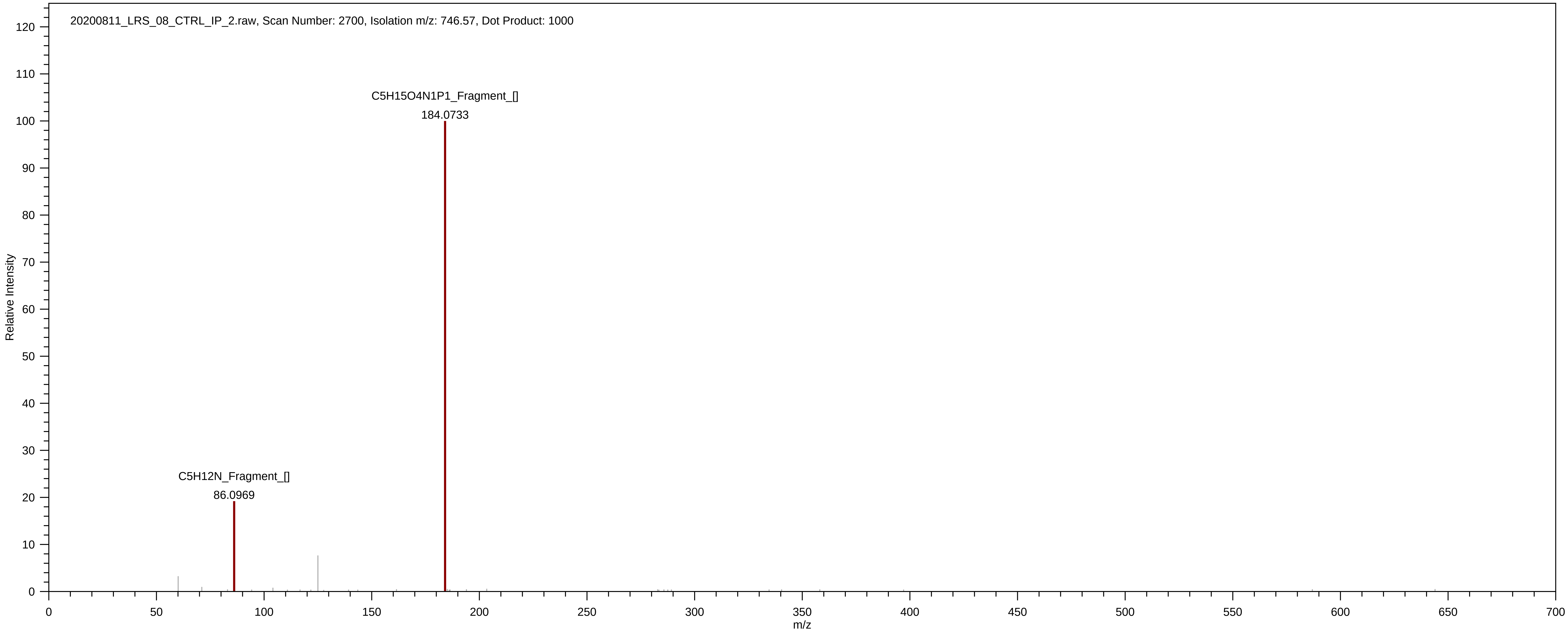

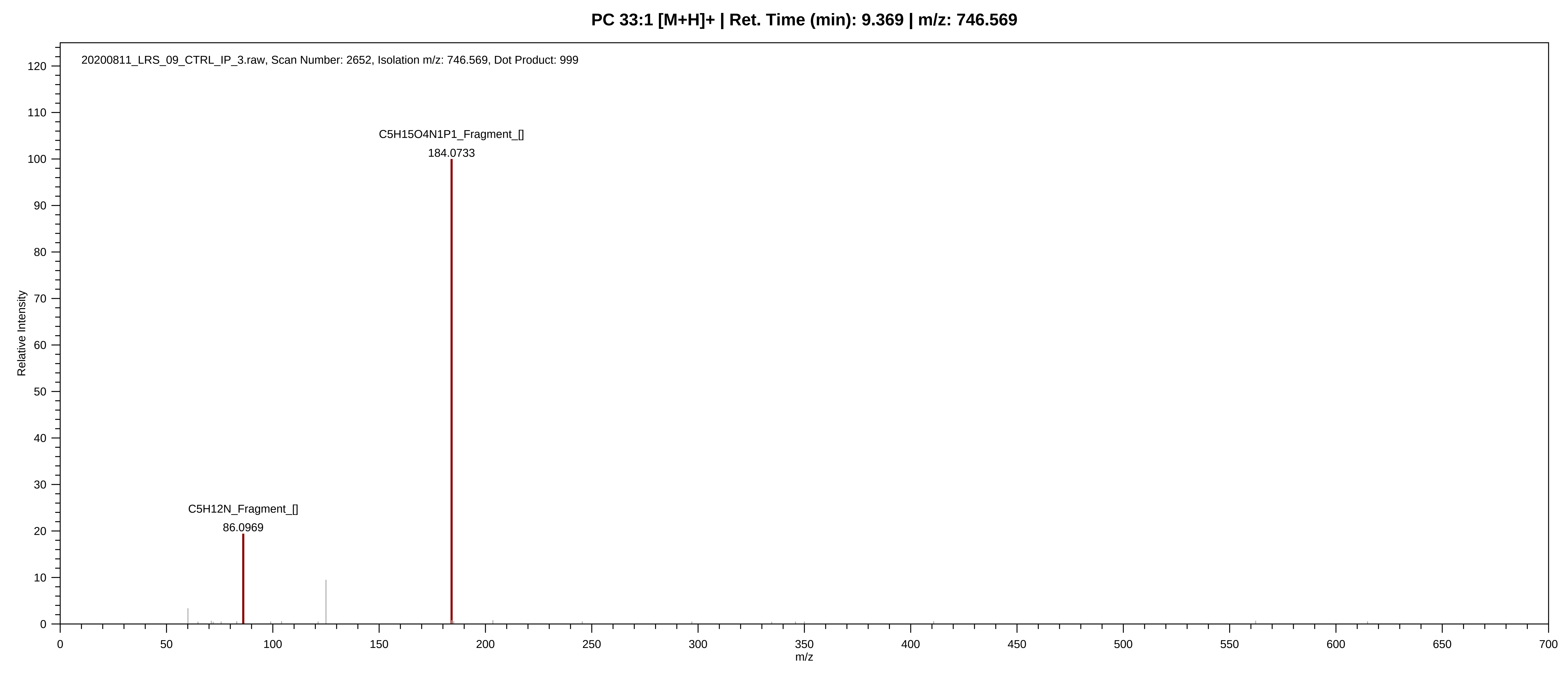
PC 33:1 [M+H]+ | Ret. Time (min): 9.369 | m/z: 746.569
20200811_LRS_09_CTRL_IP_3.raw, Scan Number: 2652, Isolation m/z: 746.569, Dot Product: 999
C5H15O4N1P1_Fragment_[]
184.0733
C5H12N_Fragment_[]
86.0969
Relative Intensity
m/z

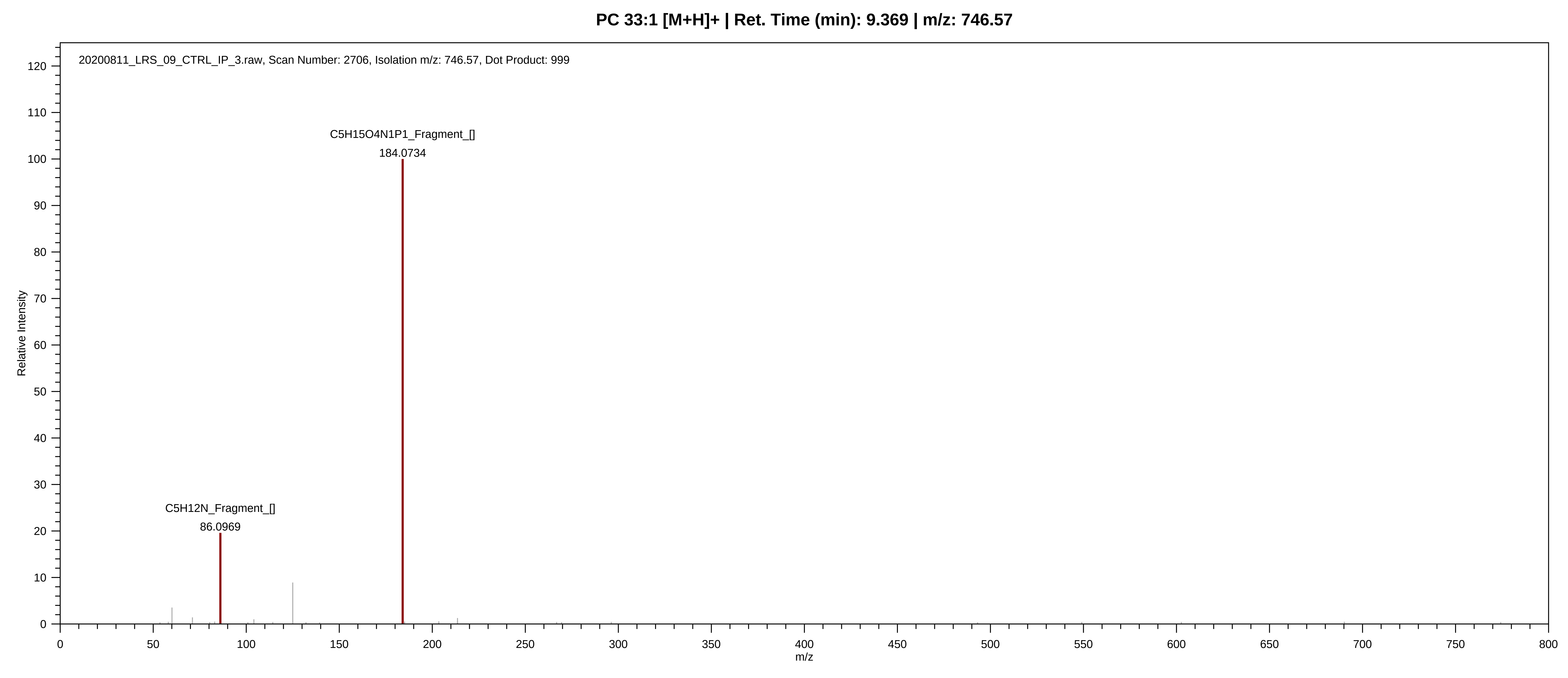

PC 33:1 [M+H]+ | Ret. Time (min): 9.369 | m/z: 746.57
20200811_LRS_09_CTRL_IP_3.raw, Scan Number: 2706, Isolation m/z: 746.57, Dot Product: 999
C5H15O4N1P1_Fragment_[]
184.0734
C5H12N_Fragment_[]
86.0969
Relative Intensity
m/z

PC 33:1 [M+H]+ | Ret. Time (min): 9.276 | m/z: 746.569

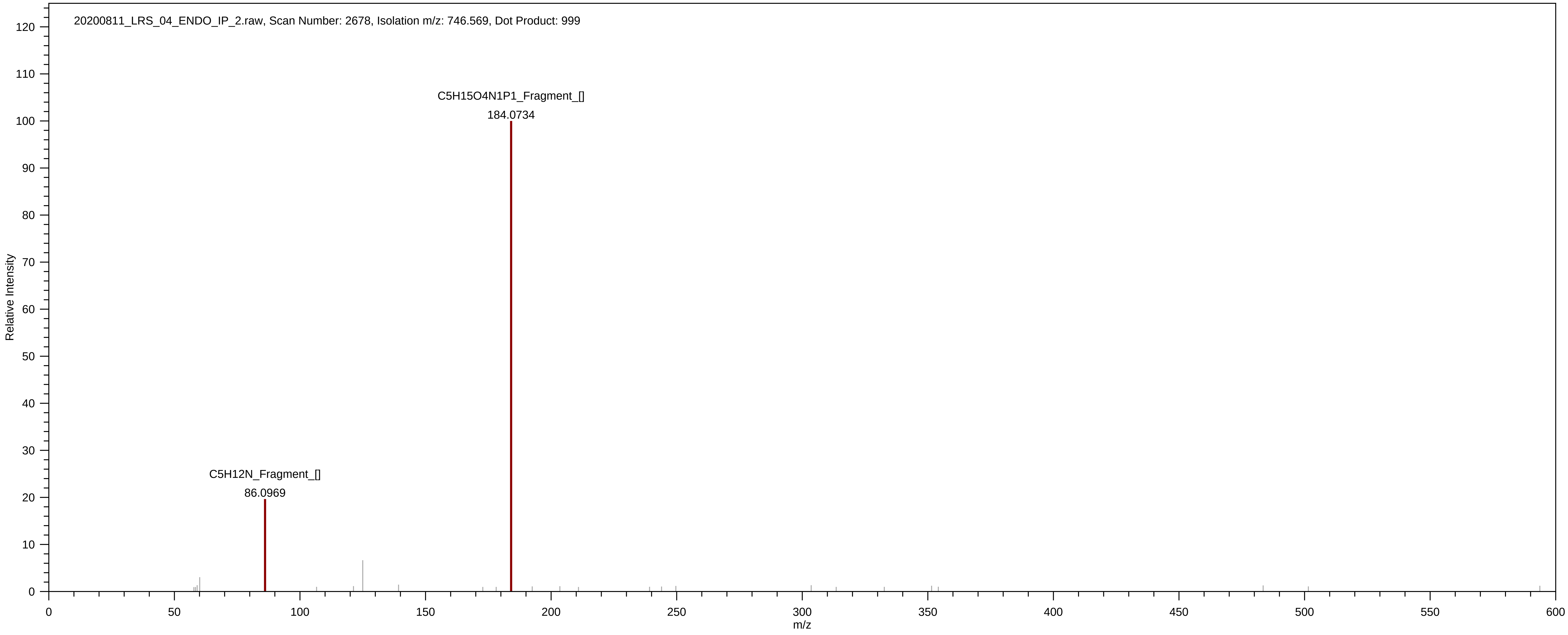

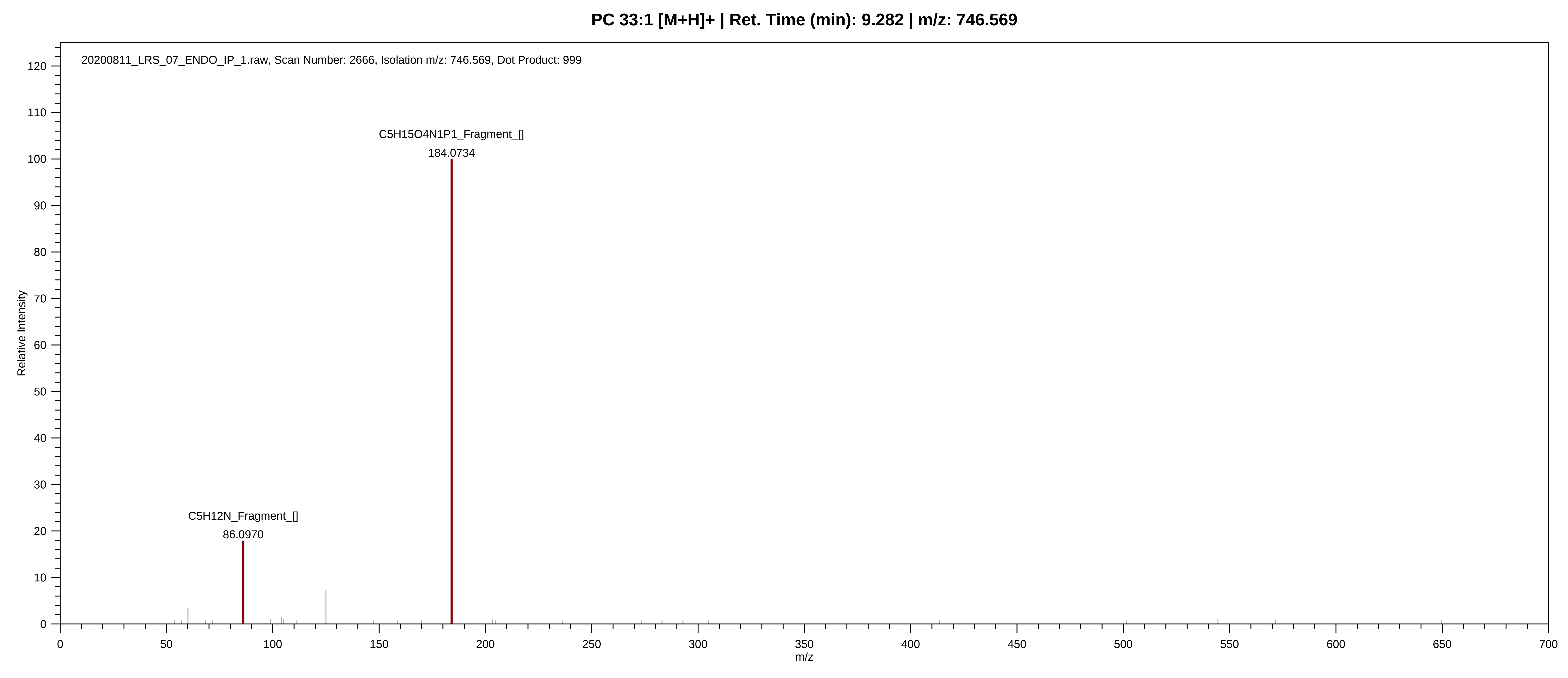
PC 33:1 [M+H]+ | Ret. Time (min): 9.282 | m/z: 746.569
20200811_LRS_07_ENDO_IP_1.raw, Scan Number: 2666, Isolation m/z: 746.569, Dot Product: 999
C5H15O4N1P1_Fragment_[]
184.0734
C5H12N_Fragment_[]
86.0970
Relative Intensity
m/z

PC 33:1 [M+H]+ | Ret. Time (min): 9.366 | m/z: 746.569

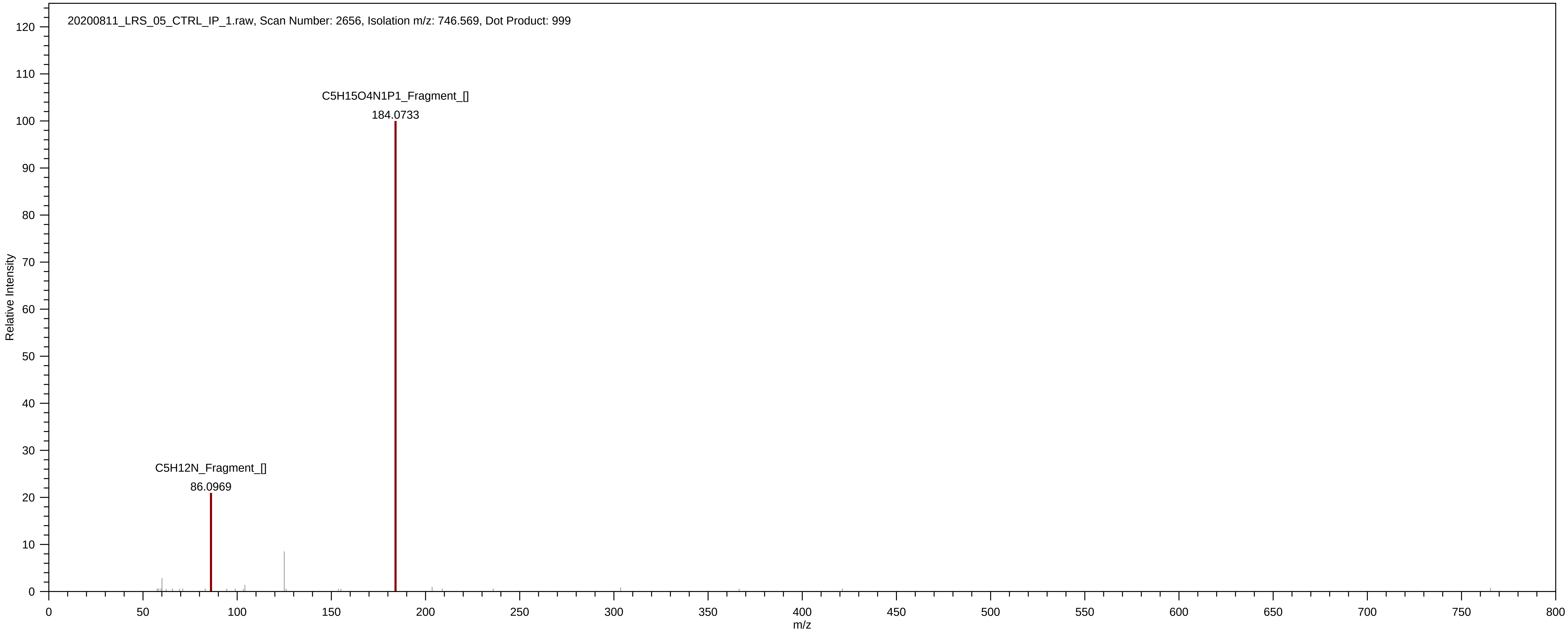

PC 33:1 [M+H]+ | Ret. Time (min): 9.366 | m/z: 746.569

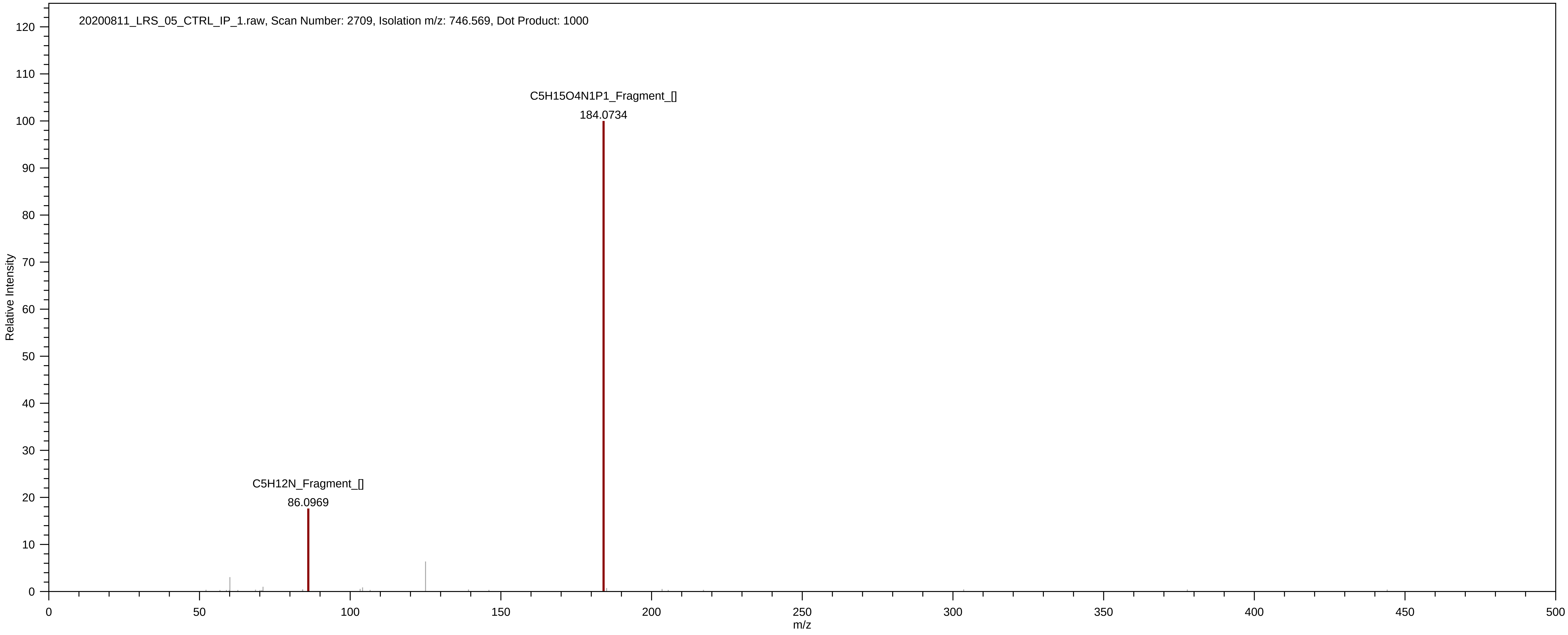

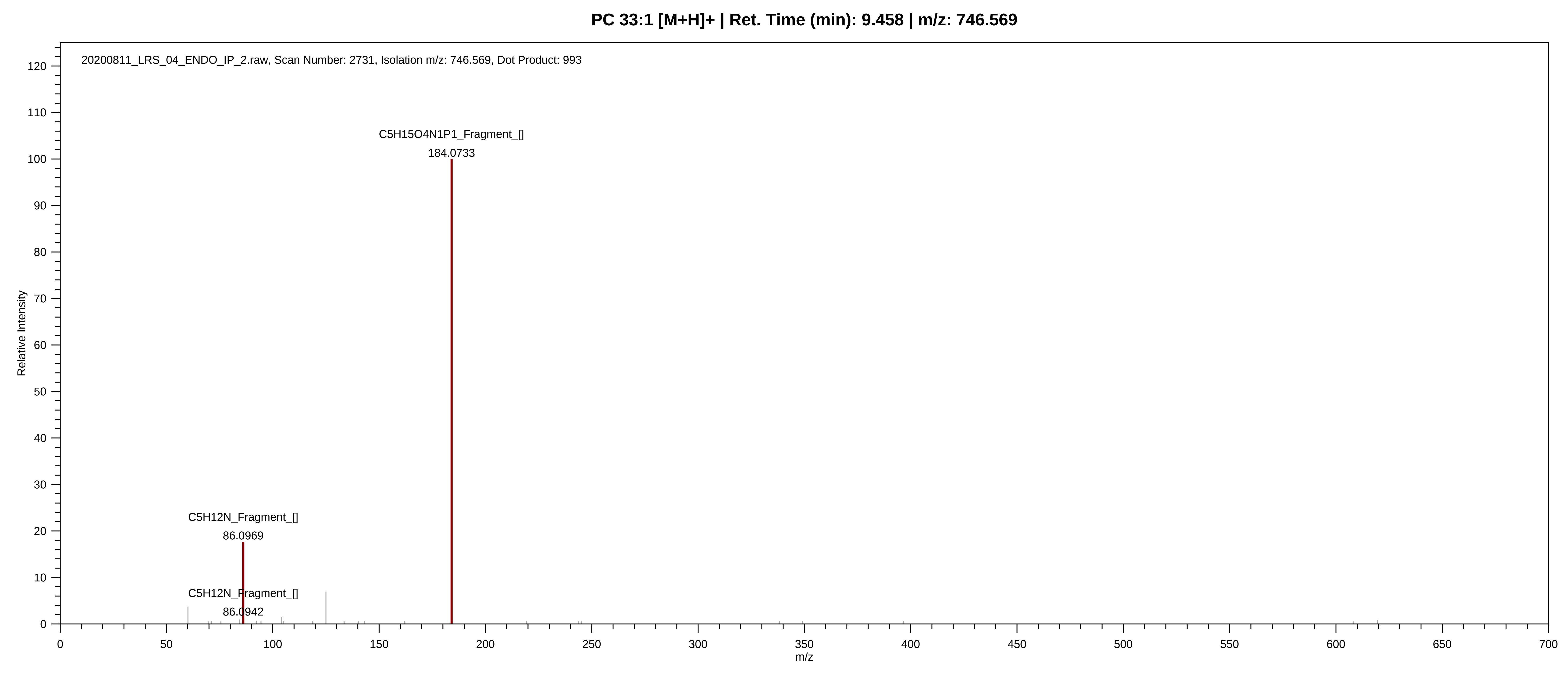
PC 33:1 [M+H]+ | Ret. Time (min): 9.458 | m/z: 746.569
20200811_LRS_04_ENDO_IP_2.raw, Scan Number: 2731, Isolation m/z: 746.569, Dot Product: 993
C5H15O4N1P1_Fragment_[]
184.0733
C5H12N_Fragment_[]
86.0969
C5H12N_Fragment_[]
86.0942
Relative Intensity
m/z

PC 33:1 [M+H]+ | Ret. Time (min): 9.462 | m/z: 746.569

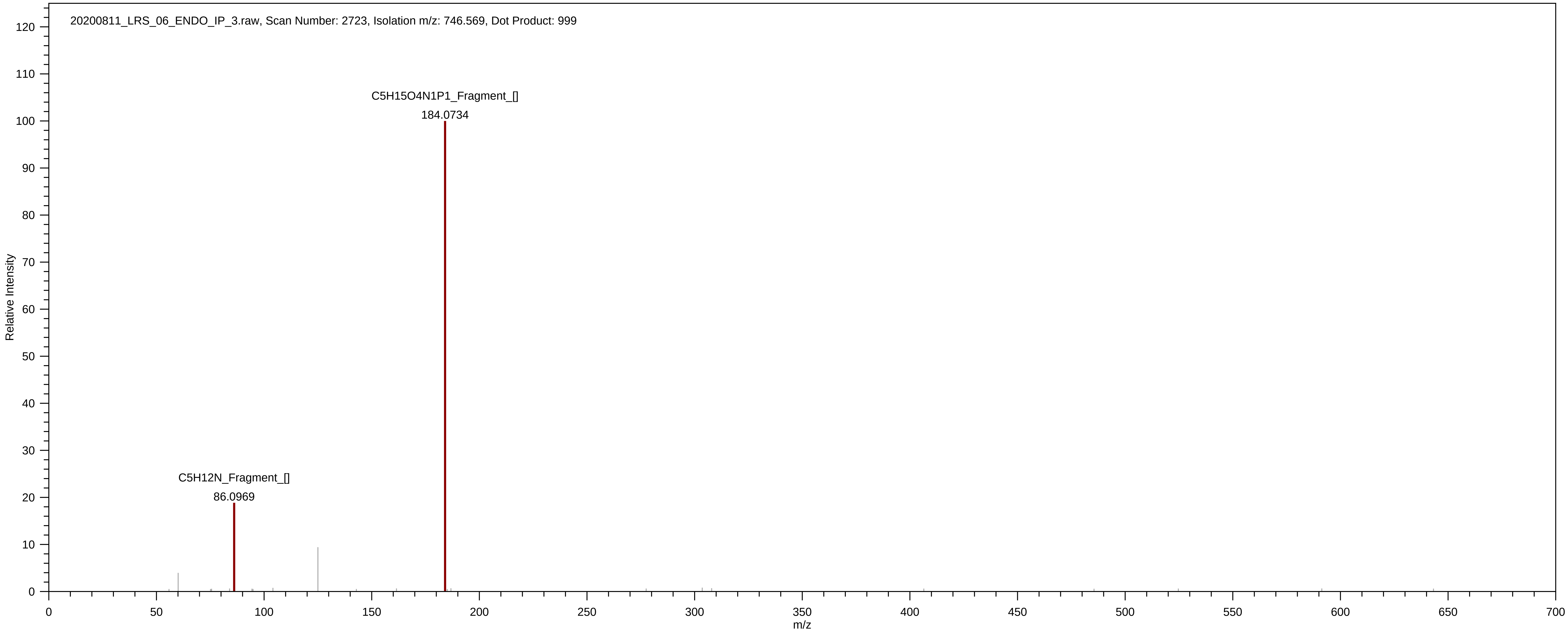

PC 33:1 [M+H]+ | Ret. Time (min): 9.463 | m/z: 746.569

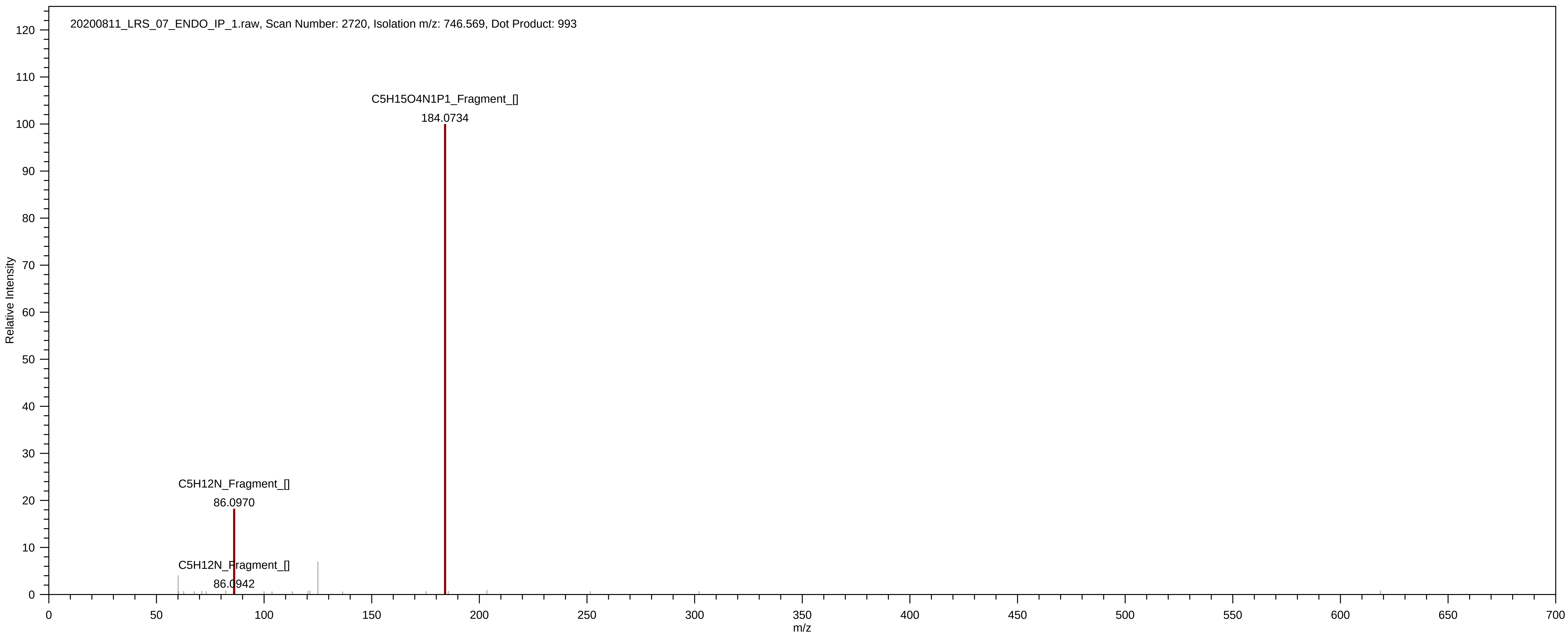

PC 33:2 [M+H]+ | Ret. Time (min): 8.577 | m/z: 744.553

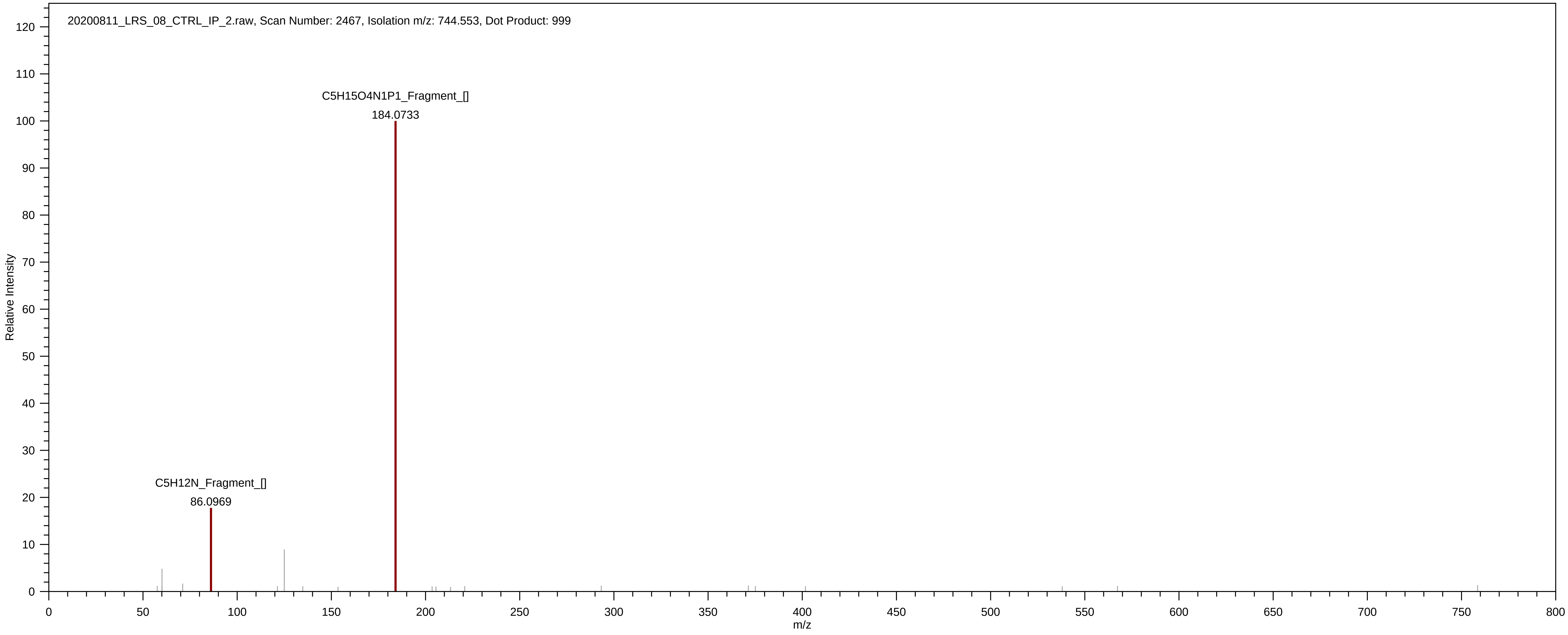

PC 33:2 [M+H]+ | Ret. Time (min): 8.597 | m/z: 744.553

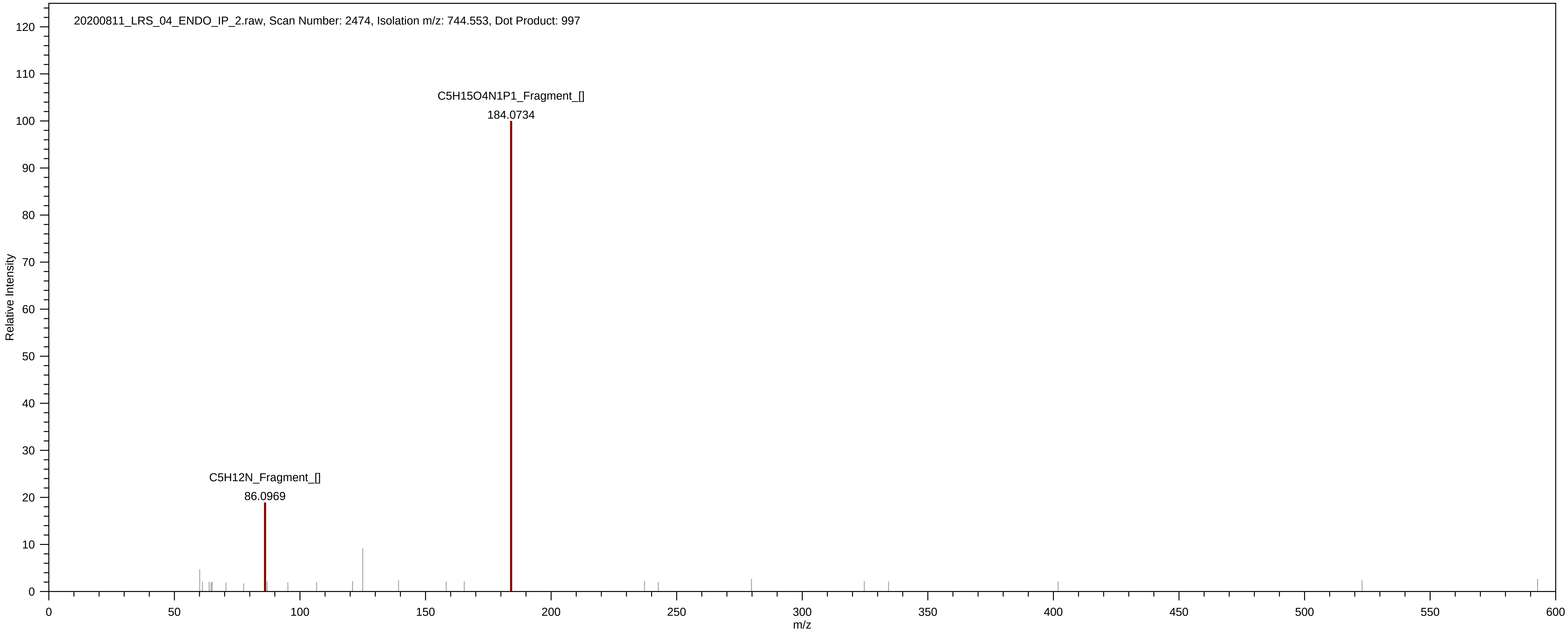

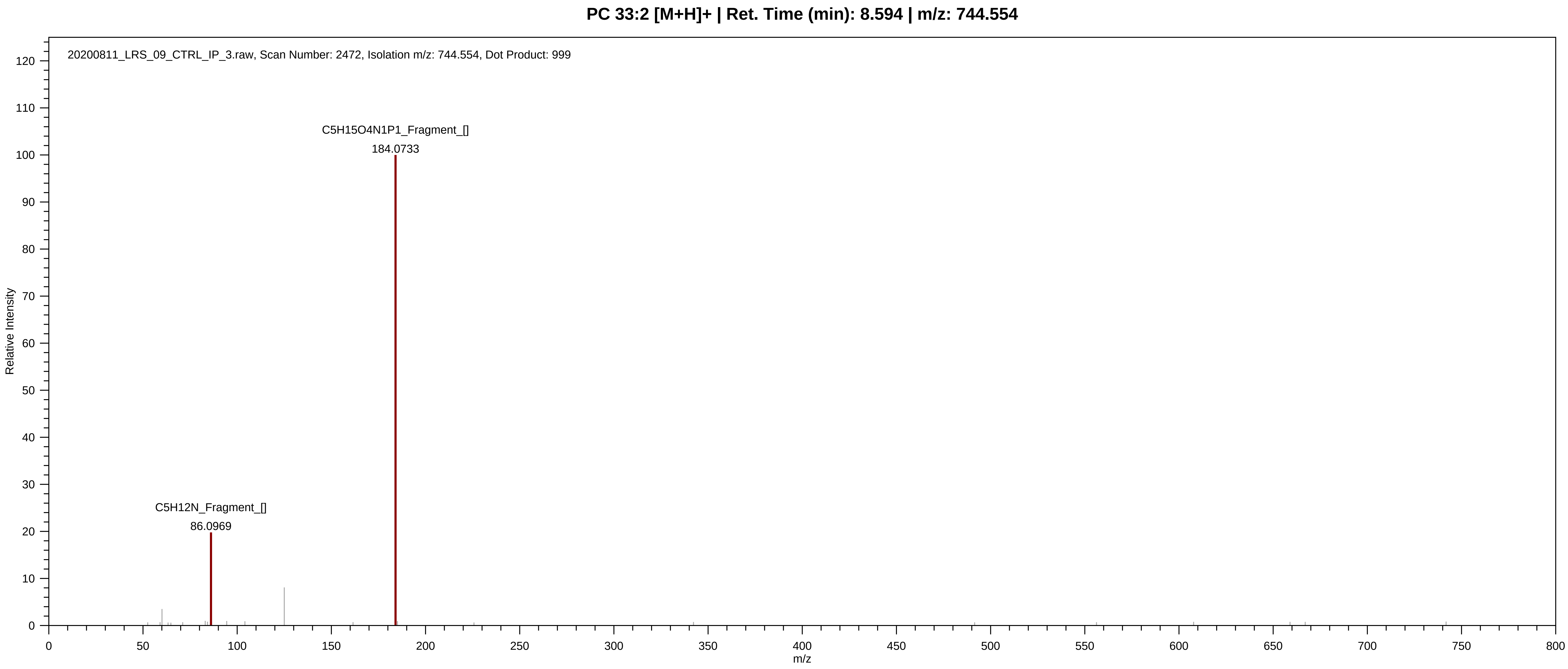
PC 33:2 [M+H]+ | Ret. Time (min): 8.594 | m/z: 744.554
20200811_LRS_09_CTRL_IP_3.raw, Scan Number: 2472, Isolation m/z: 744.554, Dot Product: 999
C5H15O4N1P1_Fragment_[]
184.0733
C5H12N_Fragment_[]
86.0969
Relative Intensity
m/z

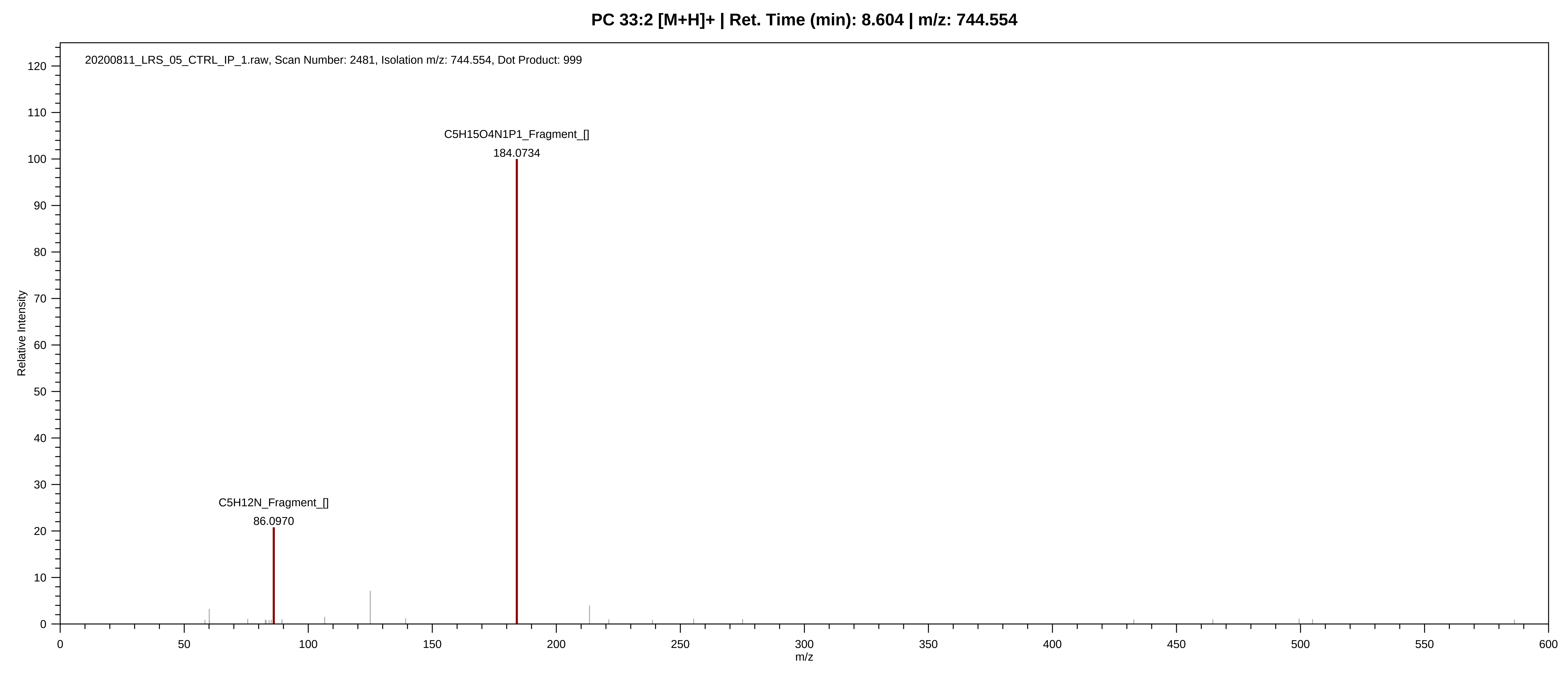

PC 33:2 [M+H]+ | Ret. Time (min): 8.604 | m/z: 744.554
20200811_LRS_05_CTRL_IP_1.raw, Scan Number: 2481, Isolation m/z: 744.554, Dot Product: 999
C5H15O4N1P1_Fragment_[]
184.0734
C5H12N_Fragment_[]
86.0970
Relative Intensity
m/z

PC 33:2 [M+H]+ | Ret. Time (min): 8.624 | m/z: 744.554

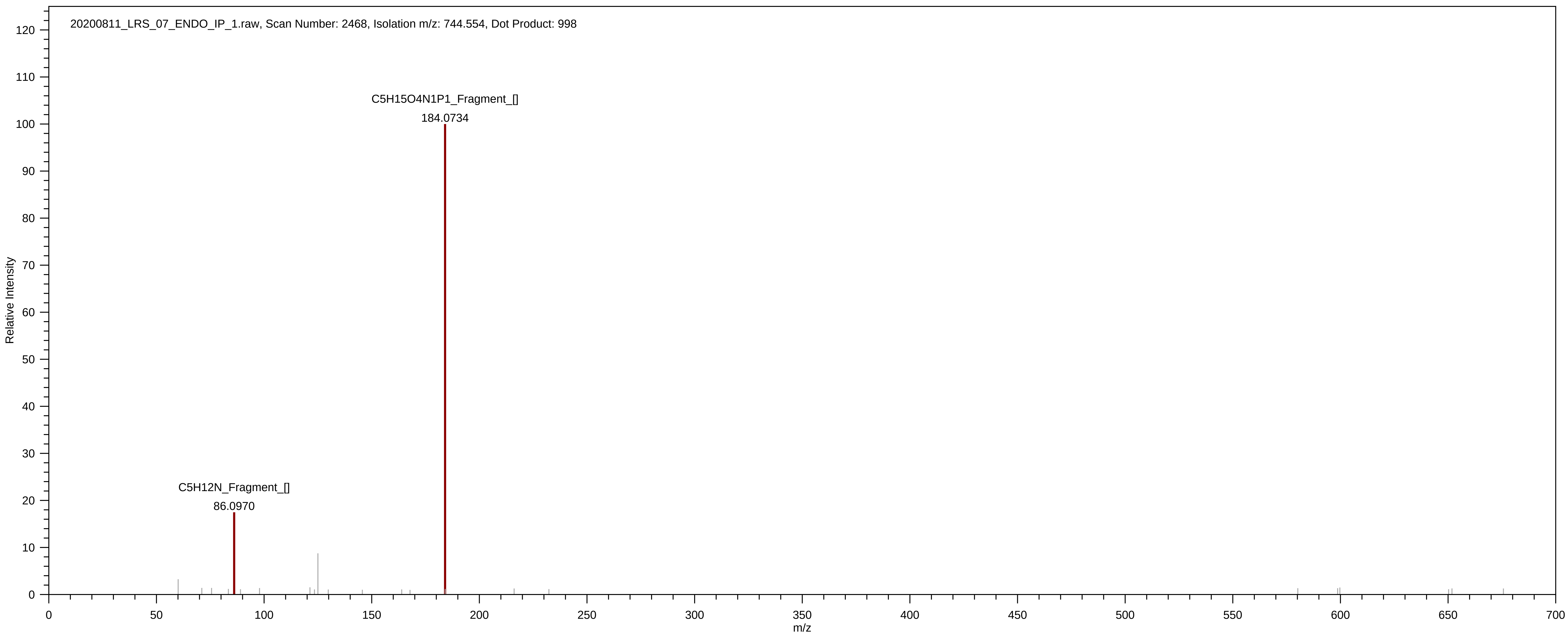

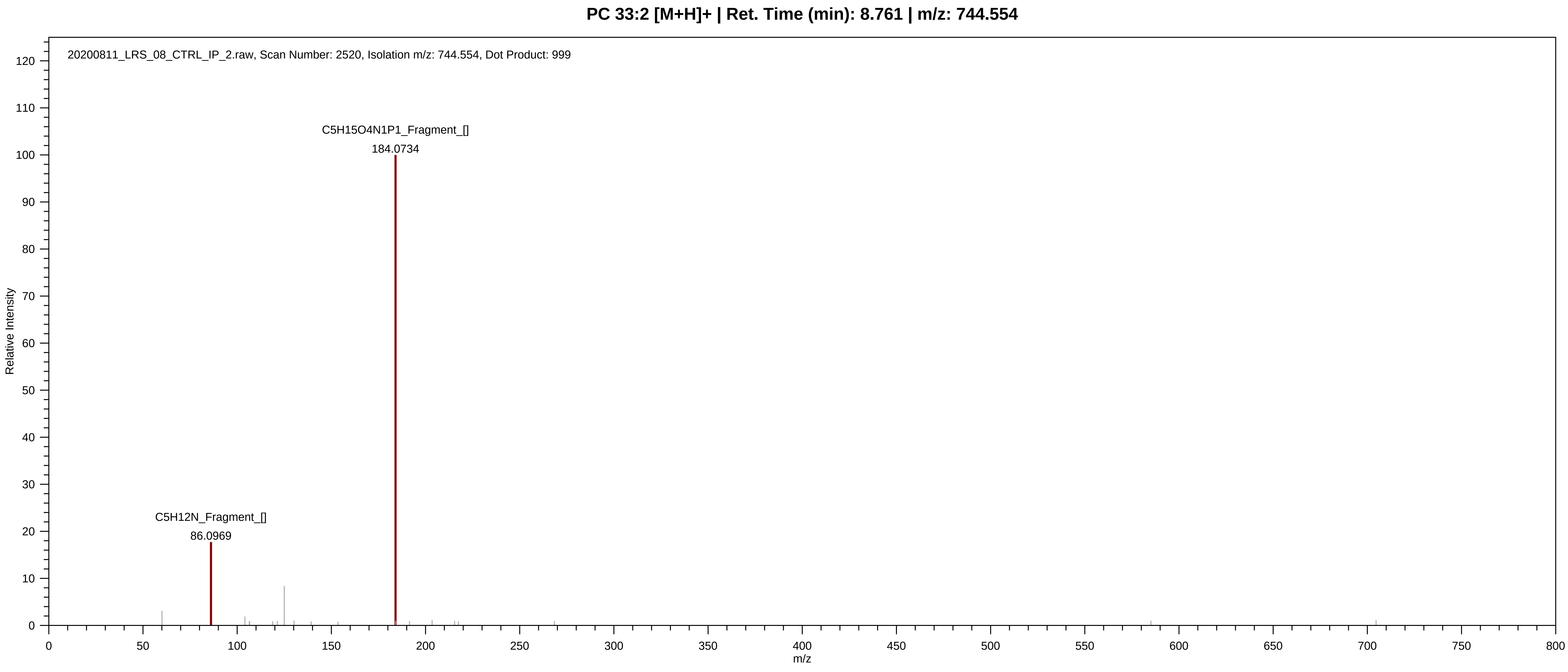
PC 33:2 [M+H]+ | Ret. Time (min): 8.761 | m/z: 744.554
20200811_LRS_08_CTRL_IP_2.raw, Scan Number: 2520, Isolation m/z: 744.554, Dot Product: 999
C5H15O4N1P1_Fragment_[]
184.0734
C5H12N_Fragment_[]
86.0969
Relative Intensity
m/z

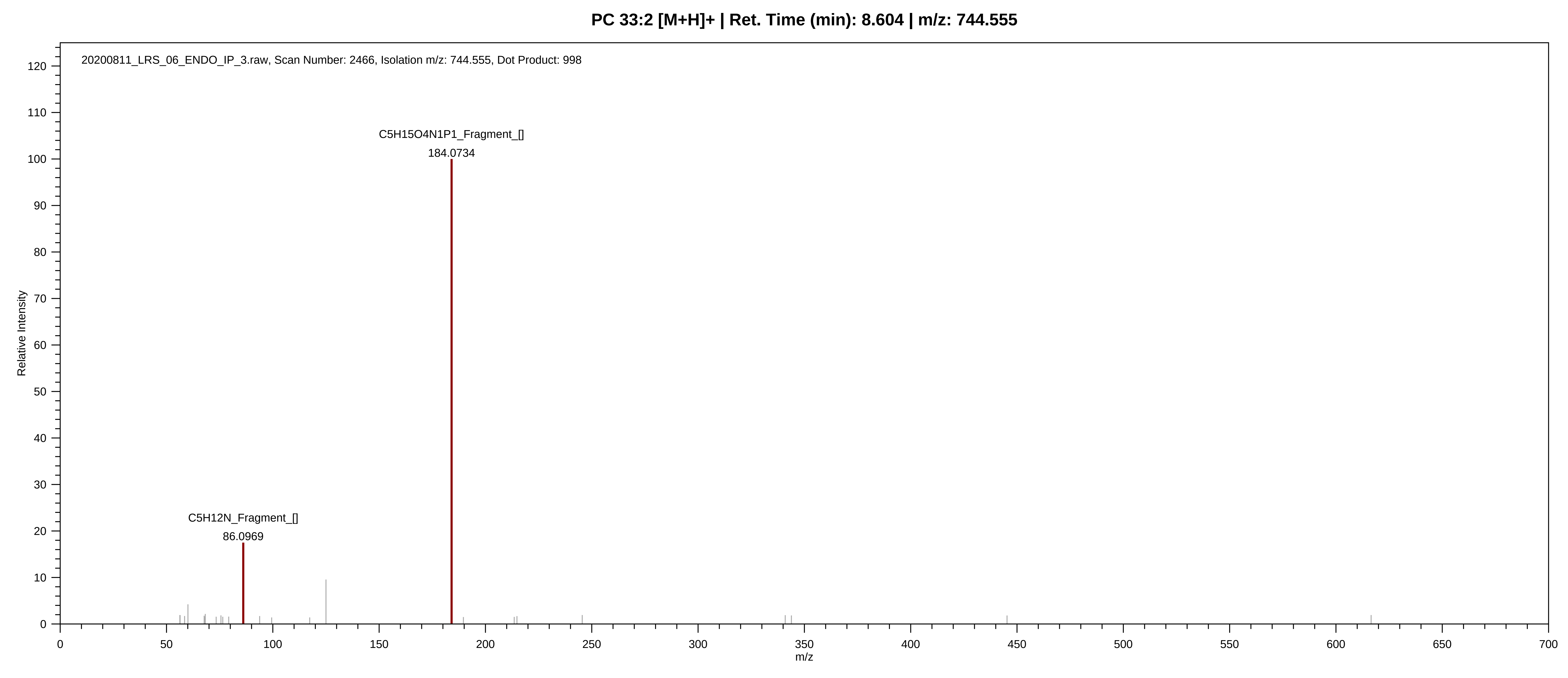

PC 33:2 [M+H]+ | Ret. Time (min): 8.604 | m/z: 744.555
20200811_LRS_06_ENDO_IP_3.raw, Scan Number: 2466, Isolation m/z: 744.555, Dot Product: 998
C5H15O4N1P1_Fragment_[]
184.0734
C5H12N_Fragment_[]
86.0969
Relative Intensity
m/z

PC 34:0 [M+H]+ | Ret. Time (min): 10.734 | m/z: 762.6

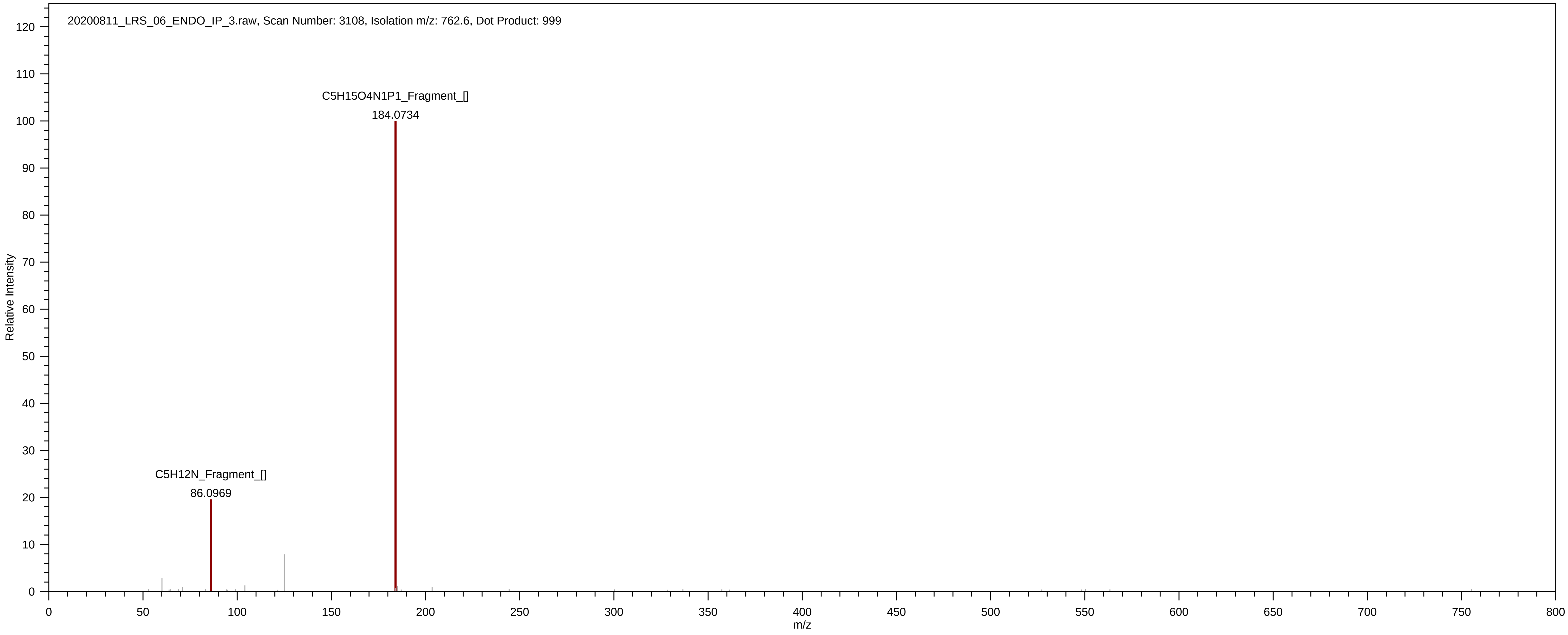

PC 34:0 [M+H]+ | Ret. Time (min): 10.915 | m/z: 762.6

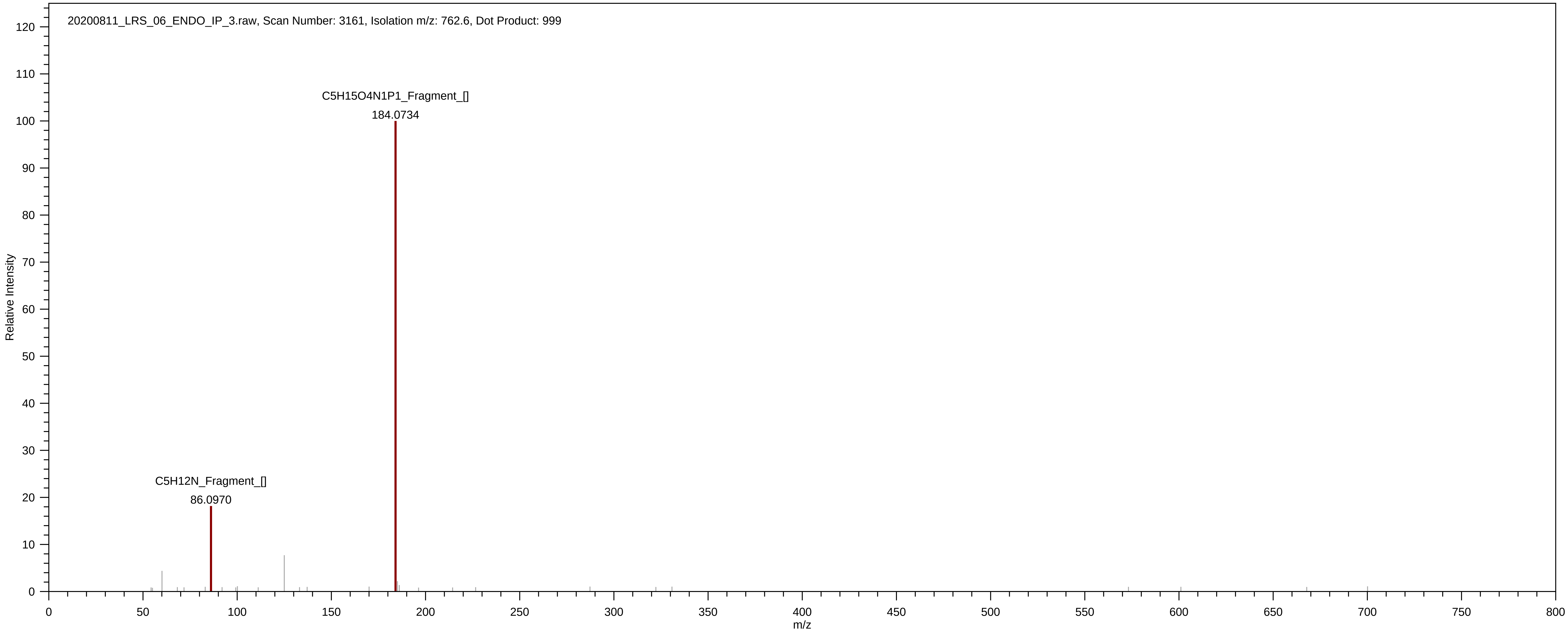

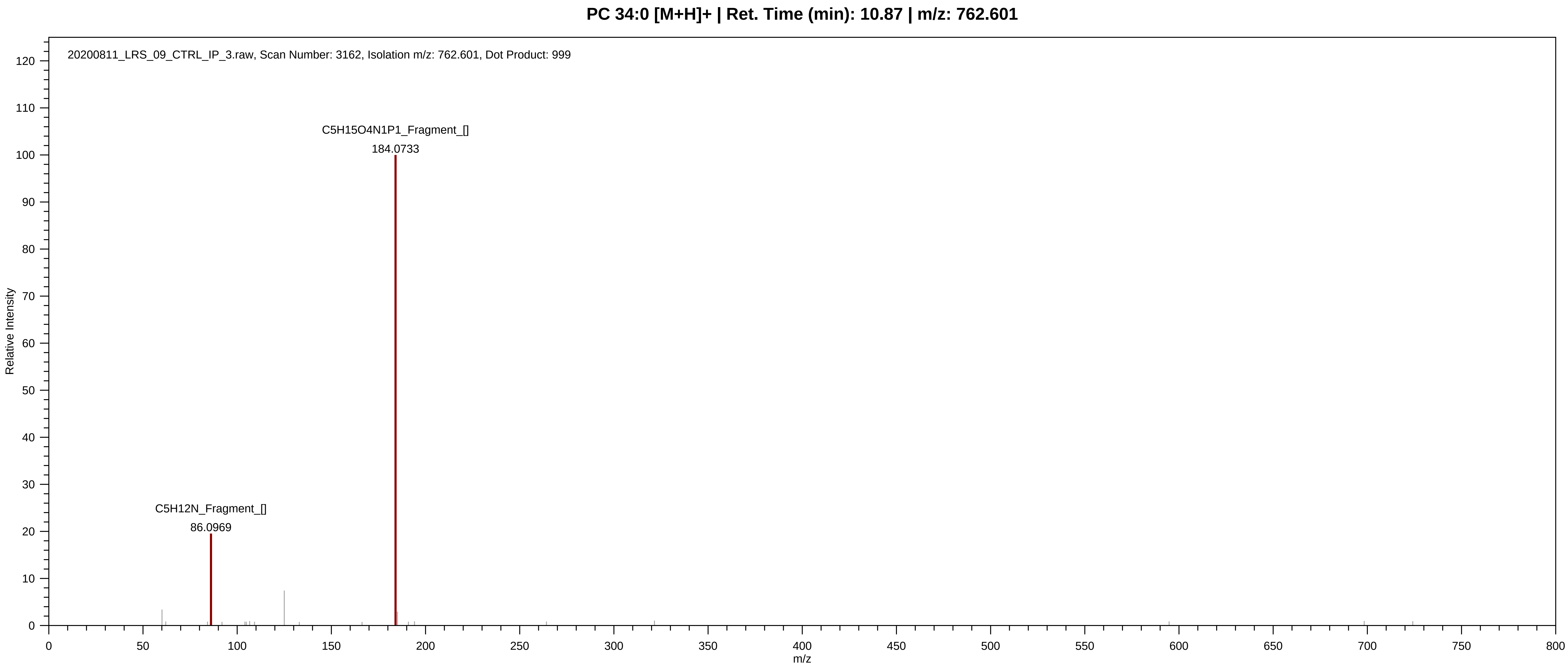

PC 34:0 [M+H]+ | Ret. Time (min): 10.87 | m/z: 762.601
20200811_LRS_09_CTRL_IP_3.raw, Scan Number: 3162, Isolation m/z: 762.601, Dot Product: 999
C5H15O4N1P1_Fragment_[]
184.0733
C5H12N_Fragment_[]
86.0969
Relative Intensity
m/z

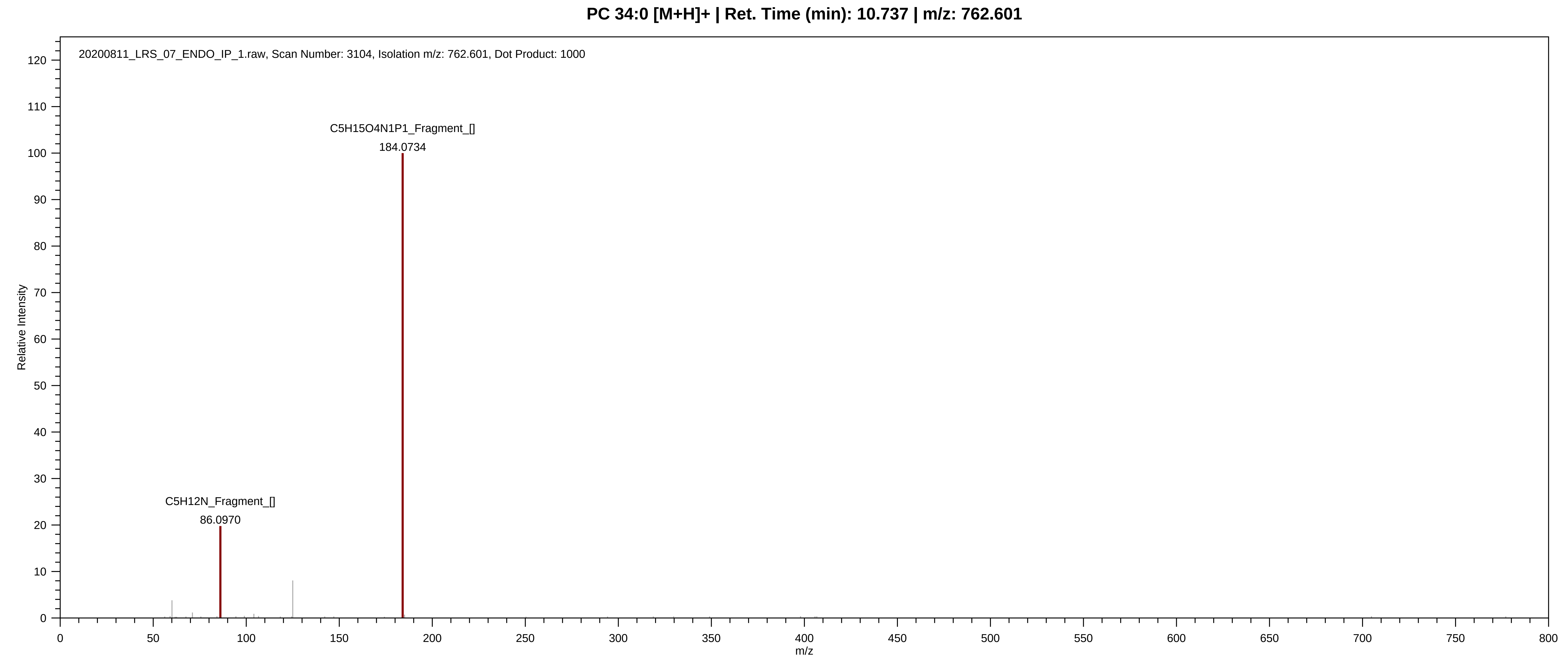
PC 34:0 [M+H]+ | Ret. Time (min): 10.737 | m/z: 762.601
20200811_LRS_07_ENDO_IP_1.raw, Scan Number: 3104, Isolation m/z: 762.601, Dot Product: 1000
C5H15O4N1P1_Fragment_[]
184.0734
C5H12N_Fragment_[]
86.0970
Relative Intensity
m/z

PC 34:0 [M+H]+ | Ret. Time (min): 10.773 | m/z: 762.601

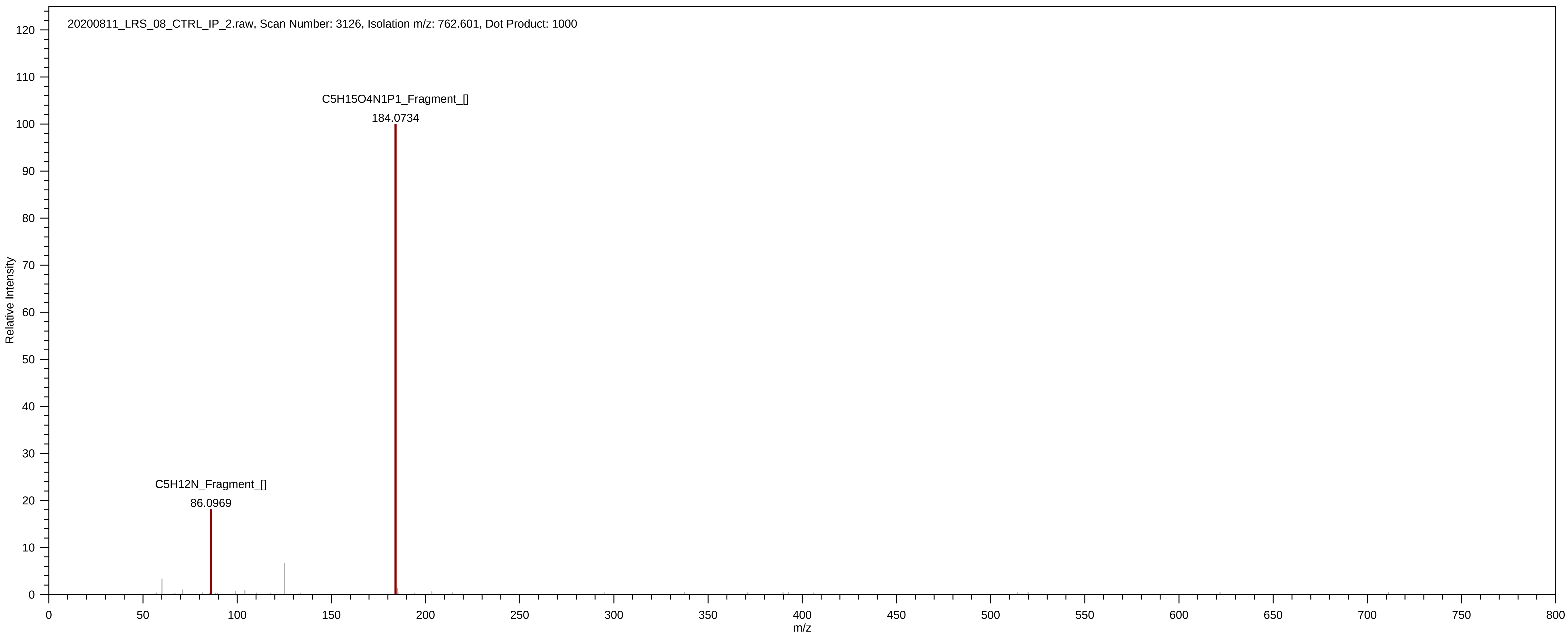

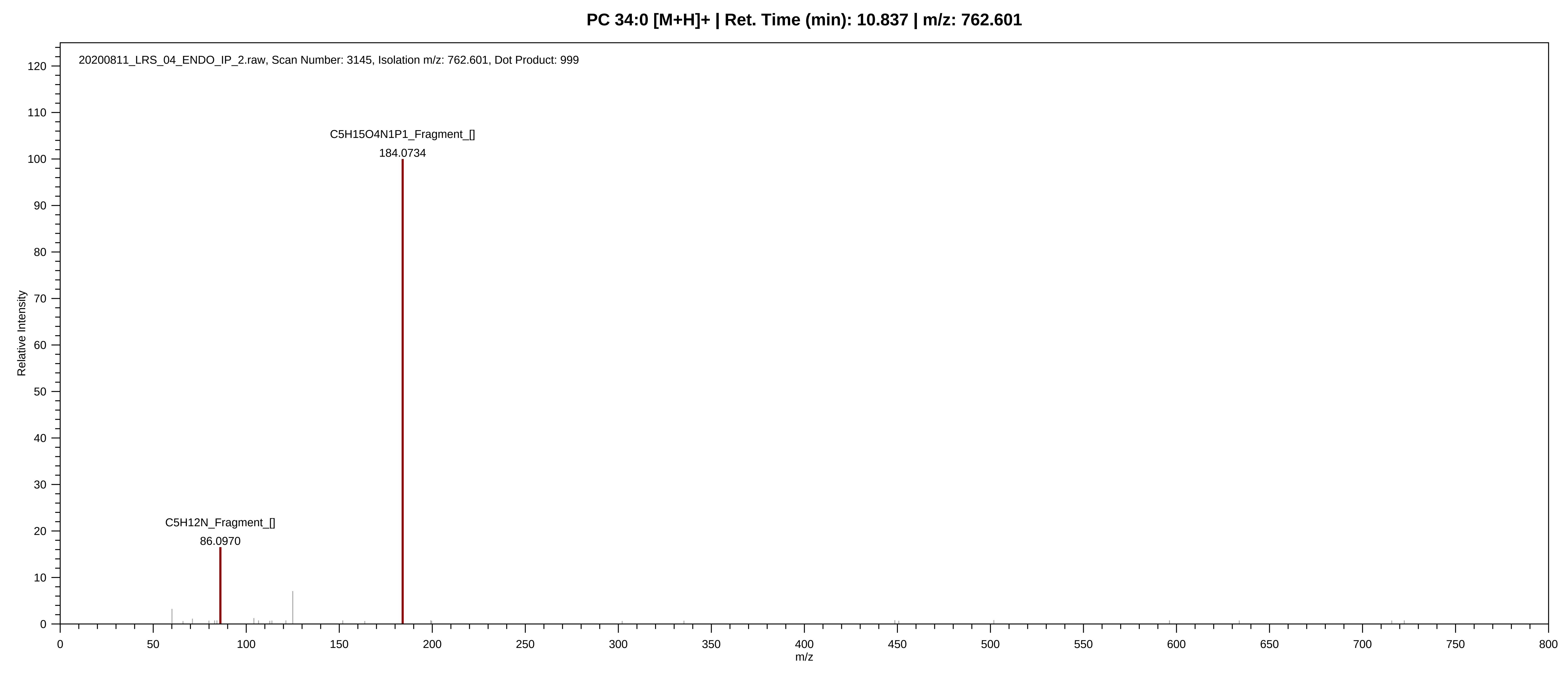

PC 34:0 [M+H]+ | Ret. Time (min): 10.837 | m/z: 762.601
20200811_LRS_04_ENDO_IP_2.raw, Scan Number: 3145, Isolation m/z: 762.601, Dot Product: 999
C5H15O4N1P1_Fragment_[]
184.0734
C5H12N_Fragment_[]
86.0970
Relative Intensity
m/z

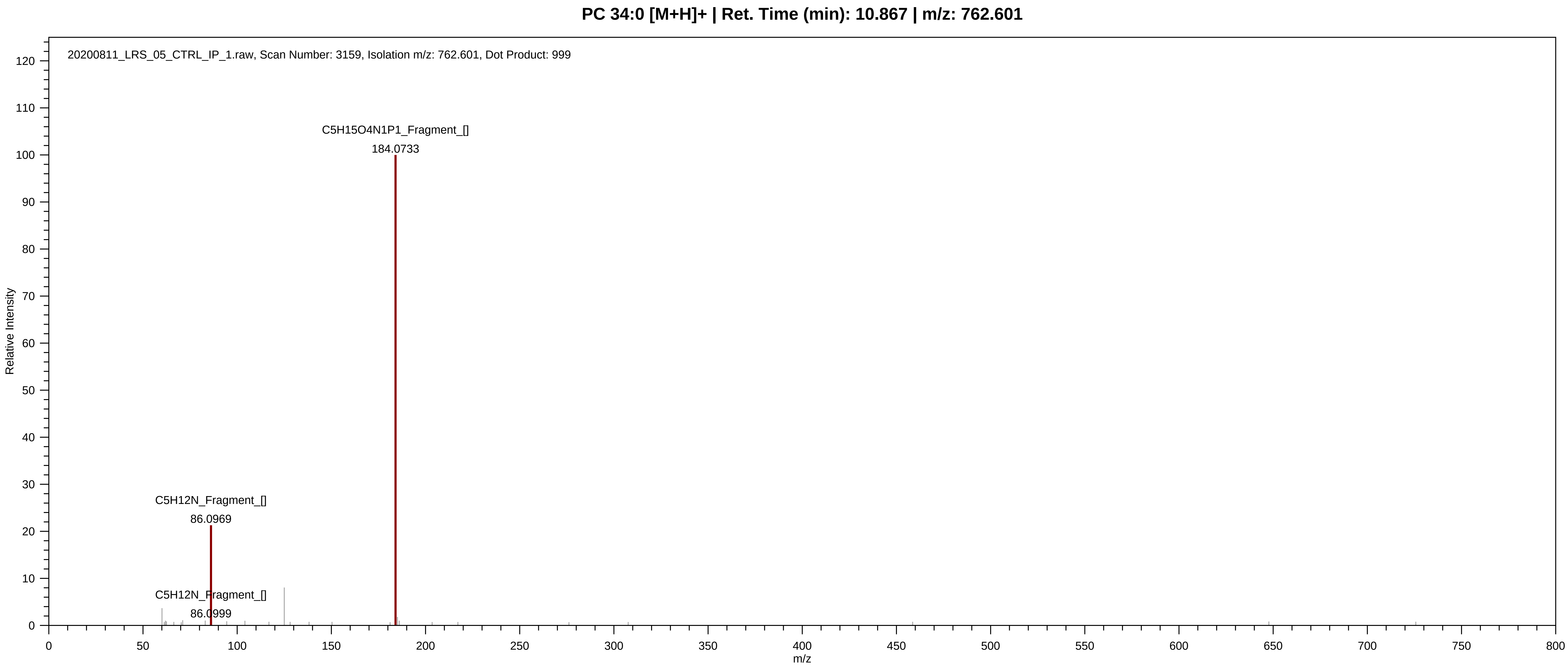
PC 34:0 [M+H]+ | Ret. Time (min): 10.867 | m/z: 762.601
20200811_LRS_05_CTRL_IP_1.raw, Scan Number: 3159, Isolation m/z: 762.601, Dot Product: 999
C5H15O4N1P1_Fragment_[]
184.0733
C5H12N_Fragment_[]
86.0969
C5H12N_Fragment_[]
86.0999
Relative Intensity
m/z

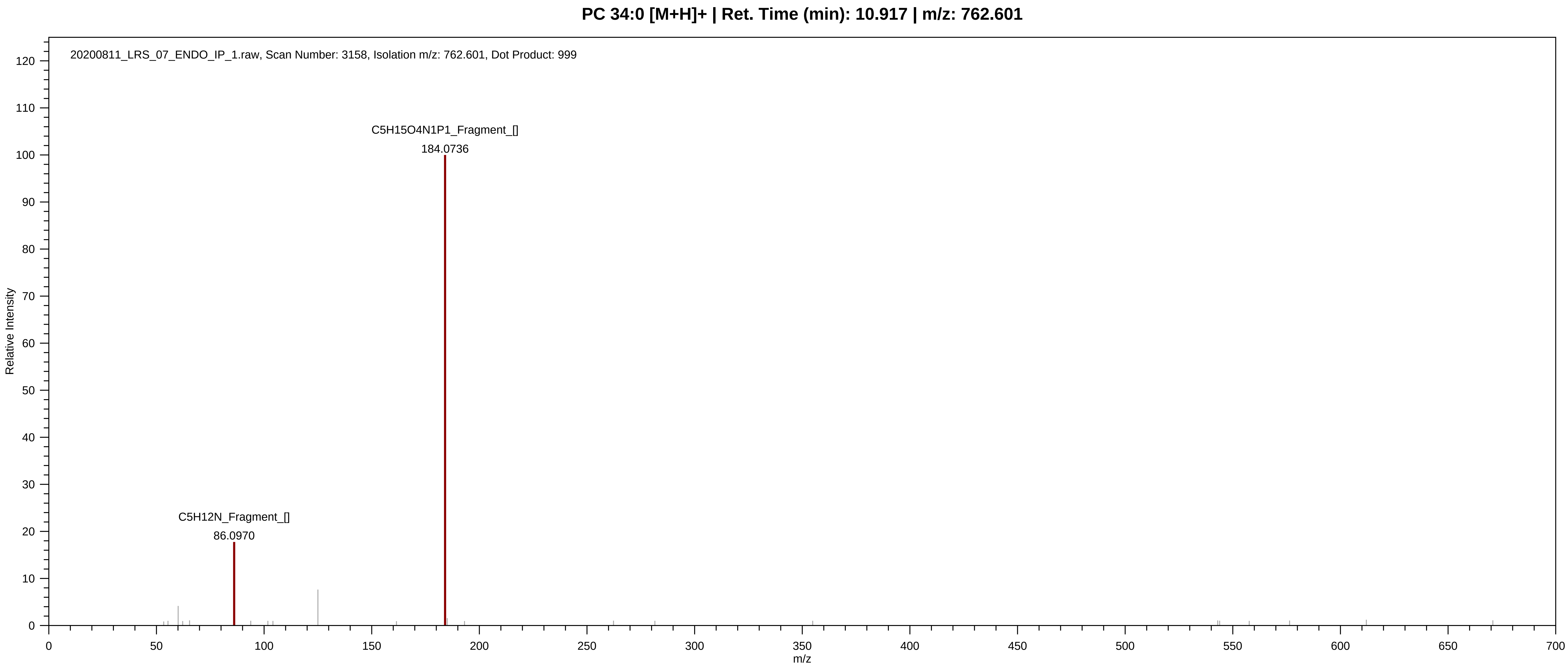
PC 34:0 [M+H]+ | Ret. Time (min): 10.917 | m/z: 762.601
20200811_LRS_07_ENDO_IP_1.raw, Scan Number: 3158, Isolation m/z: 762.601, Dot Product: 999
C5H15O4N1P1_Fragment_[]
184.0736
C5H12N_Fragment_[]
86.0970
Relative Intensity
m/z

PC 34:1 [M+H]+ | Ret. Time (min): 9.841 | m/z: 760.586

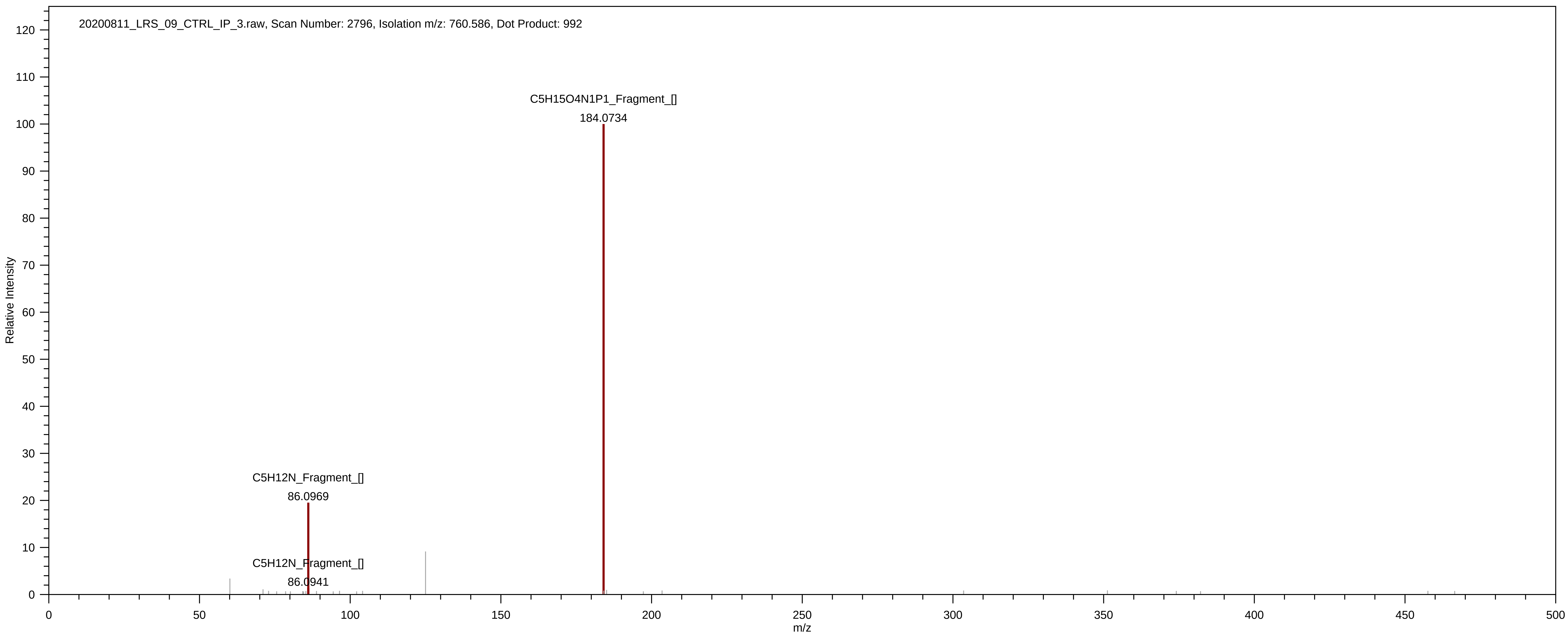

PC 34:1 [M+H]+ | Ret. Time (min): 9.841 | m/z: 760.584

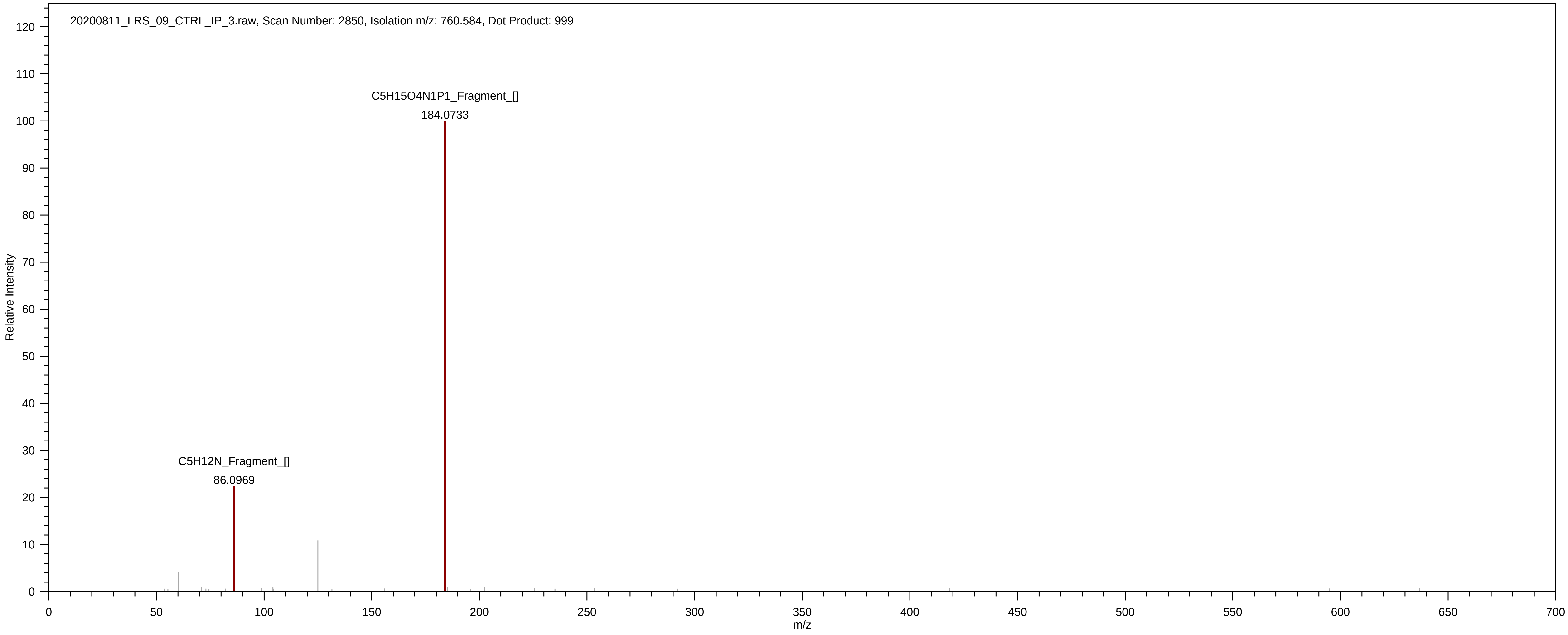

PC 34:1 [M+H]+ | Ret. Time (min): 9.844 | m/z: 760.586

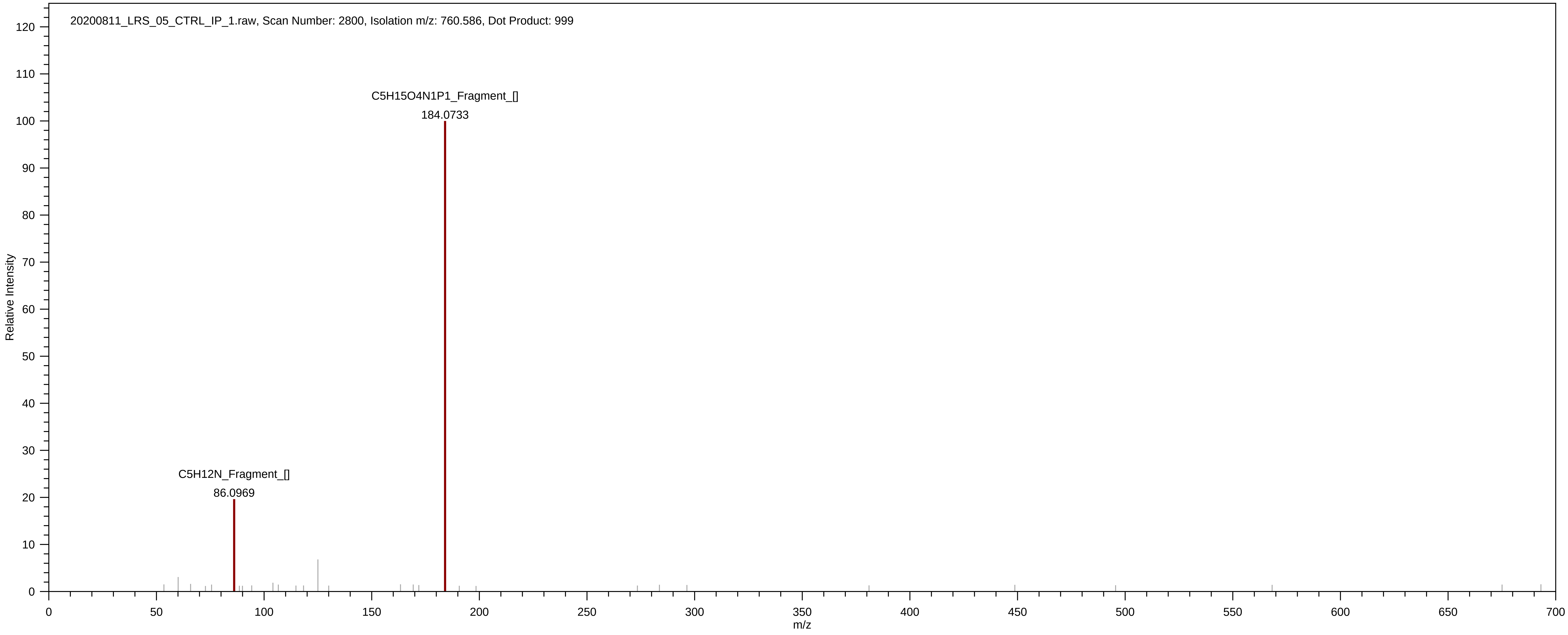

PC 34:1 [M+H]+ | Ret. Time (min): 9.844 | m/z: 760.584

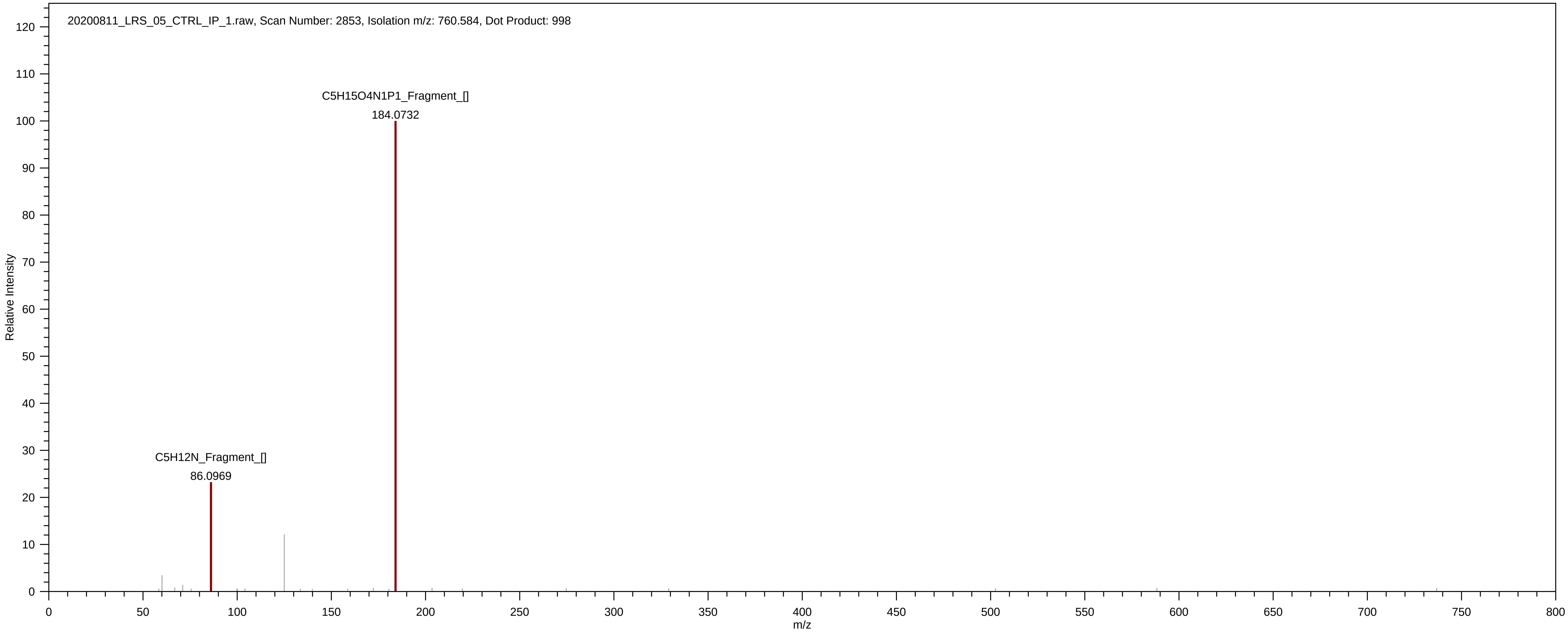

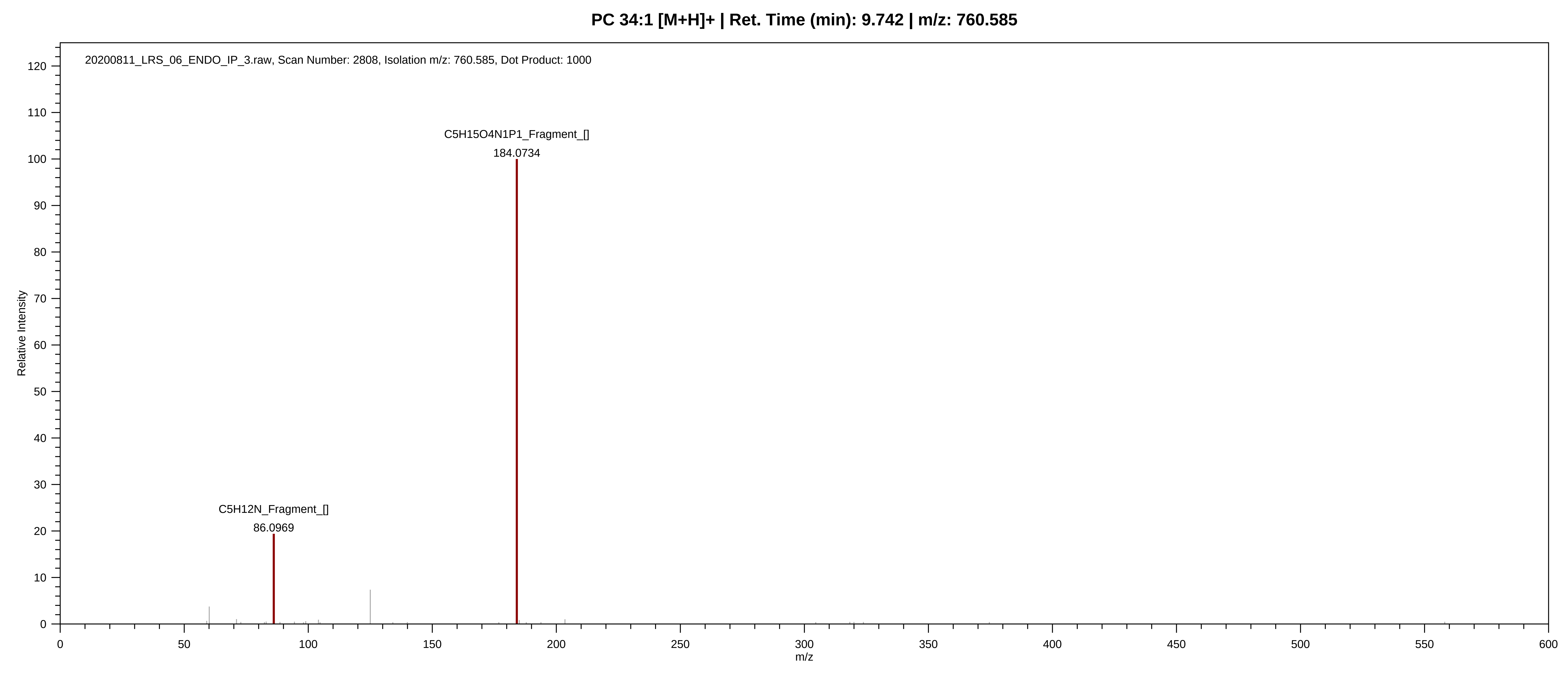

PC 34:1 [M+H]+ | Ret. Time (min): 9.742 | m/z: 760.585
20200811_LRS_06_ENDO_IP_3.raw, Scan Number: 2808, Isolation m/z: 760.585, Dot Product: 1000
C5H15O4N1P1_Fragment_[]
184.0734
C5H12N_Fragment_[]
86.0969
Relative Intensity
m/z

PC 34:1 [M+H]+ | Ret. Time (min): 9.743 | m/z: 760.585

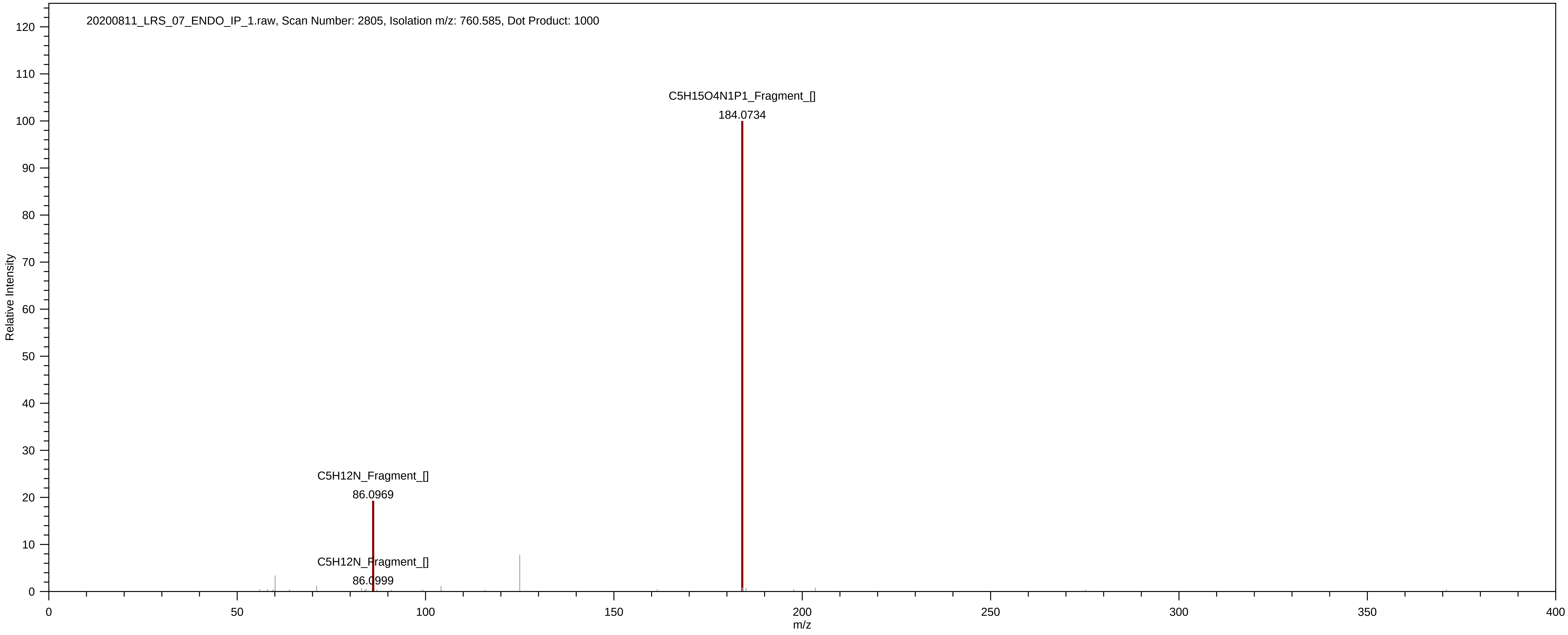

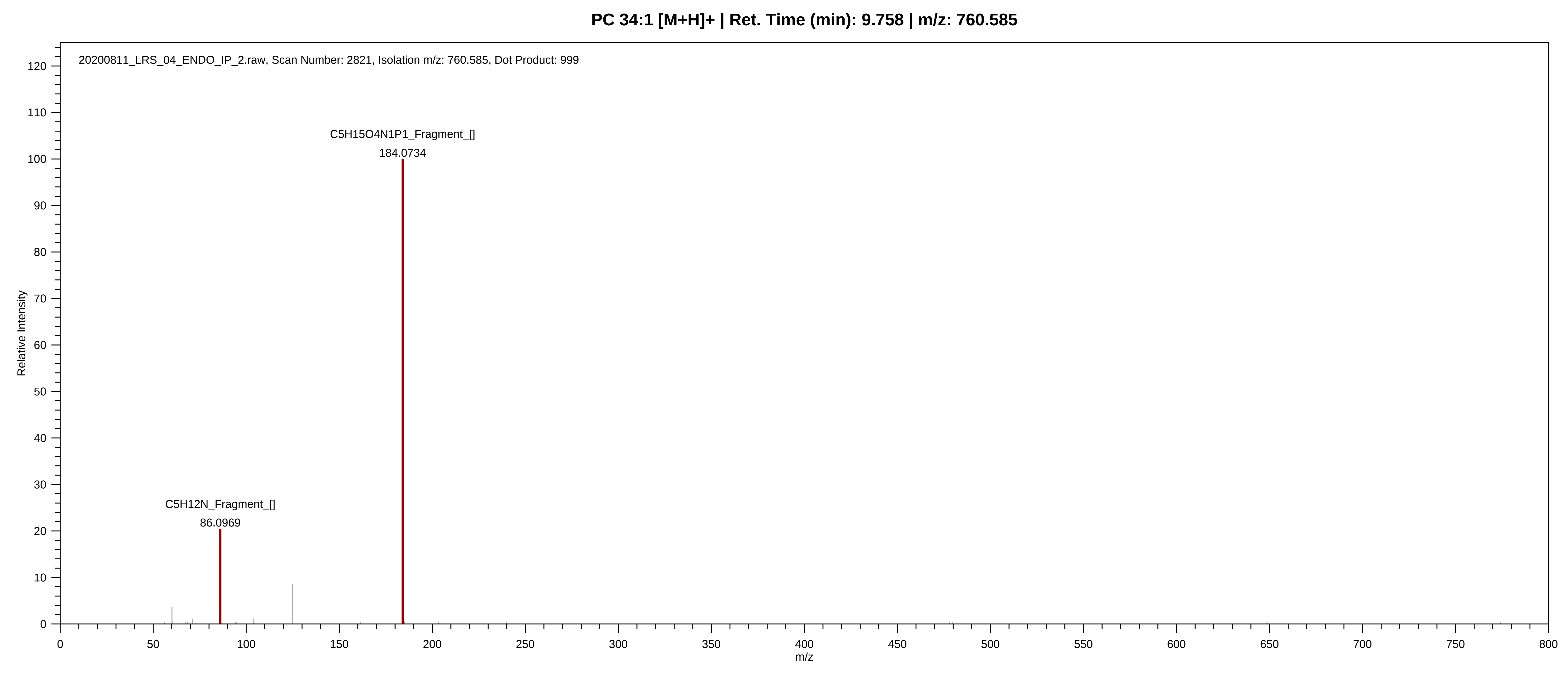
PC 34:1 [M+H]+ | Ret. Time (min): 9.758 | m/z: 760.585
20200811_LRS_04_ENDO_IP_2.raw, Scan Number: 2821, Isolation m/z: 760.585, Dot Product: 999
C5H15O4N1P1_Fragment_[]
184.0734
C5H12N_Fragment_[]
86.0969
Relative Intensity
m/z

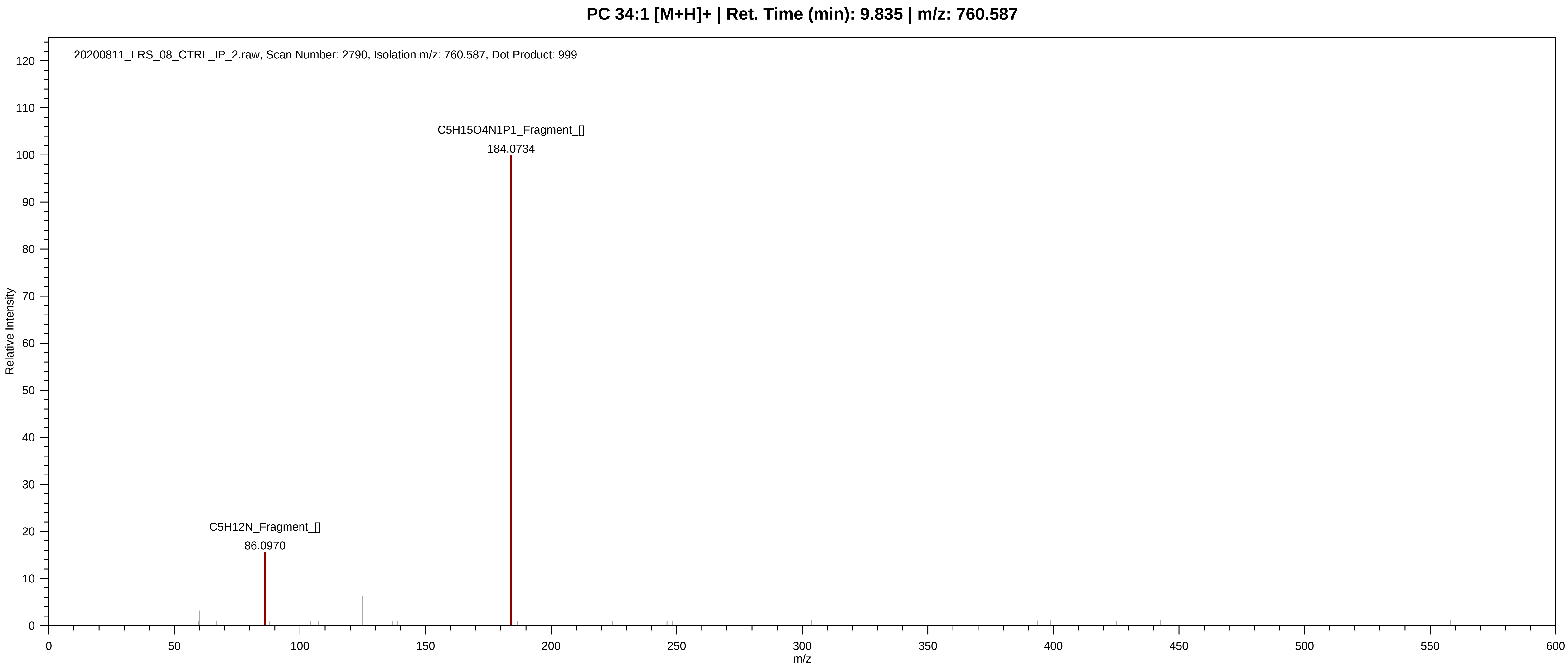

PC 34:1 [M+H]+ | Ret. Time (min): 9.835 | m/z: 760.587
20200811_LRS_08_CTRL_IP_2.raw, Scan Number: 2790, Isolation m/z: 760.587, Dot Product: 999
C5H15O4N1P1_Fragment_[]
184.0734
C5H12N_Fragment_[]
86.0970
Relative Intensity
m/z

PC 34:1 [M+H]+ | Ret. Time (min): 9.835 | m/z: 760.585

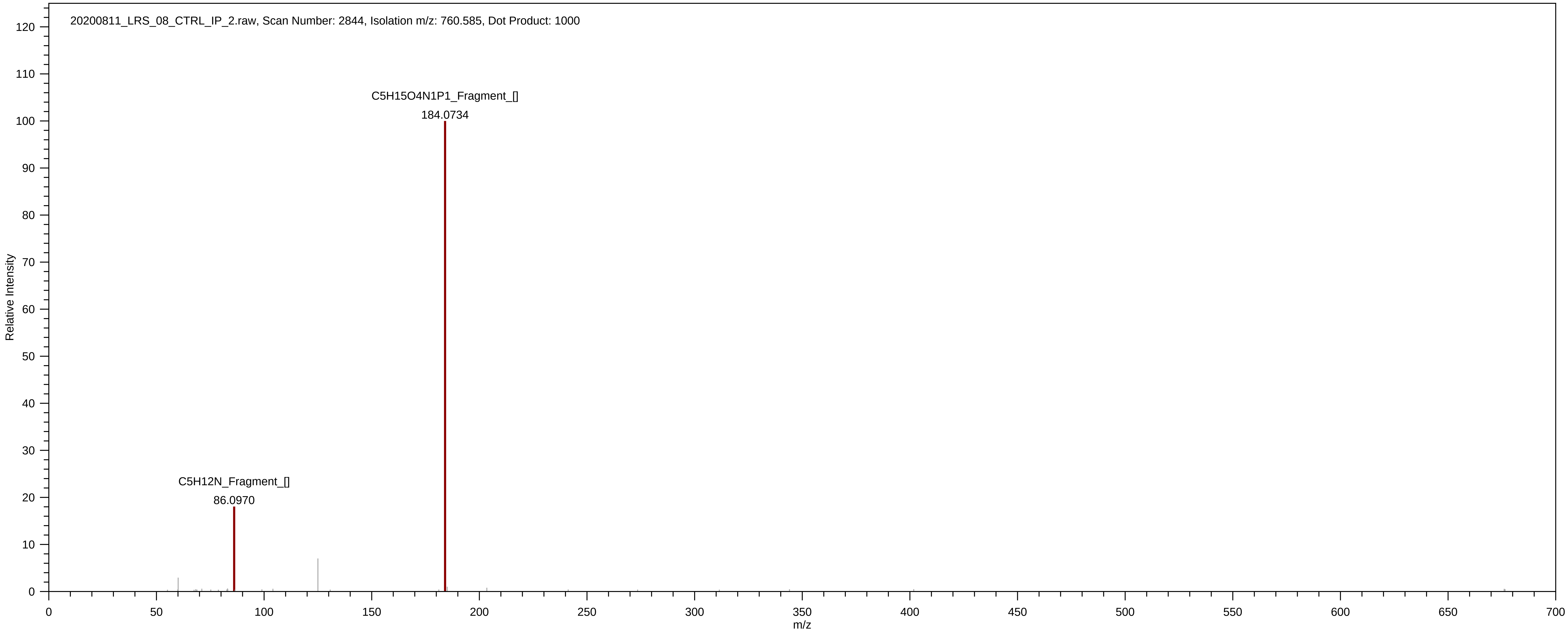

PC 34:1 [M+H]+ | Ret. Time (min): 9.933 | m/z: 760.585

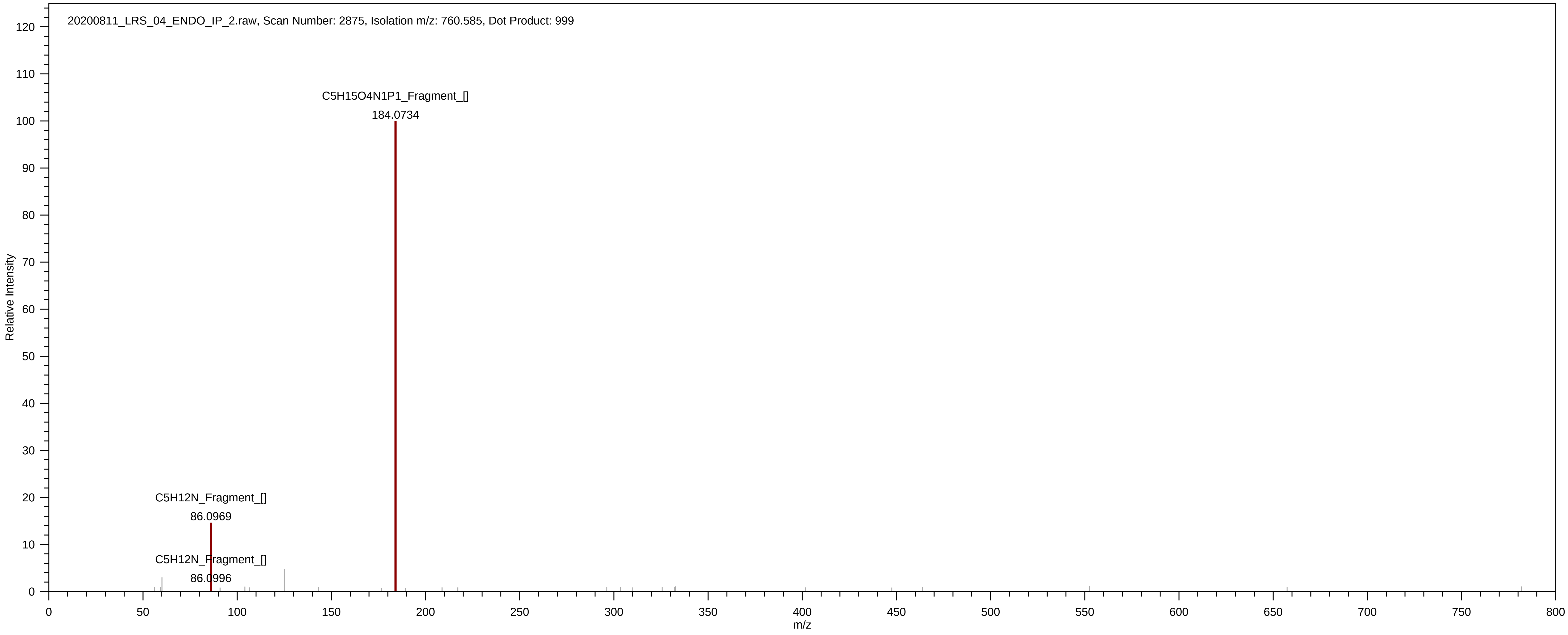

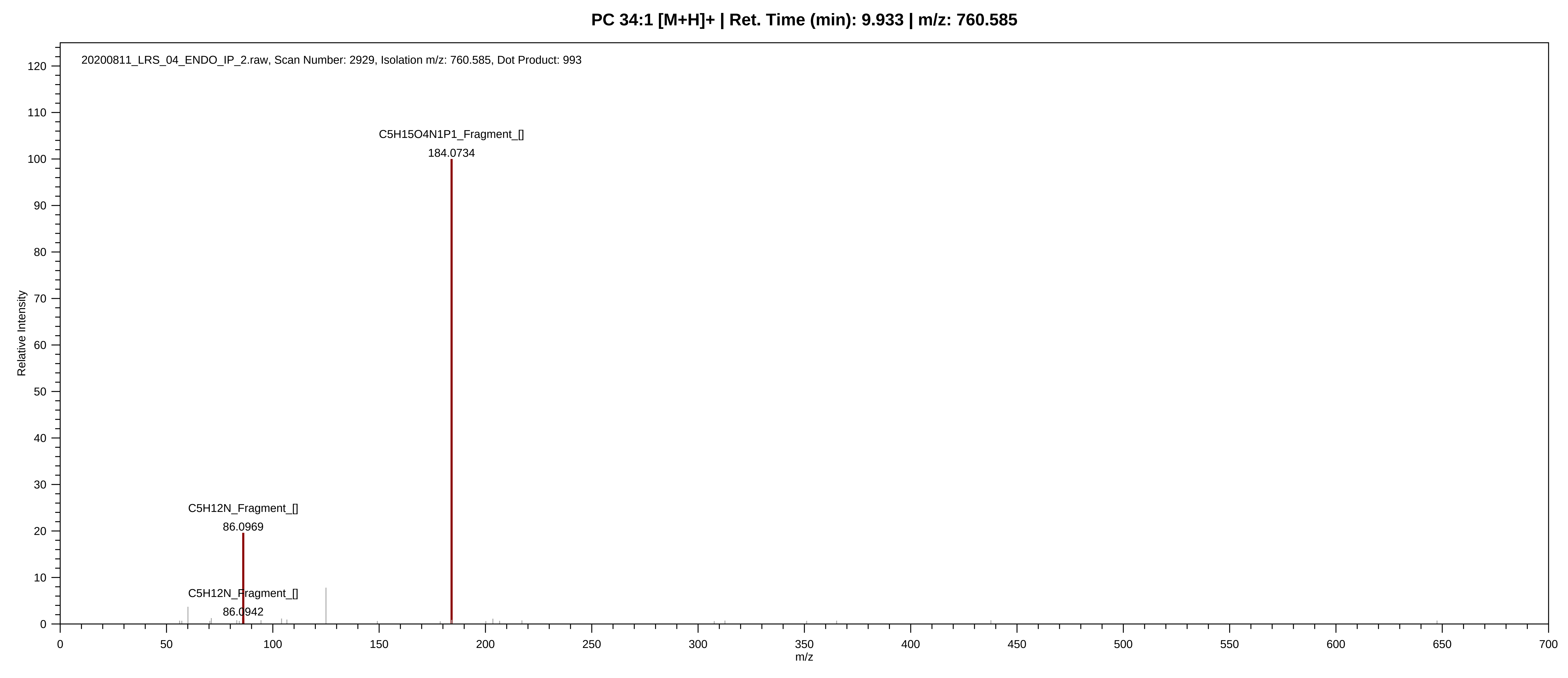
PC 34:1 [M+H]+ | Ret. Time (min): 9.933 | m/z: 760.585
20200811_LRS_04_ENDO_IP_2.raw, Scan Number: 2929, Isolation m/z: 760.585, Dot Product: 993
C5H15O4N1P1_Fragment_[]
184.0734
C5H12N_Fragment_[]
86.0969
C5H12N_Fragment_[]
86.0942
Relative Intensity
m/z

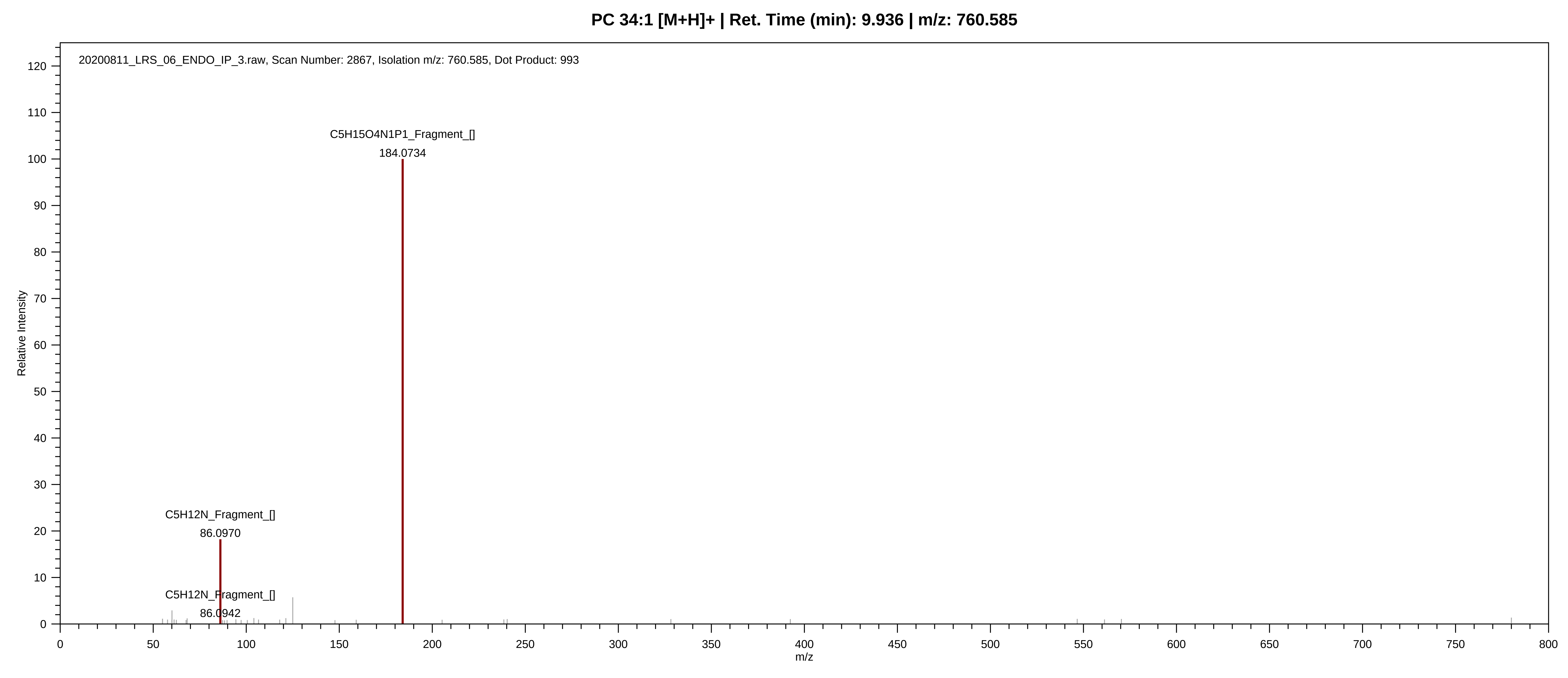
PC 34:1 [M+H]+ | Ret. Time (min): 9.936 | m/z: 760.585
20200811_LRS_06_ENDO_IP_3.raw, Scan Number: 2867, Isolation m/z: 760.585, Dot Product: 993
C5H15O4N1P1_Fragment_[]
184.0734
C5H12N_Fragment_[]
86.0970
C5H12N_Fragment_[]
86.0942
Relative Intensity
m/z

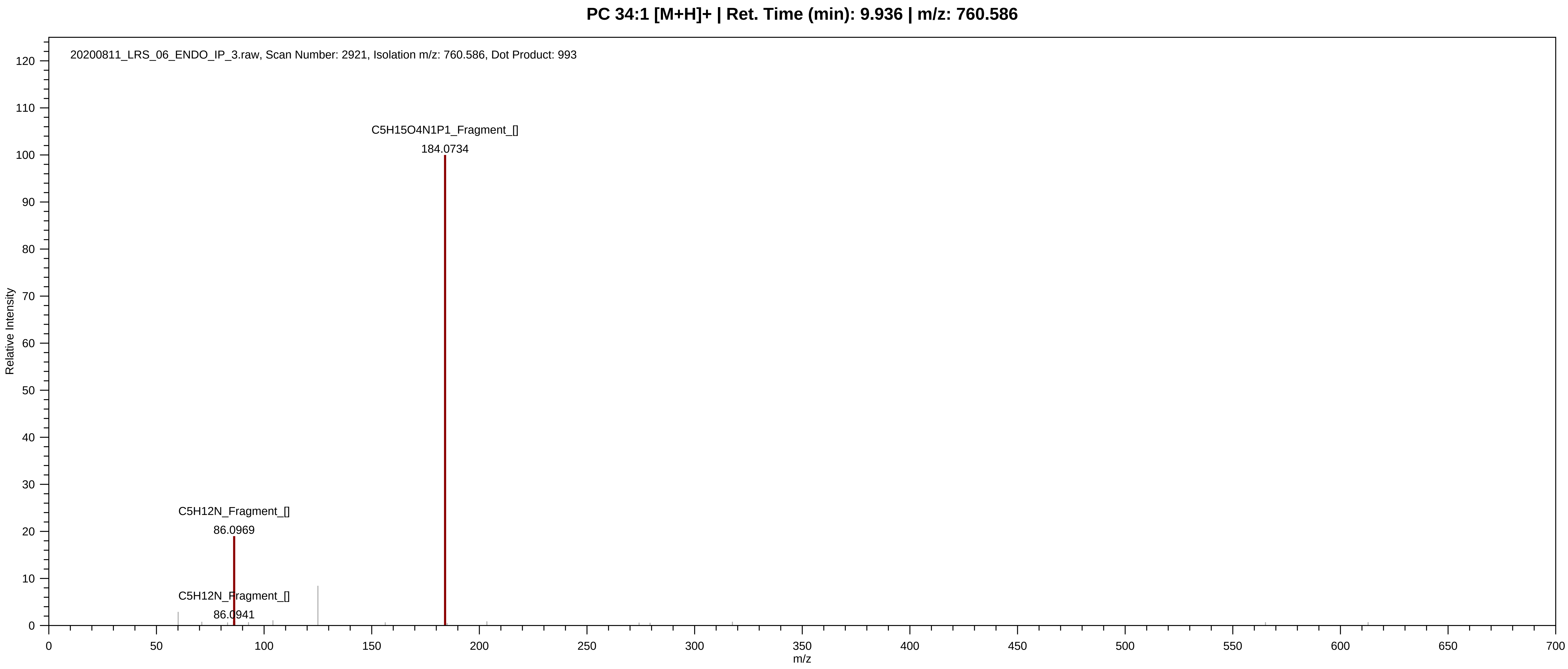

PC 34:1 [M+H]+ | Ret. Time (min): 9.936 | m/z: 760.586
20200811_LRS_06_ENDO_IP_3.raw, Scan Number: 2921, Isolation m/z: 760.586, Dot Product: 993
C5H15O4N1P1_Fragment_[]
184.0734
C5H12N_Fragment_[]
86.0969
C5H12N_Fragment_[]
86.0941
Relative Intensity
m/z

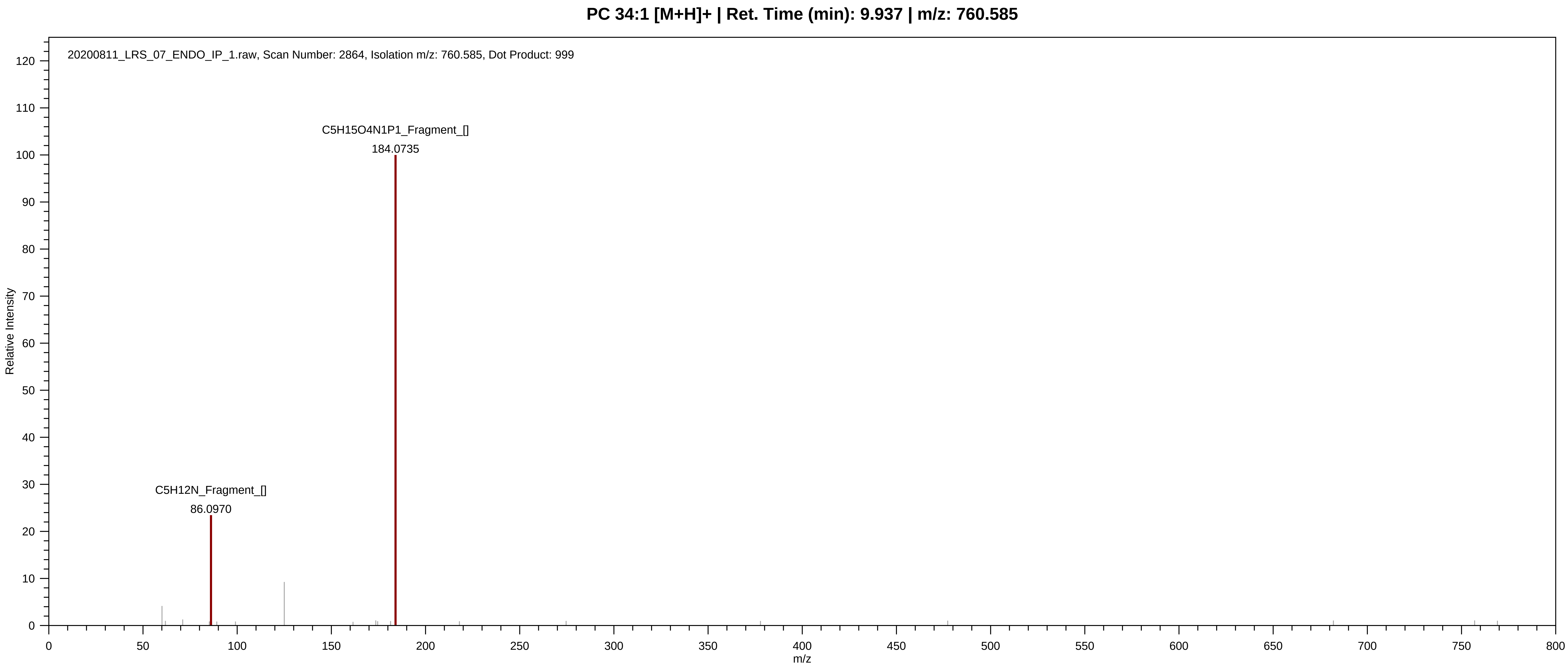
PC 34:1 [M+H]+ | Ret. Time (min): 9.937 | m/z: 760.585
20200811_LRS_07_ENDO_IP_1.raw, Scan Number: 2864, Isolation m/z: 760.585, Dot Product: 999
C5H15O4N1P1_Fragment_[]
184.0735
C5H12N_Fragment_[]
86.0970
Relative Intensity
m/z
0
10
20
30
40
50
60
70
80
90
100
110
120
0
50
100
150
200
250
300
350
400
450
500
550
600
650
700
750
800

PC 34:1 [M+H]+ | Ret. Time (min): 9.937 | m/z: 760.585

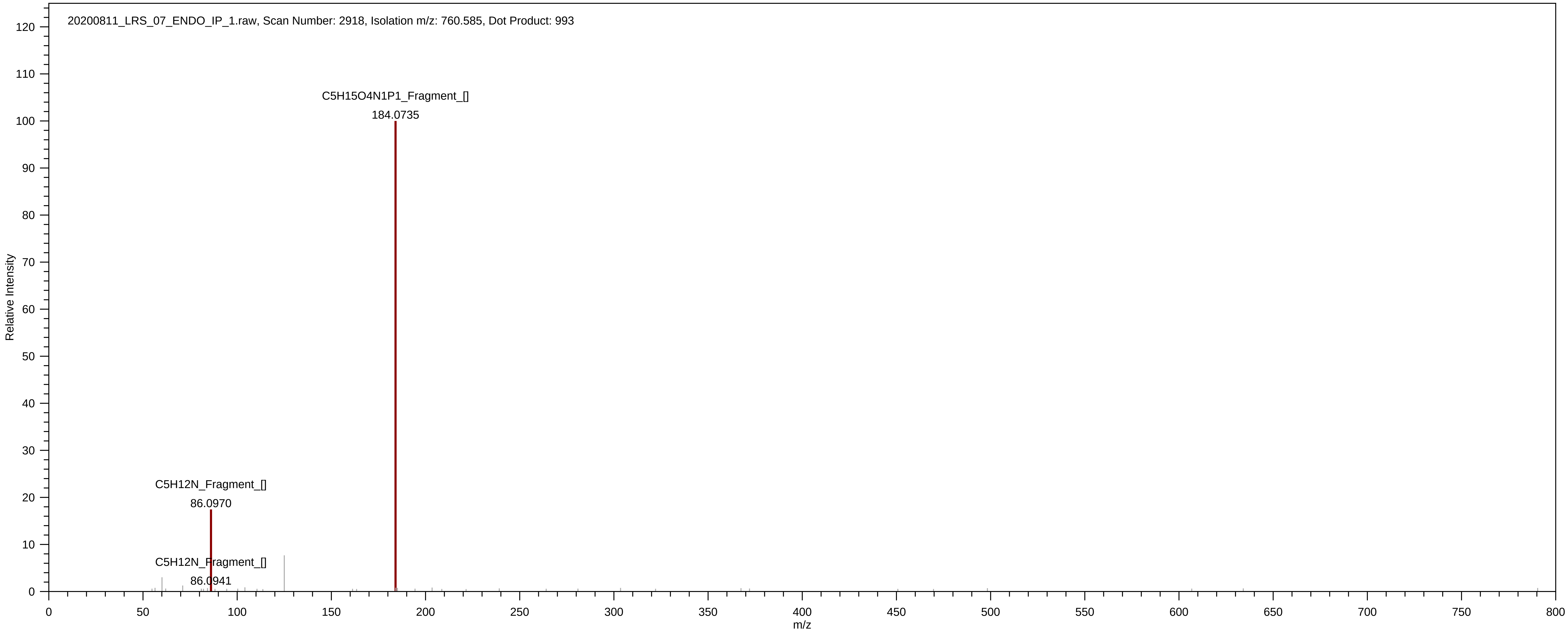

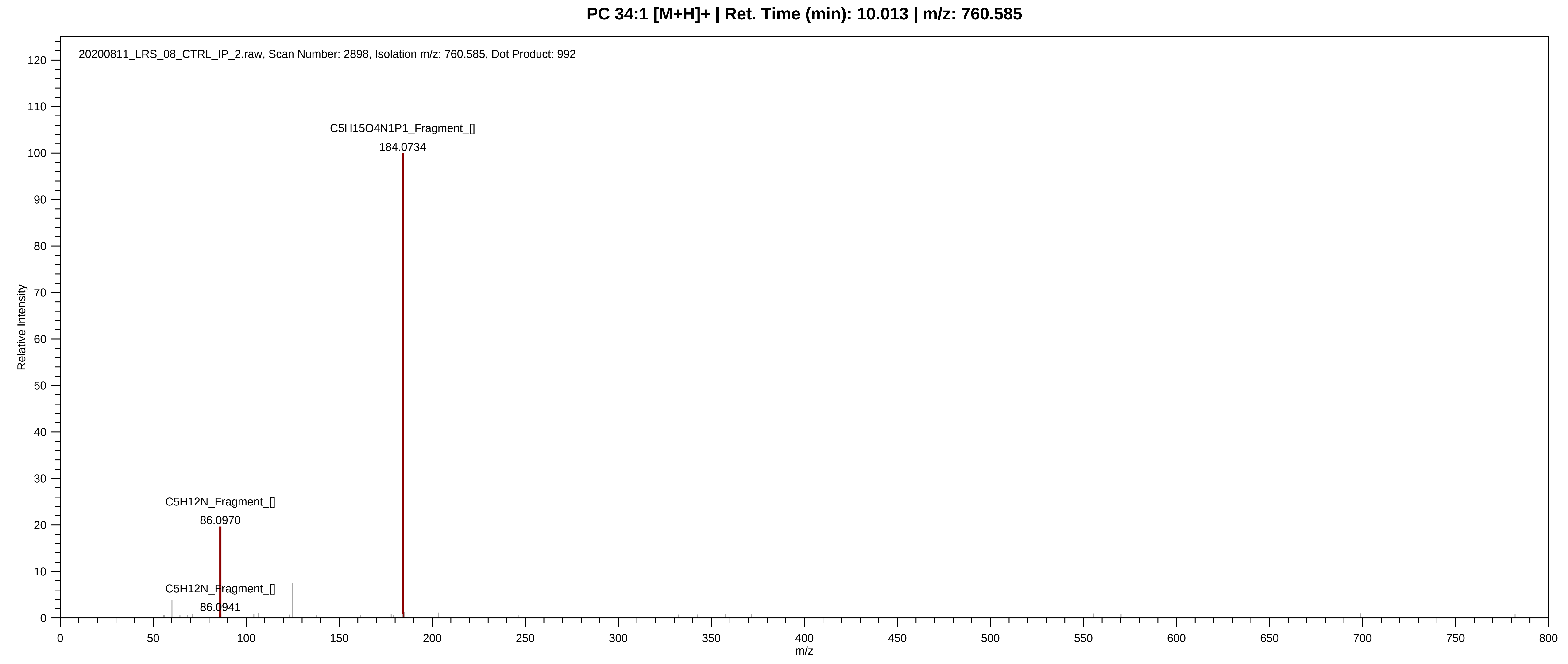

PC 34:1 [M+H]+ | Ret. Time (min): 10.013 | m/z: 760.585
20200811_LRS_08_CTRL_IP_2.raw, Scan Number: 2898, Isolation m/z: 760.585, Dot Product: 992
C5H15O4N1P1_Fragment_[]
184.0734
C5H12N_Fragment_[]
86.0970
C5H12N_Fragment_[]
86.0941
Relative Intensity
m/z

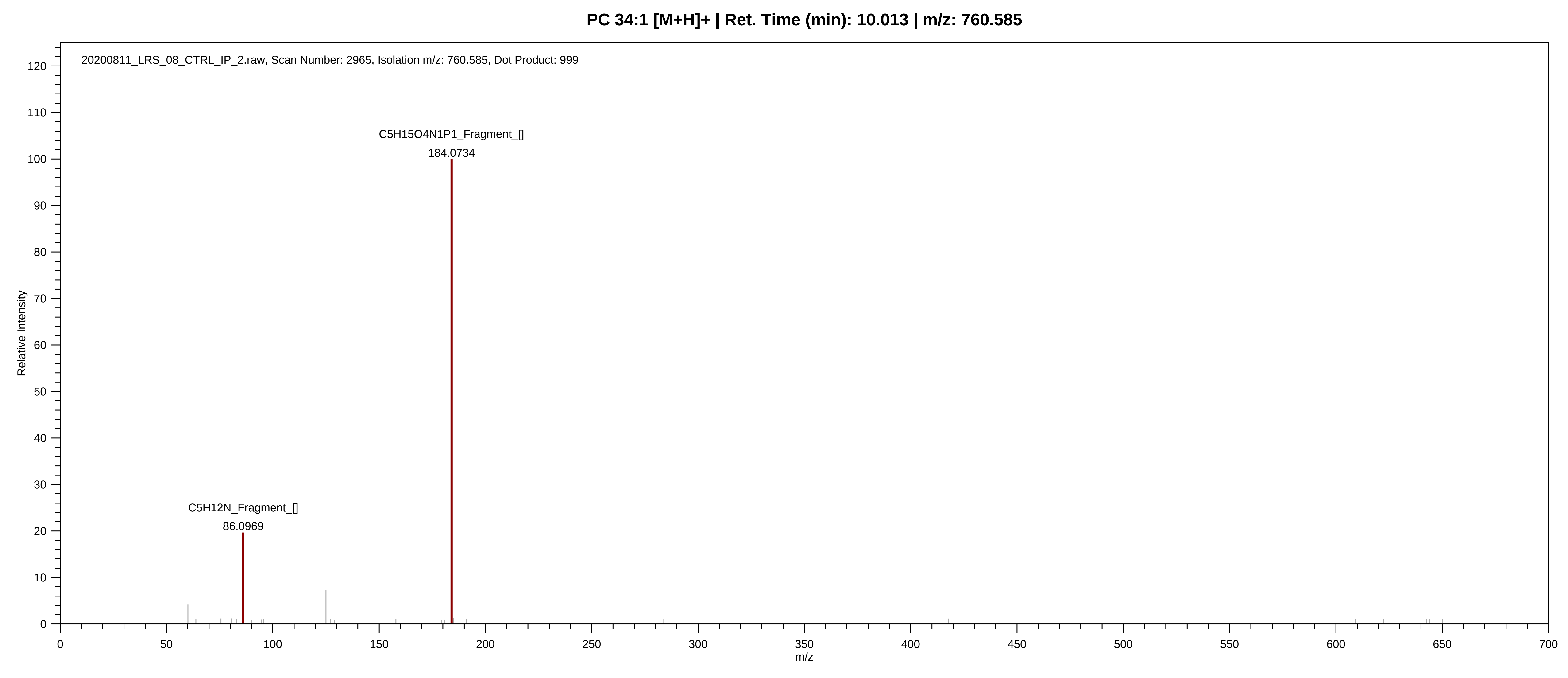

PC 34:1 [M+H]+ | Ret. Time (min): 10.013 | m/z: 760.585
20200811_LRS_08_CTRL_IP_2.raw, Scan Number: 2965, Isolation m/z: 760.585, Dot Product: 999
C5H15O4N1P1_Fragment_[]
184.0734
C5H12N_Fragment_[]
86.0969
Relative Intensity
m/z

PC 34:1 [M+H]+ | Ret. Time (min): 10.017 | m/z: 760.585

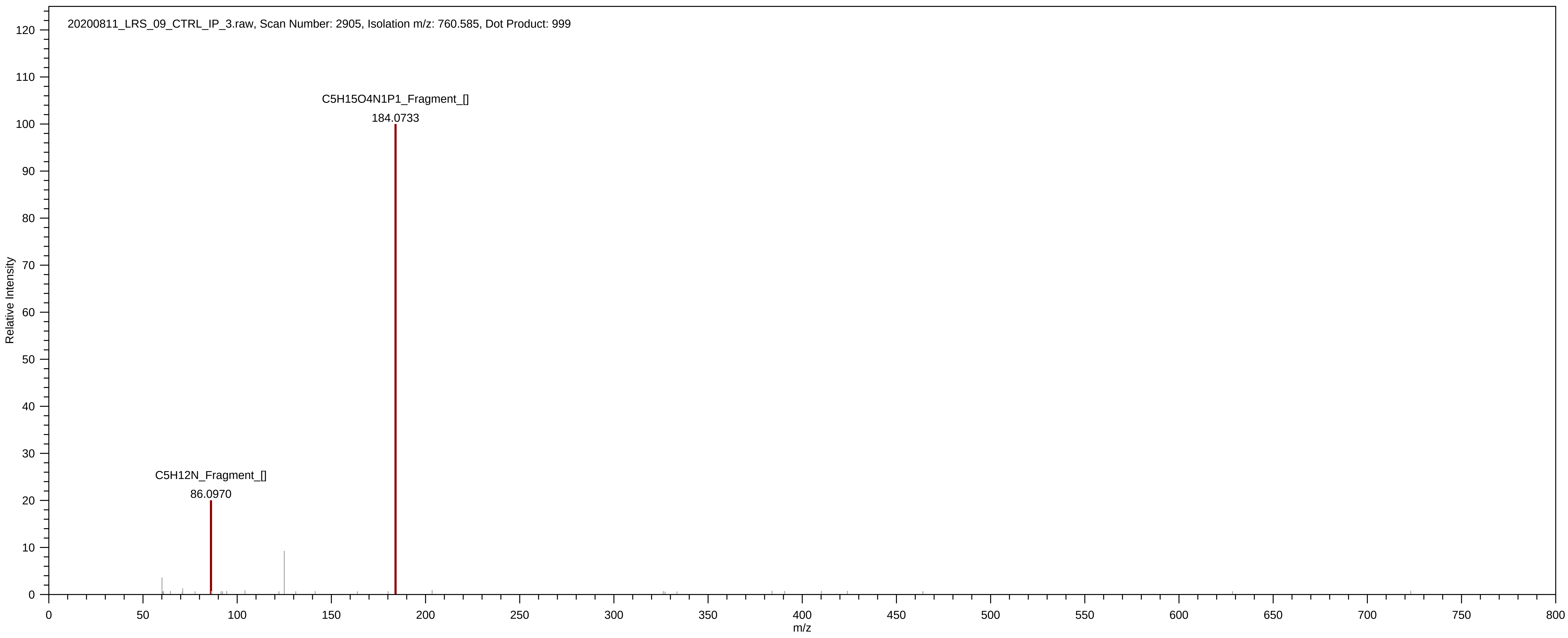

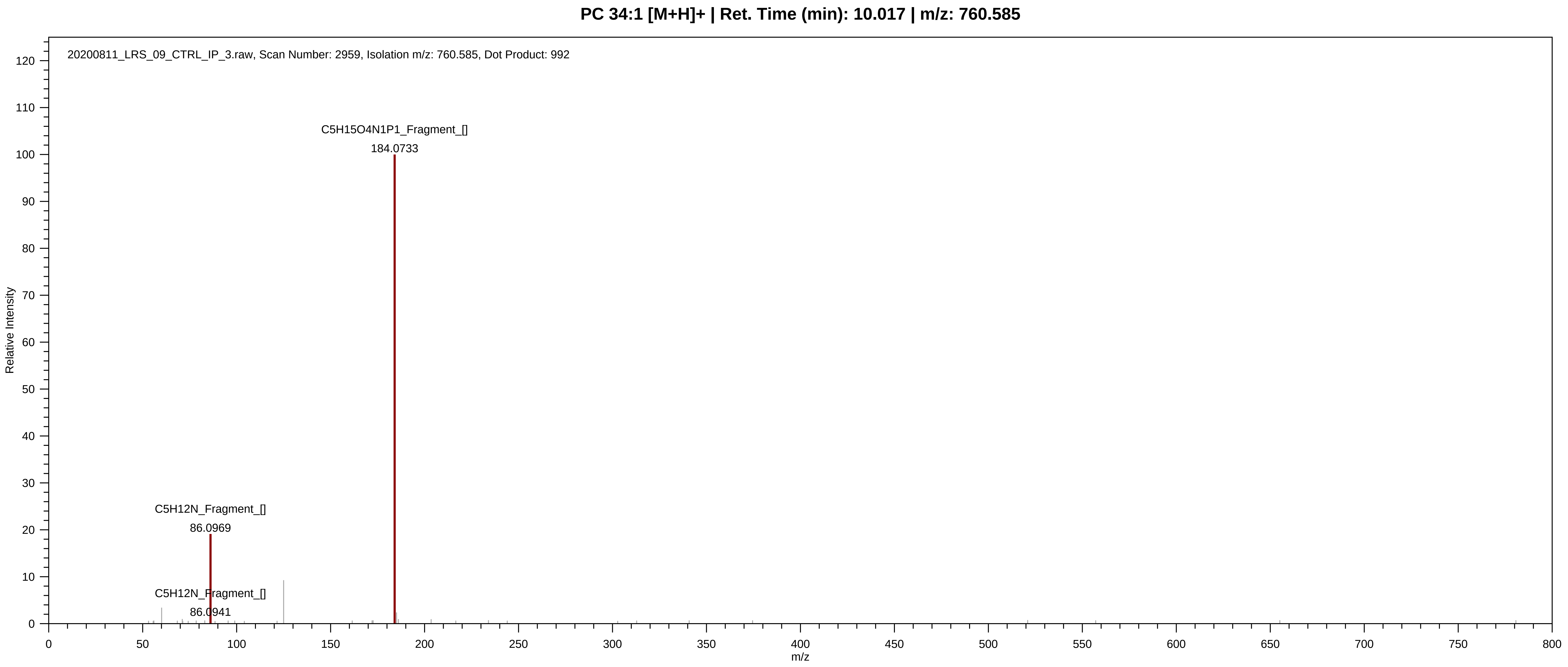
PC 34:1 [M+H]+ | Ret. Time (min): 10.017 | m/z: 760.585
20200811_LRS_09_CTRL_IP_3.raw, Scan Number: 2959, Isolation m/z: 760.585, Dot Product: 992
C5H15O4N1P1_Fragment_[]
184.0733
C5H12N_Fragment_[]
86.0969
C5H12N_Fragment_[]
86.0941
Relative Intensity
m/z

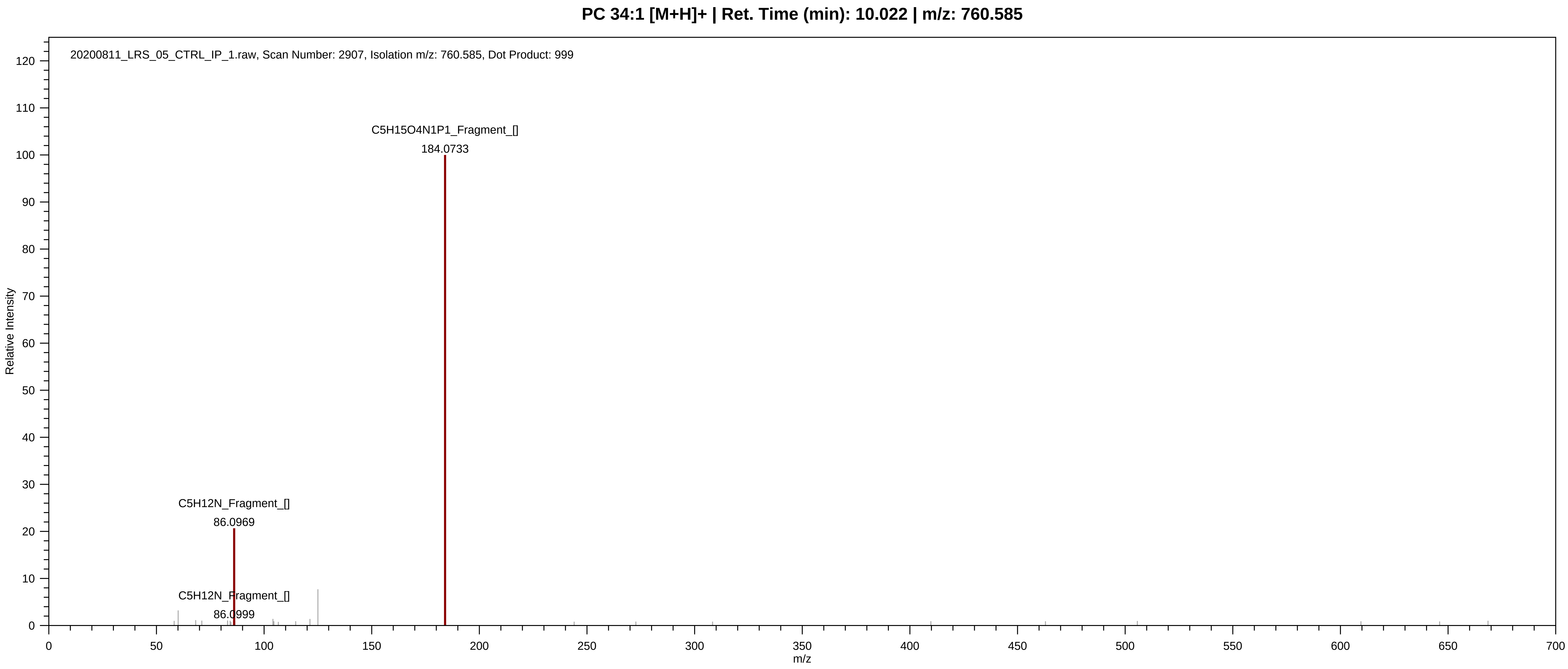

PC 34:1 [M+H]+ | Ret. Time (min): 10.022 | m/z: 760.585
20200811_LRS_05_CTRL_IP_1.raw, Scan Number: 2907, Isolation m/z: 760.585, Dot Product: 999
C5H15O4N1P1_Fragment_[]
184.0733
C5H12N_Fragment_[]
86.0969
C5H12N_Fragment_[]
86.0999
Relative Intensity
m/z

PC 34:1 [M+H]+ | Ret. Time (min): 10.022 | m/z: 760.585

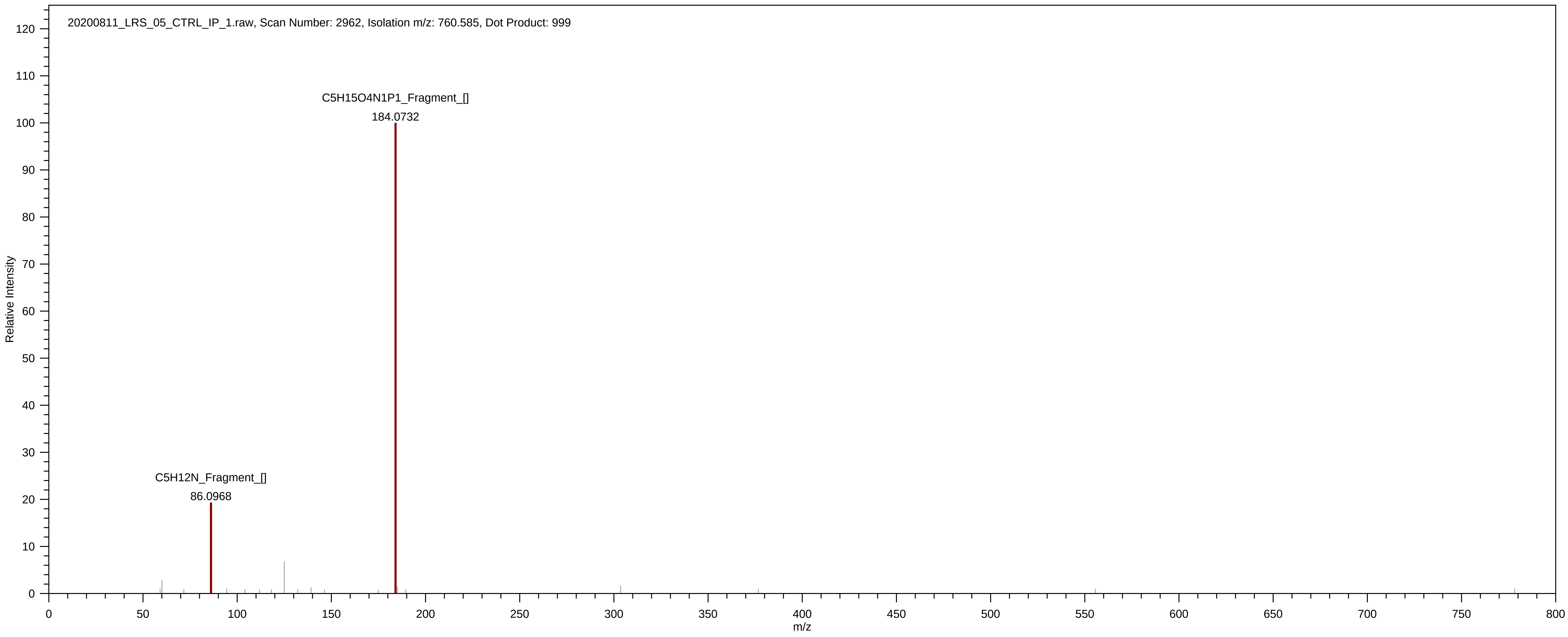

PC 34:2 [M+Ac-H]- | Ret. Time (min): 9.159 | m/z: 816.575

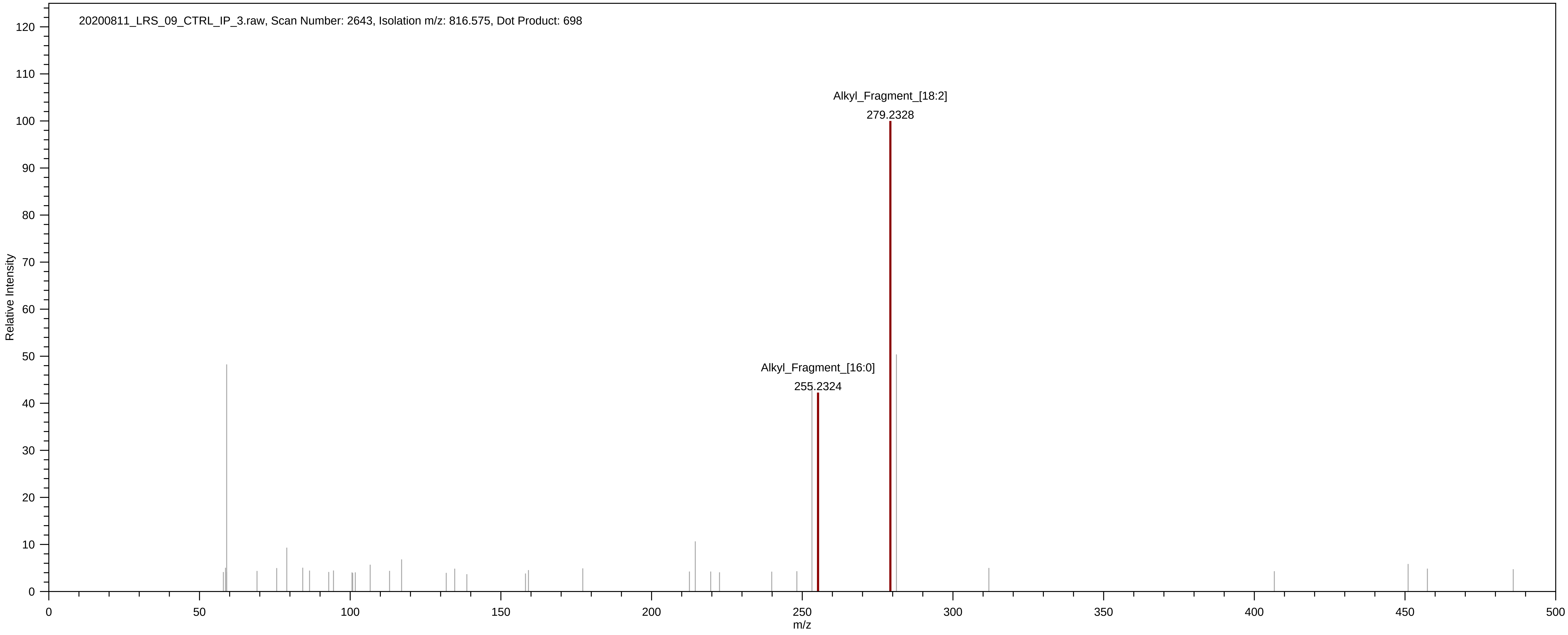

PC 34:2 [M+Ac-H]- | Ret. Time (min): 9.148 | m/z: 816.576

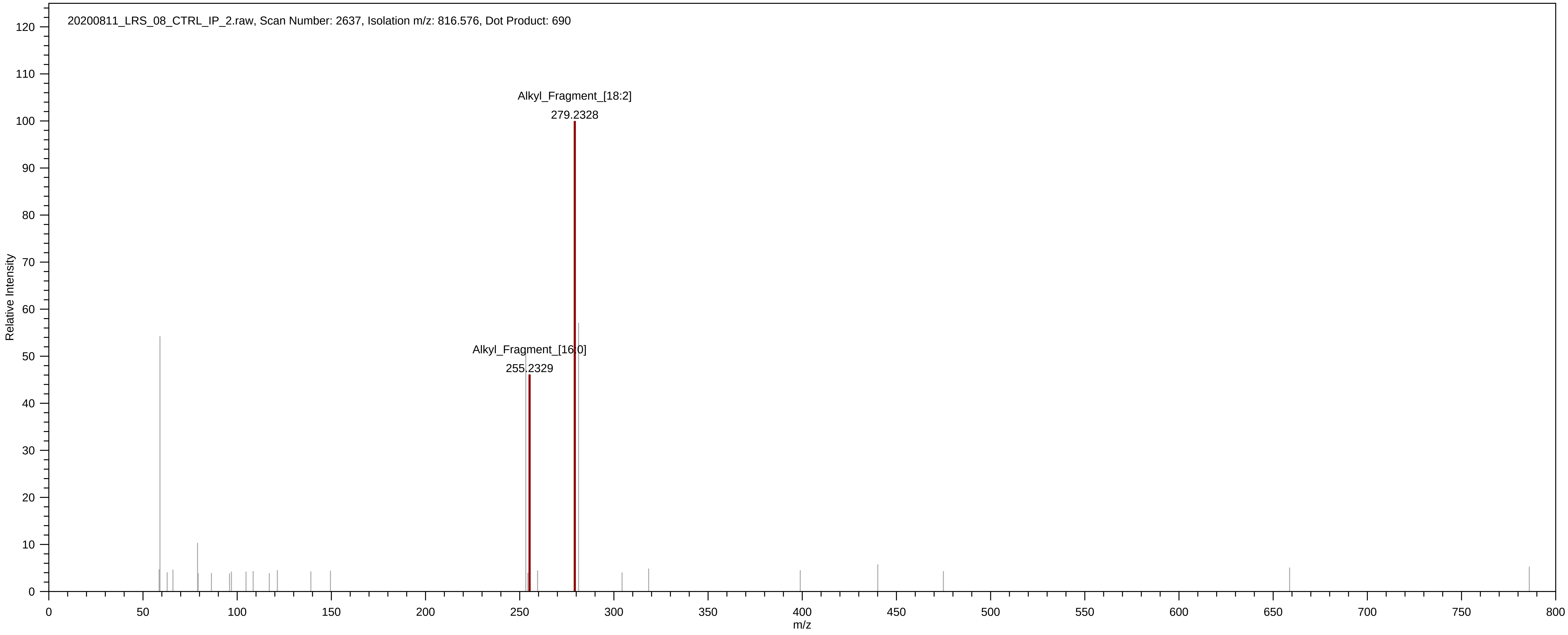

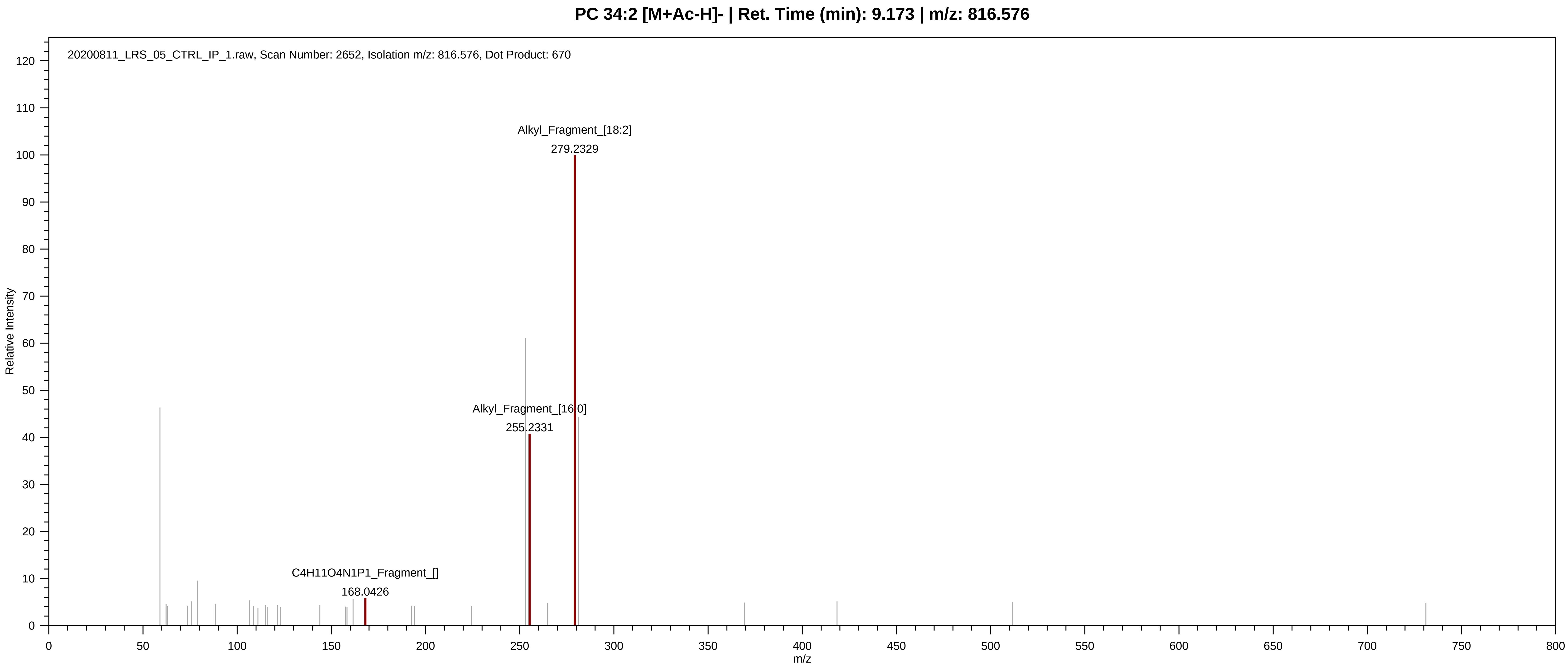

PC 34:2 [M+Ac-H]- | Ret. Time (min): 9.173 | m/z: 816.576
20200811_LRS_05_CTRL_IP_1.raw, Scan Number: 2652, Isolation m/z: 816.576, Dot Product: 670
Alkyl_Fragment_[18:2]
279.2329
Alkyl_Fragment_[16:0]
255.2331
C4H11O4N1P1_Fragment_[]
168.0426
Relative Intensity
m/z

PC 34:2 [M+H]+ | Ret. Time (min): 8.966 | m/z: 758.57

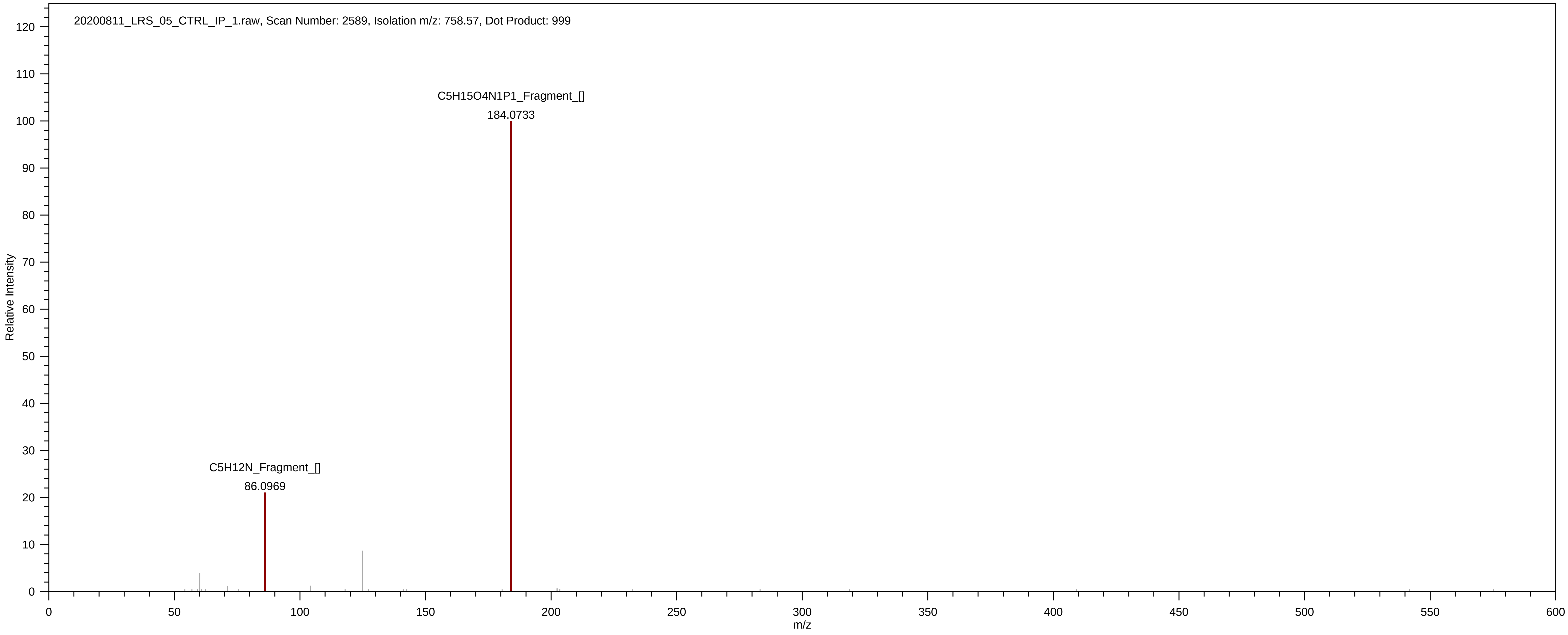

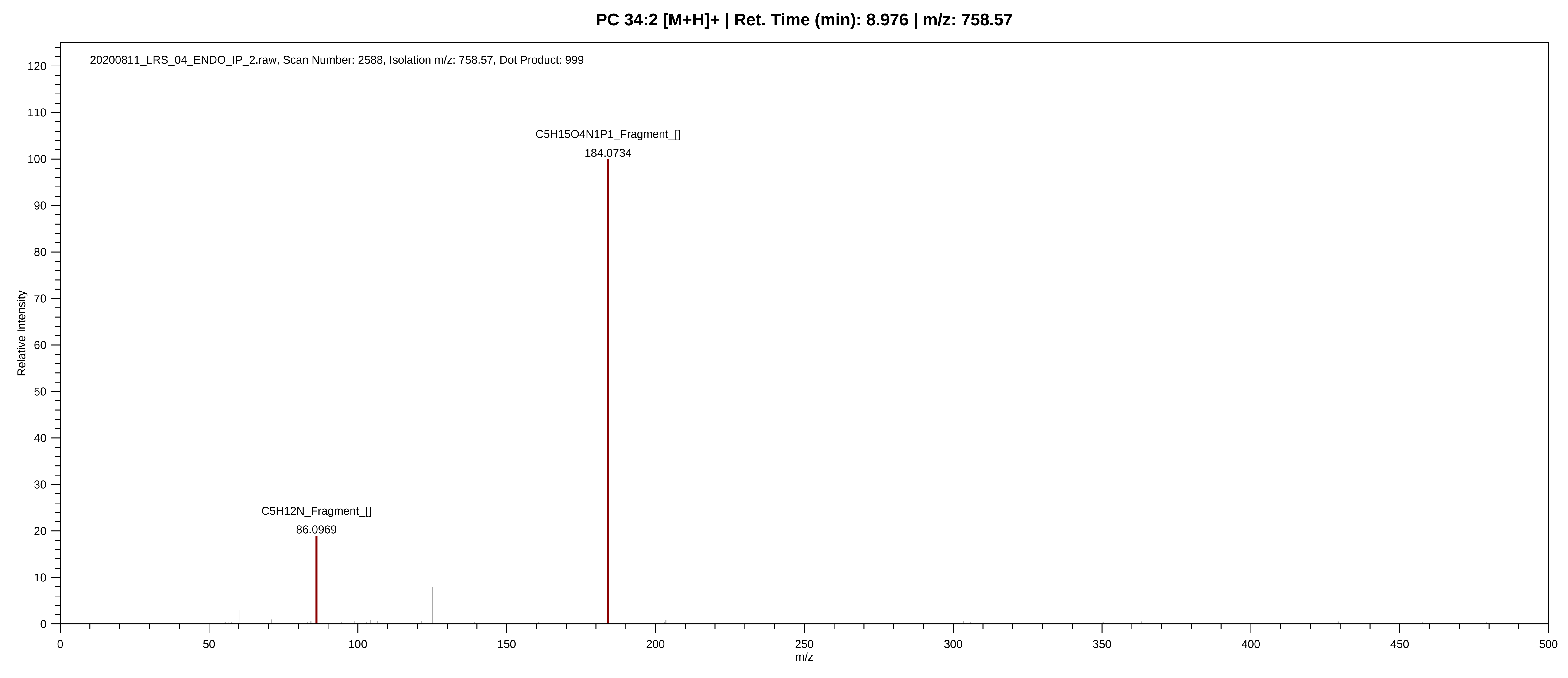
PC 34:2 [M+H]+ | Ret. Time (min): 8.976 | m/z: 758.57
20200811_LRS_04_ENDO_IP_2.raw, Scan Number: 2588, Isolation m/z: 758.57, Dot Product: 999
C5H15O4N1P1_Fragment_[]
184.0734
C5H12N_Fragment_[]
86.0969
Relative Intensity
m/z

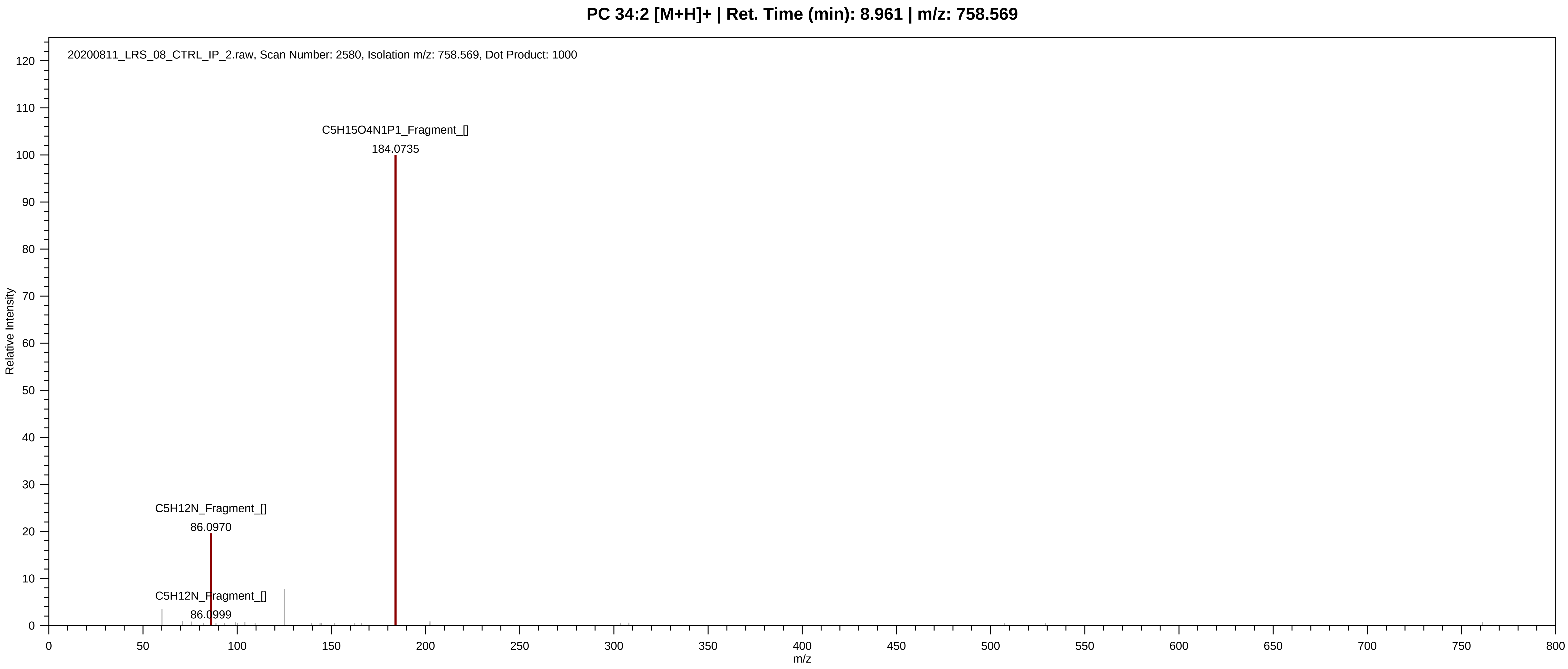
PC 34:2 [M+H]+ | Ret. Time (min): 8.961 | m/z: 758.569
20200811_LRS_08_CTRL_IP_2.raw, Scan Number: 2580, Isolation m/z: 758.569, Dot Product: 1000
C5H15O4N1P1_Fragment_[]
184.0735
C5H12N_Fragment_[]
86.0970
C5H12N_Fragment_[]
86.0999
Relative Intensity
m/z

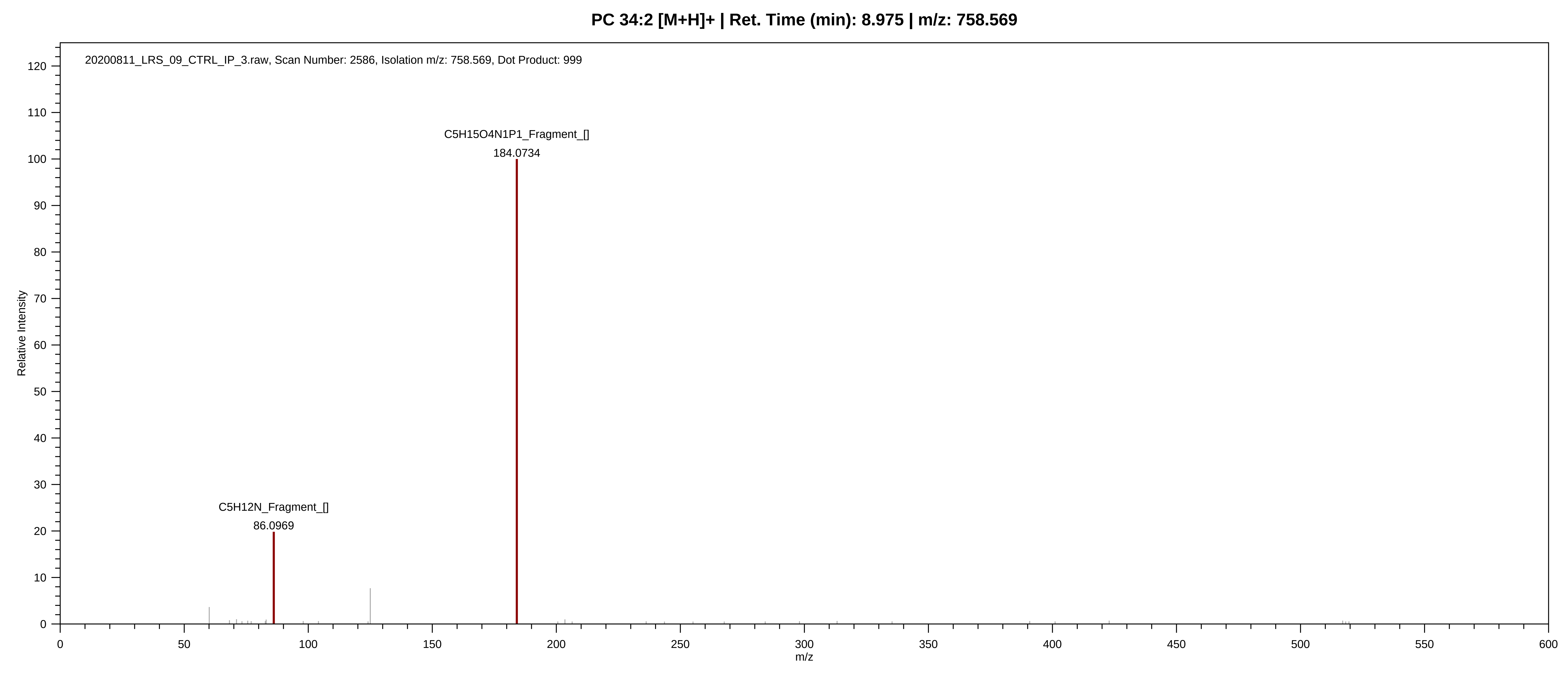

PC 34:2 [M+H]+ | Ret. Time (min): 8.975 | m/z: 758.569
20200811_LRS_09_CTRL_IP_3.raw, Scan Number: 2586, Isolation m/z: 758.569, Dot Product: 999
C5H15O4N1P1_Fragment_[]
184.0734
C5H12N_Fragment_[]
86.0969
Relative Intensity
m/z

PC 34:2 [M+H]+ | Ret. Time (min): 8.982 | m/z: 758.569

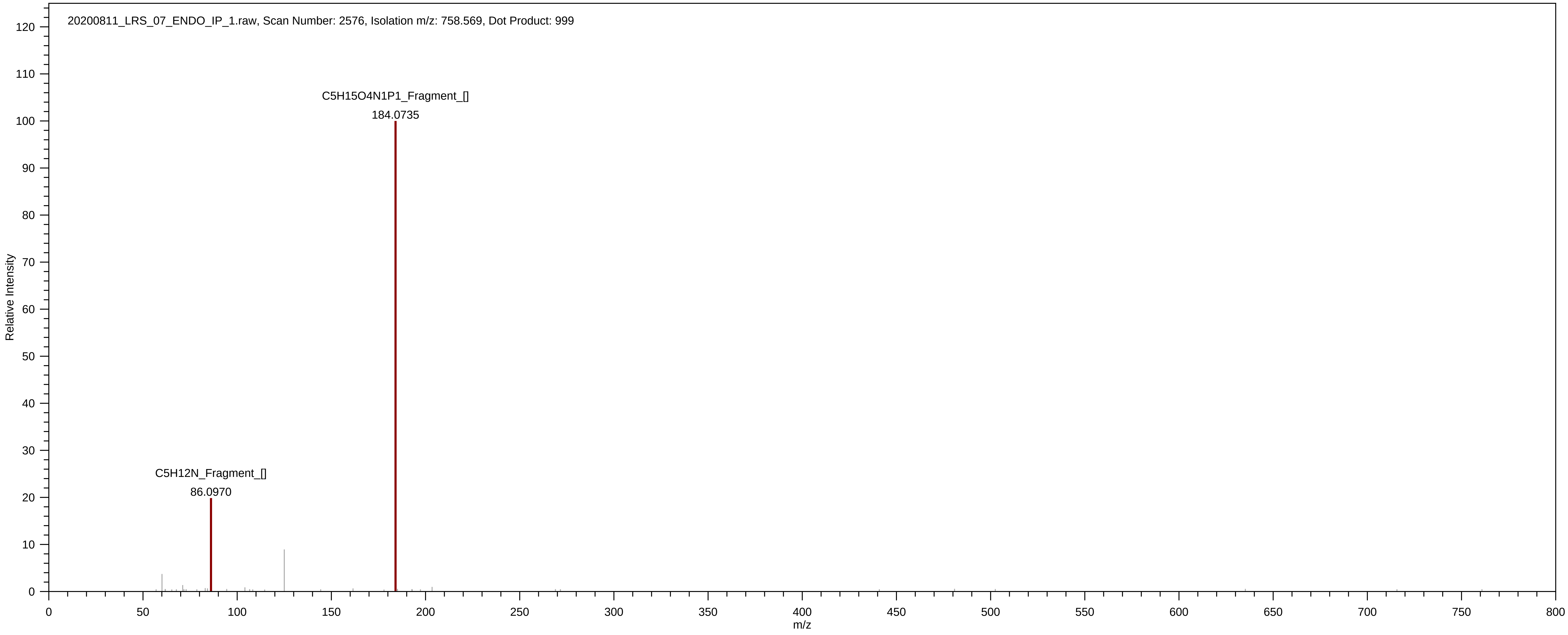

PC 34:2 [M+H]+ | Ret. Time (min): 8.983 | m/z: 758.569

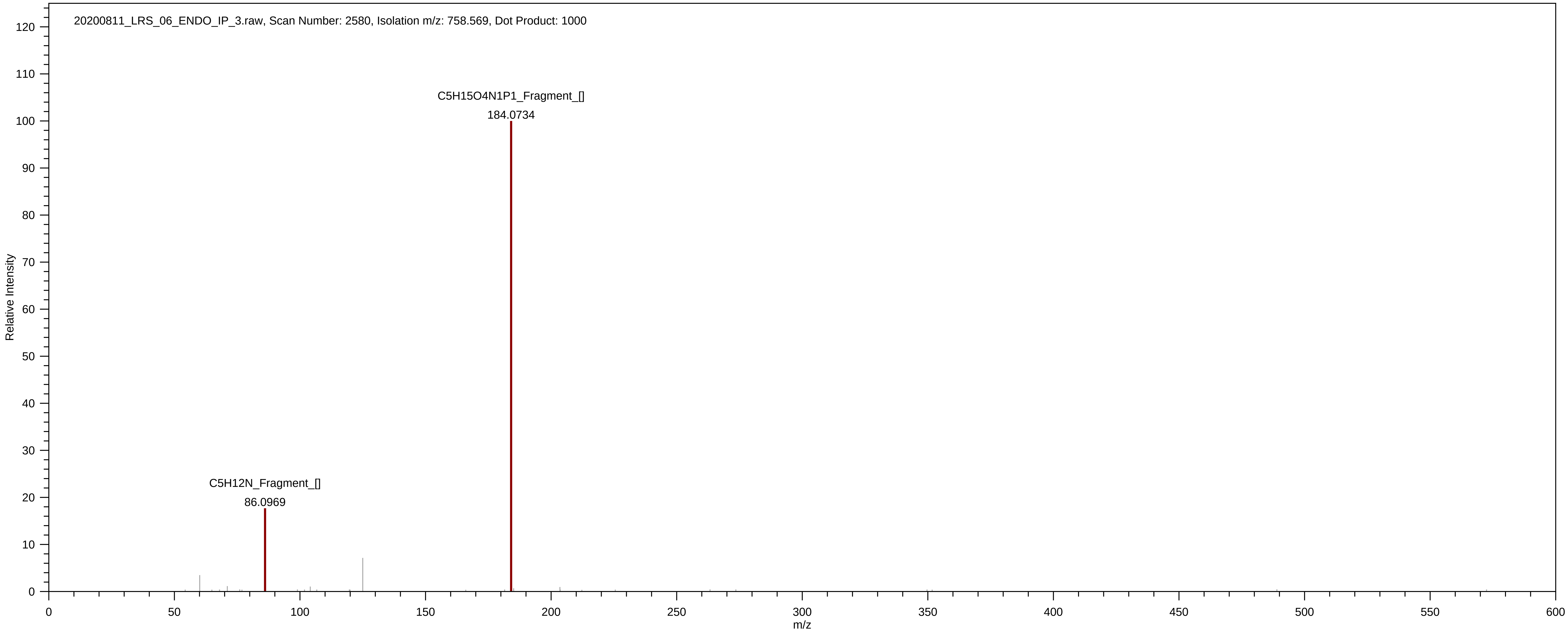

PC 34:2 [M+H]+ | Ret. Time (min): 9.16 | m/z: 758.569

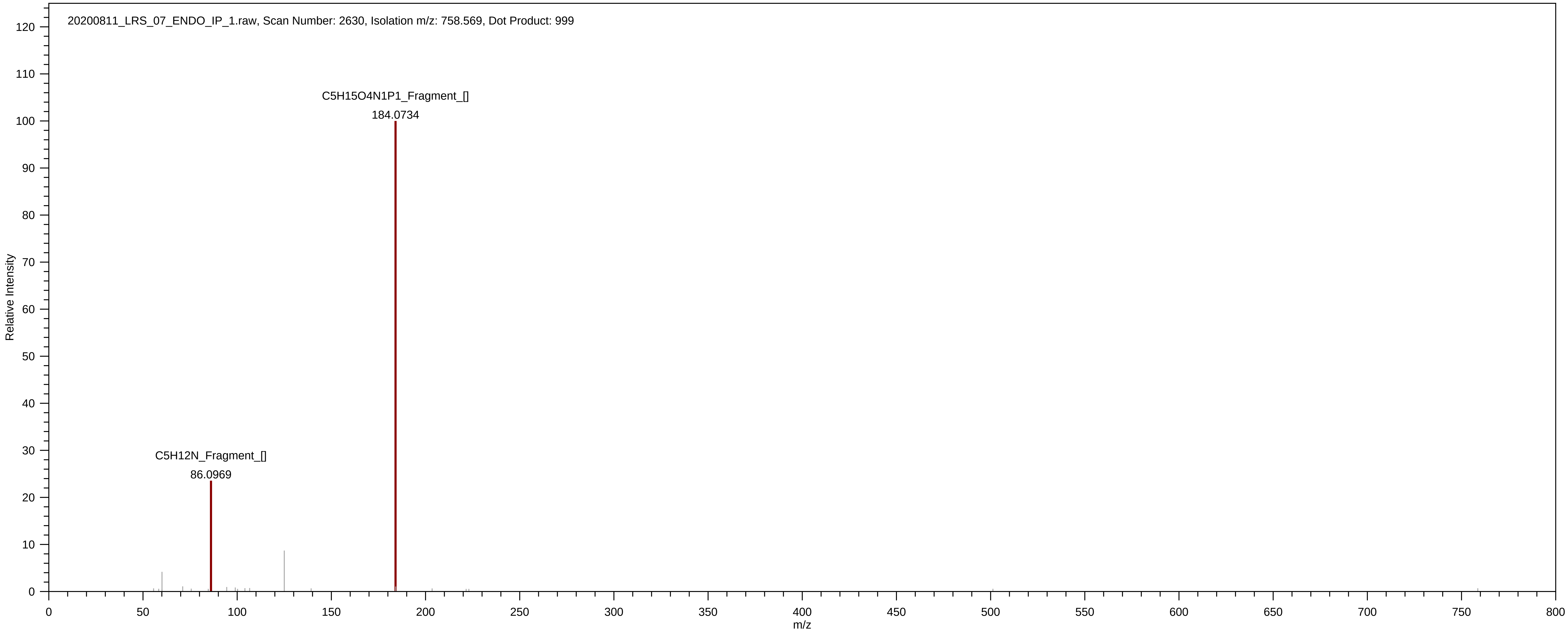

PC 34:2 [M+H]+ | Ret. Time (min): 9.139 | m/z: 758.569

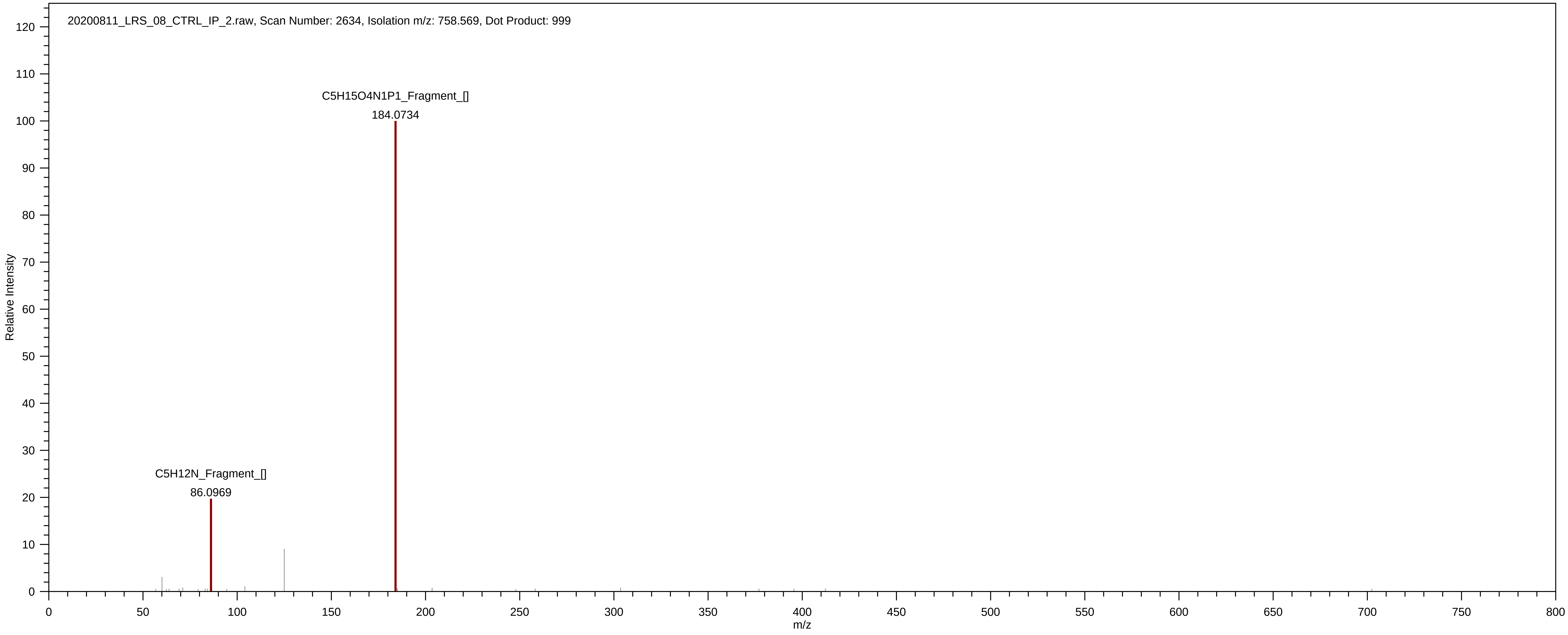

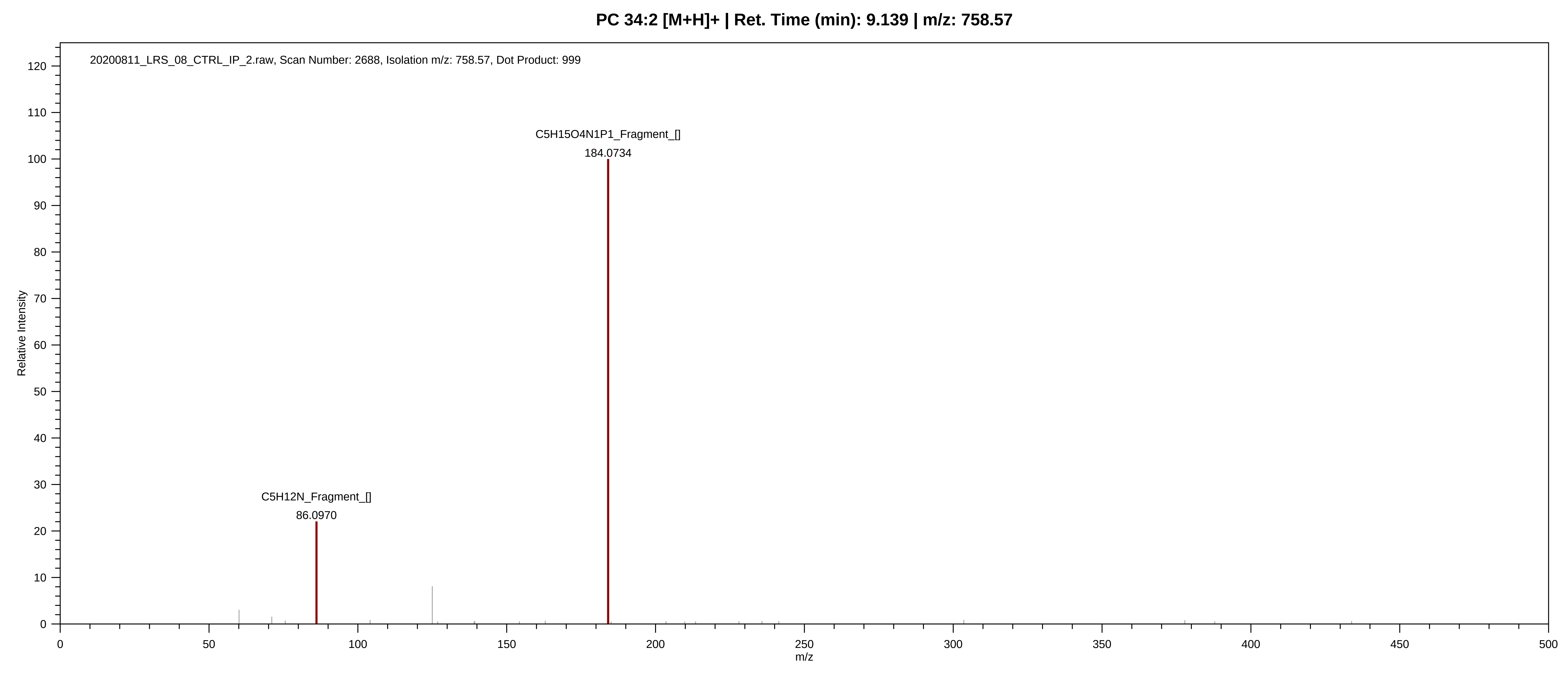

PC 34:2 [M+H]+ | Ret. Time (min): 9.139 | m/z: 758.57
20200811_LRS_08_CTRL_IP_2.raw, Scan Number: 2688, Isolation m/z: 758.57, Dot Product: 999
C5H15O4N1P1_Fragment_[]
184.0734
C5H12N_Fragment_[]
86.0970
Relative Intensity
m/z
0
10
20
30
40
50
60
70
80
90
100
110
120
0
50
100
150
200
250
300
350
400
450
500

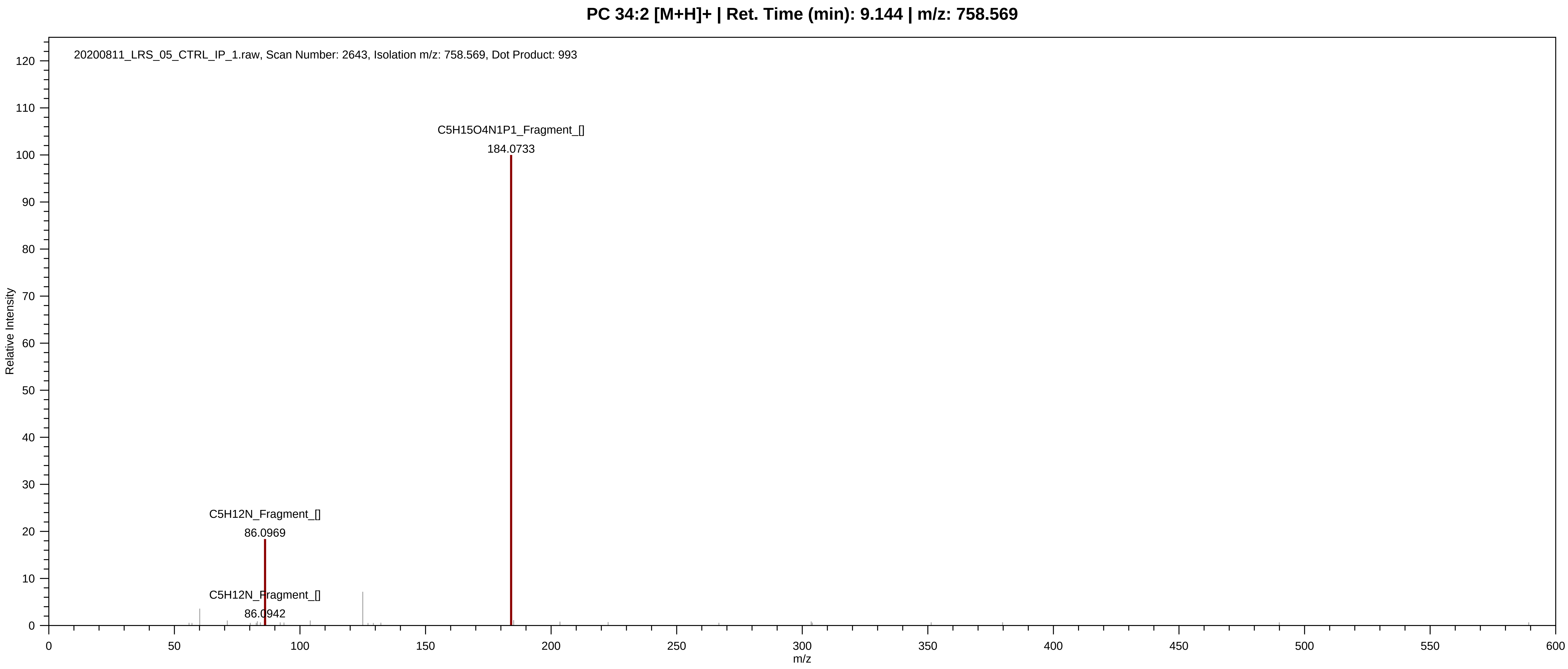

PC 34:2 [M+H]+ | Ret. Time (min): 9.144 | m/z: 758.569
20200811_LRS_05_CTRL_IP_1.raw, Scan Number: 2643, Isolation m/z: 758.569, Dot Product: 993
C5H15O4N1P1_Fragment_[]
184.0733
C5H12N_Fragment_[]
86.0969
C5H12N_Fragment_[]
86.0942
Relative Intensity
m/z

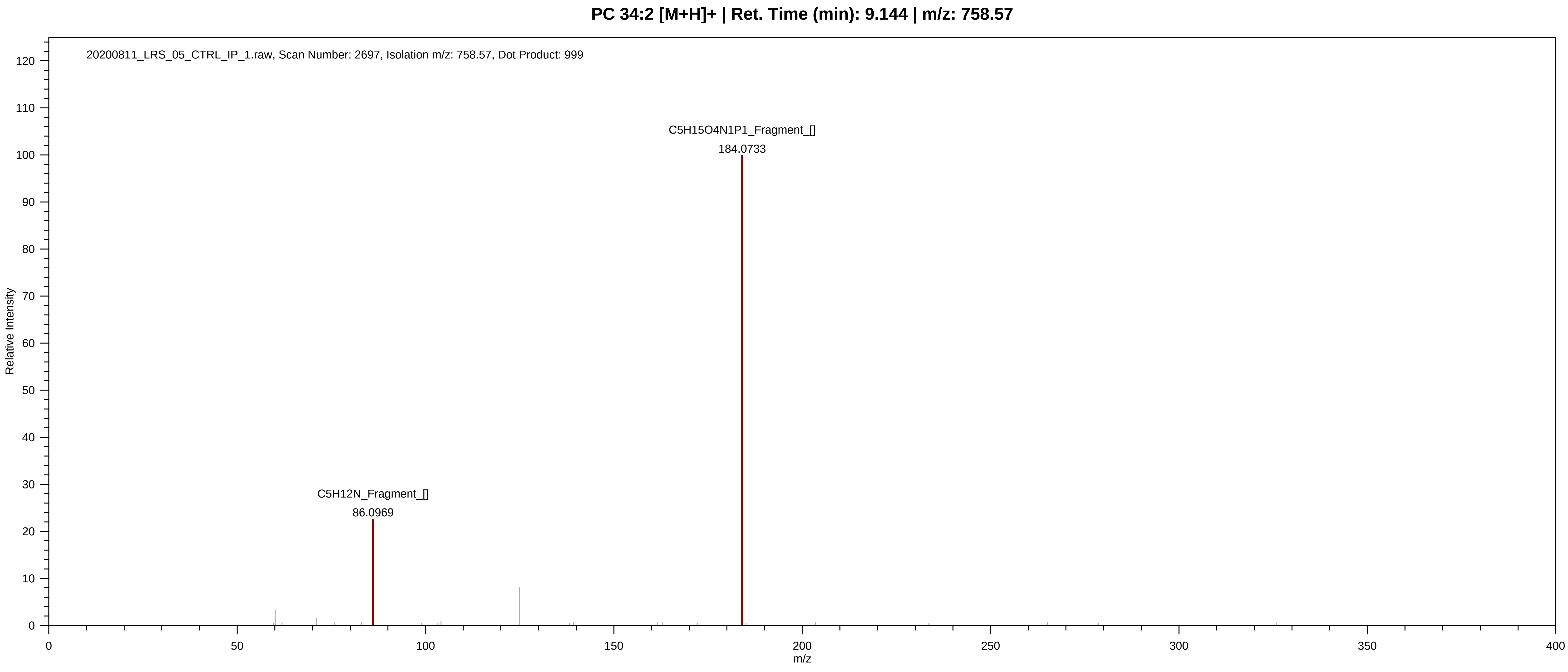
PC 34:2 [M+H]+ | Ret. Time (min): 9.144 | m/z: 758.57
20200811_LRS_05_CTRL_IP_1.raw, Scan Number: 2697, Isolation m/z: 758.57, Dot Product: 999
C5H15O4N1P1_Fragment_[]
184.0733
C5H12N_Fragment_[]
86.0969
Relative Intensity
m/z

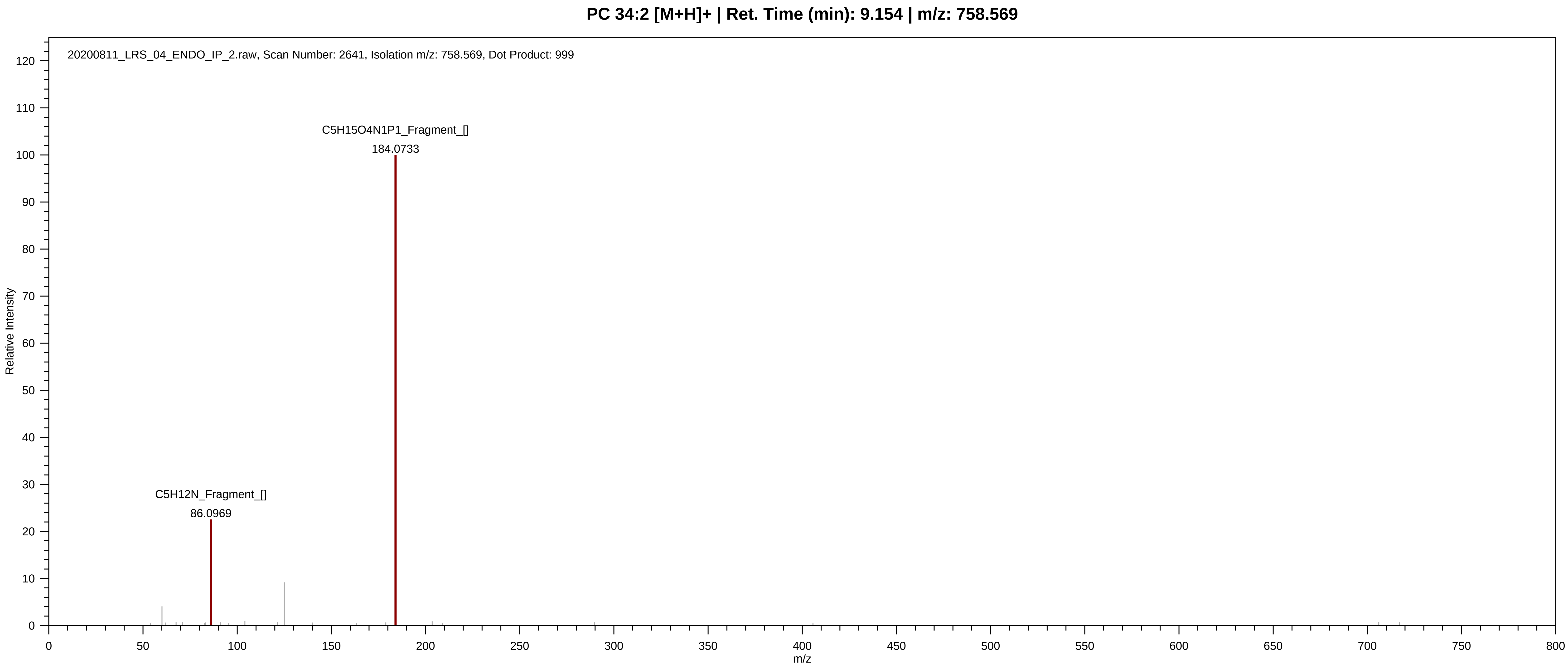
PC 34:2 [M+H]+ | Ret. Time (min): 9.154 | m/z: 758.569
20200811_LRS_04_ENDO_IP_2.raw, Scan Number: 2641, Isolation m/z: 758.569, Dot Product: 999
C5H15O4N1P1_Fragment_[]
184.0733
C5H12N_Fragment_[]
86.0969
Relative Intensity
m/z

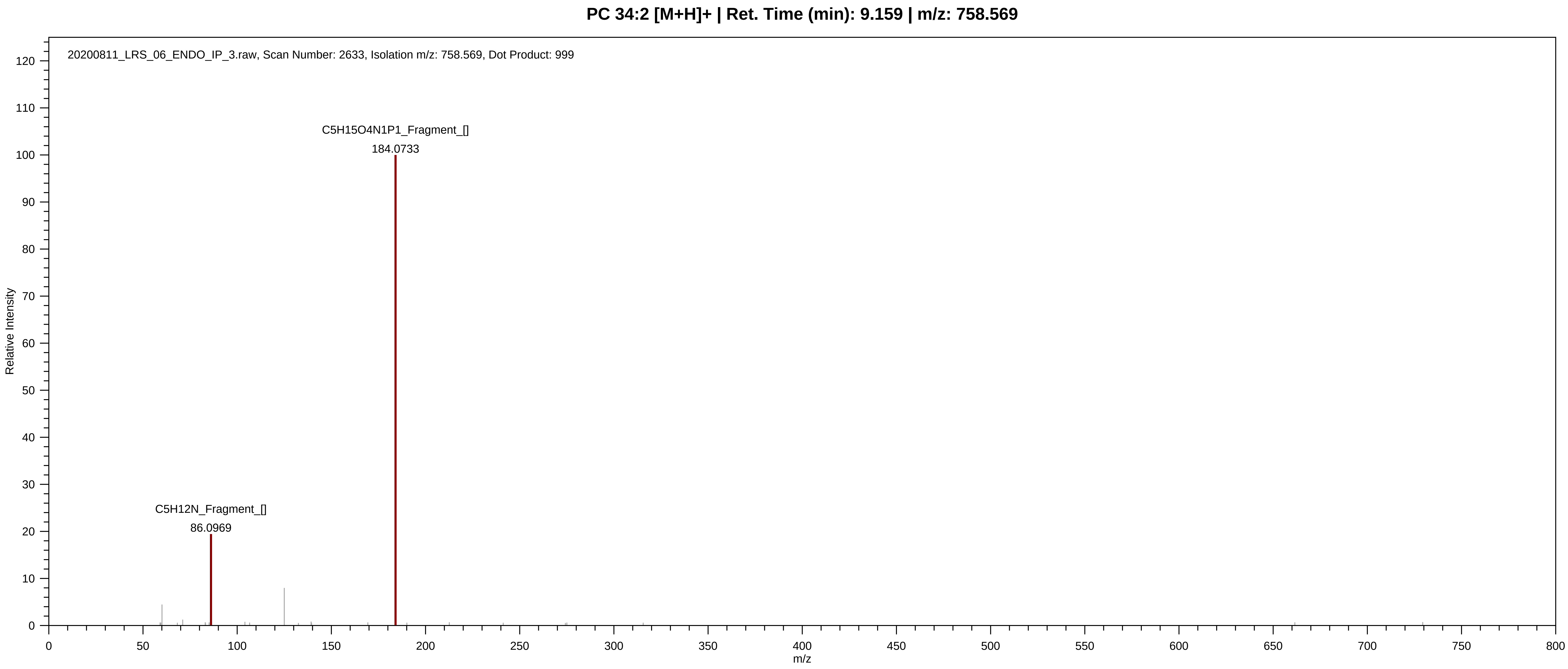
PC 34:2 [M+H]+ | Ret. Time (min): 9.159 | m/z: 758.569
20200811_LRS_06_ENDO_IP_3.raw, Scan Number: 2633, Isolation m/z: 758.569, Dot Product: 999
C5H15O4N1P1_Fragment_[]
184.0733
C5H12N_Fragment_[]
86.0969
Relative Intensity
m/z
0
10
20
30
40
50
60
70
80
90
100
110
120
0
50
100
150
200
250
300
350
400
450
500
550
600
650
700
750
800

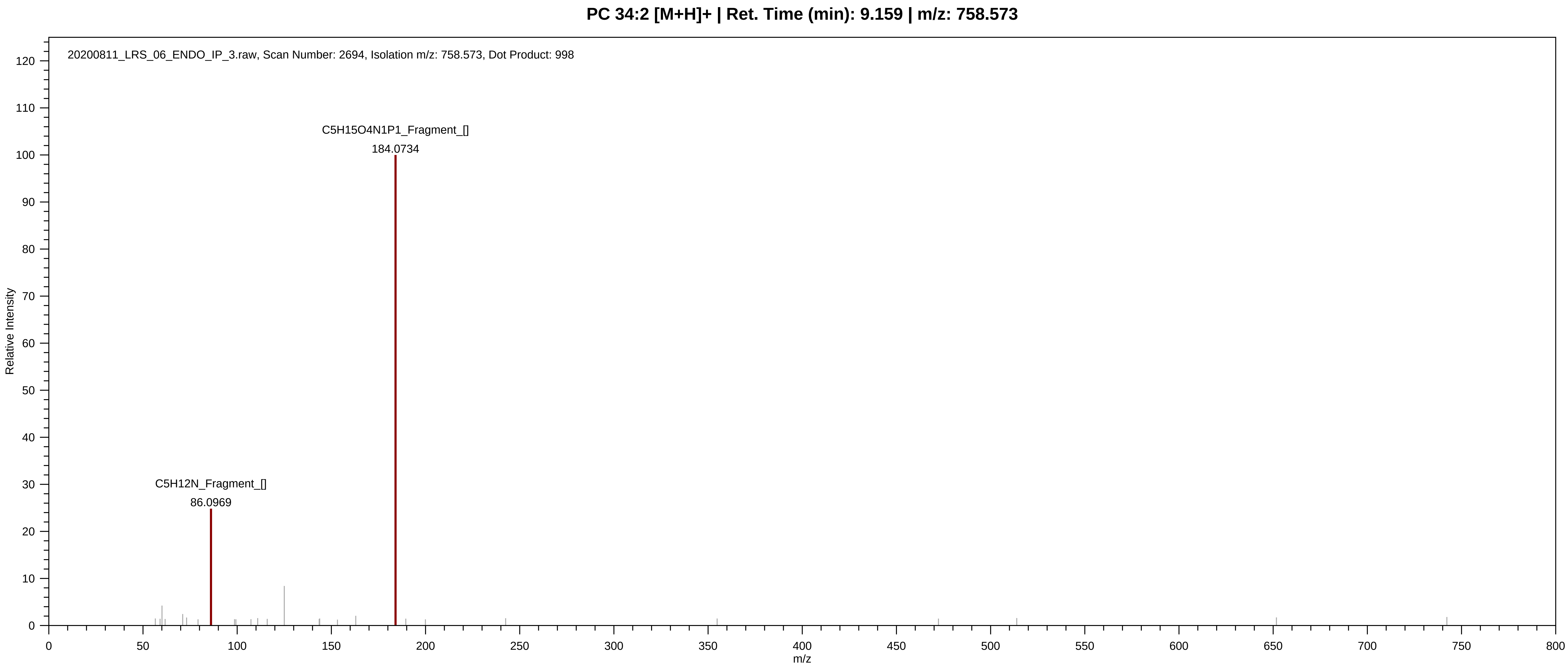

PC 34:2 [M+H]+ | Ret. Time (min): 9.159 | m/z: 758.573
20200811_LRS_06_ENDO_IP_3.raw, Scan Number: 2694, Isolation m/z: 758.573, Dot Product: 998
C5H15O4N1P1_Fragment_[]
184.0734
C5H12N_Fragment_[]
86.0969
Relative Intensity
m/z

PC 34:2 [M+H]+ | Ret. Time (min): 9.169 | m/z: 758.569

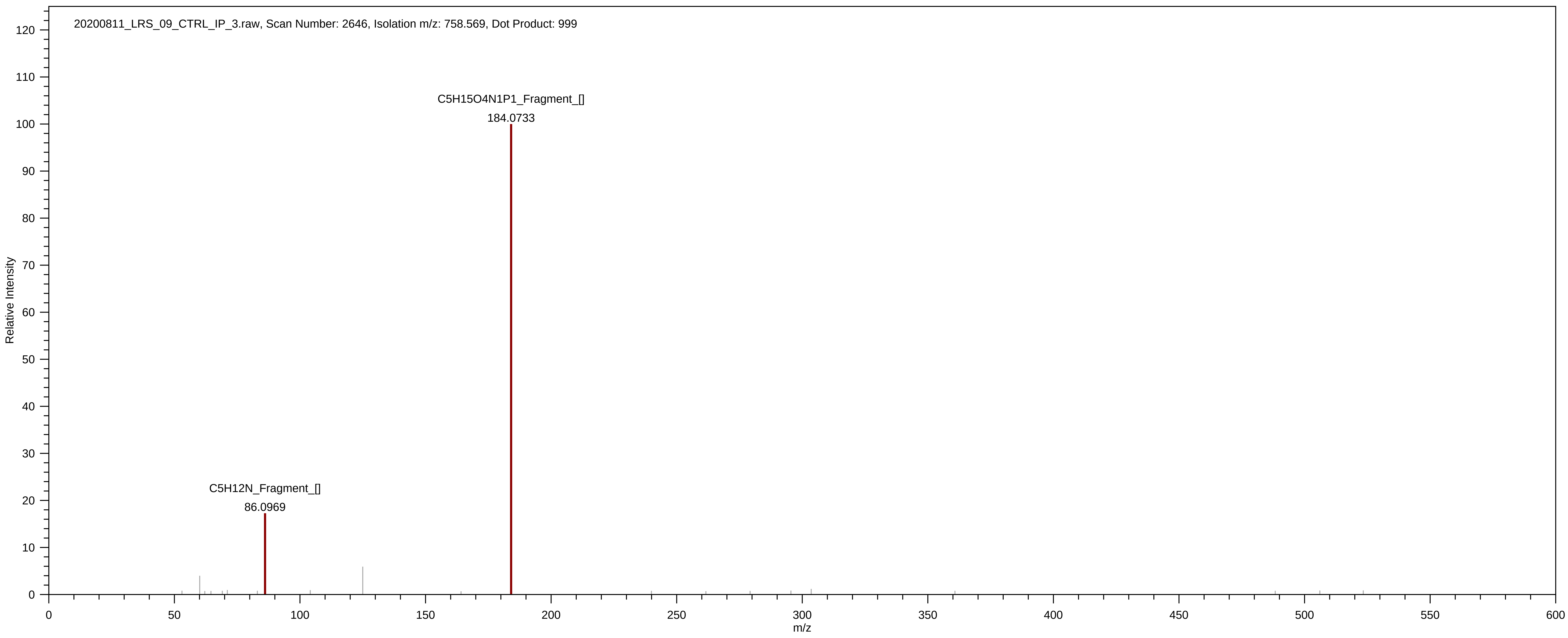

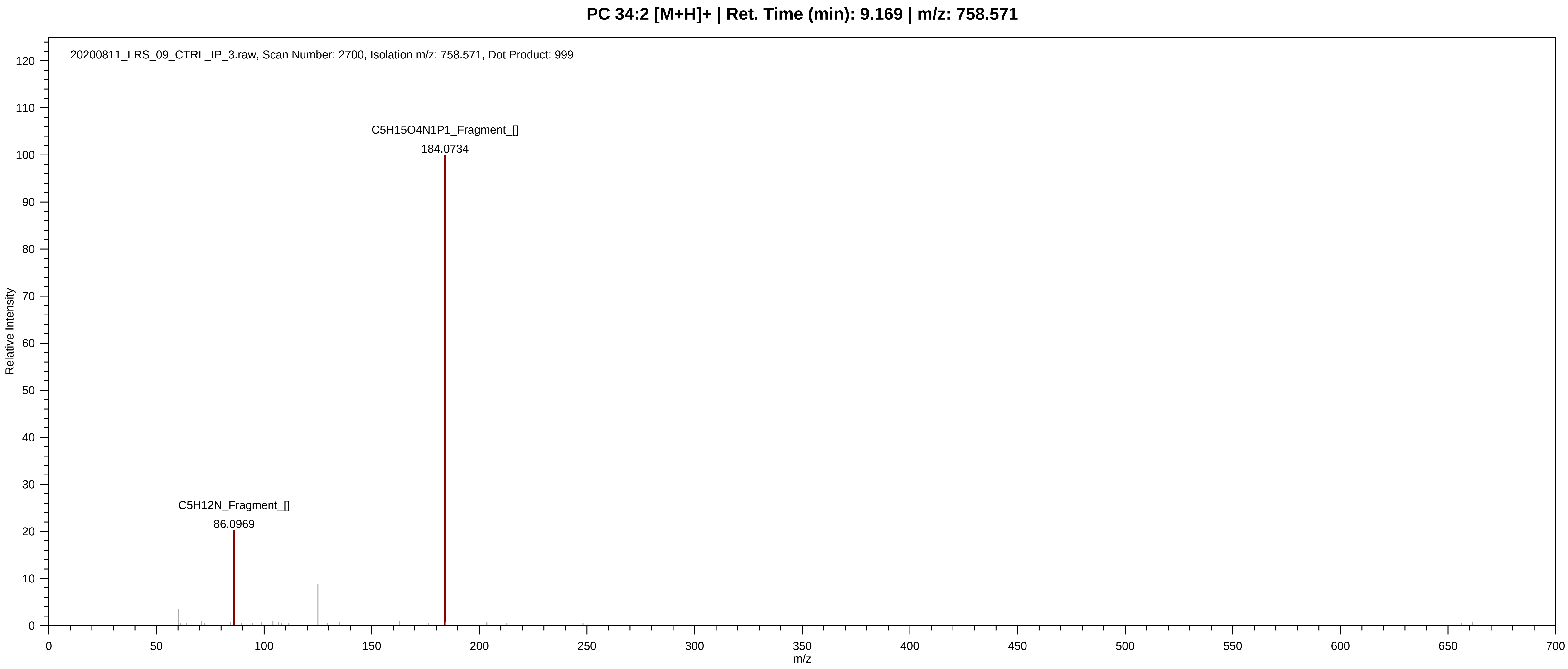
PC 34:2 [M+H]+ | Ret. Time (min): 9.169 | m/z: 758.571
20200811_LRS_09_CTRL_IP_3.raw, Scan Number: 2700, Isolation m/z: 758.571, Dot Product: 999
C5H15O4N1P1_Fragment_[]
184.0734
C5H12N_Fragment_[]
86.0969
Relative Intensity
m/z

PC 34:3 [M+H]+ | Ret. Time (min): 8.357 | m/z: 756.551

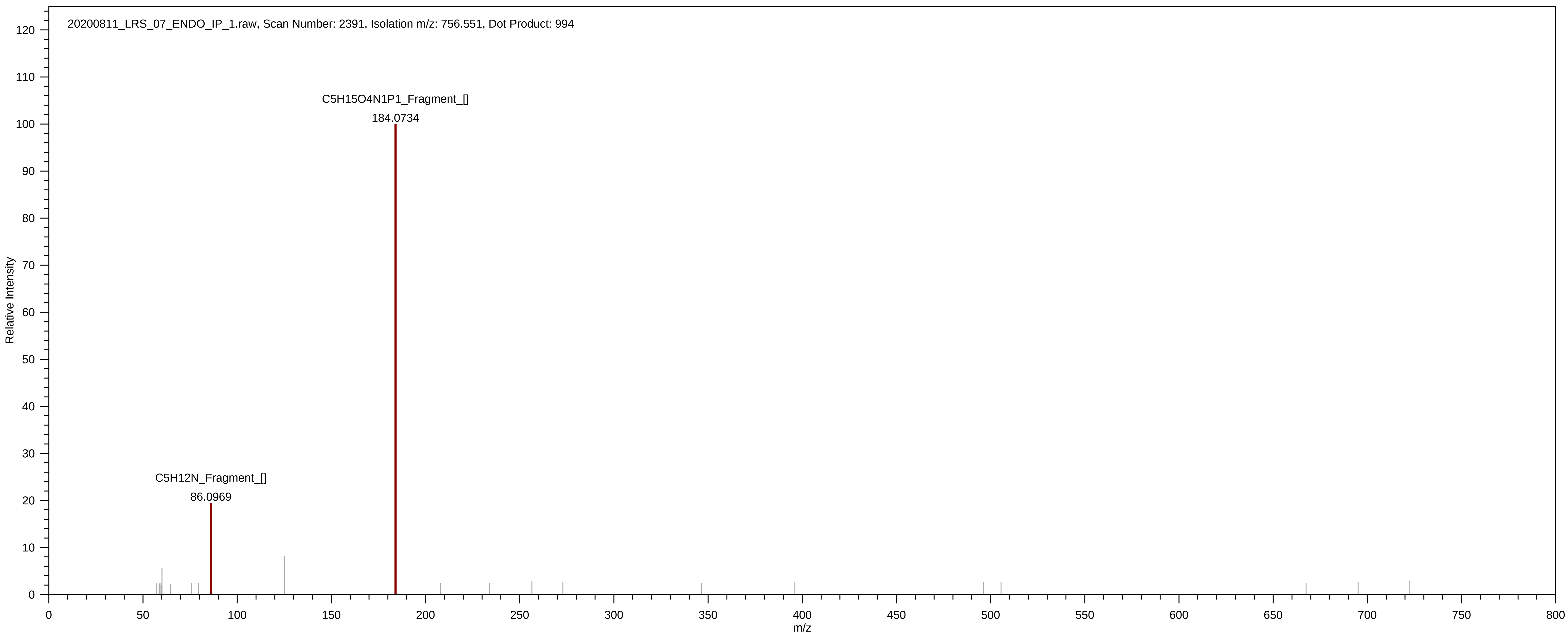

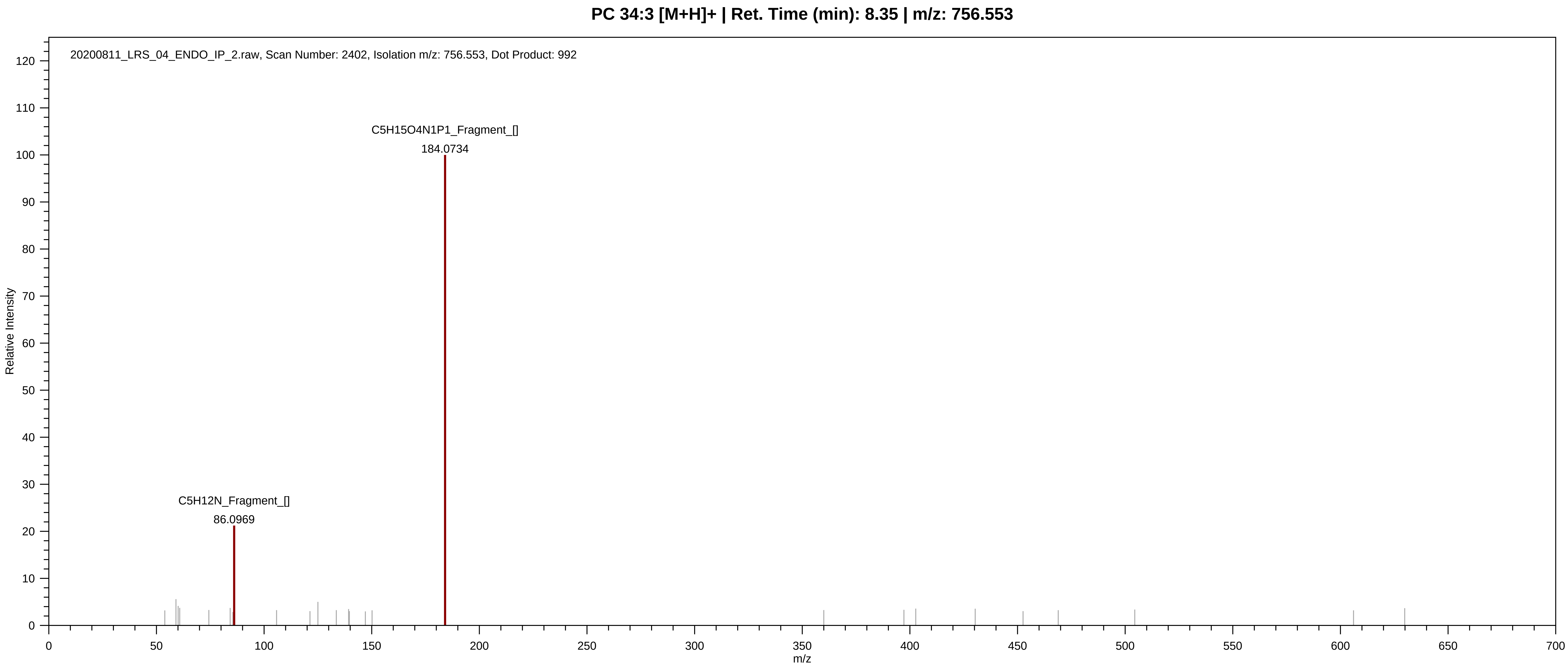
PC 34:3 [M+H]+ | Ret. Time (min): 8.35 | m/z: 756.553
20200811_LRS_04_ENDO_IP_2.raw, Scan Number: 2402, Isolation m/z: 756.553, Dot Product: 992
C5H15O4N1P1_Fragment_[]
184.0734
C5H12N_Fragment_[]
86.0969
Relative Intensity
m/z

PC 34:3 [M+H]+ | Ret. Time (min): 8.382 | m/z: 756.554

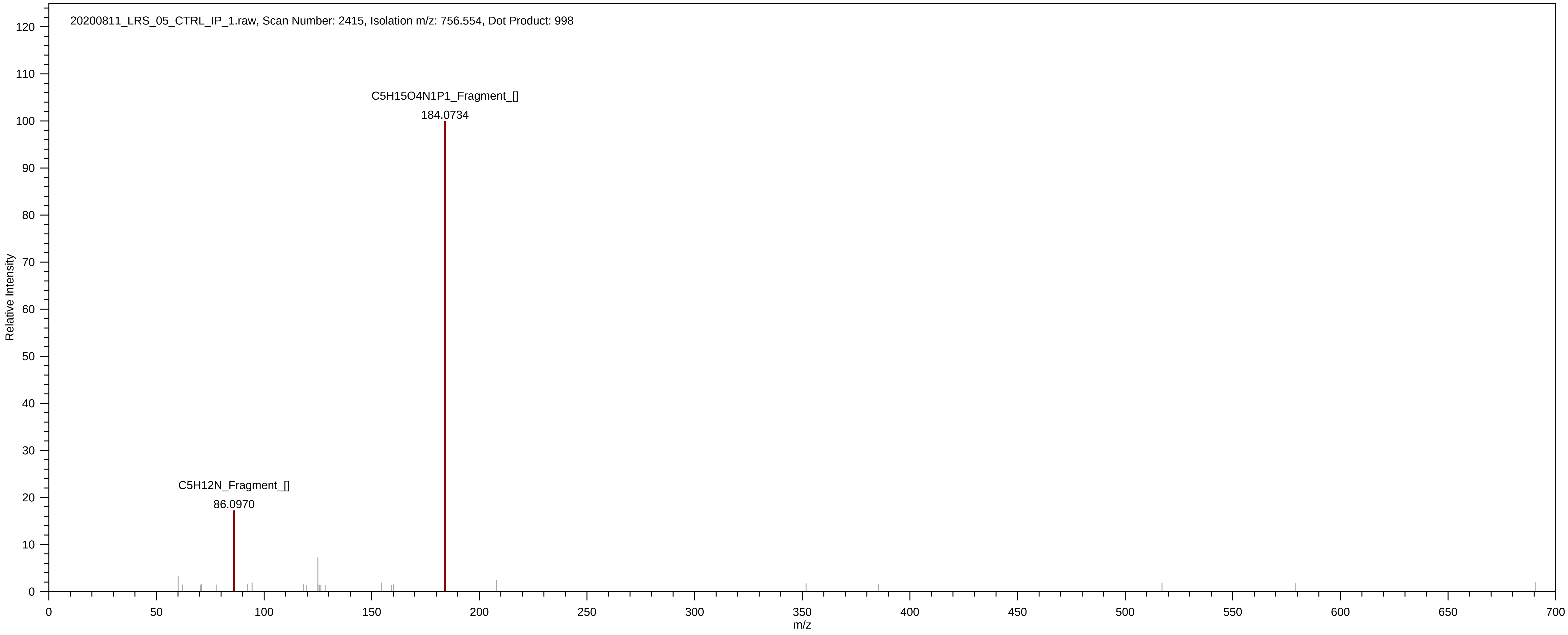

PC 34:3 [M+H]+ | Ret. Time (min): 8.396 | m/z: 756.554

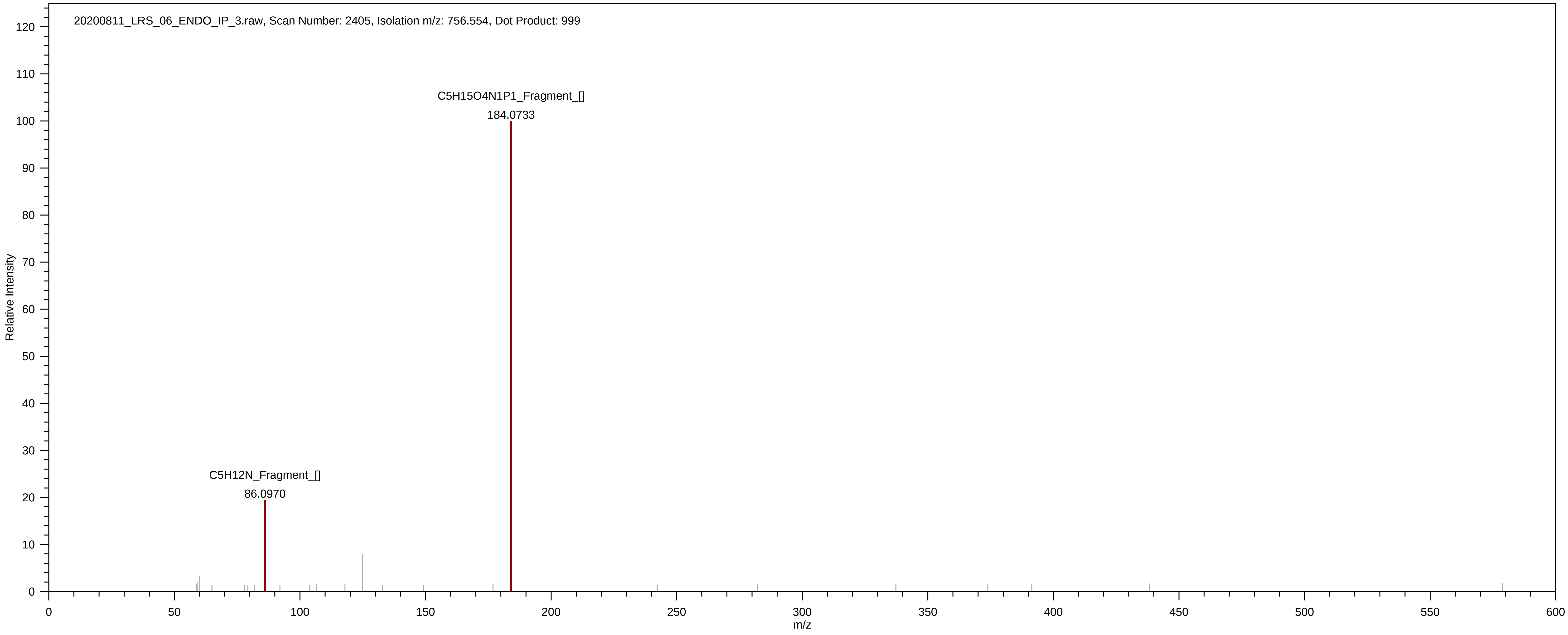

PC 34:3 [M+H]+ | Ret. Time (min): 8.534 | m/z: 756.554

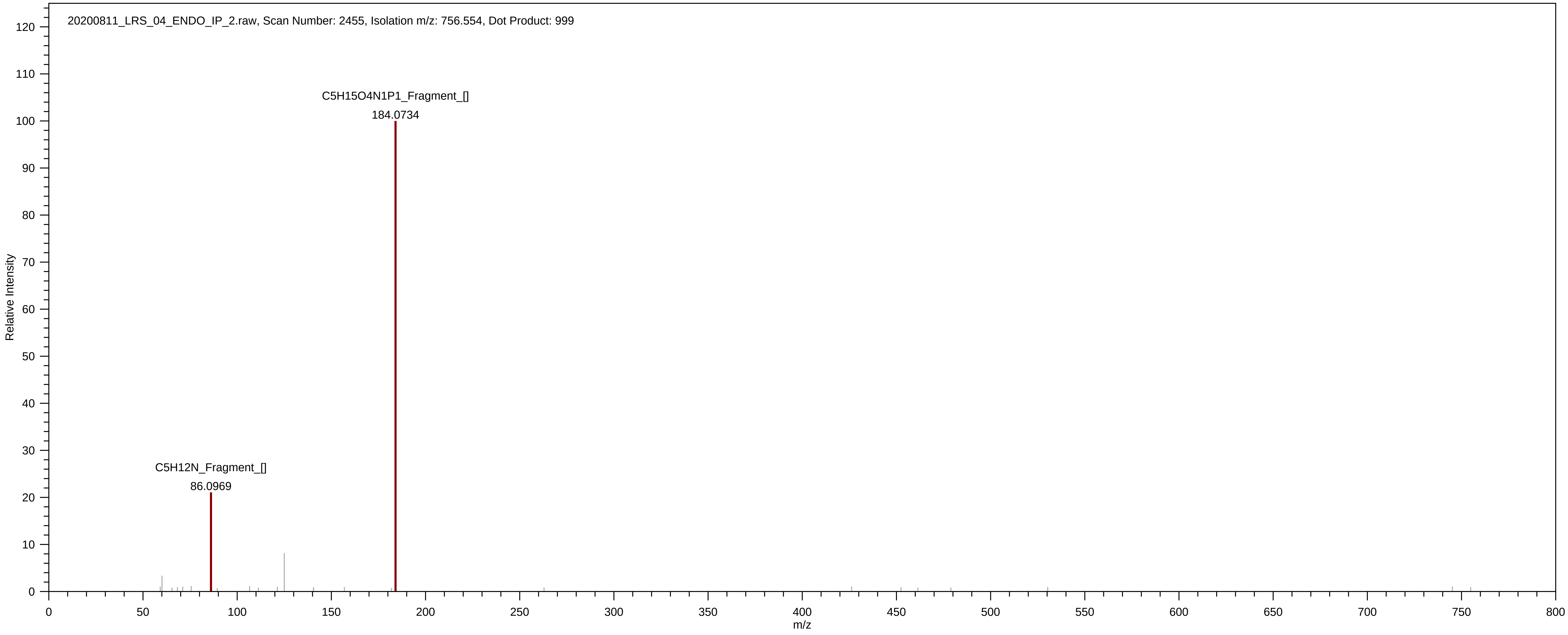

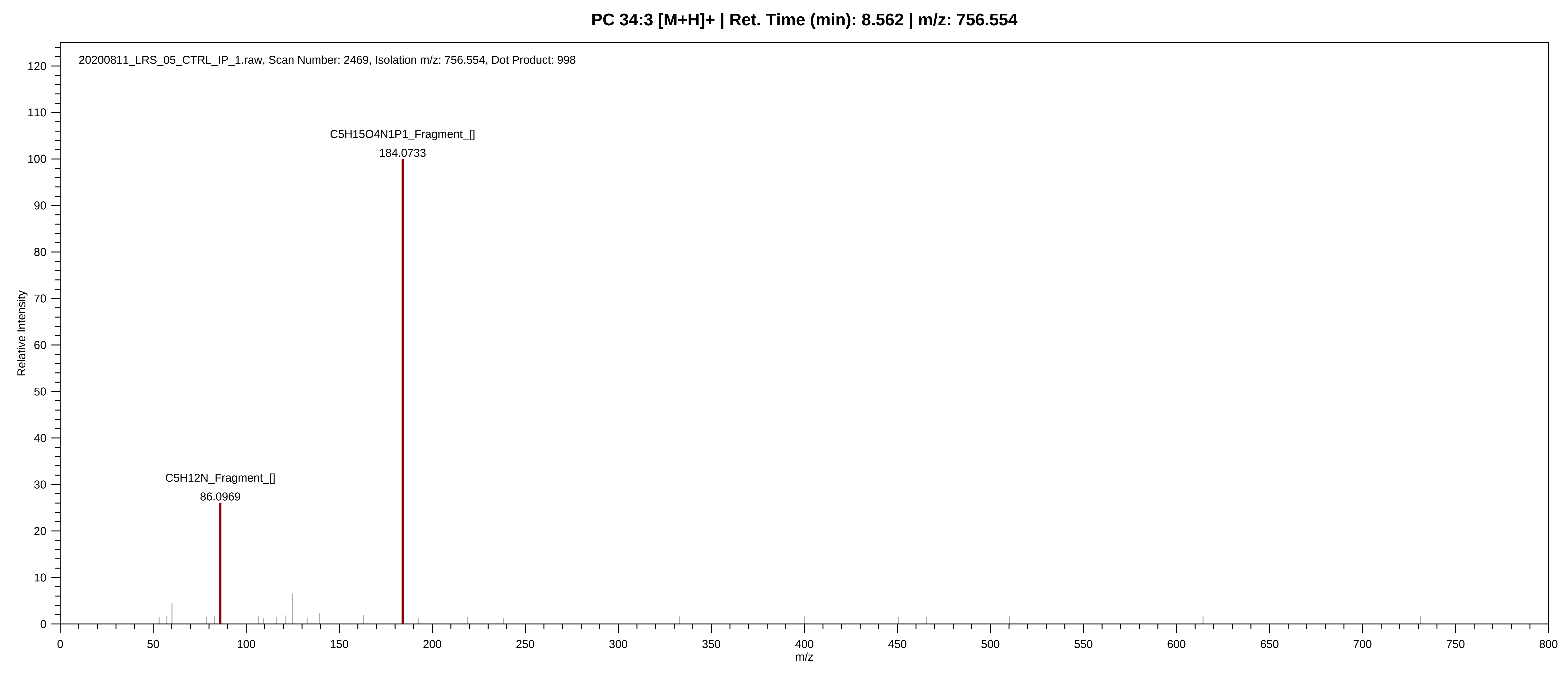
PC 34:3 [M+H]+ | Ret. Time (min): 8.562 | m/z: 756.554
20200811_LRS_05_CTRL_IP_1.raw, Scan Number: 2469, Isolation m/z: 756.554, Dot Product: 998
C5H15O4N1P1_Fragment_[]
184.0733
C5H12N_Fragment_[]
86.0969
Relative Intensity
m/z

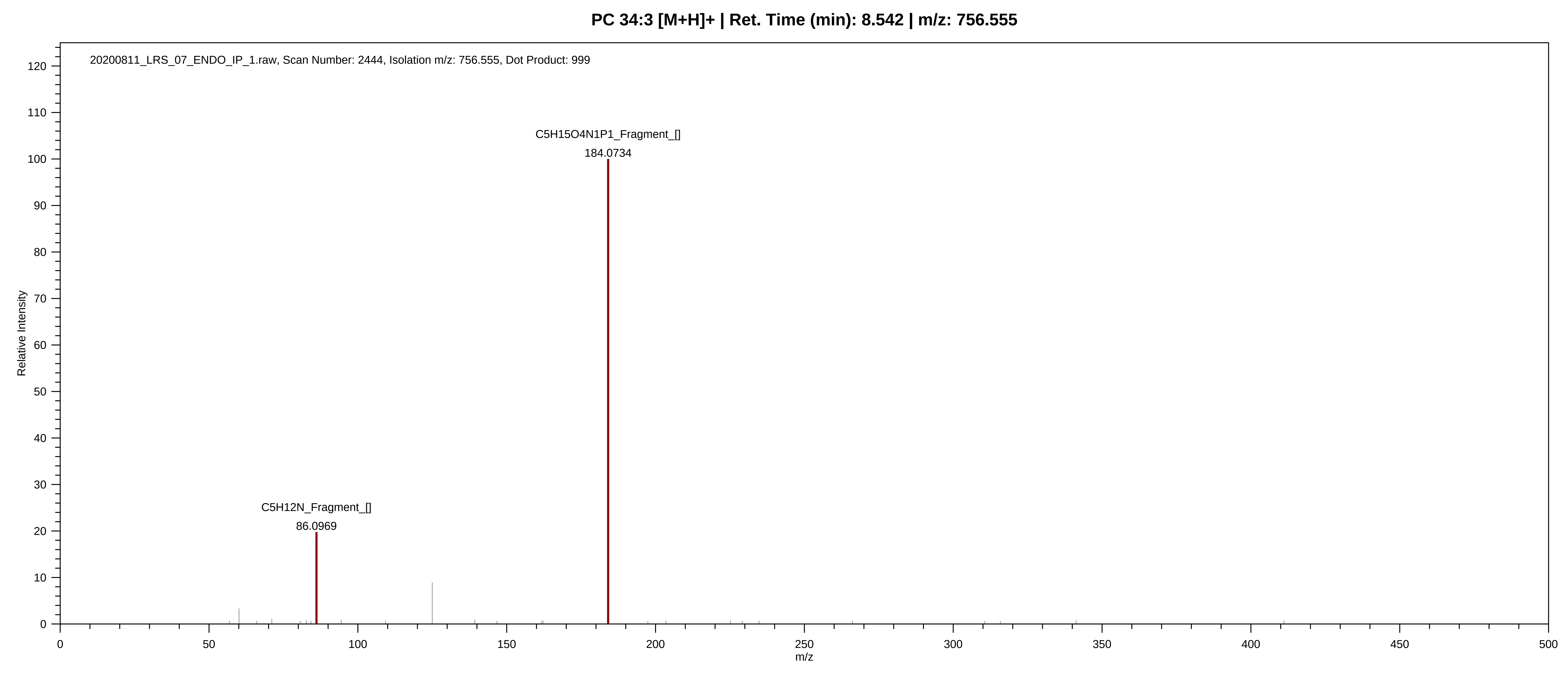
PC 34:3 [M+H]+ | Ret. Time (min): 8.542 | m/z: 756.555
20200811_LRS_07_ENDO_IP_1.raw, Scan Number: 2444, Isolation m/z: 756.555, Dot Product: 999
C5H15O4N1P1_Fragment_[]
184.0734
C5H12N_Fragment_[]
86.0969
Relative Intensity
m/z

PC 34:3 [M+H]+ | Ret. Time (min): 8.581 | m/z: 756.555

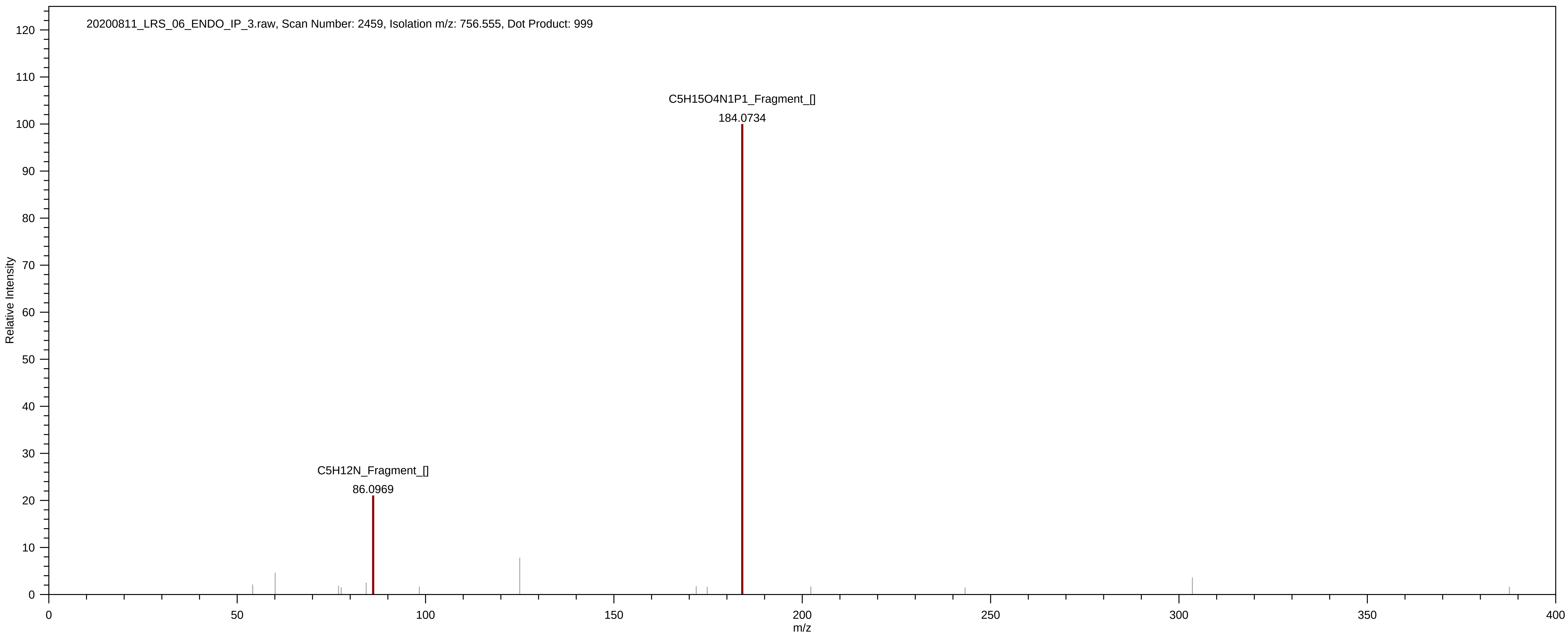

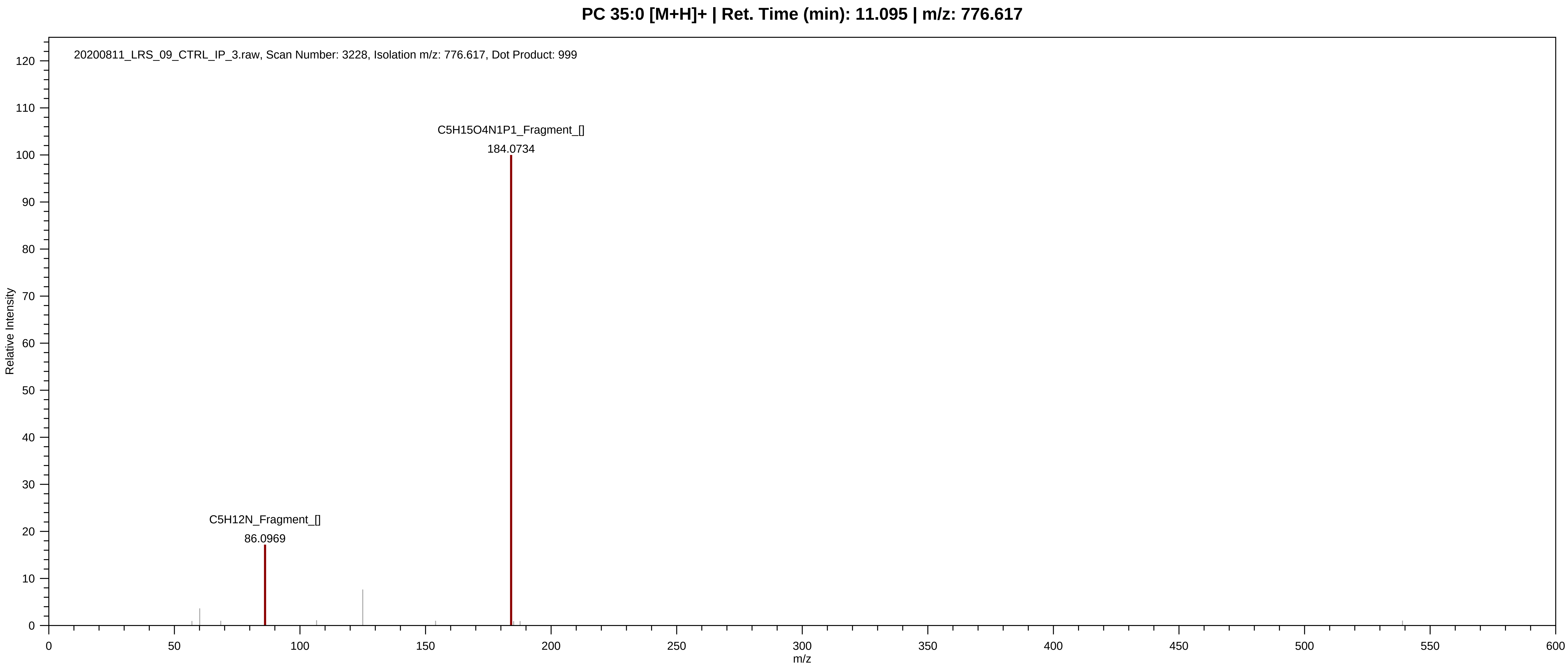

PC 35:0 [M+H]+ | Ret. Time (min): 11.095 | m/z: 776.617
20200811_LRS_09_CTRL_IP_3.raw, Scan Number: 3228, Isolation m/z: 776.617, Dot Product: 999
C5H15O4N1P1_Fragment_[]
184.0734
C5H12N_Fragment_[]
86.0969
Relative Intensity
m/z

PC 35:0 [M+H]+ | Ret. Time (min): 11.099 | m/z: 776.618

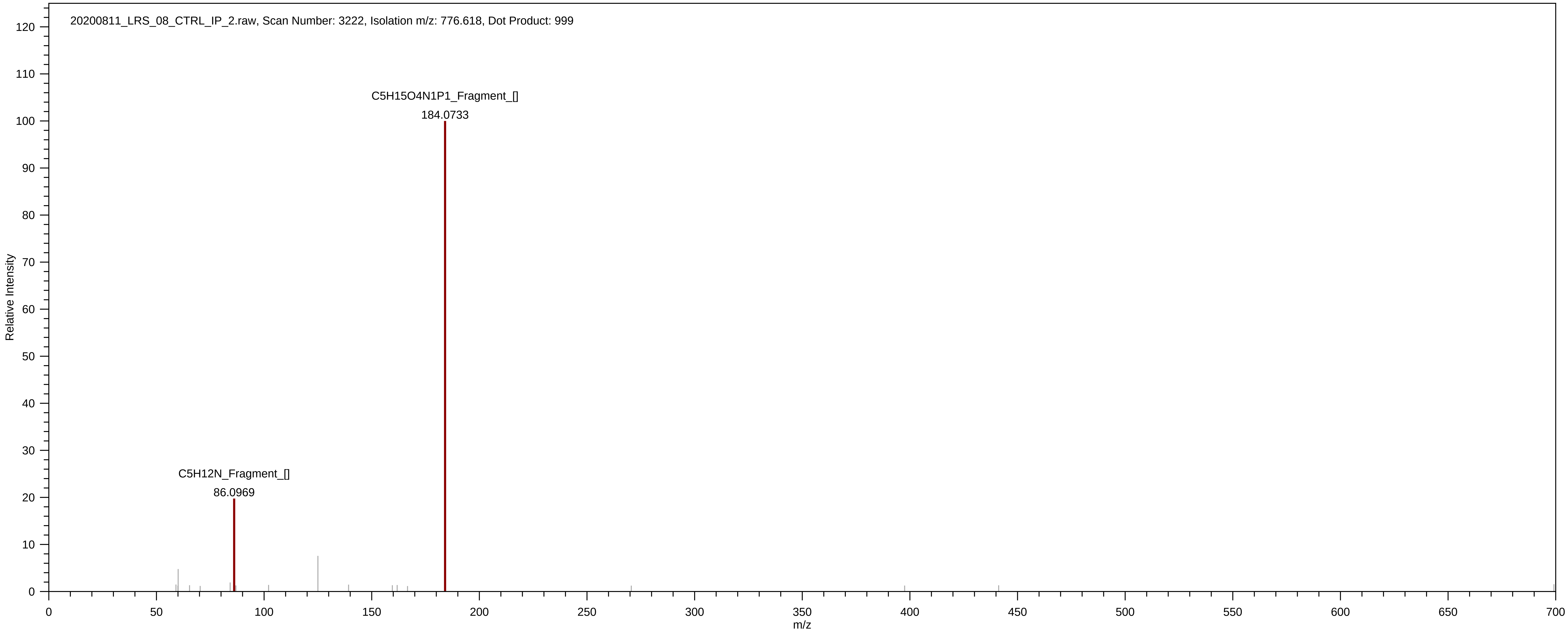

PC 35:0 [M+H]+ | Ret. Time (min): 11.093 | m/z: 776.619

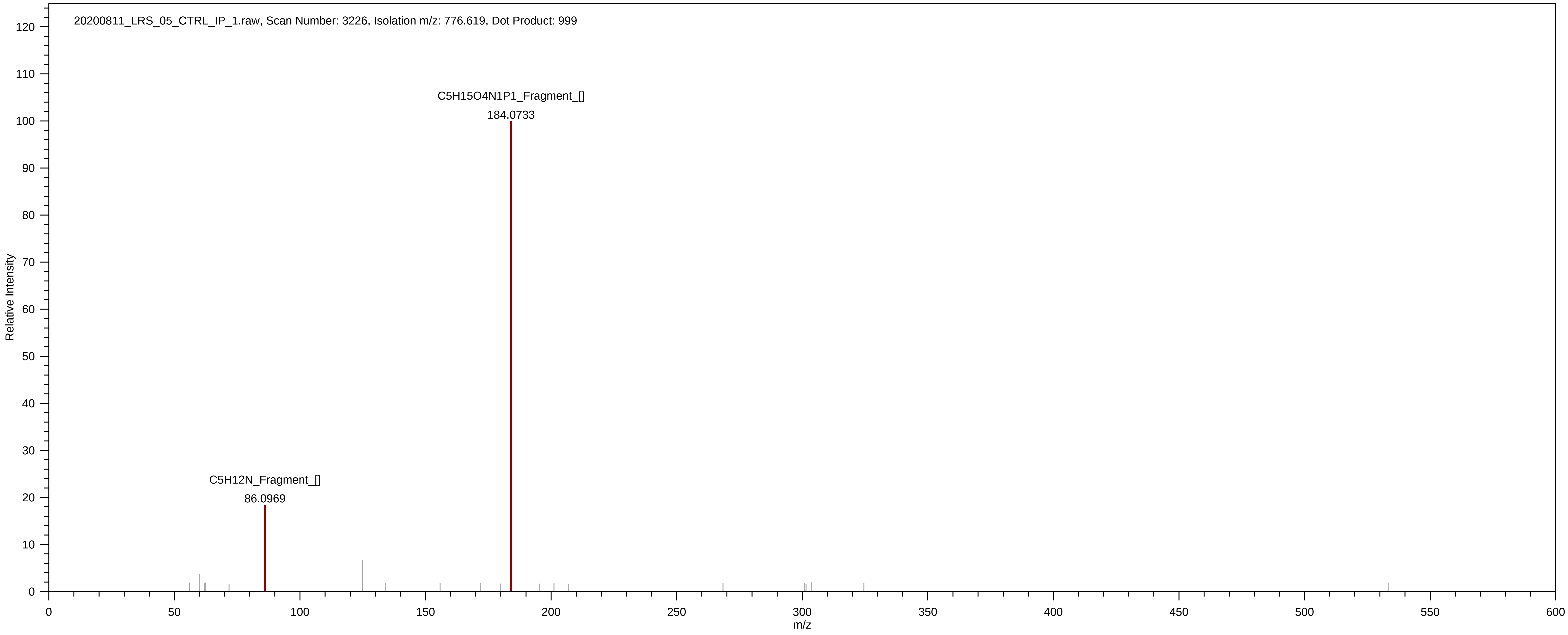

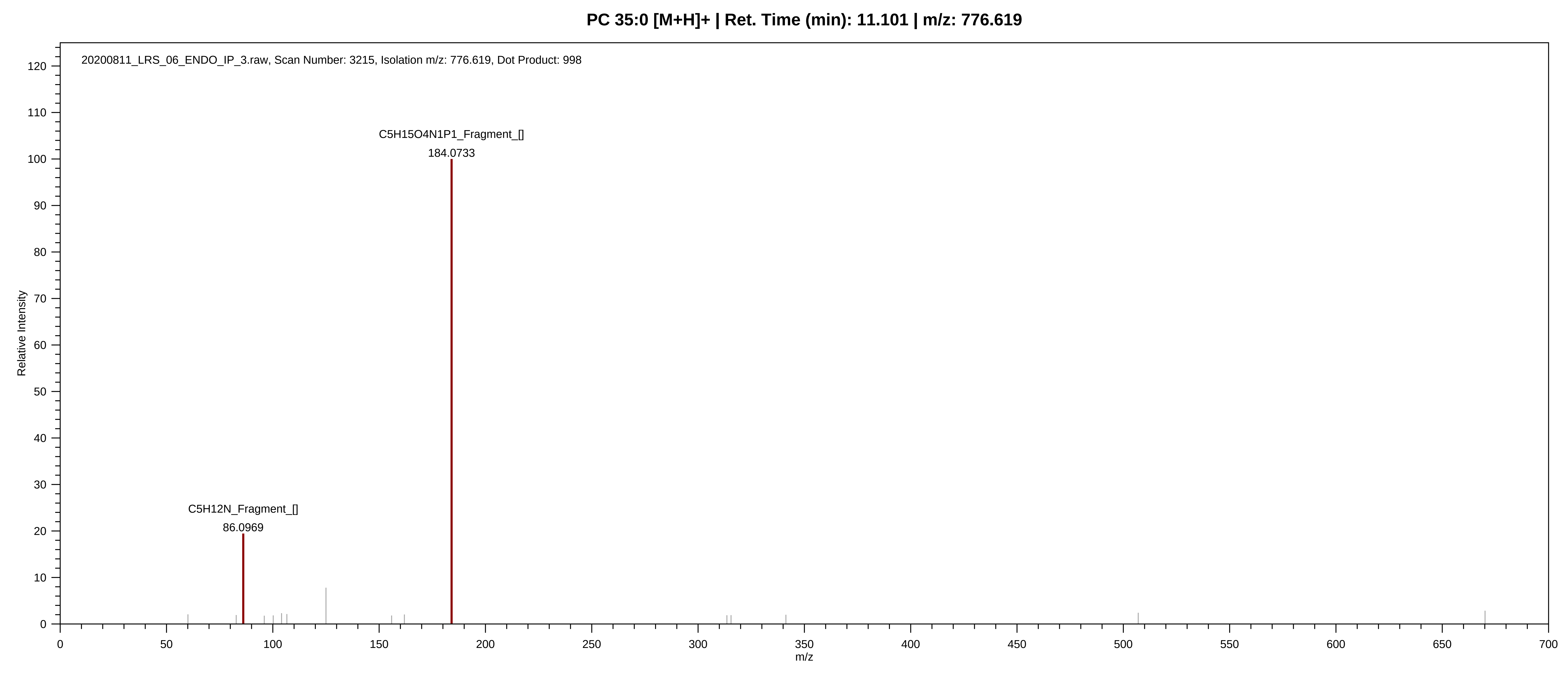

PC 35:0 [M+H]+ | Ret. Time (min): 11.101 | m/z: 776.619
20200811_LRS_06_ENDO_IP_3.raw, Scan Number: 3215, Isolation m/z: 776.619, Dot Product: 998
C5H15O4N1P1_Fragment_[]
184.0733
C5H12N_Fragment_[]
86.0969
Relative Intensity
m/z

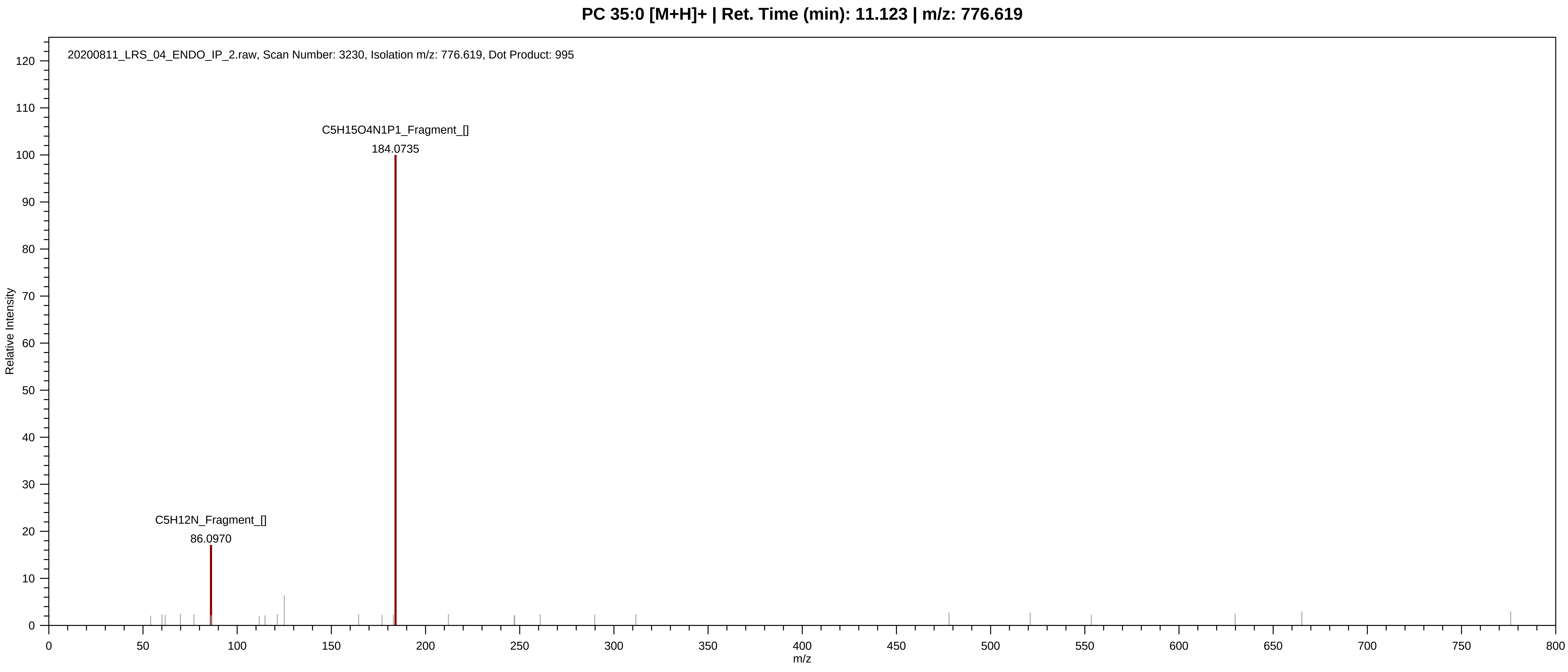
PC 35:0 [M+H]+ | Ret. Time (min): 11.123 | m/z: 776.619
20200811_LRS_04_ENDO_IP_2.raw, Scan Number: 3230, Isolation m/z: 776.619, Dot Product: 995
C5H15O4N1P1_Fragment_[]
184.0735
C5H12N_Fragment_[]
86.0970
Relative Intensity
m/z

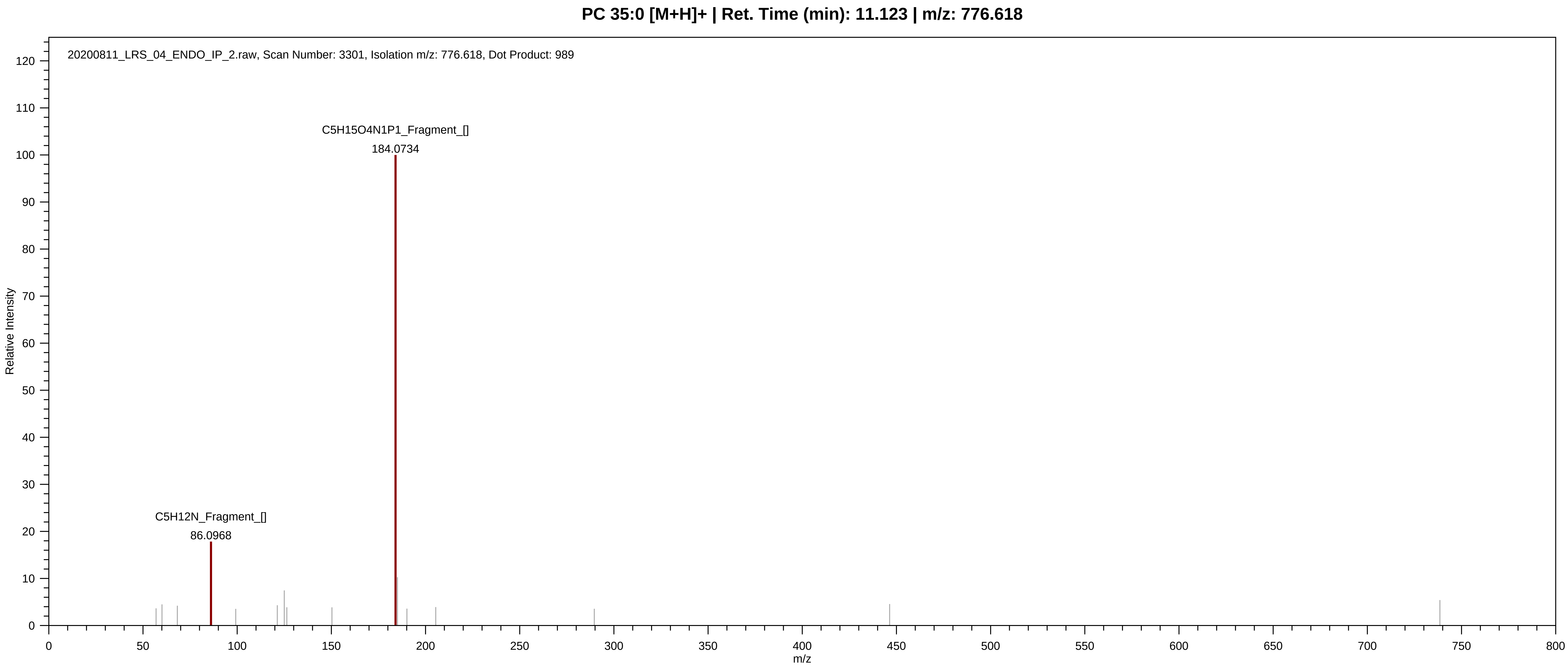
PC 35:0 [M+H]+ | Ret. Time (min): 11.123 | m/z: 776.618
20200811_LRS_04_ENDO_IP_2.raw, Scan Number: 3301, Isolation m/z: 776.618, Dot Product: 989
C5H15O4N1P1_Fragment_[]
184.0734
C5H12N_Fragment_[]
86.0968
Relative Intensity
m/z

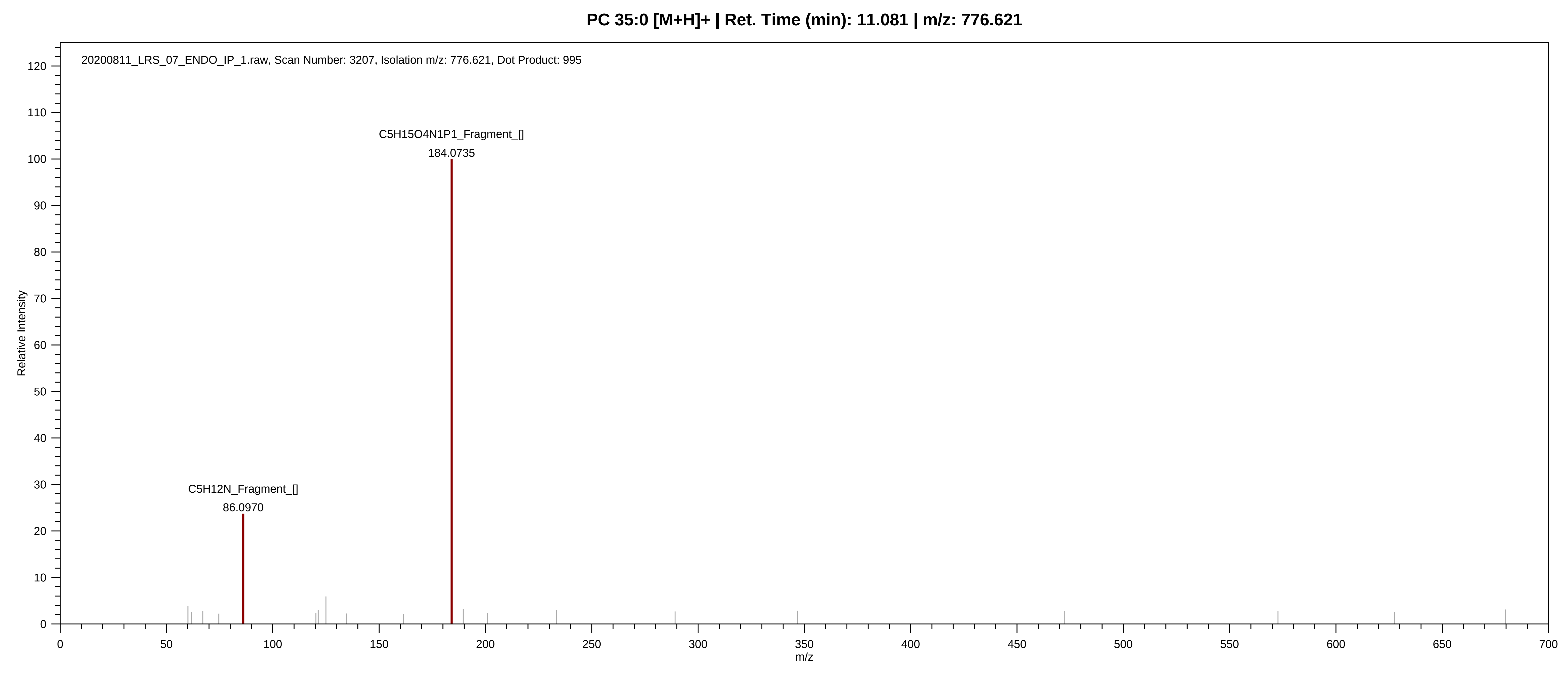
PC 35:0 [M+H]+ | Ret. Time (min): 11.081 | m/z: 776.621
20200811_LRS_07_ENDO_IP_1.raw, Scan Number: 3207, Isolation m/z: 776.621, Dot Product: 995
C5H15O4N1P1_Fragment_[]
184.0735
C5H12N_Fragment_[]
86.0970
Relative Intensity
m/z
0
10
20
30
40
50
60
70
80
90
100
110
120
0
50
100
150
200
250
300
350
400
450
500
550
600
650
700

PC 35:1 [M+Ac-H]- | Ret. Time (min): 10.365 | m/z: 832.609

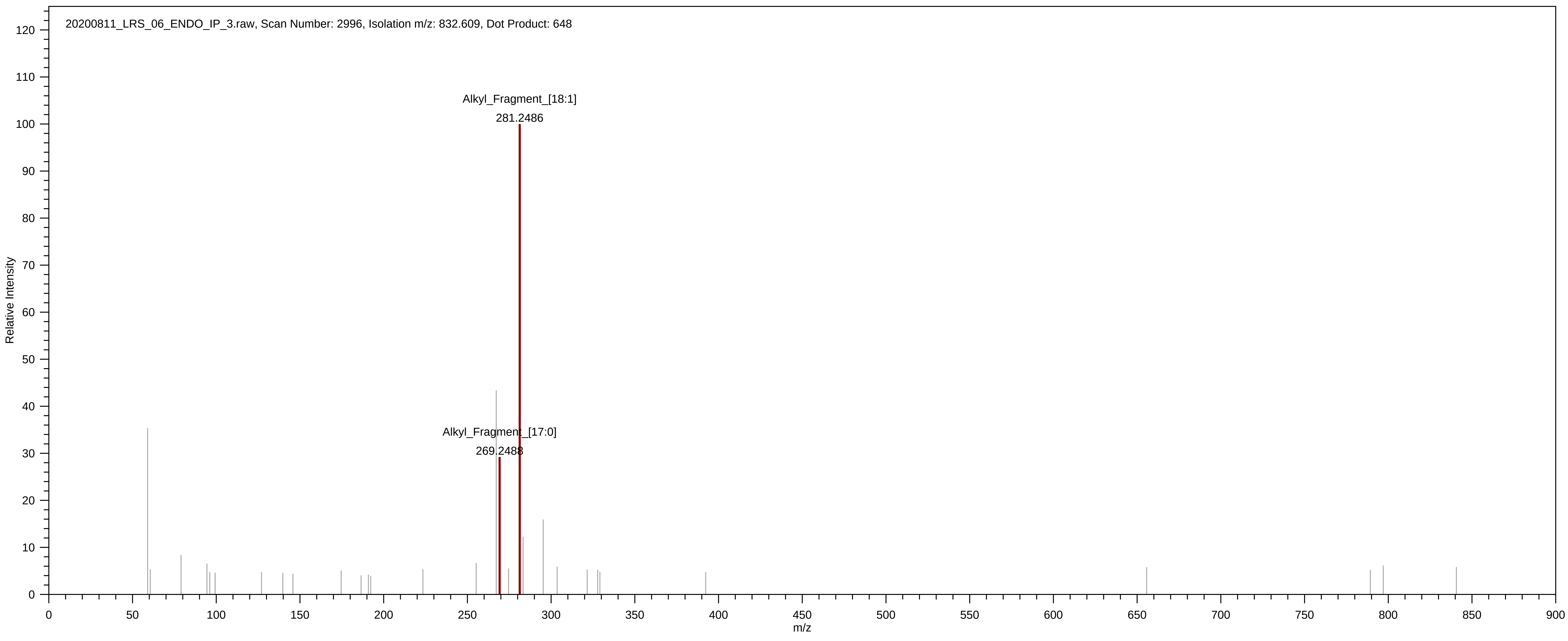

PC 35:1 [M+Ac-H]- | Ret. Time (min): 10.369 | m/z: 832.609

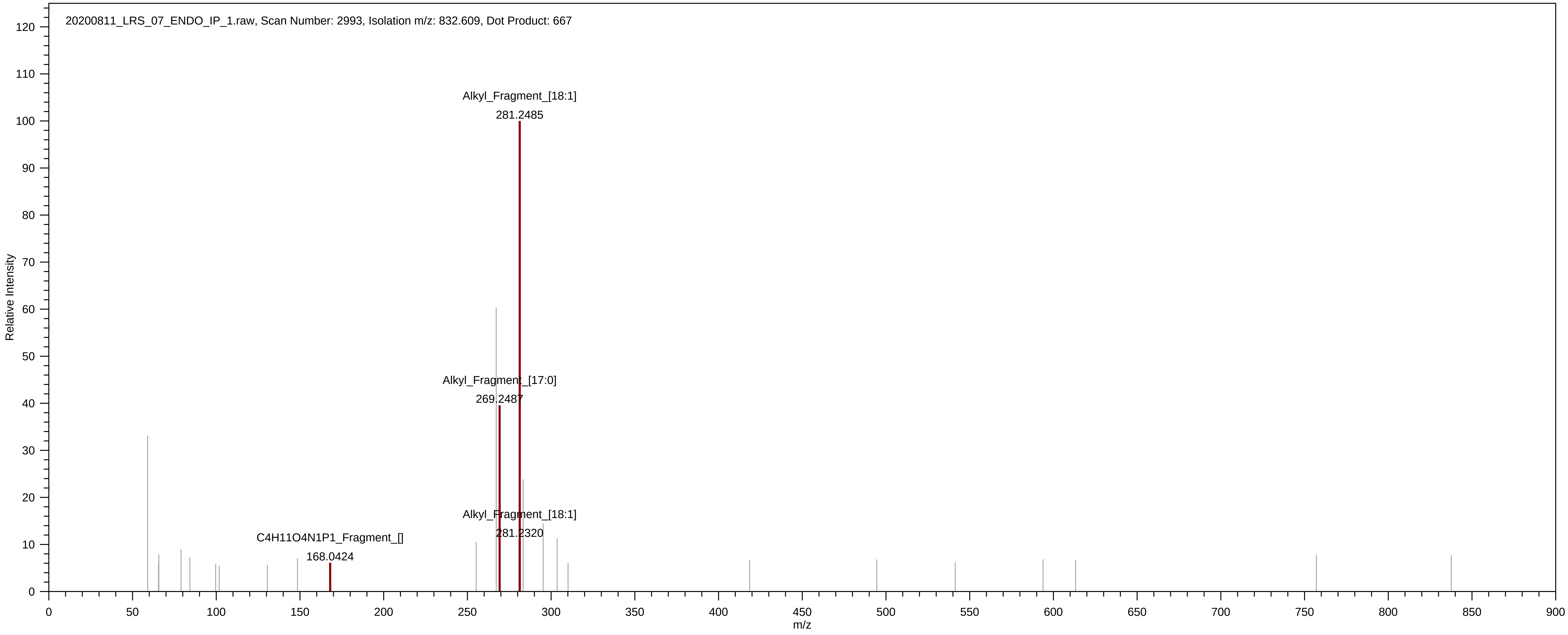

PC 35:1 [M+Ac-H]- | Ret. Time (min): 10.388 | m/z: 832.609

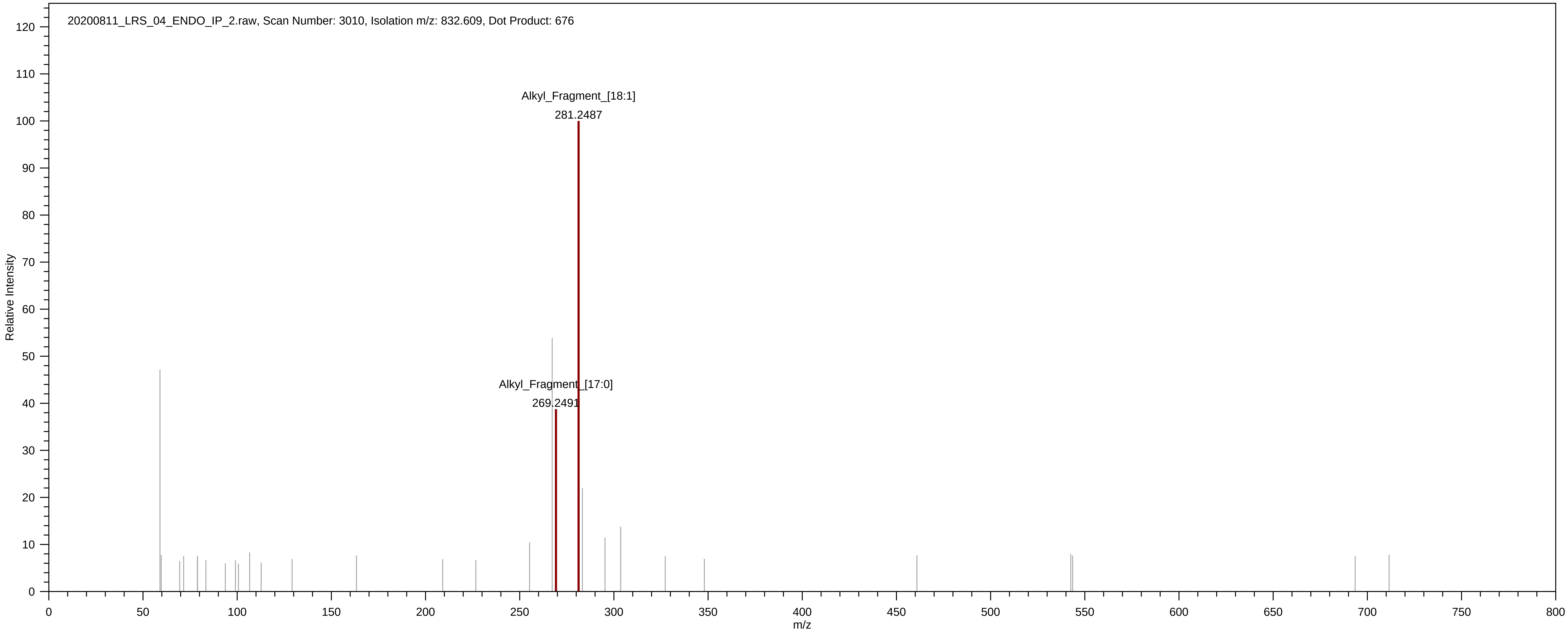

PC 35:1 [M+H]+ | Ret. Time (min): 12.121 | m/z: 774.597

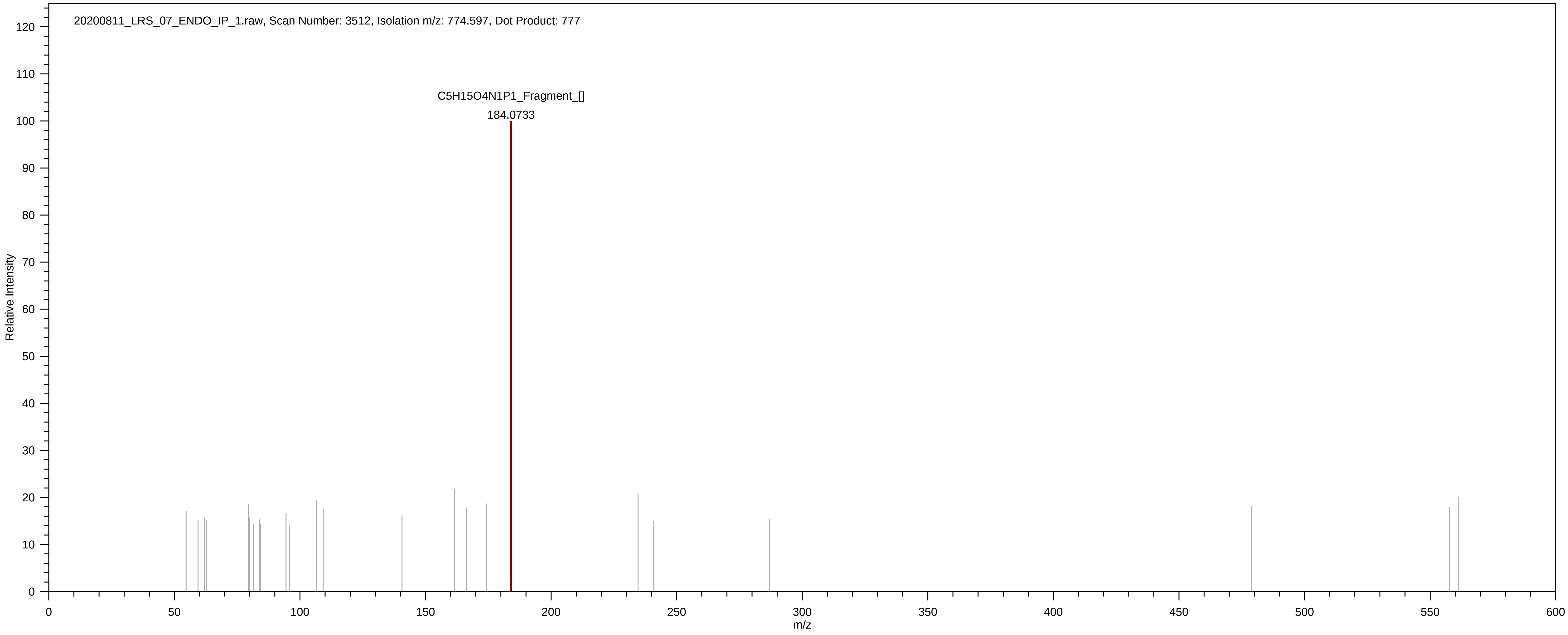

PC 35:1 [M+H]+ | Ret. Time (min): 12.139 | m/z: 774.597

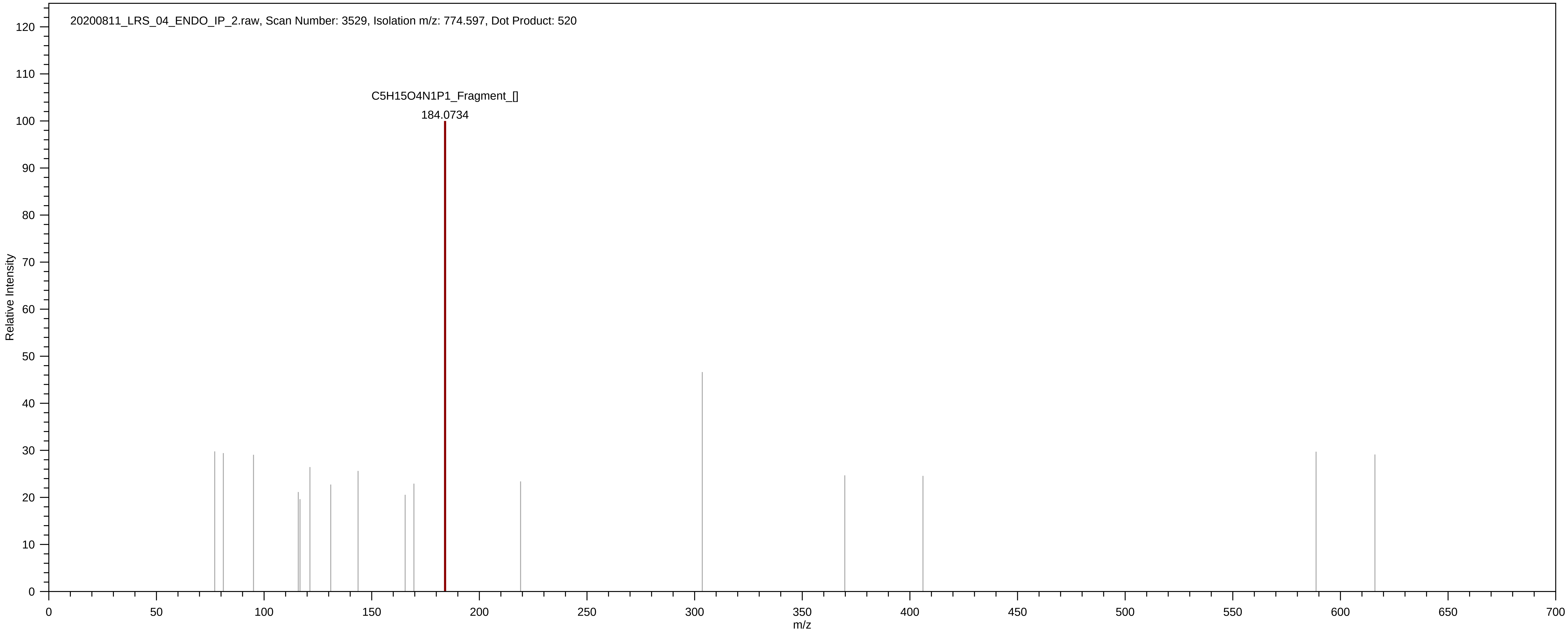

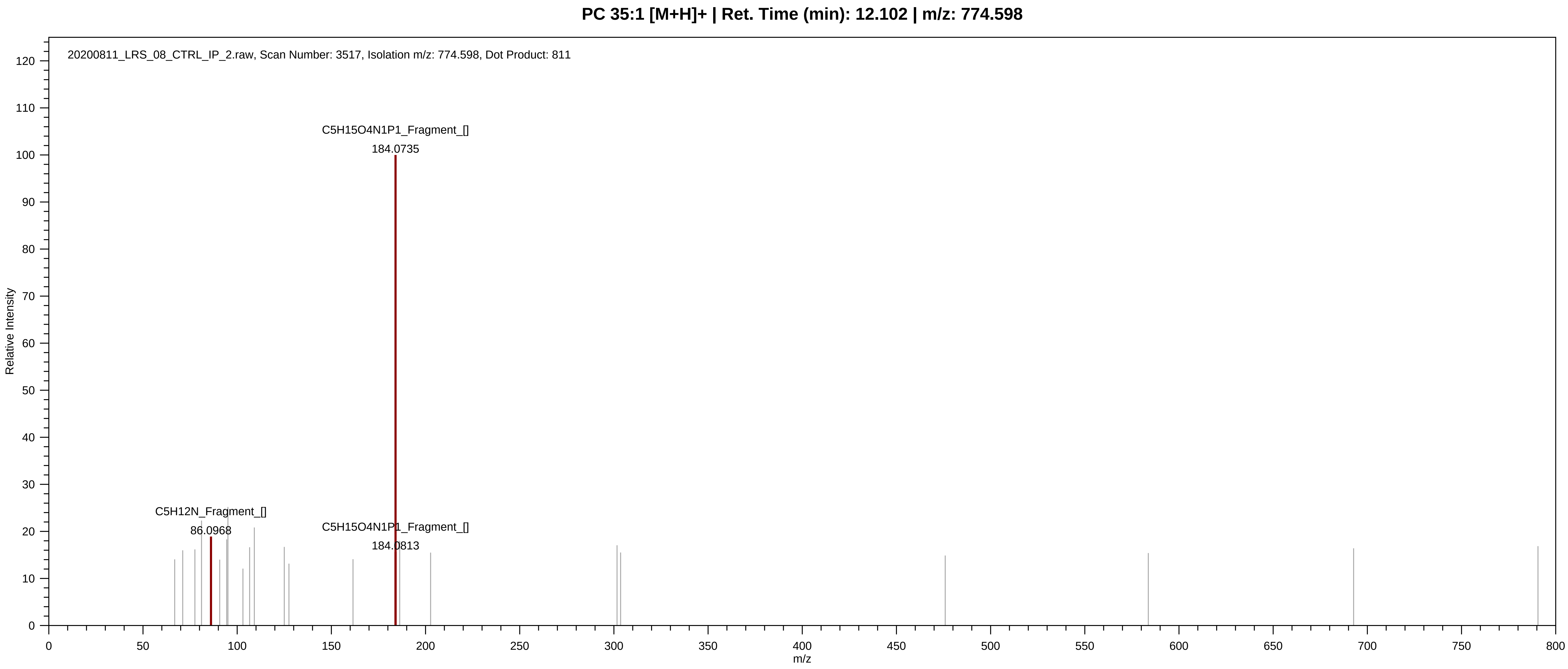
PC 35:1 [M+H]+ | Ret. Time (min): 12.102 | m/z: 774.598
20200811_LRS_08_CTRL_IP_2.raw, Scan Number: 3517, Isolation m/z: 774.598, Dot Product: 811
C5H15O4N1P1_Fragment_[]
184.0735
C5H12N_Fragment_[]
86.0968
C5H15O4N1P1_Fragment_[]
184.0813
Relative Intensity
m/z

PC 35:1 [M+H]+ | Ret. Time (min): 12.121 | m/z: 774.598

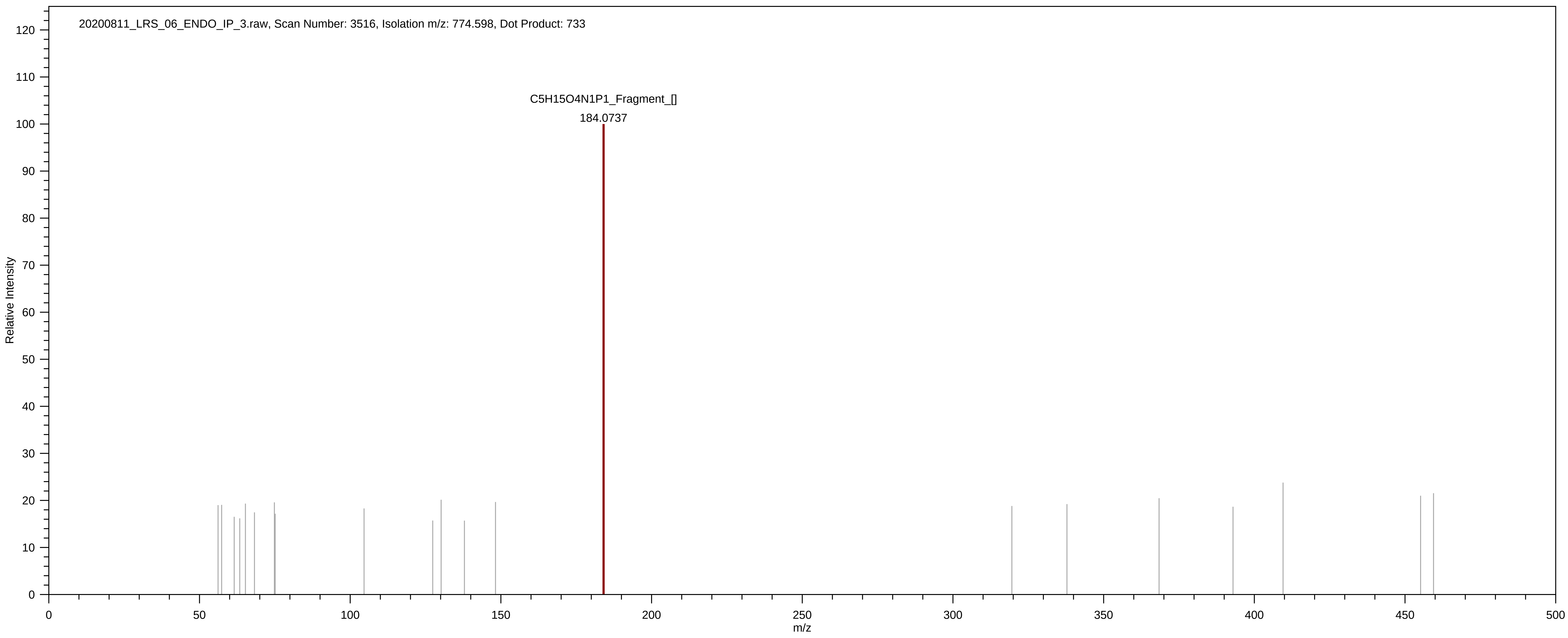

PC 35:1 [M+H]+ | Ret. Time (min): 12.119 | m/z: 774.599

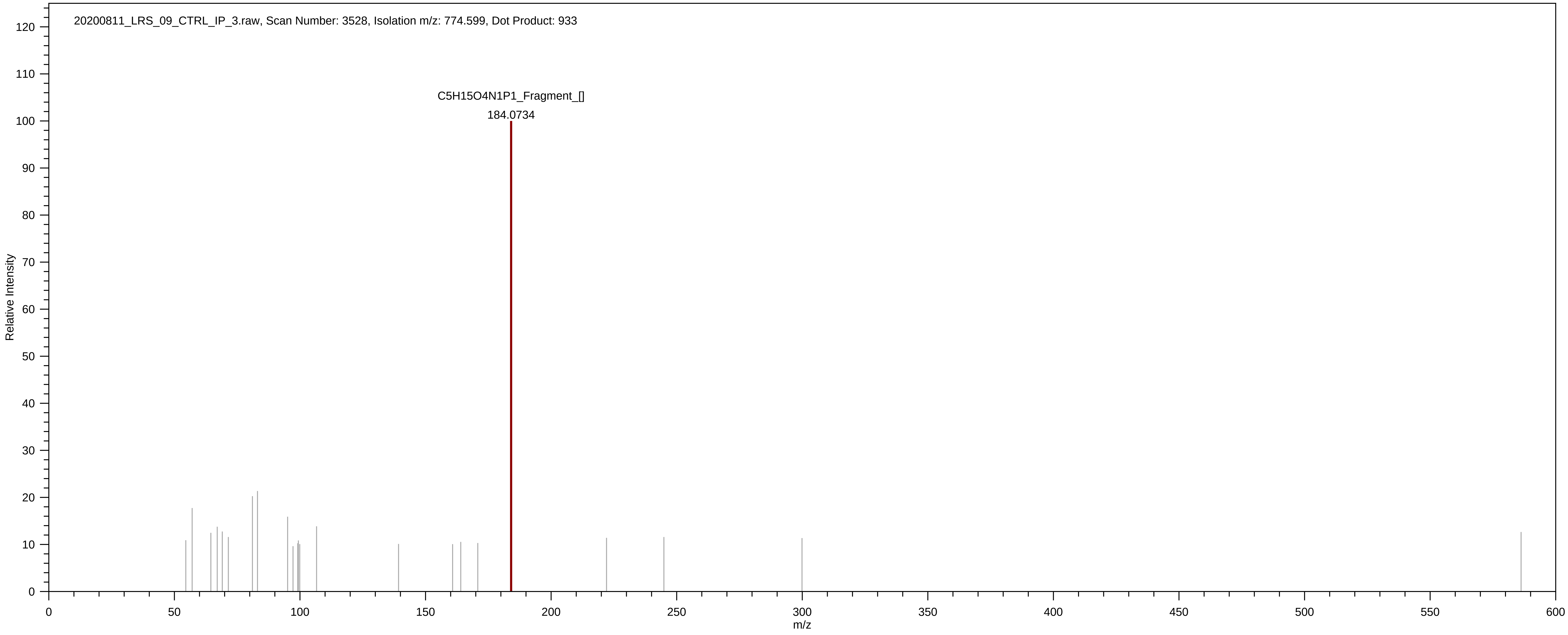

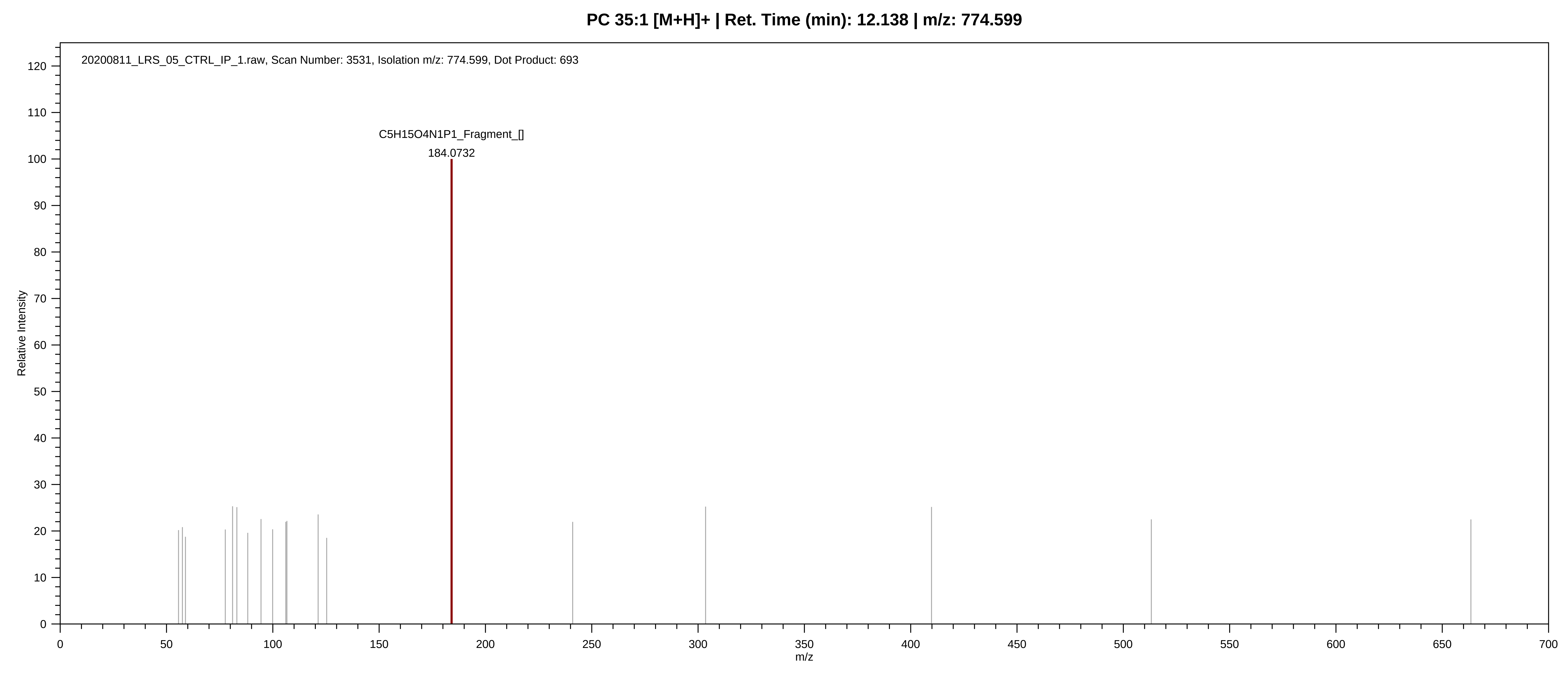

PC 35:1 [M+H]+ | Ret. Time (min): 12.138 | m/z: 774.599
20200811_LRS_05_CTRL_IP_1.raw, Scan Number: 3531, Isolation m/z: 774.599, Dot Product: 693
C5H15O4N1P1_Fragment_[]
184.0732
Relative Intensity
m/z

PC 35:1 [M+H]+ | Ret. Time (min): 10.073 | m/z: 774.601

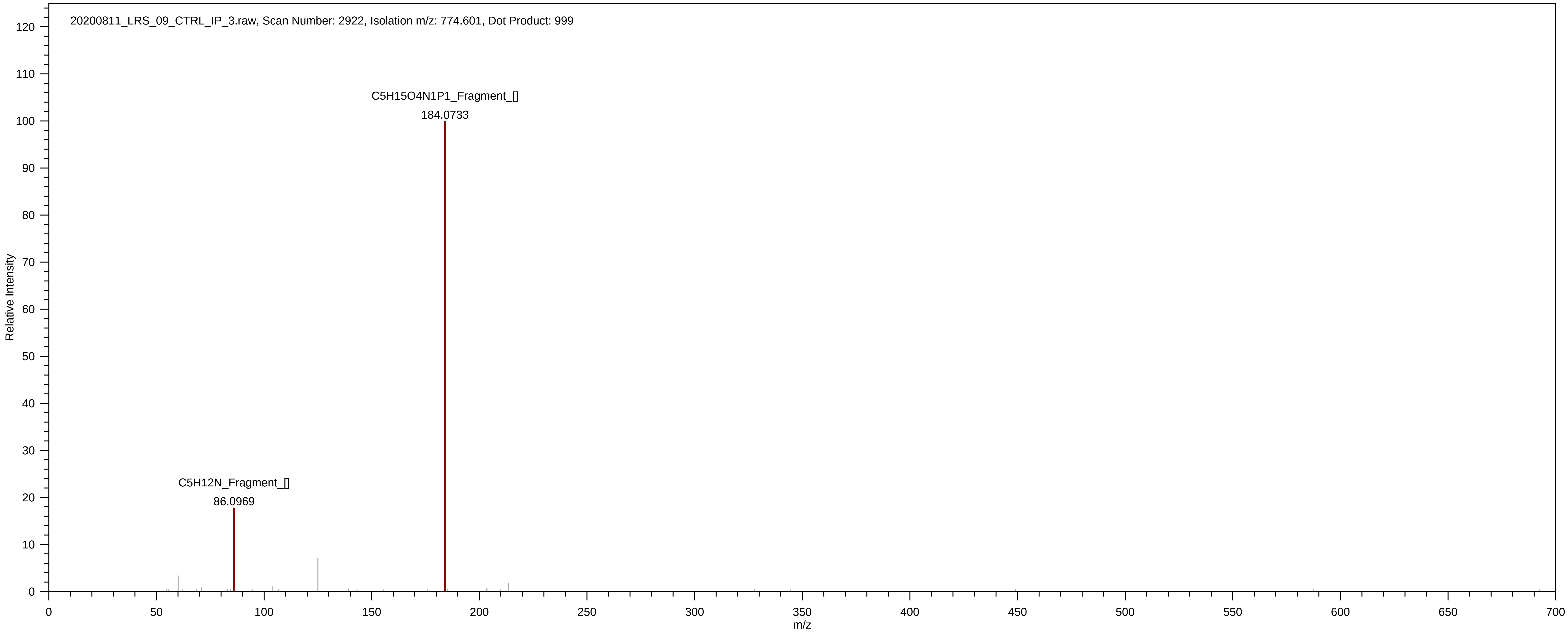

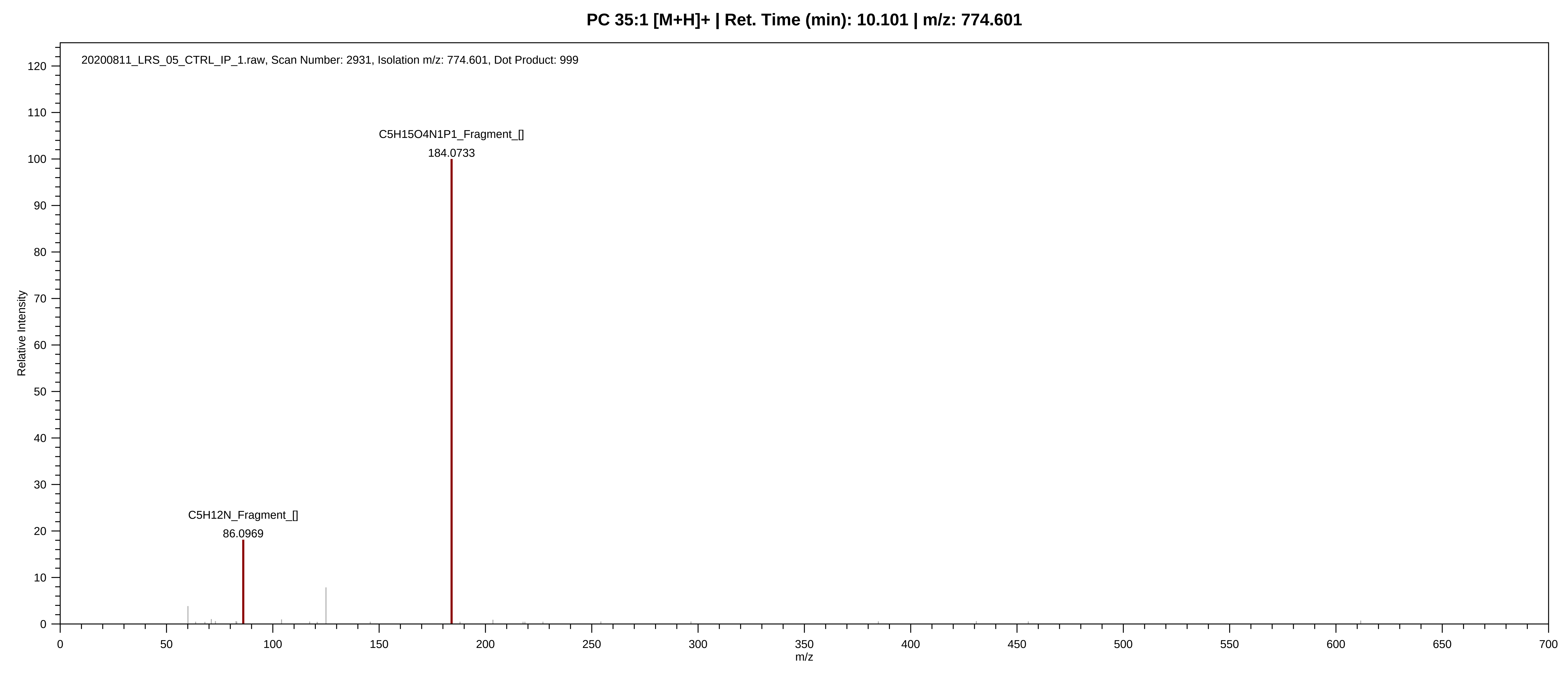
PC 35:1 [M+H]+ | Ret. Time (min): 10.101 | m/z: 774.601
20200811_LRS_05_CTRL_IP_1.raw, Scan Number: 2931, Isolation m/z: 774.601, Dot Product: 999
C5H15O4N1P1_Fragment_[]
184.0733
C5H12N_Fragment_[]
86.0969
Relative Intensity
m/z

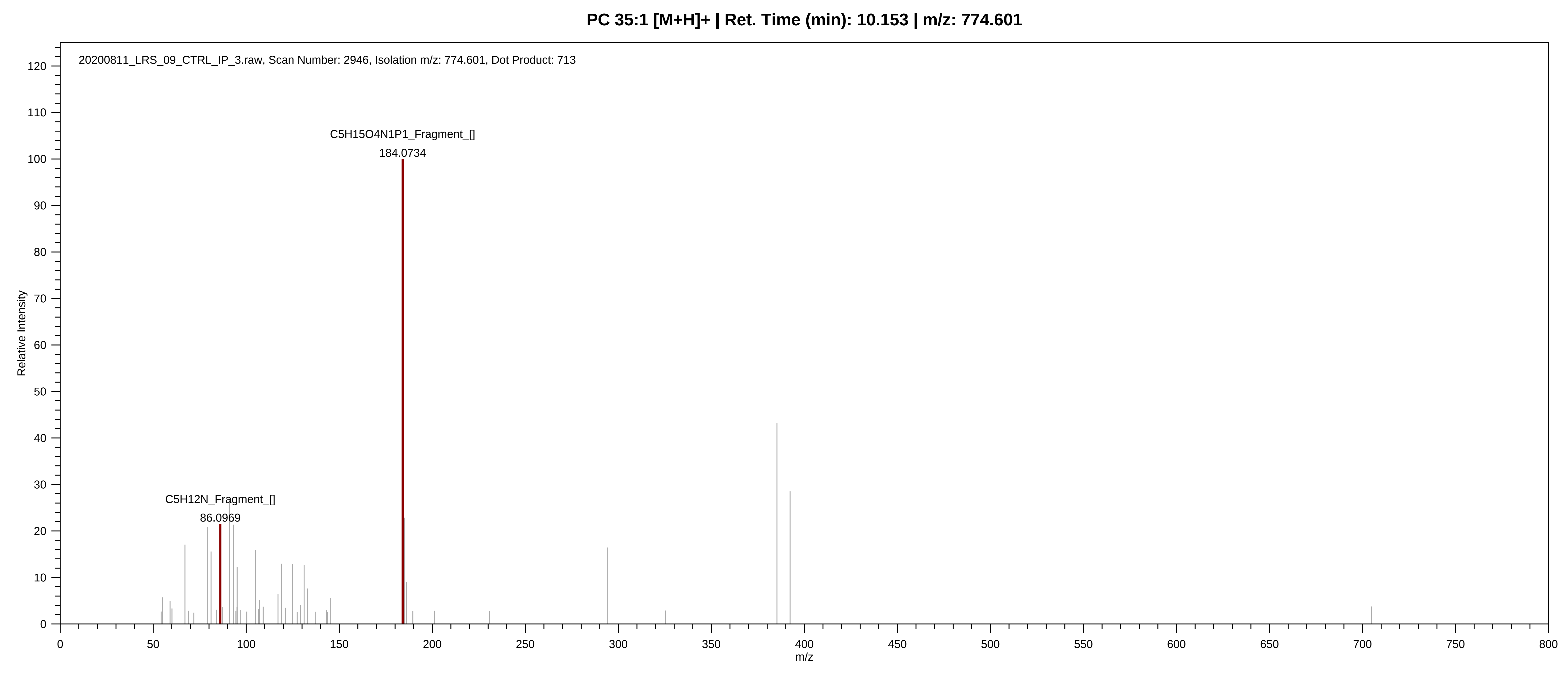
PC 35:1 [M+H]+ | Ret. Time (min): 10.153 | m/z: 774.601
20200811_LRS_09_CTRL_IP_3.raw, Scan Number: 2946, Isolation m/z: 774.601, Dot Product: 713
C5H15O4N1P1_Fragment_[]
184.0734
C5H12N_Fragment_[]
86.0969
Relative Intensity
m/z

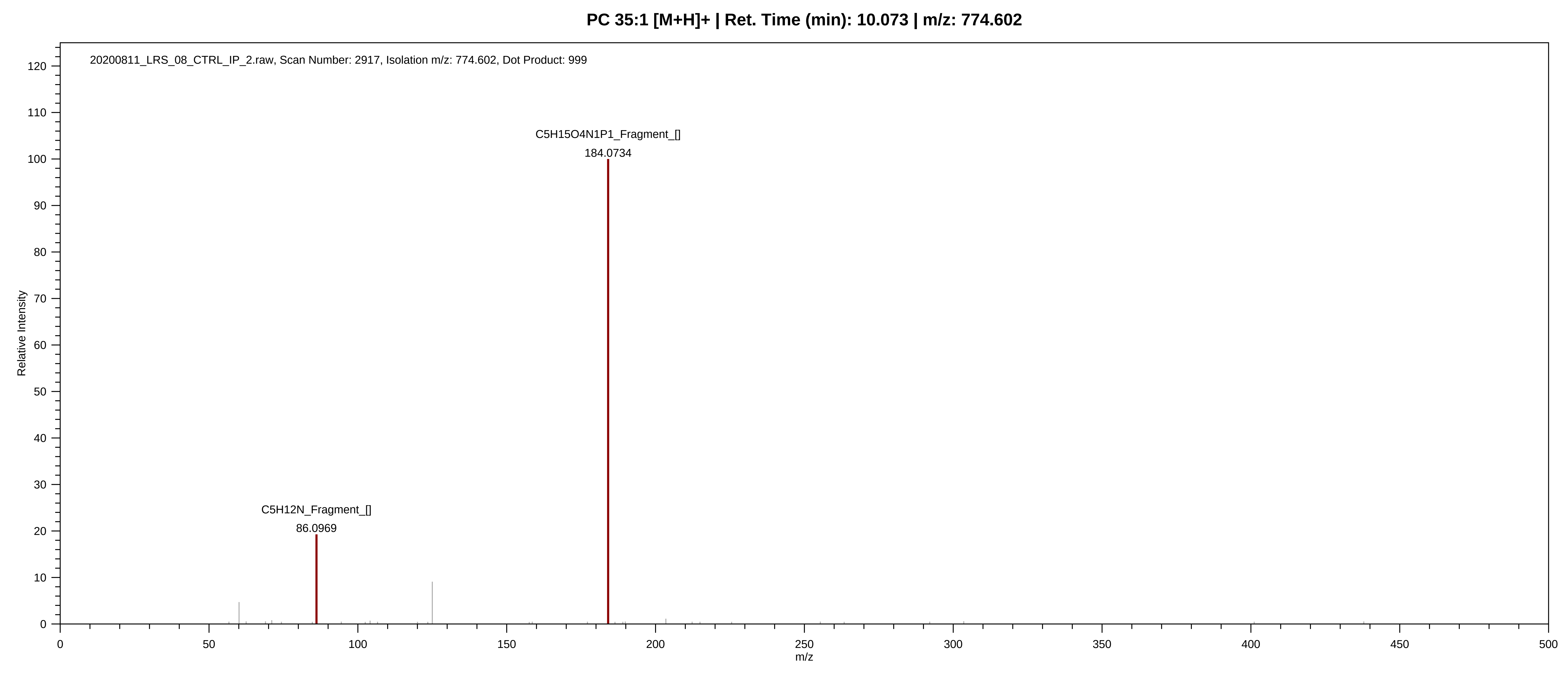

PC 35:1 [M+H]+ | Ret. Time (min): 10.073 | m/z: 774.602
20200811_LRS_08_CTRL_IP_2.raw, Scan Number: 2917, Isolation m/z: 774.602, Dot Product: 999
C5H15O4N1P1_Fragment_[]
184.0734
C5H12N_Fragment_[]
86.0969
Relative Intensity
m/z

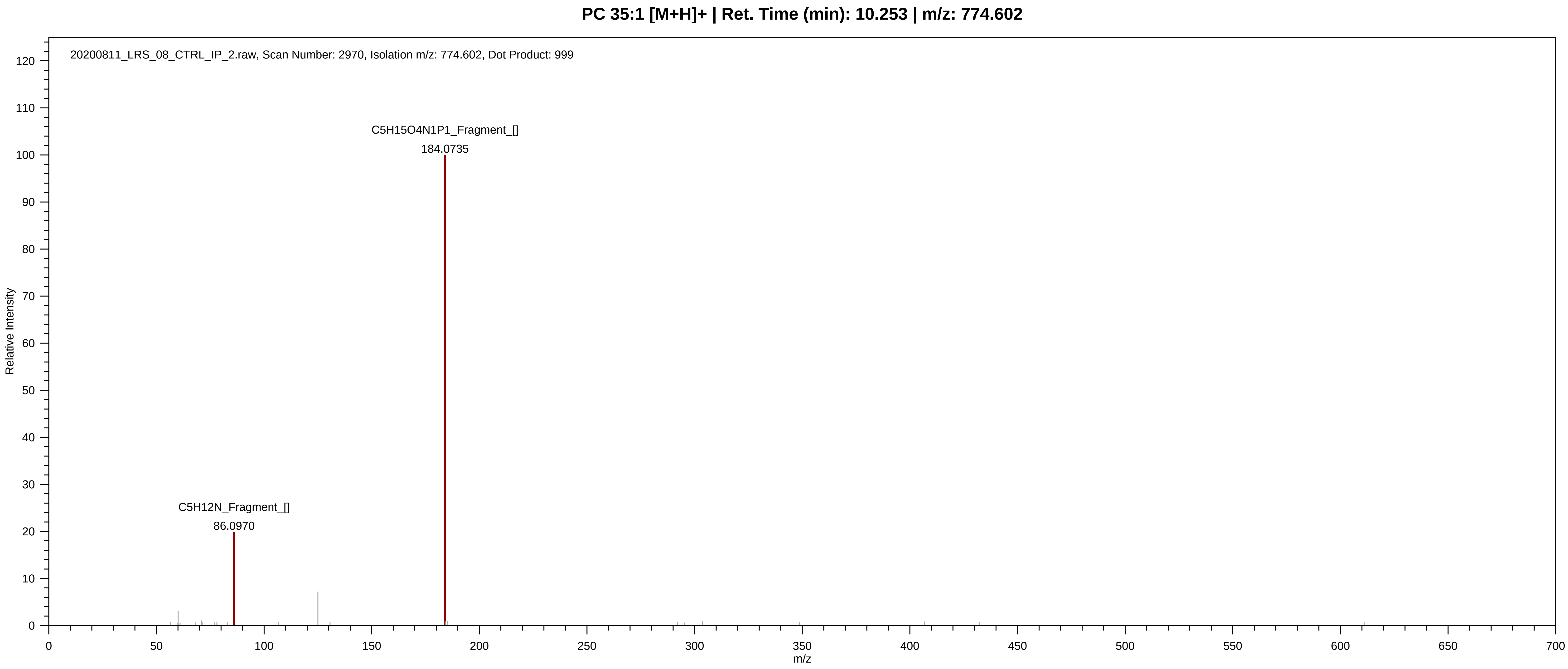
PC 35:1 [M+H]+ | Ret. Time (min): 10.253 | m/z: 774.602
20200811_LRS_08_CTRL_IP_2.raw, Scan Number: 2970, Isolation m/z: 774.602, Dot Product: 999
C5H15O4N1P1_Fragment_[]
184.0735
C5H12N_Fragment_[]
86.0970
Relative Intensity
m/z

PC 35:1 [M+H]+ | Ret. Time (min): 10.256 | m/z: 774.602

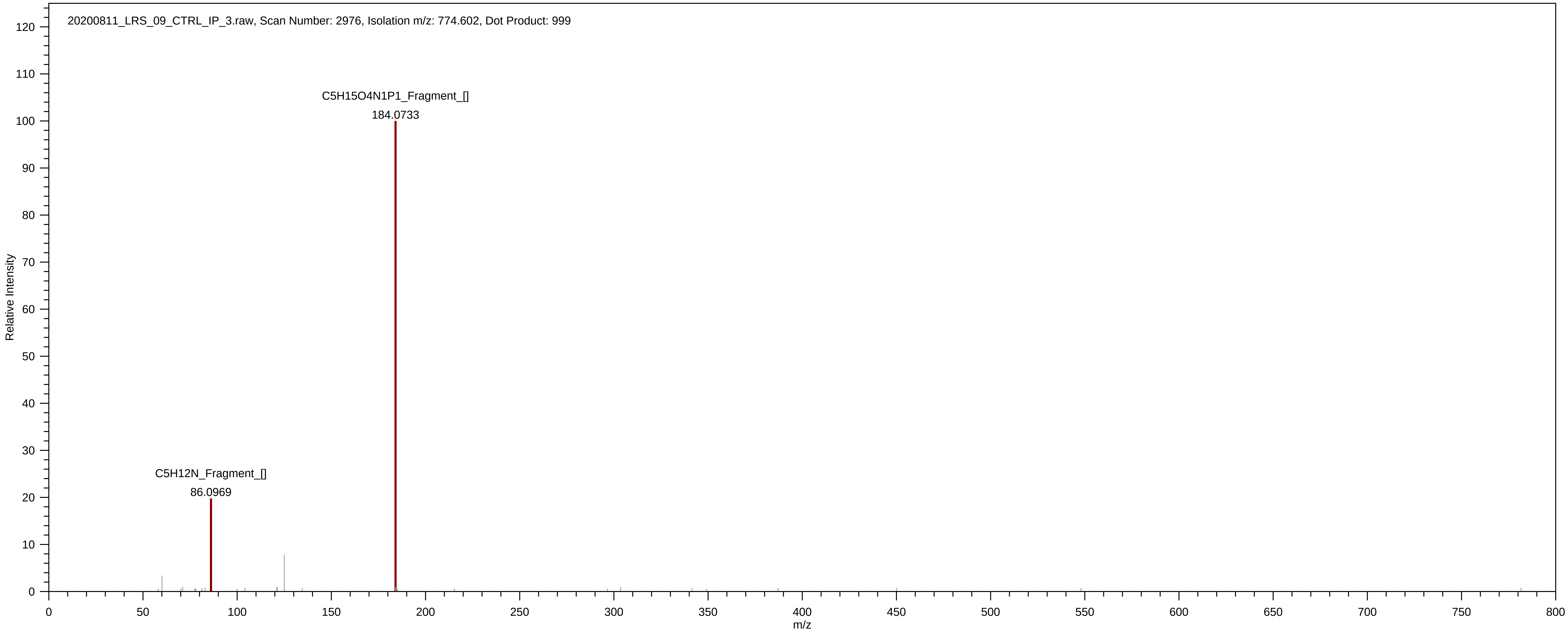

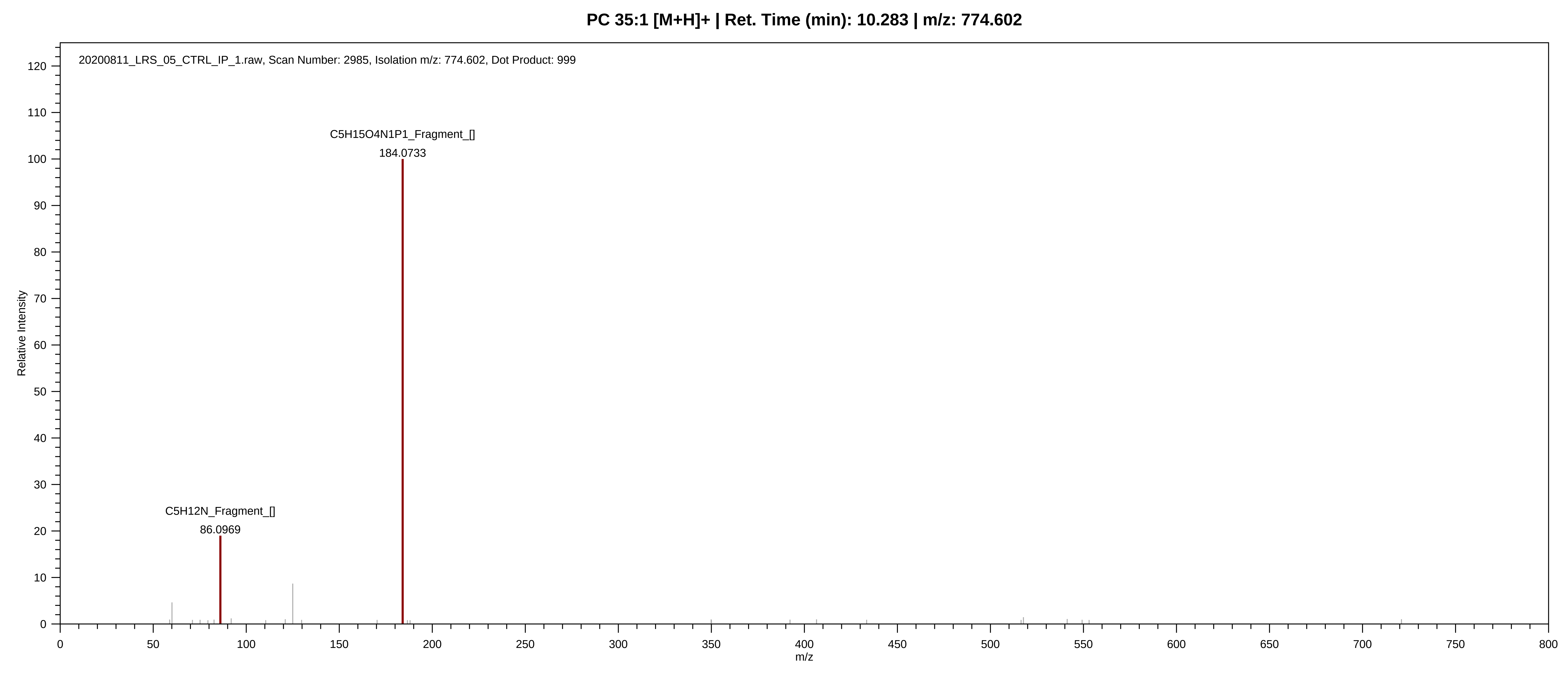

PC 35:1 [M+H]+ | Ret. Time (min): 10.283 | m/z: 774.602
20200811_LRS_05_CTRL_IP_1.raw, Scan Number: 2985, Isolation m/z: 774.602, Dot Product: 999
C5H15O4N1P1_Fragment_[]
184.0733
C5H12N_Fragment_[]
86.0969
Relative Intensity
m/z

PC 35:1 [M+H]+ | Ret. Time (min): 10.315 | m/z: 774.602

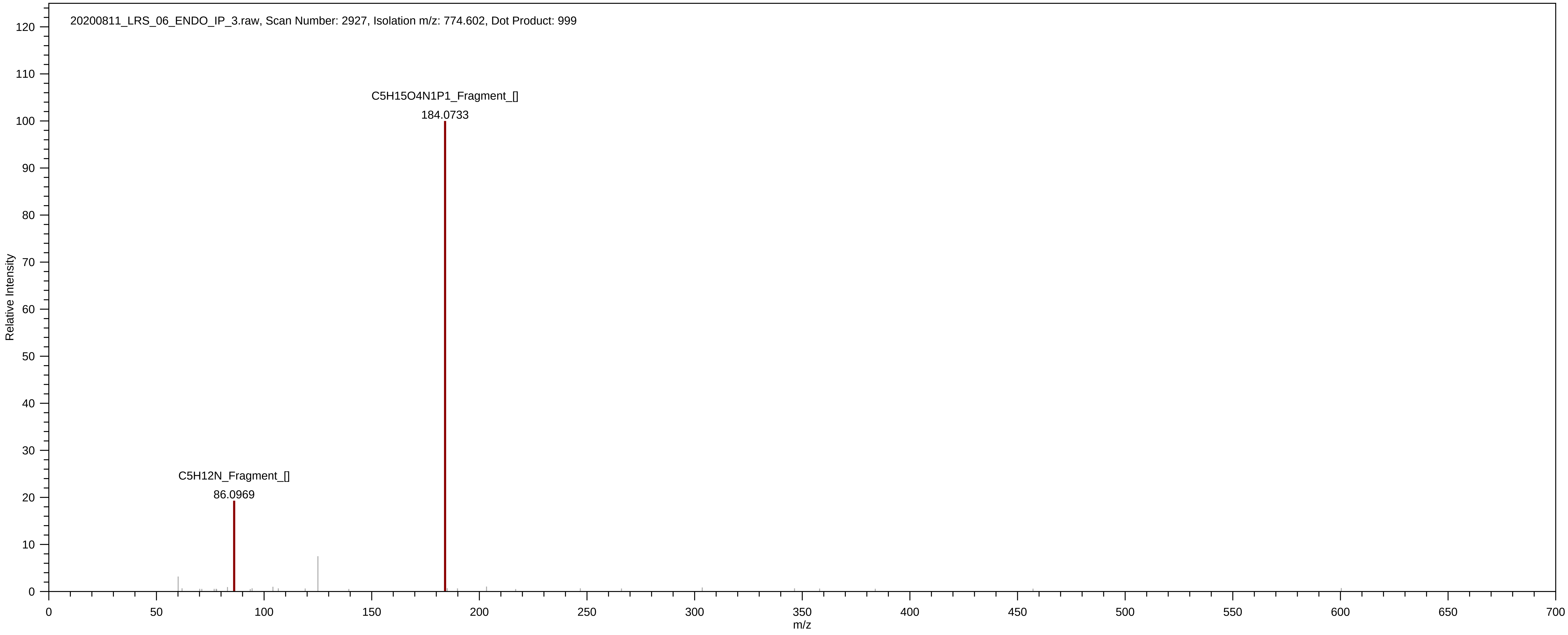

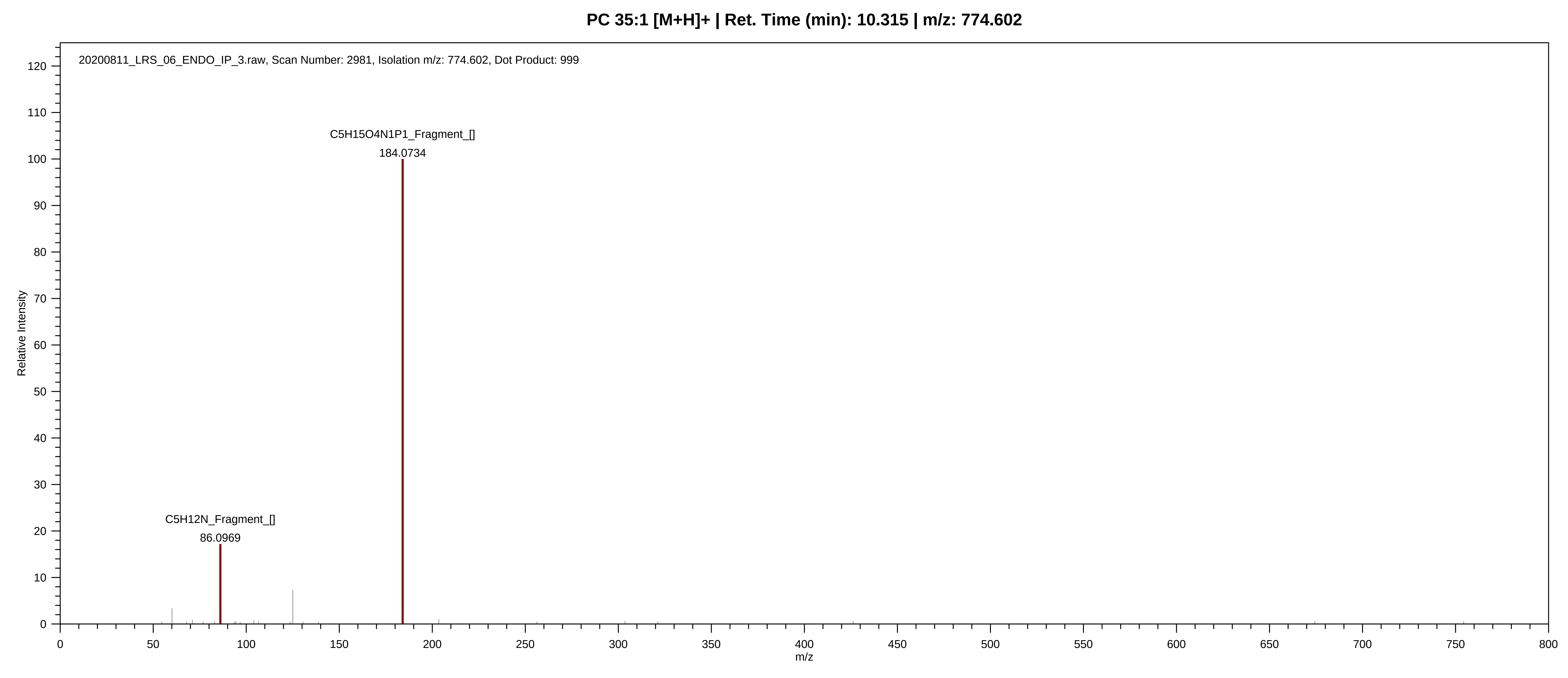
PC 35:1 [M+H]+ | Ret. Time (min): 10.315 | m/z: 774.602
20200811_LRS_06_ENDO_IP_3.raw, Scan Number: 2981, Isolation m/z: 774.602, Dot Product: 999
C5H15O4N1P1_Fragment_[]
184.0734
C5H12N_Fragment_[]
86.0969
Relative Intensity
m/z

PC 35:1 [M+H]+ | Ret. Time (min): 10.316 | m/z: 774.602

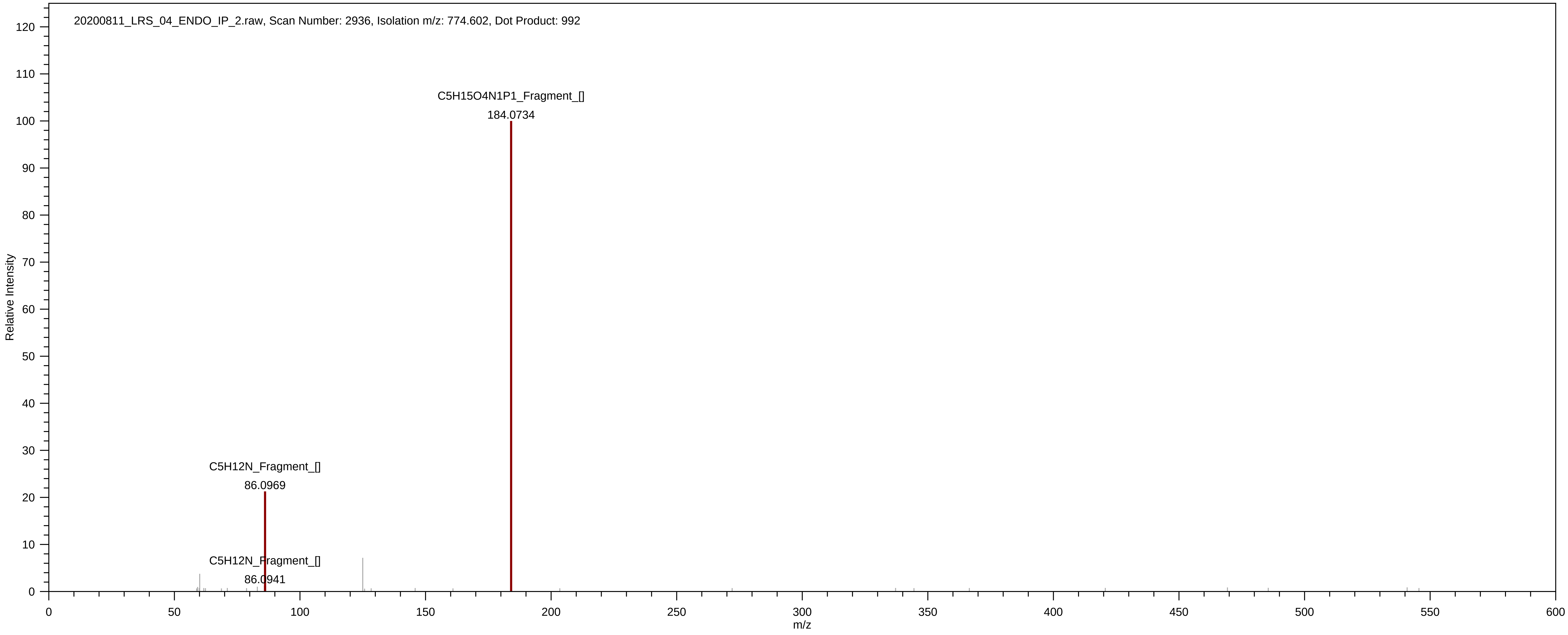

PC 35:1 [M+H]+ | Ret. Time (min): 10.316 | m/z: 774.602

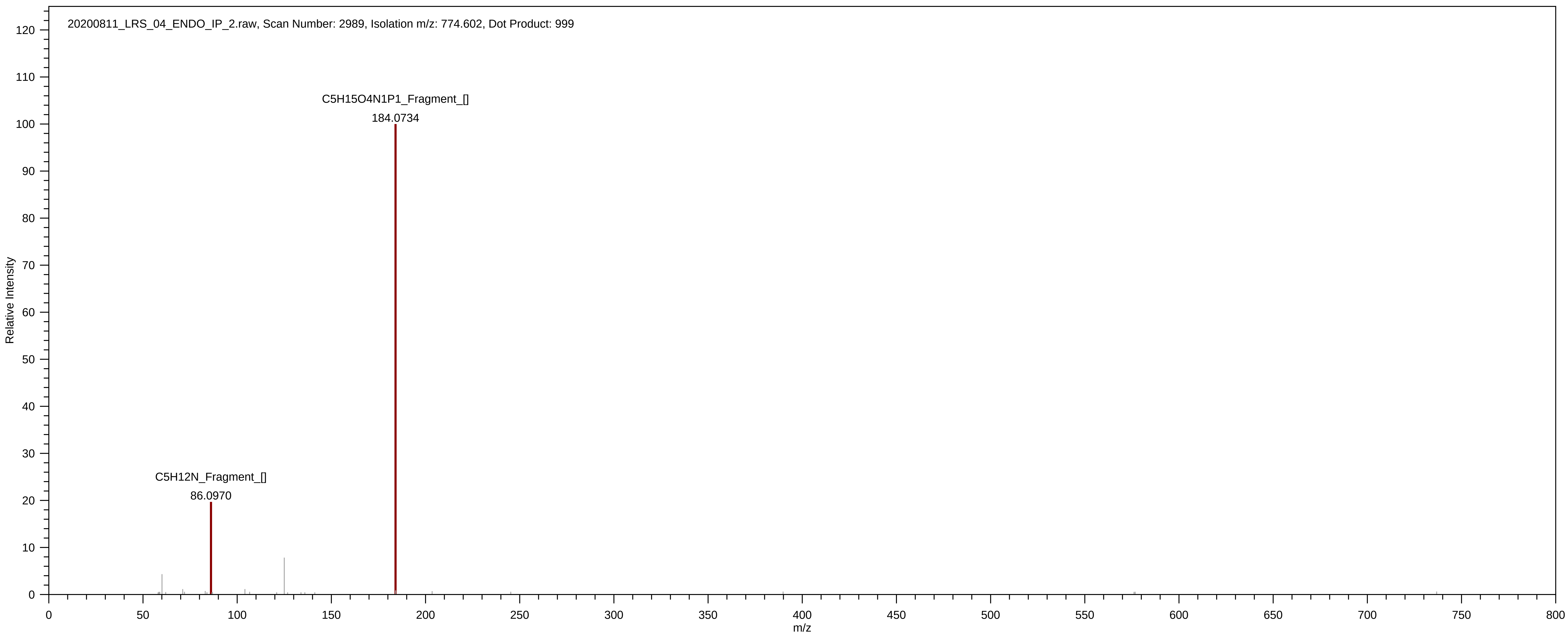

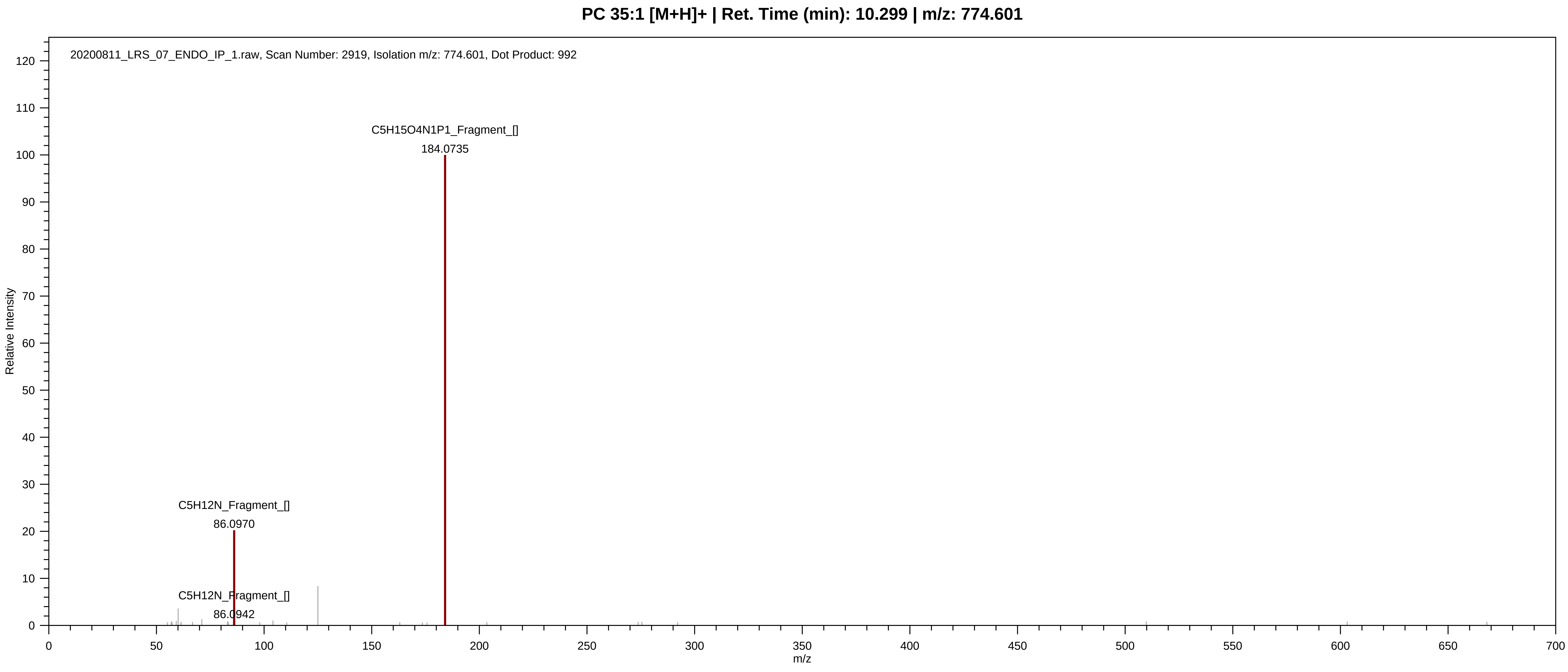
PC 35:1 [M+H]+ | Ret. Time (min): 10.299 | m/z: 774.601
20200811_LRS_07_ENDO_IP_1.raw, Scan Number: 2919, Isolation m/z: 774.601, Dot Product: 992
C5H15O4N1P1_Fragment_[]
184.0735
C5H12N_Fragment_[]
86.0970
C5H12N_Fragment_[]
86.0942
Relative Intensity
m/z

PC 35:1 [M+H]+ | Ret. Time (min): 10.299 | m/z: 774.603

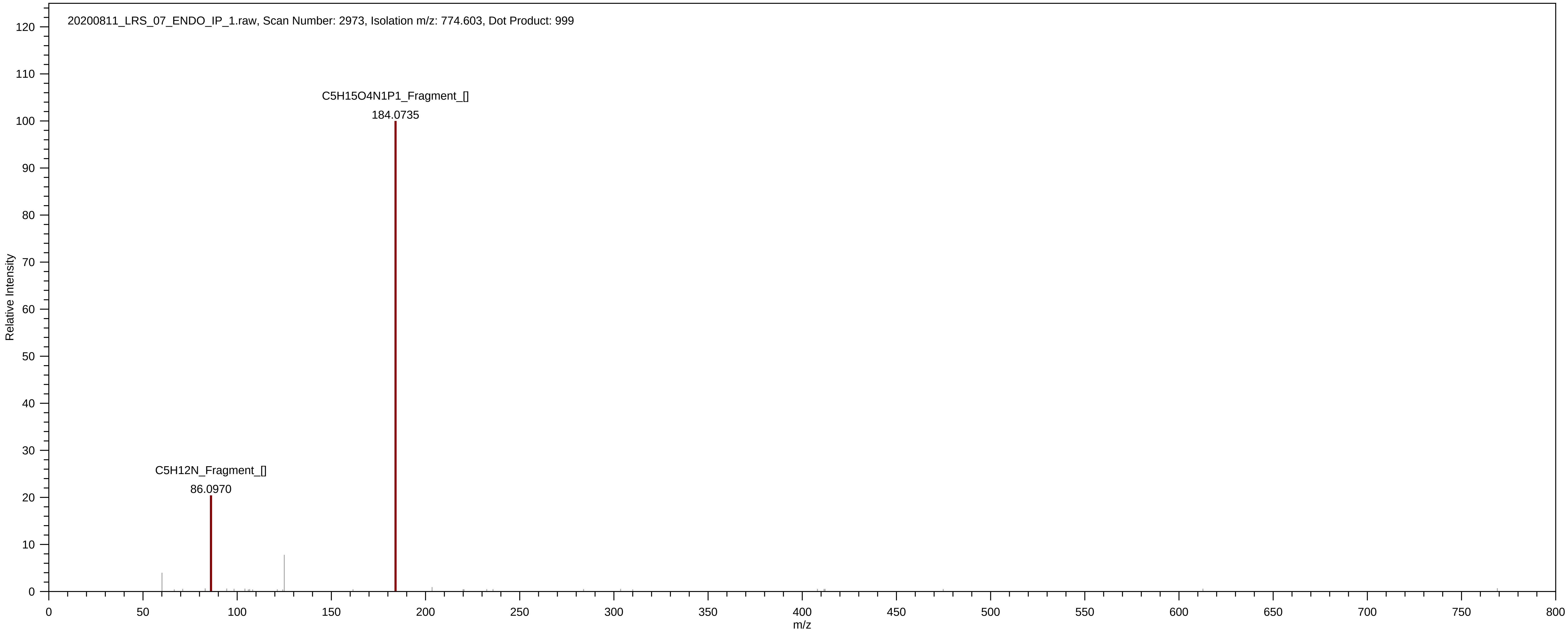

PC 35:2 [M+H]+ | Ret. Time (min): 9.597 | m/z: 772.59

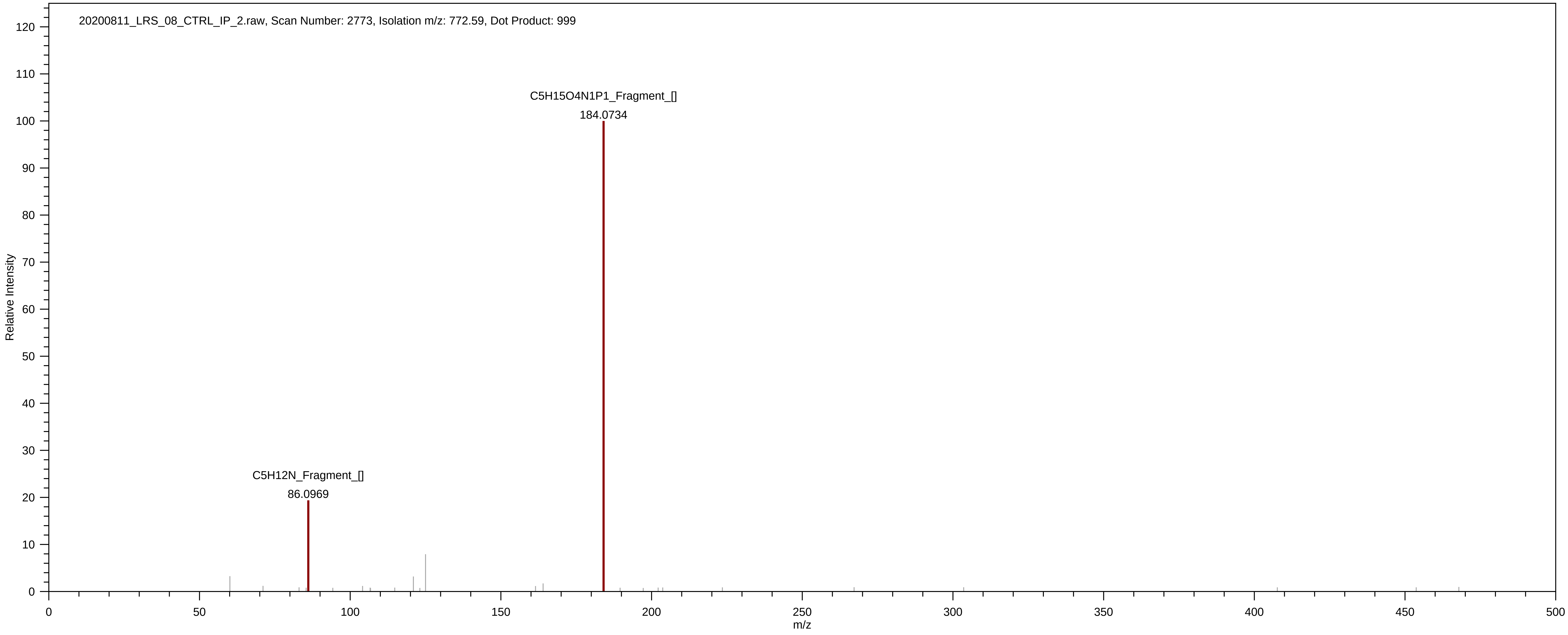

PC 35:2 [M+H]+ | Ret. Time (min): 9.422 | m/z: 772.585

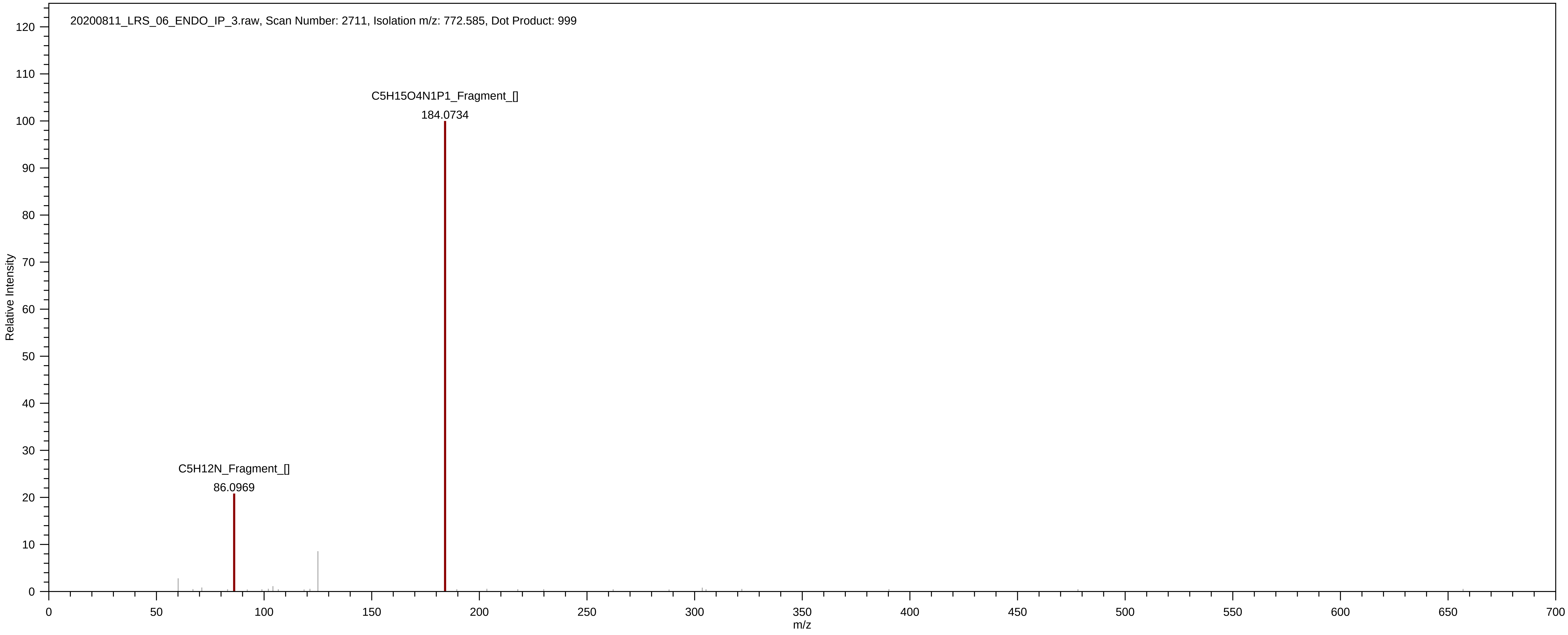

PC 35:2 [M+H]+ | Ret. Time (min): 9.4 | m/z: 772.586

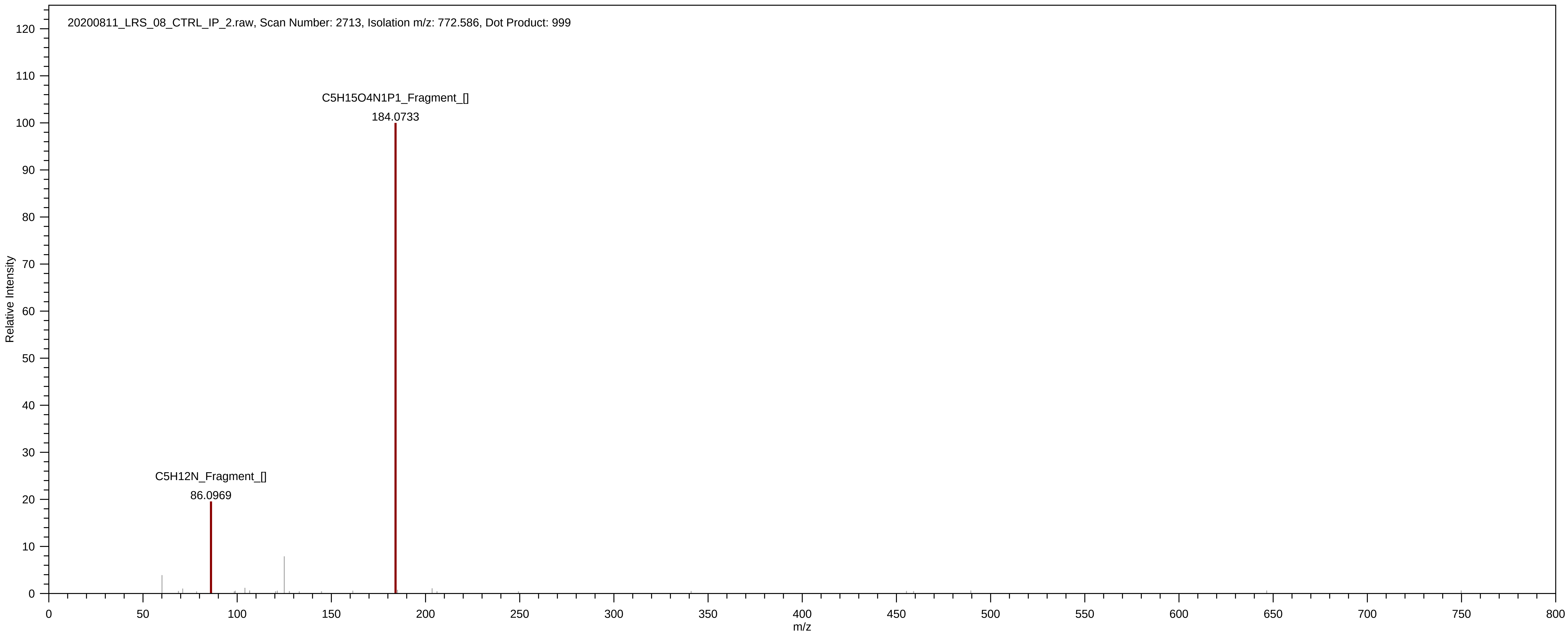

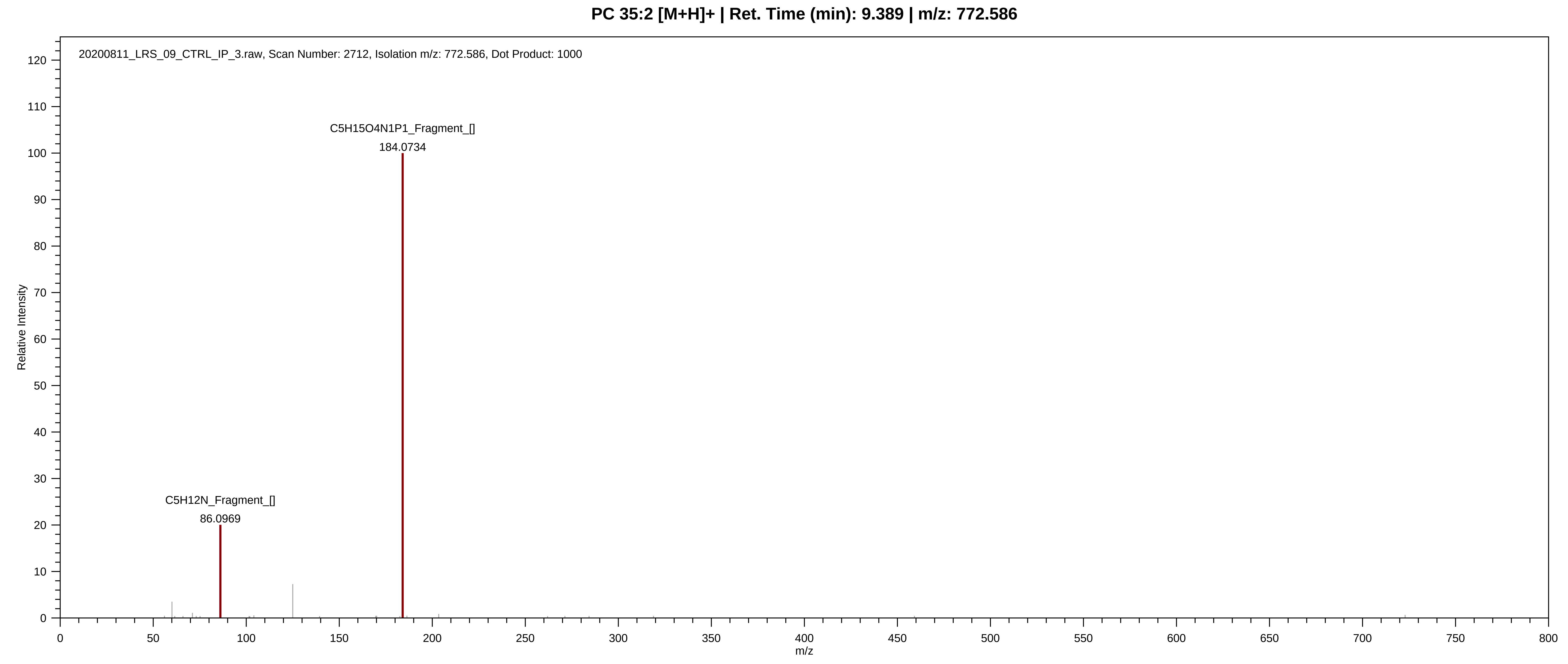
PC 35:2 [M+H]+ | Ret. Time (min): 9.389 | m/z: 772.586
20200811_LRS_09_CTRL_IP_3.raw, Scan Number: 2712, Isolation m/z: 772.586, Dot Product: 1000
C5H15O4N1P1_Fragment_[]
184.0734
C5H12N_Fragment_[]
86.0969
Relative Intensity
m/z

PC 35:2 [M+H]+ | Ret. Time (min): 9.405 | m/z: 772.586

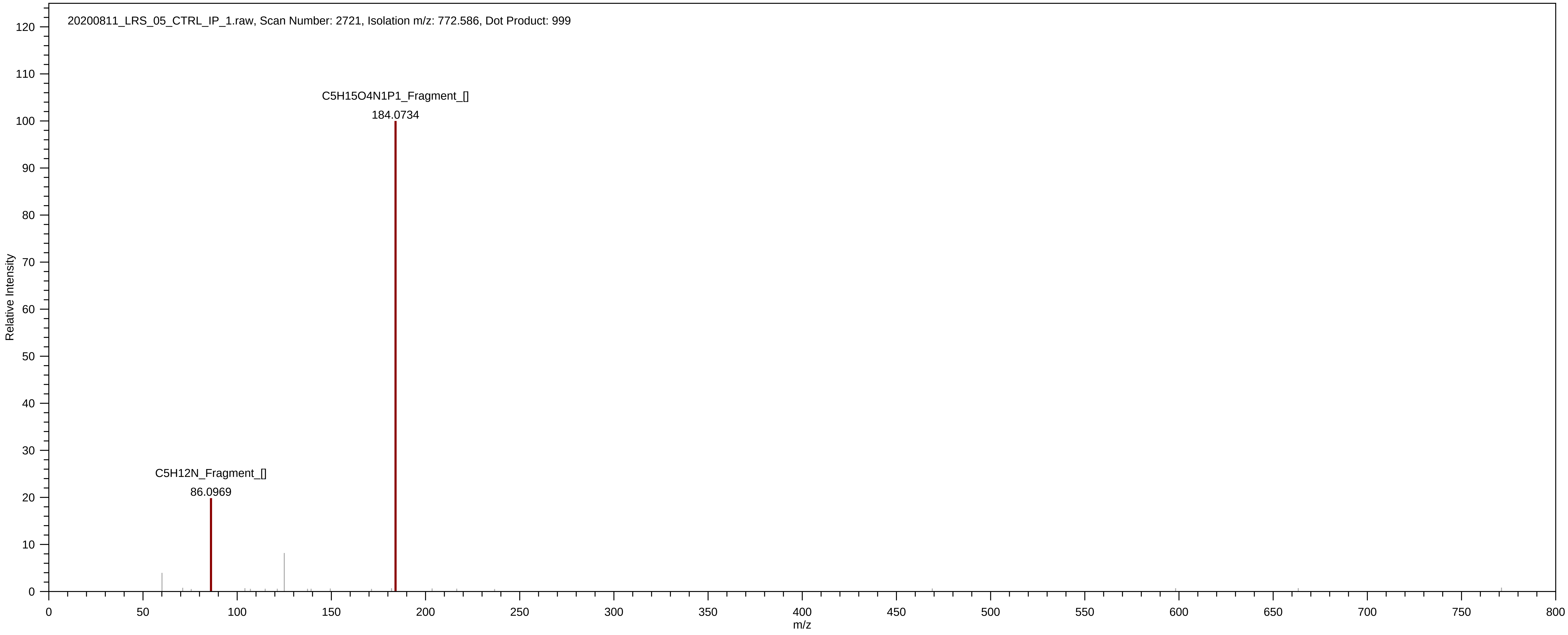

PC 35:2 [M+H]+ | Ret. Time (min): 9.423 | m/z: 772.586

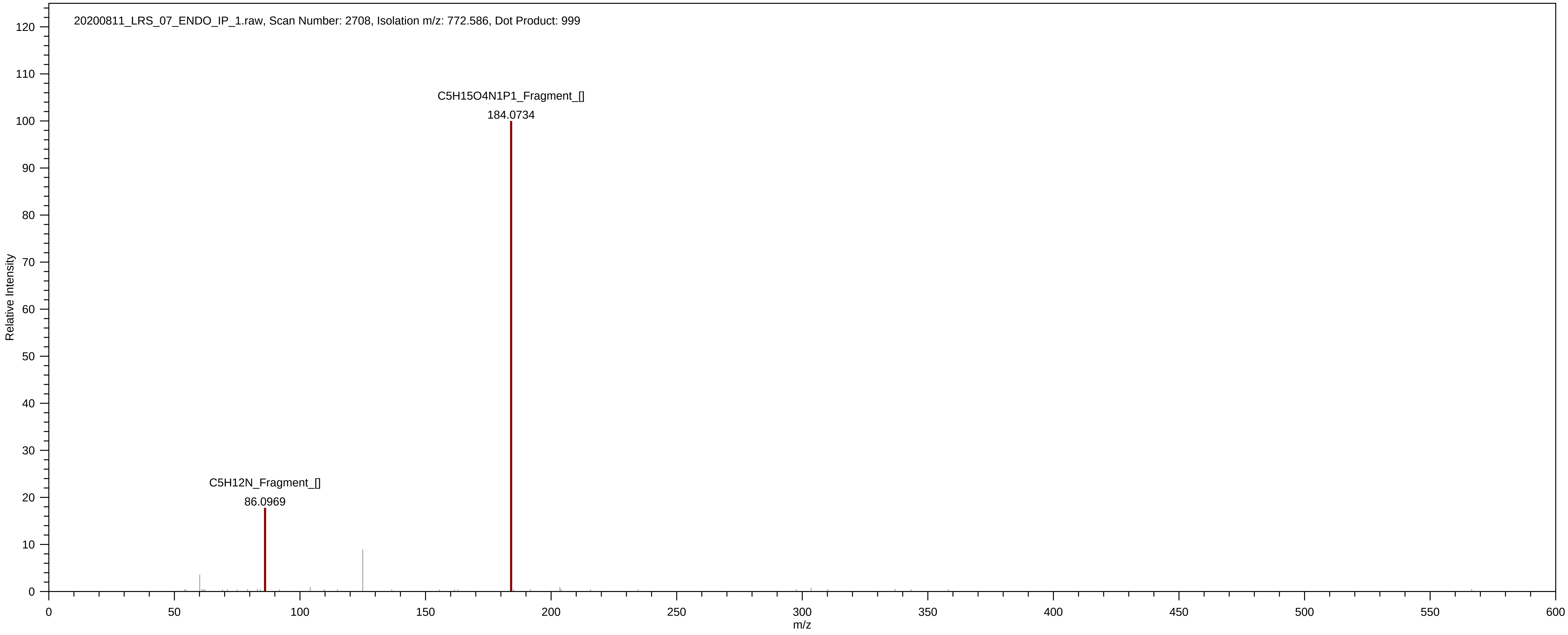

PC 35:2 [M+H]+ | Ret. Time (min): 9.437 | m/z: 772.586

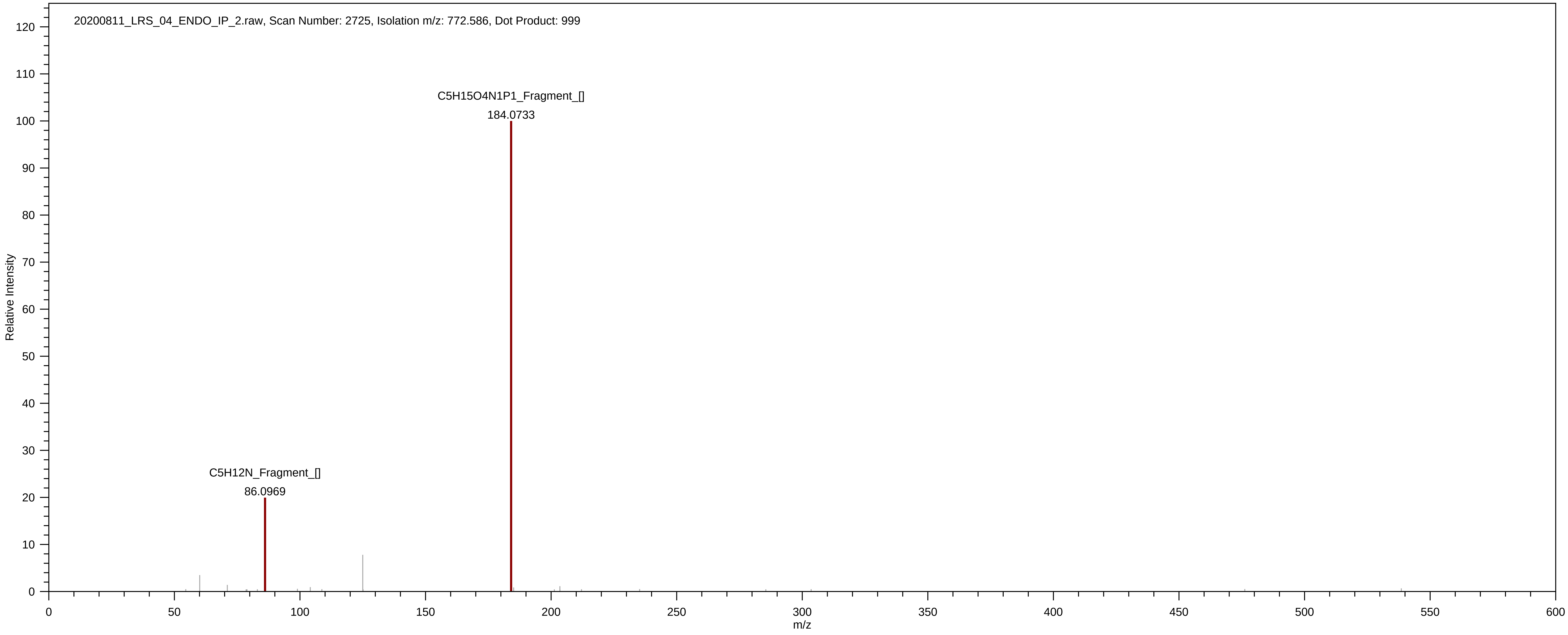

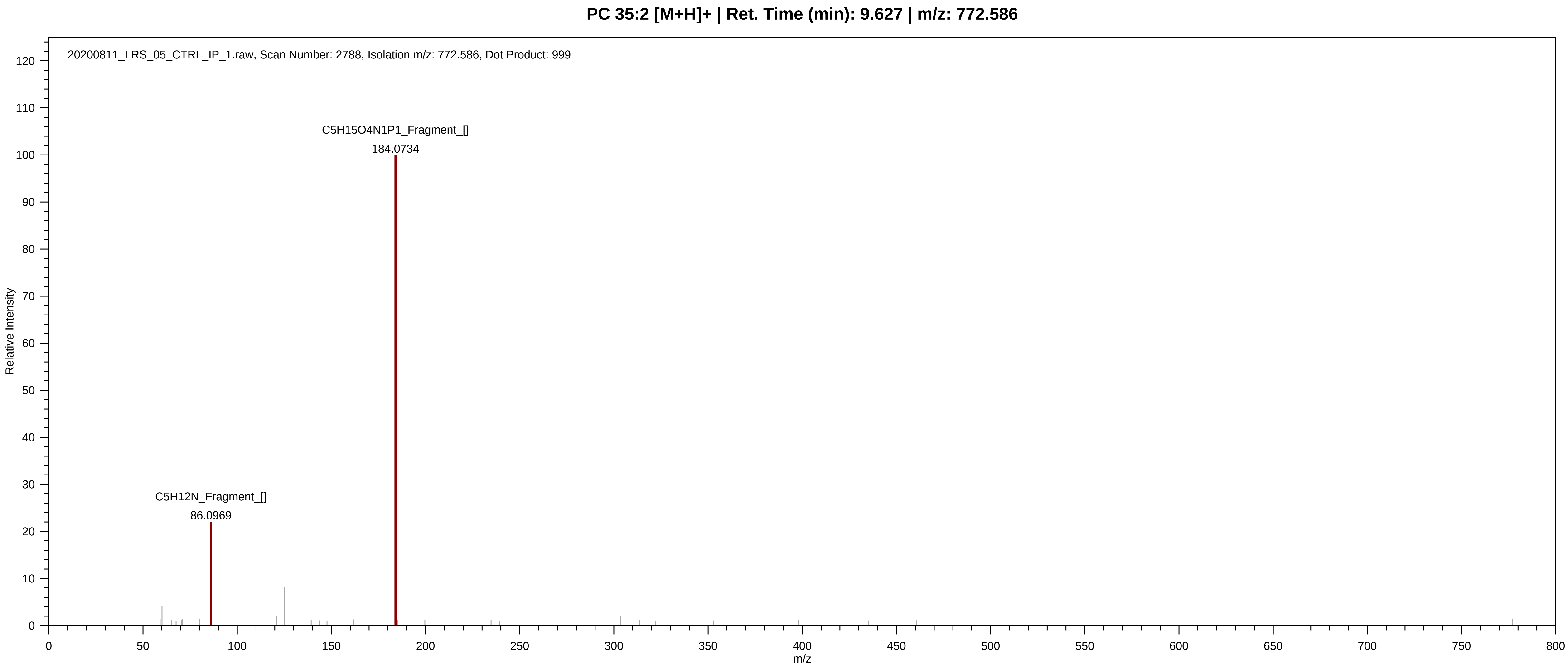
PC 35:2 [M+H]+ | Ret. Time (min): 9.627 | m/z: 772.586
20200811_LRS_05_CTRL_IP_1.raw, Scan Number: 2788, Isolation m/z: 772.586, Dot Product: 999
C5H15O4N1P1_Fragment_[]
184.0734
C5H12N_Fragment_[]
86.0969
Relative Intensity
m/z

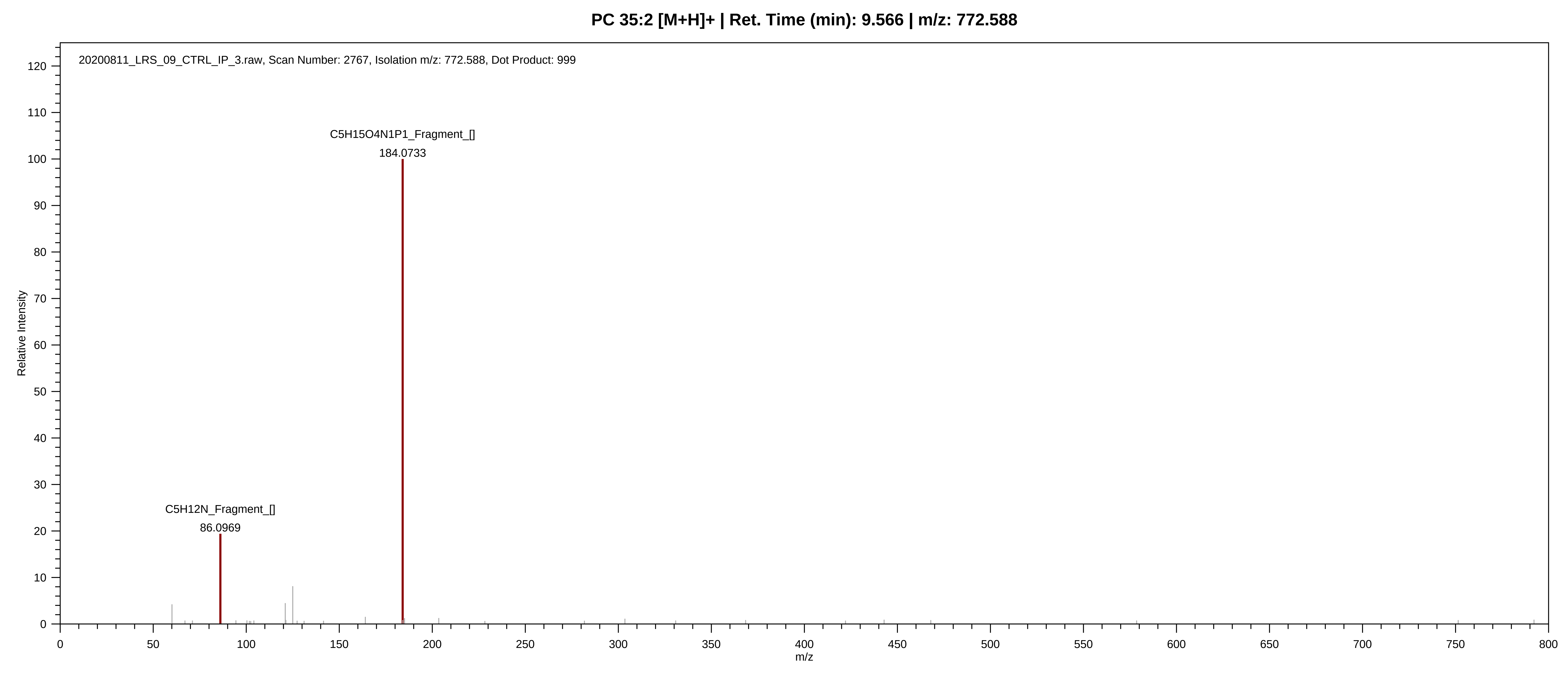

PC 35:2 [M+H]+ | Ret. Time (min): 9.566 | m/z: 772.588
20200811_LRS_09_CTRL_IP_3.raw, Scan Number: 2767, Isolation m/z: 772.588, Dot Product: 999
C5H15O4N1P1_Fragment_[]
184.0733
C5H12N_Fragment_[]
86.0969
Relative Intensity
m/z

PC 36:0 [M+H]+ | Ret. Time (min): 11.813 | m/z: 790.632

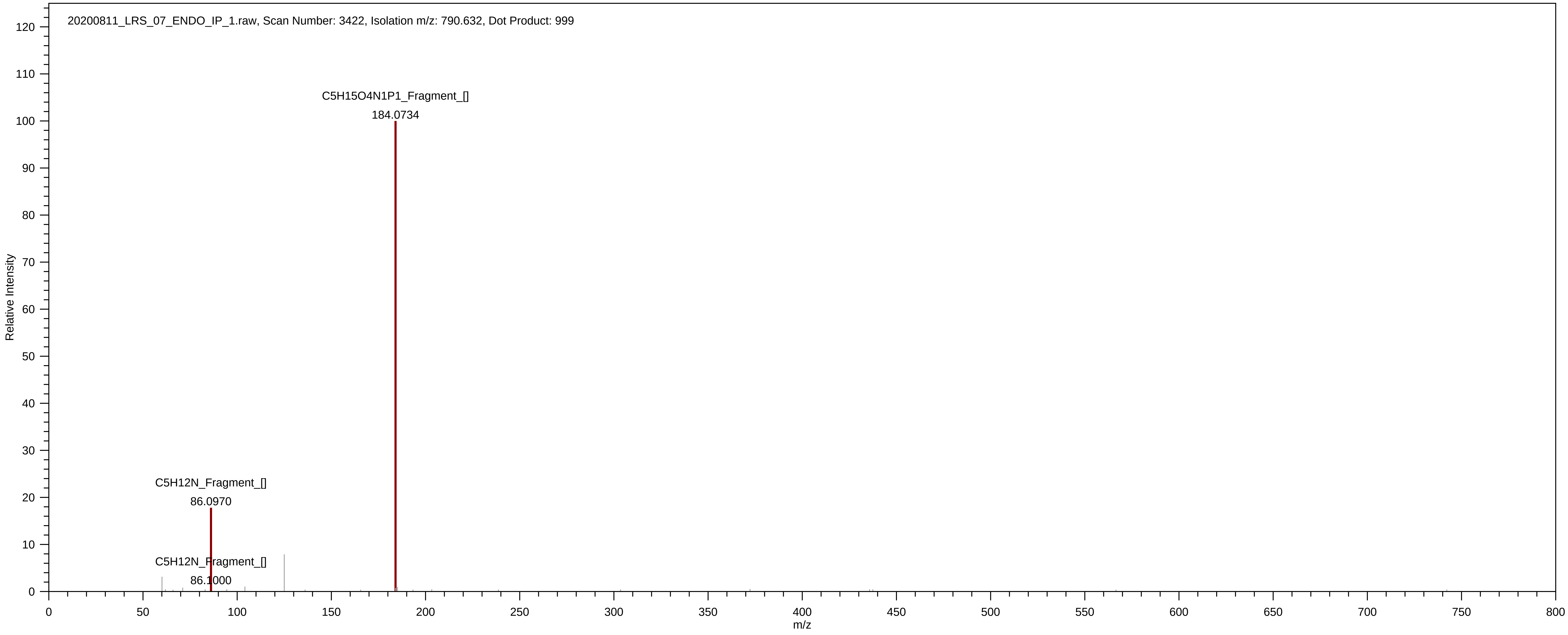

PC 36:0 [M+H]+ | Ret. Time (min): 11.813 | m/z: 790.632

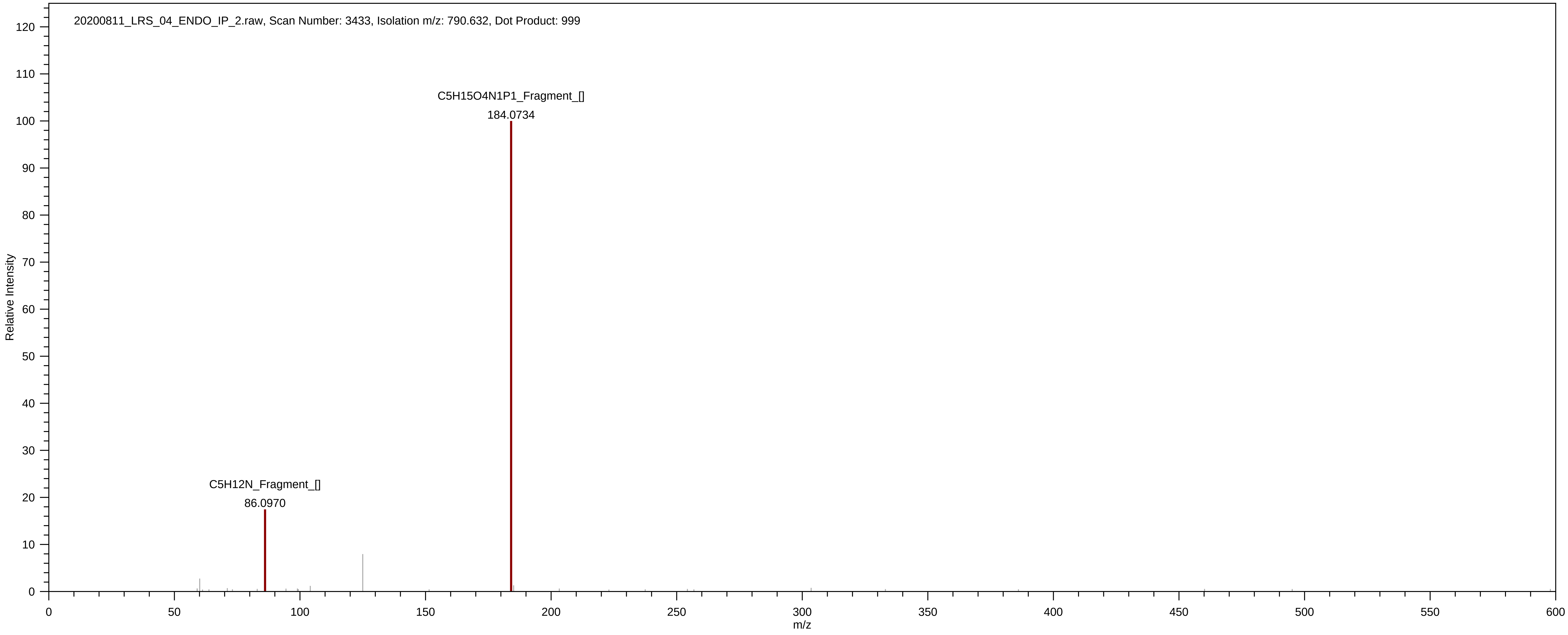

PC 36:0 [M+H]+ | Ret. Time (min): 11.832 | m/z: 790.632

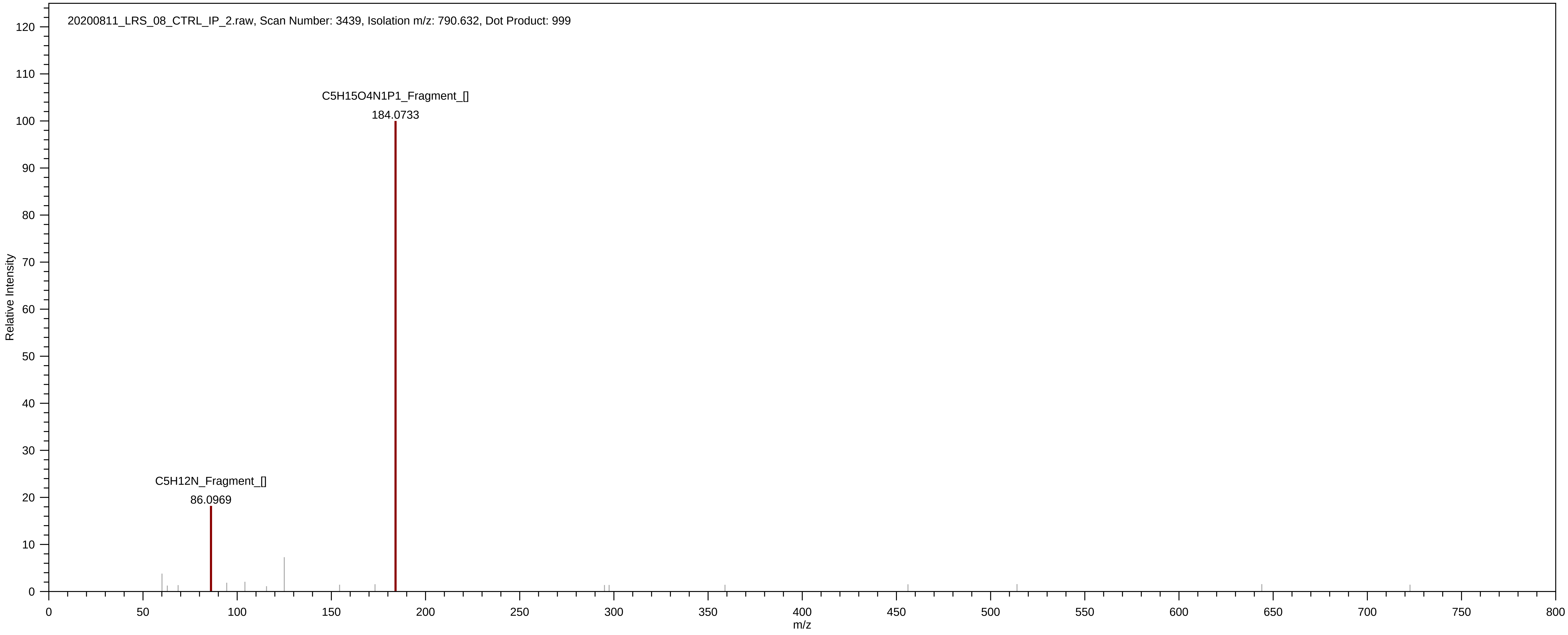

PC 36:0 [M+H]+ | Ret. Time (min): 11.795 | m/z: 790.633

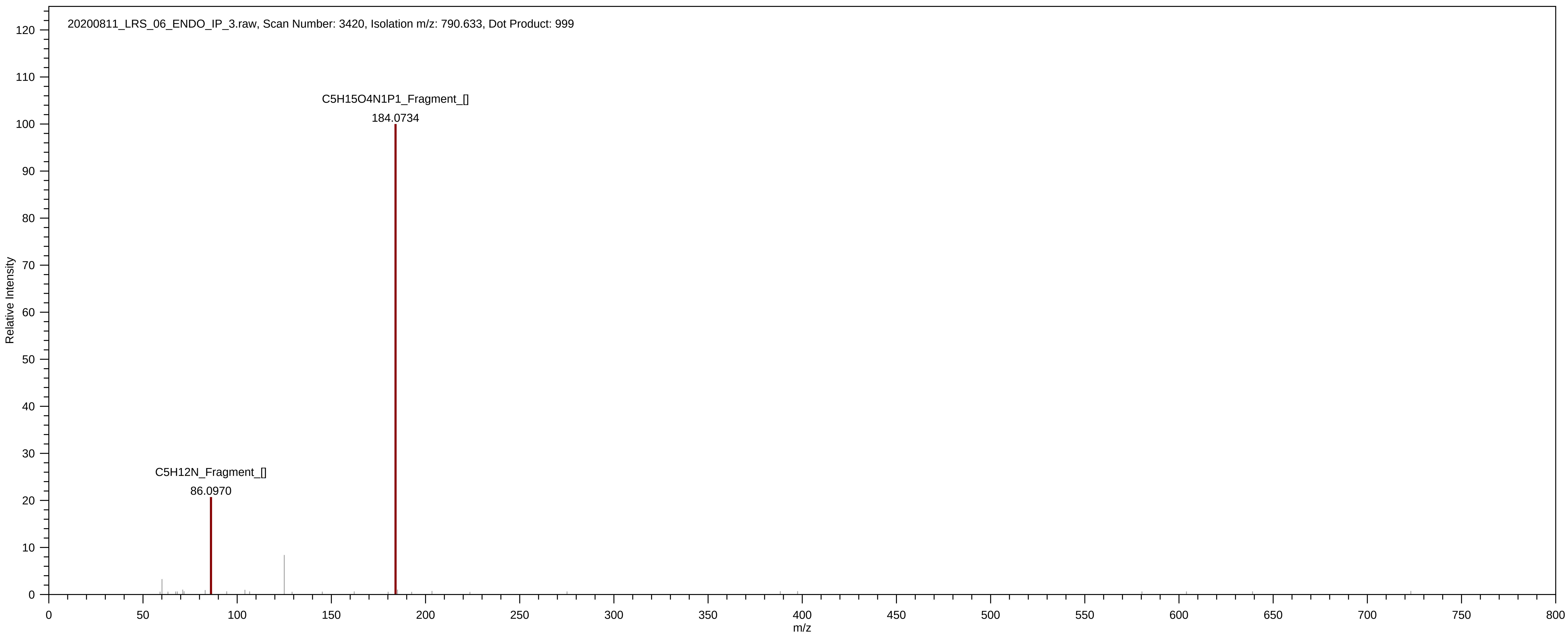

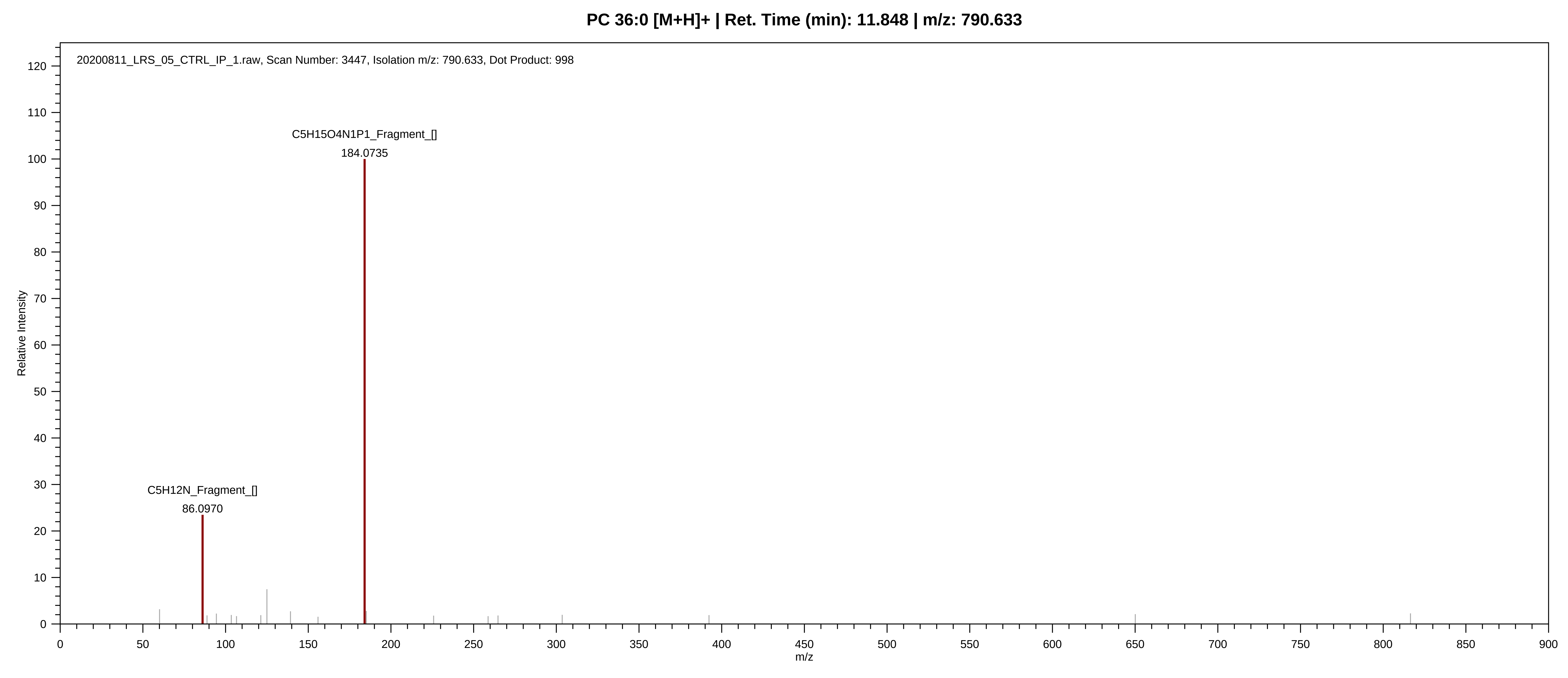

PC 36:0 [M+H]+ | Ret. Time (min): 11.848 | m/z: 790.633
20200811_LRS_05_CTRL_IP_1.raw, Scan Number: 3447, Isolation m/z: 790.633, Dot Product: 998
C5H15O4N1P1_Fragment_[]
184.0735
C5H12N_Fragment_[]
86.0970
Relative Intensity
m/z

PC 36:1 [M+Ac-H]- | Ret. Time (min): 10.761 | m/z: 846.623

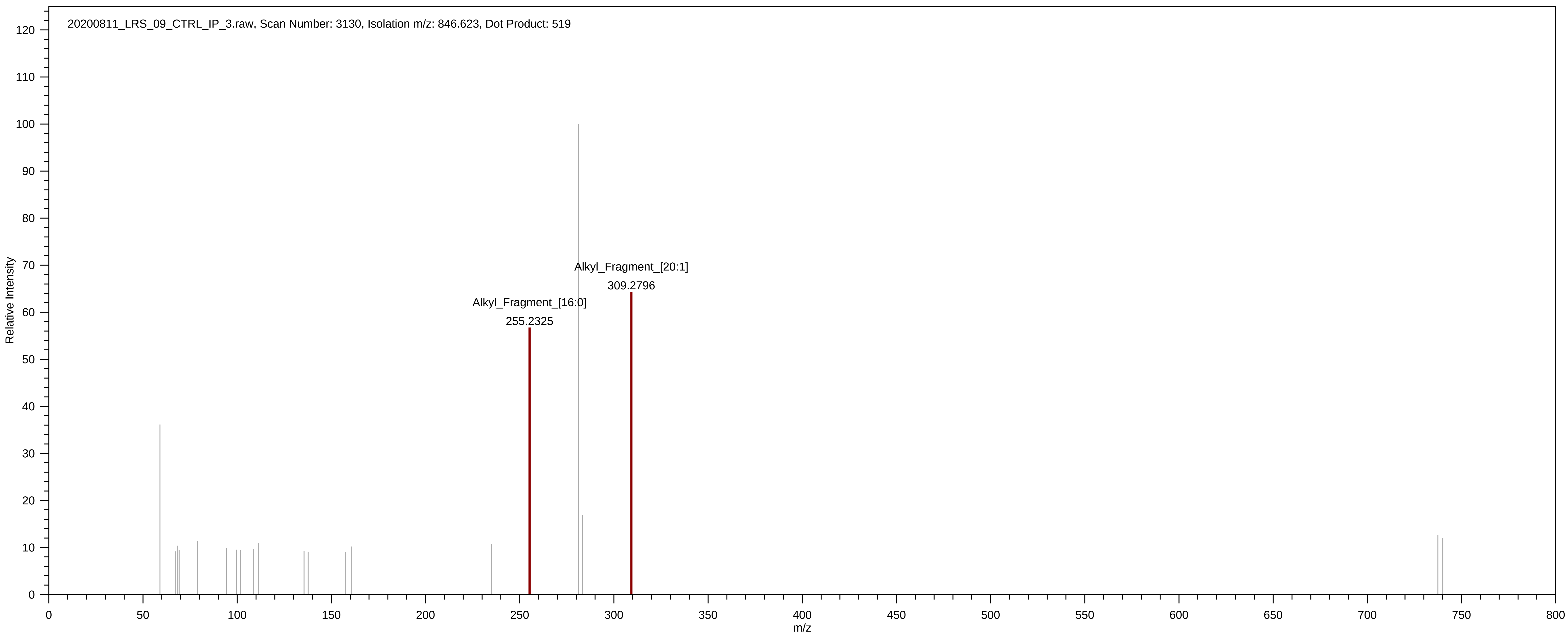

PC 36:1 [M+Ac-H]- | Ret. Time (min): 10.766 | m/z: 846.623

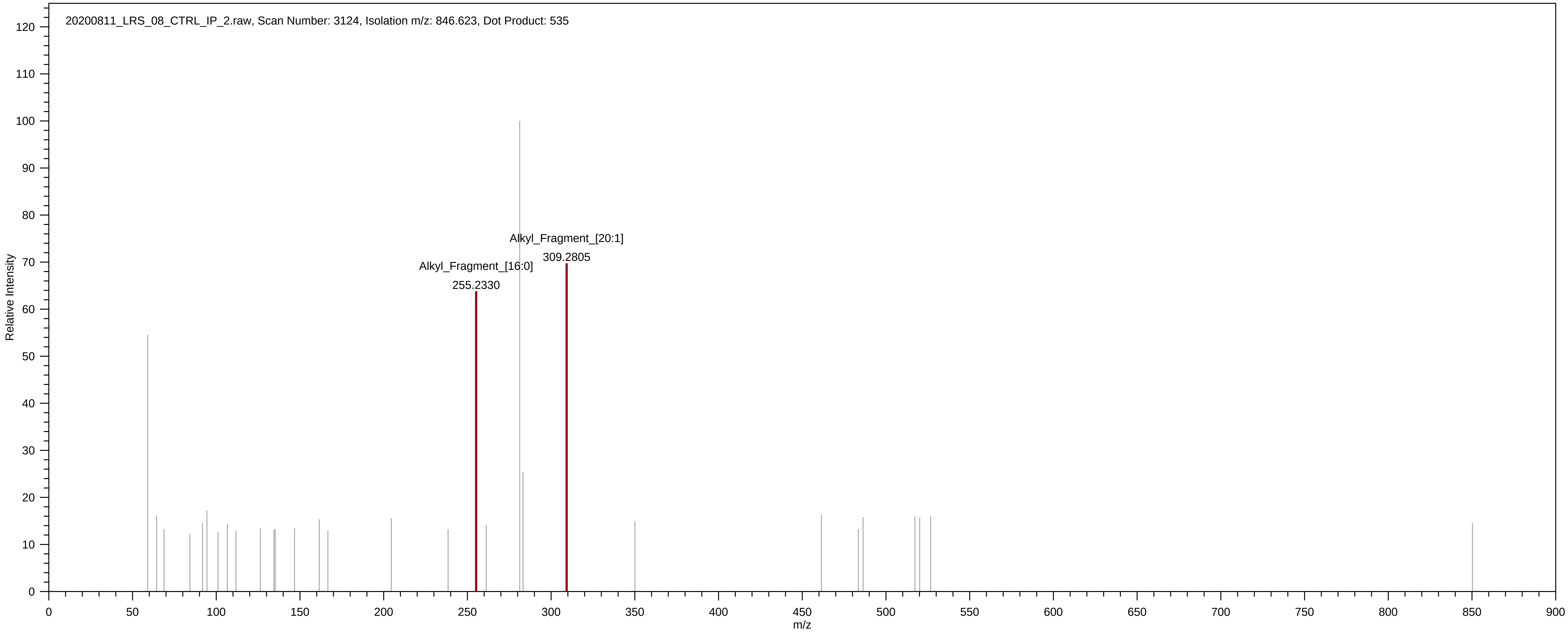

PC 36:1 [M+Ac-H]- | Ret. Time (min): 10.744 | m/z: 846.624

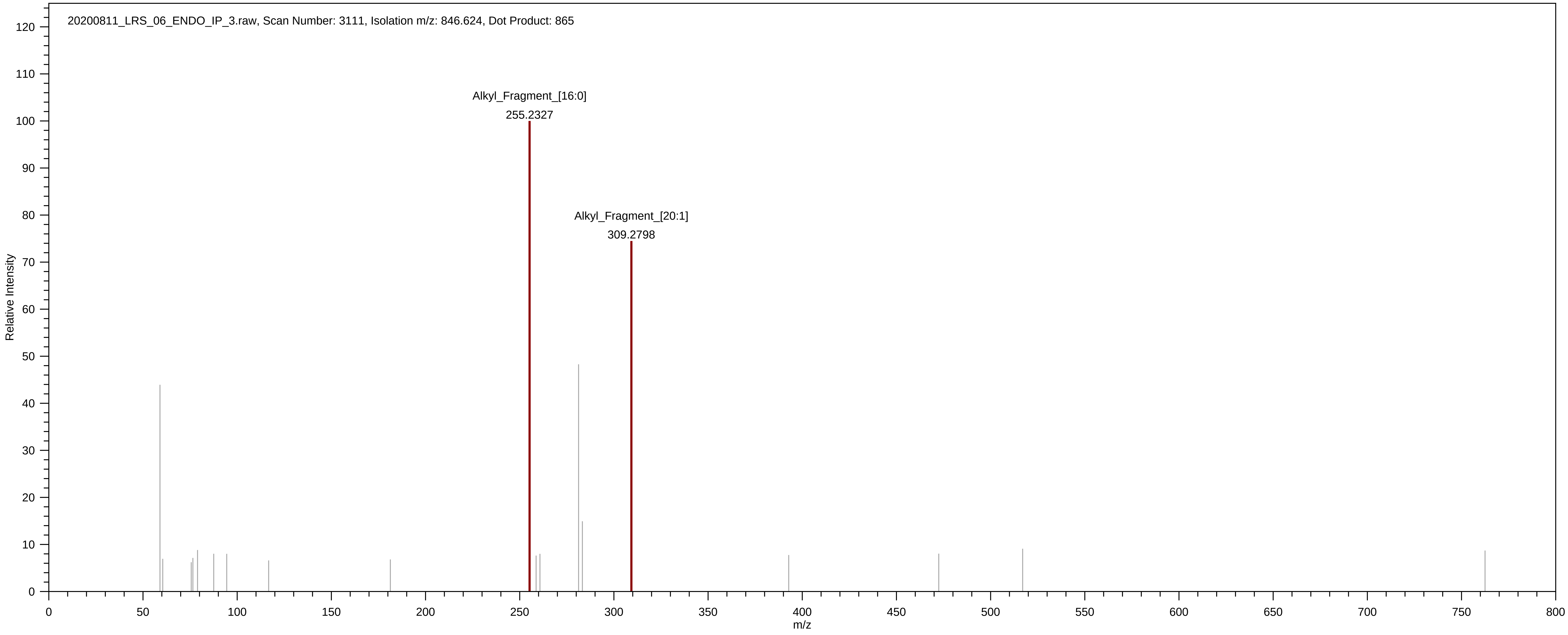

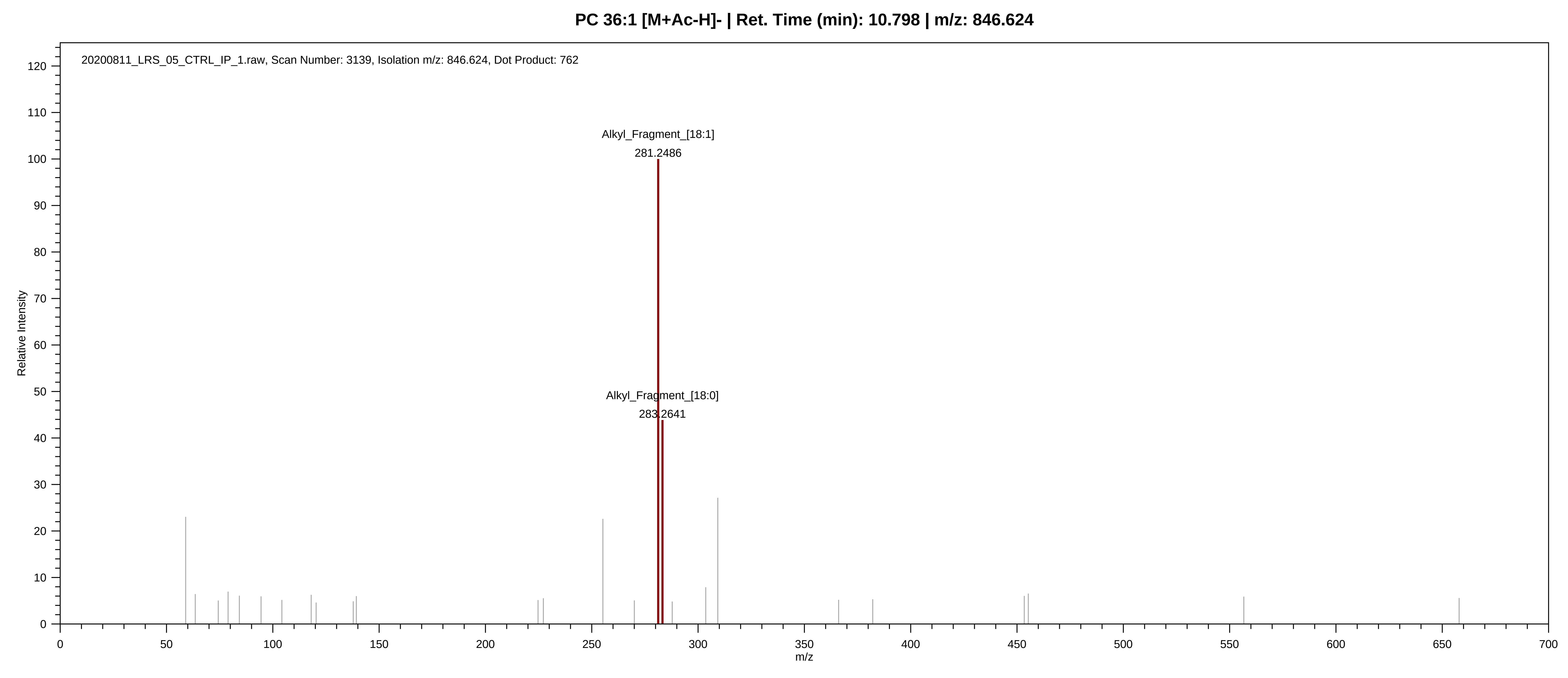
PC 36:1 [M+Ac-H]- | Ret. Time (min): 10.798 | m/z: 846.624
20200811_LRS_05_CTRL_IP_1.raw, Scan Number: 3139, Isolation m/z: 846.624, Dot Product: 762
Alkyl_Fragment_[18:1]
281.2486
Alkyl_Fragment_[18:0]
283.2641
Relative Intensity
m/z

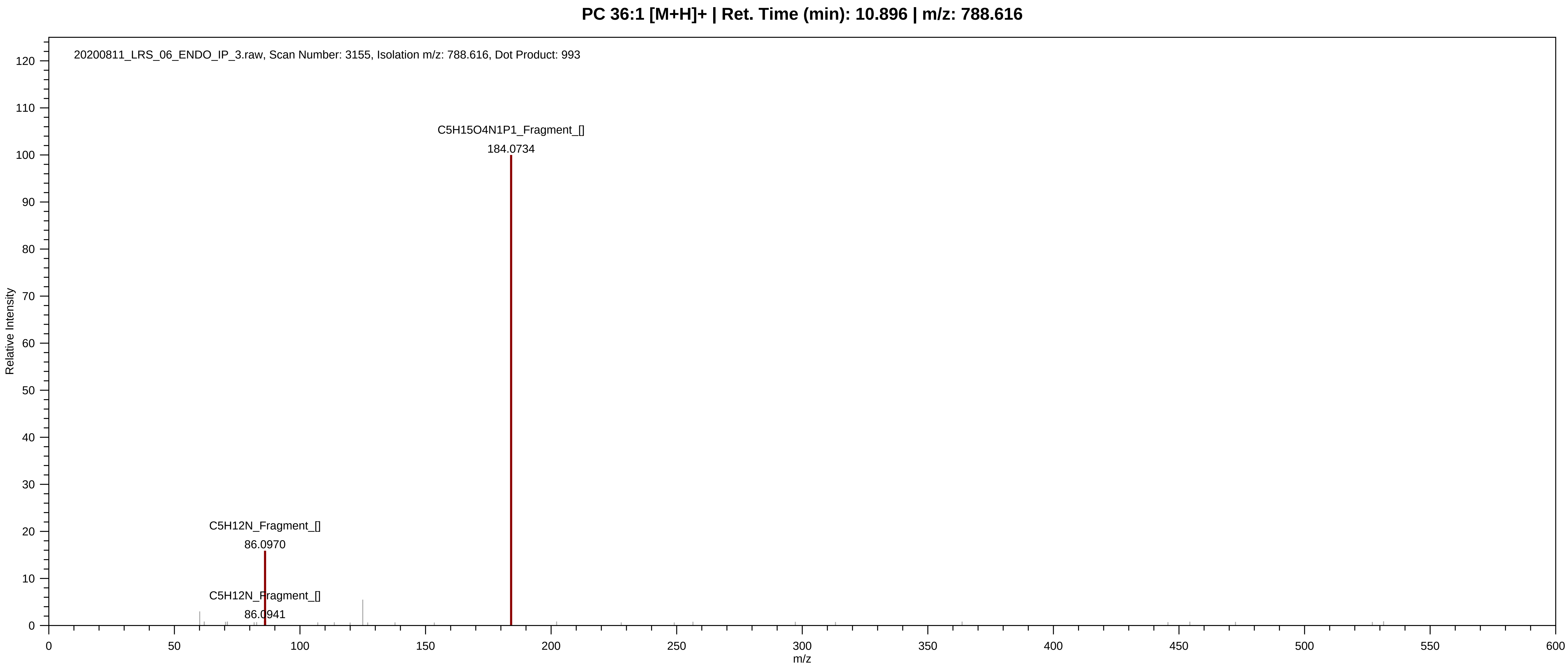

PC 36:1 [M+H]+ | Ret. Time (min): 10.896 | m/z: 788.616
20200811_LRS_06_ENDO_IP_3.raw, Scan Number: 3155, Isolation m/z: 788.616, Dot Product: 993
C5H15O4N1P1_Fragment_[]
184.0734
C5H12N_Fragment_[]
86.0970
C5H12N_Fragment_[]
86.0941
Relative Intensity
m/z

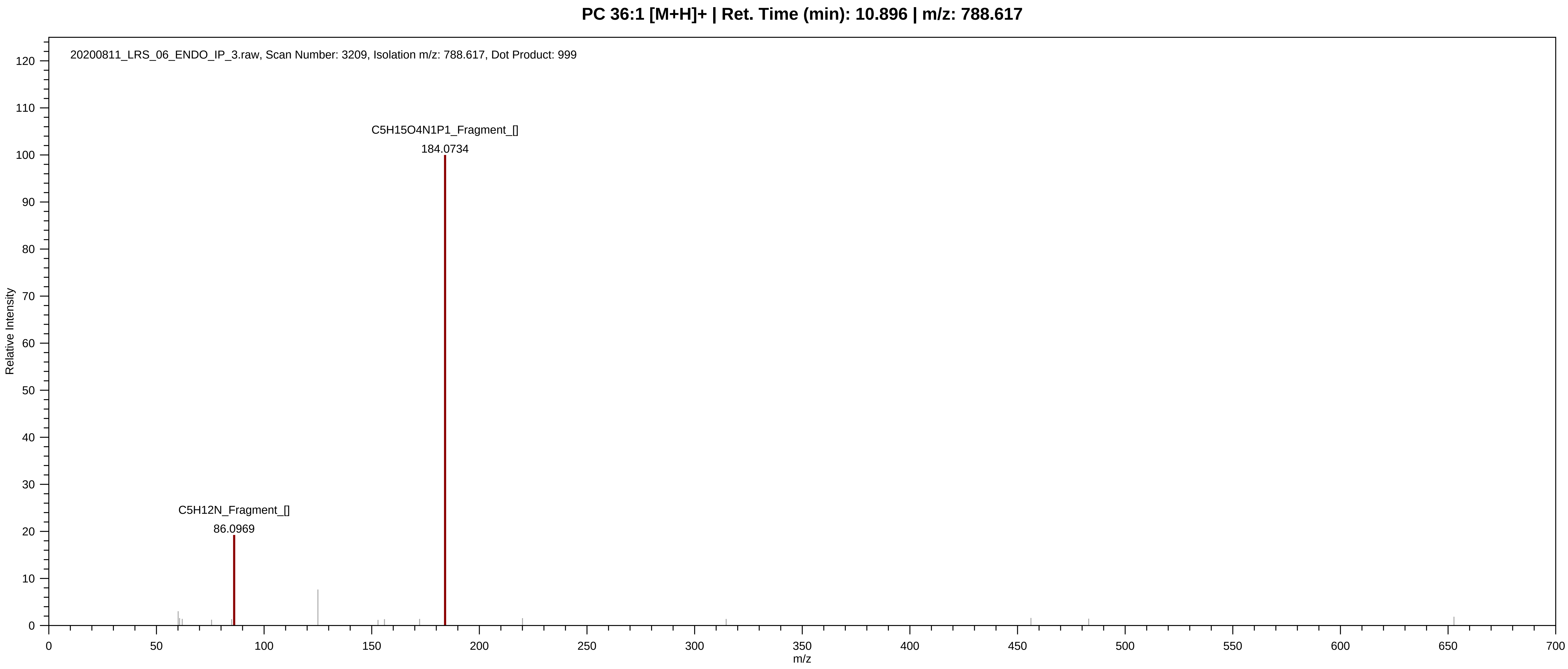
PC 36:1 [M+H]+ | Ret. Time (min): 10.896 | m/z: 788.617
20200811_LRS_06_ENDO_IP_3.raw, Scan Number: 3209, Isolation m/z: 788.617, Dot Product: 999
C5H15O4N1P1_Fragment_[]
184.0734
C5H12N_Fragment_[]
86.0969
Relative Intensity
m/z

PC 36:1 [M+H]+ | Ret. Time (min): 10.93 | m/z: 788.617

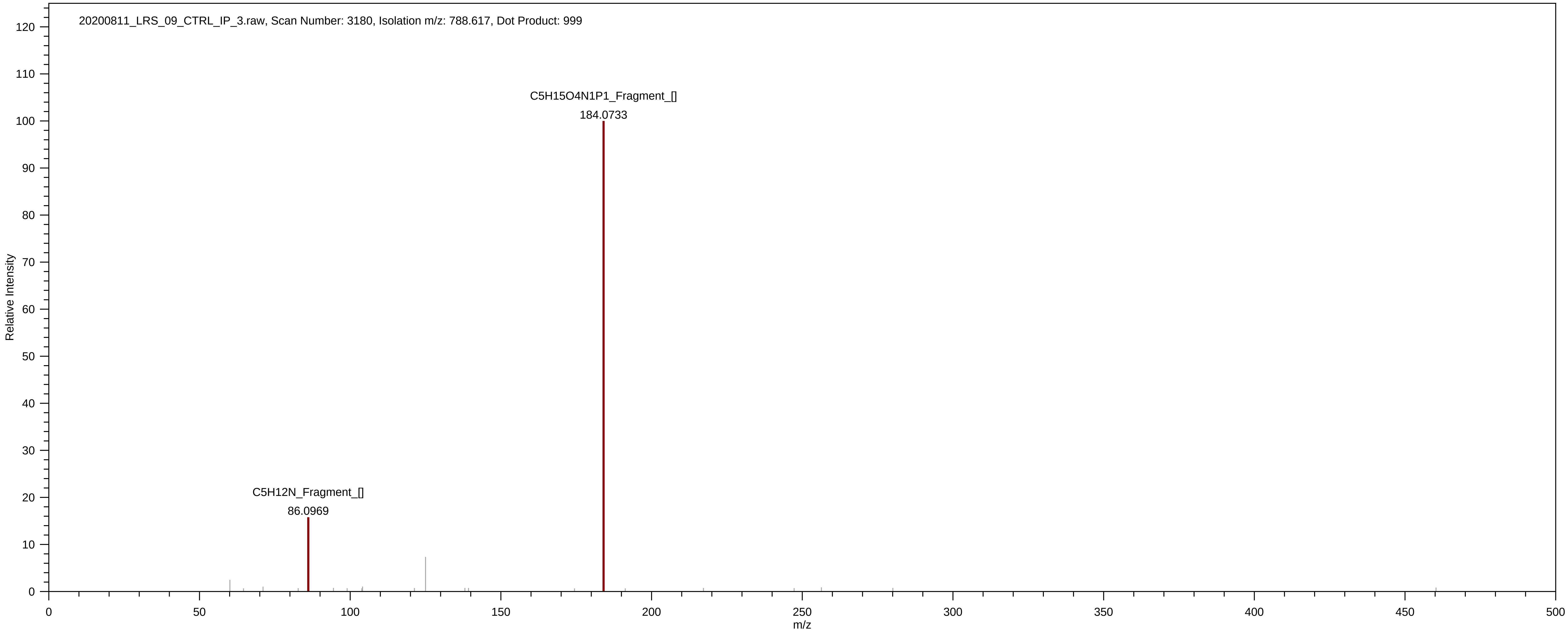

PC 36:1 [M+H]+ | Ret. Time (min): 10.714 | m/z: 788.617

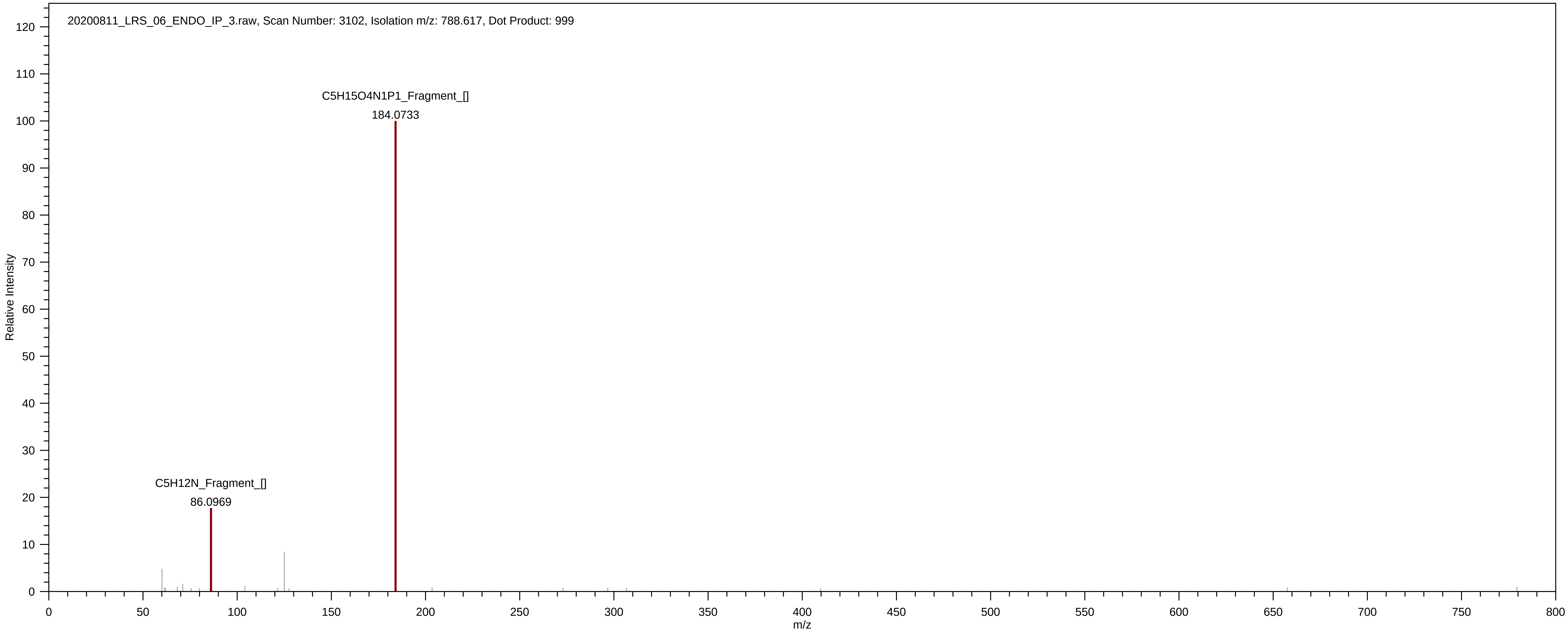

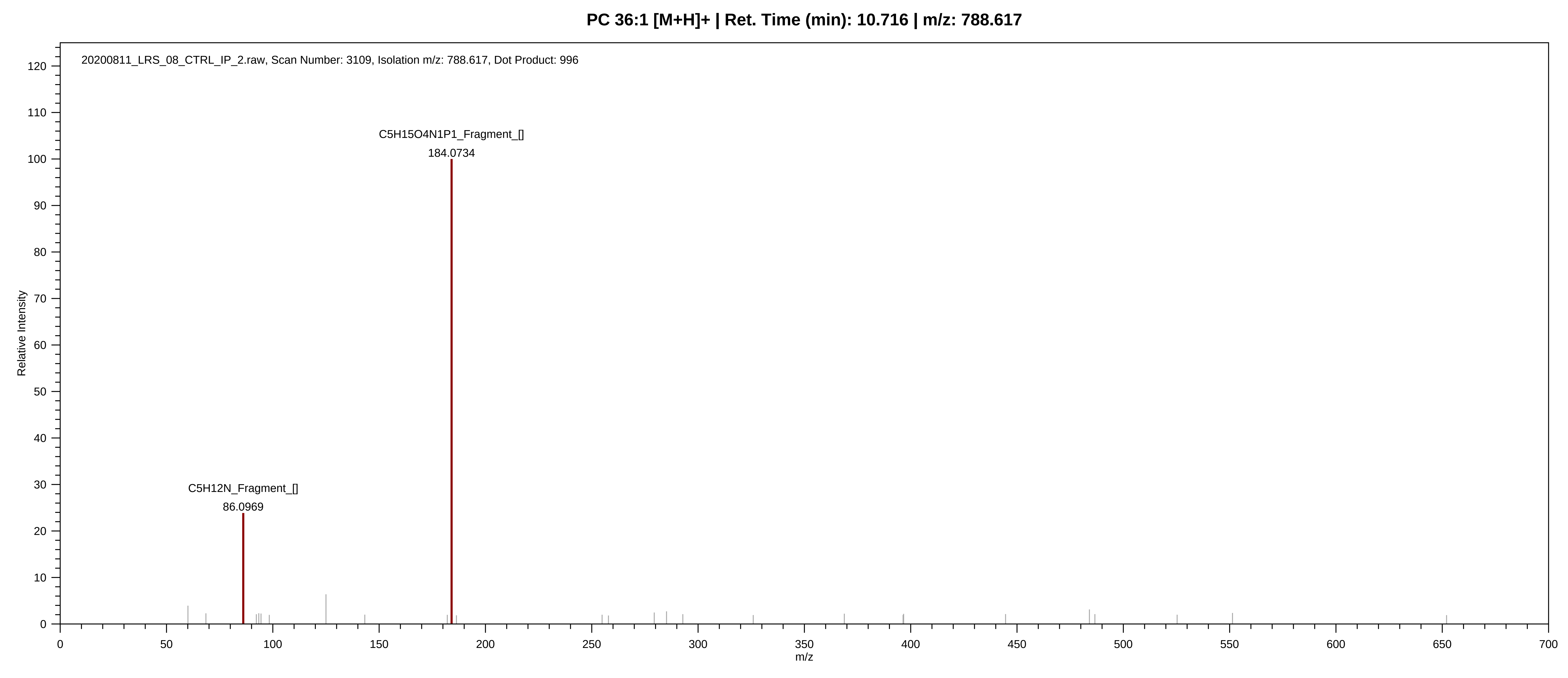
PC 36:1 [M+H]+ | Ret. Time (min): 10.716 | m/z: 788.617
20200811_LRS_08_CTRL_IP_2.raw, Scan Number: 3109, Isolation m/z: 788.617, Dot Product: 996
C5H15O4N1P1_Fragment_[]
184.0734
C5H12N_Fragment_[]
86.0969
Relative Intensity
m/z
0
10
20
30
40
50
60
70
80
90
100
110
120
0
50
100
150
200
250
300
350
400
450
500
550
600
650
700

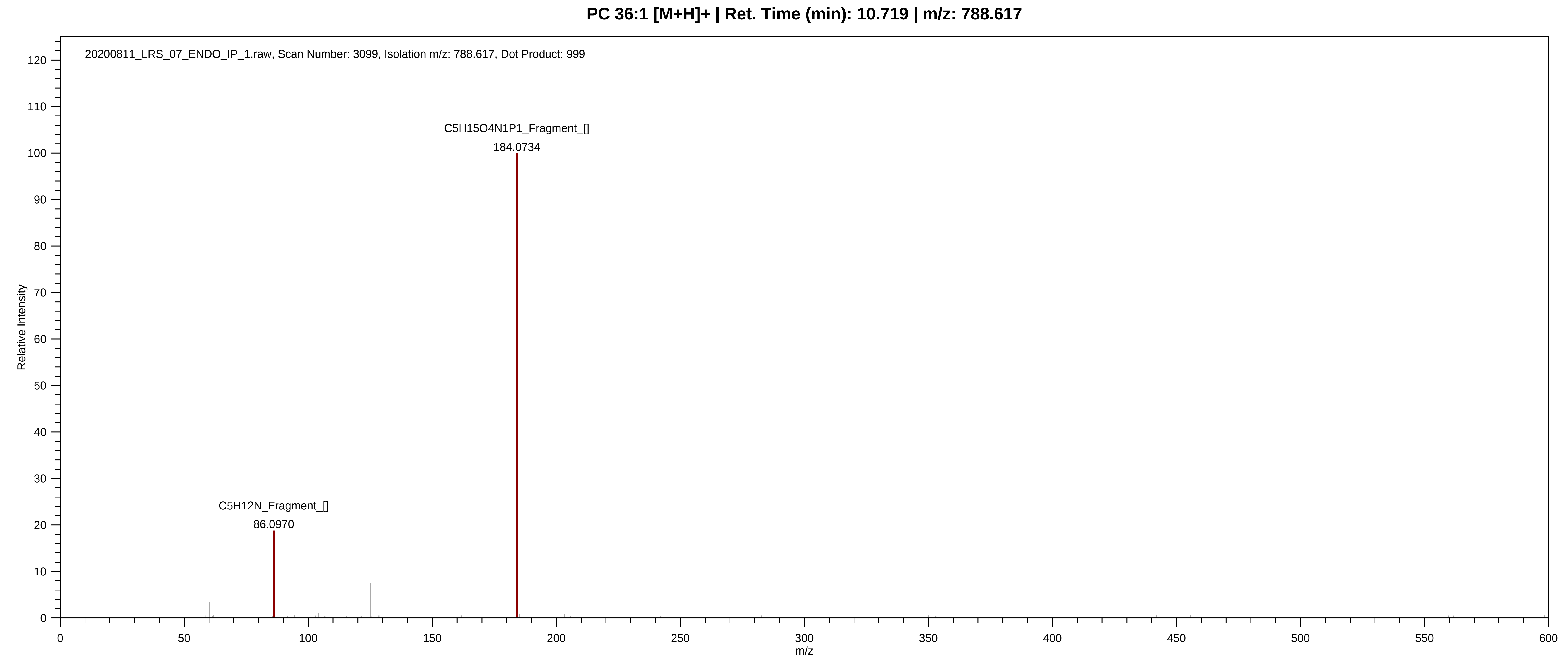

PC 36:1 [M+H]+ | Ret. Time (min): 10.719 | m/z: 788.617
20200811_LRS_07_ENDO_IP_1.raw, Scan Number: 3099, Isolation m/z: 788.617, Dot Product: 999
C5H15O4N1P1_Fragment_[]
184.0734
C5H12N_Fragment_[]
86.0970
Relative Intensity
m/z

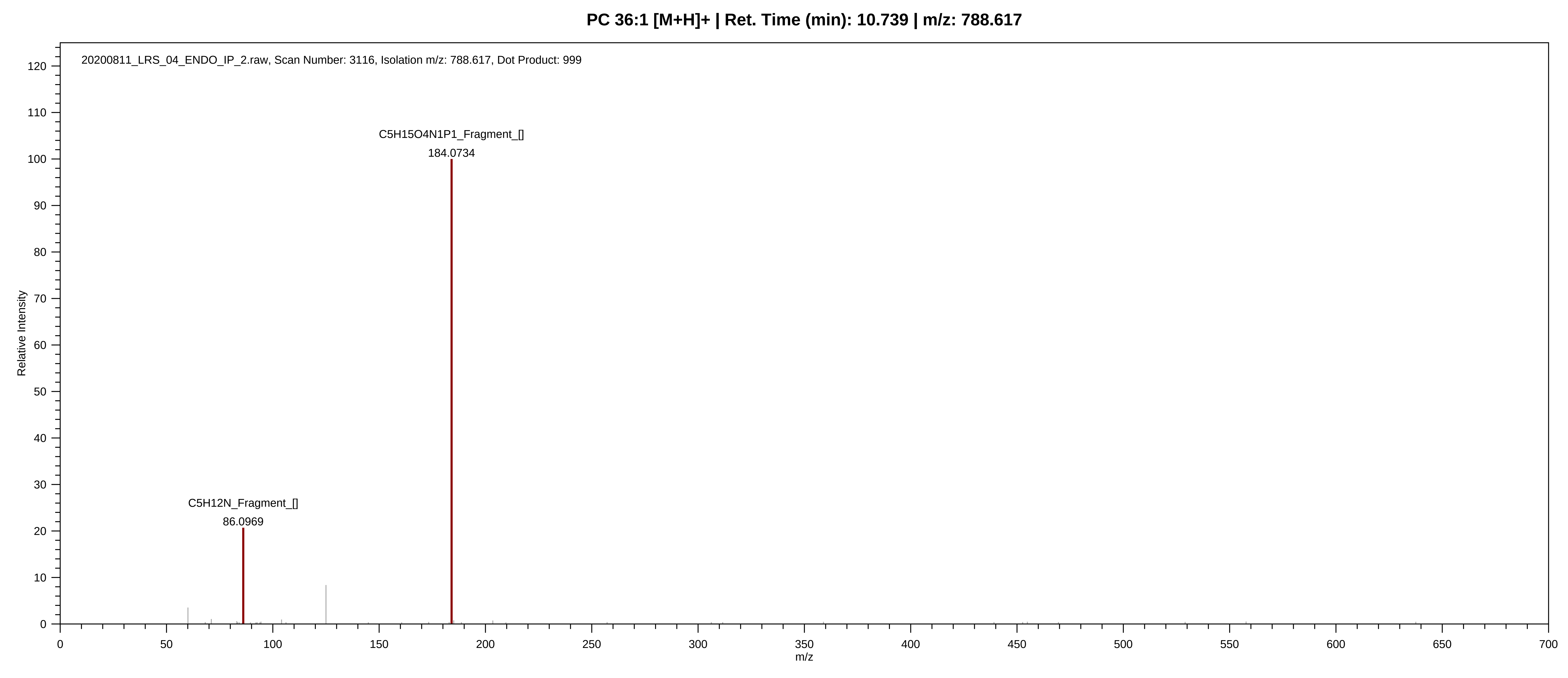

PC 36:1 [M+H]+ | Ret. Time (min): 10.739 | m/z: 788.617
20200811_LRS_04_ENDO_IP_2.raw, Scan Number: 3116, Isolation m/z: 788.617, Dot Product: 999
C5H15O4N1P1_Fragment_[]
184.0734
C5H12N_Fragment_[]
86.0969
Relative Intensity
m/z

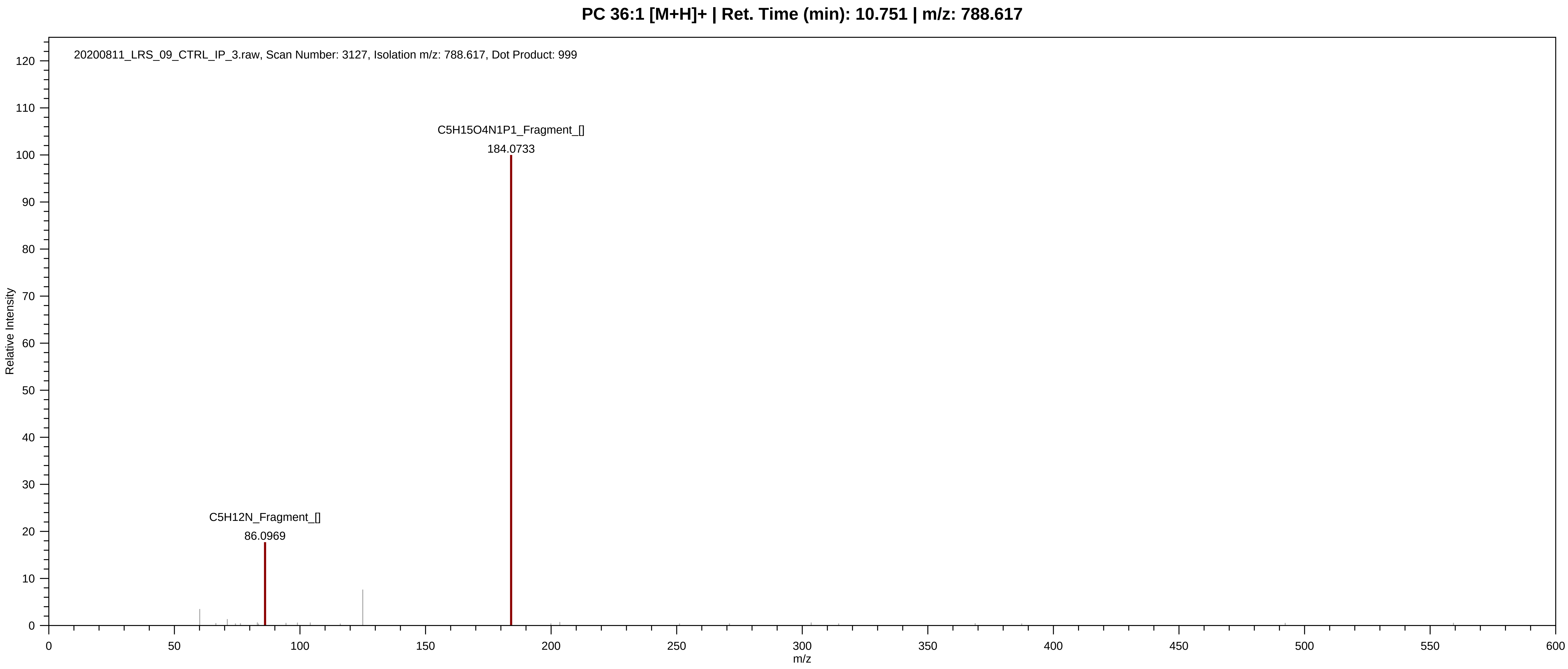
PC 36:1 [M+H]+ | Ret. Time (min): 10.751 | m/z: 788.617
20200811_LRS_09_CTRL_IP_3.raw, Scan Number: 3127, Isolation m/z: 788.617, Dot Product: 999
C5H15O4N1P1_Fragment_[]
184.0733
C5H12N_Fragment_[]
86.0969
Relative Intensity
m/z
0
10
20
30
40
50
60
70
80
90
100
110
120
0
50
100
150
200
250
300
350
400
450
500
550
600

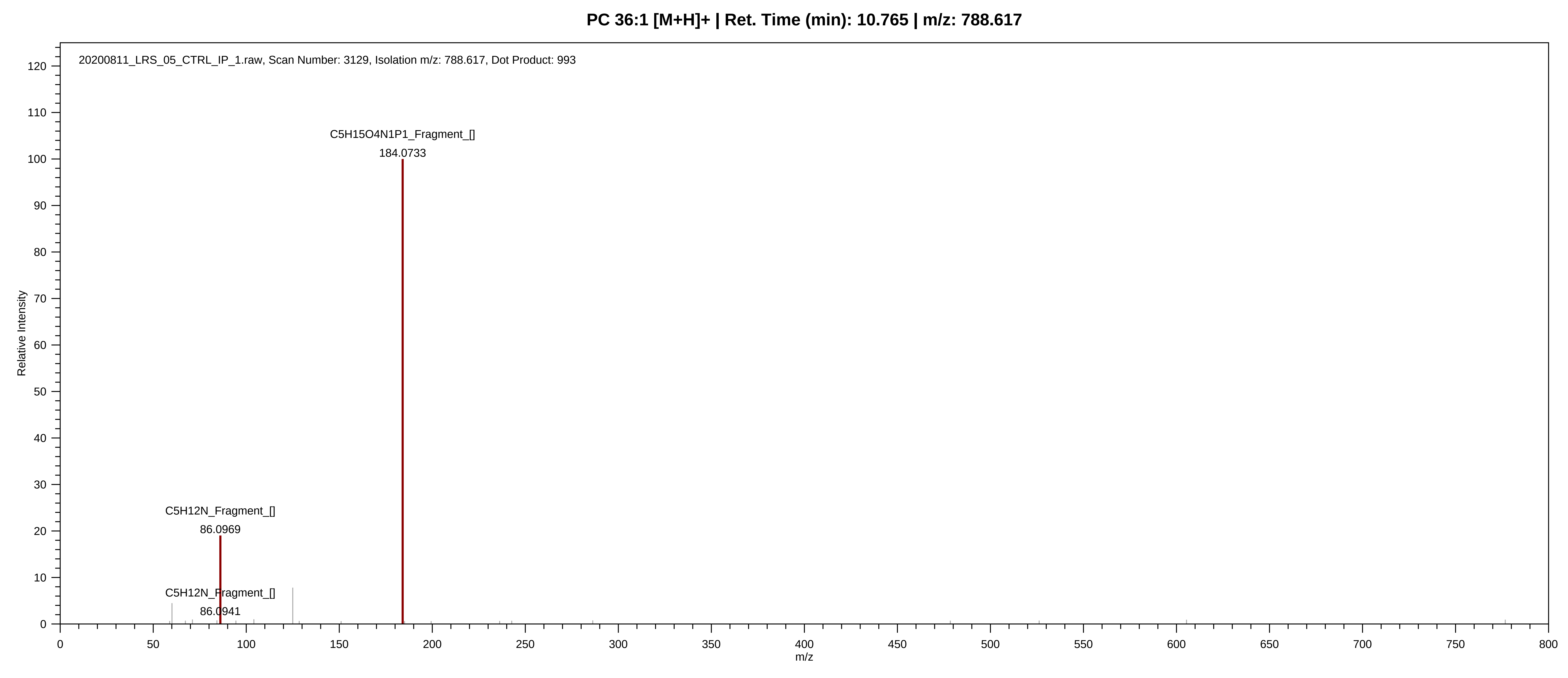

PC 36:1 [M+H]+ | Ret. Time (min): 10.765 | m/z: 788.617
20200811_LRS_05_CTRL_IP_1.raw, Scan Number: 3129, Isolation m/z: 788.617, Dot Product: 993
C5H15O4N1P1_Fragment_[]
184.0733
C5H12N_Fragment_[]
86.0969
C5H12N_Fragment_[]
86.0941
Relative Intensity
m/z

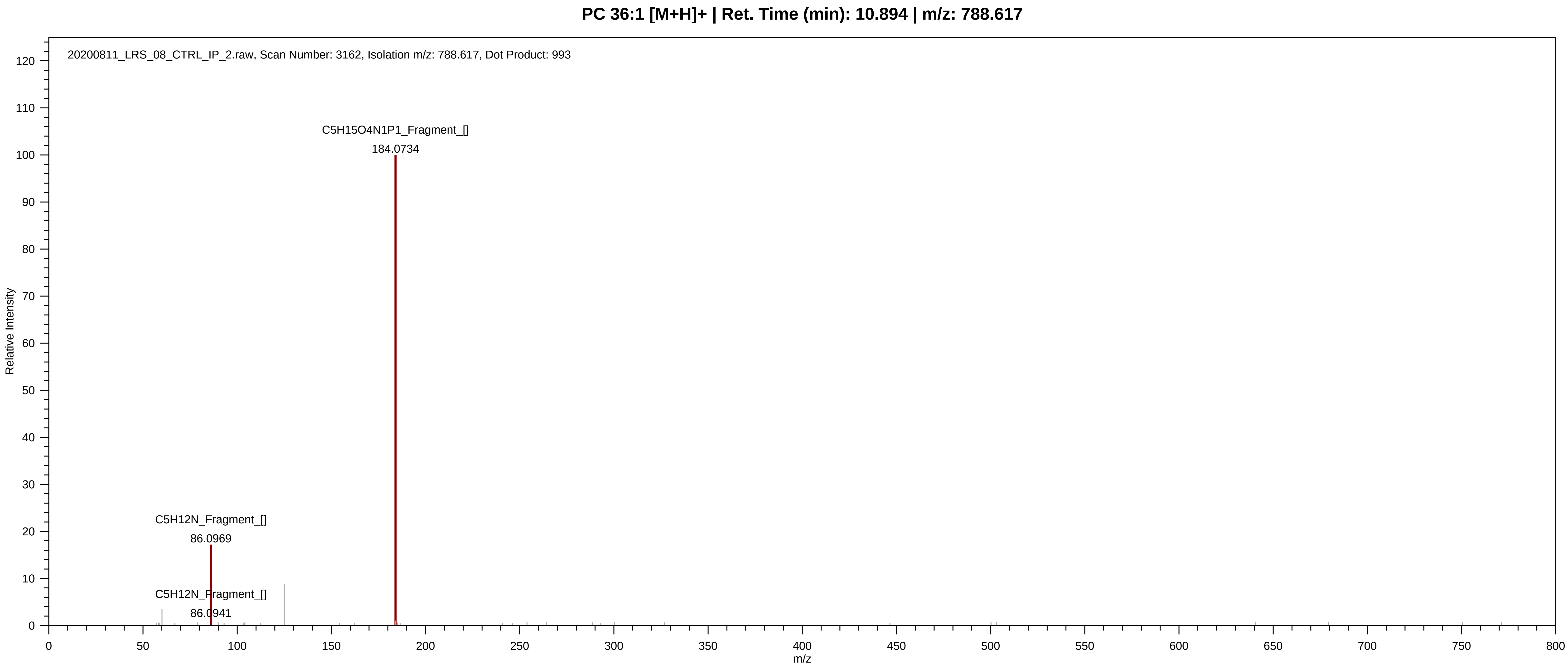

PC 36:1 [M+H]+ | Ret. Time (min): 10.894 | m/z: 788.617
20200811_LRS_08_CTRL_IP_2.raw, Scan Number: 3162, Isolation m/z: 788.617, Dot Product: 993
C5H15O4N1P1_Fragment_[]
184.0734
C5H12N_Fragment_[]
86.0969
C5H12N_Fragment_[]
86.0941
Relative Intensity
m/z

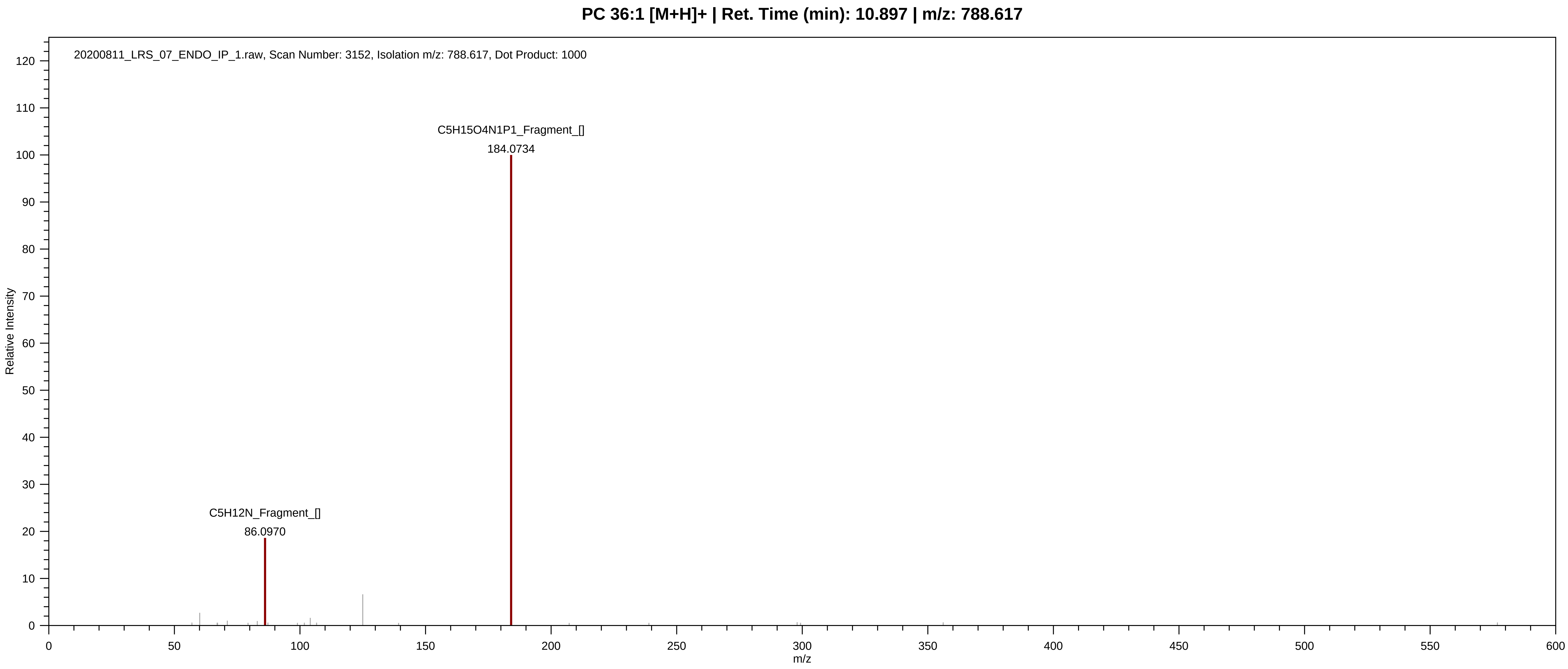
PC 36:1 [M+H]+ | Ret. Time (min): 10.897 | m/z: 788.617
20200811_LRS_07_ENDO_IP_1.raw, Scan Number: 3152, Isolation m/z: 788.617, Dot Product: 1000
C5H15O4N1P1_Fragment_[]
184.0734
C5H12N_Fragment_[]
86.0970
Relative Intensity
m/z

PC 36:1 [M+H]+ | Ret. Time (min): 10.897 | m/z: 788.617

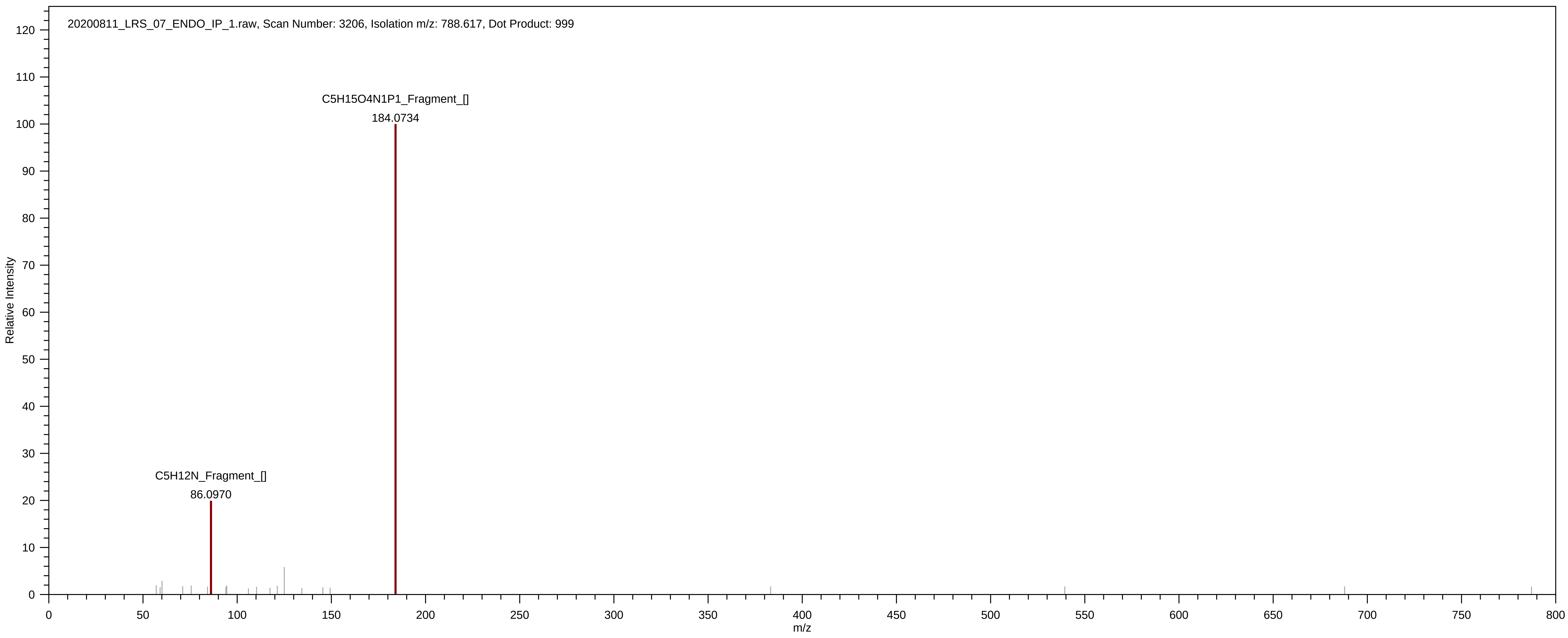

PC 36:1 [M+H]+ | Ret. Time (min): 10.916 | m/z: 788.617

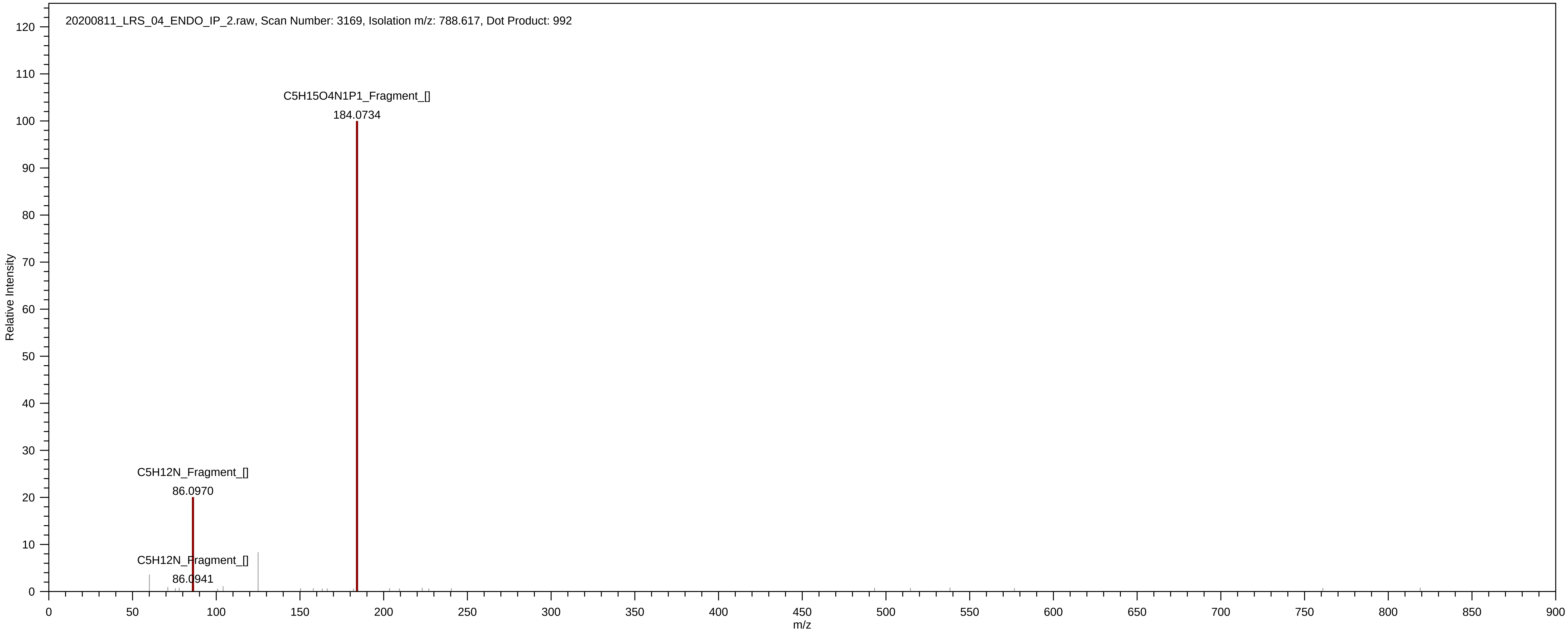

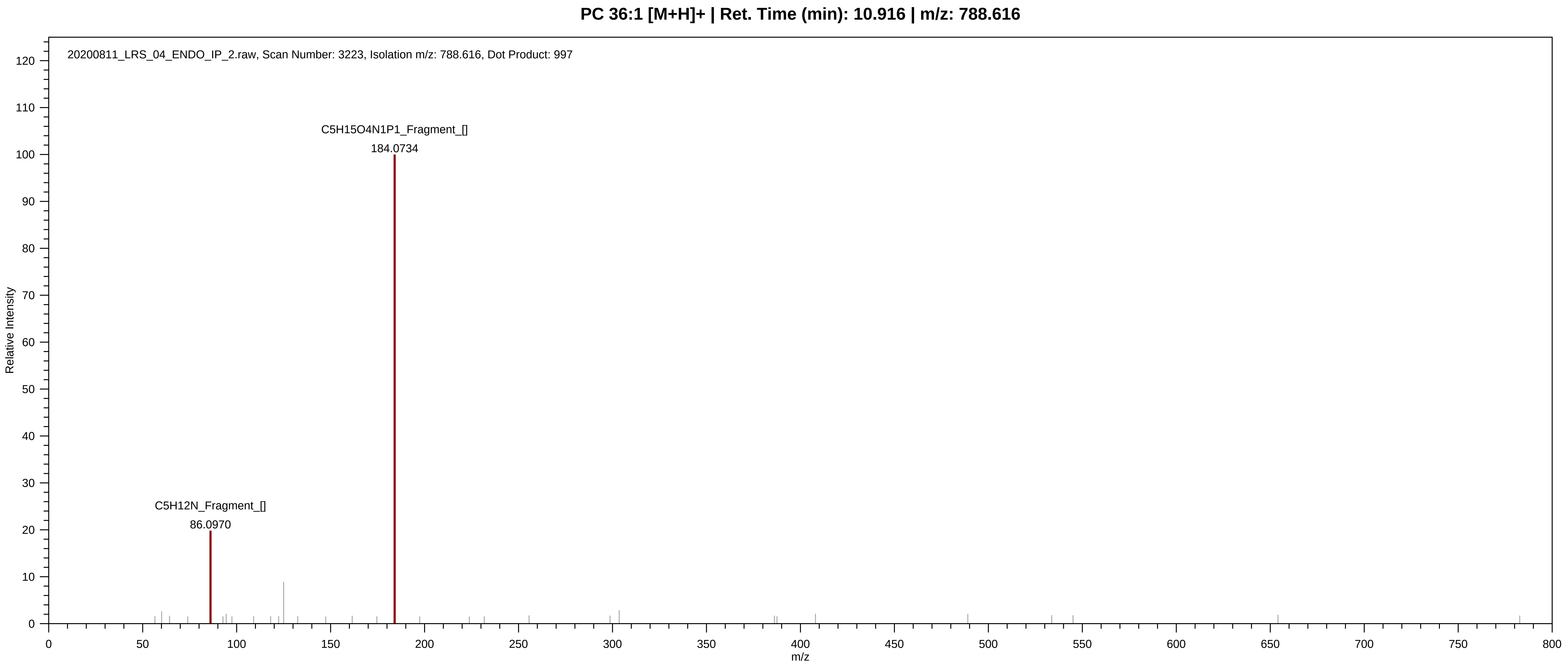
PC 36:1 [M+H]+ | Ret. Time (min): 10.916 | m/z: 788.616
20200811_LRS_04_ENDO_IP_2.raw, Scan Number: 3223, Isolation m/z: 788.616, Dot Product: 997
C5H15O4N1P1_Fragment_[]
184.0734
C5H12N_Fragment_[]
86.0970
Relative Intensity
m/z

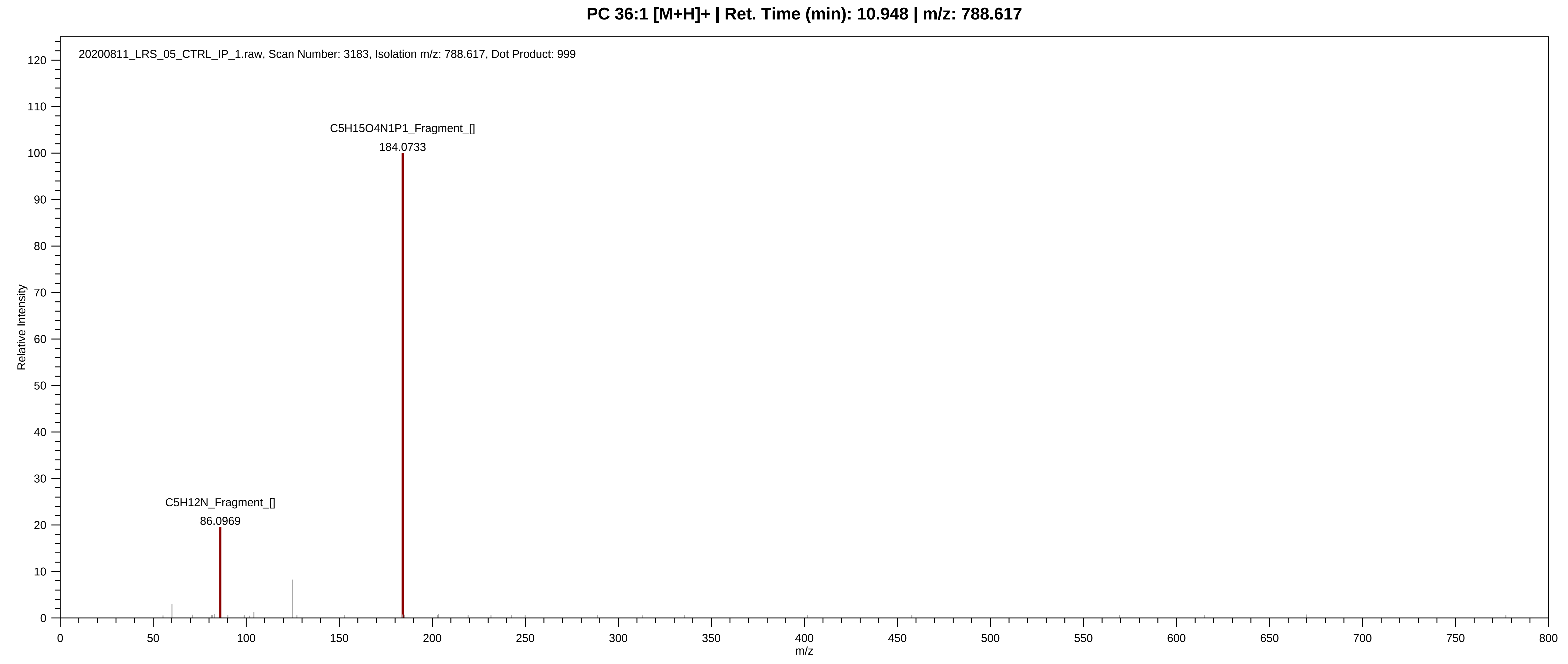
PC 36:1 [M+H]+ | Ret. Time (min): 10.948 | m/z: 788.617
20200811_LRS_05_CTRL_IP_1.raw, Scan Number: 3183, Isolation m/z: 788.617, Dot Product: 999
C5H15O4N1P1_Fragment_[]
184.0733
C5H12N_Fragment_[]
86.0969
Relative Intensity
m/z

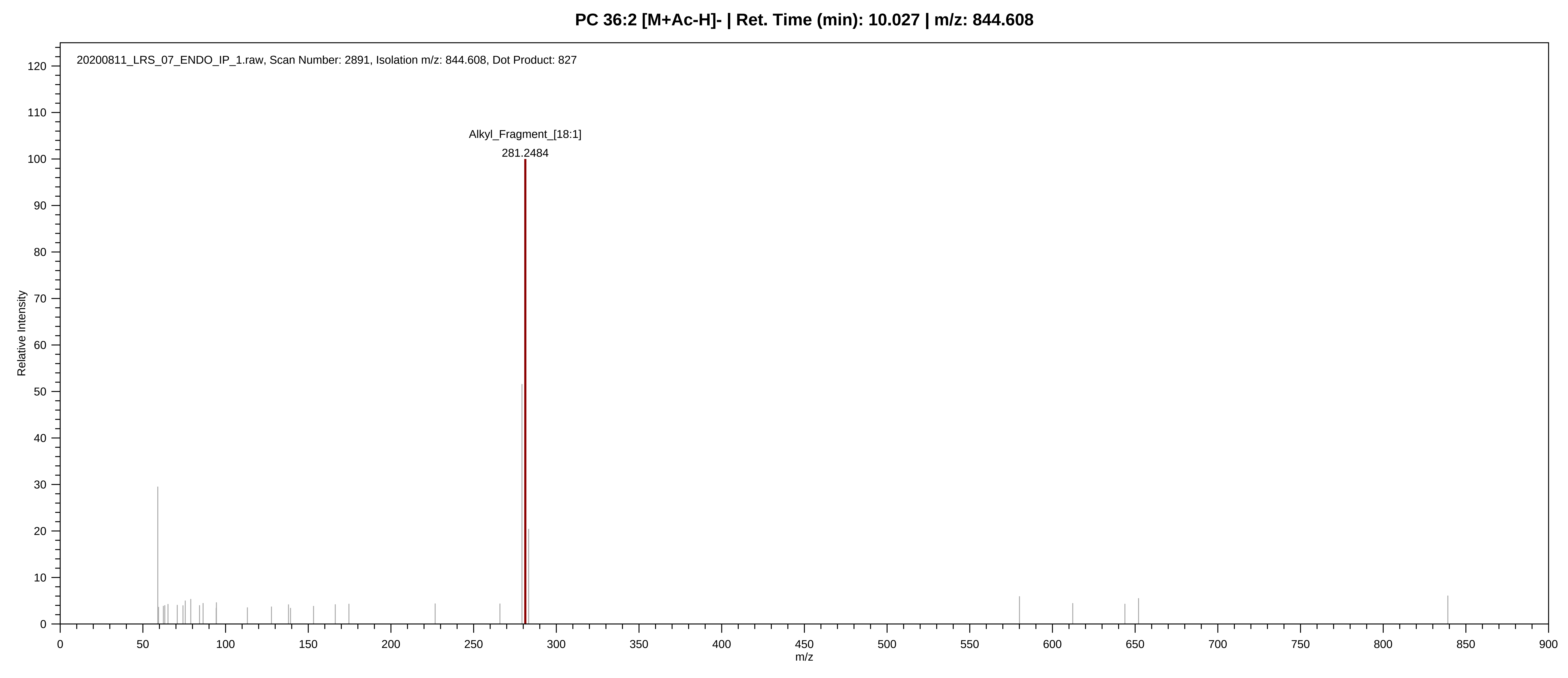
PC 36:2 [M+Ac-H]- | Ret. Time (min): 10.027 | m/z: 844.608
20200811_LRS_07_ENDO_IP_1.raw, Scan Number: 2891, Isolation m/z: 844.608, Dot Product: 827
Alkyl_Fragment_[18:1]
281.2484
Relative Intensity
m/z

PC 36:2 [M+Ac-H]- | Ret. Time (min): 10.053 | m/z: 844.608

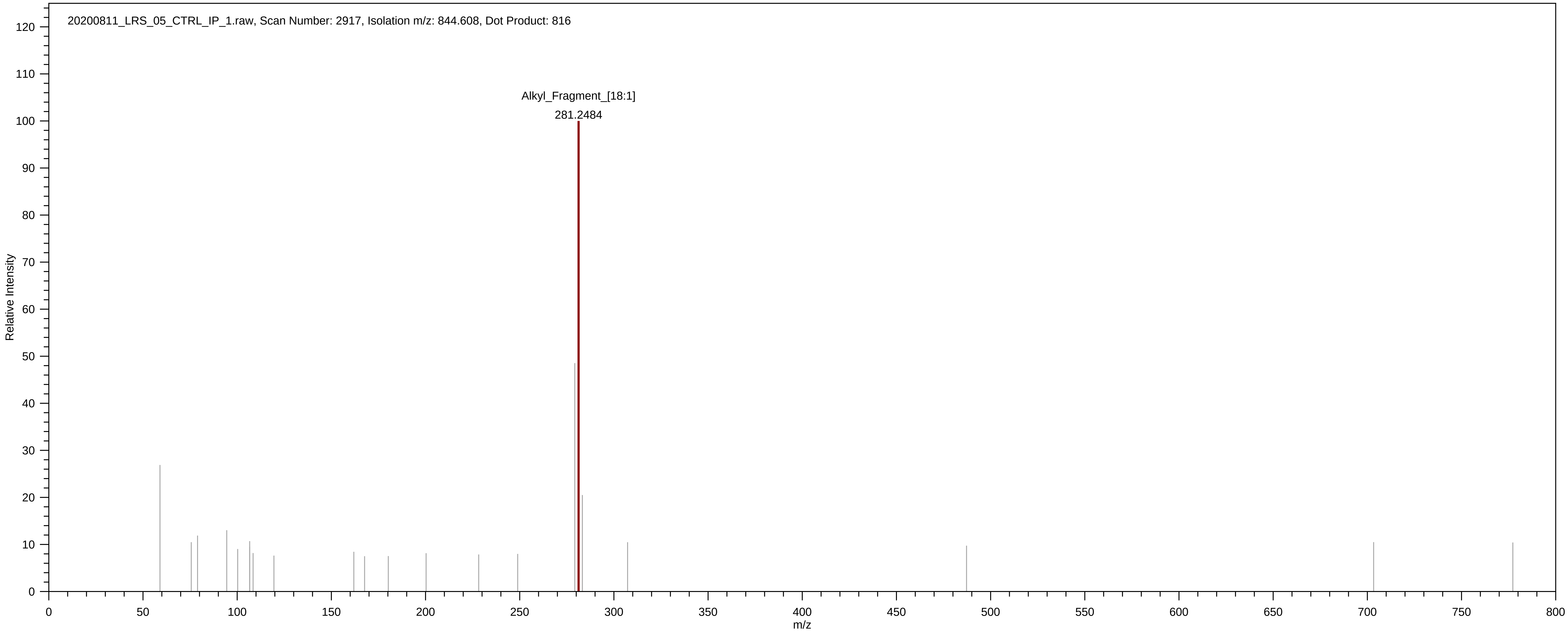

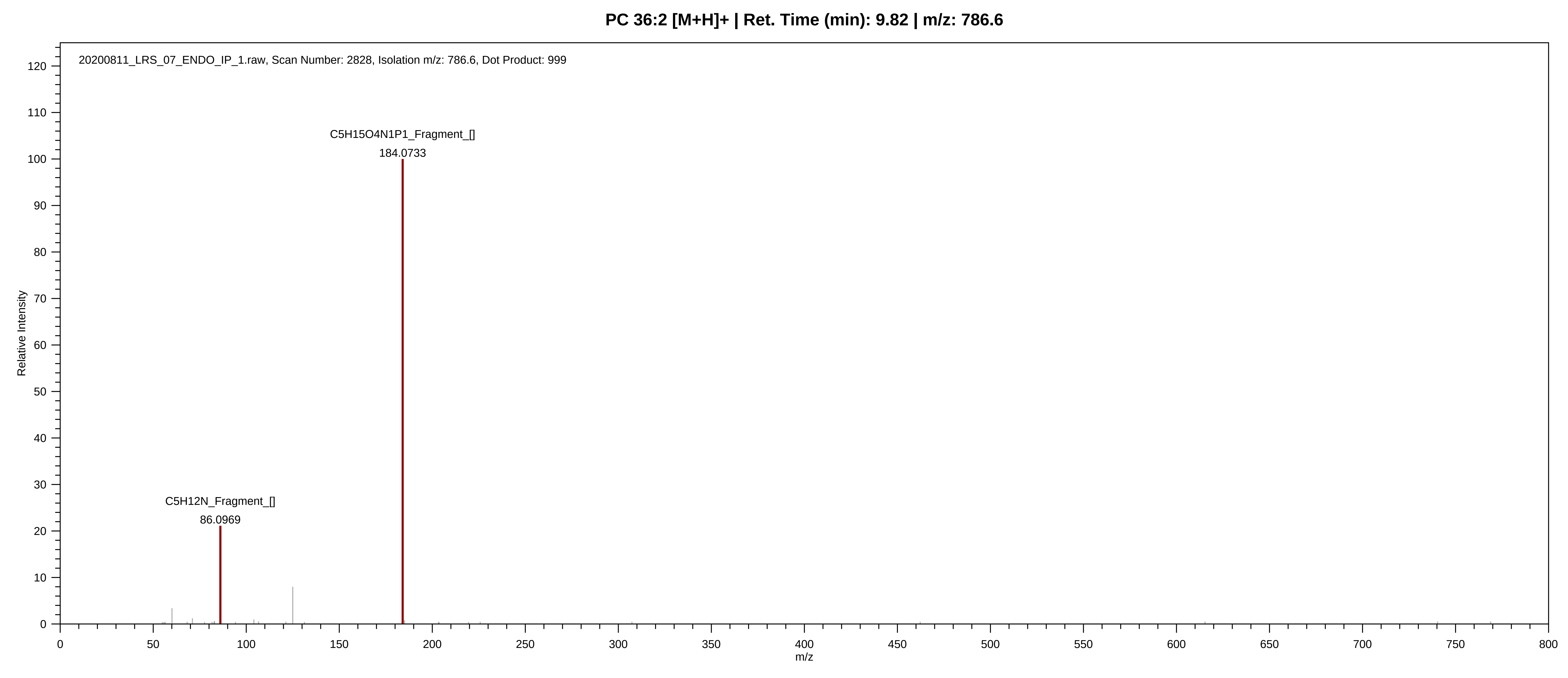
PC 36:2 [M+H]+ | Ret. Time (min): 9.82 | m/z: 786.6
20200811_LRS_07_ENDO_IP_1.raw, Scan Number: 2828, Isolation m/z: 786.6, Dot Product: 999
C5H15O4N1P1_Fragment_[]
184.0733
C5H12N_Fragment_[]
86.0969
Relative Intensity
m/z

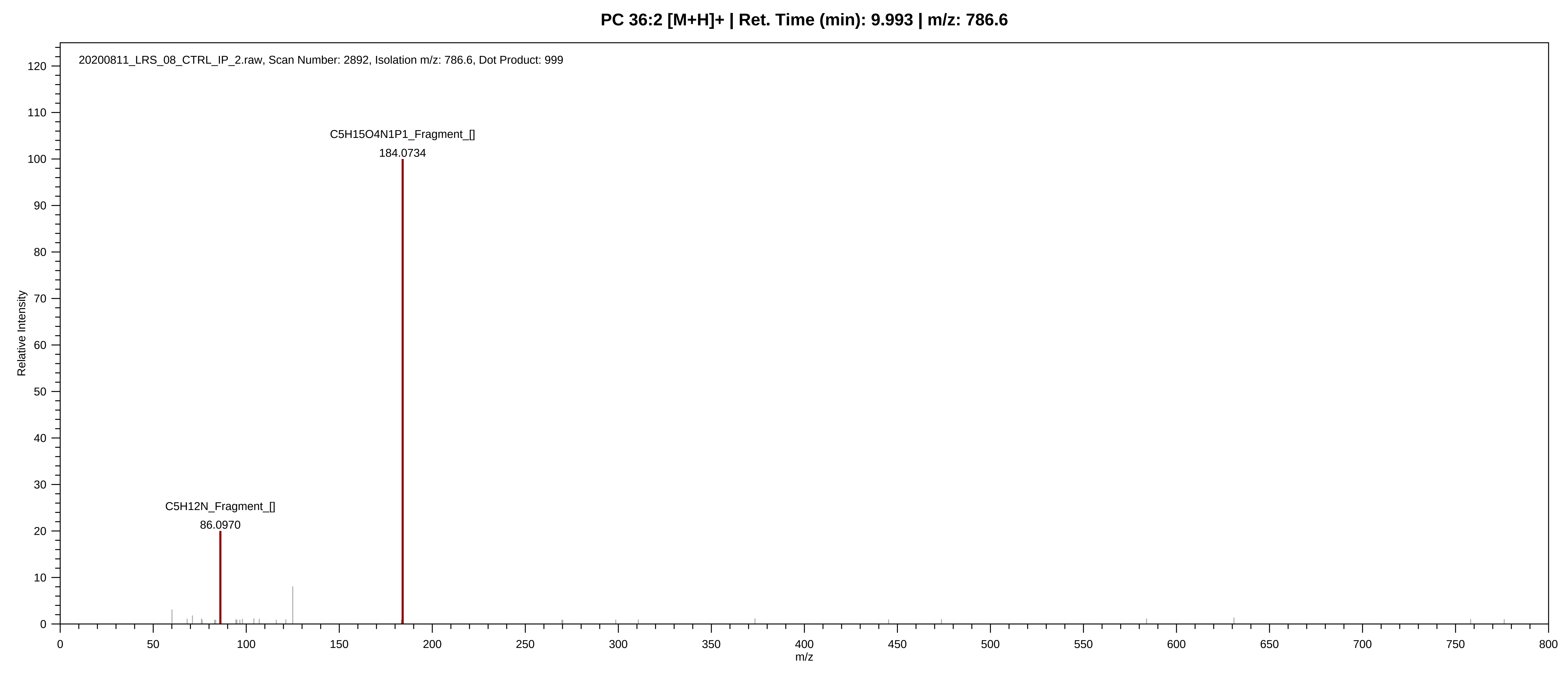
PC 36:2 [M+H]+ | Ret. Time (min): 9.993 | m/z: 786.6
20200811_LRS_08_CTRL_IP_2.raw, Scan Number: 2892, Isolation m/z: 786.6, Dot Product: 999
C5H15O4N1P1_Fragment_[]
184.0734
C5H12N_Fragment_[]
86.0970
Relative Intensity
m/z
0
10
20
30
40
50
60
70
80
90
100
110
120
0
50
100
150
200
250
300
350
400
450
500
550
600
650
700
750
800

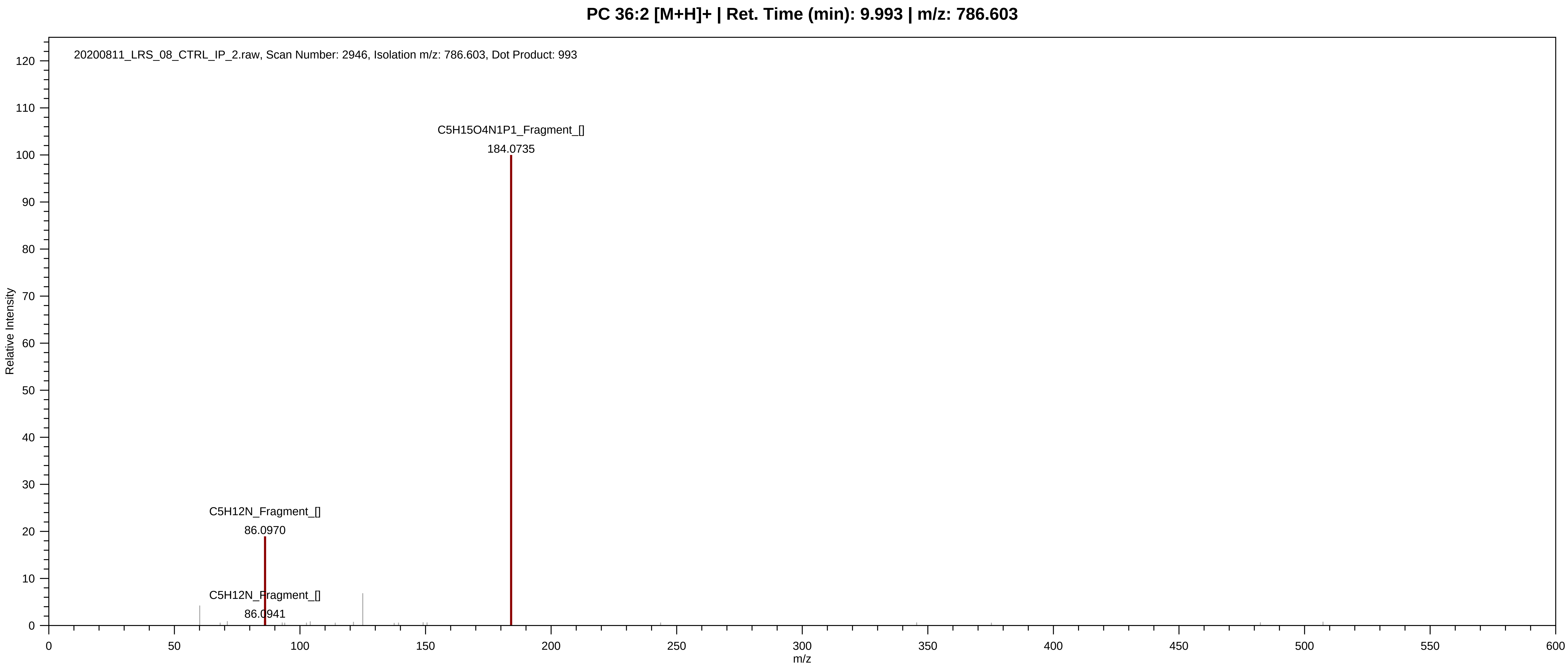

PC 36:2 [M+H]+ | Ret. Time (min): 9.993 | m/z: 786.603
20200811_LRS_08_CTRL_IP_2.raw, Scan Number: 2946, Isolation m/z: 786.603, Dot Product: 993
C5H15O4N1P1_Fragment_[]
184.0735
C5H12N_Fragment_[]
86.0970
C5H12N_Fragment_[]
86.0941
Relative Intensity
m/z

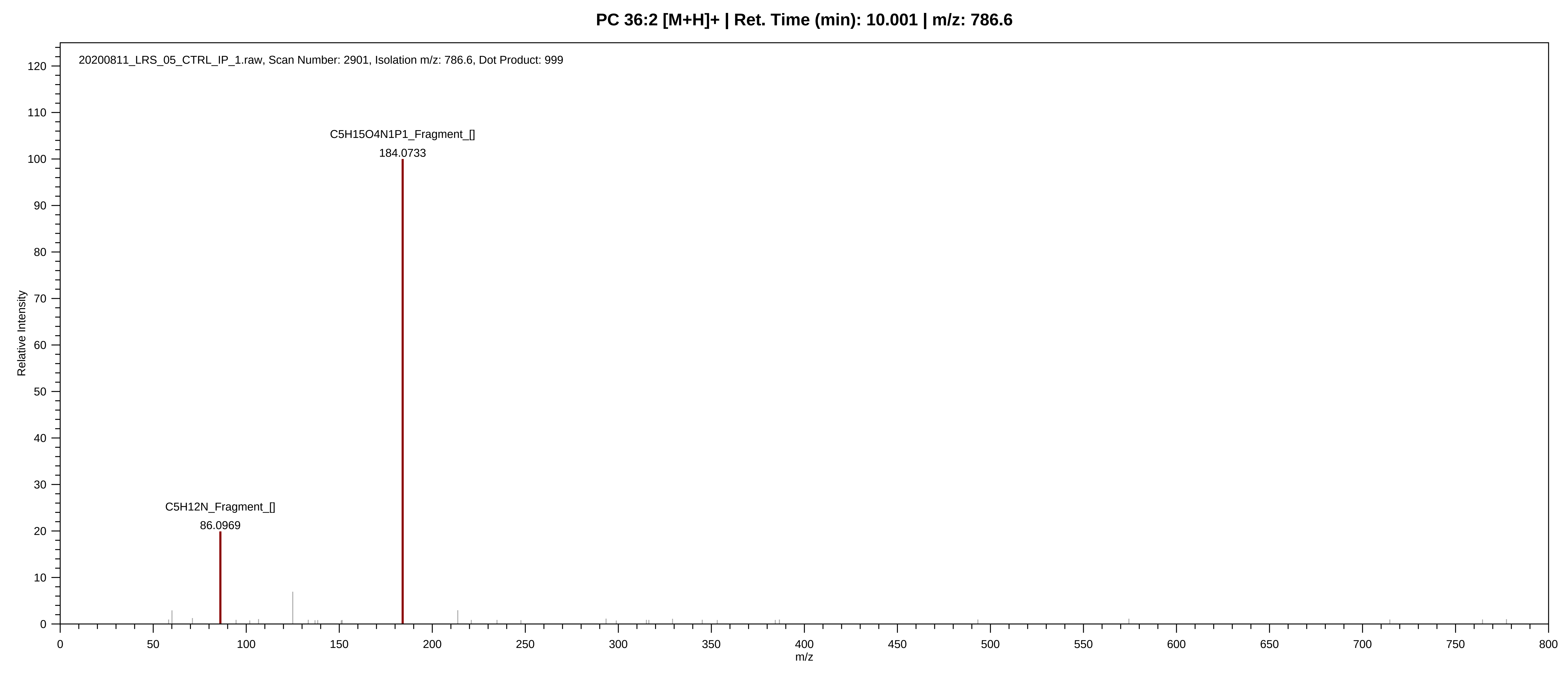

PC 36:2 [M+H]+ | Ret. Time (min): 10.001 | m/z: 786.6
20200811_LRS_05_CTRL_IP_1.raw, Scan Number: 2901, Isolation m/z: 786.6, Dot Product: 999
C5H15O4N1P1_Fragment_[]
184.0733
C5H12N_Fragment_[]
86.0969
Relative Intensity
m/z

PC 36:2 [M+H]+ | Ret. Time (min): 10.001 | m/z: 786.603

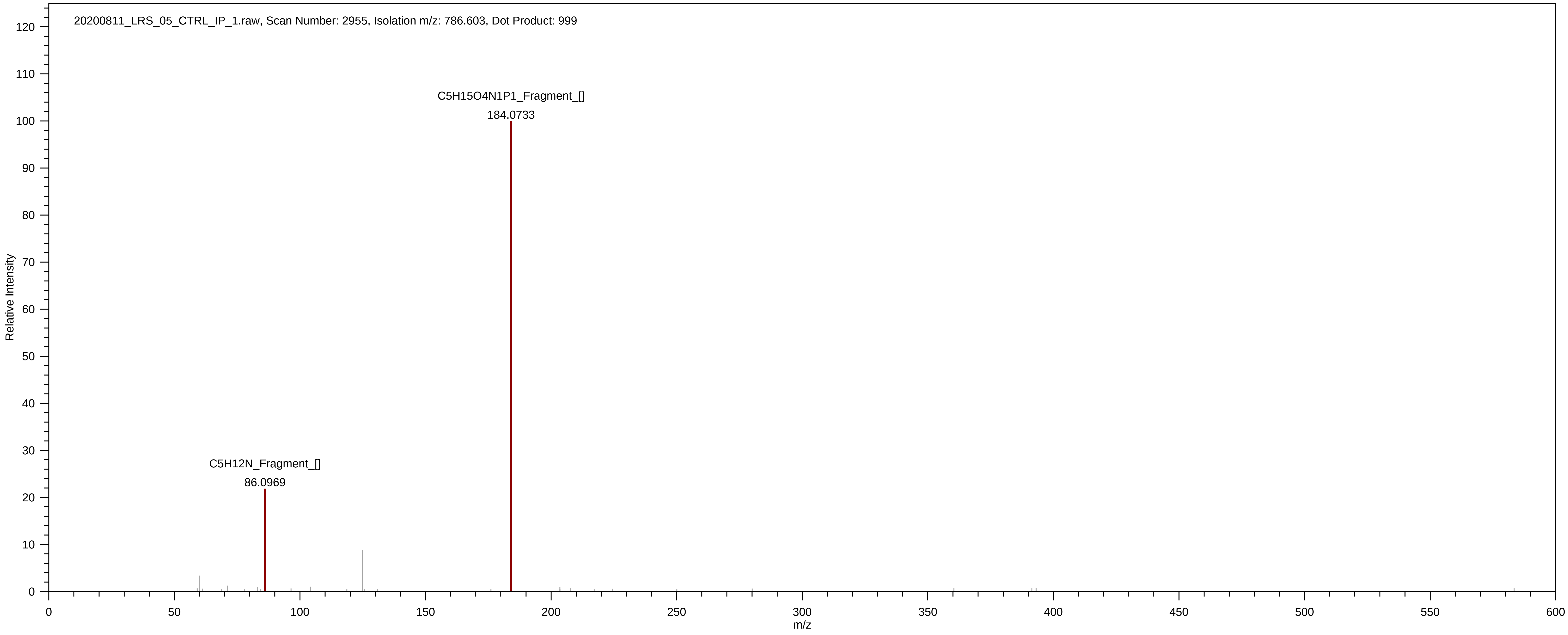

PC 36:2 [M+H]+ | Ret. Time (min): 10.016 | m/z: 786.6

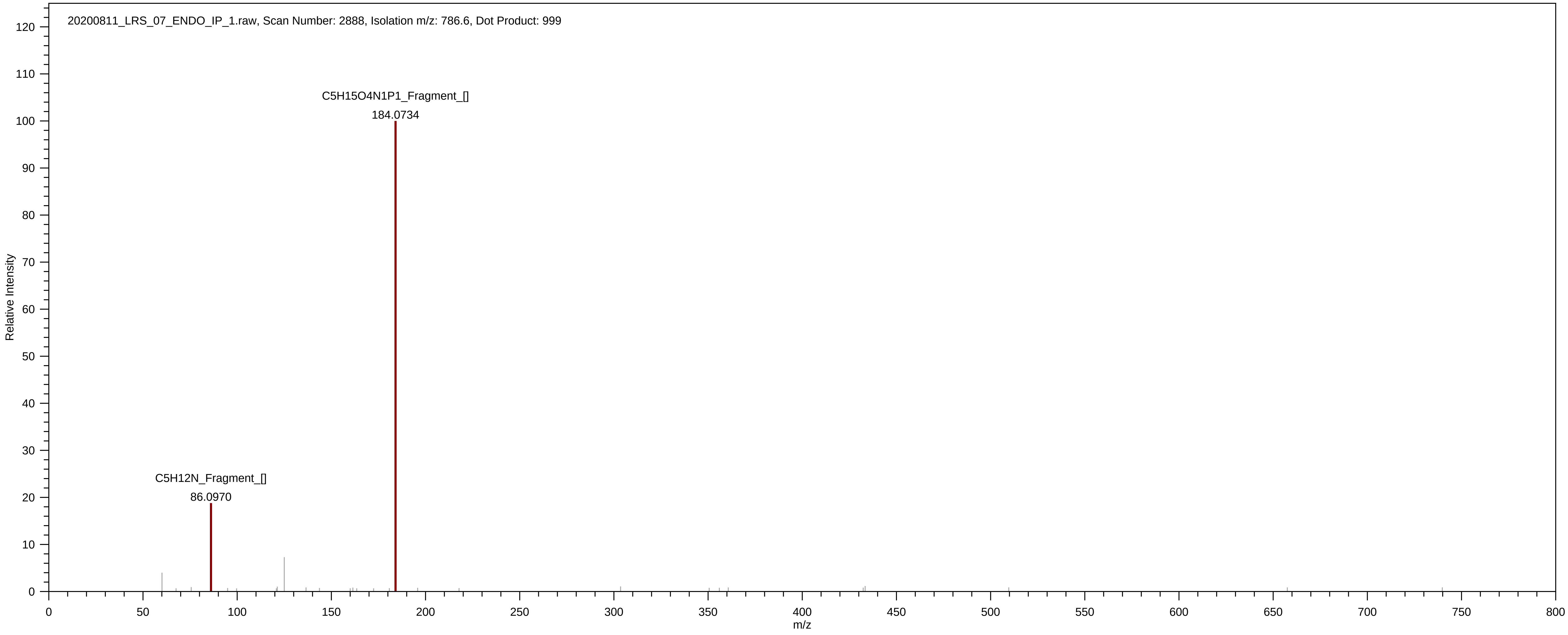

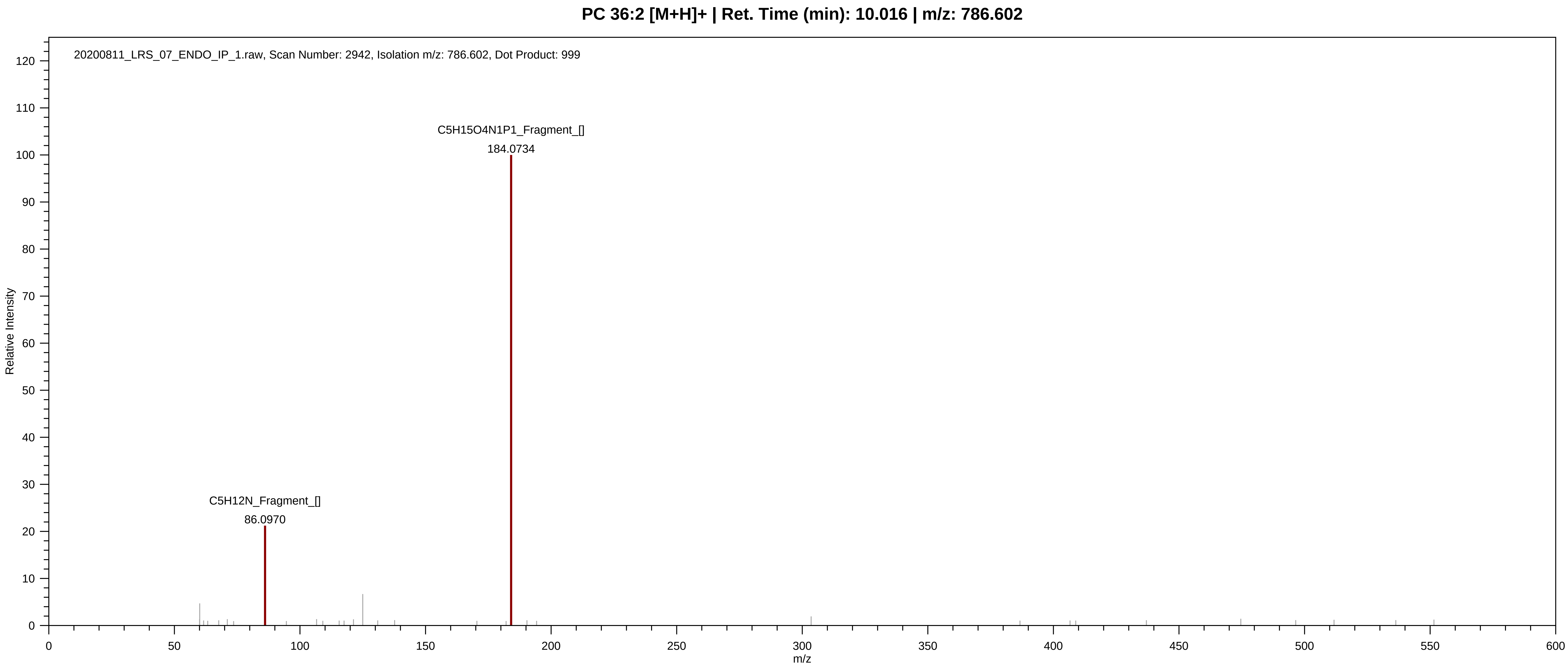
PC 36:2 [M+H]+ | Ret. Time (min): 10.016 | m/z: 786.602
20200811_LRS_07_ENDO_IP_1.raw, Scan Number: 2942, Isolation m/z: 786.602, Dot Product: 999
C5H15O4N1P1_Fragment_[]
184.0734
C5H12N_Fragment_[]
86.0970
Relative Intensity
m/z

PC 36:2 [M+H]+ | Ret. Time (min): 10.033 | m/z: 786.6

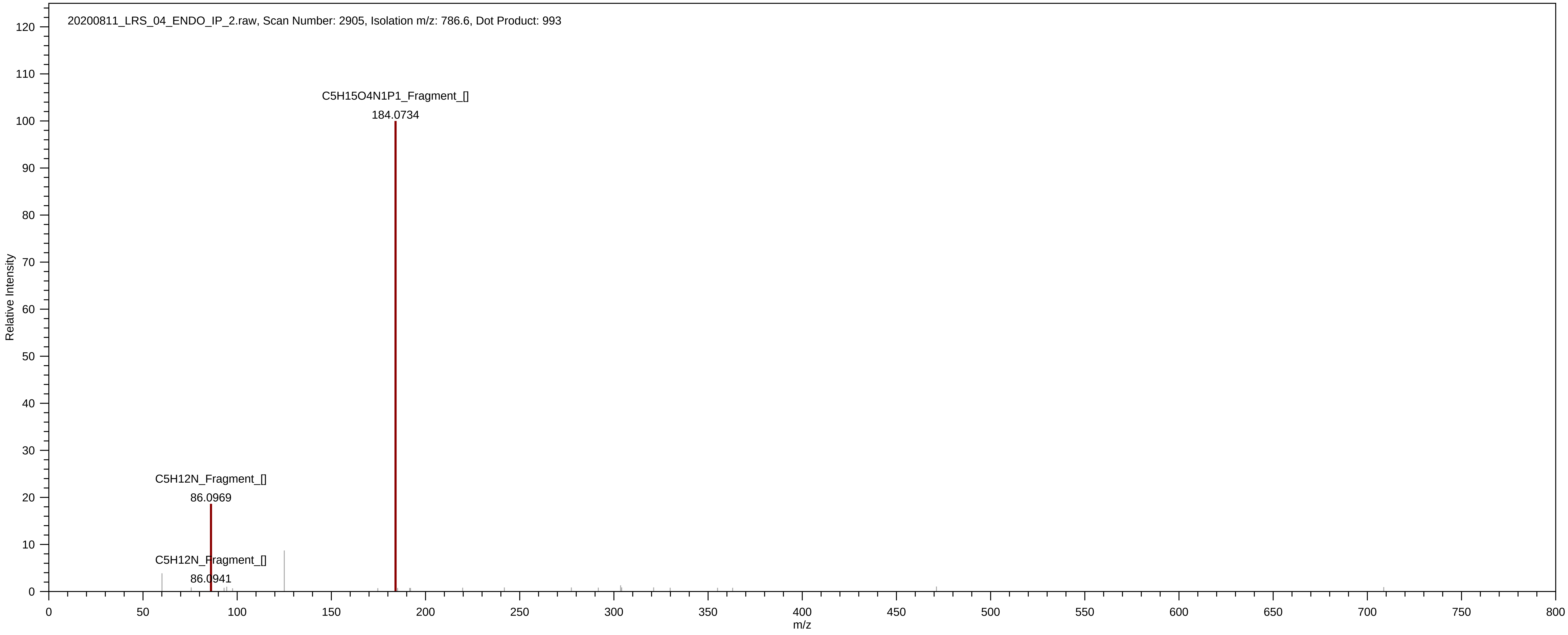

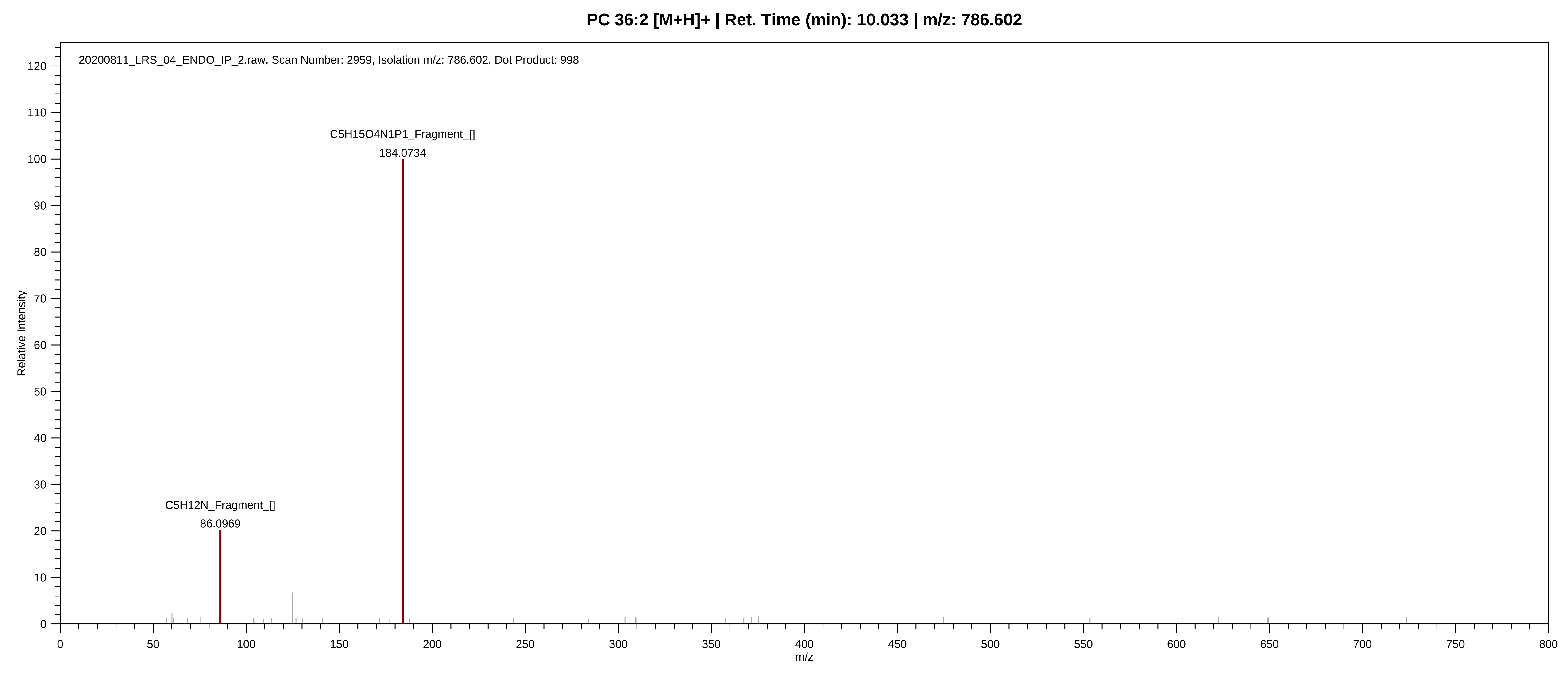
PC 36:2 [M+H]+ | Ret. Time (min): 10.033 | m/z: 786.602
20200811_LRS_04_ENDO_IP_2.raw, Scan Number: 2959, Isolation m/z: 786.602, Dot Product: 998
C5H15O4N1P1_Fragment_[]
184.0734
C5H12N_Fragment_[]
86.0969
Relative Intensity
m/z

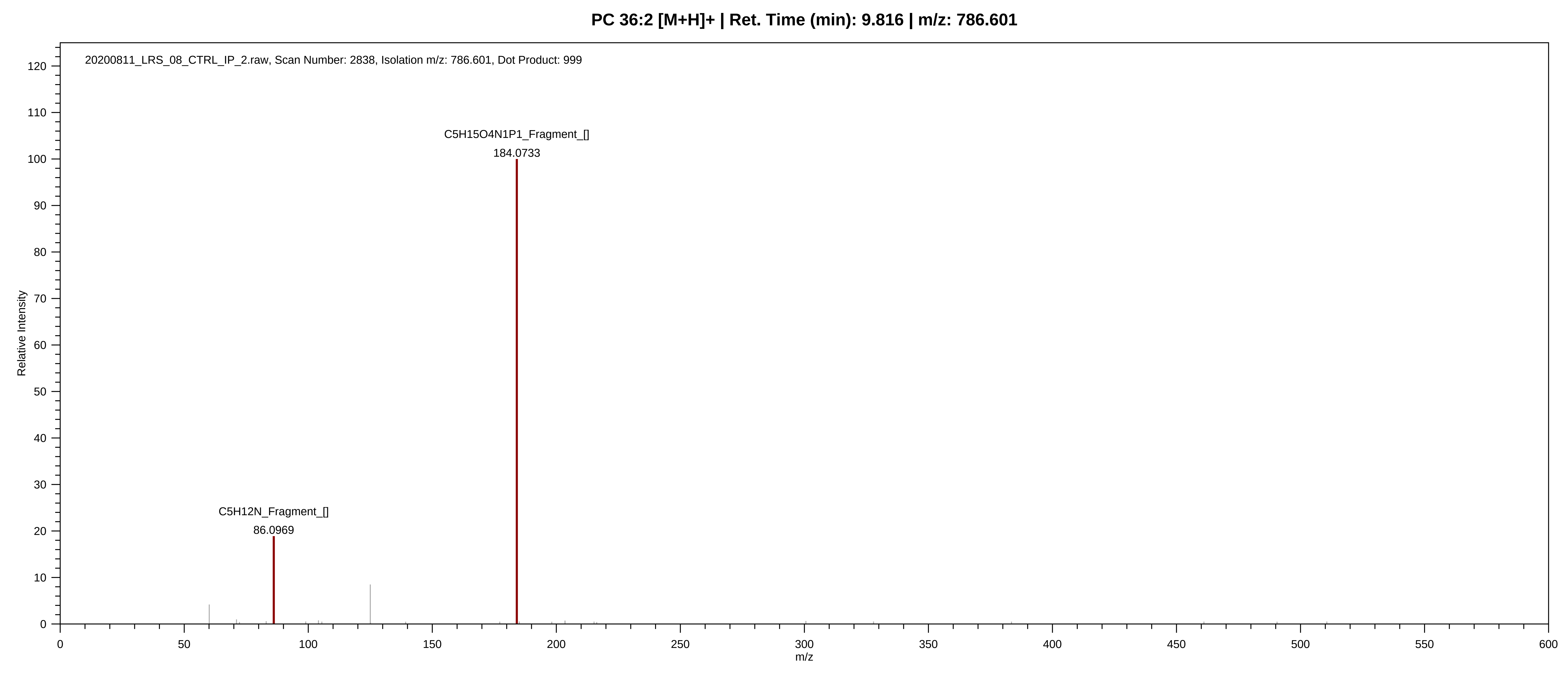
PC 36:2 [M+H]+ | Ret. Time (min): 9.816 | m/z: 786.601
20200811_LRS_08_CTRL_IP_2.raw, Scan Number: 2838, Isolation m/z: 786.601, Dot Product: 999
C5H15O4N1P1_Fragment_[]
184.0733
C5H12N_Fragment_[]
86.0969
Relative Intensity
m/z

PC 36:2 [M+H]+ | Ret. Time (min): 9.819 | m/z: 786.601

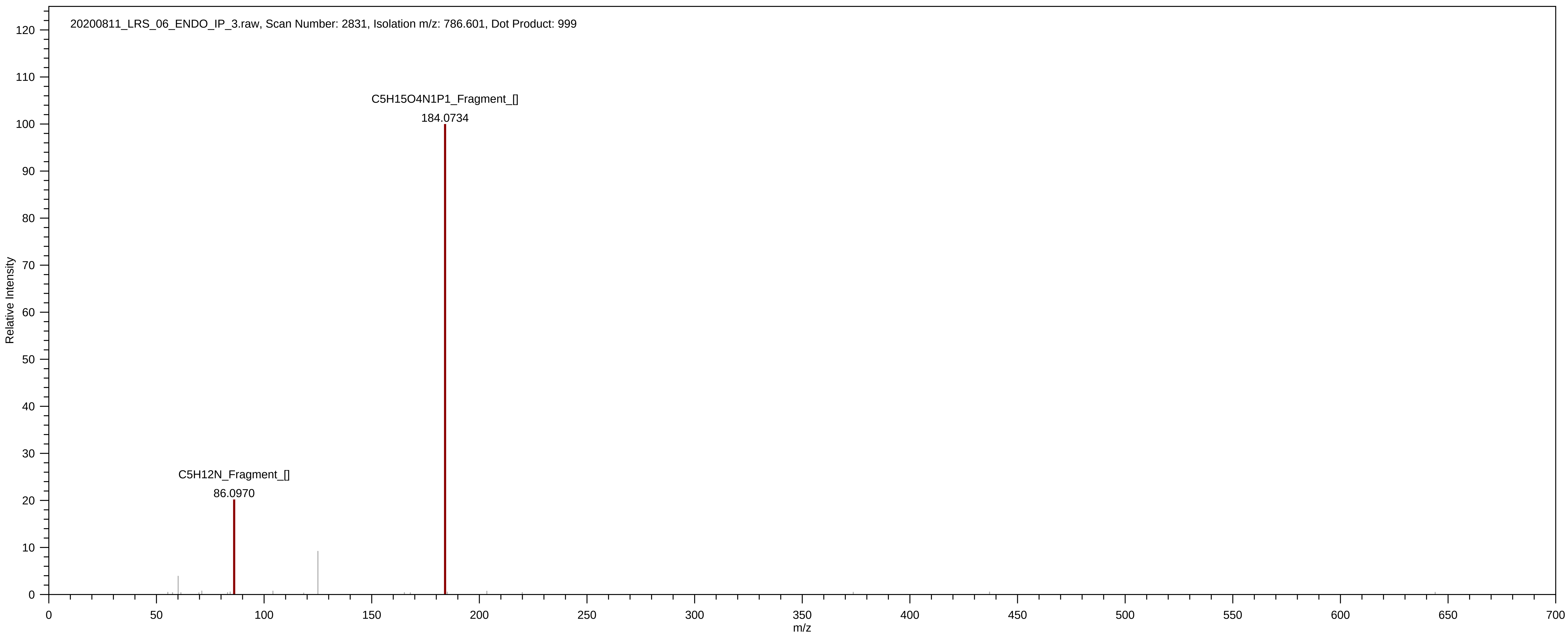

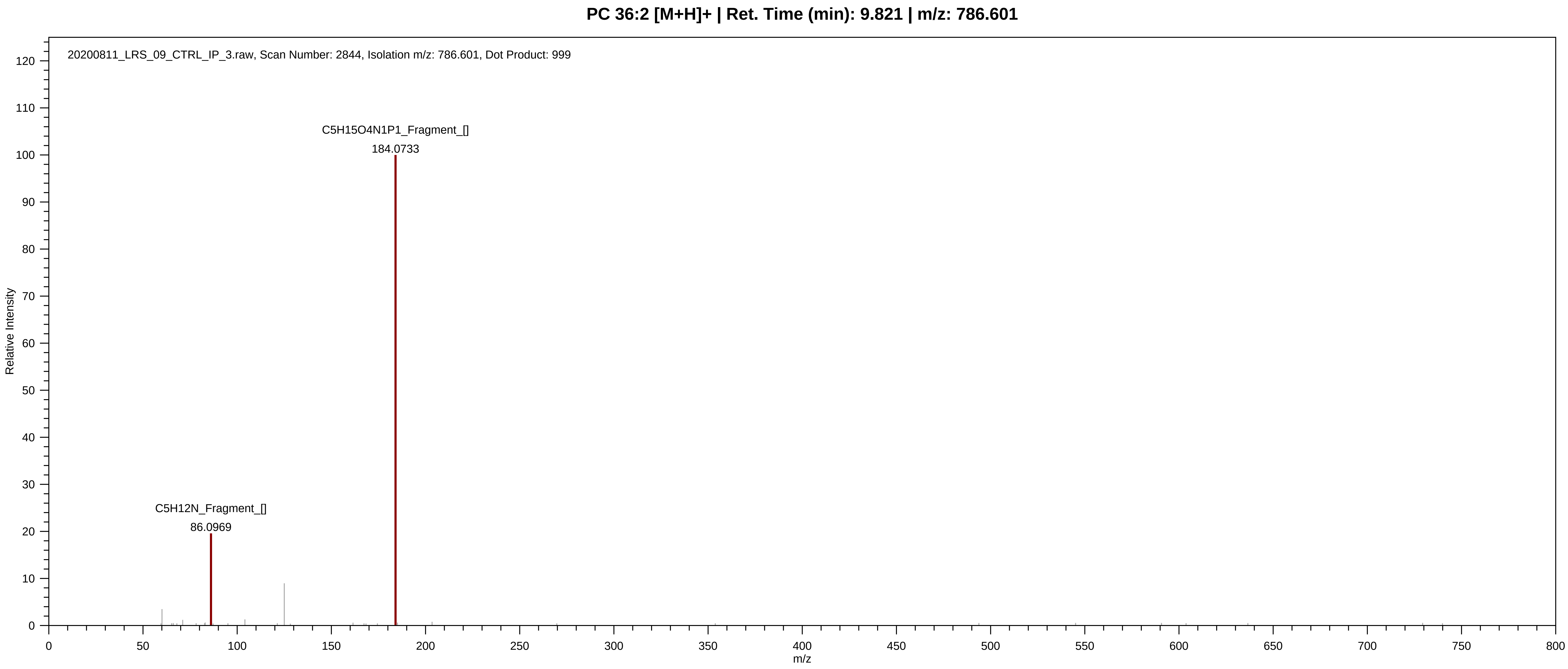
PC 36:2 [M+H]+ | Ret. Time (min): 9.821 | m/z: 786.601
20200811_LRS_09_CTRL_IP_3.raw, Scan Number: 2844, Isolation m/z: 786.601, Dot Product: 999
C5H15O4N1P1_Fragment_[]
184.0733
C5H12N_Fragment_[]
86.0969
Relative Intensity
m/z

PC 36:2 [M+H]+ | Ret. Time (min): 9.825 | m/z: 786.601

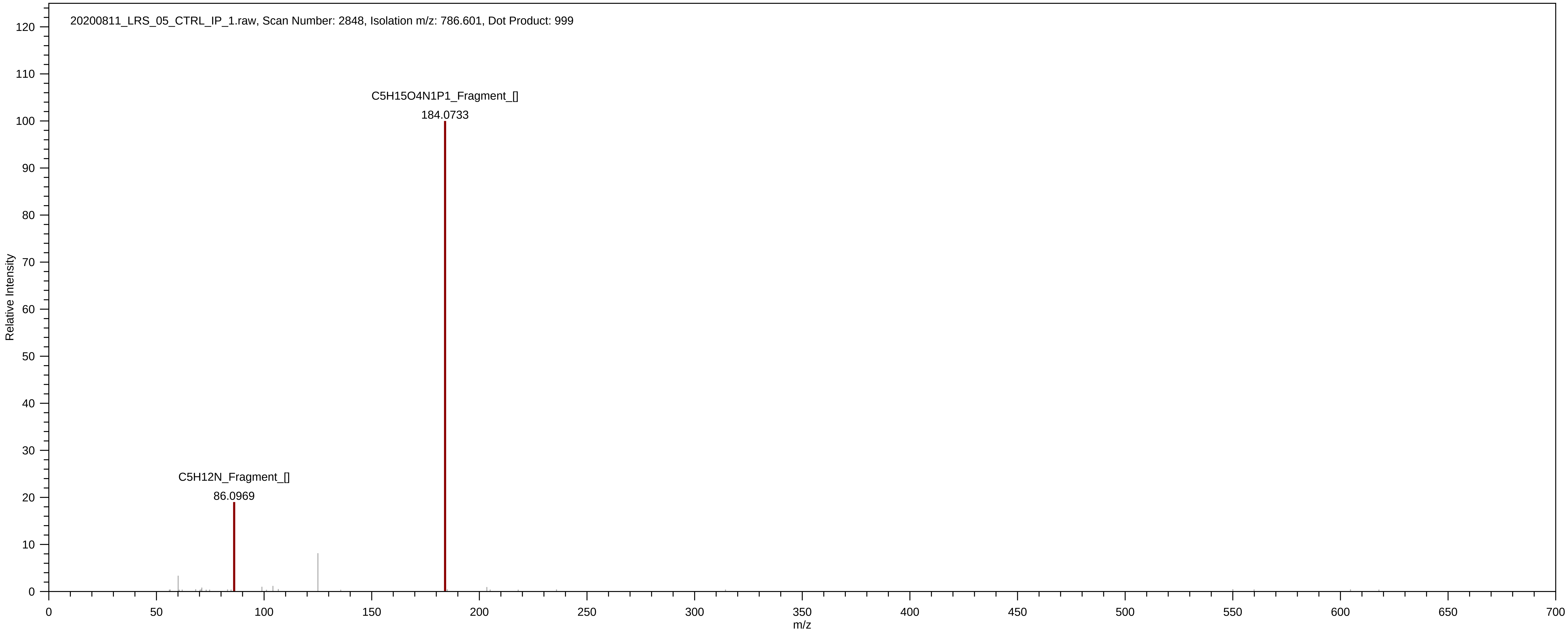

PC 36:2 [M+H]+ | Ret. Time (min): 9.836 | m/z: 786.601

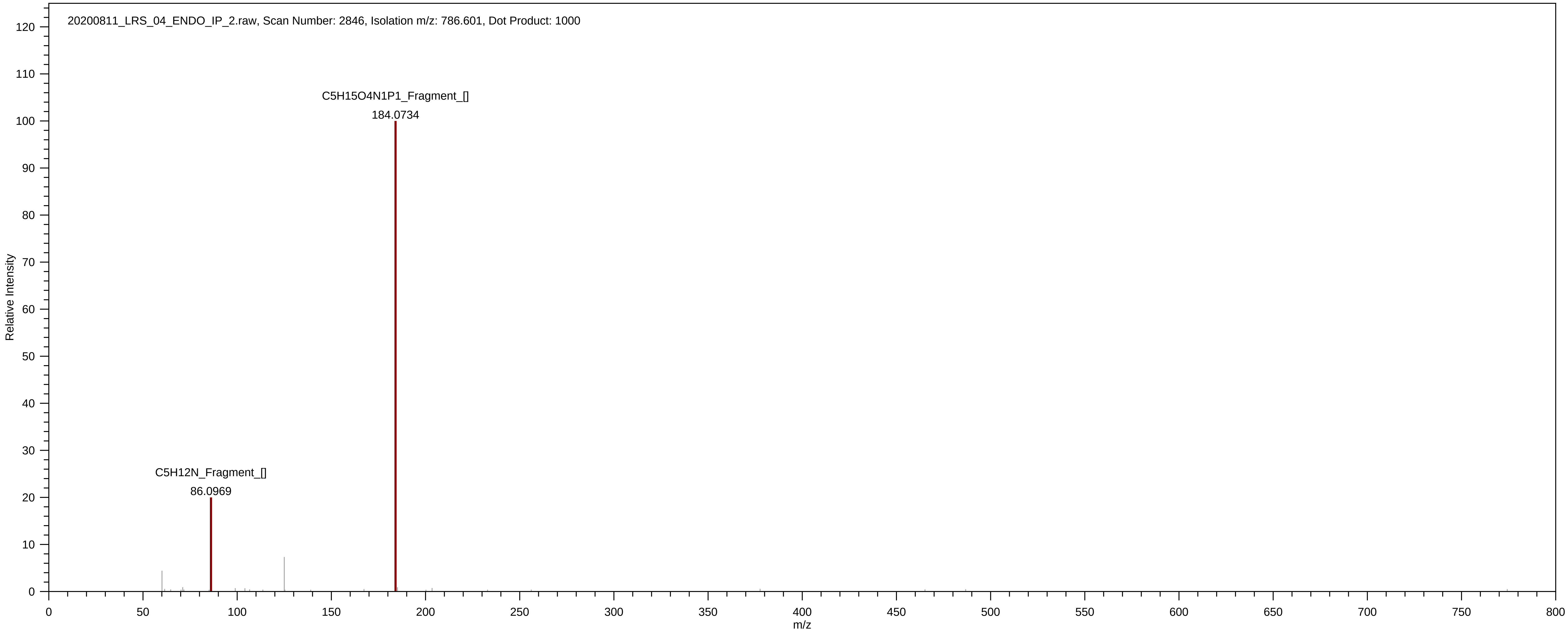

PC 36:2 [M+H]+ | Ret. Time (min): 10.015 | m/z: 786.601

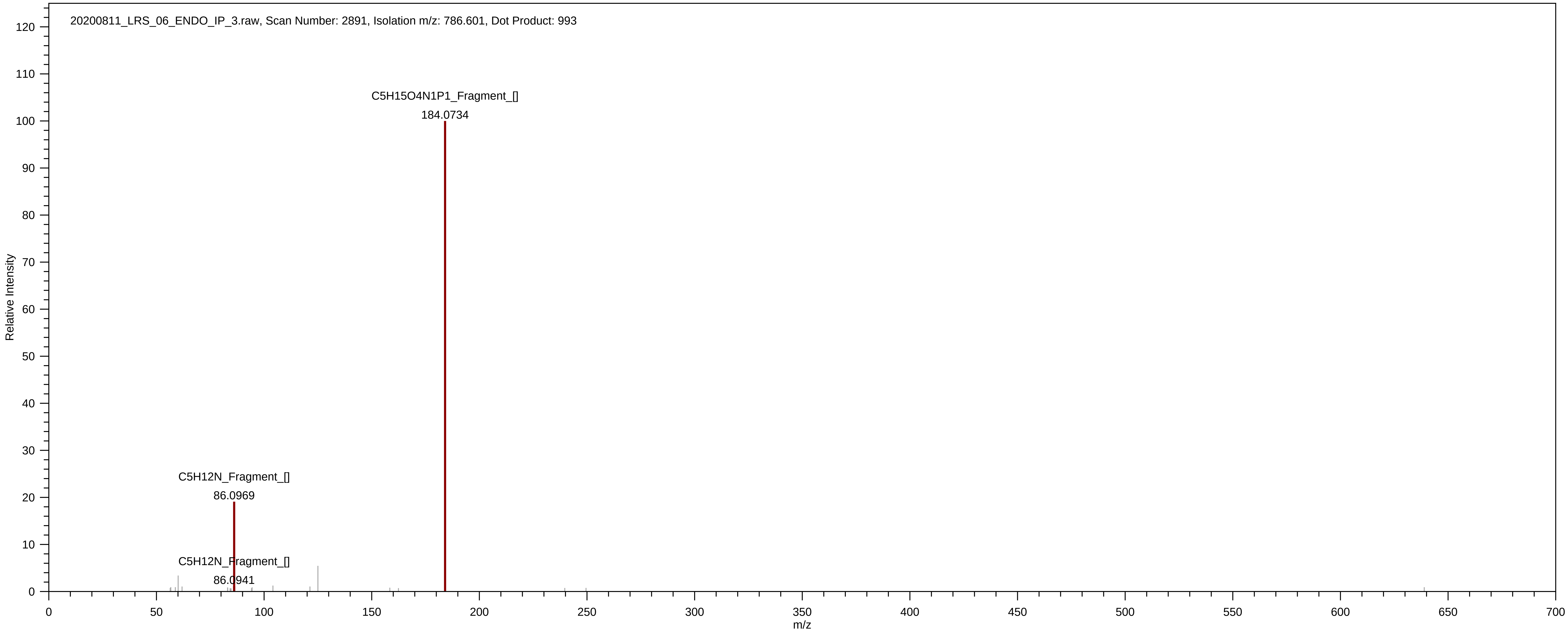

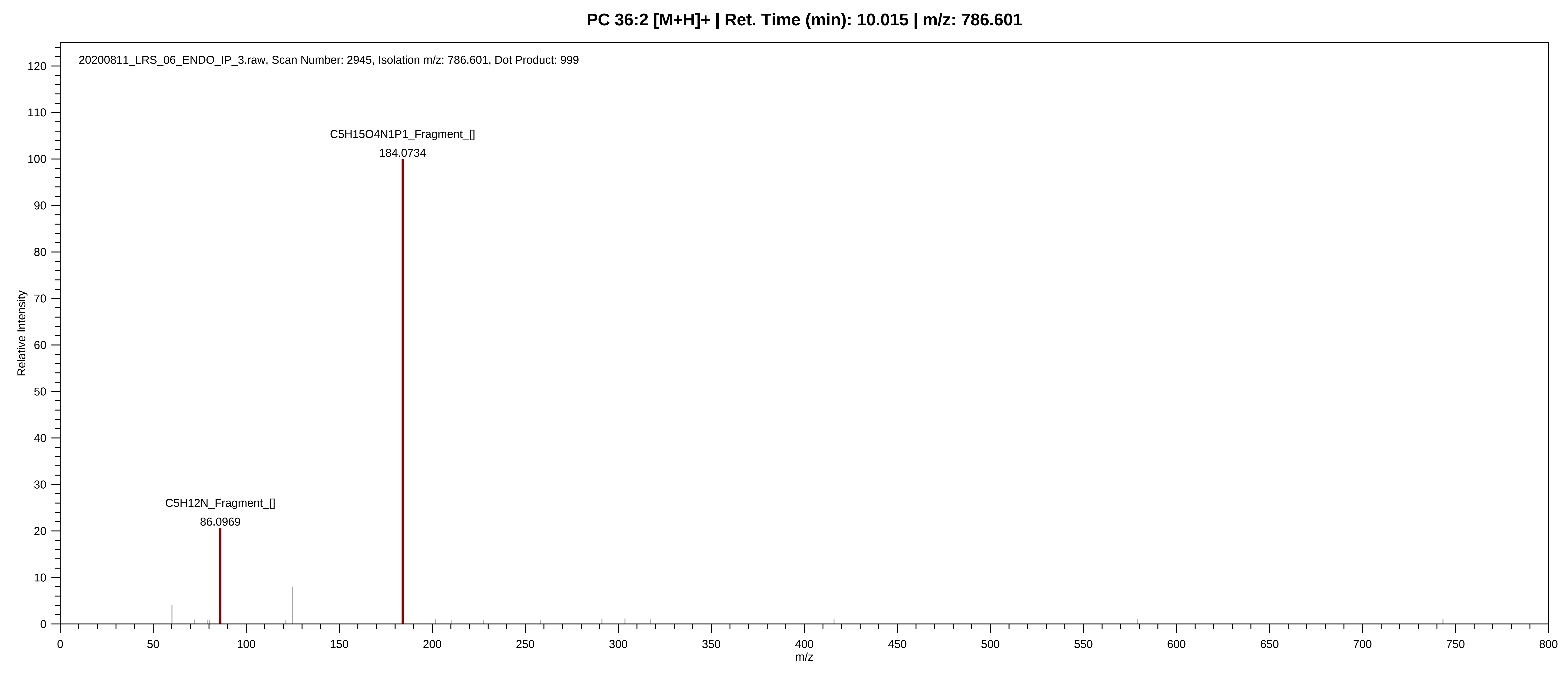
PC 36:2 [M+H]+ | Ret. Time (min): 10.015 | m/z: 786.601
20200811_LRS_06_ENDO_IP_3.raw, Scan Number: 2945, Isolation m/z: 786.601, Dot Product: 999
C5H15O4N1P1_Fragment_[]
184.0734
C5H12N_Fragment_[]
86.0969
Relative Intensity
m/z
0
10
20
30
40
50
60
70
80
90
100
110
120
0
50
100
150
200
250
300
350
400
450
500
550
600
650
700
750
800

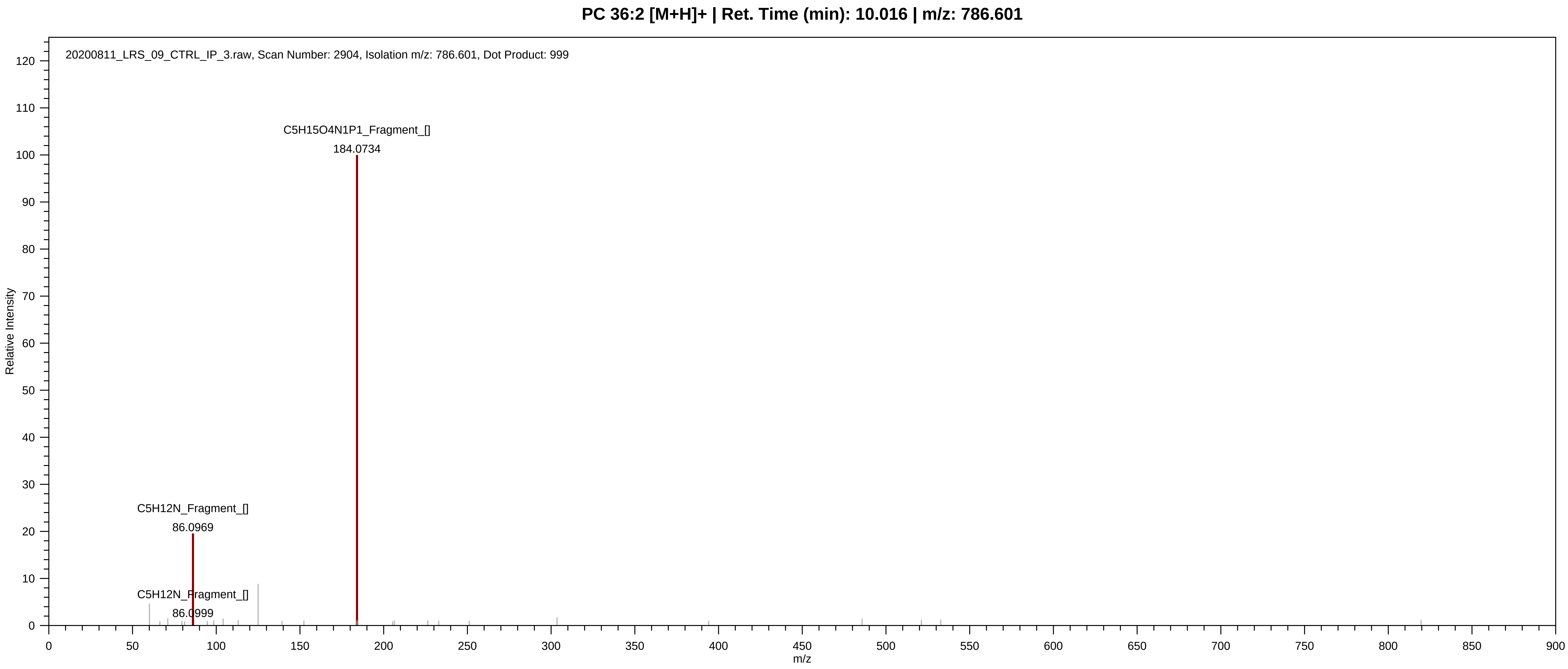
PC 36:2 [M+H]+ | Ret. Time (min): 10.016 | m/z: 786.601
20200811_LRS_09_CTRL_IP_3.raw, Scan Number: 2904, Isolation m/z: 786.601, Dot Product: 999
C5H15O4N1P1_Fragment_[]
184.0734
C5H12N_Fragment_[]
86.0969
C5H12N_Fragment_[]
86.0999
Relative Intensity
m/z
0
10
20
30
40
50
60
70
80
90
100
110
120
0
50
100
150
200
250
300
350
400
450
500
550
600
650
700
750
800
850
900

PC 36:2 [M+H]+ | Ret. Time (min): 10.016 | m/z: 786.602

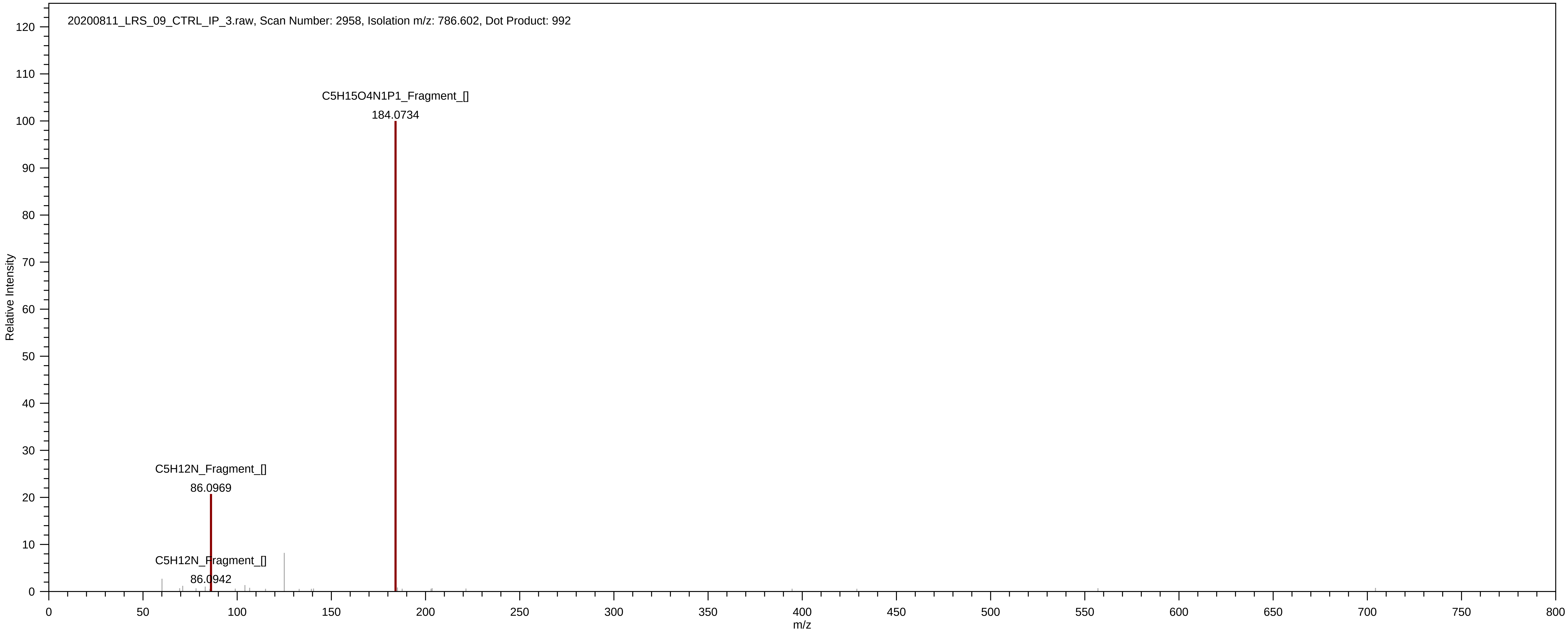

PC 36:3 [M+H]+ | Ret. Time (min): 10.773 | m/z: 784.581

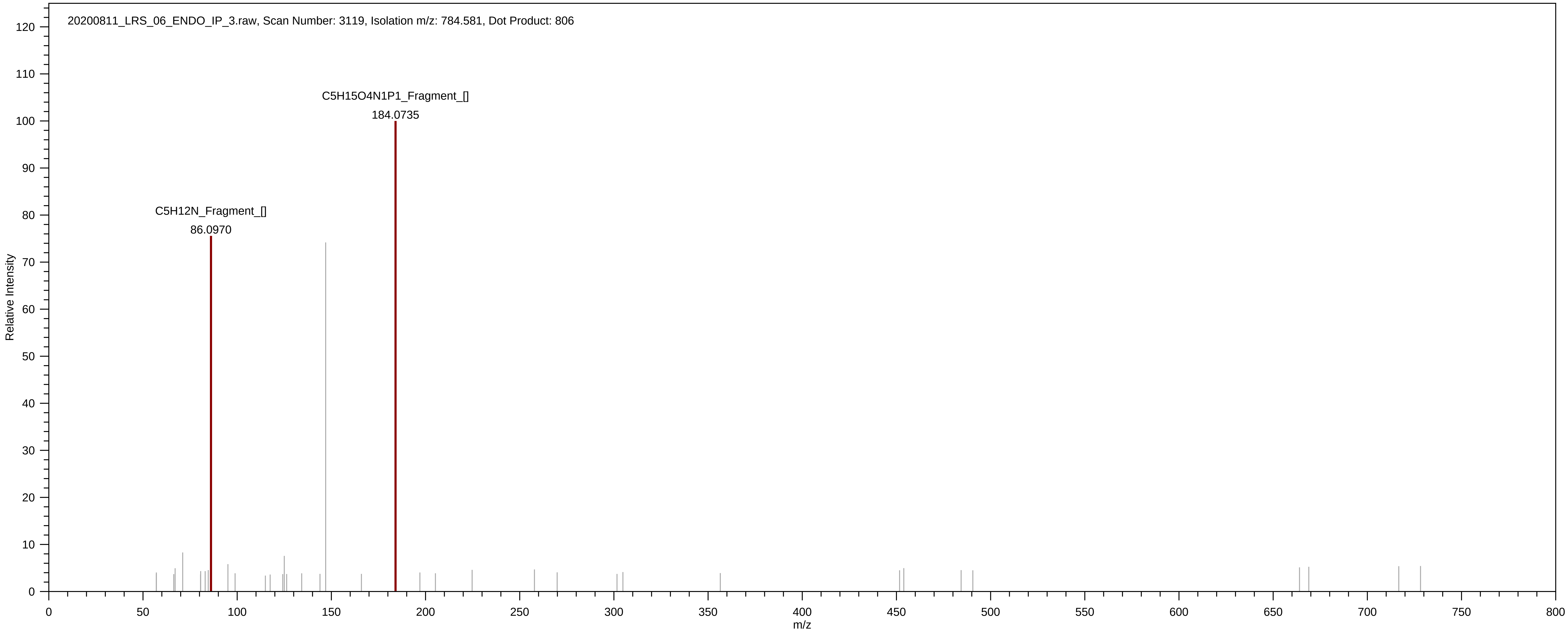

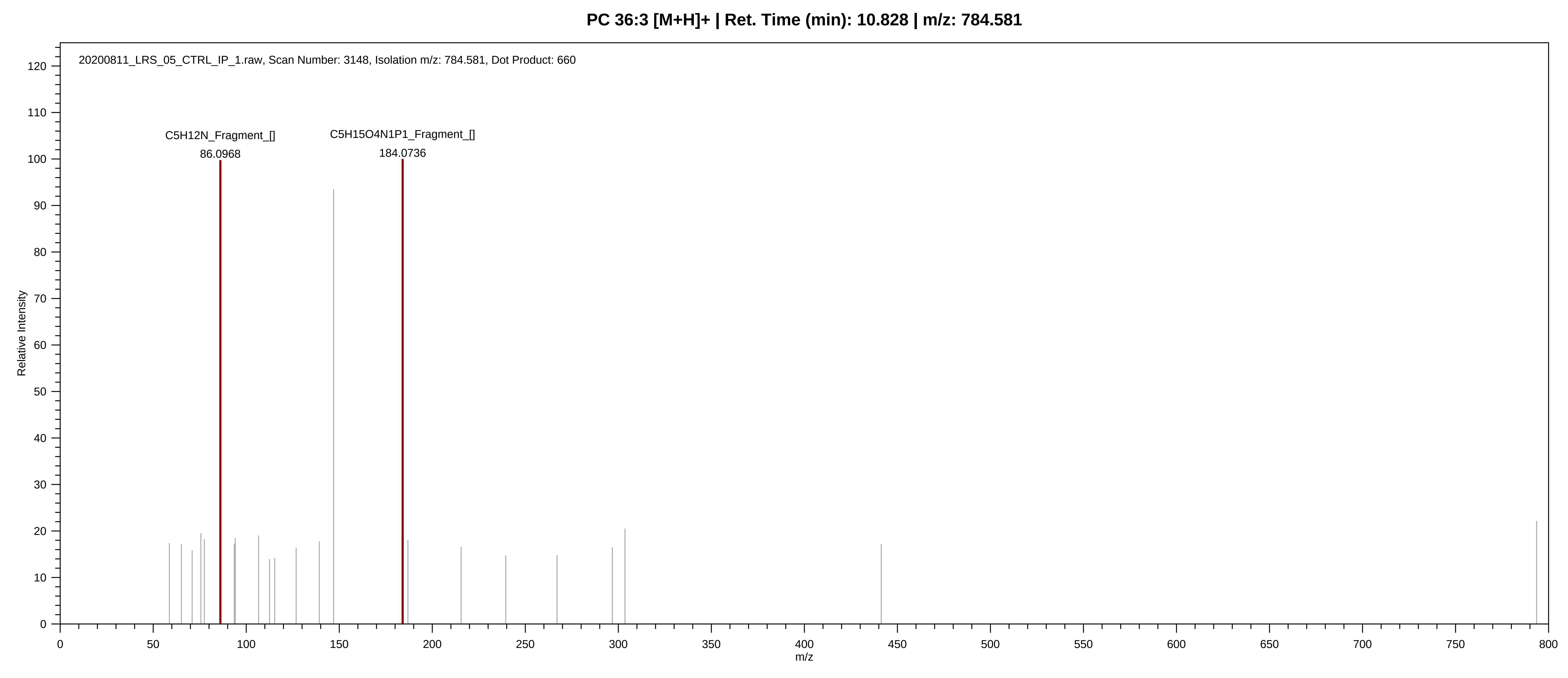

PC 36:3 [M+H]+ | Ret. Time (min): 10.828 | m/z: 784.581
20200811_LRS_05_CTRL_IP_1.raw, Scan Number: 3148, Isolation m/z: 784.581, Dot Product: 660
C5H12N_Fragment_[]
86.0968
C5H15O4N1P1_Fragment_[]
184.0736
Relative Intensity
m/z

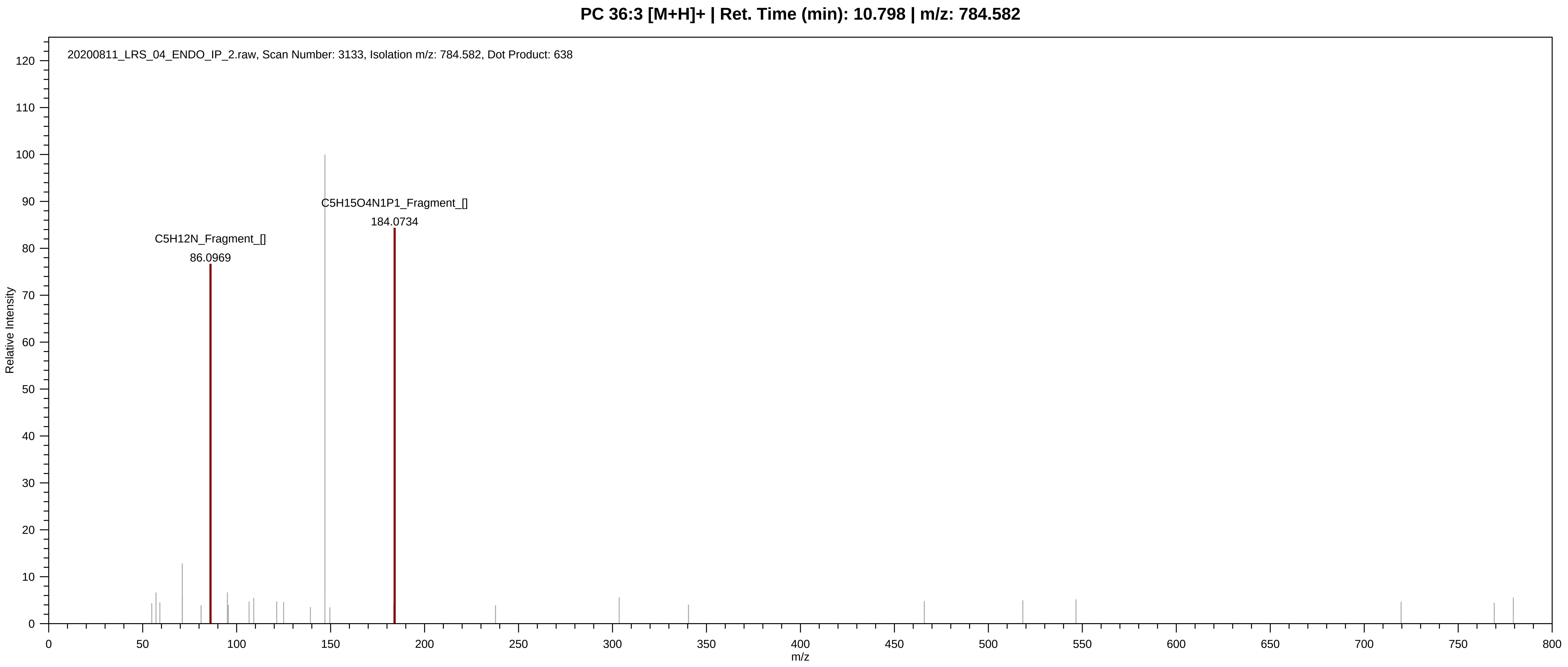

PC 36:3 [M+H]+ | Ret. Time (min): 10.798 | m/z: 784.582
20200811_LRS_04_ENDO_IP_2.raw, Scan Number: 3133, Isolation m/z: 784.582, Dot Product: 638
C5H15O4N1P1_Fragment_[]
184.0734
C5H12N_Fragment_[]
86.0969
Relative Intensity
m/z

PC 36:3 [M+H]+ | Ret. Time (min): 9.152 | m/z: 784.584

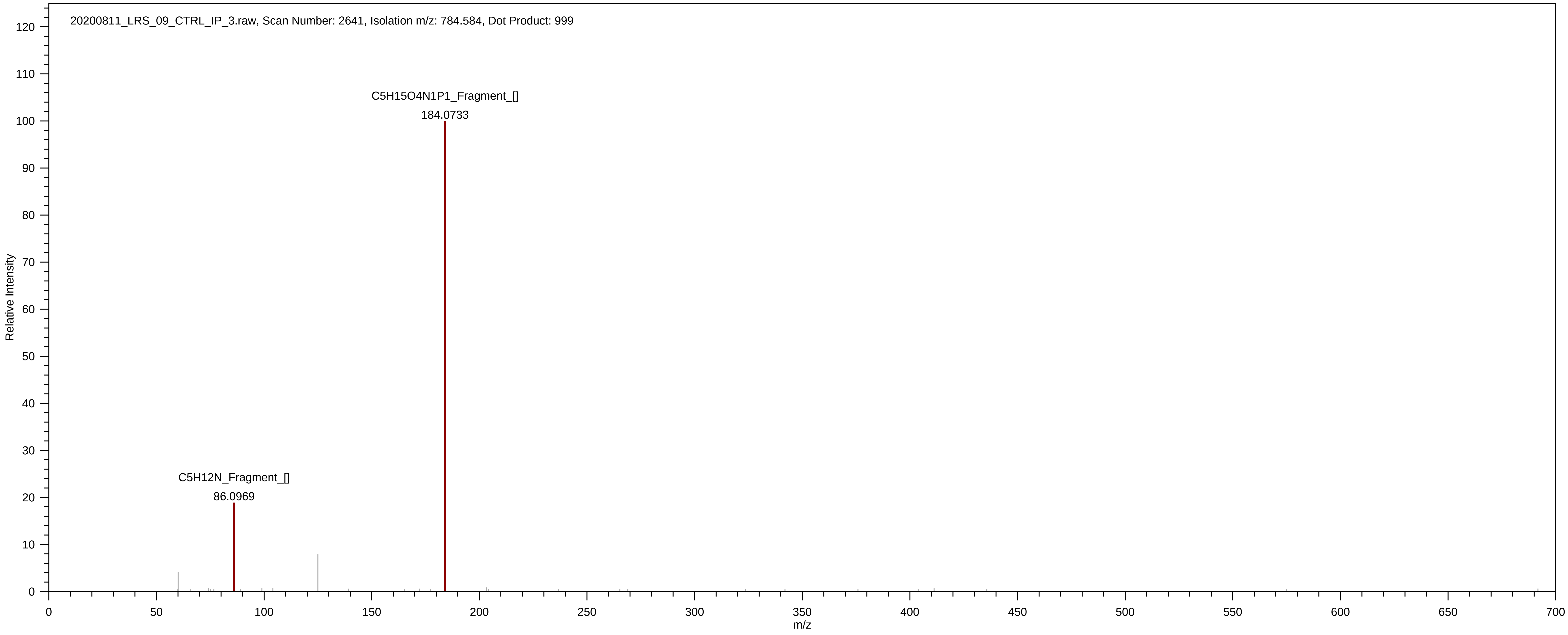

PC 36:3 [M+H]+ | Ret. Time (min): 9.159 | m/z: 784.584

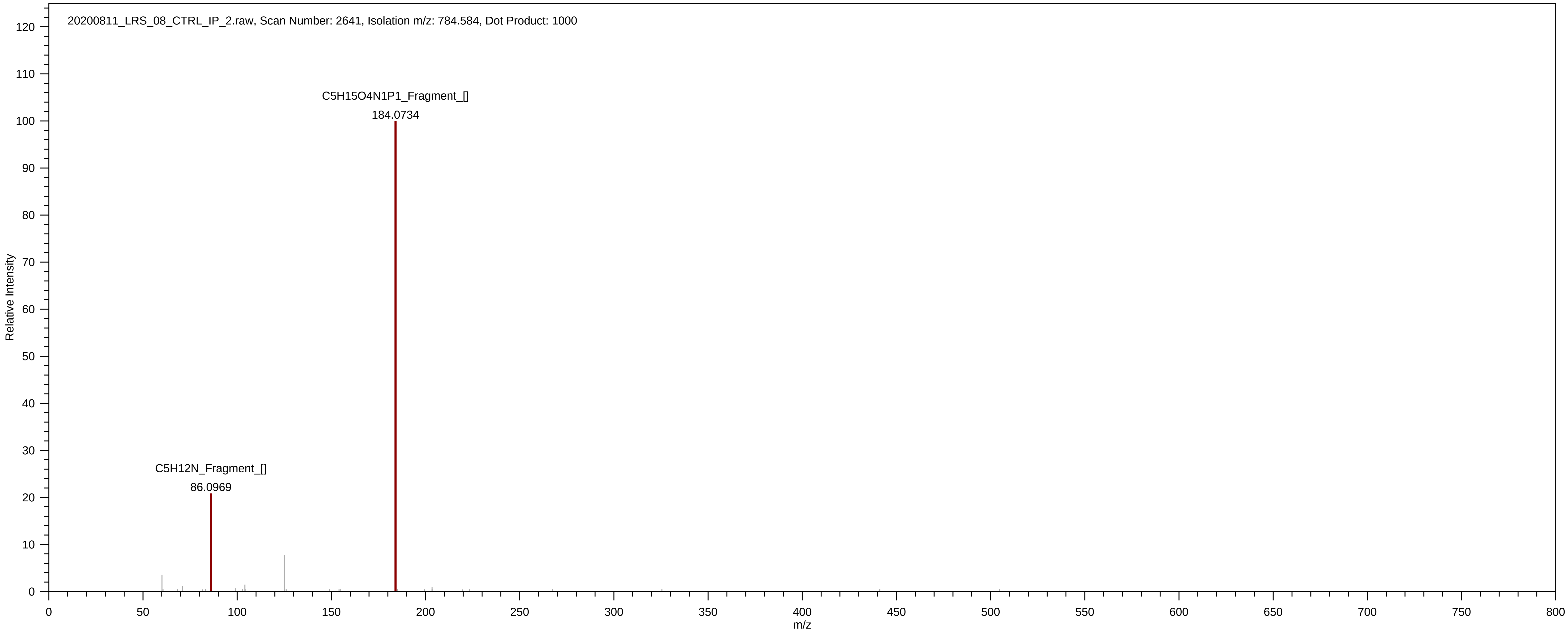

PC 36:3 [M+H]+ | Ret. Time (min): 9.161 | m/z: 784.584

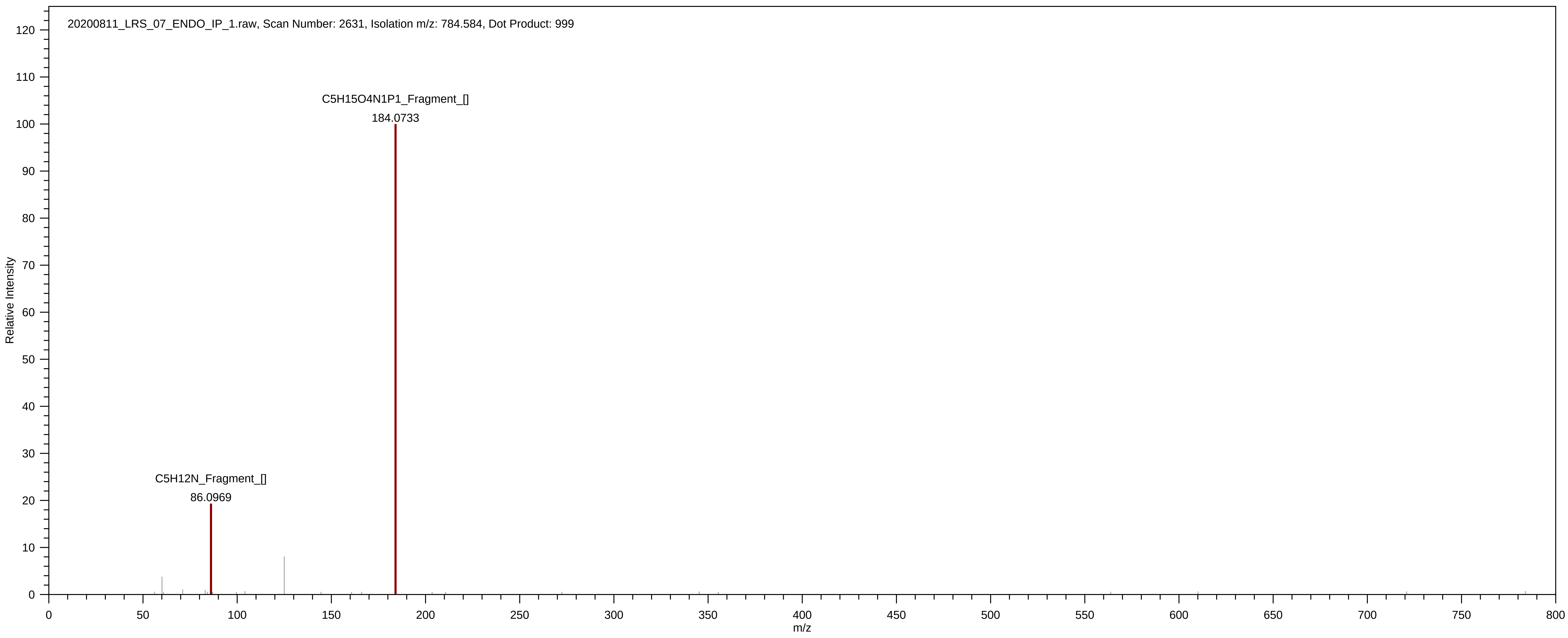

PC 36:3 [M+H]+ | Ret. Time (min): 9.174 | m/z: 784.584

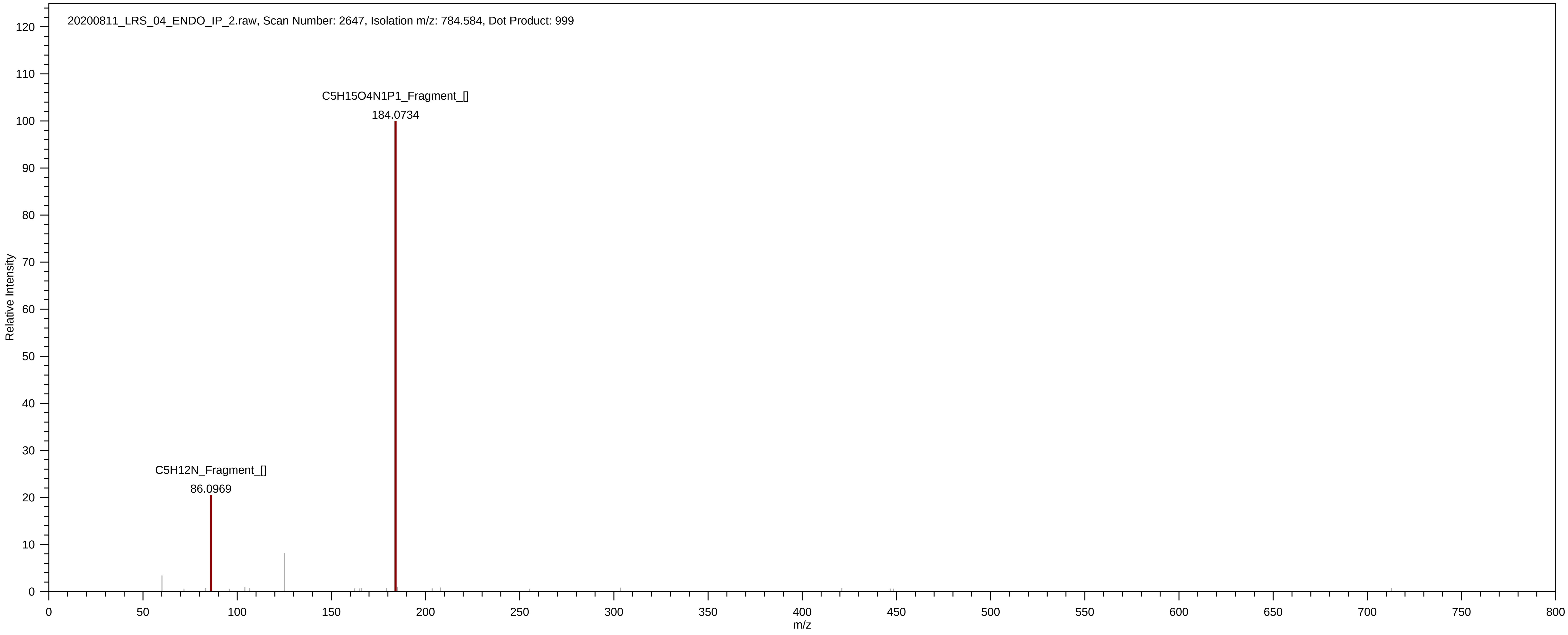

PC 36:3 [M+H]+ | Ret. Time (min): 9.16 | m/z: 784.585

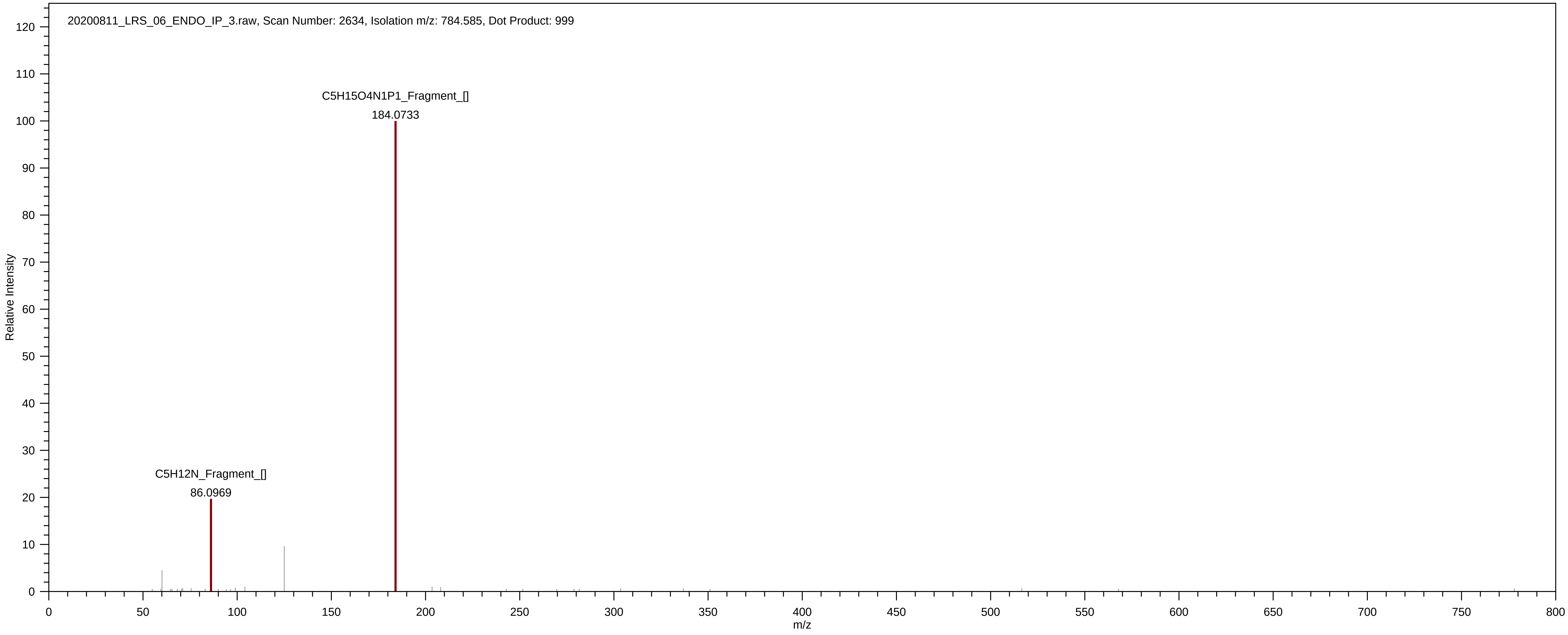

PC 36:3 [M+H]+ | Ret. Time (min): 9.184 | m/z: 784.585

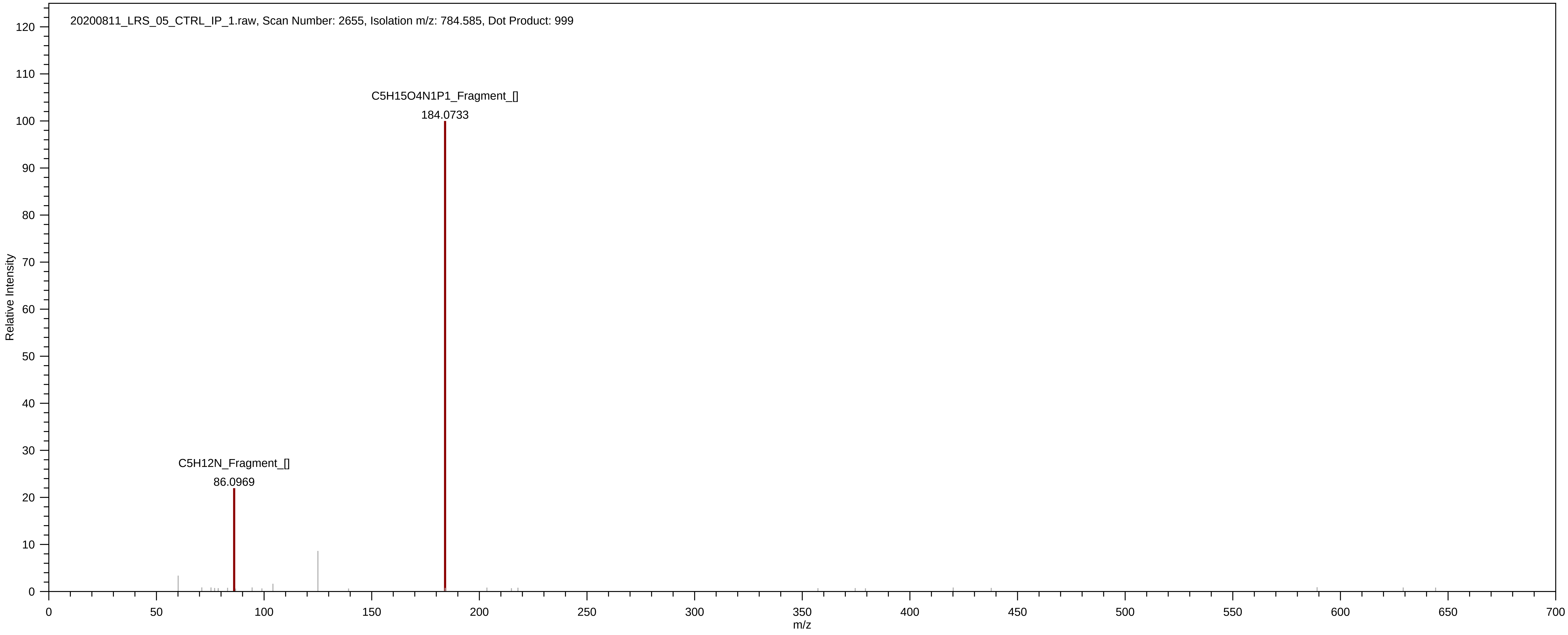

PC 36:3 [M+H]+ | Ret. Time (min): 9.341 | m/z: 784.585

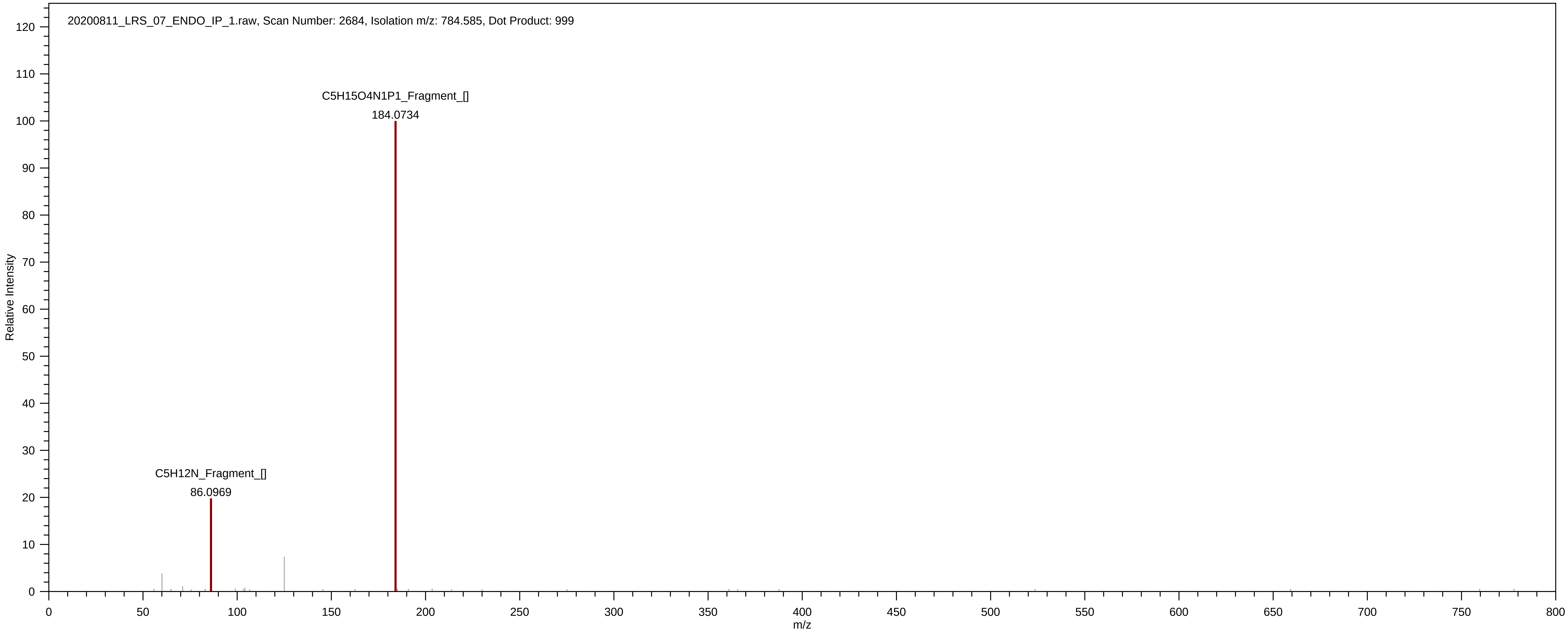

PC 36:3 [M+H]+ | Ret. Time (min): 9.341 | m/z: 784.585

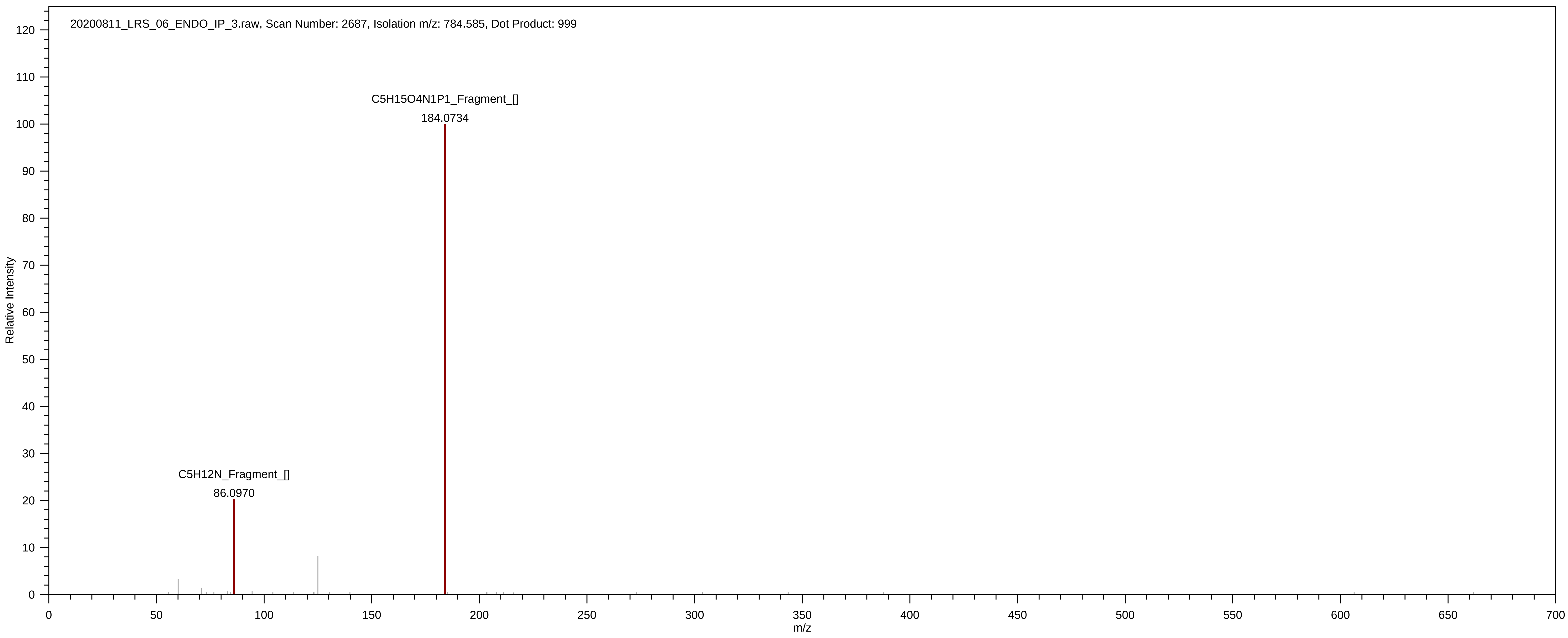

PC 36:3 [M+H]+ | Ret. Time (min): 9.355 | m/z: 784.585

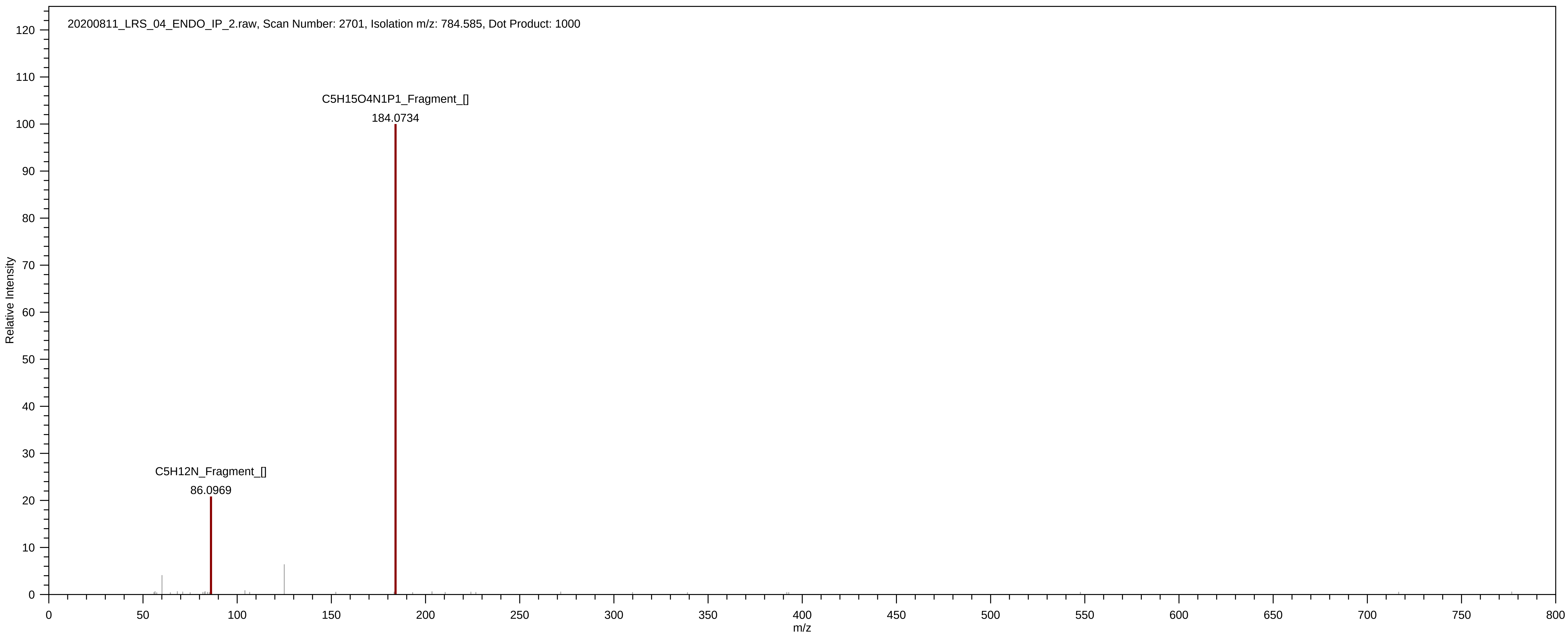

PC 36:3 [M+H]+ | Ret. Time (min): 9.33 | m/z: 784.586

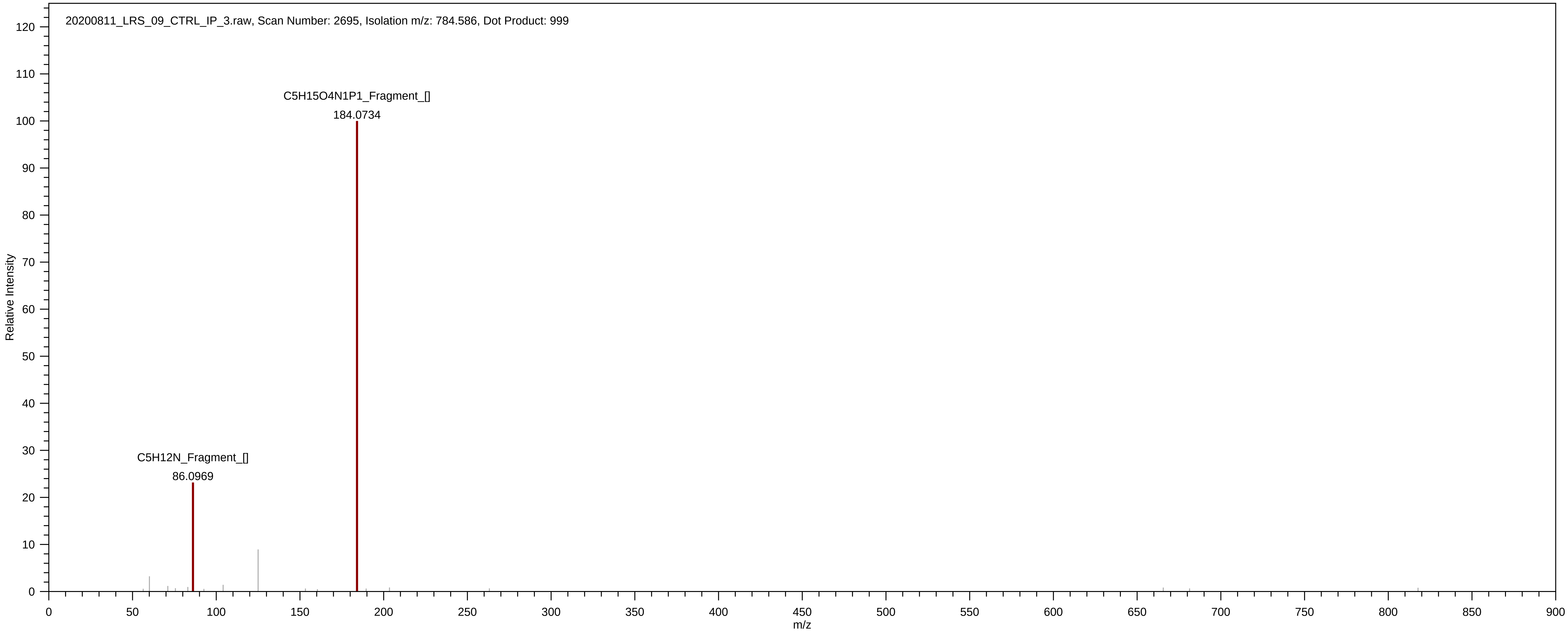

PC 36:3 [M+H]+ | Ret. Time (min): 9.339 | m/z: 784.586

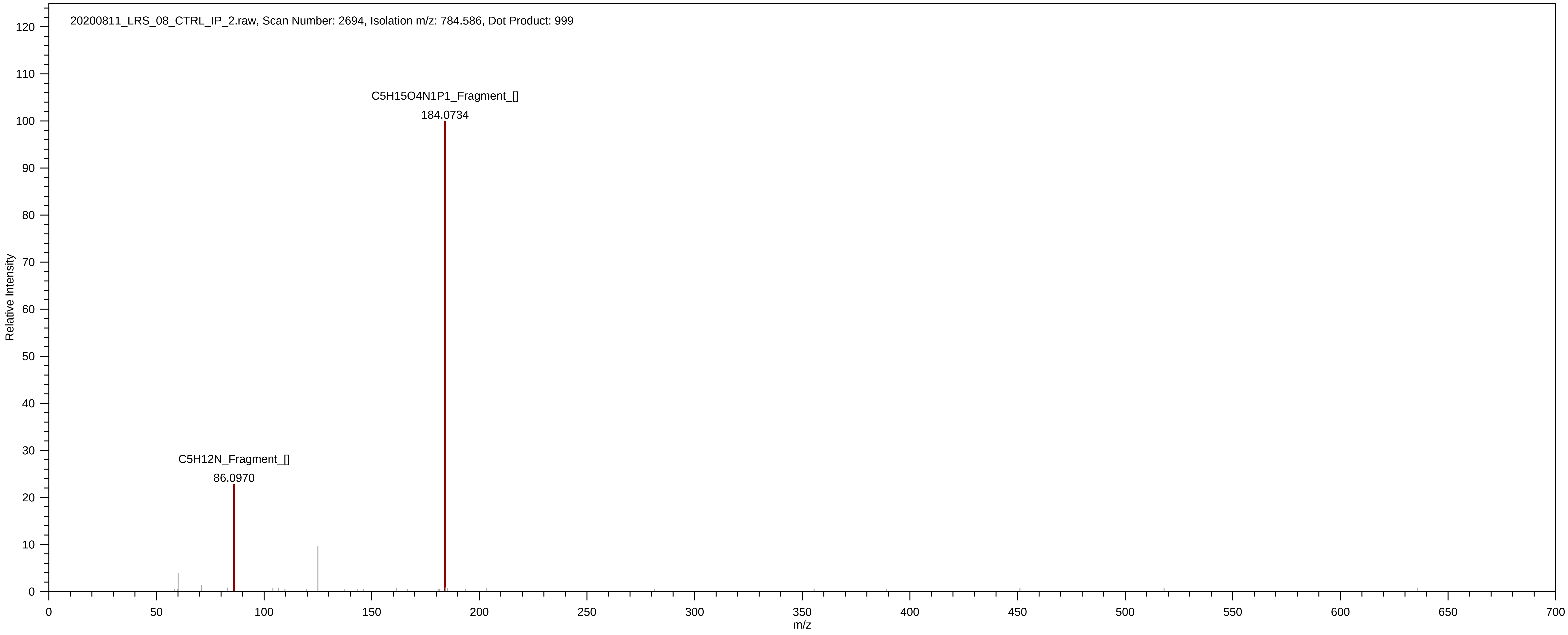

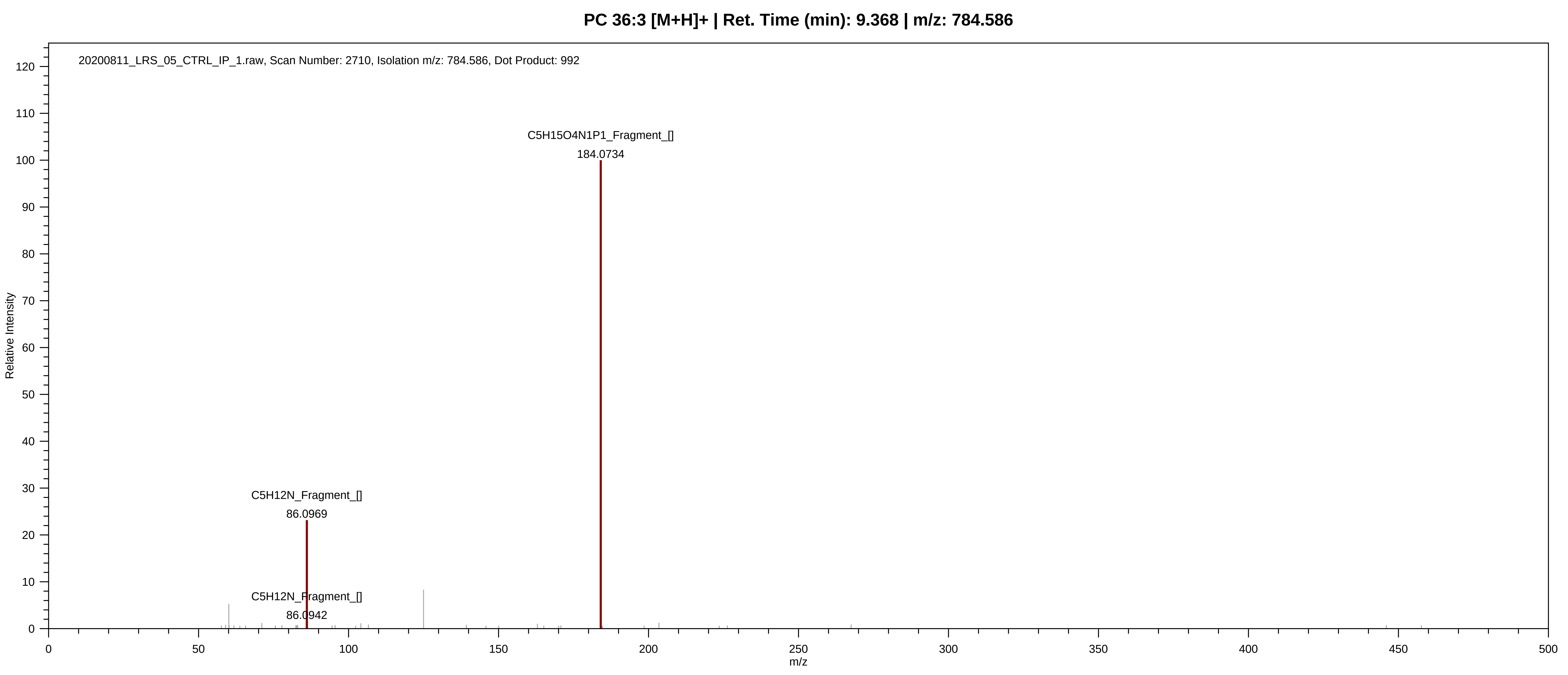

PC 36:3 [M+H]+ | Ret. Time (min): 9.368 | m/z: 784.586
20200811_LRS_05_CTRL_IP_1.raw, Scan Number: 2710, Isolation m/z: 784.586, Dot Product: 992
C5H15O4N1P1_Fragment_[]
184.0734
C5H12N_Fragment_[]
86.0969
C5H12N_Fragment_[]
86.0942
Relative Intensity
m/z

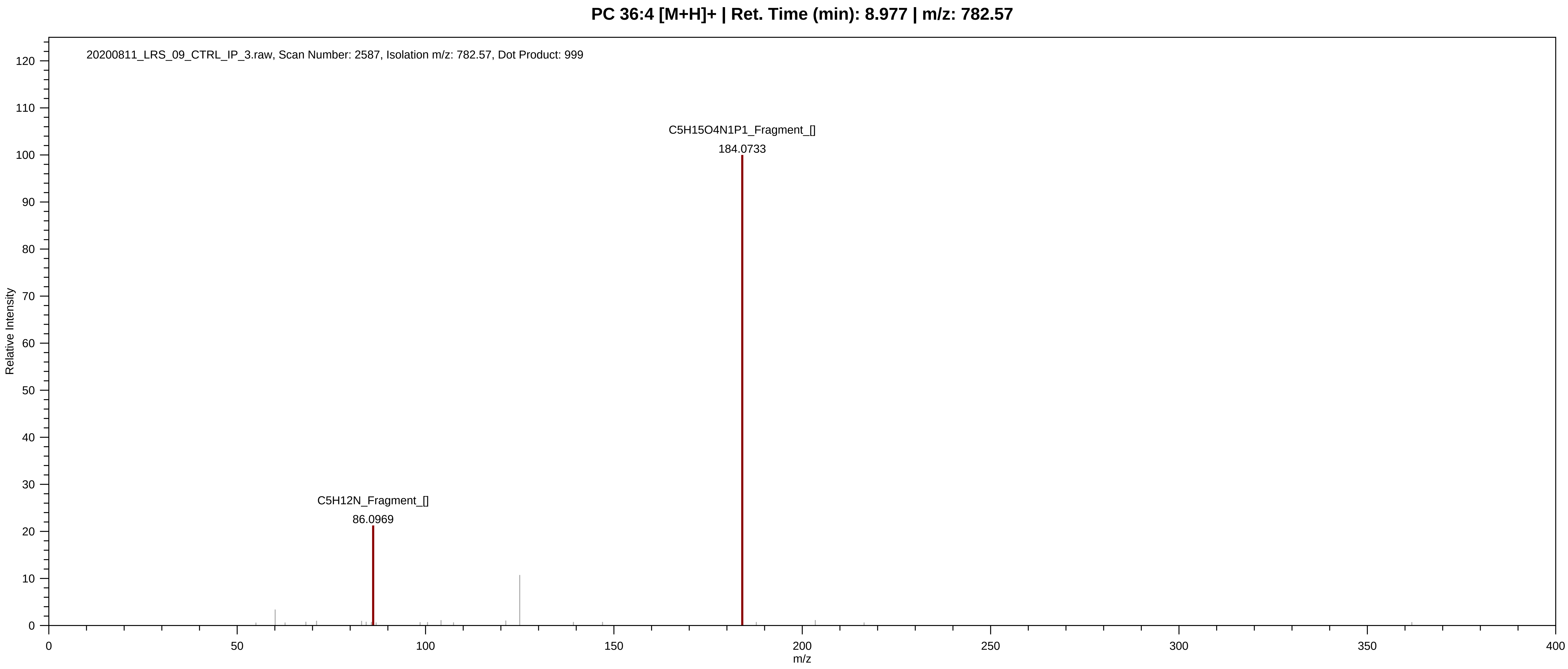

PC 36:4 [M+H]+ | Ret. Time (min): 8.977 | m/z: 782.57
20200811_LRS_09_CTRL_IP_3.raw, Scan Number: 2587, Isolation m/z: 782.57, Dot Product: 999
C5H15O4N1P1_Fragment_[]
184.0733
C5H12N_Fragment_[]
86.0969
Relative Intensity
m/z

PC 36:4 [M+H]+ | Ret. Time (min): 8.984 | m/z: 782.568

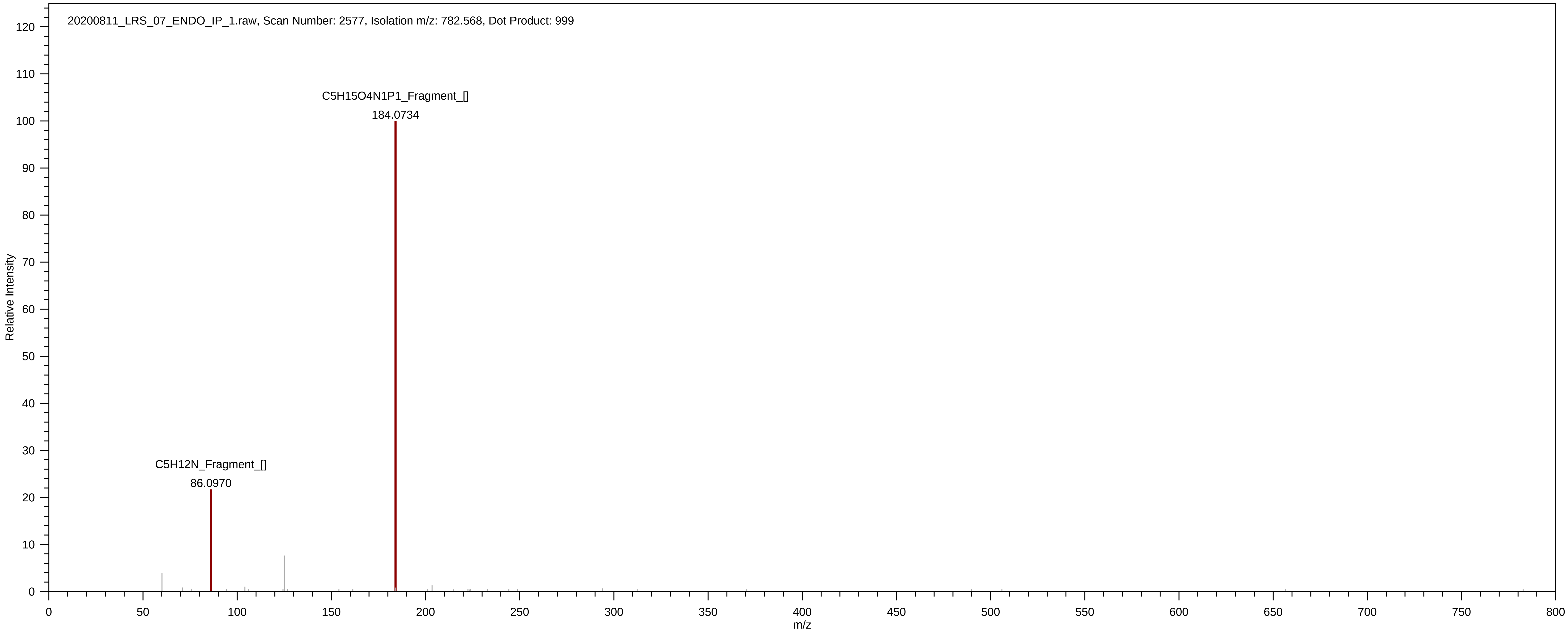

PC 36:4 [M+H]+ | Ret. Time (min): 8.994 | m/z: 782.568

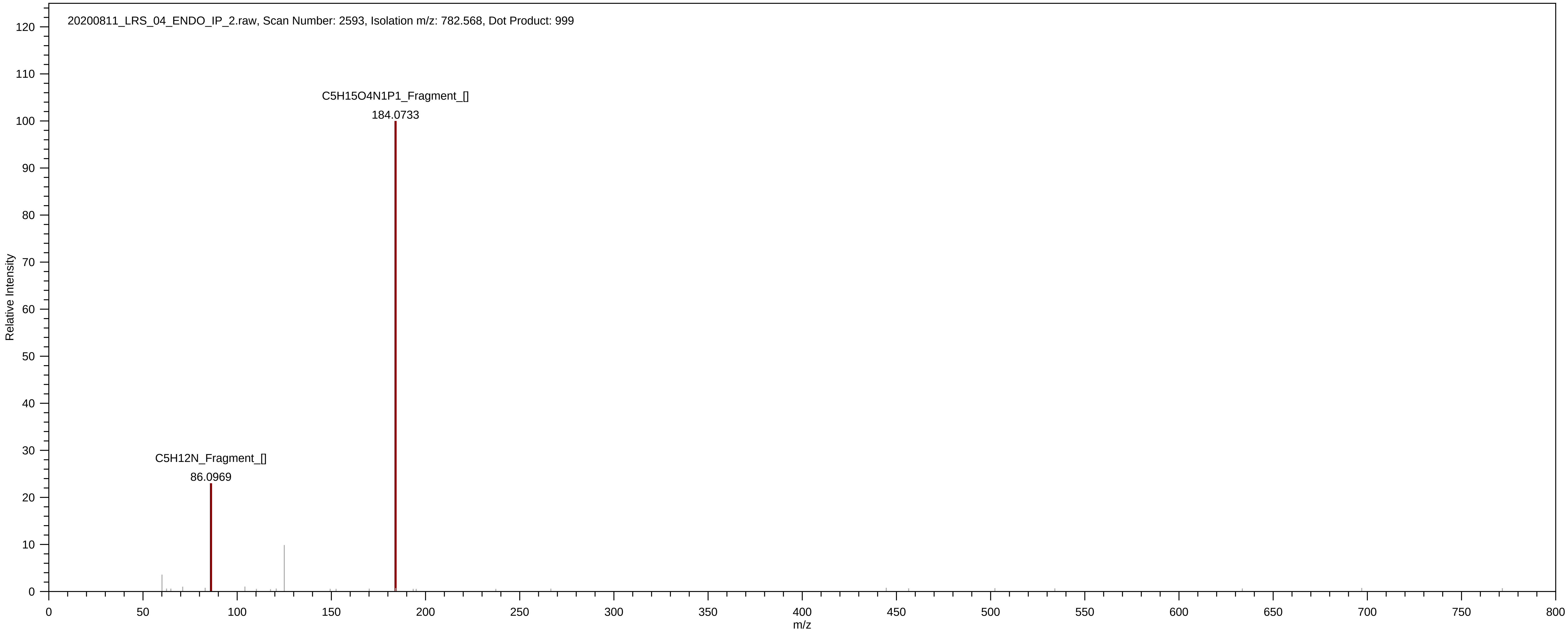

PC 36:4 [M+H]+ | Ret. Time (min): 9.002 | m/z: 782.568

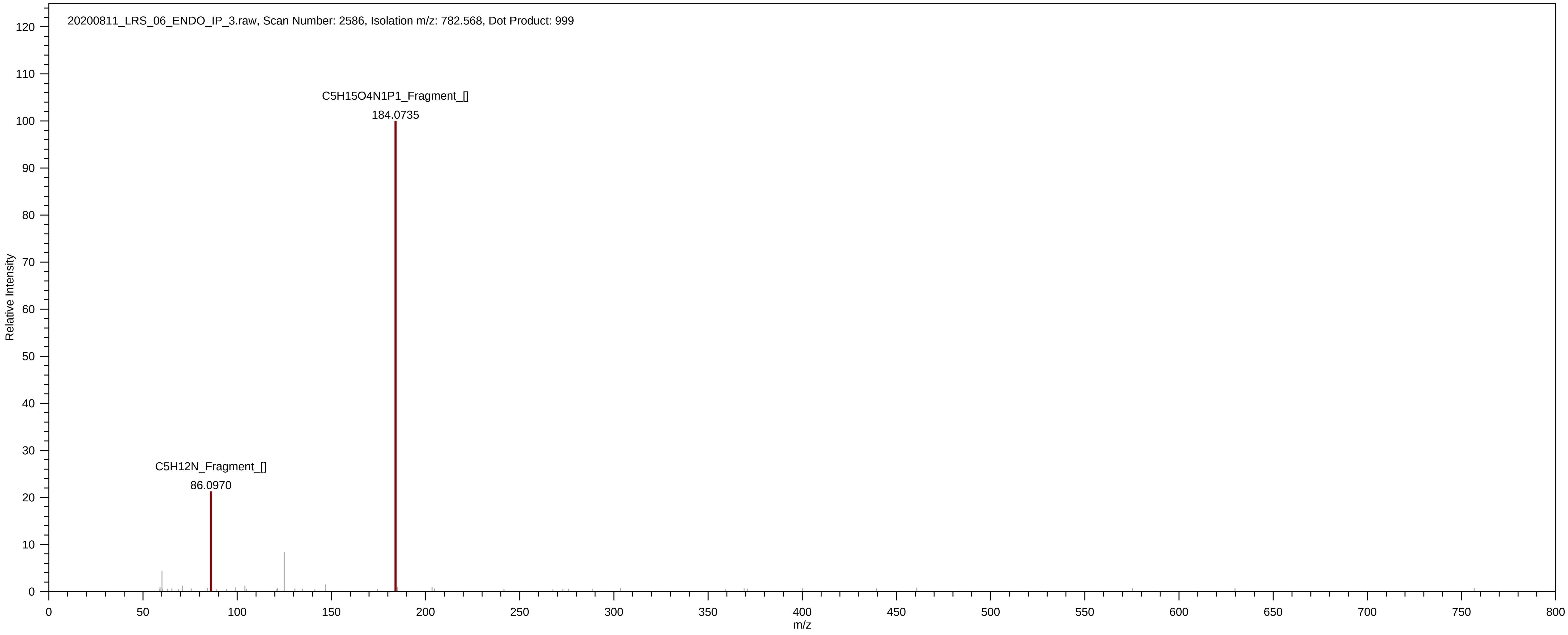

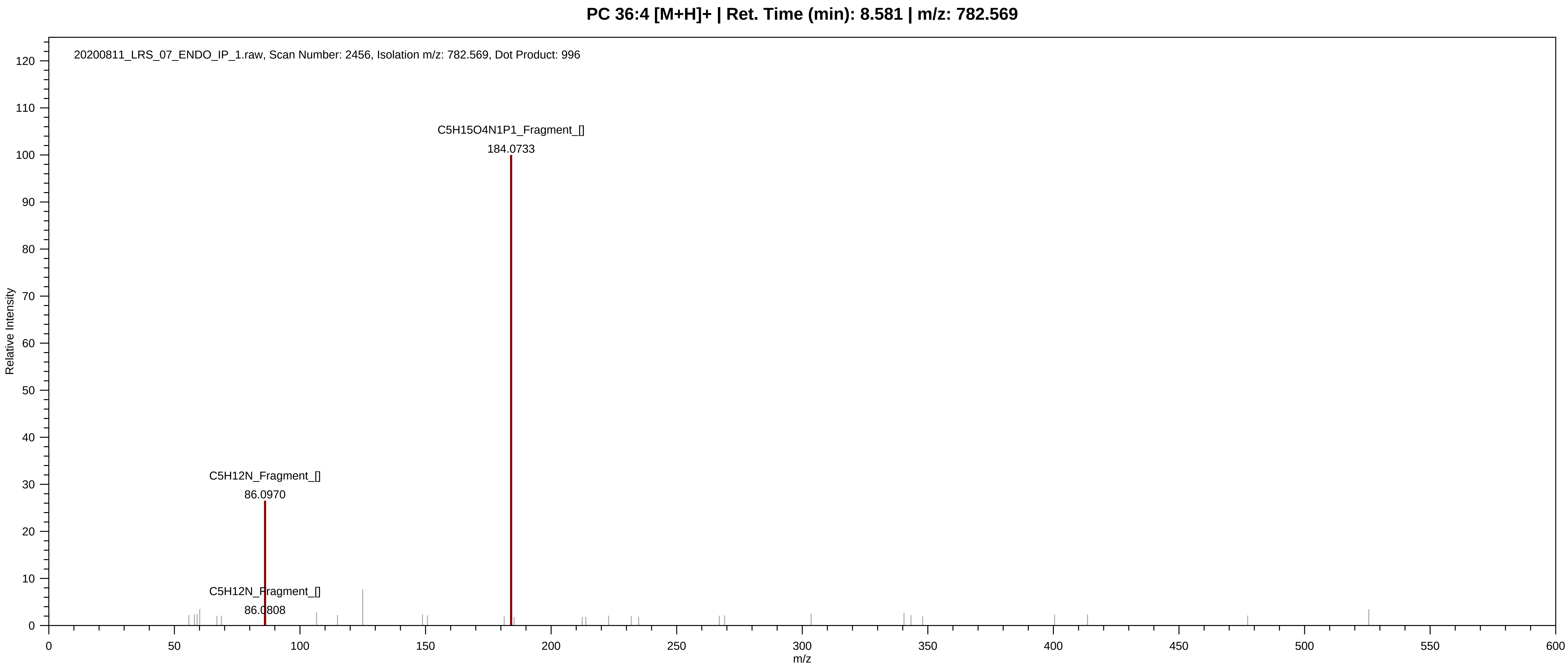
PC 36:4 [M+H]+ | Ret. Time (min): 8.581 | m/z: 782.569
20200811_LRS_07_ENDO_IP_1.raw, Scan Number: 2456, Isolation m/z: 782.569, Dot Product: 996
C5H15O4N1P1_Fragment_[]
184.0733
C5H12N_Fragment_[]
86.0970
C5H12N_Fragment_[]
86.0808
Relative Intensity
m/z

PC 36:4 [M+H]+ | Ret. Time (min): 8.595 | m/z: 782.569

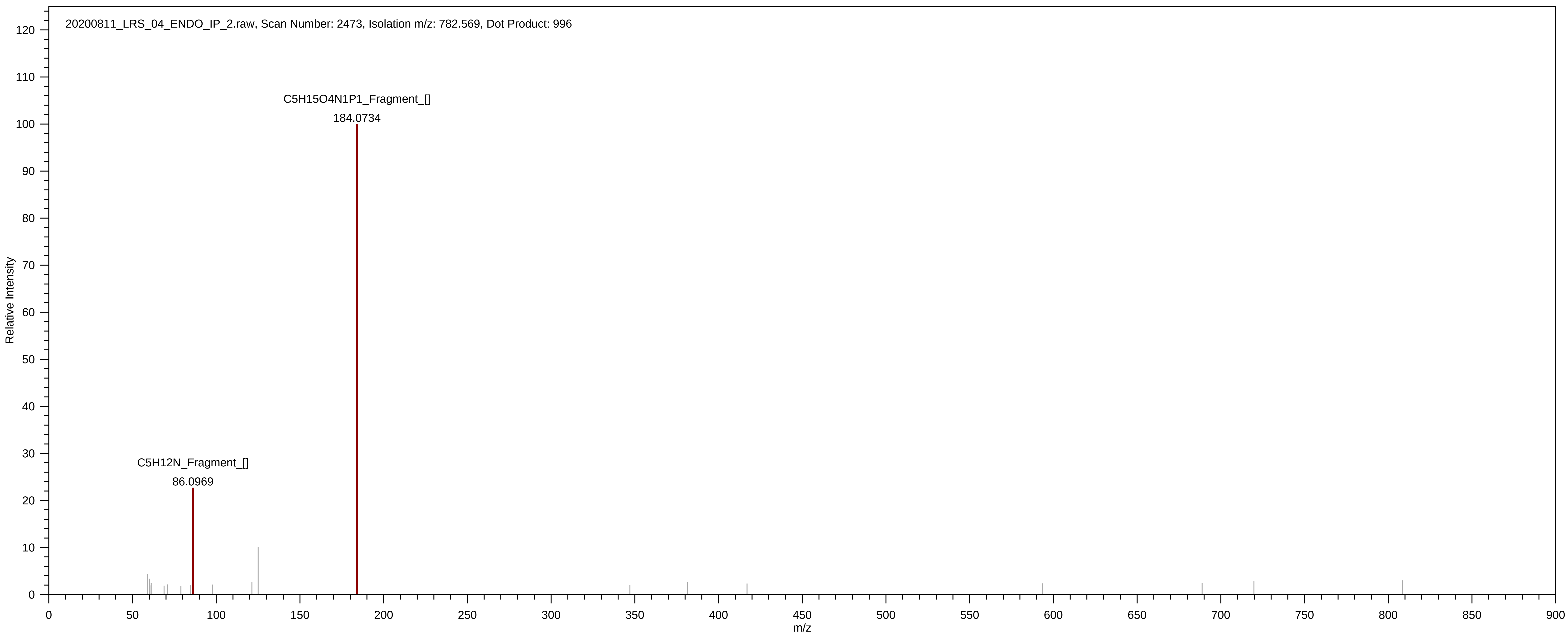

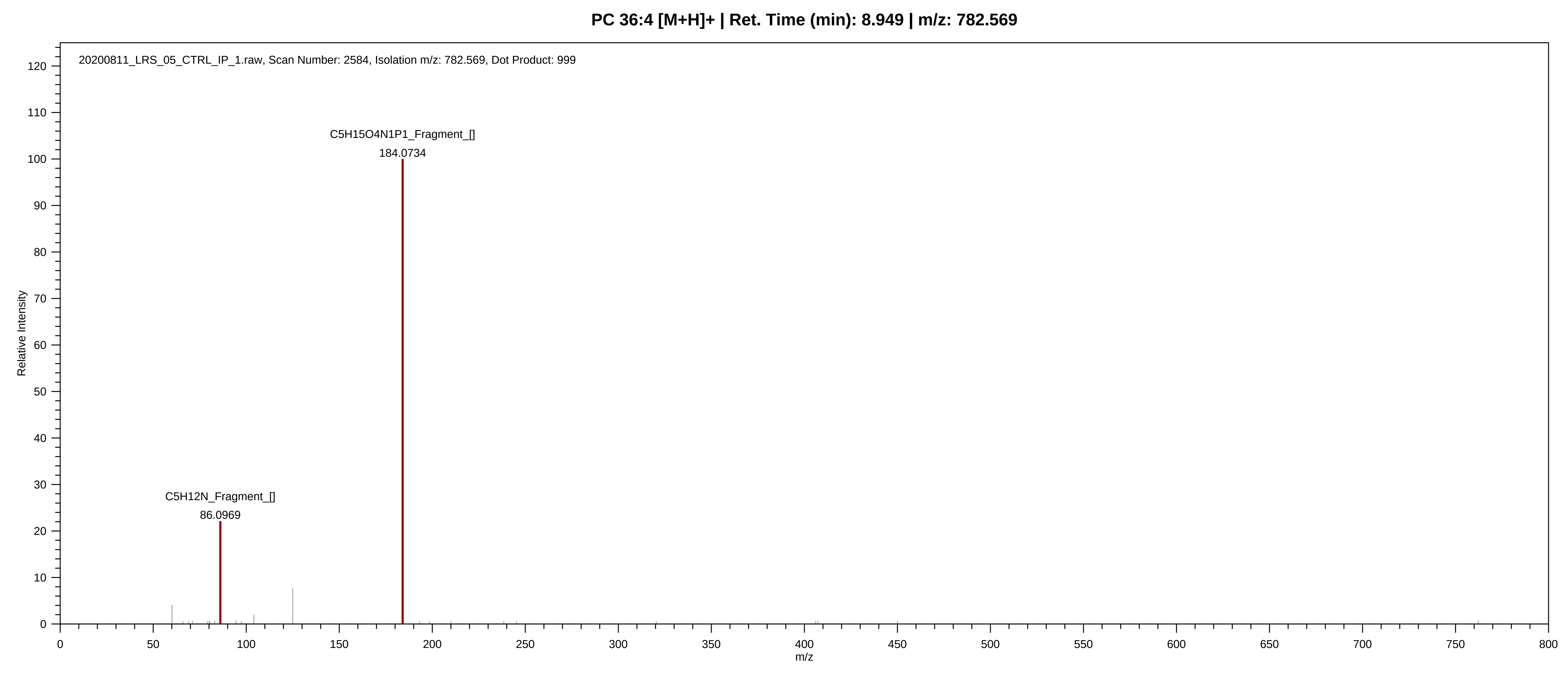
PC 36:4 [M+H]+ | Ret. Time (min): 8.949 | m/z: 782.569
20200811_LRS_05_CTRL_IP_1.raw, Scan Number: 2584, Isolation m/z: 782.569, Dot Product: 999
C5H15O4N1P1_Fragment_[]
184.0734
C5H12N_Fragment_[]
86.0969
Relative Intensity
m/z

PC 36:4 [M+H]+ | Ret. Time (min): 8.981 | m/z: 782.569

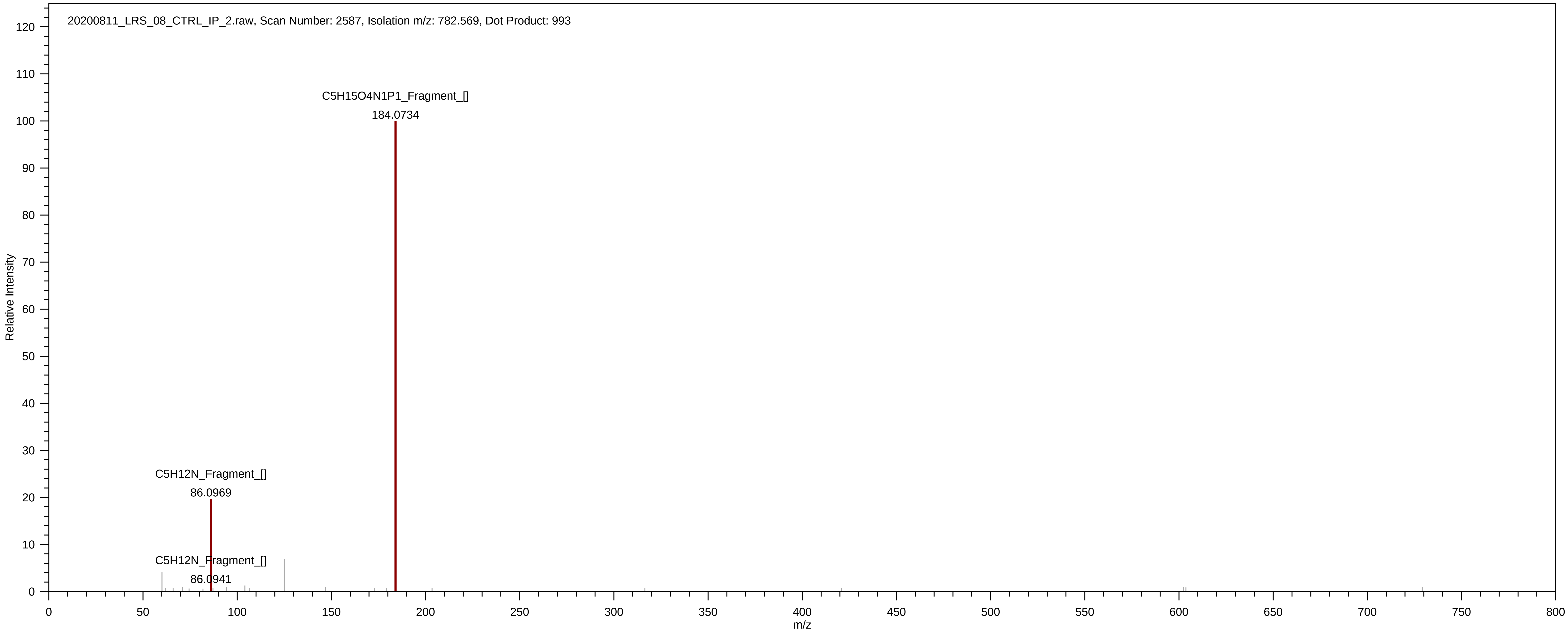

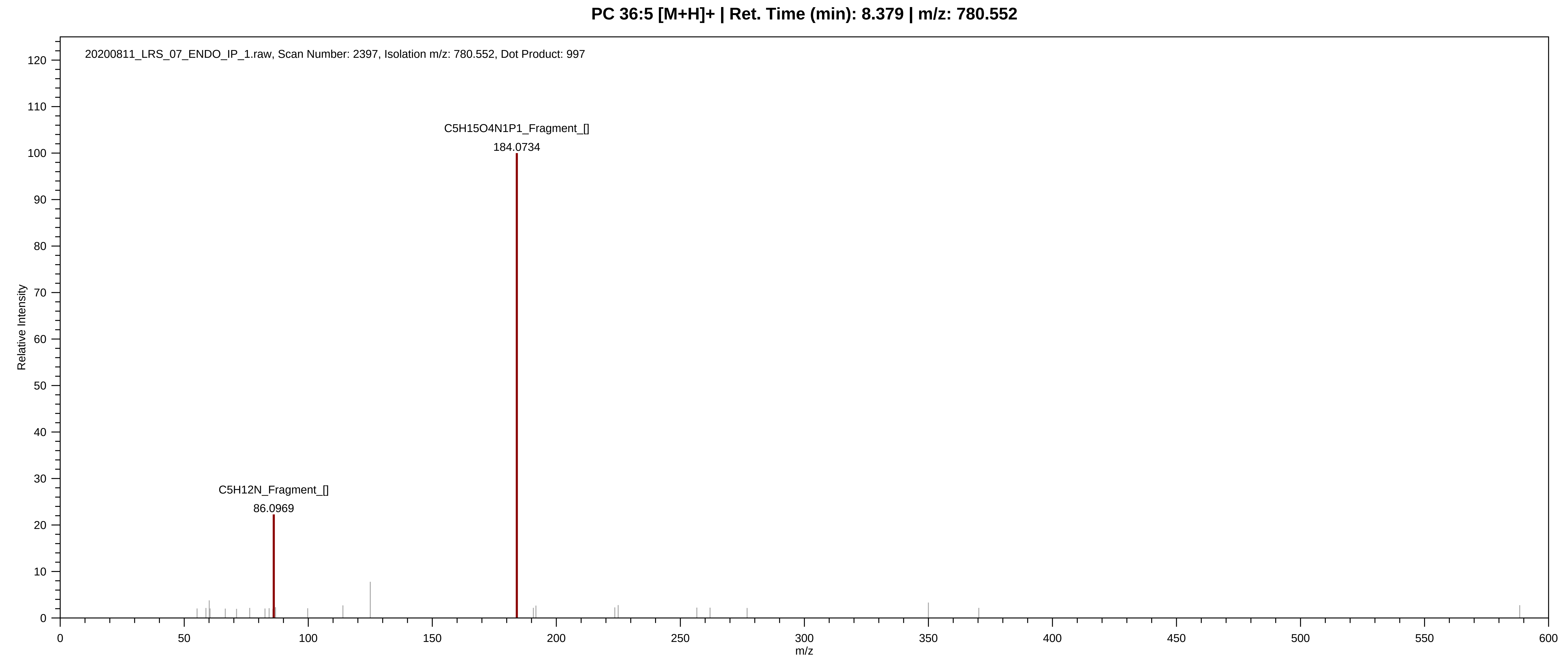
PC 36:5 [M+H]+ | Ret. Time (min): 8.379 | m/z: 780.552
20200811_LRS_07_ENDO_IP_1.raw, Scan Number: 2397, Isolation m/z: 780.552, Dot Product: 997
C5H15O4N1P1_Fragment_[]
184.0734
C5H12N_Fragment_[]
86.0969
Relative Intensity
m/z
0
10
20
30
40
50
60
70
80
90
100
110
120
0
50
100
150
200
250
300
350
400
450
500
550
600

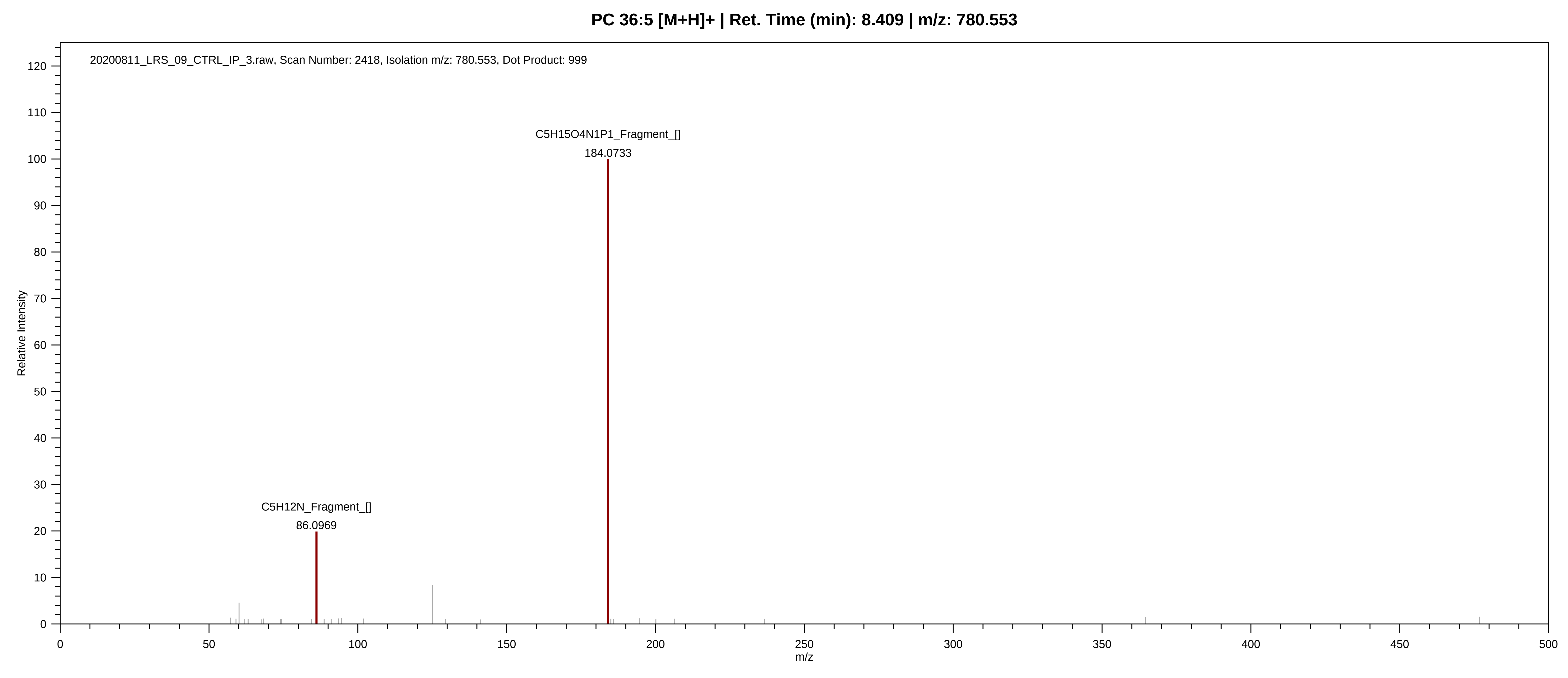

PC 36:5 [M+H]+ | Ret. Time (min): 8.409 | m/z: 780.553
20200811_LRS_09_CTRL_IP_3.raw, Scan Number: 2418, Isolation m/z: 780.553, Dot Product: 999
C5H15O4N1P1_Fragment_[]
184.0733
C5H12N_Fragment_[]
86.0969
Relative Intensity
m/z

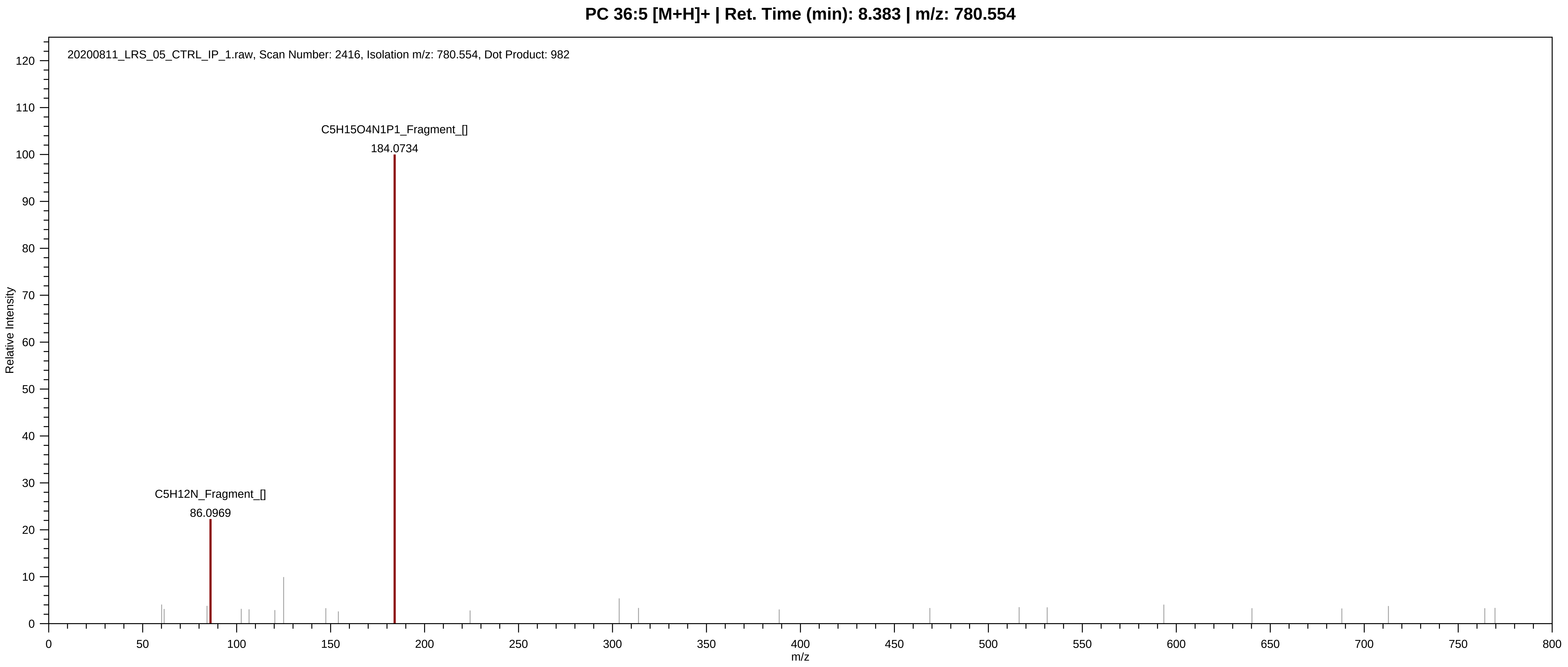
PC 36:5 [M+H]+ | Ret. Time (min): 8.383 | m/z: 780.554
20200811_LRS_05_CTRL_IP_1.raw, Scan Number: 2416, Isolation m/z: 780.554, Dot Product: 982
C5H15O4N1P1_Fragment_[]
184.0734
C5H12N_Fragment_[]
86.0969
Relative Intensity
m/z
0
10
20
30
40
50
60
70
80
90
100
110
120
0
50
100
150
200
250
300
350
400
450
500
550
600
650
700
750
800

PC 36:5 [M+H]+ | Ret. Time (min): 8.411 | m/z: 780.554

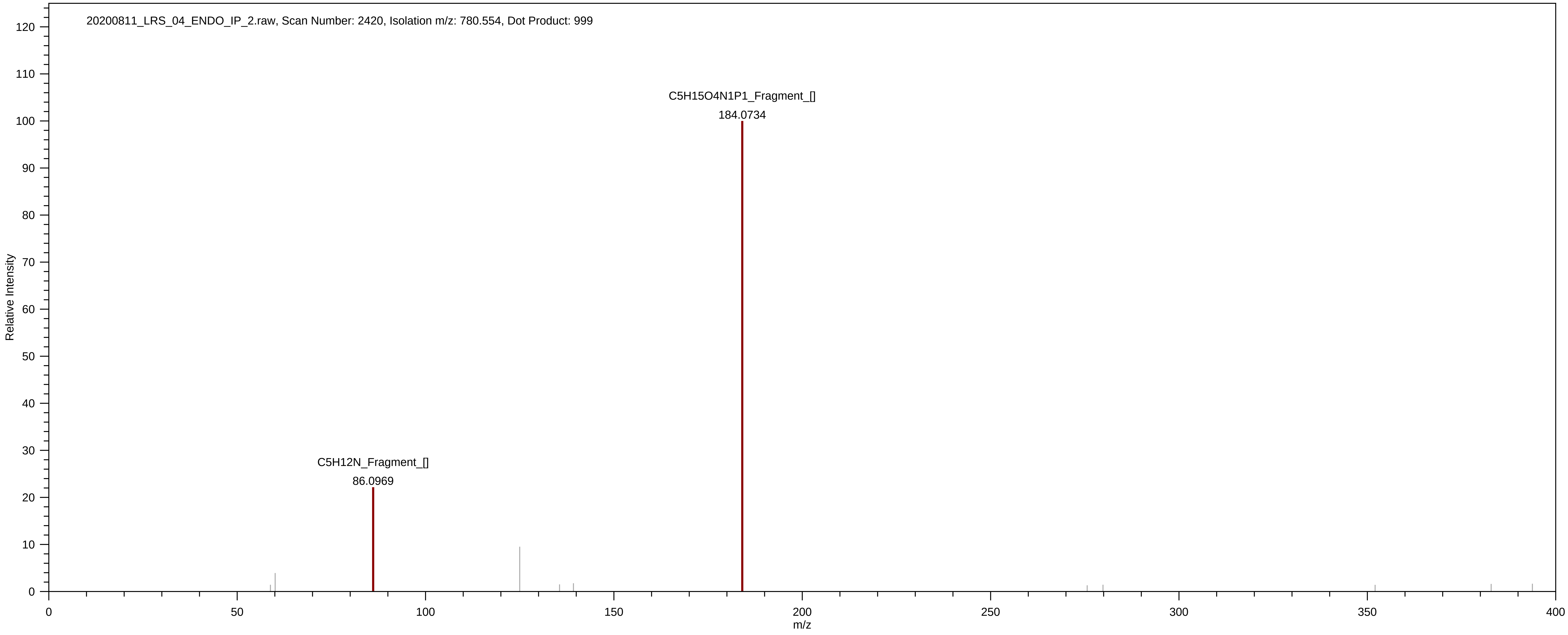

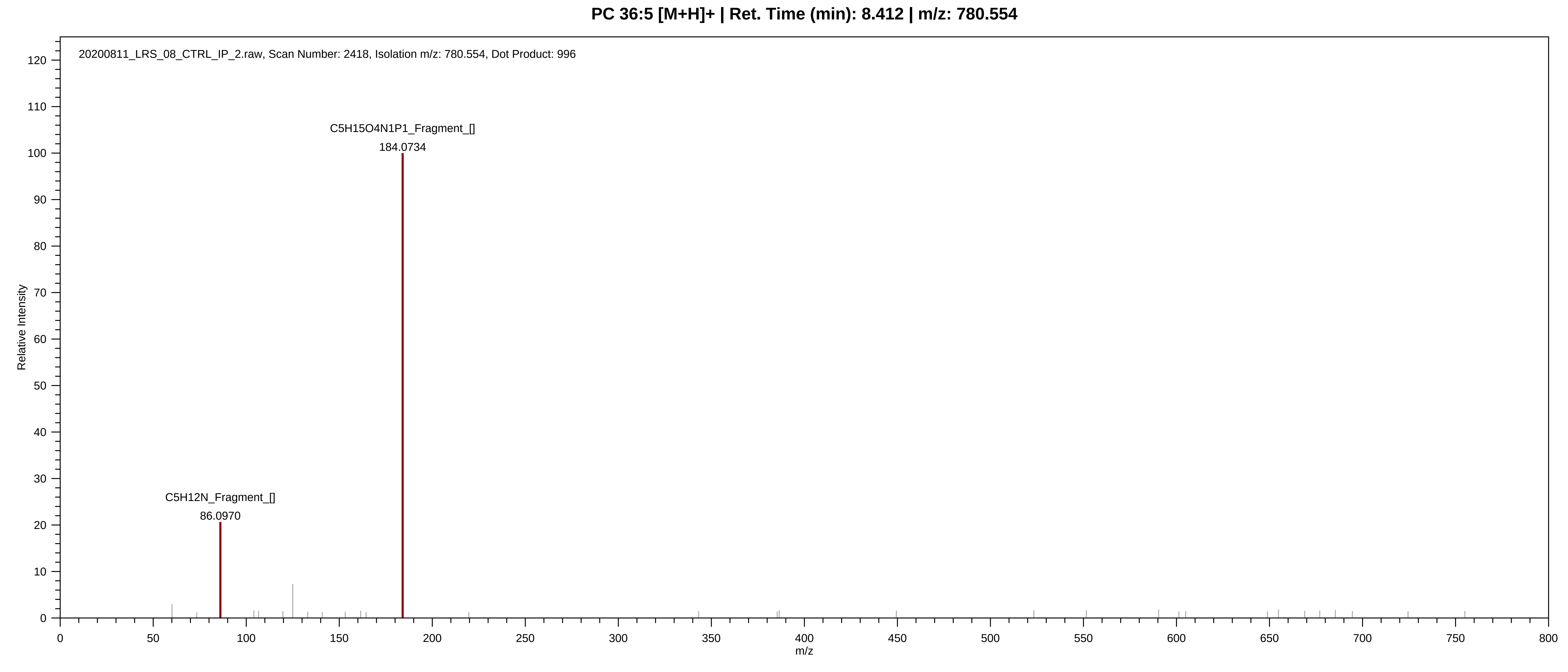

PC 36:5 [M+H]+ | Ret. Time (min): 8.412 | m/z: 780.554
20200811_LRS_08_CTRL_IP_2.raw, Scan Number: 2418, Isolation m/z: 780.554, Dot Product: 996
C5H15O4N1P1_Fragment_[]
184.0734
C5H12N_Fragment_[]
86.0970
Relative Intensity
m/z

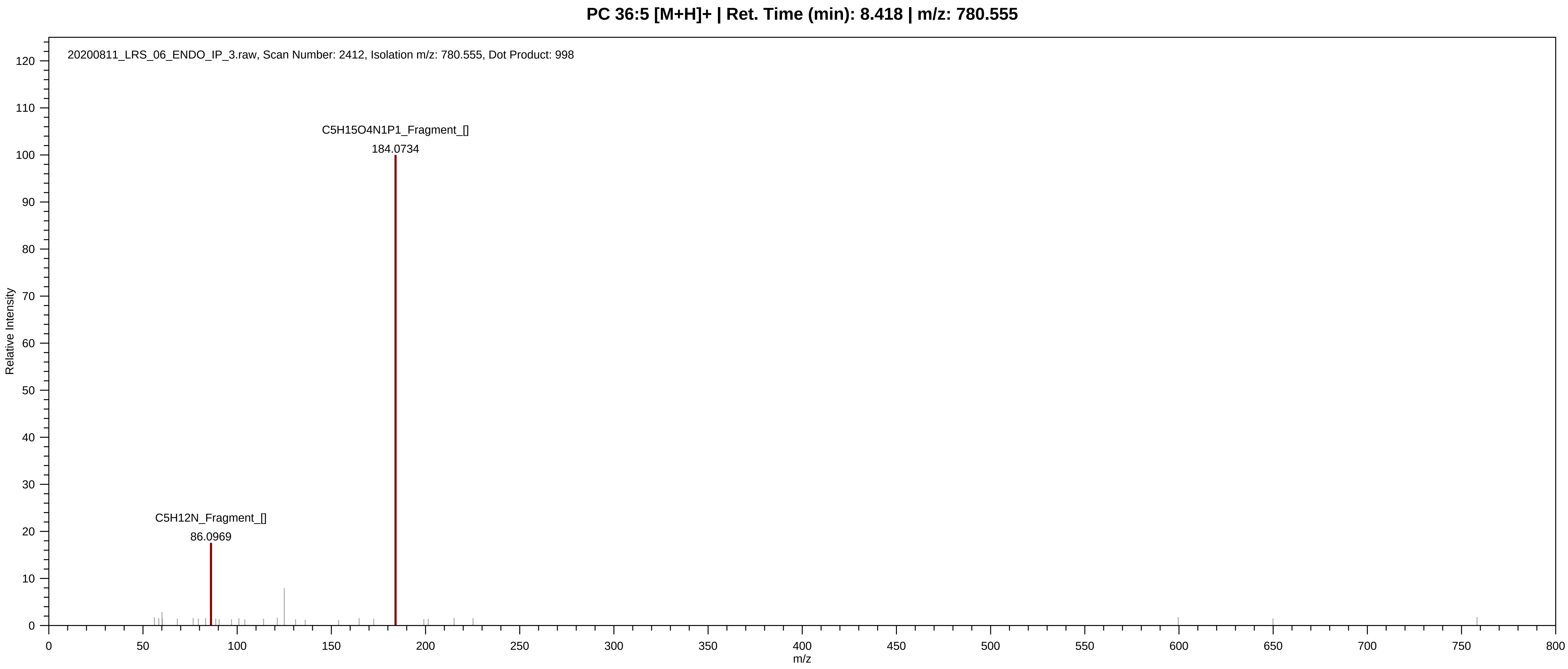

PC 36:5 [M+H]+ | Ret. Time (min): 8.418 | m/z: 780.555
20200811_LRS_06_ENDO_IP_3.raw, Scan Number: 2412, Isolation m/z: 780.555, Dot Product: 998
C5H15O4N1P1_Fragment_[]
184.0734
C5H12N_Fragment_[]
86.0969
Relative Intensity
m/z

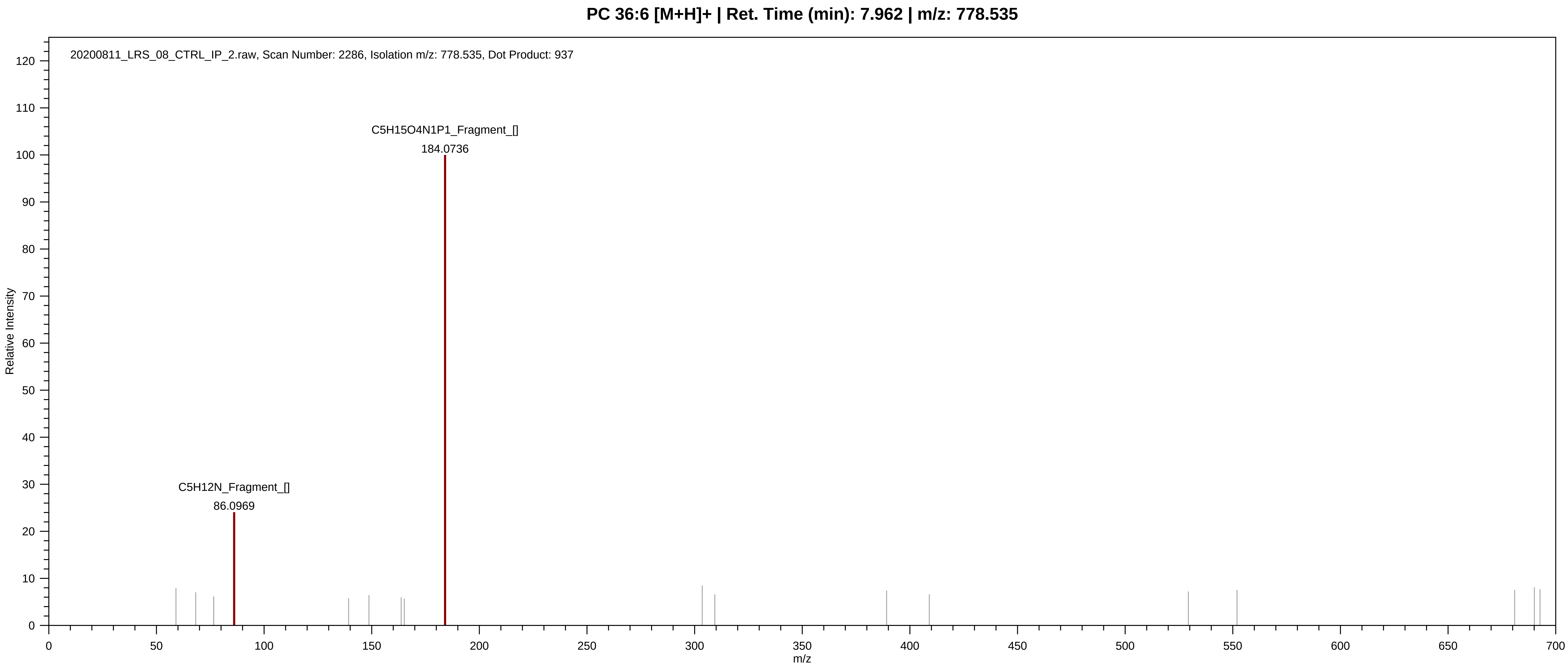
PC 36:6 [M+H]+ | Ret. Time (min): 7.962 | m/z: 778.535
20200811_LRS_08_CTRL_IP_2.raw, Scan Number: 2286, Isolation m/z: 778.535, Dot Product: 937
C5H15O4N1P1_Fragment_[]
184.0736
C5H12N_Fragment_[]
86.0969
Relative Intensity
m/z
0
10
20
30
40
50
60
70
80
90
100
110
120
0
50
100
150
200
250
300
350
400
450
500
550
600
650
700

PC 36:6 [M+H]+ | Ret. Time (min): 7.962 | m/z: 778.537

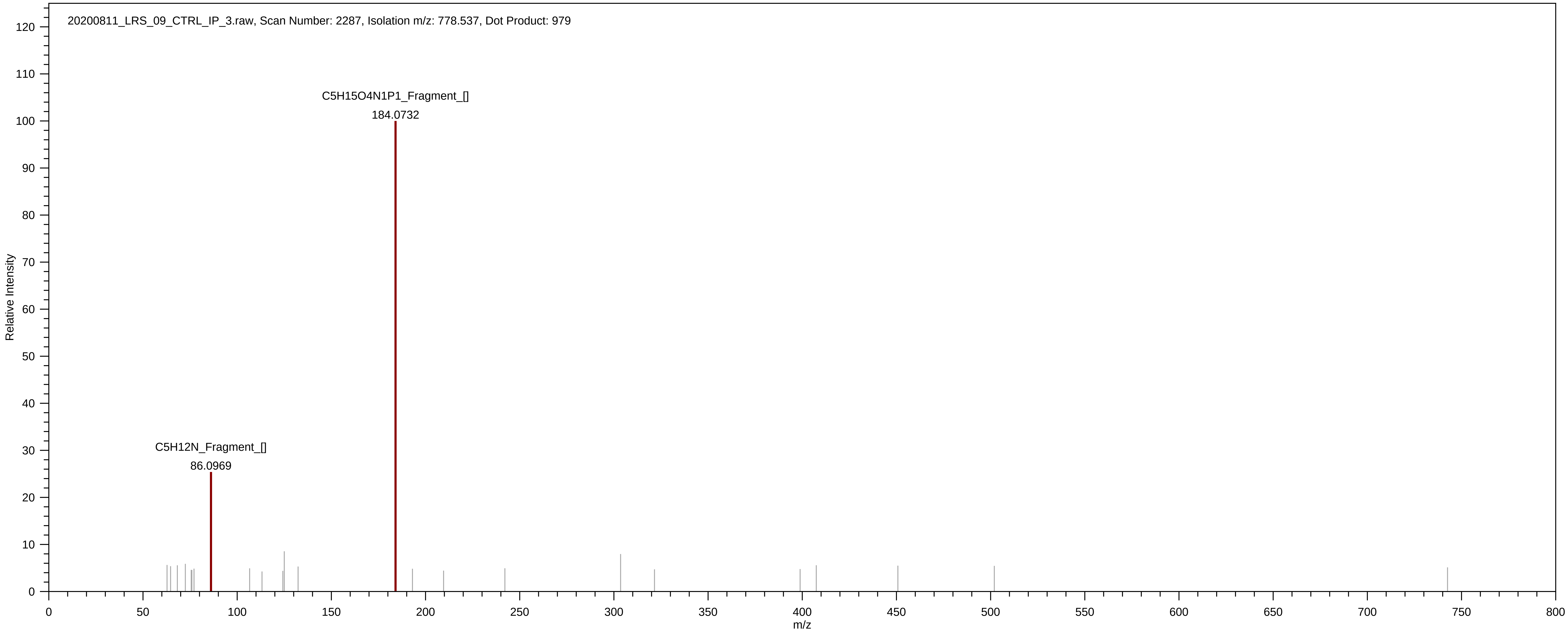

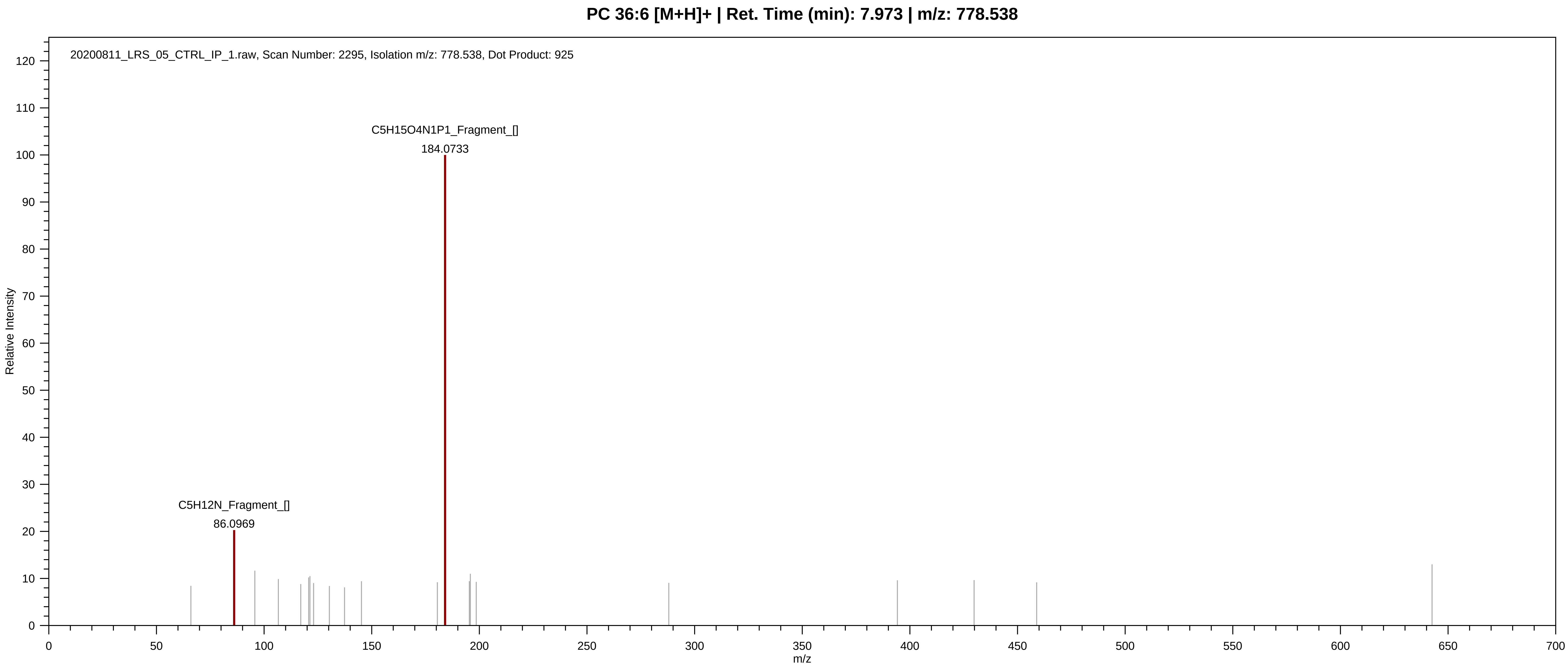
PC 36:6 [M+H]+ | Ret. Time (min): 7.973 | m/z: 778.538
20200811_LRS_05_CTRL_IP_1.raw, Scan Number: 2295, Isolation m/z: 778.538, Dot Product: 925
C5H15O4N1P1_Fragment_[]
184.0733
C5H12N_Fragment_[]
86.0969
Relative Intensity
m/z

PC 37:1 [M+H]+ | Ret. Time (min): 13.338 | m/z: 802.63

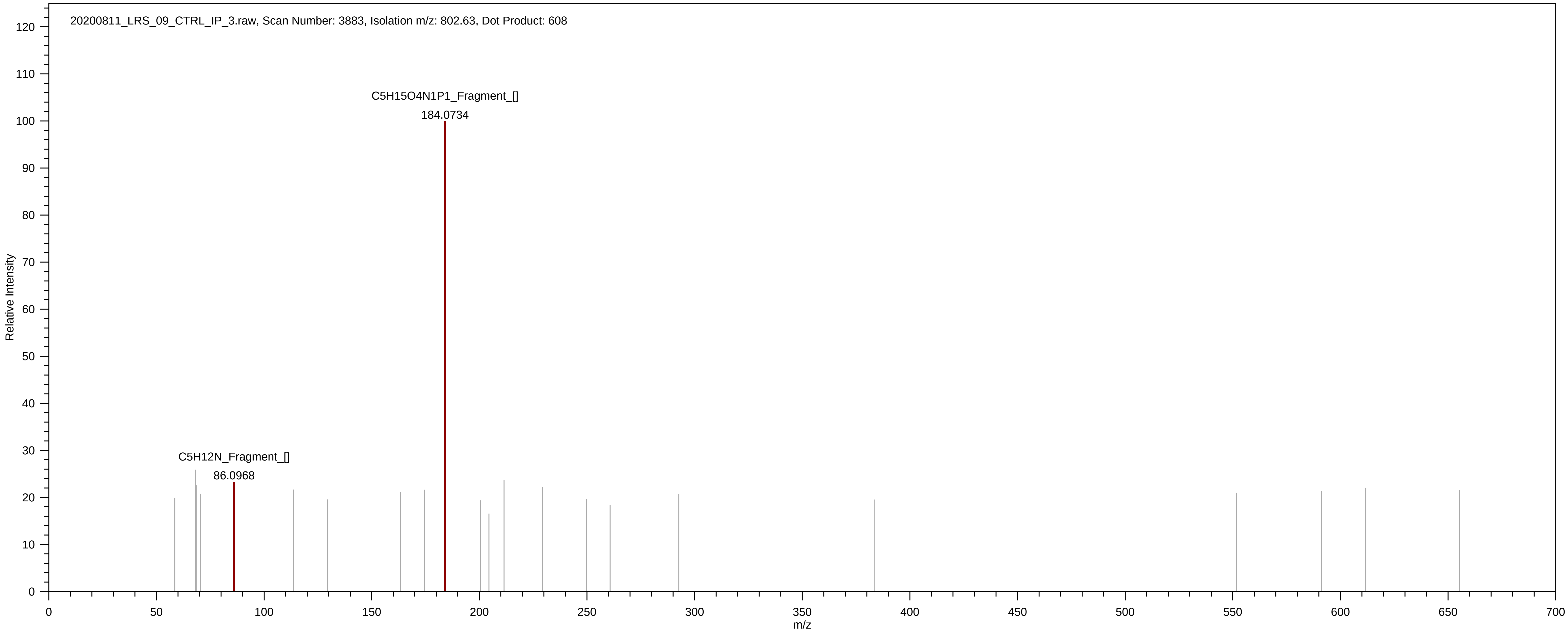

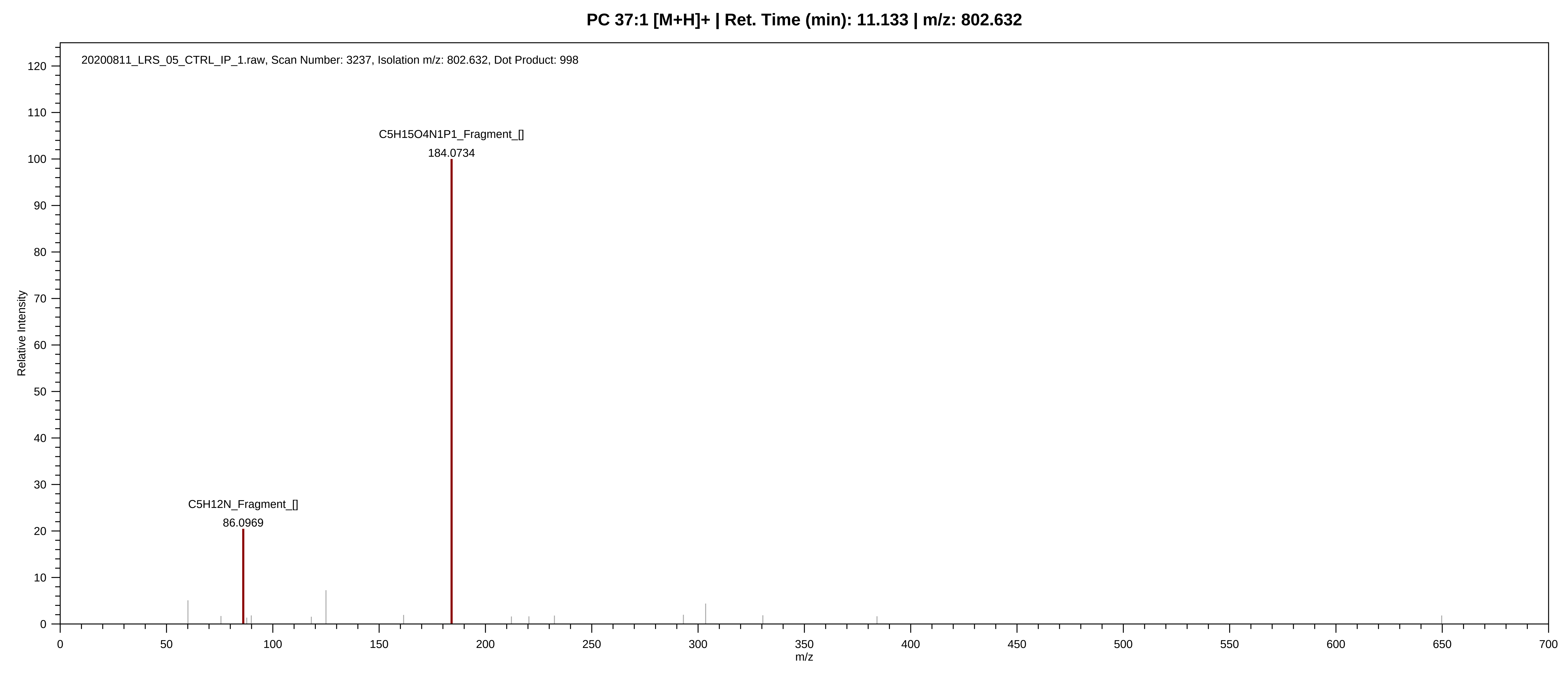
PC 37:1 [M+H]+ | Ret. Time (min): 11.133 | m/z: 802.632
20200811_LRS_05_CTRL_IP_1.raw, Scan Number: 3237, Isolation m/z: 802.632, Dot Product: 998
C5H15O4N1P1_Fragment_[]
184.0734
C5H12N_Fragment_[]
86.0969
Relative Intensity
m/z

PC 37:1 [M+H]+ | Ret. Time (min): 11.341 | m/z: 802.632

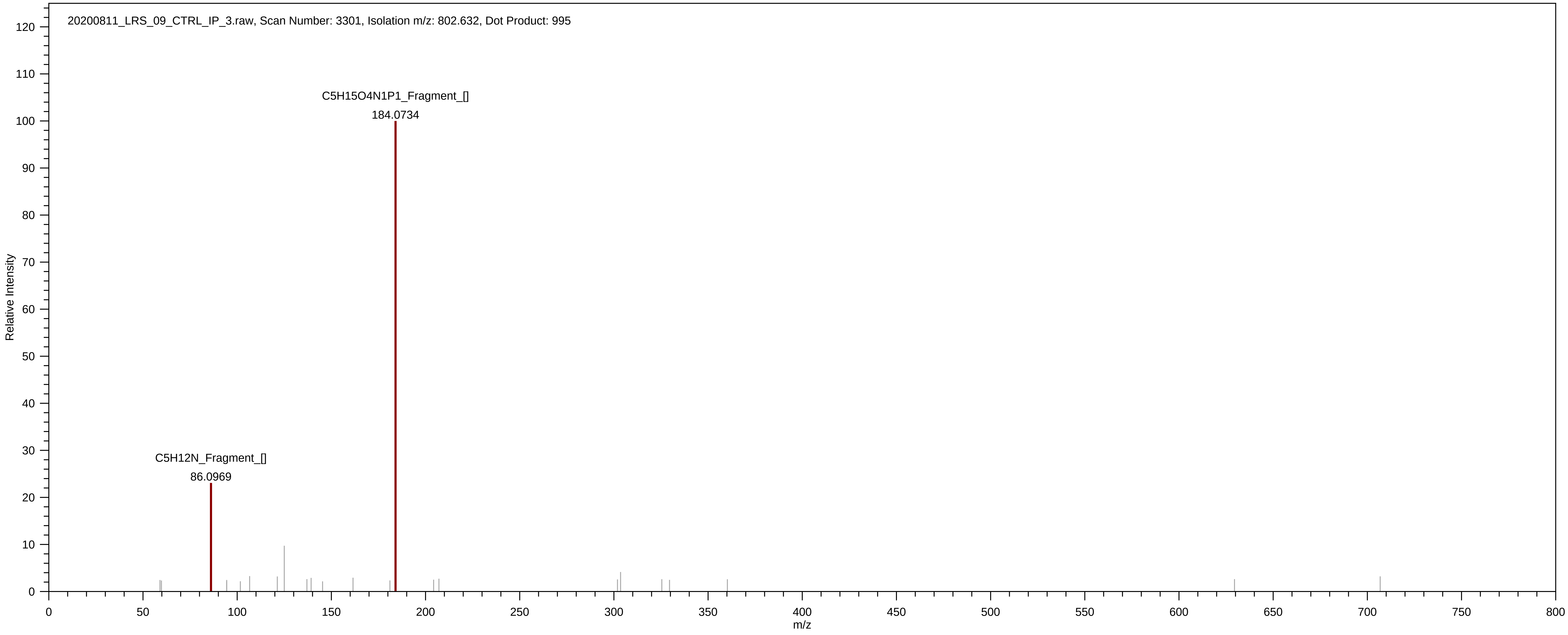

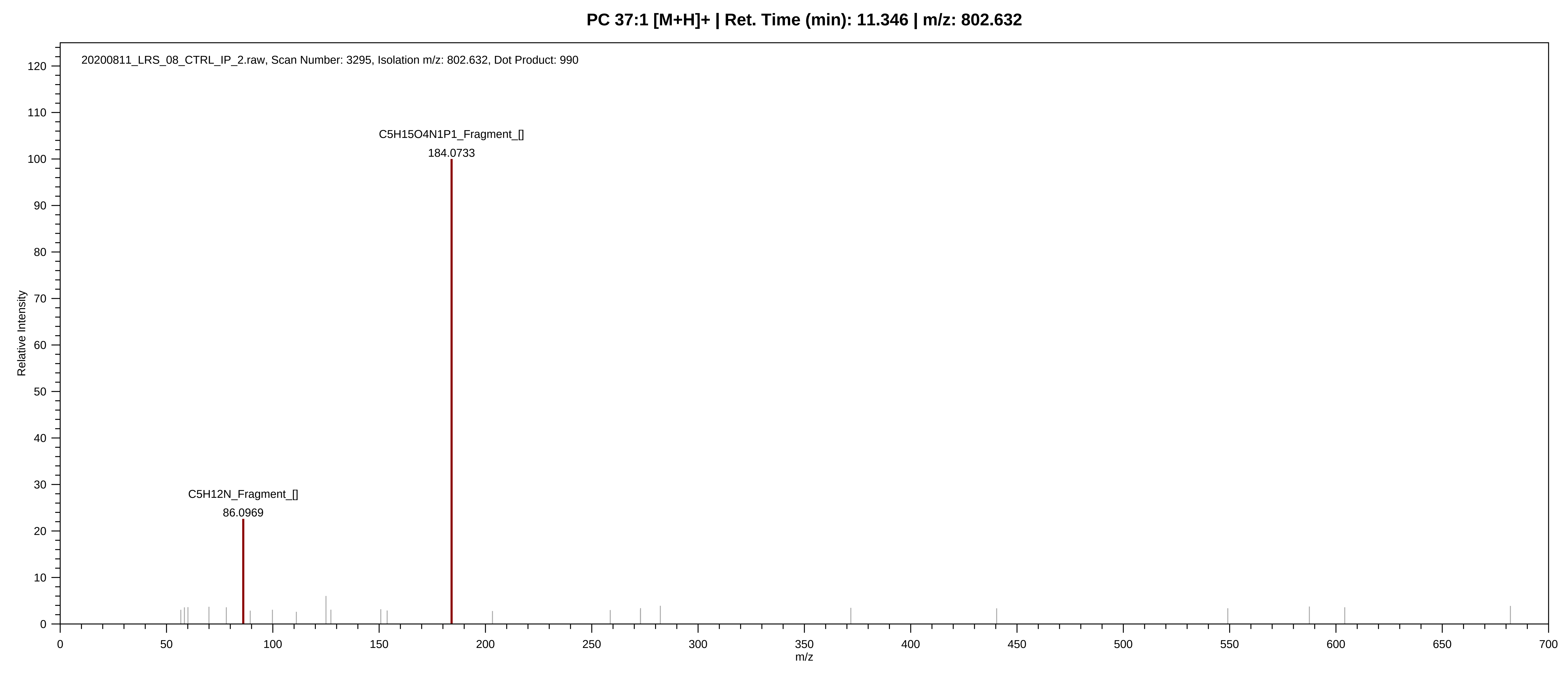

PC 37:1 [M+H]+ | Ret. Time (min): 11.346 | m/z: 802.632
20200811_LRS_08_CTRL_IP_2.raw, Scan Number: 3295, Isolation m/z: 802.632, Dot Product: 990
C5H15O4N1P1_Fragment_[]
184.0733
C5H12N_Fragment_[]
86.0969
Relative Intensity
m/z

PC 37:1 [M+H]+ | Ret. Time (min): 11.138 | m/z: 802.633

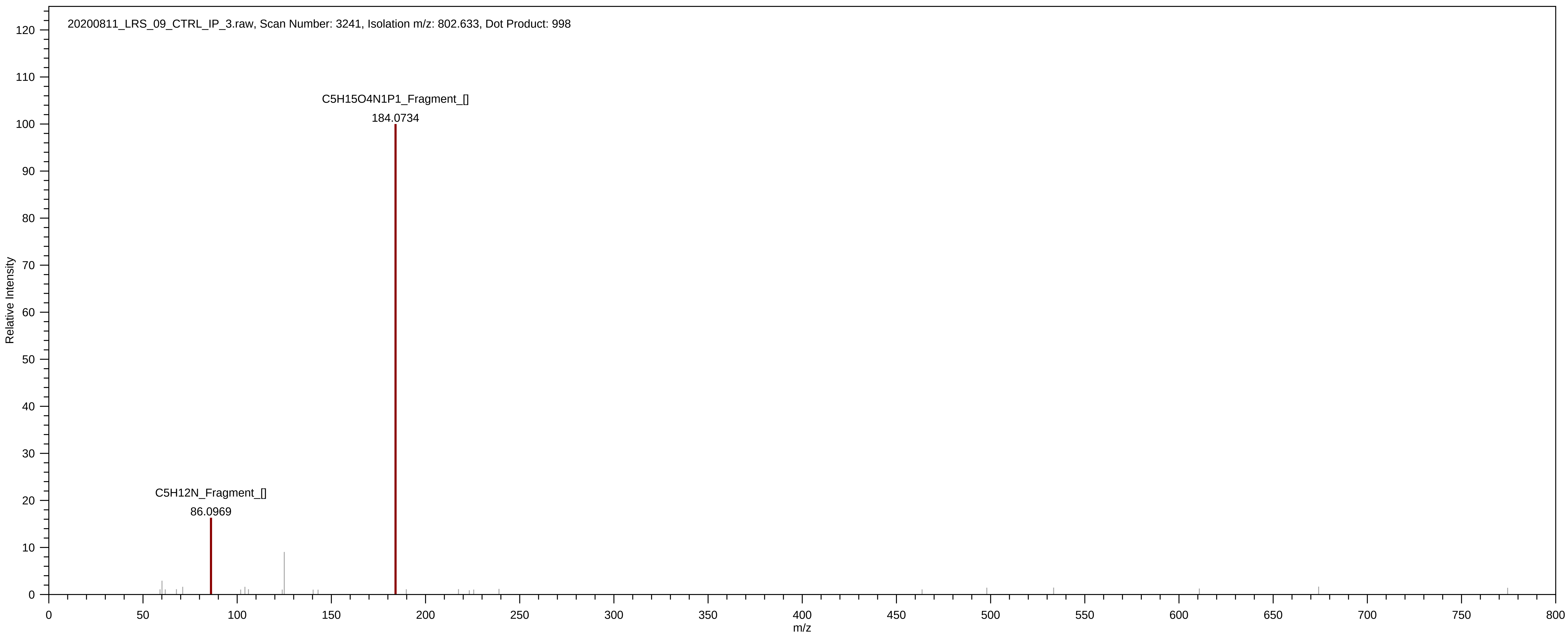

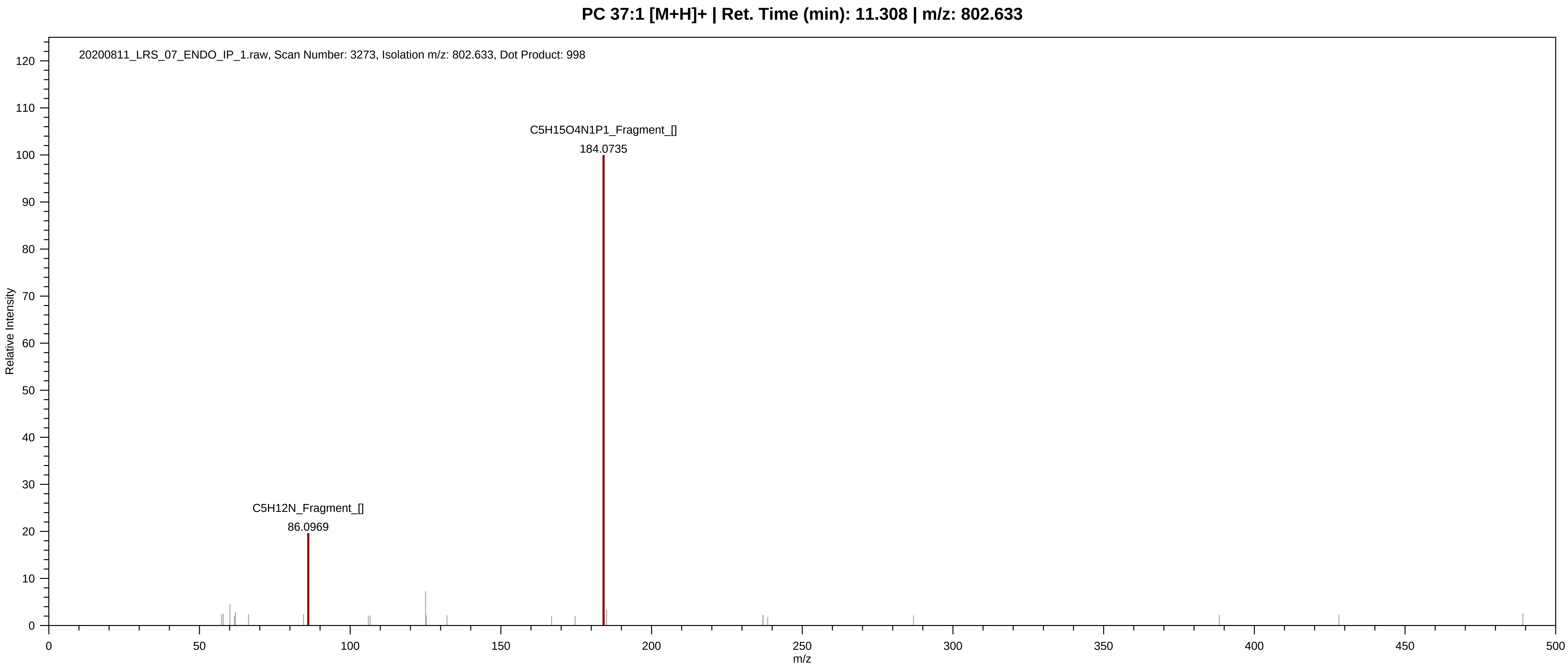
PC 37:1 [M+H]+ | Ret. Time (min): 11.308 | m/z: 802.633
20200811_LRS_07_ENDO_IP_1.raw, Scan Number: 3273, Isolation m/z: 802.633, Dot Product: 998
C5H15O4N1P1_Fragment_[]
184.0735
C5H12N_Fragment_[]
86.0969
Relative Intensity
m/z

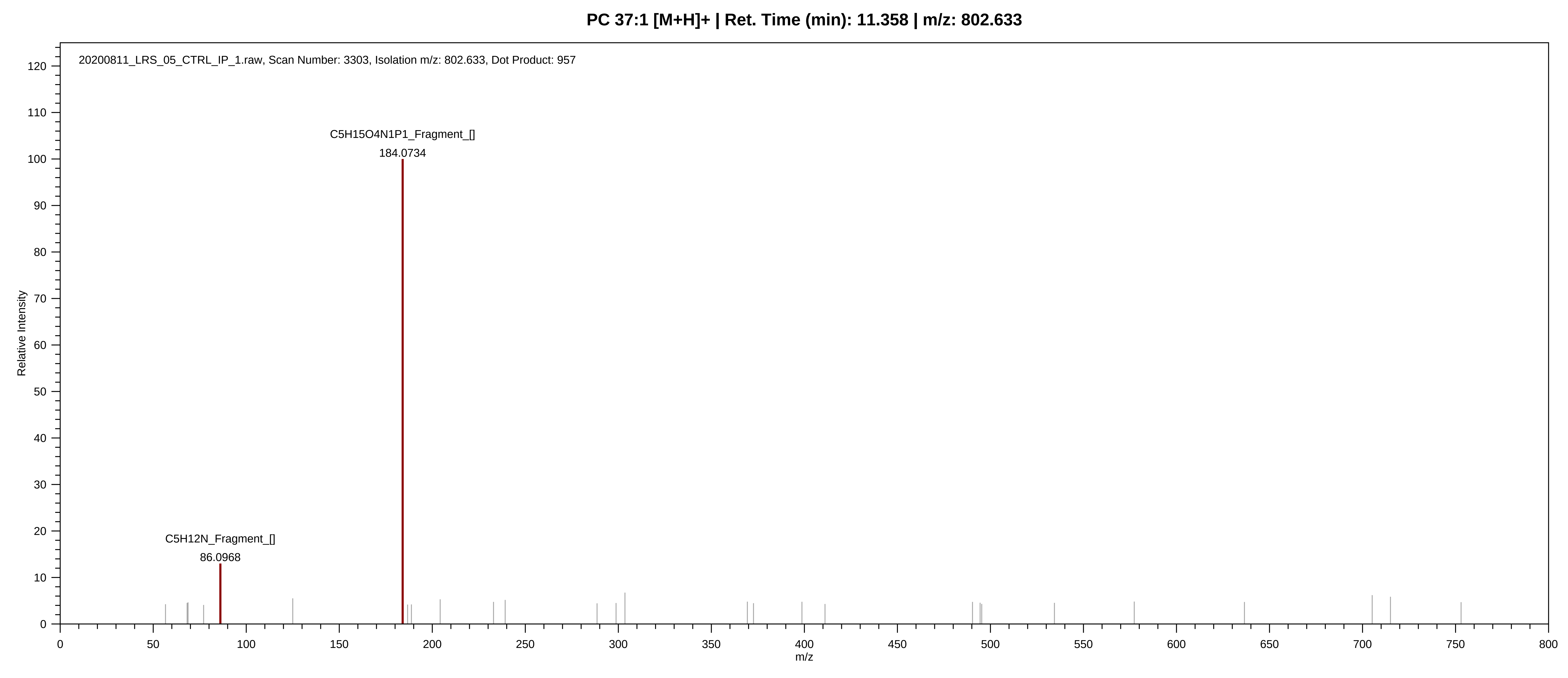
PC 37:1 [M+H]+ | Ret. Time (min): 11.358 | m/z: 802.633
20200811_LRS_05_CTRL_IP_1.raw, Scan Number: 3303, Isolation m/z: 802.633, Dot Product: 957
C5H15O4N1P1_Fragment_[]
184.0734
C5H12N_Fragment_[]
86.0968
Relative Intensity
m/z

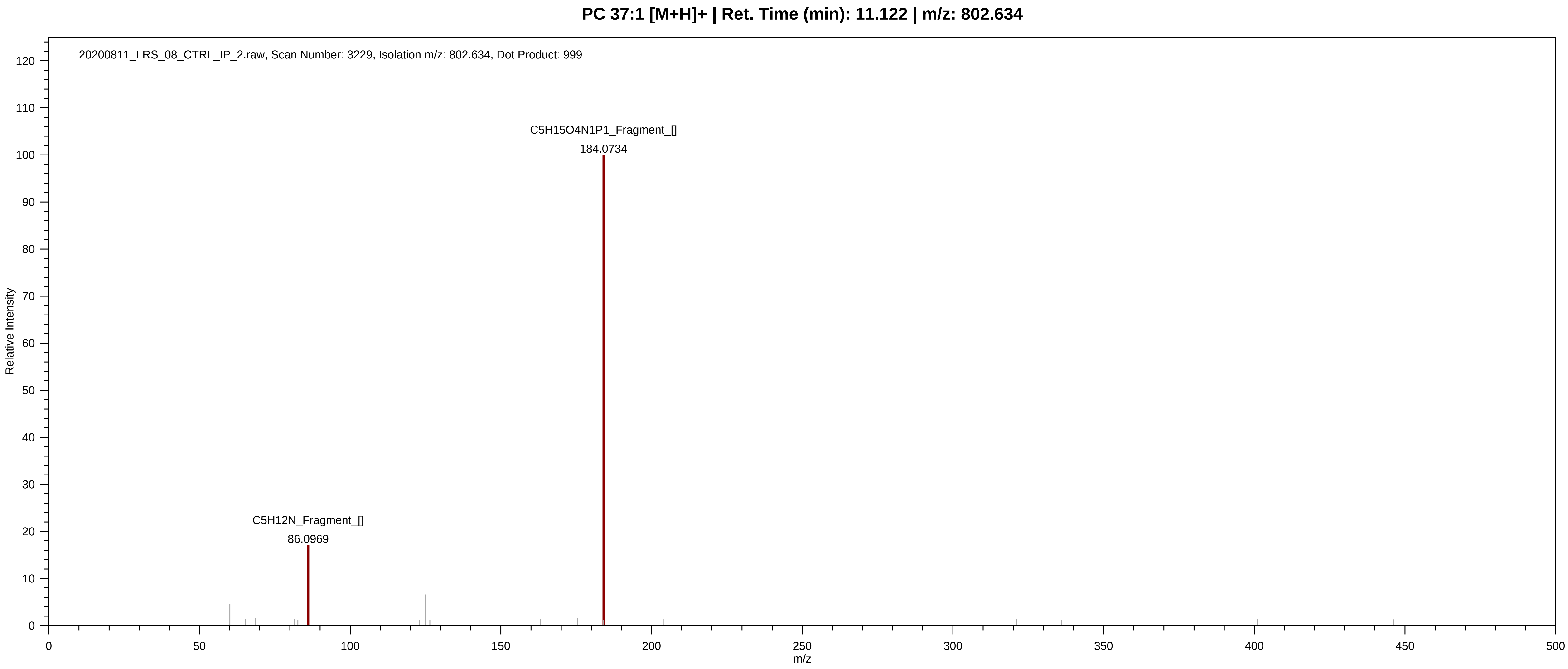

PC 37:1 [M+H]+ | Ret. Time (min): 11.122 | m/z: 802.634
20200811_LRS_08_CTRL_IP_2.raw, Scan Number: 3229, Isolation m/z: 802.634, Dot Product: 999
C5H15O4N1P1_Fragment_[]
184.0734
C5H12N_Fragment_[]
86.0969
Relative Intensity
m/z

PC 37:1 [M+H]+ | Ret. Time (min): 11.307 | m/z: 802.634

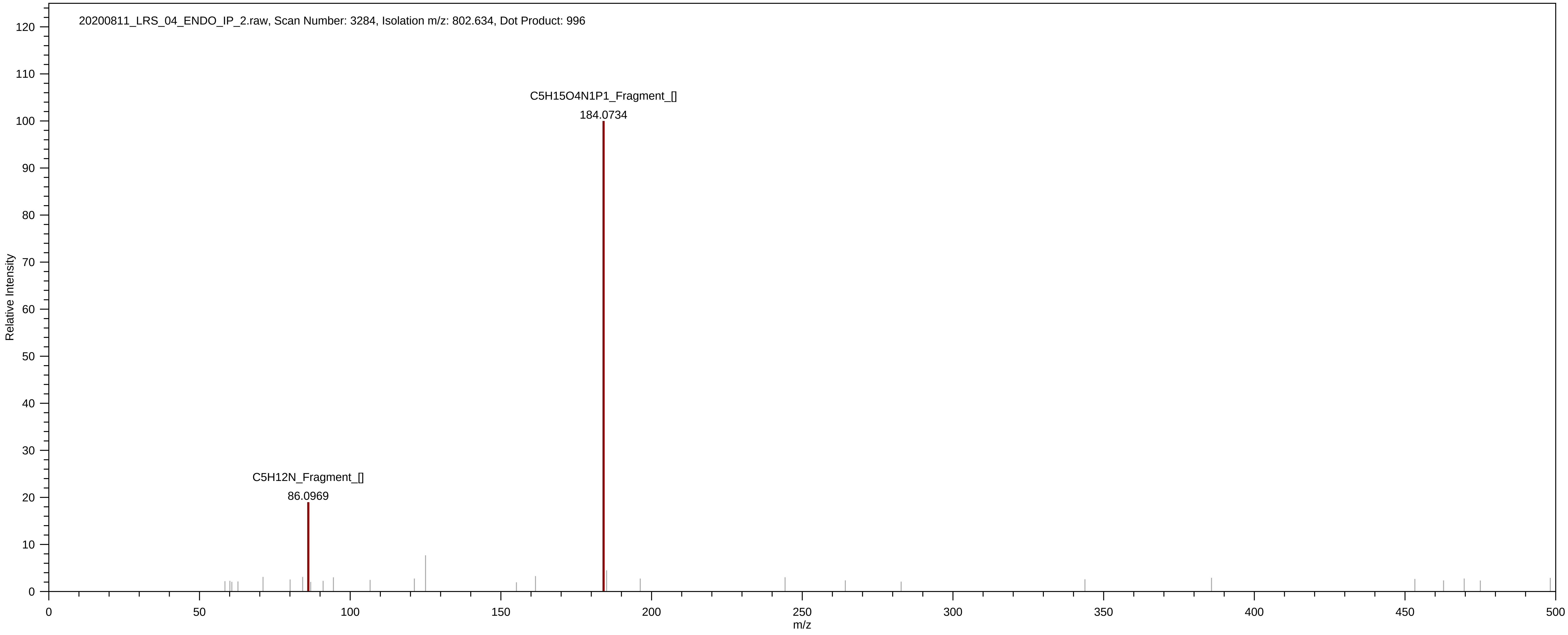

PC 37:2 [M+H]+ | Ret. Time (min): 10.355 | m/z: 800.62

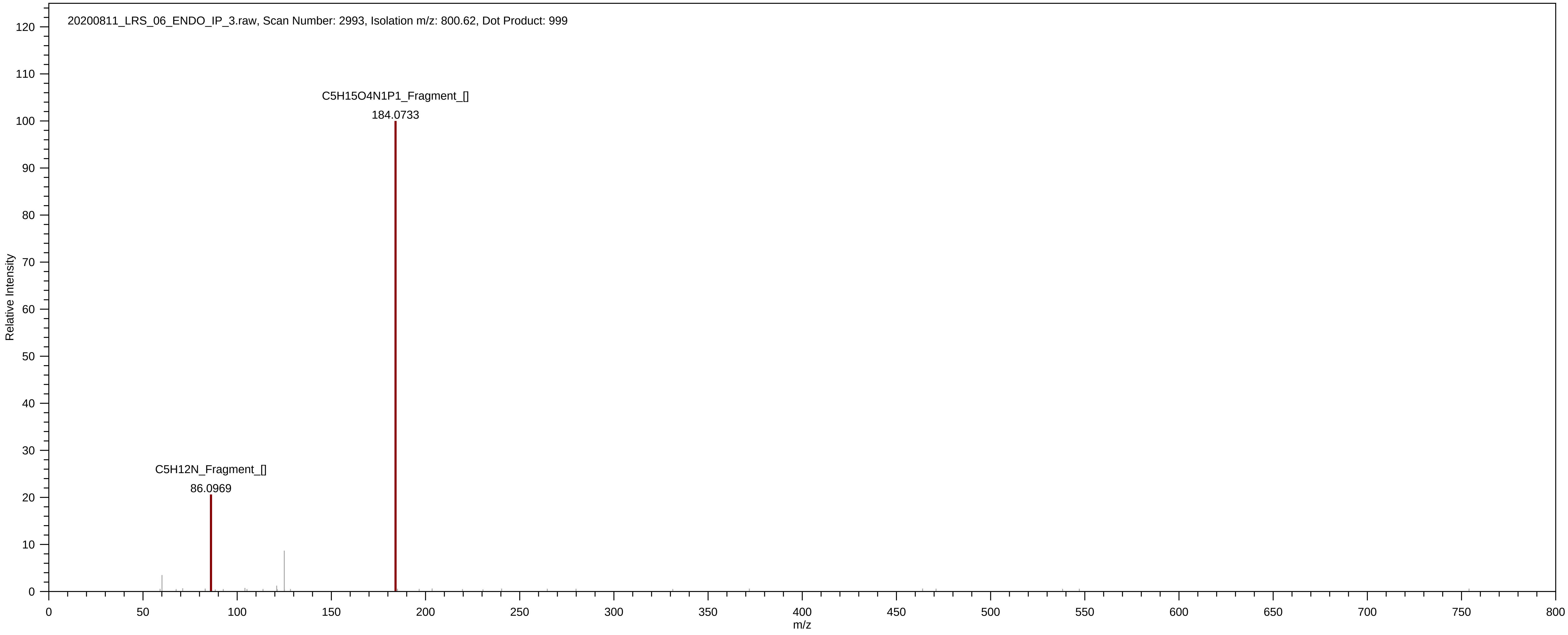

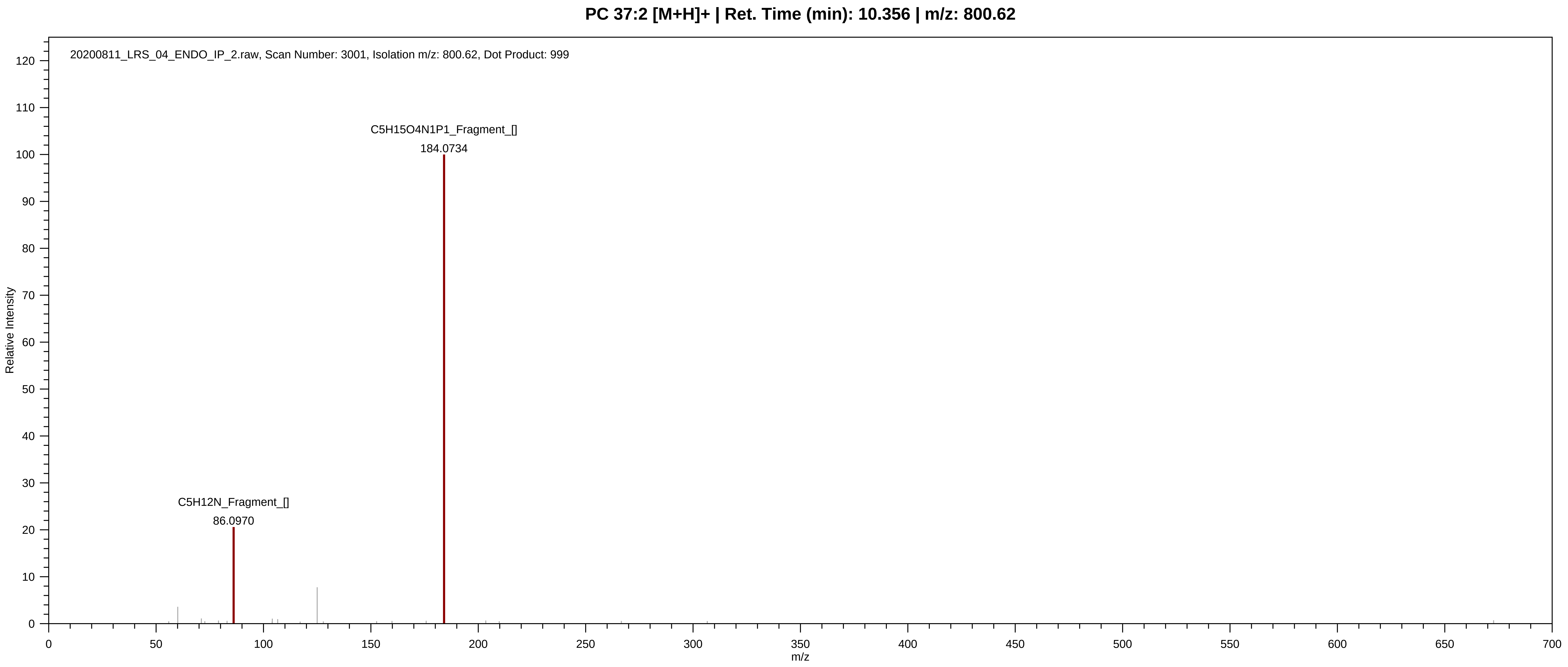
PC 37:2 [M+H]+ | Ret. Time (min): 10.356 | m/z: 800.62
20200811_LRS_04_ENDO_IP_2.raw, Scan Number: 3001, Isolation m/z: 800.62, Dot Product: 999
C5H15O4N1P1_Fragment_[]
184.0734
C5H12N_Fragment_[]
86.0970
Relative Intensity
m/z
0
10
20
30
40
50
60
70
80
90
100
110
120
0
50
100
150
200
250
300
350
400
450
500
550
600
650
700

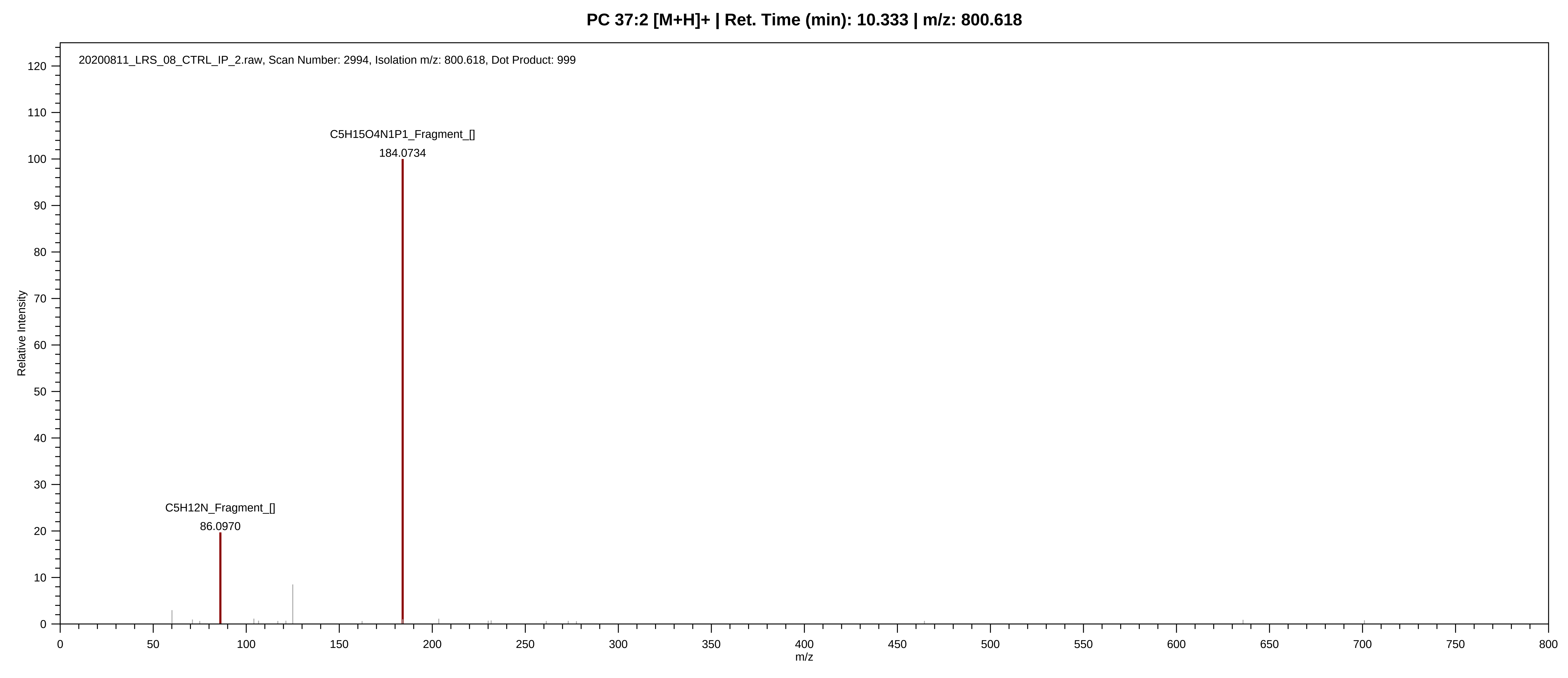
PC 37:2 [M+H]+ | Ret. Time (min): 10.333 | m/z: 800.618
20200811_LRS_08_CTRL_IP_2.raw, Scan Number: 2994, Isolation m/z: 800.618, Dot Product: 999
C5H15O4N1P1_Fragment_[]
184.0734
C5H12N_Fragment_[]
86.0970
Relative Intensity
m/z

PC 37:2 [M+H]+ | Ret. Time (min): 10.336 | m/z: 800.618

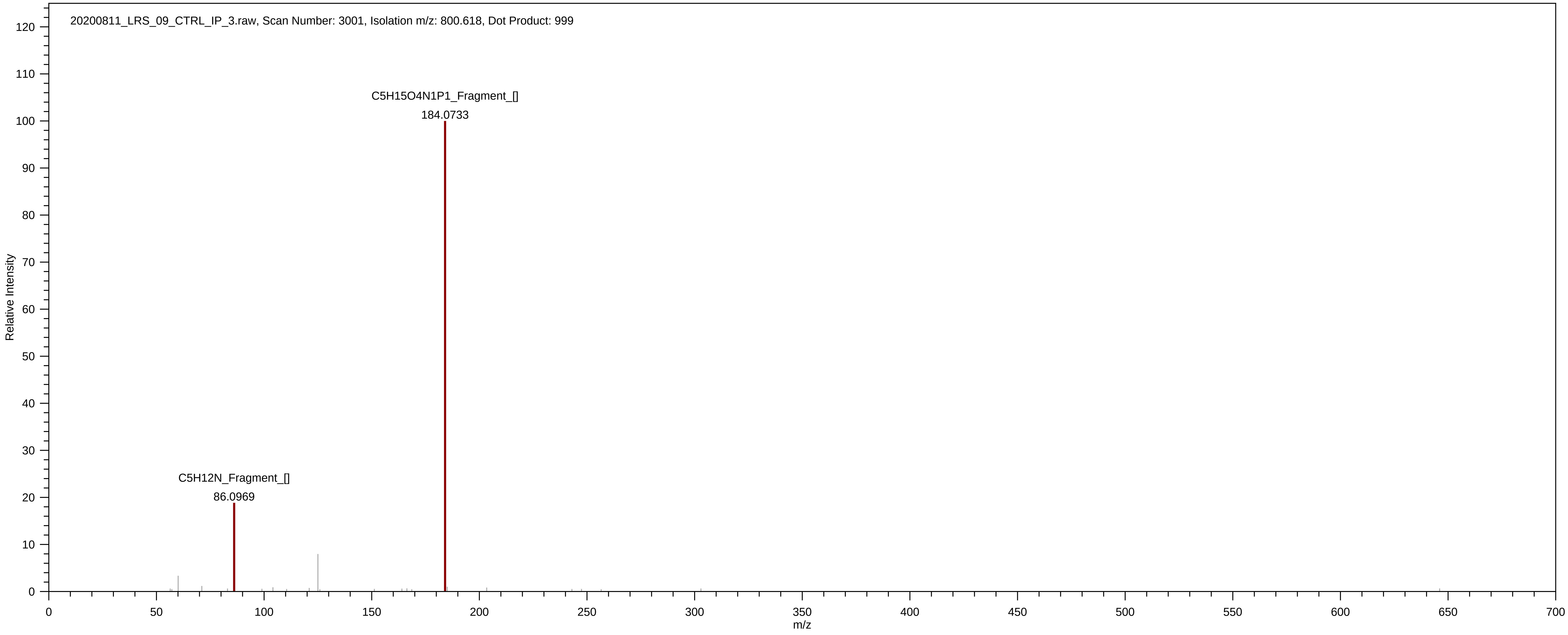

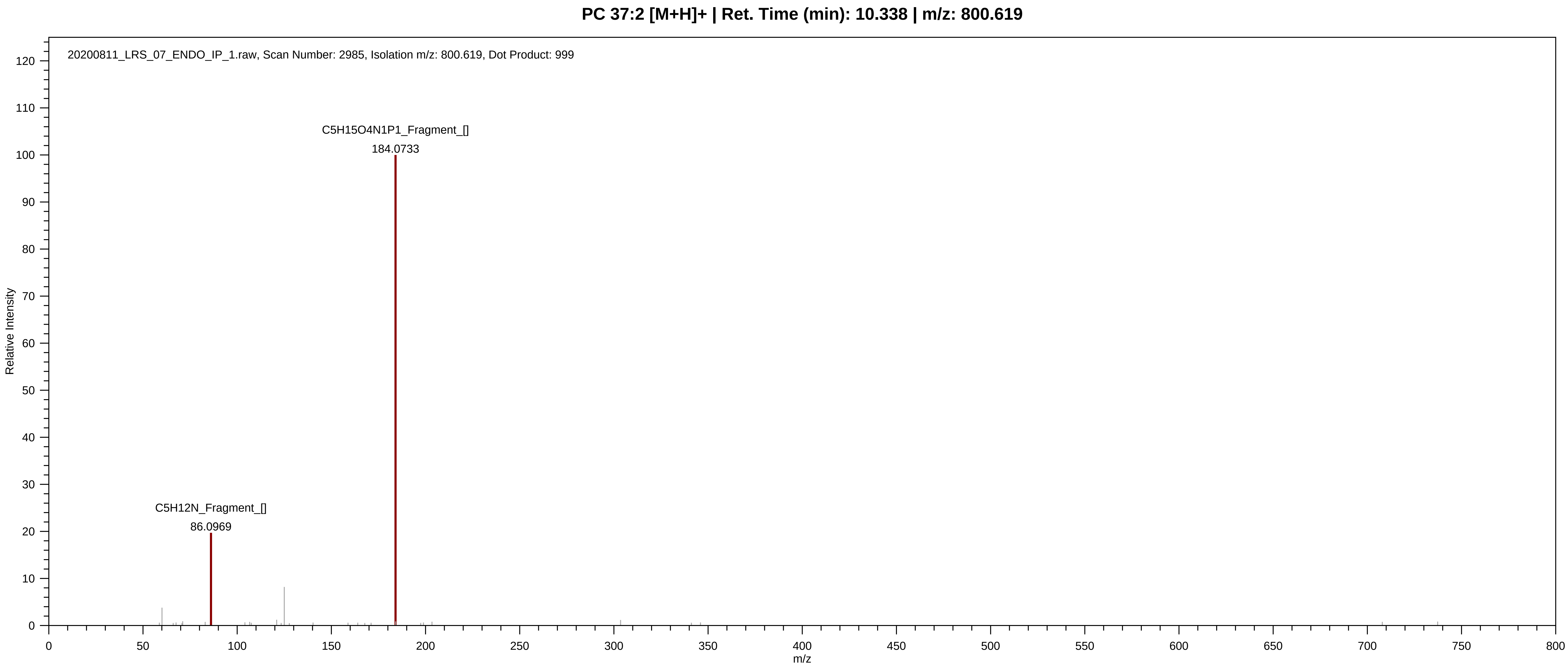
PC 37:2 [M+H]+ | Ret. Time (min): 10.338 | m/z: 800.619
20200811_LRS_07_ENDO_IP_1.raw, Scan Number: 2985, Isolation m/z: 800.619, Dot Product: 999
C5H15O4N1P1_Fragment_[]
184.0733
C5H12N_Fragment_[]
86.0969
Relative Intensity
m/z

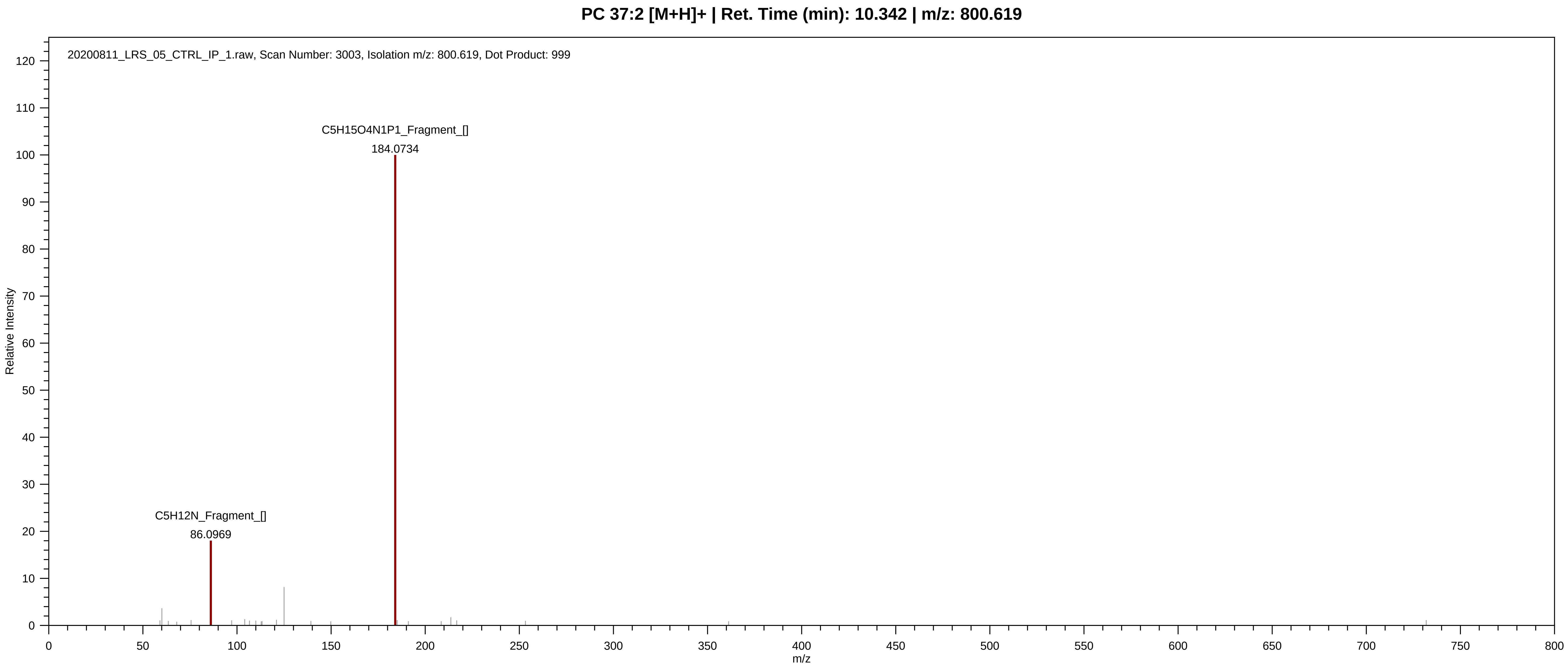
PC 37:2 [M+H]+ | Ret. Time (min): 10.342 | m/z: 800.619
20200811_LRS_05_CTRL_IP_1.raw, Scan Number: 3003, Isolation m/z: 800.619, Dot Product: 999
C5H15O4N1P1_Fragment_[]
184.0734
C5H12N_Fragment_[]
86.0969
Relative Intensity
m/z
0
10
20
30
40
50
60
70
80
90
100
110
120
0
50
100
150
200
250
300
350
400
450
500
550
600
650
700
750
800

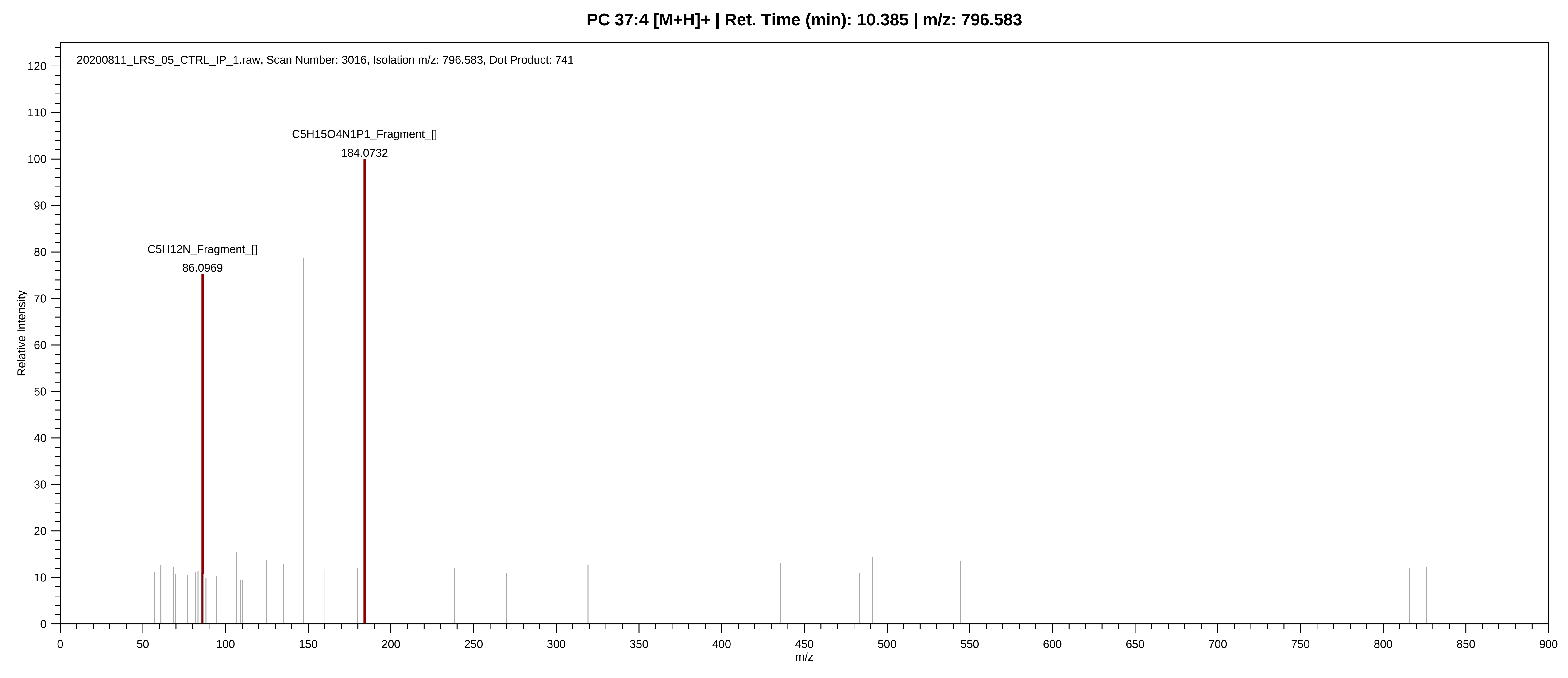

PC 37:4 [M+H]+ | Ret. Time (min): 10.385 | m/z: 796.583
20200811_LRS_05_CTRL_IP_1.raw, Scan Number: 3016, Isolation m/z: 796.583, Dot Product: 741
C5H15O4N1P1_Fragment_[]
184.0732
C5H12N_Fragment_[]
86.0969
Relative Intensity
m/z

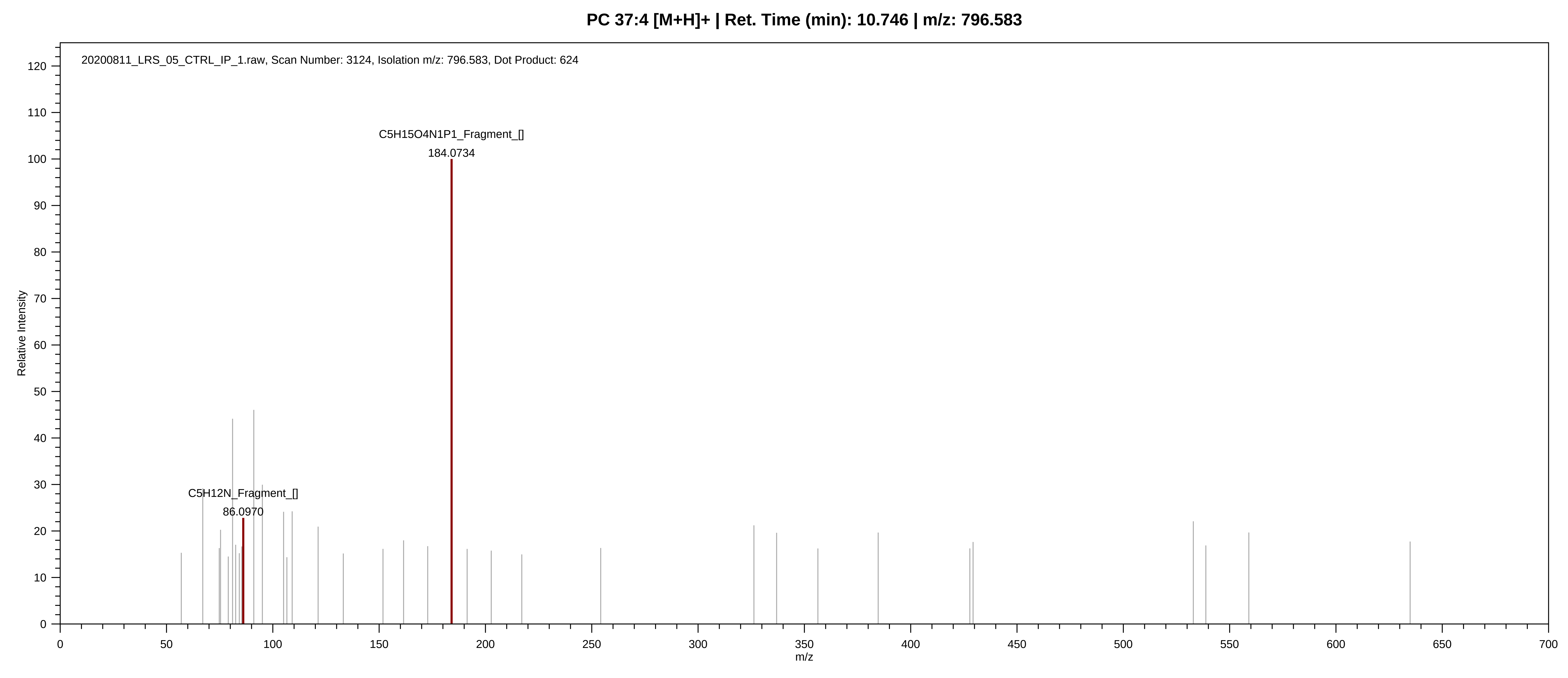
PC 37:4 [M+H]+ | Ret. Time (min): 10.746 | m/z: 796.583
20200811_LRS_05_CTRL_IP_1.raw, Scan Number: 3124, Isolation m/z: 796.583, Dot Product: 624
C5H15O4N1P1_Fragment_[]
184.0734
C5H12N_Fragment_[]
86.0970
Relative Intensity
m/z

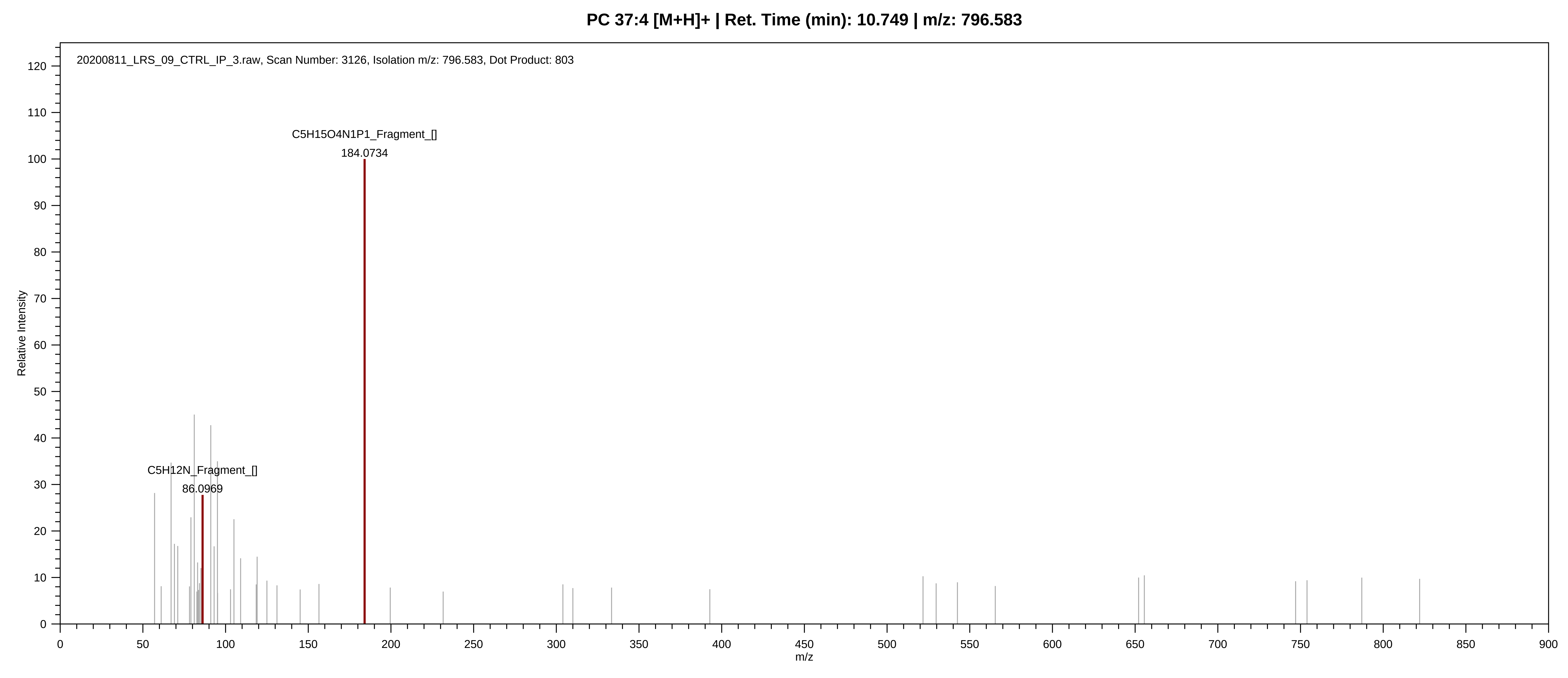
PC 37:4 [M+H]+ | Ret. Time (min): 10.749 | m/z: 796.583
20200811_LRS_09_CTRL_IP_3.raw, Scan Number: 3126, Isolation m/z: 796.583, Dot Product: 803
C5H15O4N1P1_Fragment_[]
184.0734
C5H12N_Fragment_[]
86.0969
Relative Intensity
m/z

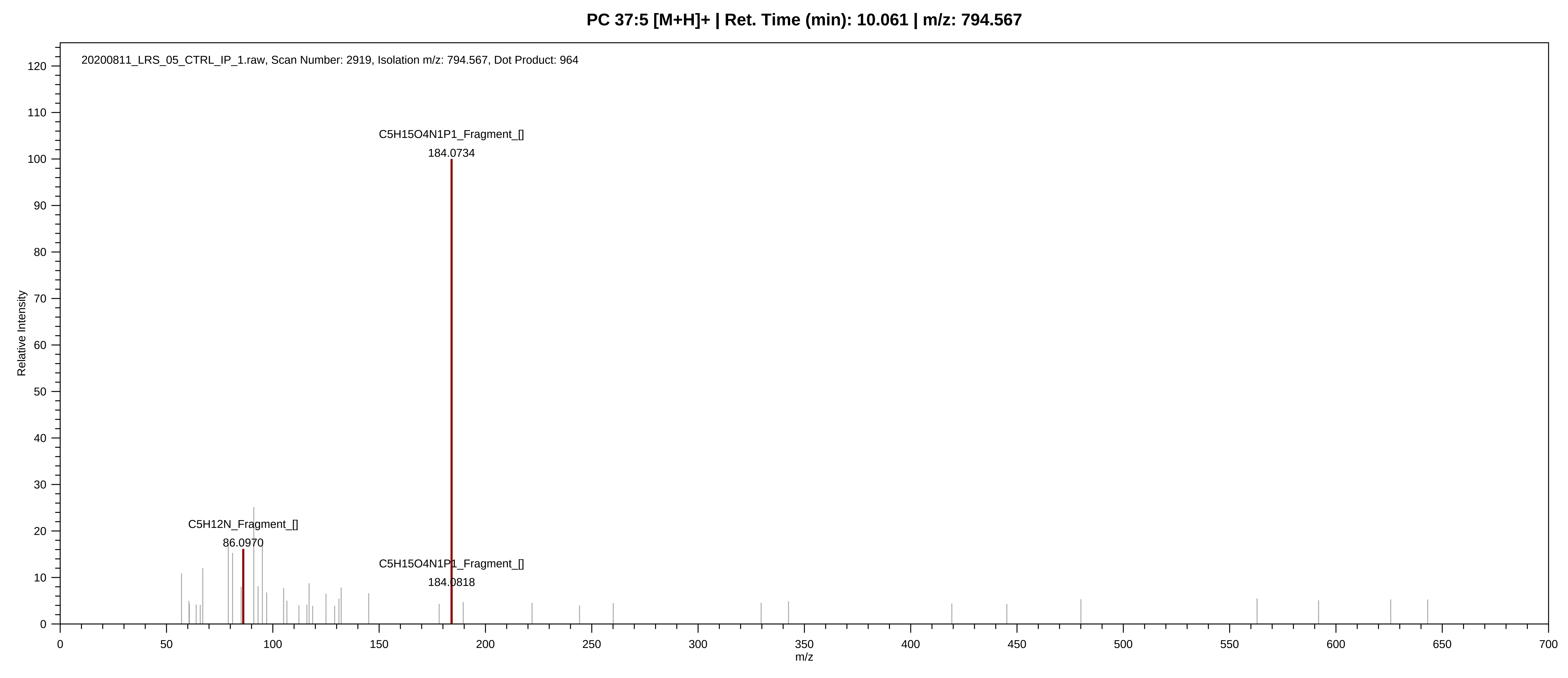
PC 37:5 [M+H]+ | Ret. Time (min): 10.061 | m/z: 794.567
20200811_LRS_05_CTRL_IP_1.raw, Scan Number: 2919, Isolation m/z: 794.567, Dot Product: 964
C5H15O4N1P1_Fragment_[]
184.0734
C5H12N_Fragment_[]
86.0970
C5H15O4N1P1_Fragment_[]
184.0818
Relative Intensity
m/z

PC 37:5 [M+H]+ | Ret. Time (min): 10.096 | m/z: 794.573

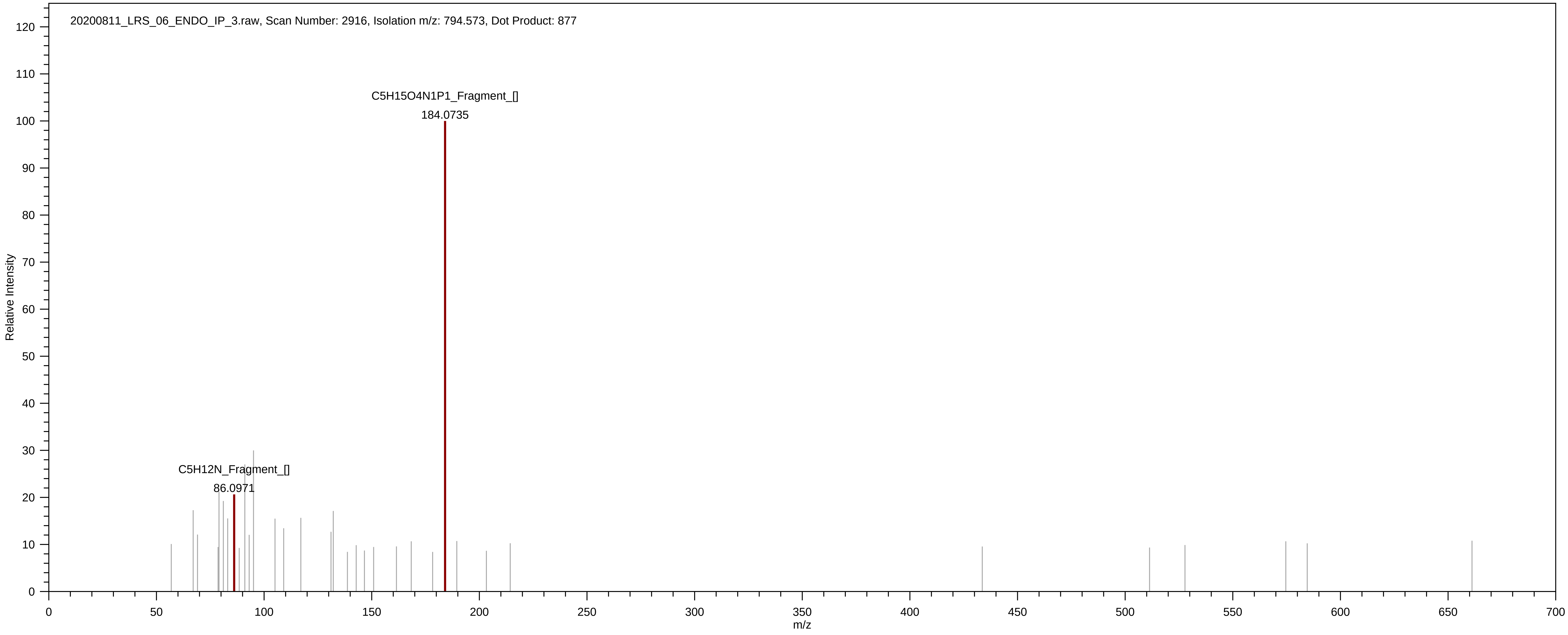

PC 38:0 [M+H]+ | Ret. Time (min): 12.863 | m/z: 818.663

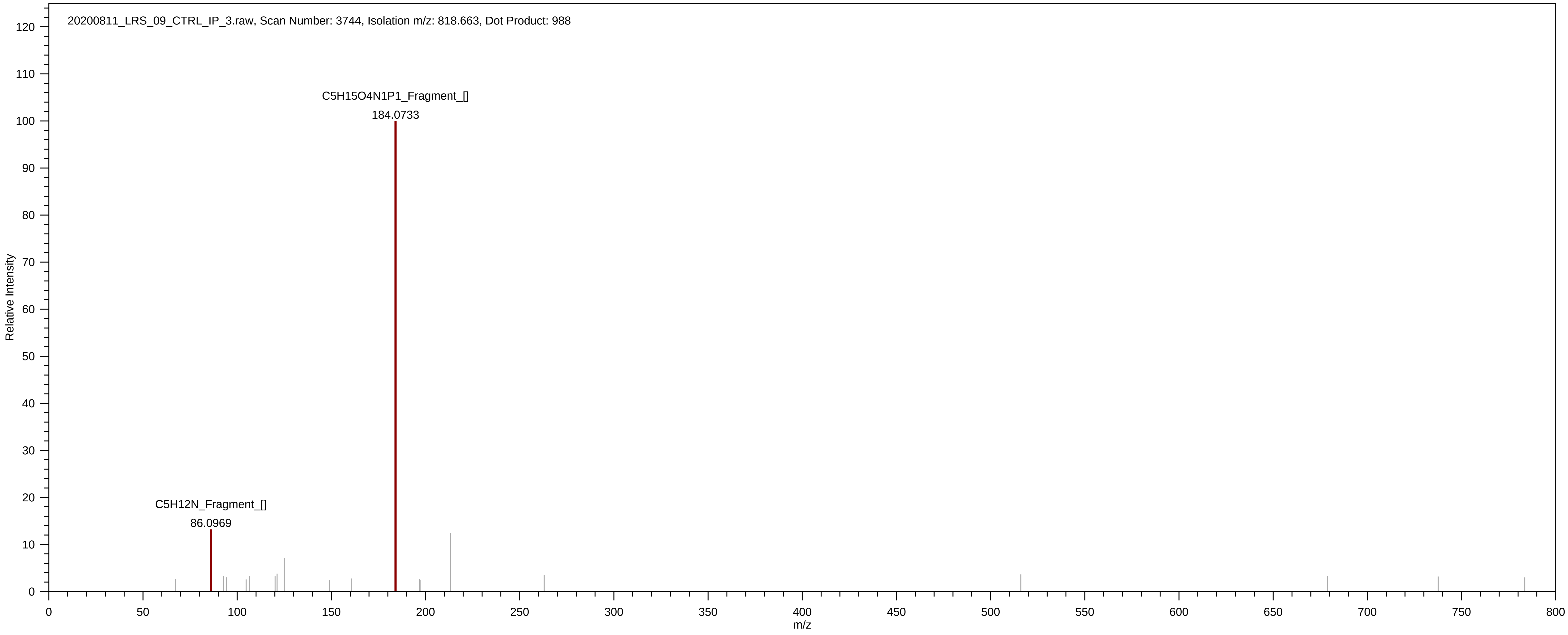

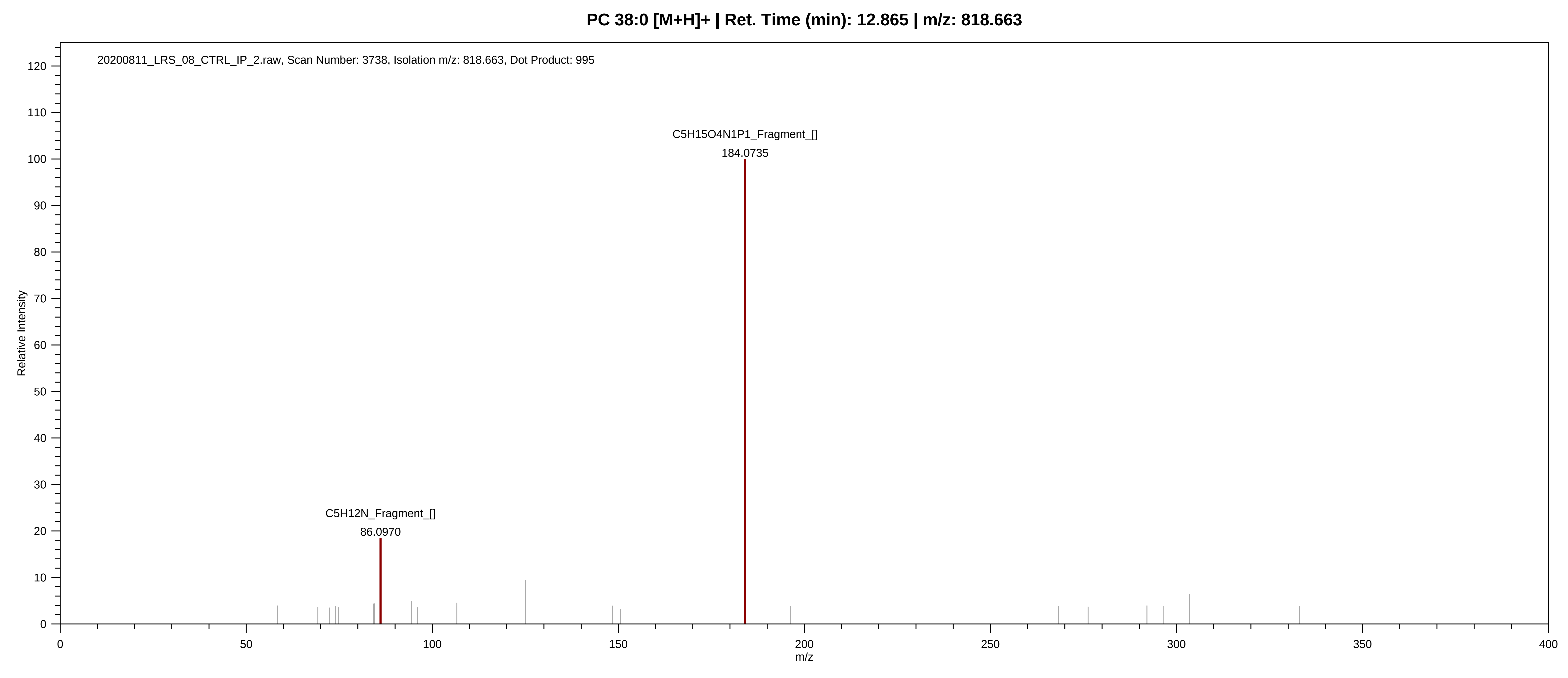

PC 38:0 [M+H]+ | Ret. Time (min): 12.865 | m/z: 818.663
20200811_LRS_08_CTRL_IP_2.raw, Scan Number: 3738, Isolation m/z: 818.663, Dot Product: 995
C5H15O4N1P1_Fragment_[]
184.0735
C5H12N_Fragment_[]
86.0970
Relative Intensity
m/z

PC 38:0 [M+H]+ | Ret. Time (min): 12.86 | m/z: 818.664

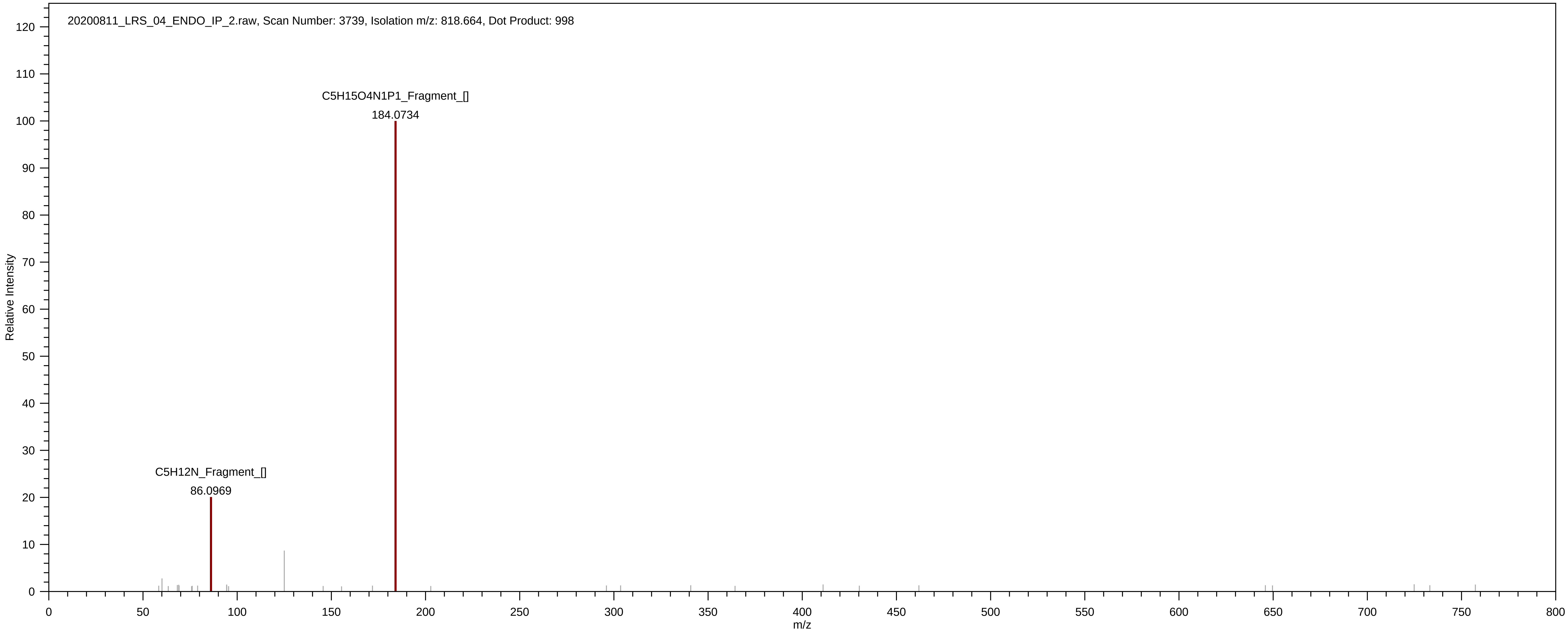

PC 38:0 [M+H]+ | Ret. Time (min): 12.88 | m/z: 818.664

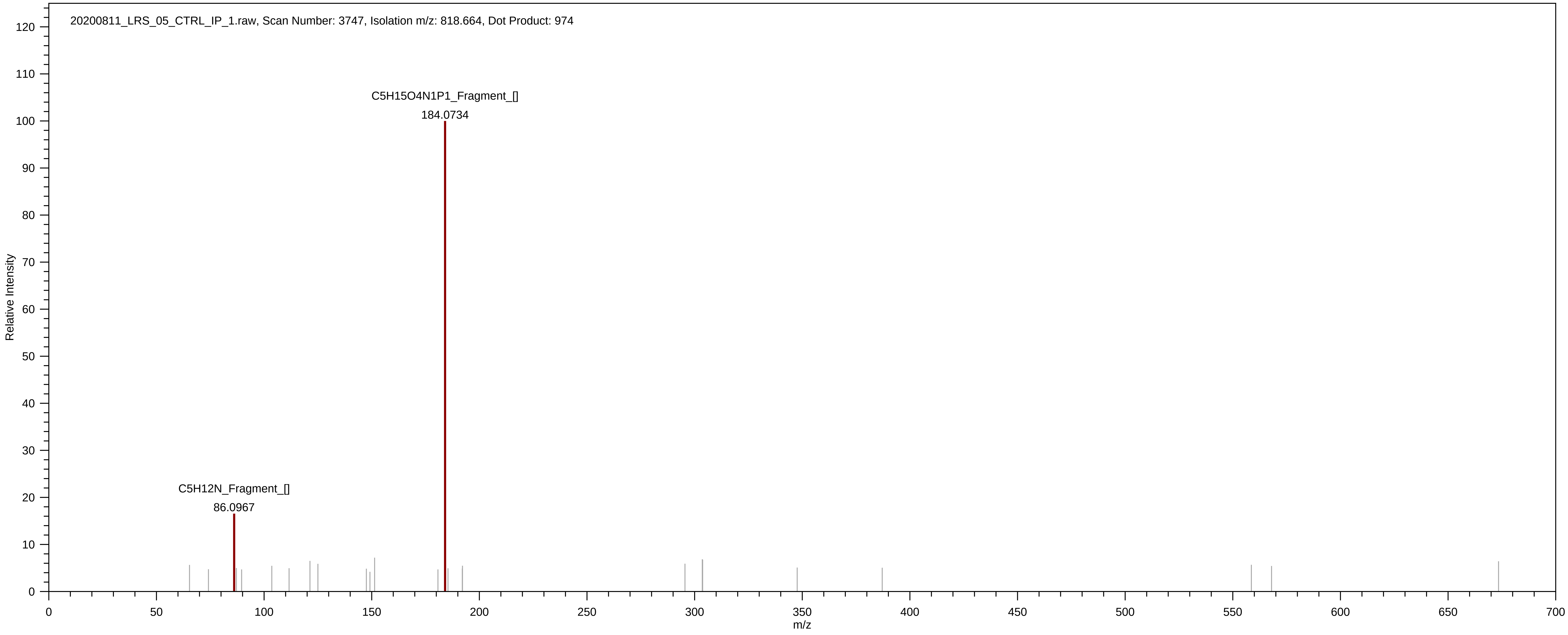

PC 38:0 [M+H]+ | Ret. Time (min): 12.841 | m/z: 818.664

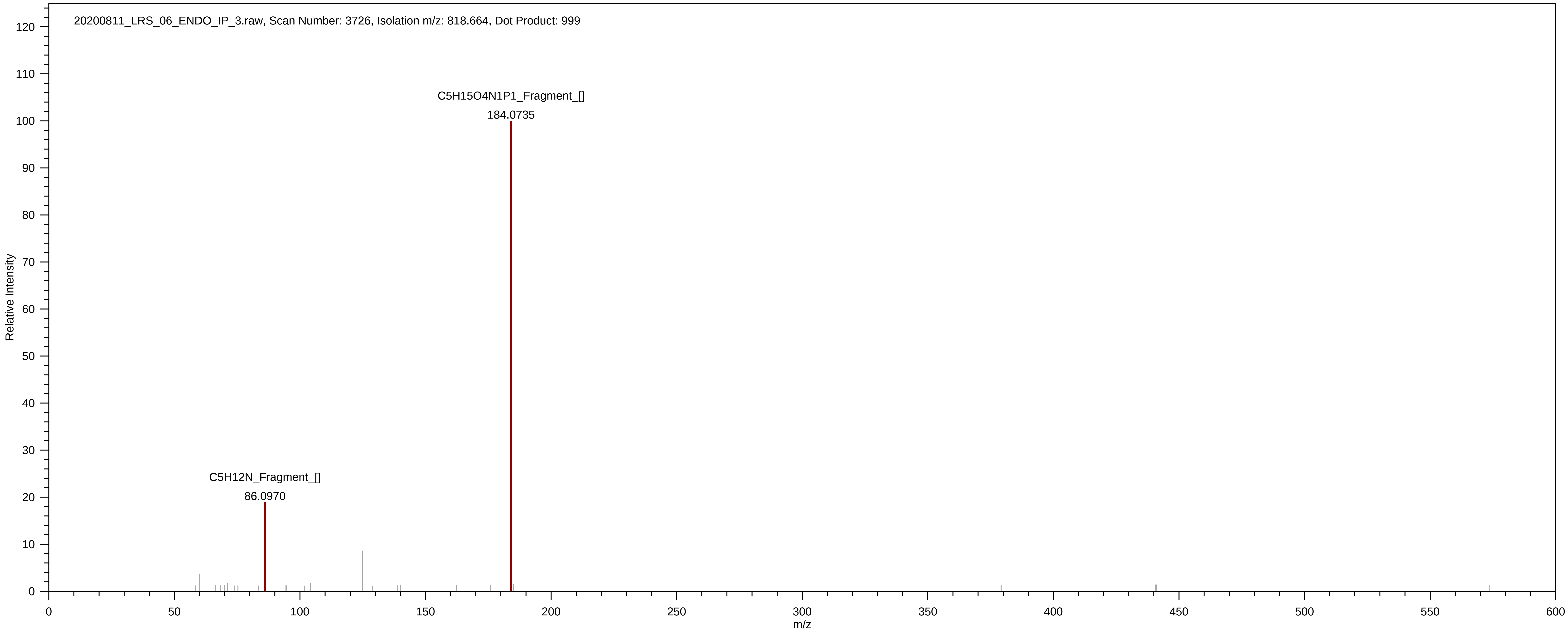

PC 38:0 [M+H]+ | Ret. Time (min): 12.842 | m/z: 818.664

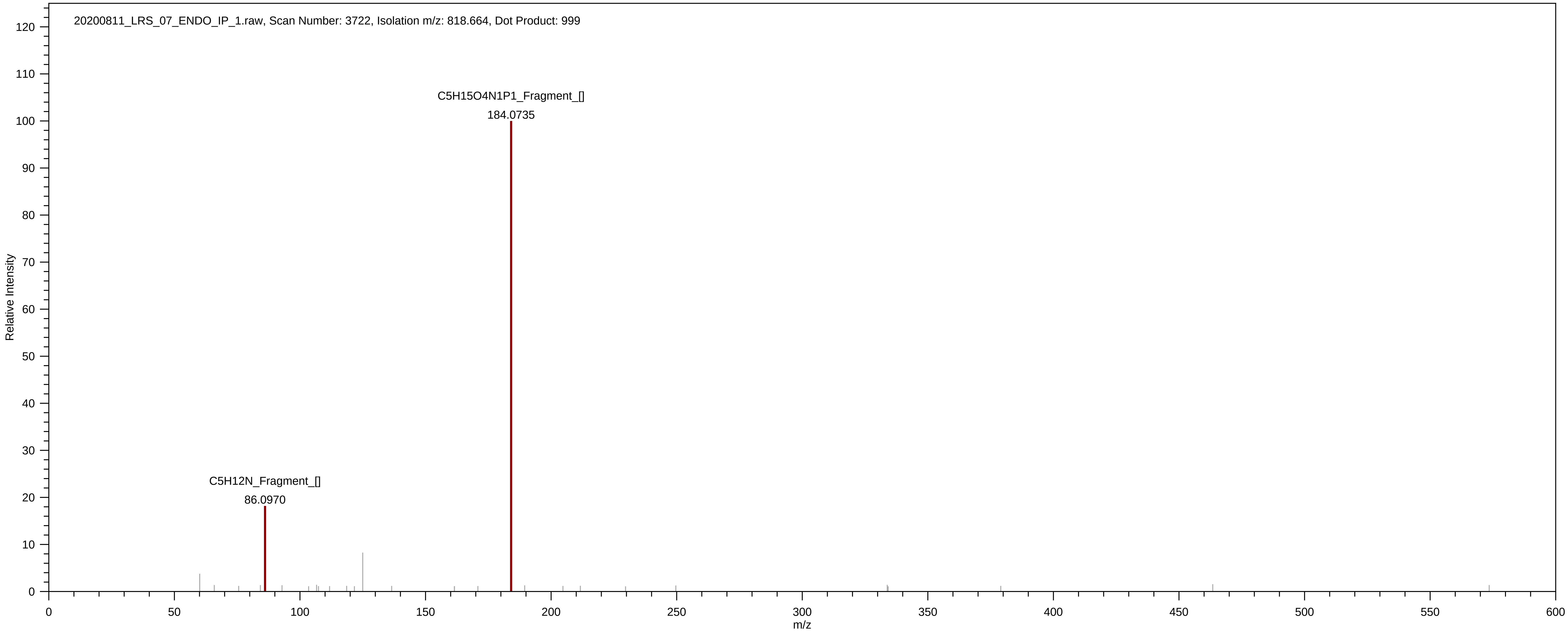

PC 38:1 [M+Ac-H]- | Ret. Time (min): 11.783 | m/z: 874.651

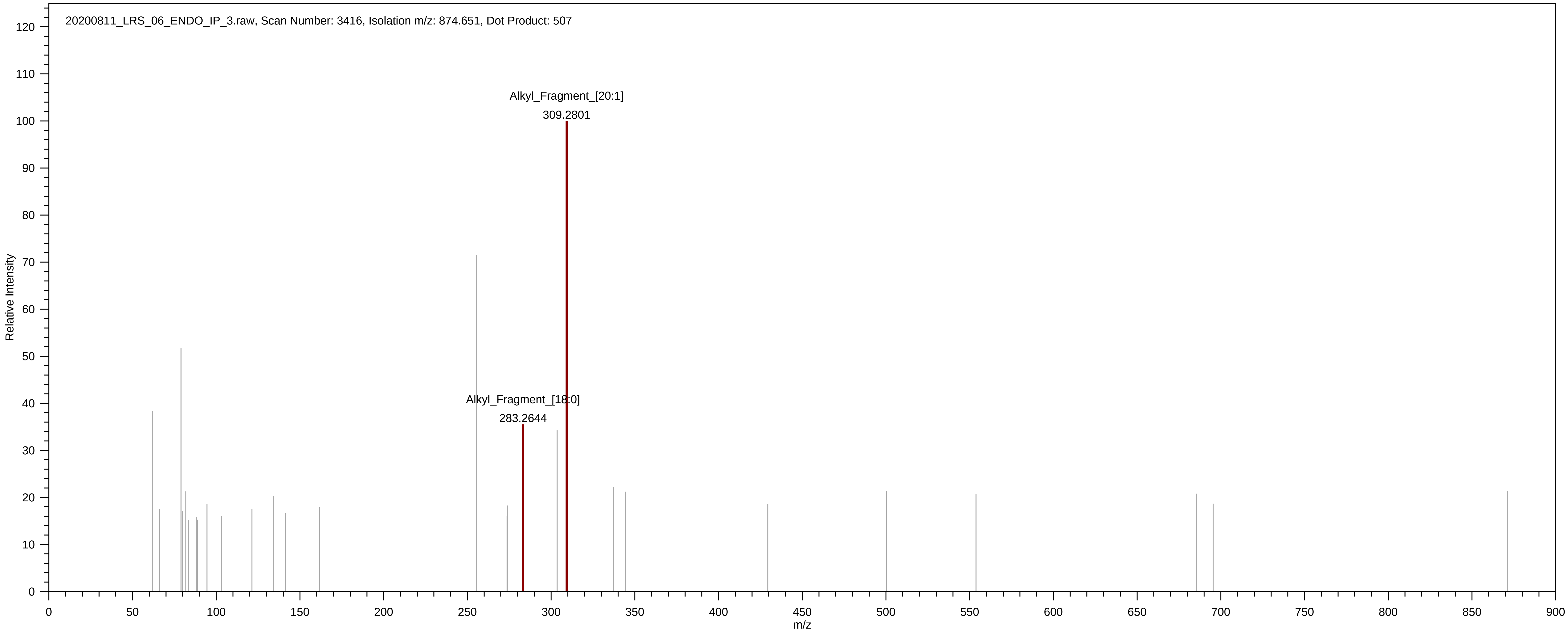

PC 38:1 [M+H]+ | Ret. Time (min): 11.87 | m/z: 816.644

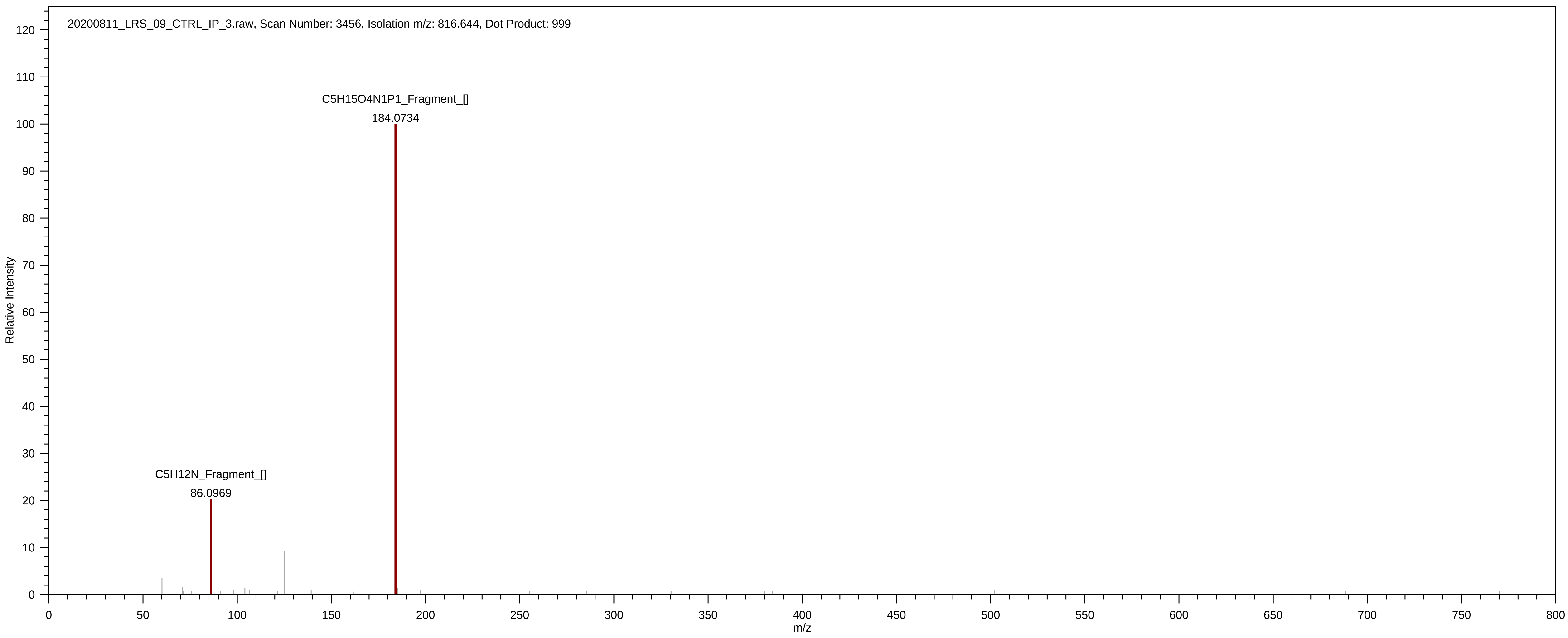

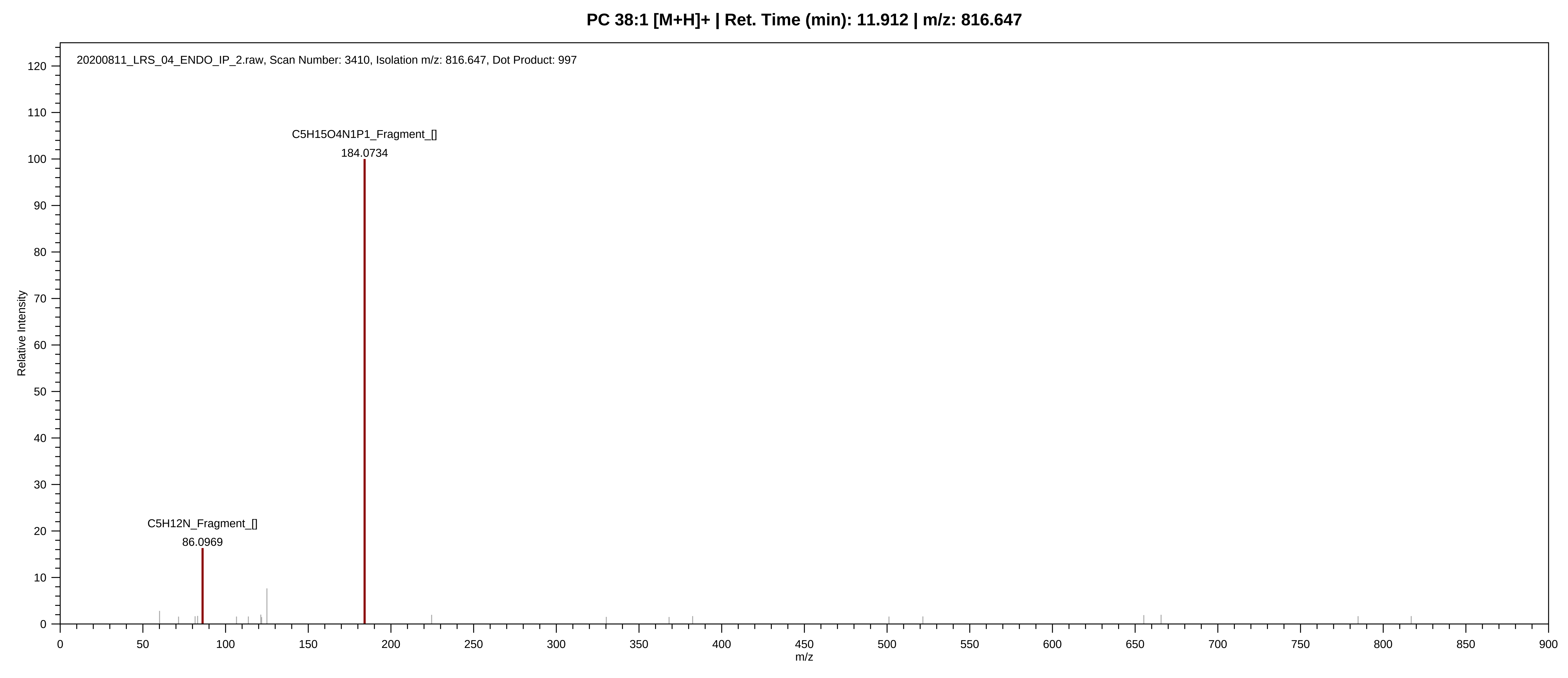
PC 38:1 [M+H]+ | Ret. Time (min): 11.912 | m/z: 816.647
20200811_LRS_04_ENDO_IP_2.raw, Scan Number: 3410, Isolation m/z: 816.647, Dot Product: 997
C5H15O4N1P1_Fragment_[]
184.0734
C5H12N_Fragment_[]
86.0969
Relative Intensity
m/z

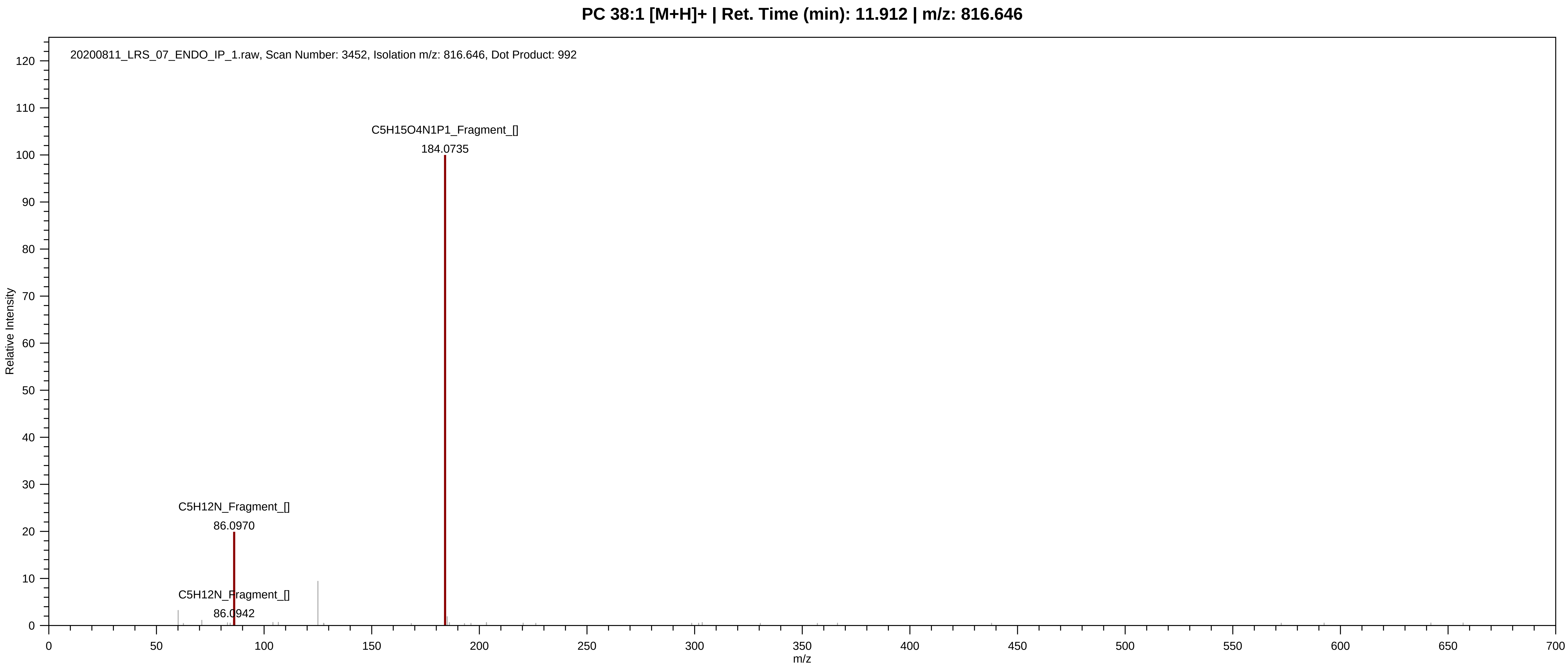

PC 38:1 [M+H]+ | Ret. Time (min): 11.912 | m/z: 816.646
20200811_LRS_07_ENDO_IP_1.raw, Scan Number: 3452, Isolation m/z: 816.646, Dot Product: 992
C5H15O4N1P1_Fragment_[]
184.0735
C5H12N_Fragment_[]
86.0970
C5H12N_Fragment_[]
86.0942
Relative Intensity
m/z

PC 38:1 [M+H]+ | Ret. Time (min): 11.912 | m/z: 816.646

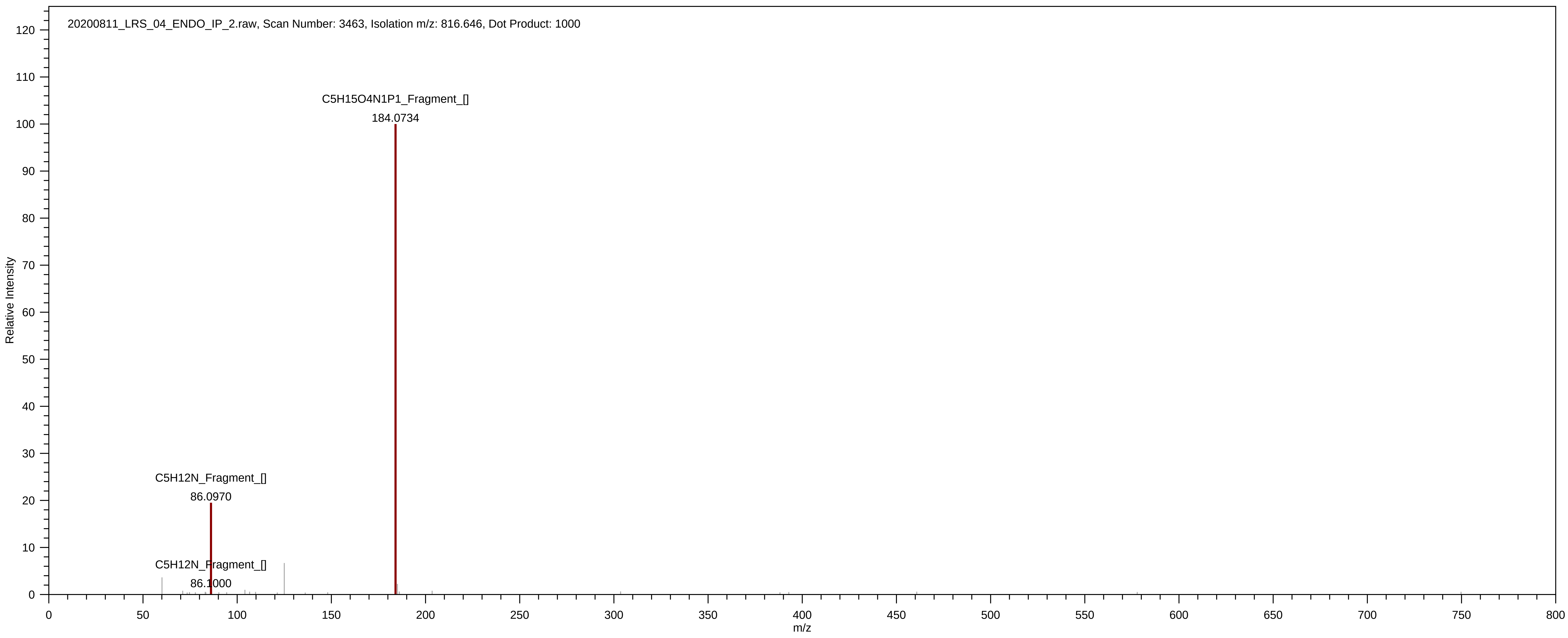

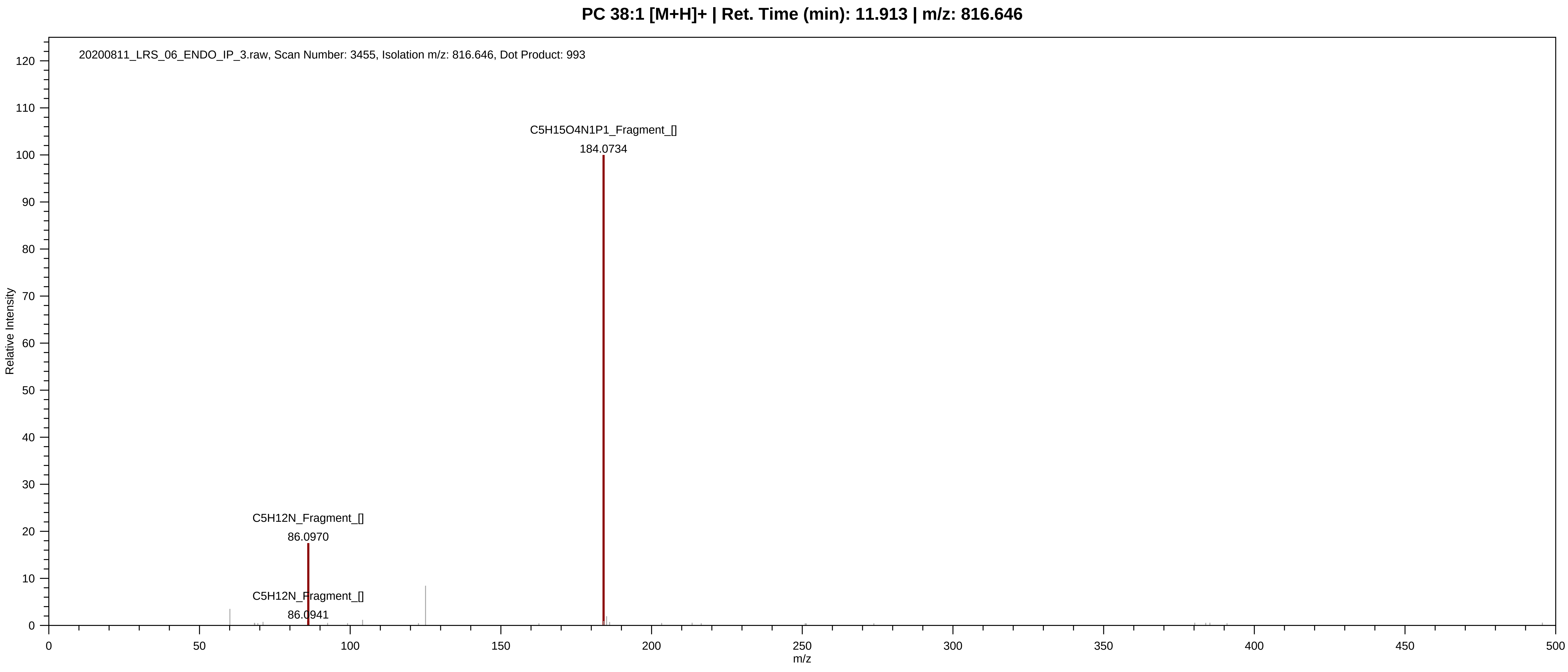
PC 38:1 [M+H]+ | Ret. Time (min): 11.913 | m/z: 816.646
20200811_LRS_06_ENDO_IP_3.raw, Scan Number: 3455, Isolation m/z: 816.646, Dot Product: 993
C5H15O4N1P1_Fragment_[]
184.0734
C5H12N_Fragment_[]
86.0970
C5H12N_Fragment_[]
86.0941
Relative Intensity
m/z

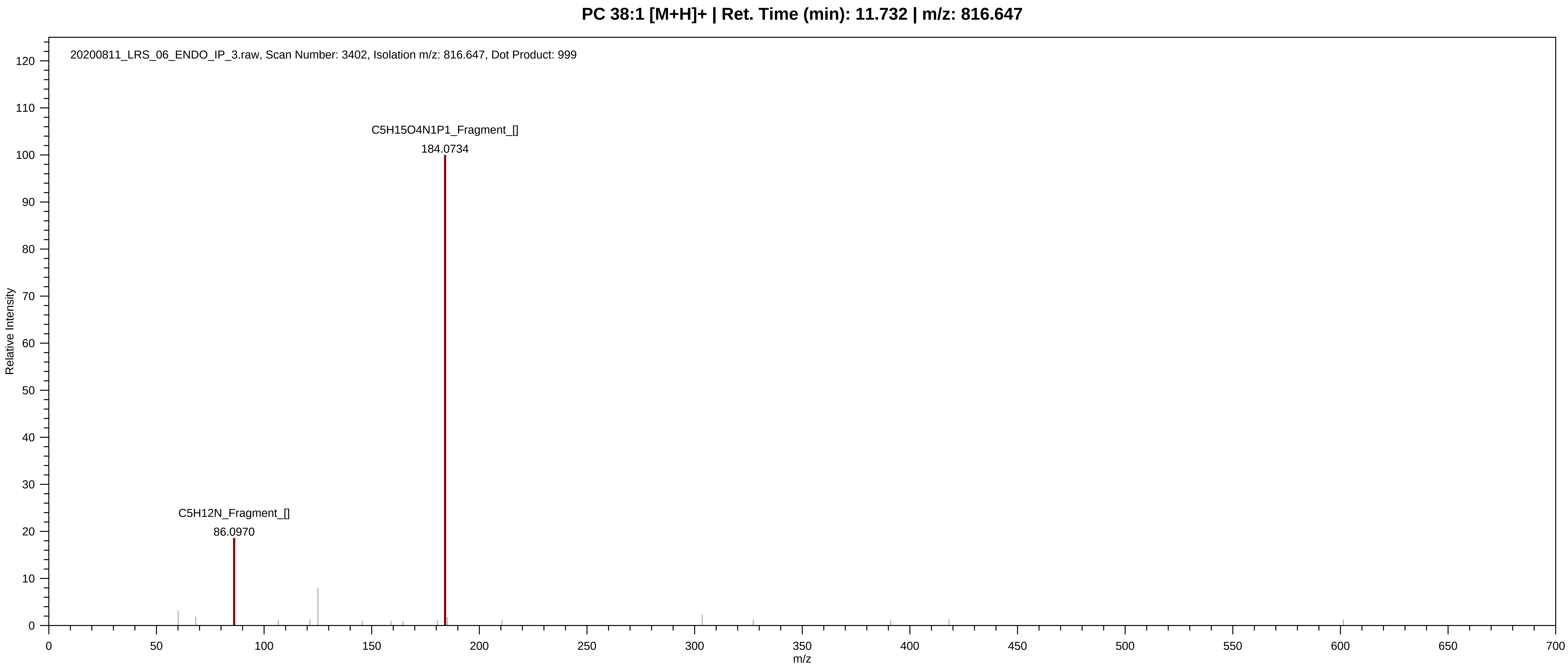
PC 38:1 [M+H]+ | Ret. Time (min): 11.732 | m/z: 816.647
20200811_LRS_06_ENDO_IP_3.raw, Scan Number: 3402, Isolation m/z: 816.647, Dot Product: 999
C5H15O4N1P1_Fragment_[]
184.0734
C5H12N_Fragment_[]
86.0970
Relative Intensity
m/z

PC 38:1 [M+H]+ | Ret. Time (min): 11.731 | m/z: 816.648

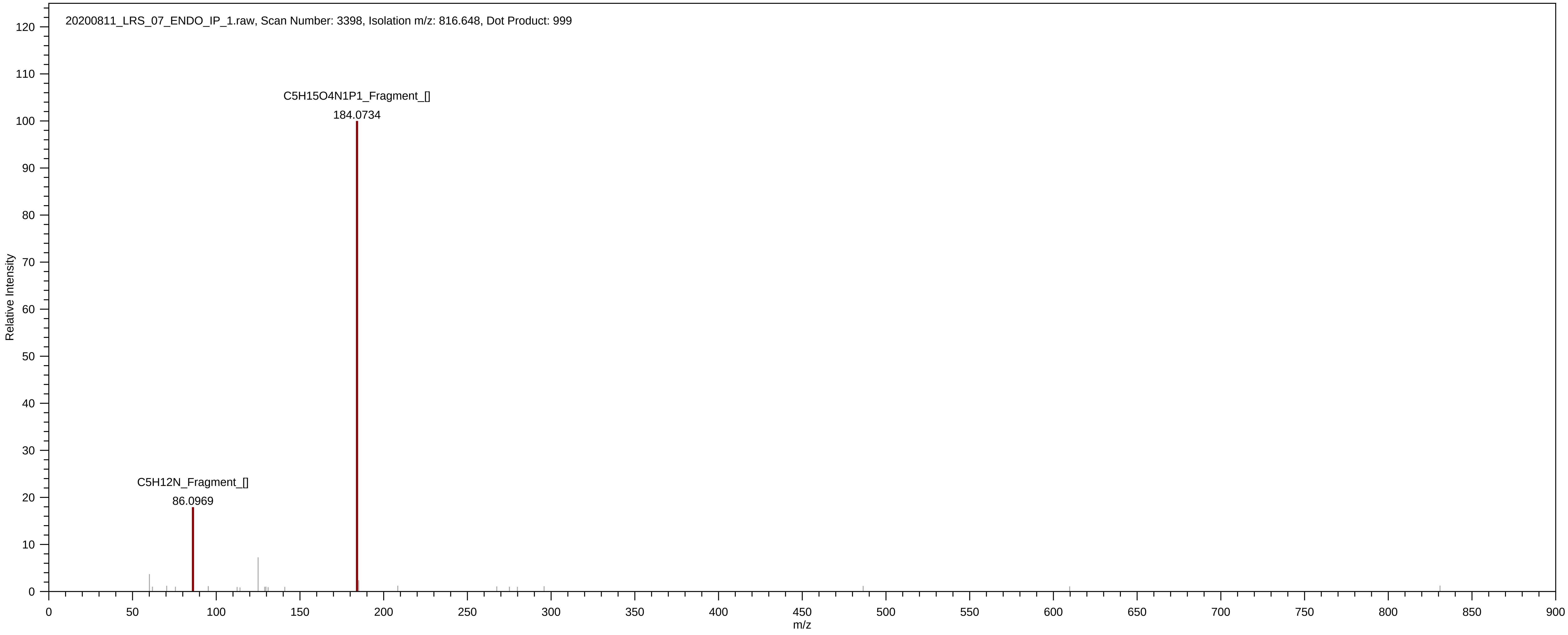

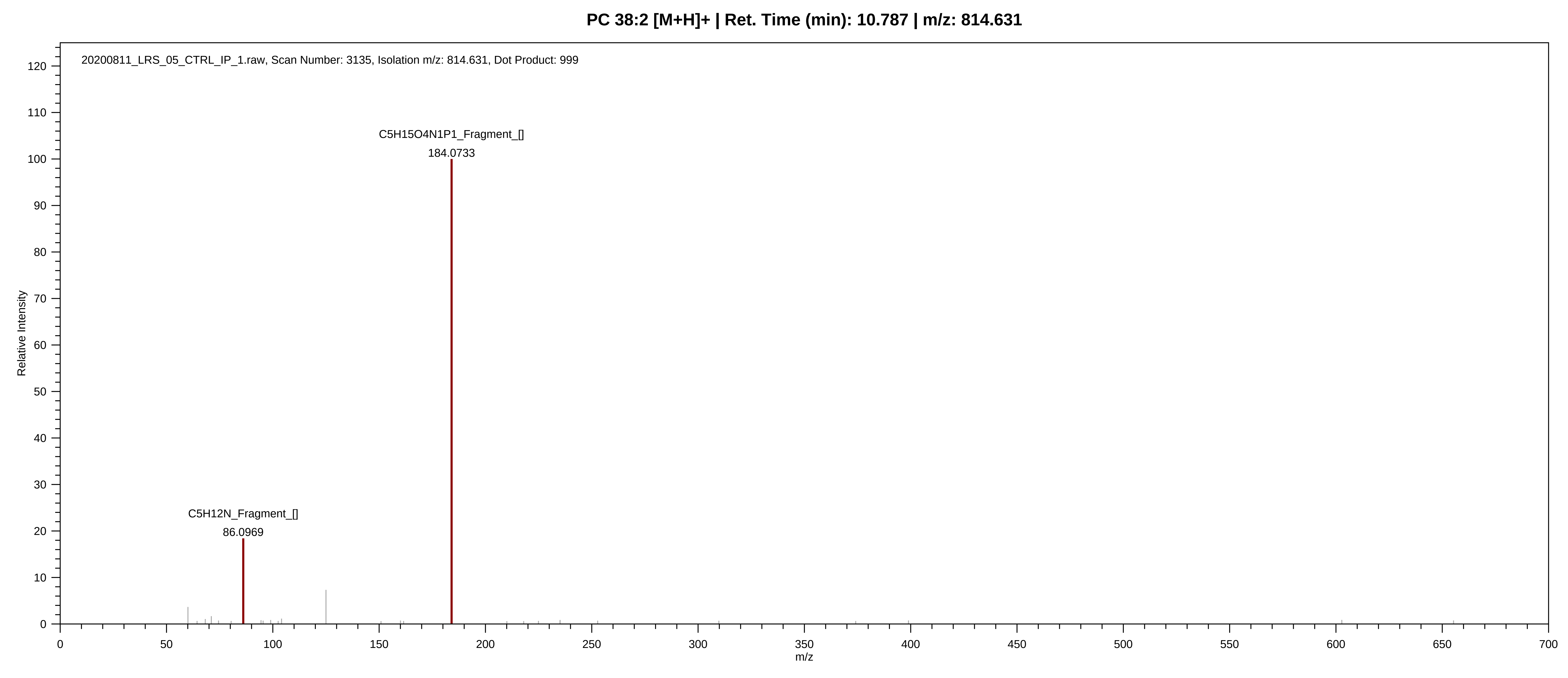
PC 38:2 [M+H]+ | Ret. Time (min): 10.787 | m/z: 814.631
20200811_LRS_05_CTRL_IP_1.raw, Scan Number: 3135, Isolation m/z: 814.631, Dot Product: 999
C5H15O4N1P1_Fragment_[]
184.0733
C5H12N_Fragment_[]
86.0969
Relative Intensity
m/z

PC 38:2 [M+H]+ | Ret. Time (min): 10.937 | m/z: 814.631

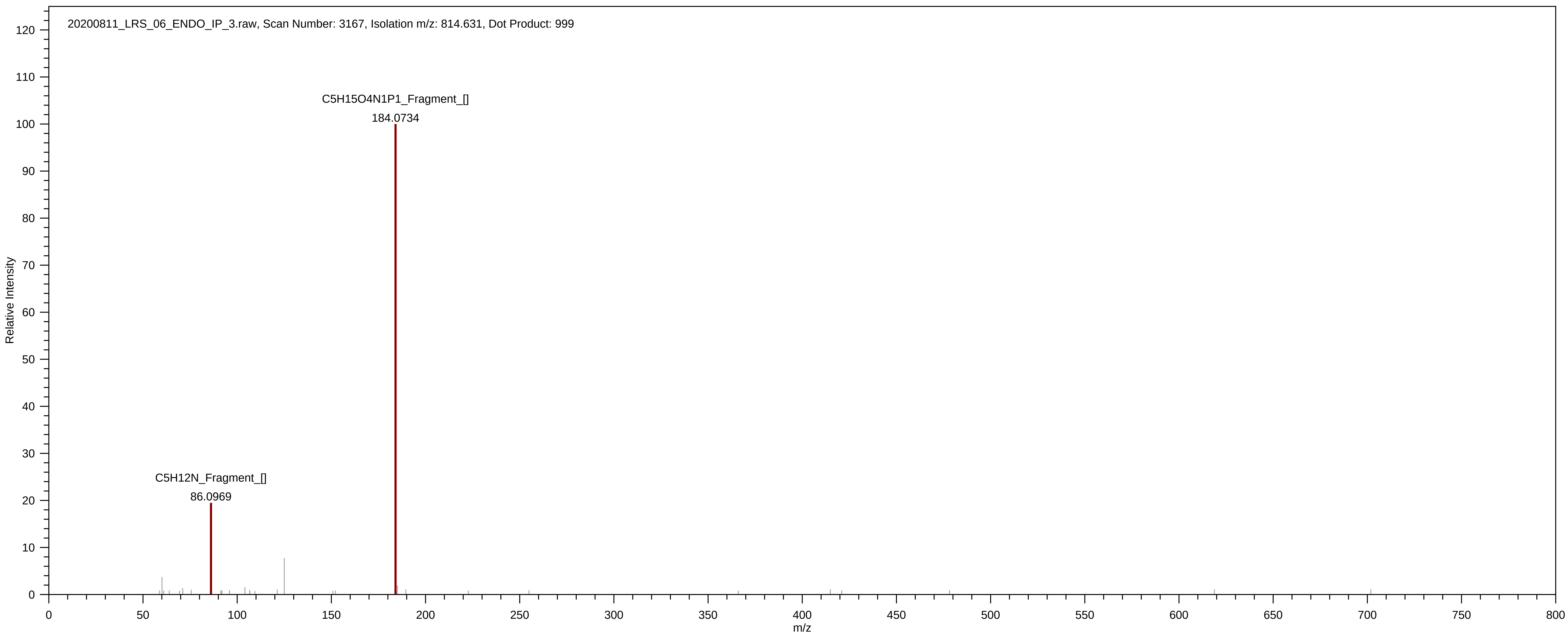

PC 38:2 [M+H]+ | Ret. Time (min): 10.938 | m/z: 814.631

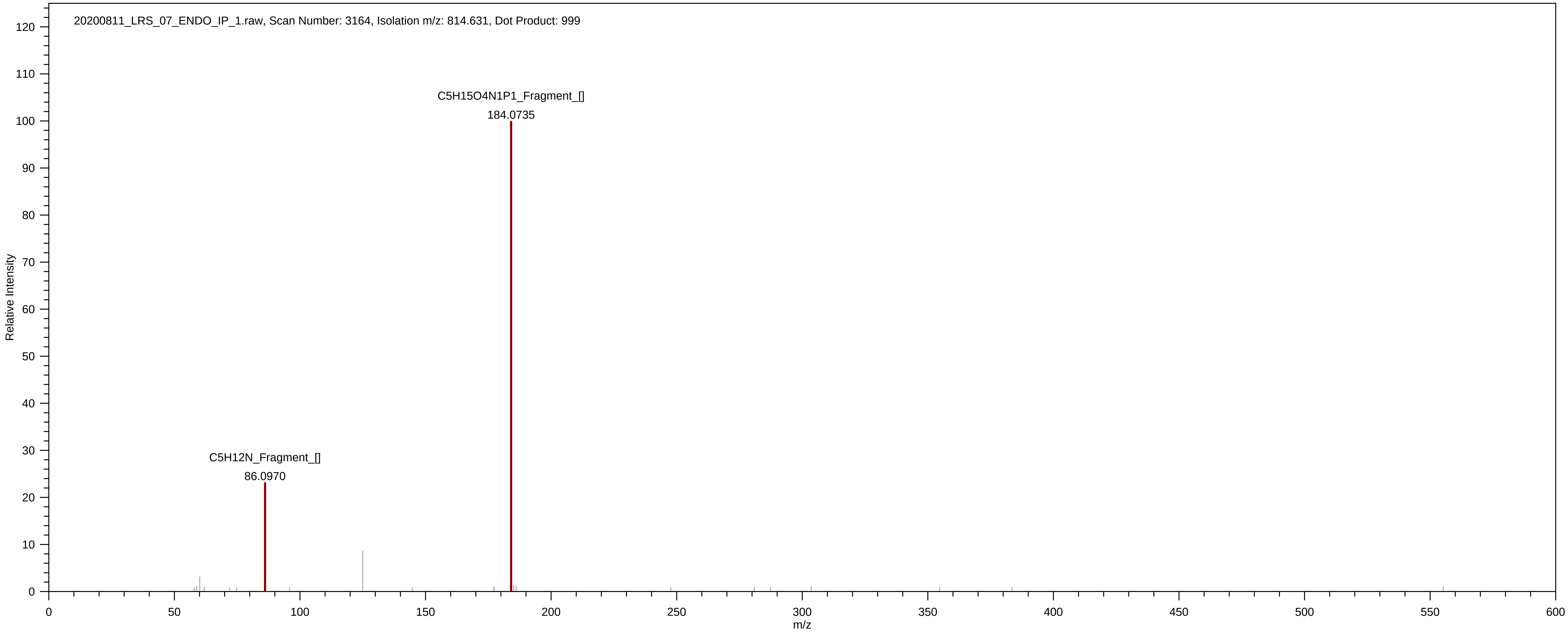

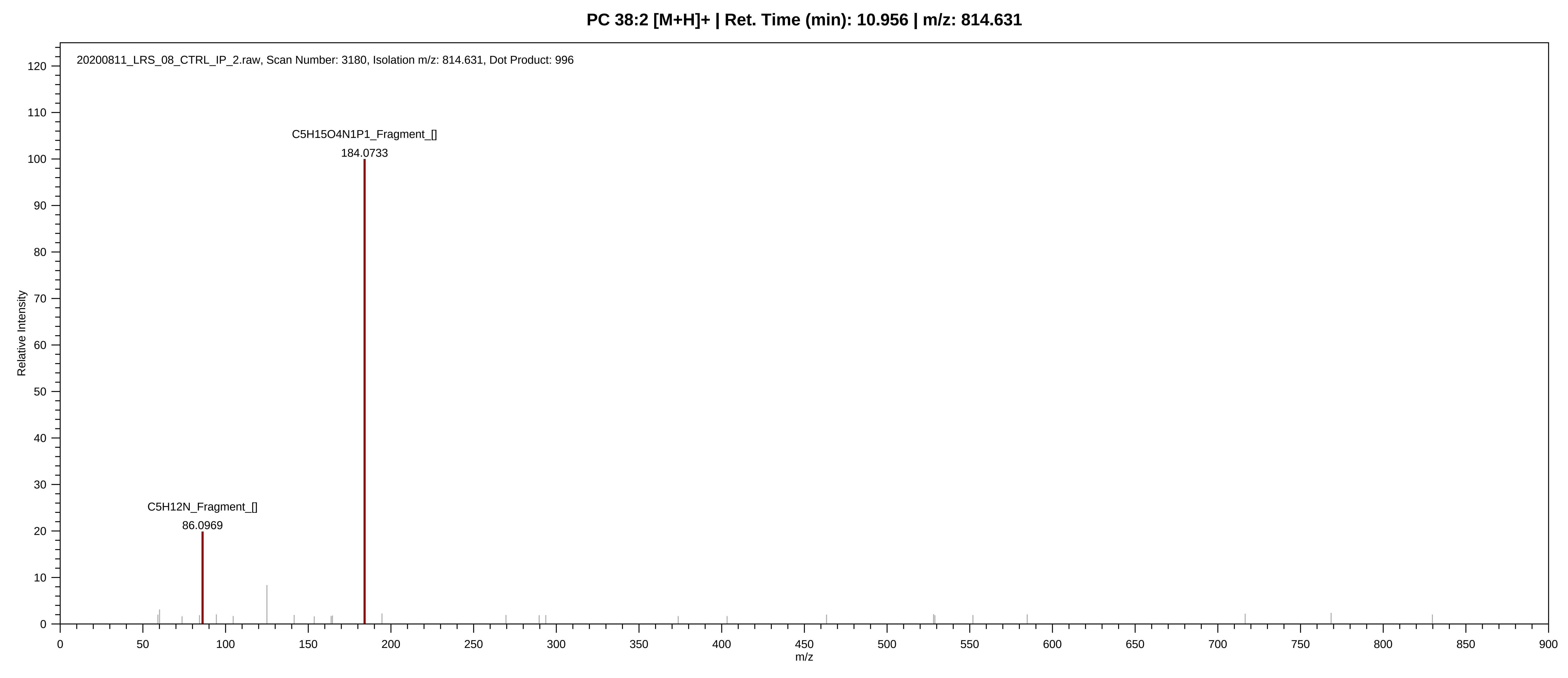
PC 38:2 [M+H]+ | Ret. Time (min): 10.956 | m/z: 814.631
20200811_LRS_08_CTRL_IP_2.raw, Scan Number: 3180, Isolation m/z: 814.631, Dot Product: 996
C5H15O4N1P1_Fragment_[]
184.0733
C5H12N_Fragment_[]
86.0969
Relative Intensity
m/z

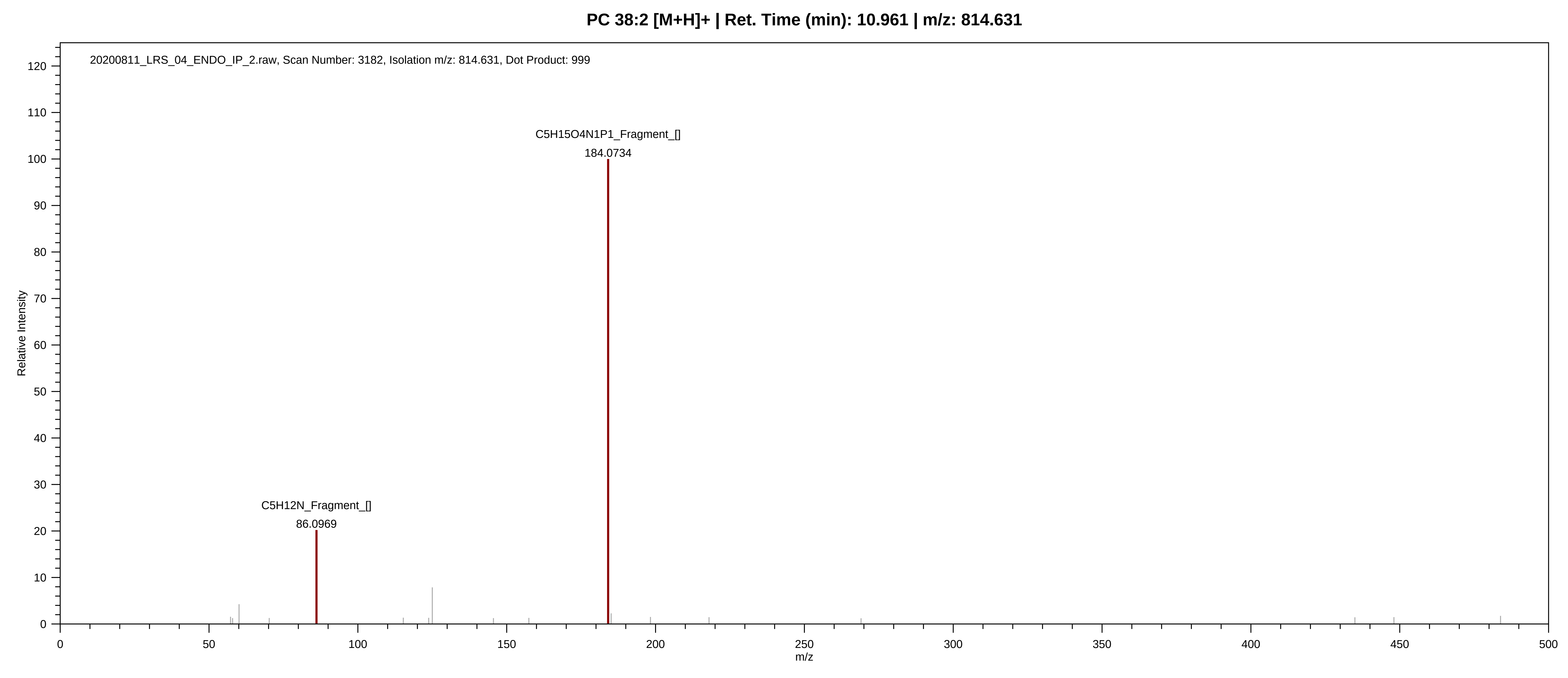

PC 38:2 [M+H]+ | Ret. Time (min): 10.961 | m/z: 814.631
20200811_LRS_04_ENDO_IP_2.raw, Scan Number: 3182, Isolation m/z: 814.631, Dot Product: 999
C5H15O4N1P1_Fragment_[]
184.0734
C5H12N_Fragment_[]
86.0969
Relative Intensity
m/z

PC 38:2 [M+H]+ | Ret. Time (min): 10.754 | m/z: 814.632

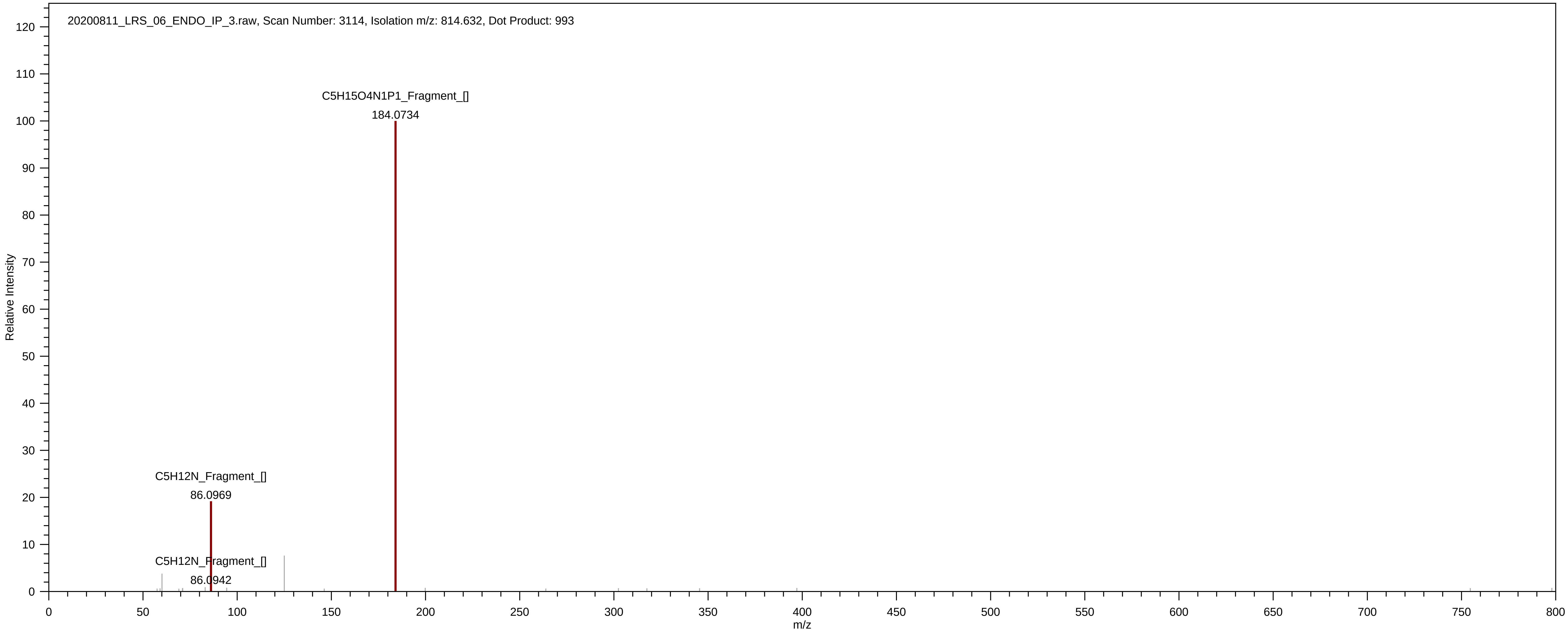

PC 38:2 [M+H]+ | Ret. Time (min): 10.757 | m/z: 814.632

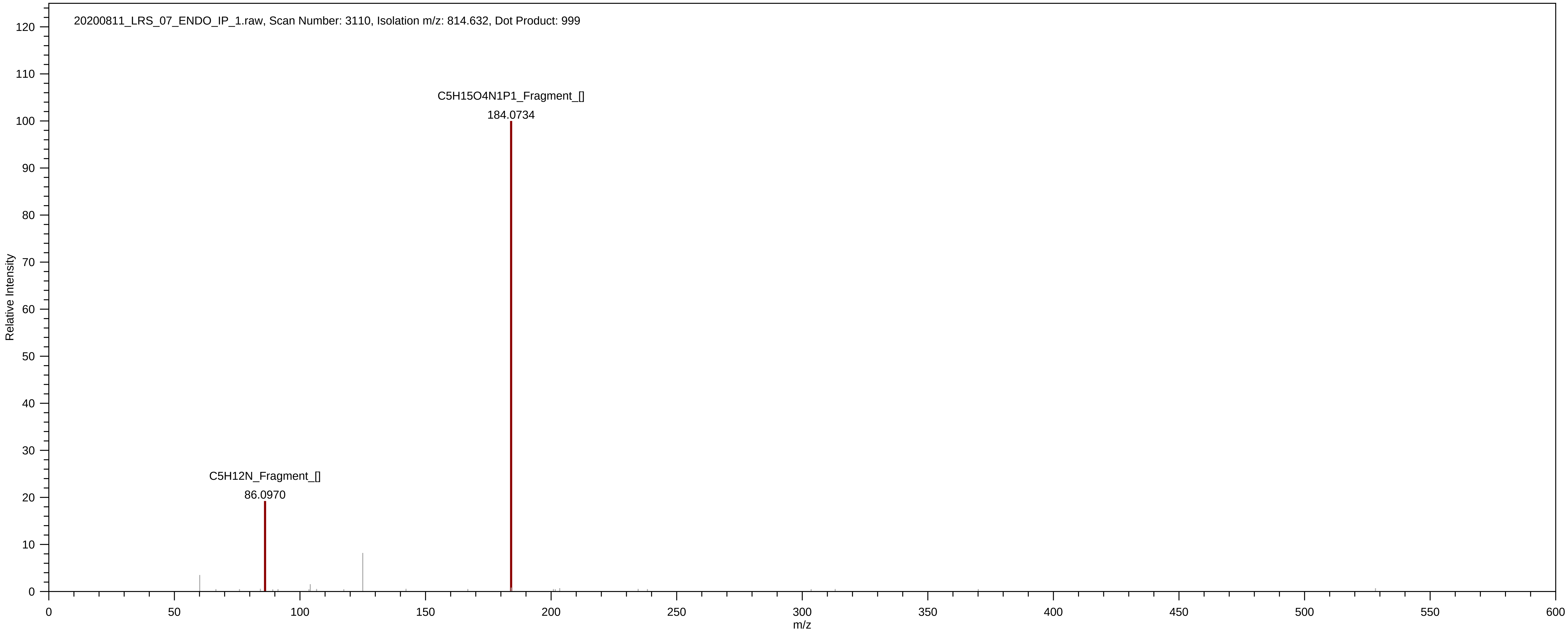

PC 38:2 [M+H]+ | Ret. Time (min): 10.771 | m/z: 814.632

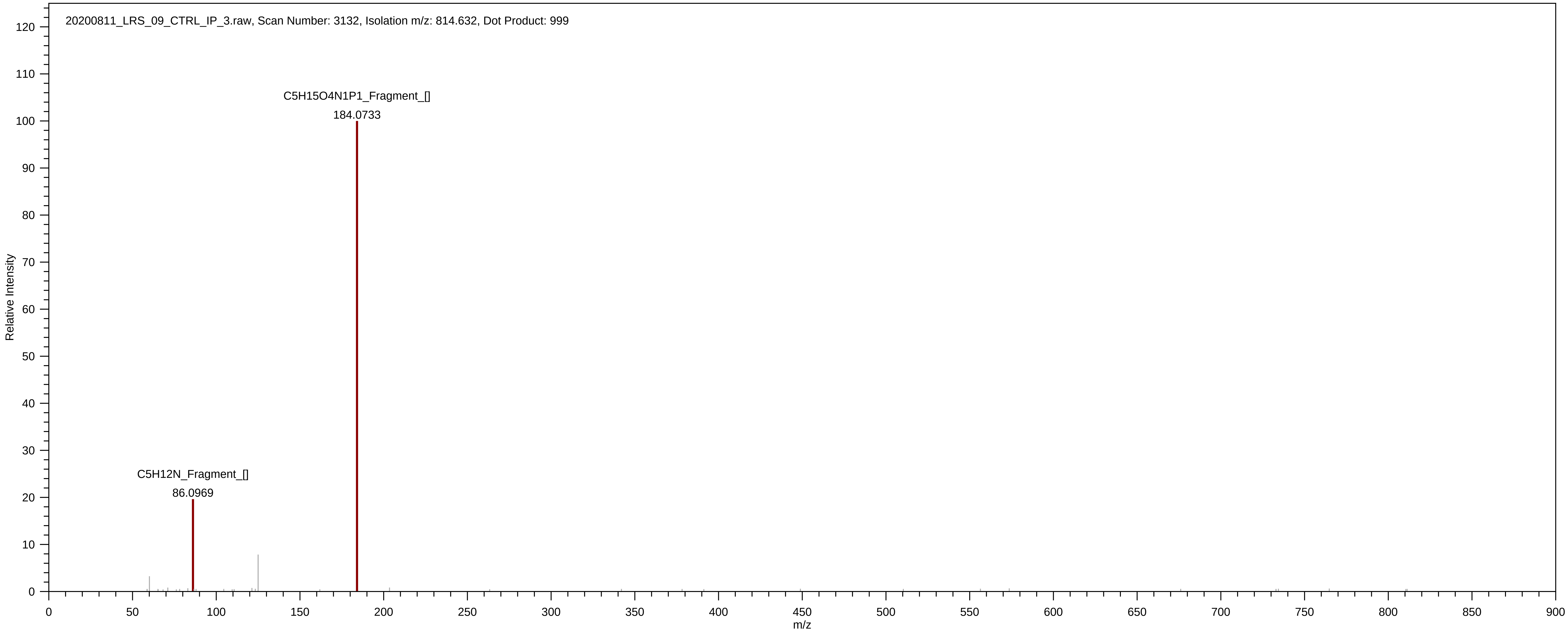

PC 38:2 [M+H]+ | Ret. Time (min): 10.775 | m/z: 814.632

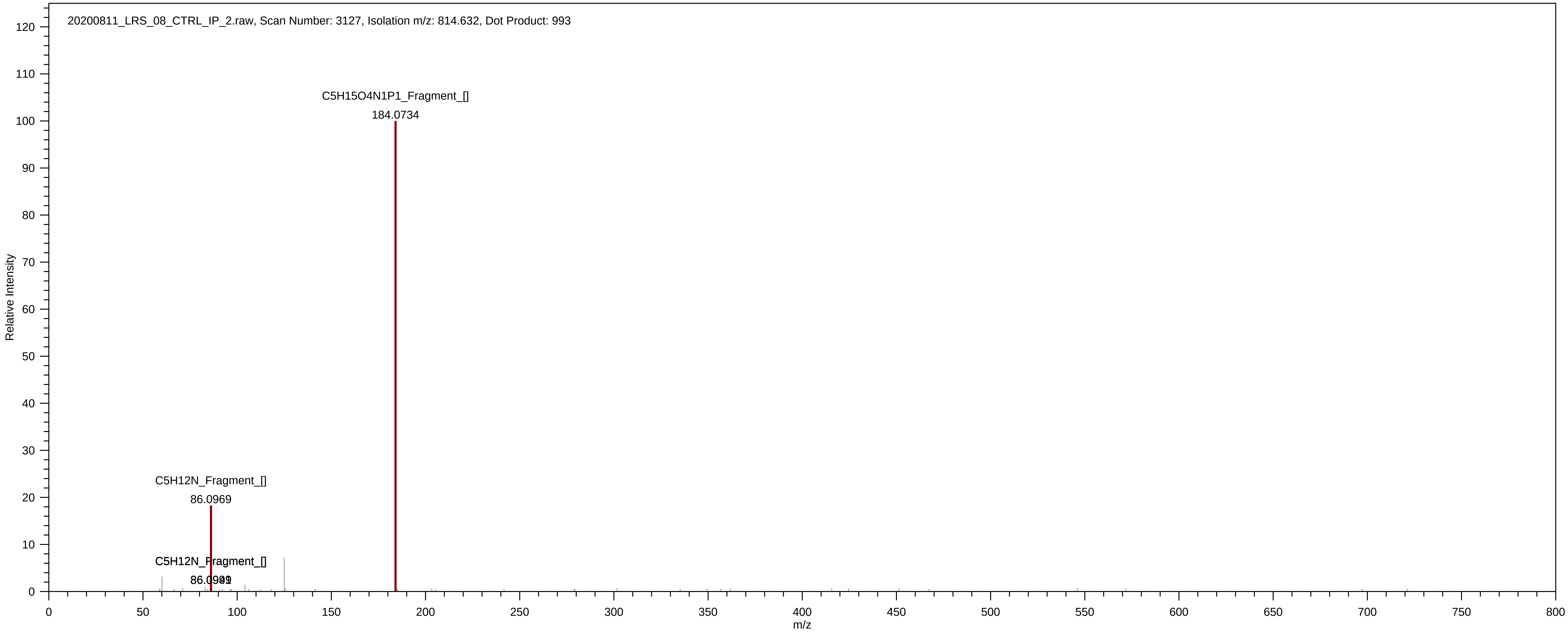

PC 38:2 [M+H]+ | Ret. Time (min): 10.778 | m/z: 814.632

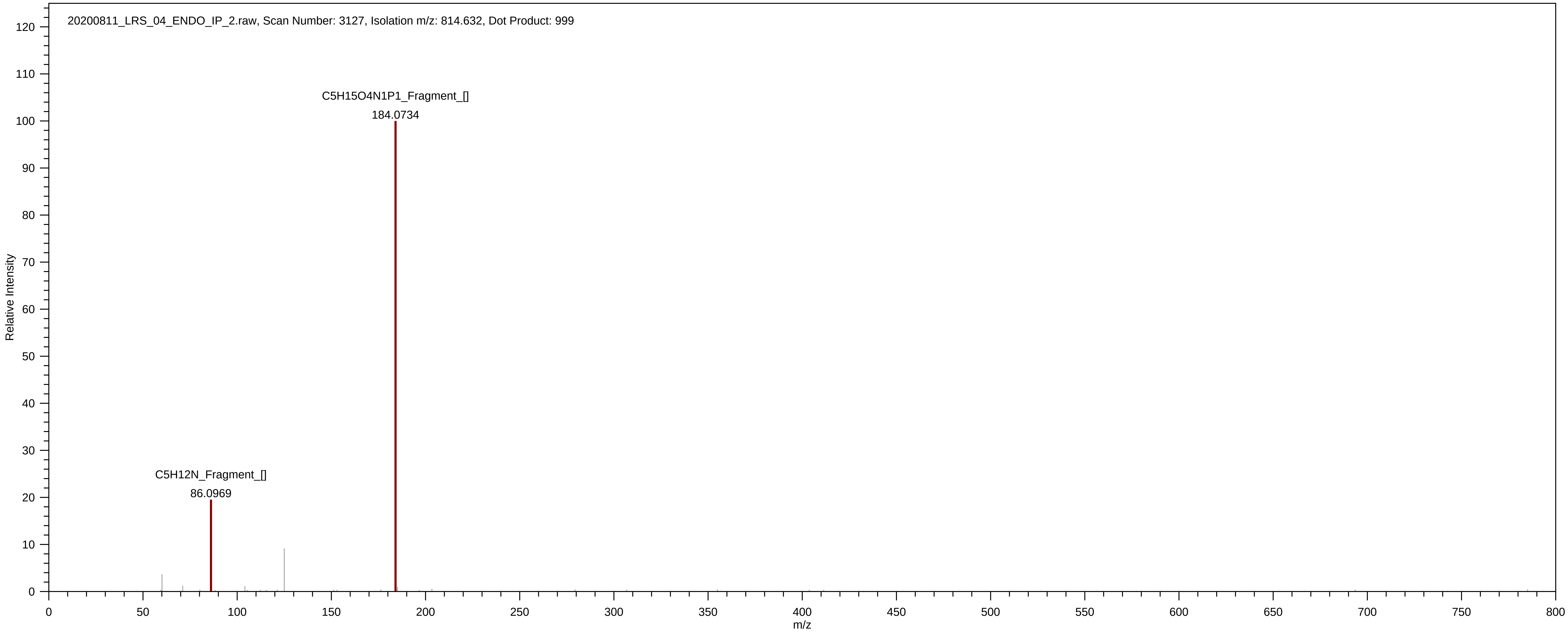

PC 38:2 [M+H]+ | Ret. Time (min): 10.952 | m/z: 814.632

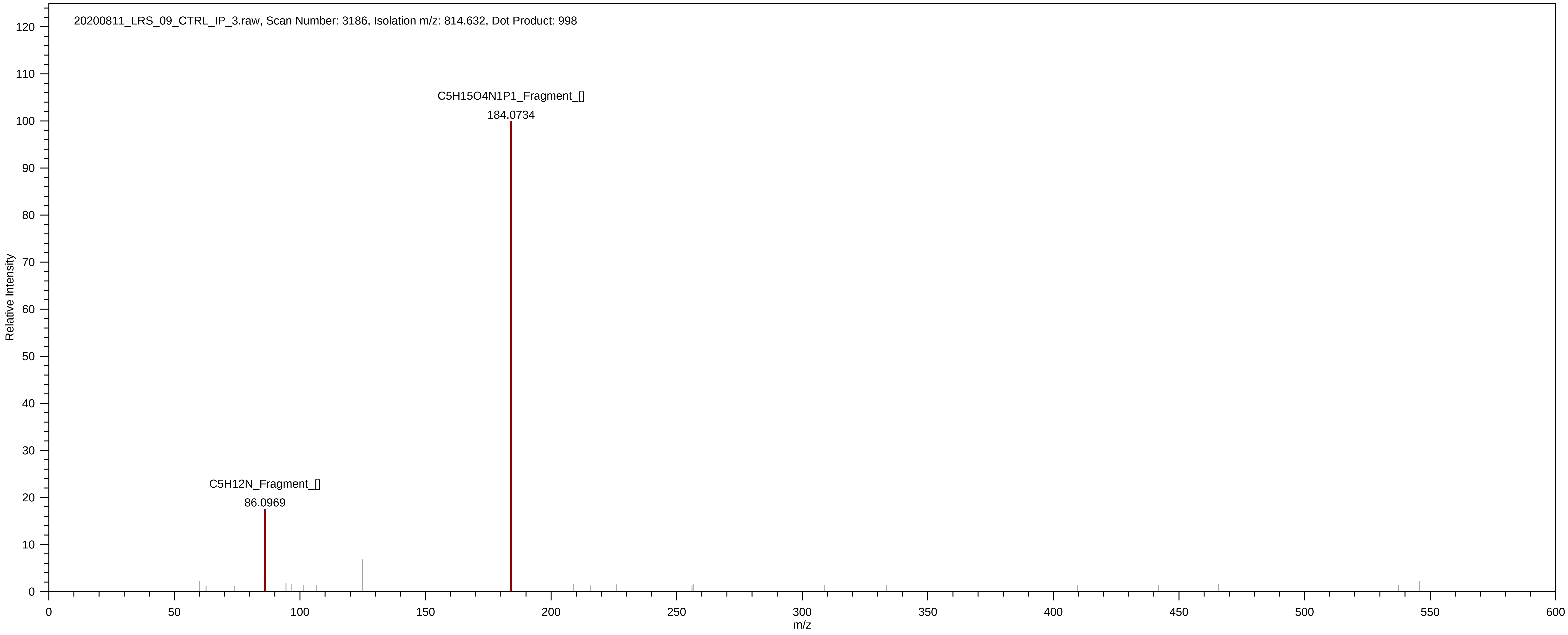

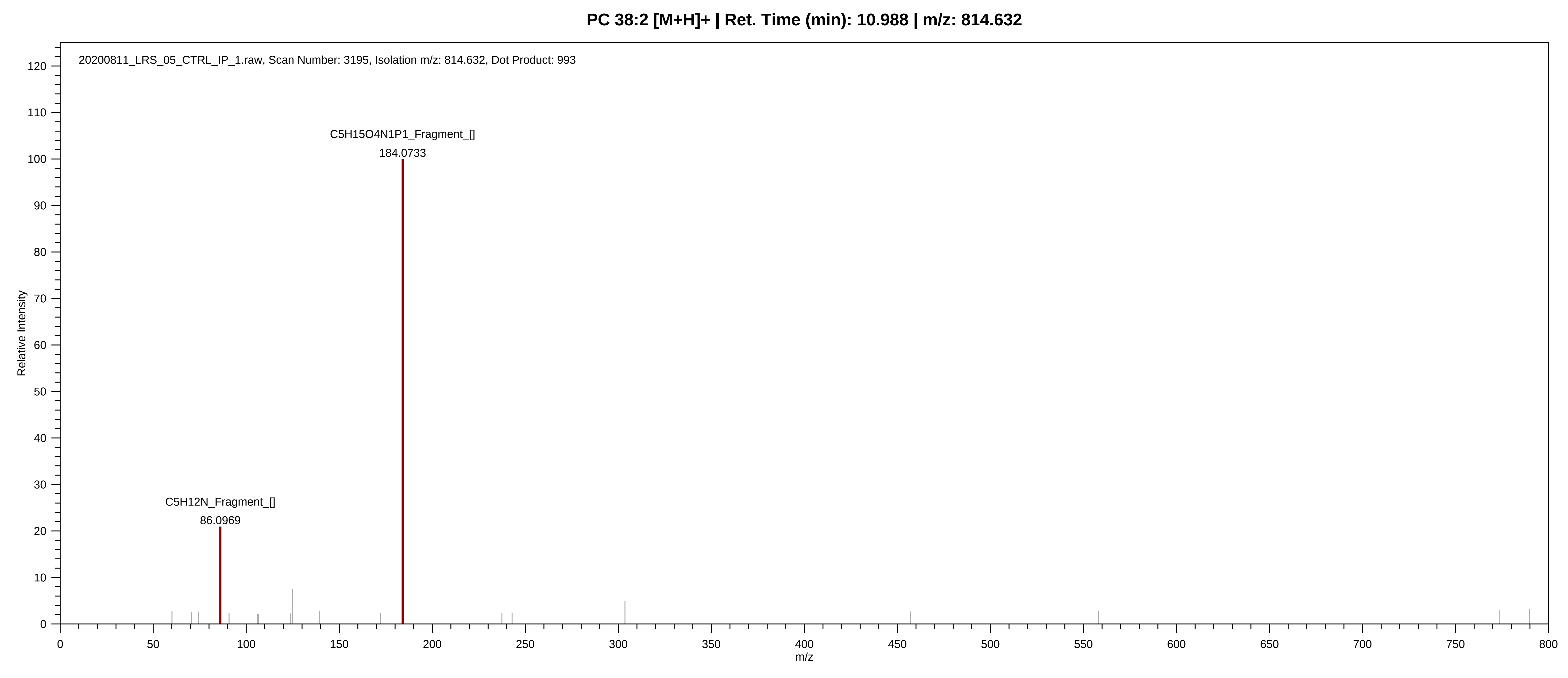

PC 38:2 [M+H]+ | Ret. Time (min): 10.988 | m/z: 814.632
20200811_LRS_05_CTRL_IP_1.raw, Scan Number: 3195, Isolation m/z: 814.632, Dot Product: 993
C5H15O4N1P1_Fragment_[]
184.0733
C5H12N_Fragment_[]
86.0969
Relative Intensity
m/z

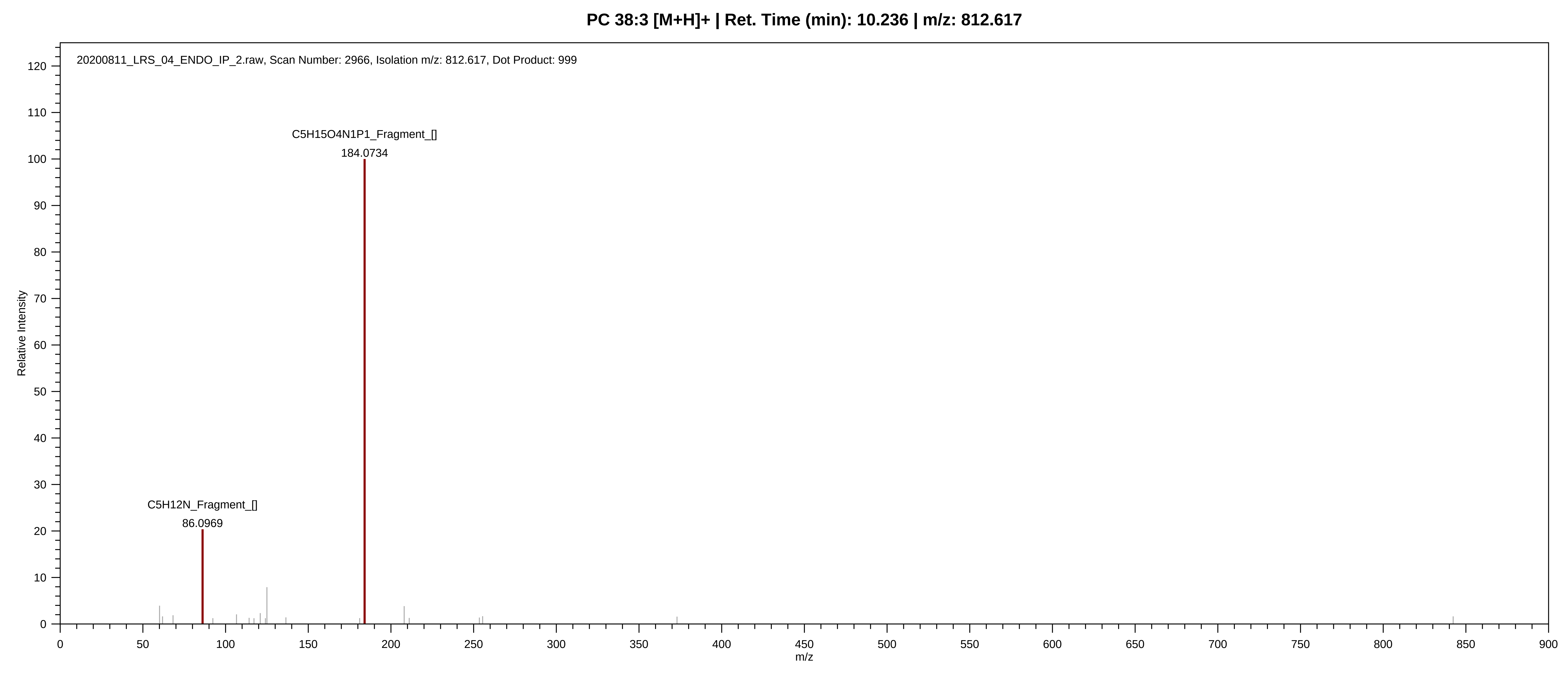
PC 38:3 [M+H]+ | Ret. Time (min): 10.236 | m/z: 812.617
20200811_LRS_04_ENDO_IP_2.raw, Scan Number: 2966, Isolation m/z: 812.617, Dot Product: 999
C5H15O4N1P1_Fragment_[]
184.0734
C5H12N_Fragment_[]
86.0969
Relative Intensity
m/z

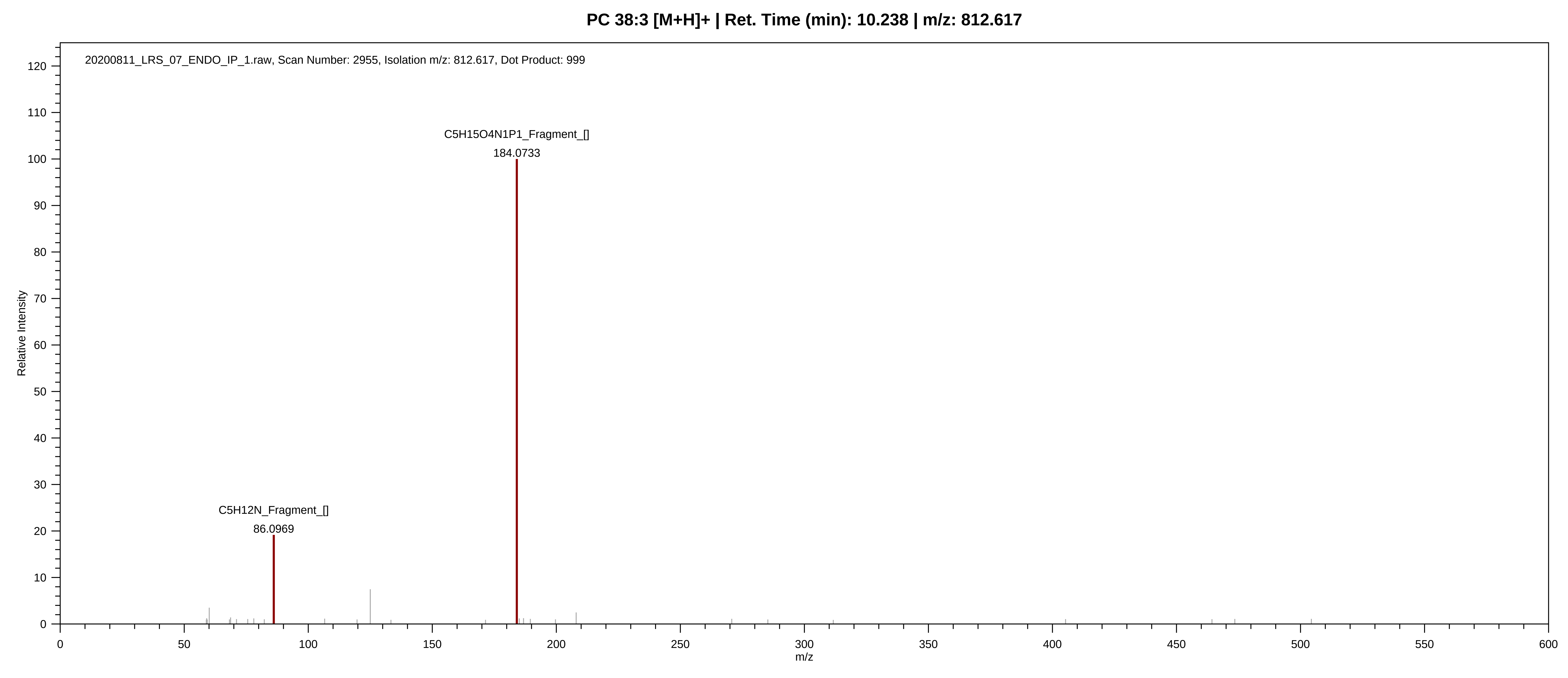

PC 38:3 [M+H]+ | Ret. Time (min): 10.238 | m/z: 812.617
20200811_LRS_07_ENDO_IP_1.raw, Scan Number: 2955, Isolation m/z: 812.617, Dot Product: 999
C5H15O4N1P1_Fragment_[]
184.0733
C5H12N_Fragment_[]
86.0969
Relative Intensity
m/z

PC 38:3 [M+H]+ | Ret. Time (min): 10.237 | m/z: 812.618

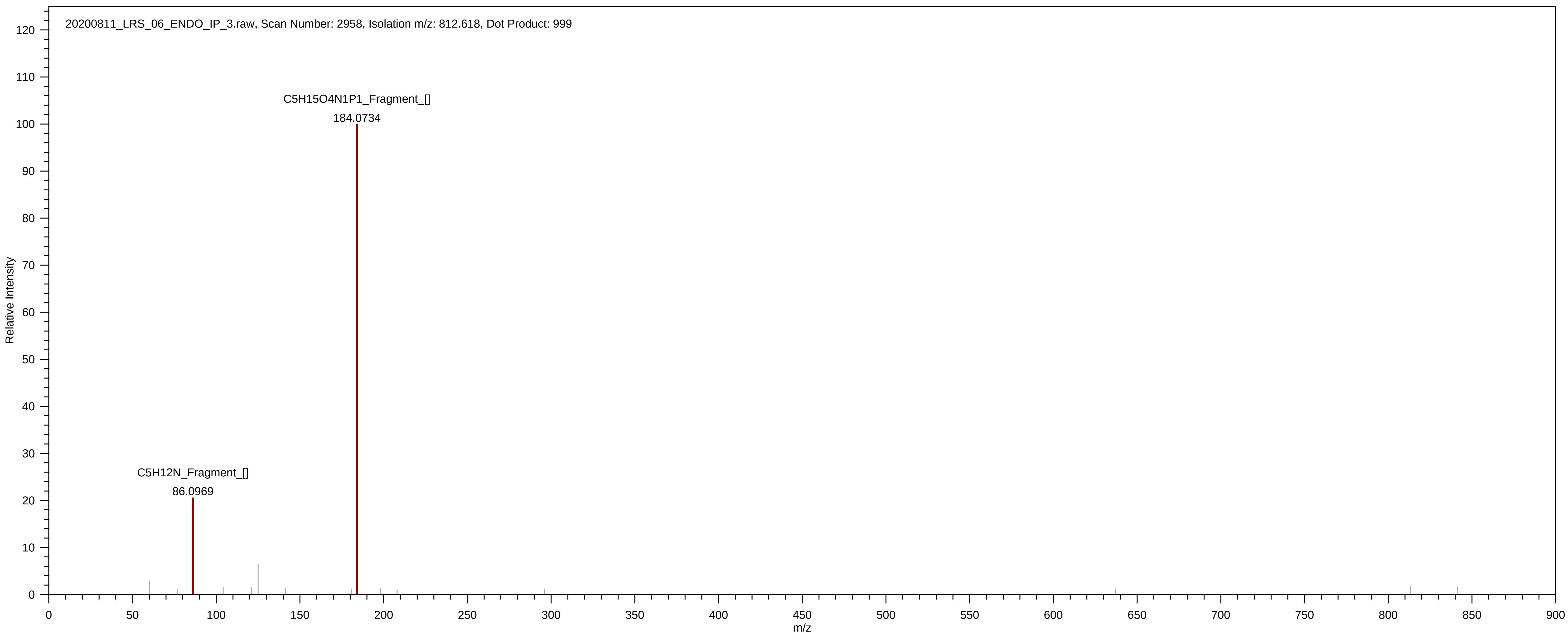

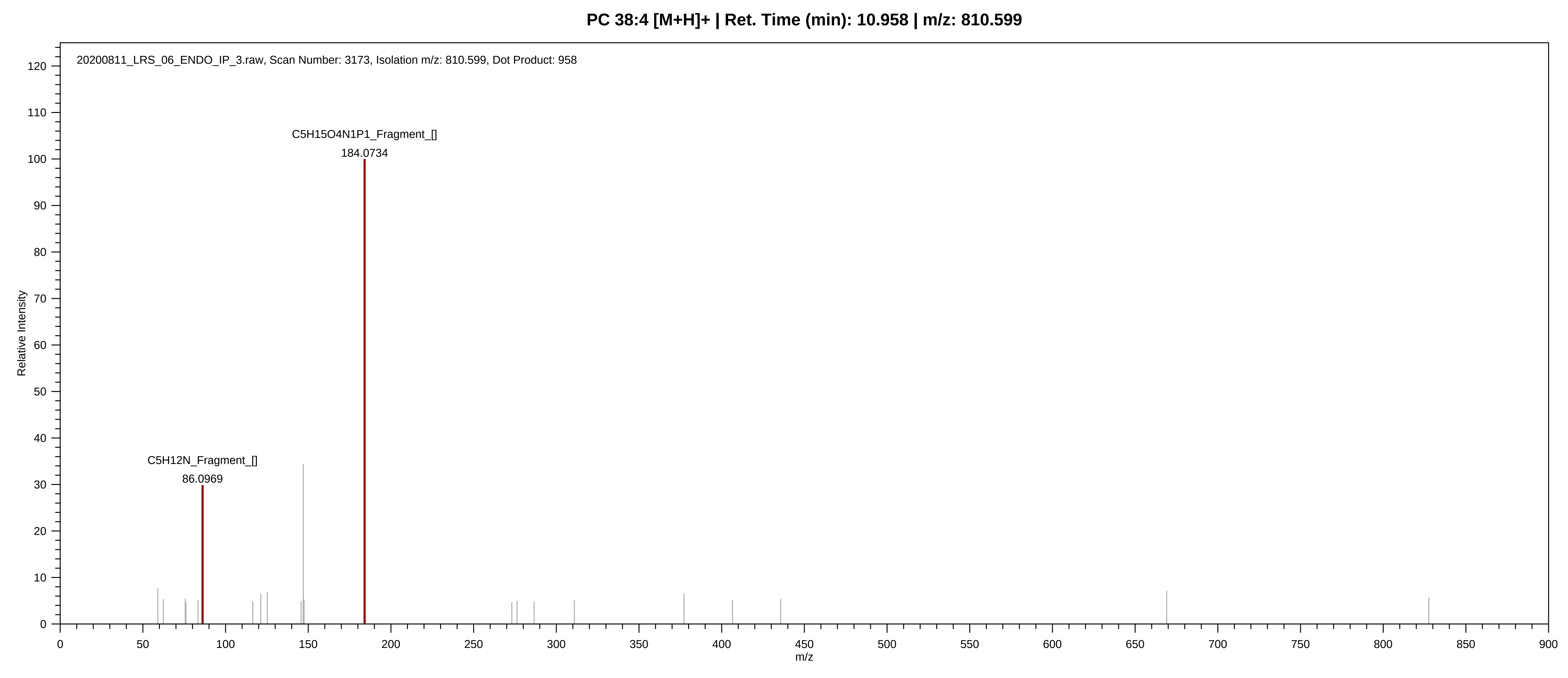

PC 38:4 [M+H]+ | Ret. Time (min): 10.958 | m/z: 810.599
20200811_LRS_06_ENDO_IP_3.raw, Scan Number: 3173, Isolation m/z: 810.599, Dot Product: 958
C5H15O4N1P1_Fragment_[]
184.0734
C5H12N_Fragment_[]
86.0969
Relative Intensity
m/z

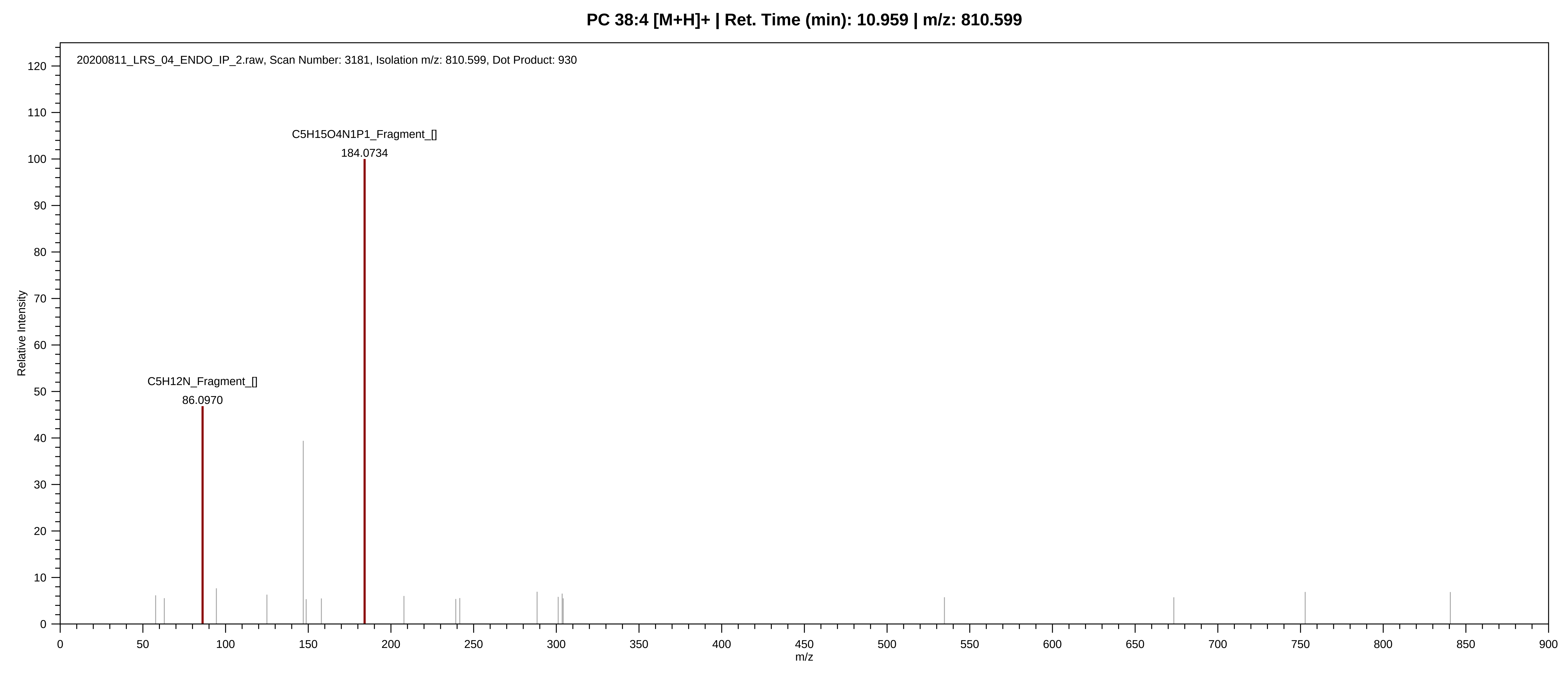
PC 38:4 [M+H]+ | Ret. Time (min): 10.959 | m/z: 810.599
20200811_LRS_04_ENDO_IP_2.raw, Scan Number: 3181, Isolation m/z: 810.599, Dot Product: 930
C5H15O4N1P1_Fragment_[]
184.0734
C5H12N_Fragment_[]
86.0970
Relative Intensity
m/z

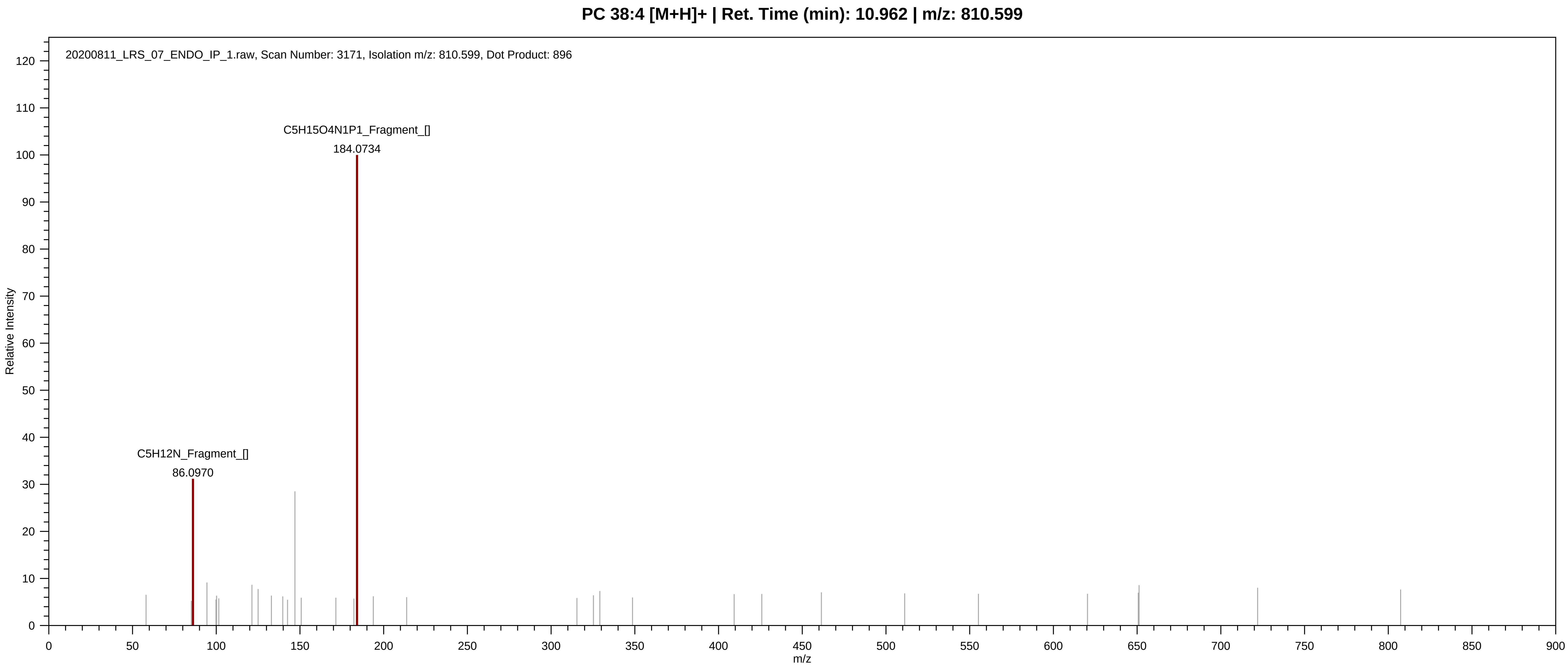
PC 38:4 [M+H]+ | Ret. Time (min): 10.962 | m/z: 810.599
20200811_LRS_07_ENDO_IP_1.raw, Scan Number: 3171, Isolation m/z: 810.599, Dot Product: 896
C5H15O4N1P1_Fragment_[]
184.0734
C5H12N_Fragment_[]
86.0970
Relative Intensity
m/z

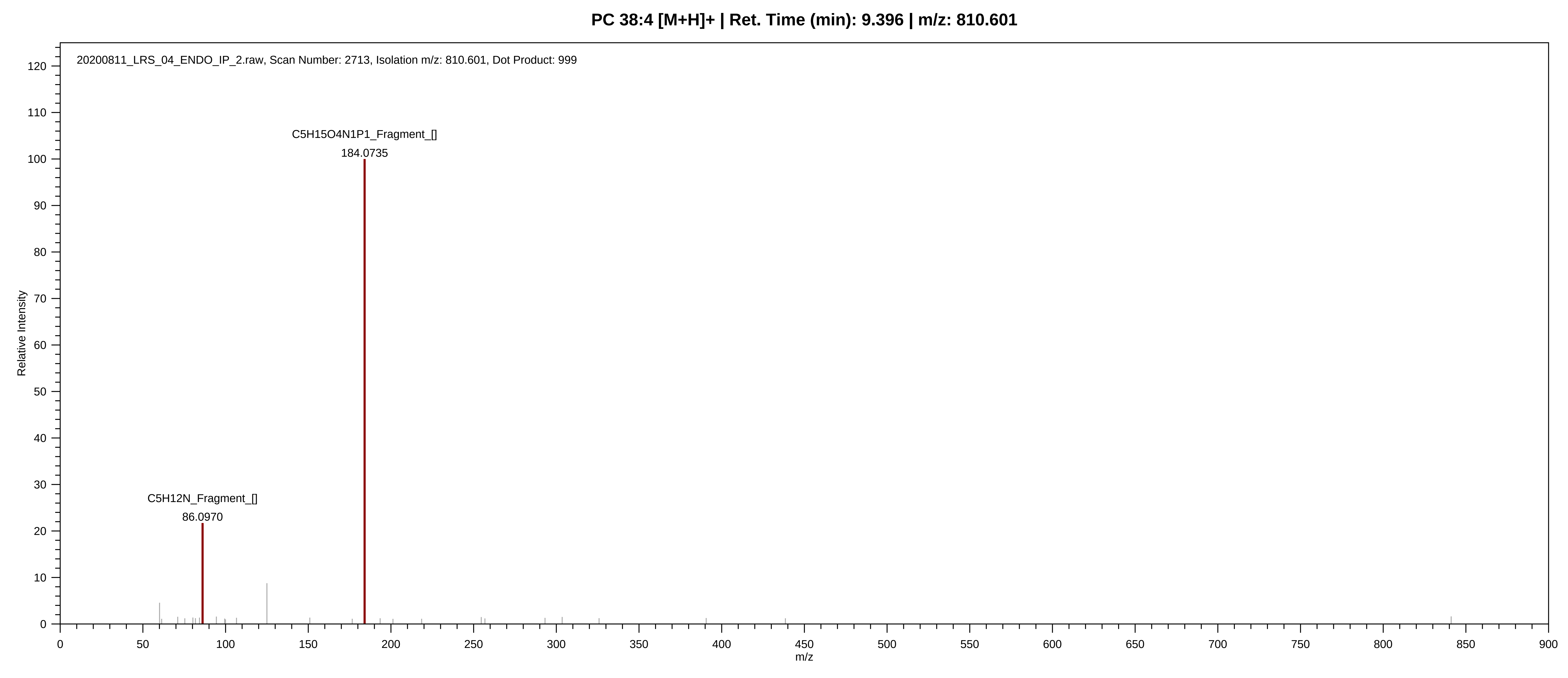
PC 38:4 [M+H]+ | Ret. Time (min): 9.396 | m/z: 810.601
20200811_LRS_04_ENDO_IP_2.raw, Scan Number: 2713, Isolation m/z: 810.601, Dot Product: 999
C5H15O4N1P1_Fragment_[]
184.0735
C5H12N_Fragment_[]
86.0970
Relative Intensity
m/z

PC 38:4 [M+H]+ | Ret. Time (min): 9.403 | m/z: 810.602

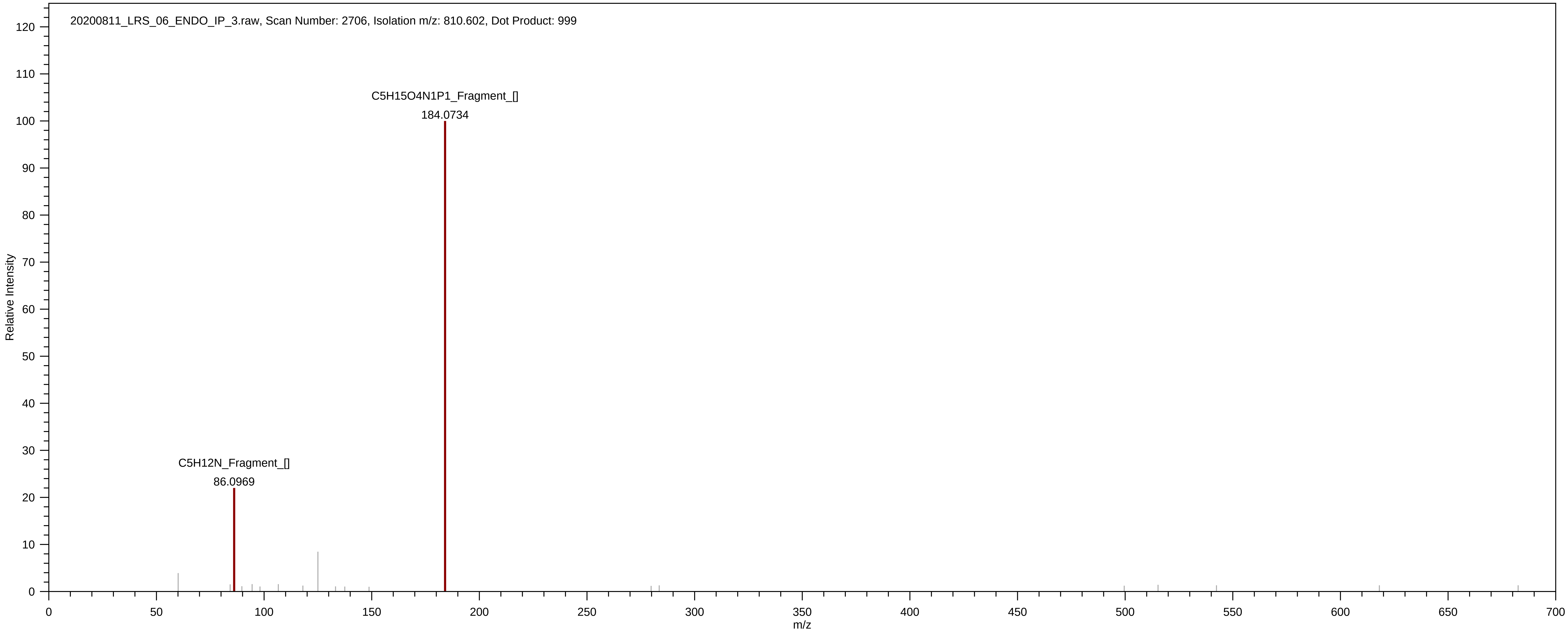

PC 38:5 [M+H]+ | Ret. Time (min): 9.001 | m/z: 808.581

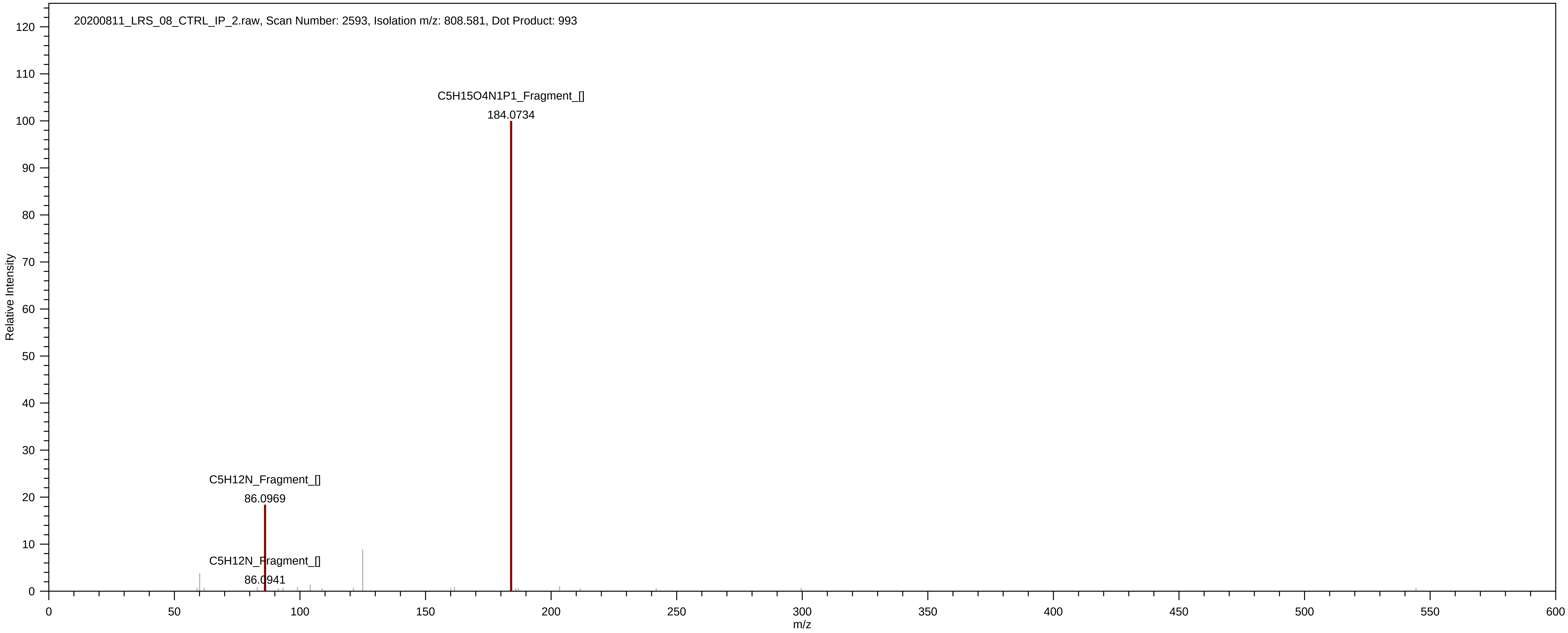

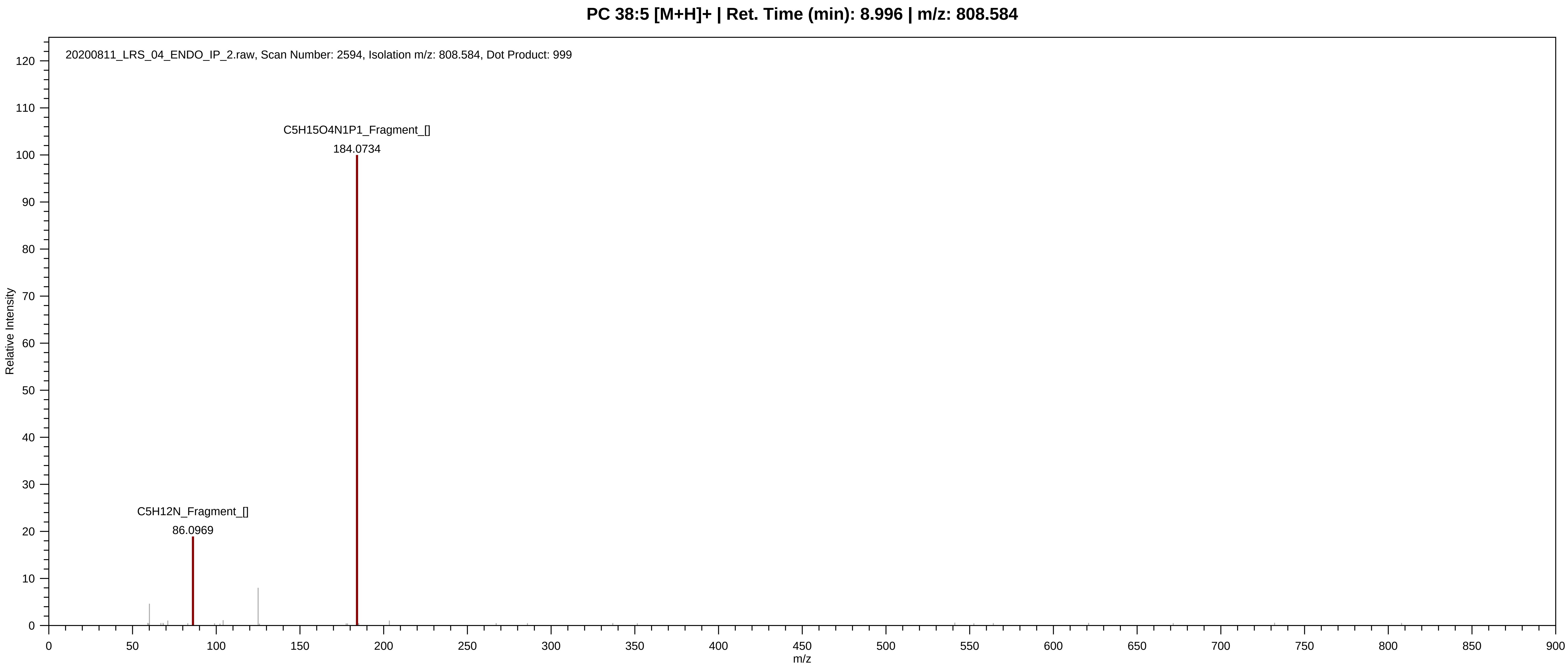
PC 38:5 [M+H]+ | Ret. Time (min): 8.996 | m/z: 808.584
20200811_LRS_04_ENDO_IP_2.raw, Scan Number: 2594, Isolation m/z: 808.584, Dot Product: 999
C5H15O4N1P1_Fragment_[]
184.0734
C5H12N_Fragment_[]
86.0969
Relative Intensity
m/z
0
10
20
30
40
50
60
70
80
90
100
110
120
0
50
100
150
200
250
300
350
400
450
500
550
600
650
700
750
800
850
900

PC 38:5 [M+H]+ | Ret. Time (min): 9.007 | m/z: 808.584

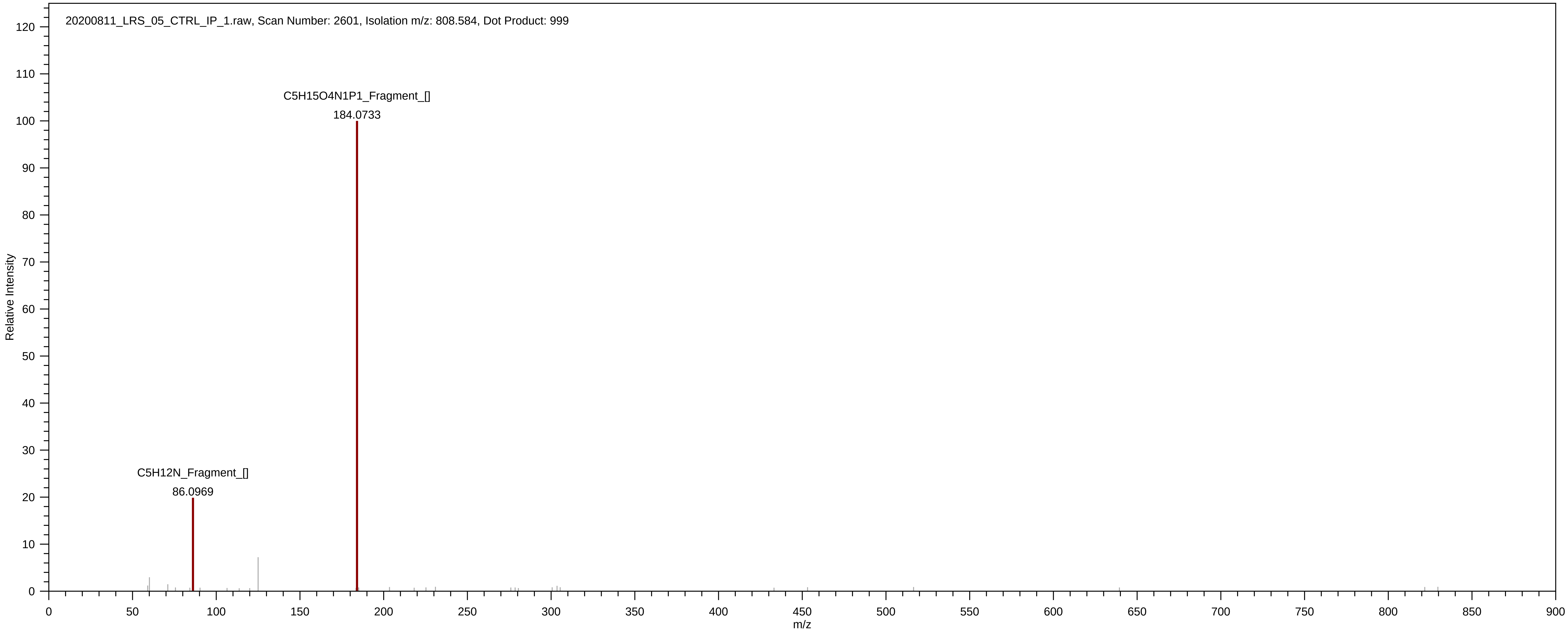

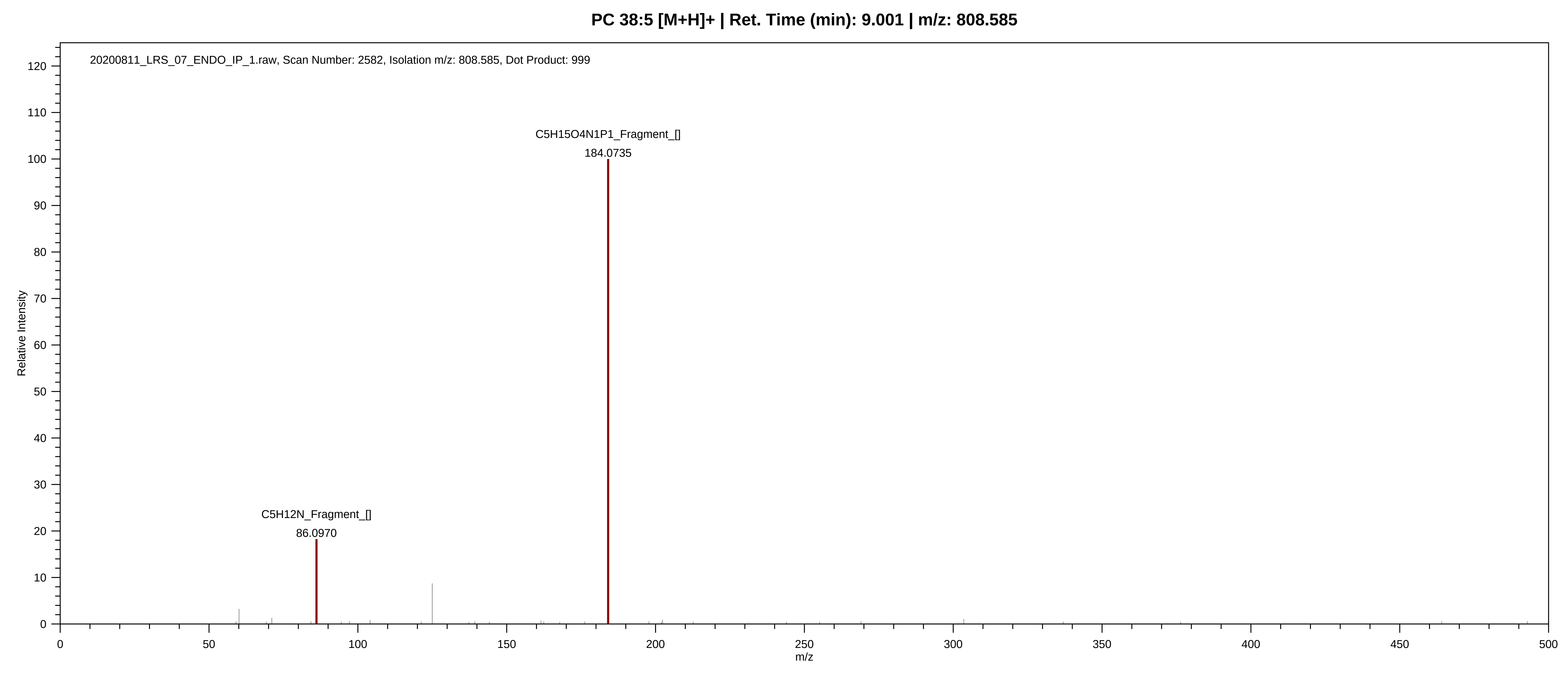
PC 38:5 [M+H]+ | Ret. Time (min): 9.001 | m/z: 808.585
20200811_LRS_07_ENDO_IP_1.raw, Scan Number: 2582, Isolation m/z: 808.585, Dot Product: 999
C5H15O4N1P1_Fragment_[]
184.0735
C5H12N_Fragment_[]
86.0970
Relative Intensity
m/z

PC 38:5 [M+H]+ | Ret. Time (min): 9 | m/z: 808.586

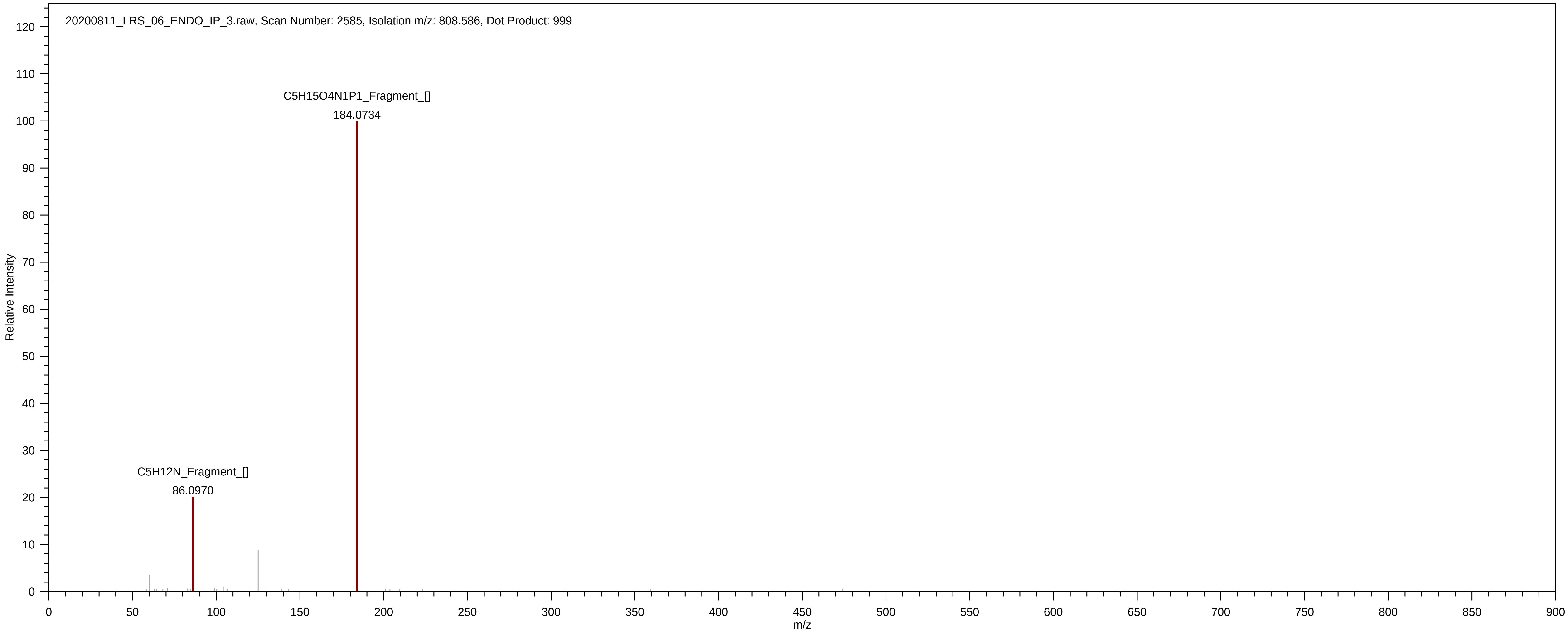

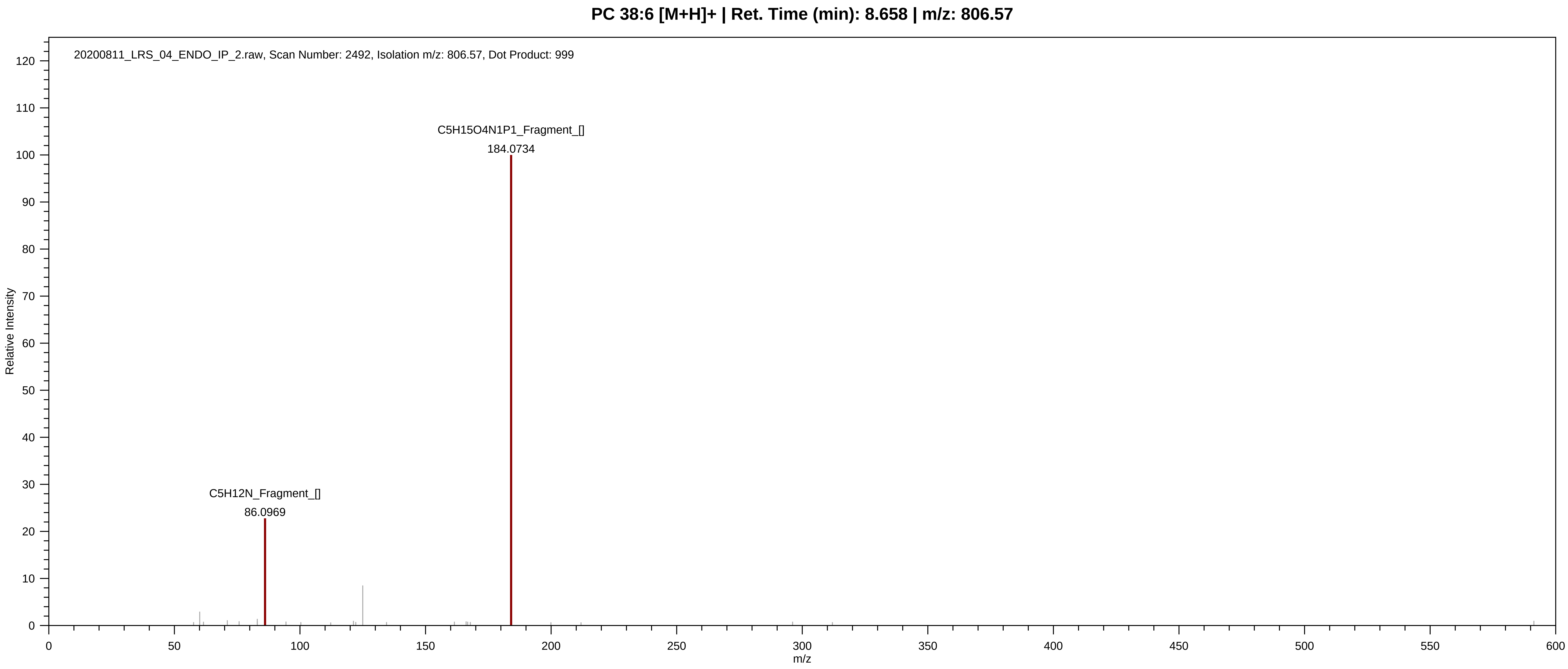

PC 38:6 [M+H]+ | Ret. Time (min): 8.658 | m/z: 806.57
20200811_LRS_04_ENDO_IP_2.raw, Scan Number: 2492, Isolation m/z: 806.57, Dot Product: 999
C5H15O4N1P1_Fragment_[]
184.0734
C5H12N_Fragment_[]
86.0969
Relative Intensity
m/z

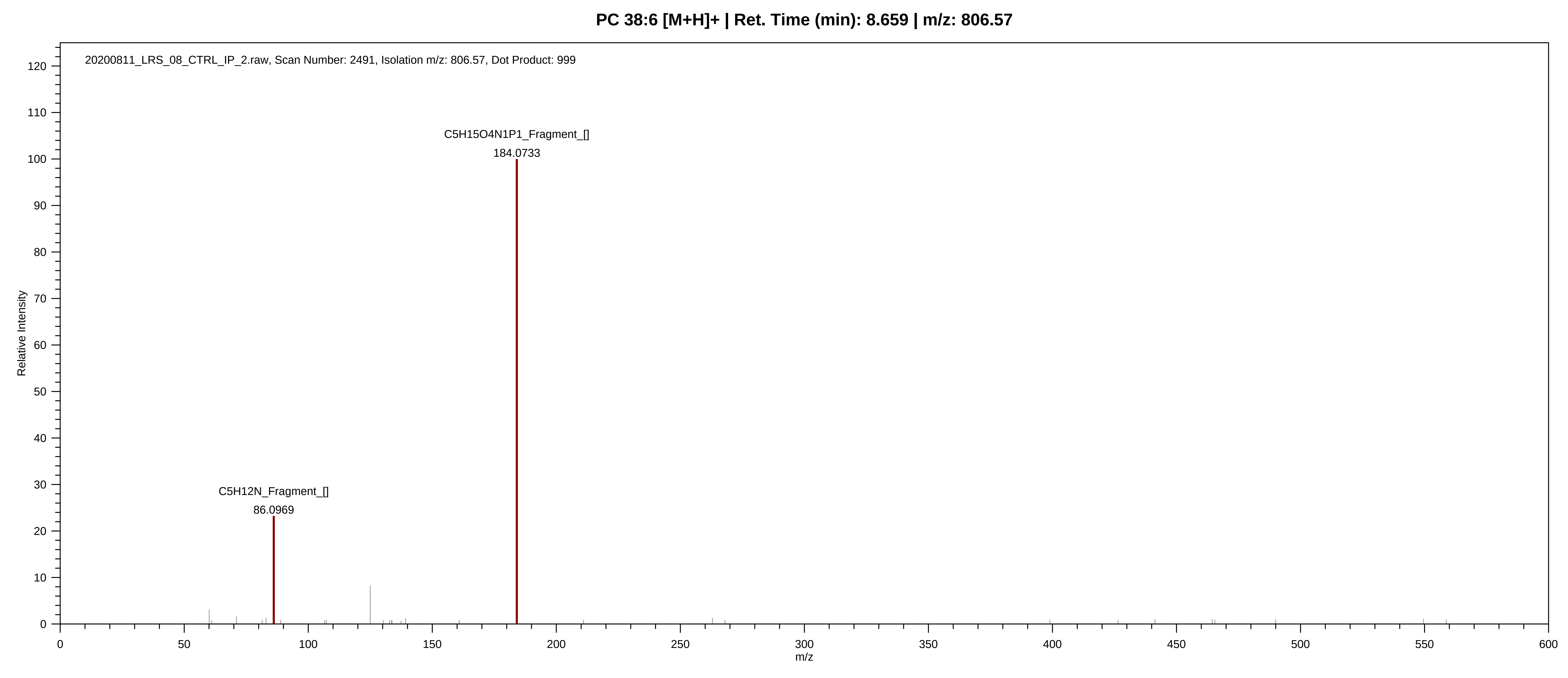
PC 38:6 [M+H]+ | Ret. Time (min): 8.659 | m/z: 806.57
20200811_LRS_08_CTRL_IP_2.raw, Scan Number: 2491, Isolation m/z: 806.57, Dot Product: 999
C5H15O4N1P1_Fragment_[]
184.0733
C5H12N_Fragment_[]
86.0969
Relative Intensity
m/z

PC 38:6 [M+H]+ | Ret. Time (min): 8.663 | m/z: 806.57

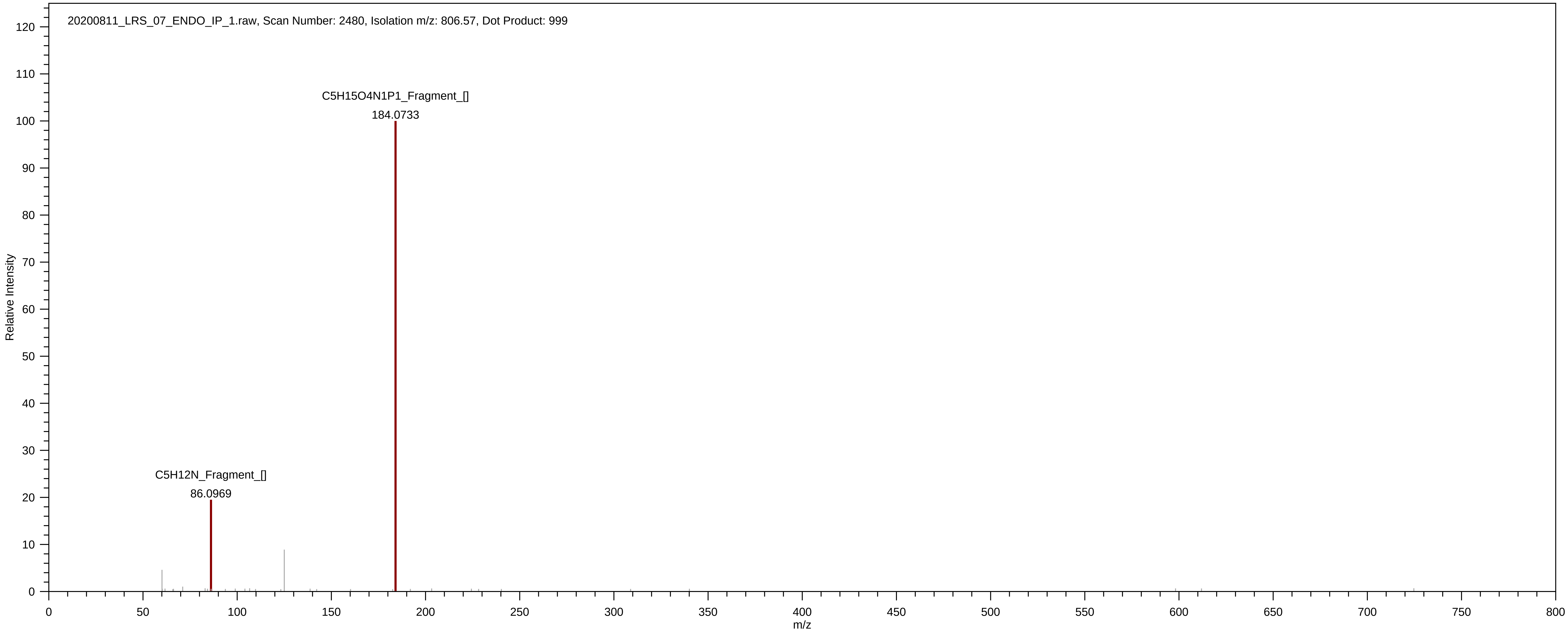

PC 38:6 [M+H]+ | Ret. Time (min): 8.663 | m/z: 806.57

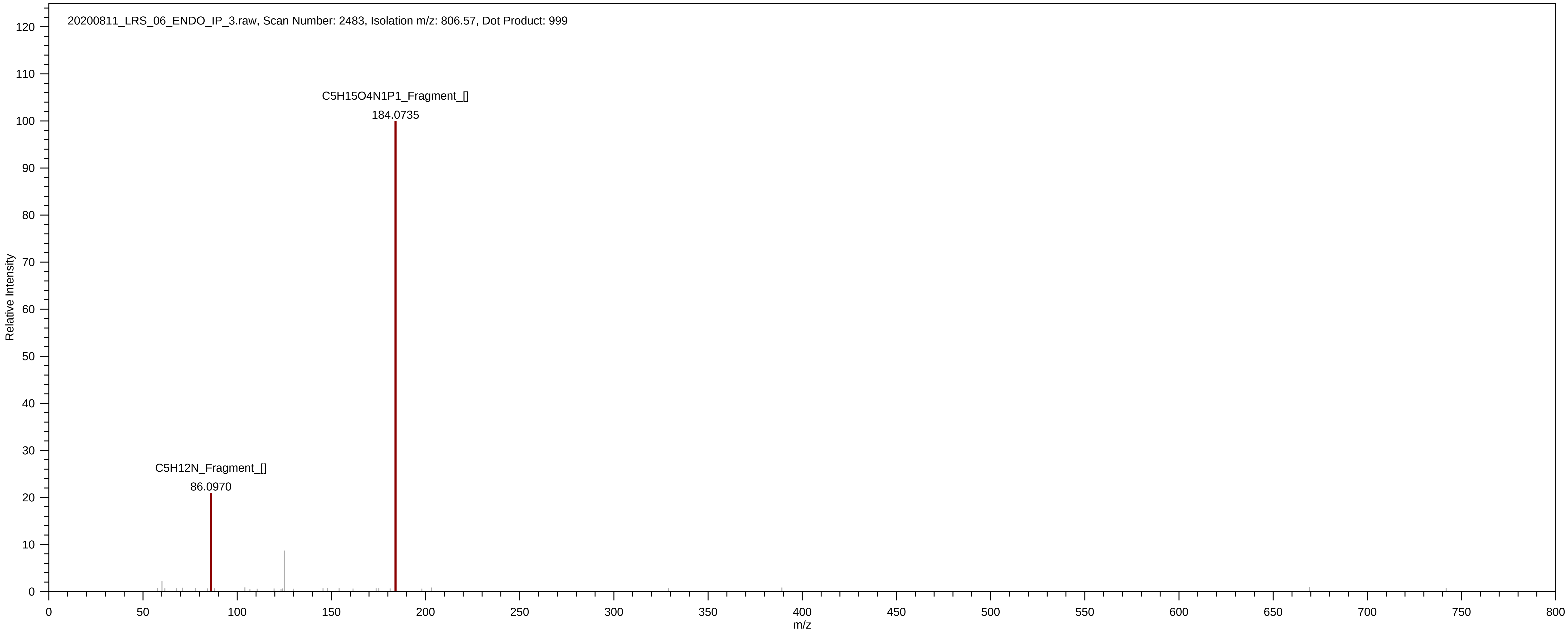

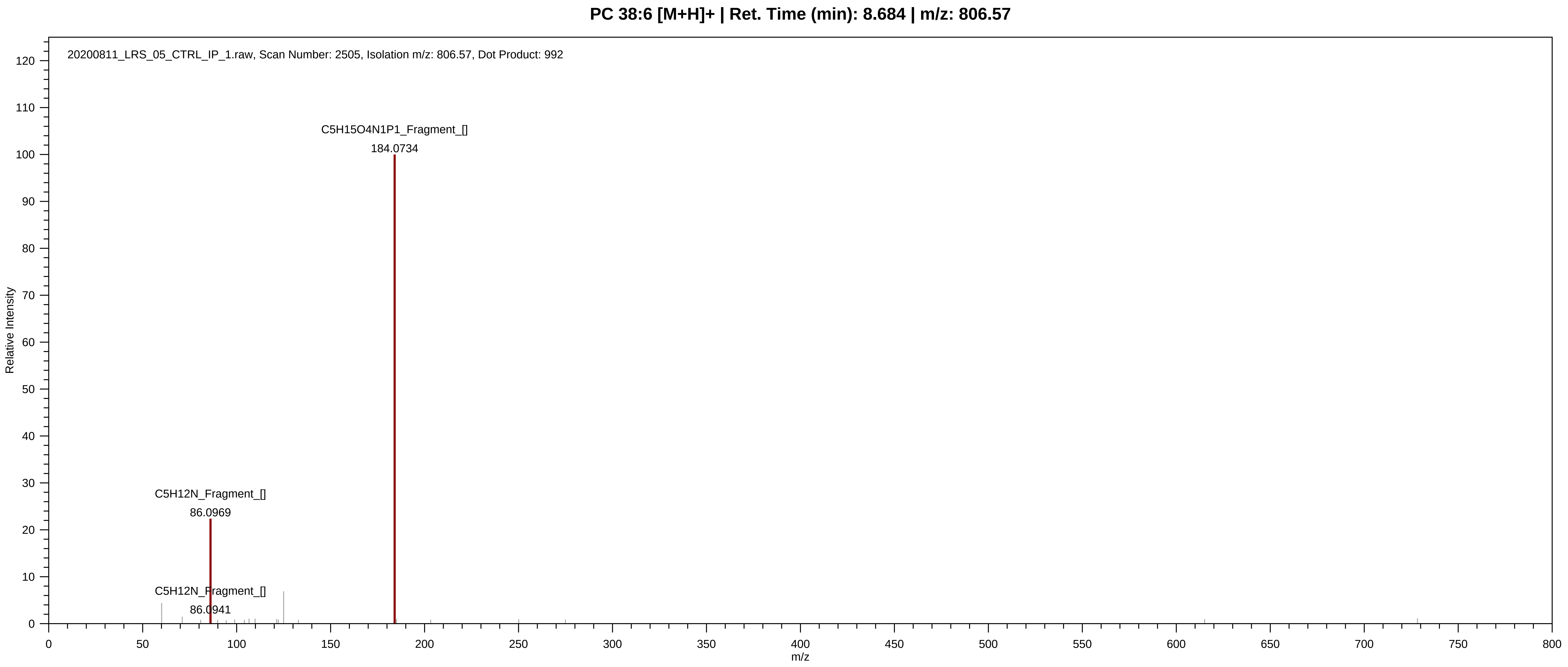
PC 38:6 [M+H]+ | Ret. Time (min): 8.684 | m/z: 806.57
20200811_LRS_05_CTRL_IP_1.raw, Scan Number: 2505, Isolation m/z: 806.57, Dot Product: 992
C5H15O4N1P1_Fragment_[]
184.0734
C5H12N_Fragment_[]
86.0969
C5H12N_Fragment_[]
86.0941
Relative Intensity
m/z

PC 38:6 [M+H]+ | Ret. Time (min): 8.656 | m/z: 806.569

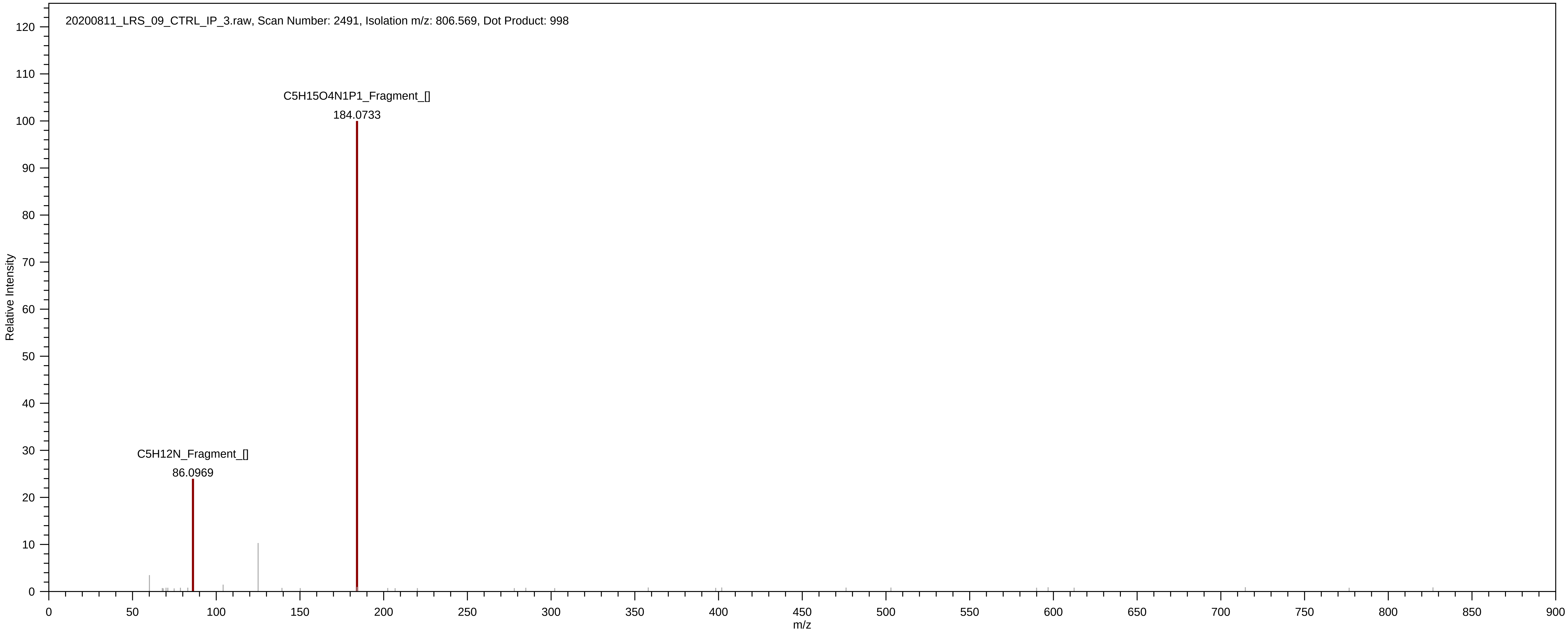

PC 38:7 [M+H]+ | Ret. Time (min): 8.001 | m/z: 804.552

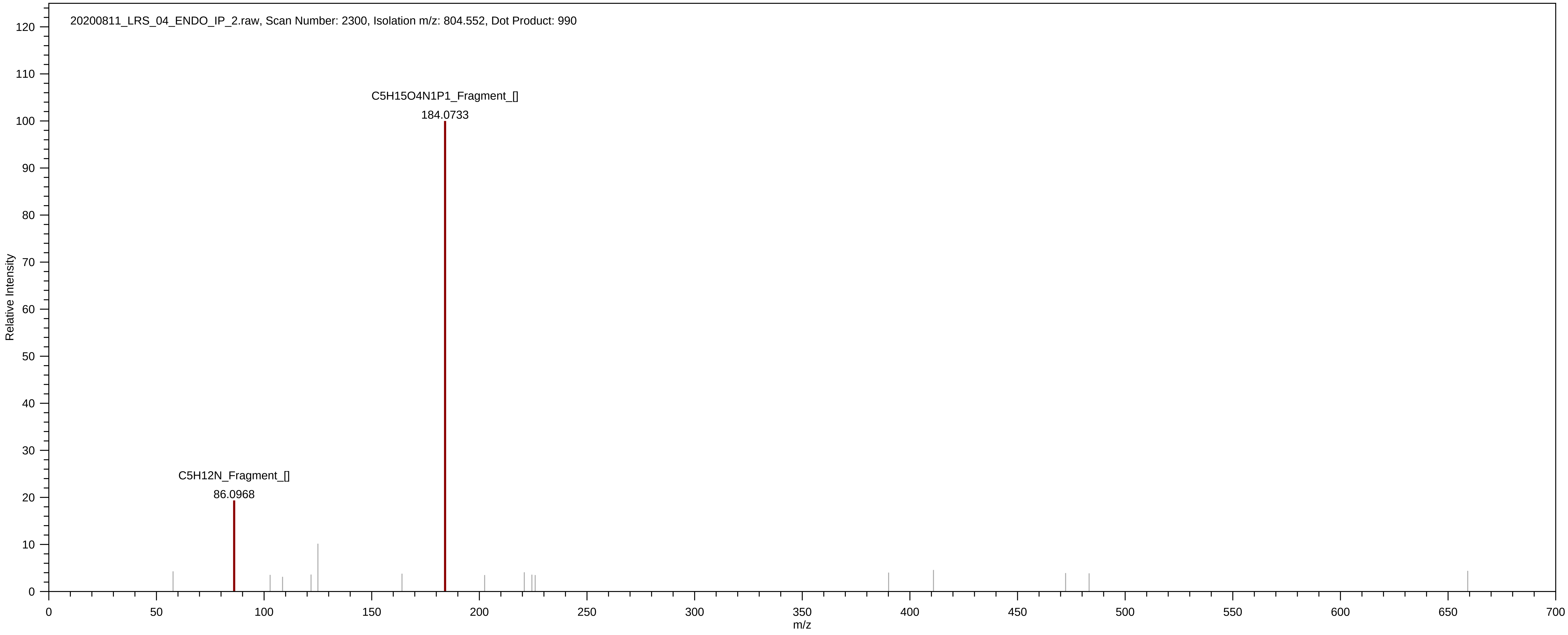

PC 39:2 [M+H]+ | Ret. Time (min): 11.327 | m/z: 828.652

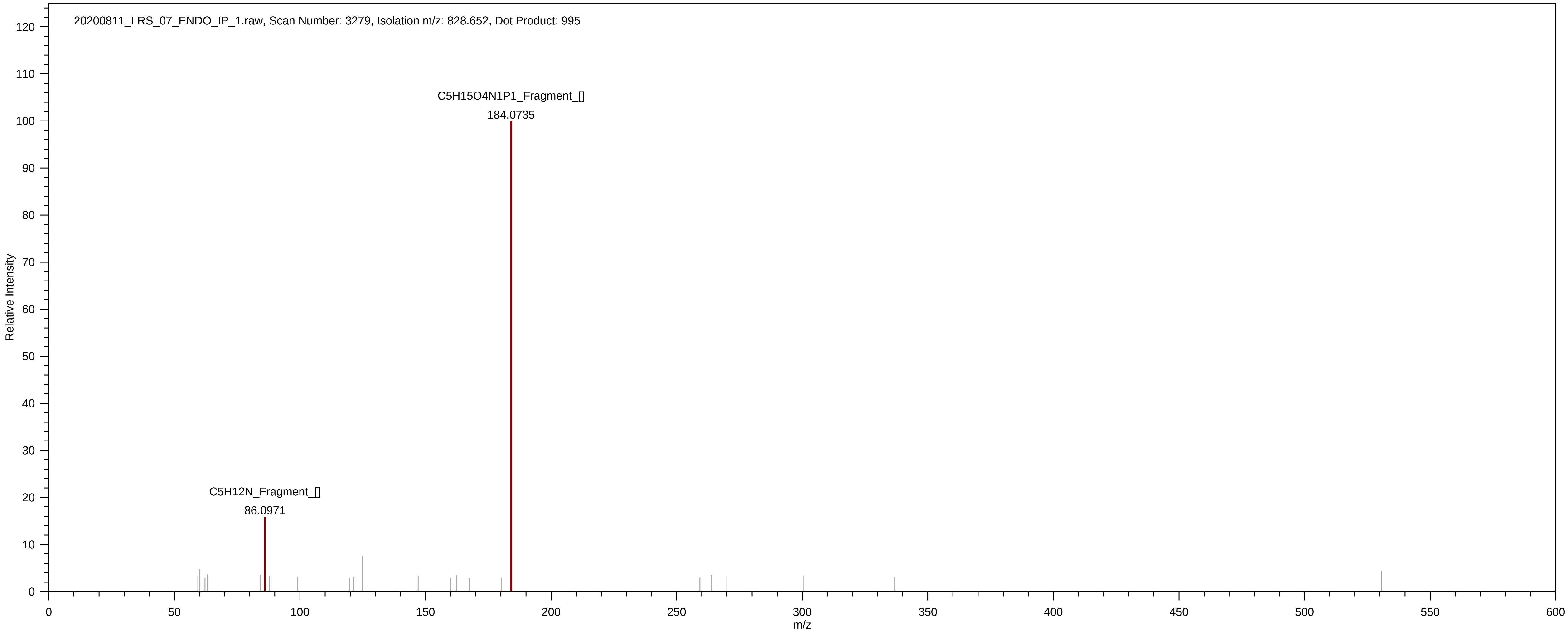

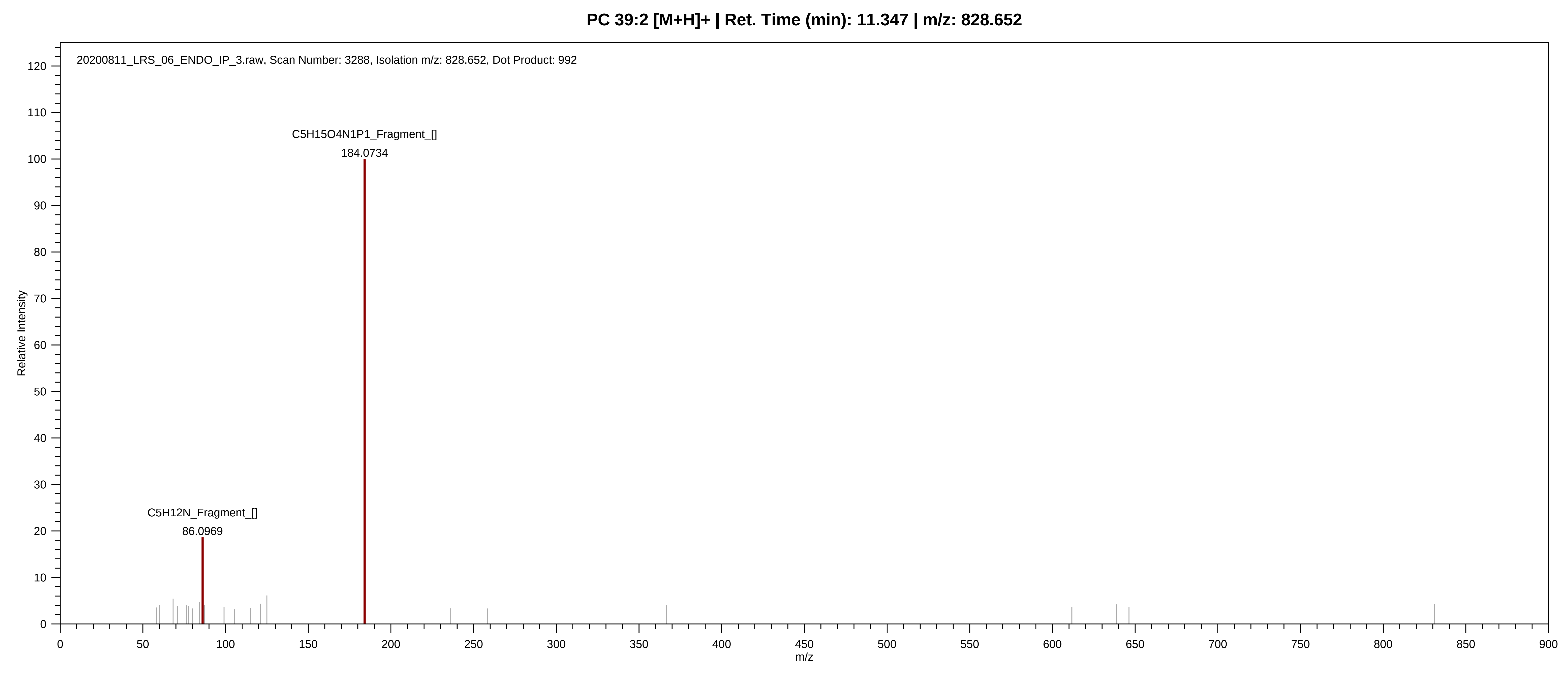

PC 39:2 [M+H]+ | Ret. Time (min): 11.347 | m/z: 828.652
20200811_LRS_06_ENDO_IP_3.raw, Scan Number: 3288, Isolation m/z: 828.652, Dot Product: 992
C5H15O4N1P1_Fragment_[]
184.0734
C5H12N_Fragment_[]
86.0969
Relative Intensity
m/z

PC 40:0 [M+H]+ | Ret. Time (min): 13.825 | m/z: 846.693

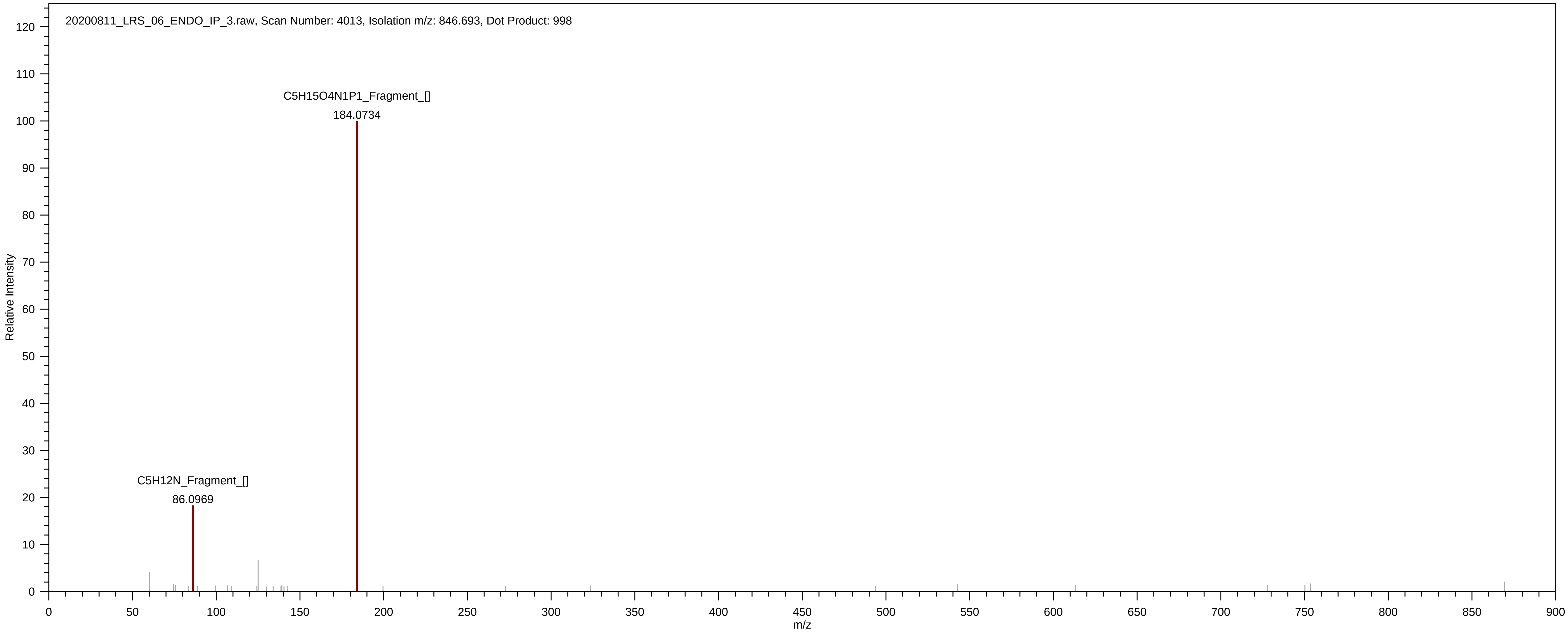

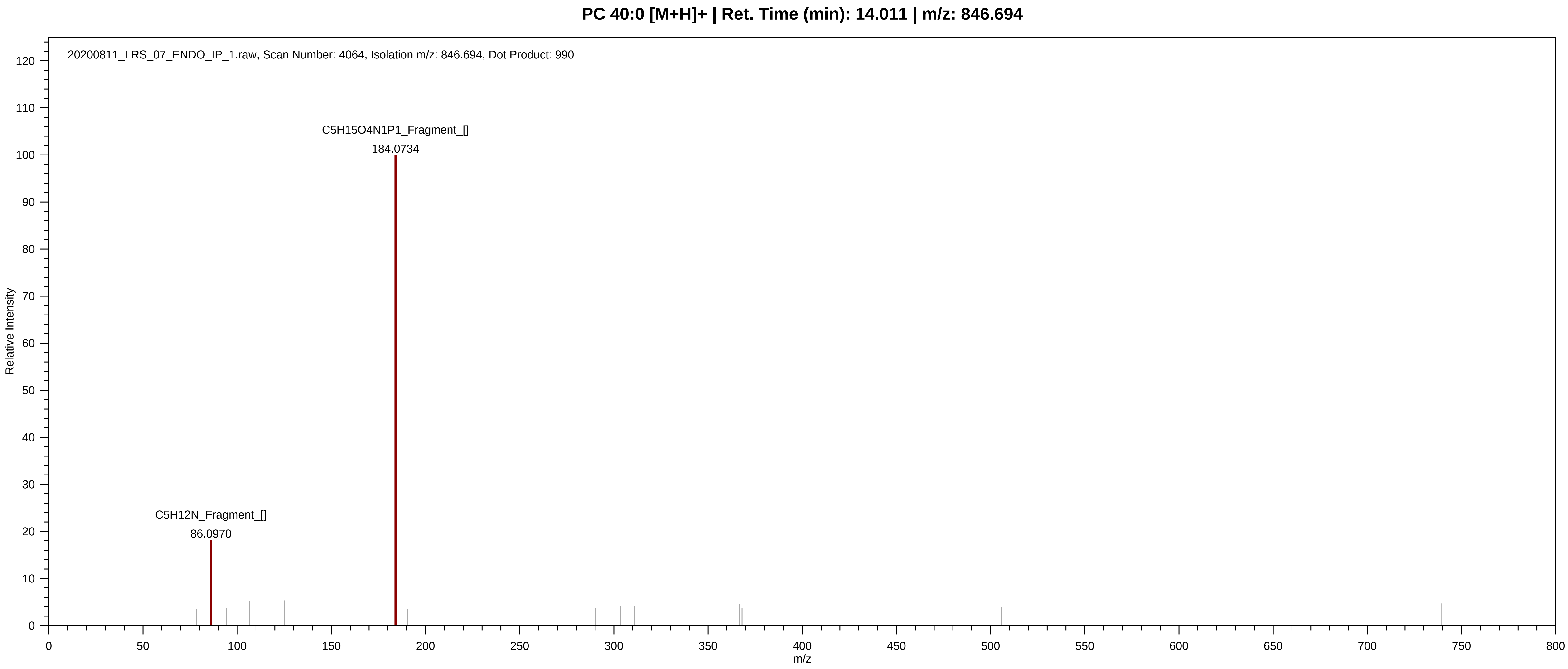
PC 40:0 [M+H]+ | Ret. Time (min): 14.011 | m/z: 846.694
20200811_LRS_07_ENDO_IP_1.raw, Scan Number: 4064, Isolation m/z: 846.694, Dot Product: 990
C5H15O4N1P1_Fragment_[]
184.0734
C5H12N_Fragment_[]
86.0970
Relative Intensity
m/z

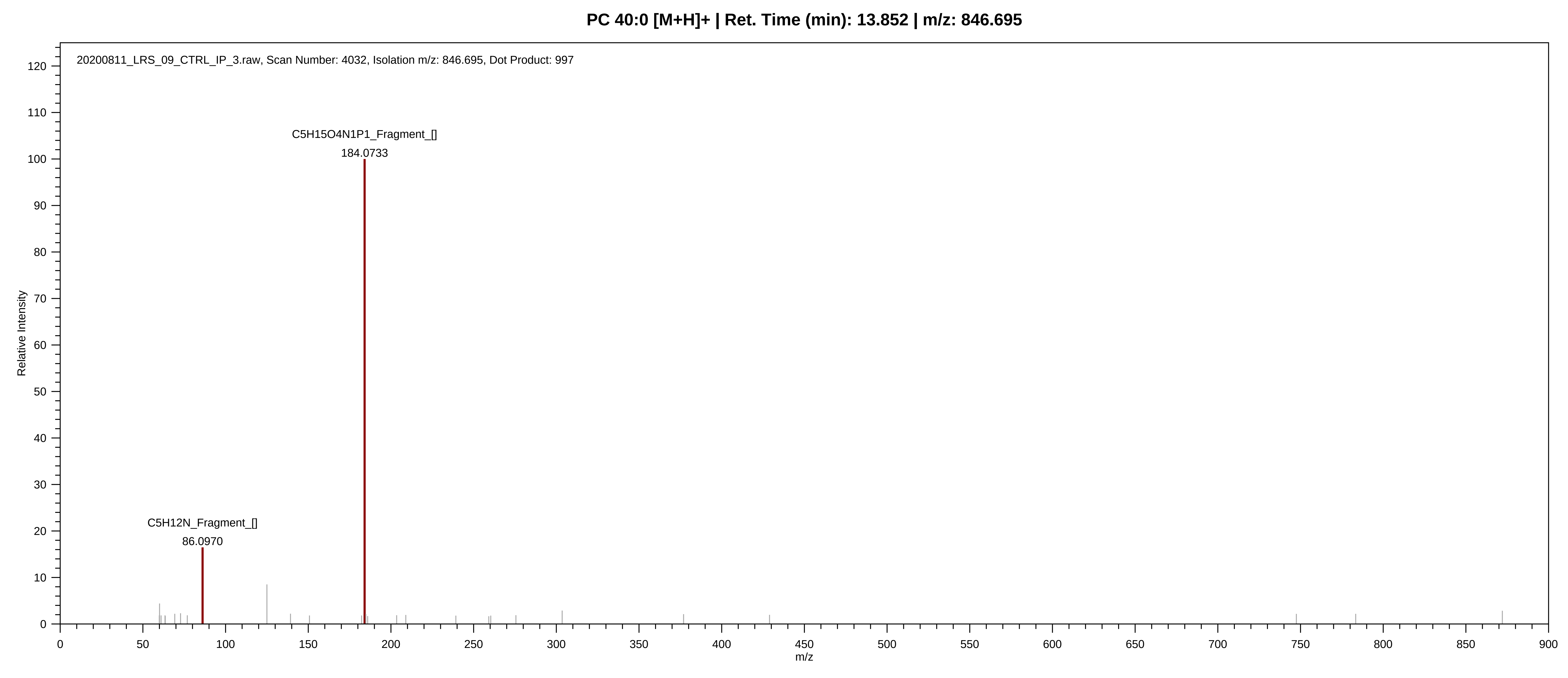
PC 40:0 [M+H]+ | Ret. Time (min): 13.852 | m/z: 846.695
20200811_LRS_09_CTRL_IP_3.raw, Scan Number: 4032, Isolation m/z: 846.695, Dot Product: 997
C5H15O4N1P1_Fragment_[]
184.0733
C5H12N_Fragment_[]
86.0970
Relative Intensity
m/z

PC 40:0 [M+H]+ | Ret. Time (min): 13.864 | m/z: 846.695

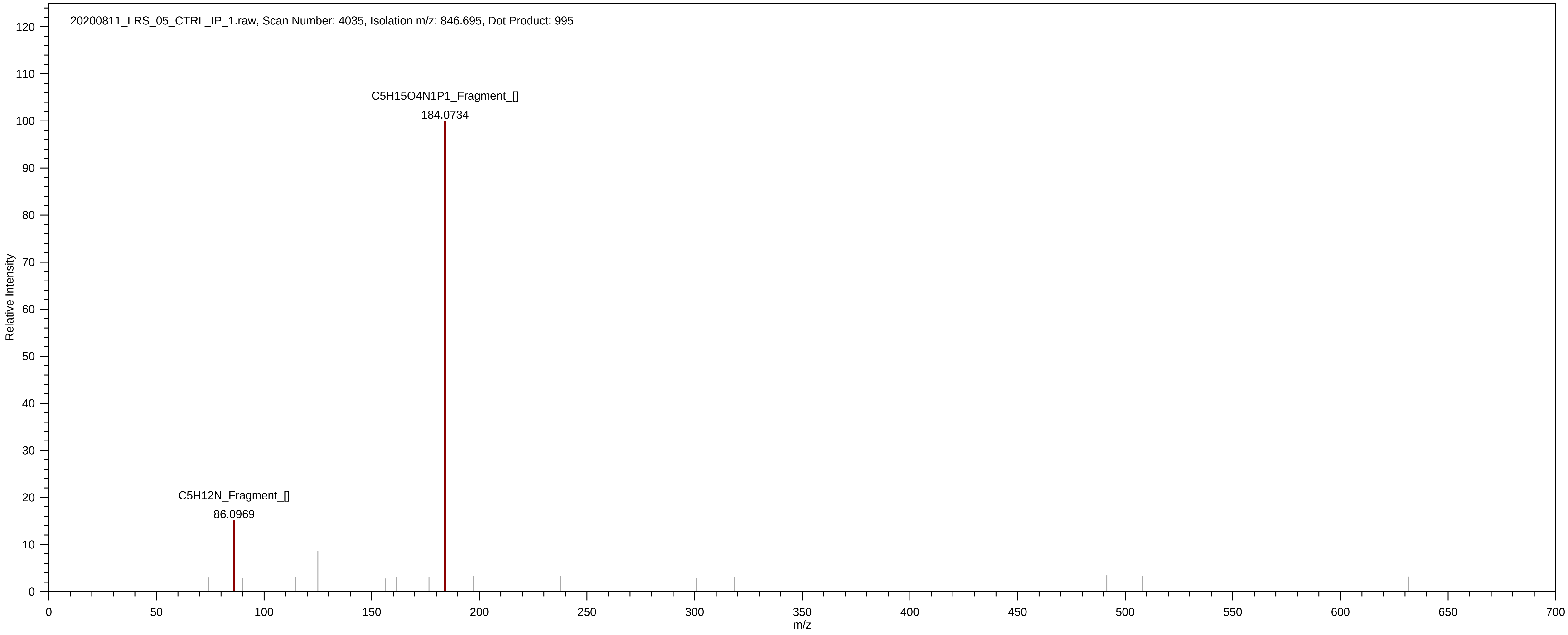

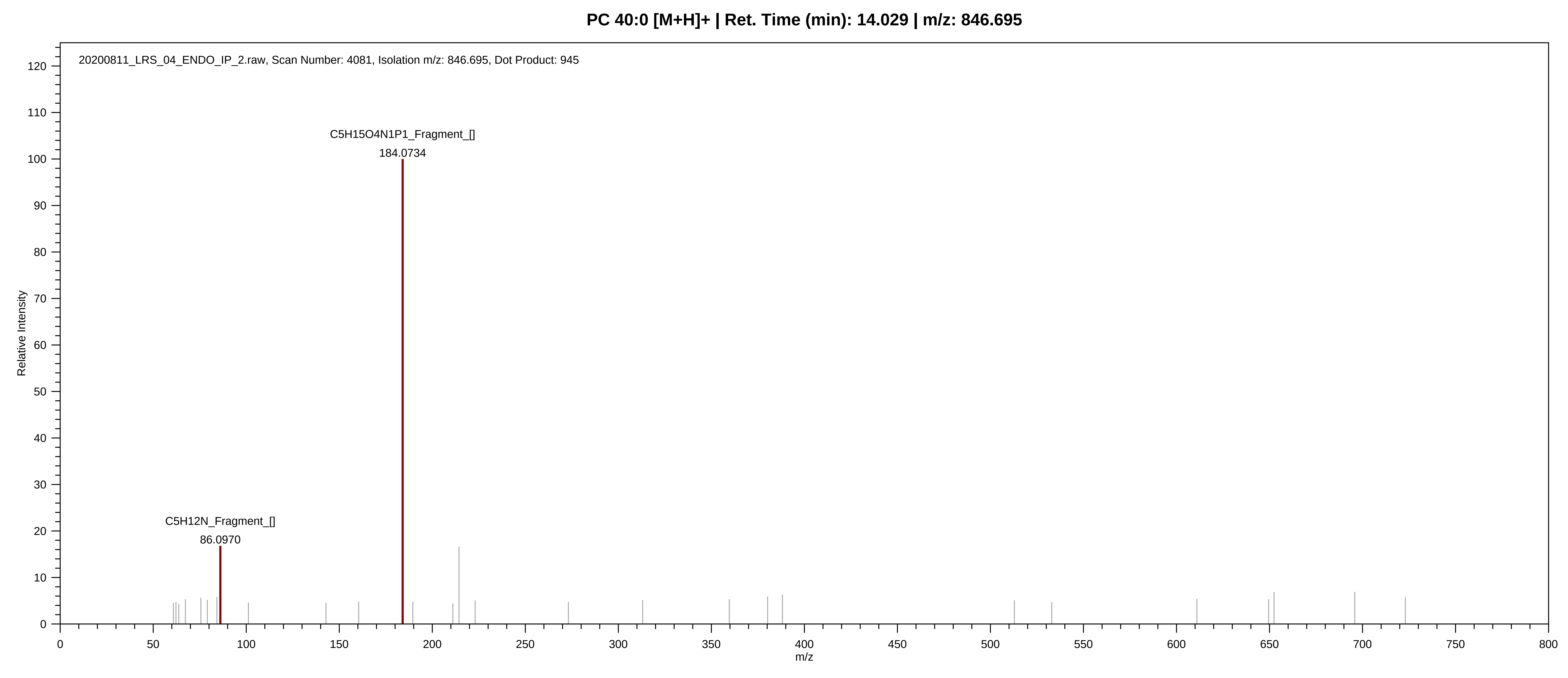
PC 40:0 [M+H]+ | Ret. Time (min): 14.029 | m/z: 846.695
20200811_LRS_04_ENDO_IP_2.raw, Scan Number: 4081, Isolation m/z: 846.695, Dot Product: 945
C5H15O4N1P1_Fragment_[]
184.0734
C5H12N_Fragment_[]
86.0970
Relative Intensity
m/z

PC 40:0 [M+H]+ | Ret. Time (min): 13.843 | m/z: 846.696

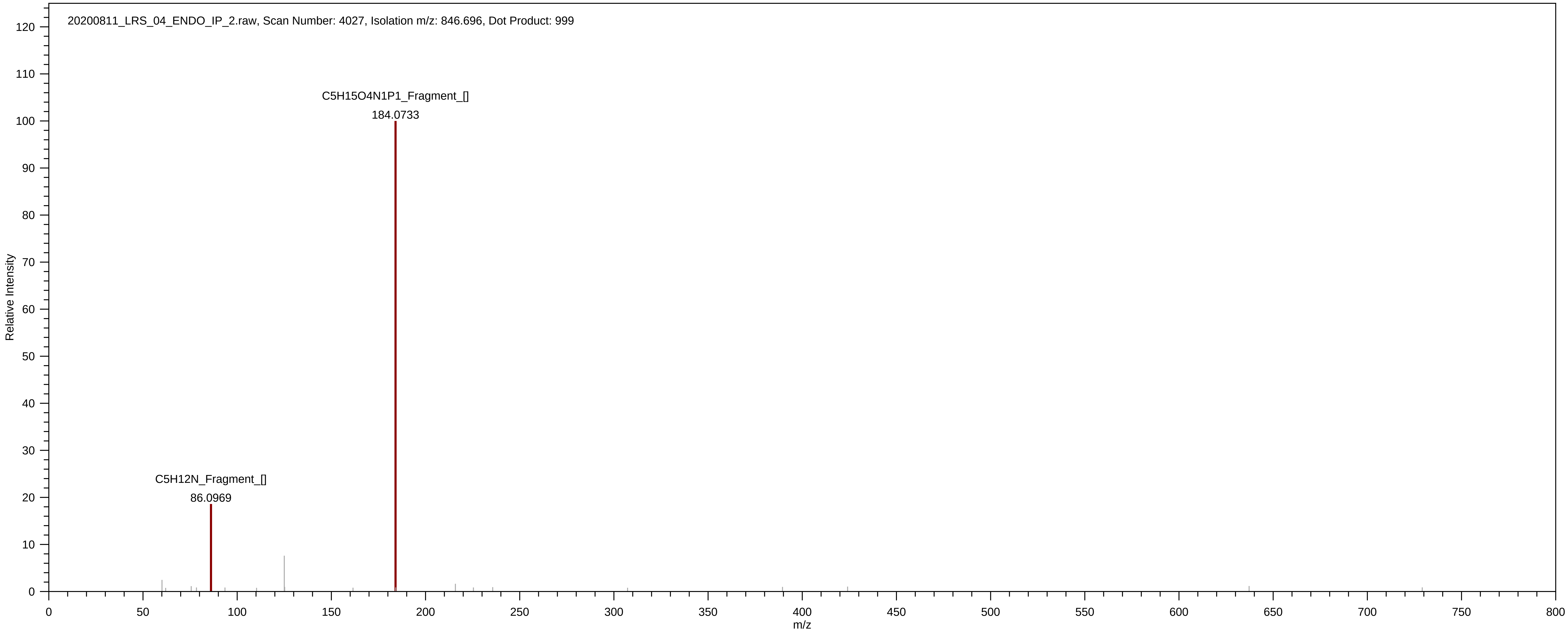

PC 40:0 [M+H]+ | Ret. Time (min): 13.849 | m/z: 846.696

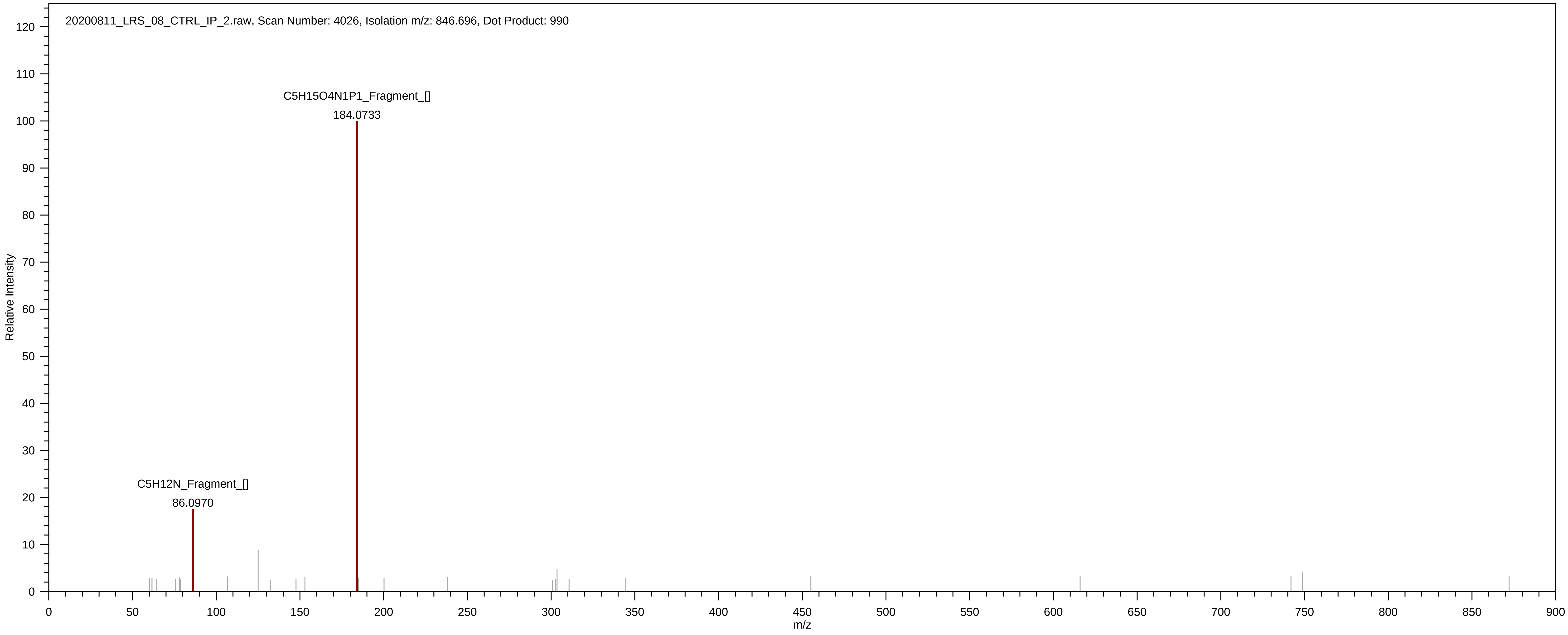

PC 40:0 [M+H]+ | Ret. Time (min): 14.011 | m/z: 846.696

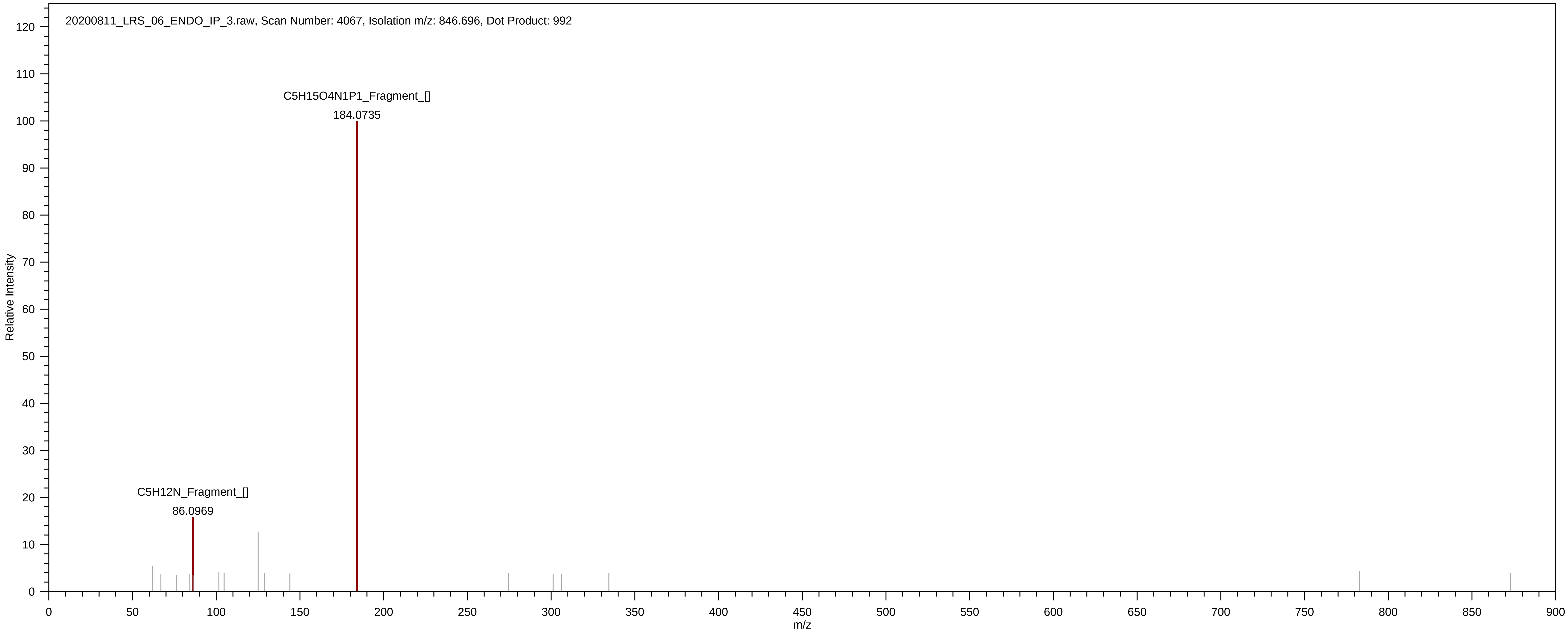

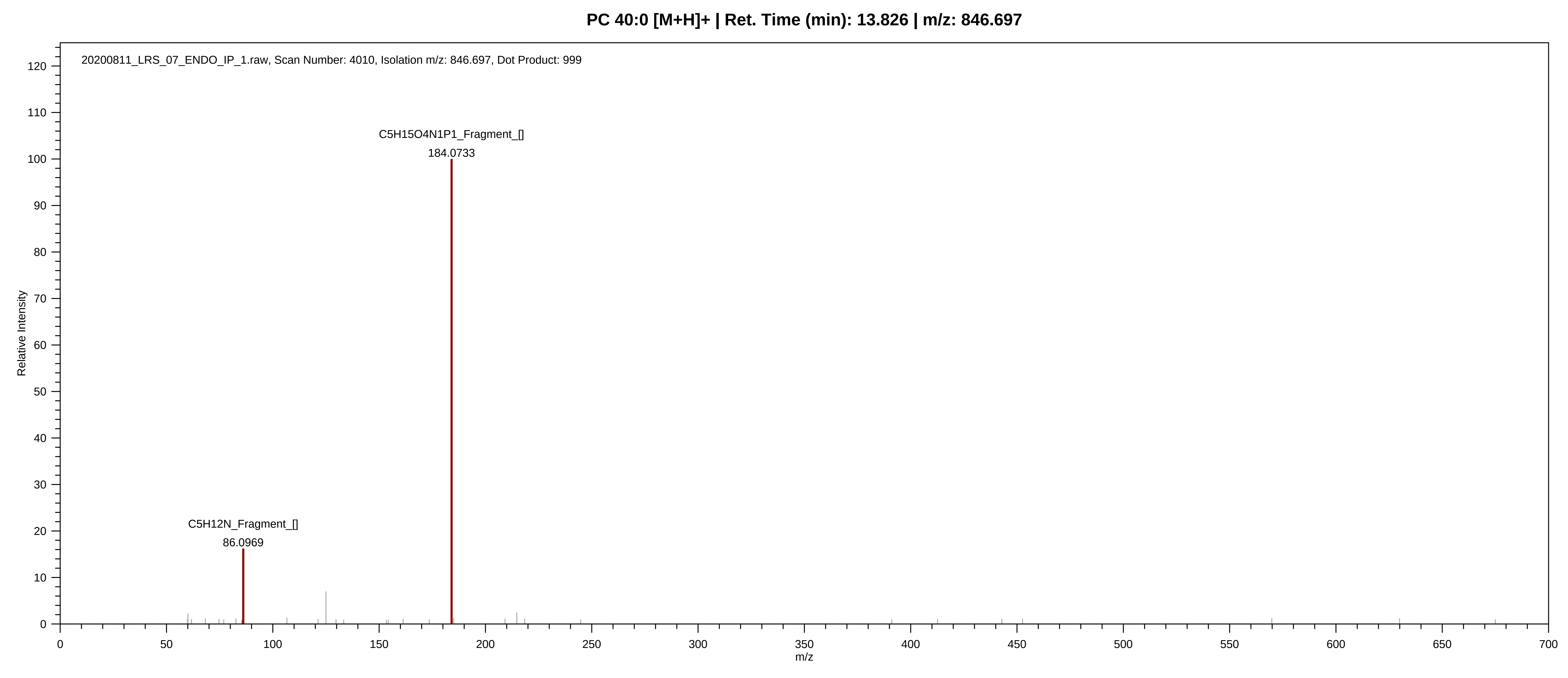

PC 40:0 [M+H]+ | Ret. Time (min): 13.826 | m/z: 846.697
20200811_LRS_07_ENDO_IP_1.raw, Scan Number: 4010, Isolation m/z: 846.697, Dot Product: 999
C5H15O4N1P1_Fragment_[]
184.0733
C5H12N_Fragment_[]
86.0969
Relative Intensity
m/z

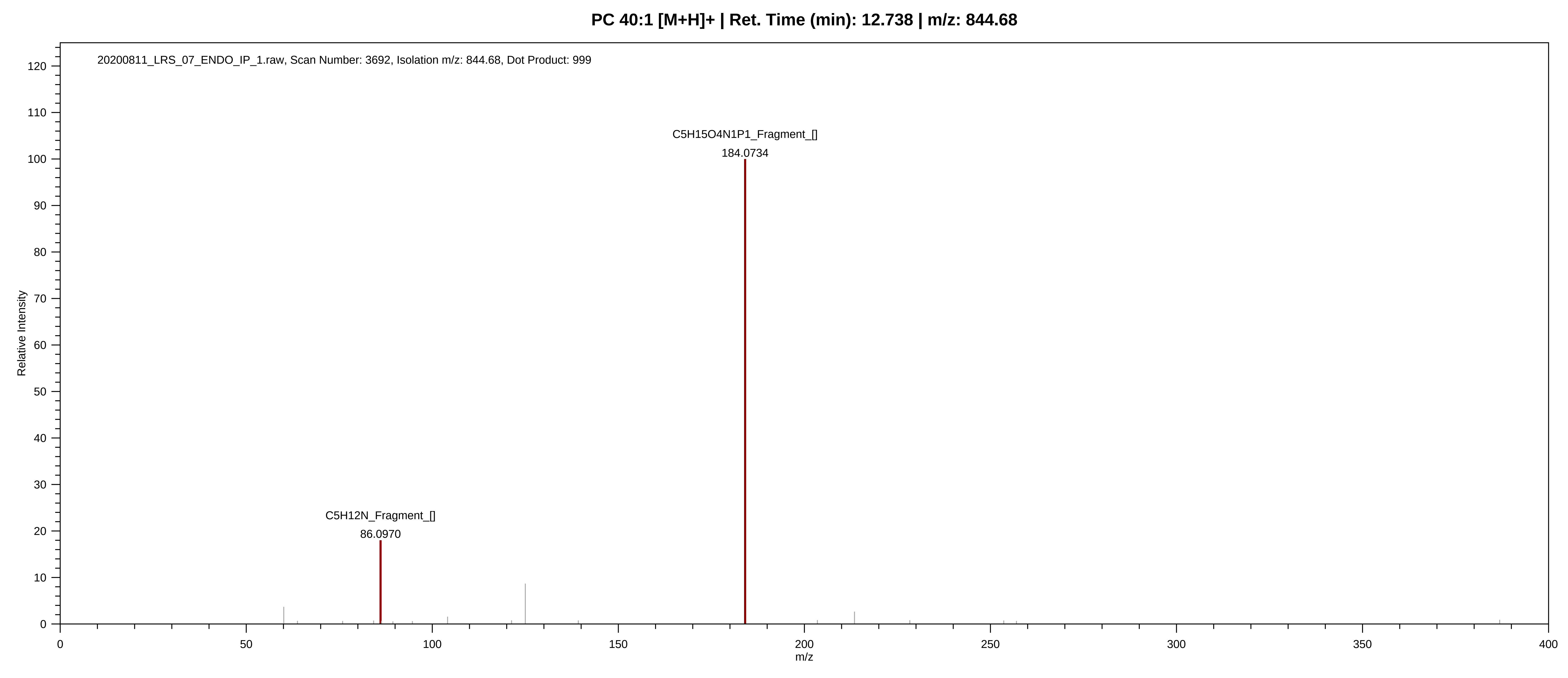

PC 40:1 [M+H]+ | Ret. Time (min): 12.738 | m/z: 844.68
20200811_LRS_07_ENDO_IP_1.raw, Scan Number: 3692, Isolation m/z: 844.68, Dot Product: 999
C5H15O4N1P1_Fragment_[]
184.0734
C5H12N_Fragment_[]
86.0970
Relative Intensity
m/z

PC 40:1 [M+H]+ | Ret. Time (min): 12.738 | m/z: 844.68

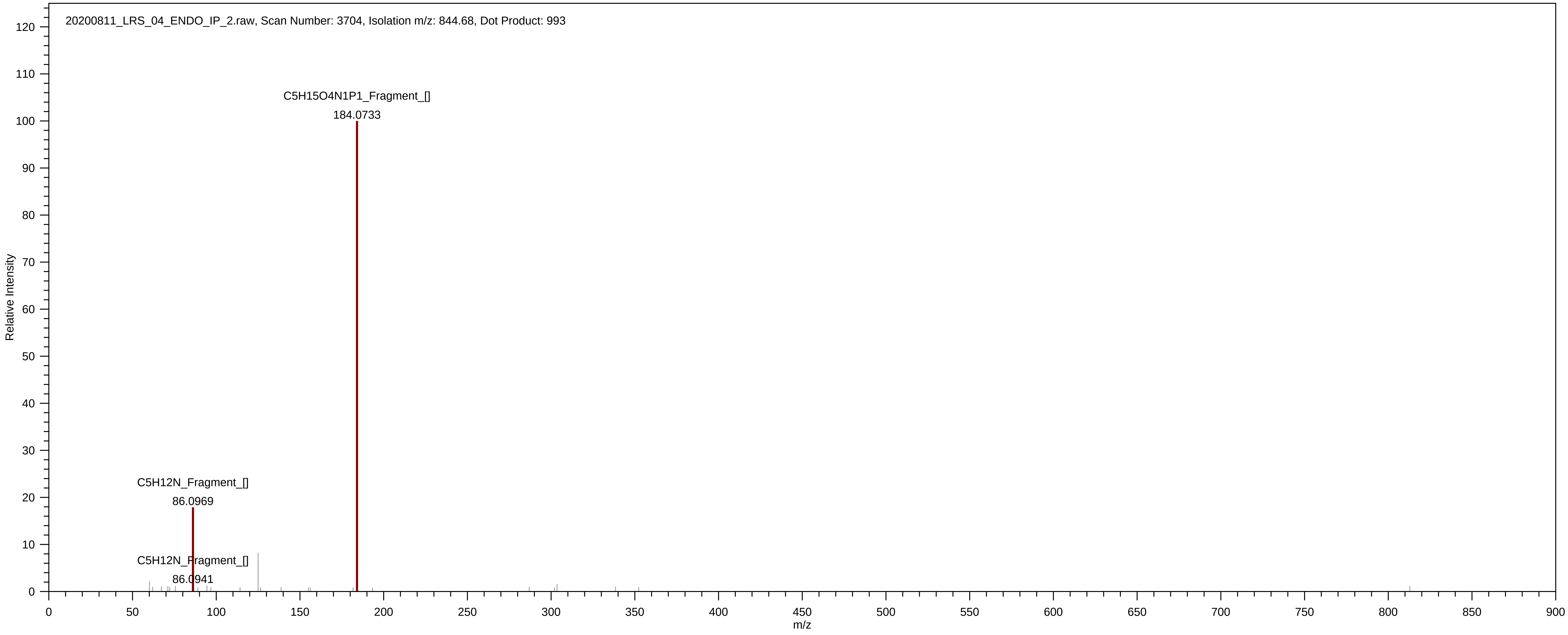

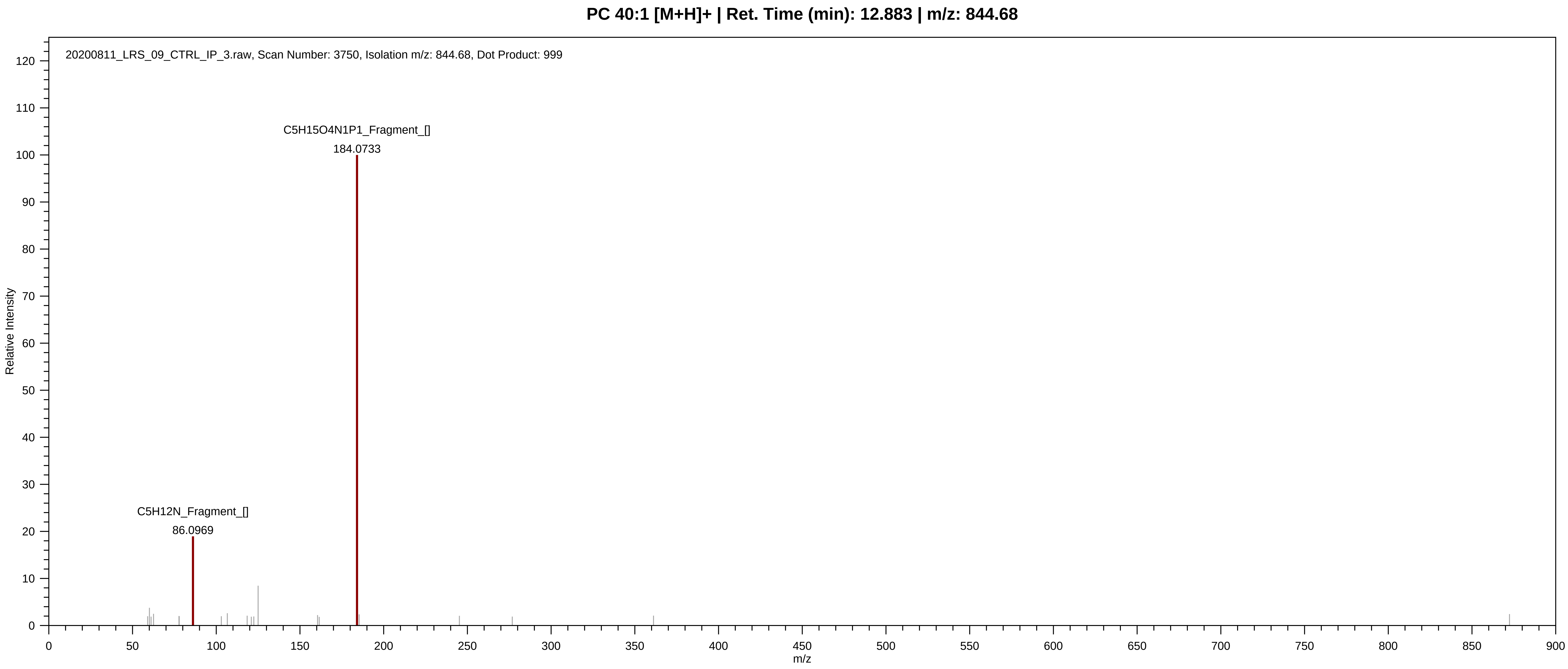

PC 40:1 [M+H]+ | Ret. Time (min): 12.883 | m/z: 844.68
20200811_LRS_09_CTRL_IP_3.raw, Scan Number: 3750, Isolation m/z: 844.68, Dot Product: 999
C5H15O4N1P1_Fragment_[]
184.0733
C5H12N_Fragment_[]
86.0969
Relative Intensity
m/z

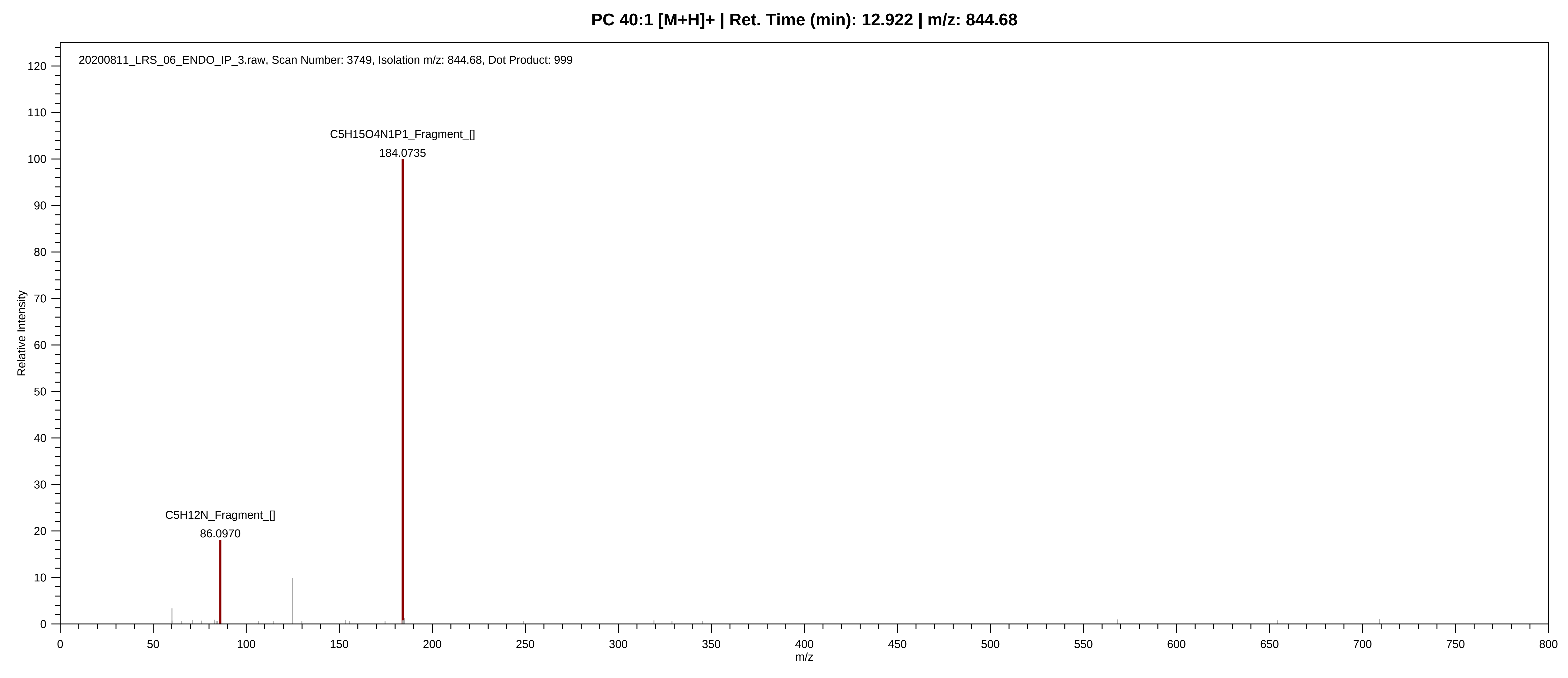
PC 40:1 [M+H]+ | Ret. Time (min): 12.922 | m/z: 844.68
20200811_LRS_06_ENDO_IP_3.raw, Scan Number: 3749, Isolation m/z: 844.68, Dot Product: 999
C5H15O4N1P1_Fragment_[]
184.0735
C5H12N_Fragment_[]
86.0970
Relative Intensity
m/z
0
10
20
30
40
50
60
70
80
90
100
110
120
0
50
100
150
200
250
300
350
400
450
500
550
600
650
700
750
800

PC 40:1 [M+H]+ | Ret. Time (min): 12.923 | m/z: 844.68

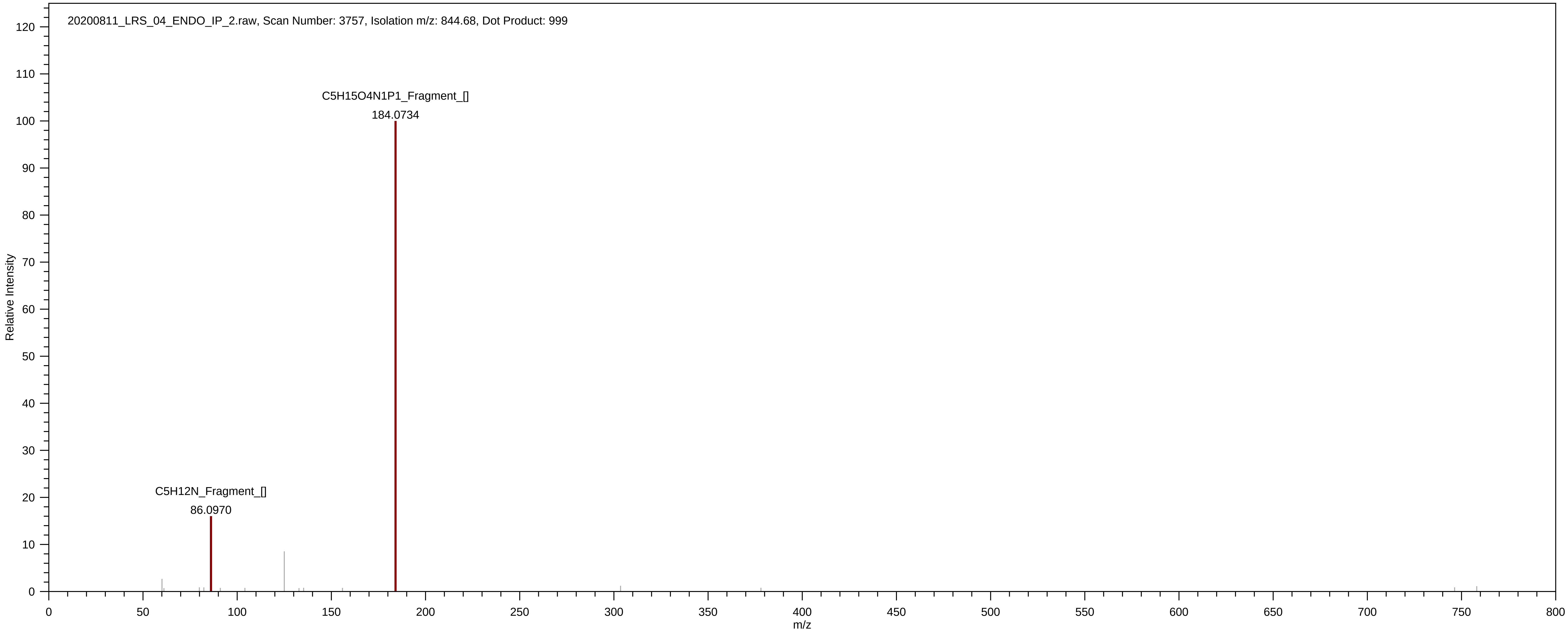

PC 40:1 [M+H]+ | Ret. Time (min): 12.925 | m/z: 844.68

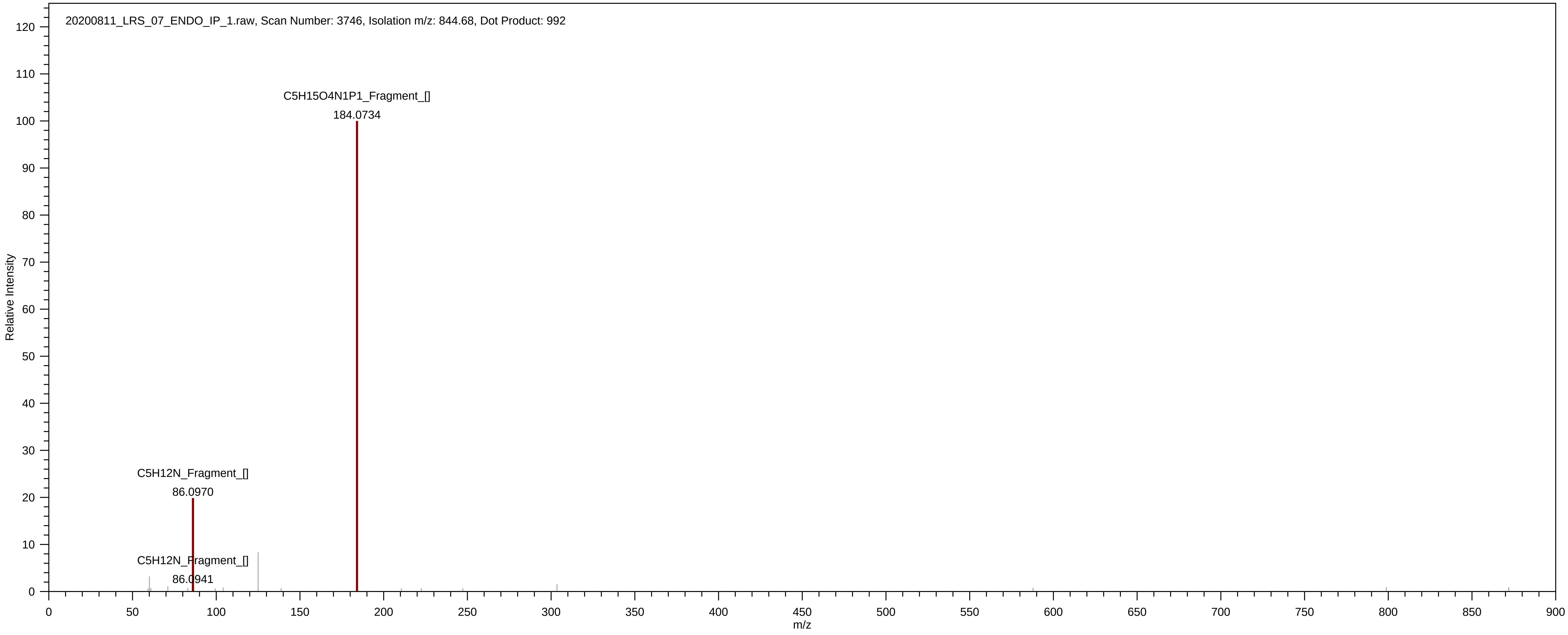

PC 40:1 [M+H]+ | Ret. Time (min): 12.9 | m/z: 844.678

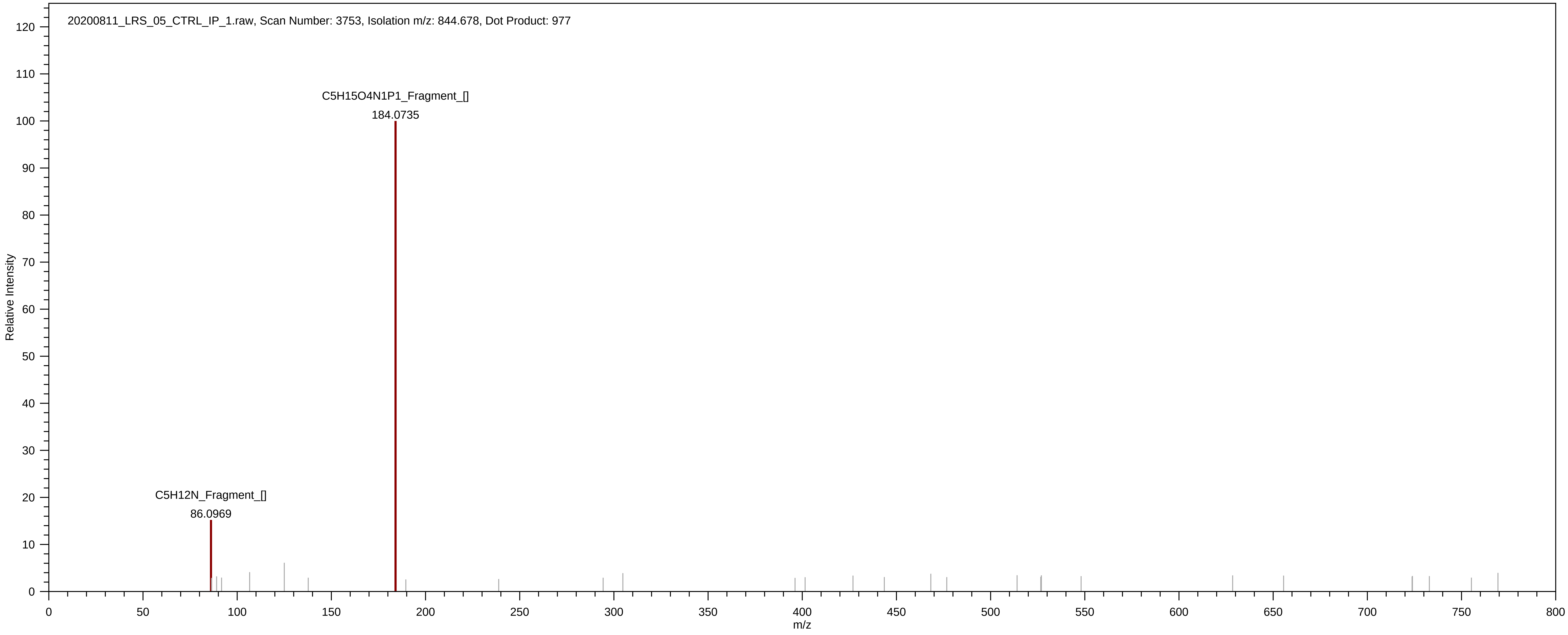

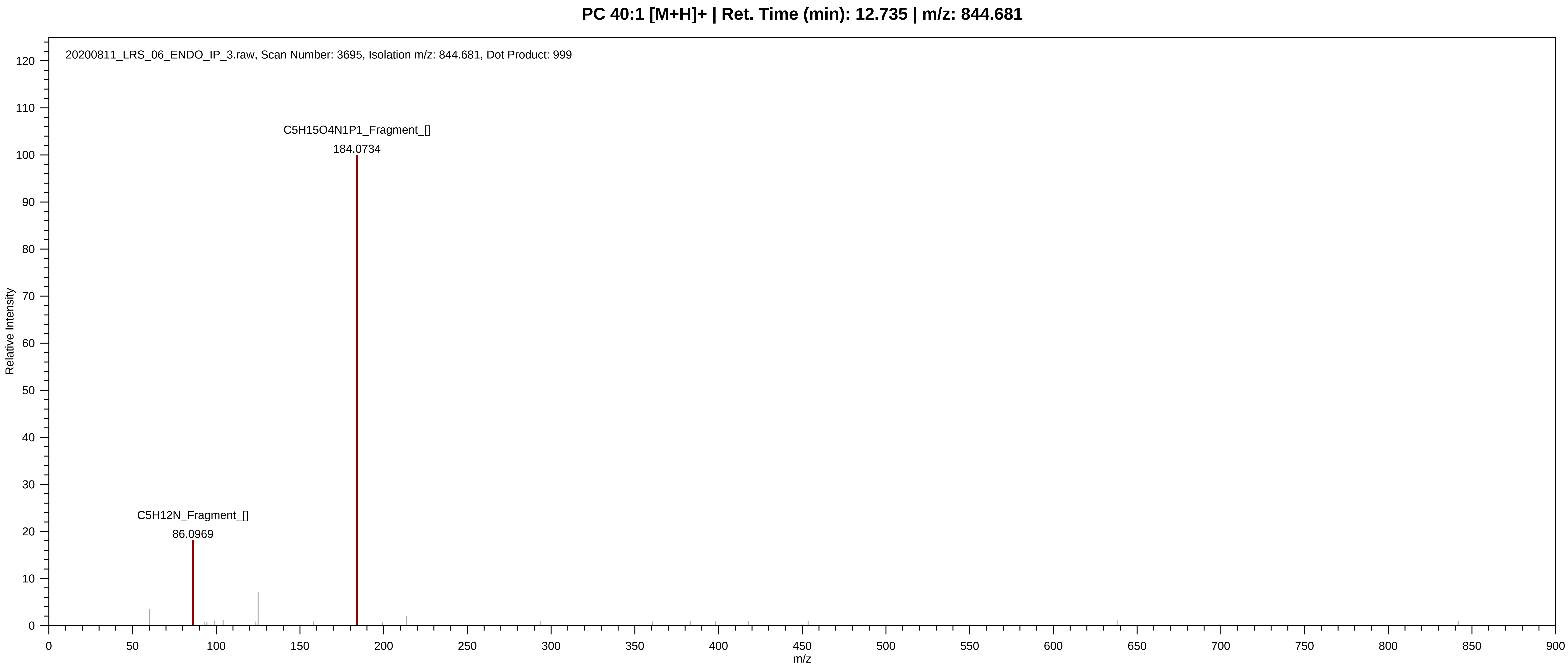
PC 40:1 [M+H]+ | Ret. Time (min): 12.735 | m/z: 844.681
20200811_LRS_06_ENDO_IP_3.raw, Scan Number: 3695, Isolation m/z: 844.681, Dot Product: 999
C5H15O4N1P1_Fragment_[]
184.0734
C5H12N_Fragment_[]
86.0969
Relative Intensity
m/z

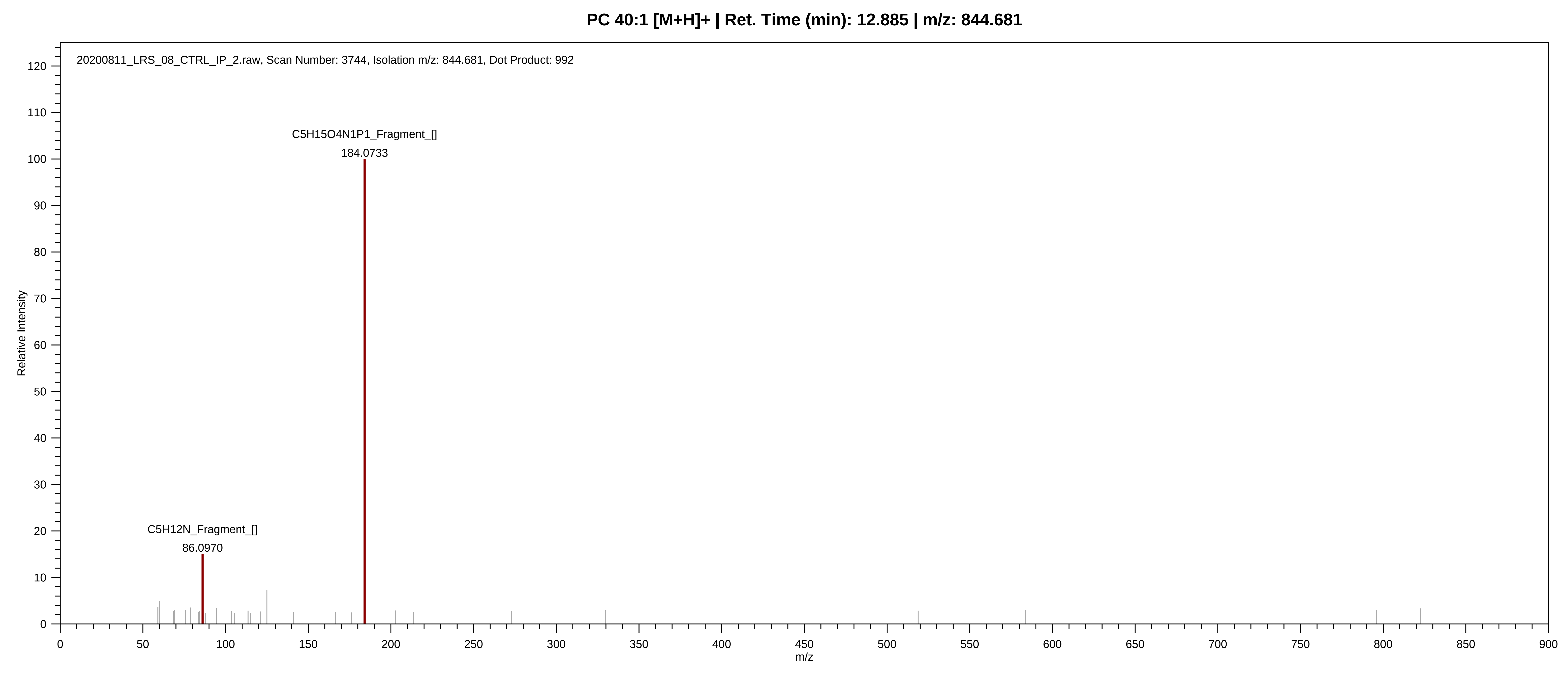
PC 40:1 [M+H]+ | Ret. Time (min): 12.885 | m/z: 844.681
20200811_LRS_08_CTRL_IP_2.raw, Scan Number: 3744, Isolation m/z: 844.681, Dot Product: 992
C5H15O4N1P1_Fragment_[]
184.0733
C5H12N_Fragment_[]
86.0970
Relative Intensity
m/z

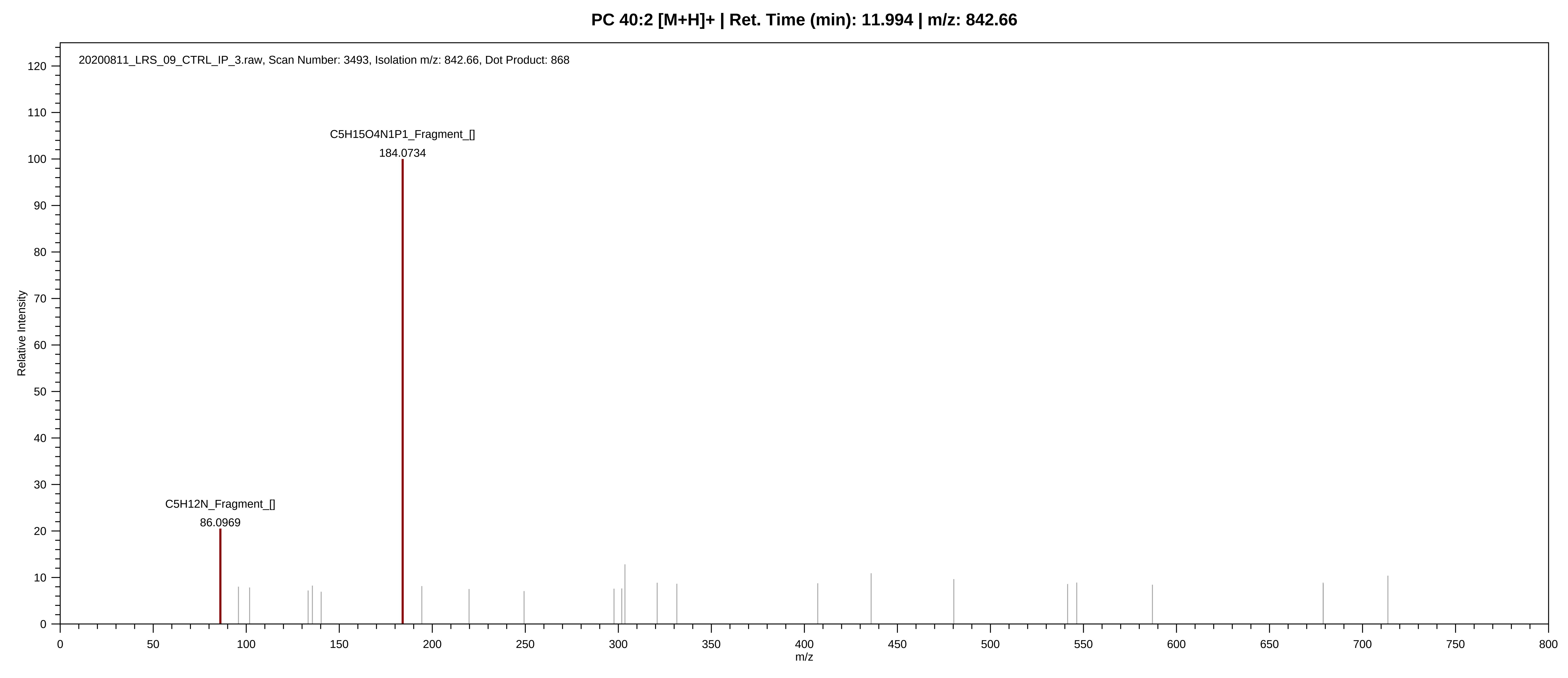
PC 40:2 [M+H]+ | Ret. Time (min): 11.994 | m/z: 842.66
20200811_LRS_09_CTRL_IP_3.raw, Scan Number: 3493, Isolation m/z: 842.66, Dot Product: 868
C5H15O4N1P1_Fragment_[]
184.0734
C5H12N_Fragment_[]
86.0969
Relative Intensity
m/z

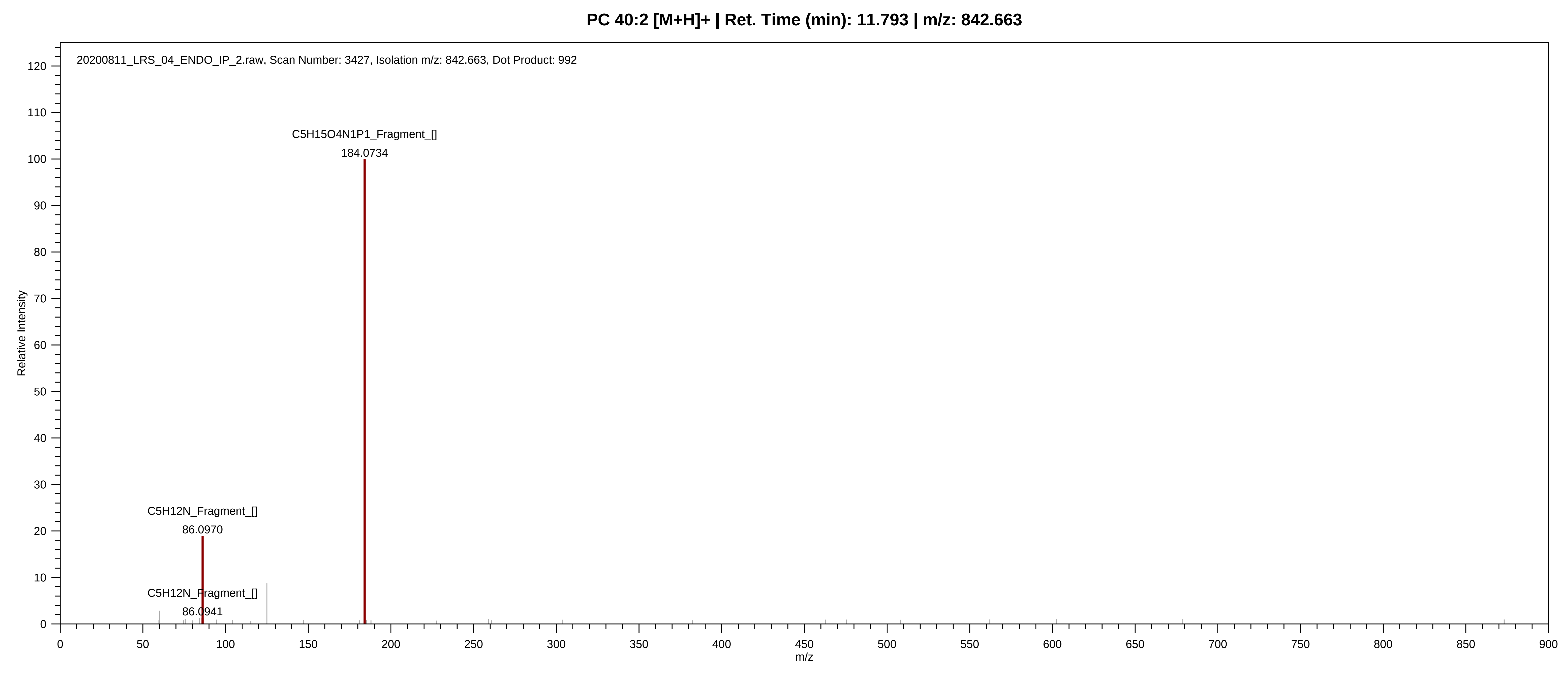
PC 40:2 [M+H]+ | Ret. Time (min): 11.793 | m/z: 842.663
20200811_LRS_04_ENDO_IP_2.raw, Scan Number: 3427, Isolation m/z: 842.663, Dot Product: 992
C5H15O4N1P1_Fragment_[]
184.0734
C5H12N_Fragment_[]
86.0970
C5H12N_Fragment_[]
86.0941
Relative Intensity
m/z

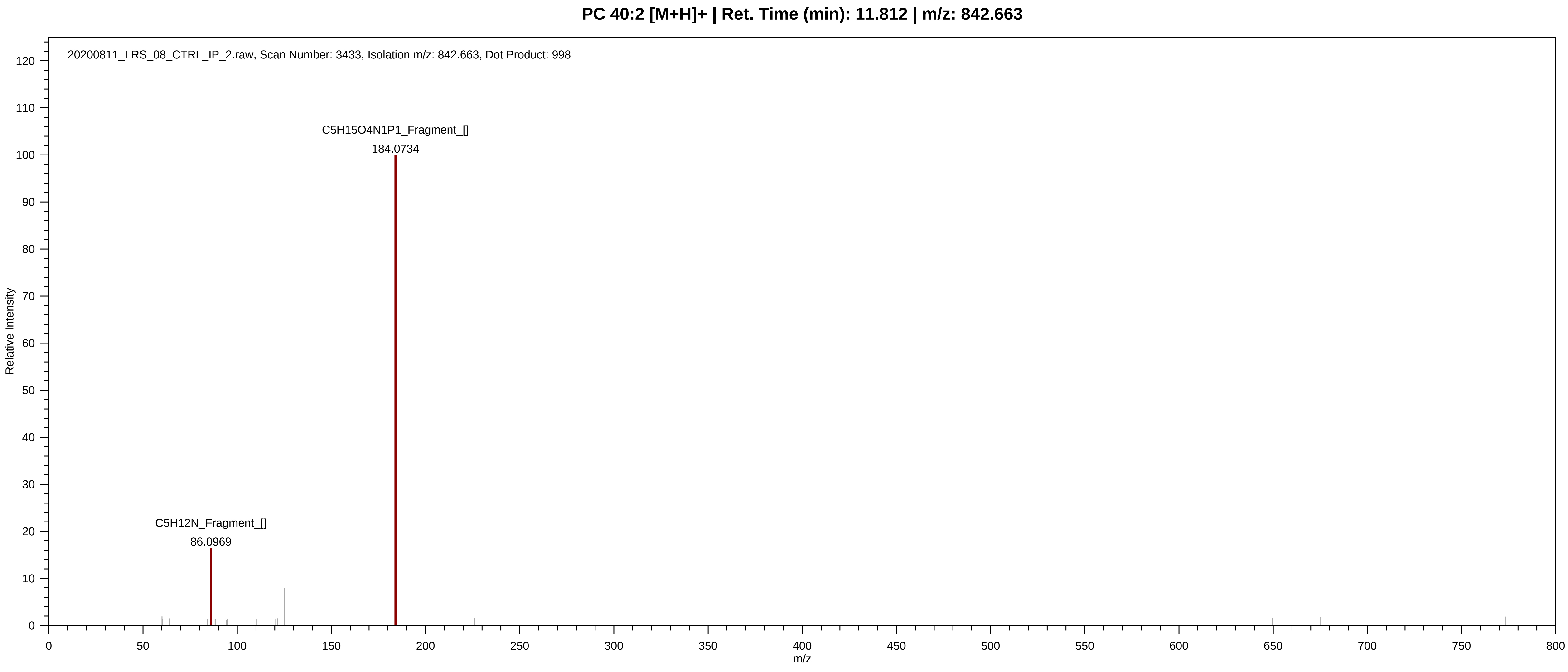

PC 40:2 [M+H]+ | Ret. Time (min): 11.812 | m/z: 842.663
20200811_LRS_08_CTRL_IP_2.raw, Scan Number: 3433, Isolation m/z: 842.663, Dot Product: 998
C5H15O4N1P1_Fragment_[]
184.0734
C5H12N_Fragment_[]
86.0969
Relative Intensity
m/z

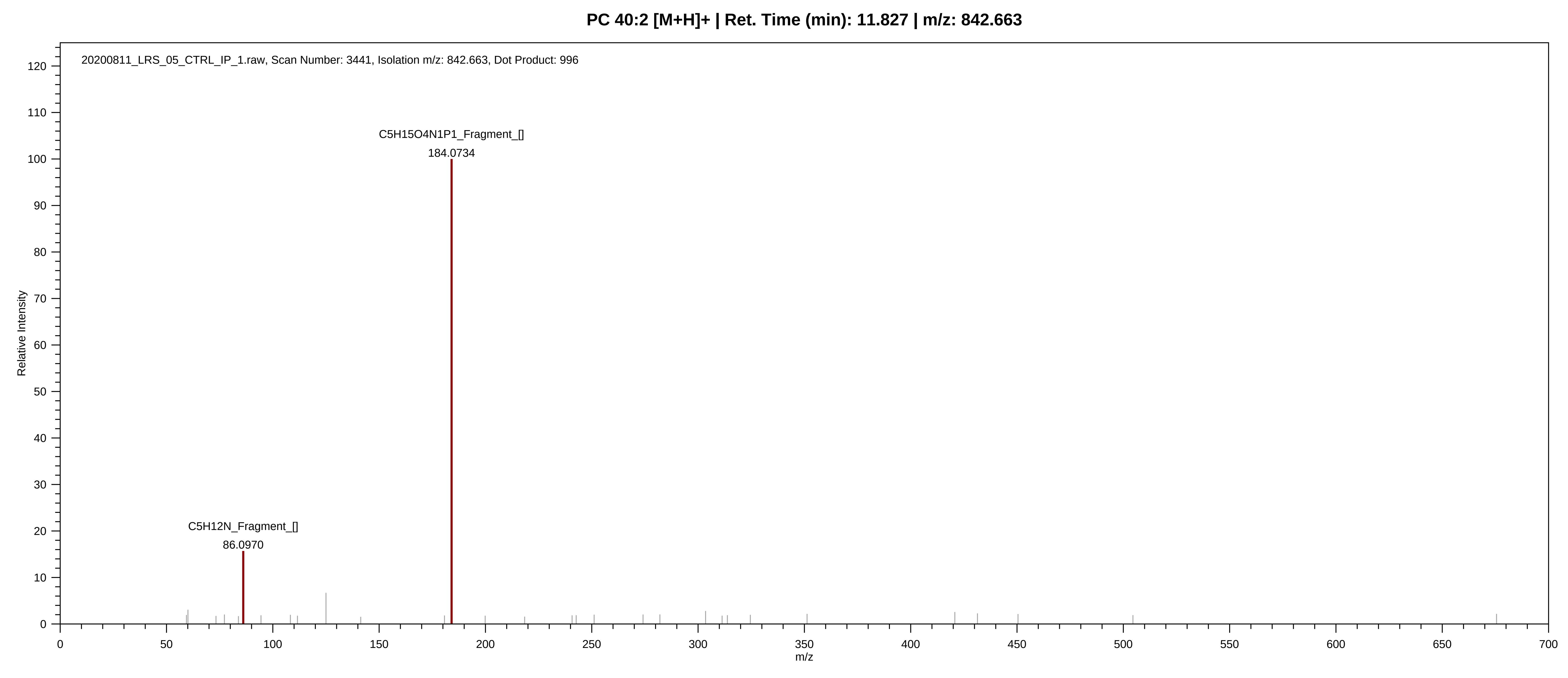
PC 40:2 [M+H]+ | Ret. Time (min): 11.827 | m/z: 842.663
20200811_LRS_05_CTRL_IP_1.raw, Scan Number: 3441, Isolation m/z: 842.663, Dot Product: 996
C5H15O4N1P1_Fragment_[]
184.0734
C5H12N_Fragment_[]
86.0970
Relative Intensity
m/z

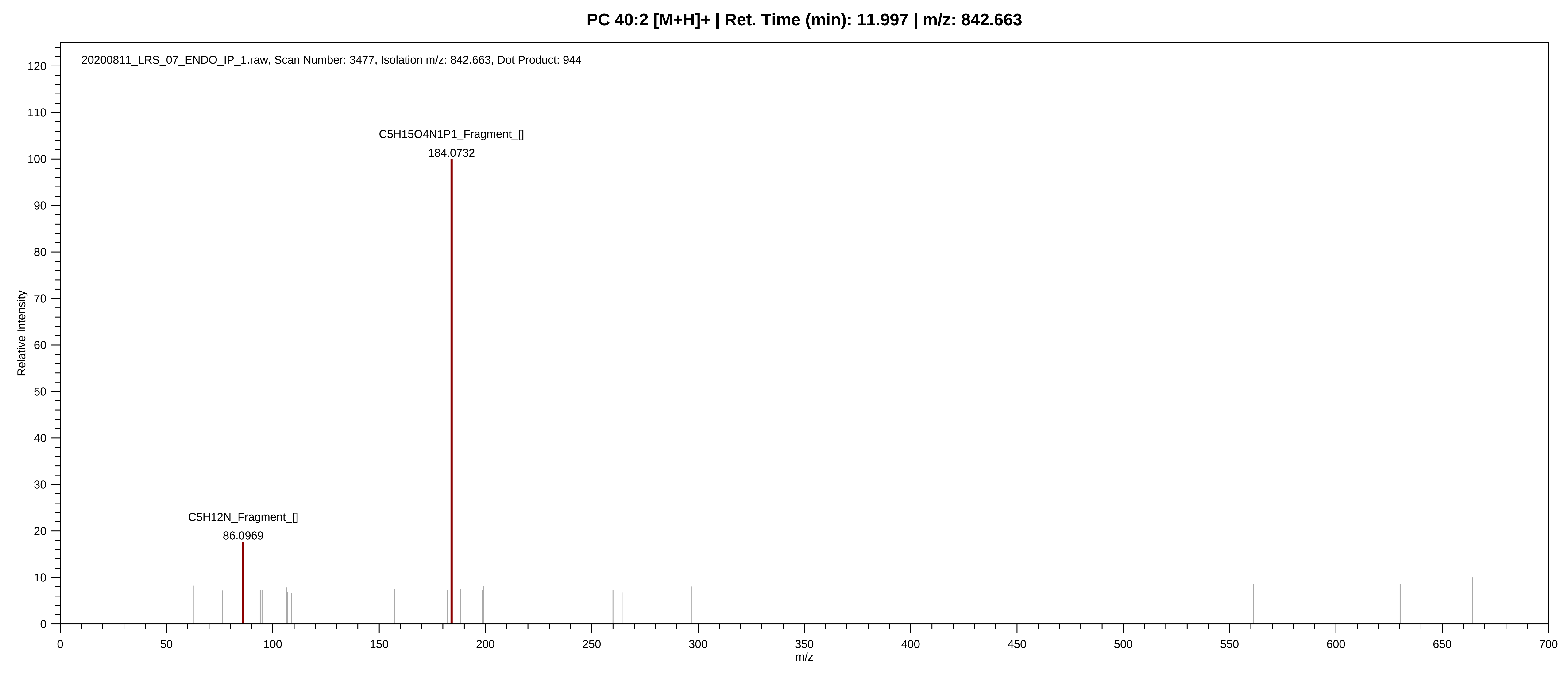
PC 40:2 [M+H]+ | Ret. Time (min): 11.997 | m/z: 842.663
20200811_LRS_07_ENDO_IP_1.raw, Scan Number: 3477, Isolation m/z: 842.663, Dot Product: 944
C5H15O4N1P1_Fragment_[]
184.0732
C5H12N_Fragment_[]
86.0969
Relative Intensity
m/z

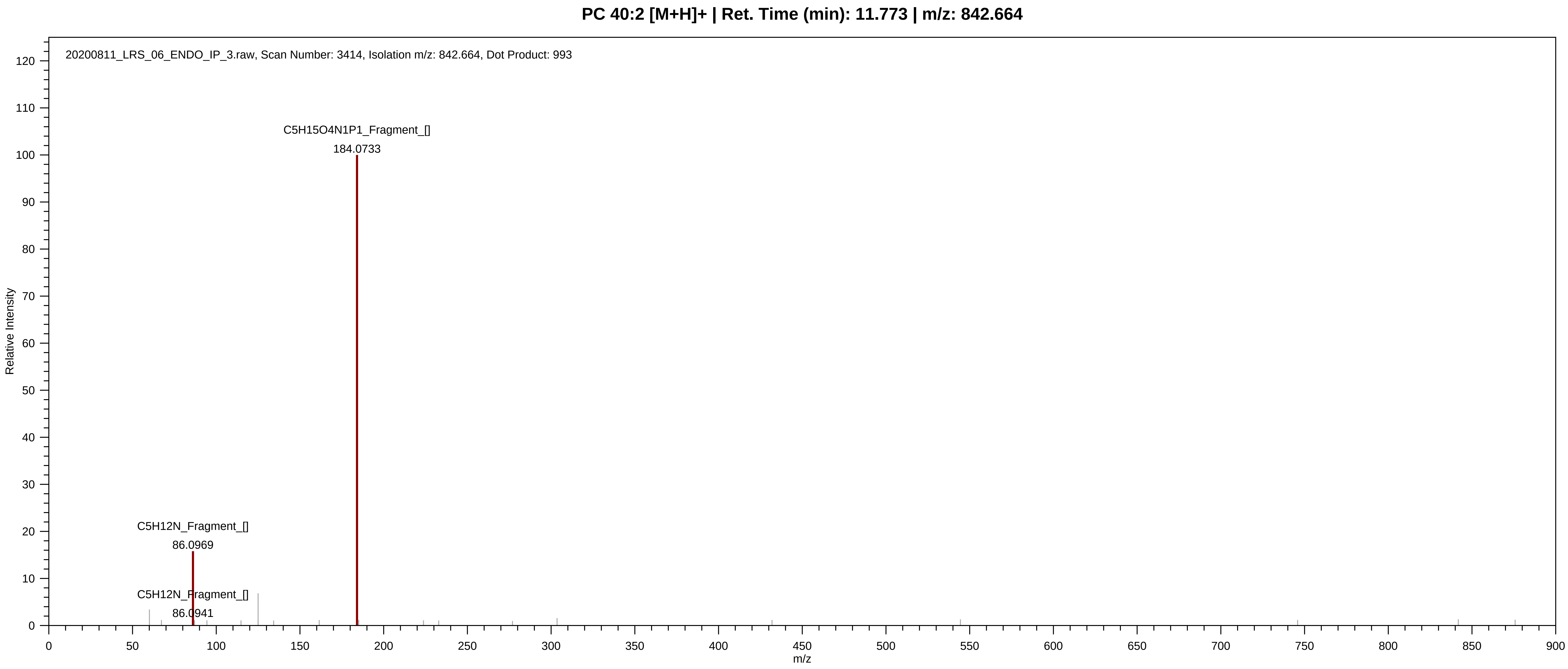

PC 40:2 [M+H]+ | Ret. Time (min): 11.773 | m/z: 842.664
20200811_LRS_06_ENDO_IP_3.raw, Scan Number: 3414, Isolation m/z: 842.664, Dot Product: 993
C5H15O4N1P1_Fragment_[]
184.0733
C5H12N_Fragment_[]
86.0969
C5H12N_Fragment_[]
86.0941
Relative Intensity
m/z

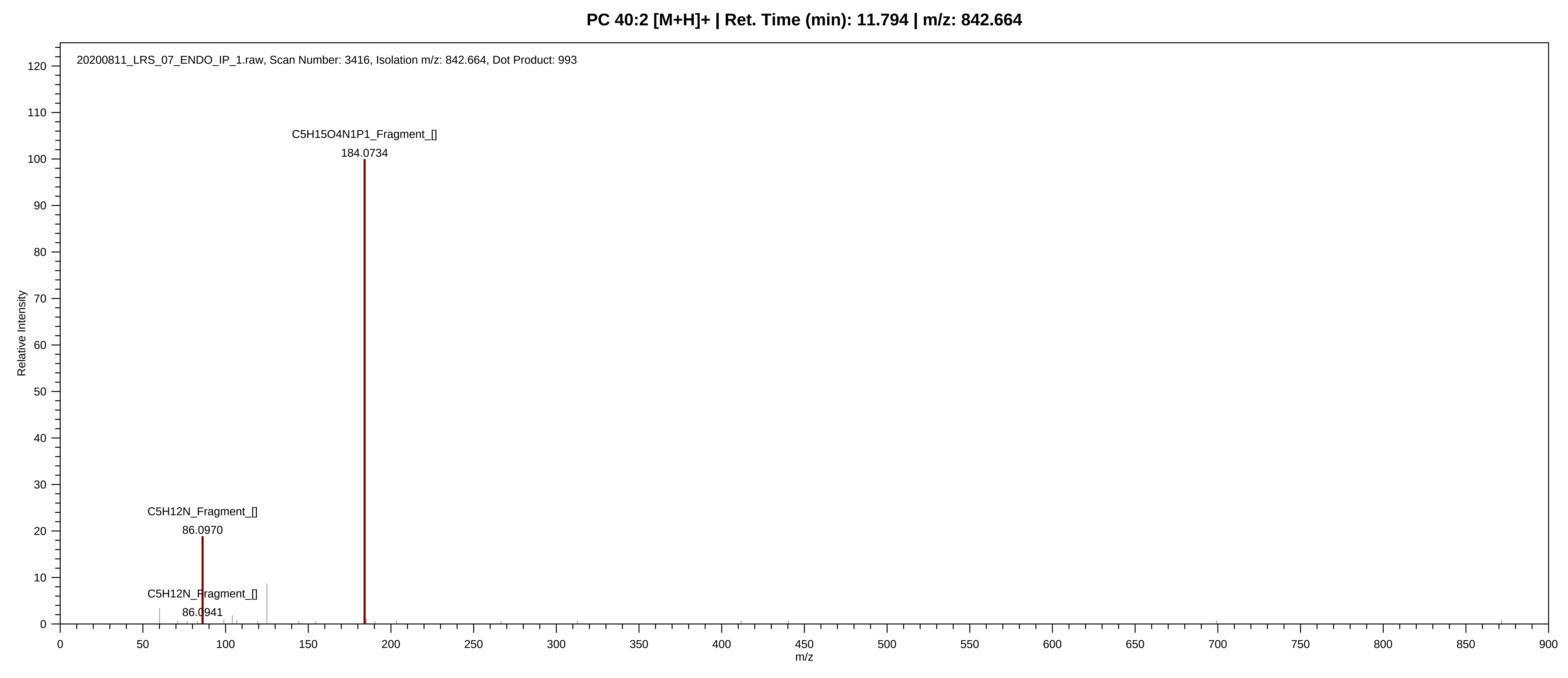

PC 40:2 [M+H]+ | Ret. Time (min): 11.794 | m/z: 842.664
20200811_LRS_07_ENDO_IP_1.raw, Scan Number: 3416, Isolation m/z: 842.664, Dot Product: 993
C5H15O4N1P1_Fragment_[]
184.0734
C5H12N_Fragment_[]
86.0970
C5H12N_Fragment_[]
86.0941
Relative Intensity
m/z

PC 40:2 [M+H]+ | Ret. Time (min): 11.807 | m/z: 842.664

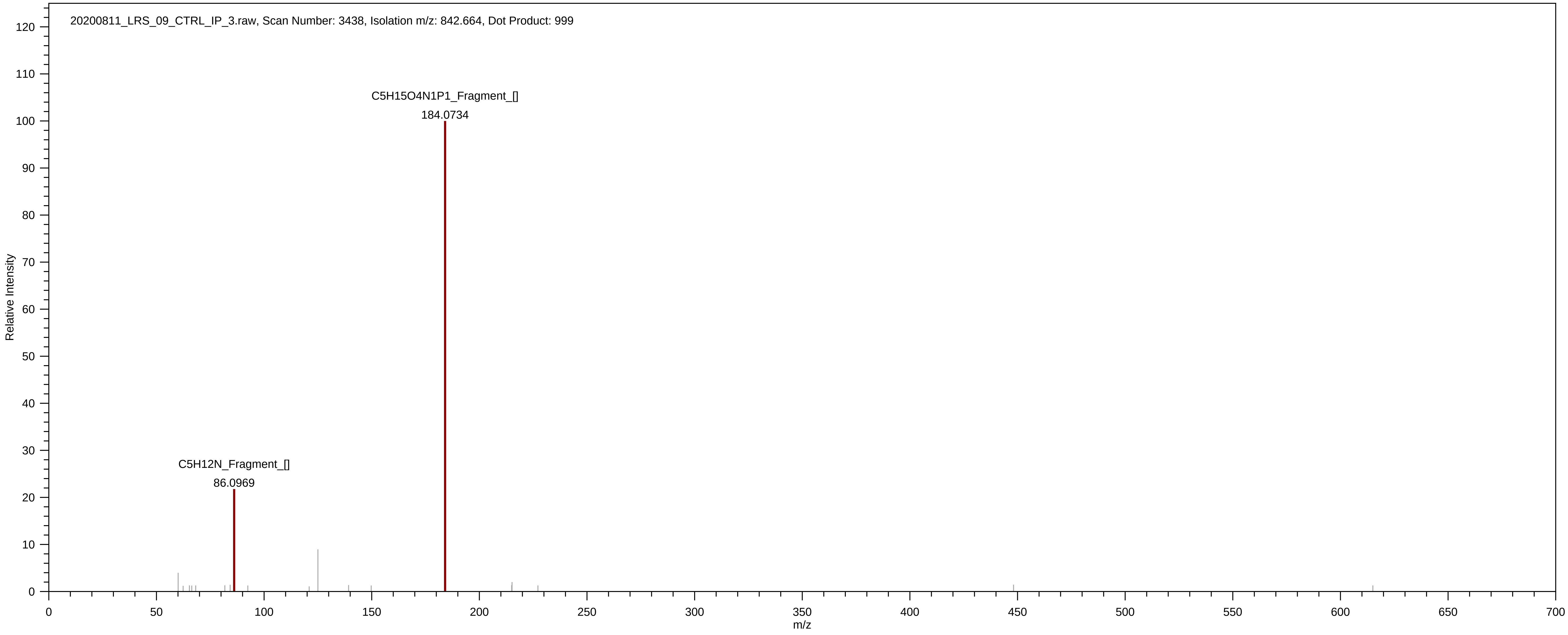

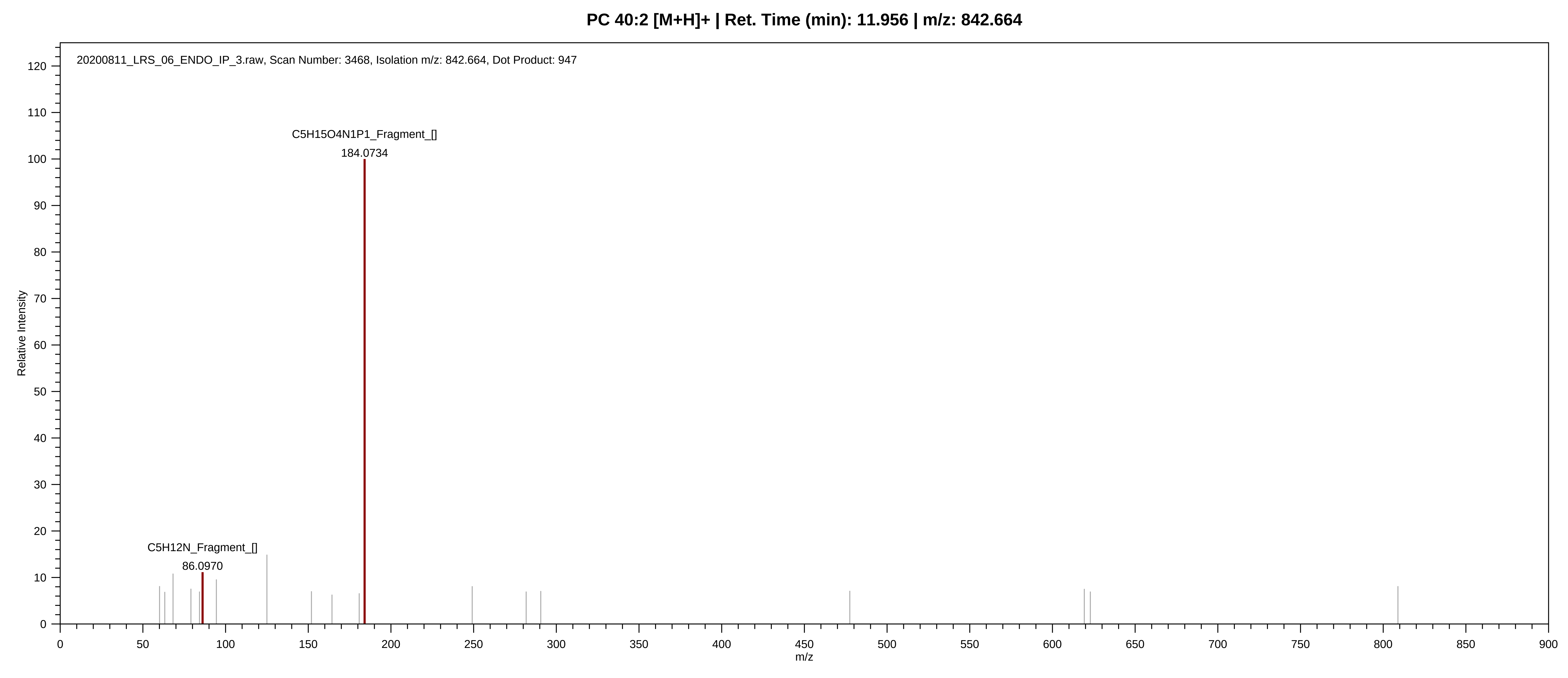
PC 40:2 [M+H]+ | Ret. Time (min): 11.956 | m/z: 842.664
20200811_LRS_06_ENDO_IP_3.raw, Scan Number: 3468, Isolation m/z: 842.664, Dot Product: 947
C5H15O4N1P1_Fragment_[]
184.0734
C5H12N_Fragment_[]
86.0970
Relative Intensity
m/z

PC 40:2 [M+H]+ | Ret. Time (min): 11.974 | m/z: 842.666

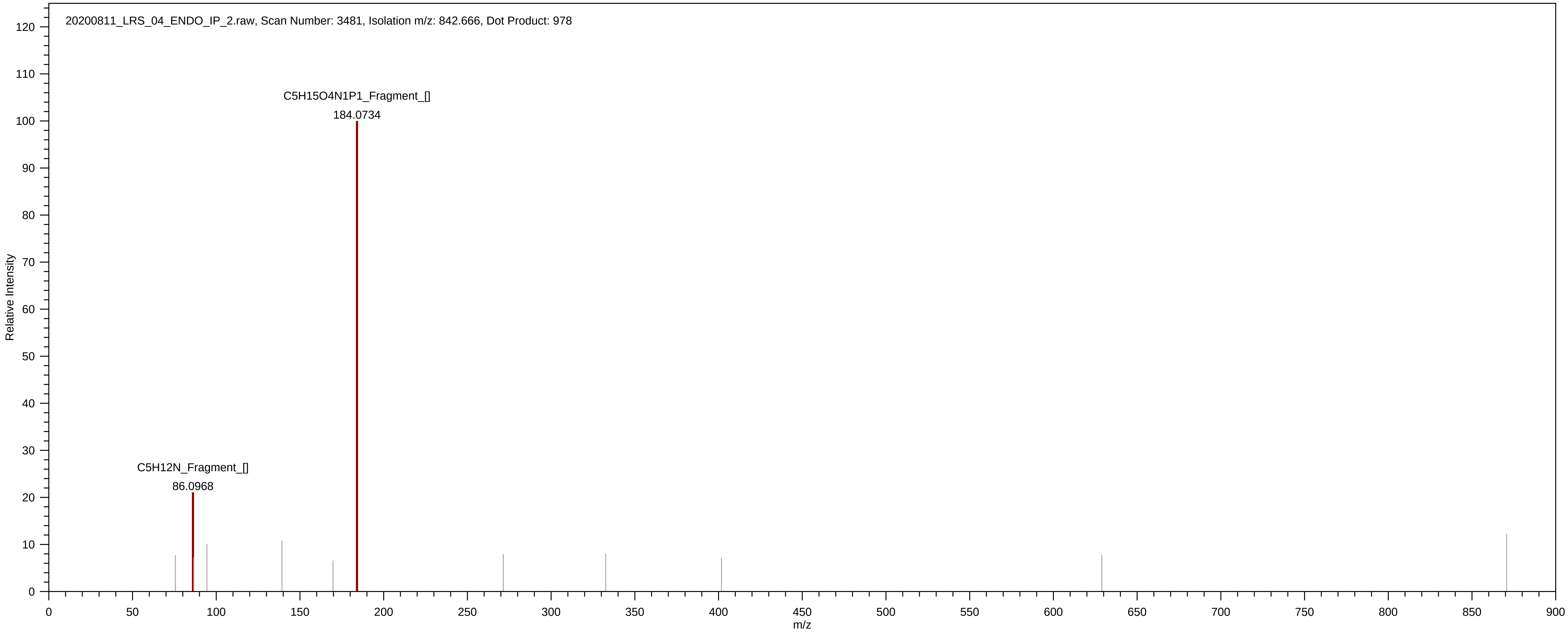

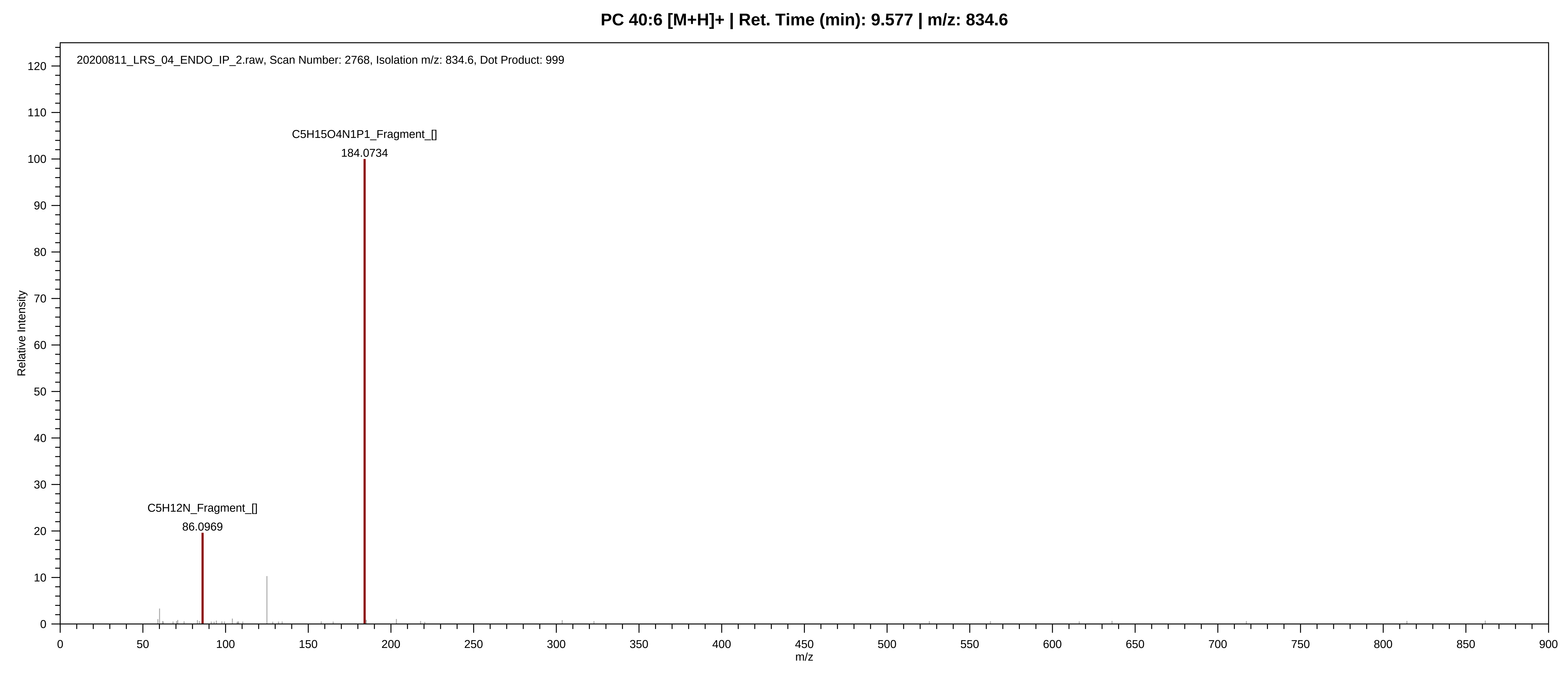
PC 40:6 [M+H]+ | Ret. Time (min): 9.577 | m/z: 834.6
20200811_LRS_04_ENDO_IP_2.raw, Scan Number: 2768, Isolation m/z: 834.6, Dot Product: 999
C5H15O4N1P1_Fragment_[]
184.0734
C5H12N_Fragment_[]
86.0969
Relative Intensity
m/z

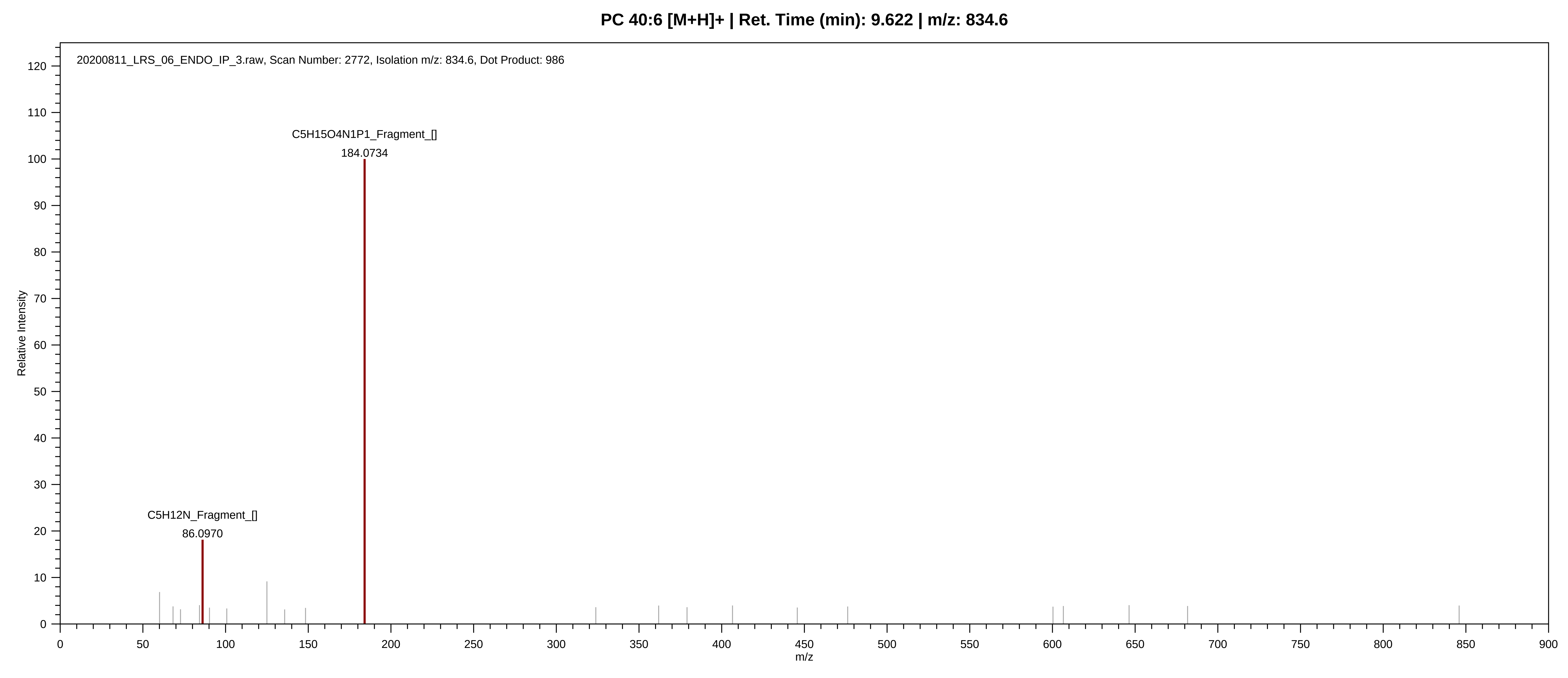
PC 40:6 [M+H]+ | Ret. Time (min): 9.622 | m/z: 834.6
20200811_LRS_06_ENDO_IP_3.raw, Scan Number: 2772, Isolation m/z: 834.6, Dot Product: 986
C5H15O4N1P1_Fragment_[]
184.0734
C5H12N_Fragment_[]
86.0970
Relative Intensity
m/z

PC 40:6 [M+H]+ | Ret. Time (min): 9.562 | m/z: 834.601

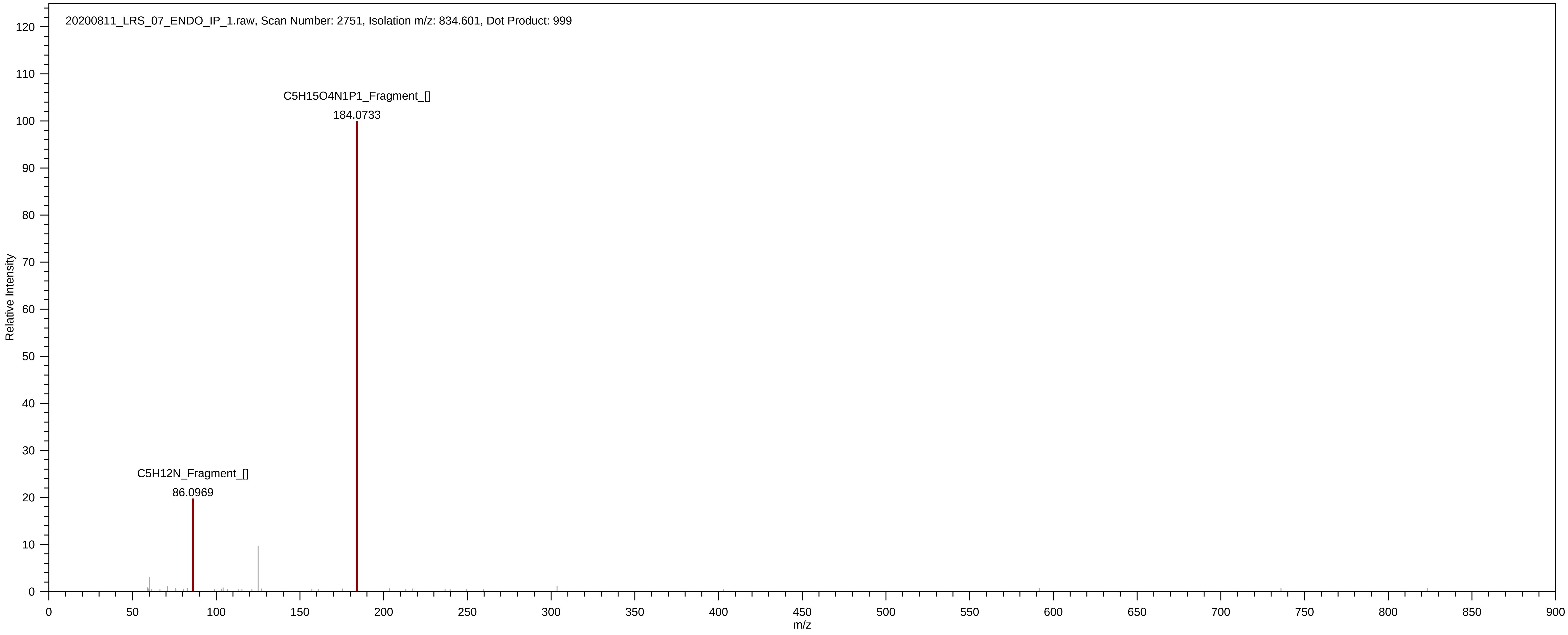

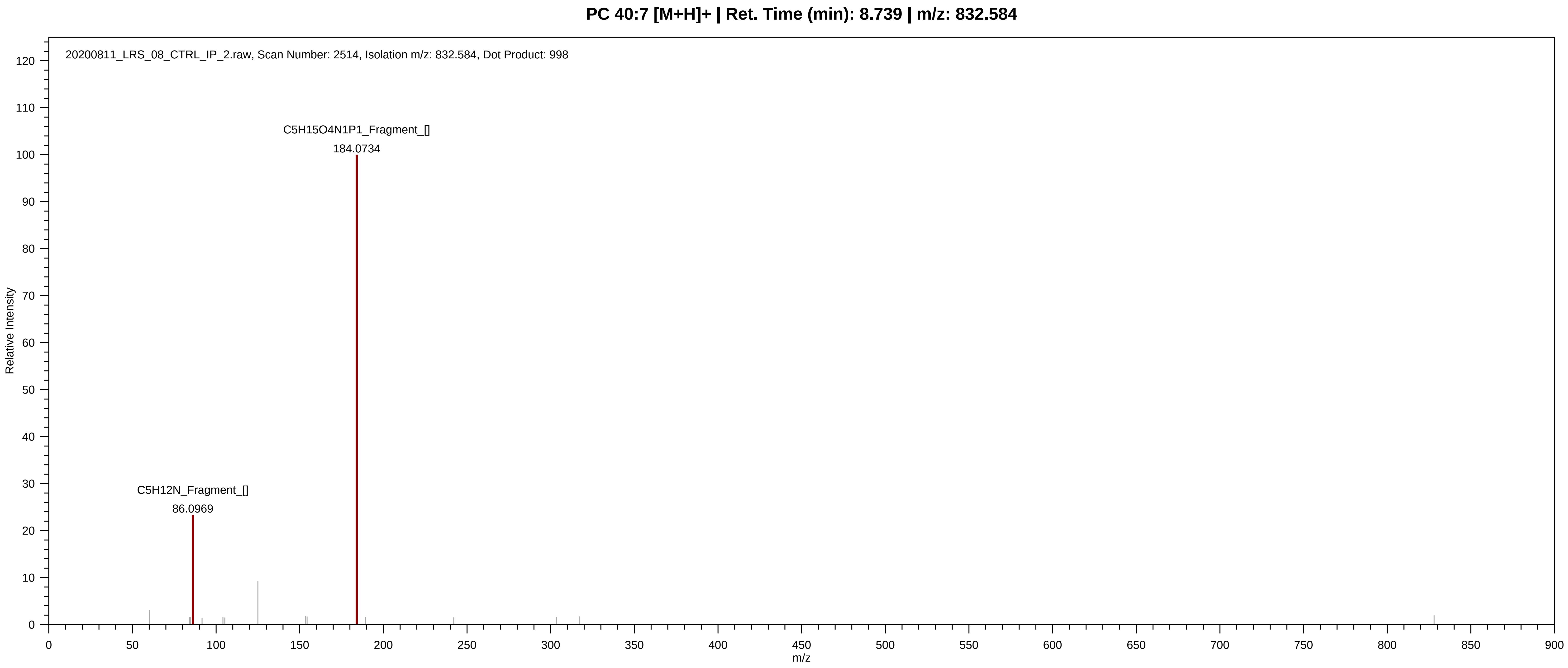

PC 40:7 [M+H]+ | Ret. Time (min): 8.739 | m/z: 832.584
20200811_LRS_08_CTRL_IP_2.raw, Scan Number: 2514, Isolation m/z: 832.584, Dot Product: 998
C5H15O4N1P1_Fragment_[]
184.0734
C5H12N_Fragment_[]
86.0969
Relative Intensity
m/z

PC 40:7 [M+H]+ | Ret. Time (min): 8.757 | m/z: 832.584

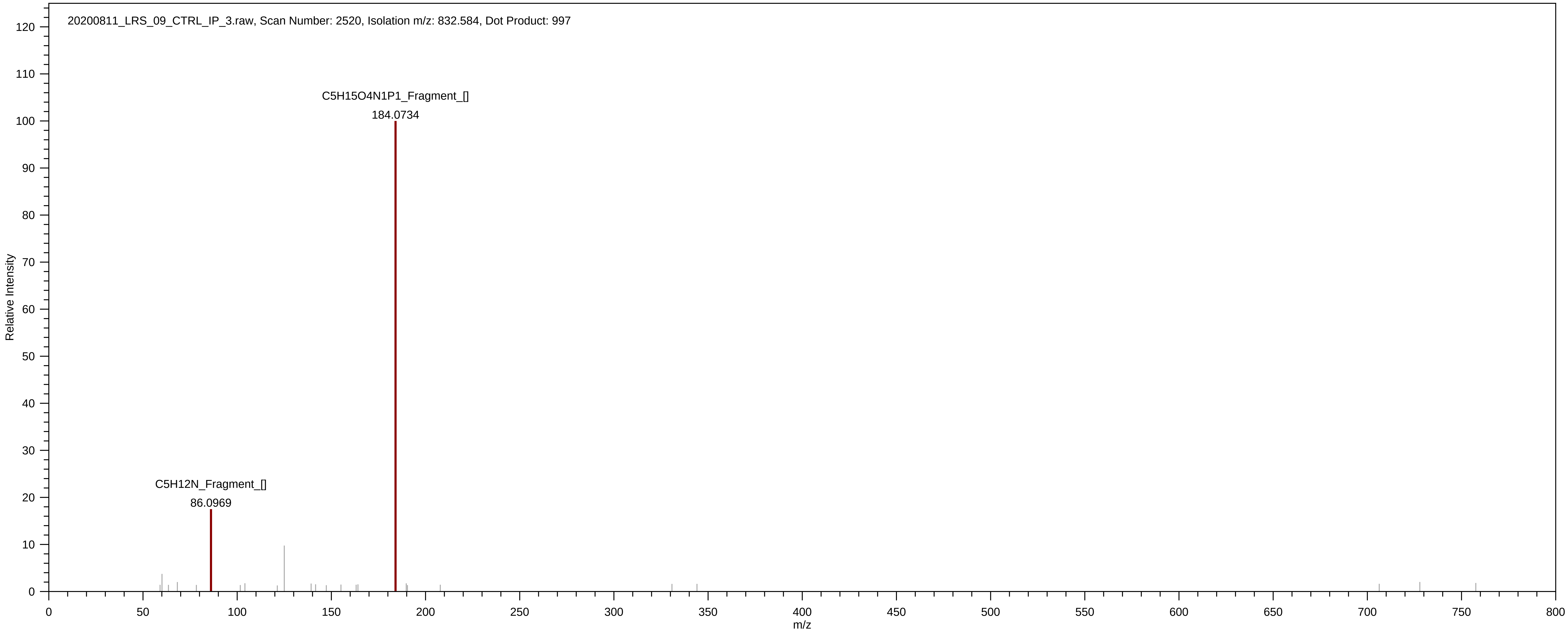

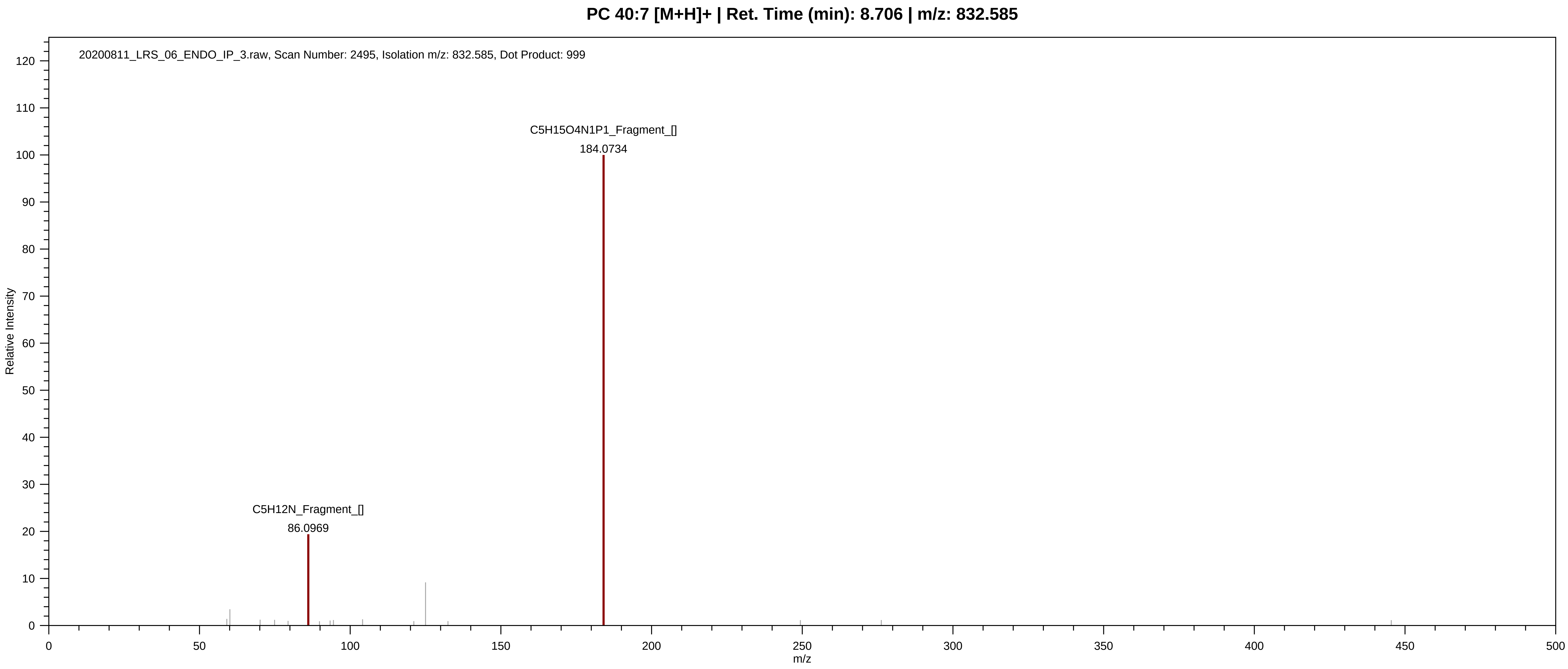
PC 40:7 [M+H]+ | Ret. Time (min): 8.706 | m/z: 832.585
20200811_LRS_06_ENDO_IP_3.raw, Scan Number: 2495, Isolation m/z: 832.585, Dot Product: 999
C5H15O4N1P1_Fragment_[]
184.0734
C5H12N_Fragment_[]
86.0969
Relative Intensity
m/z

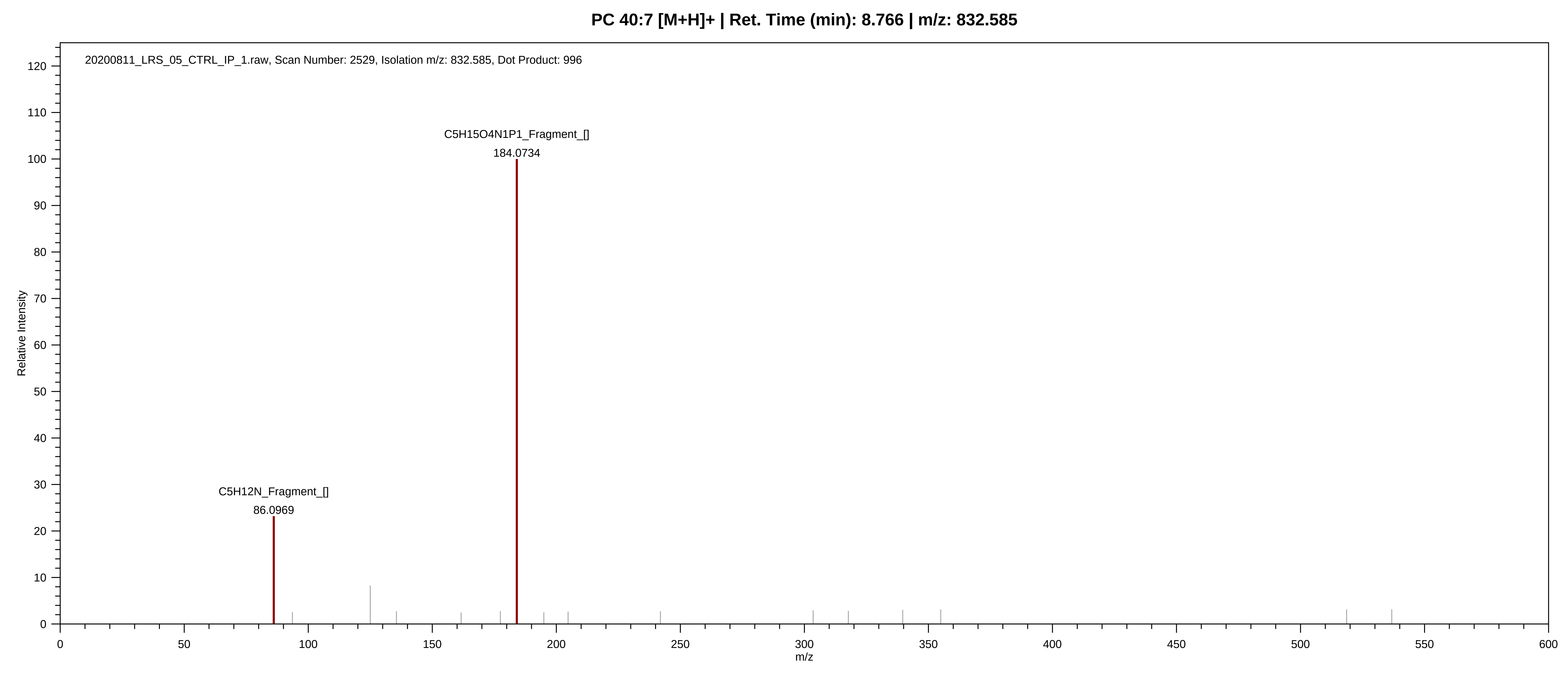
PC 40:7 [M+H]+ | Ret. Time (min): 8.766 | m/z: 832.585
20200811_LRS_05_CTRL_IP_1.raw, Scan Number: 2529, Isolation m/z: 832.585, Dot Product: 996
C5H15O4N1P1_Fragment_[]
184.0734
C5H12N_Fragment_[]
86.0969
Relative Intensity
m/z

PC 40:7 [M+H]+ | Ret. Time (min): 8.708 | m/z: 832.586

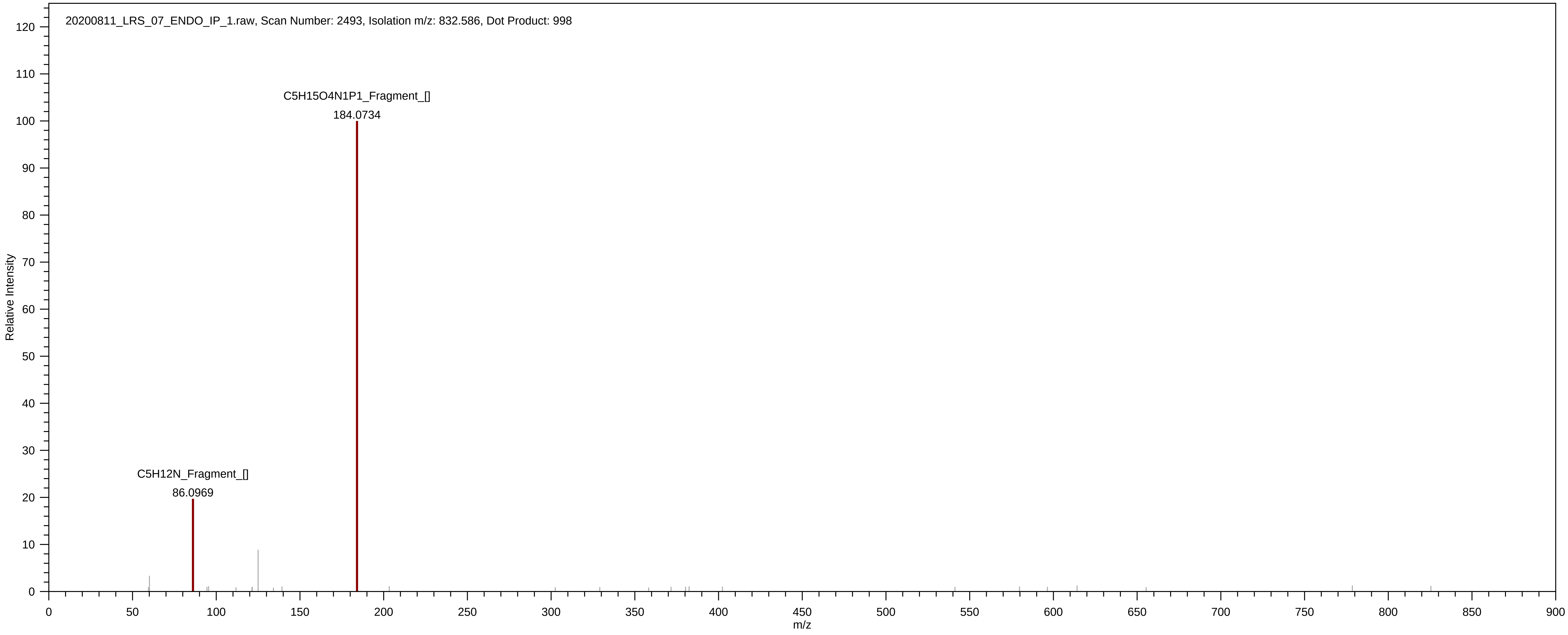

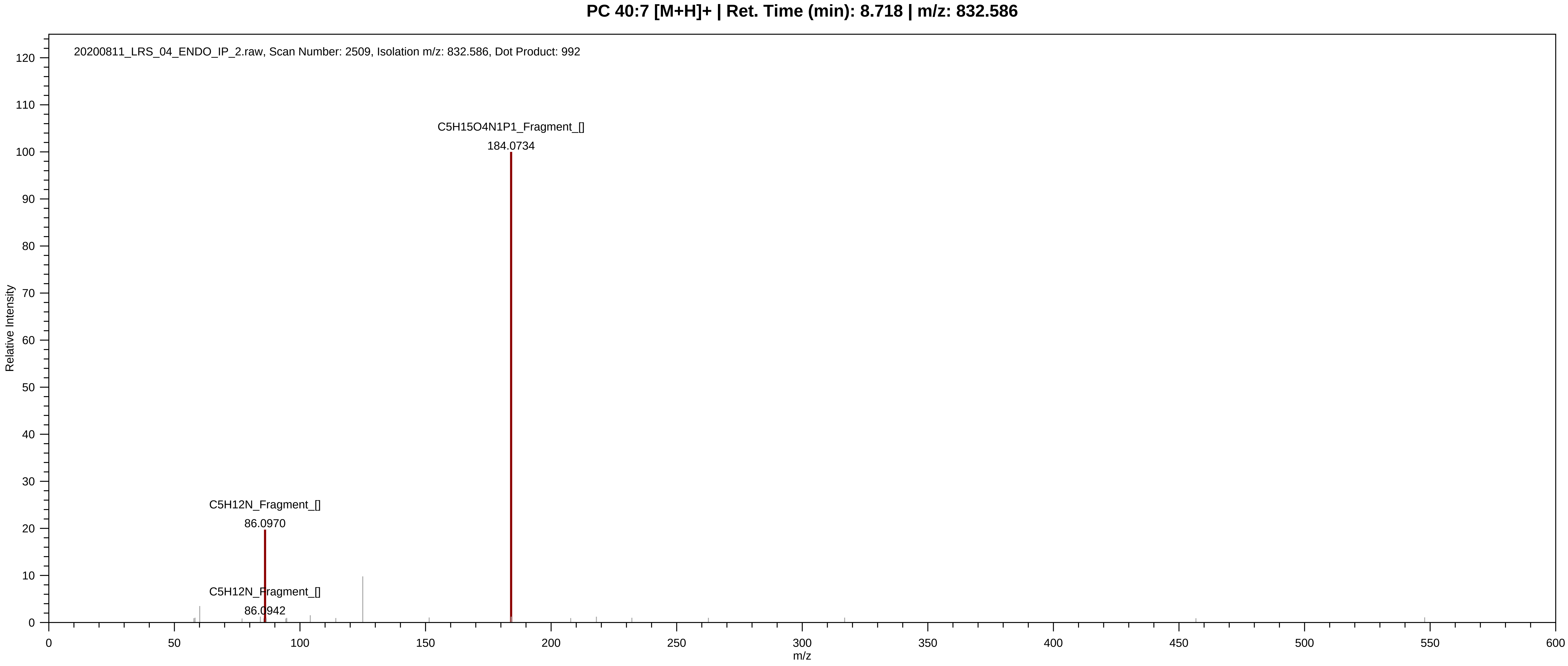

PC 40:7 [M+H]+ | Ret. Time (min): 8.718 | m/z: 832.586
20200811_LRS_04_ENDO_IP_2.raw, Scan Number: 2509, Isolation m/z: 832.586, Dot Product: 992
C5H15O4N1P1_Fragment_[]
184.0734
C5H12N_Fragment_[]
86.0970
C5H12N_Fragment_[]
86.0942
Relative Intensity
m/z

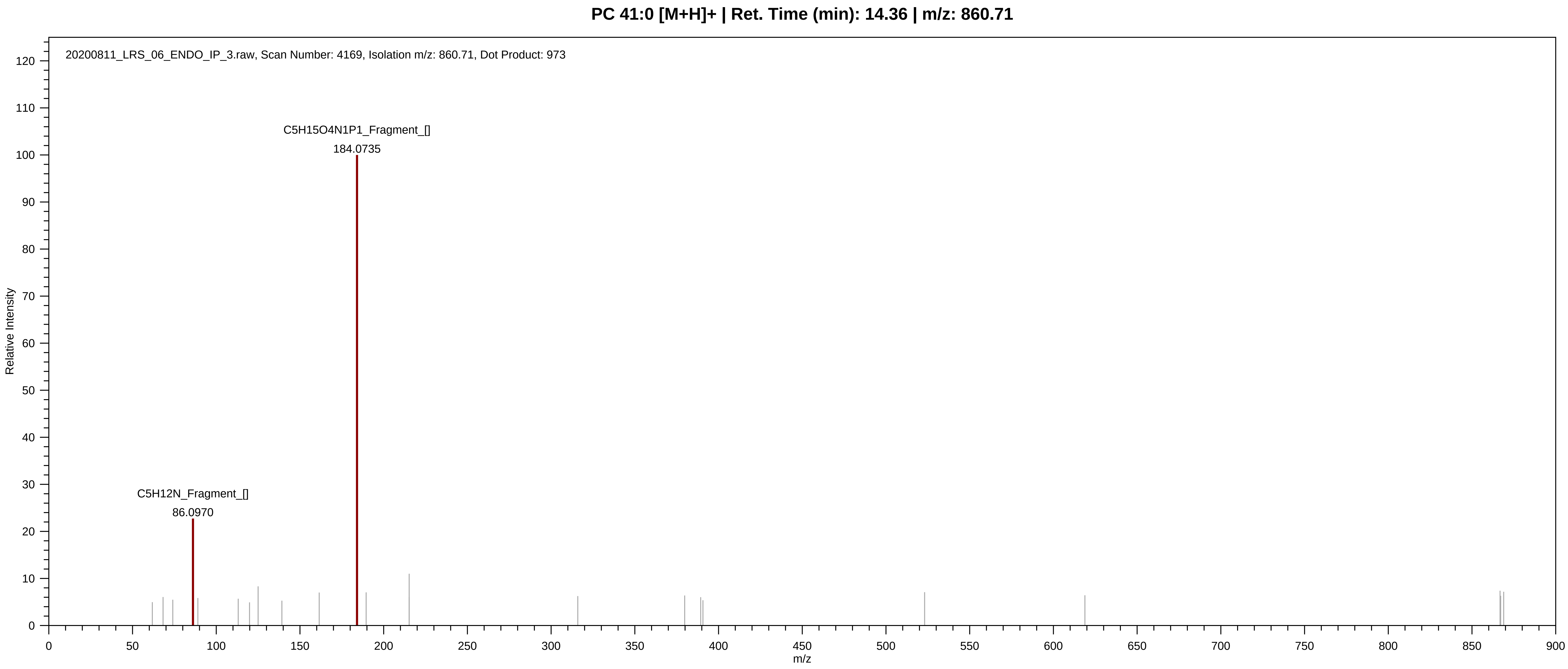

PC 41:0 [M+H]+ | Ret. Time (min): 14.36 | m/z: 860.71
20200811_LRS_06_ENDO_IP_3.raw, Scan Number: 4169, Isolation m/z: 860.71, Dot Product: 973
C5H15O4N1P1_Fragment_[]
184.0735
C5H12N_Fragment_[]
86.0970
Relative Intensity
m/z

PC 41:0 [M+H]+ | Ret. Time (min): 14.362 | m/z: 860.712

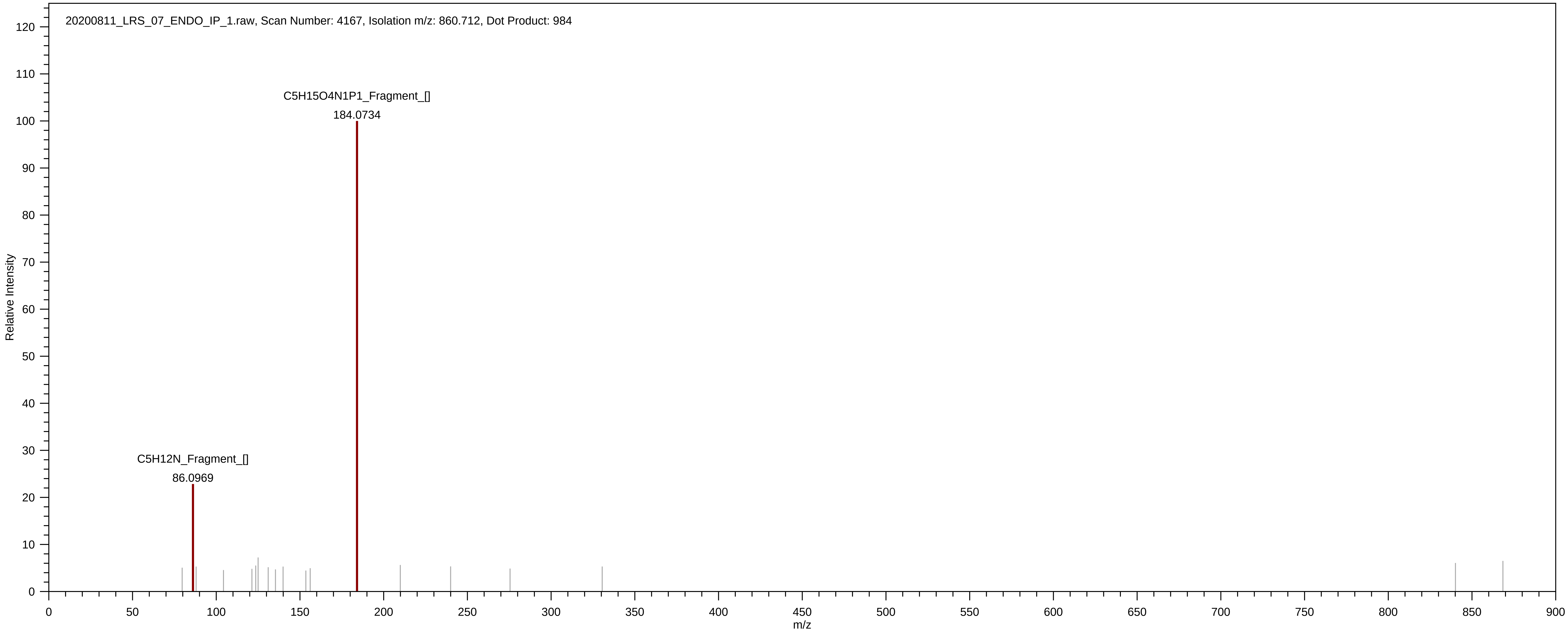

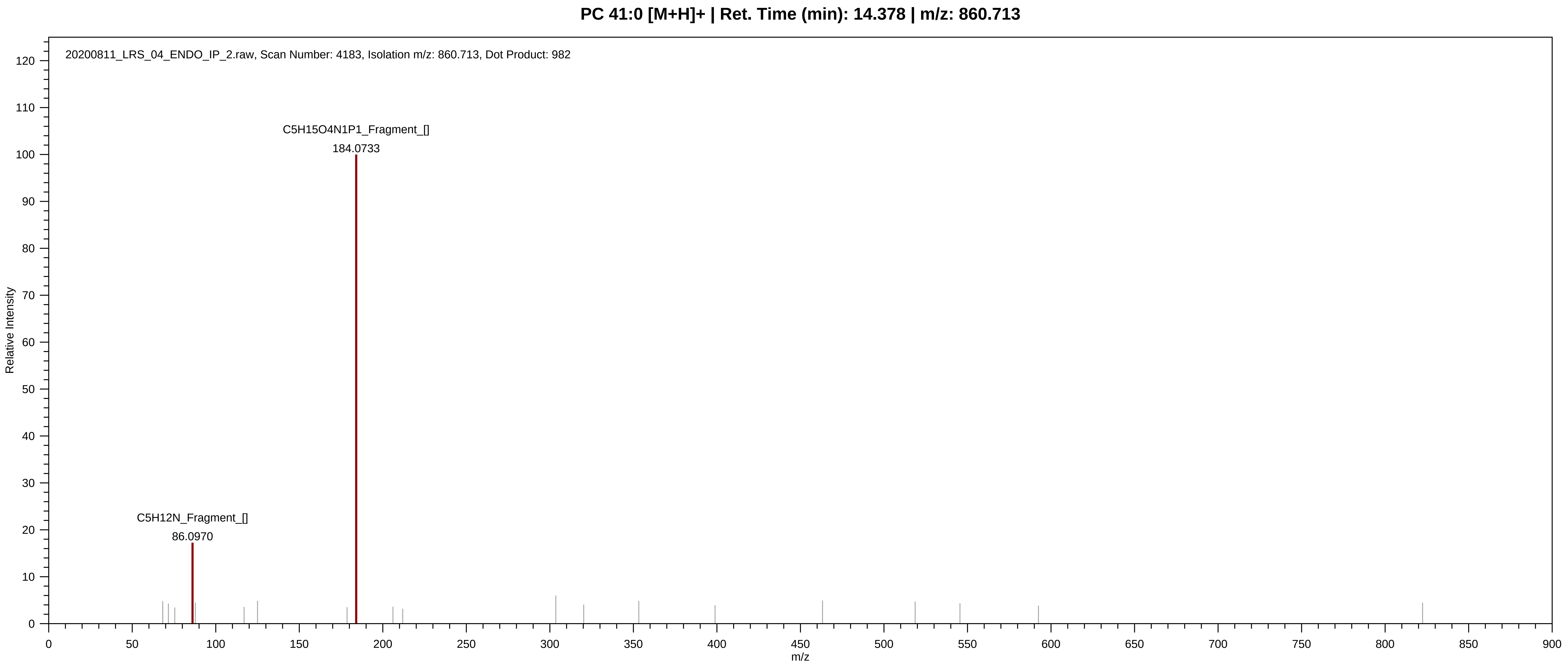

PC 41:0 [M+H]+ | Ret. Time (min): 14.378 | m/z: 860.713
20200811_LRS_04_ENDO_IP_2.raw, Scan Number: 4183, Isolation m/z: 860.713, Dot Product: 982
C5H15O4N1P1_Fragment_[]
184.0733
C5H12N_Fragment_[]
86.0970
Relative Intensity
m/z

PC 41:1 [M+H]+ | Ret. Time (min): 13.294 | m/z: 858.696

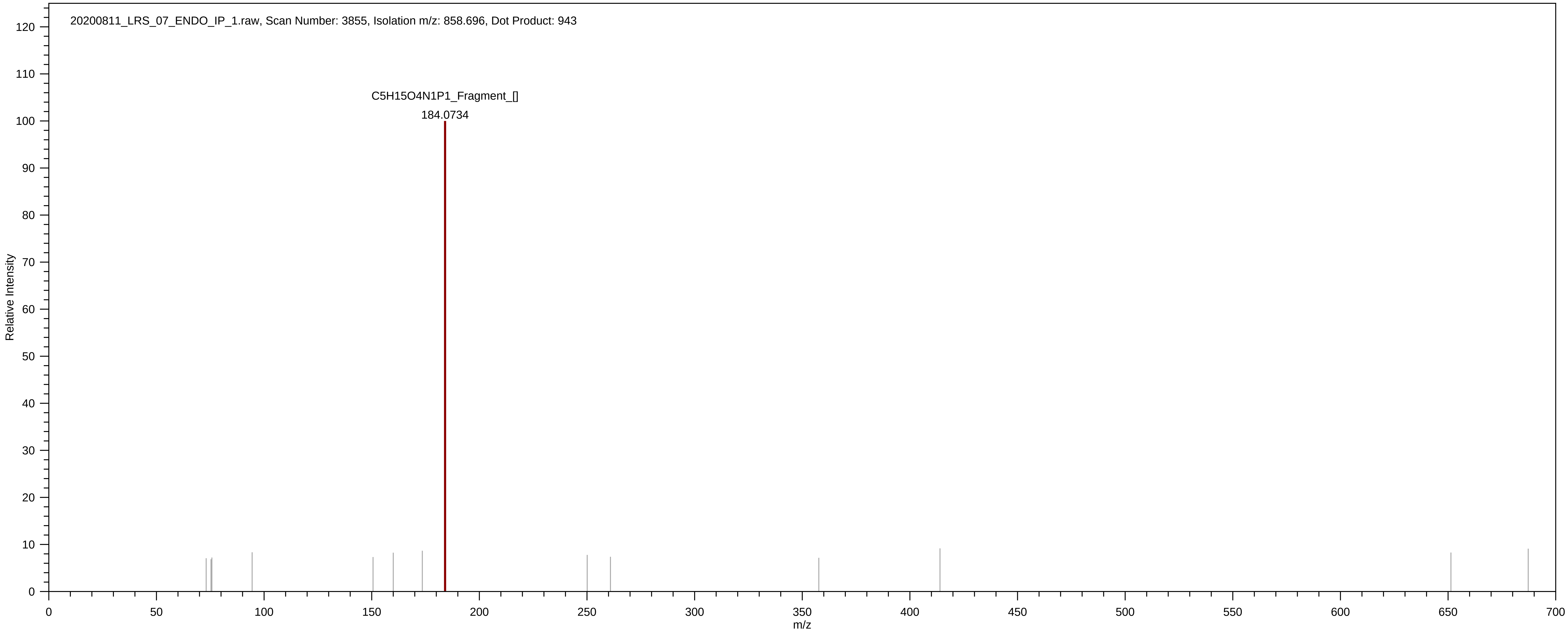

PC 42:0 [M+H]+ | Ret. Time (min): 14.817 | m/z: 874.723

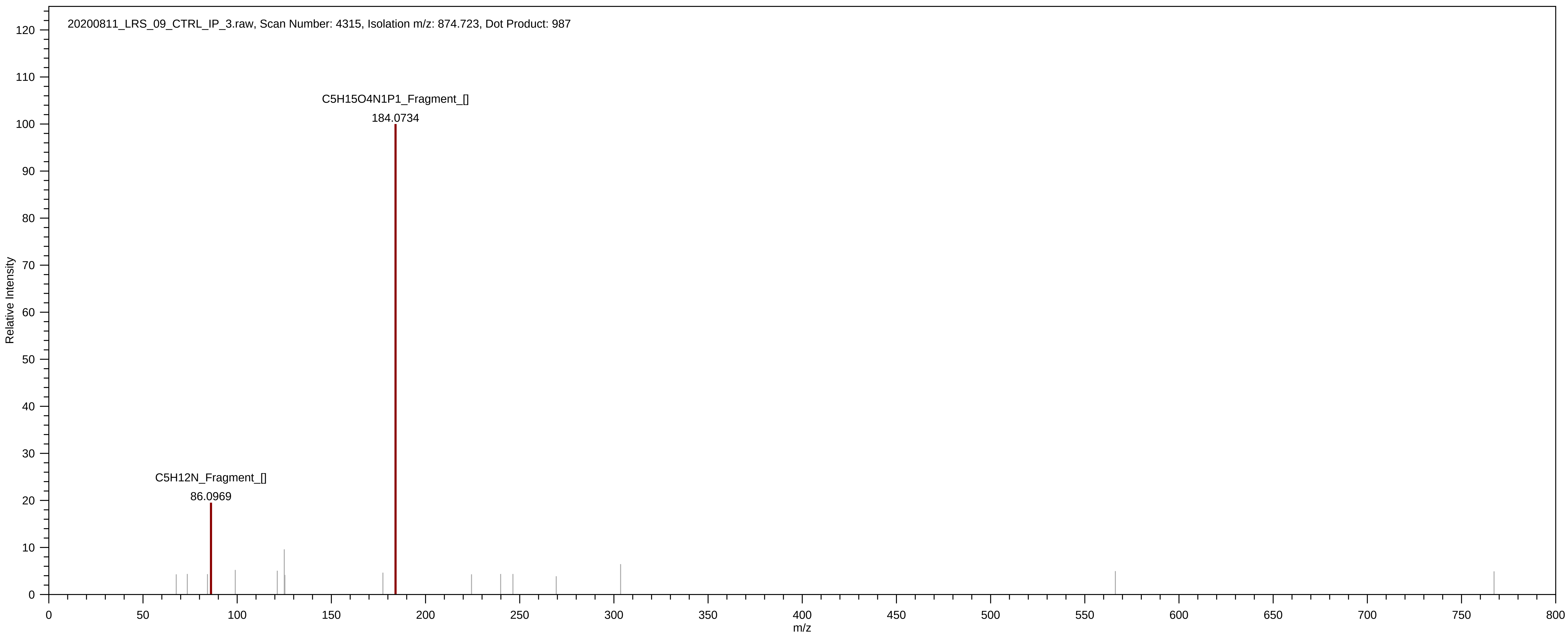

PC 42:0 [M+H]+ | Ret. Time (min): 14.832 | m/z: 874.726

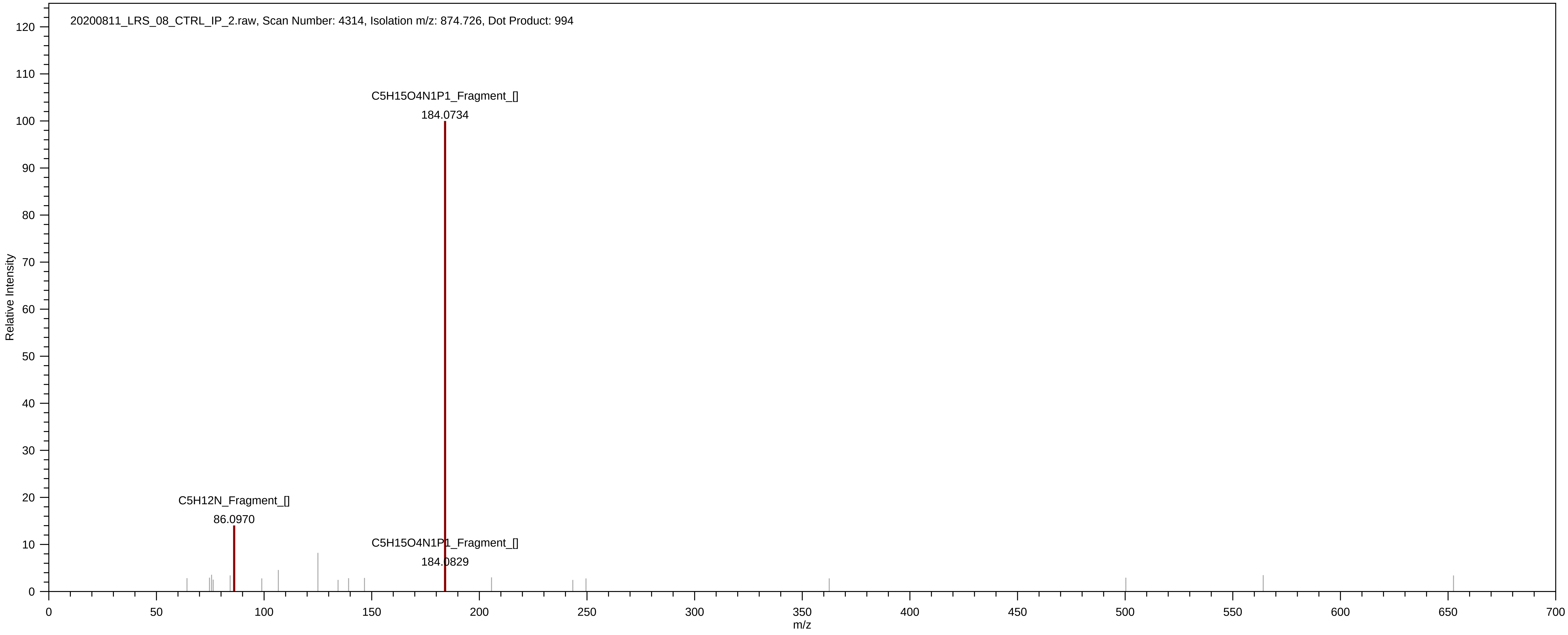

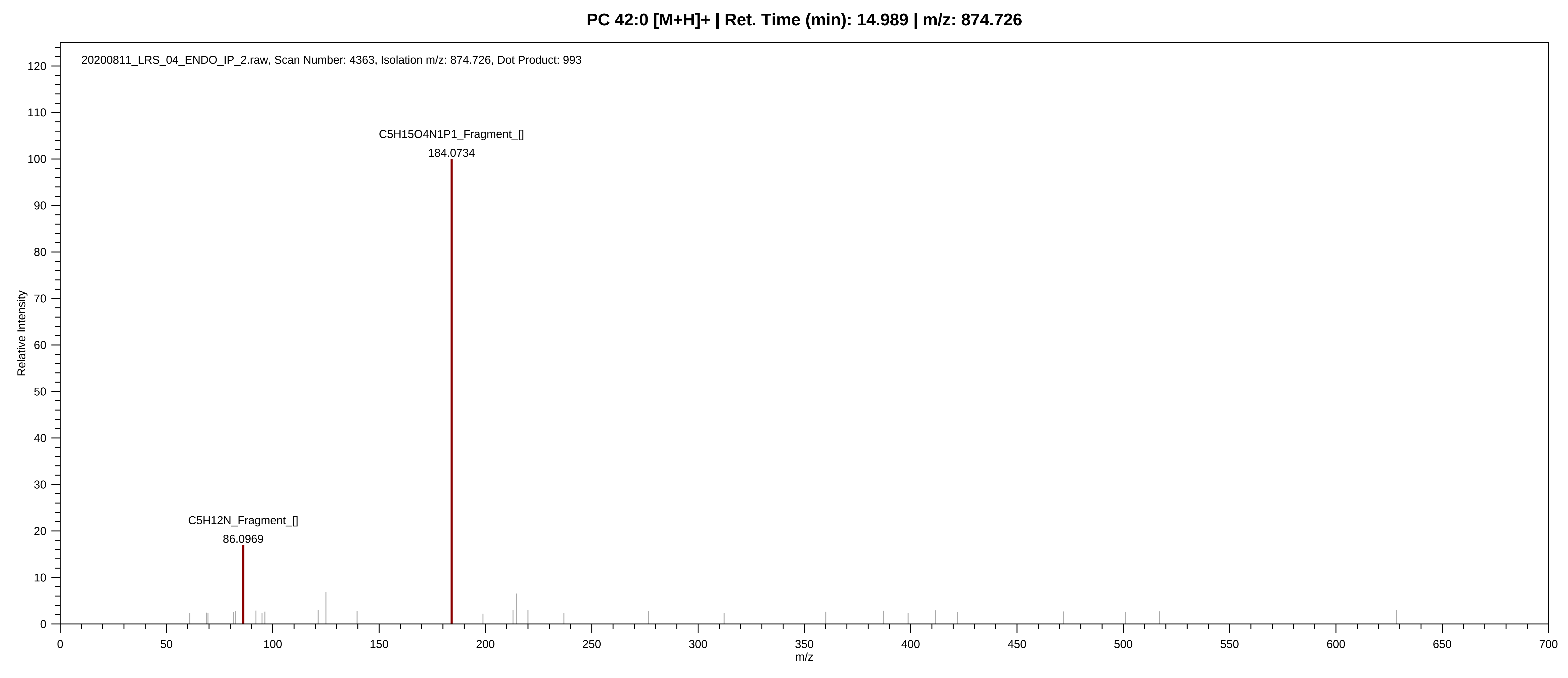
PC 42:0 [M+H]+ | Ret. Time (min): 14.989 | m/z: 874.726
20200811_LRS_04_ENDO_IP_2.raw, Scan Number: 4363, Isolation m/z: 874.726, Dot Product: 993
C5H15O4N1P1_Fragment_[]
184.0734
C5H12N_Fragment_[]
86.0969
Relative Intensity
m/z

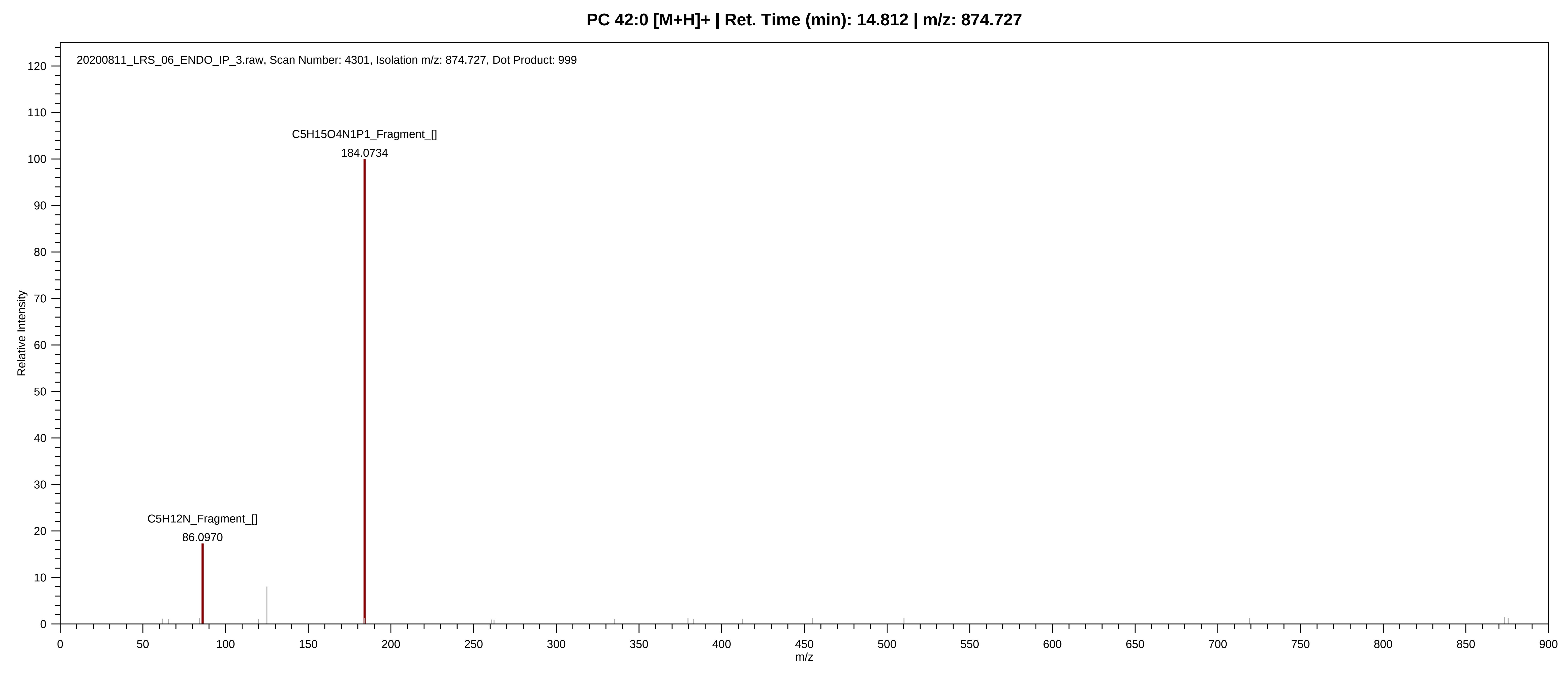
PC 42:0 [M+H]+ | Ret. Time (min): 14.812 | m/z: 874.727
20200811_LRS_06_ENDO_IP_3.raw, Scan Number: 4301, Isolation m/z: 874.727, Dot Product: 999
C5H15O4N1P1_Fragment_[]
184.0734
C5H12N_Fragment_[]
86.0970
Relative Intensity
m/z

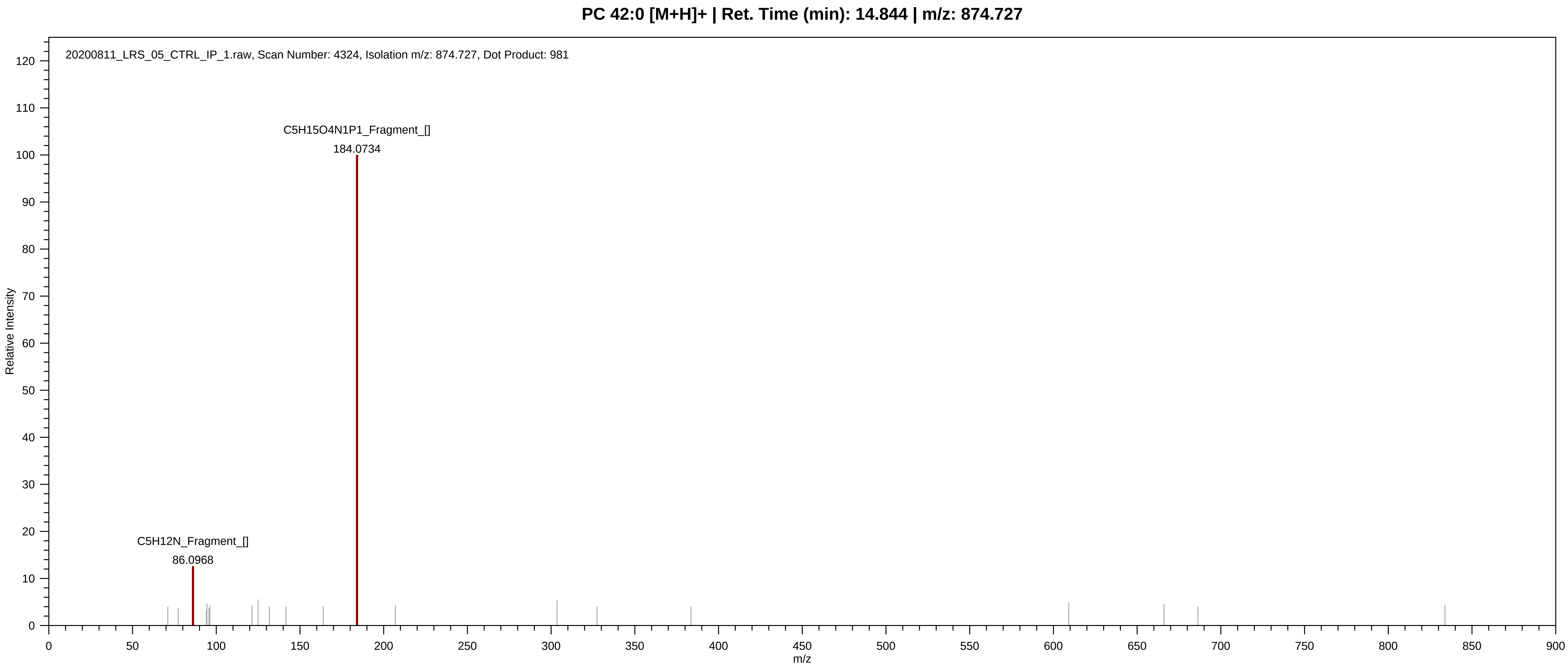
PC 42:0 [M+H]+ | Ret. Time (min): 14.844 | m/z: 874.727
20200811_LRS_05_CTRL_IP_1.raw, Scan Number: 4324, Isolation m/z: 874.727, Dot Product: 981
C5H15O4N1P1_Fragment_[]
184.0734
C5H12N_Fragment_[]
86.0968
Relative Intensity
m/z
0
10
20
30
40
50
60
70
80
90
100
110
120
0
50
100
150
200
250
300
350
400
450
500
550
600
650
700
750
800
850
900

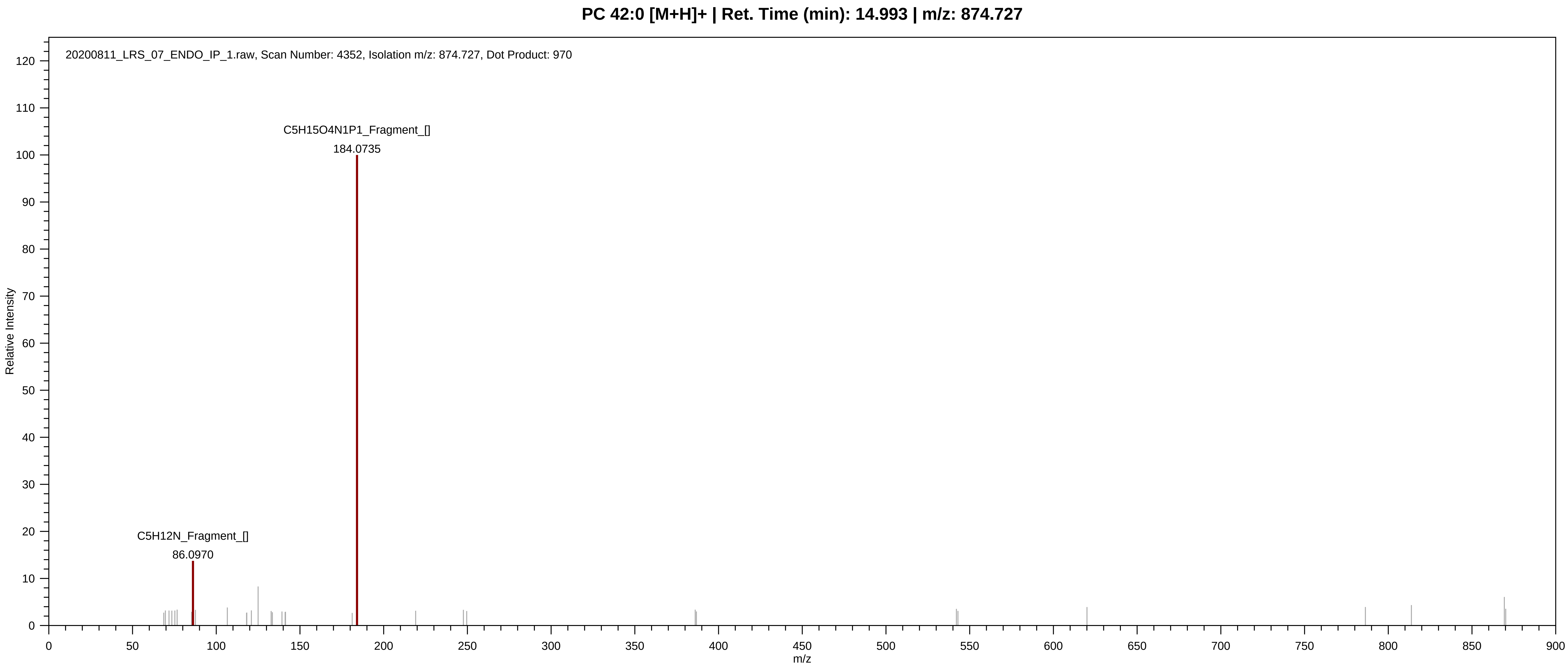
PC 42:0 [M+H]+ | Ret. Time (min): 14.993 | m/z: 874.727
20200811_LRS_07_ENDO_IP_1.raw, Scan Number: 4352, Isolation m/z: 874.727, Dot Product: 970
C5H15O4N1P1_Fragment_[]
184.0735
C5H12N_Fragment_[]
86.0970
Relative Intensity
m/z
0
10
20
30
40
50
60
70
80
90
100
110
120
0
50
100
150
200
250
300
350
400
450
500
550
600
650
700
750
800
850
900

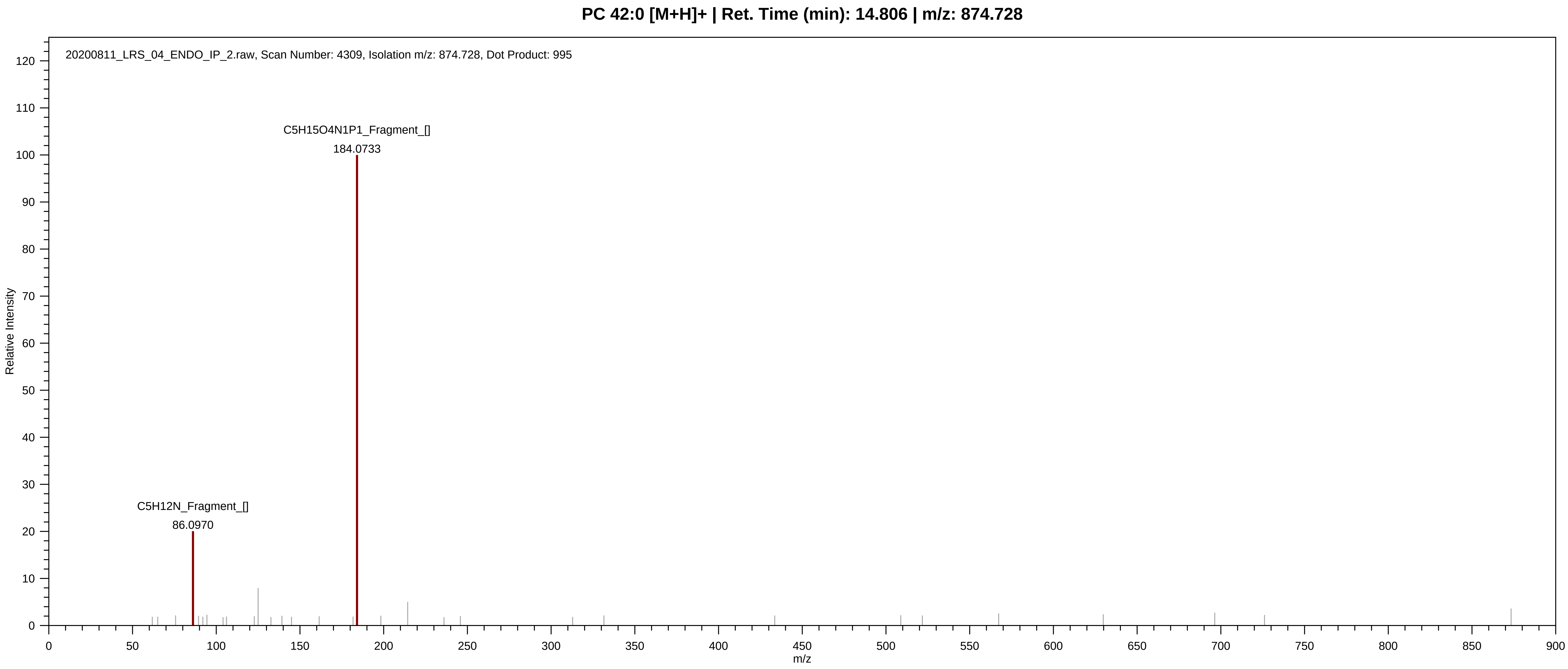
PC 42:0 [M+H]+ | Ret. Time (min): 14.806 | m/z: 874.728
20200811_LRS_04_ENDO_IP_2.raw, Scan Number: 4309, Isolation m/z: 874.728, Dot Product: 995
C5H15O4N1P1_Fragment_[]
184.0733
C5H12N_Fragment_[]
86.0970
Relative Intensity
m/z
0
10
20
30
40
50
60
70
80
90
100
110
120
0
50
100
150
200
250
300
350
400
450
500
550
600
650
700
750
800
850
900

PC 42:0 [M+H]+ | Ret. Time (min): 14.812 | m/z: 874.728

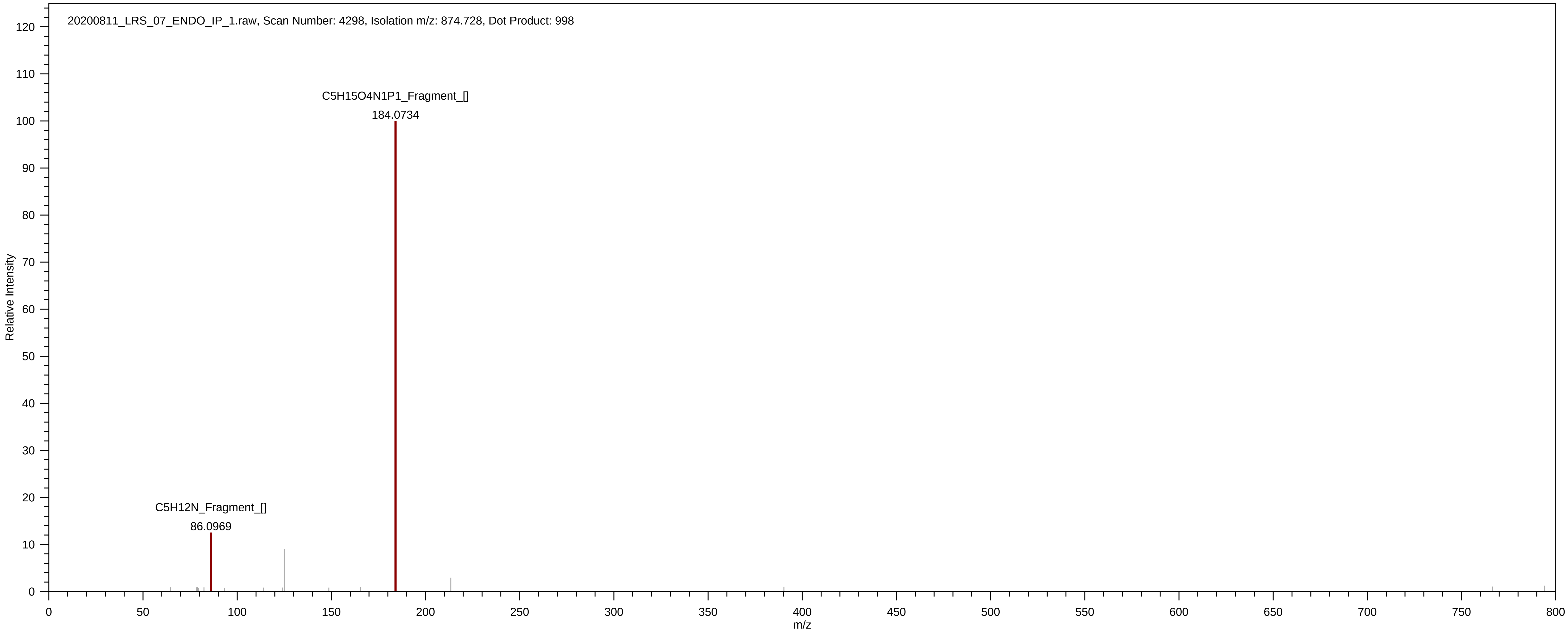

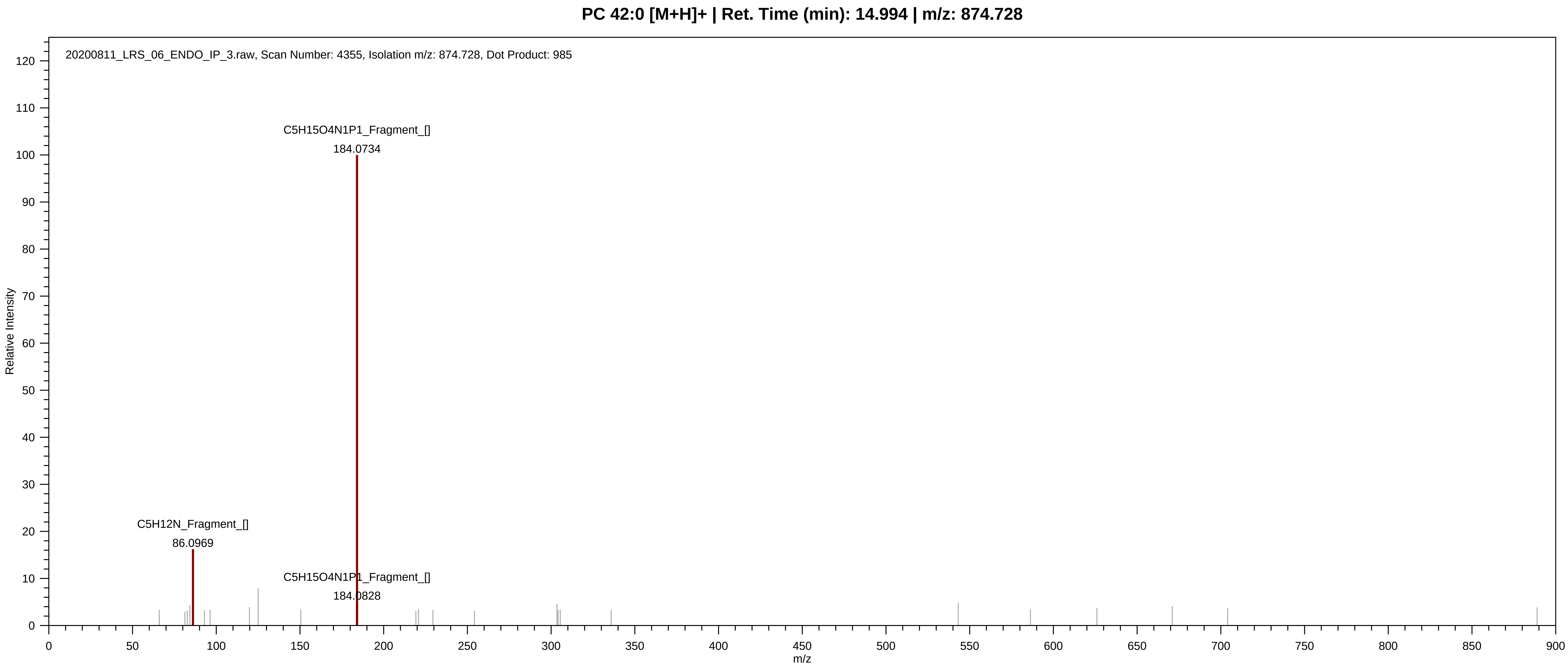
PC 42:0 [M+H]+ | Ret. Time (min): 14.994 | m/z: 874.728
20200811_LRS_06_ENDO_IP_3.raw, Scan Number: 4355, Isolation m/z: 874.728, Dot Product: 985
C5H15O4N1P1_Fragment_[]
184.0734
C5H12N_Fragment_[]
86.0969
C5H15O4N1P1_Fragment_[]
184.0828
Relative Intensity
m/z
0
10
20
30
40
50
60
70
80
90
100
110
120
0
50
100
150
200
250
300
350
400
450
500
550
600
650
700
750
800
850
900

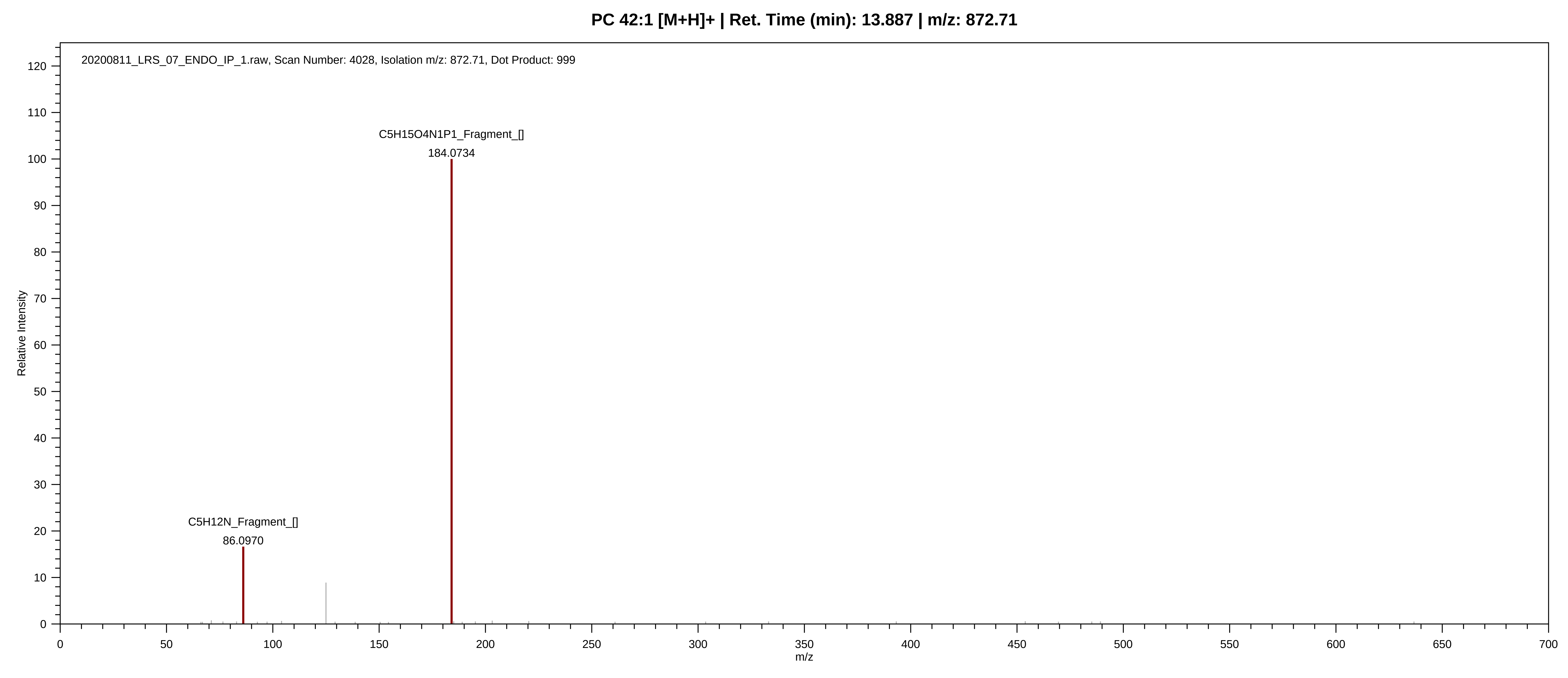

PC 42:1 [M+H]+ | Ret. Time (min): 13.887 | m/z: 872.71
20200811_LRS_07_ENDO_IP_1.raw, Scan Number: 4028, Isolation m/z: 872.71, Dot Product: 999
C5H15O4N1P1_Fragment_[]
184.0734
C5H12N_Fragment_[]
86.0970
Relative Intensity
m/z

PC 42:1 [M+H]+ | Ret. Time (min): 14.093 | m/z: 872.71

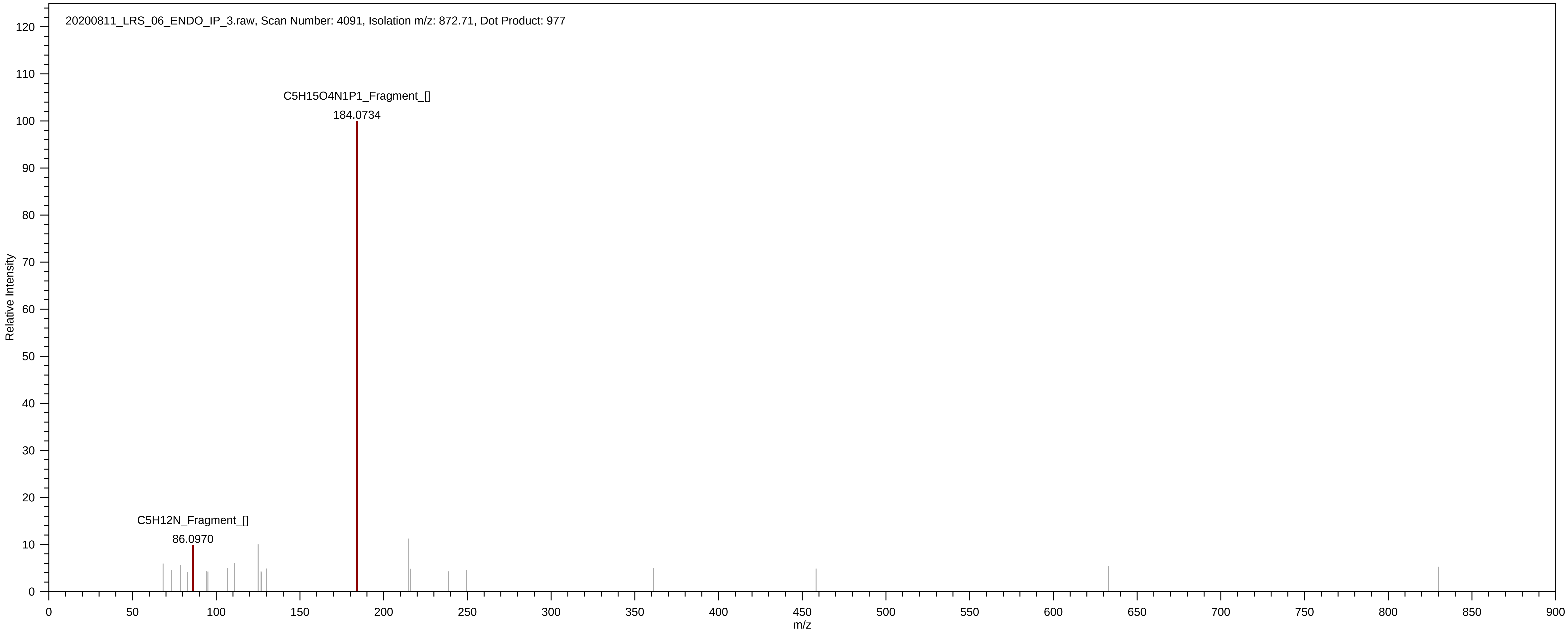

PC 42:1 [M+H]+ | Ret. Time (min): 13.83 | m/z: 872.708

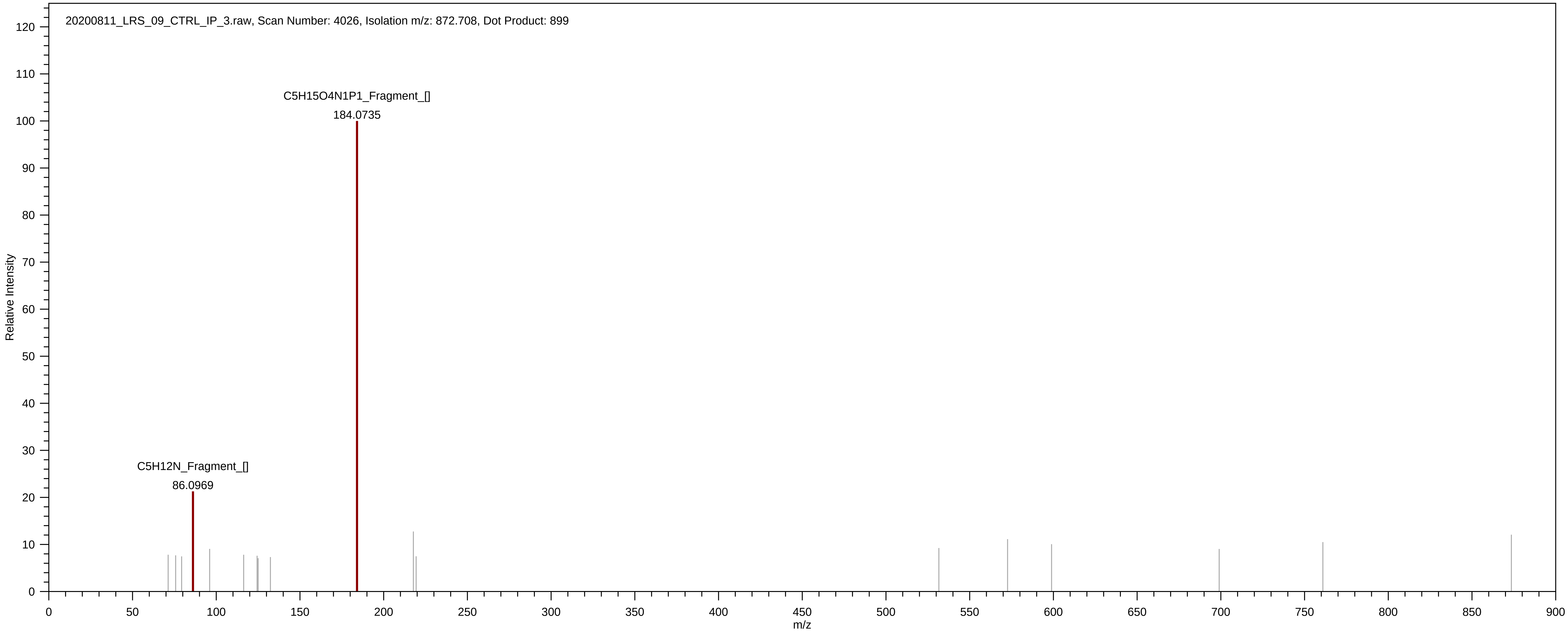

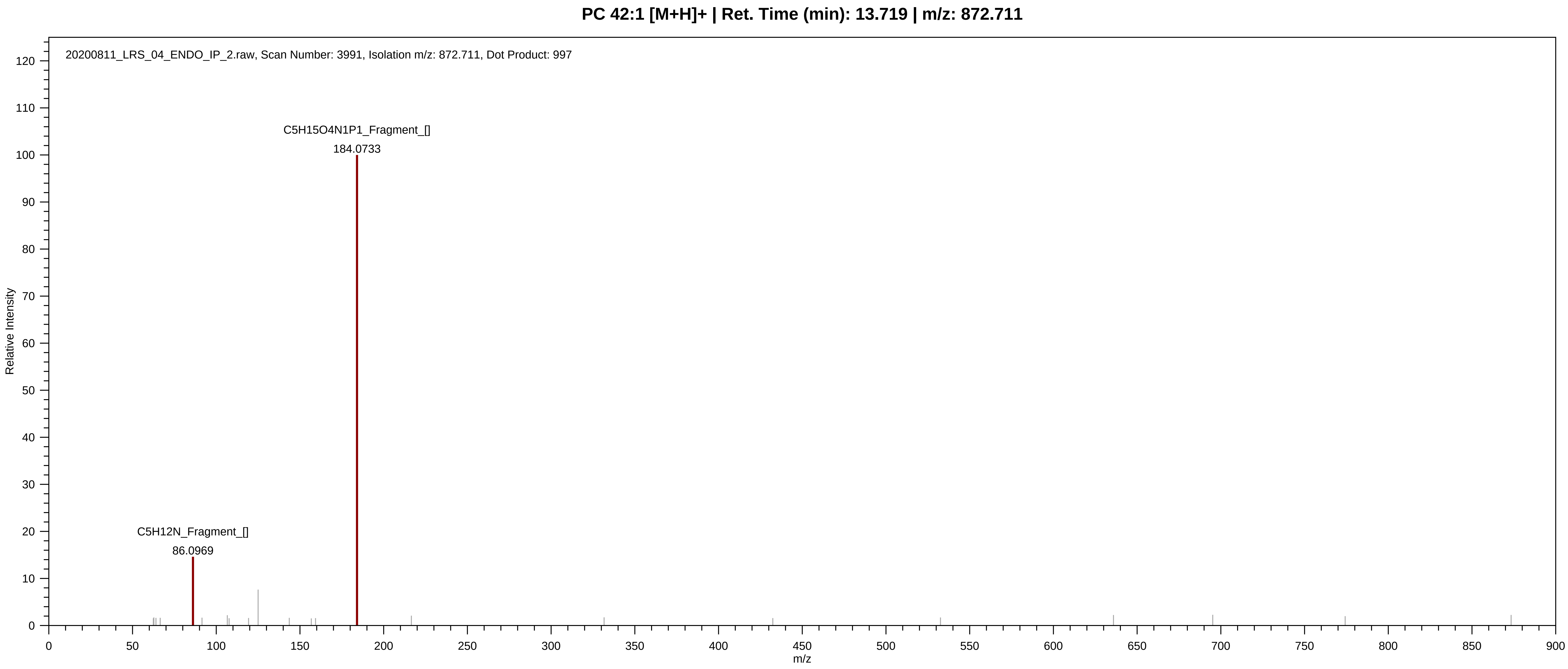
PC 42:1 [M+H]+ | Ret. Time (min): 13.719 | m/z: 872.711
20200811_LRS_04_ENDO_IP_2.raw, Scan Number: 3991, Isolation m/z: 872.711, Dot Product: 997
C5H15O4N1P1_Fragment_[]
184.0733
C5H12N_Fragment_[]
86.0969
Relative Intensity
m/z
0
10
20
30
40
50
60
70
80
90
100
110
120
0
50
100
150
200
250
300
350
400
450
500
550
600
650
700
750
800
850
900

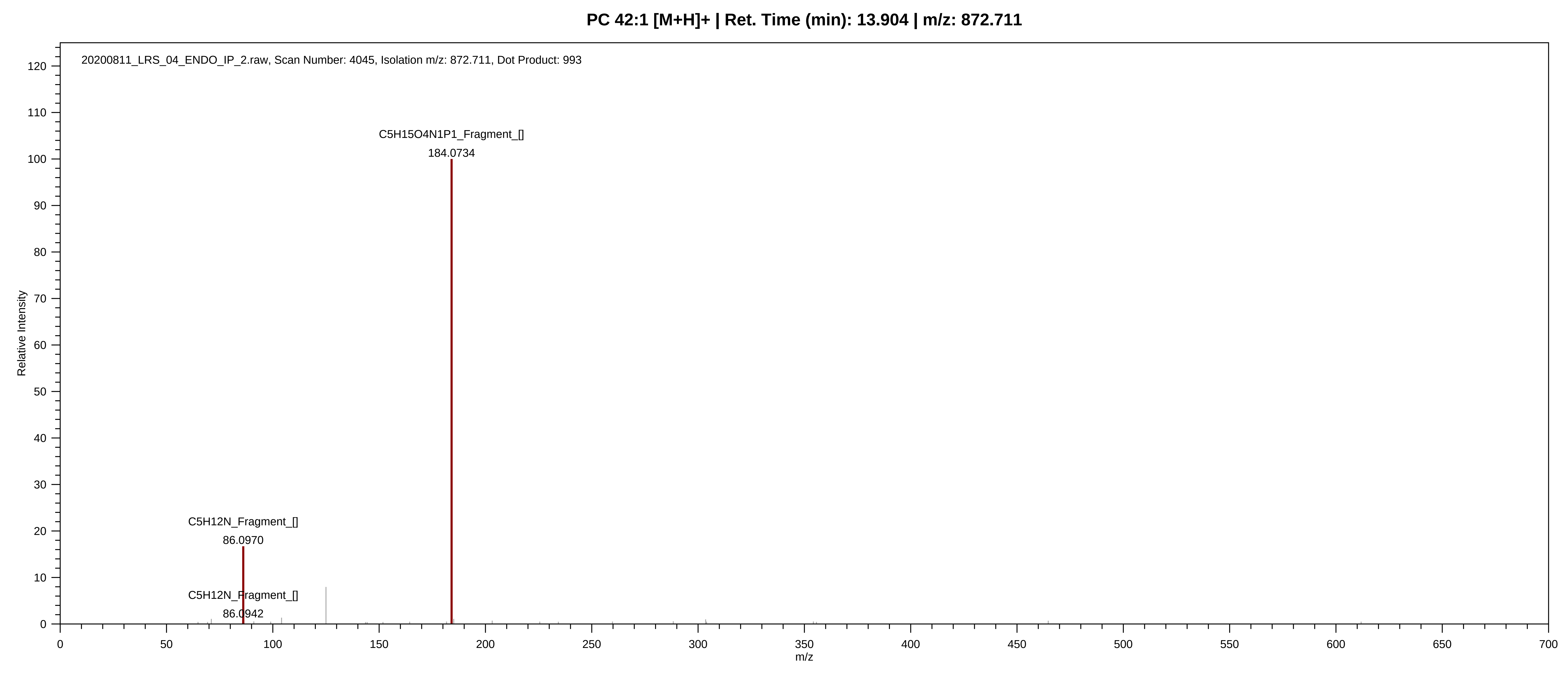
PC 42:1 [M+H]+ | Ret. Time (min): 13.904 | m/z: 872.711
20200811_LRS_04_ENDO_IP_2.raw, Scan Number: 4045, Isolation m/z: 872.711, Dot Product: 993
C5H15O4N1P1_Fragment_[]
184.0734
C5H12N_Fragment_[]
86.0970
C5H12N_Fragment_[]
86.0942
Relative Intensity
m/z
0
10
20
30
40
50
60
70
80
90
100
110
120
0
50
100
150
200
250
300
350
400
450
500
550
600
650
700

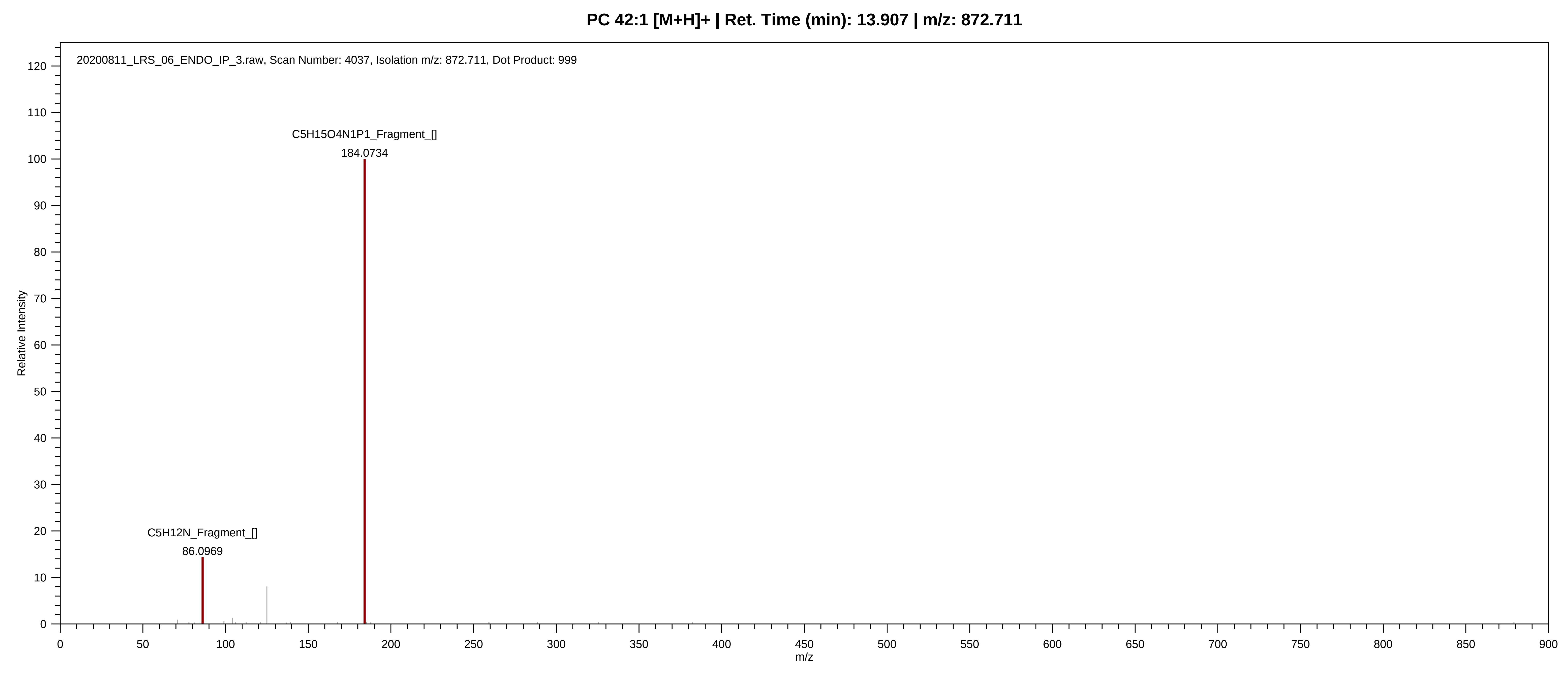

PC 42:1 [M+H]+ | Ret. Time (min): 13.907 | m/z: 872.711
20200811_LRS_06_ENDO_IP_3.raw, Scan Number: 4037, Isolation m/z: 872.711, Dot Product: 999
C5H15O4N1P1_Fragment_[]
184.0734
C5H12N_Fragment_[]
86.0969
Relative Intensity
m/z

PC 42:1 [M+H]+ | Ret. Time (min): 14.017 | m/z: 872.711

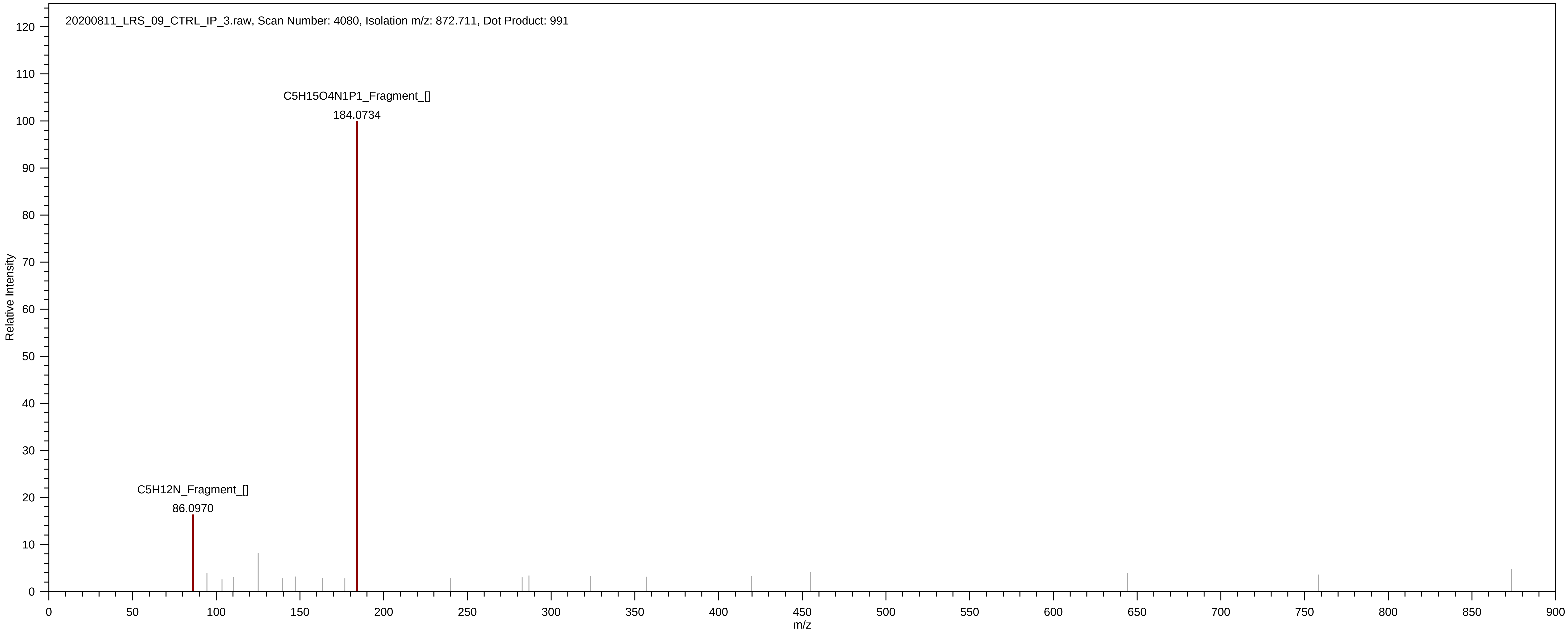

PC 42:1 [M+H]+ | Ret. Time (min): 14.072 | m/z: 872.711

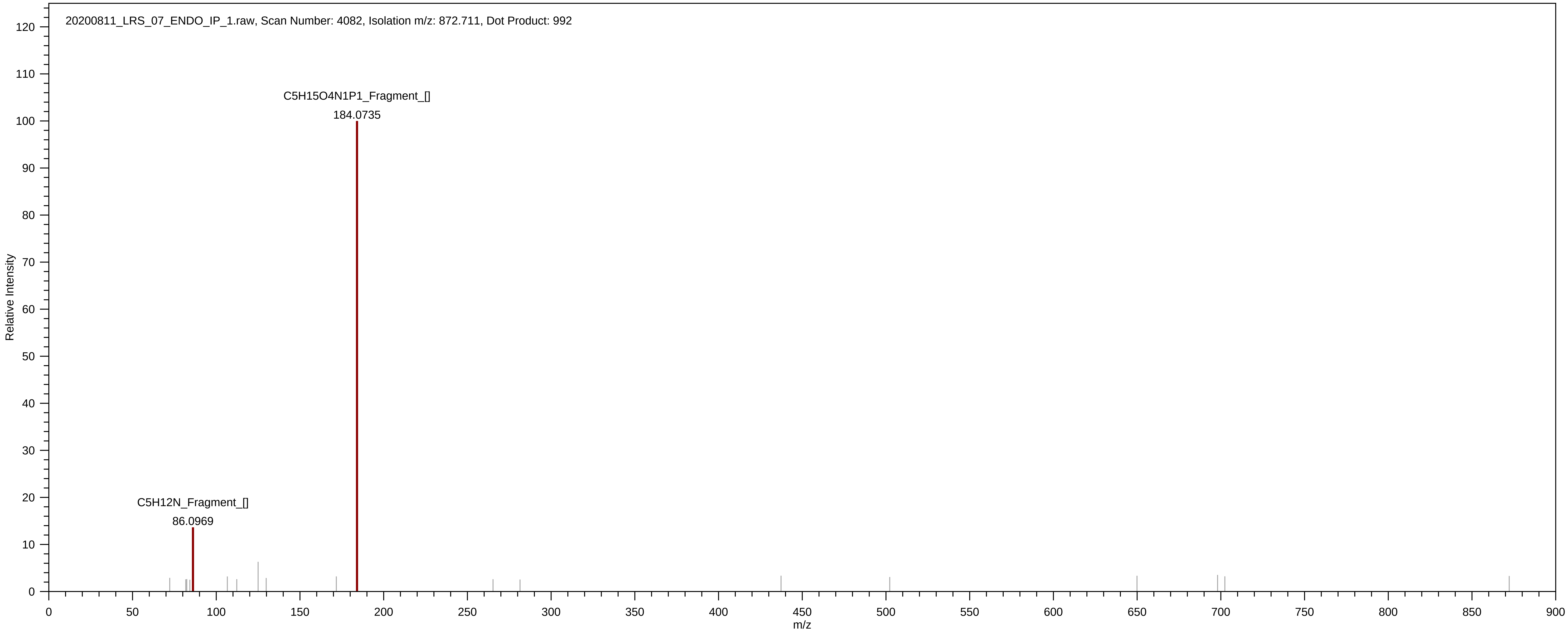

PC 42:1 [M+H]+ | Ret. Time (min): 13.706 | m/z: 872.712

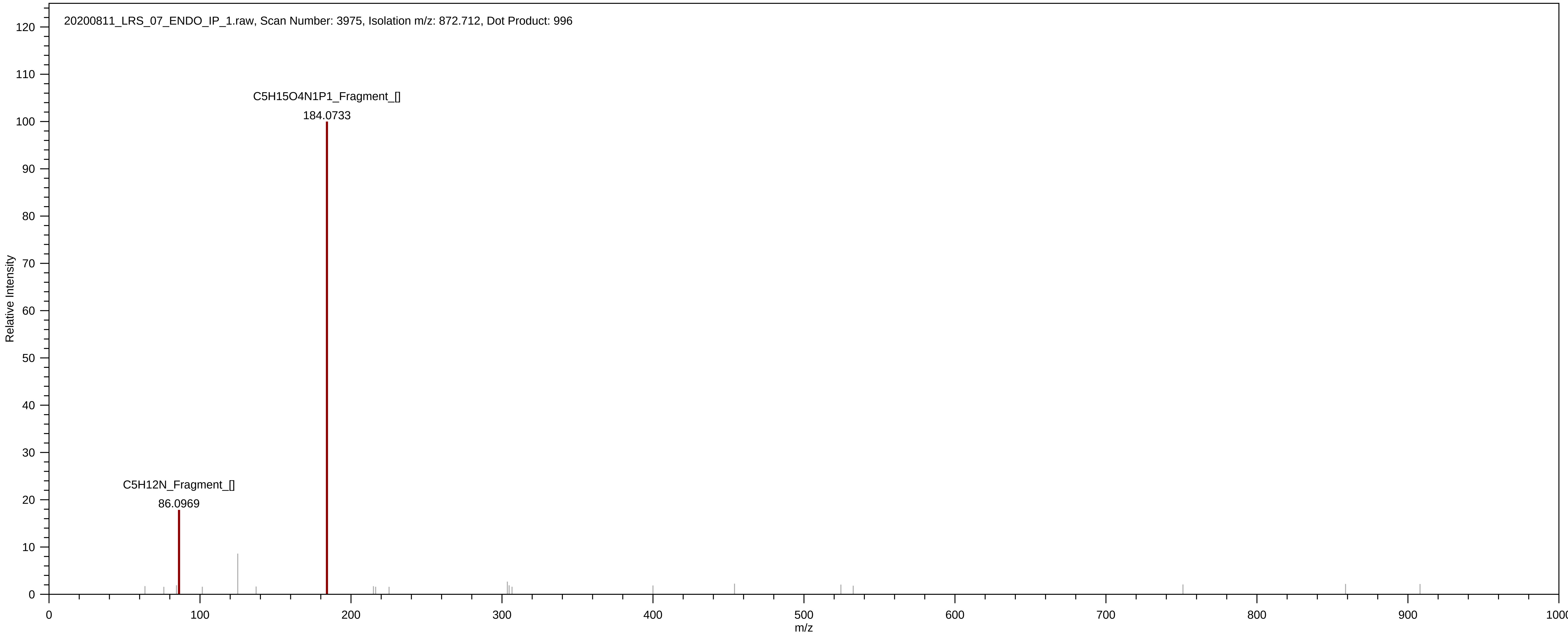

PC 42:1 [M+H]+ | Ret. Time (min): 13.722 | m/z: 872.712

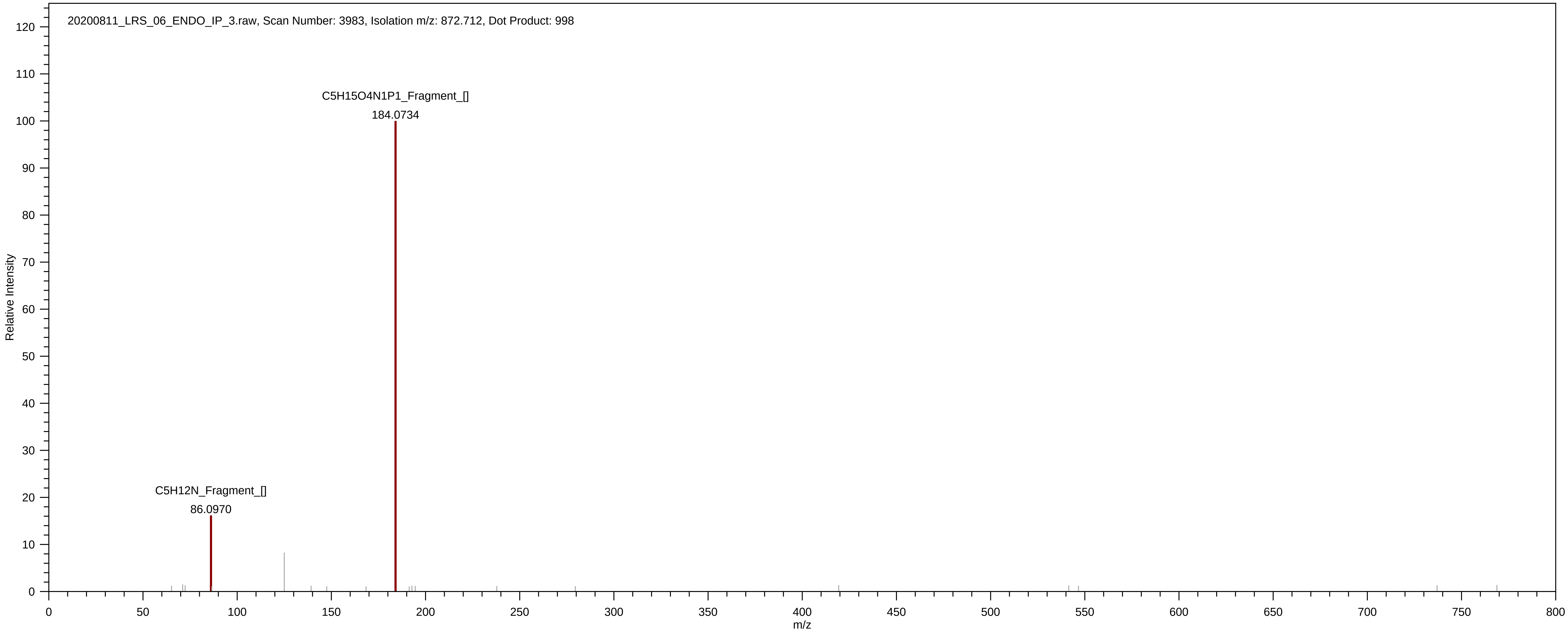

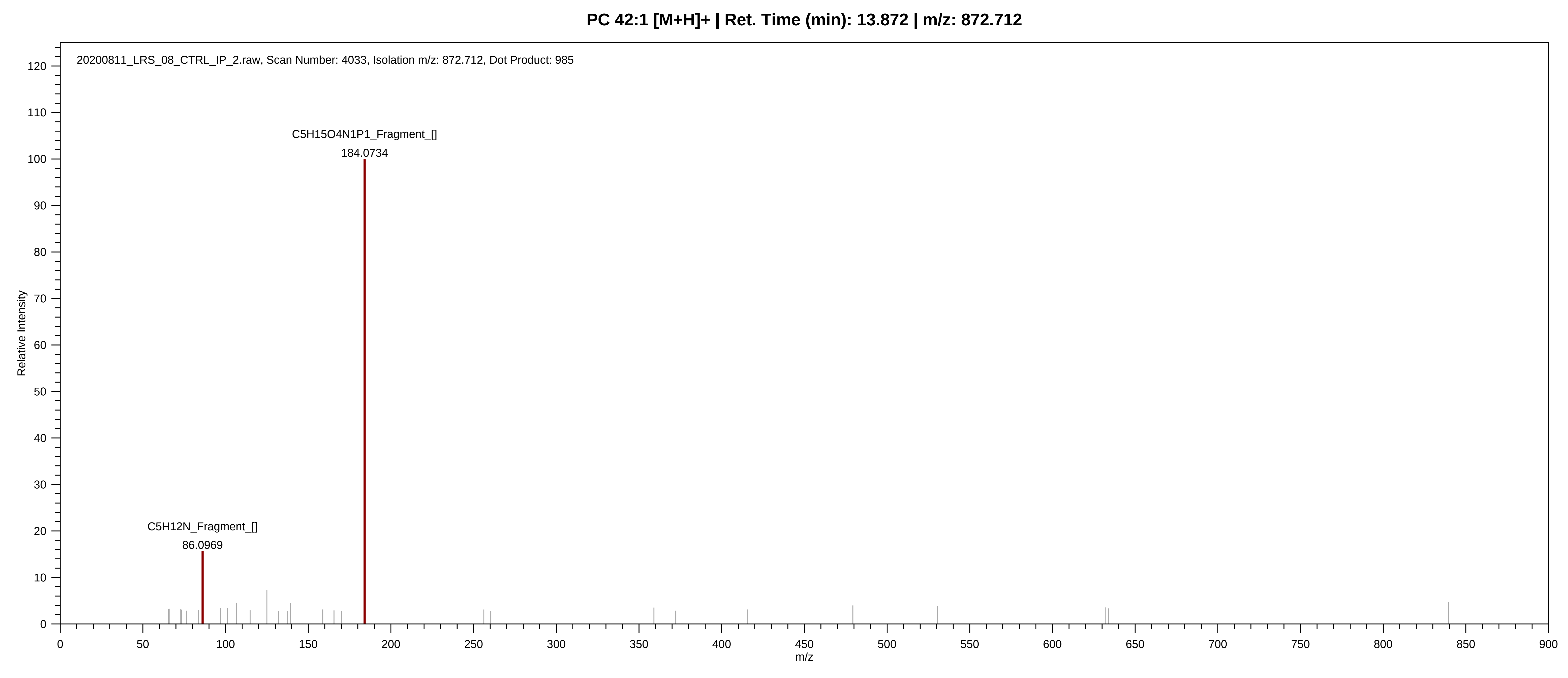
PC 42:1 [M+H]+ | Ret. Time (min): 13.872 | m/z: 872.712
20200811_LRS_08_CTRL_IP_2.raw, Scan Number: 4033, Isolation m/z: 872.712, Dot Product: 985
C5H15O4N1P1_Fragment_[]
184.0734
C5H12N_Fragment_[]
86.0969
Relative Intensity
m/z

PC 42:1 [M+H]+ | Ret. Time (min): 13.884 | m/z: 872.712

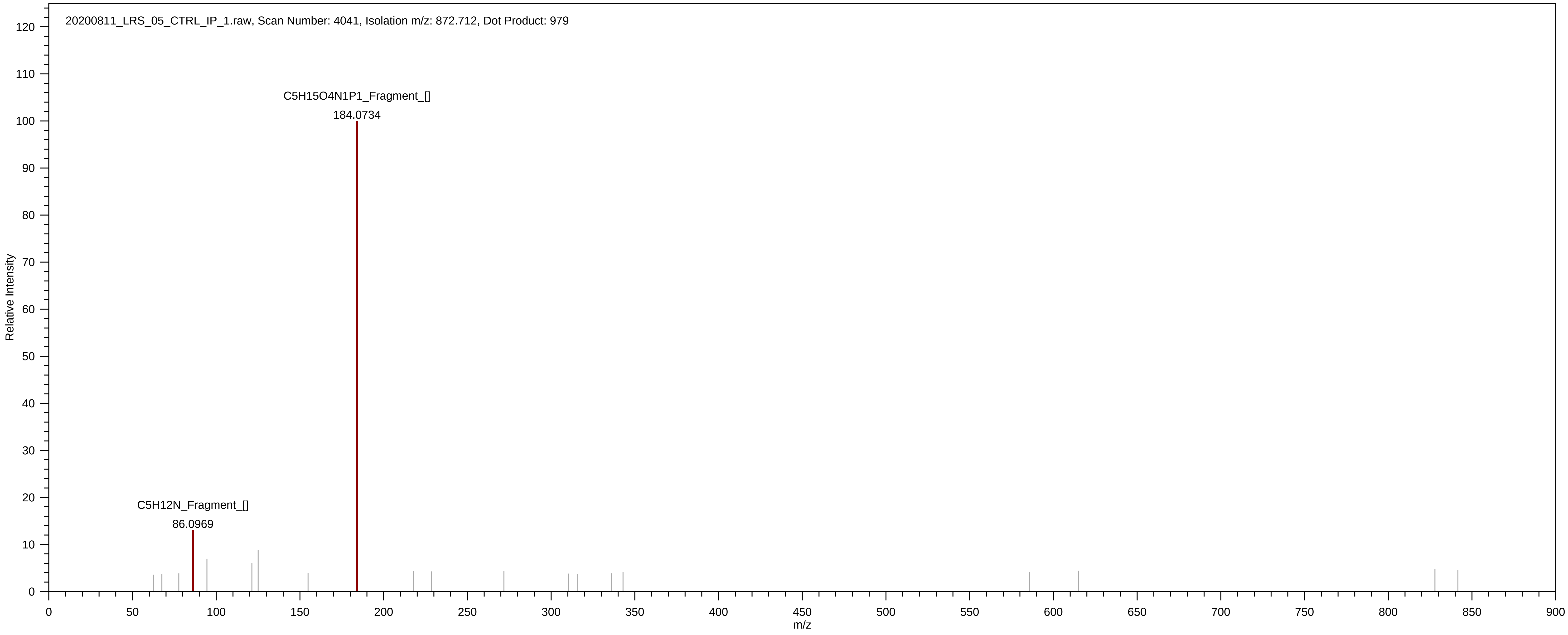

PC 42:1 [M+H]+ | Ret. Time (min): 14.09 | m/z: 872.712

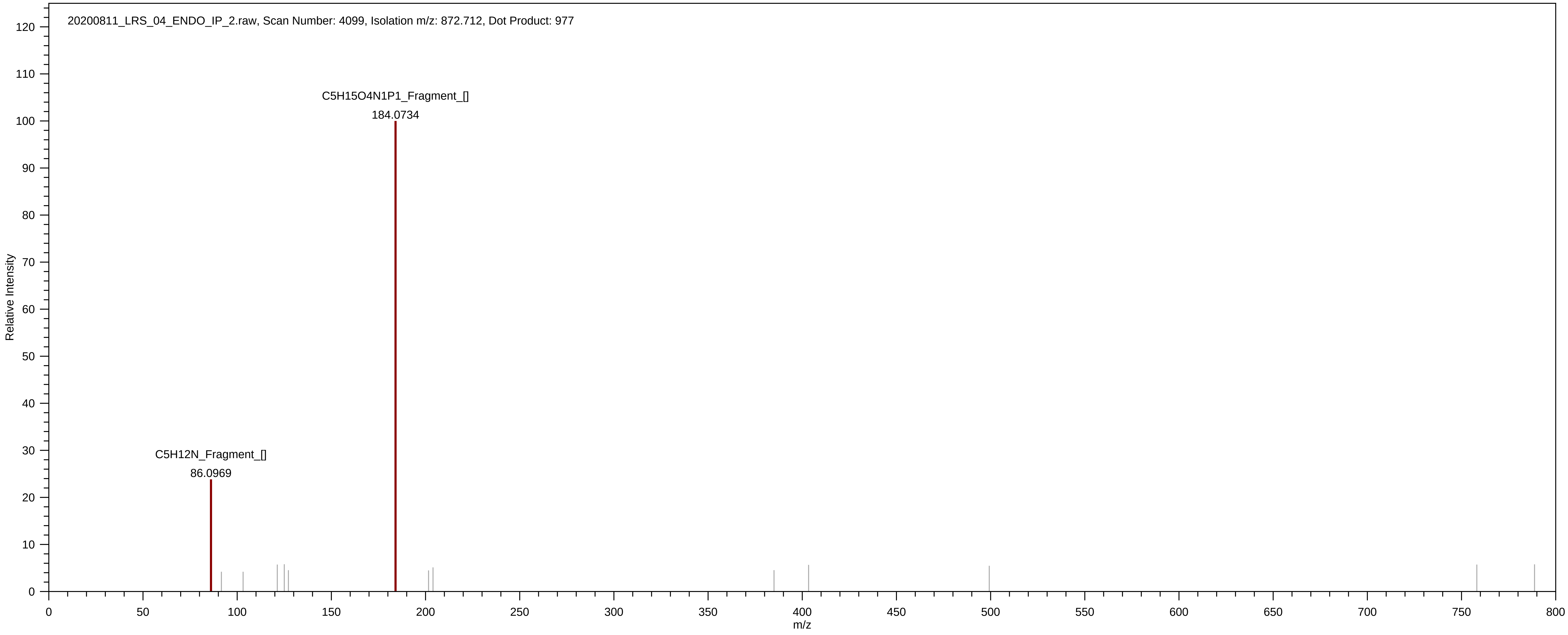

PC 42:1 [M+H]+ | Ret. Time (min): 14.055 | m/z: 872.712

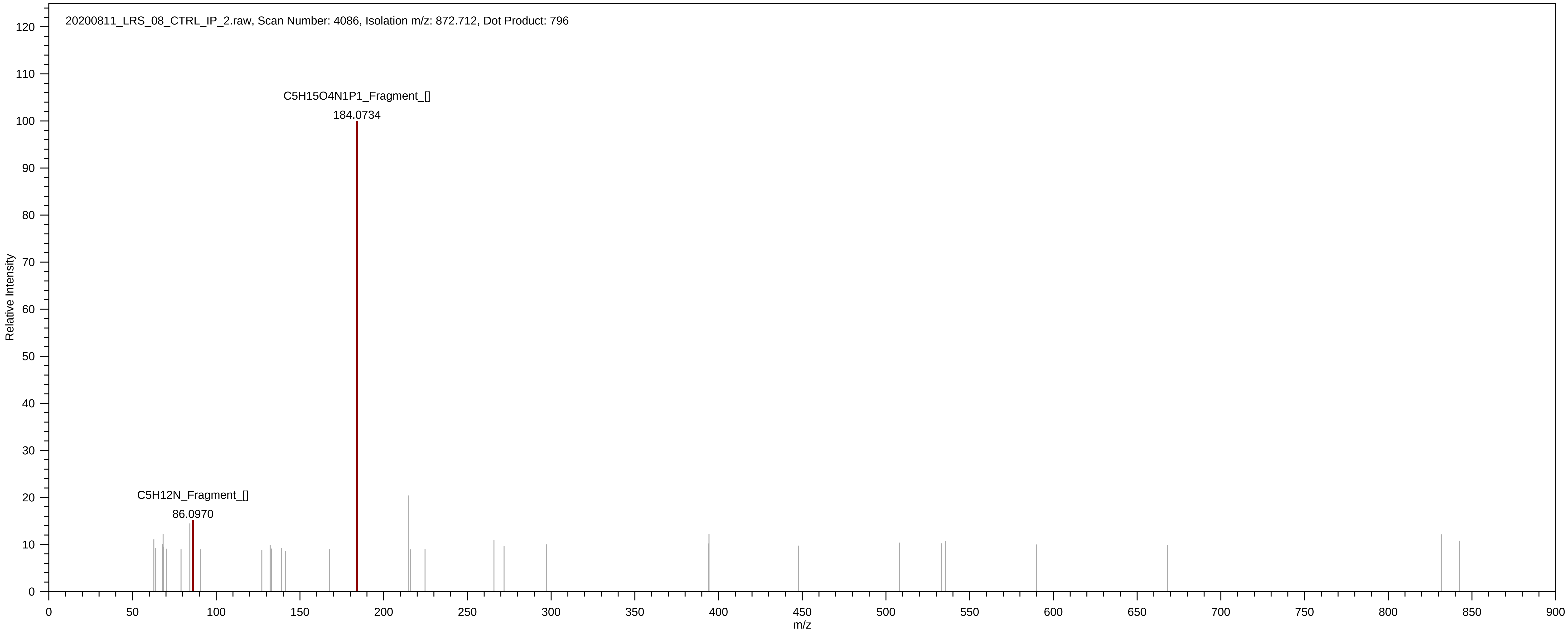

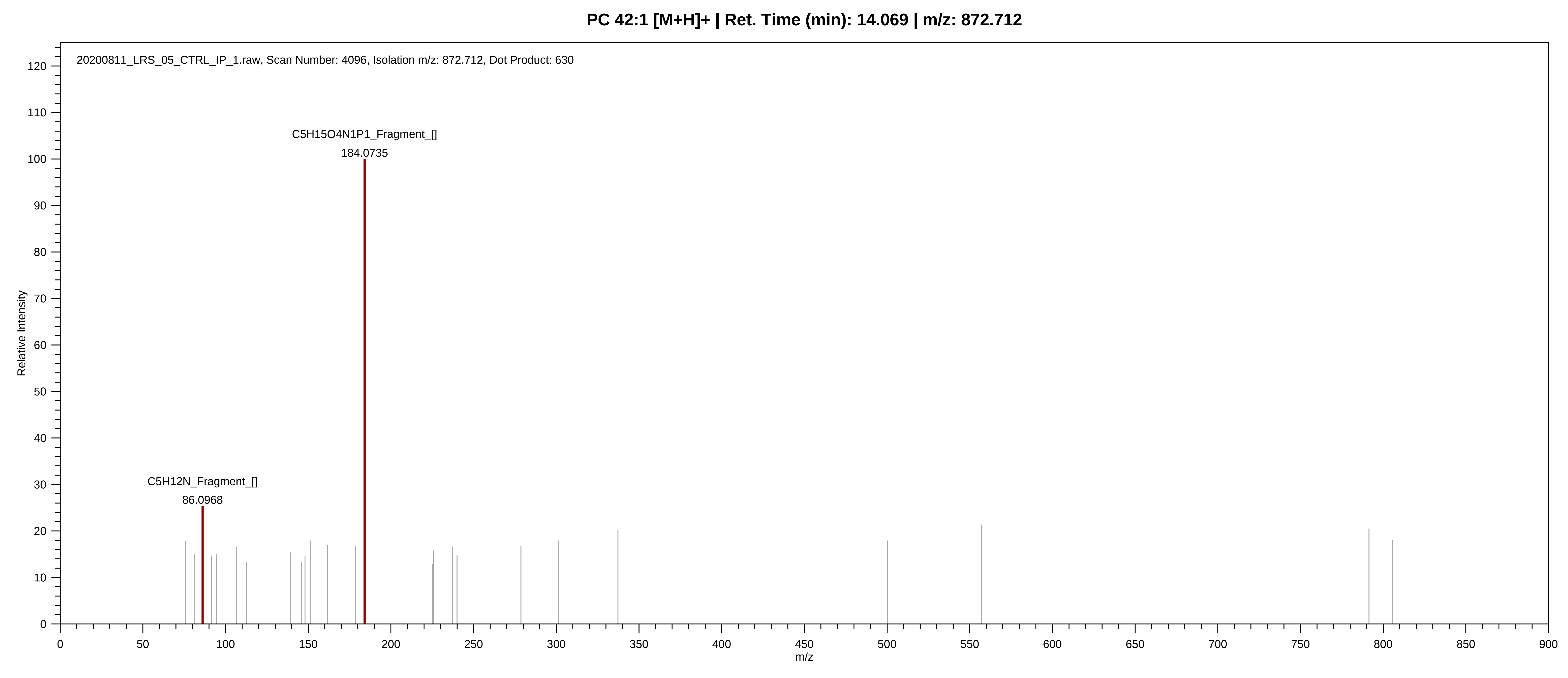
PC 42:1 [M+H]+ | Ret. Time (min): 14.069 | m/z: 872.712
20200811_LRS_05_CTRL_IP_1.raw, Scan Number: 4096, Isolation m/z: 872.712, Dot Product: 630
C5H15O4N1P1_Fragment_[]
184.0735
C5H12N_Fragment_[]
86.0968
Relative Intensity
m/z

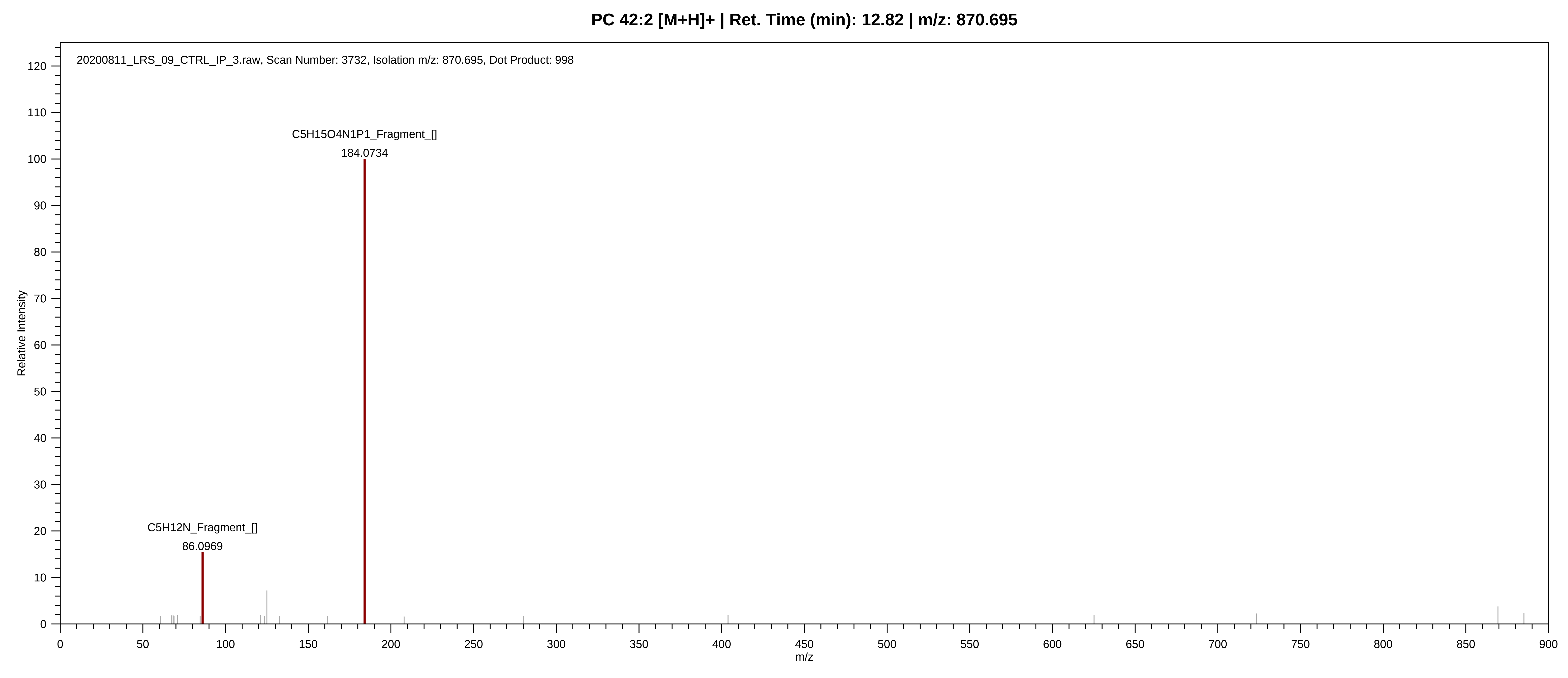
PC 42:2 [M+H]+ | Ret. Time (min): 12.82 | m/z: 870.695
20200811_LRS_09_CTRL_IP_3.raw, Scan Number: 3732, Isolation m/z: 870.695, Dot Product: 998
C5H15O4N1P1_Fragment_[]
184.0734
C5H12N_Fragment_[]
86.0969
Relative Intensity
m/z

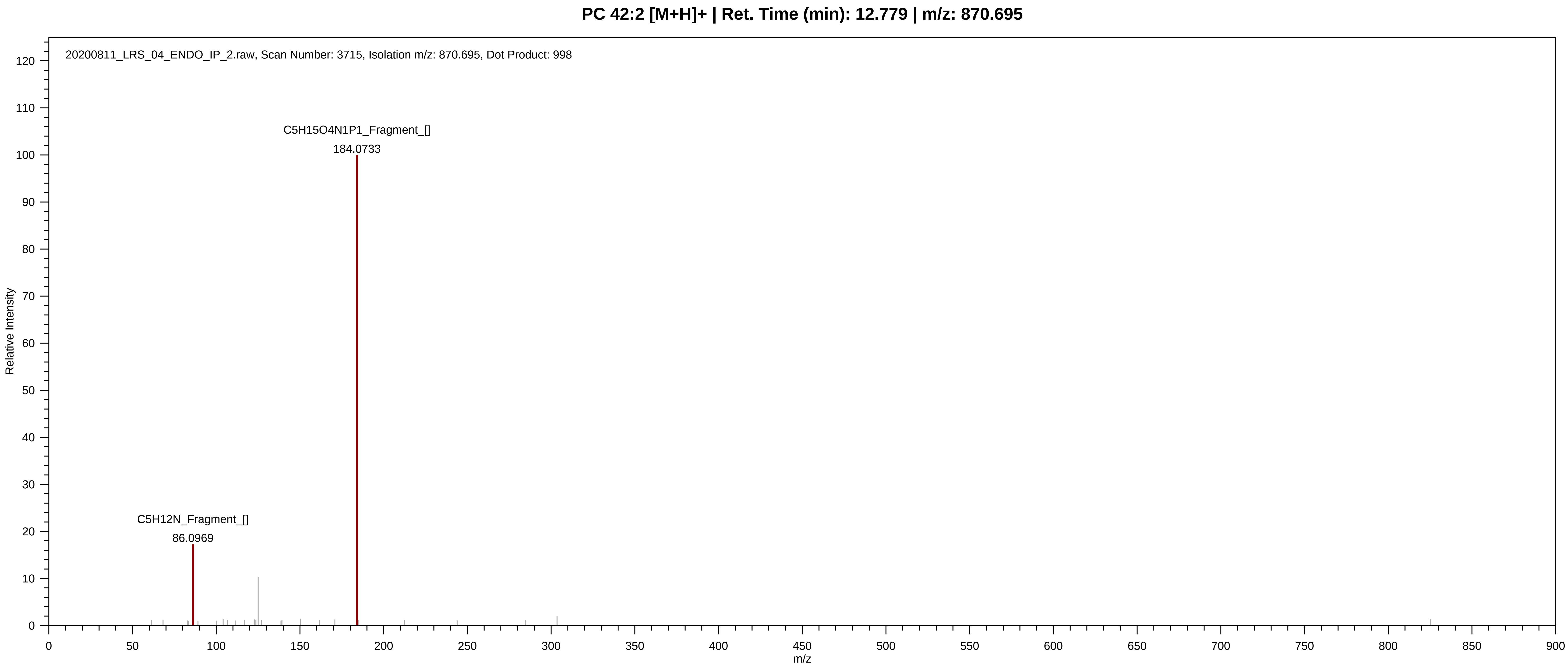

PC 42:2 [M+H]+ | Ret. Time (min): 12.779 | m/z: 870.695
20200811_LRS_04_ENDO_IP_2.raw, Scan Number: 3715, Isolation m/z: 870.695, Dot Product: 998
C5H15O4N1P1_Fragment_[]
184.0733
C5H12N_Fragment_[]
86.0969
Relative Intensity
m/z

PC 42:2 [M+H]+ | Ret. Time (min): 12.783 | m/z: 870.695

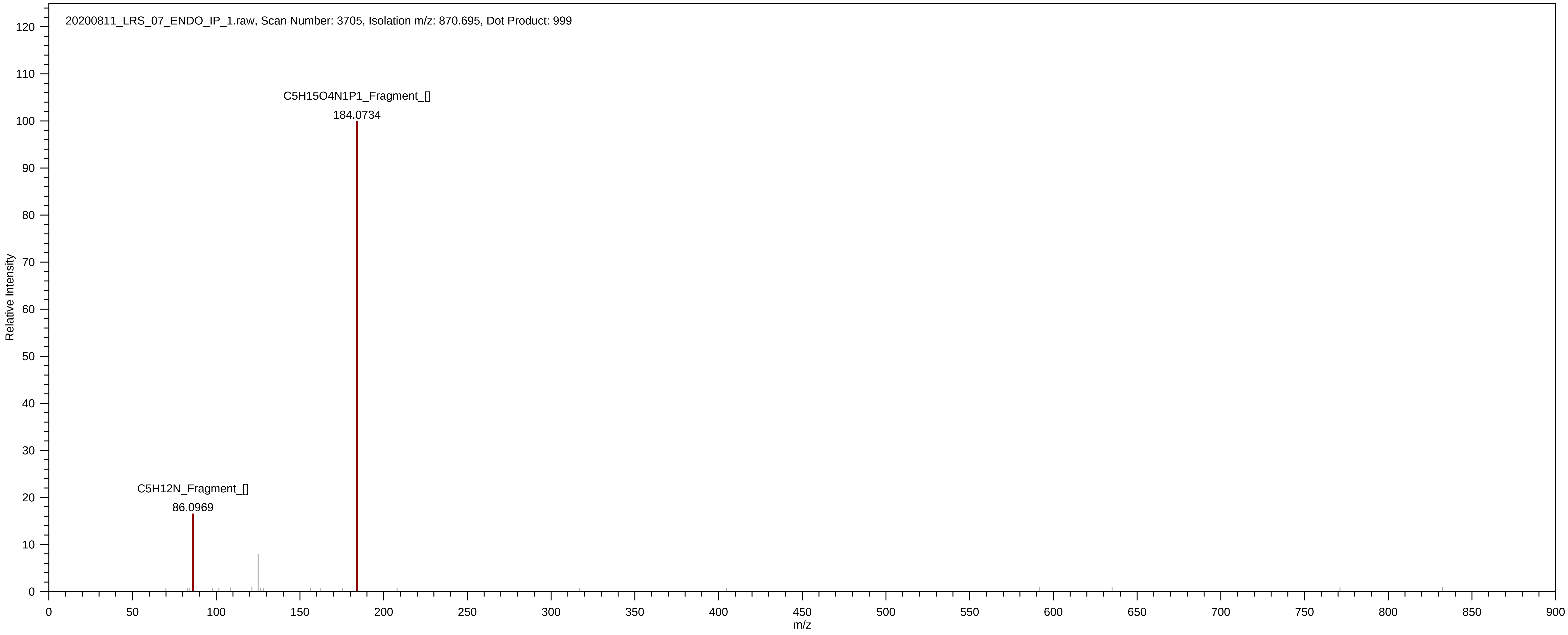

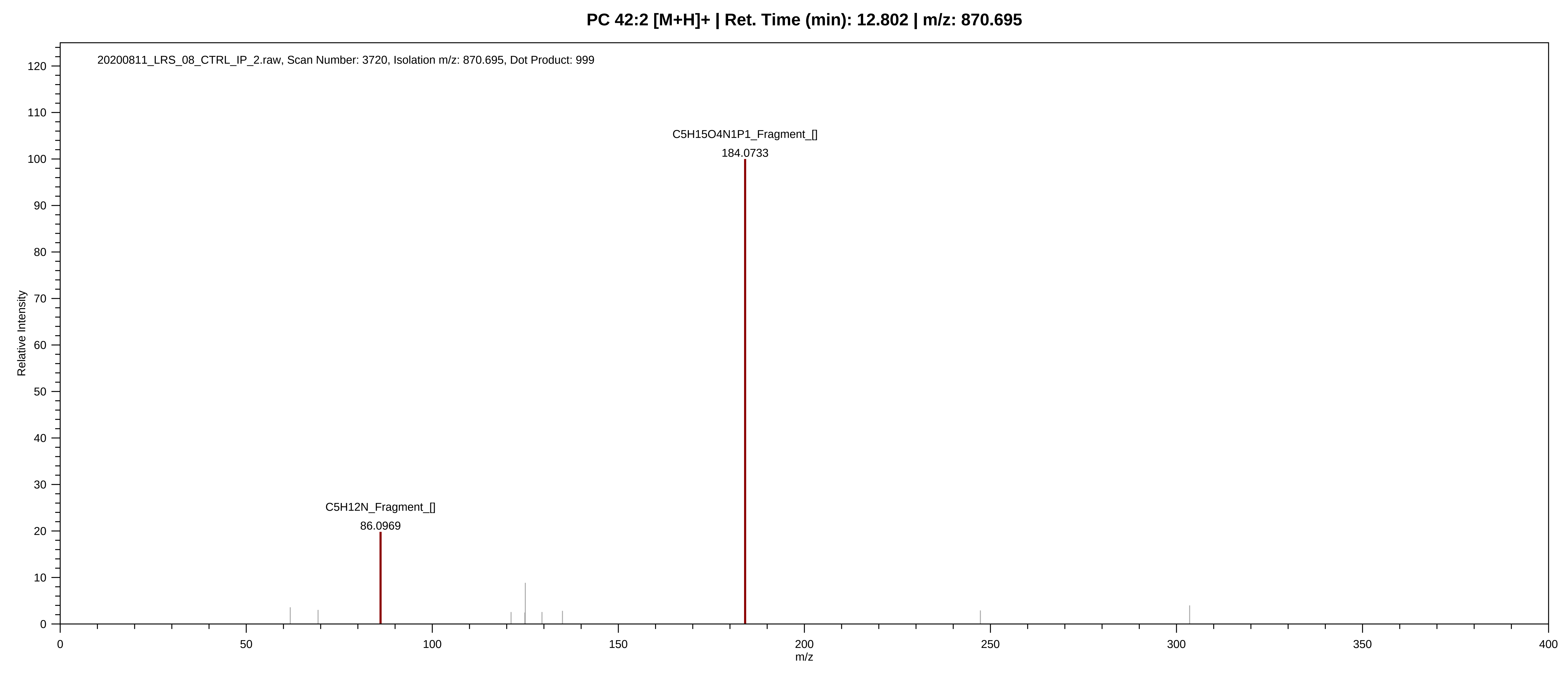

PC 42:2 [M+H]+ | Ret. Time (min): 12.802 | m/z: 870.695
20200811_LRS_08_CTRL_IP_2.raw, Scan Number: 3720, Isolation m/z: 870.695, Dot Product: 999
C5H15O4N1P1_Fragment_[]
184.0733
C5H12N_Fragment_[]
86.0969
Relative Intensity
m/z

PC 42:2 [M+H]+ | Ret. Time (min): 12.966 | m/z: 870.695

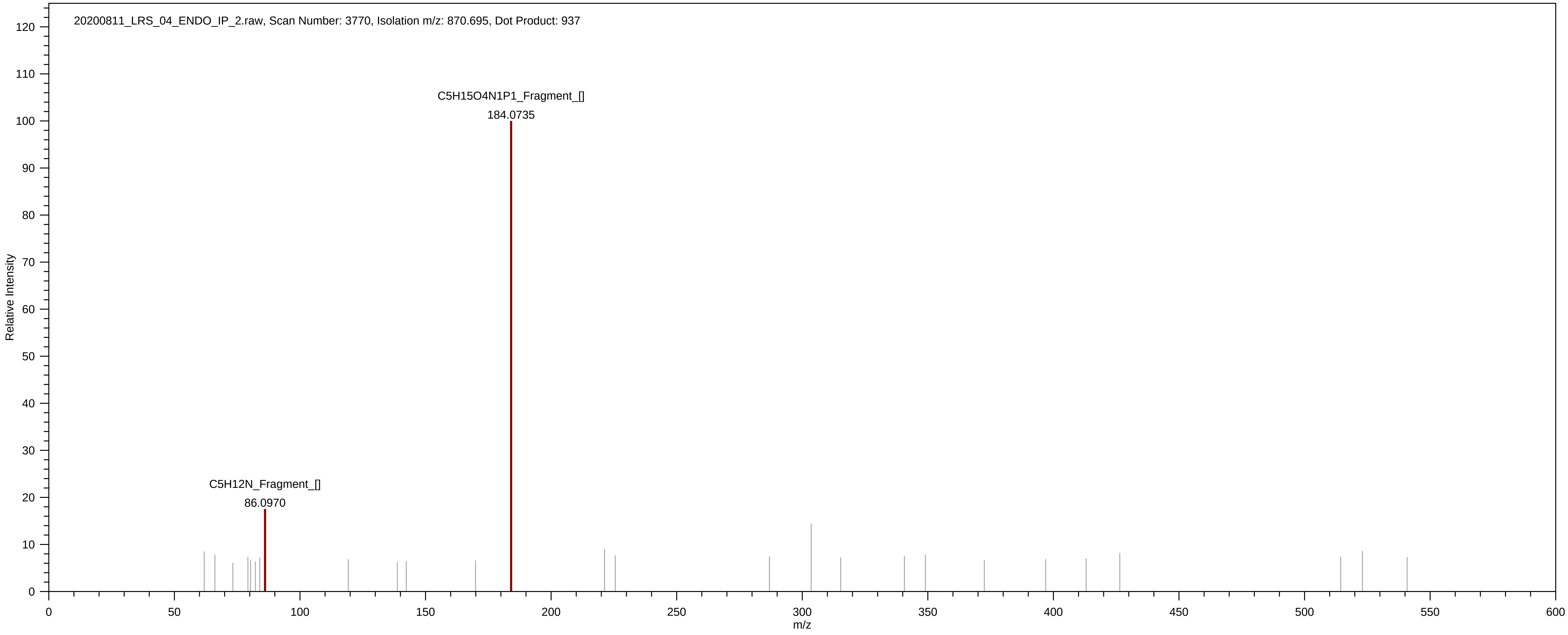

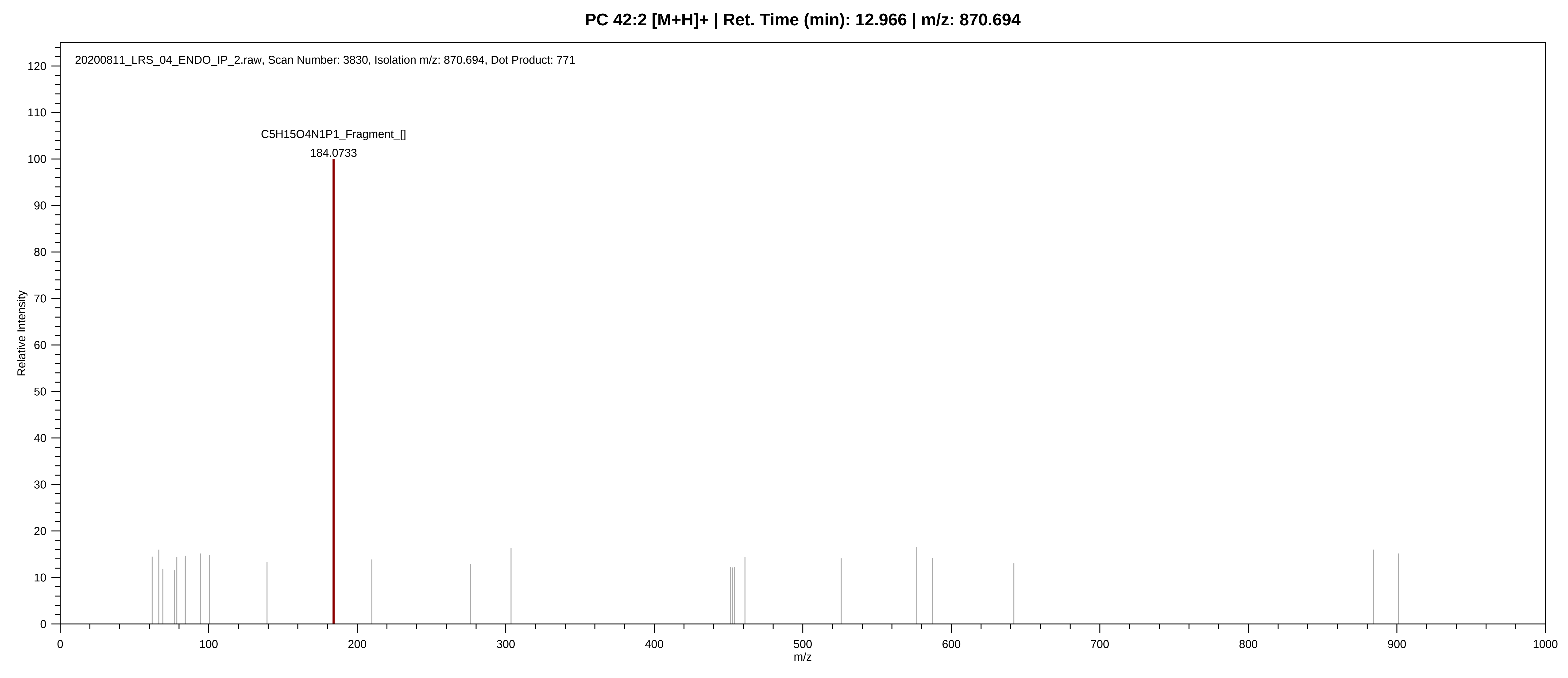

PC 42:2 [M+H]+ | Ret. Time (min): 12.966 | m/z: 870.694
20200811_LRS_04_ENDO_IP_2.raw, Scan Number: 3830, Isolation m/z: 870.694, Dot Product: 771
C5H15O4N1P1_Fragment_[]
184.0733
Relative Intensity
m/z

PC 42:2 [M+H]+ | Ret. Time (min): 12.968 | m/z: 870.695

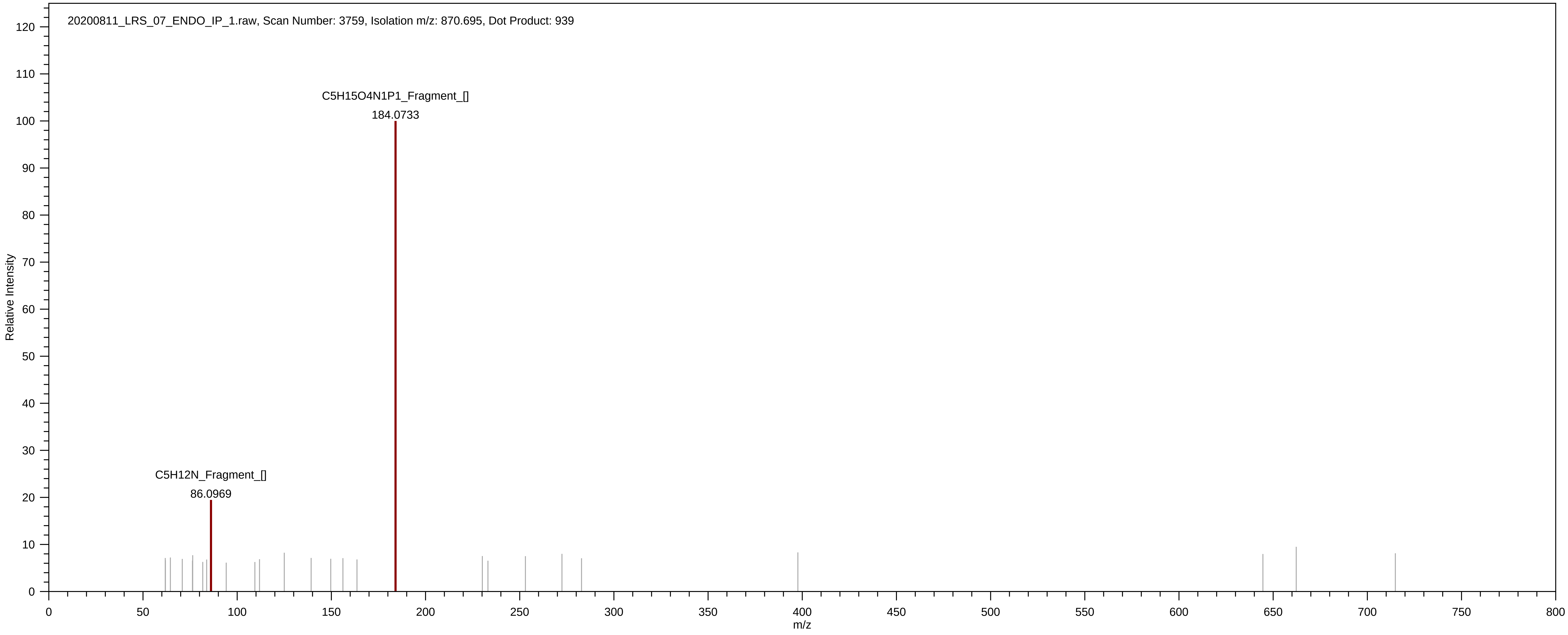

PC 42:2 [M+H]+ | Ret. Time (min): 12.759 | m/z: 870.696

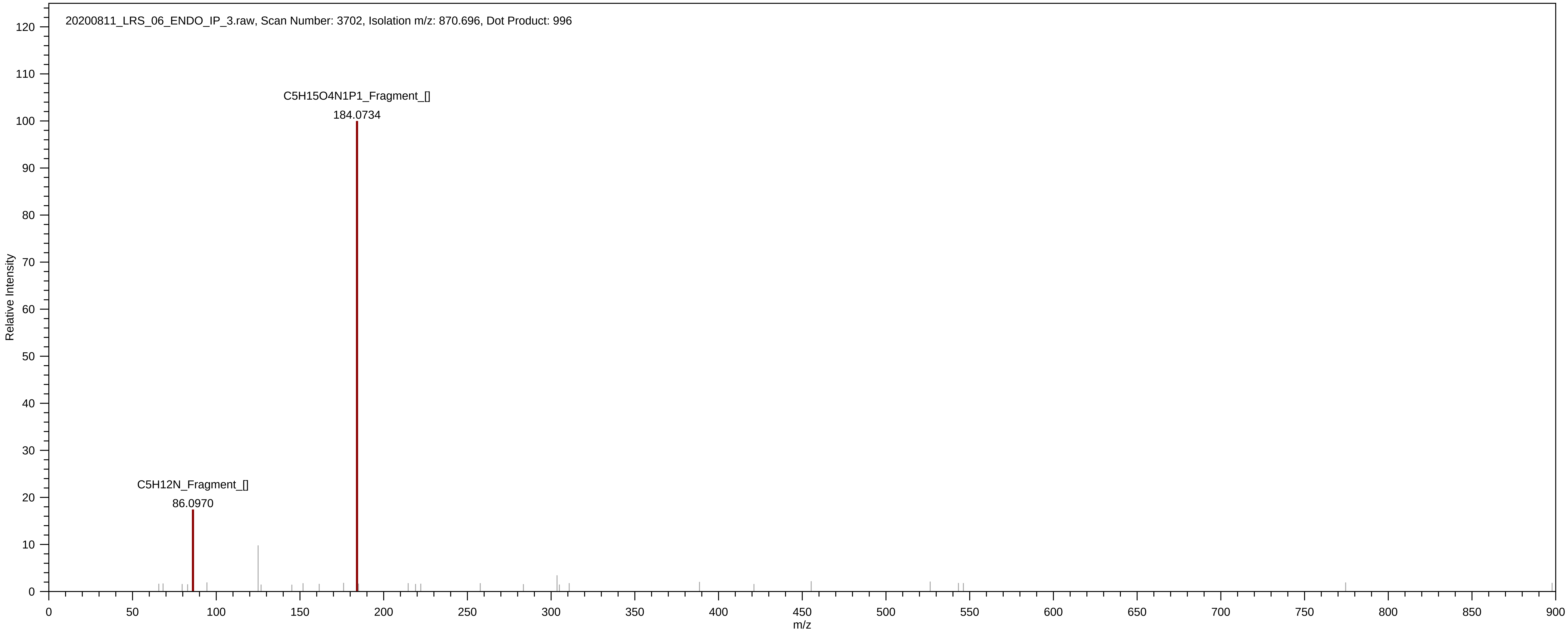

PC 42:2 [M+H]+ | Ret. Time (min): 12.942 | m/z: 870.696

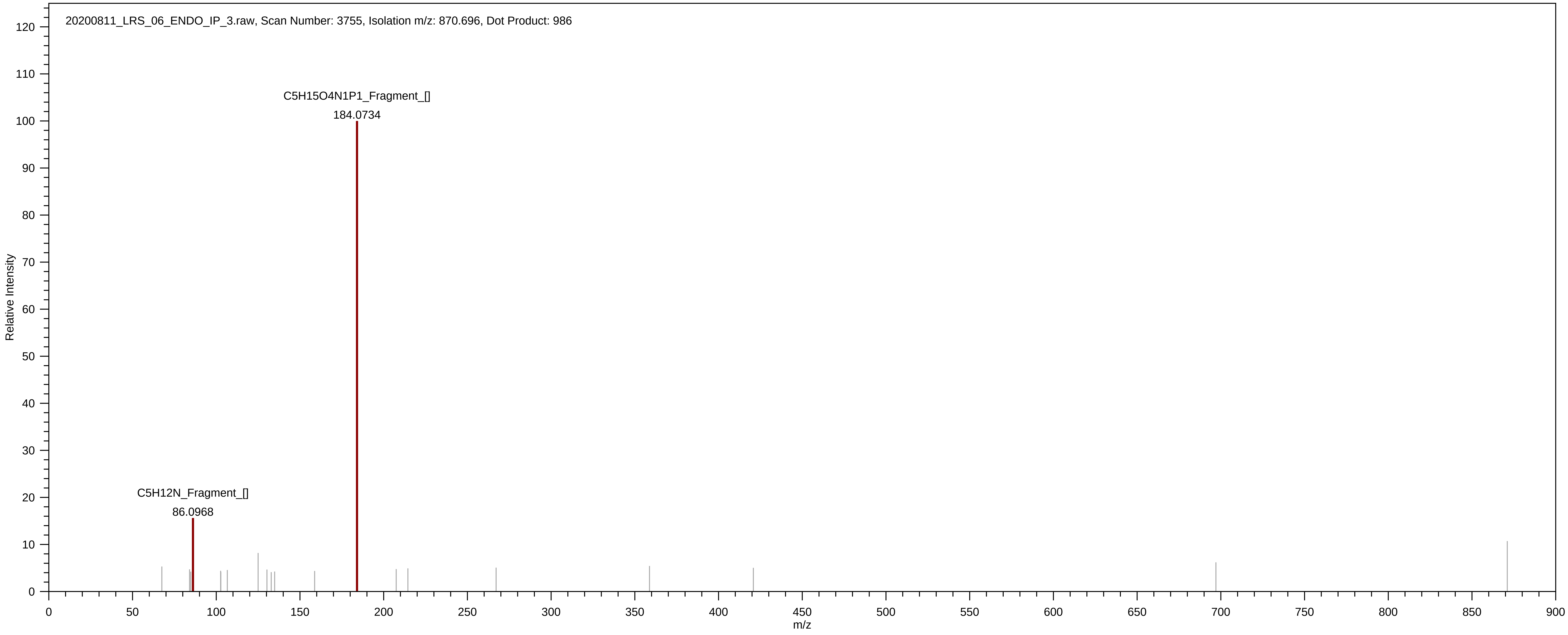

PC 42:2 [M+H]+ | Ret. Time (min): 12.942 | m/z: 870.692

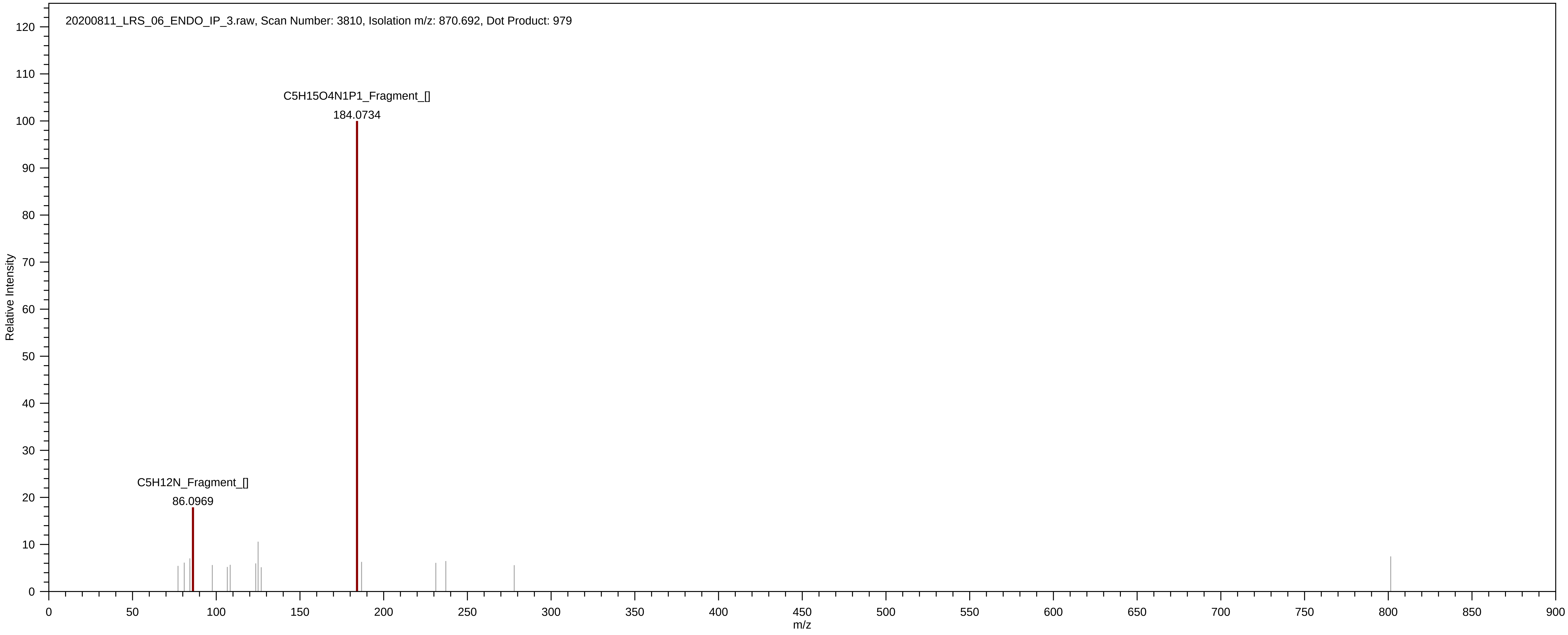

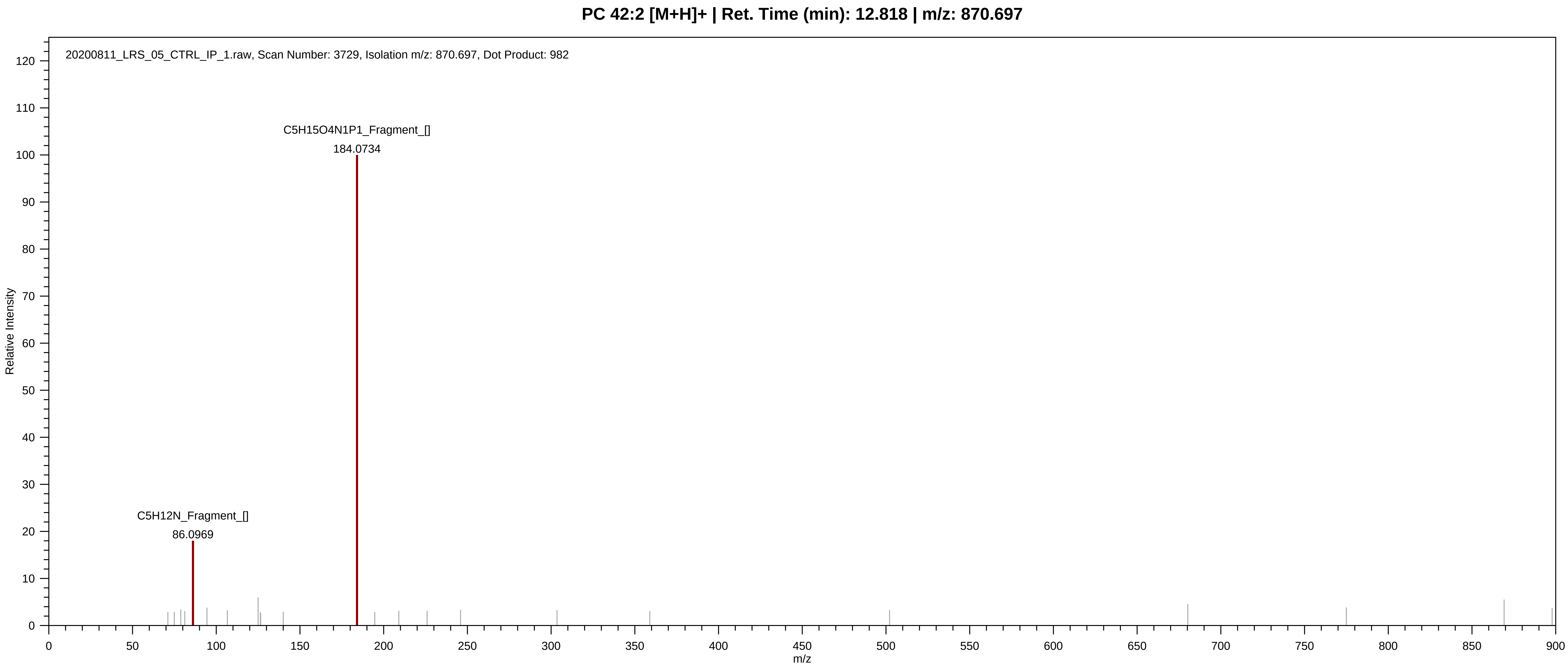
PC 42:2 [M+H]+ | Ret. Time (min): 12.818 | m/z: 870.697
20200811_LRS_05_CTRL_IP_1.raw, Scan Number: 3729, Isolation m/z: 870.697, Dot Product: 982
C5H15O4N1P1_Fragment_[]
184.0734
C5H12N_Fragment_[]
86.0969
Relative Intensity
m/z

PC 42:3 [M+H]+ | Ret. Time (min): 12.018 | m/z: 868.683

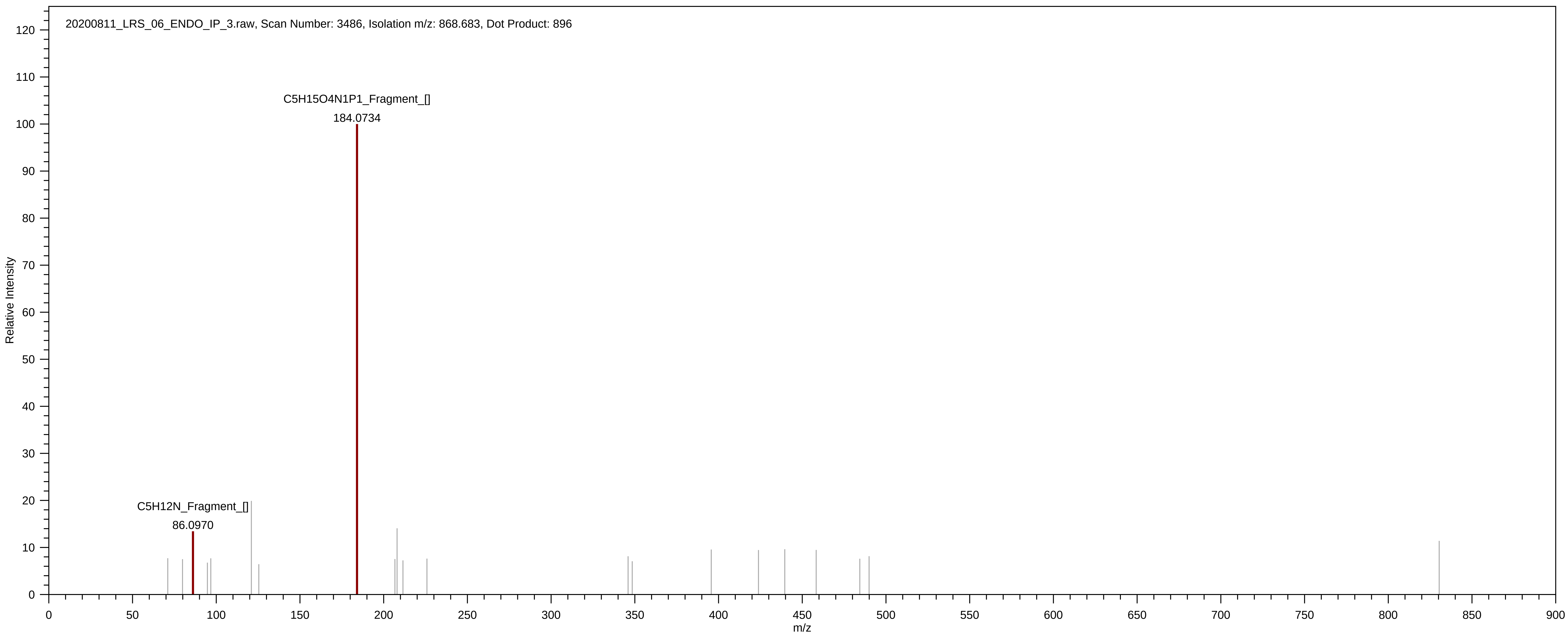

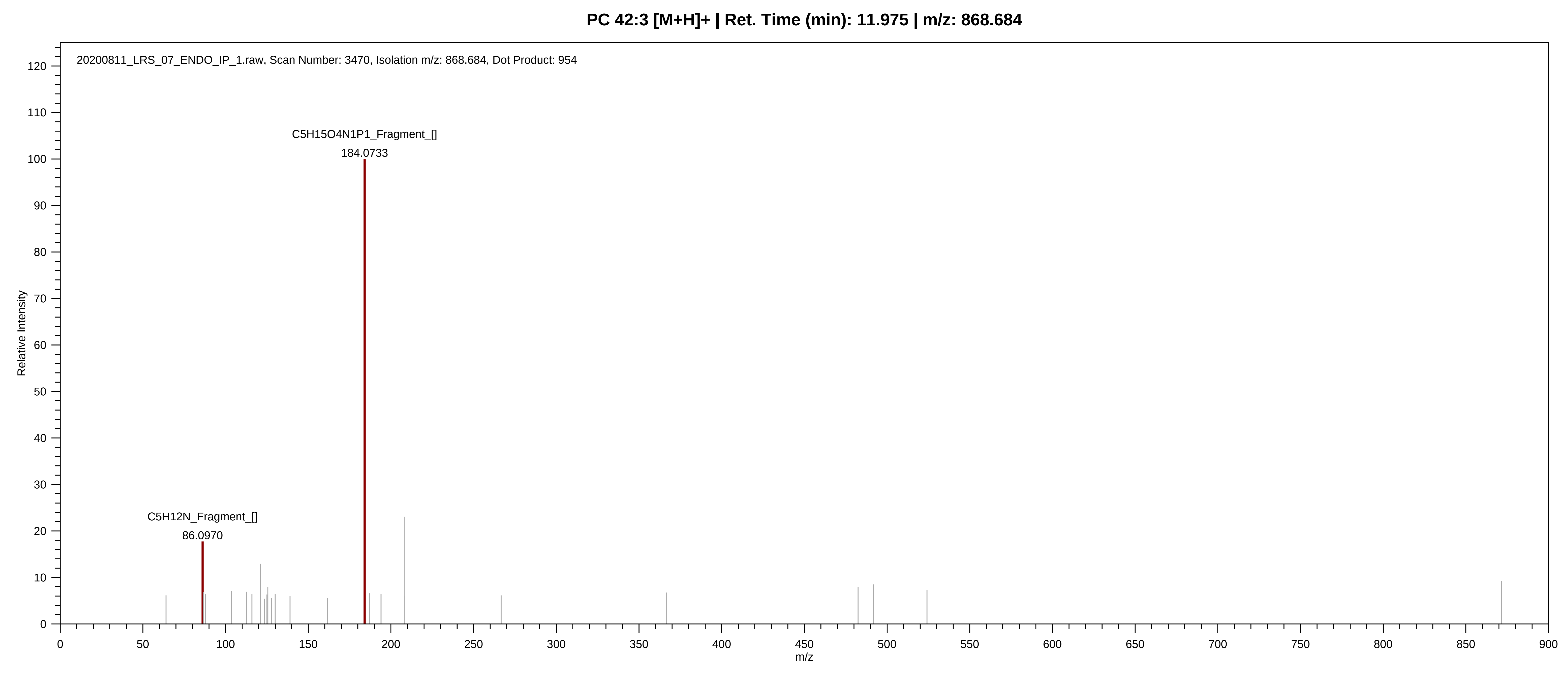
PC 42:3 [M+H]+ | Ret. Time (min): 11.975 | m/z: 868.684
20200811_LRS_07_ENDO_IP_1.raw, Scan Number: 3470, Isolation m/z: 868.684, Dot Product: 954
C5H15O4N1P1_Fragment_[]
184.0733
C5H12N_Fragment_[]
86.0970
Relative Intensity
m/z

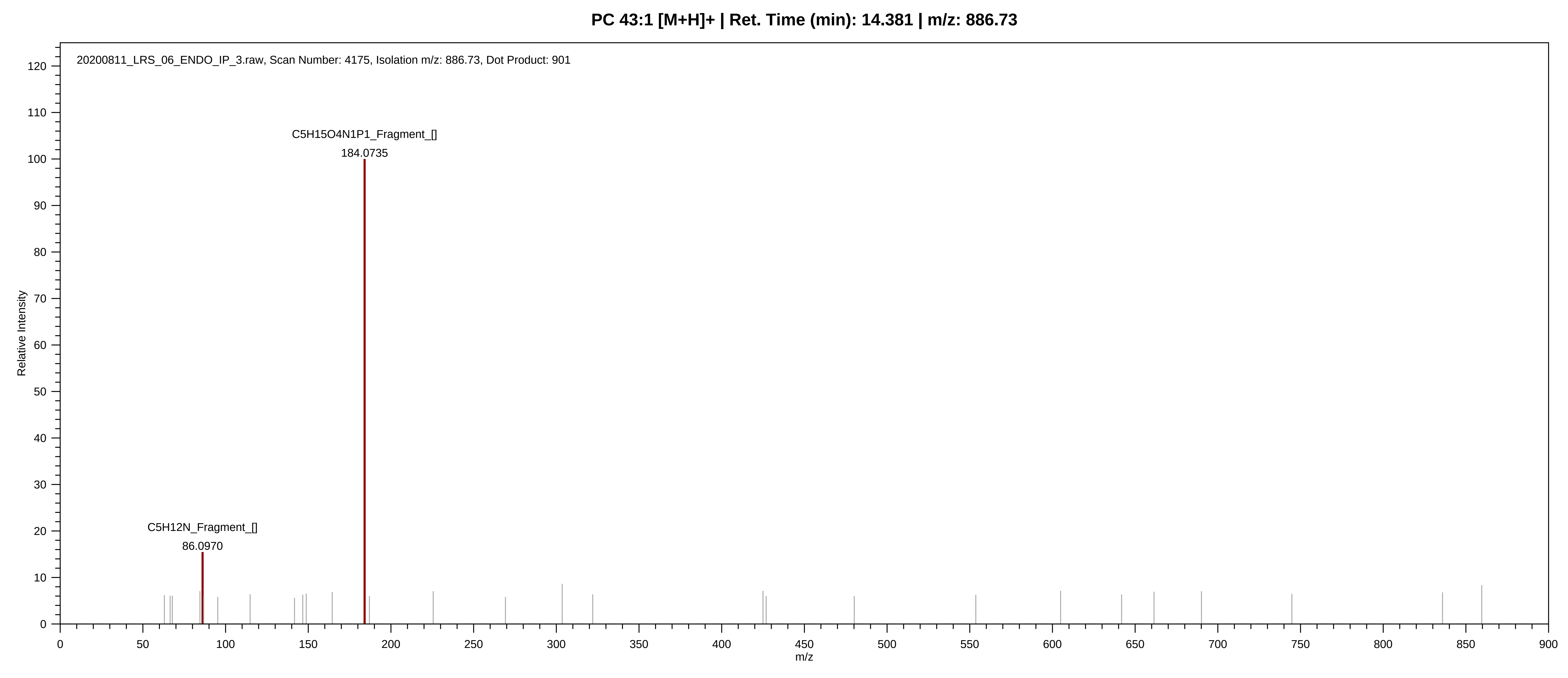

PC 43:1 [M+H]+ | Ret. Time (min): 14.381 | m/z: 886.73
20200811_LRS_06_ENDO_IP_3.raw, Scan Number: 4175, Isolation m/z: 886.73, Dot Product: 901
C5H15O4N1P1_Fragment_[]
184.0735
C5H12N_Fragment_[]
86.0970
Relative Intensity
m/z

PC 43:1 [M+H]+ | Ret. Time (min): 14.417 | m/z: 886.725

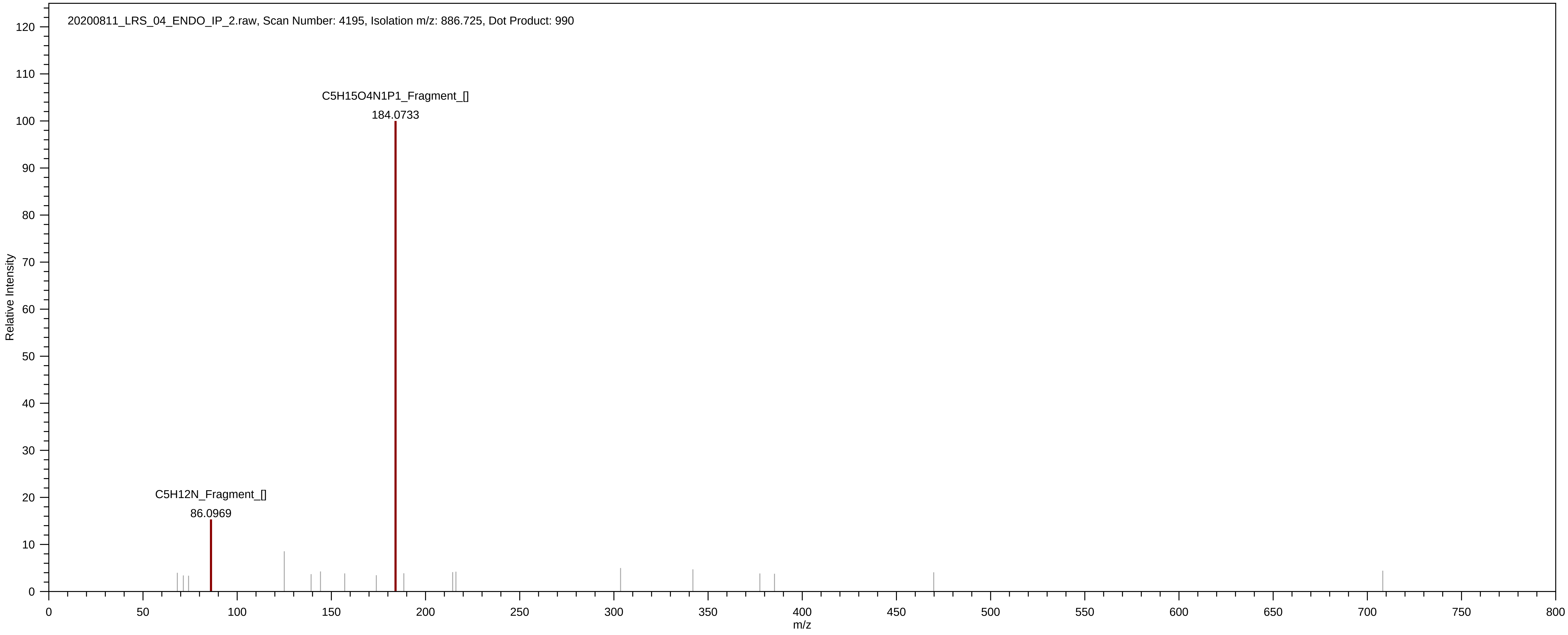

PC 43:1 [M+H]+ | Ret. Time (min): 14.381 | m/z: 886.726

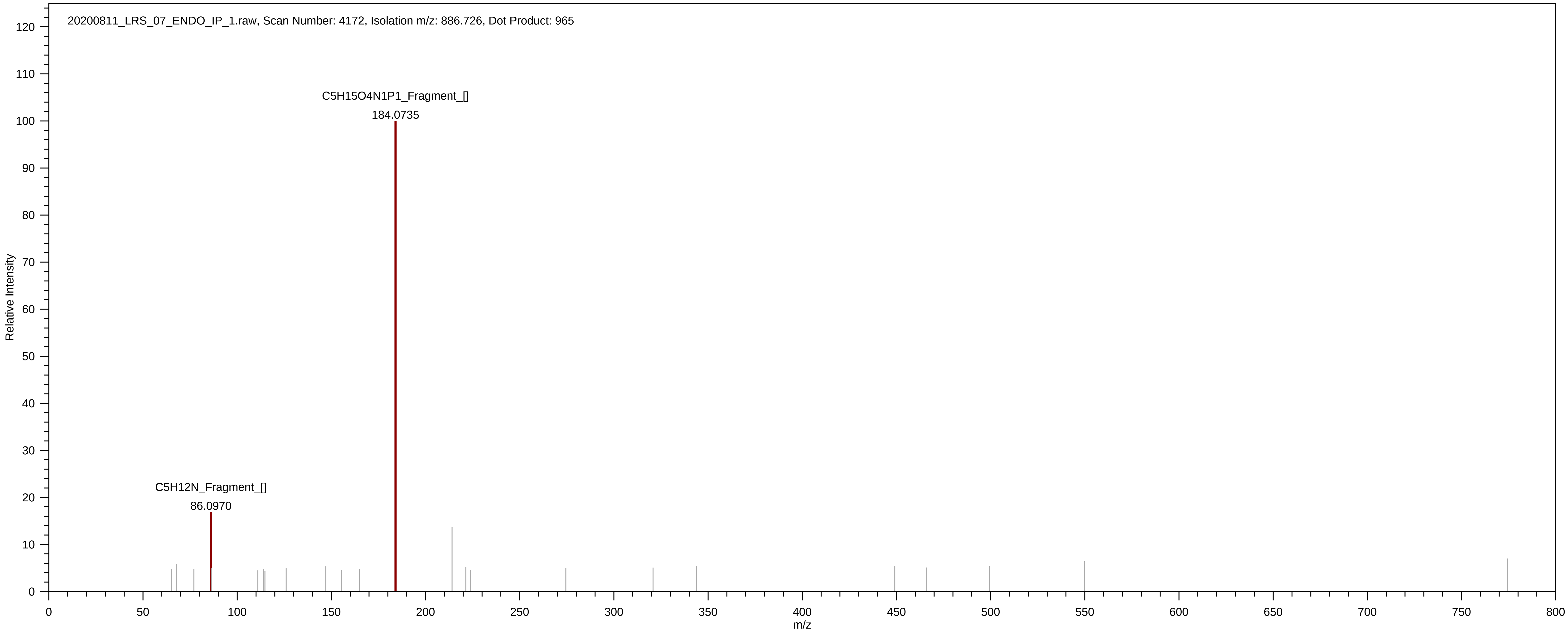

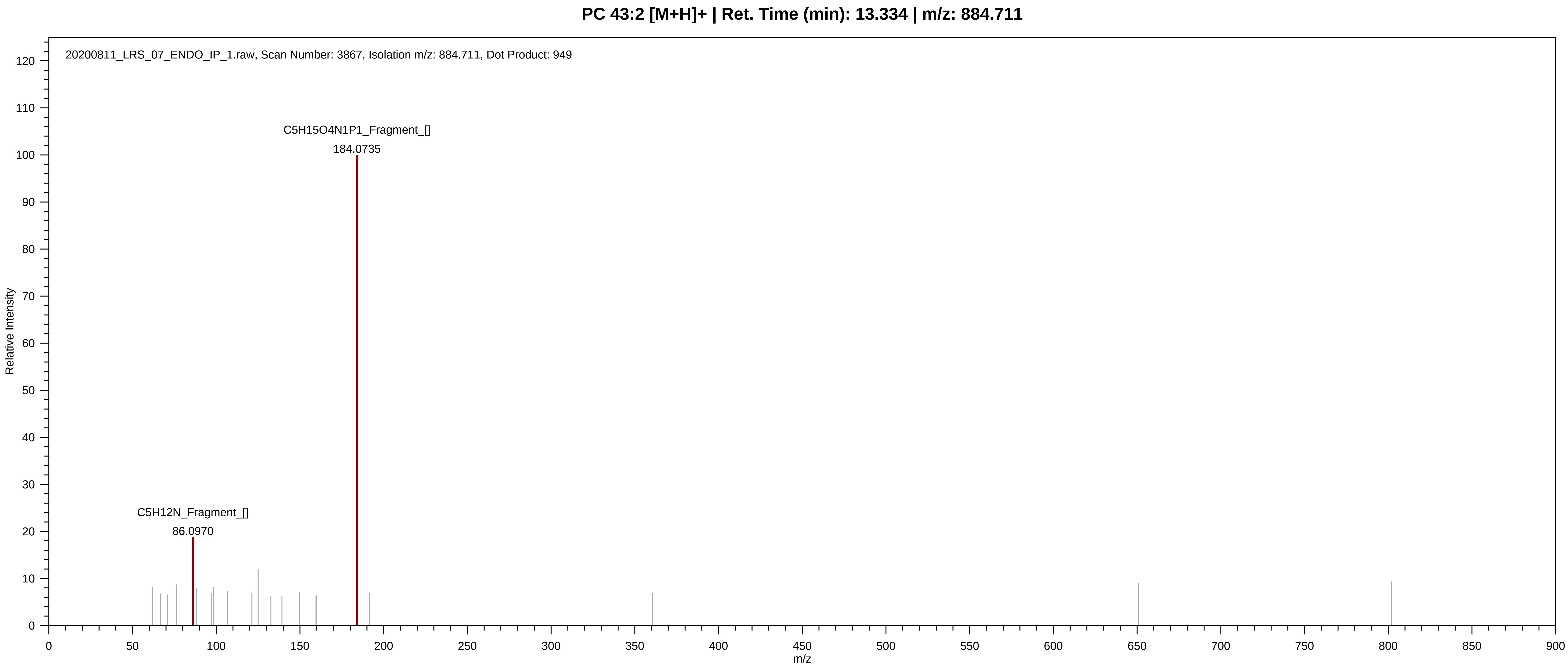

PC 43:2 [M+H]+ | Ret. Time (min): 13.334 | m/z: 884.711
20200811_LRS_07_ENDO_IP_1.raw, Scan Number: 3867, Isolation m/z: 884.711, Dot Product: 949
C5H15O4N1P1_Fragment_[]
184.0735
C5H12N_Fragment_[]
86.0970
Relative Intensity
m/z

PC 43:2 [M+H]+ | Ret. Time (min): 13.331 | m/z: 884.712

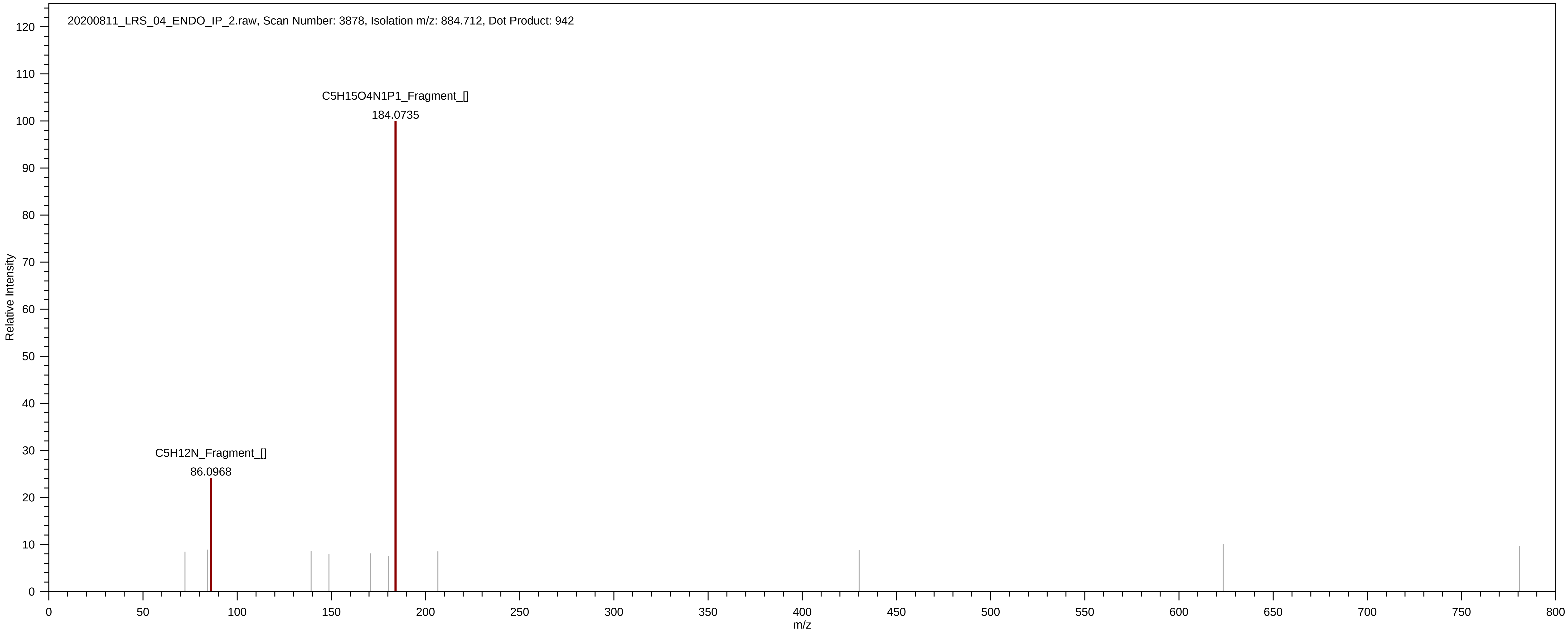

PC 44:0 [M+H]+ | Ret. Time (min): 15.761 | m/z: 902.76

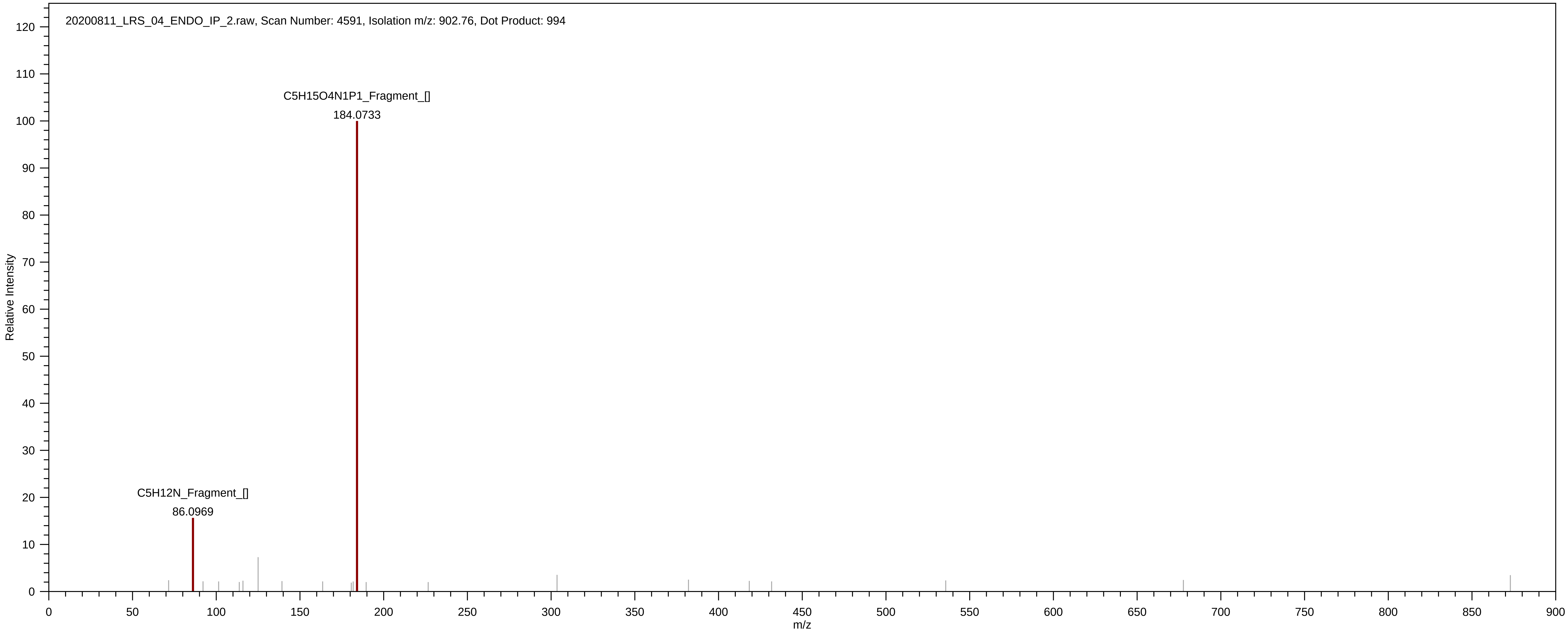

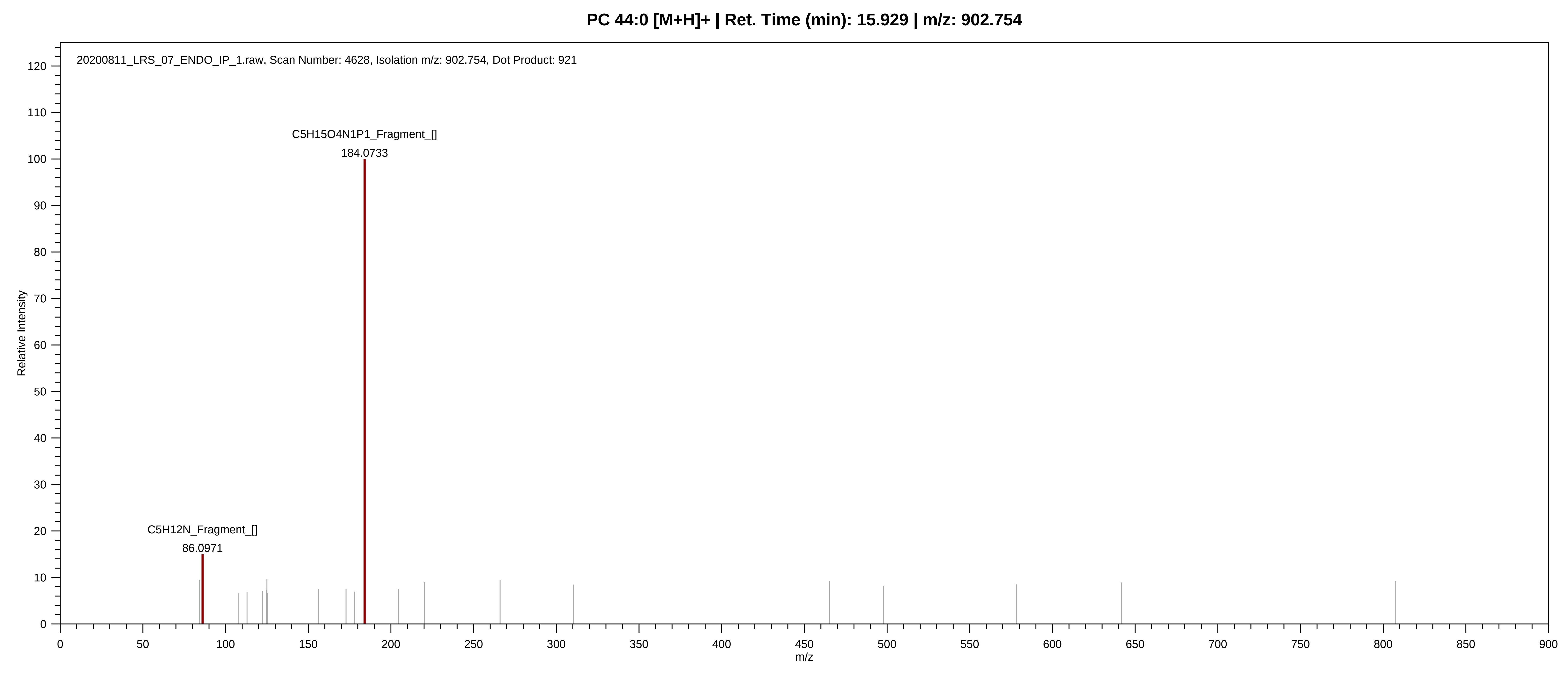
PC 44:0 [M+H]+ | Ret. Time (min): 15.929 | m/z: 902.754
20200811_LRS_07_ENDO_IP_1.raw, Scan Number: 4628, Isolation m/z: 902.754, Dot Product: 921
C5H15O4N1P1_Fragment_[]
184.0733
C5H12N_Fragment_[]
86.0971
Relative Intensity
m/z

PC 44:0 [M+H]+ | Ret. Time (min): 15.798 | m/z: 902.756

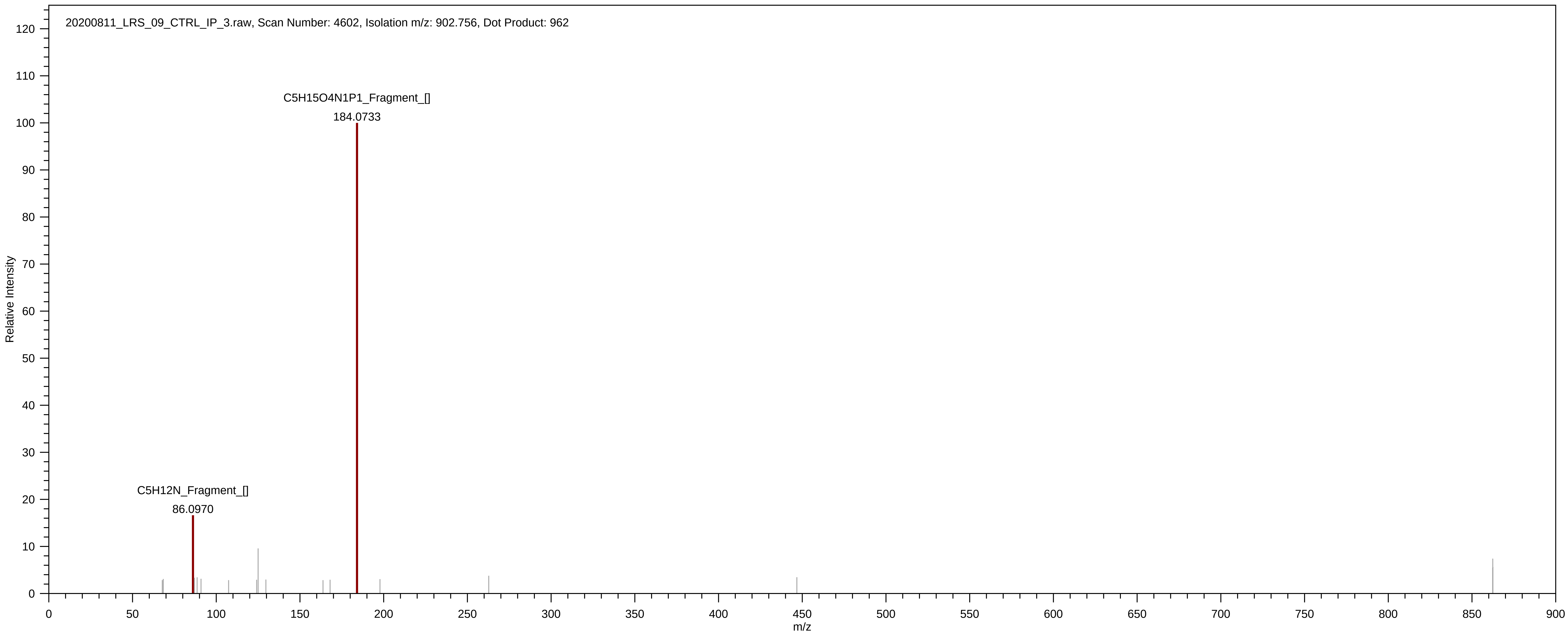

PC 44:0 [M+H]+ | Ret. Time (min): 15.942 | m/z: 902.757

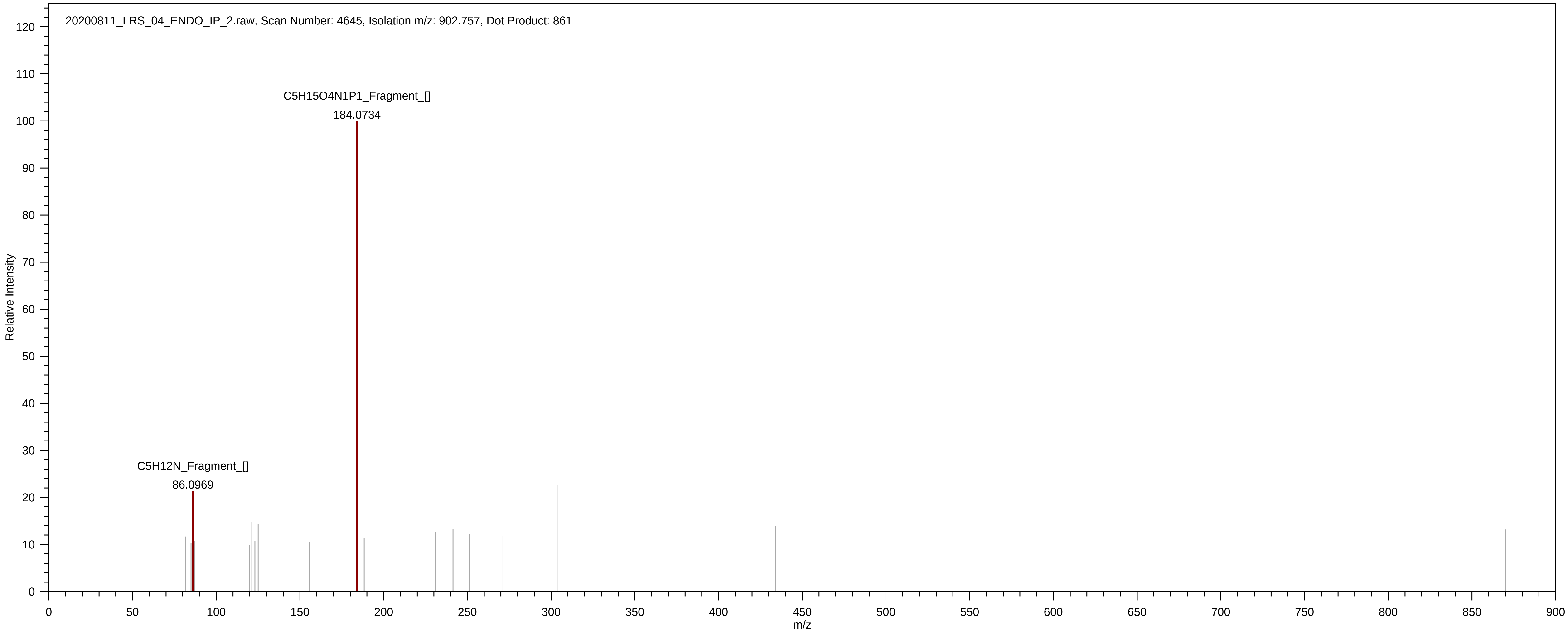

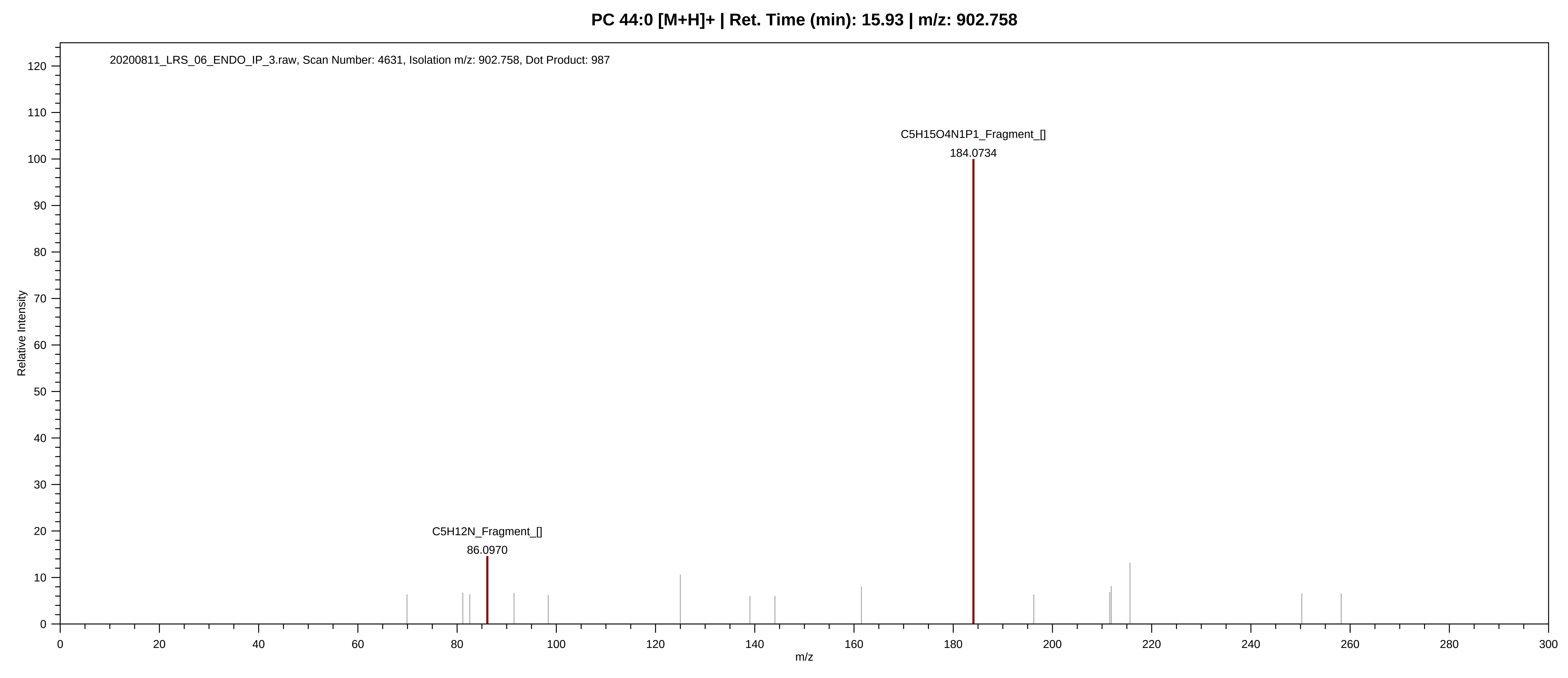

PC 44:0 [M+H]+ | Ret. Time (min): 15.93 | m/z: 902.758
20200811_LRS_06_ENDO_IP_3.raw, Scan Number: 4631, Isolation m/z: 902.758, Dot Product: 987
C5H15O4N1P1_Fragment_[]
184.0734
C5H12N_Fragment_[]
86.0970
Relative Intensity
m/z

PC 44:0 [M+H]+ | Ret. Time (min): 15.815 | m/z: 902.758

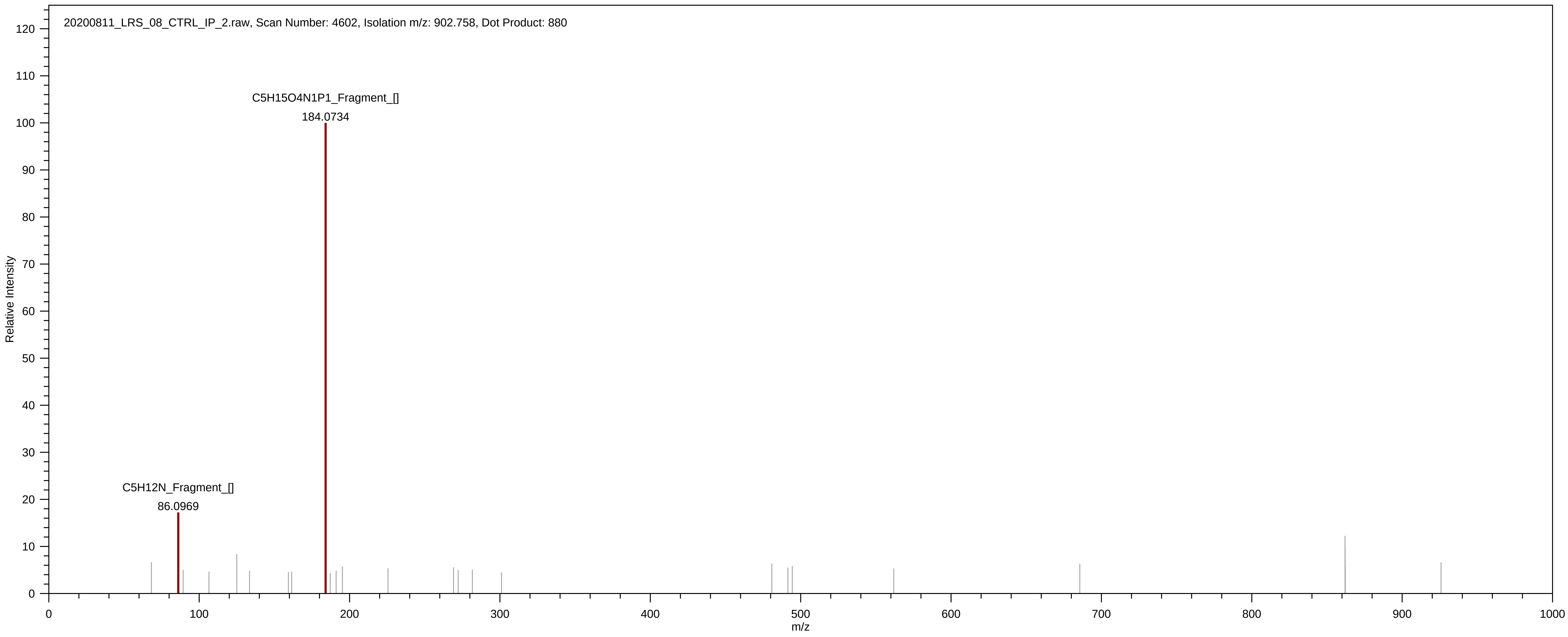

PC 44:0 [M+H]+ | Ret. Time (min): 15.747 | m/z: 902.759

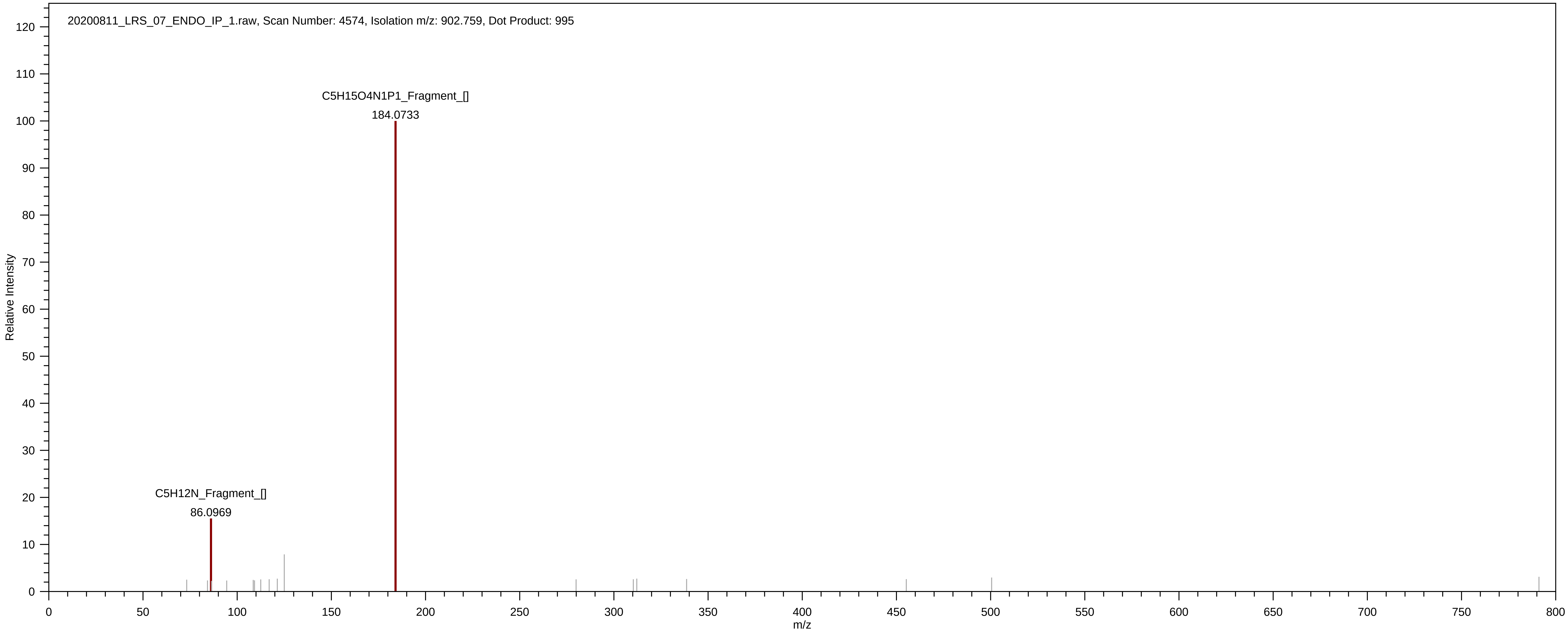

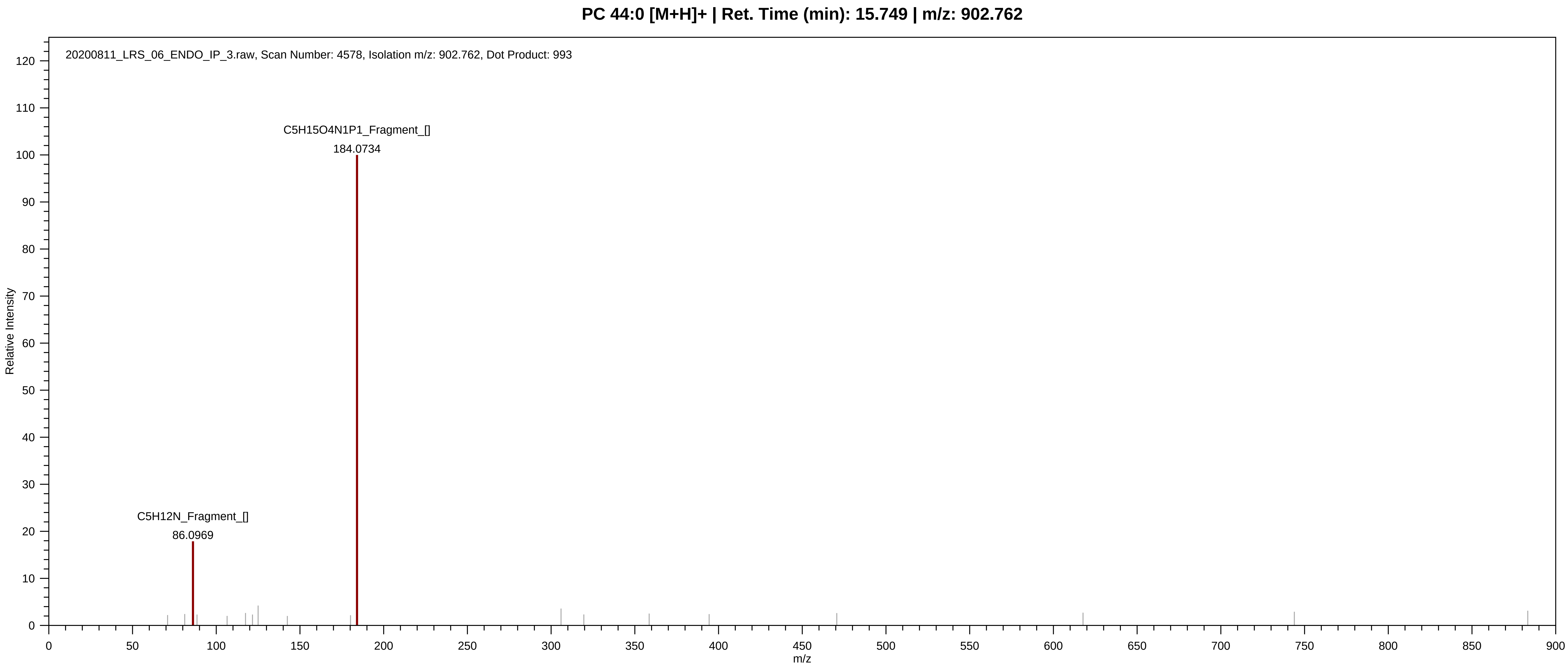
PC 44:0 [M+H]+ | Ret. Time (min): 15.749 | m/z: 902.762
20200811_LRS_06_ENDO_IP_3.raw, Scan Number: 4578, Isolation m/z: 902.762, Dot Product: 993
C5H15O4N1P1_Fragment_[]
184.0734
C5H12N_Fragment_[]
86.0969
Relative Intensity
m/z
0
10
20
30
40
50
60
70
80
90
100
110
120
0
50
100
150
200
250
300
350
400
450
500
550
600
650
700
750
800
850
900

PC 44:1 [M+H]+ | Ret. Time (min): 14.752 | m/z: 900.741

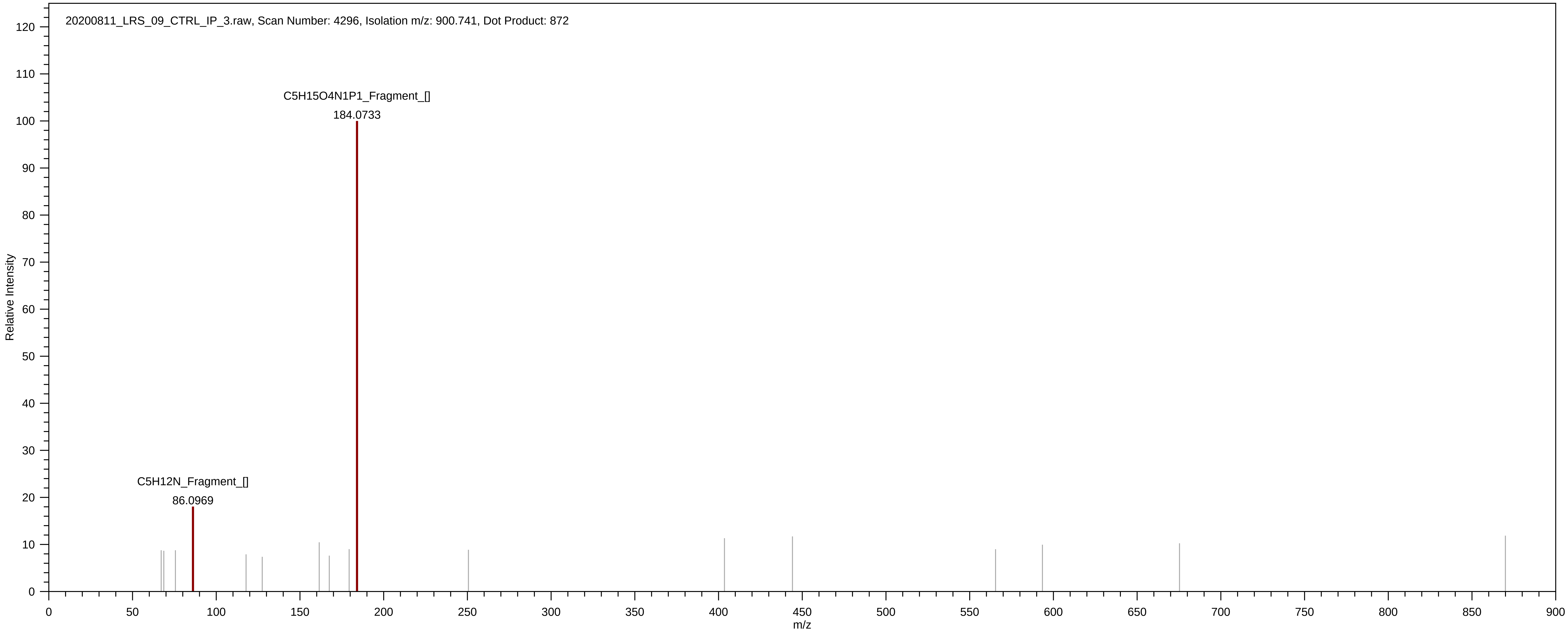

PC 44:1 [M+H]+ | Ret. Time (min): 14.873 | m/z: 900.741

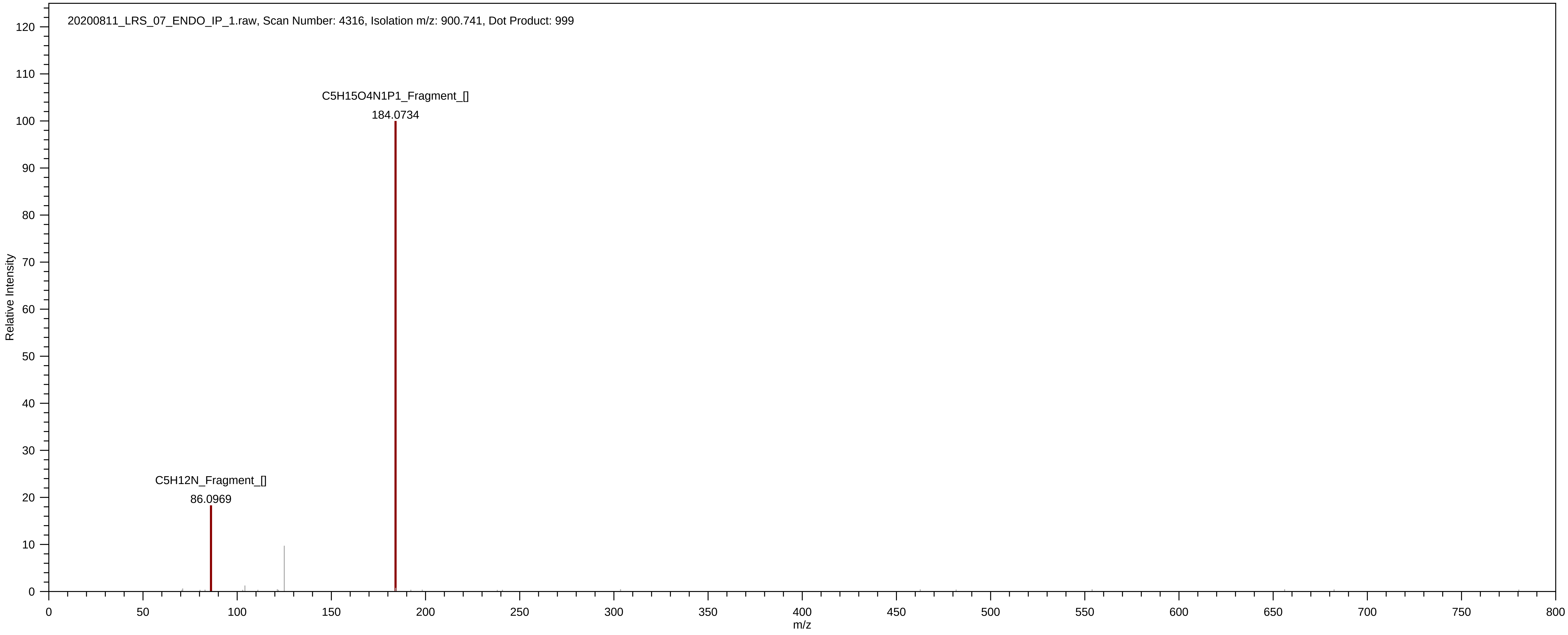

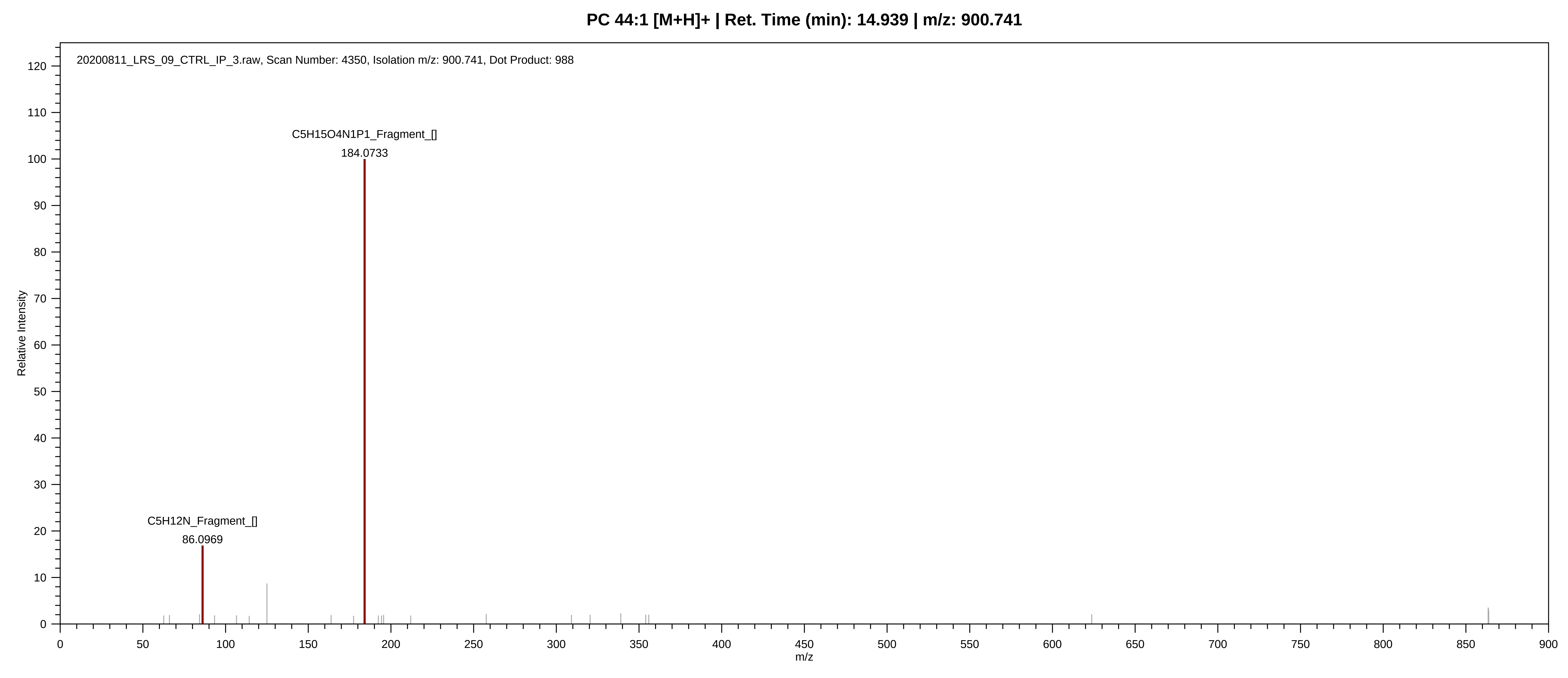
PC 44:1 [M+H]+ | Ret. Time (min): 14.939 | m/z: 900.741
20200811_LRS_09_CTRL_IP_3.raw, Scan Number: 4350, Isolation m/z: 900.741, Dot Product: 988
C5H15O4N1P1_Fragment_[]
184.0733
C5H12N_Fragment_[]
86.0969
Relative Intensity
m/z

PC 44:1 [M+H]+ | Ret. Time (min): 14.853 | m/z: 900.742

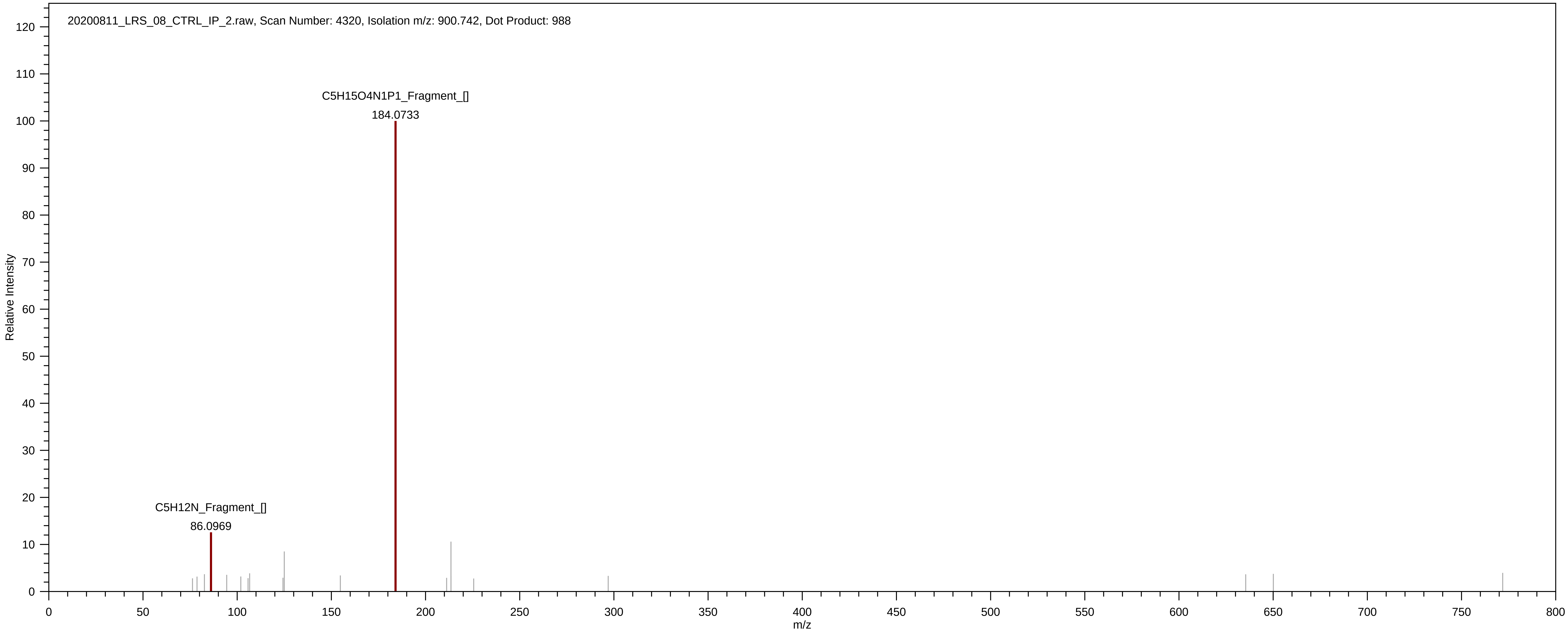

PC 44:1 [M+H]+ | Ret. Time (min): 14.874 | m/z: 900.742

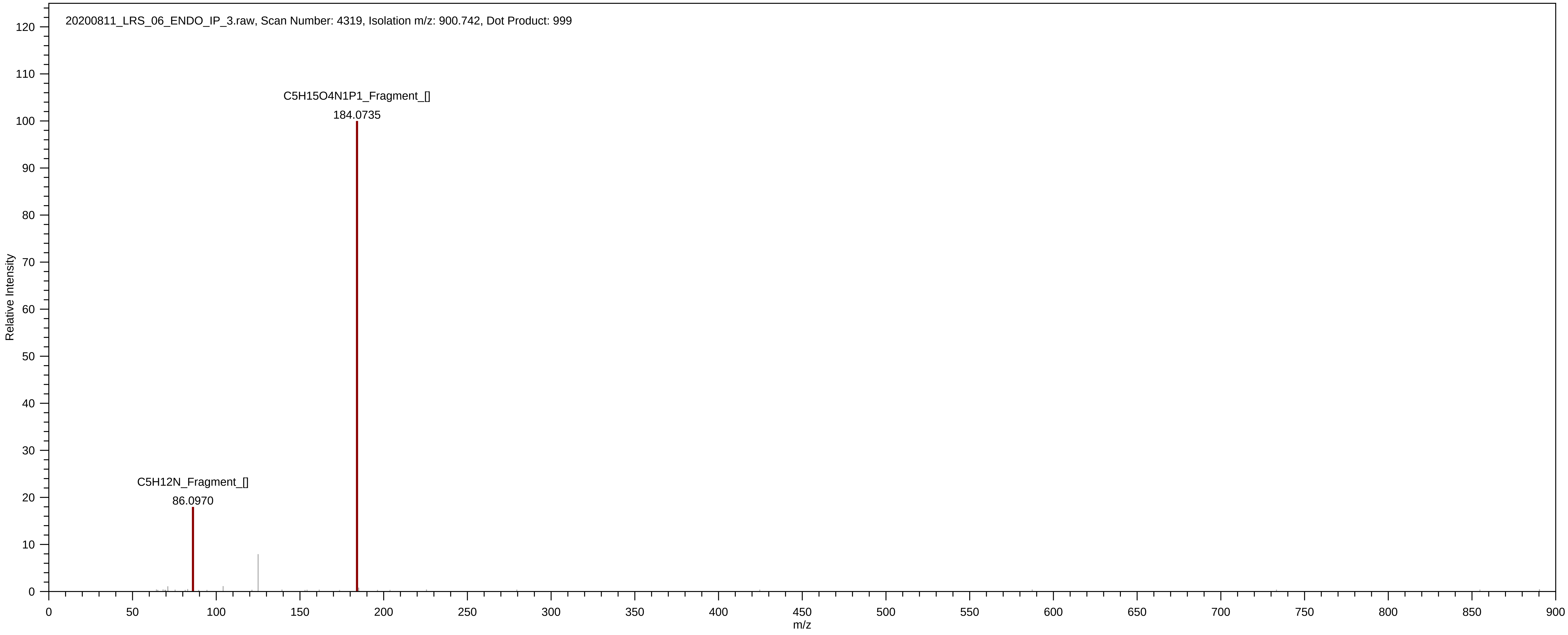

PC 44:1 [M+H]+ | Ret. Time (min): 14.888 | m/z: 900.742

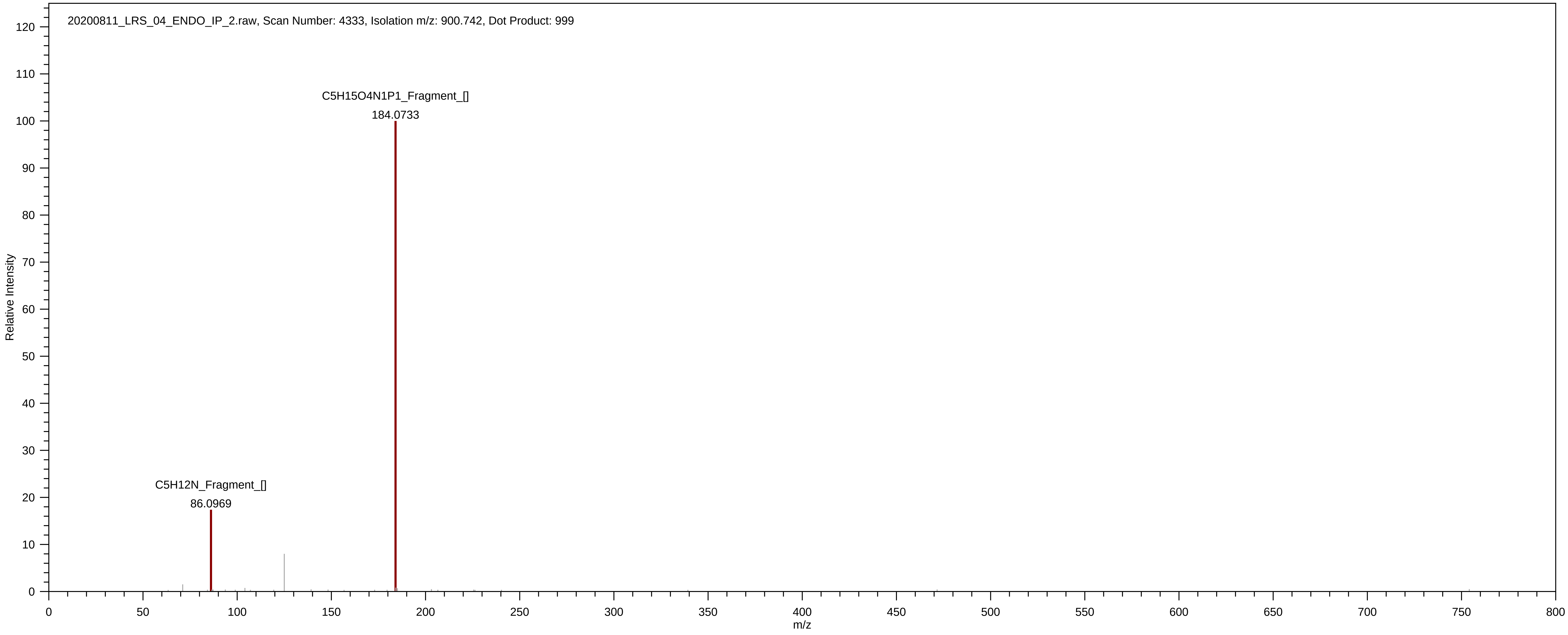

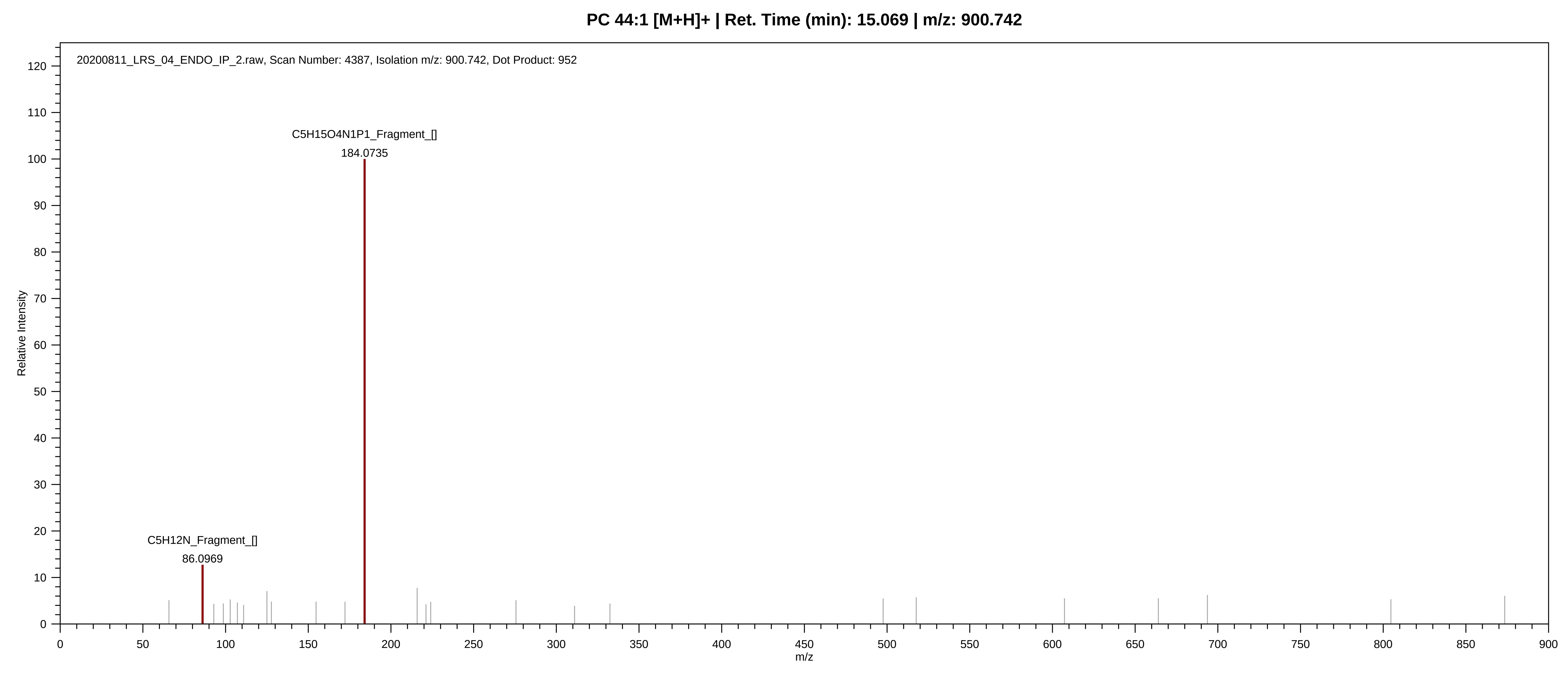

PC 44:1 [M+H]+ | Ret. Time (min): 15.069 | m/z: 900.742
20200811_LRS_04_ENDO_IP_2.raw, Scan Number: 4387, Isolation m/z: 900.742, Dot Product: 952
C5H15O4N1P1_Fragment_[]
184.0735
C5H12N_Fragment_[]
86.0969
Relative Intensity
m/z

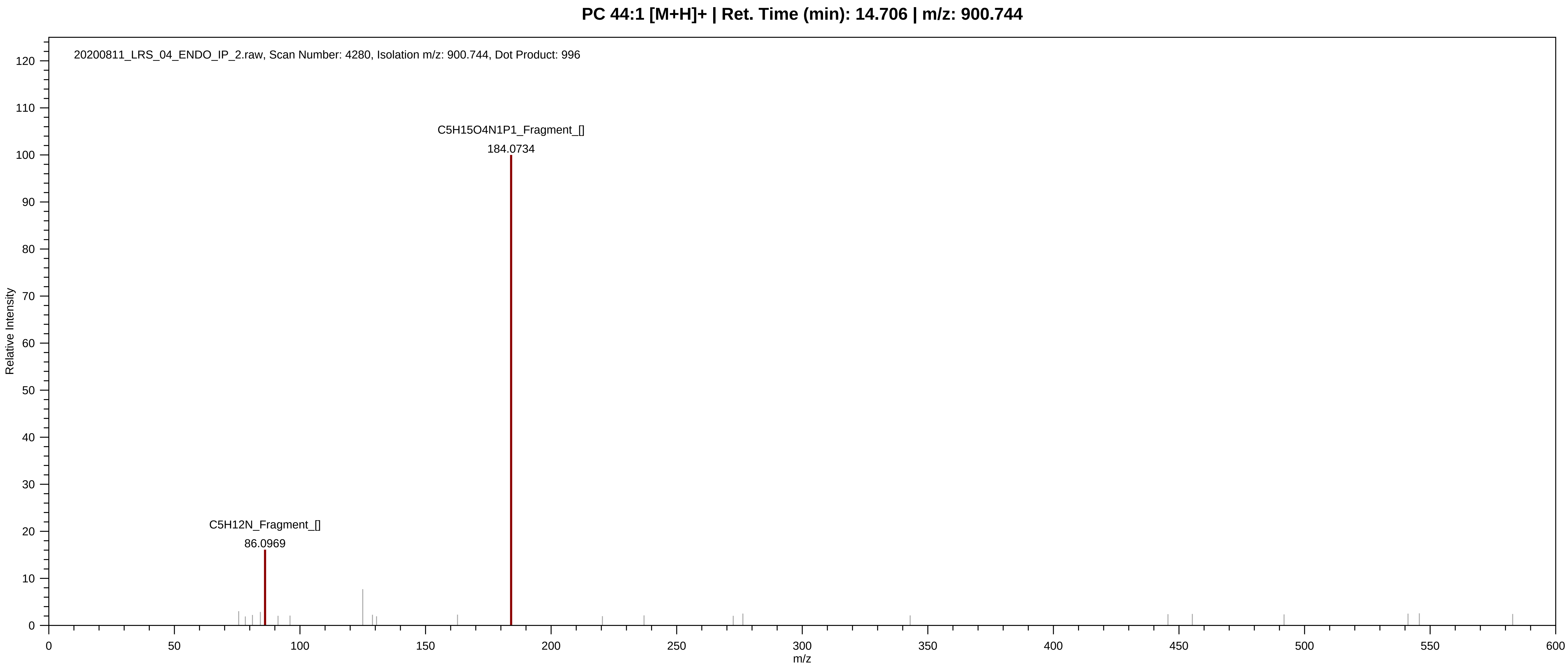

PC 44:1 [M+H]+ | Ret. Time (min): 14.706 | m/z: 900.744
20200811_LRS_04_ENDO_IP_2.raw, Scan Number: 4280, Isolation m/z: 900.744, Dot Product: 996
C5H15O4N1P1_Fragment_[]
184.0734
C5H12N_Fragment_[]
86.0969
Relative Intensity
m/z

PC 44:1 [M+H]+ | Ret. Time (min): 14.885 | m/z: 900.744

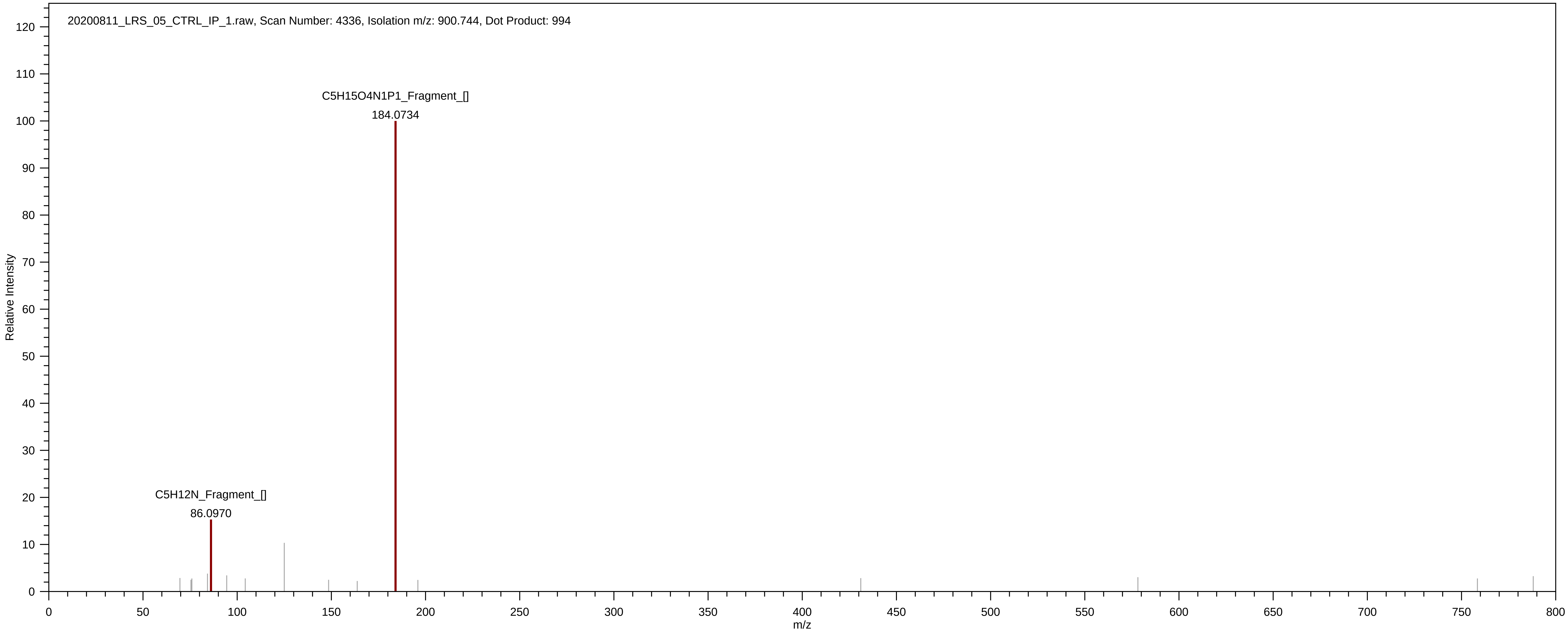

PC 44:1 [M+H]+ | Ret. Time (min): 15.056 | m/z: 900.744

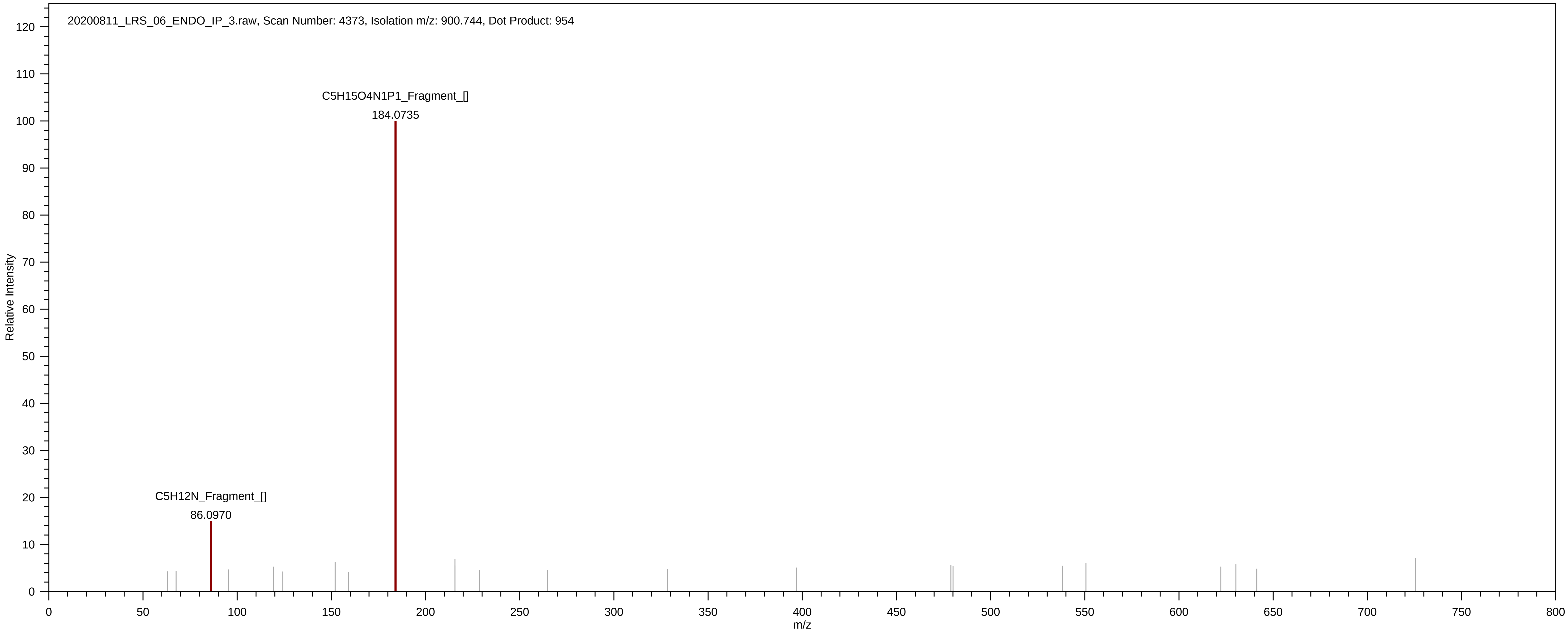

PC 44:1 [M+H]+ | Ret. Time (min): 14.689 | m/z: 900.745

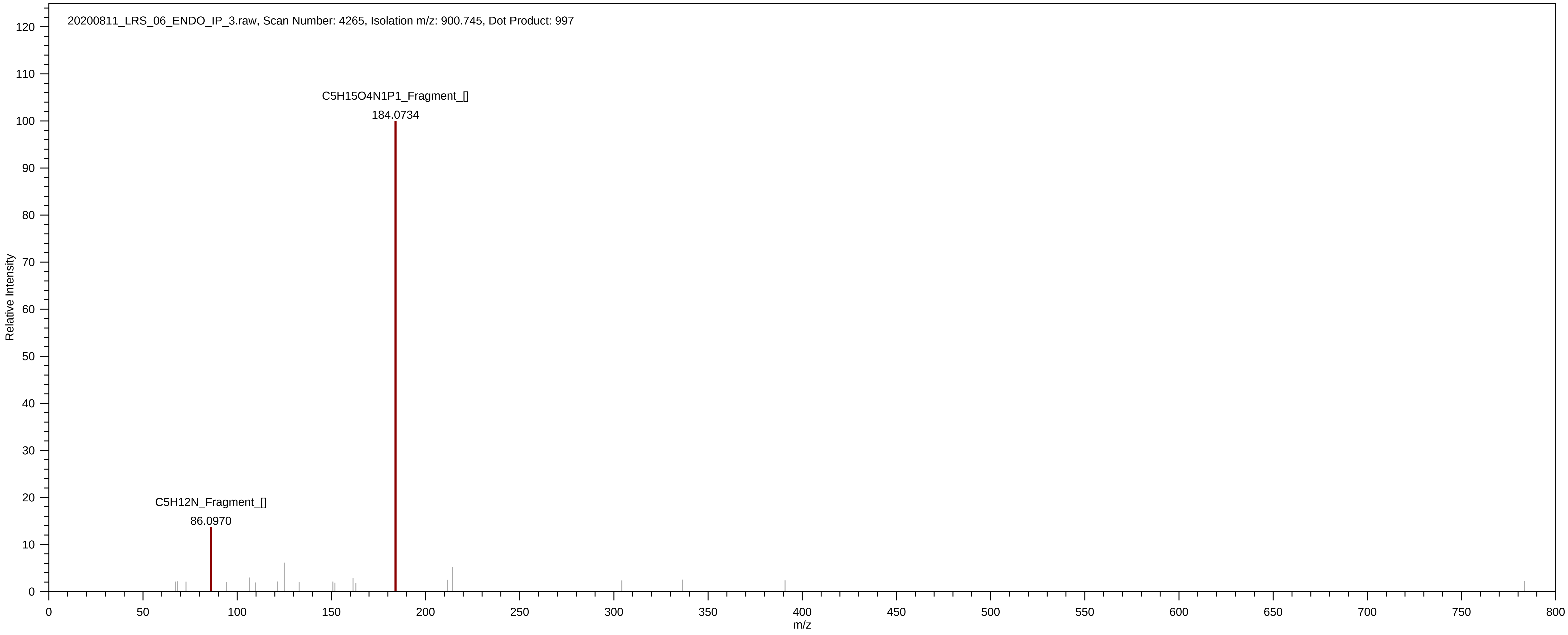

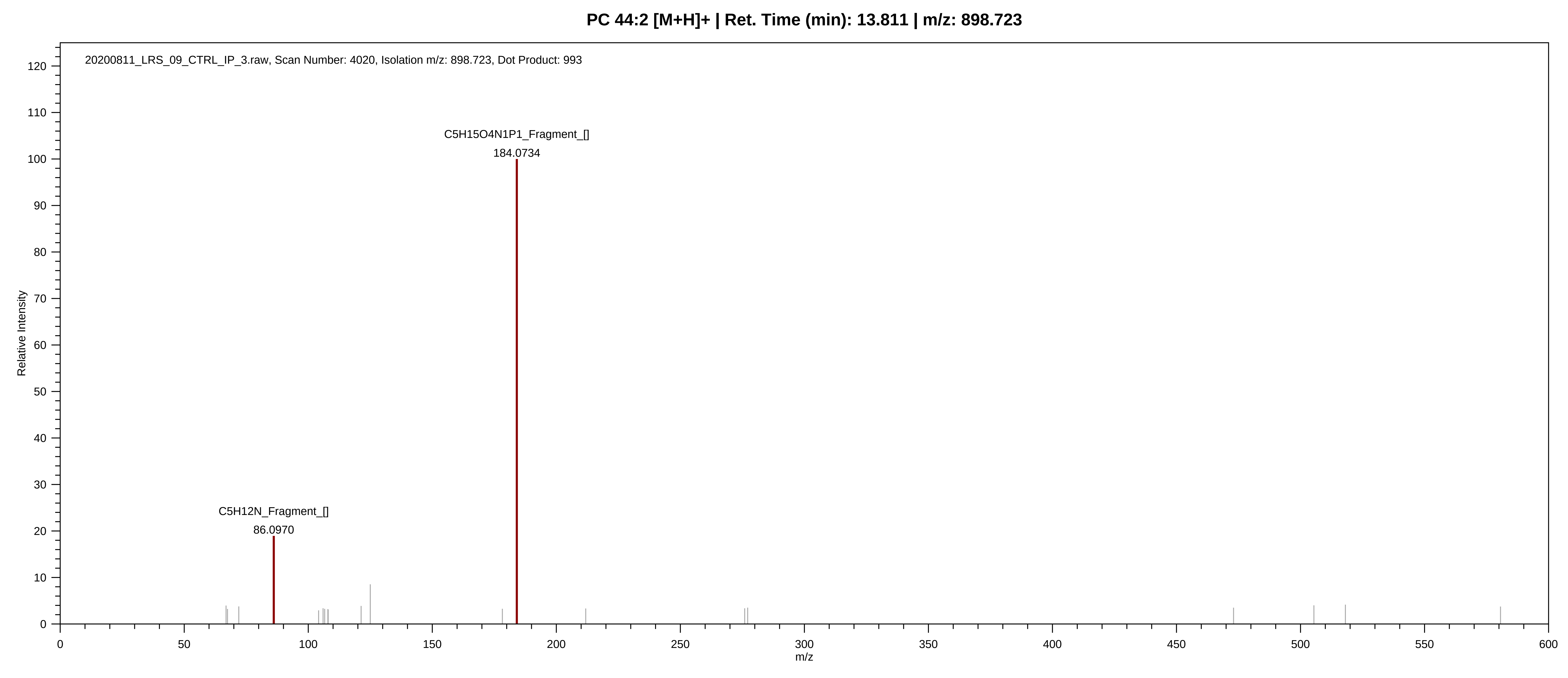
PC 44:2 [M+H]+ | Ret. Time (min): 13.811 | m/z: 898.723
20200811_LRS_09_CTRL_IP_3.raw, Scan Number: 4020, Isolation m/z: 898.723, Dot Product: 993
C5H15O4N1P1_Fragment_[]
184.0734
C5H12N_Fragment_[]
86.0970
Relative Intensity
m/z

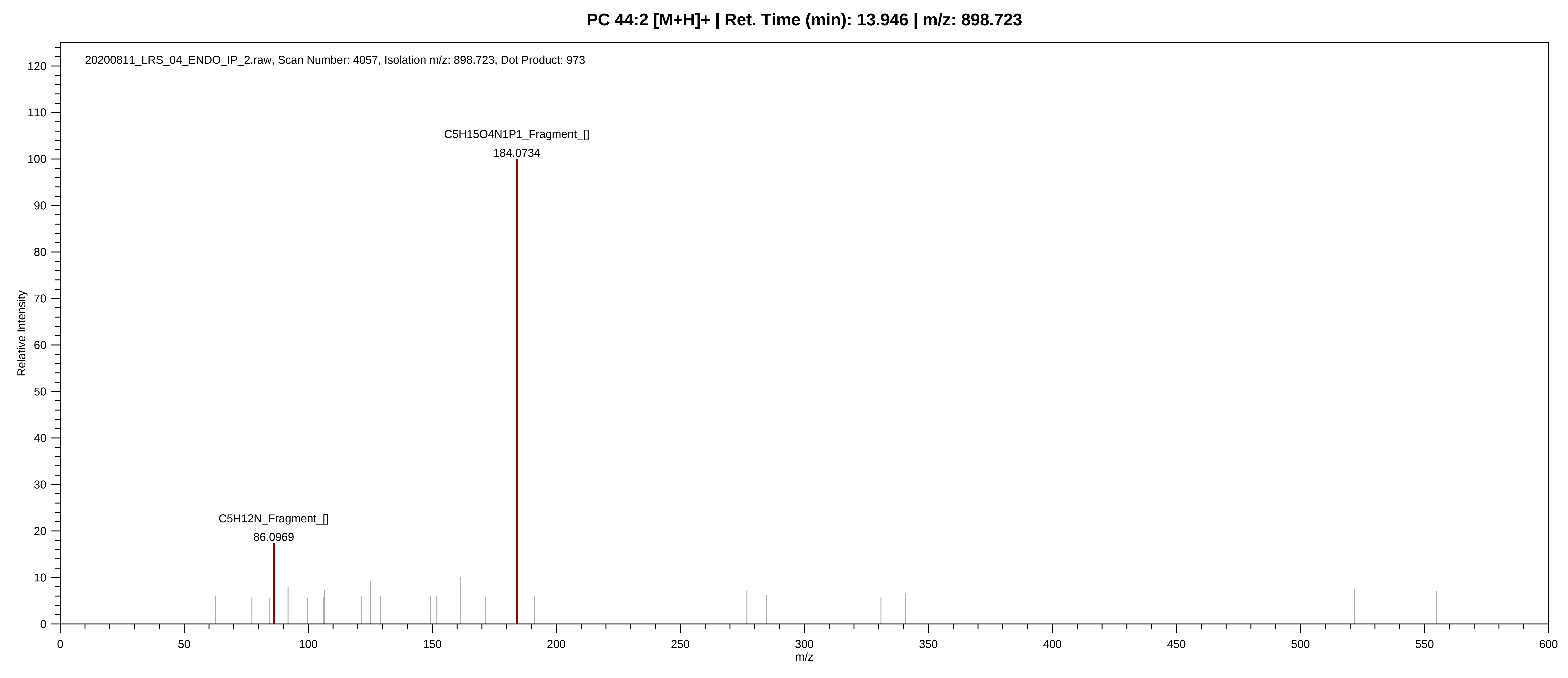
PC 44:2 [M+H]+ | Ret. Time (min): 13.946 | m/z: 898.723
20200811_LRS_04_ENDO_IP_2.raw, Scan Number: 4057, Isolation m/z: 898.723, Dot Product: 973
C5H15O4N1P1_Fragment_[]
184.0734
C5H12N_Fragment_[]
86.0969
Relative Intensity
m/z

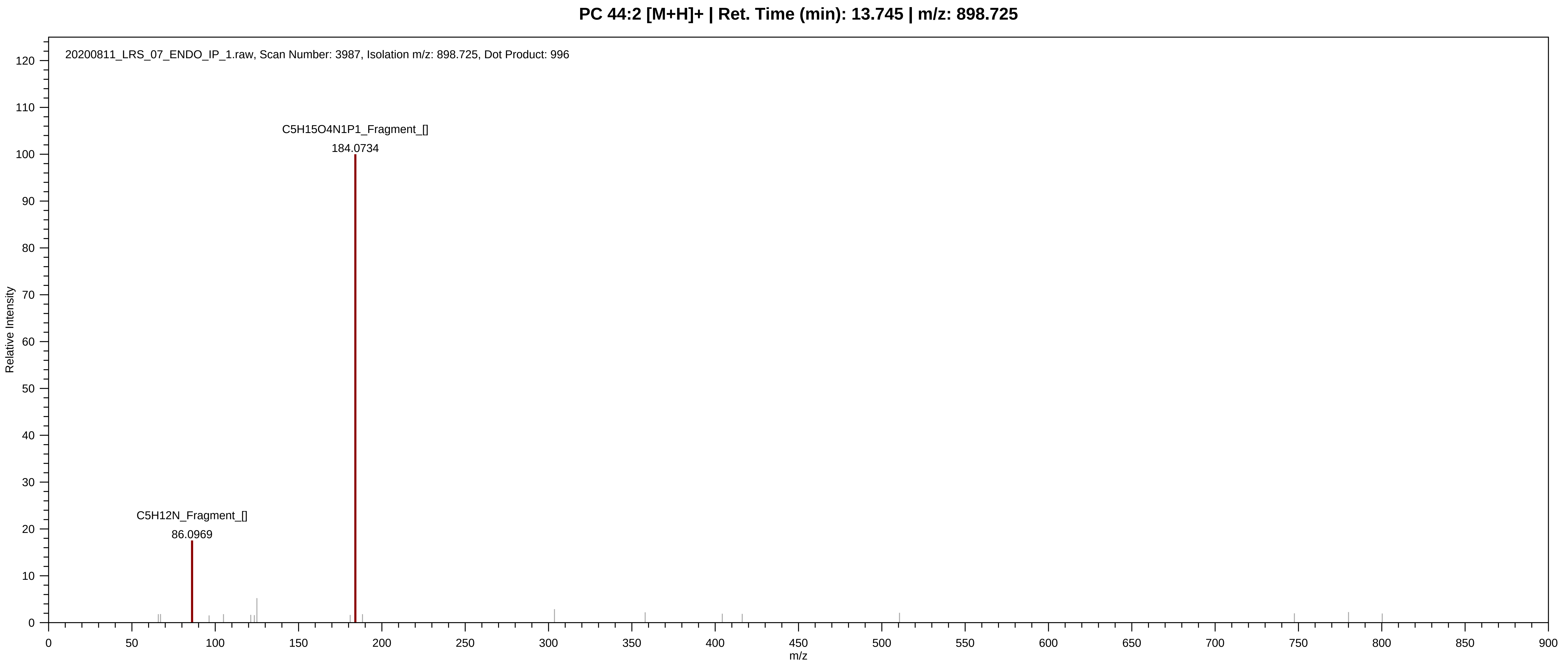
PC 44:2 [M+H]+ | Ret. Time (min): 13.745 | m/z: 898.725
20200811_LRS_07_ENDO_IP_1.raw, Scan Number: 3987, Isolation m/z: 898.725, Dot Product: 996
C5H15O4N1P1_Fragment_[]
184.0734
C5H12N_Fragment_[]
86.0969
Relative Intensity
m/z

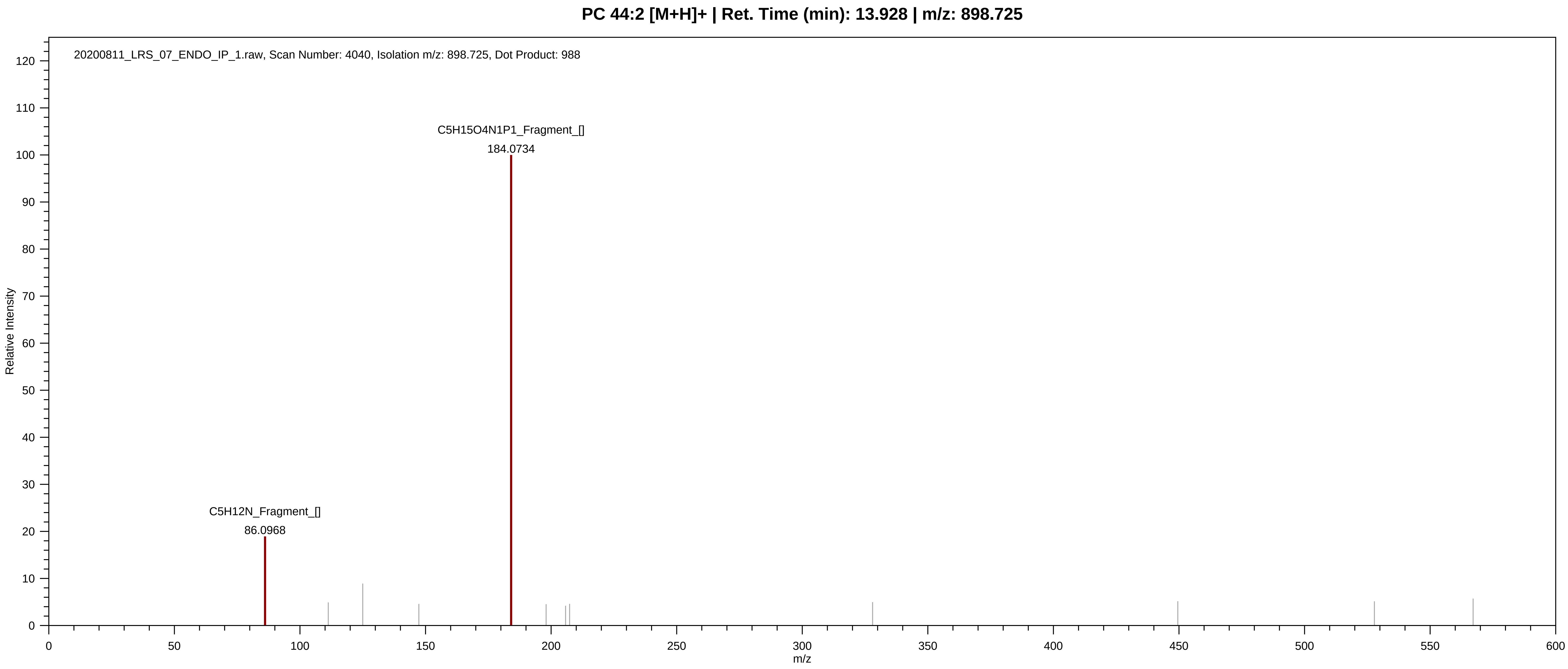
PC 44:2 [M+H]+ | Ret. Time (min): 13.928 | m/z: 898.725
20200811_LRS_07_ENDO_IP_1.raw, Scan Number: 4040, Isolation m/z: 898.725, Dot Product: 988
C5H15O4N1P1_Fragment_[]
184.0734
C5H12N_Fragment_[]
86.0968
Relative Intensity
m/z

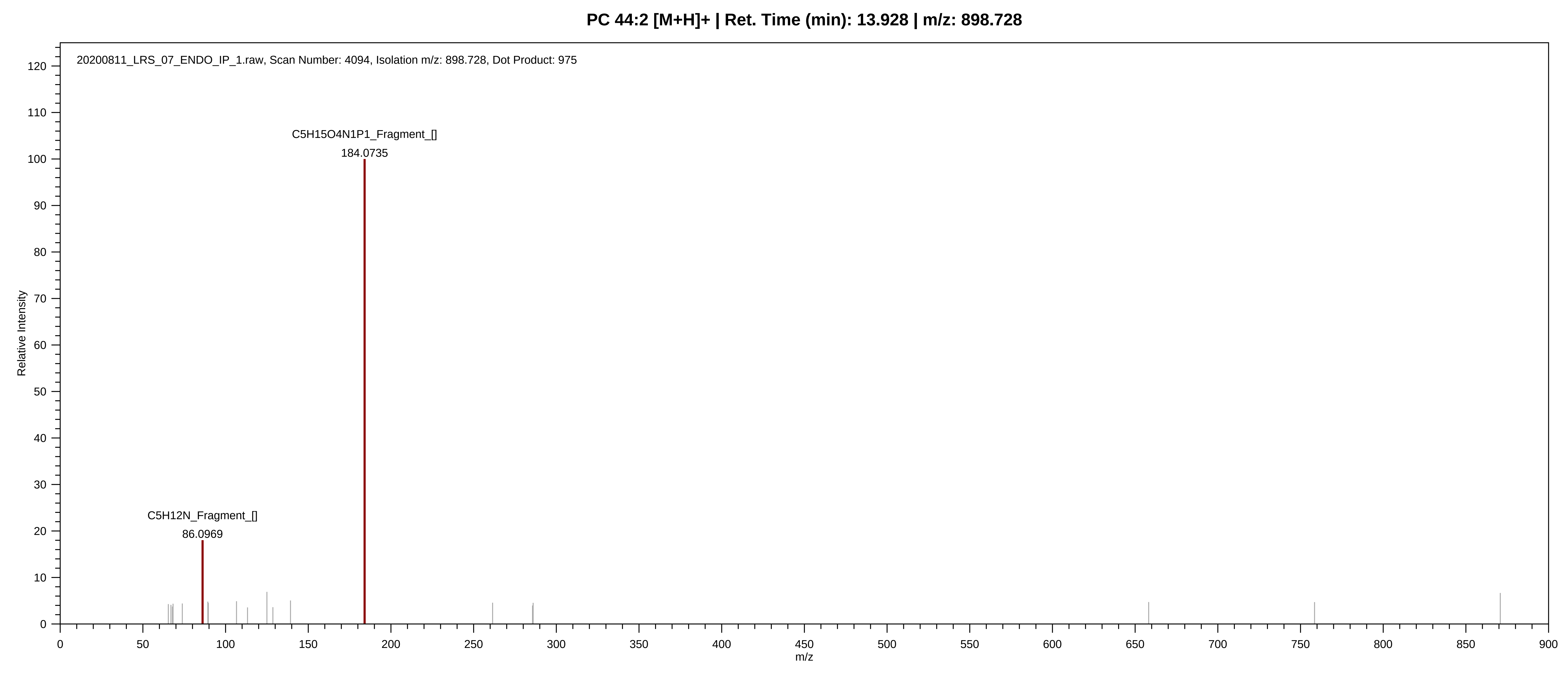
PC 44:2 [M+H]+ | Ret. Time (min): 13.928 | m/z: 898.728
20200811_LRS_07_ENDO_IP_1.raw, Scan Number: 4094, Isolation m/z: 898.728, Dot Product: 975
C5H15O4N1P1_Fragment_[]
184.0735
C5H12N_Fragment_[]
86.0969
Relative Intensity
m/z

PC 44:2 [M+H]+ | Ret. Time (min): 13.808 | m/z: 898.726

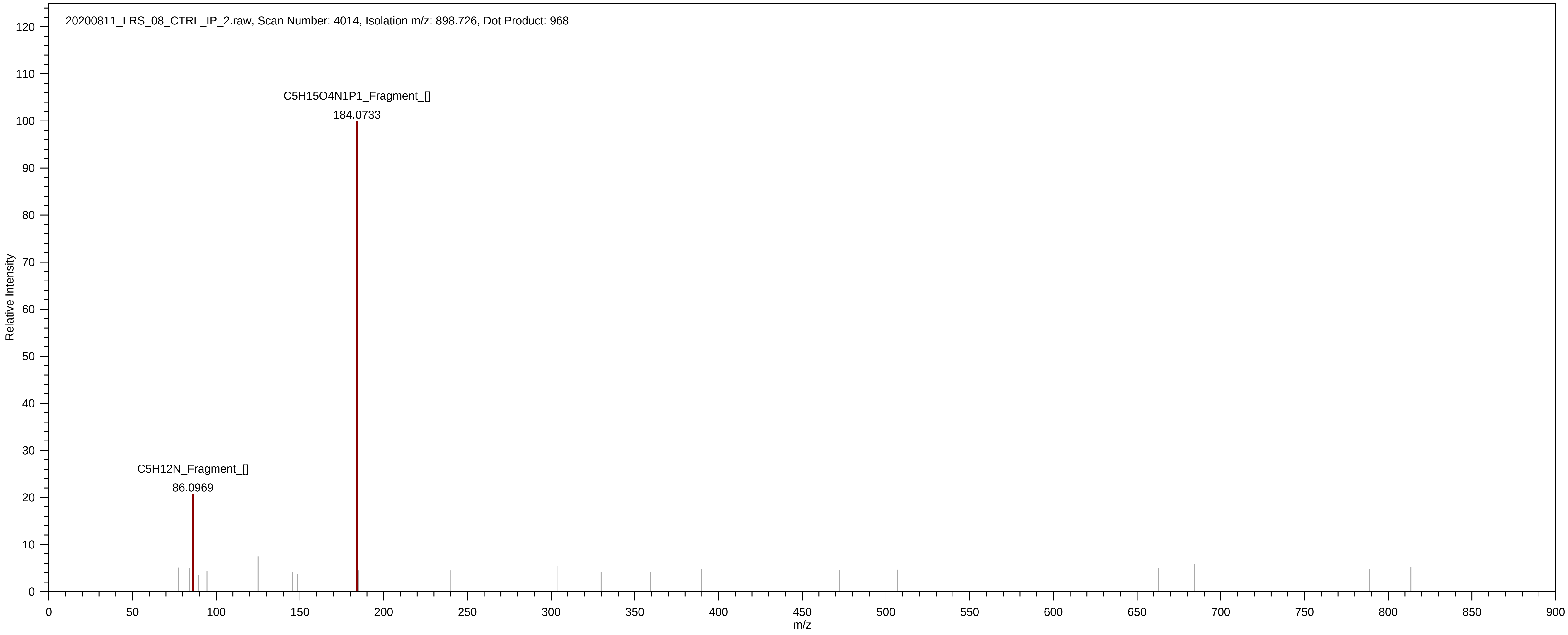

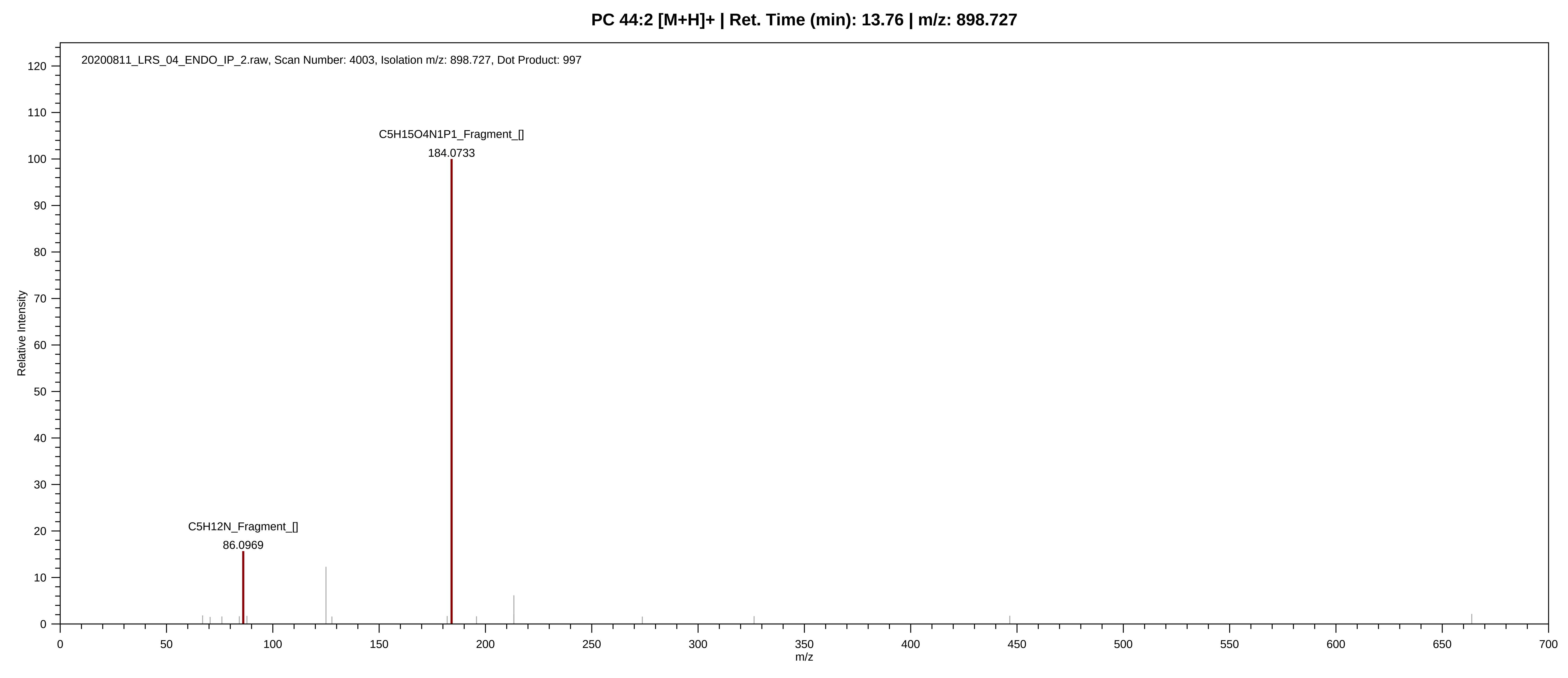
PC 44:2 [M+H]+ | Ret. Time (min): 13.76 | m/z: 898.727
20200811_LRS_04_ENDO_IP_2.raw, Scan Number: 4003, Isolation m/z: 898.727, Dot Product: 997
C5H15O4N1P1_Fragment_[]
184.0733
C5H12N_Fragment_[]
86.0969
Relative Intensity
m/z
0
10
20
30
40
50
60
70
80
90
100
110
120
0
50
100
150
200
250
300
350
400
450
500
550
600
650
700

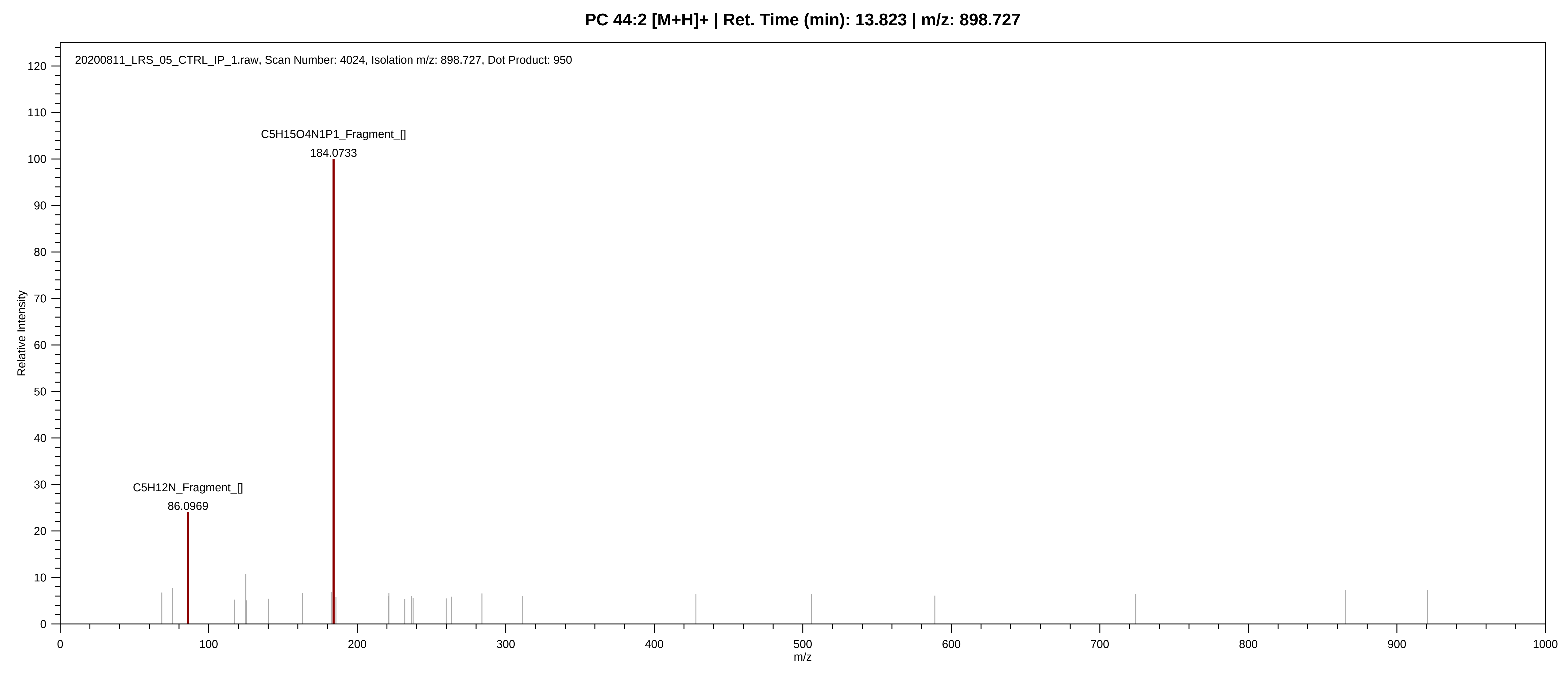

PC 44:2 [M+H]+ | Ret. Time (min): 13.823 | m/z: 898.727
20200811_LRS_05_CTRL_IP_1.raw, Scan Number: 4024, Isolation m/z: 898.727, Dot Product: 950
C5H15O4N1P1_Fragment_[]
184.0733
C5H12N_Fragment_[]
86.0969
Relative Intensity
m/z

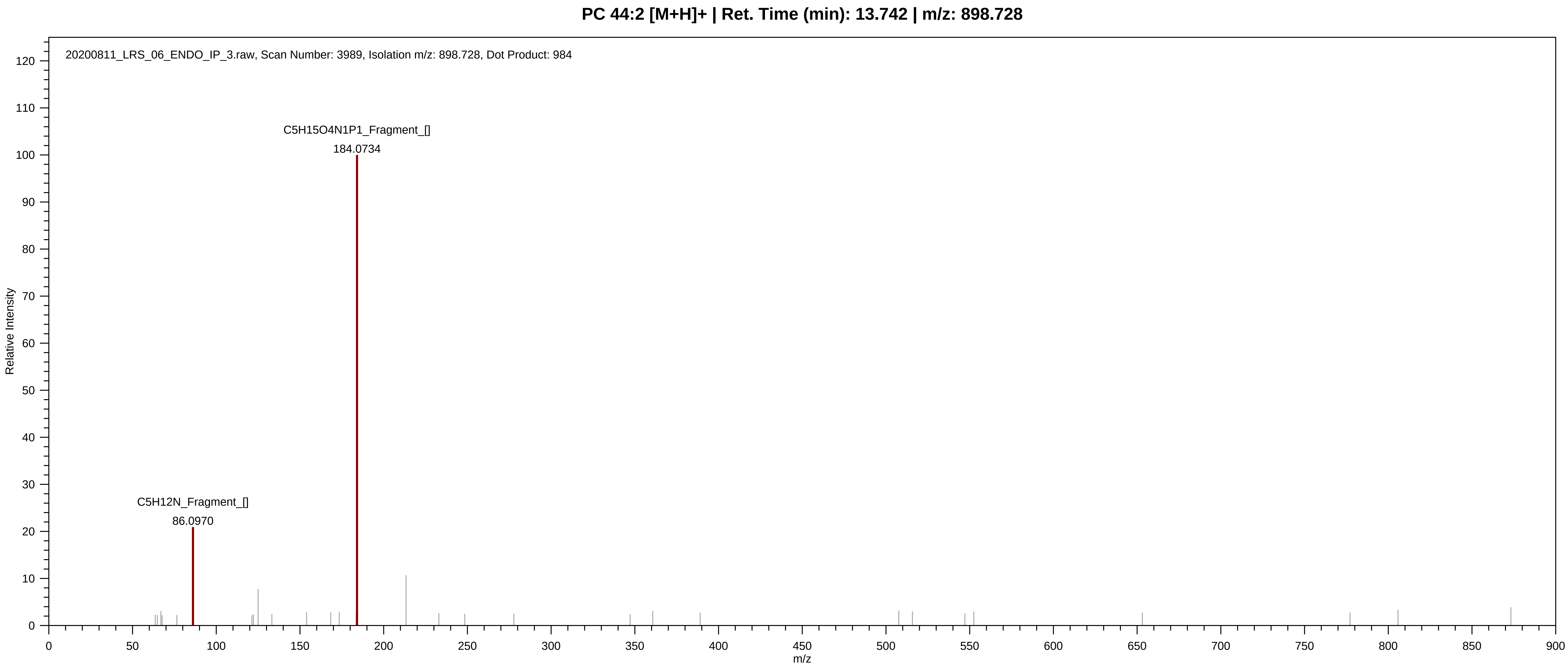
PC 44:2 [M+H]+ | Ret. Time (min): 13.742 | m/z: 898.728
20200811_LRS_06_ENDO_IP_3.raw, Scan Number: 3989, Isolation m/z: 898.728, Dot Product: 984
C5H15O4N1P1_Fragment_[]
184.0734
C5H12N_Fragment_[]
86.0970
Relative Intensity
m/z
0
10
20
30
40
50
60
70
80
90
100
110
120
0
50
100
150
200
250
300
350
400
450
500
550
600
650
700
750
800
850
900

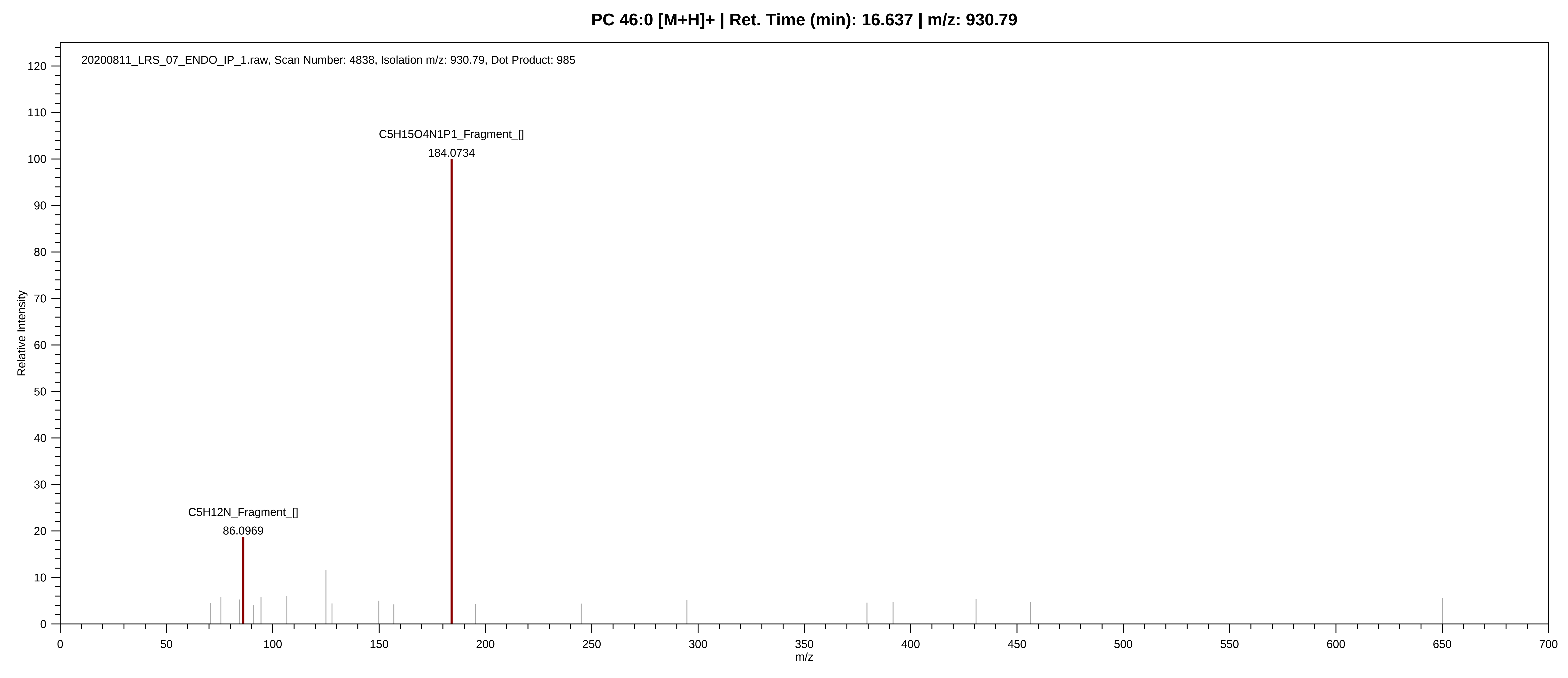
PC 46:0 [M+H]+ | Ret. Time (min): 16.637 | m/z: 930.79
20200811_LRS_07_ENDO_IP_1.raw, Scan Number: 4838, Isolation m/z: 930.79, Dot Product: 985
C5H15O4N1P1_Fragment_[]
184.0734
C5H12N_Fragment_[]
86.0969
Relative Intensity
m/z

PC 46:0 [M+H]+ | Ret. Time (min): 16.651 | m/z: 930.79

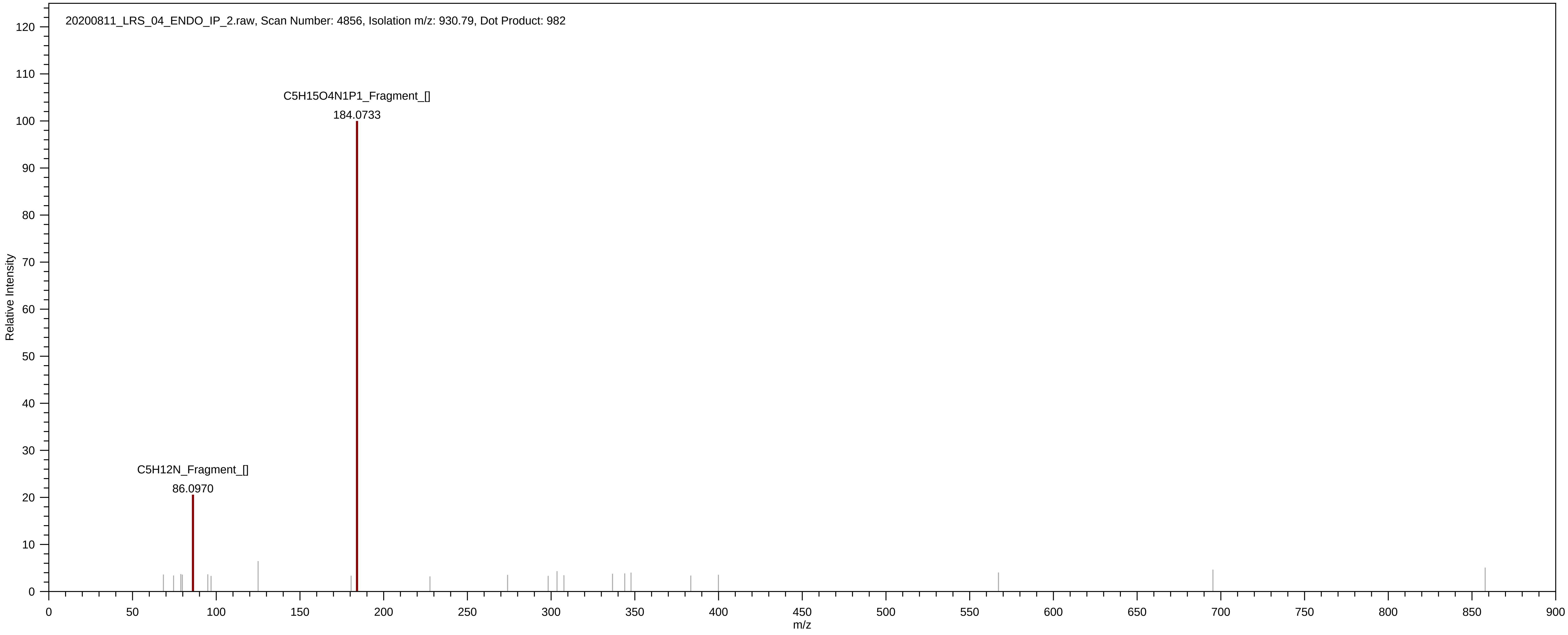

PC 46:0 [M+H]+ | Ret. Time (min): 16.678 | m/z: 930.788

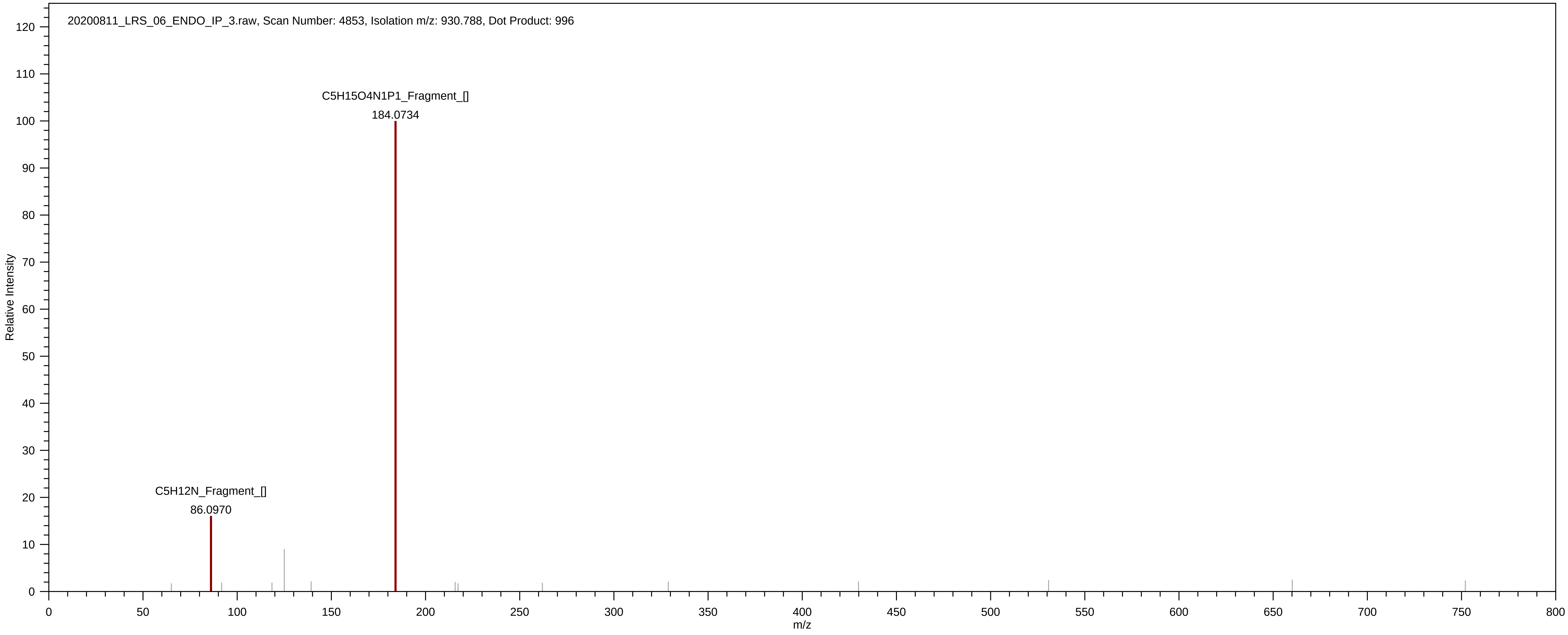

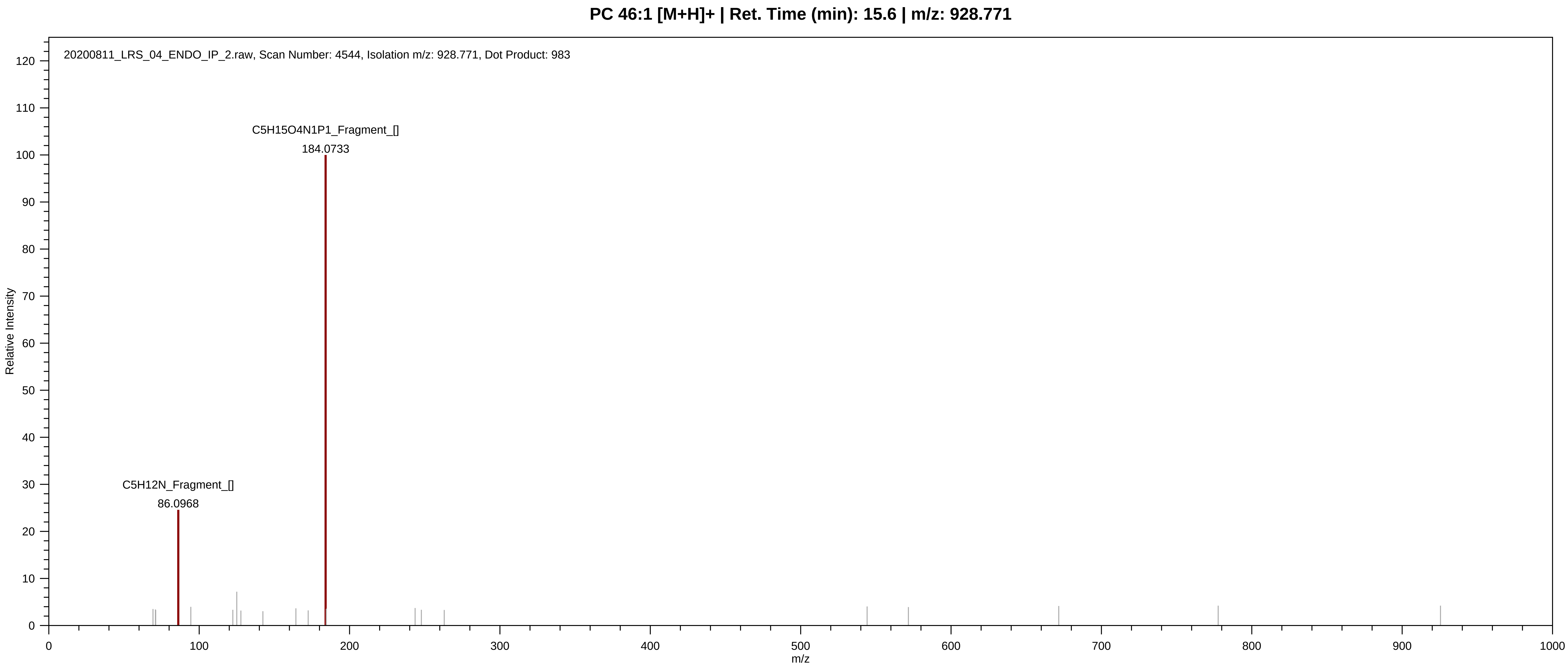
PC 46:1 [M+H]+ | Ret. Time (min): 15.6 | m/z: 928.771
20200811_LRS_04_ENDO_IP_2.raw, Scan Number: 4544, Isolation m/z: 928.771, Dot Product: 983
C5H15O4N1P1_Fragment_[]
184.0733
C5H12N_Fragment_[]
86.0968
Relative Intensity
m/z

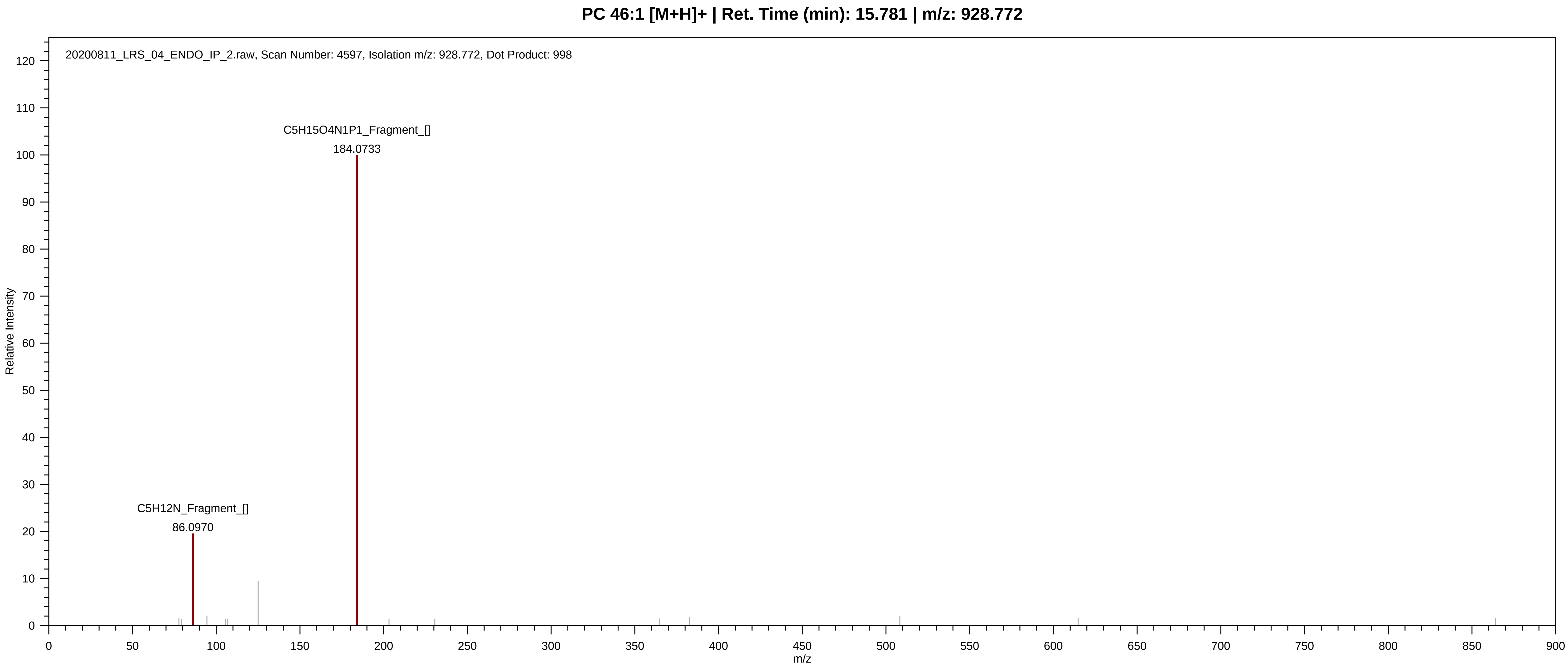
PC 46:1 [M+H]+ | Ret. Time (min): 15.781 | m/z: 928.772
20200811_LRS_04_ENDO_IP_2.raw, Scan Number: 4597, Isolation m/z: 928.772, Dot Product: 998
C5H15O4N1P1_Fragment_[]
184.0733
C5H12N_Fragment_[]
86.0970
Relative Intensity
m/z

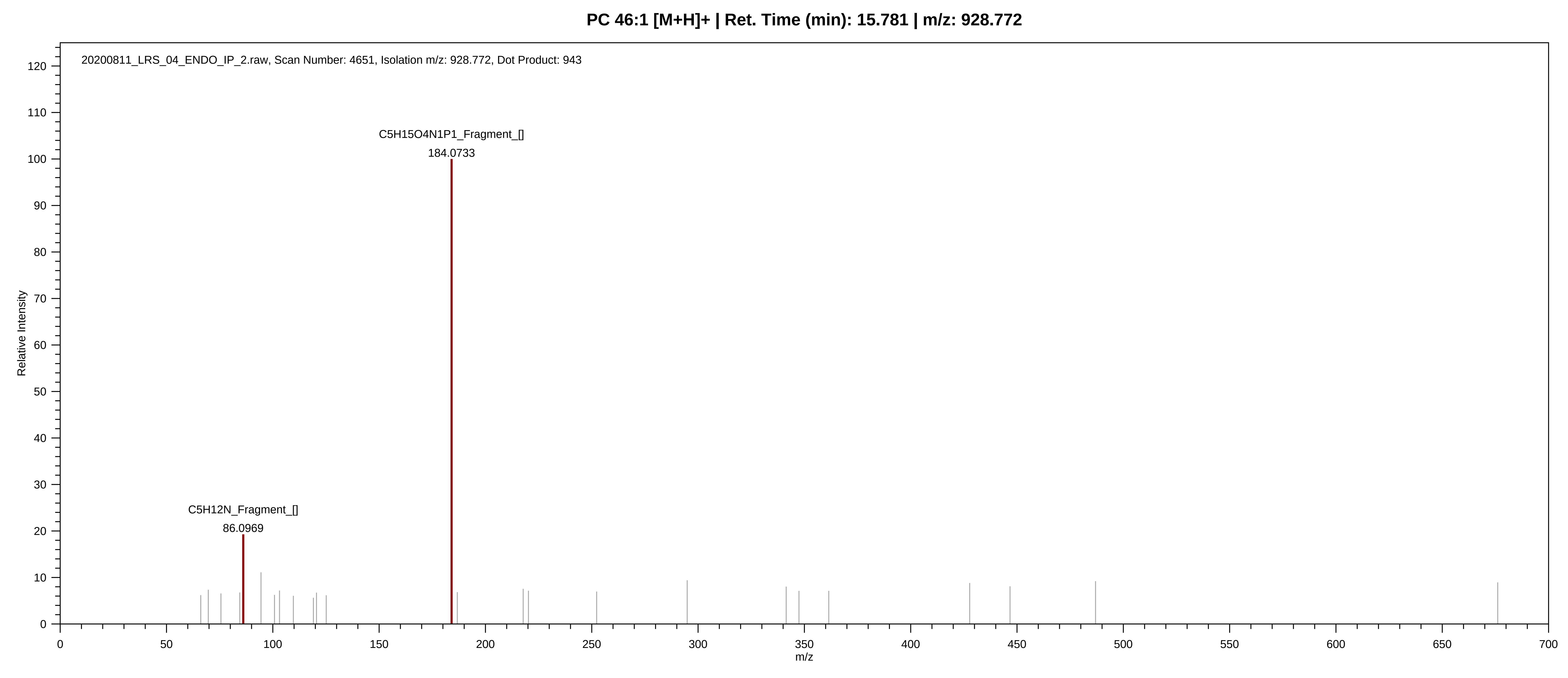

PC 46:1 [M+H]+ | Ret. Time (min): 15.781 | m/z: 928.772
20200811_LRS_04_ENDO_IP_2.raw, Scan Number: 4651, Isolation m/z: 928.772, Dot Product: 943
C5H15O4N1P1_Fragment_[]
184.0733
C5H12N_Fragment_[]
86.0969
Relative Intensity
m/z

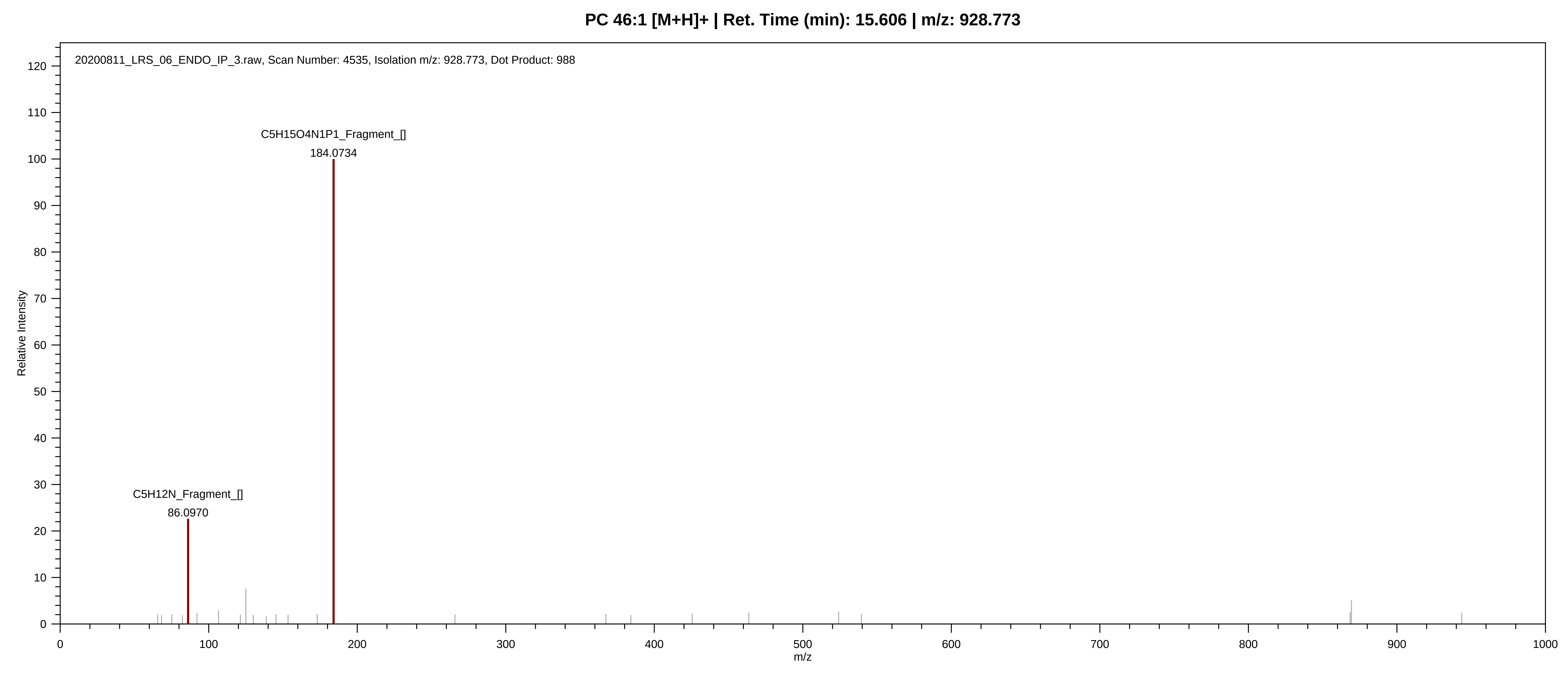

PC 46:1 [M+H]+ | Ret. Time (min): 15.606 | m/z: 928.773
20200811_LRS_06_ENDO_IP_3.raw, Scan Number: 4535, Isolation m/z: 928.773, Dot Product: 988
C5H15O4N1P1_Fragment_[]
184.0734
C5H12N_Fragment_[]
86.0970
Relative Intensity
m/z

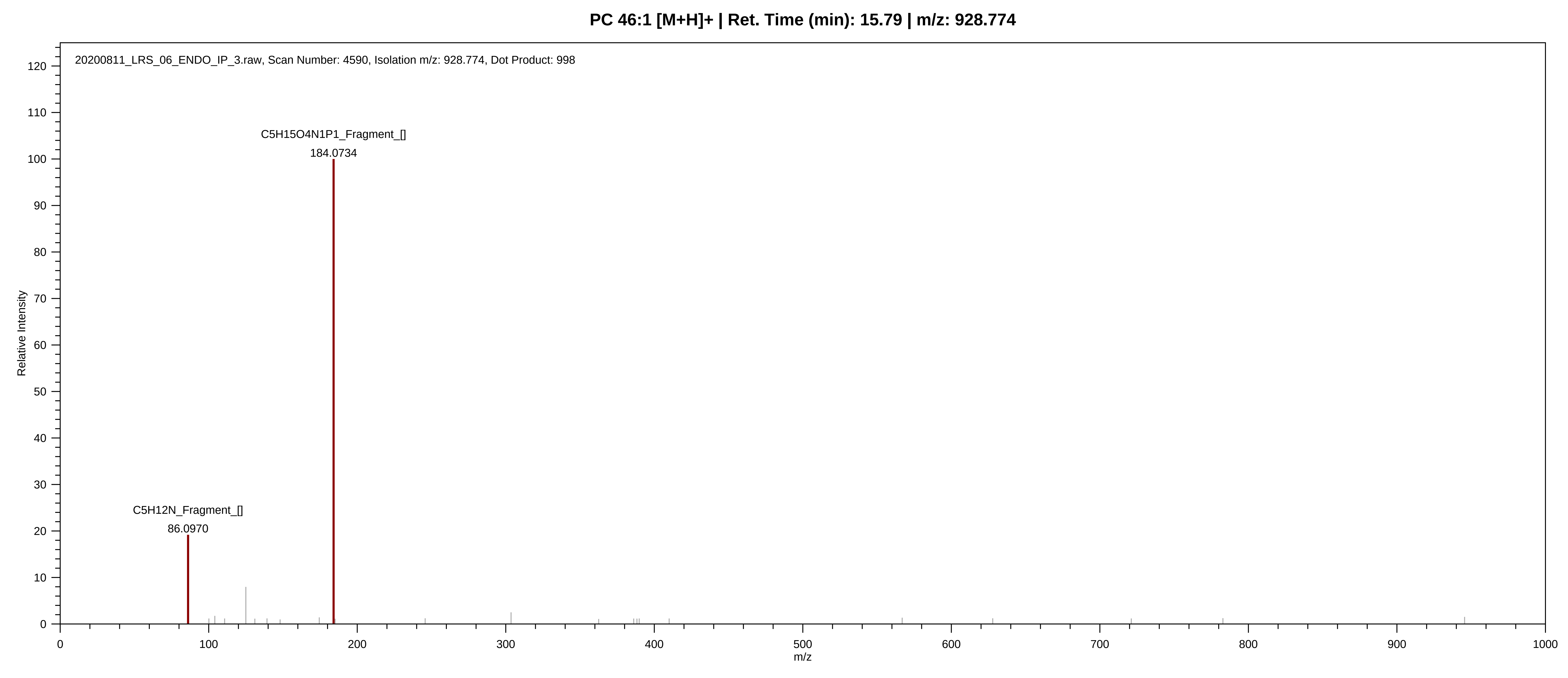
PC 46:1 [M+H]+ | Ret. Time (min): 15.79 | m/z: 928.774
20200811_LRS_06_ENDO_IP_3.raw, Scan Number: 4590, Isolation m/z: 928.774, Dot Product: 998
C5H15O4N1P1_Fragment_[]
184.0734
C5H12N_Fragment_[]
86.0970
Relative Intensity
m/z

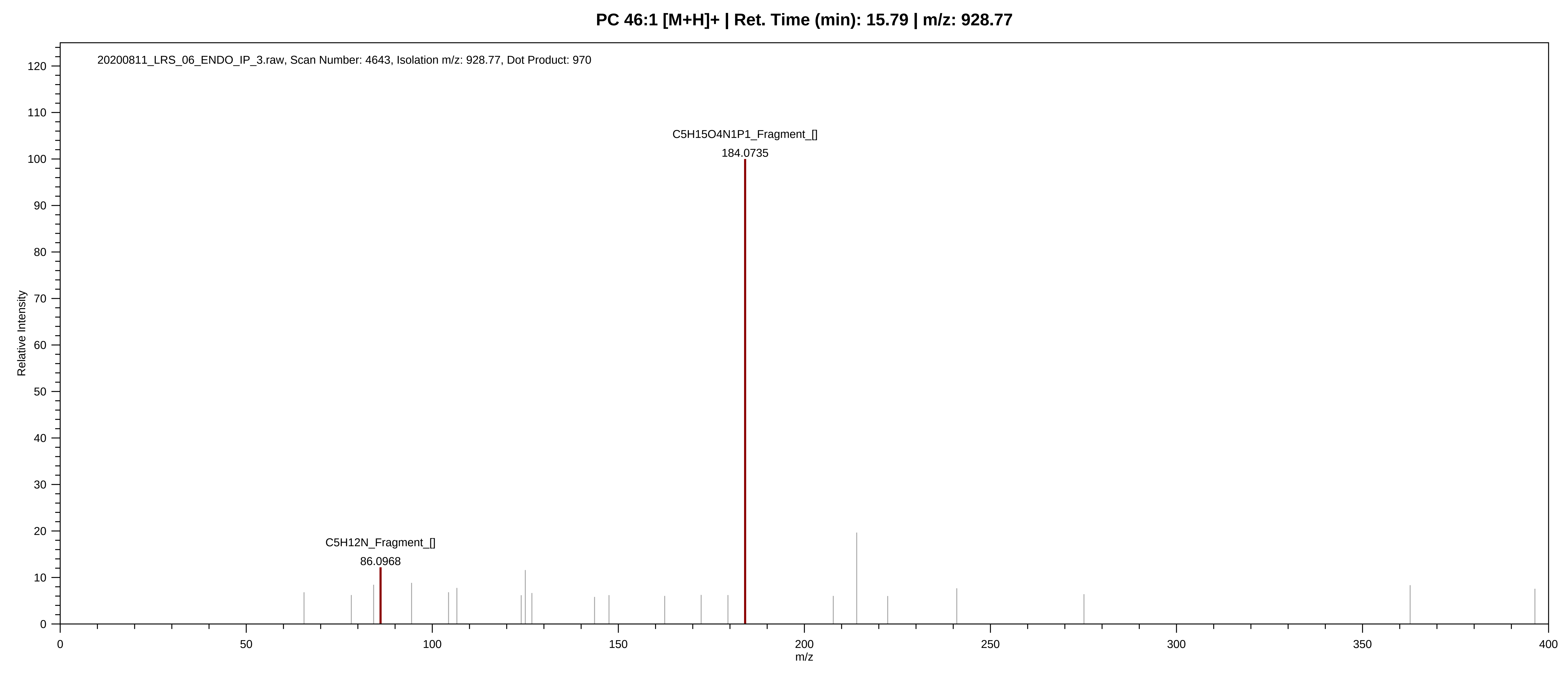
PC 46:1 [M+H]+ | Ret. Time (min): 15.79 | m/z: 928.77
20200811_LRS_06_ENDO_IP_3.raw, Scan Number: 4643, Isolation m/z: 928.77, Dot Product: 970
C5H15O4N1P1_Fragment_[]
184.0735
C5H12N_Fragment_[]
86.0968
Relative Intensity
m/z
0
10
20
30
40
50
60
70
80
90
100
110
120
0
50
100
150
200
250
300
350
400

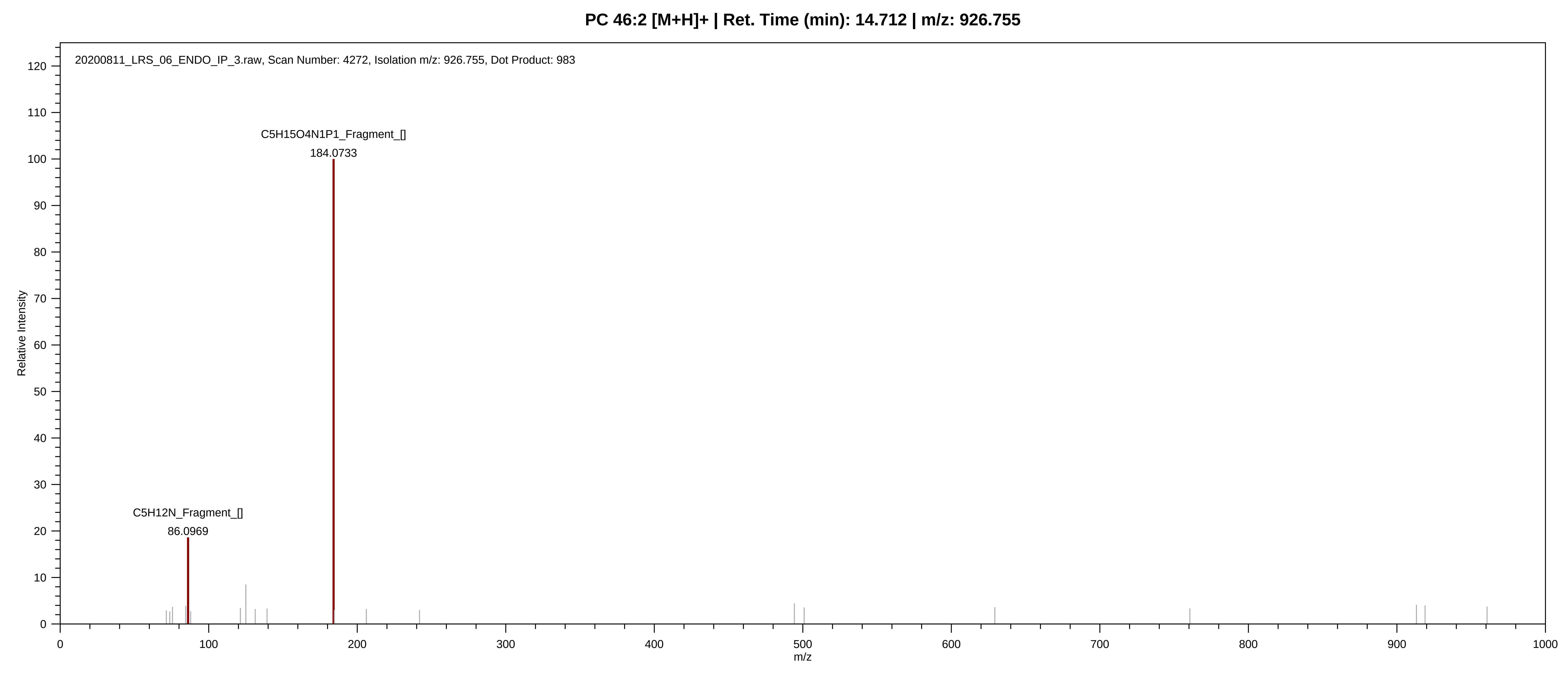

PC 46:2 [M+H]+ | Ret. Time (min): 14.712 | m/z: 926.755
20200811_LRS_06_ENDO_IP_3.raw, Scan Number: 4272, Isolation m/z: 926.755, Dot Product: 983
C5H15O4N1P1_Fragment_[]
184.0733
C5H12N_Fragment_[]
86.0969
Relative Intensity
m/z

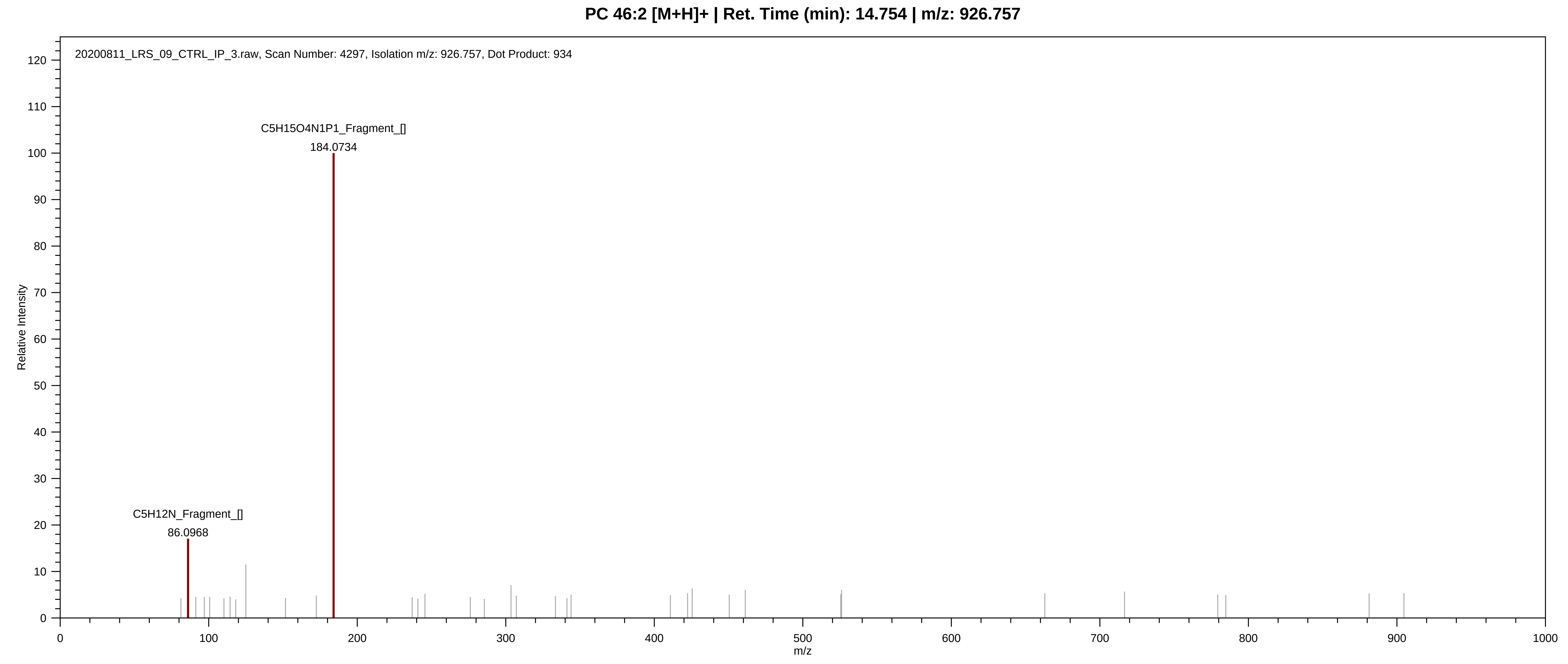

PC 46:2 [M+H]+ | Ret. Time (min): 14.754 | m/z: 926.757
20200811_LRS_09_CTRL_IP_3.raw, Scan Number: 4297, Isolation m/z: 926.757, Dot Product: 934
C5H15O4N1P1_Fragment_[]
184.0734
C5H12N_Fragment_[]
86.0968
Relative Intensity
m/z

PC 46:2 [M+H]+ | Ret. Time (min): 14.874 | m/z: 926.757

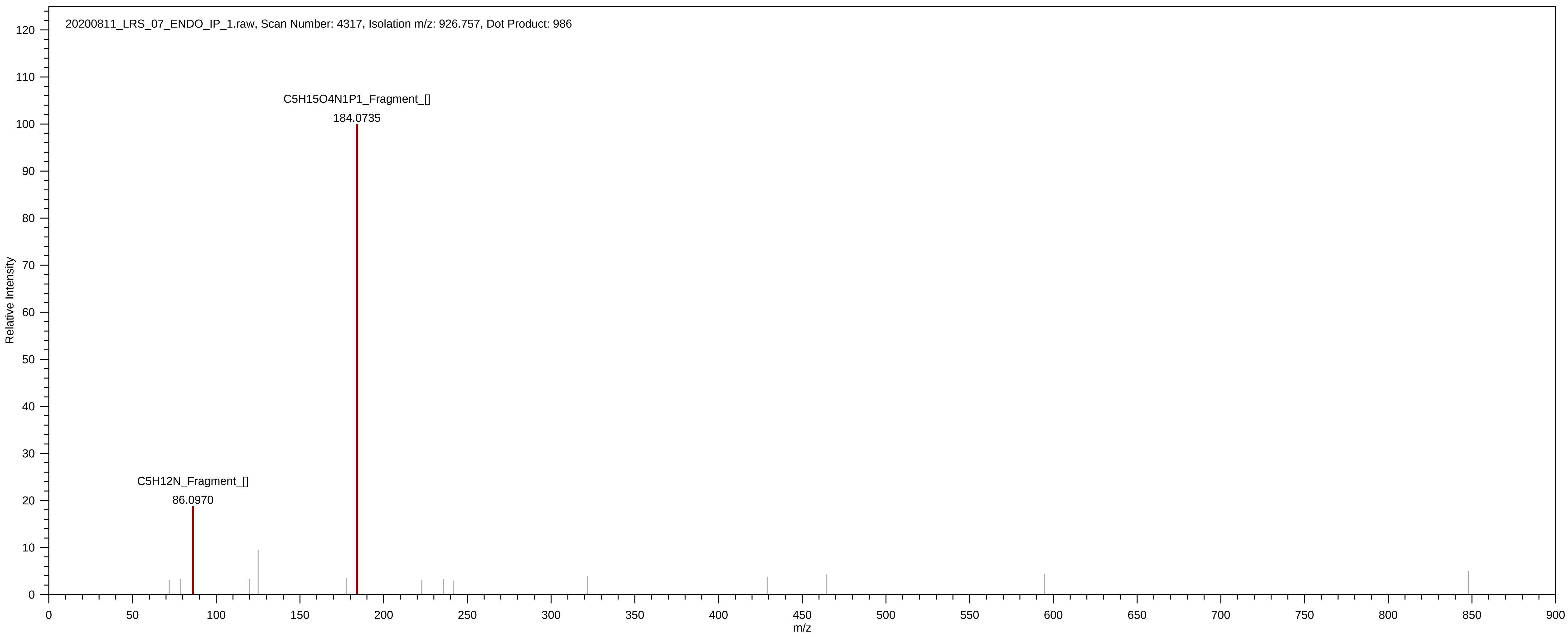

PC 46:2 [M+H]+ | Ret. Time (min): 14.69 | m/z: 926.758

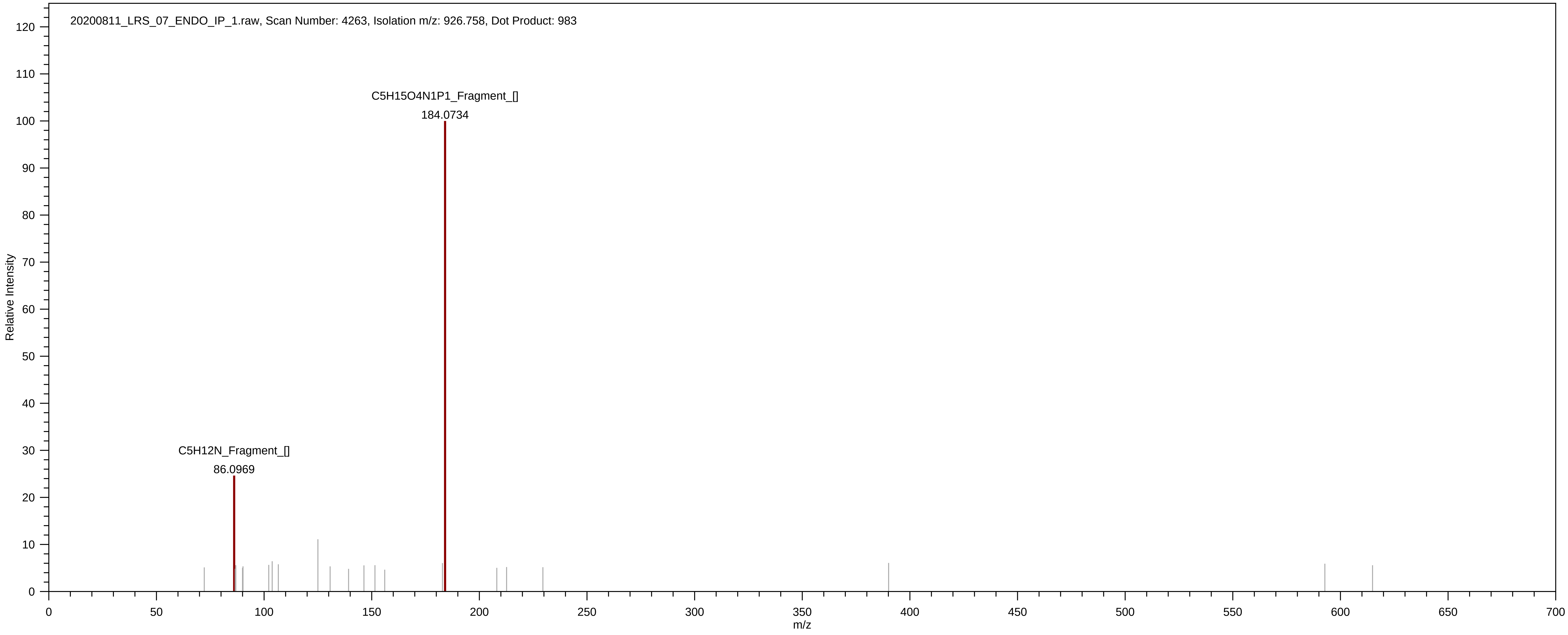

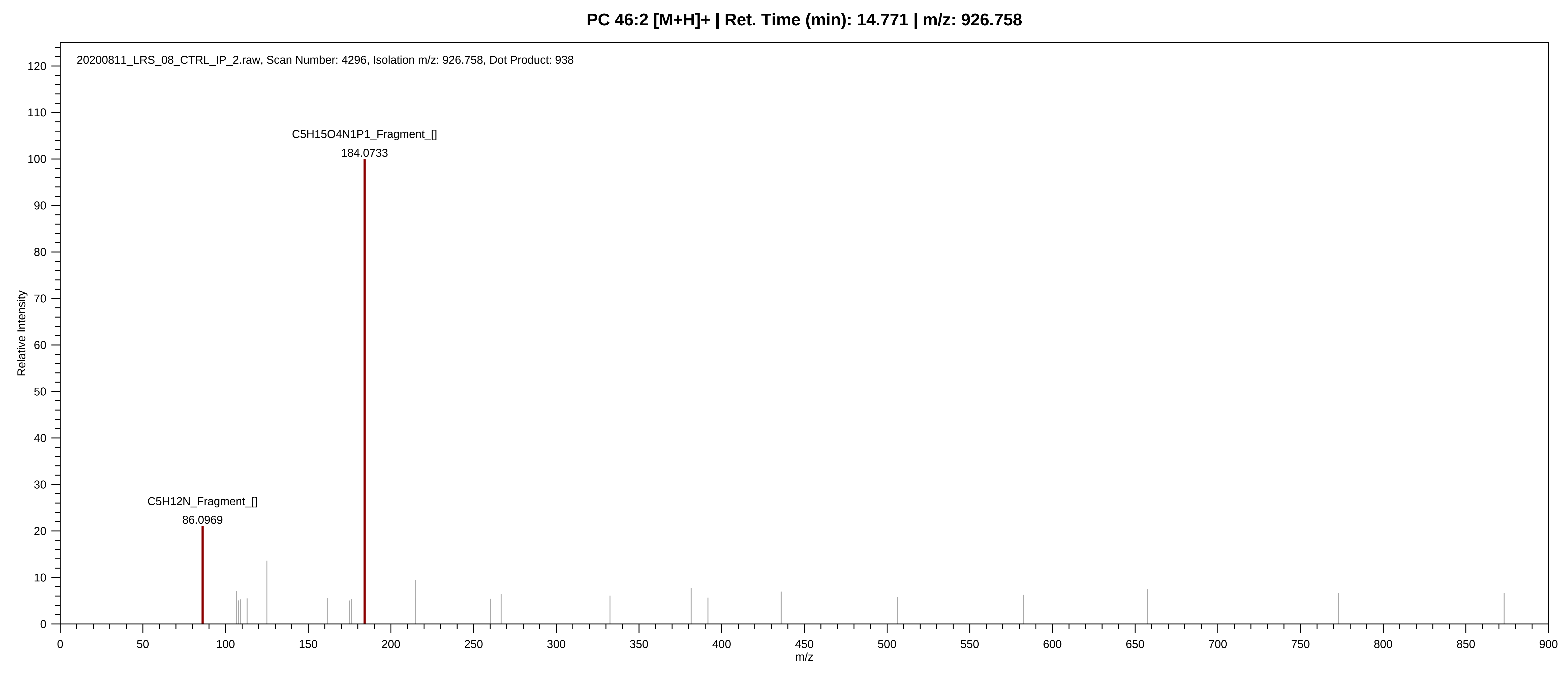
PC 46:2 [M+H]+ | Ret. Time (min): 14.771 | m/z: 926.758
20200811_LRS_08_CTRL_IP_2.raw, Scan Number: 4296, Isolation m/z: 926.758, Dot Product: 938
C5H15O4N1P1_Fragment_[]
184.0733
C5H12N_Fragment_[]
86.0969
Relative Intensity
m/z

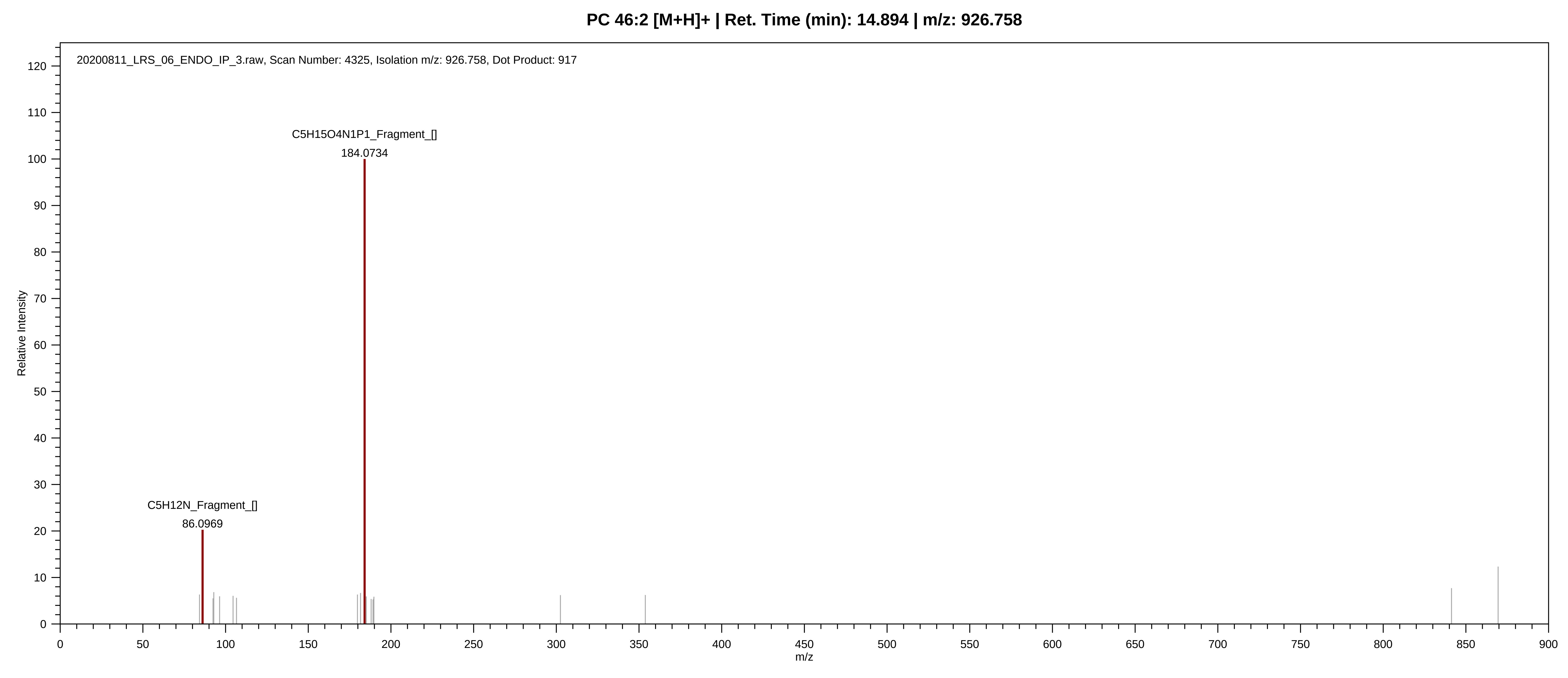

PC 46:2 [M+H]+ | Ret. Time (min): 14.894 | m/z: 926.758
20200811_LRS_06_ENDO_IP_3.raw, Scan Number: 4325, Isolation m/z: 926.758, Dot Product: 917
C5H15O4N1P1_Fragment_[]
184.0734
C5H12N_Fragment_[]
86.0969
Relative Intensity
m/z

PC 46:2 [M+H]+ | Ret. Time (min): 14.908 | m/z: 926.758

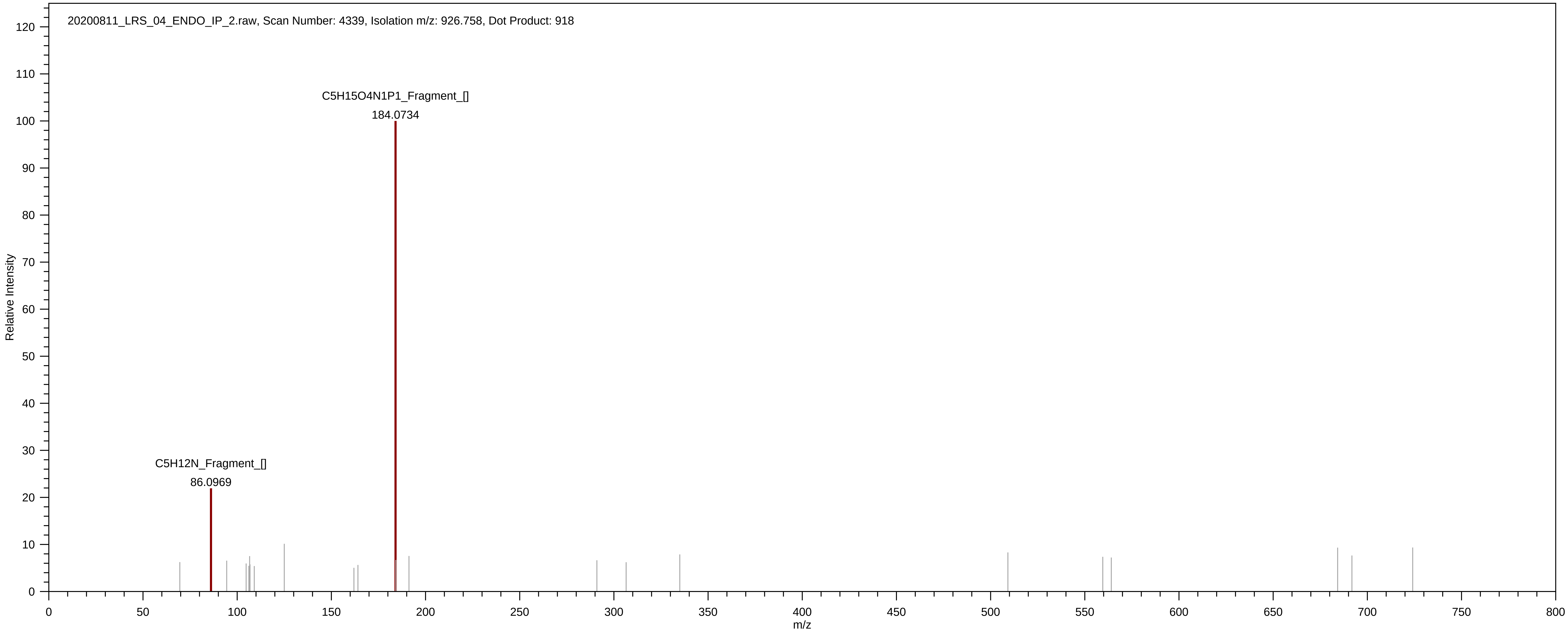

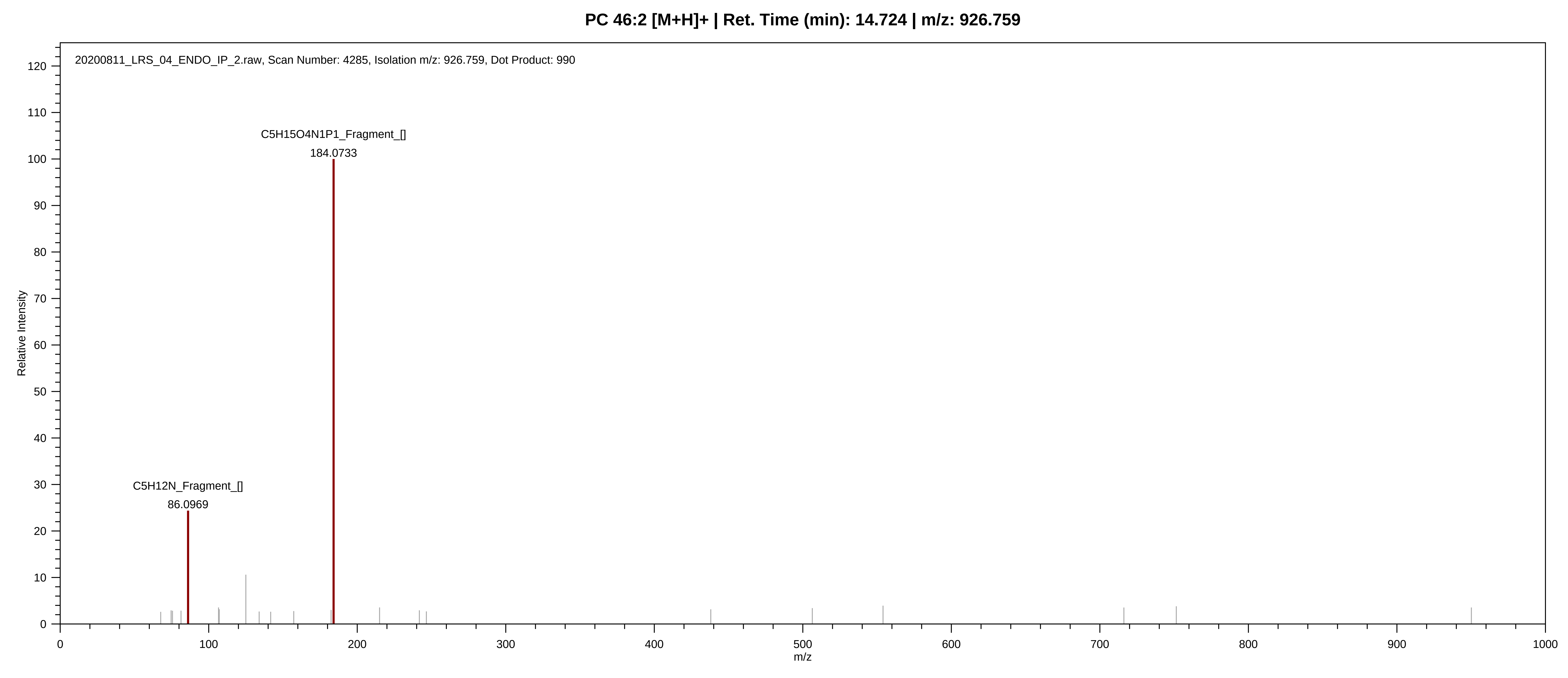
PC 46:2 [M+H]+ | Ret. Time (min): 14.724 | m/z: 926.759
20200811_LRS_04_ENDO_IP_2.raw, Scan Number: 4285, Isolation m/z: 926.759, Dot Product: 990
C5H15O4N1P1_Fragment_[]
184.0733
C5H12N_Fragment_[]
86.0969
Relative Intensity
m/z

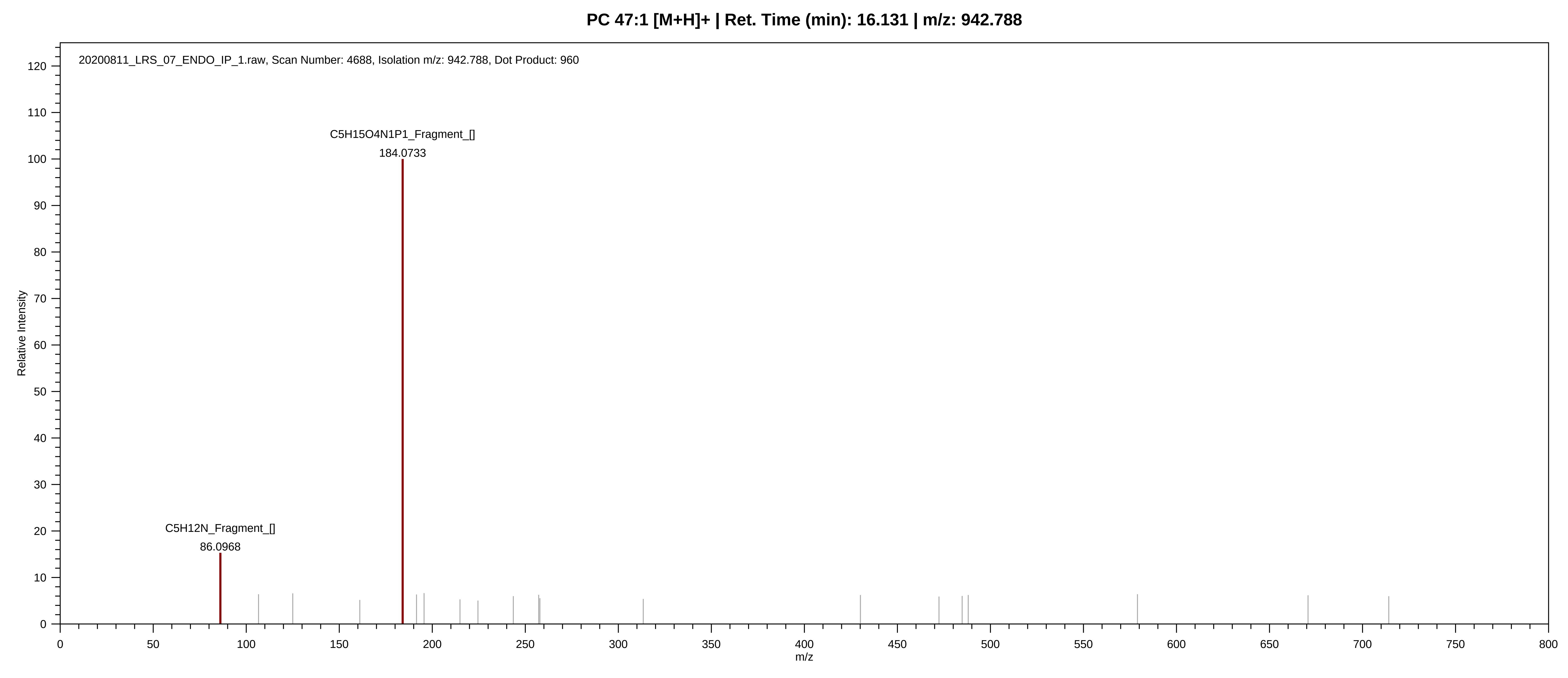

PC 47:1 [M+H]+ | Ret. Time (min): 16.131 | m/z: 942.788
20200811_LRS_07_ENDO_IP_1.raw, Scan Number: 4688, Isolation m/z: 942.788, Dot Product: 960
C5H15O4N1P1_Fragment_[]
184.0733
C5H12N_Fragment_[]
86.0968
Relative Intensity
m/z

PC 47:1 [M+H]+ | Ret. Time (min): 16.115 | m/z: 942.791

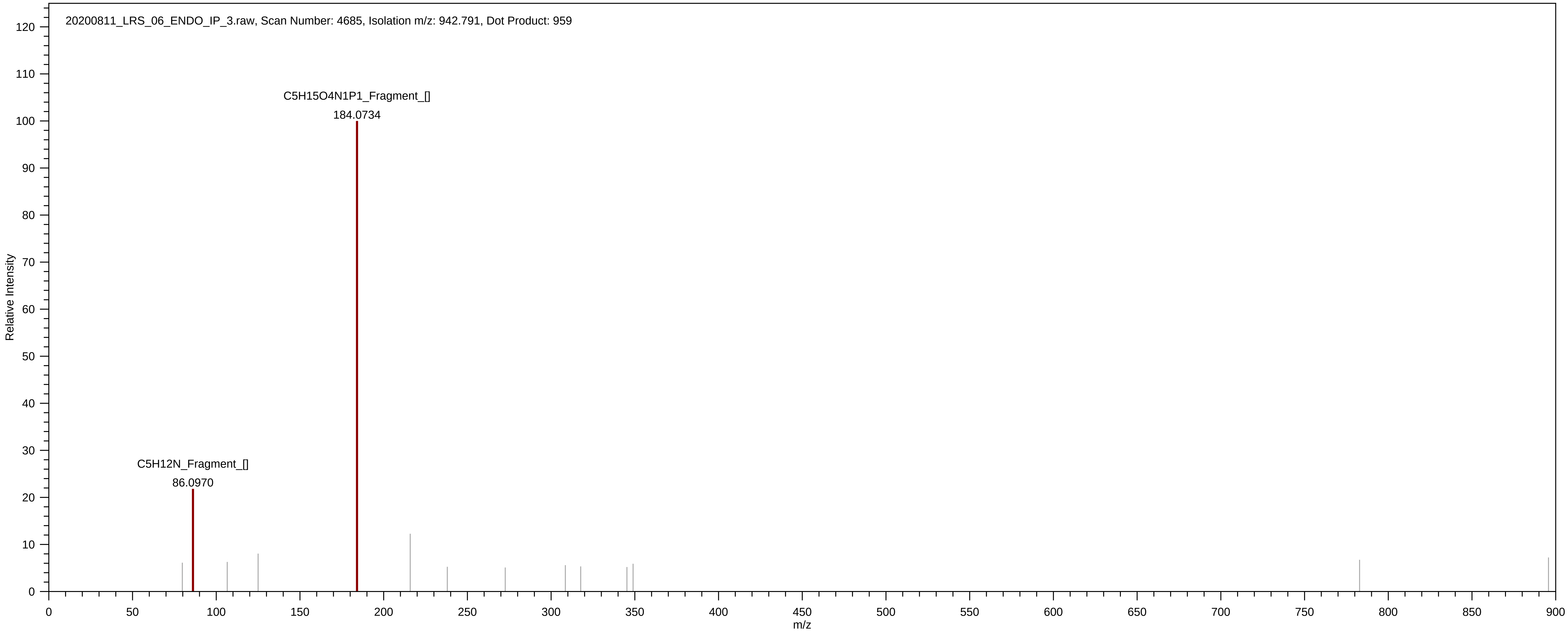

PC 47:2 [M+H]+ | Ret. Time (min): 15.272 | m/z: 940.774

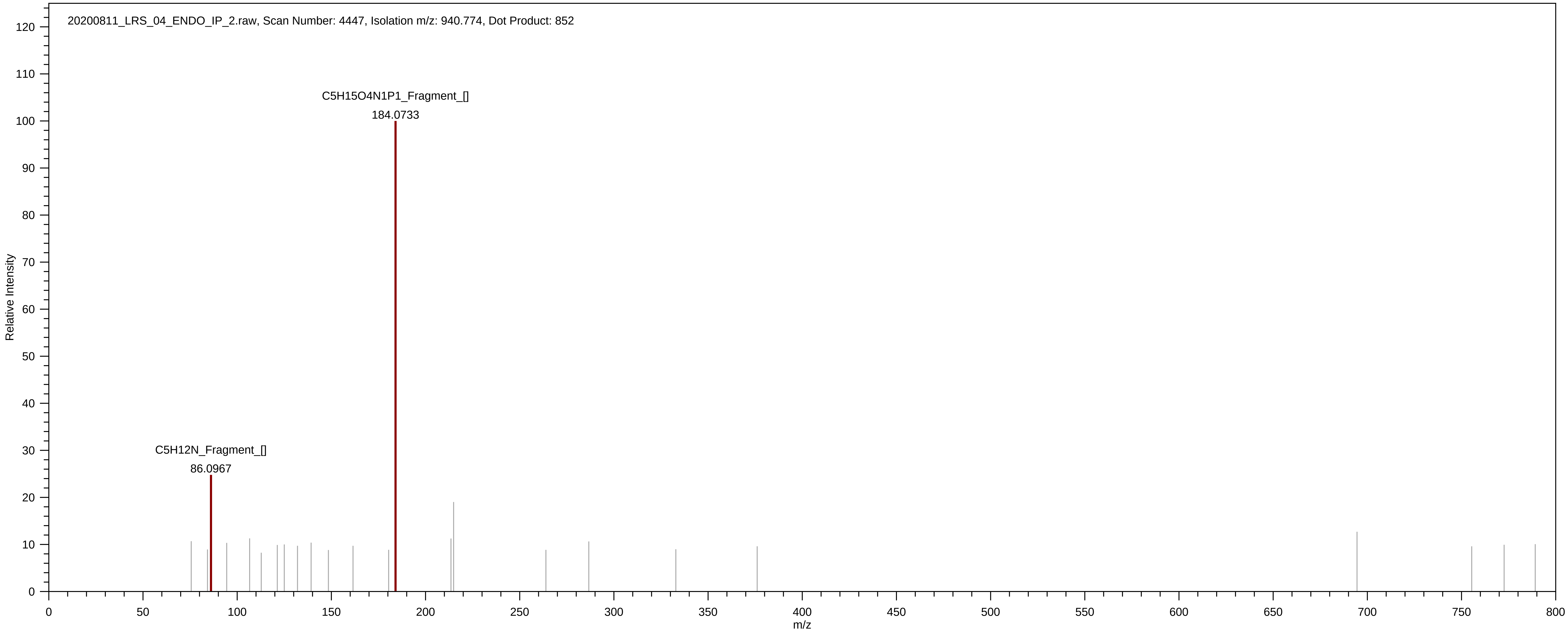

PC 48:1 [M+H]+ | Ret. Time (min): 16.761 | m/z: 956.8

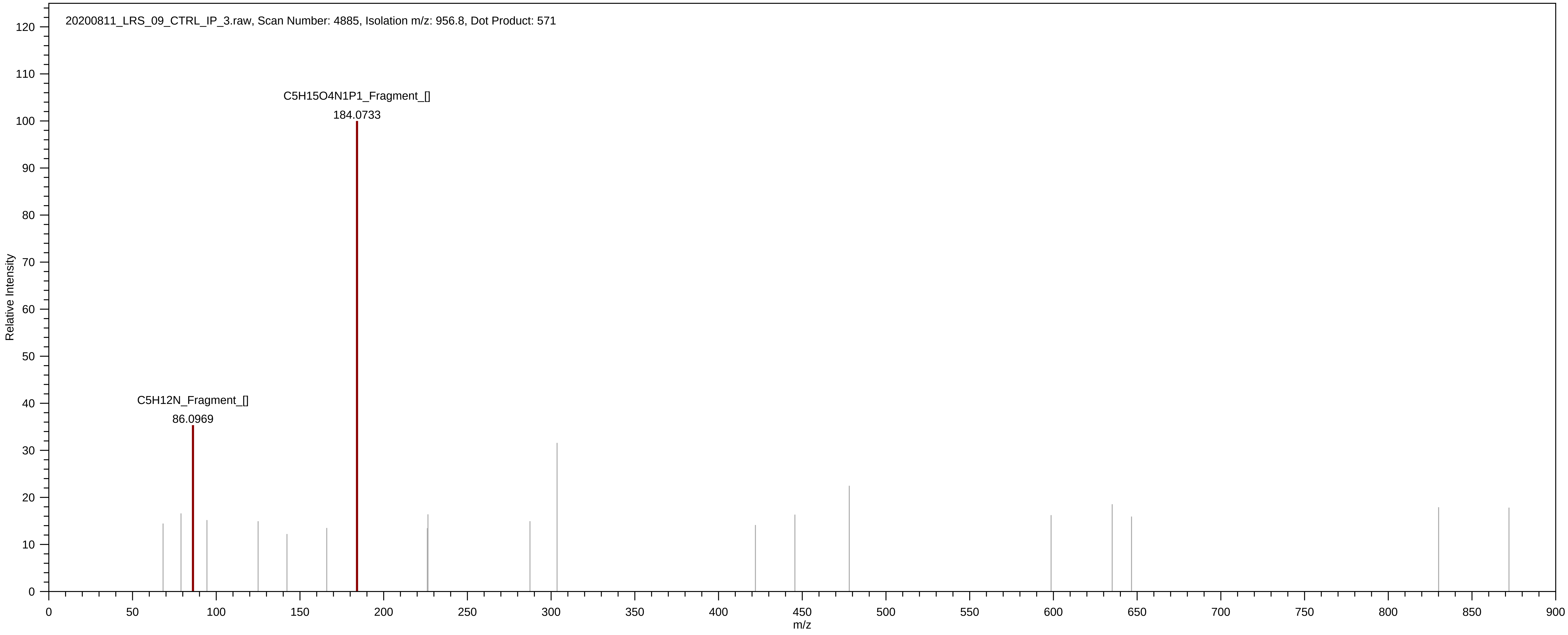

PC 48:1 [M+H]+ | Ret. Time (min): 16.507 | m/z: 956.801

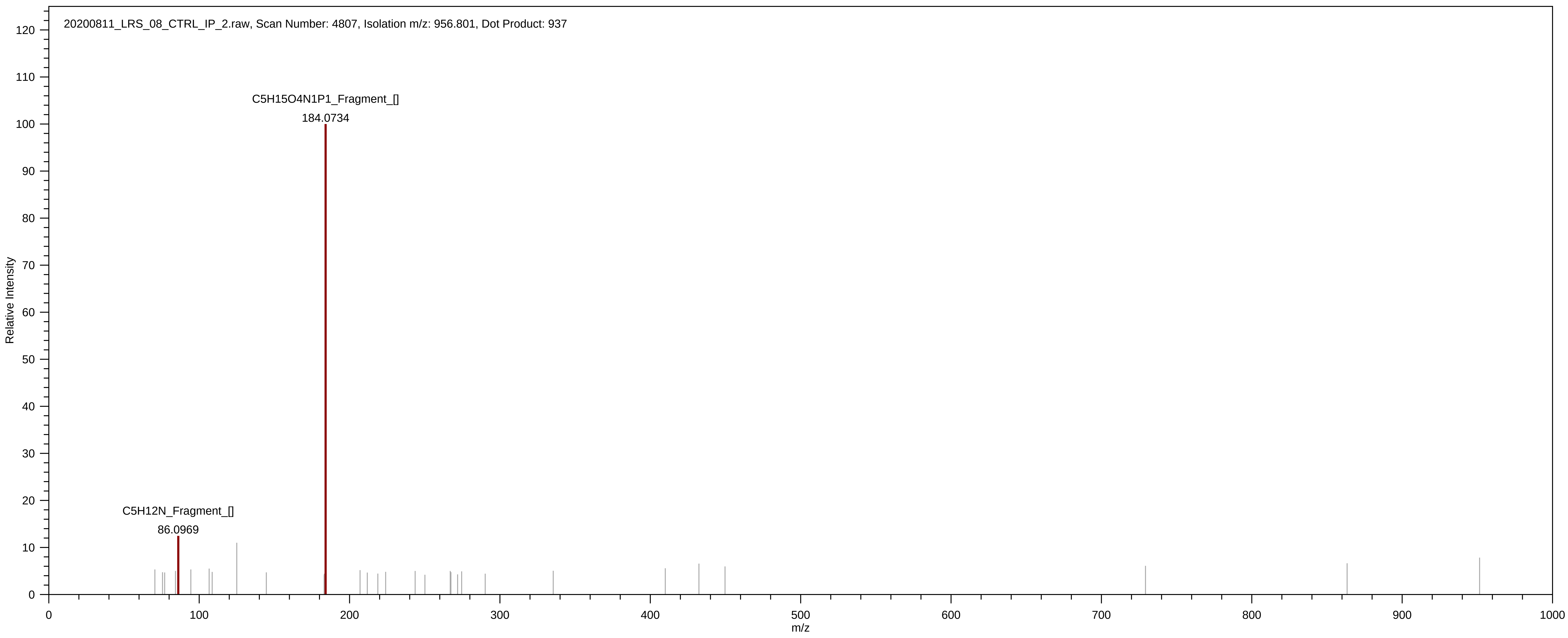

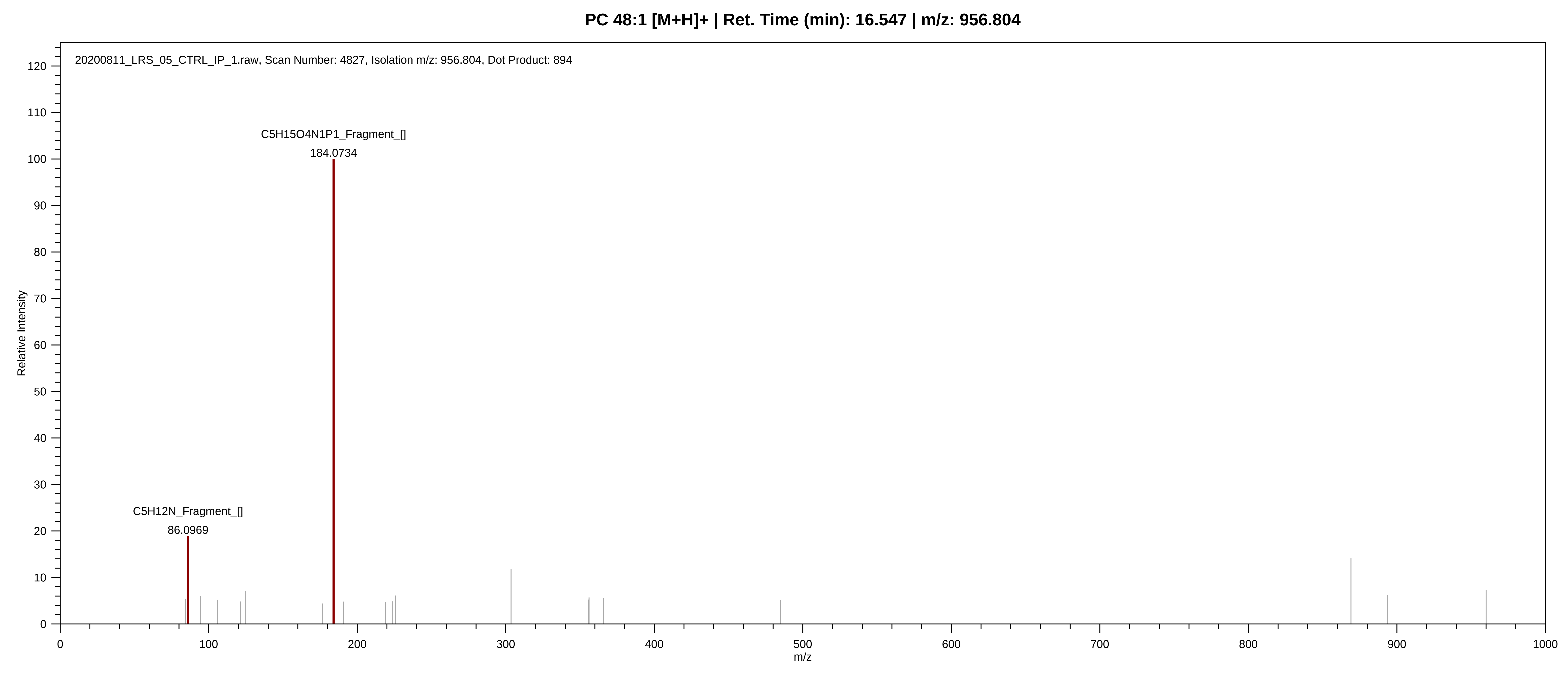
PC 48:1 [M+H]+ | Ret. Time (min): 16.547 | m/z: 956.804
20200811_LRS_05_CTRL_IP_1.raw, Scan Number: 4827, Isolation m/z: 956.804, Dot Product: 894
C5H15O4N1P1_Fragment_[]
184.0734
C5H12N_Fragment_[]
86.0969
Relative Intensity
m/z

PC 48:1 [M+H]+ | Ret. Time (min): 16.671 | m/z: 956.804

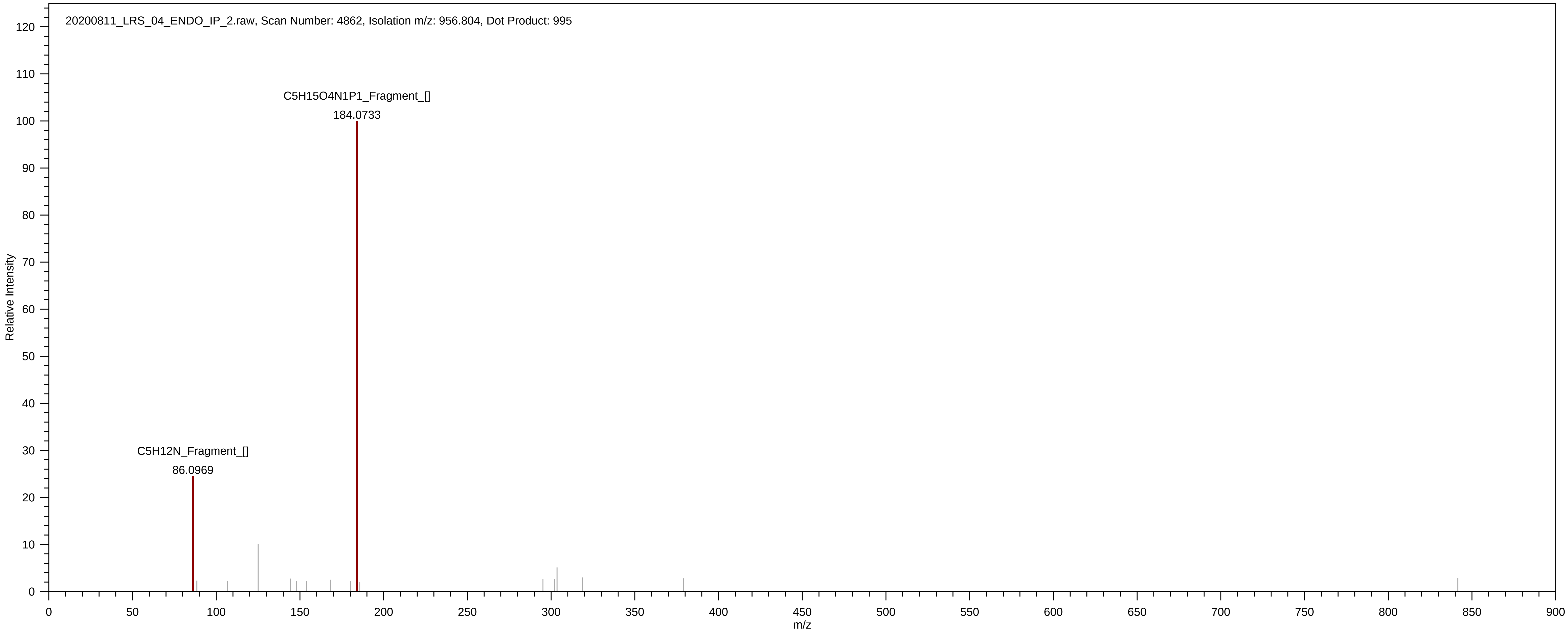

PC 48:1 [M+H]+ | Ret. Time (min): 16.66 | m/z: 956.805

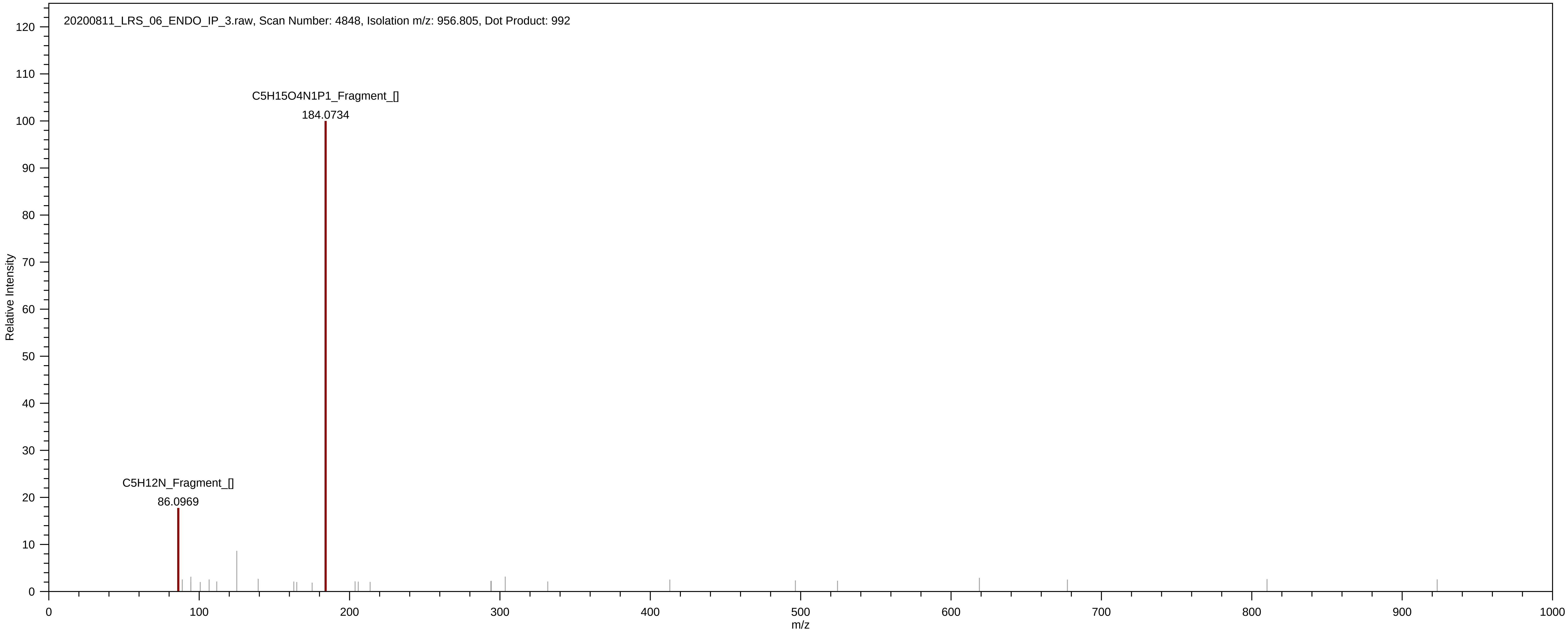

PC 48:1 [M+H]+ | Ret. Time (min): 16.66 | m/z: 956.803

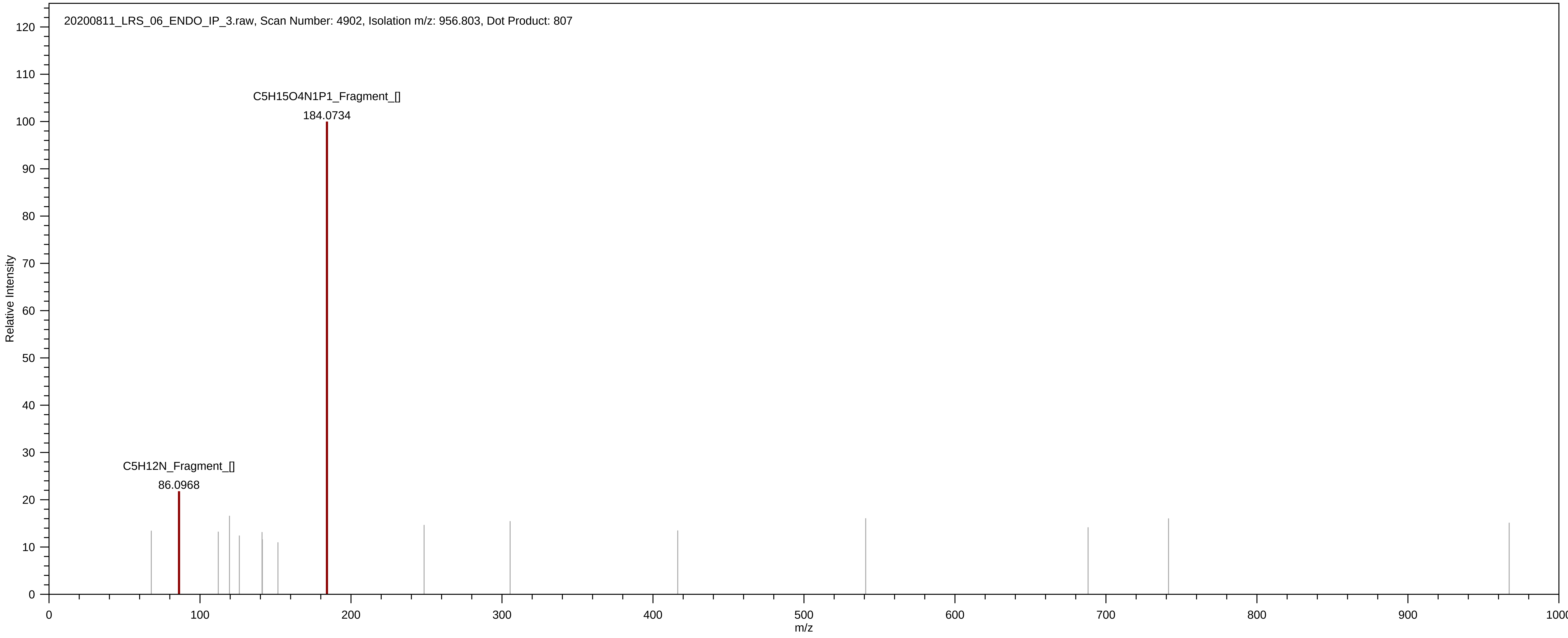

PC 48:1 [M+H]+ | Ret. Time (min): 16.476 | m/z: 956.806

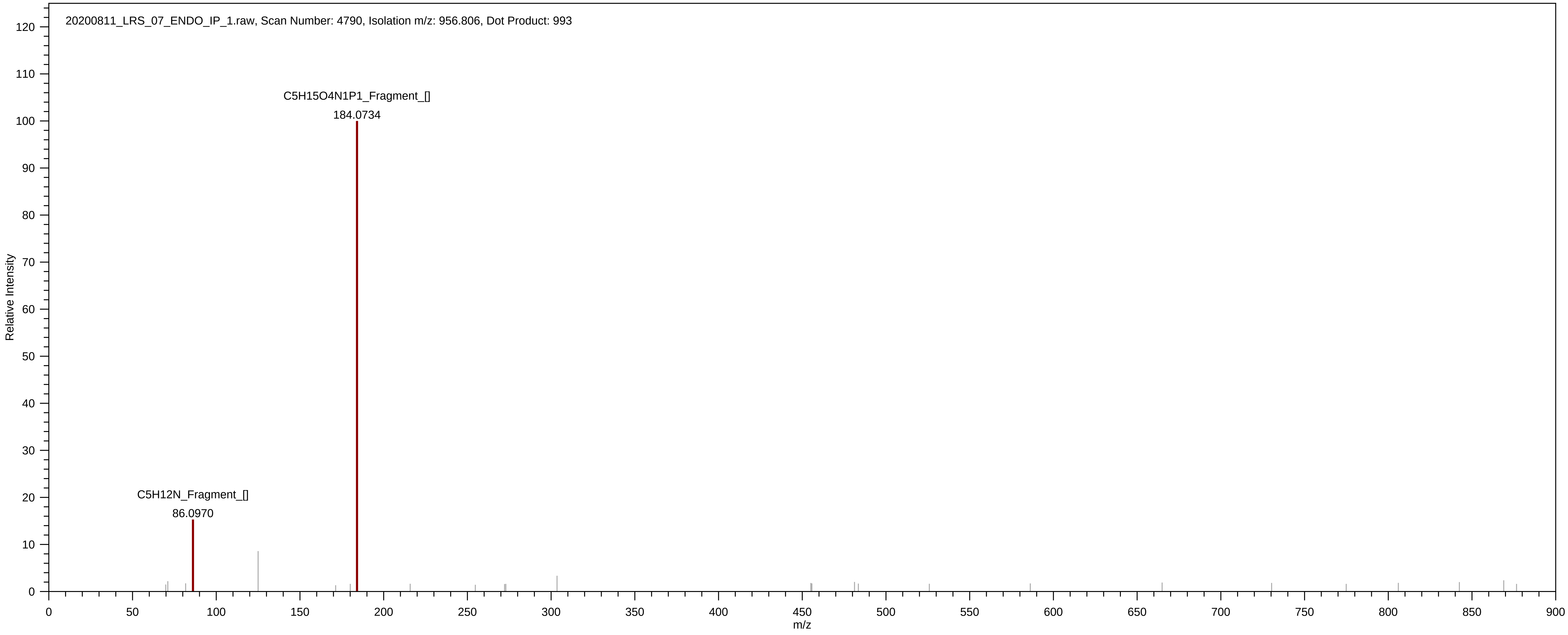

PC 48:1 [M+H]+ | Ret. Time (min): 16.659 | m/z: 956.806

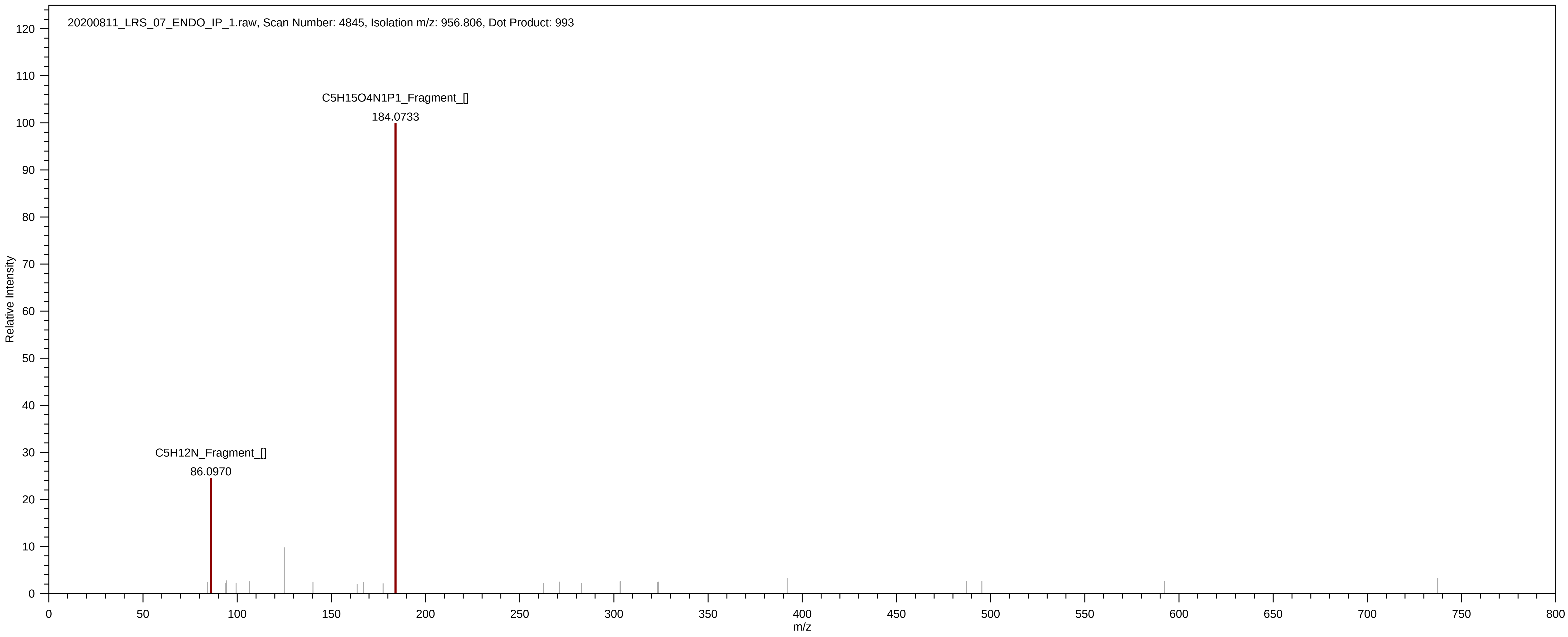

PC 48:1 [M+H]+ | Ret. Time (min): 16.659 | m/z: 956.808

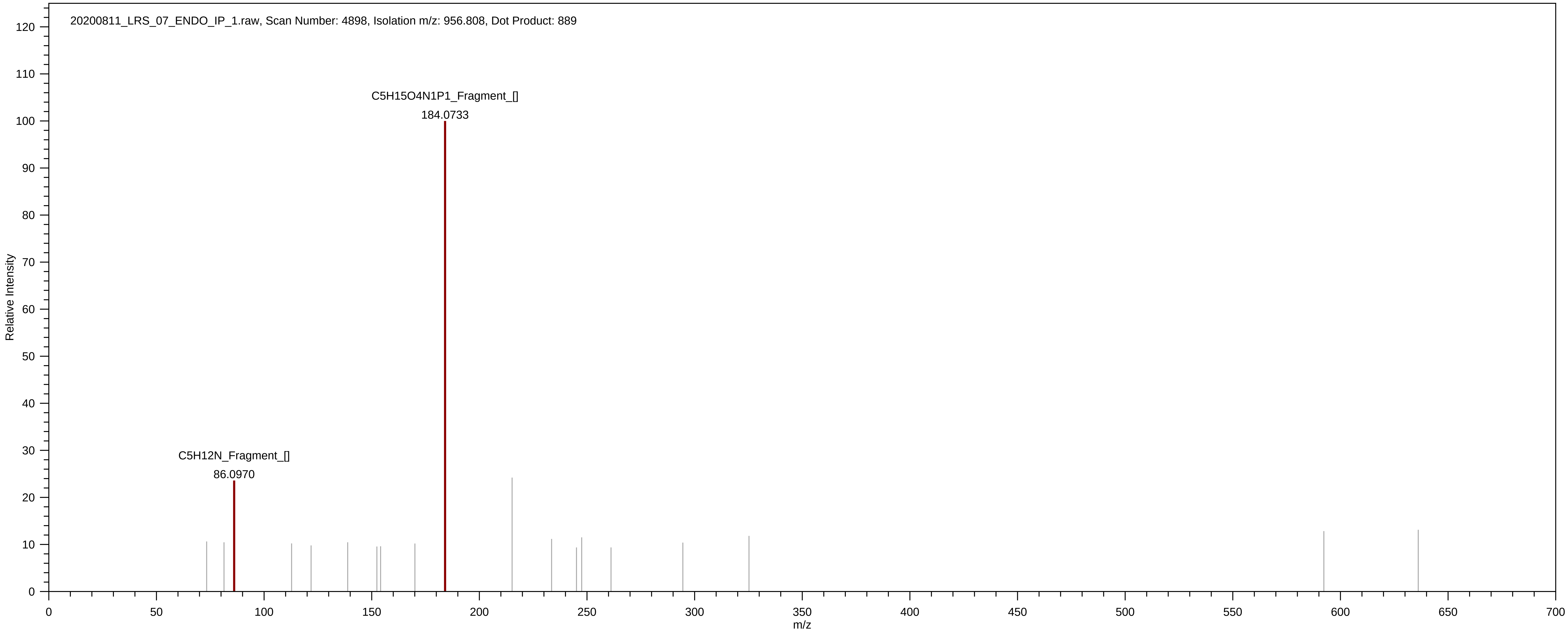

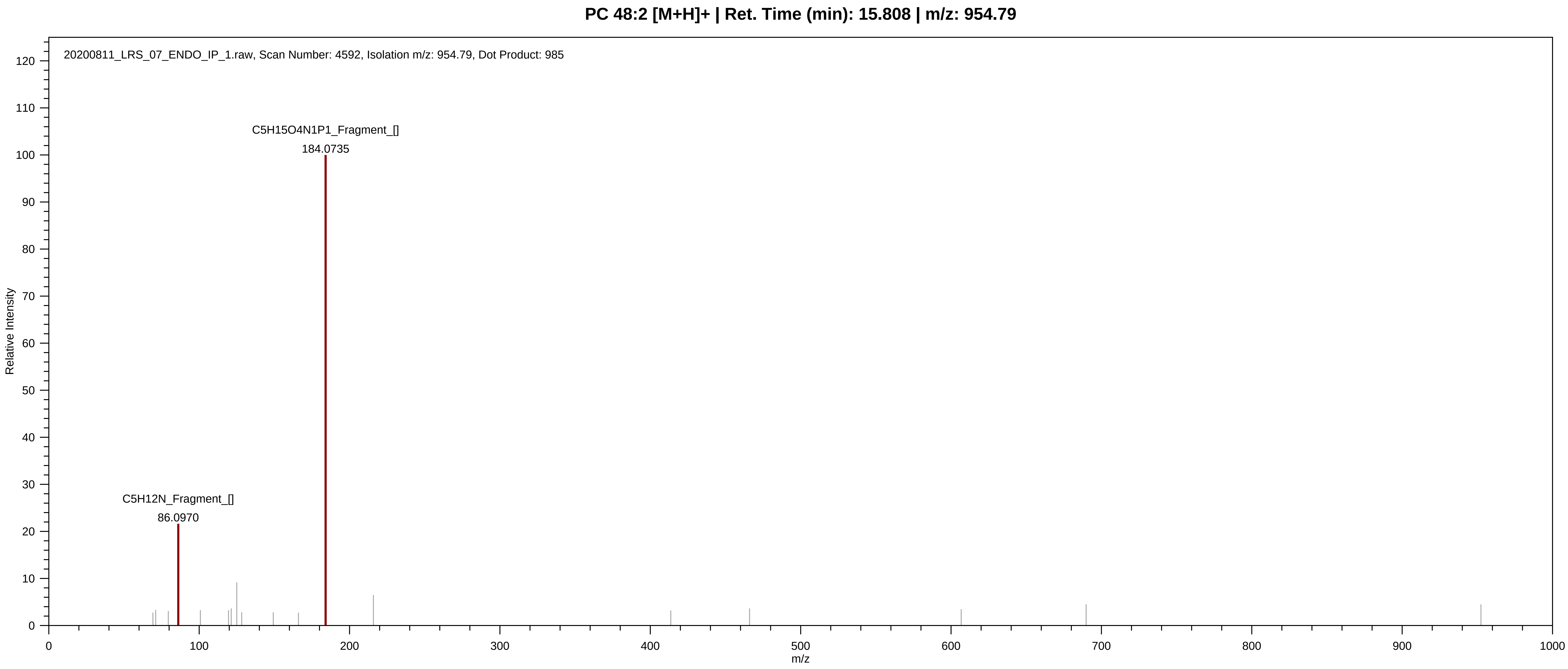
PC 48:2 [M+H]+ | Ret. Time (min): 15.808 | m/z: 954.79
20200811_LRS_07_ENDO_IP_1.raw, Scan Number: 4592, Isolation m/z: 954.79, Dot Product: 985
C5H15O4N1P1_Fragment_[]
184.0735
C5H12N_Fragment_[]
86.0970
Relative Intensity
m/z

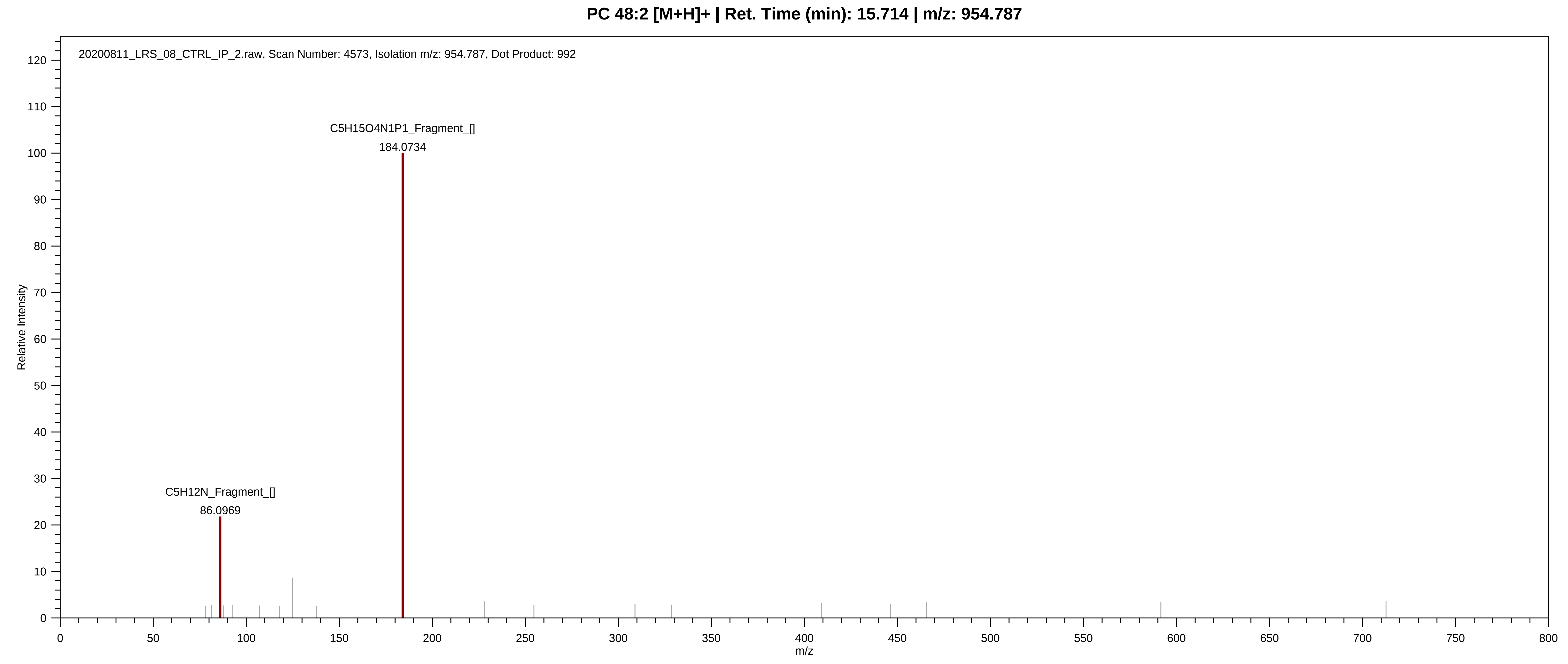

PC 48:2 [M+H]+ | Ret. Time (min): 15.714 | m/z: 954.787
20200811_LRS_08_CTRL_IP_2.raw, Scan Number: 4573, Isolation m/z: 954.787, Dot Product: 992
C5H15O4N1P1_Fragment_[]
184.0734
C5H12N_Fragment_[]
86.0969
Relative Intensity
m/z

PC 48:2 [M+H]+ | Ret. Time (min): 15.639 | m/z: 954.788

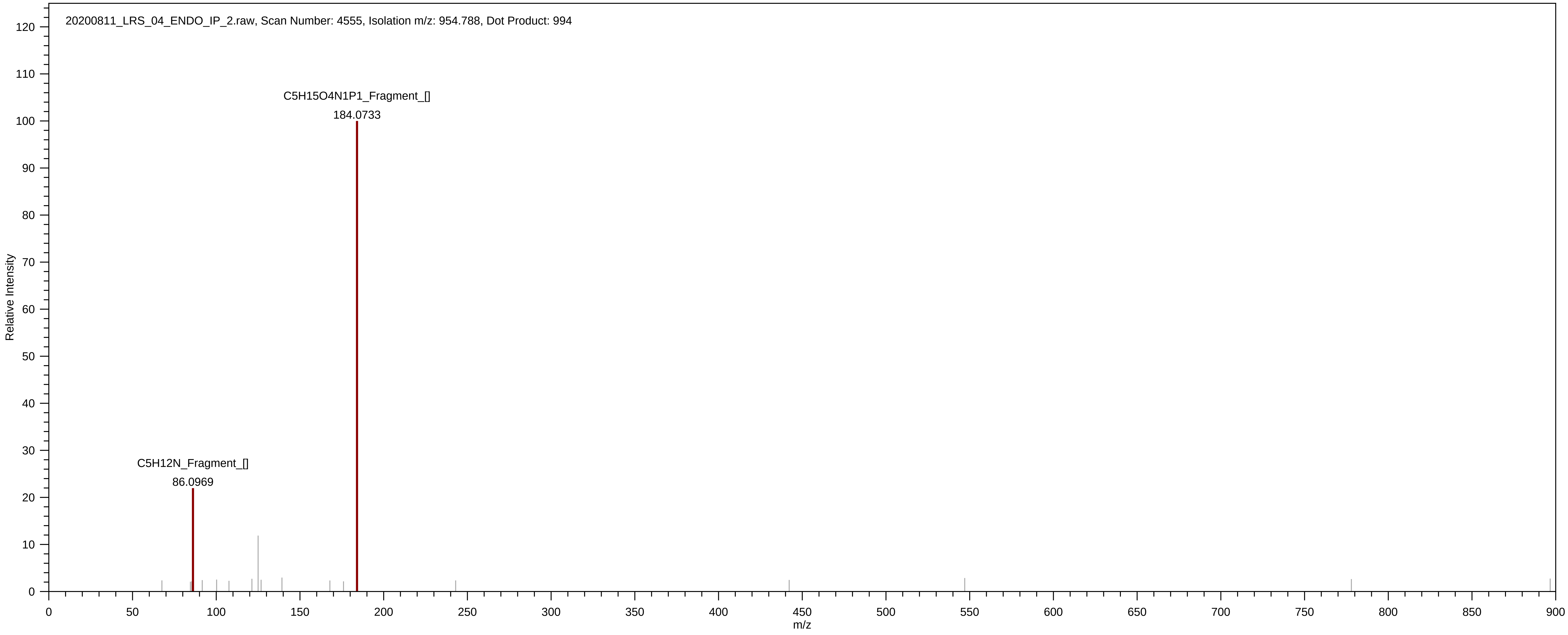

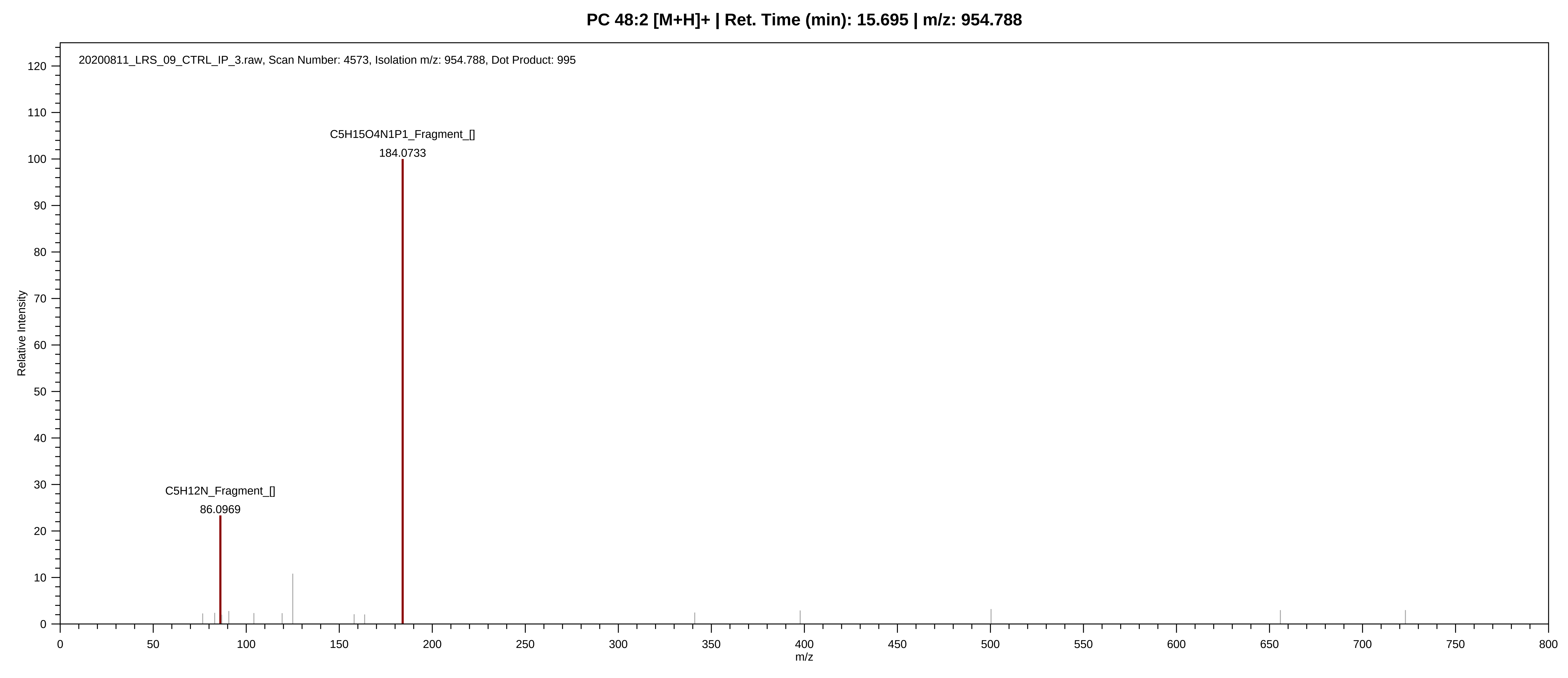
PC 48:2 [M+H]+ | Ret. Time (min): 15.695 | m/z: 954.788
20200811_LRS_09_CTRL_IP_3.raw, Scan Number: 4573, Isolation m/z: 954.788, Dot Product: 995
C5H15O4N1P1_Fragment_[]
184.0733
C5H12N_Fragment_[]
86.0969
Relative Intensity
m/z

PC 48:2 [M+H]+ | Ret. Time (min): 15.789 | m/z: 954.788

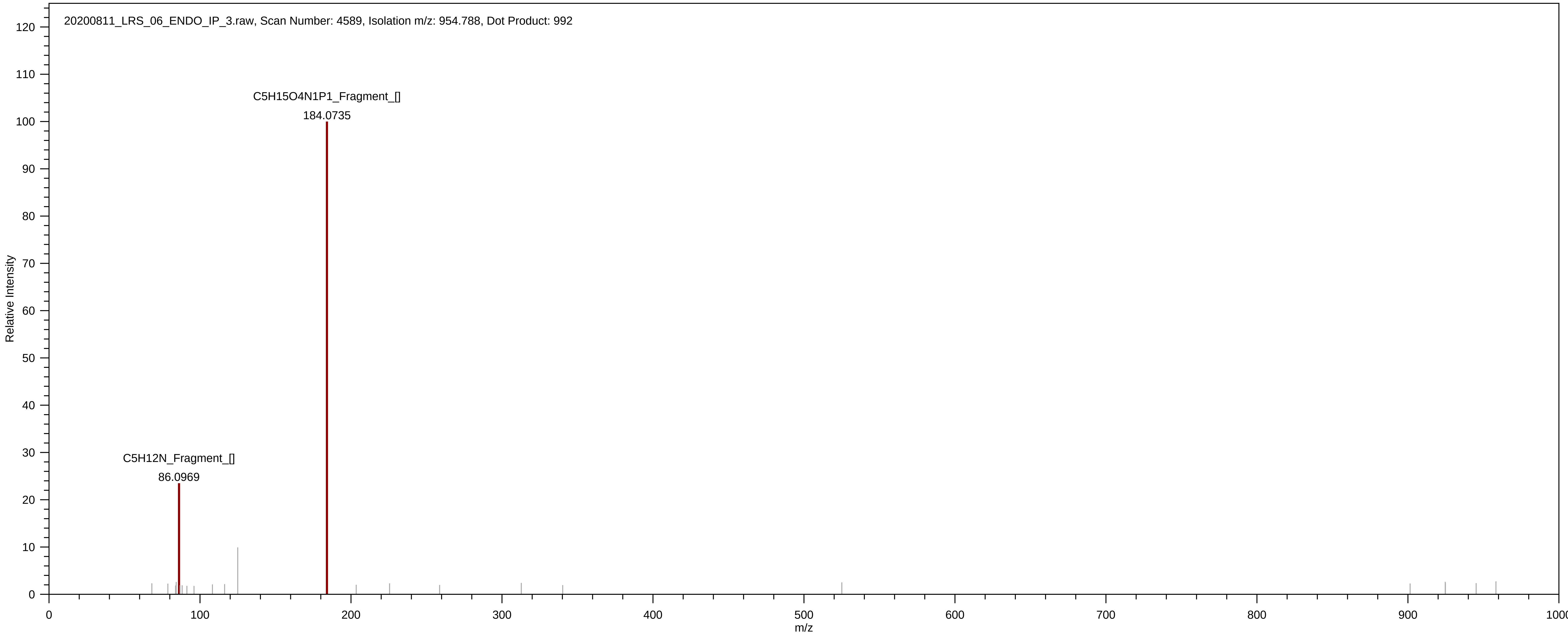

PC 48:2 [M+H]+ | Ret. Time (min): 15.72 | m/z: 954.789

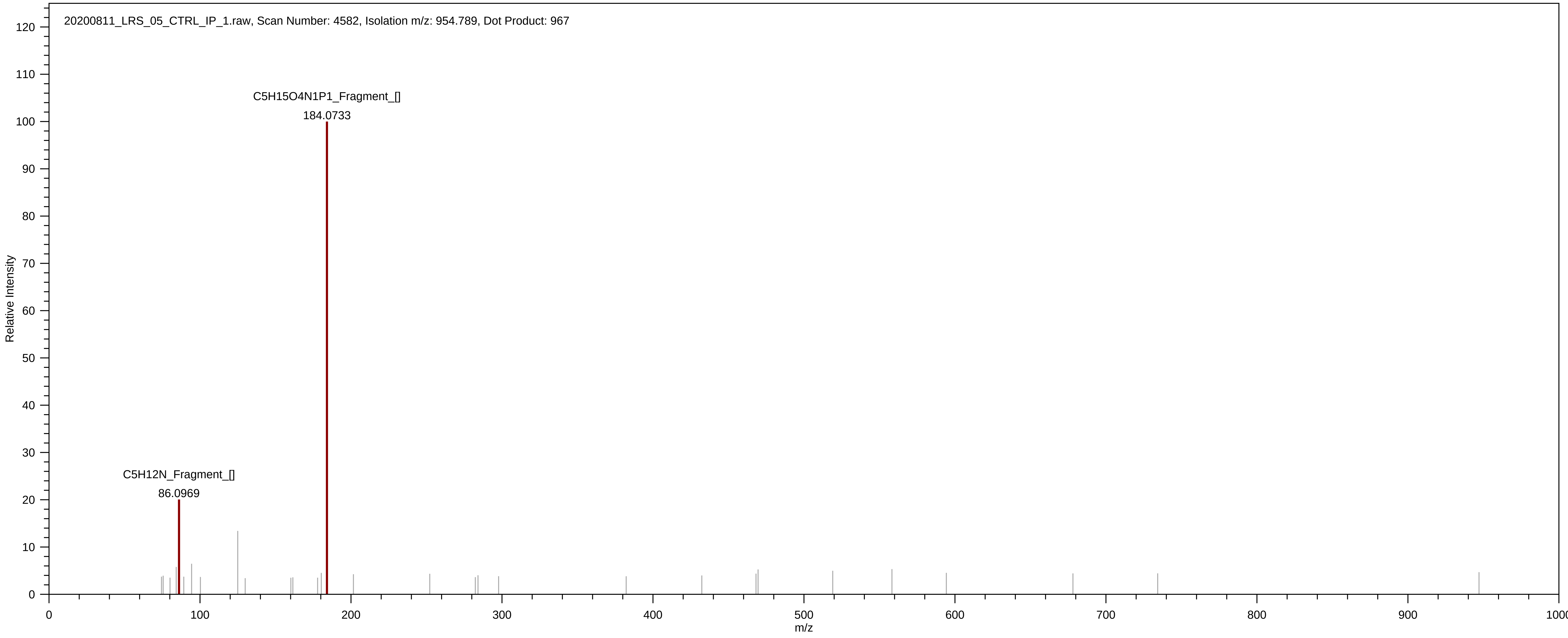

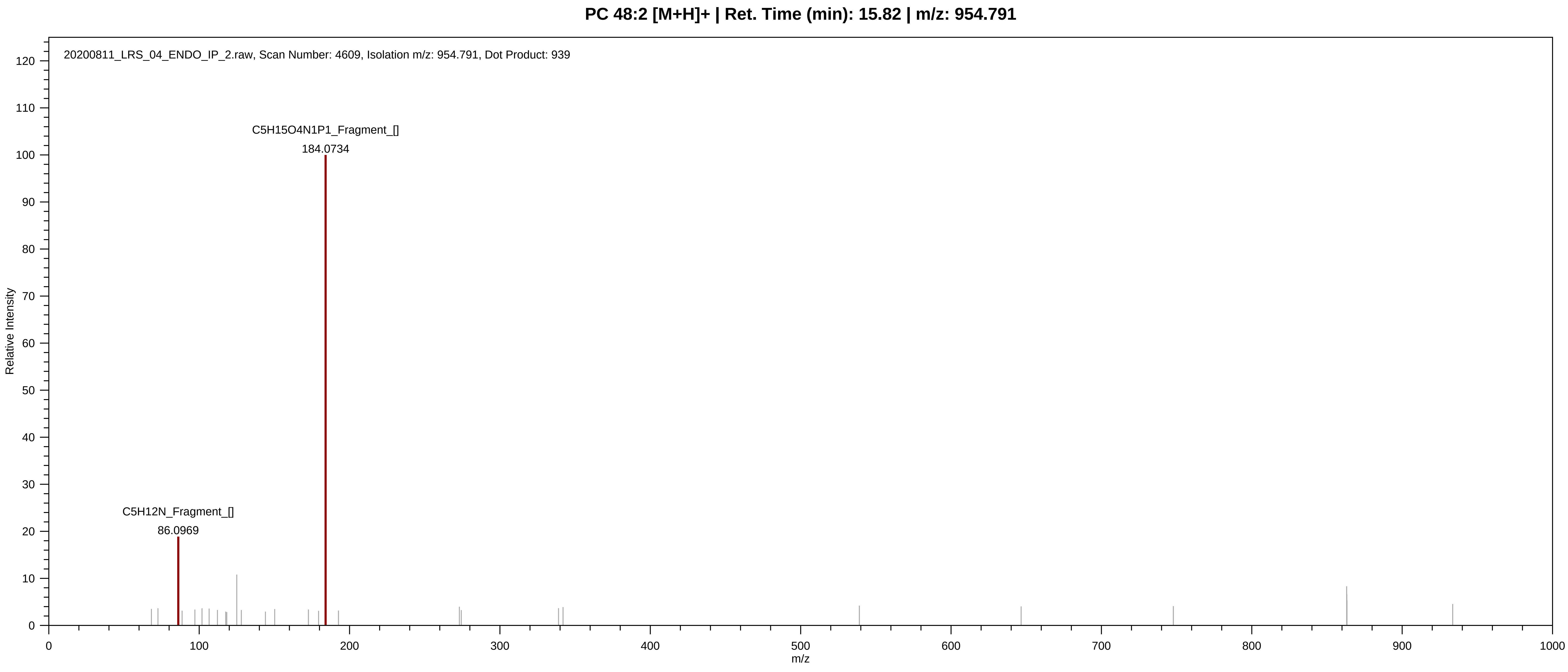
PC 48:2 [M+H]+ | Ret. Time (min): 15.82 | m/z: 954.791
20200811_LRS_04_ENDO_IP_2.raw, Scan Number: 4609, Isolation m/z: 954.791, Dot Product: 939
C5H15O4N1P1_Fragment_[]
184.0734
C5H12N_Fragment_[]
86.0969
Relative Intensity
m/z

PC 49:2 [M+H]+ | Ret. Time (min): 16.147 | m/z: 968.804

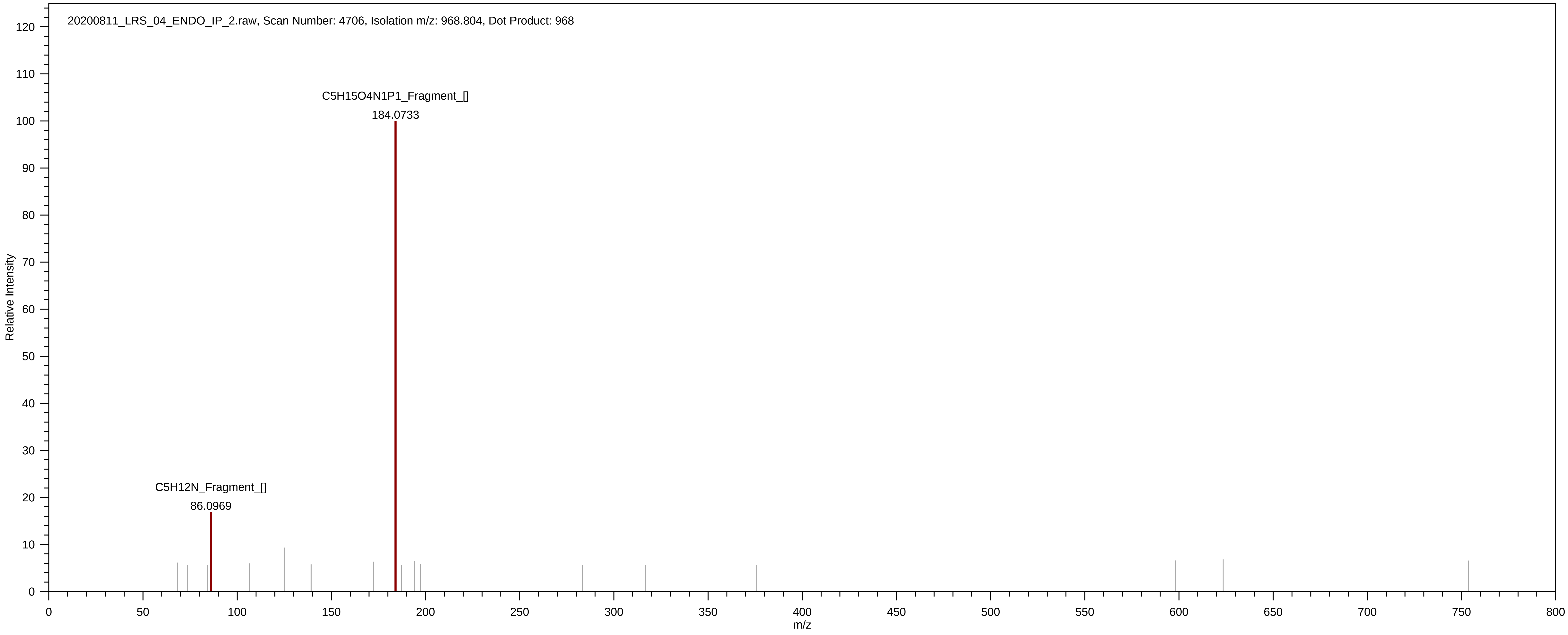

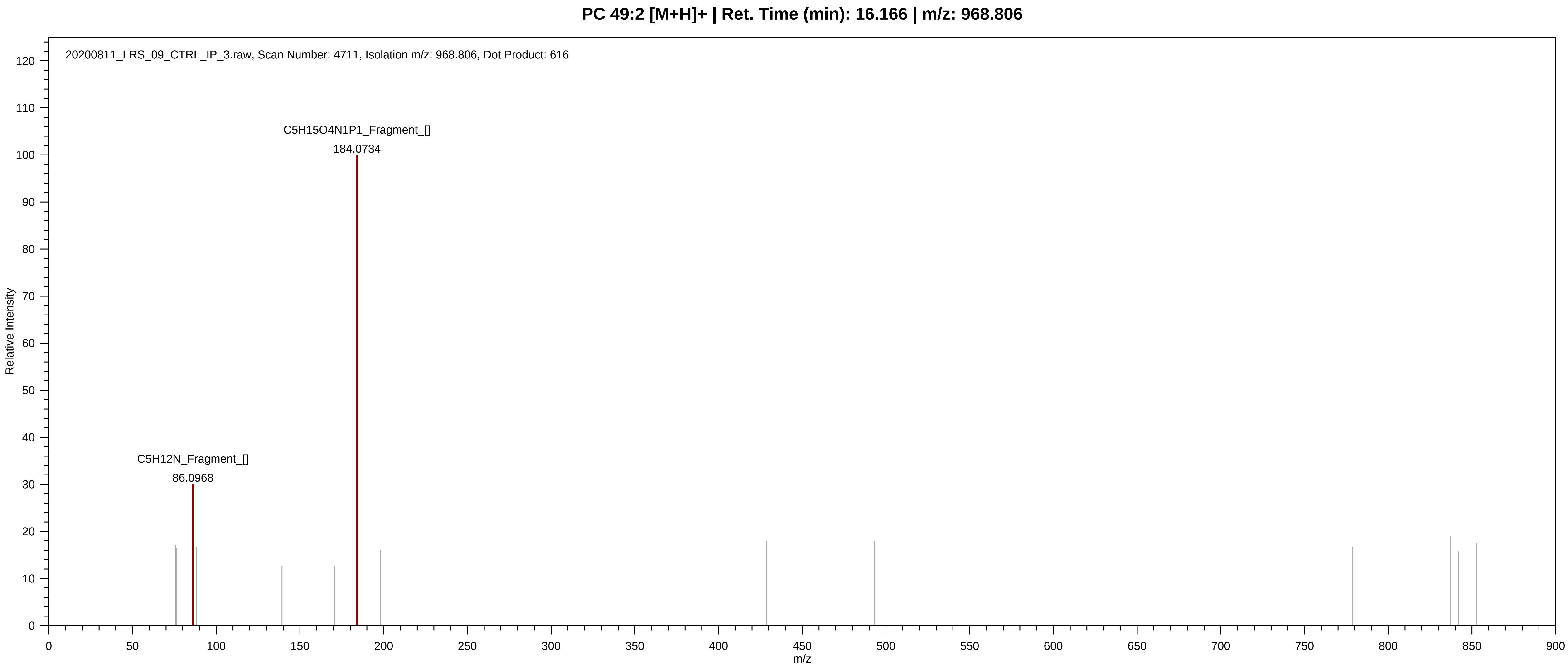
PC 49:2 [M+H]+ | Ret. Time (min): 16.166 | m/z: 968.806
20200811_LRS_09_CTRL_IP_3.raw, Scan Number: 4711, Isolation m/z: 968.806, Dot Product: 616
C5H15O4N1P1_Fragment_[]
184.0734
C5H12N_Fragment_[]
86.0968
Relative Intensity
m/z
0
10
20
30
40
50
60
70
80
90
100
110
120
0
50
100
150
200
250
300
350
400
450
500
550
600
650
700
750
800
850
900

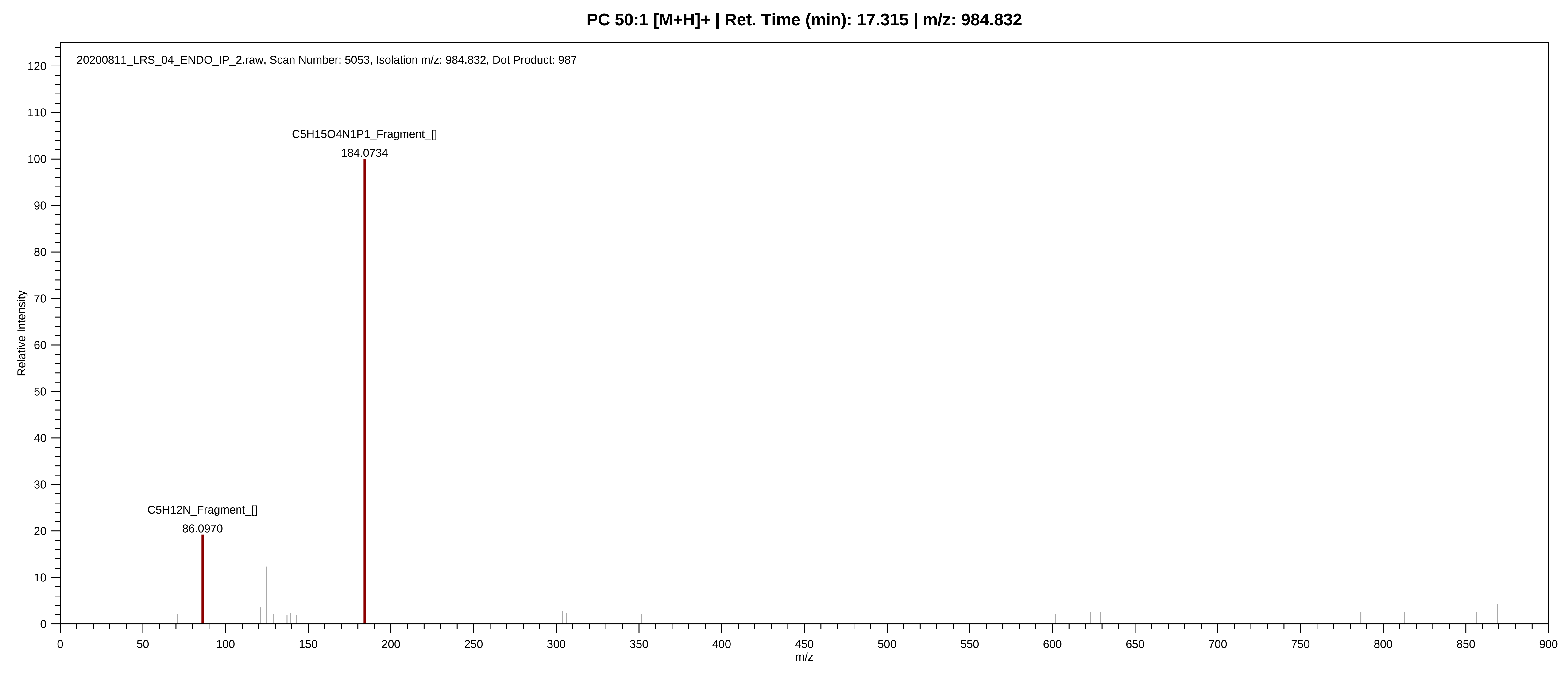
PC 50:1 [M+H]+ | Ret. Time (min): 17.315 | m/z: 984.832
20200811_LRS_04_ENDO_IP_2.raw, Scan Number: 5053, Isolation m/z: 984.832, Dot Product: 987
C5H15O4N1P1_Fragment_[]
184.0734
C5H12N_Fragment_[]
86.0970
Relative Intensity
m/z

PC 50:1 [M+H]+ | Ret. Time (min): 17.37 | m/z: 984.835

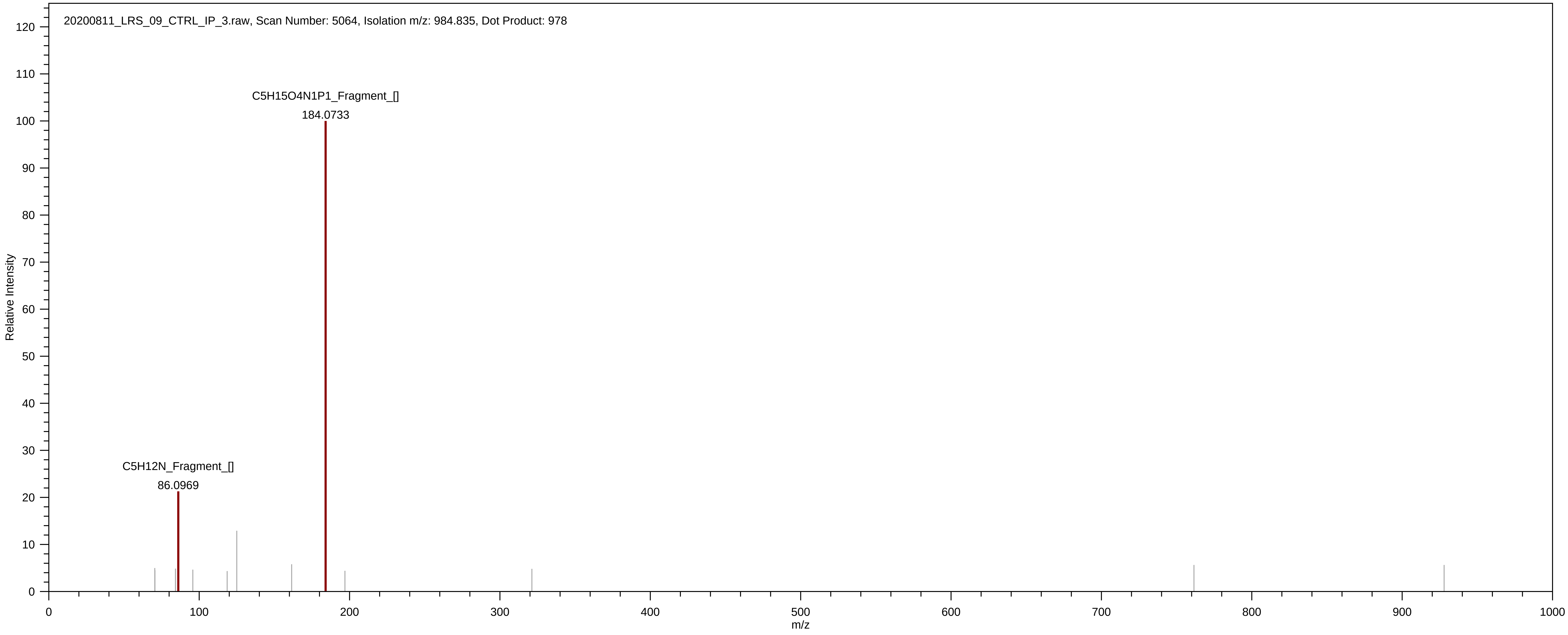

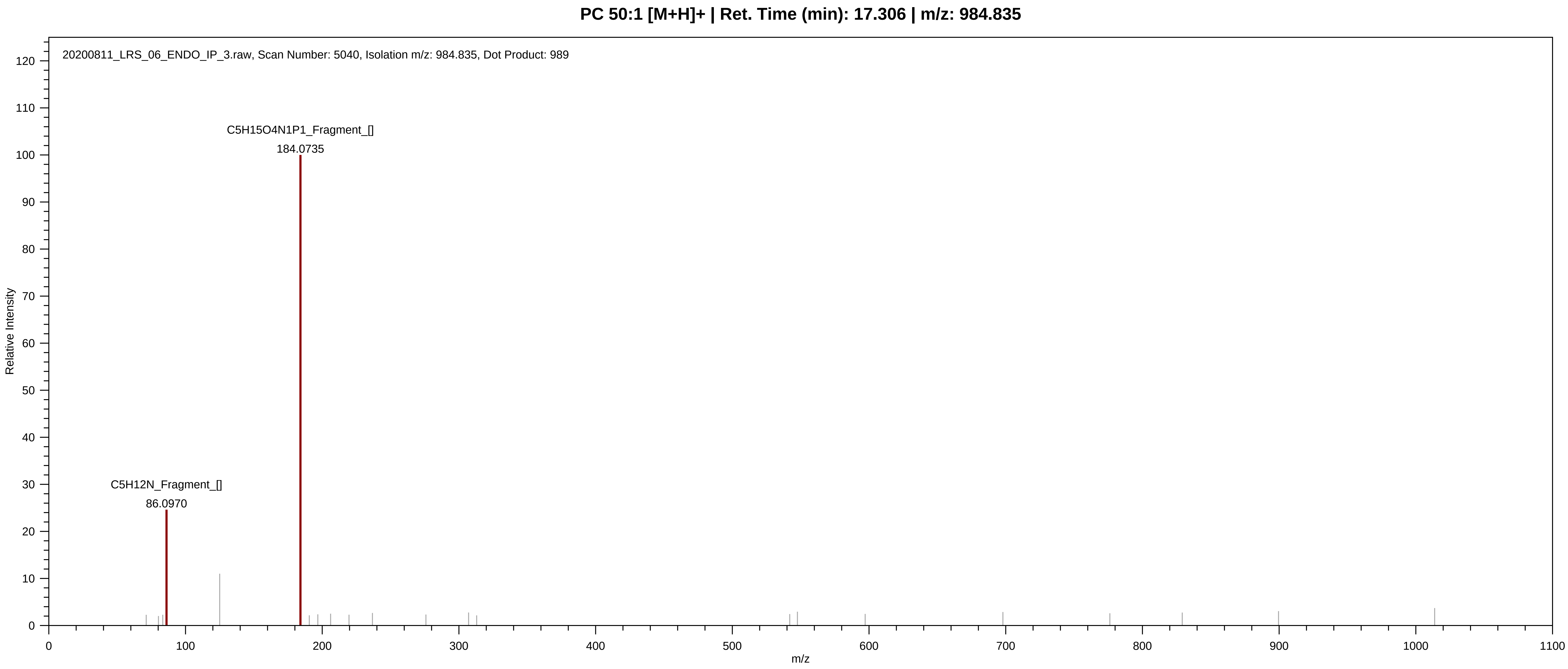

PC 50:1 [M+H]+ | Ret. Time (min): 17.306 | m/z: 984.835
20200811_LRS_06_ENDO_IP_3.raw, Scan Number: 5040, Isolation m/z: 984.835, Dot Product: 989
C5H15O4N1P1_Fragment_[]
184.0735
C5H12N_Fragment_[]
86.0970
Relative Intensity
m/z

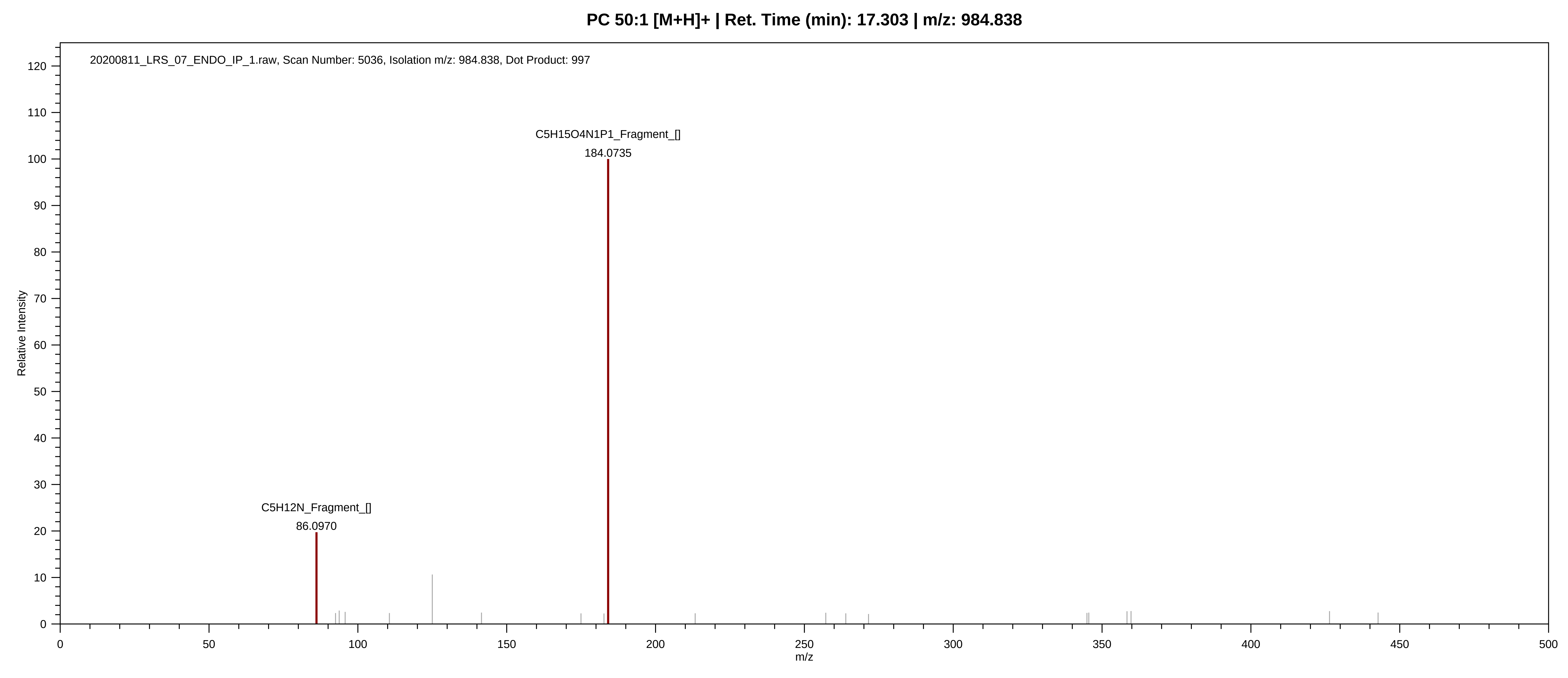

PC 50:1 [M+H]+ | Ret. Time (min): 17.303 | m/z: 984.838
20200811_LRS_07_ENDO_IP_1.raw, Scan Number: 5036, Isolation m/z: 984.838, Dot Product: 997
C5H15O4N1P1_Fragment_[]
184.0735
C5H12N_Fragment_[]
86.0970
Relative Intensity
m/z

PC 50:1 [M+H]+ | Ret. Time (min): 17.486 | m/z: 984.839

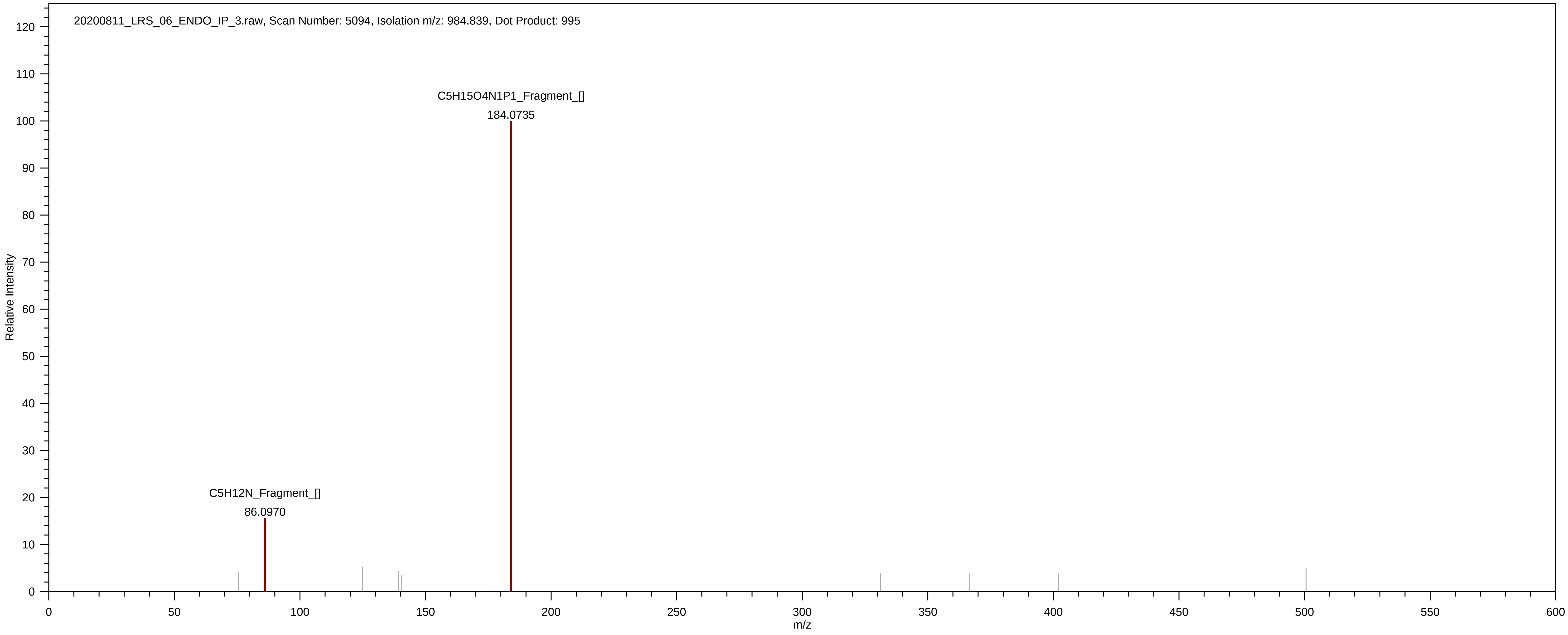

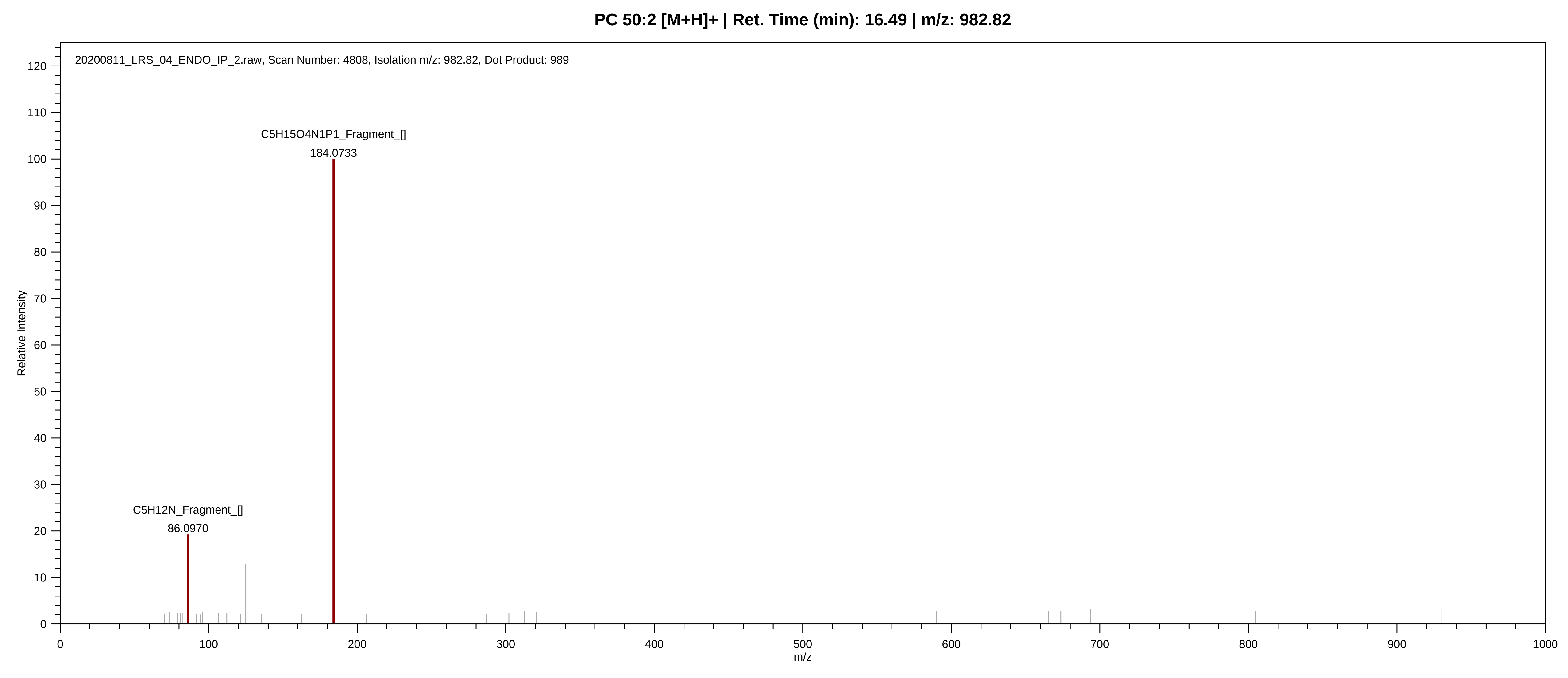

PC 50:2 [M+H]+ | Ret. Time (min): 16.49 | m/z: 982.82
20200811_LRS_04_ENDO_IP_2.raw, Scan Number: 4808, Isolation m/z: 982.82, Dot Product: 989
C5H15O4N1P1_Fragment_[]
184.0733
C5H12N_Fragment_[]
86.0970
Relative Intensity
m/z

PC 50:2 [M+H]+ | Ret. Time (min): 16.657 | m/z: 982.82

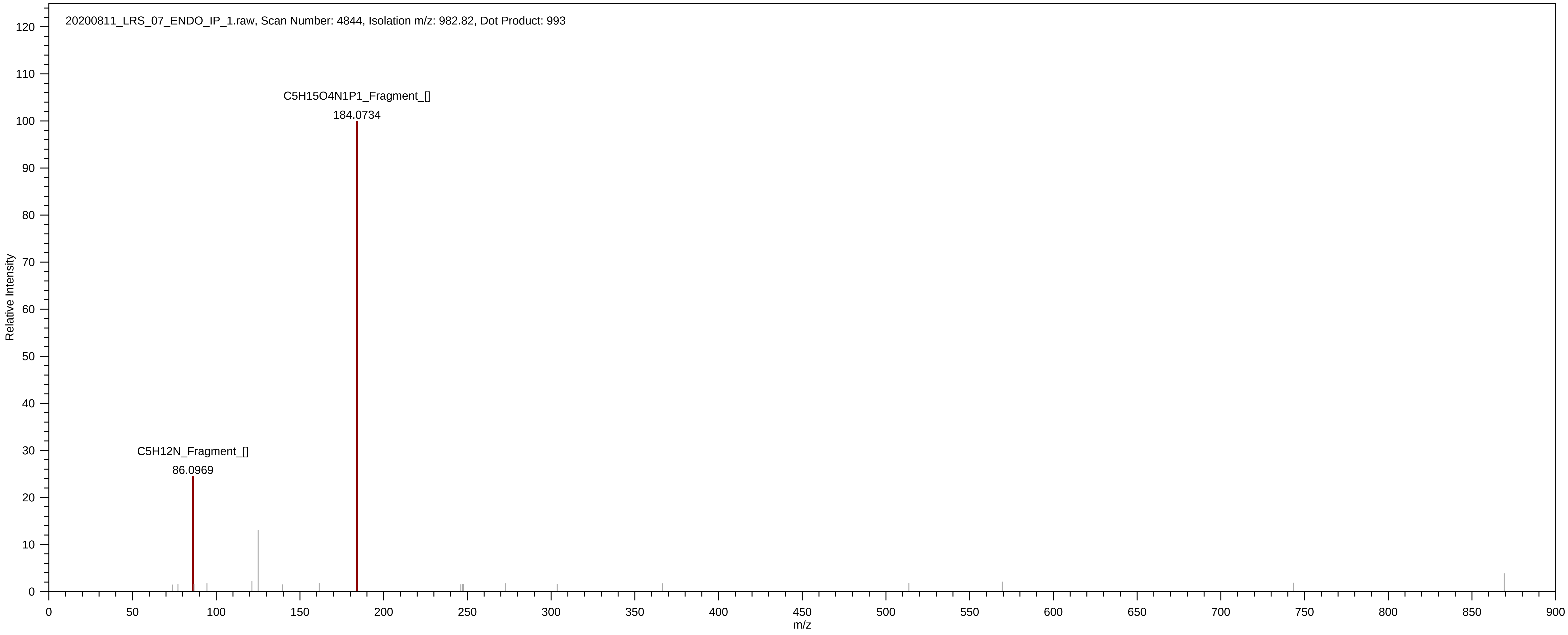

PC 50:2 [M+H]+ | Ret. Time (min): 16.659 | m/z: 982.82

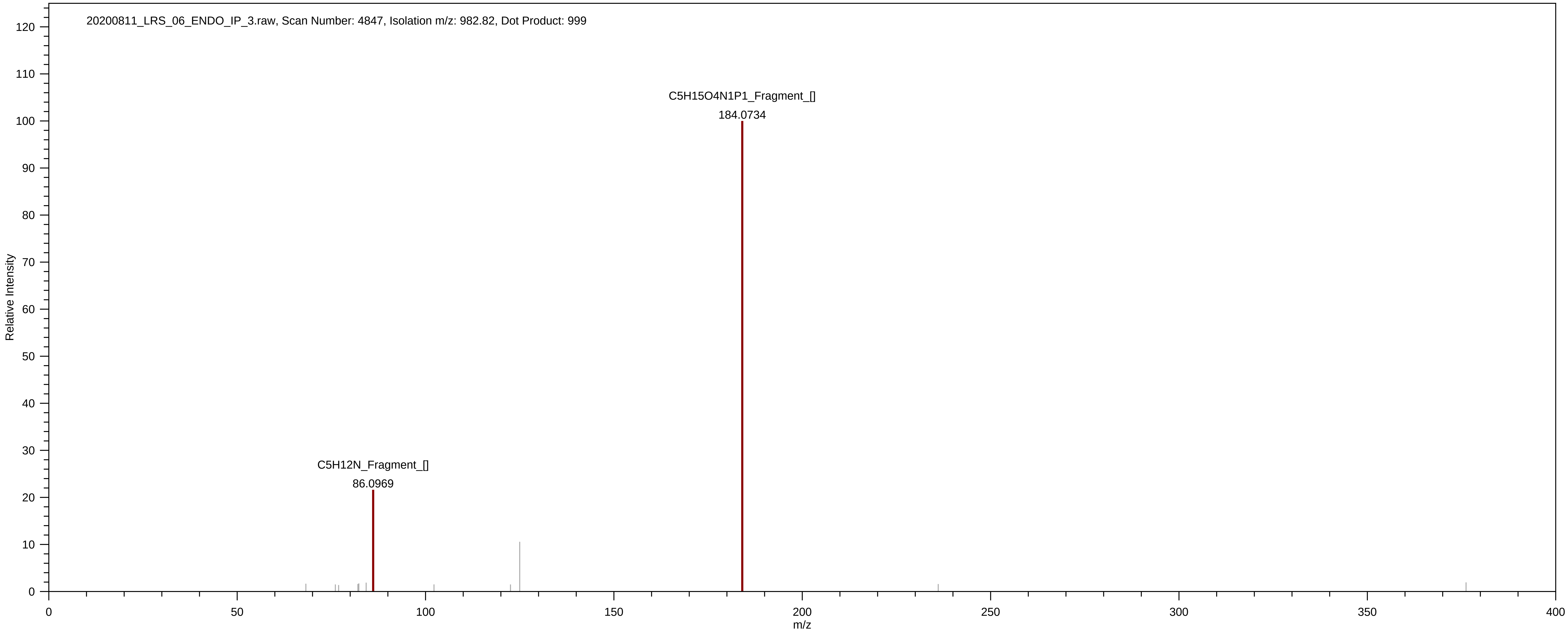

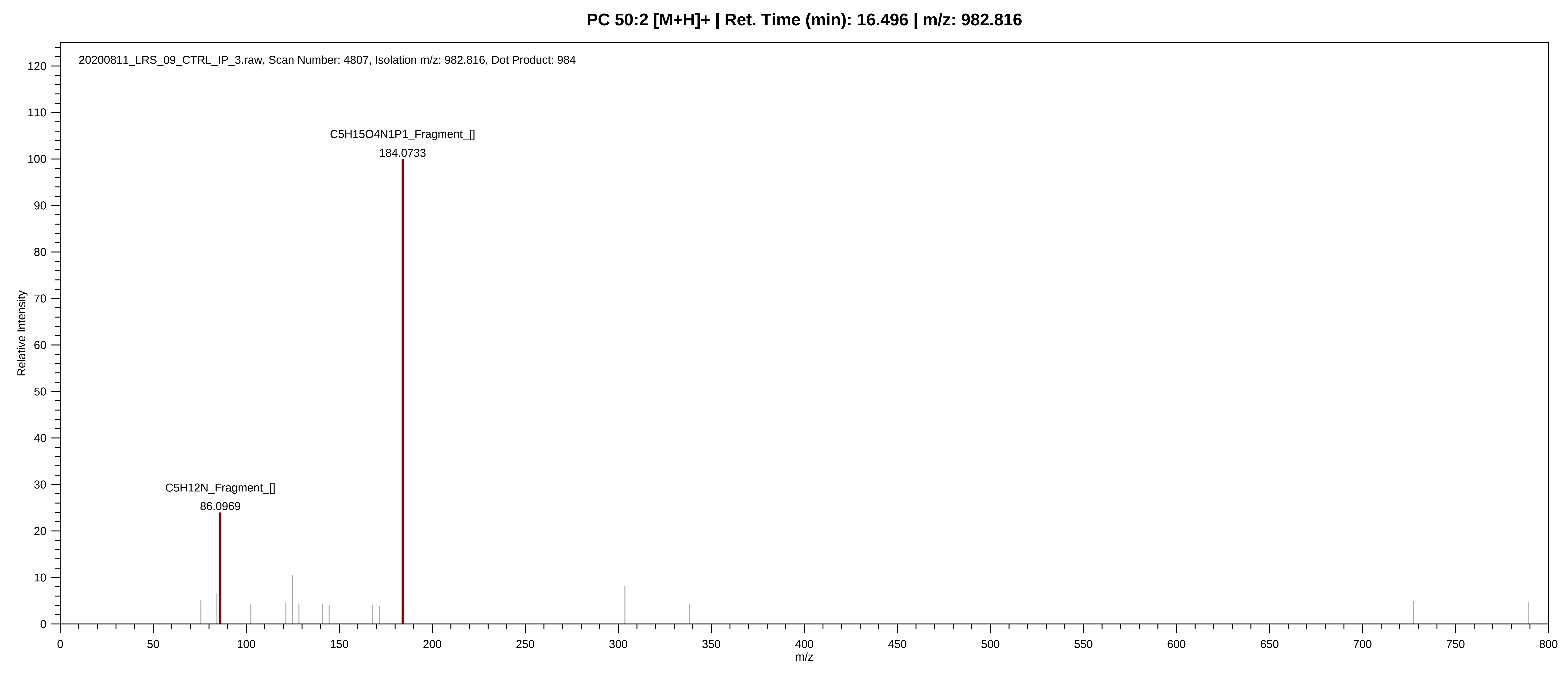

PC 50:2 [M+H]+ | Ret. Time (min): 16.496 | m/z: 982.816
20200811_LRS_09_CTRL_IP_3.raw, Scan Number: 4807, Isolation m/z: 982.816, Dot Product: 984
C5H15O4N1P1_Fragment_[]
184.0733
C5H12N_Fragment_[]
86.0969
Relative Intensity
m/z

PC 50:2 [M+H]+ | Ret. Time (min): 16.688 | m/z: 982.816

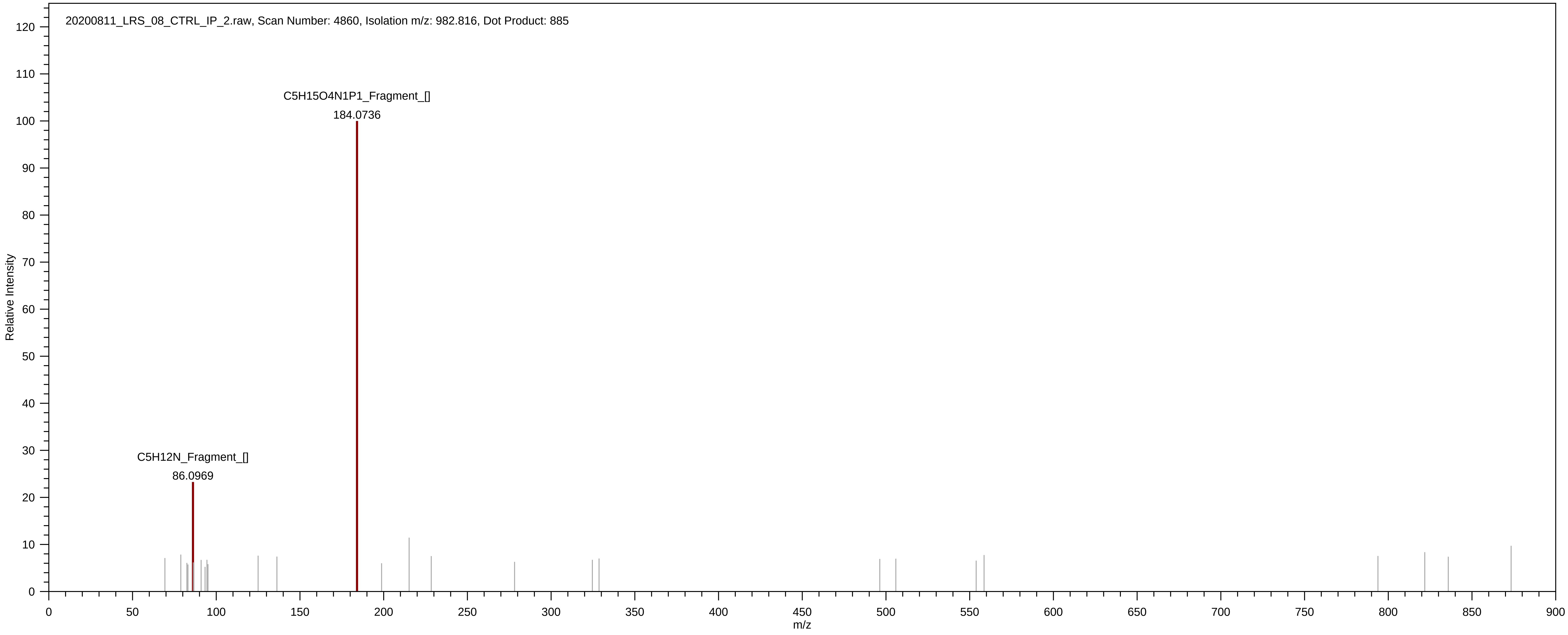

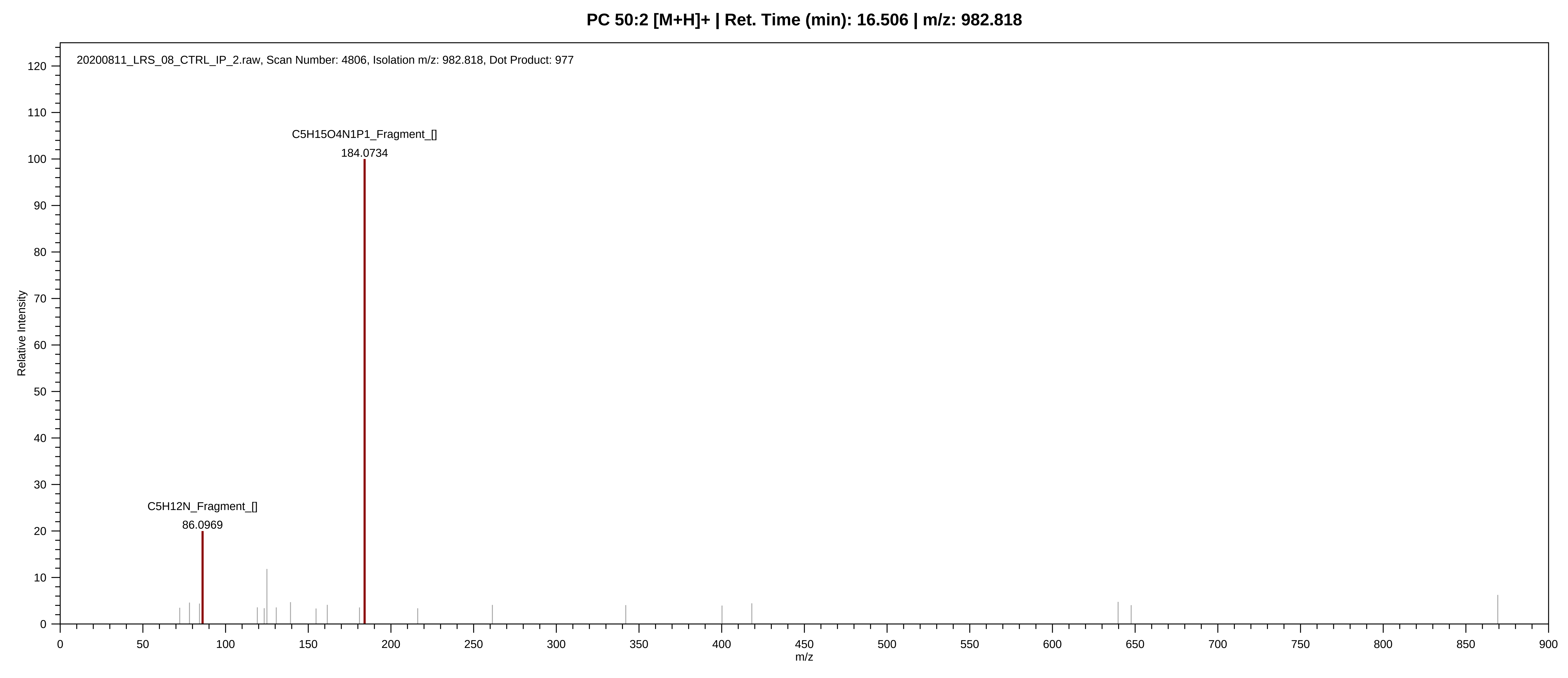

PC 50:2 [M+H]+ | Ret. Time (min): 16.506 | m/z: 982.818
20200811_LRS_08_CTRL_IP_2.raw, Scan Number: 4806, Isolation m/z: 982.818, Dot Product: 977
C5H15O4N1P1_Fragment_[]
184.0734
C5H12N_Fragment_[]
86.0969
Relative Intensity
m/z

PC 50:2 [M+H]+ | Ret. Time (min): 16.48 | m/z: 982.819

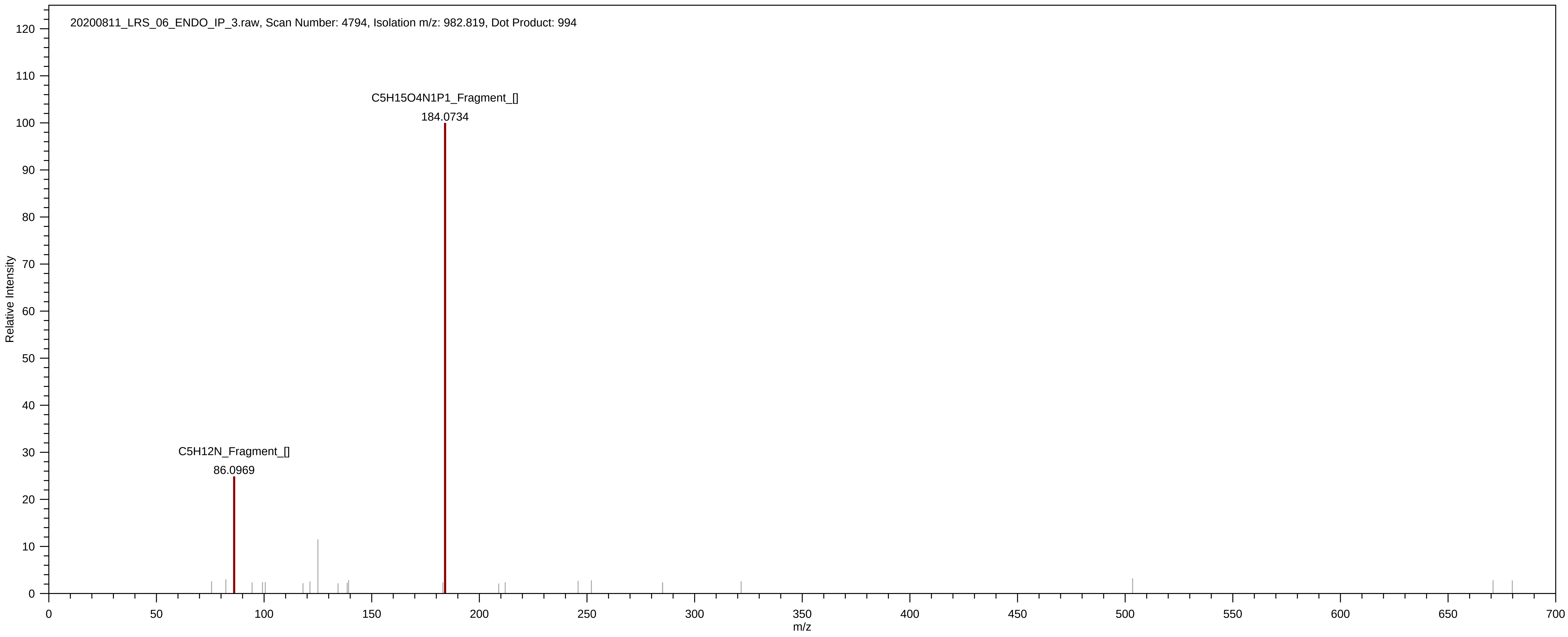

PC 50:2 [M+H]+ | Ret. Time (min): 16.669 | m/z: 982.819

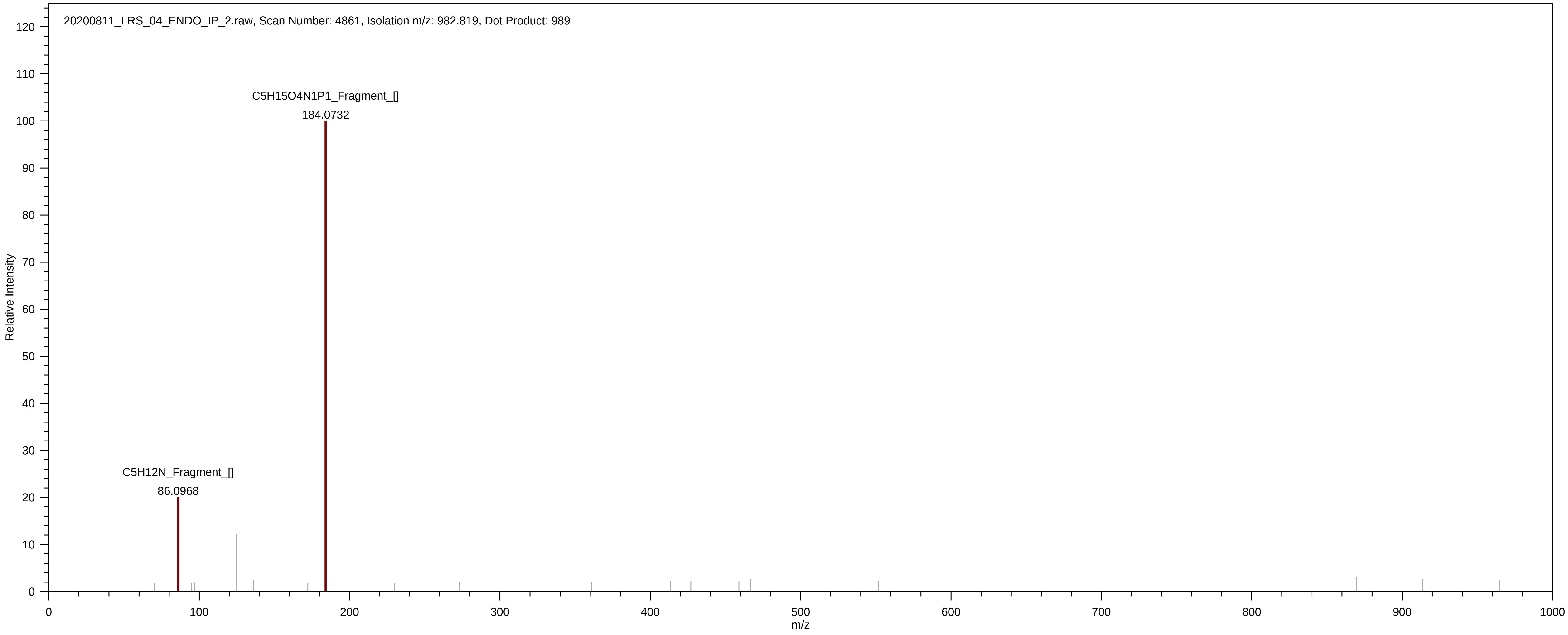

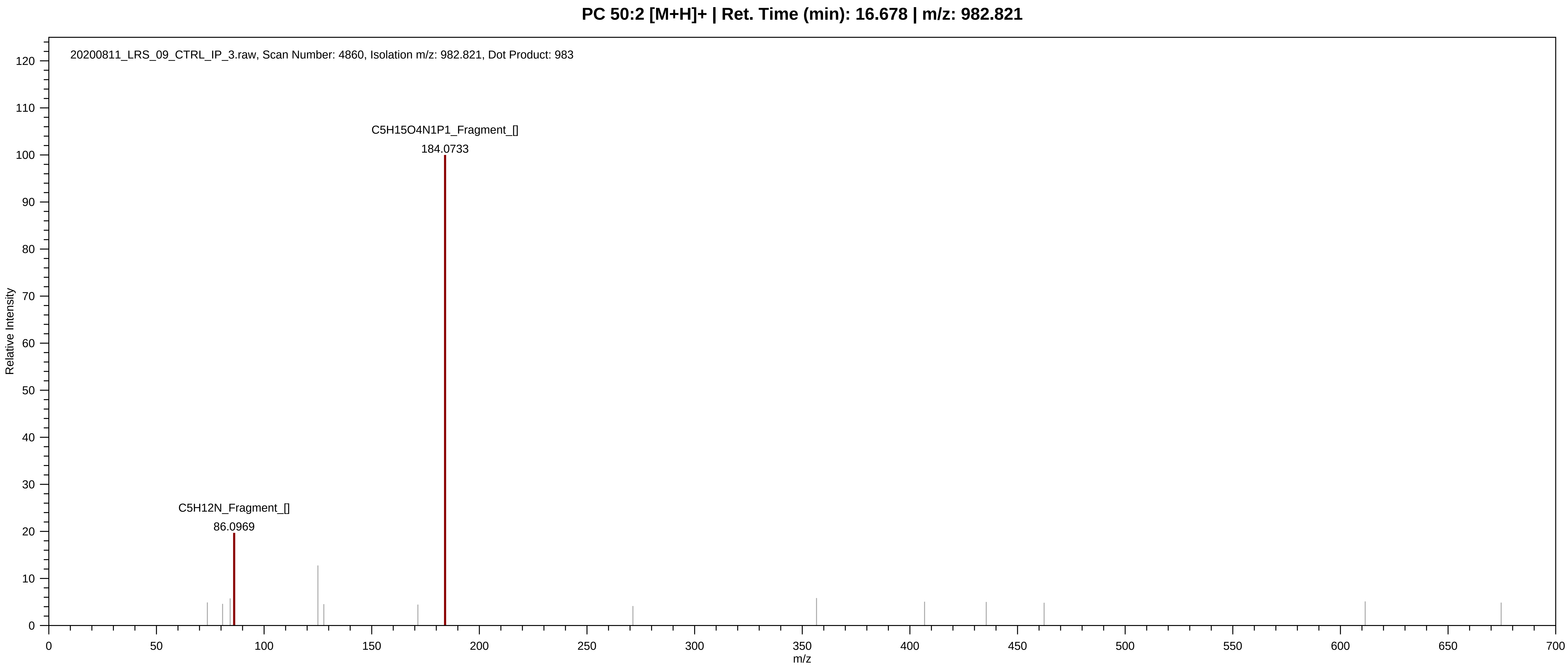

PC 50:2 [M+H]+ | Ret. Time (min): 16.678 | m/z: 982.821
20200811_LRS_09_CTRL_IP_3.raw, Scan Number: 4860, Isolation m/z: 982.821, Dot Product: 983
C5H15O4N1P1_Fragment_[]
184.0733
C5H12N_Fragment_[]
86.0969
Relative Intensity
m/z

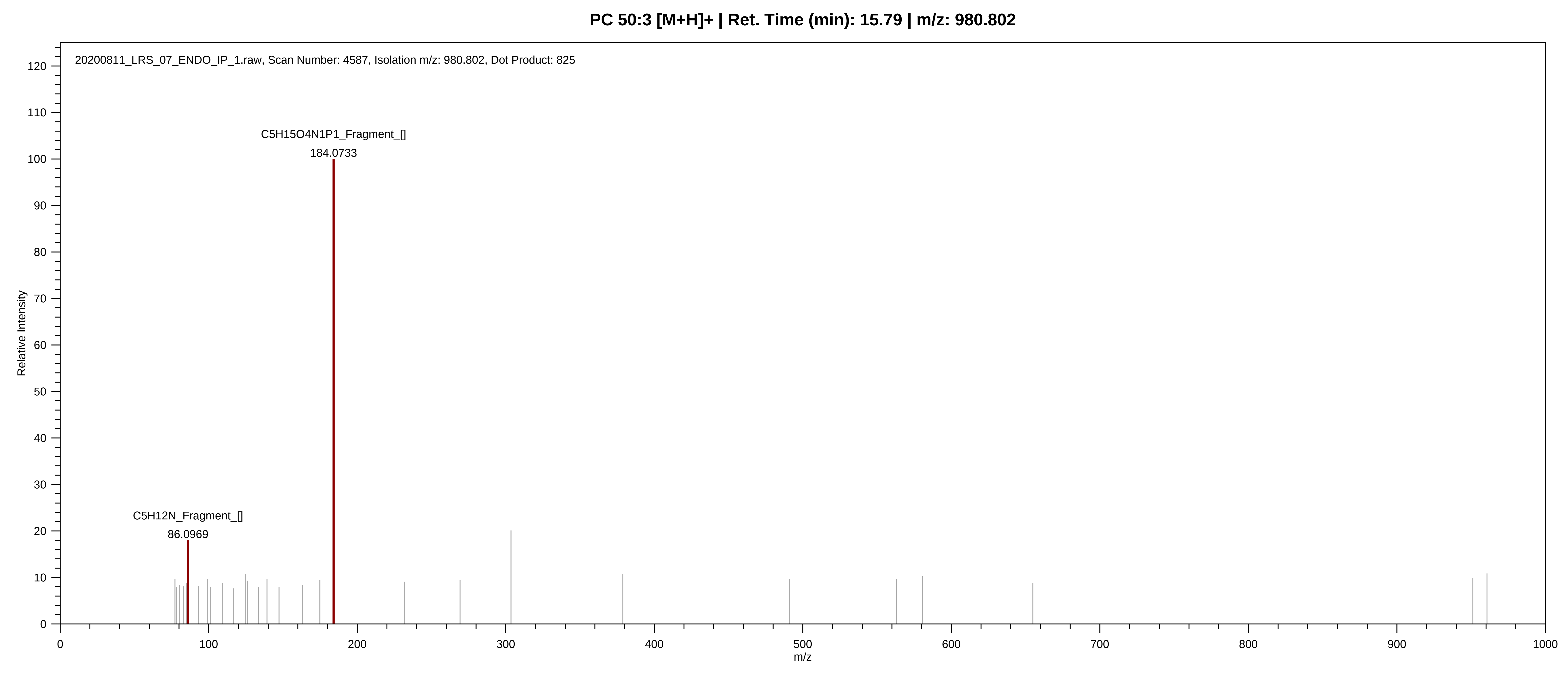
PC 50:3 [M+H]+ | Ret. Time (min): 15.79 | m/z: 980.802
20200811_LRS_07_ENDO_IP_1.raw, Scan Number: 4587, Isolation m/z: 980.802, Dot Product: 825
C5H15O4N1P1_Fragment_[]
184.0733
C5H12N_Fragment_[]
86.0969
Relative Intensity
m/z

PC 50:3 [M+H]+ | Ret. Time (min): 15.828 | m/z: 980.803

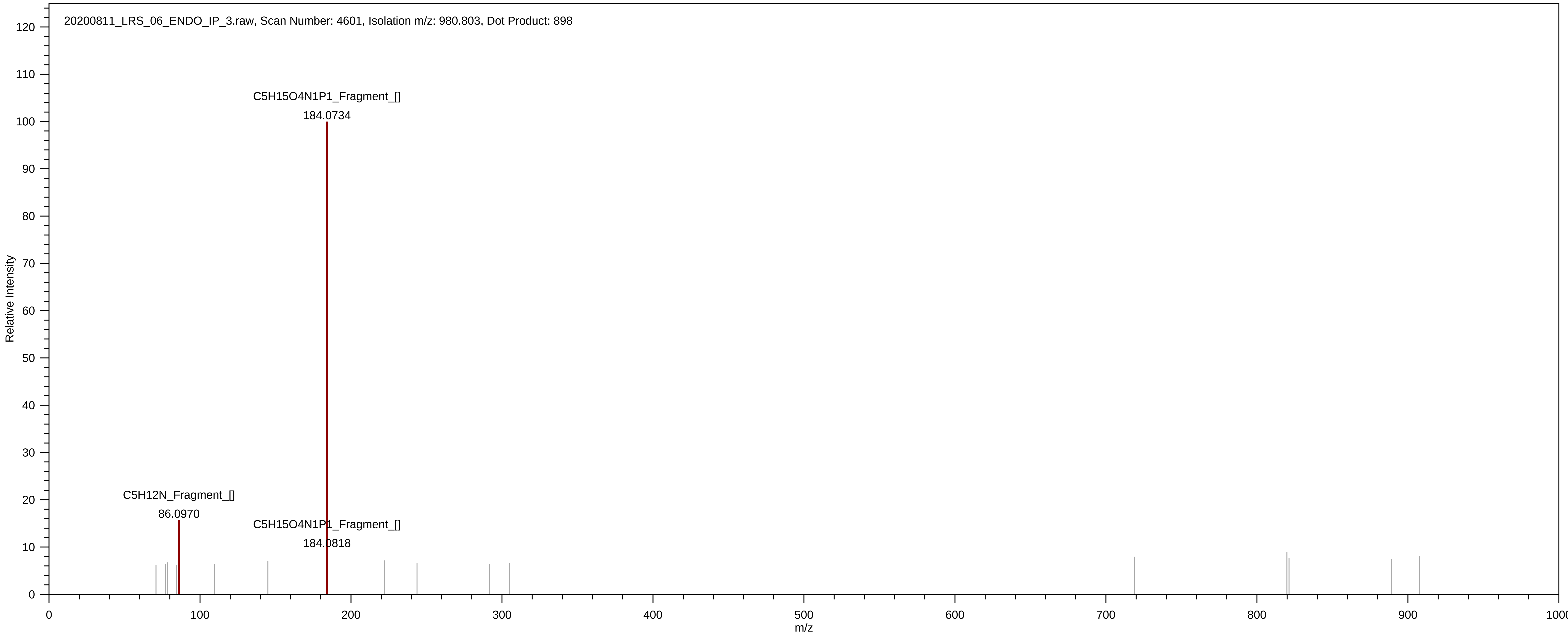

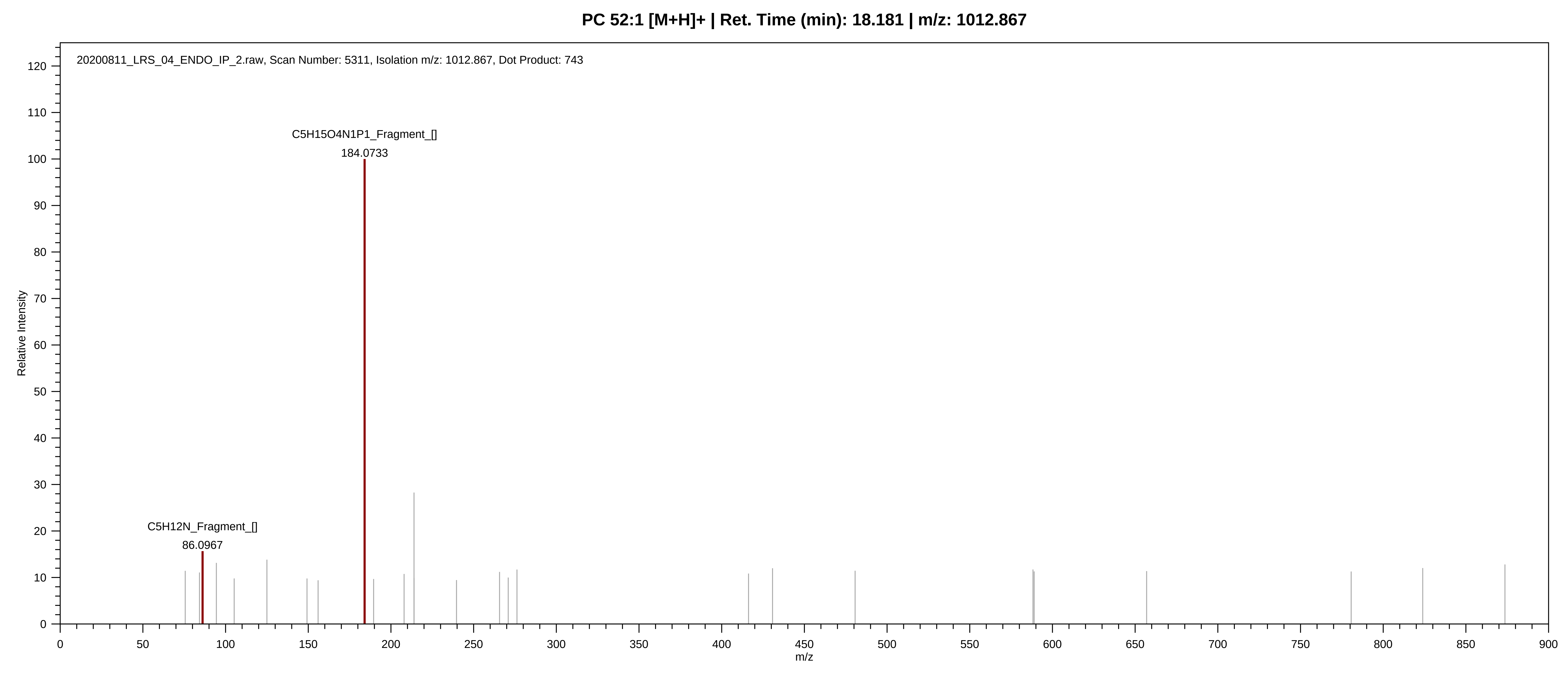
PC 52:1 [M+H]+ | Ret. Time (min): 18.181 | m/z: 1012.867
20200811_LRS_04_ENDO_IP_2.raw, Scan Number: 5311, Isolation m/z: 1012.867, Dot Product: 743
C5H15O4N1P1_Fragment_[]
184.0733
C5H12N_Fragment_[]
86.0967
Relative Intensity
m/z

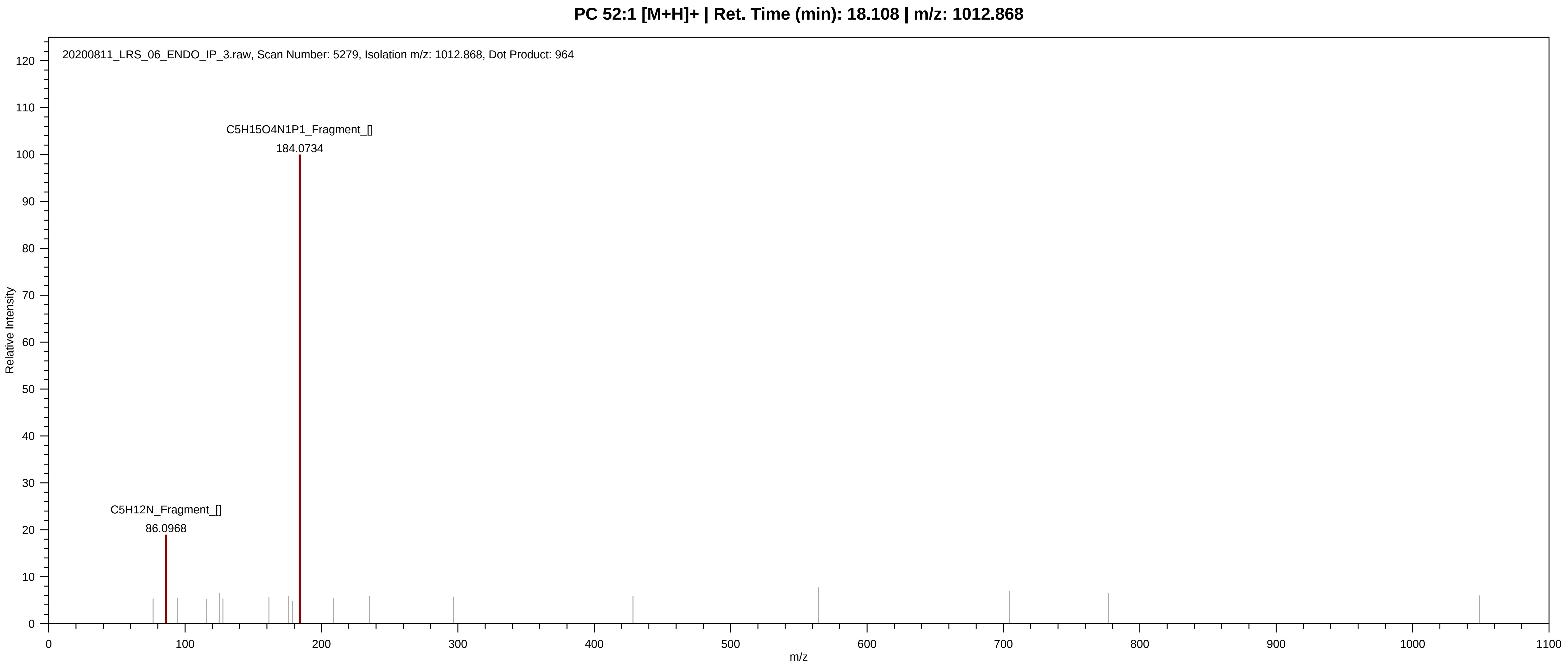
PC 52:1 [M+H]+ | Ret. Time (min): 18.108 | m/z: 1012.868
20200811_LRS_06_ENDO_IP_3.raw, Scan Number: 5279, Isolation m/z: 1012.868, Dot Product: 964
C5H15O4N1P1_Fragment_[]
184.0734
C5H12N_Fragment_[]
86.0968
Relative Intensity
m/z
0
10
20
30
40
50
60
70
80
90
100
110
120
0
100
200
300
400
500
600
700
800
900
1000
1100

PC 52:2 [M+H]+ | Ret. Time (min): 17.354 | m/z: 1010.849

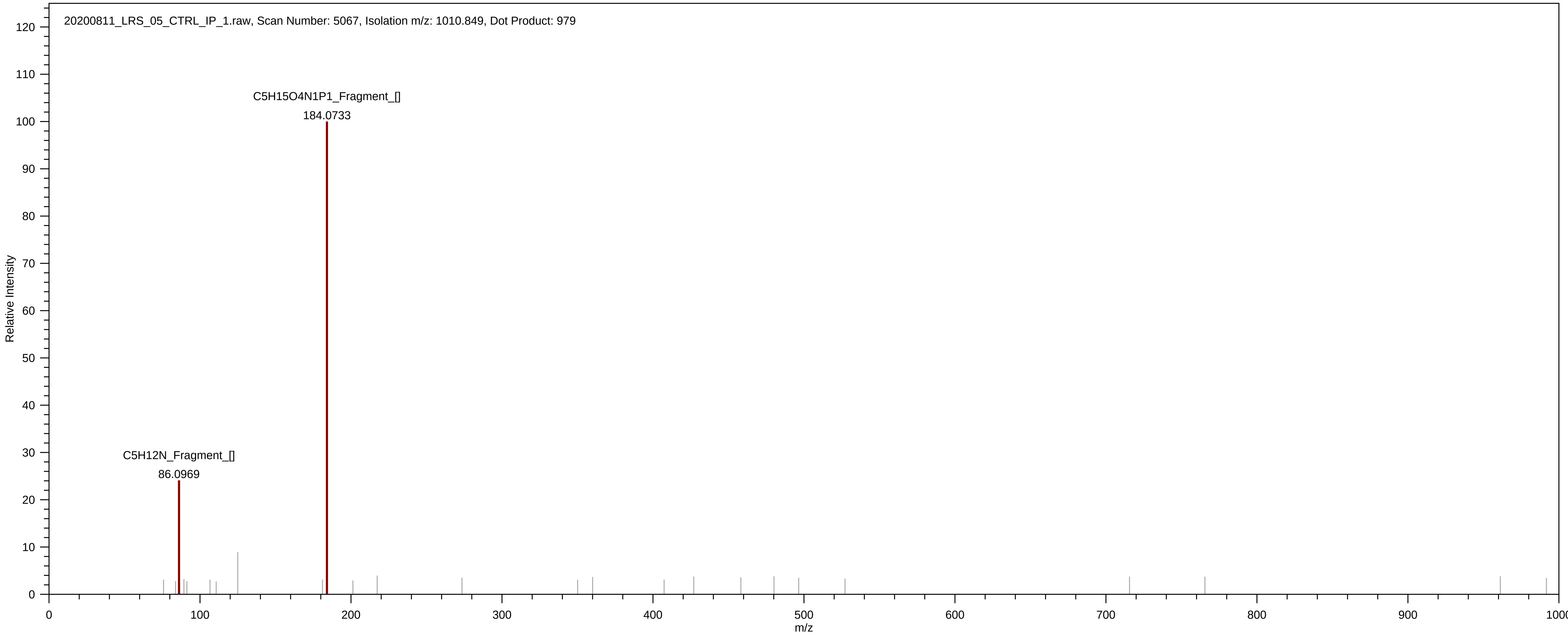

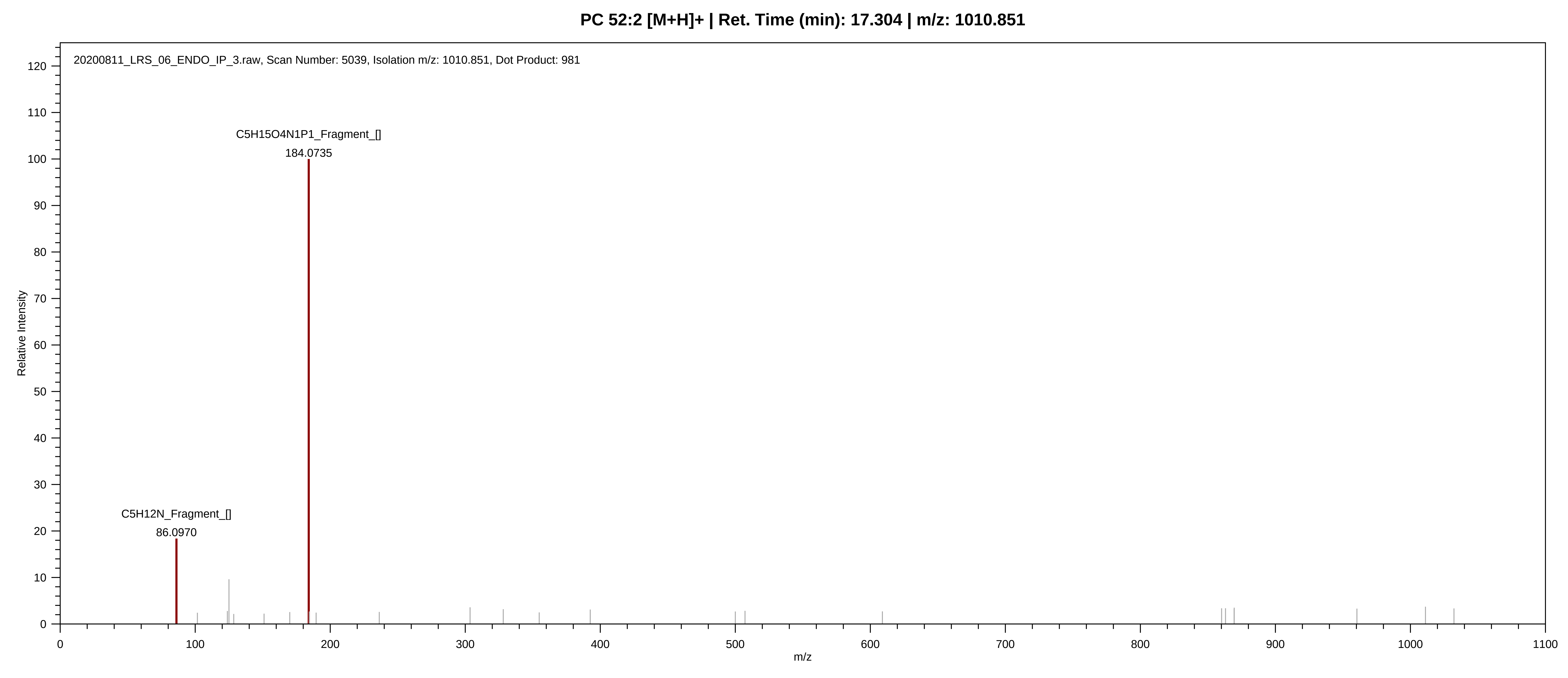
PC 52:2 [M+H]+ | Ret. Time (min): 17.304 | m/z: 1010.851
20200811_LRS_06_ENDO_IP_3.raw, Scan Number: 5039, Isolation m/z: 1010.851, Dot Product: 981
C5H15O4N1P1_Fragment_[]
184.0735
C5H12N_Fragment_[]
86.0970
Relative Intensity
m/z

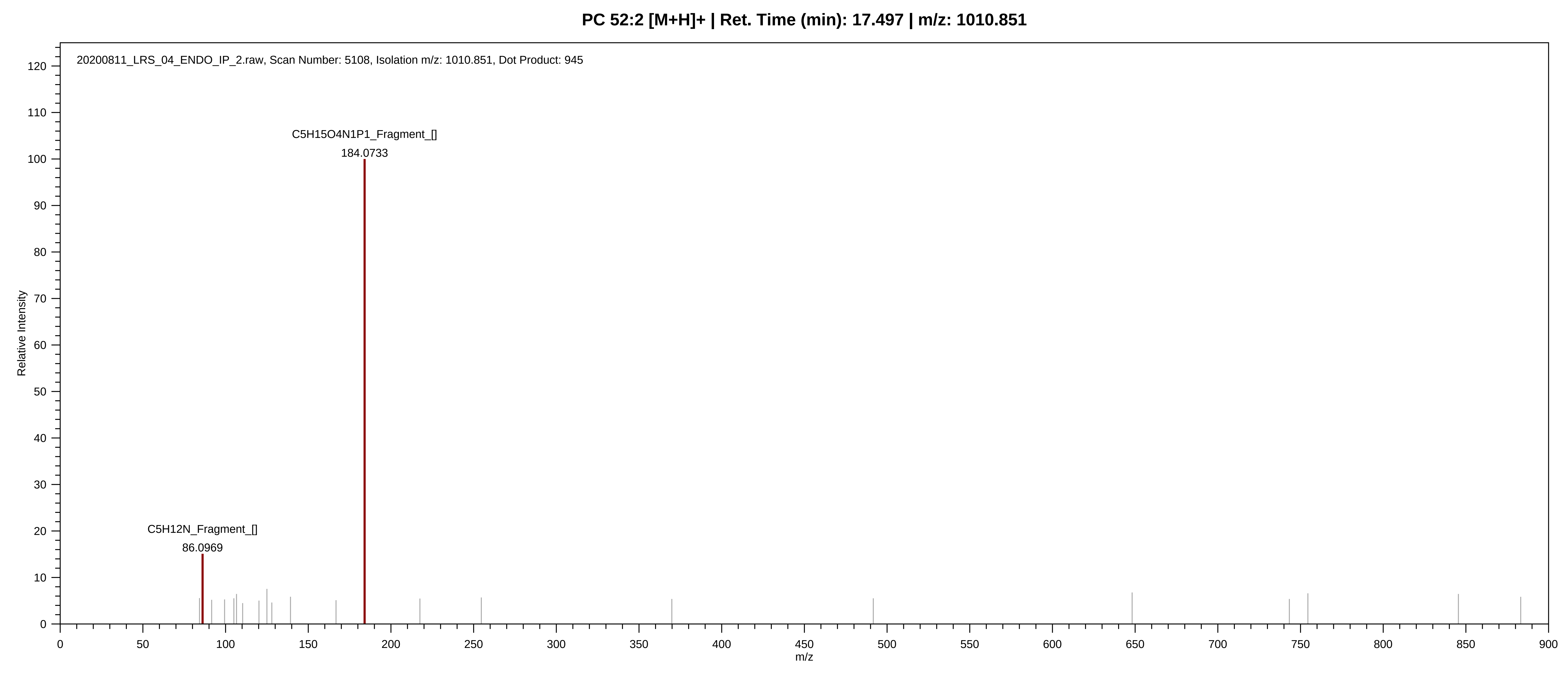
PC 52:2 [M+H]+ | Ret. Time (min): 17.497 | m/z: 1010.851
20200811_LRS_04_ENDO_IP_2.raw, Scan Number: 5108, Isolation m/z: 1010.851, Dot Product: 945
C5H15O4N1P1_Fragment_[]
184.0733
C5H12N_Fragment_[]
86.0969
Relative Intensity
m/z

PC 52:2 [M+H]+ | Ret. Time (min): 17.336 | m/z: 1010.852

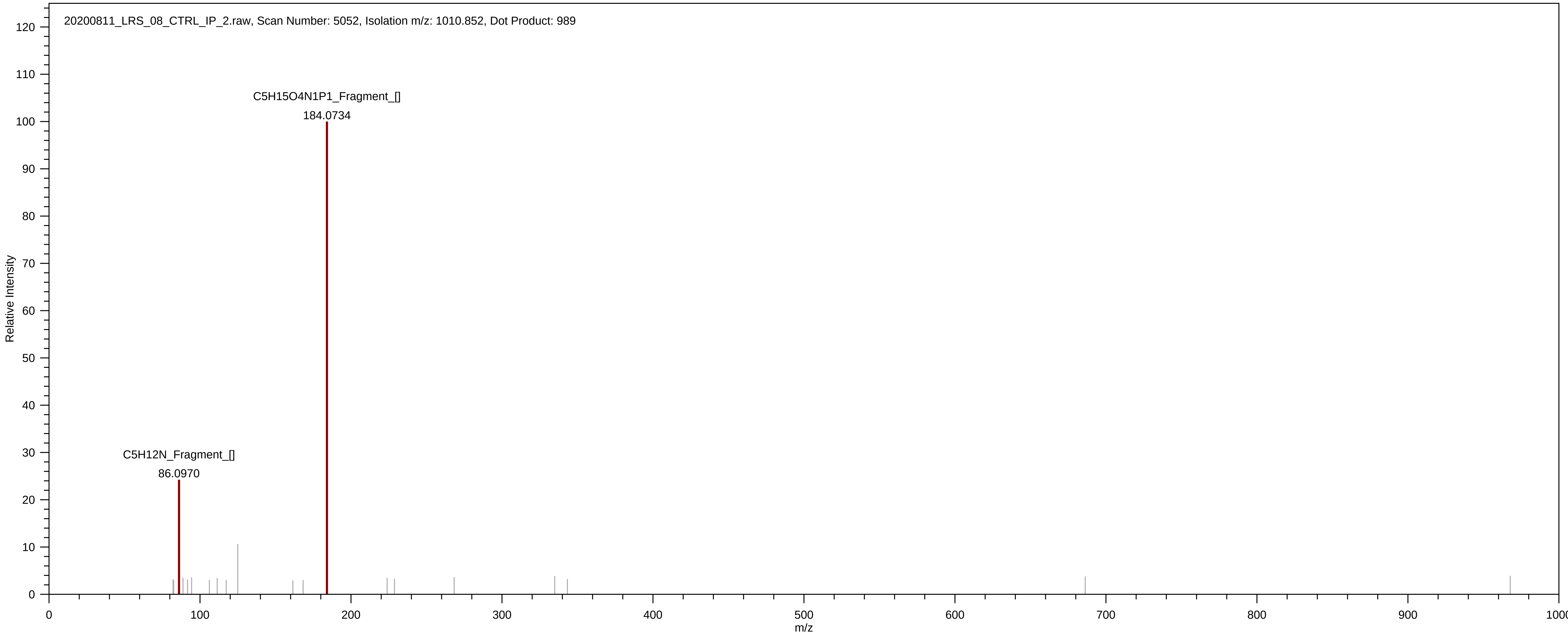

PC 52:2 [M+H]+ | Ret. Time (min): 17.305 | m/z: 1010.853

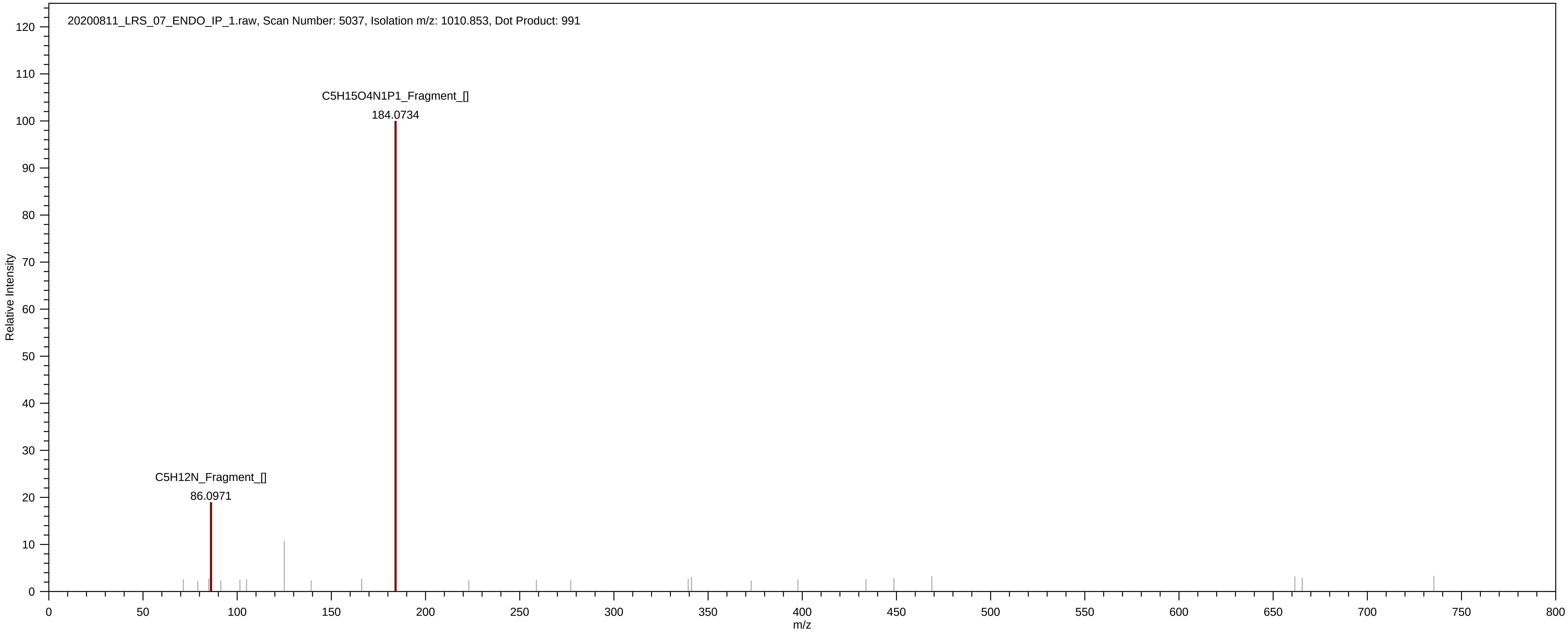

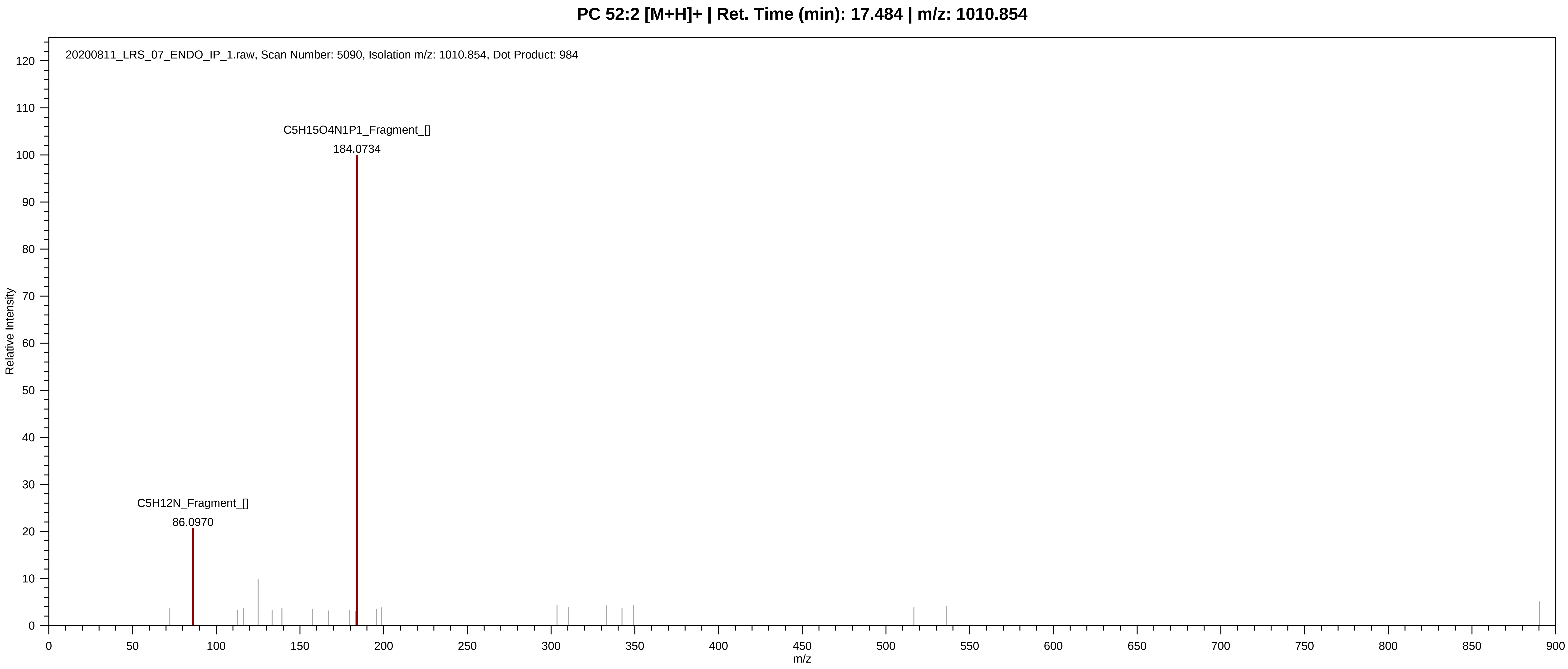
PC 52:2 [M+H]+ | Ret. Time (min): 17.484 | m/z: 1010.854
20200811_LRS_07_ENDO_IP_1.raw, Scan Number: 5090, Isolation m/z: 1010.854, Dot Product: 984
C5H15O4N1P1_Fragment_[]
184.0734
C5H12N_Fragment_[]
86.0970
Relative Intensity
m/z

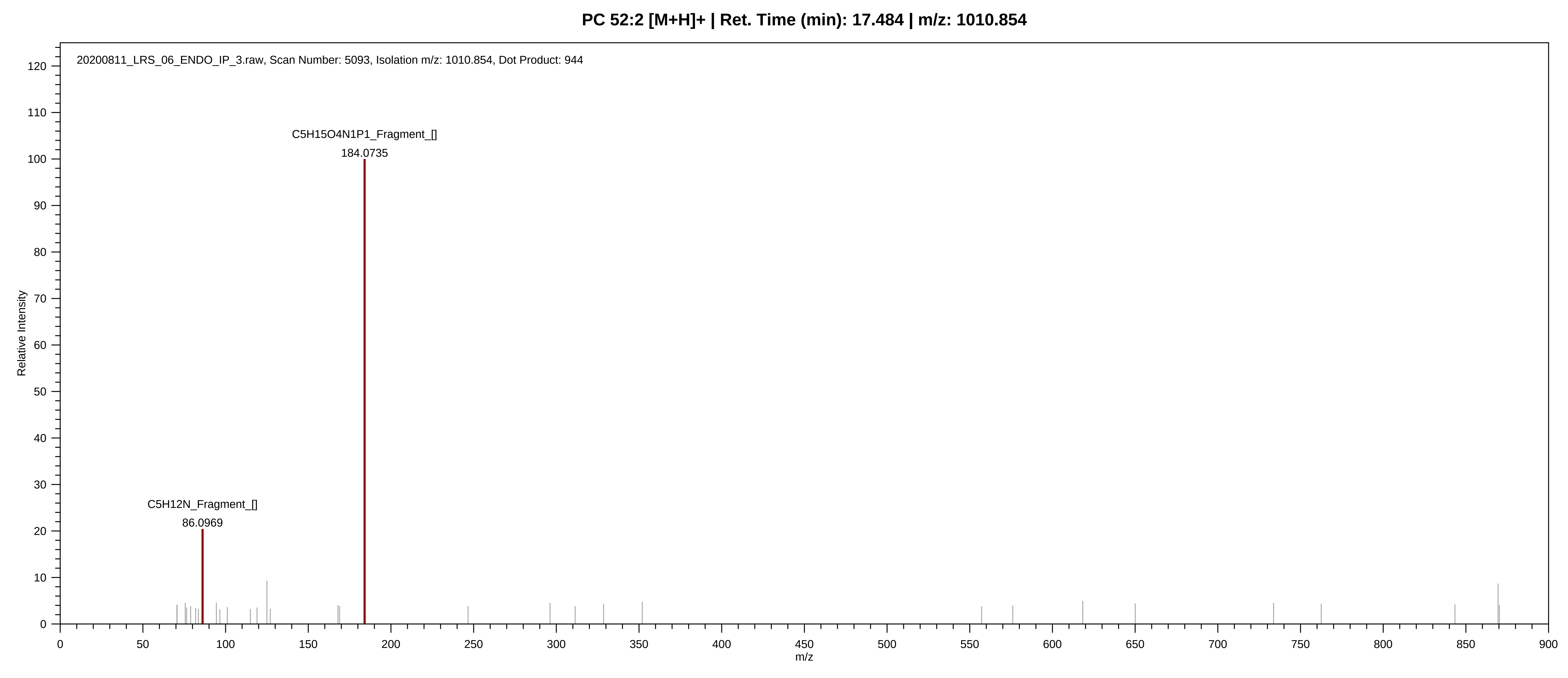

PC 52:2 [M+H]+ | Ret. Time (min): 17.484 | m/z: 1010.854
20200811_LRS_06_ENDO_IP_3.raw, Scan Number: 5093, Isolation m/z: 1010.854, Dot Product: 944
C5H15O4N1P1_Fragment_[]
184.0735
C5H12N_Fragment_[]
86.0969
Relative Intensity
m/z

PC 52:2 [M+H]+ | Ret. Time (min): 17.329 | m/z: 1010.856

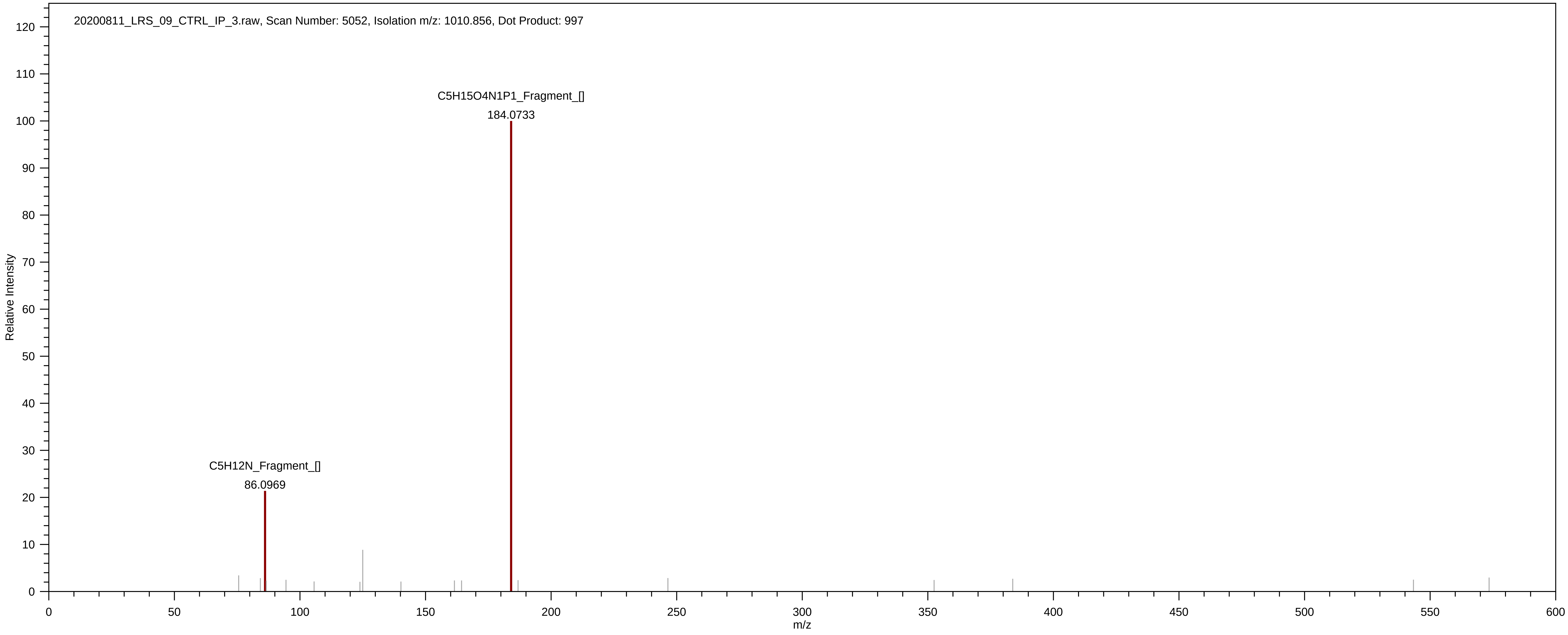

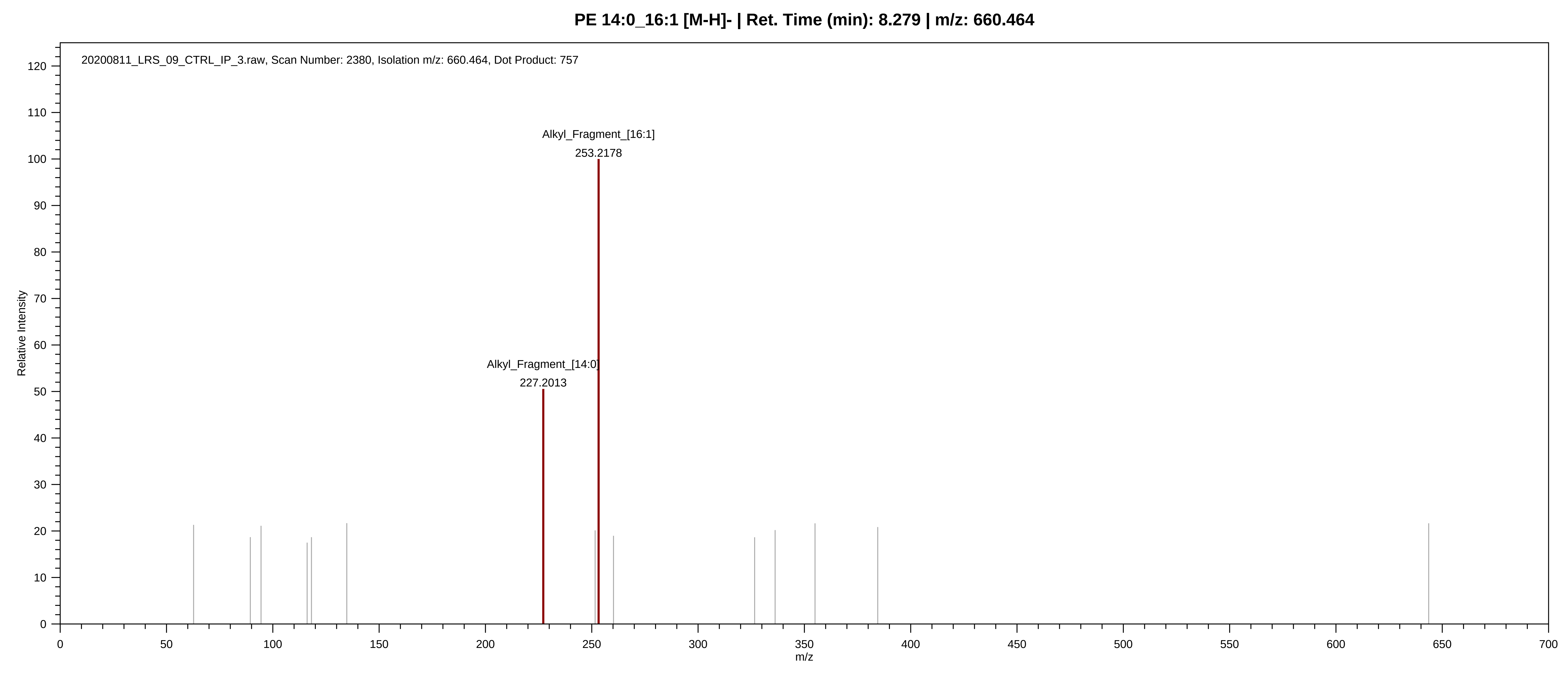

PE 14:0_16:1 [M-H]- | Ret. Time (min): 8.279 | m/z: 660.464
20200811_LRS_09_CTRL_IP_3.raw, Scan Number: 2380, Isolation m/z: 660.464, Dot Product: 757
Alkyl_Fragment_[16:1]
253.2178
Alkyl_Fragment_[14:0]
227.2013
Relative Intensity
m/z

PE 16:0_18:1 [M-H]- | Ret. Time (min): 10.026 | m/z: 716.52

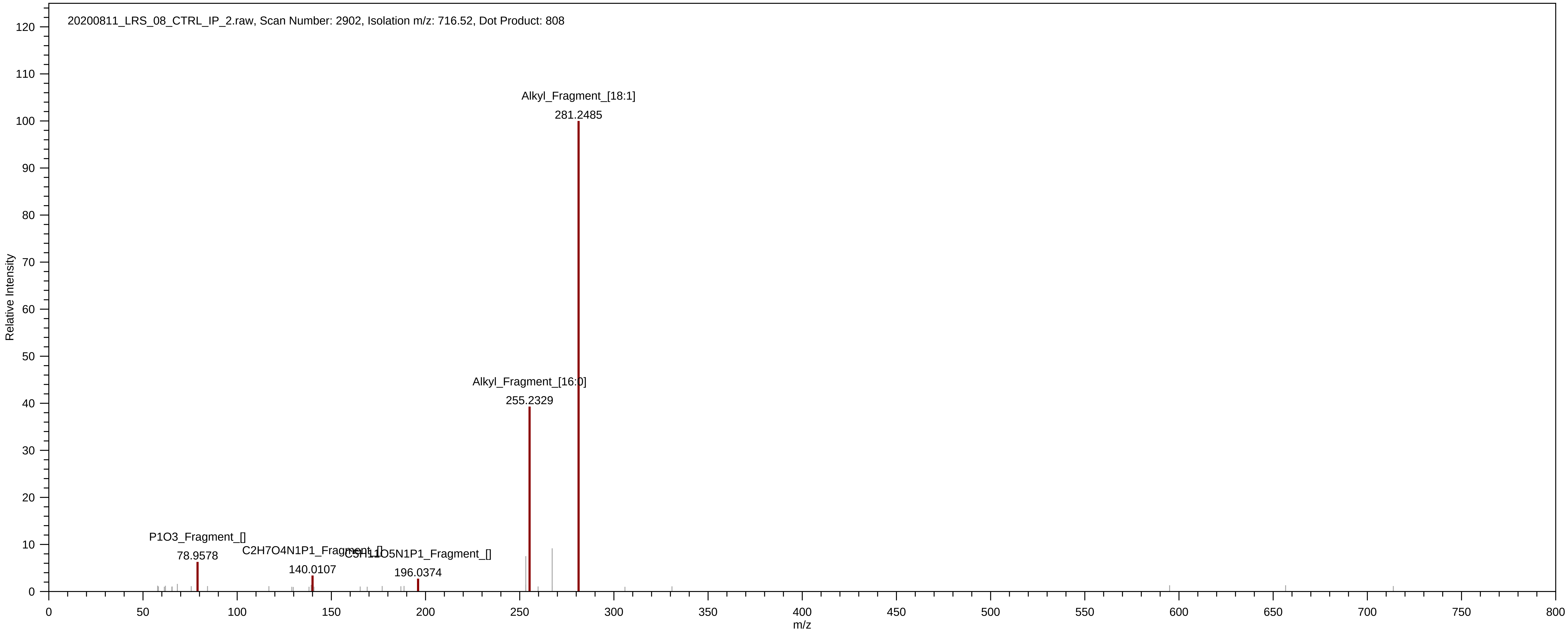

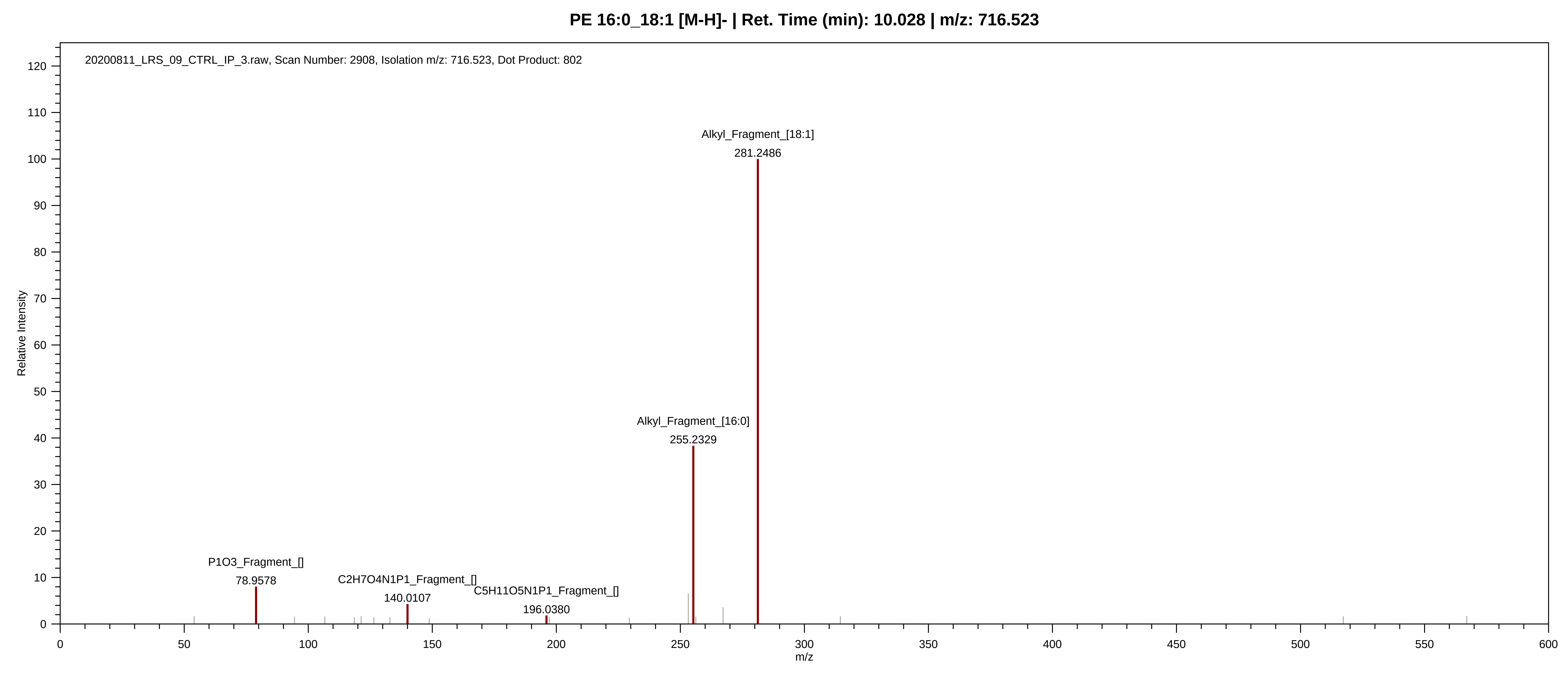
PE 16:0_18:1 [M-H]- | Ret. Time (min): 10.028 | m/z: 716.523
20200811_LRS_09_CTRL_IP_3.raw, Scan Number: 2908, Isolation m/z: 716.523, Dot Product: 802
Alkyl_Fragment_[18:1]
281.2486
Alkyl_Fragment_[16:0]
255.2329
P1O3_Fragment_[]
78.9578
C2H7O4N1P1_Fragment_[]
140.0107
C5H11O5N1P1_Fragment_[]
196.0380
Relative Intensity
m/z

PE 16:0_18:1 [M-H]- | Ret. Time (min): 10.046 | m/z: 716.523

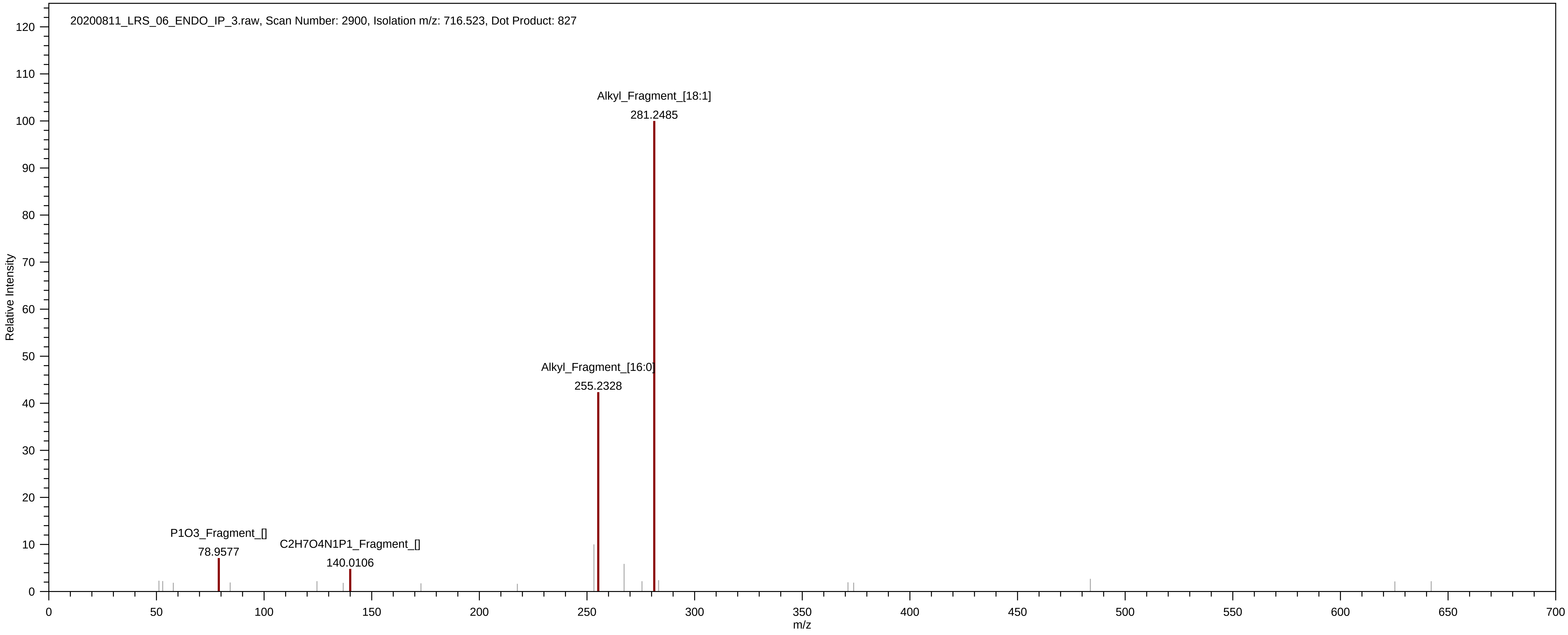

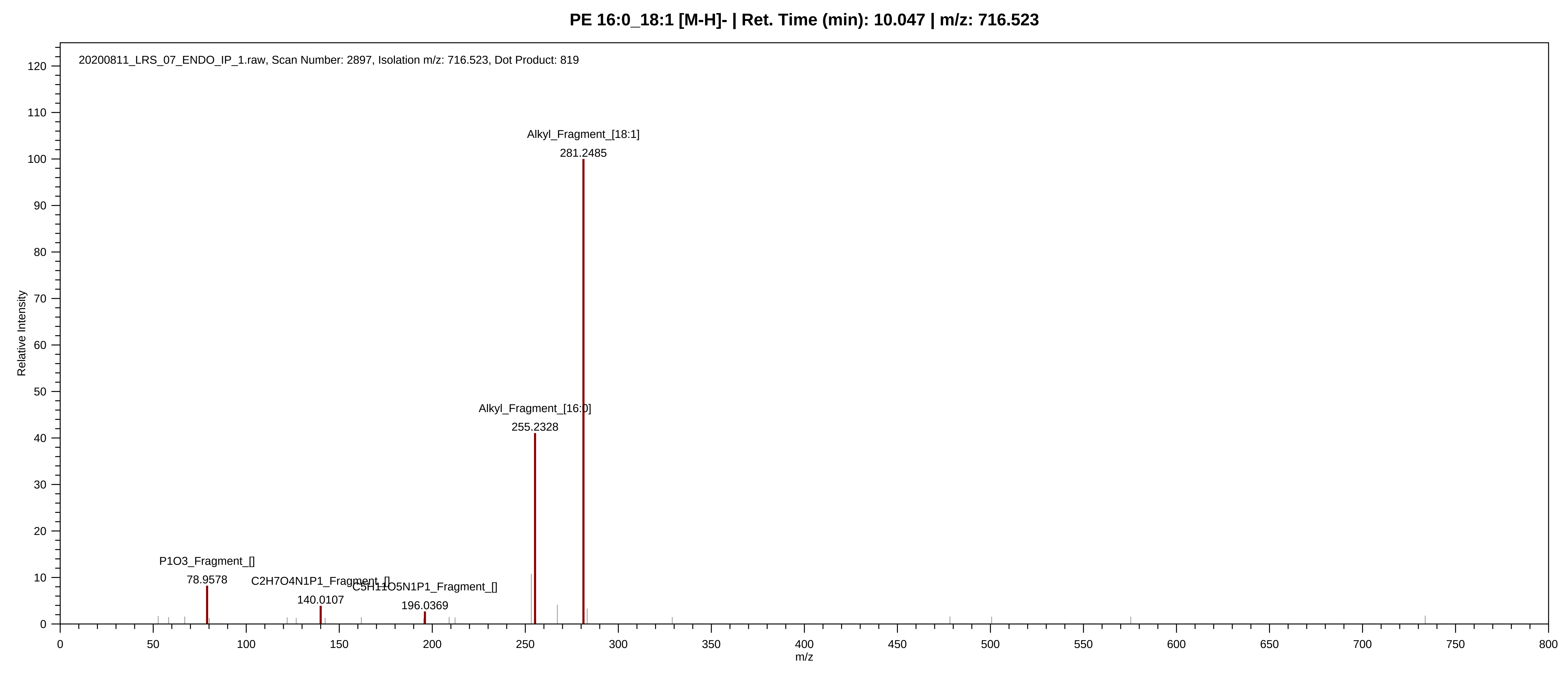

PE 16:0_18:1 [M-H]- | Ret. Time (min): 10.047 | m/z: 716.523
20200811_LRS_07_ENDO_IP_1.raw, Scan Number: 2897, Isolation m/z: 716.523, Dot Product: 819
Alkyl_Fragment_[18:1]
281.2485
Alkyl_Fragment_[16:0]
255.2328
P1O3_Fragment_[]
78.9578
C2H7O4N1P1_Fragment_[]
140.0107
C5H11O5N1P1_Fragment_[]
196.0369
Relative Intensity
m/z

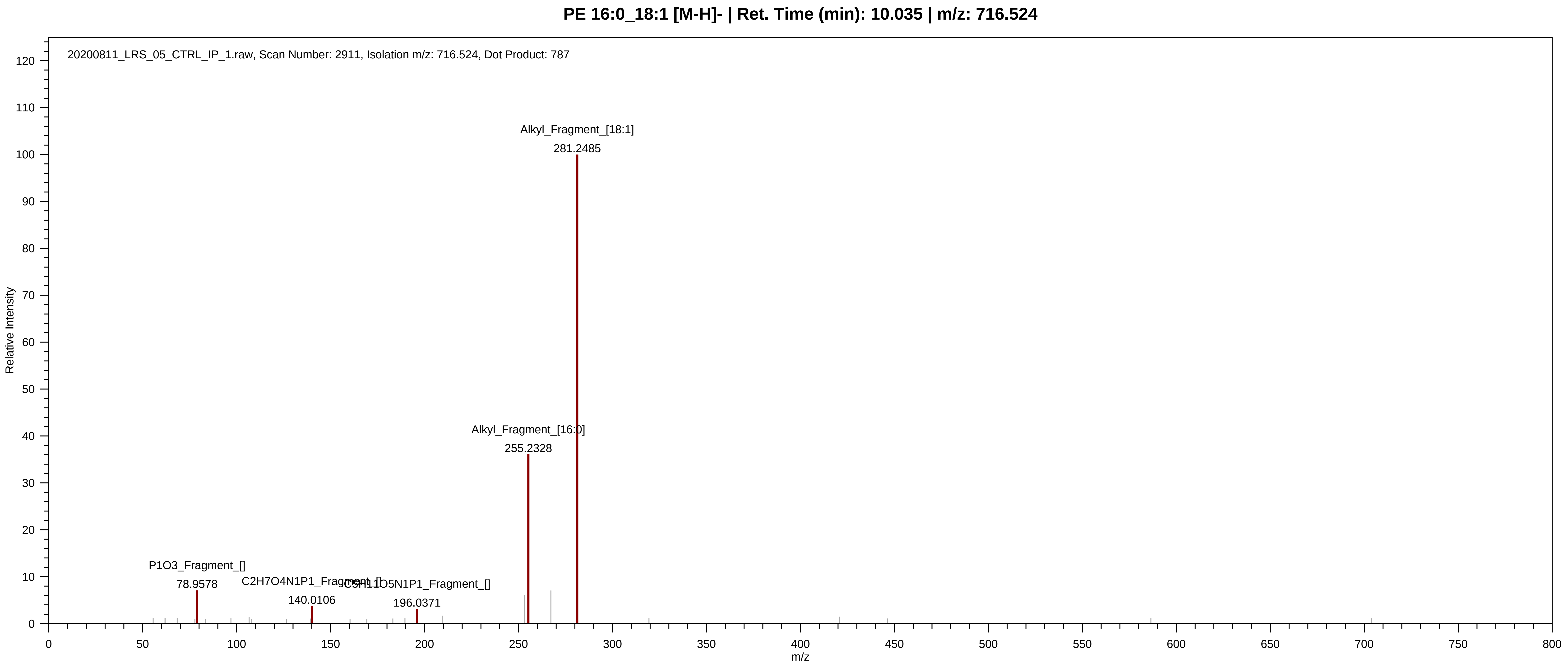

PE 16:0_18:1 [M-H]- | Ret. Time (min): 10.035 | m/z: 716.524
20200811_LRS_05_CTRL_IP_1.raw, Scan Number: 2911, Isolation m/z: 716.524, Dot Product: 787
Alkyl_Fragment_[18:1]
281.2485
Alkyl_Fragment_[16:0]
255.2328
P1O3_Fragment_[]
78.9578
C2H7O4N1P1_Fragment_[]
140.0106
C5H11O5N1P1_Fragment_[]
196.0371
Relative Intensity
m/z

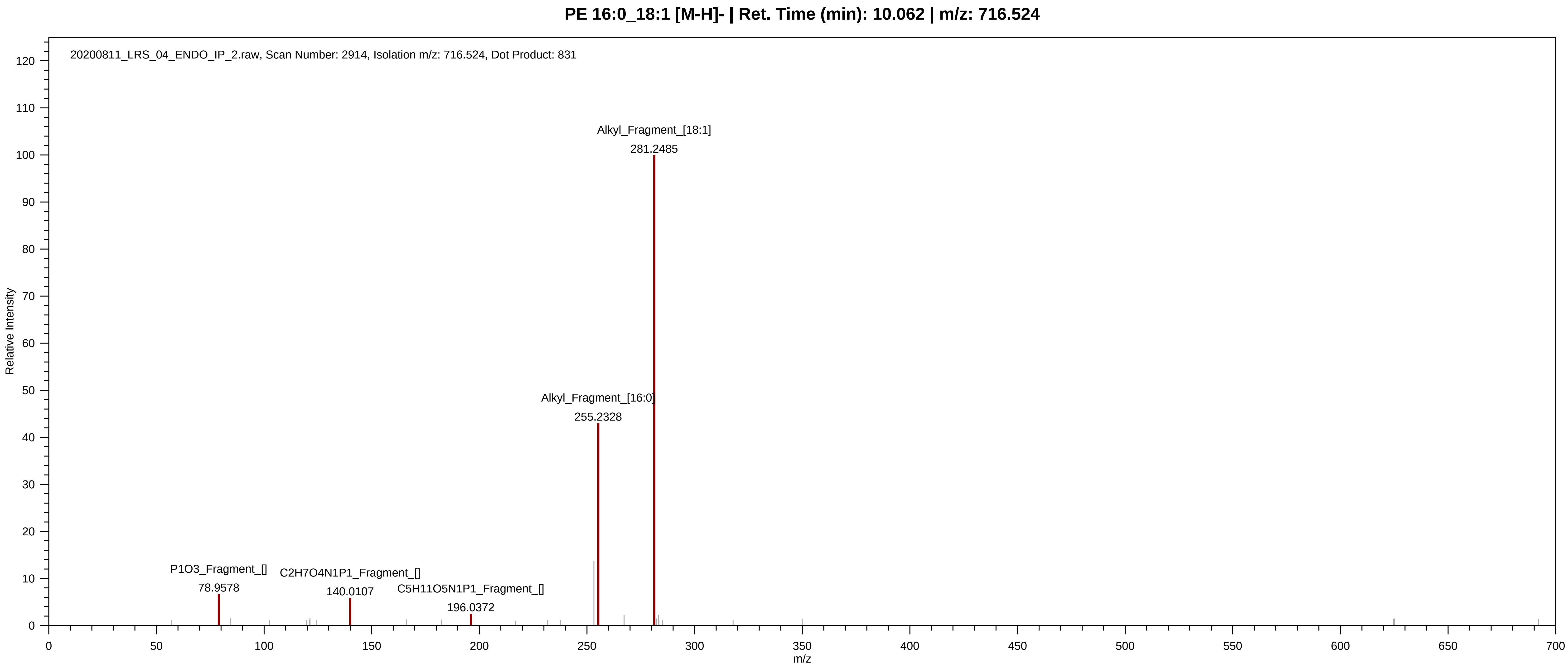

PE 16:0_18:1 [M-H]- | Ret. Time (min): 10.062 | m/z: 716.524
20200811_LRS_04_ENDO_IP_2.raw, Scan Number: 2914, Isolation m/z: 716.524, Dot Product: 831
Alkyl_Fragment_[18:1]
281.2485
Alkyl_Fragment_[16:0]
255.2328
P1O3_Fragment_[]
78.9578
C2H7O4N1P1_Fragment_[]
140.0107
C5H11O5N1P1_Fragment_[]
196.0372
Relative Intensity
m/z

PE 16:0_20:4 [M-H]- | Ret. Time (min): 9.17 | m/z: 738.506

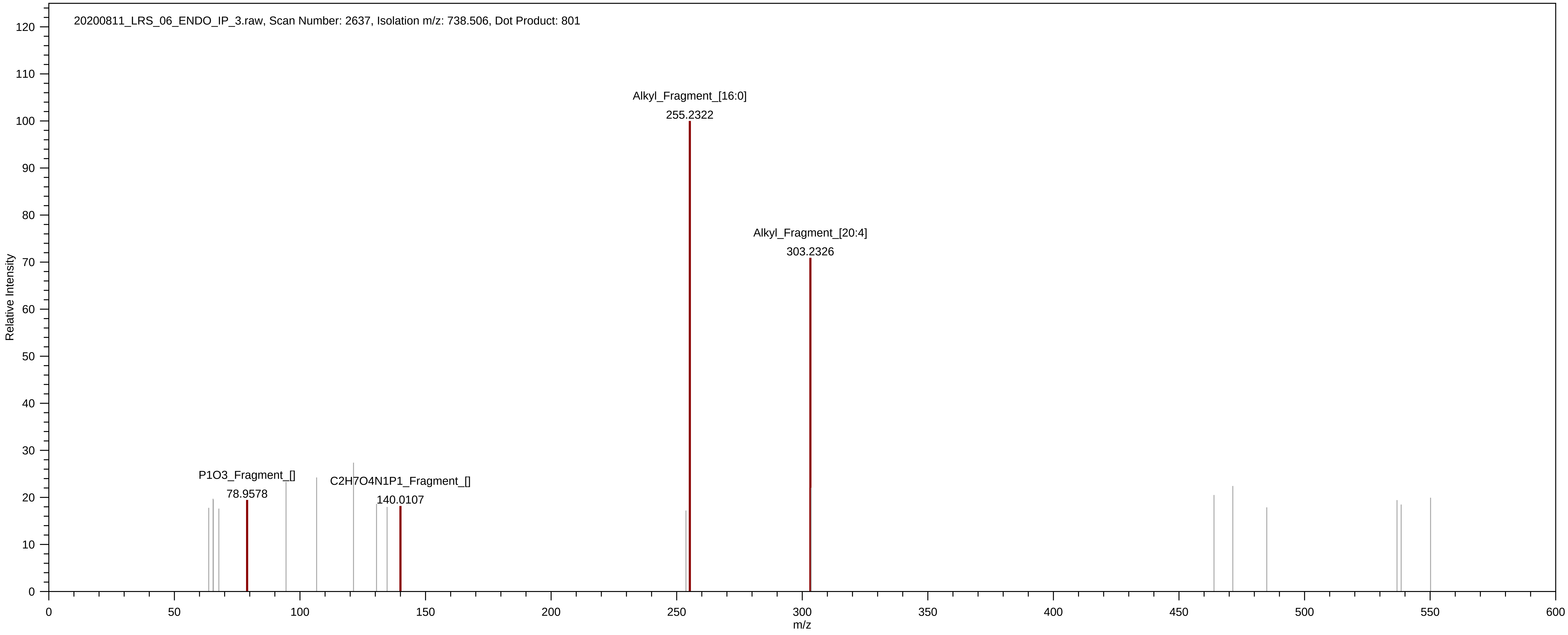

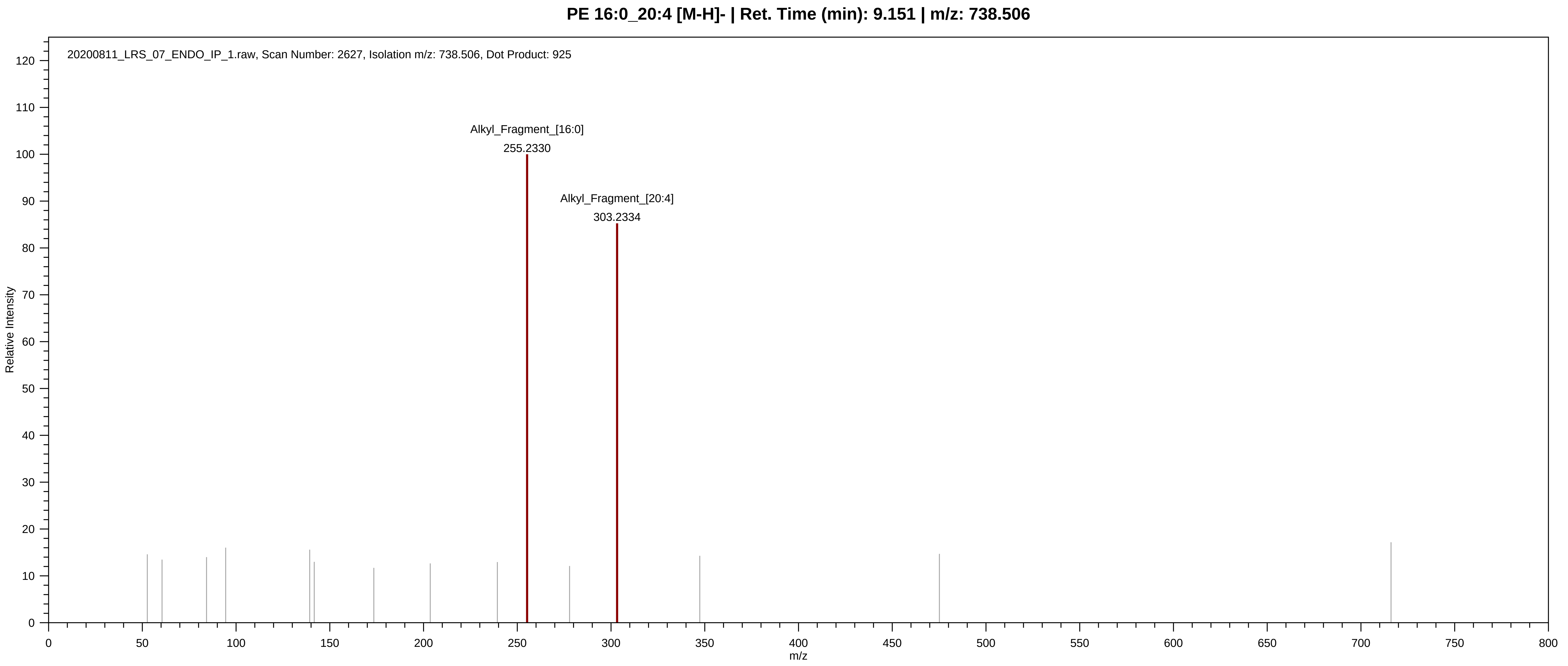

PE 16:0_20:4 [M-H]- | Ret. Time (min): 9.151 | m/z: 738.506
20200811_LRS_07_ENDO_IP_1.raw, Scan Number: 2627, Isolation m/z: 738.506, Dot Product: 925
Alkyl_Fragment_[16:0]
255.2330
Alkyl_Fragment_[20:4]
303.2334
Relative Intensity
m/z

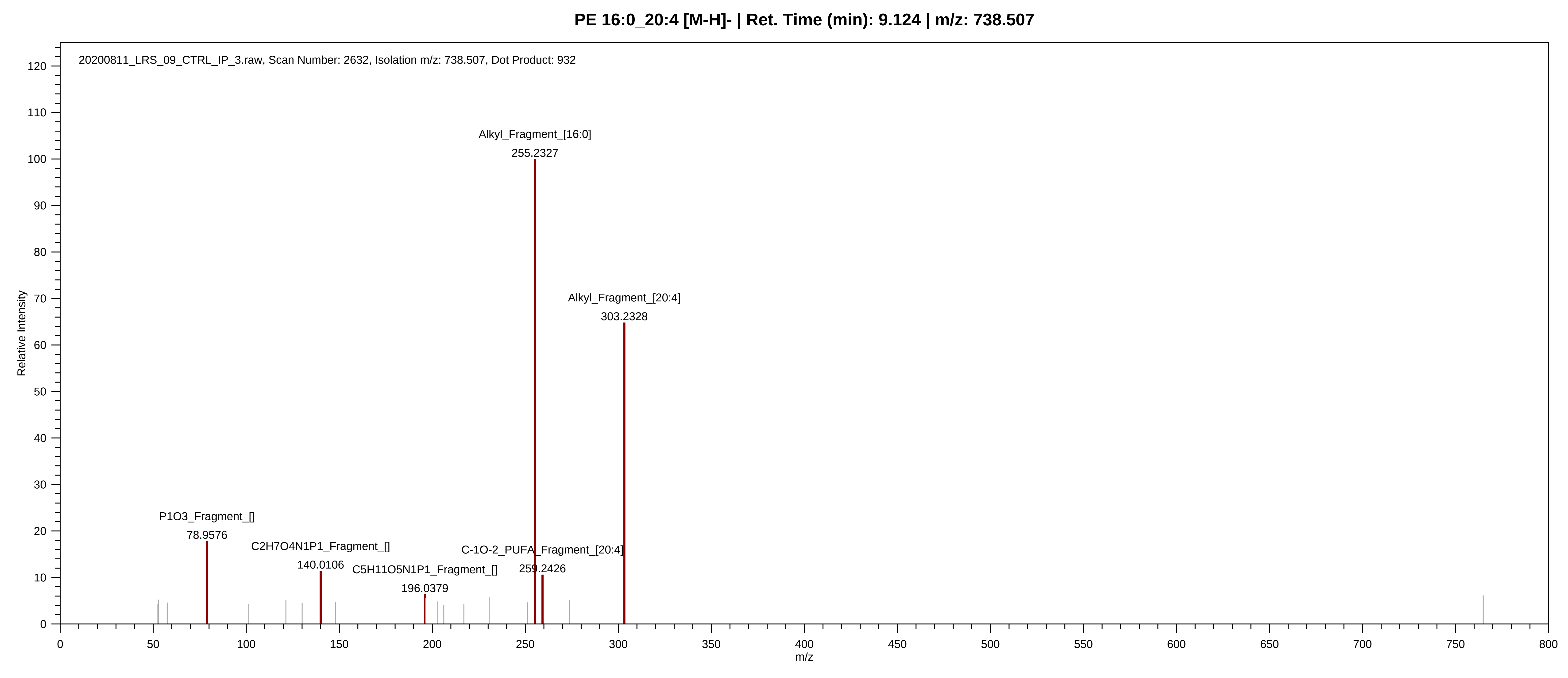
PE 16:0_20:4 [M-H]- | Ret. Time (min): 9.124 | m/z: 738.507
20200811_LRS_09_CTRL_IP_3.raw, Scan Number: 2632, Isolation m/z: 738.507, Dot Product: 932
Alkyl_Fragment_[16:0]
255.2327
Alkyl_Fragment_[20:4]
303.2328
P1O3_Fragment_[]
78.9576
C2H7O4N1P1_Fragment_[]
140.0106
C5H11O5N1P1_Fragment_[]
196.0379
C-1O-2_PUFA_Fragment_[20:4]
259.2426
Relative Intensity
m/z

PE 16:0_20:4 [M-H]- | Ret. Time (min): 9.131 | m/z: 738.507

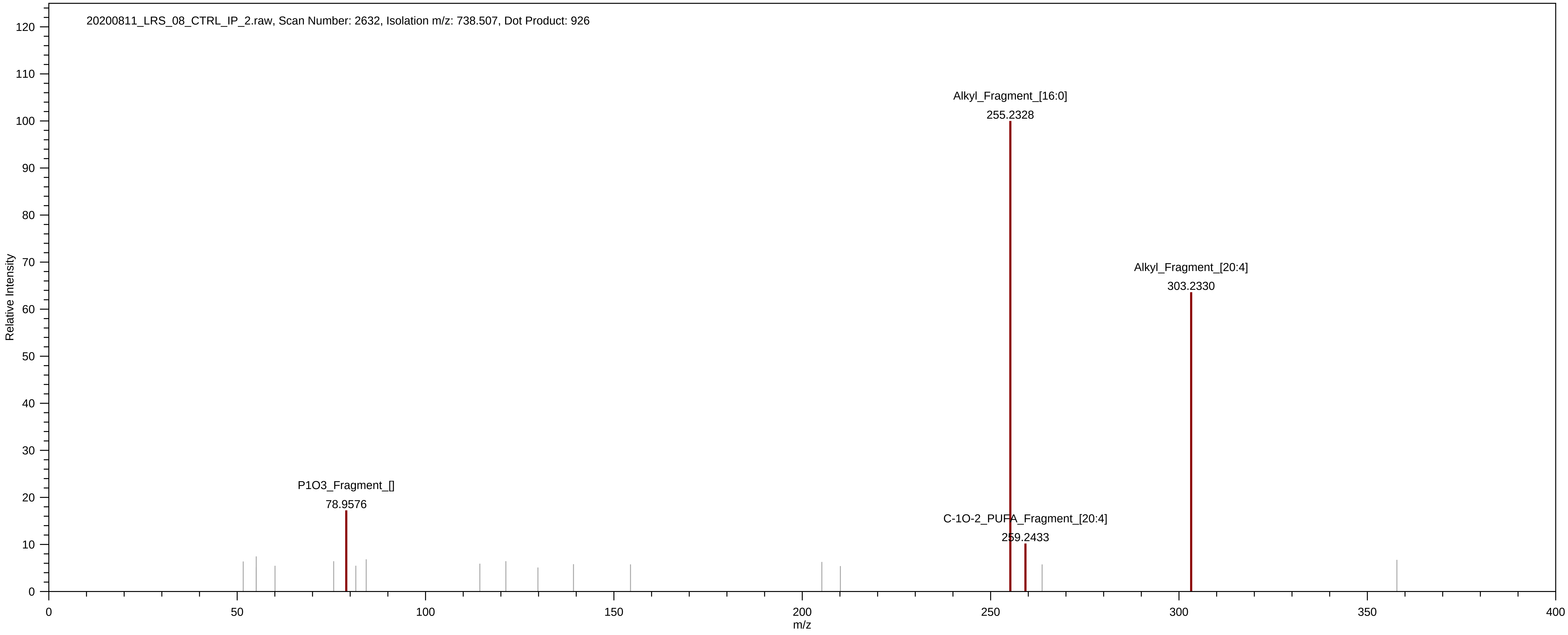

PE 16:0_20:4 [M-H]- | Ret. Time (min): 9.154 | m/z: 738.508

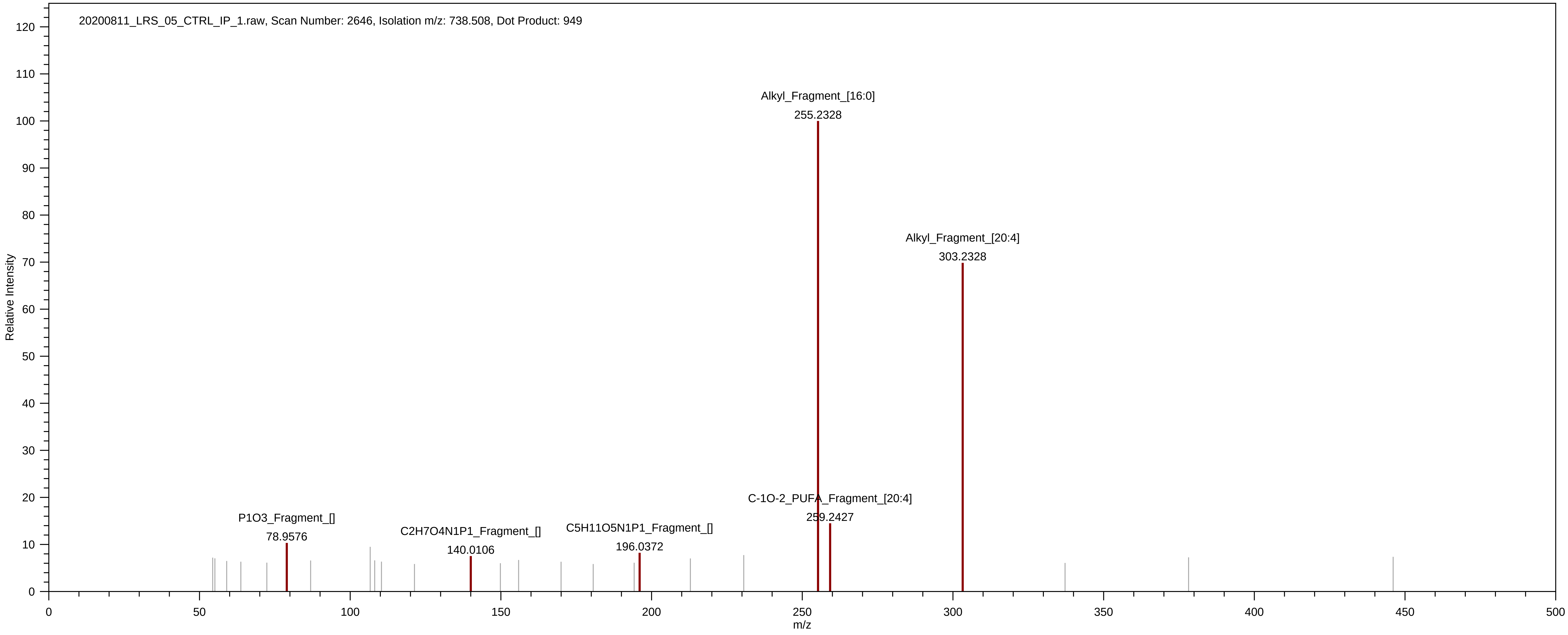

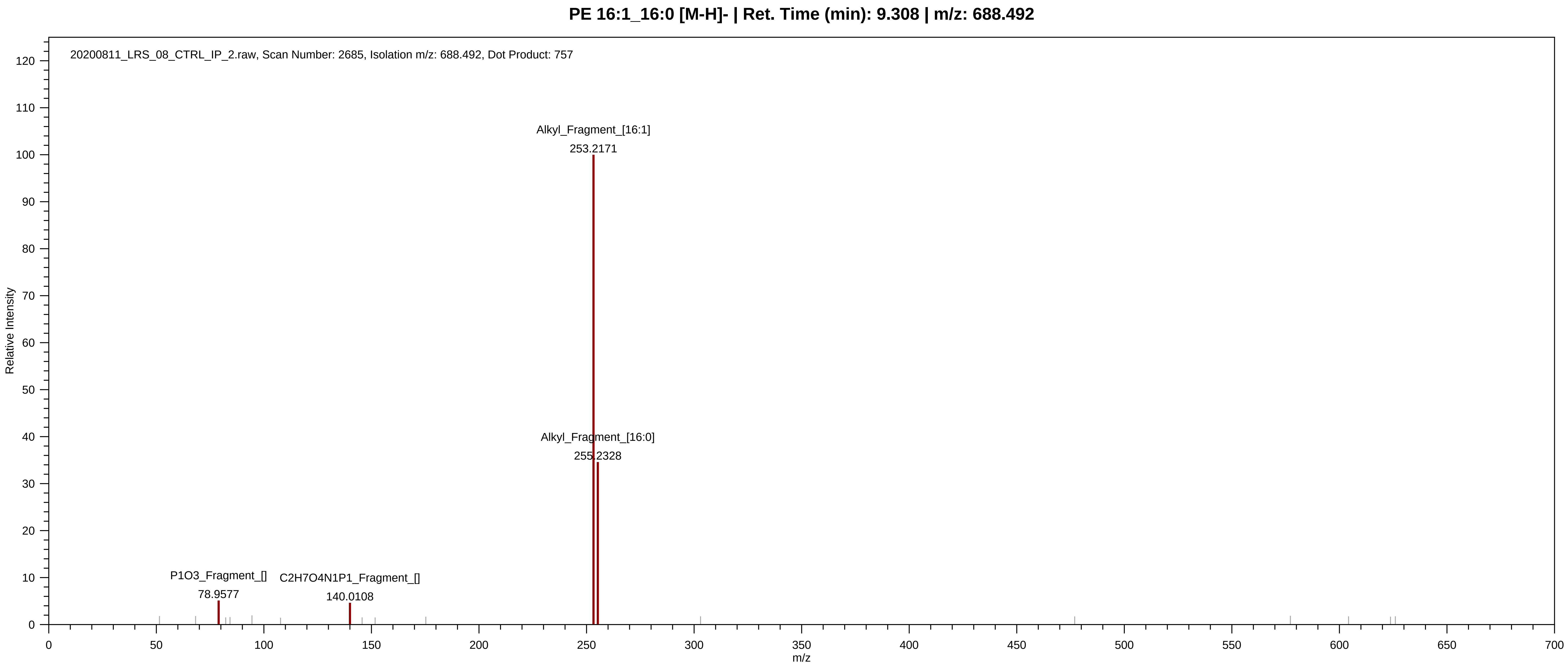
PE 16:1_16:0 [M-H]- | Ret. Time (min): 9.308 | m/z: 688.492
20200811_LRS_08_CTRL_IP_2.raw, Scan Number: 2685, Isolation m/z: 688.492, Dot Product: 757
Alkyl_Fragment_[16:1]
253.2171
Alkyl_Fragment_[16:0]
255.2328
P1O3_Fragment_[]
78.9577
C2H7O4N1P1_Fragment_[]
140.0108
Relative Intensity
m/z

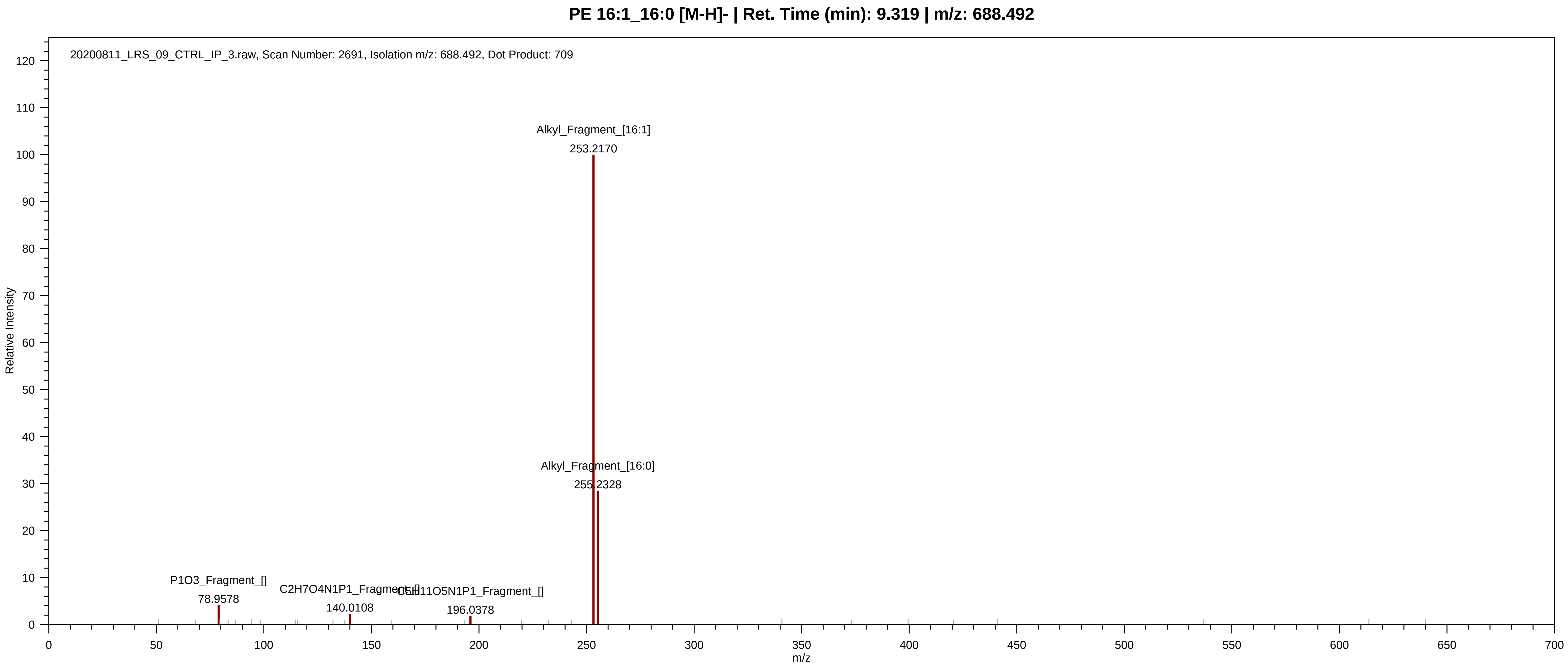
PE 16:1_16:0 [M-H]- | Ret. Time (min): 9.319 | m/z: 688.492
20200811_LRS_09_CTRL_IP_3.raw, Scan Number: 2691, Isolation m/z: 688.492, Dot Product: 709
Alkyl_Fragment_[16:1]
253.2170
Alkyl_Fragment_[16:0]
255.2328
P1O3_Fragment_[]
78.9578
C2H7O4N1P1_Fragment_[]
140.0108
C5H11O5N1P1_Fragment_[]
196.0378
Relative Intensity
m/z

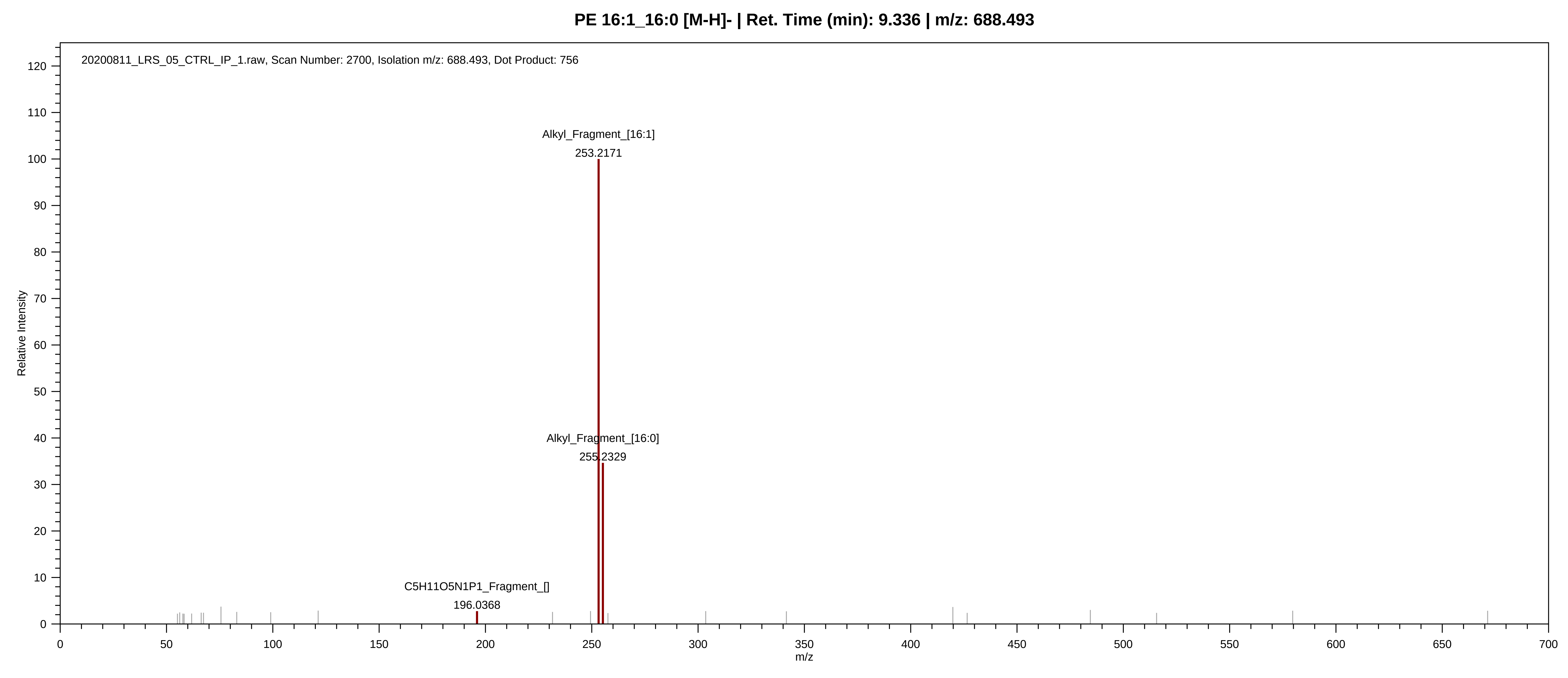
PE 16:1_16:0 [M-H]- | Ret. Time (min): 9.336 | m/z: 688.493
20200811_LRS_05_CTRL_IP_1.raw, Scan Number: 2700, Isolation m/z: 688.493, Dot Product: 756
Alkyl_Fragment_[16:1]
253.2171
Alkyl_Fragment_[16:0]
255.2329
C5H11O5N1P1_Fragment_[]
196.0368
Relative Intensity
m/z

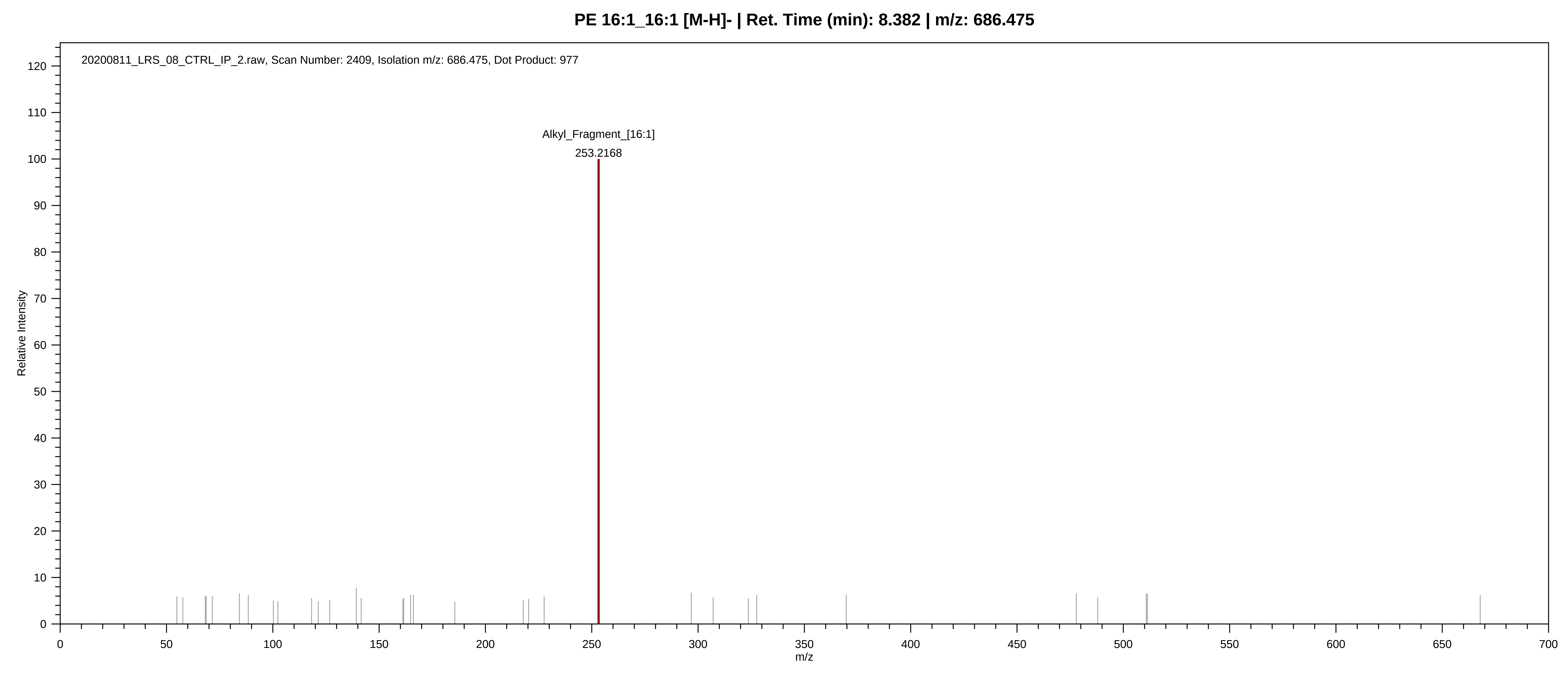
PE 16:1_16:1 [M-H]- | Ret. Time (min): 8.382 | m/z: 686.475
20200811_LRS_08_CTRL_IP_2.raw, Scan Number: 2409, Isolation m/z: 686.475, Dot Product: 977
Alkyl_Fragment_[16:1]
253.2168
Relative Intensity
m/z
0
10
20
30
40
50
60
70
80
90
100
110
120
0
50
100
150
200
250
300
350
400
450
500
550
600
650
700

PE 16:1_16:1 [M-H]- | Ret. Time (min): 8.381 | m/z: 686.476

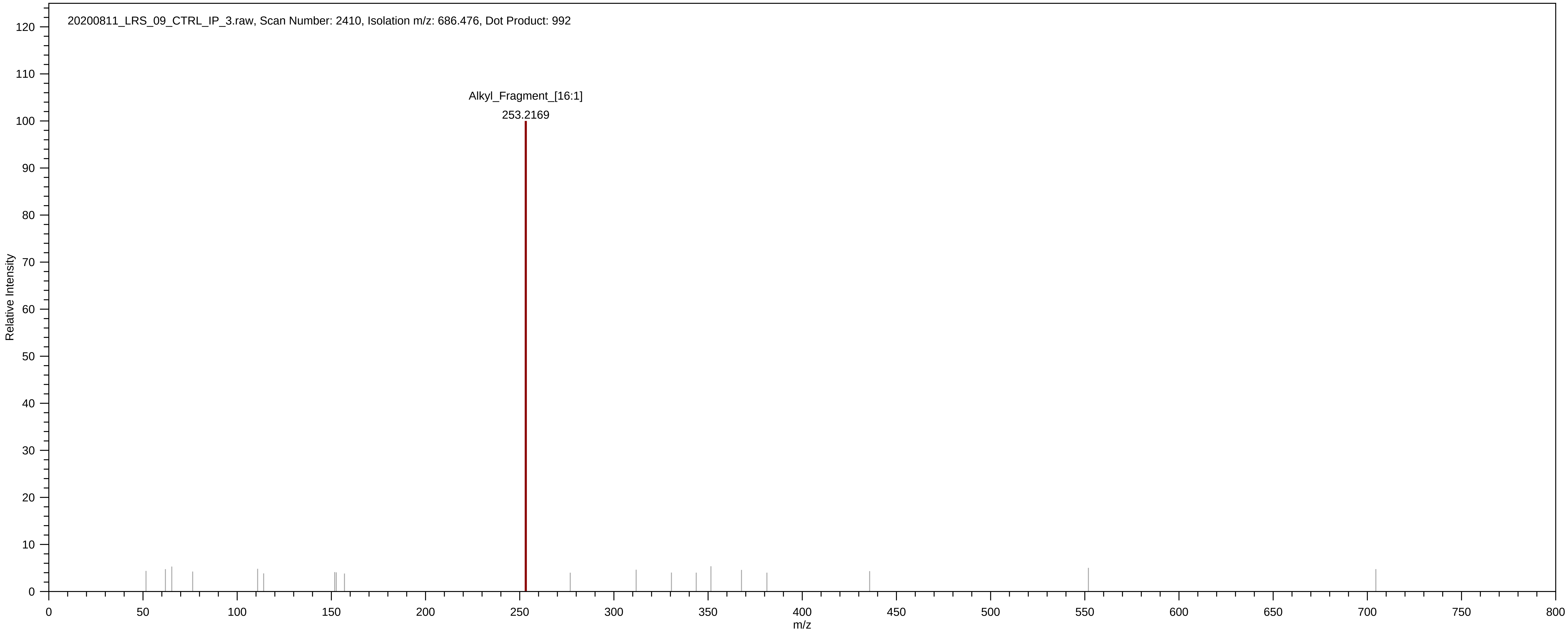

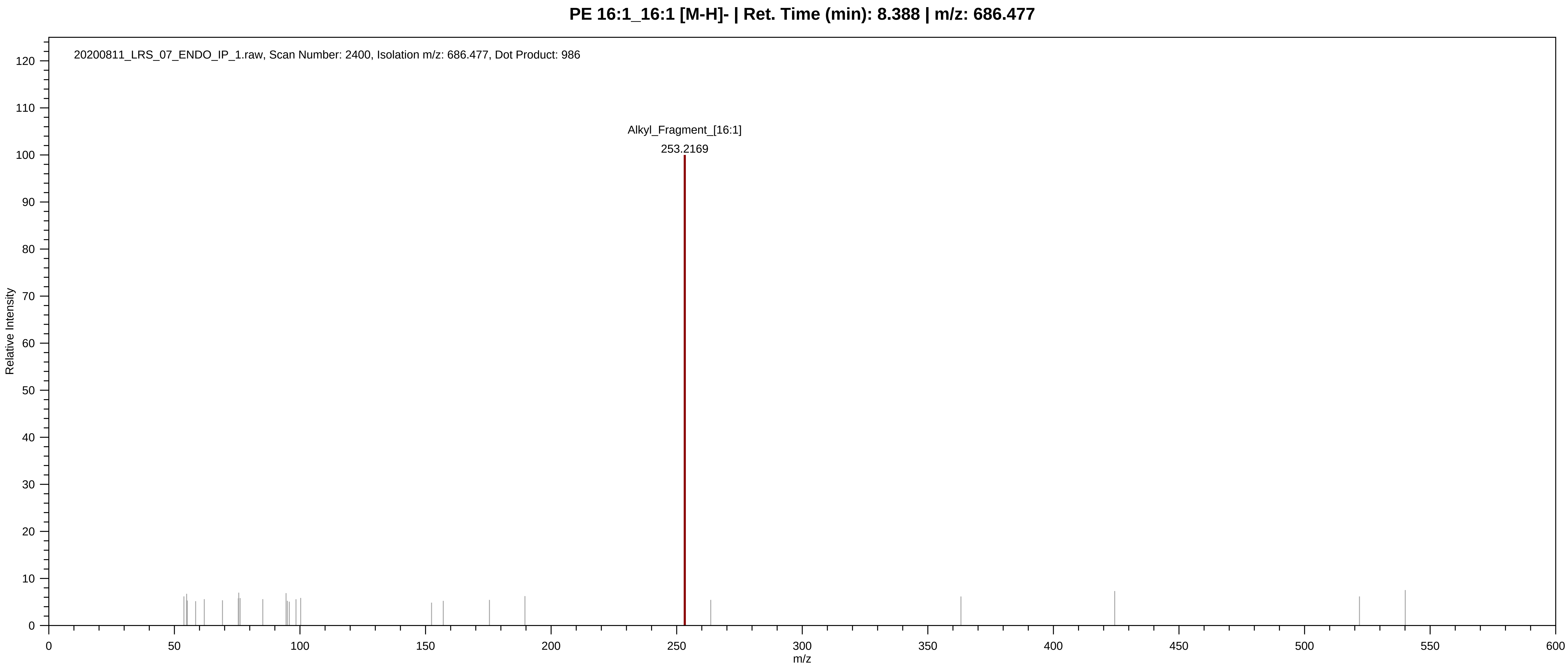
PE 16:1_16:1 [M-H]- | Ret. Time (min): 8.388 | m/z: 686.477
20200811_LRS_07_ENDO_IP_1.raw, Scan Number: 2400, Isolation m/z: 686.477, Dot Product: 986
Alkyl_Fragment_[16:1]
253.2169
Relative Intensity
m/z
0
10
20
30
40
50
60
70
80
90
100
110
120
0
50
100
150
200
250
300
350
400
450
500
550
600

PE 16:1_16:1 [M-H]- | Ret. Time (min): 8.399 | m/z: 686.477

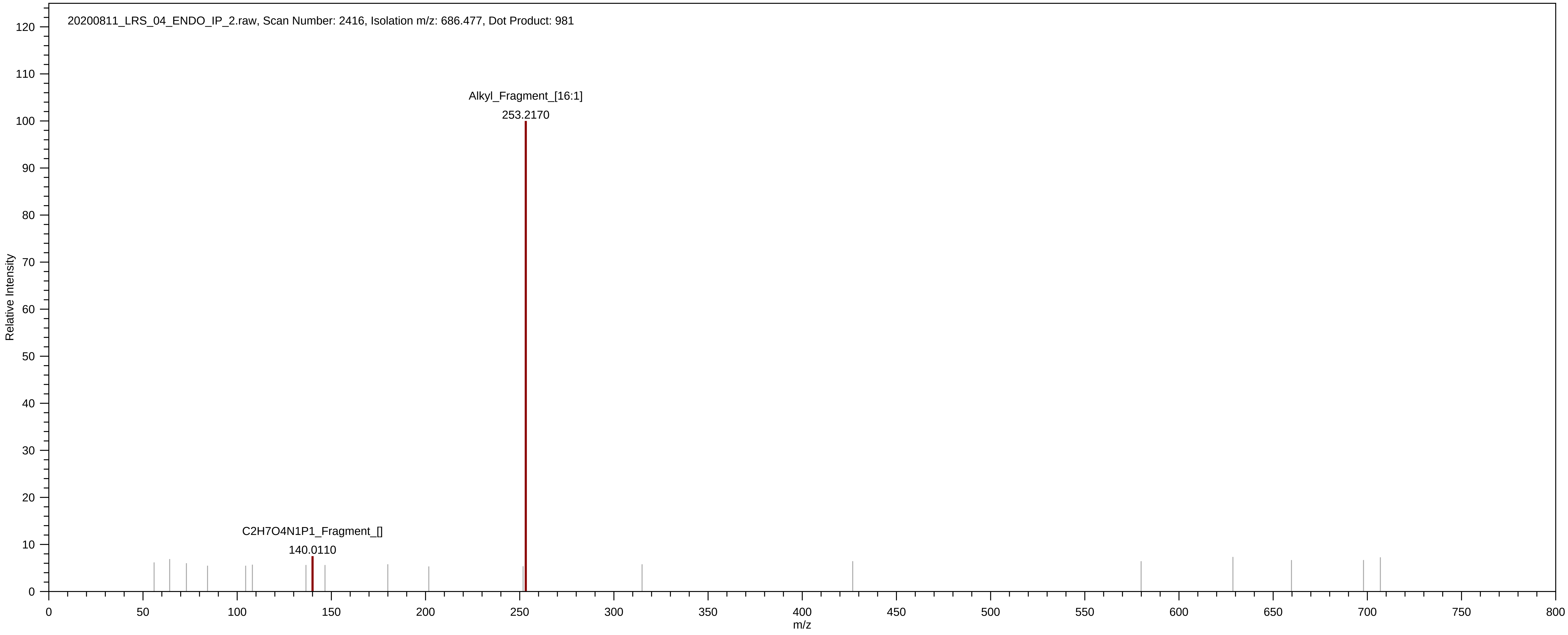

PE 16:1_16:1 [M-H]- | Ret. Time (min): 8.406 | m/z: 686.477

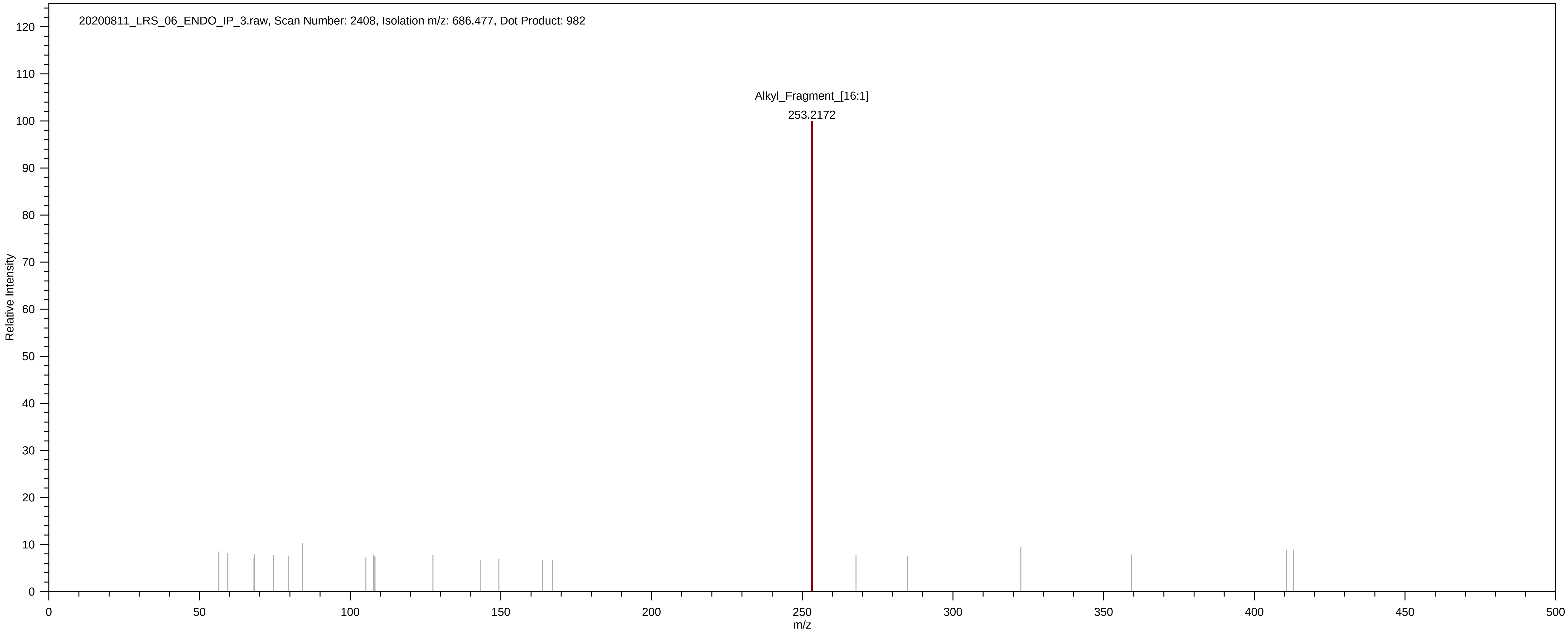

PE 16:1_16:1 [M-H]- | Ret. Time (min): 8.411 | m/z: 686.477

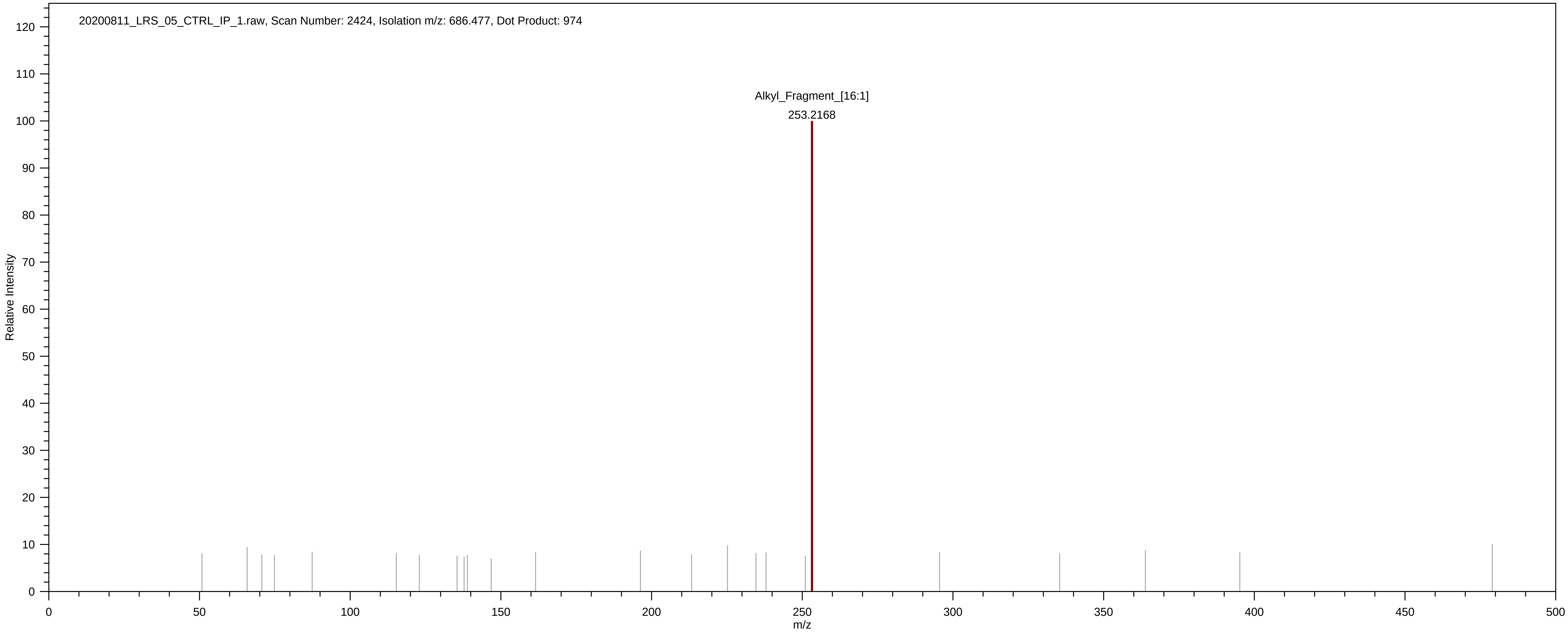

PE 16:1_18:1 [M-H]- | Ret. Time (min): 9.189 | m/z: 714.508

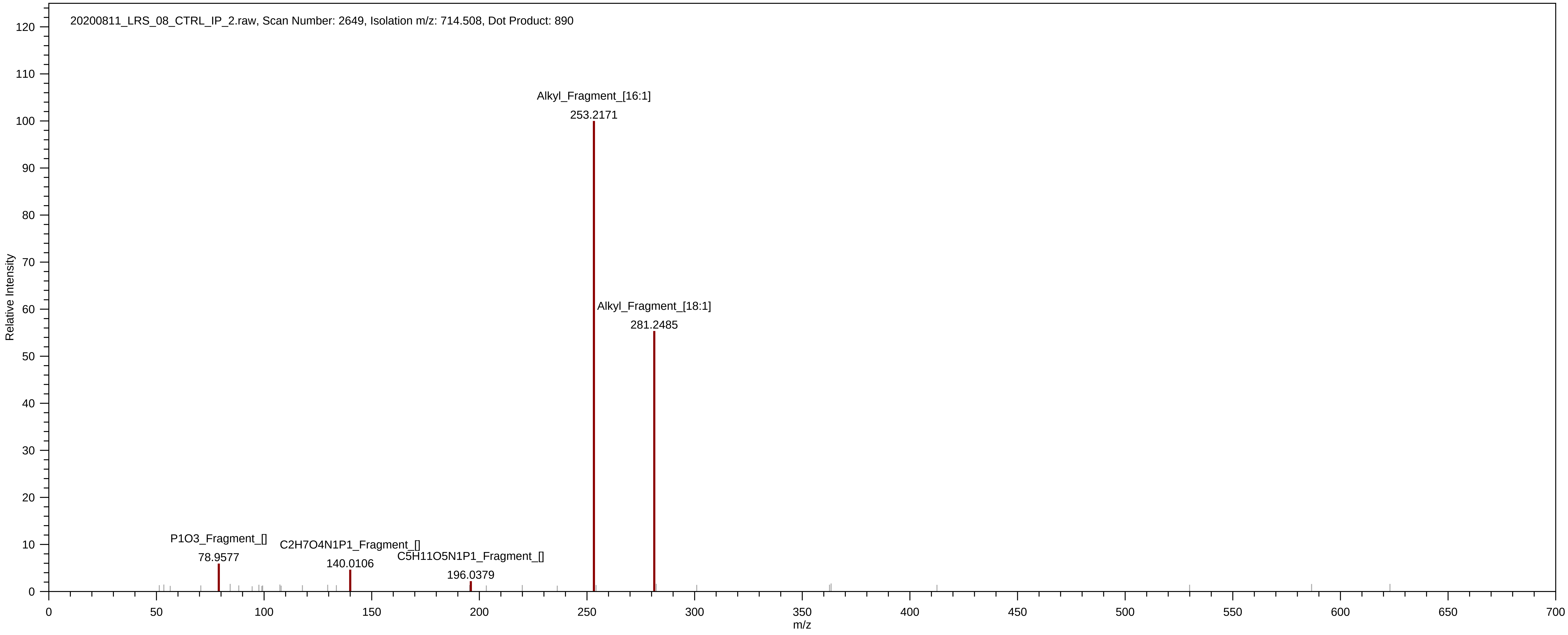

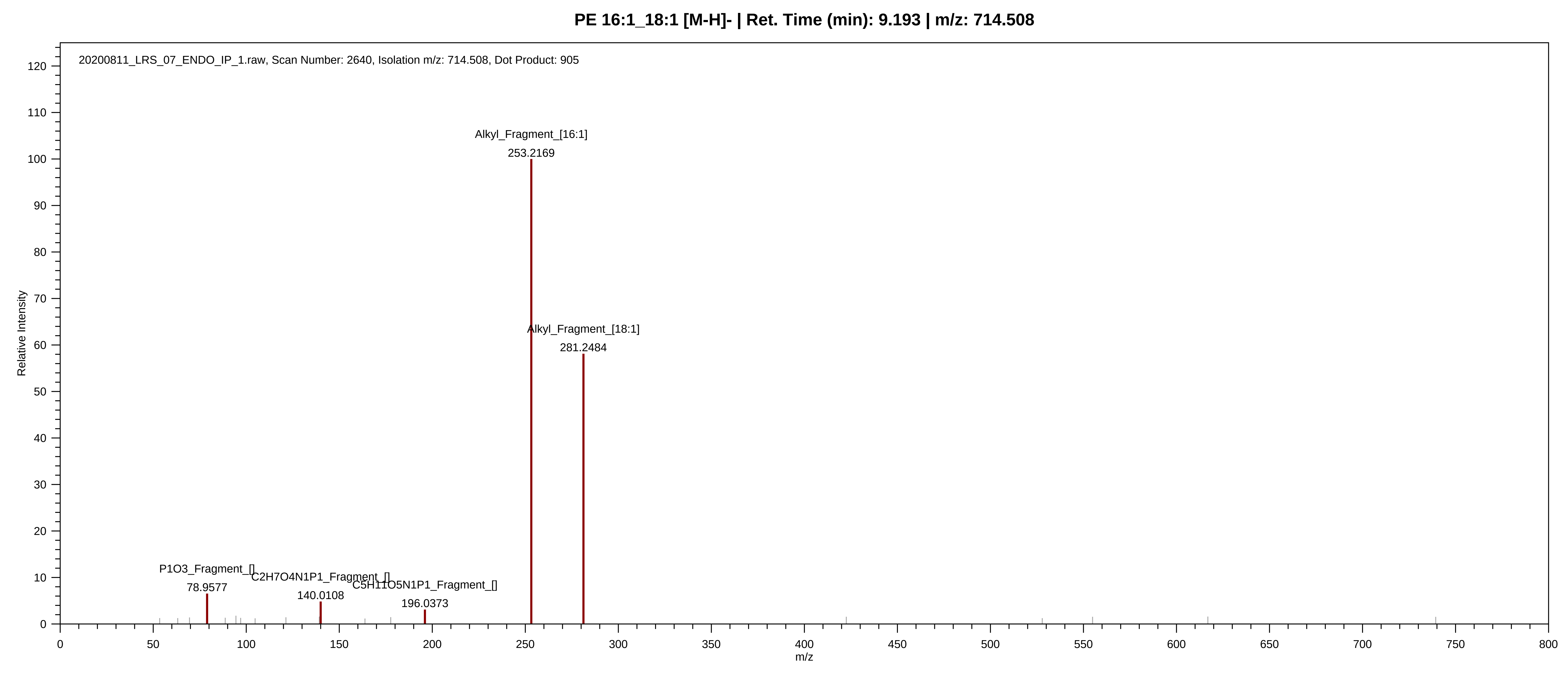
PE 16:1_18:1 [M-H]- | Ret. Time (min): 9.193 | m/z: 714.508
20200811_LRS_07_ENDO_IP_1.raw, Scan Number: 2640, Isolation m/z: 714.508, Dot Product: 905
Alkyl_Fragment_[16:1]
253.2169
Alkyl_Fragment_[18:1]
281.2484
P1O3_Fragment_[]
78.9577
C2H7O4N1P1_Fragment_[]
140.0108
C5H11O5N1P1_Fragment_[]
196.0373
Relative Intensity
m/z

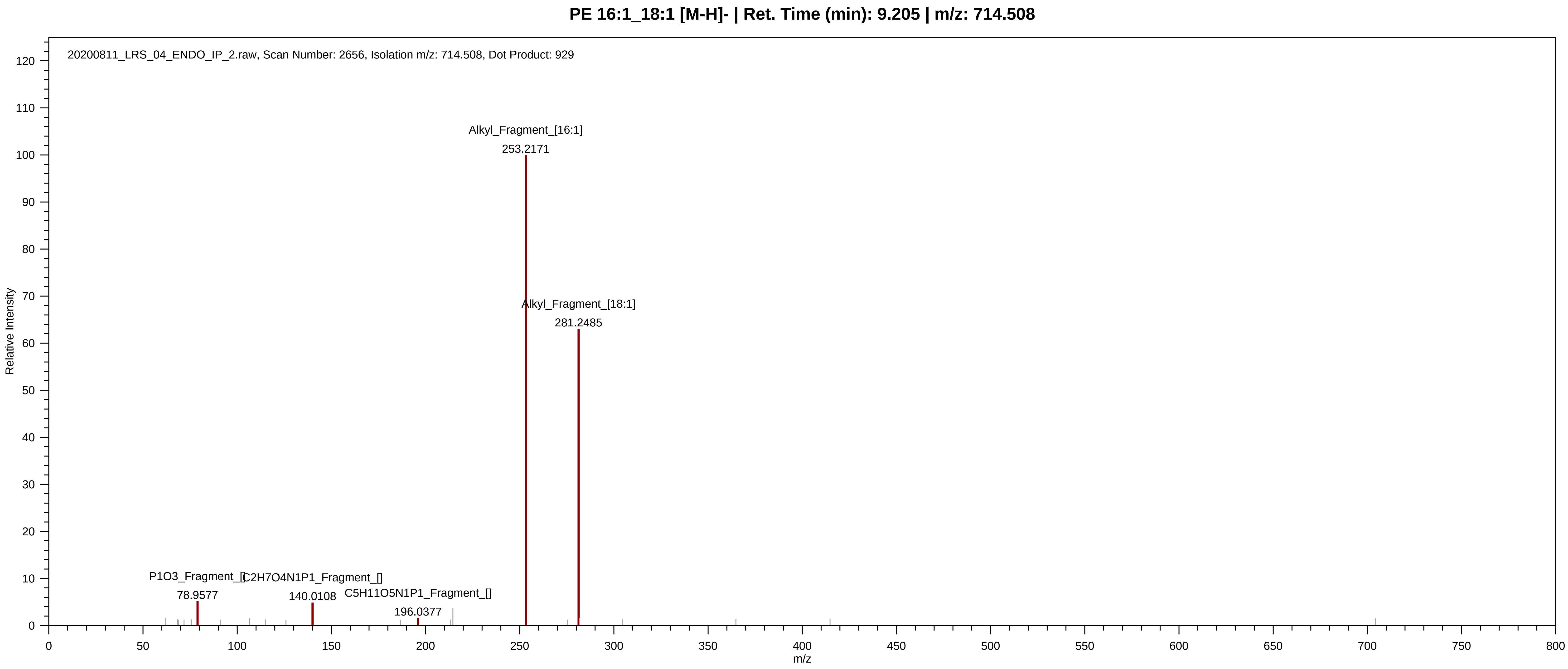
PE 16:1_18:1 [M-H]- | Ret. Time (min): 9.205 | m/z: 714.508
20200811_LRS_04_ENDO_IP_2.raw, Scan Number: 2656, Isolation m/z: 714.508, Dot Product: 929
Alkyl_Fragment_[16:1]
253.2171
Alkyl_Fragment_[18:1]
281.2485
P1O3_Fragment_[]
78.9577
C2H7O4N1P1_Fragment_[]
140.0108
C5H11O5N1P1_Fragment_[]
196.0377
Relative Intensity
m/z

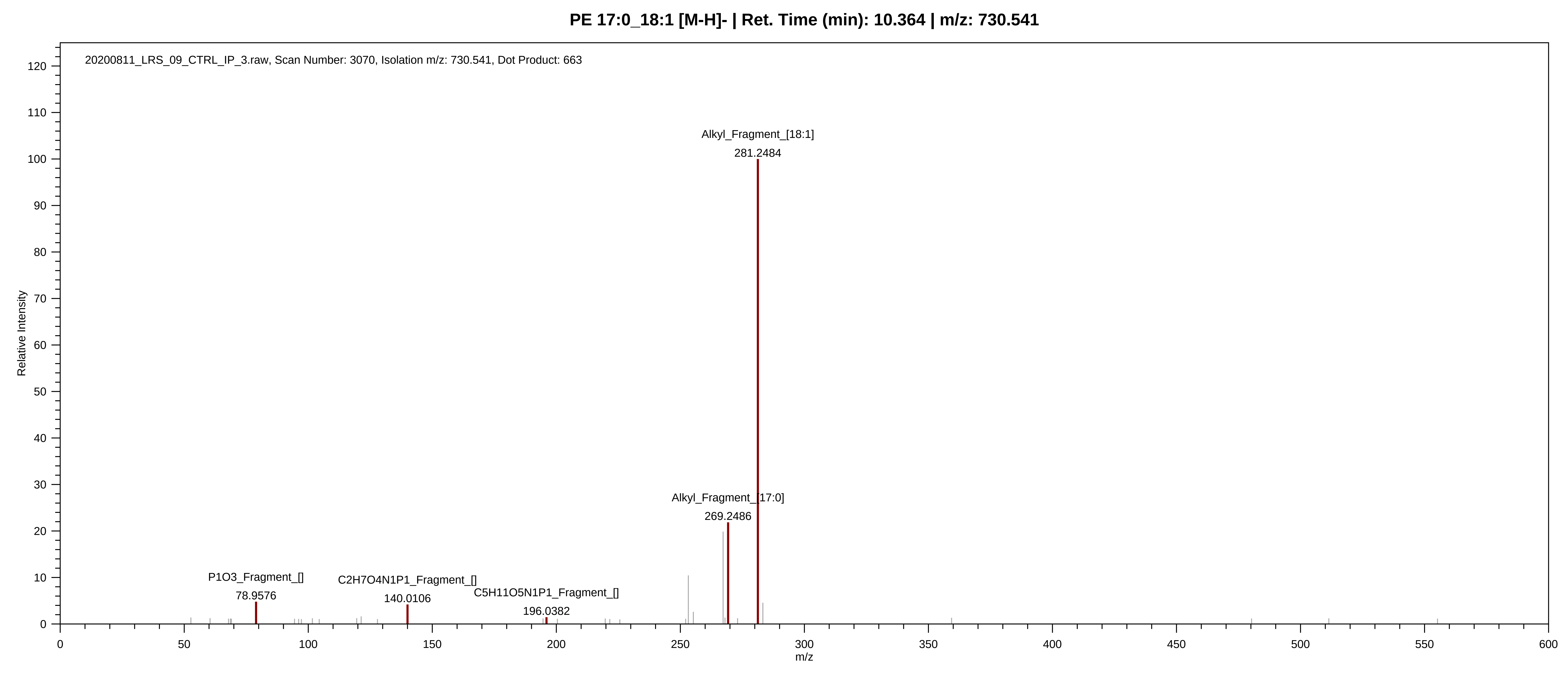

PE 17:0_18:1 [M-H]- | Ret. Time (min): 10.364 | m/z: 730.541
20200811_LRS_09_CTRL_IP_3.raw, Scan Number: 3070, Isolation m/z: 730.541, Dot Product: 663
Alkyl_Fragment_[18:1]
281.2484
Alkyl_Fragment_[17:0]
269.2486
P1O3_Fragment_[]
78.9576
C2H7O4N1P1_Fragment_[]
140.0106
C5H11O5N1P1_Fragment_[]
196.0382
Relative Intensity
m/z

PE 17:1_18:1 [M-H]- | Ret. Time (min): 9.669 | m/z: 728.522

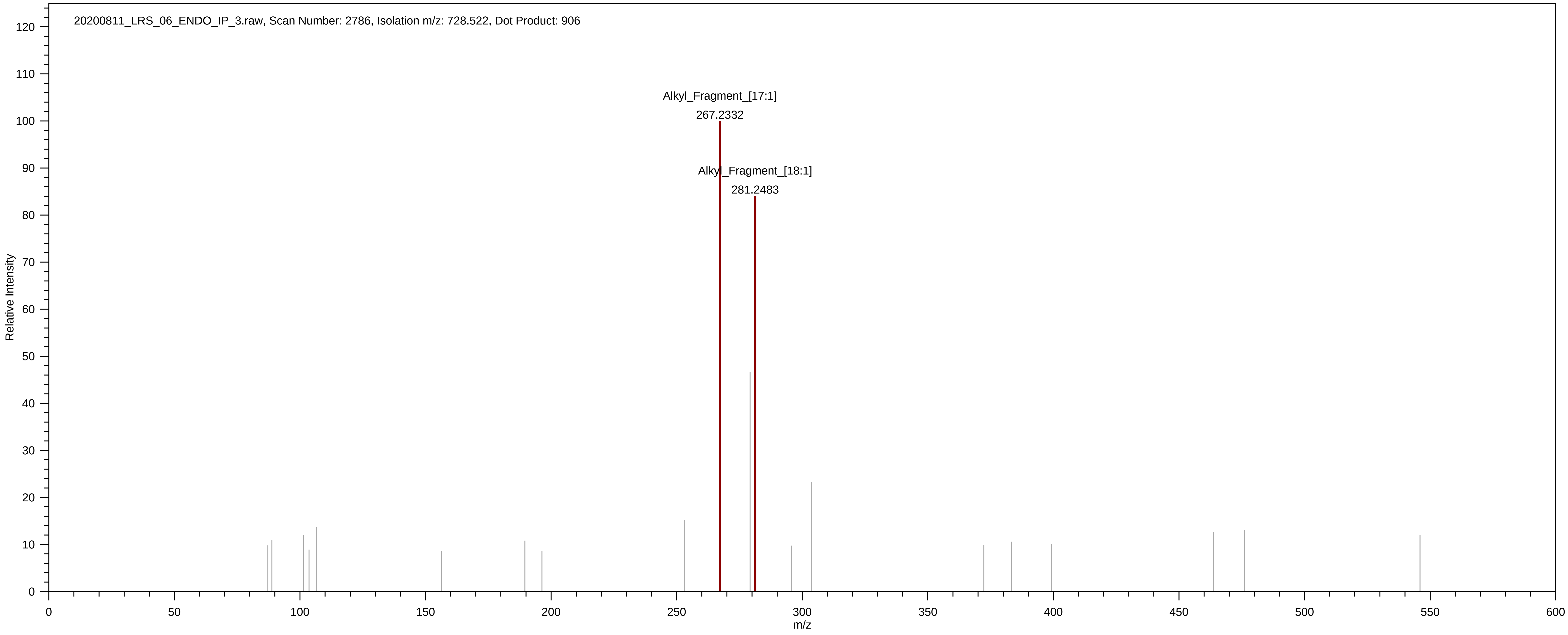

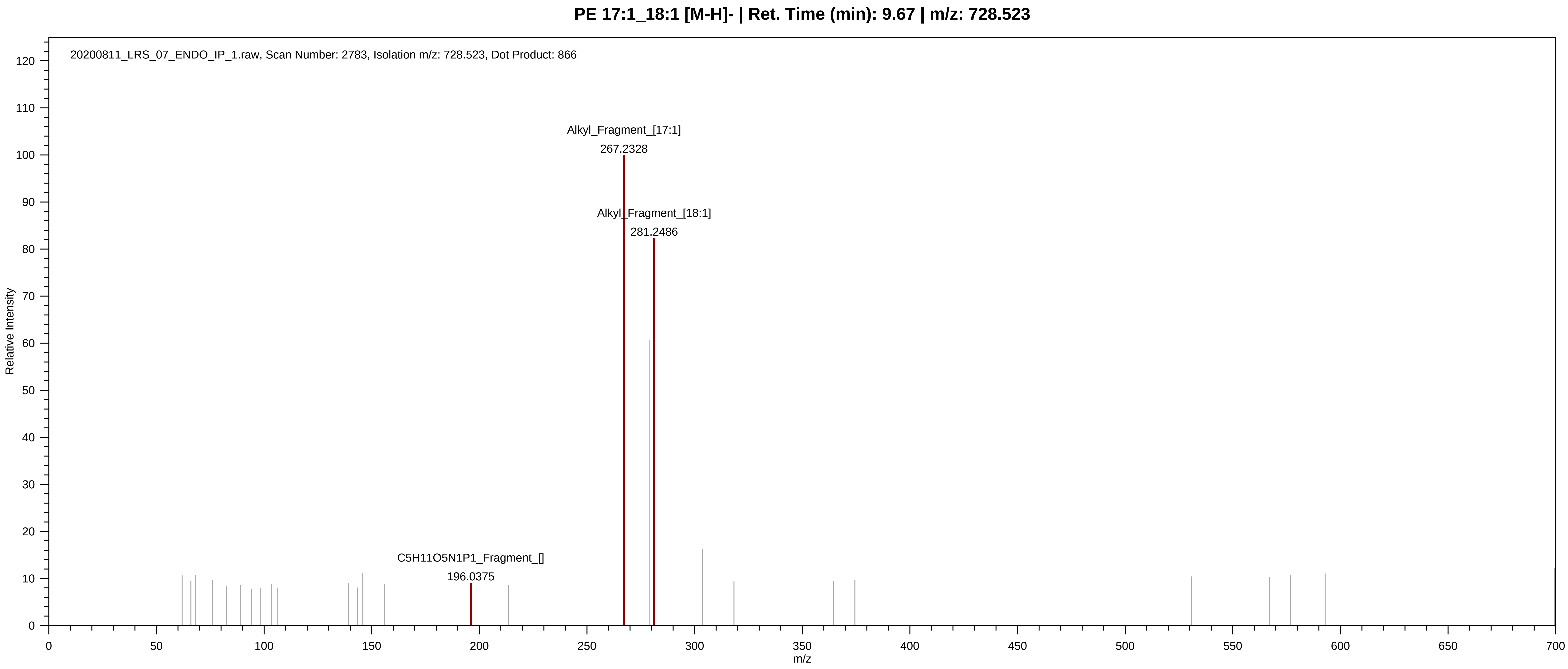

PE 17:1_18:1 [M-H]- | Ret. Time (min): 9.67 | m/z: 728.523
20200811_LRS_07_ENDO_IP_1.raw, Scan Number: 2783, Isolation m/z: 728.523, Dot Product: 866
Alkyl_Fragment_[17:1]
267.2328
Alkyl_Fragment_[18:1]
281.2486
C5H11O5N1P1_Fragment_[]
196.0375
Relative Intensity
m/z

PE 17:1_18:1 [M-H]- | Ret. Time (min): 9.666 | m/z: 728.523

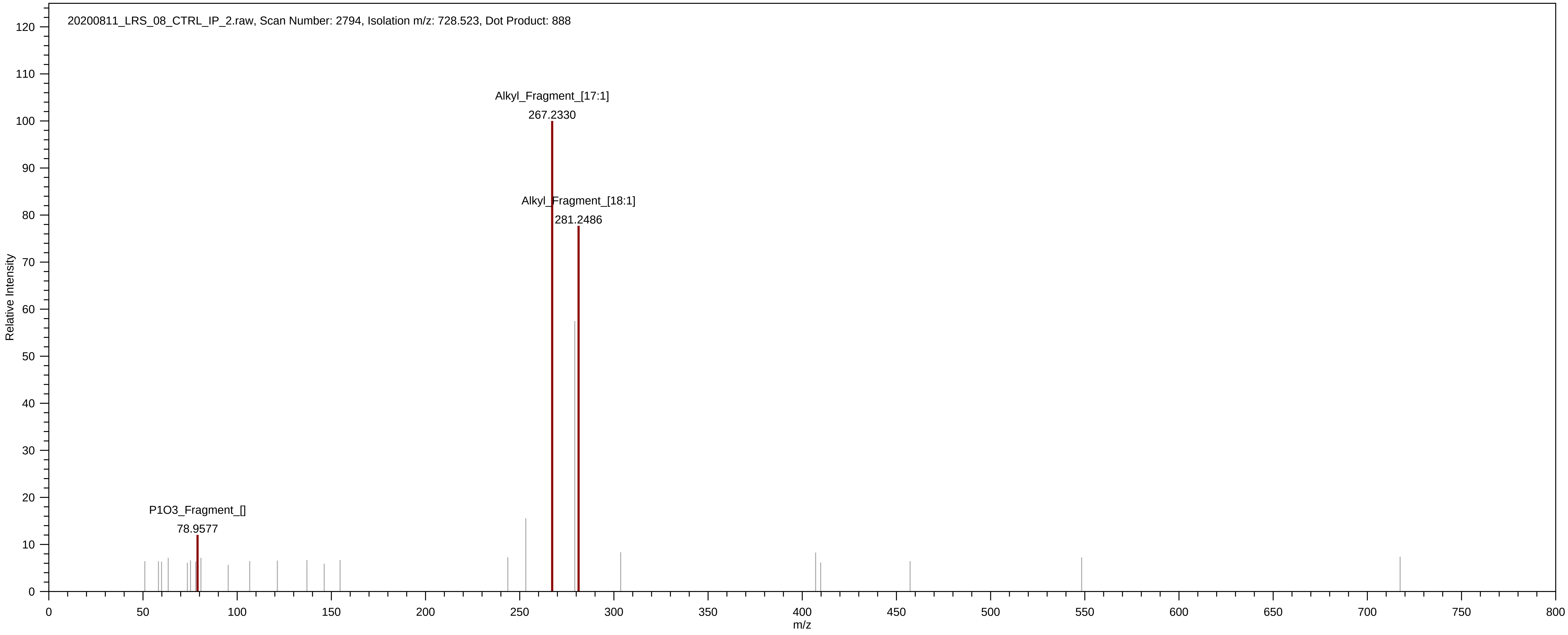

PE 17:1_18:1 [M-H]- | Ret. Time (min): 9.688 | m/z: 728.523

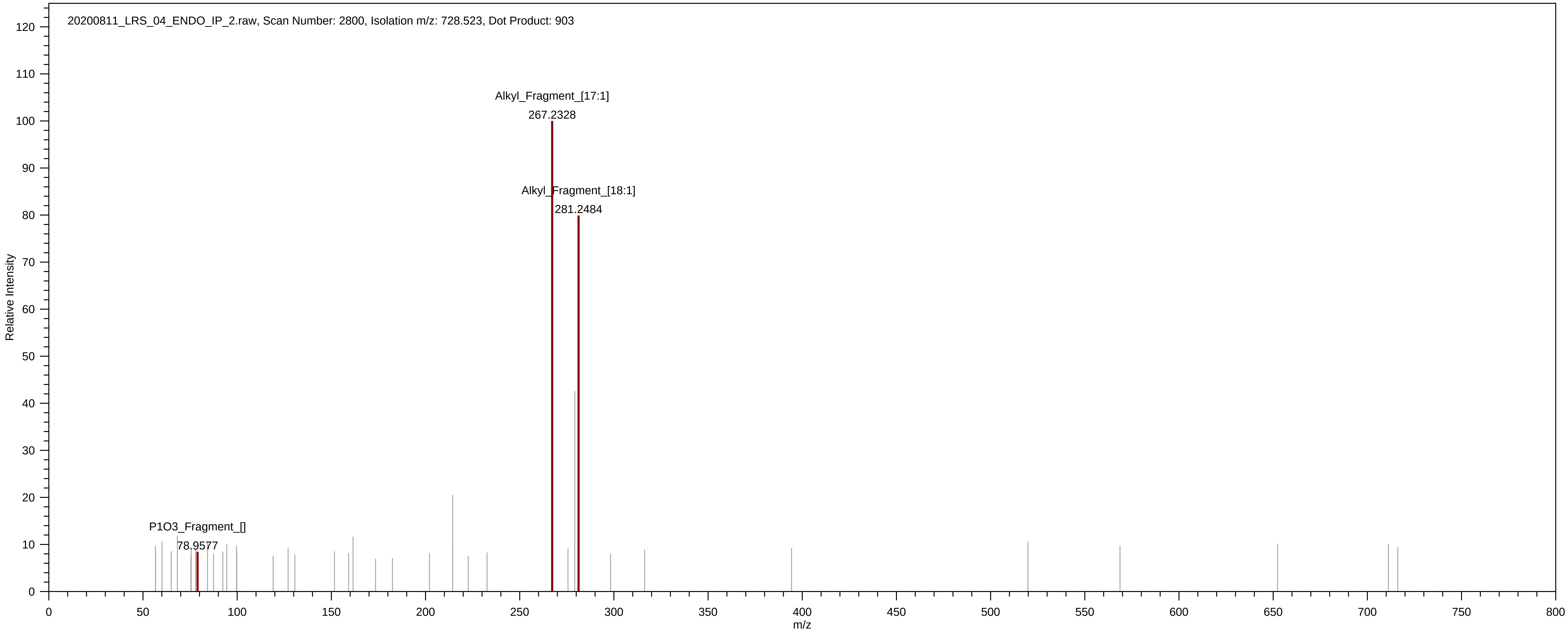

PE 18:0_20:3 [M-H]- | Ret. Time (min): 10.824 | m/z: 768.556

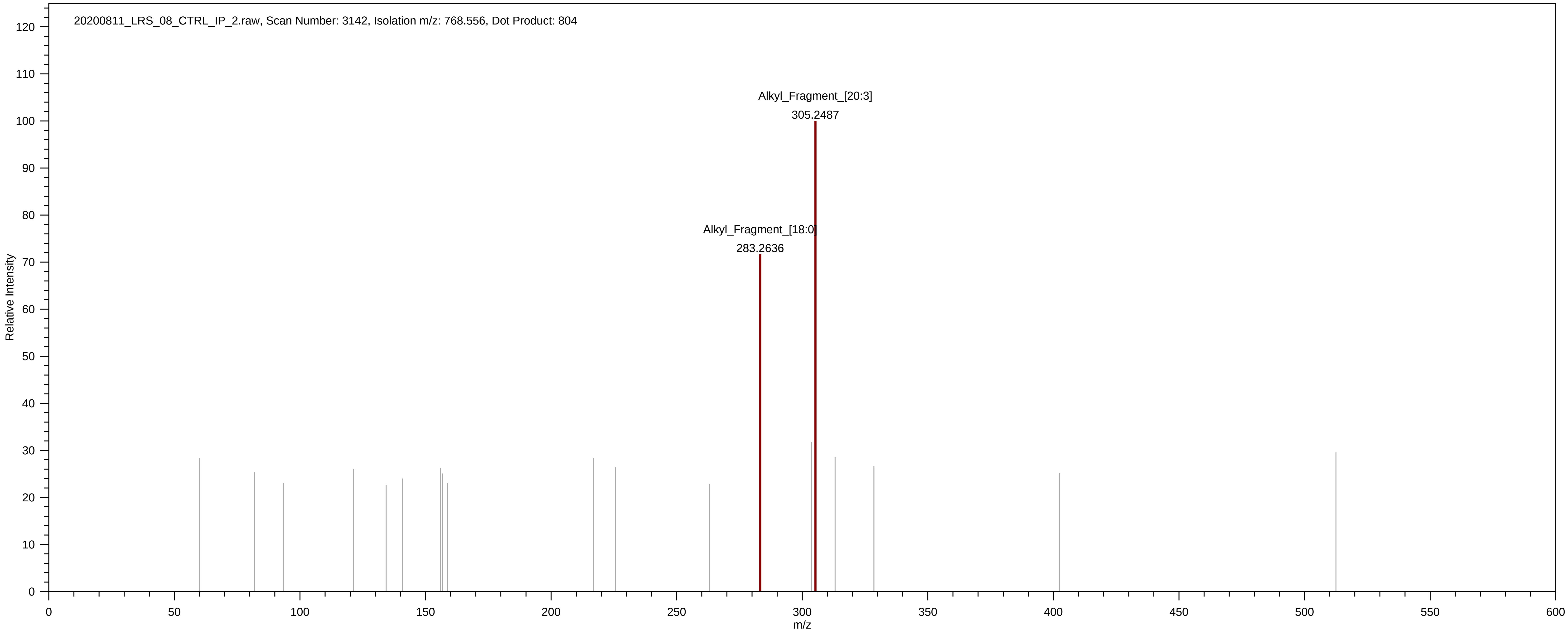

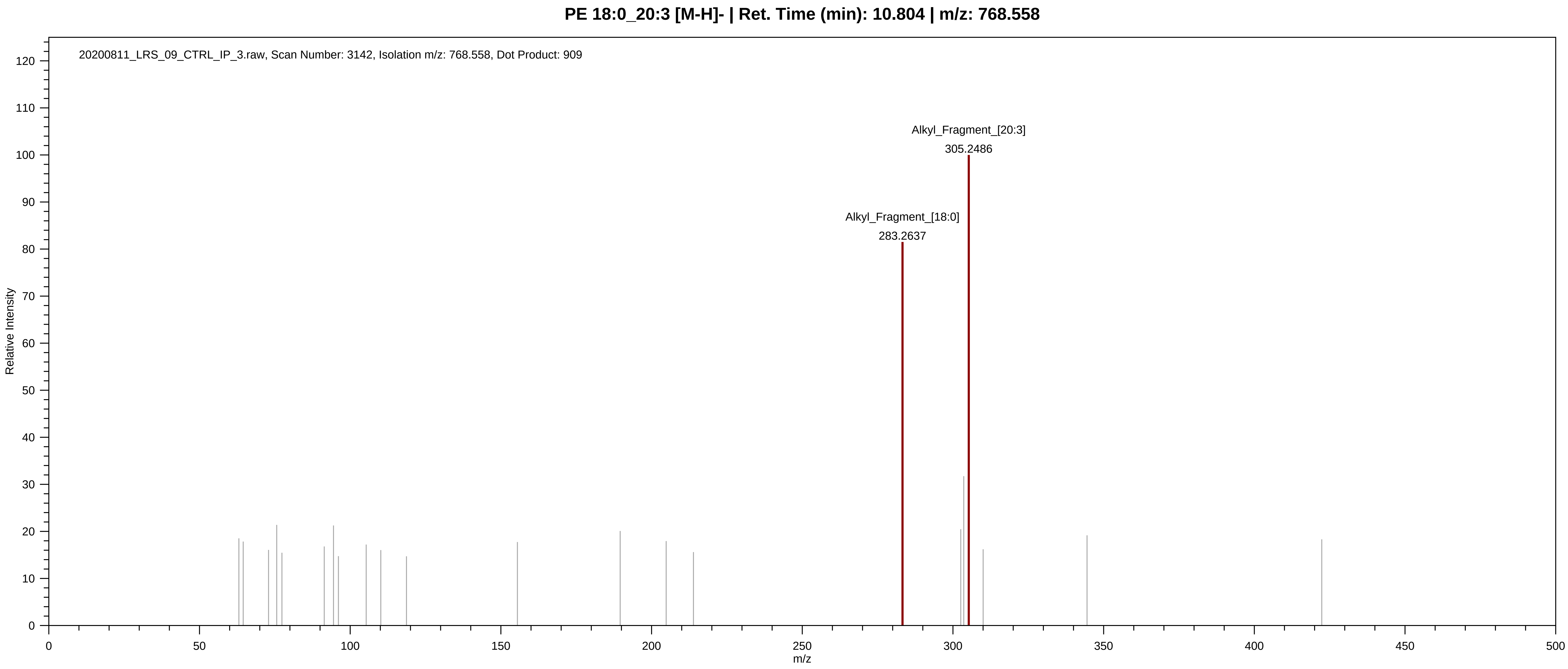
PE 18:0_20:3 [M-H]- | Ret. Time (min): 10.804 | m/z: 768.558
20200811_LRS_09_CTRL_IP_3.raw, Scan Number: 3142, Isolation m/z: 768.558, Dot Product: 909
Alkyl_Fragment_[20:3]
305.2486
Alkyl_Fragment_[18:0]
283.2637
Relative Intensity
m/z

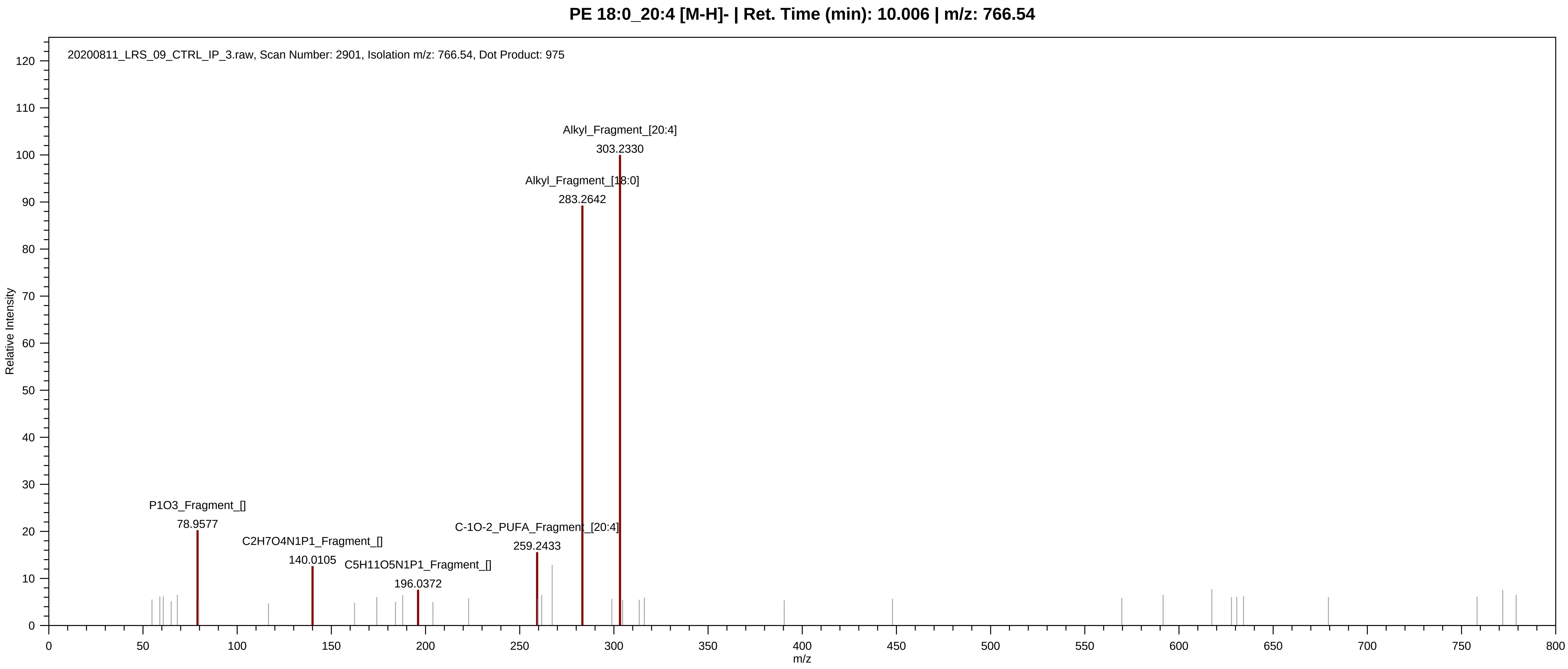
PE 18:0_20:4 [M-H]- | Ret. Time (min): 10.006 | m/z: 766.54
20200811_LRS_09_CTRL_IP_3.raw, Scan Number: 2901, Isolation m/z: 766.54, Dot Product: 975
Alkyl_Fragment_[20:4]
303.2330
Alkyl_Fragment_[18:0]
283.2642
P1O3_Fragment_[]
78.9577
C2H7O4N1P1_Fragment_[]
140.0105
C5H11O5N1P1_Fragment_[]
196.0372
C-1O-2_PUFA_Fragment_[20:4]
259.2433
Relative Intensity
m/z
0
10
20
30
40
50
60
70
80
90
100
110
120
0
50
100
150
200
250
300
350
400
450
500
550
600
650
700
750
800

PE 18:0_20:4 [M-H]- | Ret. Time (min): 10.024 | m/z: 766.54

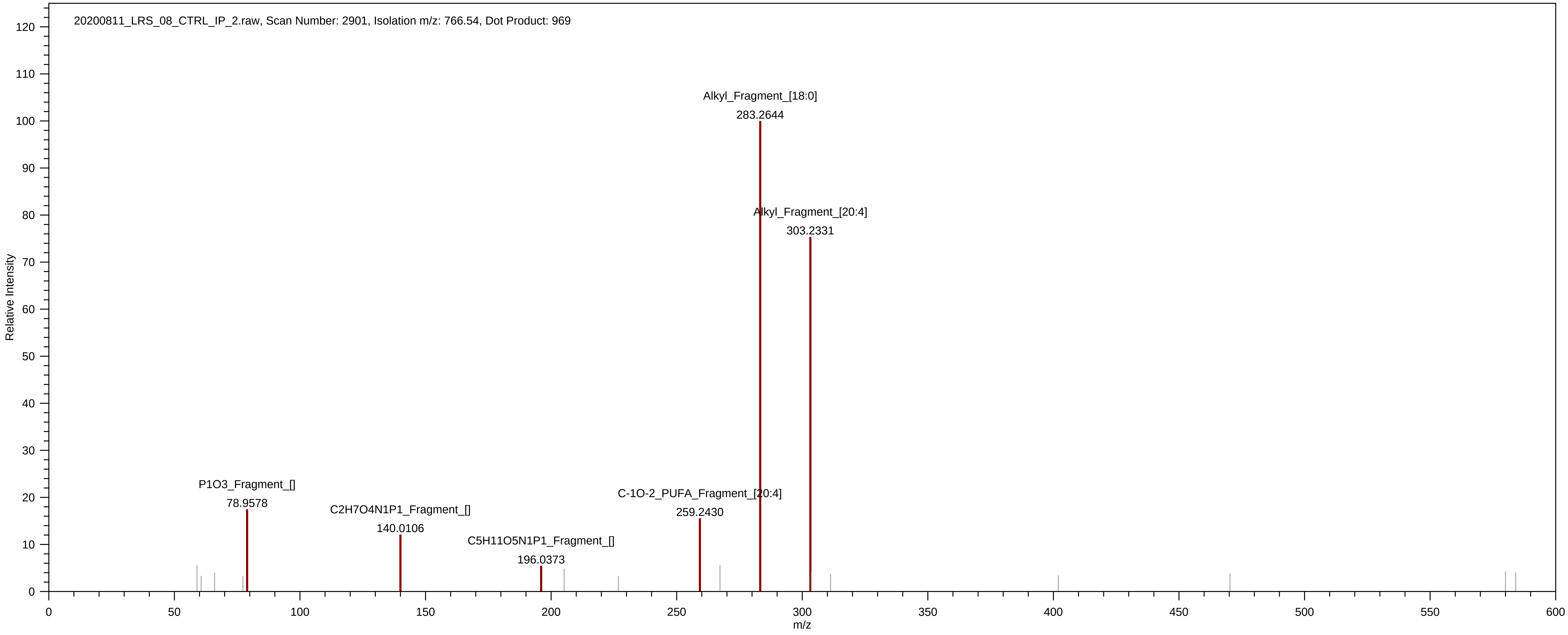

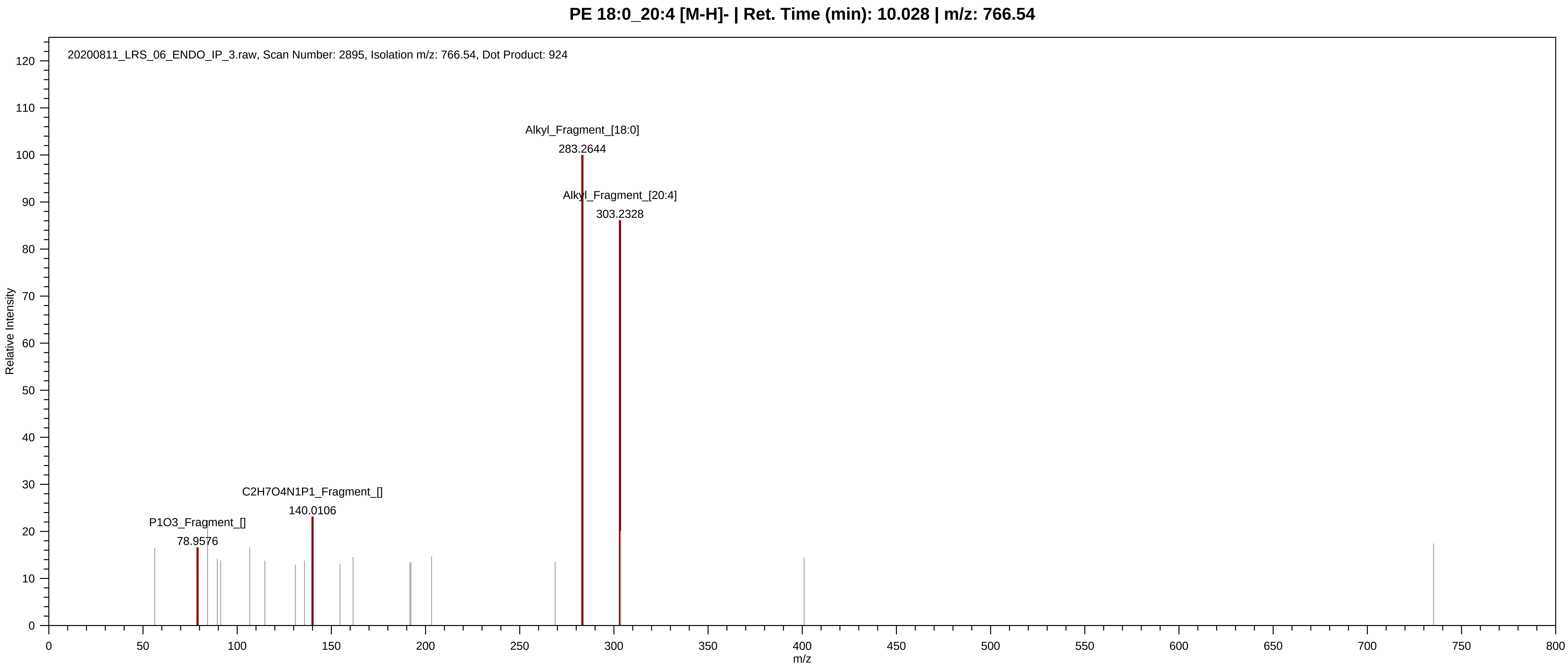
PE 18:0_20:4 [M-H]- | Ret. Time (min): 10.028 | m/z: 766.54
20200811_LRS_06_ENDO_IP_3.raw, Scan Number: 2895, Isolation m/z: 766.54, Dot Product: 924
Alkyl_Fragment_[18:0]
283.2644
Alkyl_Fragment_[20:4]
303.2328
C2H7O4N1P1_Fragment_[]
140.0106
P1O3_Fragment_[]
78.9576
Relative Intensity
m/z

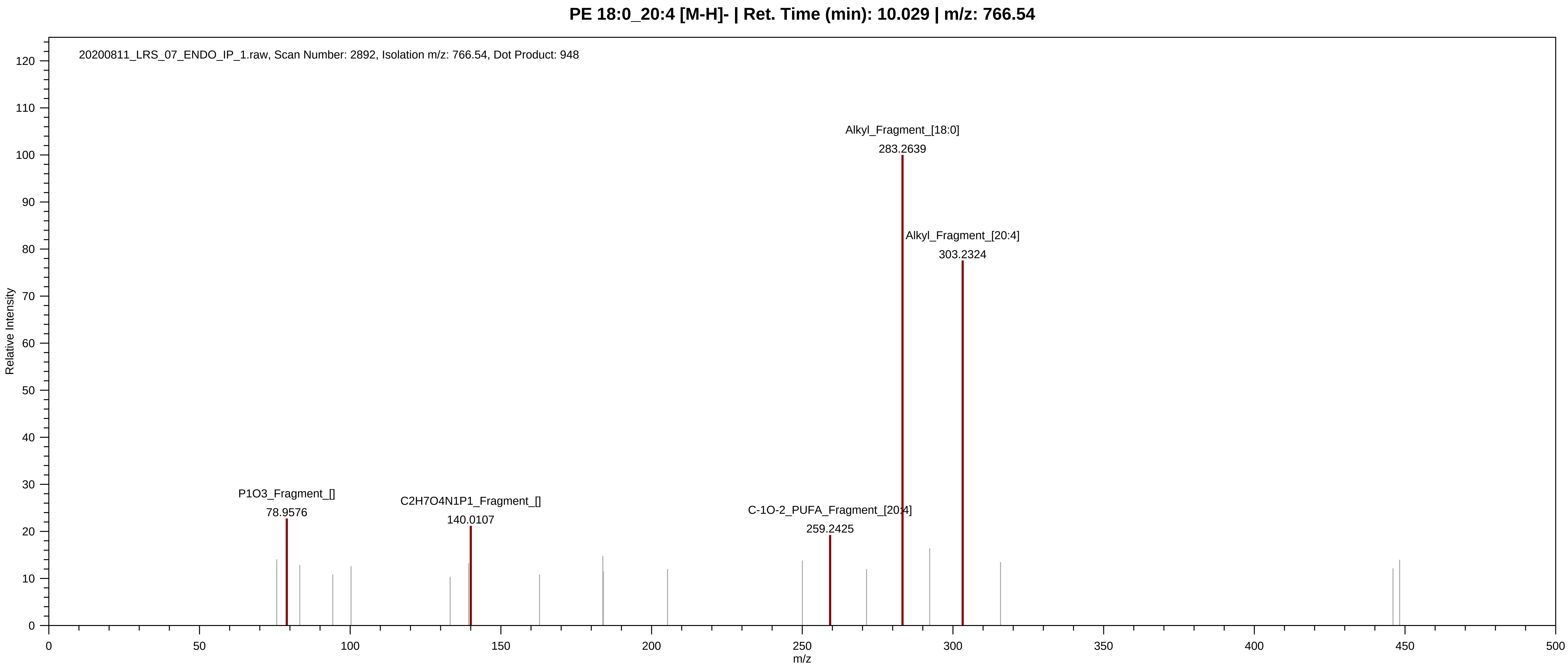
PE 18:0_20:4 [M-H]- | Ret. Time (min): 10.029 | m/z: 766.54
20200811_LRS_07_ENDO_IP_1.raw, Scan Number: 2892, Isolation m/z: 766.54, Dot Product: 948
Alkyl_Fragment_[18:0]
283.2639
Alkyl_Fragment_[20:4]
303.2324
P1O3_Fragment_[]
78.9576
C2H7O4N1P1_Fragment_[]
140.0107
C-1O-2_PUFA_Fragment_[20:4]
259.2425
Relative Intensity
m/z

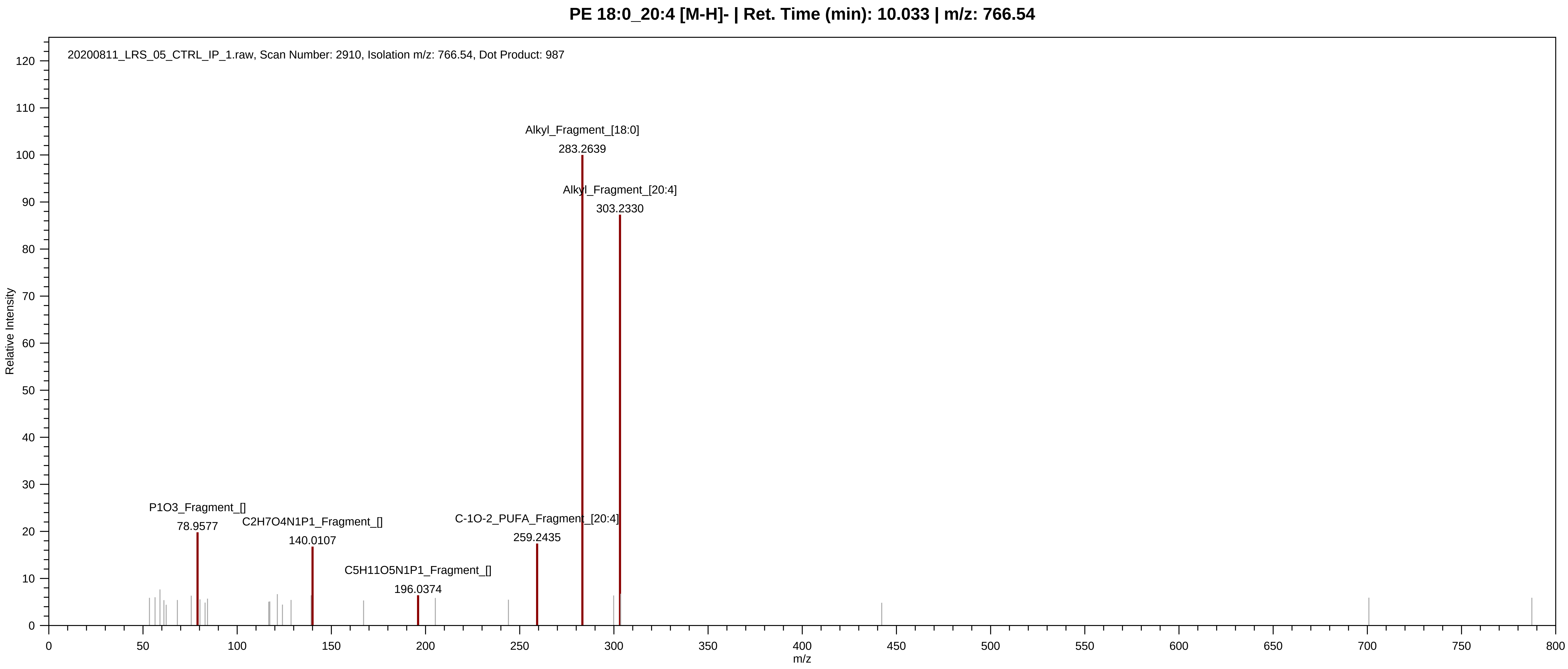
PE 18:0_20:4 [M-H]- | Ret. Time (min): 10.033 | m/z: 766.54
20200811_LRS_05_CTRL_IP_1.raw, Scan Number: 2910, Isolation m/z: 766.54, Dot Product: 987
Alkyl_Fragment_[18:0]
283.2639
Alkyl_Fragment_[20:4]
303.2330
P1O3_Fragment_[]
78.9577
C2H7O4N1P1_Fragment_[]
140.0107
C-1O-2_PUFA_Fragment_[20:4]
259.2435
C5H11O5N1P1_Fragment_[]
196.0374
Relative Intensity
m/z
0
10
20
30
40
50
60
70
80
90
100
110
120
0
50
100
150
200
250
300
350
400
450
500
550
600
650
700
750
800

PE 18:0_20:4 [M-H]- | Ret. Time (min): 10.045 | m/z: 766.54

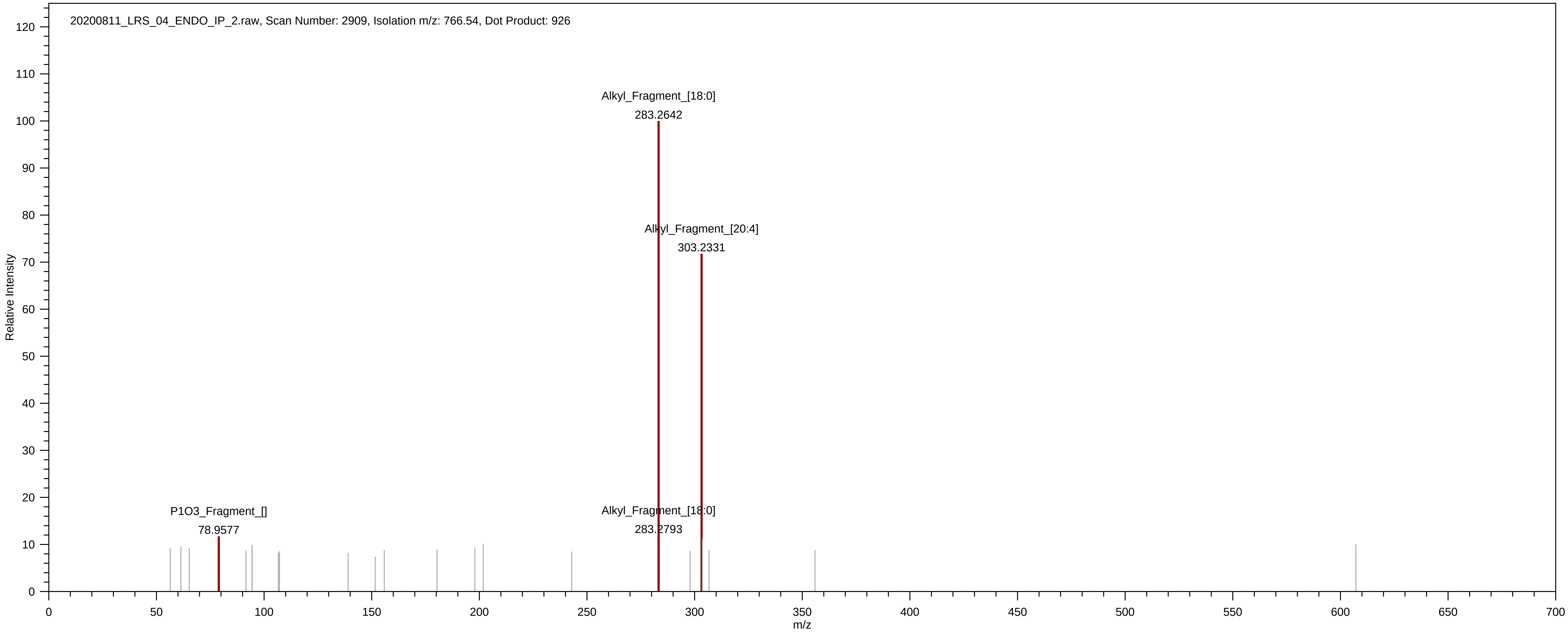

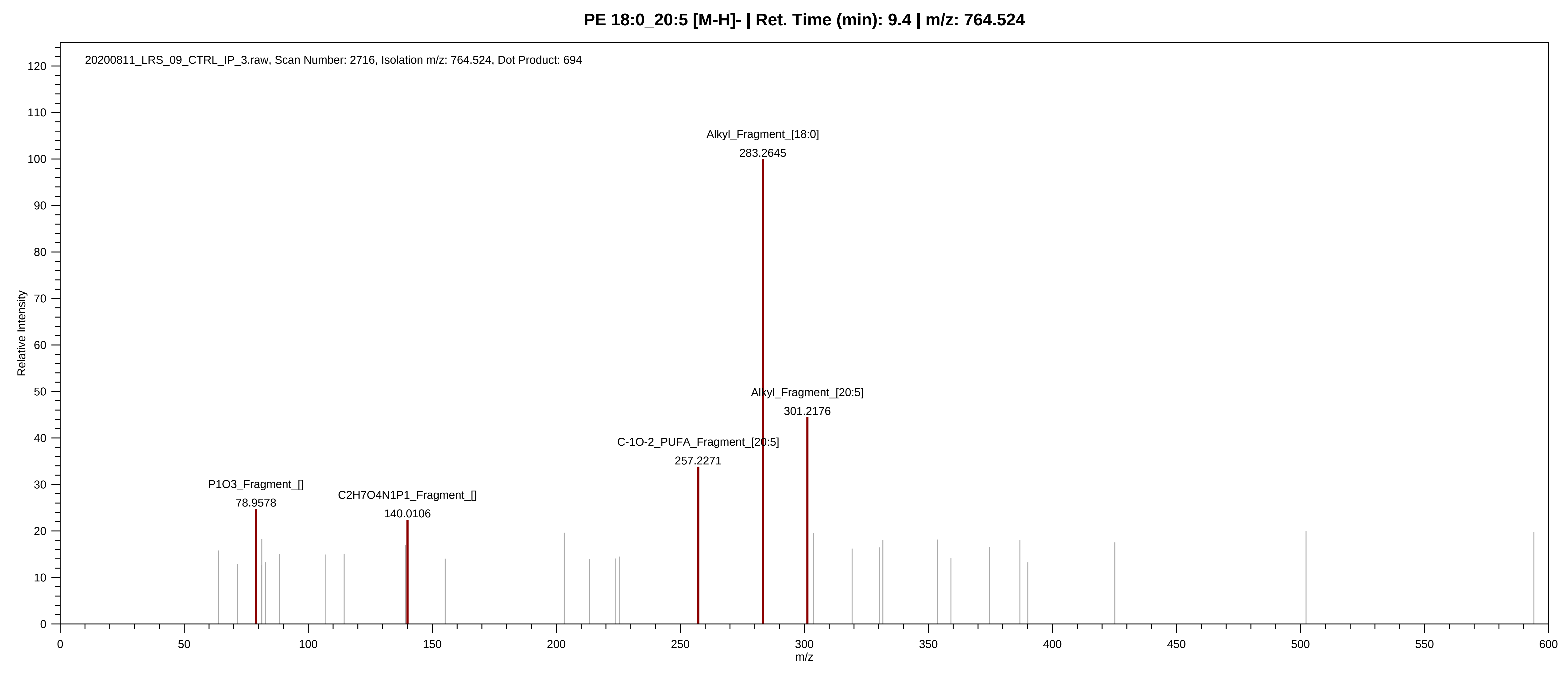

PE 18:0_20:5 [M-H]- | Ret. Time (min): 9.4 | m/z: 764.524
20200811_LRS_09_CTRL_IP_3.raw, Scan Number: 2716, Isolation m/z: 764.524, Dot Product: 694
Alkyl_Fragment_[18:0]
283.2645
Alkyl_Fragment_[20:5]
301.2176
C-1O-2_PUFA_Fragment_[20:5]
257.2271
P1O3_Fragment_[]
78.9578
C2H7O4N1P1_Fragment_[]
140.0106
Relative Intensity
m/z

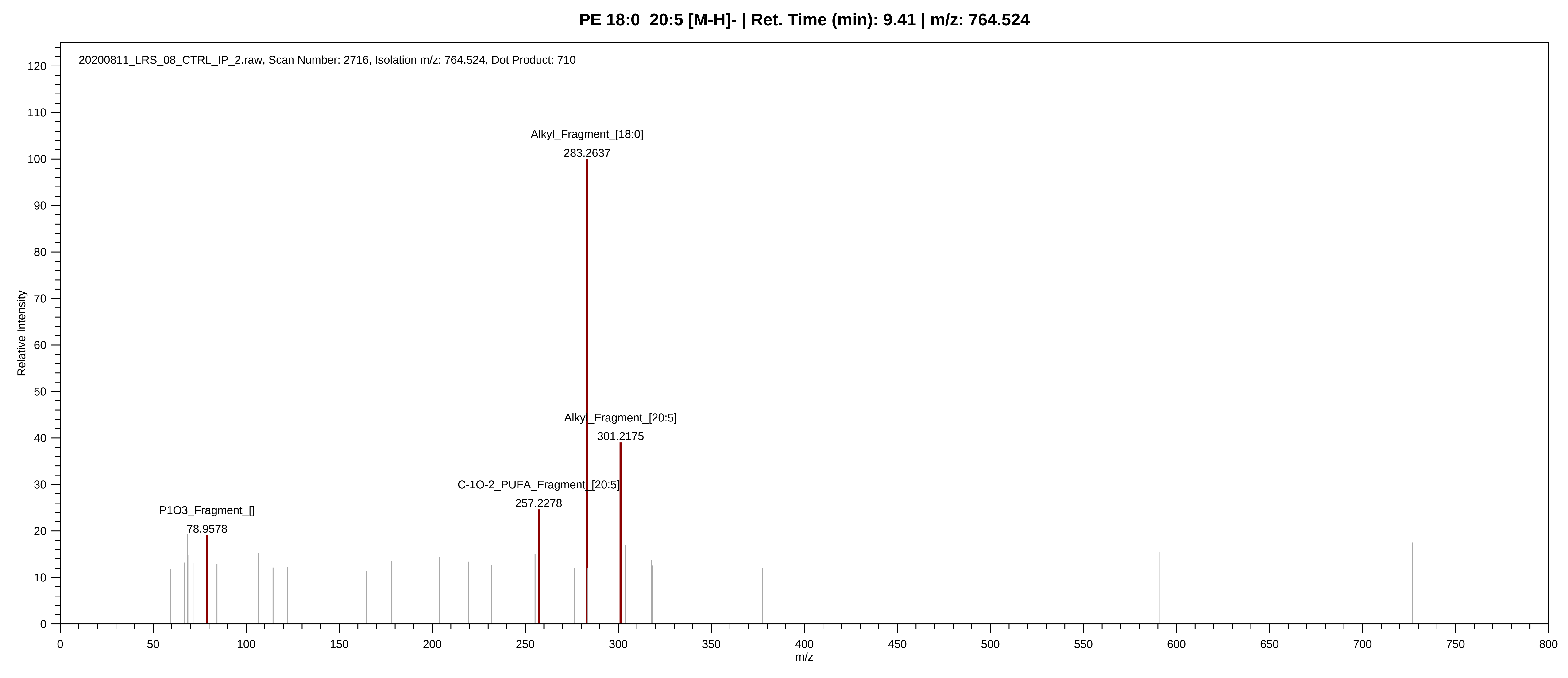
PE 18:0_20:5 [M-H]- | Ret. Time (min): 9.41 | m/z: 764.524
20200811_LRS_08_CTRL_IP_2.raw, Scan Number: 2716, Isolation m/z: 764.524, Dot Product: 710
Alkyl_Fragment_[18:0]
283.2637
Alkyl_Fragment_[20:5]
301.2175
C-1O-2_PUFA_Fragment_[20:5]
257.2278
P1O3_Fragment_[]
78.9578
Relative Intensity
m/z

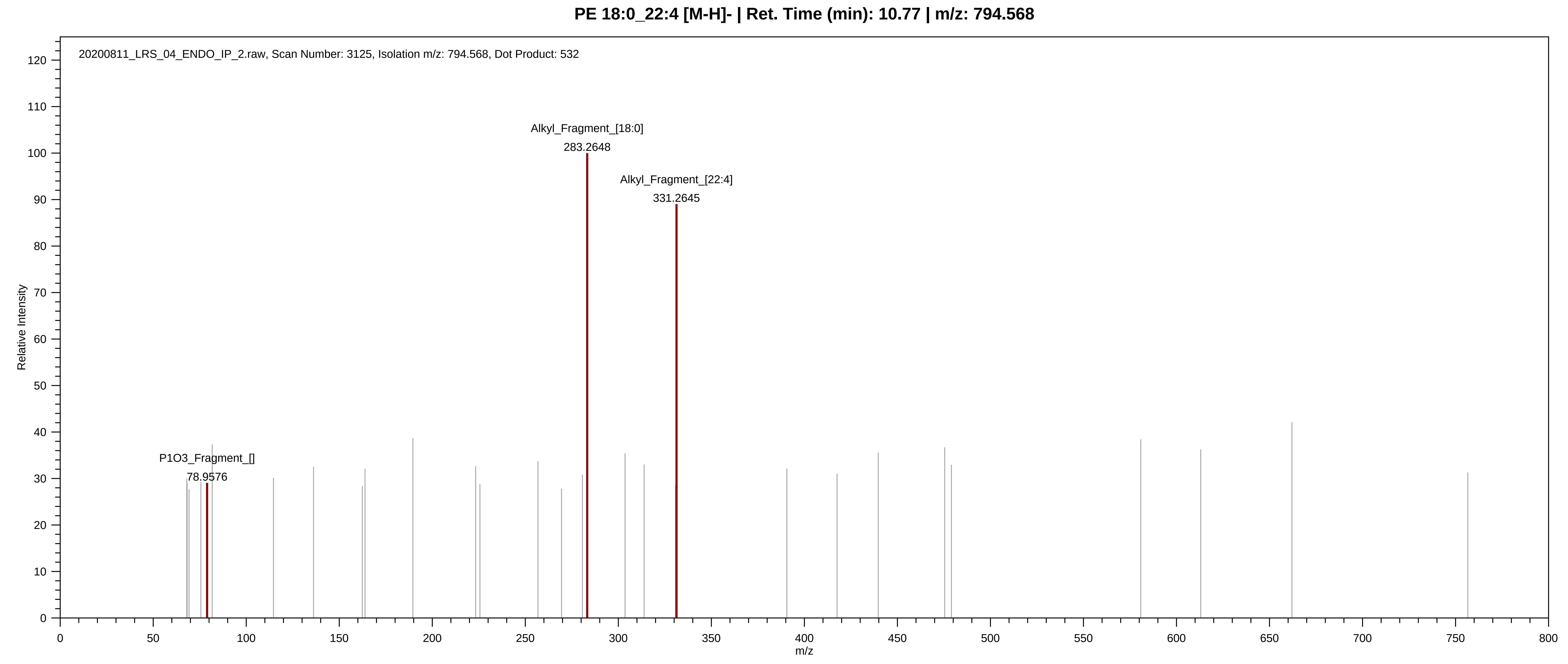

PE 18:0_22:4 [M-H]- | Ret. Time (min): 10.77 | m/z: 794.568
20200811_LRS_04_ENDO_IP_2.raw, Scan Number: 3125, Isolation m/z: 794.568, Dot Product: 532
Alkyl_Fragment_[18:0]
283.2648
Alkyl_Fragment_[22:4]
331.2645
P1O3_Fragment_[]
78.9576
Relative Intensity
m/z

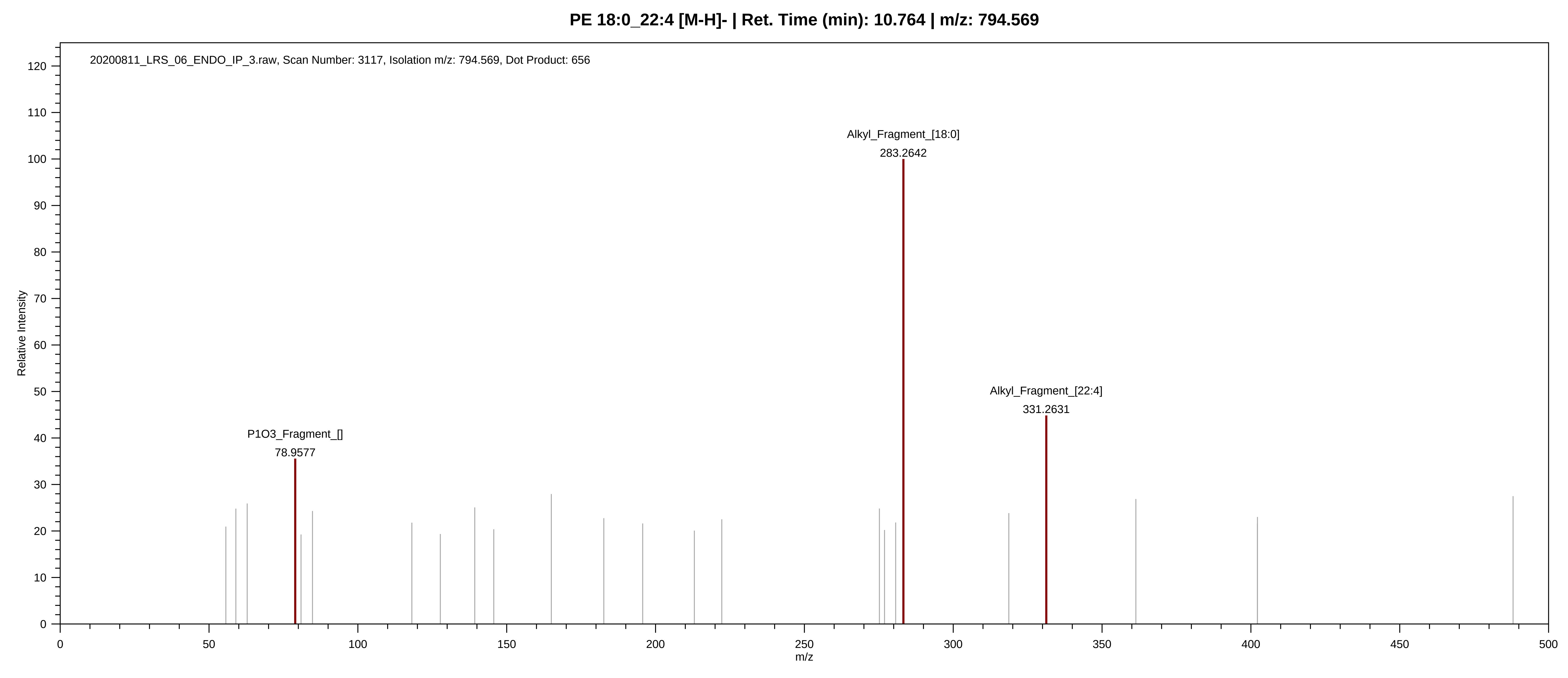

PE 18:0_22:4 [M-H]- | Ret. Time (min): 10.764 | m/z: 794.569
20200811_LRS_06_ENDO_IP_3.raw, Scan Number: 3117, Isolation m/z: 794.569, Dot Product: 656
Alkyl_Fragment_[18:0]
283.2642
Alkyl_Fragment_[22:4]
331.2631
P1O3_Fragment_[]
78.9577
Relative Intensity
m/z

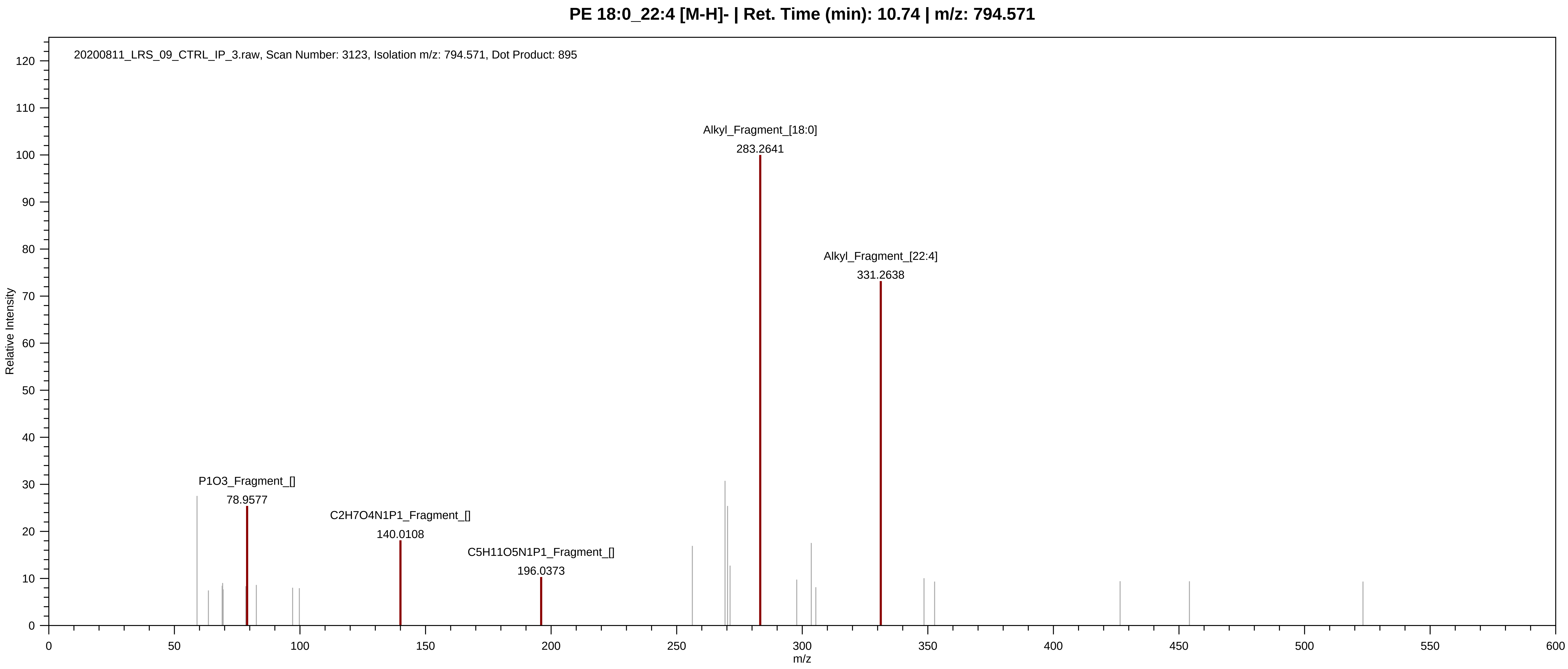

PE 18:0_22:4 [M-H]- | Ret. Time (min): 10.74 | m/z: 794.571
20200811_LRS_09_CTRL_IP_3.raw, Scan Number: 3123, Isolation m/z: 794.571, Dot Product: 895
Alkyl_Fragment_[18:0]
283.2641
Alkyl_Fragment_[22:4]
331.2638
P1O3_Fragment_[]
78.9577
C2H7O4N1P1_Fragment_[]
140.0108
C5H11O5N1P1_Fragment_[]
196.0373
Relative Intensity
m/z

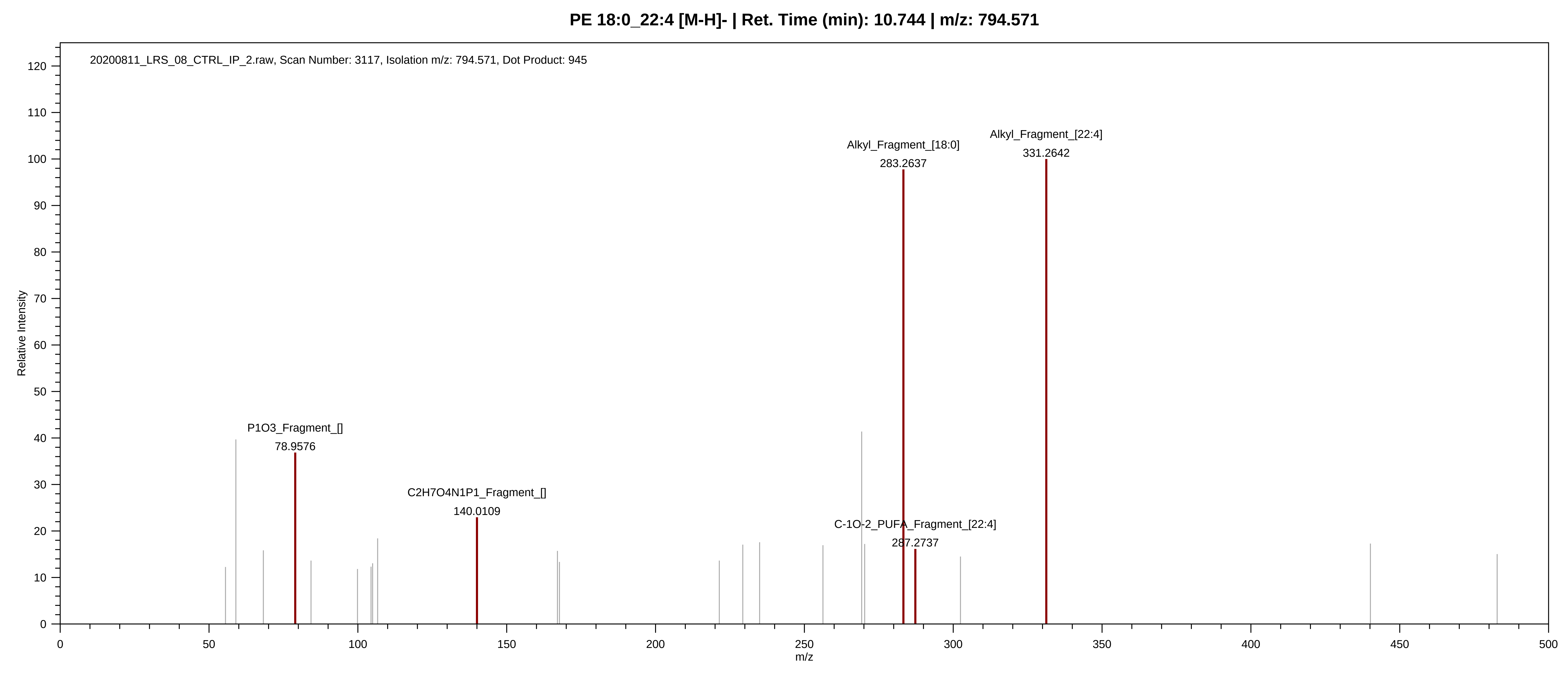

PE 18:0_22:4 [M-H]- | Ret. Time (min): 10.744 | m/z: 794.571
20200811_LRS_08_CTRL_IP_2.raw, Scan Number: 3117, Isolation m/z: 794.571, Dot Product: 945
Alkyl_Fragment_[22:4]
331.2642
Alkyl_Fragment_[18:0]
283.2637
P1O3_Fragment_[]
78.9576
C2H7O4N1P1_Fragment_[]
140.0109
C-1O-2_PUFA_Fragment_[22:4]
287.2737
Relative Intensity
m/z

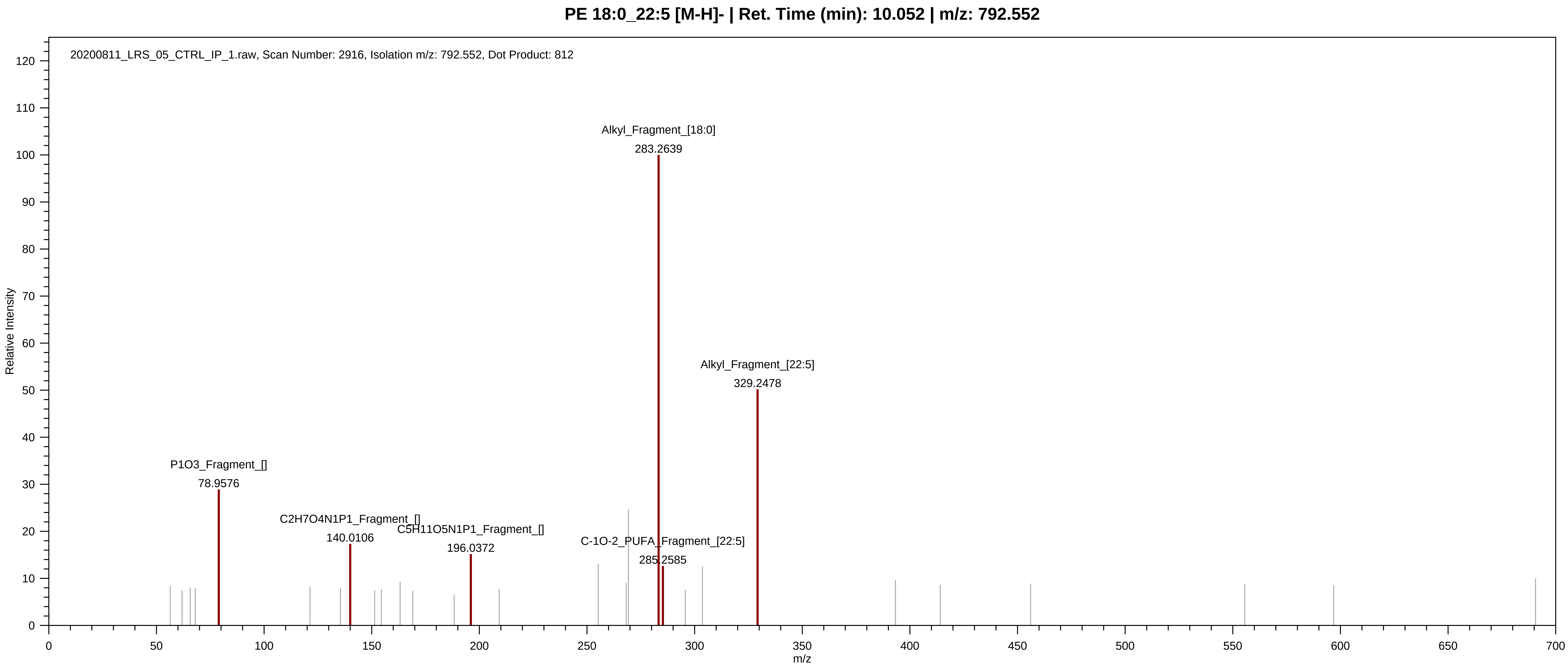

PE 18:0_22:5 [M-H]- | Ret. Time (min): 10.052 | m/z: 792.552
20200811_LRS_05_CTRL_IP_1.raw, Scan Number: 2916, Isolation m/z: 792.552, Dot Product: 812
Alkyl_Fragment_[18:0]
283.2639
Alkyl_Fragment_[22:5]
329.2478
P1O3_Fragment_[]
78.9576
C2H7O4N1P1_Fragment_[]
140.0106
C5H11O5N1P1_Fragment_[]
196.0372
C-1O-2_PUFA_Fragment_[22:5]
285.2585
Relative Intensity
m/z

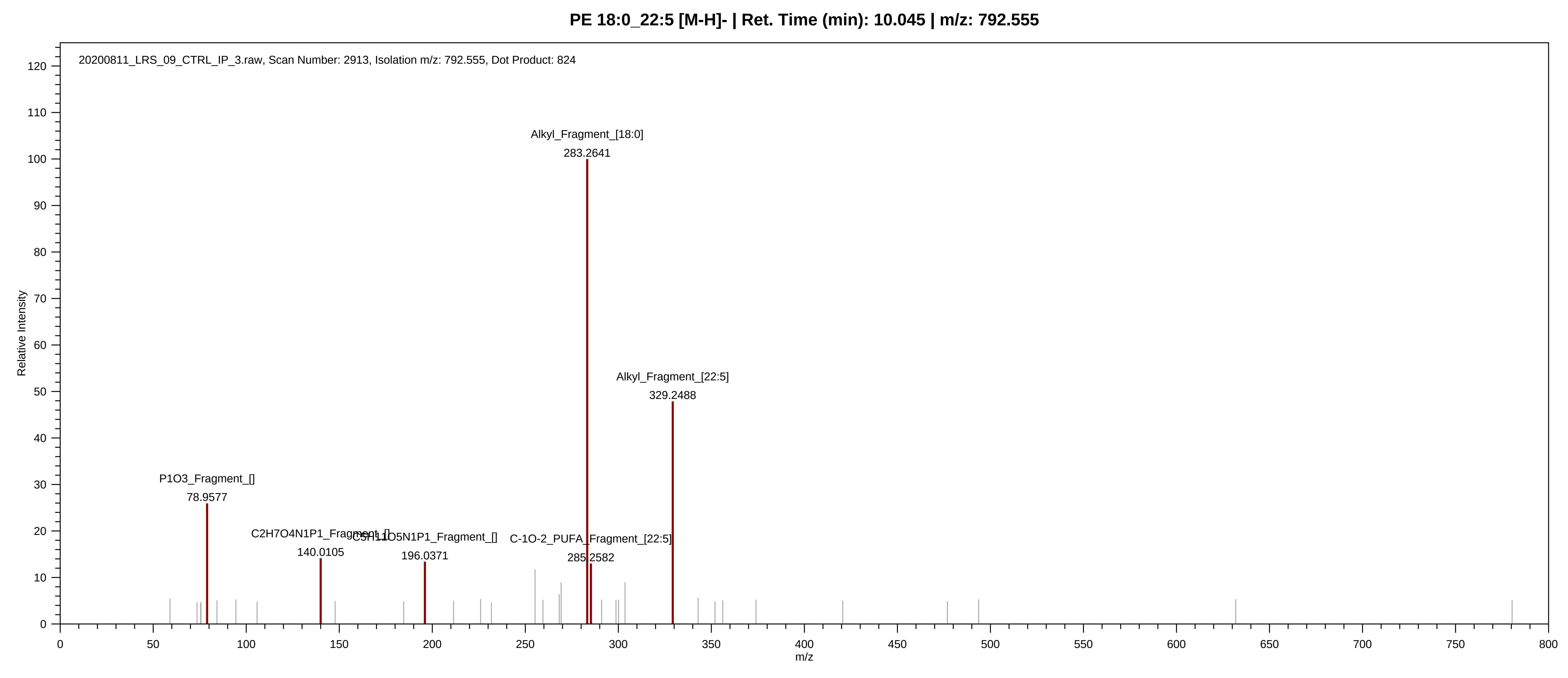
PE 18:0_22:5 [M-H]- | Ret. Time (min): 10.045 | m/z: 792.555
20200811_LRS_09_CTRL_IP_3.raw, Scan Number: 2913, Isolation m/z: 792.555, Dot Product: 824
Alkyl_Fragment_[18:0]
283.2641
Alkyl_Fragment_[22:5]
329.2488
P1O3_Fragment_[]
78.9577
C2H7O4N1P1_Fragment_[]
140.0105
C5H11O5N1P1_Fragment_[]
196.0371
C-1O-2_PUFA_Fragment_[22:5]
285.2582
Relative Intensity
m/z

PE 18:0_22:5 [M-H]- | Ret. Time (min): 10.049 | m/z: 792.555

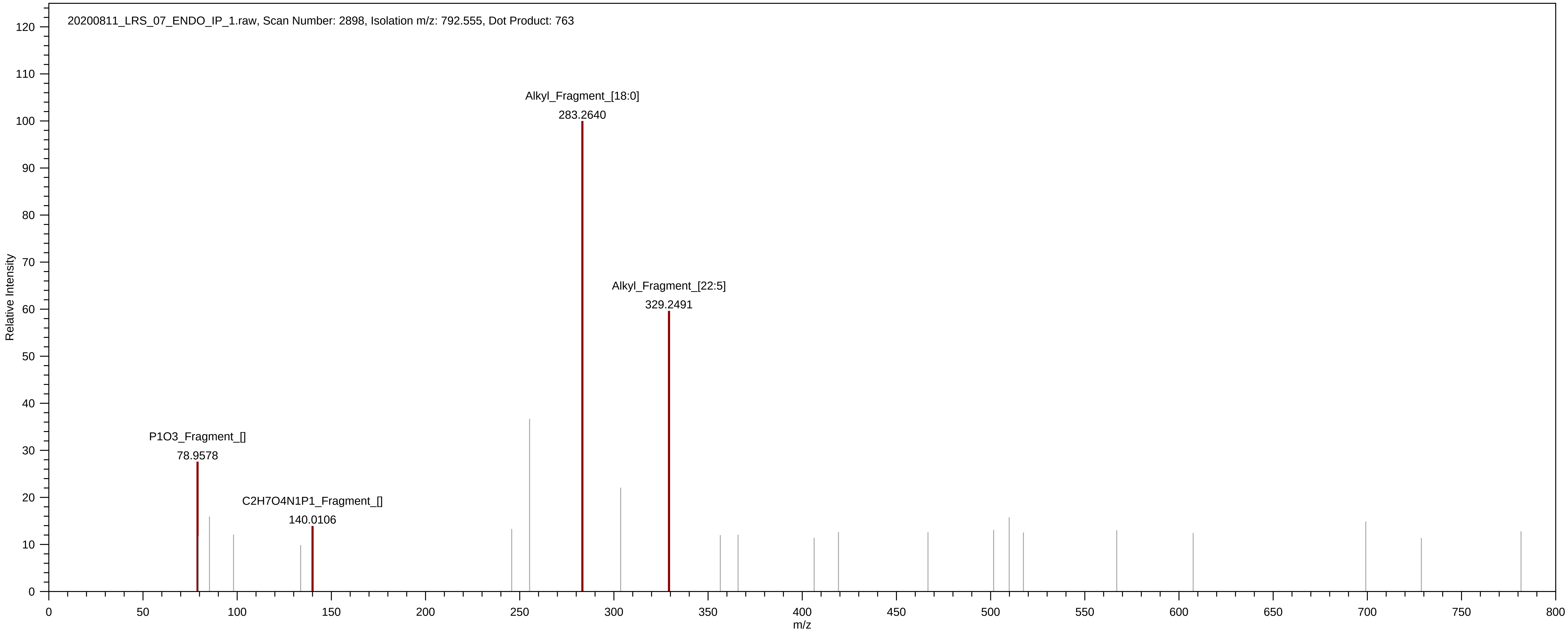

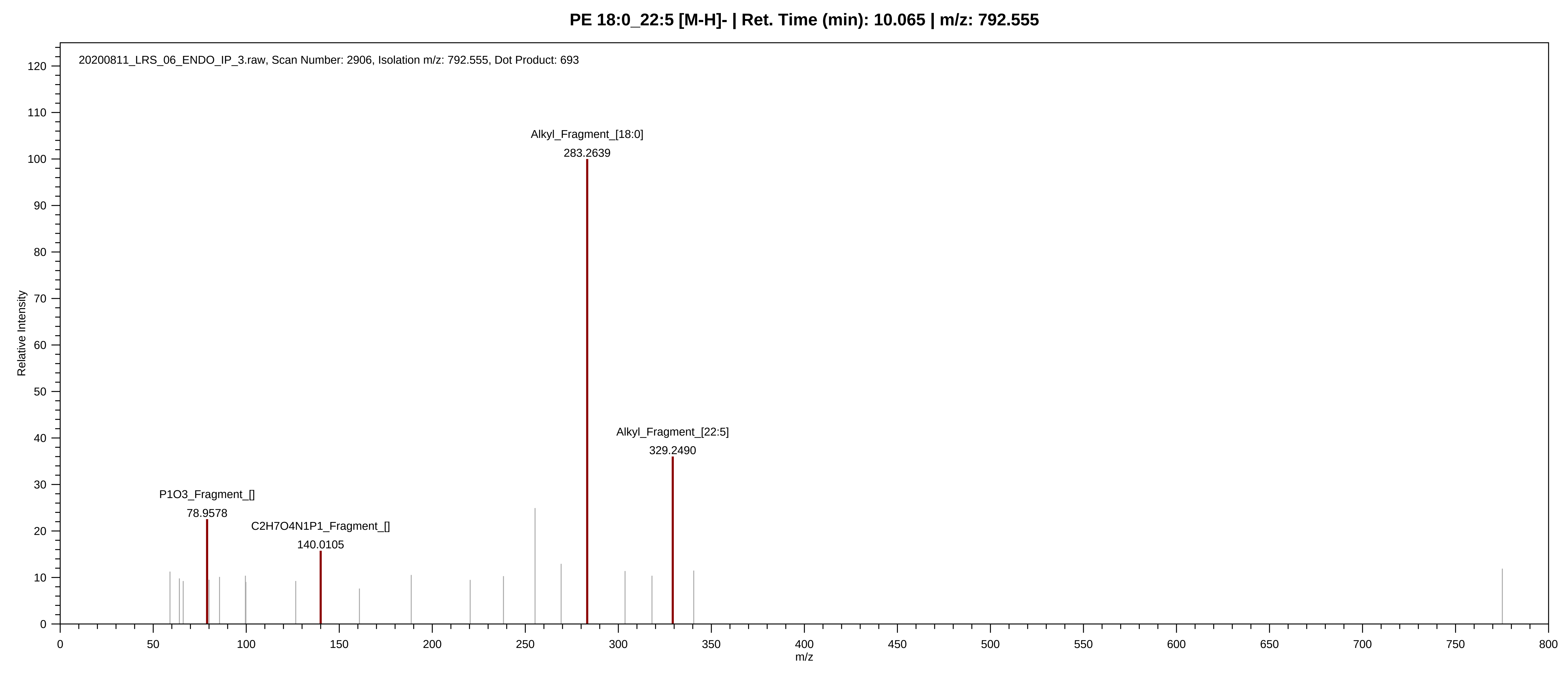
PE 18:0_22:5 [M-H]- | Ret. Time (min): 10.065 | m/z: 792.555
20200811_LRS_06_ENDO_IP_3.raw, Scan Number: 2906, Isolation m/z: 792.555, Dot Product: 693
Alkyl_Fragment_[18:0]
283.2639
Alkyl_Fragment_[22:5]
329.2490
P1O3_Fragment_[]
78.9578
C2H7O4N1P1_Fragment_[]
140.0105
Relative Intensity
m/z

PE 18:1_18:0 [M-H]- | Ret. Time (min): 11.003 | m/z: 744.551

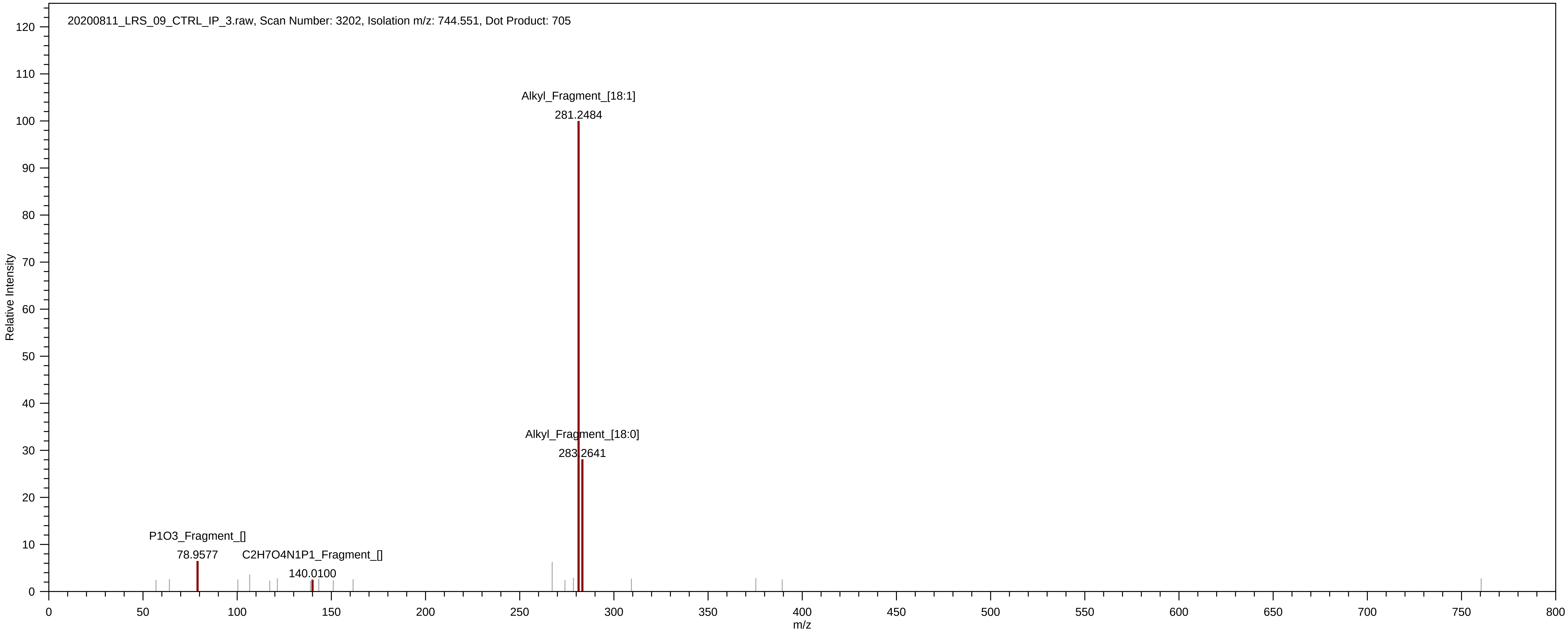

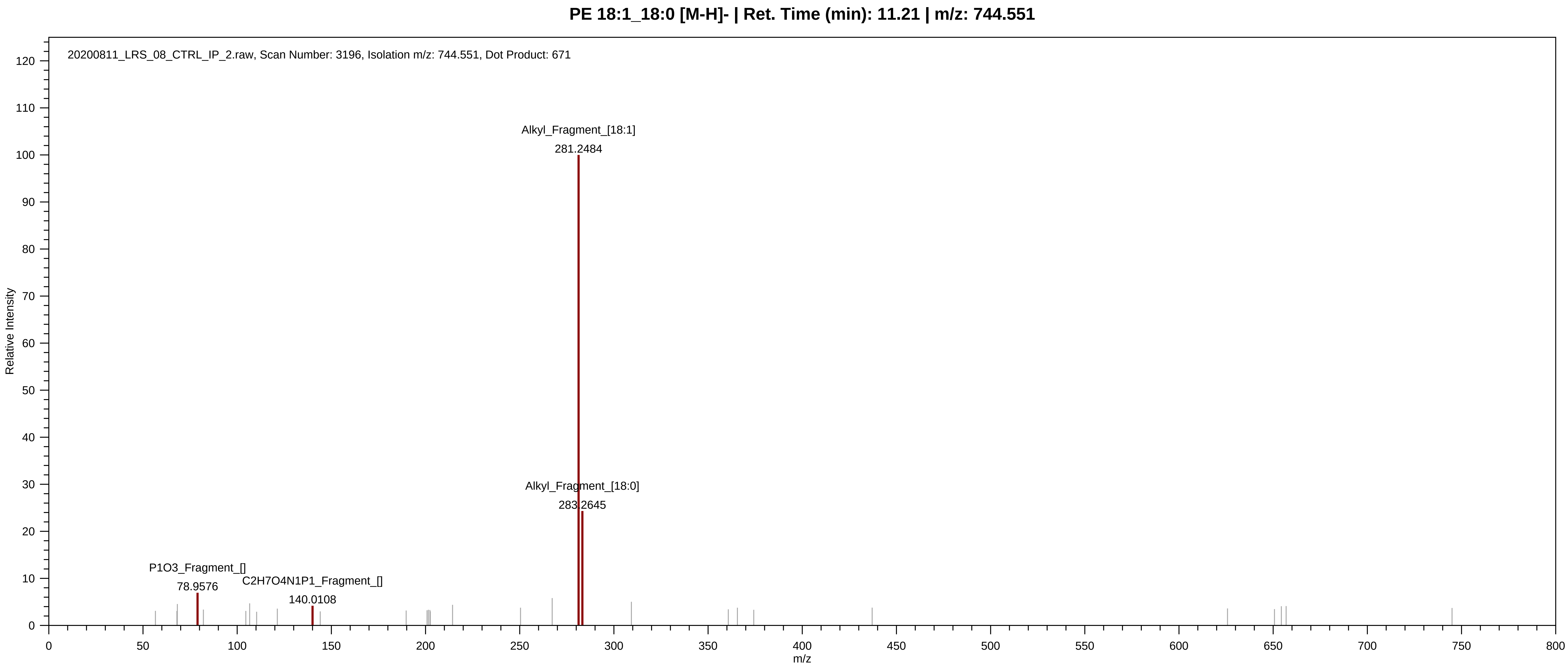

PE 18:1_18:0 [M-H]- | Ret. Time (min): 11.21 | m/z: 744.551
20200811_LRS_08_CTRL_IP_2.raw, Scan Number: 3196, Isolation m/z: 744.551, Dot Product: 671
Alkyl_Fragment_[18:1]
281.2484
Alkyl_Fragment_[18:0]
283.2645
P1O3_Fragment_[]
78.9576
C2H7O4N1P1_Fragment_[]
140.0108
Relative Intensity
m/z

PE 18:1_18:0 [M-H]- | Ret. Time (min): 11.21 | m/z: 744.555

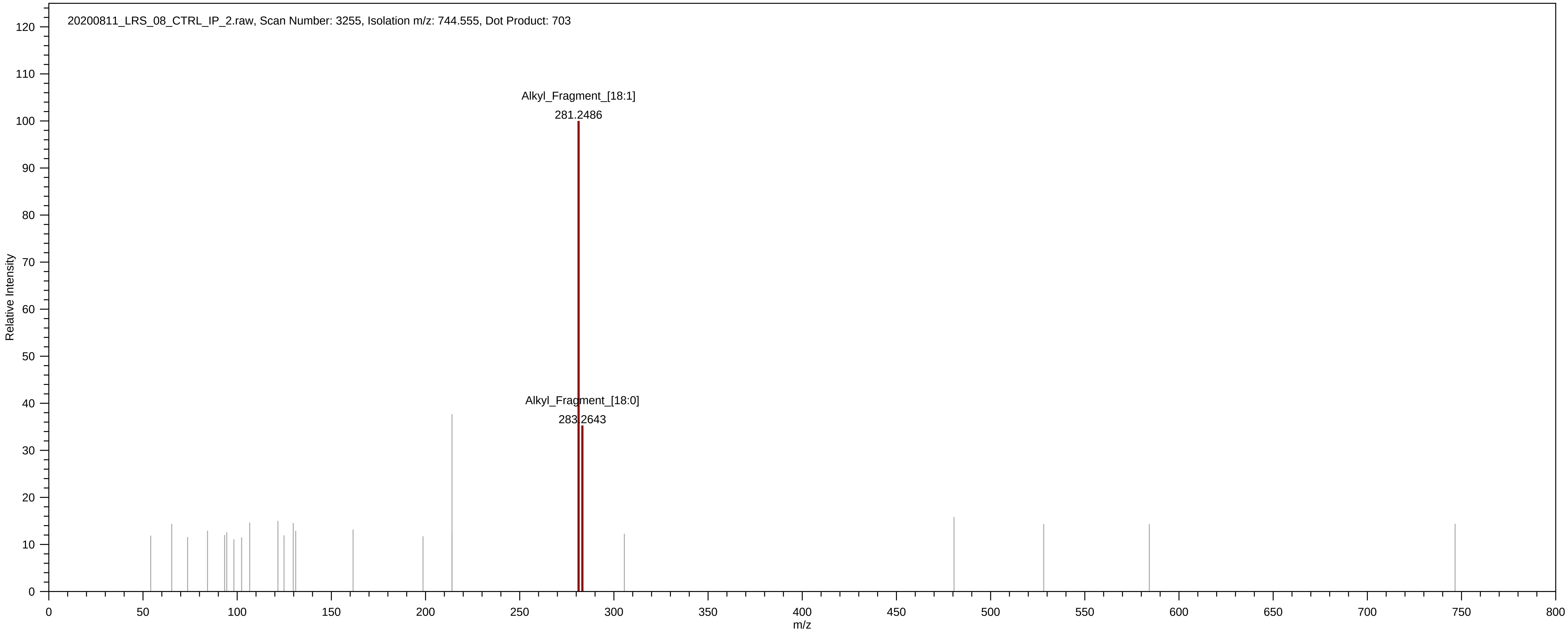

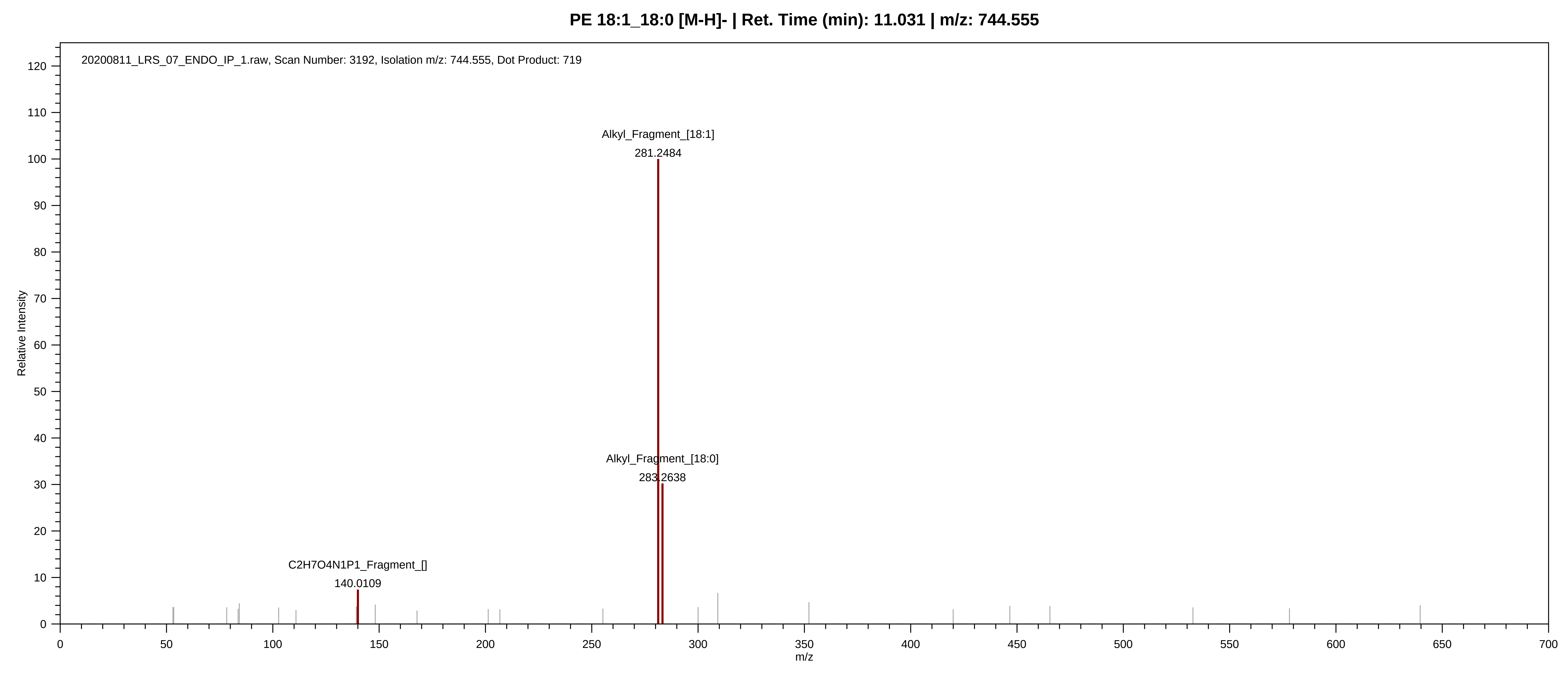

PE 18:1_18:0 [M-H]- | Ret. Time (min): 11.031 | m/z: 744.555
20200811_LRS_07_ENDO_IP_1.raw, Scan Number: 3192, Isolation m/z: 744.555, Dot Product: 719
Alkyl_Fragment_[18:1]
281.2484
Alkyl_Fragment_[18:0]
283.2638
C2H7O4N1P1_Fragment_[]
140.0109
Relative Intensity
m/z

PE 18:1_18:0 [M-H]- | Ret. Time (min): 11.031 | m/z: 744.555

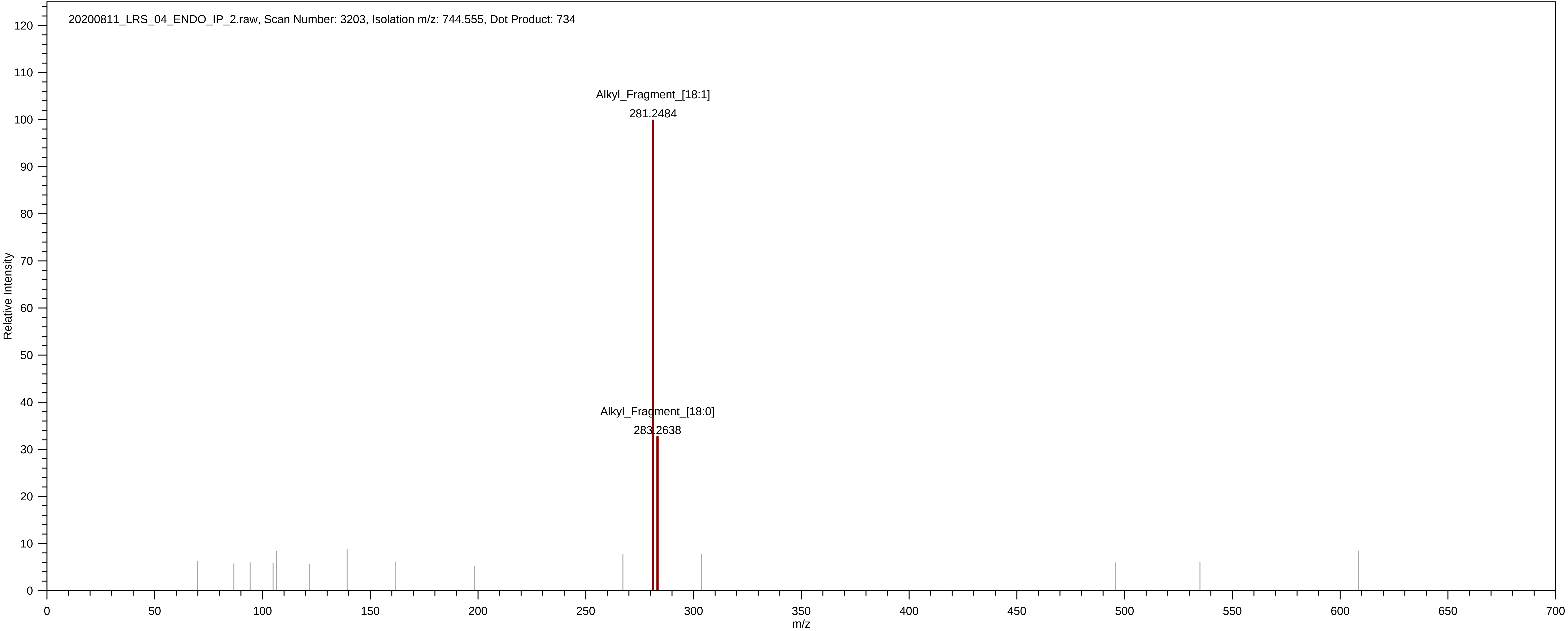

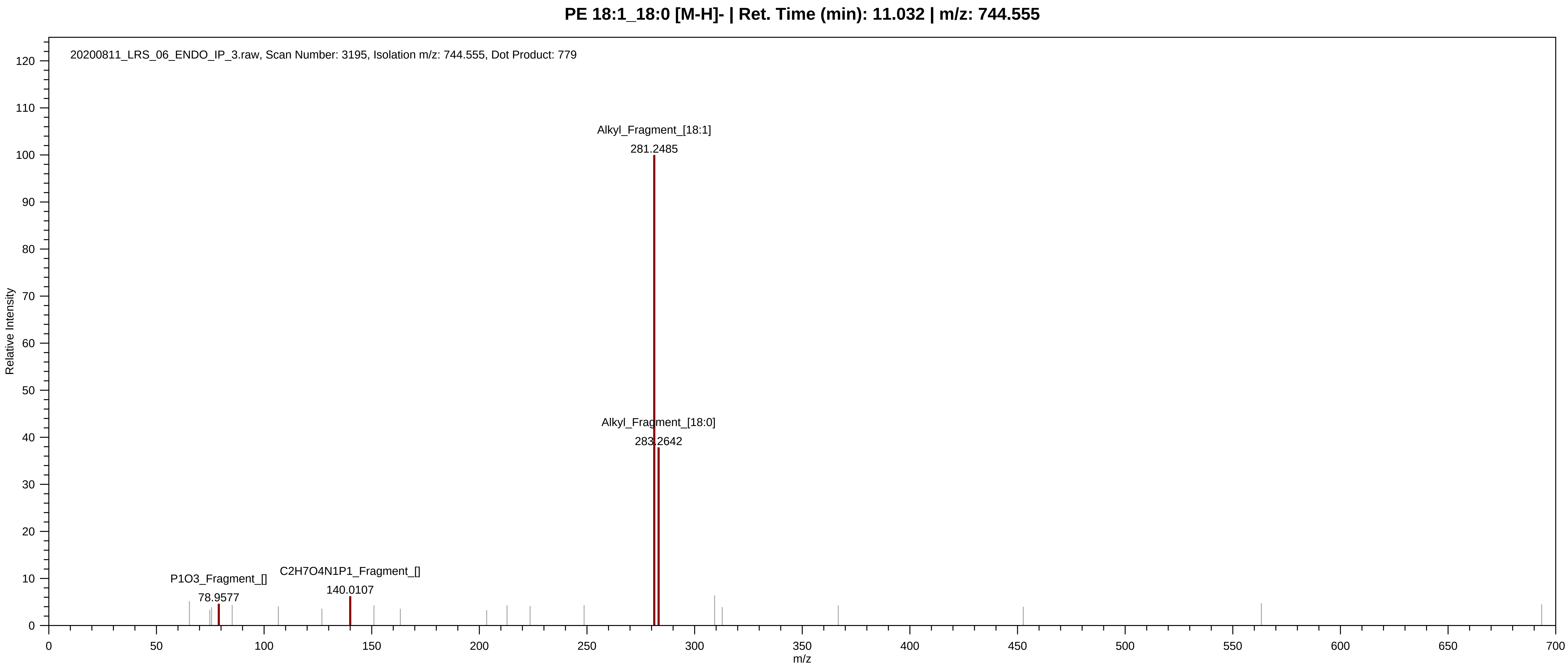

PE 18:1_18:0 [M-H]- | Ret. Time (min): 11.032 | m/z: 744.555
20200811_LRS_06_ENDO_IP_3.raw, Scan Number: 3195, Isolation m/z: 744.555, Dot Product: 779
Alkyl_Fragment_[18:1]
281.2485
Alkyl_Fragment_[18:0]
283.2642
P1O3_Fragment_[]
78.9577
C2H7O4N1P1_Fragment_[]
140.0107
Relative Intensity
m/z

PE 18:1_18:0 [M-H]- | Ret. Time (min): 11.207 | m/z: 744.556

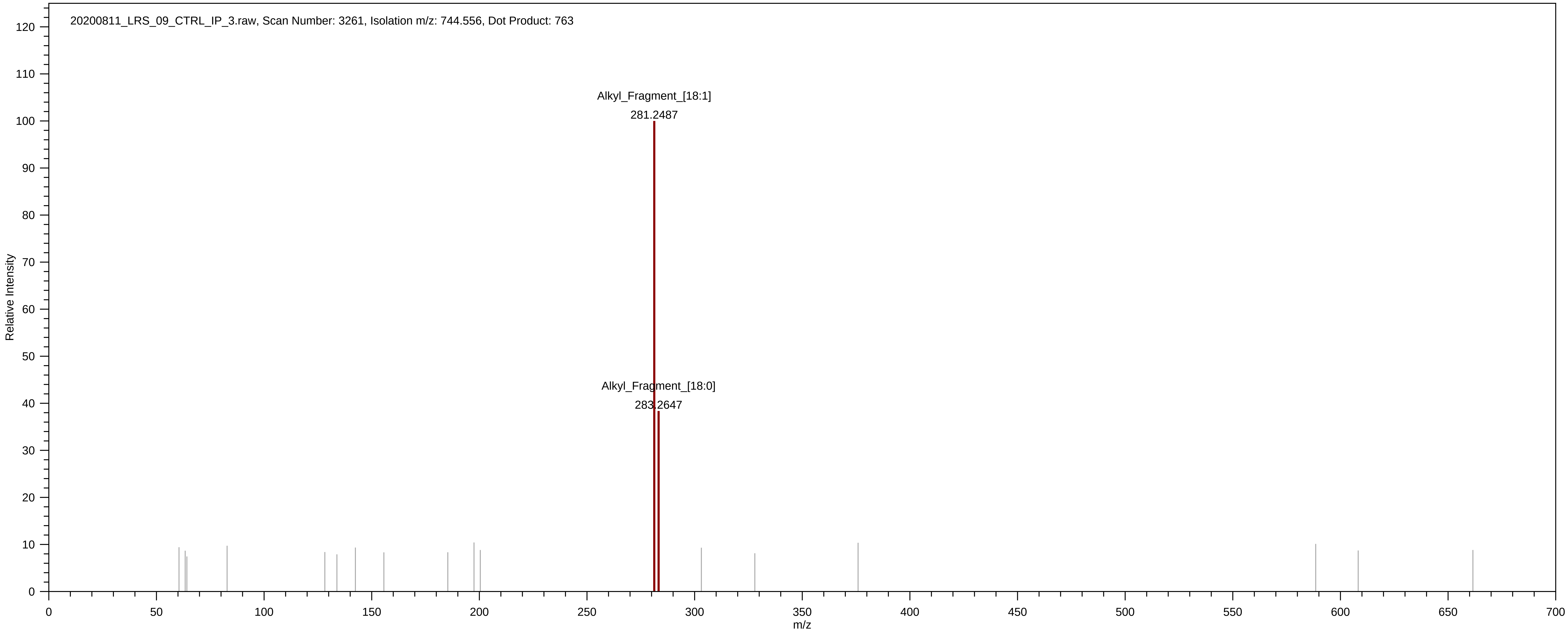

PE 18:1_18:0 [M-H]- | Ret. Time (min): 11.224 | m/z: 744.556

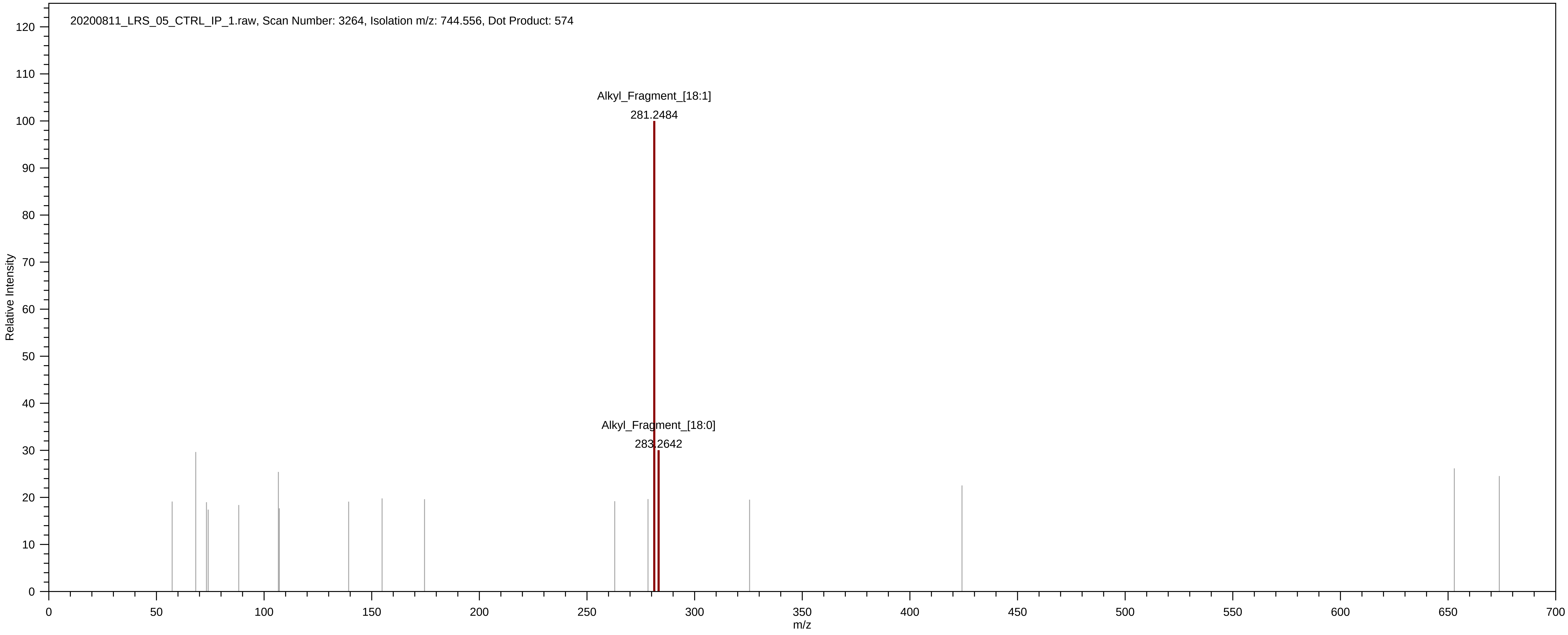

PE 18:1_18:1 [M-H]- | Ret. Time (min): 10.066 | m/z: 742.54

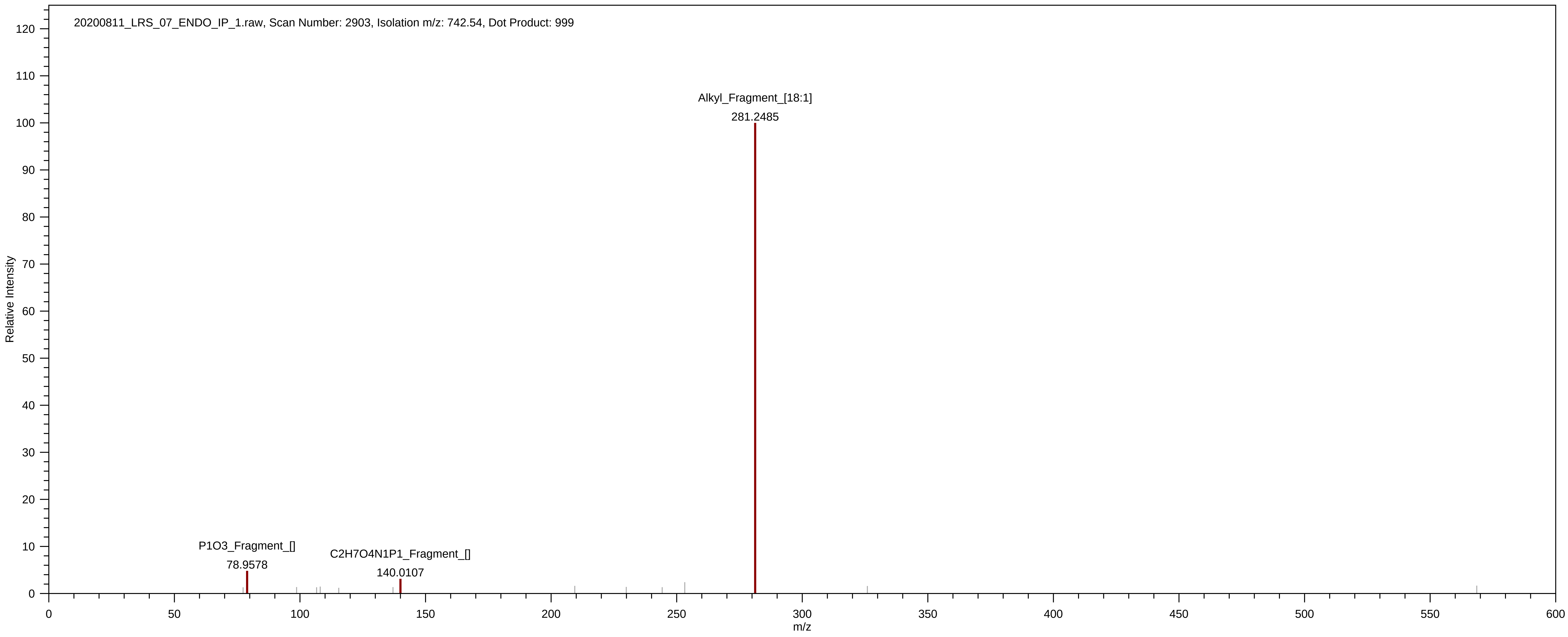

PE 18:1_18:1 [M-H]- | Ret. Time (min): 10.082 | m/z: 742.54

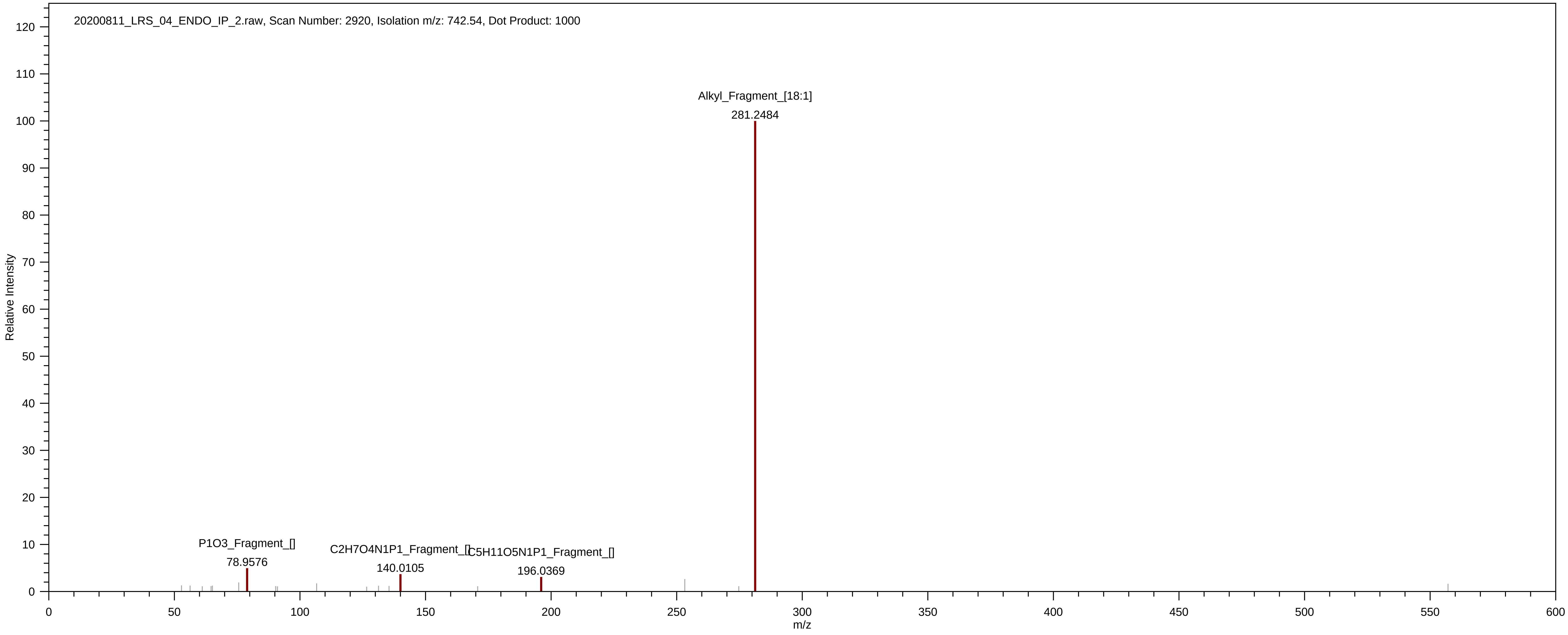

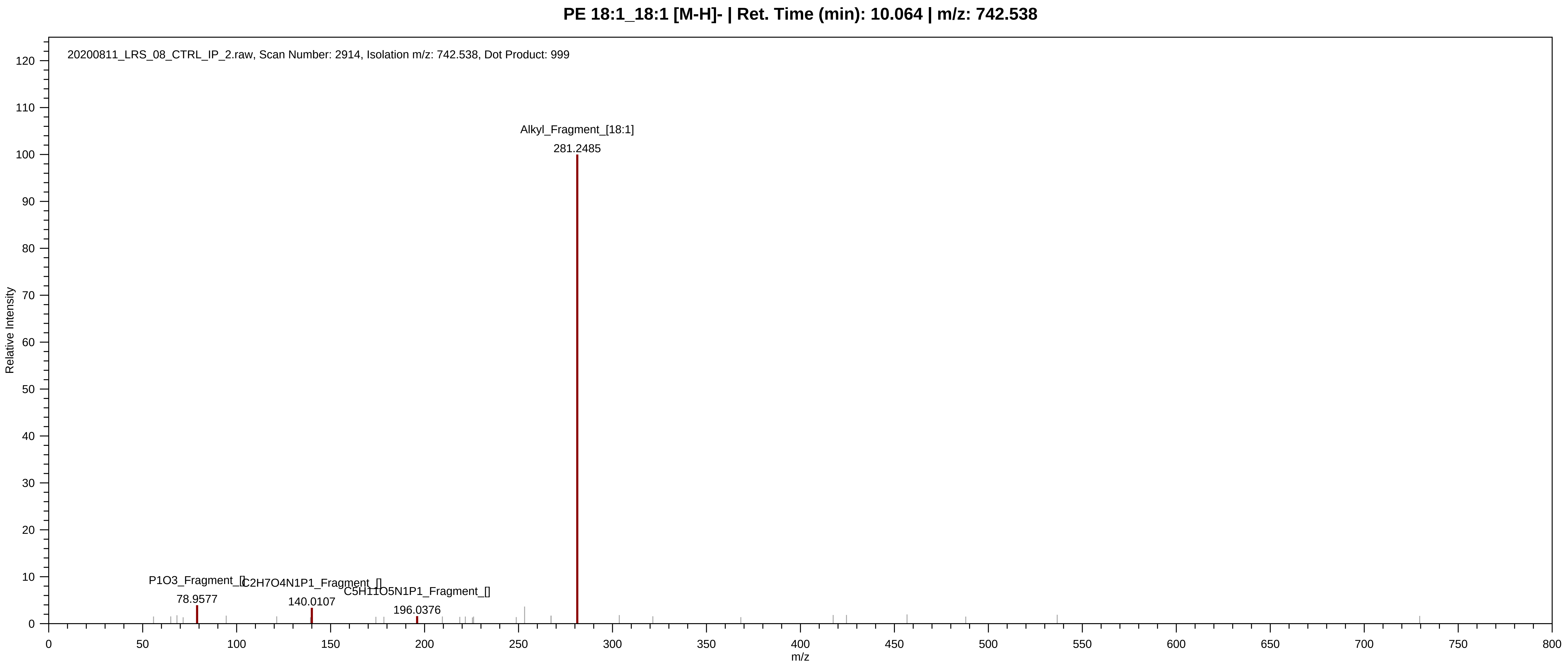

PE 18:1_18:1 [M-H]- | Ret. Time (min): 10.064 | m/z: 742.538
20200811_LRS_08_CTRL_IP_2.raw, Scan Number: 2914, Isolation m/z: 742.538, Dot Product: 999
Alkyl_Fragment_[18:1]
281.2485
P1O3_Fragment_[]
78.9577
C2H7O4N1P1_Fragment_[]
140.0107
C5H11O5N1P1_Fragment_[]
196.0376
Relative Intensity
m/z

PE 18:1_18:1 [M-H]- | Ret. Time (min): 10.09 | m/z: 742.539

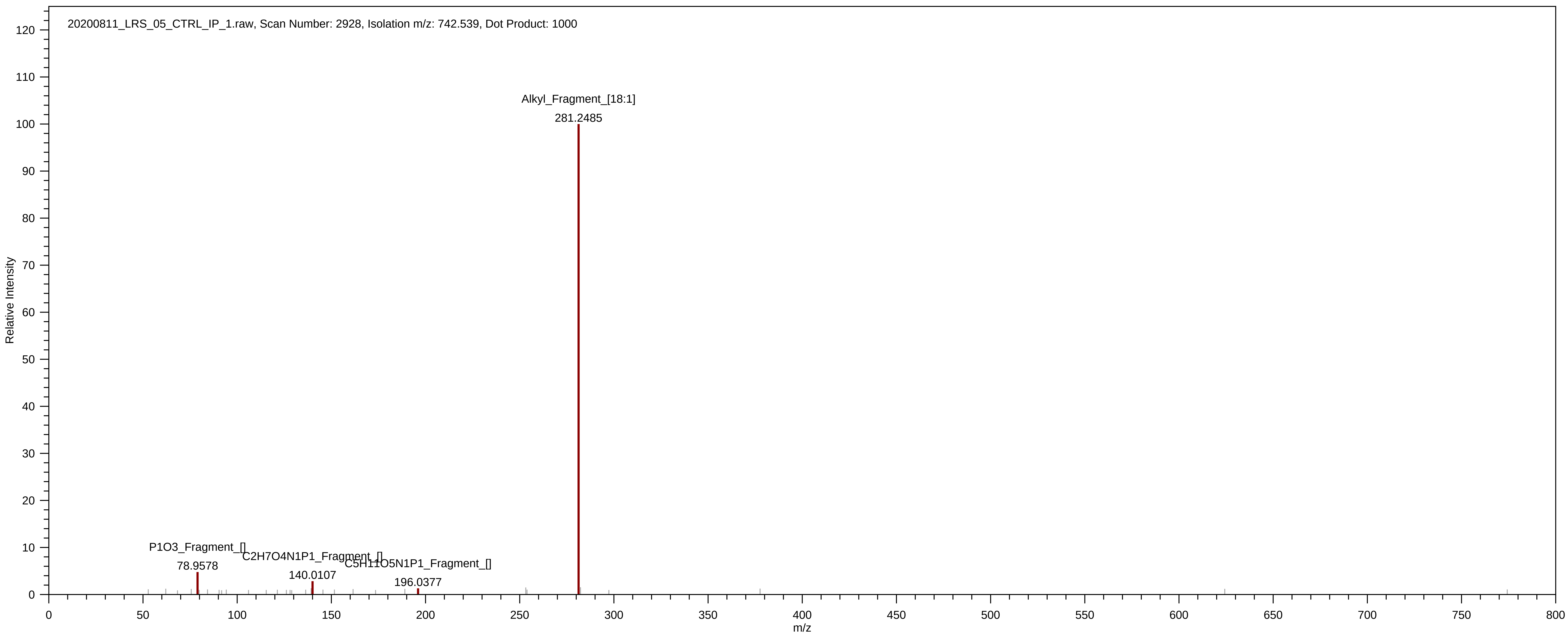

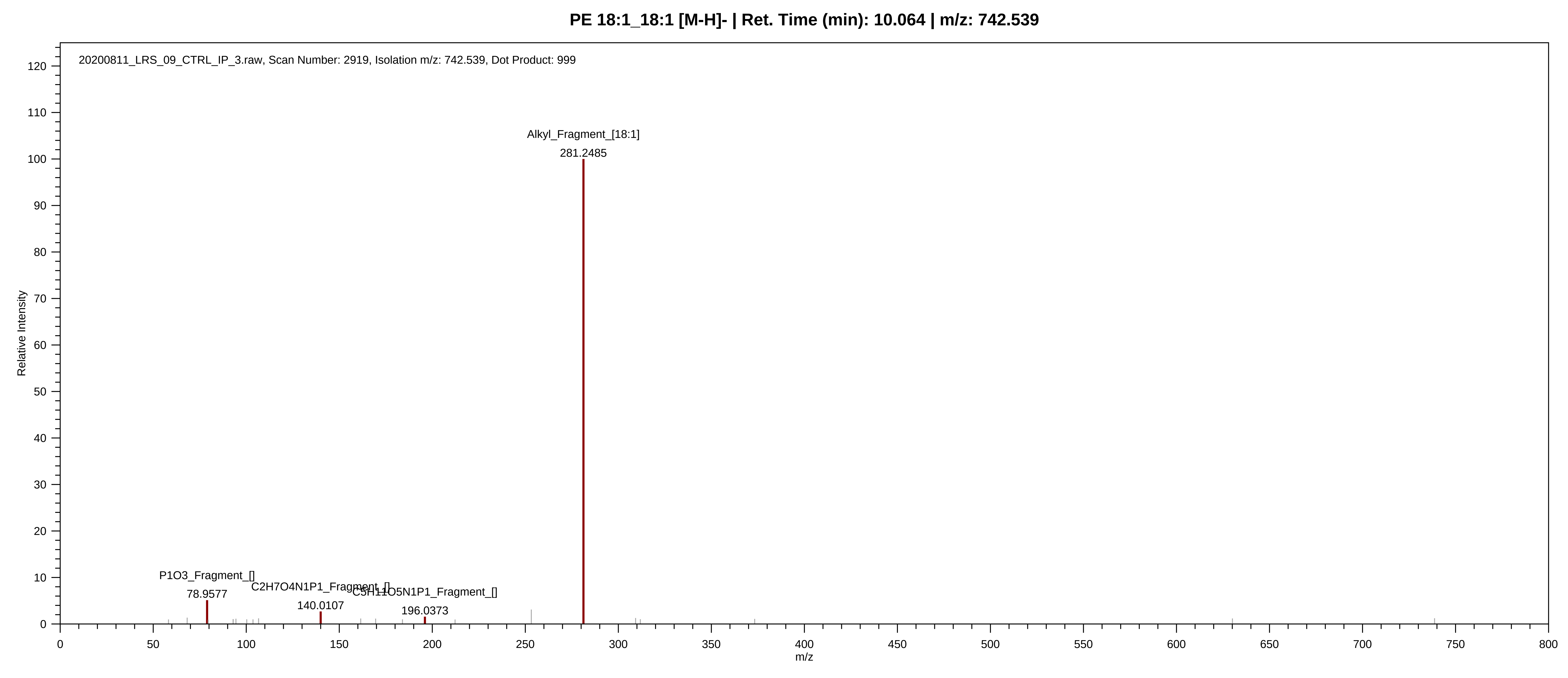
PE 18:1_18:1 [M-H]- | Ret. Time (min): 10.064 | m/z: 742.539
20200811_LRS_09_CTRL_IP_3.raw, Scan Number: 2919, Isolation m/z: 742.539, Dot Product: 999
Alkyl_Fragment_[18:1]
281.2485
P1O3_Fragment_[]
78.9577
C2H7O4N1P1_Fragment_[]
140.0107
C5H11O5N1P1_Fragment_[]
196.0373
Relative Intensity
m/z

PE 18:1_18:1 [M-H]- | Ret. Time (min): 10.066 | m/z: 742.539

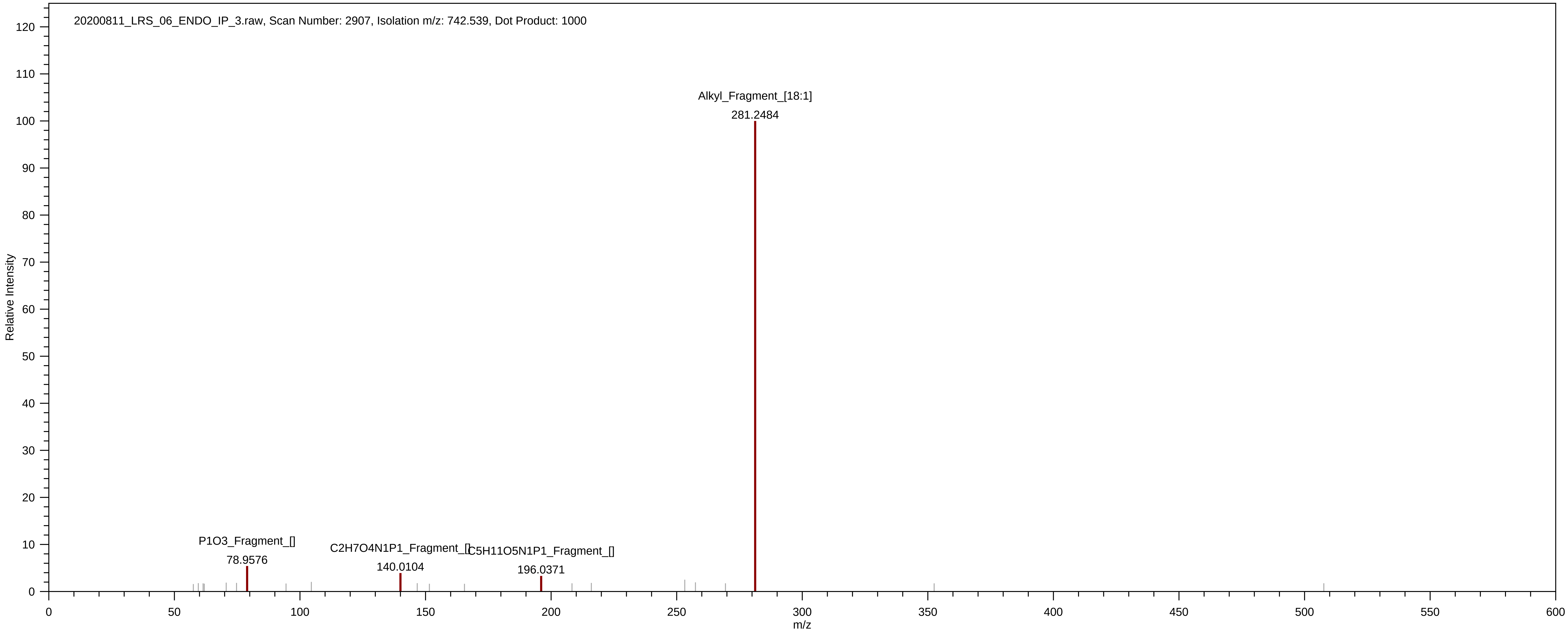

PE 18:1_20:1 [M-H]- | Ret. Time (min): 11.049 | m/z: 770.571

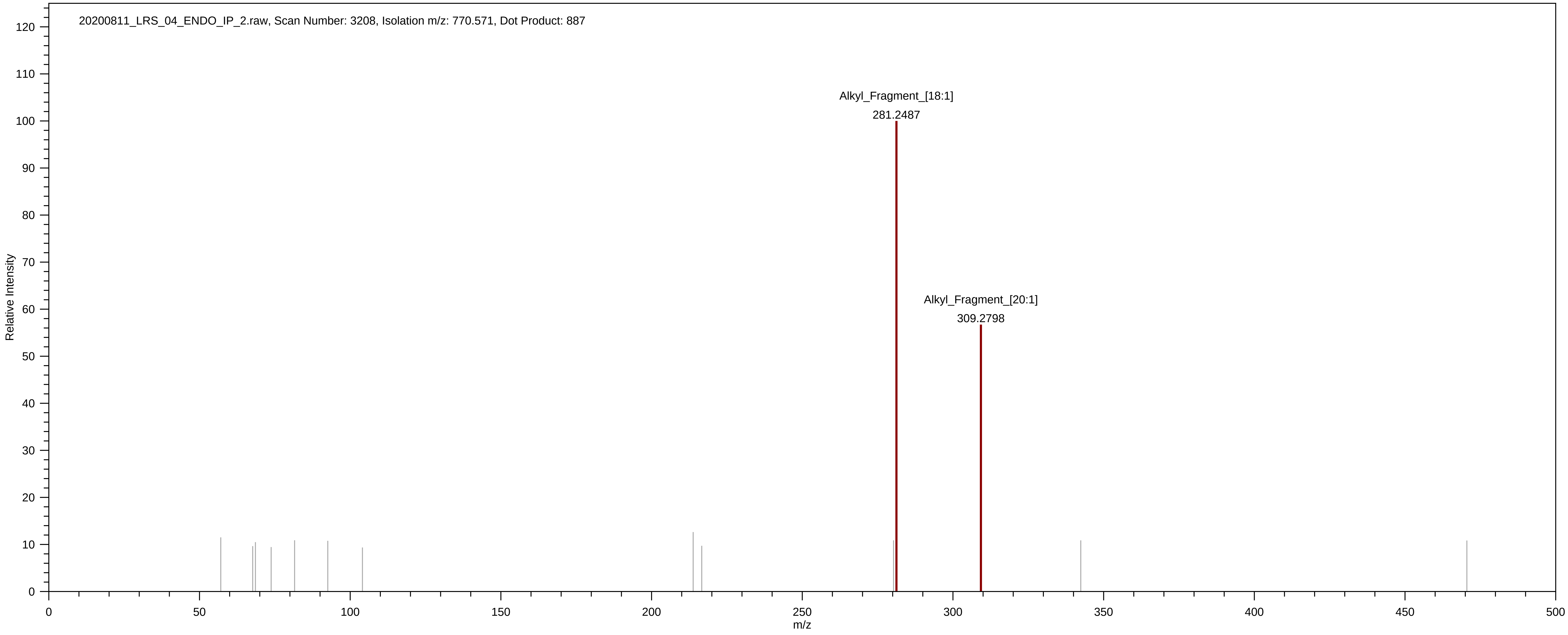

PE 18:1_20:4 [M-H]- | Ret. Time (min): 9.192 | m/z: 764.524

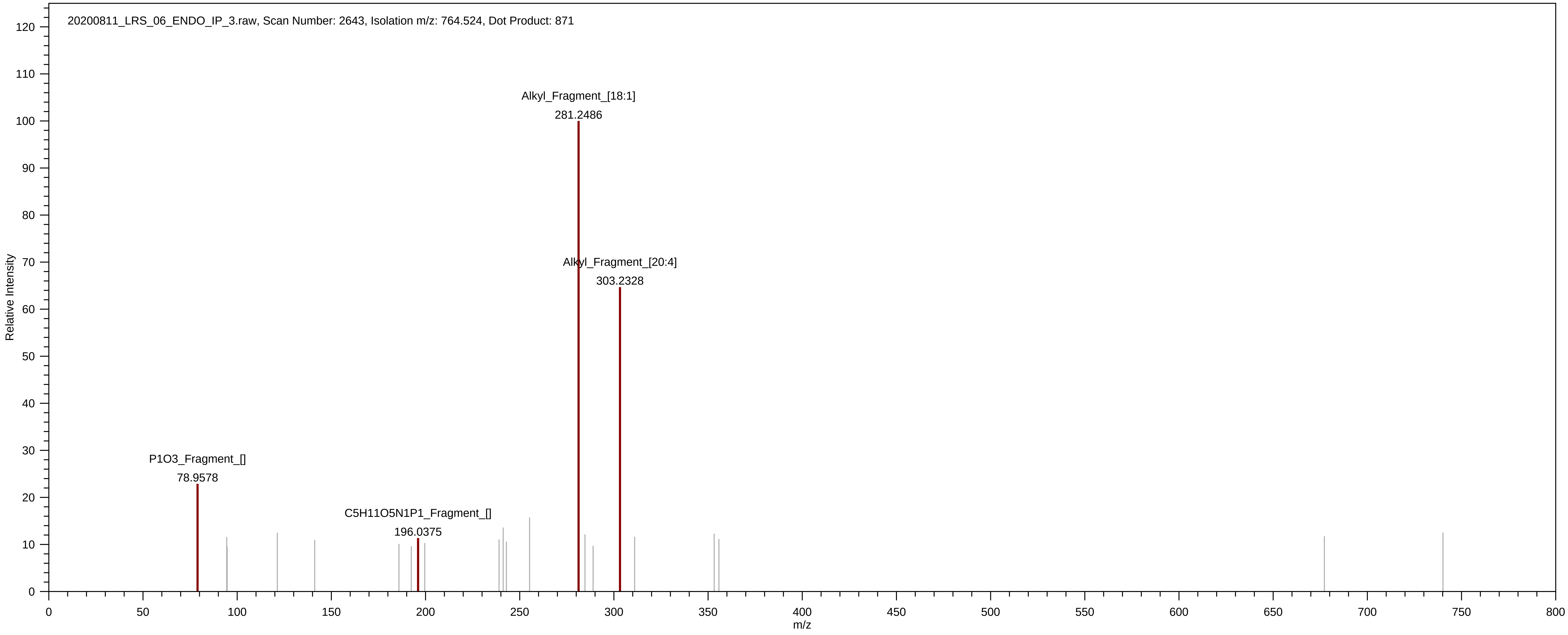

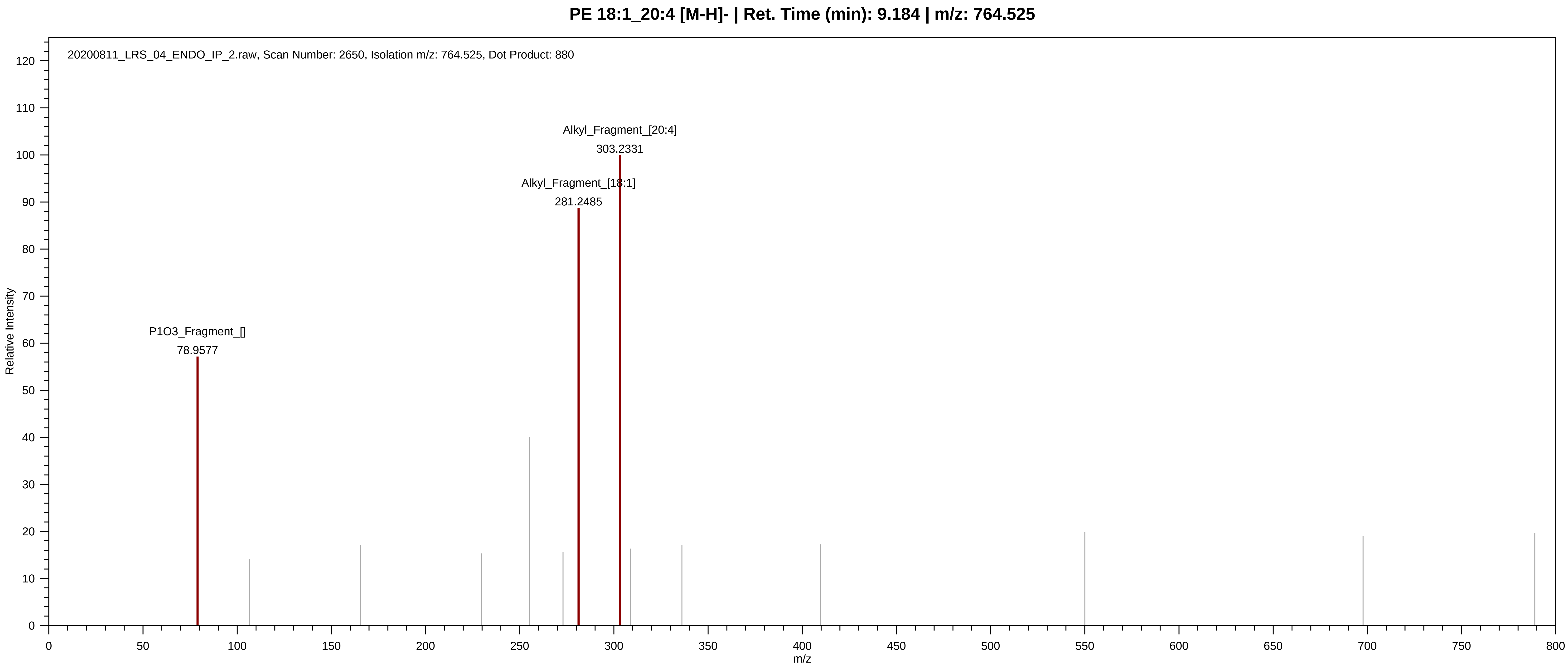

PE 18:1_20:4 [M-H]- | Ret. Time (min): 9.184 | m/z: 764.525
20200811_LRS_04_ENDO_IP_2.raw, Scan Number: 2650, Isolation m/z: 764.525, Dot Product: 880
Alkyl_Fragment_[20:4]
303.2331
Alkyl_Fragment_[18:1]
281.2485
P1O3_Fragment_[]
78.9577
Relative Intensity
m/z

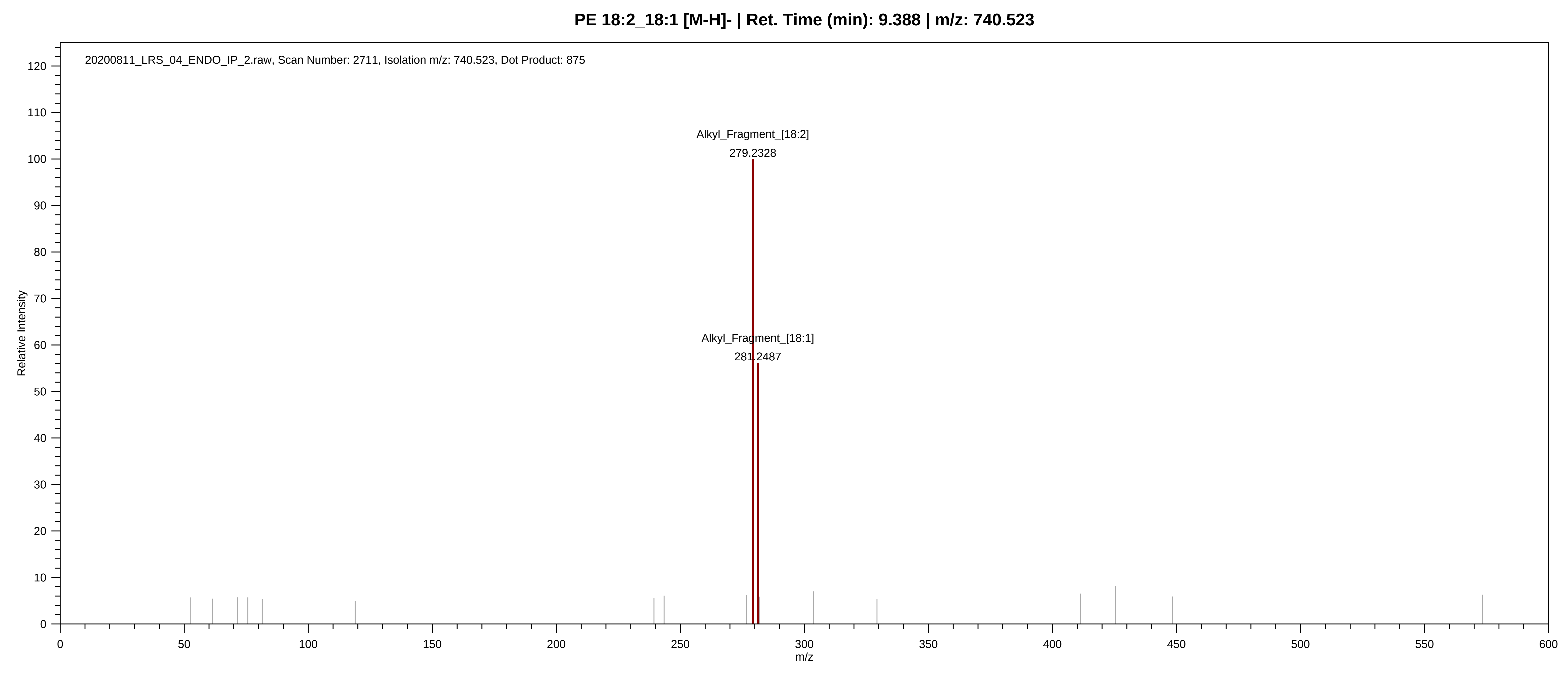
PE 18:2_18:1 [M-H]- | Ret. Time (min): 9.388 | m/z: 740.523
20200811_LRS_04_ENDO_IP_2.raw, Scan Number: 2711, Isolation m/z: 740.523, Dot Product: 875
Alkyl_Fragment_[18:2]
279.2328
Alkyl_Fragment_[18:1]
281.2487
Relative Intensity
m/z

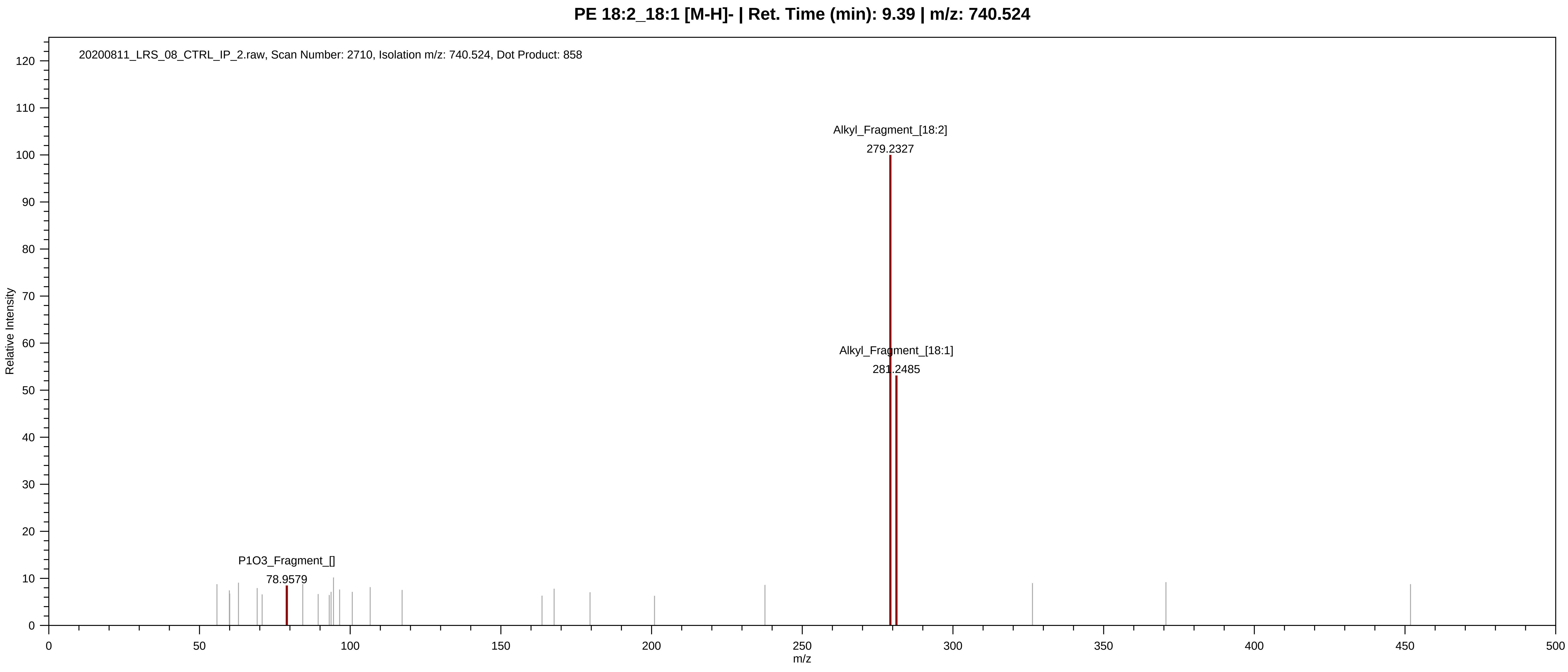

PE 18:2_18:1 [M-H]- | Ret. Time (min): 9.39 | m/z: 740.524
20200811_LRS_08_CTRL_IP_2.raw, Scan Number: 2710, Isolation m/z: 740.524, Dot Product: 858
Alkyl_Fragment_[18:2]
279.2327
Alkyl_Fragment_[18:1]
281.2485
P1O3_Fragment_[]
78.9579
Relative Intensity
m/z

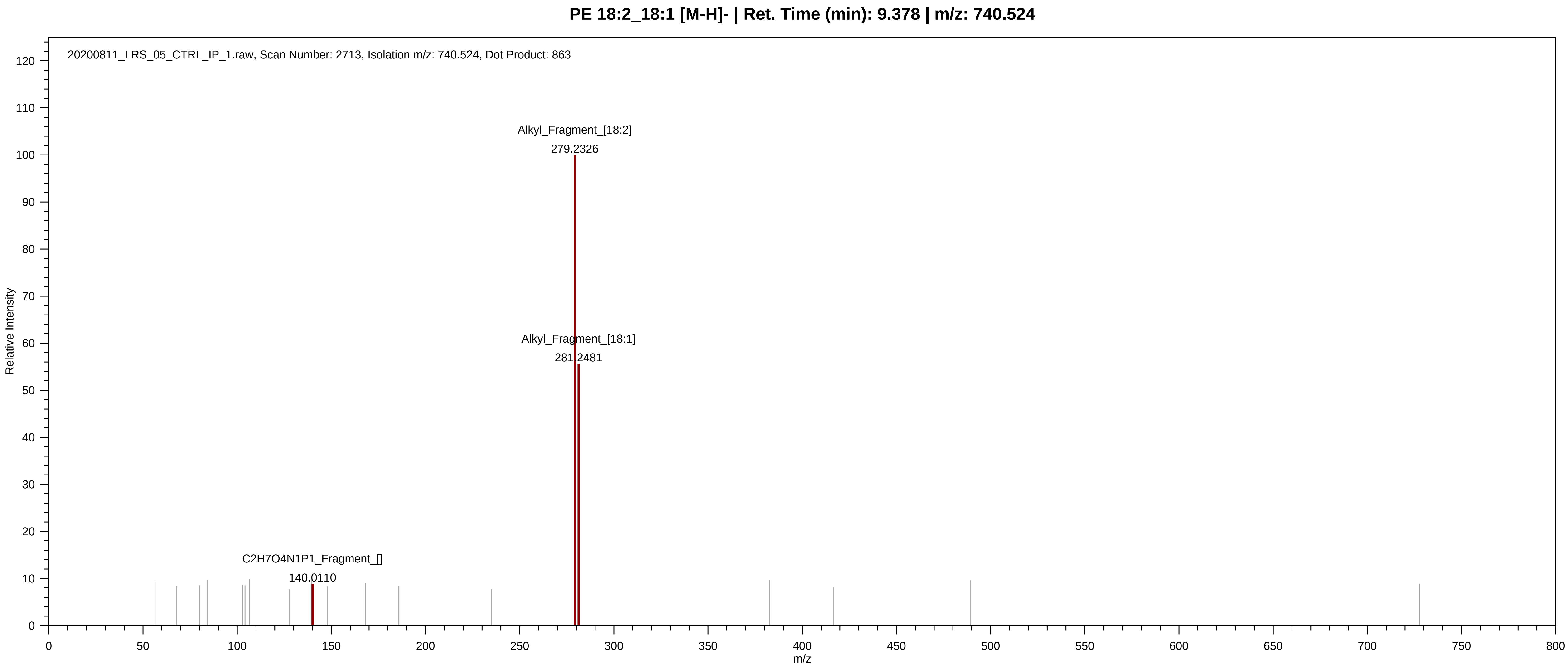

PE 18:2_18:1 [M-H]- | Ret. Time (min): 9.378 | m/z: 740.524
20200811_LRS_05_CTRL_IP_1.raw, Scan Number: 2713, Isolation m/z: 740.524, Dot Product: 863
Alkyl_Fragment_[18:2]
279.2326
Alkyl_Fragment_[18:1]
281.2481
C2H7O4N1P1_Fragment_[]
140.0110
Relative Intensity
m/z

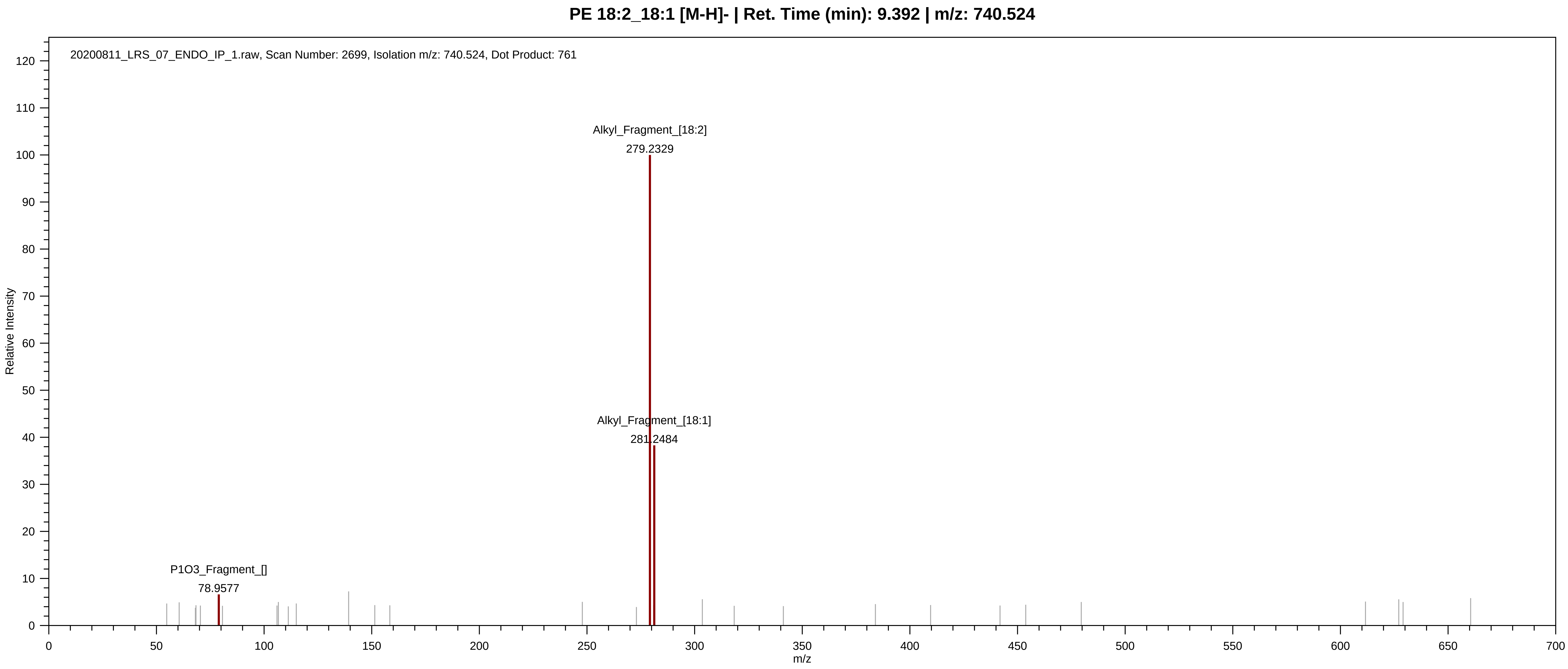

PE 18:2_18:1 [M-H]- | Ret. Time (min): 9.392 | m/z: 740.524
20200811_LRS_07_ENDO_IP_1.raw, Scan Number: 2699, Isolation m/z: 740.524, Dot Product: 761
Alkyl_Fragment_[18:2]
279.2329
Alkyl_Fragment_[18:1]
281.2484
P1O3_Fragment_[]
78.9577
Relative Intensity
m/z

PE 18:2_18:1 [M-H]- | Ret. Time (min): 9.392 | m/z: 740.524

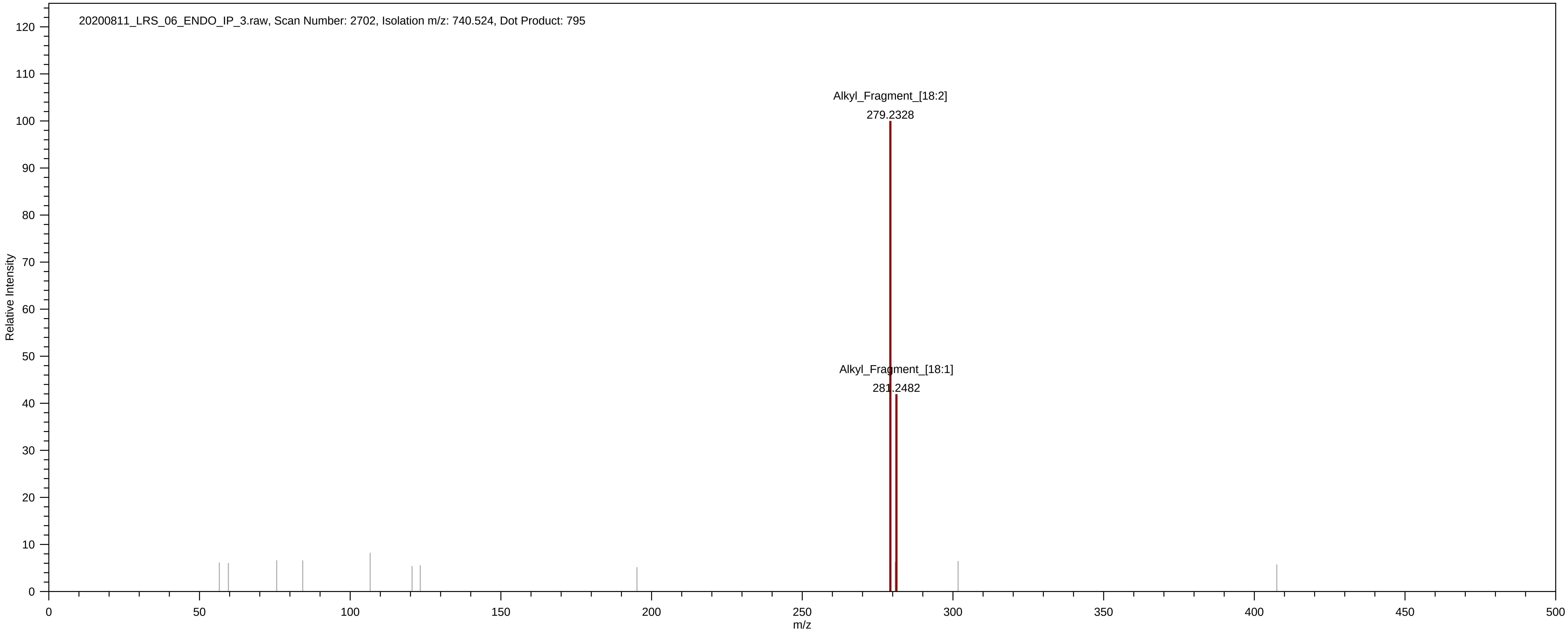

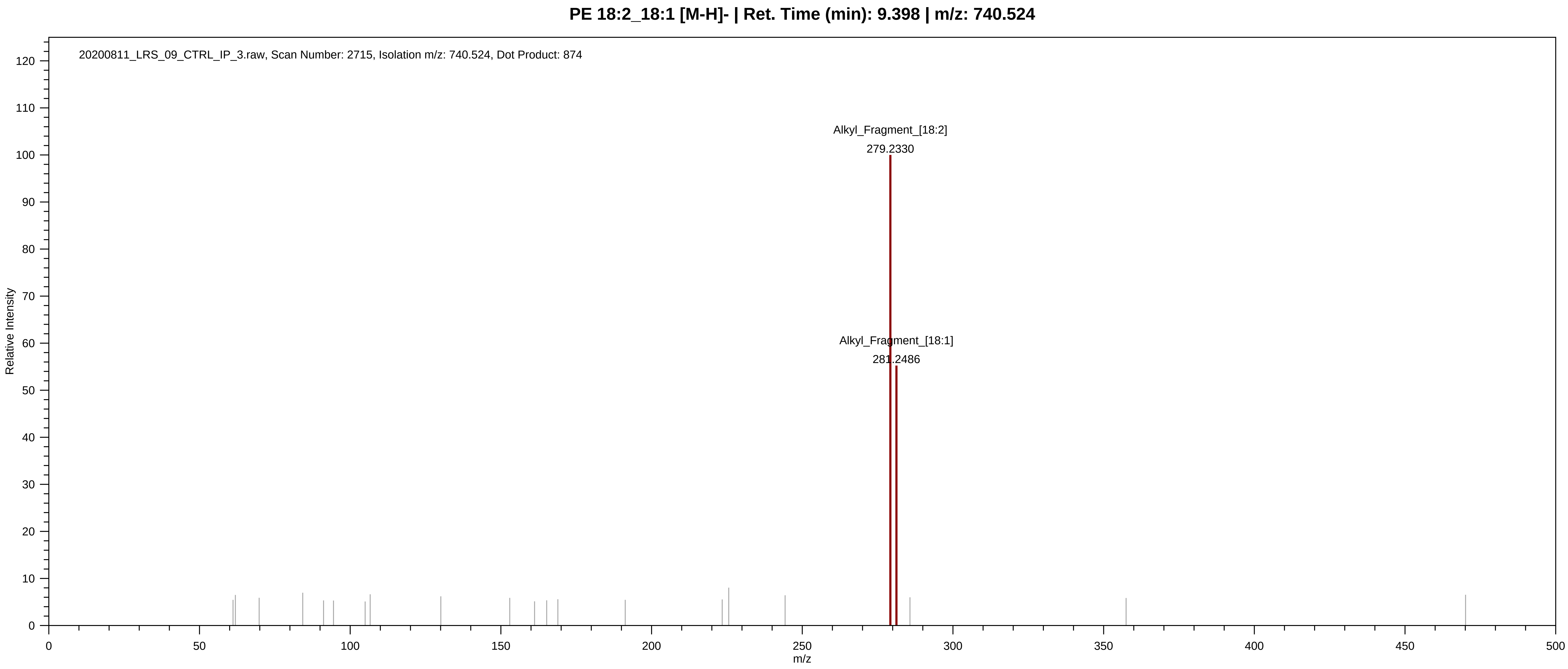
PE 18:2_18:1 [M-H]- | Ret. Time (min): 9.398 | m/z: 740.524
20200811_LRS_09_CTRL_IP_3.raw, Scan Number: 2715, Isolation m/z: 740.524, Dot Product: 874
Alkyl_Fragment_[18:2]
279.2330
Alkyl_Fragment_[18:1]
281.2486
Relative Intensity
m/z
0
10
20
30
40
50
60
70
80
90
100
110
120
0
50
100
150
200
250
300
350
400
450
500

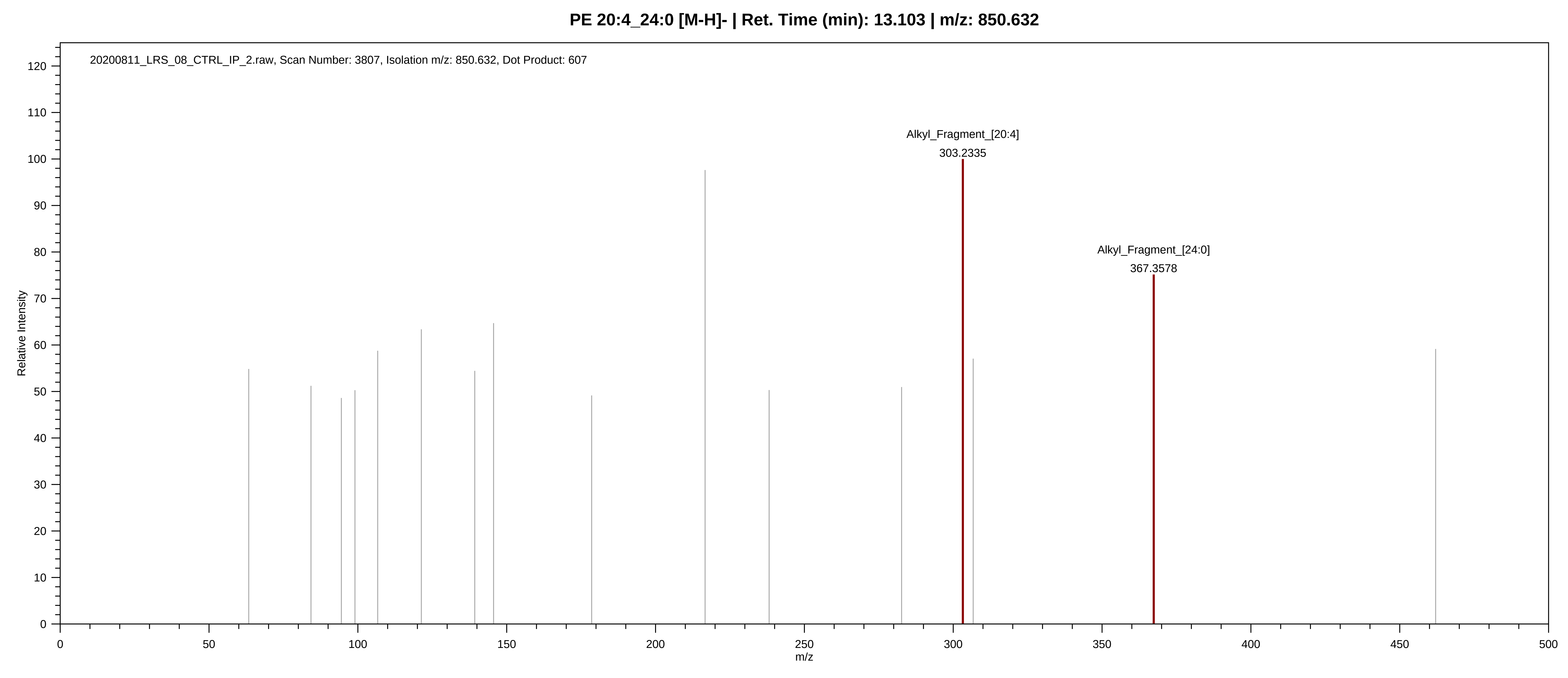

PE 20:4_24:0 [M-H]- | Ret. Time (min): 13.103 | m/z: 850.632
20200811_LRS_08_CTRL_IP_2.raw, Scan Number: 3807, Isolation m/z: 850.632, Dot Product: 607
Alkyl_Fragment_[20:4]
303.2335
Alkyl_Fragment_[24:0]
367.3578
Relative Intensity
m/z

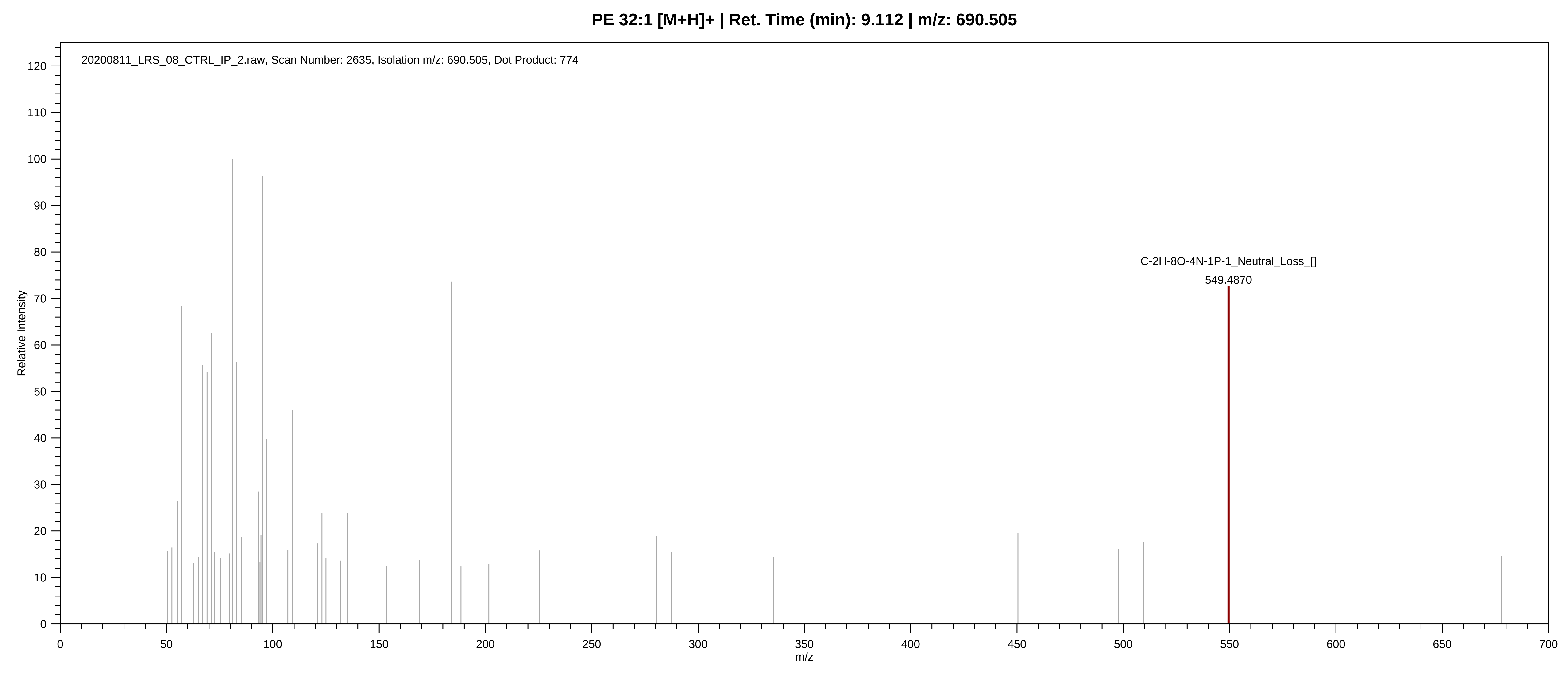

PE 32:1 [M+H]+ | Ret. Time (min): 9.112 | m/z: 690.505
20200811_LRS_08_CTRL_IP_2.raw, Scan Number: 2635, Isolation m/z: 690.505, Dot Product: 774
C-2H-8O-4N-1P-1_Neutral_Loss_[]
549.4870
Relative Intensity
m/z

PE 32:1 [M+H]+ | Ret. Time (min): 9.123 | m/z: 690.506

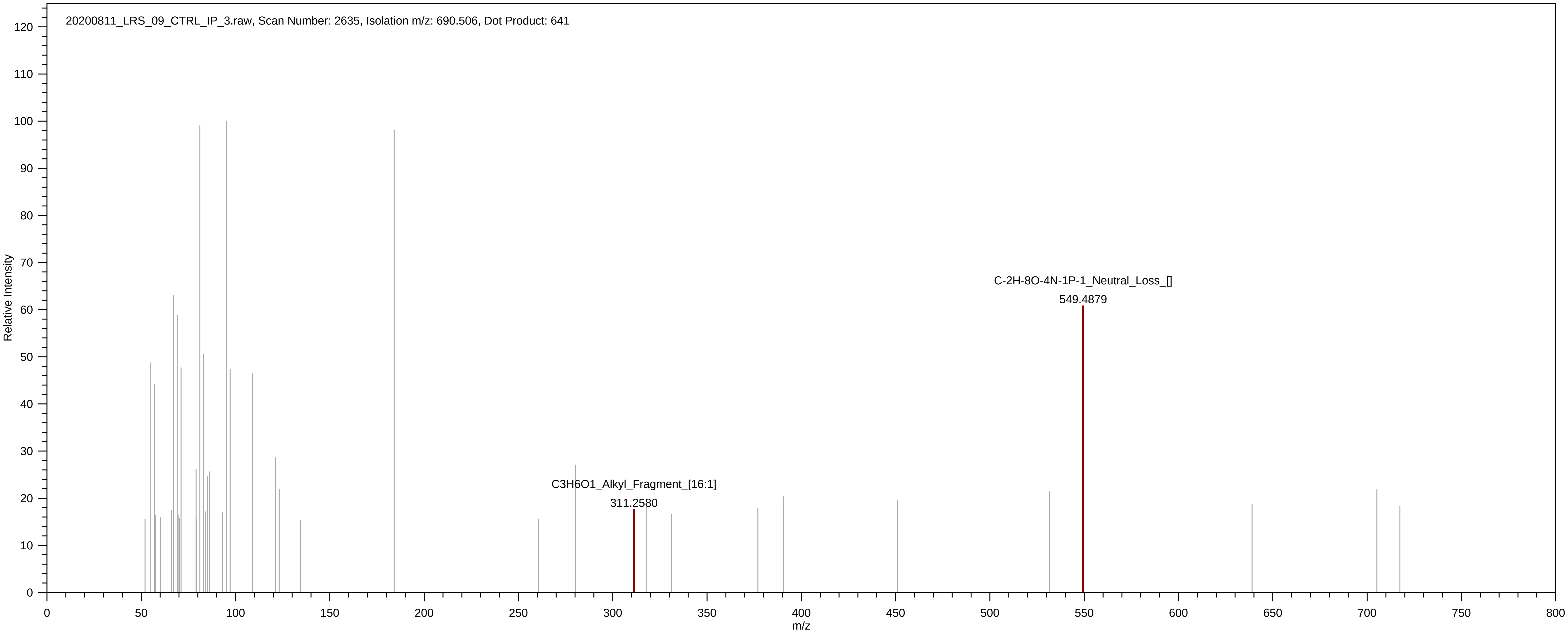

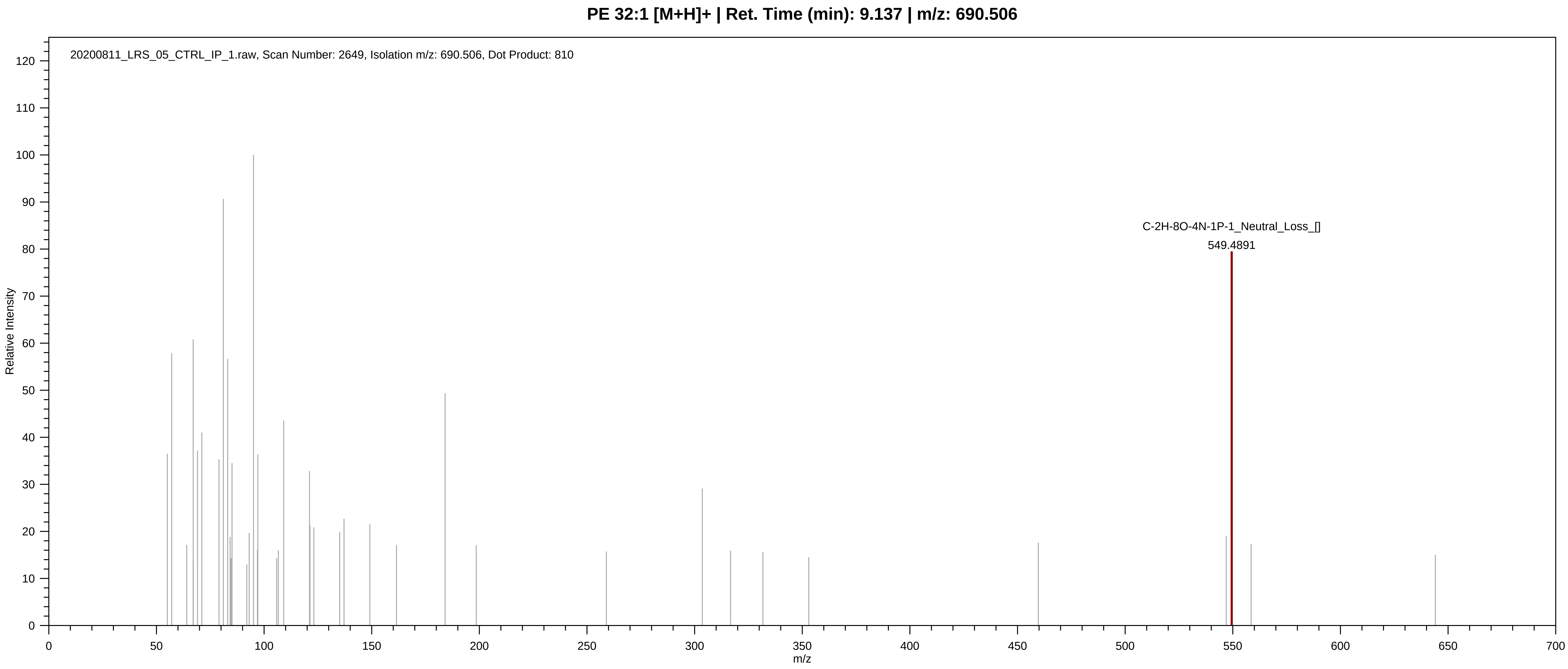

PE 32:1 [M+H]+ | Ret. Time (min): 9.137 | m/z: 690.506
20200811_LRS_05_CTRL_IP_1.raw, Scan Number: 2649, Isolation m/z: 690.506, Dot Product: 810
C-2H-8O-4N-1P-1_Neutral_Loss_[]
549.4891
Relative Intensity
m/z
0
10
20
30
40
50
60
70
80
90
100
110
120
0
50
100
150
200
250
300
350
400
450
500
550
600
650
700

PE 32:1 [M+H]+ | Ret. Time (min): 9.146 | m/z: 690.506

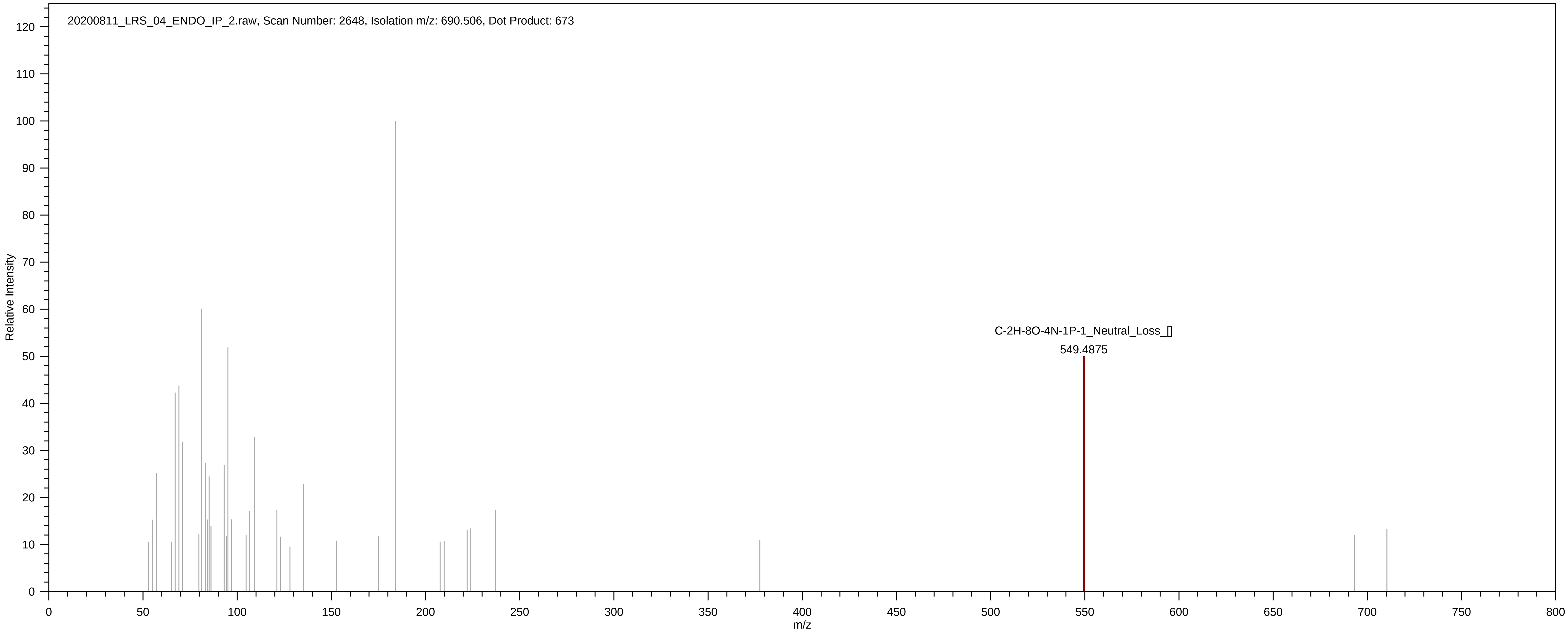

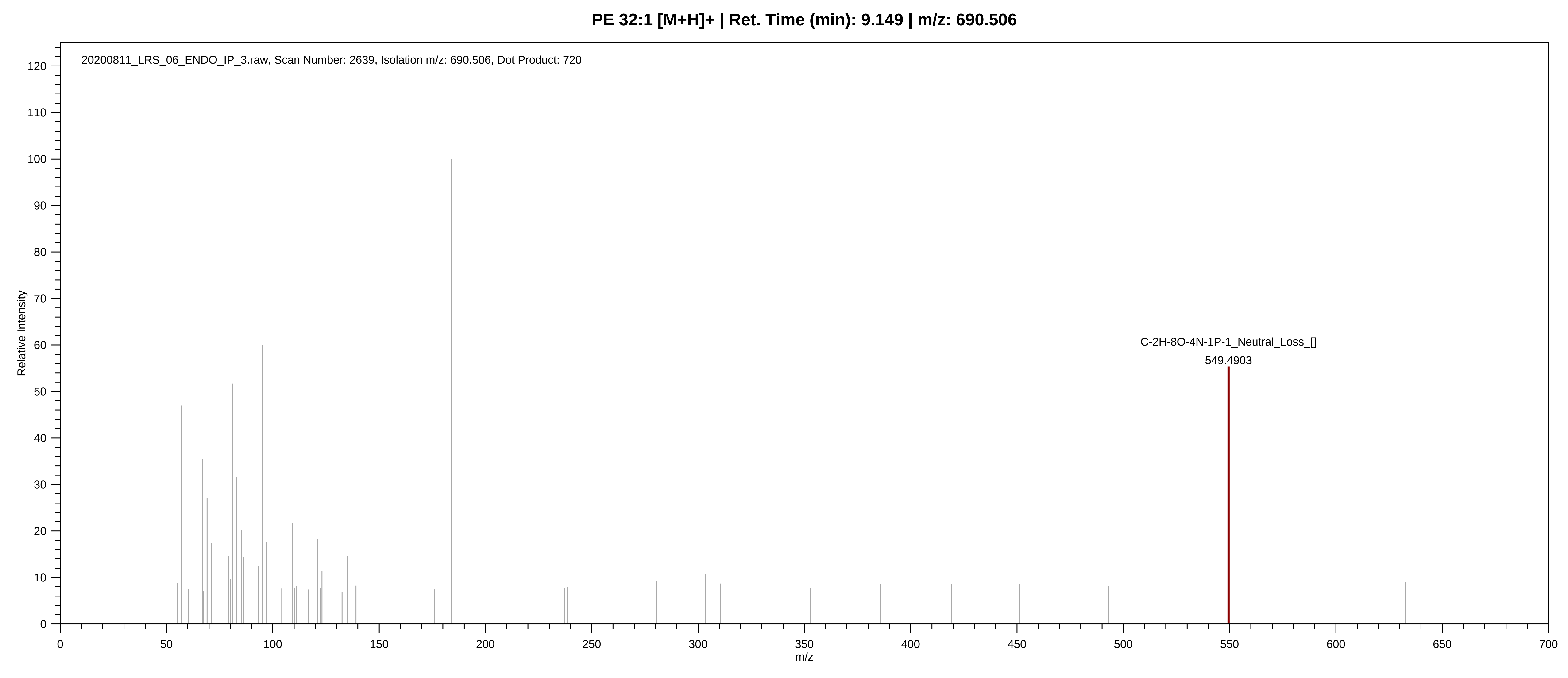
PE 32:1 [M+H]+ | Ret. Time (min): 9.149 | m/z: 690.506
20200811_LRS_06_ENDO_IP_3.raw, Scan Number: 2639, Isolation m/z: 690.506, Dot Product: 720
C-2H-8O-4N-1P-1_Neutral_Loss_[]
549.4903
Relative Intensity
m/z

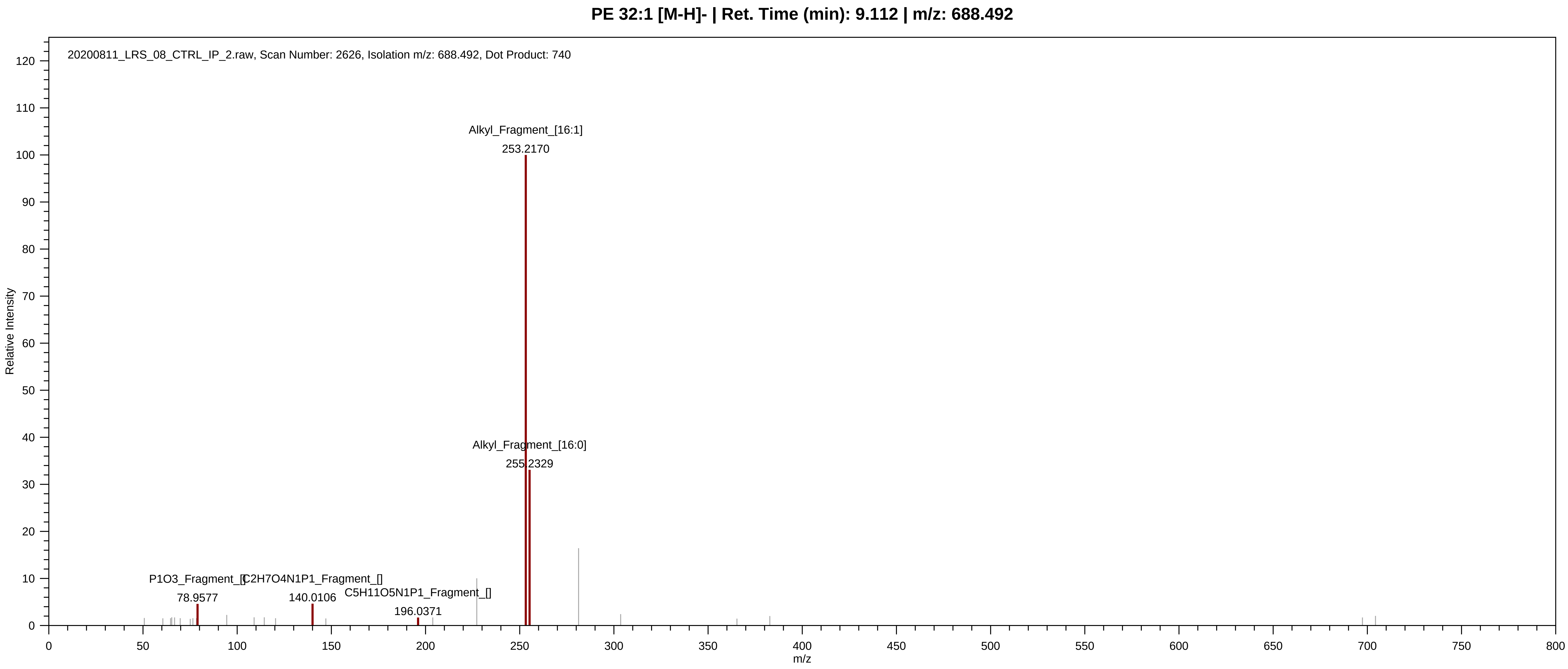

PE 32:1 [M-H]- | Ret. Time (min): 9.112 | m/z: 688.492
20200811_LRS_08_CTRL_IP_2.raw, Scan Number: 2626, Isolation m/z: 688.492, Dot Product: 740
Relative Intensity
m/z
Alkyl_Fragment_[16:1]
253.2170
Alkyl_Fragment_[16:0]
255.2329
P1O3_Fragment_[]
78.9577
C2H7O4N1P1_Fragment_[]
140.0106
C5H11O5N1P1_Fragment_[]
196.0371

PE 32:1 [M-H]- | Ret. Time (min): 9.123 | m/z: 688.492

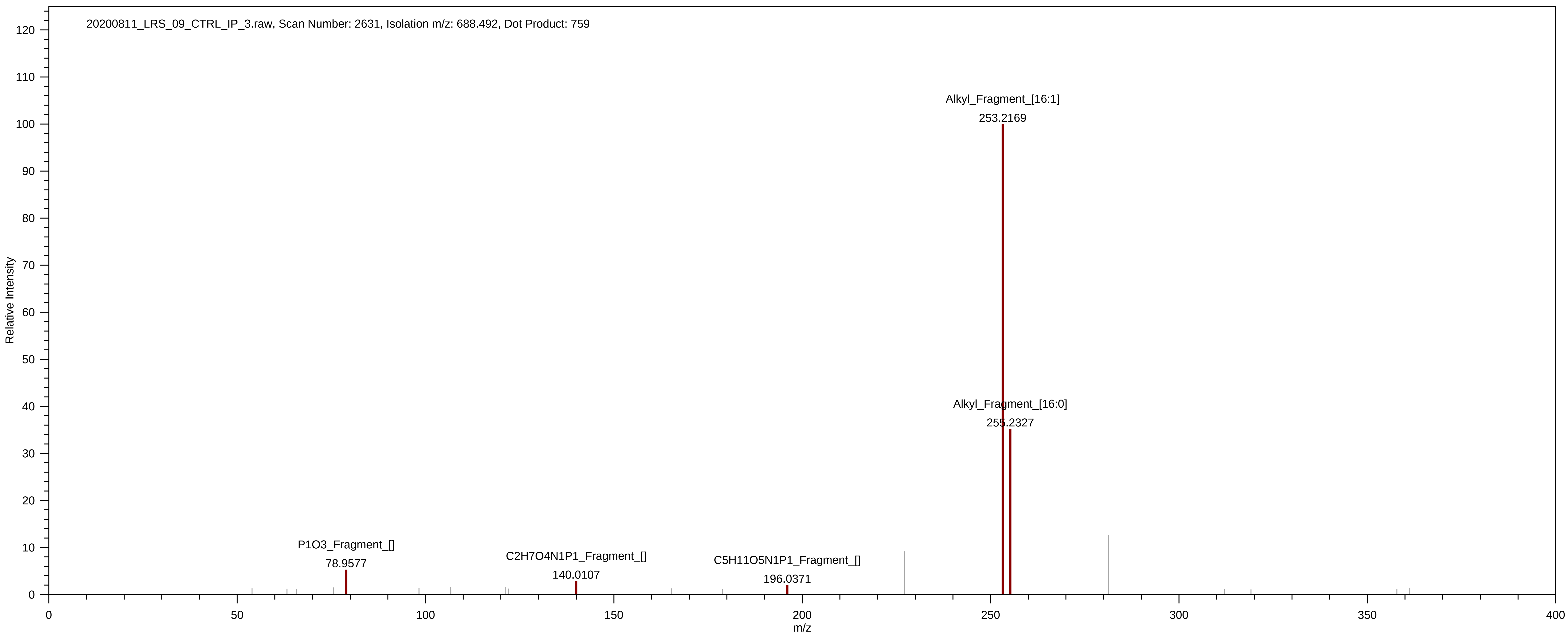

PE 32:1 [M-H]- | Ret. Time (min): 9.133 | m/z: 688.492

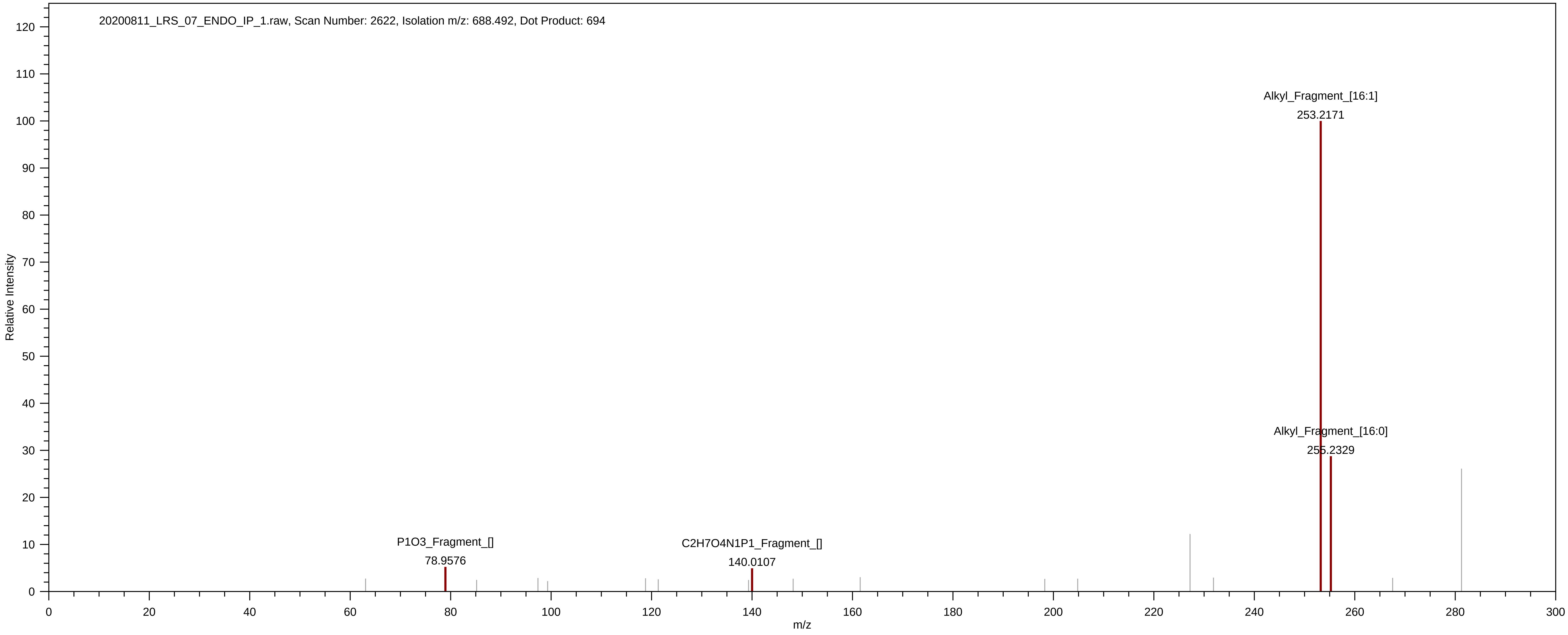

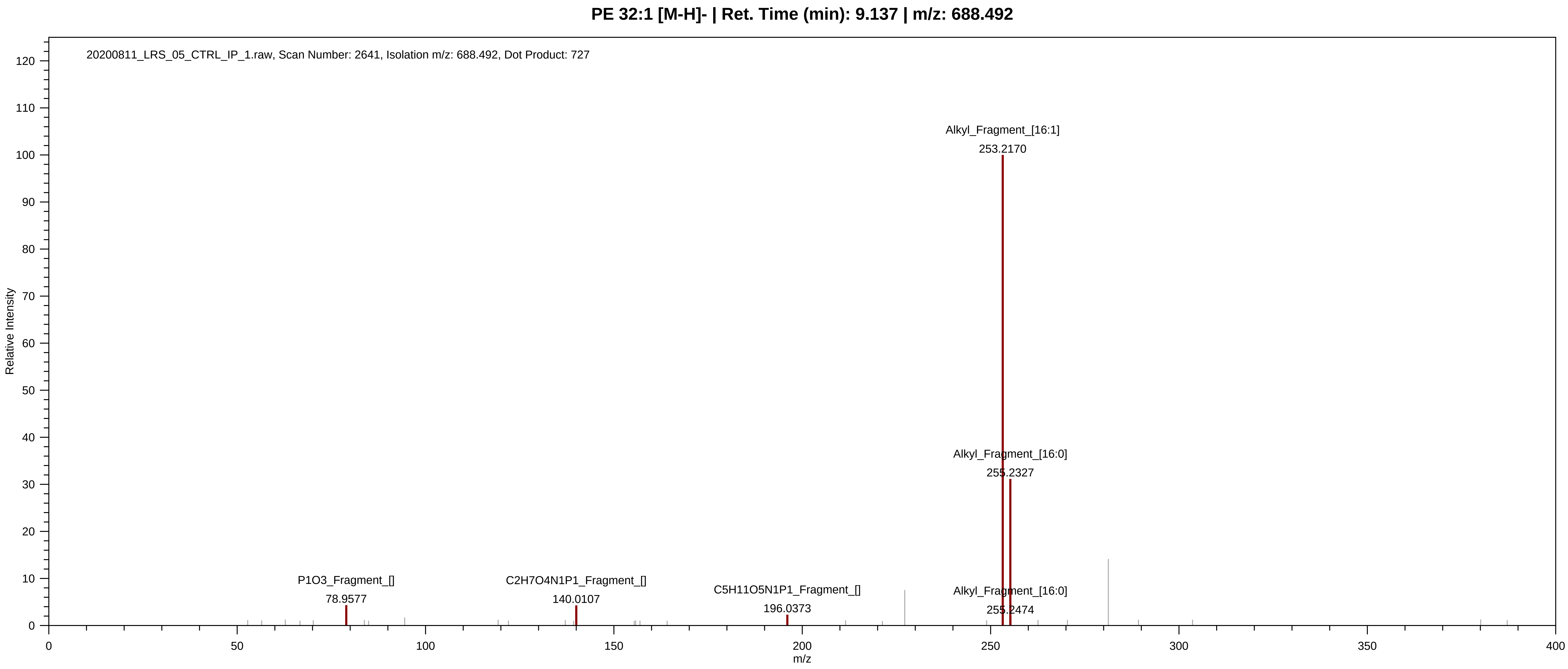
PE 32:1 [M-H]- | Ret. Time (min): 9.137 | m/z: 688.492
20200811_LRS_05_CTRL_IP_1.raw, Scan Number: 2641, Isolation m/z: 688.492, Dot Product: 727
Relative Intensity
m/z
Alkyl_Fragment_[16:1]
253.2170
Alkyl_Fragment_[16:0]
255.2327
P1O3_Fragment_[]
78.9577
C2H7O4N1P1_Fragment_[]
140.0107
C5H11O5N1P1_Fragment_[]
196.0373
Alkyl_Fragment_[16:0]
255.2474

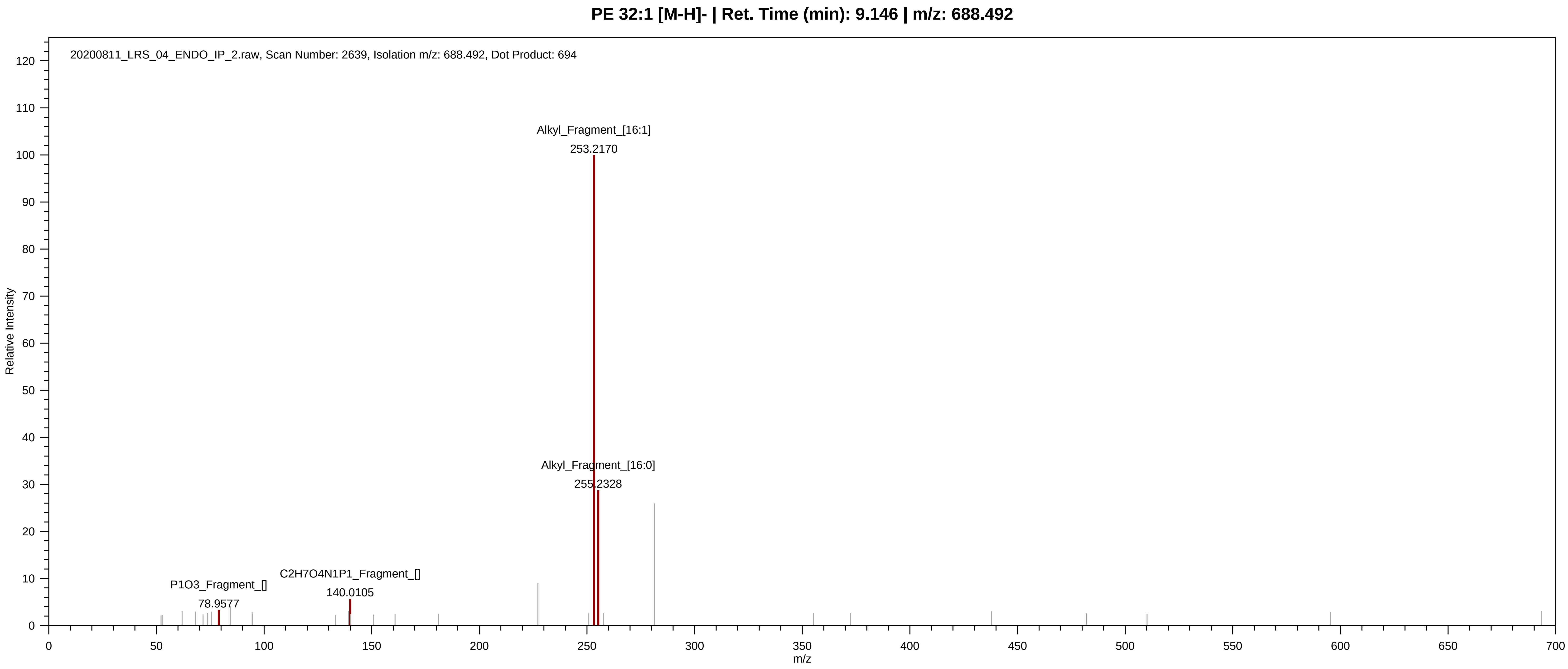

PE 32:1 [M-H]- | Ret. Time (min): 9.146 | m/z: 688.492
20200811_LRS_04_ENDO_IP_2.raw, Scan Number: 2639, Isolation m/z: 688.492, Dot Product: 694
Alkyl_Fragment_[16:1]
253.2170
Alkyl_Fragment_[16:0]
255.2328
C2H7O4N1P1_Fragment_[]
140.0105
P1O3_Fragment_[]
78.9577
Relative Intensity
m/z

PE 32:1 [M-H]- | Ret. Time (min): 9.149 | m/z: 688.492

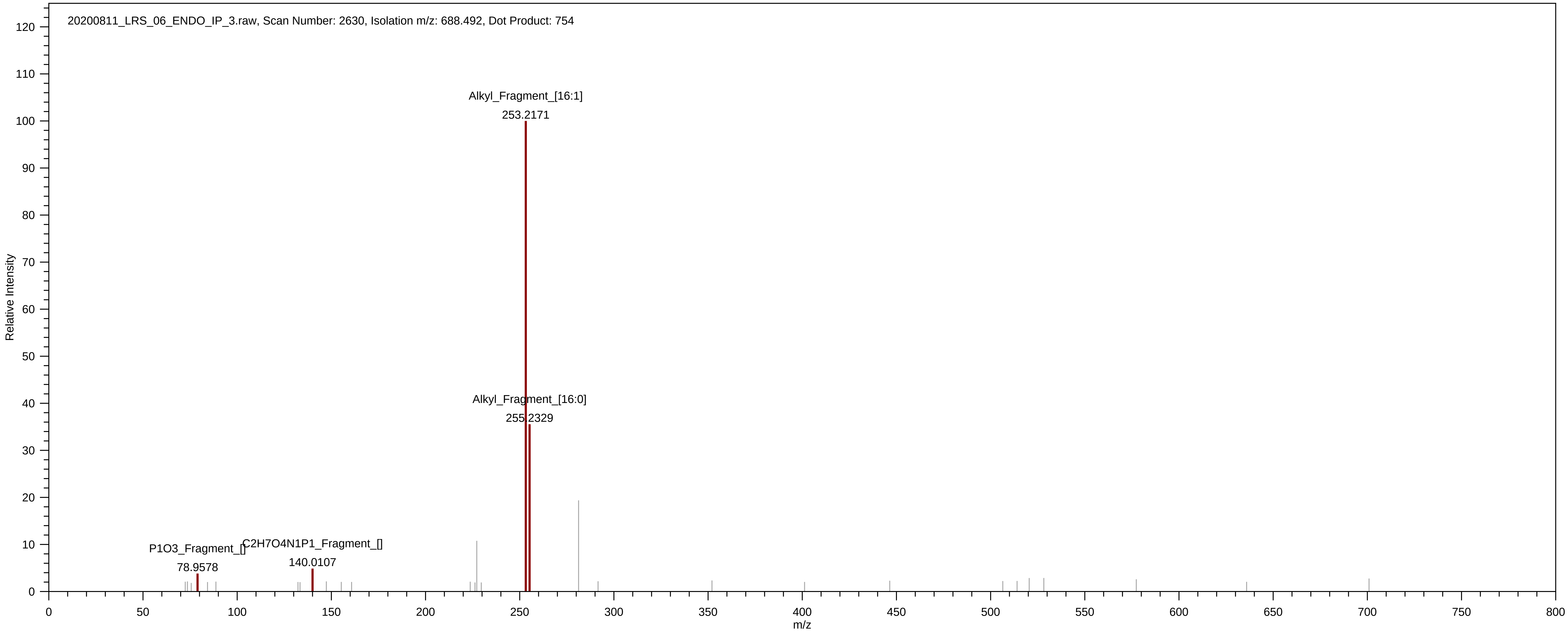

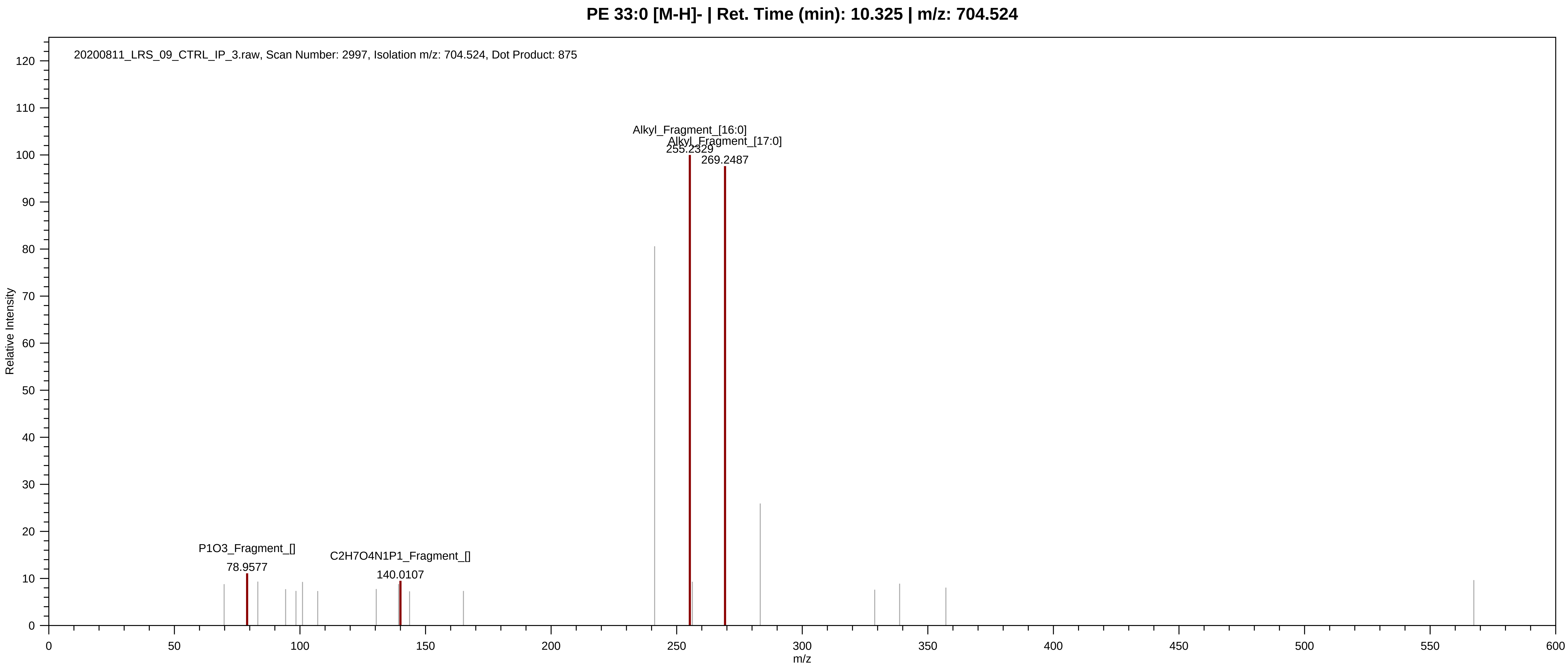

PE 33:0 [M-H]- | Ret. Time (min): 10.325 | m/z: 704.524
20200811_LRS_09_CTRL_IP_3.raw, Scan Number: 2997, Isolation m/z: 704.524, Dot Product: 875
Alkyl_Fragment_[16:0]
255.2329
Alkyl_Fragment_[17:0]
269.2487
P1O3_Fragment_[]
78.9577
C2H7O4N1P1_Fragment_[]
140.0107
Relative Intensity
m/z

PE 34:1 [M-H]- | Ret. Time (min): 10.224 | m/z: 716.524

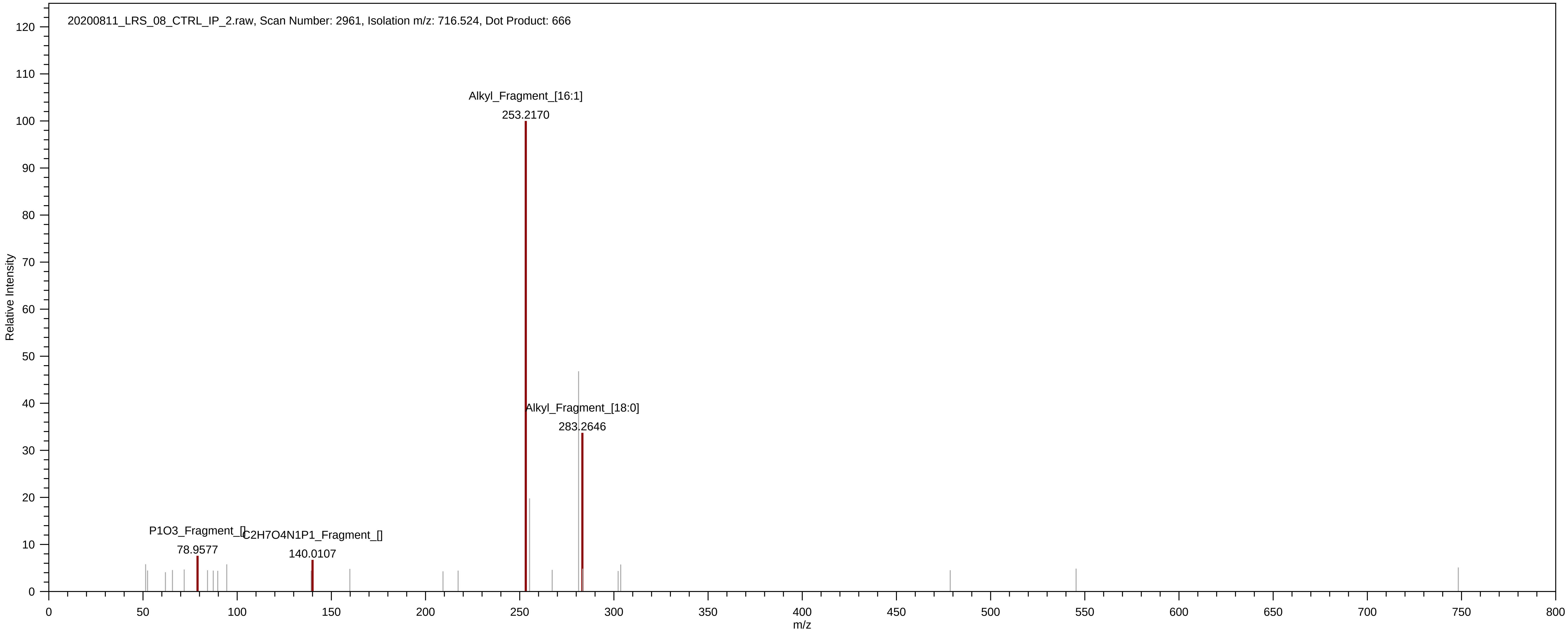

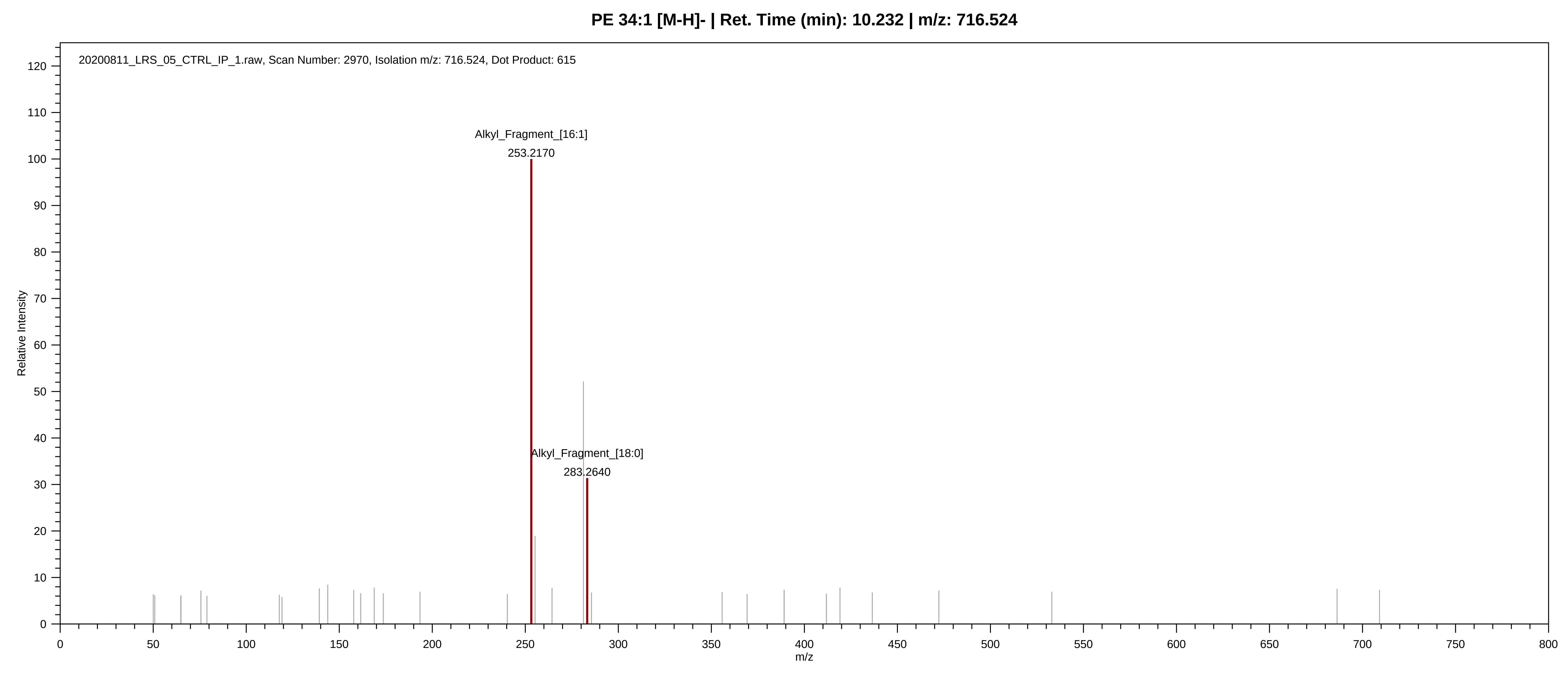

PE 34:1 [M-H]- | Ret. Time (min): 10.232 | m/z: 716.524
20200811_LRS_05_CTRL_IP_1.raw, Scan Number: 2970, Isolation m/z: 716.524, Dot Product: 615
Alkyl_Fragment_[16:1]
253.2170
Alkyl_Fragment_[18:0]
283.2640
Relative Intensity
m/z

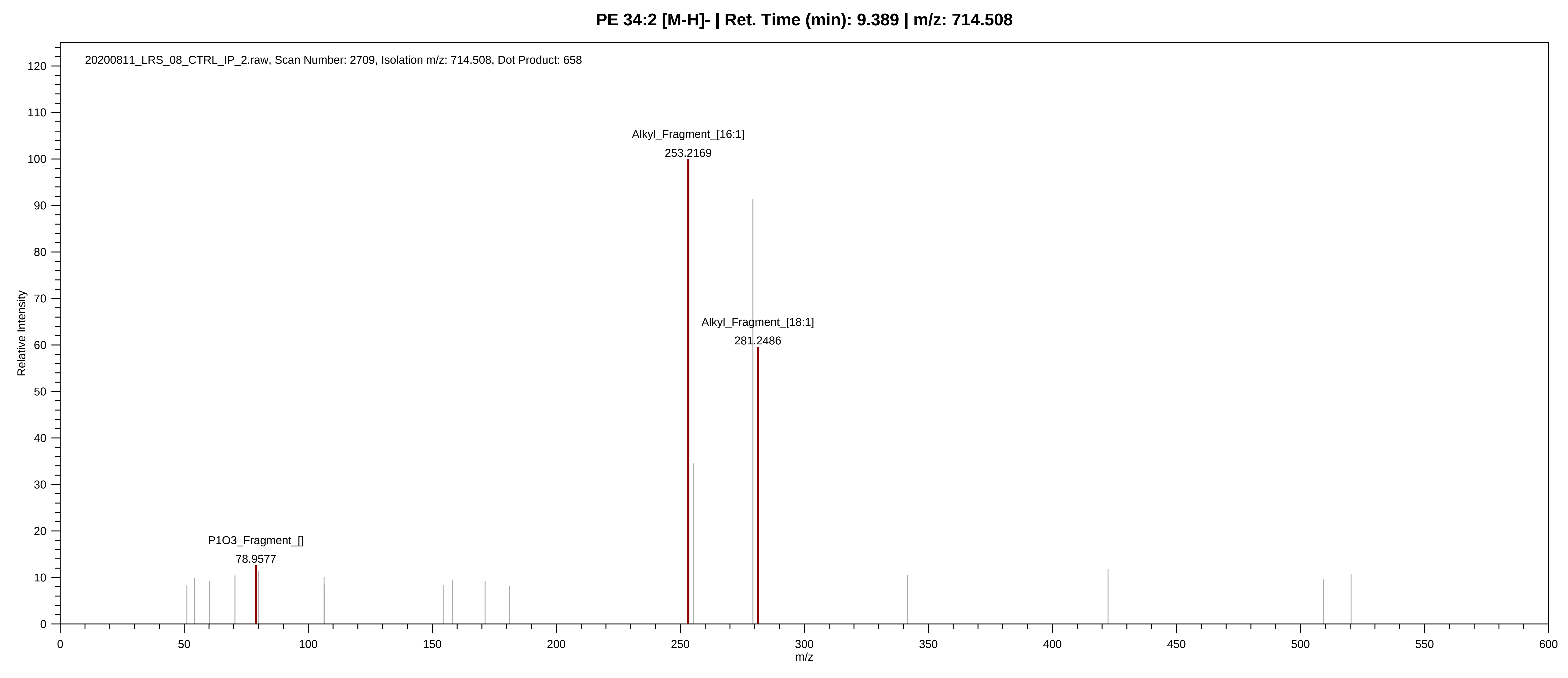
PE 34:2 [M-H]- | Ret. Time (min): 9.389 | m/z: 714.508
20200811_LRS_08_CTRL_IP_2.raw, Scan Number: 2709, Isolation m/z: 714.508, Dot Product: 658
Alkyl_Fragment_[16:1]
253.2169
Alkyl_Fragment_[18:1]
281.2486
P1O3_Fragment_[]
78.9577
Relative Intensity
m/z

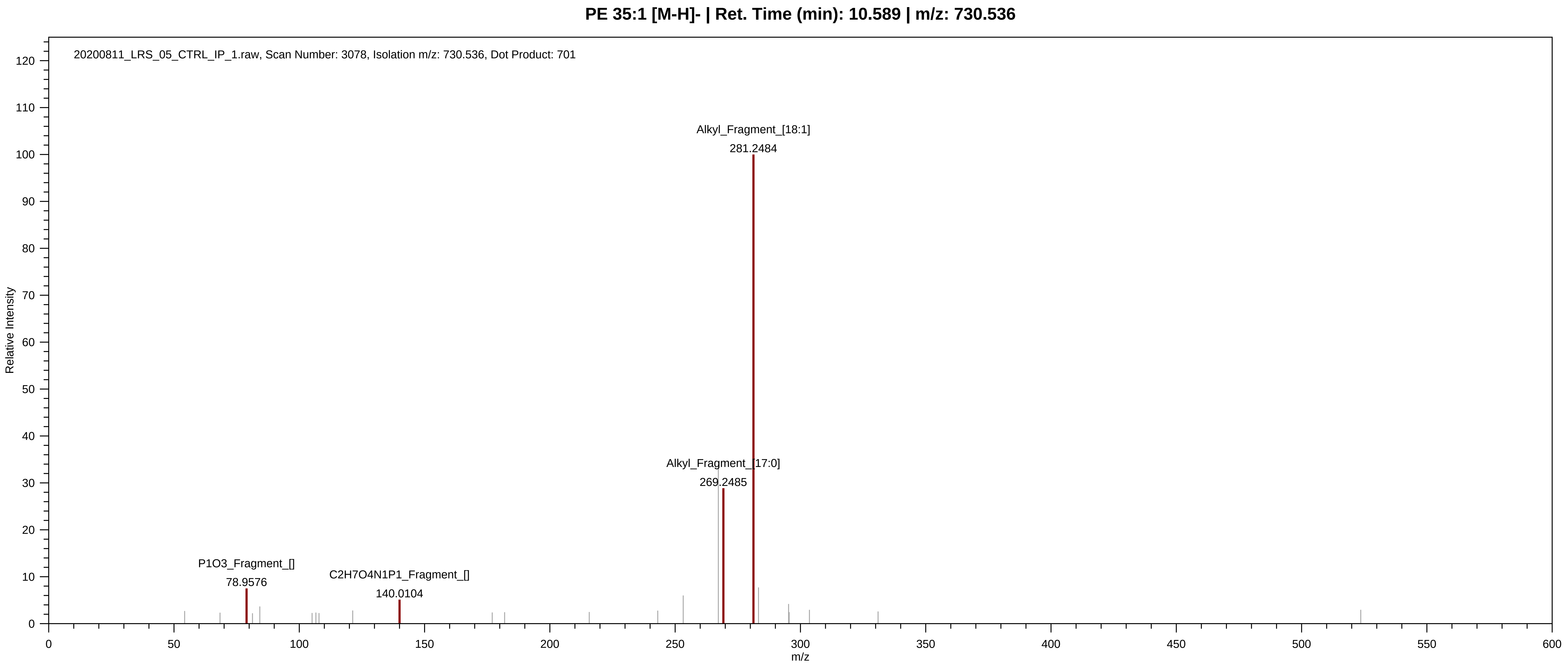

PE 35:1 [M-H]- | Ret. Time (min): 10.589 | m/z: 730.536
20200811_LRS_05_CTRL_IP_1.raw, Scan Number: 3078, Isolation m/z: 730.536, Dot Product: 701
Alkyl_Fragment_[18:1]
281.2484
Alkyl_Fragment_[17:0]
269.2485
P1O3_Fragment_[]
78.9576
C2H7O4N1P1_Fragment_[]
140.0104
Relative Intensity
m/z

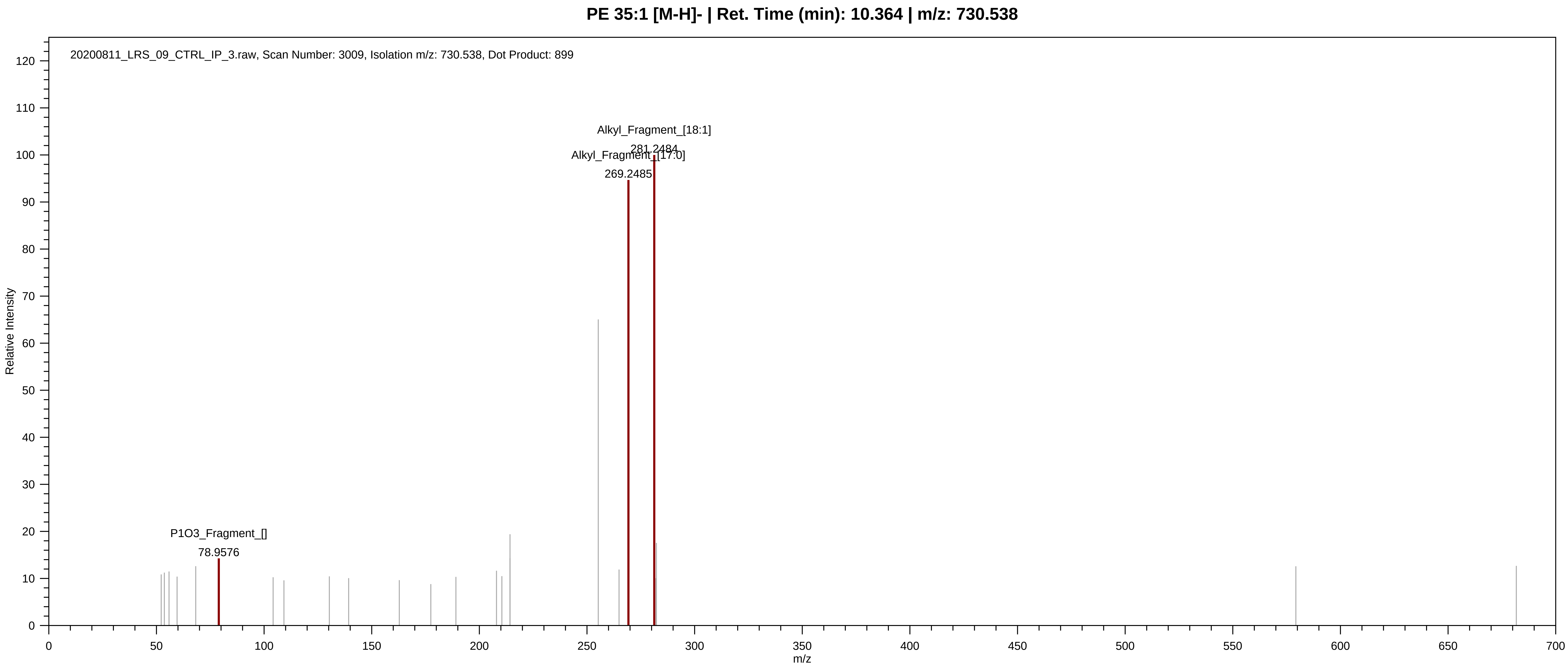

PE 35:1 [M-H]- | Ret. Time (min): 10.364 | m/z: 730.538
20200811_LRS_09_CTRL_IP_3.raw, Scan Number: 3009, Isolation m/z: 730.538, Dot Product: 899
Alkyl_Fragment_[18:1]
281.2484
Alkyl_Fragment_[17:0]
269.2485
P1O3_Fragment_[]
78.9576
Relative Intensity
m/z

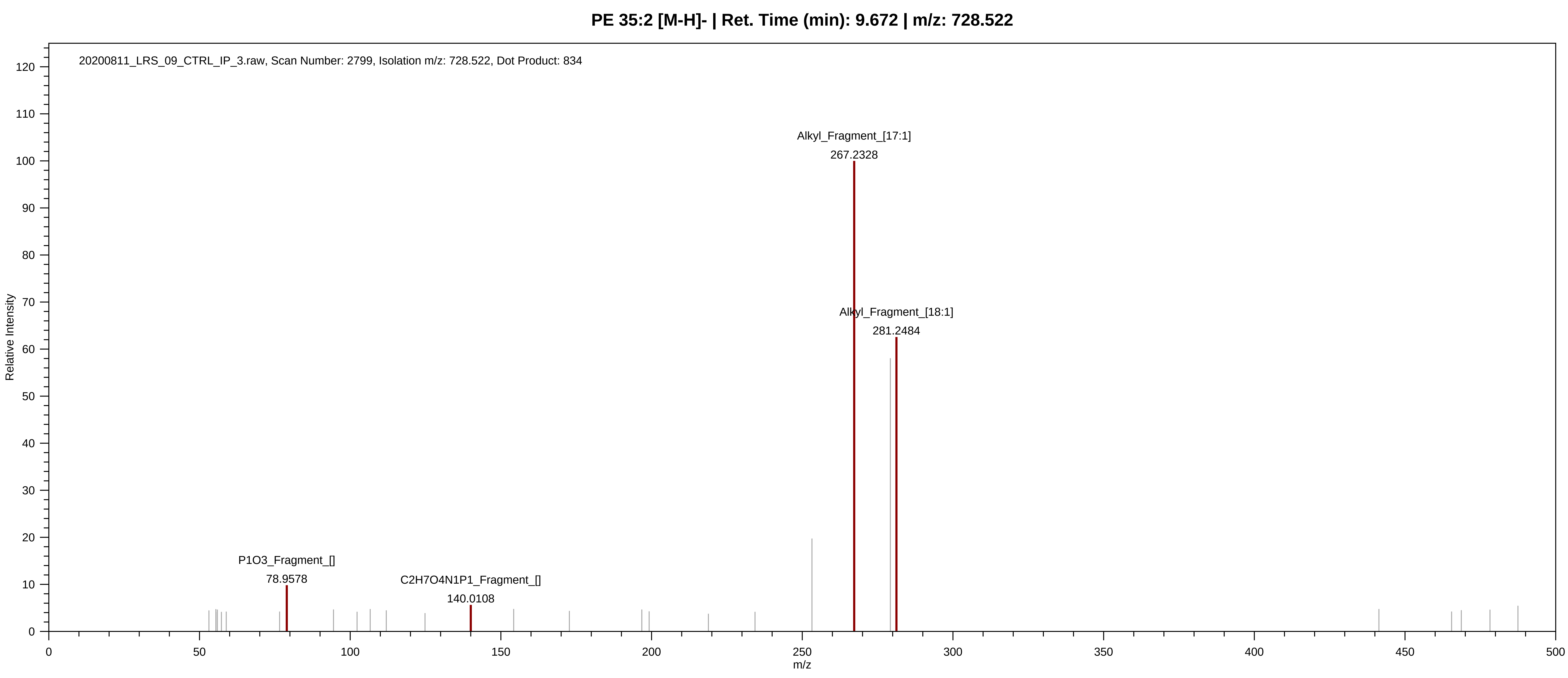
PE 35:2 [M-H]- | Ret. Time (min): 9.672 | m/z: 728.522
20200811_LRS_09_CTRL_IP_3.raw, Scan Number: 2799, Isolation m/z: 728.522, Dot Product: 834
Alkyl_Fragment_[17:1]
267.2328
Alkyl_Fragment_[18:1]
281.2484
P1O3_Fragment_[]
78.9578
C2H7O4N1P1_Fragment_[]
140.0108
Relative Intensity
m/z

PE 35:2 [M-H]- | Ret. Time (min): 9.675 | m/z: 728.522

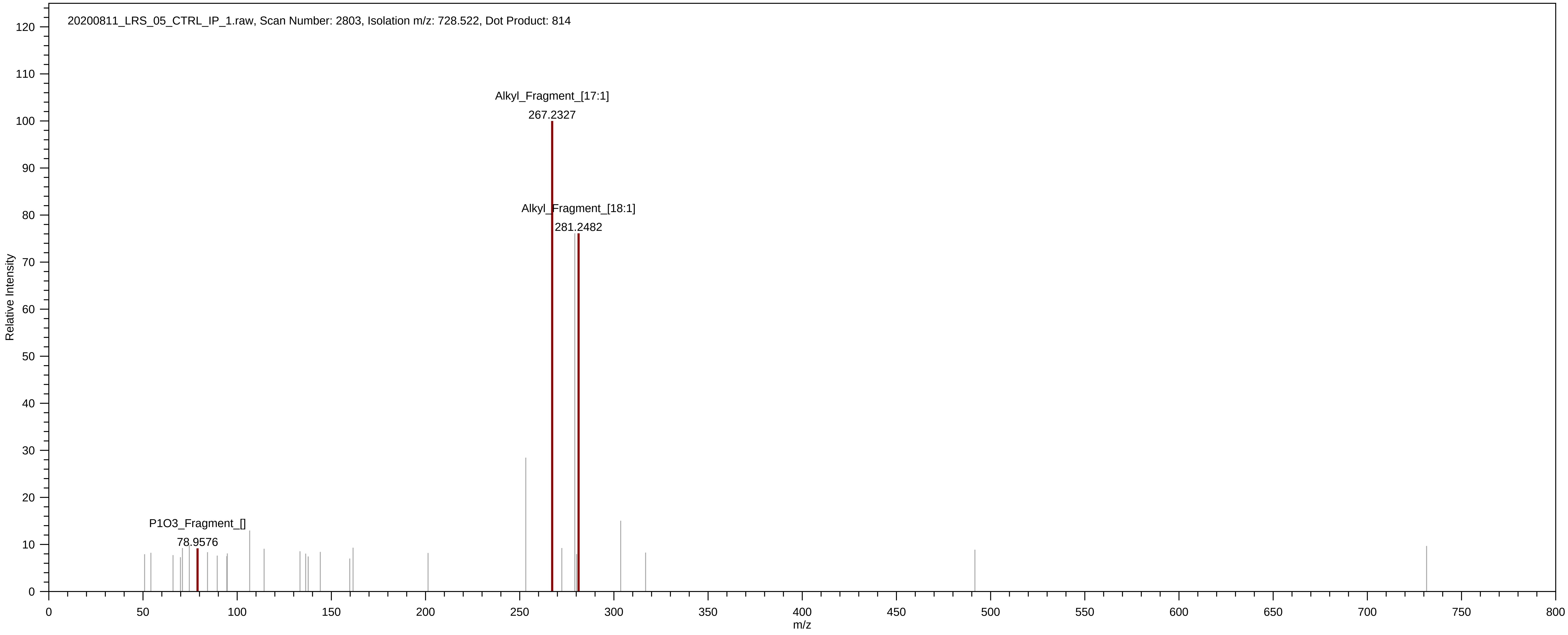

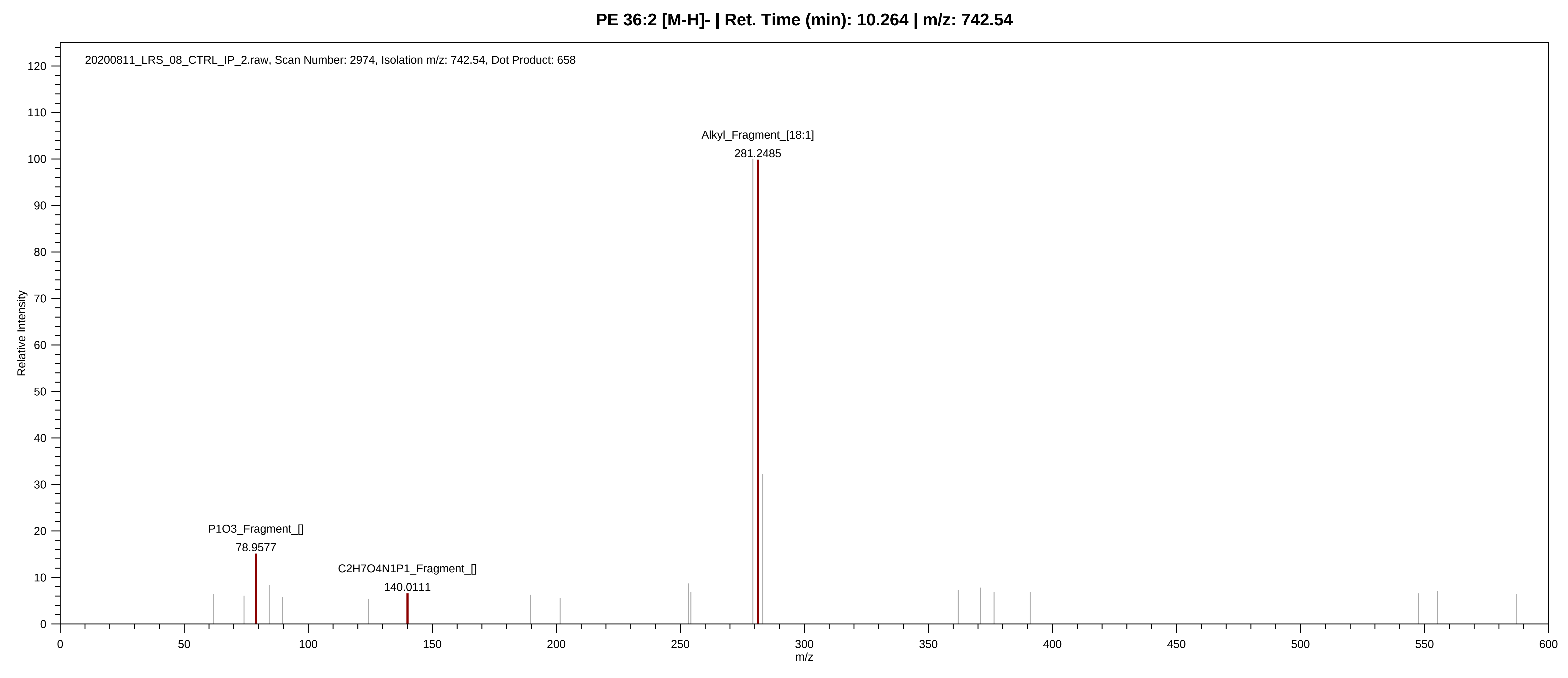

PE 36:2 [M-H]- | Ret. Time (min): 10.264 | m/z: 742.54
20200811_LRS_08_CTRL_IP_2.raw, Scan Number: 2974, Isolation m/z: 742.54, Dot Product: 658
Alkyl_Fragment_[18:1]
281.2485
P1O3_Fragment_[]
78.9577
C2H7O4N1P1_Fragment_[]
140.0111
Relative Intensity
m/z

PE 36:2 [M-H]- | Ret. Time (min): 10.266 | m/z: 742.54

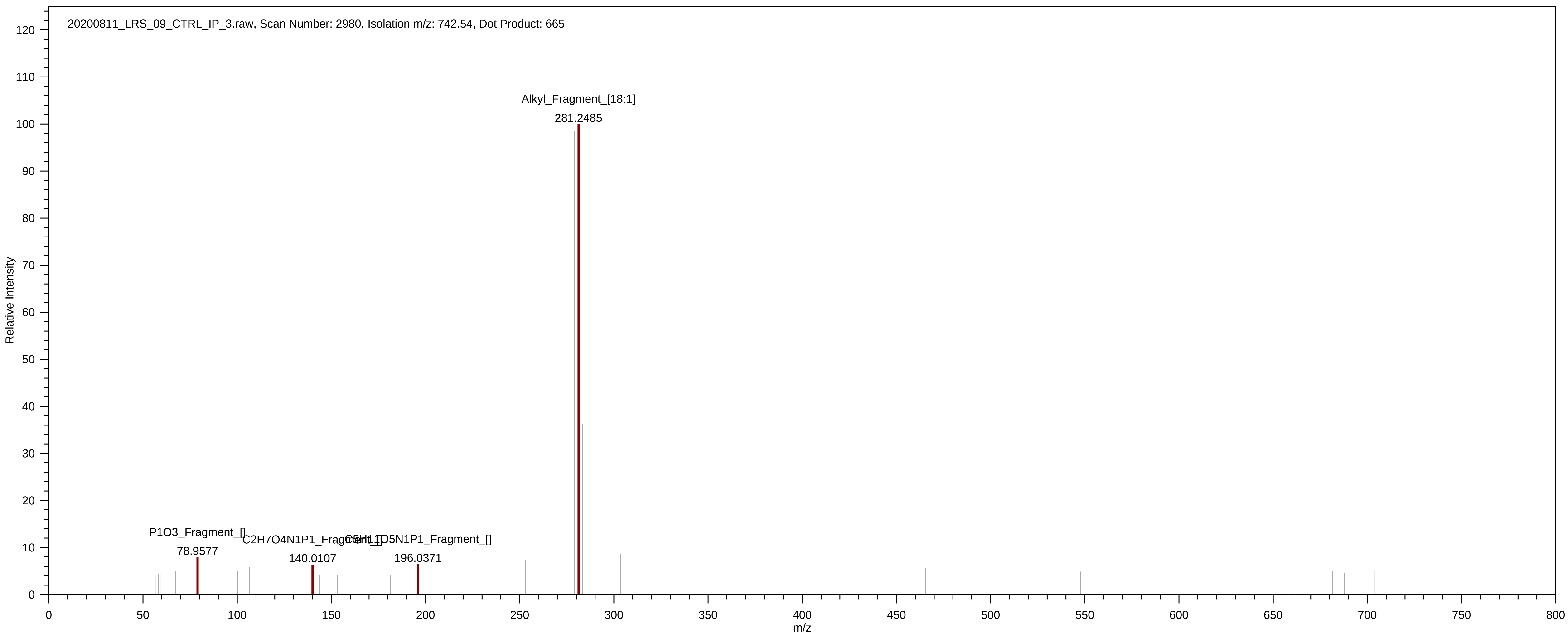

PE 36:2 [M-H]- | Ret. Time (min): 10.286 | m/z: 742.54

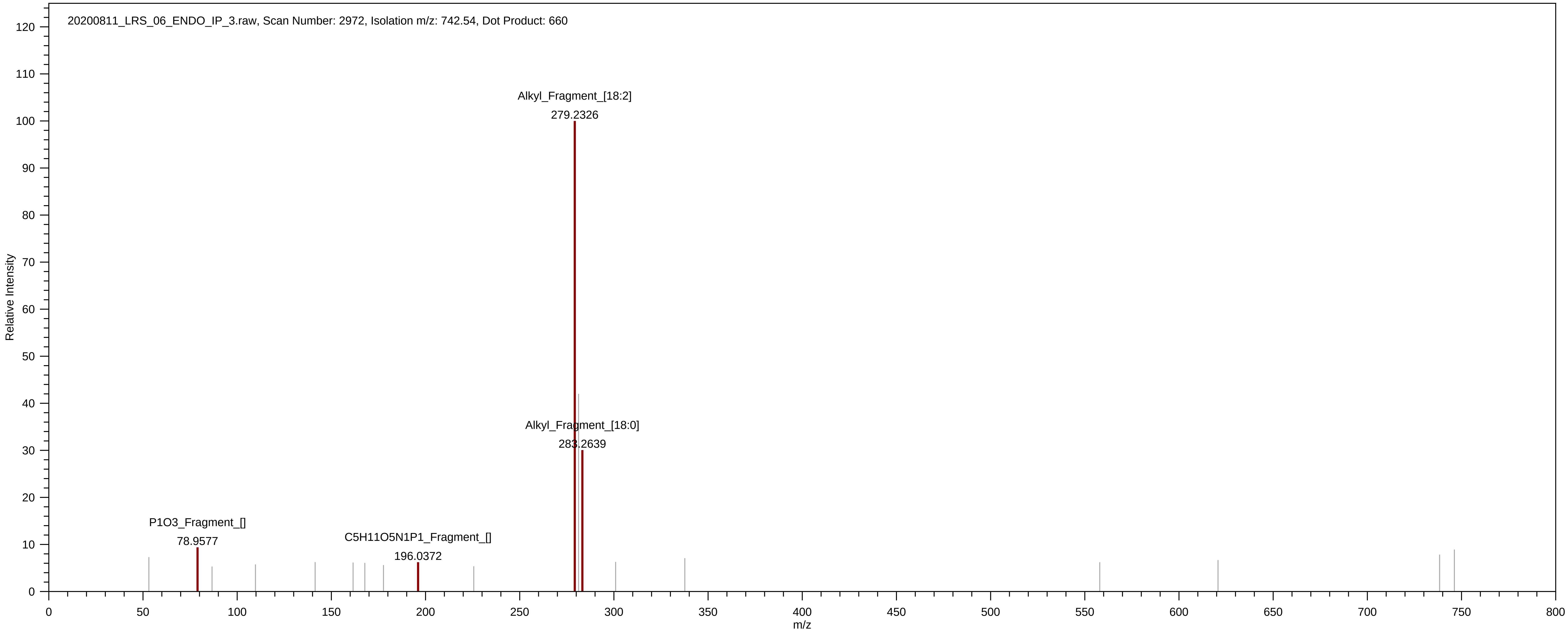

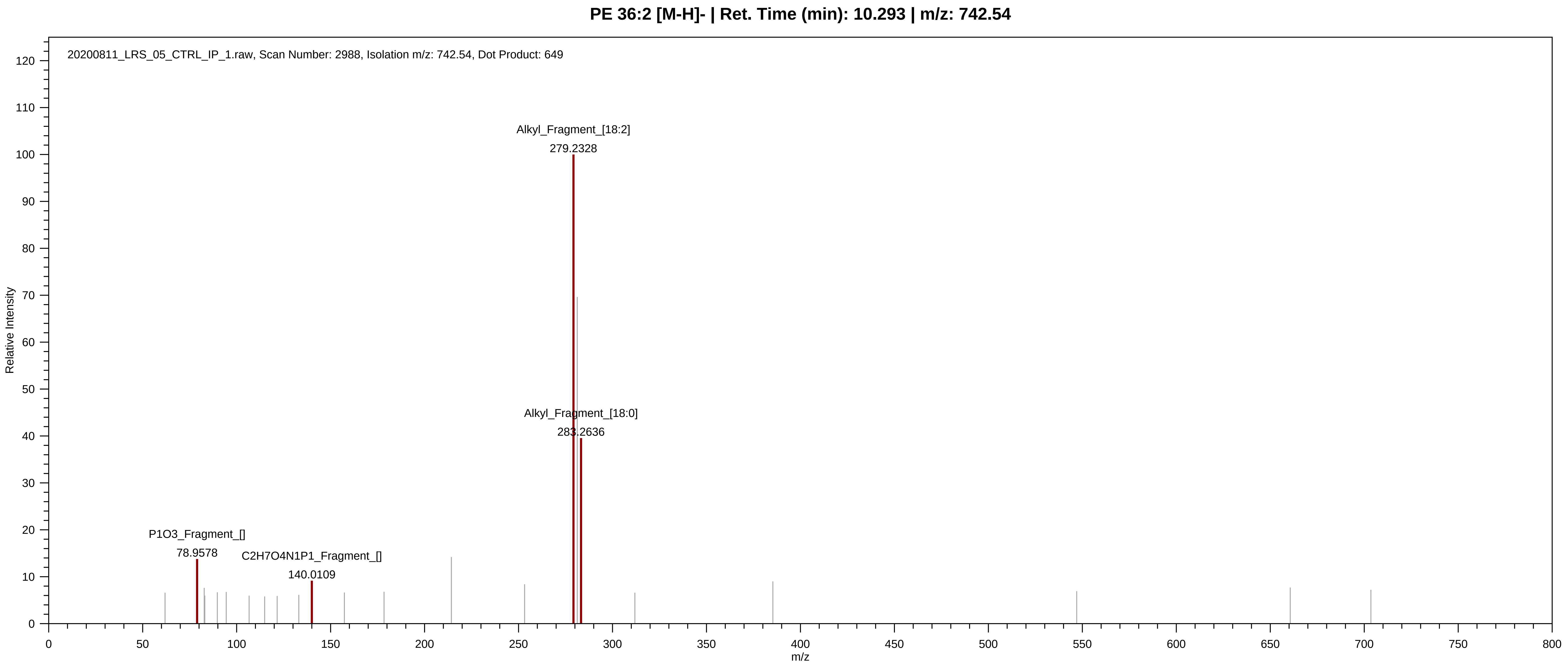
PE 36:2 [M-H]- | Ret. Time (min): 10.293 | m/z: 742.54
20200811_LRS_05_CTRL_IP_1.raw, Scan Number: 2988, Isolation m/z: 742.54, Dot Product: 649
Alkyl_Fragment_[18:2]
279.2328
Alkyl_Fragment_[18:0]
283.2636
P1O3_Fragment_[]
78.9578
C2H7O4N1P1_Fragment_[]
140.0109
Relative Intensity
m/z

PE 36:2 [M-H]- | Ret. Time (min): 10.289 | m/z: 742.541

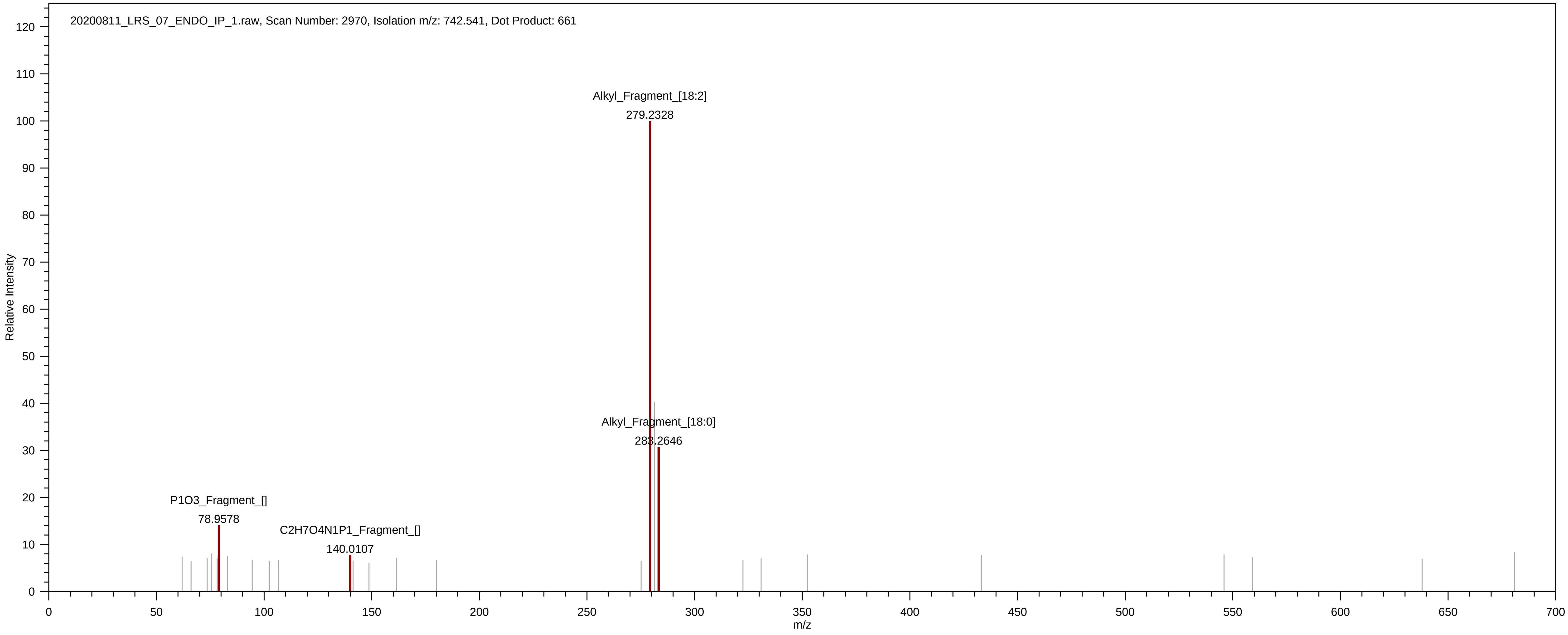

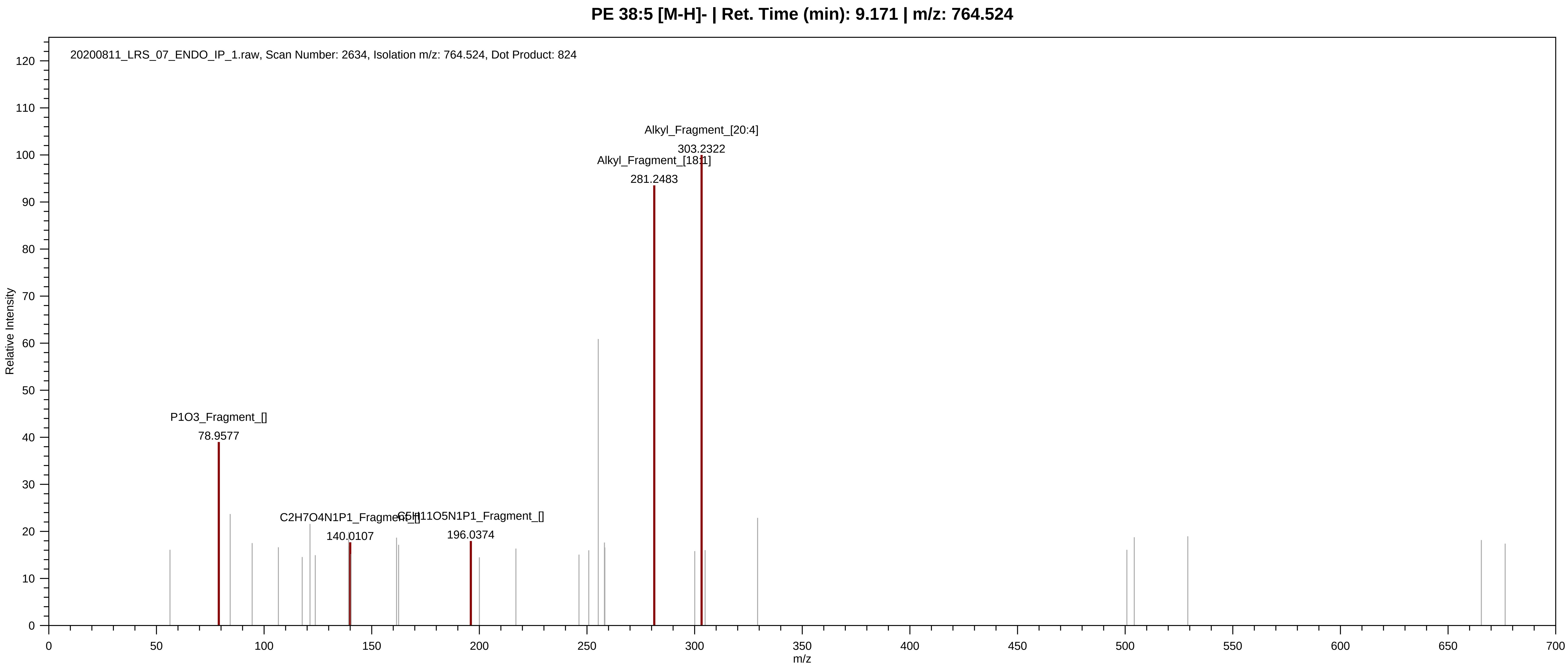

PE 38:5 [M-H]- | Ret. Time (min): 9.171 | m/z: 764.524
20200811_LRS_07_ENDO_IP_1.raw, Scan Number: 2634, Isolation m/z: 764.524, Dot Product: 824
Alkyl_Fragment_[20:4]
303.2322
Alkyl_Fragment_[18:1]
281.2483
P1O3_Fragment_[]
78.9577
C2H7O4N1P1_Fragment_[]
140.0107
C5H11O5N1P1_Fragment_[]
196.0374
Relative Intensity
m/z

PE 38:5 [M-H]- | Ret. Time (min): 9.141 | m/z: 764.525

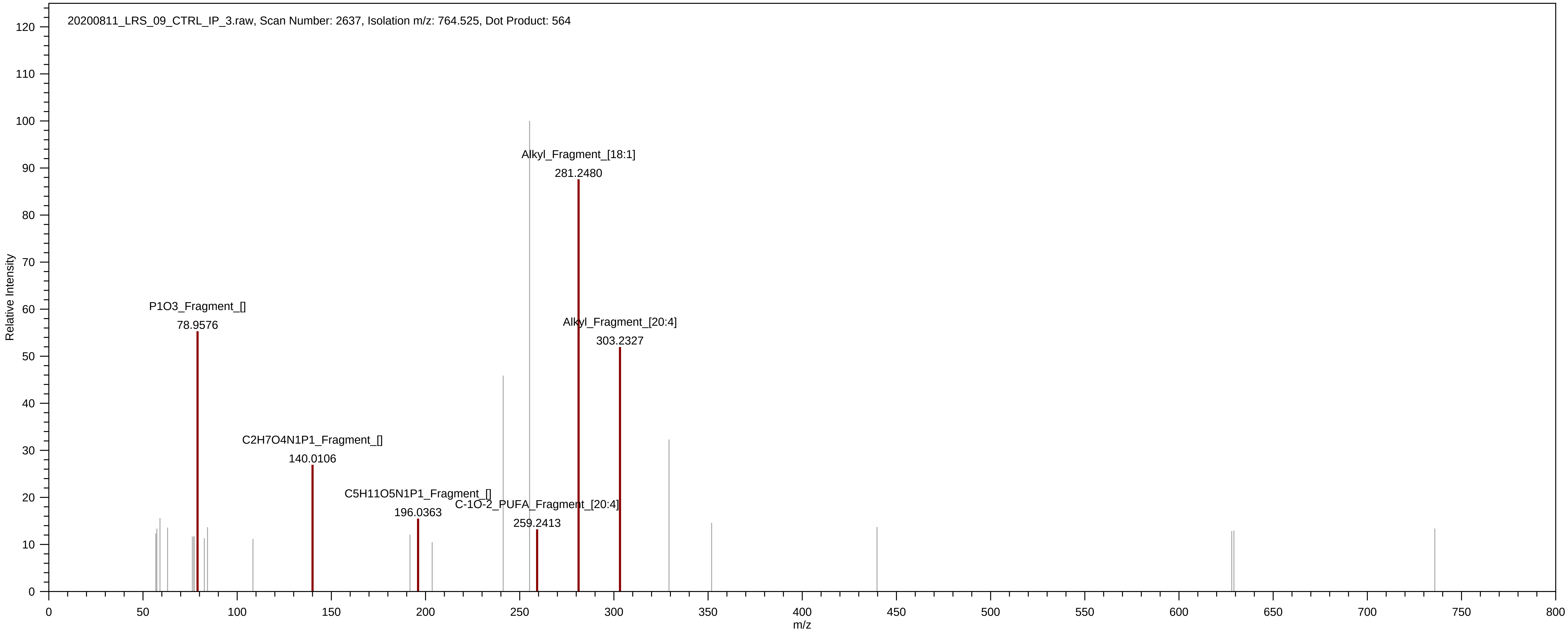

PE 38:5 [M-H]- | Ret. Time (min): 9.167 | m/z: 764.526

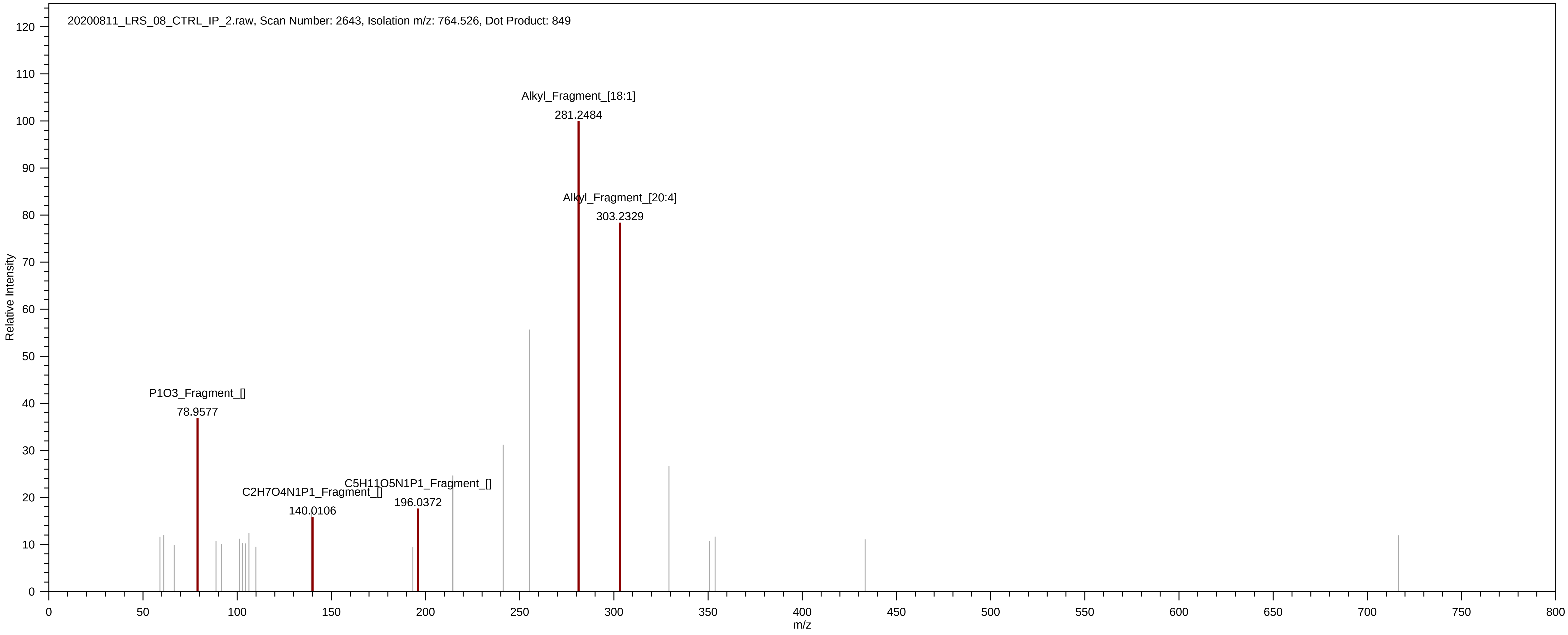

PE 38:5 [M-H]- | Ret. Time (min): 9.174 | m/z: 764.526

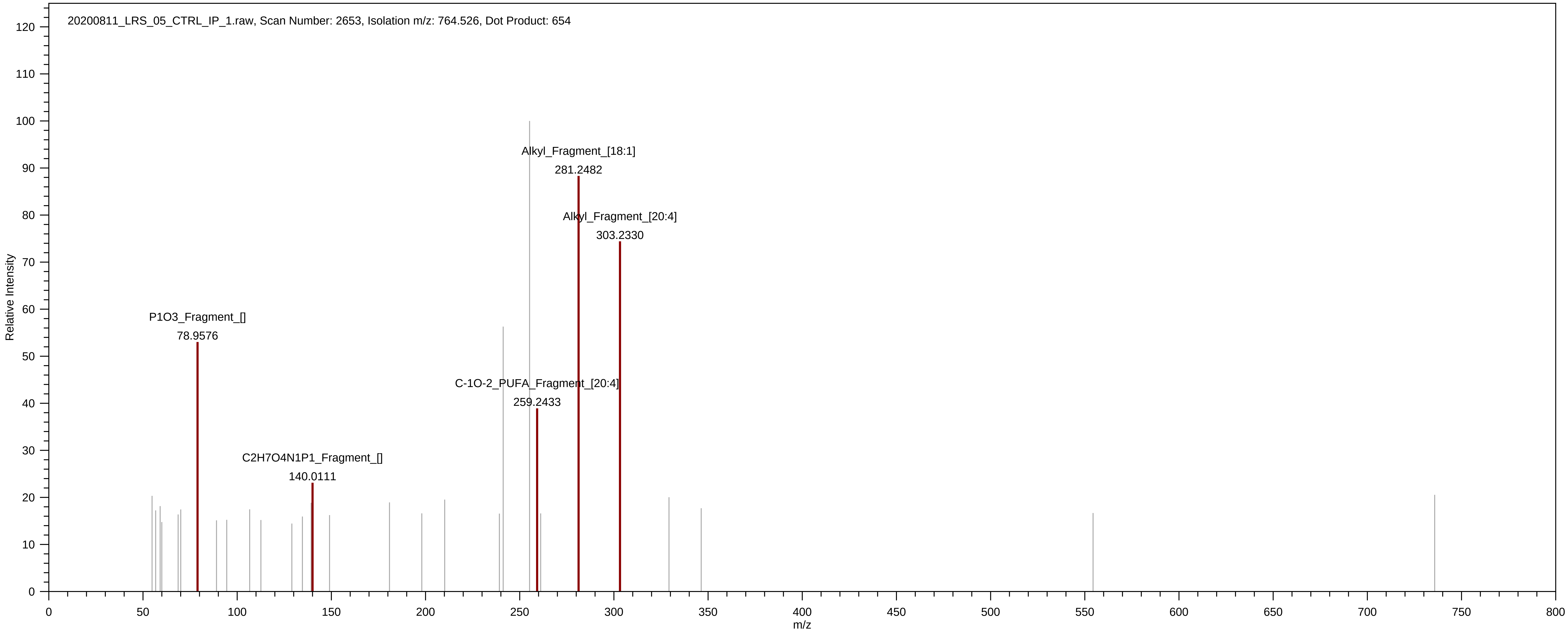

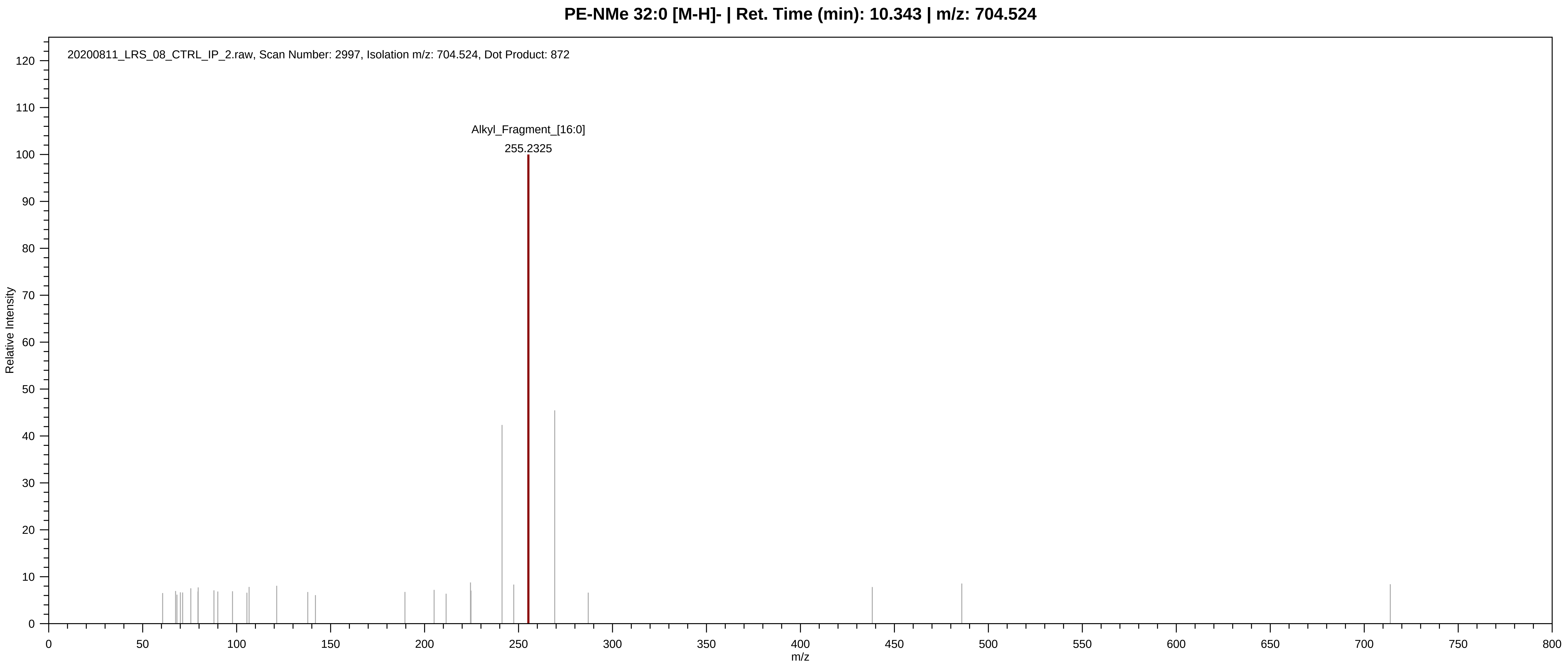
PE-NMe 32:0 [M-H]- | Ret. Time (min): 10.343 | m/z: 704.524
20200811_LRS_08_CTRL_IP_2.raw, Scan Number: 2997, Isolation m/z: 704.524, Dot Product: 872
Alkyl_Fragment_[16:0]
255.2325
Relative Intensity
m/z

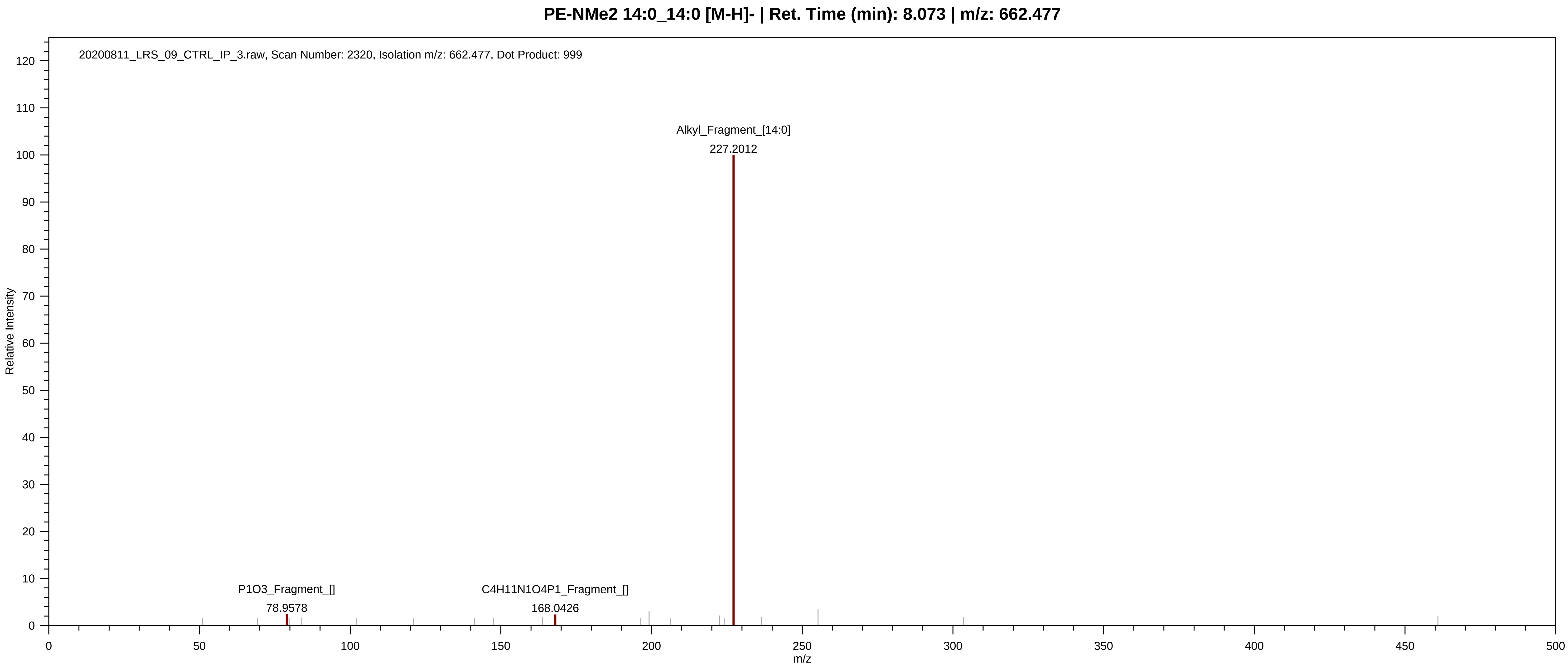

PE-NMe2 14:0_14:0 [M-H]- | Ret. Time (min): 8.073 | m/z: 662.477
20200811_LRS_09_CTRL_IP_3.raw, Scan Number: 2320, Isolation m/z: 662.477, Dot Product: 999
Alkyl_Fragment_[14:0]
227.2012
P1O3_Fragment_[]
78.9578
C4H11N1O4P1_Fragment_[]
168.0426
Relative Intensity
m/z

PE-NMe2 14:0_16:1 [M-H]- | Ret. Time (min): 8.175 | m/z: 688.491

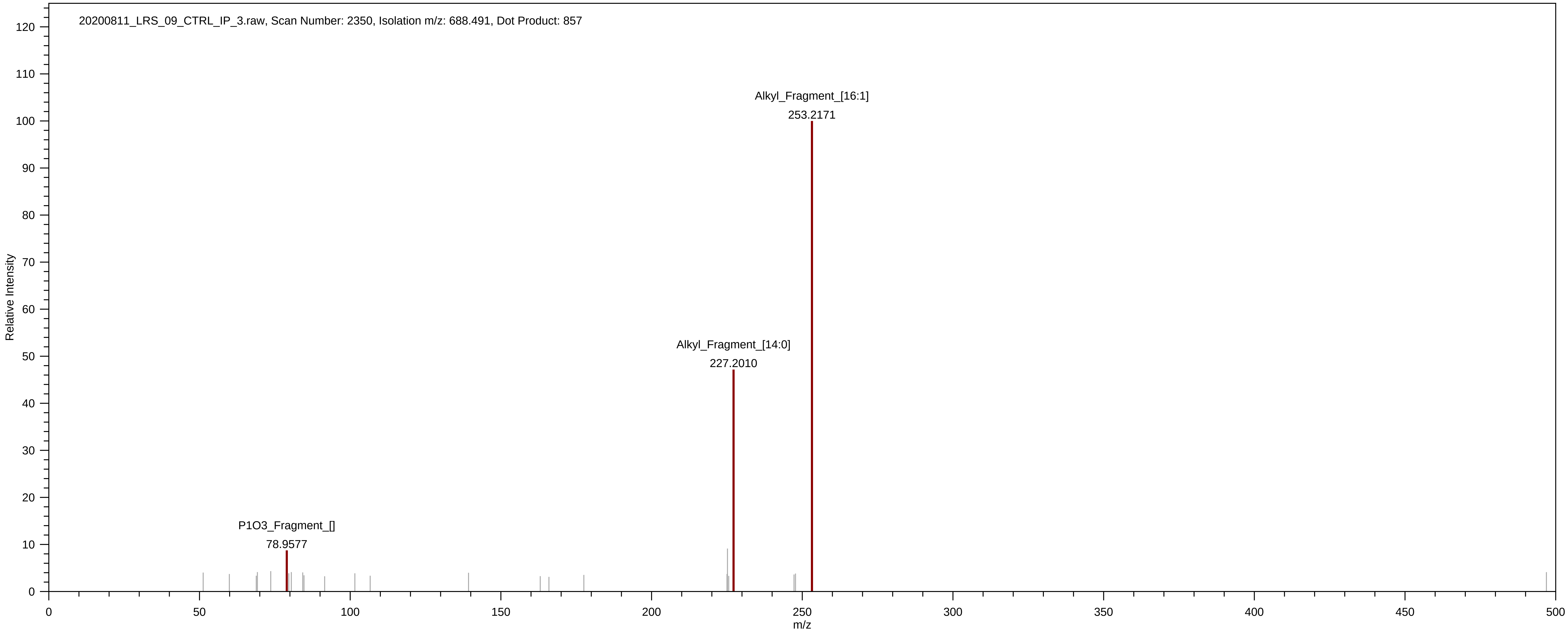

PE-NMe2 14:0_16:1 [M-H]- | Ret. Time (min): 8.176 | m/z: 688.493

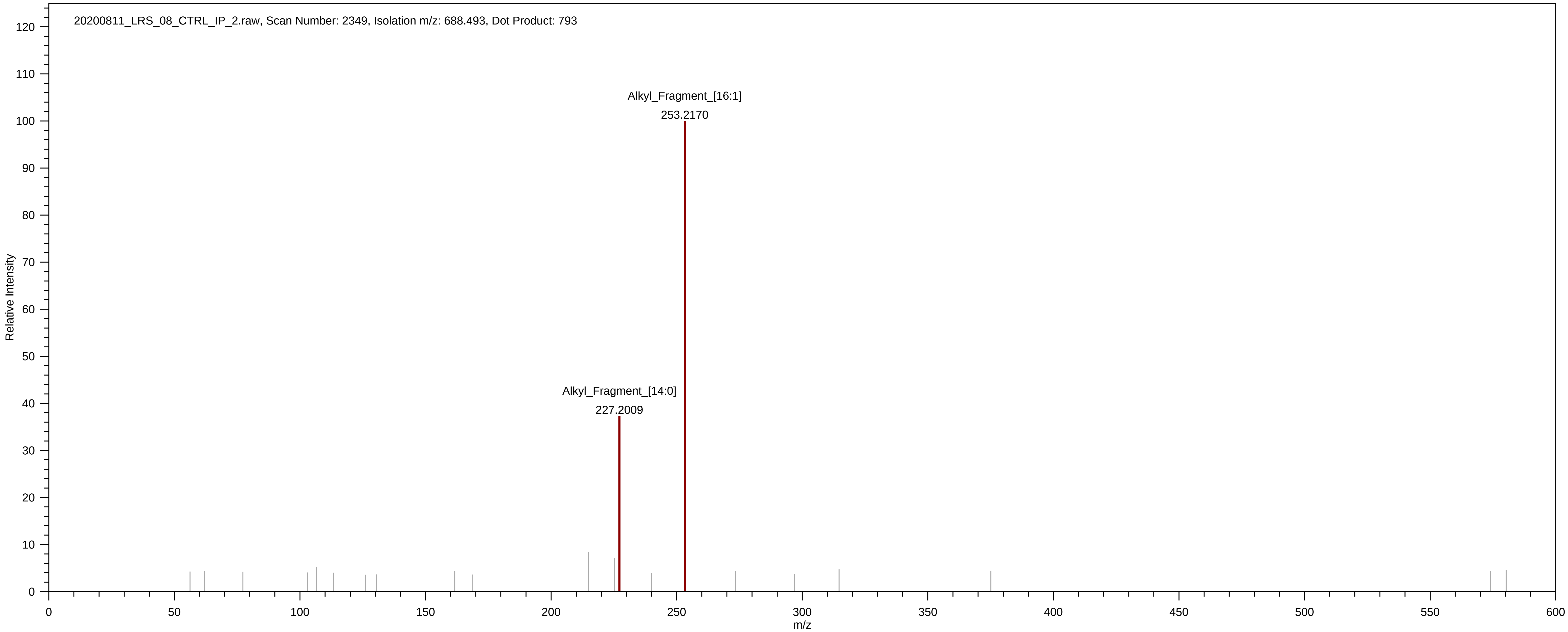

PE-NMe2 16:0_16:0 [M-H]- | Ret. Time (min): 9.83 | m/z: 718.54

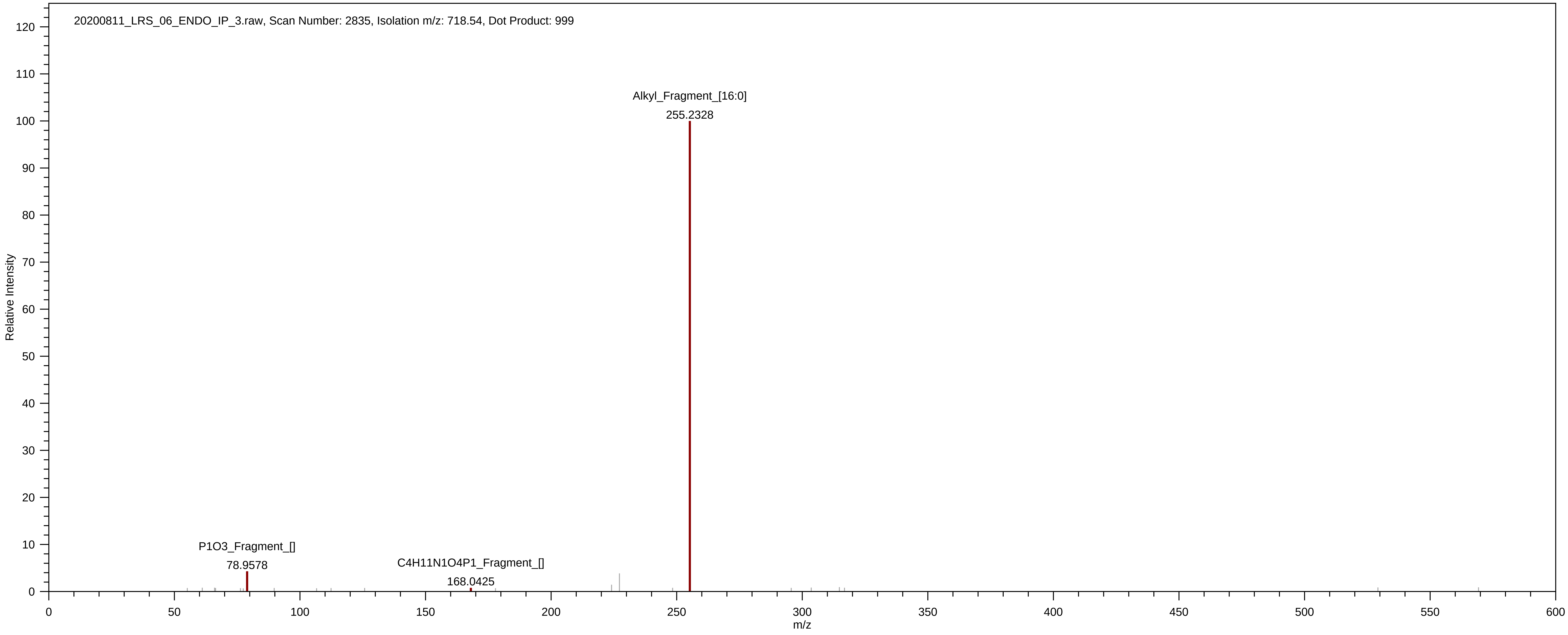

PE-NMe2 16:0_16:0 [M-H]- | Ret. Time (min): 11.048 | m/z: 718.54

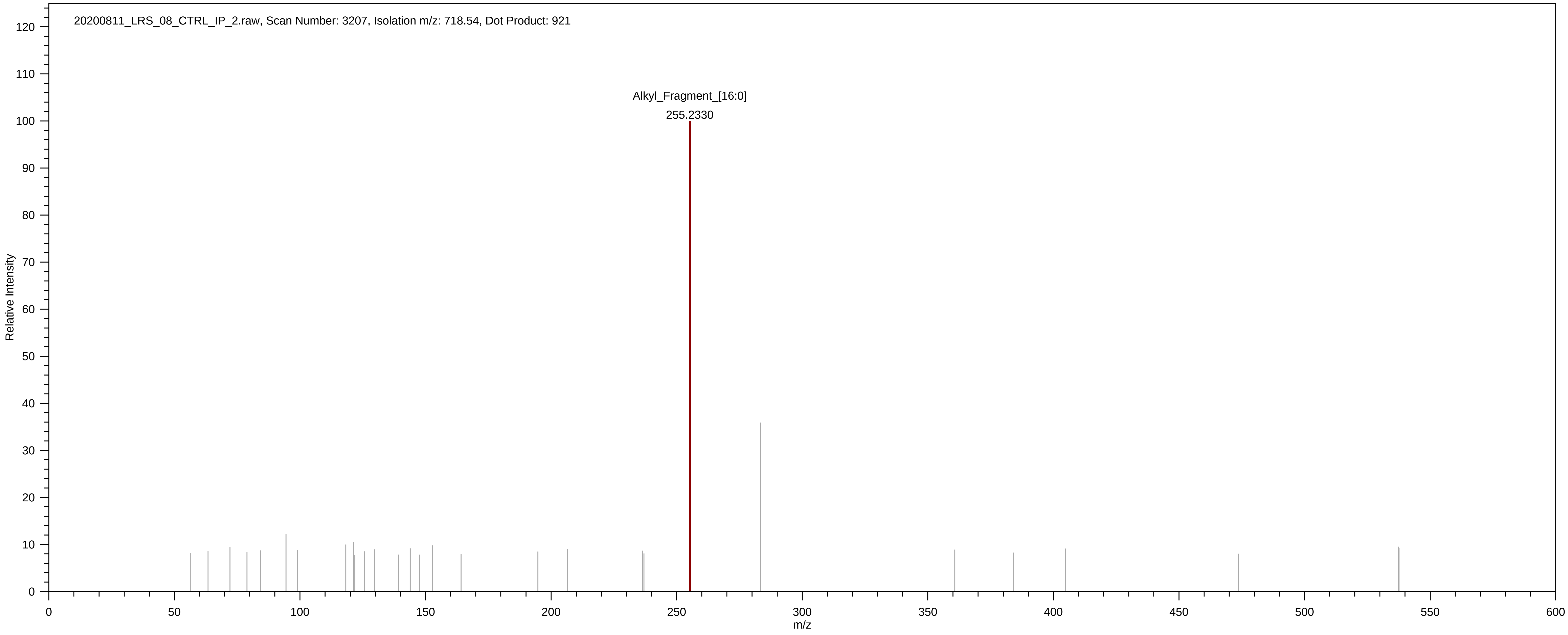

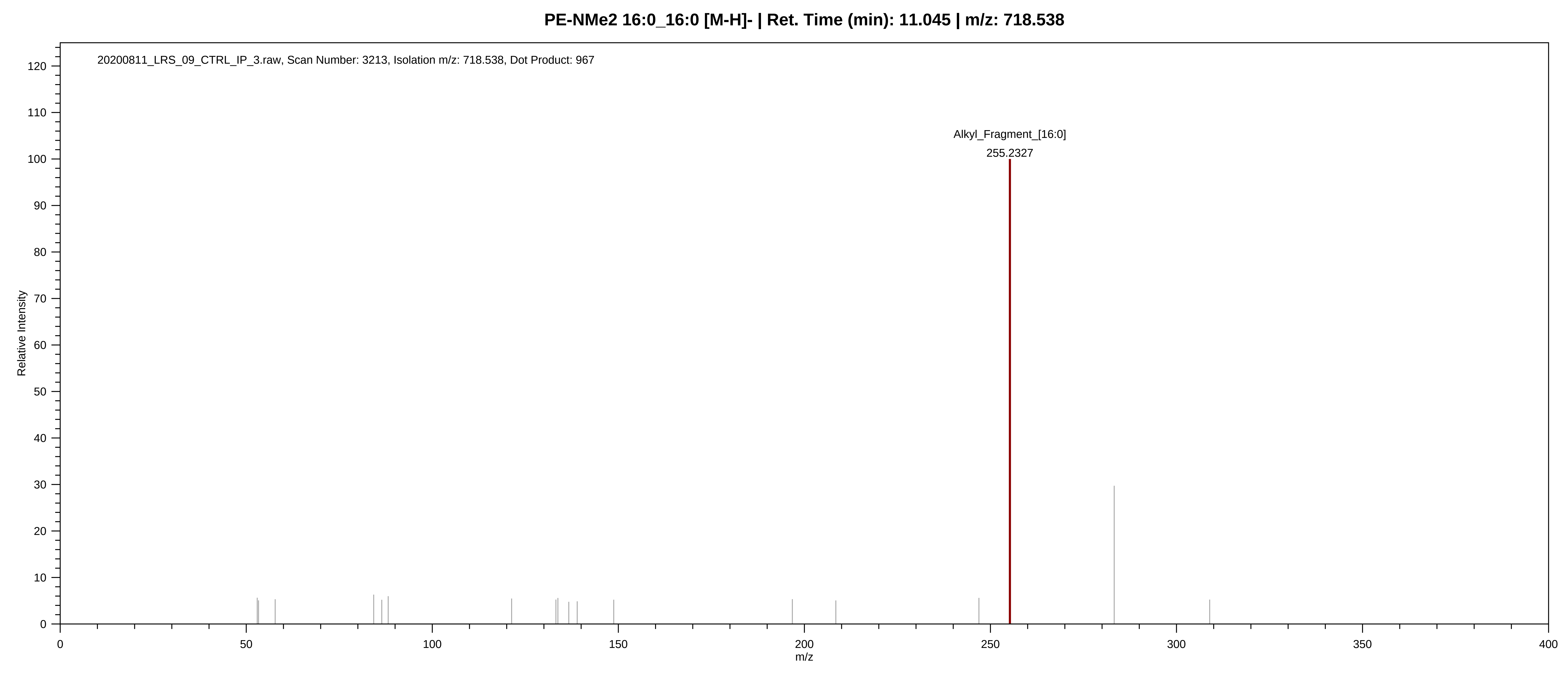

PE-NMe2 16:0_16:0 [M-H]- | Ret. Time (min): 11.045 | m/z: 718.538
20200811_LRS_09_CTRL_IP_3.raw, Scan Number: 3213, Isolation m/z: 718.538, Dot Product: 967
Alkyl_Fragment_[16:0]
255.2327
Relative Intensity
m/z
0
10
20
30
40
50
60
70
80
90
100
110
120
0
50
100
150
200
250
300
350
400

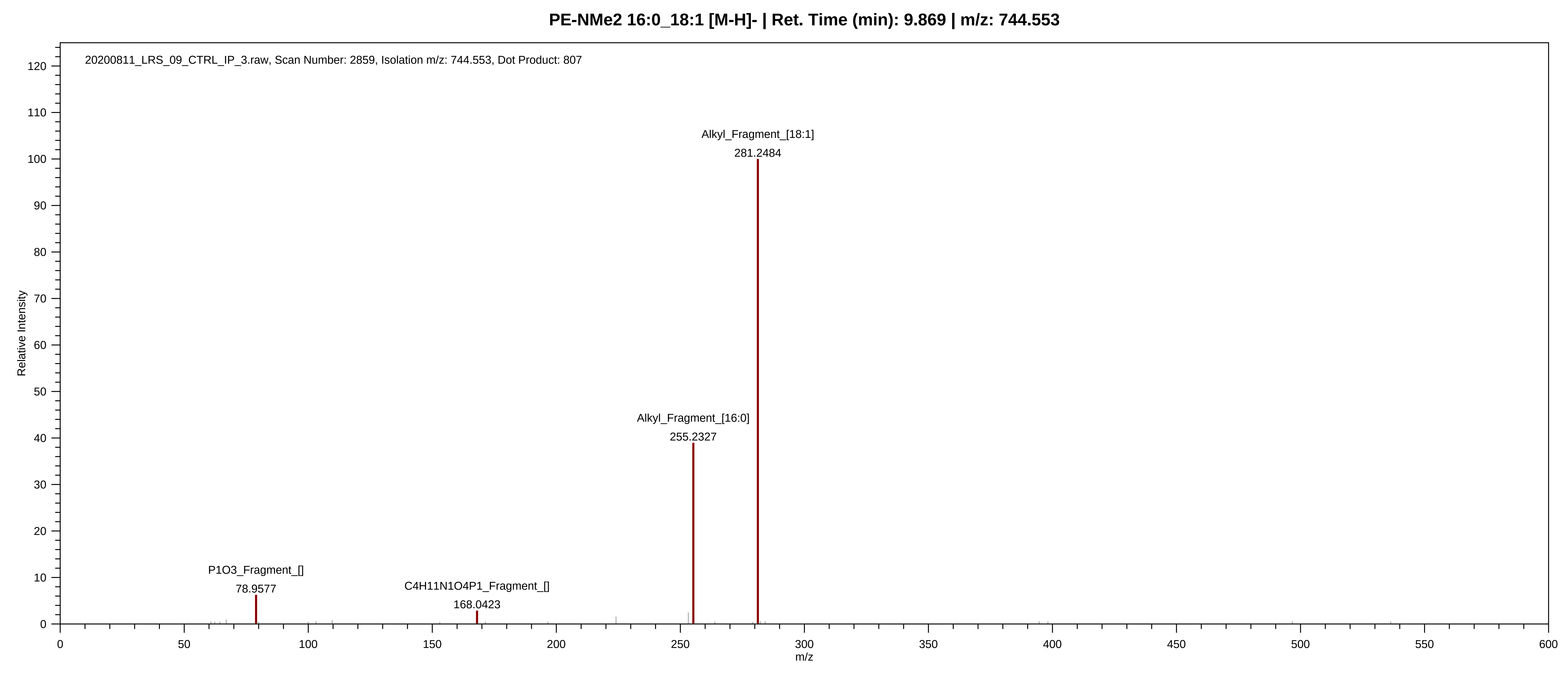
PE-NMe2 16:0_18:1 [M-H]- | Ret. Time (min): 9.869 | m/z: 744.553
20200811_LRS_09_CTRL_IP_3.raw, Scan Number: 2859, Isolation m/z: 744.553, Dot Product: 807
Alkyl_Fragment_[18:1]
281.2484
Alkyl_Fragment_[16:0]
255.2327
P1O3_Fragment_[]
78.9577
C4H11N1O4P1_Fragment_[]
168.0423
Relative Intensity
m/z

PE-NMe2 16:0_18:1 [M-H]- | Ret. Time (min): 9.85 | m/z: 744.554

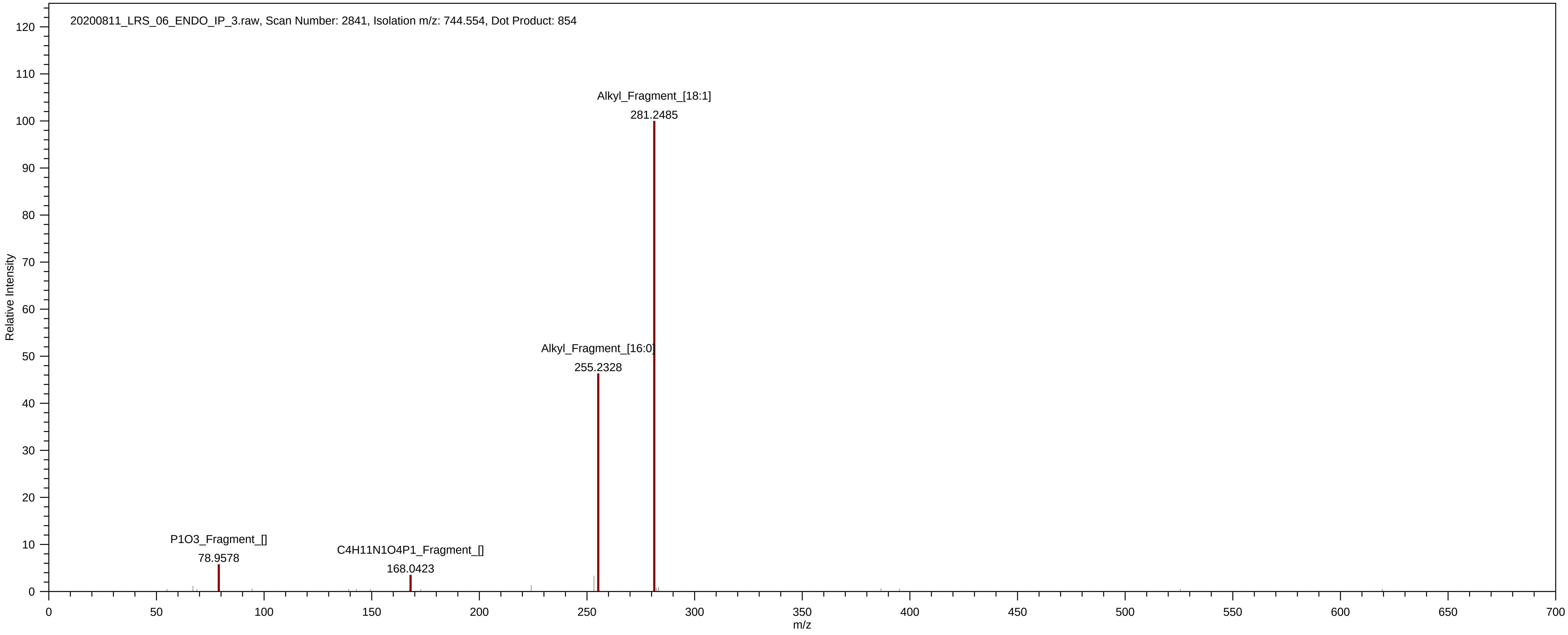

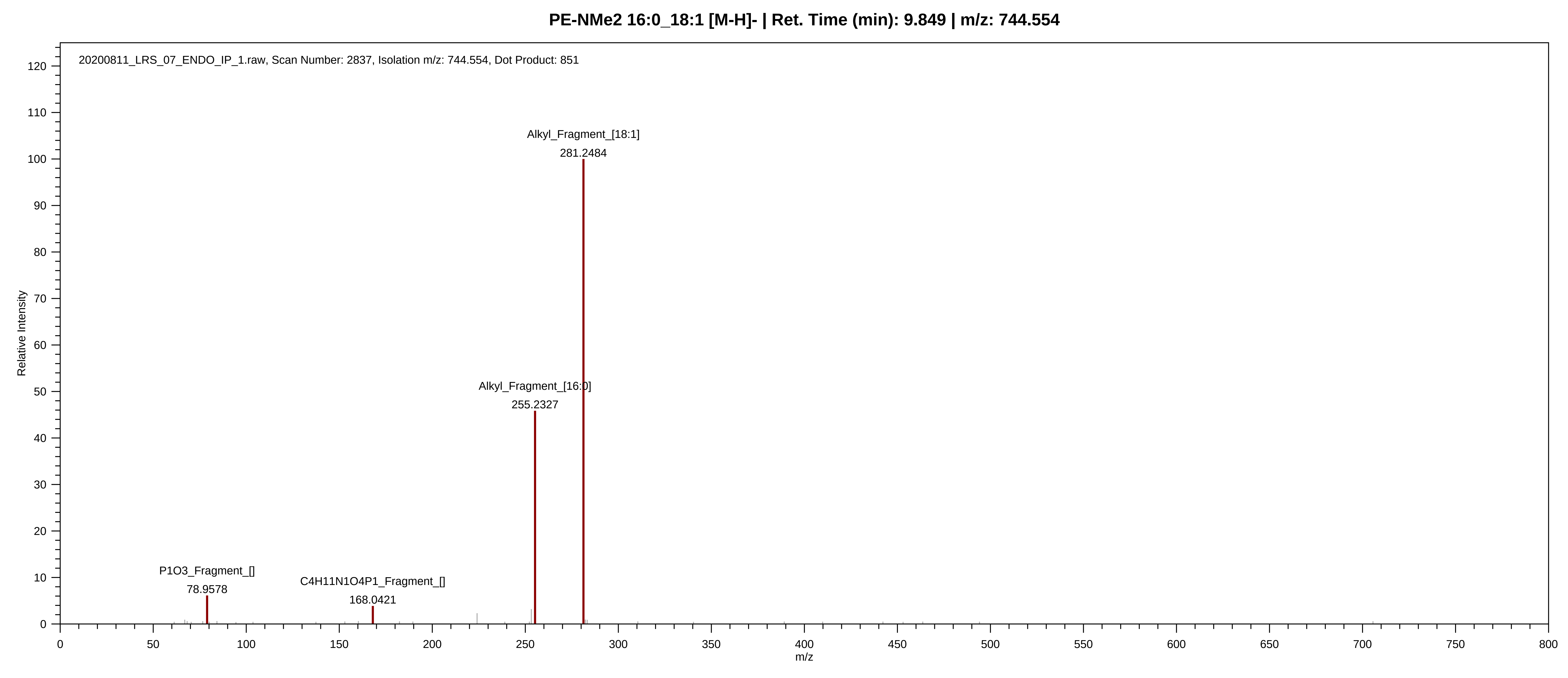

PE-NMe2 16:0_18:1 [M-H]- | Ret. Time (min): 9.849 | m/z: 744.554
20200811_LRS_07_ENDO_IP_1.raw, Scan Number: 2837, Isolation m/z: 744.554, Dot Product: 851
Alkyl_Fragment_[18:1]
281.2484
Alkyl_Fragment_[16:0]
255.2327
P1O3_Fragment_[]
78.9578
C4H11N1O4P1_Fragment_[]
168.0421
Relative Intensity
m/z

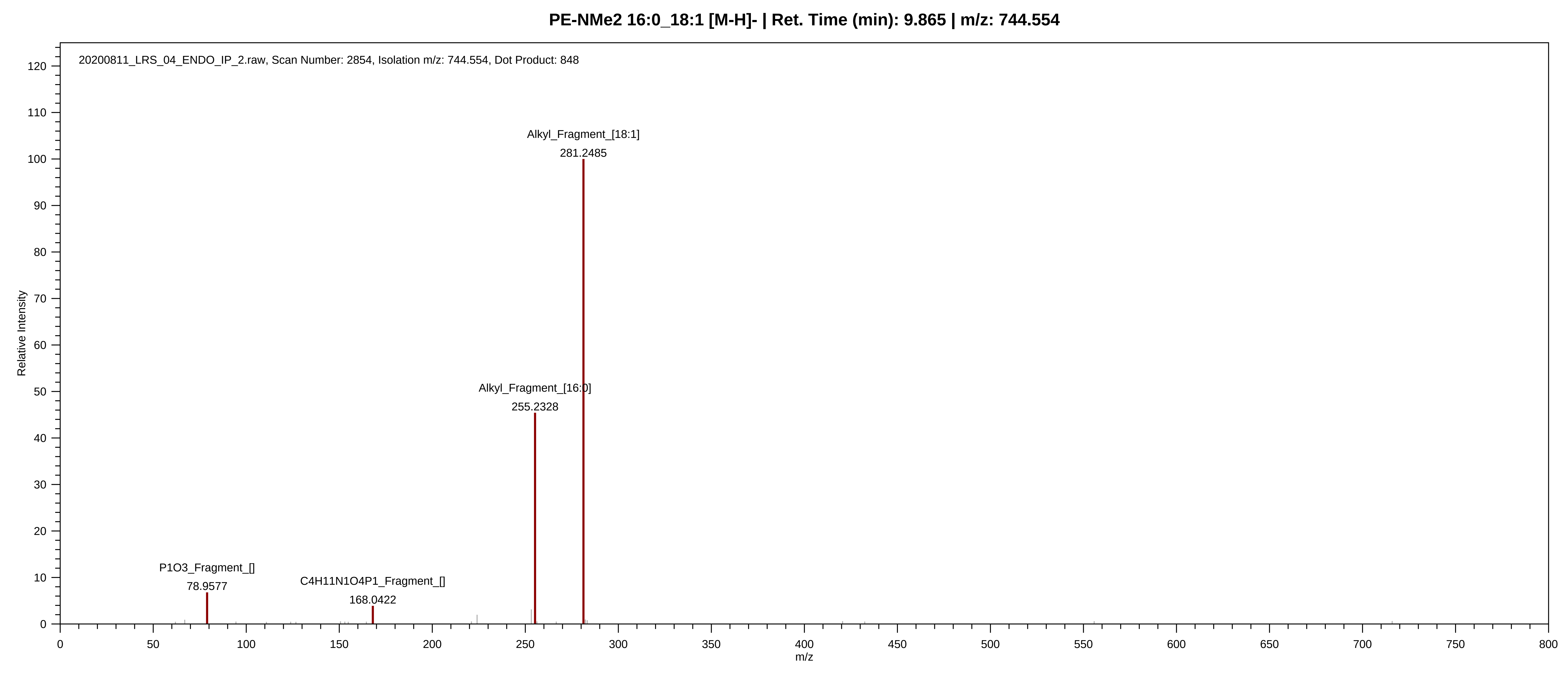

PE-NMe2 16:0_18:1 [M-H]- | Ret. Time (min): 9.865 | m/z: 744.554
20200811_LRS_04_ENDO_IP_2.raw, Scan Number: 2854, Isolation m/z: 744.554, Dot Product: 848
Alkyl_Fragment_[18:1]
281.2485
Alkyl_Fragment_[16:0]
255.2328
P1O3_Fragment_[]
78.9577
C4H11N1O4P1_Fragment_[]
168.0422
Relative Intensity
m/z

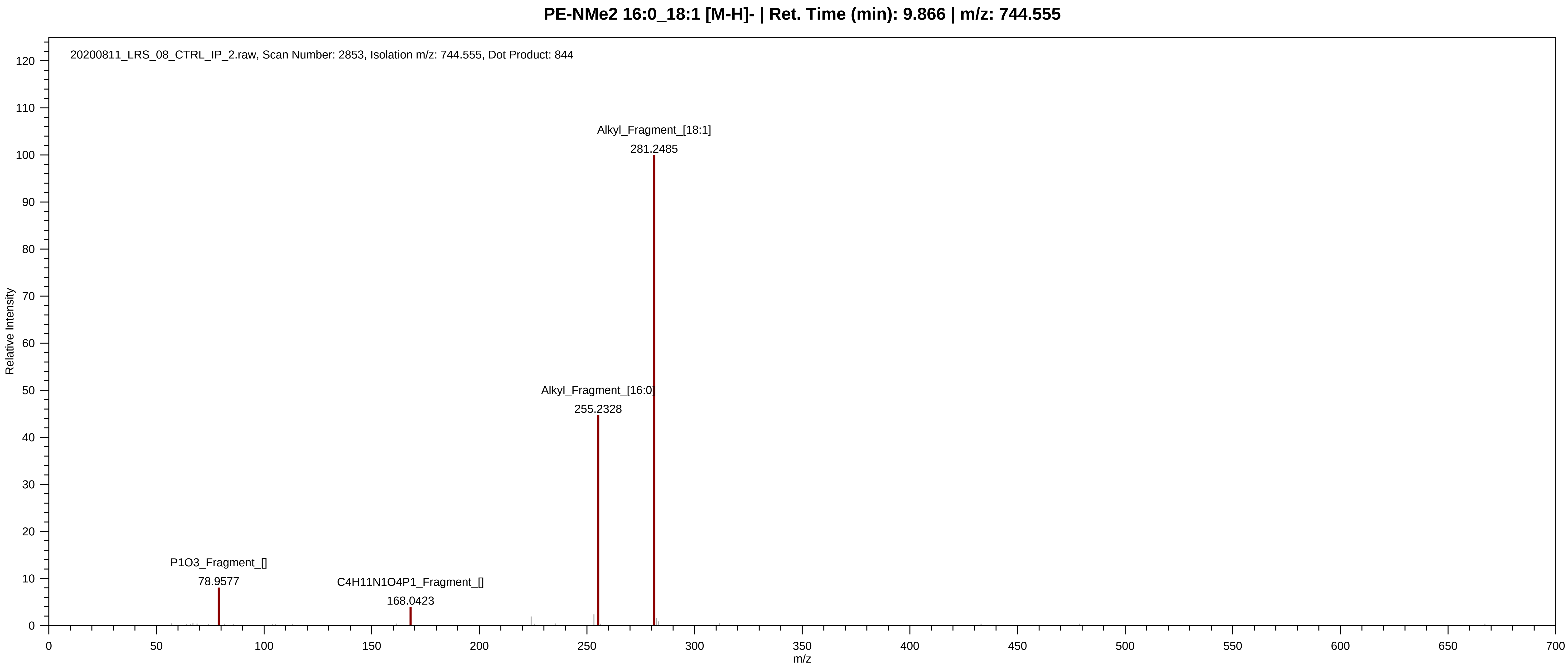
PE-NMe2 16:0_18:1 [M-H]- | Ret. Time (min): 9.866 | m/z: 744.555
20200811_LRS_08_CTRL_IP_2.raw, Scan Number: 2853, Isolation m/z: 744.555, Dot Product: 844
Alkyl_Fragment_[18:1]
281.2485
Alkyl_Fragment_[16:0]
255.2328
P1O3_Fragment_[]
78.9577
C4H11N1O4P1_Fragment_[]
168.0423
Relative Intensity
m/z

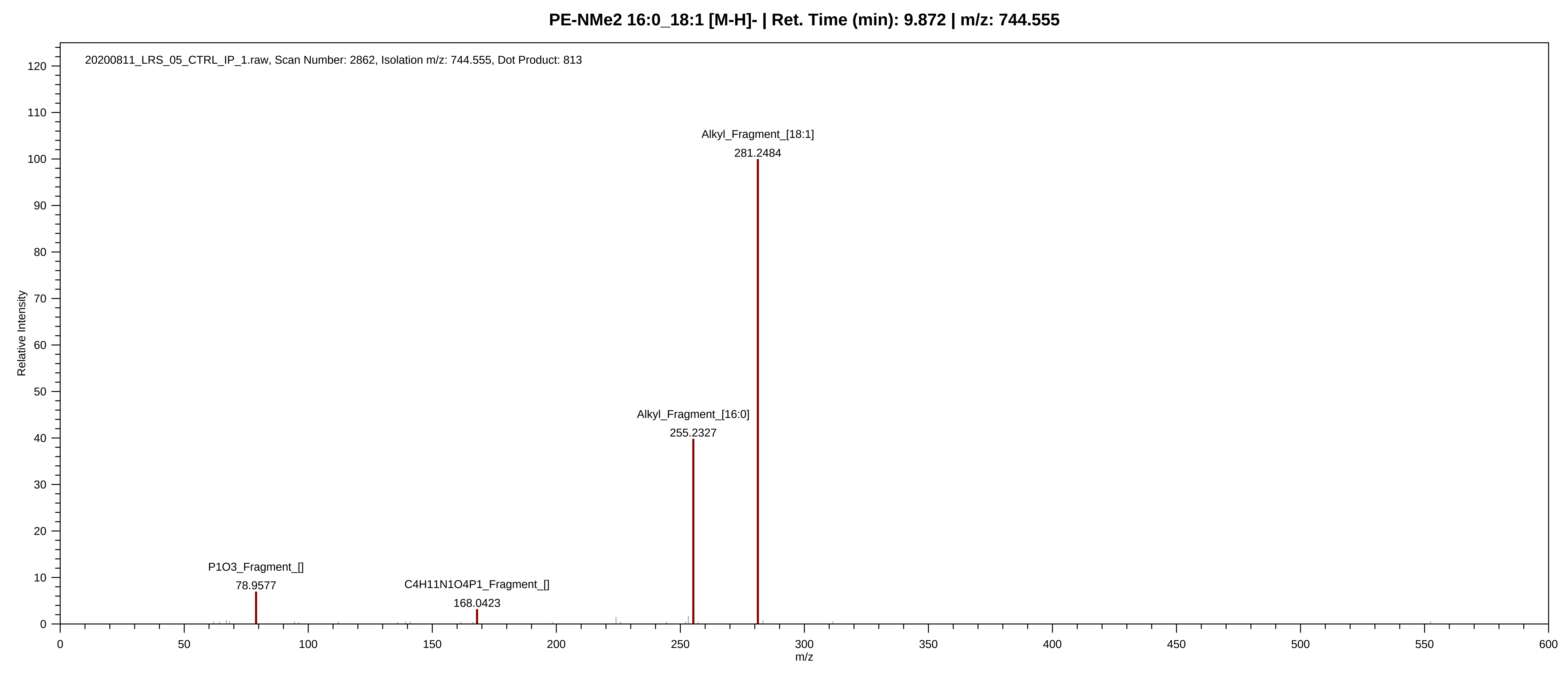

PE-NMe2 16:0_18:1 [M-H]- | Ret. Time (min): 9.872 | m/z: 744.555
20200811_LRS_05_CTRL_IP_1.raw, Scan Number: 2862, Isolation m/z: 744.555, Dot Product: 813
Alkyl_Fragment_[18:1]
281.2484
Alkyl_Fragment_[16:0]
255.2327
P1O3_Fragment_[]
78.9577
C4H11N1O4P1_Fragment_[]
168.0423
Relative Intensity
m/z

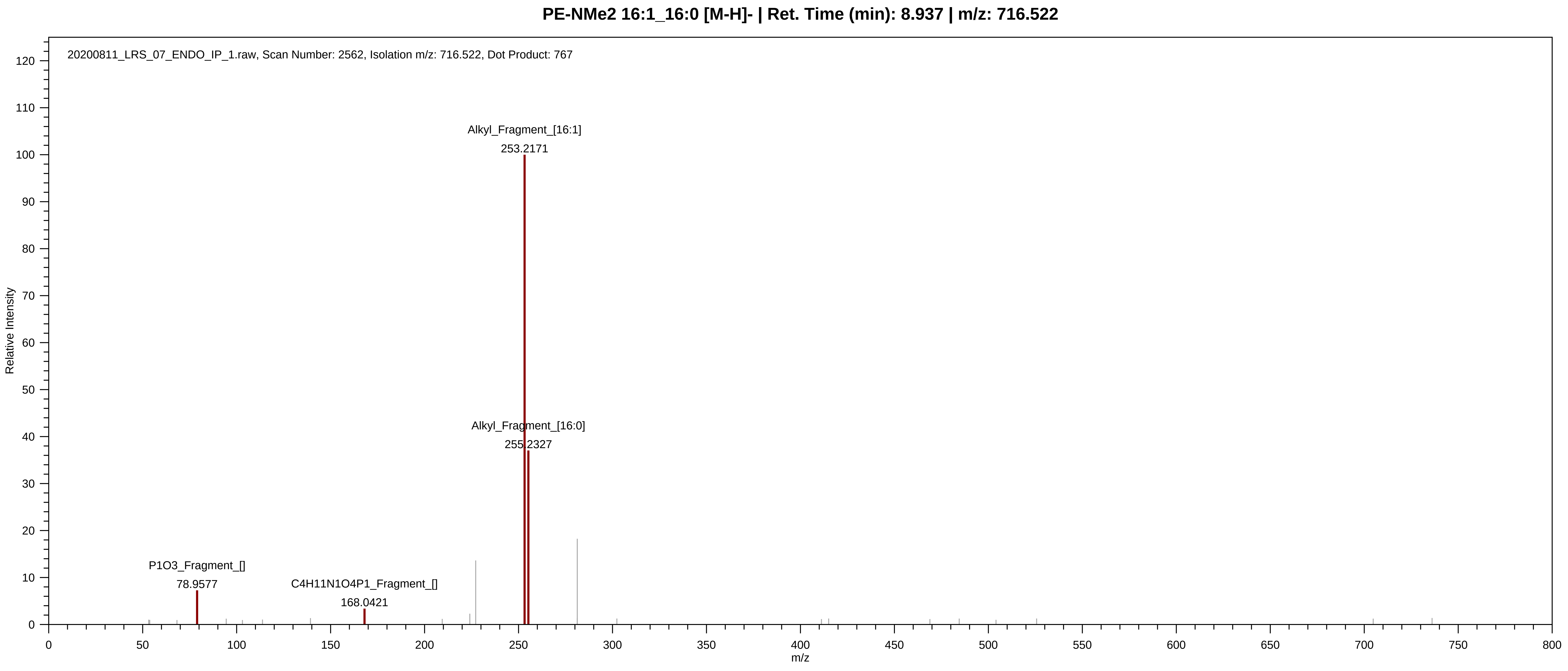

PE-NMe2 16:1_16:0 [M-H]- | Ret. Time (min): 8.937 | m/z: 716.522
20200811_LRS_07_ENDO_IP_1.raw, Scan Number: 2562, Isolation m/z: 716.522, Dot Product: 767
Alkyl_Fragment_[16:1]
253.2171
Alkyl_Fragment_[16:0]
255.2327
P1O3_Fragment_[]
78.9577
C4H11N1O4P1_Fragment_[]
168.0421
Relative Intensity
m/z

PE-NMe2 16:1_16:0 [M-H]- | Ret. Time (min): 8.97 | m/z: 716.523

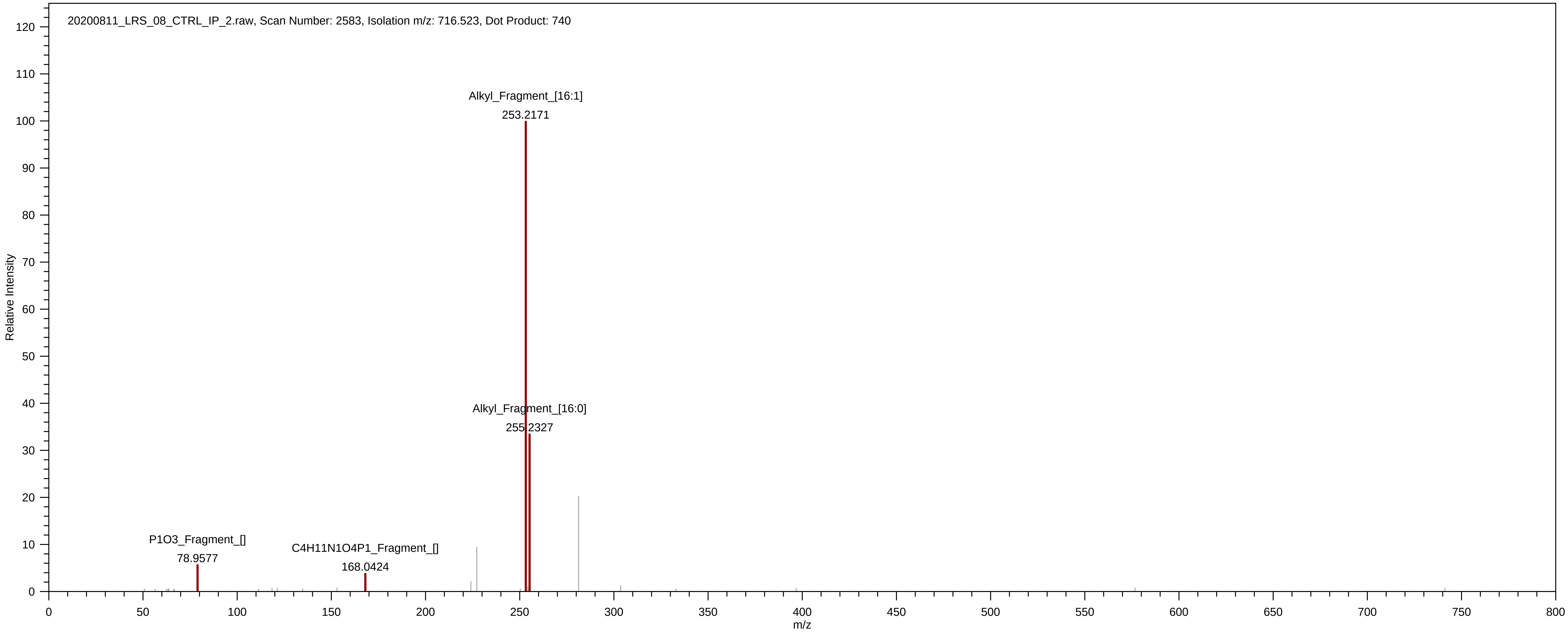

PE-NMe2 16:1_16:0 [M-H]- | Ret. Time (min): 8.966 | m/z: 716.523

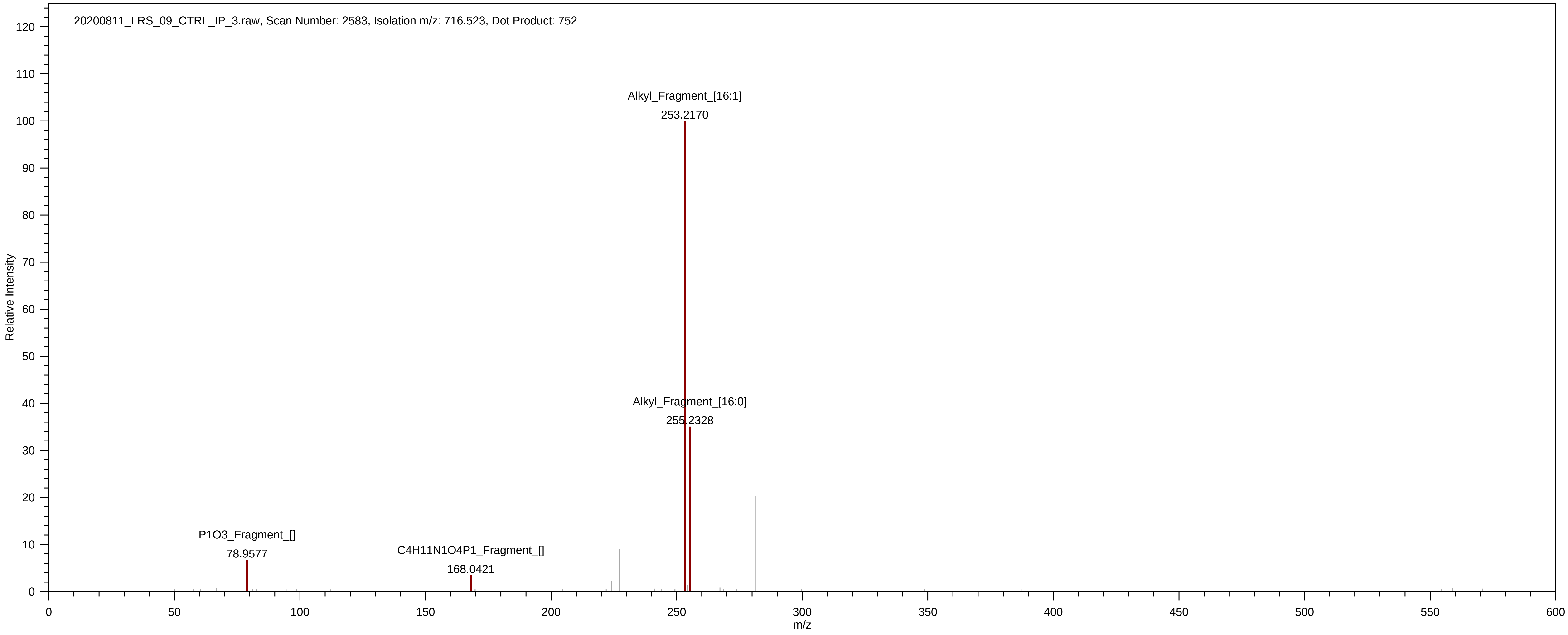

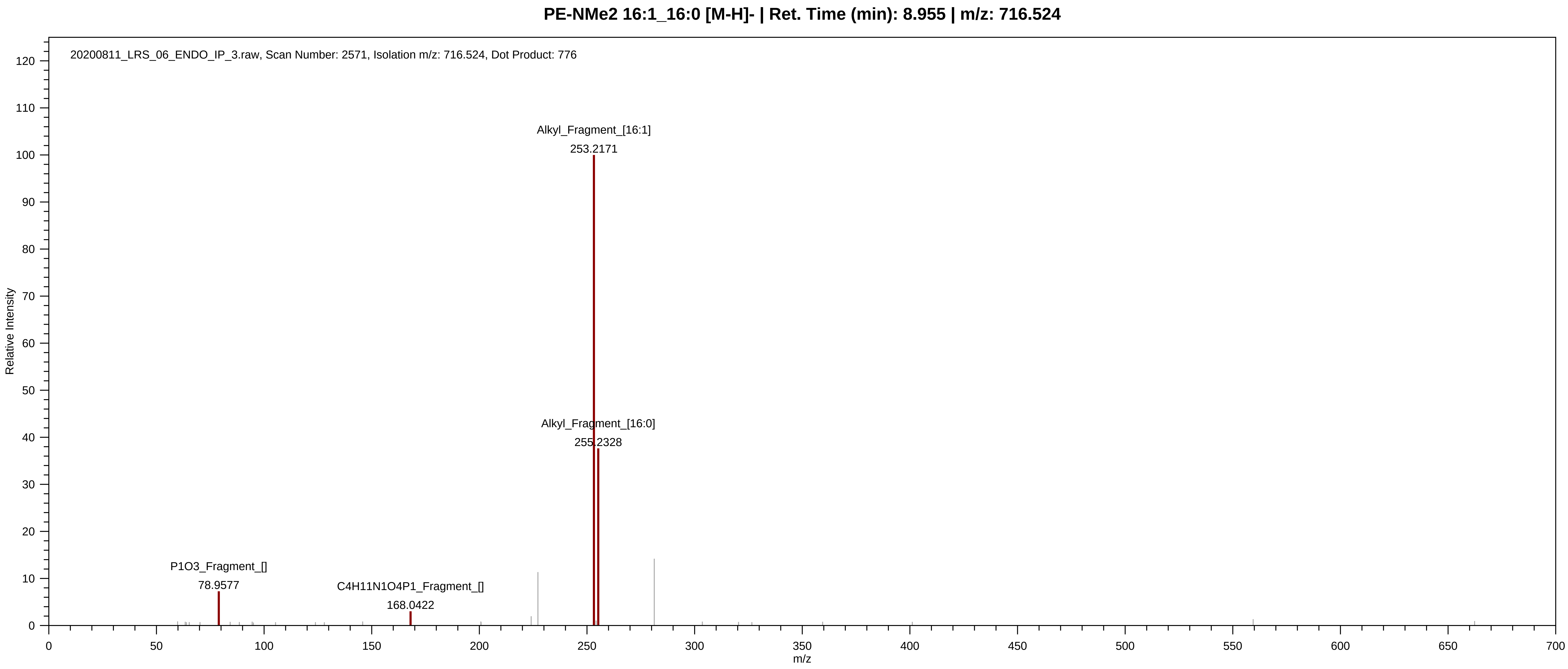

PE-NMe2 16:1_16:0 [M-H]- | Ret. Time (min): 8.955 | m/z: 716.524
20200811_LRS_06_ENDO_IP_3.raw, Scan Number: 2571, Isolation m/z: 716.524, Dot Product: 776
Alkyl_Fragment_[16:1]
253.2171
Alkyl_Fragment_[16:0]
255.2328
P1O3_Fragment_[]
78.9577
C4H11N1O4P1_Fragment_[]
168.0422
Relative Intensity
m/z

PE-NMe2 16:1_16:0 [M-H]- | Ret. Time (min): 8.978 | m/z: 716.524

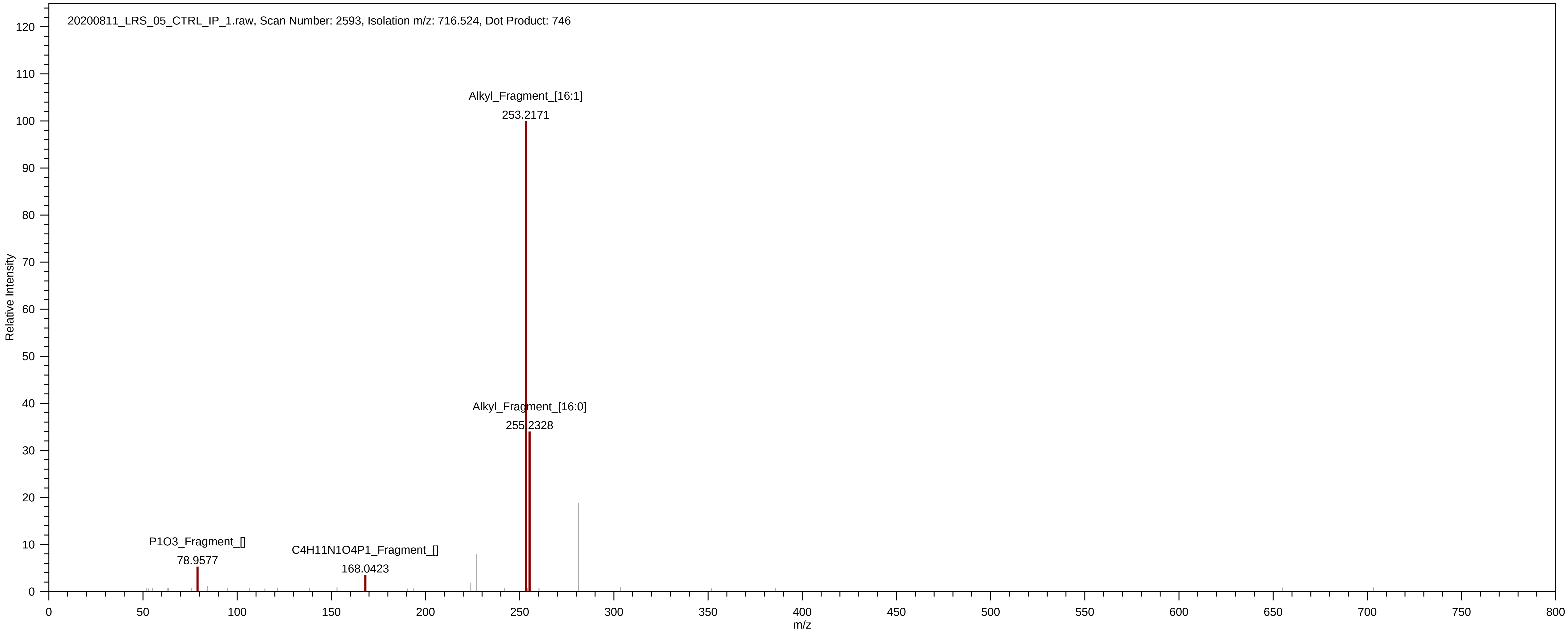

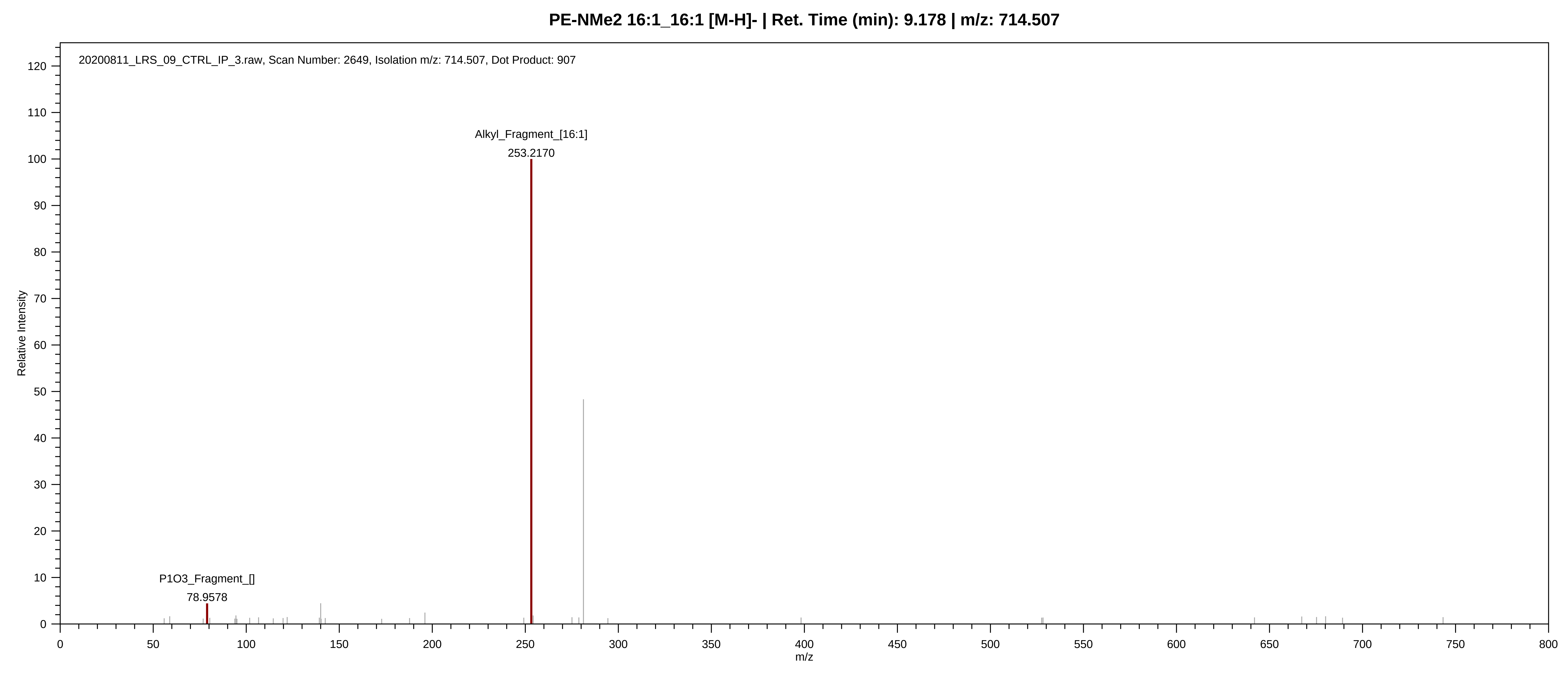
PE-NMe2 16:1_16:1 [M-H]- | Ret. Time (min): 9.178 | m/z: 714.507
20200811_LRS_09_CTRL_IP_3.raw, Scan Number: 2649, Isolation m/z: 714.507, Dot Product: 907
Alkyl_Fragment_[16:1]
253.2170
P1O3_Fragment_[]
78.9578
Relative Intensity
m/z

PE-NMe2 16:1_16:1 [M-H]- | Ret. Time (min): 9.191 | m/z: 714.508

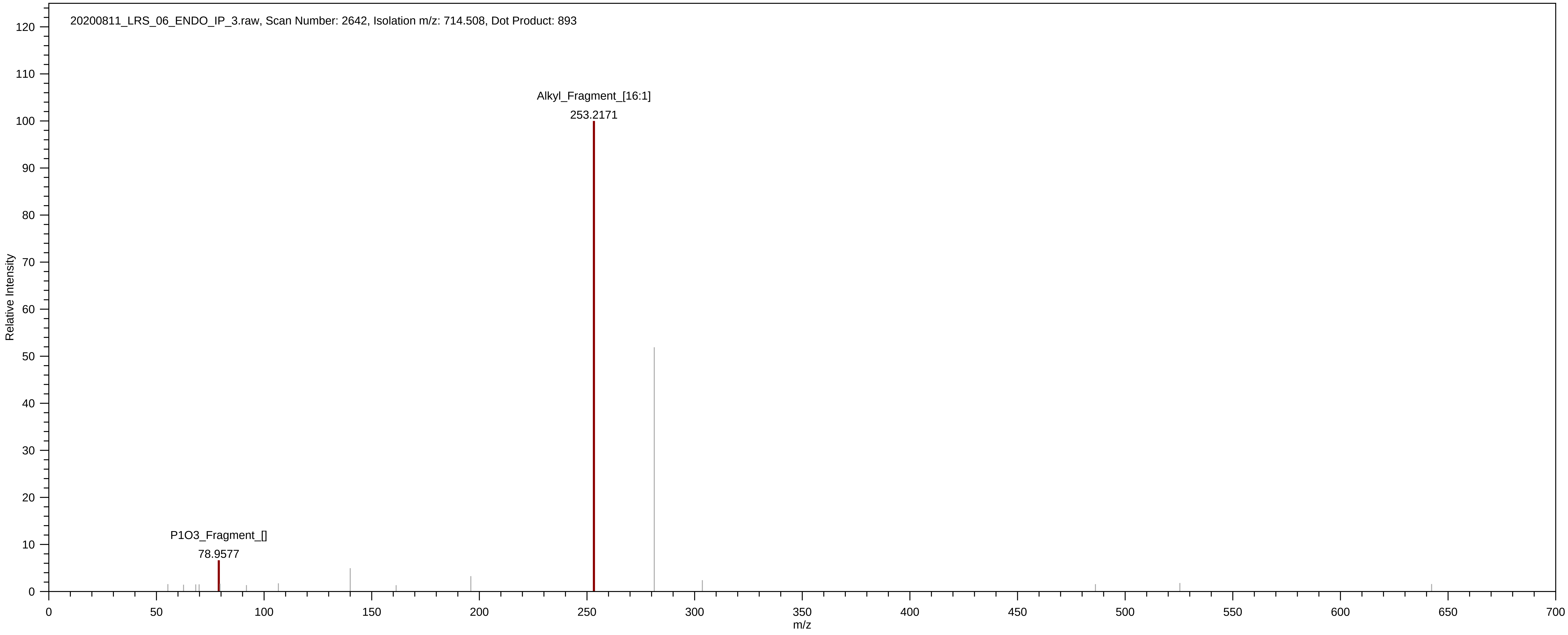

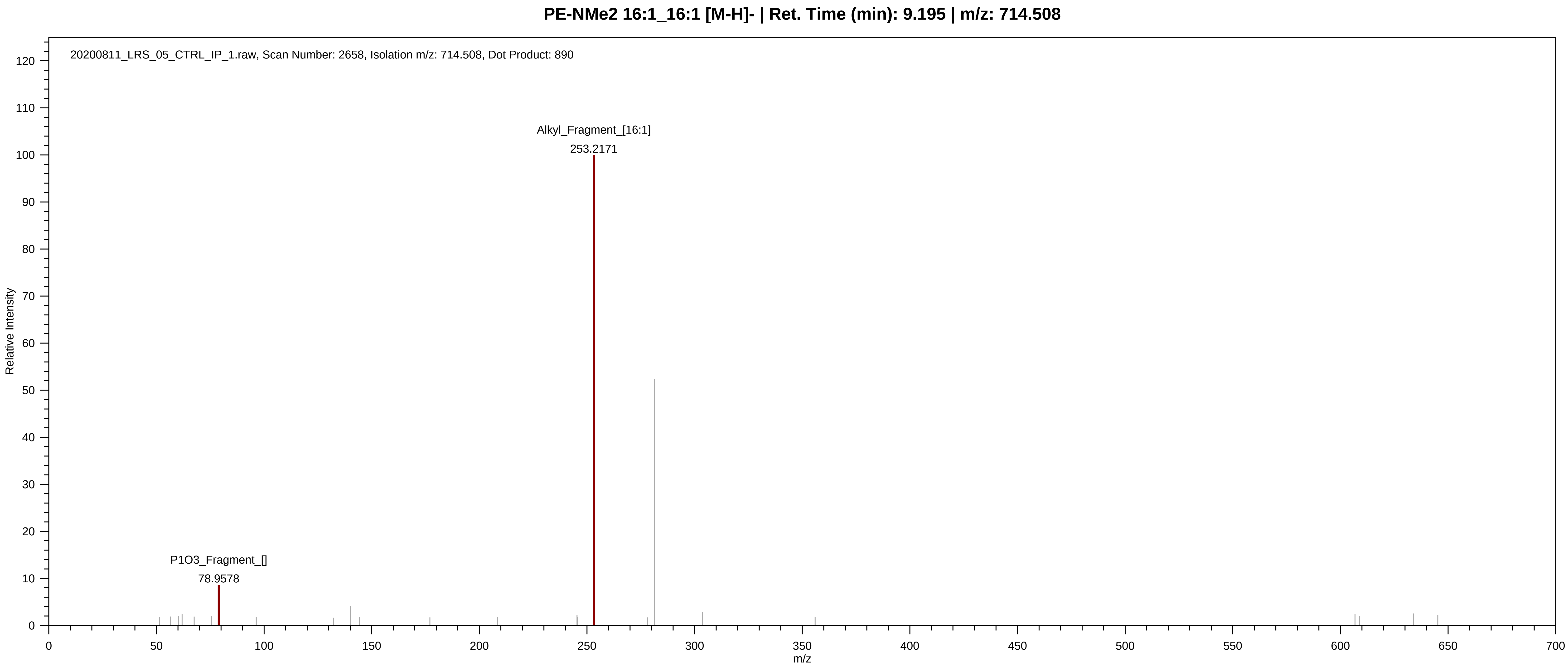
PE-NMe2 16:1_16:1 [M-H]- | Ret. Time (min): 9.195 | m/z: 714.508
20200811_LRS_05_CTRL_IP_1.raw, Scan Number: 2658, Isolation m/z: 714.508, Dot Product: 890
Alkyl_Fragment_[16:1]
253.2171
P1O3_Fragment_[]
78.9578
Relative Intensity
m/z
0
10
20
30
40
50
60
70
80
90
100
110
120
0
50
100
150
200
250
300
350
400
450
500
550
600
650
700

PE-NMe2 18:1_18:0 [M-H]- | Ret. Time (min): 10.849 | m/z: 772.586

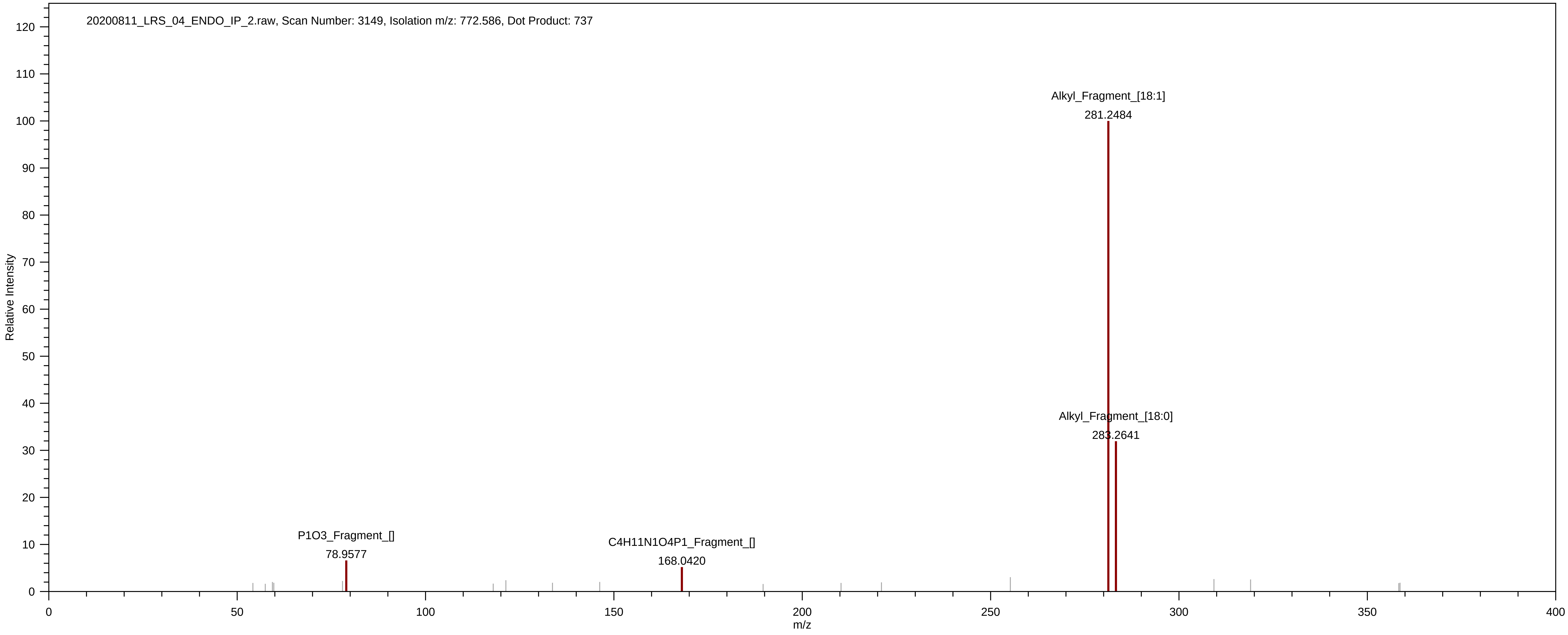

PE-NMe2 18:1_18:0 [M-H]- | Ret. Time (min): 10.868 | m/z: 772.586

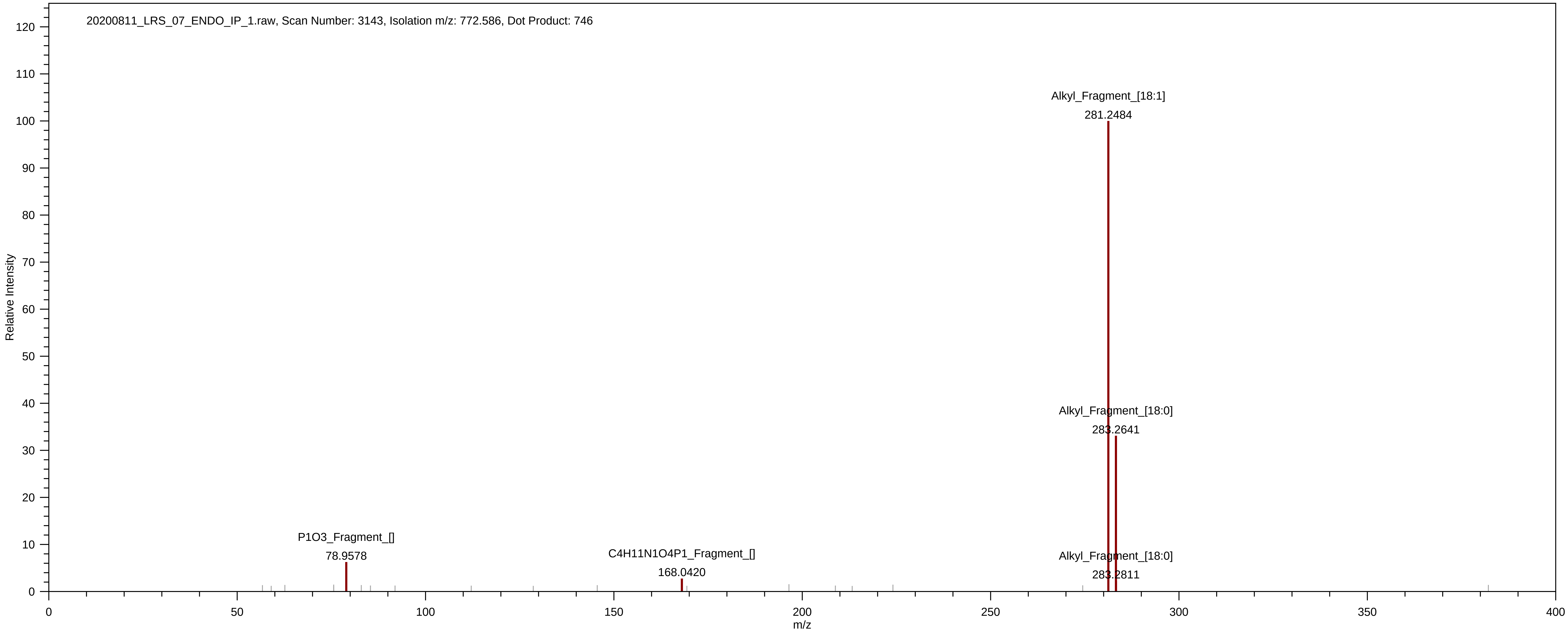

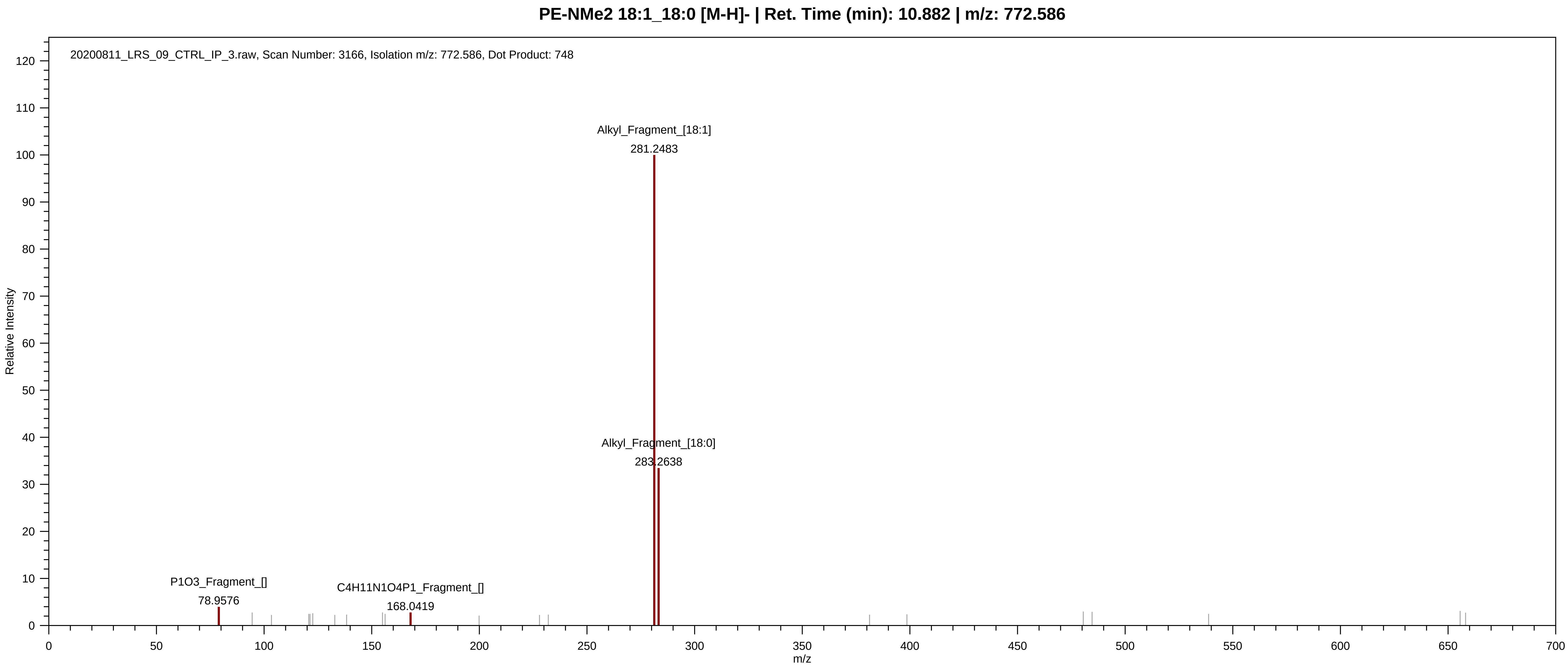

PE-NMe2 18:1_18:0 [M-H]- | Ret. Time (min): 10.882 | m/z: 772.586
20200811_LRS_09_CTRL_IP_3.raw, Scan Number: 3166, Isolation m/z: 772.586, Dot Product: 748
Alkyl_Fragment_[18:1]
281.2483
Alkyl_Fragment_[18:0]
283.2638
P1O3_Fragment_[]
78.9576
C4H11N1O4P1_Fragment_[]
168.0419
Relative Intensity
m/z

PE-NMe2 18:1_18:0 [M-H]- | Ret. Time (min): 10.867 | m/z: 772.587

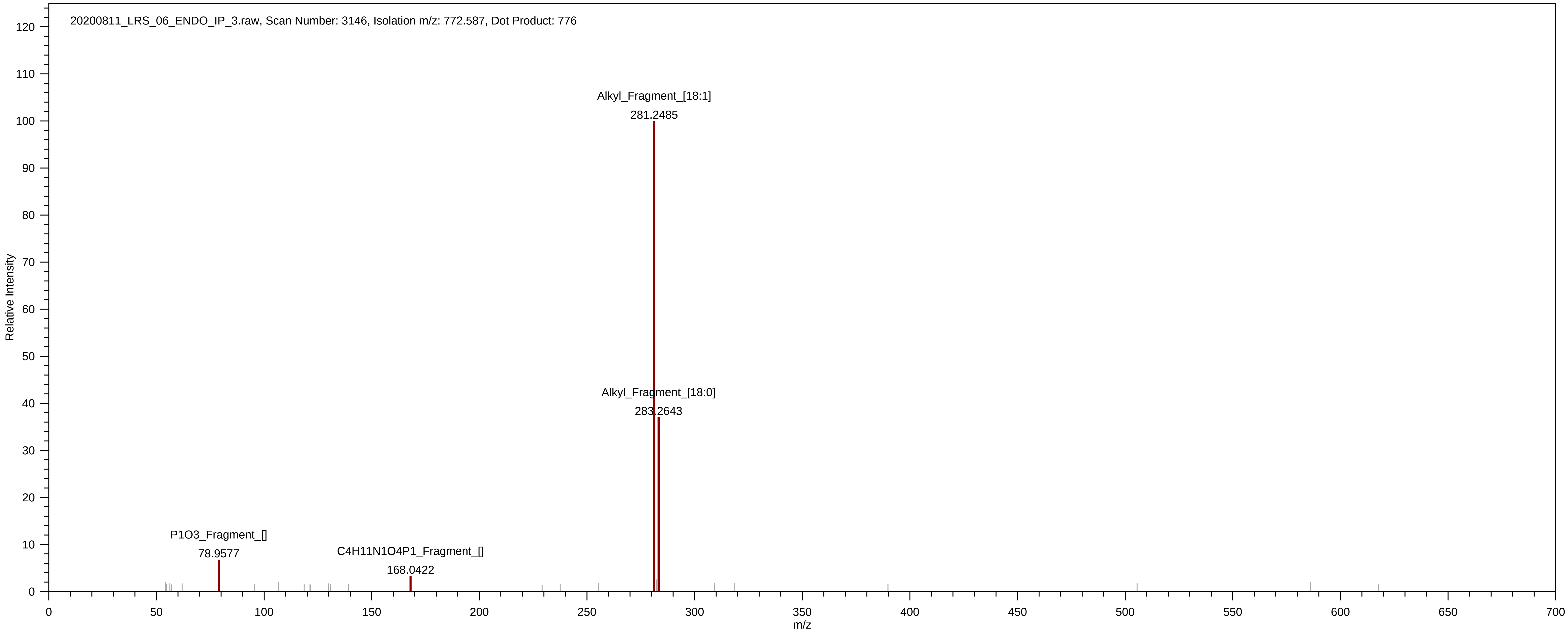

PE-NMe2 18:1_18:1 [M-H]- | Ret. Time (min): 9.944 | m/z: 770.57

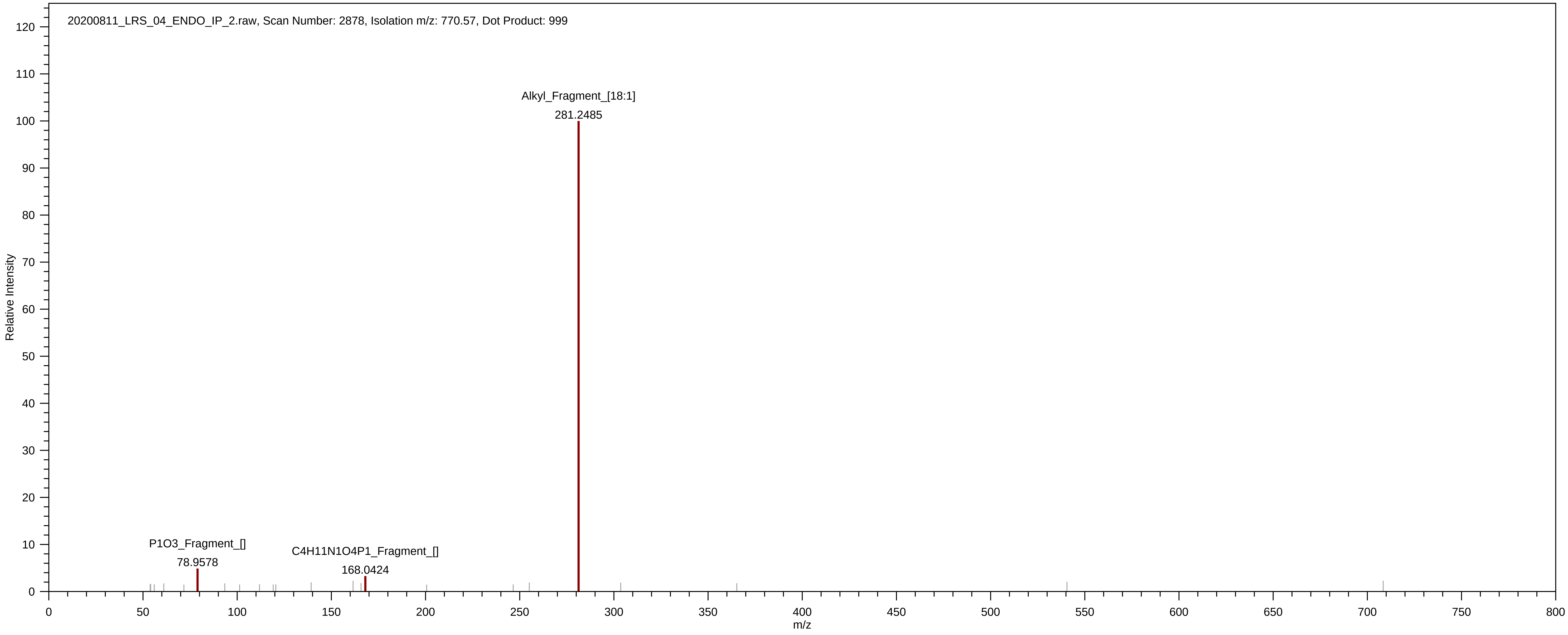

PE-NMe2 18:1_18:1 [M-H]- | Ret. Time (min): 9.946 | m/z: 770.57

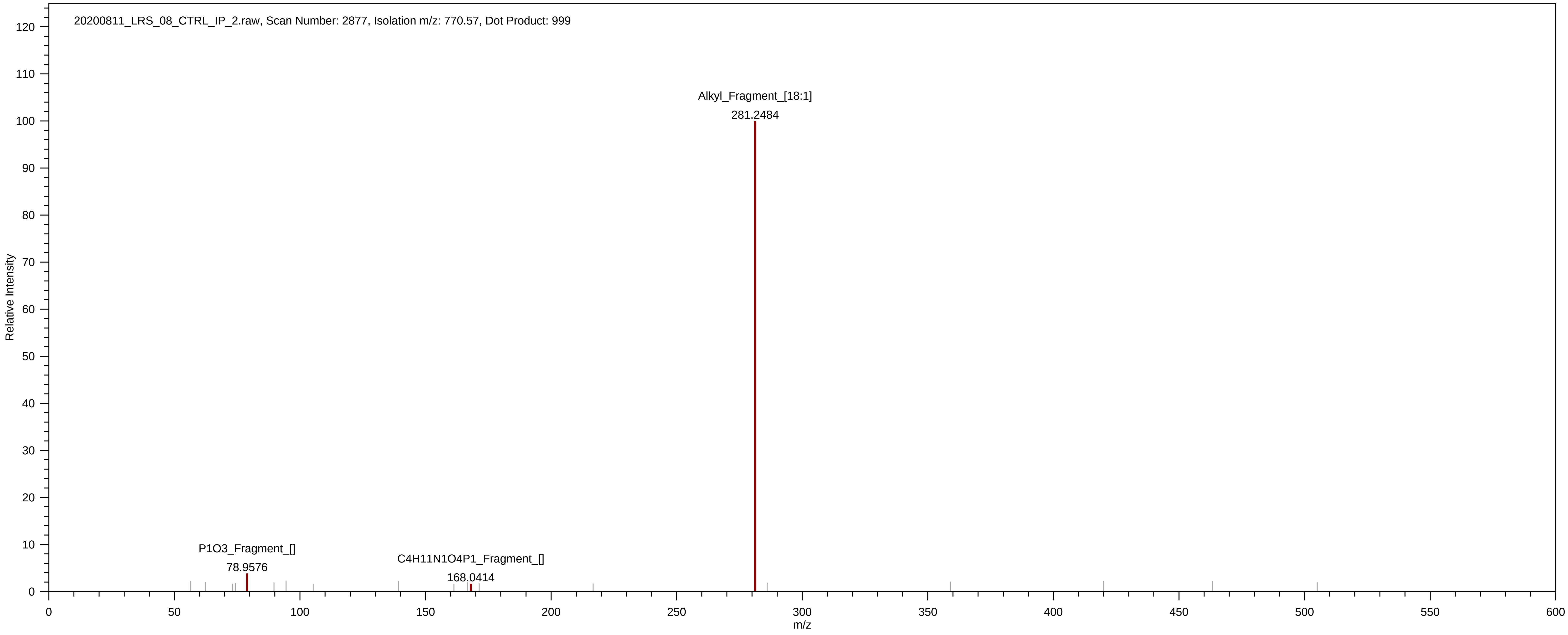

PE-NMe2 18:1_18:1 [M-H]- | Ret. Time (min): 9.929 | m/z: 770.568

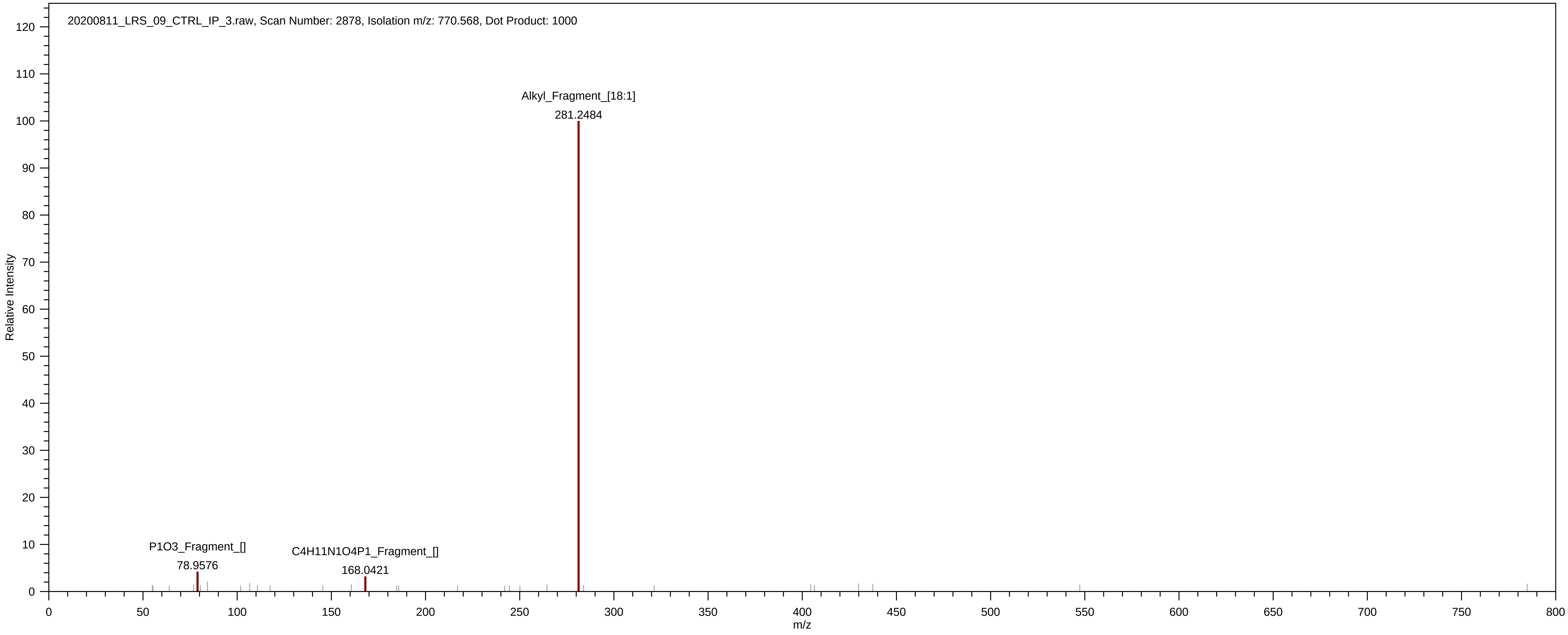

PE-NMe2 18:1_18:1 [M-H]- | Ret. Time (min): 11.03 | m/z: 770.571

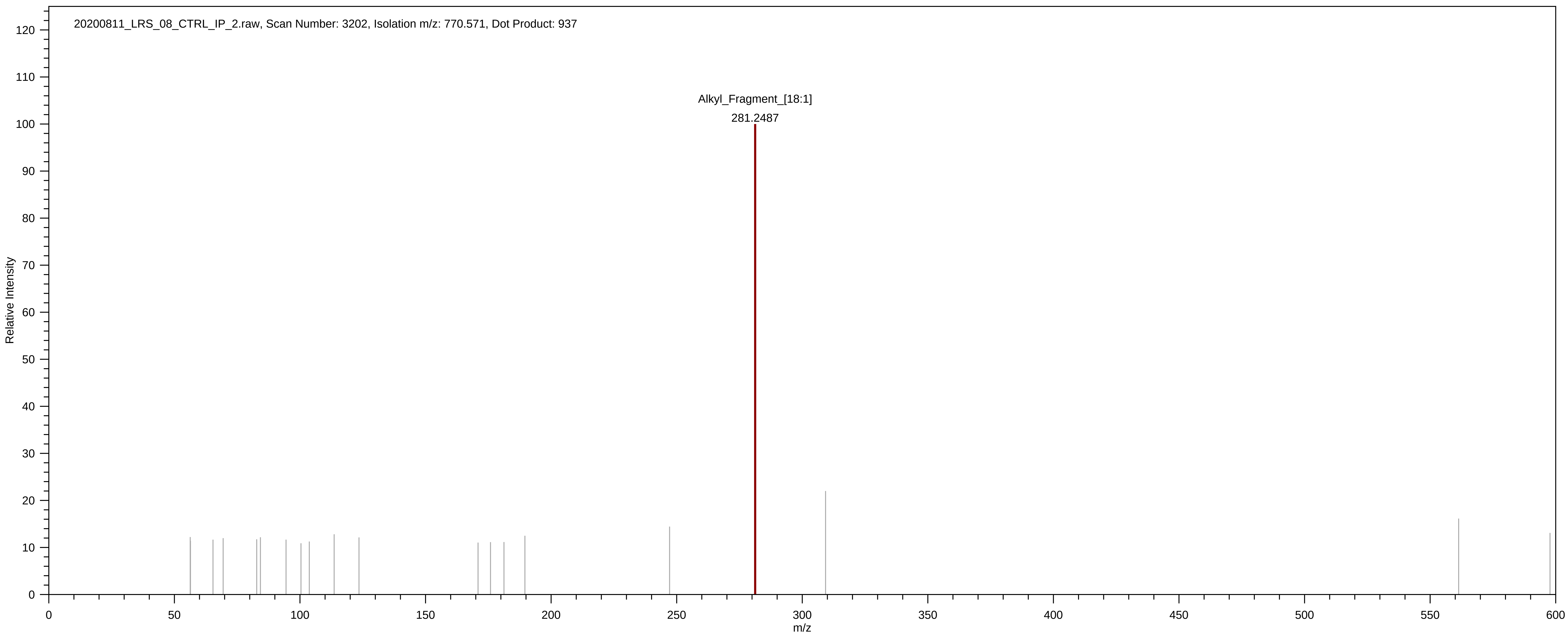

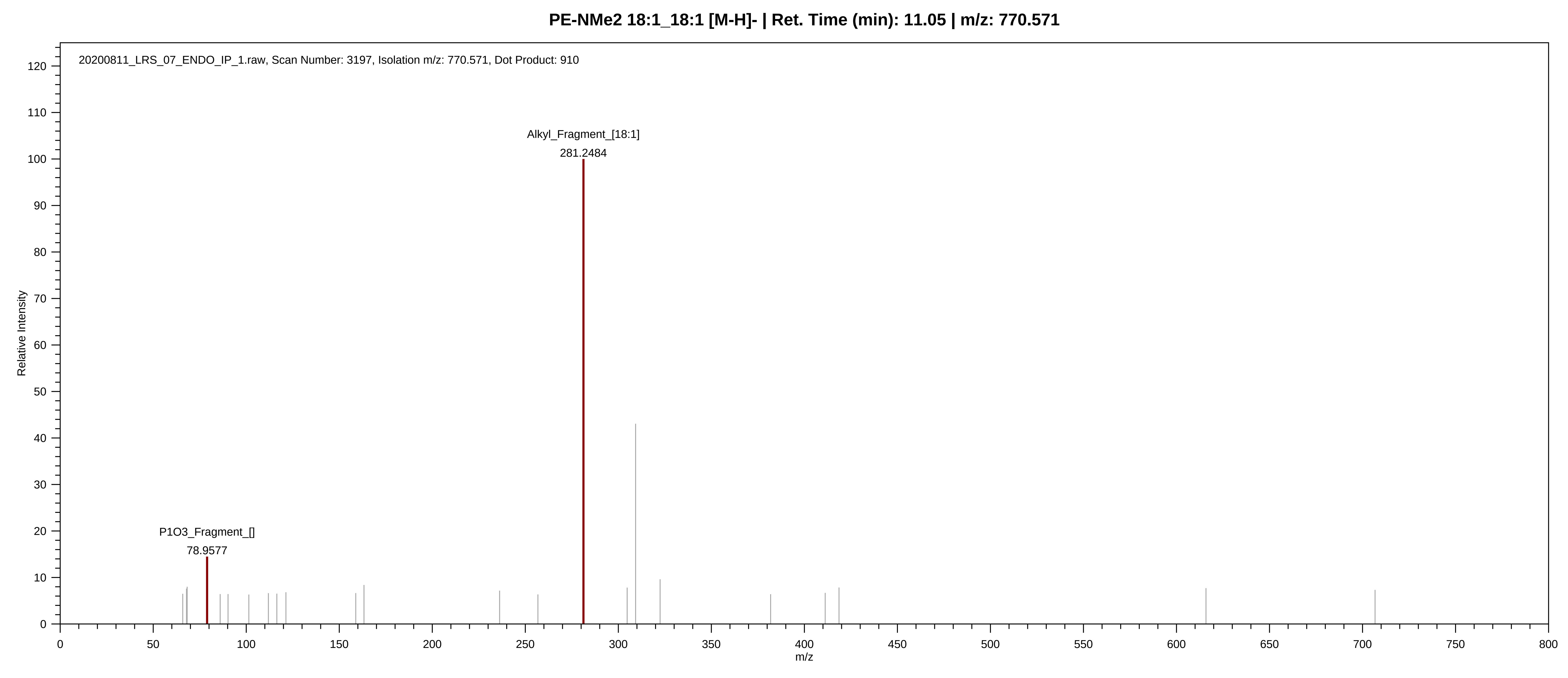
PE-NMe2 18:1_18:1 [M-H]- | Ret. Time (min): 11.05 | m/z: 770.571
20200811_LRS_07_ENDO_IP_1.raw, Scan Number: 3197, Isolation m/z: 770.571, Dot Product: 910
Alkyl_Fragment_[18:1]
281.2484
P1O3_Fragment_[]
78.9577
Relative Intensity
m/z

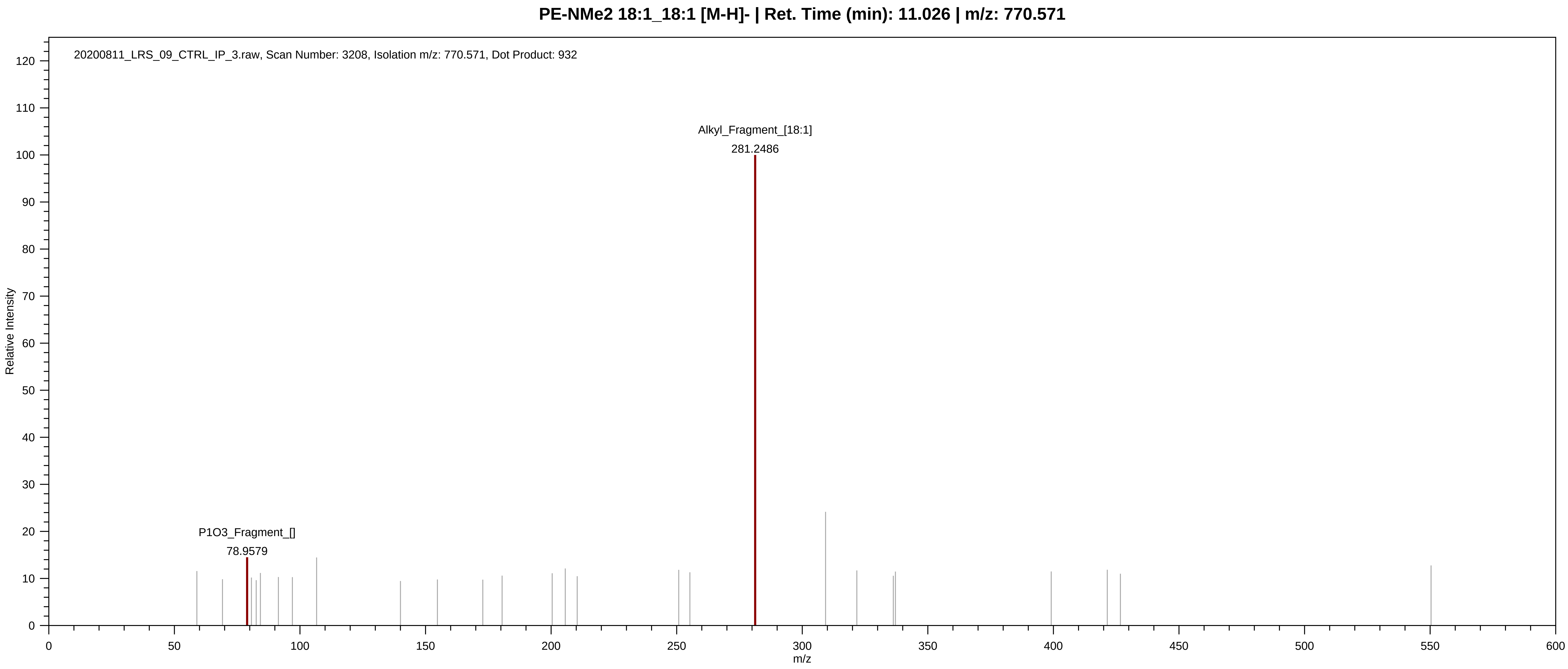
PE-NMe2 18:1_18:1 [M-H]- | Ret. Time (min): 11.026 | m/z: 770.571
20200811_LRS_09_CTRL_IP_3.raw, Scan Number: 3208, Isolation m/z: 770.571, Dot Product: 932
Alkyl_Fragment_[18:1]
281.2486
P1O3_Fragment_[]
78.9579
Relative Intensity
m/z

PE-NMe2 18:1_18:1 [M-H]- | Ret. Time (min): 11.062 | m/z: 770.571

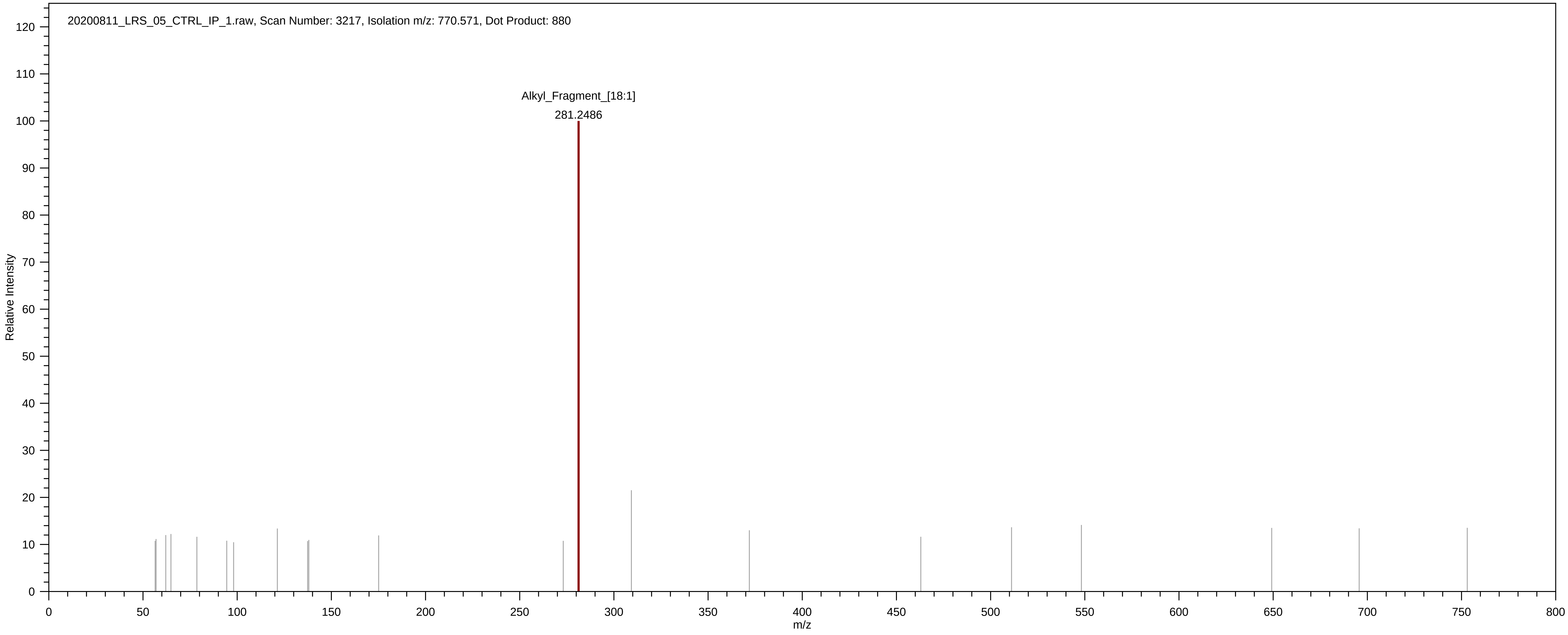

PE-NMe2 32:2 [M-H]- | Ret. Time (min): 9.379 | m/z: 714.508

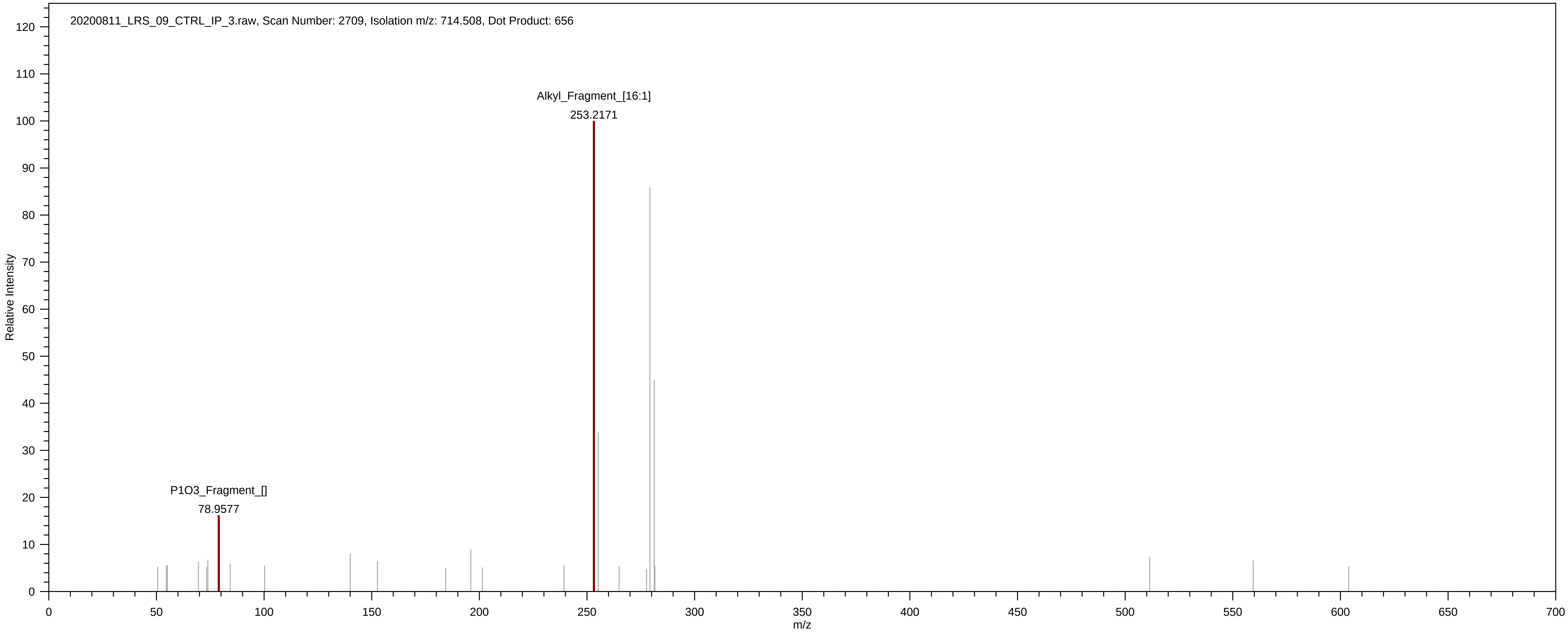

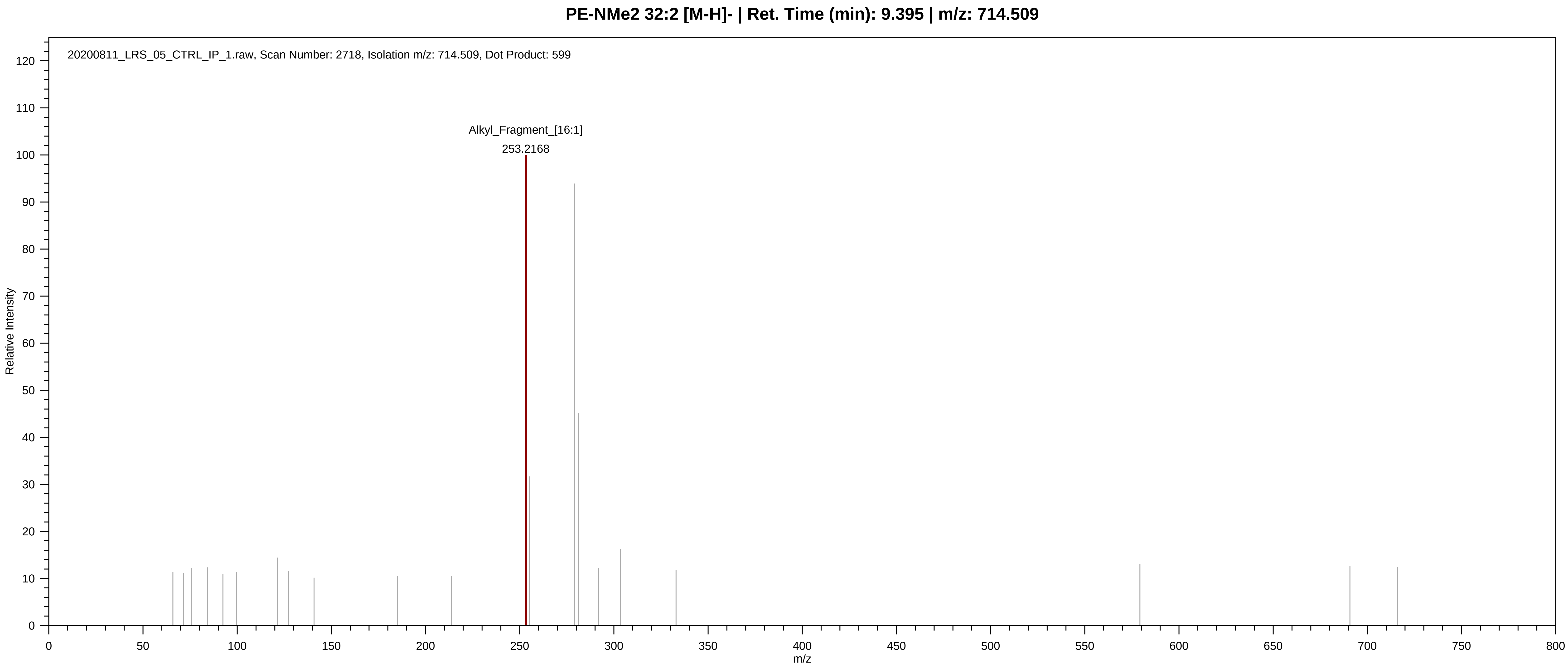
PE-NMe2 32:2 [M-H]- | Ret. Time (min): 9.395 | m/z: 714.509
20200811_LRS_05_CTRL_IP_1.raw, Scan Number: 2718, Isolation m/z: 714.509, Dot Product: 599
Alkyl_Fragment_[16:1]
253.2168
Relative Intensity
m/z

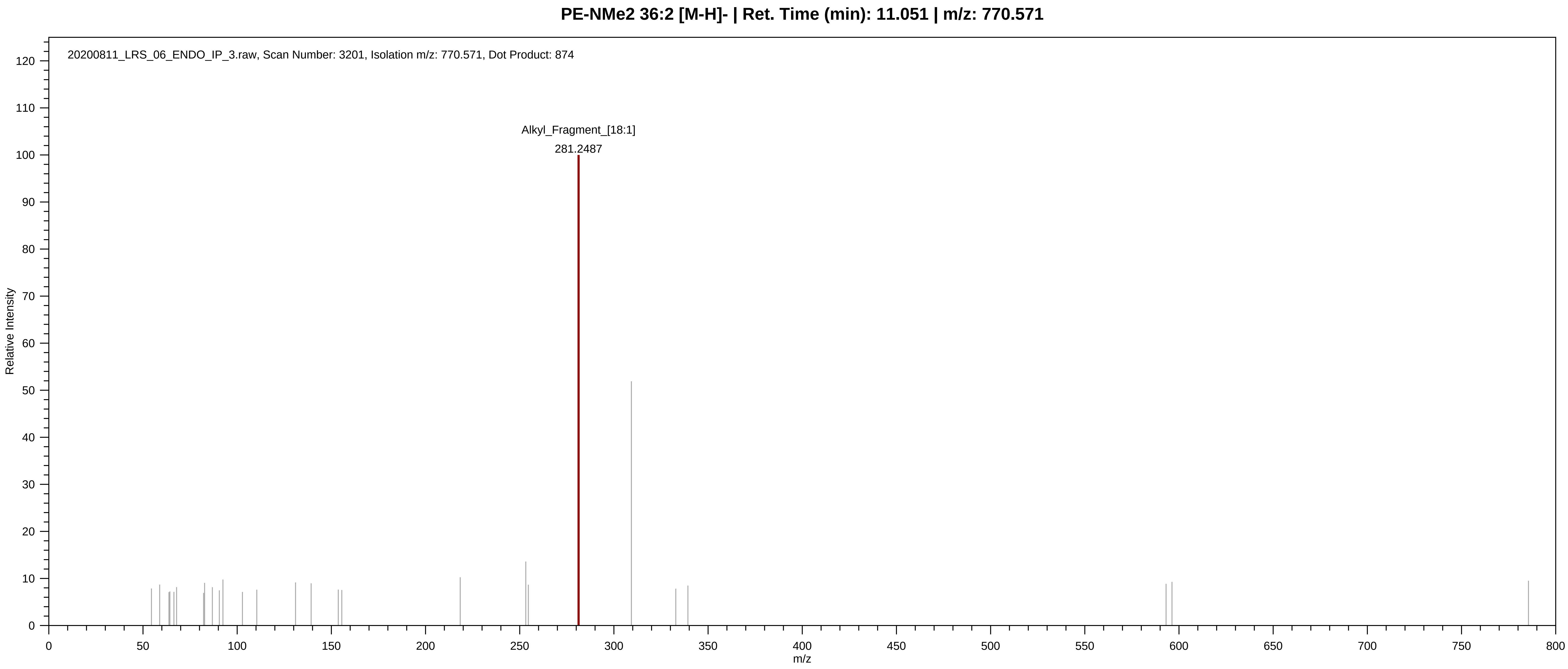
PE-NMe2 36:2 [M-H]- | Ret. Time (min): 11.051 | m/z: 770.571
20200811_LRS_06_ENDO_IP_3.raw, Scan Number: 3201, Isolation m/z: 770.571, Dot Product: 874
Alkyl_Fragment_[18:1]
281.2487
Relative Intensity
m/z
0
10
20
30
40
50
60
70
80
90
100
110
120
0
50
100
150
200
250
300
350
400
450
500
550
600
650
700
750
800

PG 14:0_16:0 [M-H]- | Ret. Time (min): 7.606 | m/z: 693.47

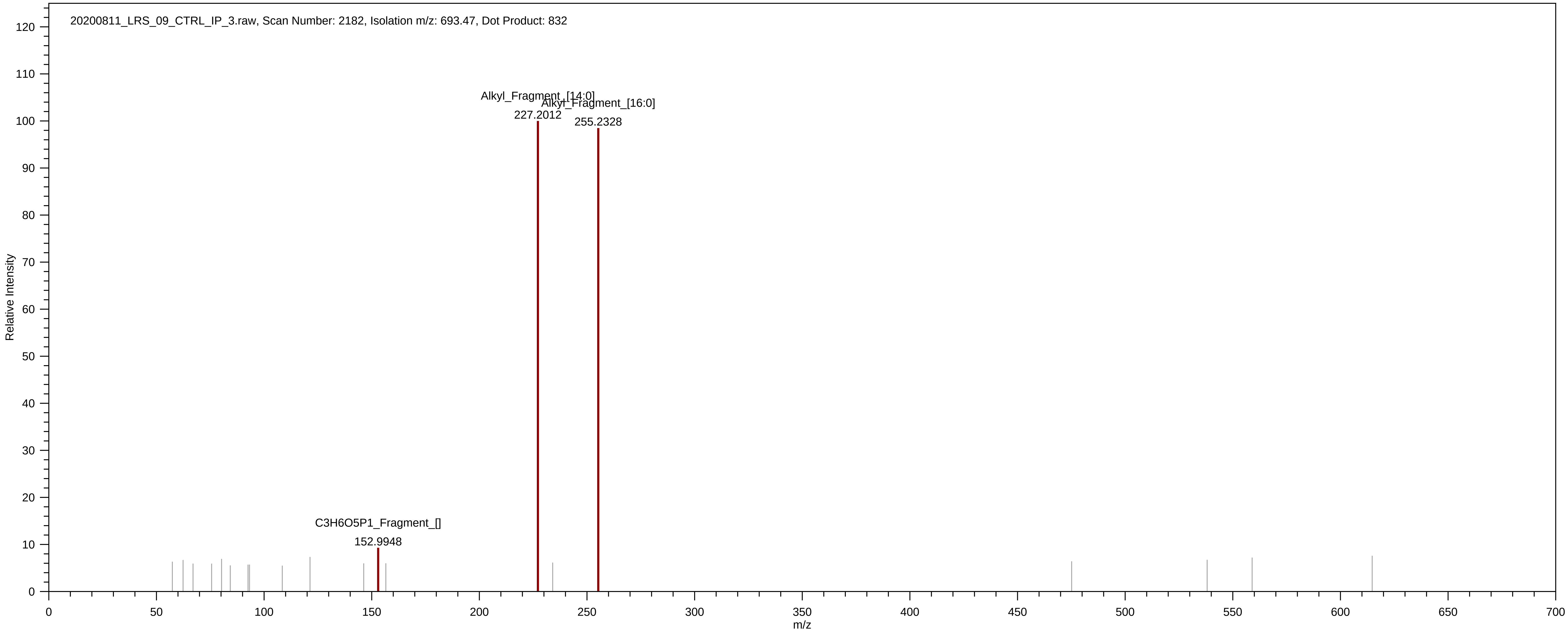

PG 14:0_16:0 [M-H]- | Ret. Time (min): 7.611 | m/z: 693.472

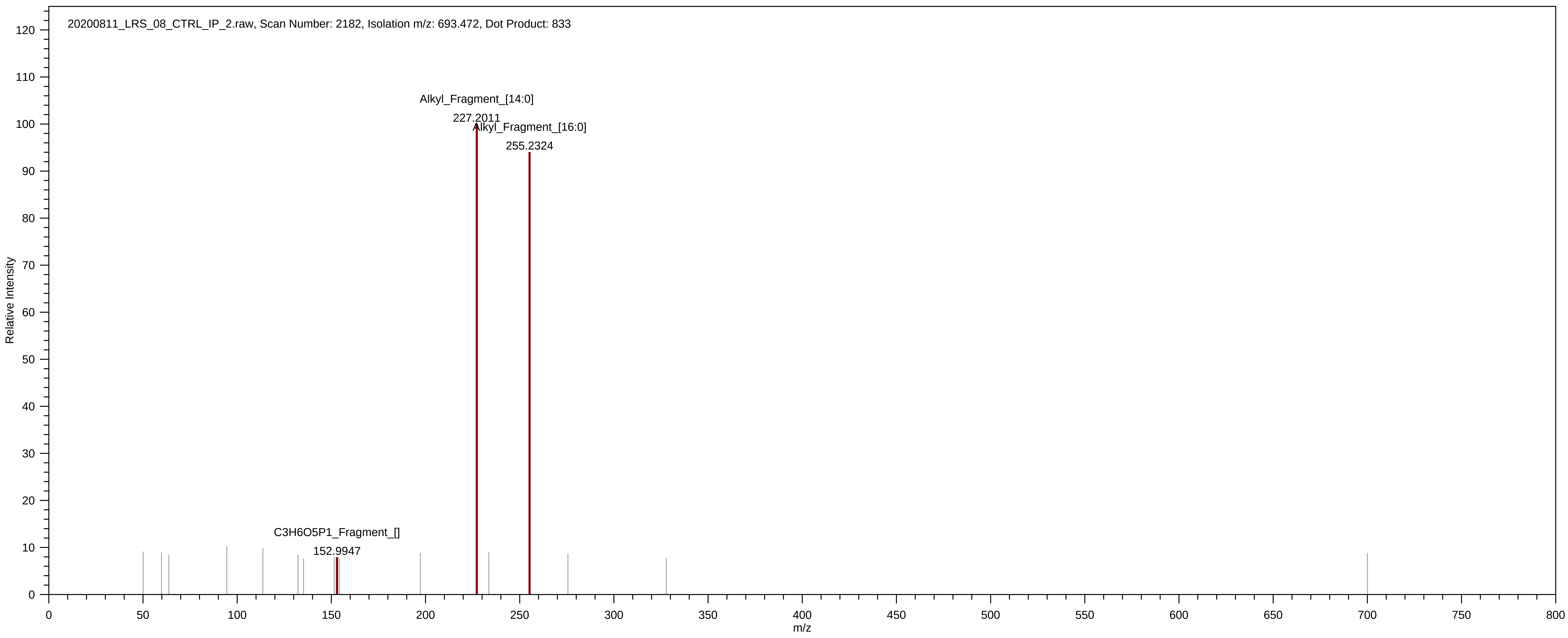

PG 14:0_18:1 [M-H]- | Ret. Time (min): 7.674 | m/z: 719.486

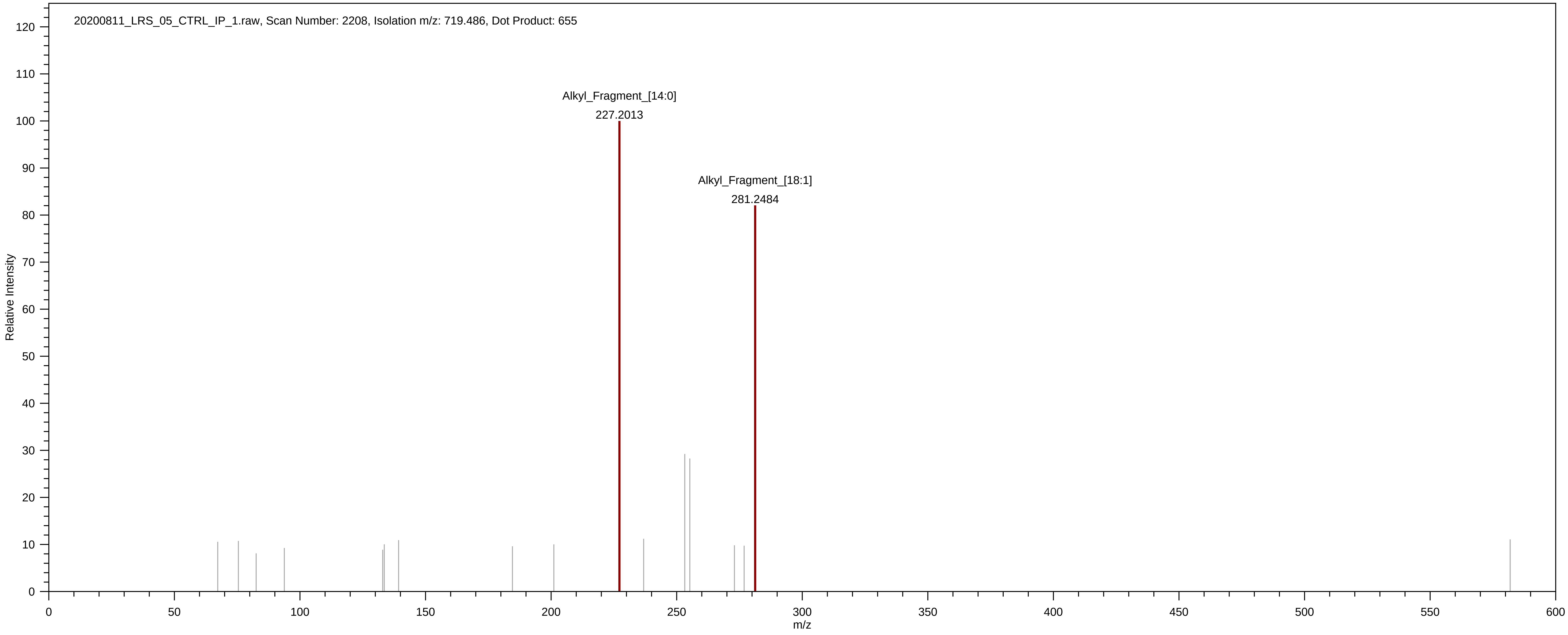

PG 16:0_16:0 [M-H]- | Ret. Time (min): 7.439 | m/z: 721.501

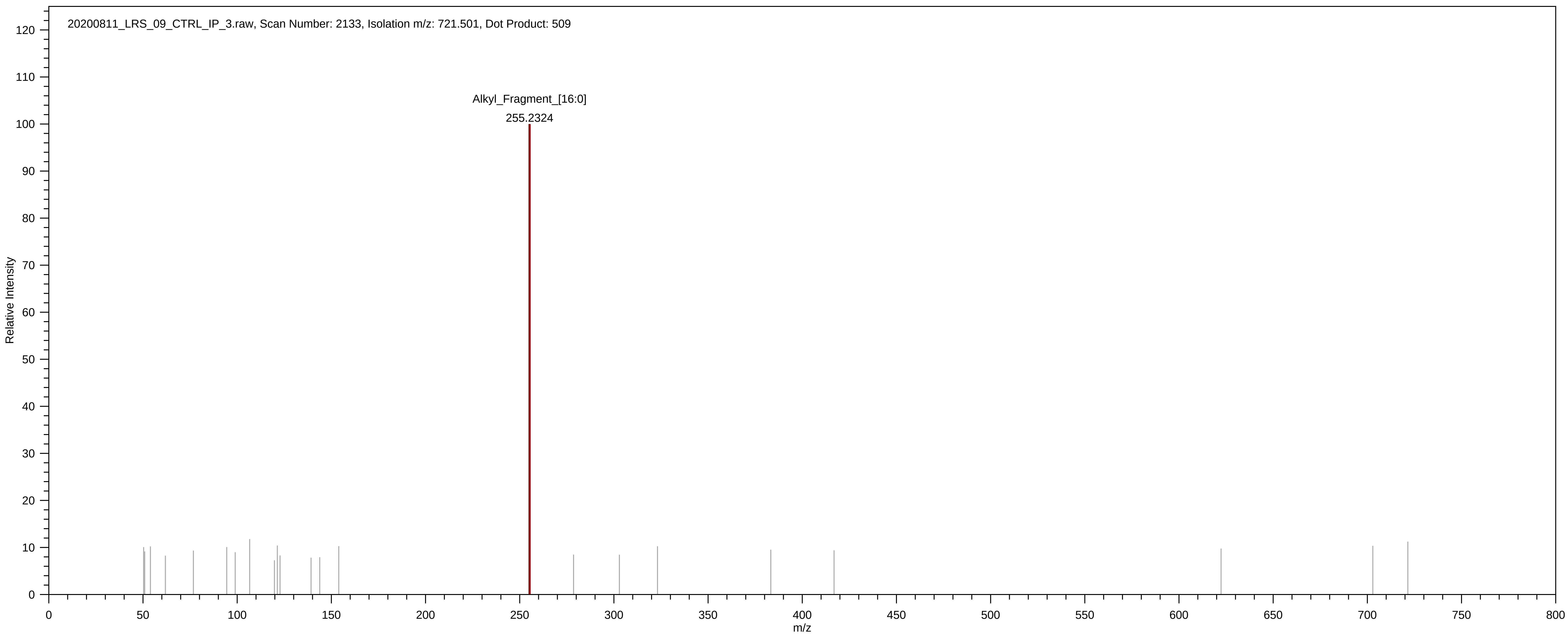

PG 16:0_16:0 [M-H]- | Ret. Time (min): 8.237 | m/z: 721.502

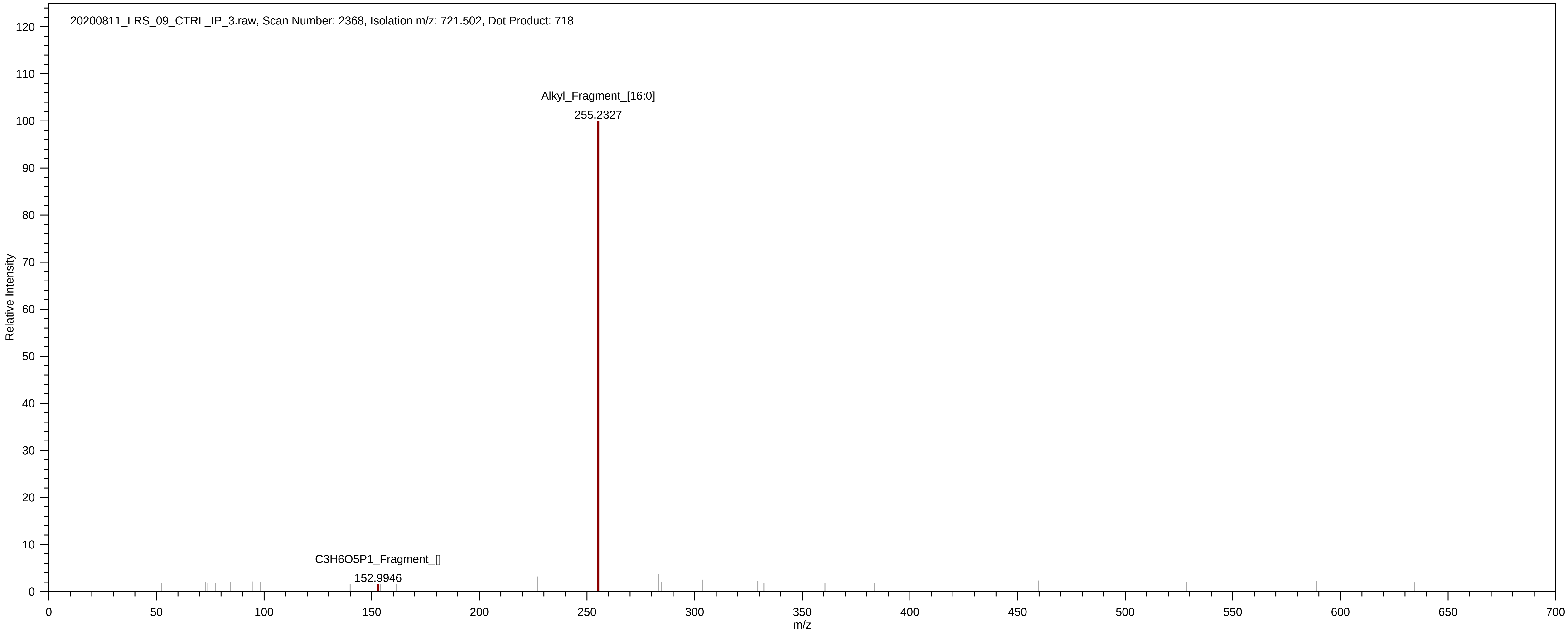

PG 16:0_16:0 [M-H]- | Ret. Time (min): 8.238 | m/z: 721.503

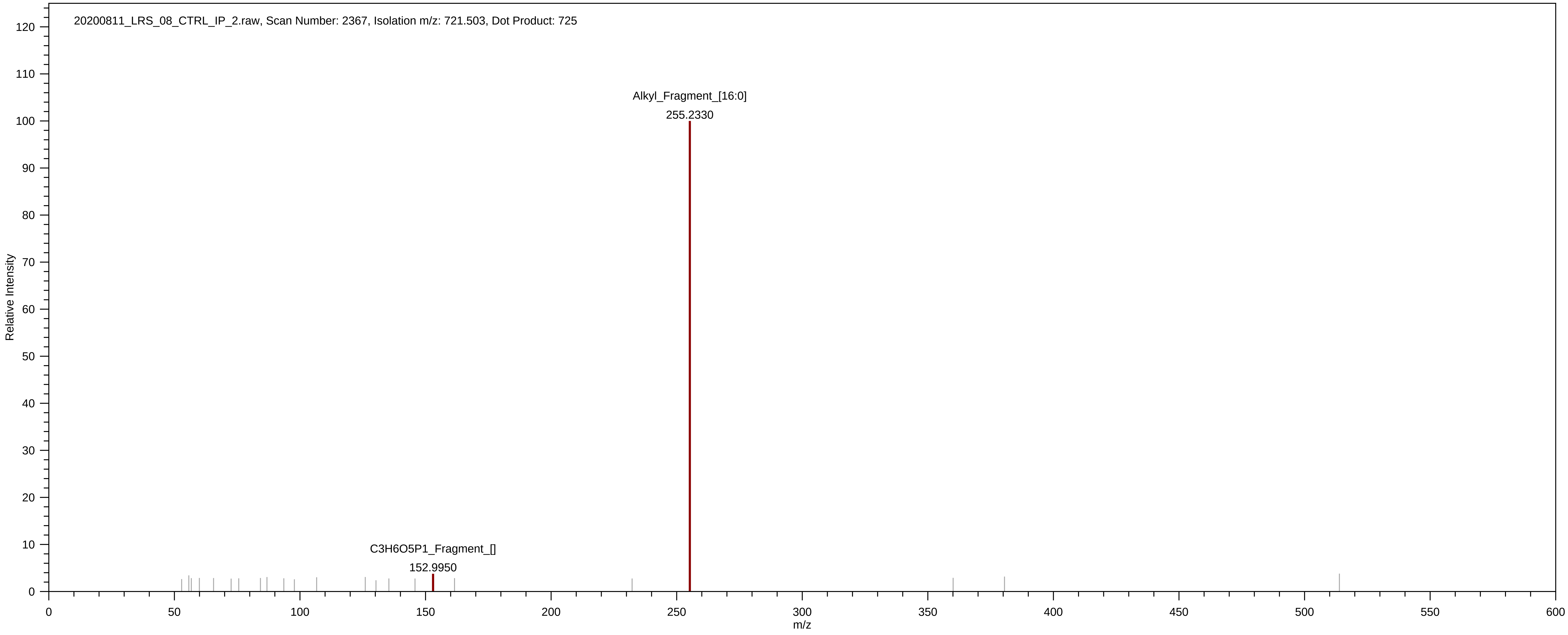

PG 16:0_16:0 [M-H]- | Ret. Time (min): 8.261 | m/z: 721.503

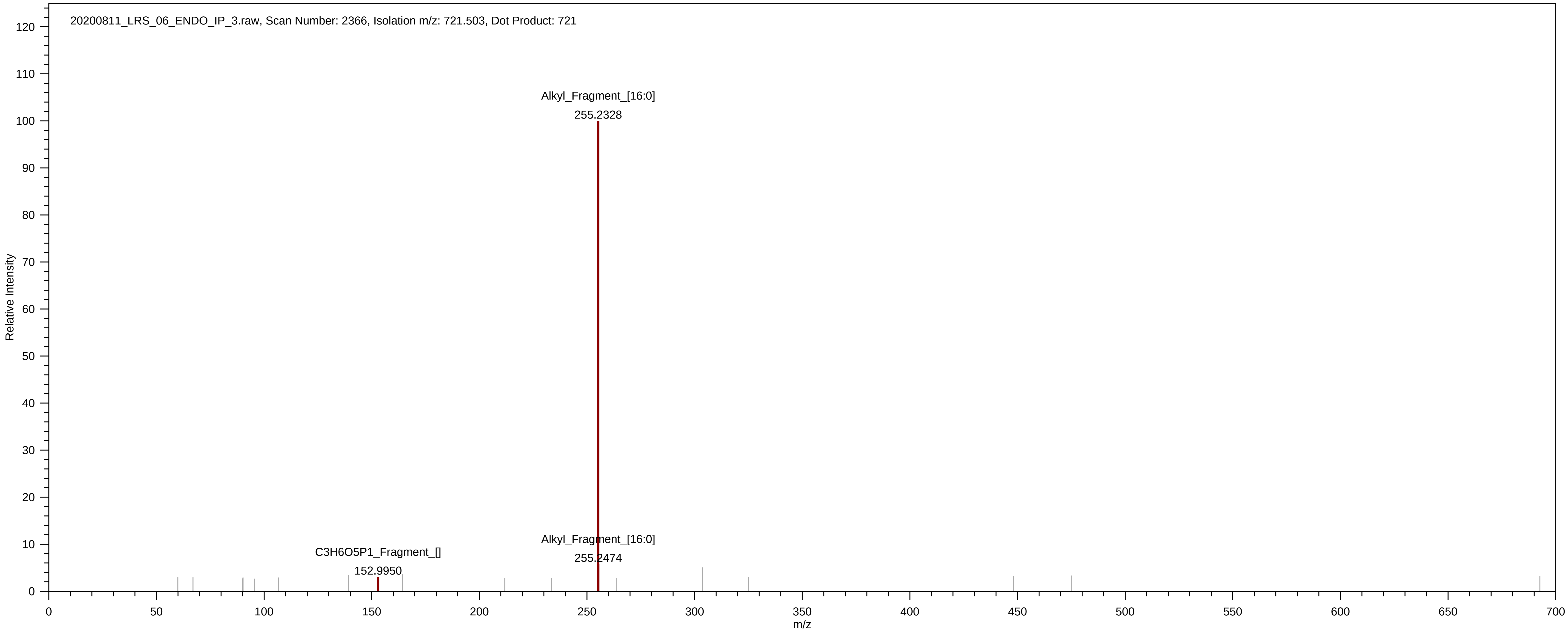

PG 16:0_16:0 [M-H]- | Ret. Time (min): 8.279 | m/z: 721.503

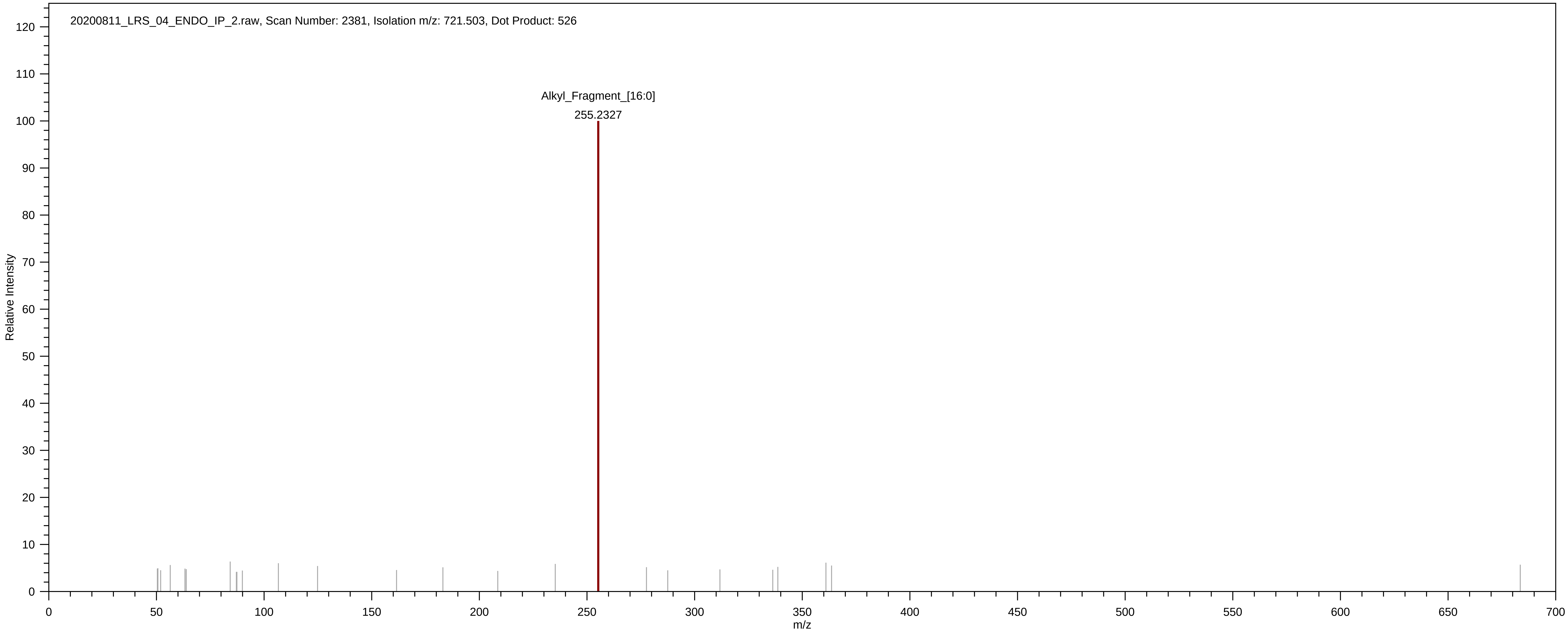

PG 16:0_16:0 [M-H]- | Ret. Time (min): 8.27 | m/z: 721.504

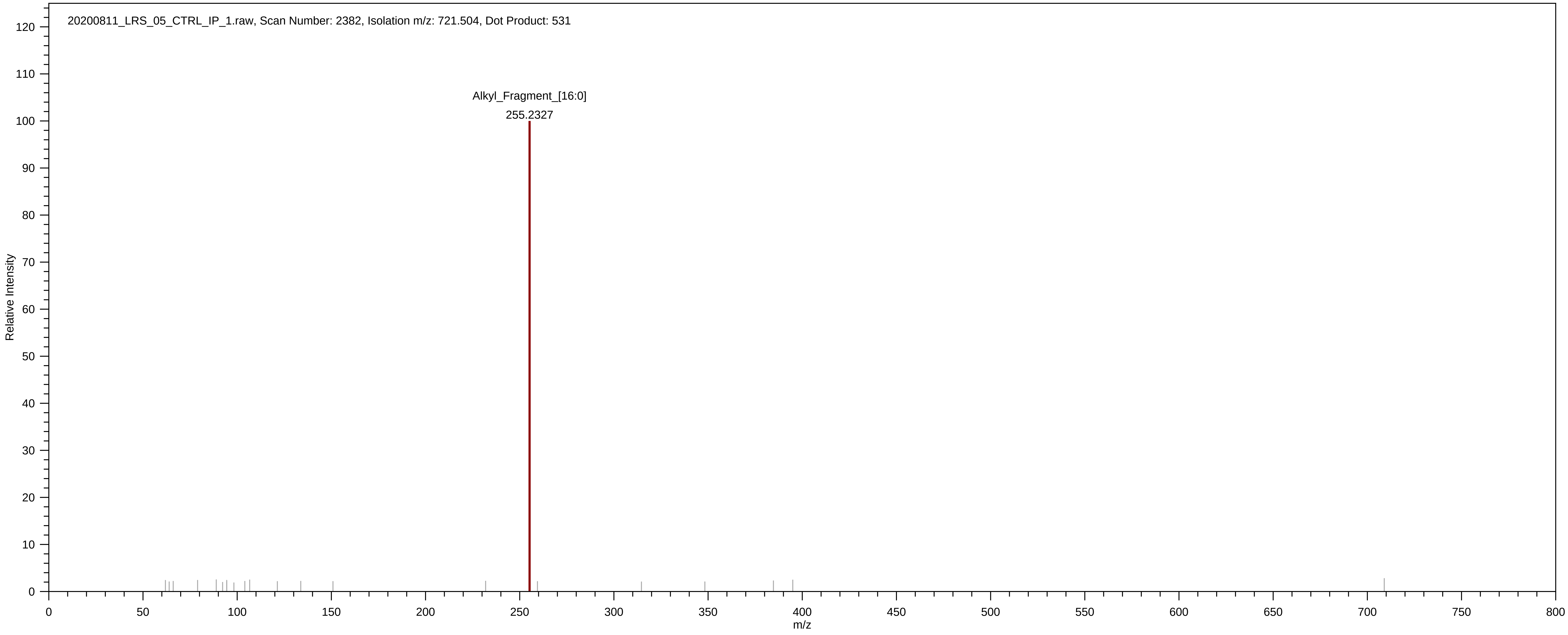

PG 16:0_18:0 [M-H]- | Ret. Time (min): 9.032 | m/z: 749.534

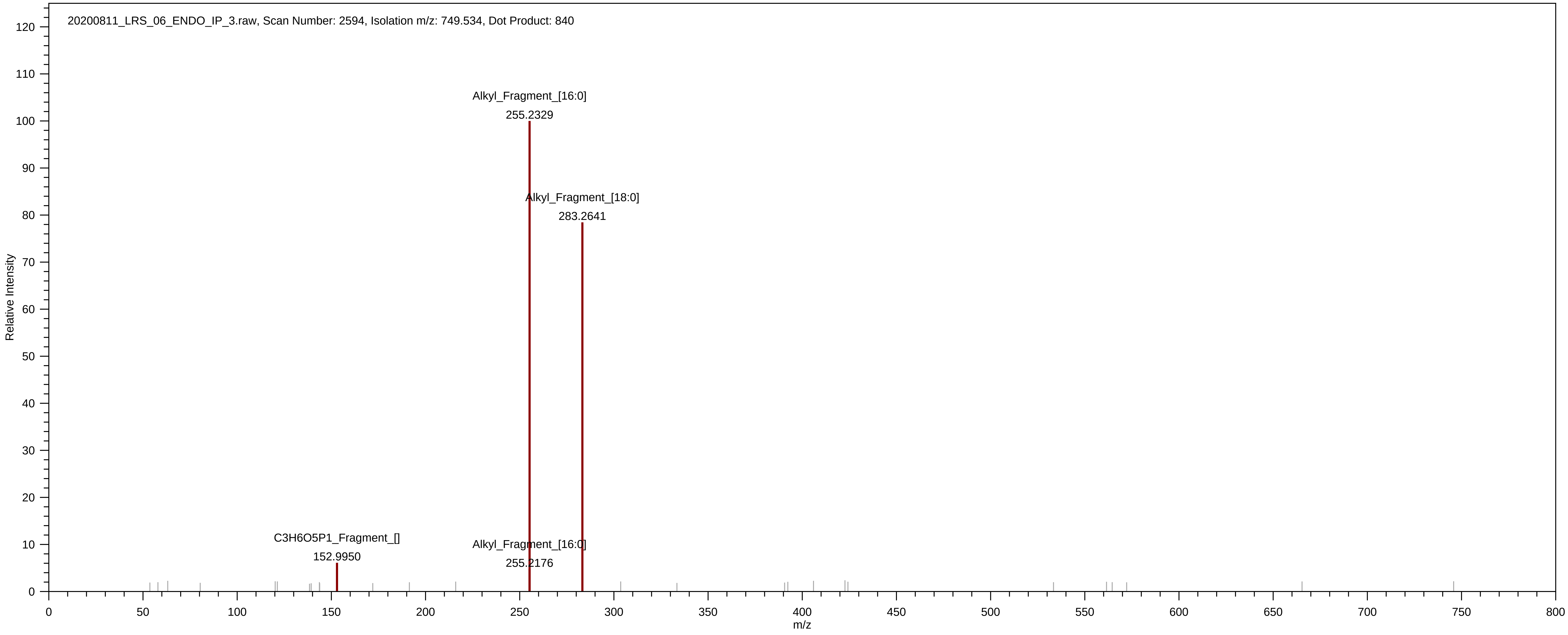

PG 16:0_18:0 [M-H]- | Ret. Time (min): 9.033 | m/z: 749.534

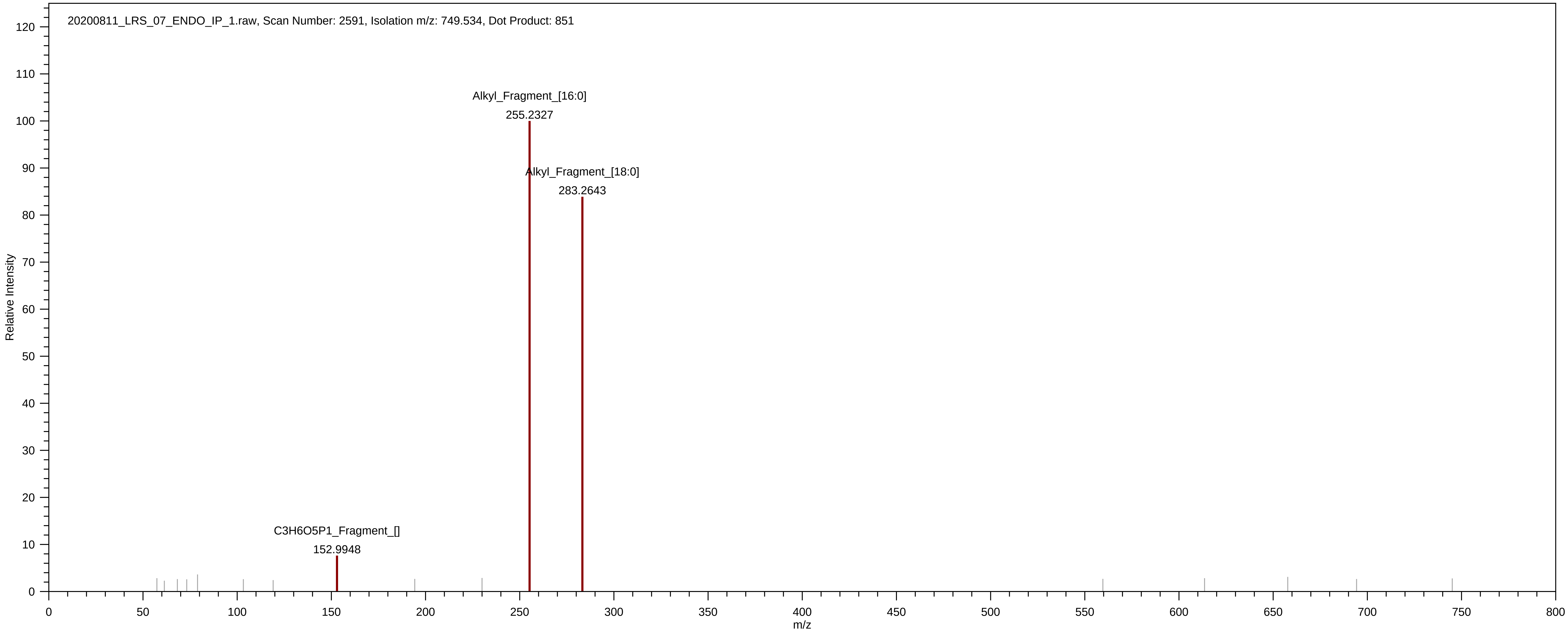

PG 16:0_18:1 [M-H]- | Ret. Time (min): 7.491 | m/z: 747.52

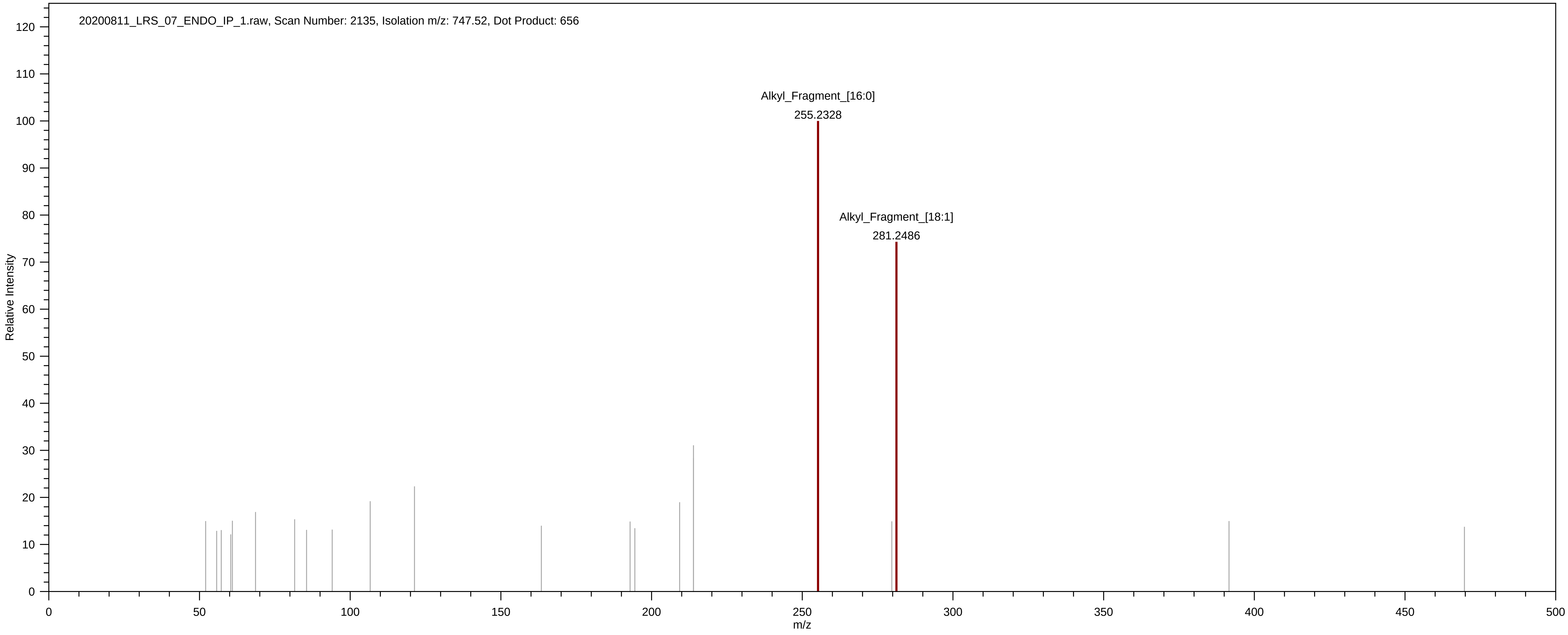

PG 16:0_18:1 [M-H]- | Ret. Time (min): 8.317 | m/z: 747.514

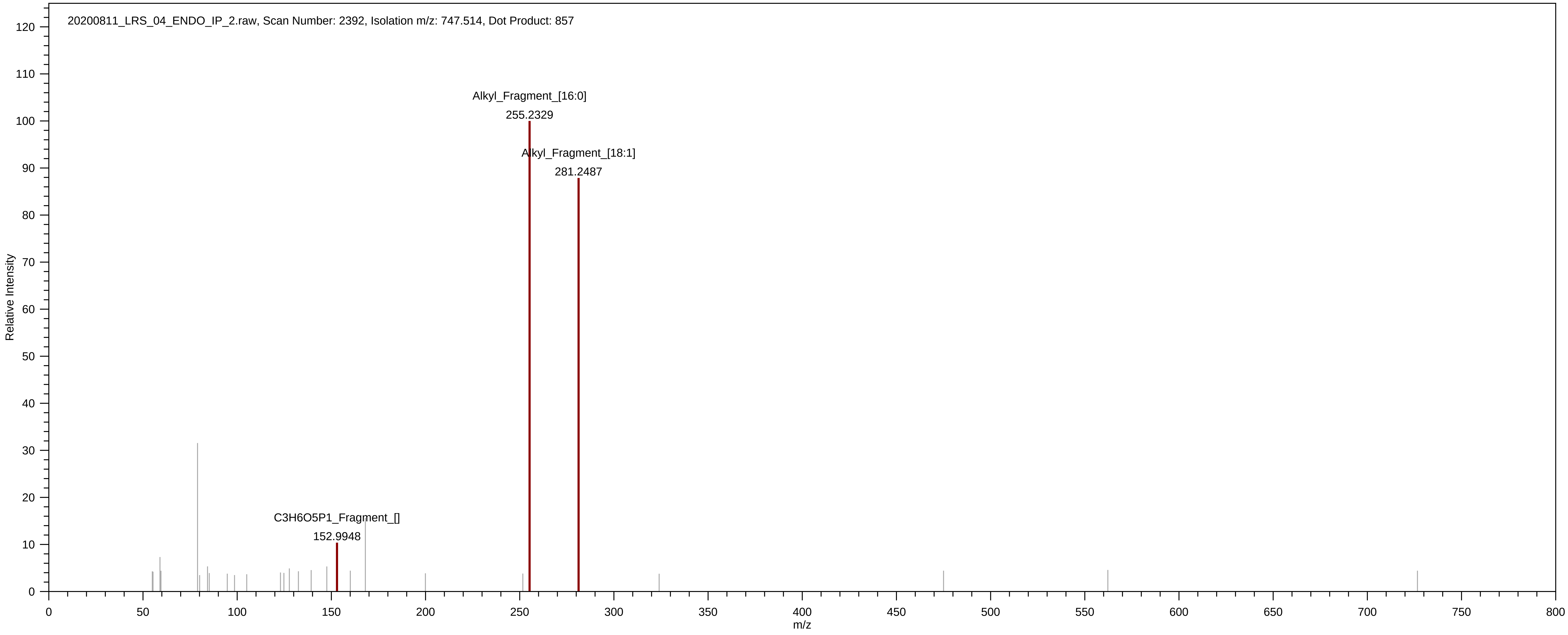

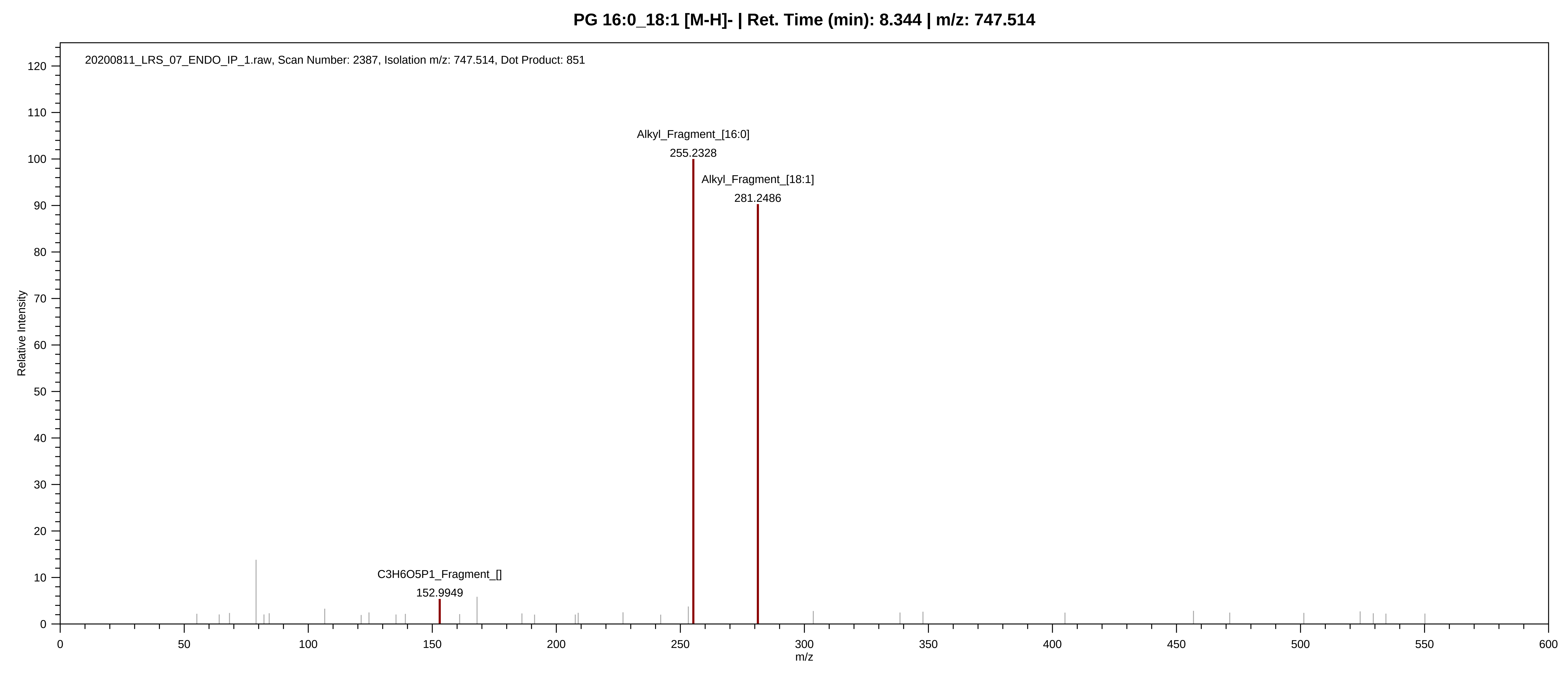
PG 16:0_18:1 [M-H]- | Ret. Time (min): 8.344 | m/z: 747.514
20200811_LRS_07_ENDO_IP_1.raw, Scan Number: 2387, Isolation m/z: 747.514, Dot Product: 851
Alkyl_Fragment_[16:0]
255.2328
Alkyl_Fragment_[18:1]
281.2486
C3H6O5P1_Fragment_[]
152.9949
Relative Intensity
m/z

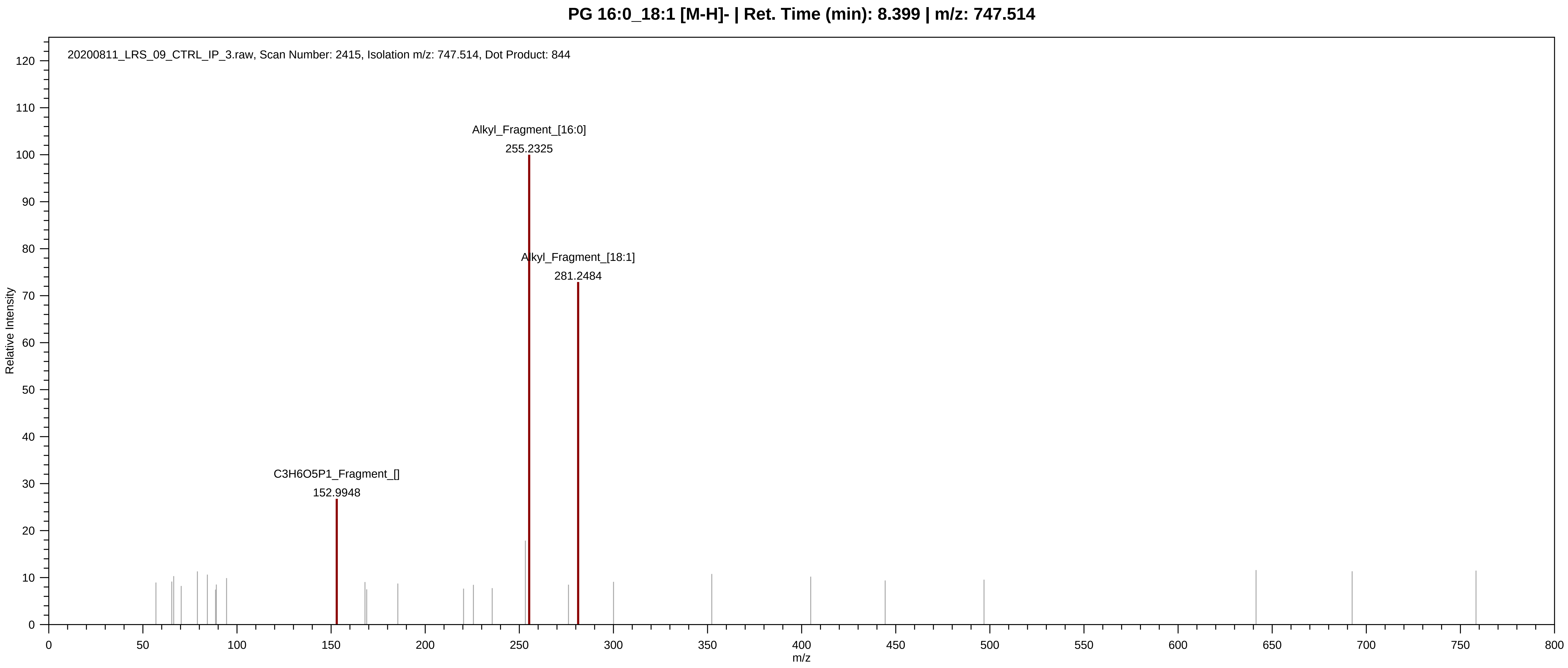
PG 16:0_18:1 [M-H]- | Ret. Time (min): 8.399 | m/z: 747.514
20200811_LRS_09_CTRL_IP_3.raw, Scan Number: 2415, Isolation m/z: 747.514, Dot Product: 844
Alkyl_Fragment_[16:0]
255.2325
Alkyl_Fragment_[18:1]
281.2484
C3H6O5P1_Fragment_[]
152.9948
Relative Intensity
m/z

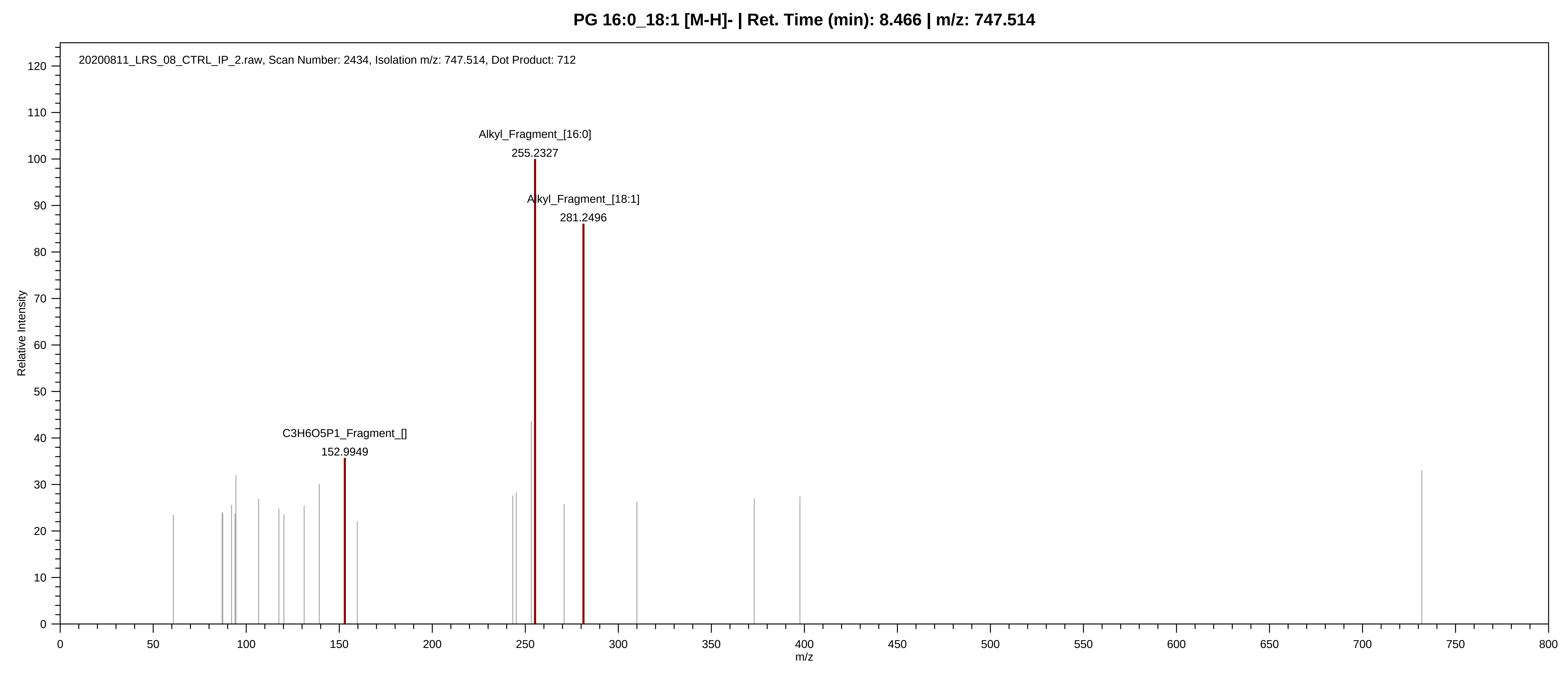

PG 16:0_18:1 [M-H]- | Ret. Time (min): 8.466 | m/z: 747.514
20200811_LRS_08_CTRL_IP_2.raw, Scan Number: 2434, Isolation m/z: 747.514, Dot Product: 712
Alkyl_Fragment_[16:0]
255.2327
Alkyl_Fragment_[18:1]
281.2496
C3H6O5P1_Fragment_[]
152.9949
Relative Intensity
m/z

PG 16:0_18:1 [M-H]- | Ret. Time (min): 8.87 | m/z: 747.516

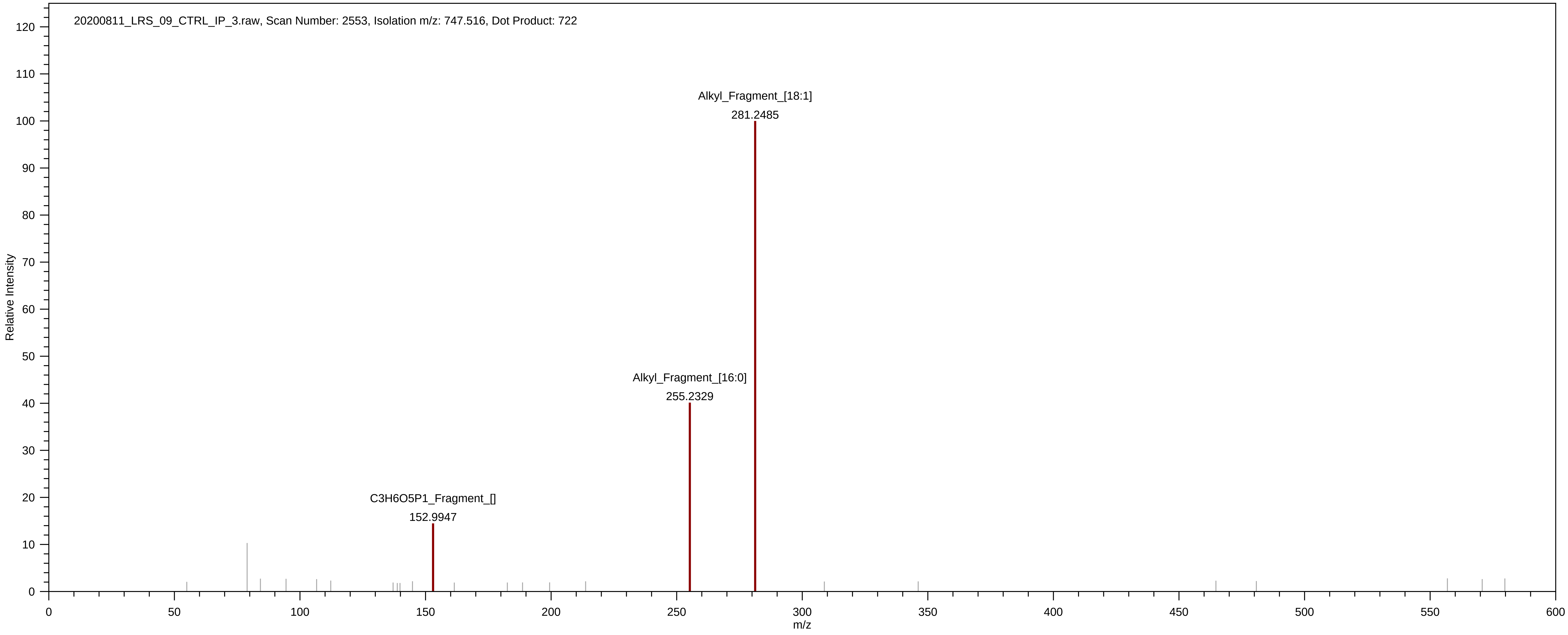

PG 16:0_18:1 [M-H]- | Ret. Time (min): 8.196 | m/z: 747.516

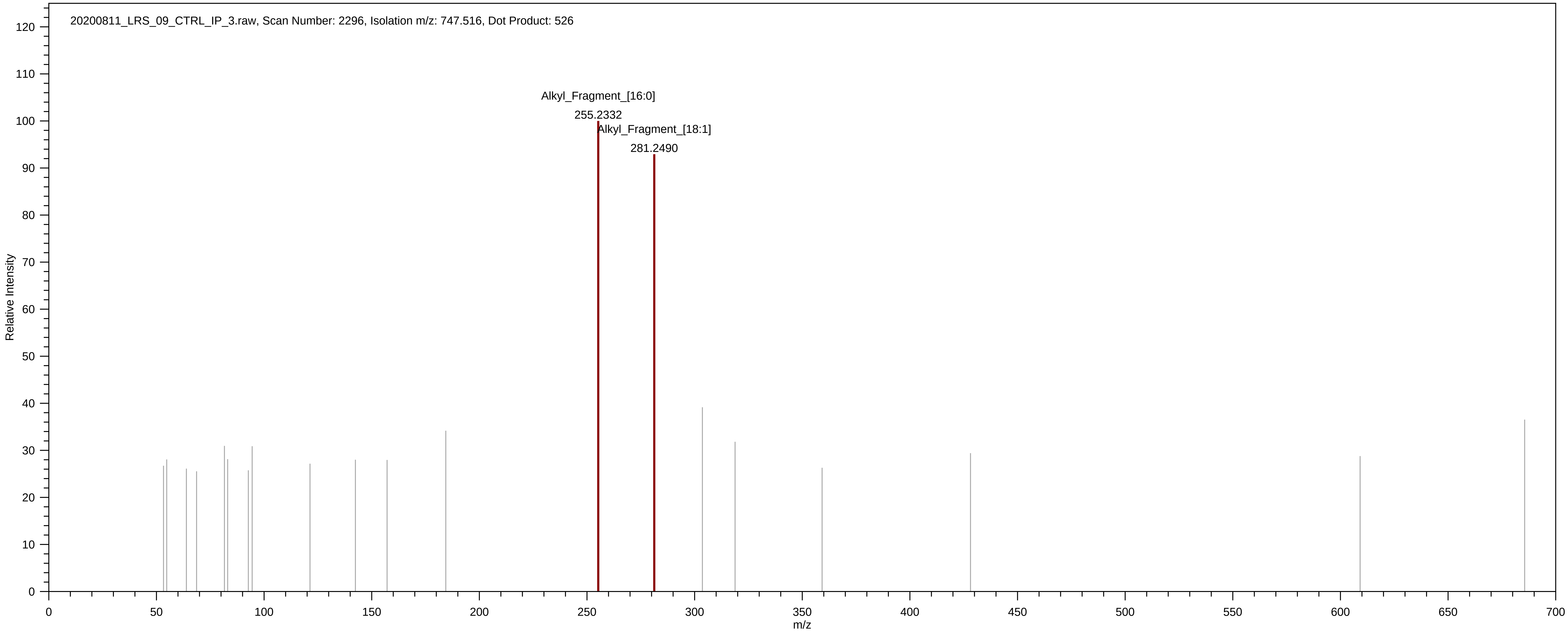

PG 16:0_18:1 [M-H]- | Ret. Time (min): 8.196 | m/z: 747.516

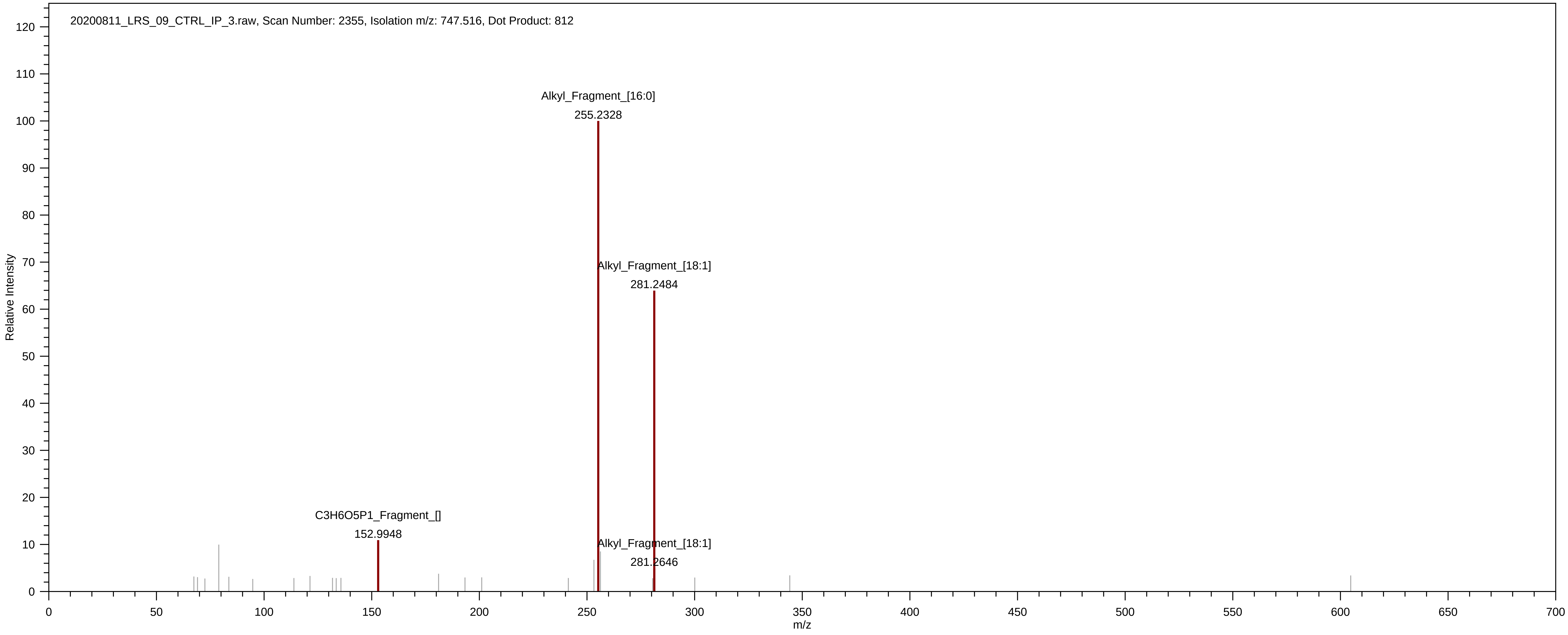

PG 16:0_18:1 [M-H]- | Ret. Time (min): 8.304 | m/z: 747.516

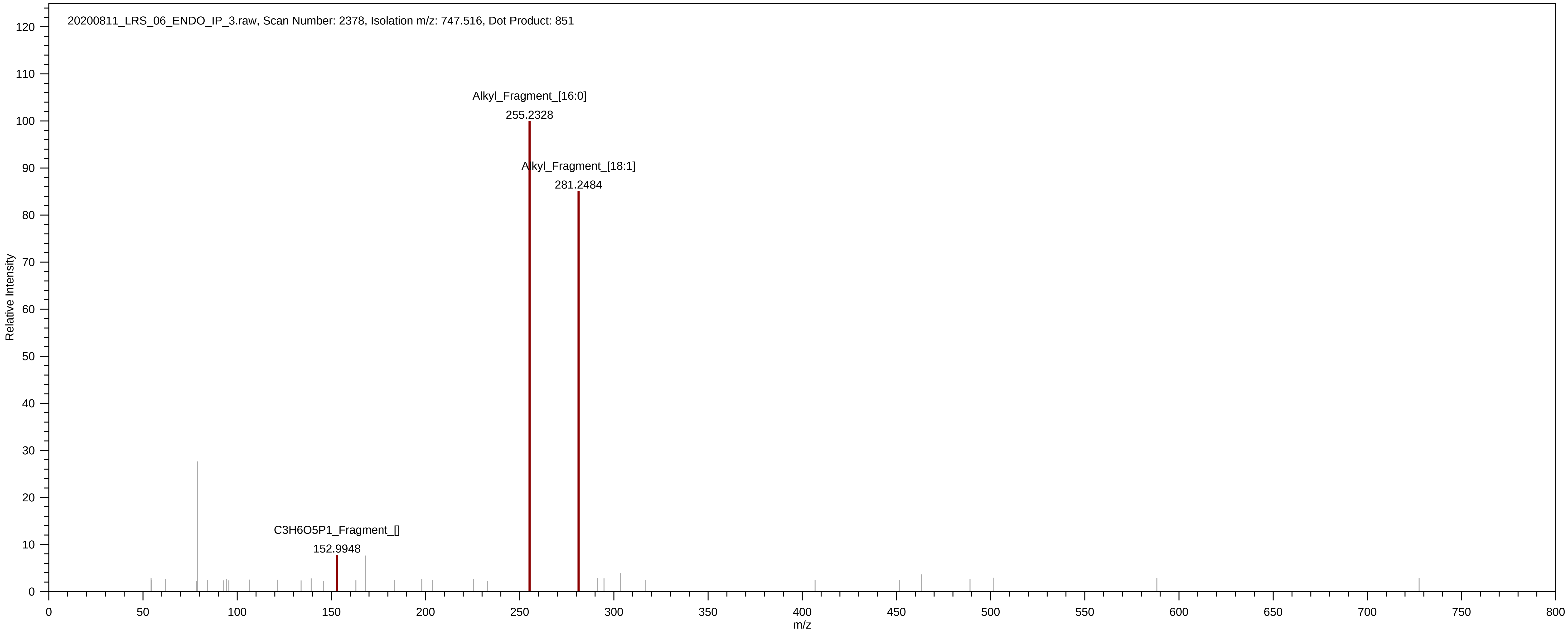

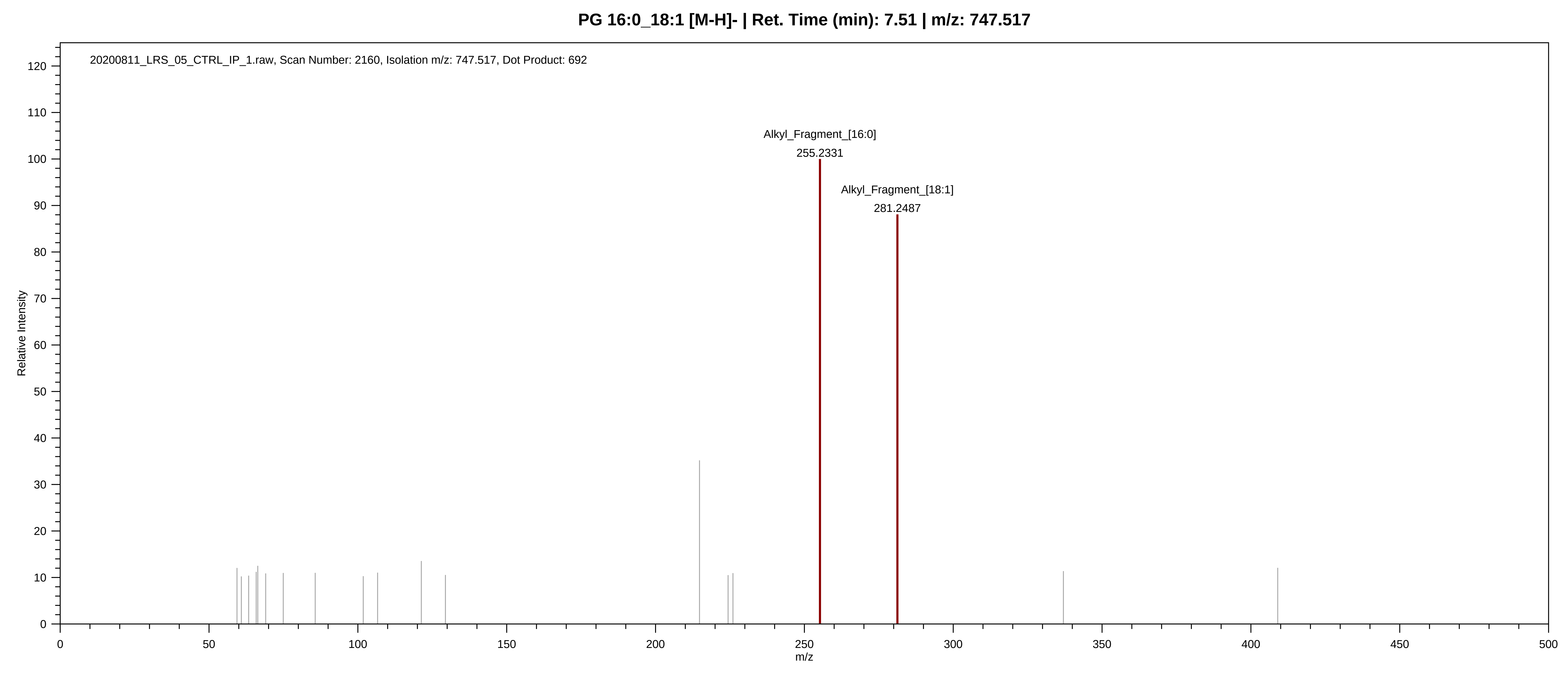
PG 16:0_18:1 [M-H]- | Ret. Time (min): 7.51 | m/z: 747.517
20200811_LRS_05_CTRL_IP_1.raw, Scan Number: 2160, Isolation m/z: 747.517, Dot Product: 692
Alkyl_Fragment_[16:0]
255.2331
Alkyl_Fragment_[18:1]
281.2487
Relative Intensity
m/z

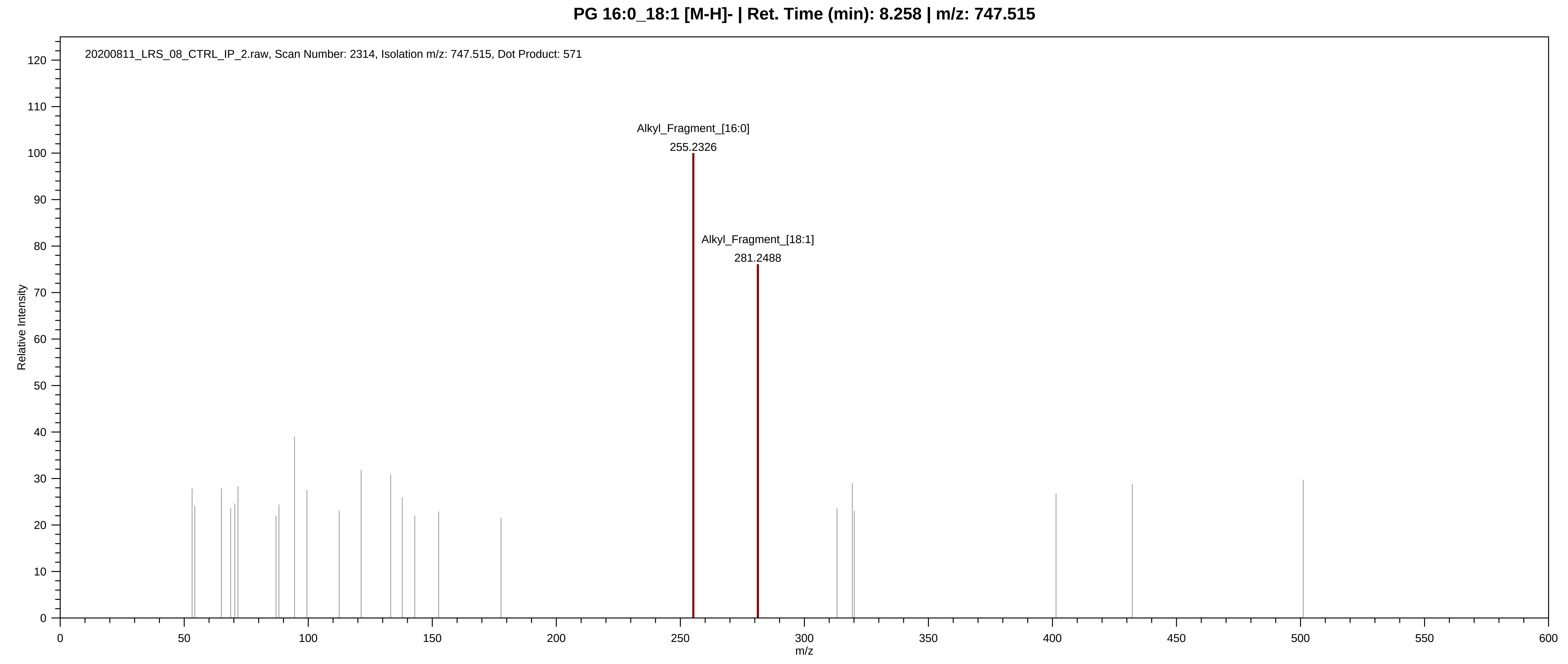

PG 16:0_18:1 [M-H]- | Ret. Time (min): 8.258 | m/z: 747.515
20200811_LRS_08_CTRL_IP_2.raw, Scan Number: 2314, Isolation m/z: 747.515, Dot Product: 571
Alkyl_Fragment_[16:0]
255.2326
Alkyl_Fragment_[18:1]
281.2488
Relative Intensity
m/z

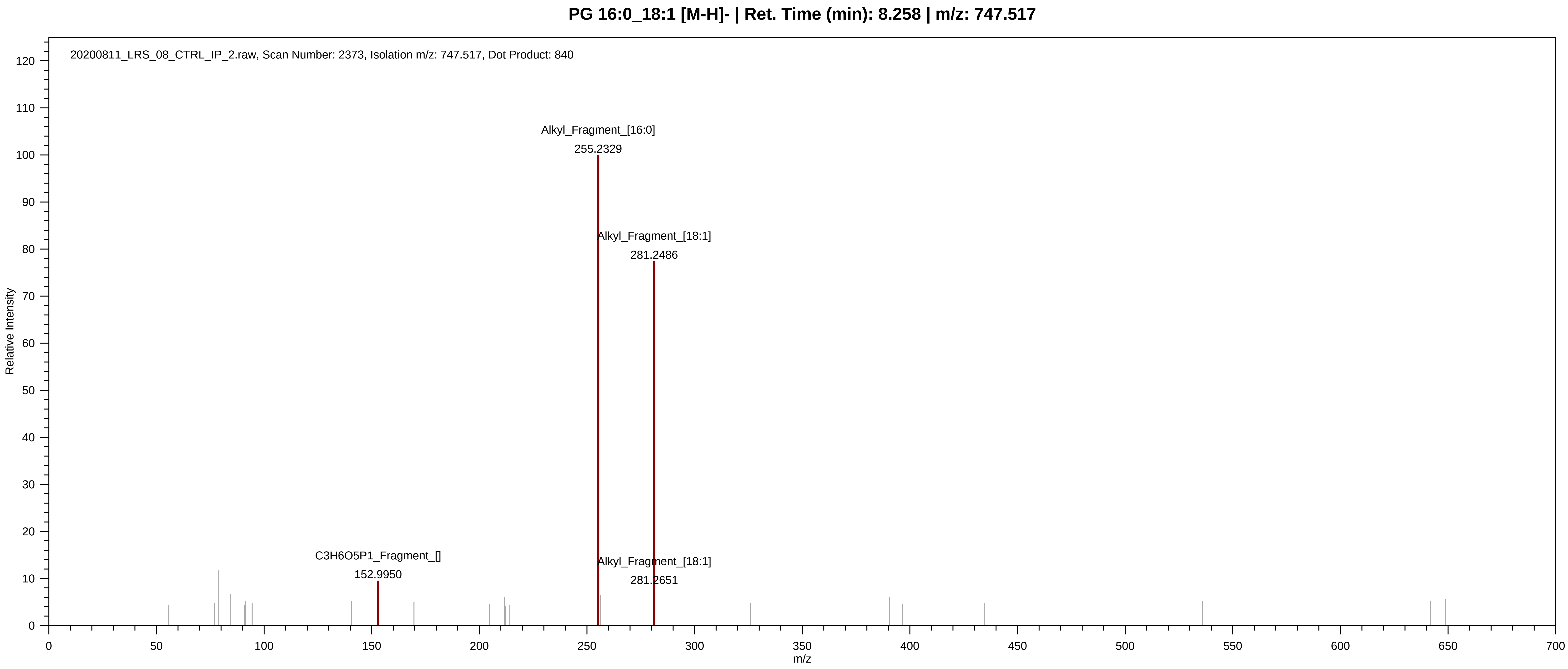

PG 16:0_18:1 [M-H]- | Ret. Time (min): 8.258 | m/z: 747.517
20200811_LRS_08_CTRL_IP_2.raw, Scan Number: 2373, Isolation m/z: 747.517, Dot Product: 840
Alkyl_Fragment_[16:0]
255.2329
Alkyl_Fragment_[18:1]
281.2486
C3H6O5P1_Fragment_[]
152.9950
Alkyl_Fragment_[18:1]
281.2651
Relative Intensity
m/z

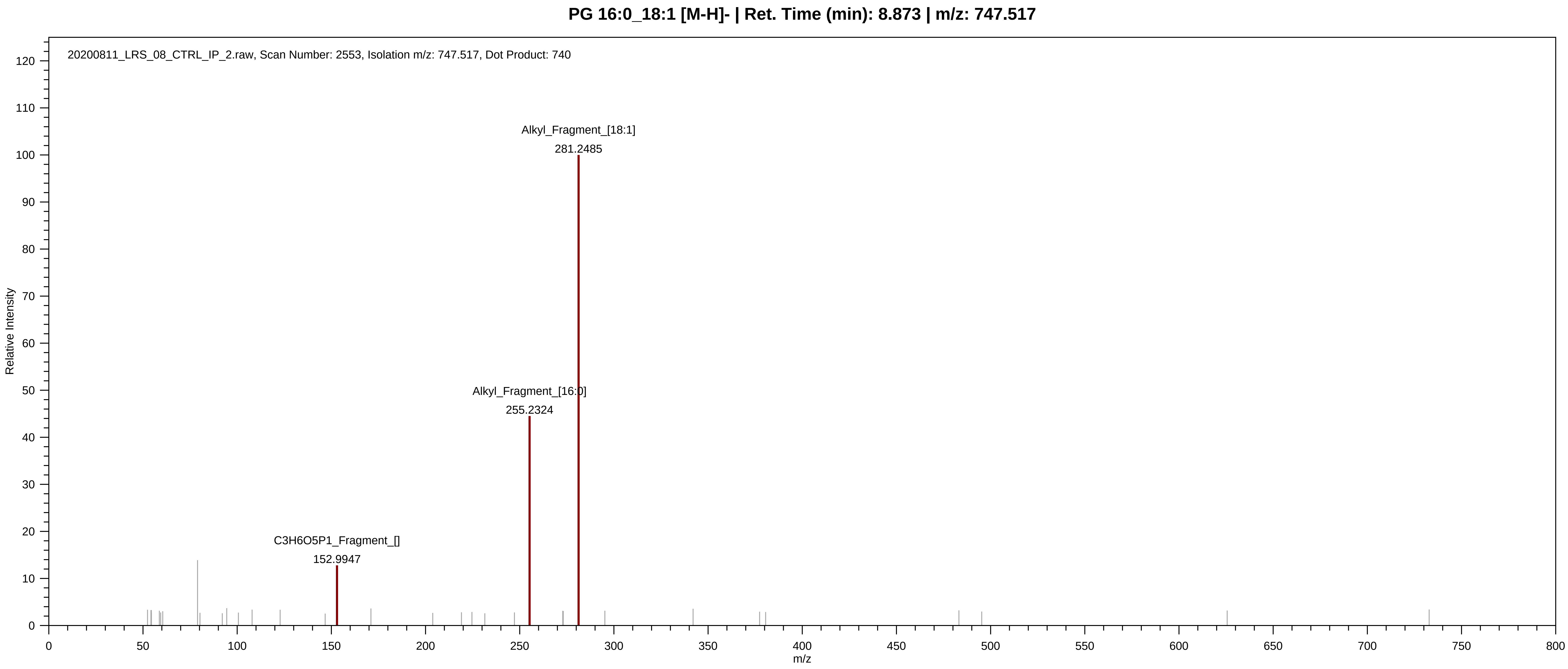
PG 16:0_18:1 [M-H]- | Ret. Time (min): 8.873 | m/z: 747.517
20200811_LRS_08_CTRL_IP_2.raw, Scan Number: 2553, Isolation m/z: 747.517, Dot Product: 740
Alkyl_Fragment_[18:1]
281.2485
Alkyl_Fragment_[16:0]
255.2324
C3H6O5P1_Fragment_[]
152.9947
Relative Intensity
m/z

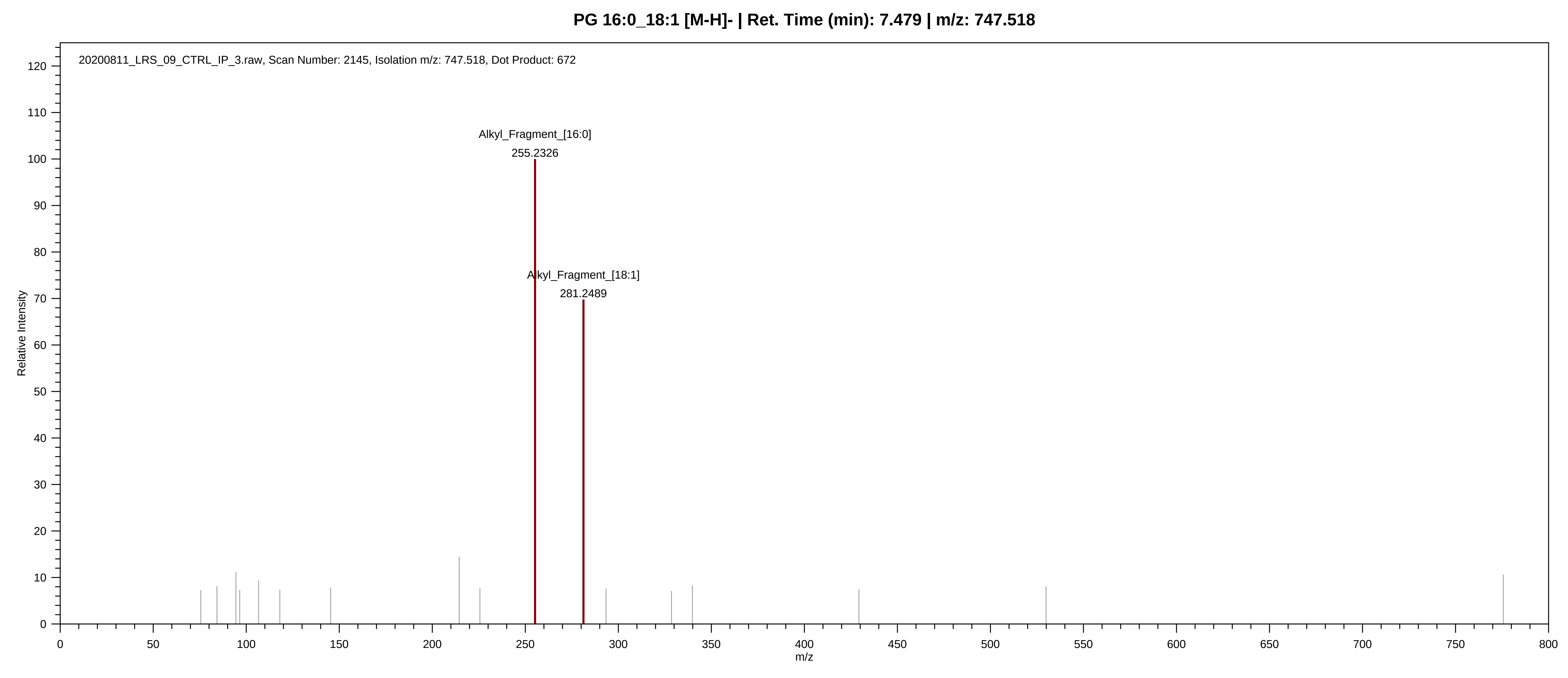

PG 16:0_18:1 [M-H]- | Ret. Time (min): 7.479 | m/z: 747.518
20200811_LRS_09_CTRL_IP_3.raw, Scan Number: 2145, Isolation m/z: 747.518, Dot Product: 672
Alkyl_Fragment_[16:0]
255.2326
Alkyl_Fragment_[18:1]
281.2489
Relative Intensity
m/z

PG 16:0_18:1 [M-H]- | Ret. Time (min): 7.522 | m/z: 747.518

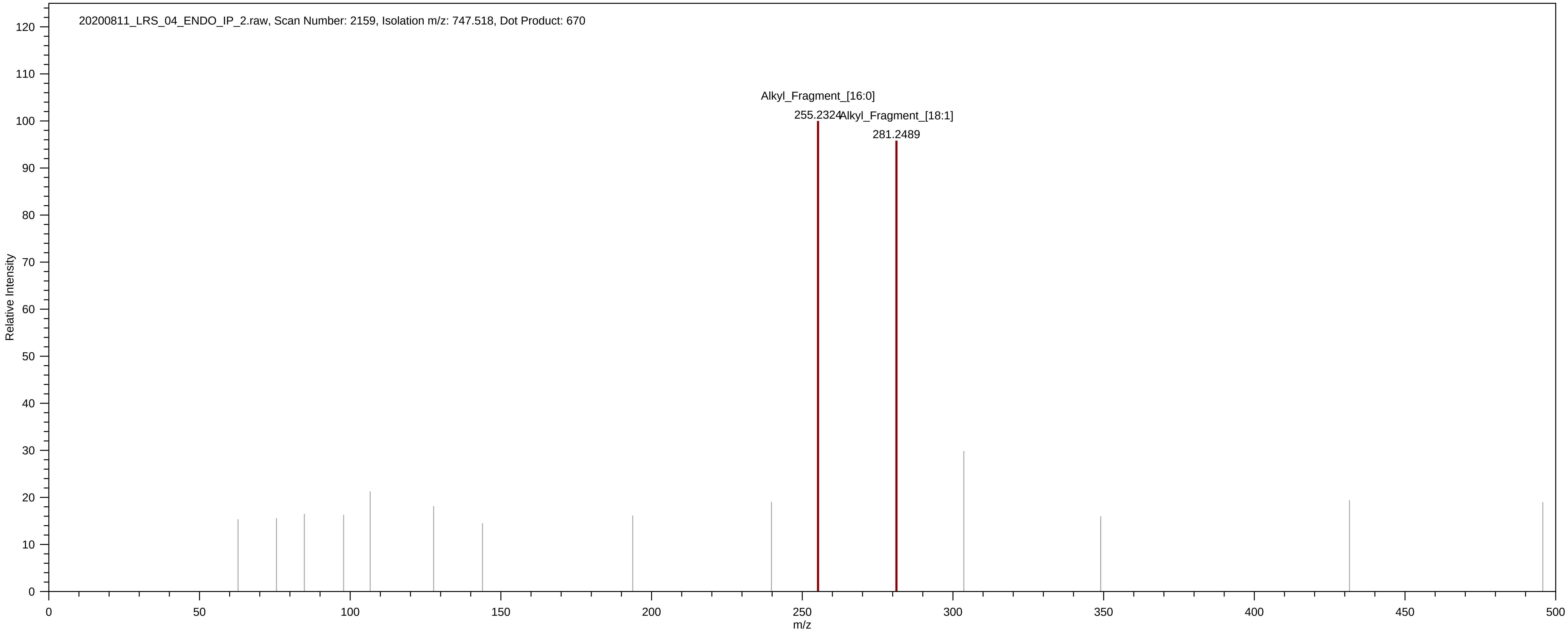

PG 16:0_18:1 [M-H]- | Ret. Time (min): 7.487 | m/z: 747.519

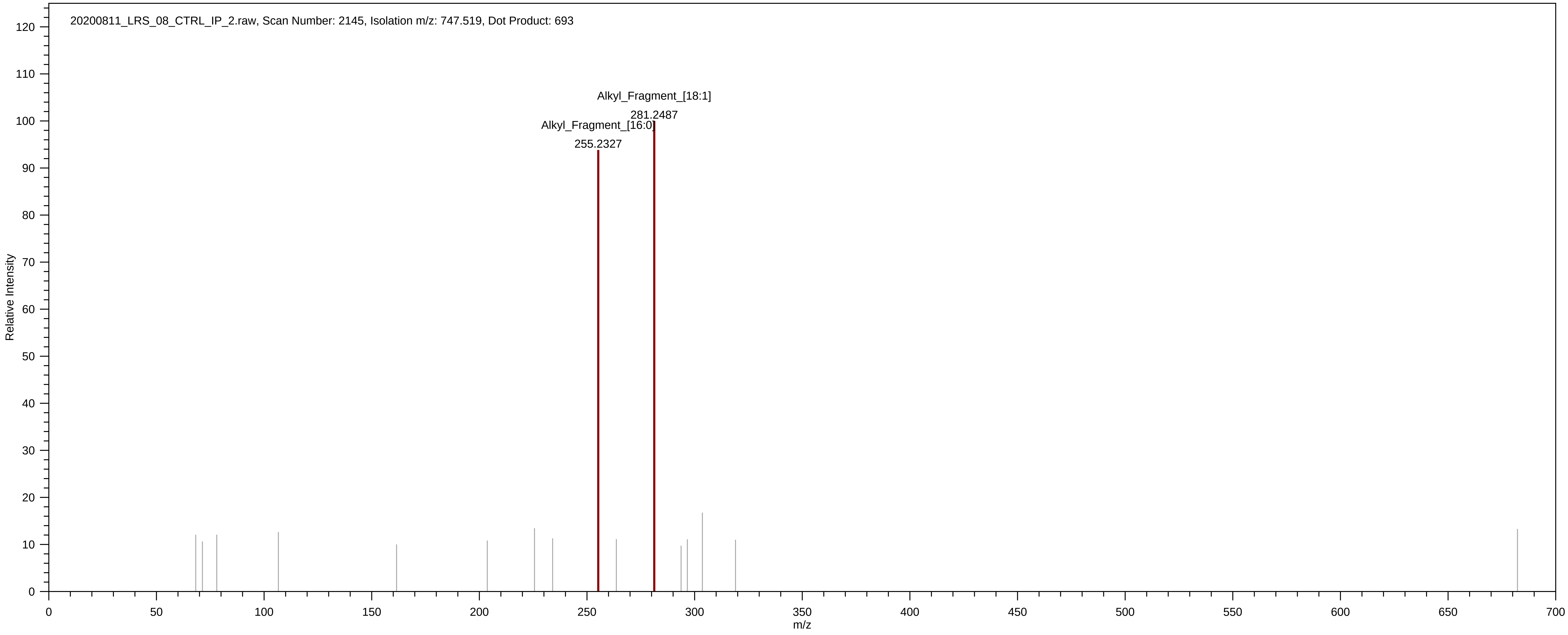

PG 16:0_18:1 [M-H]- | Ret. Time (min): 8.917 | m/z: 747.519

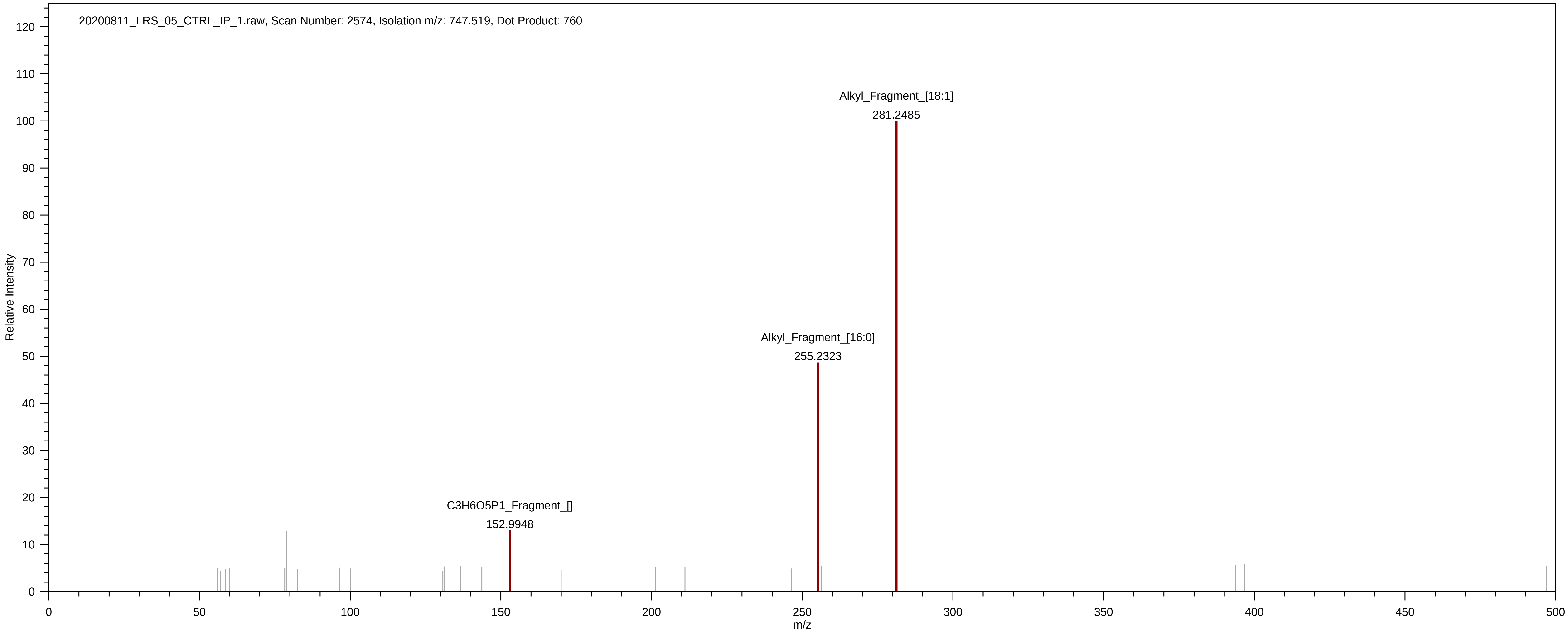

PG 16:1_18:1 [M-H]- | Ret. Time (min): 7.073 | m/z: 745.501

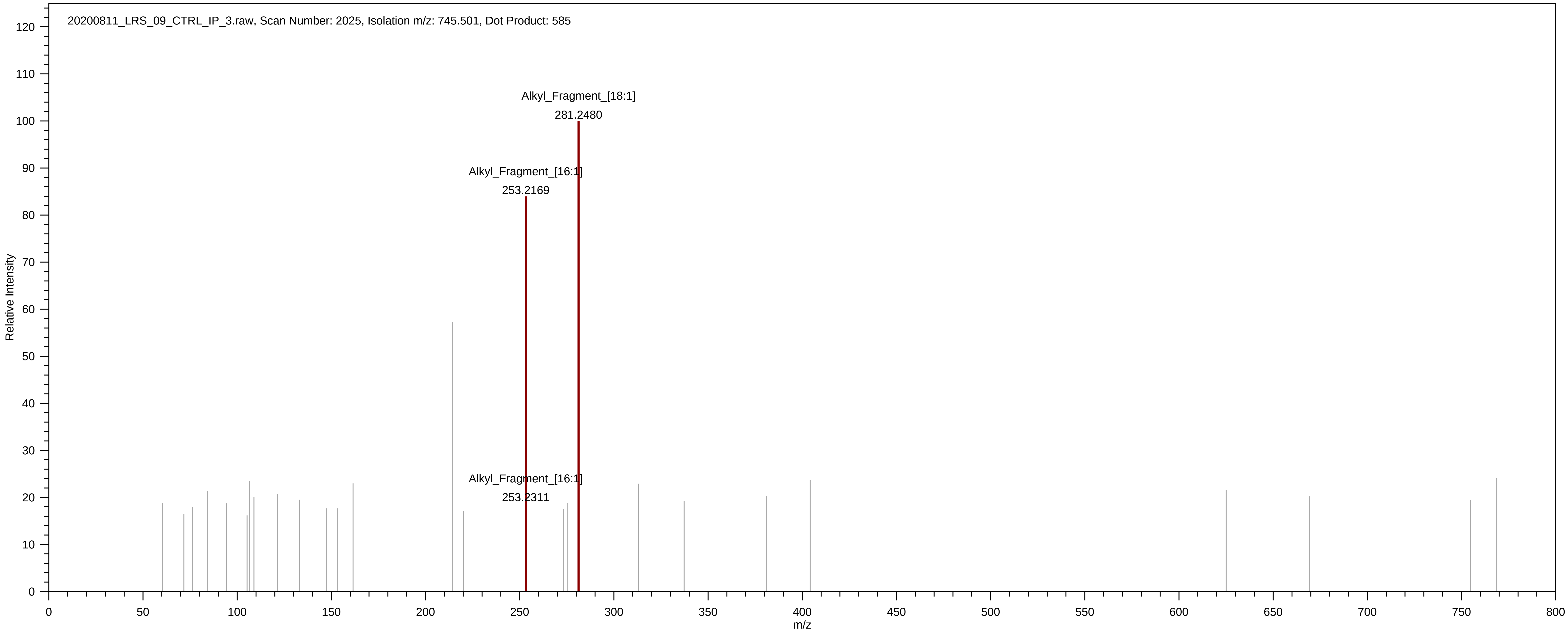

PG 16:1_18:1 [M-H]- | Ret. Time (min): 7.635 | m/z: 745.501

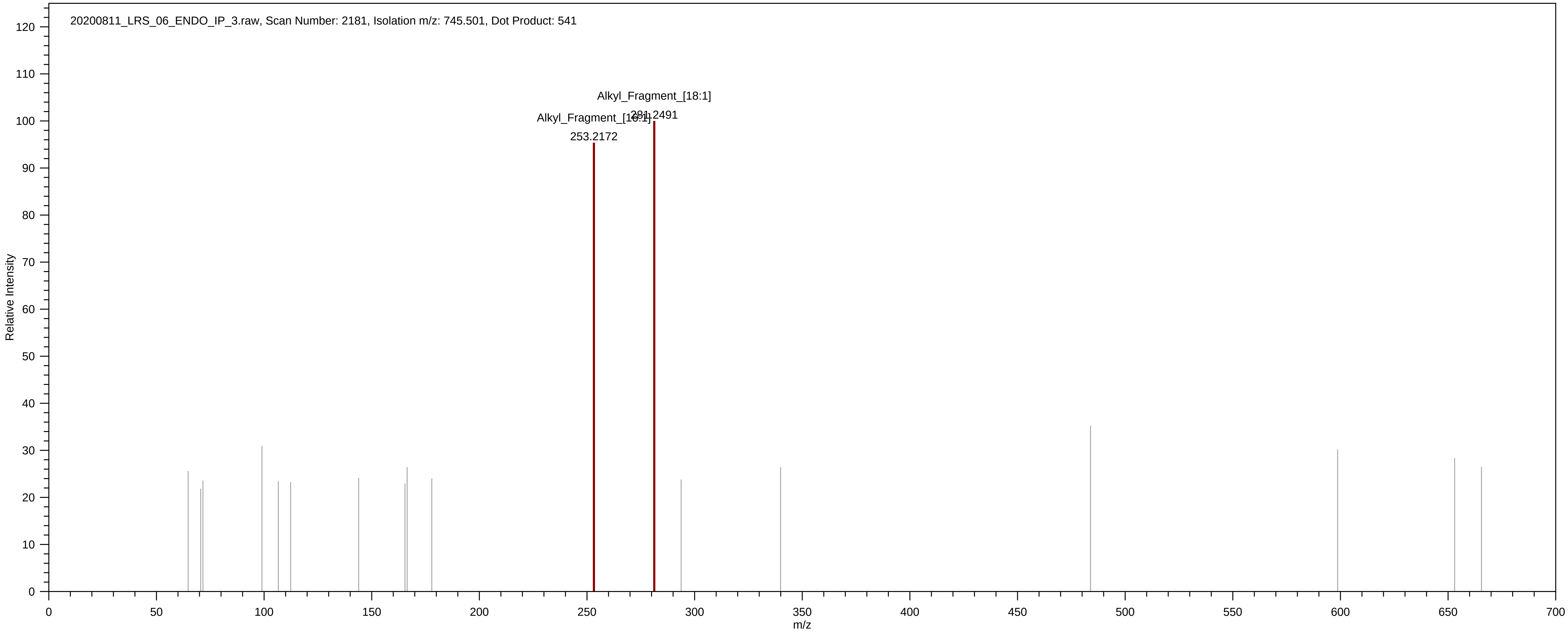

PG 16:1_18:1 [M-H]- | Ret. Time (min): 7.114 | m/z: 745.503

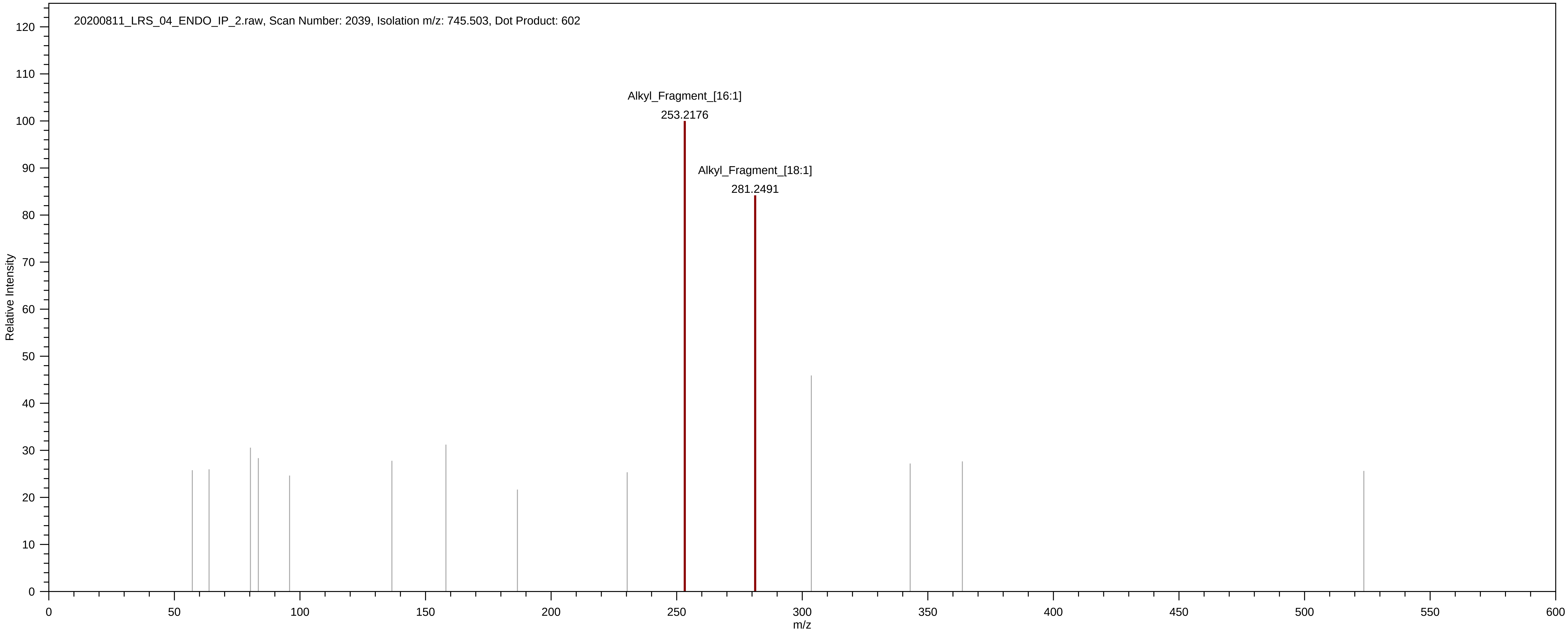

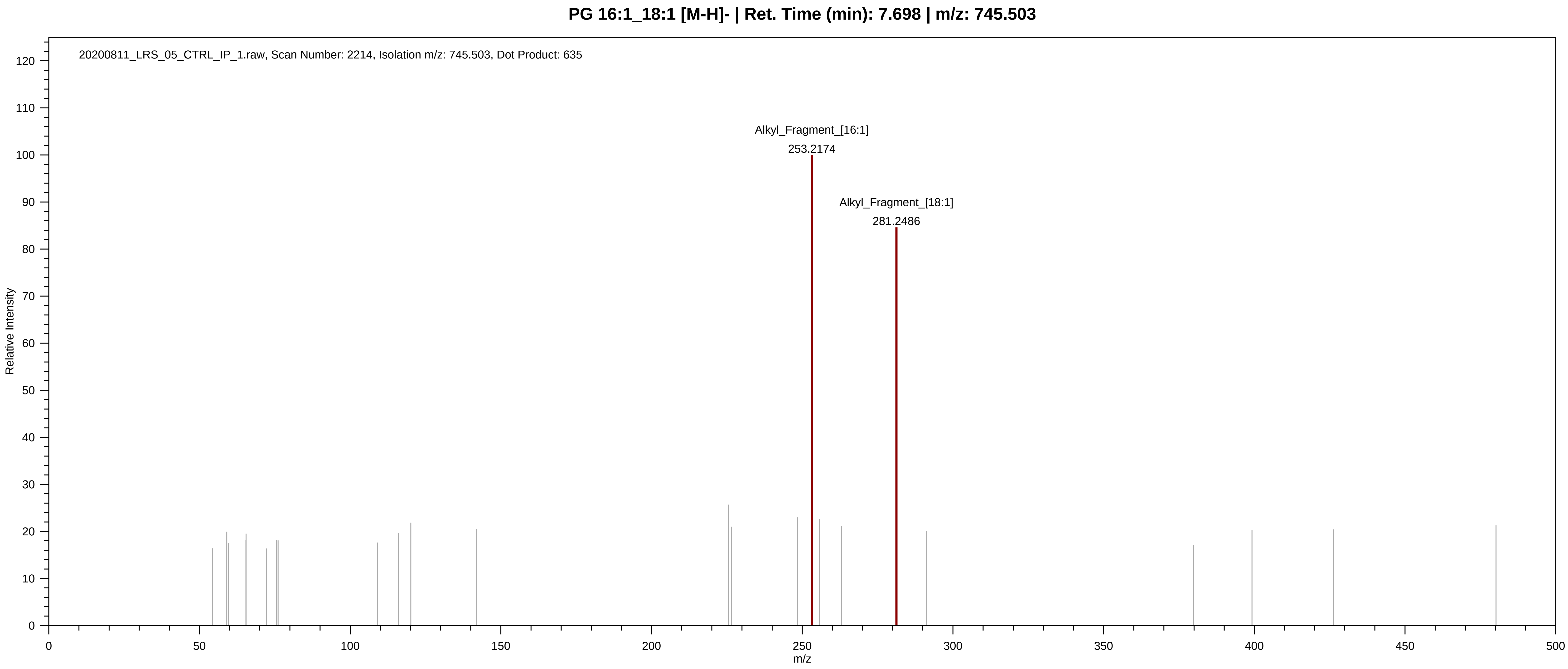
PG 16:1_18:1 [M-H]- | Ret. Time (min): 7.698 | m/z: 745.503
20200811_LRS_05_CTRL_IP_1.raw, Scan Number: 2214, Isolation m/z: 745.503, Dot Product: 635
Alkyl_Fragment_[16:1]
253.2174
Alkyl_Fragment_[18:1]
281.2486
Relative Intensity
m/z
0
10
20
30
40
50
60
70
80
90
100
110
120
0
50
100
150
200
250
300
350
400
450
500

PG 16:1_18:1 [M-H]- | Ret. Time (min): 7.787 | m/z: 745.503

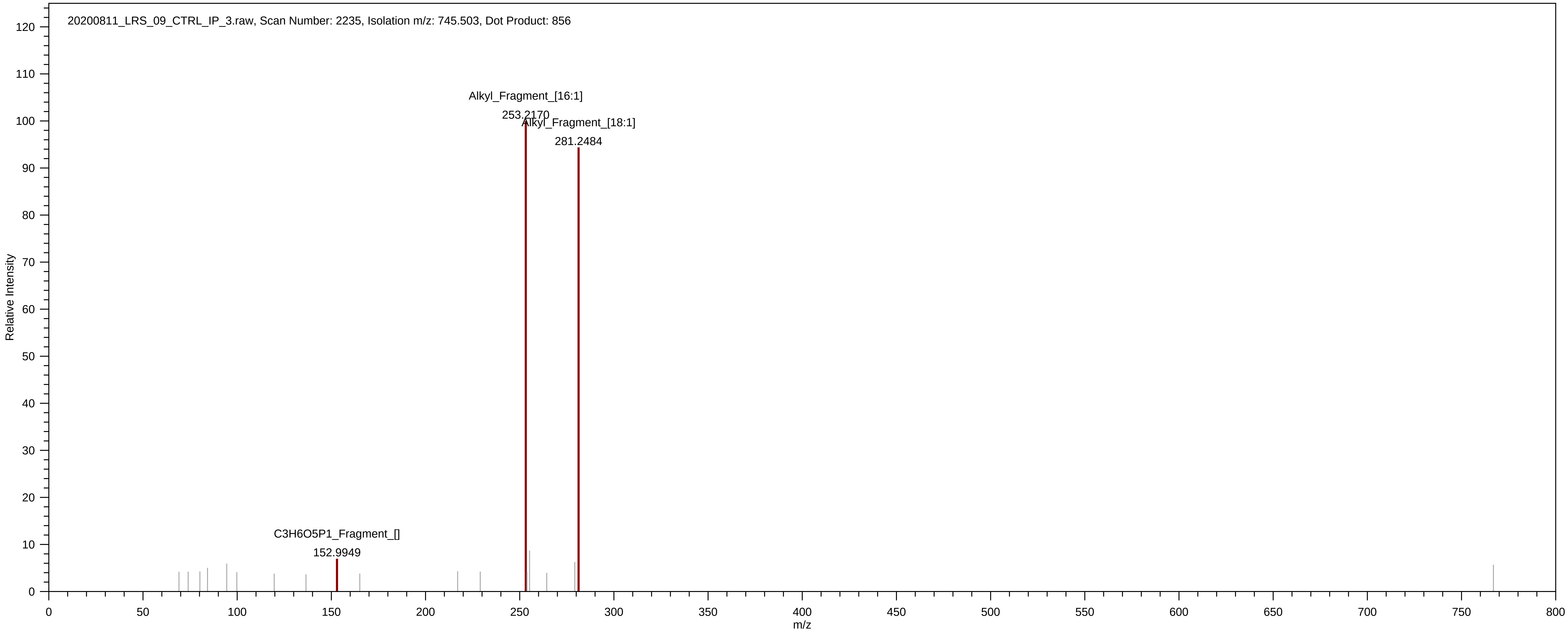

PG 16:1_18:1 [M-H]- | Ret. Time (min): 7.791 | m/z: 745.503

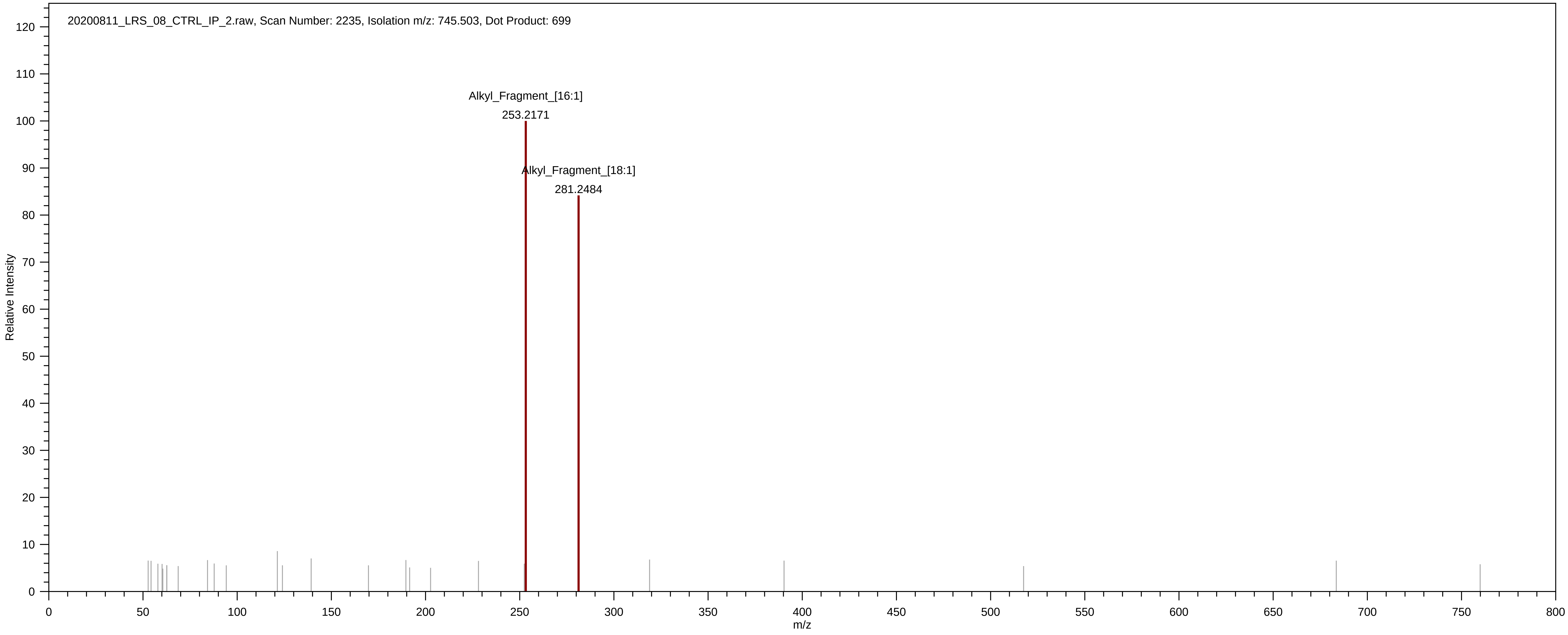

PG 16:1_18:1 [M-H]- | Ret. Time (min): 7.837 | m/z: 745.503

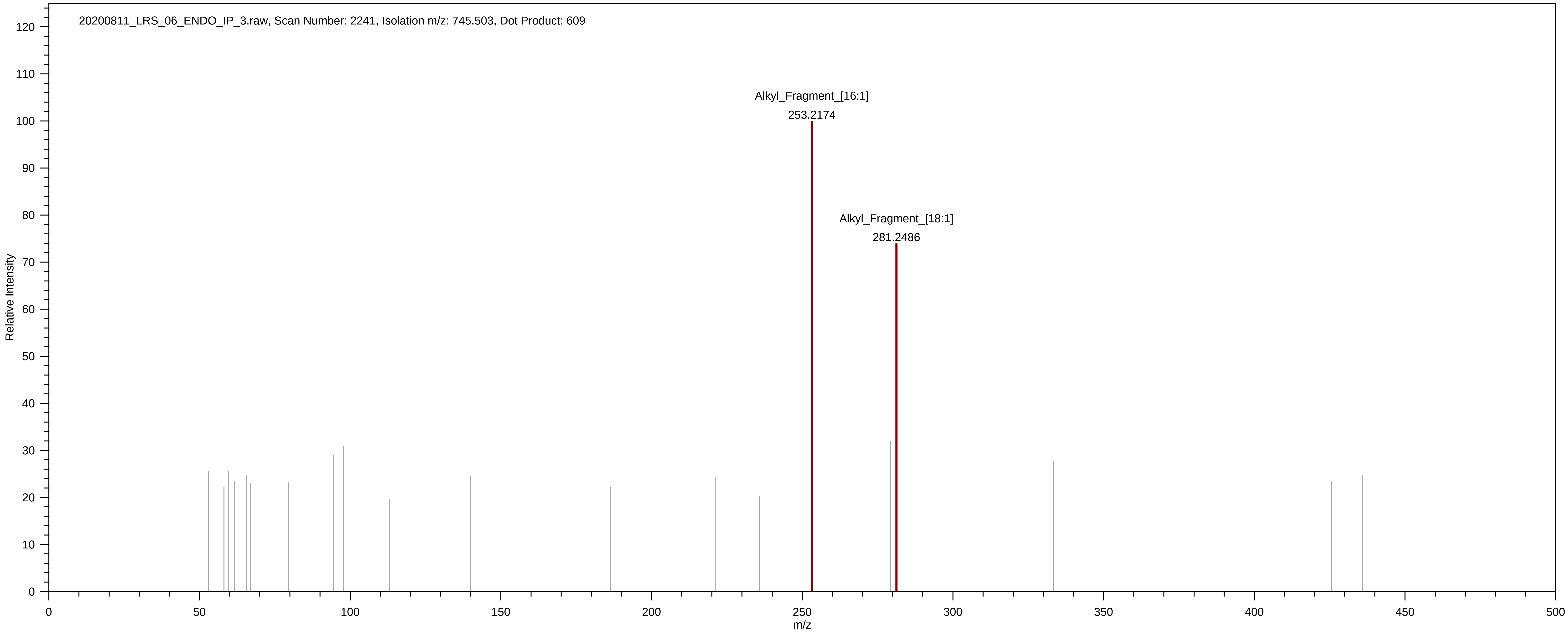

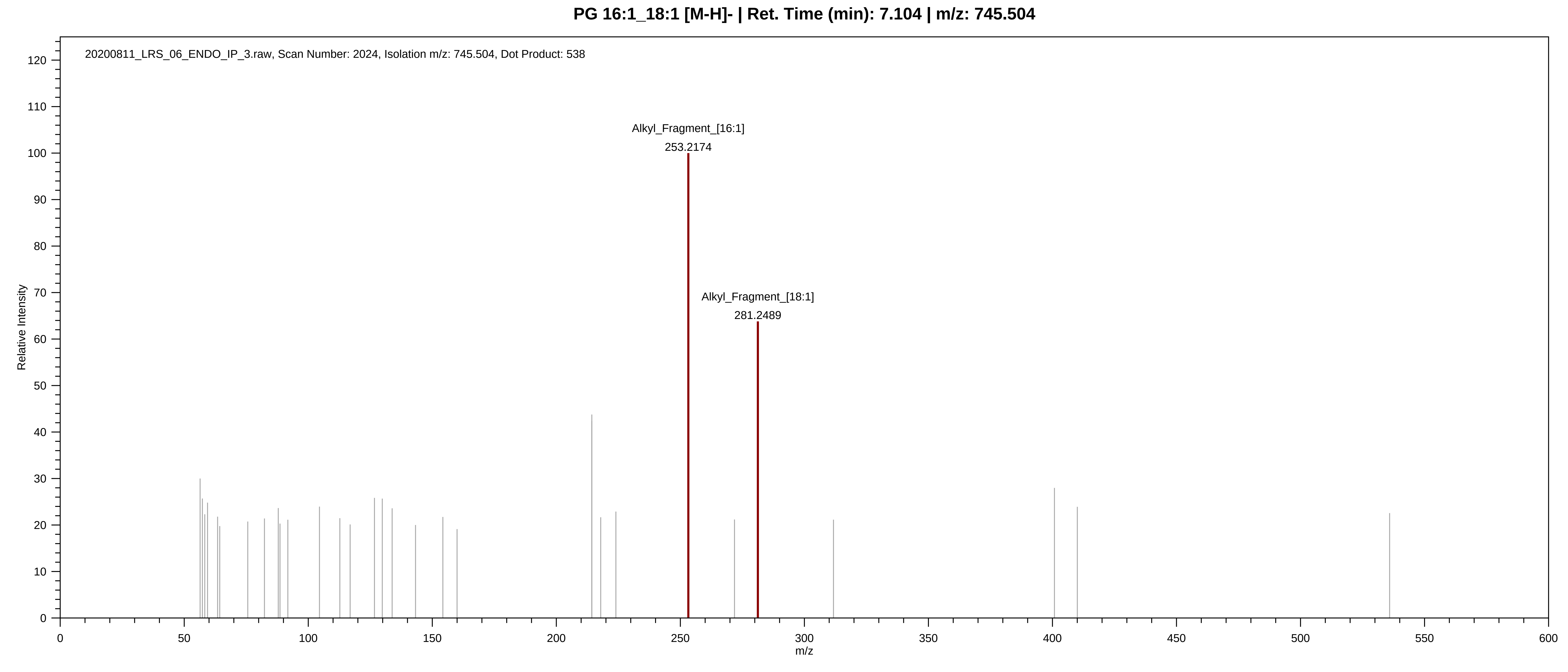

PG 16:1_18:1 [M-H]- | Ret. Time (min): 7.104 | m/z: 745.504
20200811_LRS_06_ENDO_IP_3.raw, Scan Number: 2024, Isolation m/z: 745.504, Dot Product: 538
Alkyl_Fragment_[16:1]
253.2174
Alkyl_Fragment_[18:1]
281.2489
Relative Intensity
m/z

PG 16:1_18:1 [M-H]- | Ret. Time (min): 7.857 | m/z: 745.505

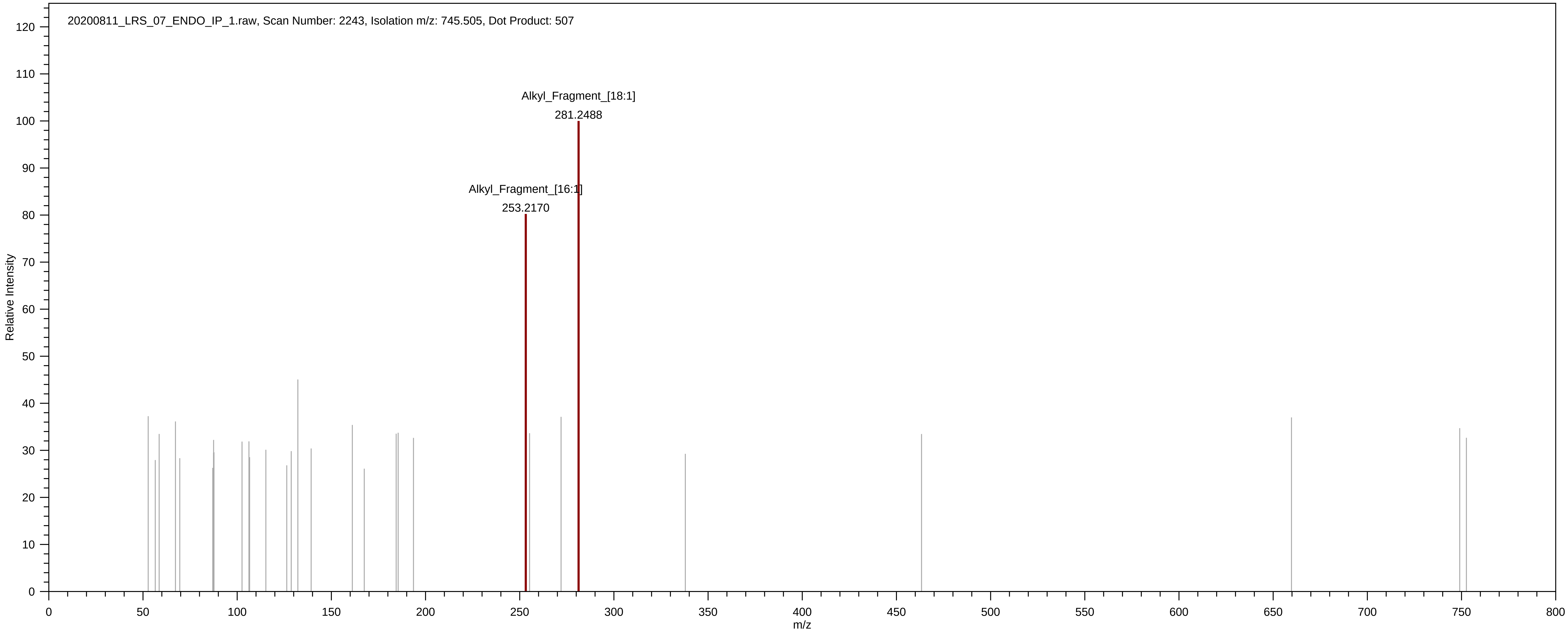

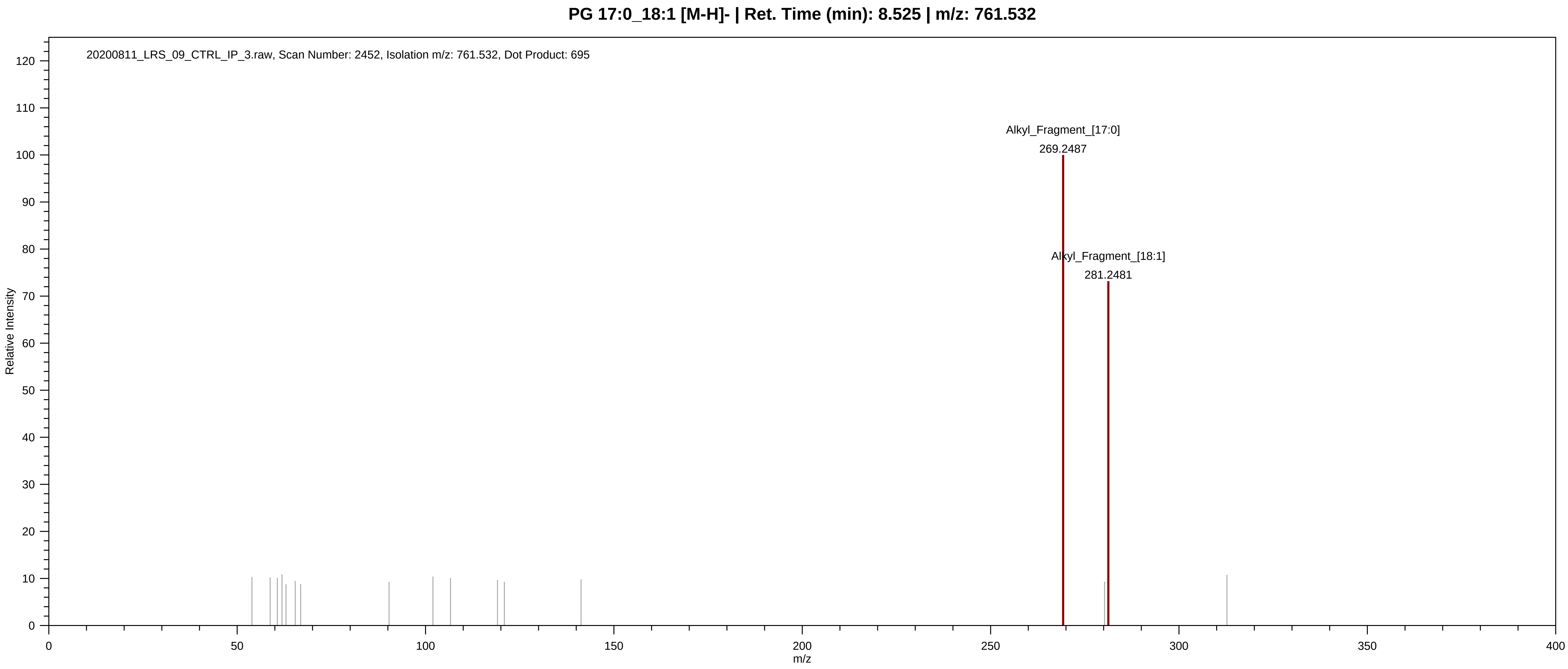

PG 17:0_18:1 [M-H]- | Ret. Time (min): 8.525 | m/z: 761.532
20200811_LRS_09_CTRL_IP_3.raw, Scan Number: 2452, Isolation m/z: 761.532, Dot Product: 695
Alkyl_Fragment_[17:0]
269.2487
Alkyl_Fragment_[18:1]
281.2481
Relative Intensity
m/z
0
10
20
30
40
50
60
70
80
90
100
110
120
0
50
100
150
200
250
300
350
400

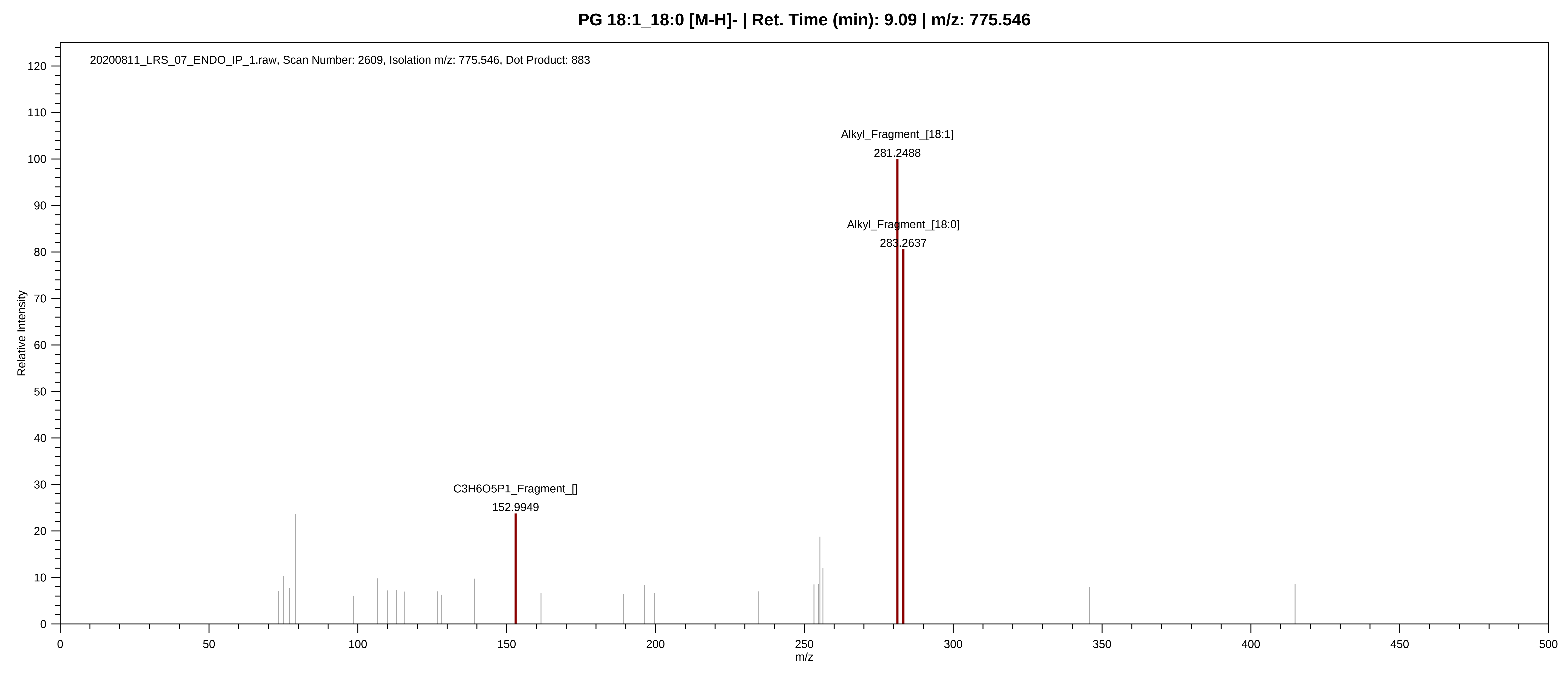

PG 18:1_18:0 [M-H]- | Ret. Time (min): 9.09 | m/z: 775.546
20200811_LRS_07_ENDO_IP_1.raw, Scan Number: 2609, Isolation m/z: 775.546, Dot Product: 883
Alkyl_Fragment_[18:1]
281.2488
Alkyl_Fragment_[18:0]
283.2637
C3H6O5P1_Fragment_[]
152.9949
Relative Intensity
m/z

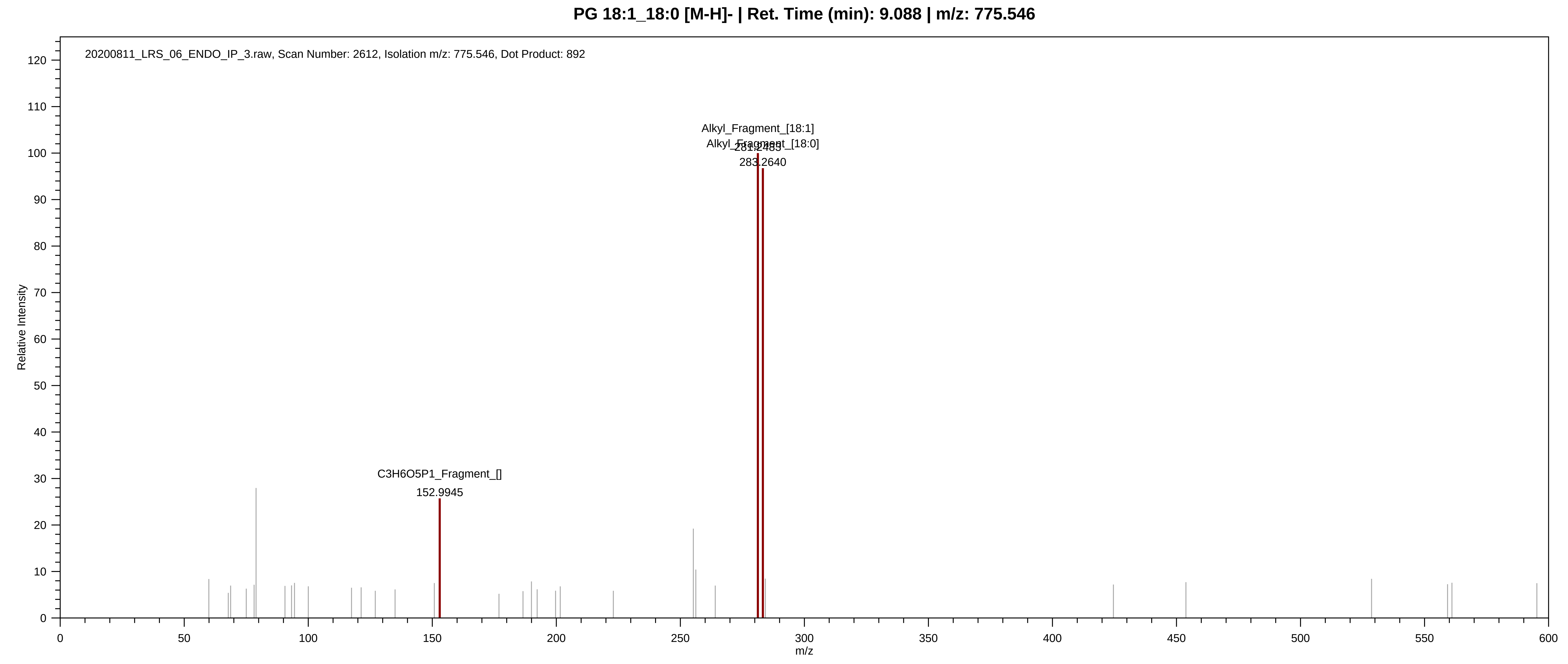

PG 18:1_18:0 [M-H]- | Ret. Time (min): 9.088 | m/z: 775.546
20200811_LRS_06_ENDO_IP_3.raw, Scan Number: 2612, Isolation m/z: 775.546, Dot Product: 892
Alkyl_Fragment_[18:1]
281.2483
Alkyl_Fragment_[18:0]
283.2640
C3H6O5P1_Fragment_[]
152.9945
Relative Intensity
m/z

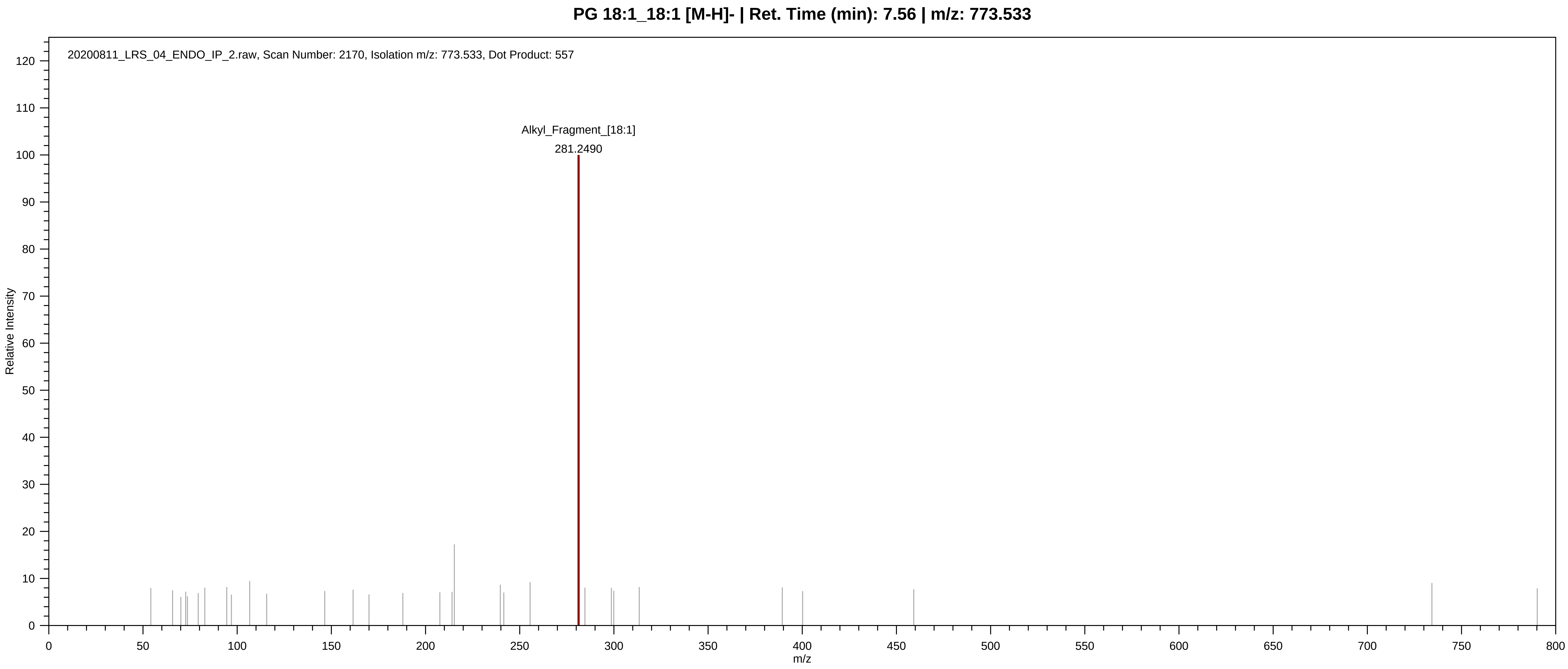
PG 18:1_18:1 [M-H]- | Ret. Time (min): 7.56 | m/z: 773.533
20200811_LRS_04_ENDO_IP_2.raw, Scan Number: 2170, Isolation m/z: 773.533, Dot Product: 557
Alkyl_Fragment_[18:1]
281.2490
Relative Intensity
m/z

PG 18:1_18:1 [M-H]- | Ret. Time (min): 8.367 | m/z: 773.533

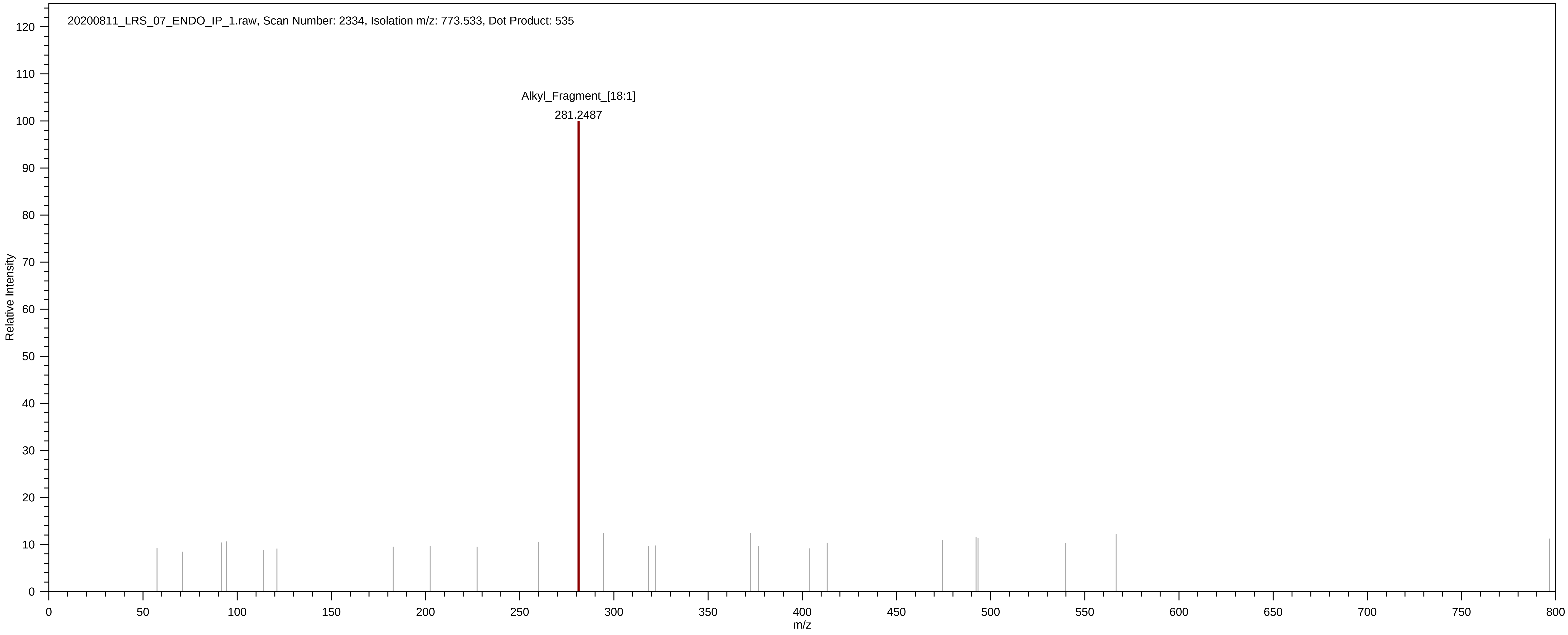

PG 18:1_18:1 [M-H]- | Ret. Time (min): 8.367 | m/z: 773.533

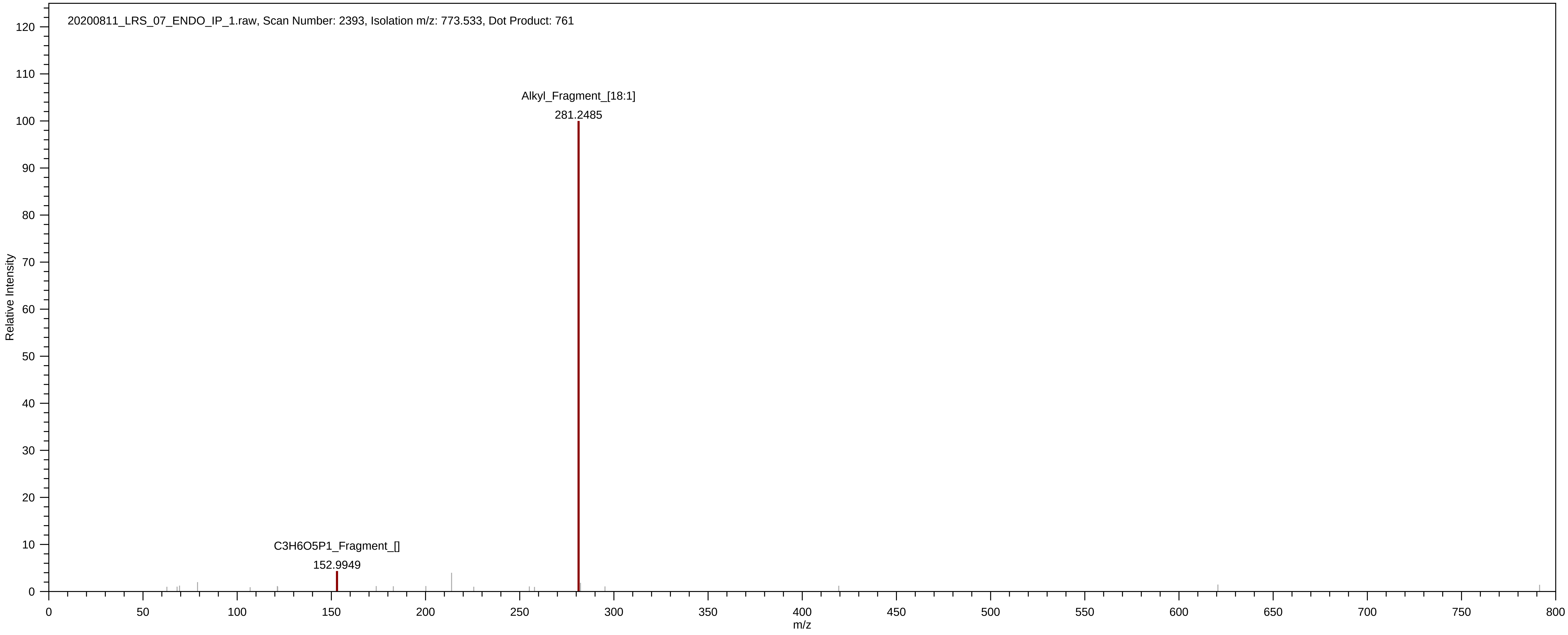

PG 18:1_18:1 [M-H]- | Ret. Time (min): 7.534 | m/z: 773.534

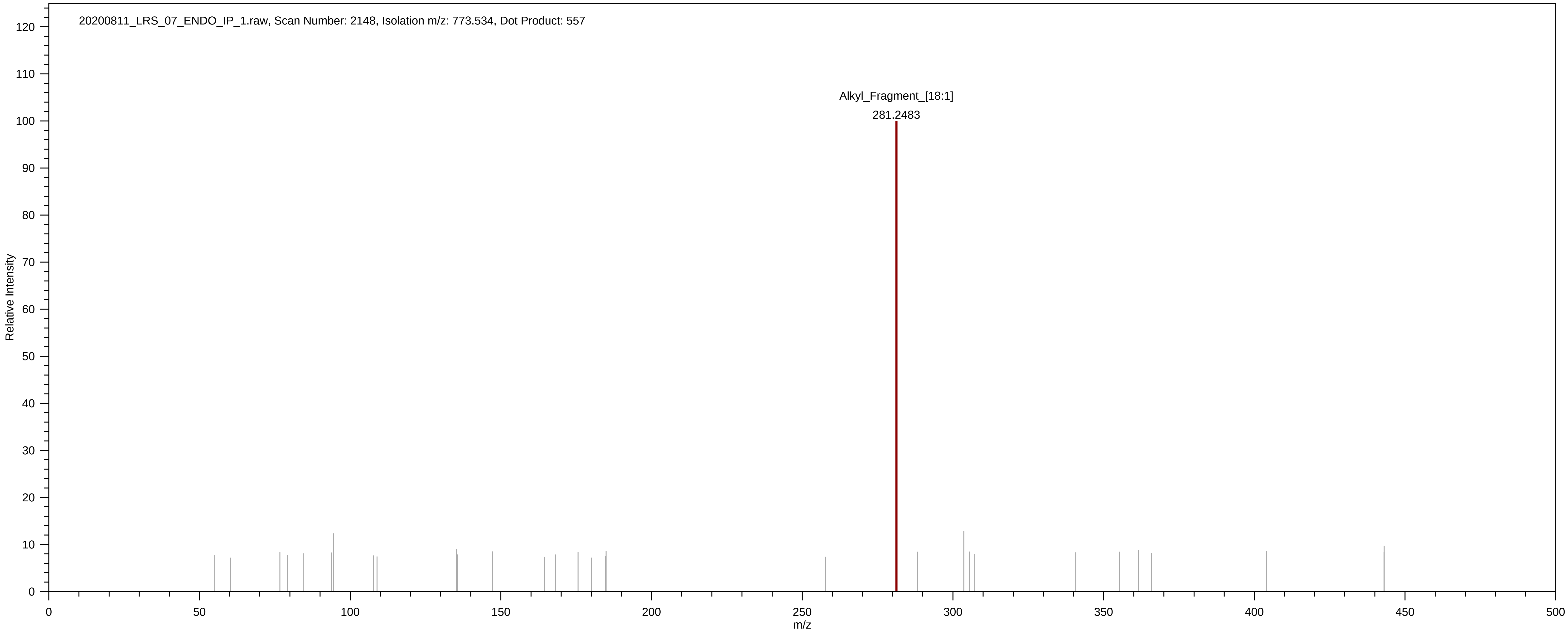

PG 18:1_18:1 [M-H]- | Ret. Time (min): 8.218 | m/z: 773.534

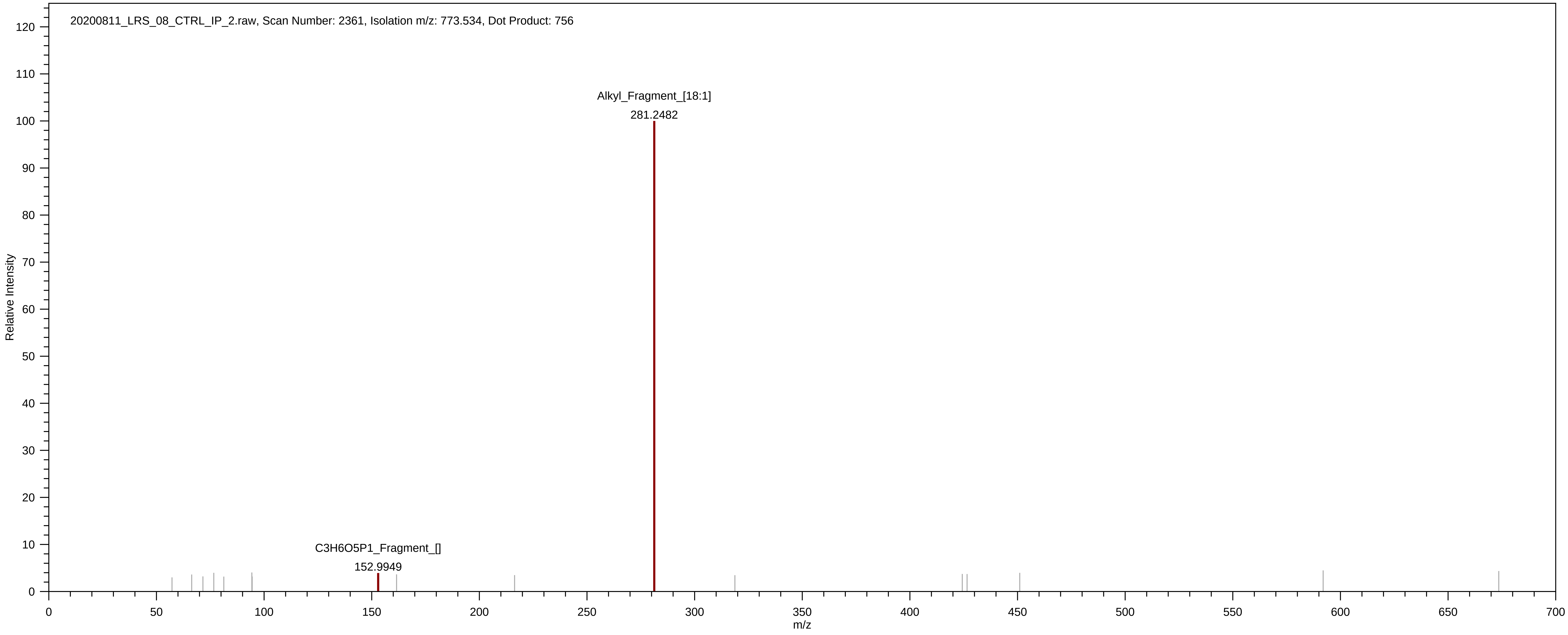

PG 18:1_18:1 [M-H]- | Ret. Time (min): 8.255 | m/z: 773.534

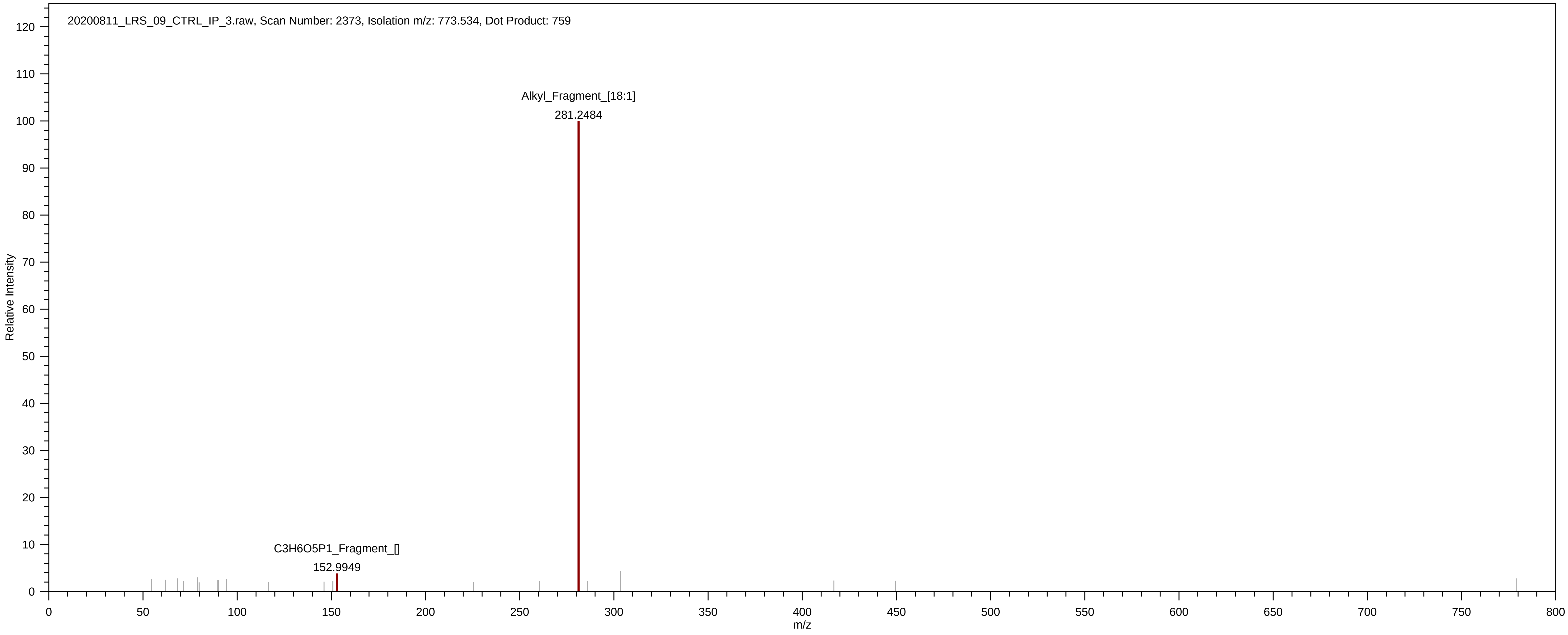

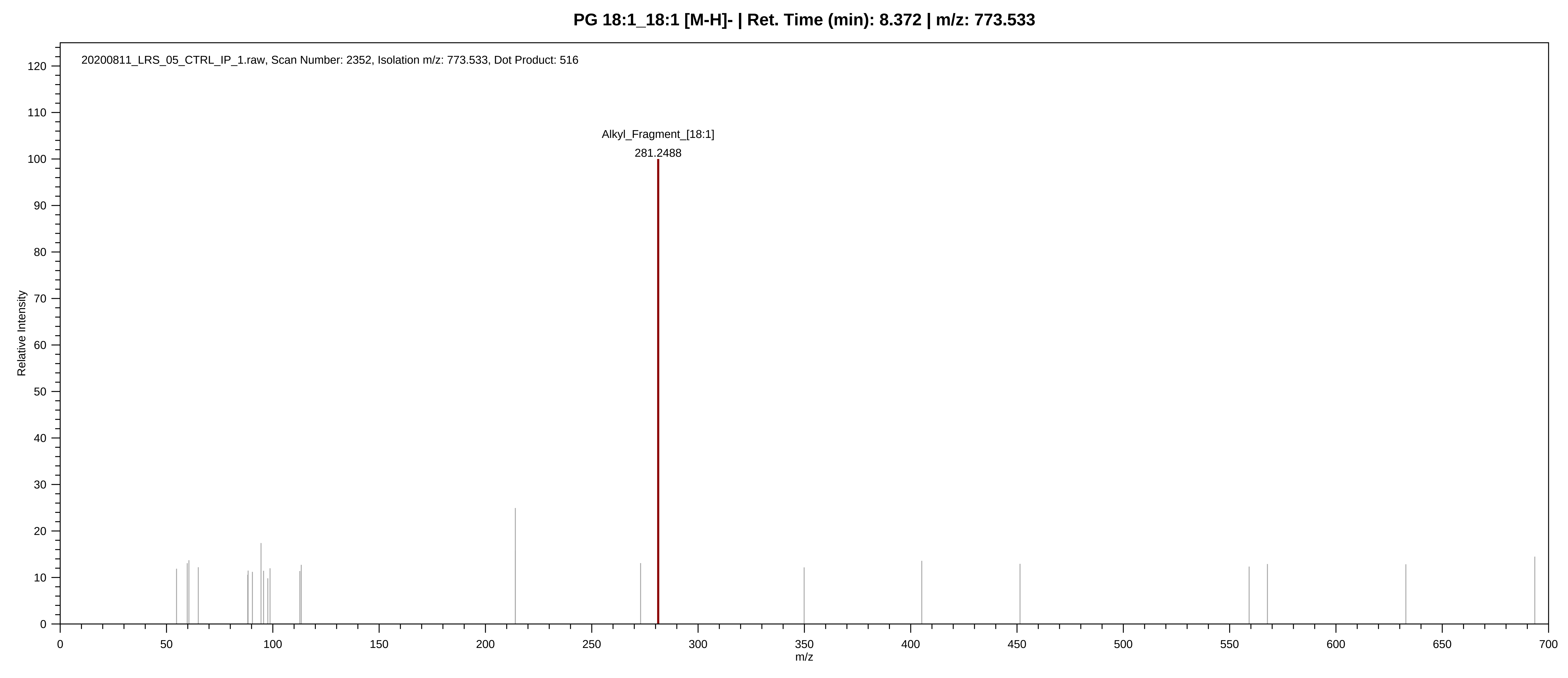

PG 18:1_18:1 [M-H]- | Ret. Time (min): 8.372 | m/z: 773.533
20200811_LRS_05_CTRL_IP_1.raw, Scan Number: 2352, Isolation m/z: 773.533, Dot Product: 516
Alkyl_Fragment_[18:1]
281.2488
Relative Intensity
m/z

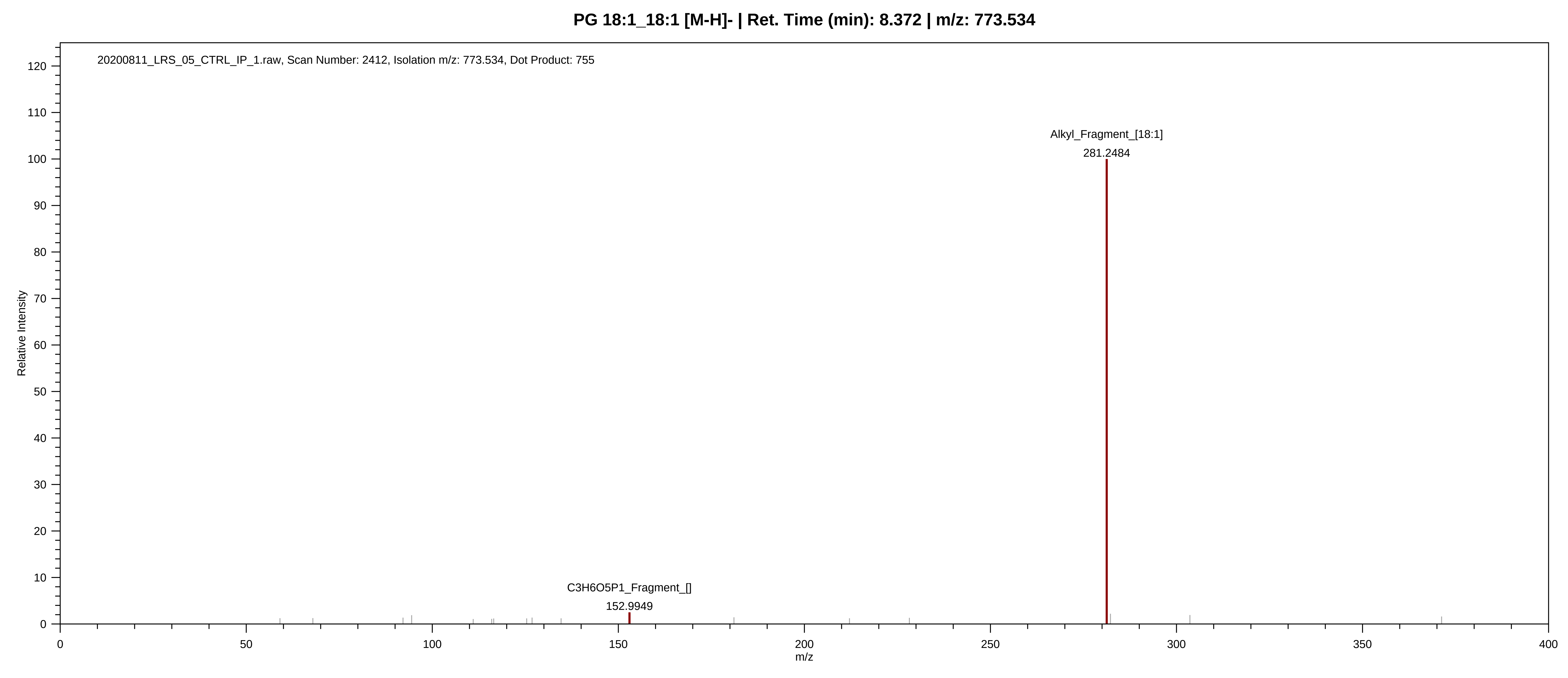

PG 18:1_18:1 [M-H]- | Ret. Time (min): 8.372 | m/z: 773.534
20200811_LRS_05_CTRL_IP_1.raw, Scan Number: 2412, Isolation m/z: 773.534, Dot Product: 755
Alkyl_Fragment_[18:1]
281.2484
C3H6O5P1_Fragment_[]
152.9949
Relative Intensity
m/z

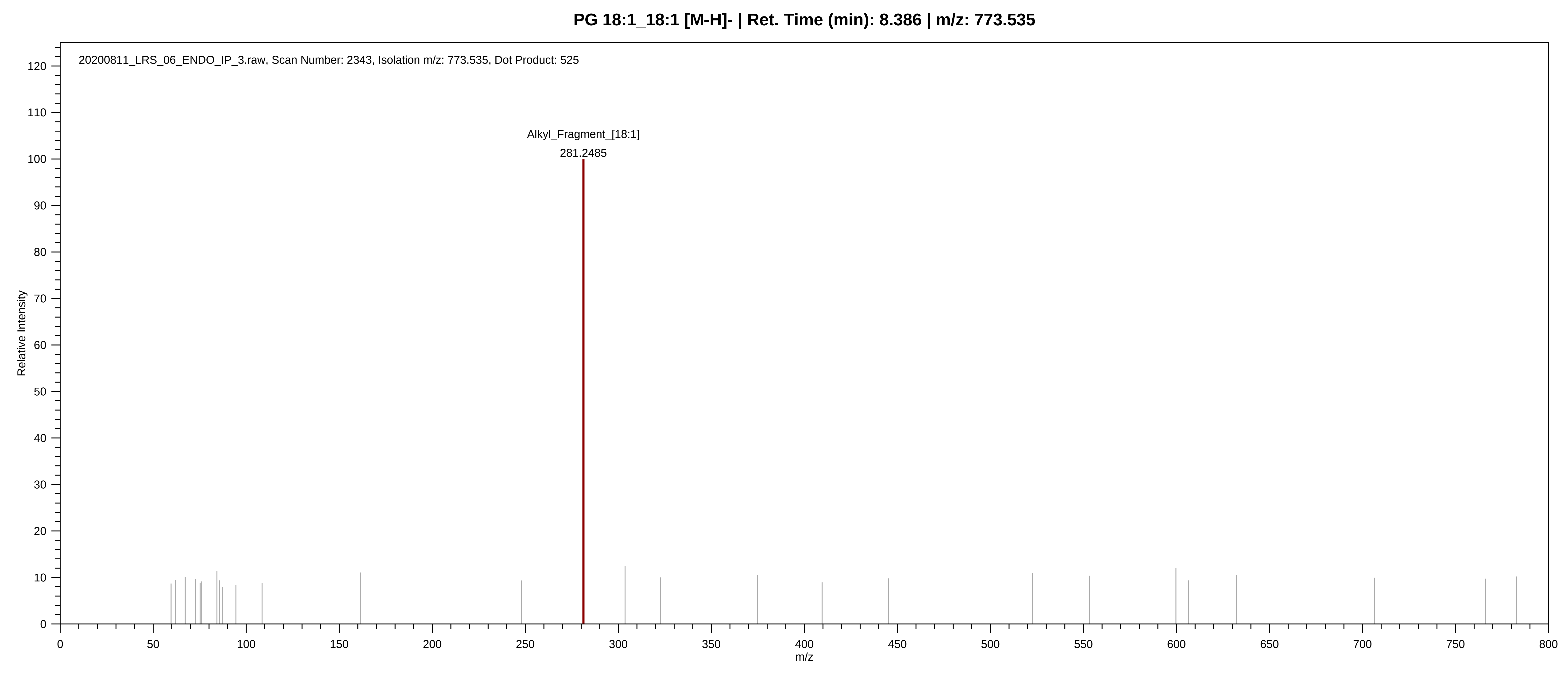

PG 18:1_18:1 [M-H]- | Ret. Time (min): 8.386 | m/z: 773.535
20200811_LRS_06_ENDO_IP_3.raw, Scan Number: 2343, Isolation m/z: 773.535, Dot Product: 525
Alkyl_Fragment_[18:1]
281.2485
Relative Intensity
m/z

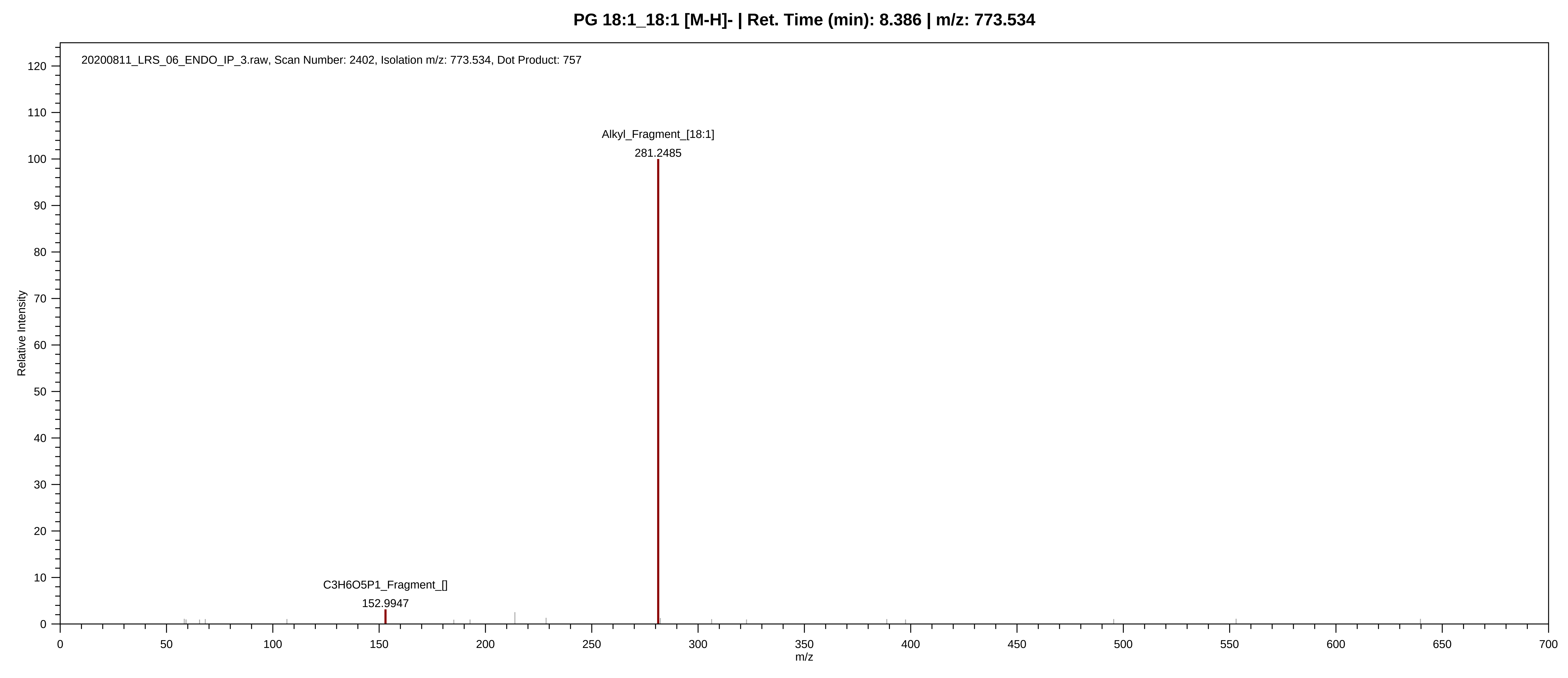
PG 18:1_18:1 [M-H]- | Ret. Time (min): 8.386 | m/z: 773.534
20200811_LRS_06_ENDO_IP_3.raw, Scan Number: 2402, Isolation m/z: 773.534, Dot Product: 757
Alkyl_Fragment_[18:1]
281.2485
C3H6O5P1_Fragment_[]
152.9947
Relative Intensity
m/z

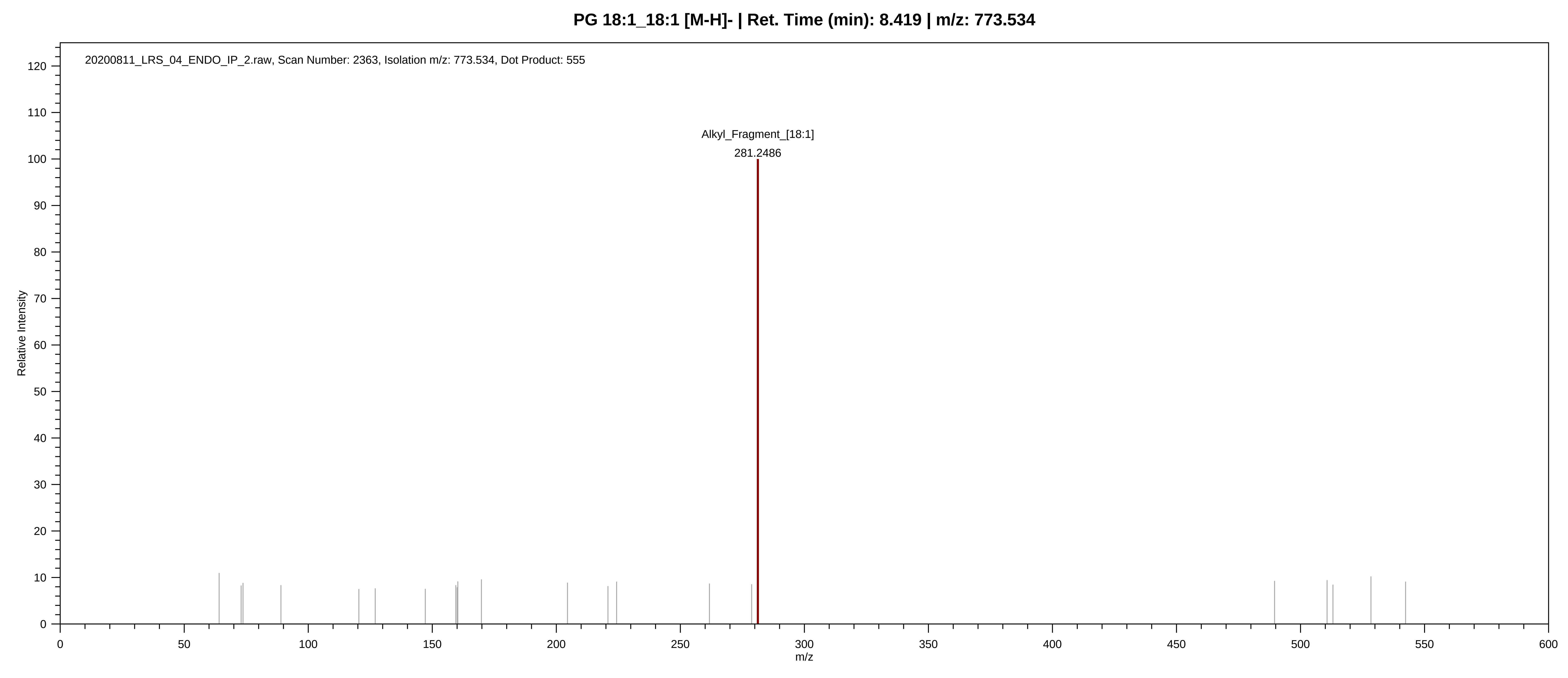

PG 18:1_18:1 [M-H]- | Ret. Time (min): 8.419 | m/z: 773.534
20200811_LRS_04_ENDO_IP_2.raw, Scan Number: 2363, Isolation m/z: 773.534, Dot Product: 555
Alkyl_Fragment_[18:1]
281.2486
Relative Intensity
m/z

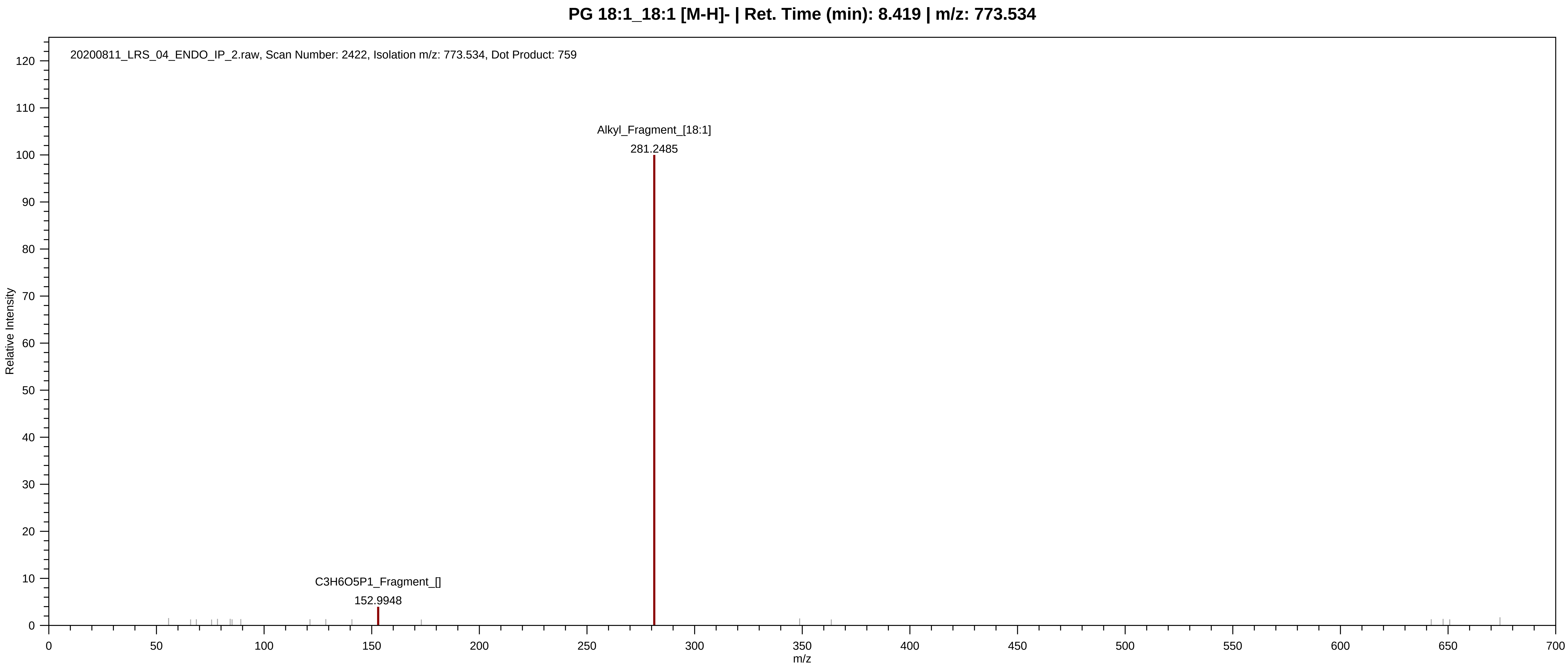

PG 18:1_18:1 [M-H]- | Ret. Time (min): 8.419 | m/z: 773.534
20200811_LRS_04_ENDO_IP_2.raw, Scan Number: 2422, Isolation m/z: 773.534, Dot Product: 759
Alkyl_Fragment_[18:1]
281.2485
C3H6O5P1_Fragment_[]
152.9948
Relative Intensity
m/z

PG 18:1_18:1 [M-H]- | Ret. Time (min): 8.421 | m/z: 773.534

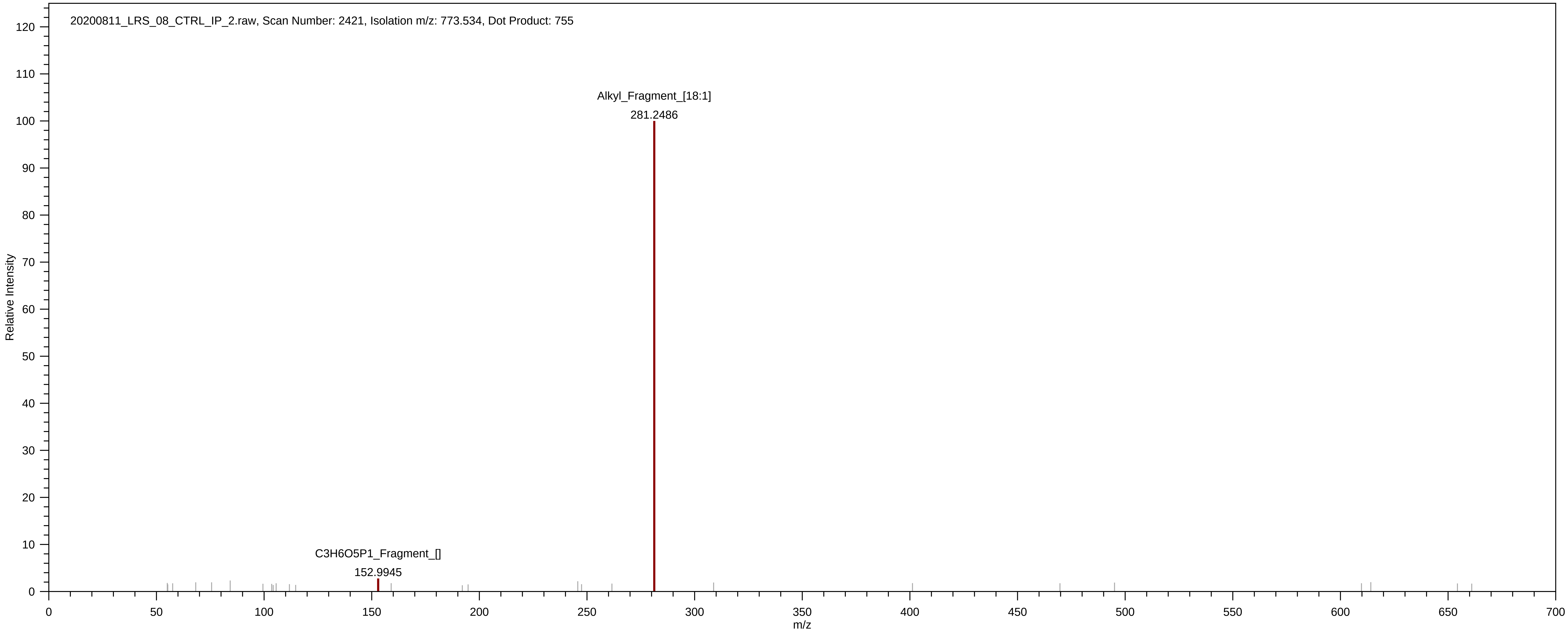

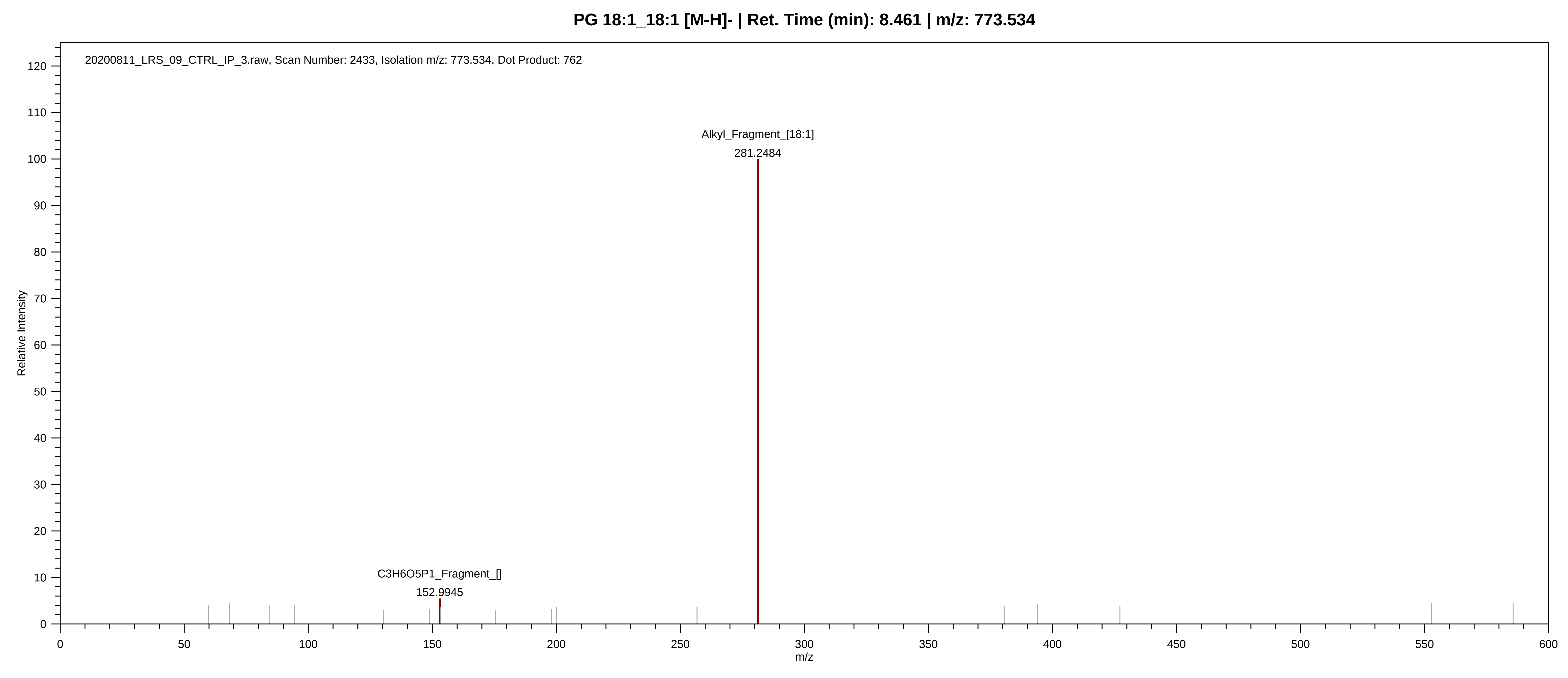
PG 18:1_18:1 [M-H]- | Ret. Time (min): 8.461 | m/z: 773.534
20200811_LRS_09_CTRL_IP_3.raw, Scan Number: 2433, Isolation m/z: 773.534, Dot Product: 762
Alkyl_Fragment_[18:1]
281.2484
C3H6O5P1_Fragment_[]
152.9945
Relative Intensity
m/z

PG 18:1_18:1 [M-H]- | Ret. Time (min): 7.53 | m/z: 773.535

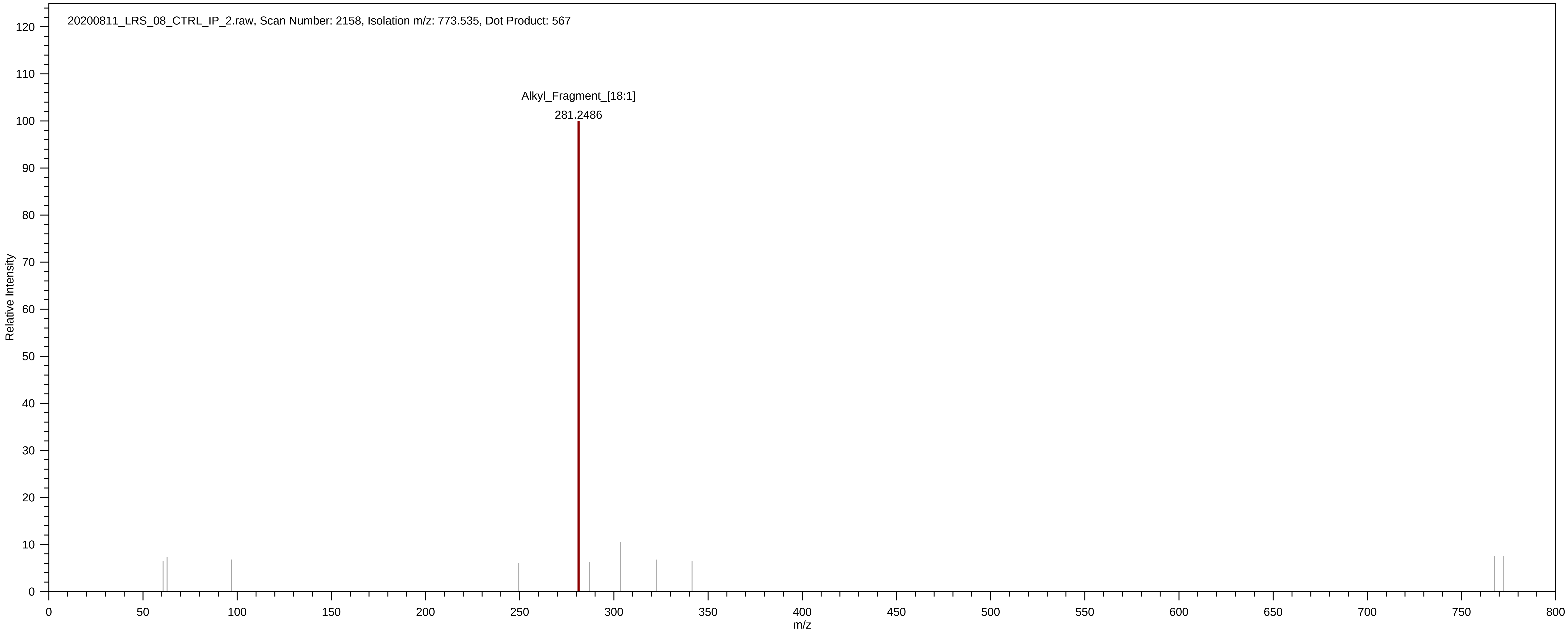

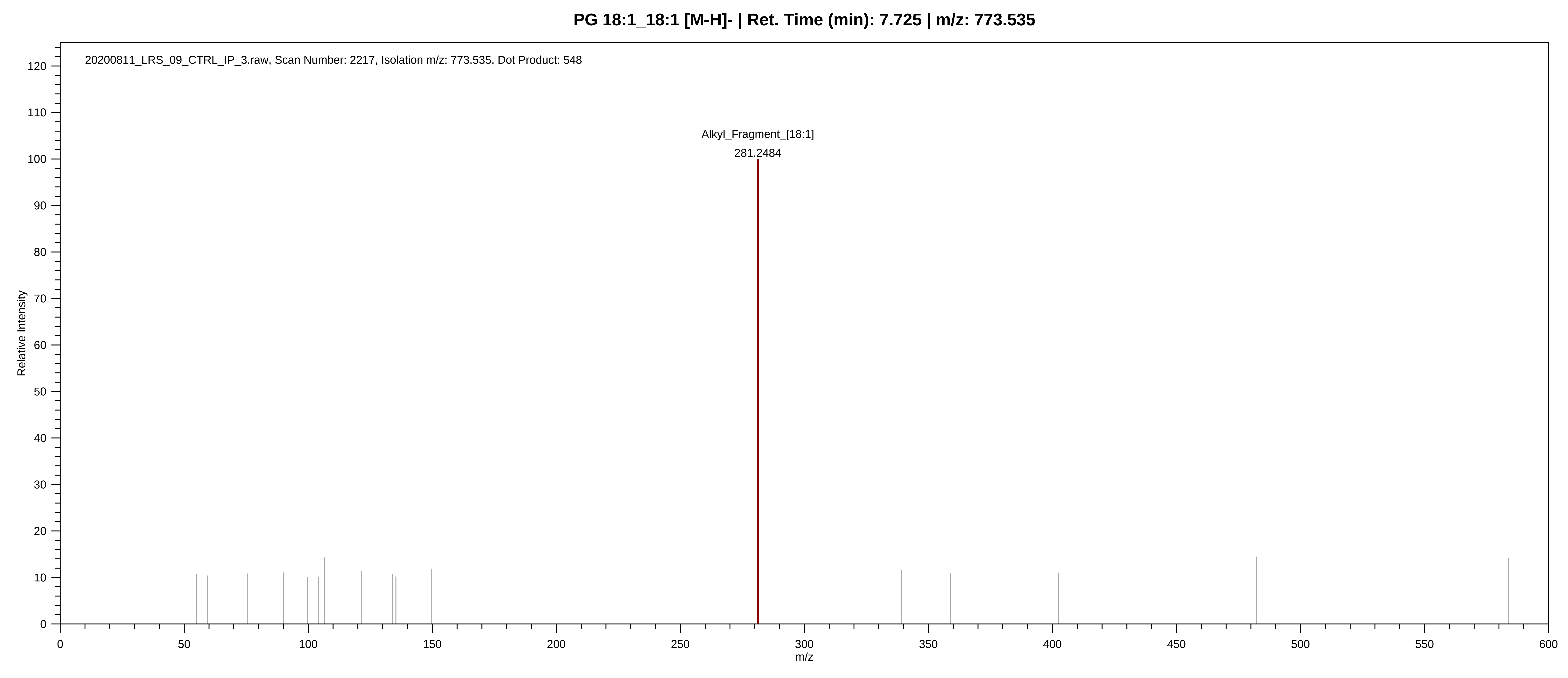

PG 18:1_18:1 [M-H]- | Ret. Time (min): 7.725 | m/z: 773.535
20200811_LRS_09_CTRL_IP_3.raw, Scan Number: 2217, Isolation m/z: 773.535, Dot Product: 548
Alkyl_Fragment_[18:1]
281.2484
Relative Intensity
m/z

PG 18:1_18:1 [M-H]- | Ret. Time (min): 7.522 | m/z: 773.536

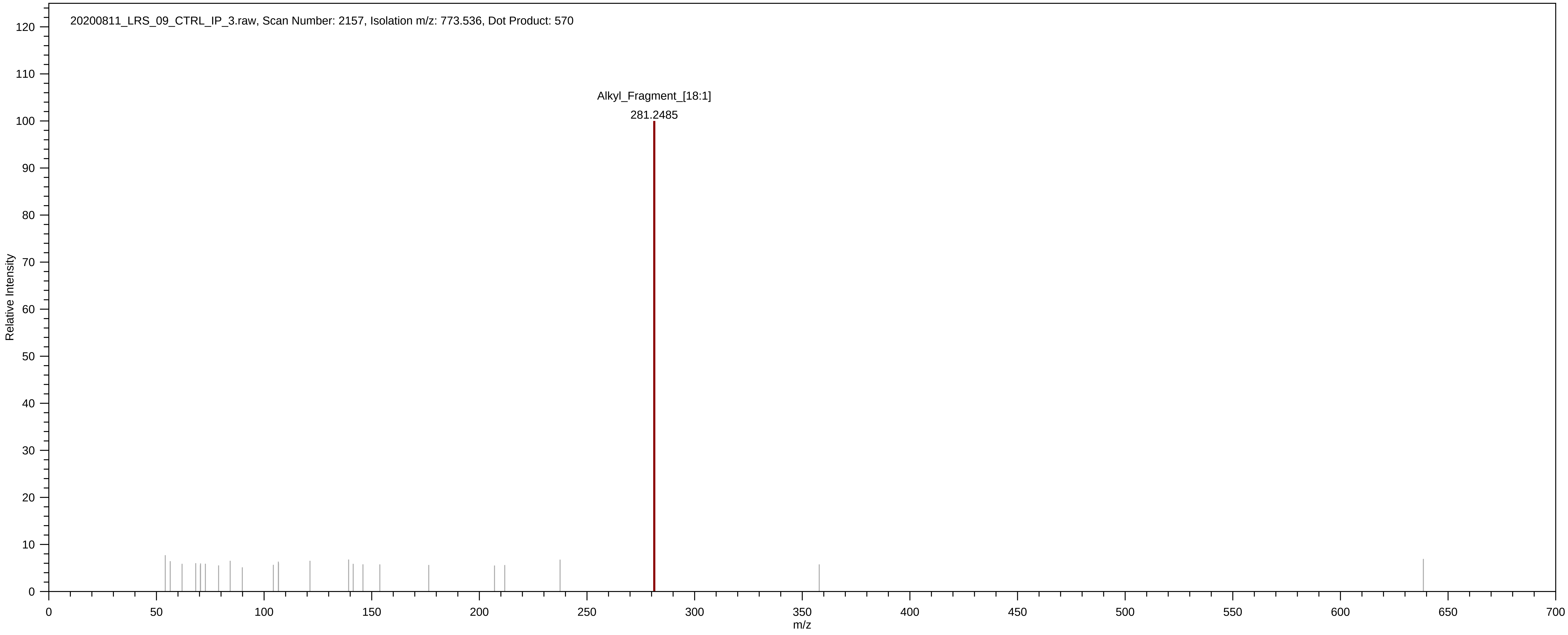

PG 18:1_20:3 [M-H]- | Ret. Time (min): 7.939 | m/z: 797.533

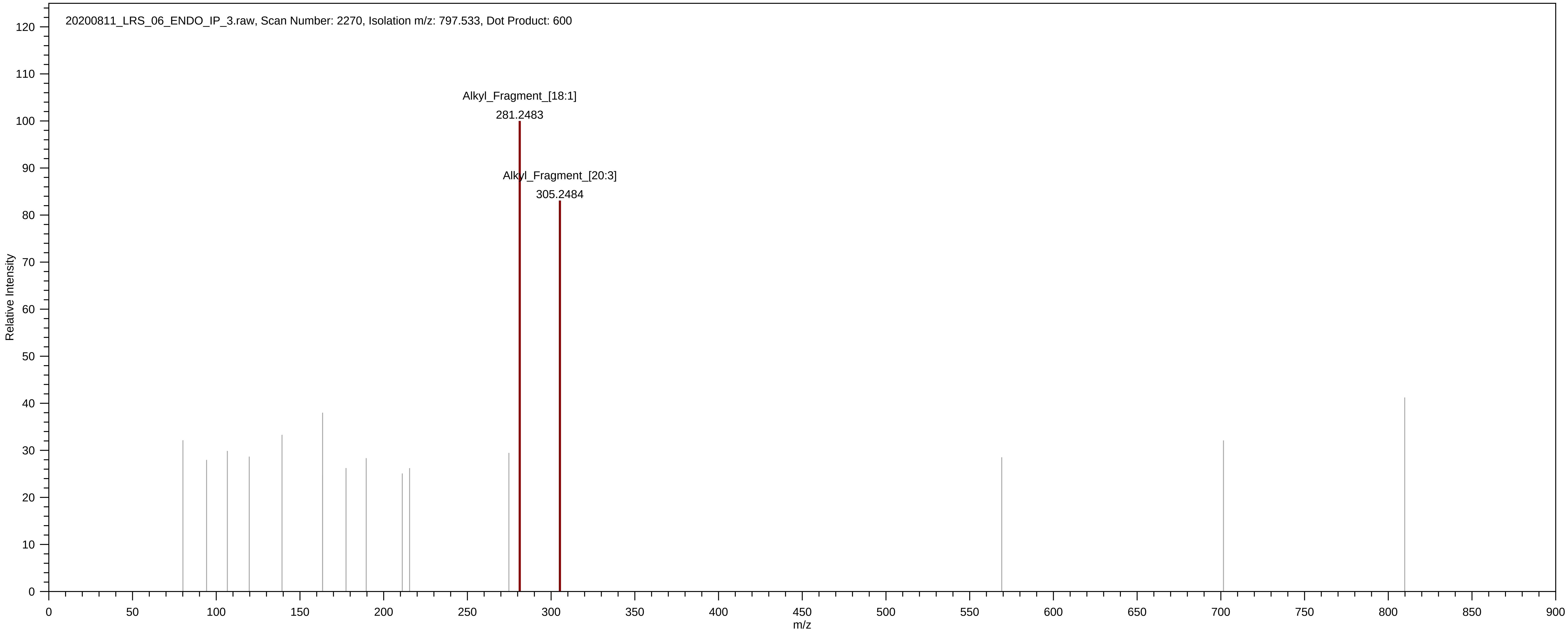

PG 18:1_20:3 [M-H]- | Ret. Time (min): 7.907 | m/z: 797.534

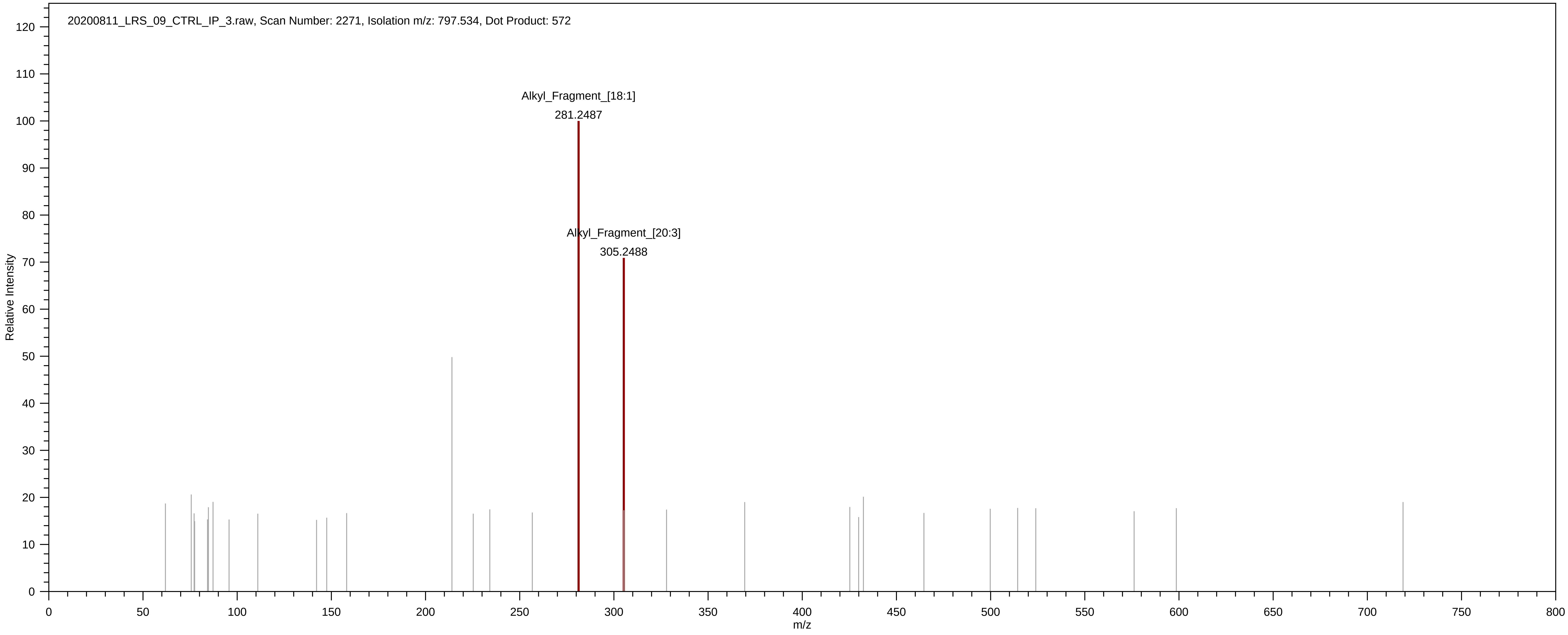

PG 32:1 [M-H]- | Ret. Time (min): 7.751 | m/z: 719.486

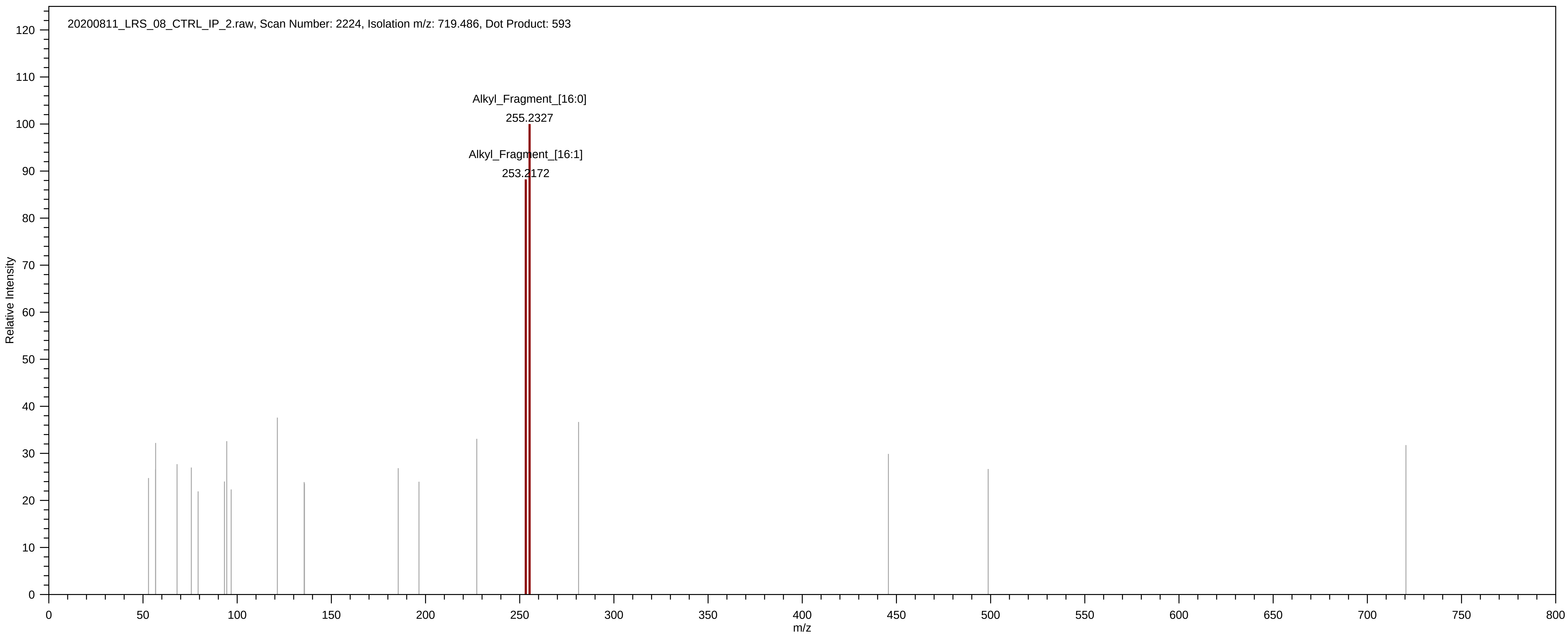

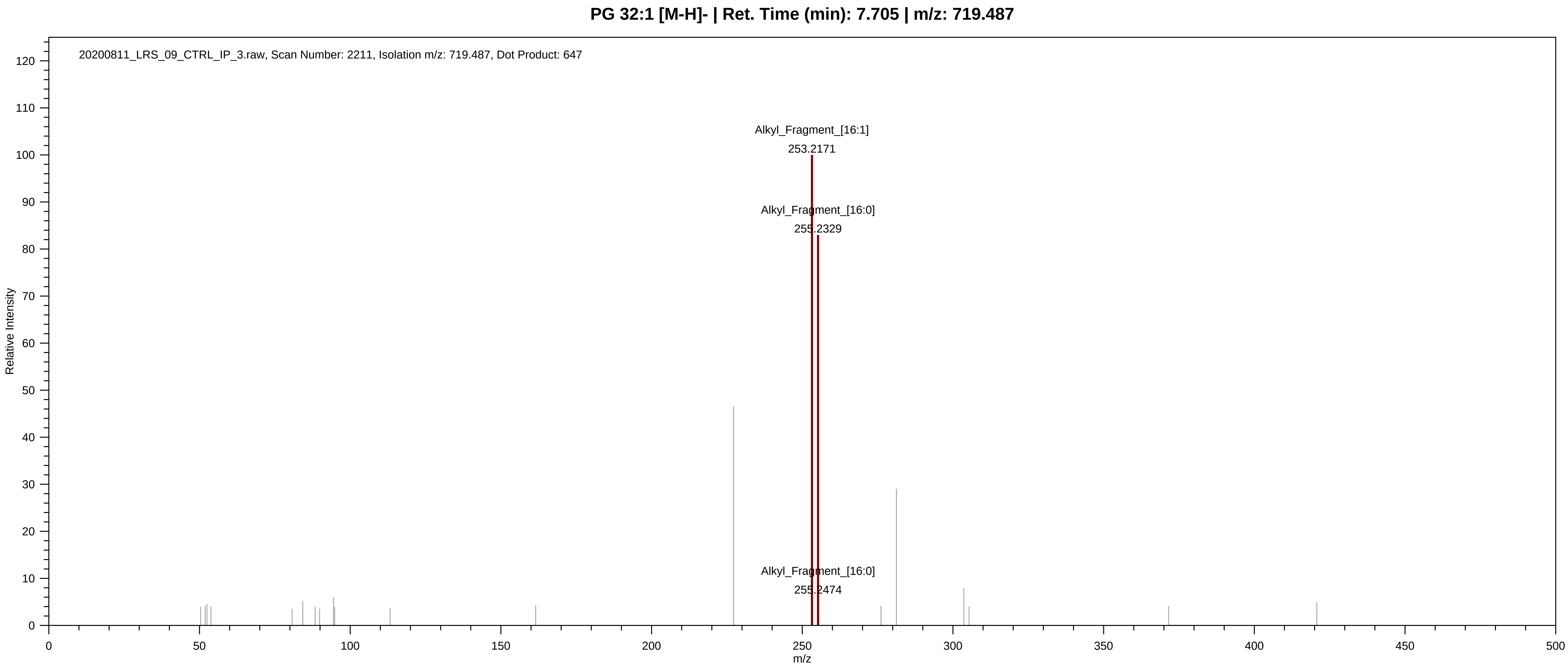

PG 32:1 [M-H]- | Ret. Time (min): 7.705 | m/z: 719.487
20200811_LRS_09_CTRL_IP_3.raw, Scan Number: 2211, Isolation m/z: 719.487, Dot Product: 647
Alkyl_Fragment_[16:1]
253.2171
Alkyl_Fragment_[16:0]
255.2329
Alkyl_Fragment_[16:0]
255.2474
Relative Intensity
m/z

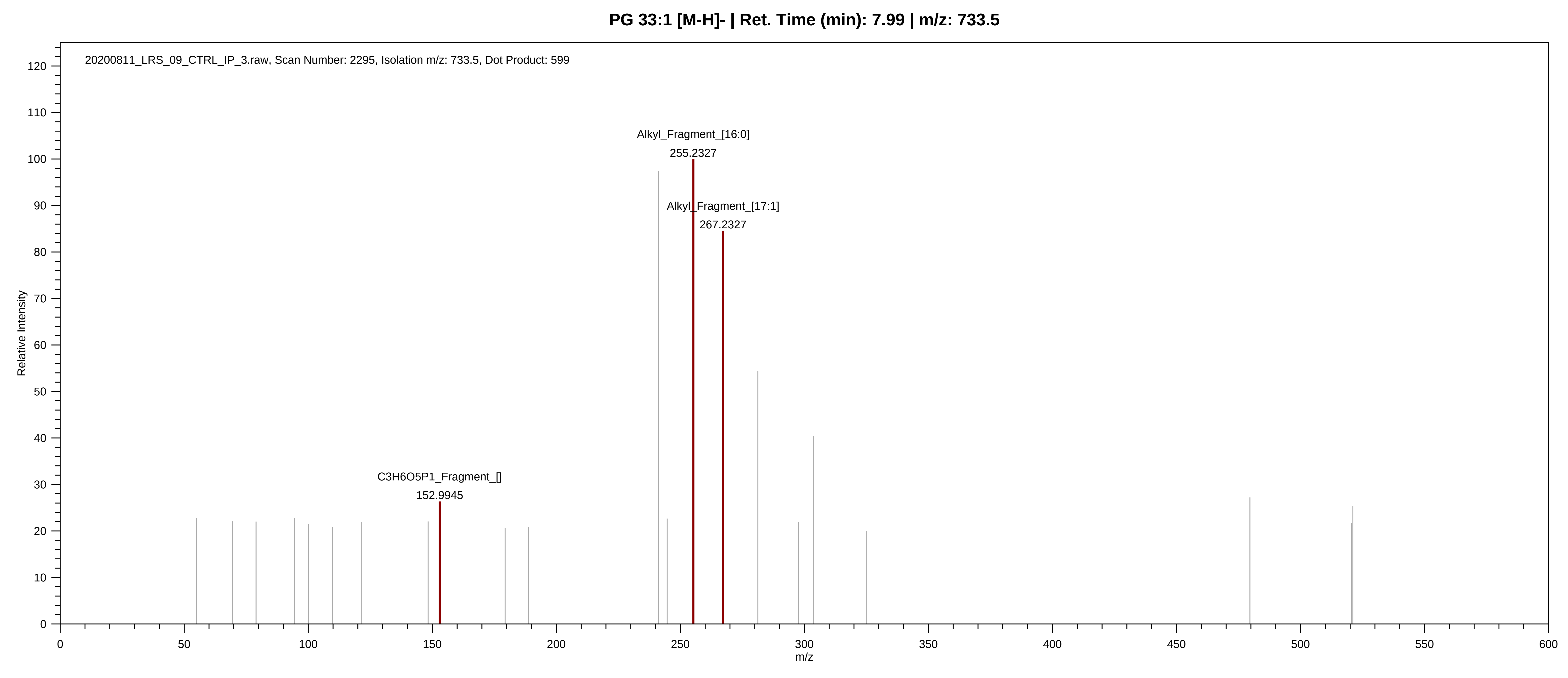
PG 33:1 [M-H]- | Ret. Time (min): 7.99 | m/z: 733.5
20200811_LRS_09_CTRL_IP_3.raw, Scan Number: 2295, Isolation m/z: 733.5, Dot Product: 599
Alkyl_Fragment_[16:0]
255.2327
Alkyl_Fragment_[17:1]
267.2327
C3H6O5P1_Fragment_[]
152.9945
Relative Intensity
m/z

PI 16:0_16:0 [M-H]- | Ret. Time (min): 8.635 | m/z: 809.52

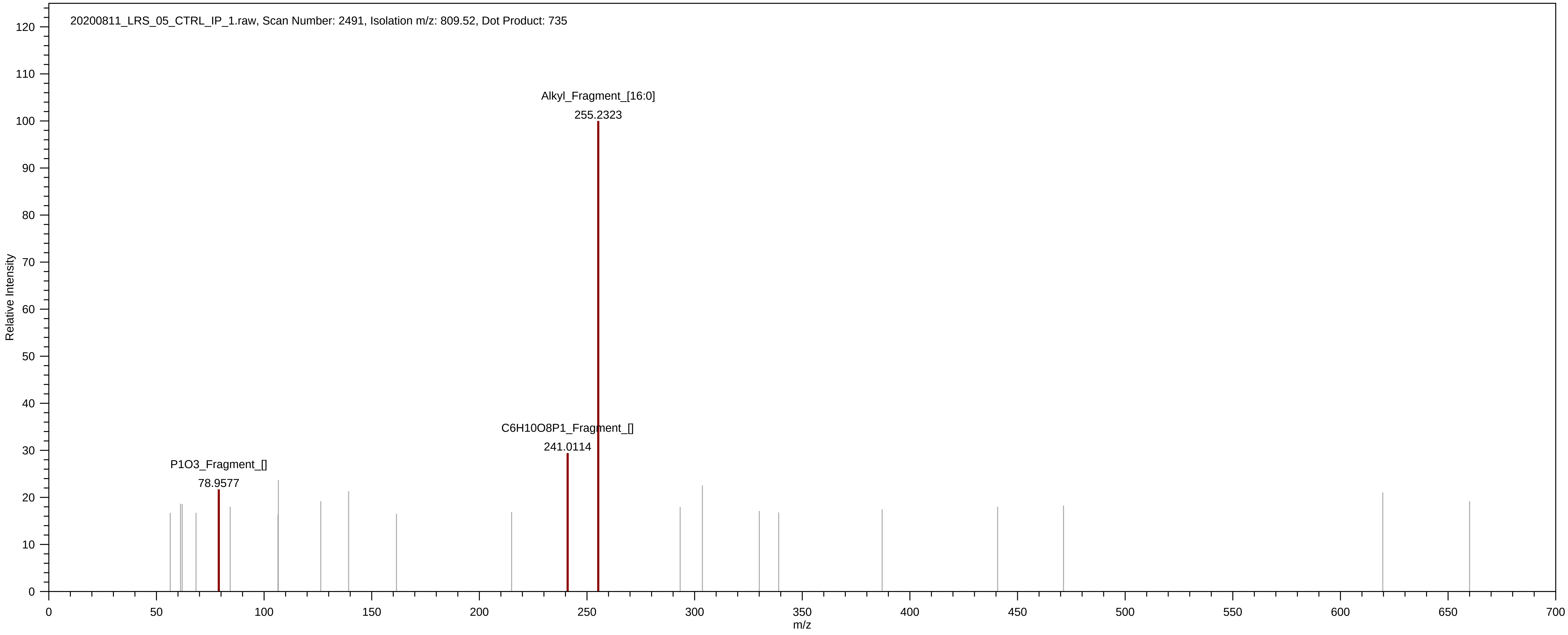

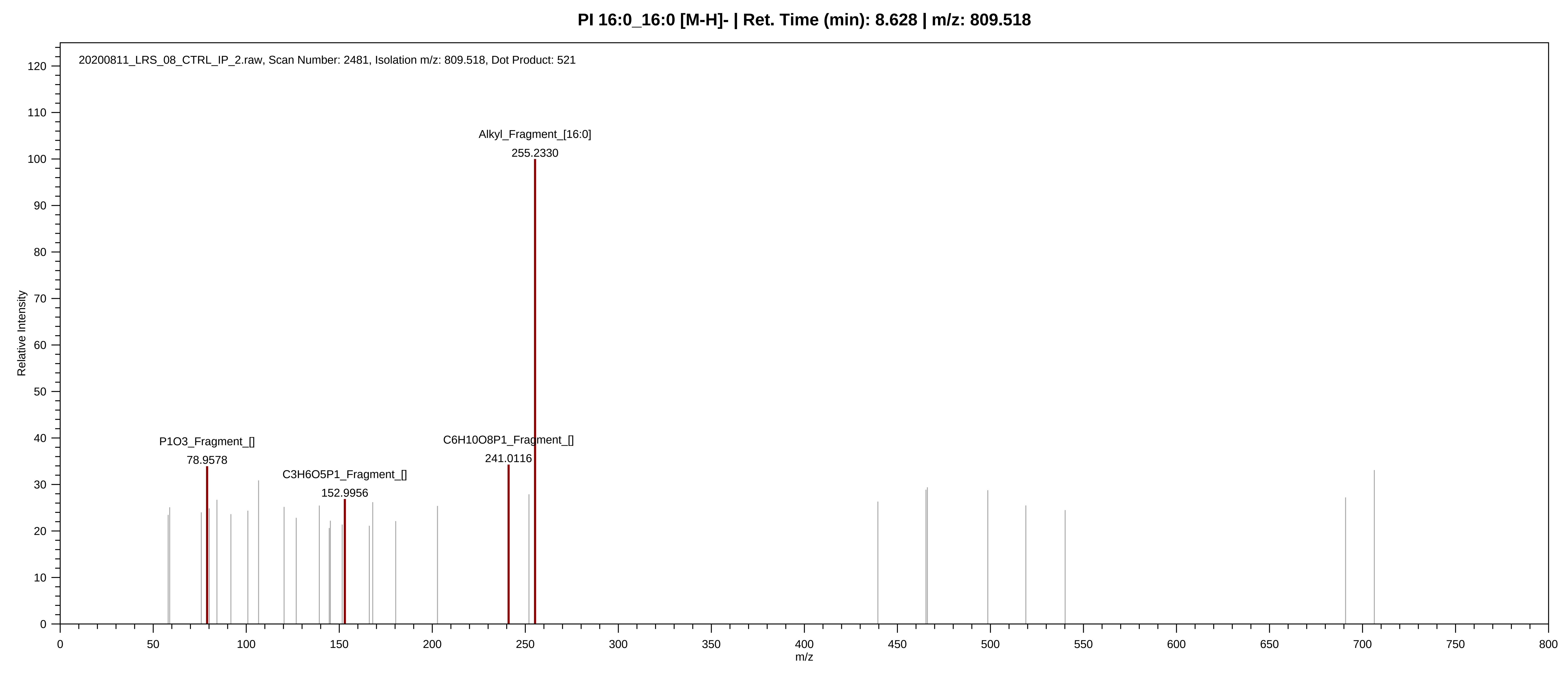

PI 16:0_16:0 [M-H]- | Ret. Time (min): 8.628 | m/z: 809.518
20200811_LRS_08_CTRL_IP_2.raw, Scan Number: 2481, Isolation m/z: 809.518, Dot Product: 521
Alkyl_Fragment_[16:0]
255.2330
P1O3_Fragment_[]
78.9578
C3H6O5P1_Fragment_[]
152.9956
C6H10O8P1_Fragment_[]
241.0116
Relative Intensity
m/z

PI 16:0_16:0 [M-H]- | Ret. Time (min): 8.636 | m/z: 809.519

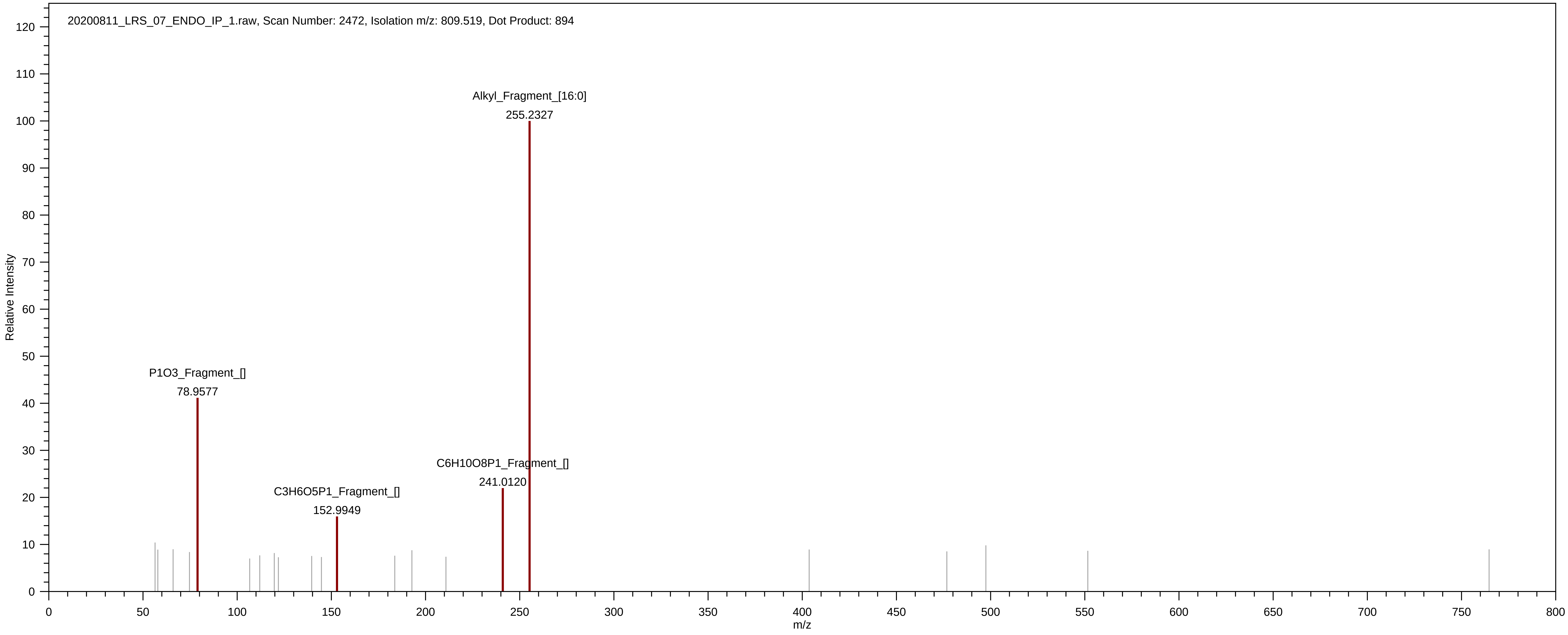

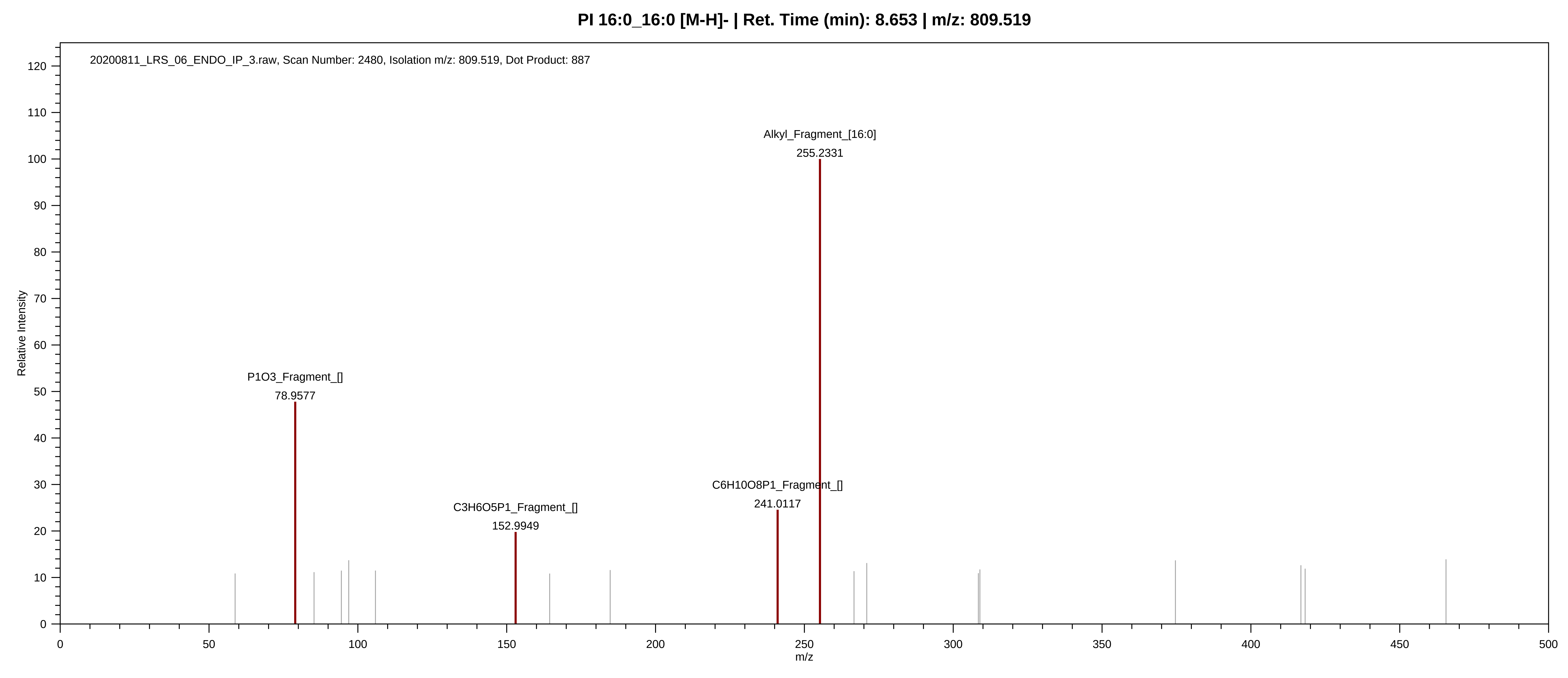

PI 16:0_16:0 [M-H]- | Ret. Time (min): 8.653 | m/z: 809.519
20200811_LRS_06_ENDO_IP_3.raw, Scan Number: 2480, Isolation m/z: 809.519, Dot Product: 887
Alkyl_Fragment_[16:0]
255.2331
P1O3_Fragment_[]
78.9577
C3H6O5P1_Fragment_[]
152.9949
C6H10O8P1_Fragment_[]
241.0117
Relative Intensity
m/z

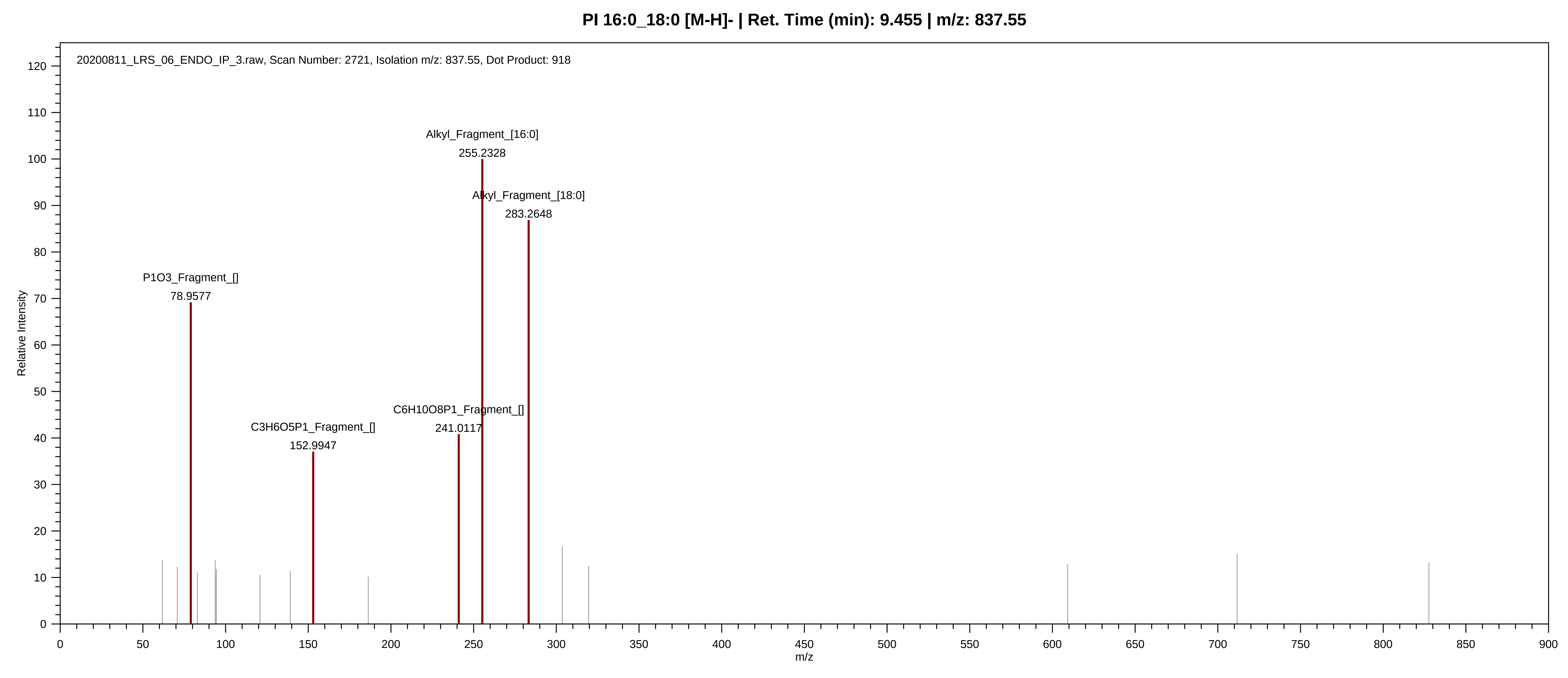
PI 16:0_18:0 [M-H]- | Ret. Time (min): 9.455 | m/z: 837.55
20200811_LRS_06_ENDO_IP_3.raw, Scan Number: 2721, Isolation m/z: 837.55, Dot Product: 918
Alkyl_Fragment_[16:0]
255.2328
Alkyl_Fragment_[18:0]
283.2648
P1O3_Fragment_[]
78.9577
C6H10O8P1_Fragment_[]
241.0117
C3H6O5P1_Fragment_[]
152.9947
Relative Intensity
m/z

PI 16:0_18:1 [M-H]- | Ret. Time (min): 8.634 | m/z: 835.534

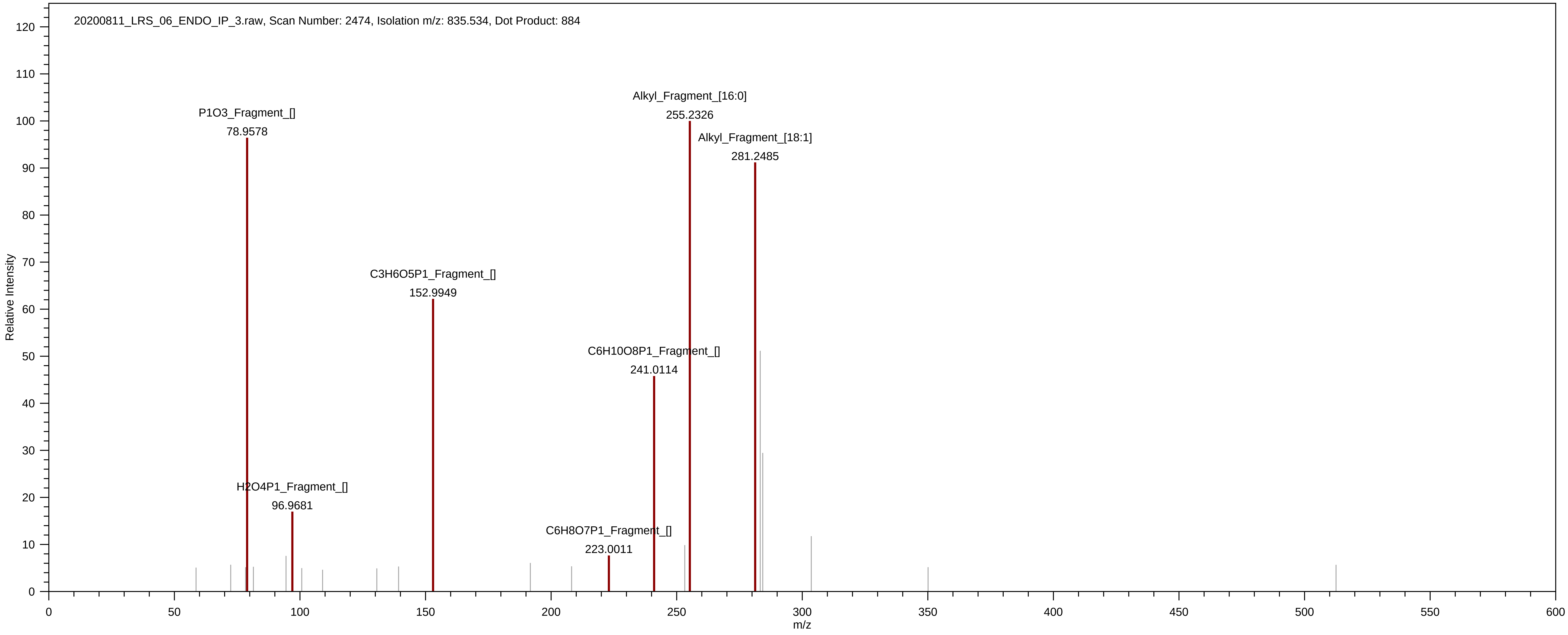

PI 16:0_18:1 [M-H]- | Ret. Time (min): 7.683 | m/z: 835.535

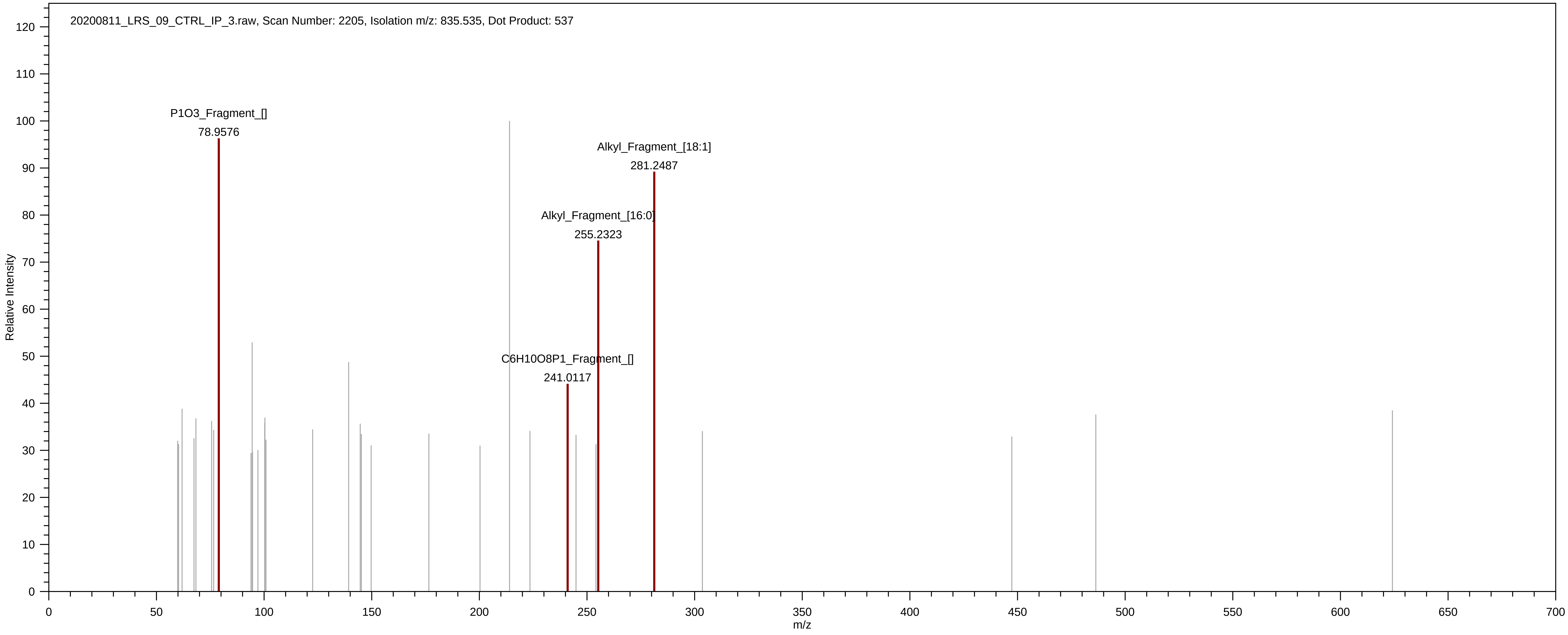

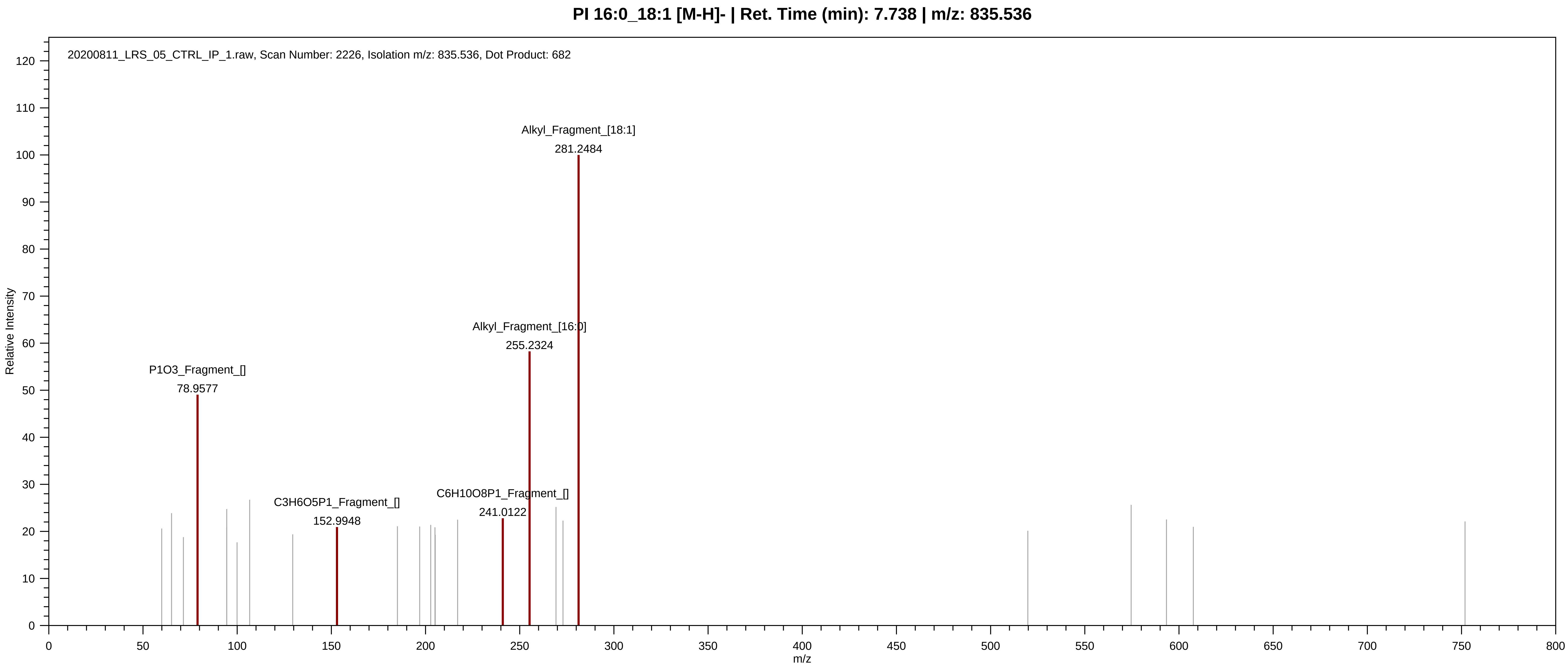
PI 16:0_18:1 [M-H]- | Ret. Time (min): 7.738 | m/z: 835.536
20200811_LRS_05_CTRL_IP_1.raw, Scan Number: 2226, Isolation m/z: 835.536, Dot Product: 682
Alkyl_Fragment_[18:1]
281.2484
Alkyl_Fragment_[16:0]
255.2324
P1O3_Fragment_[]
78.9577
C3H6O5P1_Fragment_[]
152.9948
C6H10O8P1_Fragment_[]
241.0122
Relative Intensity
m/z

PI 16:0_18:1 [M-H]- | Ret. Time (min): 7.725 | m/z: 835.537

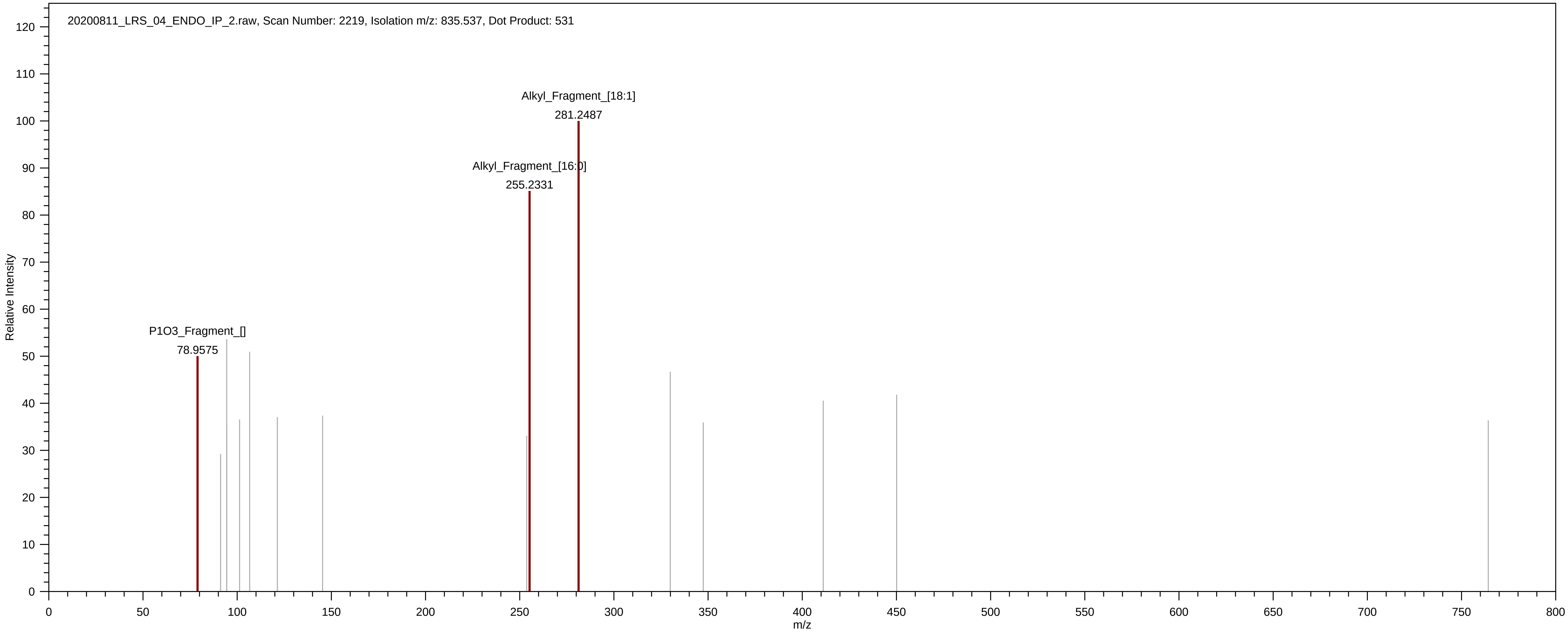

PI 17:0_18:1 [M-H]- | Ret. Time (min): 9.027 | m/z: 849.549

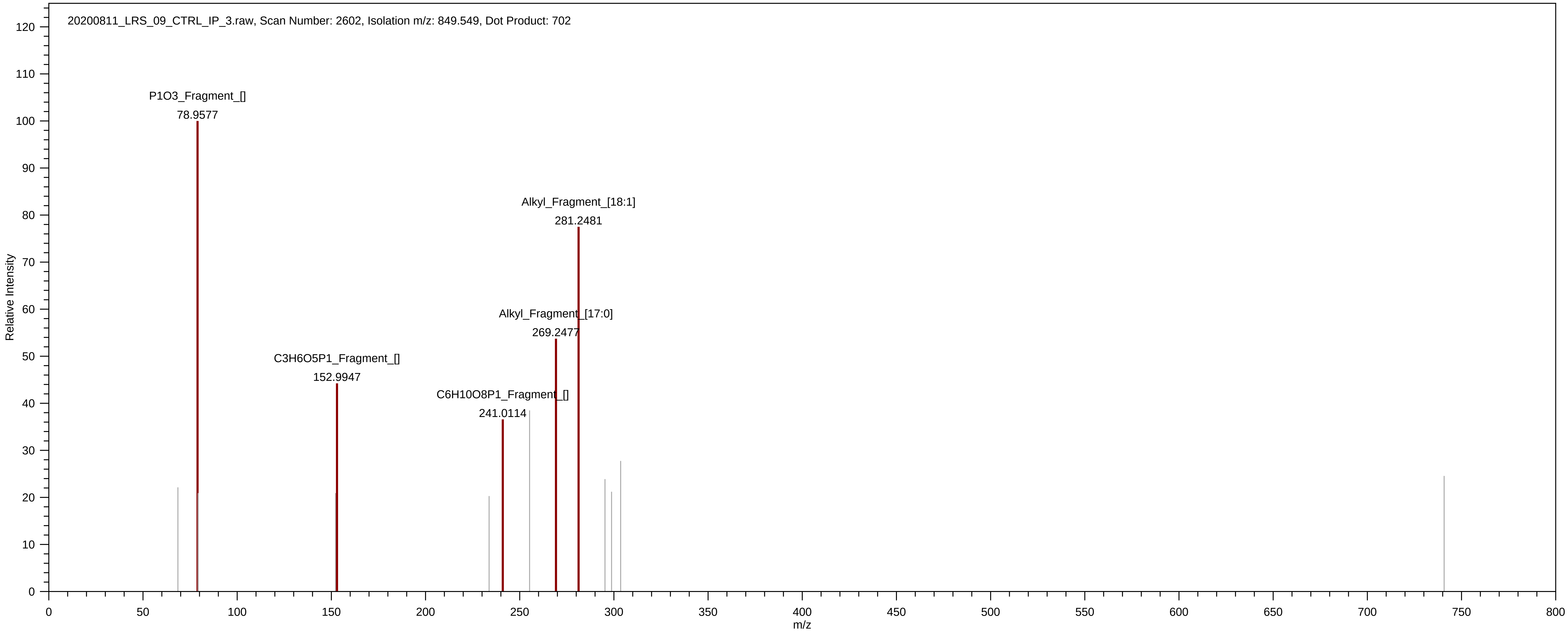

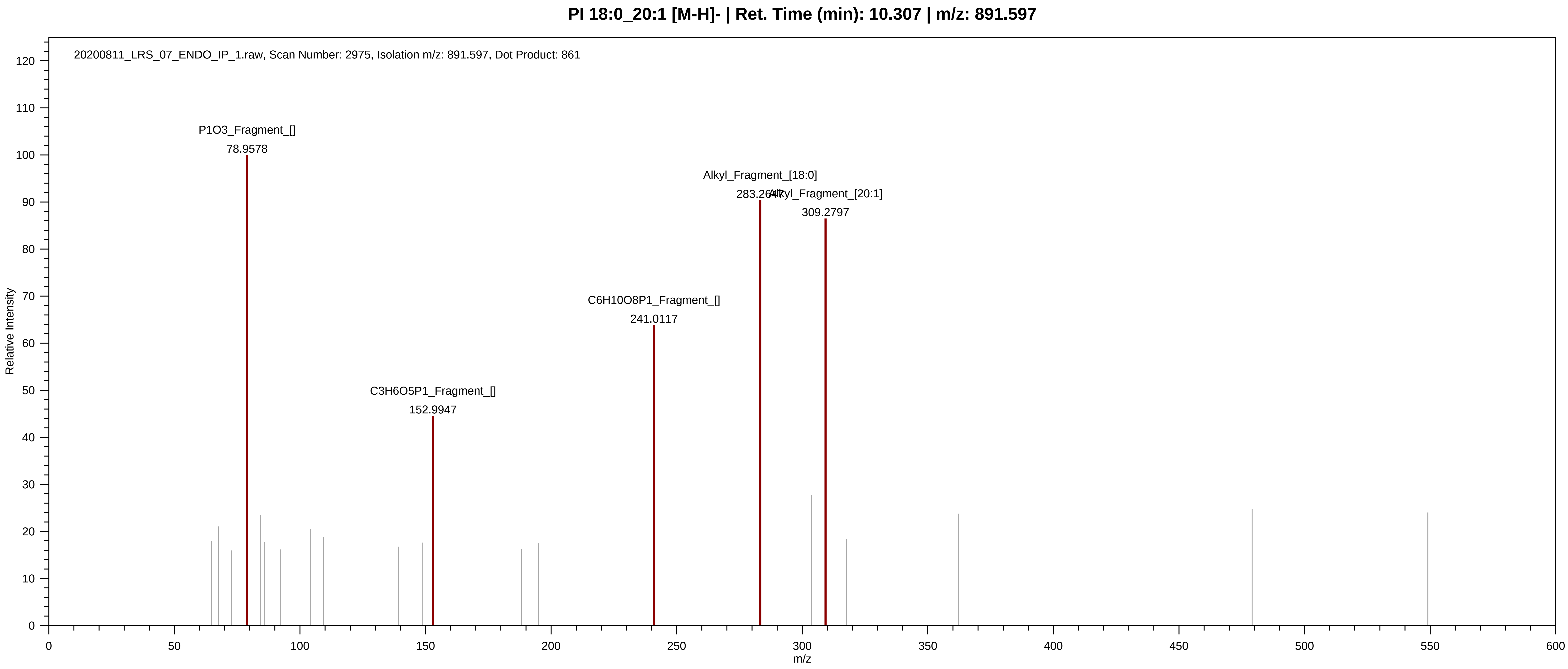
PI 18:0_20:1 [M-H]- | Ret. Time (min): 10.307 | m/z: 891.597
20200811_LRS_07_ENDO_IP_1.raw, Scan Number: 2975, Isolation m/z: 891.597, Dot Product: 861
P1O3_Fragment_[]
78.9578
C3H6O5P1_Fragment_[]
152.9947
C6H10O8P1_Fragment_[]
241.0117
Alkyl_Fragment_[18:0]
283.2647
Alkyl_Fragment_[20:1]
309.2797
Relative Intensity
m/z

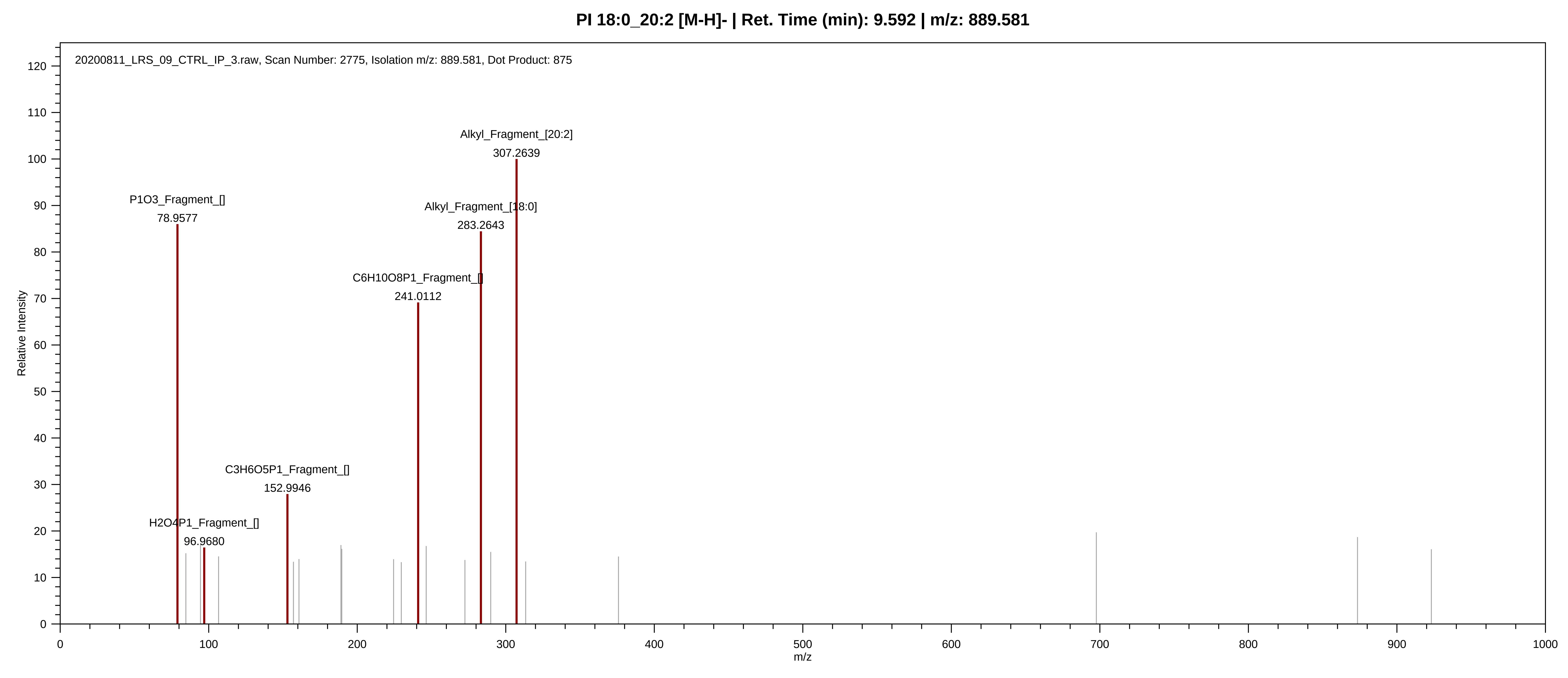

PI 18:0_20:2 [M-H]- | Ret. Time (min): 9.592 | m/z: 889.581
20200811_LRS_09_CTRL_IP_3.raw, Scan Number: 2775, Isolation m/z: 889.581, Dot Product: 875
Alkyl_Fragment_[20:2]
307.2639
P1O3_Fragment_[]
78.9577
Alkyl_Fragment_[18:0]
283.2643
C6H10O8P1_Fragment_[]
241.0112
C3H6O5P1_Fragment_[]
152.9946
H2O4P1_Fragment_[]
96.9680
Relative Intensity
m/z

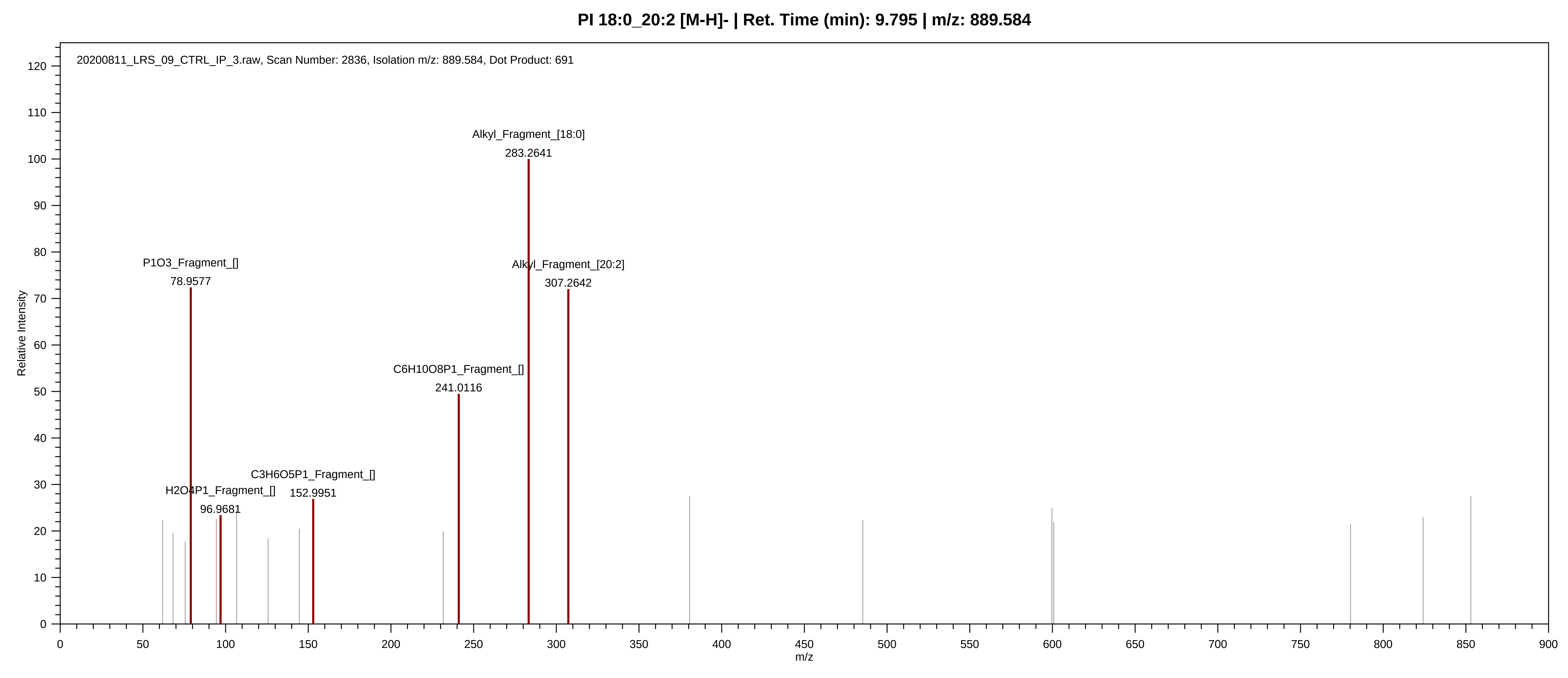
PI 18:0_20:2 [M-H]- | Ret. Time (min): 9.795 | m/z: 889.584
20200811_LRS_09_CTRL_IP_3.raw, Scan Number: 2836, Isolation m/z: 889.584, Dot Product: 691
Alkyl_Fragment_[18:0]
283.2641
P1O3_Fragment_[]
78.9577
Alkyl_Fragment_[20:2]
307.2642
C6H10O8P1_Fragment_[]
241.0116
C3H6O5P1_Fragment_[]
152.9951
H2O4P1_Fragment_[]
96.9681
Relative Intensity
m/z
0
10
20
30
40
50
60
70
80
90
100
110
120
0
50
100
150
200
250
300
350
400
450
500
550
600
650
700
750
800
850
900

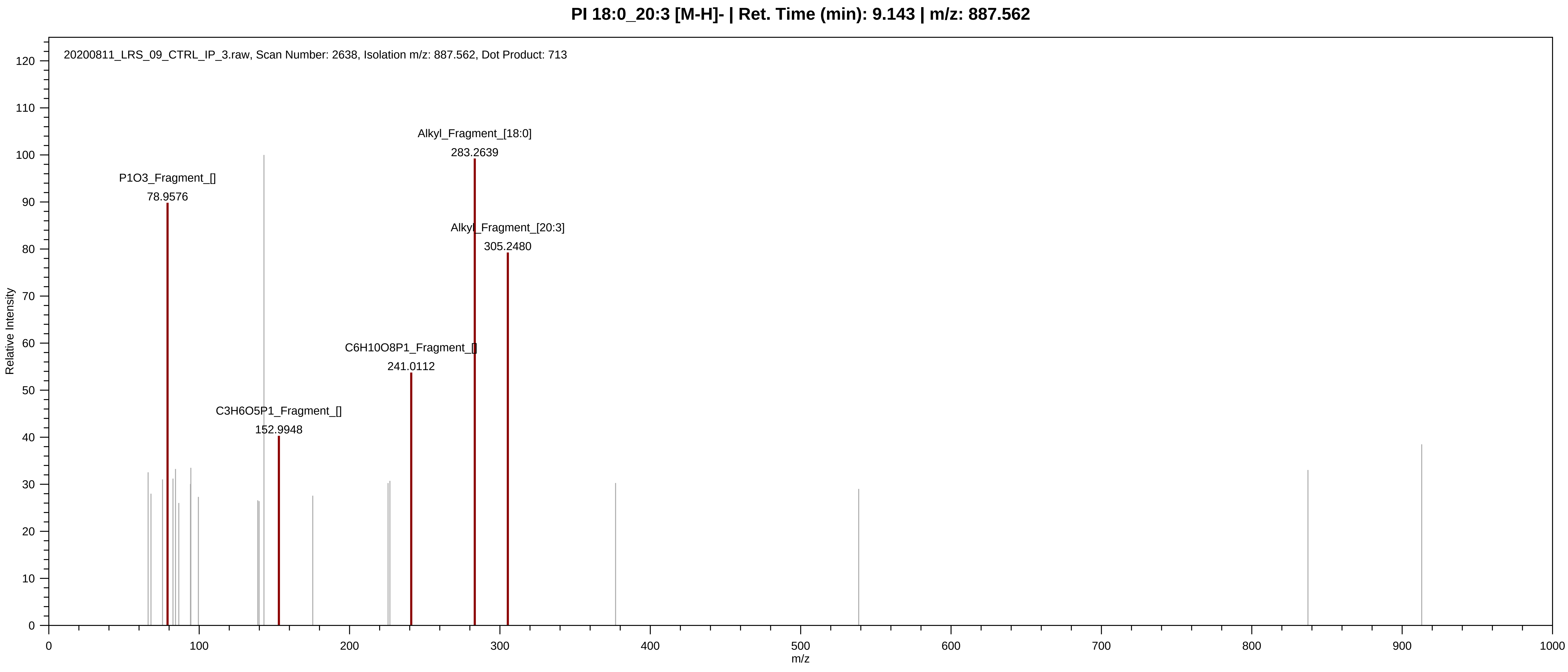

PI 18:0_20:3 [M-H]- | Ret. Time (min): 9.143 | m/z: 887.562
20200811_LRS_09_CTRL_IP_3.raw, Scan Number: 2638, Isolation m/z: 887.562, Dot Product: 713
P1O3_Fragment_[]
78.9576
C3H6O5P1_Fragment_[]
152.9948
C6H10O8P1_Fragment_[]
241.0112
Alkyl_Fragment_[18:0]
283.2639
Alkyl_Fragment_[20:3]
305.2480
Relative Intensity
m/z

PI 18:0_20:3 [M-H]- | Ret. Time (min): 9.033 | m/z: 887.565

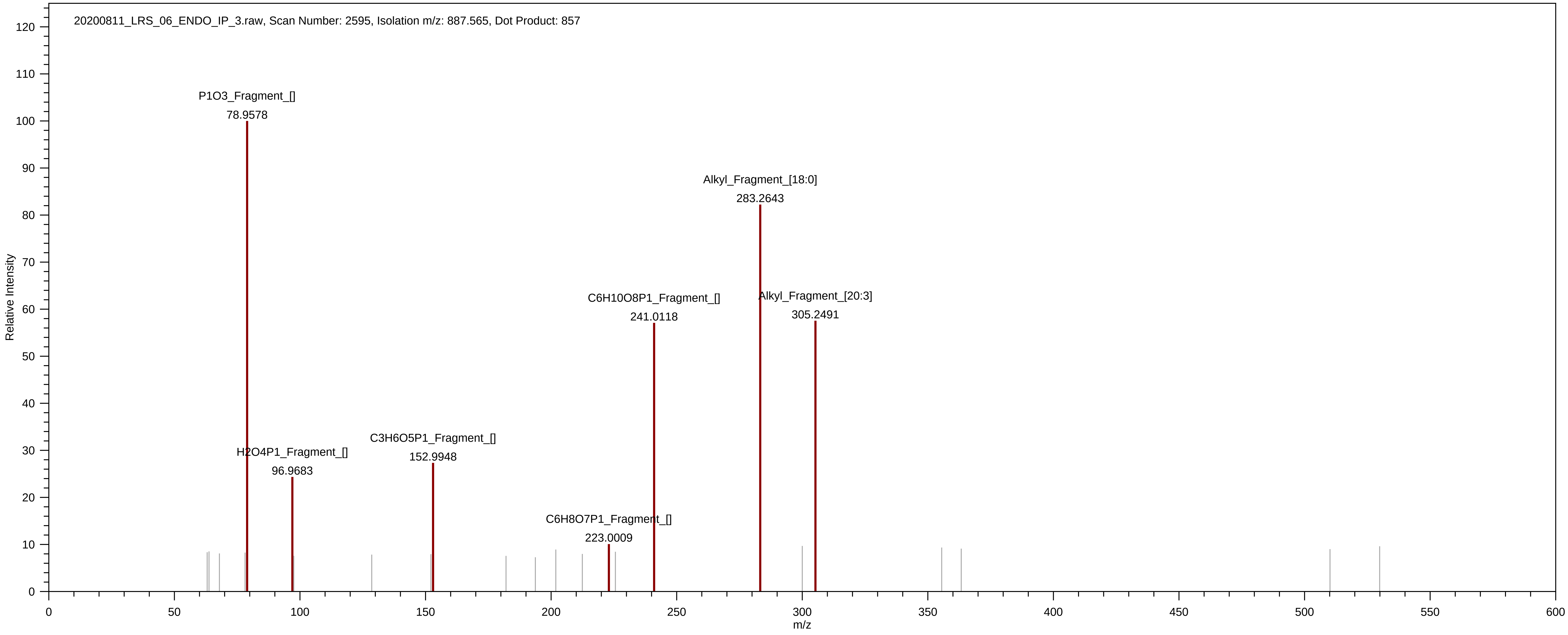

PI 18:0_20:3 [M-H]- | Ret. Time (min): 9.338 | m/z: 887.565

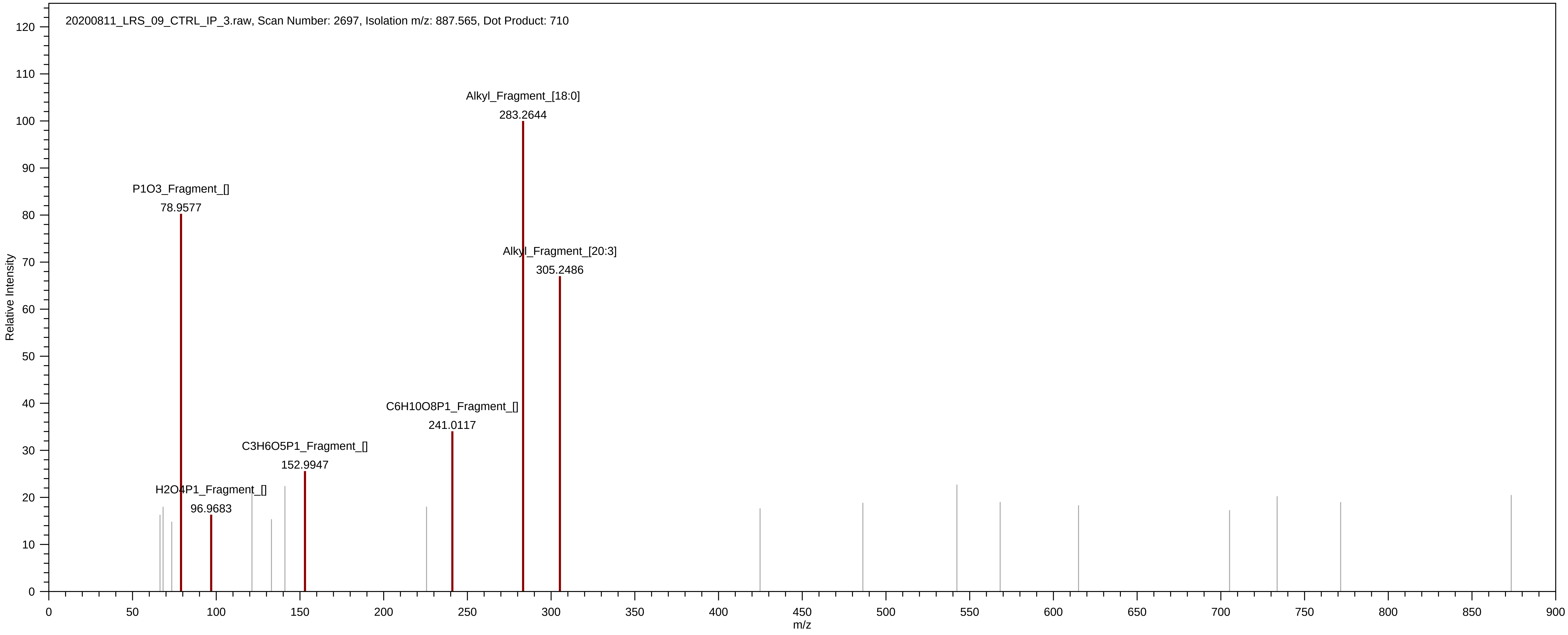

PI 18:0_20:3 [M-H]- | Ret. Time (min): 9.011 | m/z: 887.566

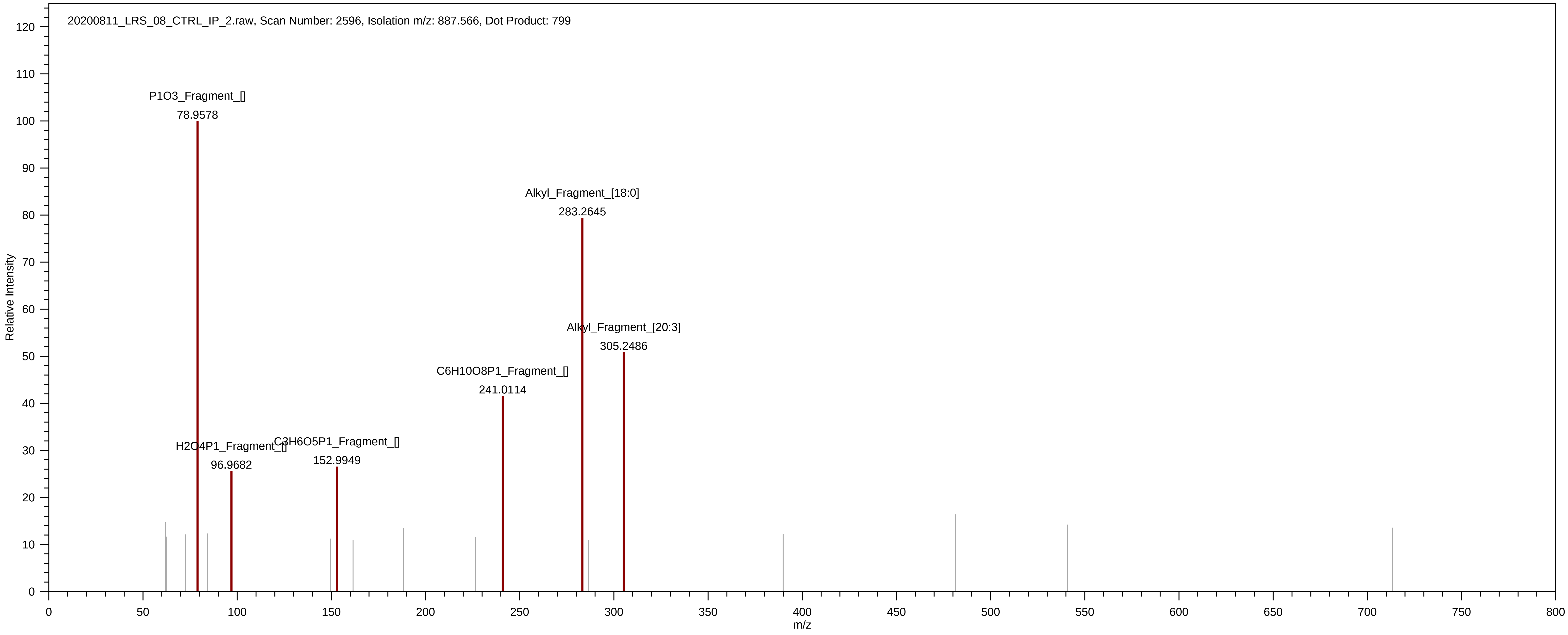

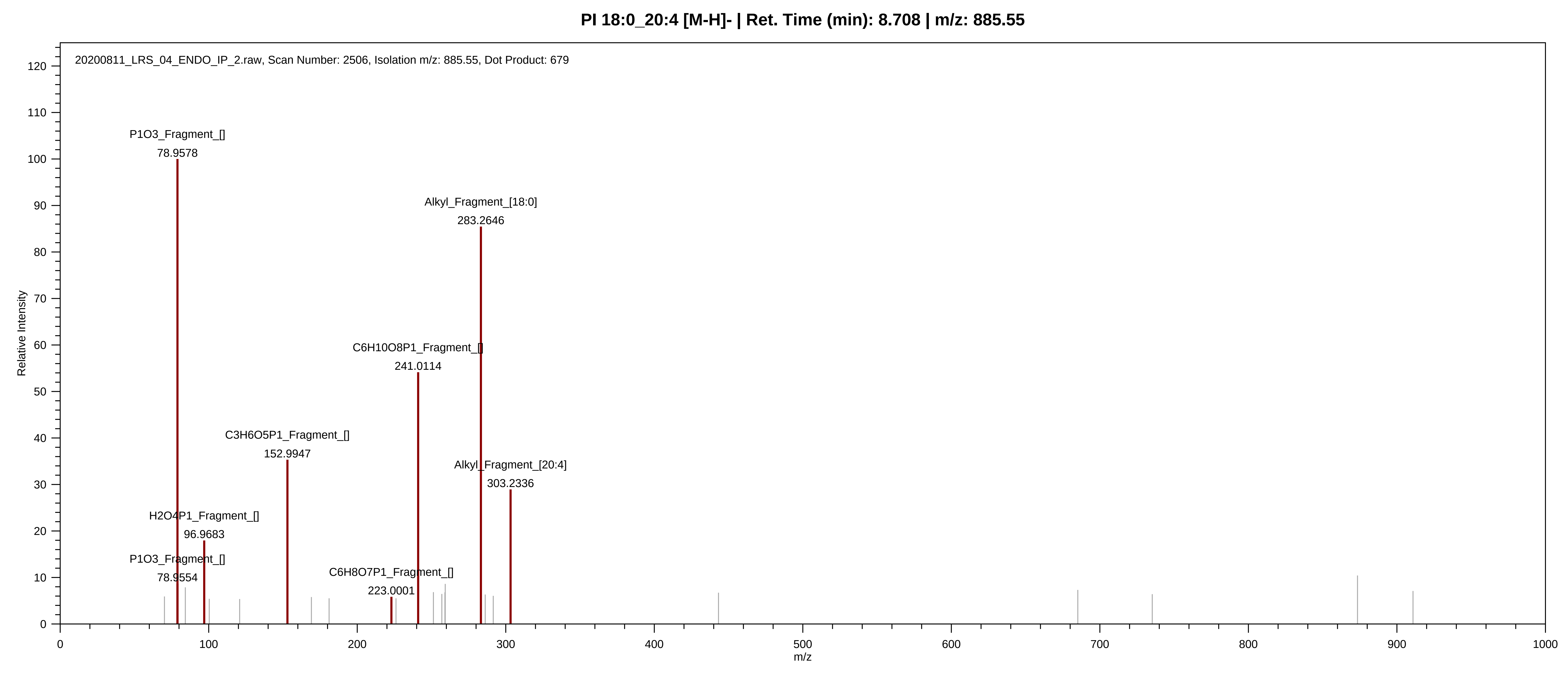

PI 18:0_20:4 [M-H]- | Ret. Time (min): 8.708 | m/z: 885.55
20200811_LRS_04_ENDO_IP_2.raw, Scan Number: 2506, Isolation m/z: 885.55, Dot Product: 679
P1O3_Fragment_[]
78.9578
Alkyl_Fragment_[18:0]
283.2646
C6H10O8P1_Fragment_[]
241.0114
C3H6O5P1_Fragment_[]
152.9947
Alkyl_Fragment_[20:4]
303.2336
H2O4P1_Fragment_[]
96.9683
P1O3_Fragment_[]
78.9554
C6H8O7P1_Fragment_[]
223.0001
Relative Intensity
m/z

PI 18:1_18:0 [M-H]- | Ret. Time (min): 8.387 | m/z: 863.565

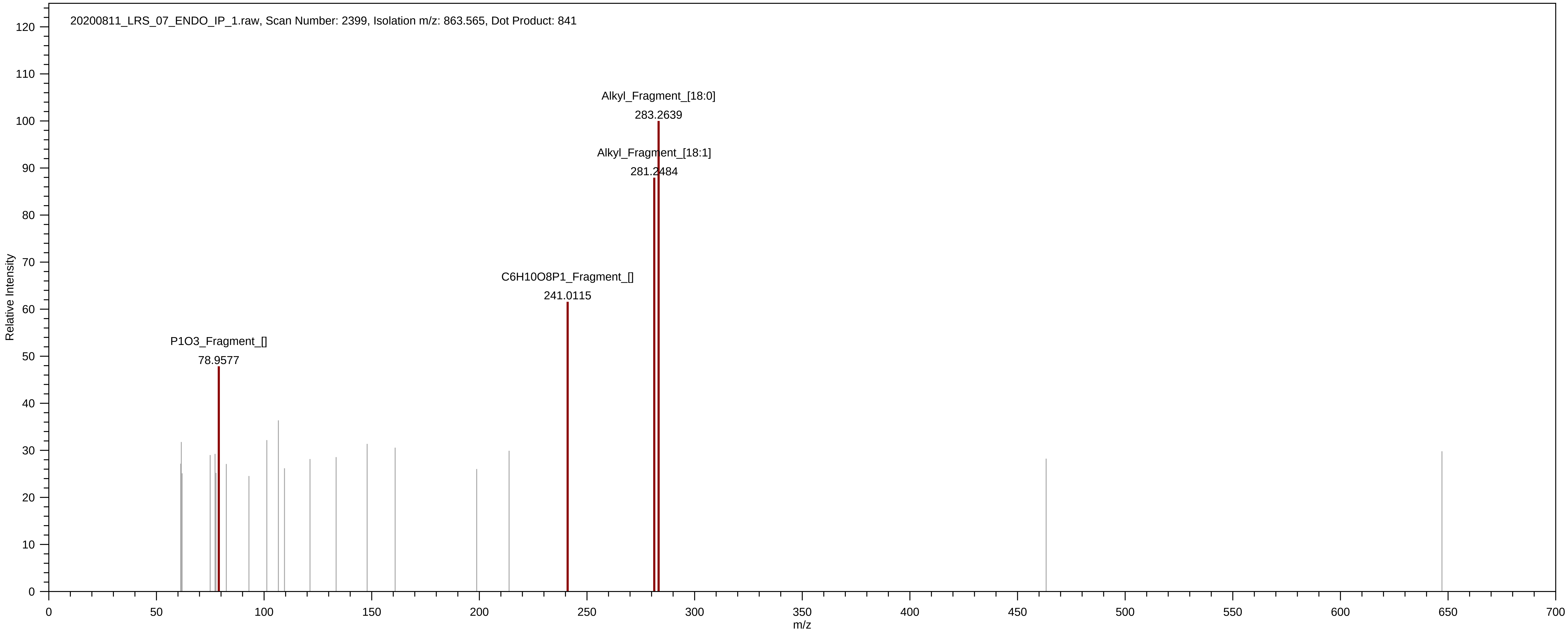

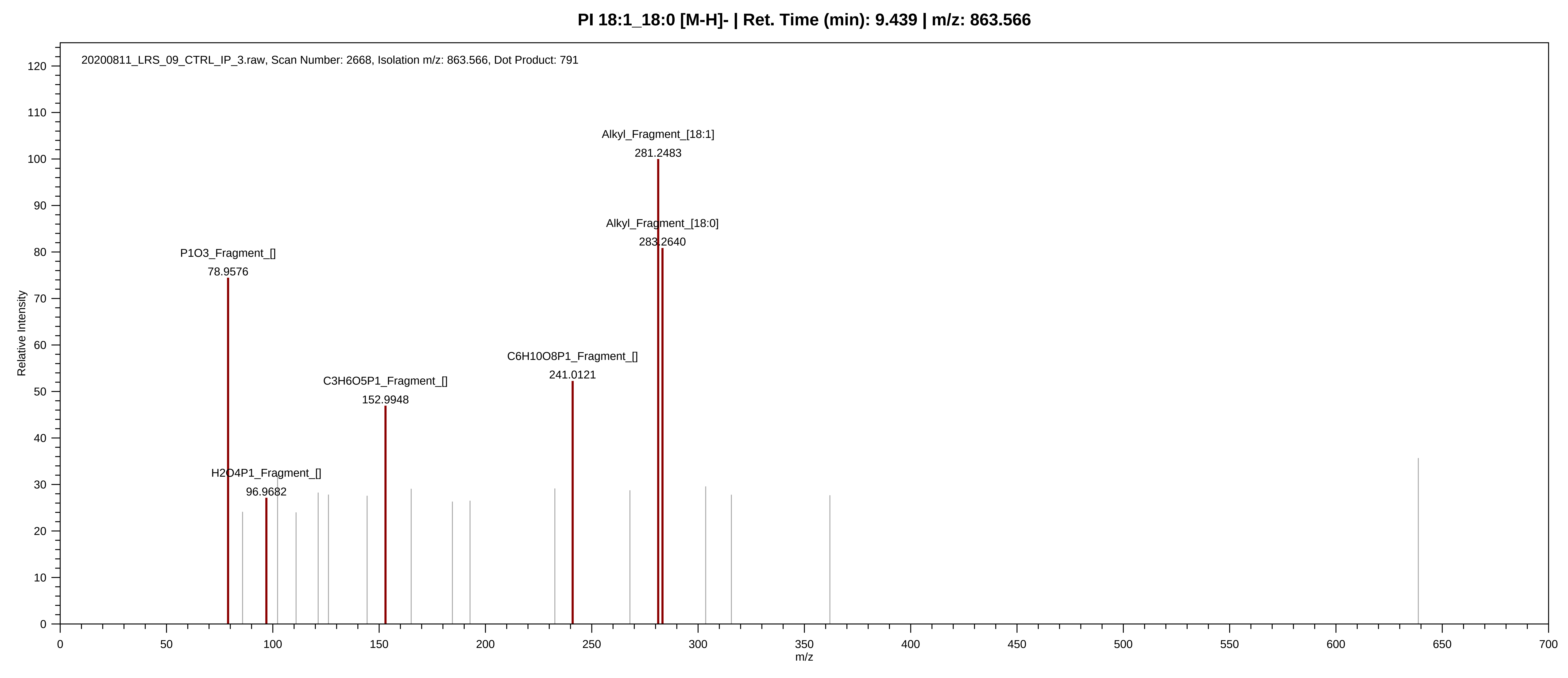
PI 18:1_18:0 [M-H]- | Ret. Time (min): 9.439 | m/z: 863.566
20200811_LRS_09_CTRL_IP_3.raw, Scan Number: 2668, Isolation m/z: 863.566, Dot Product: 791
Alkyl_Fragment_[18:1]
281.2483
Alkyl_Fragment_[18:0]
283.2640
P1O3_Fragment_[]
78.9576
C6H10O8P1_Fragment_[]
241.0121
C3H6O5P1_Fragment_[]
152.9948
H2O4P1_Fragment_[]
96.9682
Relative Intensity
m/z

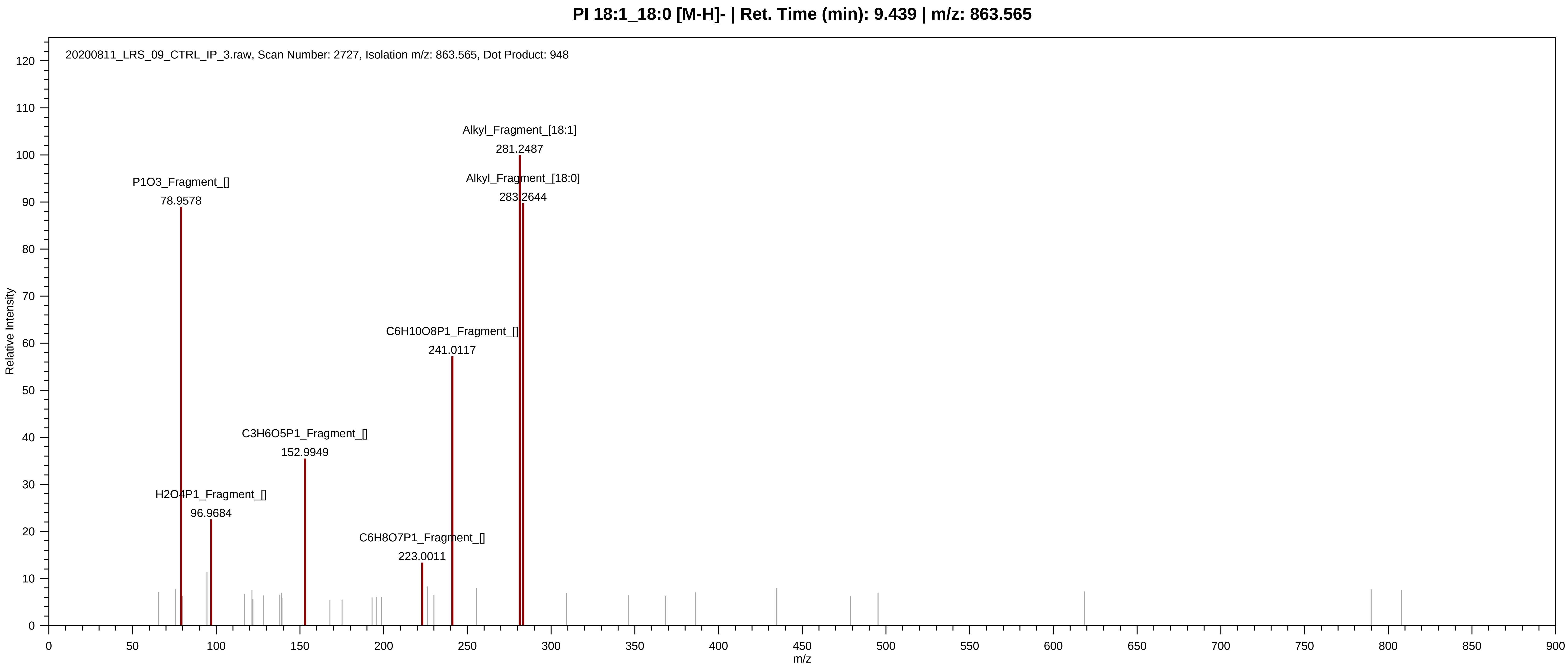
PI 18:1_18:0 [M-H]- | Ret. Time (min): 9.439 | m/z: 863.565
20200811_LRS_09_CTRL_IP_3.raw, Scan Number: 2727, Isolation m/z: 863.565, Dot Product: 948
Alkyl_Fragment_[18:1]
281.2487
Alkyl_Fragment_[18:0]
283.2644
P1O3_Fragment_[]
78.9578
C6H10O8P1_Fragment_[]
241.0117
C3H6O5P1_Fragment_[]
152.9949
H2O4P1_Fragment_[]
96.9684
C6H8O7P1_Fragment_[]
223.0011
Relative Intensity
m/z

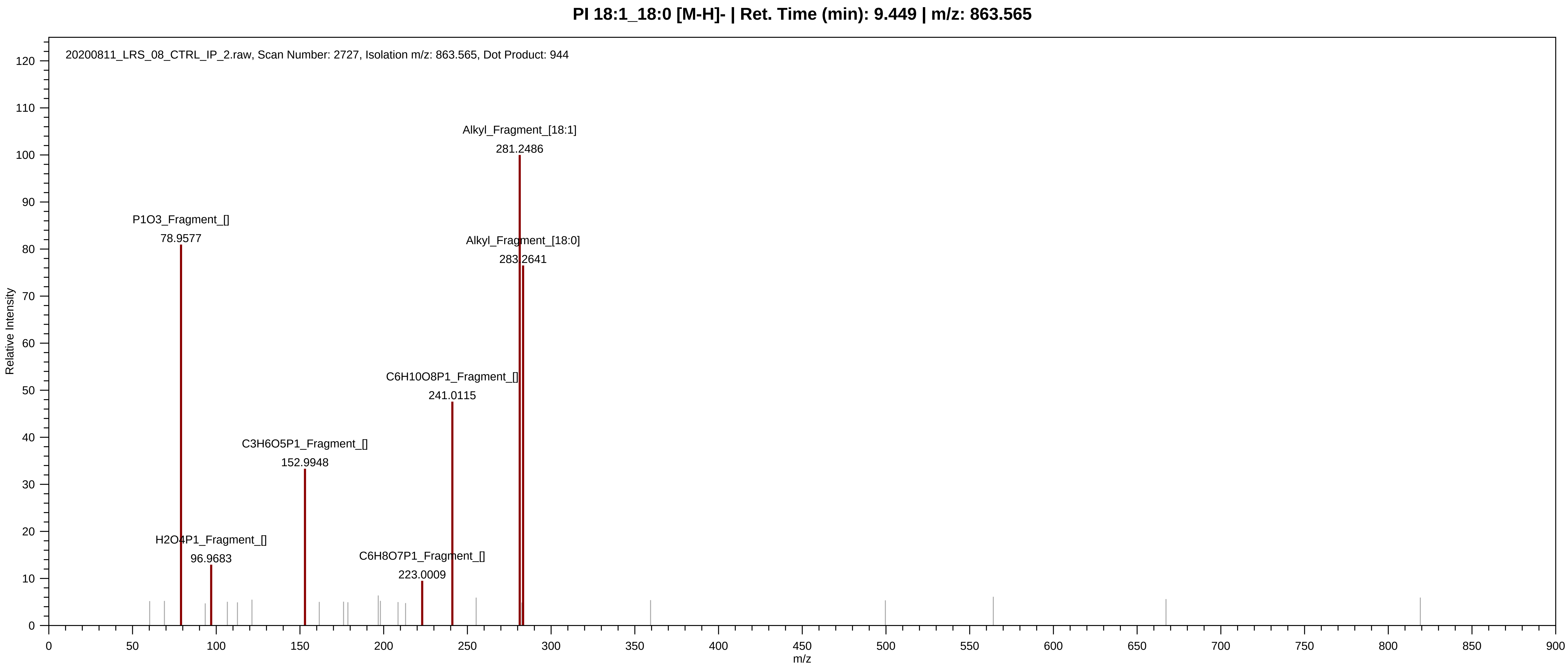
PI 18:1_18:0 [M-H]- | Ret. Time (min): 9.449 | m/z: 863.565
20200811_LRS_08_CTRL_IP_2.raw, Scan Number: 2727, Isolation m/z: 863.565, Dot Product: 944
Alkyl_Fragment_[18:1]
281.2486
Alkyl_Fragment_[18:0]
283.2641
P1O3_Fragment_[]
78.9577
C6H10O8P1_Fragment_[]
241.0115
C3H6O5P1_Fragment_[]
152.9948
H2O4P1_Fragment_[]
96.9683
C6H8O7P1_Fragment_[]
223.0009
Relative Intensity
m/z

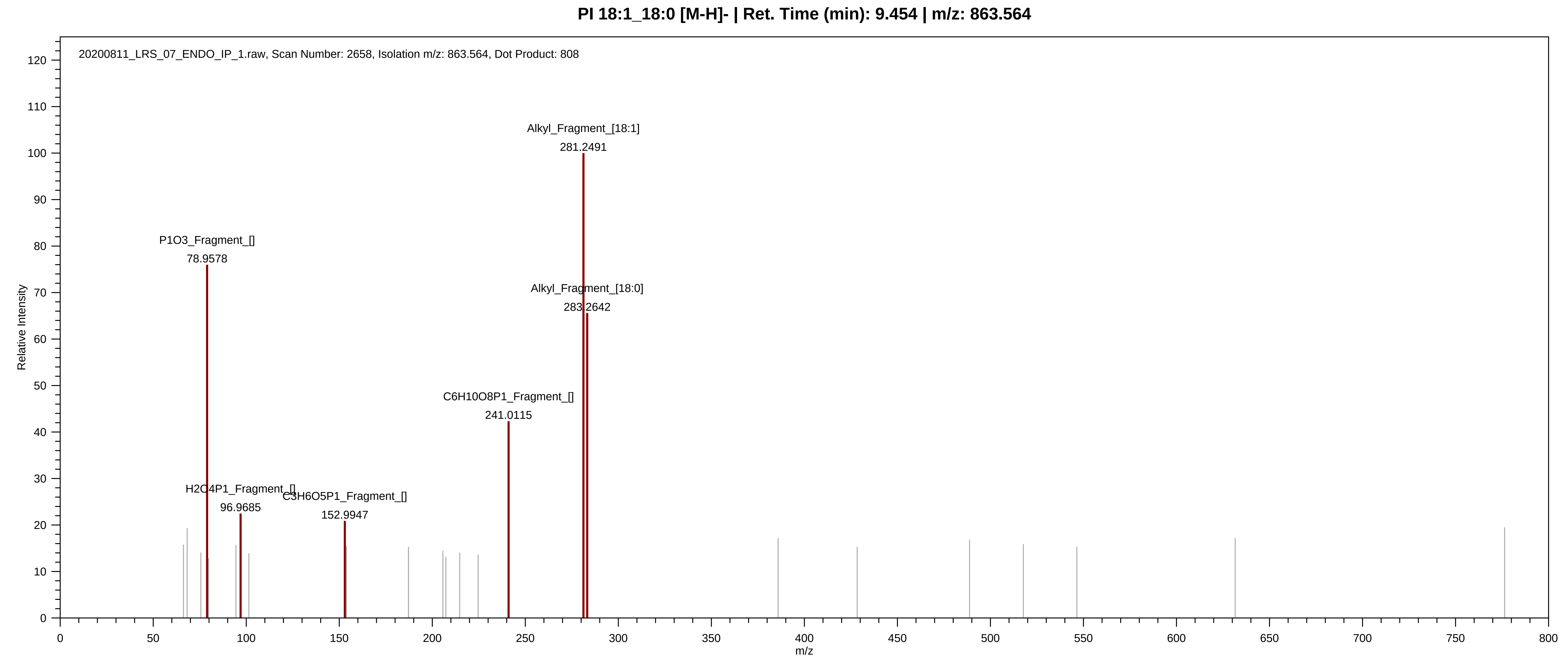

PI 18:1_18:0 [M-H]- | Ret. Time (min): 9.454 | m/z: 863.564
20200811_LRS_07_ENDO_IP_1.raw, Scan Number: 2658, Isolation m/z: 863.564, Dot Product: 808
Alkyl_Fragment_[18:1]
281.2491
P1O3_Fragment_[]
78.9578
Alkyl_Fragment_[18:0]
283.2642
C6H10O8P1_Fragment_[]
241.0115
H2O4P1_Fragment_[]
96.9685
C3H6O5P1_Fragment_[]
152.9947
Relative Intensity
m/z

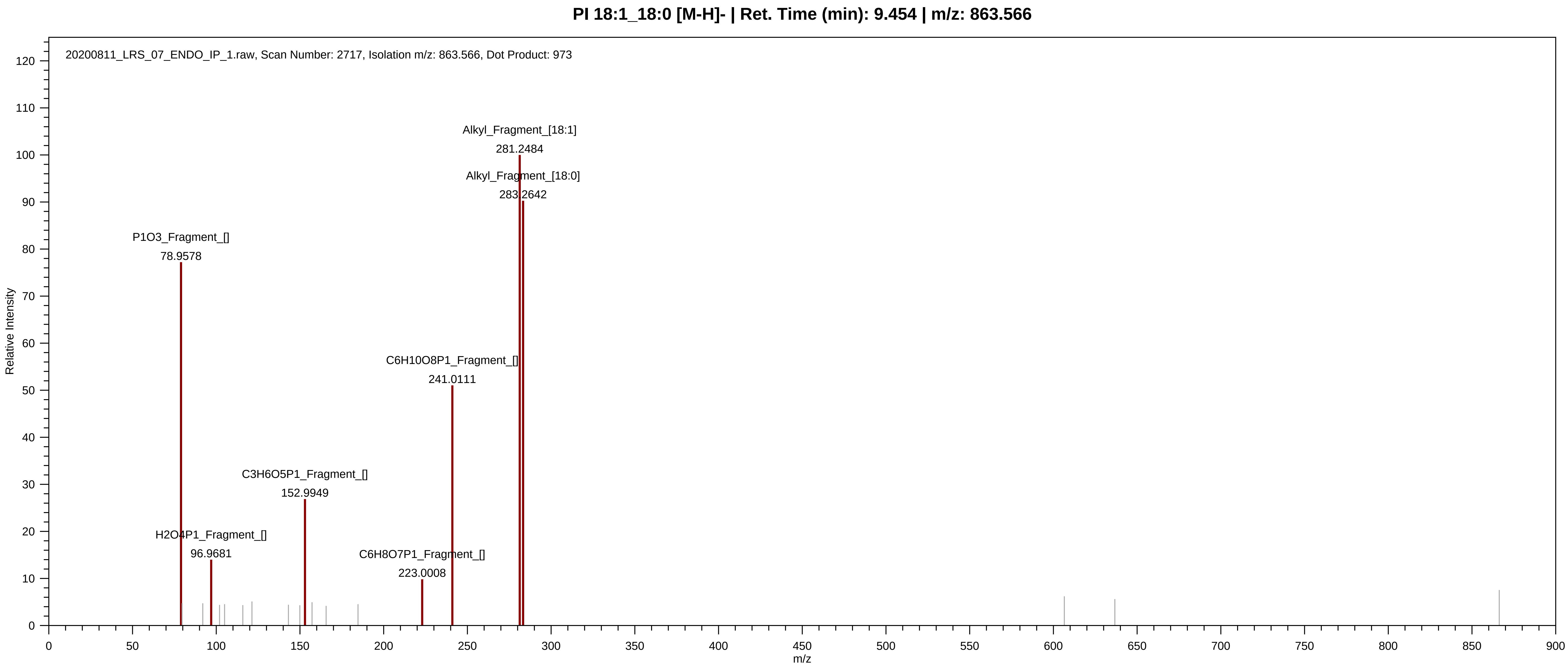
PI 18:1_18:0 [M-H]- | Ret. Time (min): 9.454 | m/z: 863.566
20200811_LRS_07_ENDO_IP_1.raw, Scan Number: 2717, Isolation m/z: 863.566, Dot Product: 973
Alkyl_Fragment_[18:1]
281.2484
Alkyl_Fragment_[18:0]
283.2642
P1O3_Fragment_[]
78.9578
C6H10O8P1_Fragment_[]
241.0111
C3H6O5P1_Fragment_[]
152.9949
H2O4P1_Fragment_[]
96.9681
C6H8O7P1_Fragment_[]
223.0008
Relative Intensity
m/z

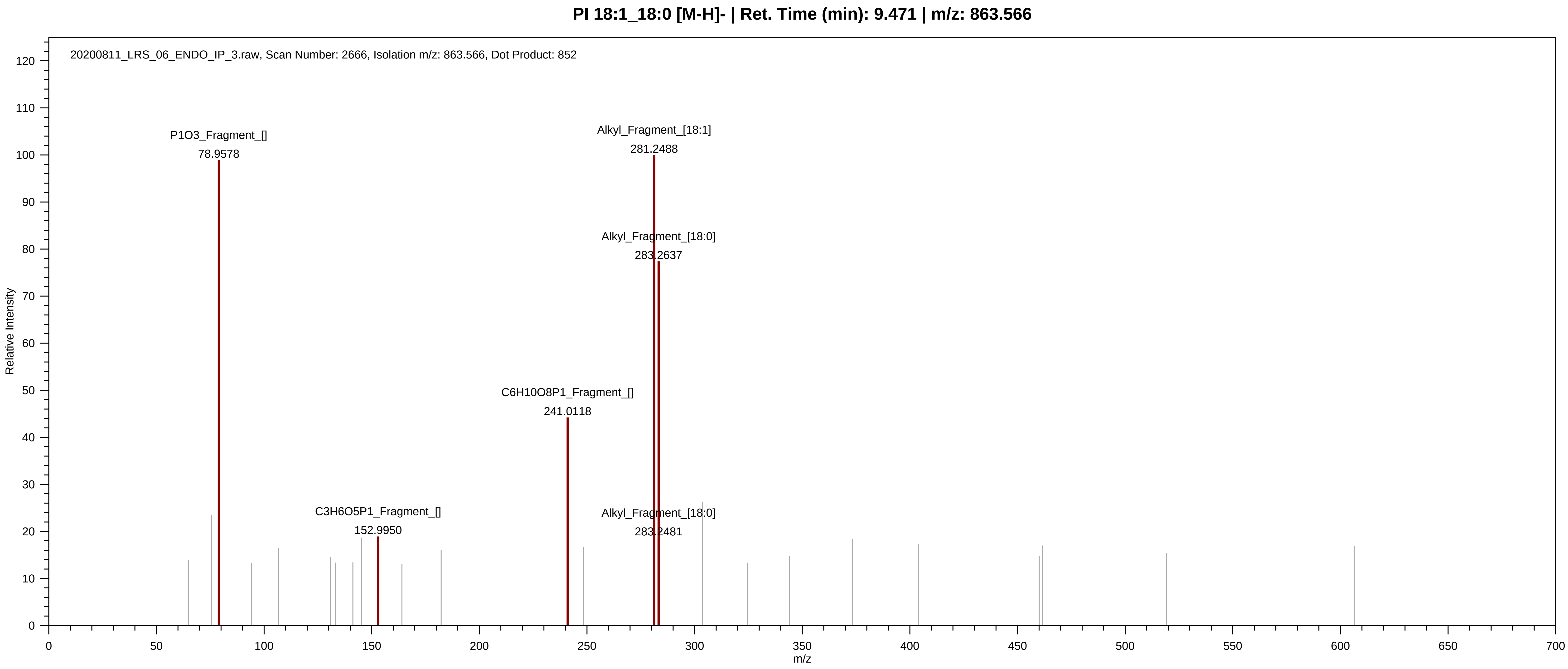
PI 18:1_18:0 [M-H]- | Ret. Time (min): 9.471 | m/z: 863.566
20200811_LRS_06_ENDO_IP_3.raw, Scan Number: 2666, Isolation m/z: 863.566, Dot Product: 852
P1O3_Fragment_[]
78.9578
Alkyl_Fragment_[18:1]
281.2488
Alkyl_Fragment_[18:0]
283.2637
C6H10O8P1_Fragment_[]
241.0118
C3H6O5P1_Fragment_[]
152.9950
Alkyl_Fragment_[18:0]
283.2481
Relative Intensity
m/z
0
10
20
30
40
50
60
70
80
90
100
110
120
0
50
100
150
200
250
300
350
400
450
500
550
600
650
700

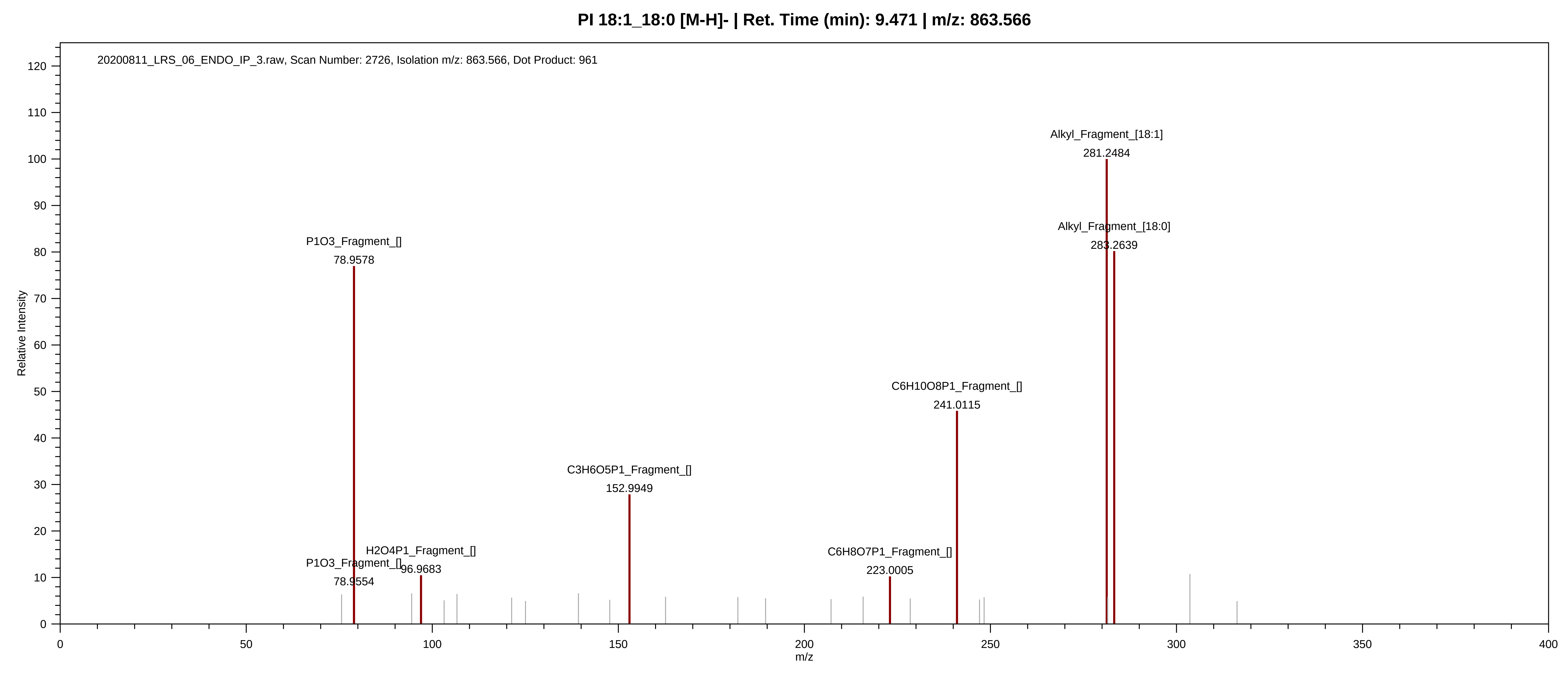
PI 18:1_18:0 [M-H]- | Ret. Time (min): 9.471 | m/z: 863.566
20200811_LRS_06_ENDO_IP_3.raw, Scan Number: 2726, Isolation m/z: 863.566, Dot Product: 961
Alkyl_Fragment_[18:1]
281.2484
Alkyl_Fragment_[18:0]
283.2639
P1O3_Fragment_[]
78.9578
C6H10O8P1_Fragment_[]
241.0115
C3H6O5P1_Fragment_[]
152.9949
H2O4P1_Fragment_[]
96.9683
P1O3_Fragment_[]
78.9554
C6H8O7P1_Fragment_[]
223.0005
Relative Intensity
m/z

PI 18:1_18:0 [M-H]- | Ret. Time (min): 9.477 | m/z: 863.566

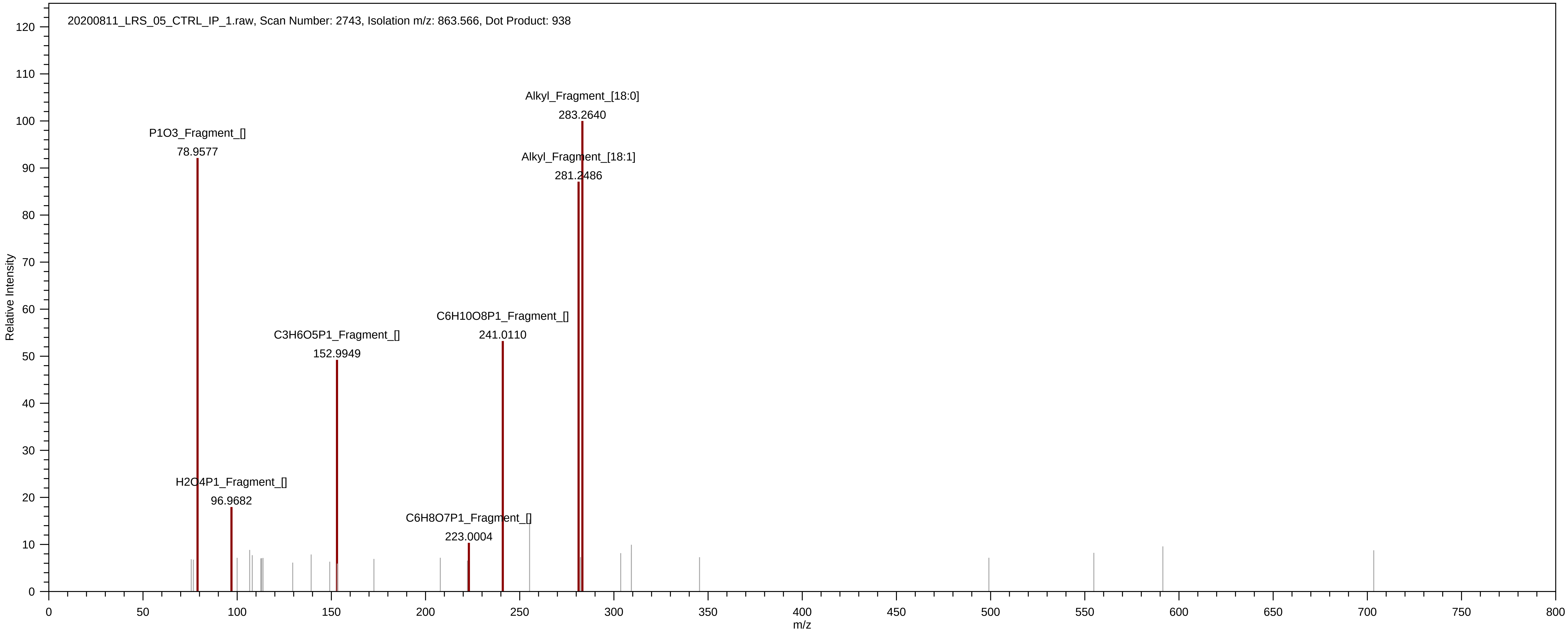

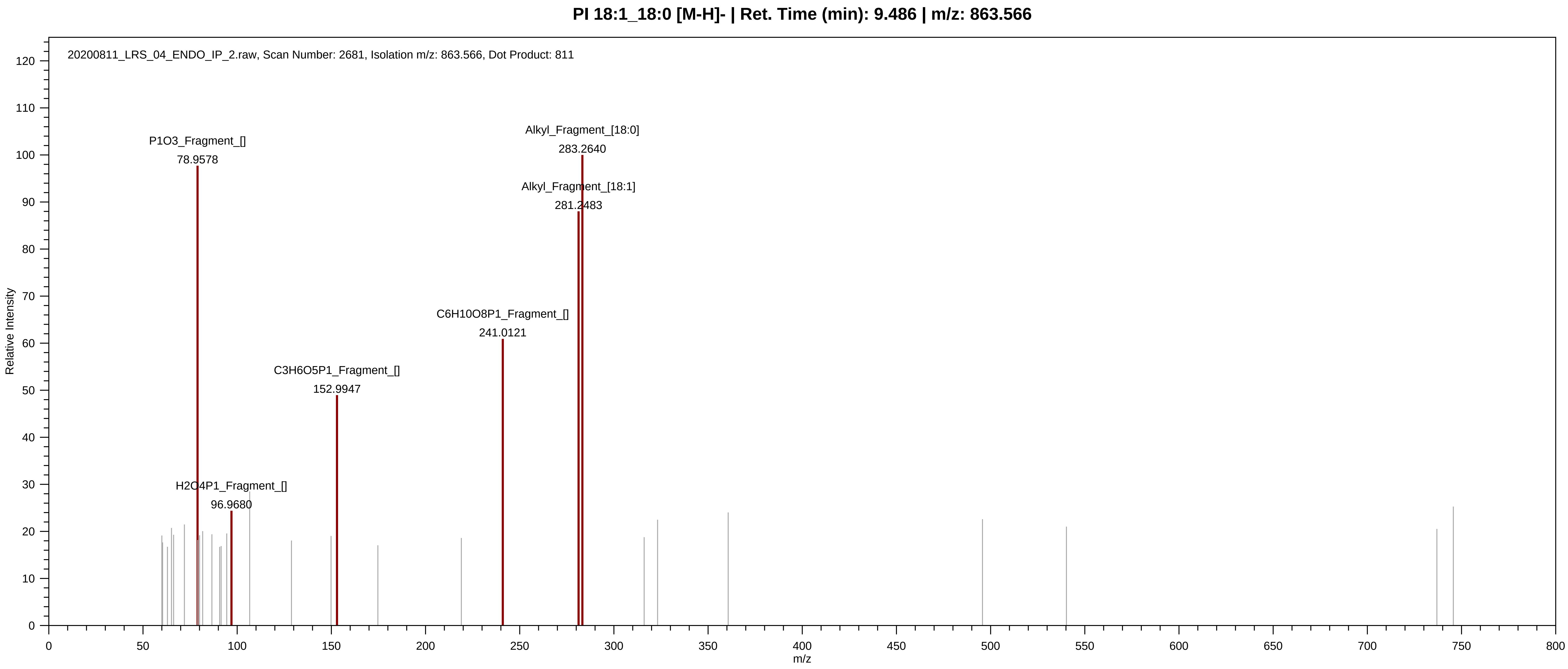
PI 18:1_18:0 [M-H]- | Ret. Time (min): 9.486 | m/z: 863.566
20200811_LRS_04_ENDO_IP_2.raw, Scan Number: 2681, Isolation m/z: 863.566, Dot Product: 811
P1O3_Fragment_[]
78.9578
Alkyl_Fragment_[18:0]
283.2640
Alkyl_Fragment_[18:1]
281.2483
C6H10O8P1_Fragment_[]
241.0121
C3H6O5P1_Fragment_[]
152.9947
H2O4P1_Fragment_[]
96.9680
Relative Intensity
m/z

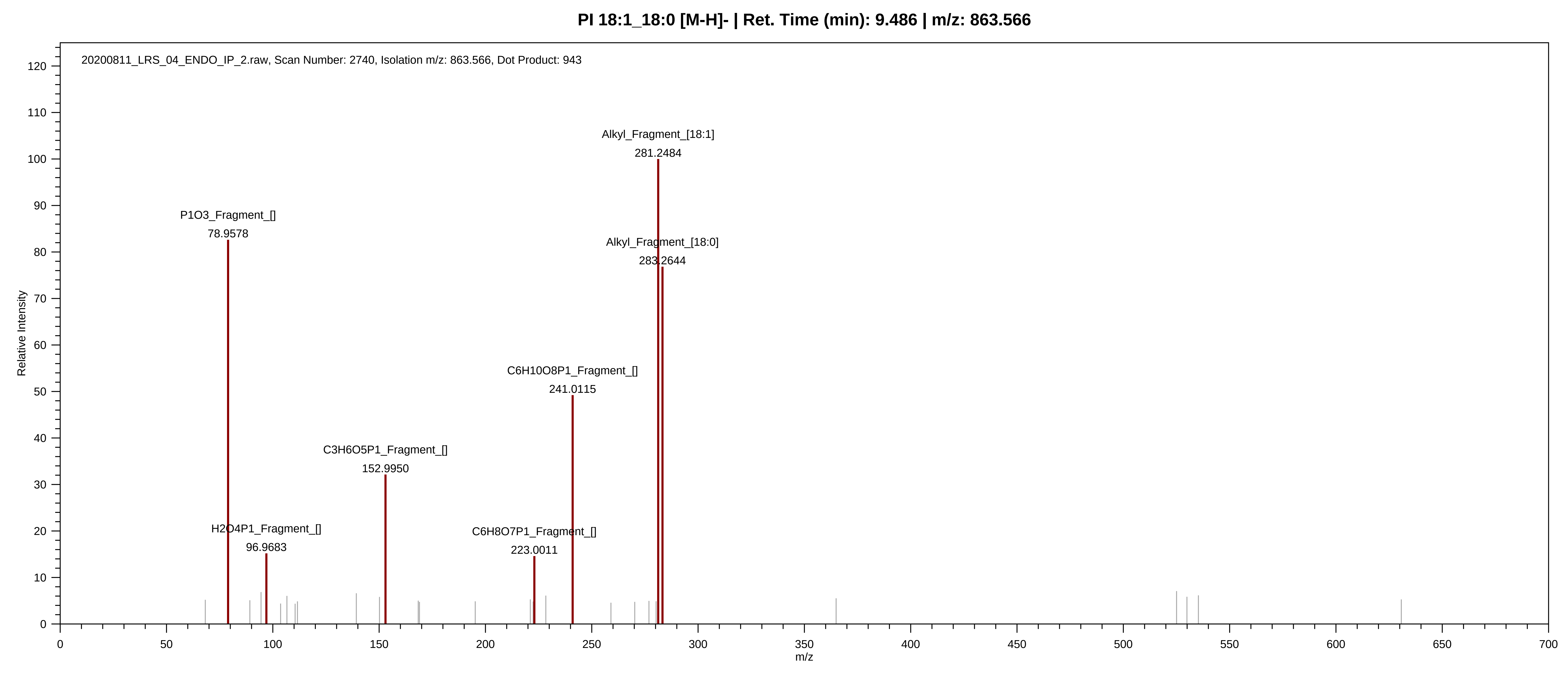
PI 18:1_18:0 [M-H]- | Ret. Time (min): 9.486 | m/z: 863.566
20200811_LRS_04_ENDO_IP_2.raw, Scan Number: 2740, Isolation m/z: 863.566, Dot Product: 943
Relative Intensity
m/z
P1O3_Fragment_[]
78.9578
H2O4P1_Fragment_[]
96.9683
C3H6O5P1_Fragment_[]
152.9950
C6H8O7P1_Fragment_[]
223.0011
C6H10O8P1_Fragment_[]
241.0115
Alkyl_Fragment_[18:1]
281.2484
Alkyl_Fragment_[18:0]
283.2644

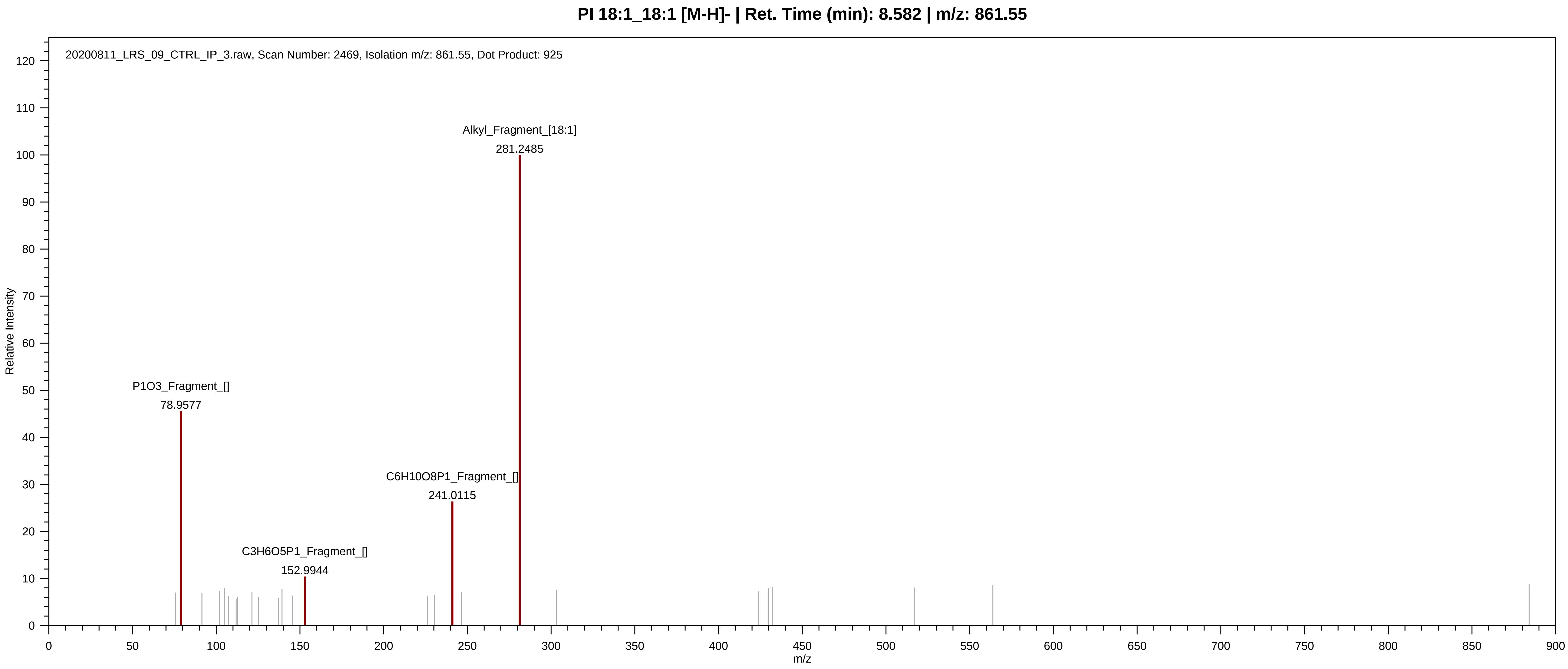

PI 18:1_18:1 [M-H]- | Ret. Time (min): 8.582 | m/z: 861.55
20200811_LRS_09_CTRL_IP_3.raw, Scan Number: 2469, Isolation m/z: 861.55, Dot Product: 925
Alkyl_Fragment_[18:1]
281.2485
P1O3_Fragment_[]
78.9577
C6H10O8P1_Fragment_[]
241.0115
C3H6O5P1_Fragment_[]
152.9944
Relative Intensity
m/z

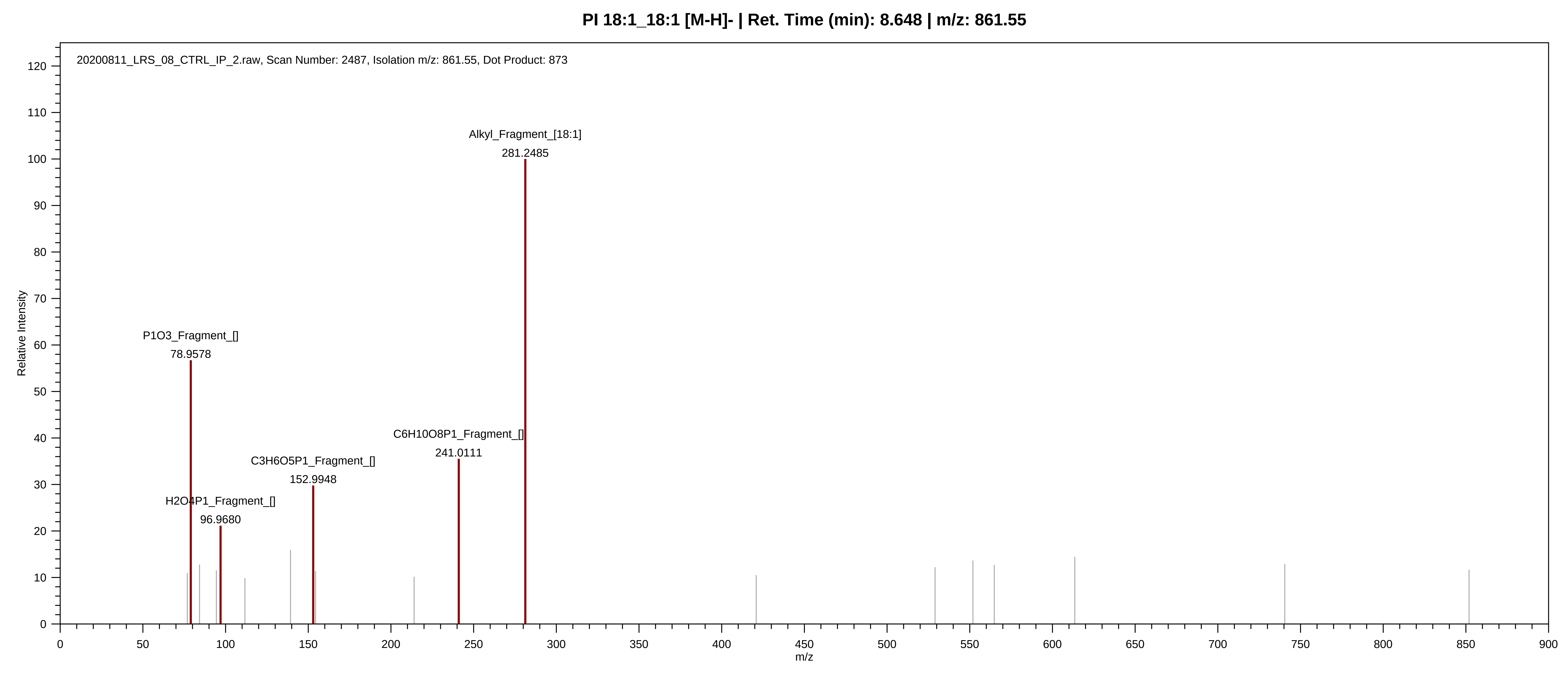
PI 18:1_18:1 [M-H]- | Ret. Time (min): 8.648 | m/z: 861.55
20200811_LRS_08_CTRL_IP_2.raw, Scan Number: 2487, Isolation m/z: 861.55, Dot Product: 873
Alkyl_Fragment_[18:1]
281.2485
P1O3_Fragment_[]
78.9578
C6H10O8P1_Fragment_[]
241.0111
C3H6O5P1_Fragment_[]
152.9948
H2O4P1_Fragment_[]
96.9680
Relative Intensity
m/z

PI 18:1_18:1 [M-H]- | Ret. Time (min): 8.77 | m/z: 861.547

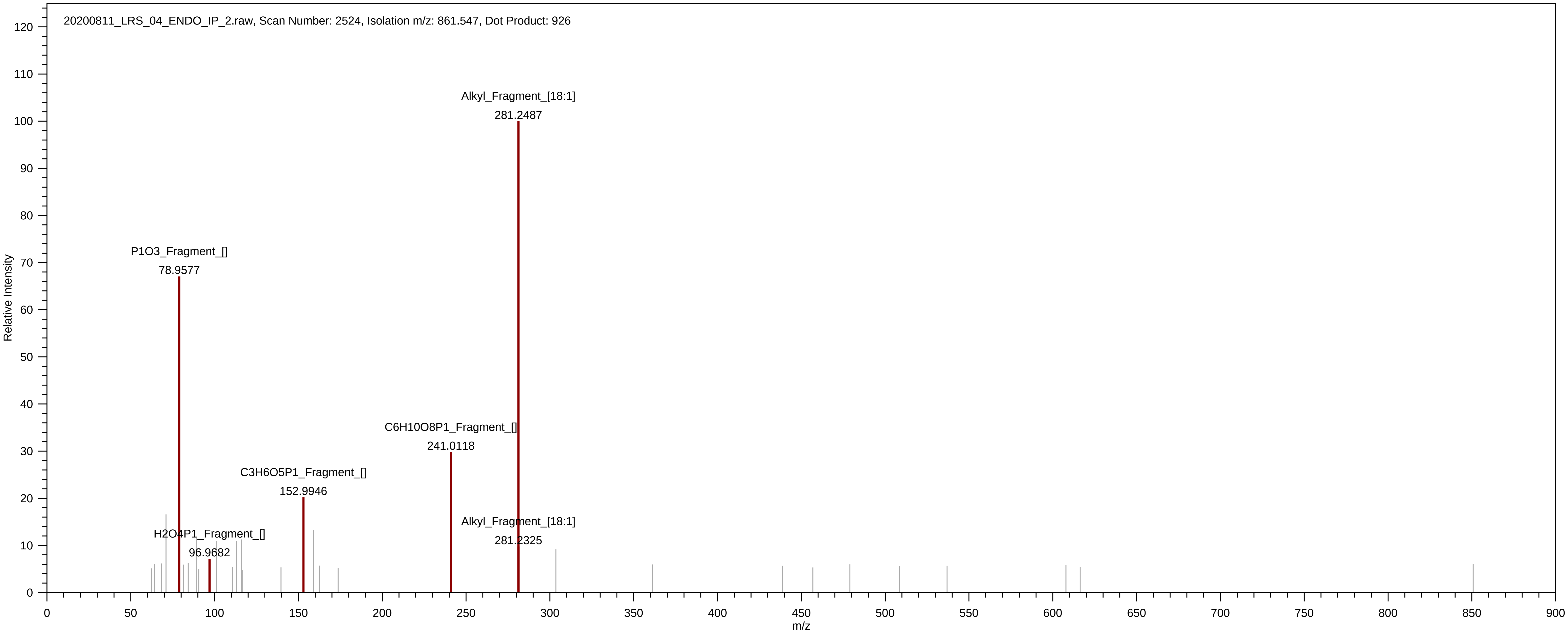

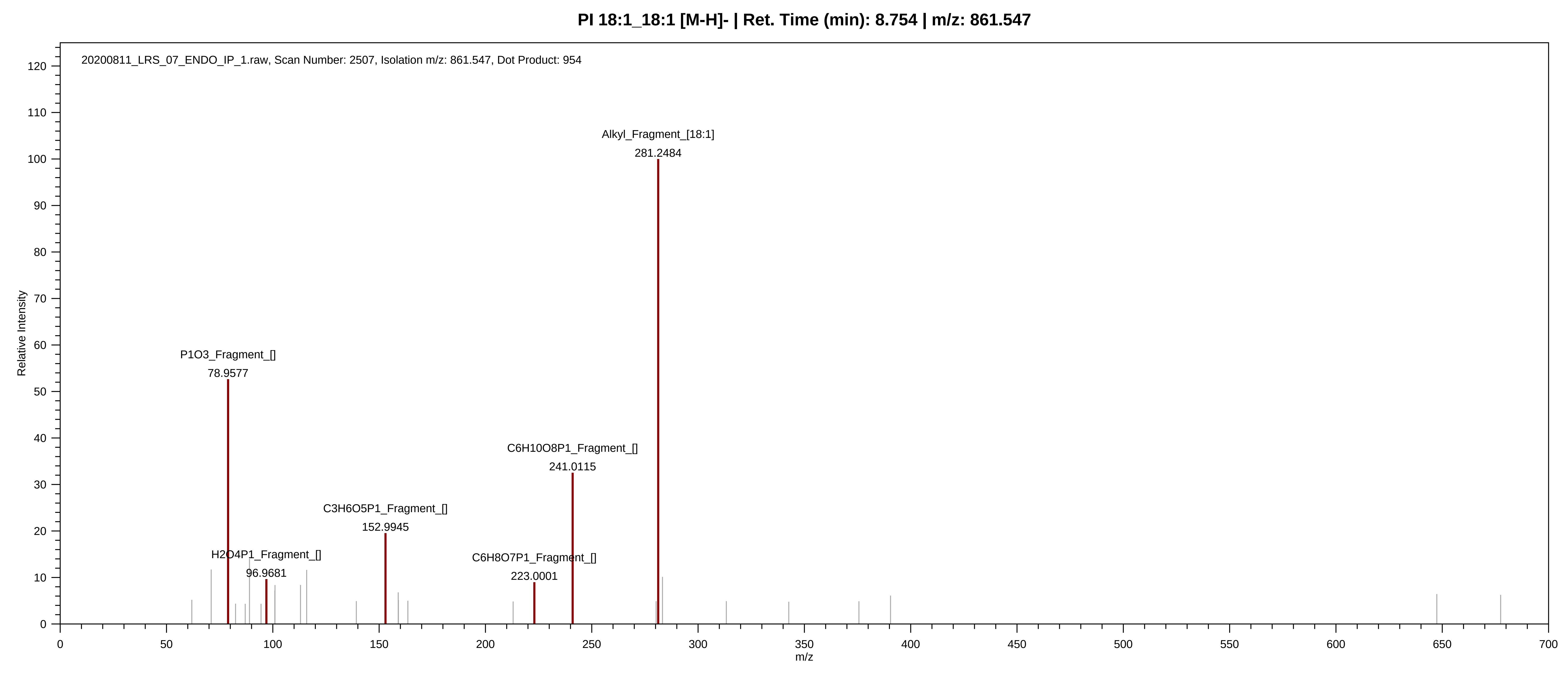
PI 18:1_18:1 [M-H]- | Ret. Time (min): 8.754 | m/z: 861.547
20200811_LRS_07_ENDO_IP_1.raw, Scan Number: 2507, Isolation m/z: 861.547, Dot Product: 954
Alkyl_Fragment_[18:1]
281.2484
P1O3_Fragment_[]
78.9577
C6H10O8P1_Fragment_[]
241.0115
C3H6O5P1_Fragment_[]
152.9945
H2O4P1_Fragment_[]
96.9681
C6H8O7P1_Fragment_[]
223.0001
Relative Intensity
m/z
0
10
20
30
40
50
60
70
80
90
100
110
120
0
50
100
150
200
250
300
350
400
450
500
550
600
650
700

PI 18:1_18:1 [M-H]- | Ret. Time (min): 8.777 | m/z: 861.547

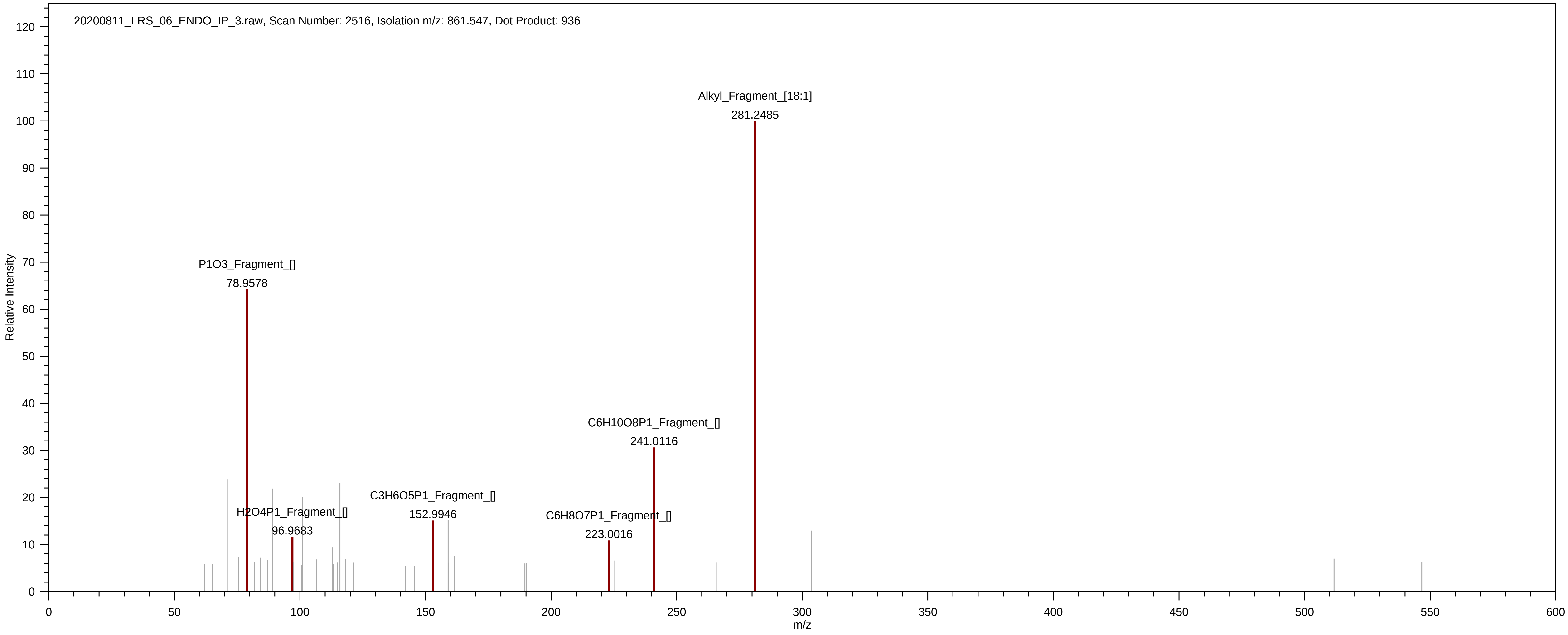

PI 18:1_18:1 [M-H]- | Ret. Time (min): 8.554 | m/z: 861.549

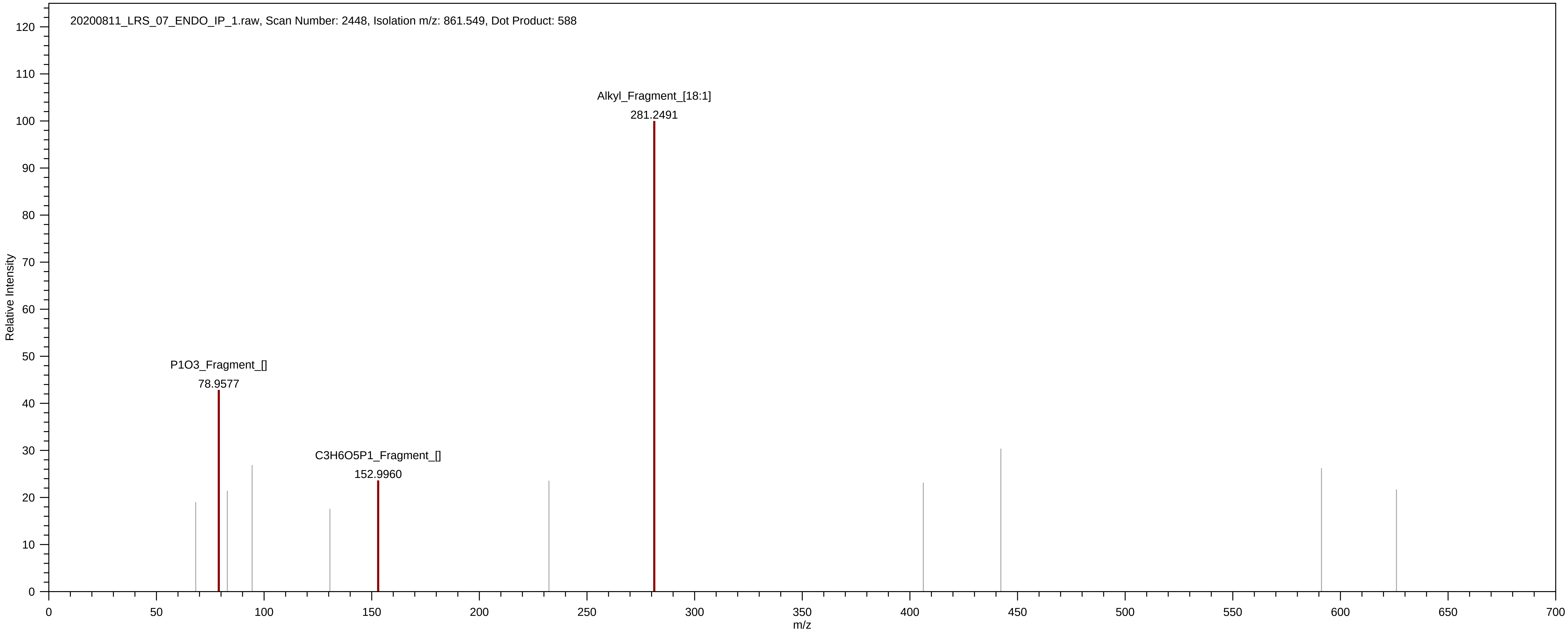

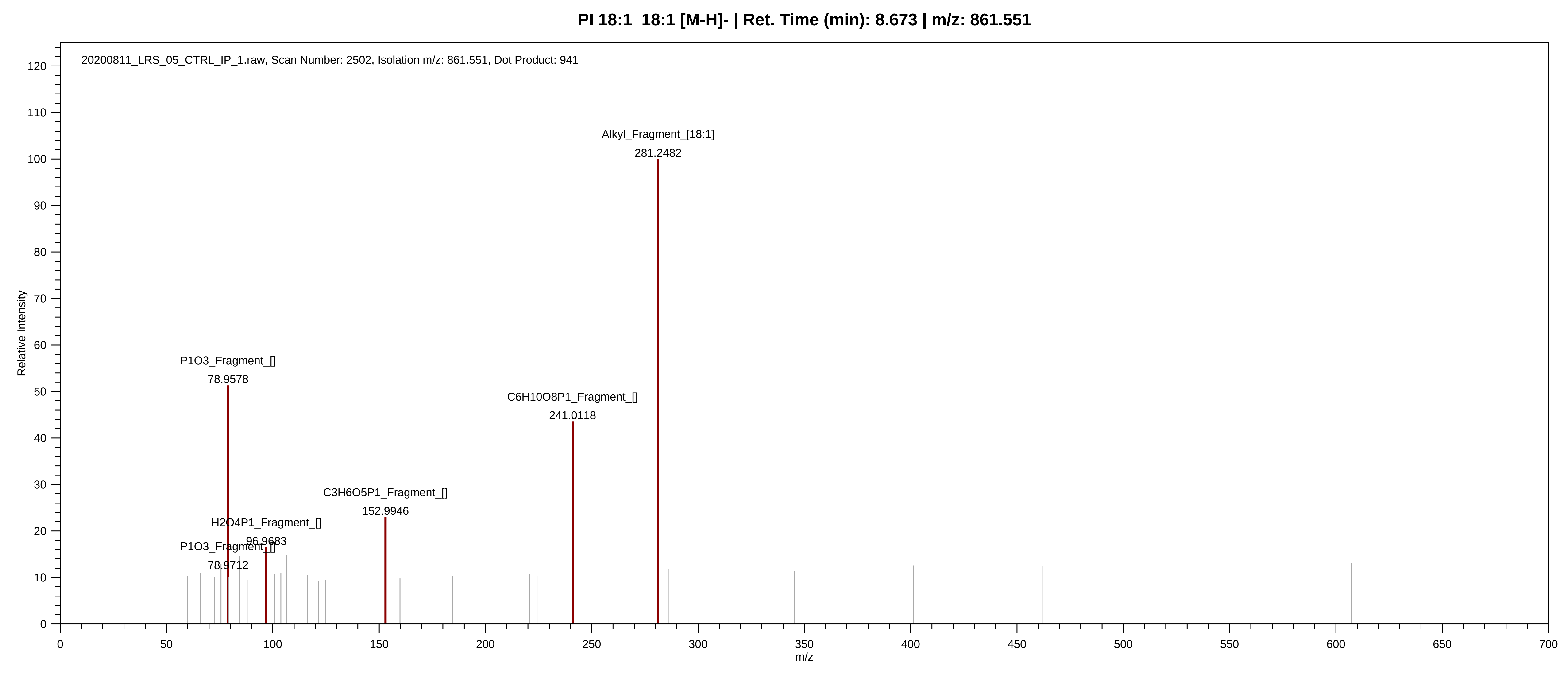

PI 18:1_18:1 [M-H]- | Ret. Time (min): 8.673 | m/z: 861.551
20200811_LRS_05_CTRL_IP_1.raw, Scan Number: 2502, Isolation m/z: 861.551, Dot Product: 941
Alkyl_Fragment_[18:1]
281.2482
P1O3_Fragment_[]
78.9578
C6H10O8P1_Fragment_[]
241.0118
C3H6O5P1_Fragment_[]
152.9946
H2O4P1_Fragment_[]
96.9683
P1O3_Fragment_[]
78.9712
Relative Intensity
m/z

PI 18:2_18:0 [M-H]- | Ret. Time (min): 8.985 | m/z: 861.549

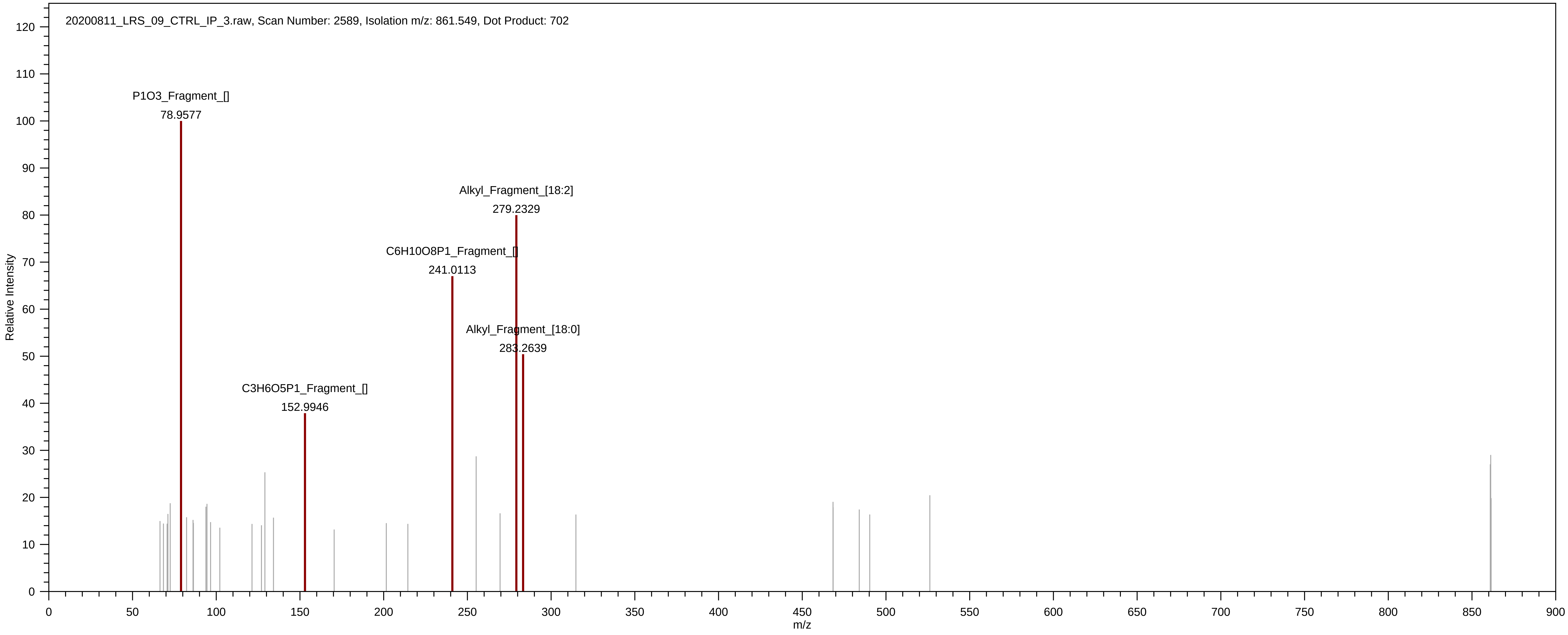

PI 36:2 [M-H]- | Ret. Time (min): 8.878 | m/z: 861.55

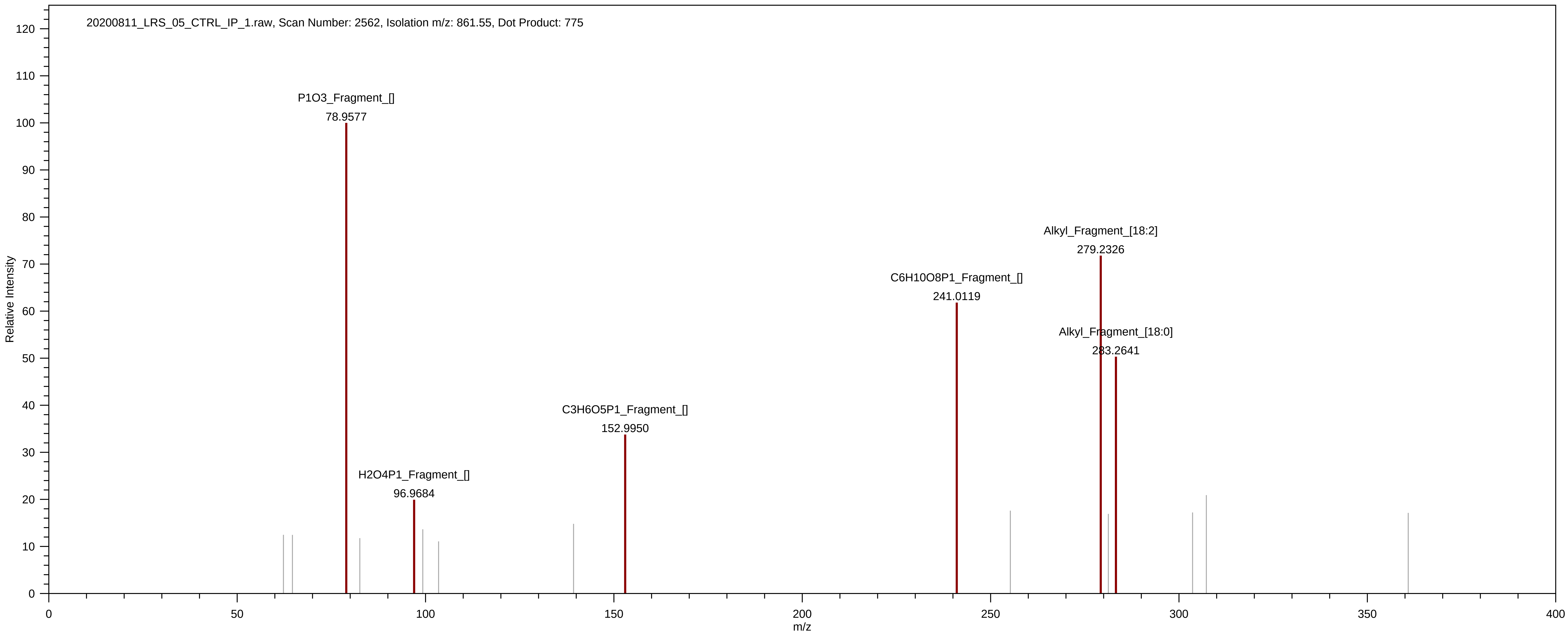

PI 36:2 [M-H]- | Ret. Time (min): 8.788 | m/z: 861.547

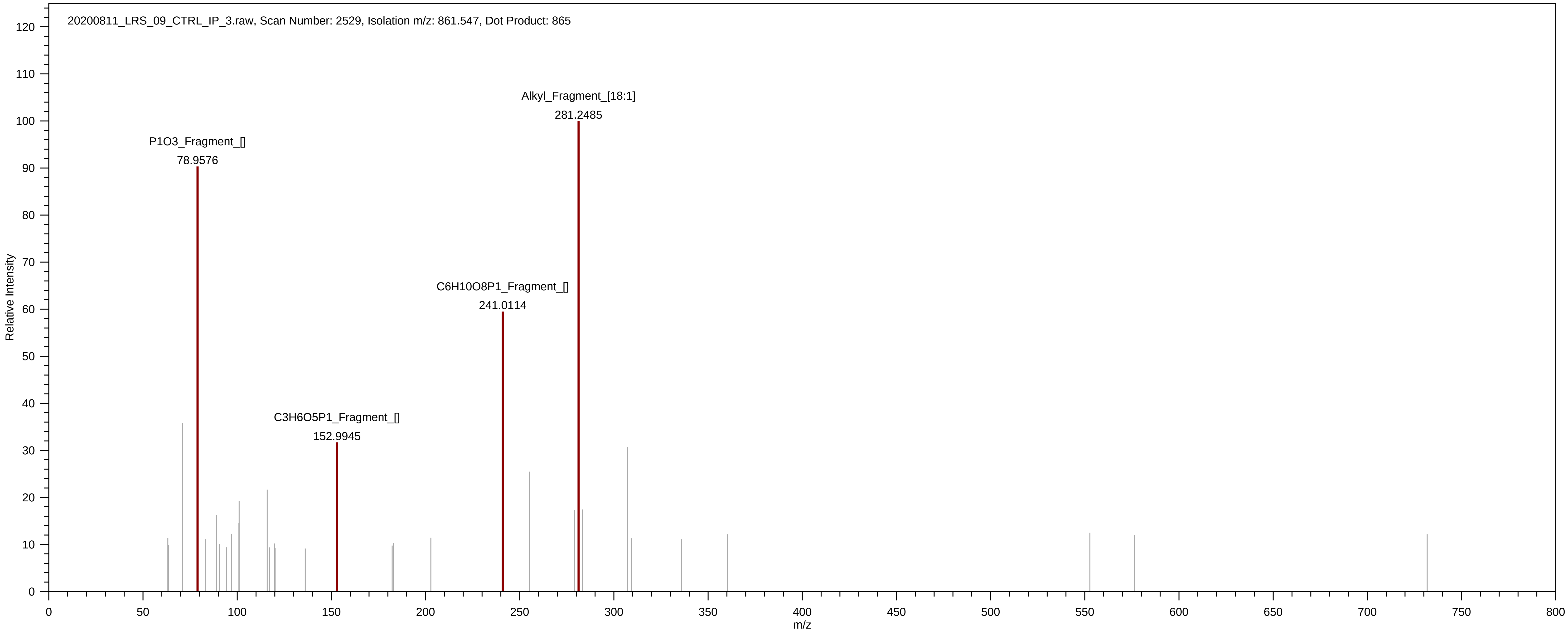

PI 36:2 [M-H]- | Ret. Time (min): 8.854 | m/z: 861.549

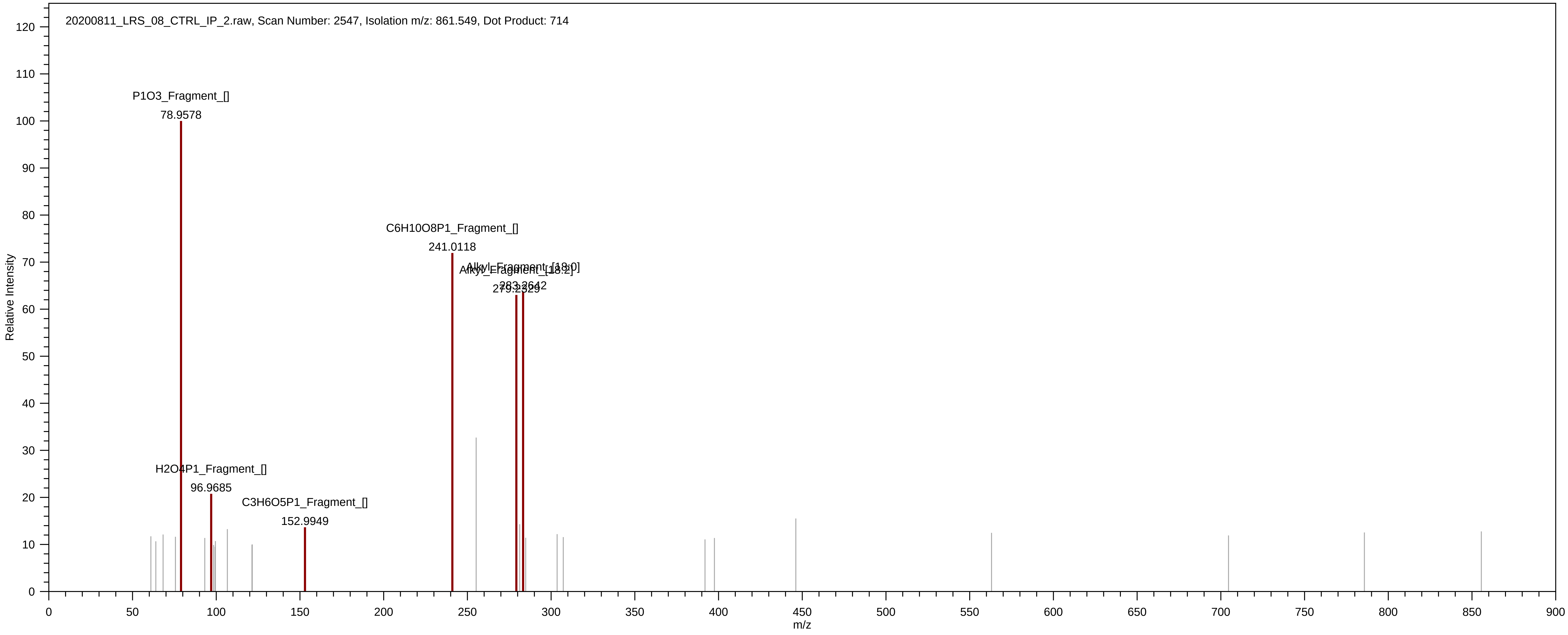

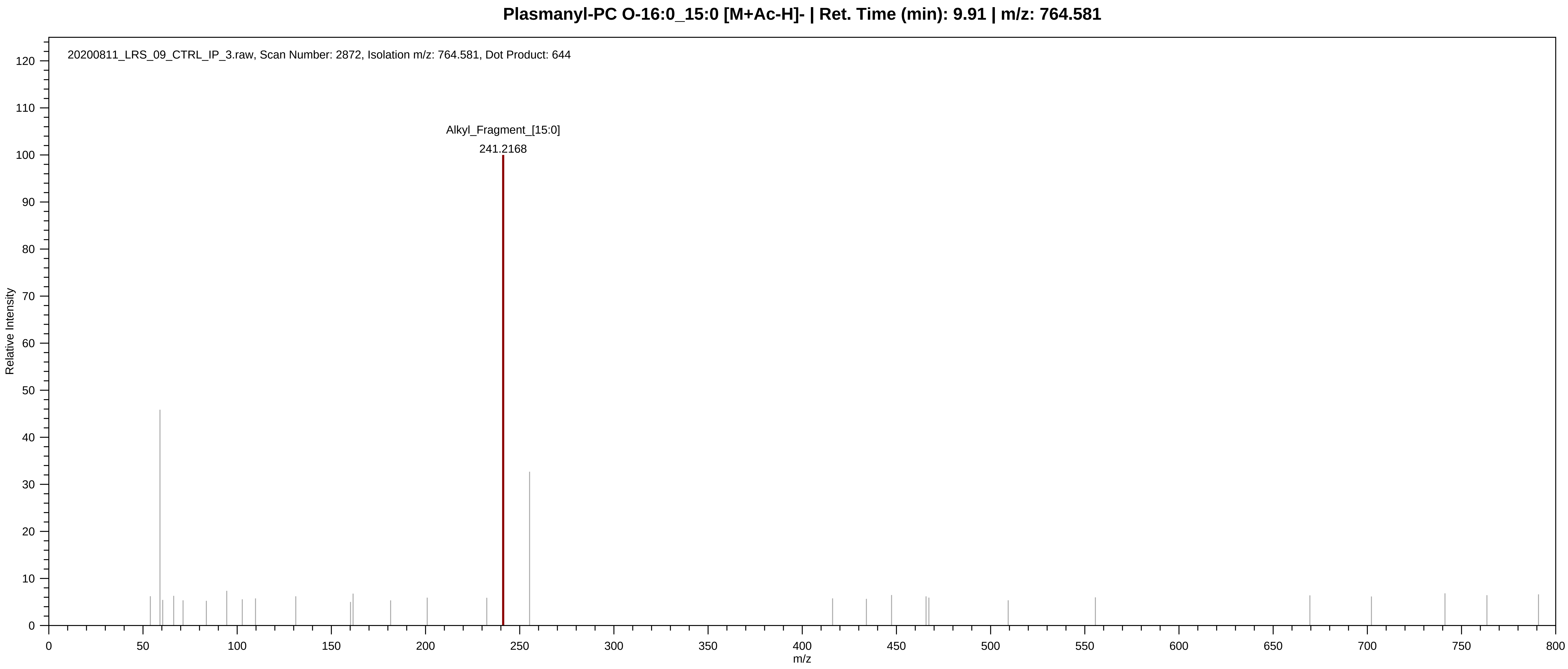
Plasmanyl-PC O-16:0_15:0 [M+Ac-H]- | Ret. Time (min): 9.91 | m/z: 764.581
20200811_LRS_09_CTRL_IP_3.raw, Scan Number: 2872, Isolation m/z: 764.581, Dot Product: 644
Alkyl_Fragment_[15:0]
241.2168
Relative Intensity
m/z
0
10
20
30
40
50
60
70
80
90
100
110
120
0
50
100
150
200
250
300
350
400
450
500
550
600
650
700
750
800

Plasmanyl-PC O-16:0_15:0 [M+Ac-H]- | Ret. Time (min): 9.689 | m/z: 764.581

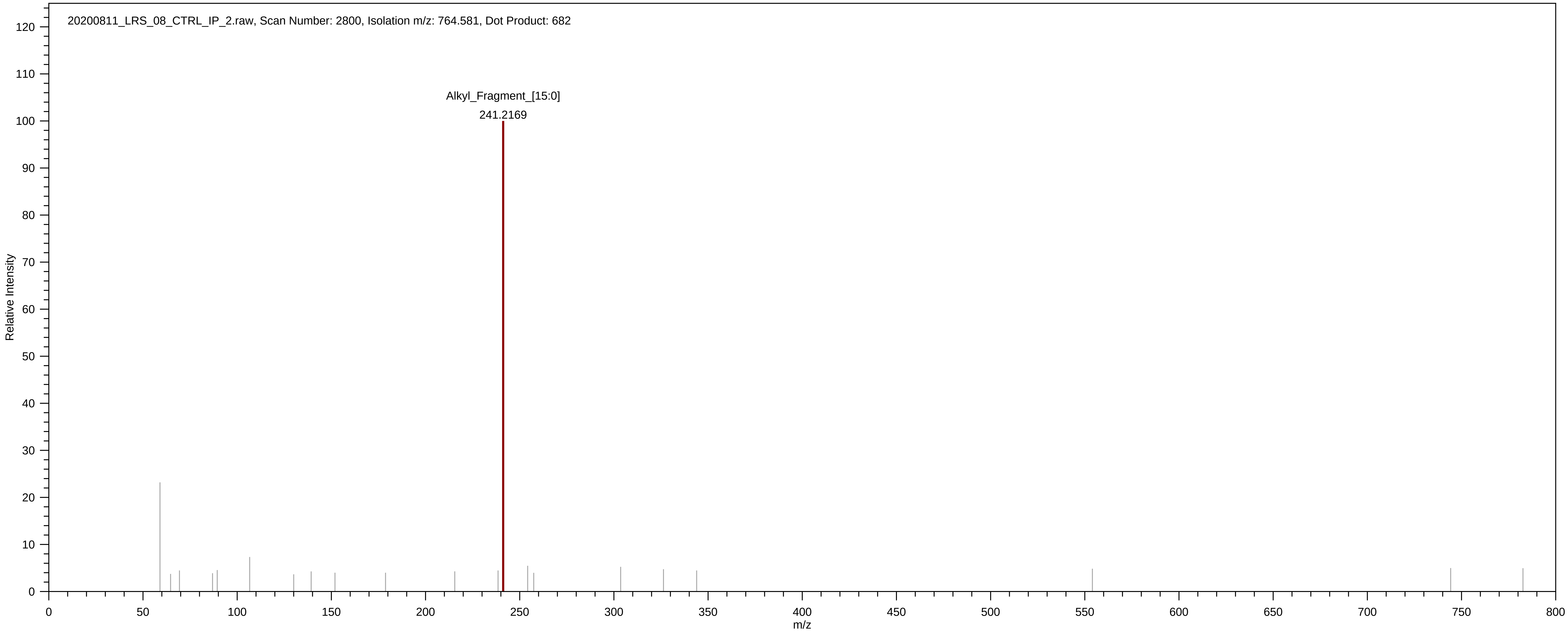

Plasmanyl-PC O-16:0_15:0 [M+Ac-H]- | Ret. Time (min): 9.694 | m/z: 764.581

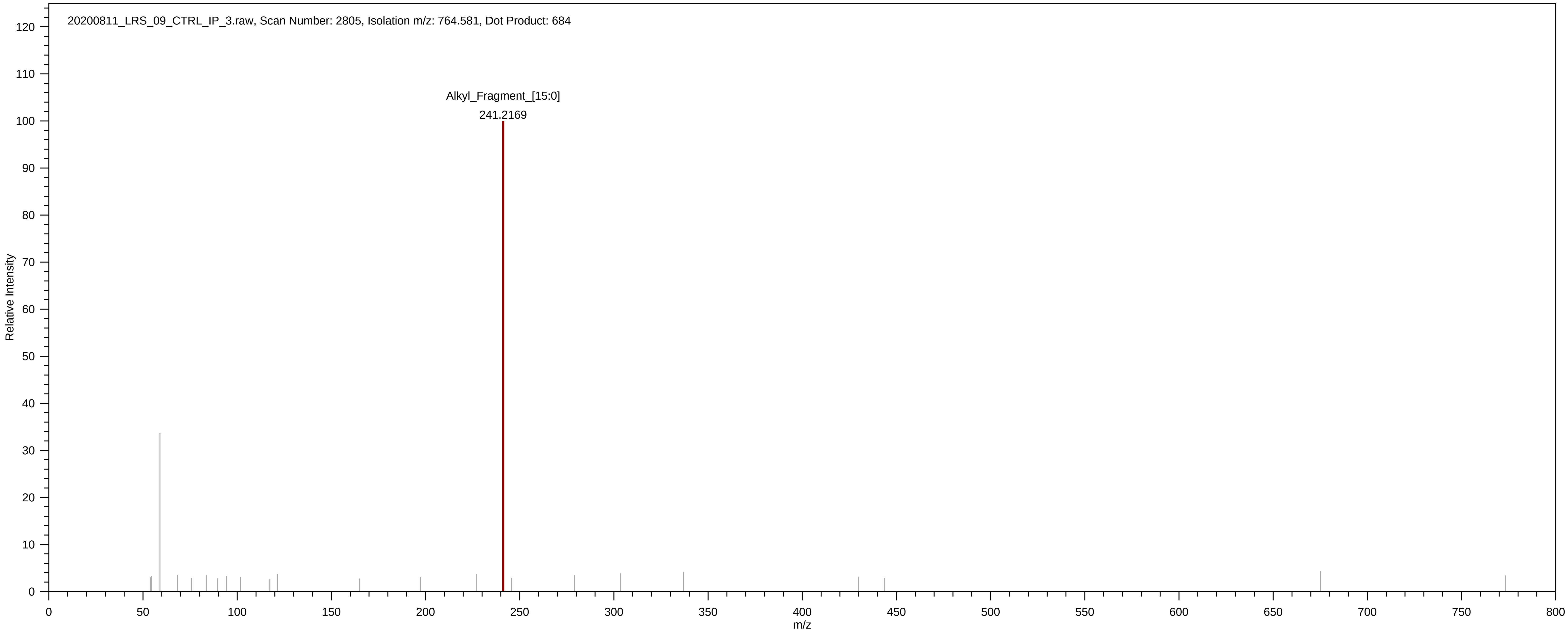

Plasmanyl-PC O-16:0_15:0 [M+Ac-H]- | Ret. Time (min): 9.696 | m/z: 764.582

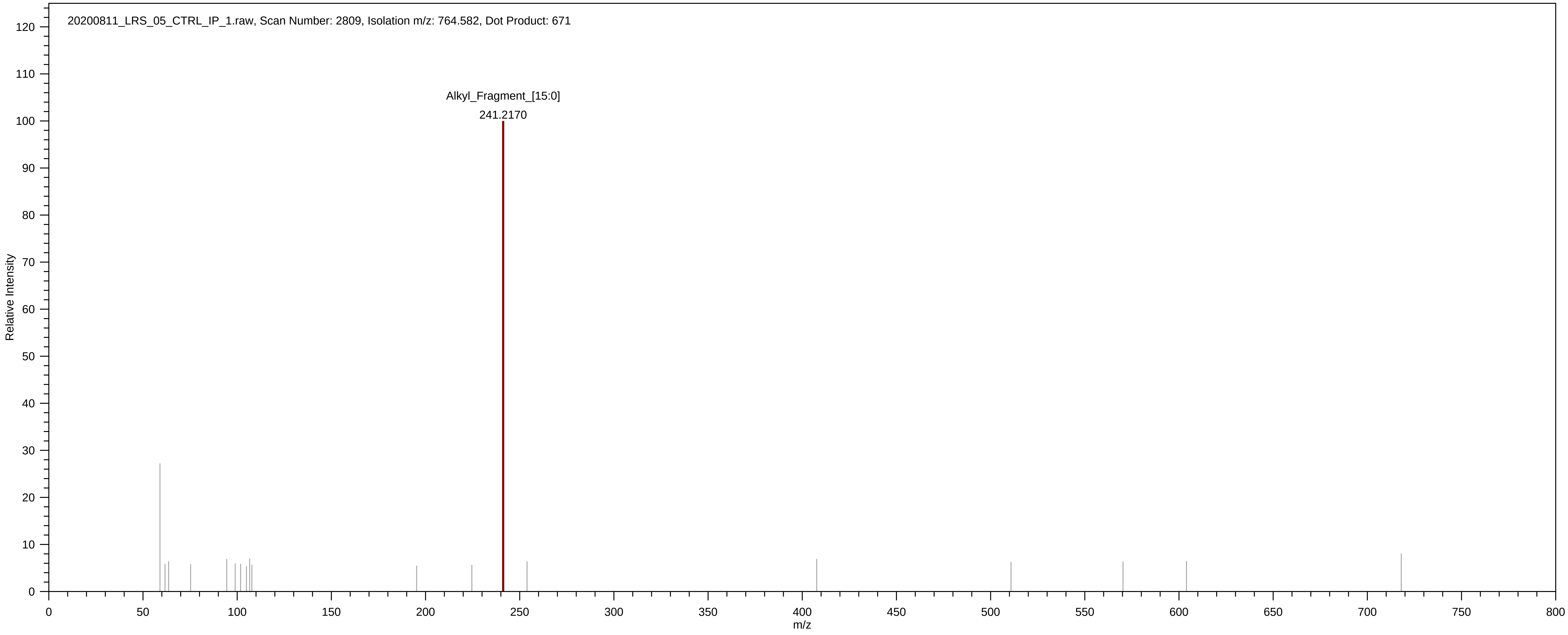

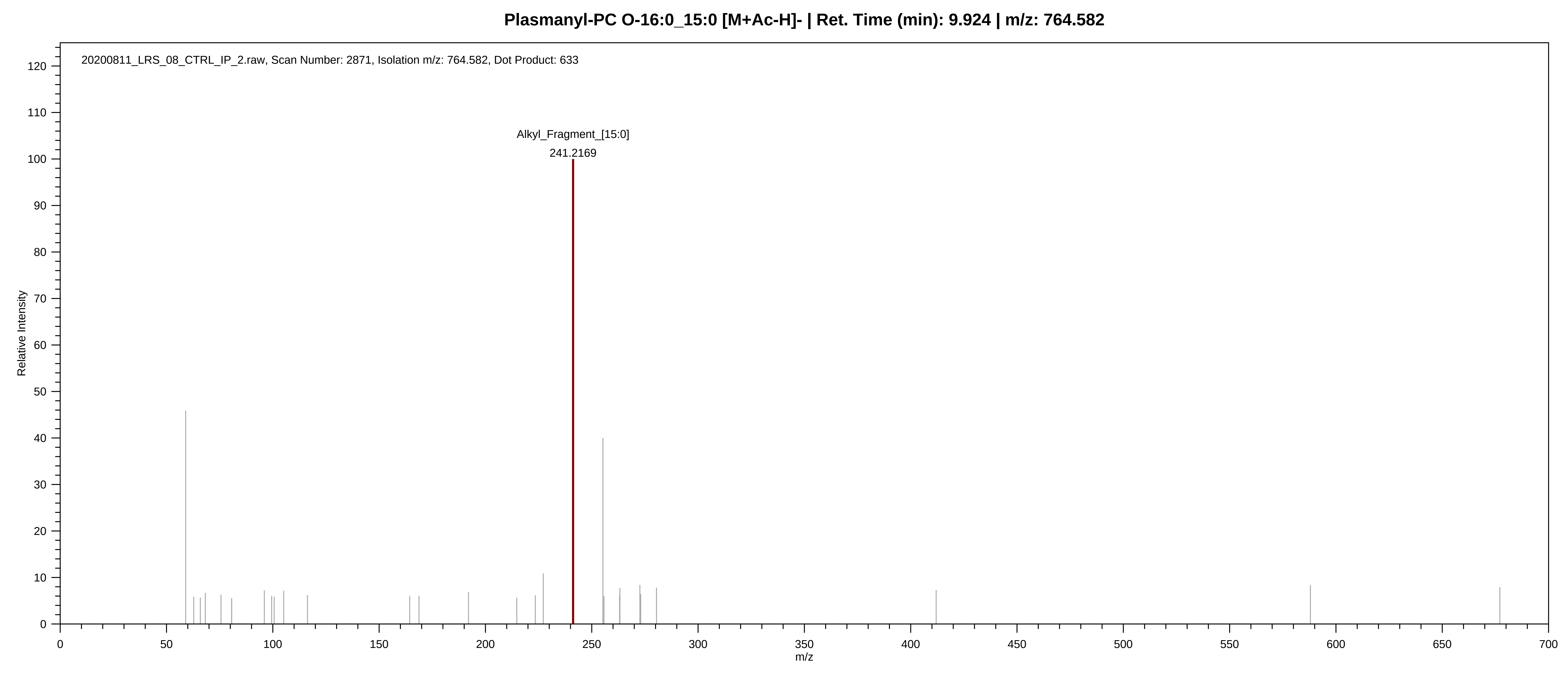
Plasmanyl-PC O-16:0_15:0 [M+Ac-H]- | Ret. Time (min): 9.924 | m/z: 764.582
20200811_LRS_08_CTRL_IP_2.raw, Scan Number: 2871, Isolation m/z: 764.582, Dot Product: 633
Alkyl_Fragment_[15:0]
241.2169
Relative Intensity
m/z

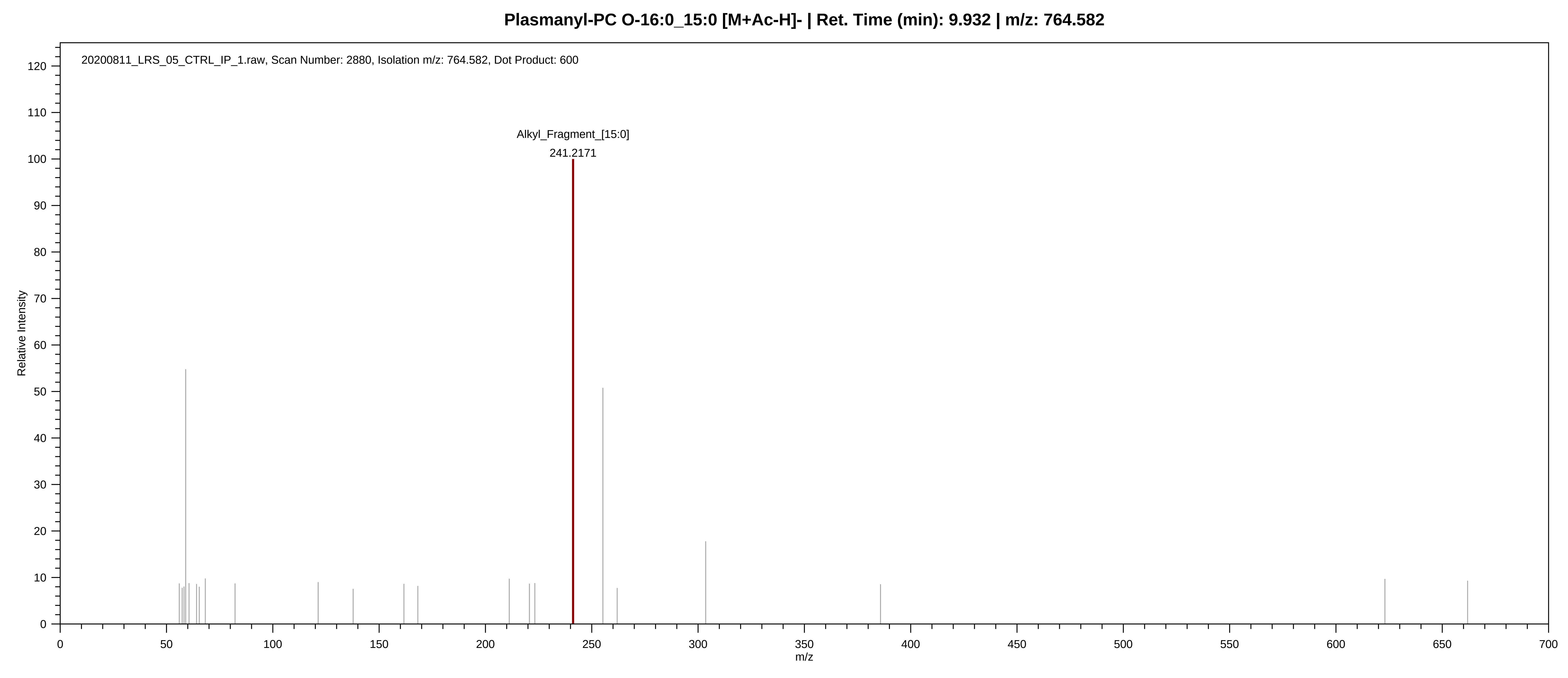
Plasmanyl-PC O-16:0_15:0 [M+Ac-H]- | Ret. Time (min): 9.932 | m/z: 764.582
20200811_LRS_05_CTRL_IP_1.raw, Scan Number: 2880, Isolation m/z: 764.582, Dot Product: 600
Alkyl_Fragment_[15:0]
241.2171
Relative Intensity
m/z
0
10
20
30
40
50
60
70
80
90
100
110
120
0
50
100
150
200
250
300
350
400
450
500
550
600
650
700

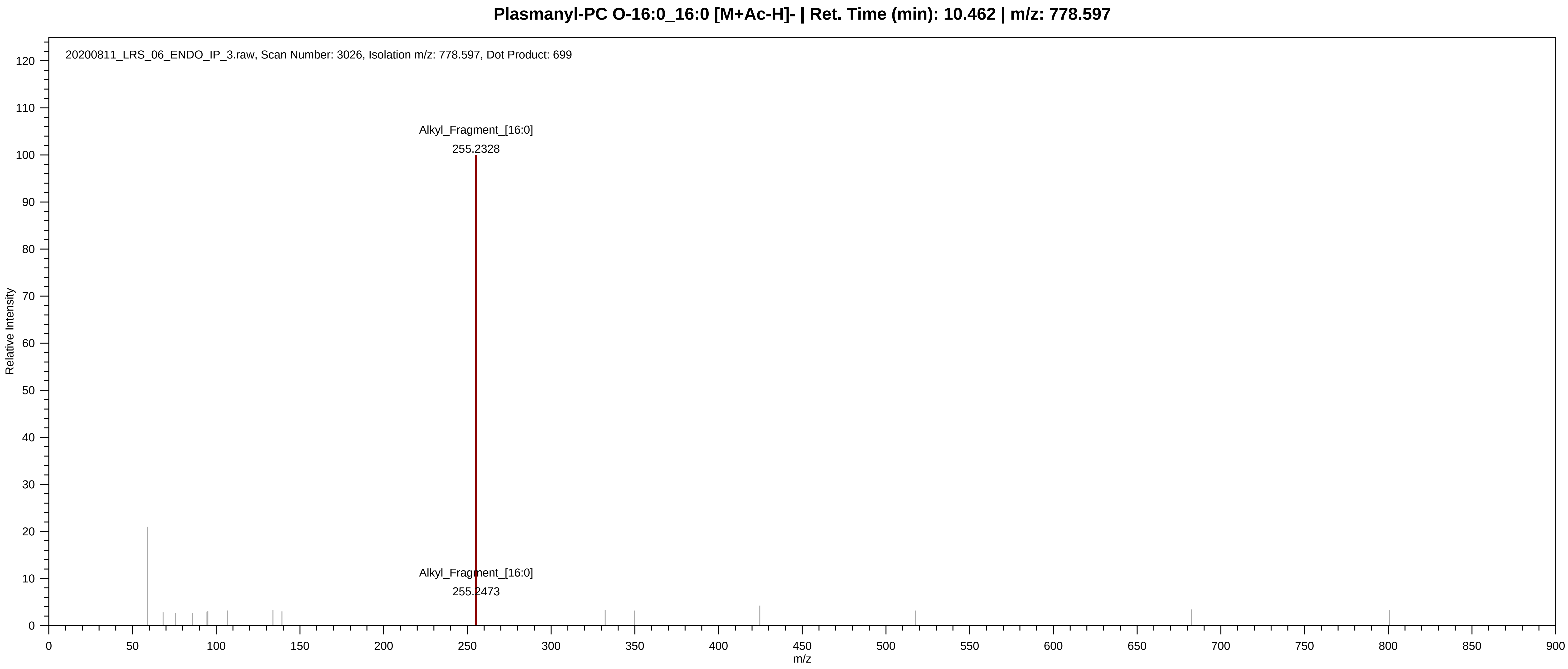
Plasmanyl-PC O-16:0_16:0 [M+Ac-H]- | Ret. Time (min): 10.462 | m/z: 778.597
20200811_LRS_06_ENDO_IP_3.raw, Scan Number: 3026, Isolation m/z: 778.597, Dot Product: 699
Alkyl_Fragment_[16:0]
255.2328
Alkyl_Fragment_[16:0]
255.2473
Relative Intensity
m/z

Plasmanyl-PC O-16:0_16:0 [M+Ac-H]- | Ret. Time (min): 10.462 | m/z: 778.597

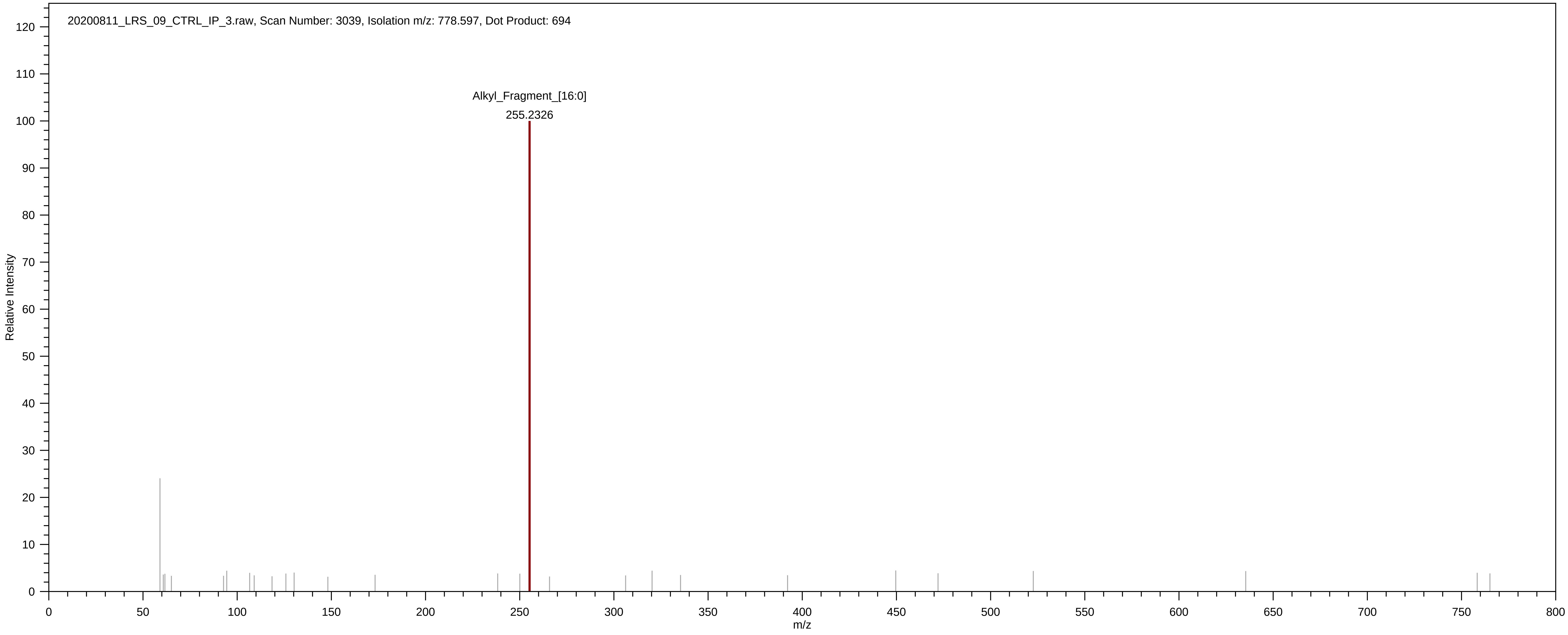

Plasmanyl-PC O-16:0_16:0 [M+Ac-H]- | Ret. Time (min): 10.462 | m/z: 778.596

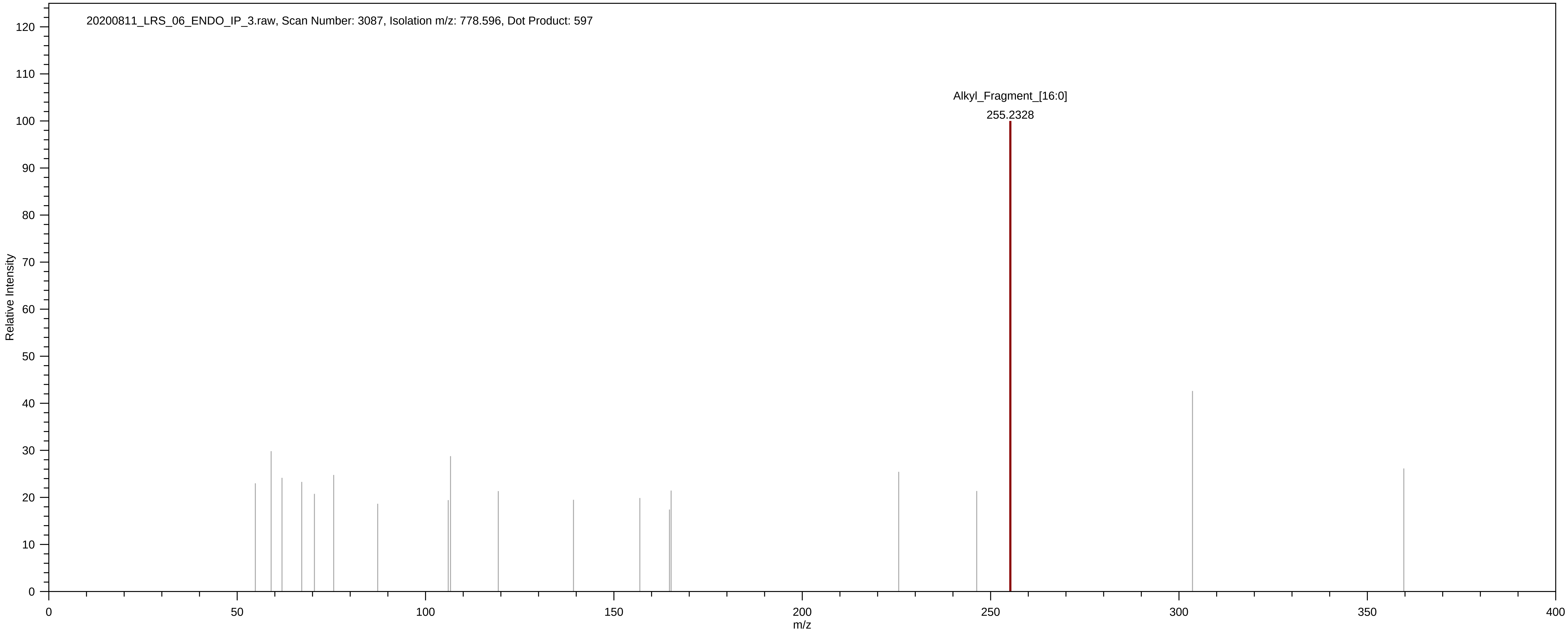

Plasmanyl-PC O-16:0_16:0 [M+Ac-H]- | Ret. Time (min): 10.462 | m/z: 778.597

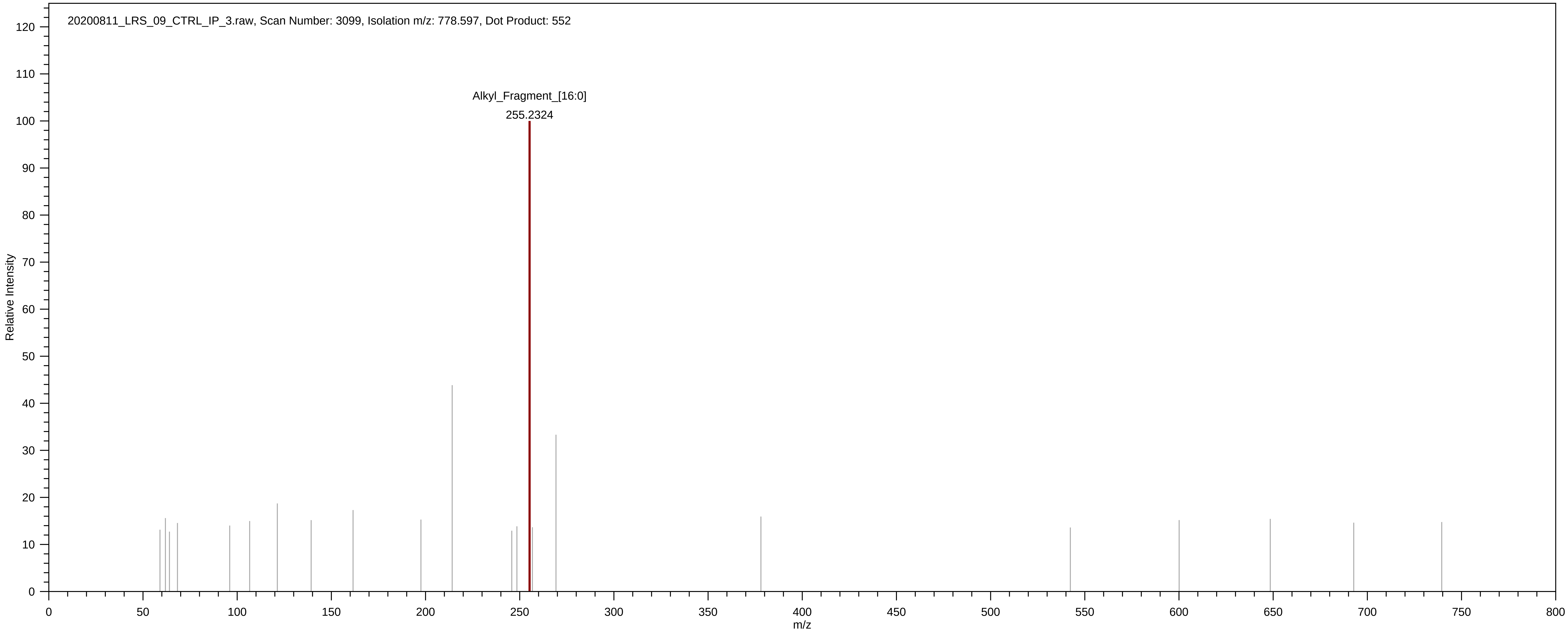

Plasmanyl-PC O-16:0_16:0 [M+Ac-H]- | Ret. Time (min): 10.464 | m/z: 778.597

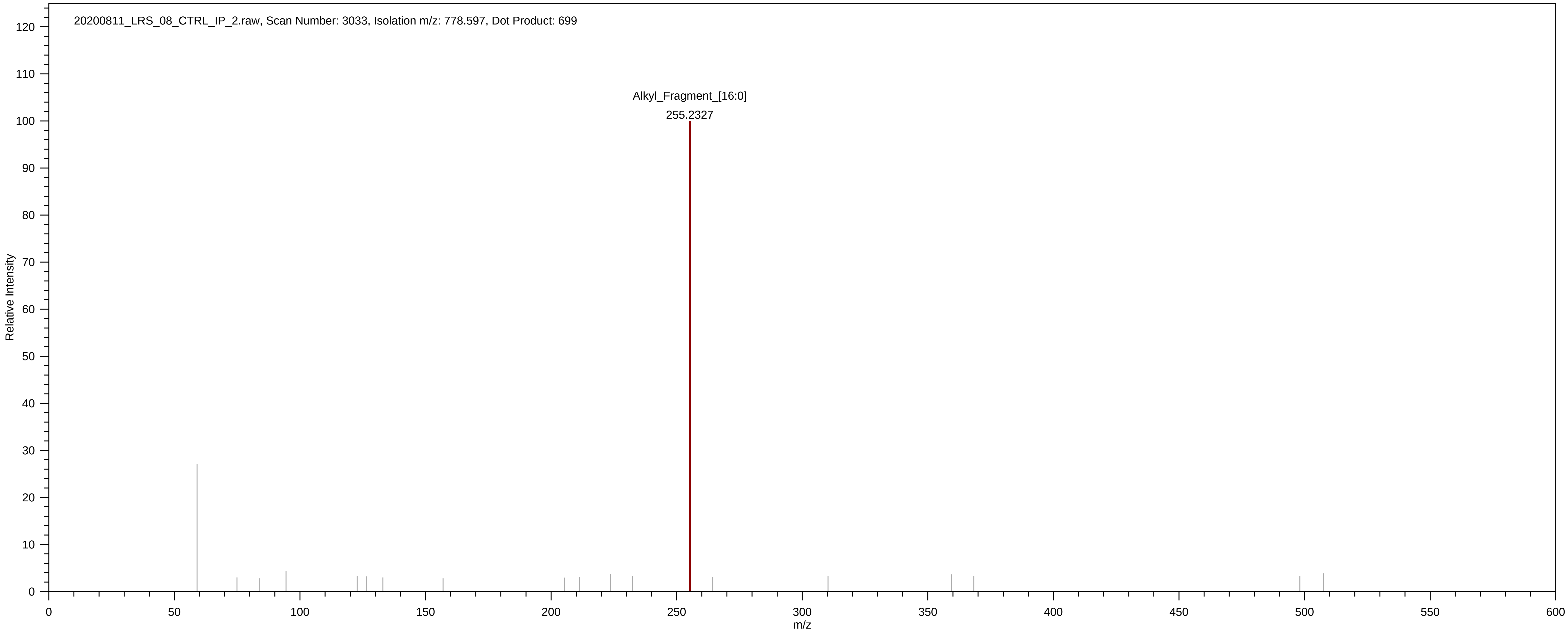

Plasmanyl-PC O-16:0_16:0 [M+Ac-H]- | Ret. Time (min): 10.466 | m/z: 778.597

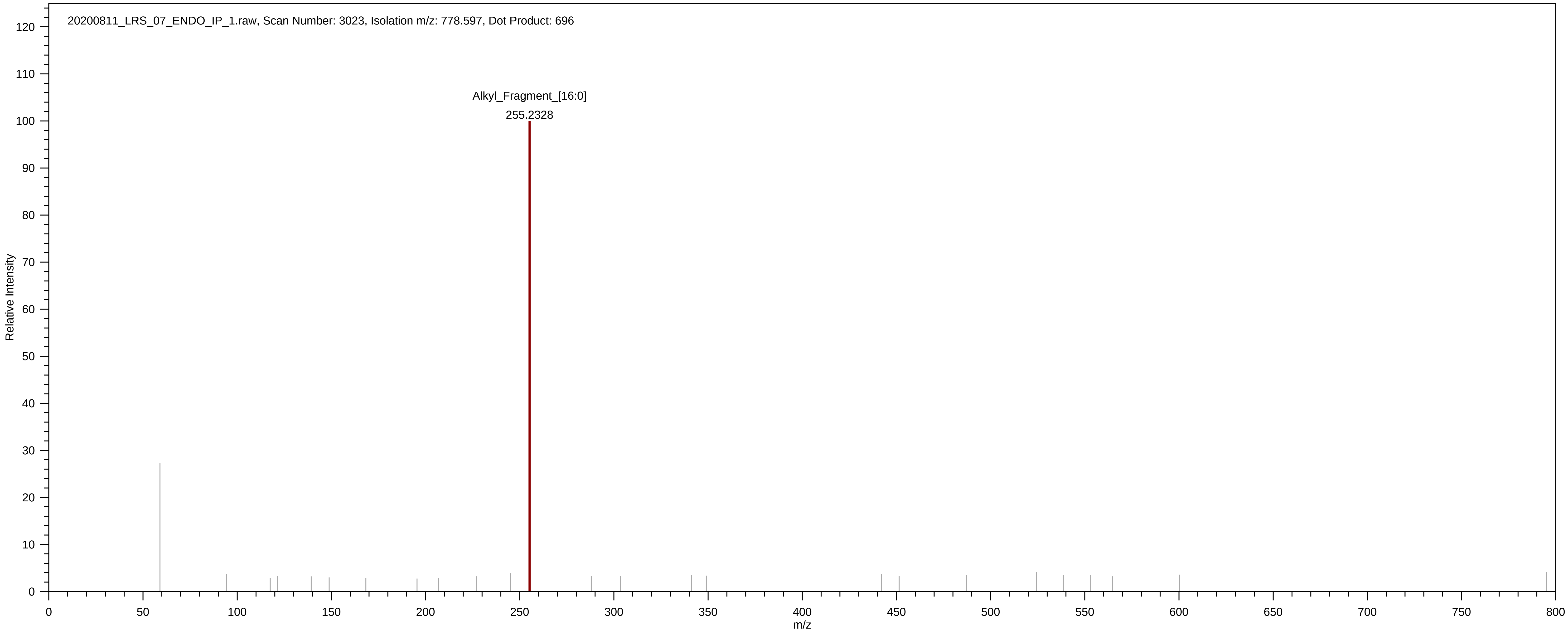

Plasmanyl-PC O-16:0_16:0 [M+Ac-H]- | Ret. Time (min): 10.466 | m/z: 778.597

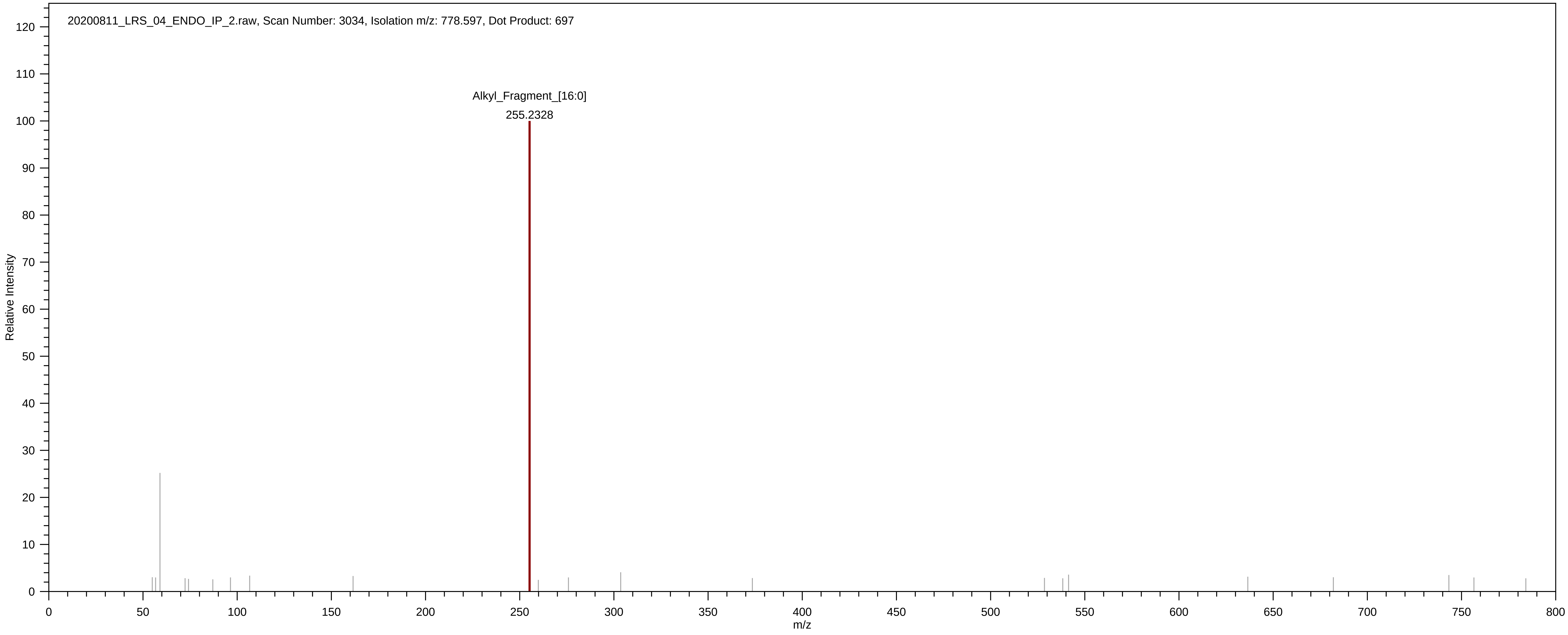

Plasmanyl-PC O-16:0_16:0 [M+Ac-H]- | Ret. Time (min): 10.466 | m/z: 778.594

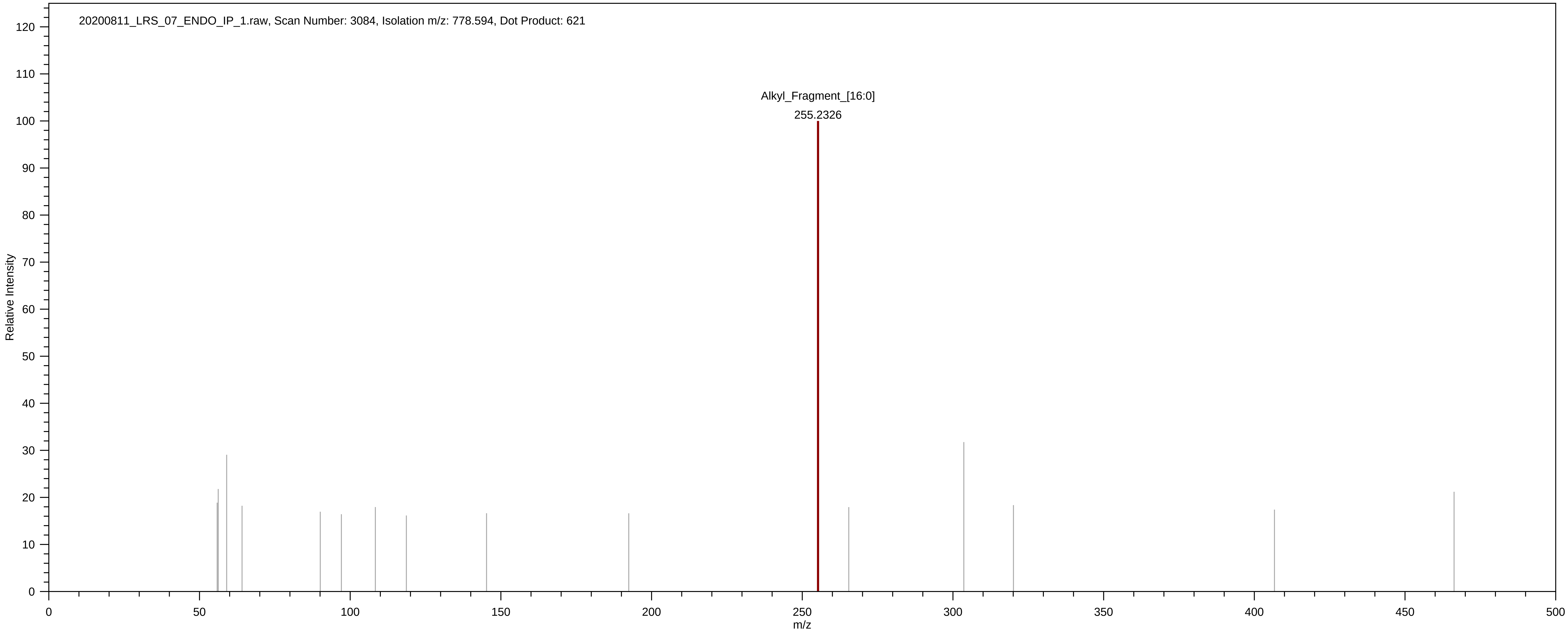

Plasmanyl-PC O-16:0_16:0 [M+Ac-H]- | Ret. Time (min): 10.466 | m/z: 778.596

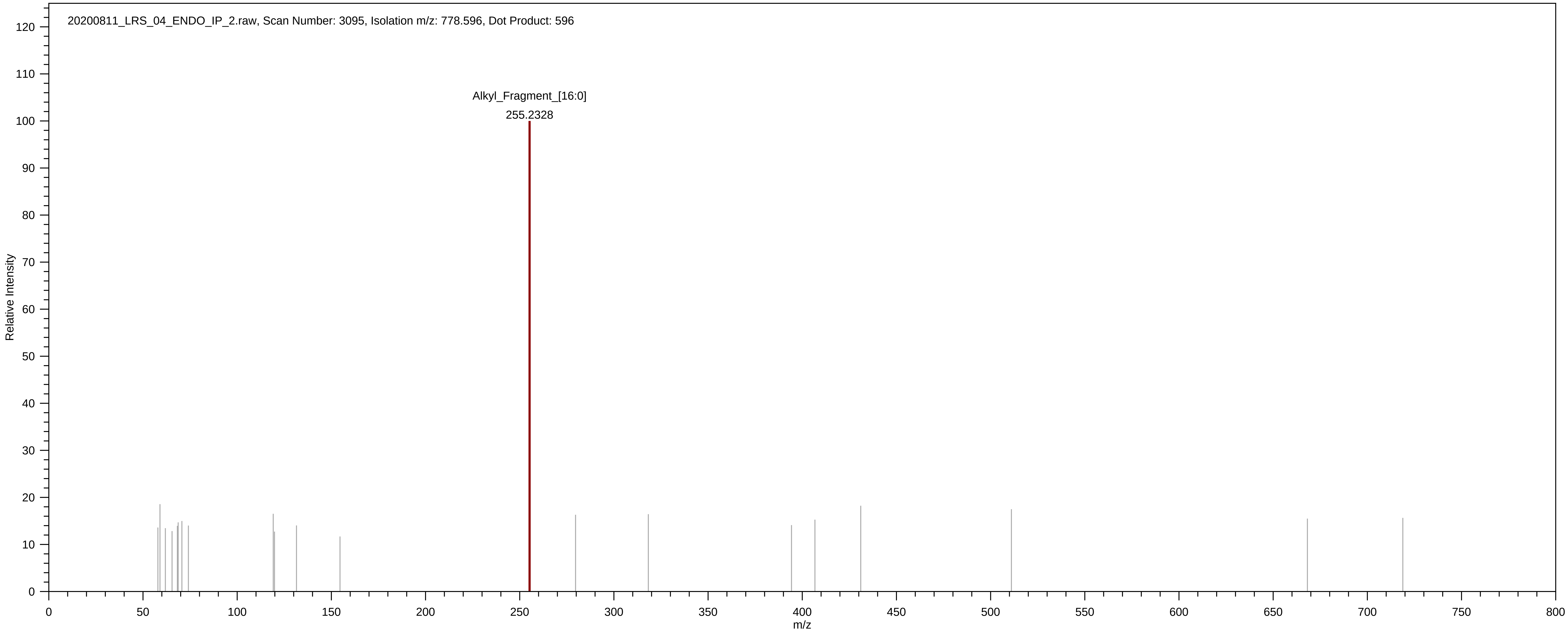

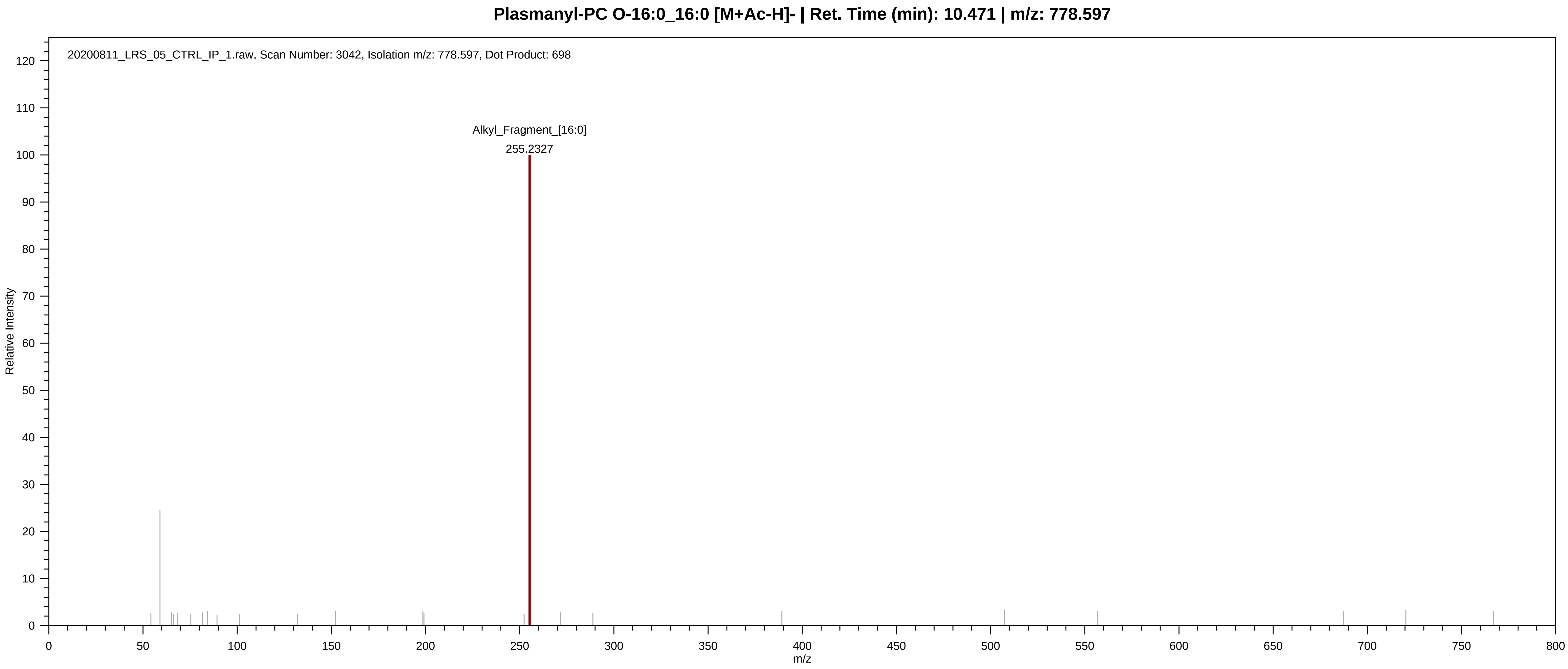

Plasmanyl-PC O-16:0_16:0 [M+Ac-H]- | Ret. Time (min): 10.471 | m/z: 778.597
20200811_LRS_05_CTRL_IP_1.raw, Scan Number: 3042, Isolation m/z: 778.597, Dot Product: 698
Alkyl_Fragment_[16:0]
255.2327
Relative Intensity
m/z
0
10
20
30
40
50
60
70
80
90
100
110
120
0
50
100
150
200
250
300
350
400
450
500
550
600
650
700
750
800

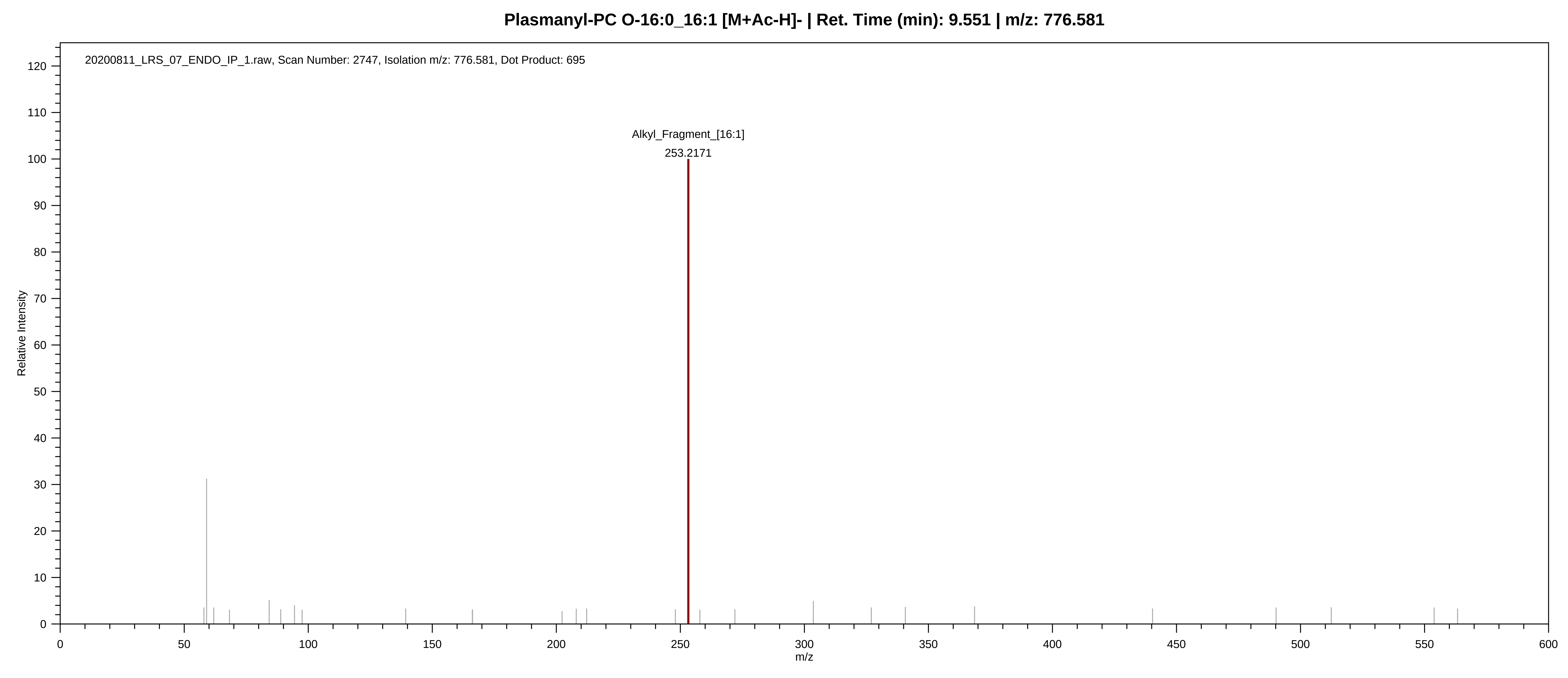

Plasmanyl-PC O-16:0_16:1 [M+Ac-H]- | Ret. Time (min): 9.551 | m/z: 776.581
20200811_LRS_07_ENDO_IP_1.raw, Scan Number: 2747, Isolation m/z: 776.581, Dot Product: 695
Alkyl_Fragment_[16:1]
253.2171
Relative Intensity
m/z

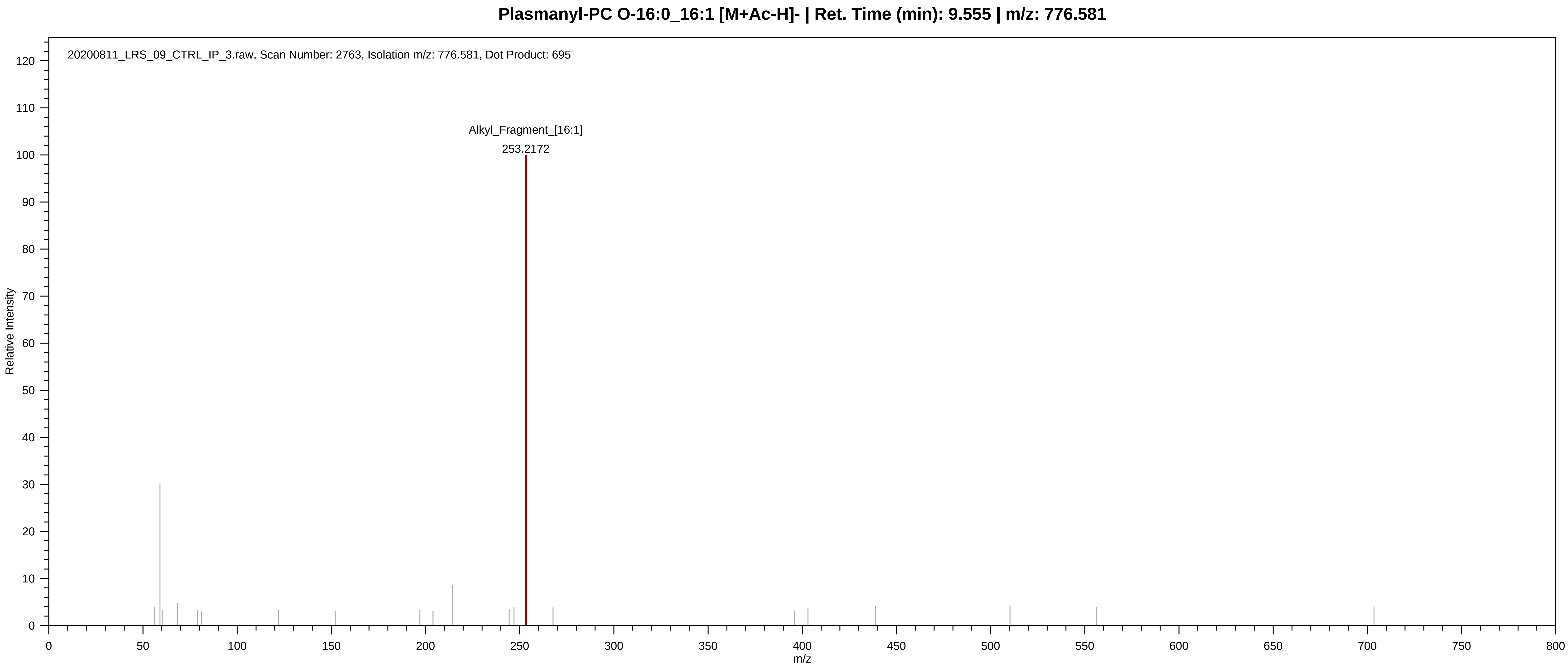
Plasmanyl-PC O-16:0_16:1 [M+Ac-H]- | Ret. Time (min): 9.555 | m/z: 776.581
20200811_LRS_09_CTRL_IP_3.raw, Scan Number: 2763, Isolation m/z: 776.581, Dot Product: 695
Alkyl_Fragment_[16:1]
253.2172
Relative Intensity
m/z
0
10
20
30
40
50
60
70
80
90
100
110
120
0
50
100
150
200
250
300
350
400
450
500
550
600
650
700
750
800

Plasmanyl-PC O-16:0_16:1 [M+Ac-H]- | Ret. Time (min): 9.555 | m/z: 776.582

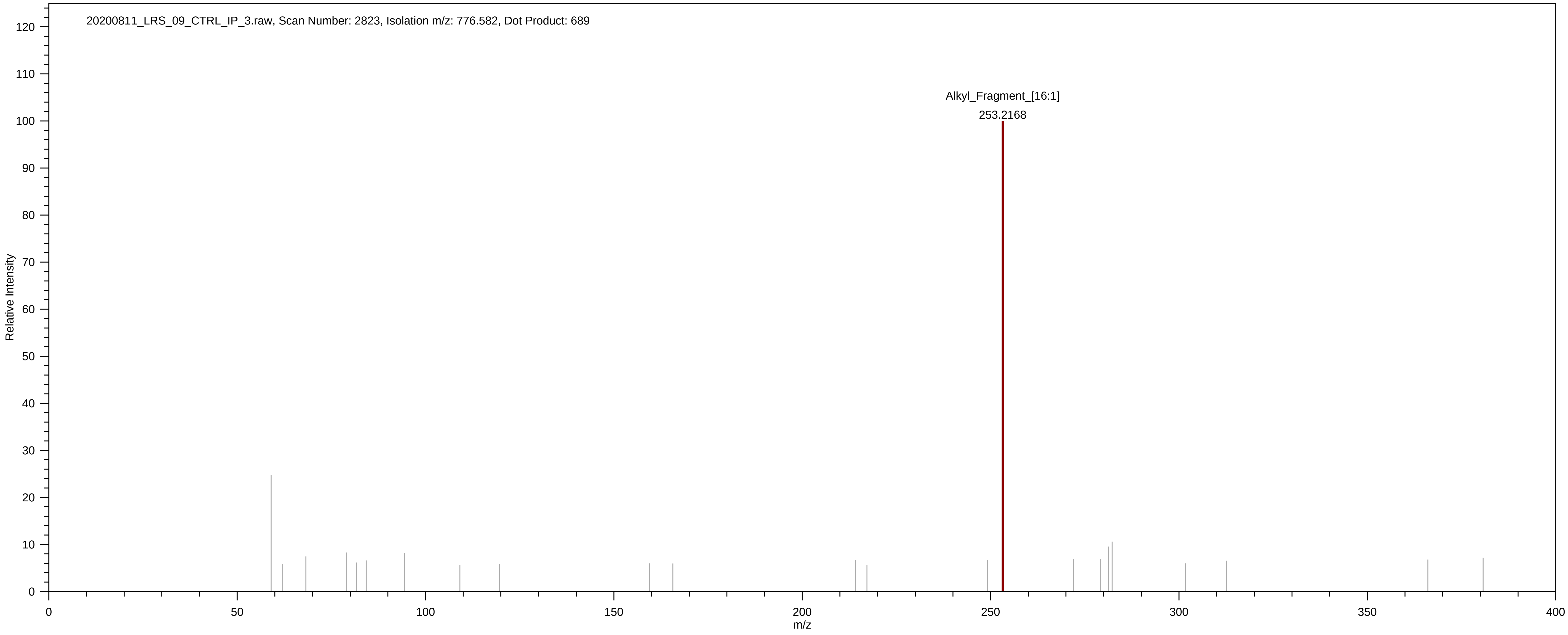

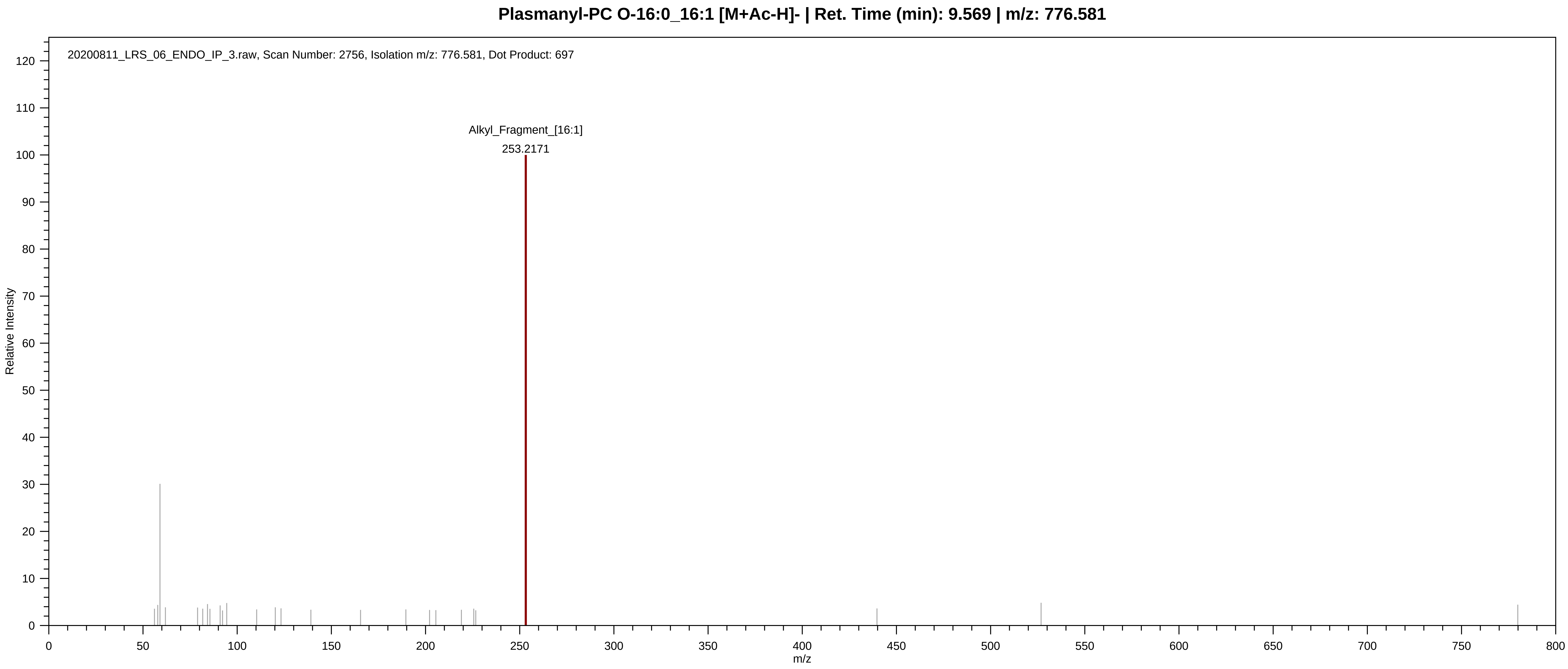
Plasmanyl-PC O-16:0_16:1 [M+Ac-H]- | Ret. Time (min): 9.569 | m/z: 776.581
20200811_LRS_06_ENDO_IP_3.raw, Scan Number: 2756, Isolation m/z: 776.581, Dot Product: 697
Alkyl_Fragment_[16:1]
253.2171
Relative Intensity
m/z
0
10
20
30
40
50
60
70
80
90
100
110
120
0
50
100
150
200
250
300
350
400
450
500
550
600
650
700
750
800

Plasmanyl-PC O-16:0_16:1 [M+Ac-H]- | Ret. Time (min): 9.451 | m/z: 776.582

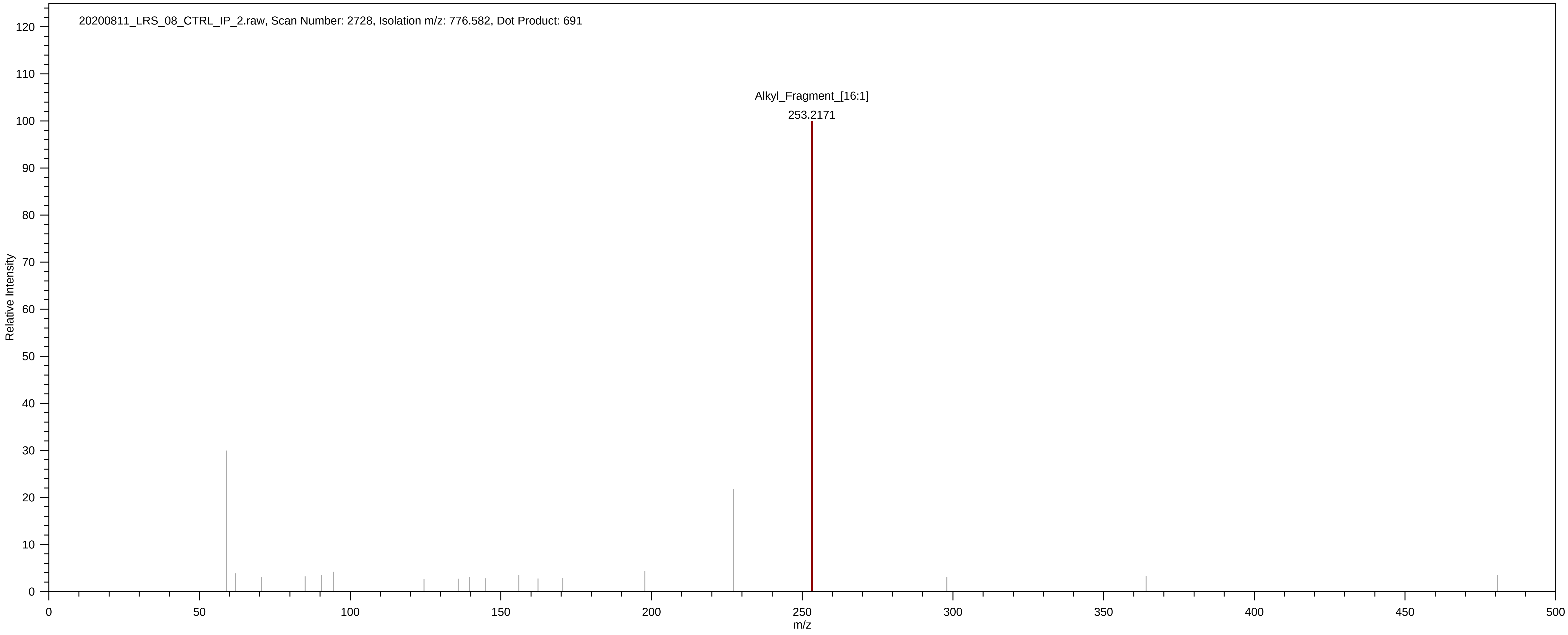

Plasmanyl-PC O-16:0_16:1 [M+Ac-H]- | Ret. Time (min): 9.494 | m/z: 776.582

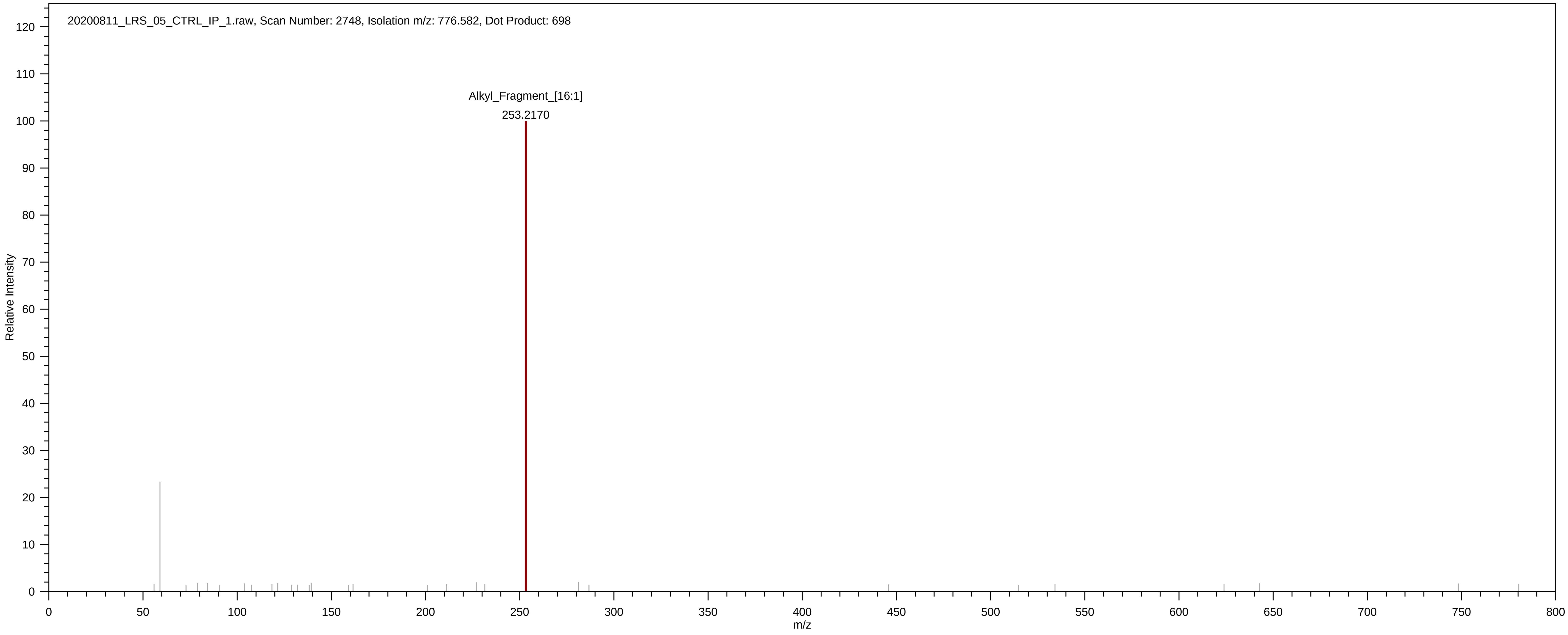

Plasmanyl-PC O-16:0_16:1 [M+Ac-H]- | Ret. Time (min): 9.566 | m/z: 776.582

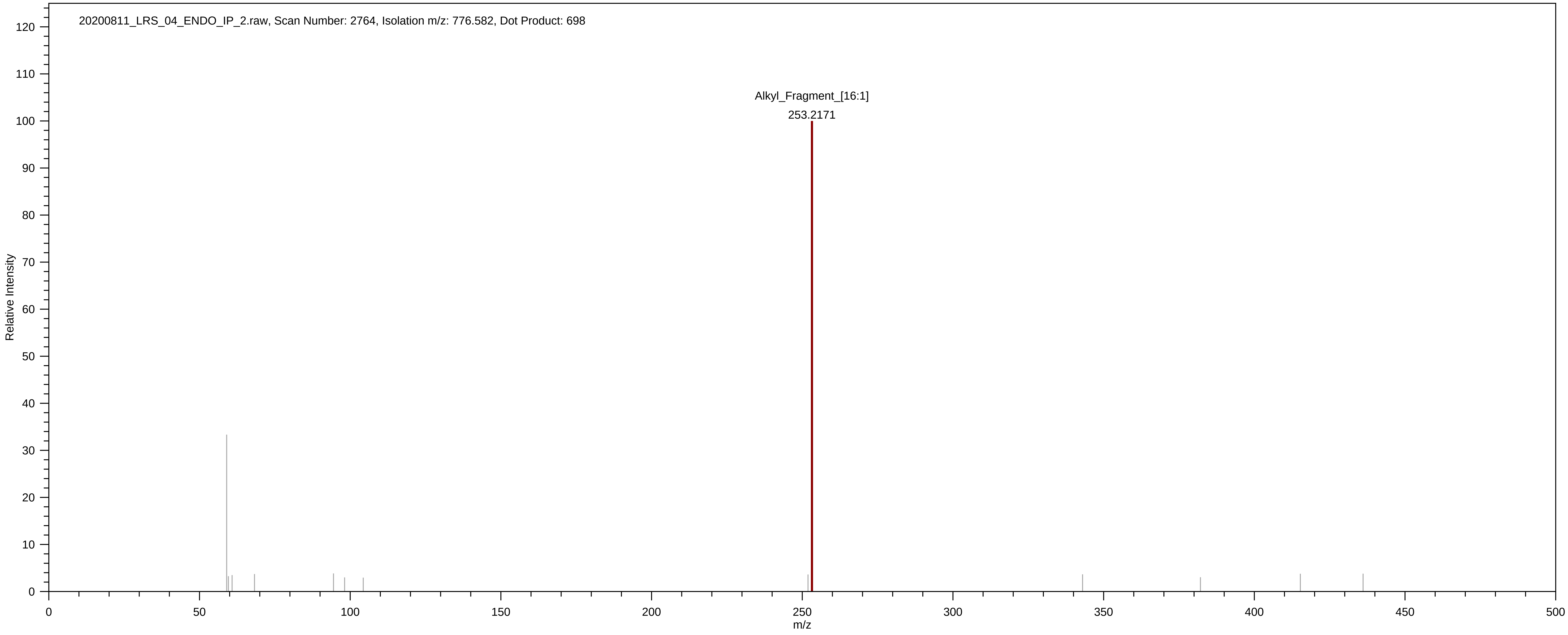

Plasmanyl-PC O-16:0_16:1 [M+Ac-H]- | Ret. Time (min): 9.645 | m/z: 776.582

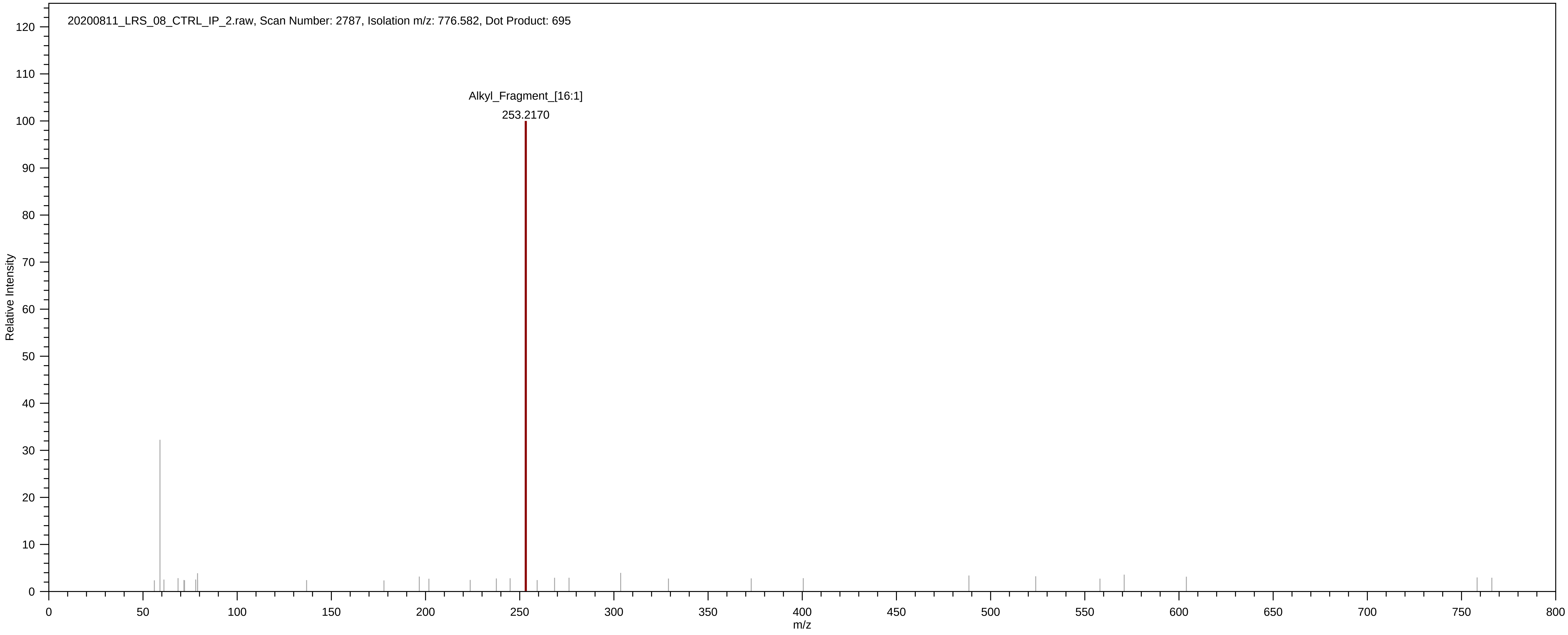

Plasmanyl-PC O-16:0_16:1 [M+Ac-H]- | Ret. Time (min): 9.695 | m/z: 776.582

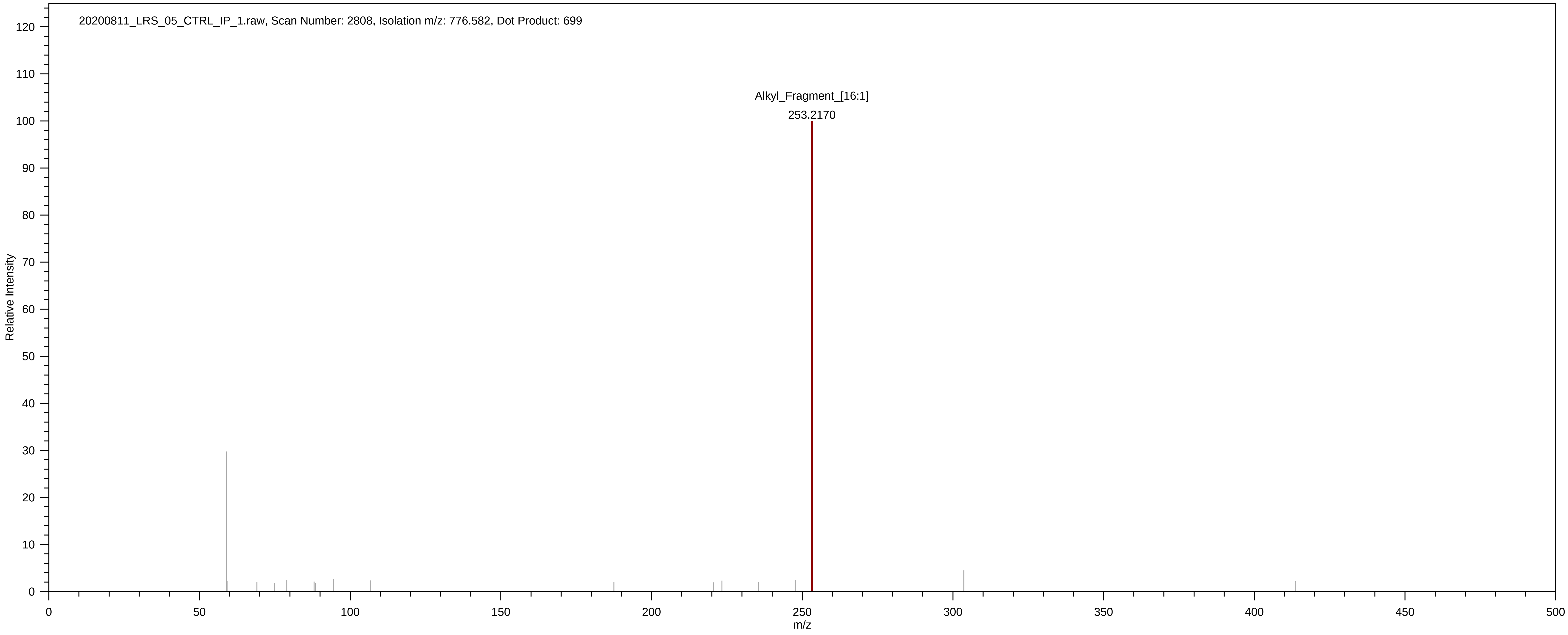

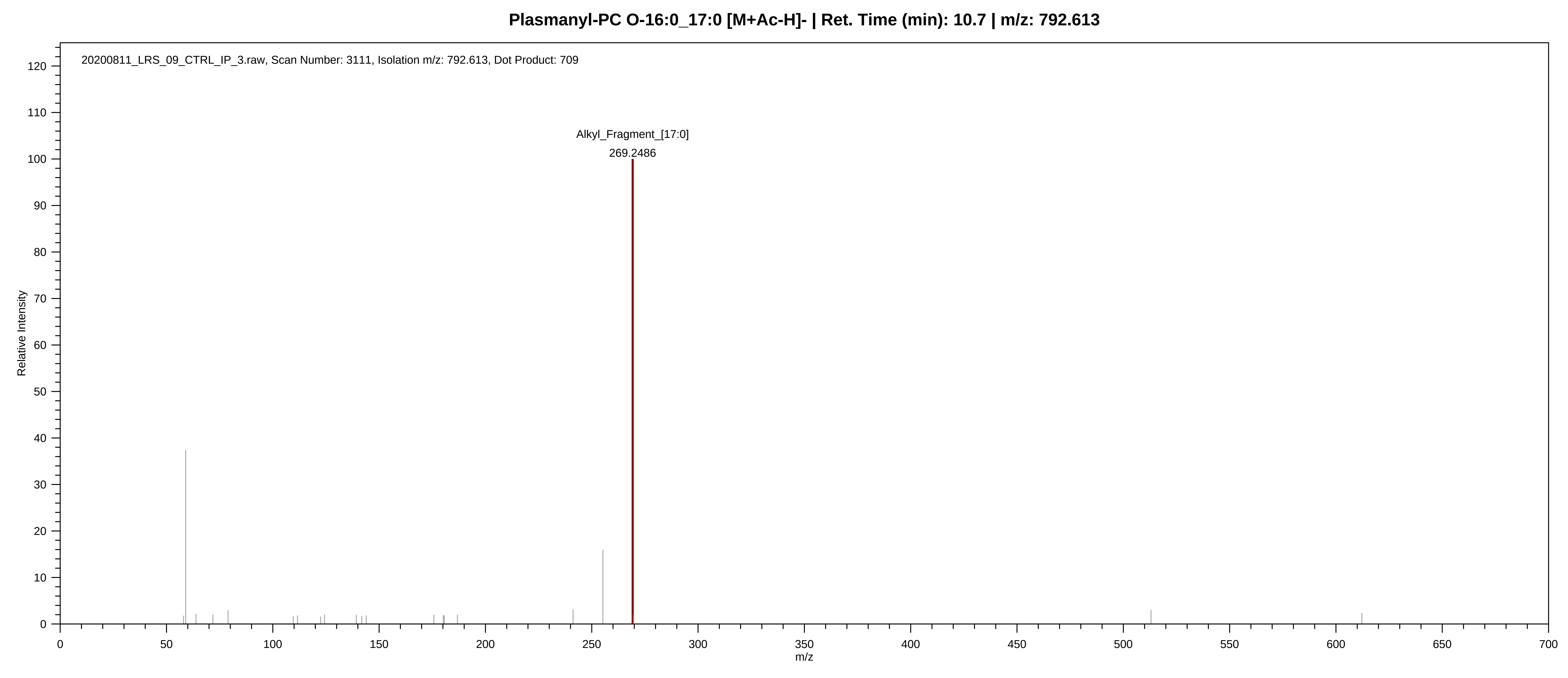

Plasmanyl-PC O-16:0_17:0 [M+Ac-H]- | Ret. Time (min): 10.7 | m/z: 792.613
20200811_LRS_09_CTRL_IP_3.raw, Scan Number: 3111, Isolation m/z: 792.613, Dot Product: 709
Alkyl_Fragment_[17:0]
269.2486
Relative Intensity
m/z

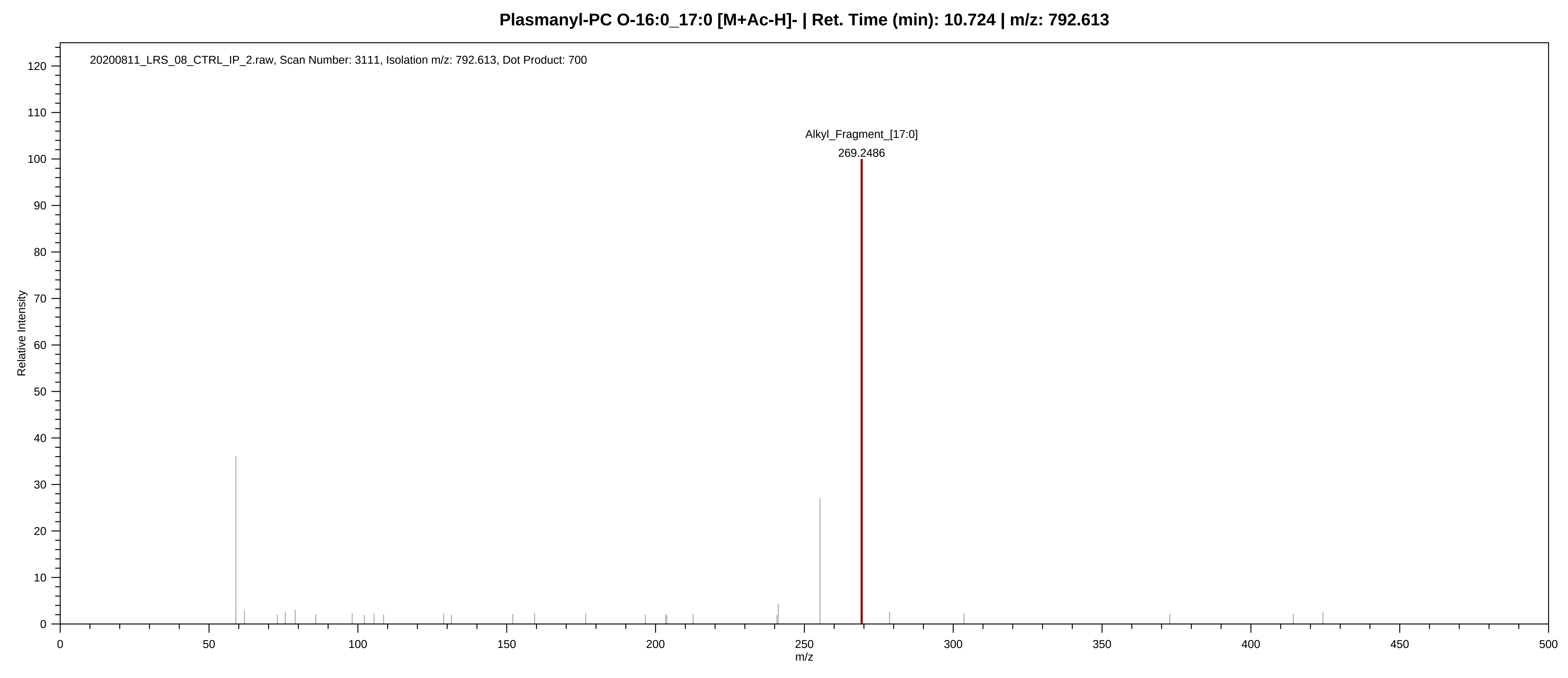
Plasmanyl-PC O-16:0_17:0 [M+Ac-H]- | Ret. Time (min): 10.724 | m/z: 792.613
20200811_LRS_08_CTRL_IP_2.raw, Scan Number: 3111, Isolation m/z: 792.613, Dot Product: 700
Alkyl_Fragment_[17:0]
269.2486
Relative Intensity
m/z

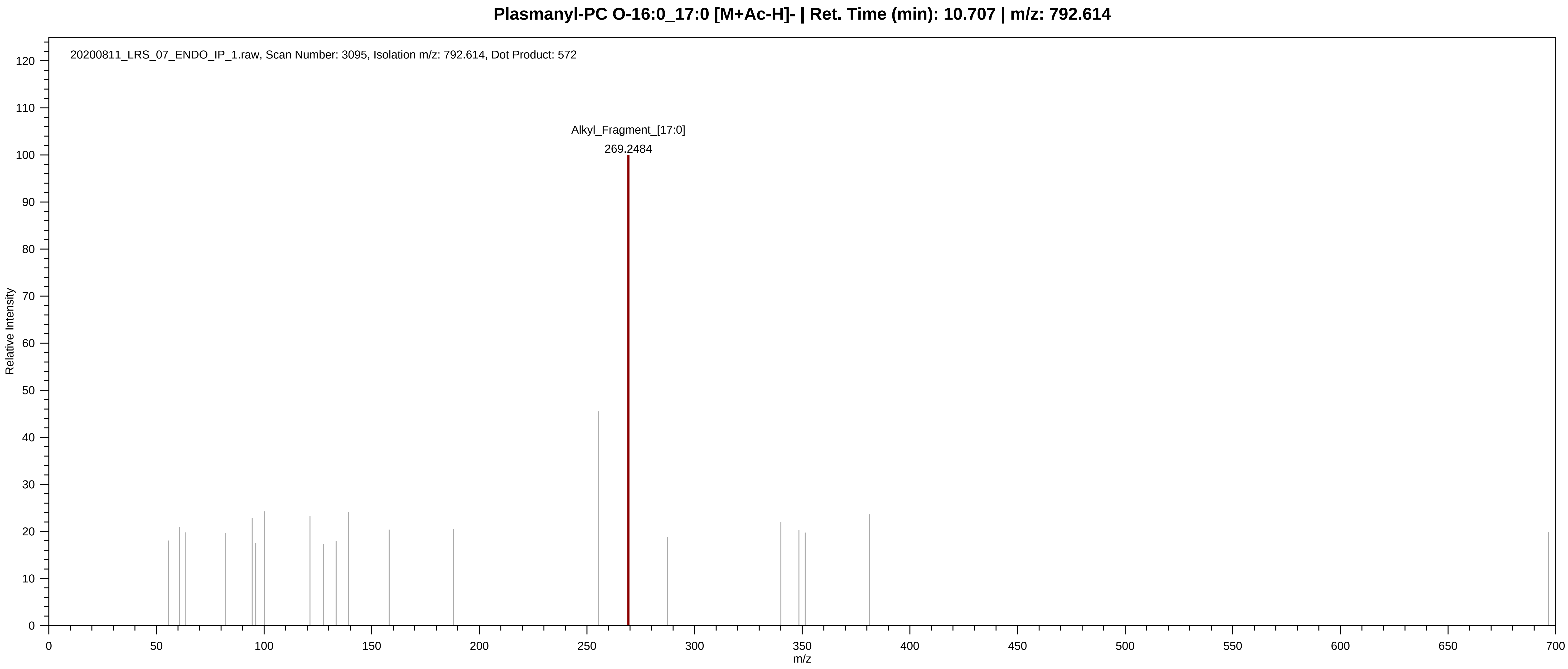
Plasmanyl-PC O-16:0_17:0 [M+Ac-H]- | Ret. Time (min): 10.707 | m/z: 792.614
20200811_LRS_07_ENDO_IP_1.raw, Scan Number: 3095, Isolation m/z: 792.614, Dot Product: 572
Alkyl_Fragment_[17:0]
269.2484
Relative Intensity
m/z
0
10
20
30
40
50
60
70
80
90
100
110
120
0
50
100
150
200
250
300
350
400
450
500
550
600
650
700

Plasmanyl-PC O-16:0_17:0 [M+Ac-H]- | Ret. Time (min): 10.702 | m/z: 792.615

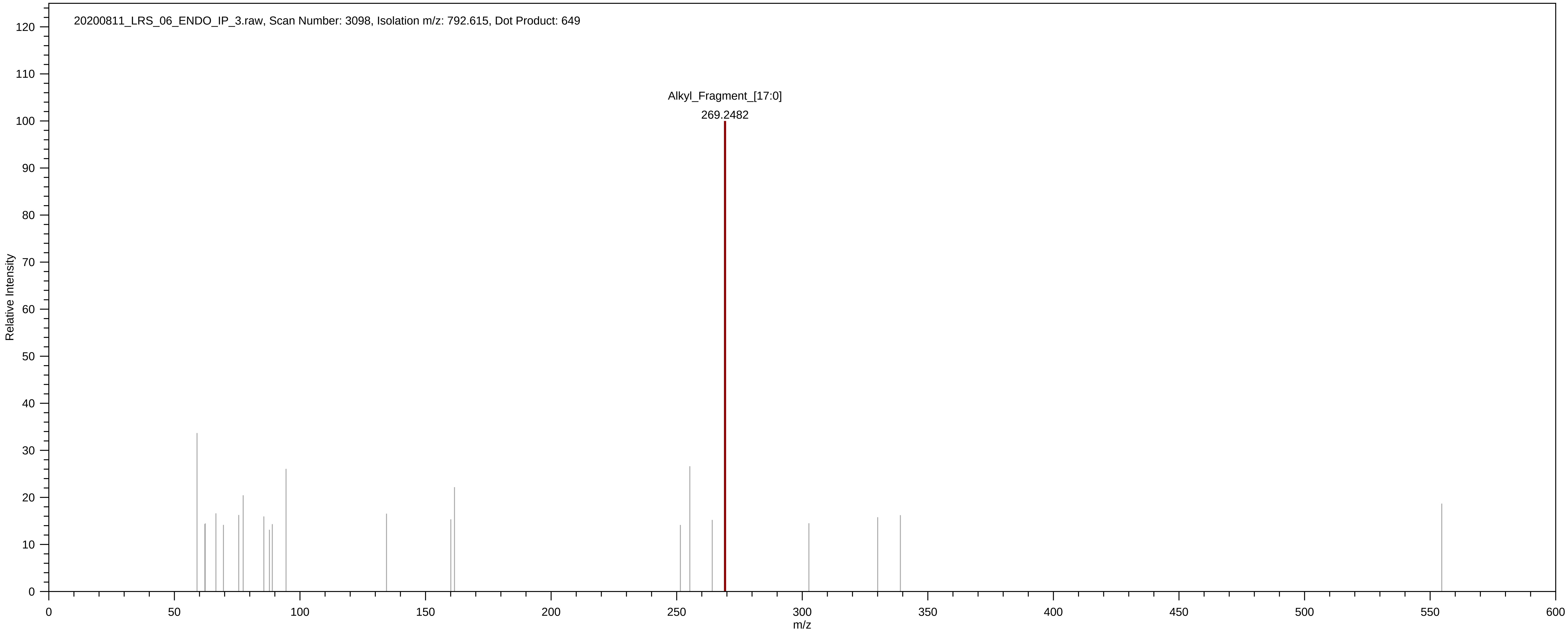

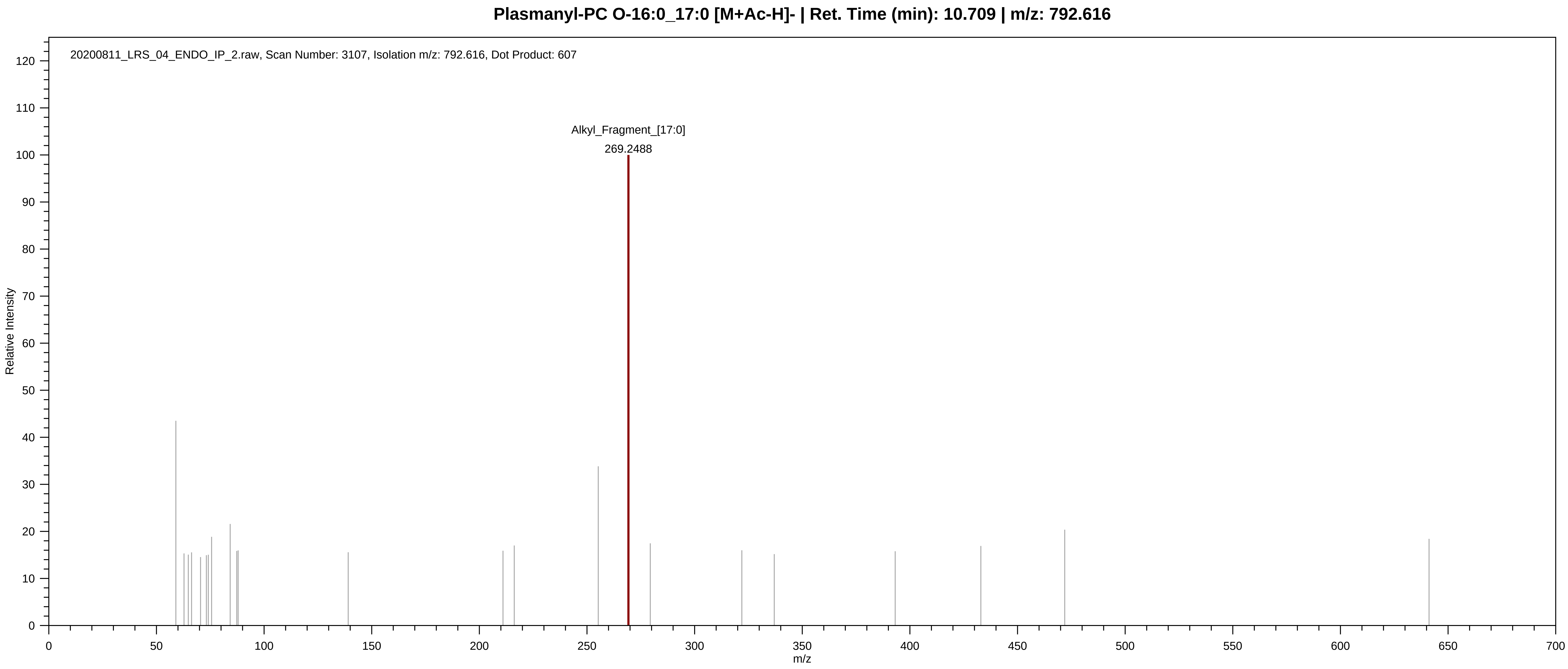
Plasmanyl-PC O-16:0_17:0 [M+Ac-H]- | Ret. Time (min): 10.709 | m/z: 792.616
20200811_LRS_04_ENDO_IP_2.raw, Scan Number: 3107, Isolation m/z: 792.616, Dot Product: 607
Alkyl_Fragment_[17:0]
269.2488
Relative Intensity
m/z
0
10
20
30
40
50
60
70
80
90
100
110
120
0
50
100
150
200
250
300
350
400
450
500
550
600
650
700

Plasmanyl-PC O-16:0_17:1 [M+Ac-H]- | Ret. Time (min): 9.947 | m/z: 790.6

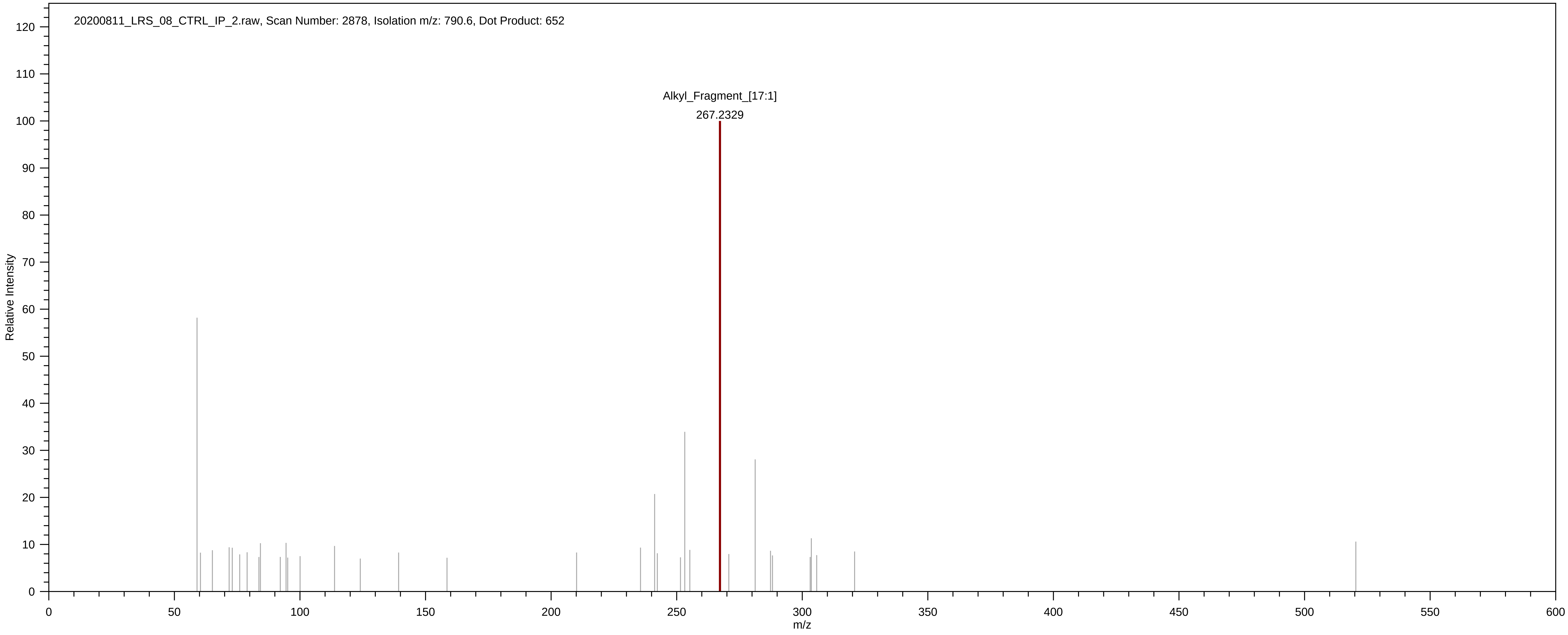

Plasmanyl-PC O-16:0_17:1 [M+Ac-H]- | Ret. Time (min): 9.947 | m/z: 790.601

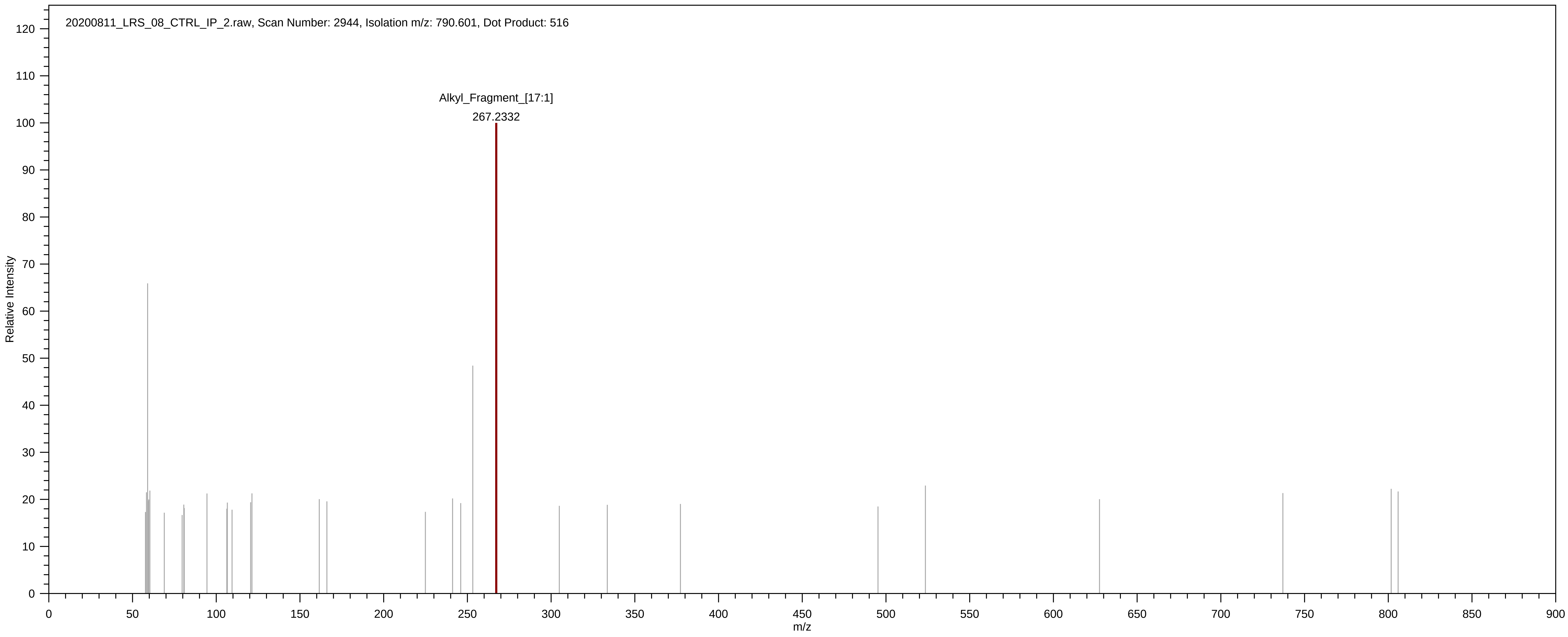

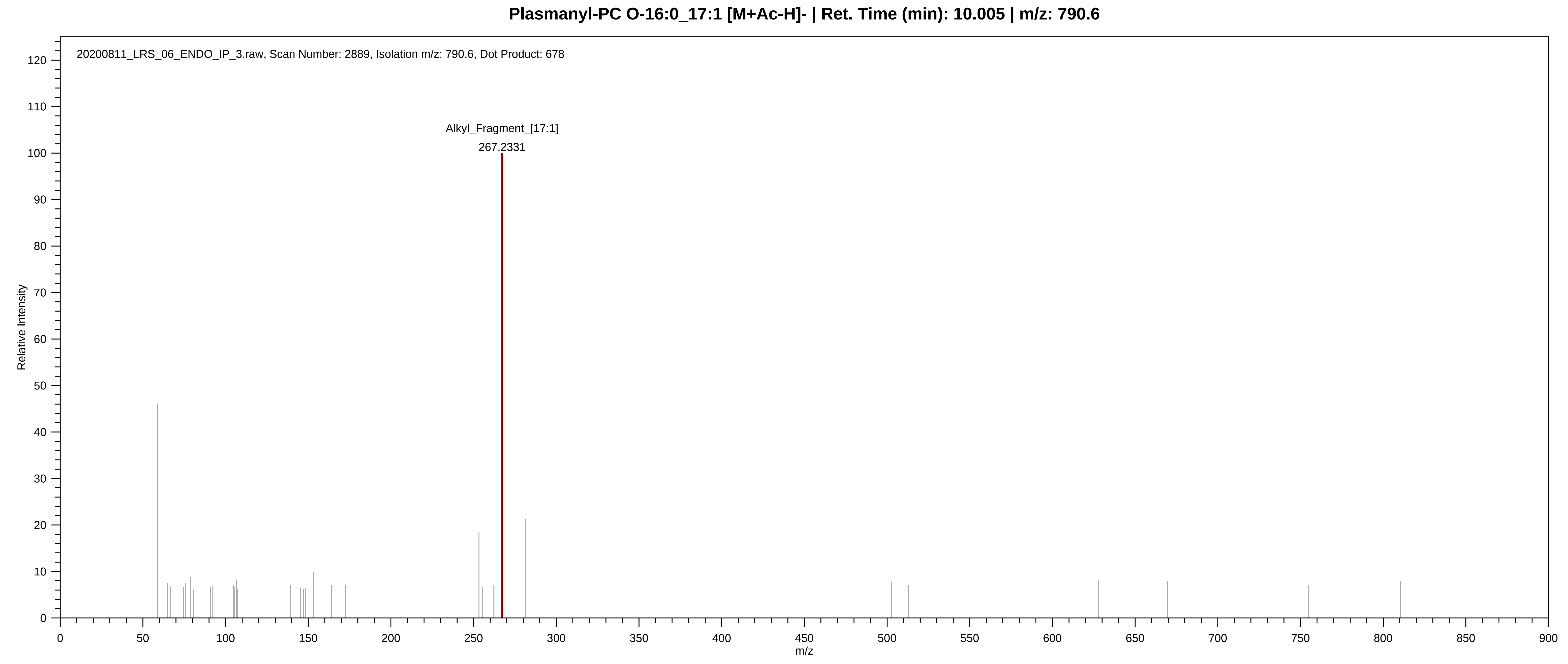
Plasmanyl-PC O-16:0_17:1 [M+Ac-H]- | Ret. Time (min): 10.005 | m/z: 790.6
20200811_LRS_06_ENDO_IP_3.raw, Scan Number: 2889, Isolation m/z: 790.6, Dot Product: 678
Alkyl_Fragment_[17:1]
267.2331
Relative Intensity
m/z

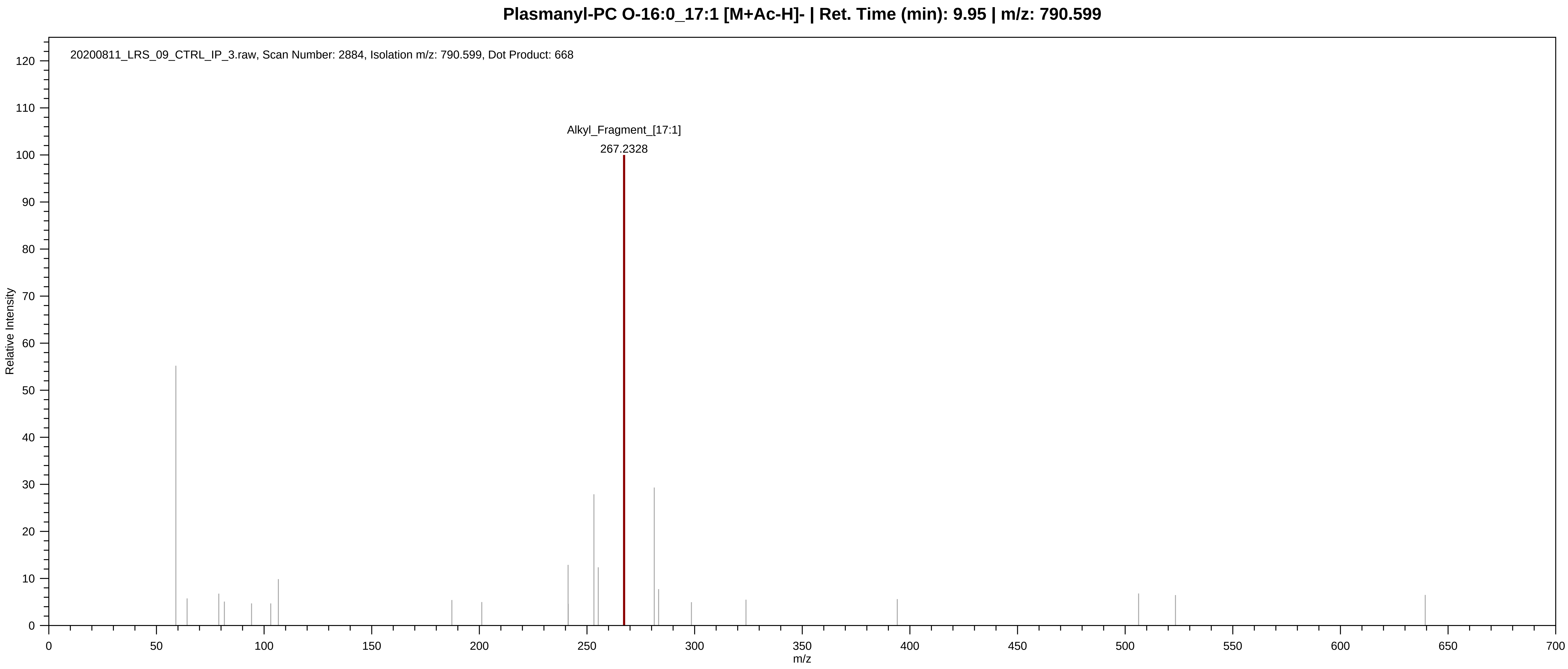

Plasmanyl-PC O-16:0_17:1 [M+Ac-H]- | Ret. Time (min): 9.95 | m/z: 790.599
20200811_LRS_09_CTRL_IP_3.raw, Scan Number: 2884, Isolation m/z: 790.599, Dot Product: 668
Alkyl_Fragment_[17:1]
267.2328
Relative Intensity
m/z

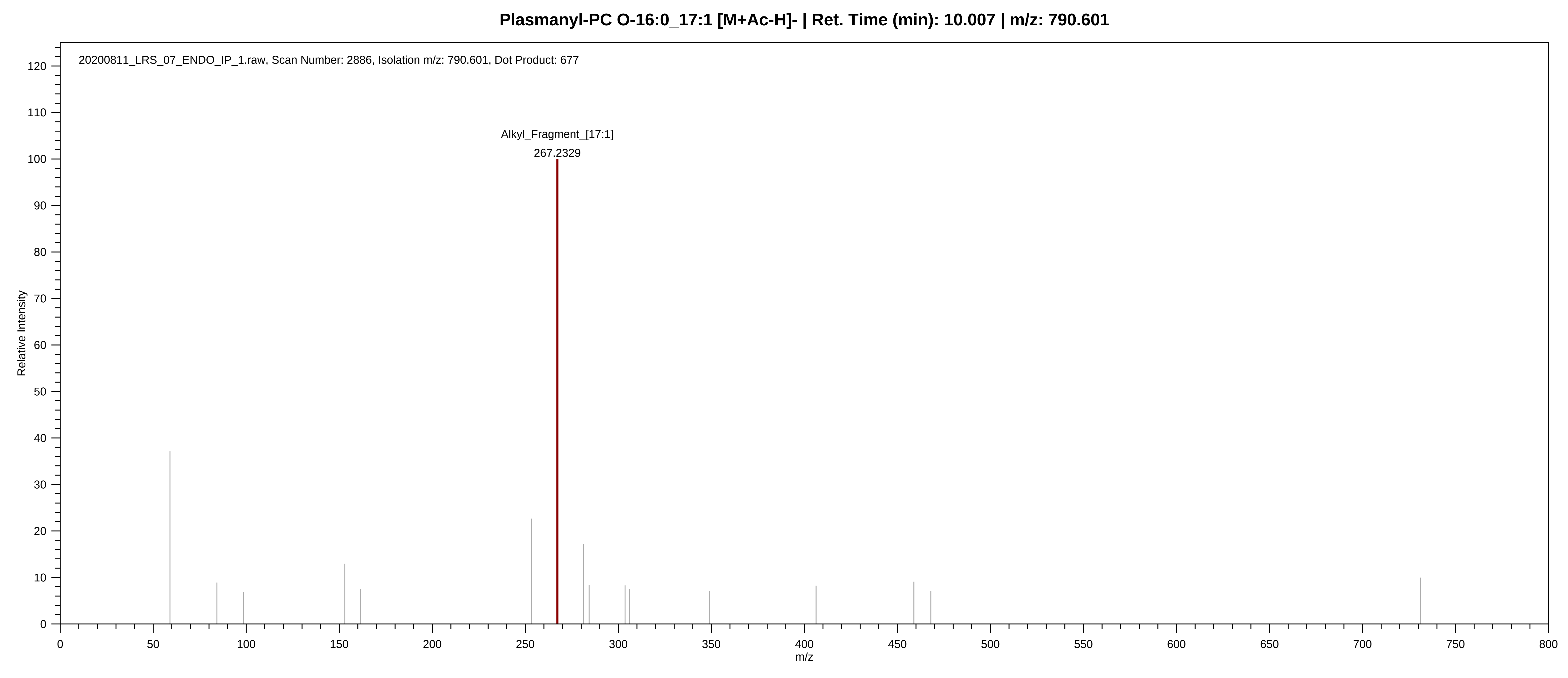
Plasmanyl-PC O-16:0_17:1 [M+Ac-H]- | Ret. Time (min): 10.007 | m/z: 790.601
20200811_LRS_07_ENDO_IP_1.raw, Scan Number: 2886, Isolation m/z: 790.601, Dot Product: 677
Alkyl_Fragment_[17:1]
267.2329
Relative Intensity
m/z
0
10
20
30
40
50
60
70
80
90
100
110
120
0
50
100
150
200
250
300
350
400
450
500
550
600
650
700
750
800

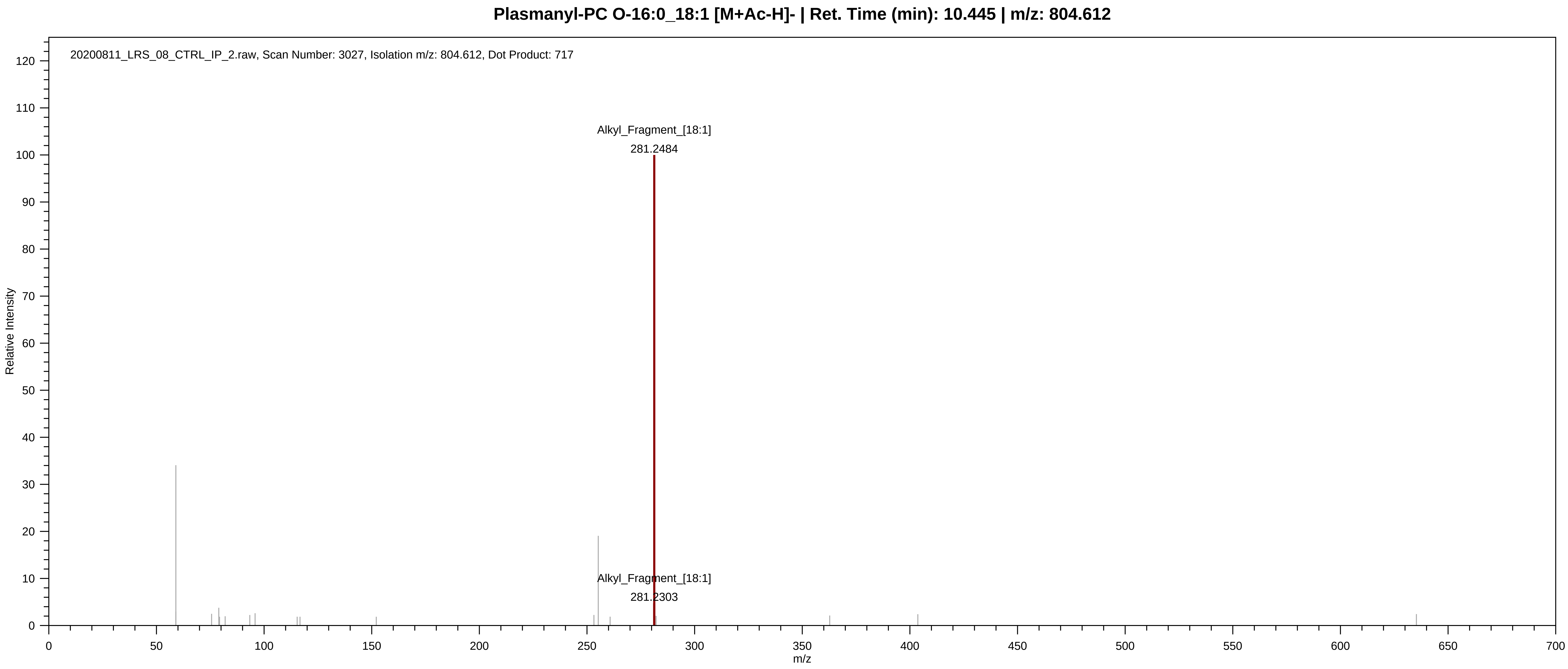

Plasmanyl-PC O-16:0_18:1 [M+Ac-H]- | Ret. Time (min): 10.445 | m/z: 804.612
20200811_LRS_08_CTRL_IP_2.raw, Scan Number: 3027, Isolation m/z: 804.612, Dot Product: 717
Alkyl_Fragment_[18:1]
281.2484
Alkyl_Fragment_[18:1]
281.2303
Relative Intensity
m/z
0
10
20
30
40
50
60
70
80
90
100
110
120
0
50
100
150
200
250
300
350
400
450
500
550
600
650
700

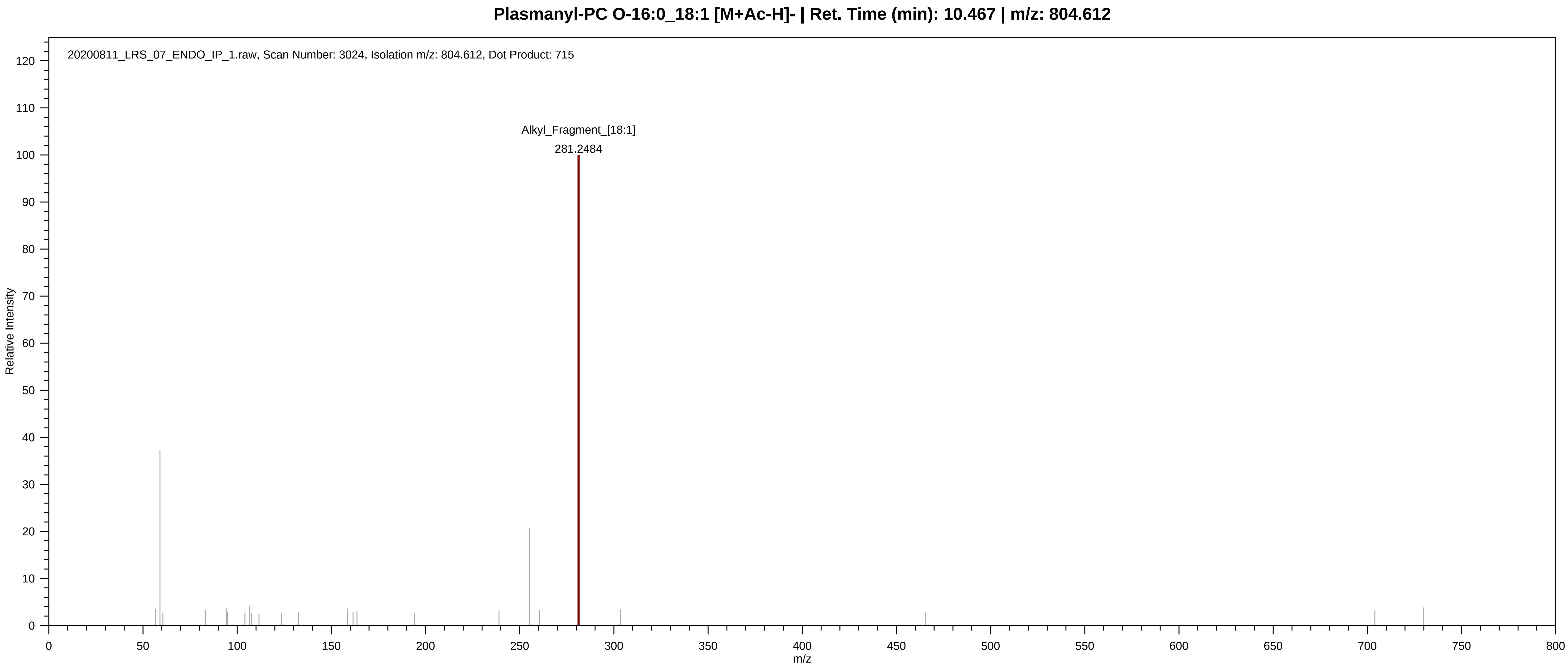

Plasmanyl-PC O-16:0_18:1 [M+Ac-H]- | Ret. Time (min): 10.467 | m/z: 804.612
20200811_LRS_07_ENDO_IP_1.raw, Scan Number: 3024, Isolation m/z: 804.612, Dot Product: 715
Alkyl_Fragment_[18:1]
281.2484
Relative Intensity
m/z
0
10
20
30
40
50
60
70
80
90
100
110
120
0
50
100
150
200
250
300
350
400
450
500
550
600
650
700
750
800

Plasmanyl-PC O-16:0_18:1 [M+Ac-H]- | Ret. Time (min): 10.444 | m/z: 804.613

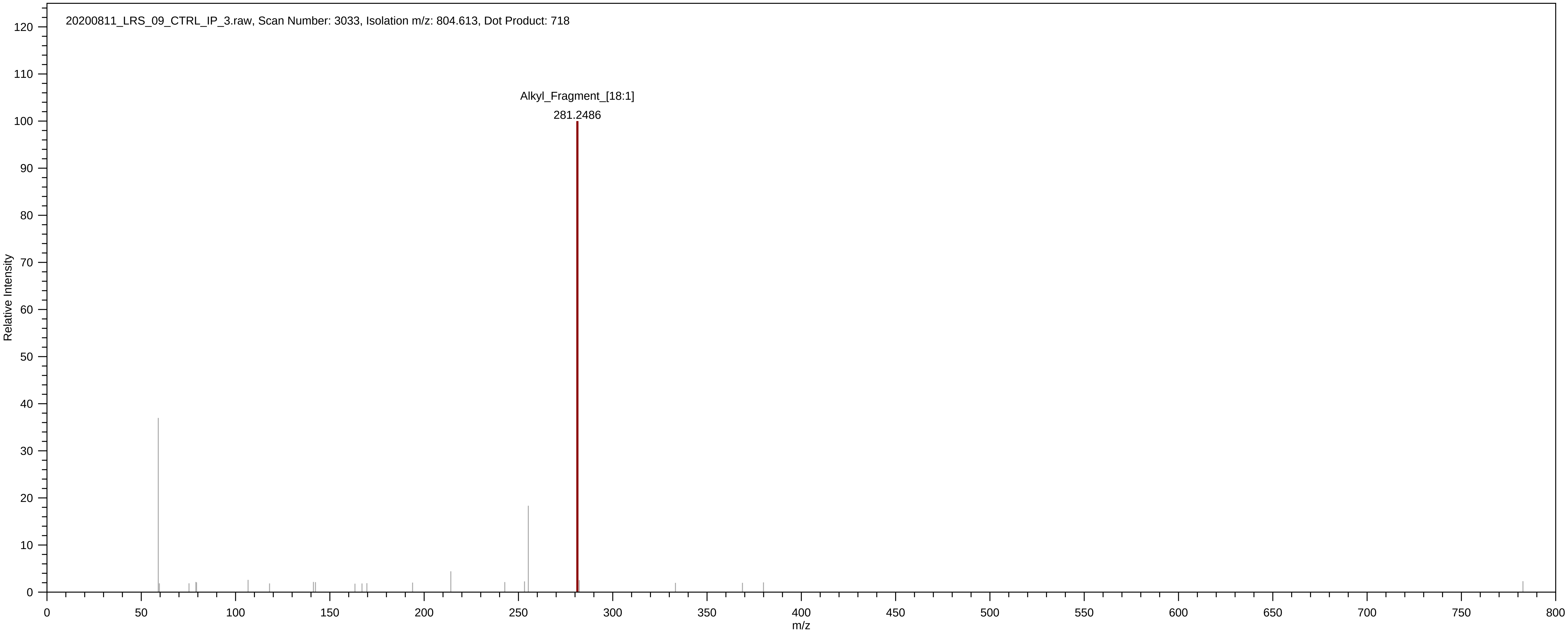

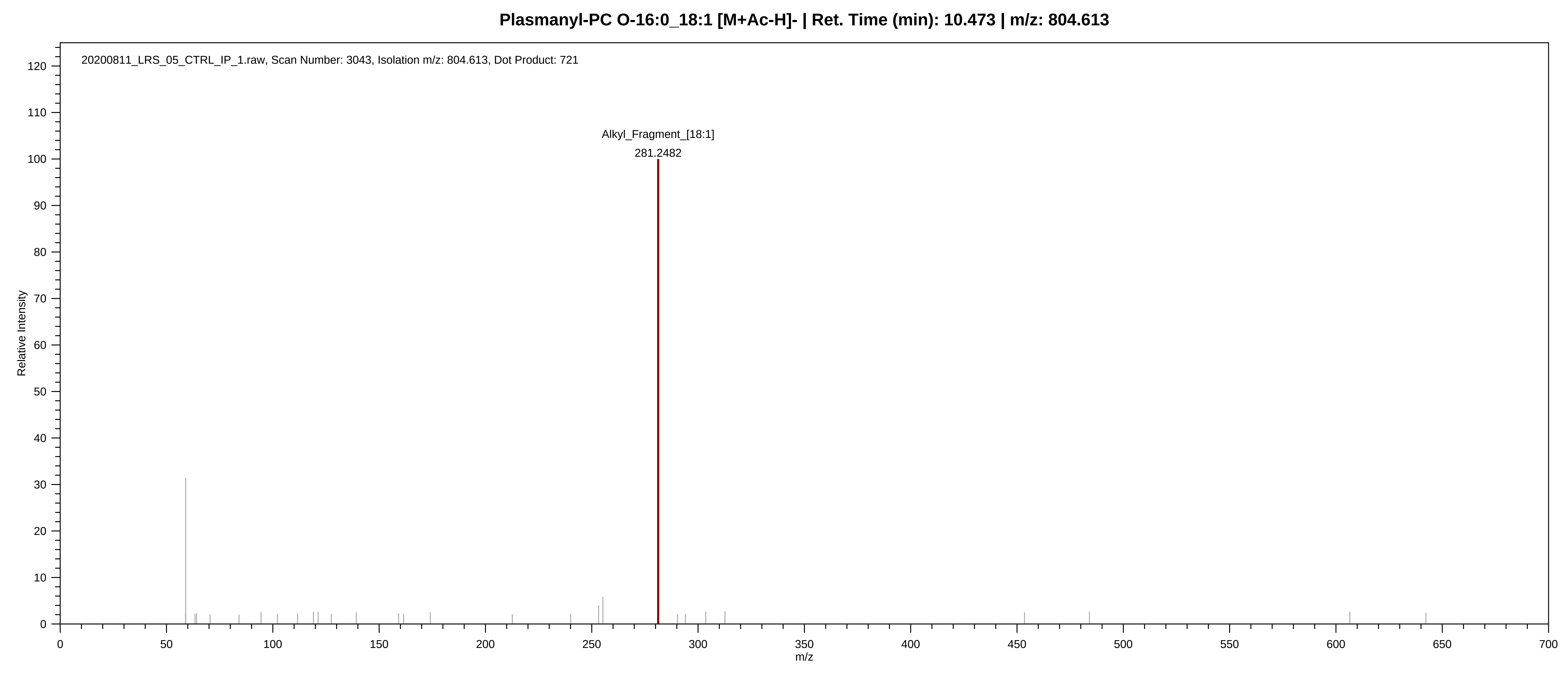
Plasmanyl-PC O-16:0_18:1 [M+Ac-H]- | Ret. Time (min): 10.473 | m/z: 804.613
20200811_LRS_05_CTRL_IP_1.raw, Scan Number: 3043, Isolation m/z: 804.613, Dot Product: 721
Alkyl_Fragment_[18:1]
281.2482
Relative Intensity
m/z

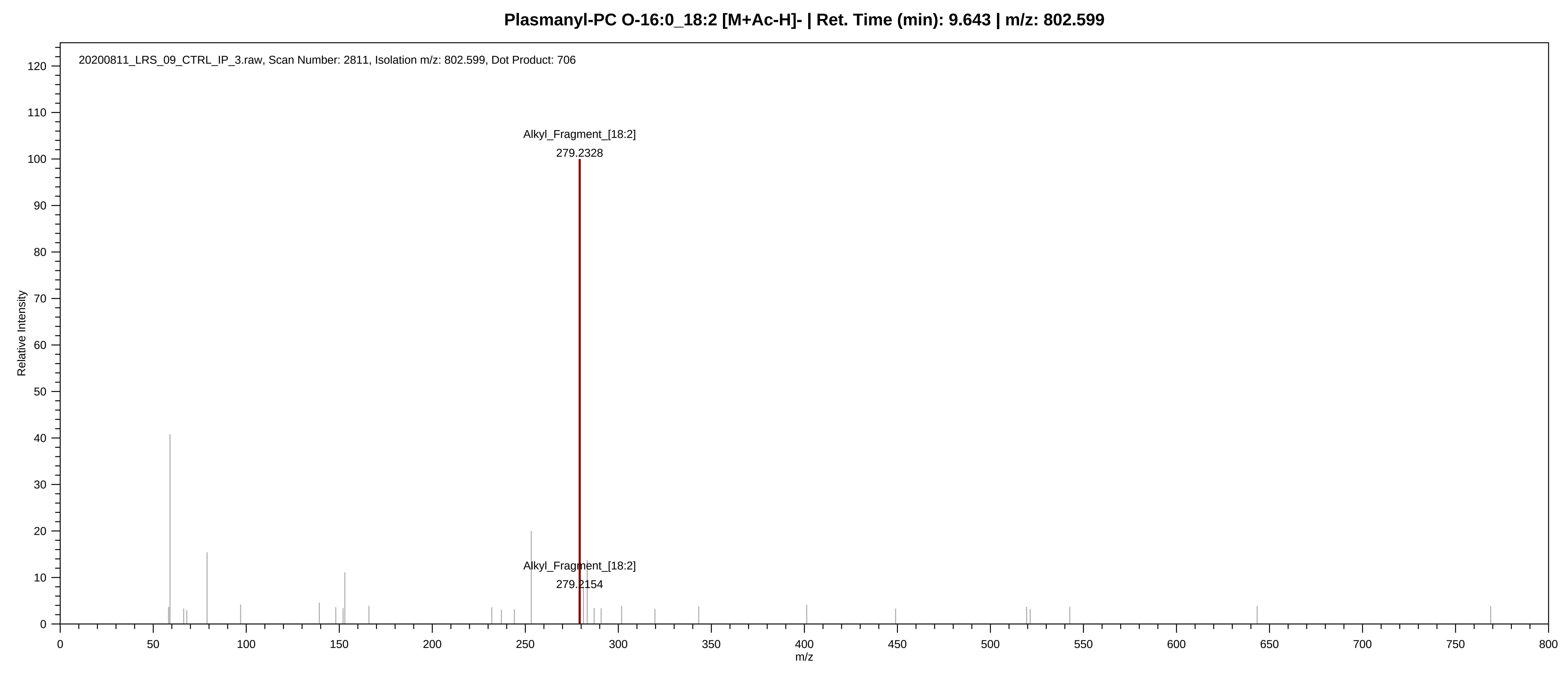

Plasmanyl-PC O-16:0_18:2 [M+Ac-H]- | Ret. Time (min): 9.643 | m/z: 802.599
20200811_LRS_09_CTRL_IP_3.raw, Scan Number: 2811, Isolation m/z: 802.599, Dot Product: 706
Alkyl_Fragment_[18:2]
279.2328
Alkyl_Fragment_[18:2]
279.2154
Relative Intensity
m/z

Plasmanyl-PC O-16:0_18:2 [M+Ac-H]- | Ret. Time (min): 9.734 | m/z: 802.598

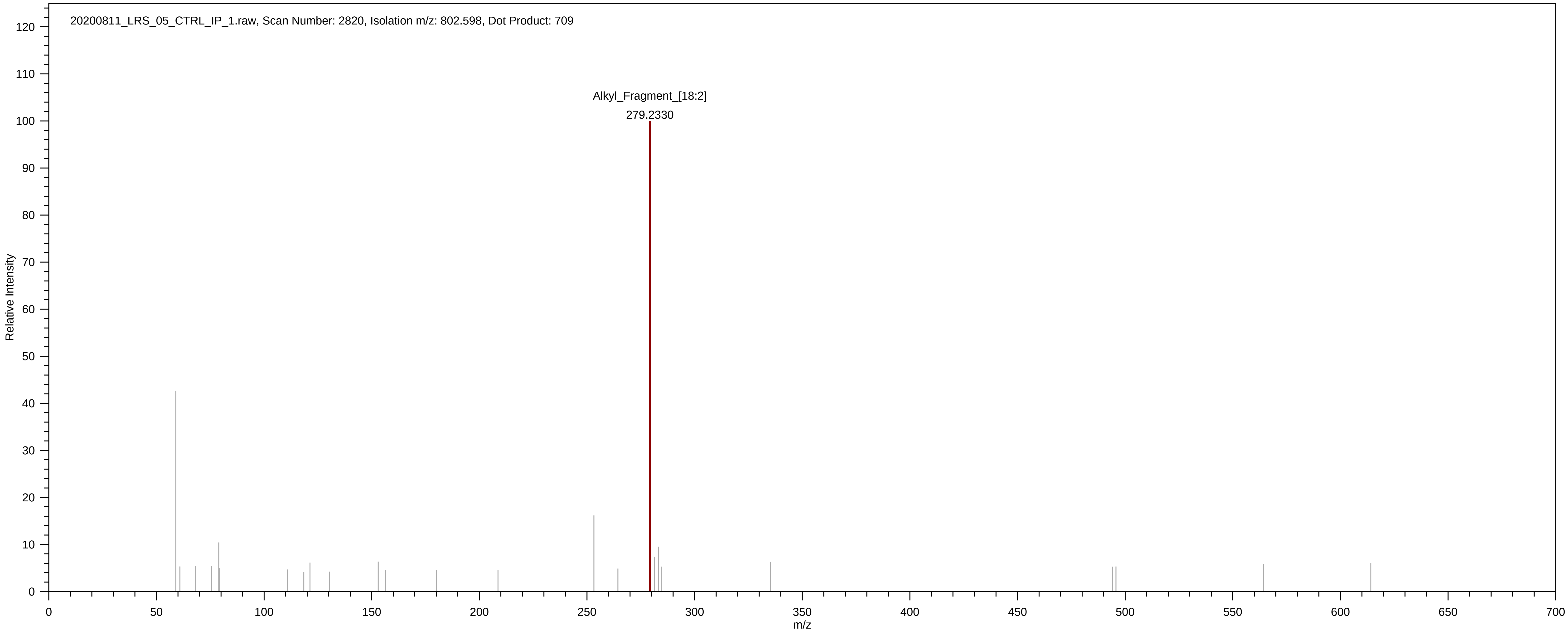

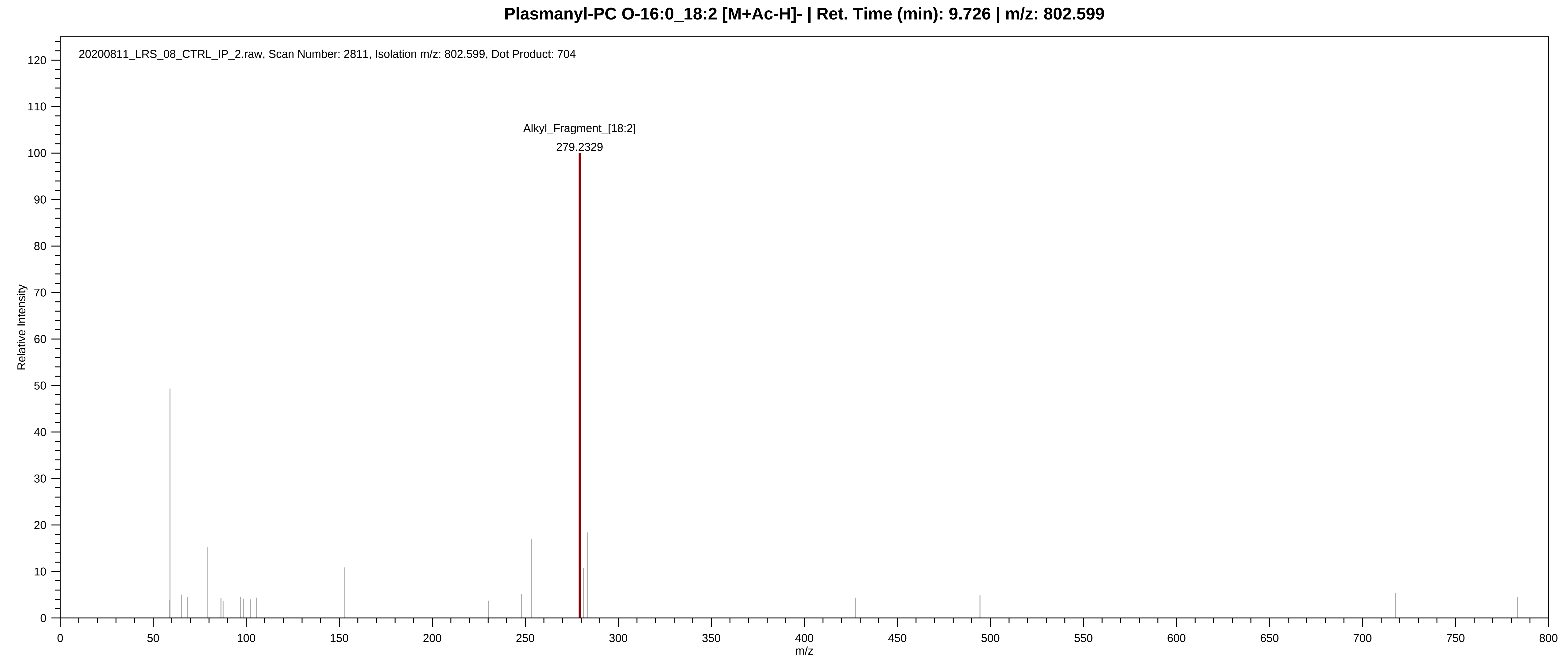

Plasmanyl-PC O-16:0_18:2 [M+Ac-H]- | Ret. Time (min): 9.726 | m/z: 802.599
20200811_LRS_08_CTRL_IP_2.raw, Scan Number: 2811, Isolation m/z: 802.599, Dot Product: 704
Alkyl_Fragment_[18:2]
279.2329
Relative Intensity
m/z

Plasmanyl-PC O-16:0_20:1 [M+Ac-H]- | Ret. Time (min): 11.44 | m/z: 832.645

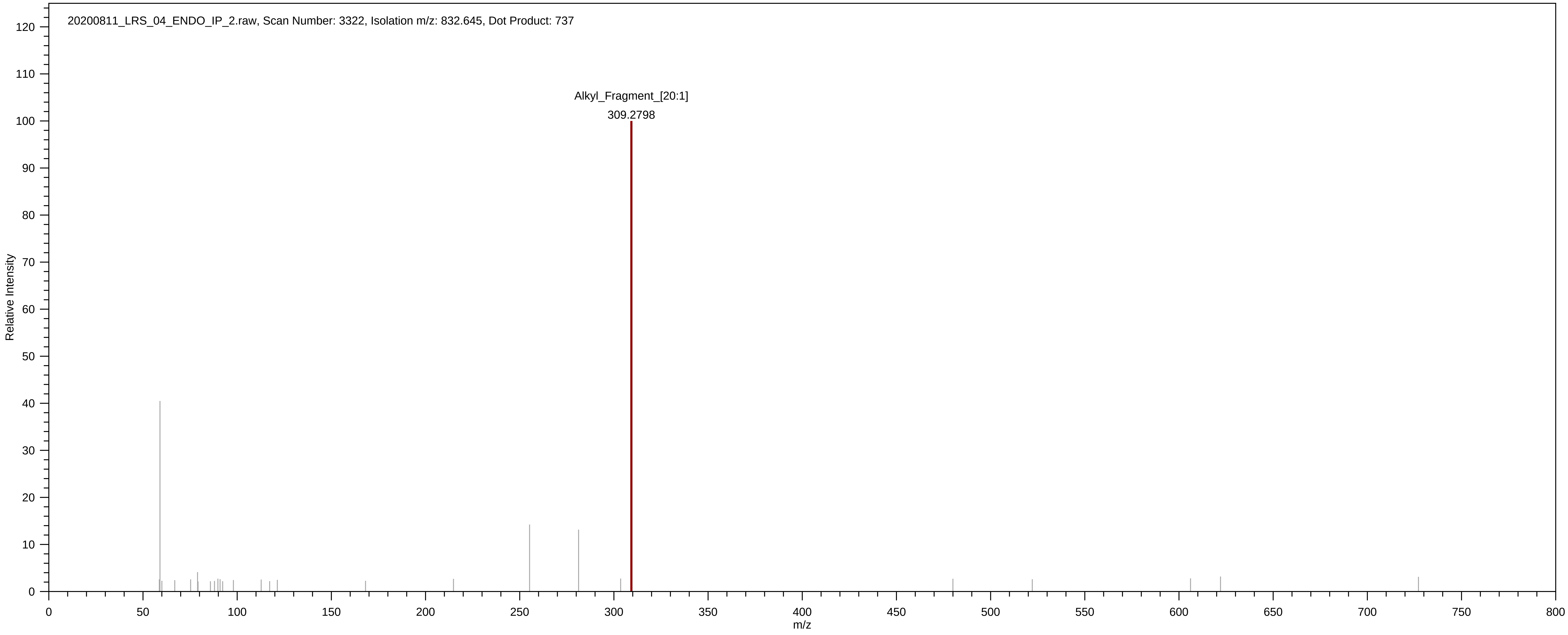

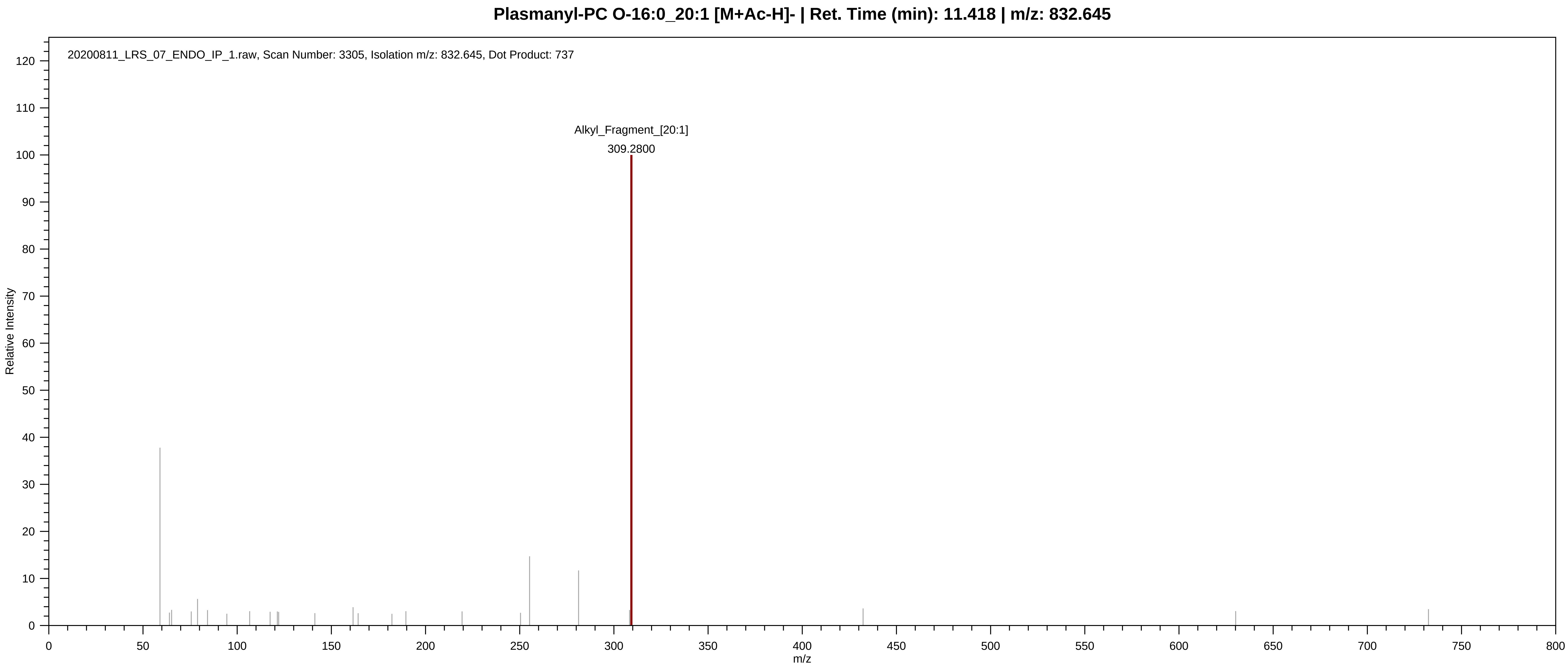
Plasmanyl-PC O-16:0_20:1 [M+Ac-H]- | Ret. Time (min): 11.418 | m/z: 832.645
20200811_LRS_07_ENDO_IP_1.raw, Scan Number: 3305, Isolation m/z: 832.645, Dot Product: 737
Alkyl_Fragment_[20:1]
309.2800
Relative Intensity
m/z

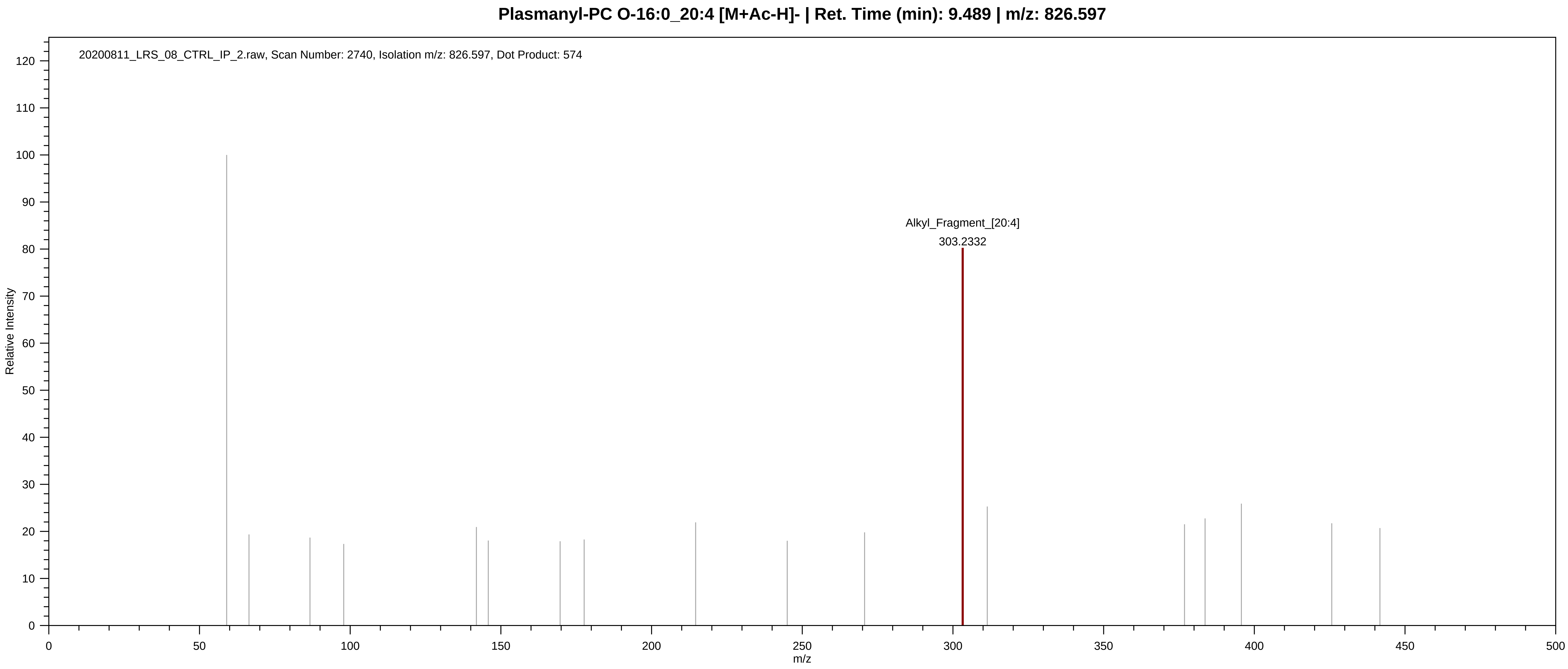
Plasmanyl-PC O-16:0_20:4 [M+Ac-H]- | Ret. Time (min): 9.489 | m/z: 826.597
20200811_LRS_08_CTRL_IP_2.raw, Scan Number: 2740, Isolation m/z: 826.597, Dot Product: 574
Alkyl_Fragment_[20:4]
303.2332
Relative Intensity
m/z
0
10
20
30
40
50
60
70
80
90
100
110
120
0
50
100
150
200
250
300
350
400
450
500

Plasmanyl-PC O-18:0_16:0 [M+Ac-H]- | Ret. Time (min): 11.599 | m/z: 806.628

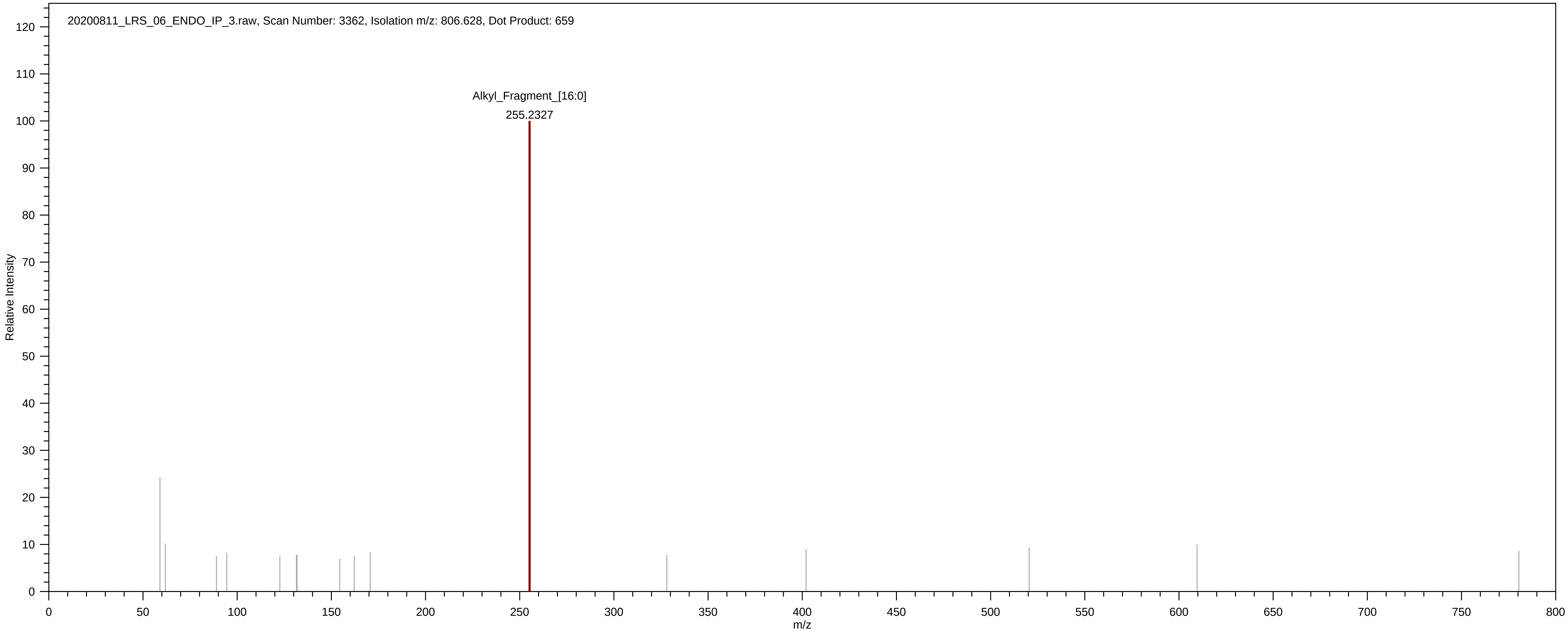

Plasmanyl-PC O-18:0_16:0 [M+Ac-H]- | Ret. Time (min): 11.602 | m/z: 806.628

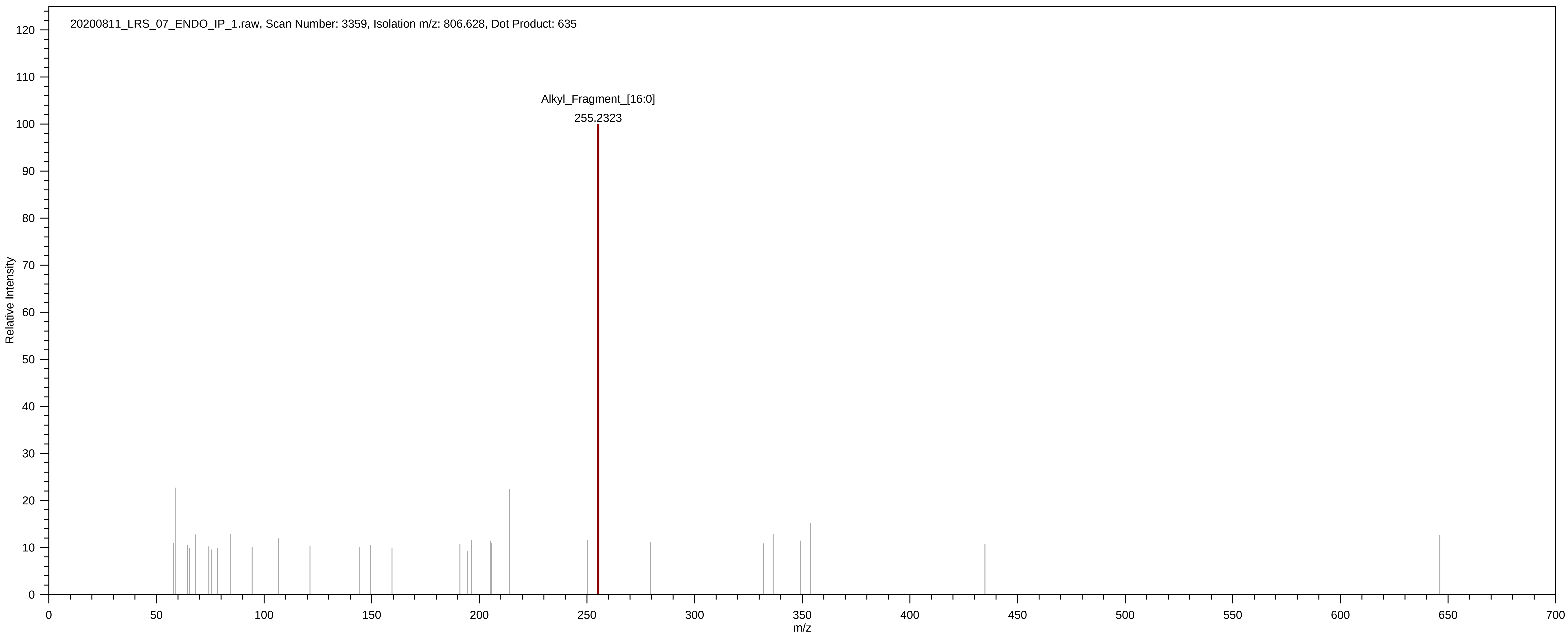

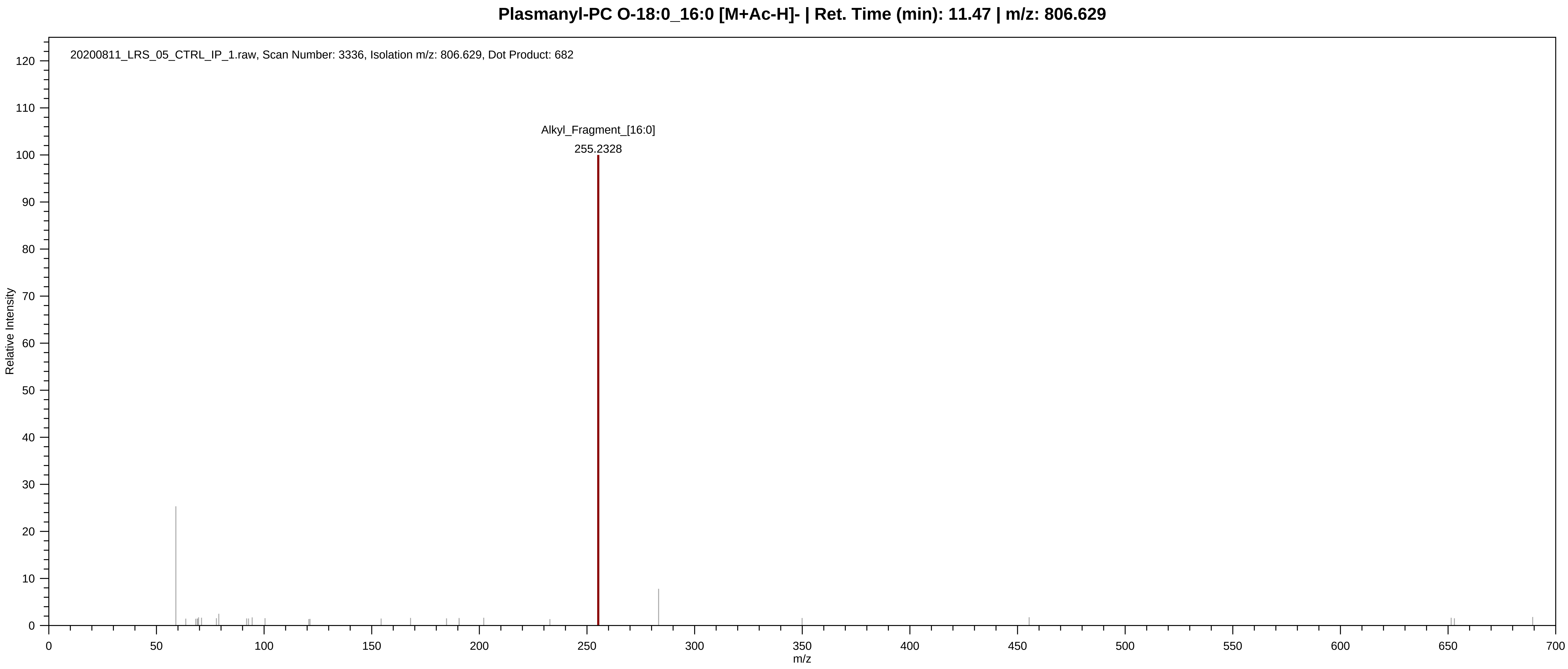
Plasmanyl-PC O-18:0_16:0 [M+Ac-H]- | Ret. Time (min): 11.47 | m/z: 806.629
20200811_LRS_05_CTRL_IP_1.raw, Scan Number: 3336, Isolation m/z: 806.629, Dot Product: 682
Alkyl_Fragment_[16:0]
255.2328
Relative Intensity
m/z

Plasmanyl-PC O-18:0_16:0 [M+Ac-H]- | Ret. Time (min): 11.472 | m/z: 806.629

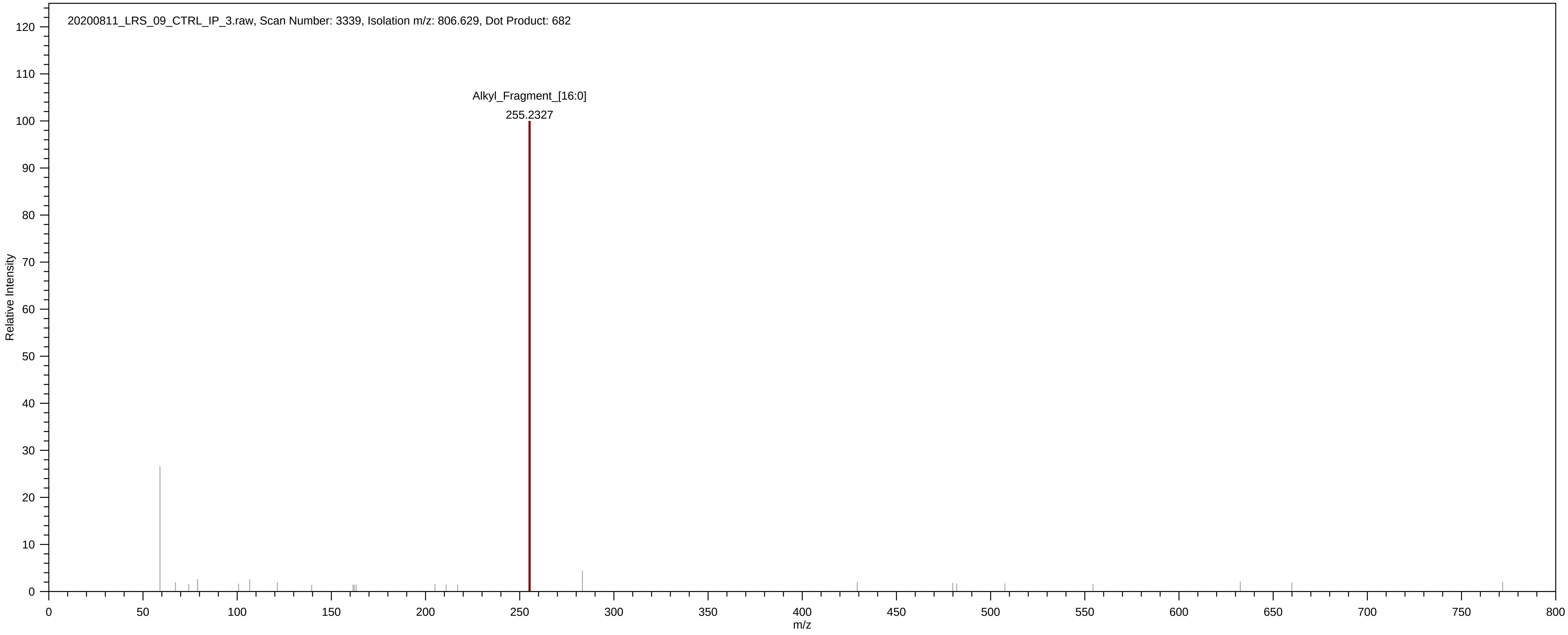

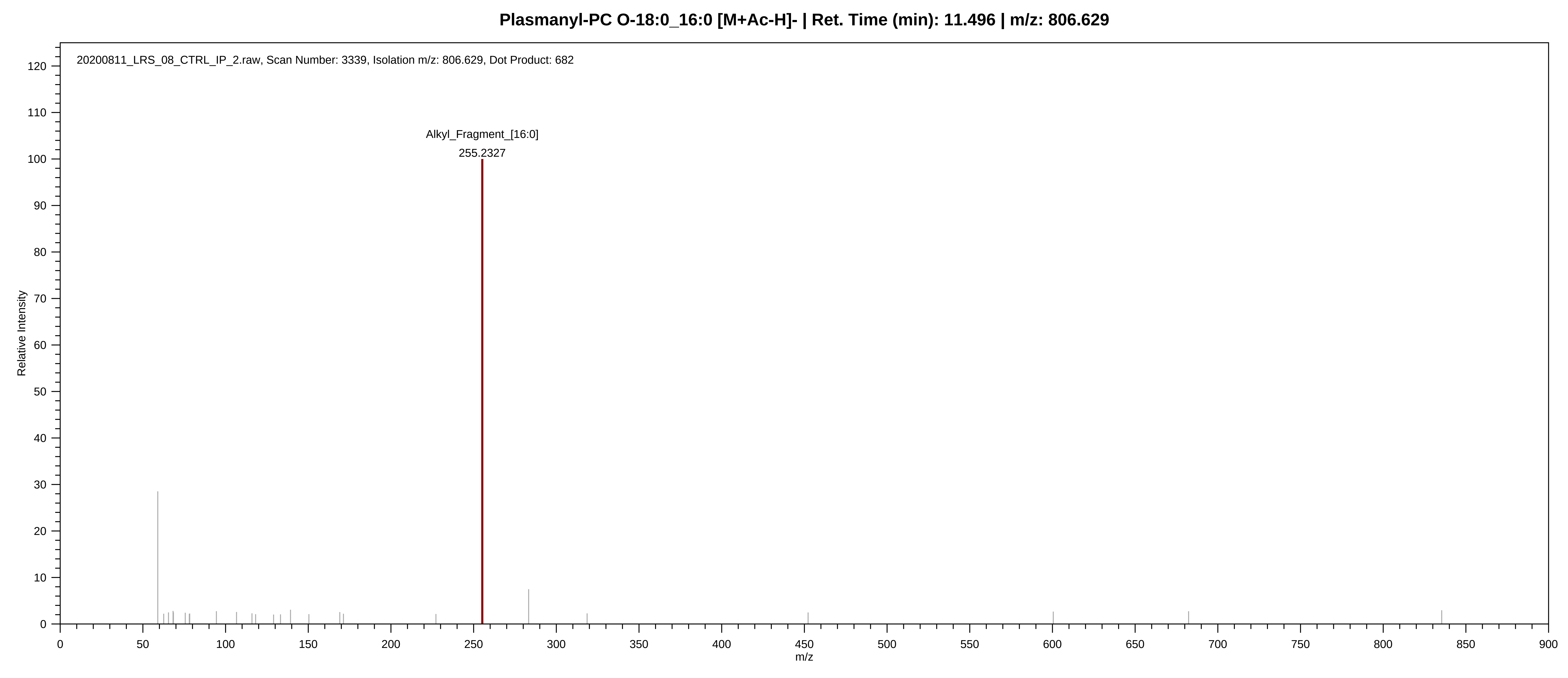
Plasmanyl-PC O-18:0_16:0 [M+Ac-H]- | Ret. Time (min): 11.496 | m/z: 806.629
20200811_LRS_08_CTRL_IP_2.raw, Scan Number: 3339, Isolation m/z: 806.629, Dot Product: 682
Alkyl_Fragment_[16:0]
255.2327
Relative Intensity
m/z

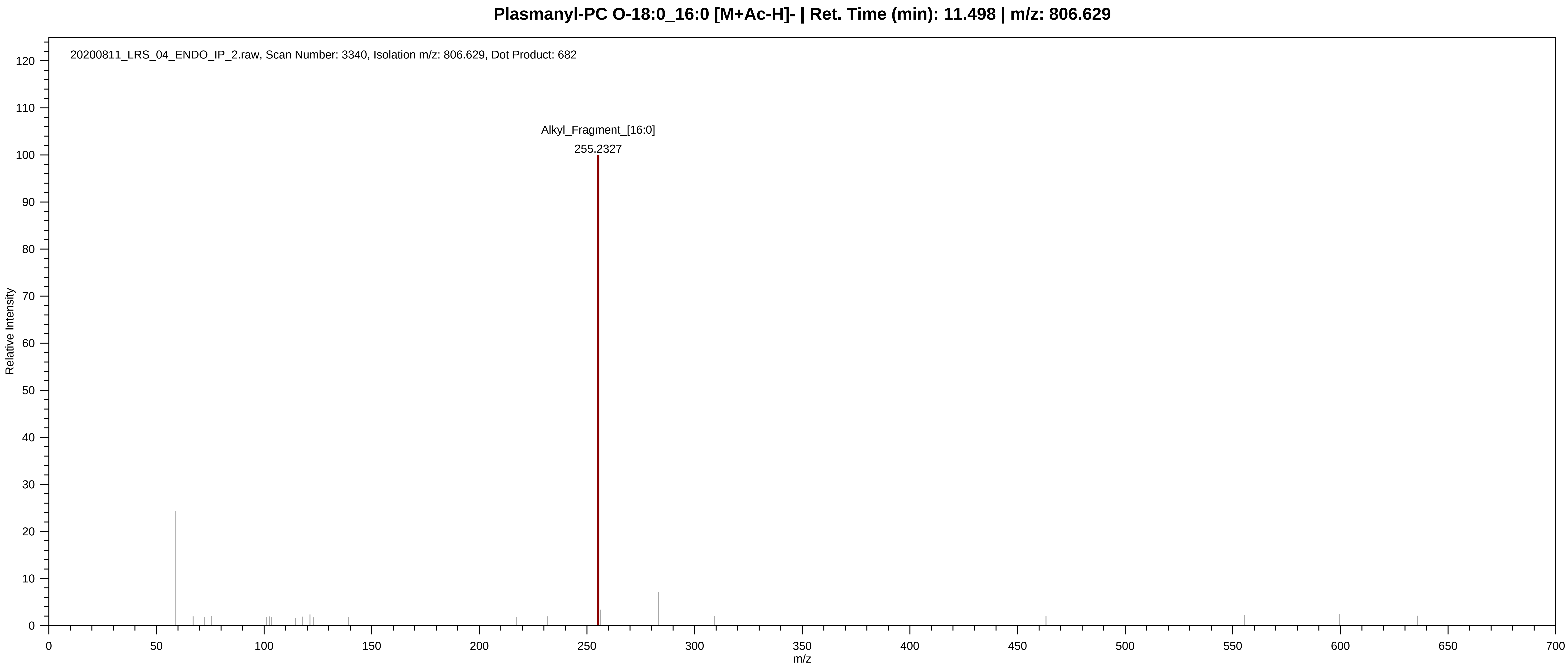

Plasmanyl-PC O-18:0_16:0 [M+Ac-H]- | Ret. Time (min): 11.498 | m/z: 806.629
20200811_LRS_04_ENDO_IP_2.raw, Scan Number: 3340, Isolation m/z: 806.629, Dot Product: 682
Alkyl_Fragment_[16:0]
255.2327
Relative Intensity
m/z

Plasmanyl-PC O-18:0_18:1 [M+Ac-H]- | Ret. Time (min): 11.49 | m/z: 832.644

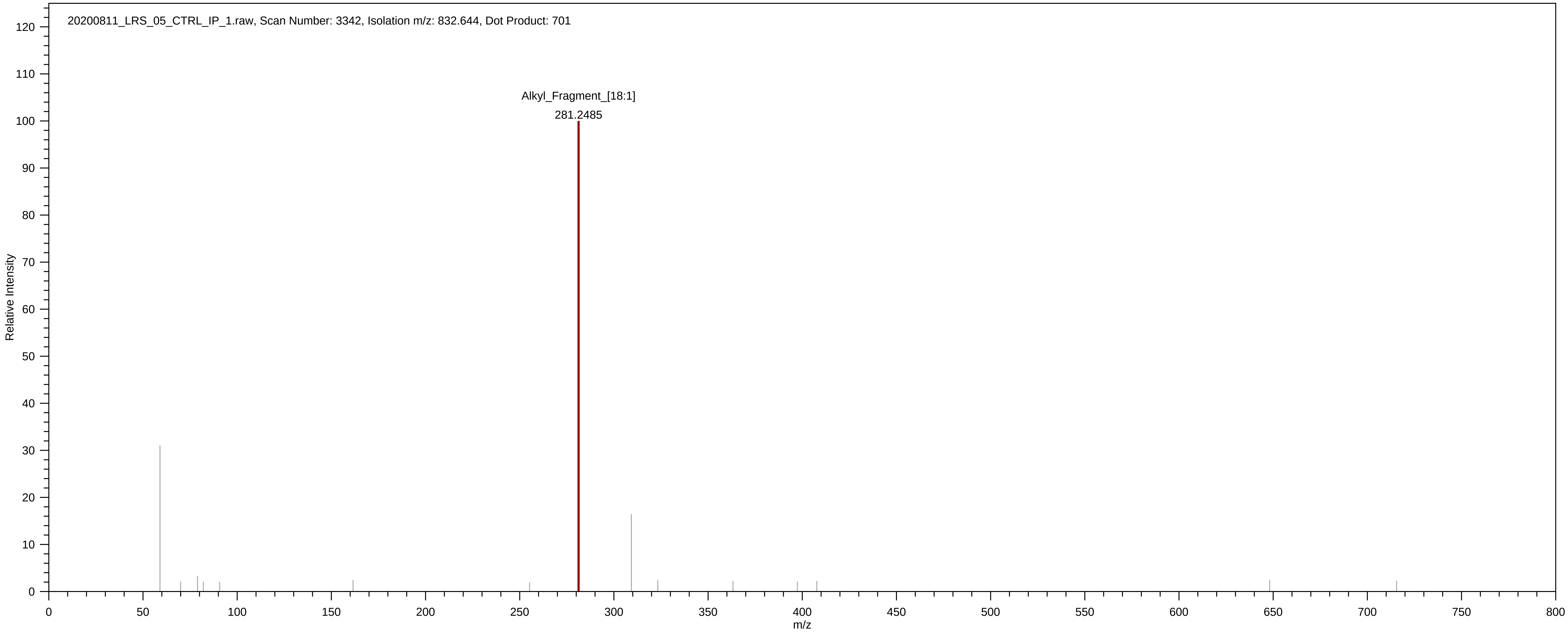

Plasmanyl-PC O-18:0_18:1 [M+Ac-H]- | Ret. Time (min): 11.473 | m/z: 832.644

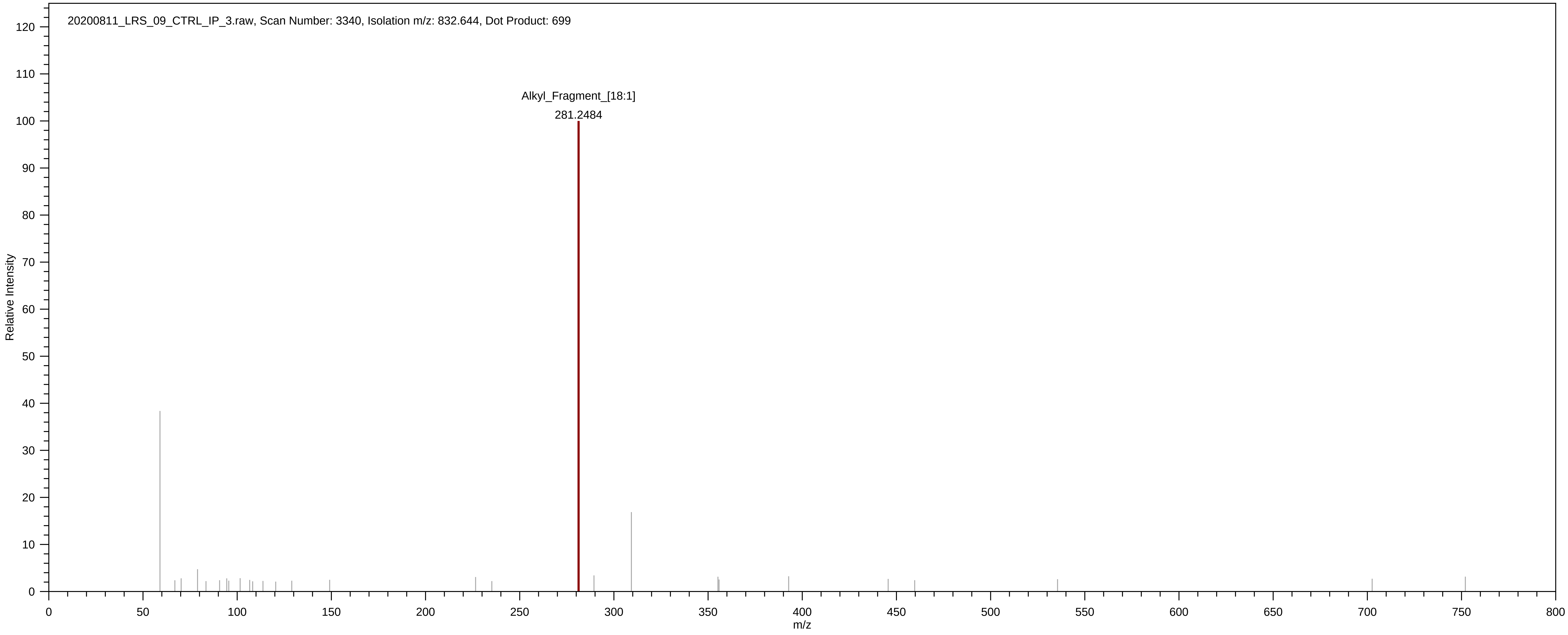

Plasmanyl-PC O-18:0_18:1 [M+Ac-H]- | Ret. Time (min): 11.476 | m/z: 832.645

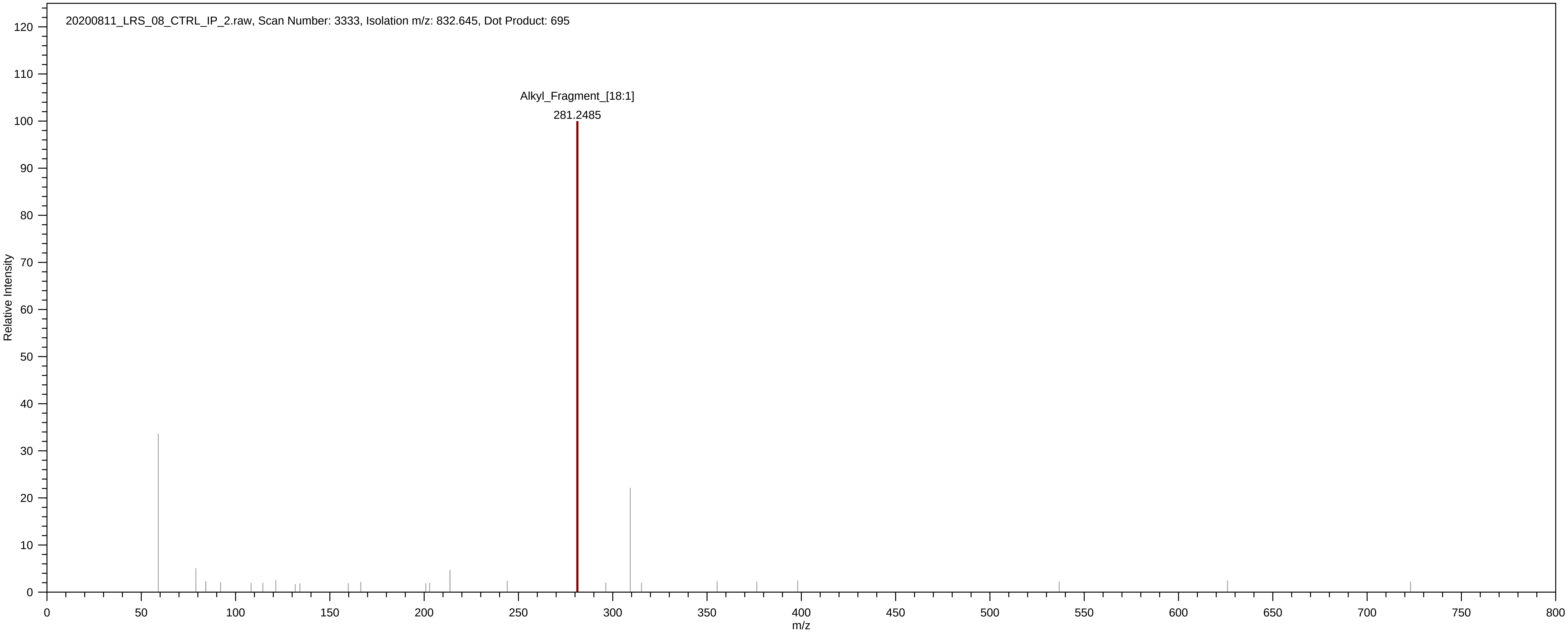

Plasmanyl-PC O-20:0_18:1 [M+Ac-H]- | Ret. Time (min): 12.607 | m/z: 860.675

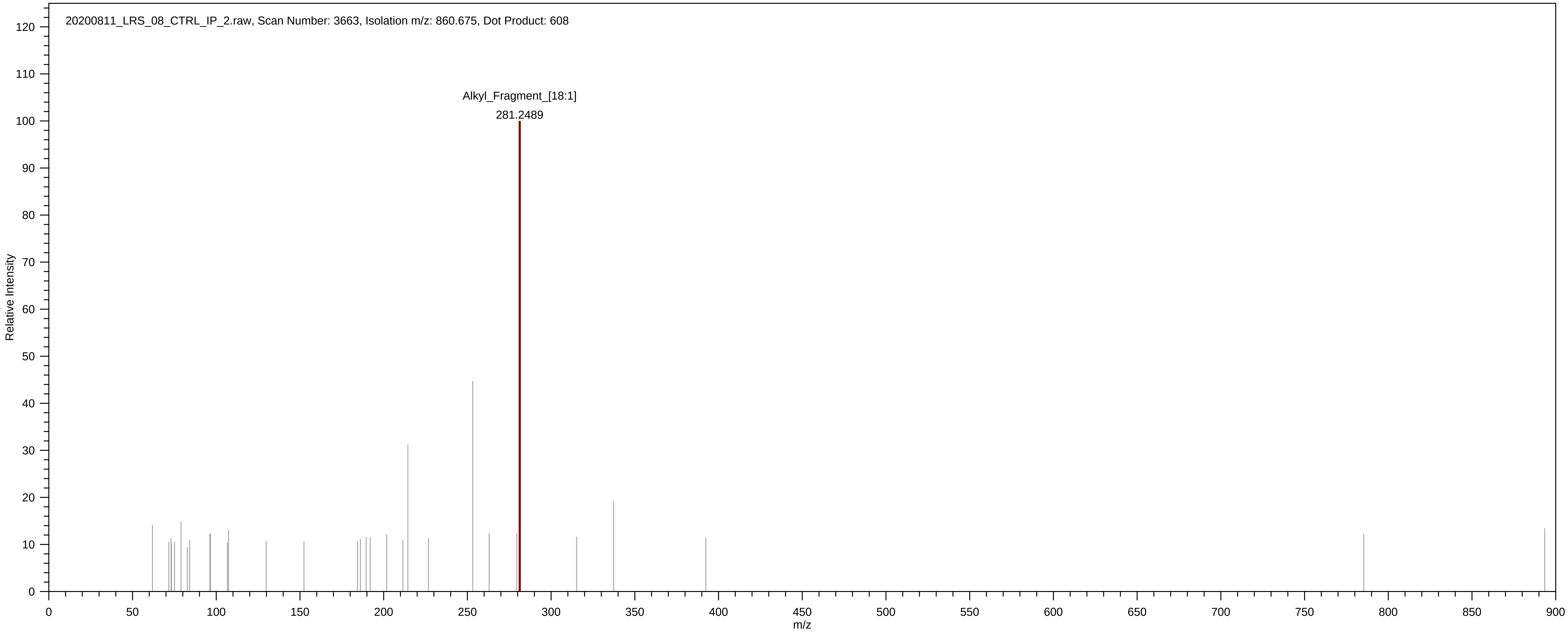

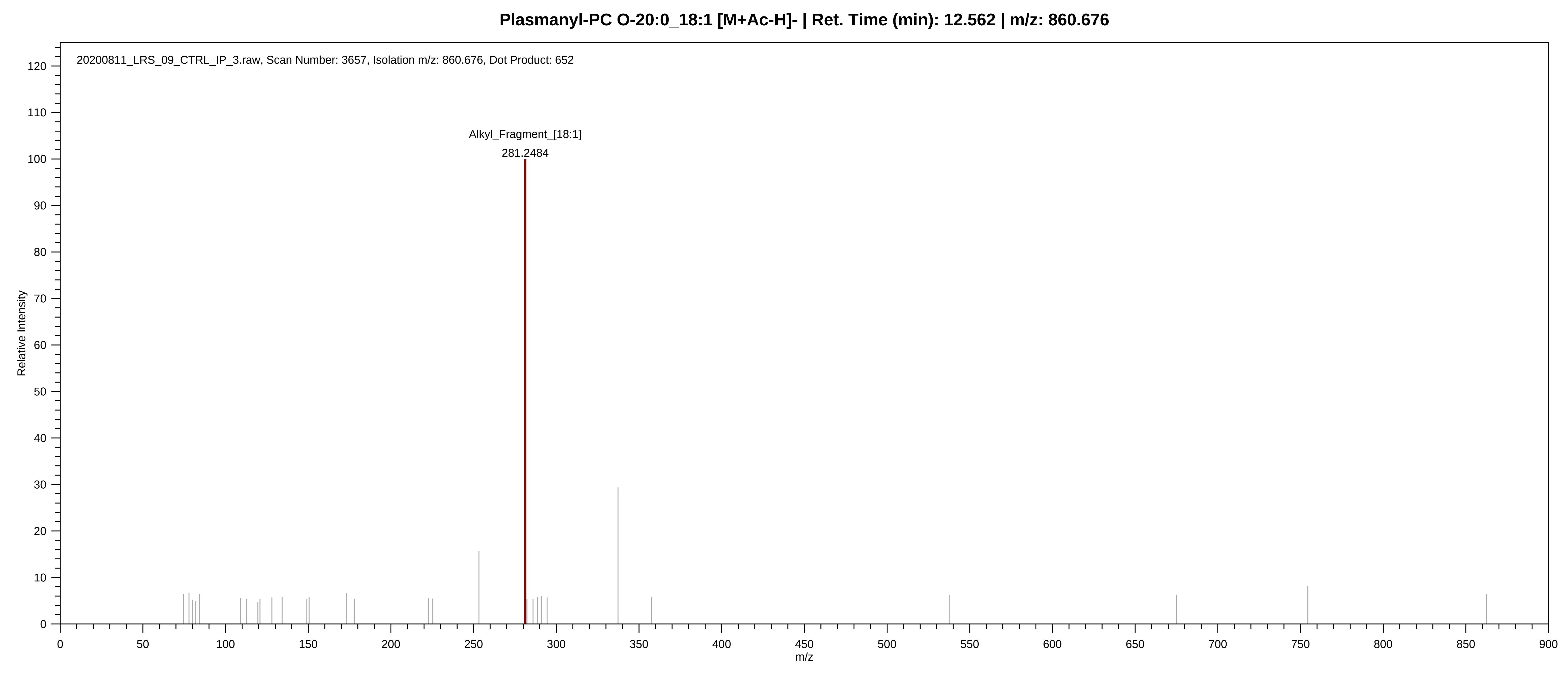

Plasmanyl-PC O-20:0_18:1 [M+Ac-H]- | Ret. Time (min): 12.562 | m/z: 860.676
20200811_LRS_09_CTRL_IP_3.raw, Scan Number: 3657, Isolation m/z: 860.676, Dot Product: 652
Alkyl_Fragment_[18:1]
281.2484
Relative Intensity
m/z

Plasmanyl-PC O-28:0 [M+H]+ | Ret. Time (min): 8.494 | m/z: 664.526

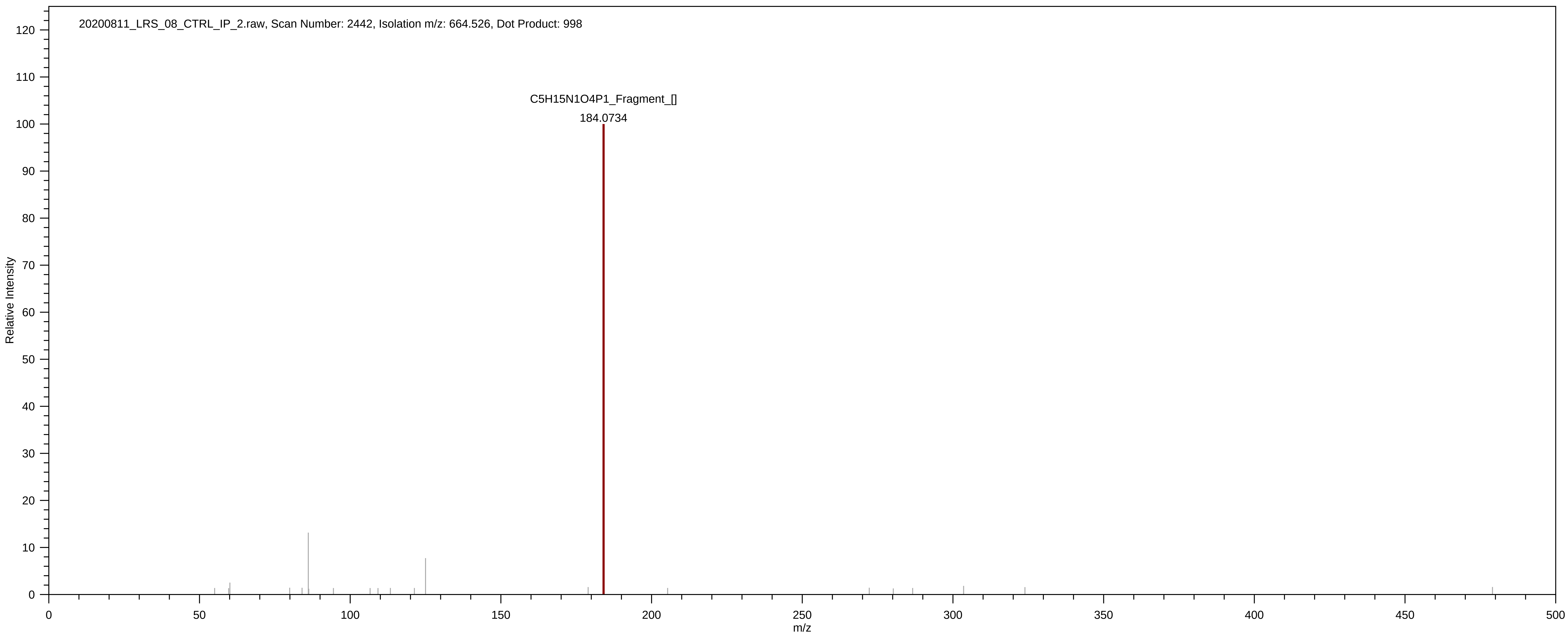

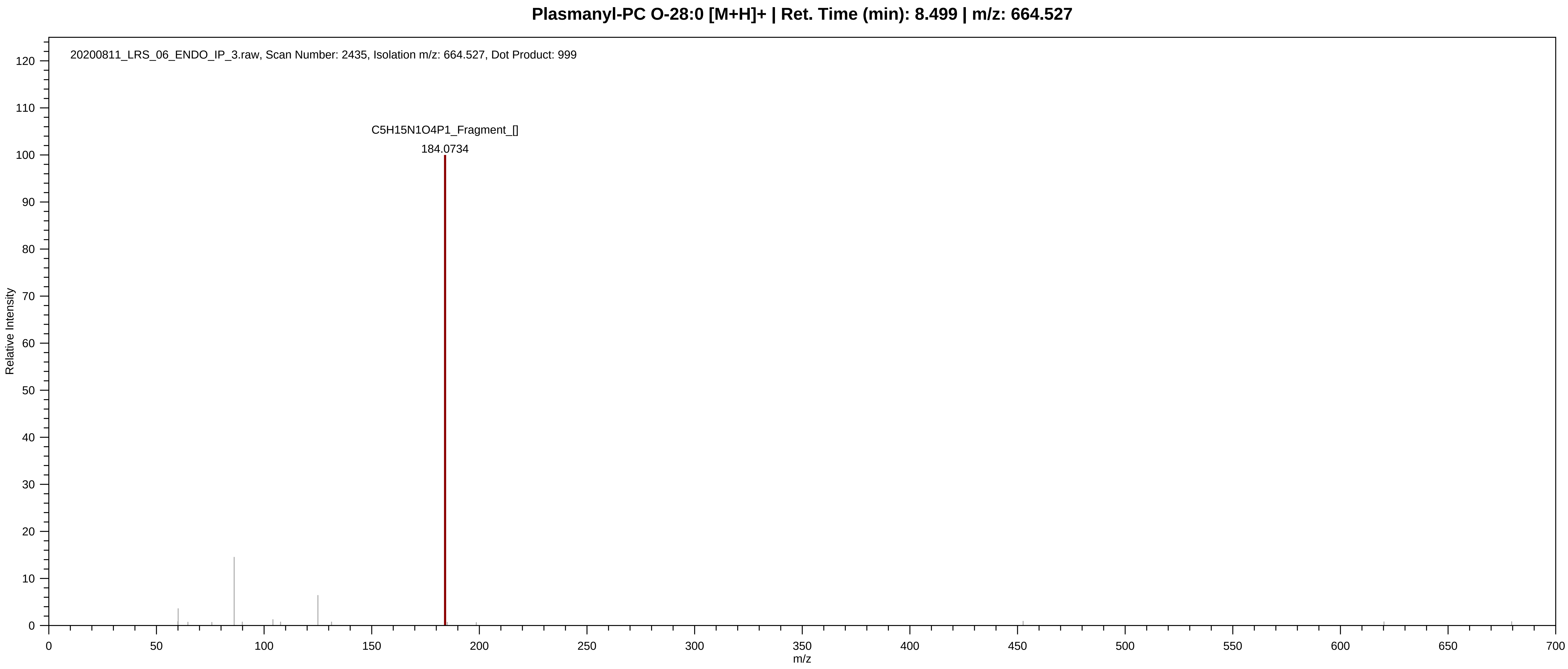
Plasmanyl-PC O-28:0 [M+H]+ | Ret. Time (min): 8.499 | m/z: 664.527
20200811_LRS_06_ENDO_IP_3.raw, Scan Number: 2435, Isolation m/z: 664.527, Dot Product: 999
C5H15N1O4P1_Fragment_[]
184.0734
Relative Intensity
m/z

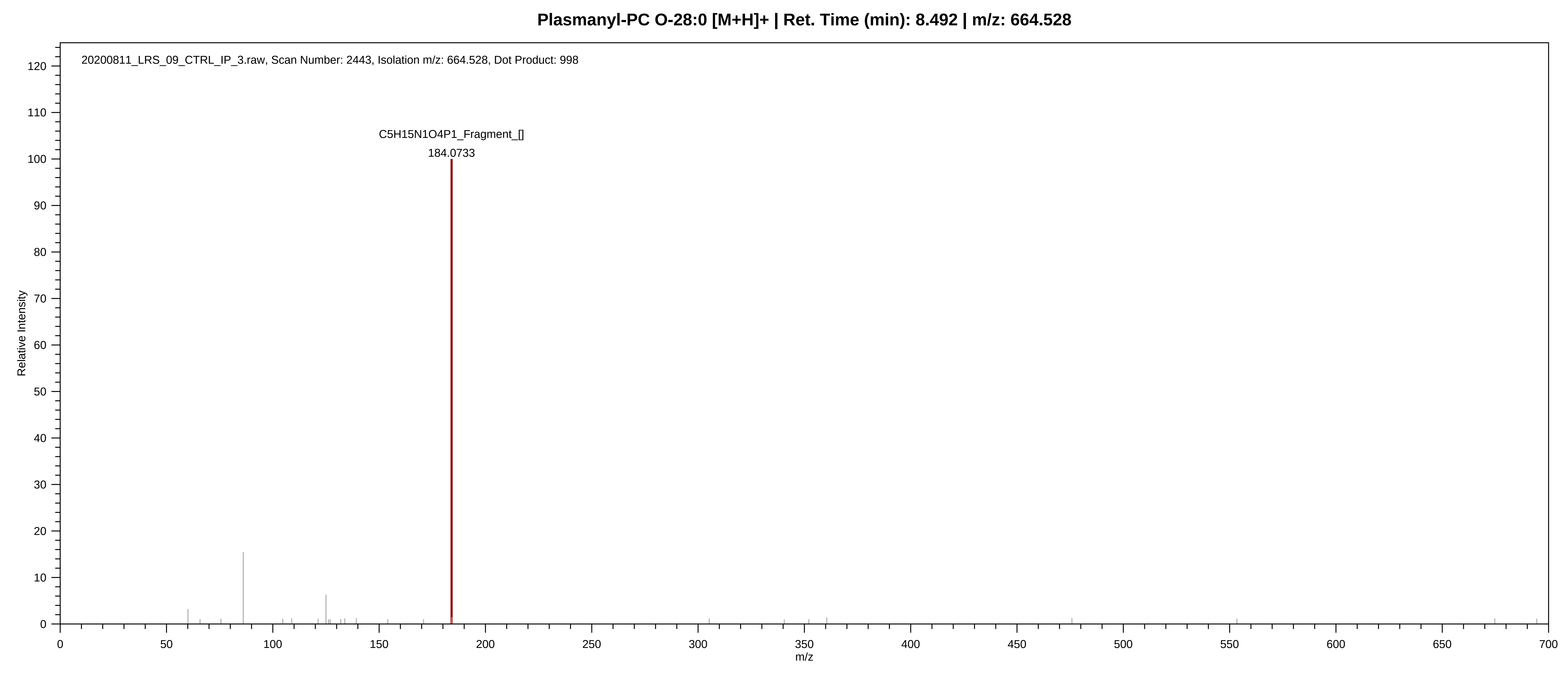

Plasmanyl-PC O-28:0 [M+H]+ | Ret. Time (min): 8.492 | m/z: 664.528
20200811_LRS_09_CTRL_IP_3.raw, Scan Number: 2443, Isolation m/z: 664.528, Dot Product: 998
C5H15N1O4P1_Fragment_[]
184.0733
Relative Intensity
m/z

Plasmanyl-PC O-28:0 [M+H]+ | Ret. Time (min): 8.493 | m/z: 664.528

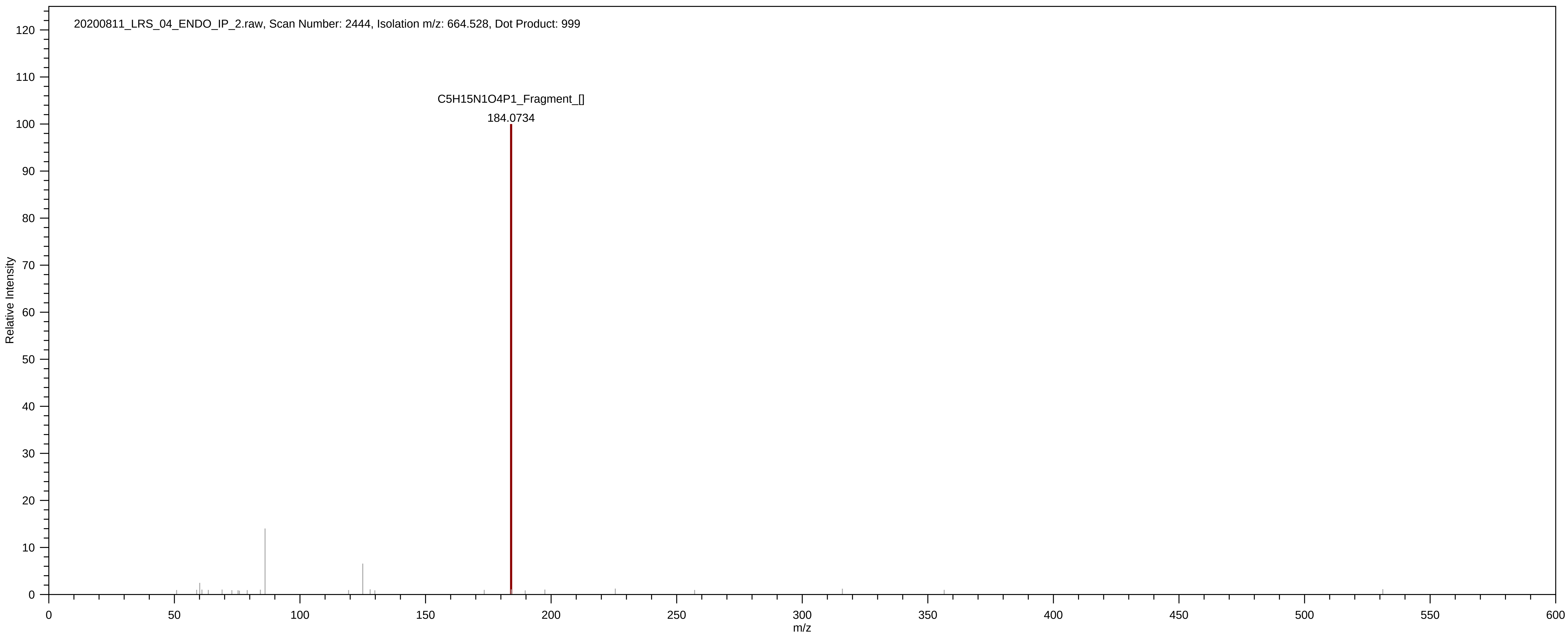

Plasmanyl-PC O-28:0 [M+H]+ | Ret. Time (min): 8.499 | m/z: 664.528

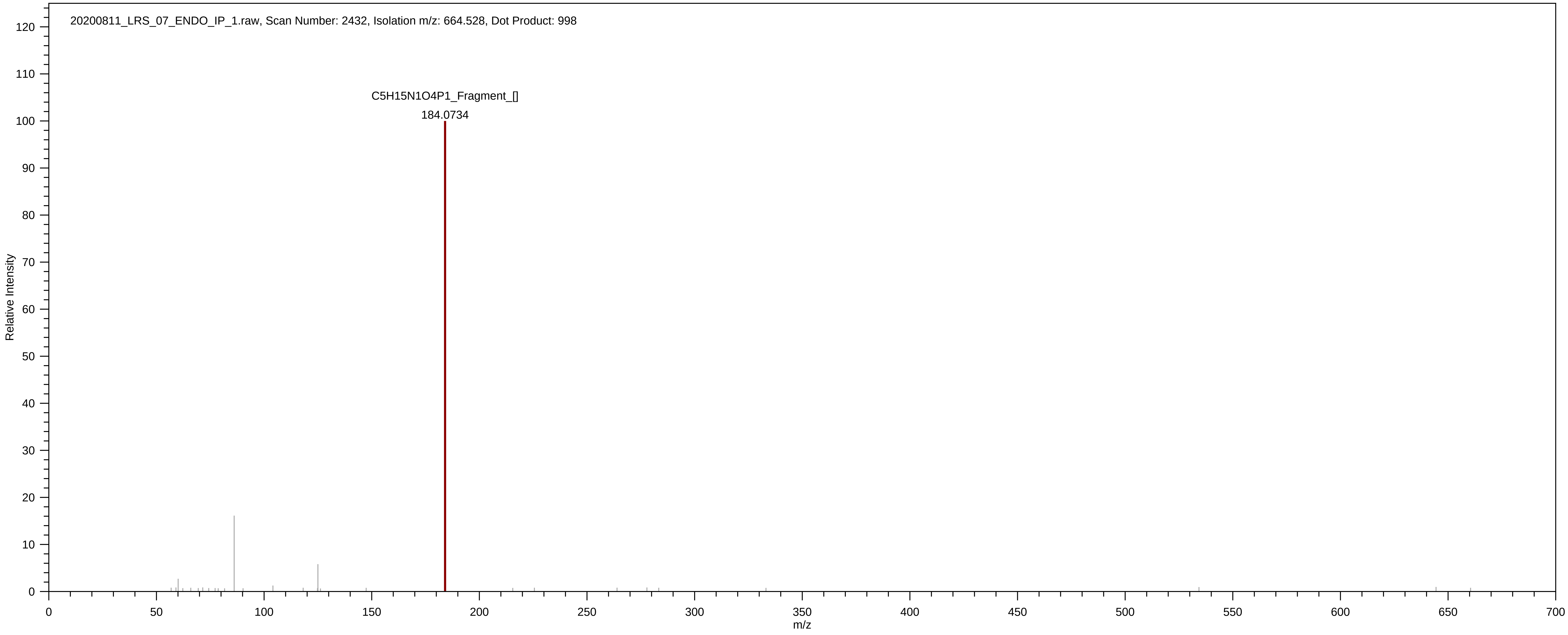

Plasmanyl-PC O-28:0 [M+H]+ | Ret. Time (min): 8.504 | m/z: 664.528

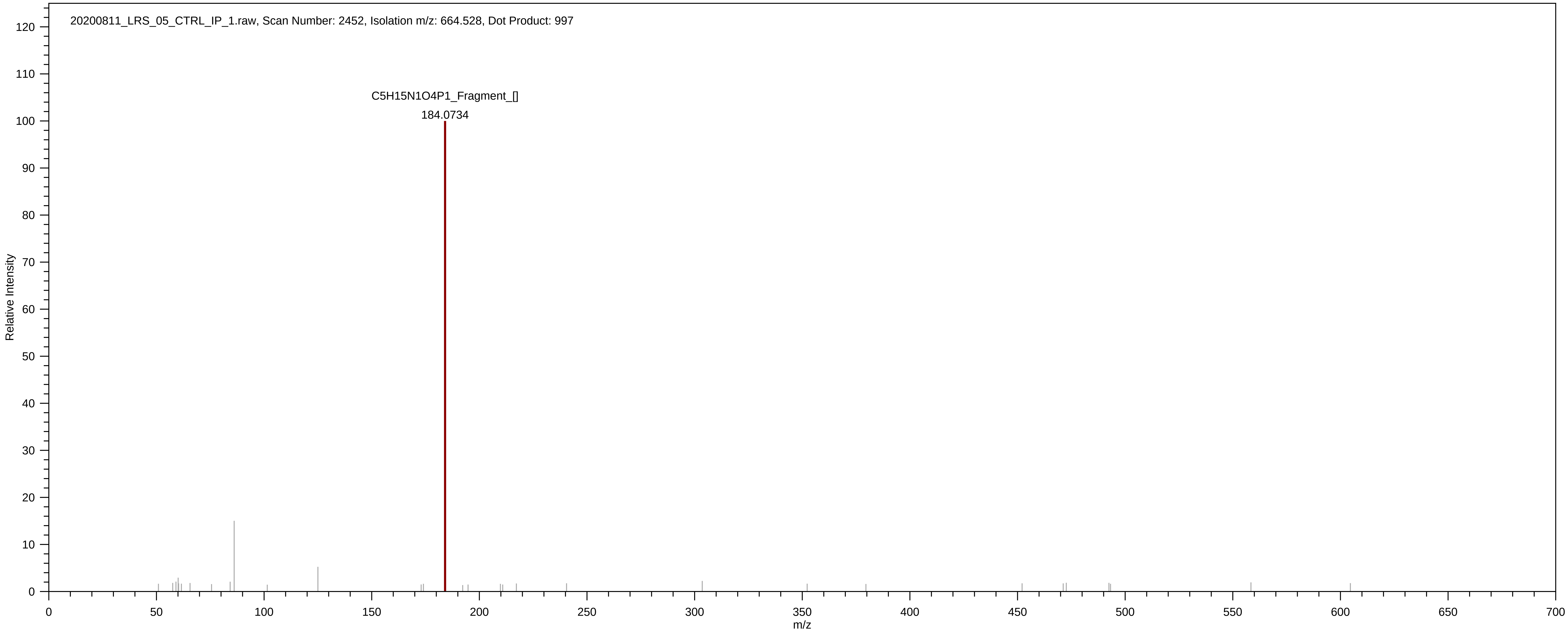

Plasmanyl-PC O-30:0 [M+H]+ | Ret. Time (min): 9.382 | m/z: 692.56

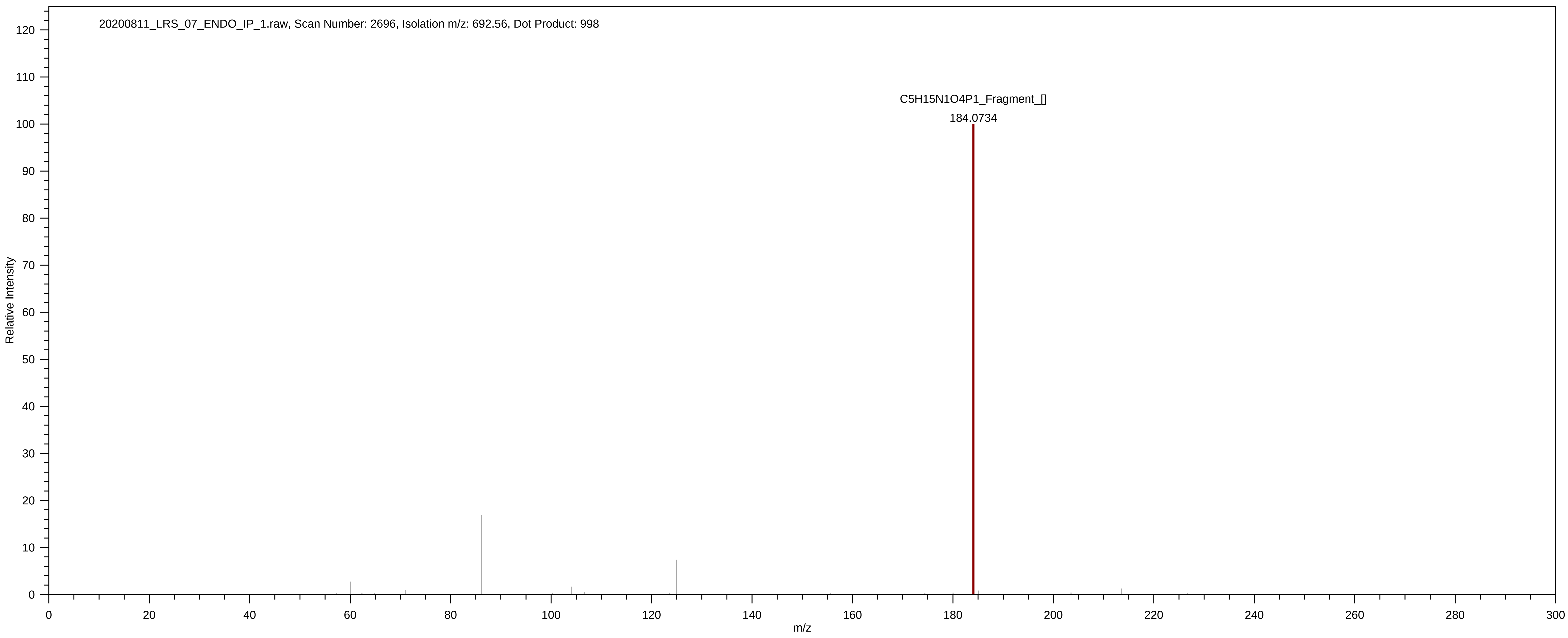

Plasmanyl-PC O-30:0 [M+H]+ | Ret. Time (min): 9.385 | m/z: 692.56

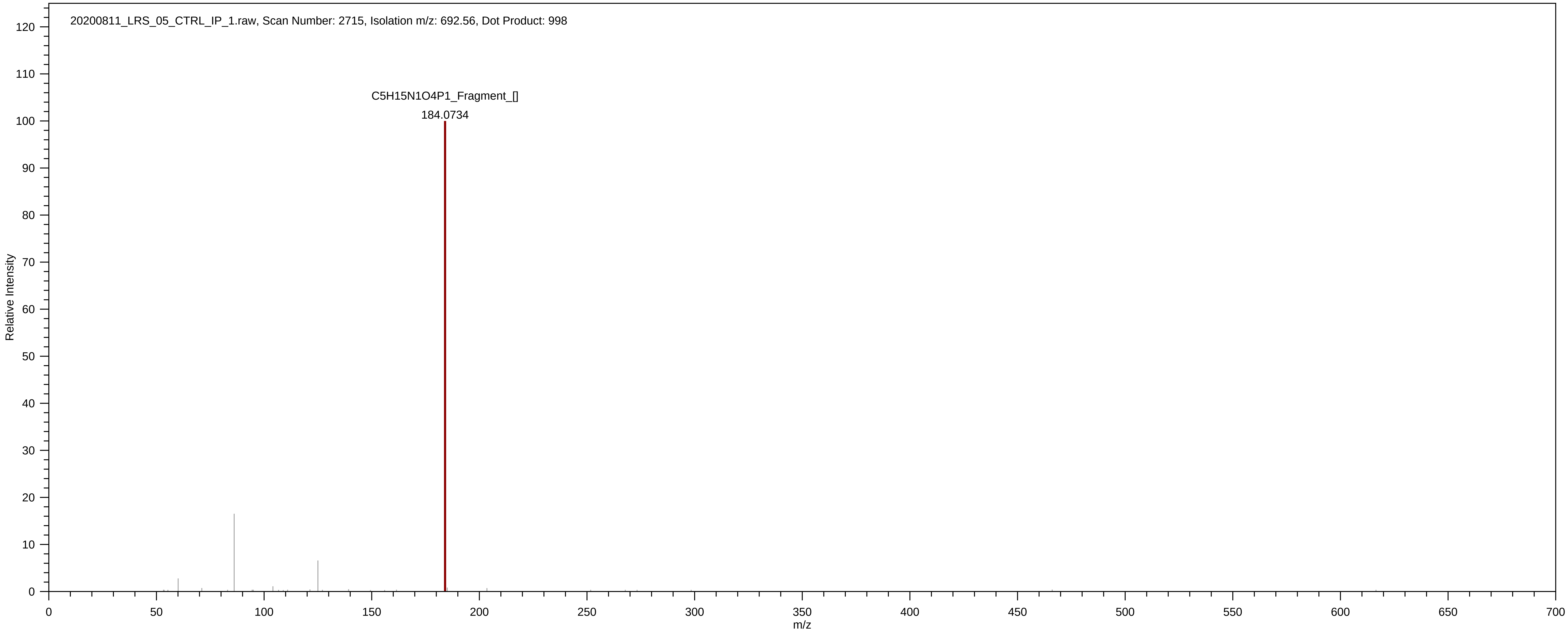

Plasmanyl-PC O-30:0 [M+H]+ | Ret. Time (min): 9.442 | m/z: 692.559

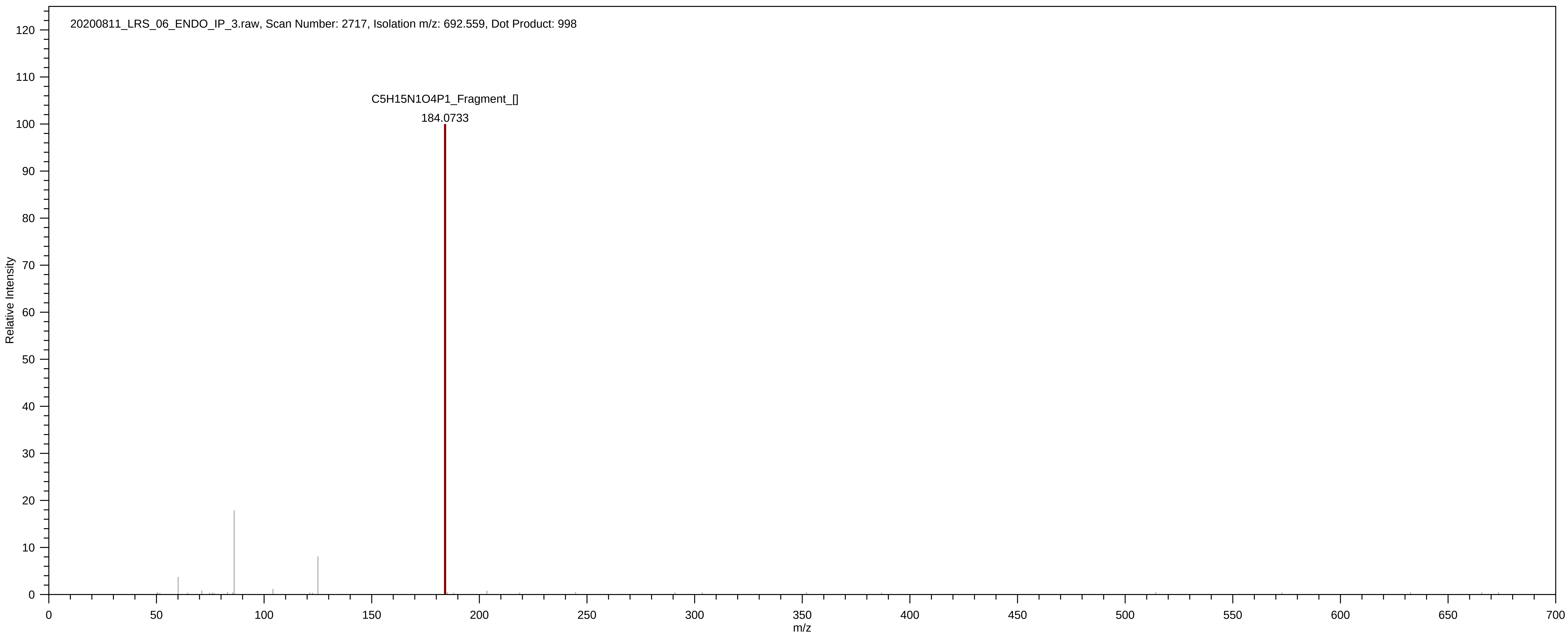

Plasmanyl-PC O-30:0 [M+H]+ | Ret. Time (min): 9.449 | m/z: 692.559

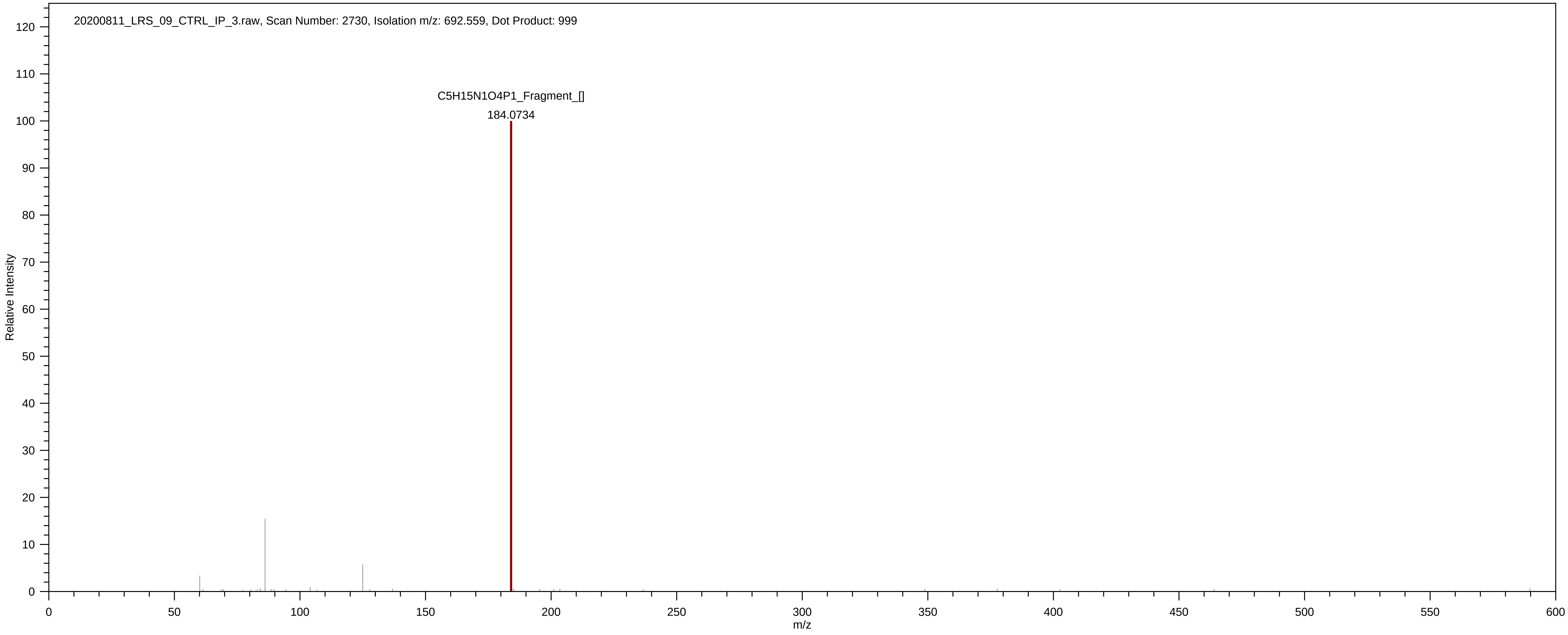

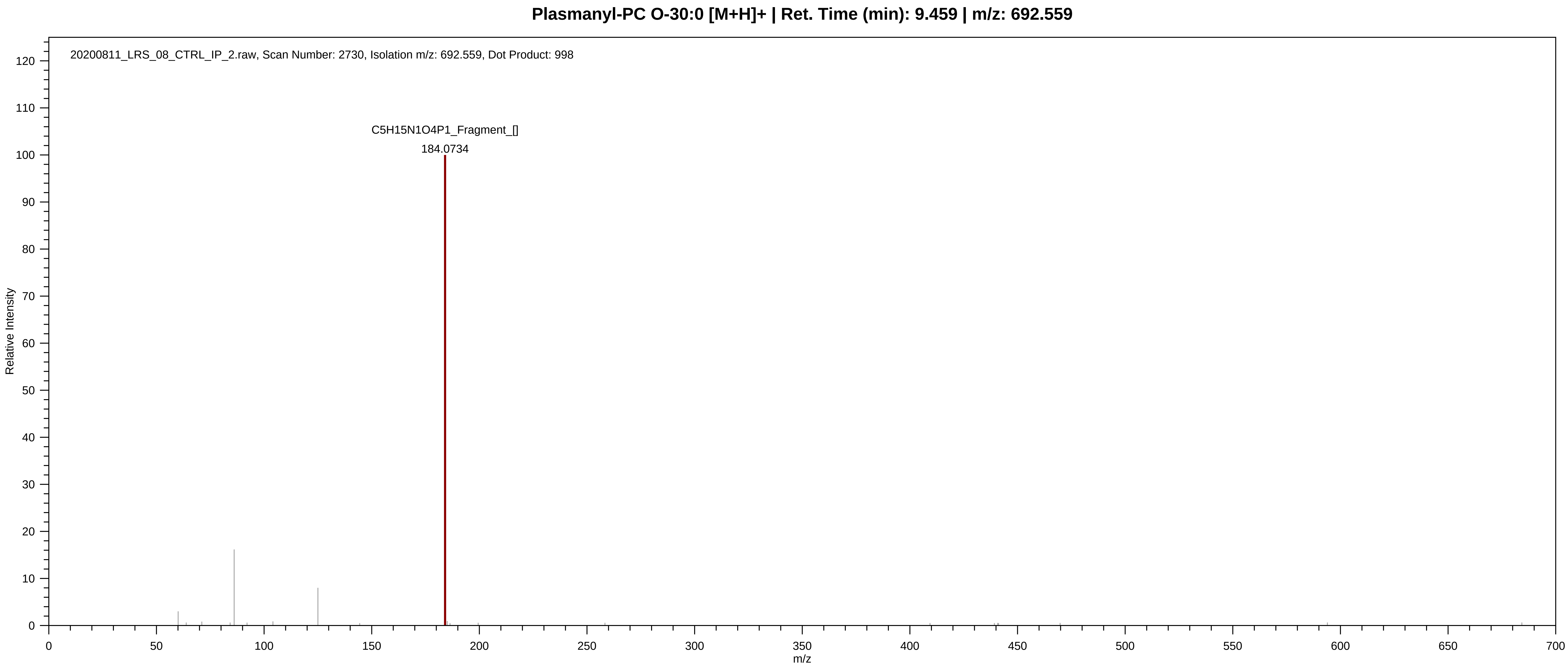
Plasmanyl-PC O-30:0 [M+H]+ | Ret. Time (min): 9.459 | m/z: 692.559
20200811_LRS_08_CTRL_IP_2.raw, Scan Number: 2730, Isolation m/z: 692.559, Dot Product: 998
C5H15N1O4P1_Fragment_[]
184.0734
Relative Intensity
m/z
0
10
20
30
40
50
60
70
80
90
100
110
120
0
50
100
150
200
250
300
350
400
450
500
550
600
650
700

Plasmanyl-PC O-30:0 [M+H]+ | Ret. Time (min): 9.459 | m/z: 692.559

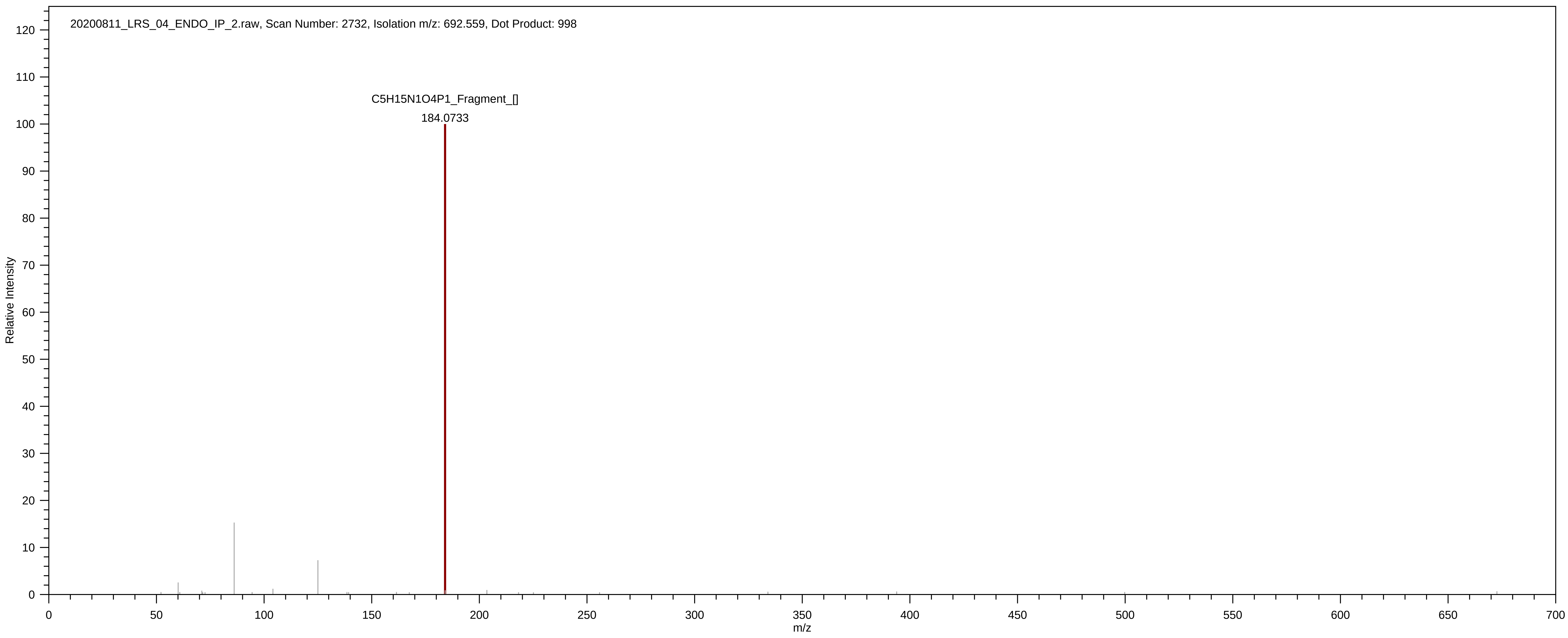

Plasmanyl-PC O-30:1 [M+H]+ | Ret. Time (min): 8.802 | m/z: 690.542

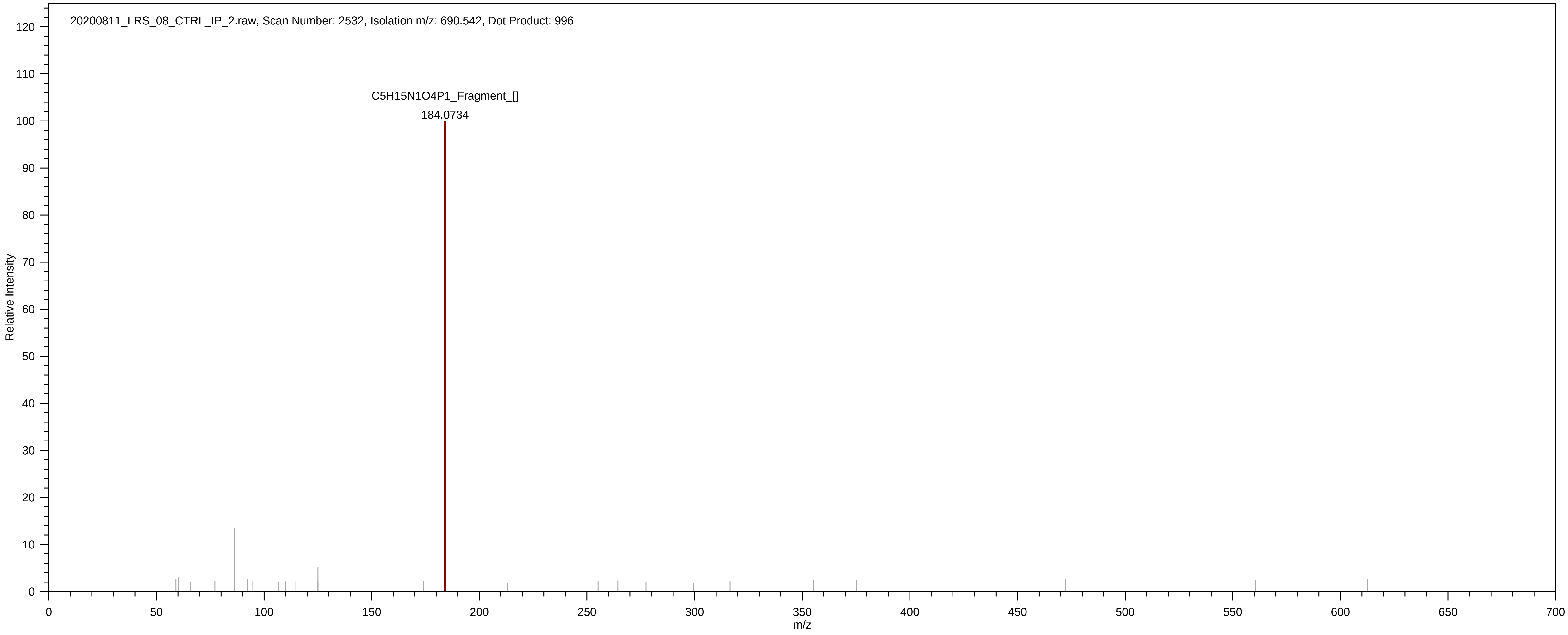

Plasmanyl-PC O-30:1 [M+H]+ | Ret. Time (min): 8.603 | m/z: 690.543

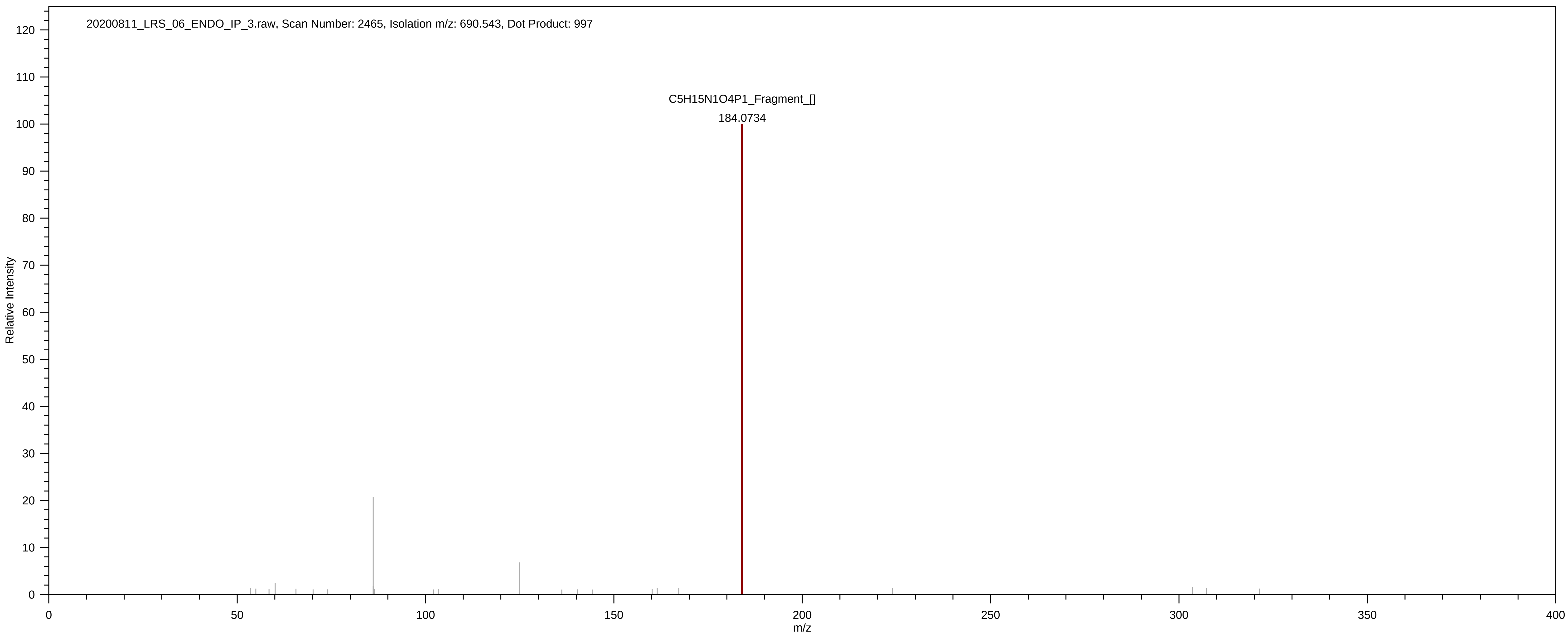

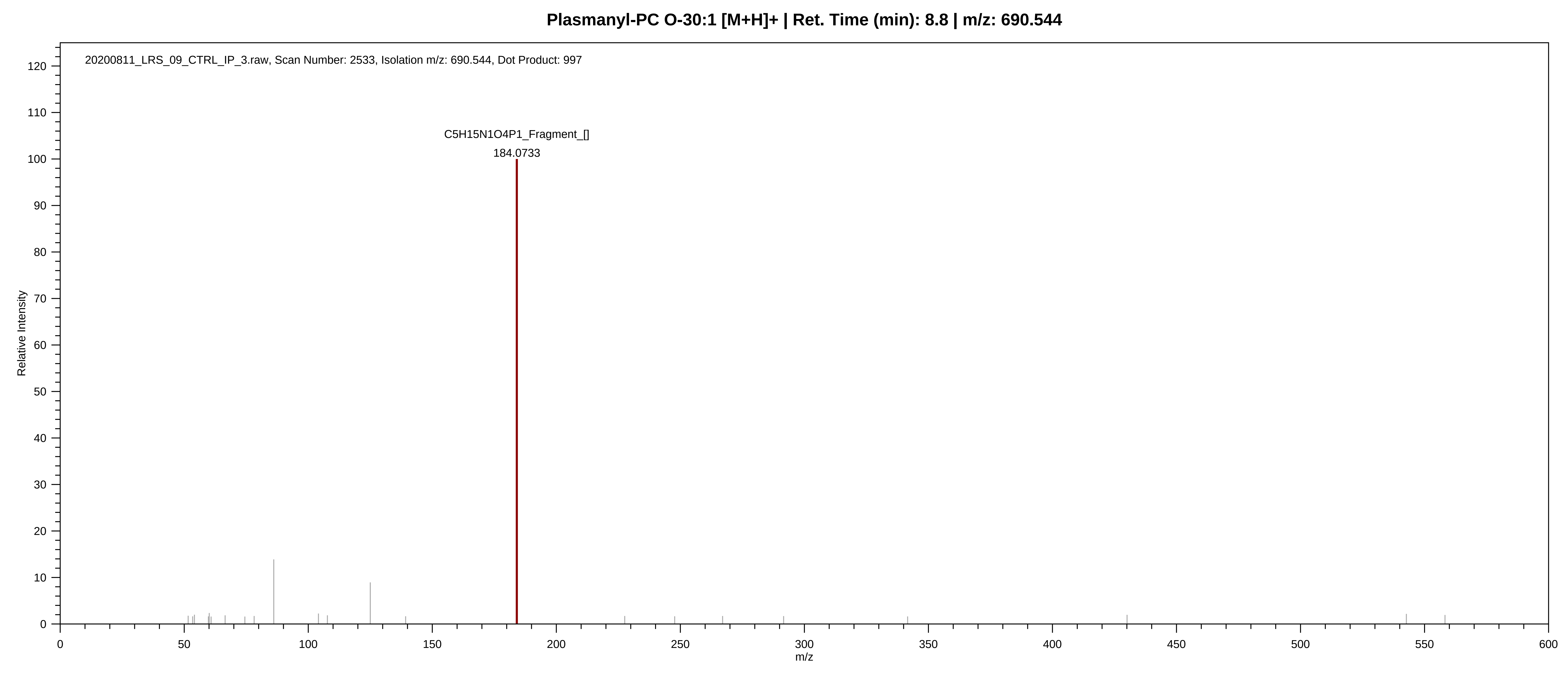

Plasmanyl-PC O-30:1 [M+H]+ | Ret. Time (min): 8.8 | m/z: 690.544
20200811_LRS_09_CTRL_IP_3.raw, Scan Number: 2533, Isolation m/z: 690.544, Dot Product: 997
C5H15N1O4P1_Fragment_[]
184.0733
Relative Intensity
m/z

Plasmanyl-PC O-30:1 [M+H]+ | Ret. Time (min): 8.596 | m/z: 690.544

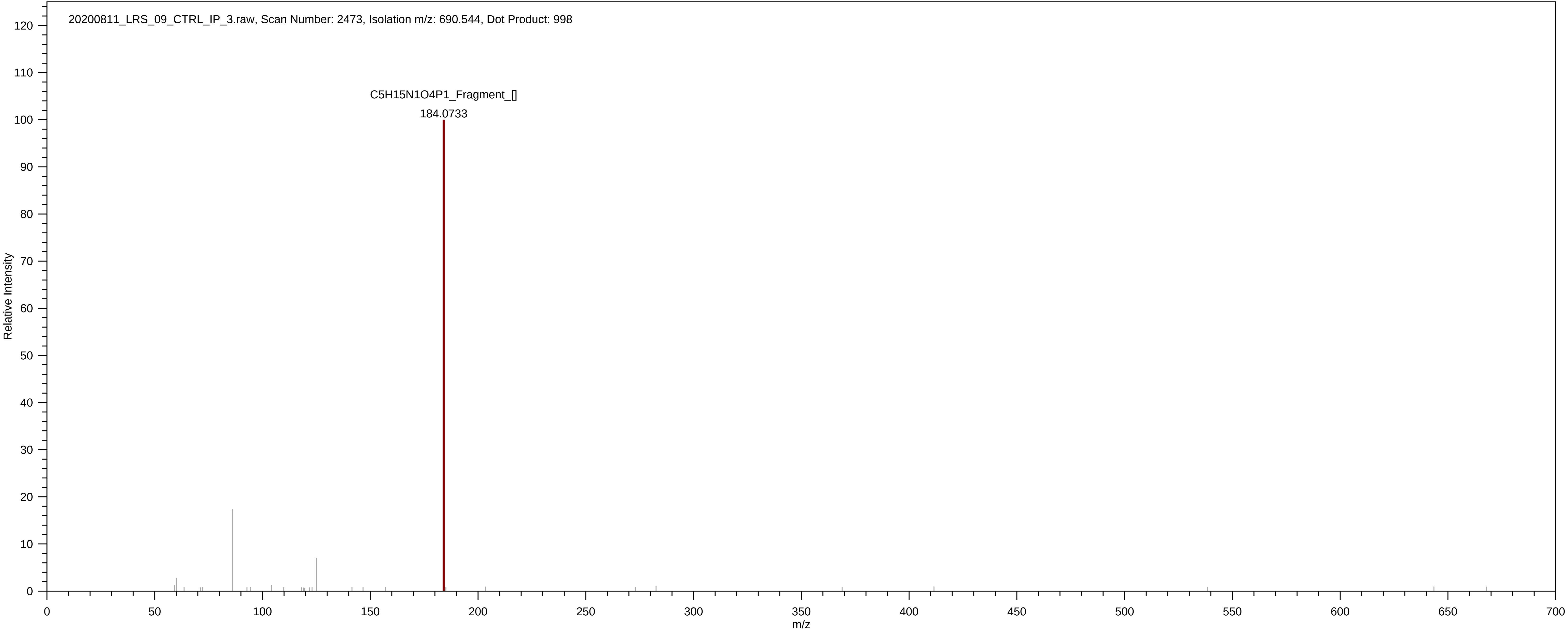

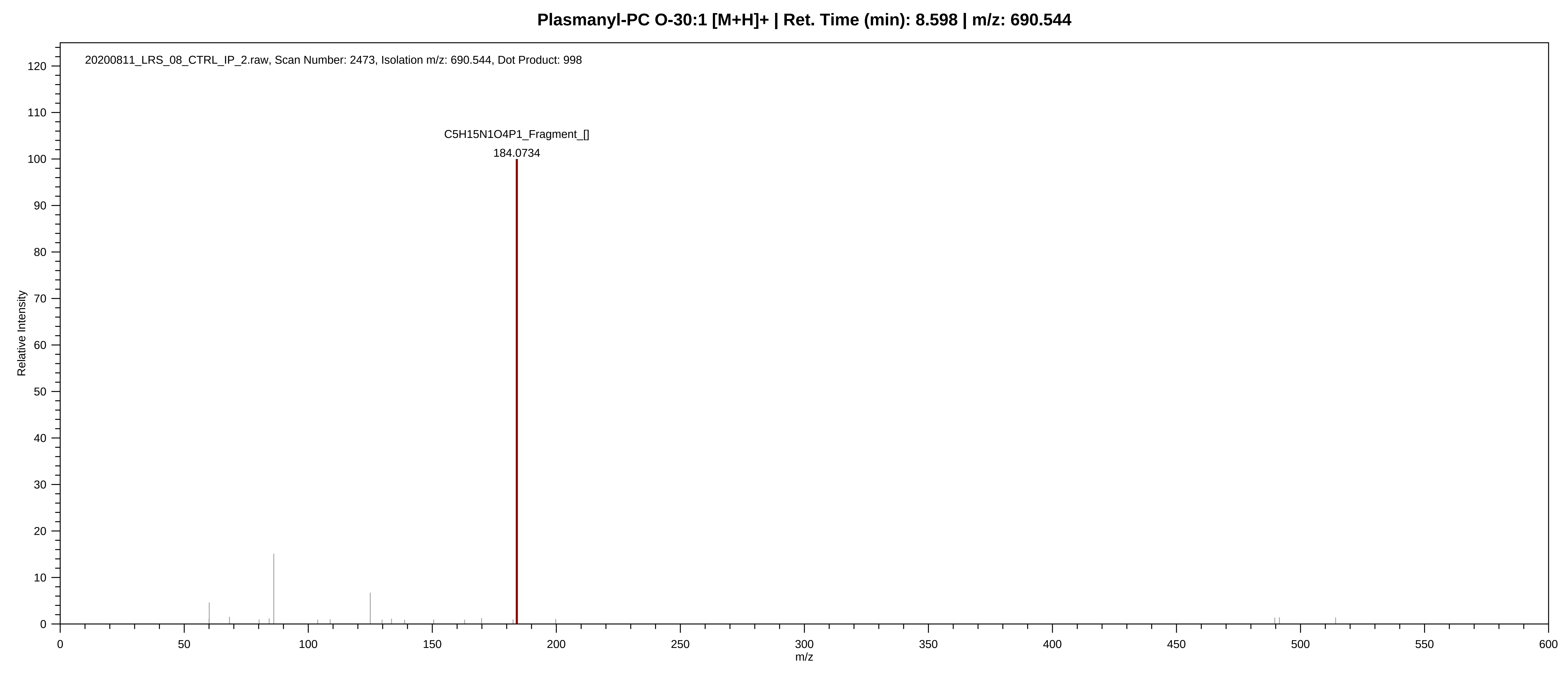

Plasmanyl-PC O-30:1 [M+H]+ | Ret. Time (min): 8.598 | m/z: 690.544
20200811_LRS_08_CTRL_IP_2.raw, Scan Number: 2473, Isolation m/z: 690.544, Dot Product: 998
C5H15N1O4P1_Fragment_[]
184.0734
Relative Intensity
m/z

Plasmanyl-PC O-30:1 [M+H]+ | Ret. Time (min): 8.623 | m/z: 690.544

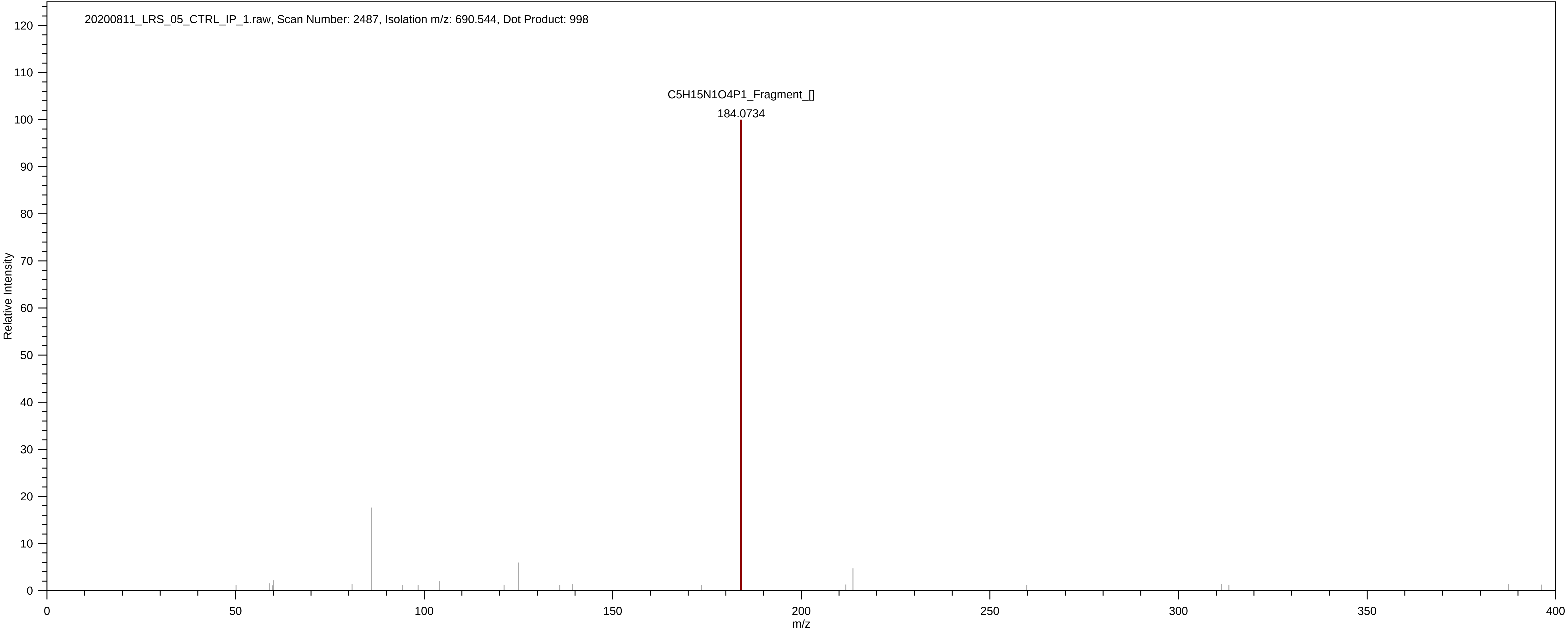

Plasmanyl-PC O-30:1 [M+H]+ | Ret. Time (min): 9.279 | m/z: 690.544

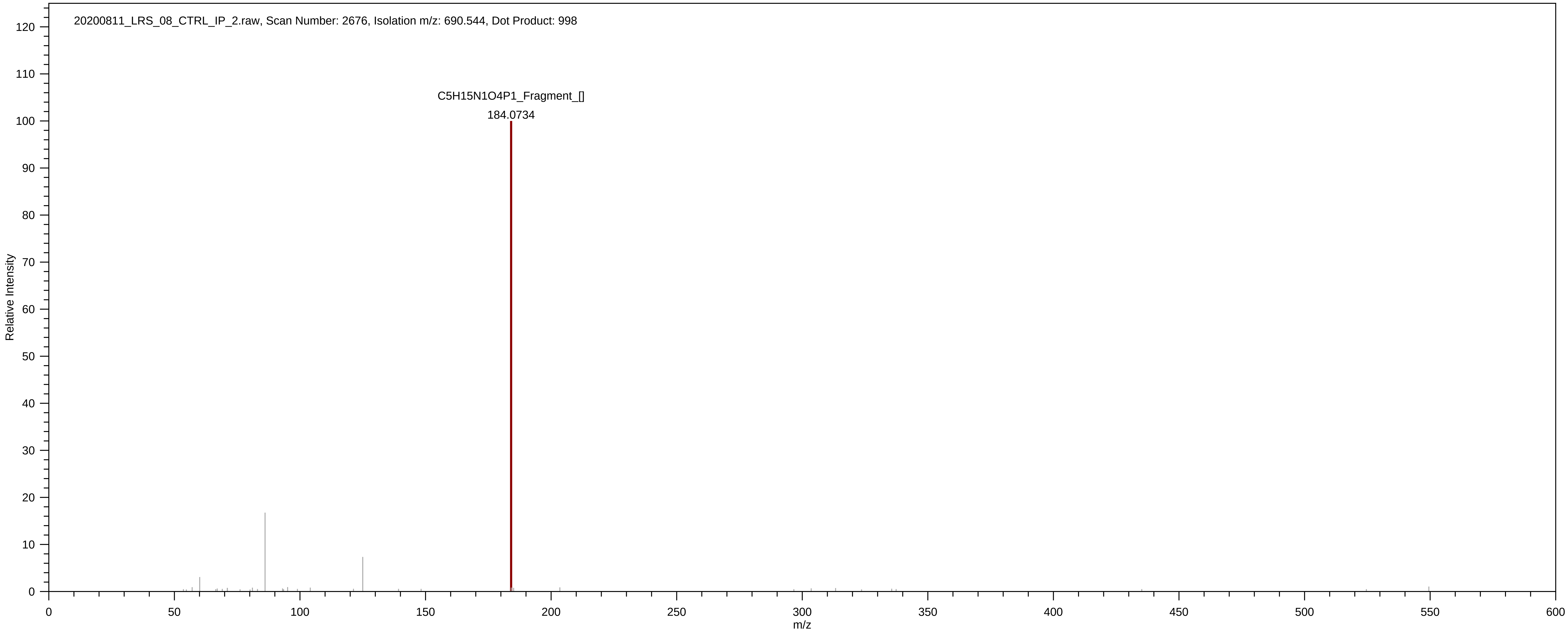

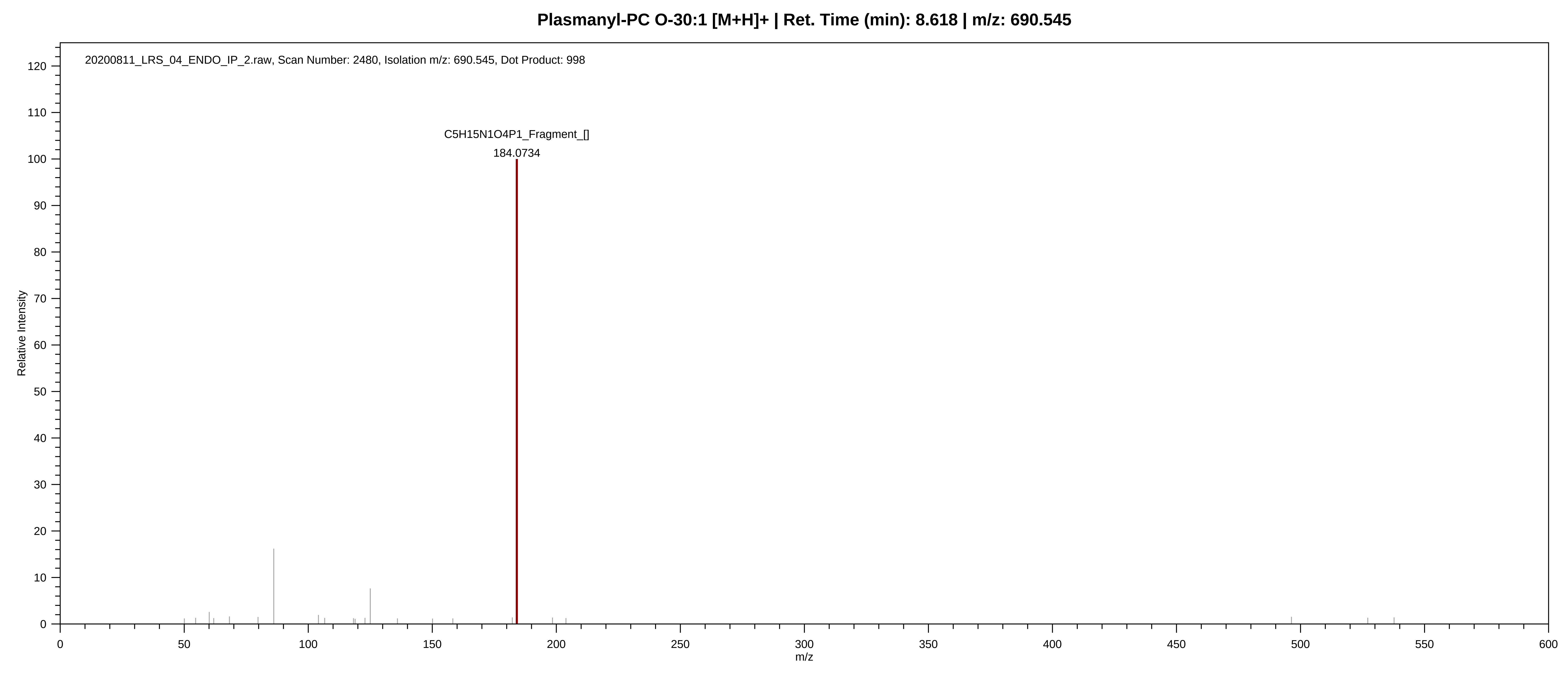
Plasmanyl-PC O-30:1 [M+H]+ | Ret. Time (min): 8.618 | m/z: 690.545
20200811_LRS_04_ENDO_IP_2.raw, Scan Number: 2480, Isolation m/z: 690.545, Dot Product: 998
C5H15N1O4P1_Fragment_[]
184.0734
Relative Intensity
m/z

Plasmanyl-PC O-30:1 [M+H]+ | Ret. Time (min): 9.274 | m/z: 690.545

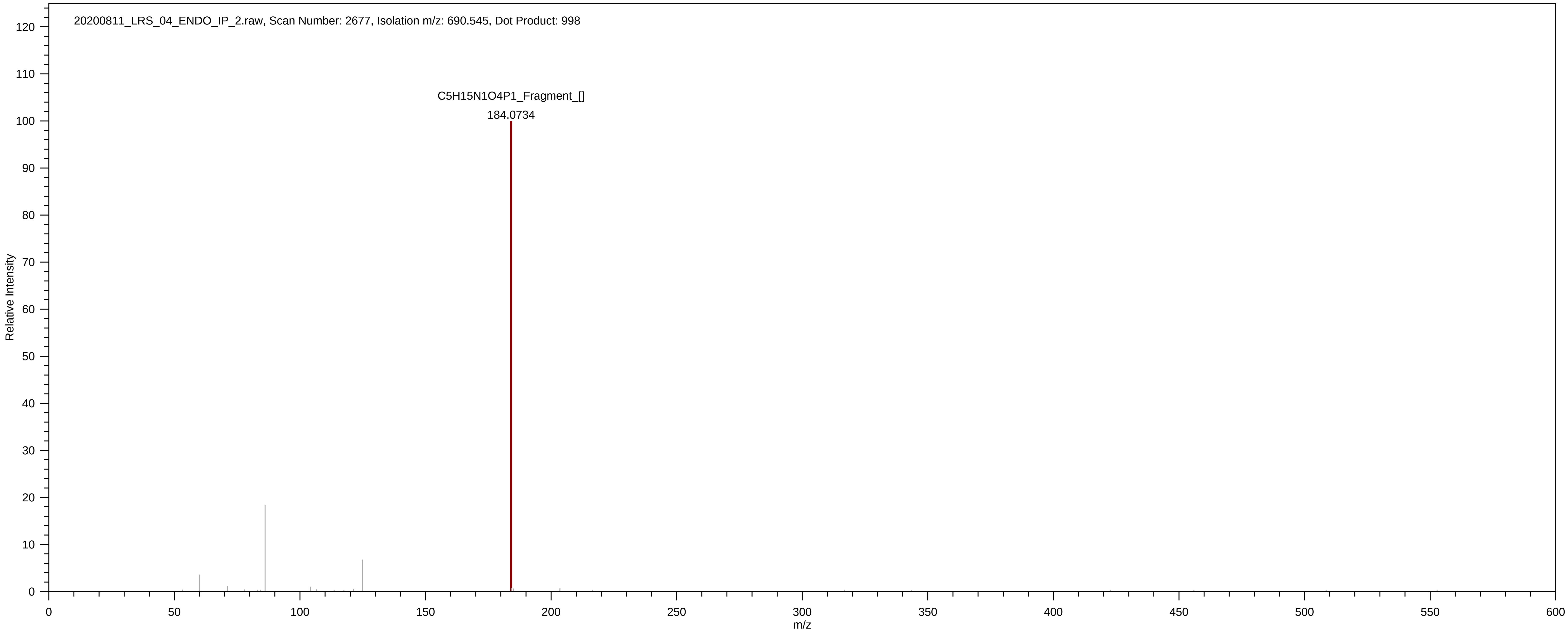

Plasmanyl-PC O-30:1 [M+H]+ | Ret. Time (min): 9.26 | m/z: 690.547

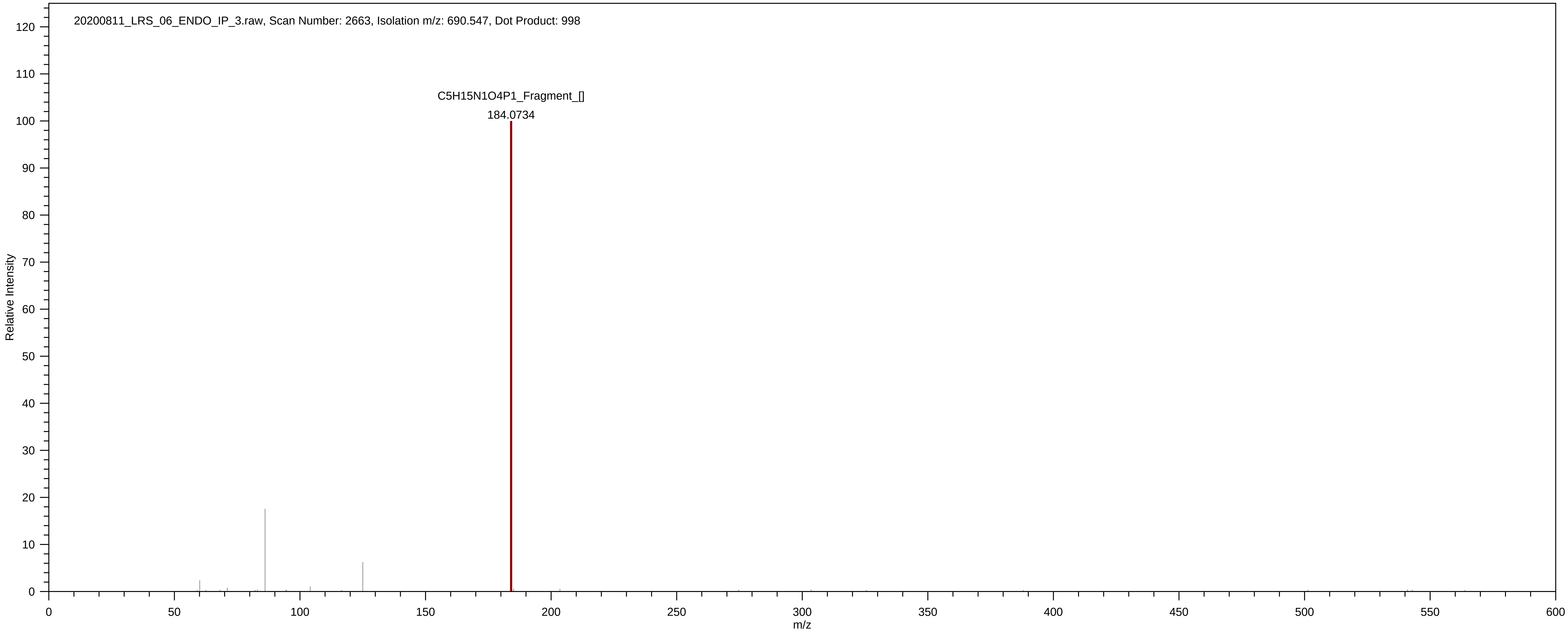

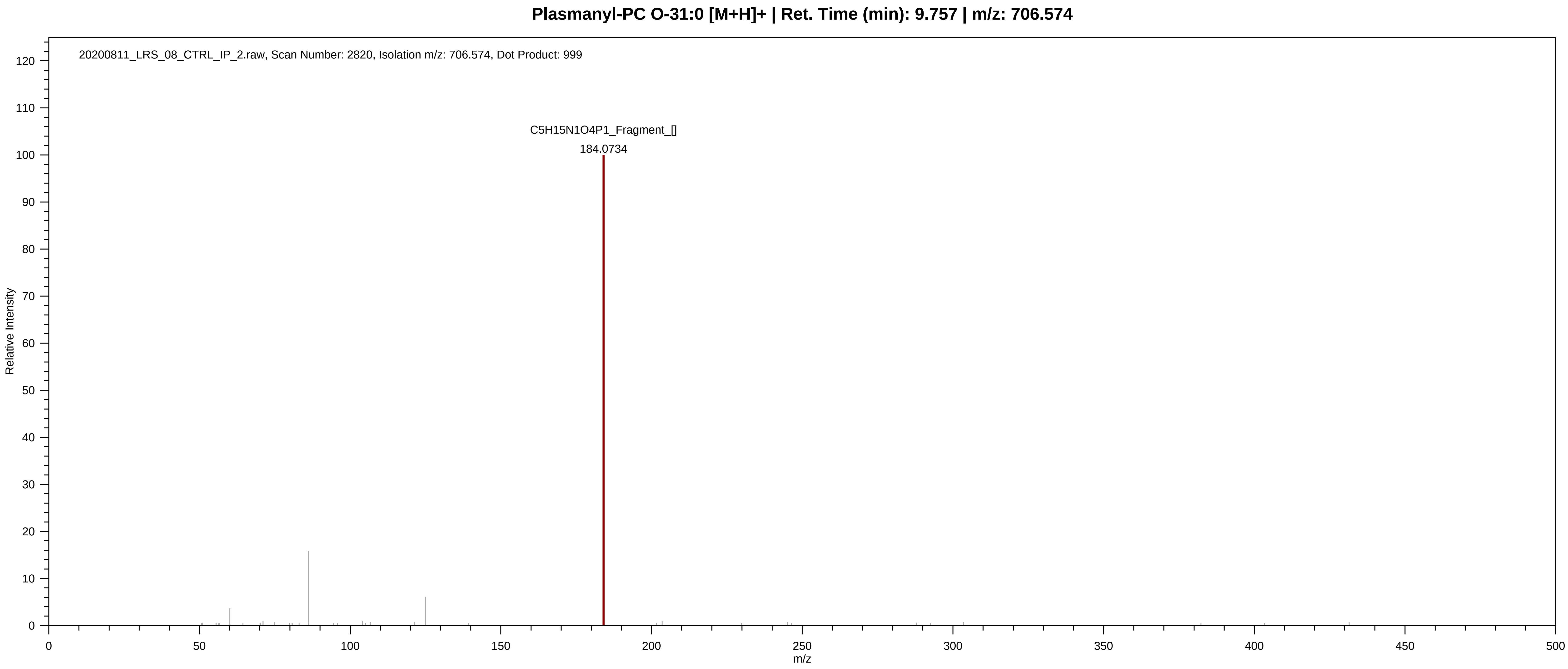

Plasmanyl-PC O-31:0 [M+H]+ | Ret. Time (min): 9.757 | m/z: 706.574
20200811_LRS_08_CTRL_IP_2.raw, Scan Number: 2820, Isolation m/z: 706.574, Dot Product: 999
C5H15N1O4P1_Fragment_[]
184.0734
Relative Intensity
m/z

Plasmanyl-PC O-31:0 [M+H]+ | Ret. Time (min): 9.764 | m/z: 706.574

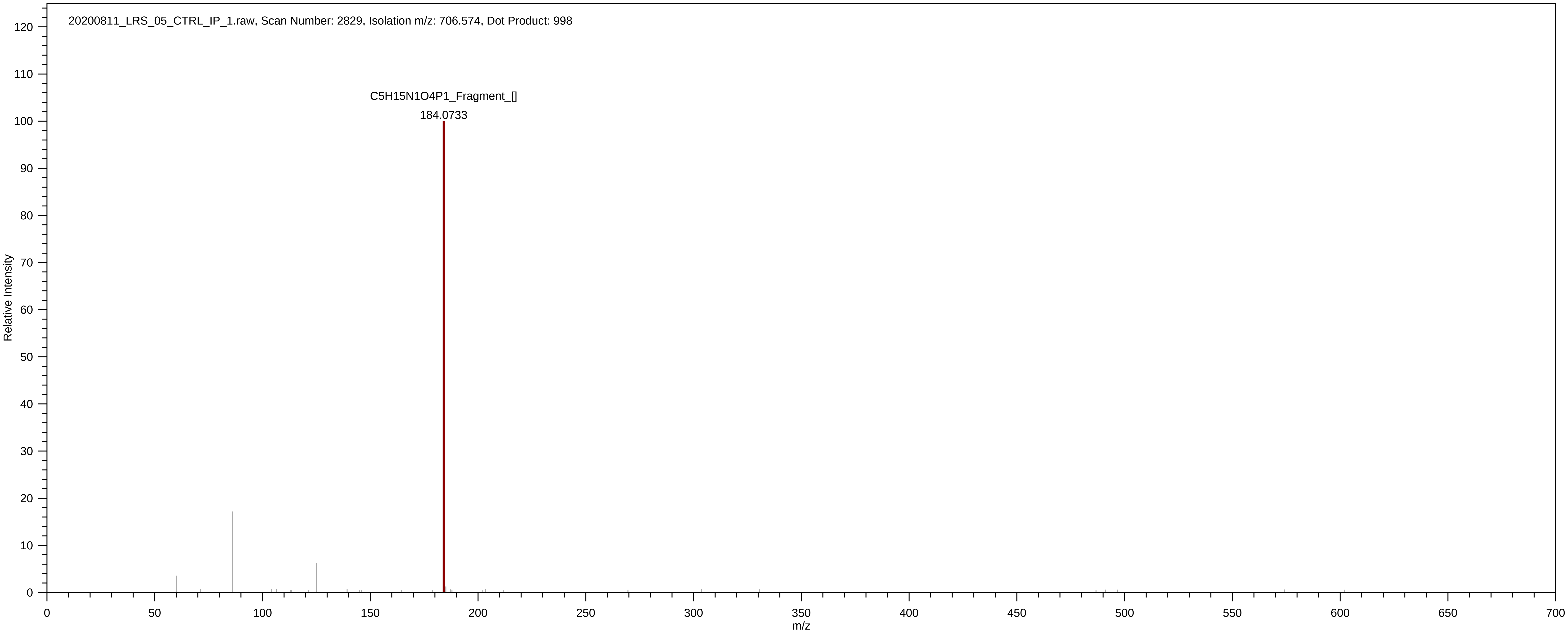

Plasmanyl-PC O-31:0 [M+H]+ | Ret. Time (min): 9.784 | m/z: 706.574

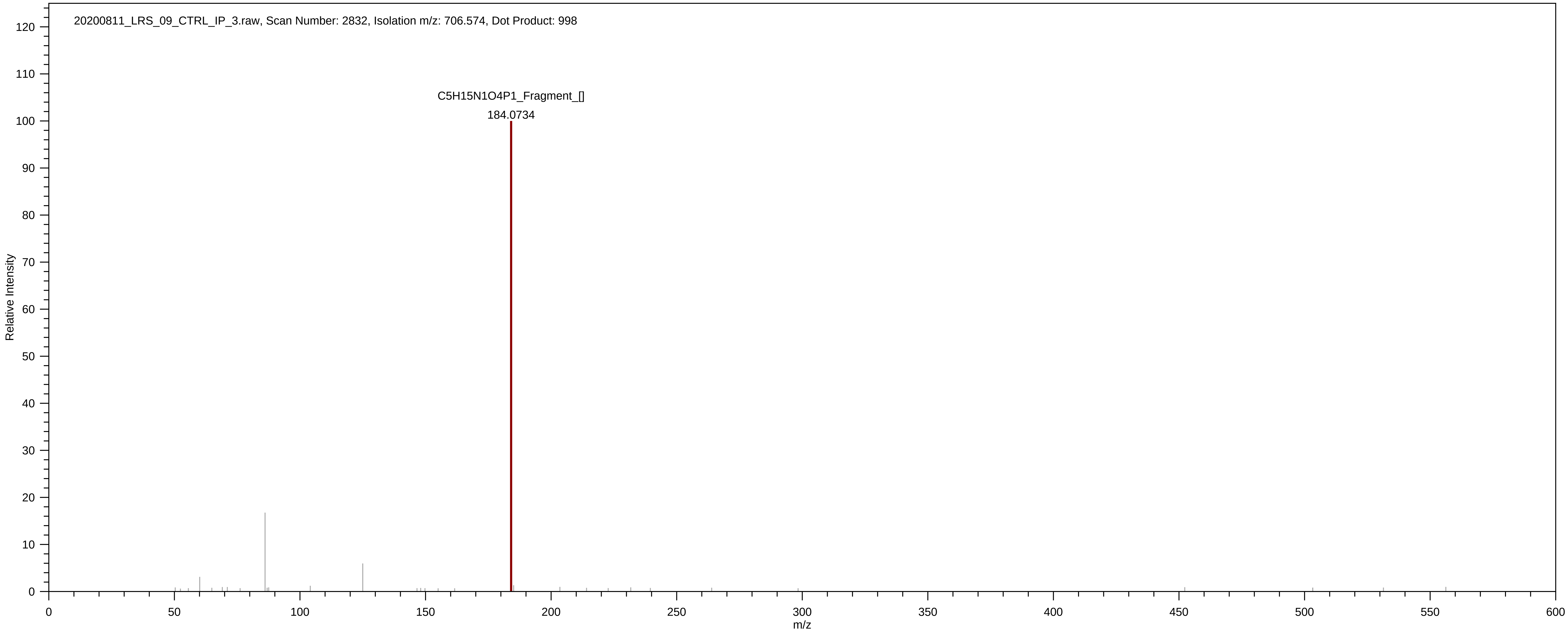

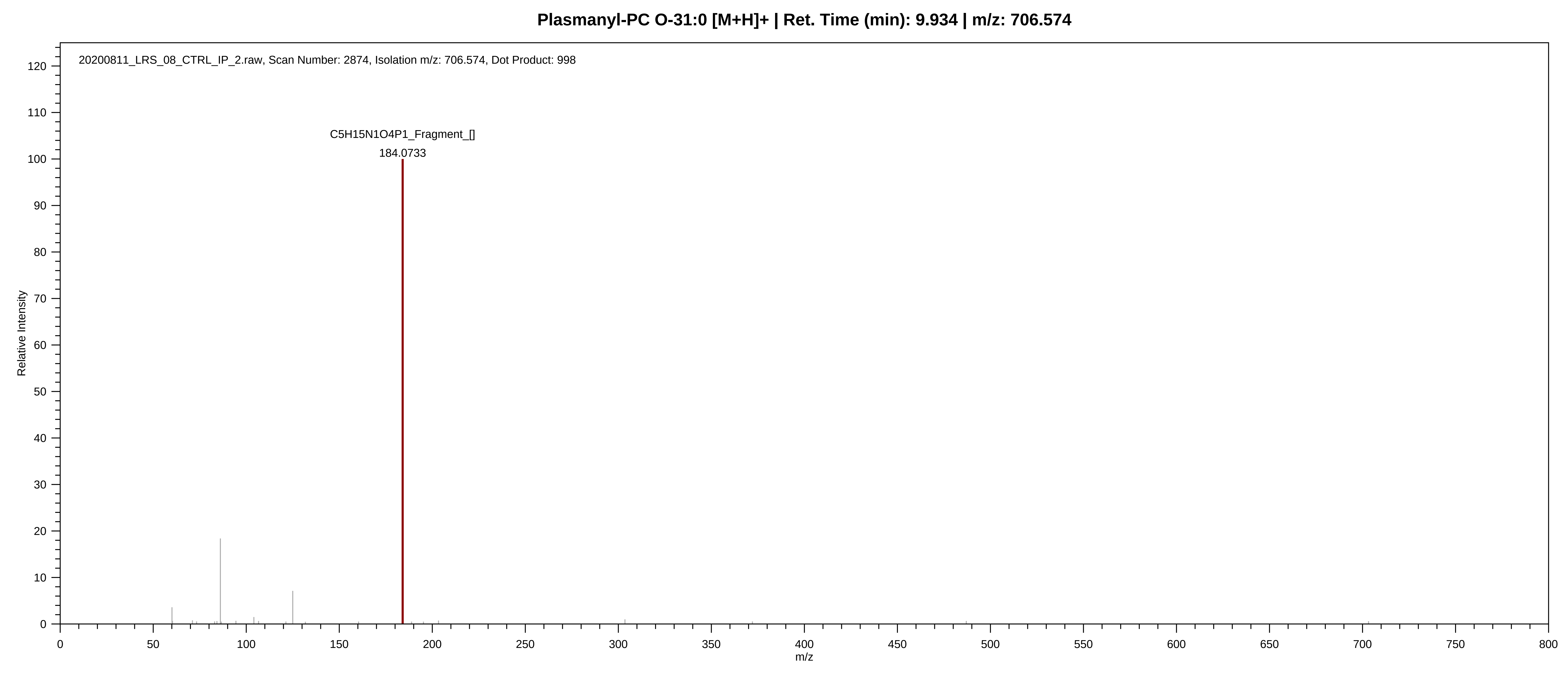

Plasmanyl-PC O-31:0 [M+H]+ | Ret. Time (min): 9.934 | m/z: 706.574
20200811_LRS_08_CTRL_IP_2.raw, Scan Number: 2874, Isolation m/z: 706.574, Dot Product: 998
C5H15N1O4P1_Fragment_[]
184.0733
Relative Intensity
m/z

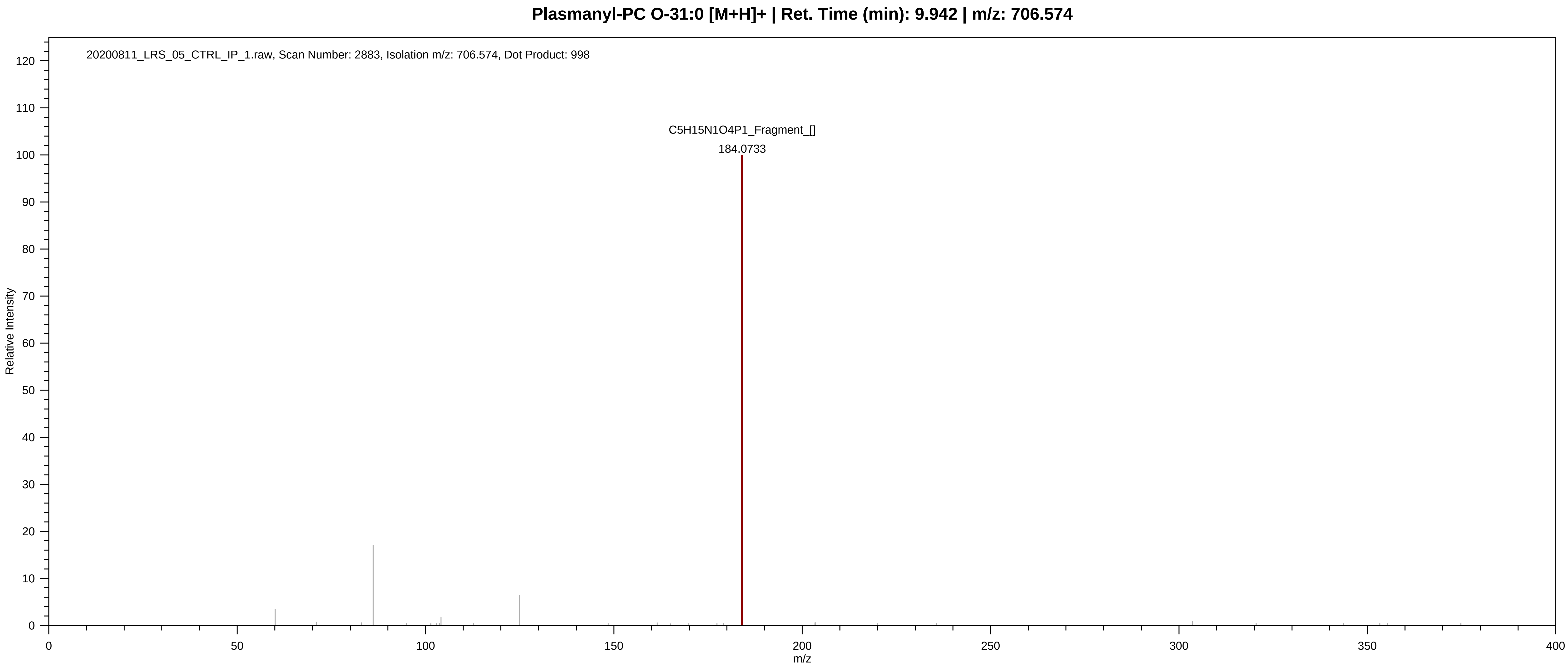

Plasmanyl-PC O-31:0 [M+H]+ | Ret. Time (min): 9.942 | m/z: 706.574
20200811_LRS_05_CTRL_IP_1.raw, Scan Number: 2883, Isolation m/z: 706.574, Dot Product: 998
C5H15N1O4P1_Fragment_[]
184.0733
Relative Intensity
m/z
0
10
20
30
40
50
60
70
80
90
100
110
120
0
50
100
150
200
250
300
350
400

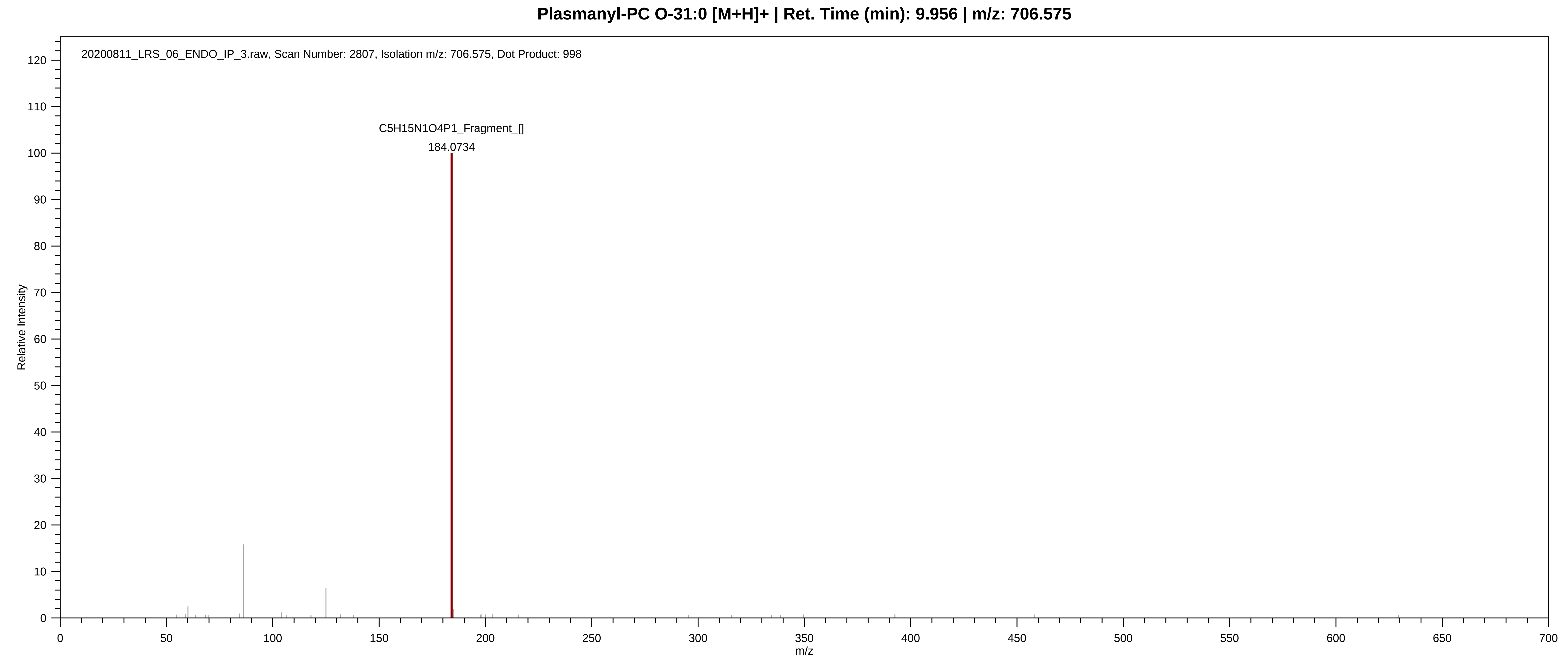
Plasmanyl-PC O-31:0 [M+H]+ | Ret. Time (min): 9.956 | m/z: 706.575
20200811_LRS_06_ENDO_IP_3.raw, Scan Number: 2807, Isolation m/z: 706.575, Dot Product: 998
C5H15N1O4P1_Fragment_[]
184.0734
Relative Intensity
m/z

Plasmanyl-PC O-31:0 [M+H]+ | Ret. Time (min): 9.956 | m/z: 706.574

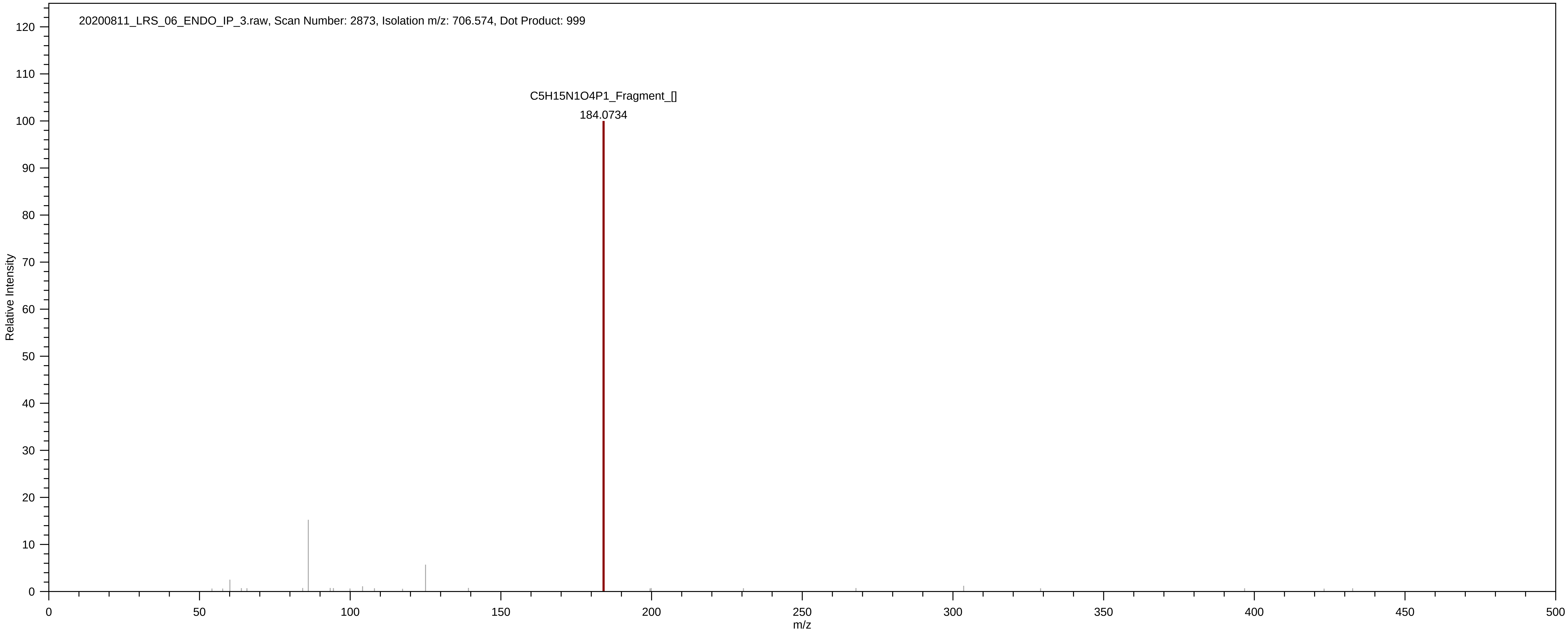

Plasmanyl-PC O-31:0 [M+H]+ | Ret. Time (min): 9.974 | m/z: 706.574

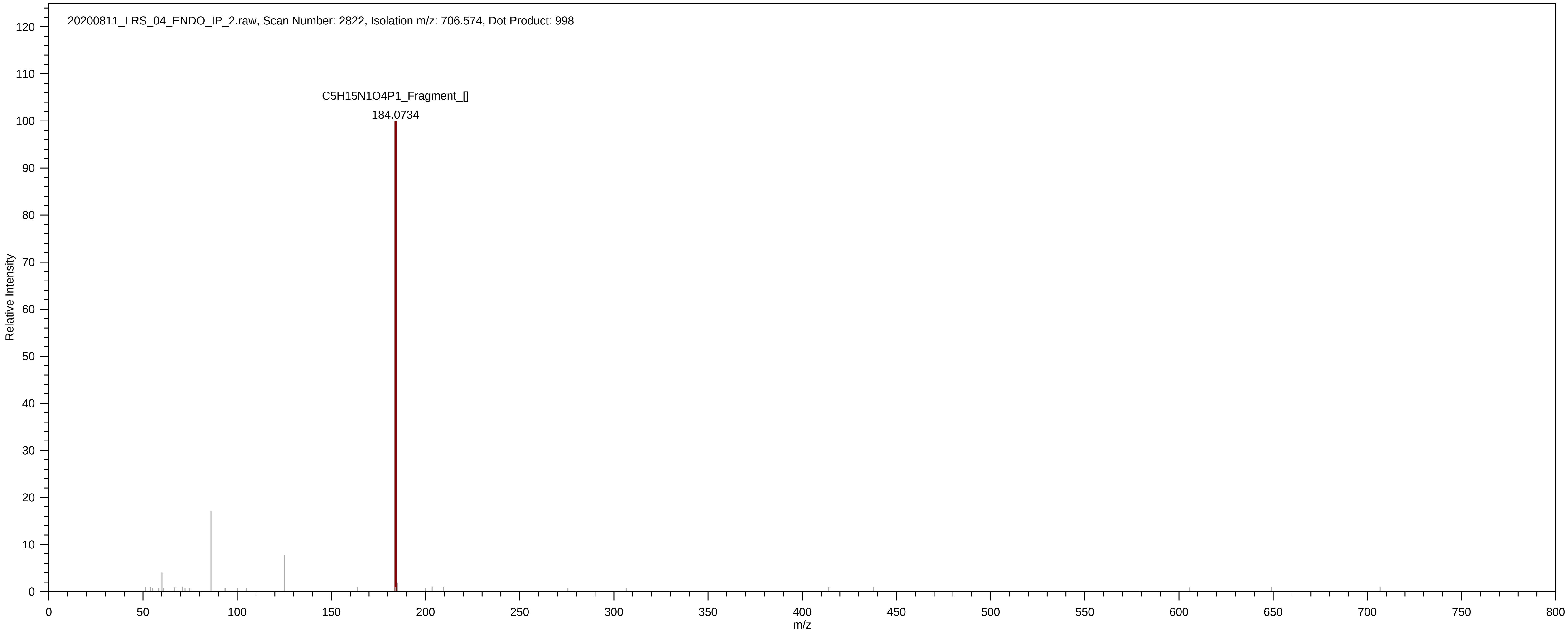

Plasmanyl-PC O-31:0 [M+H]+ | Ret. Time (min): 9.974 | m/z: 706.574

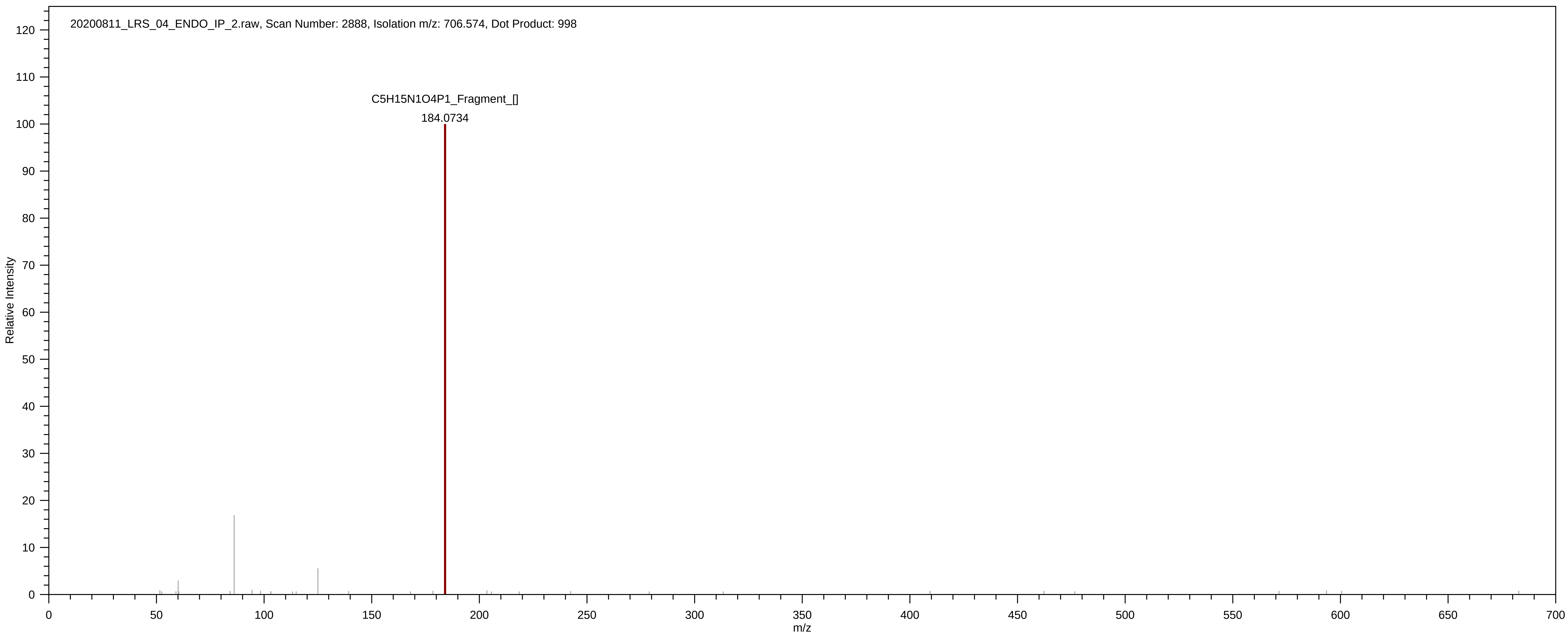

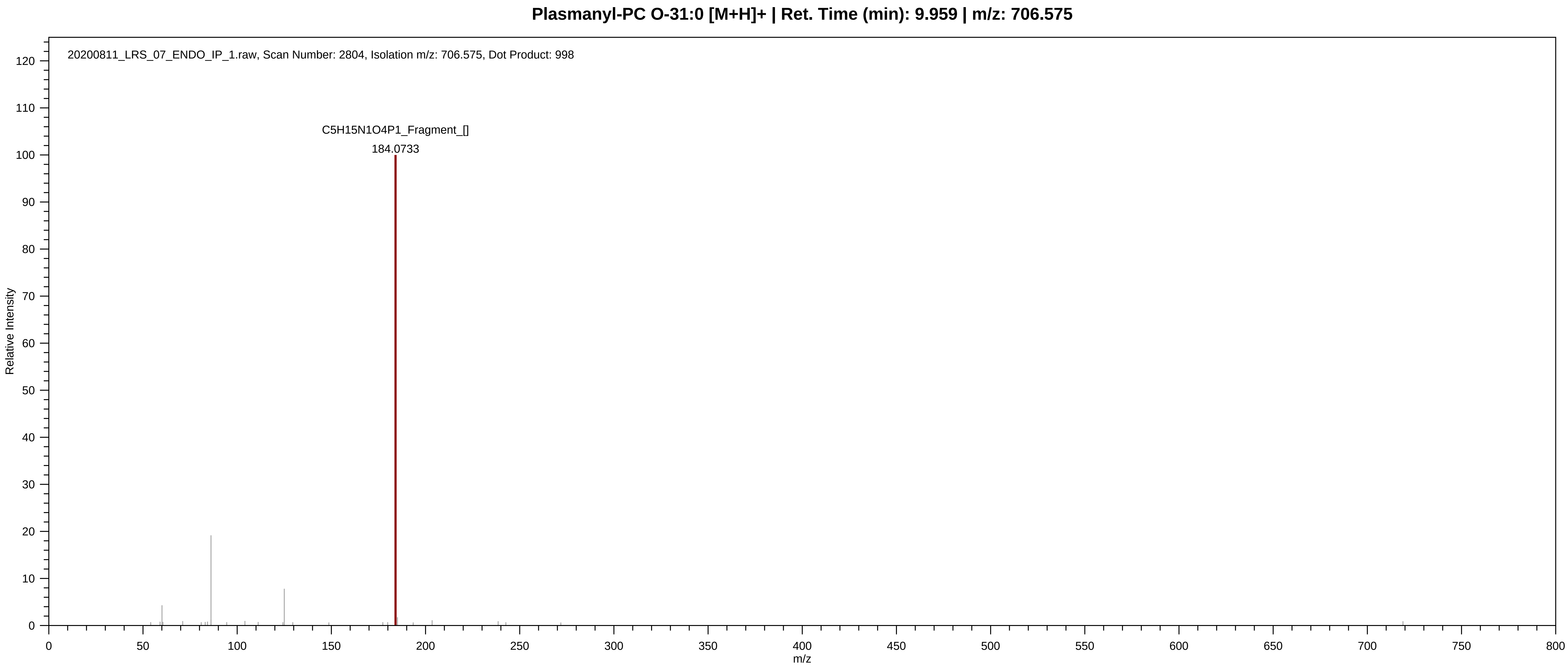
Plasmanyl-PC O-31:0 [M+H]+ | Ret. Time (min): 9.959 | m/z: 706.575
20200811_LRS_07_ENDO_IP_1.raw, Scan Number: 2804, Isolation m/z: 706.575, Dot Product: 998
C5H15N1O4P1_Fragment_[]
184.0733
Relative Intensity
m/z
0
10
20
30
40
50
60
70
80
90
100
110
120
0
50
100
150
200
250
300
350
400
450
500
550
600
650
700
750
800

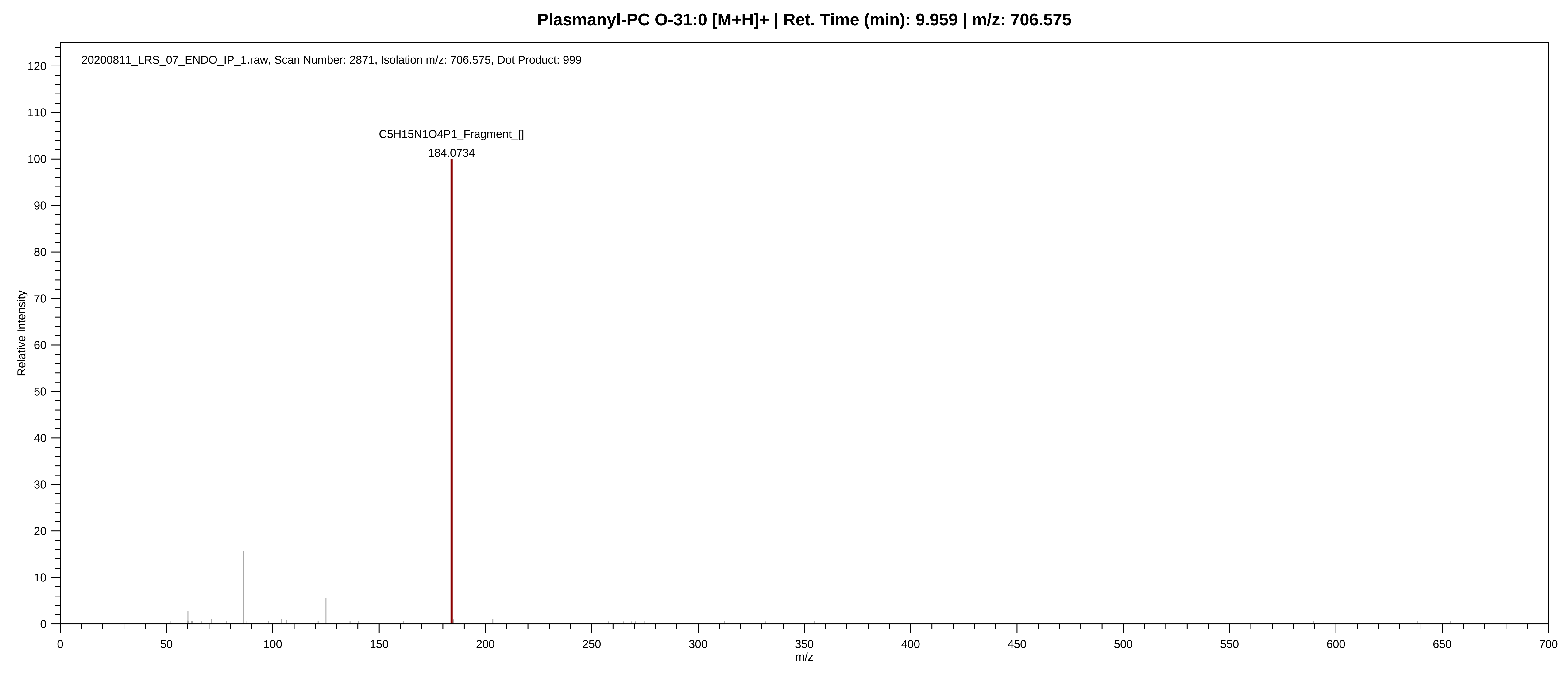
Plasmanyl-PC O-31:0 [M+H]+ | Ret. Time (min): 9.959 | m/z: 706.575
20200811_LRS_07_ENDO_IP_1.raw, Scan Number: 2871, Isolation m/z: 706.575, Dot Product: 999
C5H15N1O4P1_Fragment_[]
184.0734
Relative Intensity
m/z

Plasmanyl-PC O-31:0 [M+H]+ | Ret. Time (min): 9.977 | m/z: 706.575

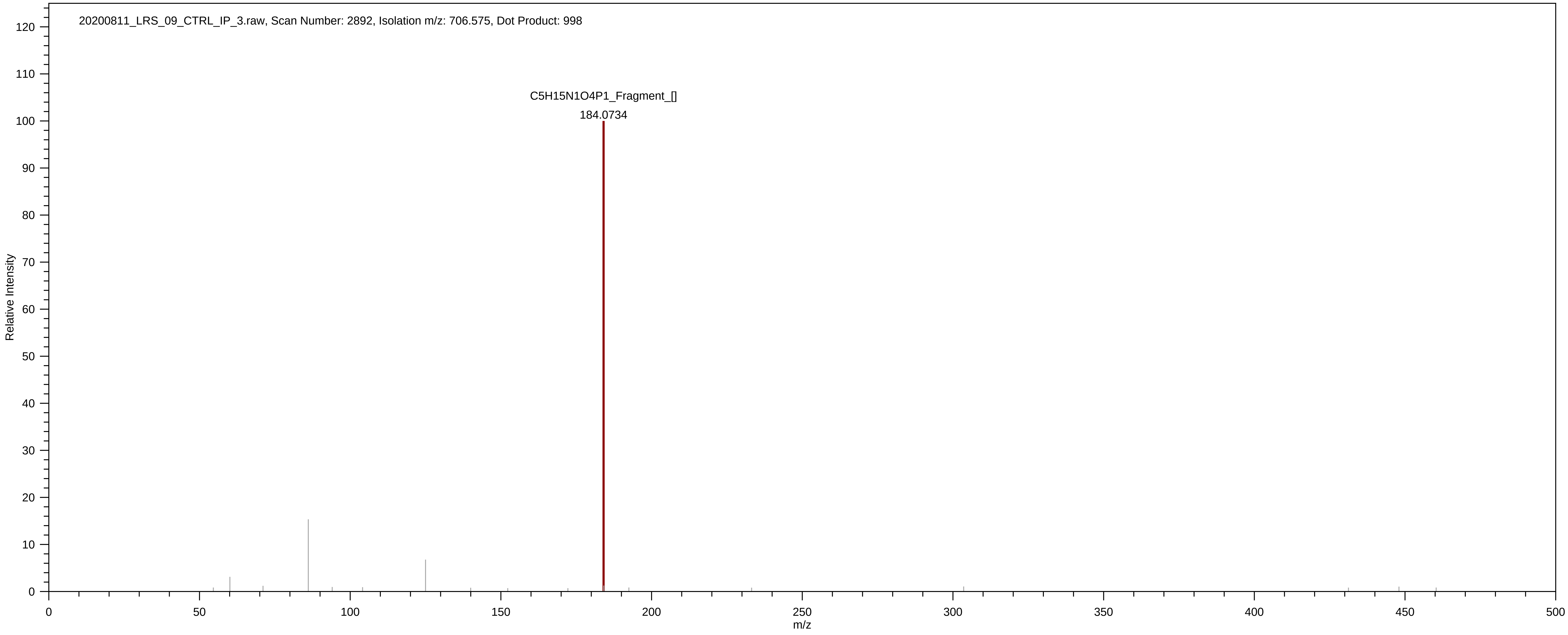

Plasmanyl-PC O-31:1 [M+H]+ | Ret. Time (min): 9.042 | m/z: 704.556

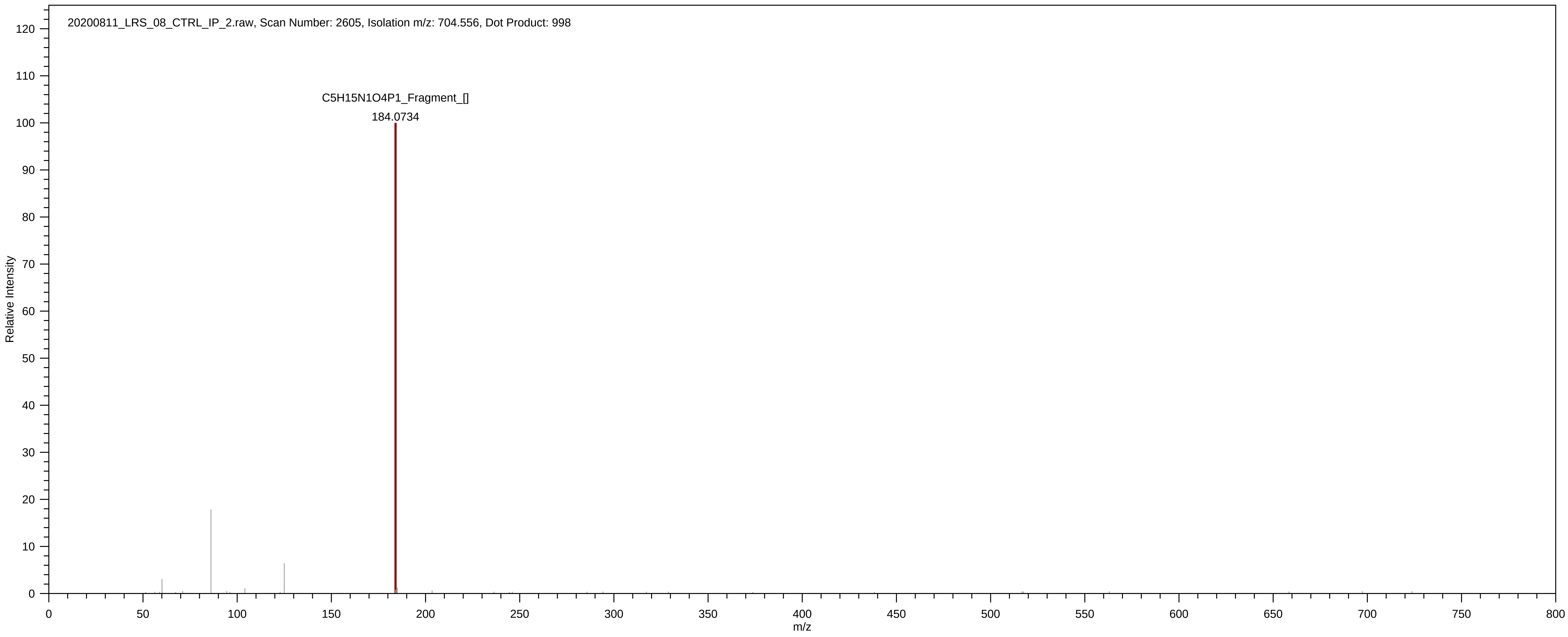

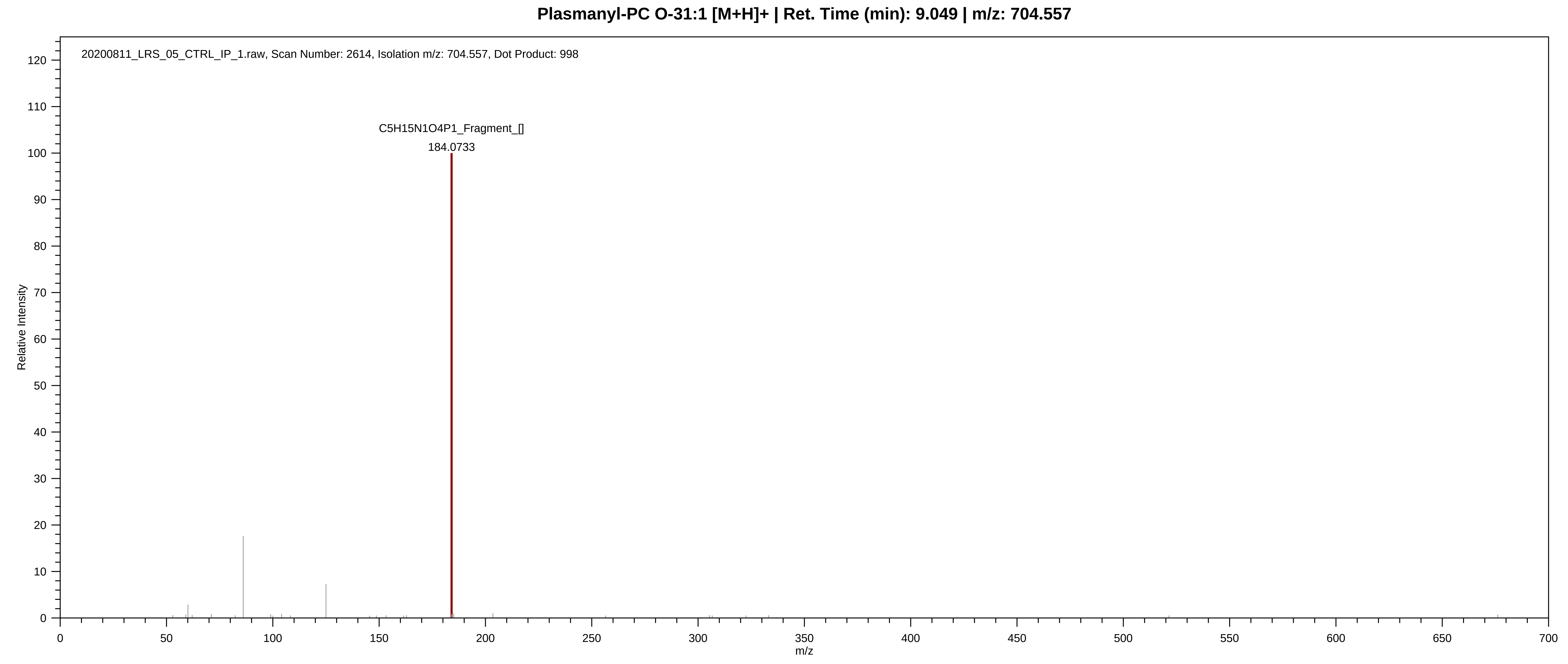
Plasmanyl-PC O-31:1 [M+H]+ | Ret. Time (min): 9.049 | m/z: 704.557
20200811_LRS_05_CTRL_IP_1.raw, Scan Number: 2614, Isolation m/z: 704.557, Dot Product: 998
C5H15N1O4P1_Fragment_[]
184.0733
Relative Intensity
m/z

Plasmanyl-PC O-31:1 [M+H]+ | Ret. Time (min): 9.054 | m/z: 704.559

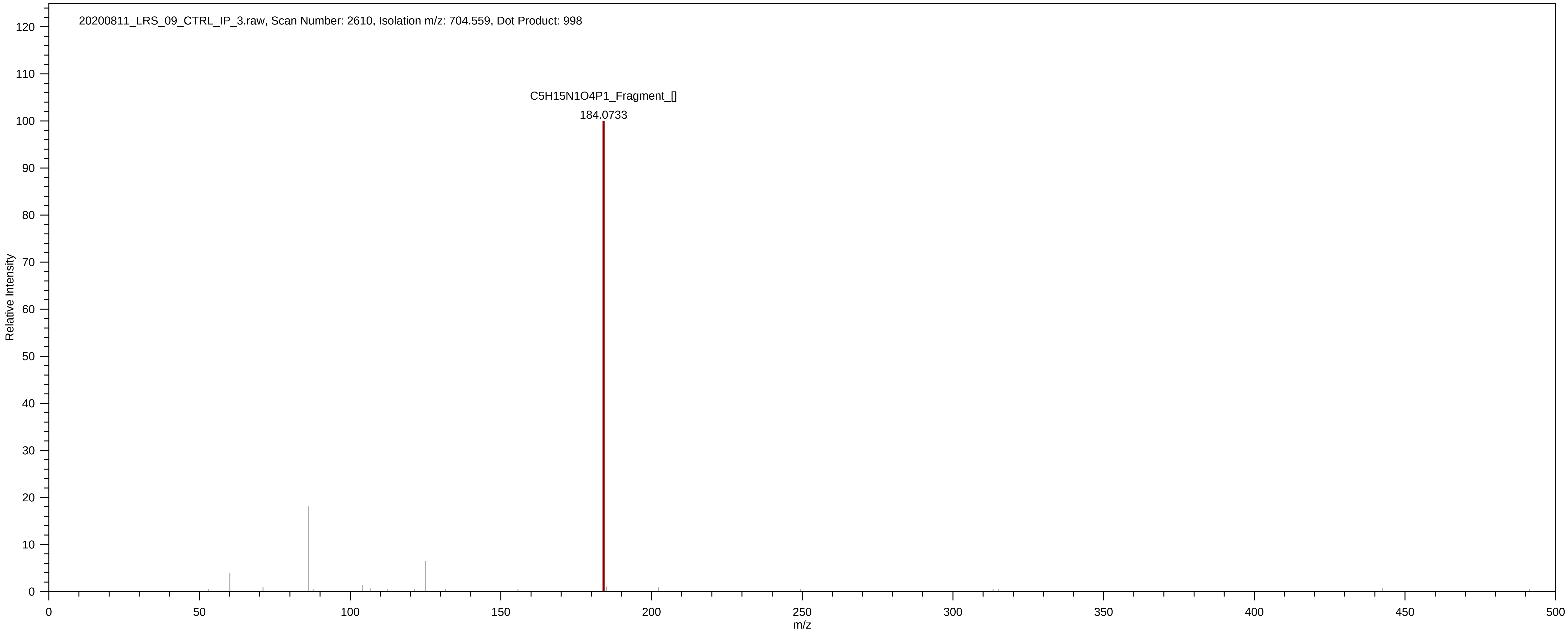

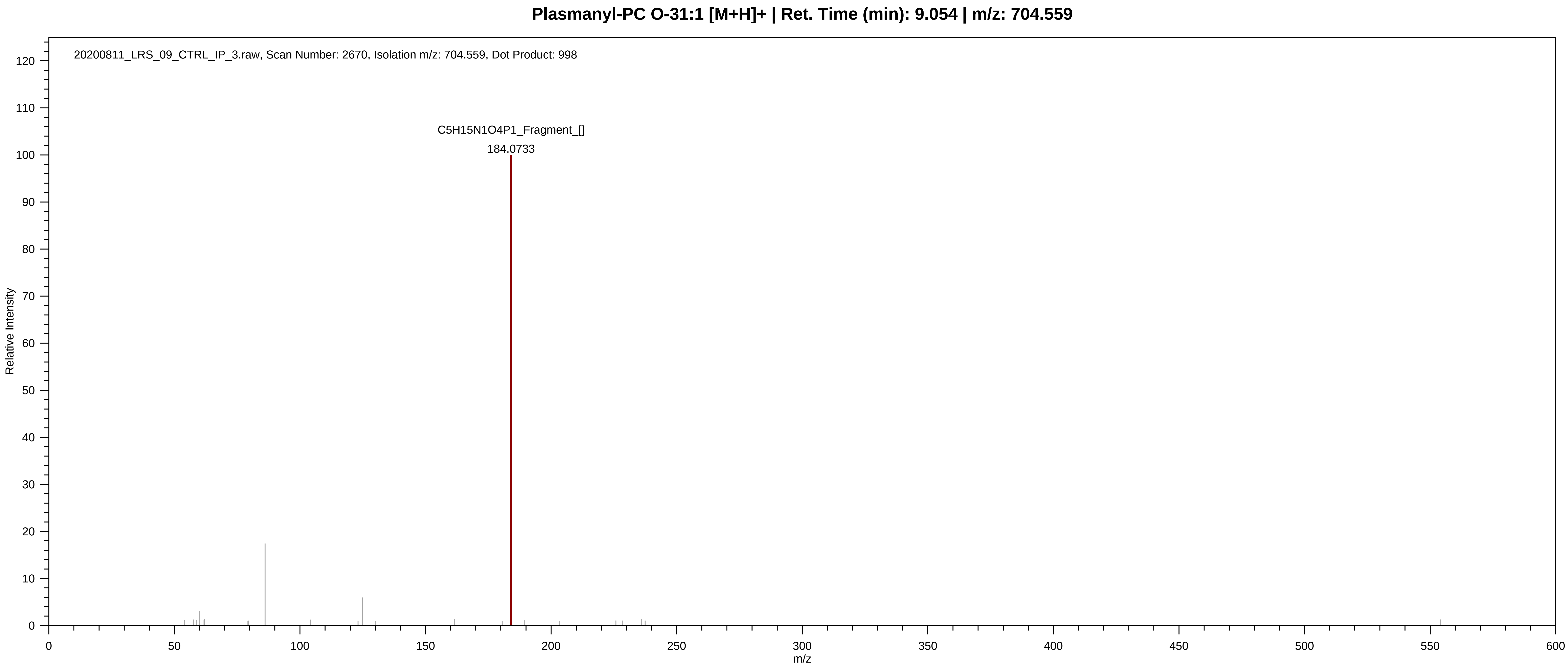
Plasmanyl-PC O-31:1 [M+H]+ | Ret. Time (min): 9.054 | m/z: 704.559
20200811_LRS_09_CTRL_IP_3.raw, Scan Number: 2670, Isolation m/z: 704.559, Dot Product: 998
C5H15N1O4P1_Fragment_[]
184.0733
Relative Intensity
m/z
0
10
20
30
40
50
60
70
80
90
100
110
120
0
50
100
150
200
250
300
350
400
450
500
550
600

Plasmanyl-PC O-31:1 [M+H]+ | Ret. Time (min): 9.78 | m/z: 704.559

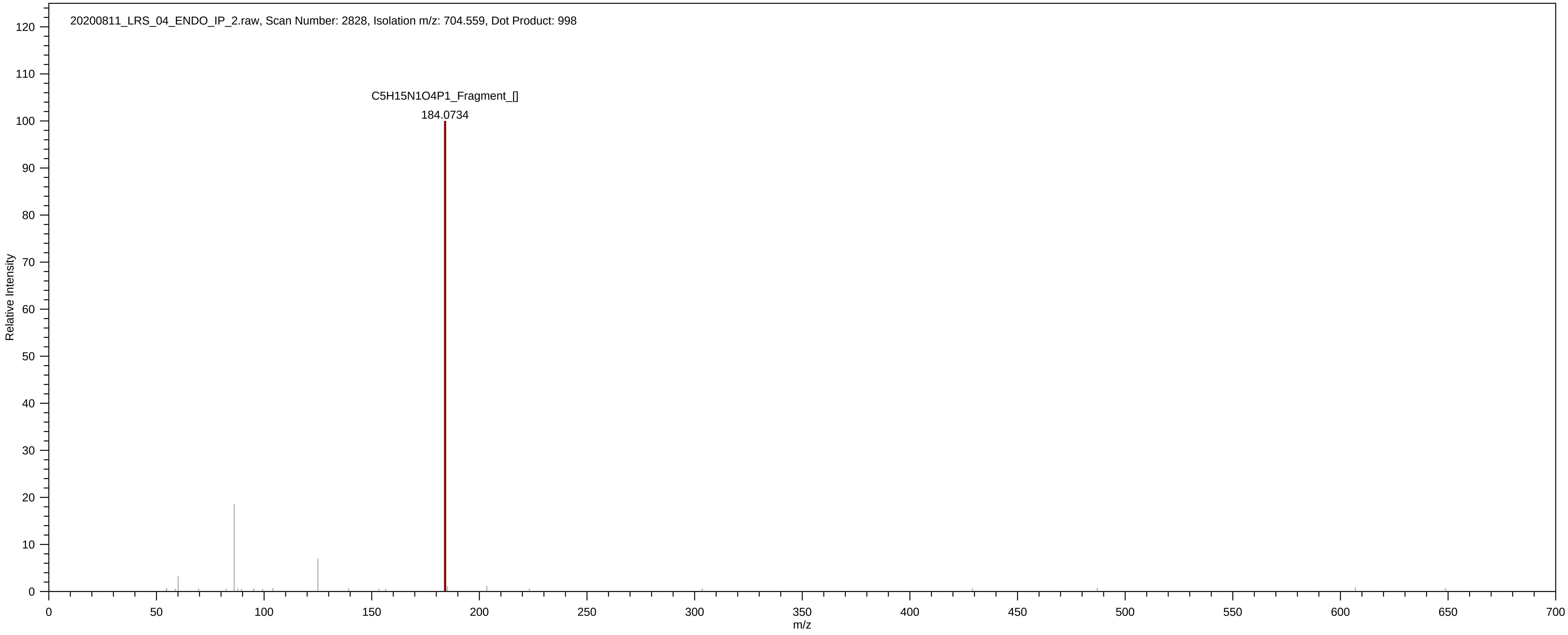

Plasmanyl-PC O-31:1 [M+H]+ | Ret. Time (min): 9.218 | m/z: 704.559

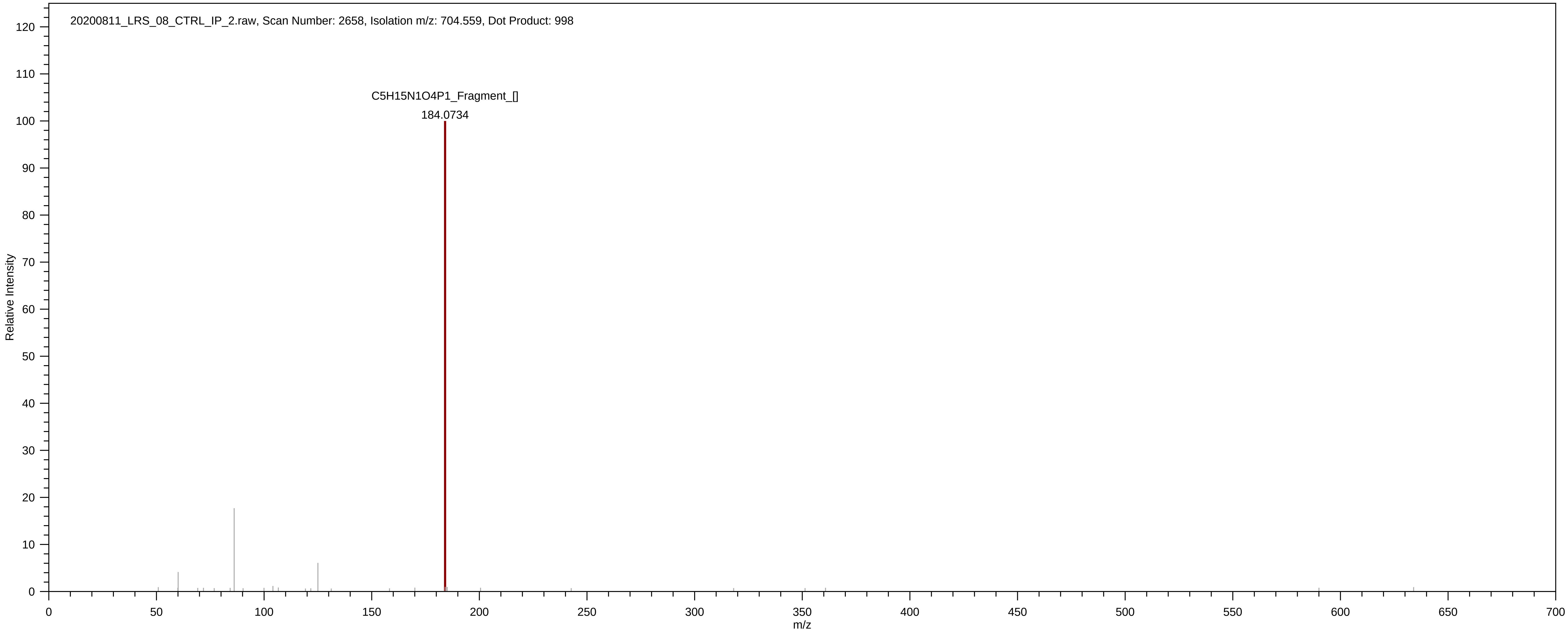

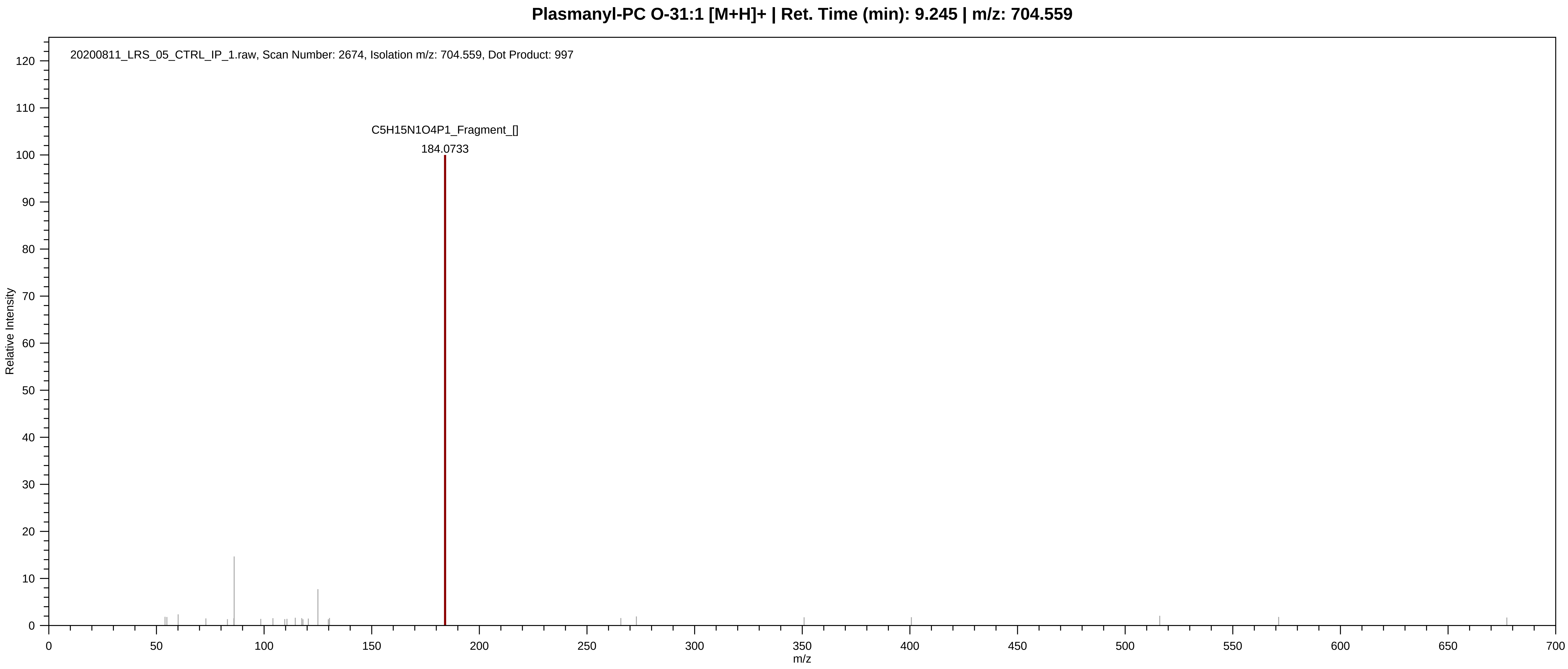
Plasmanyl-PC O-31:1 [M+H]+ | Ret. Time (min): 9.245 | m/z: 704.559
20200811_LRS_05_CTRL_IP_1.raw, Scan Number: 2674, Isolation m/z: 704.559, Dot Product: 997
C5H15N1O4P1_Fragment_[]
184.0733
Relative Intensity
m/z
0
10
20
30
40
50
60
70
80
90
100
110
120
0
50
100
150
200
250
300
350
400
450
500
550
600
650
700

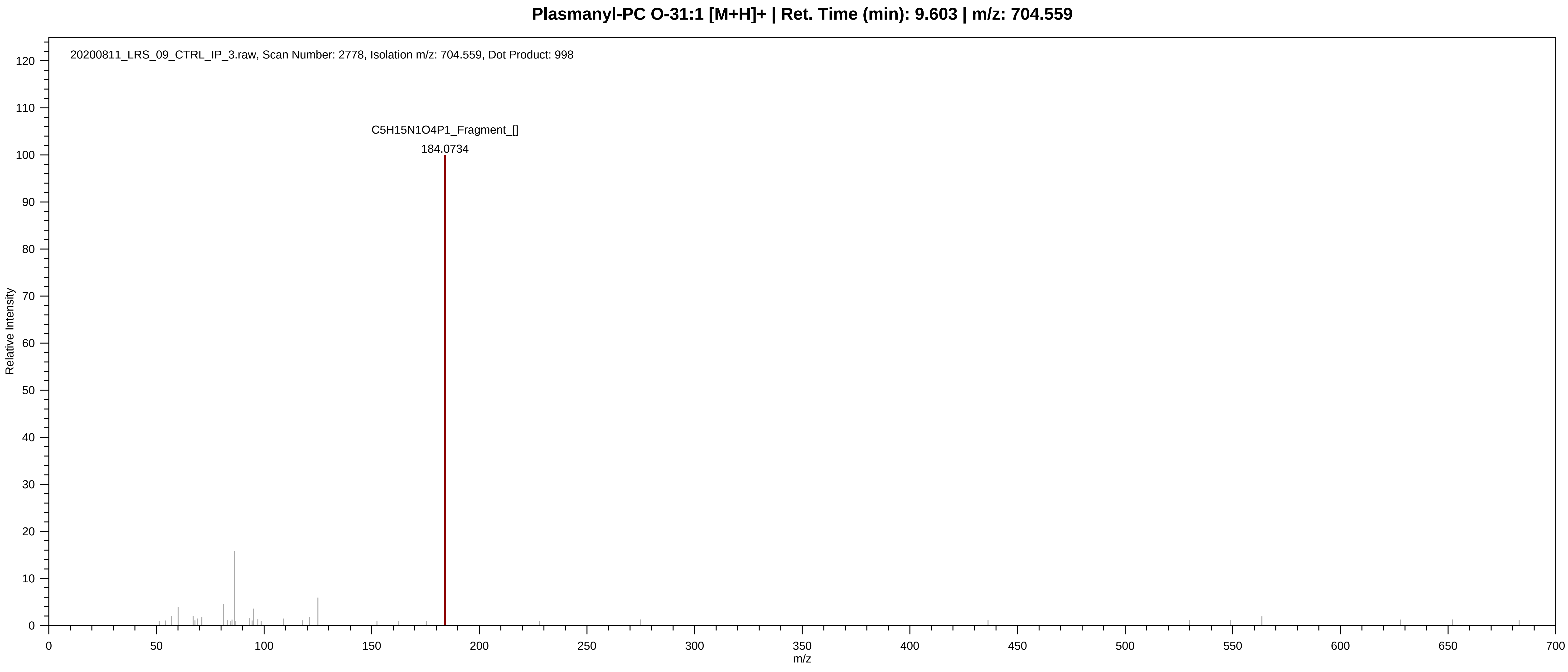
Plasmanyl-PC O-31:1 [M+H]+ | Ret. Time (min): 9.603 | m/z: 704.559
20200811_LRS_09_CTRL_IP_3.raw, Scan Number: 2778, Isolation m/z: 704.559, Dot Product: 998
C5H15N1O4P1_Fragment_[]
184.0734
Relative Intensity
m/z
0
10
20
30
40
50
60
70
80
90
100
110
120
0
50
100
150
200
250
300
350
400
450
500
550
600
650
700

Plasmanyl-PC O-31:1 [M+H]+ | Ret. Time (min): 9.603 | m/z: 704.562

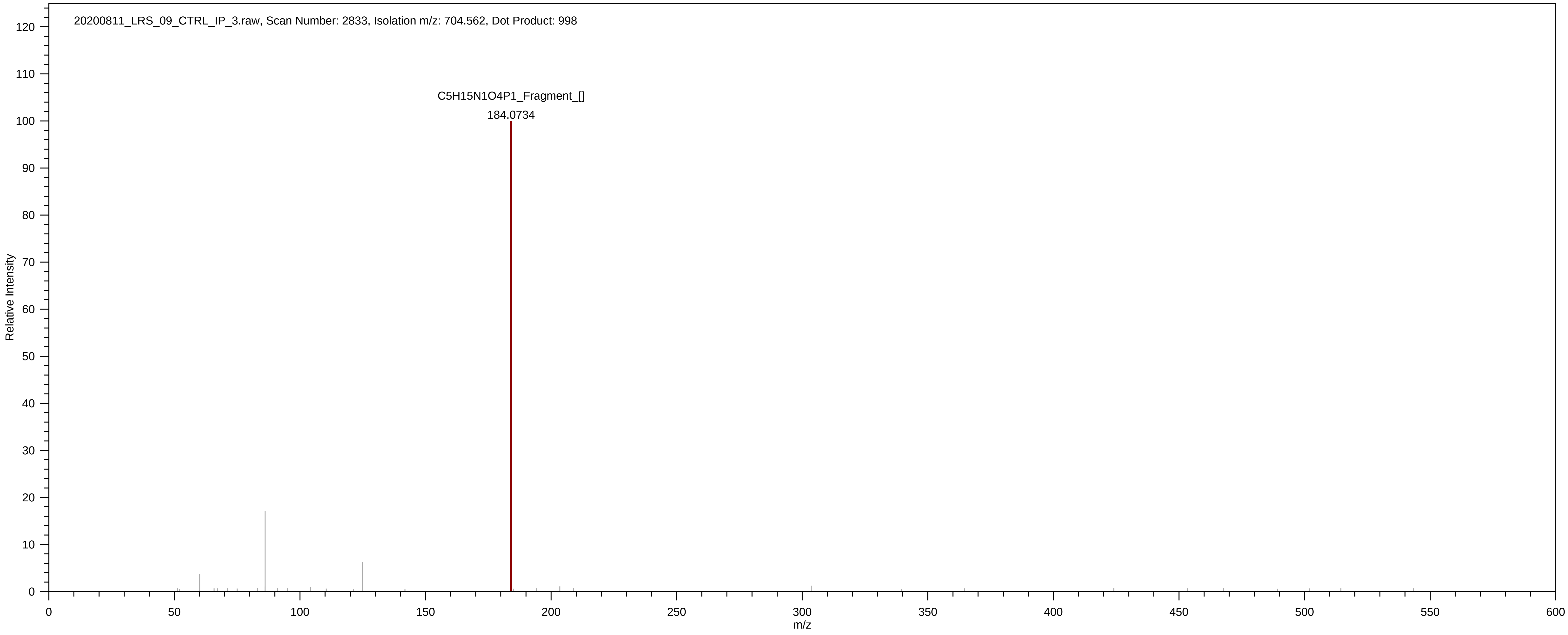

Plasmanyl-PC O-31:1 [M+H]+ | Ret. Time (min): 9.759 | m/z: 704.563

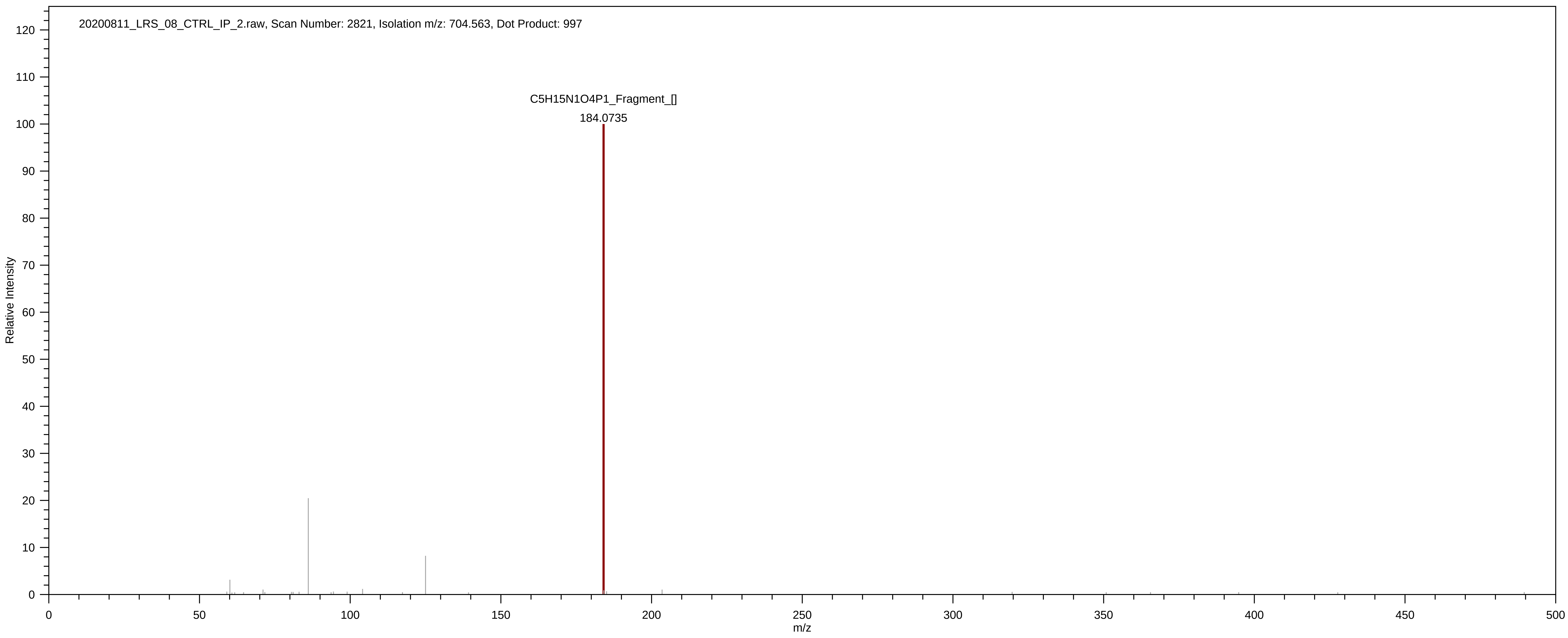

Plasmanyl-PC O-32:0 [M+H]+ | Ret. Time (min): 10.394 | m/z: 720.59

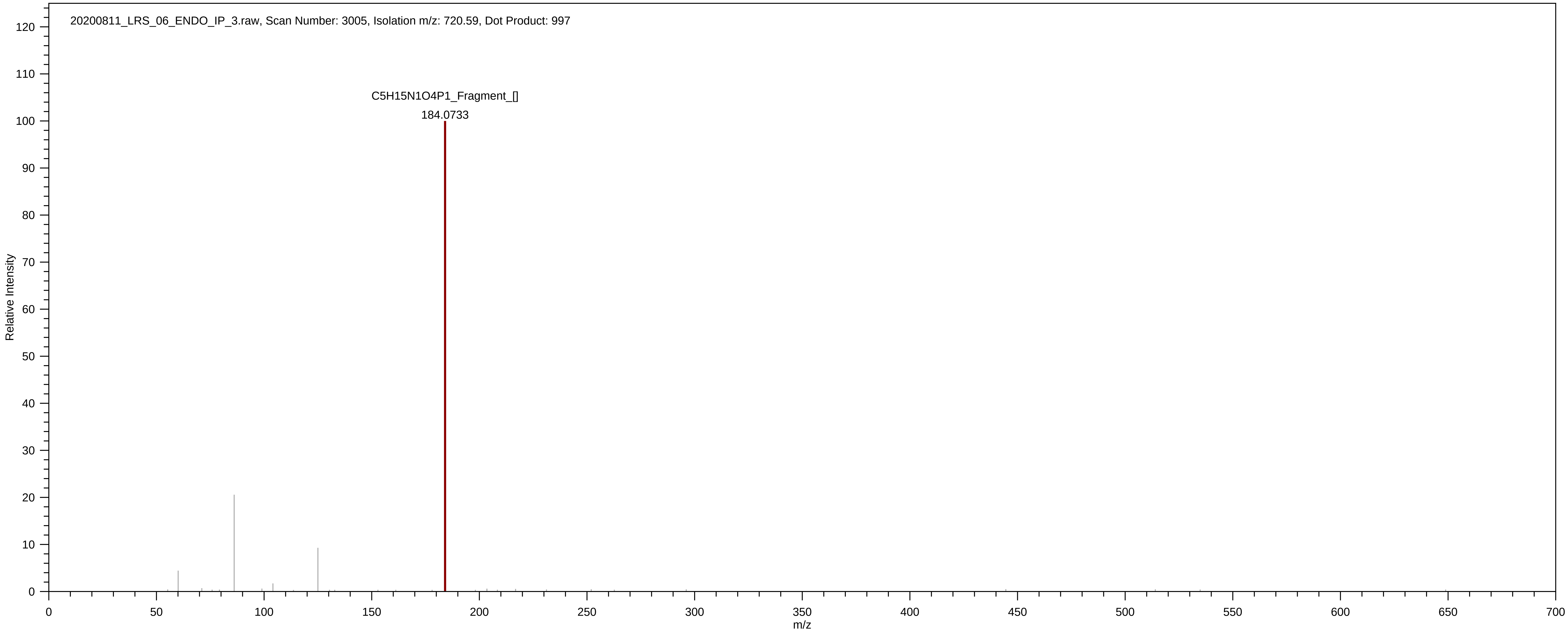

Plasmanyl-PC O-32:0 [M+H]+ | Ret. Time (min): 10.397 | m/z: 720.59

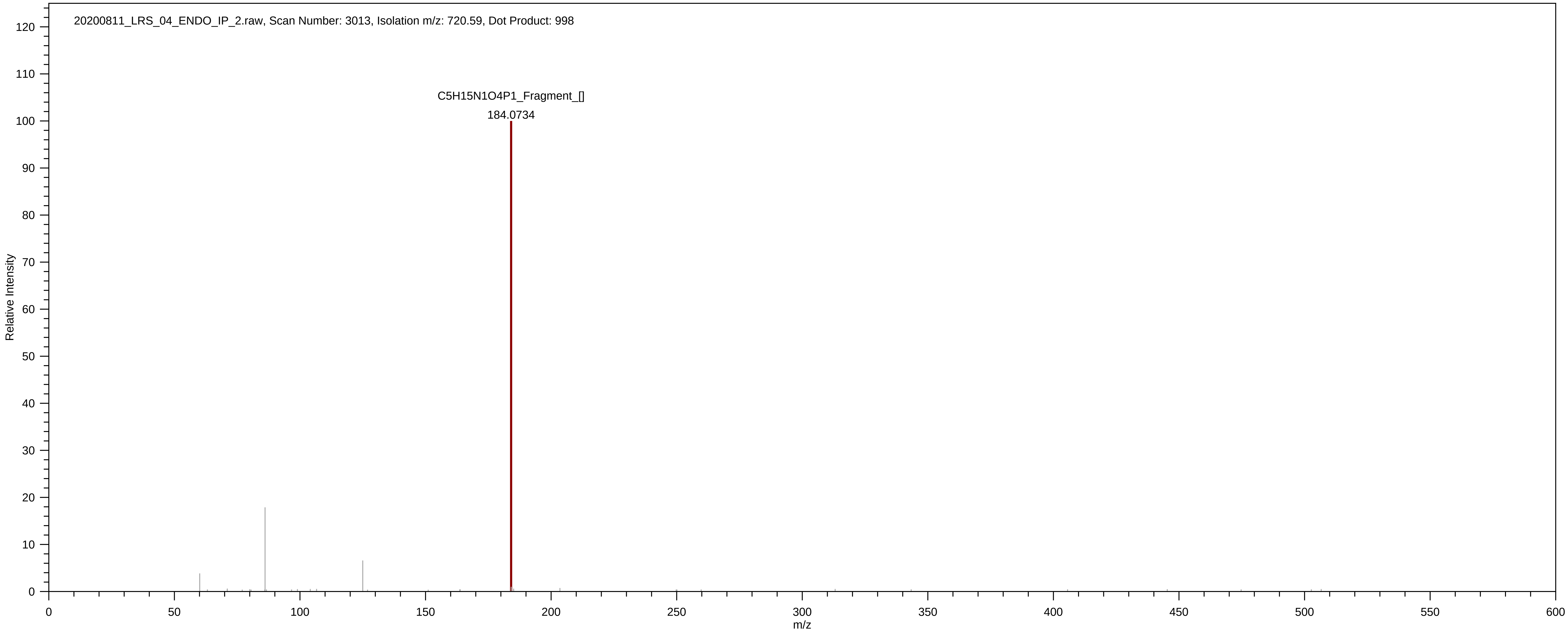

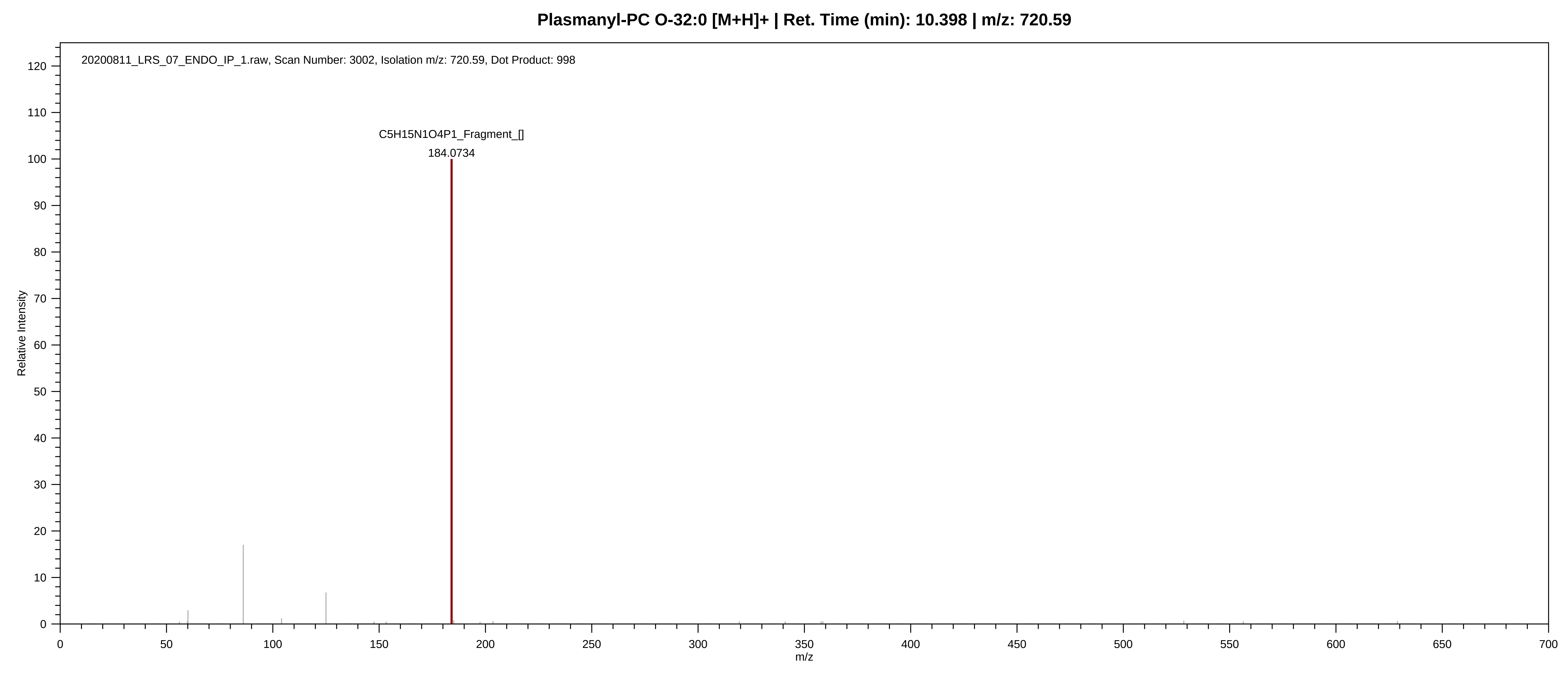
Plasmanyl-PC O-32:0 [M+H]+ | Ret. Time (min): 10.398 | m/z: 720.59
20200811_LRS_07_ENDO_IP_1.raw, Scan Number: 3002, Isolation m/z: 720.59, Dot Product: 998
C5H15N1O4P1_Fragment_[]
184.0734
Relative Intensity
m/z

Plasmanyl-PC O-32:0 [M+H]+ | Ret. Time (min): 10.413 | m/z: 720.59

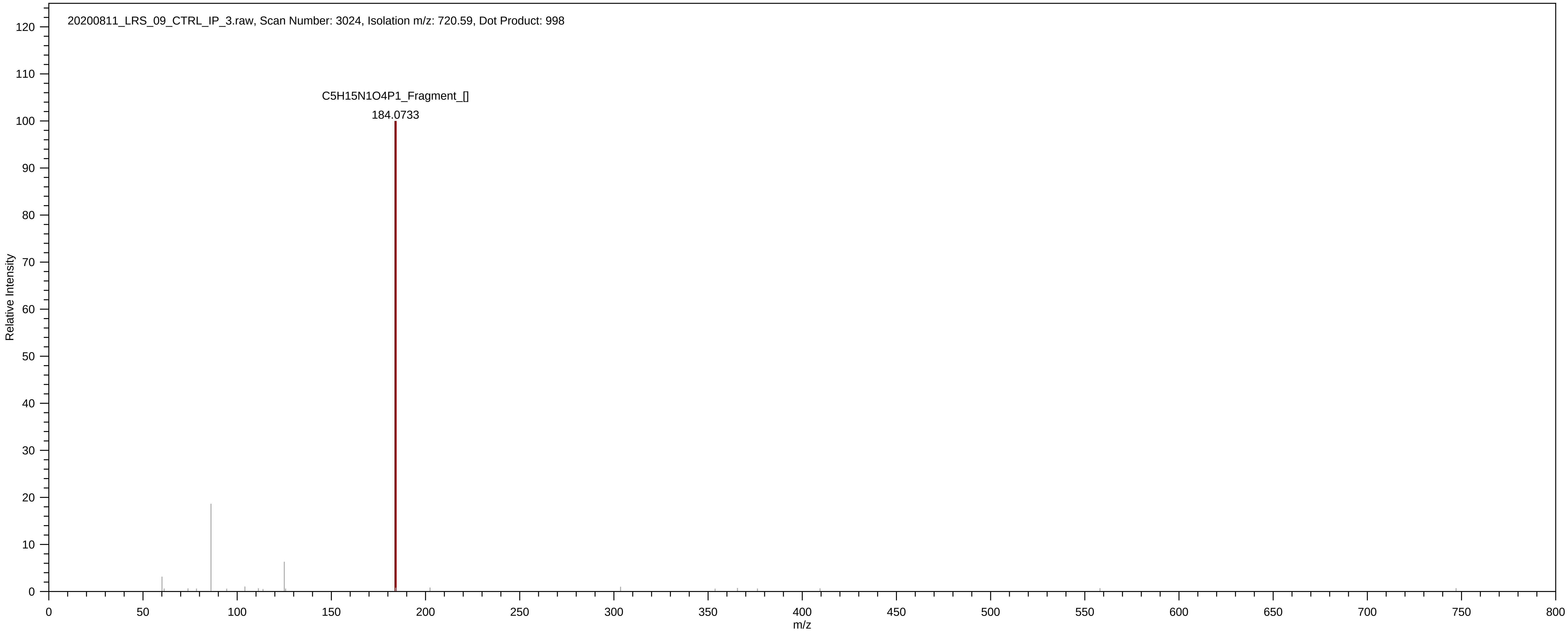

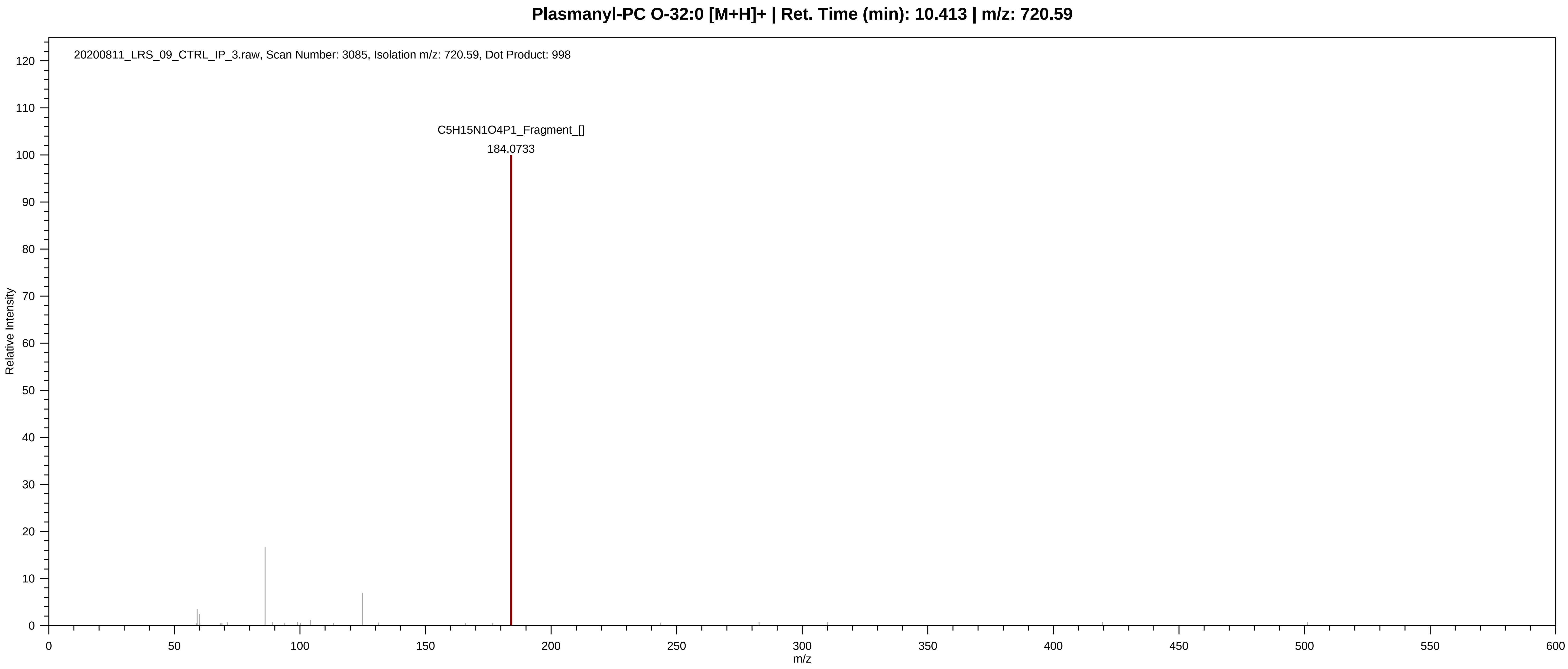

Plasmanyl-PC O-32:0 [M+H]+ | Ret. Time (min): 10.413 | m/z: 720.59
20200811_LRS_09_CTRL_IP_3.raw, Scan Number: 3085, Isolation m/z: 720.59, Dot Product: 998
C5H15N1O4P1_Fragment_[]
184.0733
Relative Intensity
m/z

Plasmanyl-PC O-32:0 [M+H]+ | Ret. Time (min): 10.414 | m/z: 720.59

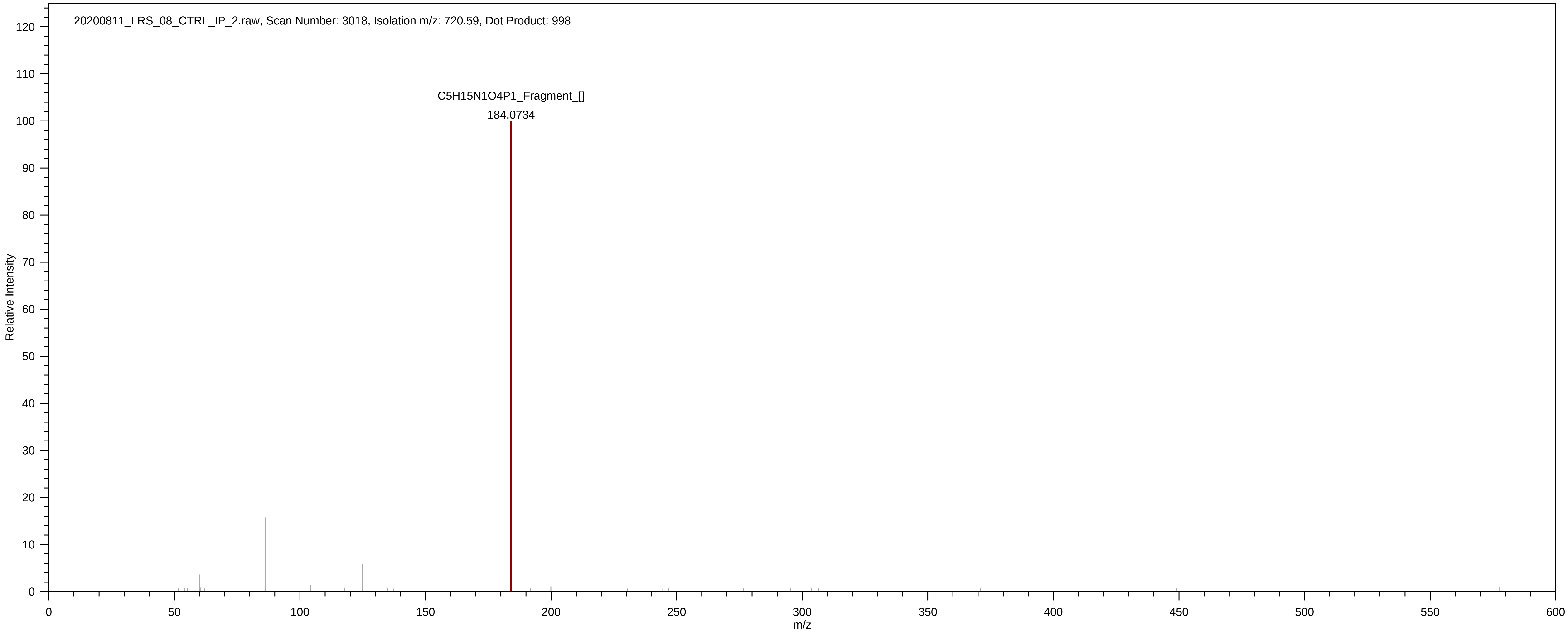

Plasmanyl-PC O-32:0 [M+H]+ | Ret. Time (min): 10.414 | m/z: 720.591

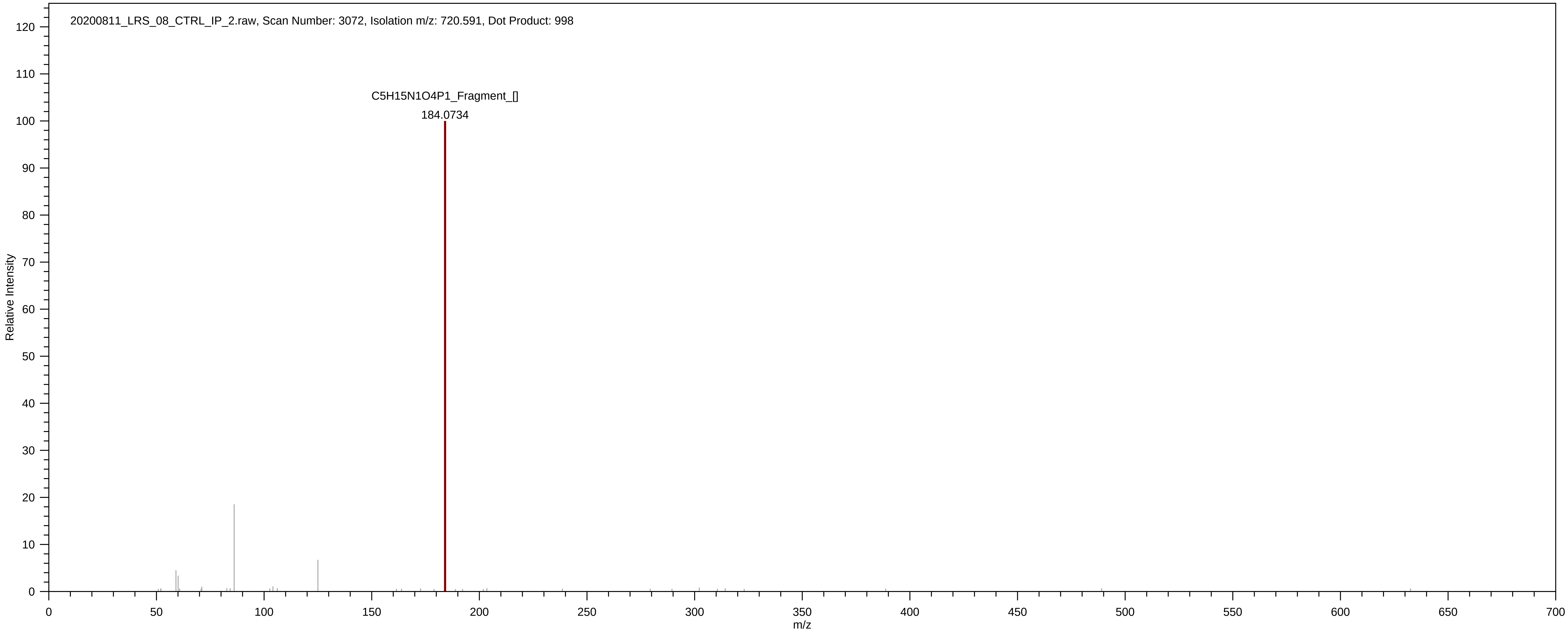

Plasmanyl-PC O-32:0 [M+H]+ | Ret. Time (min): 10.423 | m/z: 720.593

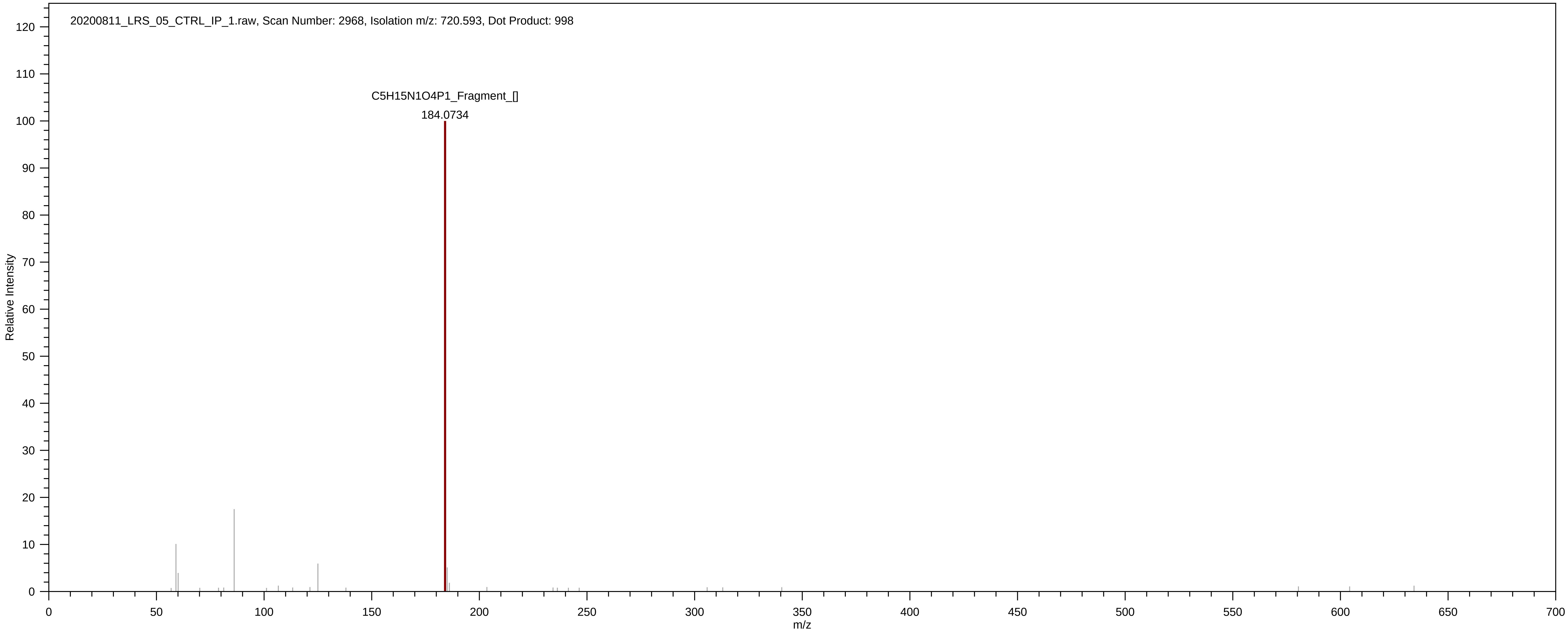

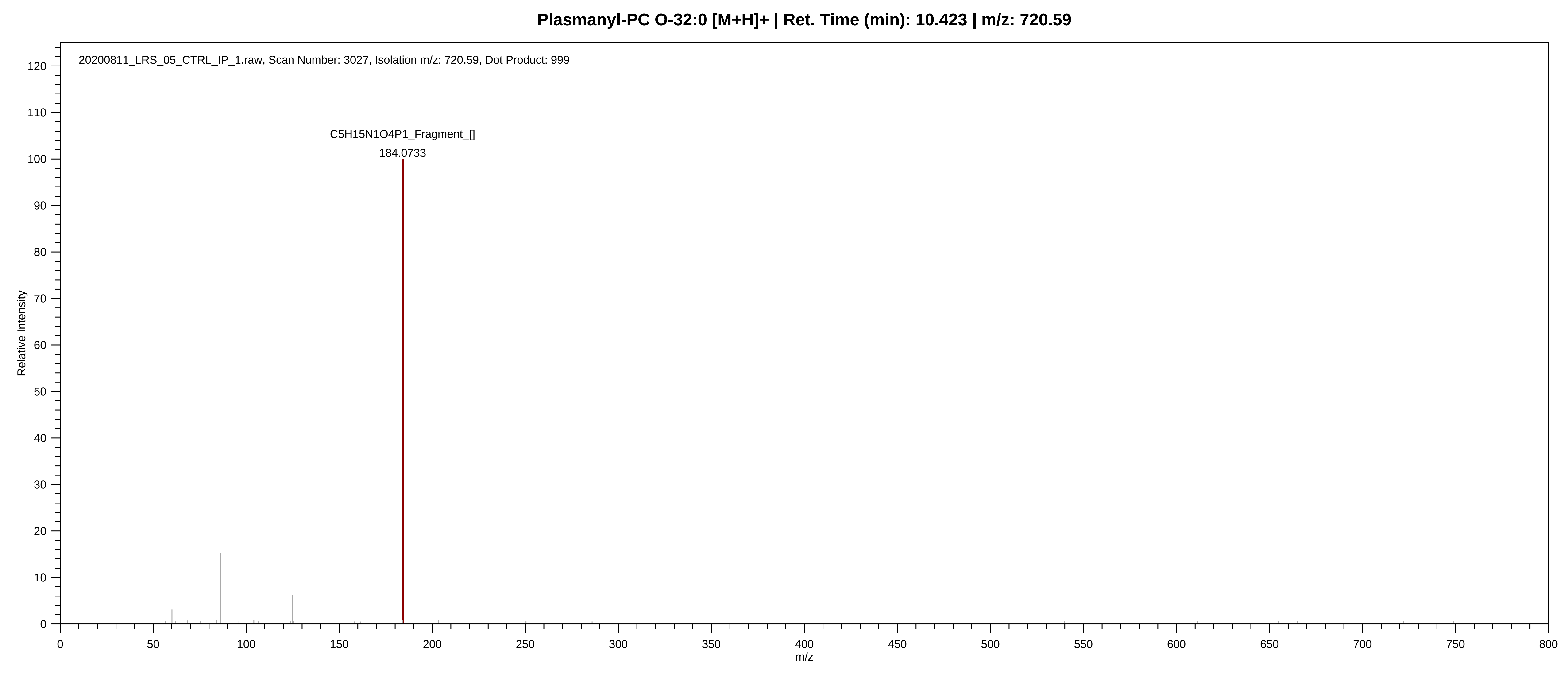
Plasmanyl-PC O-32:0 [M+H]+ | Ret. Time (min): 10.423 | m/z: 720.59
20200811_LRS_05_CTRL_IP_1.raw, Scan Number: 3027, Isolation m/z: 720.59, Dot Product: 999
C5H15N1O4P1_Fragment_[]
184.0733
Relative Intensity
m/z

Plasmanyl-PC O-32:0 [M+H]+ | Ret. Time (min): 10.423 | m/z: 720.591

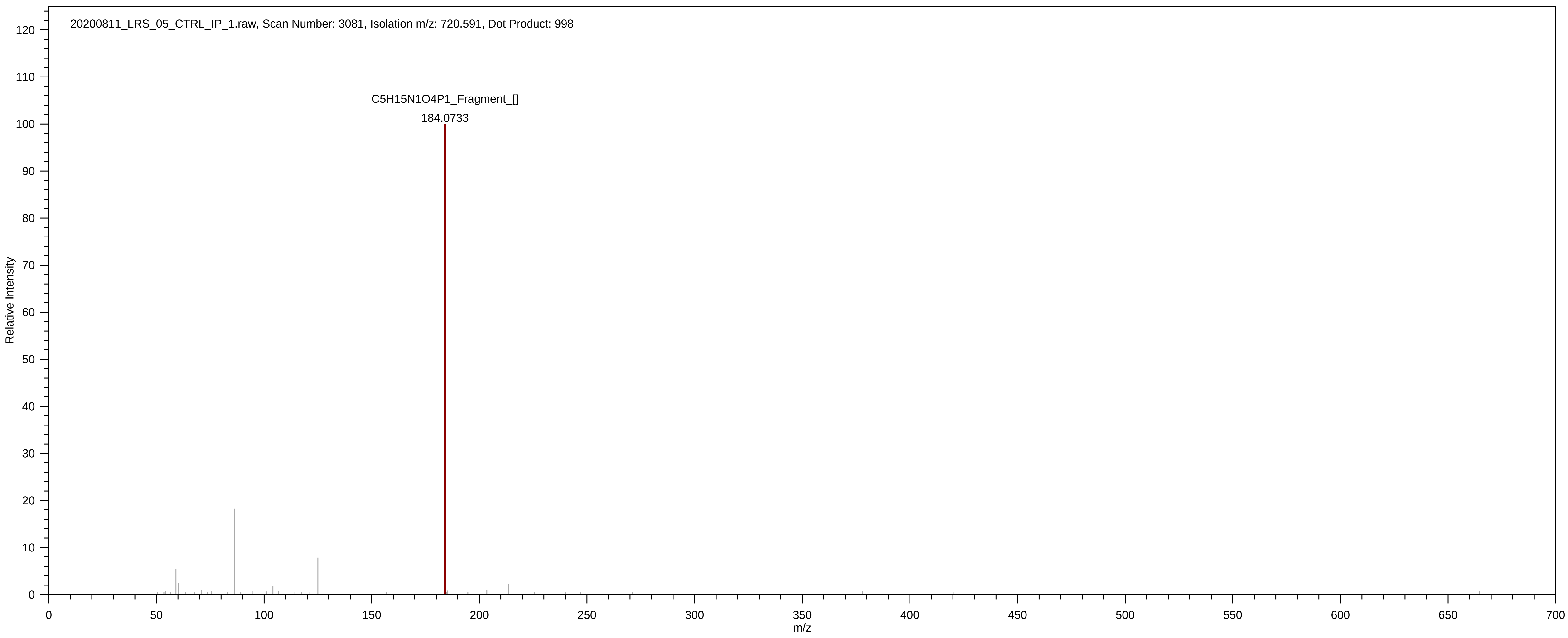

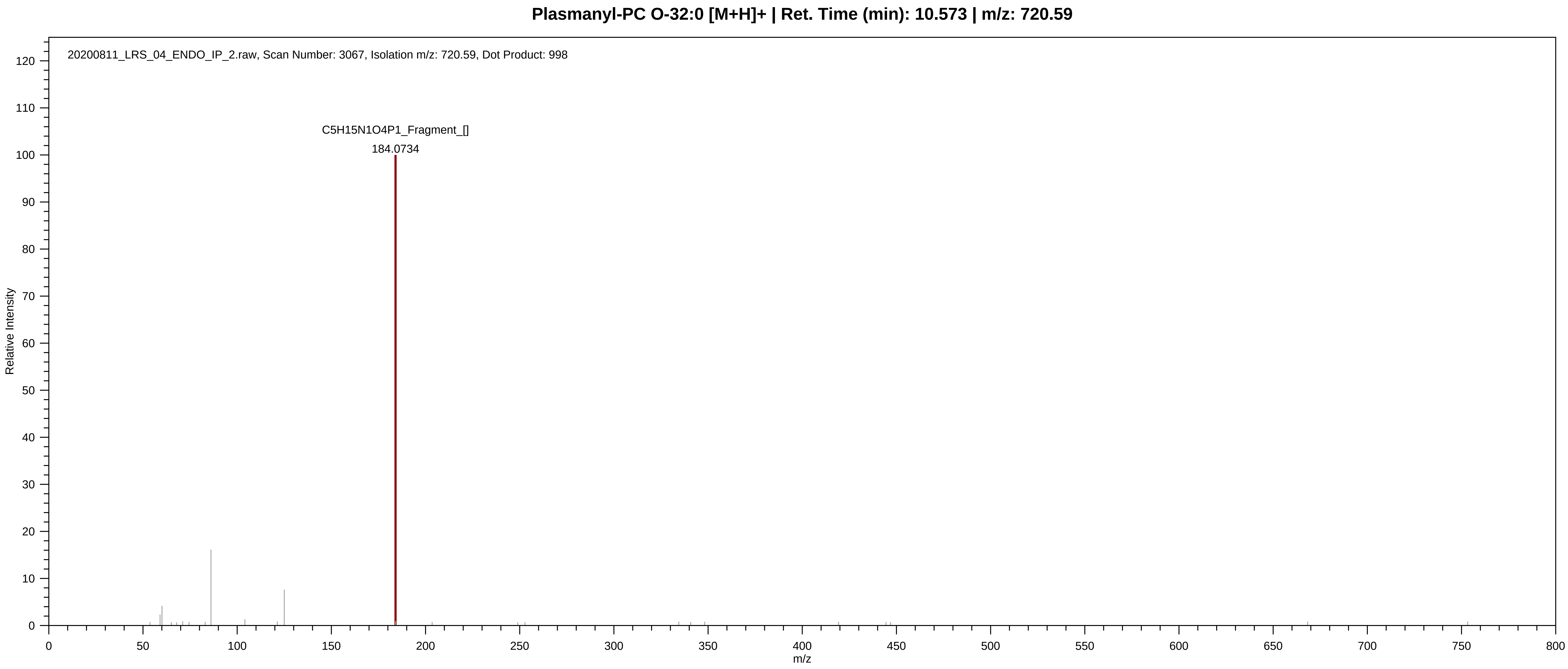

Plasmanyl-PC O-32:0 [M+H]+ | Ret. Time (min): 10.573 | m/z: 720.59
20200811_LRS_04_ENDO_IP_2.raw, Scan Number: 3067, Isolation m/z: 720.59, Dot Product: 998
C5H15N1O4P1_Fragment_[]
184.0734
Relative Intensity
m/z

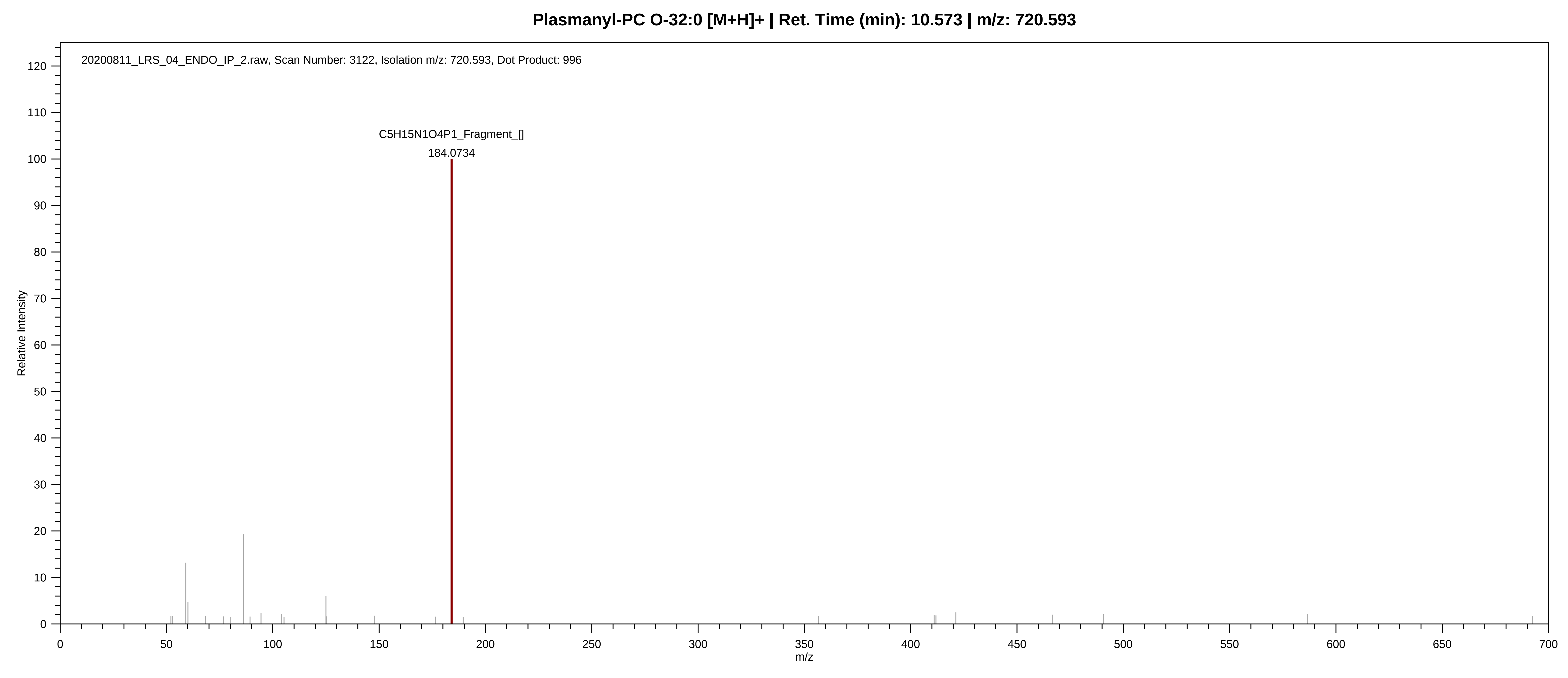
Plasmanyl-PC O-32:0 [M+H]+ | Ret. Time (min): 10.573 | m/z: 720.593
20200811_LRS_04_ENDO_IP_2.raw, Scan Number: 3122, Isolation m/z: 720.593, Dot Product: 996
C5H15N1O4P1_Fragment_[]
184.0734
Relative Intensity
m/z

Plasmanyl-PC O-32:0 [M+H]+ | Ret. Time (min): 10.589 | m/z: 720.59

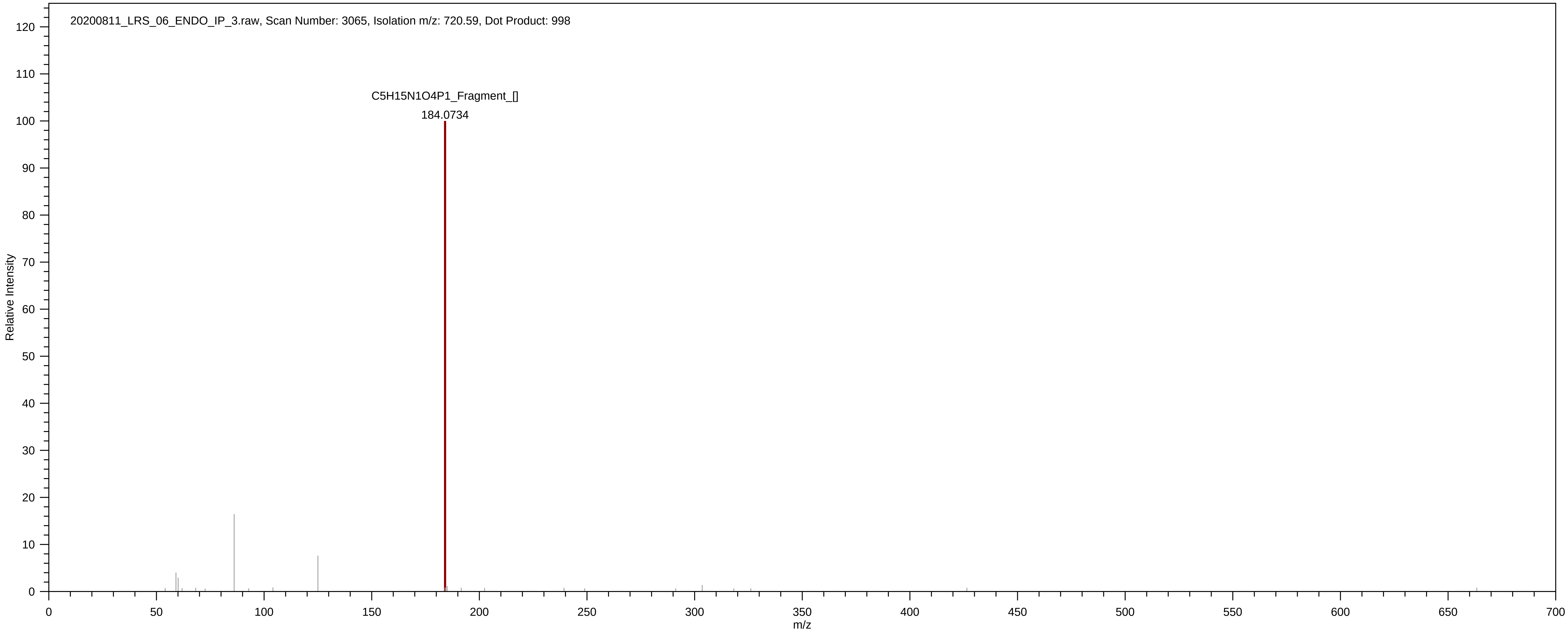

Plasmanyl-PC O-32:0 [M+H]+ | Ret. Time (min): 10.594 | m/z: 720.59

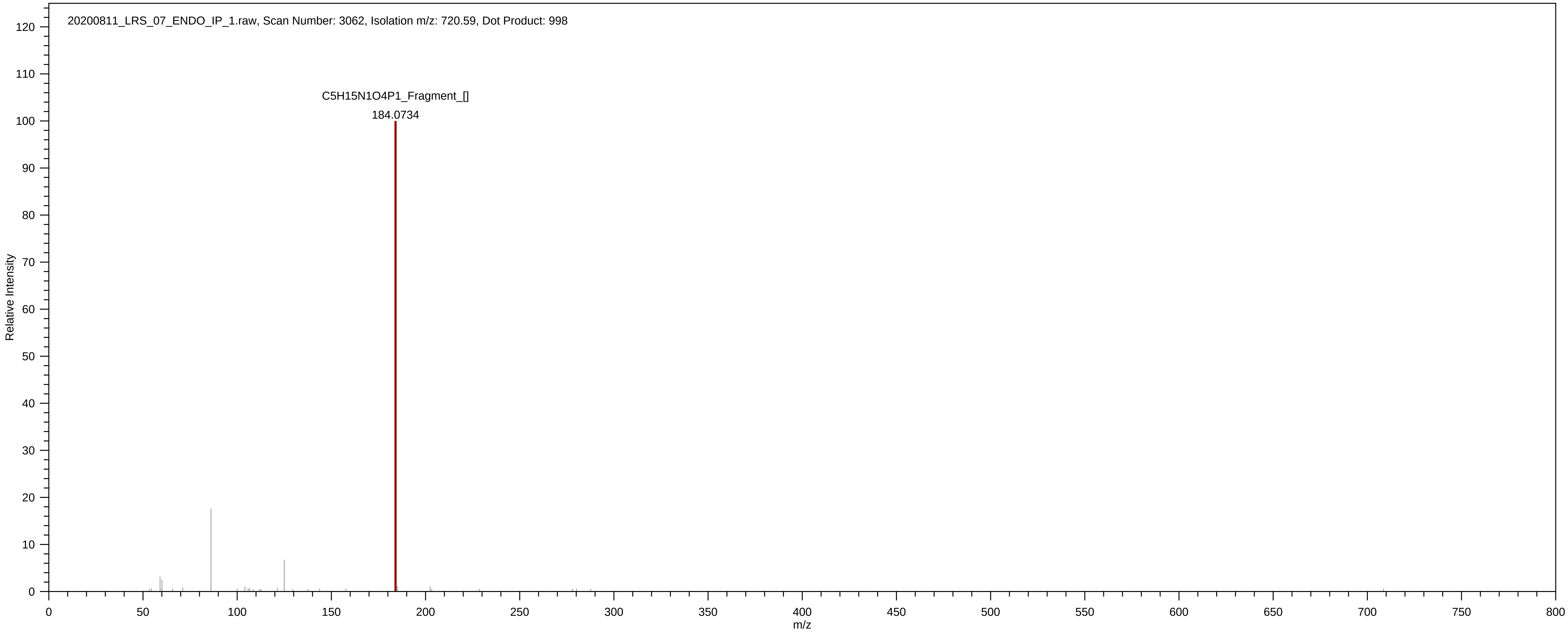

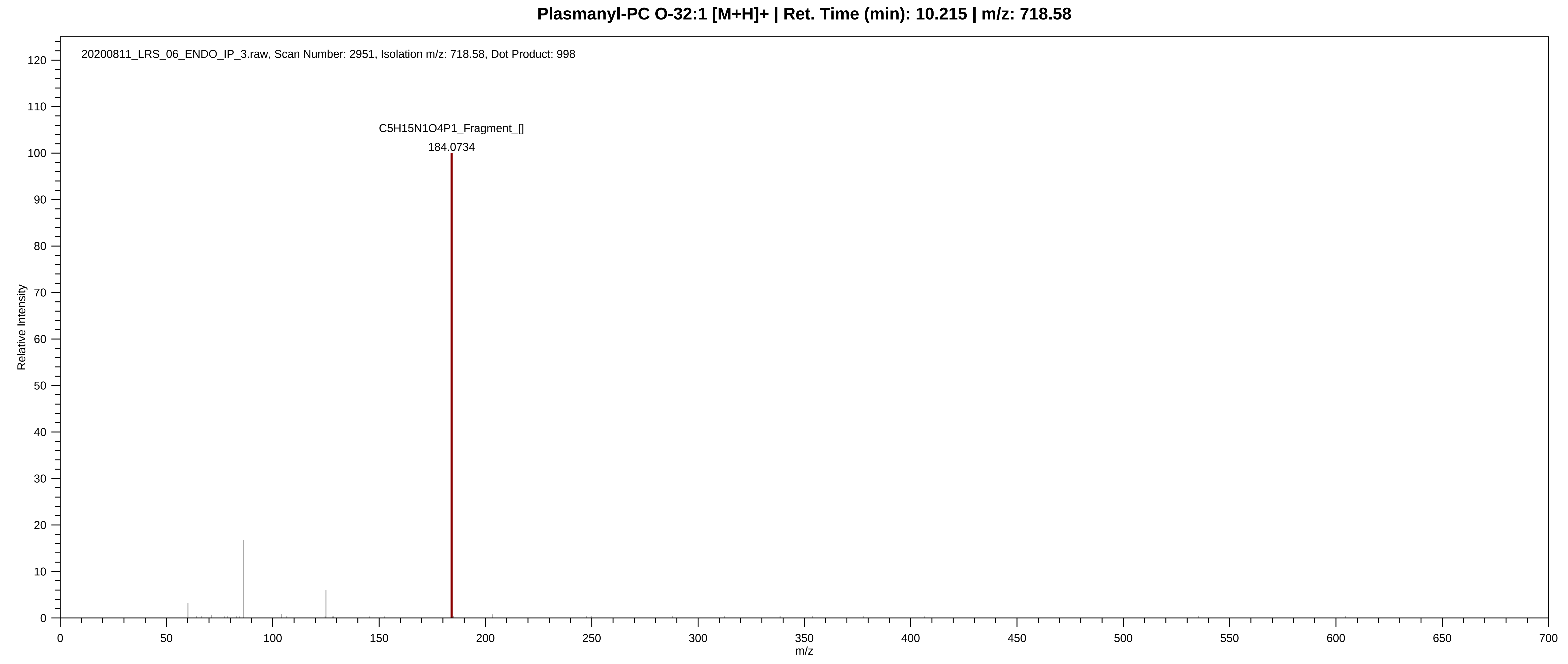
Plasmanyl-PC O-32:1 [M+H]+ | Ret. Time (min): 10.215 | m/z: 718.58
20200811_LRS_06_ENDO_IP_3.raw, Scan Number: 2951, Isolation m/z: 718.58, Dot Product: 998
C5H15N1O4P1_Fragment_[]
184.0734
Relative Intensity
m/z

Plasmanyl-PC O-32:1 [M+H]+ | Ret. Time (min): 10.216 | m/z: 718.572

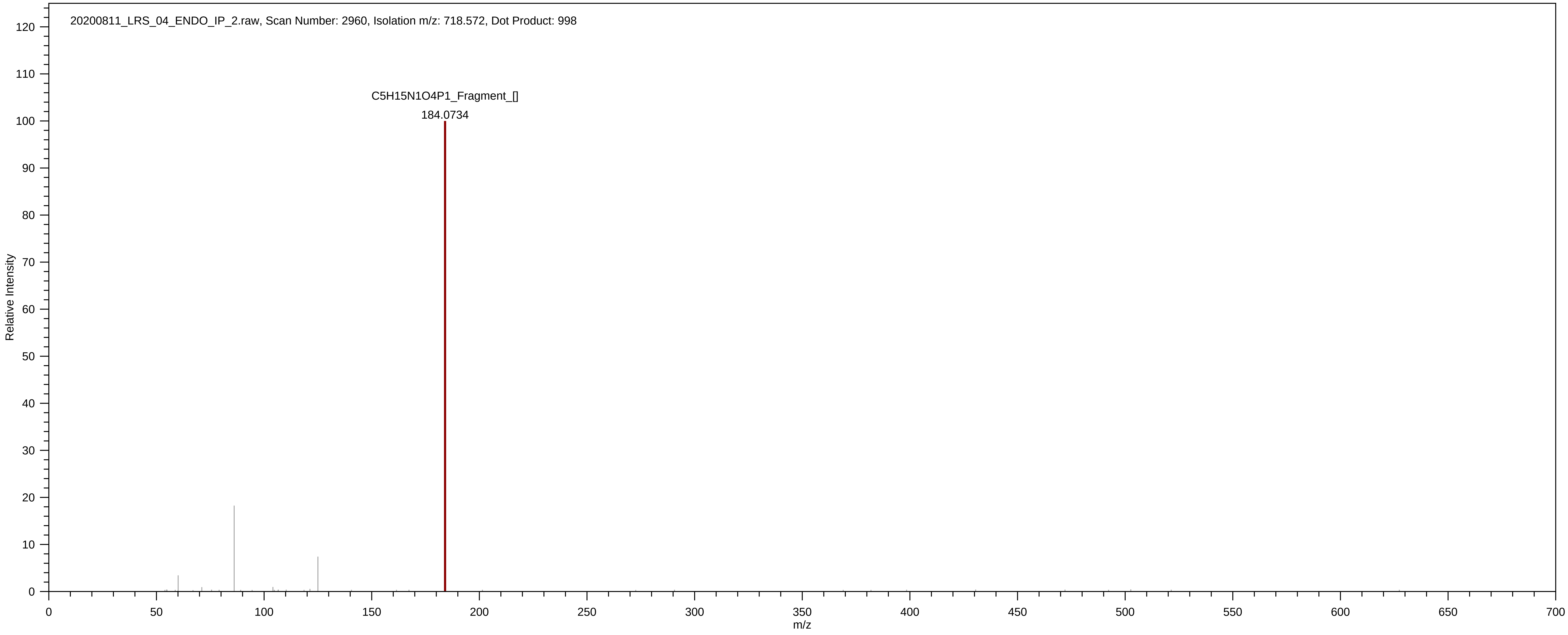

Plasmanyl-PC O-32:1 [M+H]+ | Ret. Time (min): 10.233 | m/z: 718.573

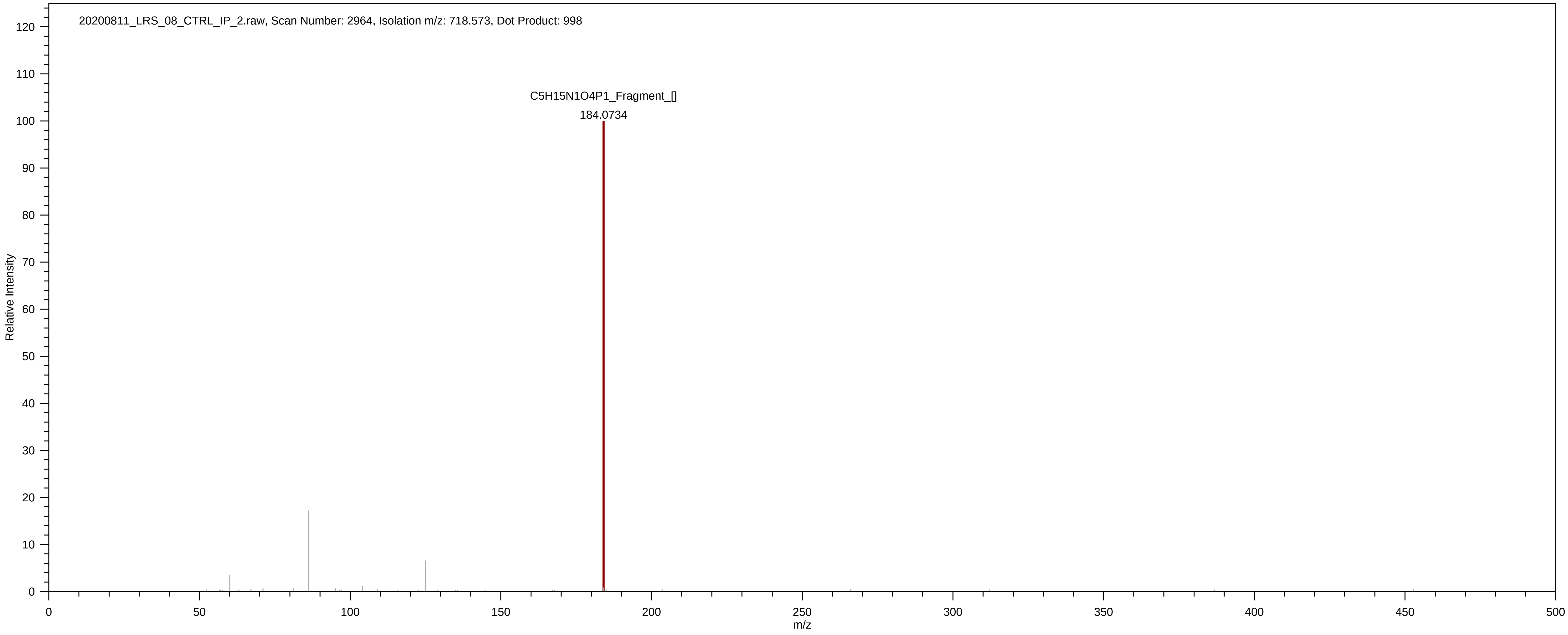

Plasmanyl-PC O-32:1 [M+H]+ | Ret. Time (min): 10.242 | m/z: 718.573

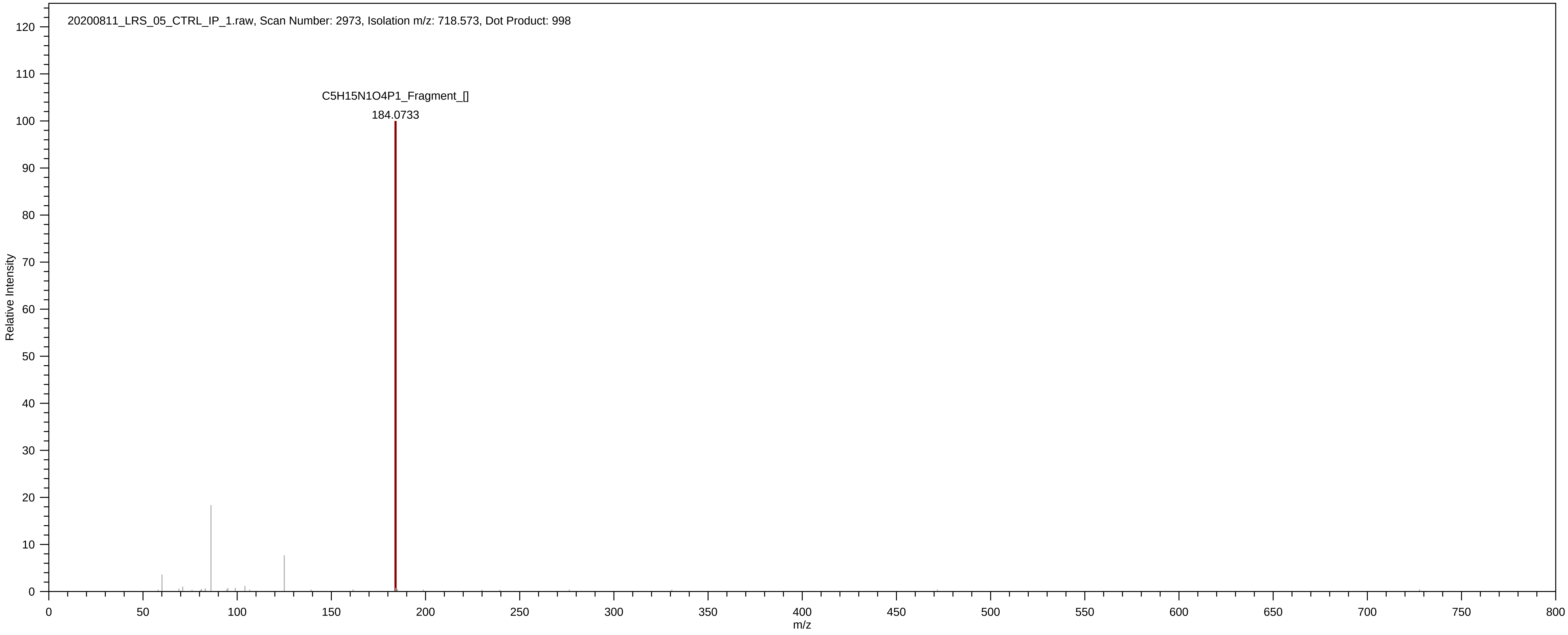

Plasmanyl-PC O-32:1 [M+H]+ | Ret. Time (min): 9.44 | m/z: 718.574

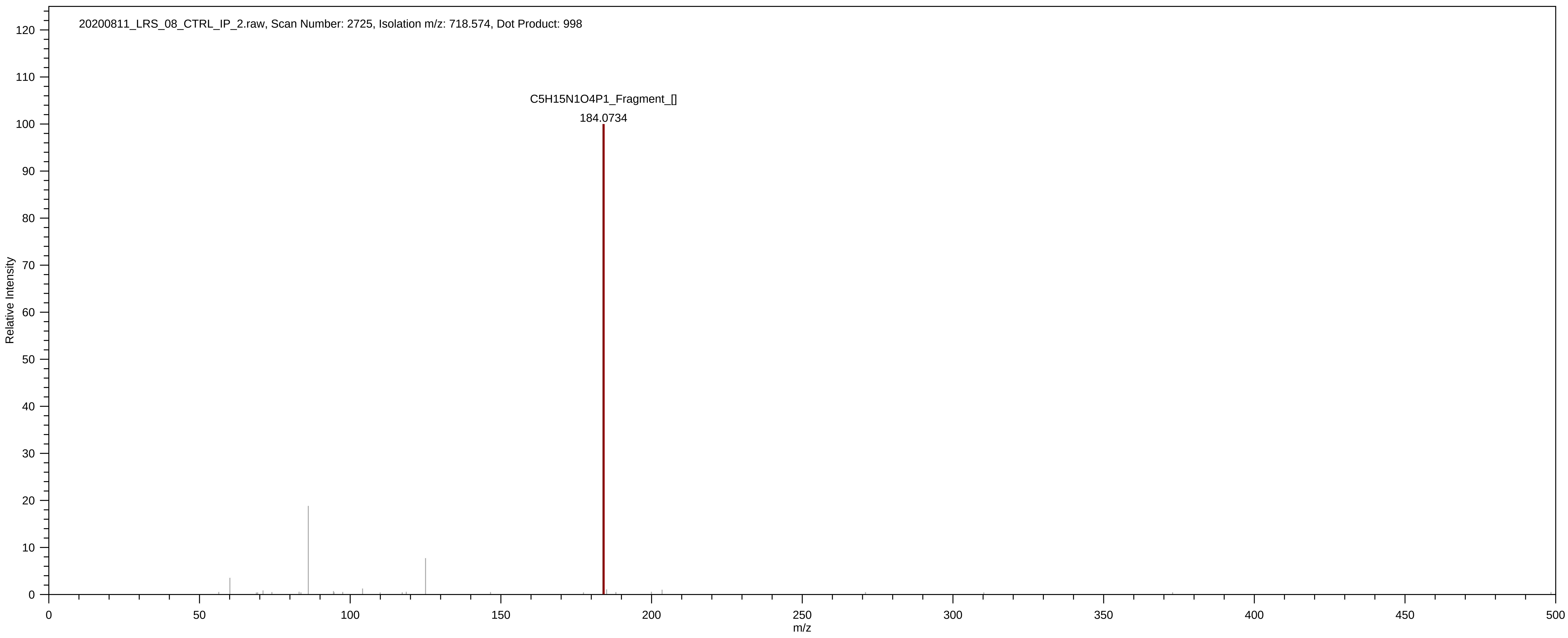

Plasmanyl-PC O-32:1 [M+H]+ | Ret. Time (min): 9.464 | m/z: 718.574

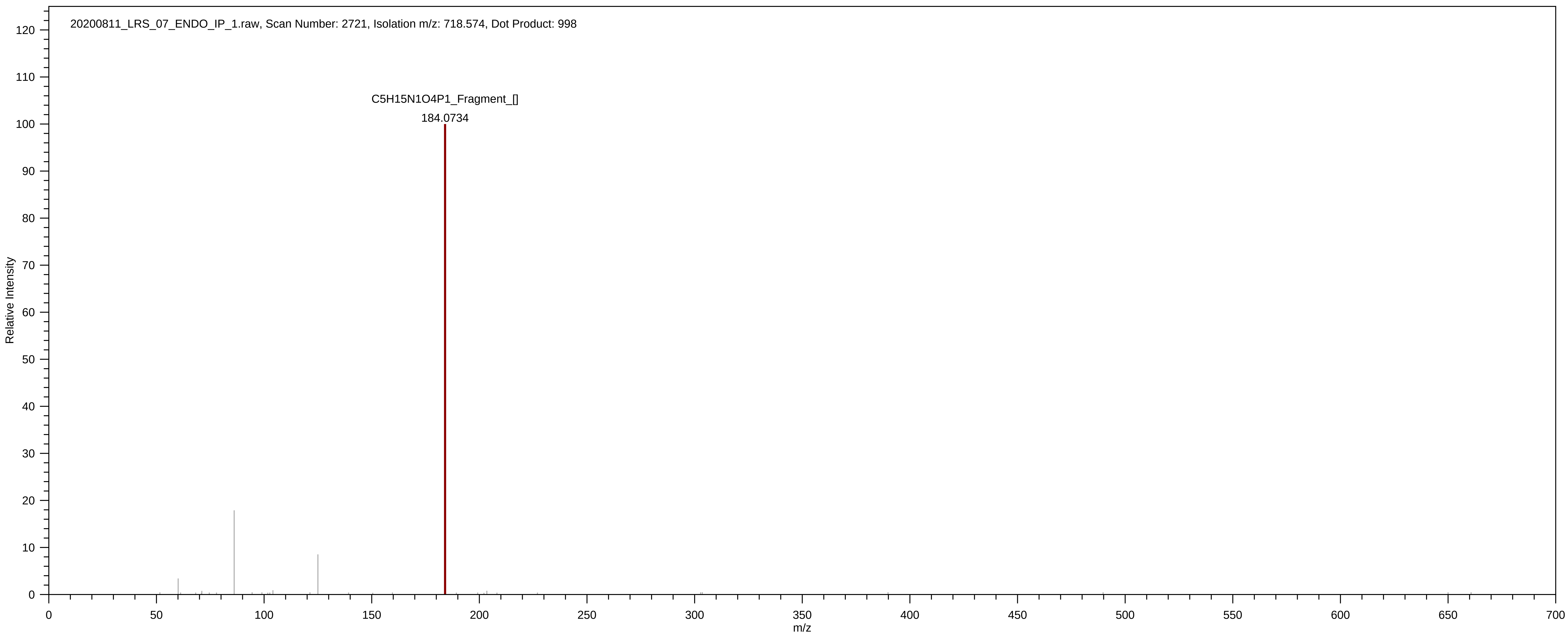

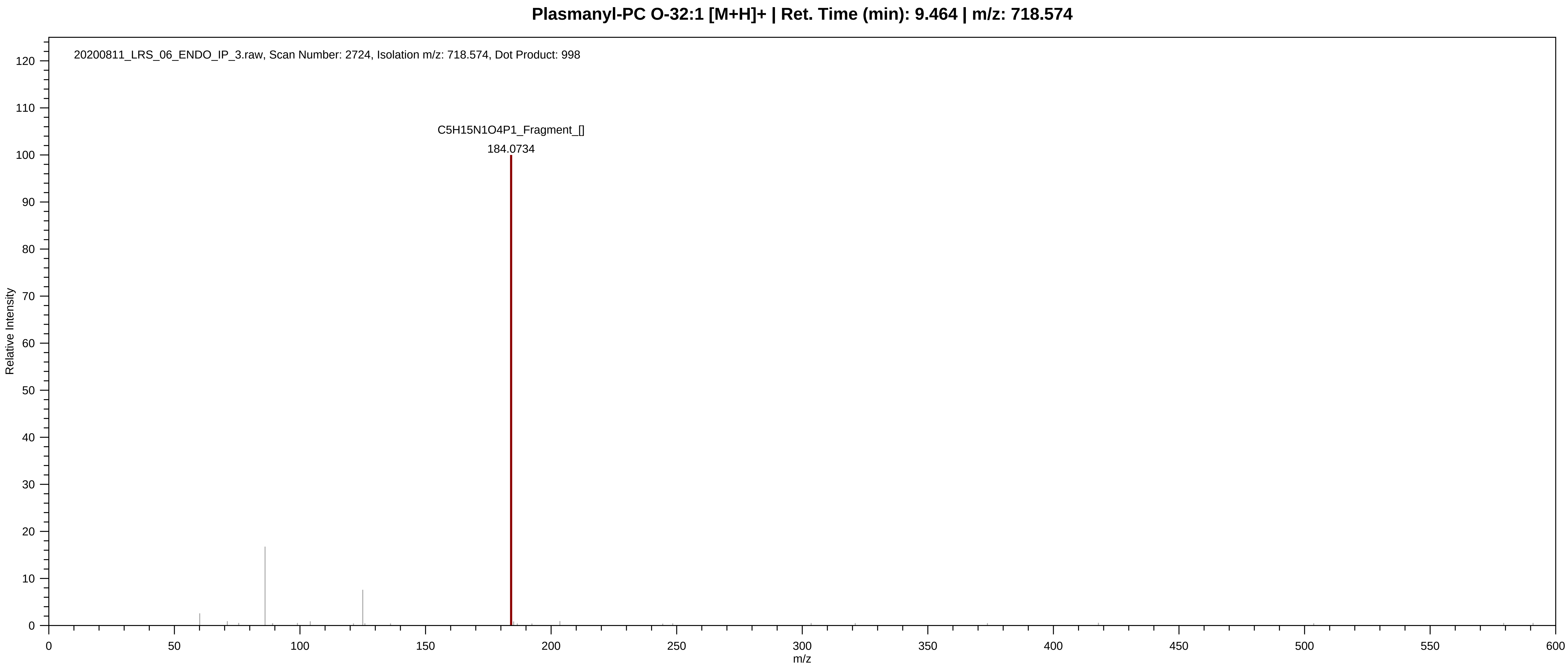
Plasmanyl-PC O-32:1 [M+H]+ | Ret. Time (min): 9.464 | m/z: 718.574
20200811_LRS_06_ENDO_IP_3.raw, Scan Number: 2724, Isolation m/z: 718.574, Dot Product: 998
C5H15N1O4P1_Fragment_[]
184.0734
Relative Intensity
m/z
0
10
20
30
40
50
60
70
80
90
100
110
120
0
50
100
150
200
250
300
350
400
450
500
550
600

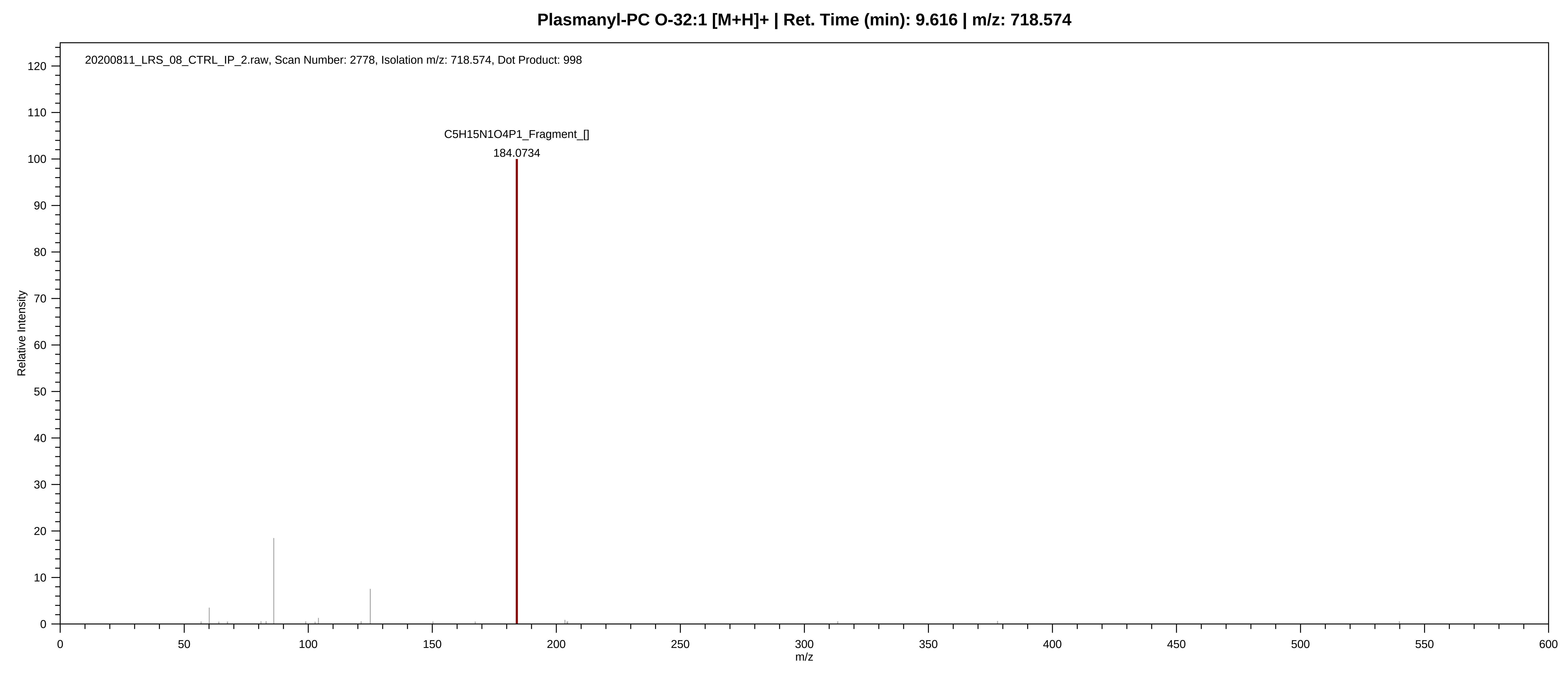

Plasmanyl-PC O-32:1 [M+H]+ | Ret. Time (min): 9.616 | m/z: 718.574
20200811_LRS_08_CTRL_IP_2.raw, Scan Number: 2778, Isolation m/z: 718.574, Dot Product: 998
C5H15N1O4P1_Fragment_[]
184.0734
Relative Intensity
m/z
0
10
20
30
40
50
60
70
80
90
100
110
120
0
50
100
150
200
250
300
350
400
450
500
550
600

Plasmanyl-PC O-32:1 [M+H]+ | Ret. Time (min): 9.616 | m/z: 718.575

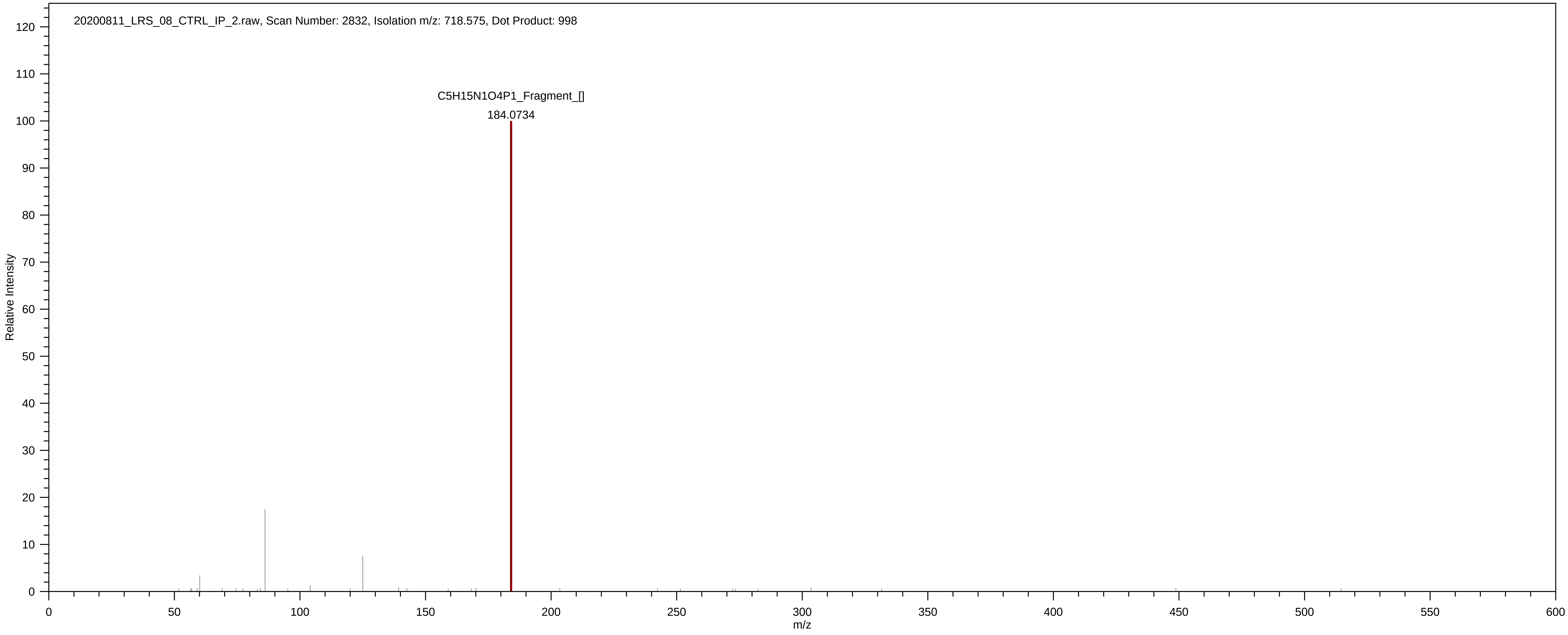

Plasmanyl-PC O-32:1 [M+H]+ | Ret. Time (min): 9.639 | m/z: 718.574

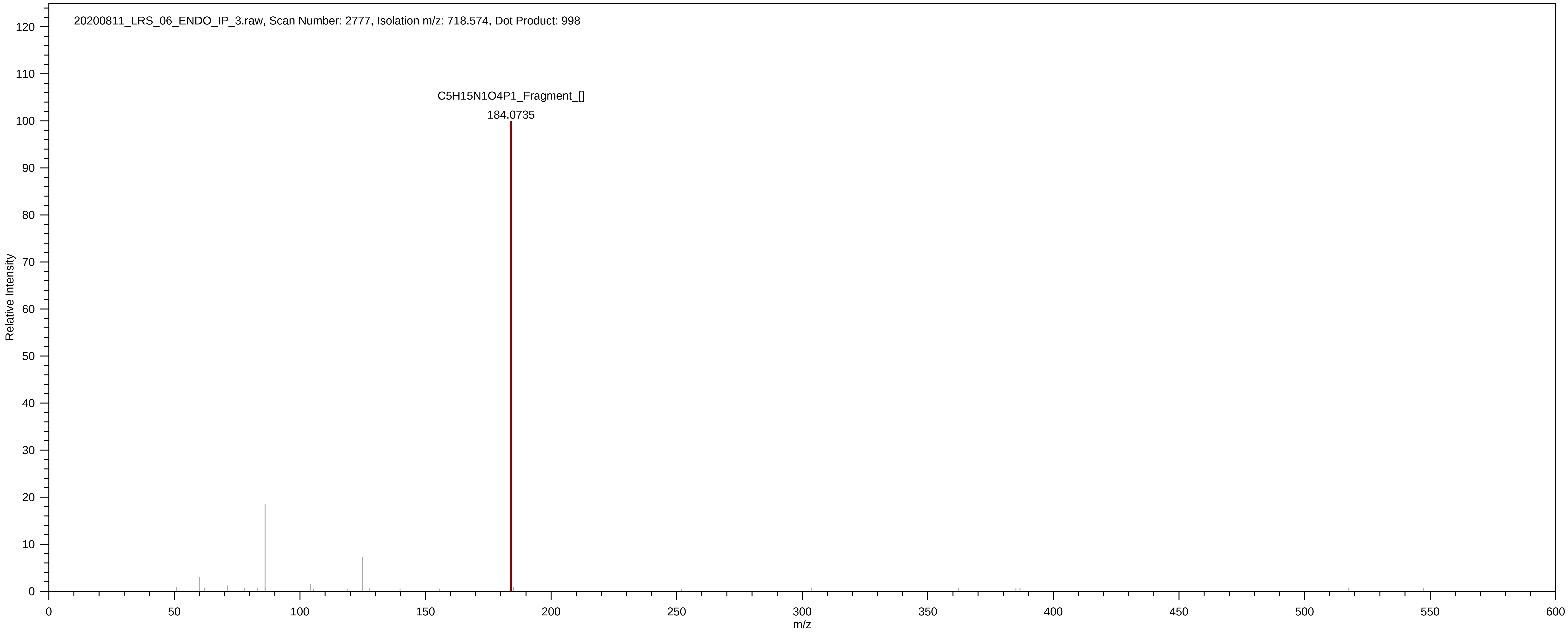

Plasmanyl-PC O-32:1 [M+H]+ | Ret. Time (min): 9.683 | m/z: 718.574

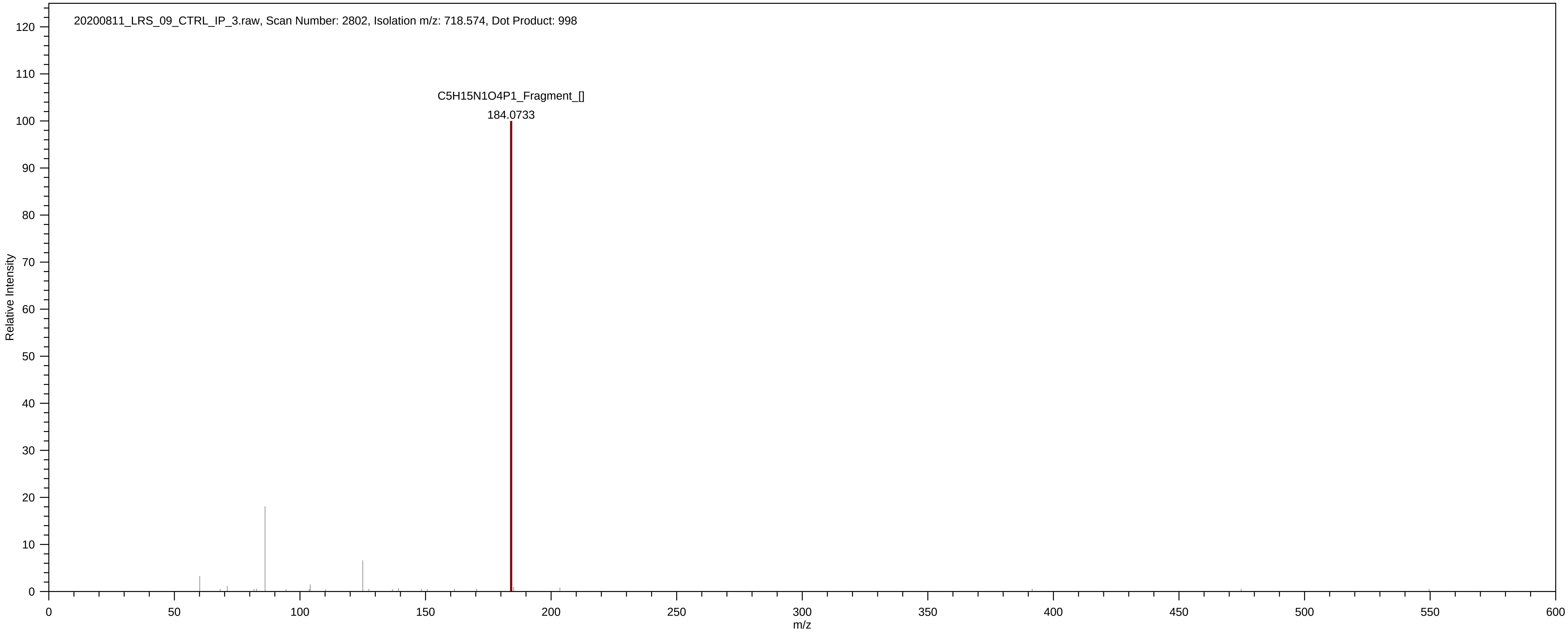

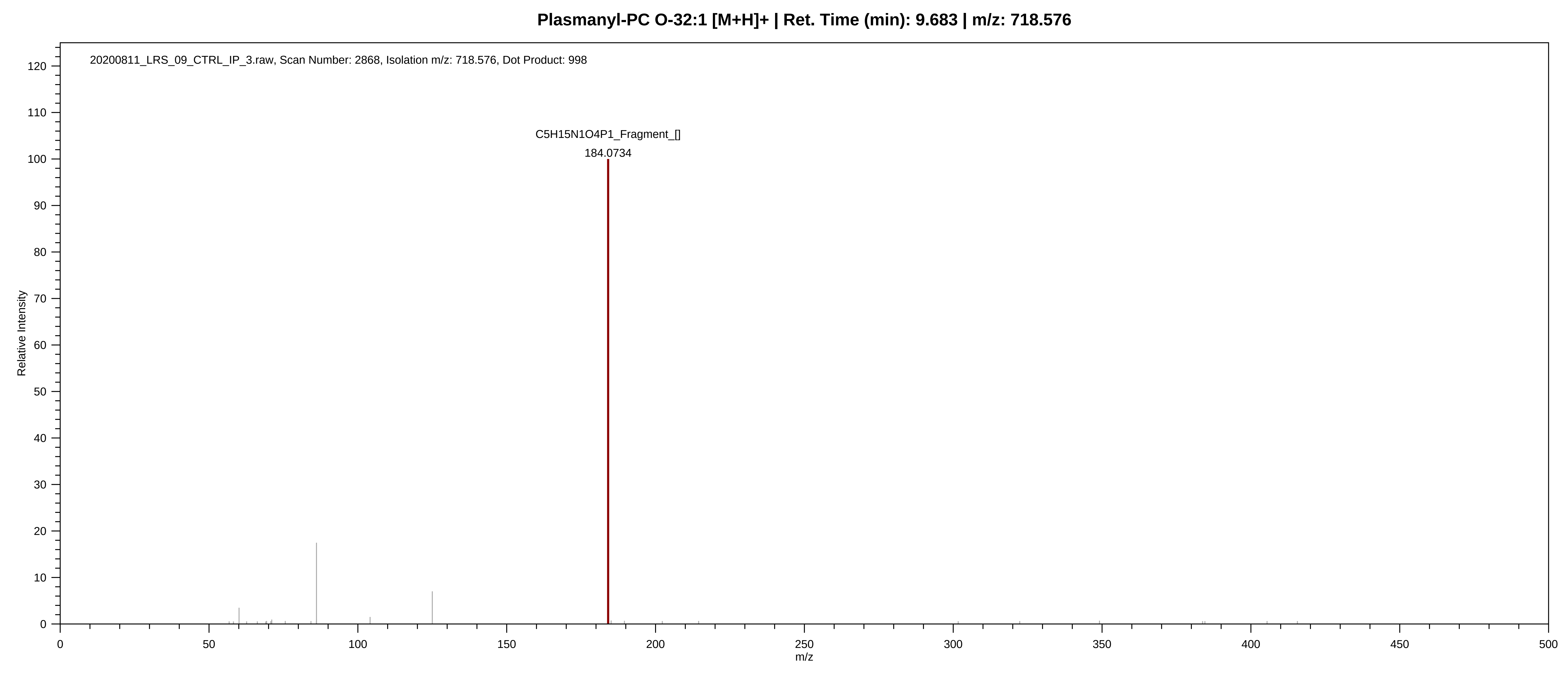

Plasmanyl-PC O-32:1 [M+H]+ | Ret. Time (min): 9.683 | m/z: 718.576
20200811_LRS_09_CTRL_IP_3.raw, Scan Number: 2868, Isolation m/z: 718.576, Dot Product: 998
C5H15N1O4P1_Fragment_[]
184.0734
Relative Intensity
m/z

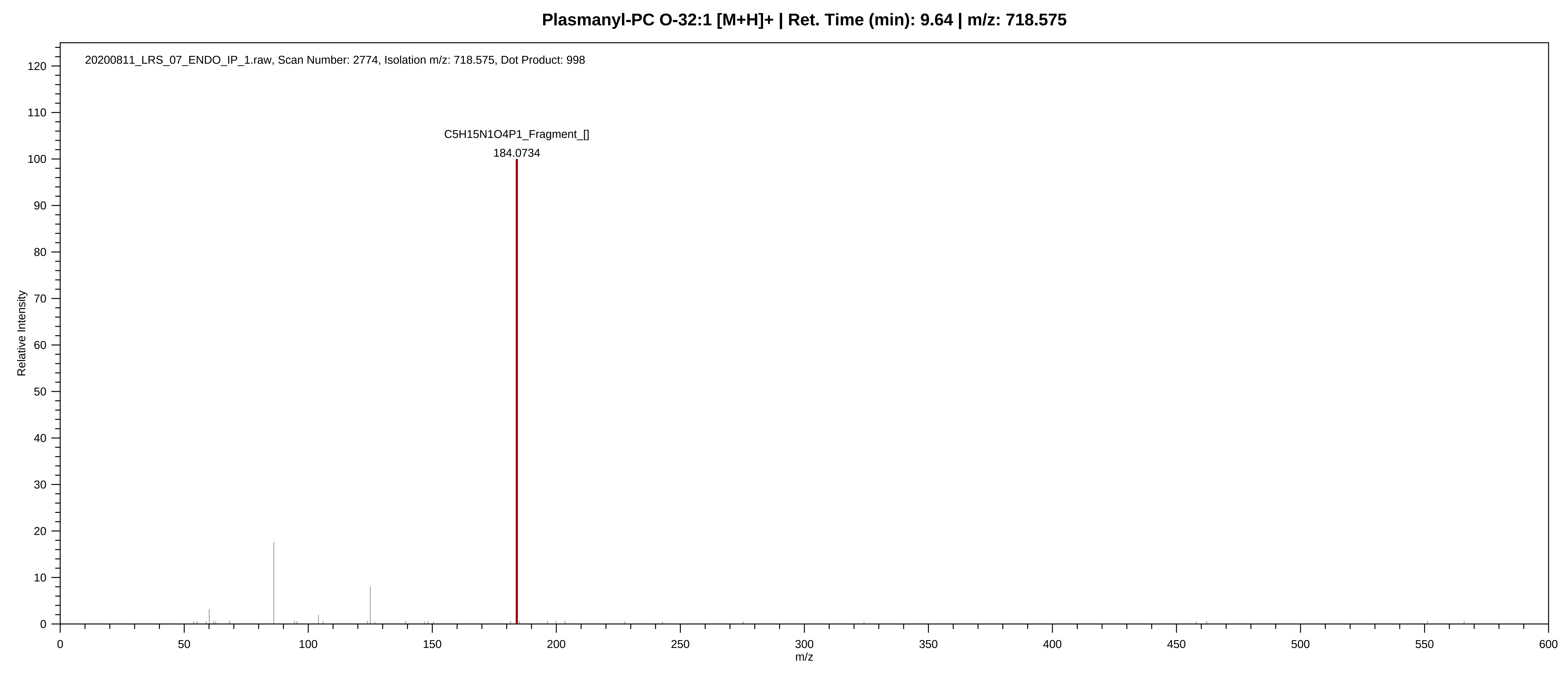
Plasmanyl-PC O-32:1 [M+H]+ | Ret. Time (min): 9.64 | m/z: 718.575
20200811_LRS_07_ENDO_IP_1.raw, Scan Number: 2774, Isolation m/z: 718.575, Dot Product: 998
C5H15N1O4P1_Fragment_[]
184.0734
Relative Intensity
m/z
0
10
20
30
40
50
60
70
80
90
100
110
120
0
50
100
150
200
250
300
350
400
450
500
550
600

Plasmanyl-PC O-32:1 [M+H]+ | Ret. Time (min): 9.466 | m/z: 718.575

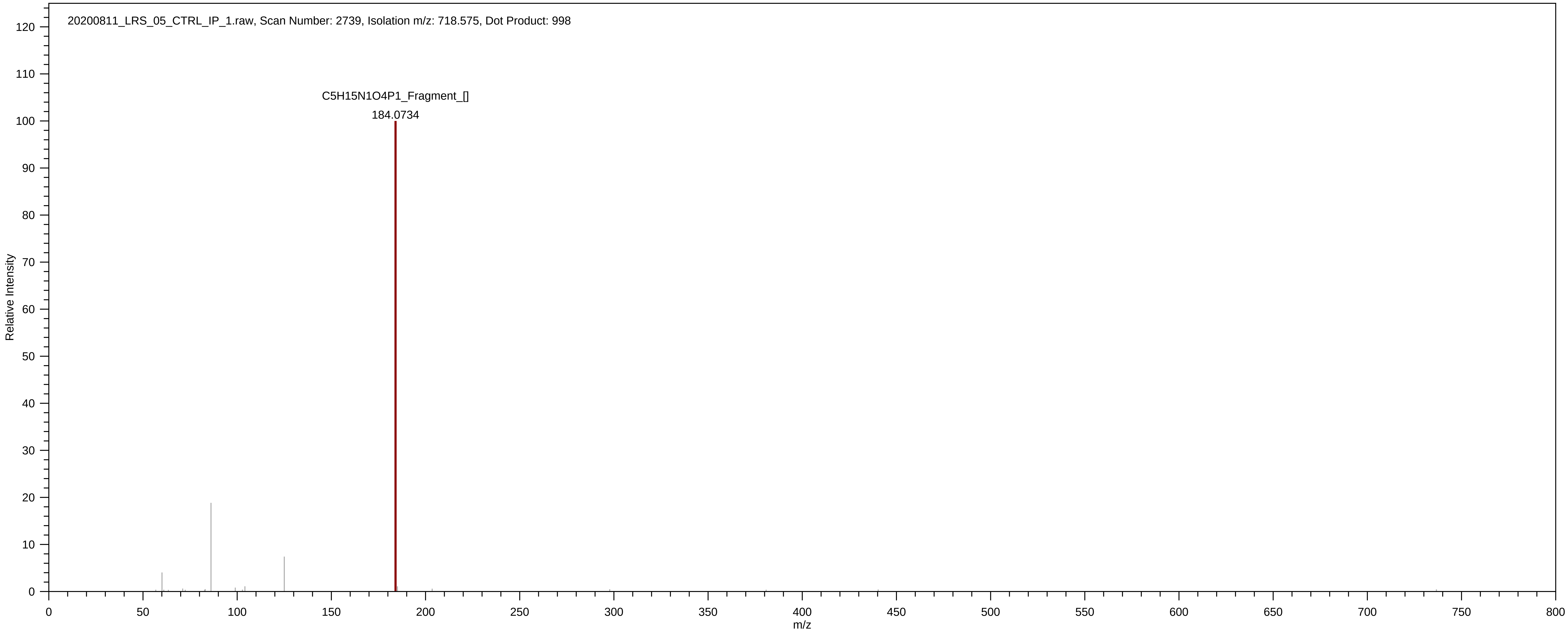

Plasmanyl-PC O-32:1 [M+H]+ | Ret. Time (min): 9.507 | m/z: 718.575

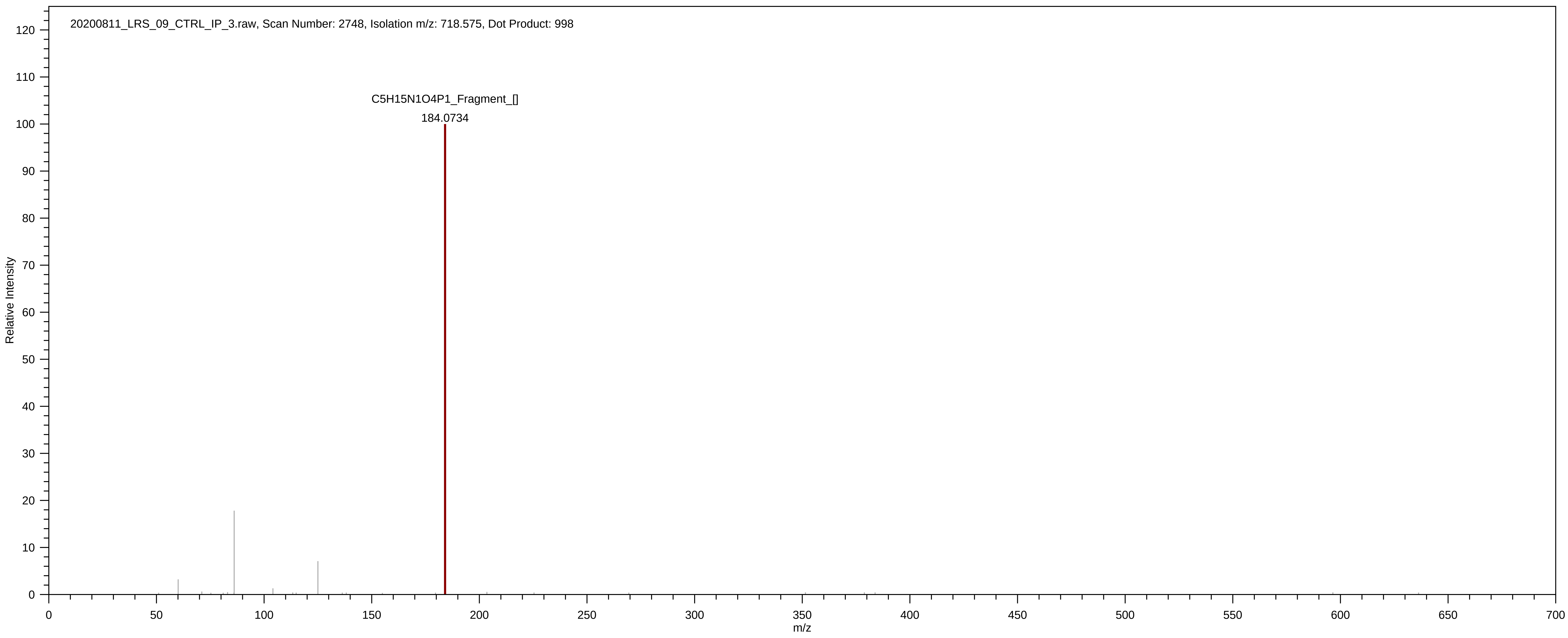

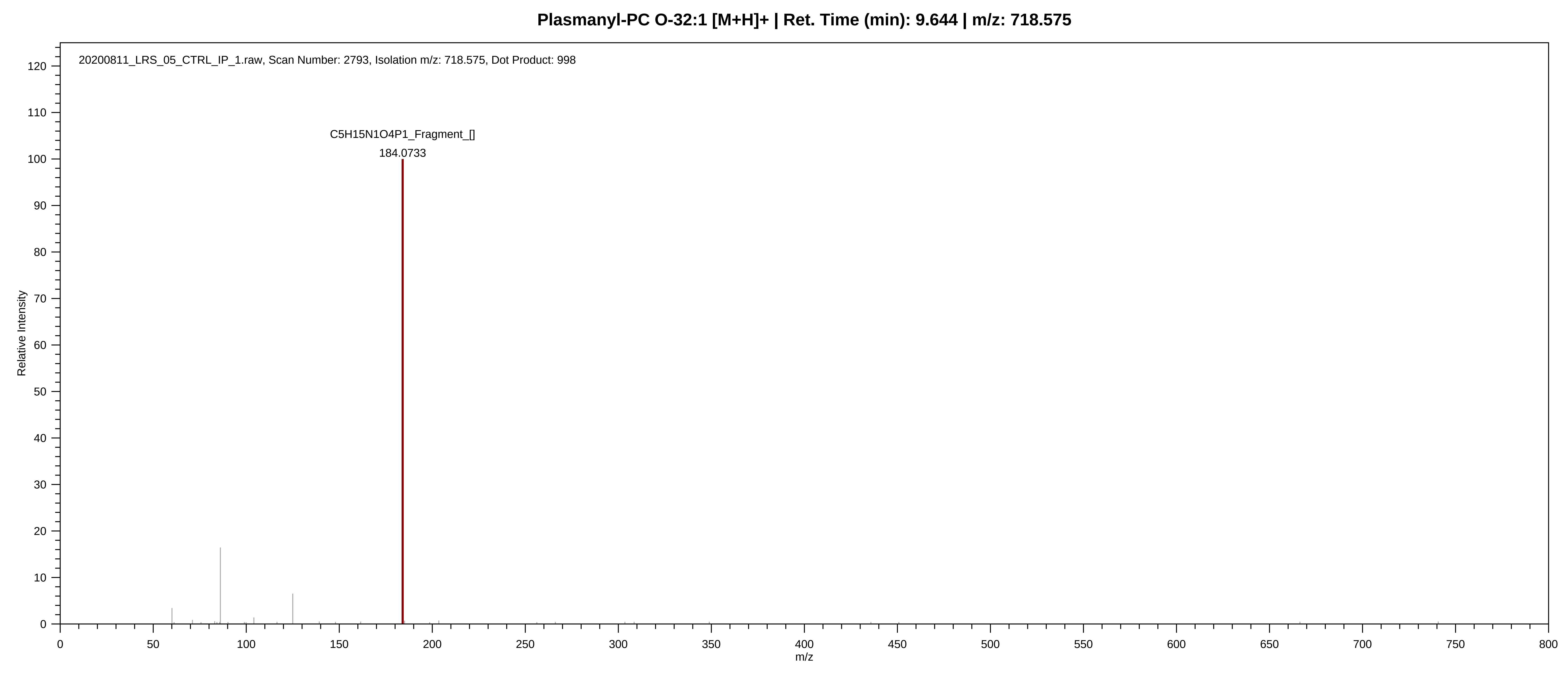
Plasmanyl-PC O-32:1 [M+H]+ | Ret. Time (min): 9.644 | m/z: 718.575
20200811_LRS_05_CTRL_IP_1.raw, Scan Number: 2793, Isolation m/z: 718.575, Dot Product: 998
C5H15N1O4P1_Fragment_[]
184.0733
Relative Intensity
m/z
0
10
20
30
40
50
60
70
80
90
100
110
120
0
50
100
150
200
250
300
350
400
450
500
550
600
650
700
750
800

Plasmanyl-PC O-32:1 [M+H]+ | Ret. Time (min): 9.655 | m/z: 718.575

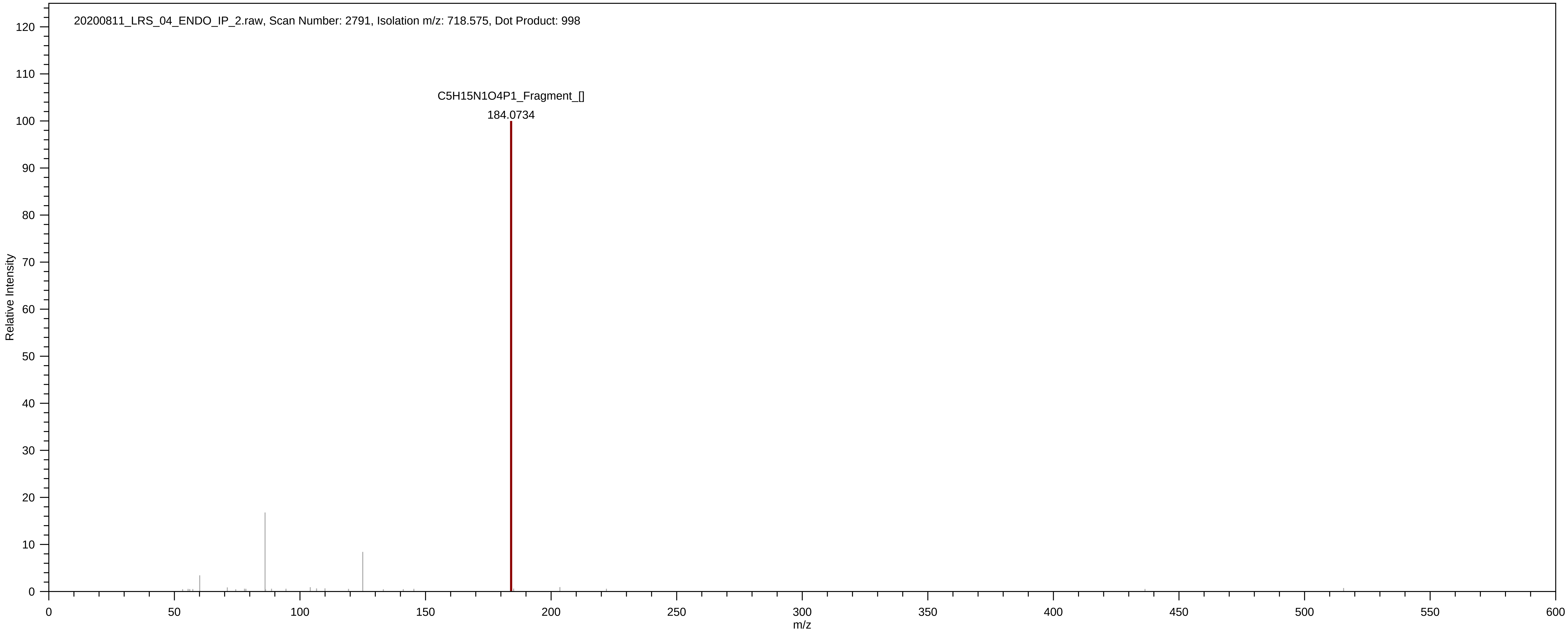

Plasmanyl-PC O-32:1 [M+H]+ | Ret. Time (min): 10.395 | m/z: 718.575

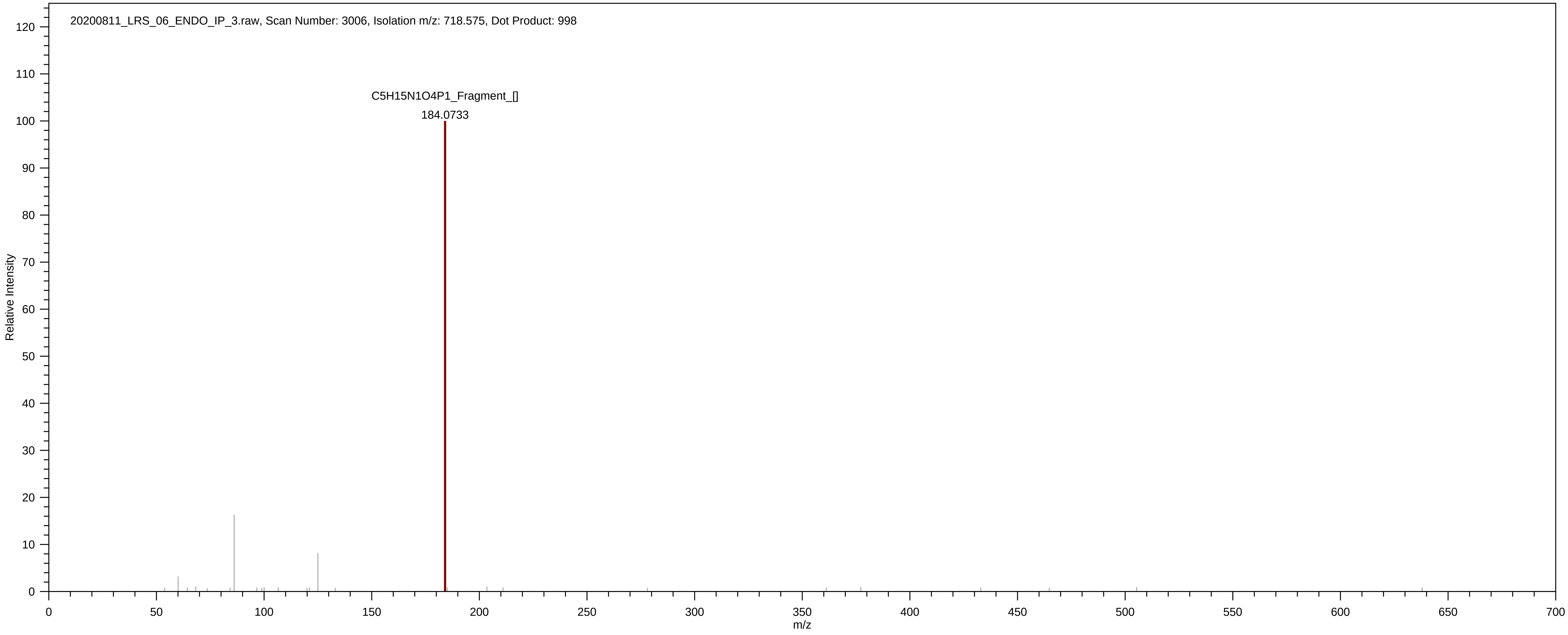

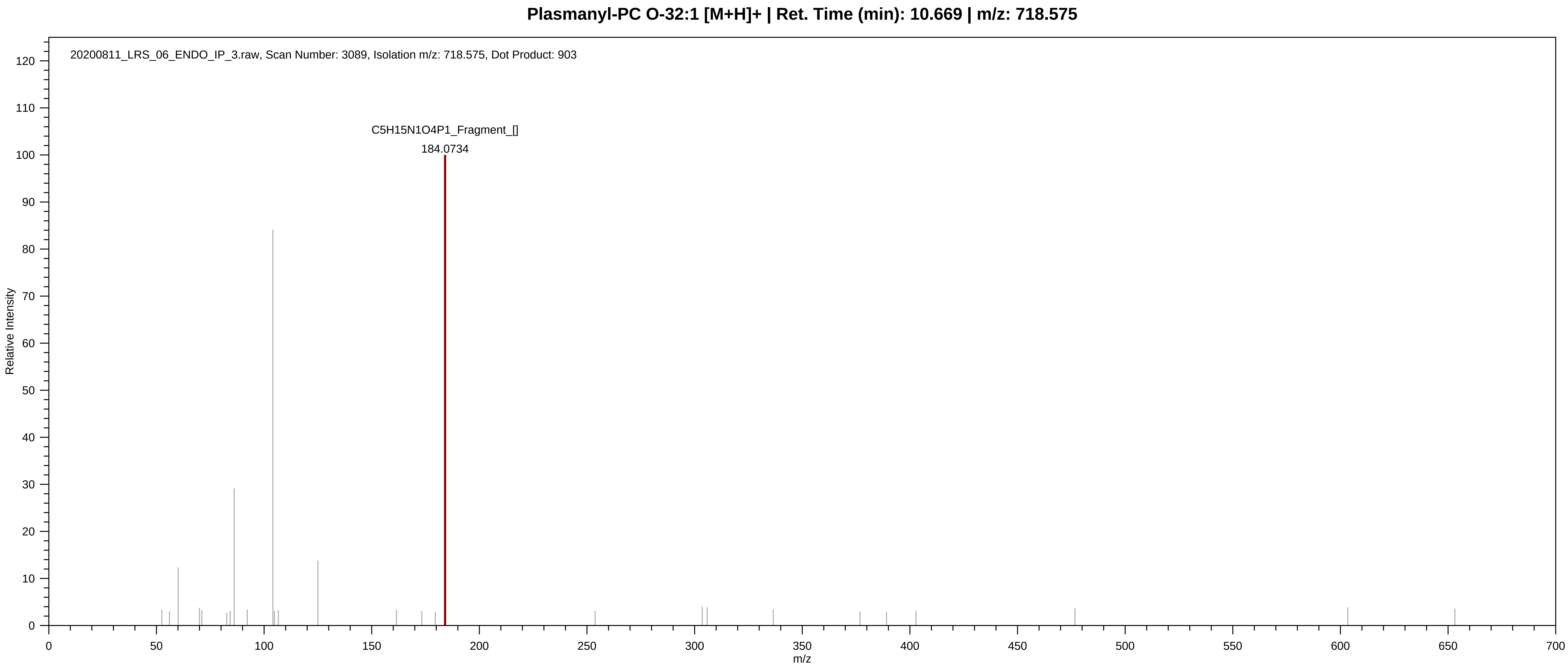
Plasmanyl-PC O-32:1 [M+H]+ | Ret. Time (min): 10.669 | m/z: 718.575
20200811_LRS_06_ENDO_IP_3.raw, Scan Number: 3089, Isolation m/z: 718.575, Dot Product: 903
C5H15N1O4P1_Fragment_[]
184.0734
Relative Intensity
m/z

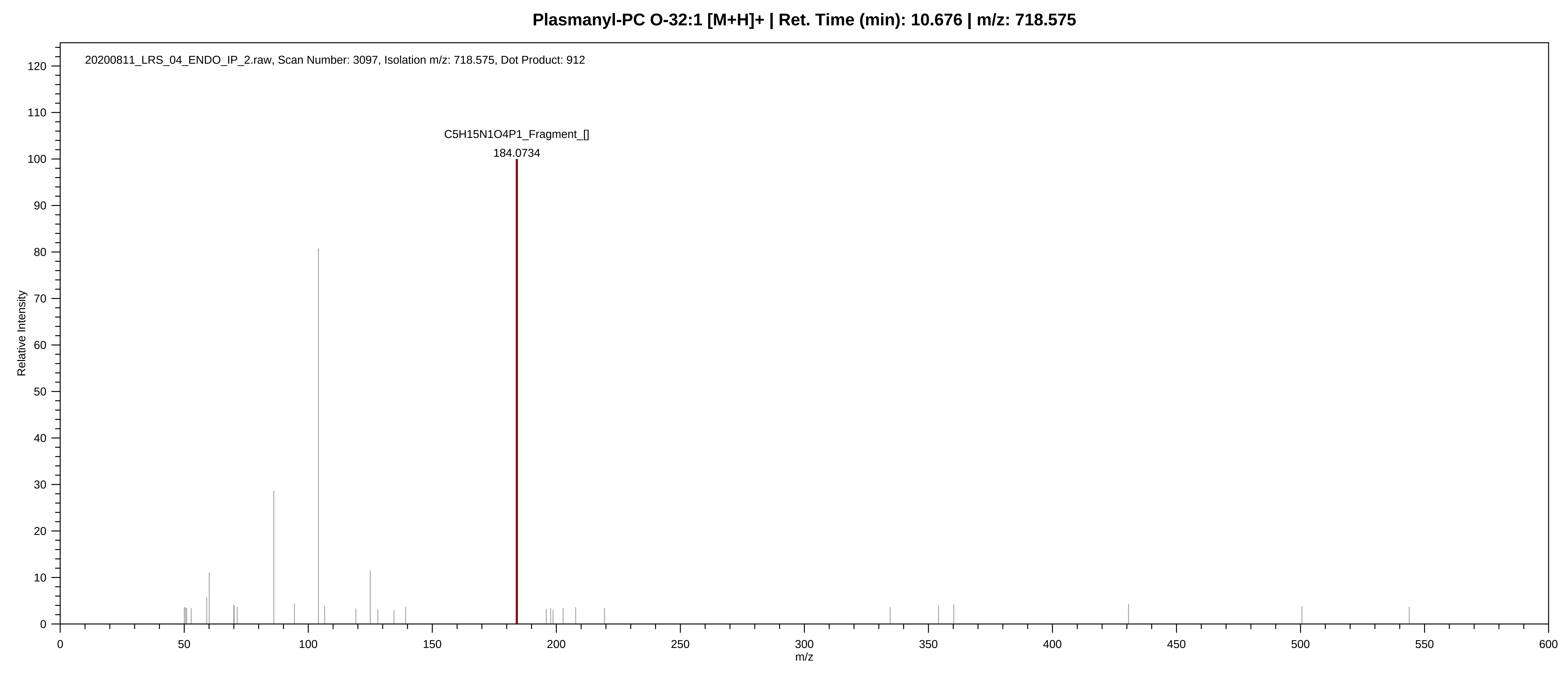
Plasmanyl-PC O-32:1 [M+H]+ | Ret. Time (min): 10.676 | m/z: 718.575
20200811_LRS_04_ENDO_IP_2.raw, Scan Number: 3097, Isolation m/z: 718.575, Dot Product: 912
C5H15N1O4P1_Fragment_[]
184.0734
Relative Intensity
m/z

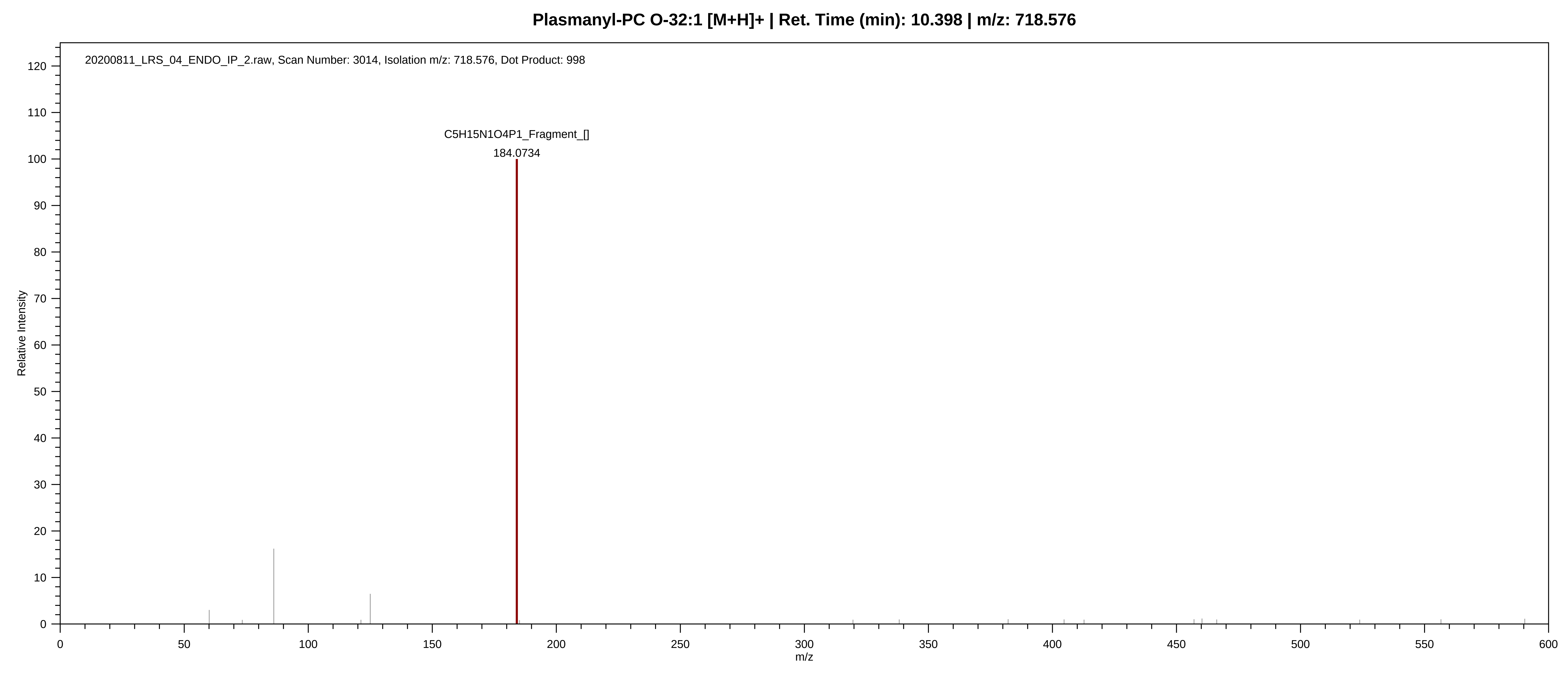
Plasmanyl-PC O-32:1 [M+H]+ | Ret. Time (min): 10.398 | m/z: 718.576
20200811_LRS_04_ENDO_IP_2.raw, Scan Number: 3014, Isolation m/z: 718.576, Dot Product: 998
C5H15N1O4P1_Fragment_[]
184.0734
Relative Intensity
m/z
0
10
20
30
40
50
60
70
80
90
100
110
120
0
50
100
150
200
250
300
350
400
450
500
550
600

Plasmanyl-PC O-32:1 [M+H]+ | Ret. Time (min): 10.217 | m/z: 718.578

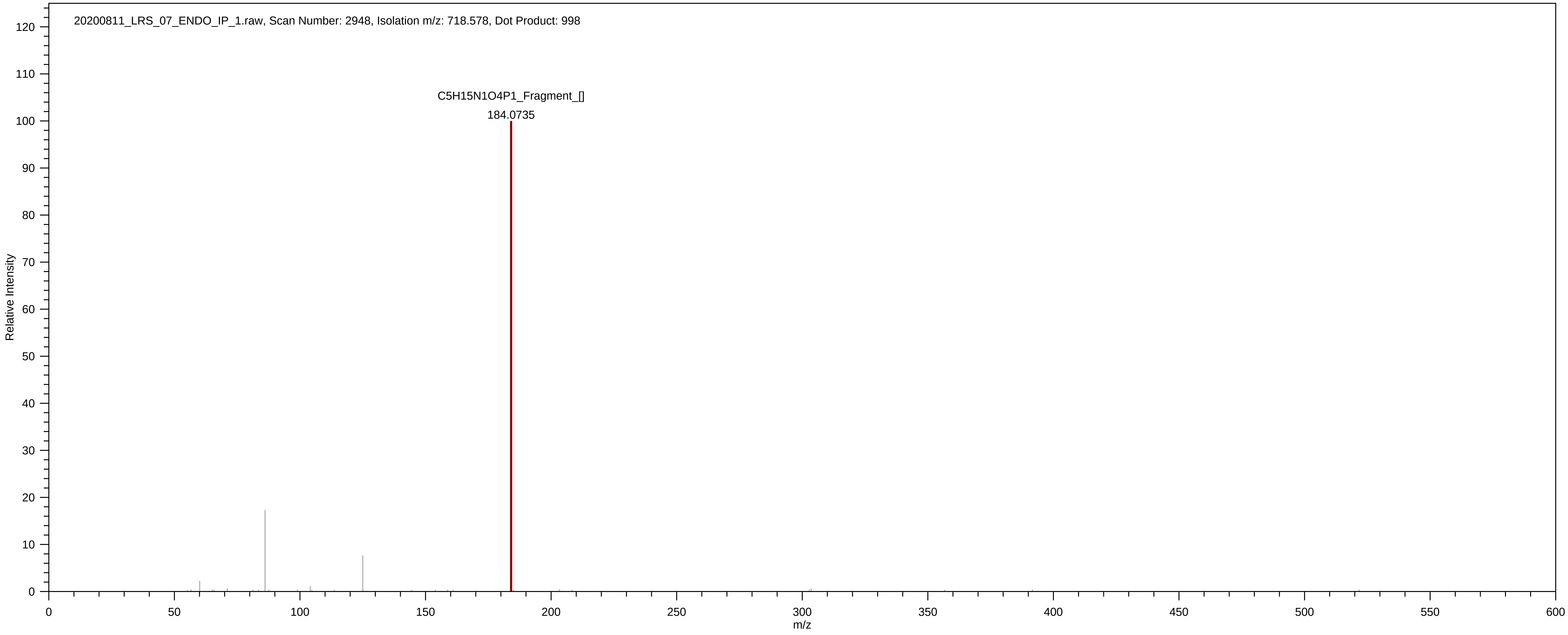

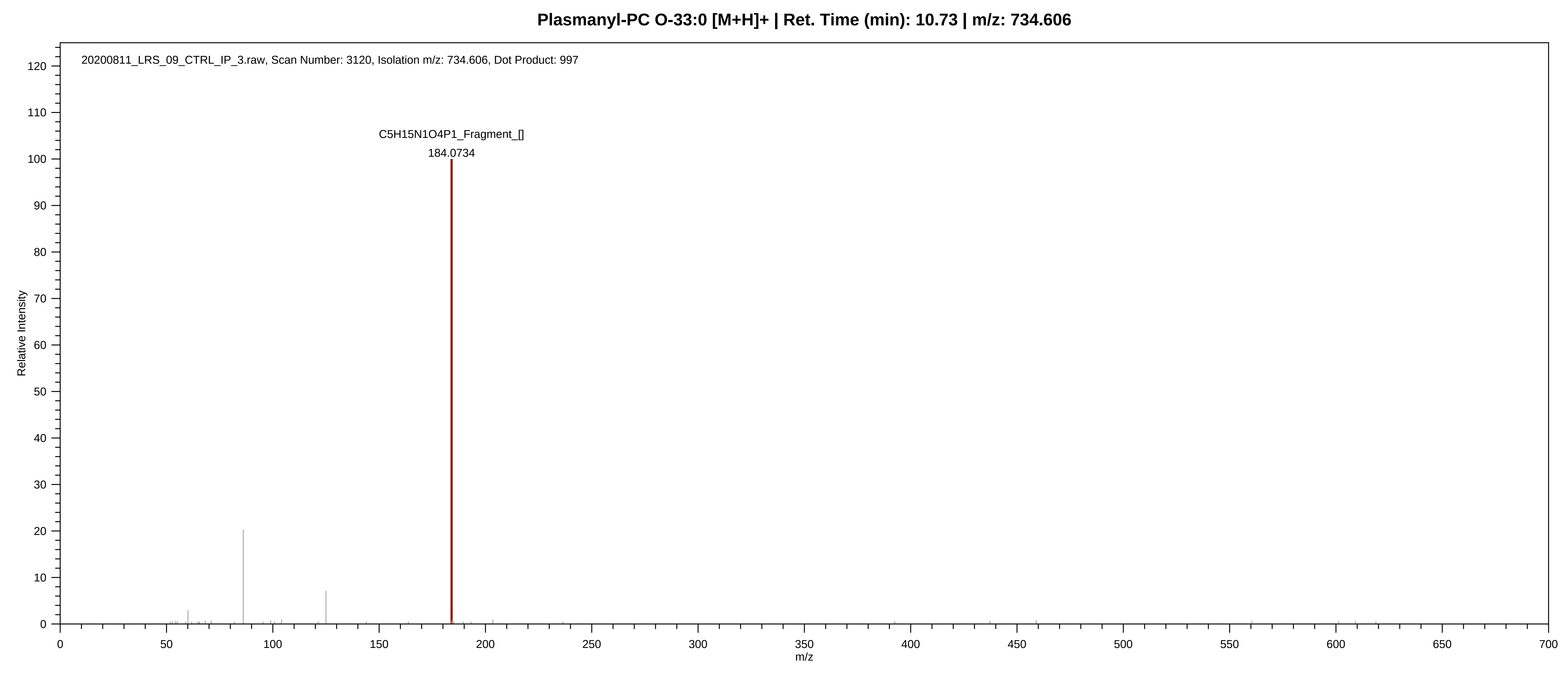

Plasmanyl-PC O-33:0 [M+H]+ | Ret. Time (min): 10.73 | m/z: 734.606
20200811_LRS_09_CTRL_IP_3.raw, Scan Number: 3120, Isolation m/z: 734.606, Dot Product: 997
C5H15N1O4P1_Fragment_[]
184.0734
Relative Intensity
m/z

Plasmanyl-PC O-33:0 [M+H]+ | Ret. Time (min): 10.909 | m/z: 734.606

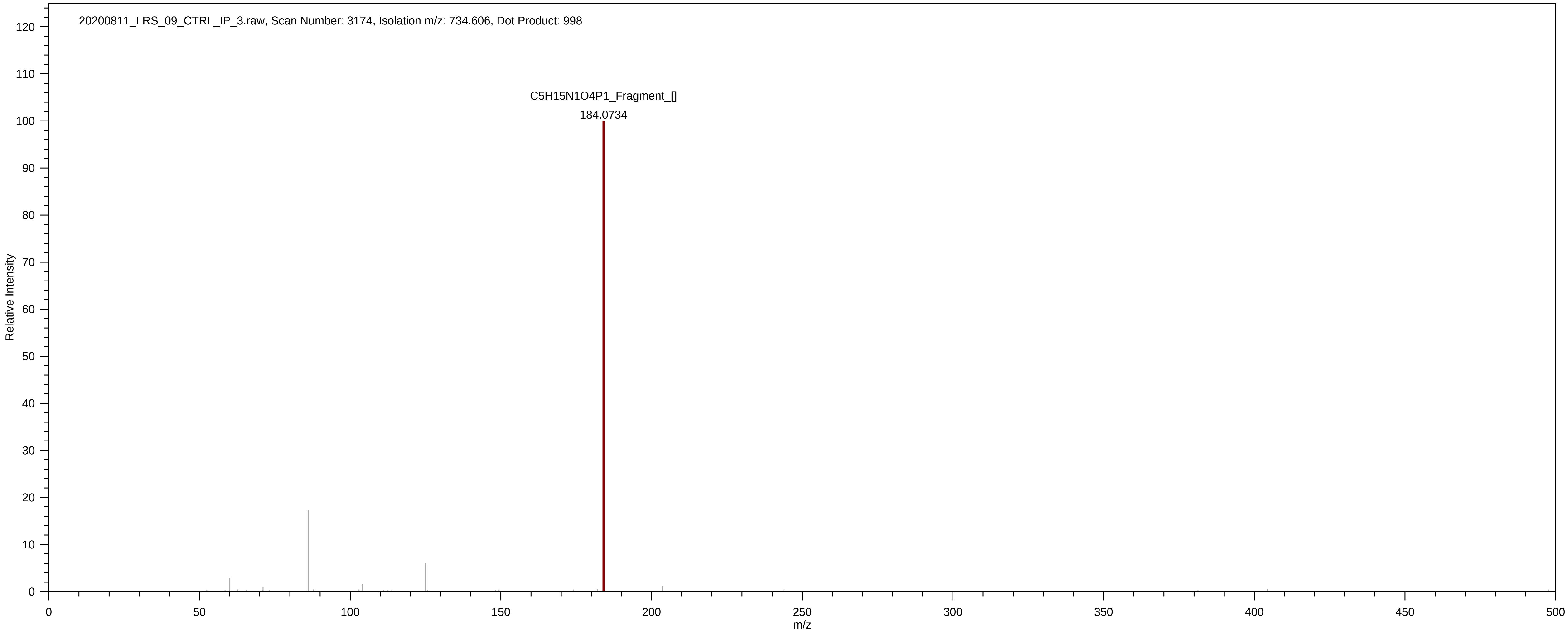

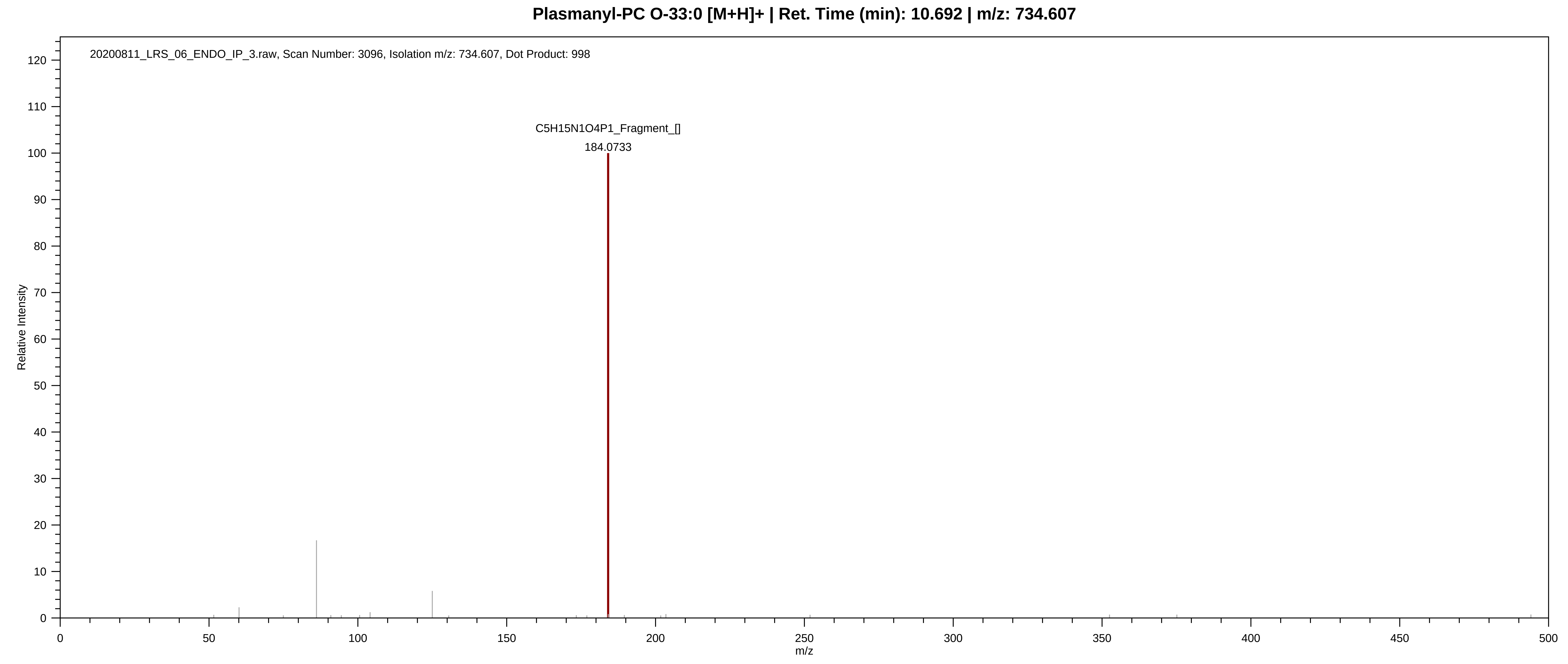

Plasmanyl-PC O-33:0 [M+H]+ | Ret. Time (min): 10.692 | m/z: 734.607
20200811_LRS_06_ENDO_IP_3.raw, Scan Number: 3096, Isolation m/z: 734.607, Dot Product: 998
C5H15N1O4P1_Fragment_[]
184.0733
Relative Intensity
m/z

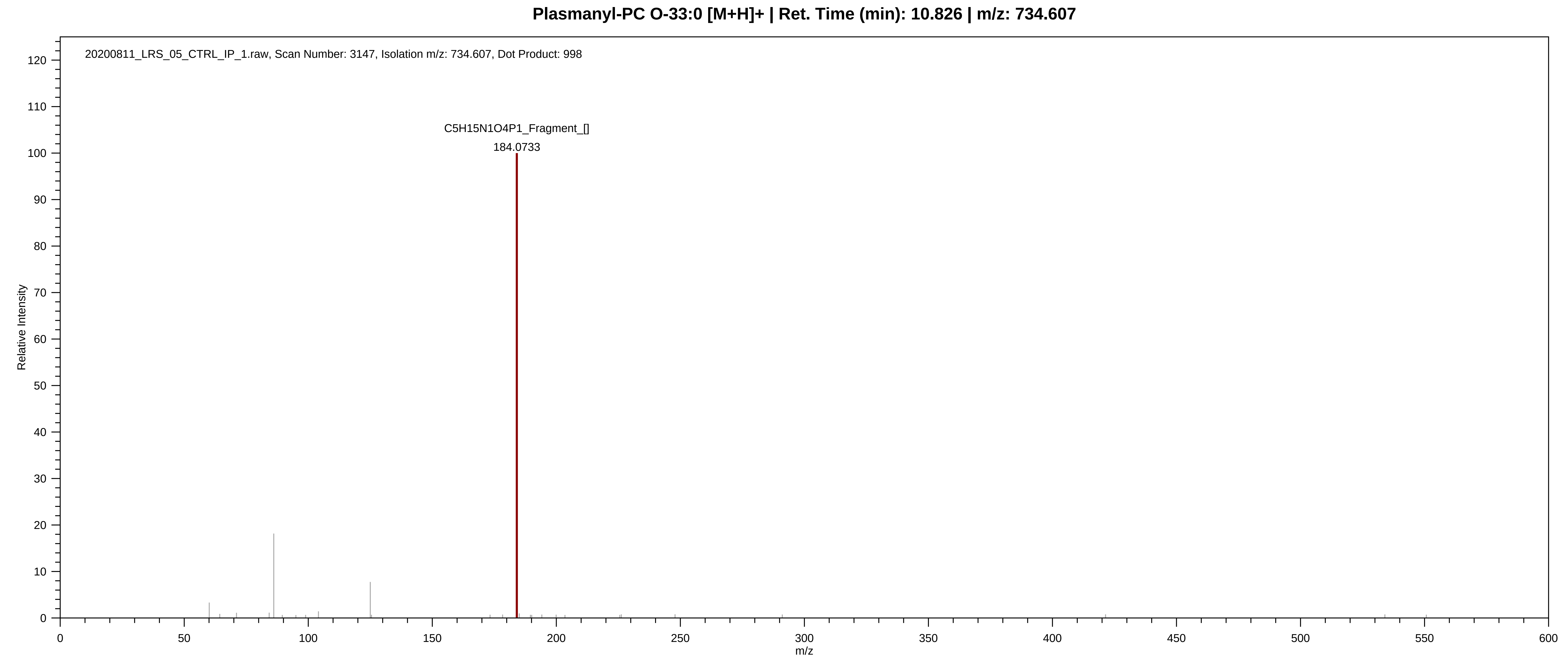
Plasmanyl-PC O-33:0 [M+H]+ | Ret. Time (min): 10.826 | m/z: 734.607
20200811_LRS_05_CTRL_IP_1.raw, Scan Number: 3147, Isolation m/z: 734.607, Dot Product: 998
C5H15N1O4P1_Fragment_[]
184.0733
Relative Intensity
m/z

Plasmanyl-PC O-33:0 [M+H]+ | Ret. Time (min): 10.826 | m/z: 734.607

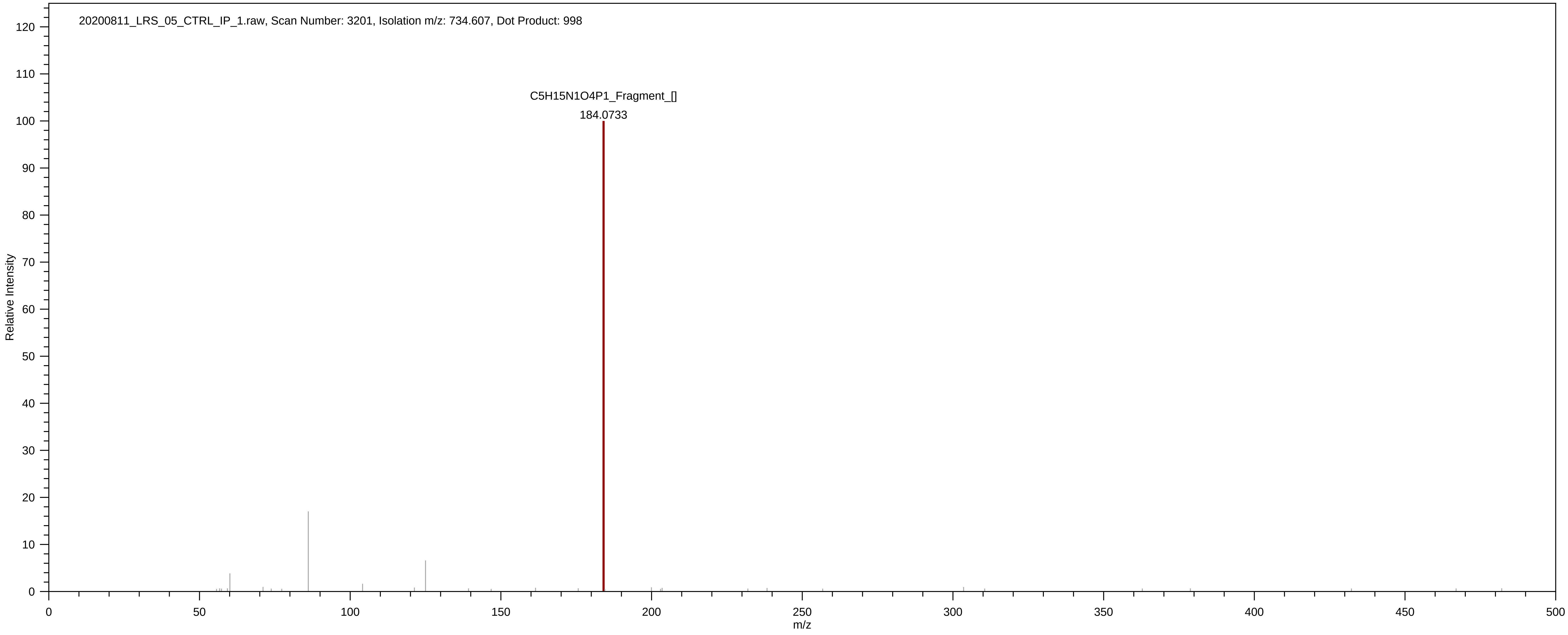

Plasmanyl-PC O-33:0 [M+H]+ | Ret. Time (min): 10.917 | m/z: 734.607

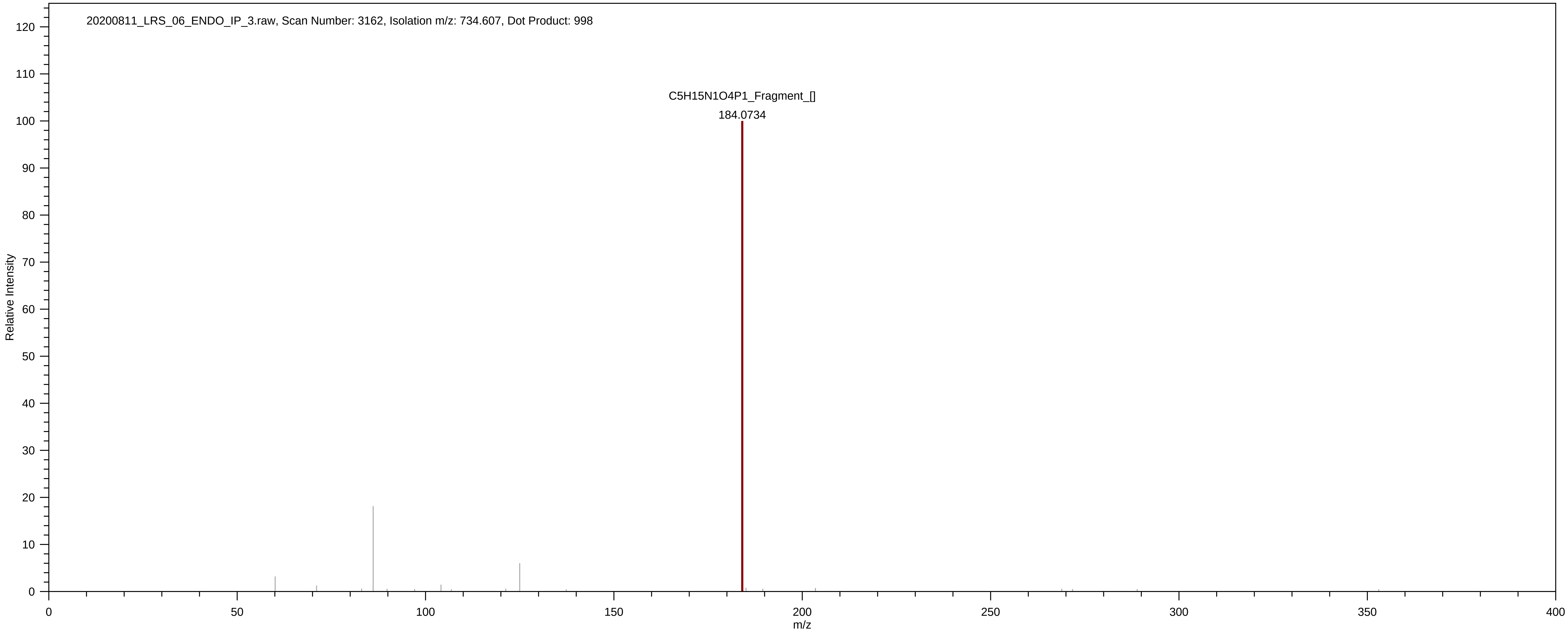

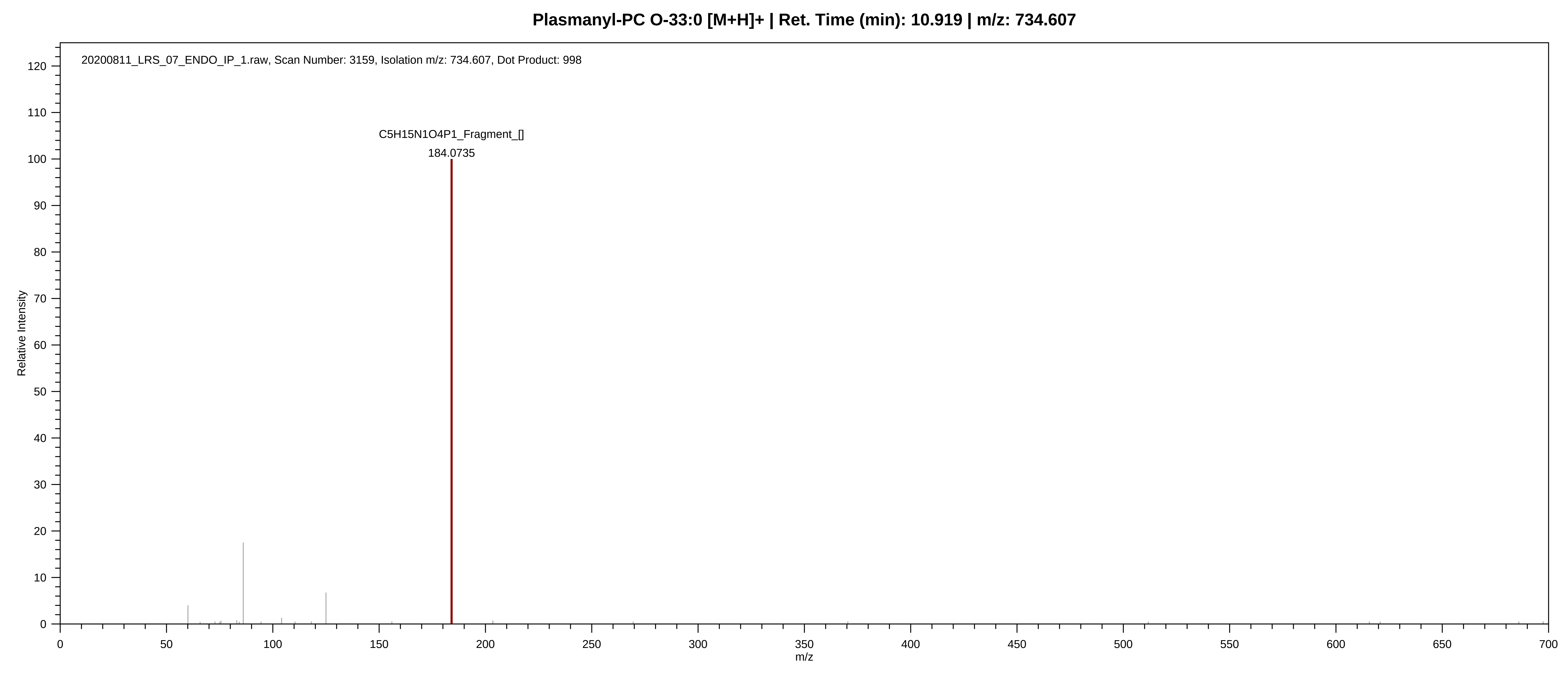

Plasmanyl-PC O-33:0 [M+H]+ | Ret. Time (min): 10.919 | m/z: 734.607
20200811_LRS_07_ENDO_IP_1.raw, Scan Number: 3159, Isolation m/z: 734.607, Dot Product: 998
C5H15N1O4P1_Fragment_[]
184.0735
Relative Intensity
m/z

Plasmanyl-PC O-33:0 [M+H]+ | Ret. Time (min): 10.699 | m/z: 734.608

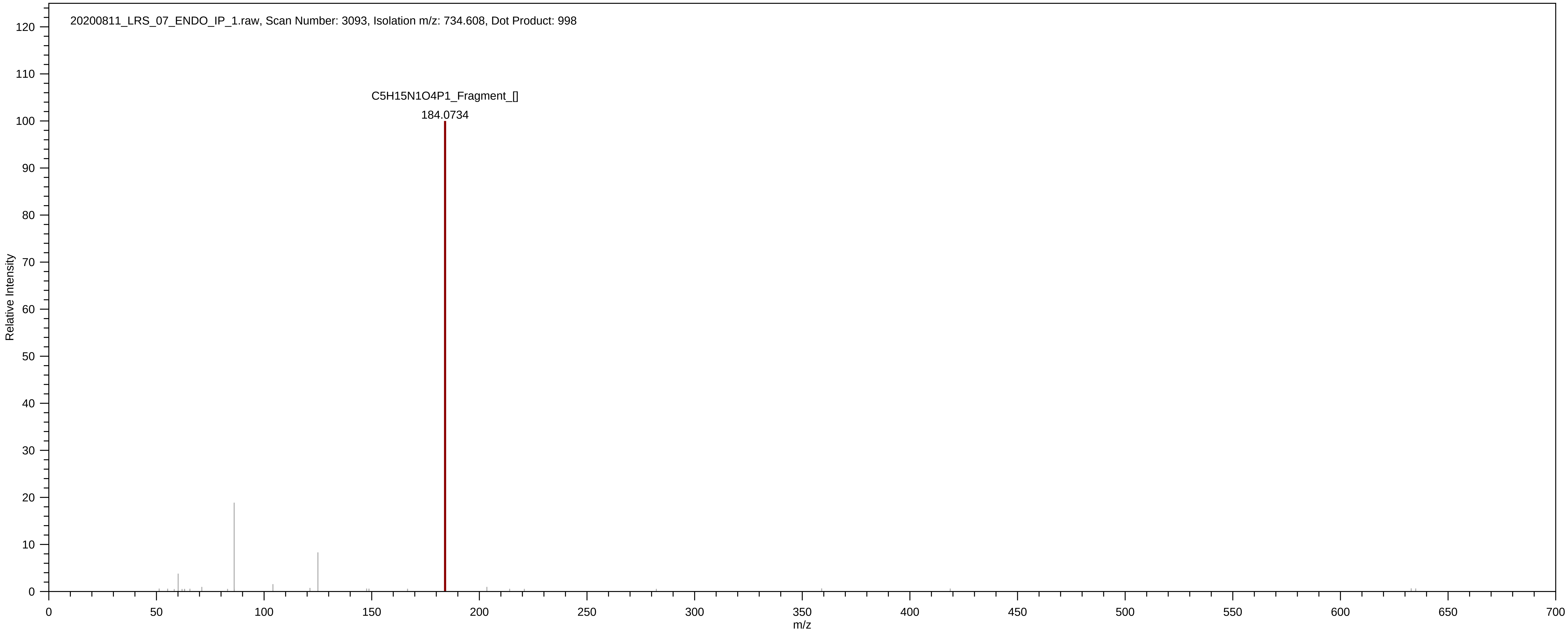

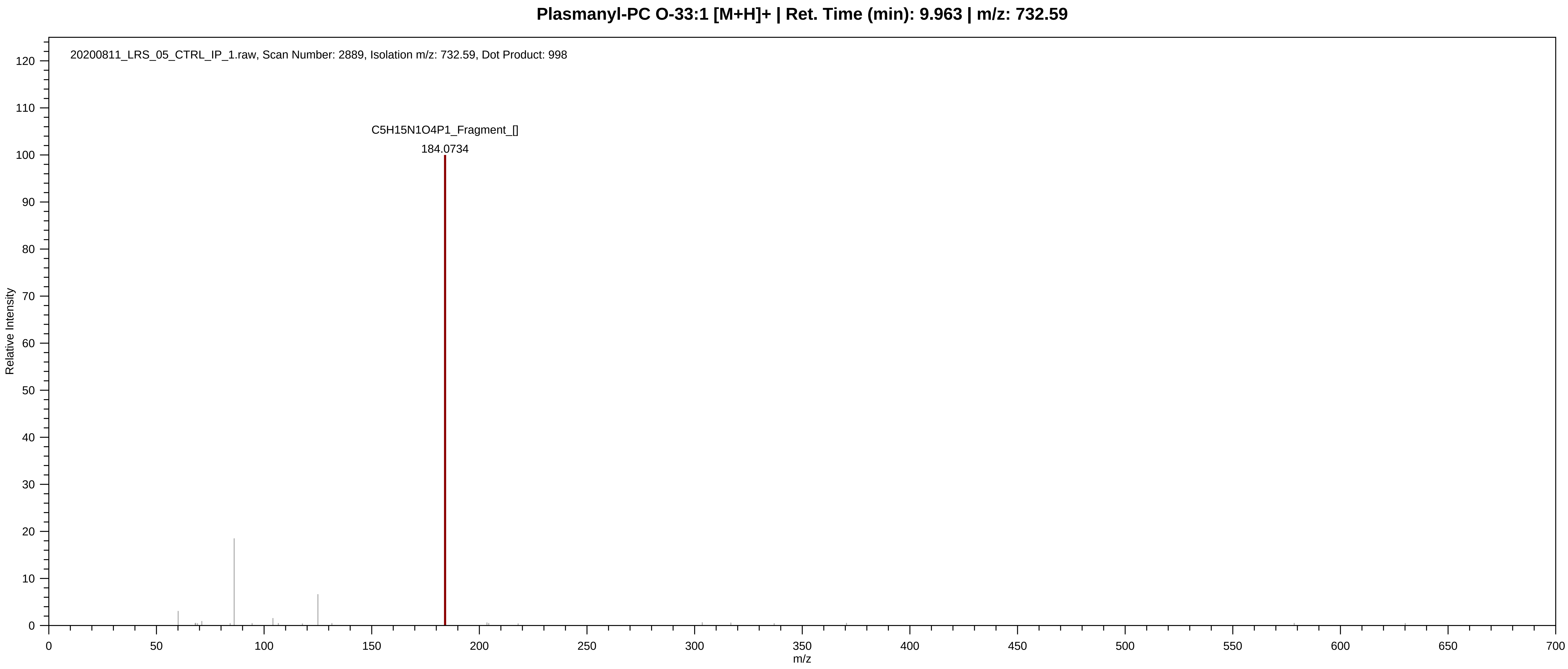
Plasmanyl-PC O-33:1 [M+H]+ | Ret. Time (min): 9.963 | m/z: 732.59
20200811_LRS_05_CTRL_IP_1.raw, Scan Number: 2889, Isolation m/z: 732.59, Dot Product: 998
C5H15N1O4P1_Fragment_[]
184.0734
Relative Intensity
m/z
0
10
20
30
40
50
60
70
80
90
100
110
120
0
50
100
150
200
250
300
350
400
450
500
550
600
650
700

Plasmanyl-PC O-33:1 [M+H]+ | Ret. Time (min): 9.972 | m/z: 732.59

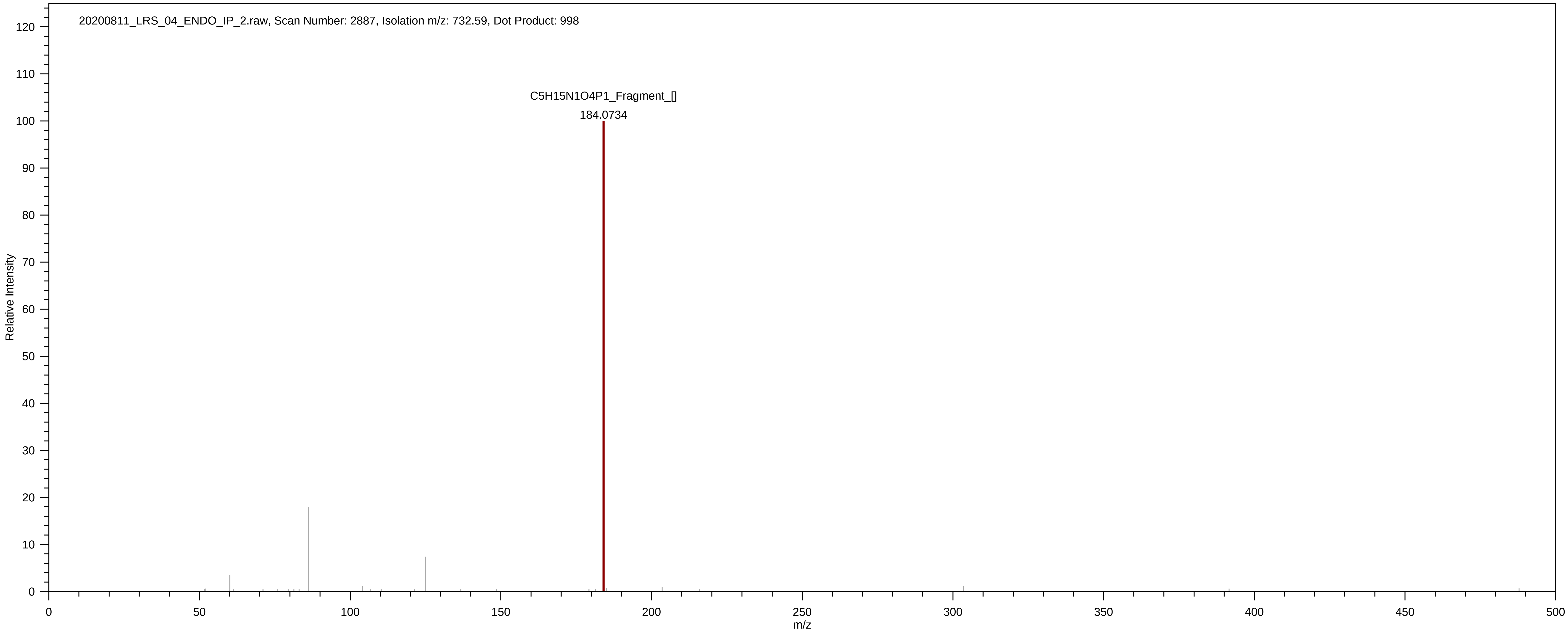

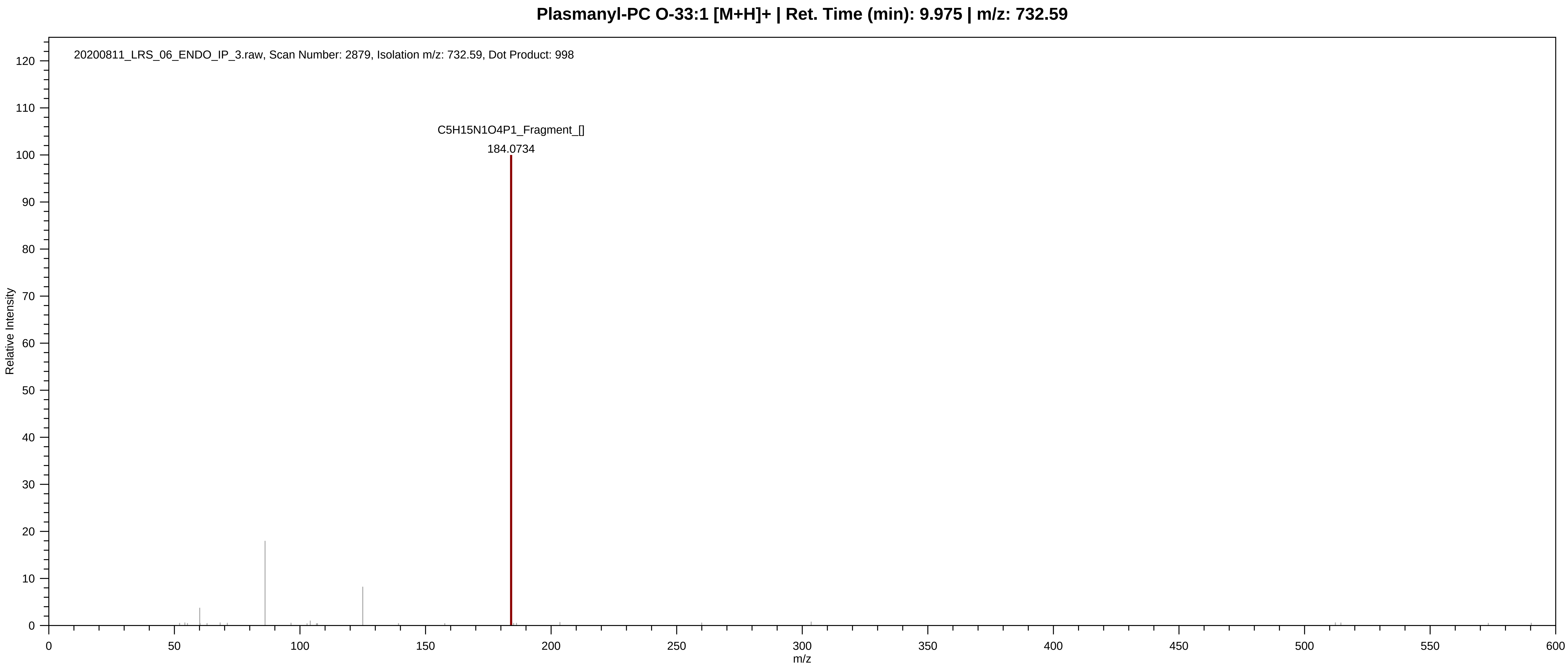

Plasmanyl-PC O-33:1 [M+H]+ | Ret. Time (min): 9.975 | m/z: 732.59
20200811_LRS_06_ENDO_IP_3.raw, Scan Number: 2879, Isolation m/z: 732.59, Dot Product: 998
C5H15N1O4P1_Fragment_[]
184.0734
Relative Intensity
m/z

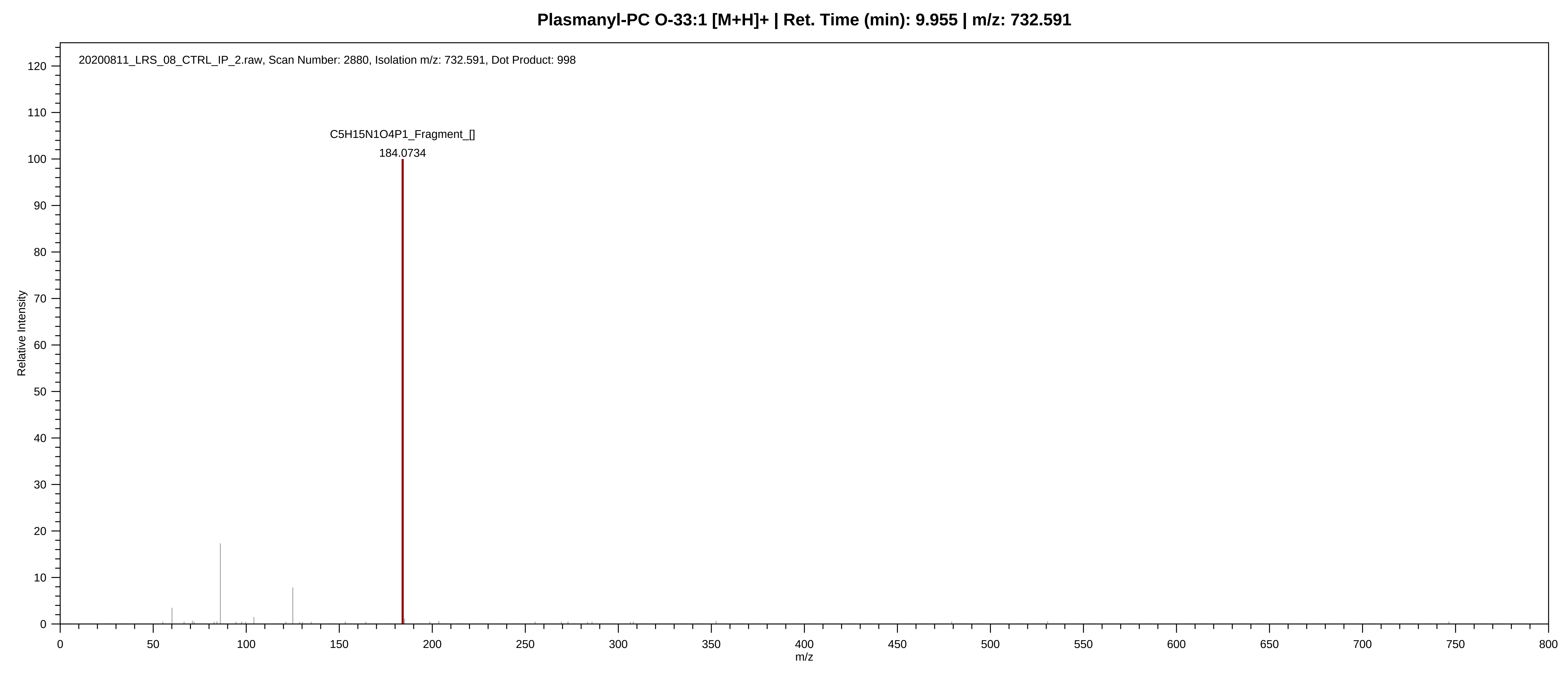

Plasmanyl-PC O-33:1 [M+H]+ | Ret. Time (min): 9.955 | m/z: 732.591
20200811_LRS_08_CTRL_IP_2.raw, Scan Number: 2880, Isolation m/z: 732.591, Dot Product: 998
C5H15N1O4P1_Fragment_[]
184.0734
Relative Intensity
m/z

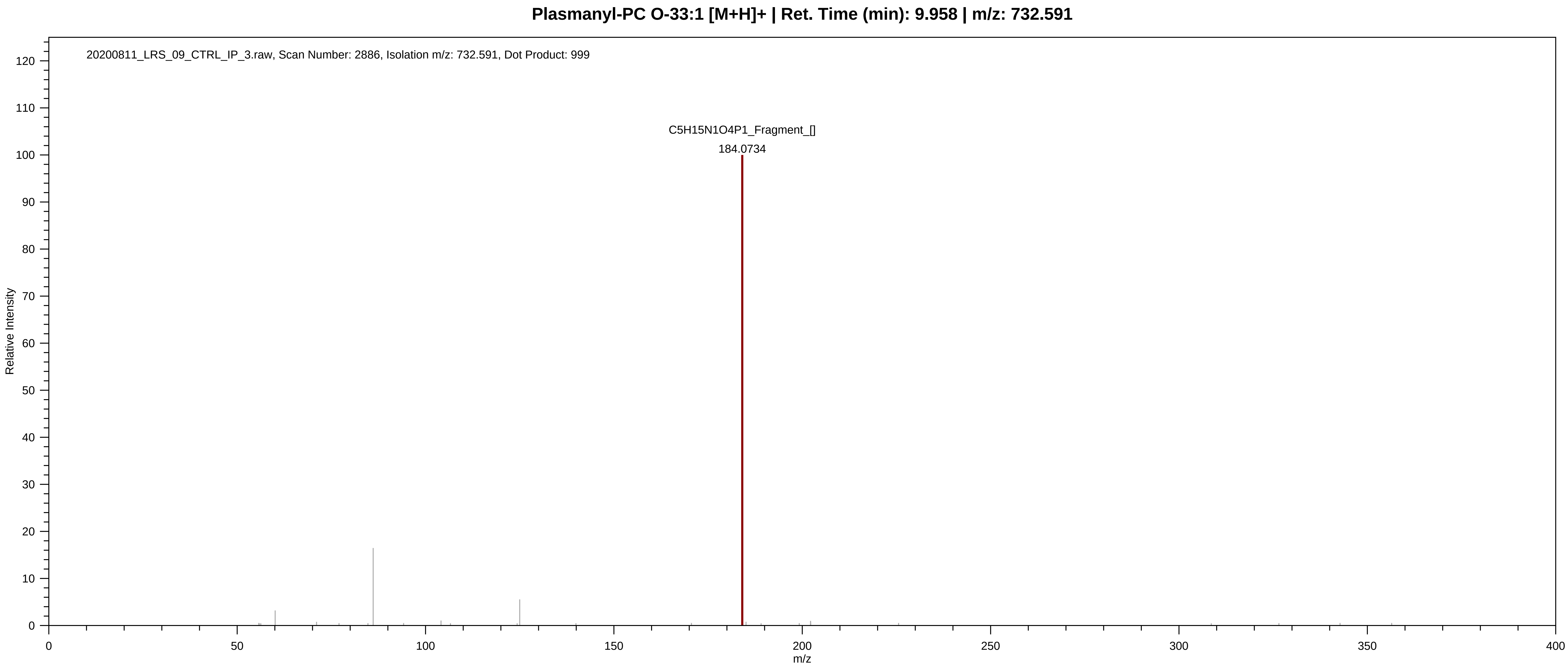

Plasmanyl-PC O-33:1 [M+H]+ | Ret. Time (min): 9.958 | m/z: 732.591
20200811_LRS_09_CTRL_IP_3.raw, Scan Number: 2886, Isolation m/z: 732.591, Dot Product: 999
C5H15N1O4P1_Fragment_[]
184.0734
Relative Intensity
m/z

Plasmanyl-PC O-33:1 [M+H]+ | Ret. Time (min): 9.976 | m/z: 732.591

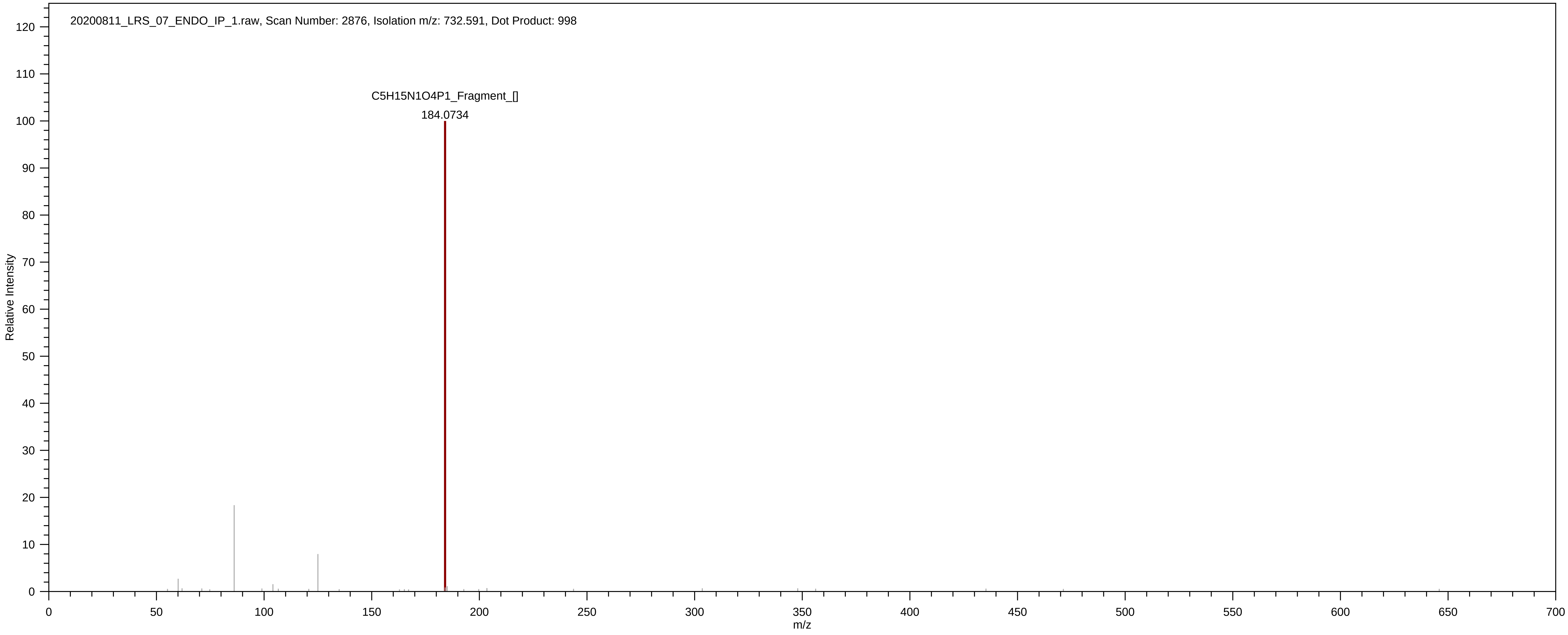

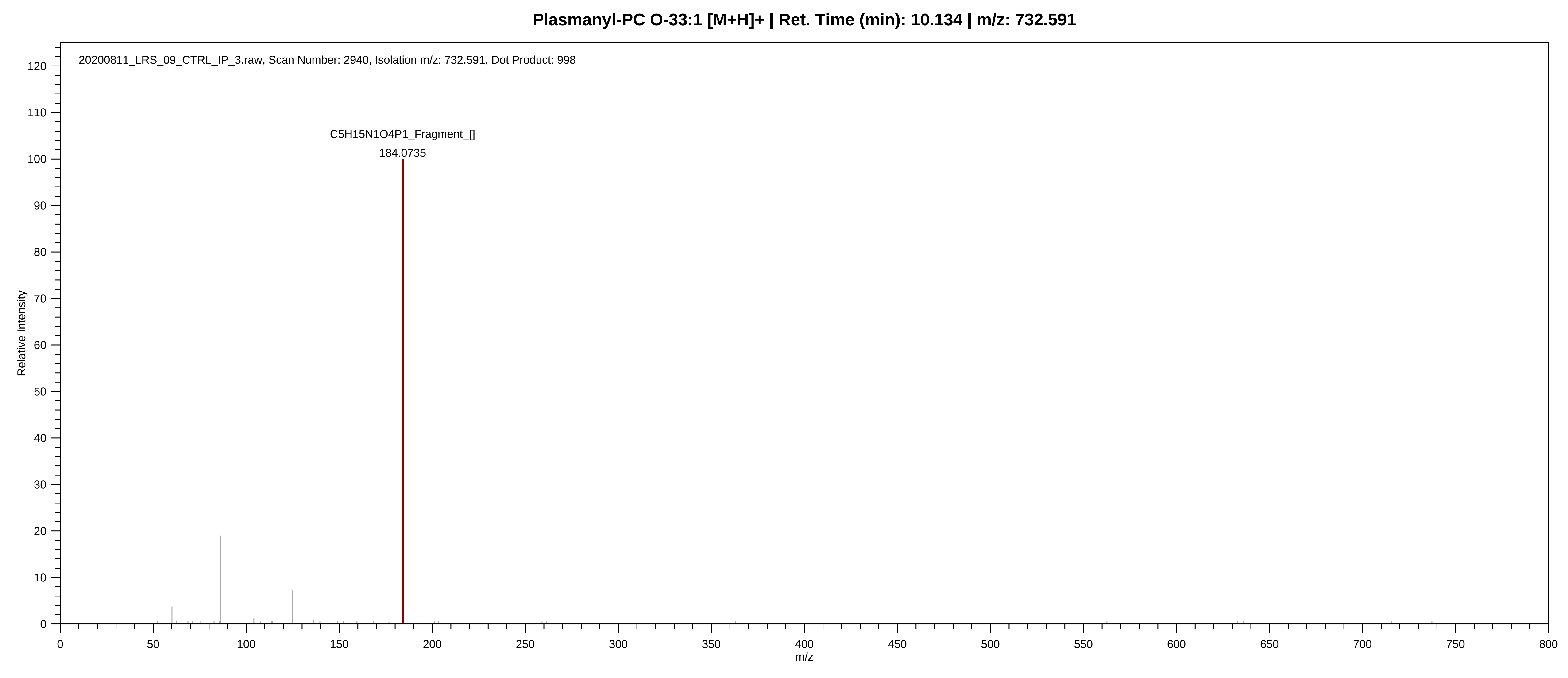
Plasmanyl-PC O-33:1 [M+H]+ | Ret. Time (min): 10.134 | m/z: 732.591
20200811_LRS_09_CTRL_IP_3.raw, Scan Number: 2940, Isolation m/z: 732.591, Dot Product: 998
C5H15N1O4P1_Fragment_[]
184.0735
Relative Intensity
m/z

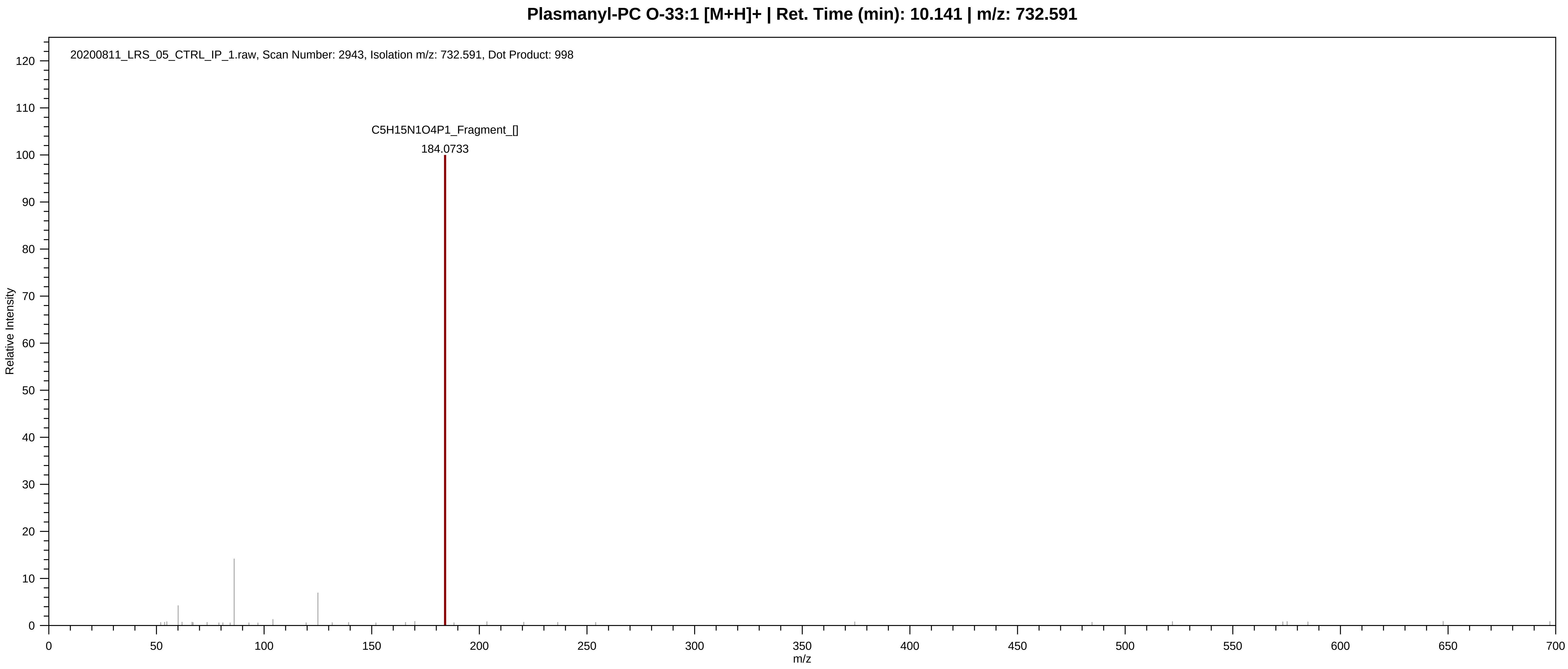

Plasmanyl-PC O-33:1 [M+H]+ | Ret. Time (min): 10.141 | m/z: 732.591
20200811_LRS_05_CTRL_IP_1.raw, Scan Number: 2943, Isolation m/z: 732.591, Dot Product: 998
C5H15N1O4P1_Fragment_[]
184.0733
Relative Intensity
m/z

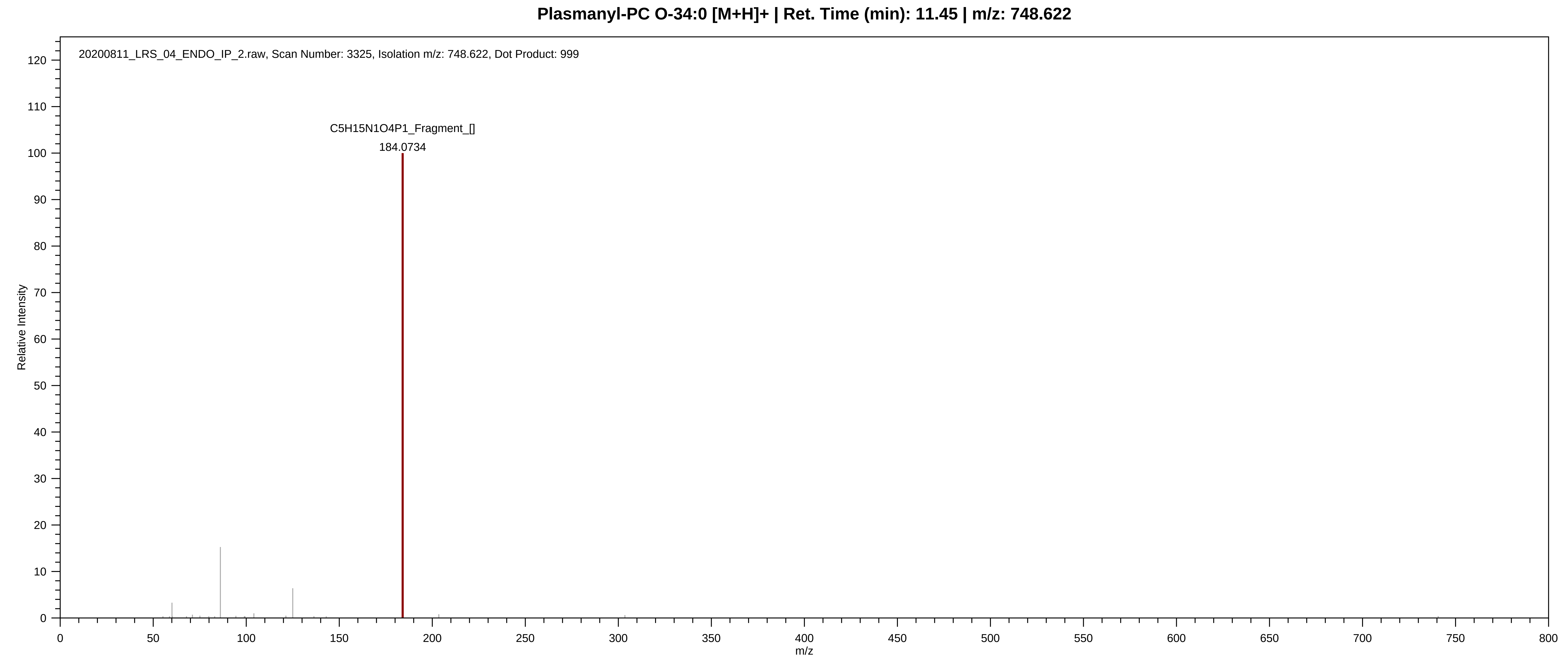
Plasmanyl-PC O-34:0 [M+H]+ | Ret. Time (min): 11.45 | m/z: 748.622
20200811_LRS_04_ENDO_IP_2.raw, Scan Number: 3325, Isolation m/z: 748.622, Dot Product: 999
C5H15N1O4P1_Fragment_[]
184.0734
Relative Intensity
m/z

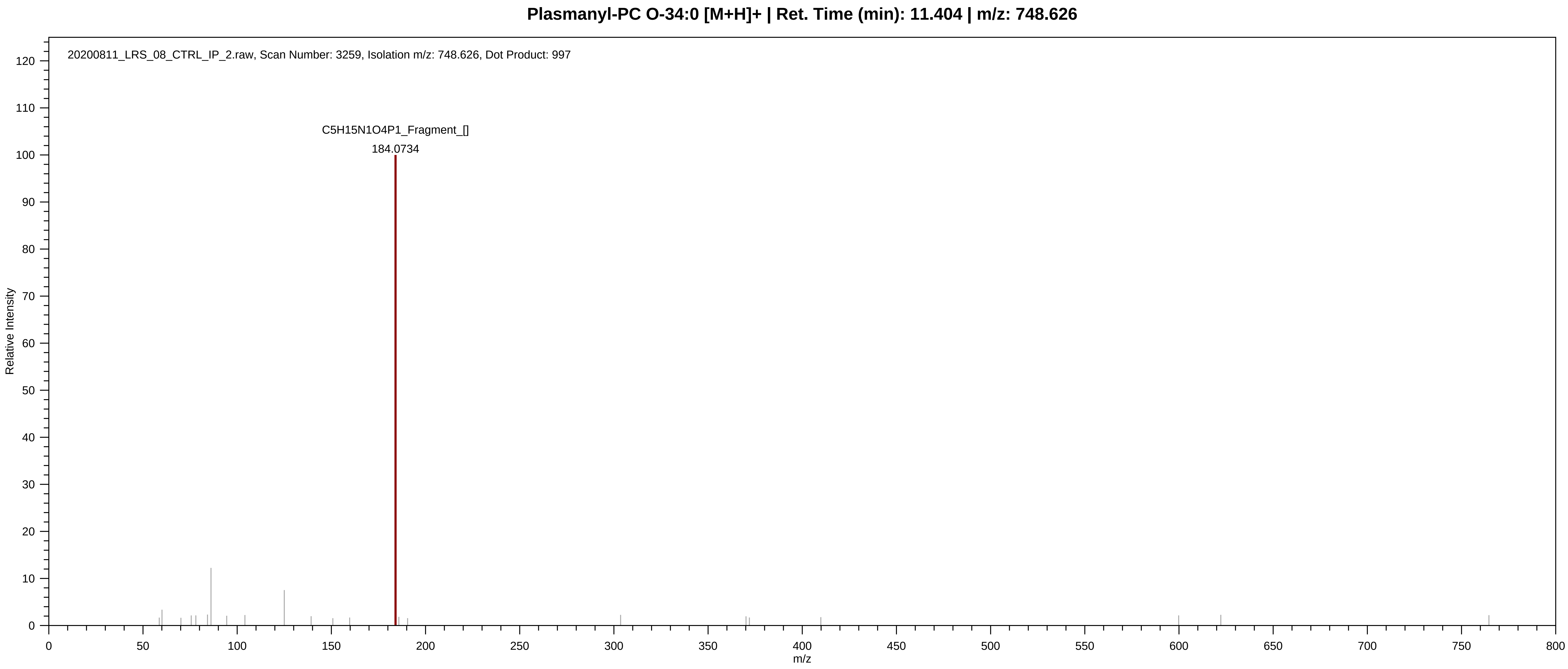
Plasmanyl-PC O-34:0 [M+H]+ | Ret. Time (min): 11.404 | m/z: 748.626
20200811_LRS_08_CTRL_IP_2.raw, Scan Number: 3259, Isolation m/z: 748.626, Dot Product: 997
C5H15N1O4P1_Fragment_[]
184.0734
Relative Intensity
m/z

Plasmanyl-PC O-34:0 [M+H]+ | Ret. Time (min): 11.404 | m/z: 748.622

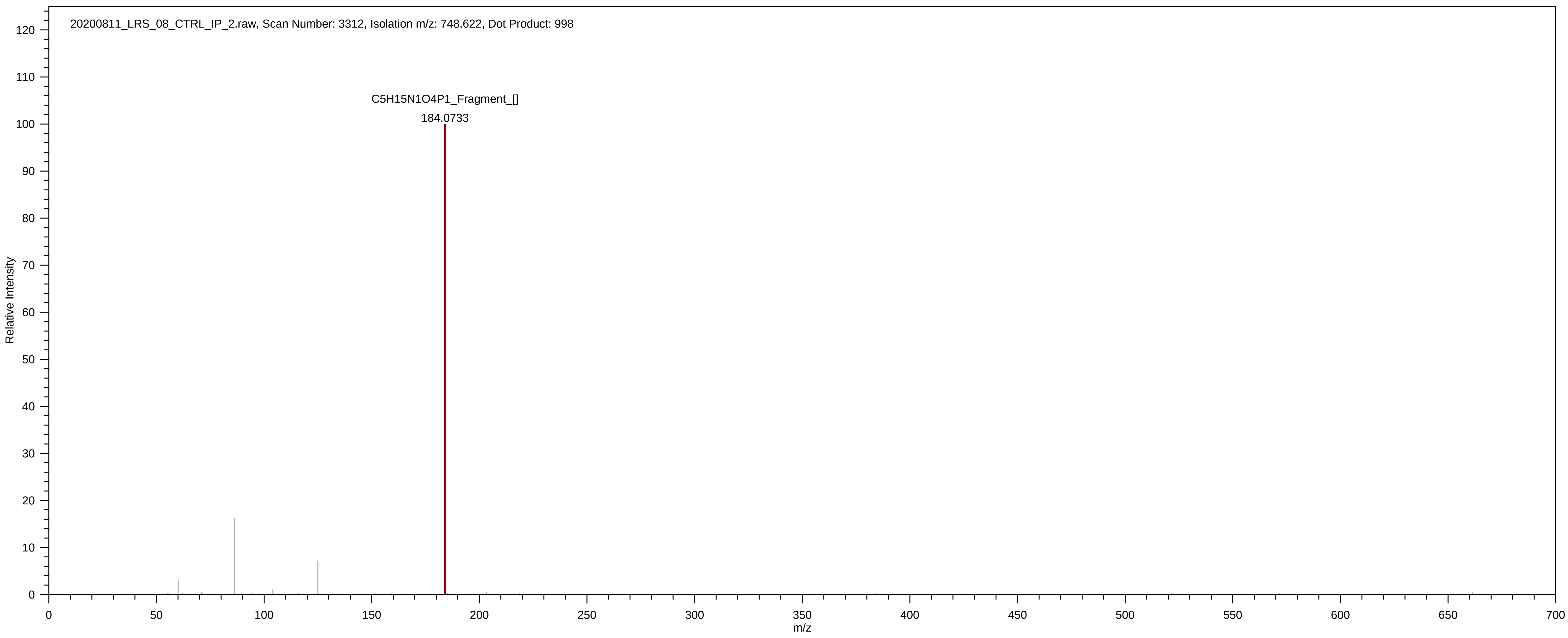

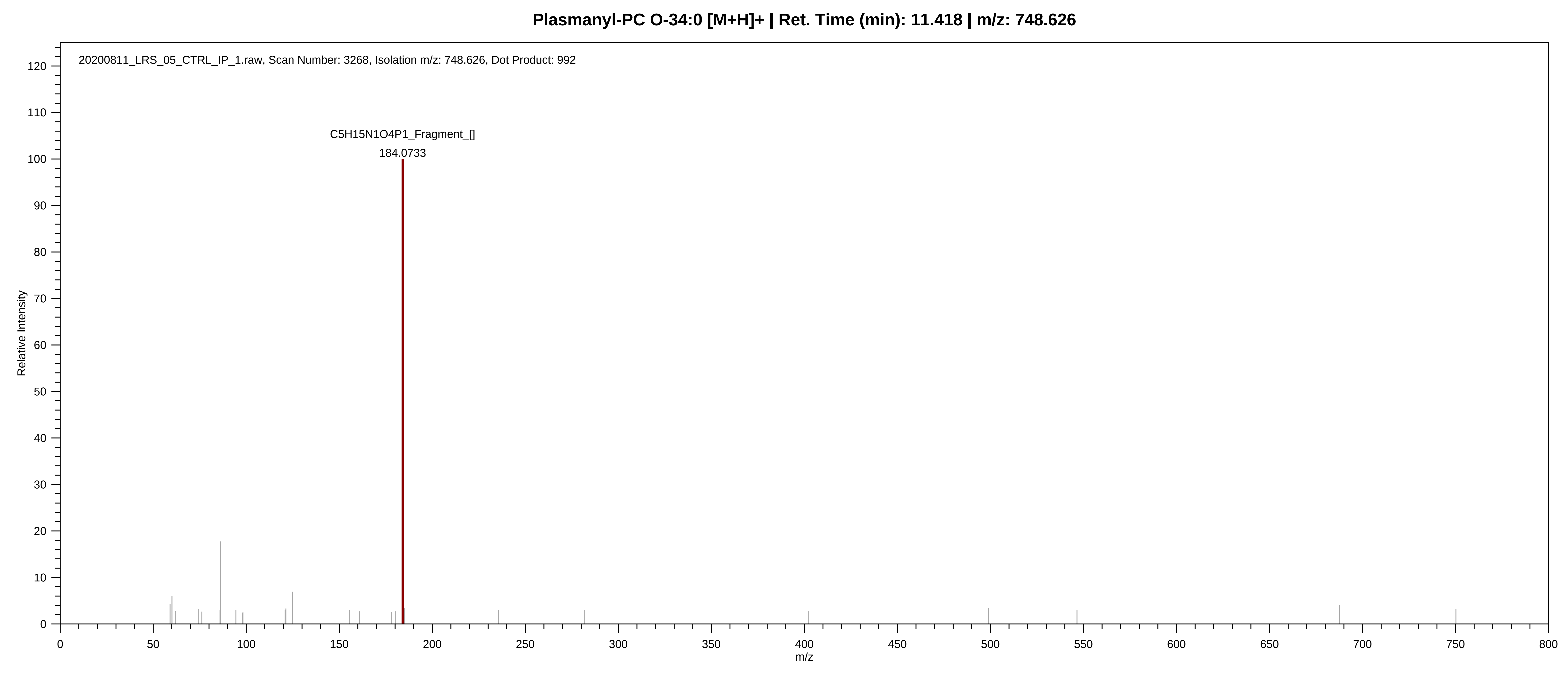
Plasmanyl-PC O-34:0 [M+H]+ | Ret. Time (min): 11.418 | m/z: 748.626
20200811_LRS_05_CTRL_IP_1.raw, Scan Number: 3268, Isolation m/z: 748.626, Dot Product: 992
C5H15N1O4P1_Fragment_[]
184.0733
Relative Intensity
m/z

Plasmanyl-PC O-34:0 [M+H]+ | Ret. Time (min): 11.418 | m/z: 748.622

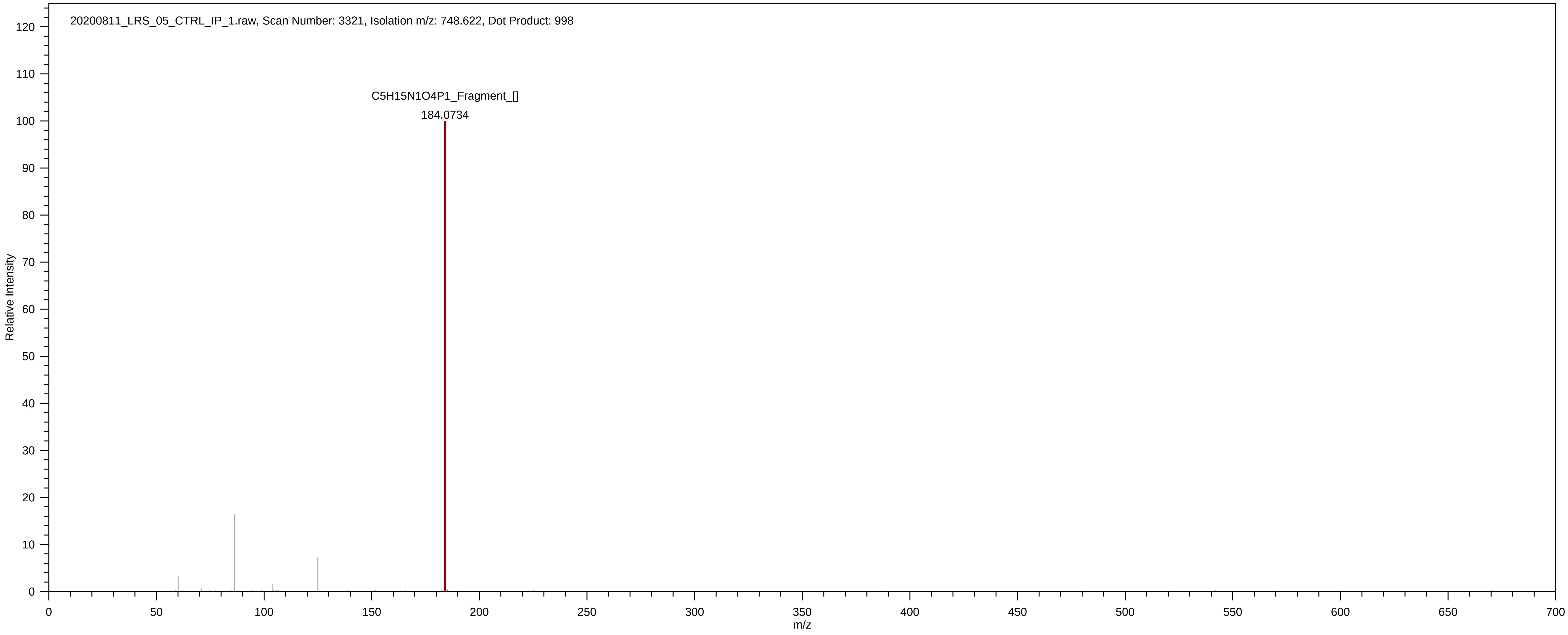

Plasmanyl-PC O-34:0 [M+H]+ | Ret. Time (min): 11.429 | m/z: 748.622

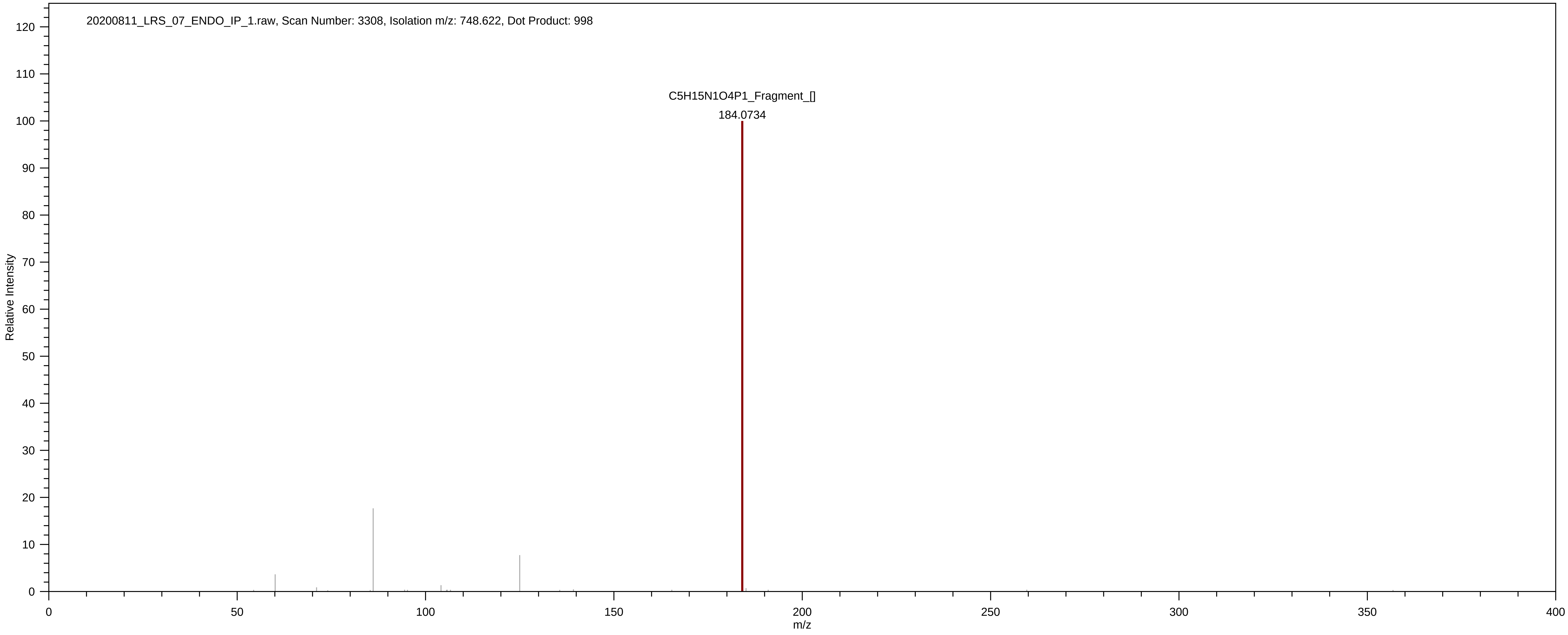

Plasmanyl-PC O-34:0 [M+H]+ | Ret. Time (min): 11.449 | m/z: 748.622

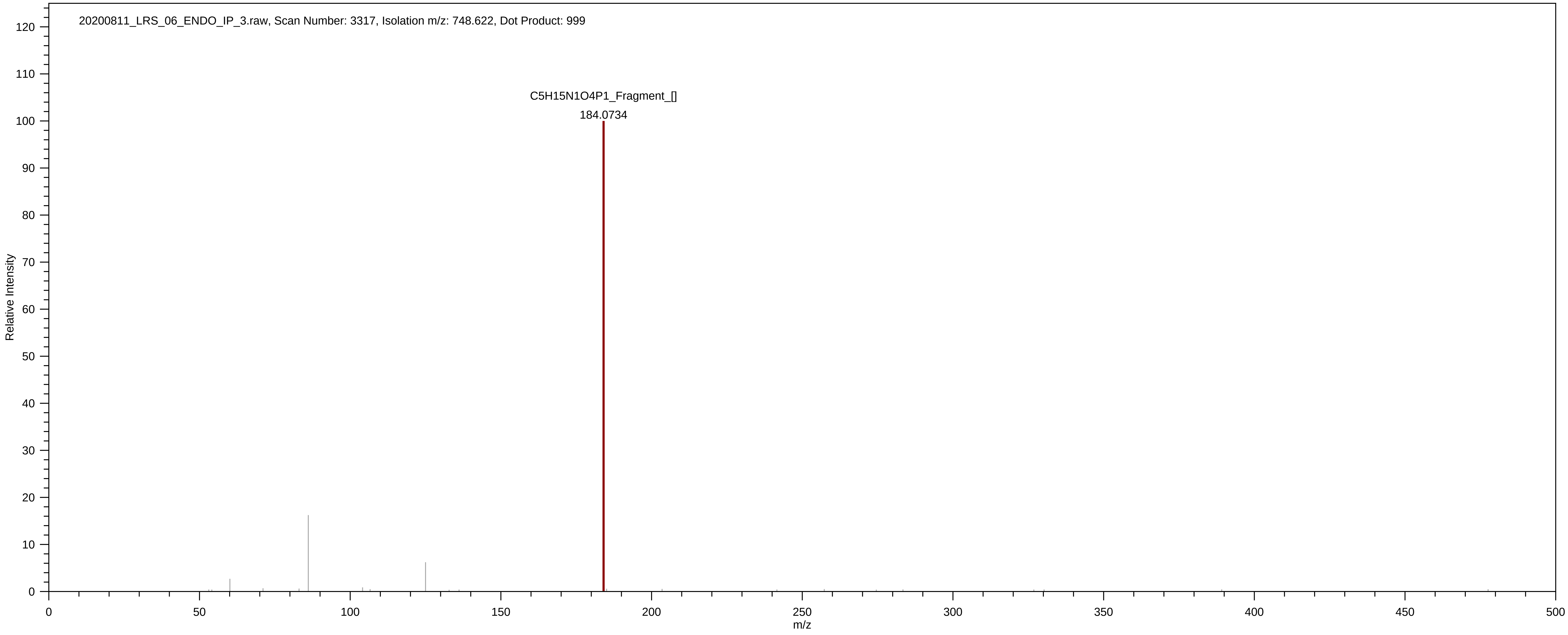

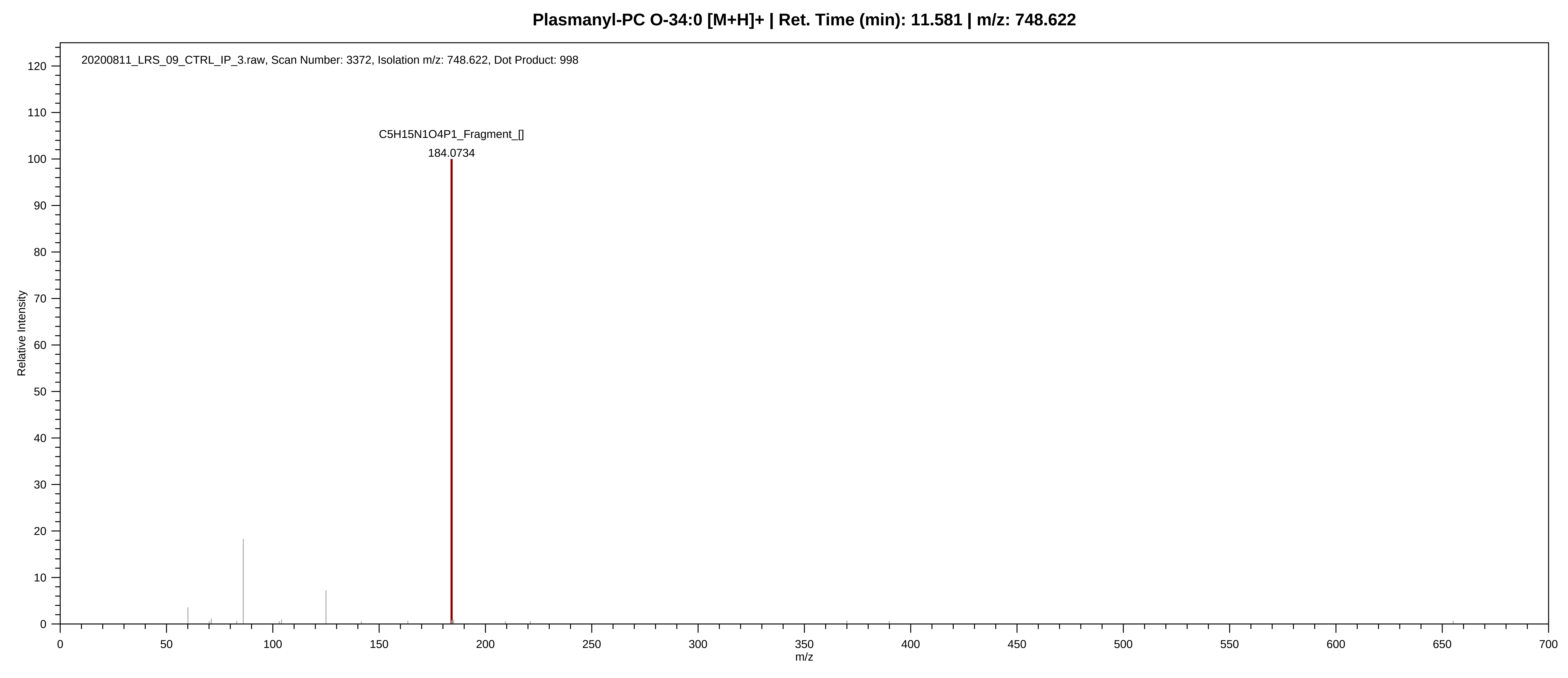
Plasmanyl-PC O-34:0 [M+H]+ | Ret. Time (min): 11.581 | m/z: 748.622
20200811_LRS_09_CTRL_IP_3.raw, Scan Number: 3372, Isolation m/z: 748.622, Dot Product: 998
C5H15N1O4P1_Fragment_[]
184.0734
Relative Intensity
m/z

Plasmanyl-PC O-34:0 [M+H]+ | Ret. Time (min): 11.584 | m/z: 748.622

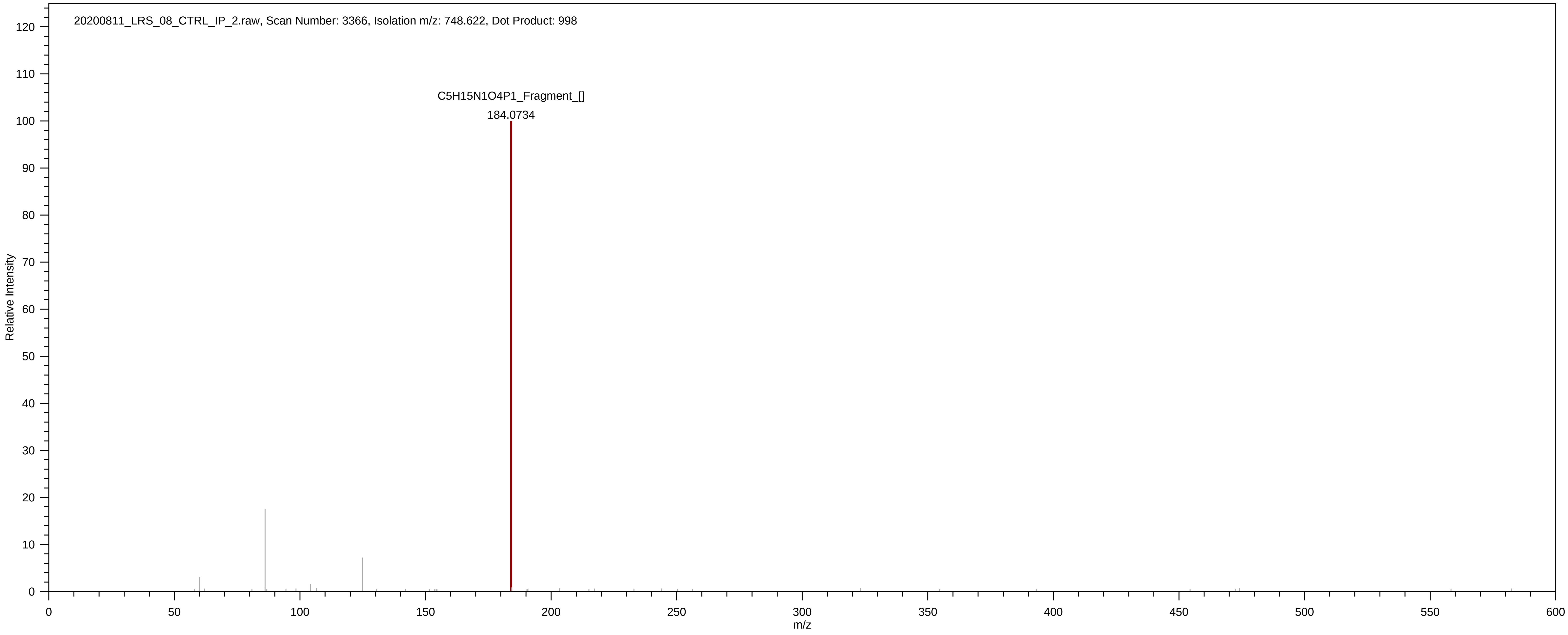

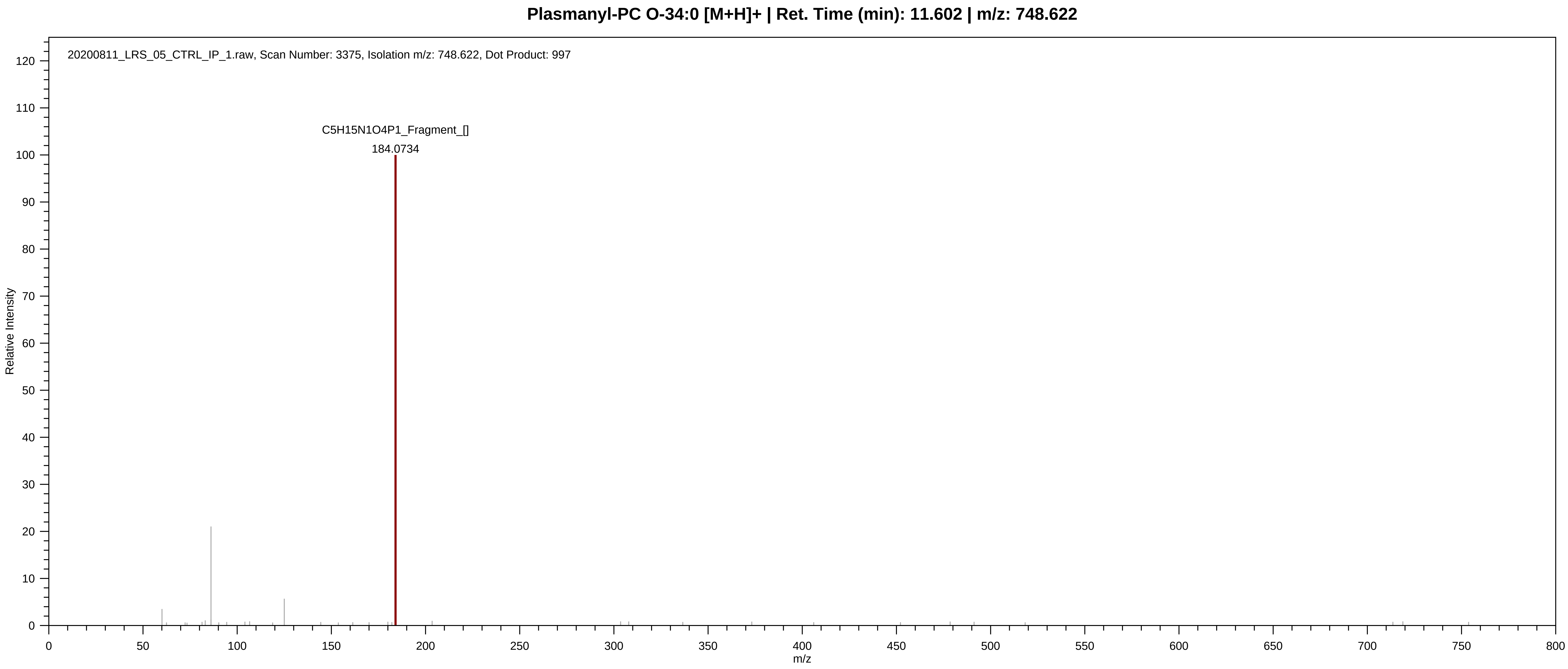
Plasmanyl-PC O-34:0 [M+H]+ | Ret. Time (min): 11.602 | m/z: 748.622
20200811_LRS_05_CTRL_IP_1.raw, Scan Number: 3375, Isolation m/z: 748.622, Dot Product: 997
C5H15N1O4P1_Fragment_[]
184.0734
Relative Intensity
m/z

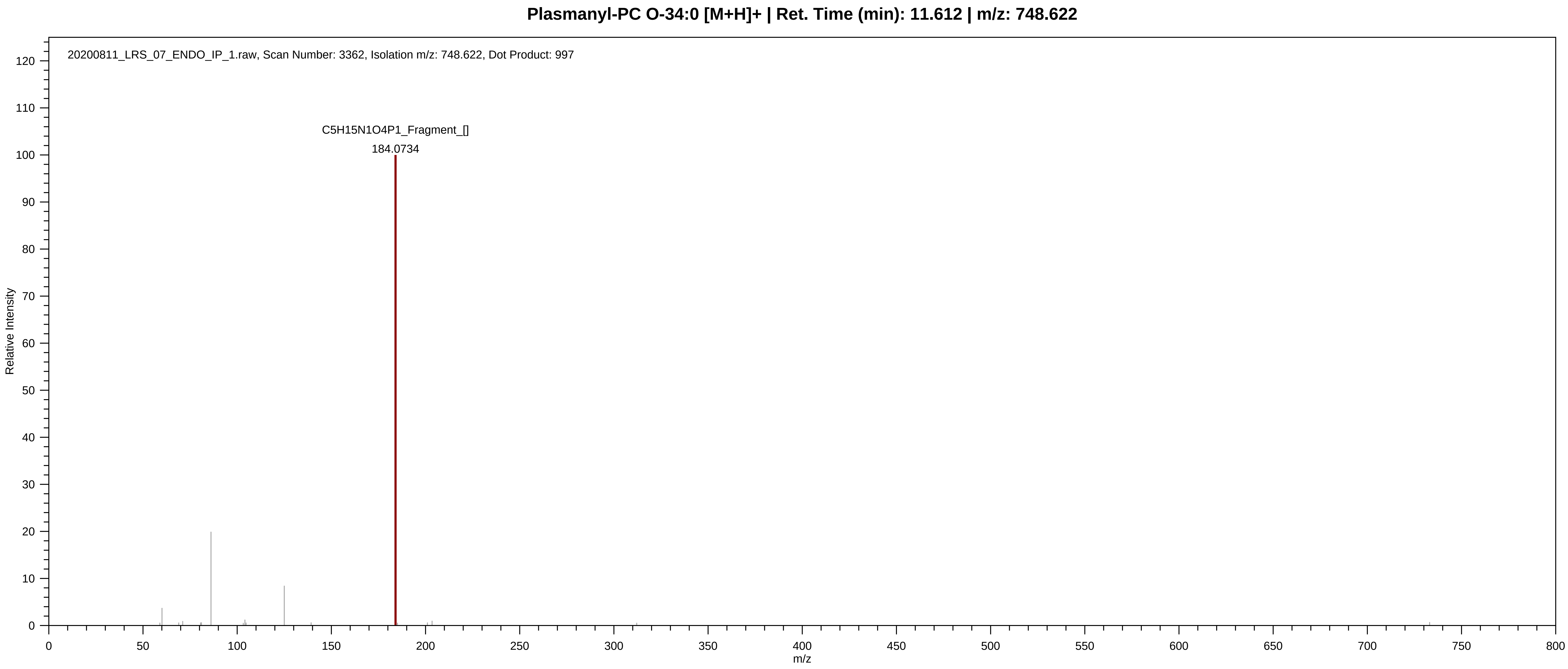
Plasmanyl-PC O-34:0 [M+H]+ | Ret. Time (min): 11.612 | m/z: 748.622
20200811_LRS_07_ENDO_IP_1.raw, Scan Number: 3362, Isolation m/z: 748.622, Dot Product: 997
C5H15N1O4P1_Fragment_[]
184.0734
Relative Intensity
m/z
0
10
20
30
40
50
60
70
80
90
100
110
120
0
50
100
150
200
250
300
350
400
450
500
550
600
650
700
750
800

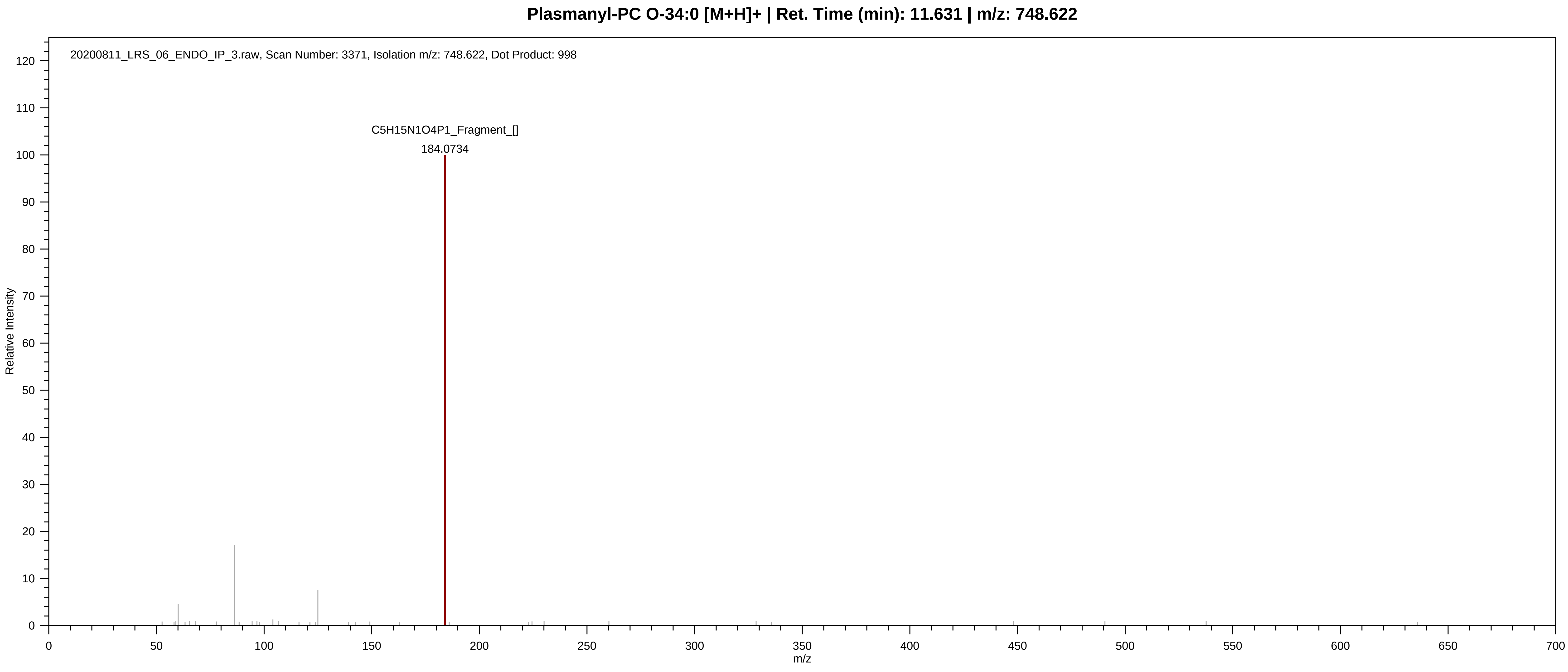
Plasmanyl-PC O-34:0 [M+H]+ | Ret. Time (min): 11.631 | m/z: 748.622
20200811_LRS_06_ENDO_IP_3.raw, Scan Number: 3371, Isolation m/z: 748.622, Dot Product: 998
C5H15N1O4P1_Fragment_[]
184.0734
Relative Intensity
m/z

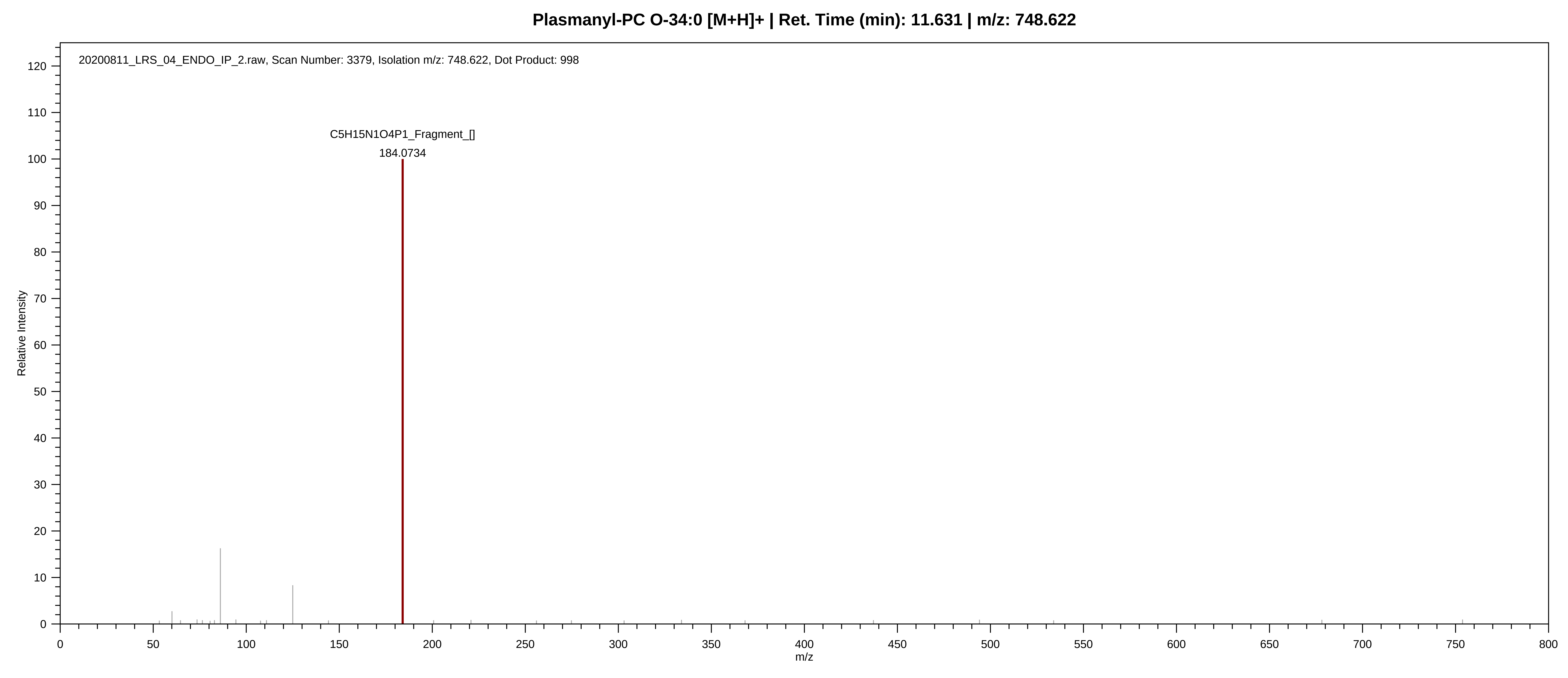

Plasmanyl-PC O-34:0 [M+H]+ | Ret. Time (min): 11.631 | m/z: 748.622
20200811_LRS_04_ENDO_IP_2.raw, Scan Number: 3379, Isolation m/z: 748.622, Dot Product: 998
C5H15N1O4P1_Fragment_[]
184.0734
Relative Intensity
m/z

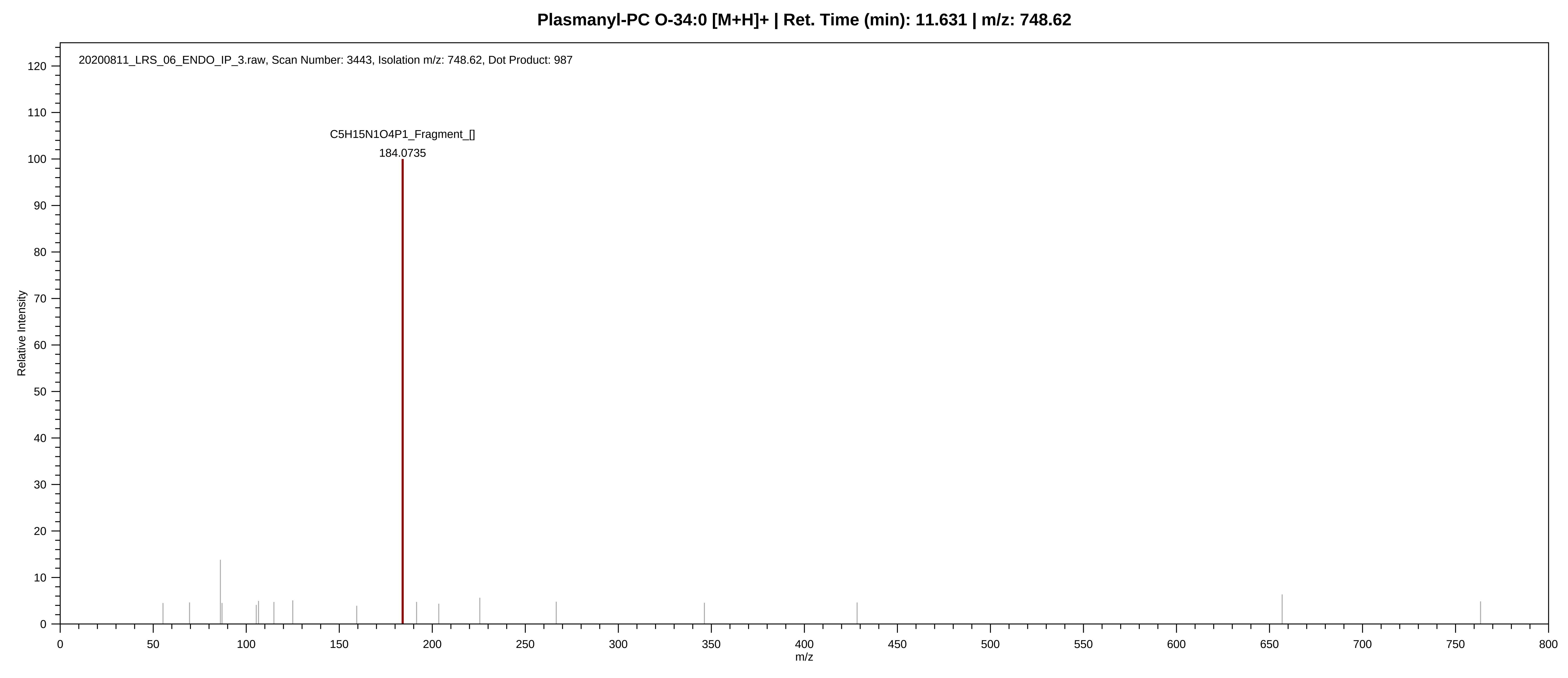

Plasmanyl-PC O-34:0 [M+H]+ | Ret. Time (min): 11.631 | m/z: 748.62
20200811_LRS_06_ENDO_IP_3.raw, Scan Number: 3443, Isolation m/z: 748.62, Dot Product: 987
C5H15N1O4P1_Fragment_[]
184.0735
Relative Intensity
m/z

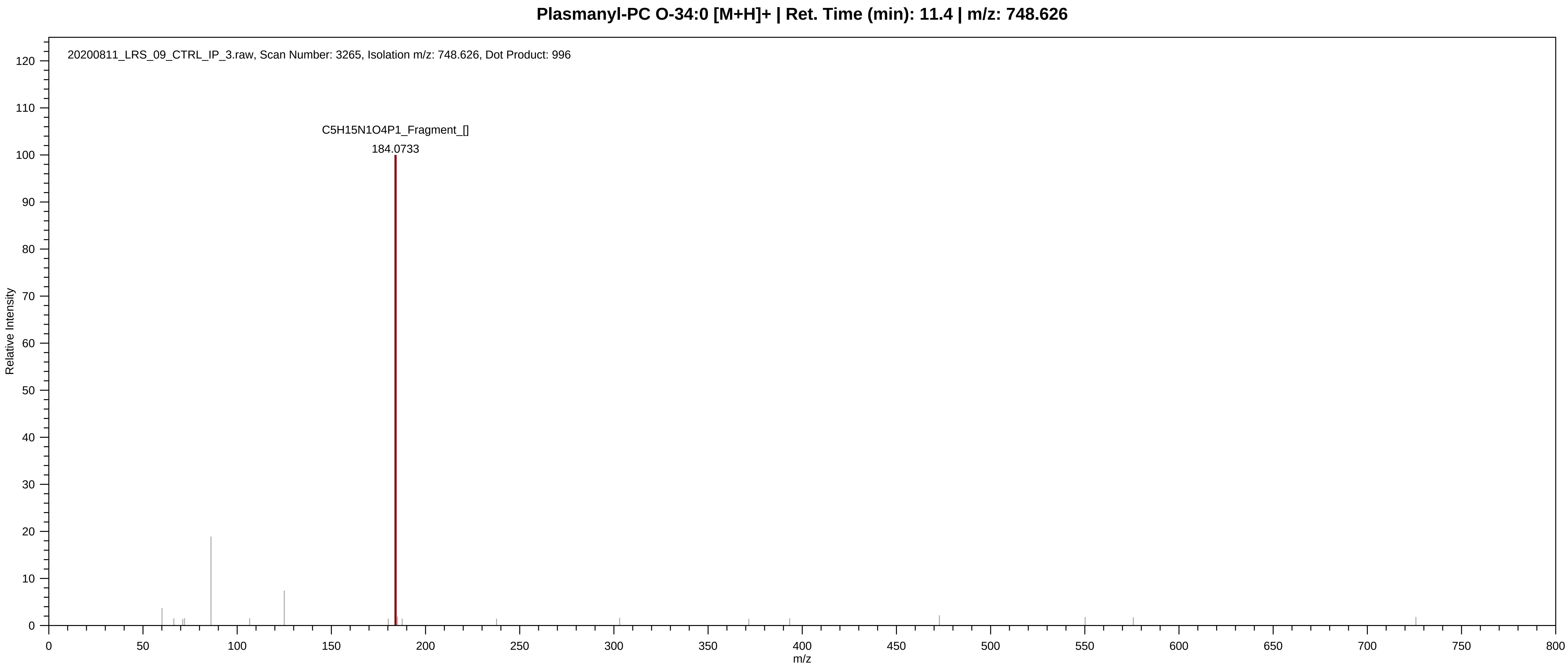
Plasmanyl-PC O-34:0 [M+H]+ | Ret. Time (min): 11.4 | m/z: 748.626
20200811_LRS_09_CTRL_IP_3.raw, Scan Number: 3265, Isolation m/z: 748.626, Dot Product: 996
C5H15N1O4P1_Fragment_[]
184.0733
Relative Intensity
m/z
0
10
20
30
40
50
60
70
80
90
100
110
120
0
50
100
150
200
250
300
350
400
450
500
550
600
650
700
750
800

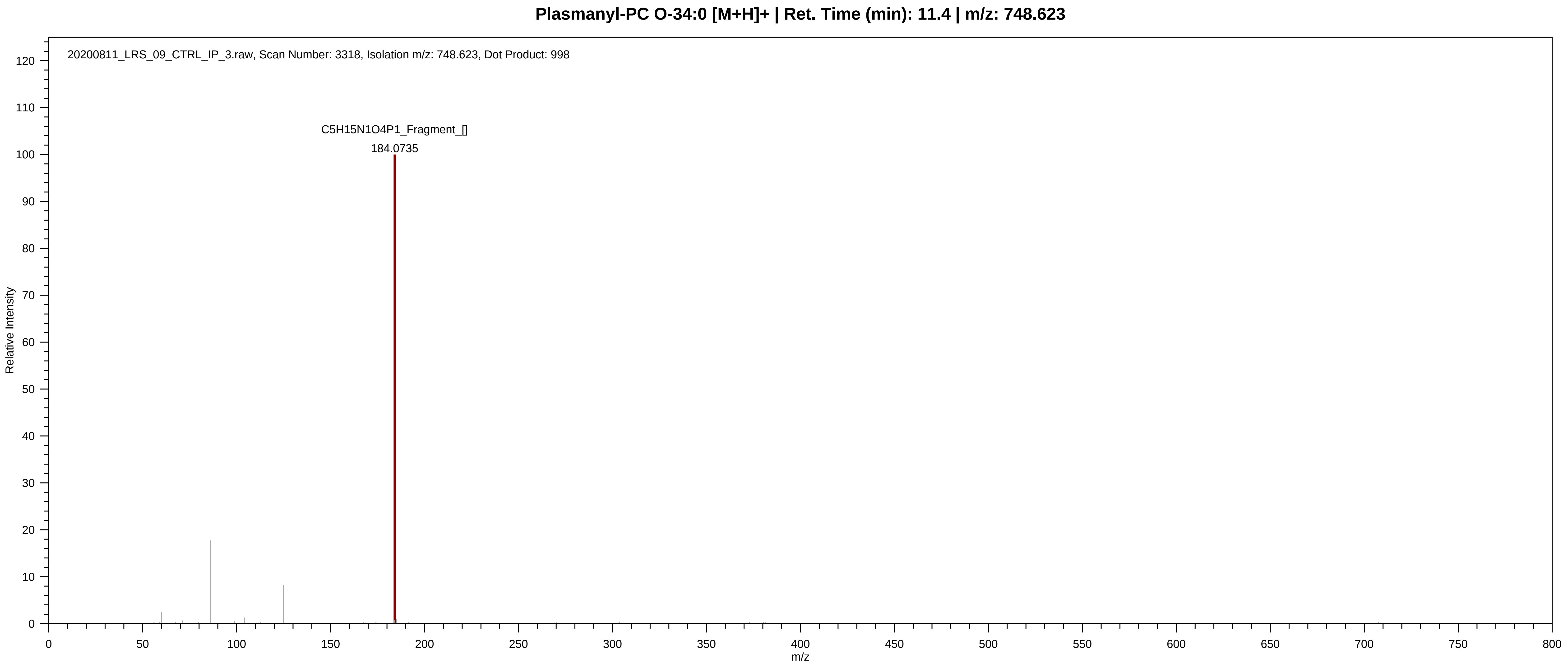
Plasmanyl-PC O-34:0 [M+H]+ | Ret. Time (min): 11.4 | m/z: 748.623
20200811_LRS_09_CTRL_IP_3.raw, Scan Number: 3318, Isolation m/z: 748.623, Dot Product: 998
C5H15N1O4P1_Fragment_[]
184.0735
Relative Intensity
m/z
0
10
20
30
40
50
60
70
80
90
100
110
120
0
50
100
150
200
250
300
350
400
450
500
550
600
650
700
750
800

Plasmanyl-PC O-34:1 [M+Ac-H]- | Ret. Time (min): 10.64 | m/z: 804.613

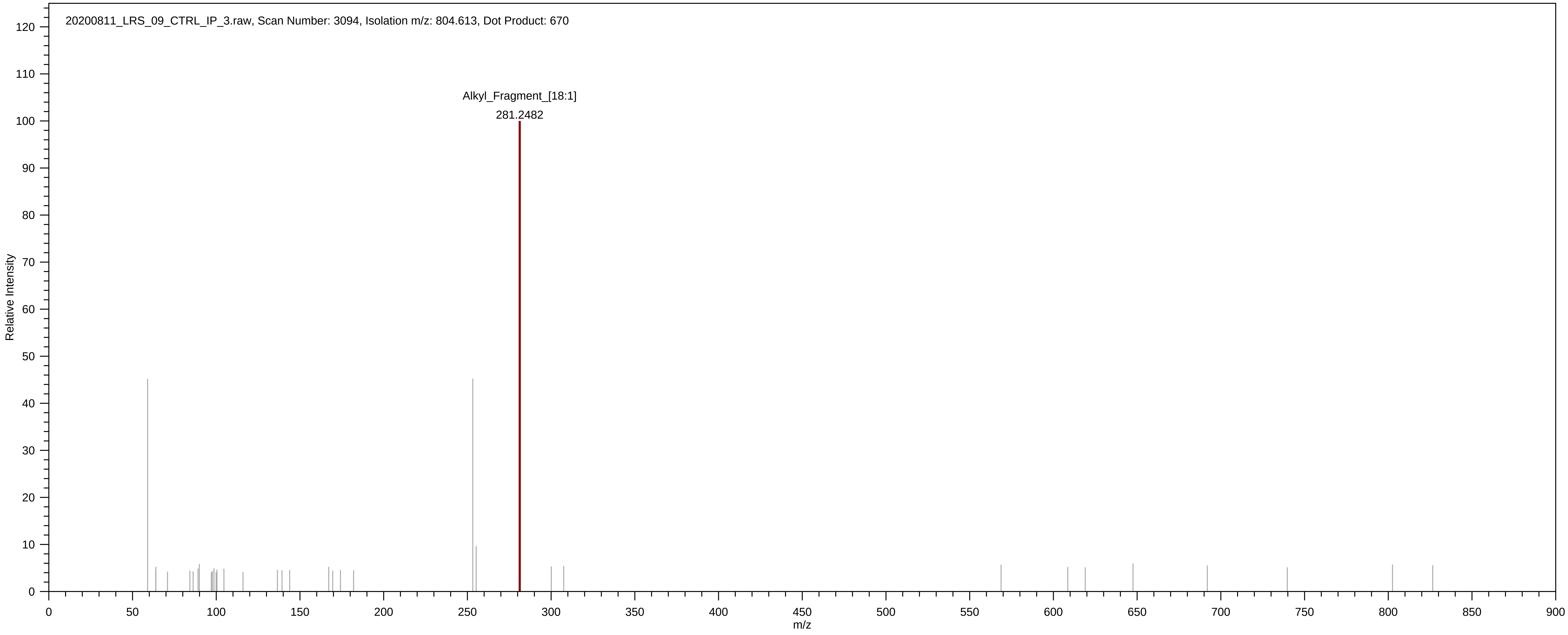

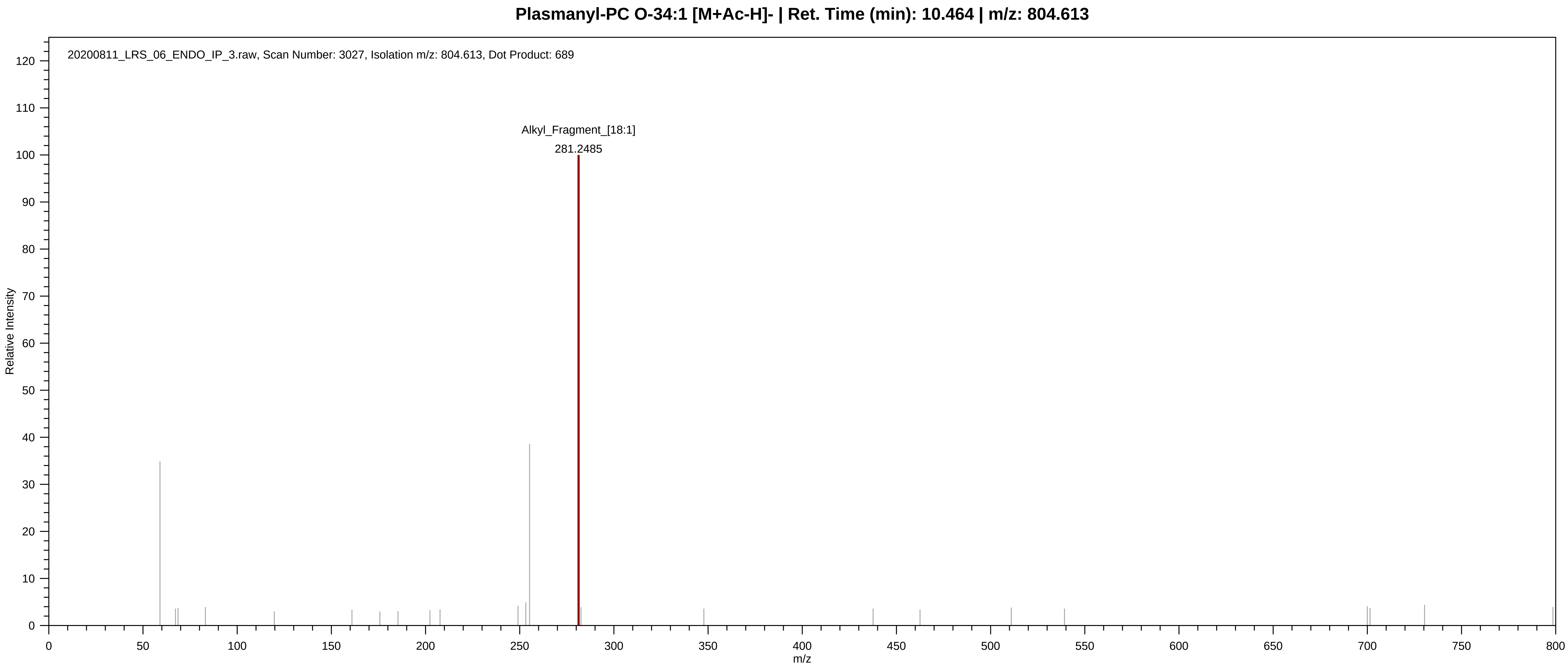

Plasmanyl-PC O-34:1 [M+Ac-H]- | Ret. Time (min): 10.464 | m/z: 804.613
20200811_LRS_06_ENDO_IP_3.raw, Scan Number: 3027, Isolation m/z: 804.613, Dot Product: 689
Alkyl_Fragment_[18:1]
281.2485
Relative Intensity
m/z

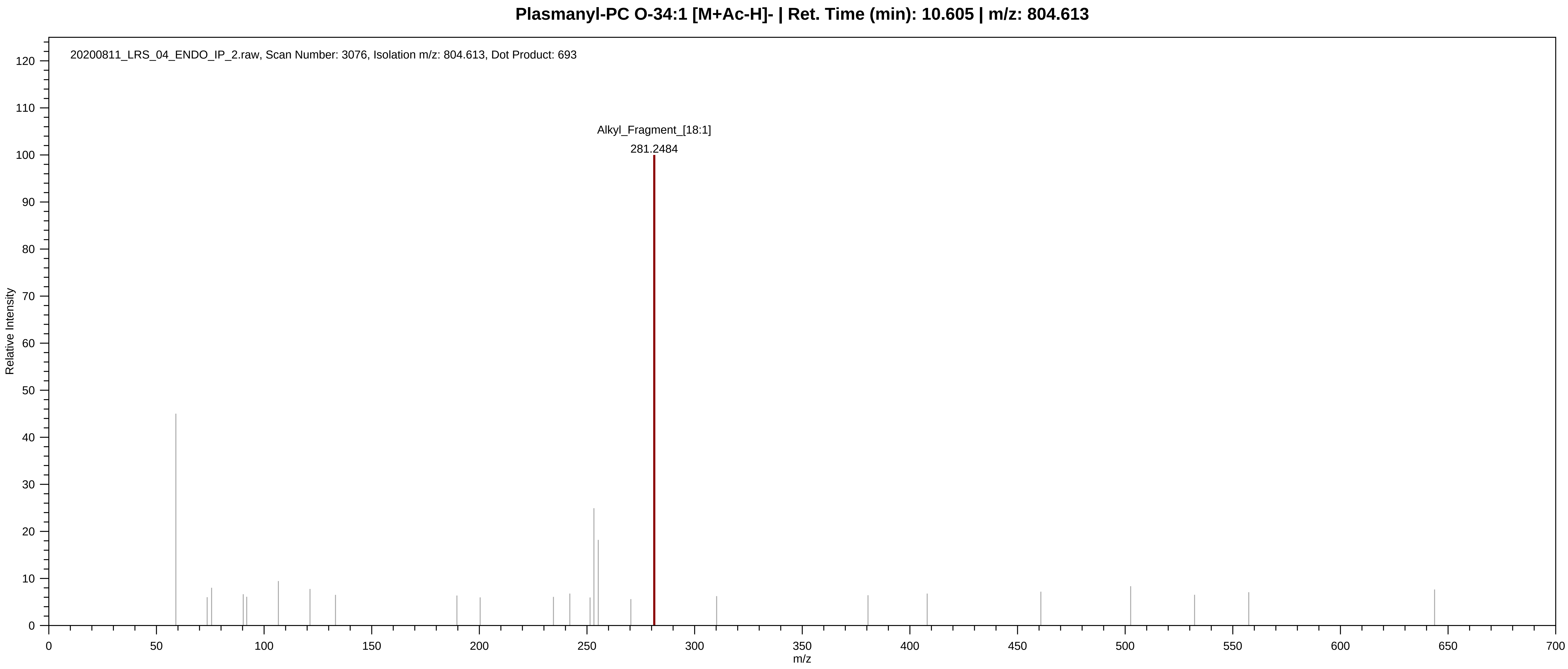
Plasmanyl-PC O-34:1 [M+Ac-H]- | Ret. Time (min): 10.605 | m/z: 804.613
20200811_LRS_04_ENDO_IP_2.raw, Scan Number: 3076, Isolation m/z: 804.613, Dot Product: 693
Alkyl_Fragment_[18:1]
281.2484
Relative Intensity
m/z
0
10
20
30
40
50
60
70
80
90
100
110
120
0
50
100
150
200
250
300
350
400
450
500
550
600
650
700

Plasmanyl-PC O-34:1 [M+Ac-H]- | Ret. Time (min): 10.642 | m/z: 804.613

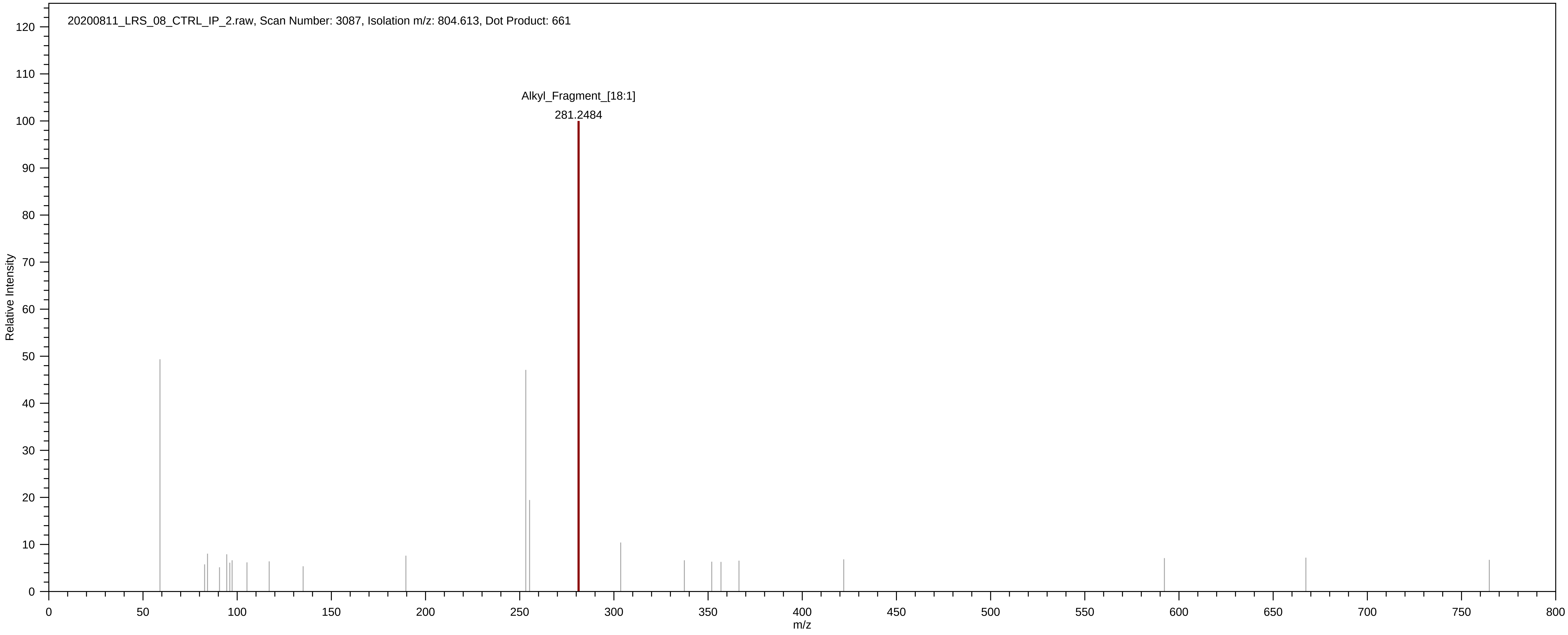

Plasmanyl-PC O-34:1 [M+Ac-H]- | Ret. Time (min): 10.659 | m/z: 804.614

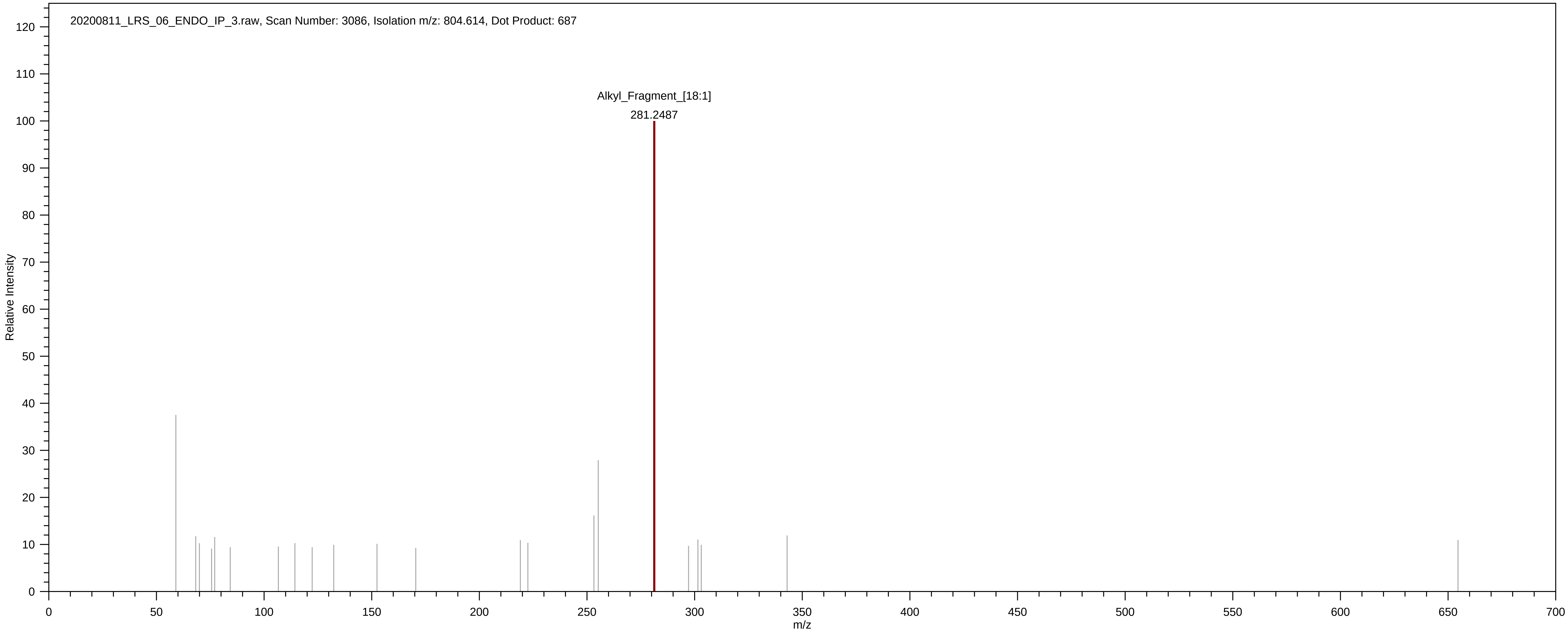

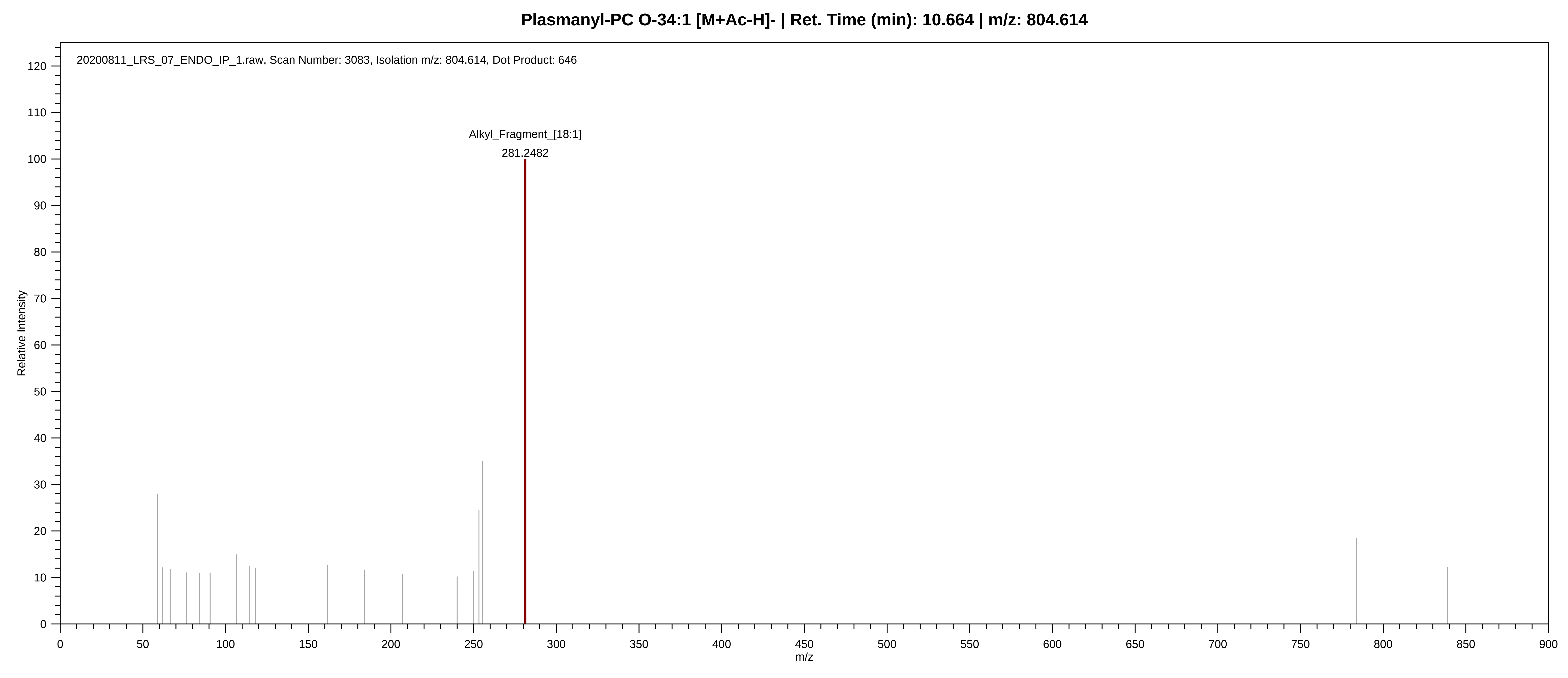

Plasmanyl-PC O-34:1 [M+Ac-H]- | Ret. Time (min): 10.664 | m/z: 804.614
20200811_LRS_07_ENDO_IP_1.raw, Scan Number: 3083, Isolation m/z: 804.614, Dot Product: 646
Alkyl_Fragment_[18:1]
281.2482
Relative Intensity
m/z

Plasmanyl-PC O-34:1 [M+Ac-H]- | Ret. Time (min): 10.671 | m/z: 804.614

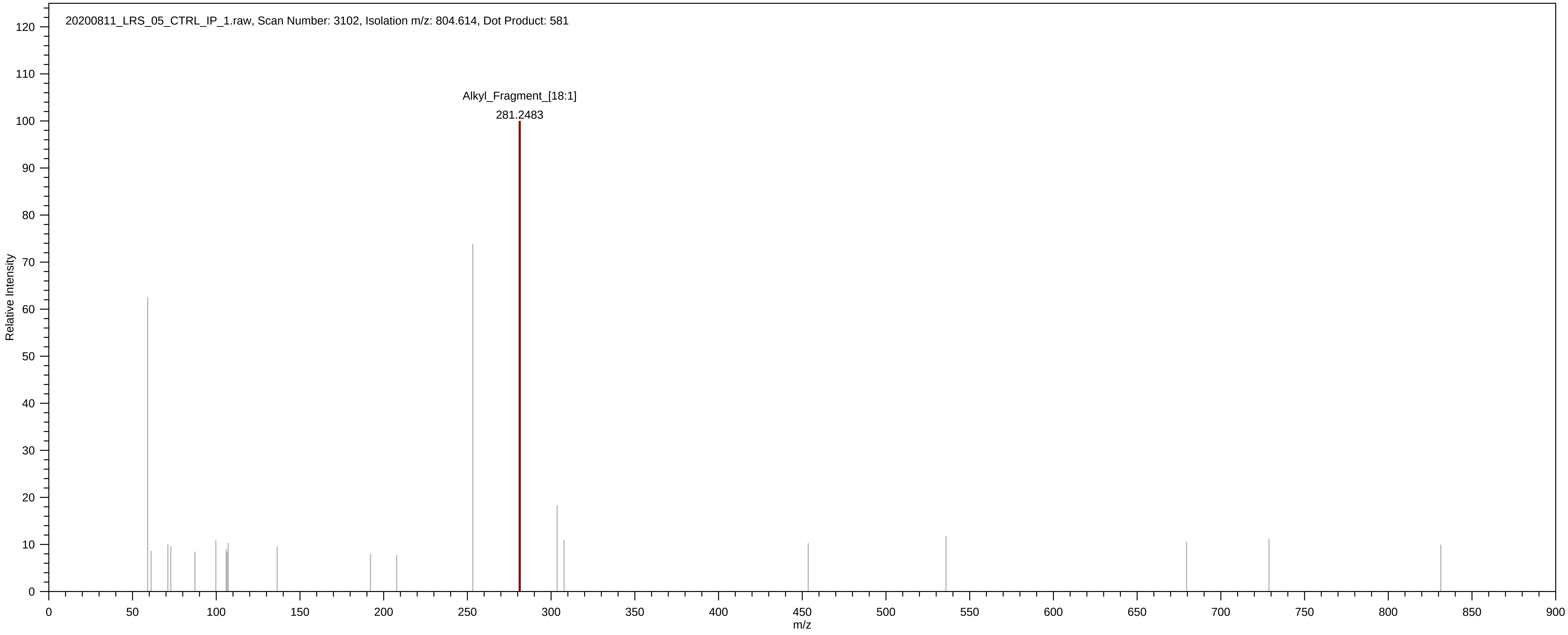

Plasmanyl-PC O-34:1 [M+H]+ | Ret. Time (min): 10.433 | m/z: 746.605

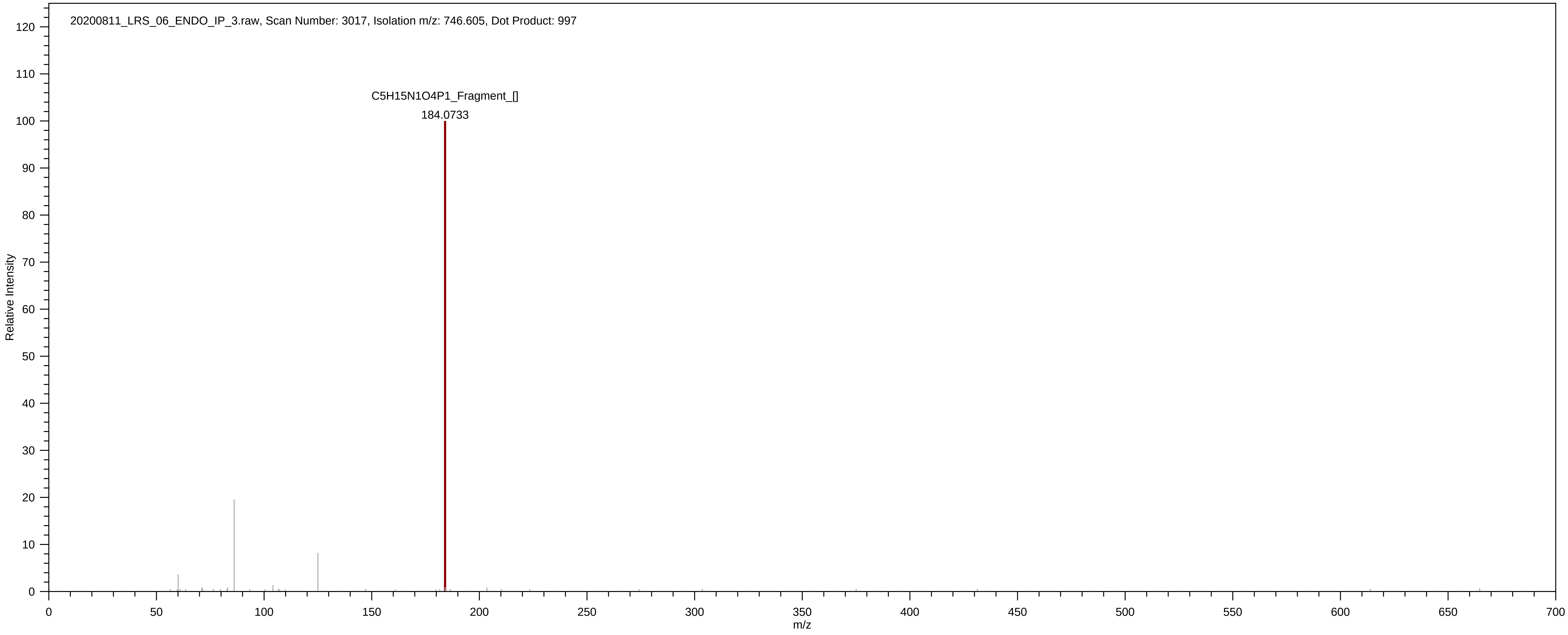

Plasmanyl-PC O-34:1 [M+H]+ | Ret. Time (min): 10.433 | m/z: 746.605

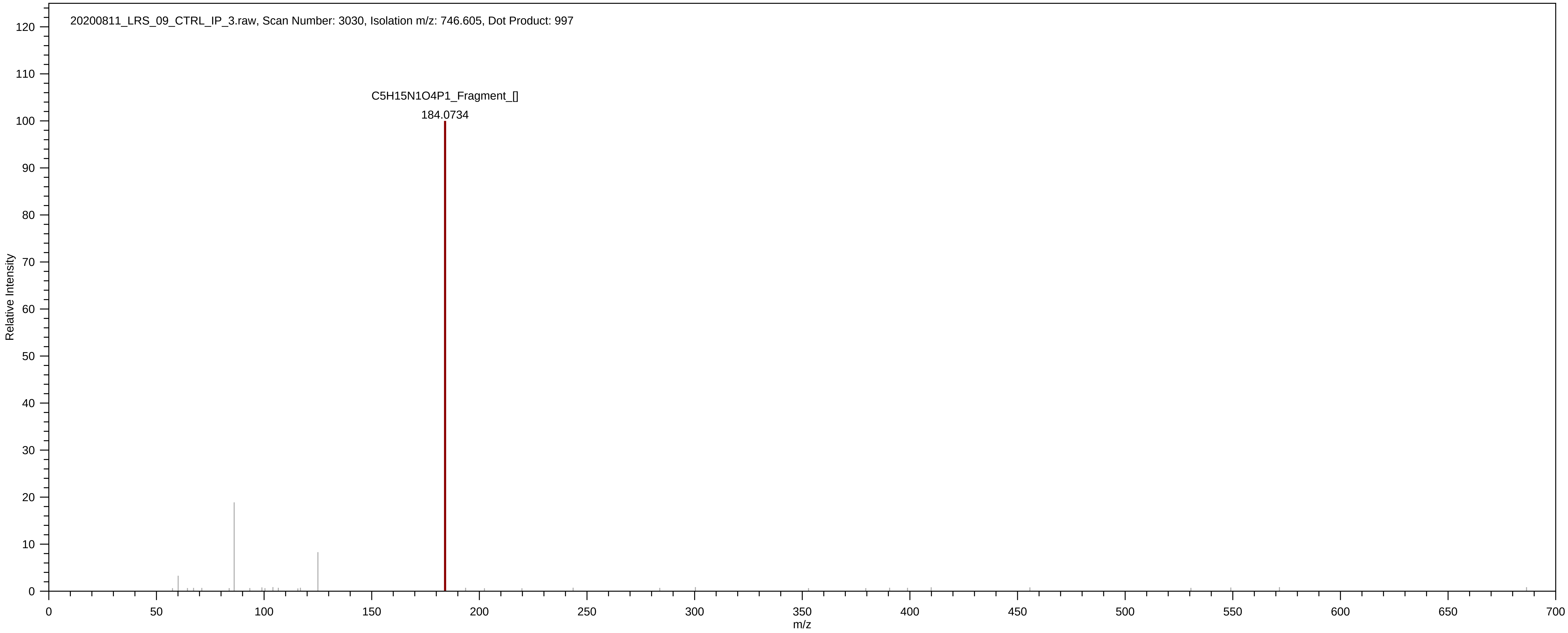

Plasmanyl-PC O-34:1 [M+H]+ | Ret. Time (min): 10.435 | m/z: 746.605

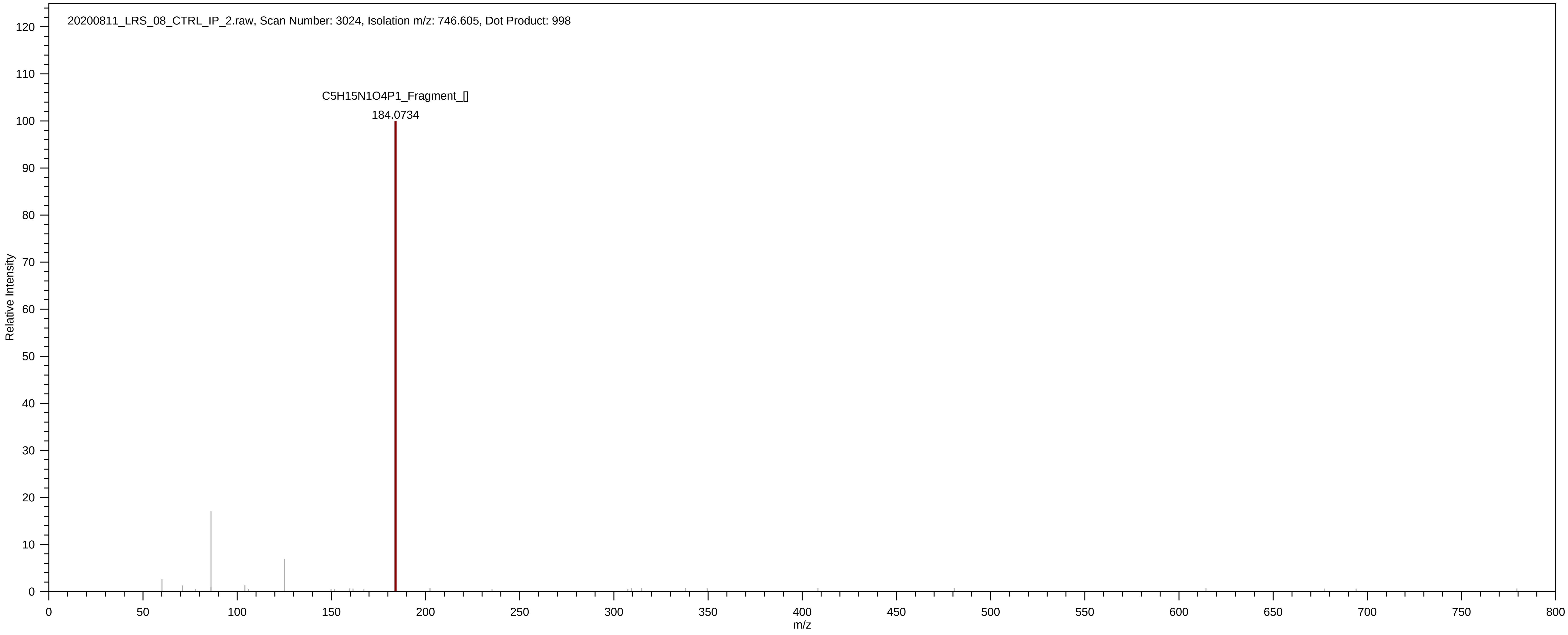

Plasmanyl-PC O-34:1 [M+H]+ | Ret. Time (min): 10.436 | m/z: 746.605

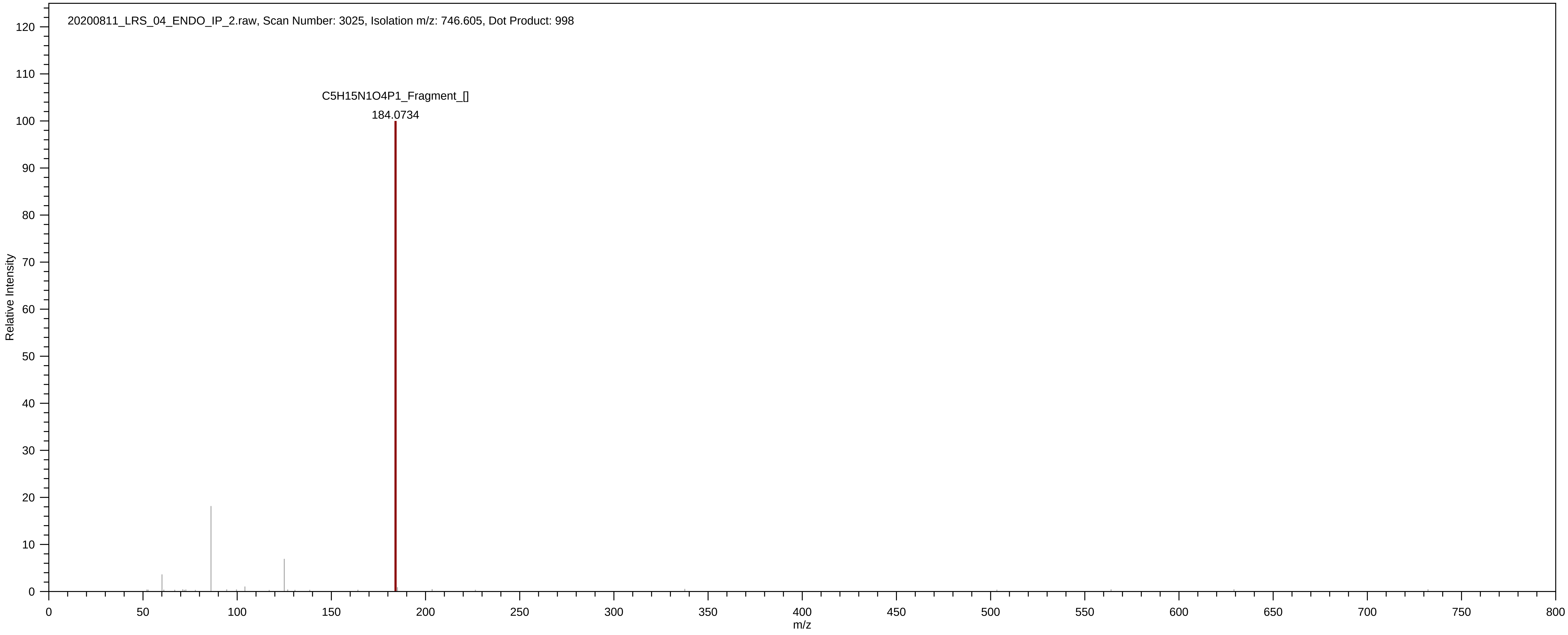

Plasmanyl-PC O-34:1 [M+H]+ | Ret. Time (min): 10.437 | m/z: 746.605

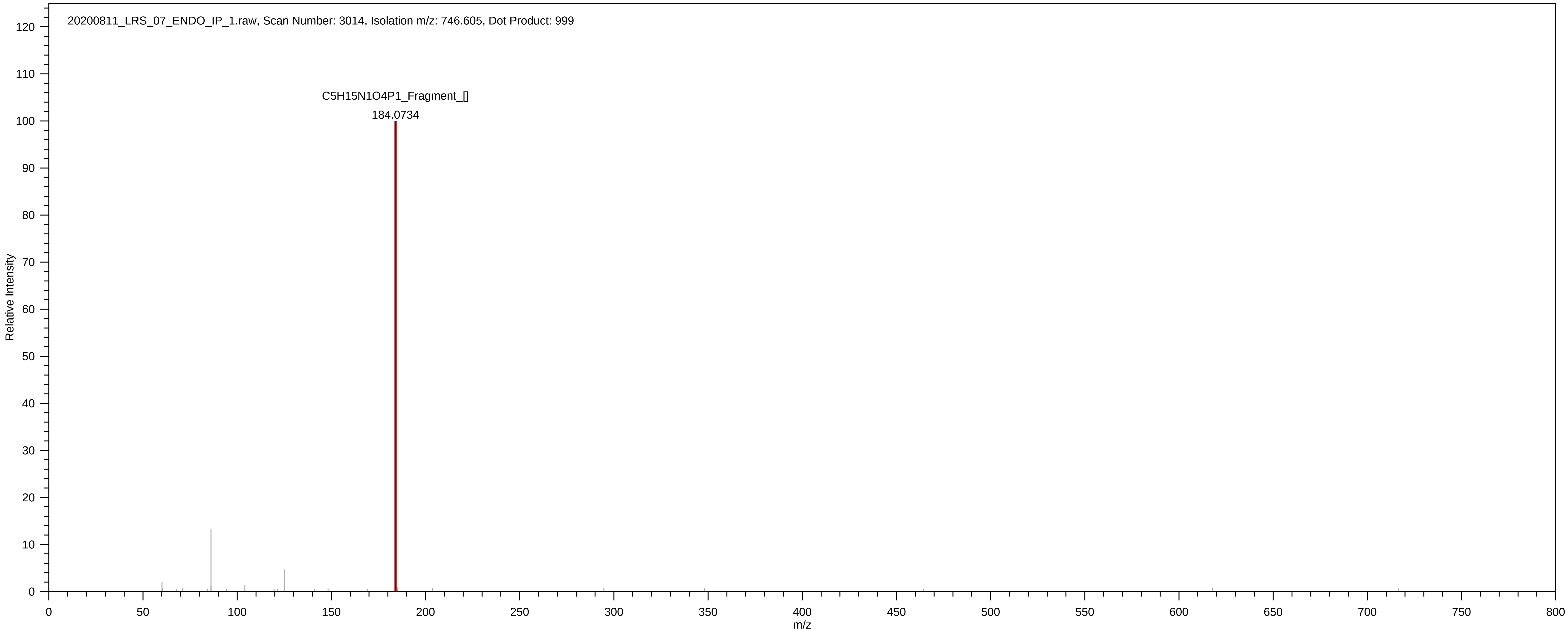

Plasmanyl-PC O-34:1 [M+H]+ | Ret. Time (min): 10.442 | m/z: 746.605

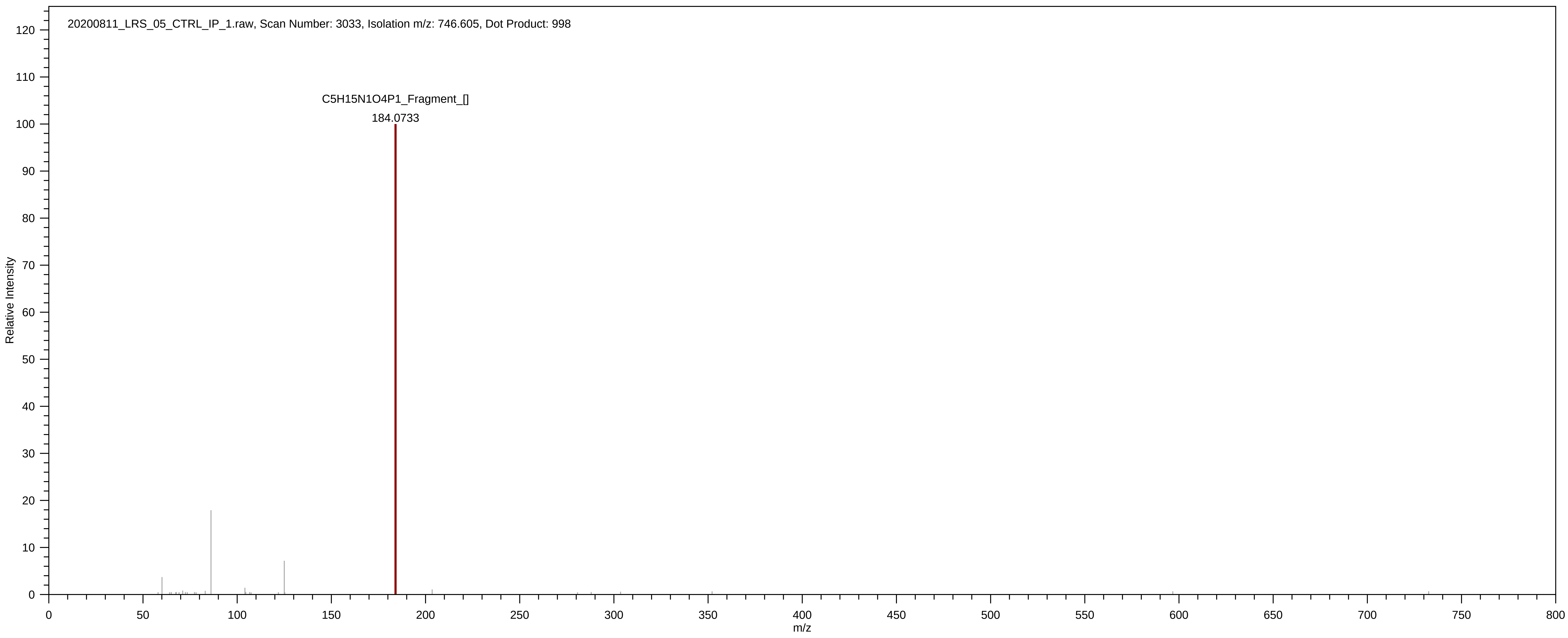

Plasmanyl-PC O-34:1 [M+H]+ | Ret. Time (min): 10.608 | m/z: 746.605

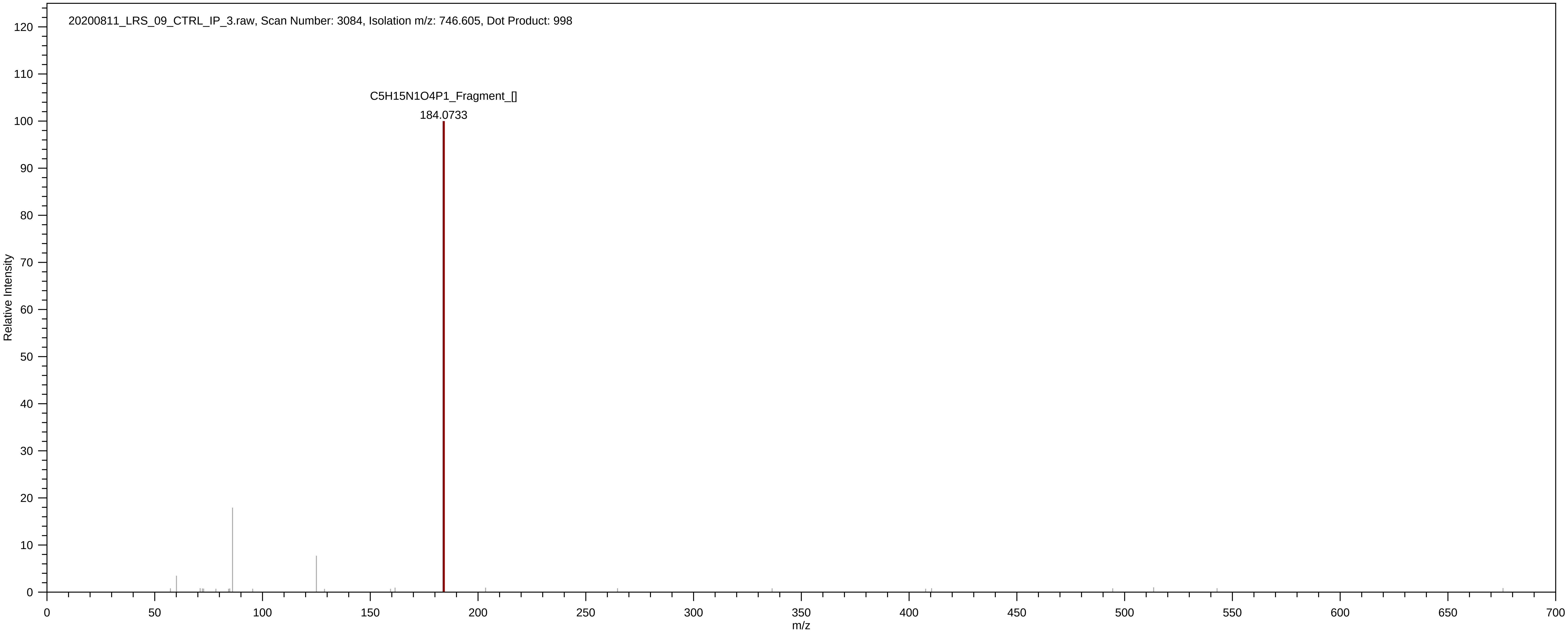

Plasmanyl-PC O-34:1 [M+H]+ | Ret. Time (min): 10.608 | m/z: 746.606

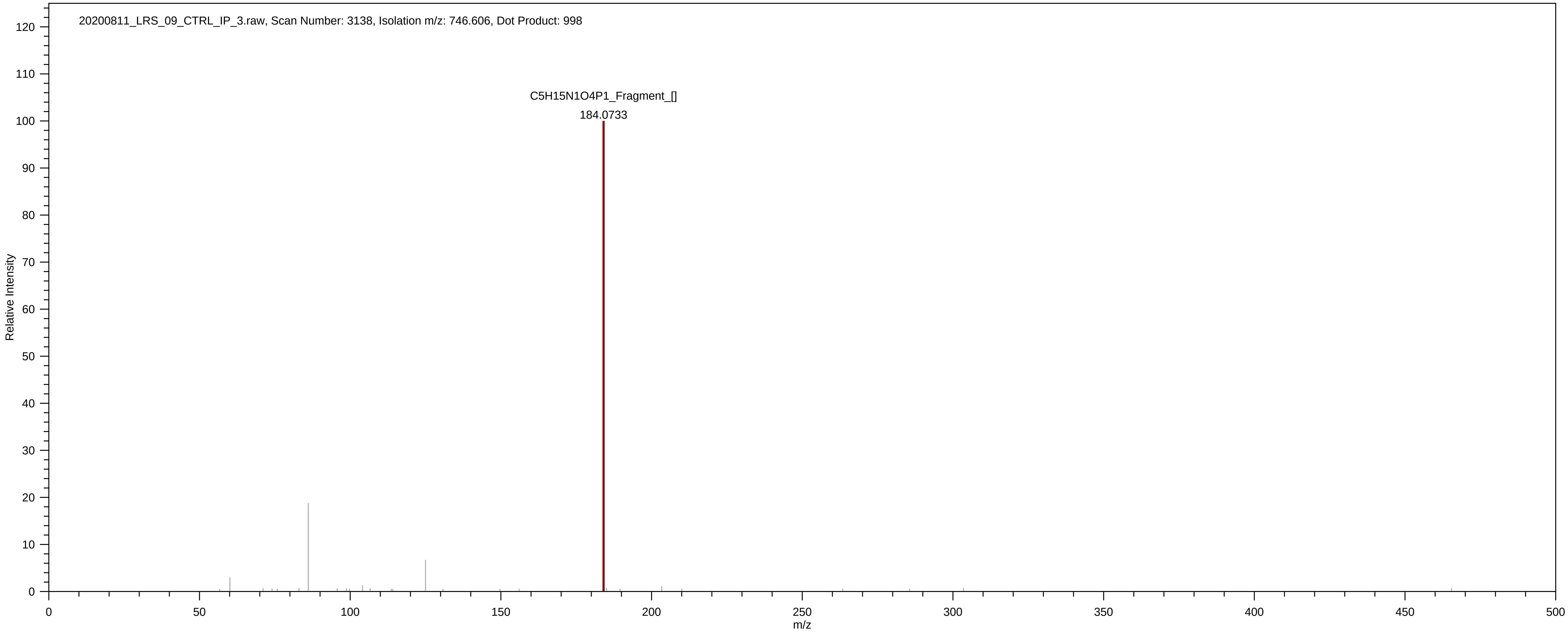

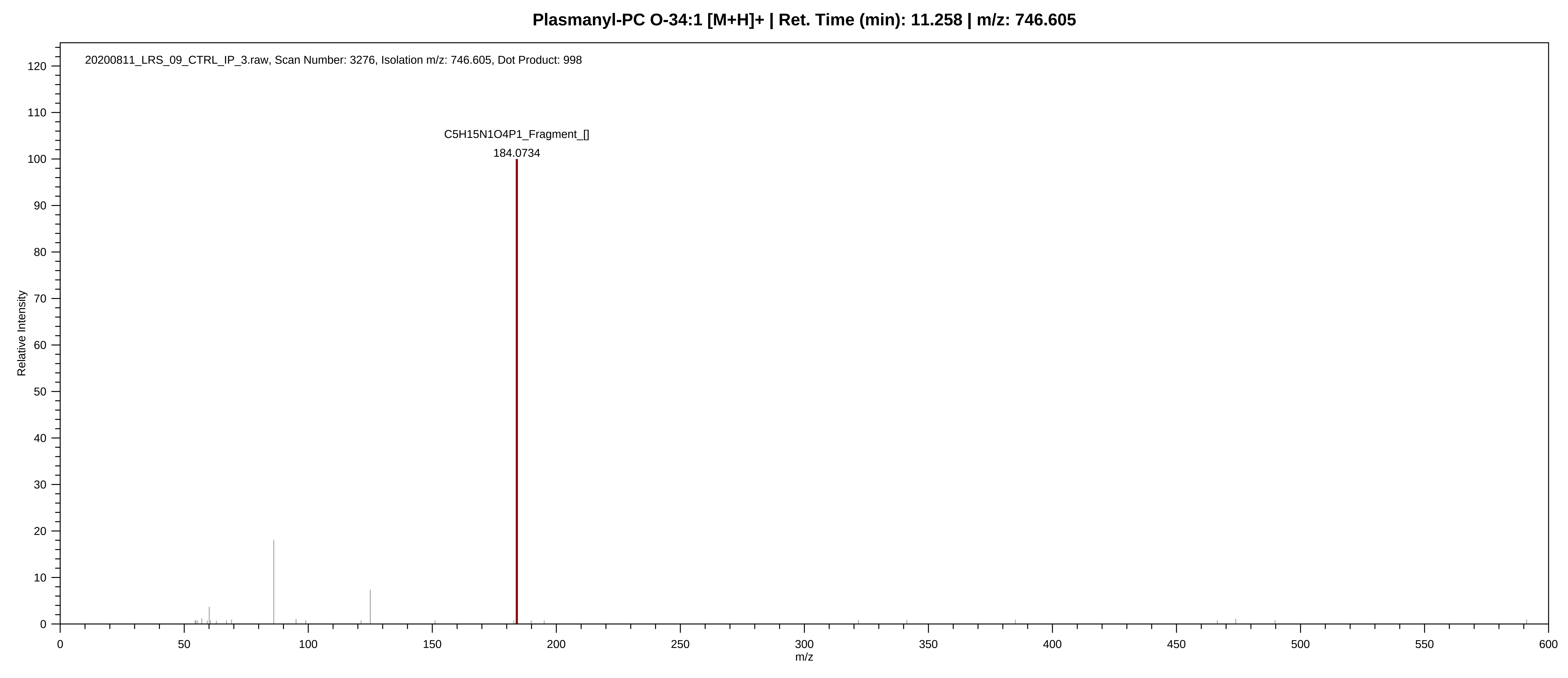

Plasmanyl-PC O-34:1 [M+H]+ | Ret. Time (min): 11.258 | m/z: 746.605
20200811_LRS_09_CTRL_IP_3.raw, Scan Number: 3276, Isolation m/z: 746.605, Dot Product: 998
C5H15N1O4P1_Fragment_[]
184.0734
Relative Intensity
m/z

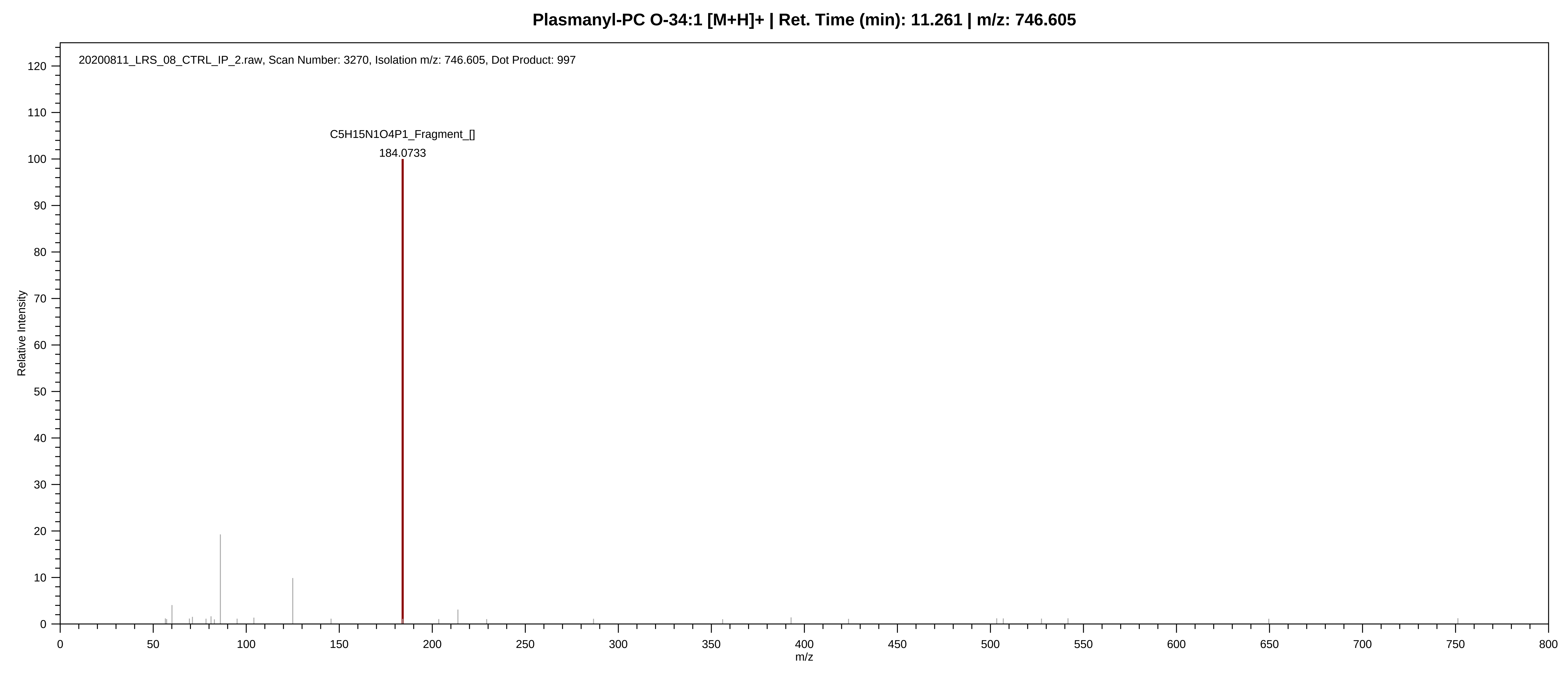

Plasmanyl-PC O-34:1 [M+H]+ | Ret. Time (min): 11.261 | m/z: 746.605
20200811_LRS_08_CTRL_IP_2.raw, Scan Number: 3270, Isolation m/z: 746.605, Dot Product: 997
C5H15N1O4P1_Fragment_[]
184.0733
Relative Intensity
m/z

Plasmanyl-PC O-34:1 [M+H]+ | Ret. Time (min): 10.608 | m/z: 746.606

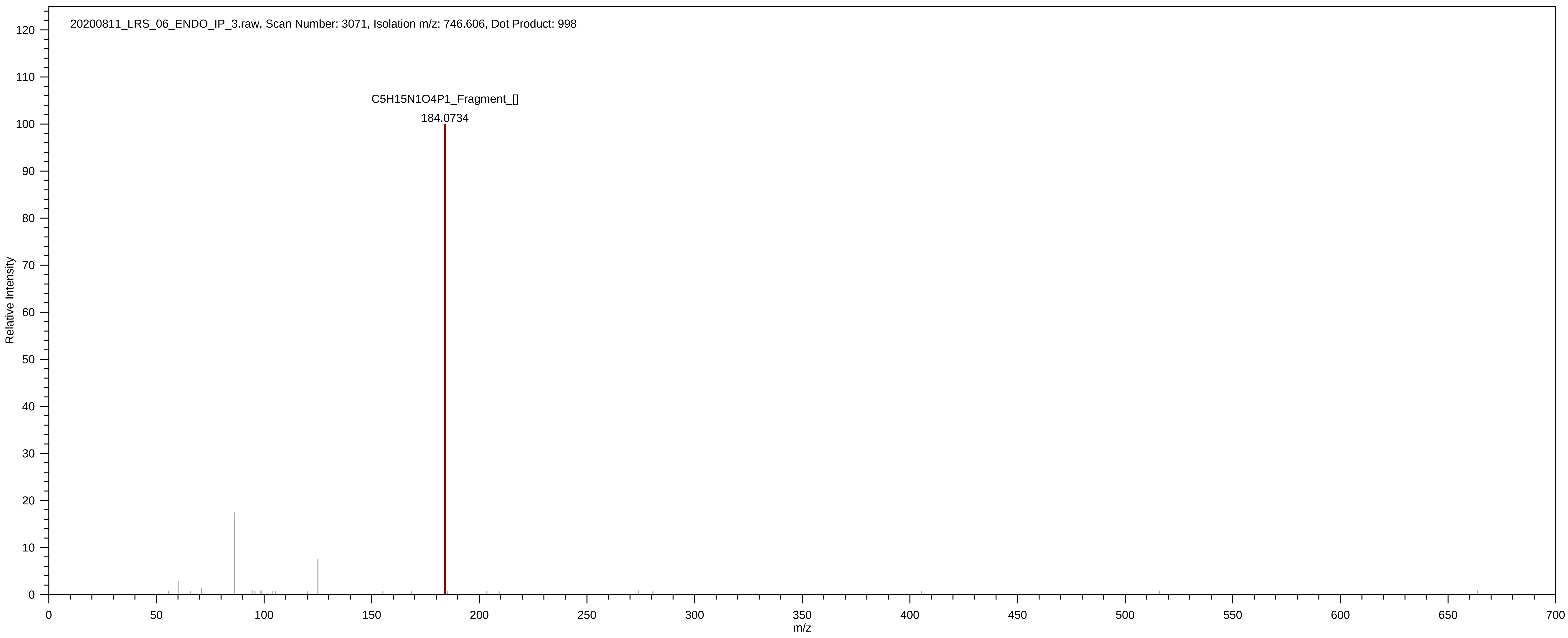

Plasmanyl-PC O-34:1 [M+H]+ | Ret. Time (min): 10.608 | m/z: 746.606

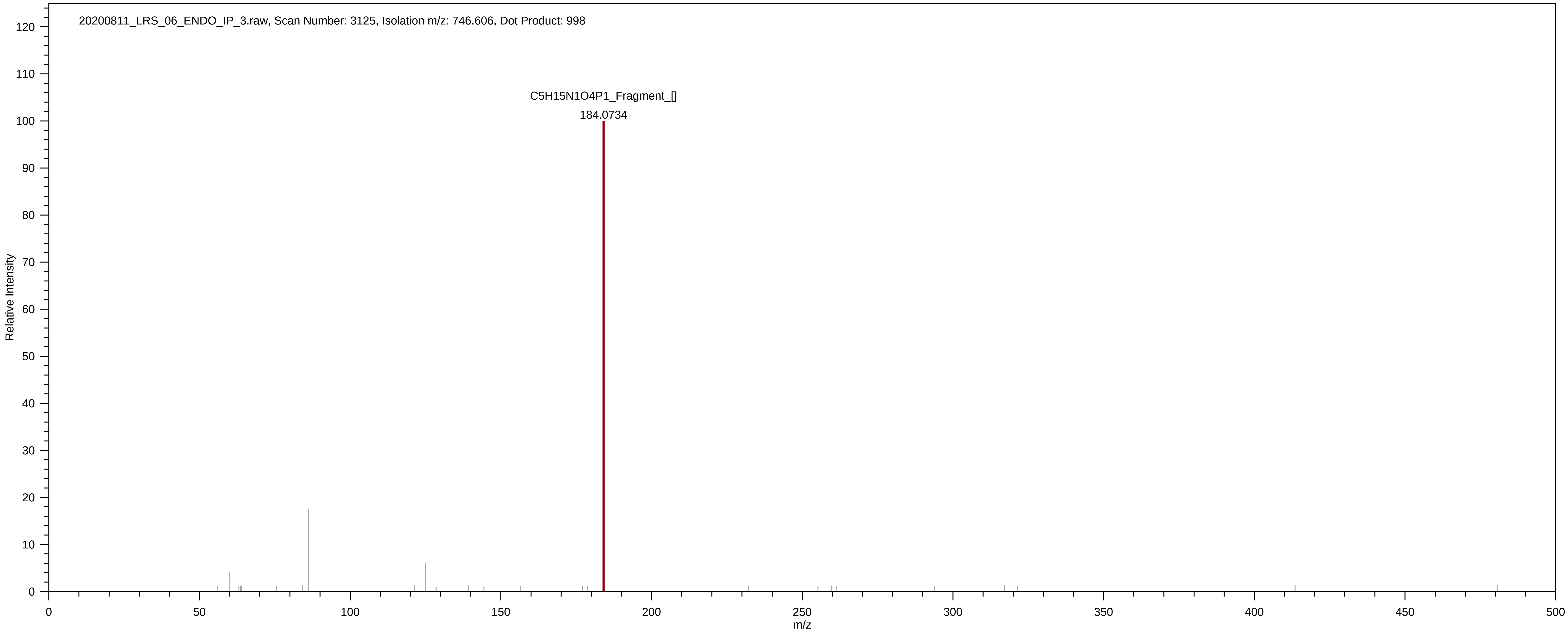

Plasmanyl-PC O-34:1 [M+H]+ | Ret. Time (min): 10.613 | m/z: 746.606

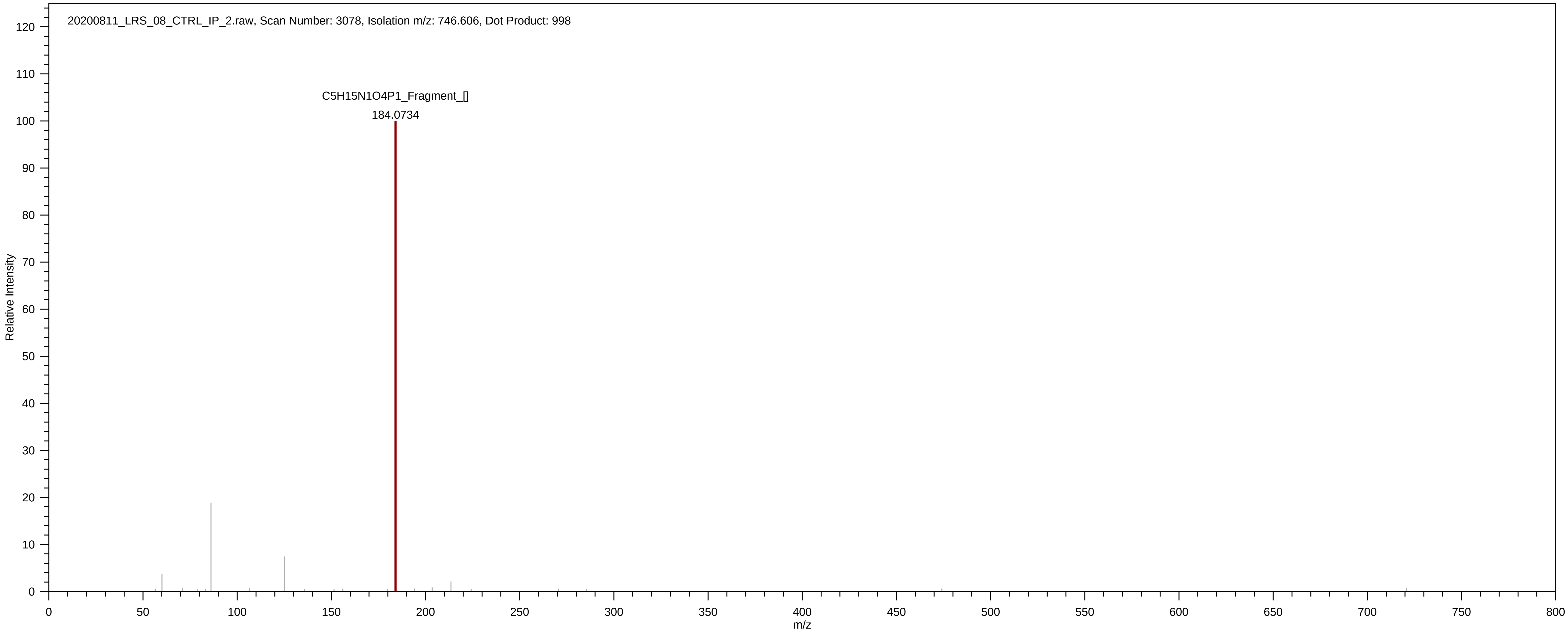

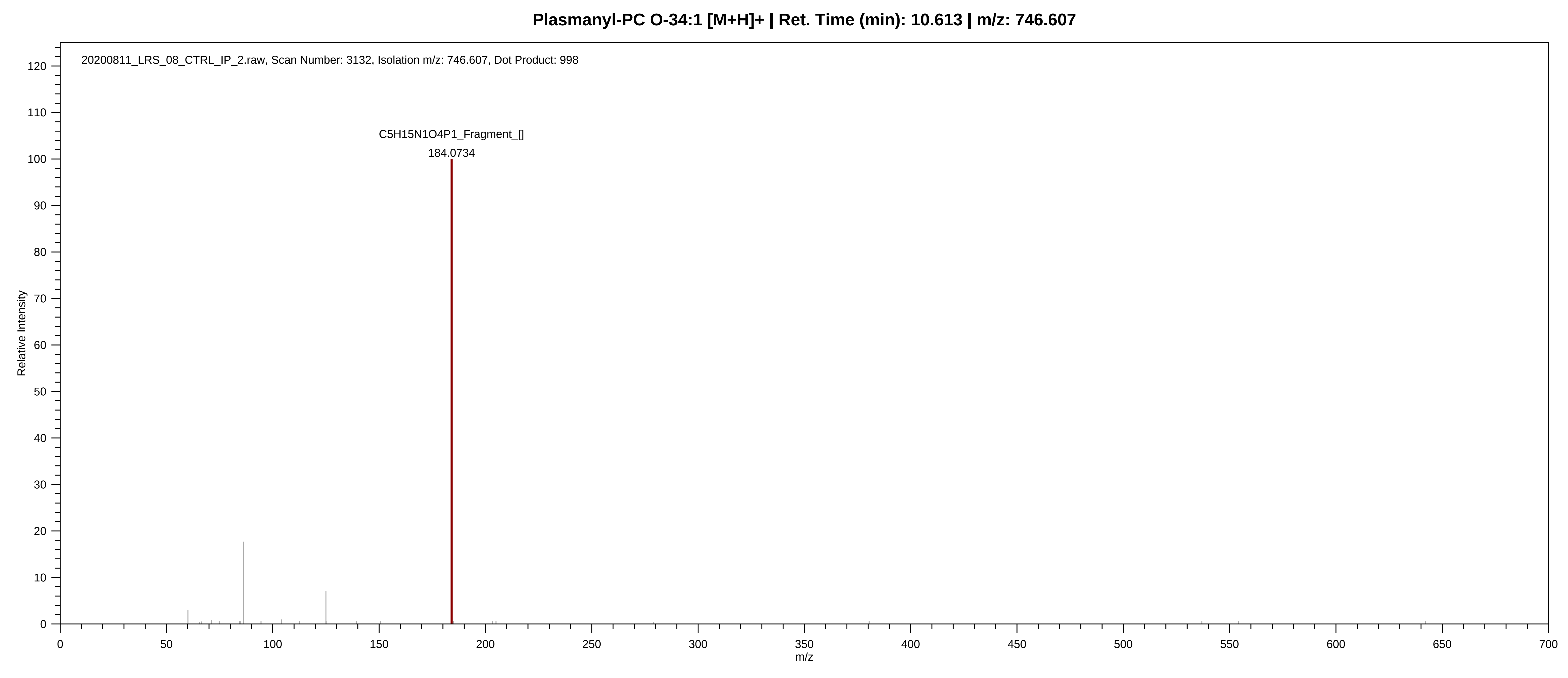
Plasmanyl-PC O-34:1 [M+H]+ | Ret. Time (min): 10.613 | m/z: 746.607
20200811_LRS_08_CTRL_IP_2.raw, Scan Number: 3132, Isolation m/z: 746.607, Dot Product: 998
C5H15N1O4P1_Fragment_[]
184.0734
Relative Intensity
m/z

Plasmanyl-PC O-34:1 [M+H]+ | Ret. Time (min): 10.615 | m/z: 746.606

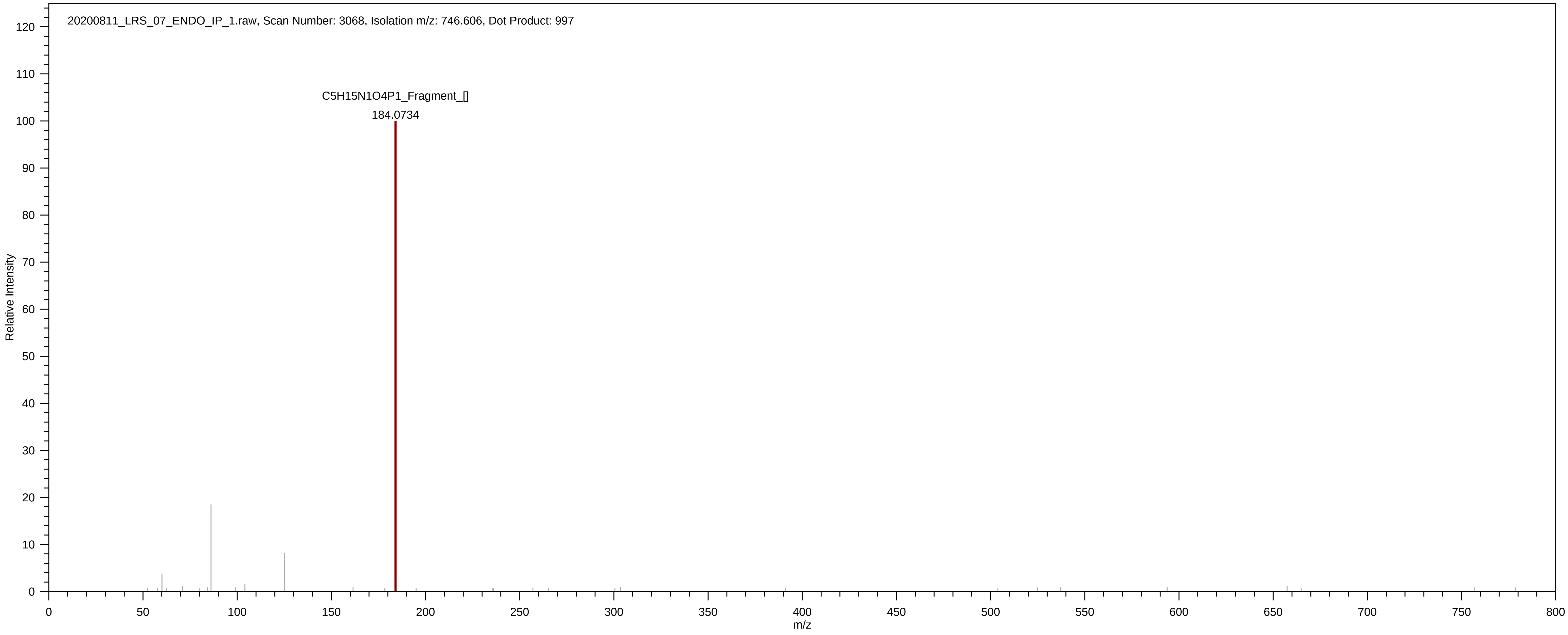

Plasmanyl-PC O-34:1 [M+H]+ | Ret. Time (min): 10.615 | m/z: 746.606

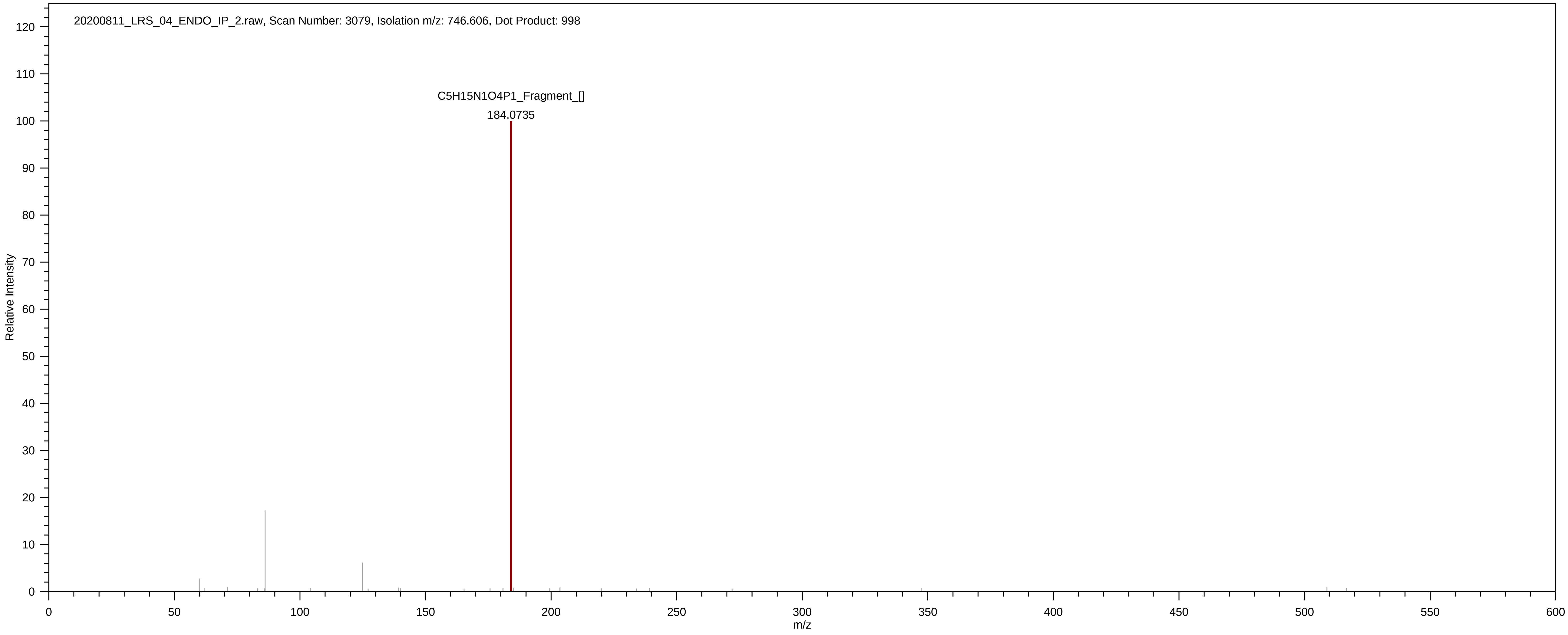

Plasmanyl-PC O-34:1 [M+H]+ | Ret. Time (min): 10.615 | m/z: 746.606

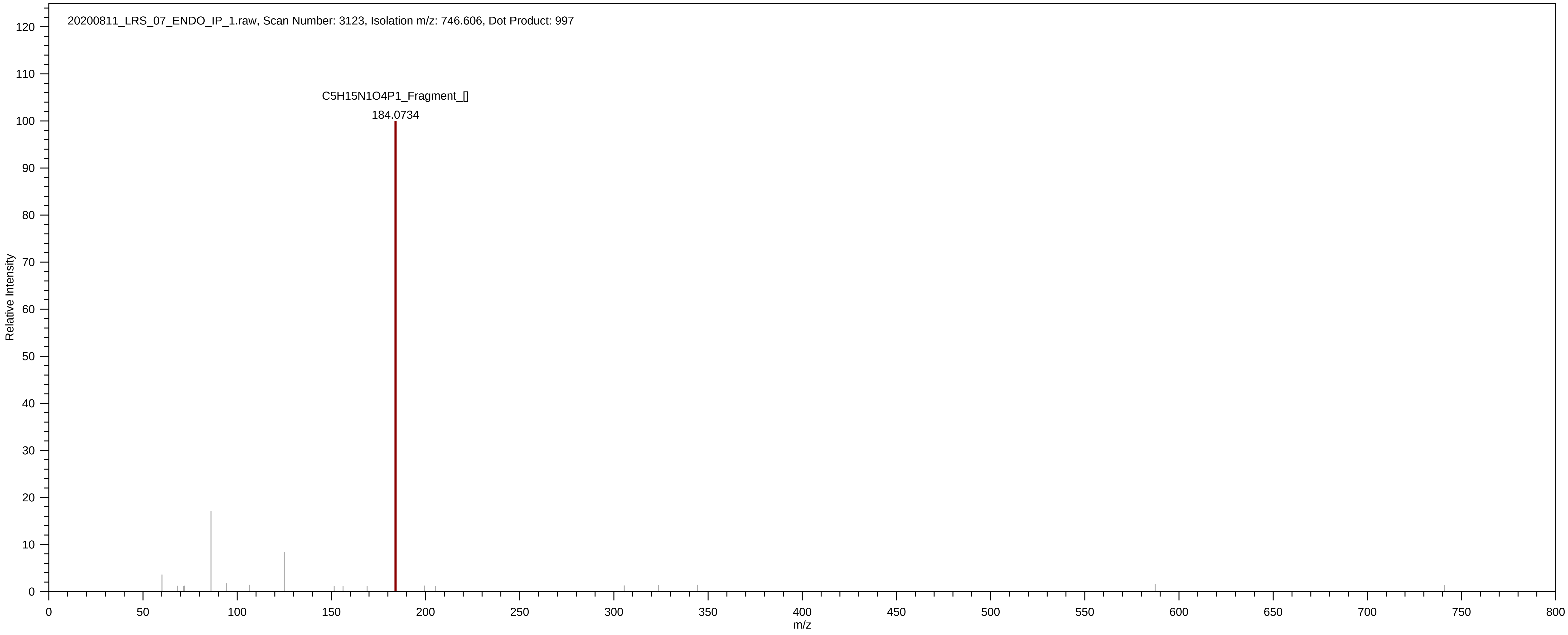

Plasmanyl-PC O-34:1 [M+H]+ | Ret. Time (min): 10.622 | m/z: 746.606

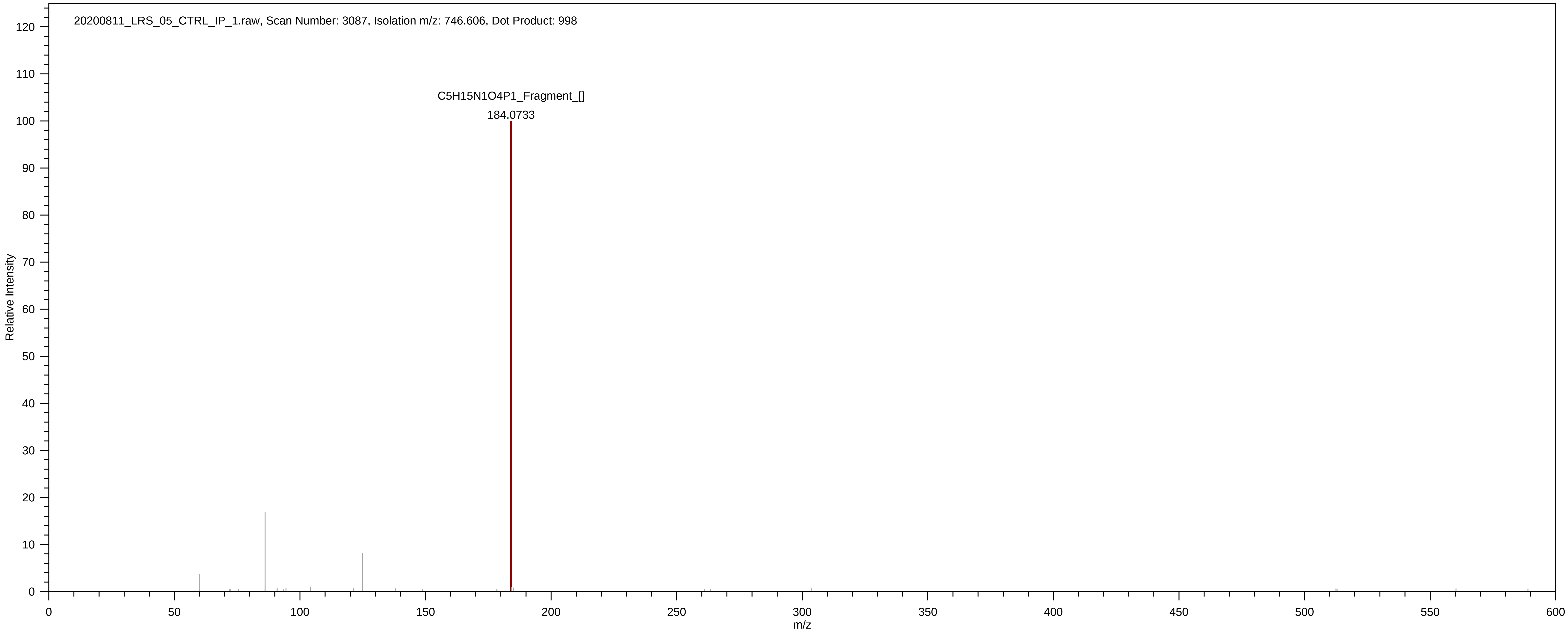

Plasmanyl-PC O-34:1 [M+H]+ | Ret. Time (min): 10.622 | m/z: 746.606

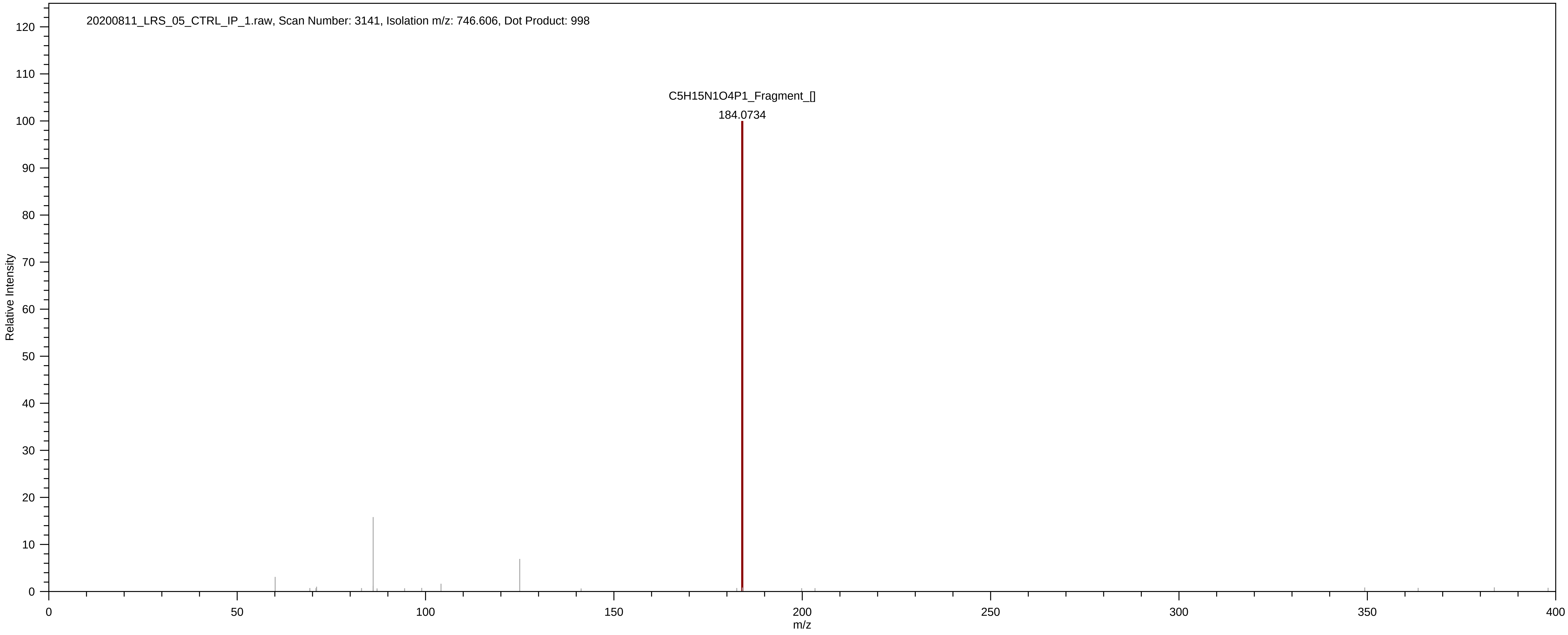

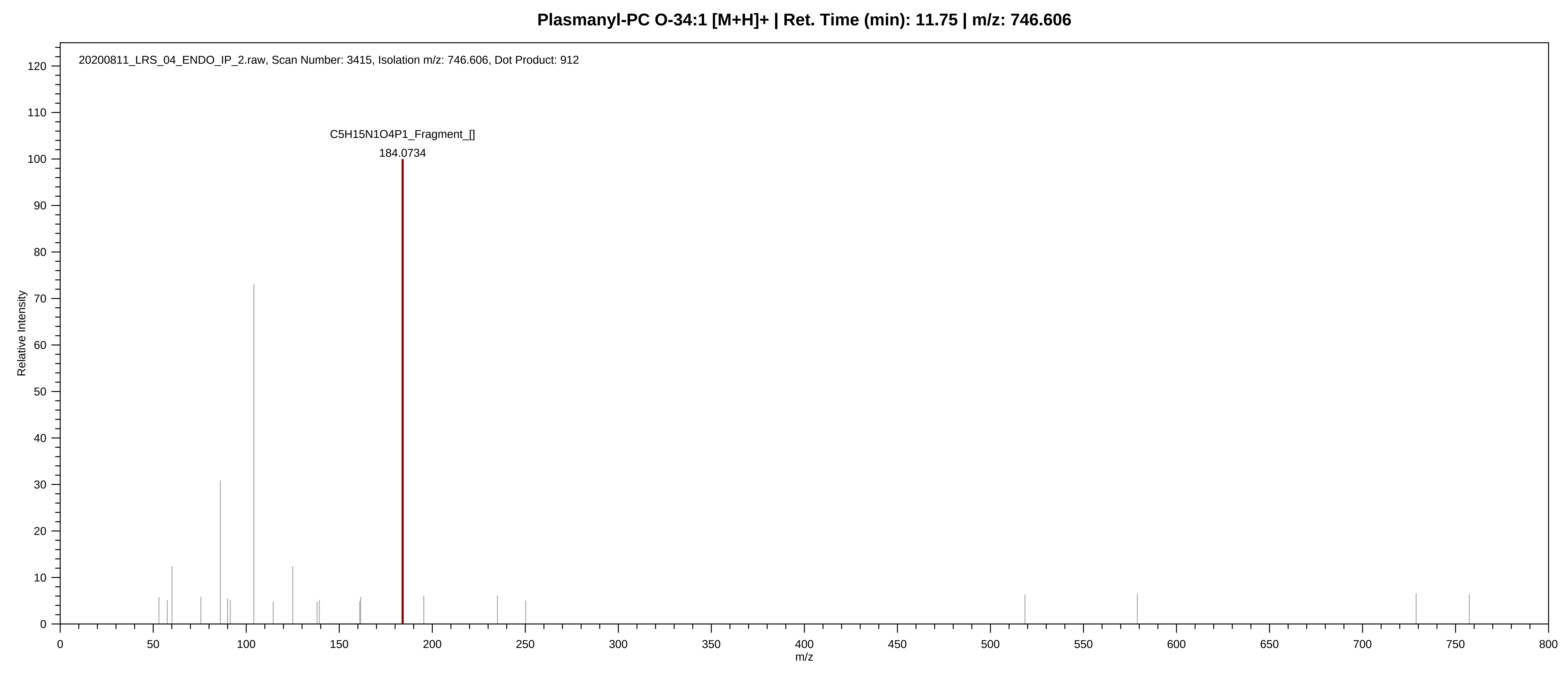

Plasmanyl-PC O-34:1 [M+H]+ | Ret. Time (min): 11.75 | m/z: 746.606
20200811_LRS_04_ENDO_IP_2.raw, Scan Number: 3415, Isolation m/z: 746.606, Dot Product: 912
C5H15N1O4P1_Fragment_[]
184.0734
Relative Intensity
m/z

Plasmanyl-PC O-34:1 [M+H]+ | Ret. Time (min): 11.75 | m/z: 746.607

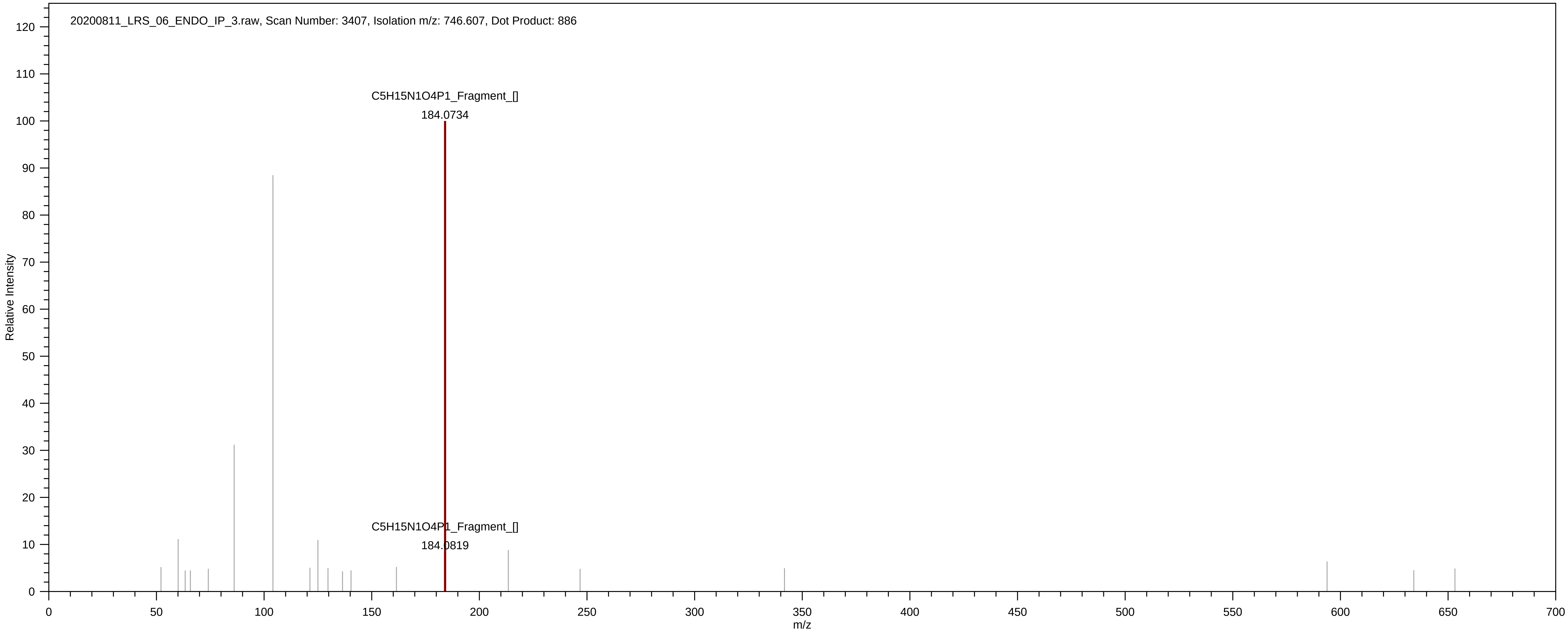

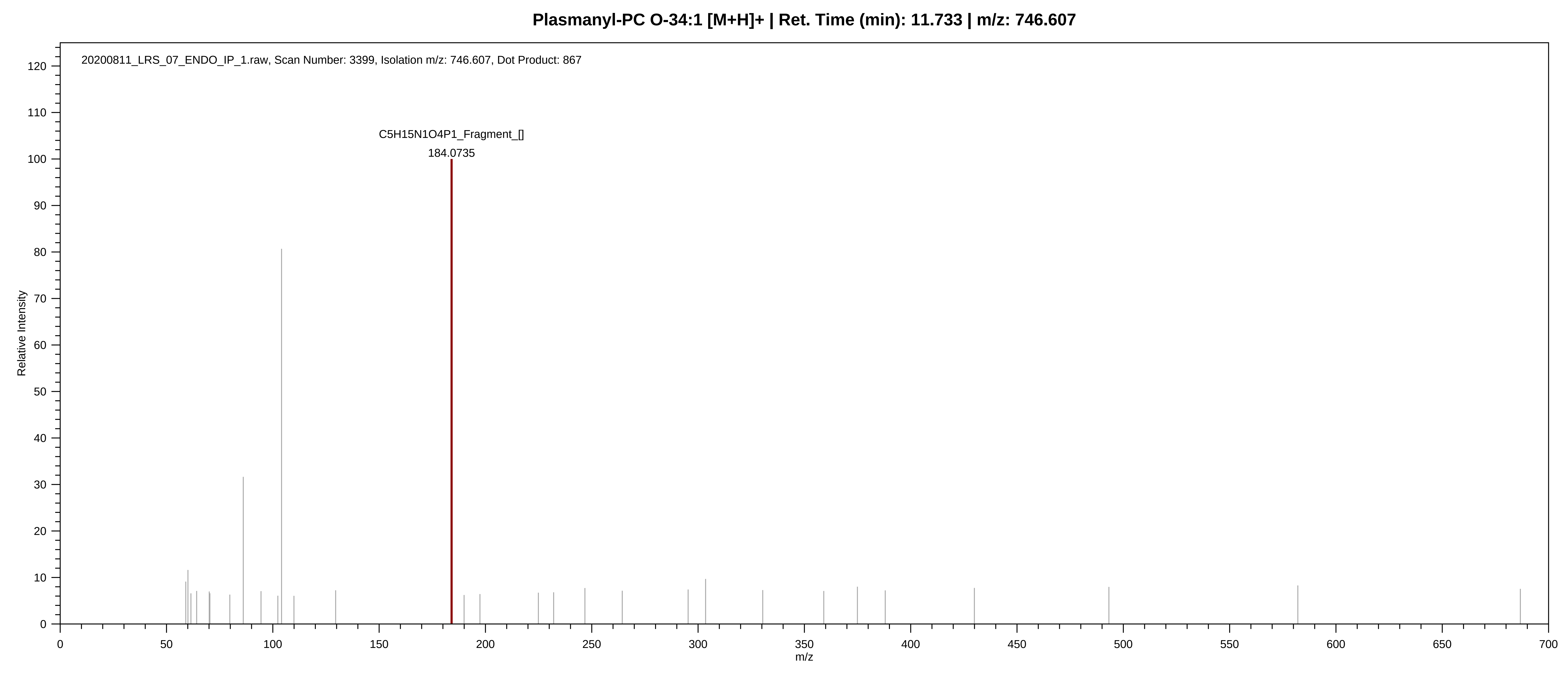
Plasmanyl-PC O-34:1 [M+H]+ | Ret. Time (min): 11.733 | m/z: 746.607
20200811_LRS_07_ENDO_IP_1.raw, Scan Number: 3399, Isolation m/z: 746.607, Dot Product: 867
C5H15N1O4P1_Fragment_[]
184.0735
Relative Intensity
m/z

Plasmanyl-PC O-34:1 [M+H]+ | Ret. Time (min): 11.264 | m/z: 746.609

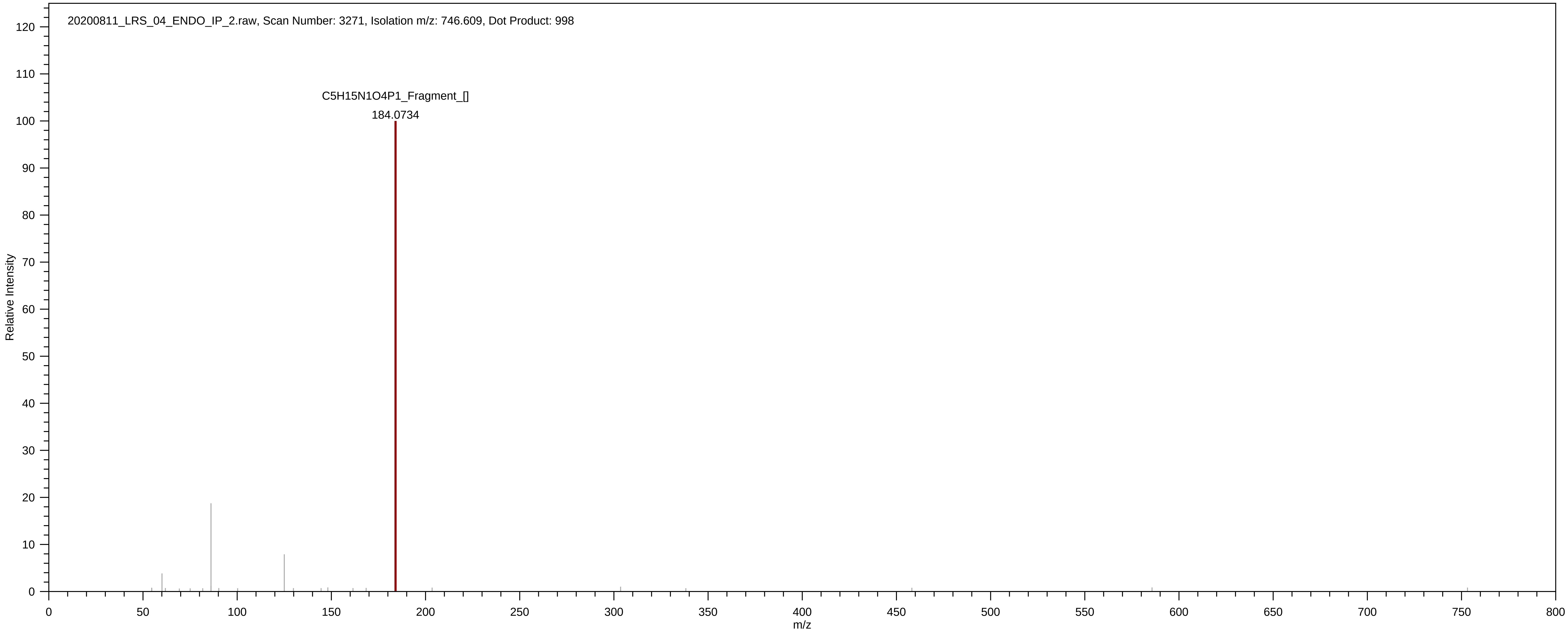

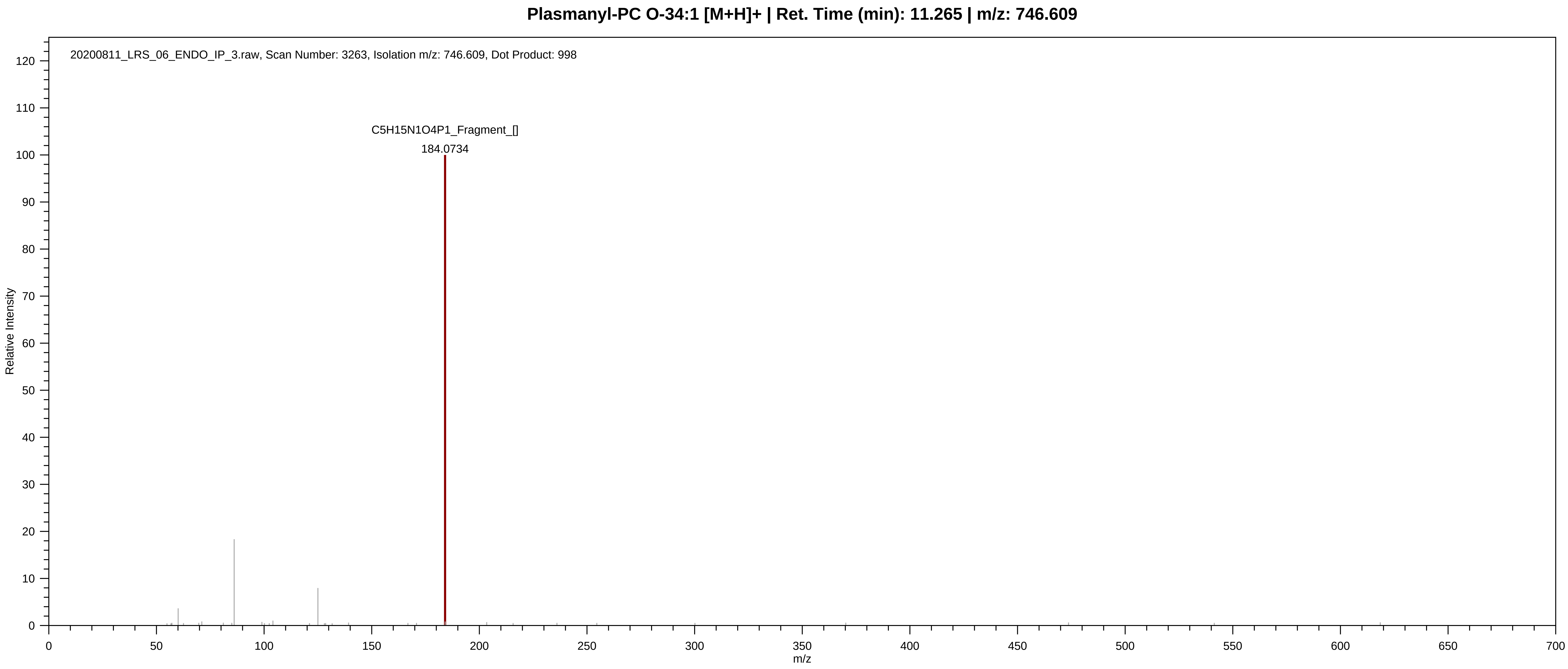
Plasmanyl-PC O-34:1 [M+H]+ | Ret. Time (min): 11.265 | m/z: 746.609
20200811_LRS_06_ENDO_IP_3.raw, Scan Number: 3263, Isolation m/z: 746.609, Dot Product: 998
C5H15N1O4P1_Fragment_[]
184.0734
Relative Intensity
m/z
0
10
20
30
40
50
60
70
80
90
100
110
120
0
50
100
150
200
250
300
350
400
450
500
550
600
650
700

Plasmanyl-PC O-34:2 [M+H]+ | Ret. Time (min): 9.517 | m/z: 744.59

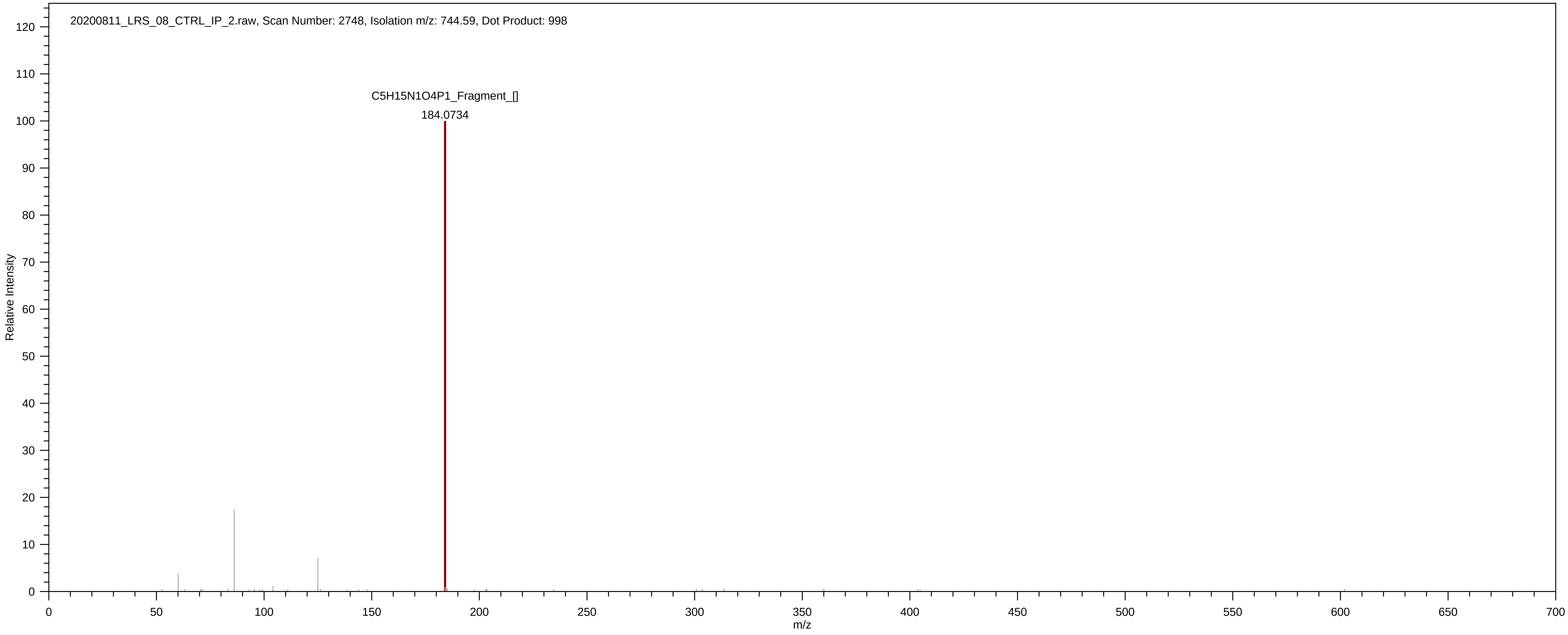

Plasmanyl-PC O-34:2 [M+H]+ | Ret. Time (min): 9.524 | m/z: 744.59

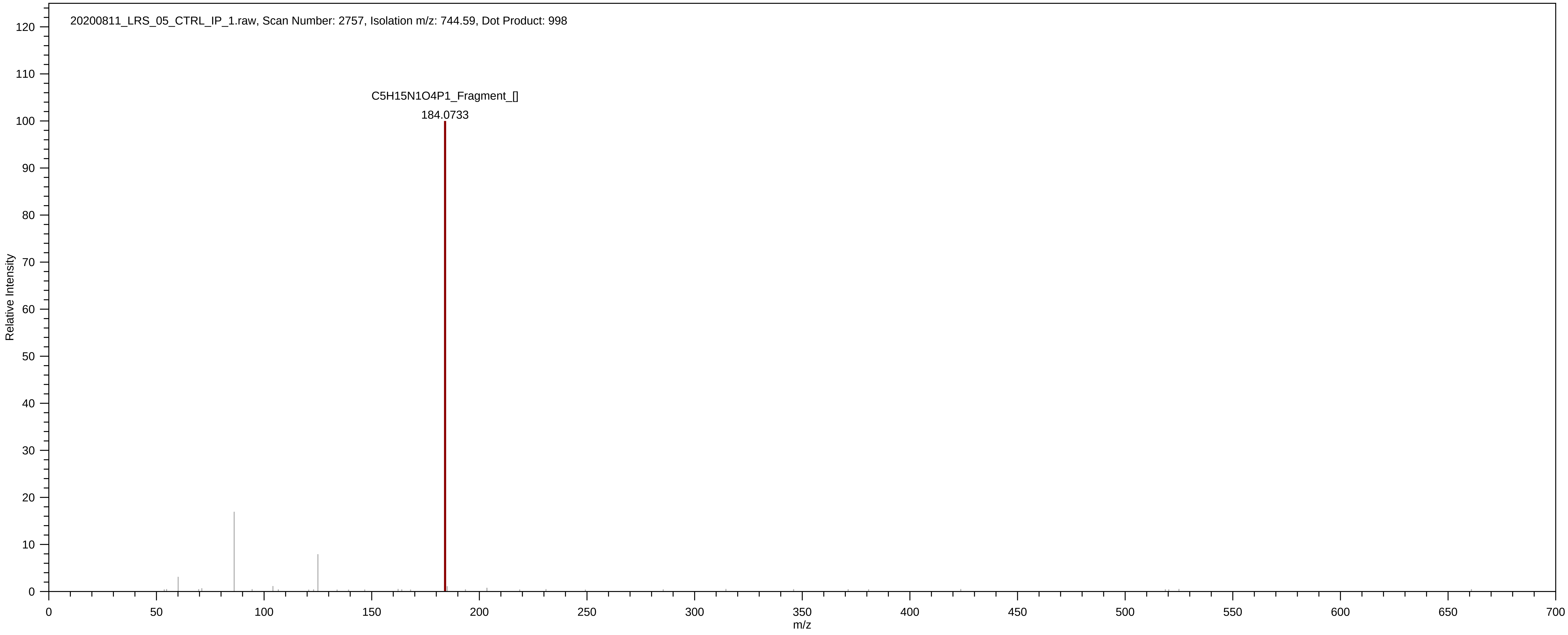

Plasmanyl-PC O-34:2 [M+H]+ | Ret. Time (min): 9.643 | m/z: 744.59

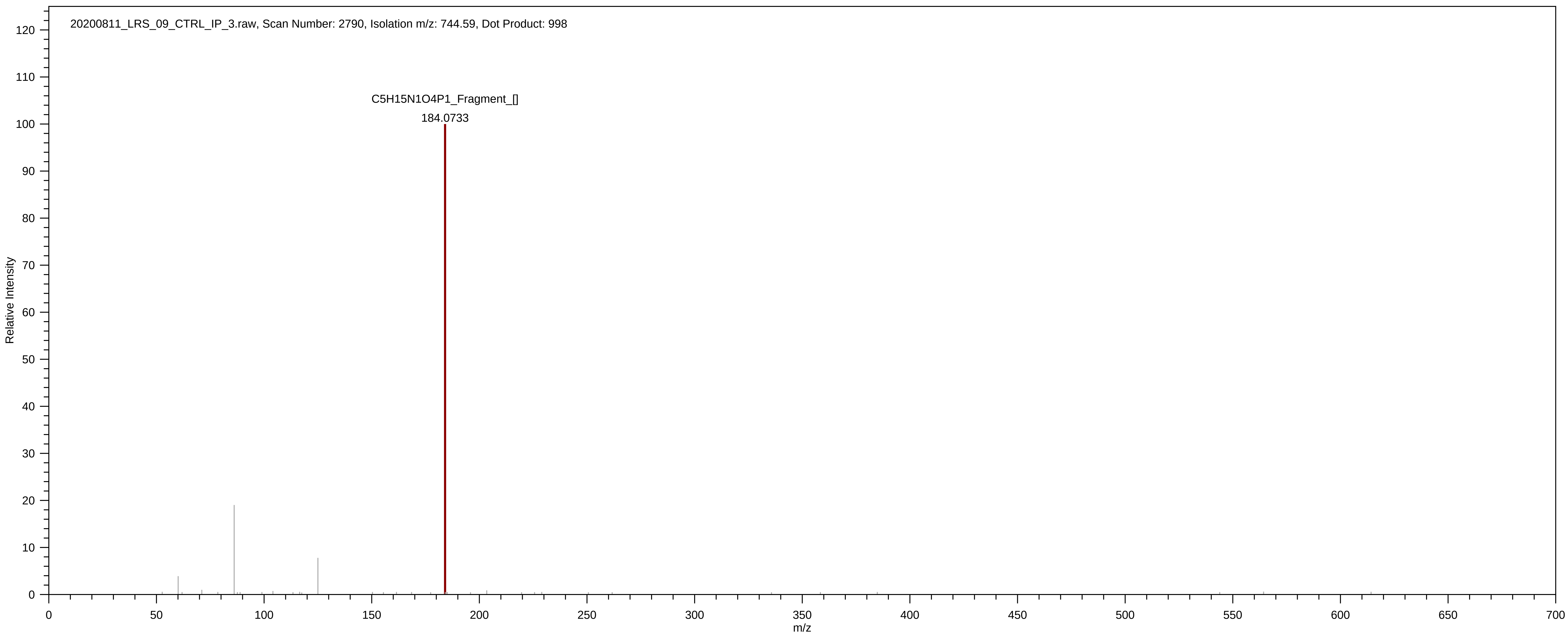

Plasmanyl-PC O-34:2 [M+H]+ | Ret. Time (min): 9.697 | m/z: 744.59

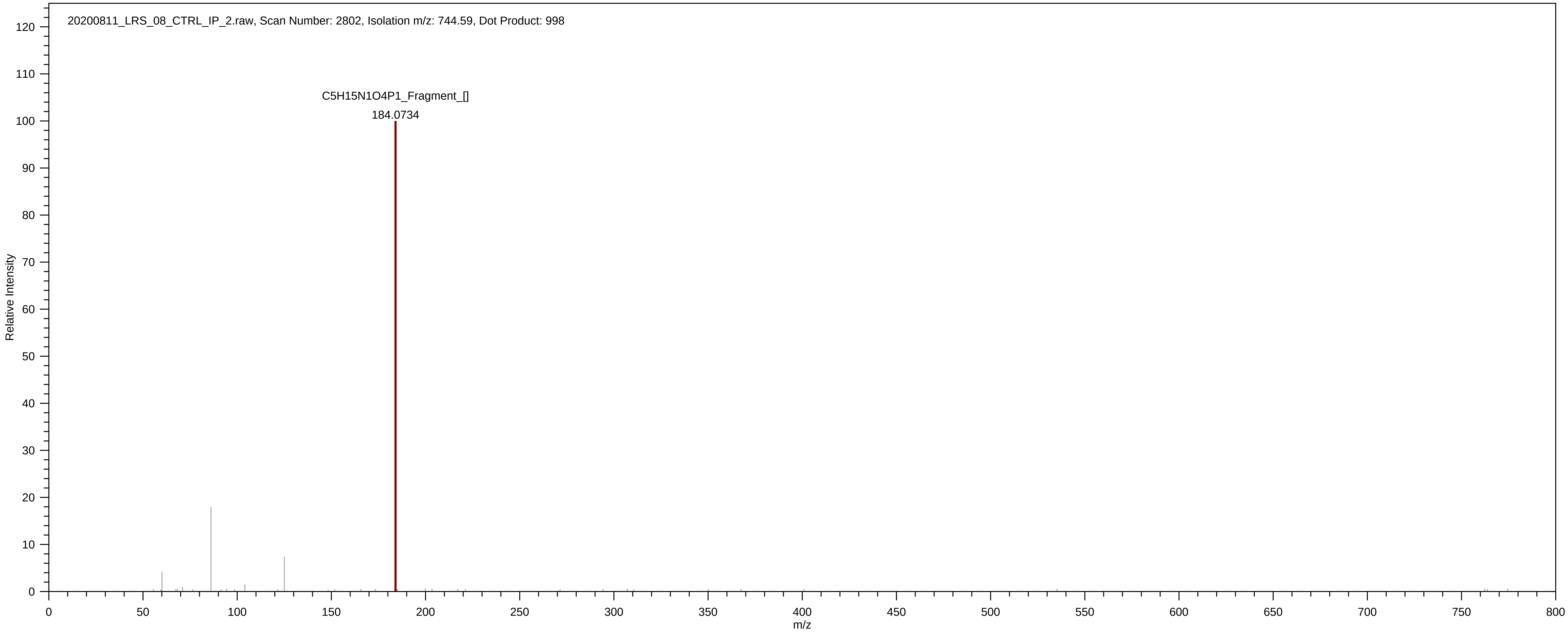

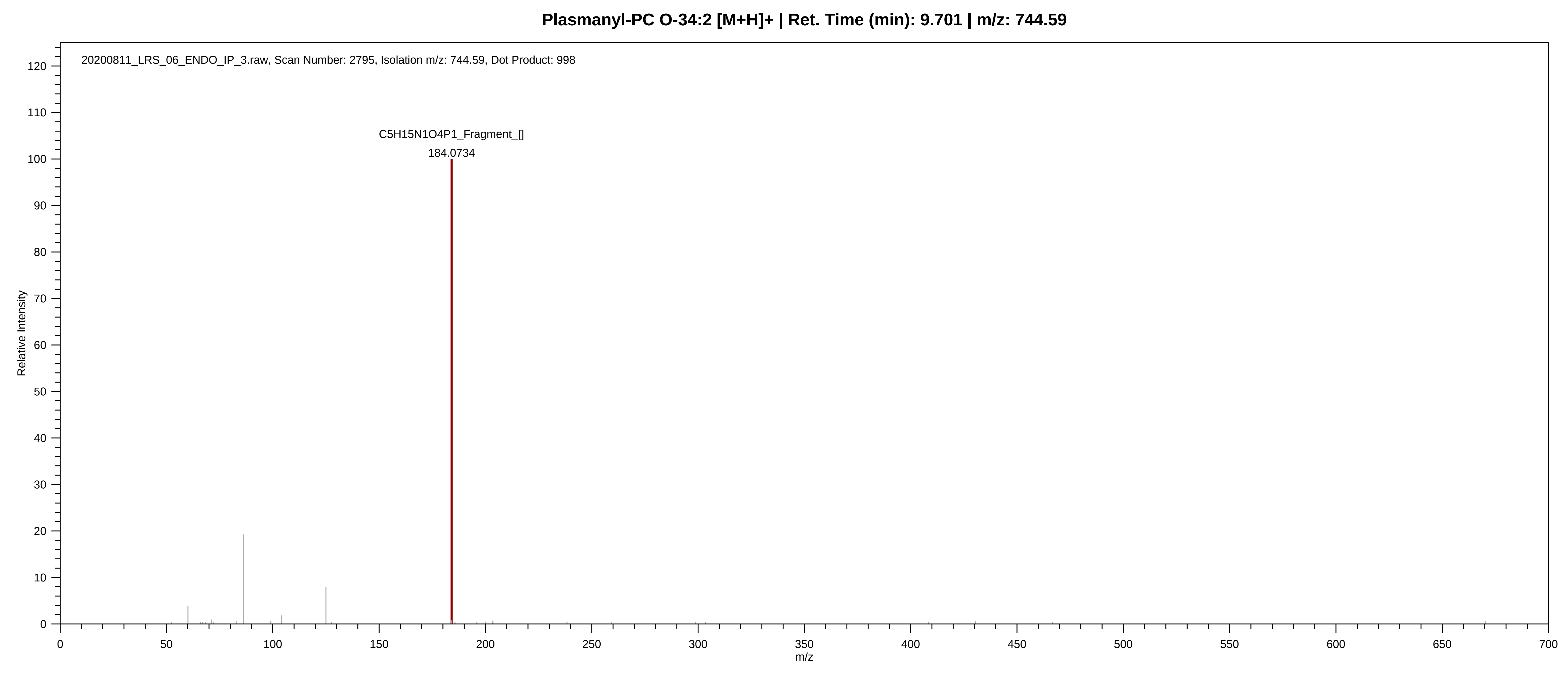

Plasmanyl-PC O-34:2 [M+H]+ | Ret. Time (min): 9.701 | m/z: 744.59
20200811_LRS_06_ENDO_IP_3.raw, Scan Number: 2795, Isolation m/z: 744.59, Dot Product: 998
C5H15N1O4P1_Fragment_[]
184.0734
Relative Intensity
m/z

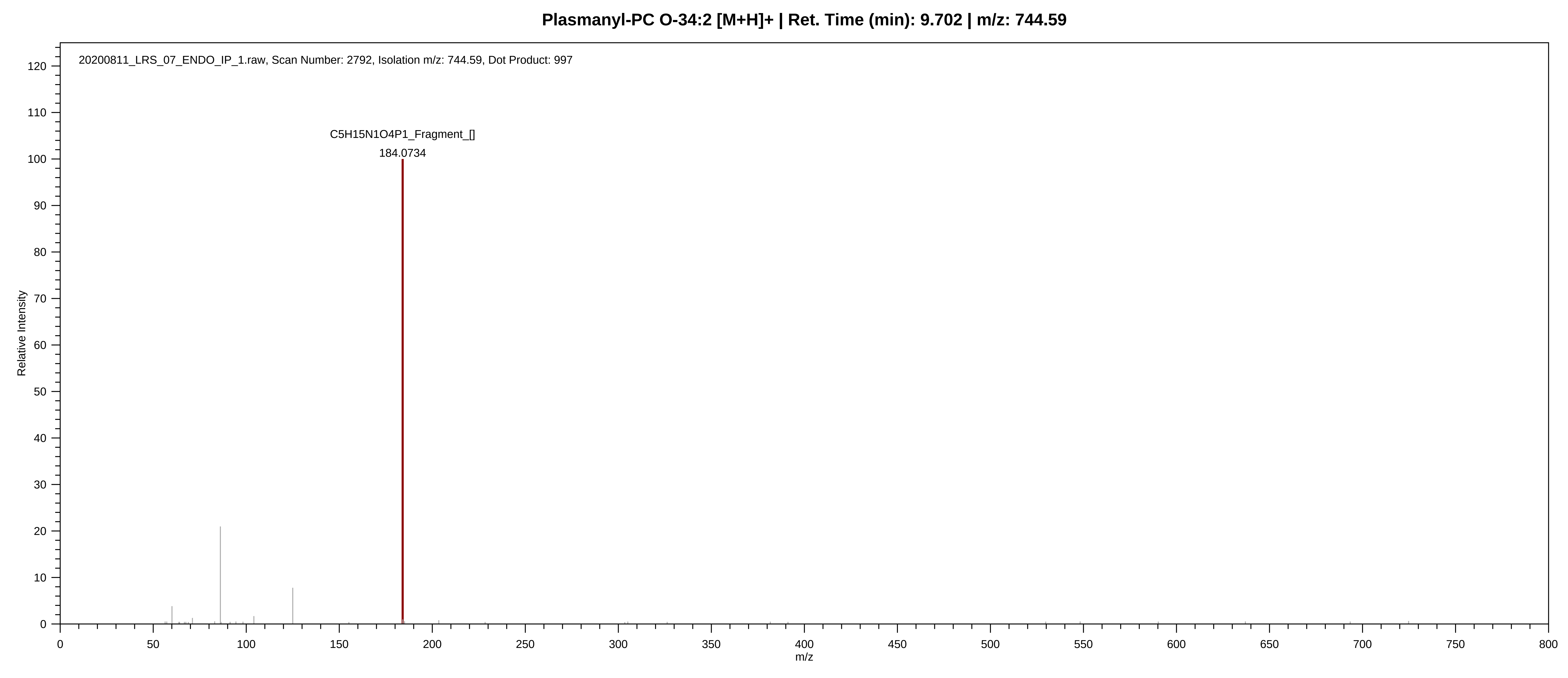
Plasmanyl-PC O-34:2 [M+H]+ | Ret. Time (min): 9.702 | m/z: 744.59
20200811_LRS_07_ENDO_IP_1.raw, Scan Number: 2792, Isolation m/z: 744.59, Dot Product: 997
C5H15N1O4P1_Fragment_[]
184.0734
Relative Intensity
m/z

Plasmanyl-PC O-34:2 [M+H]+ | Ret. Time (min): 9.704 | m/z: 744.59

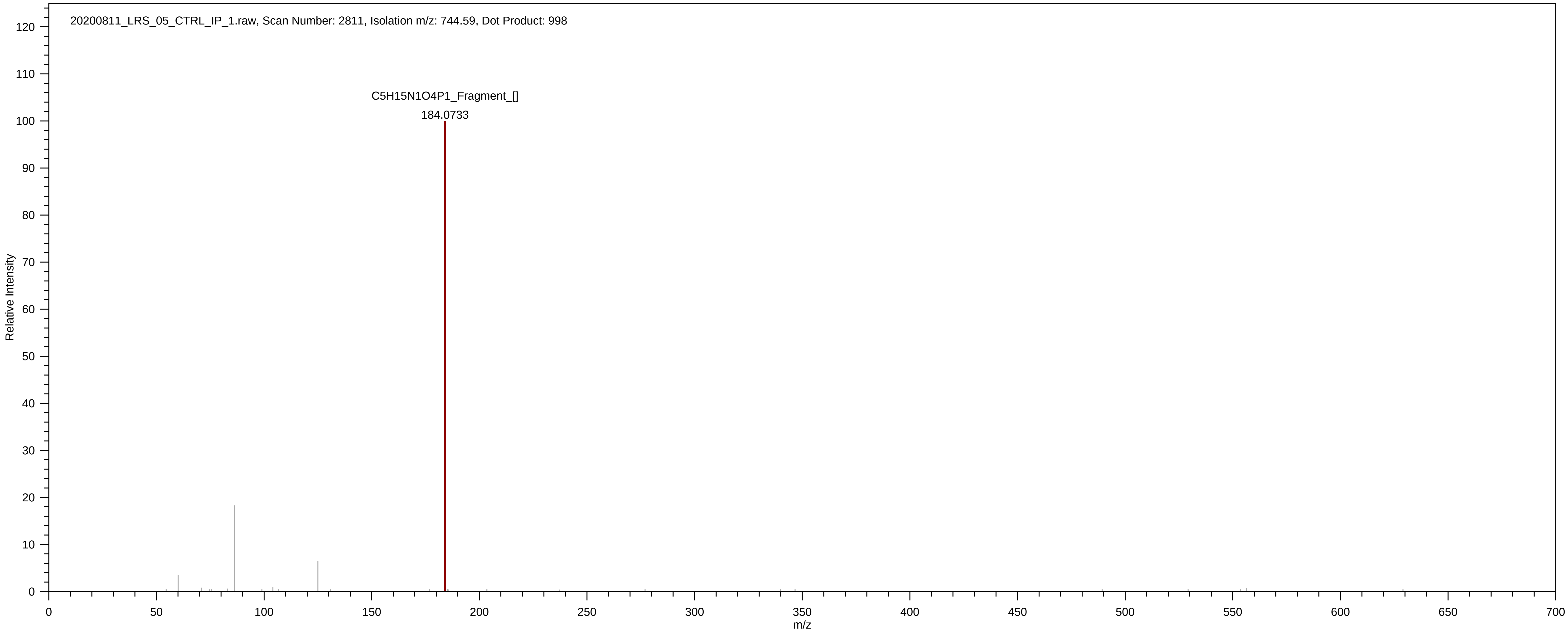

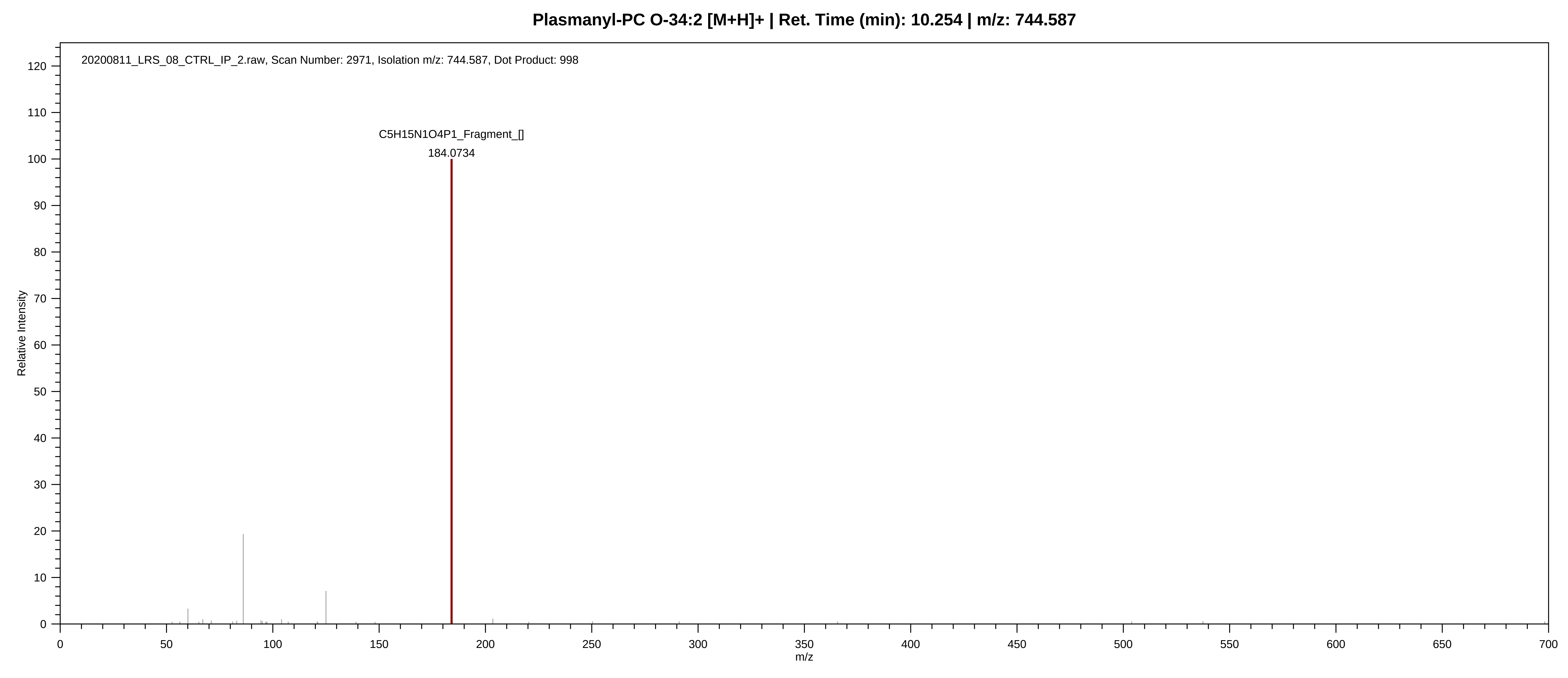
Plasmanyl-PC O-34:2 [M+H]+ | Ret. Time (min): 10.254 | m/z: 744.587
20200811_LRS_08_CTRL_IP_2.raw, Scan Number: 2971, Isolation m/z: 744.587, Dot Product: 998
C5H15N1O4P1_Fragment_[]
184.0734
Relative Intensity
m/z

Plasmanyl-PC O-34:2 [M+H]+ | Ret. Time (min): 10.263 | m/z: 744.587

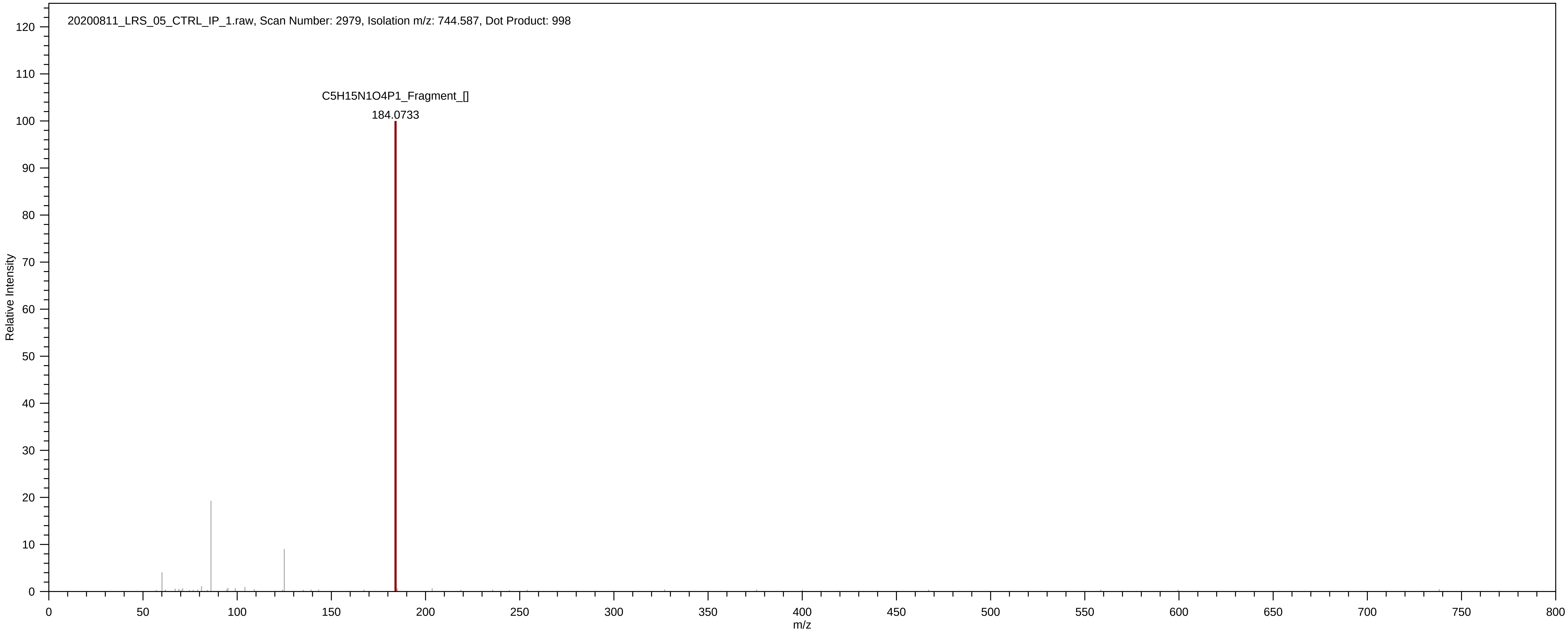

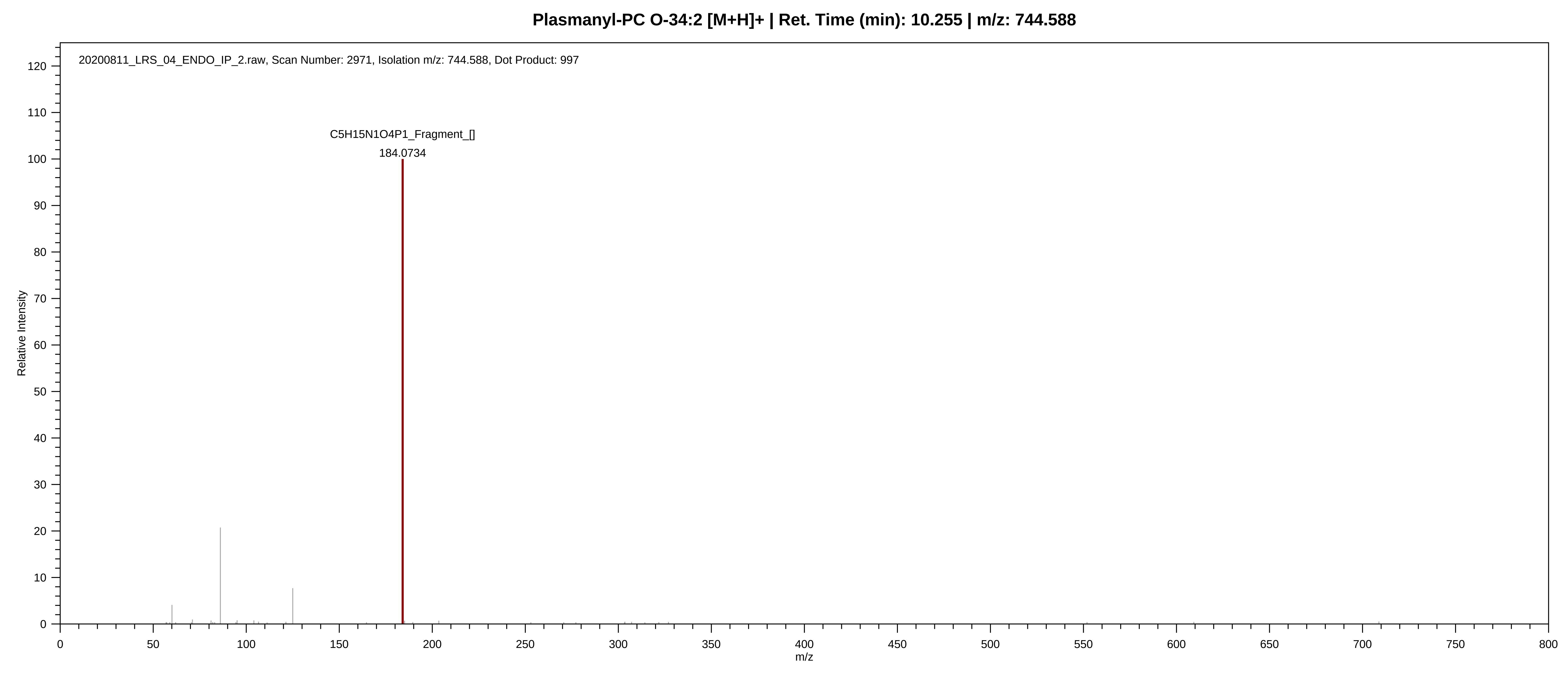

Plasmanyl-PC O-34:2 [M+H]+ | Ret. Time (min): 10.255 | m/z: 744.588
20200811_LRS_04_ENDO_IP_2.raw, Scan Number: 2971, Isolation m/z: 744.588, Dot Product: 997
C5H15N1O4P1_Fragment_[]
184.0734
Relative Intensity
m/z

Plasmanyl-PC O-34:2 [M+H]+ | Ret. Time (min): 9.522 | m/z: 744.589

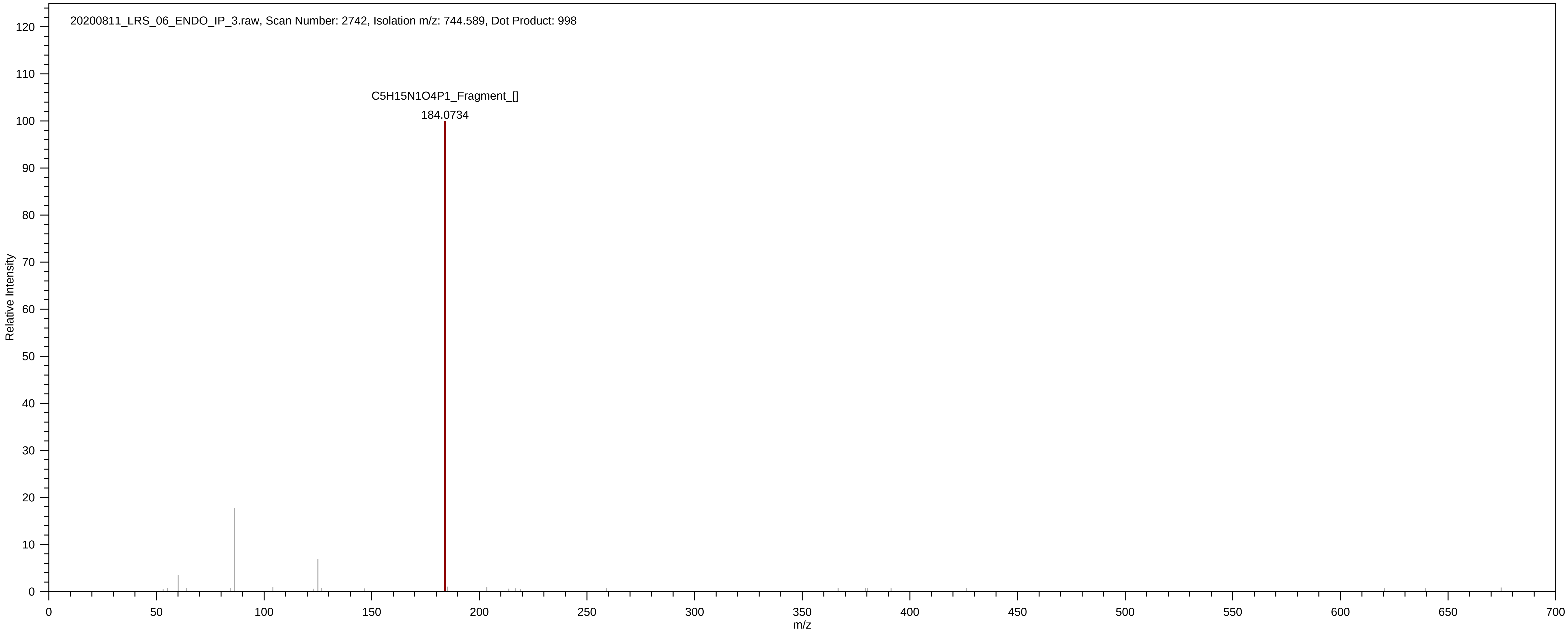

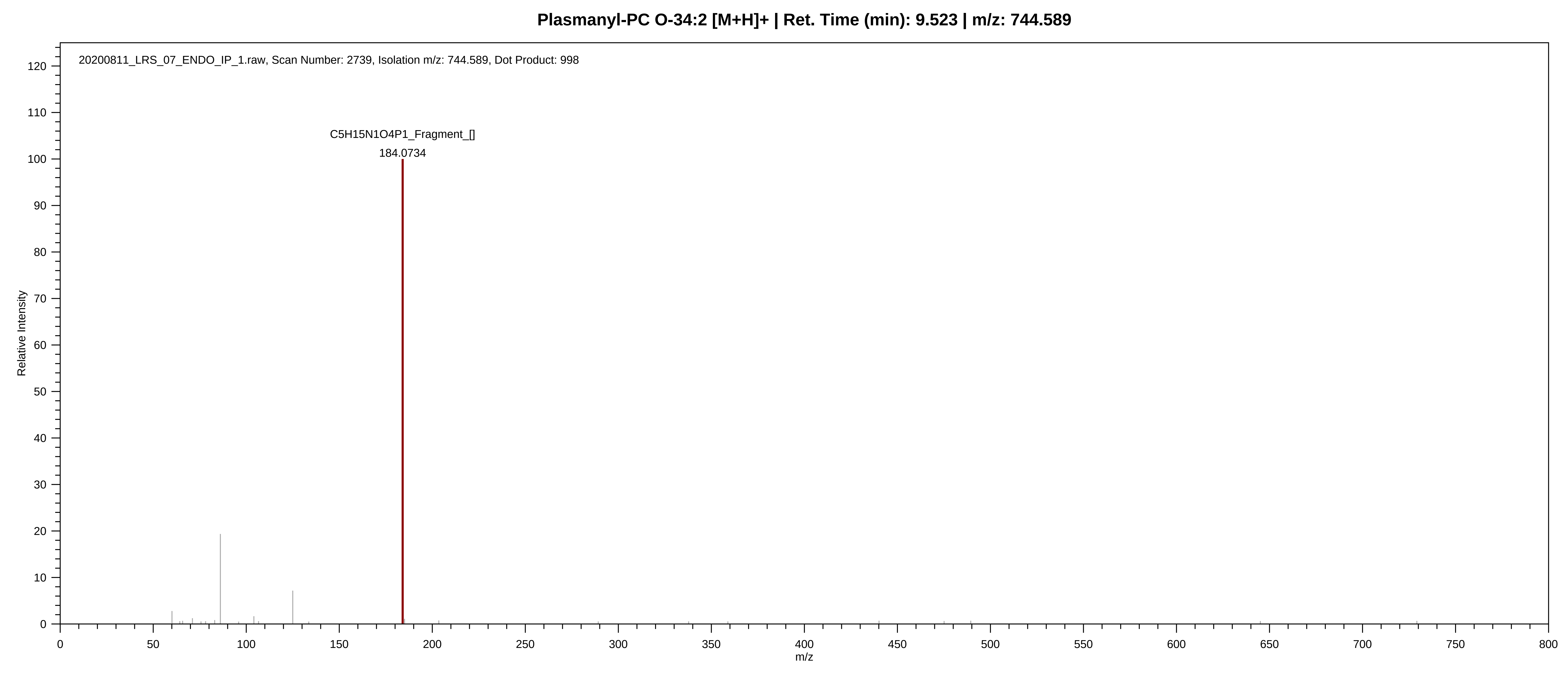
Plasmanyl-PC O-34:2 [M+H]+ | Ret. Time (min): 9.523 | m/z: 744.589
20200811_LRS_07_ENDO_IP_1.raw, Scan Number: 2739, Isolation m/z: 744.589, Dot Product: 998
C5H15N1O4P1_Fragment_[]
184.0734
Relative Intensity
m/z

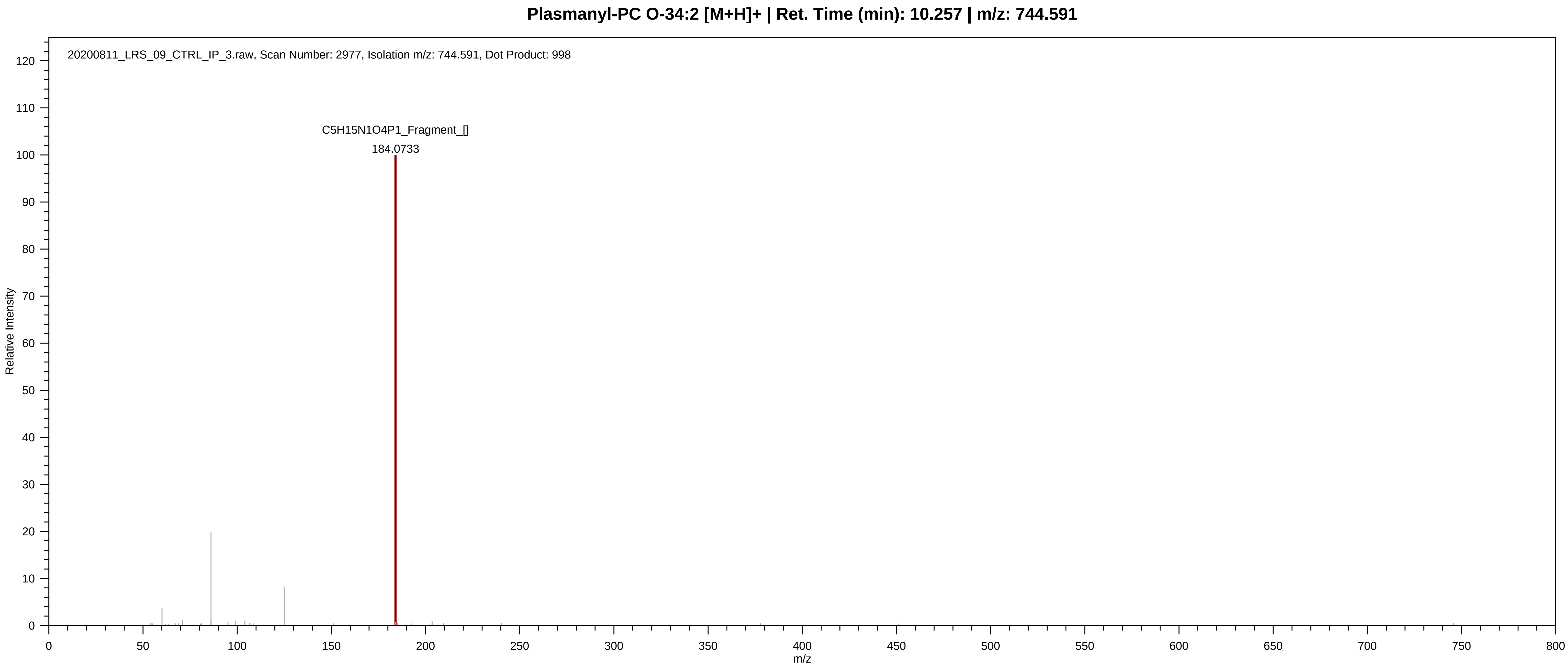
Plasmanyl-PC O-34:2 [M+H]+ | Ret. Time (min): 10.257 | m/z: 744.591
20200811_LRS_09_CTRL_IP_3.raw, Scan Number: 2977, Isolation m/z: 744.591, Dot Product: 998
C5H15N1O4P1_Fragment_[]
184.0733
Relative Intensity
m/z
0
10
20
30
40
50
60
70
80
90
100
110
120
0
50
100
150
200
250
300
350
400
450
500
550
600
650
700
750
800

Plasmanyl-PC O-34:3 [M+H]+ | Ret. Time (min): 9.45 | m/z: 742.572

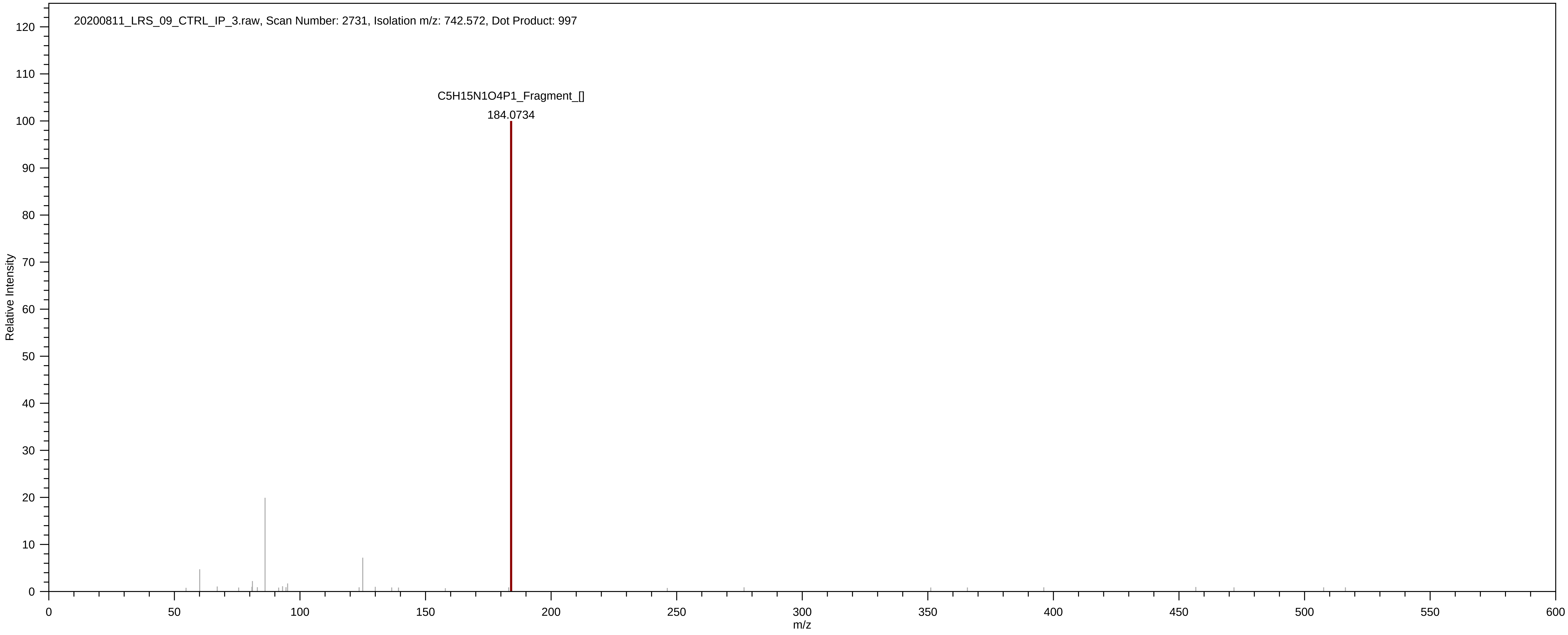

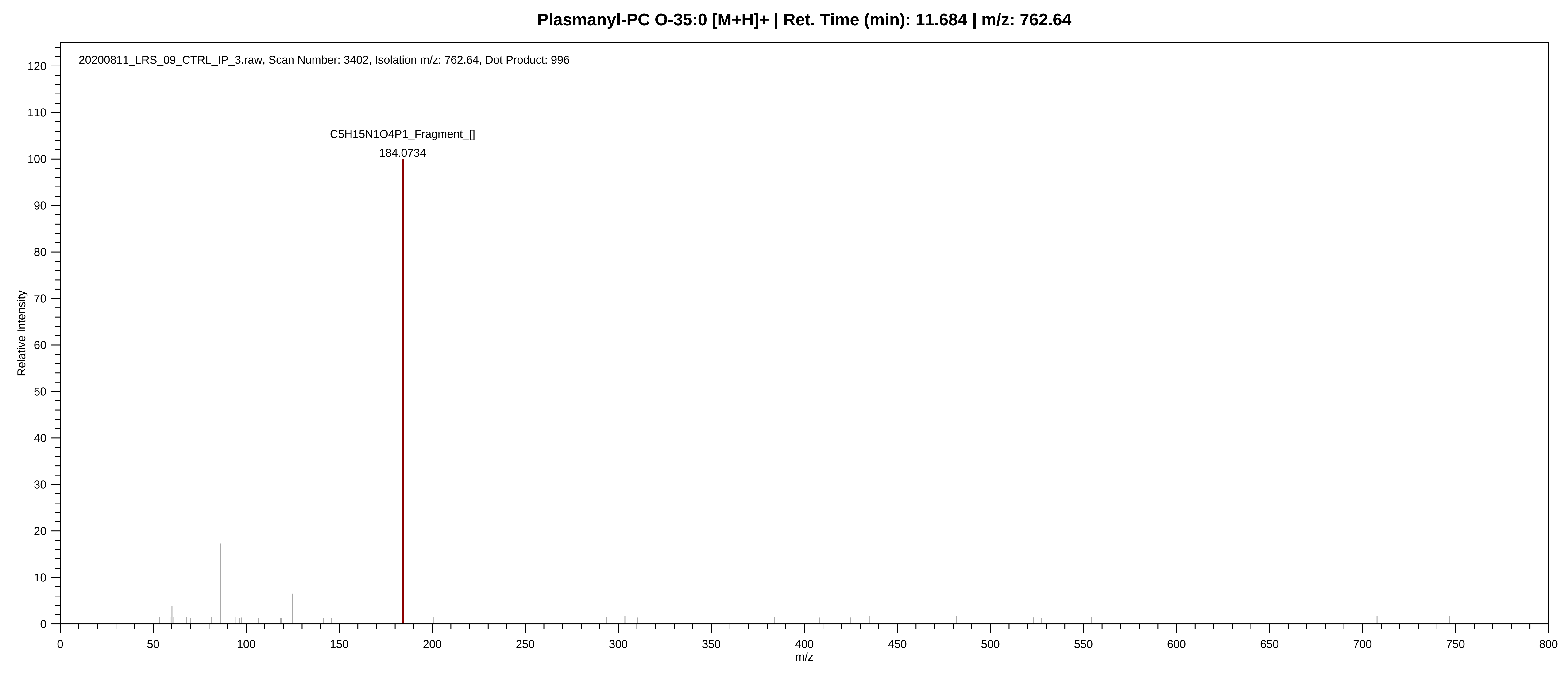

Plasmanyl-PC O-35:0 [M+H]+ | Ret. Time (min): 11.684 | m/z: 762.64
20200811_LRS_09_CTRL_IP_3.raw, Scan Number: 3402, Isolation m/z: 762.64, Dot Product: 996
C5H15N1O4P1_Fragment_[]
184.0734
Relative Intensity
m/z

Plasmanyl-PC O-35:0 [M+H]+ | Ret. Time (min): 11.704 | m/z: 762.64

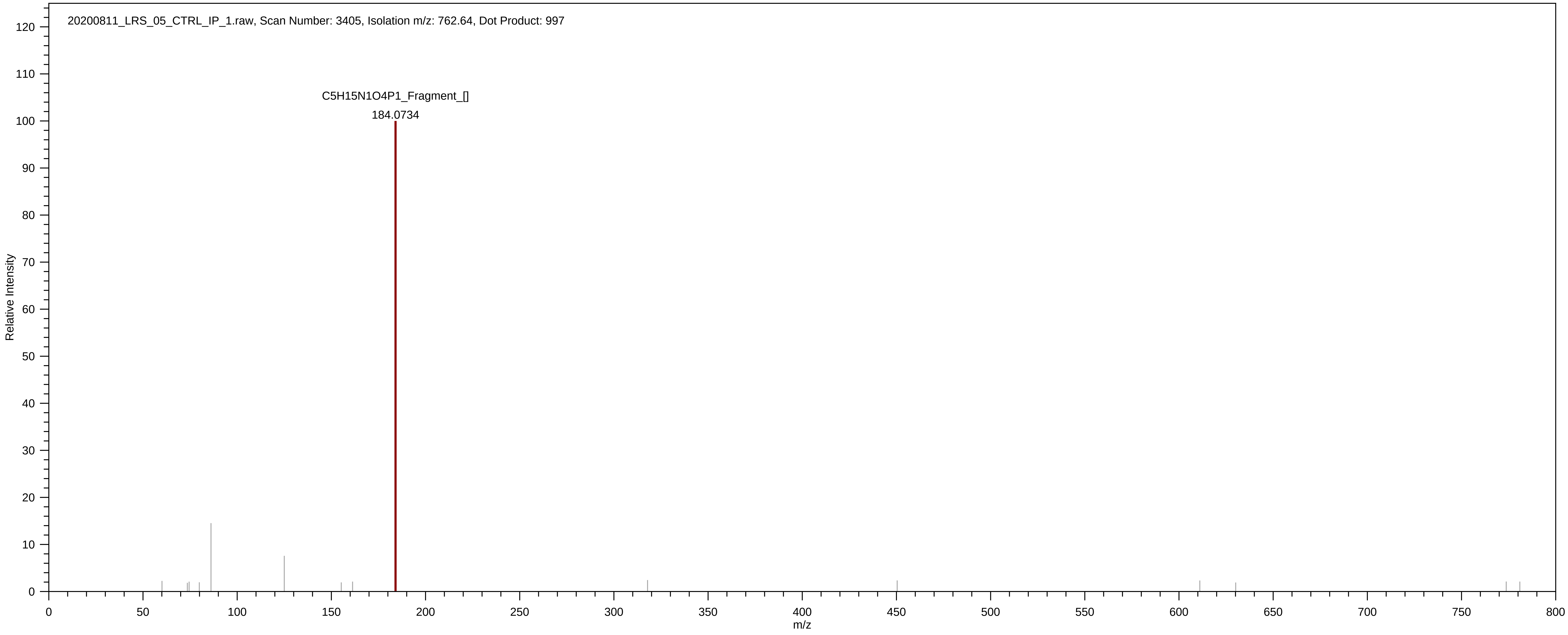

Plasmanyl-PC O-35:0 [M+H]+ | Ret. Time (min): 11.814 | m/z: 762.638

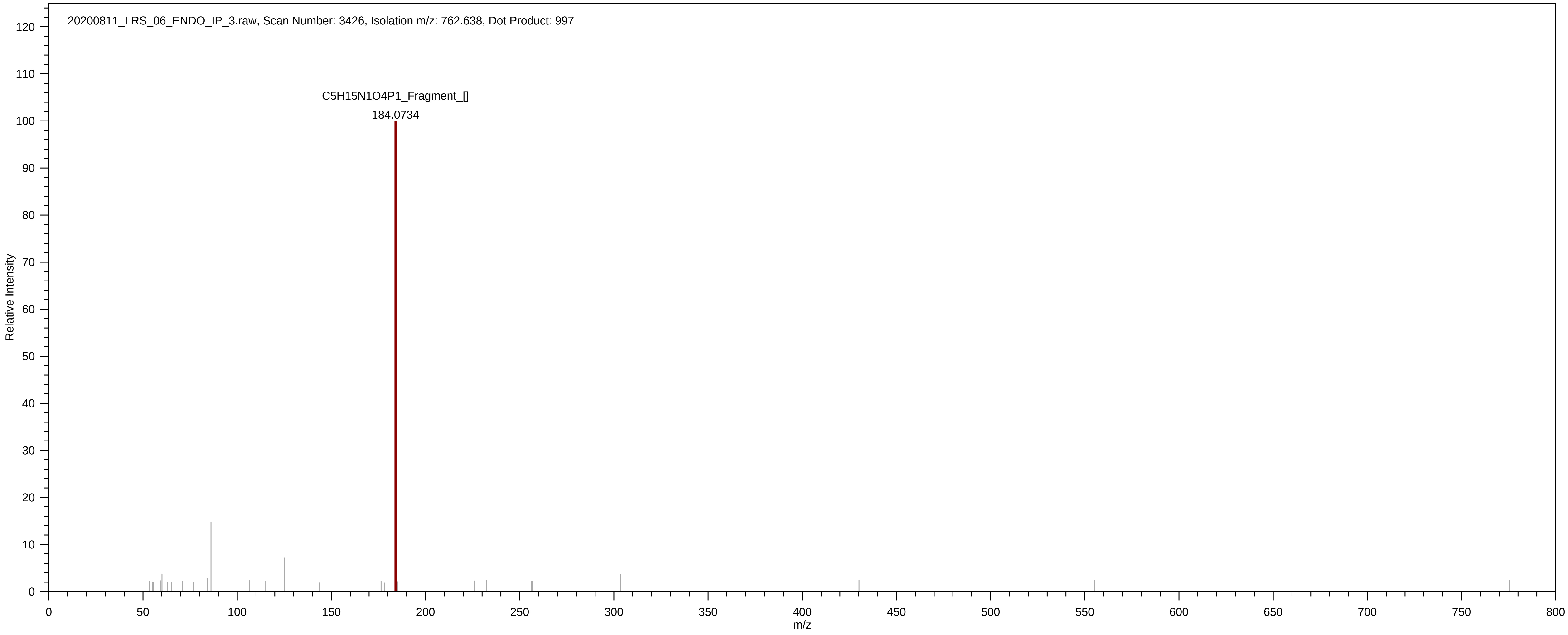

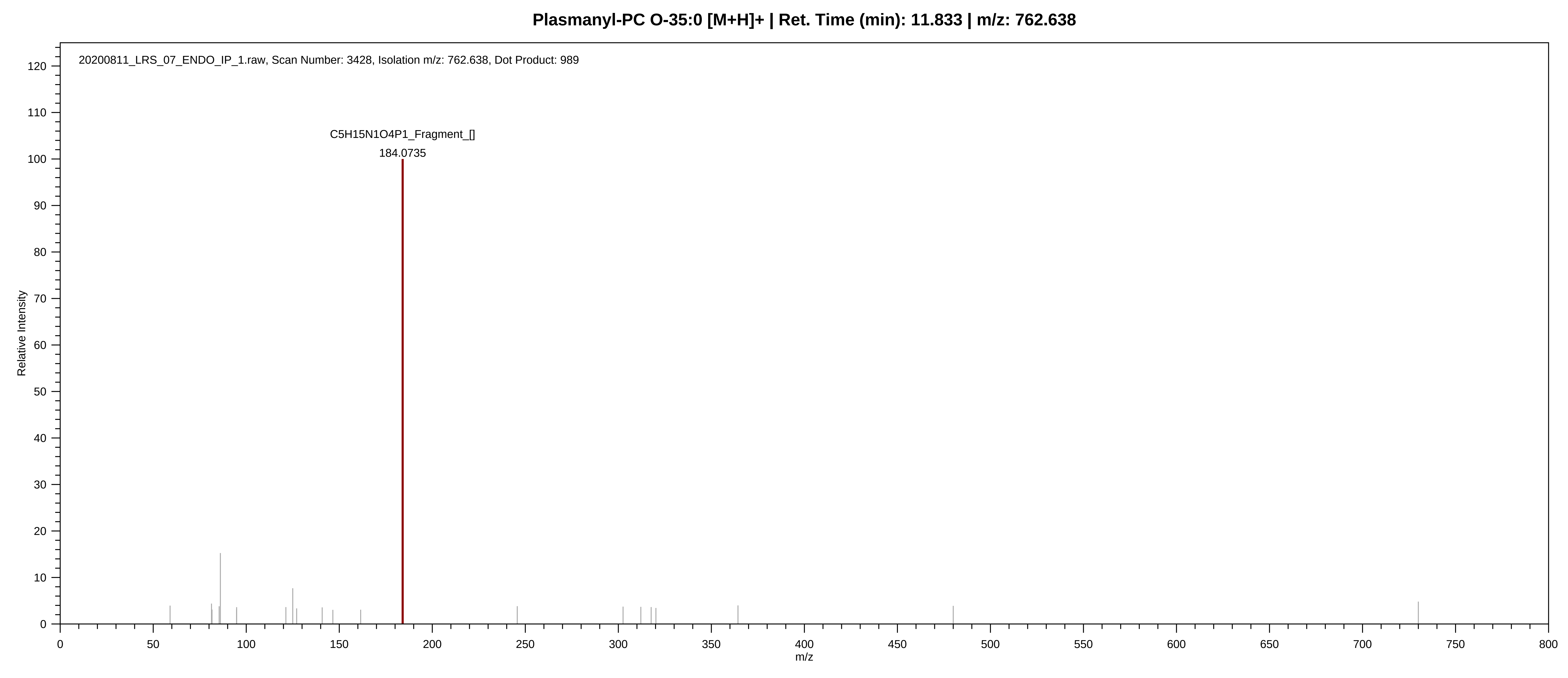

Plasmanyl-PC O-35:0 [M+H]+ | Ret. Time (min): 11.833 | m/z: 762.638
20200811_LRS_07_ENDO_IP_1.raw, Scan Number: 3428, Isolation m/z: 762.638, Dot Product: 989
C5H15N1O4P1_Fragment_[]
184.0735
Relative Intensity
m/z

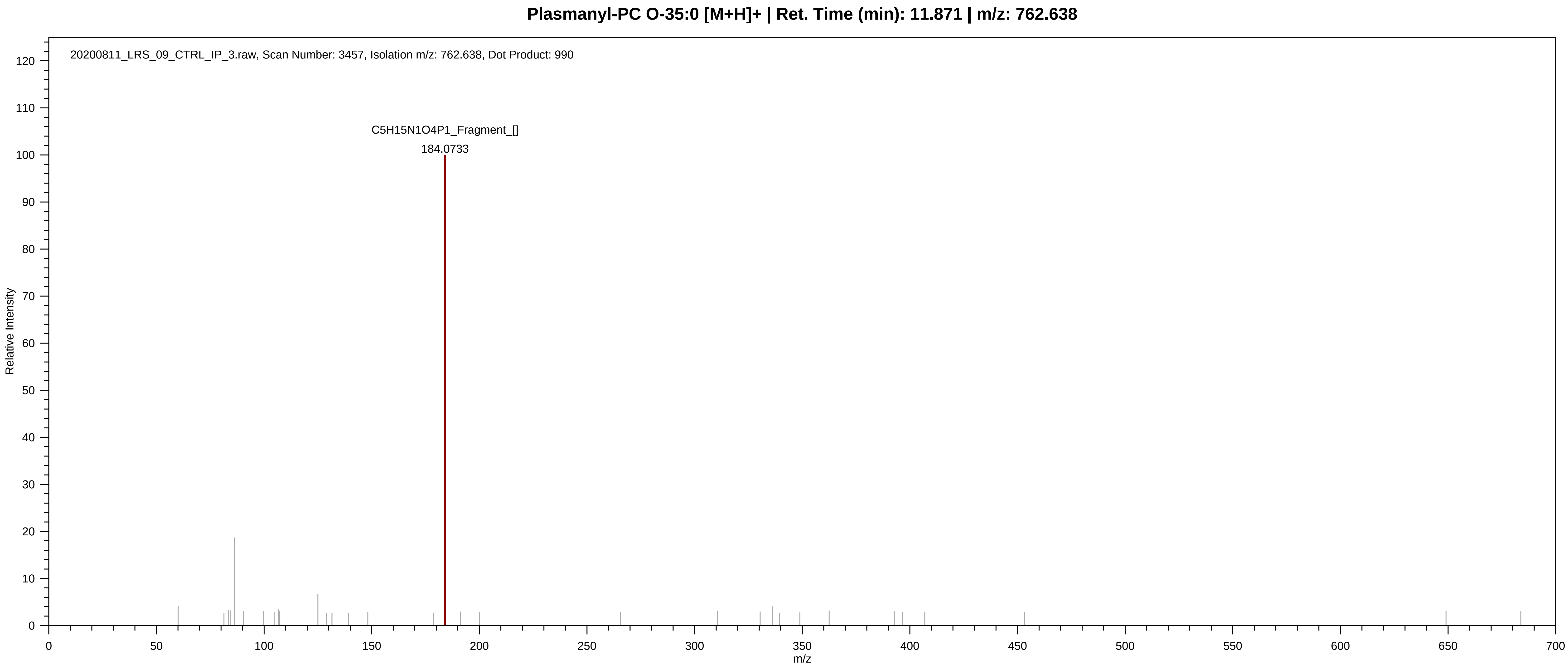
Plasmanyl-PC O-35:0 [M+H]+ | Ret. Time (min): 11.871 | m/z: 762.638
20200811_LRS_09_CTRL_IP_3.raw, Scan Number: 3457, Isolation m/z: 762.638, Dot Product: 990
C5H15N1O4P1_Fragment_[]
184.0733
Relative Intensity
m/z
0
10
20
30
40
50
60
70
80
90
100
110
120
0
50
100
150
200
250
300
350
400
450
500
550
600
650
700

Plasmanyl-PC O-35:0 [M+H]+ | Ret. Time (min): 11.895 | m/z: 762.638

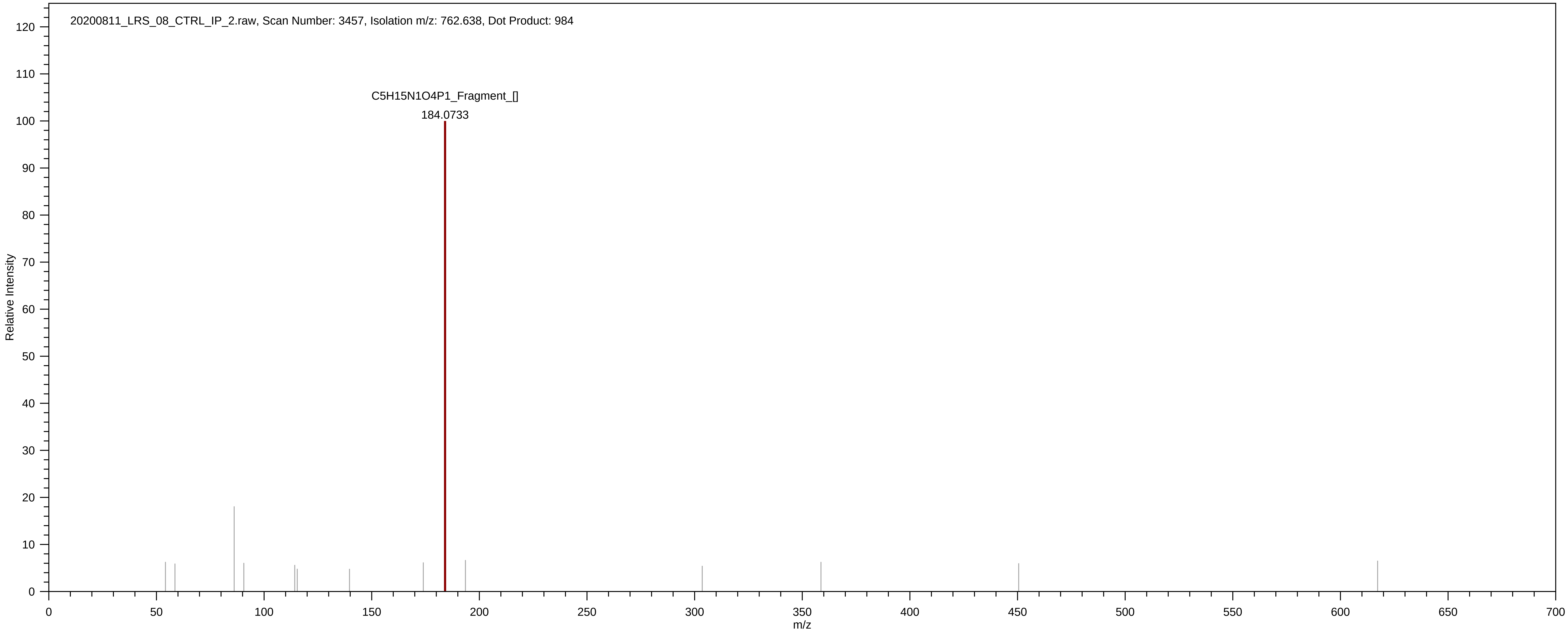

Plasmanyl-PC O-35:0 [M+H]+ | Ret. Time (min): 11.954 | m/z: 762.638

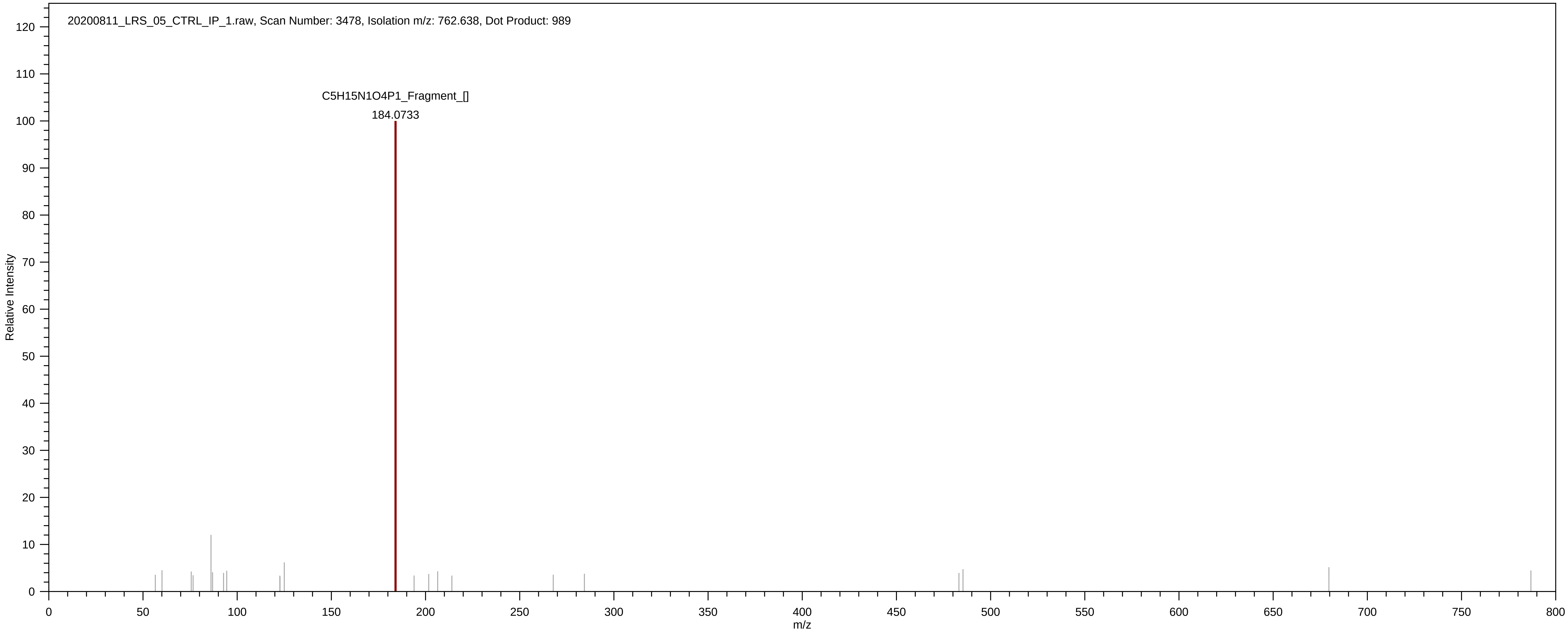

Plasmanyl-PC O-35:0 [M+H]+ | Ret. Time (min): 12.017 | m/z: 762.638

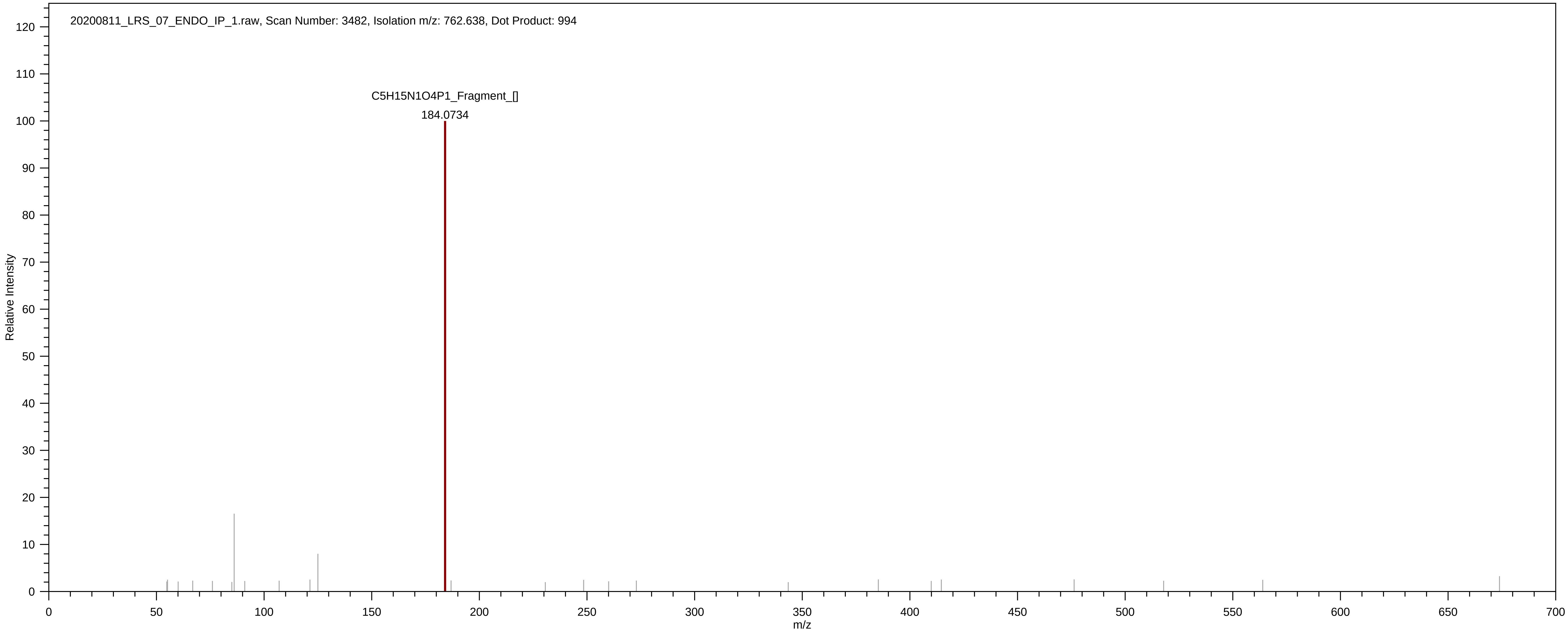

Plasmanyl-PC O-35:0 [M+H]+ | Ret. Time (min): 12.056 | m/z: 762.638

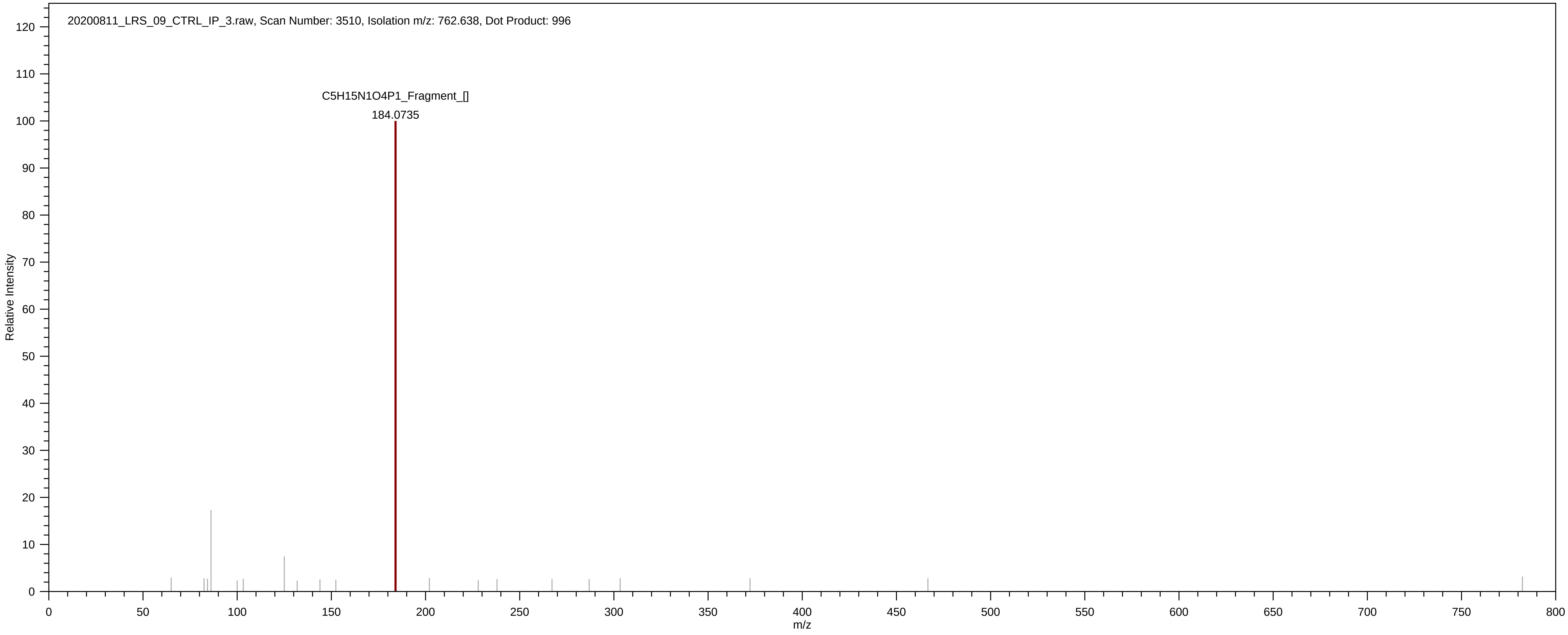

Plasmanyl-PC O-35:0 [M+H]+ | Ret. Time (min): 11.708 | m/z: 762.639

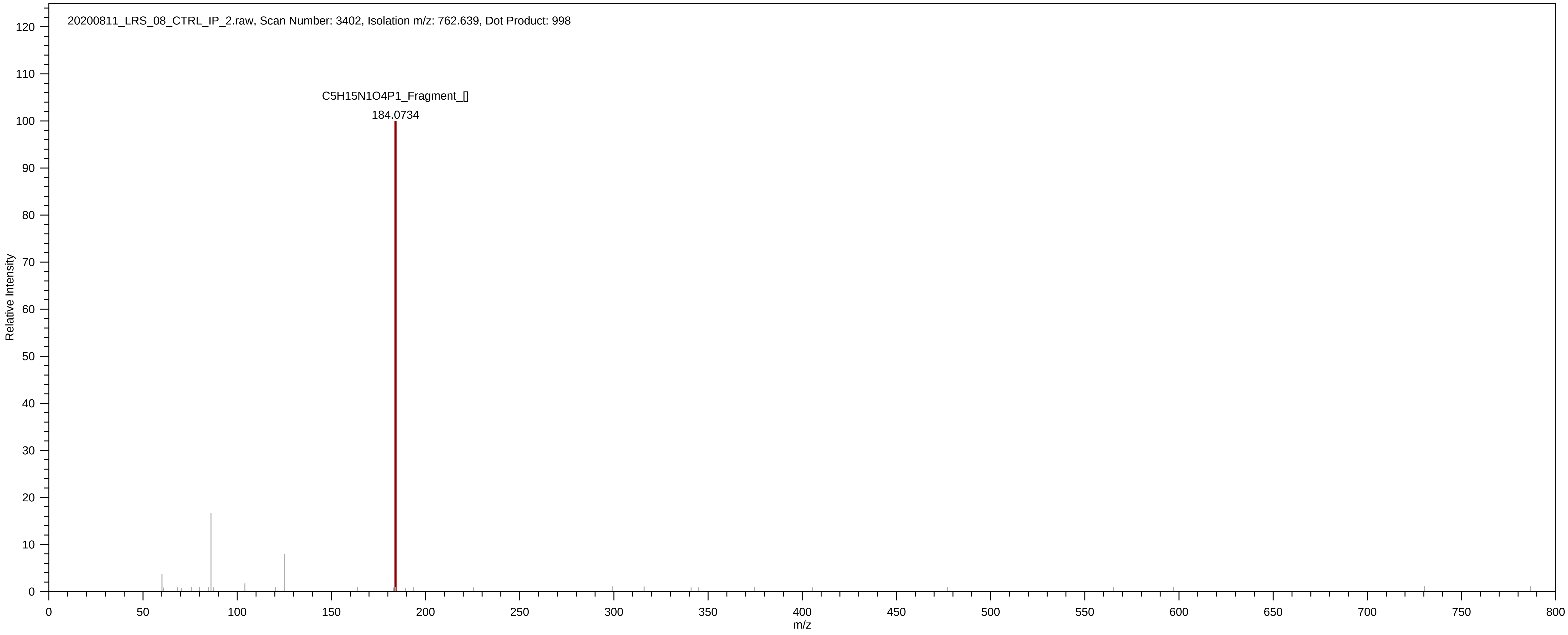

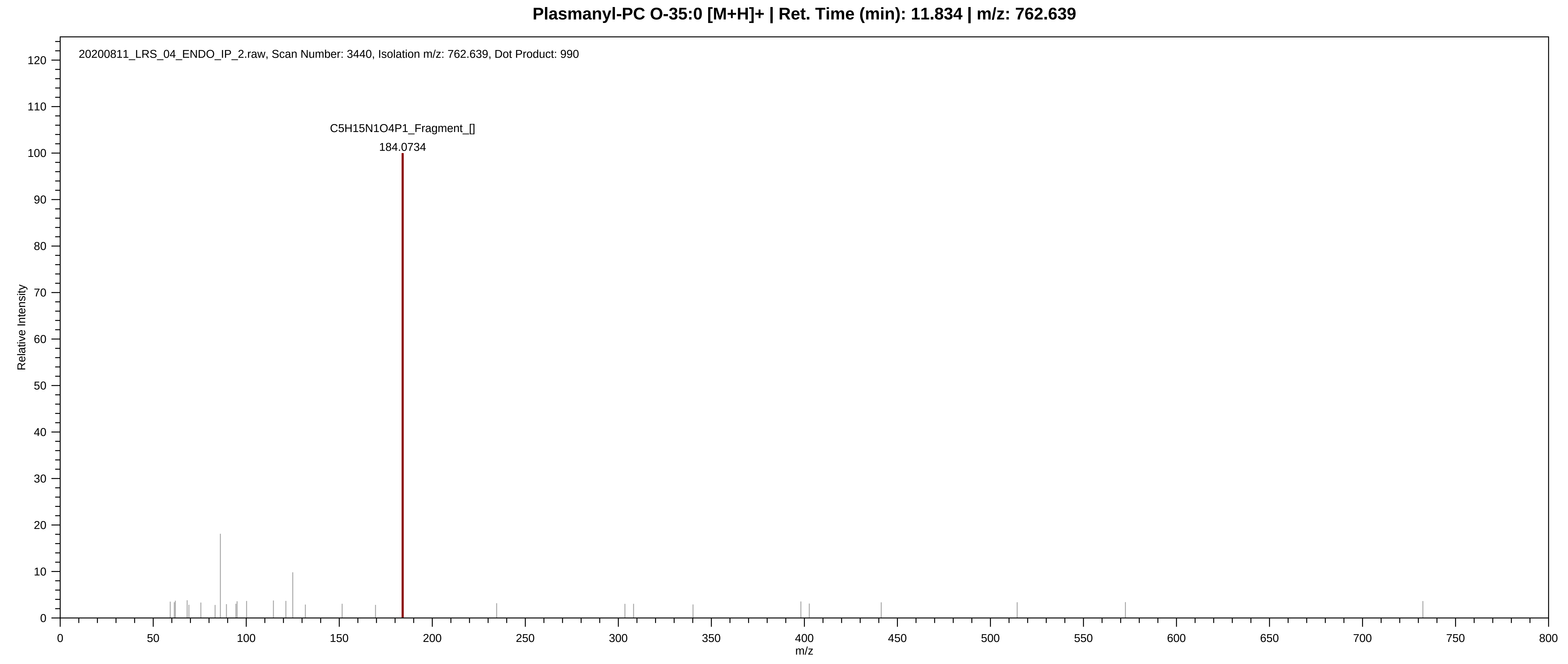

Plasmanyl-PC O-35:0 [M+H]+ | Ret. Time (min): 11.834 | m/z: 762.639
20200811_LRS_04_ENDO_IP_2.raw, Scan Number: 3440, Isolation m/z: 762.639, Dot Product: 990
C5H15N1O4P1_Fragment_[]
184.0734
Relative Intensity
m/z

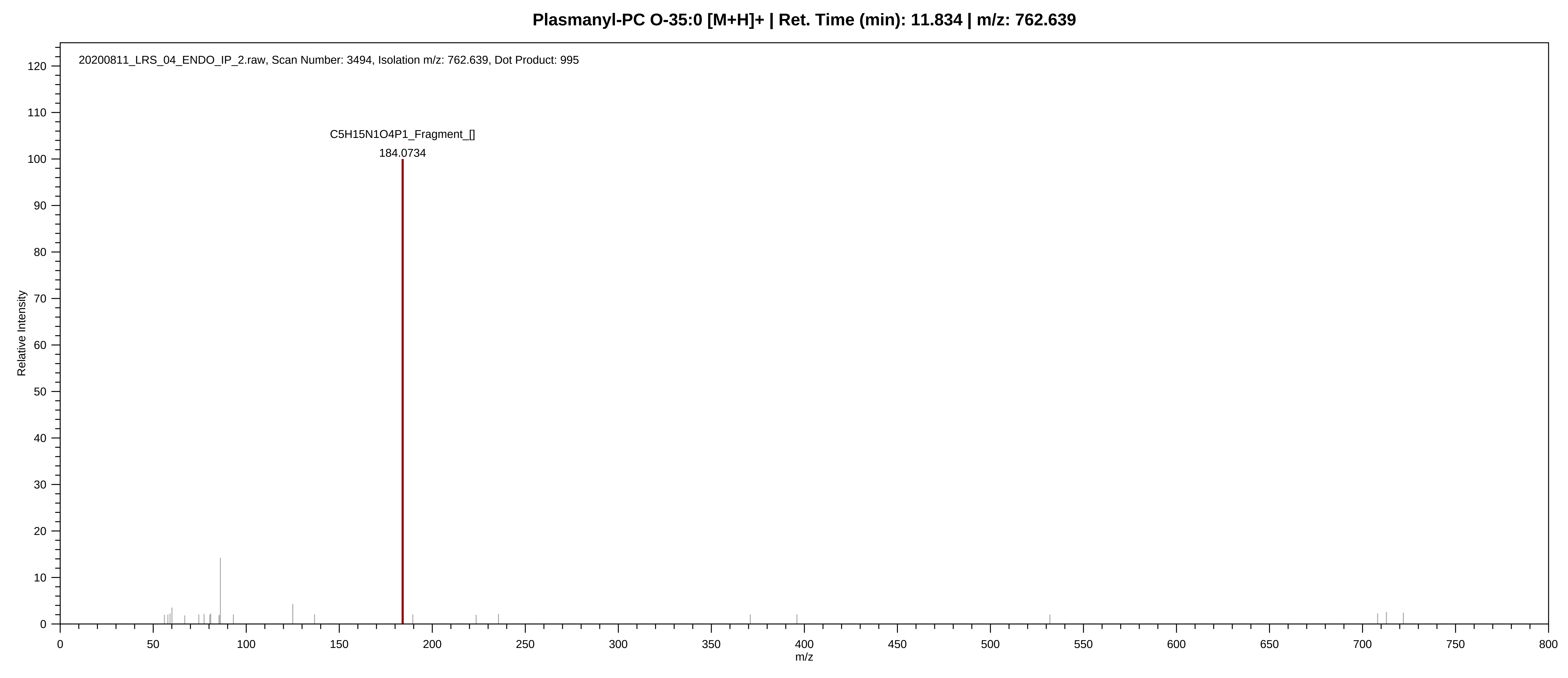

Plasmanyl-PC O-35:0 [M+H]+ | Ret. Time (min): 11.834 | m/z: 762.639
20200811_LRS_04_ENDO_IP_2.raw, Scan Number: 3494, Isolation m/z: 762.639, Dot Product: 995
C5H15N1O4P1_Fragment_[]
184.0734
Relative Intensity
m/z
0
10
20
30
40
50
60
70
80
90
100
110
120
0
50
100
150
200
250
300
350
400
450
500
550
600
650
700
750
800

Plasmanyl-PC O-35:0 [M+H]+ | Ret. Time (min): 11.994 | m/z: 762.639

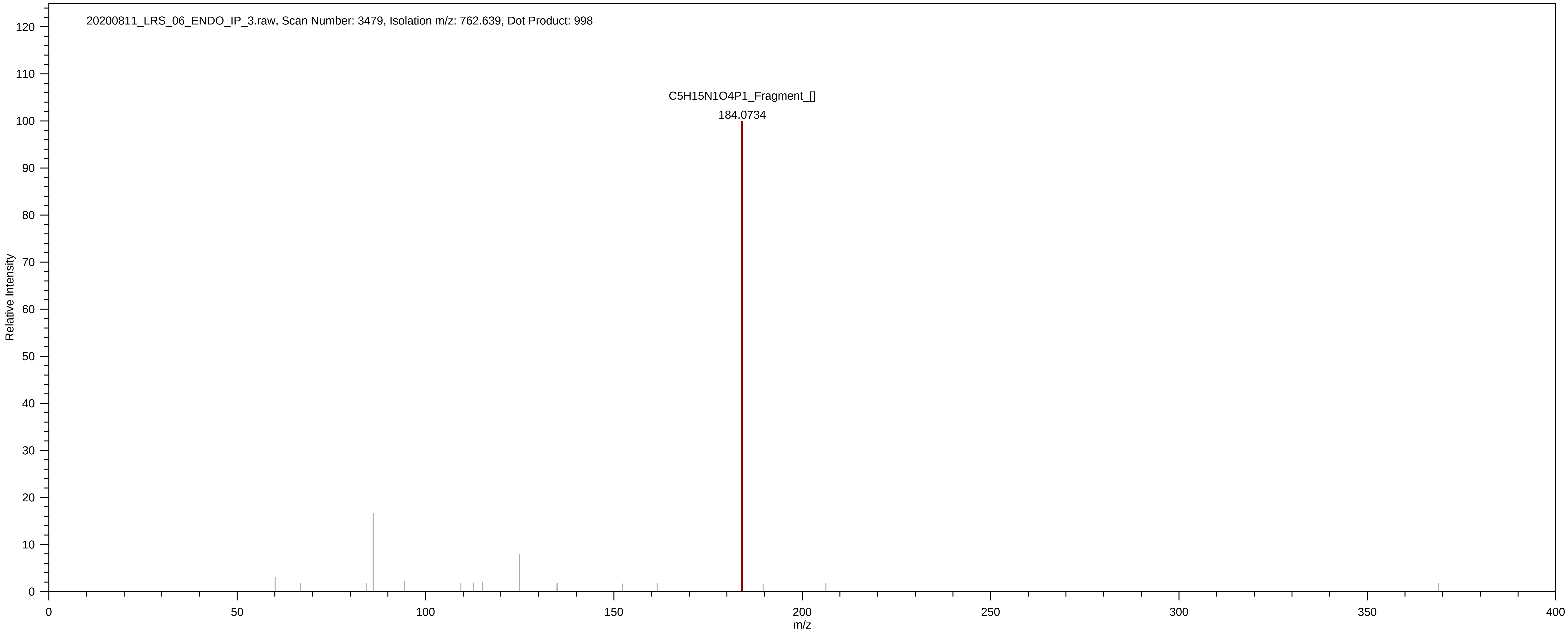

Plasmanyl-PC O-35:1 [M+H]+ | Ret. Time (min): 10.869 | m/z: 760.627

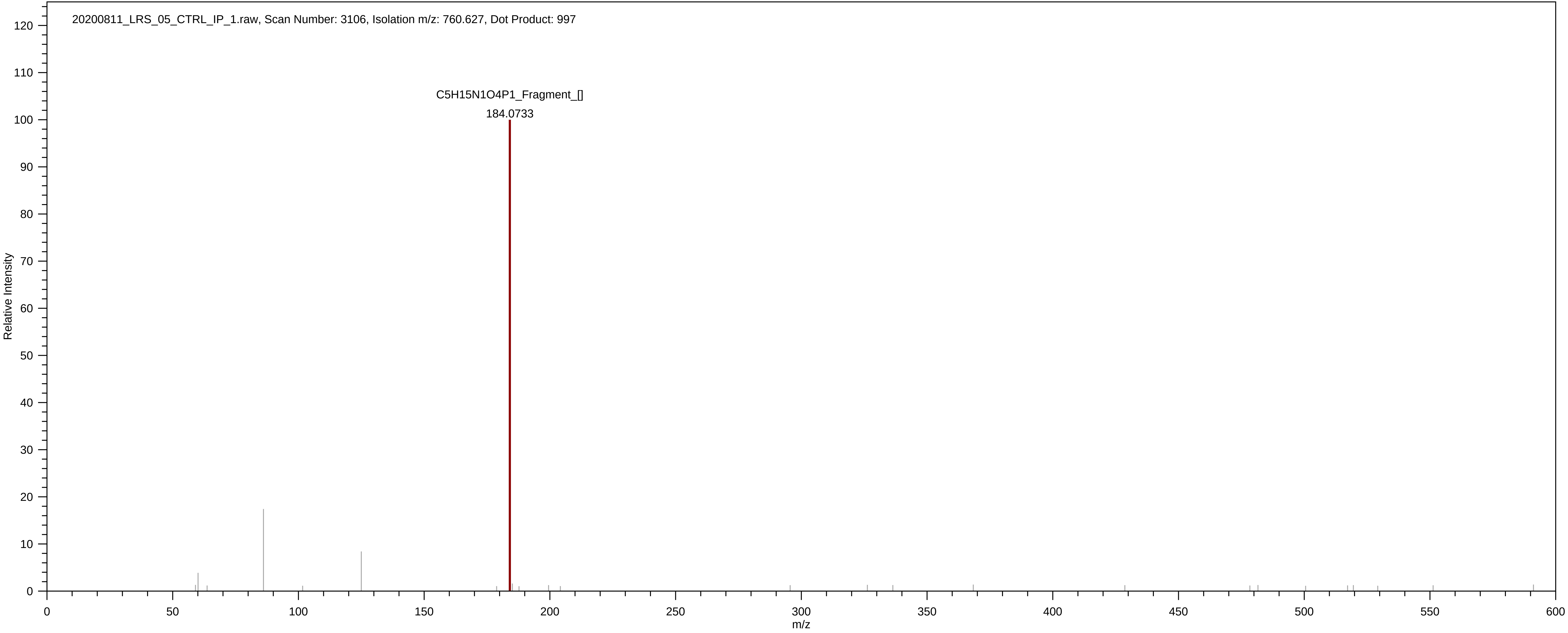

Plasmanyl-PC O-35:1 [M+H]+ | Ret. Time (min): 10.869 | m/z: 760.622

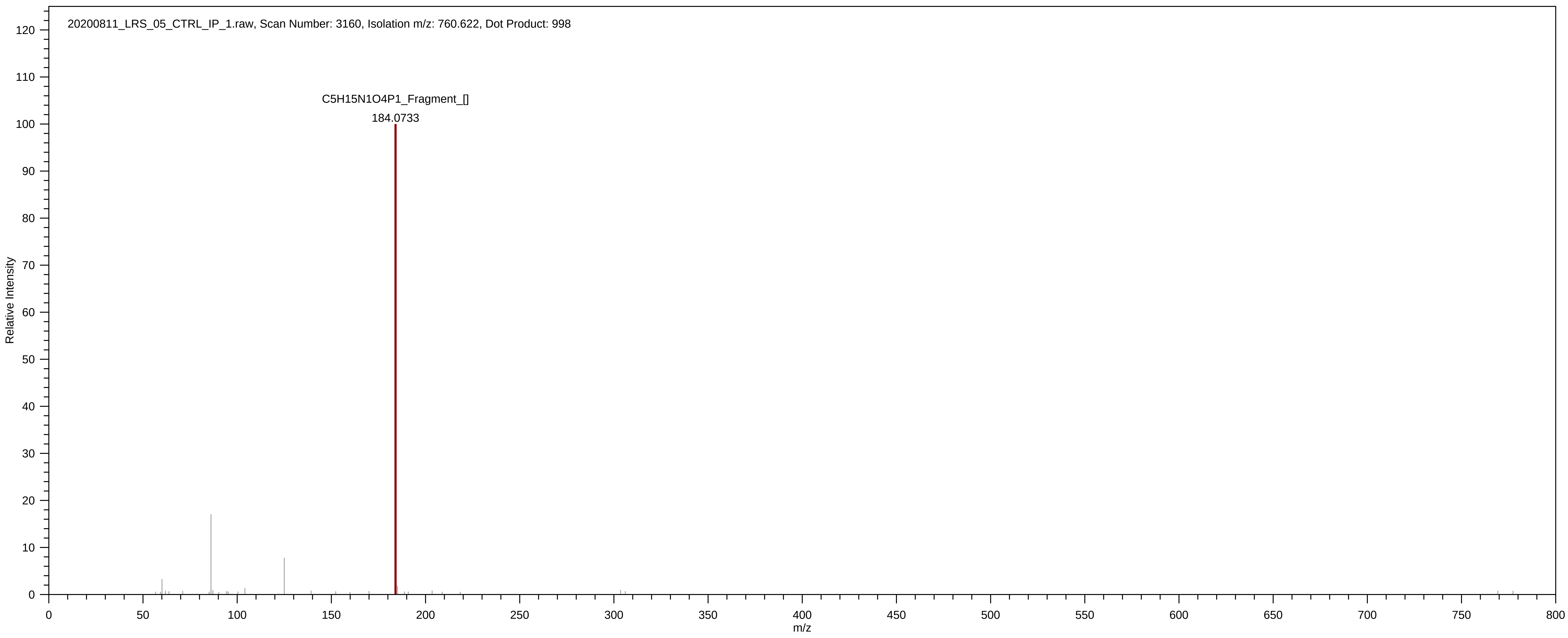

Plasmanyl-PC O-35:1 [M+H]+ | Ret. Time (min): 10.872 | m/z: 760.626

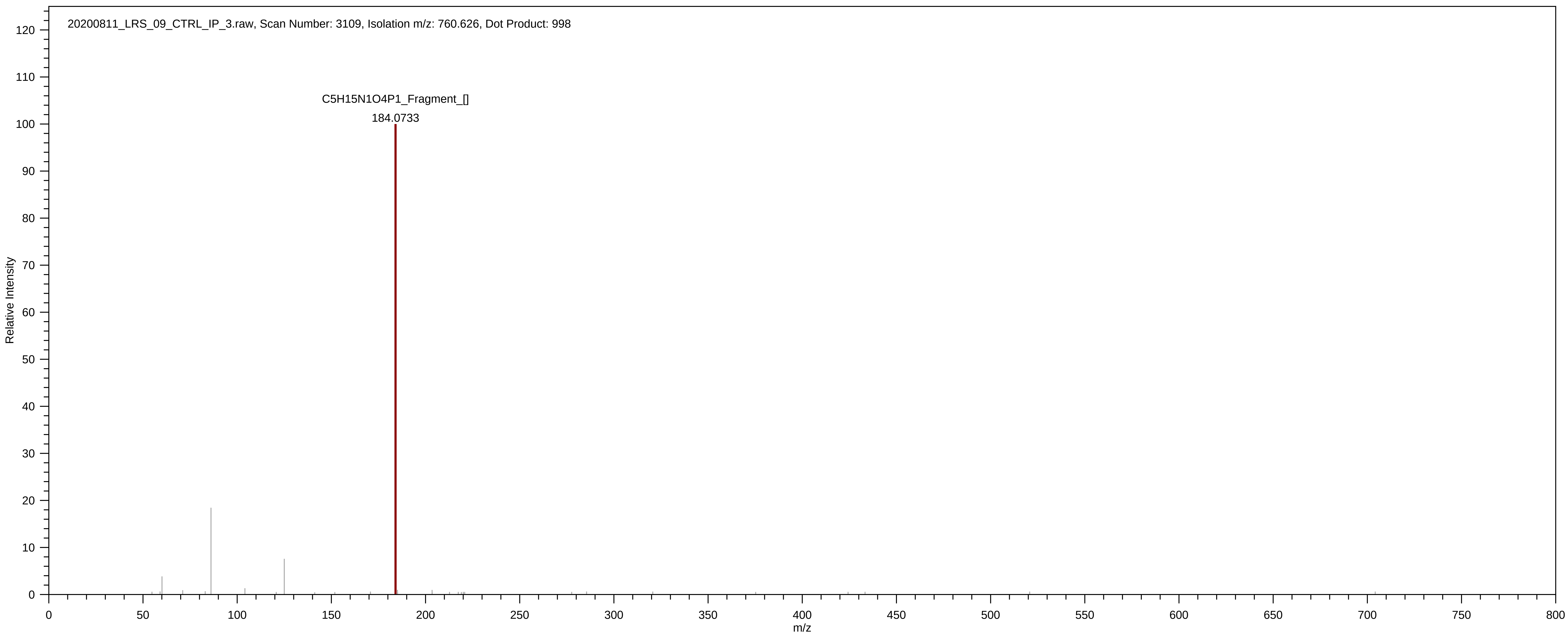

Plasmanyl-PC O-35:1 [M+H]+ | Ret. Time (min): 10.872 | m/z: 760.622

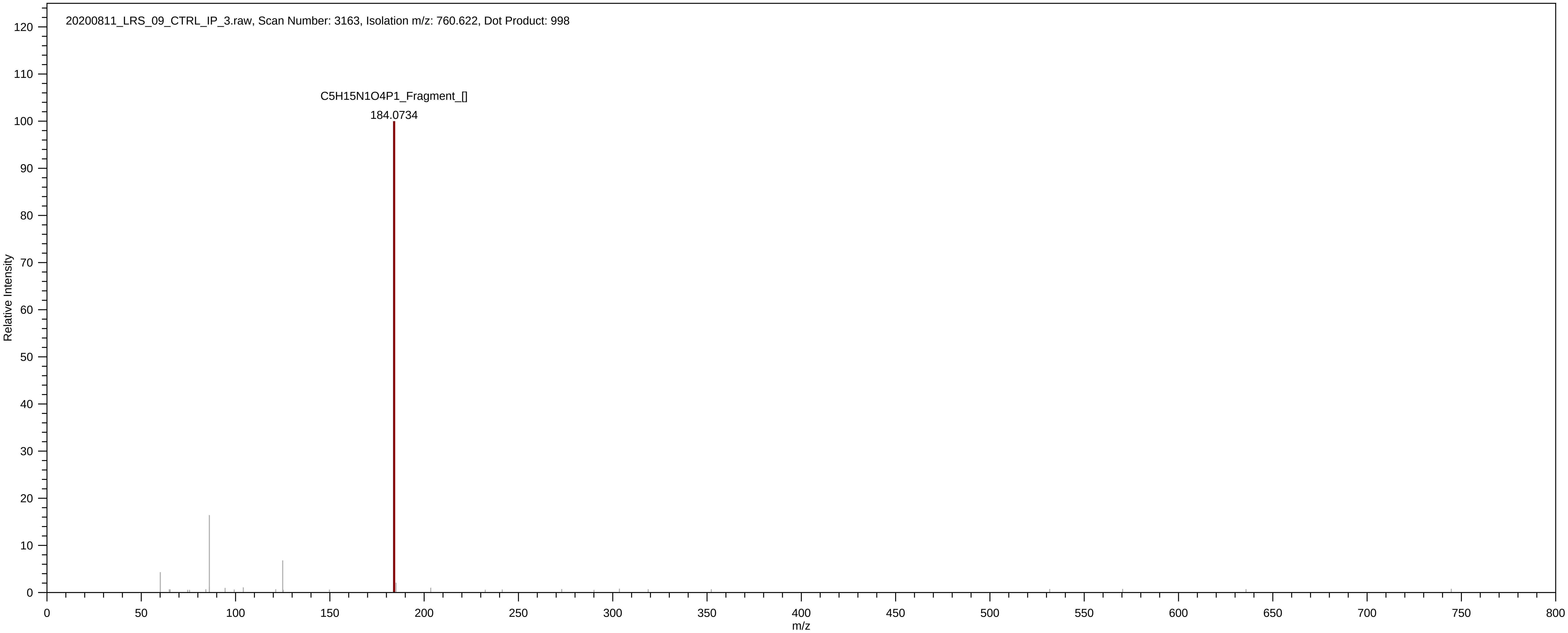

Plasmanyl-PC O-35:1 [M+H]+ | Ret. Time (min): 10.875 | m/z: 760.626

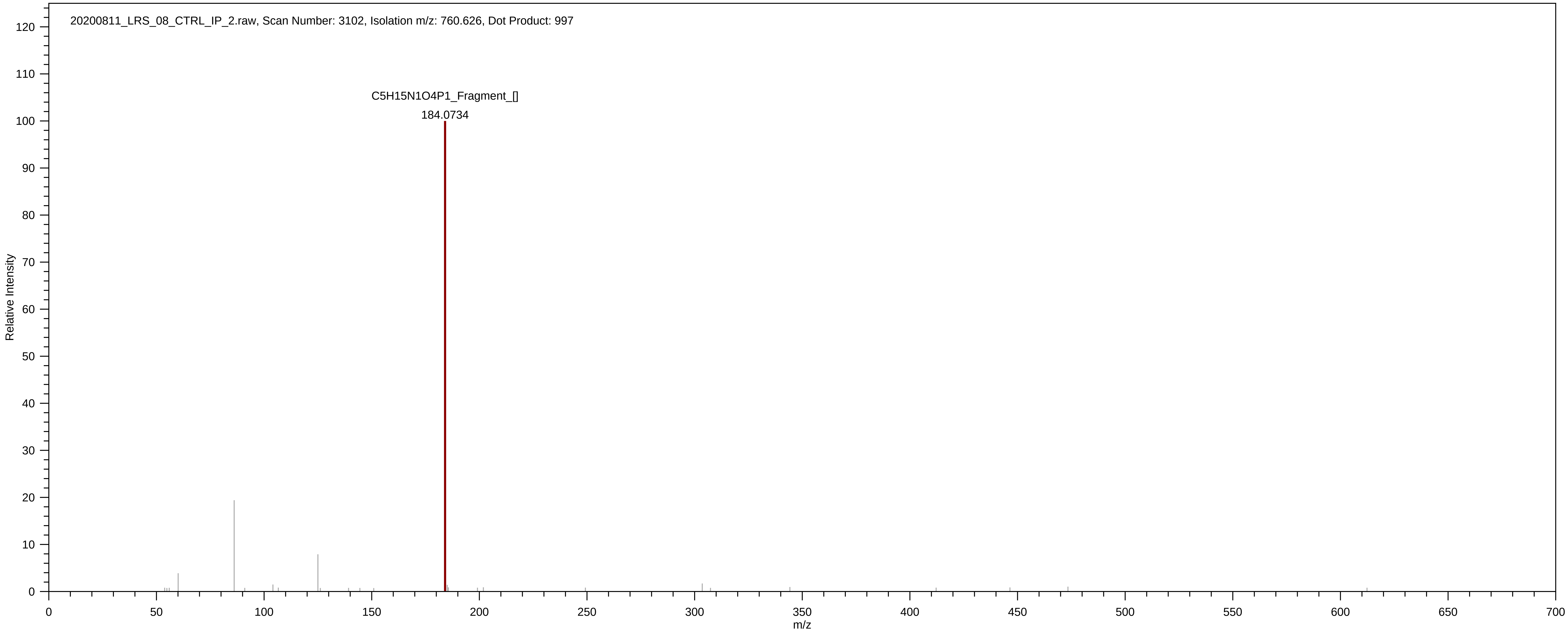

Plasmanyl-PC O-35:1 [M+H]+ | Ret. Time (min): 10.875 | m/z: 760.622

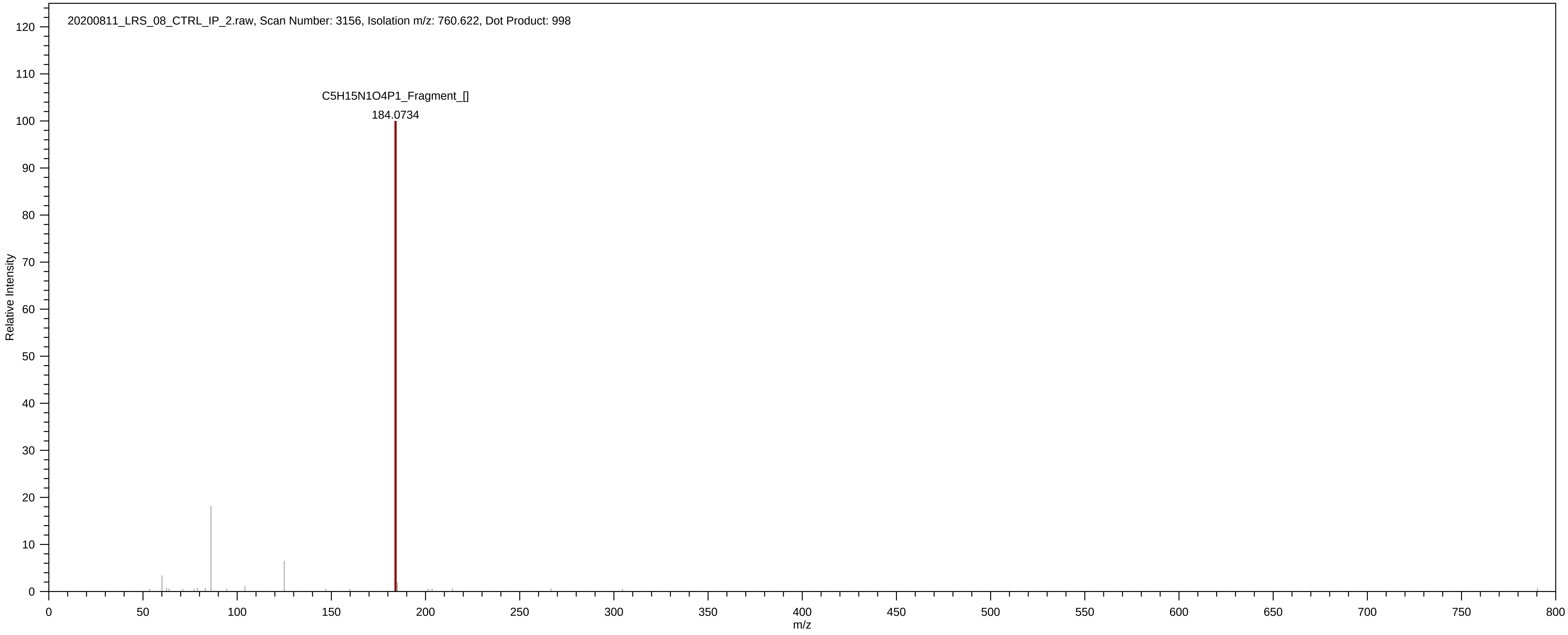

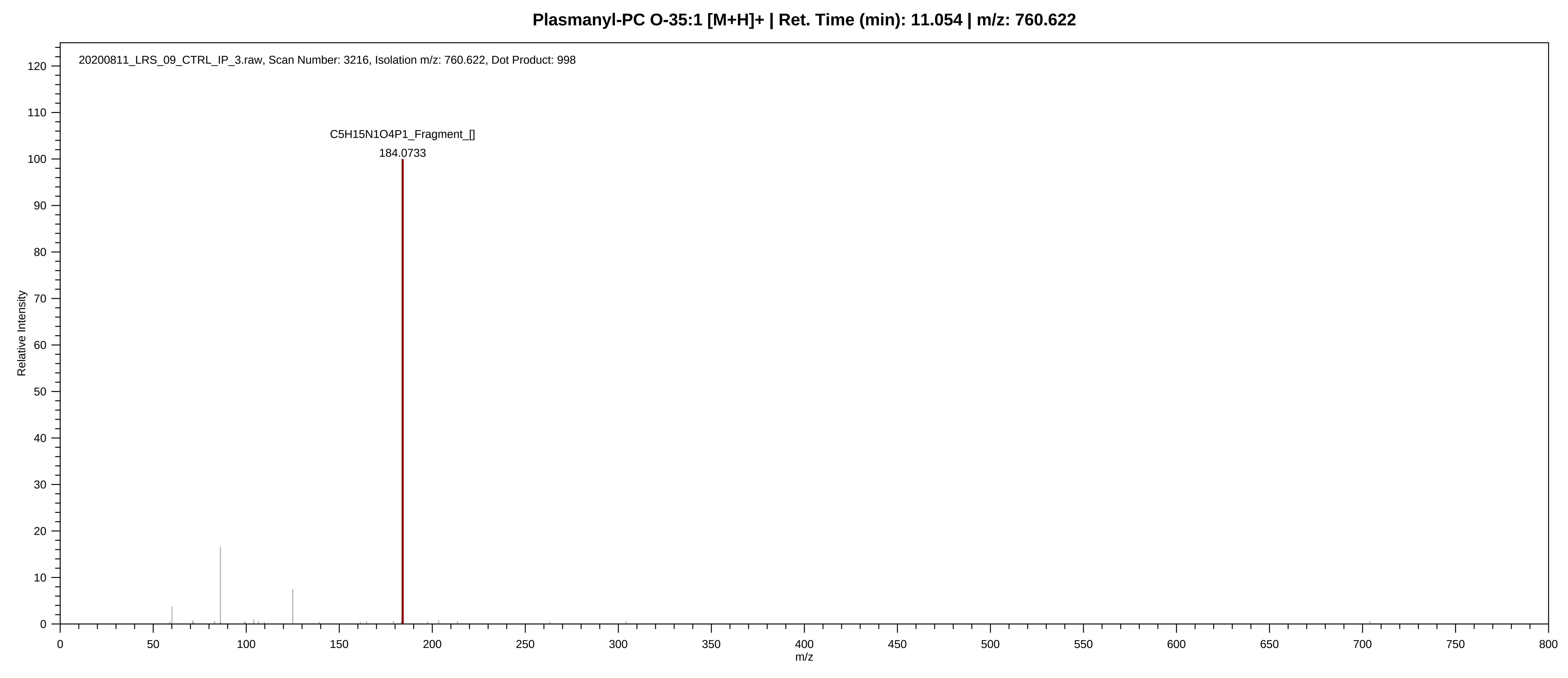
Plasmanyl-PC O-35:1 [M+H]+ | Ret. Time (min): 11.054 | m/z: 760.622
20200811_LRS_09_CTRL_IP_3.raw, Scan Number: 3216, Isolation m/z: 760.622, Dot Product: 998
C5H15N1O4P1_Fragment_[]
184.0733
Relative Intensity
m/z
0
10
20
30
40
50
60
70
80
90
100
110
120
0
50
100
150
200
250
300
350
400
450
500
550
600
650
700
750
800

Plasmanyl-PC O-35:1 [M+H]+ | Ret. Time (min): 11.05 | m/z: 760.623

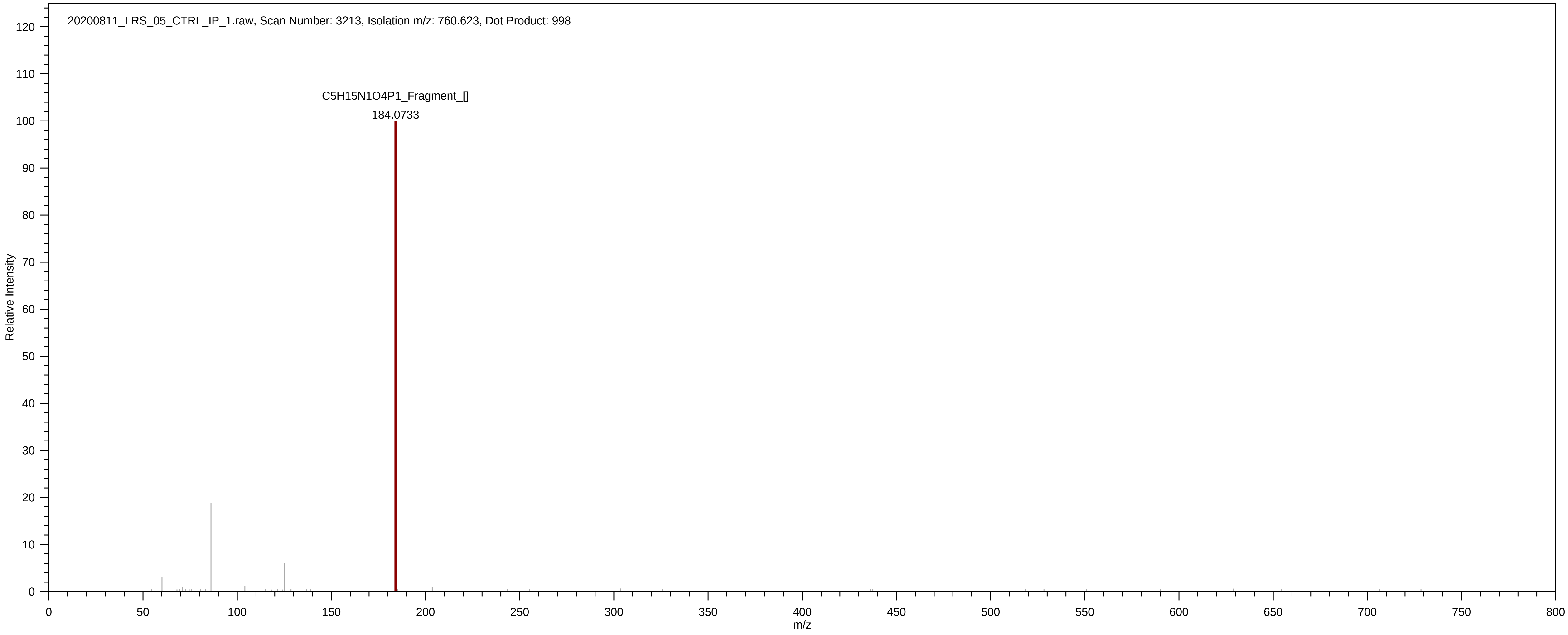

Plasmanyl-PC O-35:1 [M+H]+ | Ret. Time (min): 11.058 | m/z: 760.623

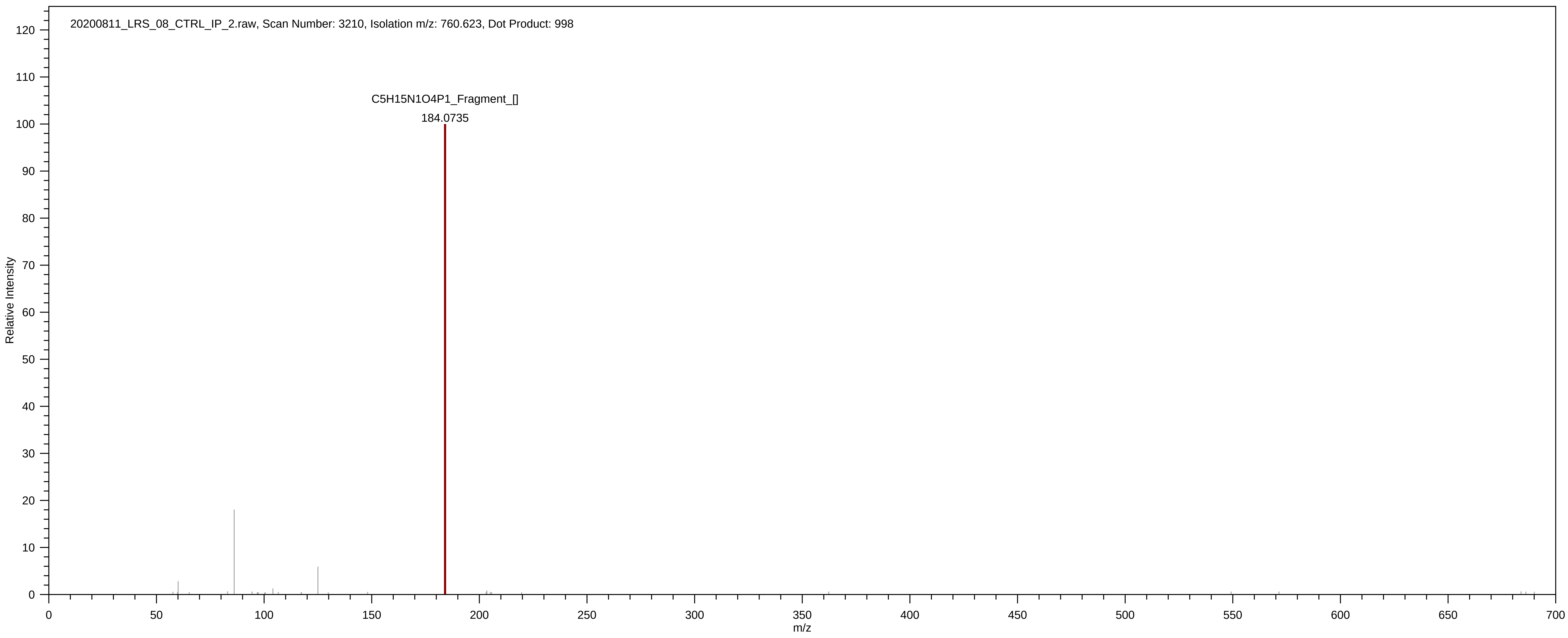

Plasmanyl-PC O-35:1 [M+H]+ | Ret. Time (min): 10.816 | m/z: 760.624

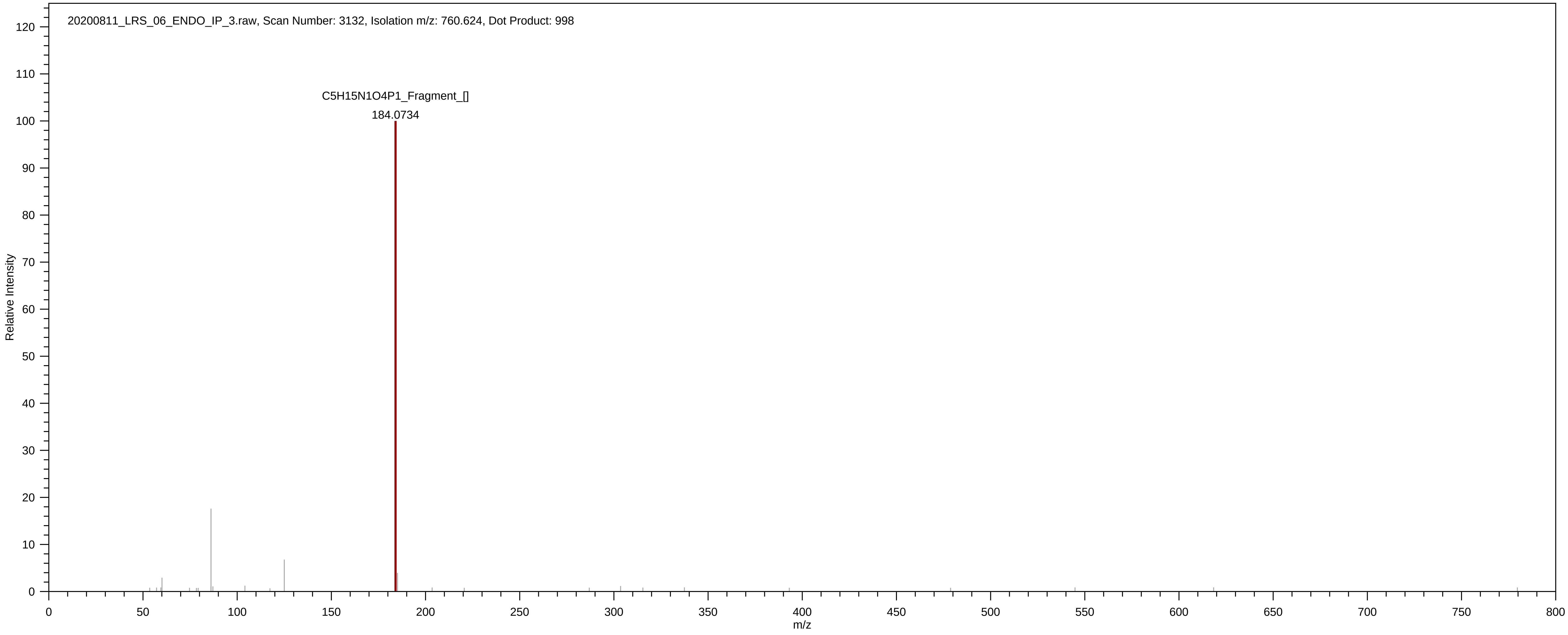

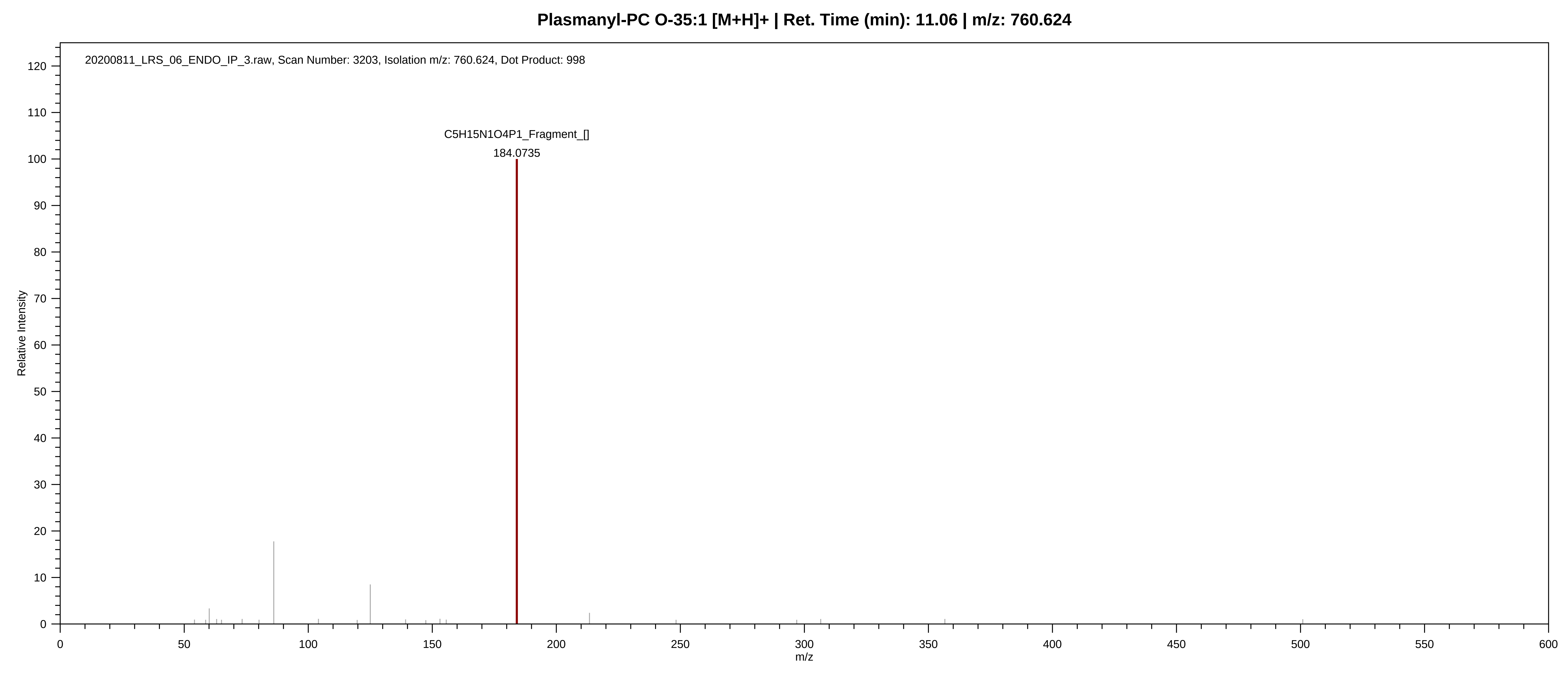

Plasmanyl-PC O-35:1 [M+H]+ | Ret. Time (min): 11.06 | m/z: 760.624
20200811_LRS_06_ENDO_IP_3.raw, Scan Number: 3203, Isolation m/z: 760.624, Dot Product: 998
C5H15N1O4P1_Fragment_[]
184.0735
Relative Intensity
m/z

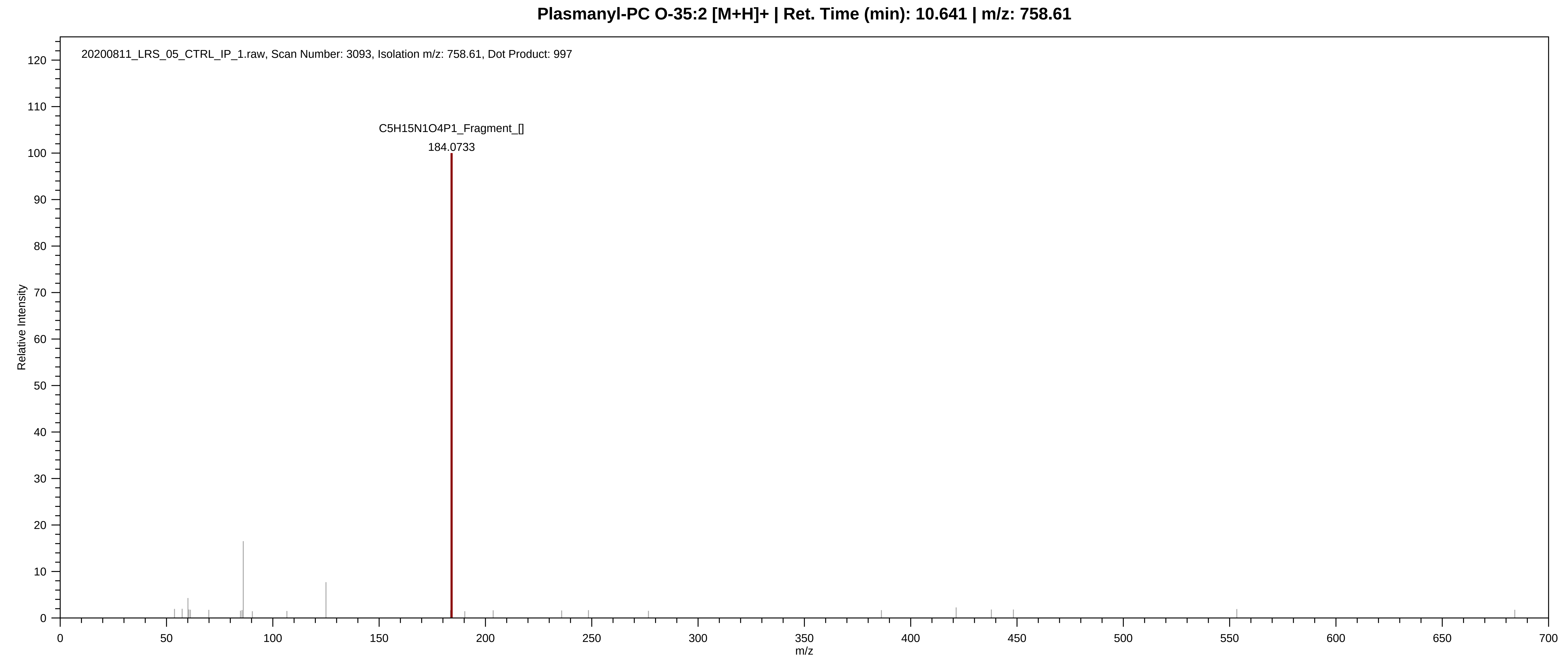

Plasmanyl-PC O-35:2 [M+H]+ | Ret. Time (min): 10.641 | m/z: 758.61
20200811_LRS_05_CTRL_IP_1.raw, Scan Number: 3093, Isolation m/z: 758.61, Dot Product: 997
C5H15N1O4P1_Fragment_[]
184.0733
Relative Intensity
m/z

Plasmanyl-PC O-35:2 [M+H]+ | Ret. Time (min): 10.653 | m/z: 758.61

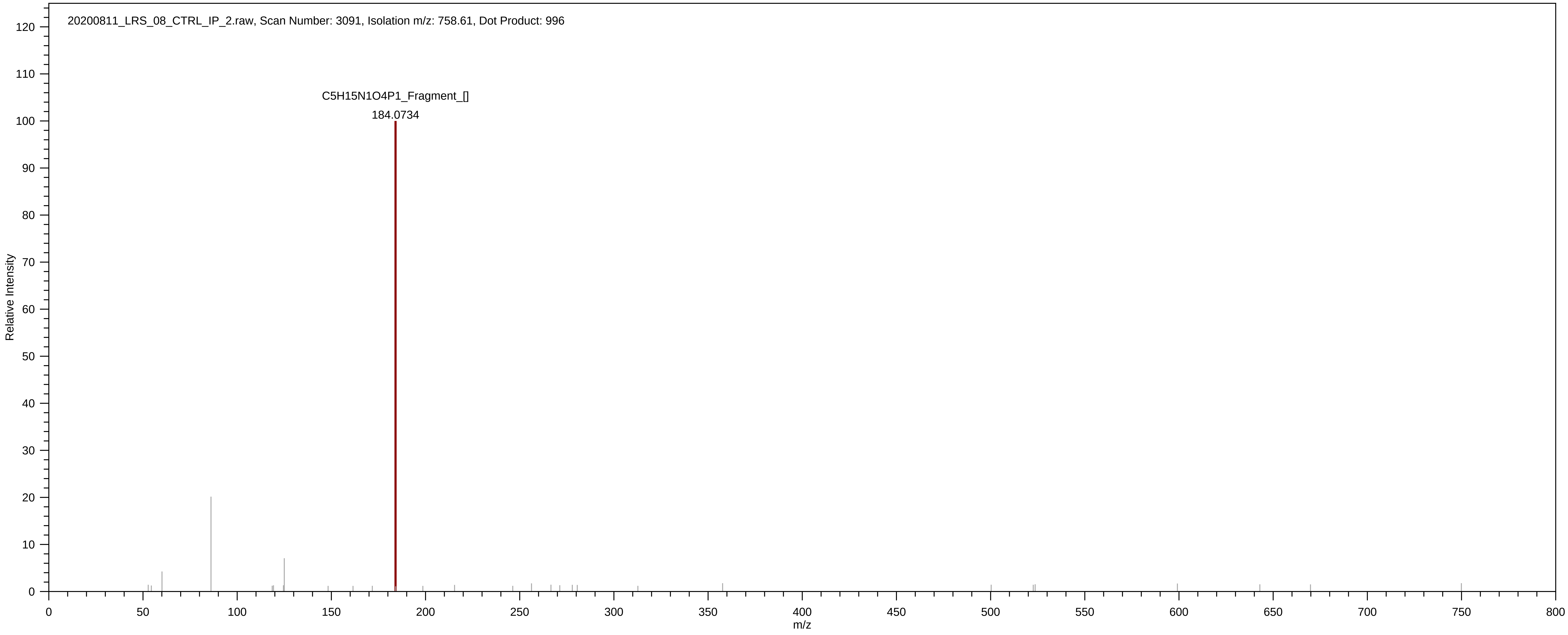

Plasmanyl-PC O-35:2 [M+H]+ | Ret. Time (min): 9.997 | m/z: 758.606

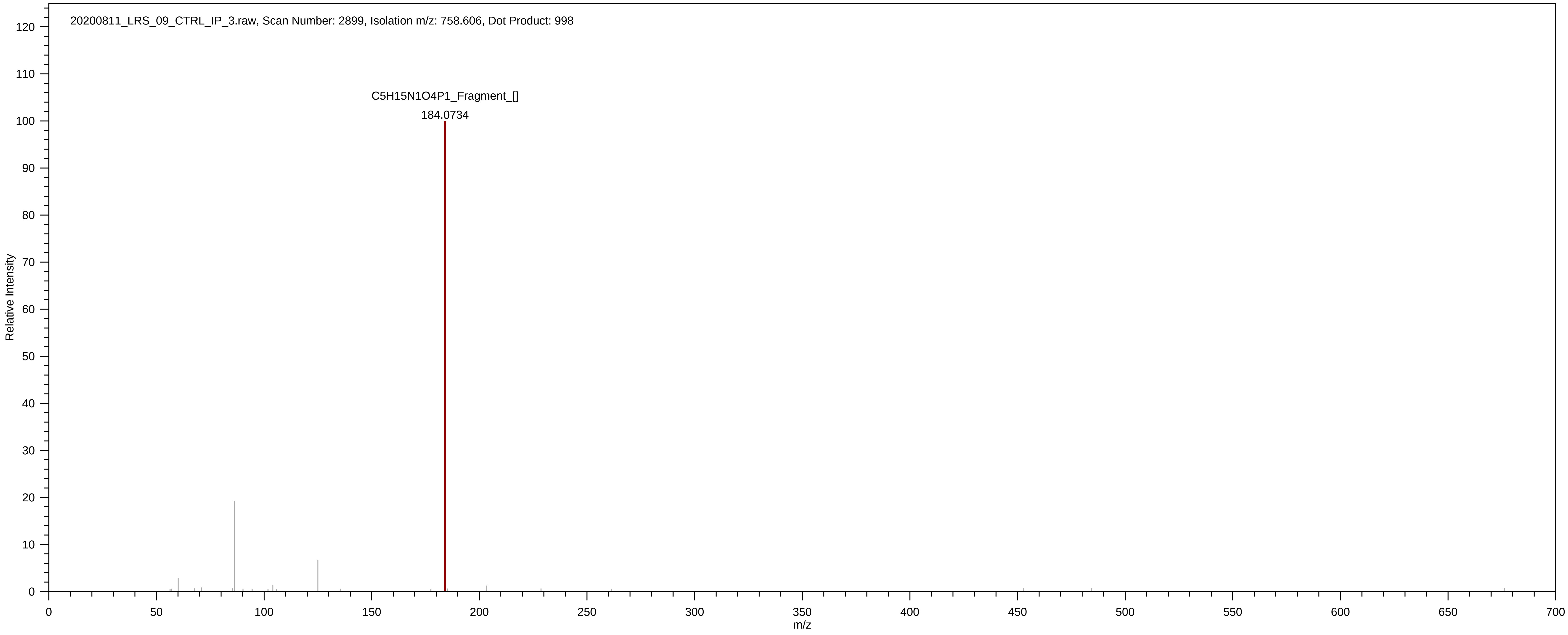

Plasmanyl-PC O-35:2 [M+H]+ | Ret. Time (min): 10.054 | m/z: 758.607

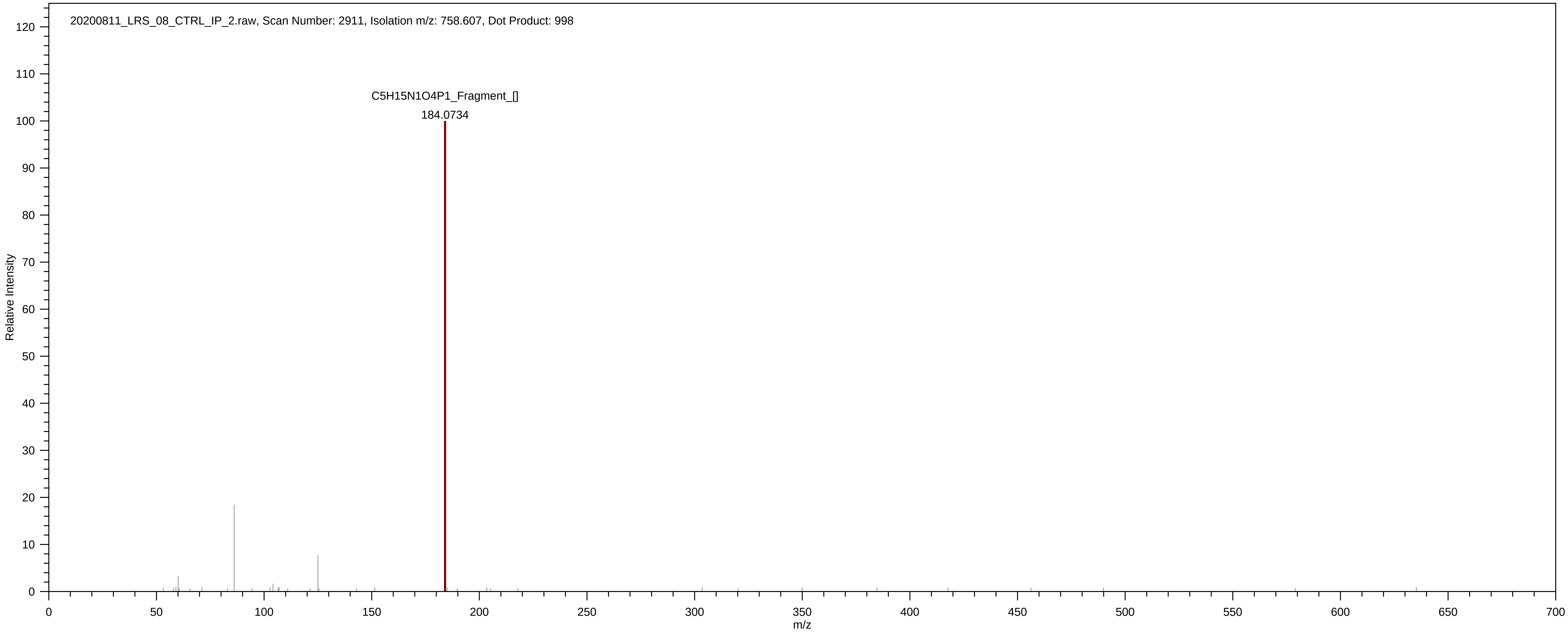

Plasmanyl-PC O-36:0 [M+Ac-H]- | Ret. Time (min): 12.479 | m/z: 834.66

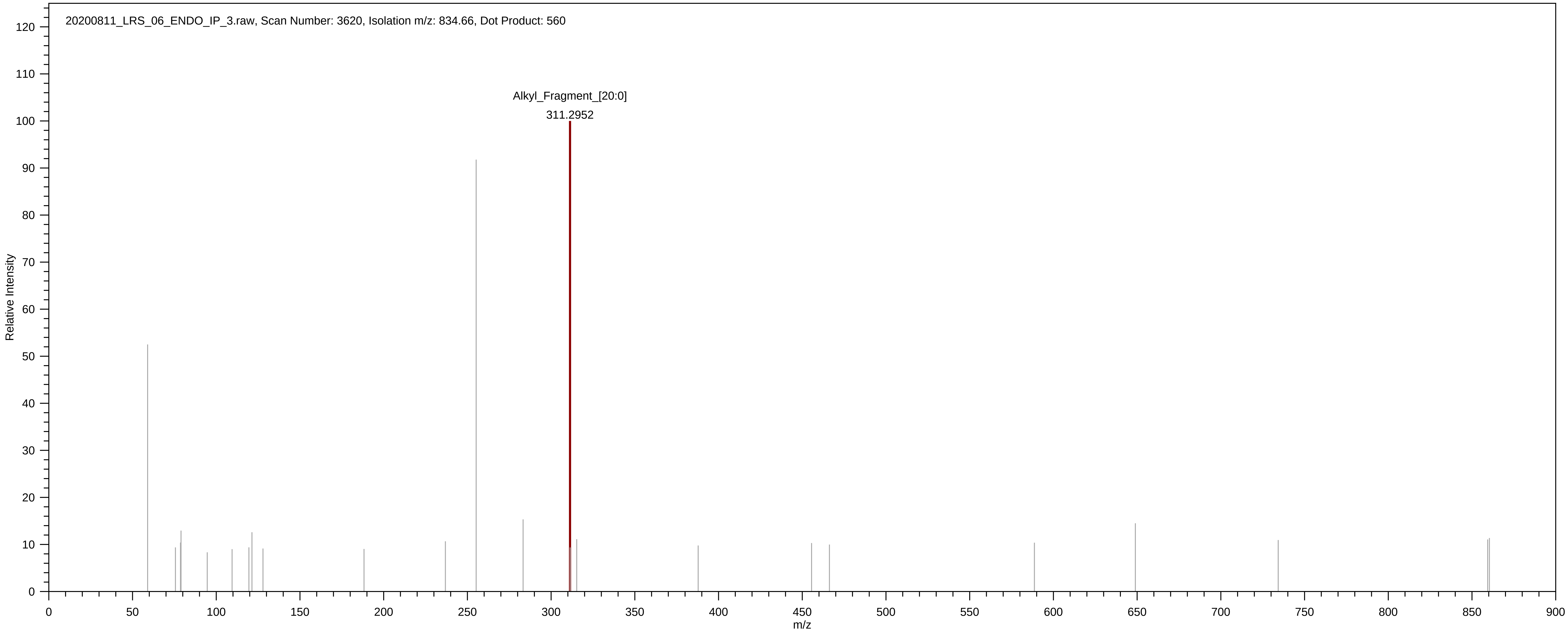

Plasmanyl-PC O-36:0 [M+Ac-H]- | Ret. Time (min): 12.482 | m/z: 834.66

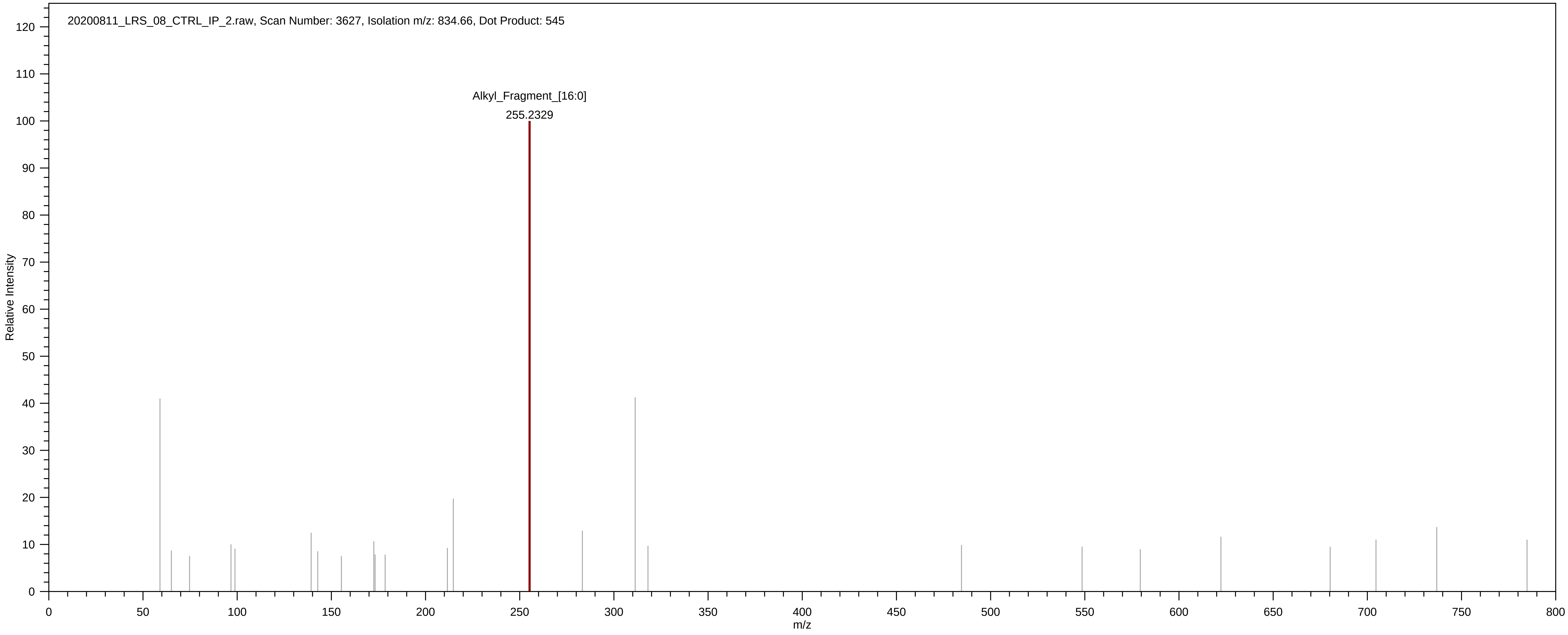

Plasmanyl-PC O-36:0 [M+Ac-H]- | Ret. Time (min): 12.478 | m/z: 834.659

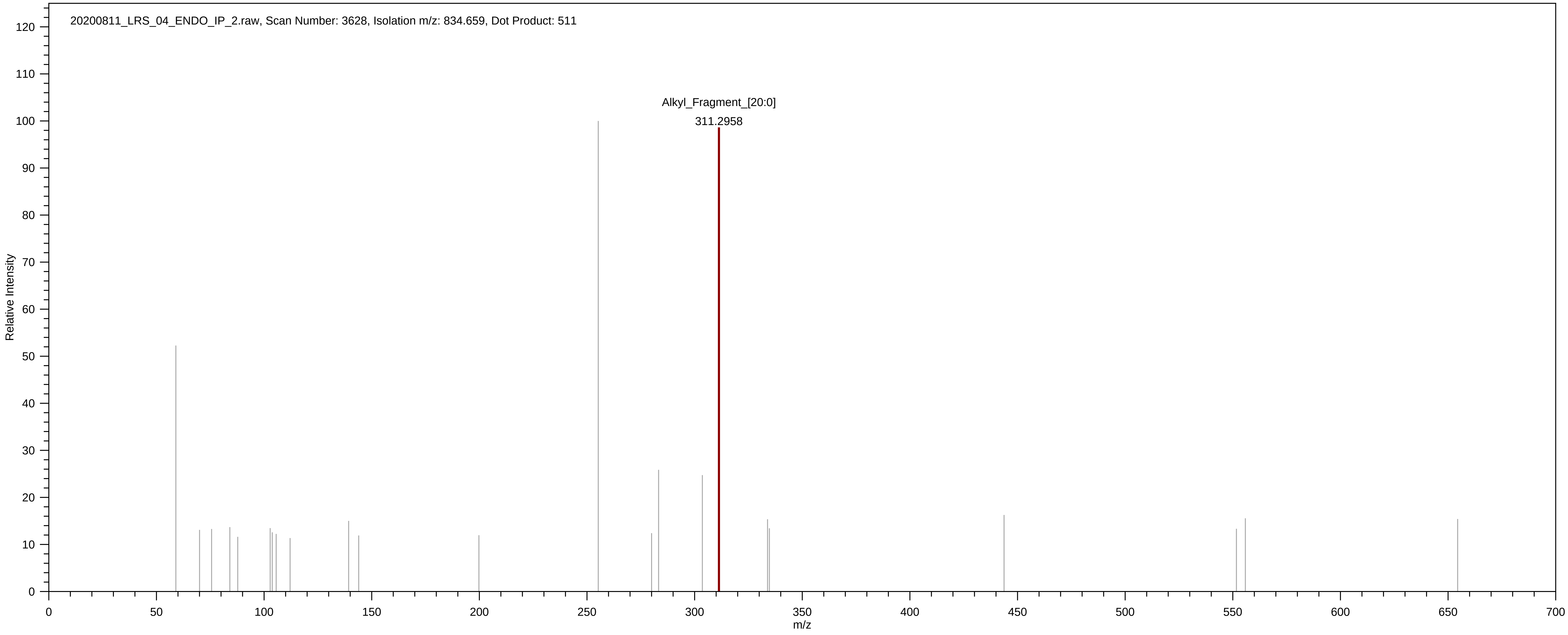

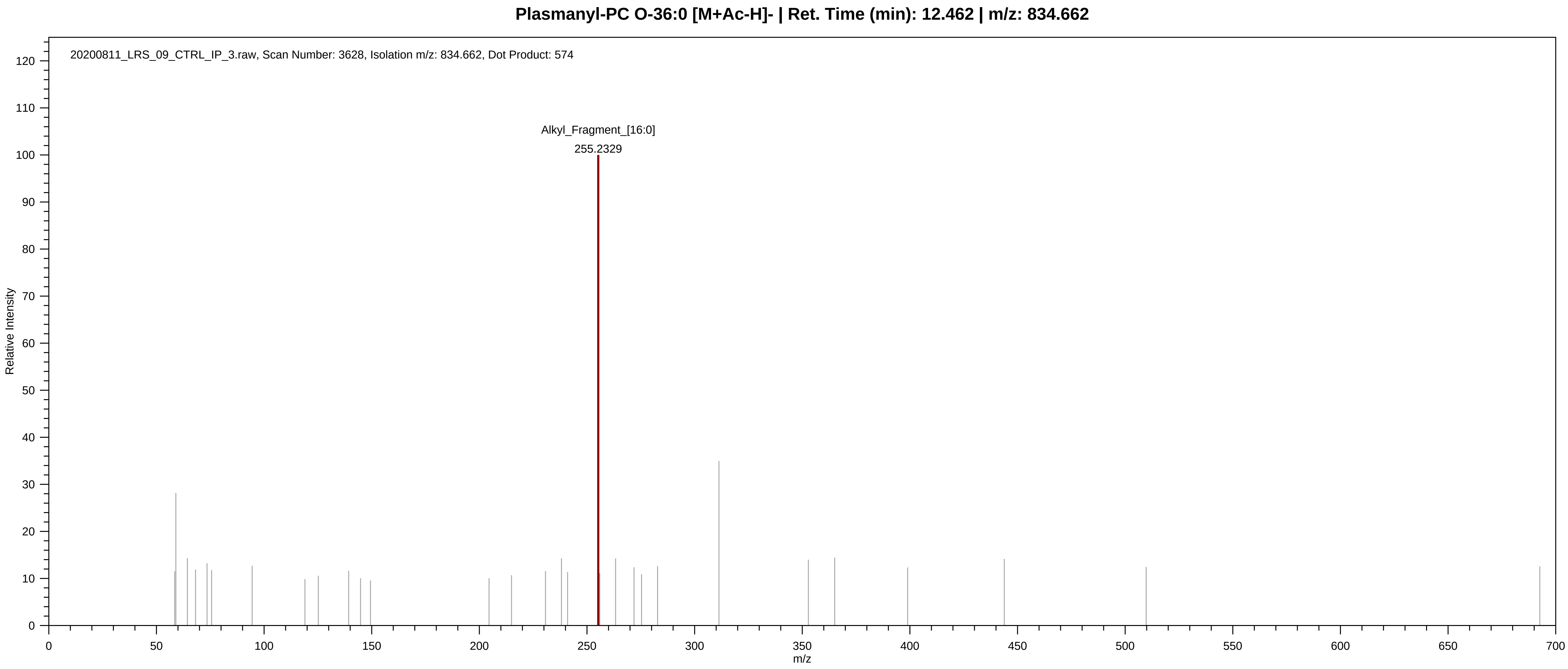

Plasmanyl-PC O-36:0 [M+Ac-H]- | Ret. Time (min): 12.462 | m/z: 834.662
20200811_LRS_09_CTRL_IP_3.raw, Scan Number: 3628, Isolation m/z: 834.662, Dot Product: 574
Alkyl_Fragment_[16:0]
255.2329
Relative Intensity
m/z

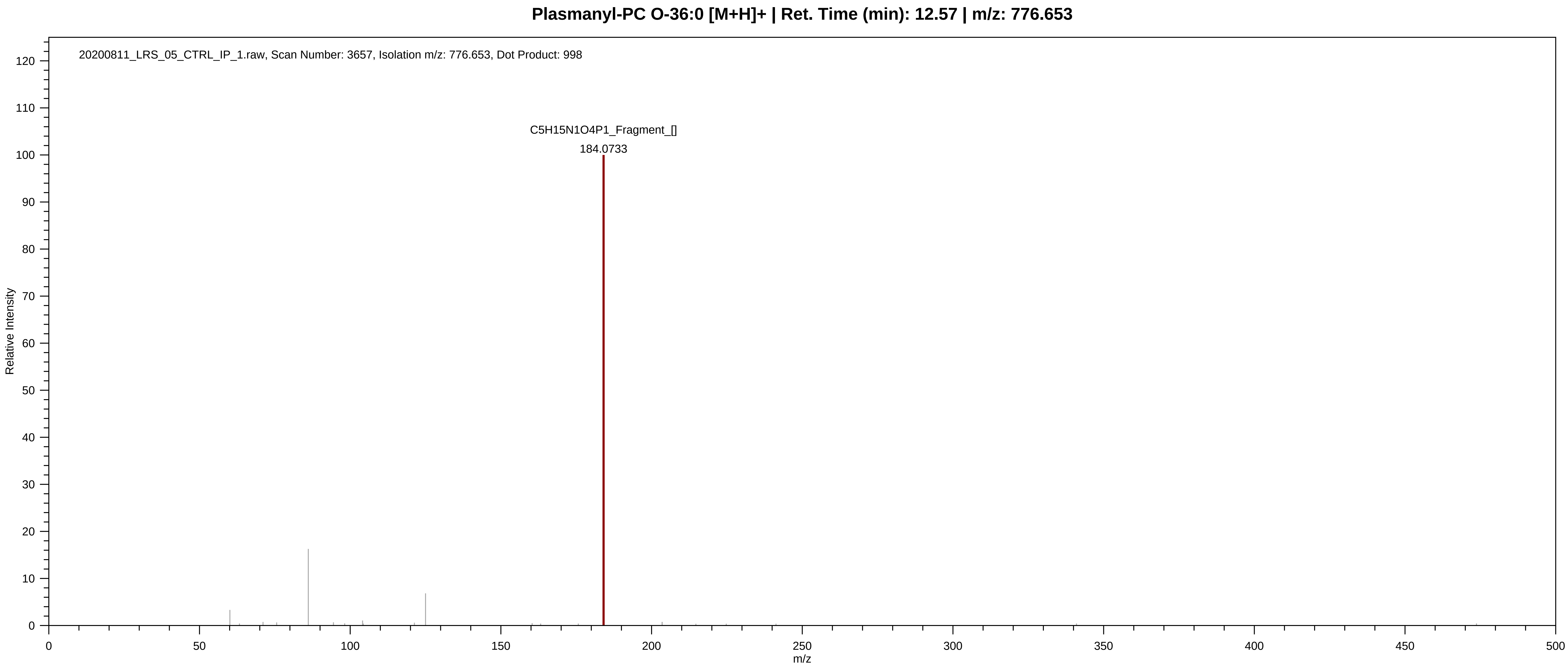
Plasmanyl-PC O-36:0 [M+H]+ | Ret. Time (min): 12.57 | m/z: 776.653
20200811_LRS_05_CTRL_IP_1.raw, Scan Number: 3657, Isolation m/z: 776.653, Dot Product: 998
C5H15N1O4P1_Fragment_[]
184.0733
Relative Intensity
m/z
0
10
20
30
40
50
60
70
80
90
100
110
120
0
50
100
150
200
250
300
350
400
450
500

Plasmanyl-PC O-36:0 [M+H]+ | Ret. Time (min): 12.511 | m/z: 776.653

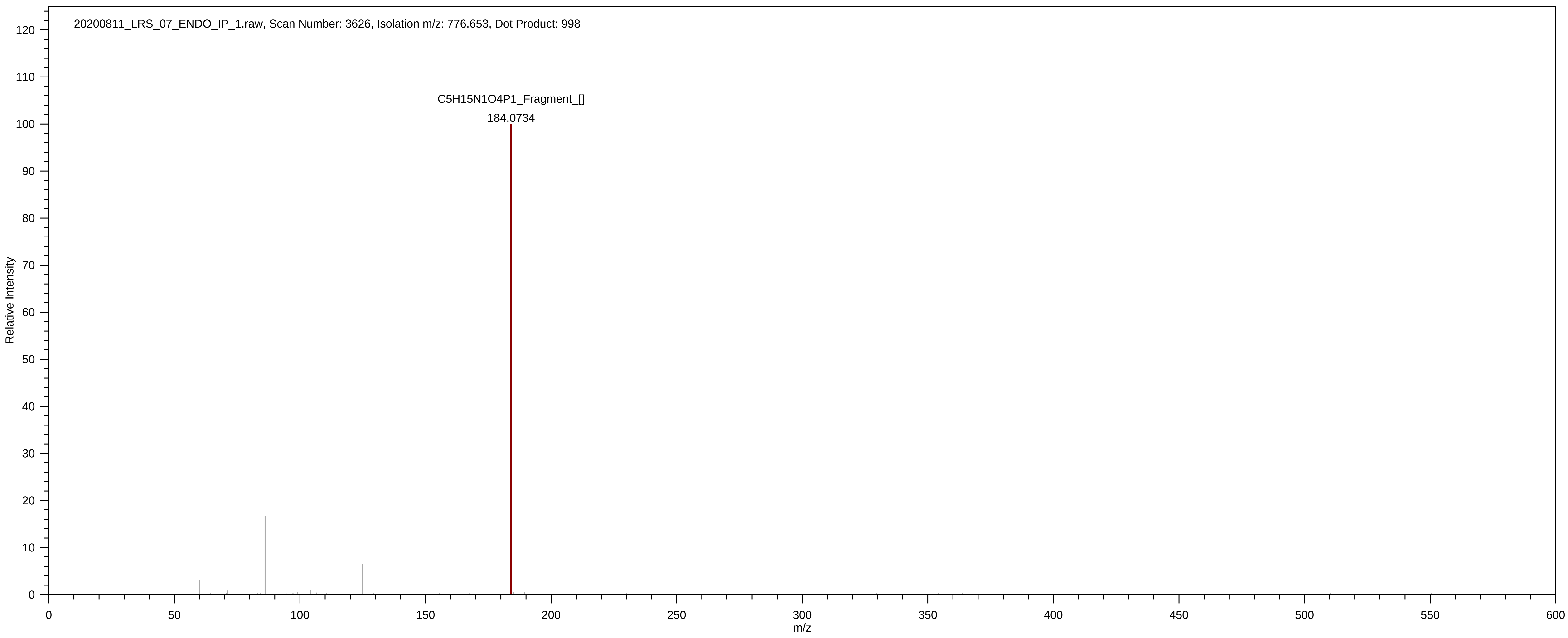

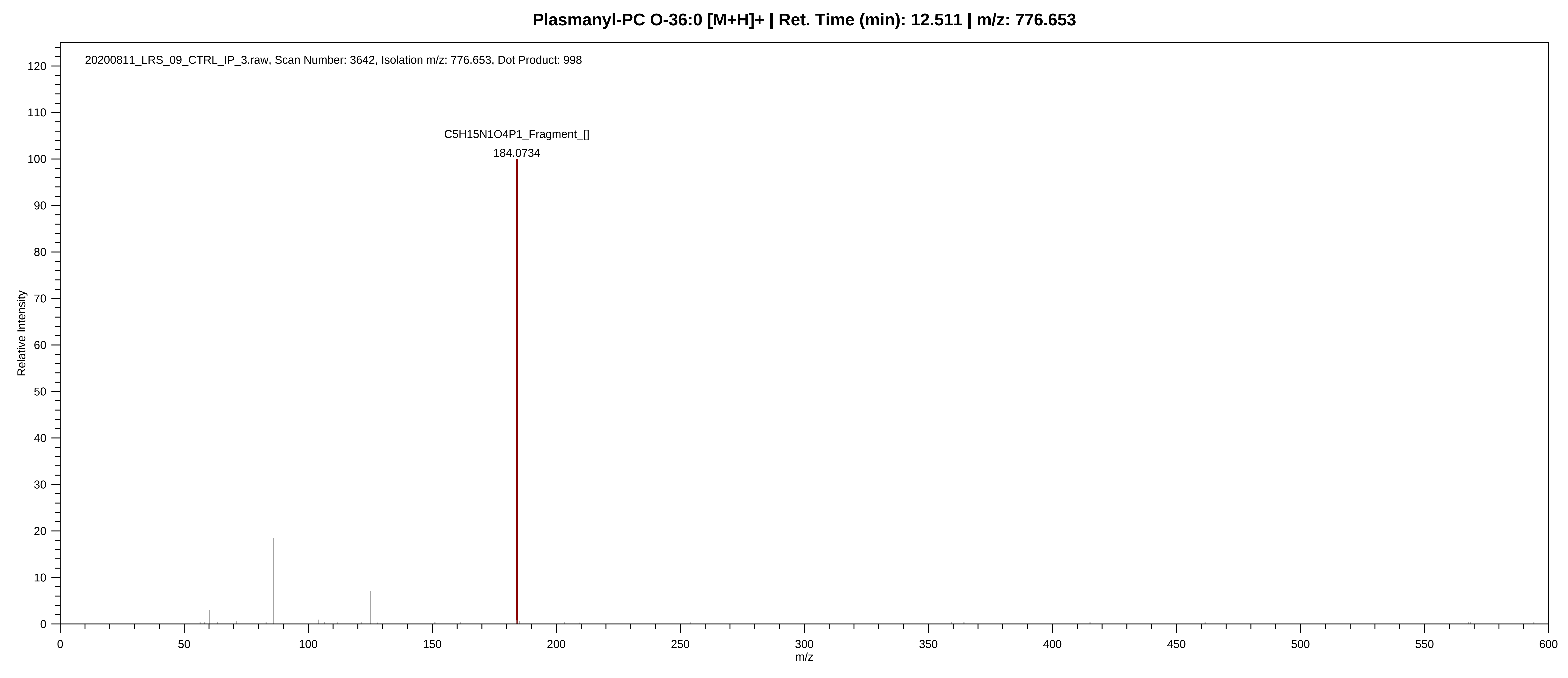
Plasmanyl-PC O-36:0 [M+H]+ | Ret. Time (min): 12.511 | m/z: 776.653
20200811_LRS_09_CTRL_IP_3.raw, Scan Number: 3642, Isolation m/z: 776.653, Dot Product: 998
C5H15N1O4P1_Fragment_[]
184.0734
Relative Intensity
m/z

Plasmanyl-PC O-36:0 [M+H]+ | Ret. Time (min): 12.531 | m/z: 776.653

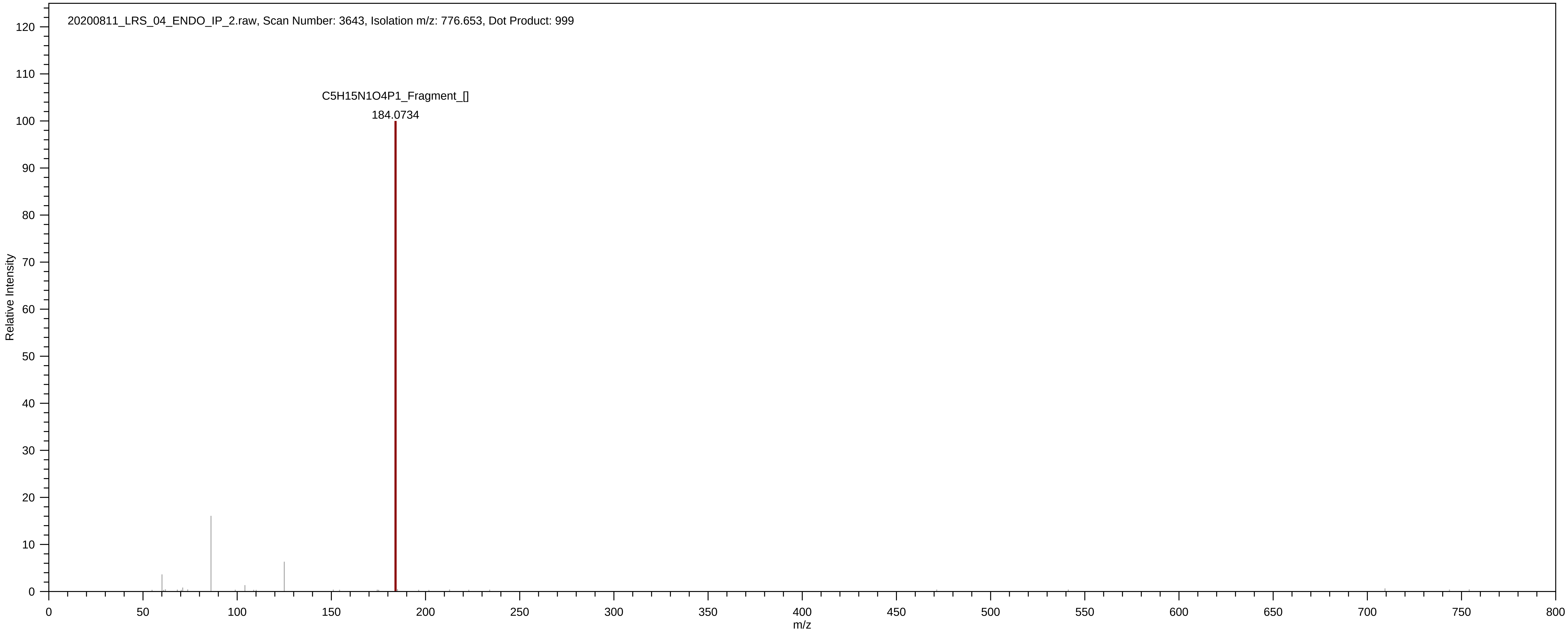

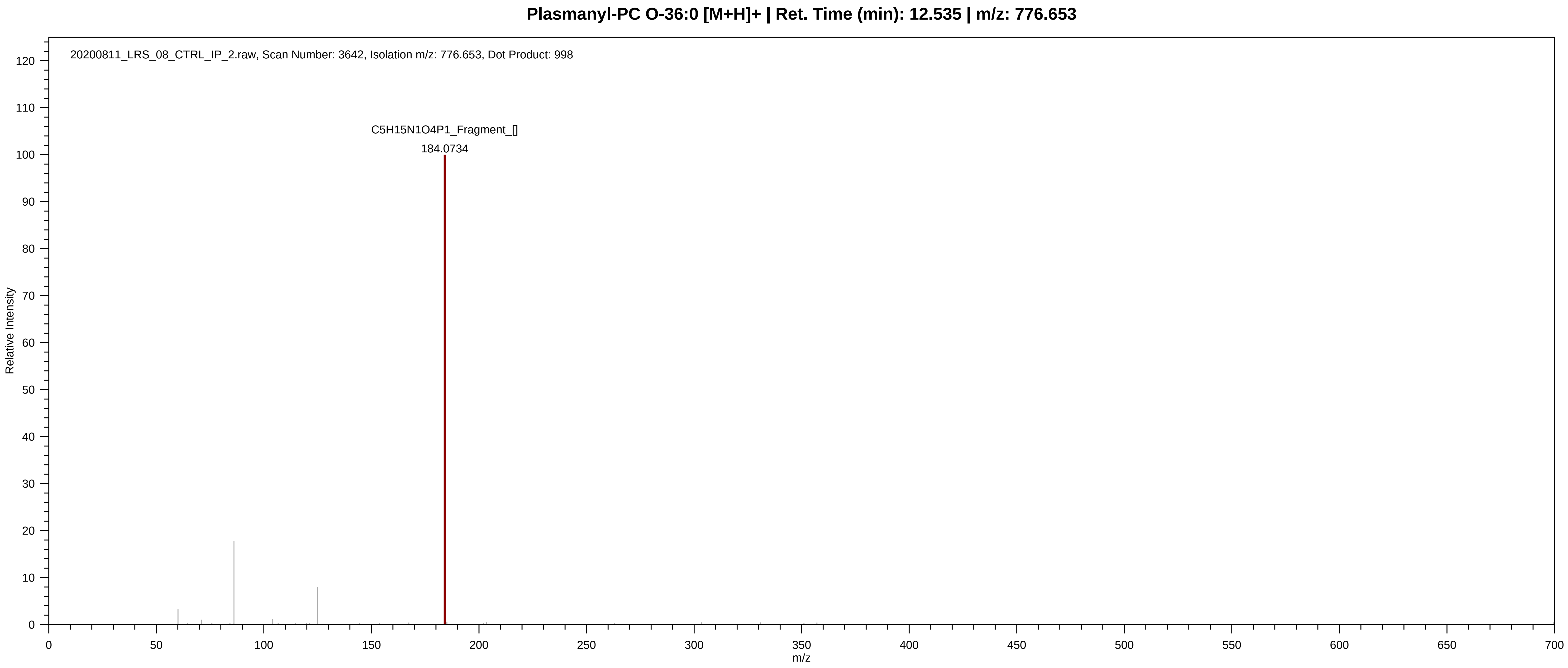

Plasmanyl-PC O-36:0 [M+H]+ | Ret. Time (min): 12.535 | m/z: 776.653
20200811_LRS_08_CTRL_IP_2.raw, Scan Number: 3642, Isolation m/z: 776.653, Dot Product: 998
C5H15N1O4P1_Fragment_[]
184.0734
Relative Intensity
m/z

Plasmanyl-PC O-36:0 [M+H]+ | Ret. Time (min): 12.694 | m/z: 776.653

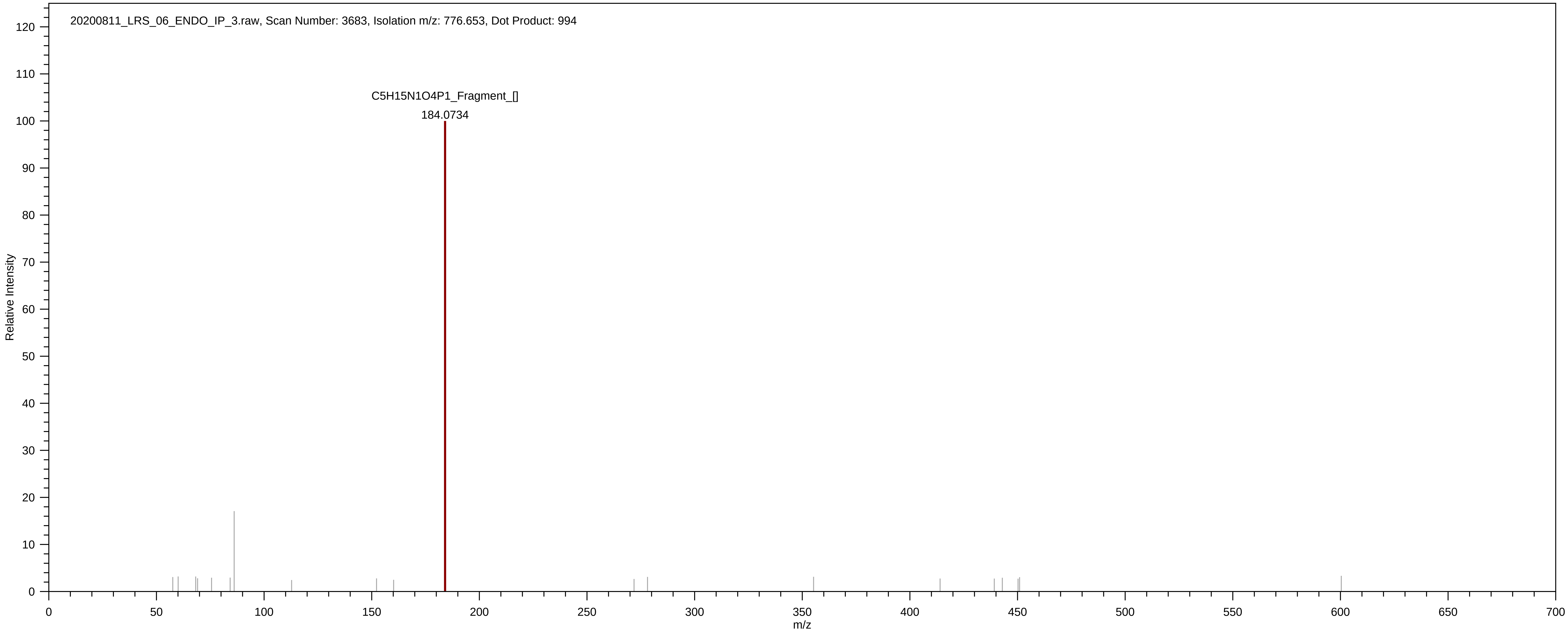

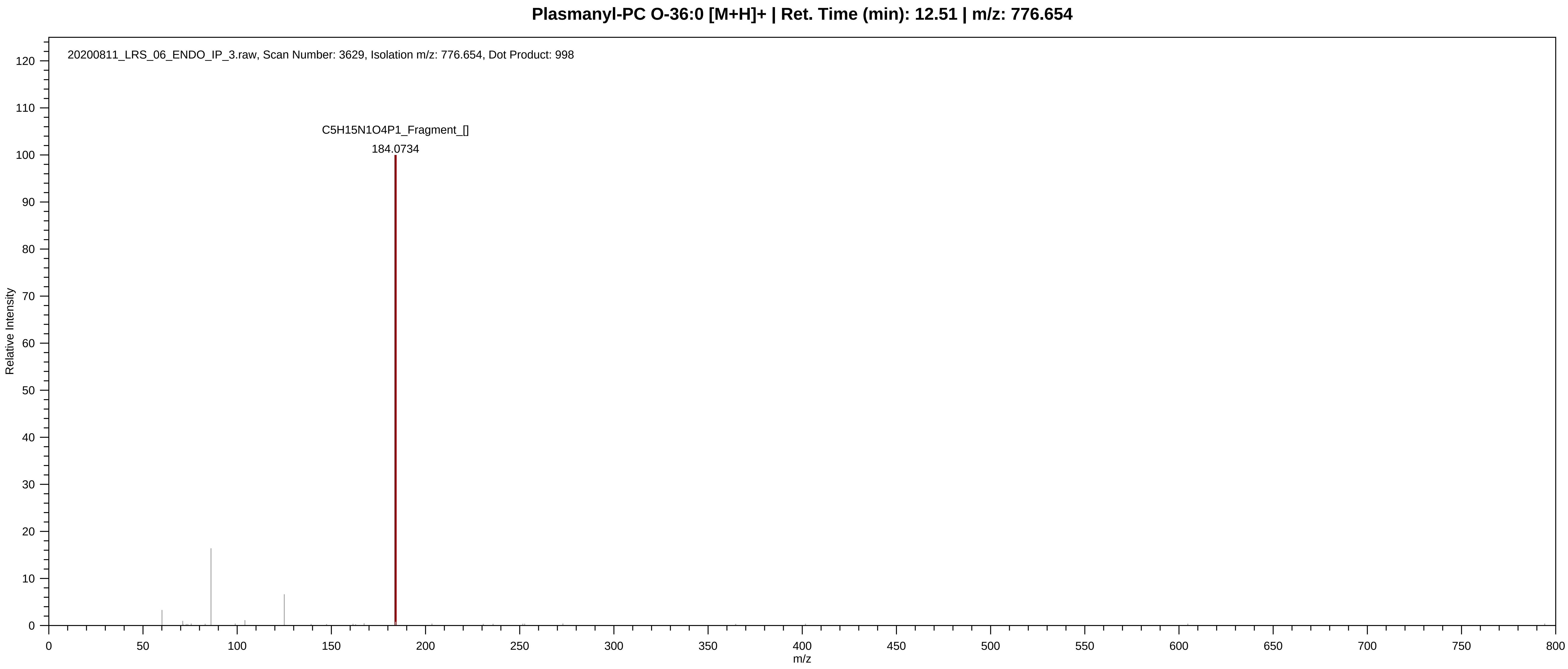
Plasmanyl-PC O-36:0 [M+H]+ | Ret. Time (min): 12.51 | m/z: 776.654
20200811_LRS_06_ENDO_IP_3.raw, Scan Number: 3629, Isolation m/z: 776.654, Dot Product: 998
C5H15N1O4P1_Fragment_[]
184.0734
Relative Intensity
m/z

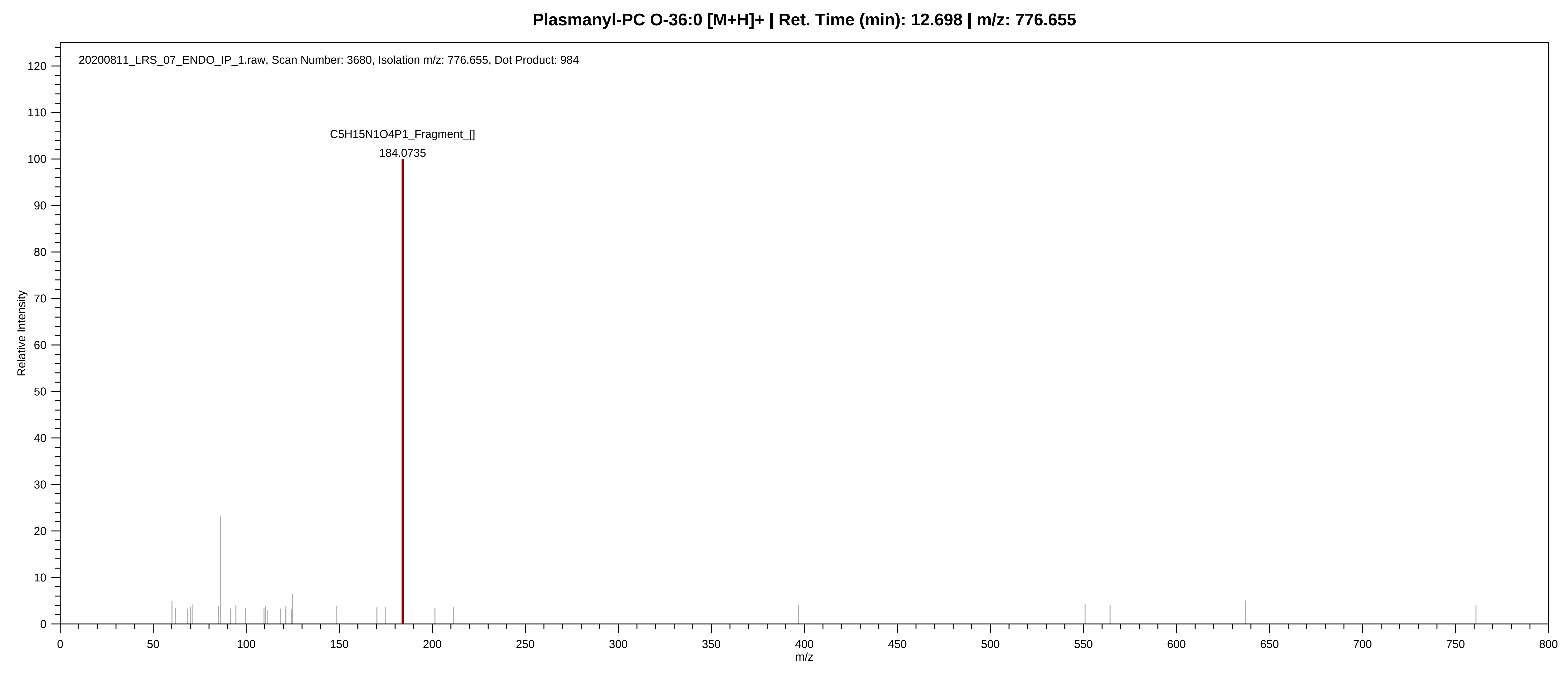

Plasmanyl-PC O-36:0 [M+H]+ | Ret. Time (min): 12.698 | m/z: 776.655
20200811_LRS_07_ENDO_IP_1.raw, Scan Number: 3680, Isolation m/z: 776.655, Dot Product: 984
C5H15N1O4P1_Fragment_[]
184.0735
Relative Intensity
m/z

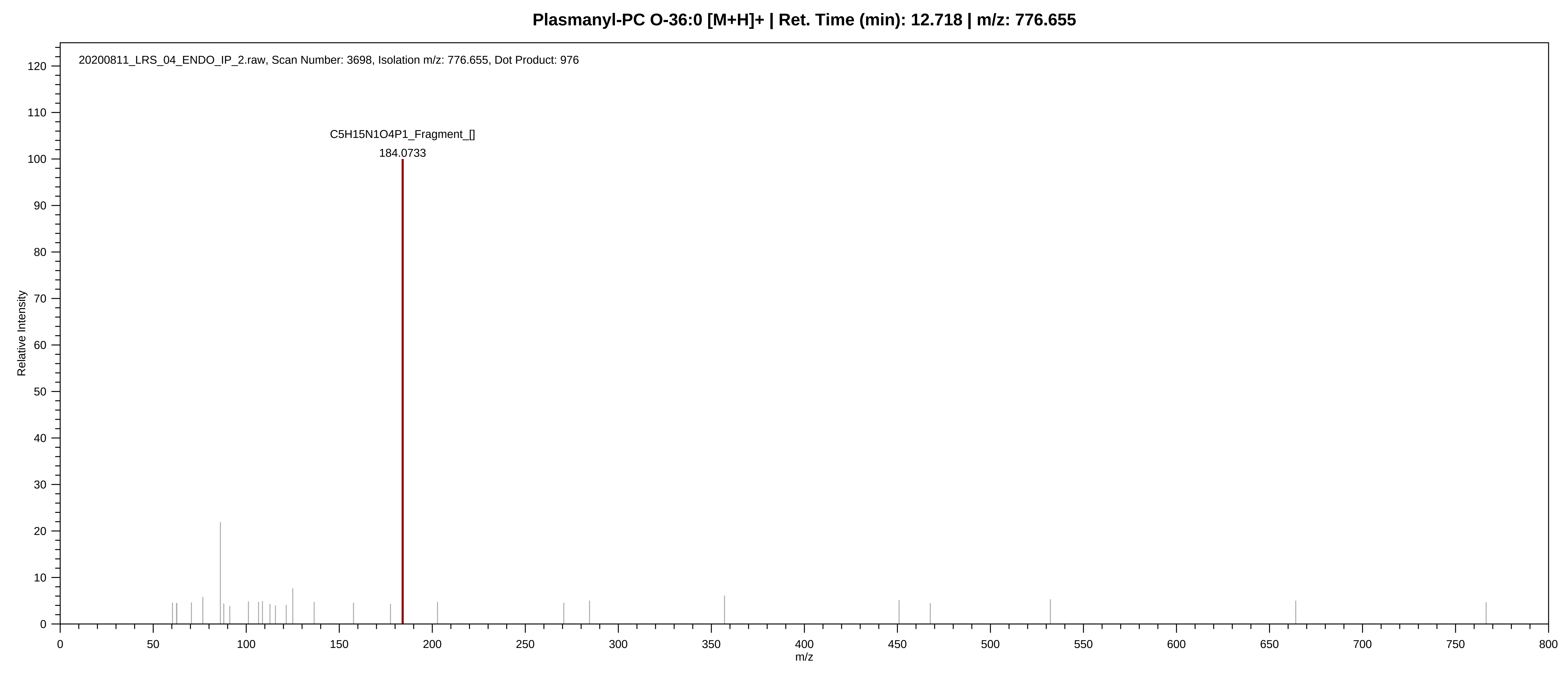

Plasmanyl-PC O-36:0 [M+H]+ | Ret. Time (min): 12.718 | m/z: 776.655
20200811_LRS_04_ENDO_IP_2.raw, Scan Number: 3698, Isolation m/z: 776.655, Dot Product: 976
C5H15N1O4P1_Fragment_[]
184.0733
Relative Intensity
m/z

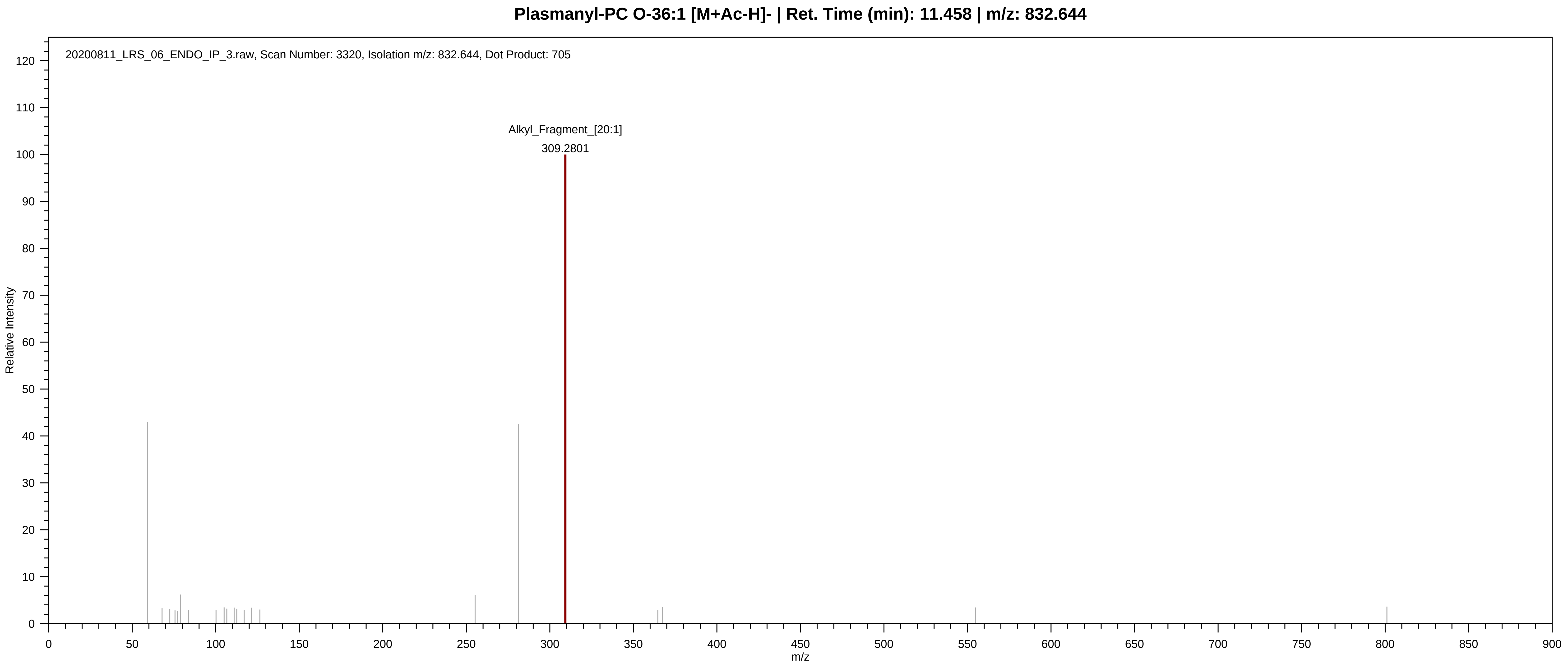
Plasmanyl-PC O-36:1 [M+Ac-H]- | Ret. Time (min): 11.458 | m/z: 832.644
20200811_LRS_06_ENDO_IP_3.raw, Scan Number: 3320, Isolation m/z: 832.644, Dot Product: 705
Alkyl_Fragment_[20:1]
309.2801
Relative Intensity
m/z
0
10
20
30
40
50
60
70
80
90
100
110
120
0
50
100
150
200
250
300
350
400
450
500
550
600
650
700
750
800
850
900

Plasmanyl-PC O-36:1 [M+Ac-H]- | Ret. Time (min): 11.66 | m/z: 832.645

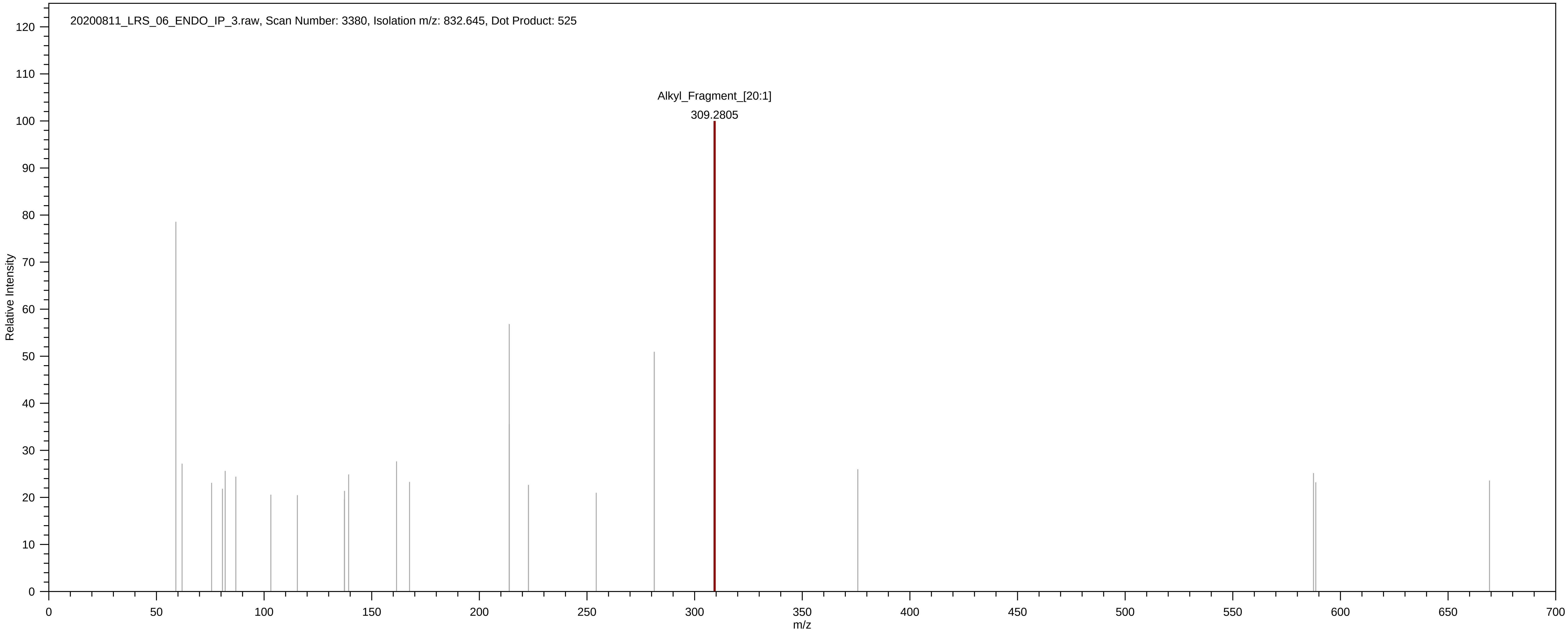

Plasmanyl-PC O-36:1 [M+Ac-H]- | Ret. Time (min): 11.673 | m/z: 832.645

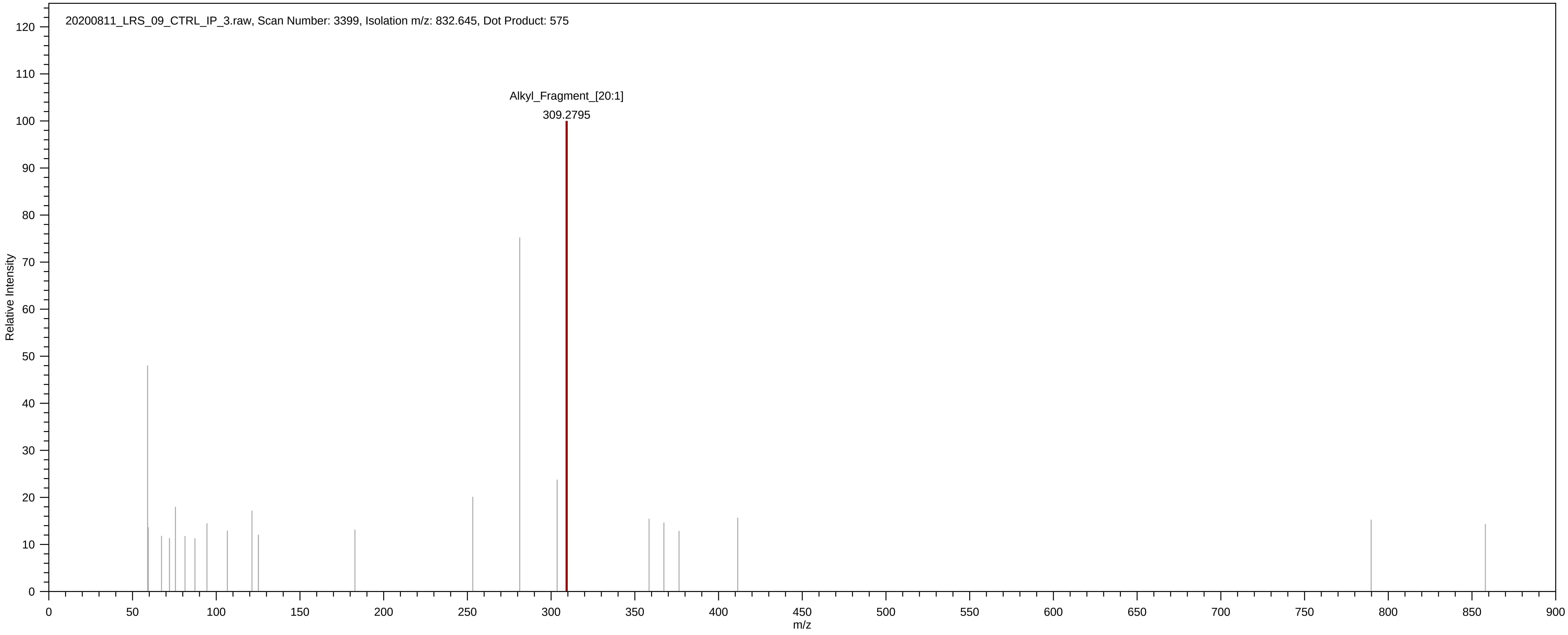

Plasmanyl-PC O-36:1 [M+H]+ | Ret. Time (min): 12.324 | m/z: 774.64

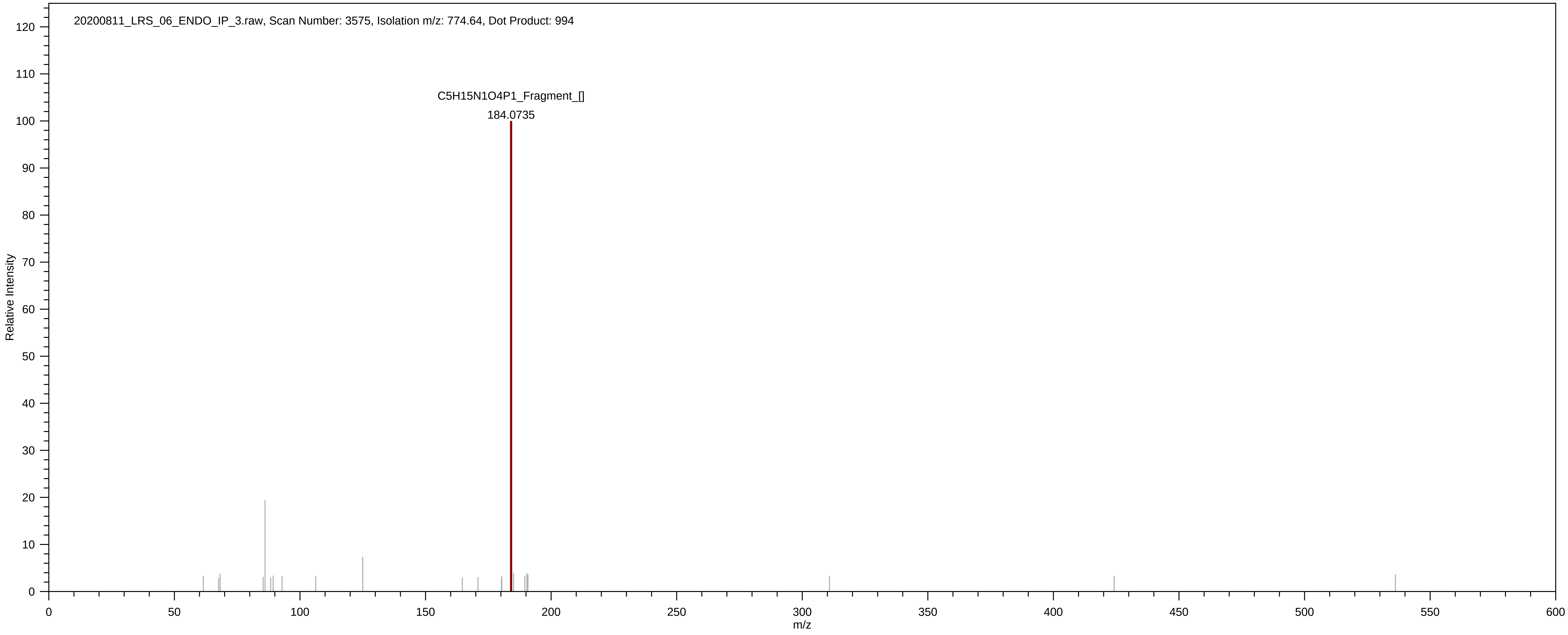

Plasmanyl-PC O-36:1 [M+H]+ | Ret. Time (min): 12.327 | m/z: 774.64

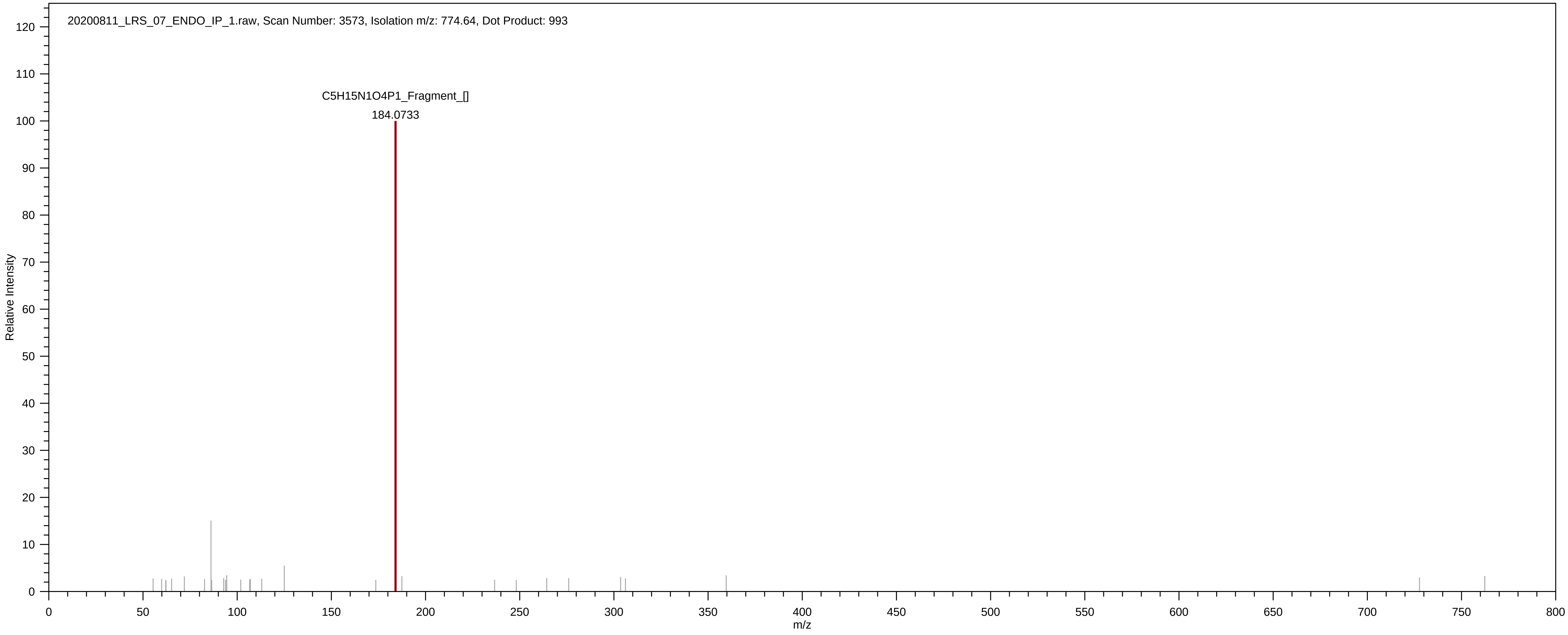

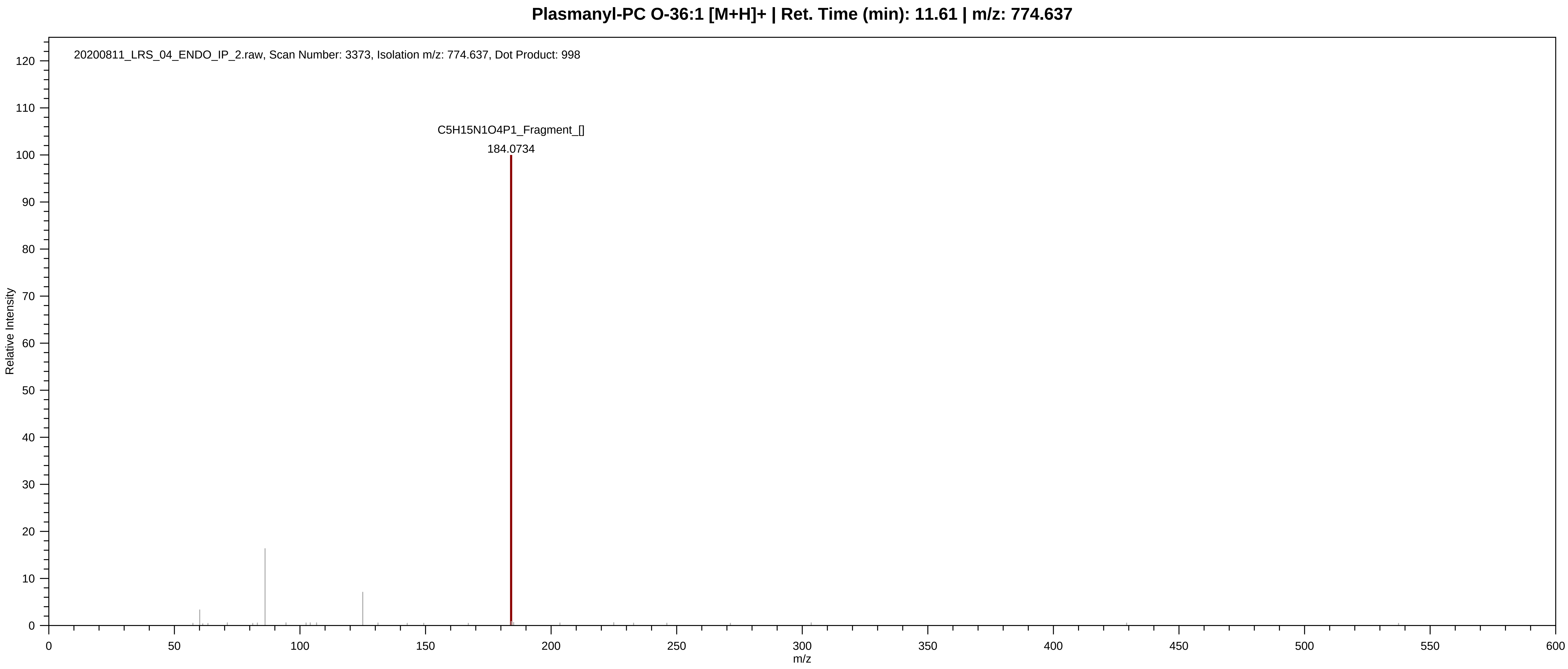
Plasmanyl-PC O-36:1 [M+H]+ | Ret. Time (min): 11.61 | m/z: 774.637
20200811_LRS_04_ENDO_IP_2.raw, Scan Number: 3373, Isolation m/z: 774.637, Dot Product: 998
C5H15N1O4P1_Fragment_[]
184.0734
Relative Intensity
m/z
0
10
20
30
40
50
60
70
80
90
100
110
120
0
50
100
150
200
250
300
350
400
450
500
550
600

Plasmanyl-PC O-36:1 [M+H]+ | Ret. Time (min): 11.426 | m/z: 774.637

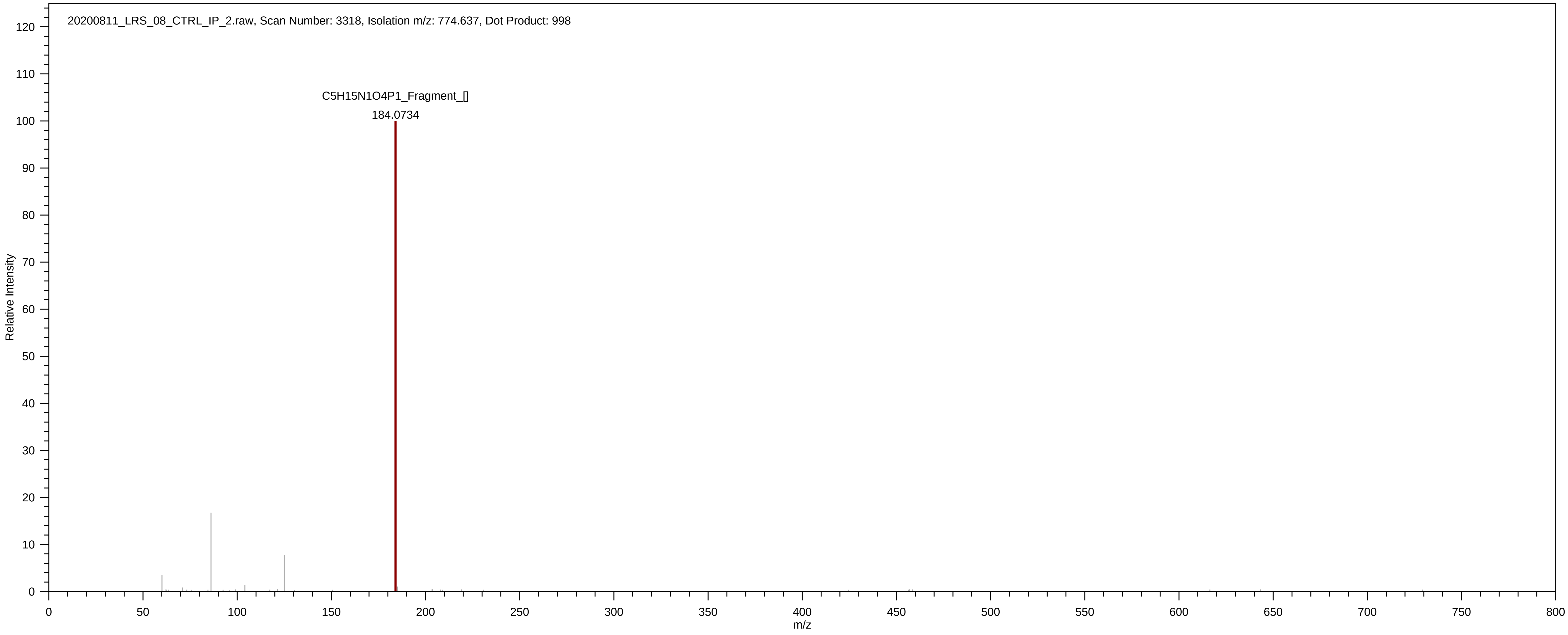

Plasmanyl-PC O-36:1 [M+H]+ | Ret. Time (min): 11.591 | m/z: 774.637

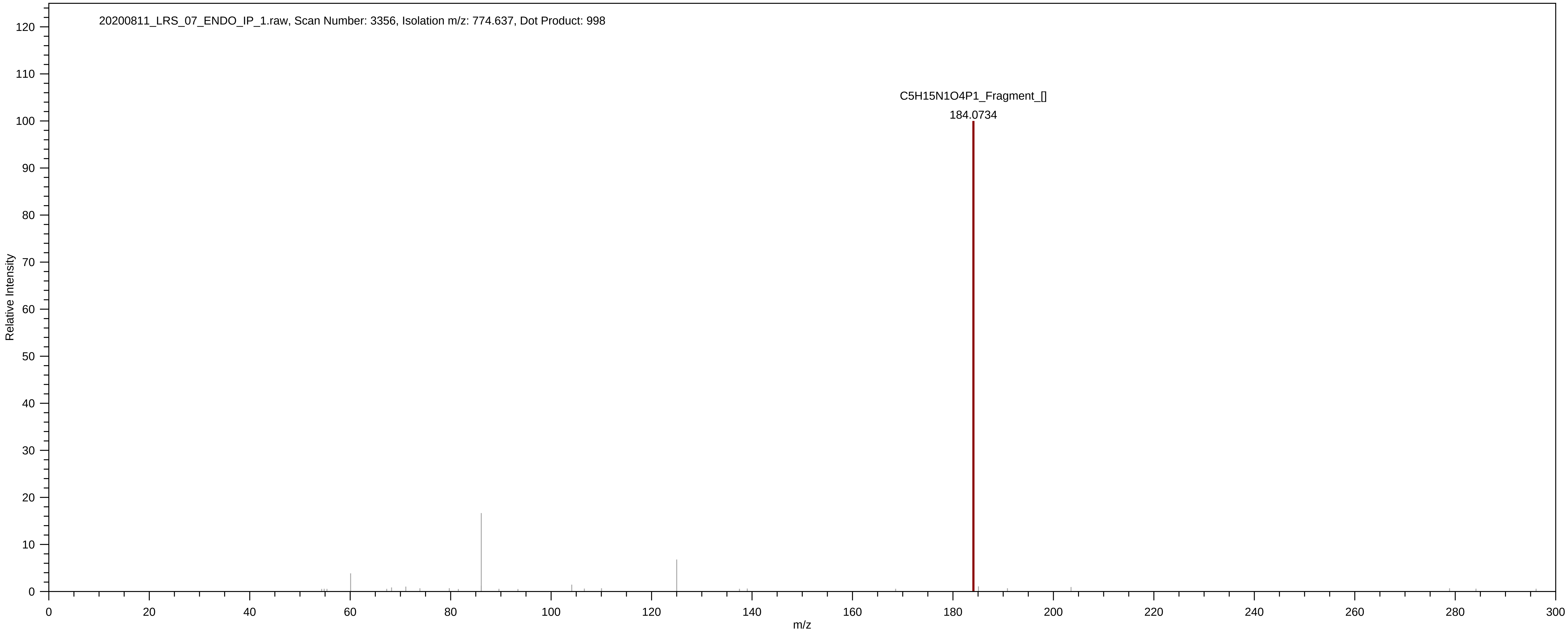

Plasmanyl-PC O-36:1 [M+H]+ | Ret. Time (min): 11.622 | m/z: 774.637

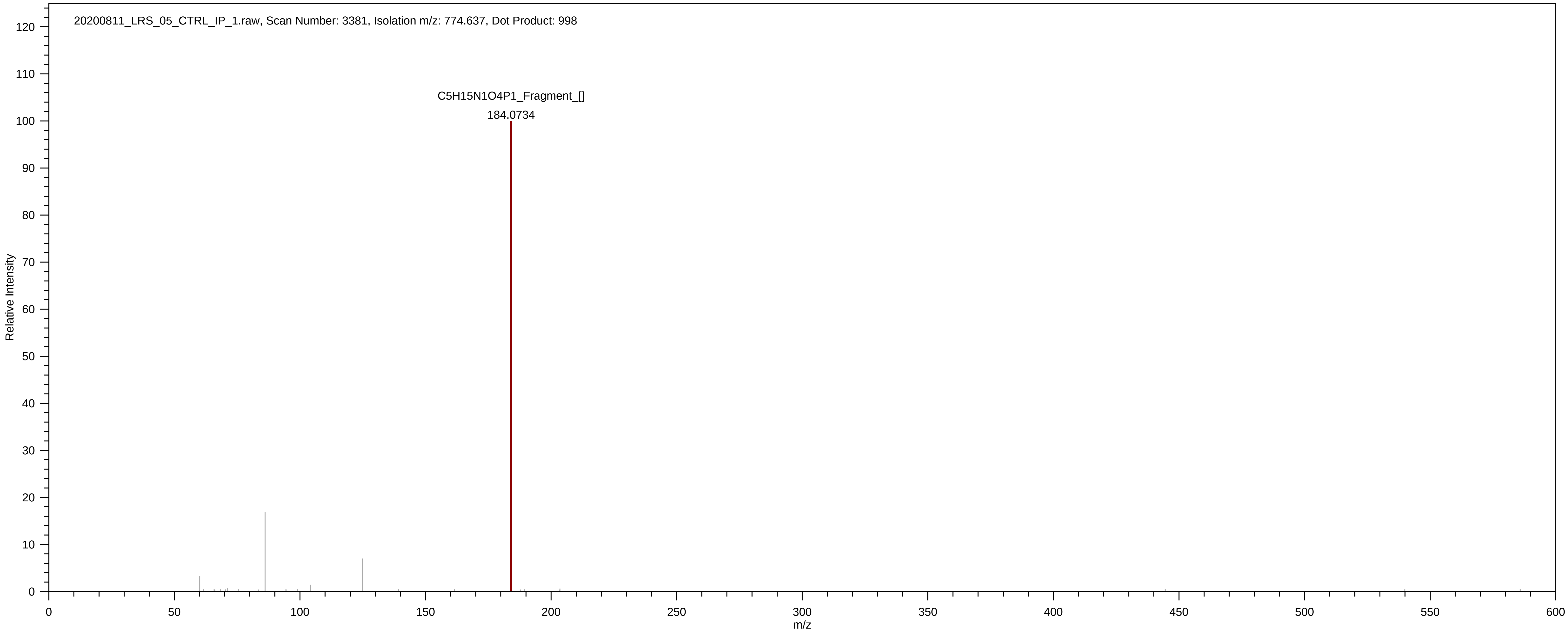

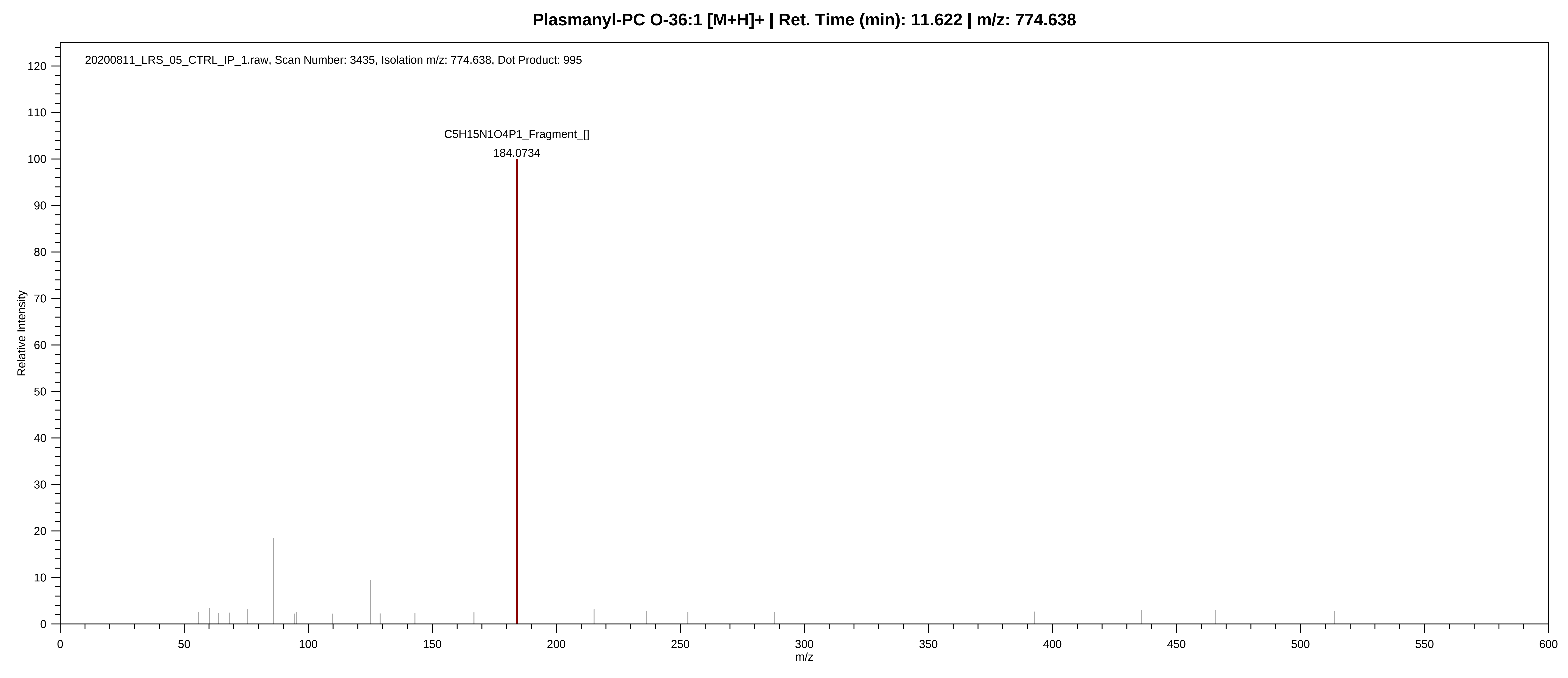
Plasmanyl-PC O-36:1 [M+H]+ | Ret. Time (min): 11.622 | m/z: 774.638
20200811_LRS_05_CTRL_IP_1.raw, Scan Number: 3435, Isolation m/z: 774.638, Dot Product: 995
C5H15N1O4P1_Fragment_[]
184.0734
Relative Intensity
m/z

Plasmanyl-PC O-36:1 [M+H]+ | Ret. Time (min): 11.42 | m/z: 774.638

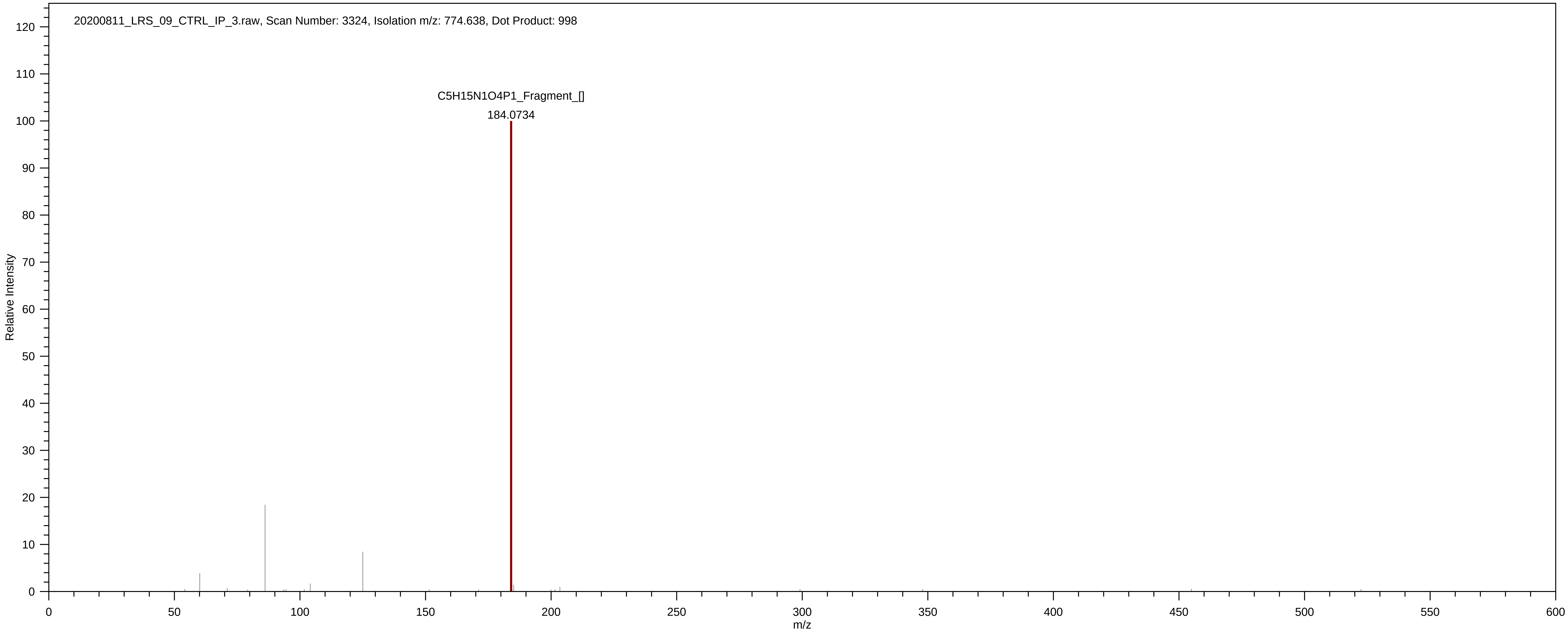

Plasmanyl-PC O-36:1 [M+H]+ | Ret. Time (min): 11.44 | m/z: 774.638

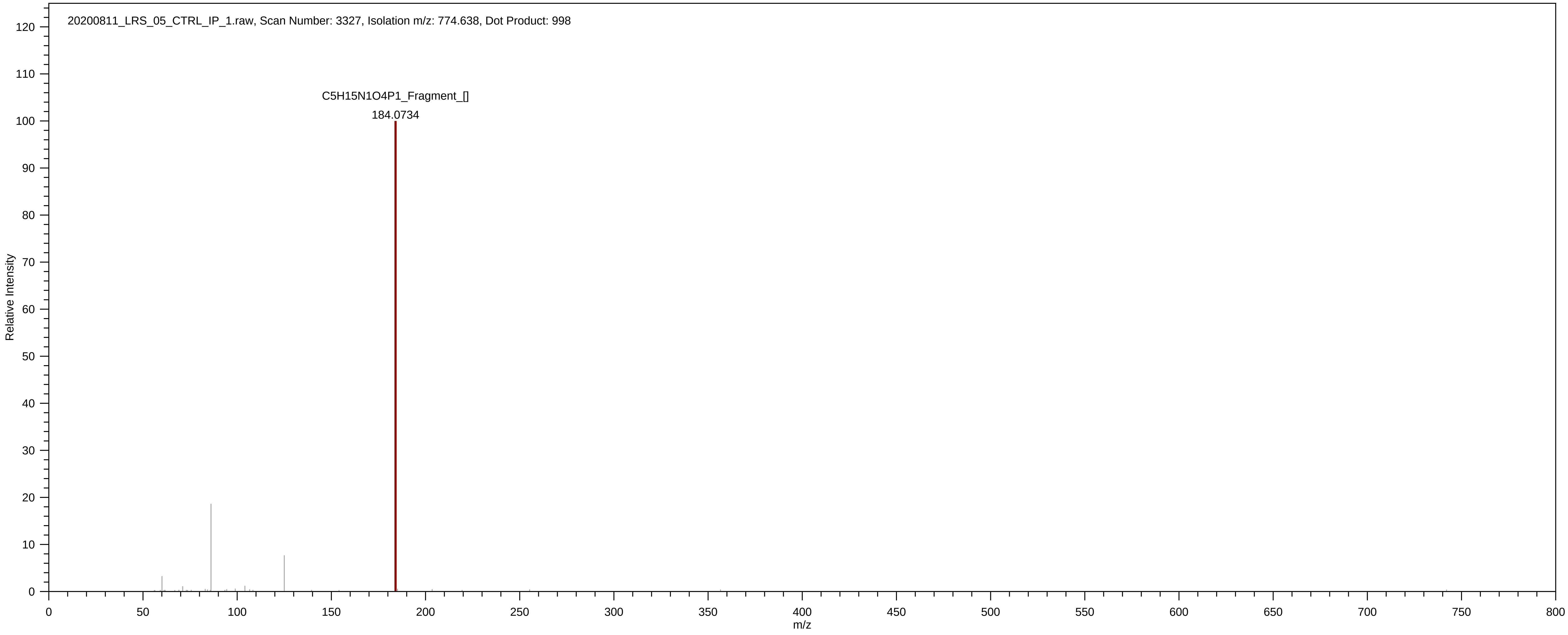

Plasmanyl-PC O-36:1 [M+H]+ | Ret. Time (min): 11.406 | m/z: 774.638

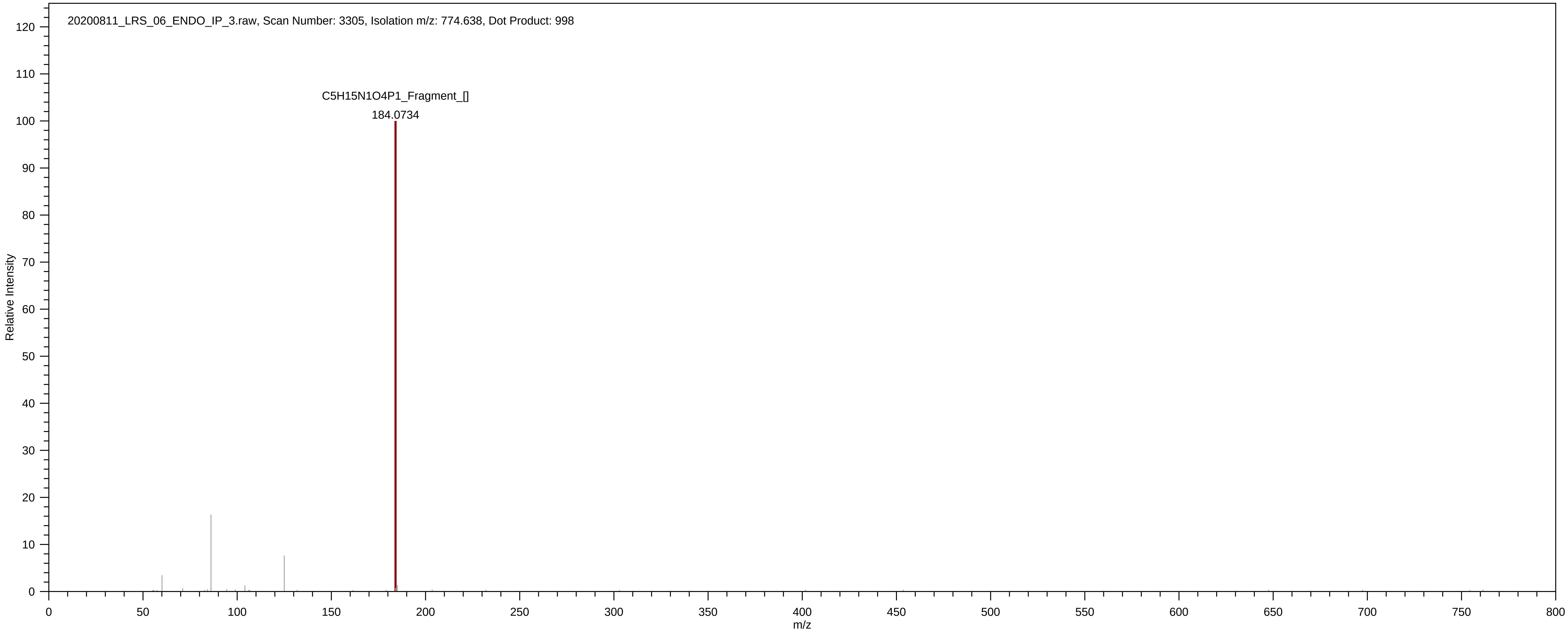

Plasmanyl-PC O-36:1 [M+H]+ | Ret. Time (min): 11.408 | m/z: 774.638

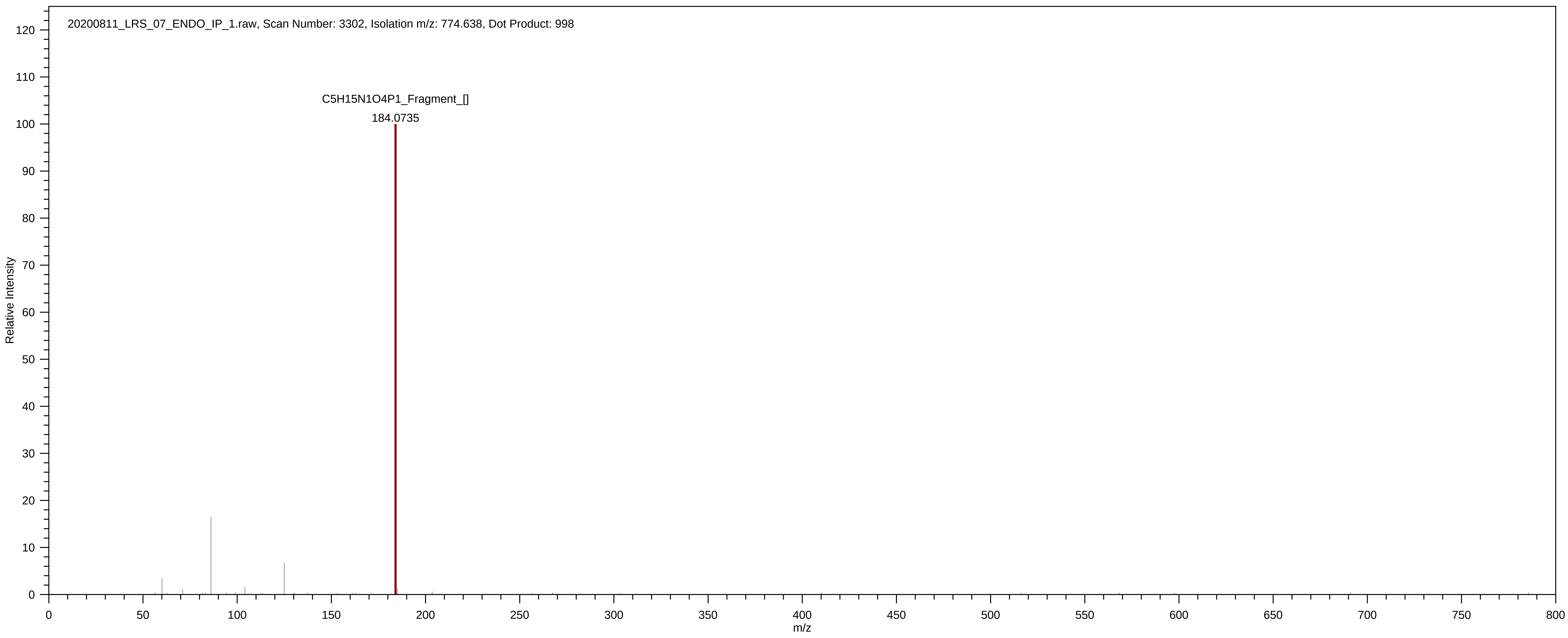

Plasmanyl-PC O-36:1 [M+H]+ | Ret. Time (min): 11.429 | m/z: 774.638

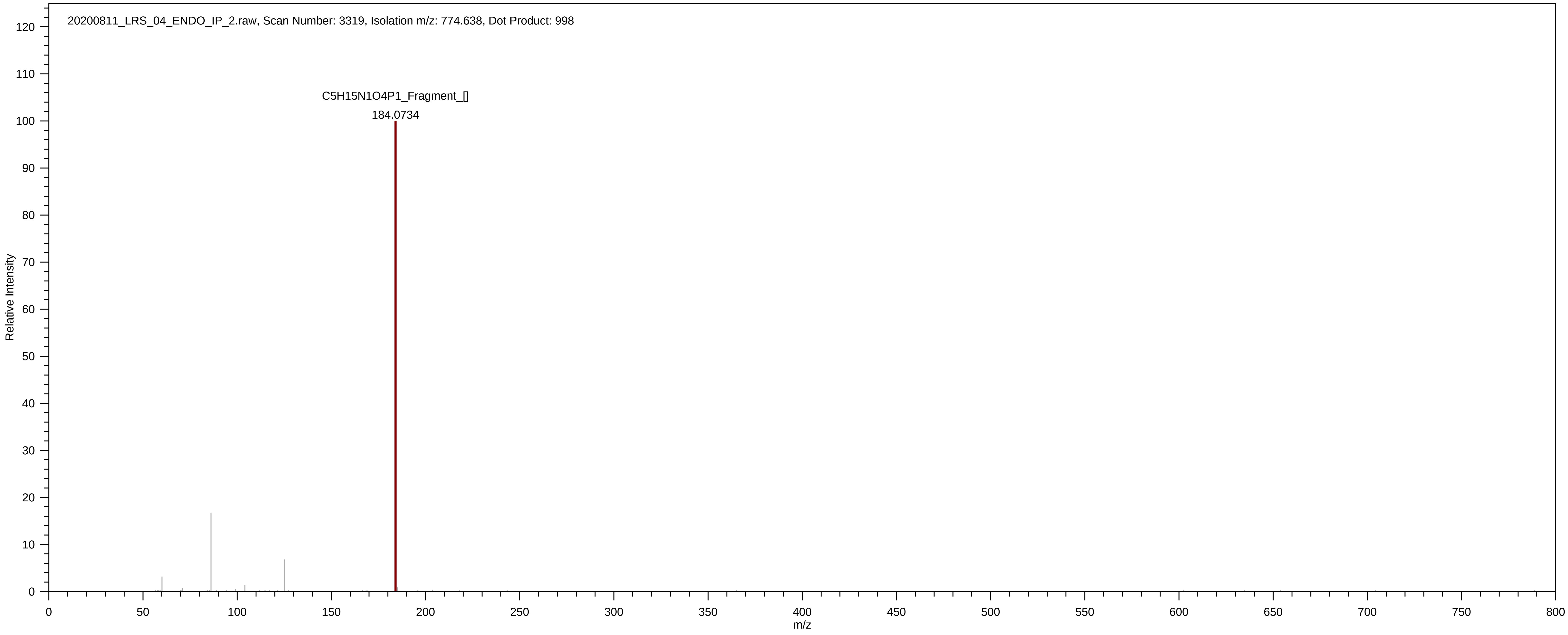

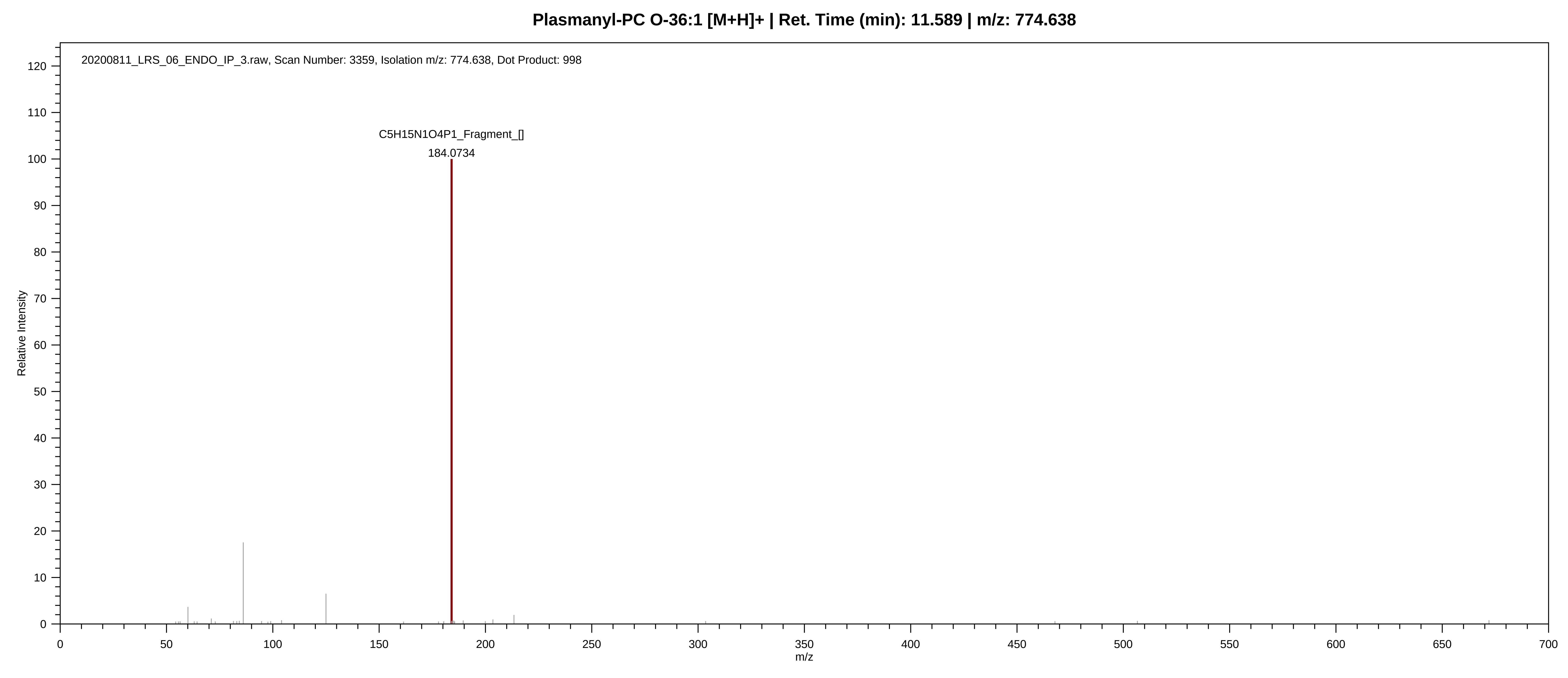
Plasmanyl-PC O-36:1 [M+H]+ | Ret. Time (min): 11.589 | m/z: 774.638
20200811_LRS_06_ENDO_IP_3.raw, Scan Number: 3359, Isolation m/z: 774.638, Dot Product: 998
C5H15N1O4P1_Fragment_[]
184.0734
Relative Intensity
m/z
0
10
20
30
40
50
60
70
80
90
100
110
120
0
50
100
150
200
250
300
350
400
450
500
550
600
650
700

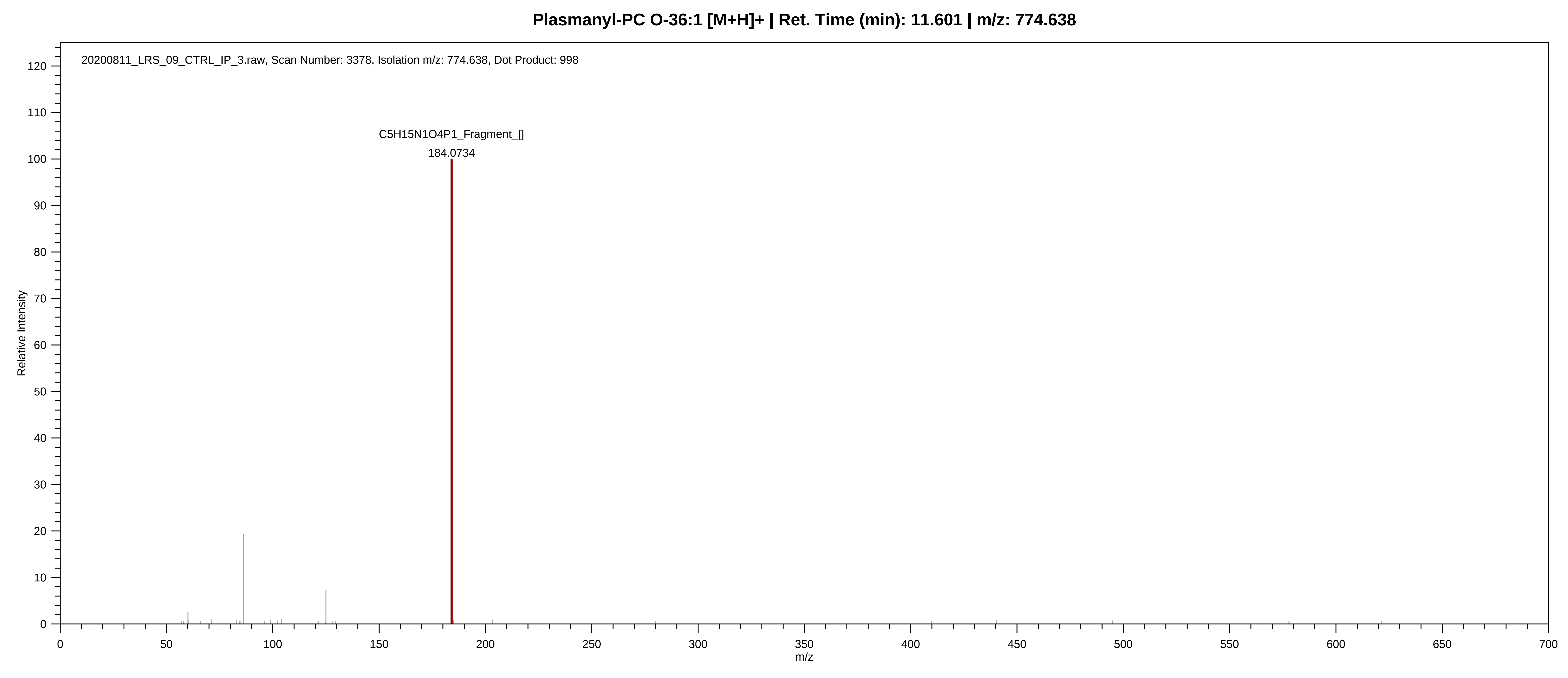

Plasmanyl-PC O-36:1 [M+H]+ | Ret. Time (min): 11.601 | m/z: 774.638
20200811_LRS_09_CTRL_IP_3.raw, Scan Number: 3378, Isolation m/z: 774.638, Dot Product: 998
C5H15N1O4P1_Fragment_[]
184.0734
Relative Intensity
m/z

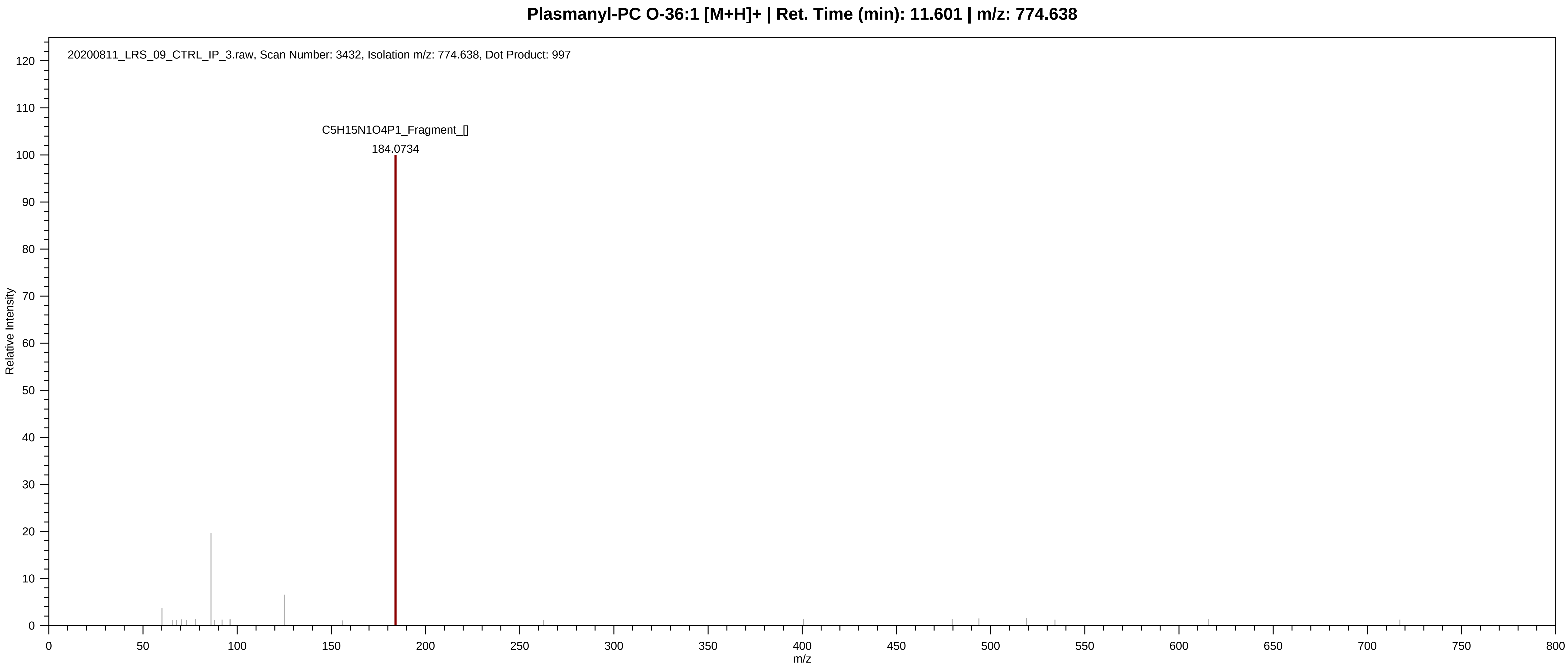

Plasmanyl-PC O-36:1 [M+H]+ | Ret. Time (min): 11.601 | m/z: 774.638
20200811_LRS_09_CTRL_IP_3.raw, Scan Number: 3432, Isolation m/z: 774.638, Dot Product: 997
C5H15N1O4P1_Fragment_[]
184.0734
Relative Intensity
m/z

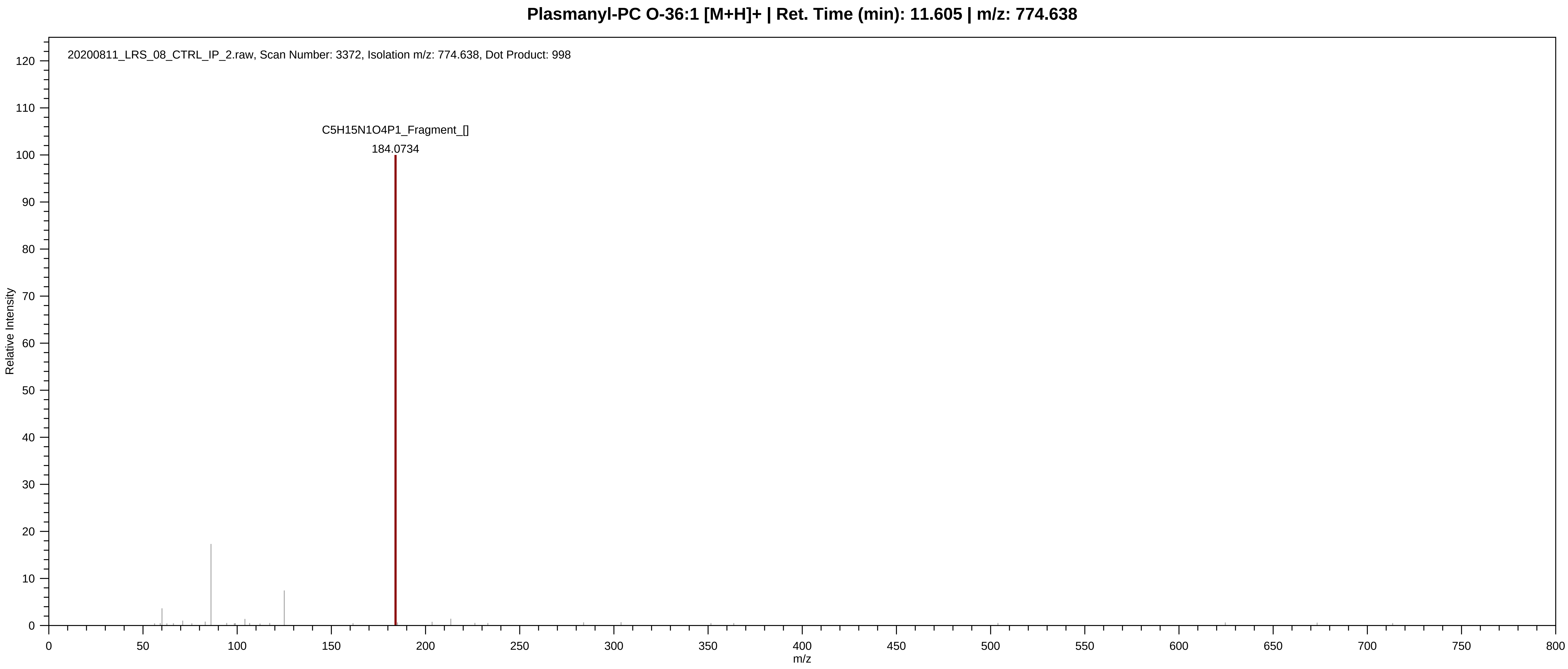
Plasmanyl-PC O-36:1 [M+H]+ | Ret. Time (min): 11.605 | m/z: 774.638
20200811_LRS_08_CTRL_IP_2.raw, Scan Number: 3372, Isolation m/z: 774.638, Dot Product: 998
C5H15N1O4P1_Fragment_[]
184.0734
Relative Intensity
m/z
0
10
20
30
40
50
60
70
80
90
100
110
120
0
50
100
150
200
250
300
350
400
450
500
550
600
650
700
750
800

Plasmanyl-PC O-36:1 [M+H]+ | Ret. Time (min): 11.605 | m/z: 774.638

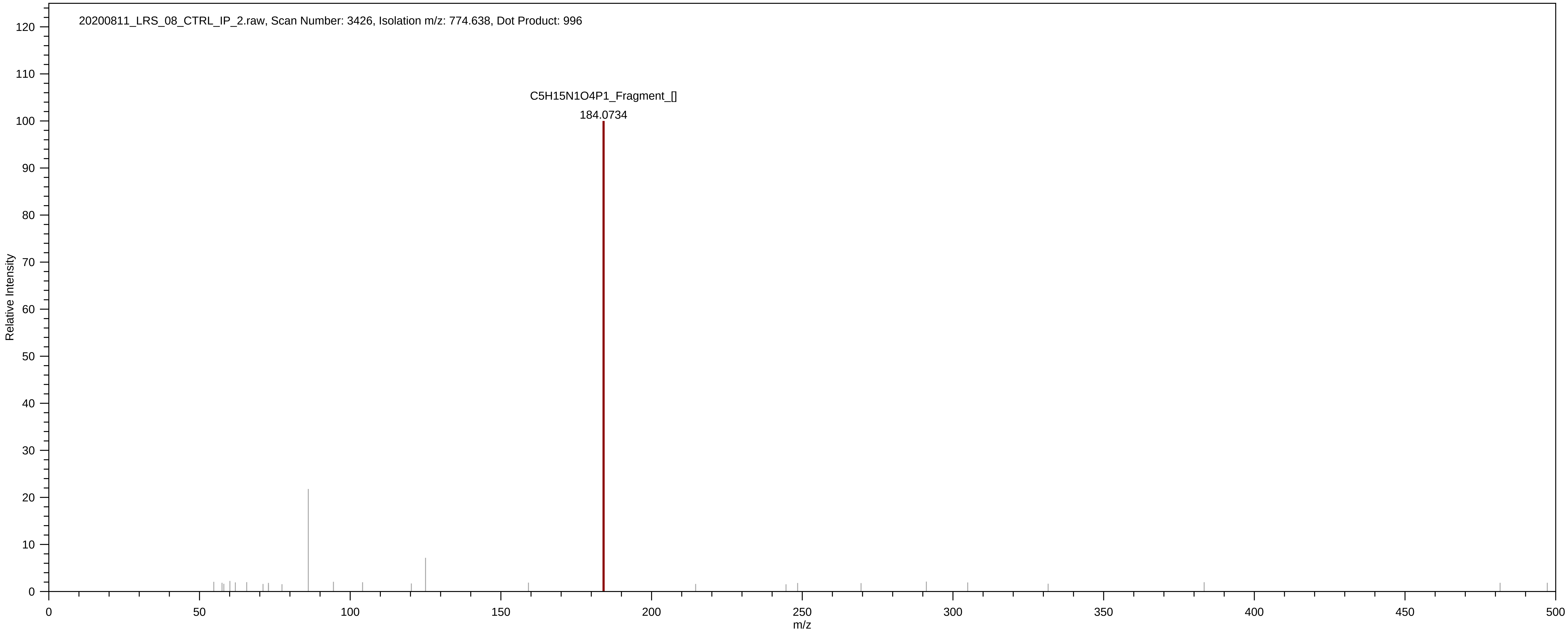

Plasmanyl-PC O-36:1 [M+H]+ | Ret. Time (min): 12.345 | m/z: 774.639

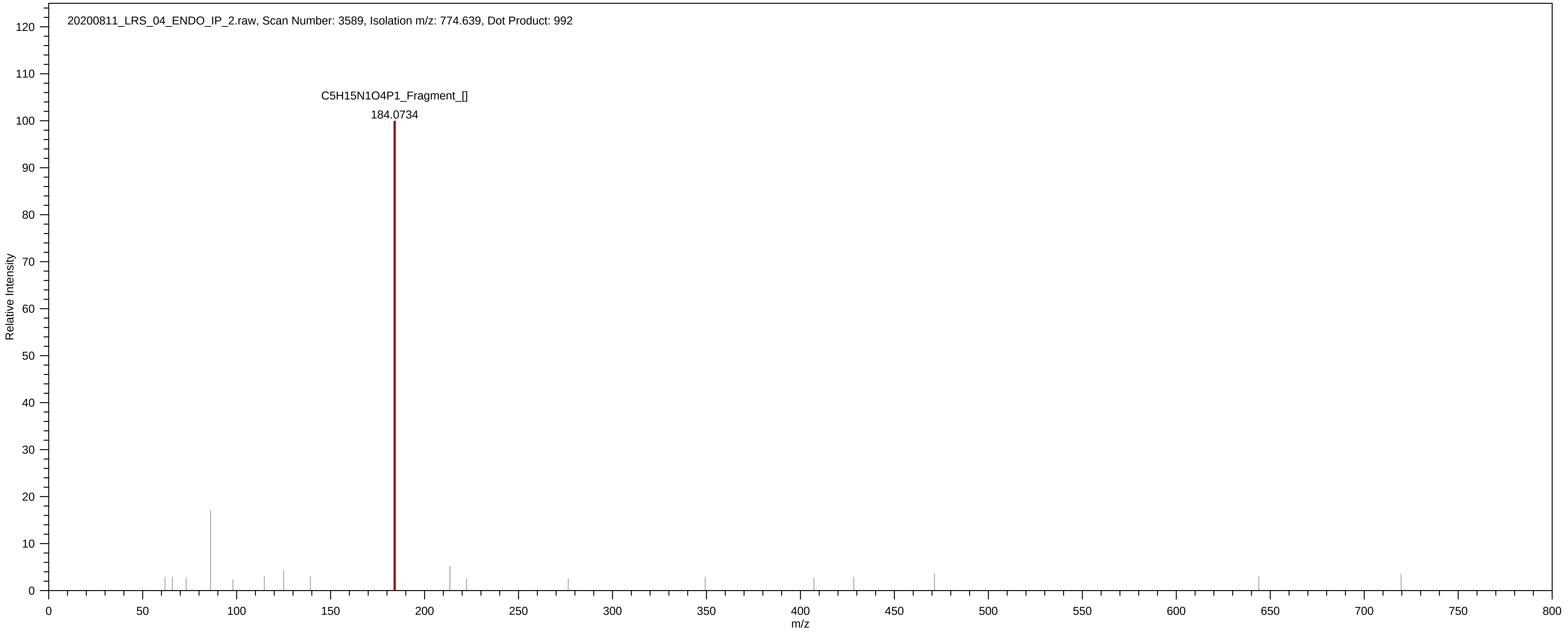

Plasmanyl-PC O-36:1 [M+H]+ | Ret. Time (min): 12.904 | m/z: 774.641

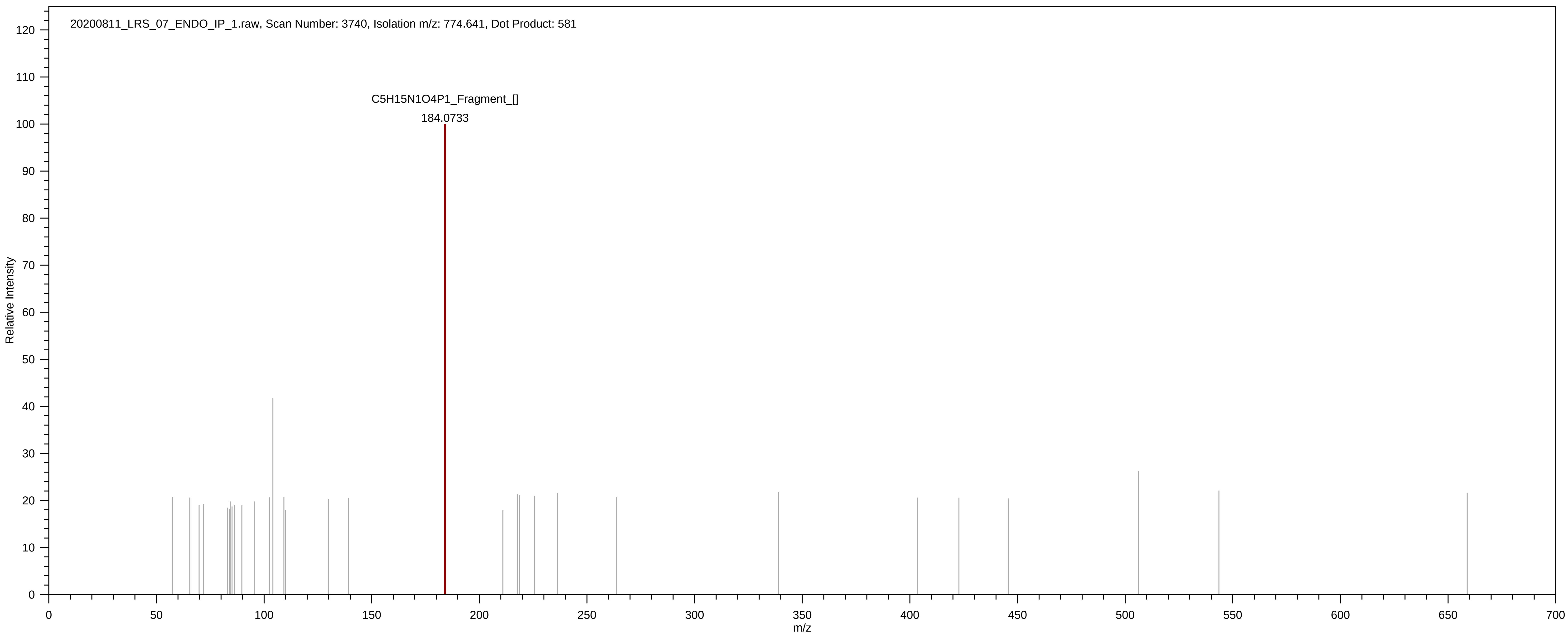

Plasmanyl-PC O-36:2 [M+Ac-H]- | Ret. Time (min): 10.632 | m/z: 830.629

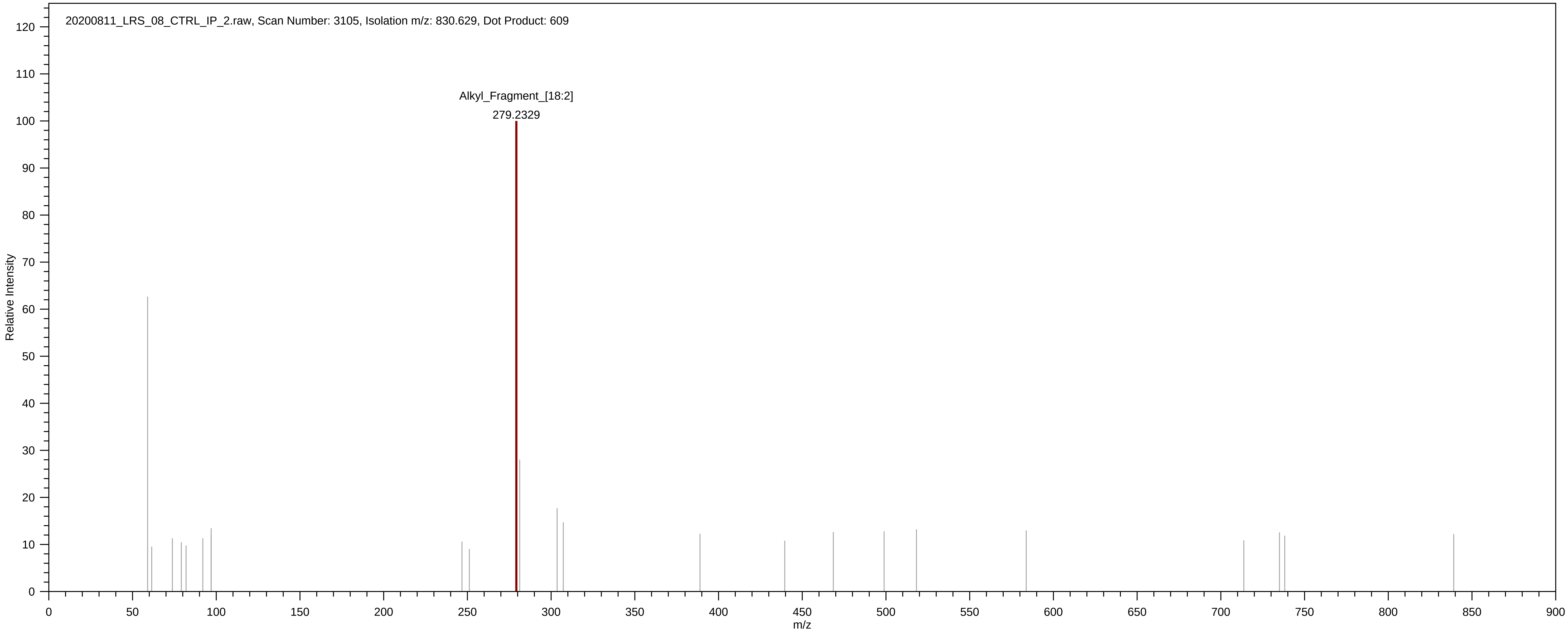

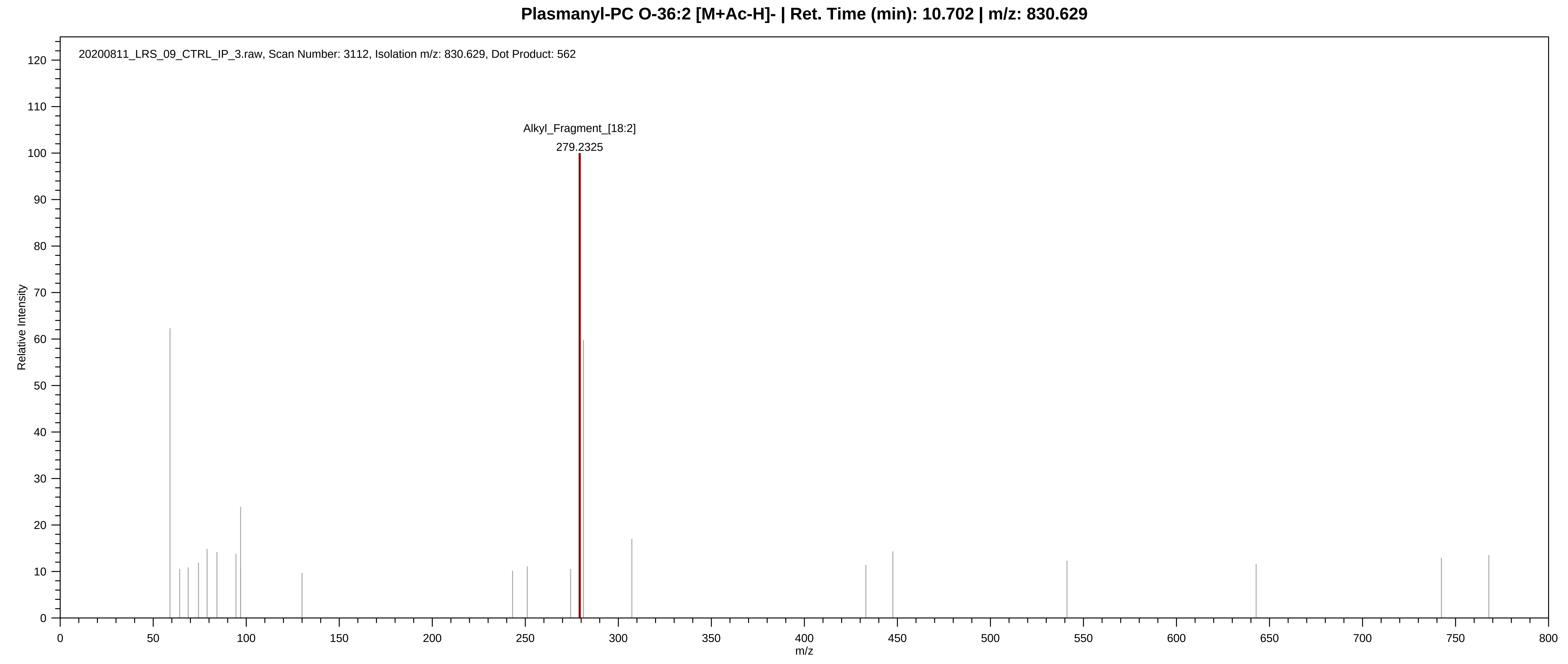

Plasmanyl-PC O-36:2 [M+Ac-H]- | Ret. Time (min): 10.702 | m/z: 830.629
20200811_LRS_09_CTRL_IP_3.raw, Scan Number: 3112, Isolation m/z: 830.629, Dot Product: 562
Alkyl_Fragment_[18:2]
279.2325
Relative Intensity
m/z

Plasmanyl-PC O-36:2 [M+H]+ | Ret. Time (min): 11.238 | m/z: 772.62

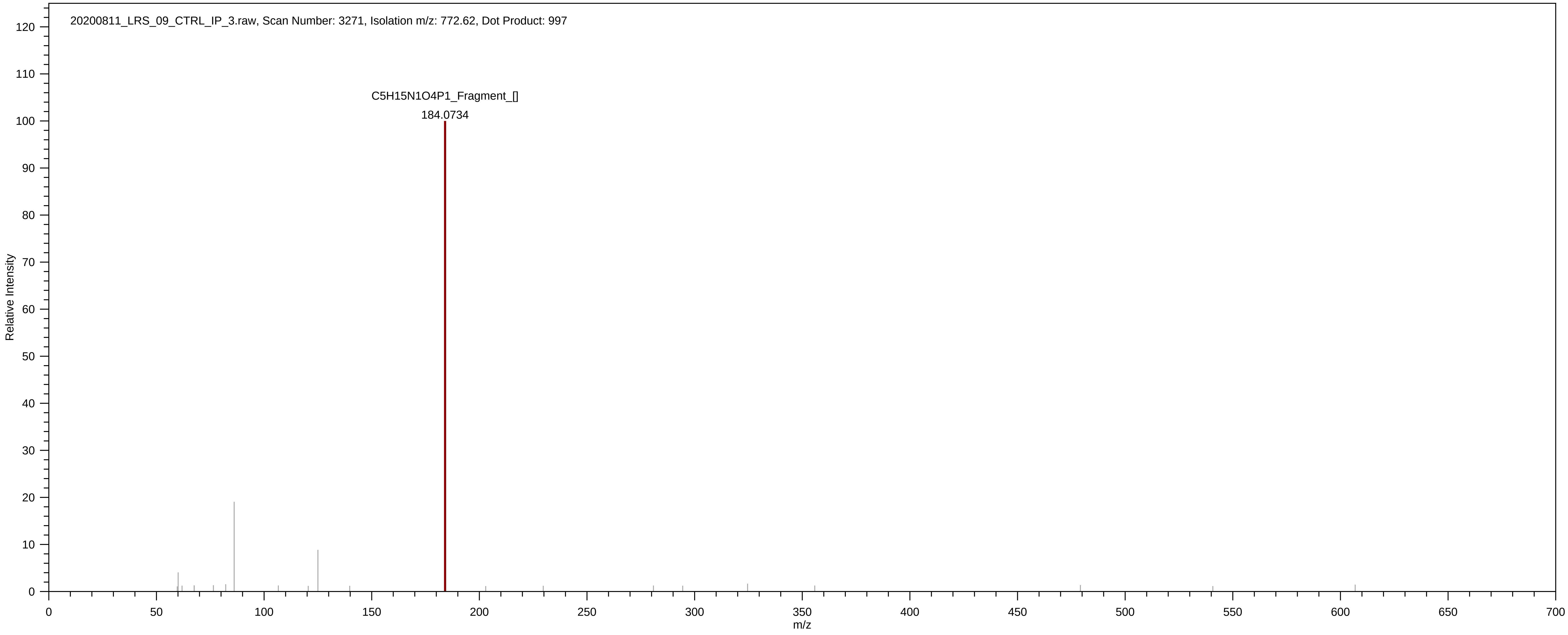

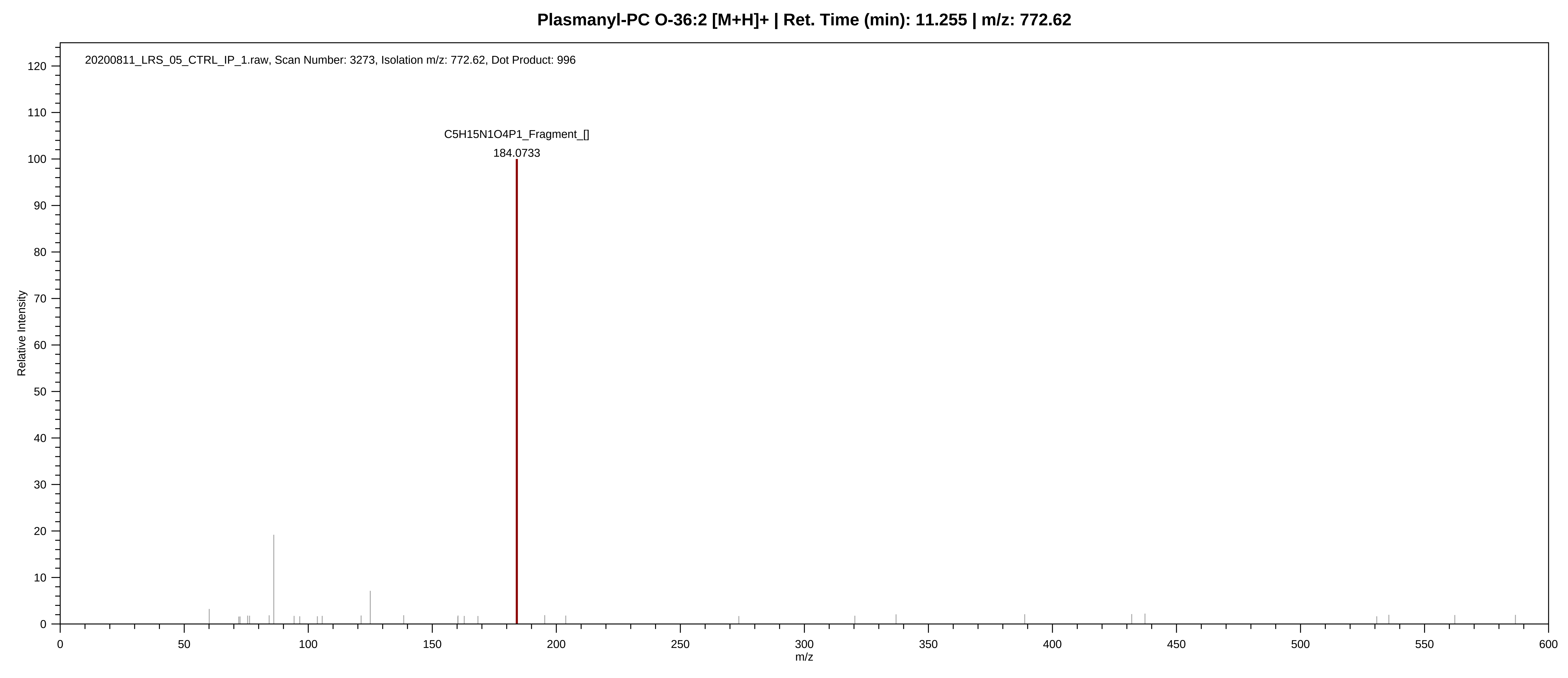
Plasmanyl-PC O-36:2 [M+H]+ | Ret. Time (min): 11.255 | m/z: 772.62
20200811_LRS_05_CTRL_IP_1.raw, Scan Number: 3273, Isolation m/z: 772.62, Dot Product: 996
C5H15N1O4P1_Fragment_[]
184.0733
Relative Intensity
m/z
0
10
20
30
40
50
60
70
80
90
100
110
120
0
50
100
150
200
250
300
350
400
450
500
550
600

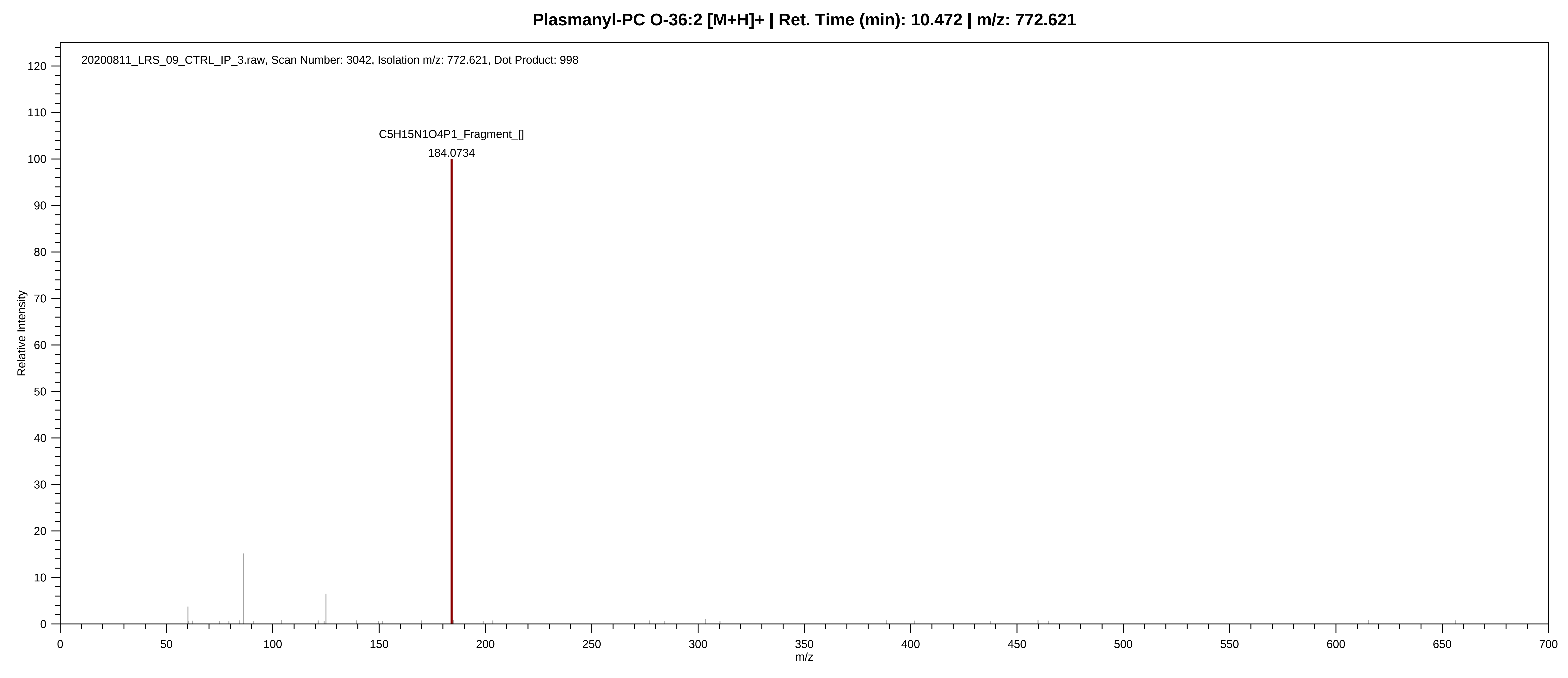

Plasmanyl-PC O-36:2 [M+H]+ | Ret. Time (min): 10.472 | m/z: 772.621
20200811_LRS_09_CTRL_IP_3.raw, Scan Number: 3042, Isolation m/z: 772.621, Dot Product: 998
C5H15N1O4P1_Fragment_[]
184.0734
Relative Intensity
m/z
0
10
20
30
40
50
60
70
80
90
100
110
120
0
50
100
150
200
250
300
350
400
450
500
550
600
650
700

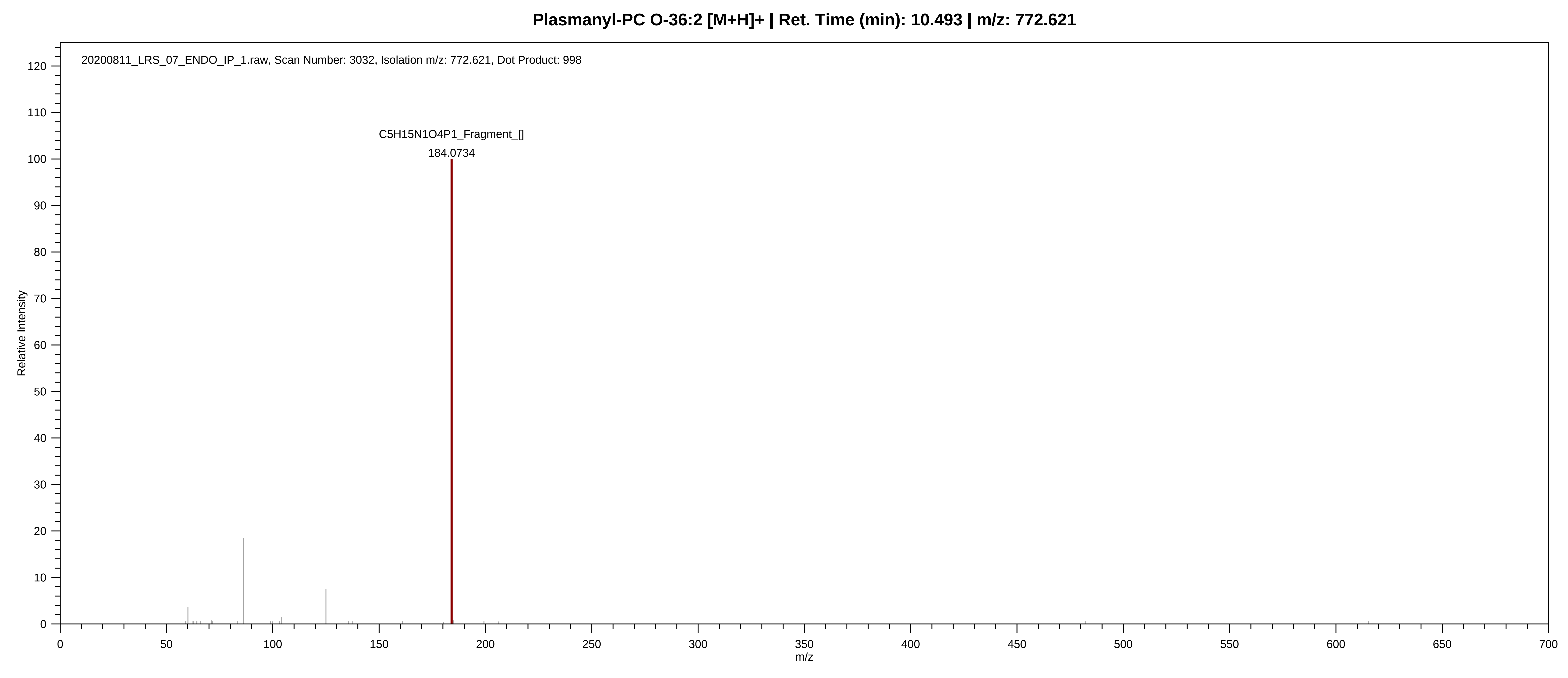
Plasmanyl-PC O-36:2 [M+H]+ | Ret. Time (min): 10.493 | m/z: 772.621
20200811_LRS_07_ENDO_IP_1.raw, Scan Number: 3032, Isolation m/z: 772.621, Dot Product: 998
C5H15N1O4P1_Fragment_[]
184.0734
Relative Intensity
m/z

Plasmanyl-PC O-36:2 [M+H]+ | Ret. Time (min): 10.511 | m/z: 772.621

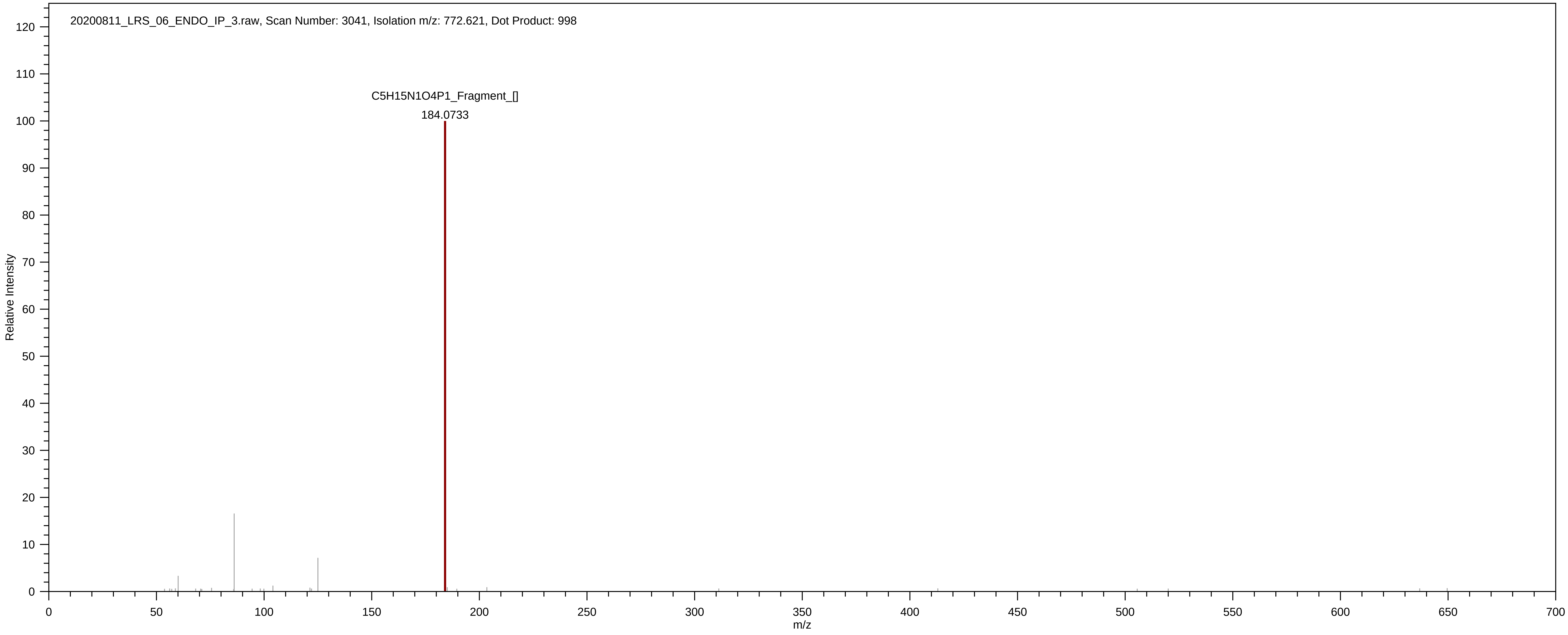

Plasmanyl-PC O-36:2 [M+H]+ | Ret. Time (min): 10.515 | m/z: 772.621

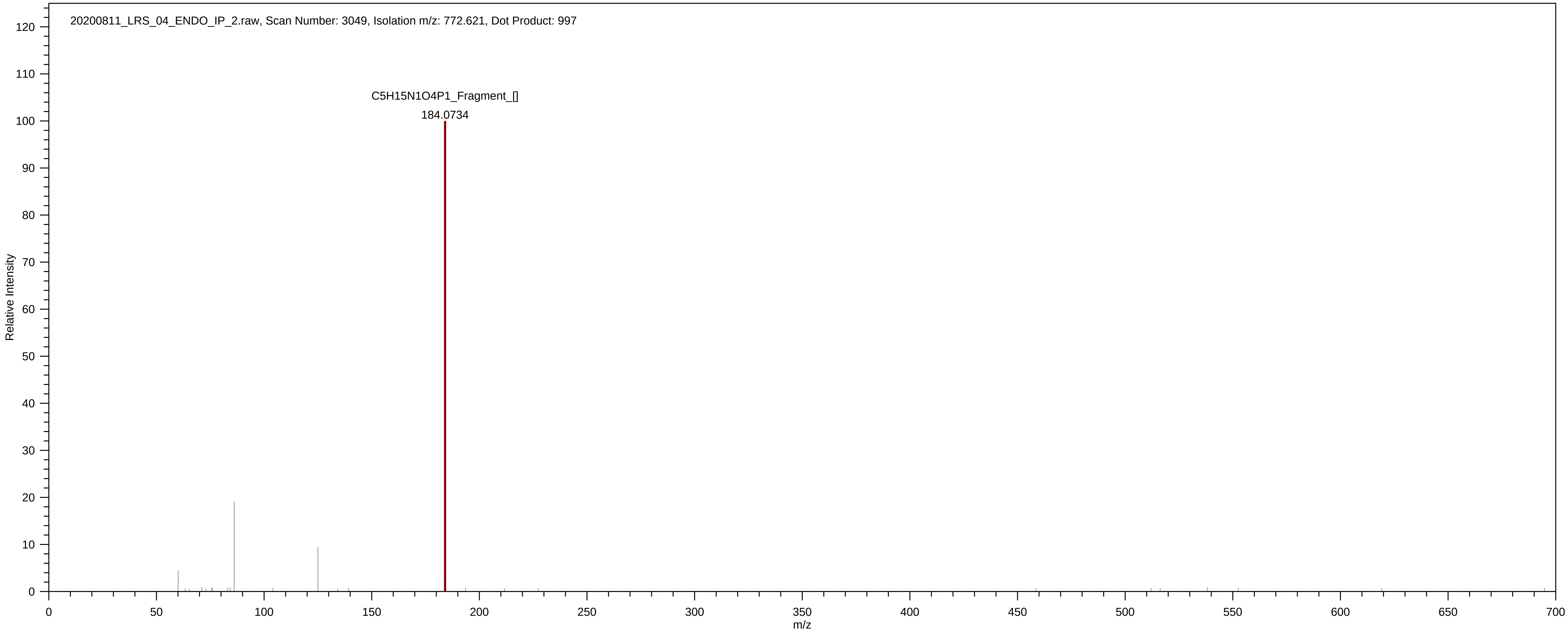

Plasmanyl-PC O-36:2 [M+H]+ | Ret. Time (min): 11.241 | m/z: 772.621

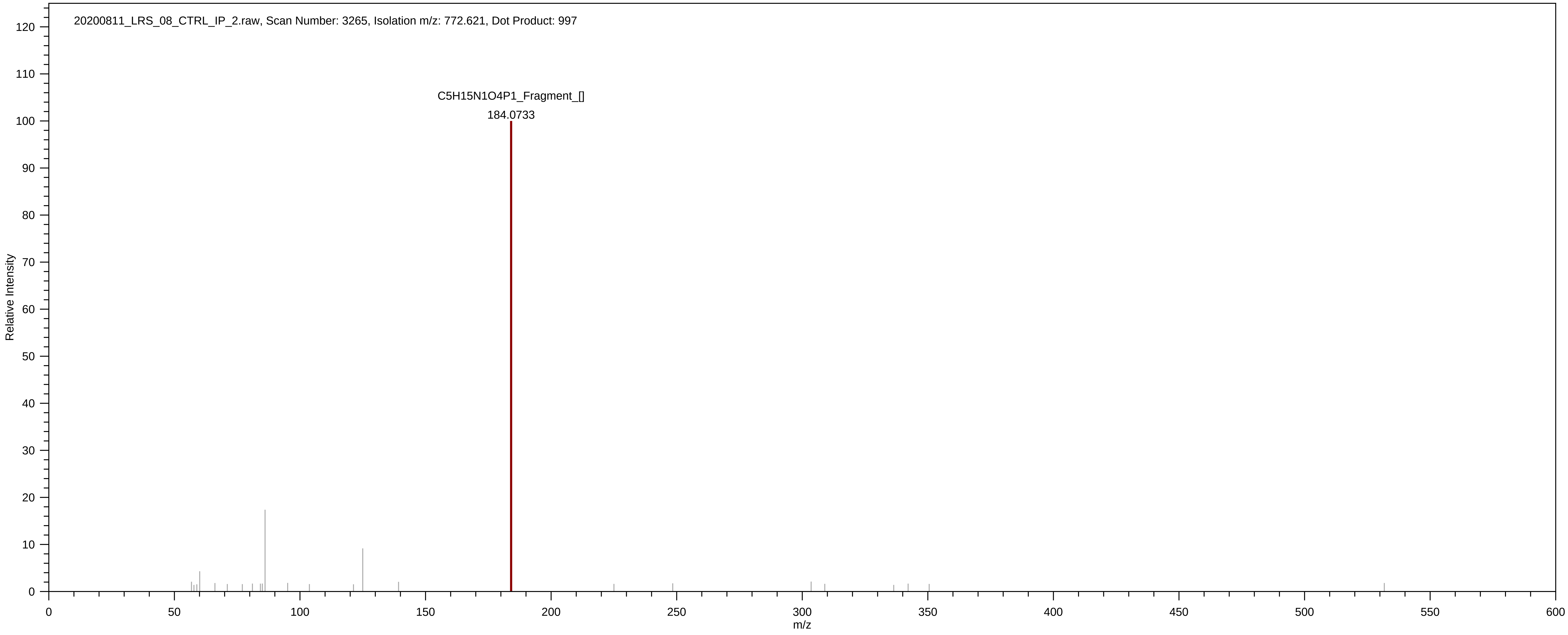

Plasmanyl-PC O-36:2 [M+H]+ | Ret. Time (min): 10.48 | m/z: 772.622

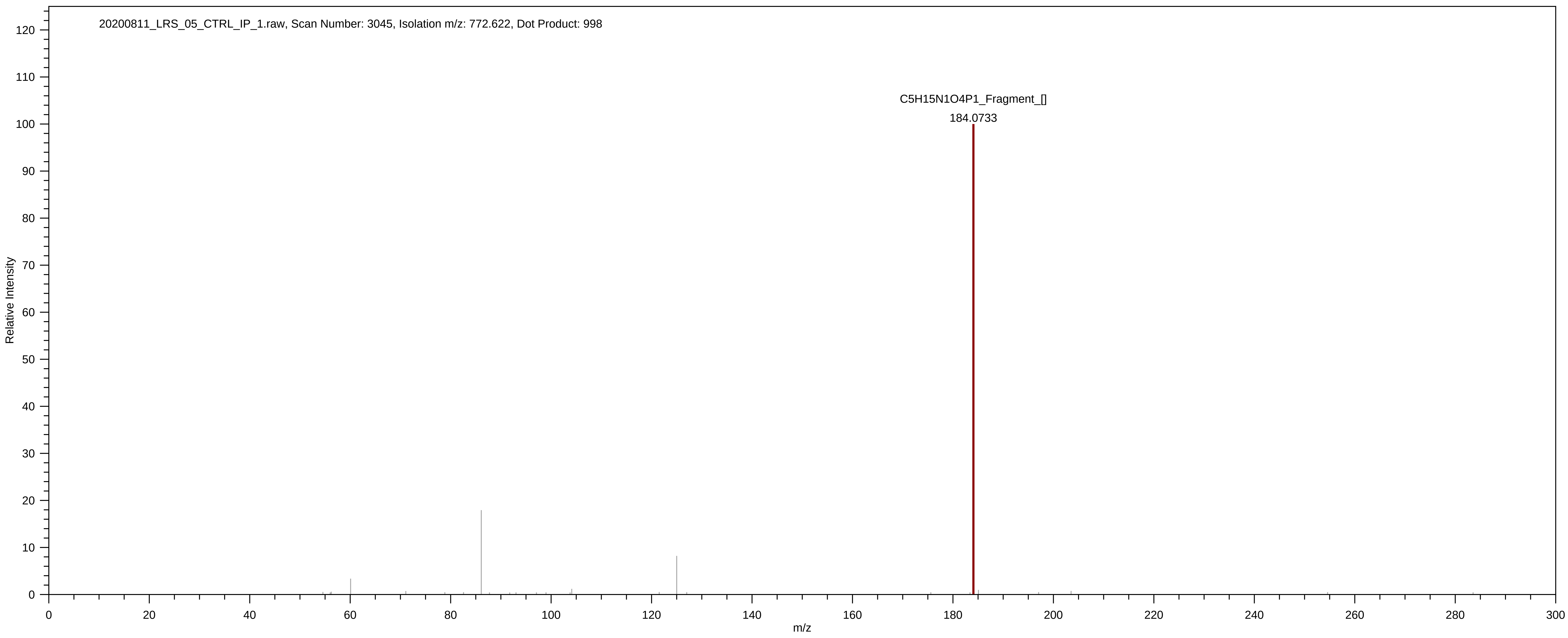

Plasmanyl-PC O-36:2 [M+H]+ | Ret. Time (min): 10.455 | m/z: 772.622

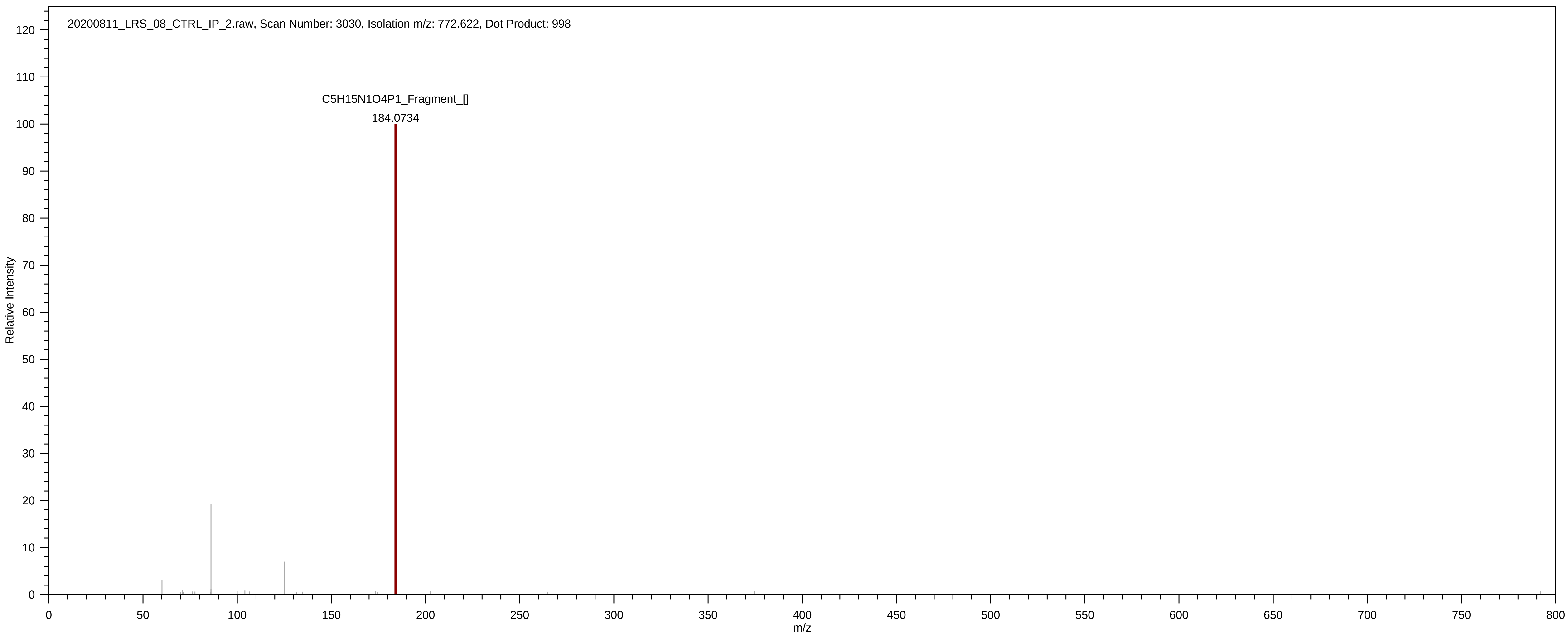

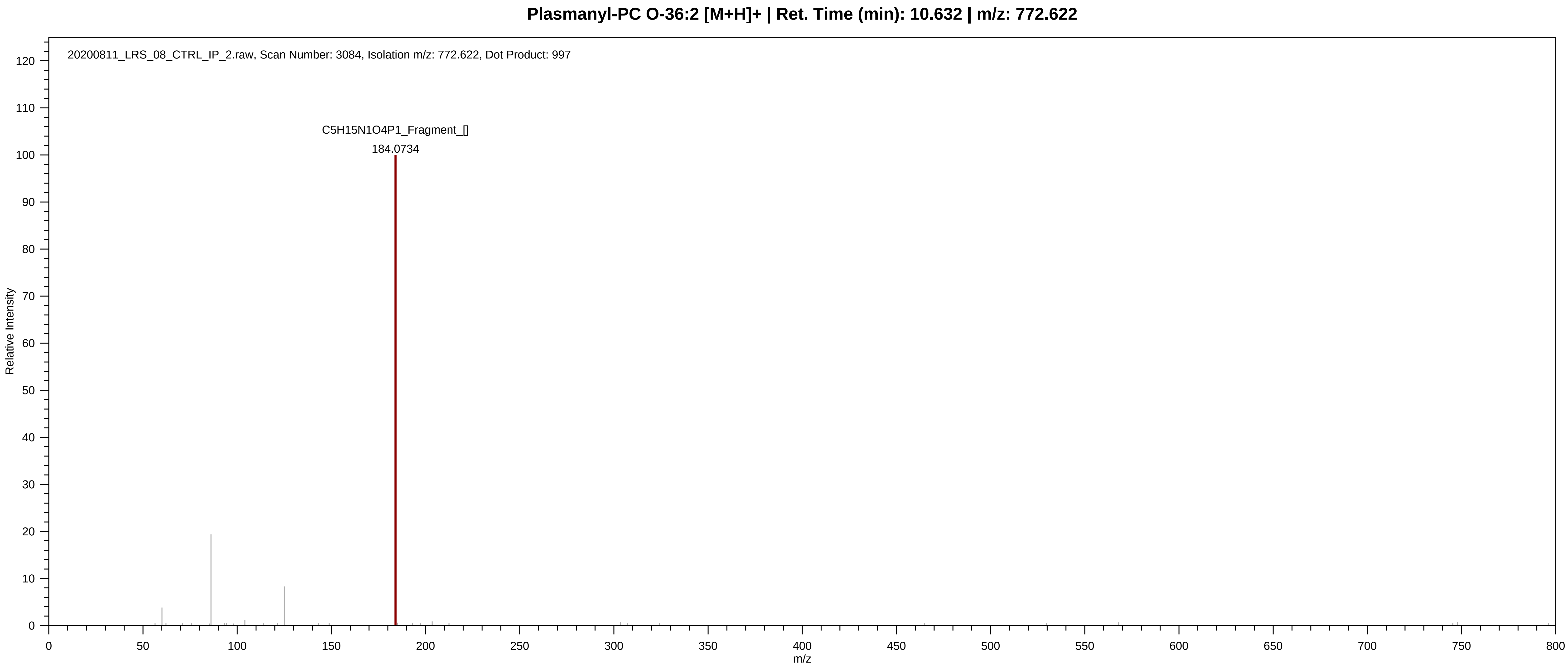
Plasmanyl-PC O-36:2 [M+H]+ | Ret. Time (min): 10.632 | m/z: 772.622
20200811_LRS_08_CTRL_IP_2.raw, Scan Number: 3084, Isolation m/z: 772.622, Dot Product: 997
C5H15N1O4P1_Fragment_[]
184.0734
Relative Intensity
m/z
0
10
20
30
40
50
60
70
80
90
100
110
120
0
50
100
150
200
250
300
350
400
450
500
550
600
650
700
750
800

Plasmanyl-PC O-36:2 [M+H]+ | Ret. Time (min): 10.648 | m/z: 772.622

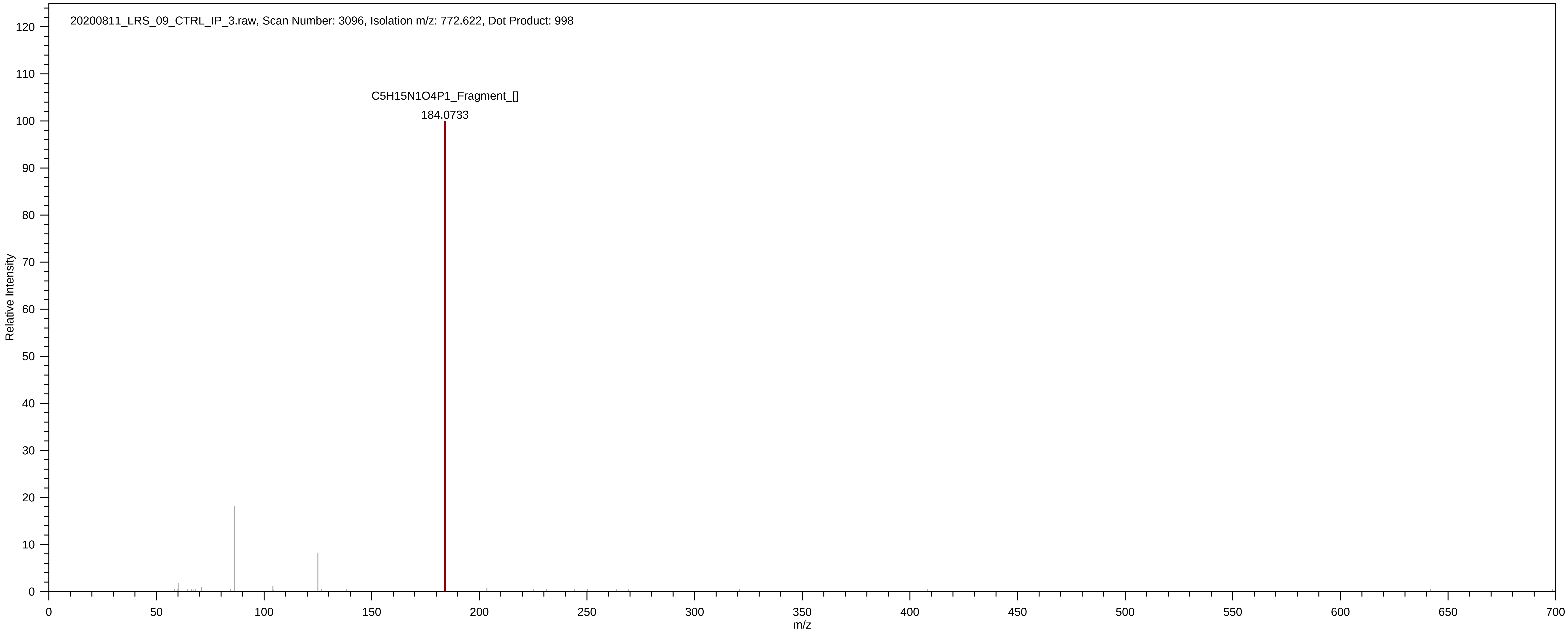

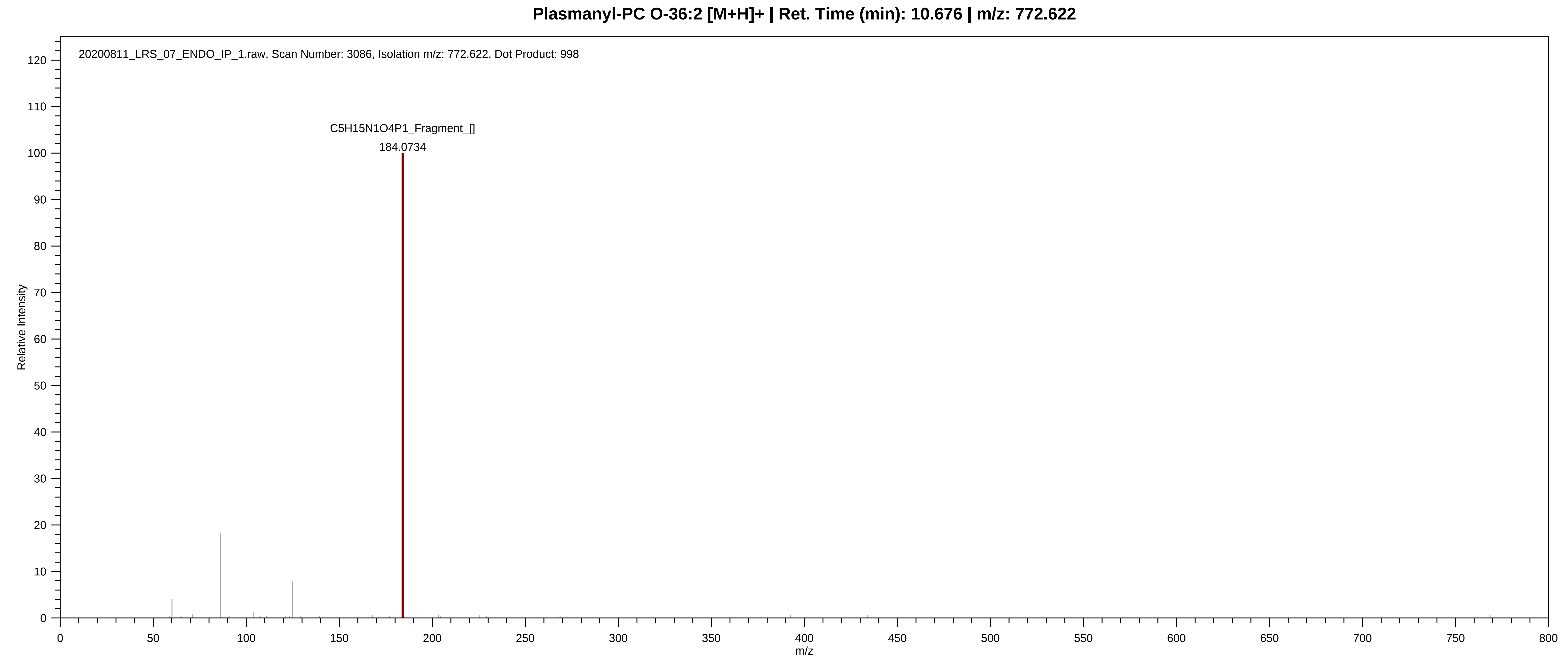
Plasmanyl-PC O-36:2 [M+H]+ | Ret. Time (min): 10.676 | m/z: 772.622
20200811_LRS_07_ENDO_IP_1.raw, Scan Number: 3086, Isolation m/z: 772.622, Dot Product: 998
C5H15N1O4P1_Fragment_[]
184.0734
Relative Intensity
m/z

Plasmanyl-PC O-36:2 [M+H]+ | Ret. Time (min): 10.691 | m/z: 772.622

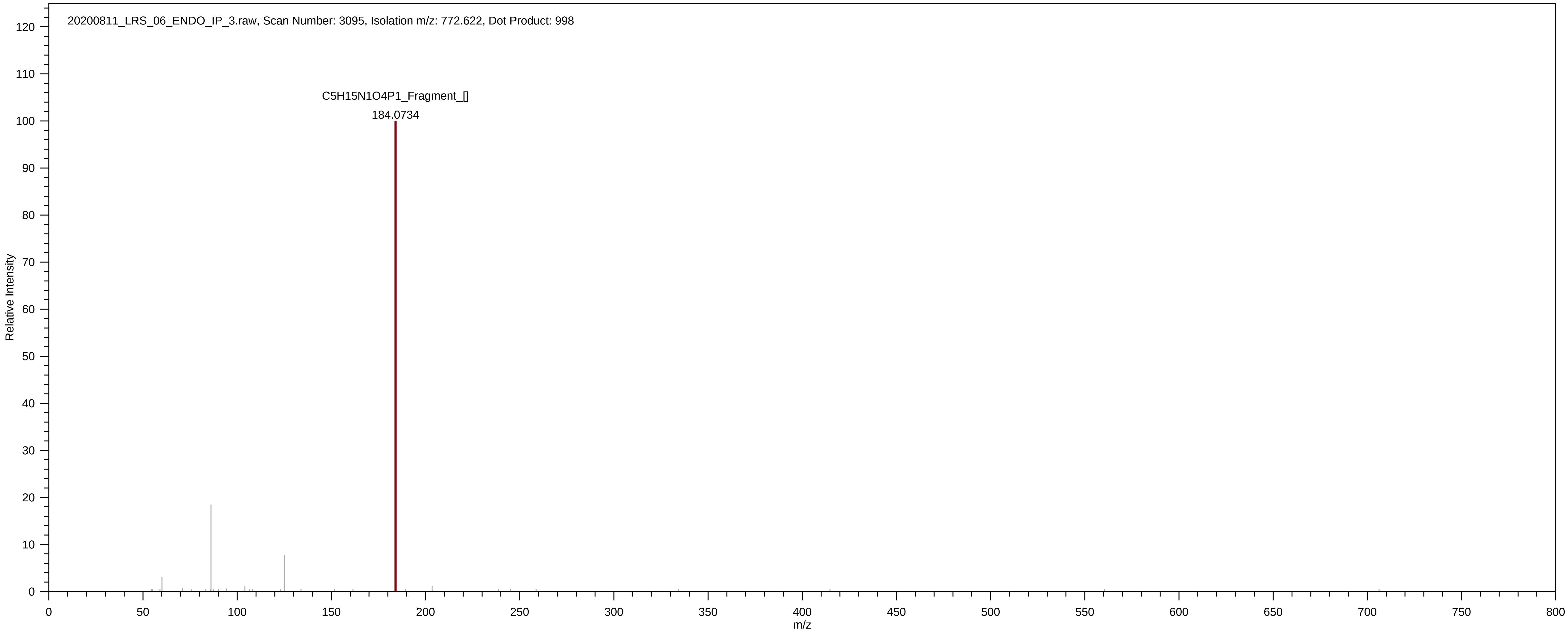

Plasmanyl-PC O-36:2 [M+H]+ | Ret. Time (min): 10.697 | m/z: 772.622

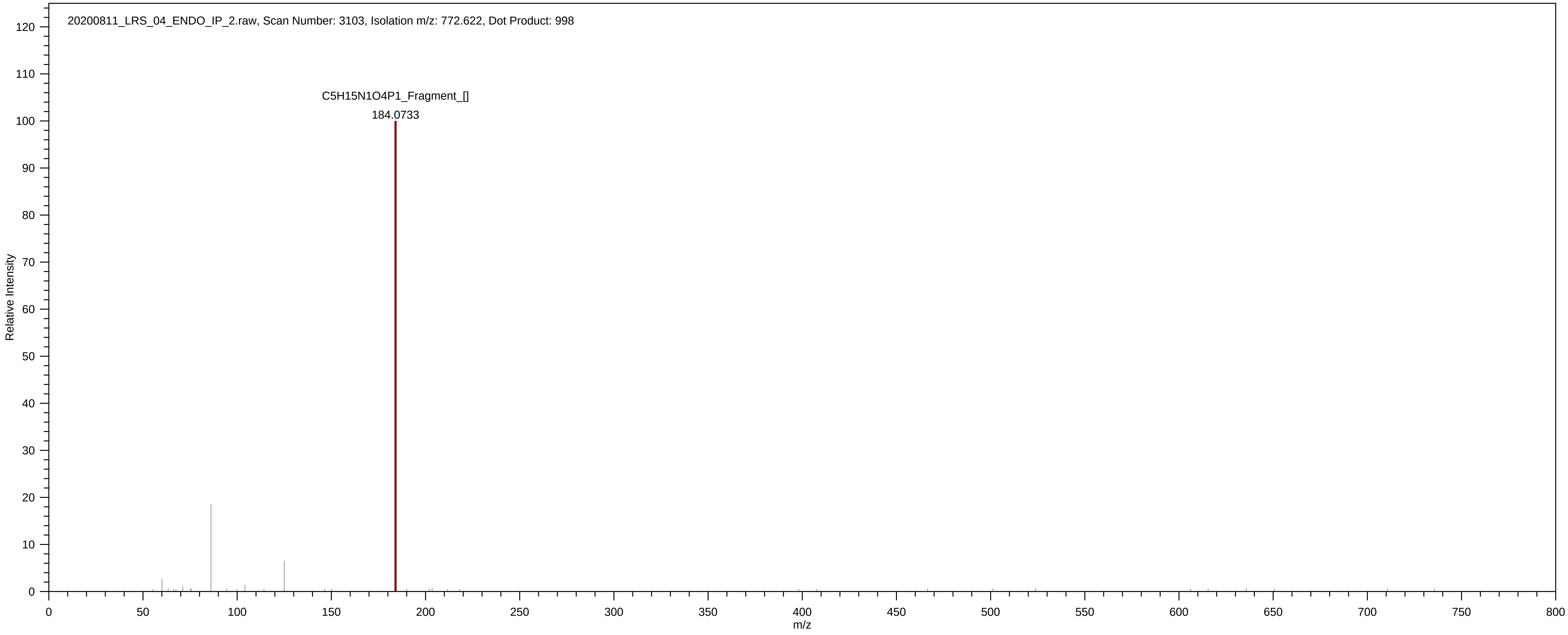

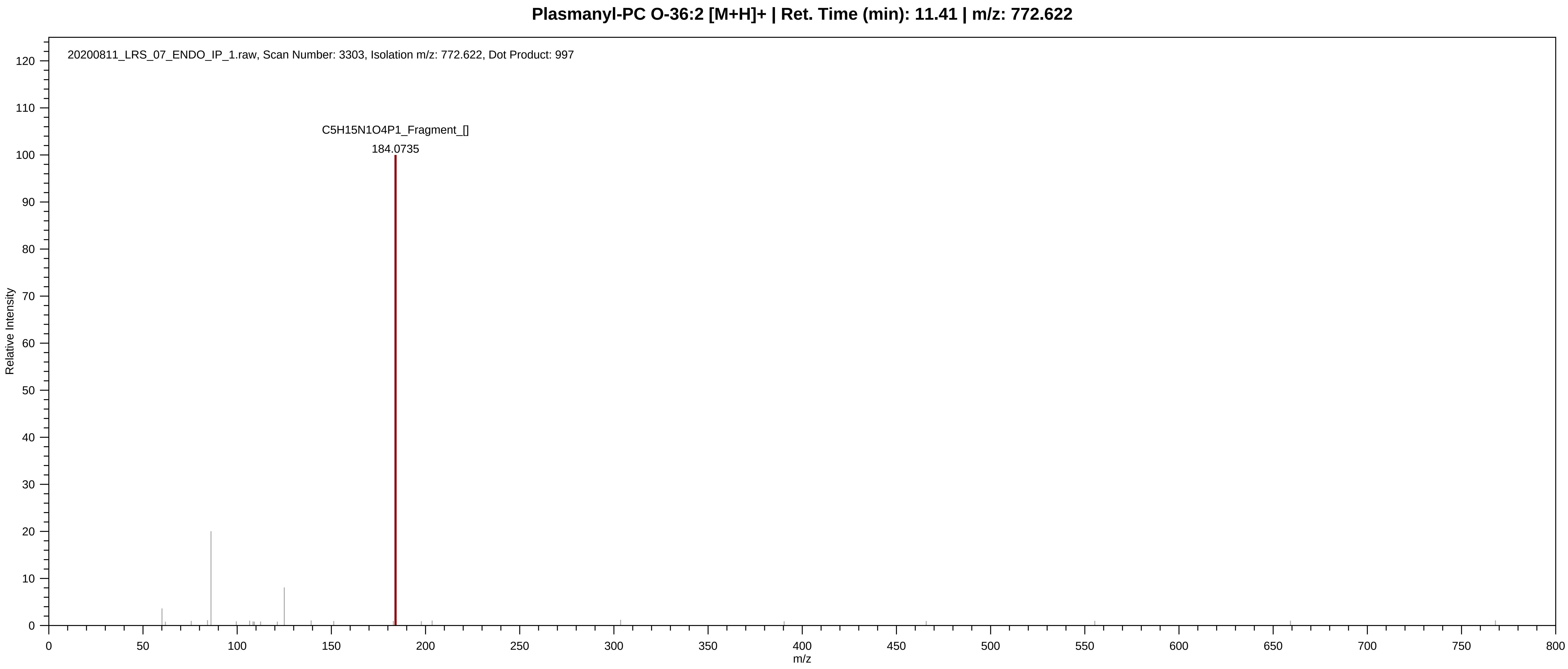
Plasmanyl-PC O-36:2 [M+H]+ | Ret. Time (min): 11.41 | m/z: 772.622
20200811_LRS_07_ENDO_IP_1.raw, Scan Number: 3303, Isolation m/z: 772.622, Dot Product: 997
C5H15N1O4P1_Fragment_[]
184.0735
Relative Intensity
m/z

Plasmanyl-PC O-36:2 [M+H]+ | Ret. Time (min): 11.43 | m/z: 772.622

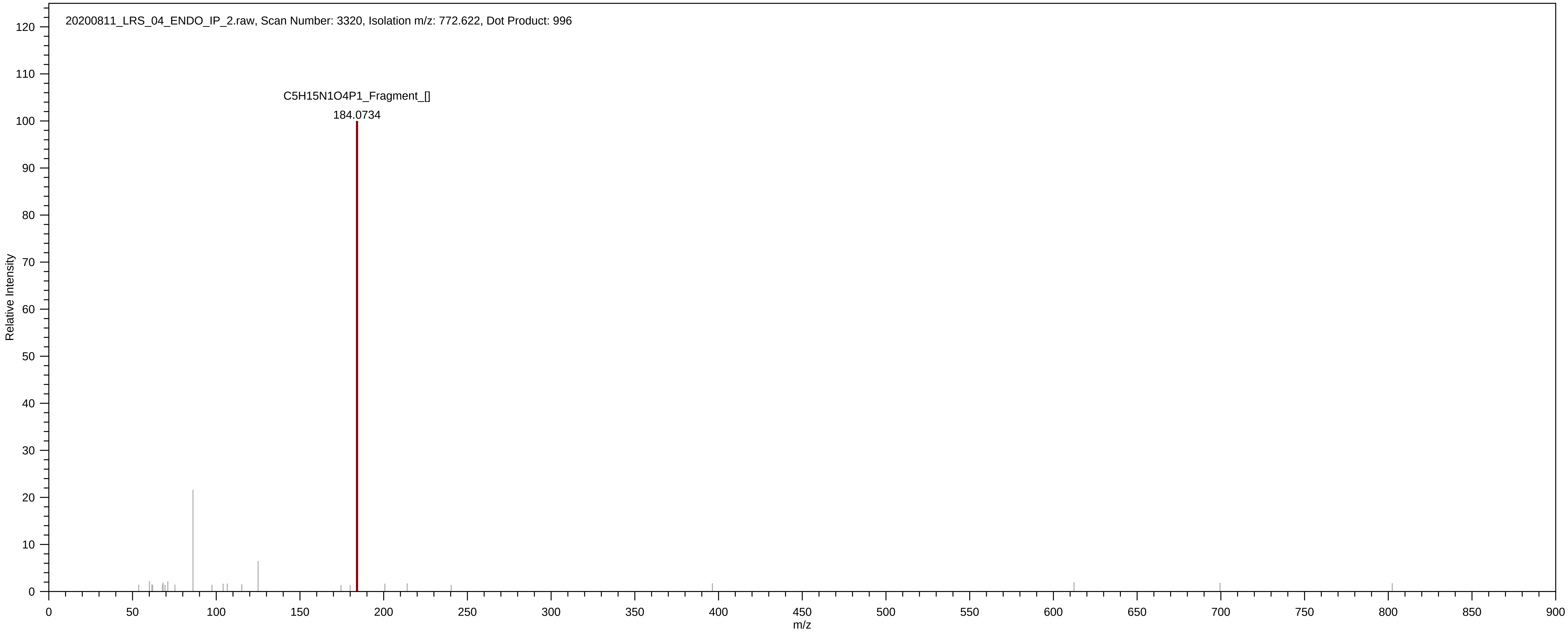

Plasmanyl-PC O-36:2 [M+H]+ | Ret. Time (min): 11.408 | m/z: 772.622

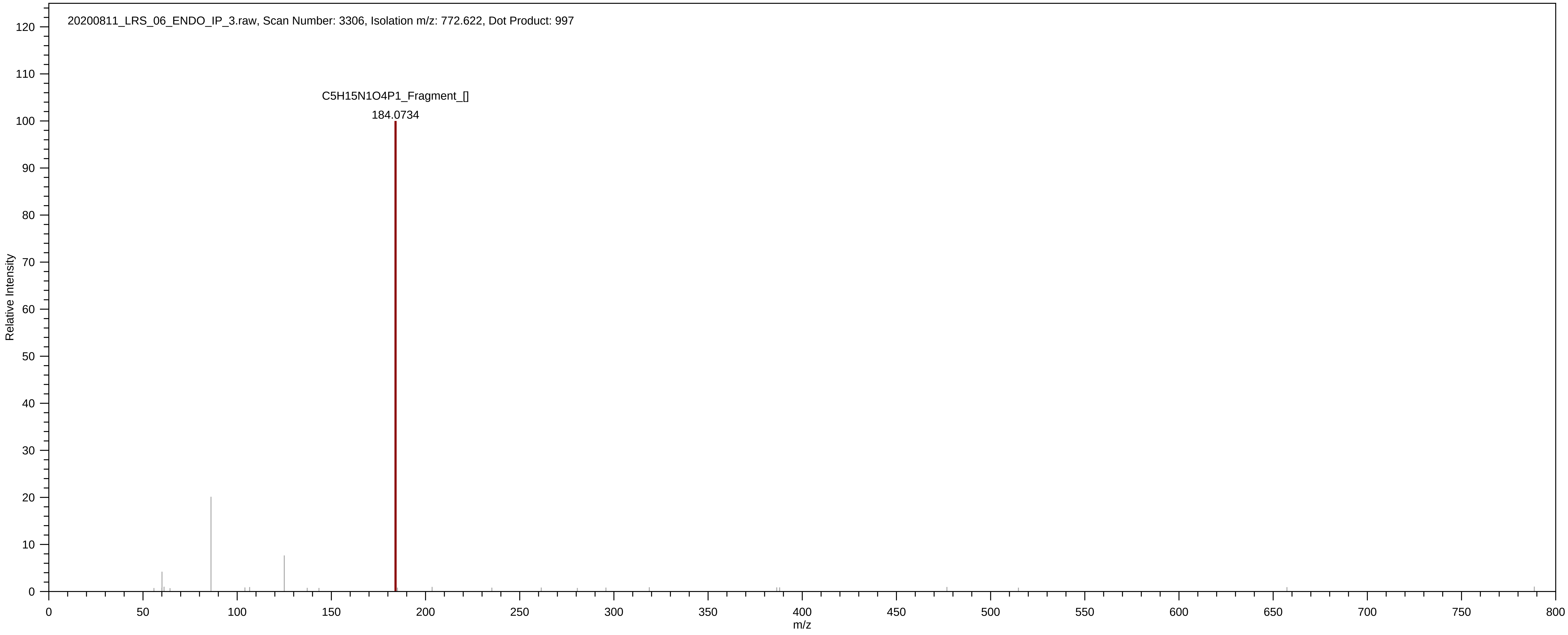

Plasmanyl-PC O-36:2 [M+H]+ | Ret. Time (min): 11.441 | m/z: 772.622

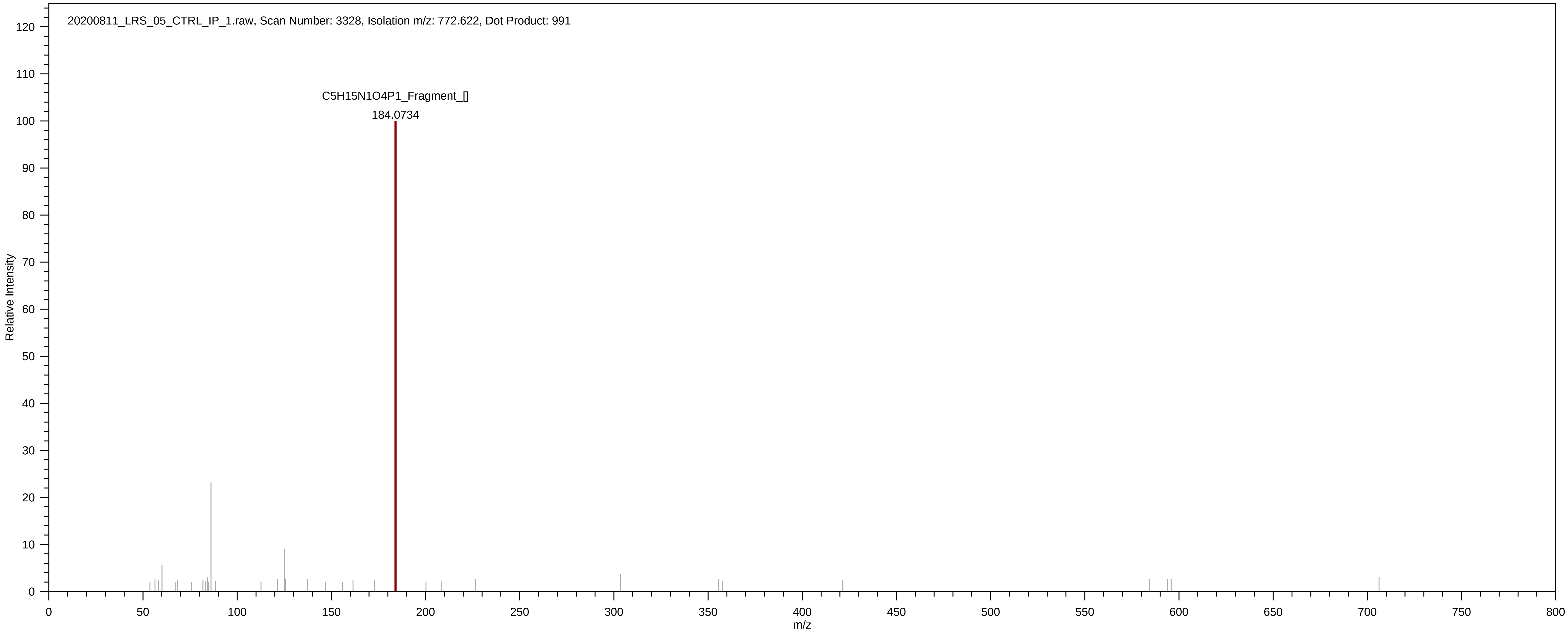

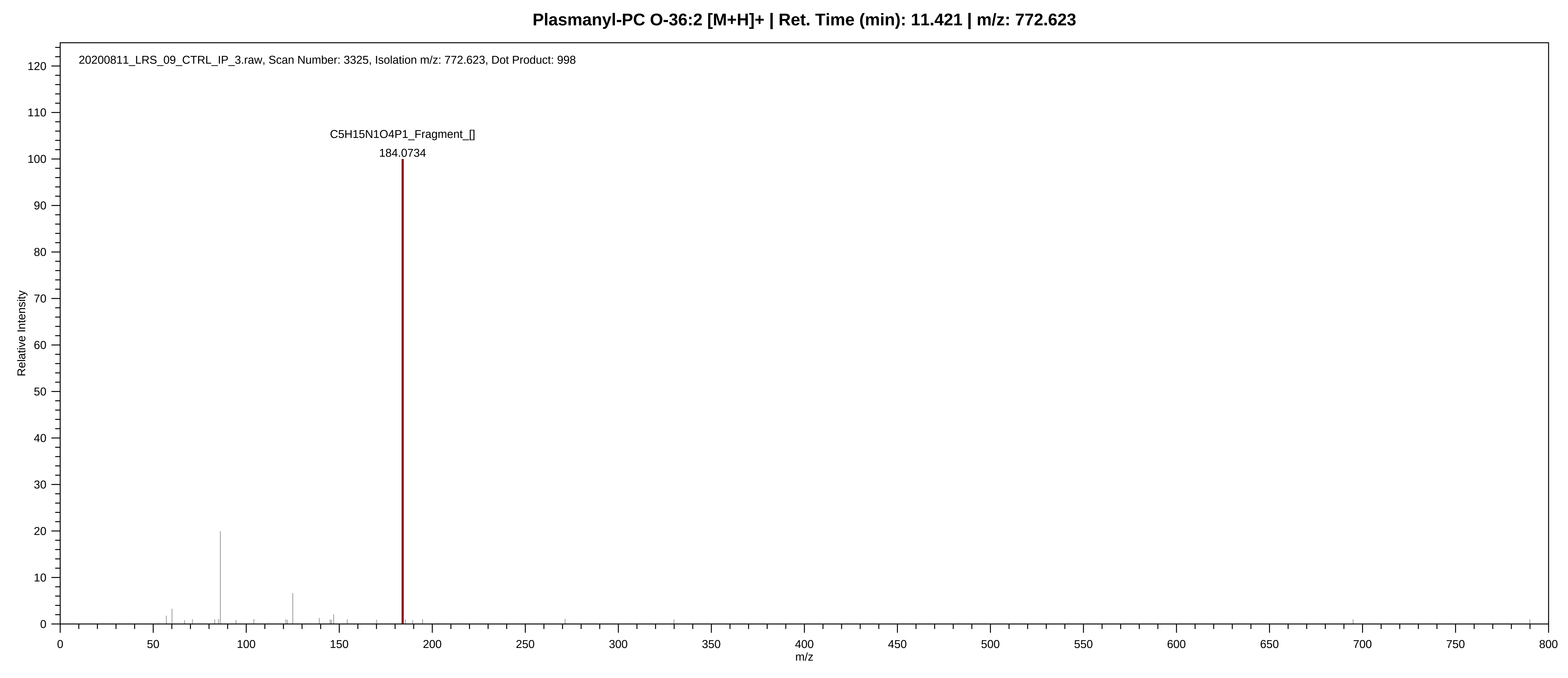
Plasmanyl-PC O-36:2 [M+H]+ | Ret. Time (min): 11.421 | m/z: 772.623
20200811_LRS_09_CTRL_IP_3.raw, Scan Number: 3325, Isolation m/z: 772.623, Dot Product: 998
C5H15N1O4P1_Fragment_[]
184.0734
Relative Intensity
m/z

Plasmanyl-PC O-36:2 [M+H]+ | Ret. Time (min): 11.427 | m/z: 772.623

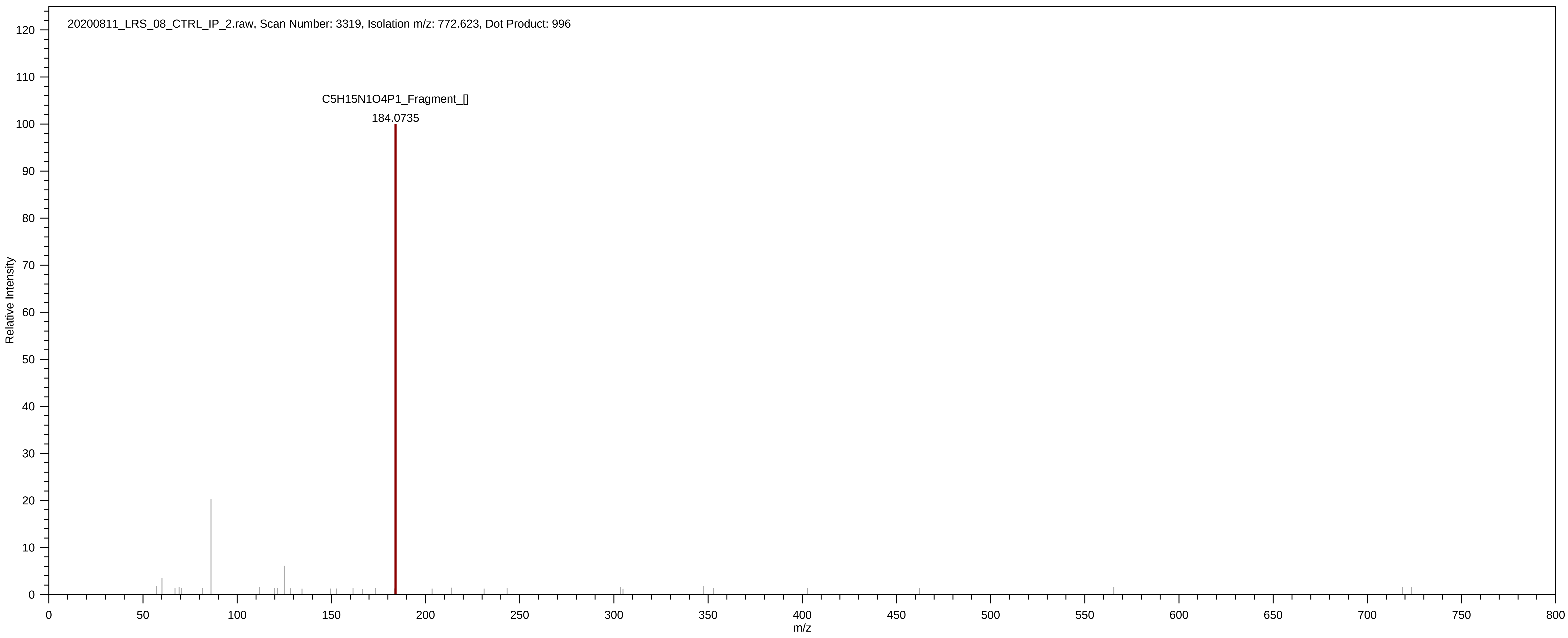

Plasmanyl-PC O-36:2 [M+H]+ | Ret. Time (min): 11.225 | m/z: 772.624

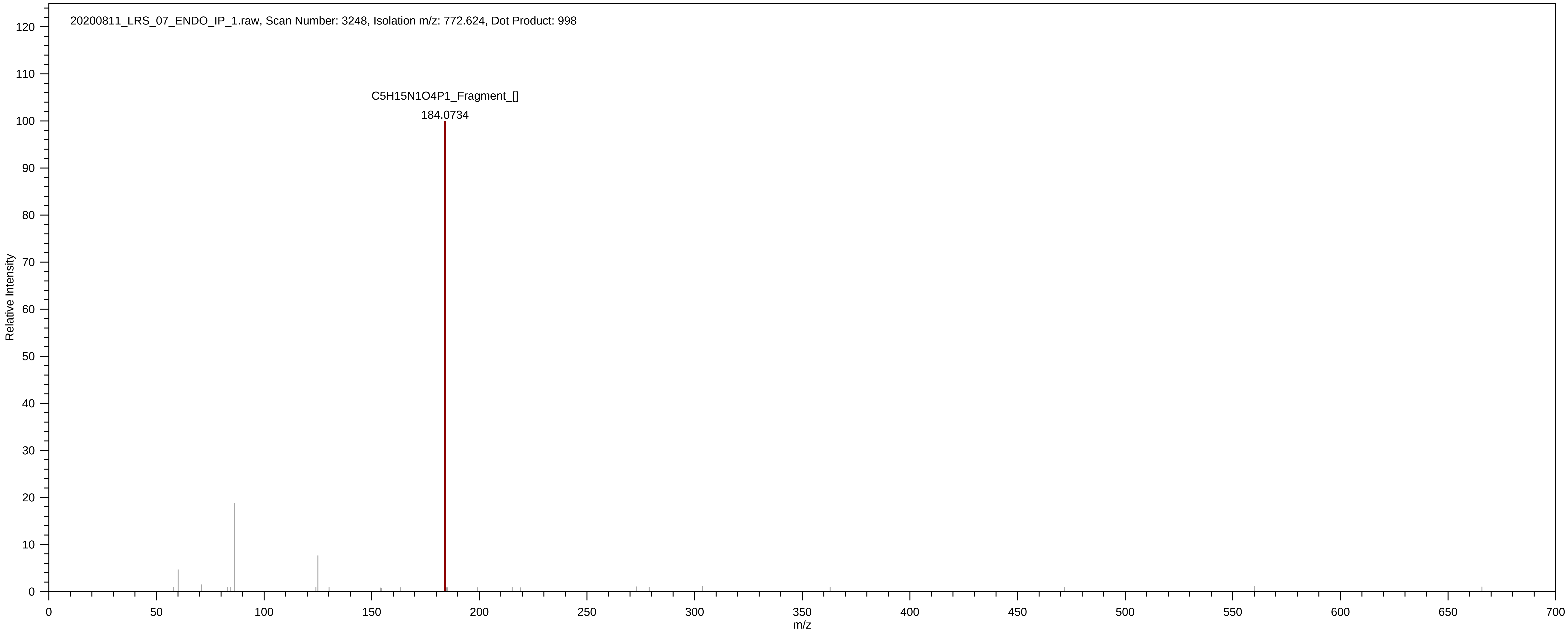

Plasmanyl-PC O-36:2 [M+H]+ | Ret. Time (min): 11.227 | m/z: 772.624

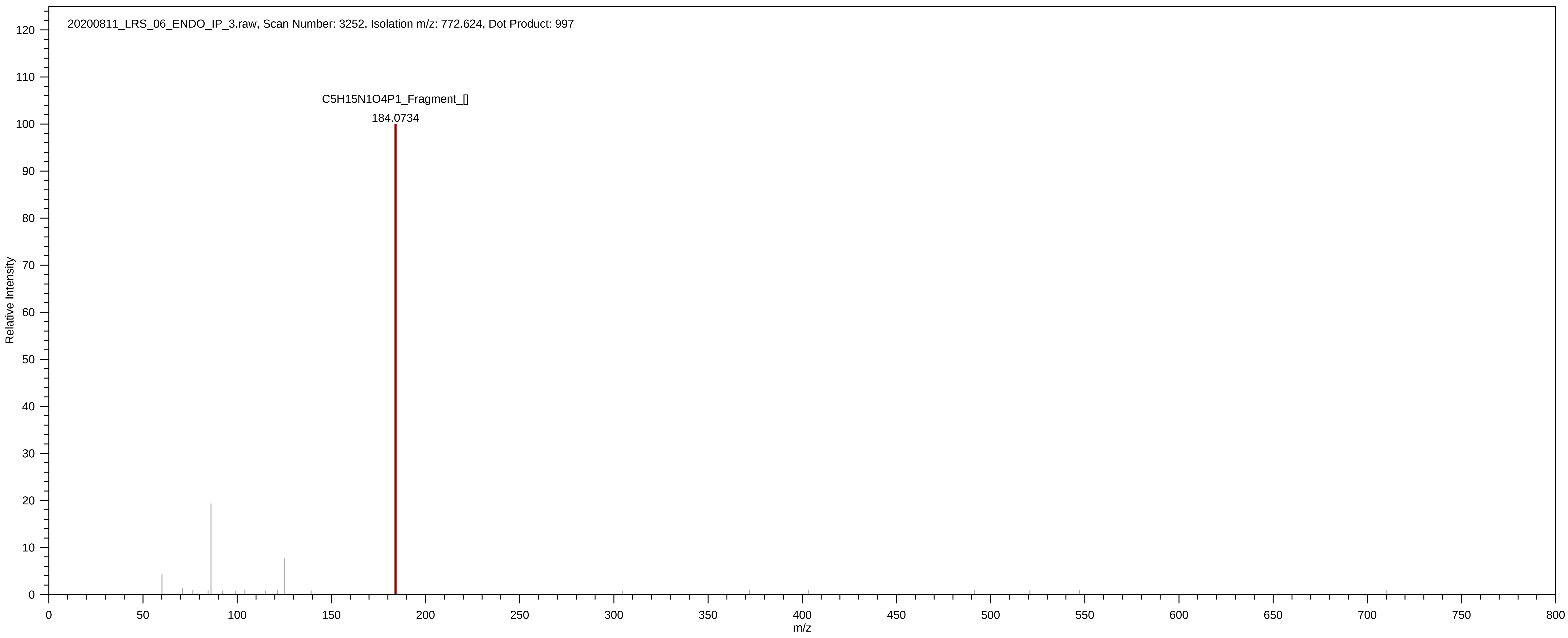

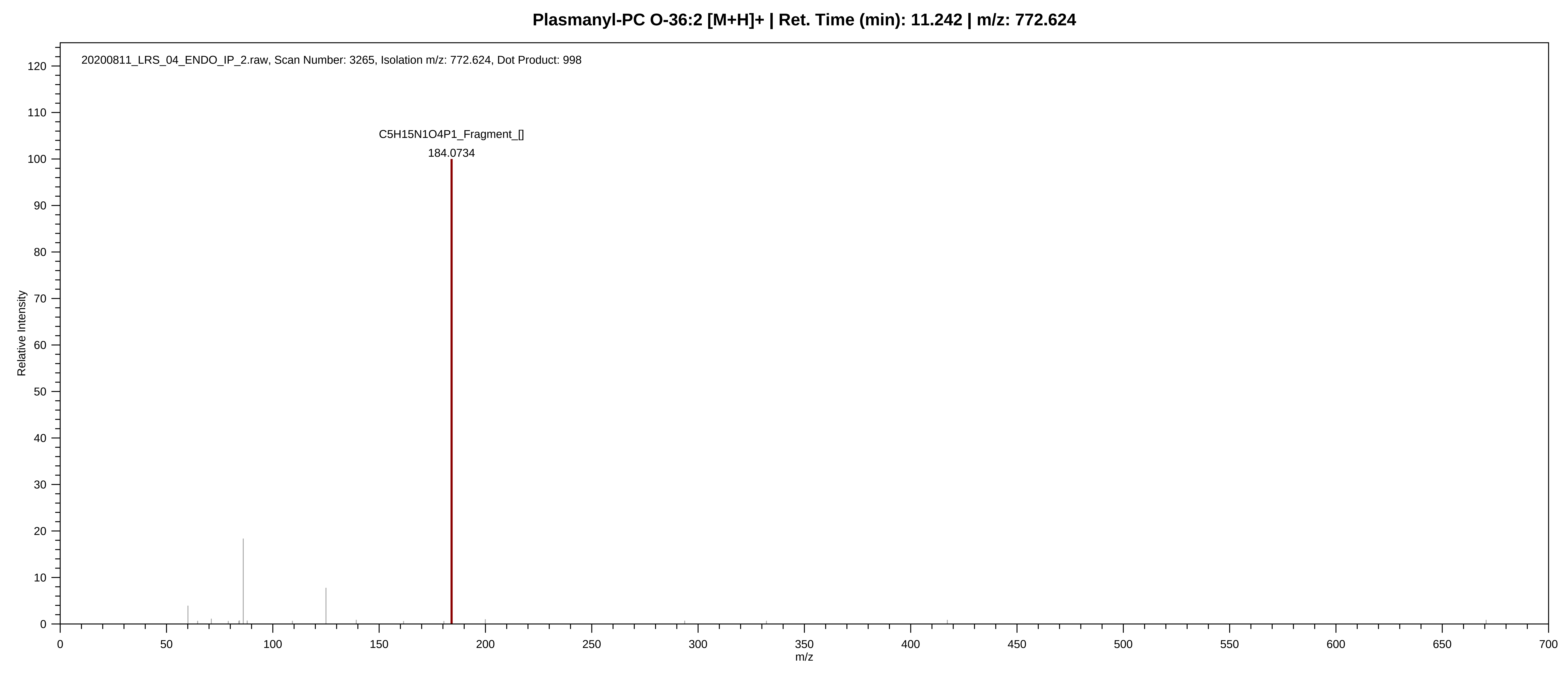

Plasmanyl-PC O-36:2 [M+H]+ | Ret. Time (min): 11.242 | m/z: 772.624
20200811_LRS_04_ENDO_IP_2.raw, Scan Number: 3265, Isolation m/z: 772.624, Dot Product: 998
C5H15N1O4P1_Fragment_[]
184.0734
Relative Intensity
m/z

Plasmanyl-PC O-36:2 [M+H]+ | Ret. Time (min): 10.702 | m/z: 772.623

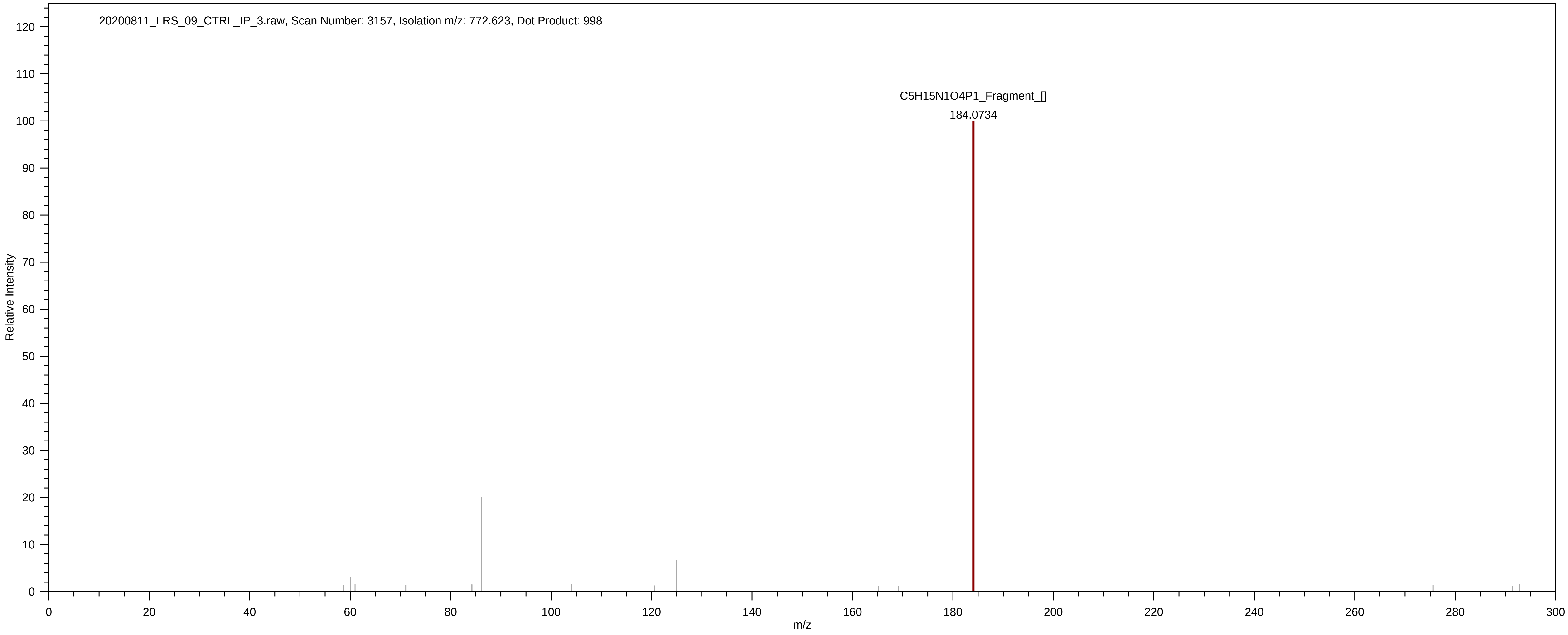

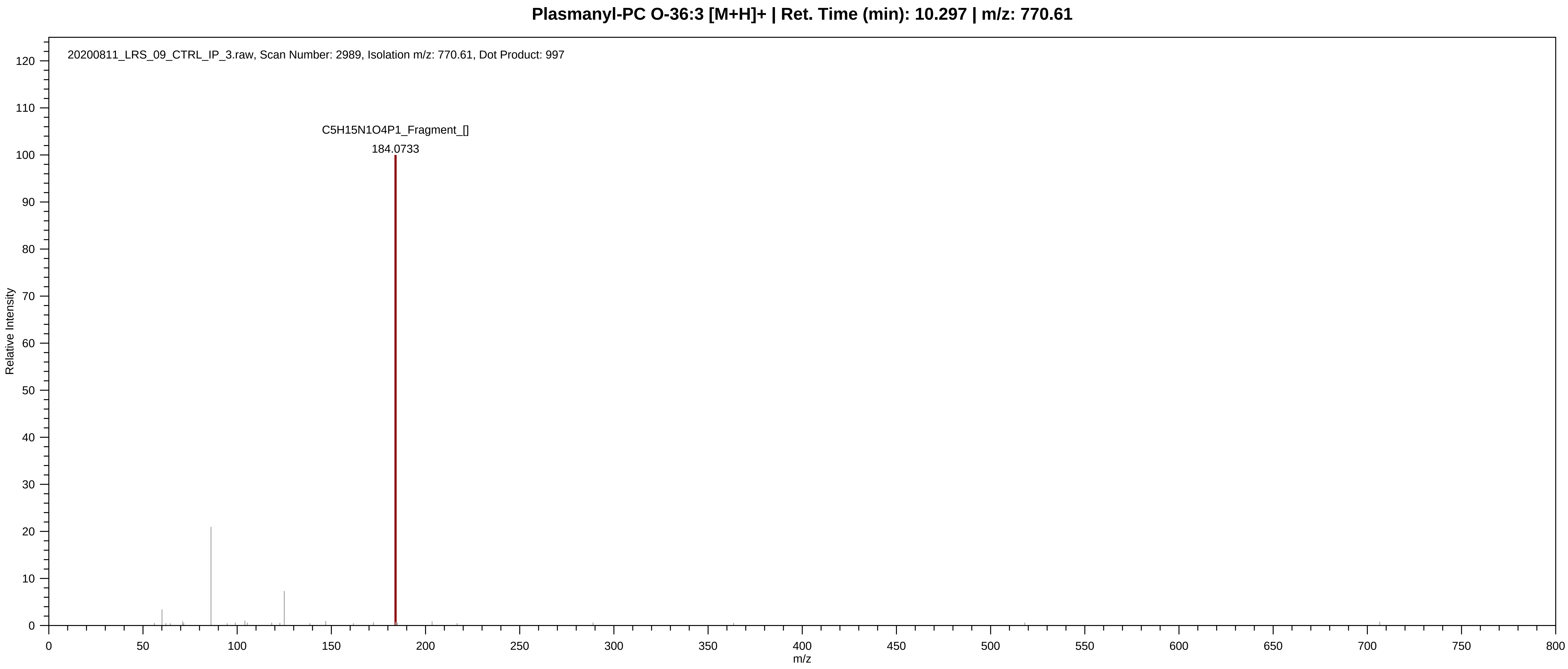

Plasmanyl-PC O-36:3 [M+H]+ | Ret. Time (min): 10.297 | m/z: 770.61
20200811_LRS_09_CTRL_IP_3.raw, Scan Number: 2989, Isolation m/z: 770.61, Dot Product: 997
C5H15N1O4P1_Fragment_[]
184.0733
Relative Intensity
m/z

Plasmanyl-PC O-36:3 [M+H]+ | Ret. Time (min): 9.687 | m/z: 770.606

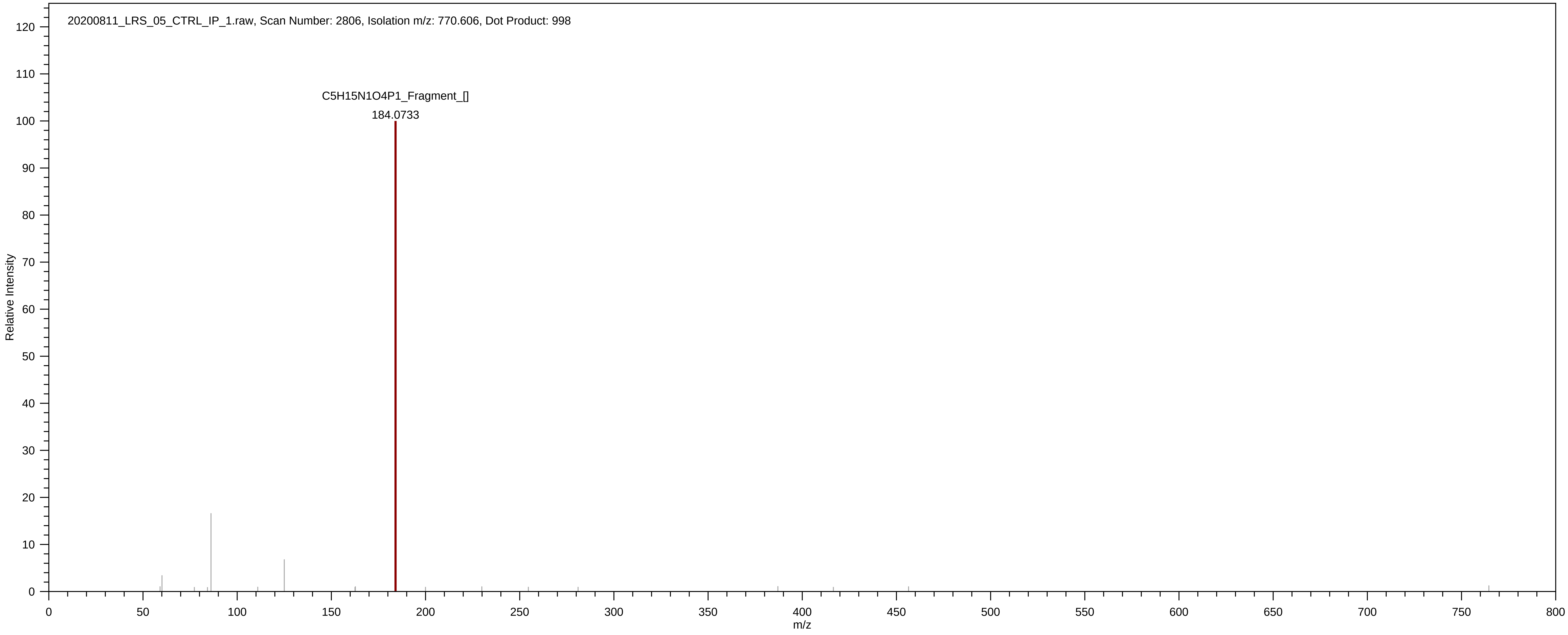

Plasmanyl-PC O-36:3 [M+H]+ | Ret. Time (min): 9.703 | m/z: 770.606

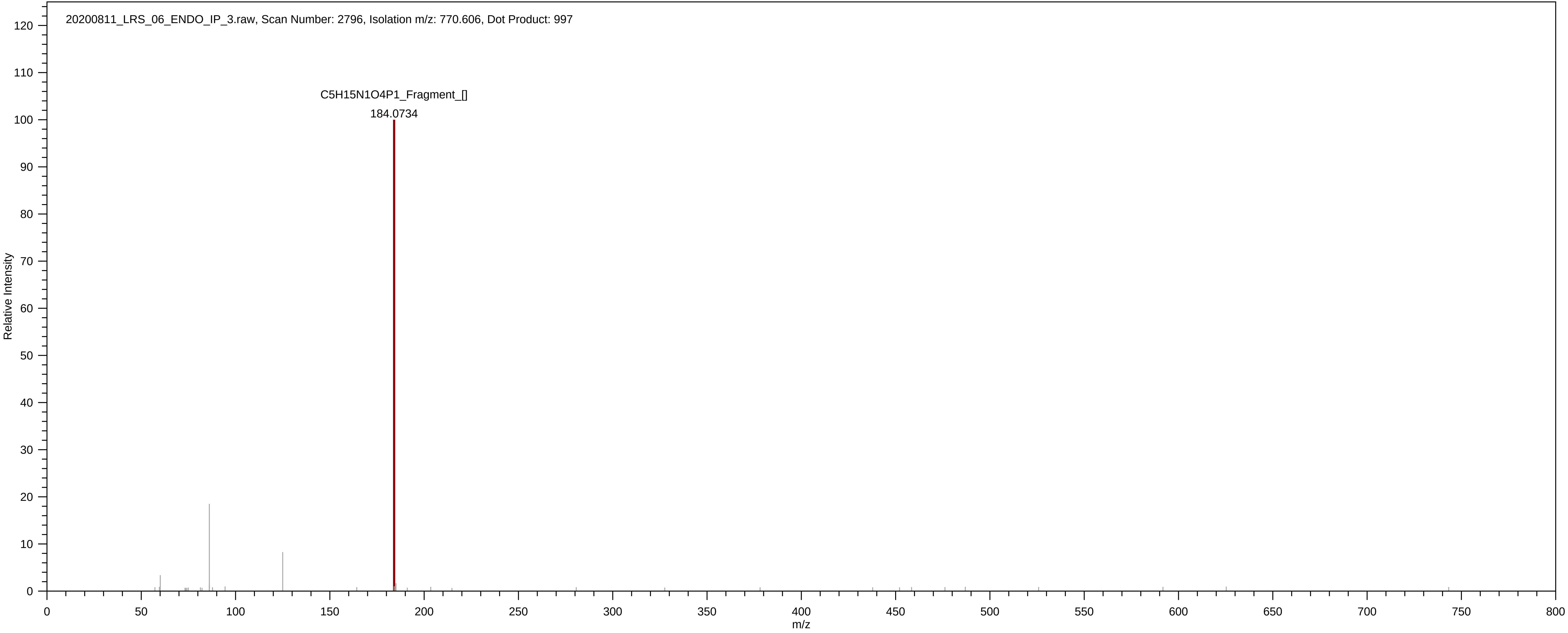

Plasmanyl-PC O-36:3 [M+H]+ | Ret. Time (min): 9.704 | m/z: 770.606

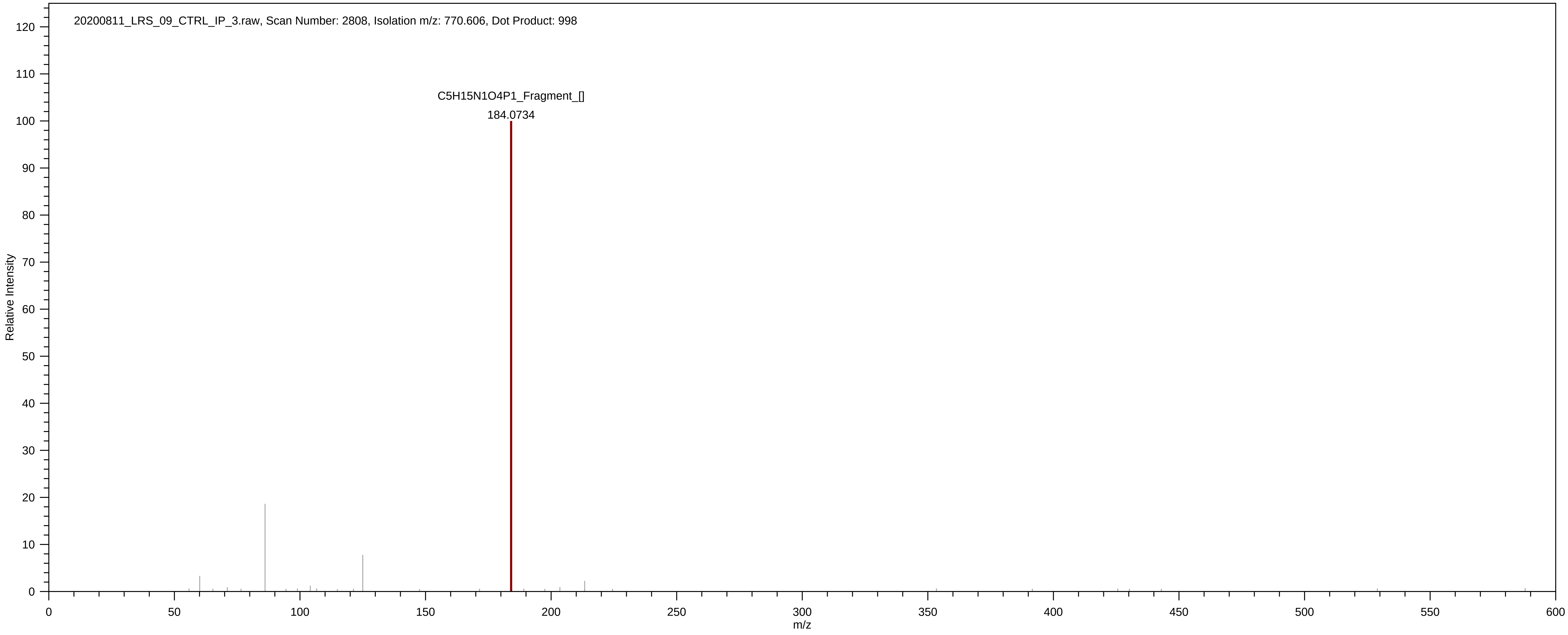

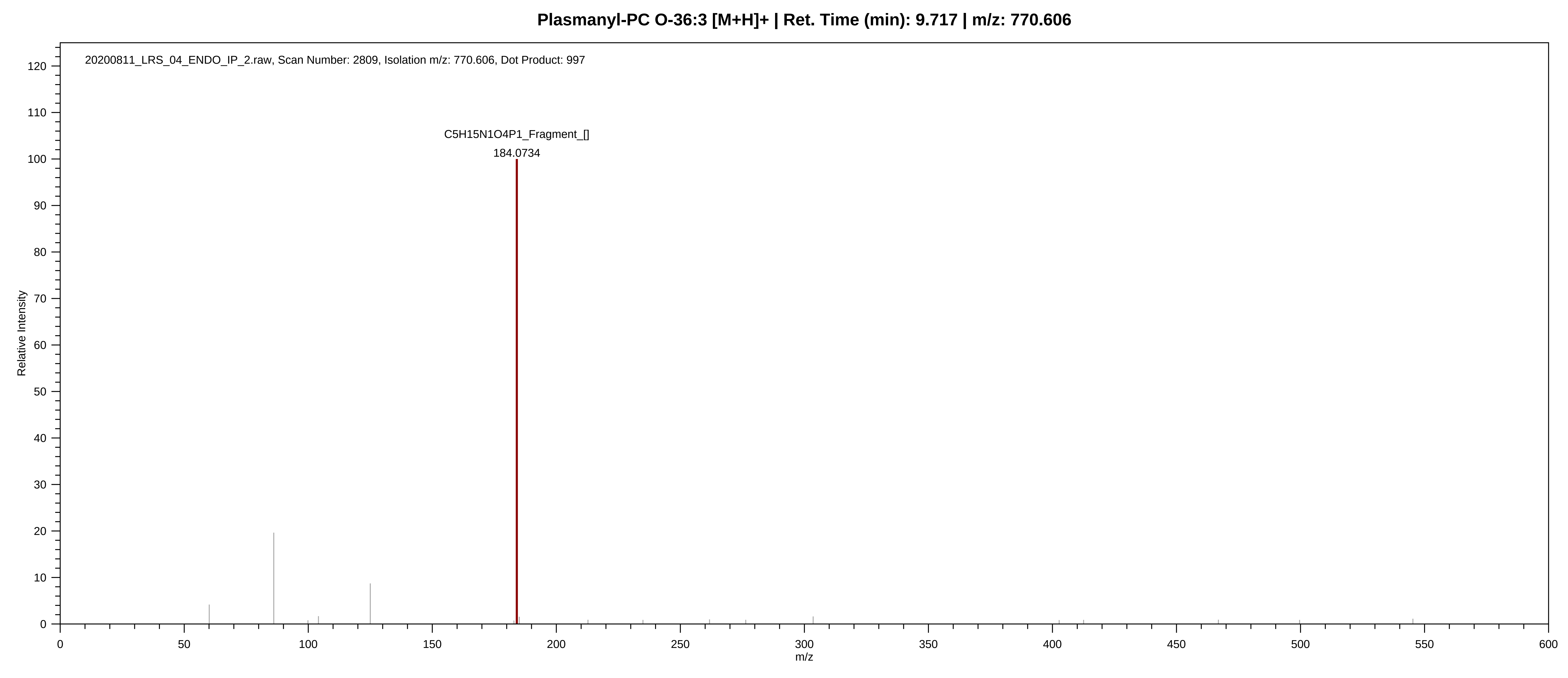

Plasmanyl-PC O-36:3 [M+H]+ | Ret. Time (min): 9.717 | m/z: 770.606
20200811_LRS_04_ENDO_IP_2.raw, Scan Number: 2809, Isolation m/z: 770.606, Dot Product: 997
C5H15N1O4P1_Fragment_[]
184.0734
Relative Intensity
m/z

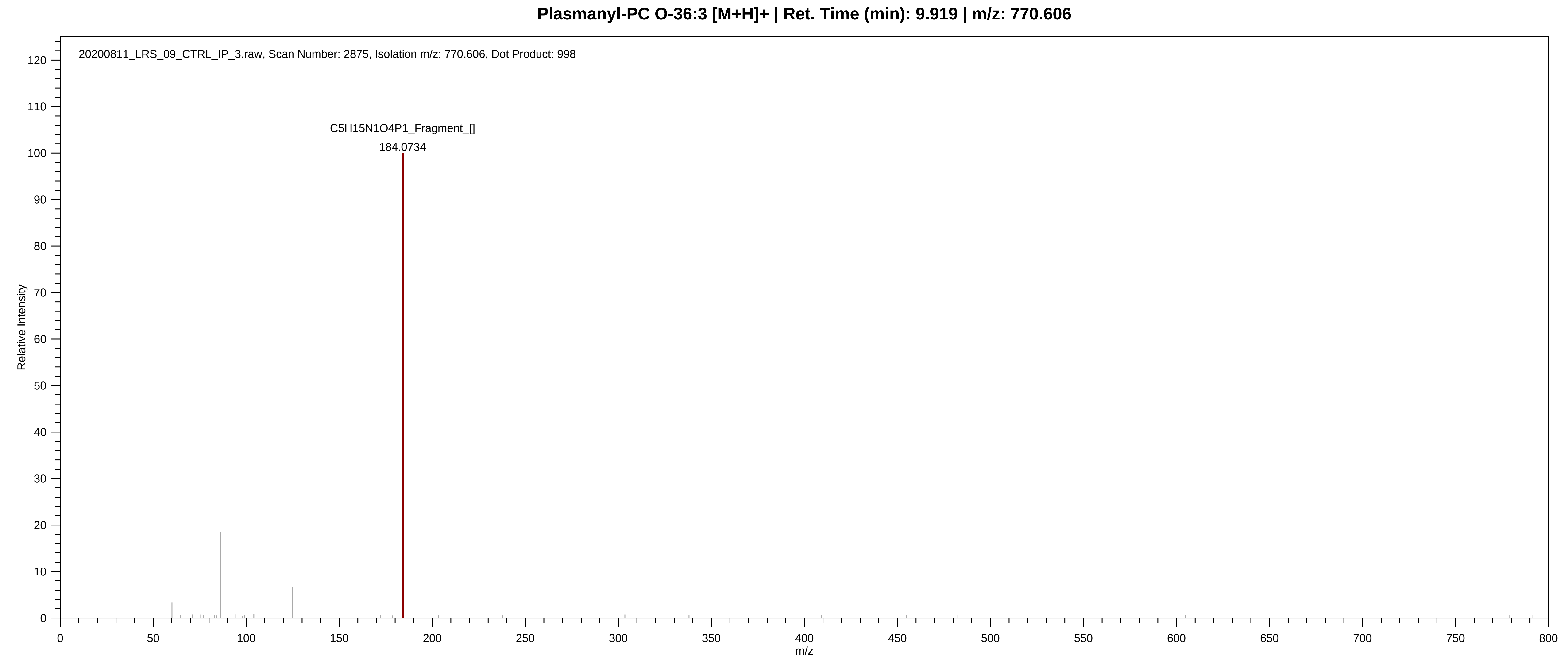

Plasmanyl-PC O-36:3 [M+H]+ | Ret. Time (min): 9.919 | m/z: 770.606
20200811_LRS_09_CTRL_IP_3.raw, Scan Number: 2875, Isolation m/z: 770.606, Dot Product: 998
C5H15N1O4P1_Fragment_[]
184.0734
Relative Intensity
m/z

Plasmanyl-PC O-36:3 [M+H]+ | Ret. Time (min): 10.317 | m/z: 770.607

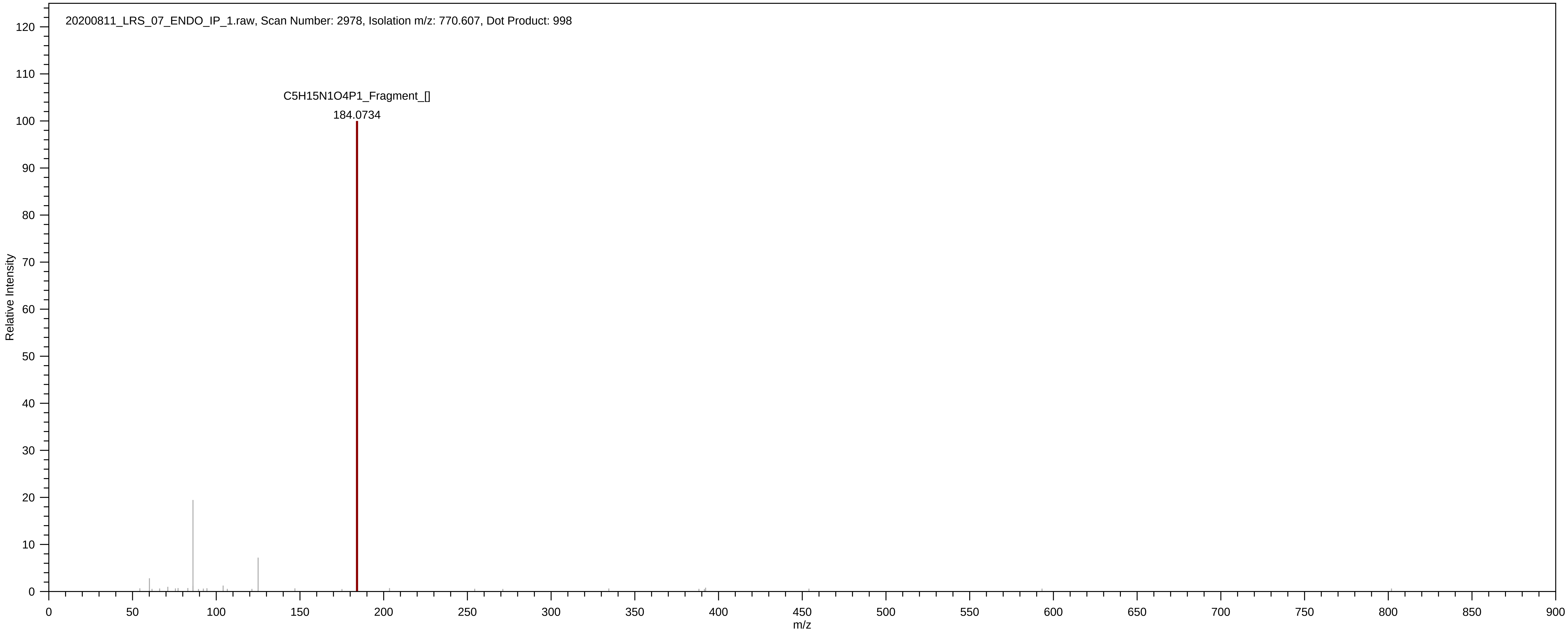

Plasmanyl-PC O-36:3 [M+H]+ | Ret. Time (min): 10.335 | m/z: 770.607

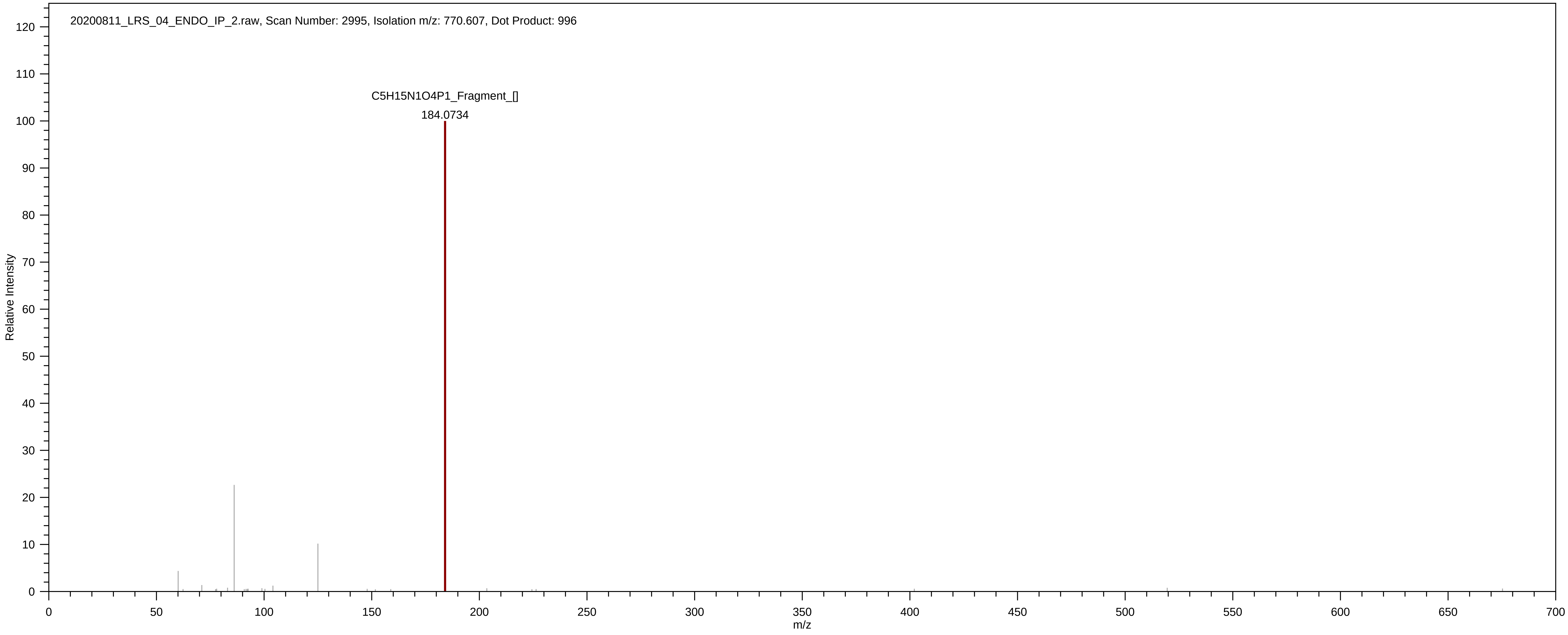

Plasmanyl-PC O-36:3 [M+H]+ | Ret. Time (min): 10.275 | m/z: 770.611

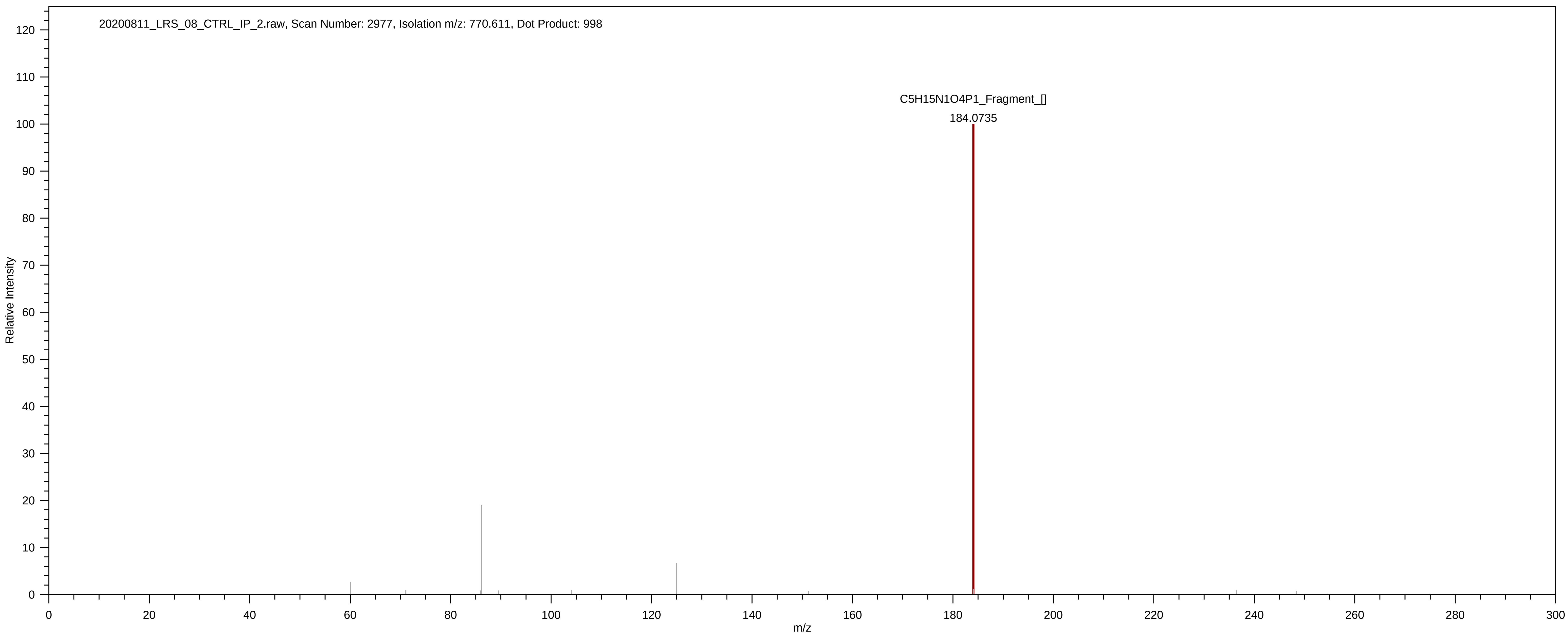

Plasmanyl-PC O-36:3 [M+H]+ | Ret. Time (min): 10.304 | m/z: 770.611

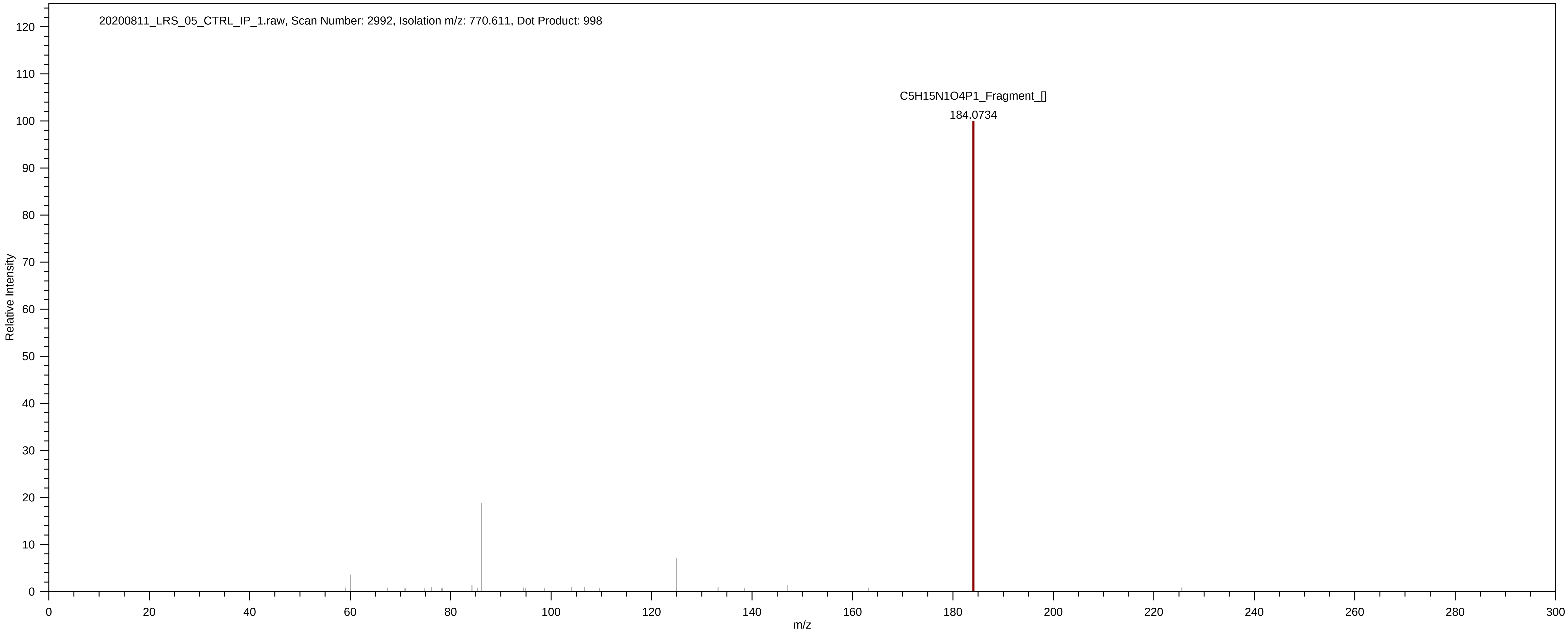

Plasmanyl-PC O-36:4 [M+H]+ | Ret. Time (min): 9.52 | m/z: 768.591

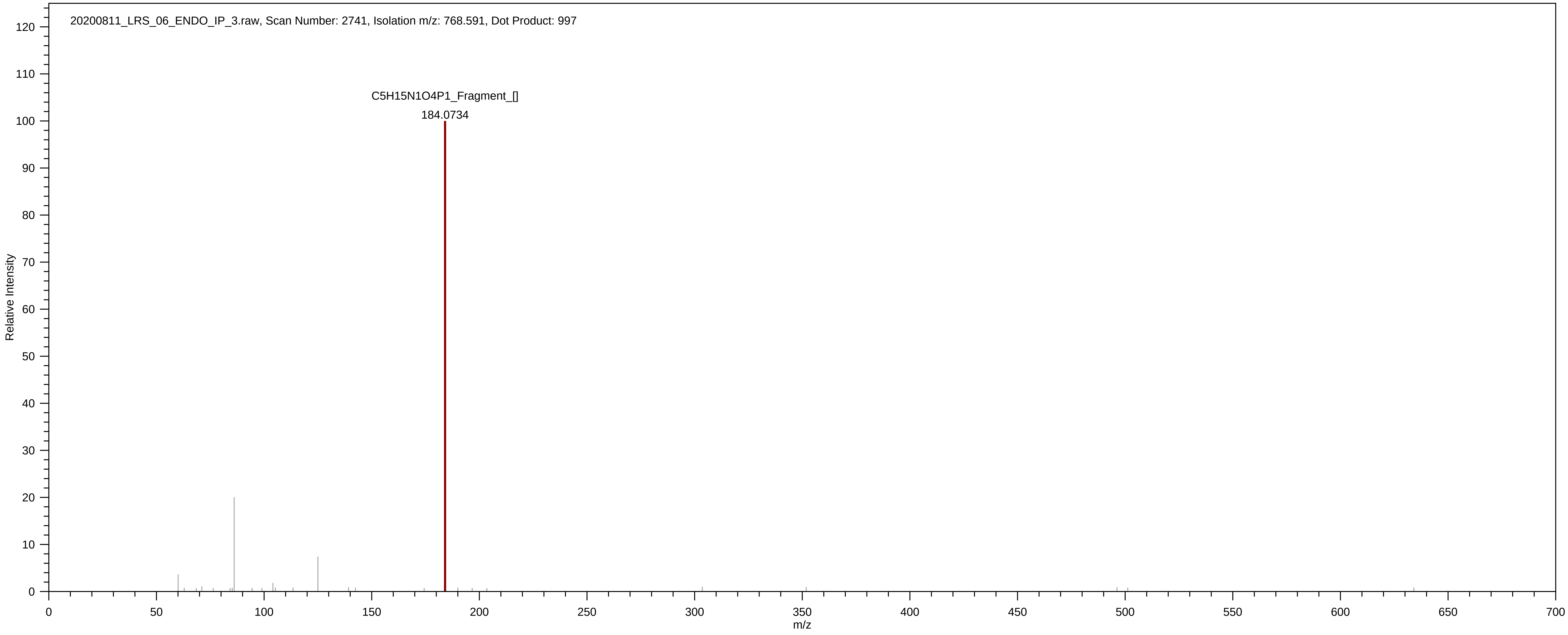

Plasmanyl-PC O-36:4 [M+H]+ | Ret. Time (min): 9.522 | m/z: 768.591

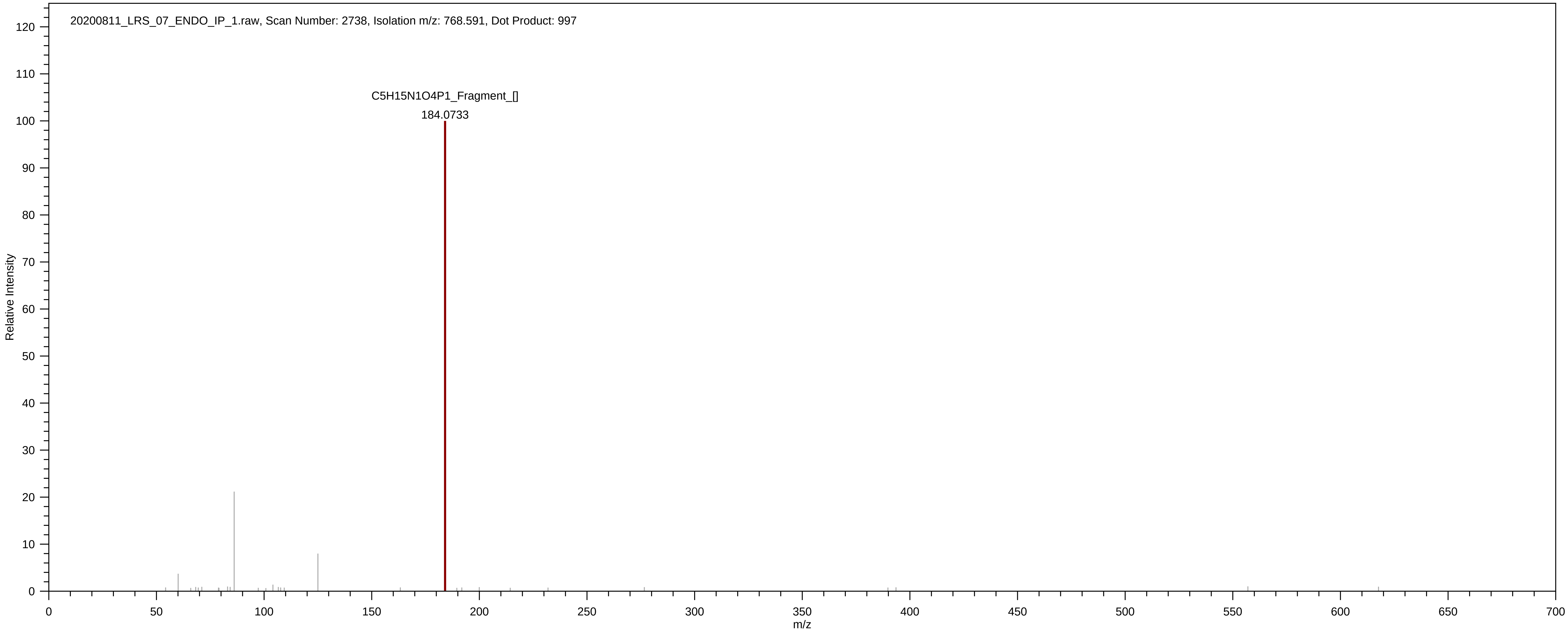

Plasmanyl-PC O-36:4 [M+H]+ | Ret. Time (min): 9.557 | m/z: 768.592

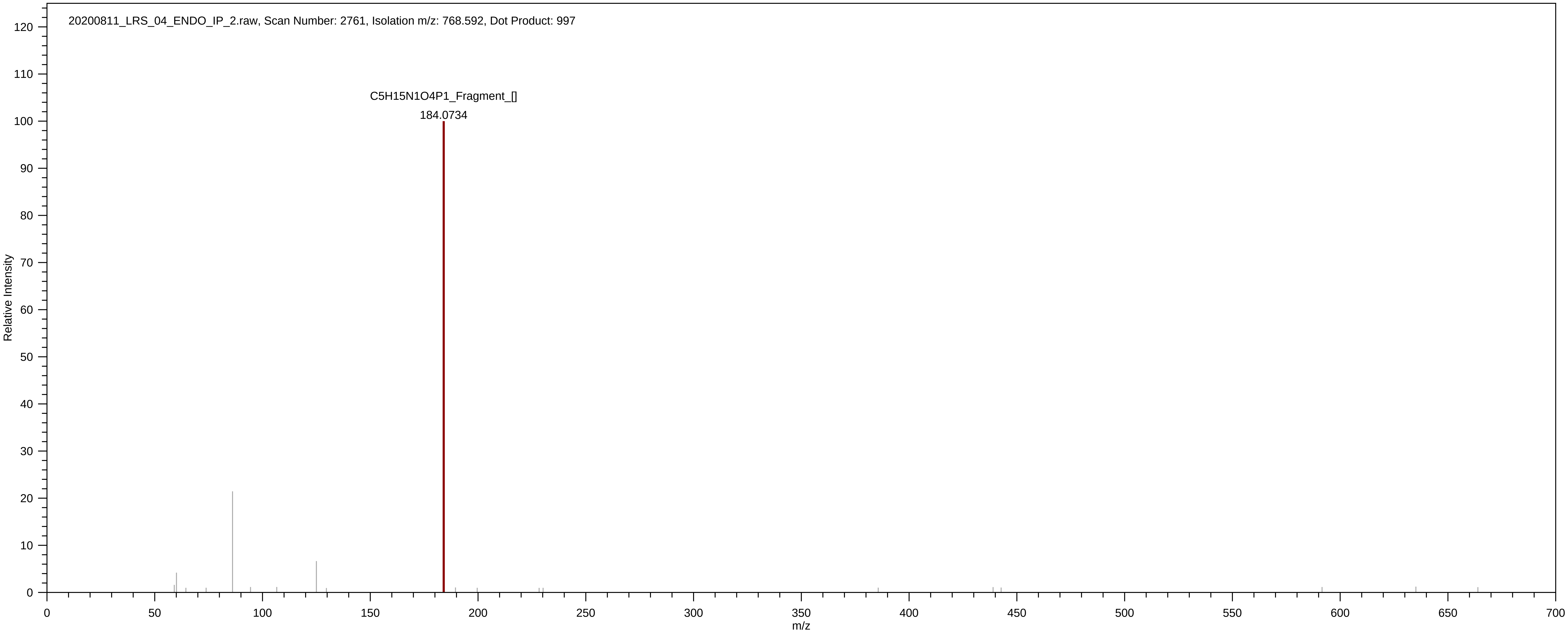

Plasmanyl-PC O-36:4 [M+H]+ | Ret. Time (min): 9.487 | m/z: 768.595

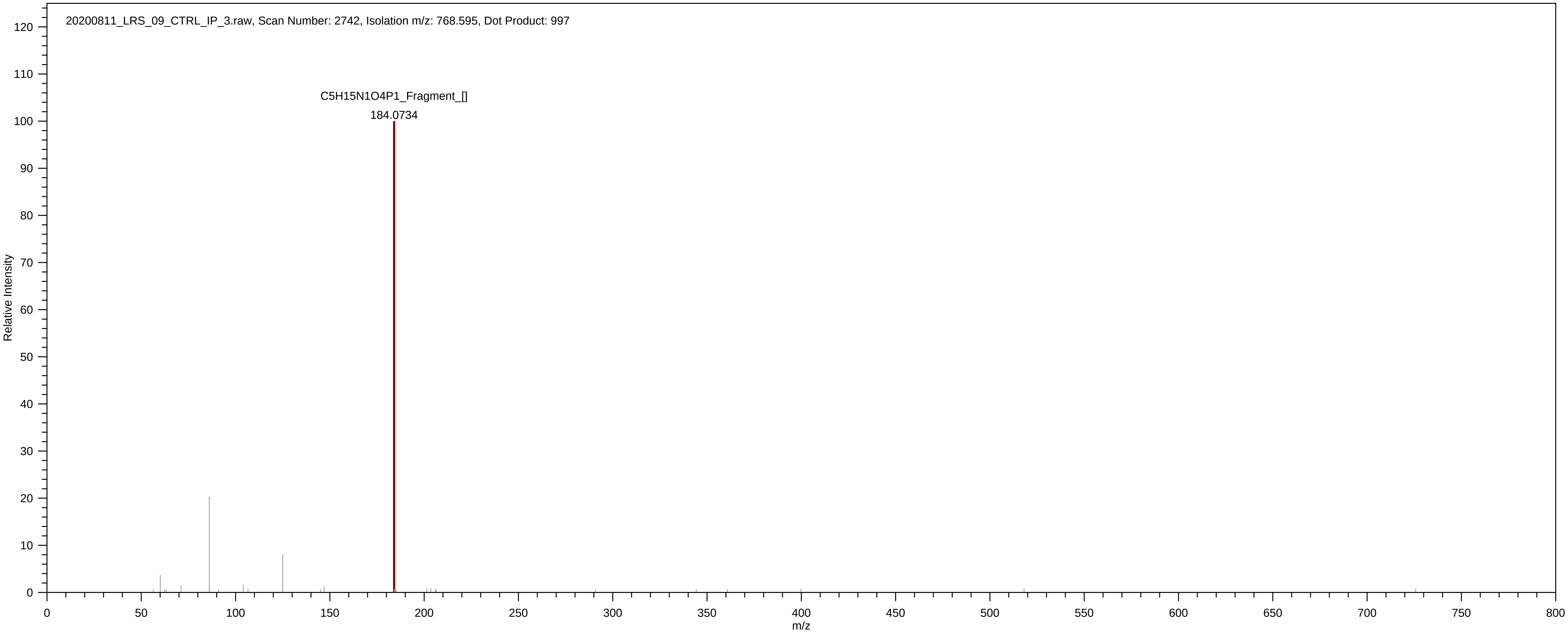

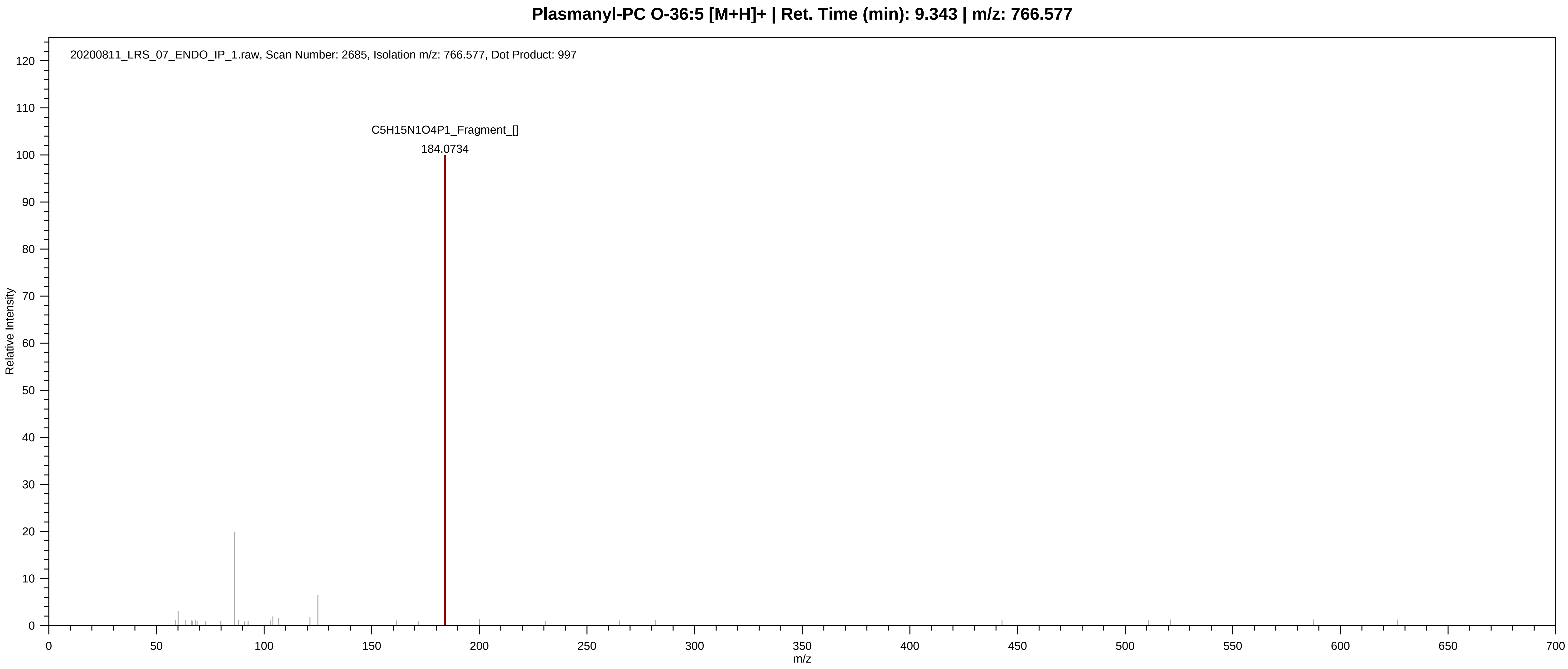

Plasmanyl-PC O-36:5 [M+H]+ | Ret. Time (min): 9.343 | m/z: 766.577
20200811_LRS_07_ENDO_IP_1.raw, Scan Number: 2685, Isolation m/z: 766.577, Dot Product: 997
C5H15N1O4P1_Fragment_[]
184.0734
Relative Intensity
m/z

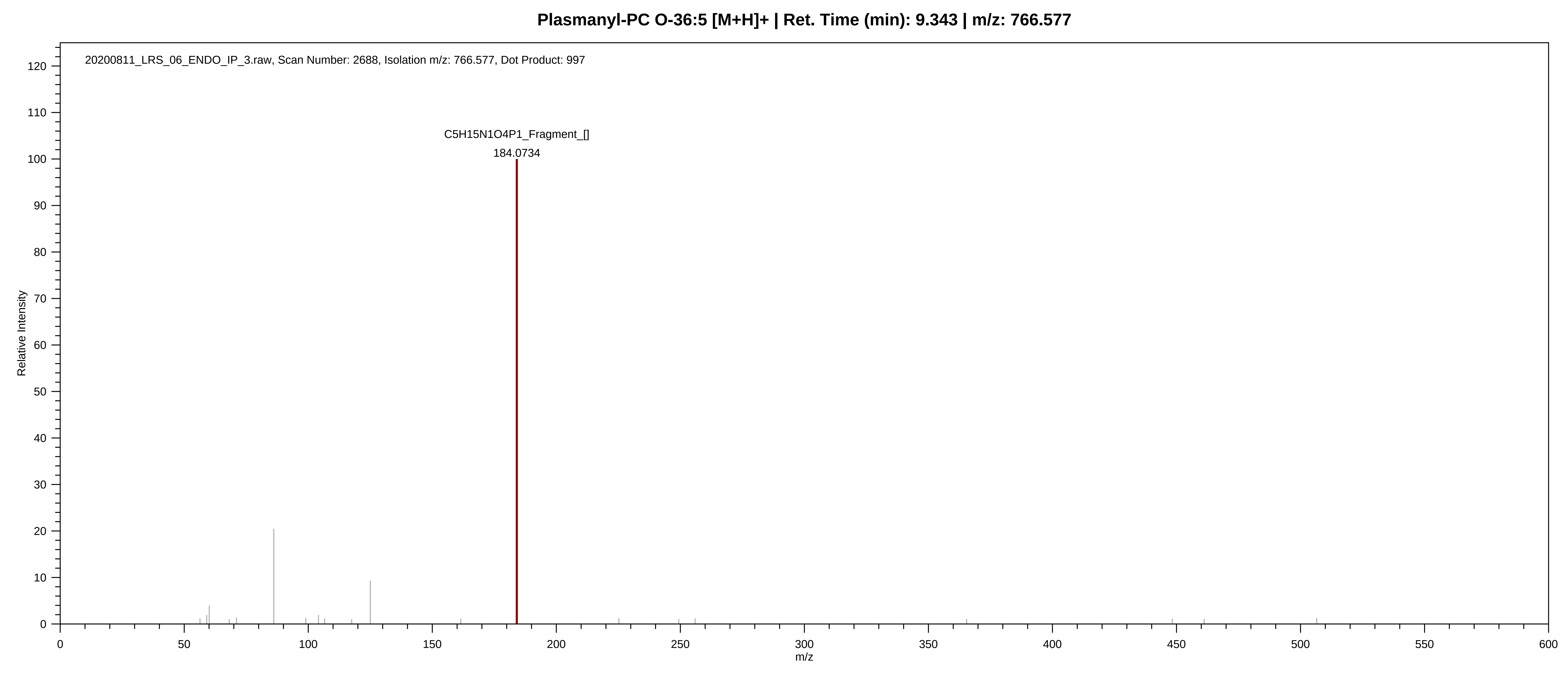
Plasmanyl-PC O-36:5 [M+H]+ | Ret. Time (min): 9.343 | m/z: 766.577
20200811_LRS_06_ENDO_IP_3.raw, Scan Number: 2688, Isolation m/z: 766.577, Dot Product: 997
C5H15N1O4P1_Fragment_[]
184.0734
Relative Intensity
m/z

Plasmanyl-PC O-36:5 [M+H]+ | Ret. Time (min): 9.378 | m/z: 766.577

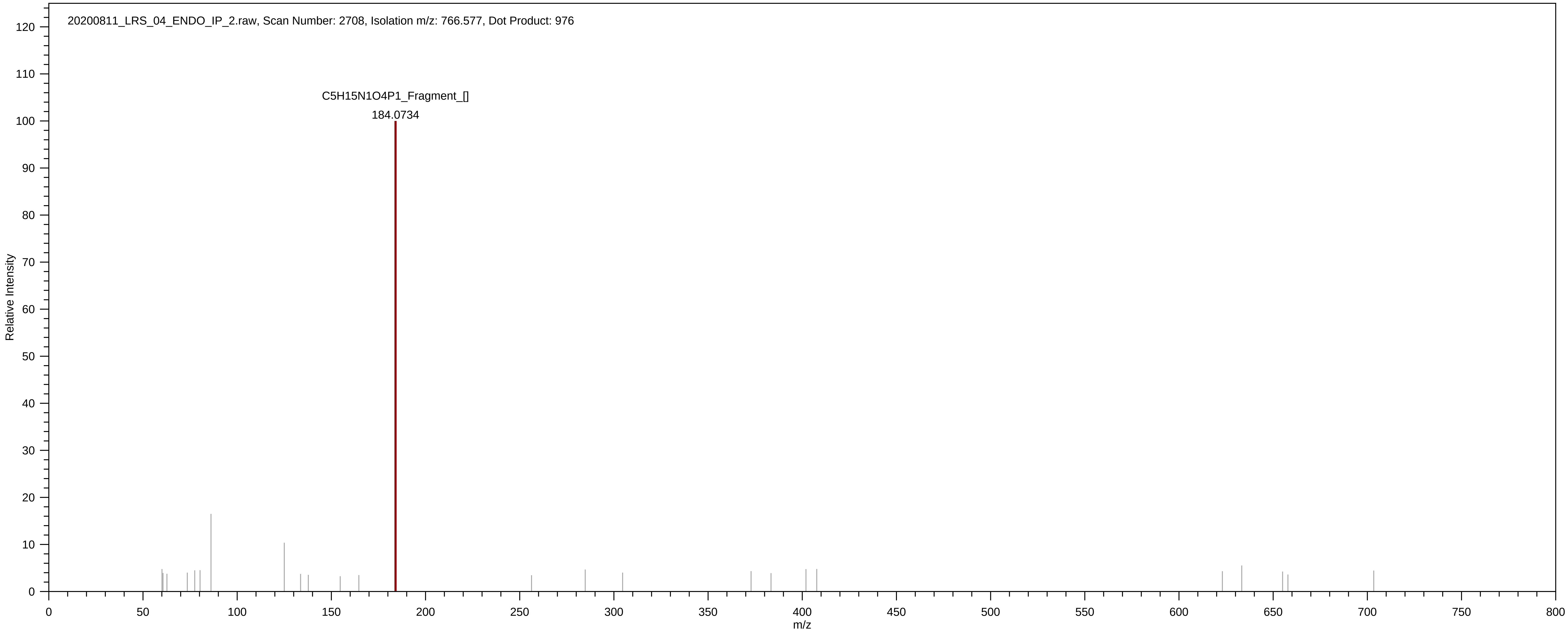

Plasmanyl-PC O-37:0 [M+H]+ | Ret. Time (min): 13.027 | m/z: 790.671

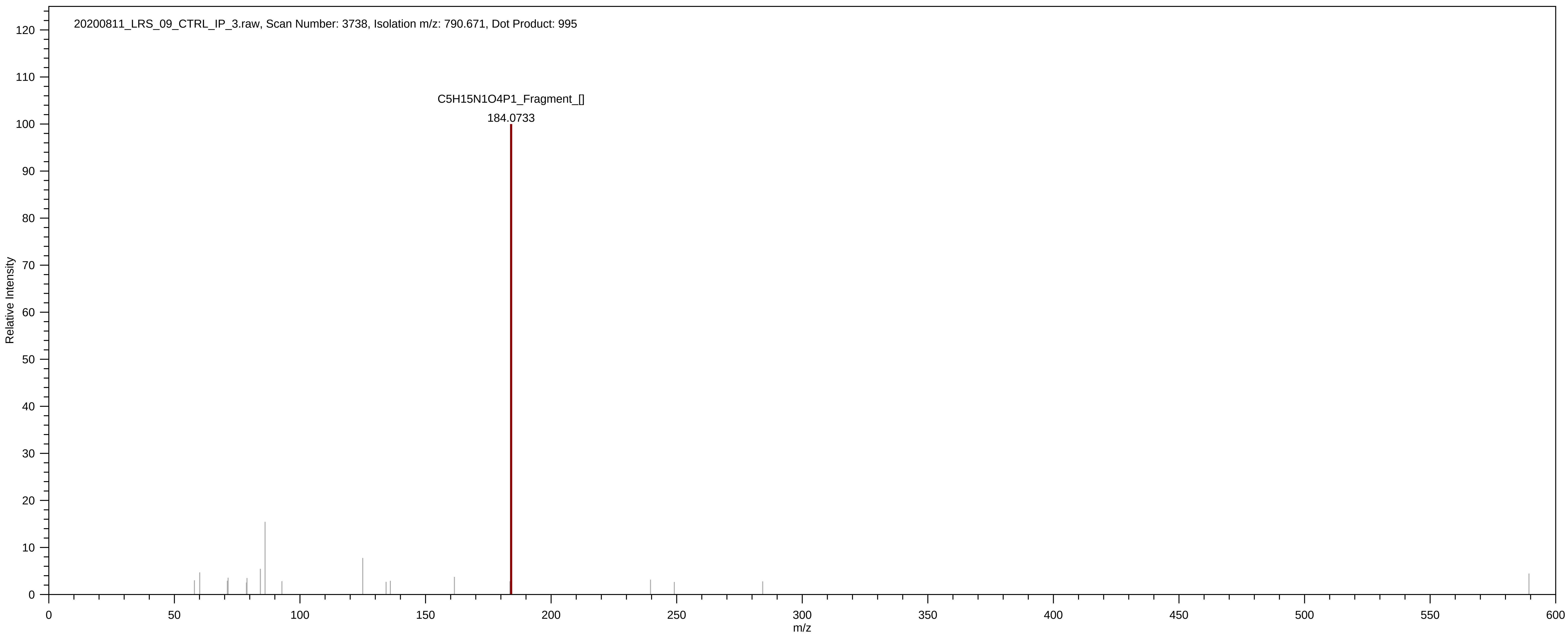

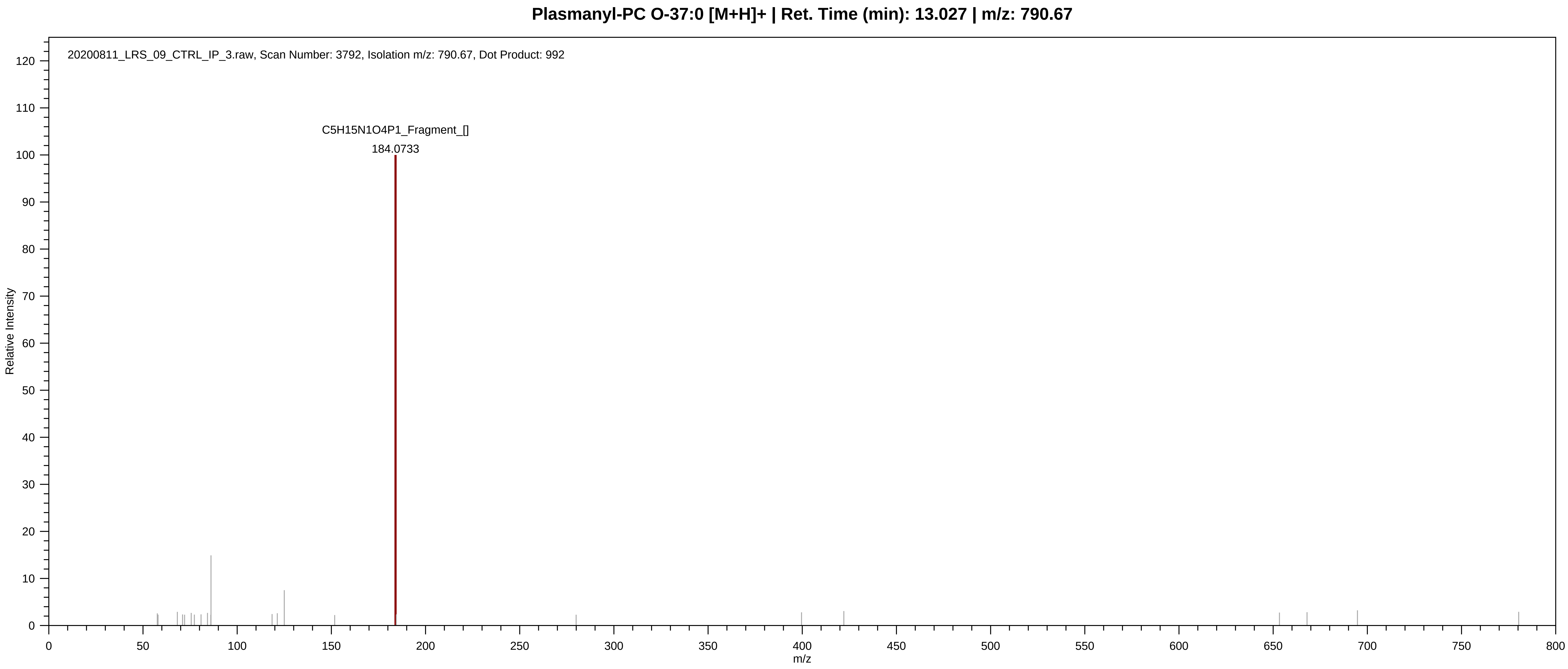

Plasmanyl-PC O-37:0 [M+H]+ | Ret. Time (min): 13.027 | m/z: 790.67
20200811_LRS_09_CTRL_IP_3.raw, Scan Number: 3792, Isolation m/z: 790.67, Dot Product: 992
C5H15N1O4P1_Fragment_[]
184.0733
Relative Intensity
m/z

Plasmanyl-PC O-37:0 [M+H]+ | Ret. Time (min): 13.045 | m/z: 790.668

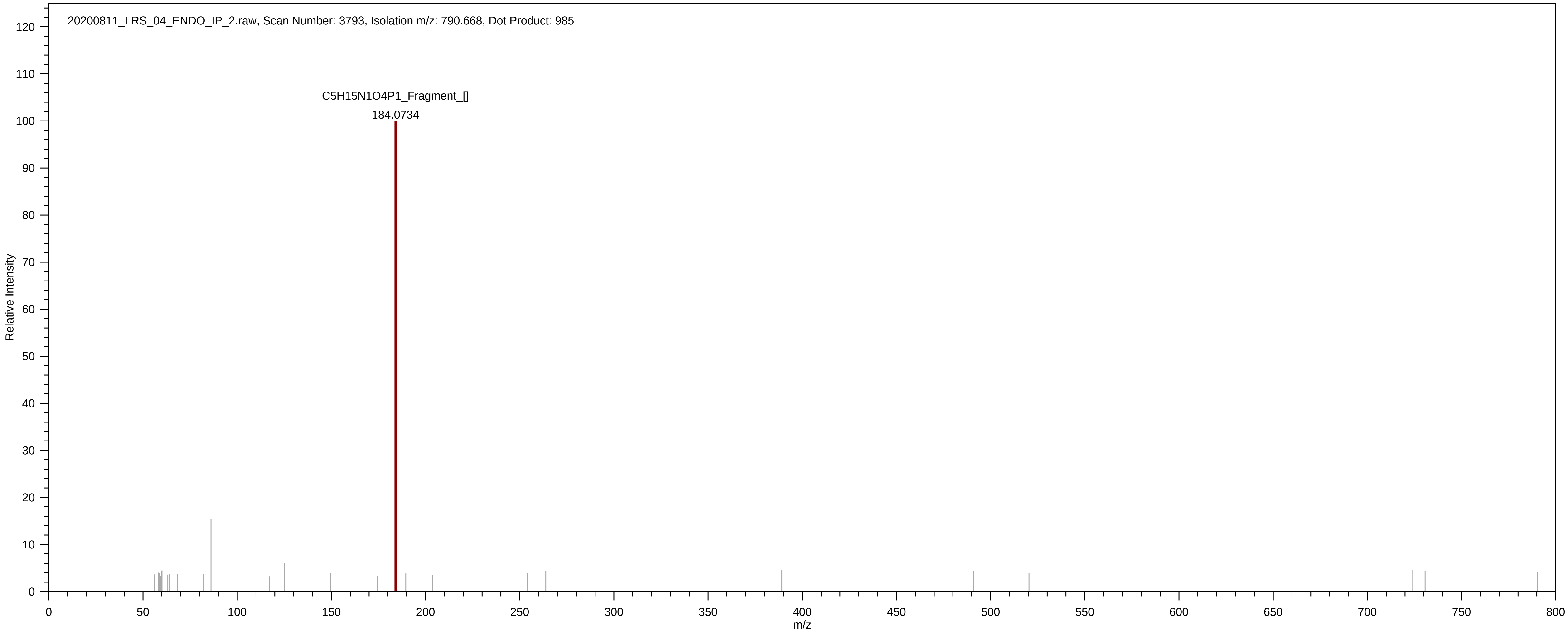

Plasmanyl-PC O-38:0 [M+H]+ | Ret. Time (min): 13.5 | m/z: 804.682

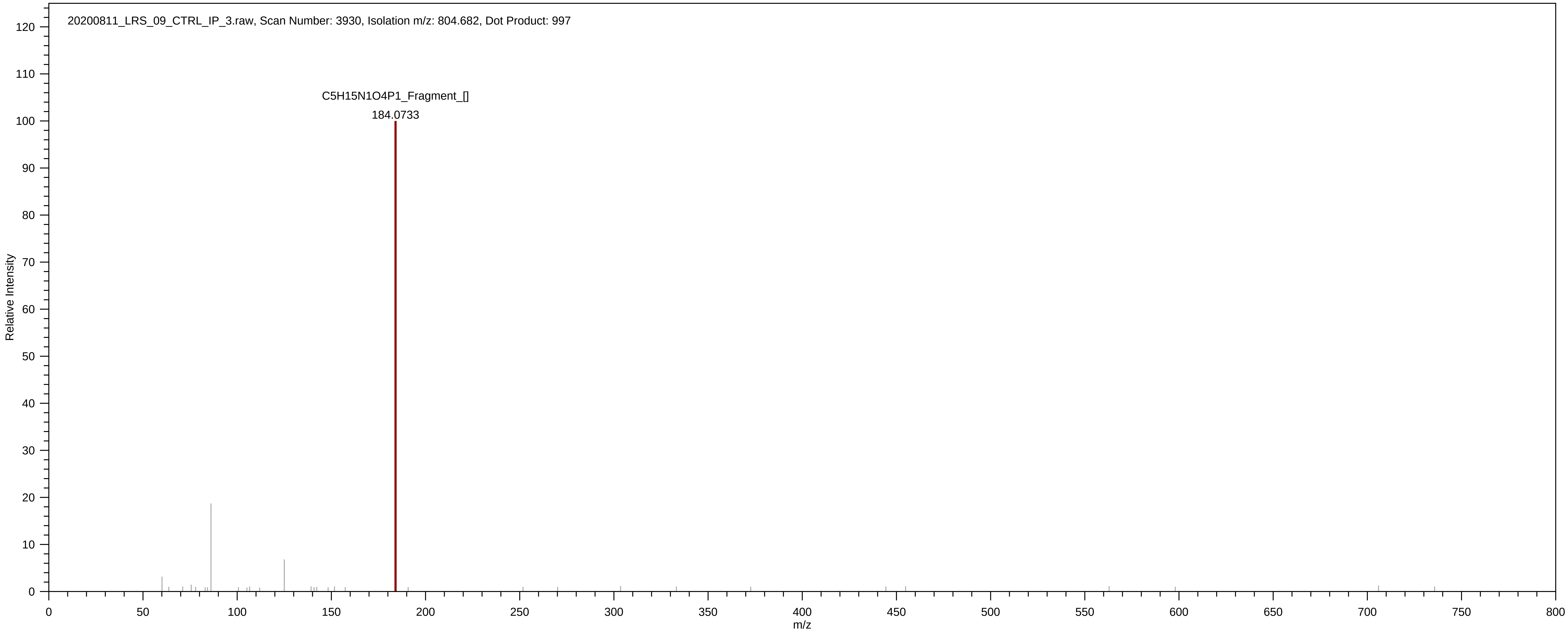

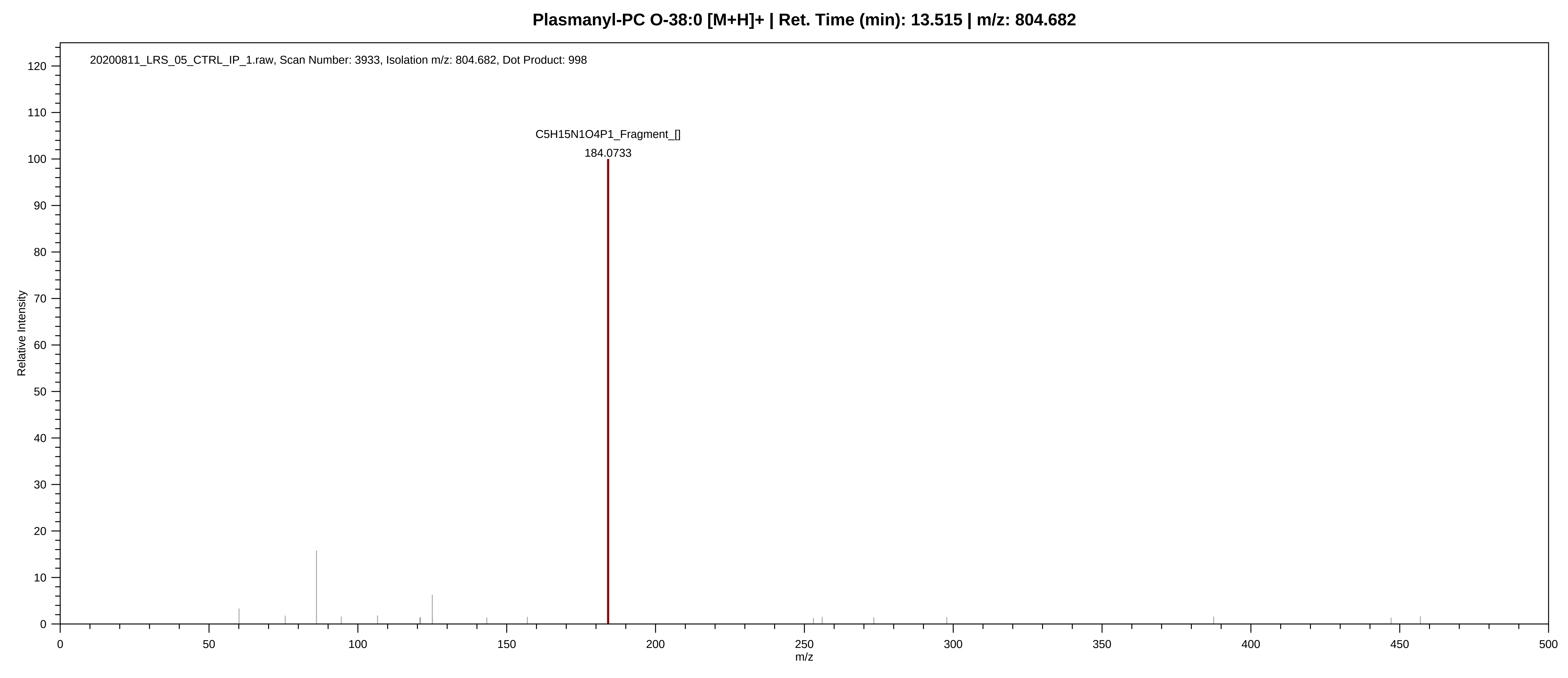
Plasmanyl-PC O-38:0 [M+H]+ | Ret. Time (min): 13.515 | m/z: 804.682
20200811_LRS_05_CTRL_IP_1.raw, Scan Number: 3933, Isolation m/z: 804.682, Dot Product: 998
C5H15N1O4P1_Fragment_[]
184.0733
Relative Intensity
m/z
0
10
20
30
40
50
60
70
80
90
100
110
120
0
50
100
150
200
250
300
350
400
450
500

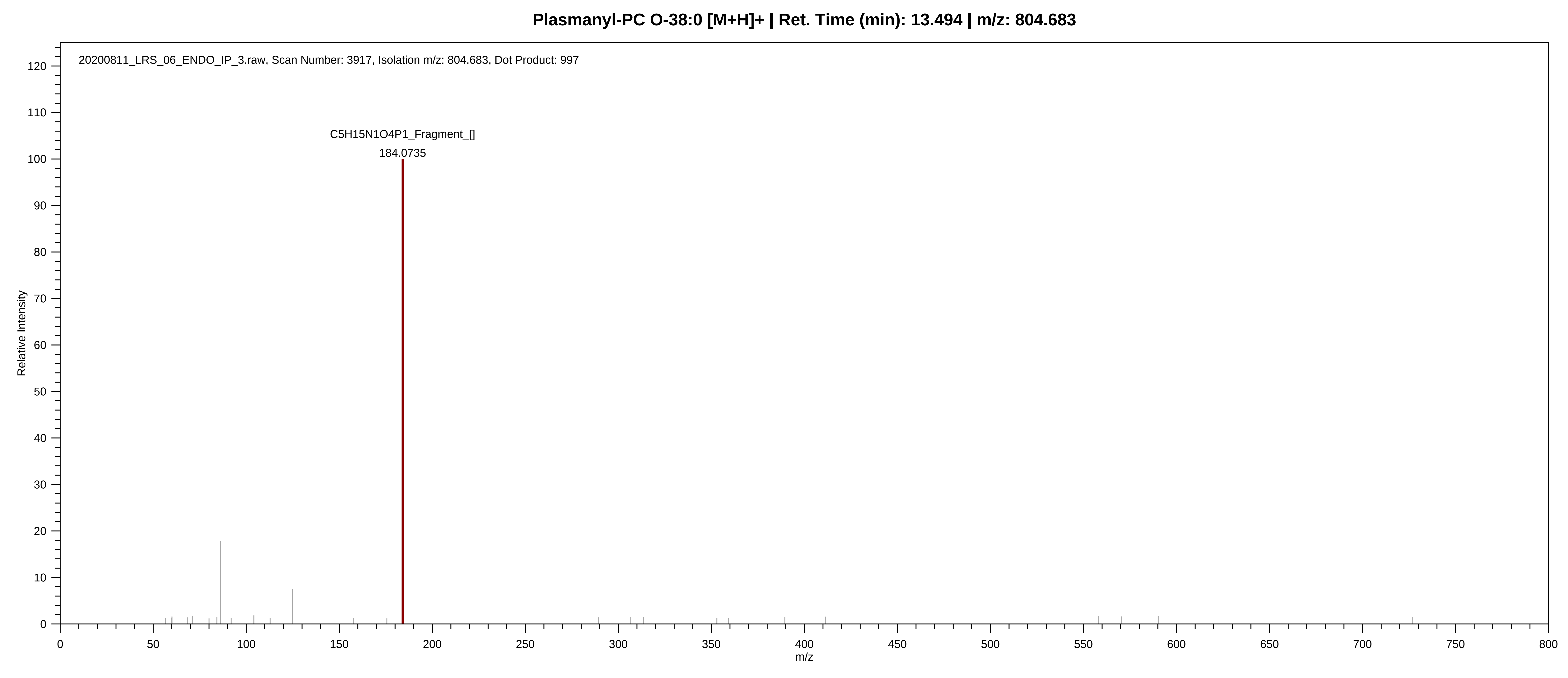

Plasmanyl-PC O-38:0 [M+H]+ | Ret. Time (min): 13.494 | m/z: 804.683
20200811_LRS_06_ENDO_IP_3.raw, Scan Number: 3917, Isolation m/z: 804.683, Dot Product: 997
C5H15N1O4P1_Fragment_[]
184.0735
Relative Intensity
m/z

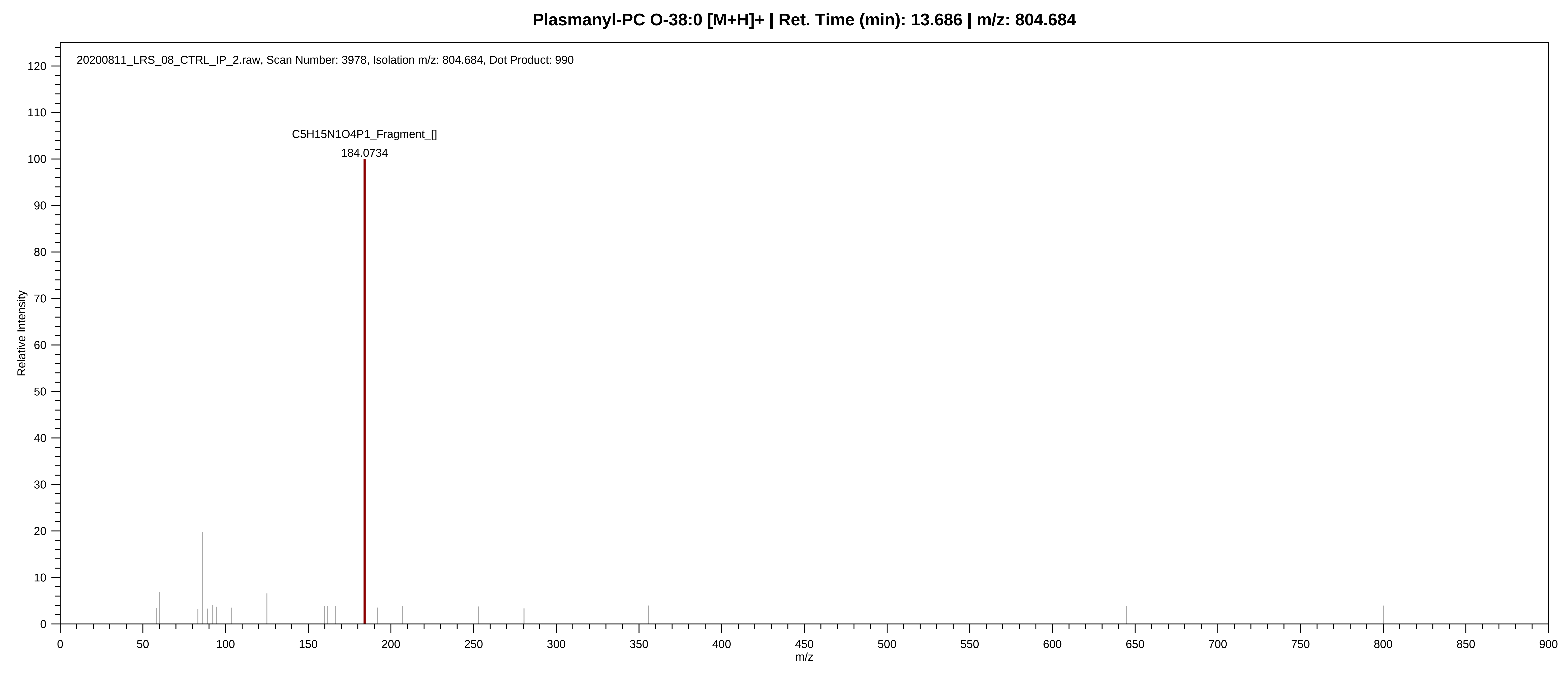

Plasmanyl-PC O-38:0 [M+H]+ | Ret. Time (min): 13.686 | m/z: 804.684
20200811_LRS_08_CTRL_IP_2.raw, Scan Number: 3978, Isolation m/z: 804.684, Dot Product: 990
C5H15N1O4P1_Fragment_[]
184.0734
Relative Intensity
m/z

Plasmanyl-PC O-38:0 [M+H]+ | Ret. Time (min): 13.7 | m/z: 804.685

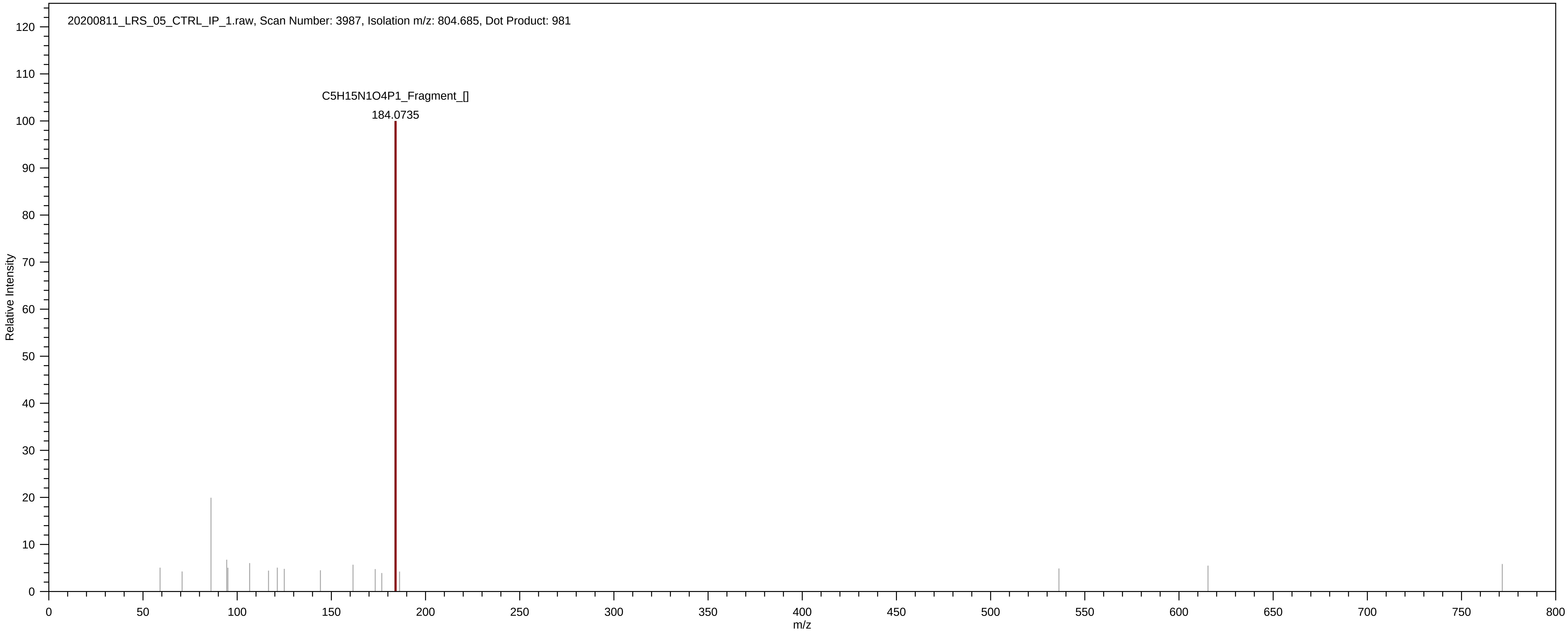

Plasmanyl-PC O-38:0 [M+H]+ | Ret. Time (min): 13.514 | m/z: 804.685

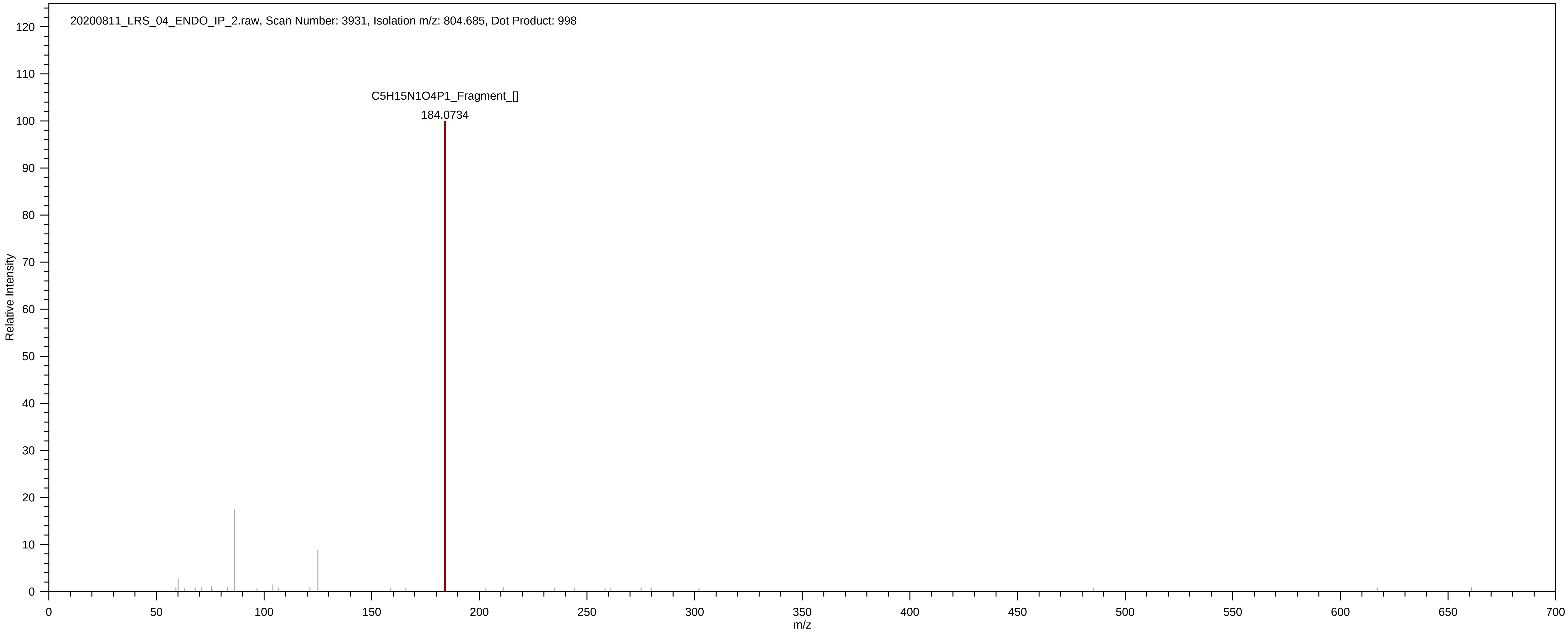

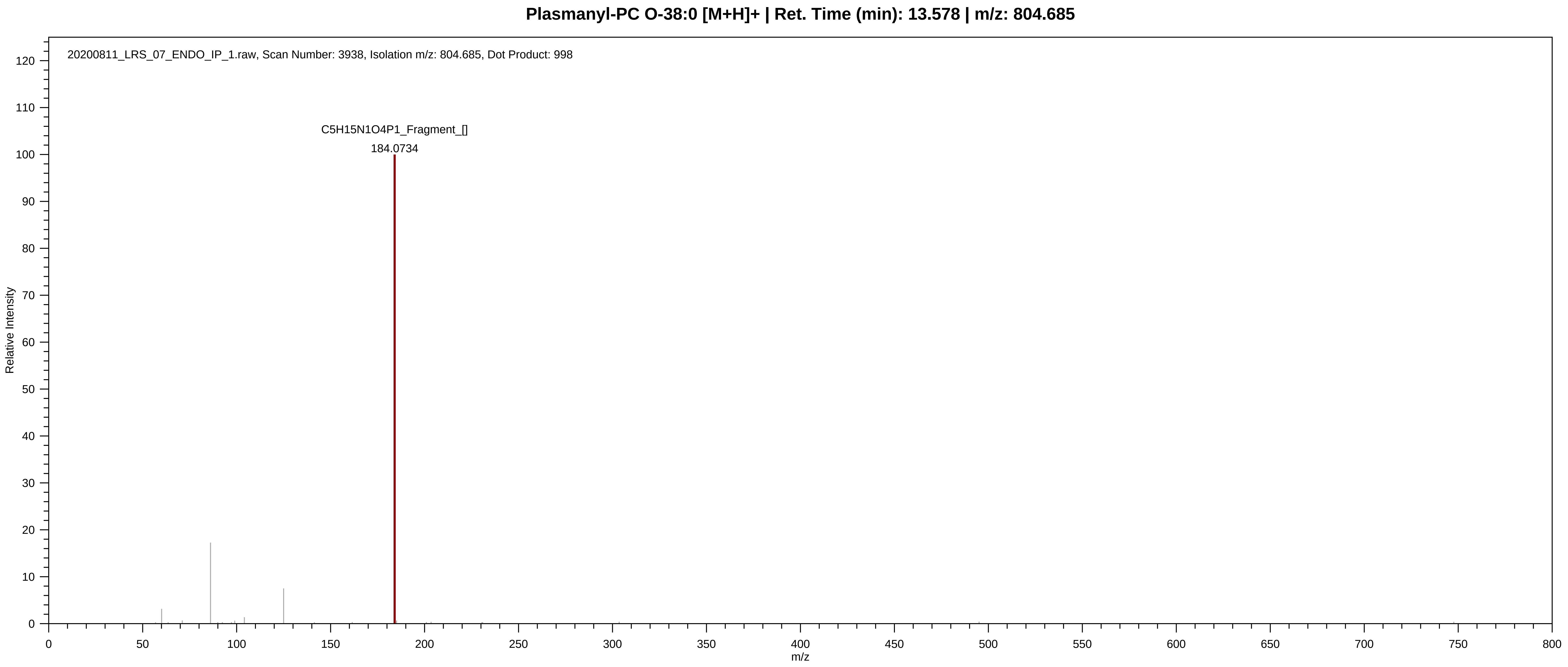

Plasmanyl-PC O-38:0 [M+H]+ | Ret. Time (min): 13.578 | m/z: 804.685
20200811_LRS_07_ENDO_IP_1.raw, Scan Number: 3938, Isolation m/z: 804.685, Dot Product: 998
C5H15N1O4P1_Fragment_[]
184.0734
Relative Intensity
m/z

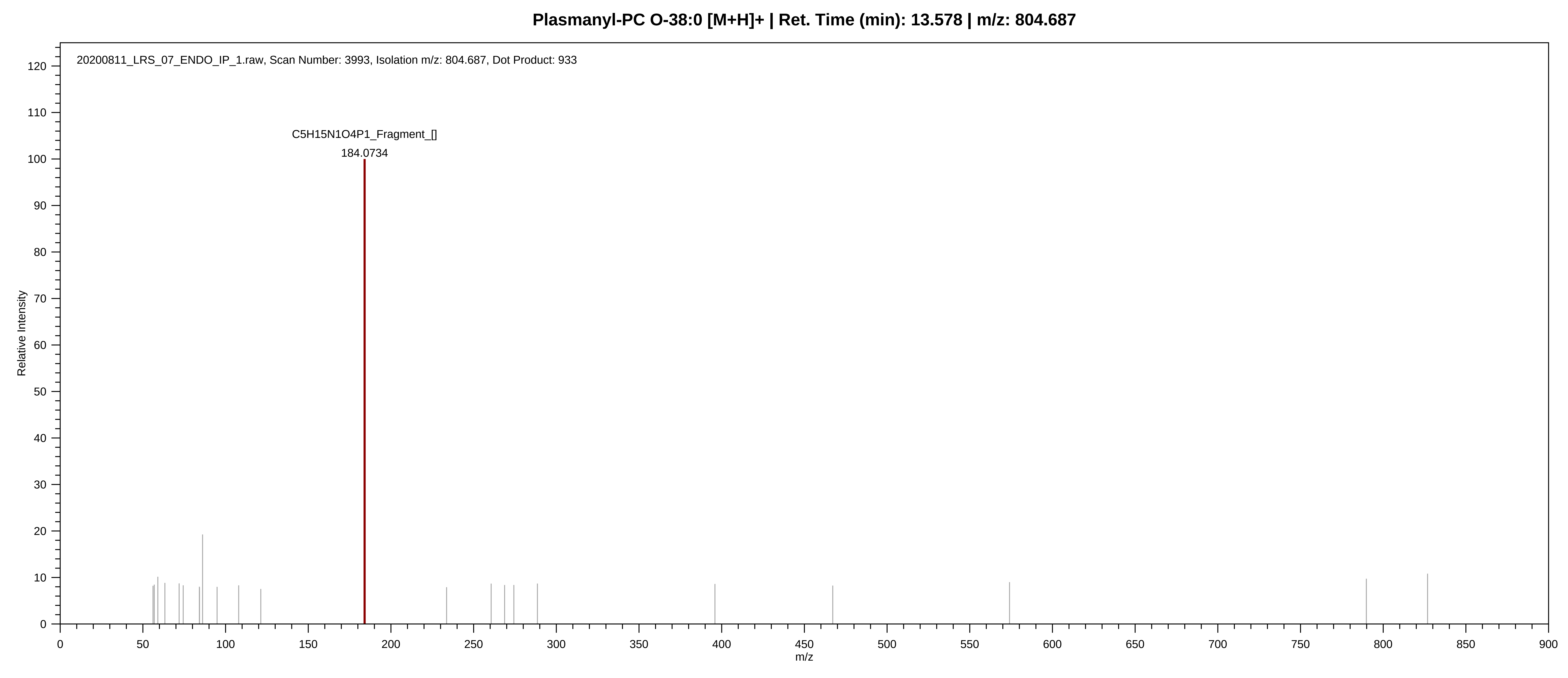

Plasmanyl-PC O-38:0 [M+H]+ | Ret. Time (min): 13.578 | m/z: 804.687
20200811_LRS_07_ENDO_IP_1.raw, Scan Number: 3993, Isolation m/z: 804.687, Dot Product: 933
C5H15N1O4P1_Fragment_[]
184.0734
Relative Intensity
m/z
0
10
20
30
40
50
60
70
80
90
100
110
120
0
50
100
150
200
250
300
350
400
450
500
550
600
650
700
750
800
850
900

Plasmanyl-PC O-38:0 [M+H]+ | Ret. Time (min): 13.681 | m/z: 804.685

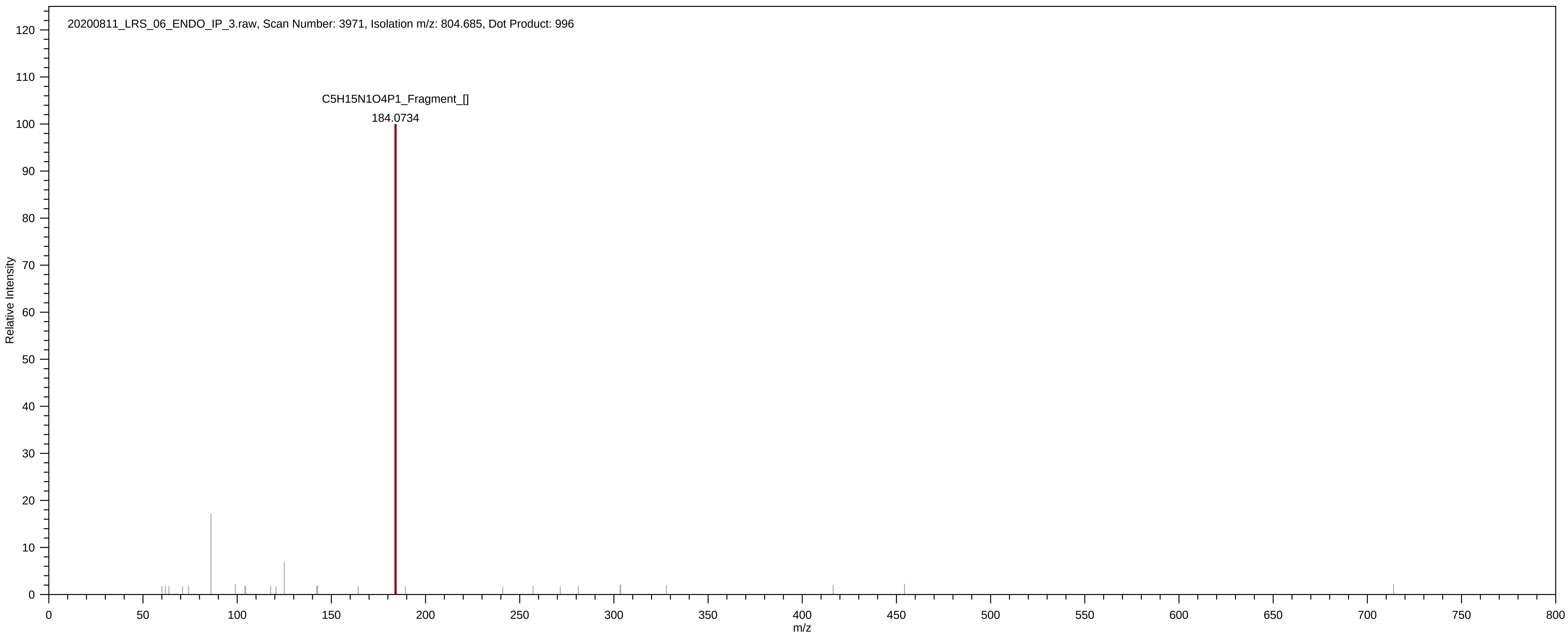

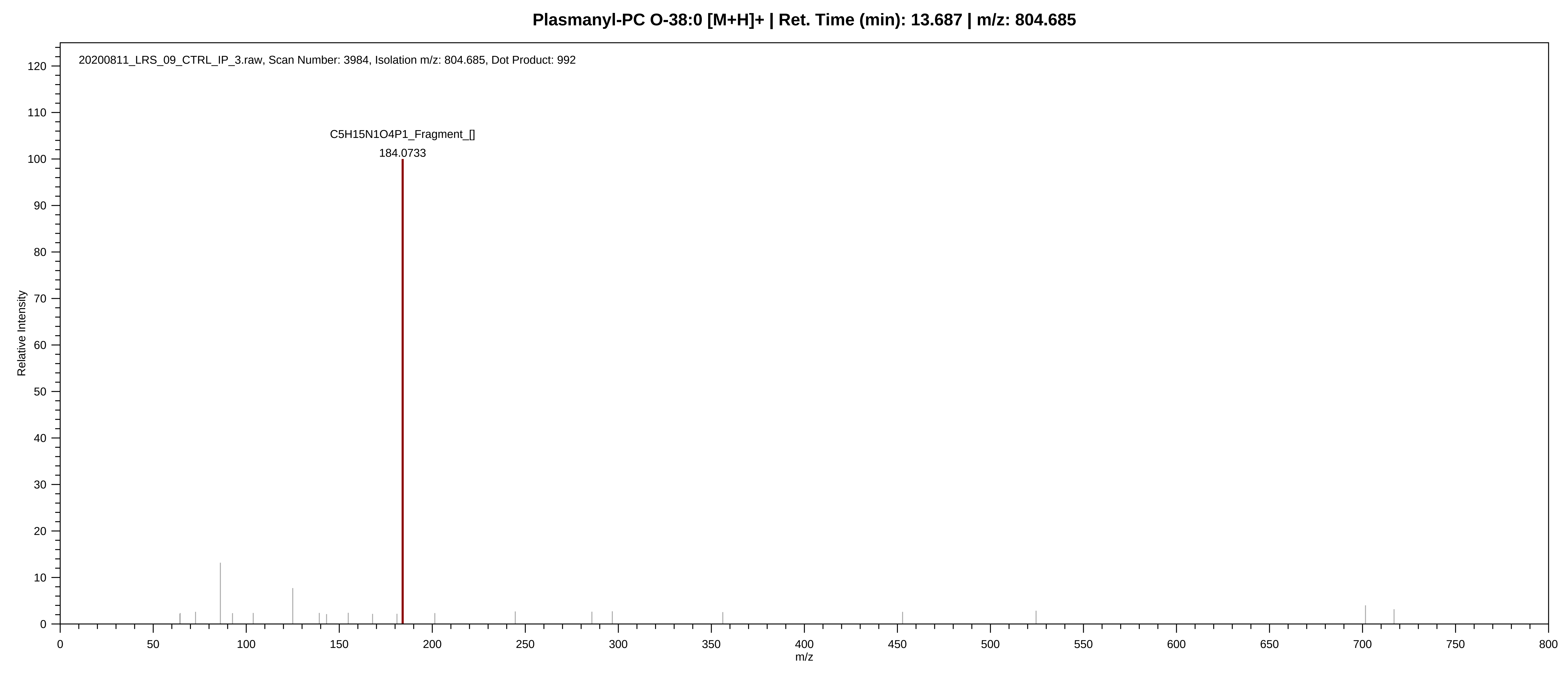
Plasmanyl-PC O-38:0 [M+H]+ | Ret. Time (min): 13.687 | m/z: 804.685
20200811_LRS_09_CTRL_IP_3.raw, Scan Number: 3984, Isolation m/z: 804.685, Dot Product: 992
C5H15N1O4P1_Fragment_[]
184.0733
Relative Intensity
m/z

Plasmanyl-PC O-38:0 [M+H]+ | Ret. Time (min): 13.699 | m/z: 804.685

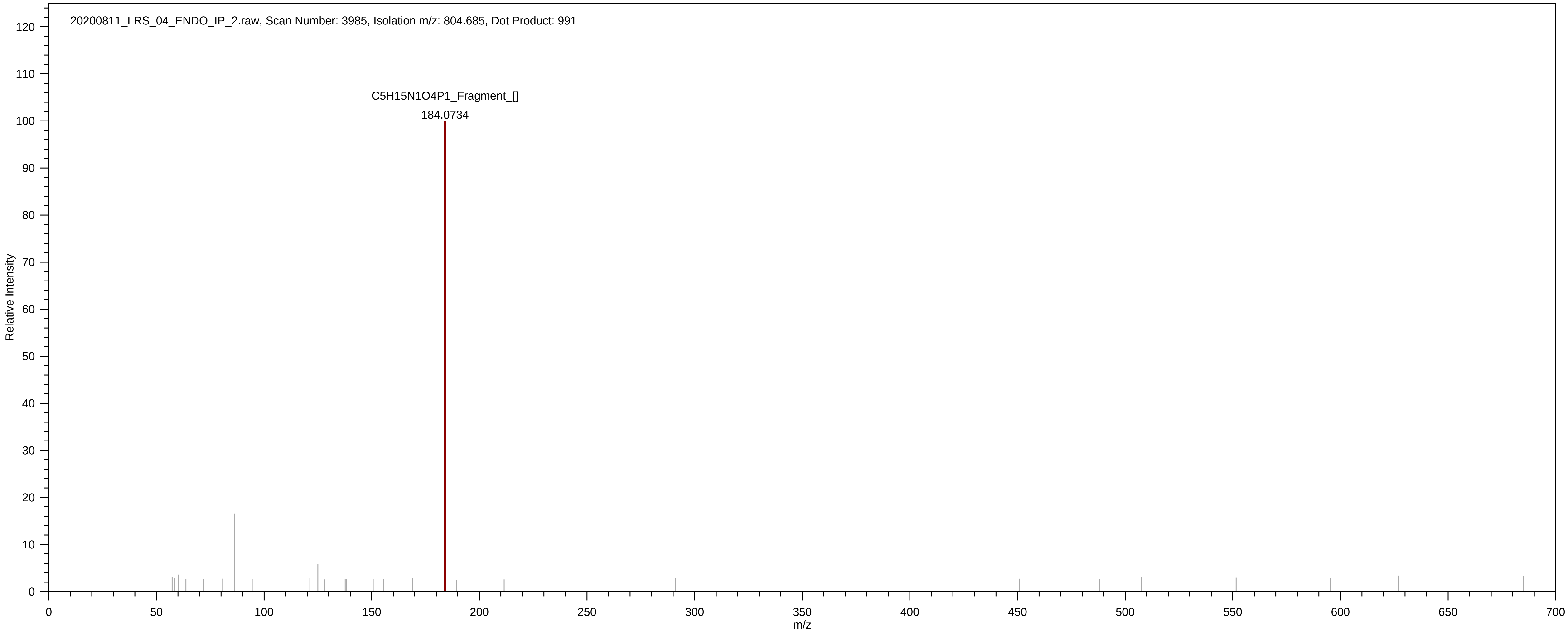

Plasmanyl-PC O-38:0 [M+H]+ | Ret. Time (min): 13.501 | m/z: 804.686

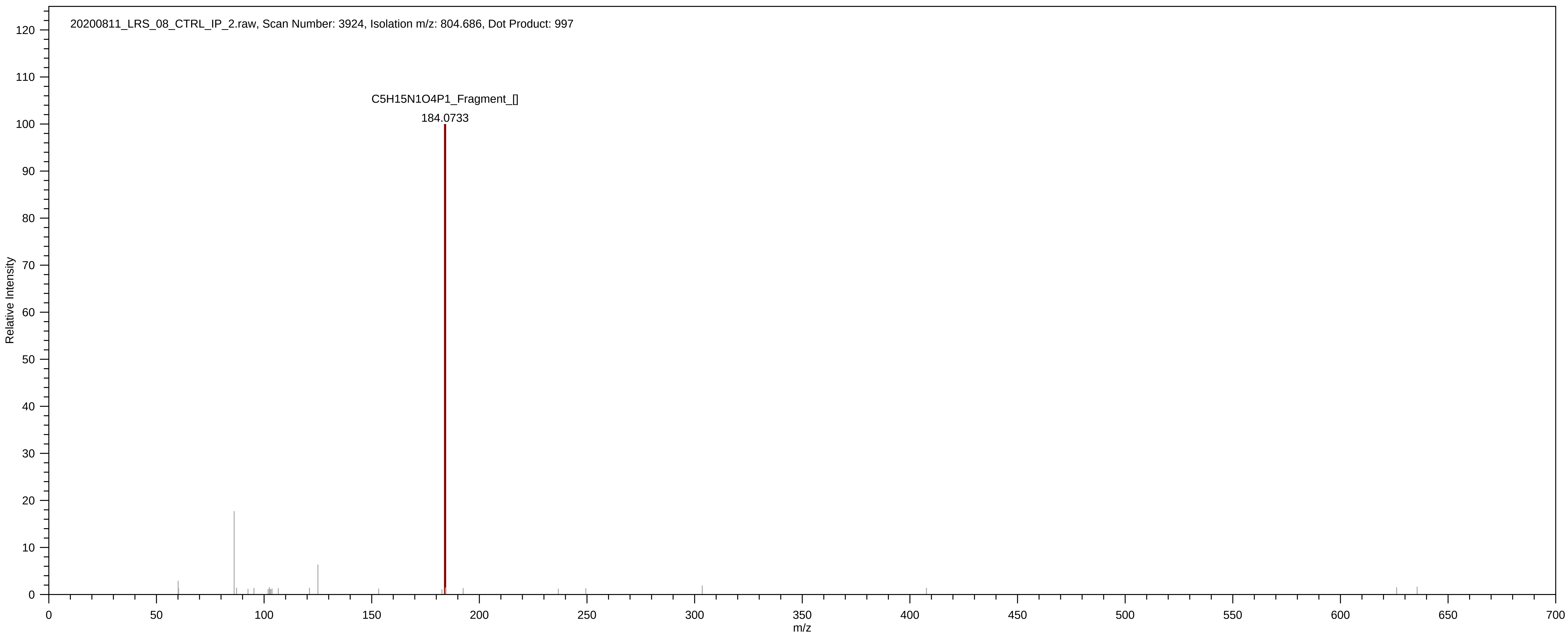

Plasmanyl-PC O-38:1 [M+Ac-H]- | Ret. Time (min): 12.522 | m/z: 860.679

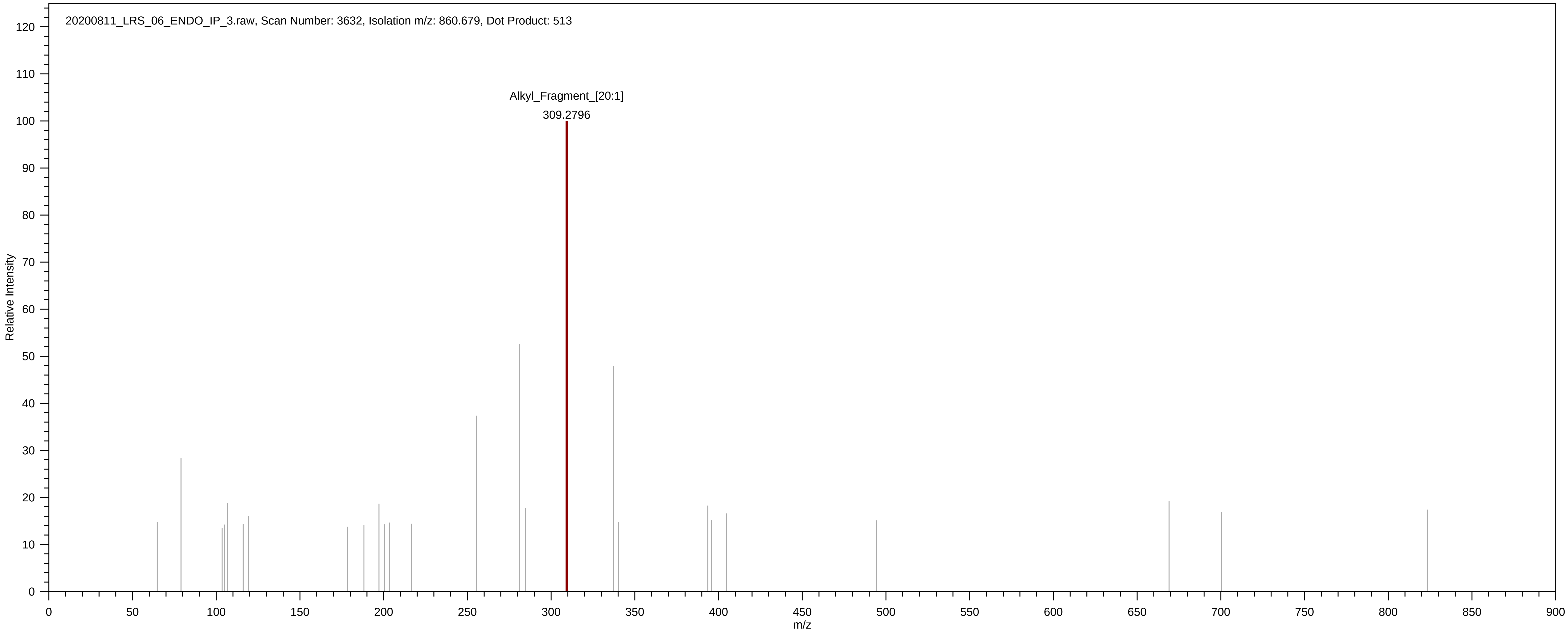

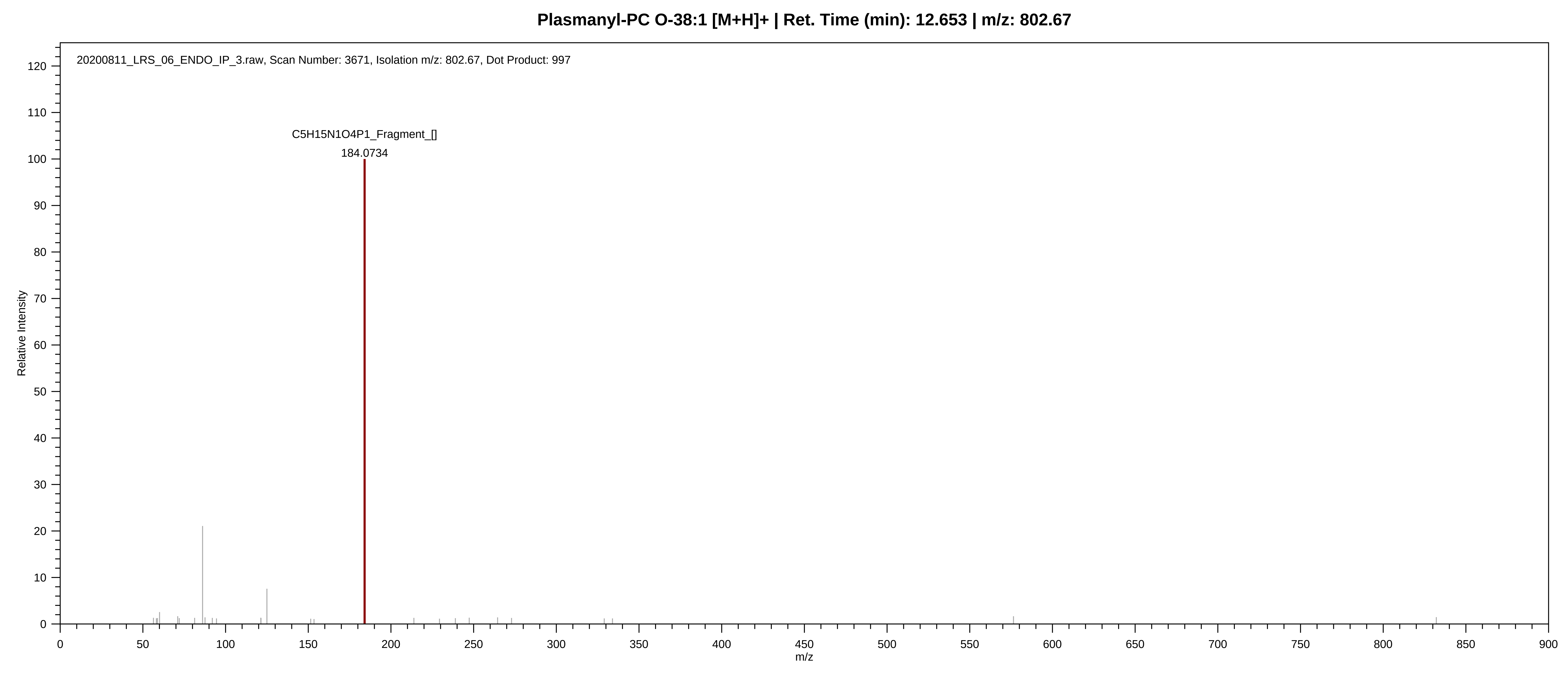

Plasmanyl-PC O-38:1 [M+H]+ | Ret. Time (min): 12.653 | m/z: 802.67
20200811_LRS_06_ENDO_IP_3.raw, Scan Number: 3671, Isolation m/z: 802.67, Dot Product: 997
C5H15N1O4P1_Fragment_[]
184.0734
Relative Intensity
m/z

Plasmanyl-PC O-38:1 [M+H]+ | Ret. Time (min): 12.676 | m/z: 802.668

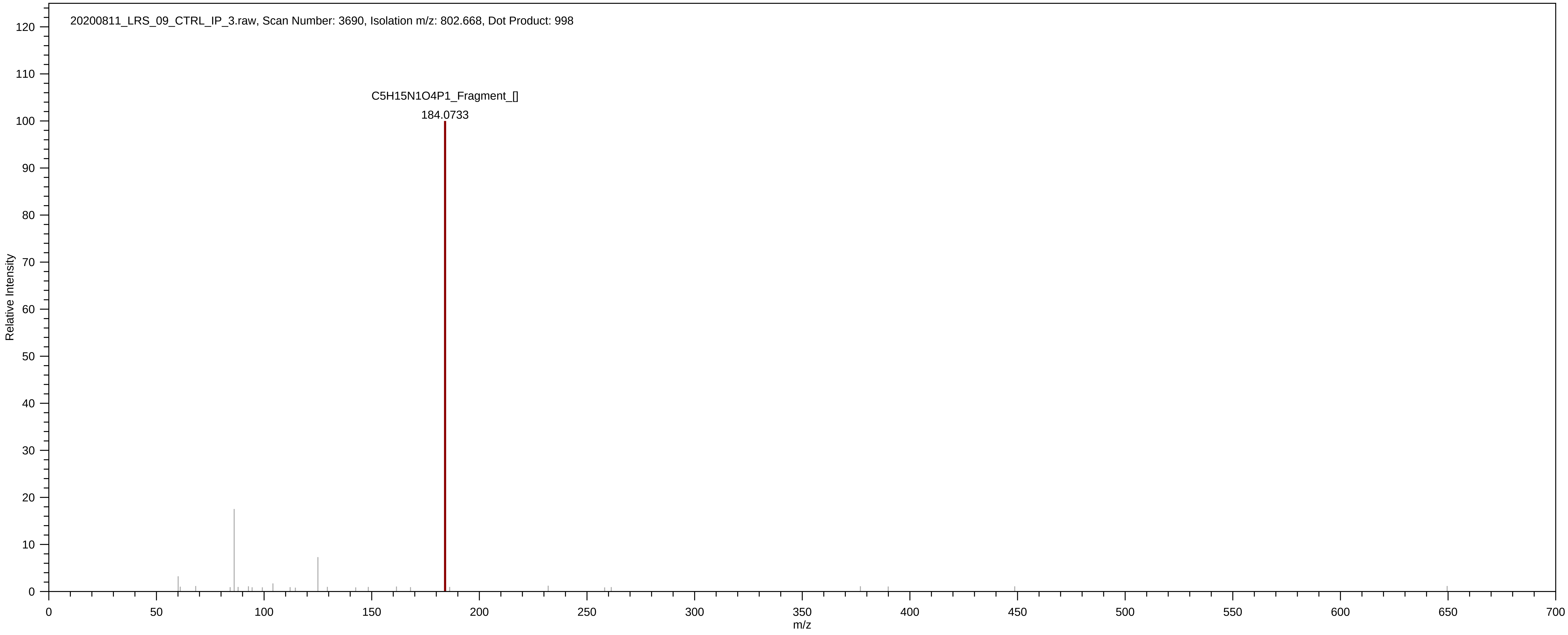

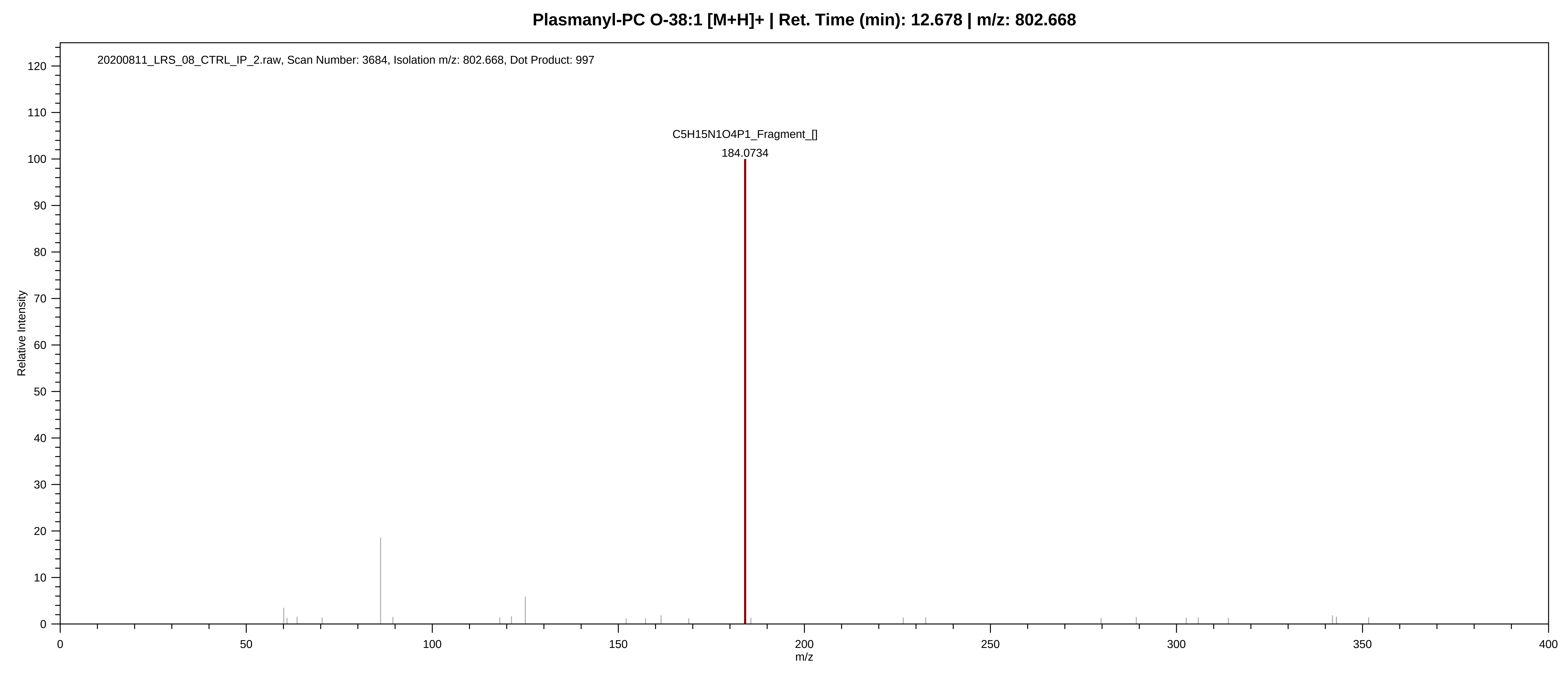

Plasmanyl-PC O-38:1 [M+H]+ | Ret. Time (min): 12.678 | m/z: 802.668
20200811_LRS_08_CTRL_IP_2.raw, Scan Number: 3684, Isolation m/z: 802.668, Dot Product: 997
C5H15N1O4P1_Fragment_[]
184.0734
Relative Intensity
m/z

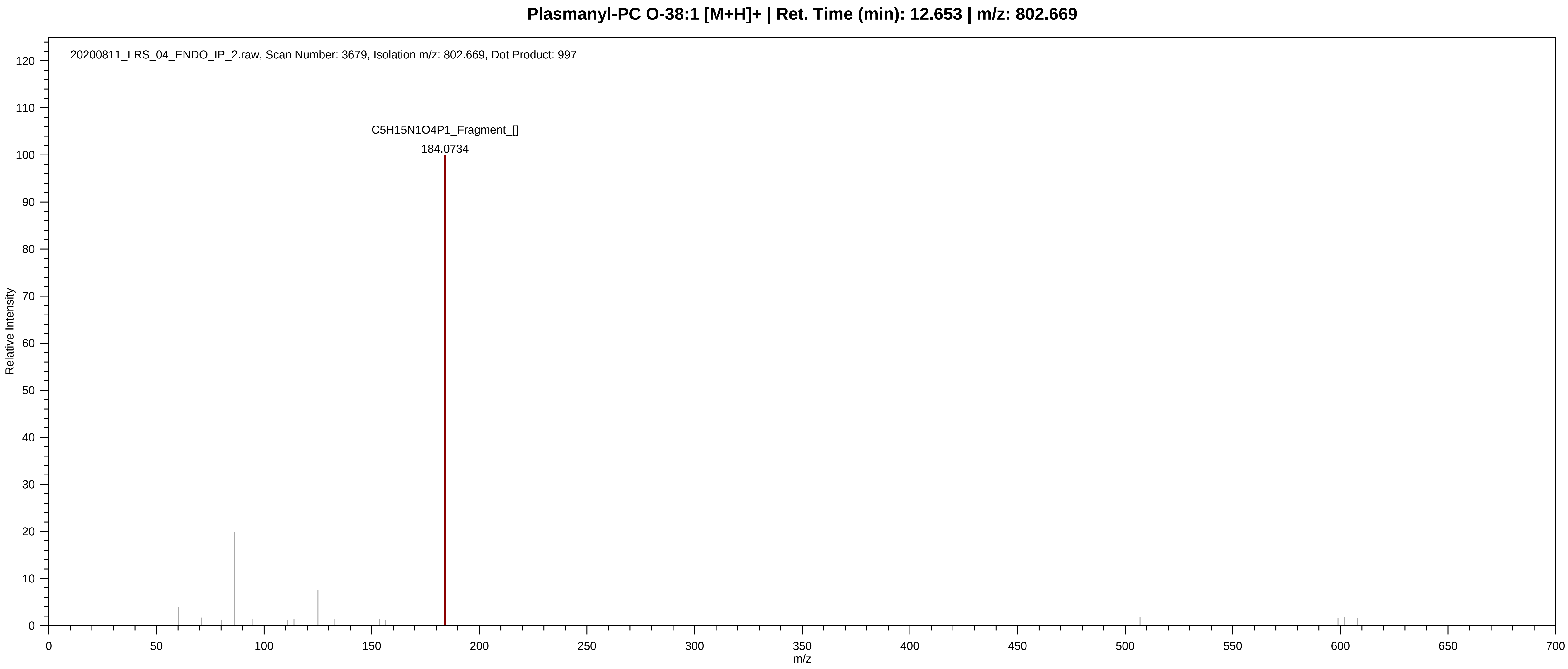

Plasmanyl-PC O-38:1 [M+H]+ | Ret. Time (min): 12.653 | m/z: 802.669
20200811_LRS_04_ENDO_IP_2.raw, Scan Number: 3679, Isolation m/z: 802.669, Dot Product: 997
C5H15N1O4P1_Fragment_[]
184.0734
Relative Intensity
m/z

Plasmanyl-PC O-38:1 [M+H]+ | Ret. Time (min): 12.492 | m/z: 802.671

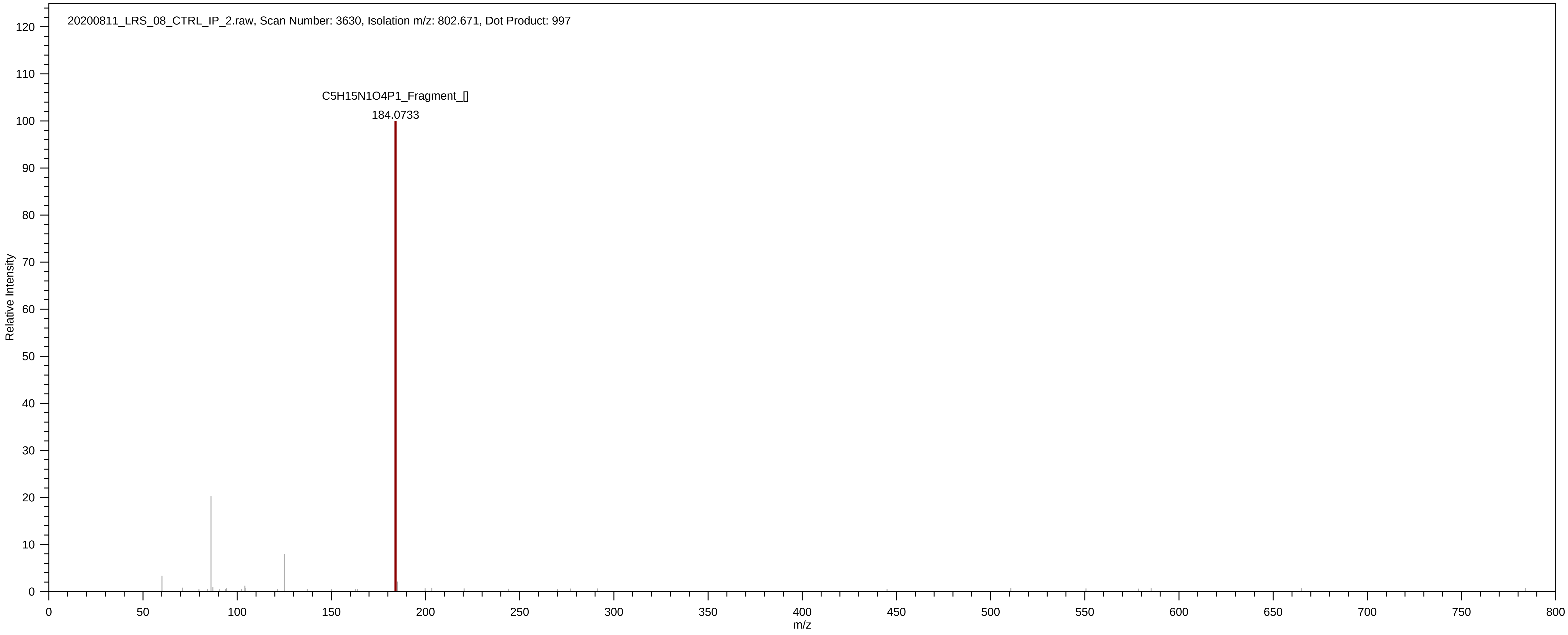

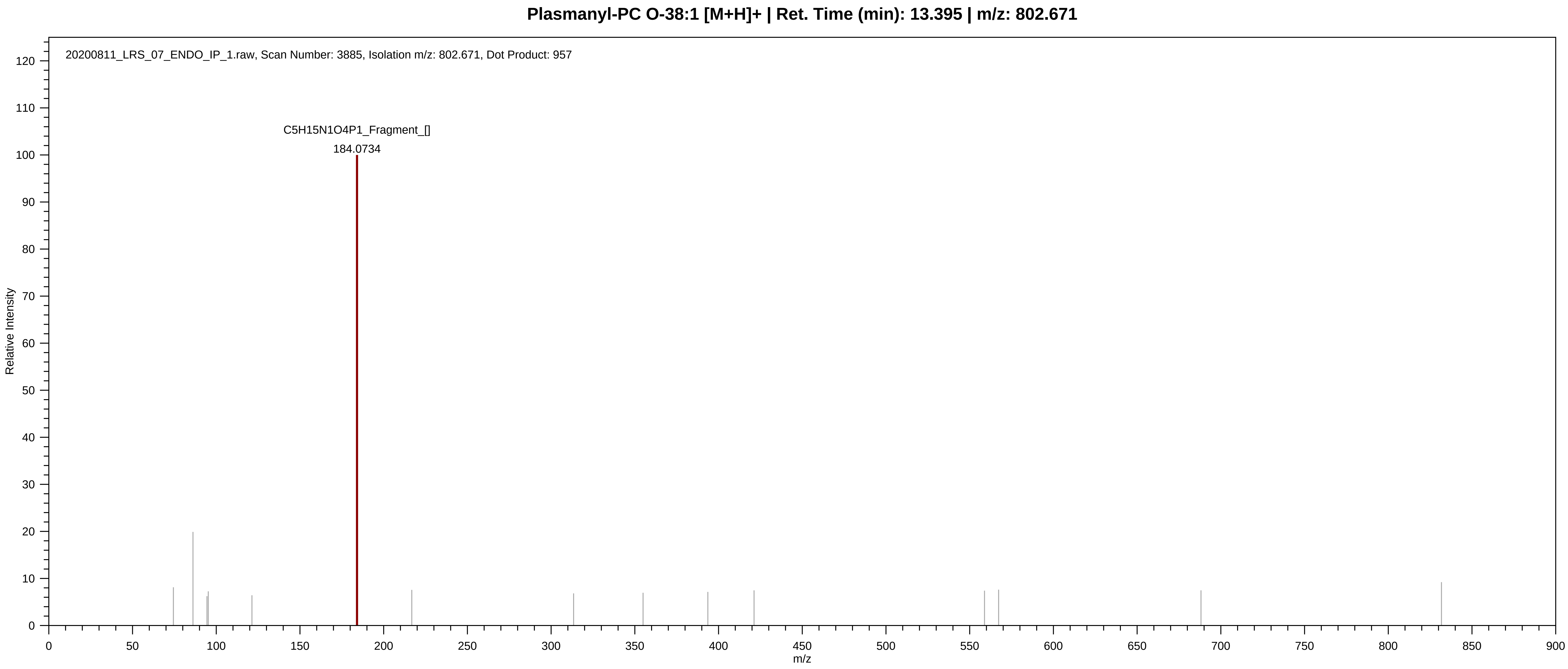
Plasmanyl-PC O-38:1 [M+H]+ | Ret. Time (min): 13.395 | m/z: 802.671
20200811_LRS_07_ENDO_IP_1.raw, Scan Number: 3885, Isolation m/z: 802.671, Dot Product: 957
C5H15N1O4P1_Fragment_[]
184.0734
Relative Intensity
m/z
0
10
20
30
40
50
60
70
80
90
100
110
120
0
50
100
150
200
250
300
350
400
450
500
550
600
650
700
750
800
850
900

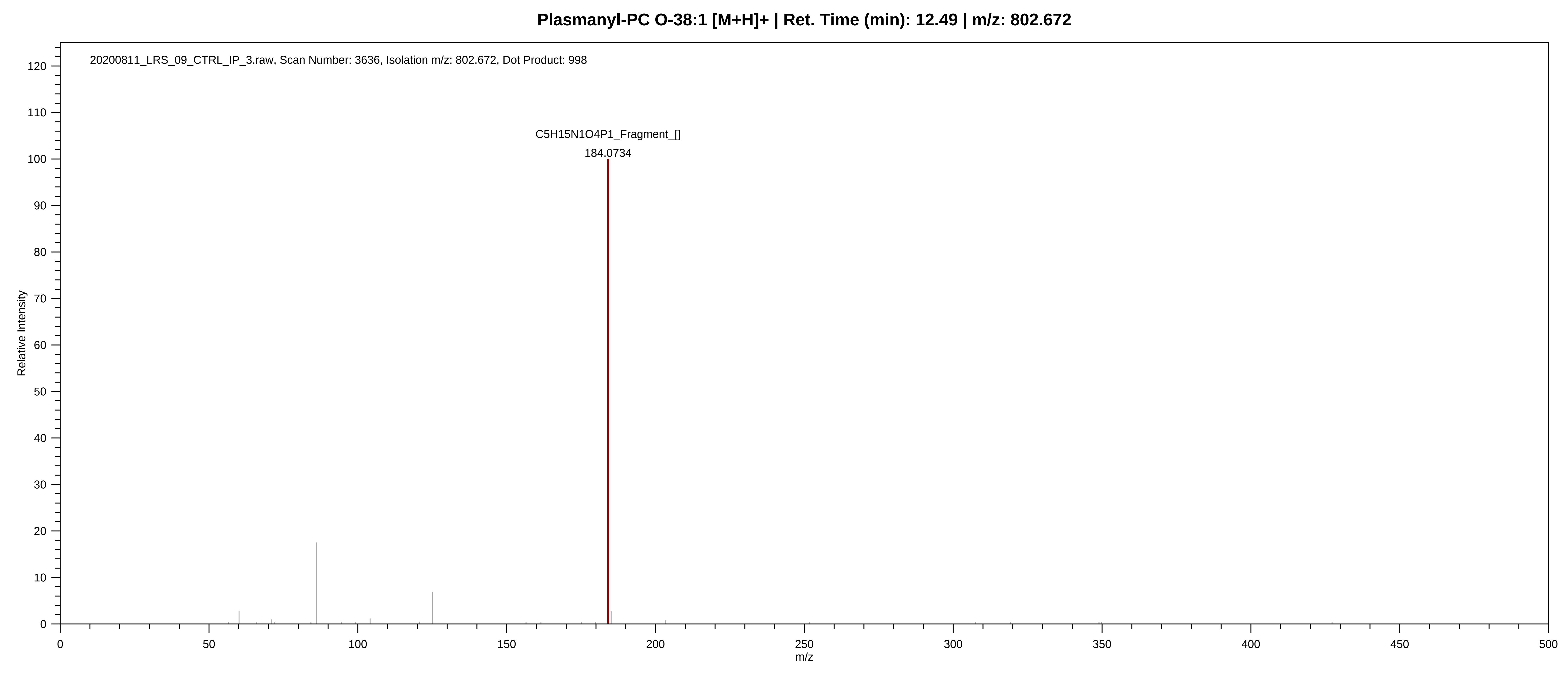
Plasmanyl-PC O-38:1 [M+H]+ | Ret. Time (min): 12.49 | m/z: 802.672
20200811_LRS_09_CTRL_IP_3.raw, Scan Number: 3636, Isolation m/z: 802.672, Dot Product: 998
C5H15N1O4P1_Fragment_[]
184.0734
Relative Intensity
m/z
0
10
20
30
40
50
60
70
80
90
100
110
120
0
50
100
150
200
250
300
350
400
450
500

Plasmanyl-PC O-38:1 [M+H]+ | Ret. Time (min): 12.469 | m/z: 802.672

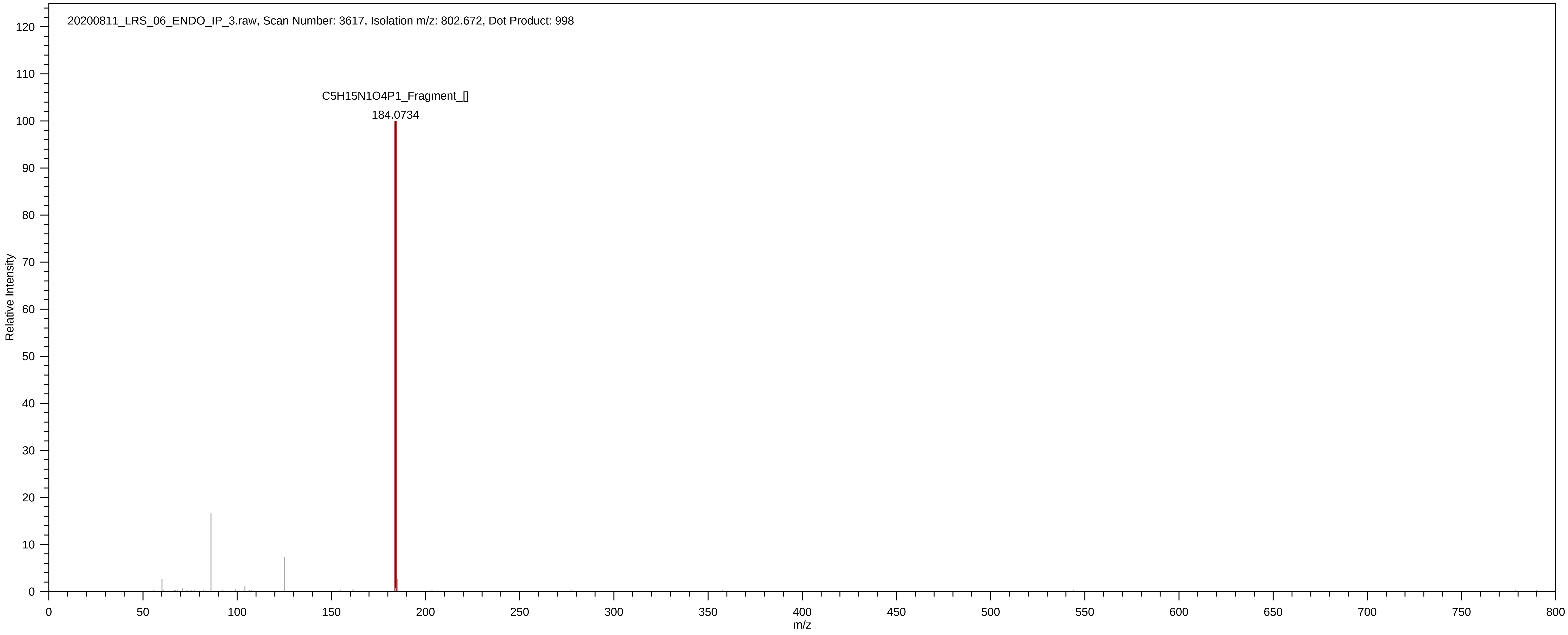

Plasmanyl-PC O-38:1 [M+H]+ | Ret. Time (min): 12.469 | m/z: 802.672

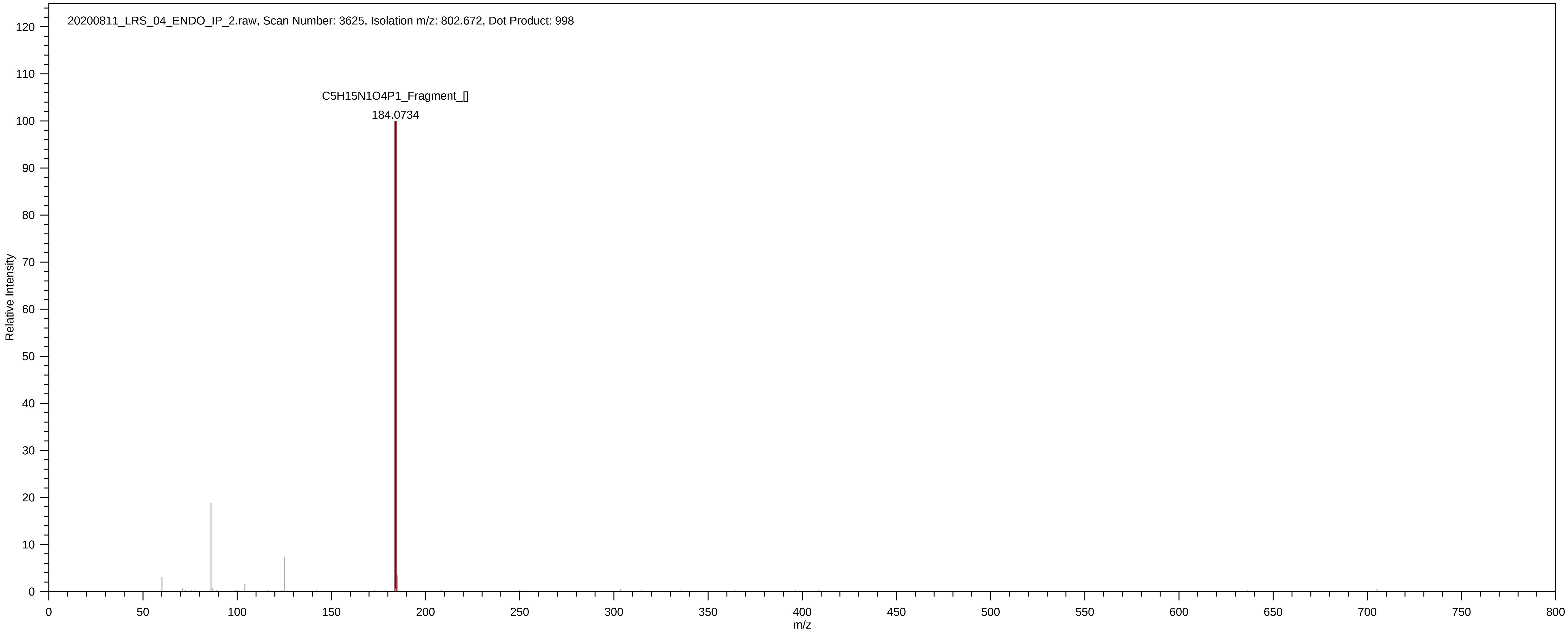

Plasmanyl-PC O-38:1 [M+H]+ | Ret. Time (min): 12.471 | m/z: 802.672

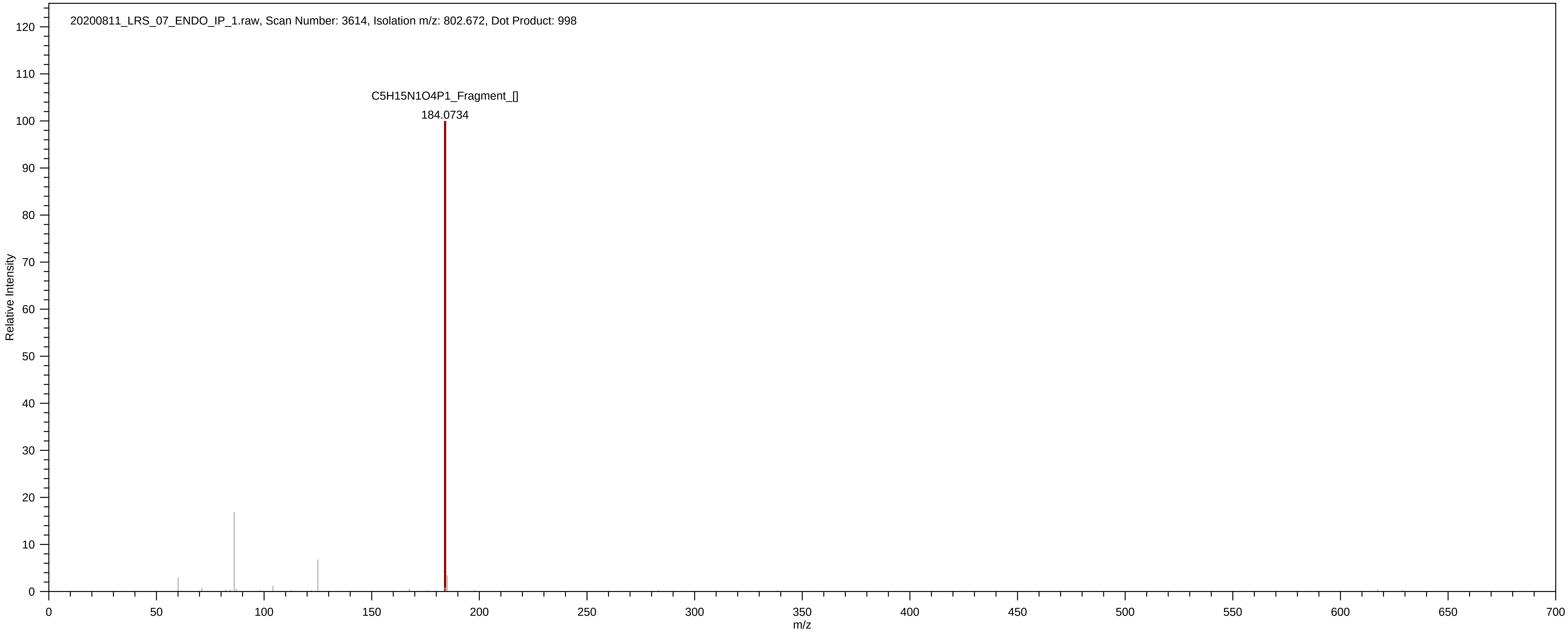

Plasmanyl-PC O-38:2 [M+H]+ | Ret. Time (min): 11.59 | m/z: 800.652

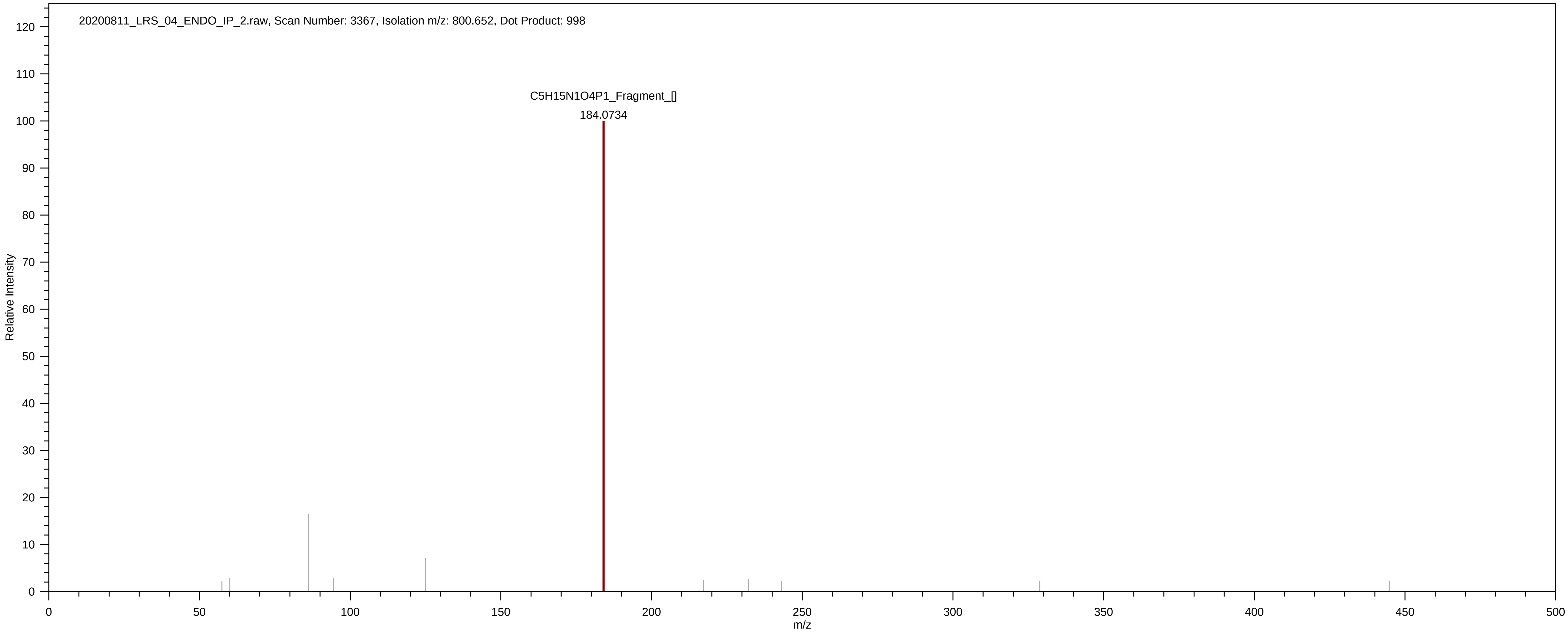

Plasmanyl-PC O-38:2 [M+H]+ | Ret. Time (min): 11.428 | m/z: 800.653

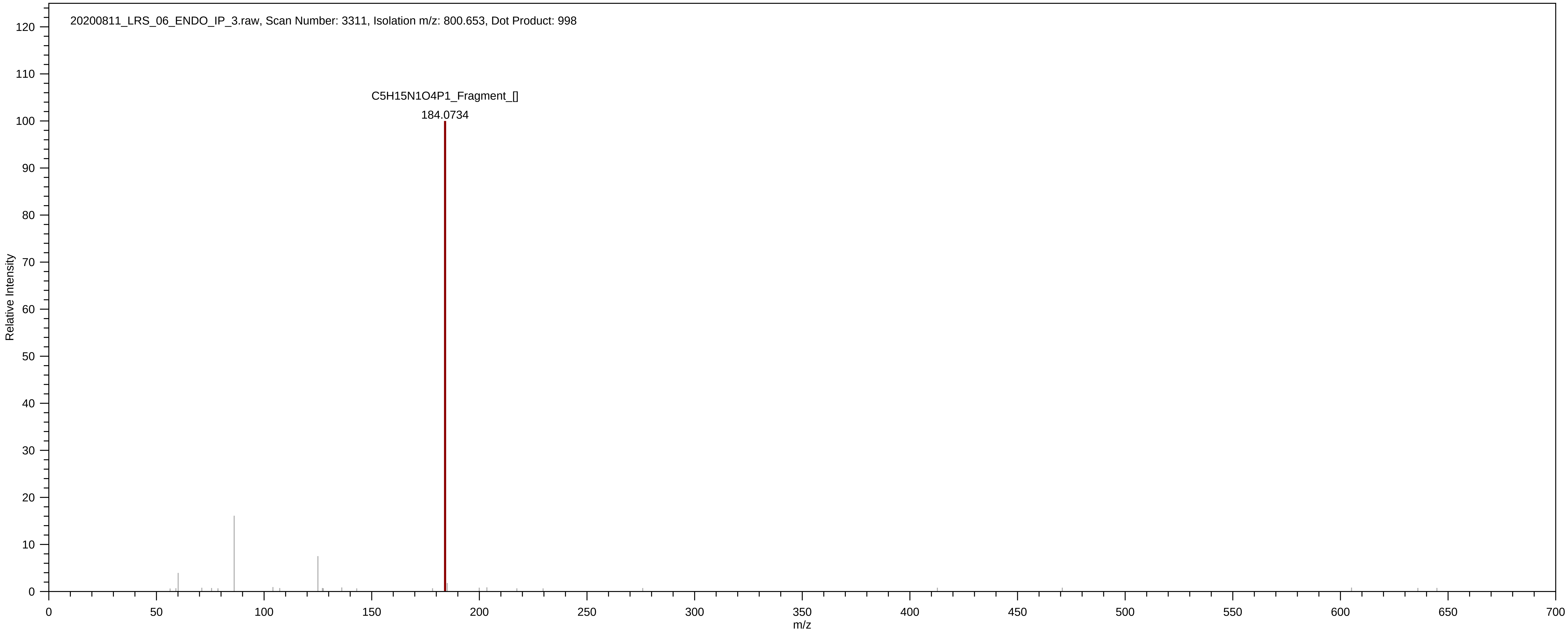

Plasmanyl-PC O-38:2 [M+H]+ | Ret. Time (min): 11.452 | m/z: 800.653

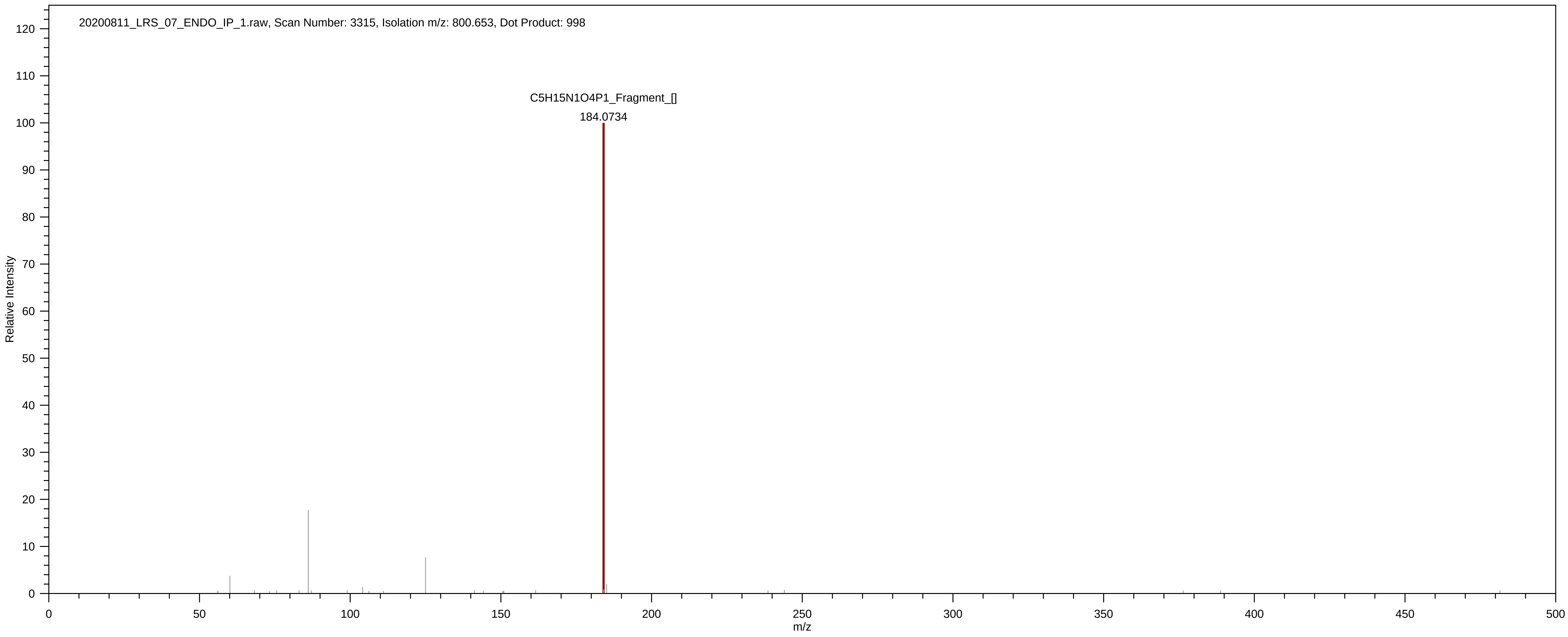

Plasmanyl-PC O-38:2 [M+H]+ | Ret. Time (min): 11.463 | m/z: 800.653

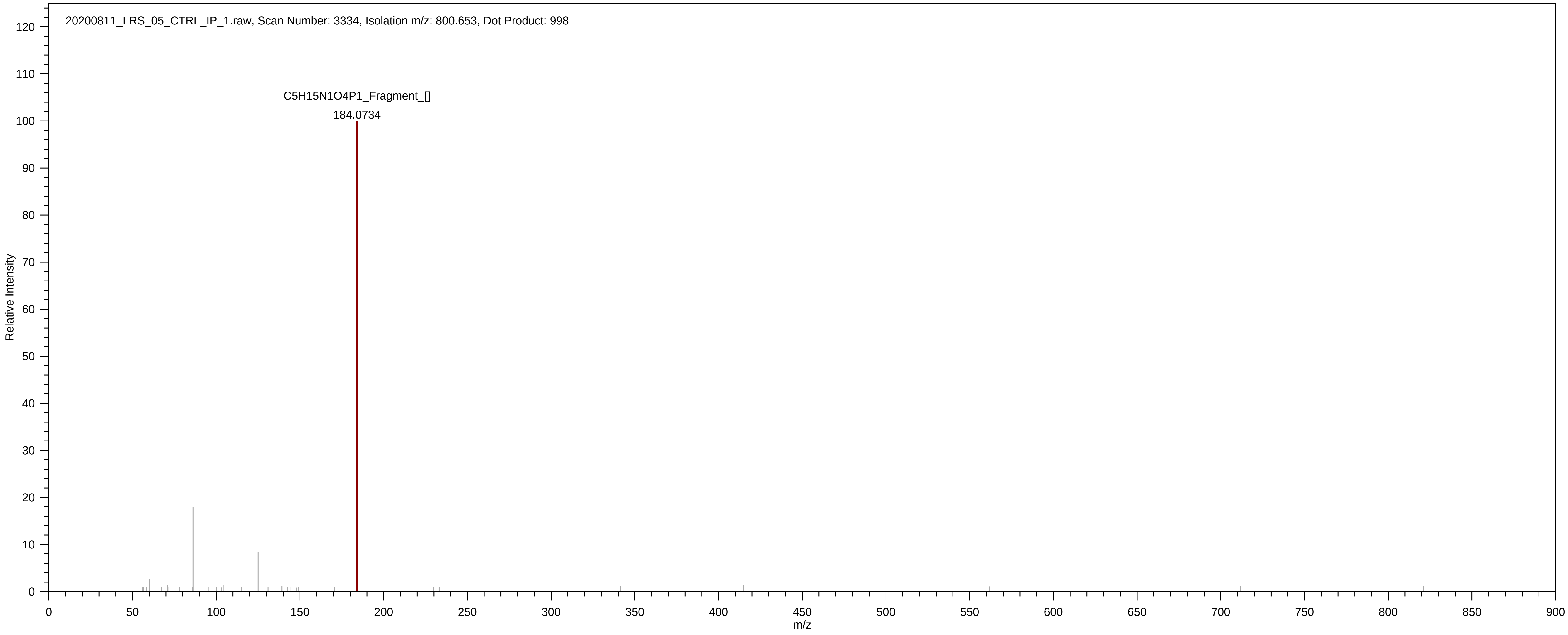

Plasmanyl-PC O-38:2 [M+H]+ | Ret. Time (min): 11.586 | m/z: 800.653

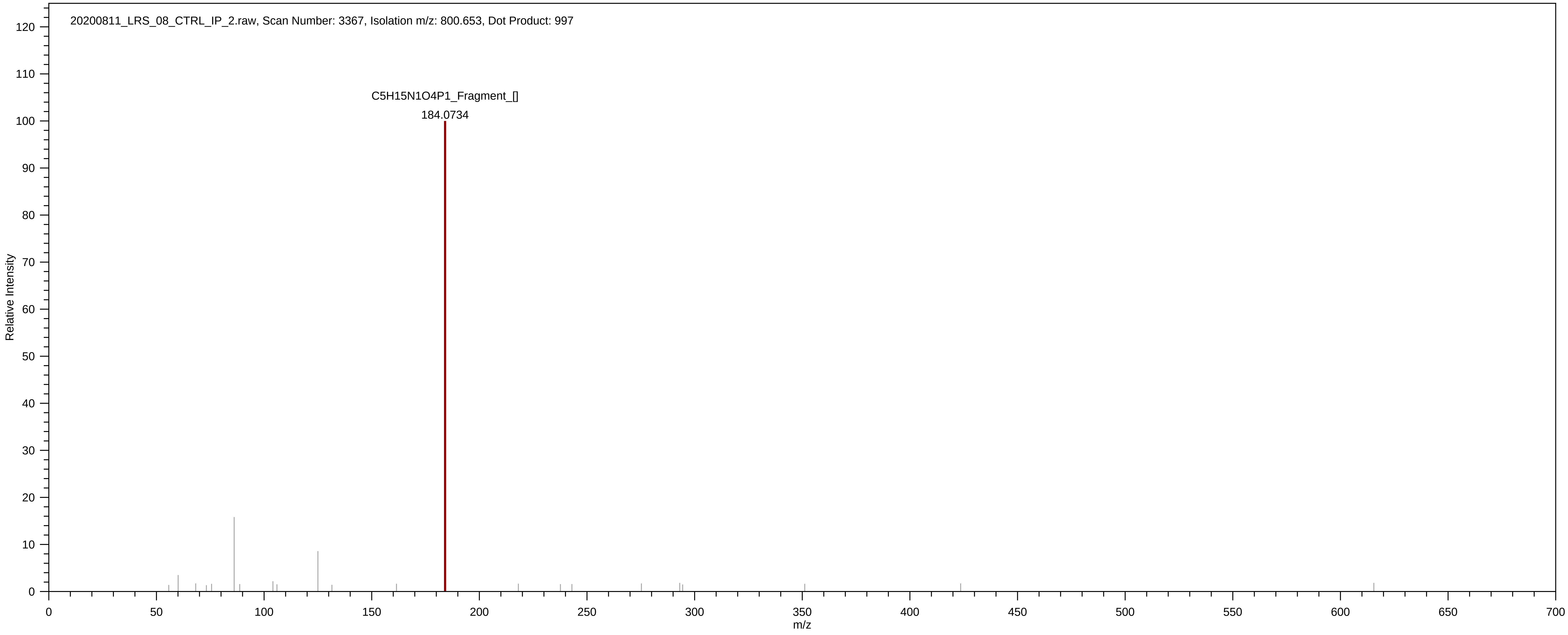

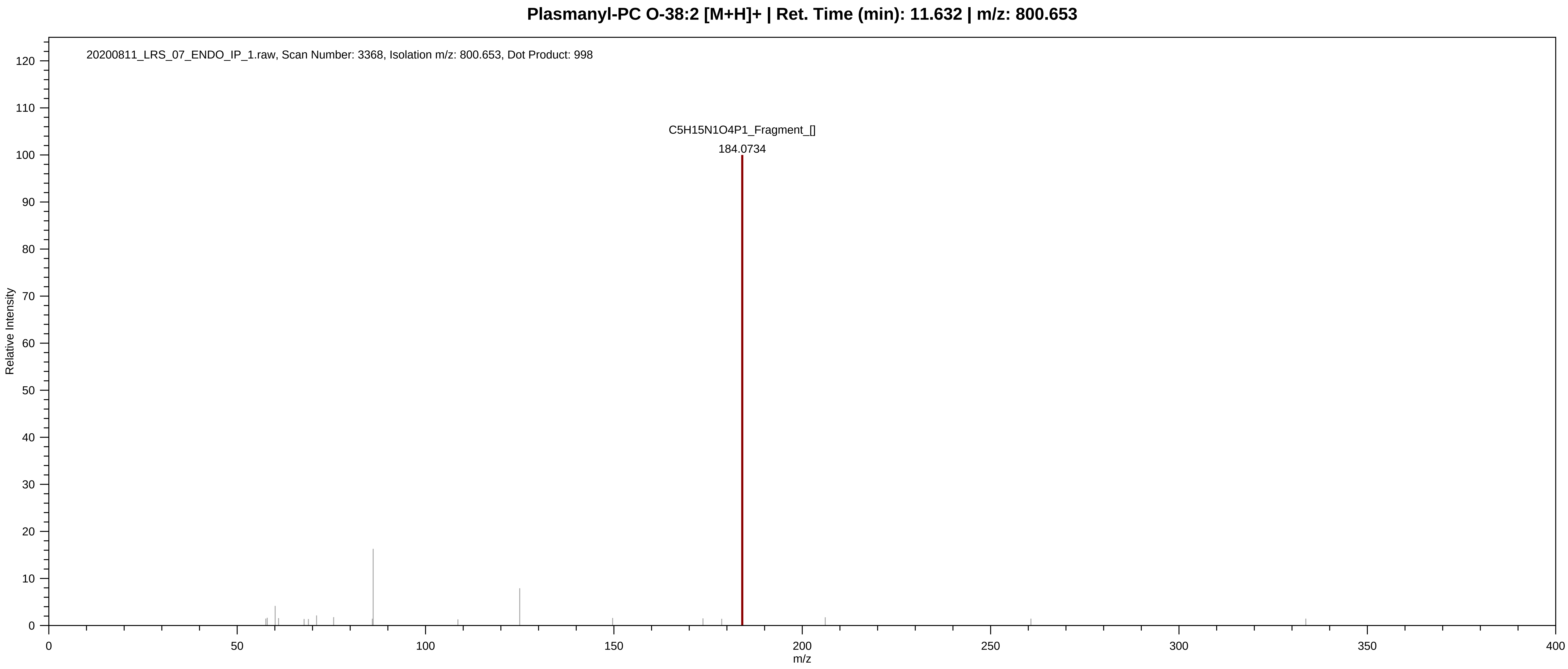
Plasmanyl-PC O-38:2 [M+H]+ | Ret. Time (min): 11.632 | m/z: 800.653
20200811_LRS_07_ENDO_IP_1.raw, Scan Number: 3368, Isolation m/z: 800.653, Dot Product: 998
C5H15N1O4P1_Fragment_[]
184.0734
Relative Intensity
m/z
0
10
20
30
40
50
60
70
80
90
100
110
120
0
50
100
150
200
250
300
350
400

Plasmanyl-PC O-38:2 [M+H]+ | Ret. Time (min): 11.642 | m/z: 800.653

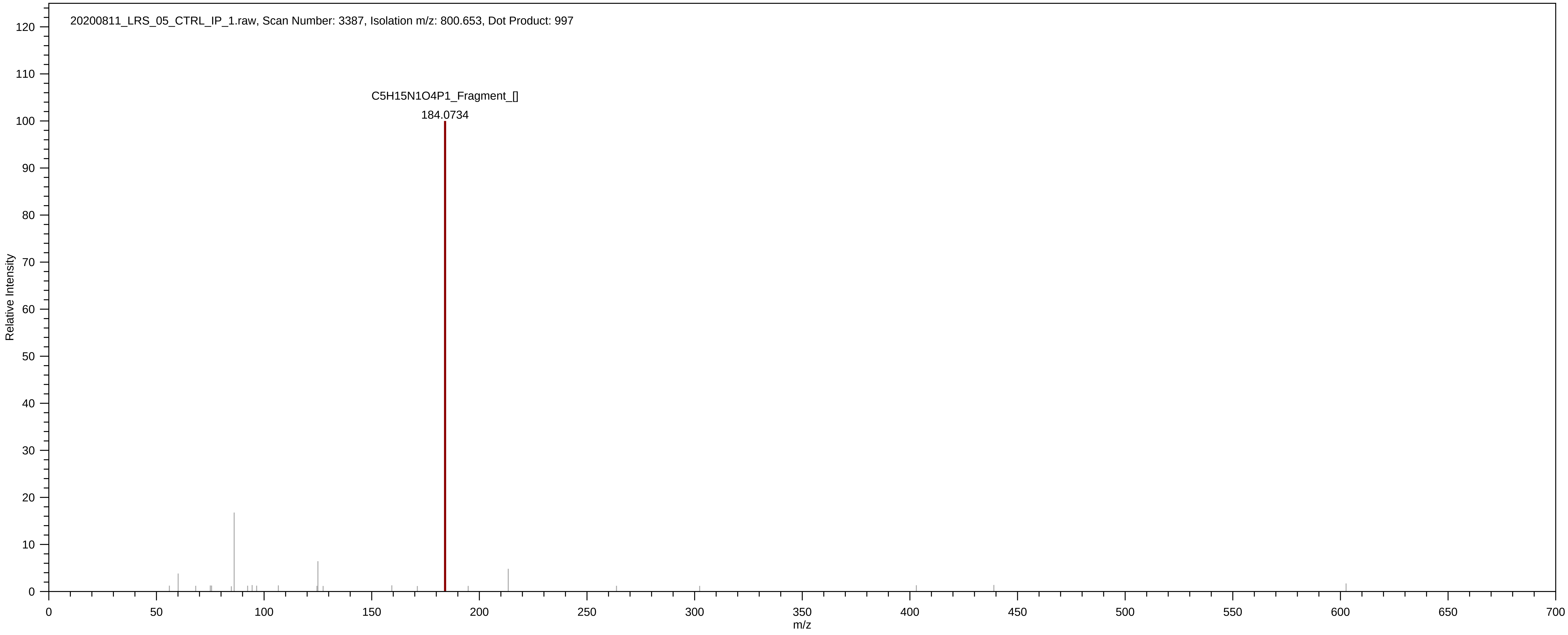

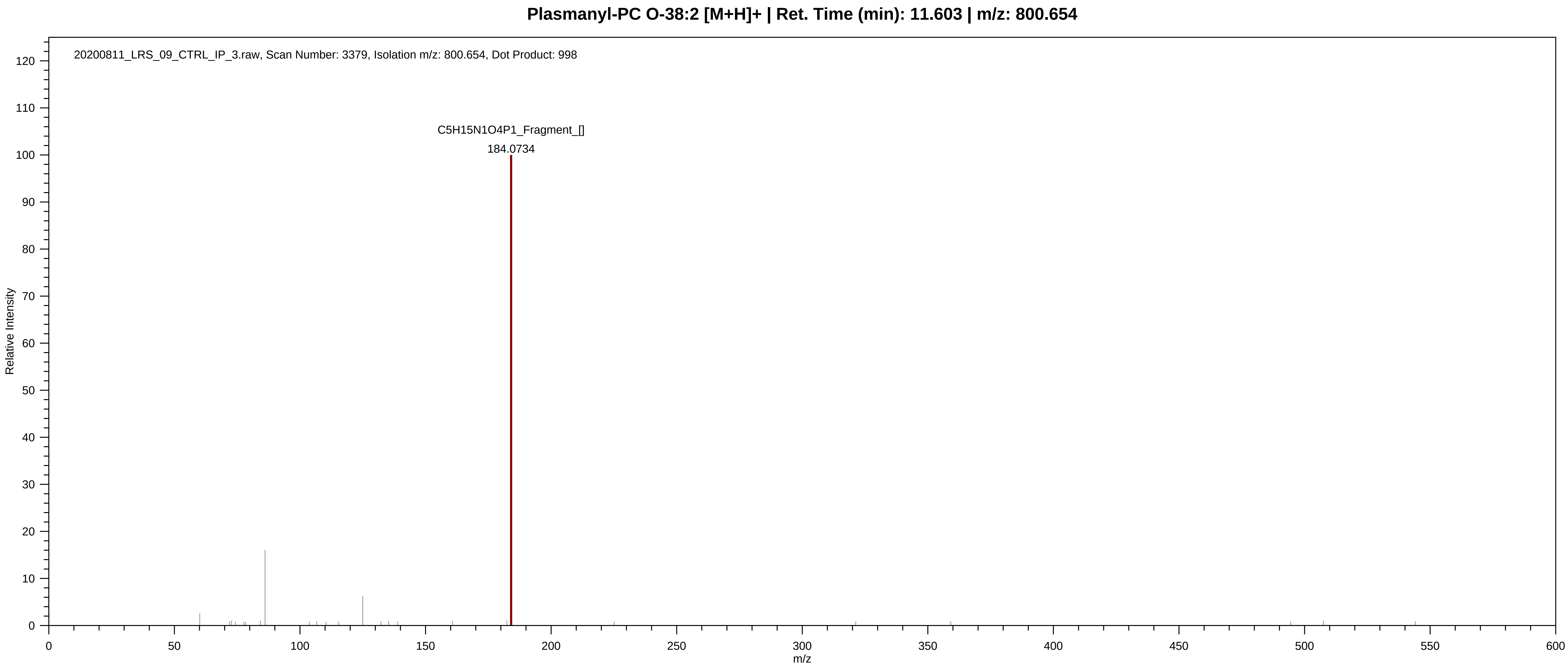

Plasmanyl-PC O-38:2 [M+H]+ | Ret. Time (min): 11.603 | m/z: 800.654
20200811_LRS_09_CTRL_IP_3.raw, Scan Number: 3379, Isolation m/z: 800.654, Dot Product: 998
C5H15N1O4P1_Fragment_[]
184.0734
Relative Intensity
m/z
0
10
20
30
40
50
60
70
80
90
100
110
120
0
50
100
150
200
250
300
350
400
450
500
550
600

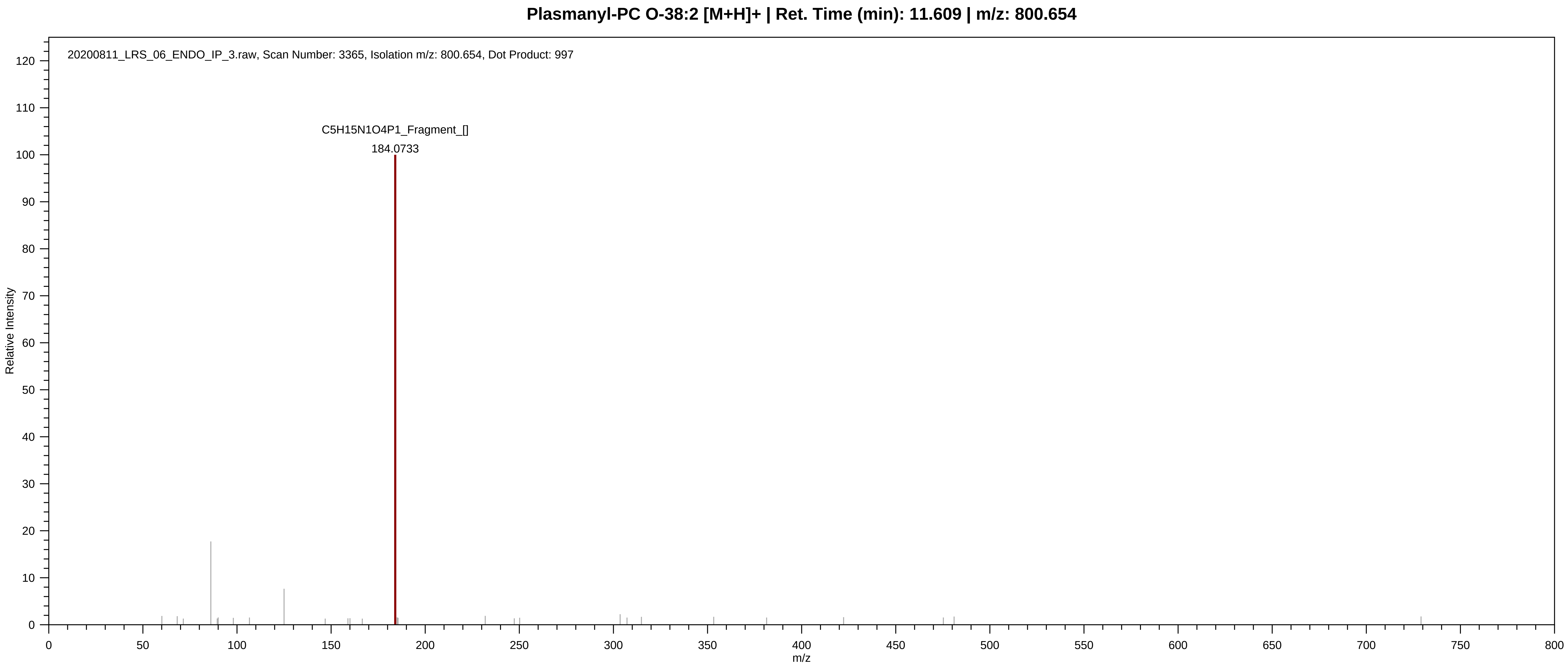

Plasmanyl-PC O-38:2 [M+H]+ | Ret. Time (min): 11.609 | m/z: 800.654
20200811_LRS_06_ENDO_IP_3.raw, Scan Number: 3365, Isolation m/z: 800.654, Dot Product: 997
C5H15N1O4P1_Fragment_[]
184.0733
Relative Intensity
m/z
0
10
20
30
40
50
60
70
80
90
100
110
120
0
50
100
150
200
250
300
350
400
450
500
550
600
650
700
750
800

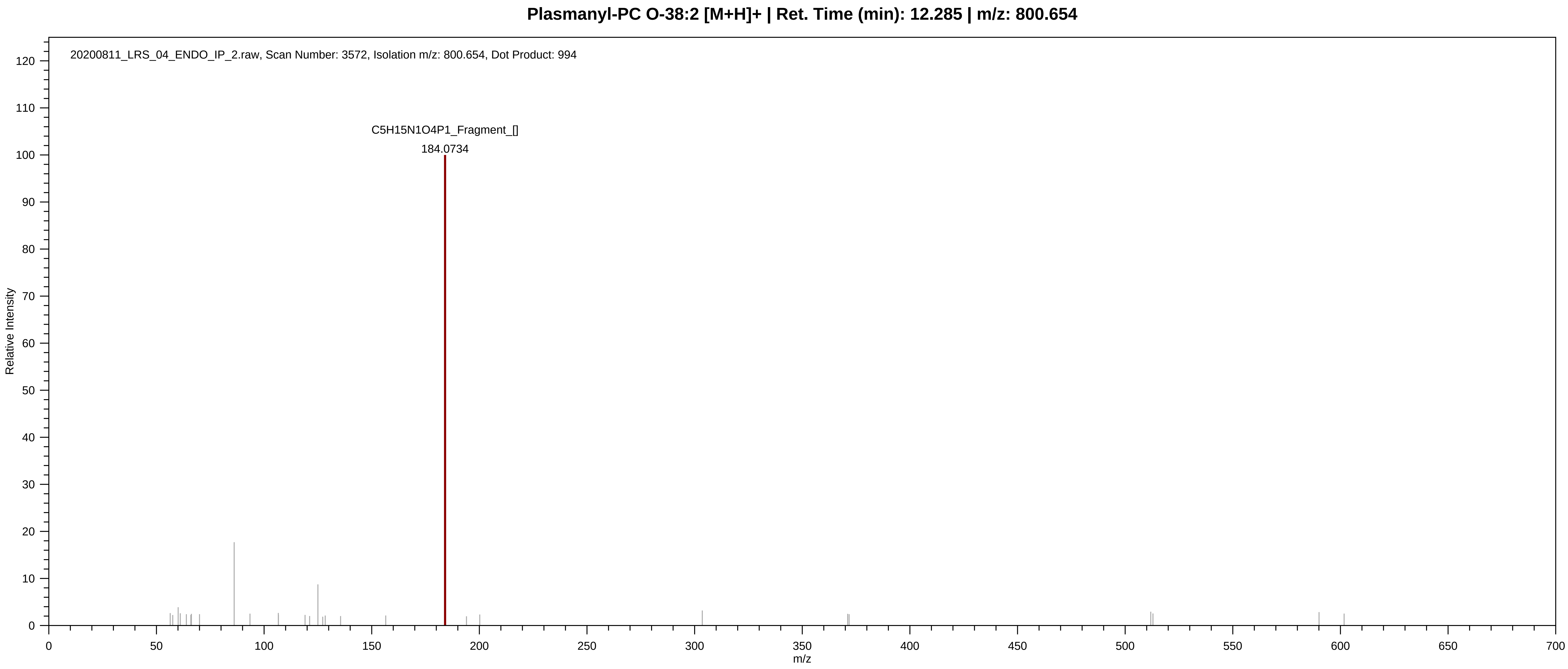
Plasmanyl-PC O-38:2 [M+H]+ | Ret. Time (min): 12.285 | m/z: 800.654
20200811_LRS_04_ENDO_IP_2.raw, Scan Number: 3572, Isolation m/z: 800.654, Dot Product: 994
C5H15N1O4P1_Fragment_[]
184.0734
Relative Intensity
m/z
0
10
20
30
40
50
60
70
80
90
100
110
120
0
50
100
150
200
250
300
350
400
450
500
550
600
650
700

Plasmanyl-PC O-38:2 [M+H]+ | Ret. Time (min): 11.406 | m/z: 800.655

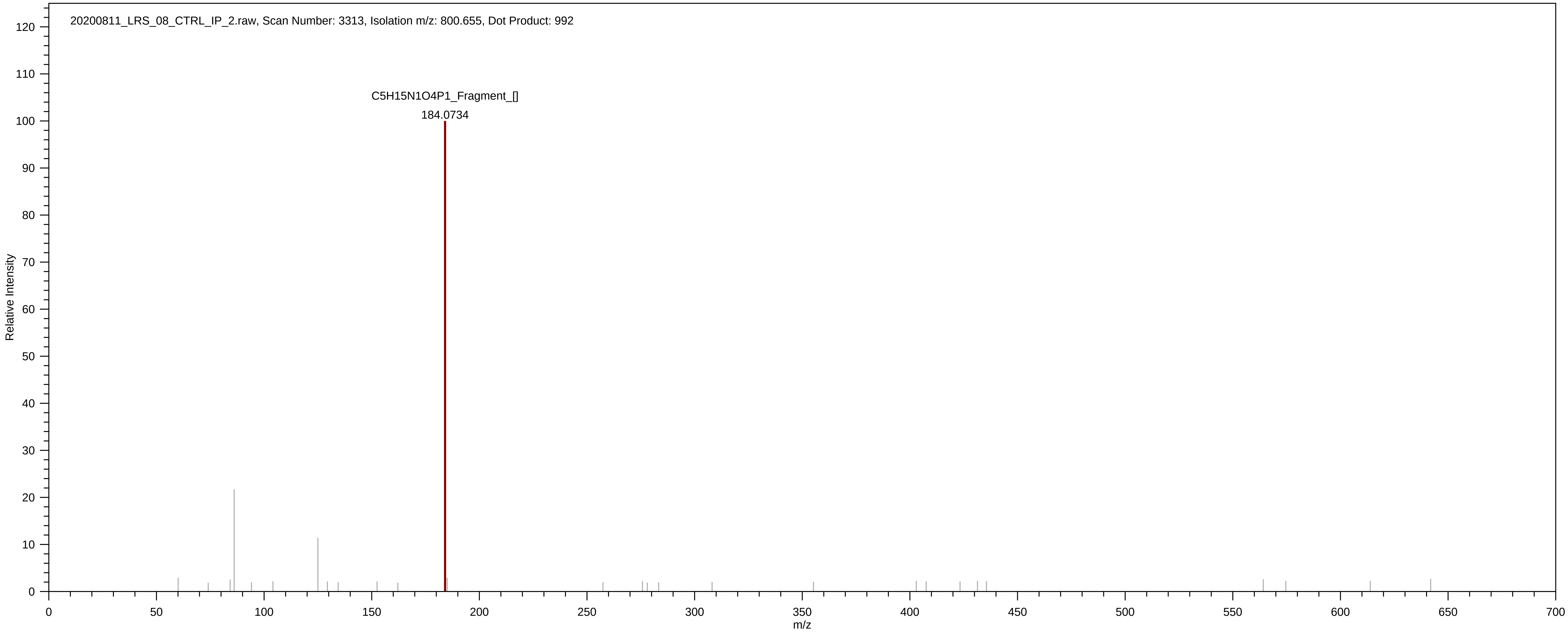

Plasmanyl-PC O-38:2 [M+H]+ | Ret. Time (min): 12.285 | m/z: 800.655

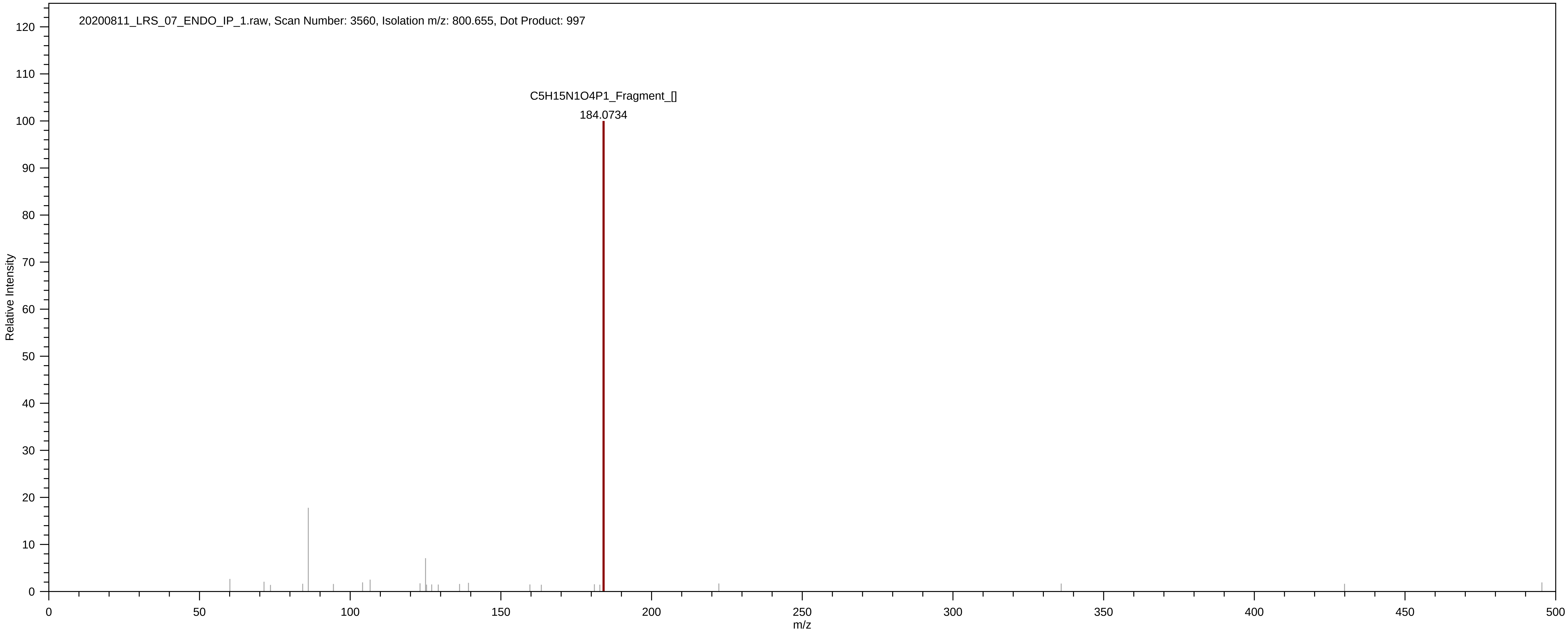

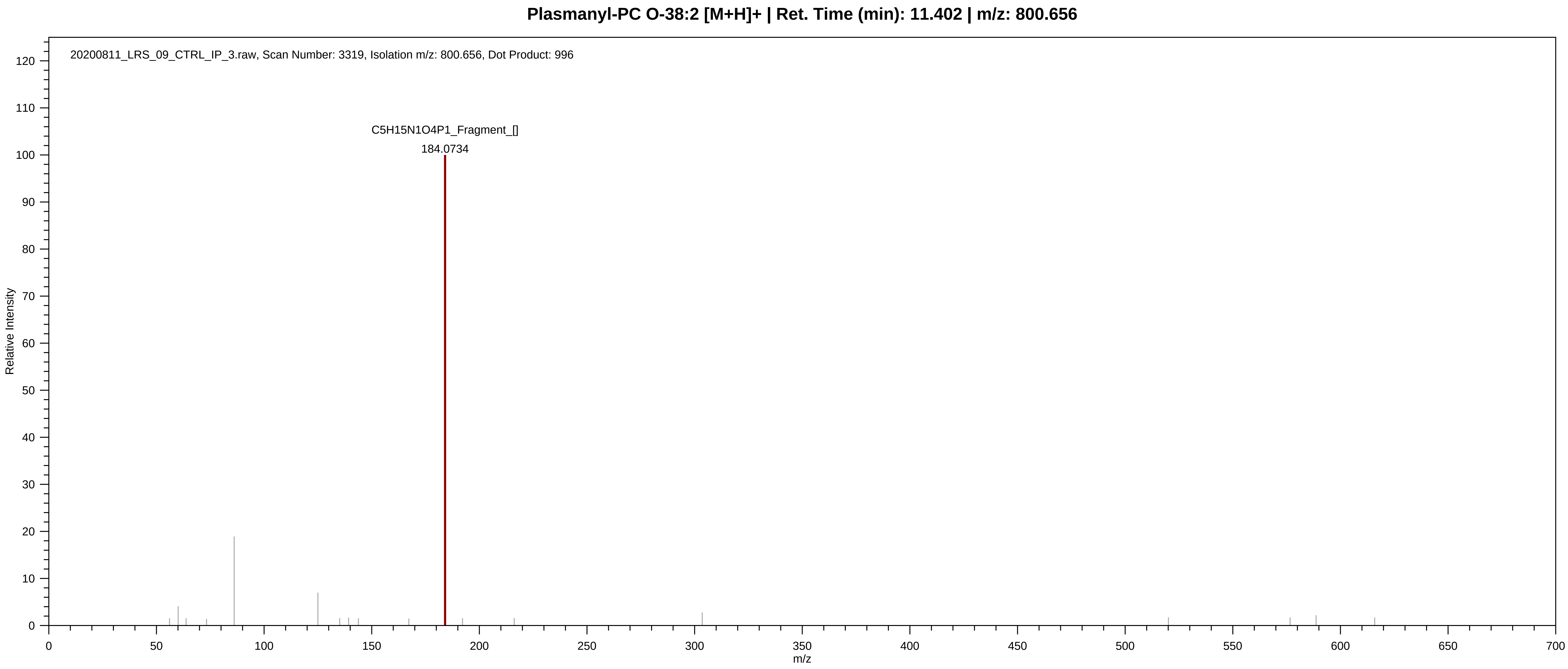
Plasmanyl-PC O-38:2 [M+H]+ | Ret. Time (min): 11.402 | m/z: 800.656
20200811_LRS_09_CTRL_IP_3.raw, Scan Number: 3319, Isolation m/z: 800.656, Dot Product: 996
C5H15N1O4P1_Fragment_[]
184.0734
Relative Intensity
m/z

Plasmanyl-PC O-38:2 [M+H]+ | Ret. Time (min): 12.47 | m/z: 800.656

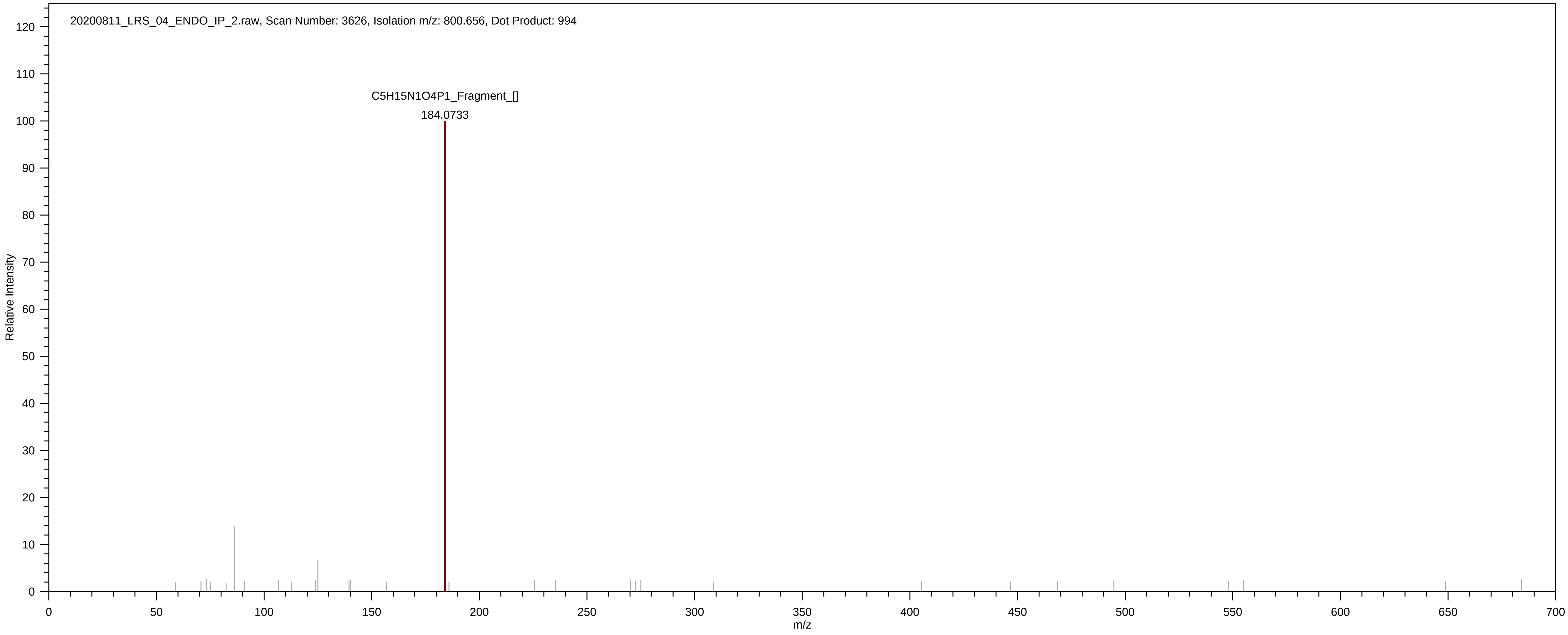

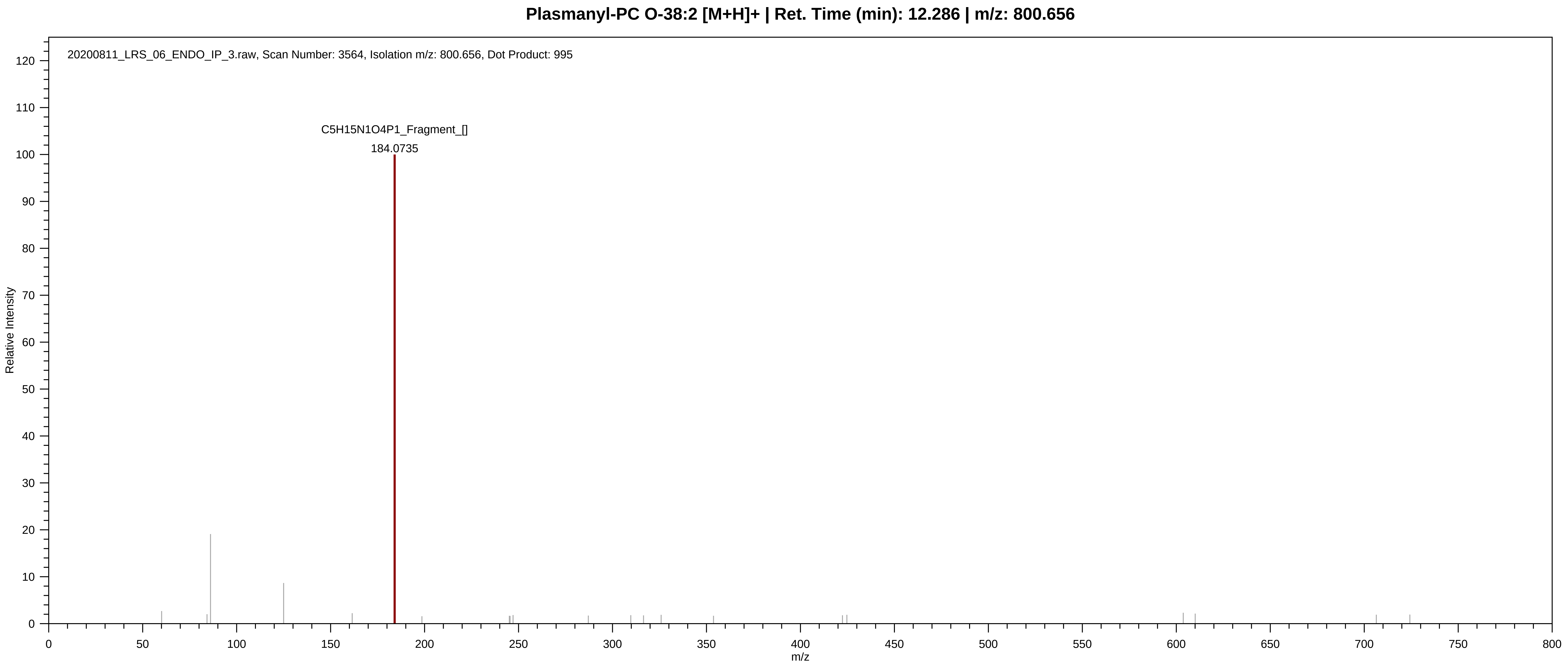
Plasmanyl-PC O-38:2 [M+H]+ | Ret. Time (min): 12.286 | m/z: 800.656
20200811_LRS_06_ENDO_IP_3.raw, Scan Number: 3564, Isolation m/z: 800.656, Dot Product: 995
C5H15N1O4P1_Fragment_[]
184.0735
Relative Intensity
m/z

Plasmanyl-PC O-38:2 [M+H]+ | Ret. Time (min): 12.325 | m/z: 800.656

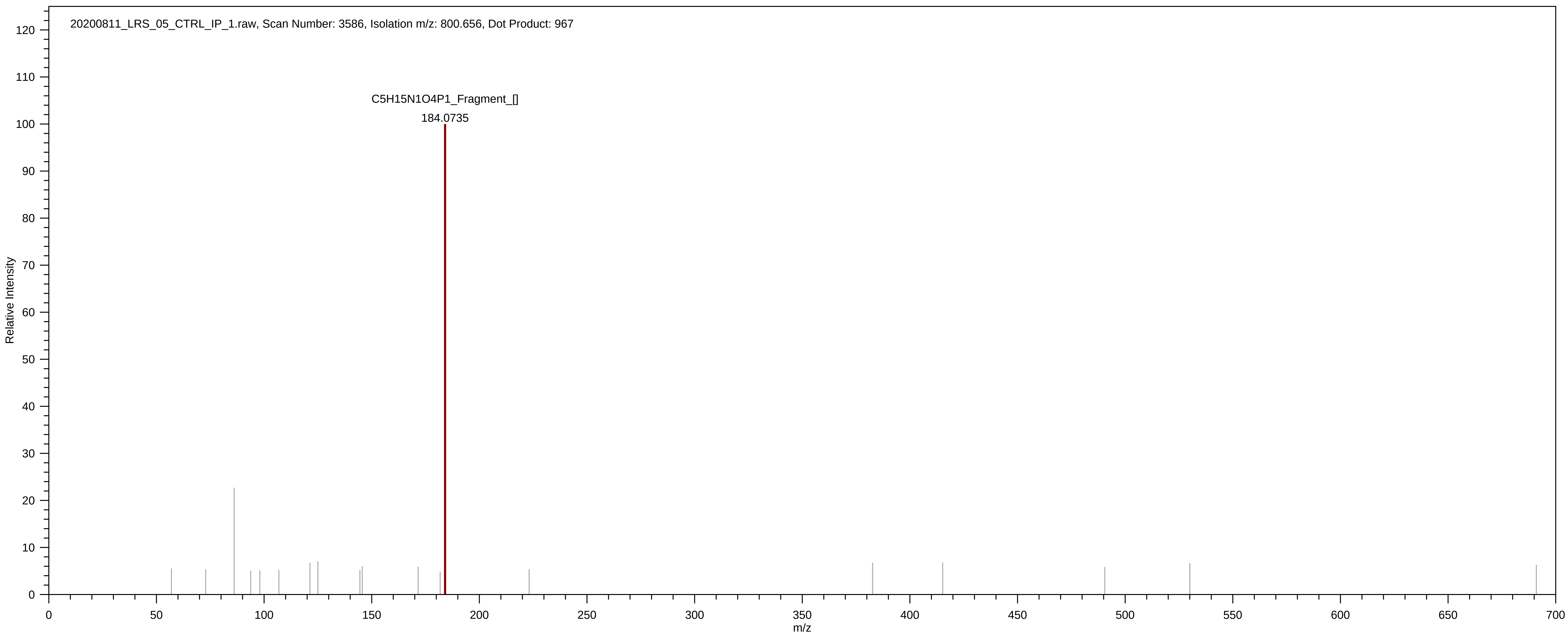

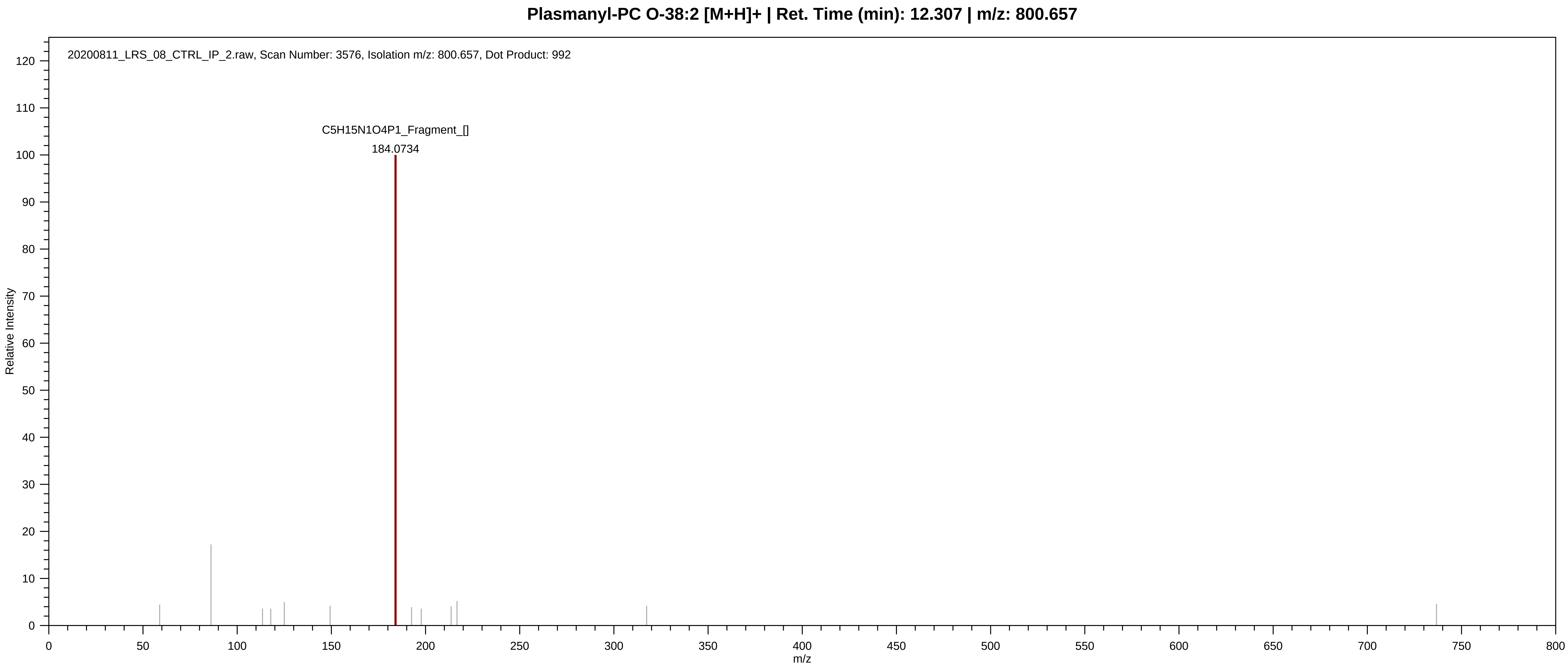
Plasmanyl-PC O-38:2 [M+H]+ | Ret. Time (min): 12.307 | m/z: 800.657
20200811_LRS_08_CTRL_IP_2.raw, Scan Number: 3576, Isolation m/z: 800.657, Dot Product: 992
C5H15N1O4P1_Fragment_[]
184.0734
Relative Intensity
m/z

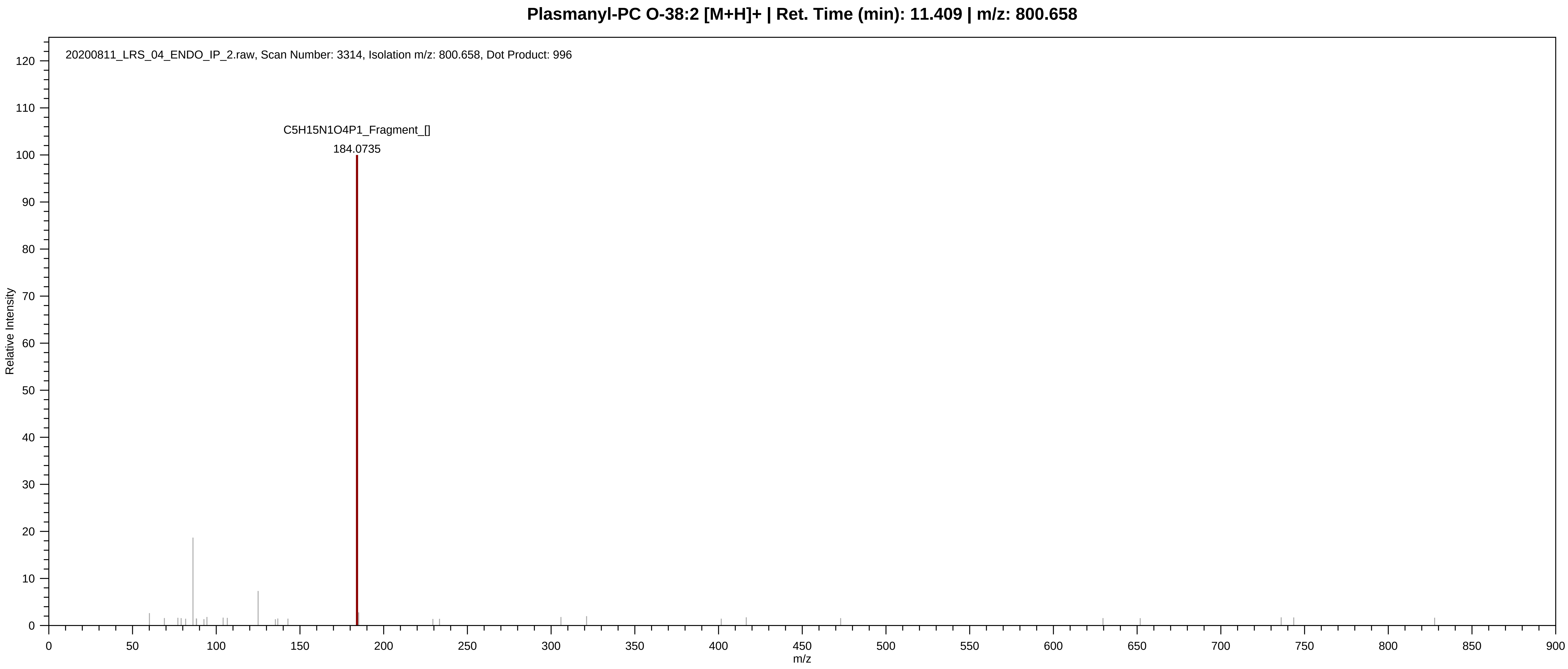
Plasmanyl-PC O-38:2 [M+H]+ | Ret. Time (min): 11.409 | m/z: 800.658
20200811_LRS_04_ENDO_IP_2.raw, Scan Number: 3314, Isolation m/z: 800.658, Dot Product: 996
C5H15N1O4P1_Fragment_[]
184.0735
Relative Intensity
m/z

Plasmanyl-PC O-38:2 [M+H]+ | Ret. Time (min): 12.304 | m/z: 800.658

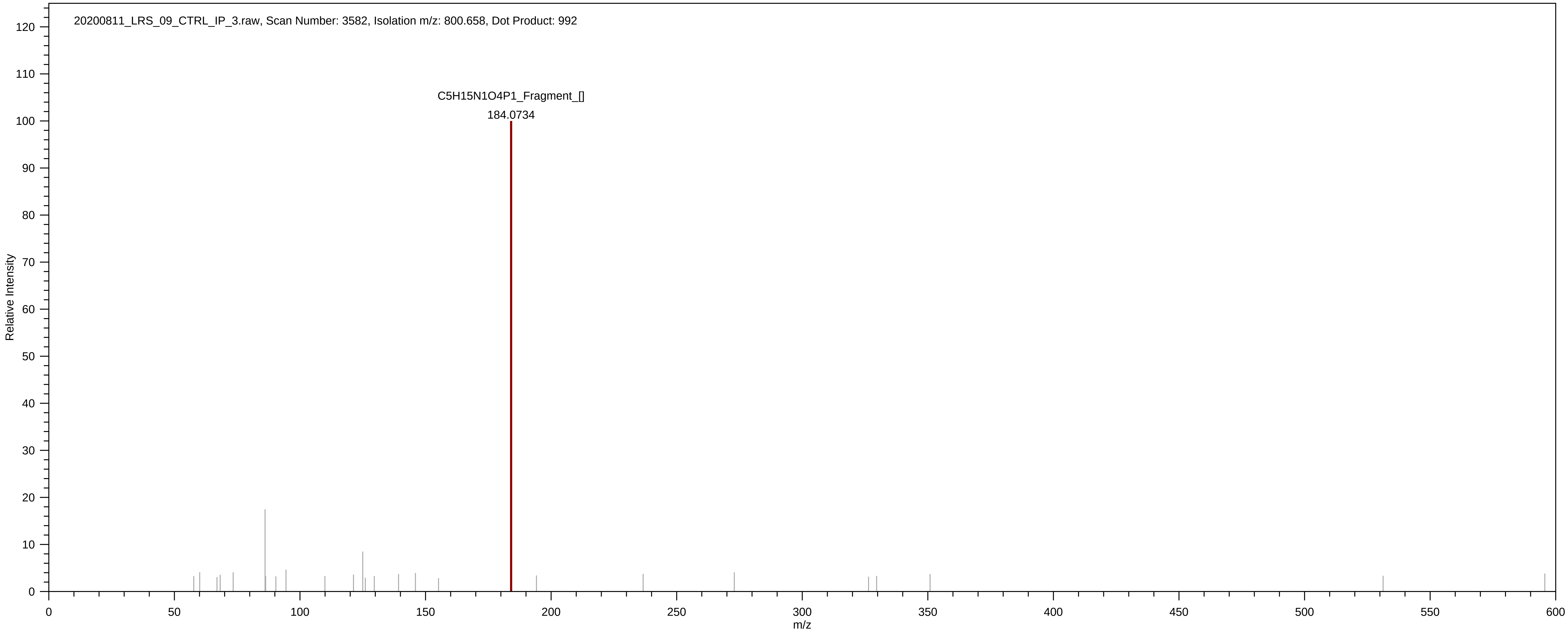

Plasmanyl-PC O-38:3 [M+H]+ | Ret. Time (min): 10.929 | m/z: 798.64

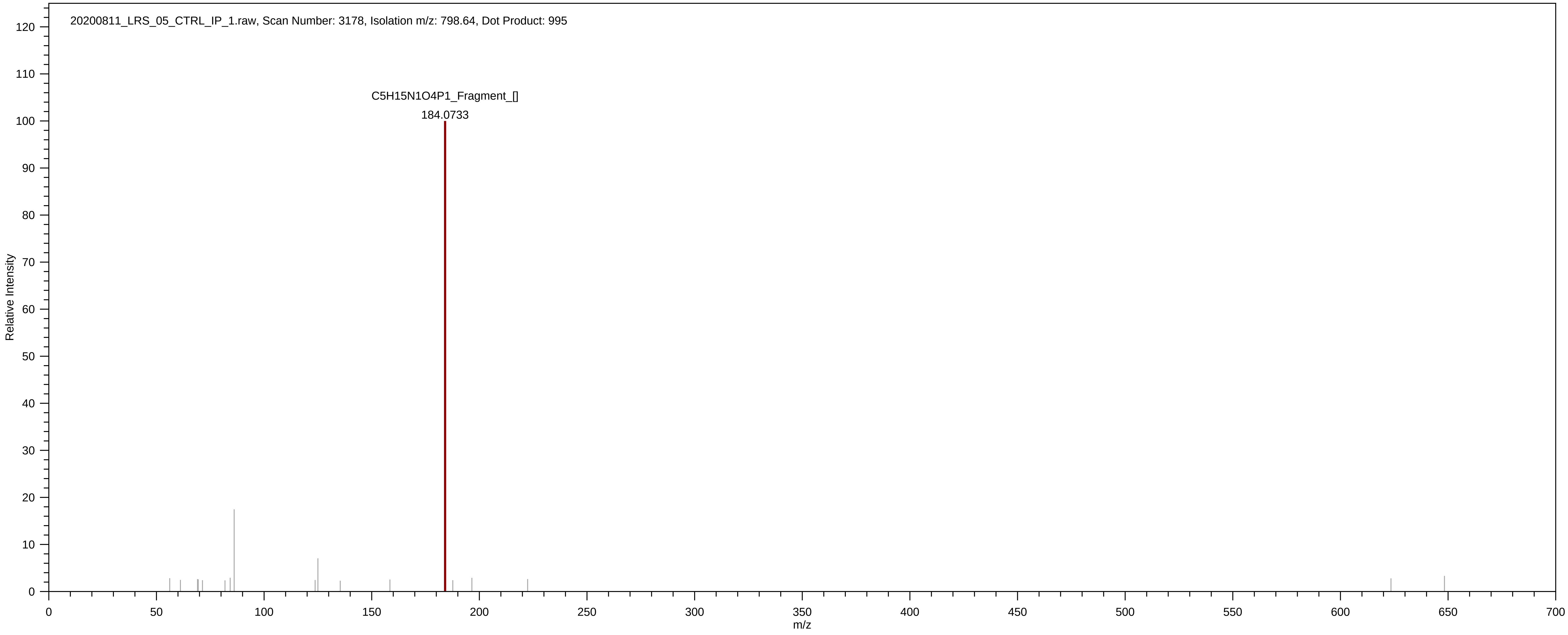

Plasmanyl-PC O-38:3 [M+H]+ | Ret. Time (min): 10.932 | m/z: 798.641

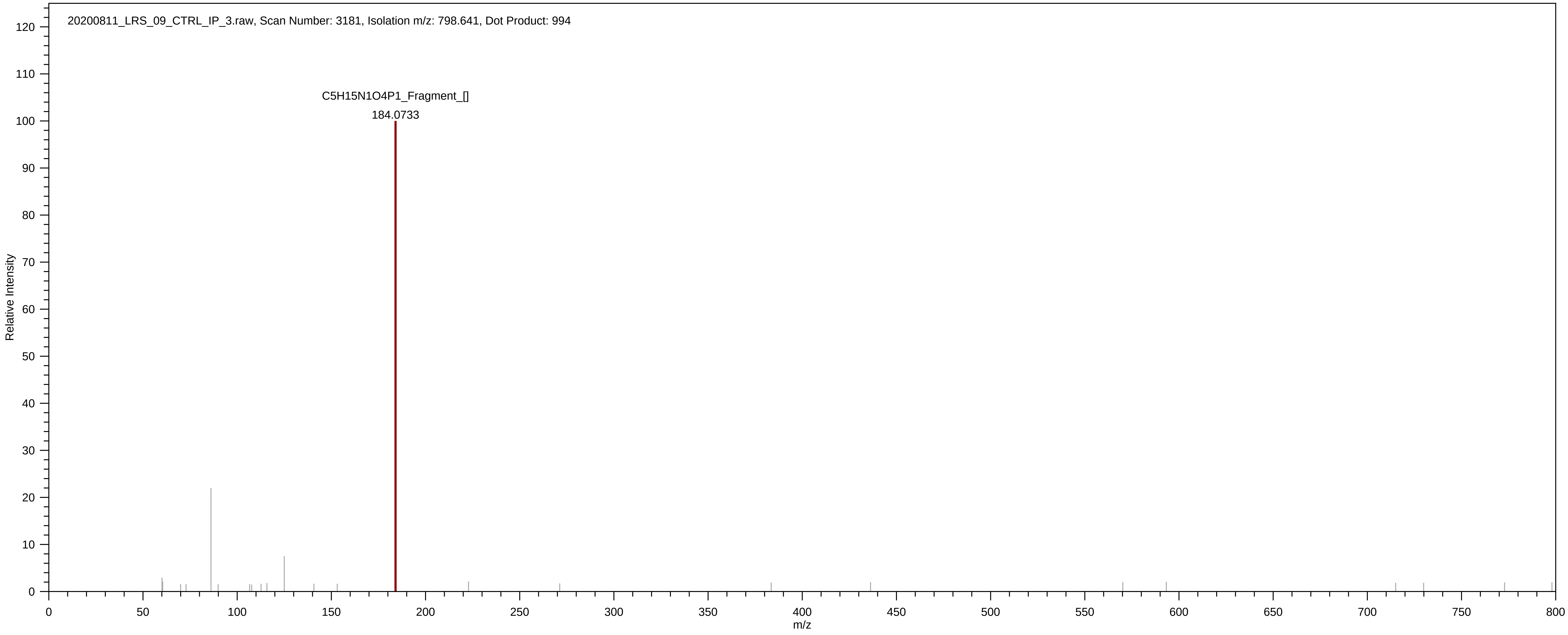

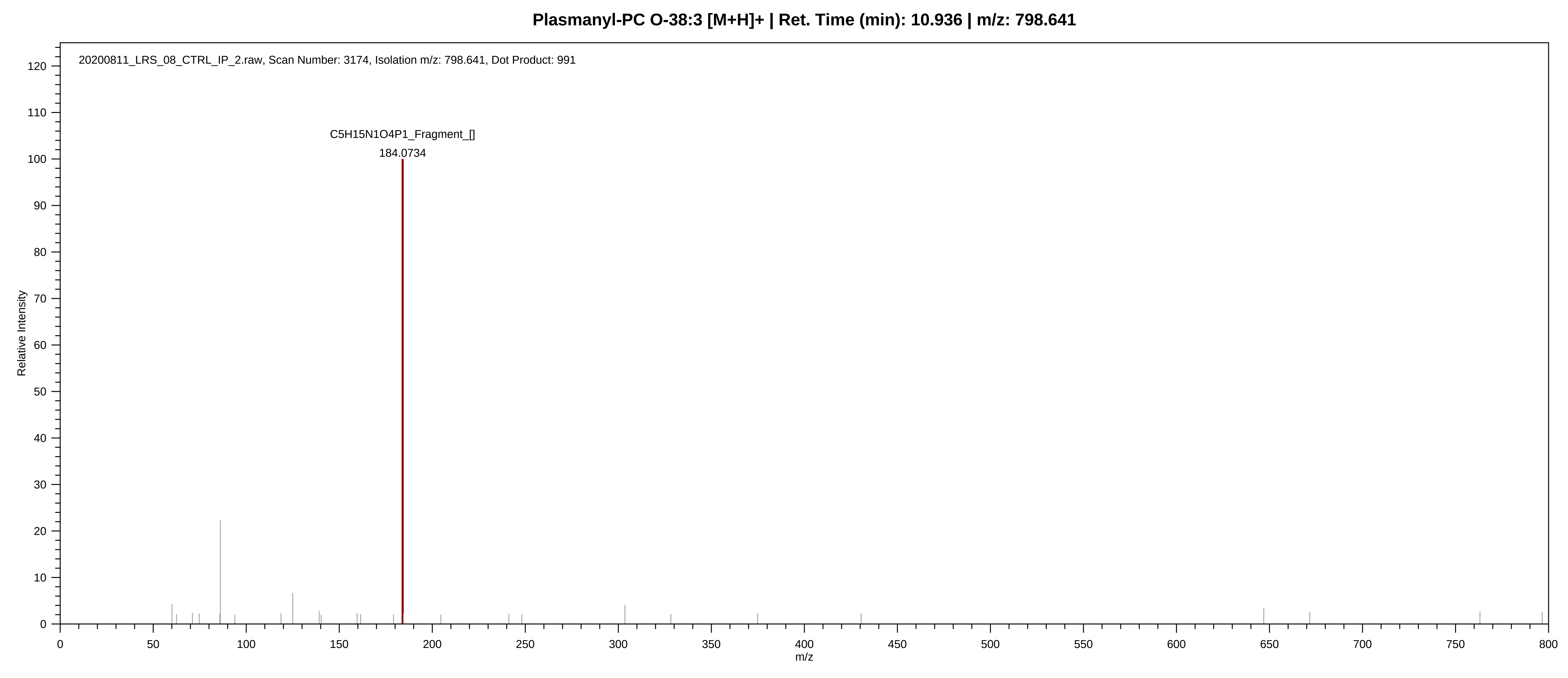
Plasmanyl-PC O-38:3 [M+H]+ | Ret. Time (min): 10.936 | m/z: 798.641
20200811_LRS_08_CTRL_IP_2.raw, Scan Number: 3174, Isolation m/z: 798.641, Dot Product: 991
C5H15N1O4P1_Fragment_[]
184.0734
Relative Intensity
m/z

Plasmanyl-PC O-38:5 [M+H]+ | Ret. Time (min): 9.537 | m/z: 794.606

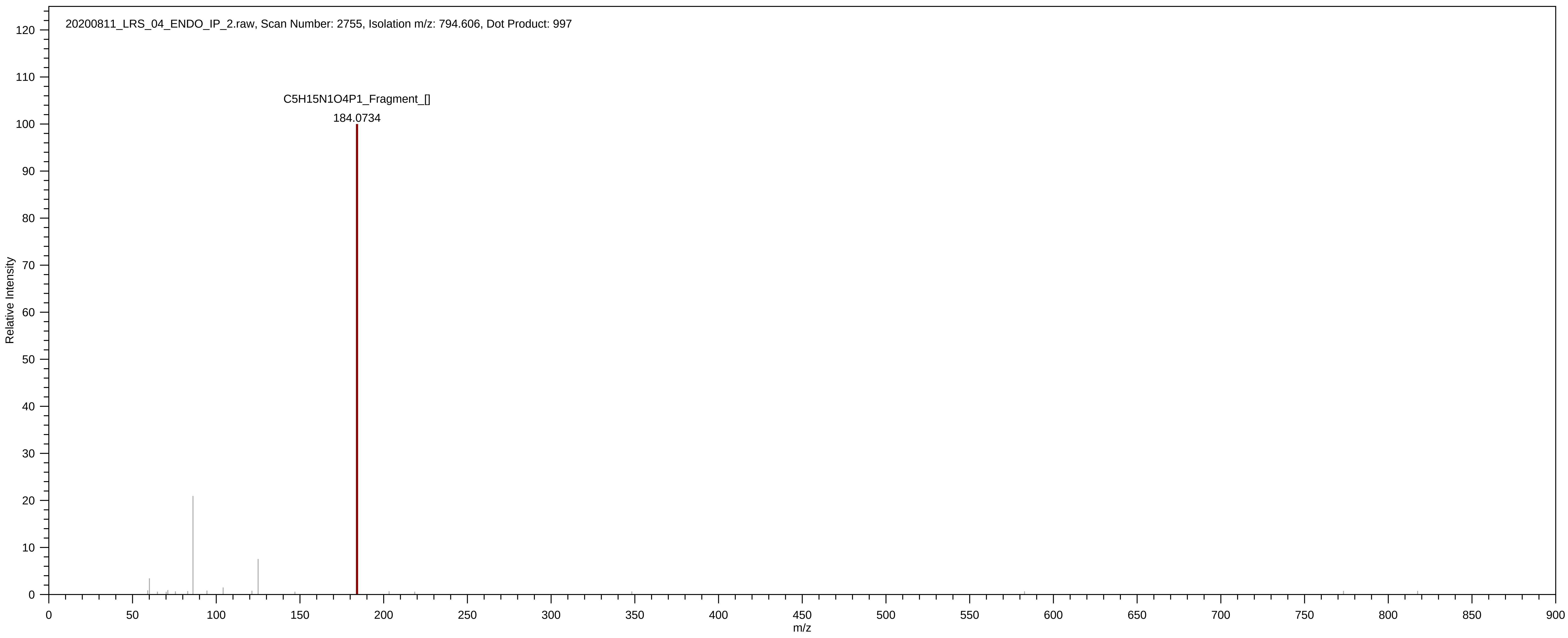

Plasmanyl-PC O-38:5 [M+H]+ | Ret. Time (min): 9.542 | m/z: 794.606

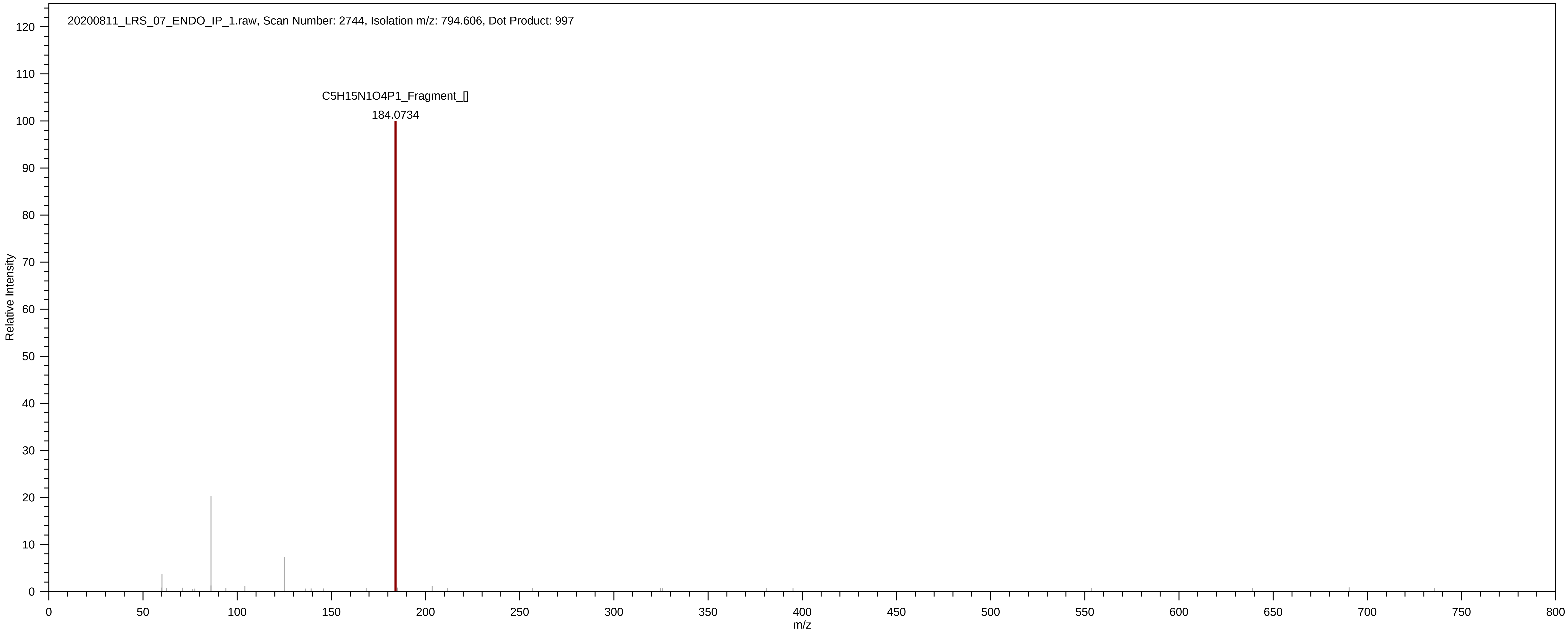

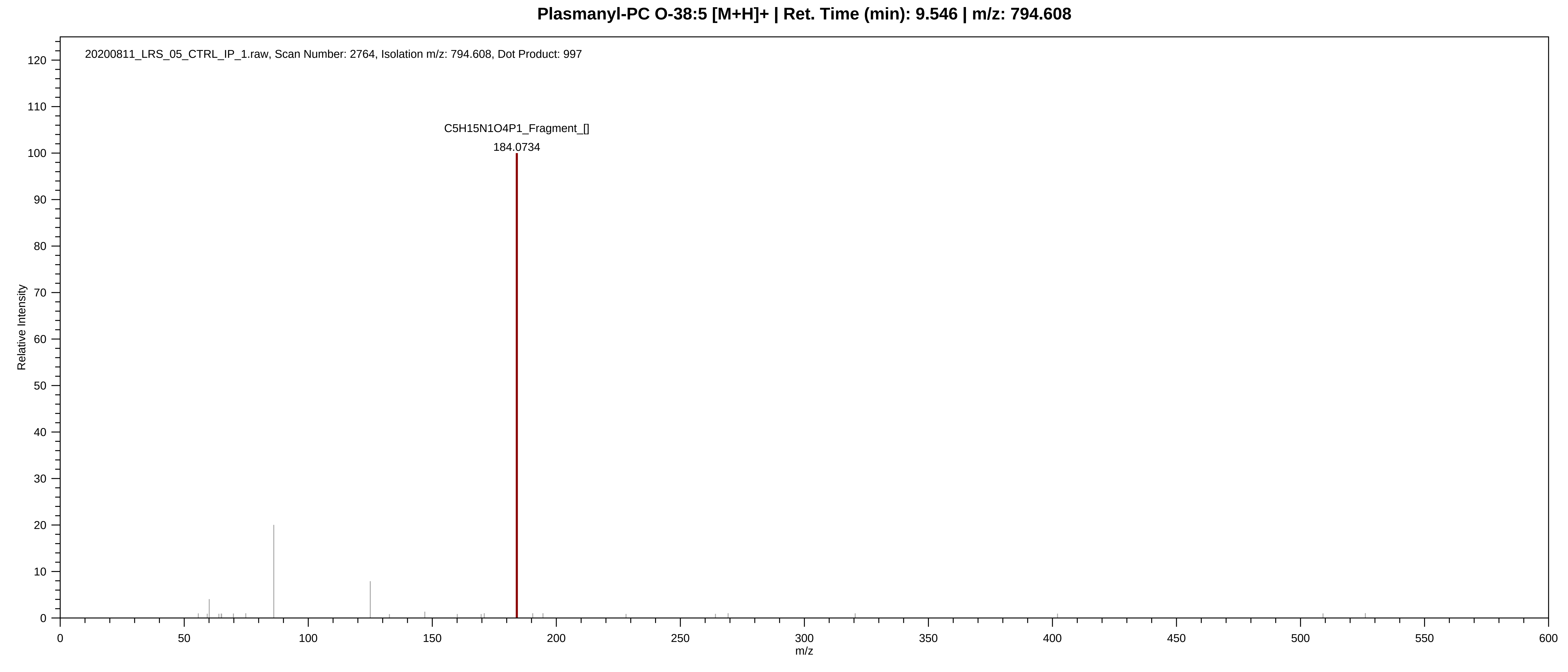

Plasmanyl-PC O-38:5 [M+H]+ | Ret. Time (min): 9.546 | m/z: 794.608
20200811_LRS_05_CTRL_IP_1.raw, Scan Number: 2764, Isolation m/z: 794.608, Dot Product: 997
C5H15N1O4P1_Fragment_[]
184.0734
Relative Intensity
m/z

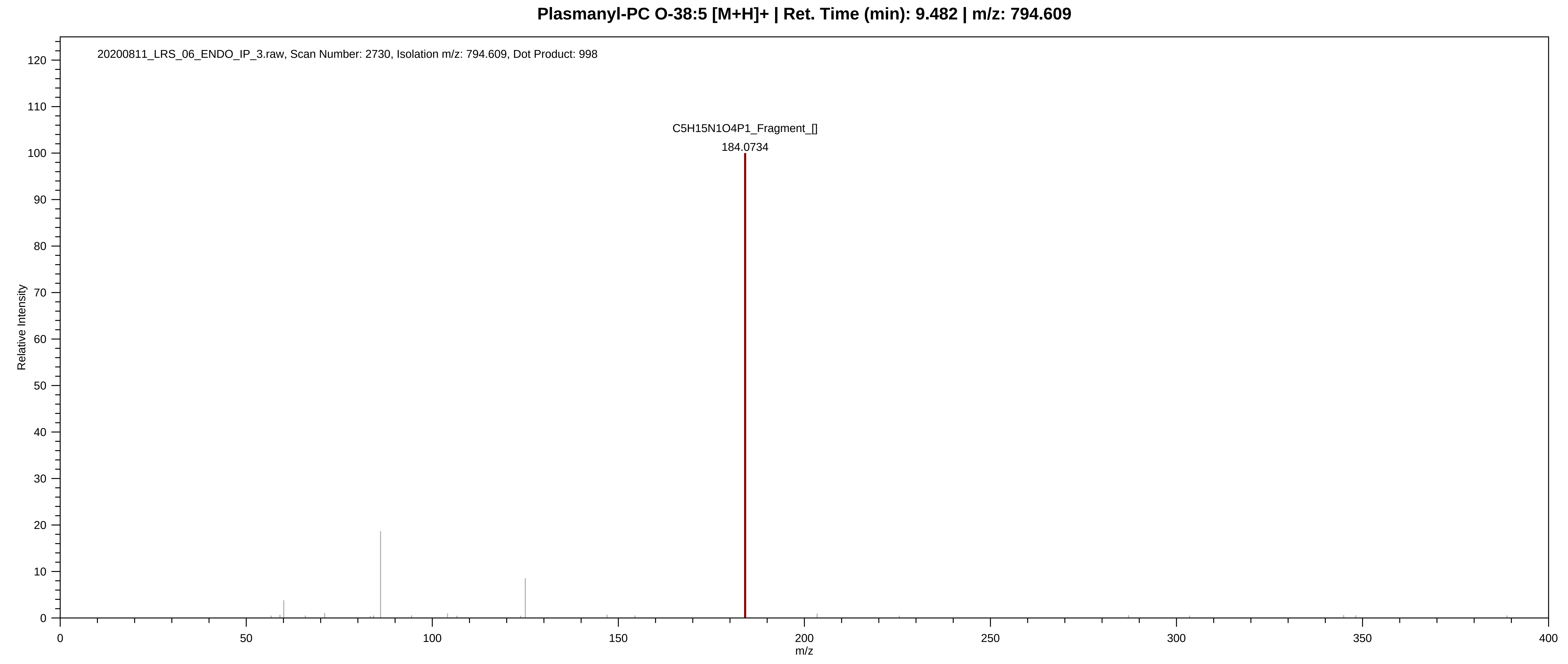
Plasmanyl-PC O-38:5 [M+H]+ | Ret. Time (min): 9.482 | m/z: 794.609
20200811_LRS_06_ENDO_IP_3.raw, Scan Number: 2730, Isolation m/z: 794.609, Dot Product: 998
C5H15N1O4P1_Fragment_[]
184.0734
Relative Intensity
m/z
0
10
20
30
40
50
60
70
80
90
100
110
120
0
50
100
150
200
250
300
350
400

Plasmanyl-PC O-38:6 [M+H]+ | Ret. Time (min): 9.357 | m/z: 792.59

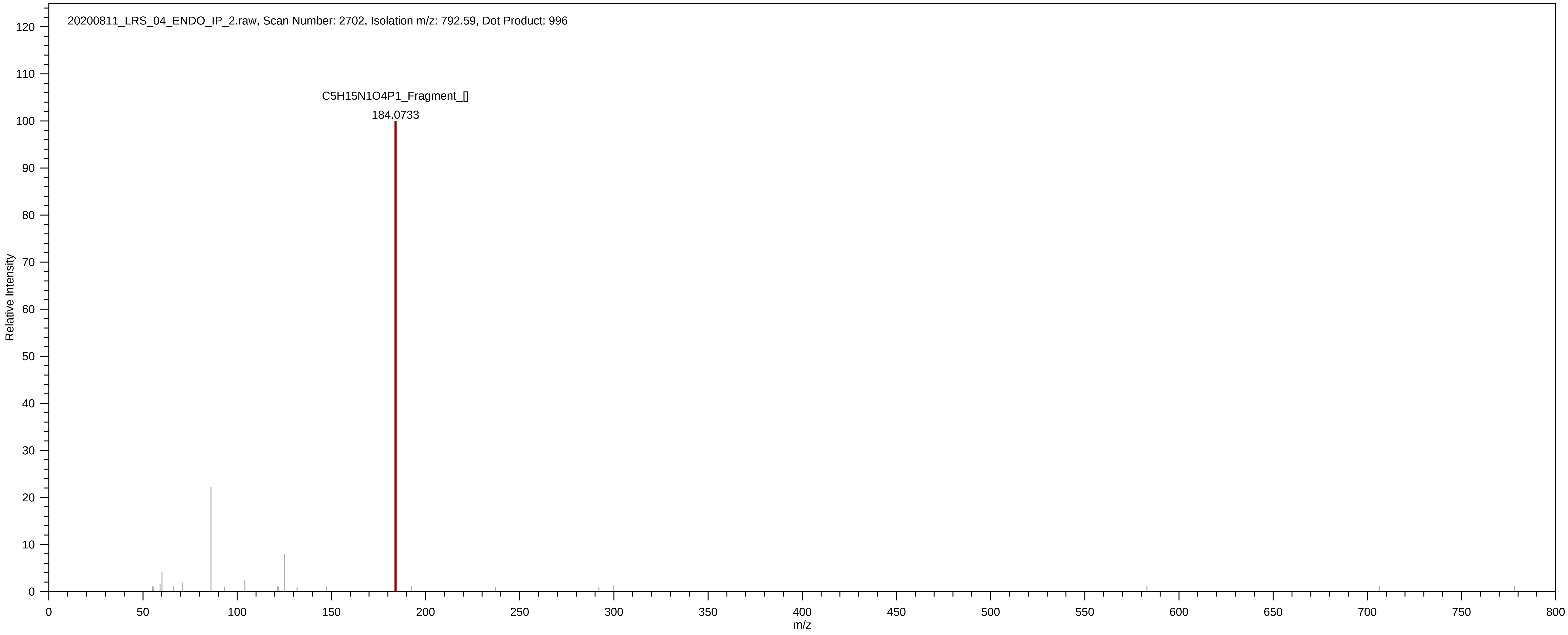

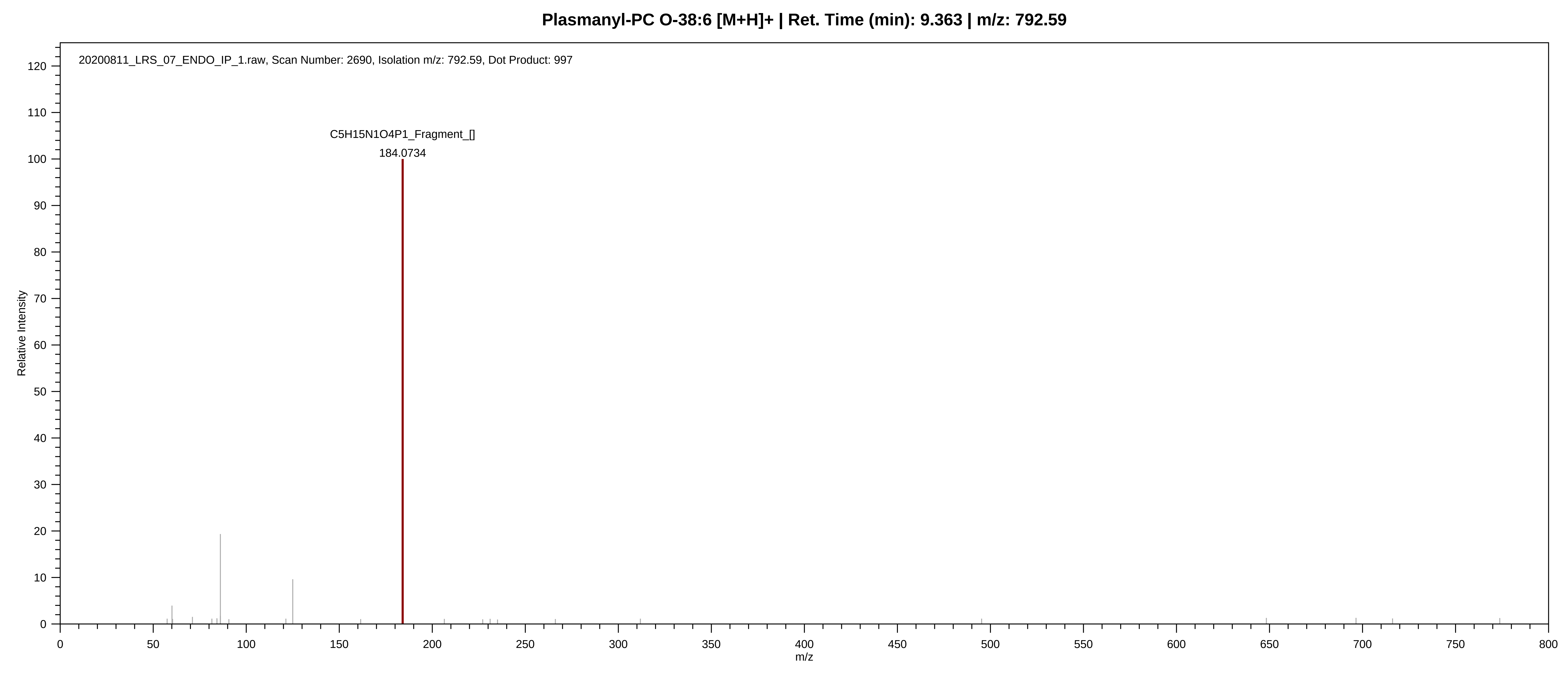

Plasmanyl-PC O-38:6 [M+H]+ | Ret. Time (min): 9.363 | m/z: 792.59
20200811_LRS_07_ENDO_IP_1.raw, Scan Number: 2690, Isolation m/z: 792.59, Dot Product: 997
C5H15N1O4P1_Fragment_[]
184.0734
Relative Intensity
m/z

Plasmanyl-PC O-38:6 [M+H]+ | Ret. Time (min): 9.18 | m/z: 792.593

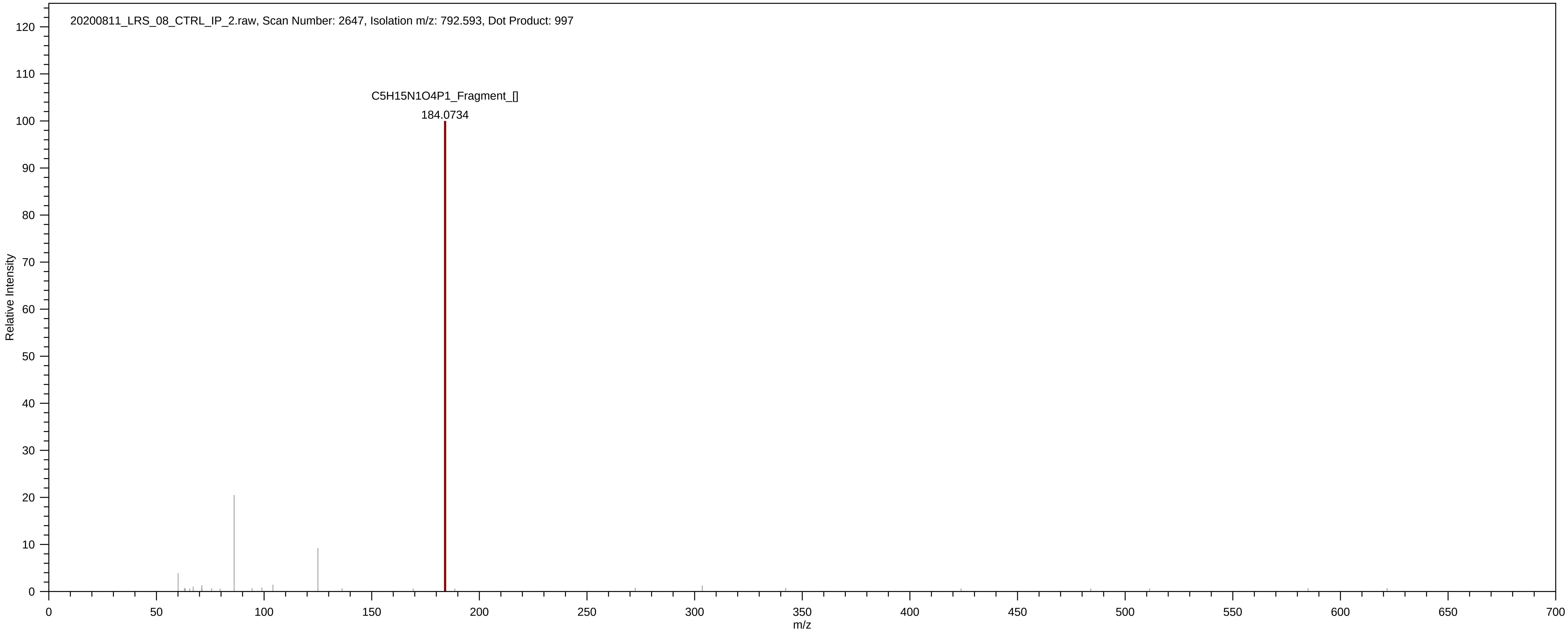

Plasmanyl-PC O-38:6 [M+H]+ | Ret. Time (min): 9.18 | m/z: 792.592

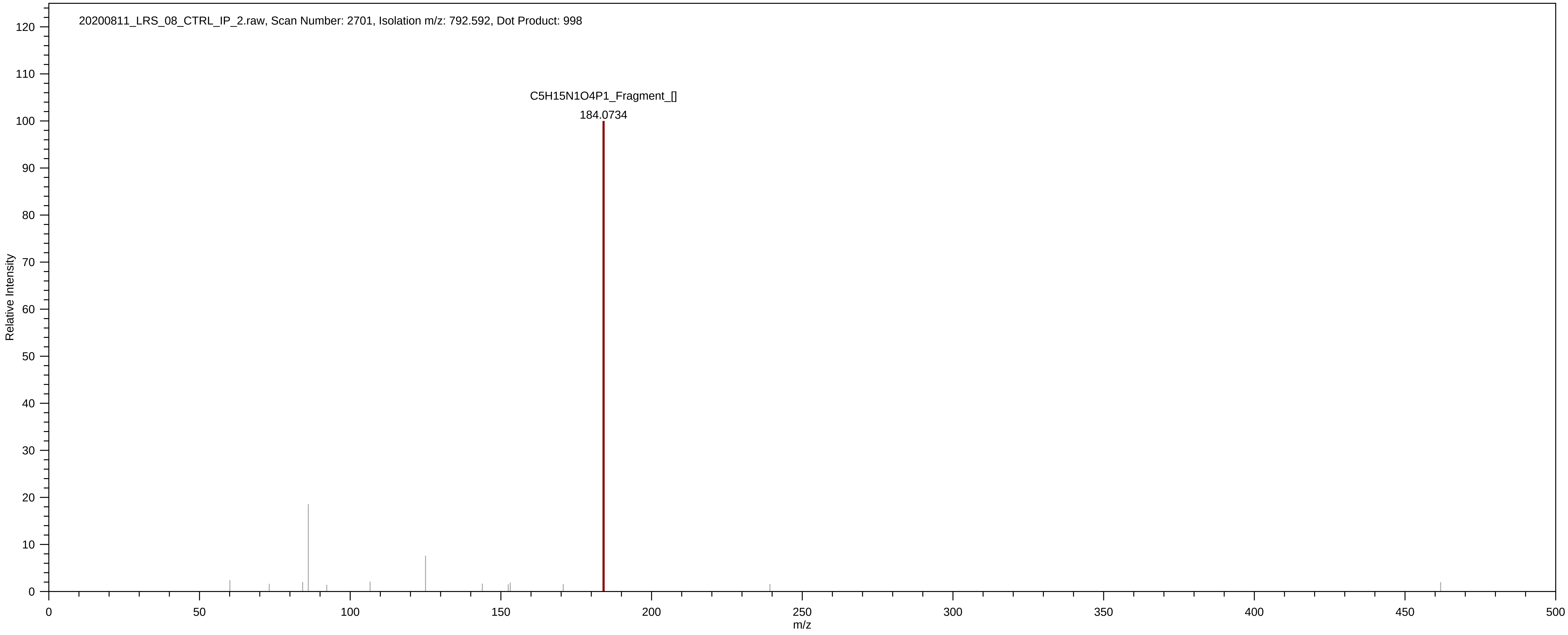

Plasmanyl-PC O-38:6 [M+H]+ | Ret. Time (min): 9.21 | m/z: 792.593

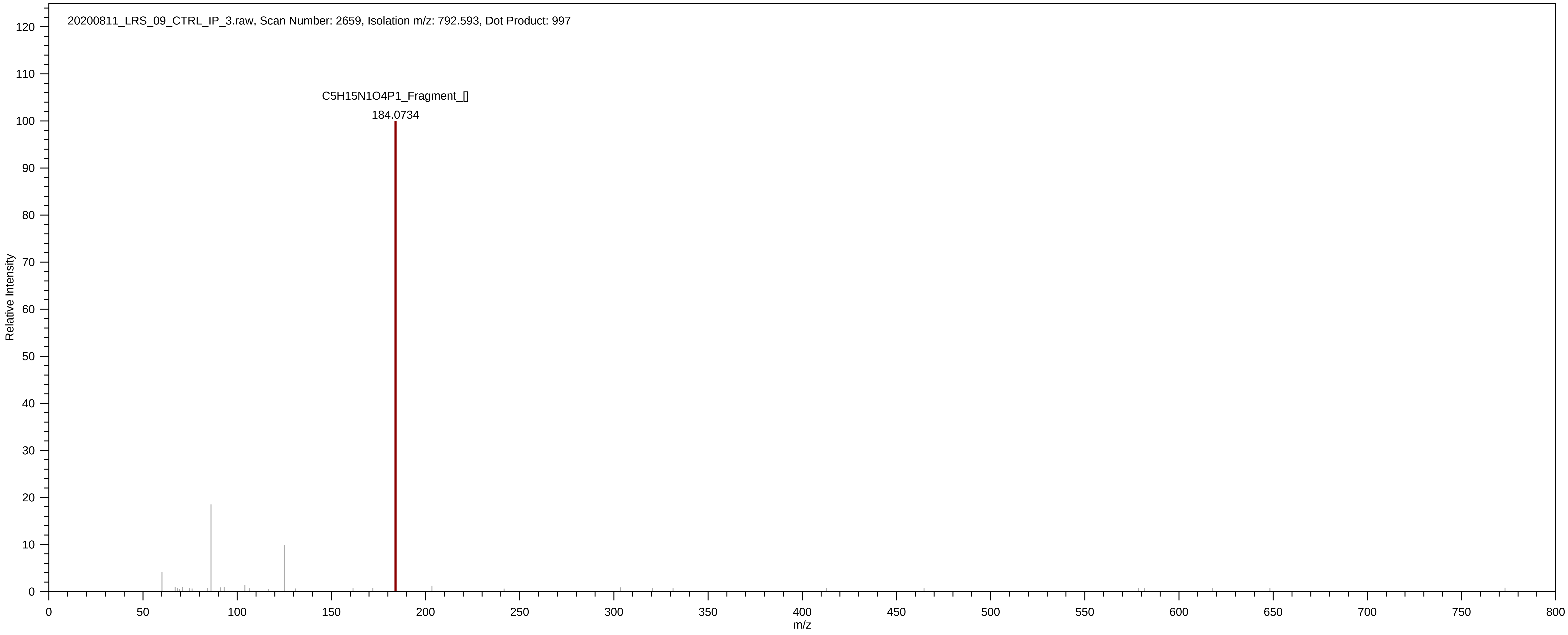

Plasmanyl-PC O-38:6 [M+H]+ | Ret. Time (min): 9.182 | m/z: 792.593

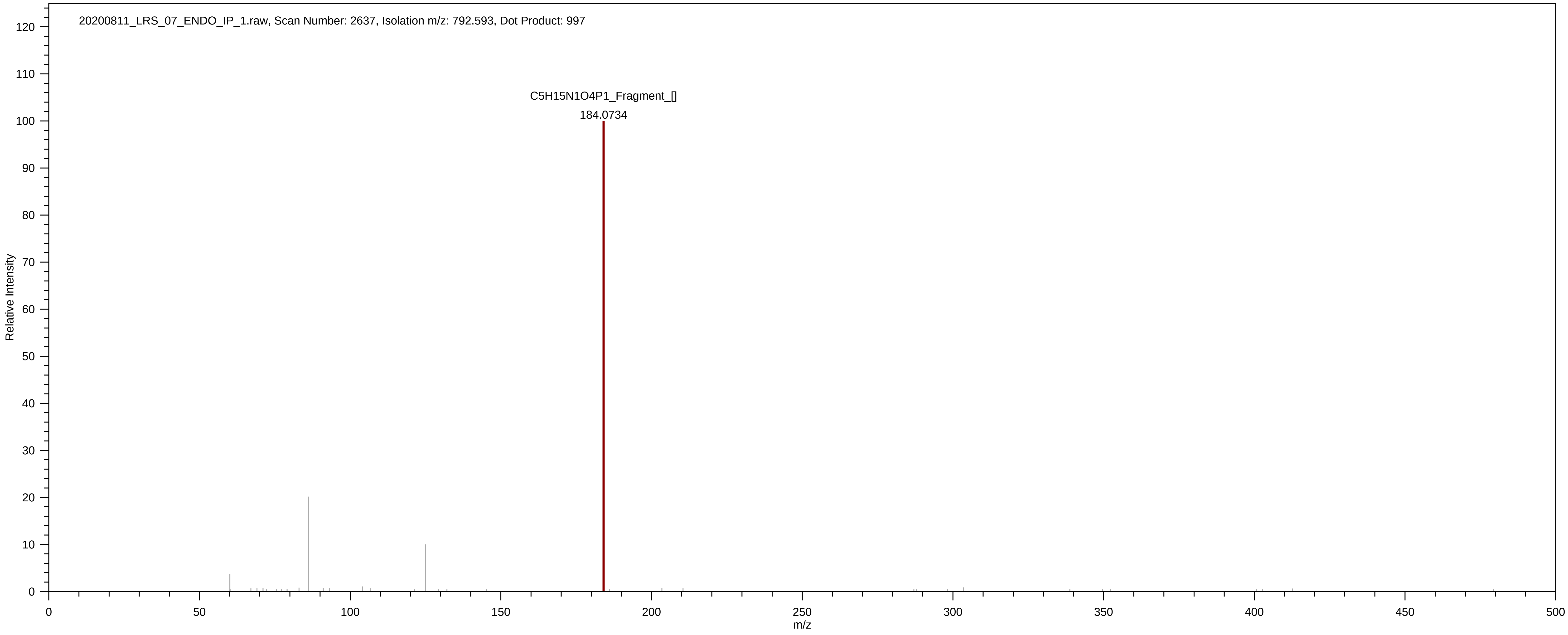

Plasmanyl-PC O-38:6 [M+H]+ | Ret. Time (min): 9.205 | m/z: 792.594

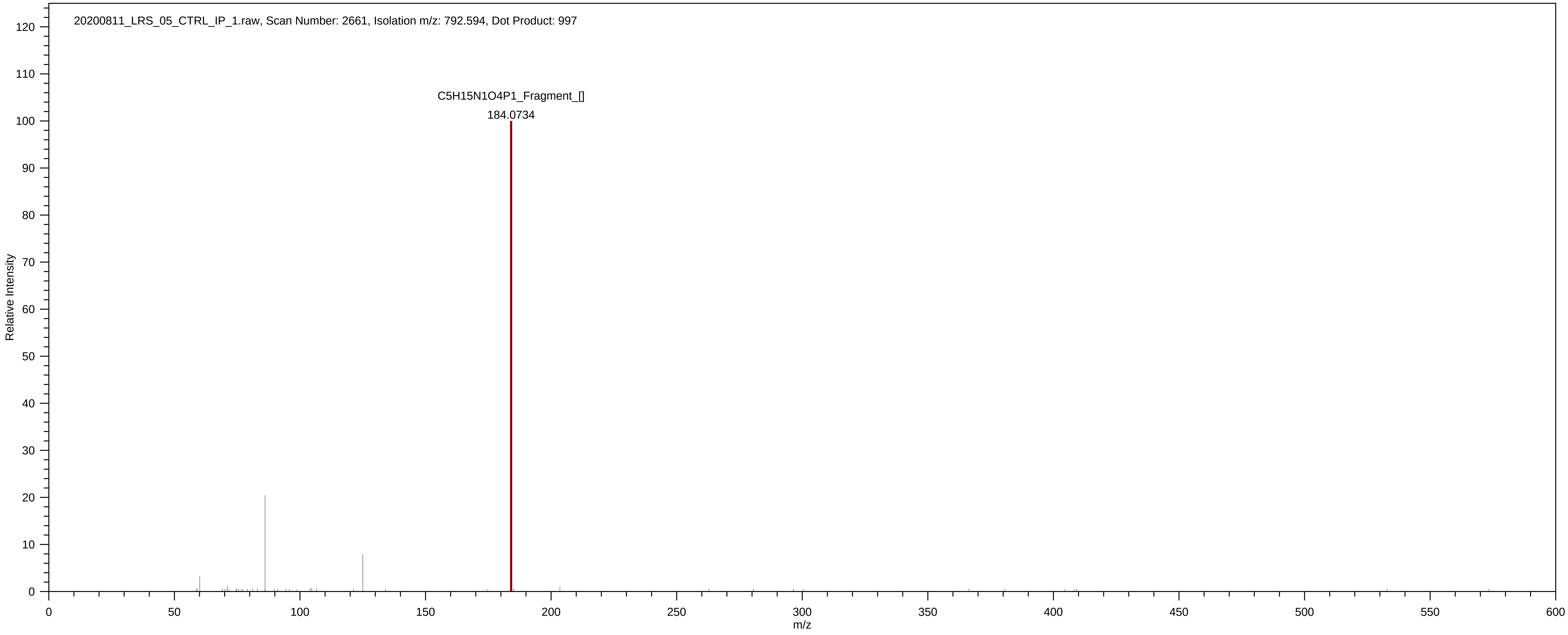

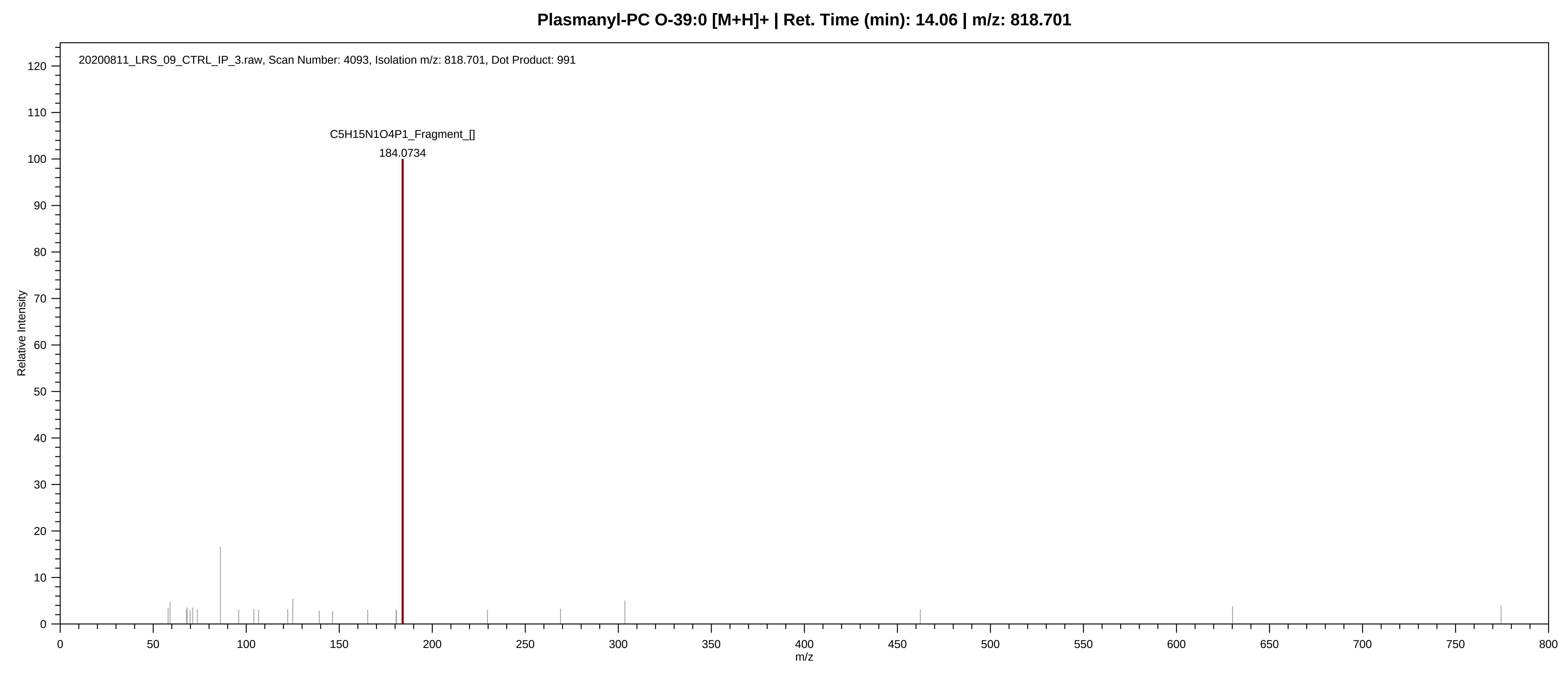

Plasmanyl-PC O-39:0 [M+H]+ | Ret. Time (min): 14.06 | m/z: 818.701
20200811_LRS_09_CTRL_IP_3.raw, Scan Number: 4093, Isolation m/z: 818.701, Dot Product: 991
C5H15N1O4P1_Fragment_[]
184.0734
Relative Intensity
m/z

Plasmanyl-PC O-39:0 [M+H]+ | Ret. Time (min): 14.091 | m/z: 818.701

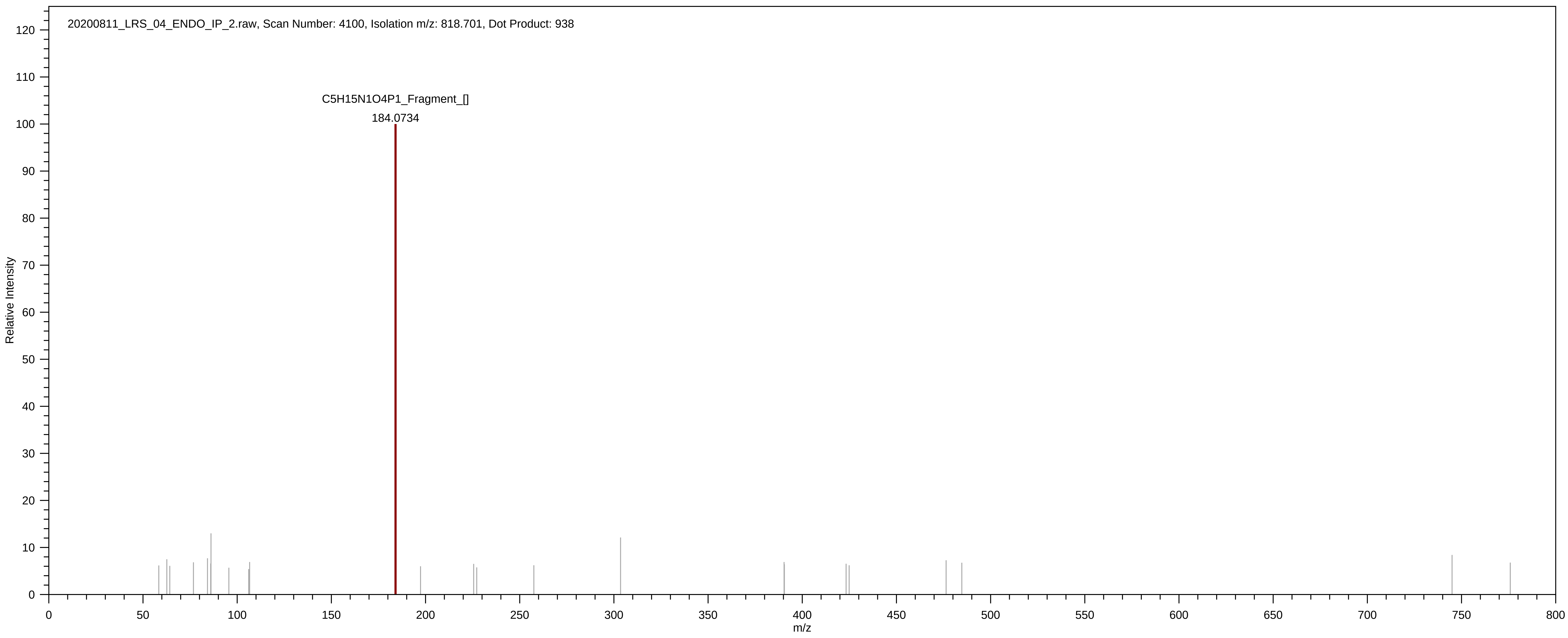

Plasmanyl-PC O-39:0 [M+H]+ | Ret. Time (min): 13.854 | m/z: 818.702

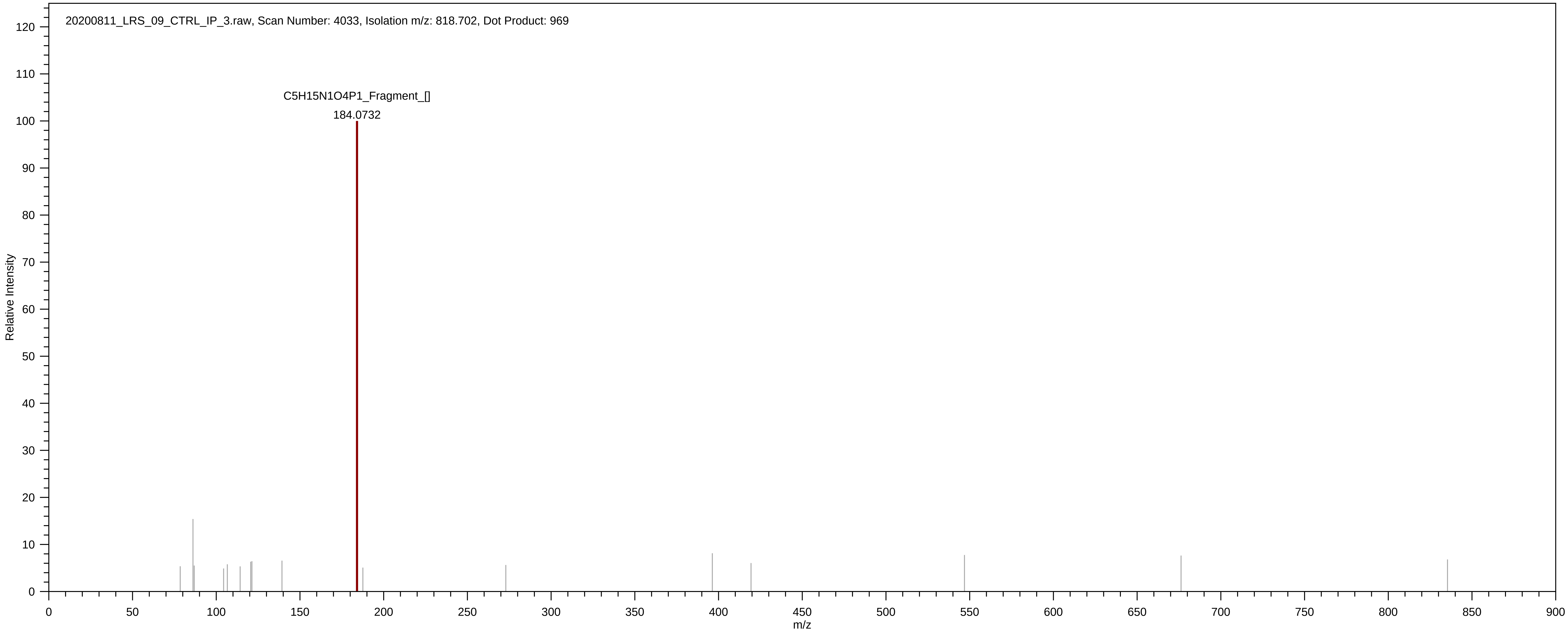

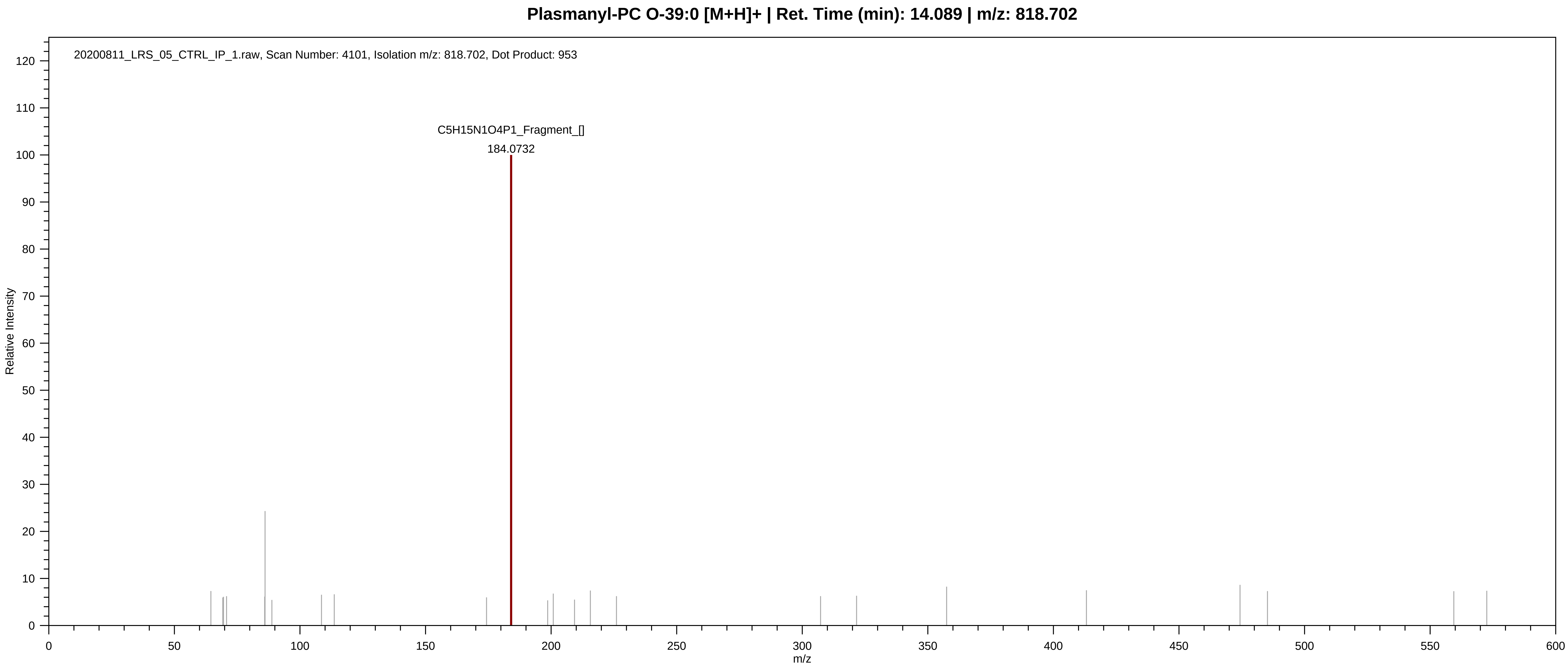

Plasmanyl-PC O-39:0 [M+H]+ | Ret. Time (min): 14.089 | m/z: 818.702
20200811_LRS_05_CTRL_IP_1.raw, Scan Number: 4101, Isolation m/z: 818.702, Dot Product: 953
C5H15N1O4P1_Fragment_[]
184.0732
Relative Intensity
m/z
0
10
20
30
40
50
60
70
80
90
100
110
120
0
50
100
150
200
250
300
350
400
450
500
550
600

Plasmanyl-PC O-40:0 [M+H]+ | Ret. Time (min): 14.52 | m/z: 832.714

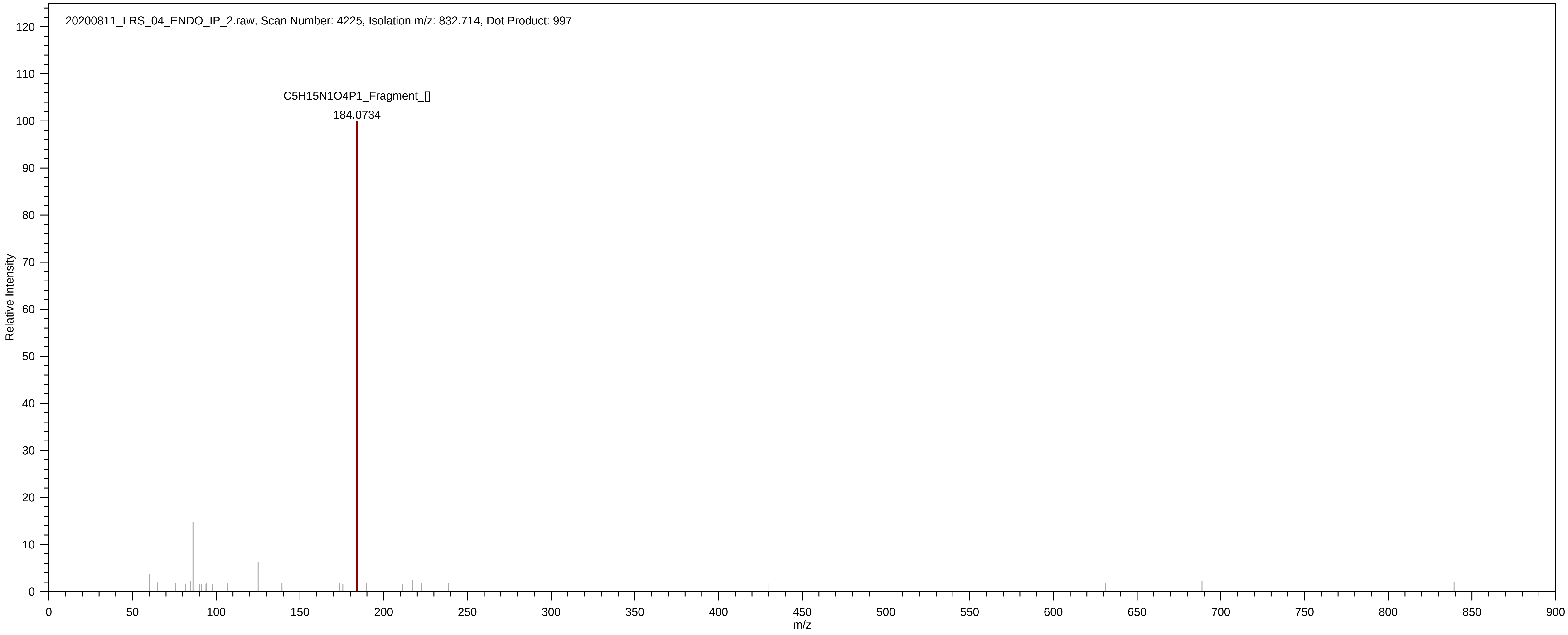

Plasmanyl-PC O-40:0 [M+H]+ | Ret. Time (min): 14.524 | m/z: 832.714

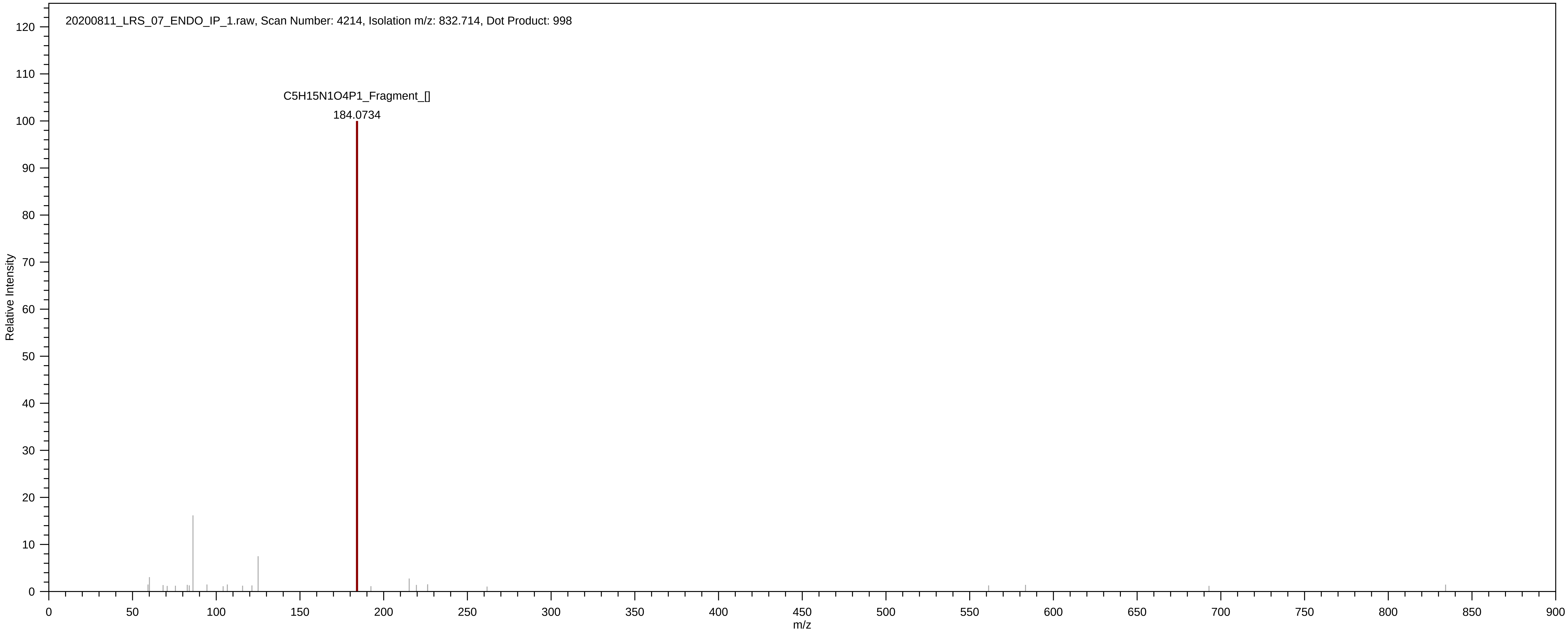

Plasmanyl-PC O-40:0 [M+H]+ | Ret. Time (min): 14.704 | m/z: 832.714

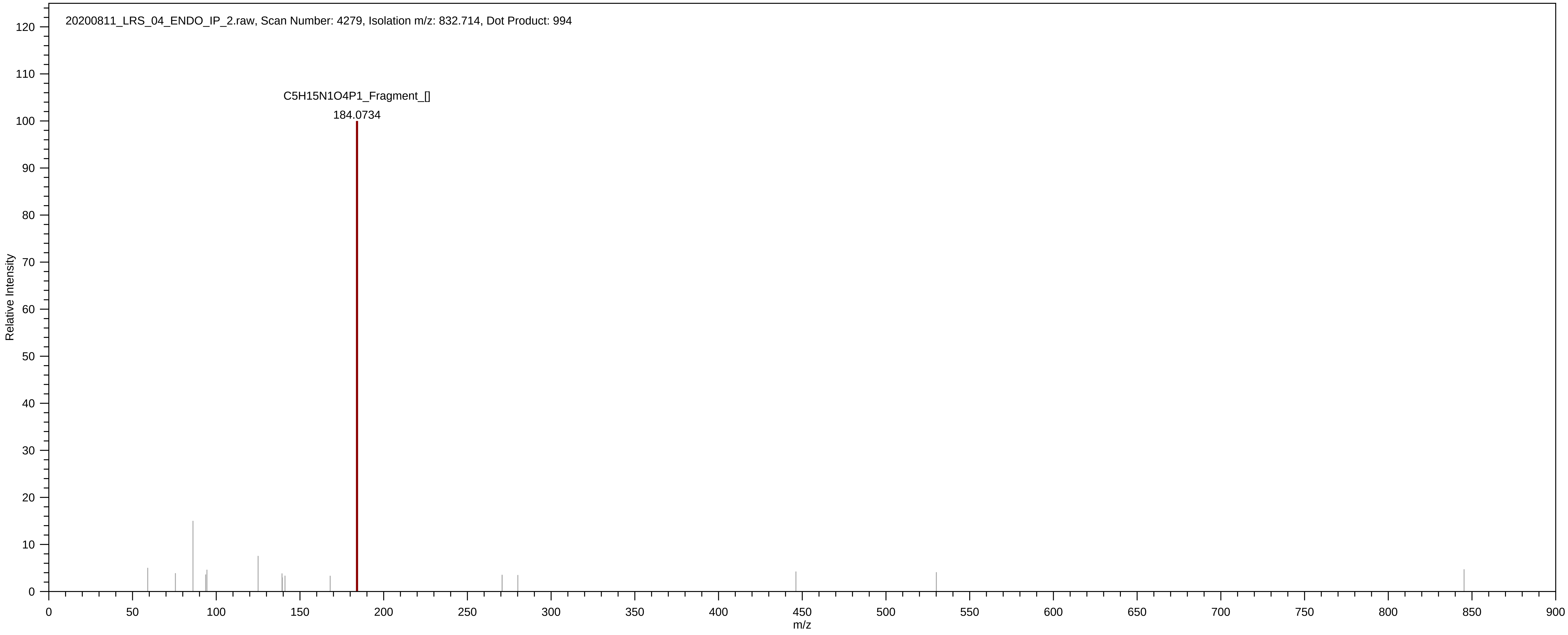

Plasmanyl-PC O-40:0 [M+H]+ | Ret. Time (min): 14.693 | m/z: 832.715

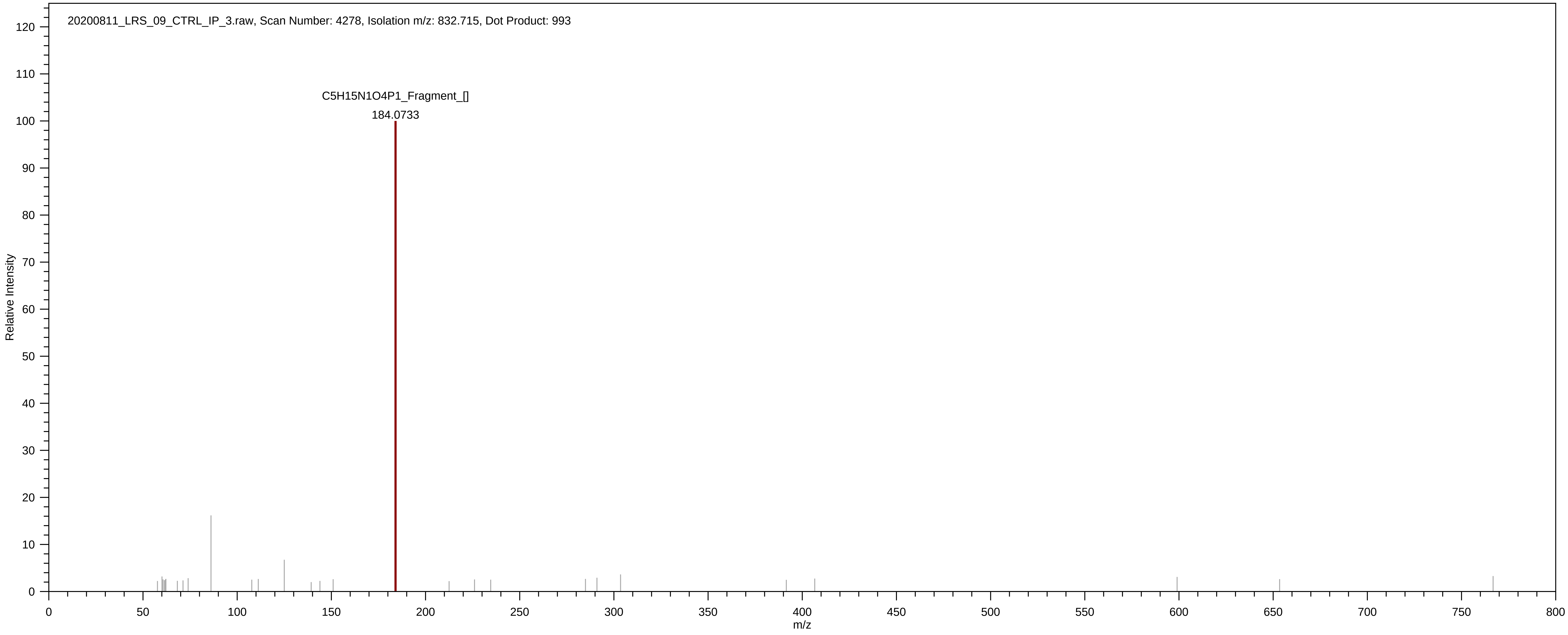

Plasmanyl-PC O-40:0 [M+H]+ | Ret. Time (min): 14.71 | m/z: 832.716

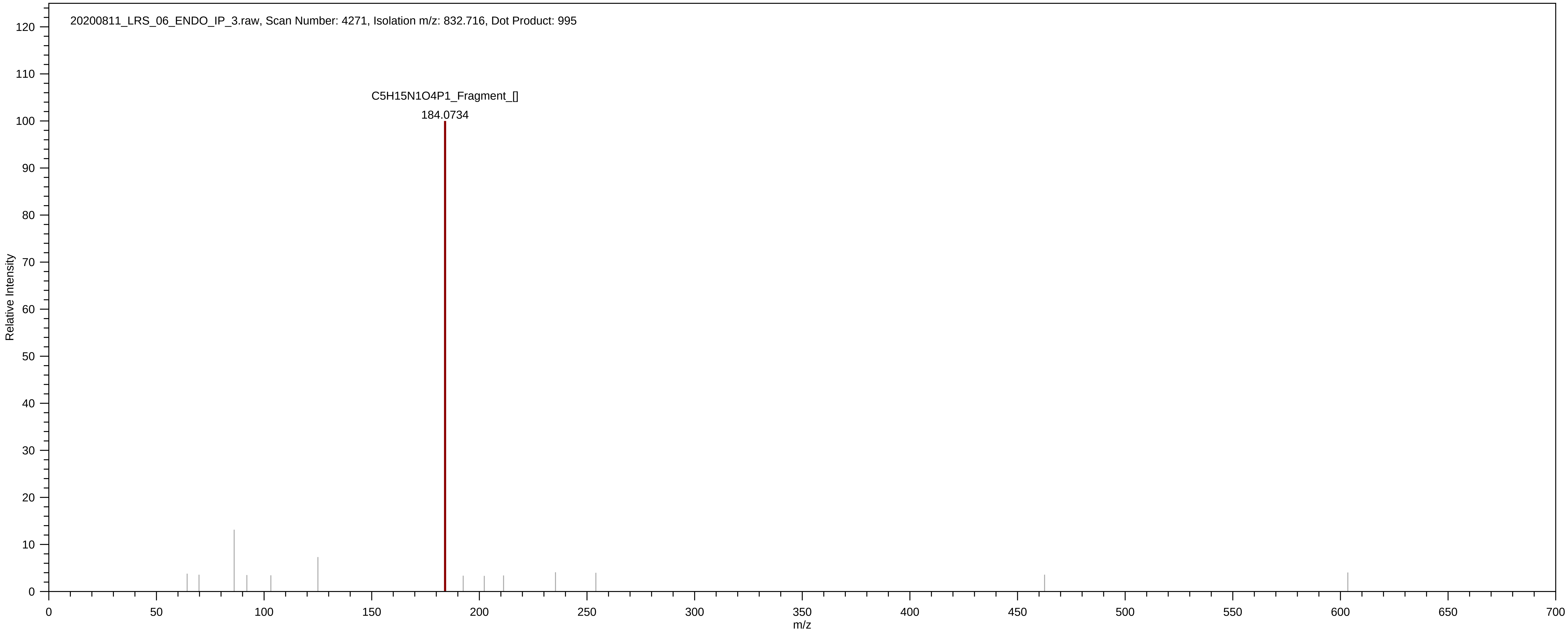

Plasmanyl-PC O-40:0 [M+H]+ | Ret. Time (min): 14.524 | m/z: 832.716

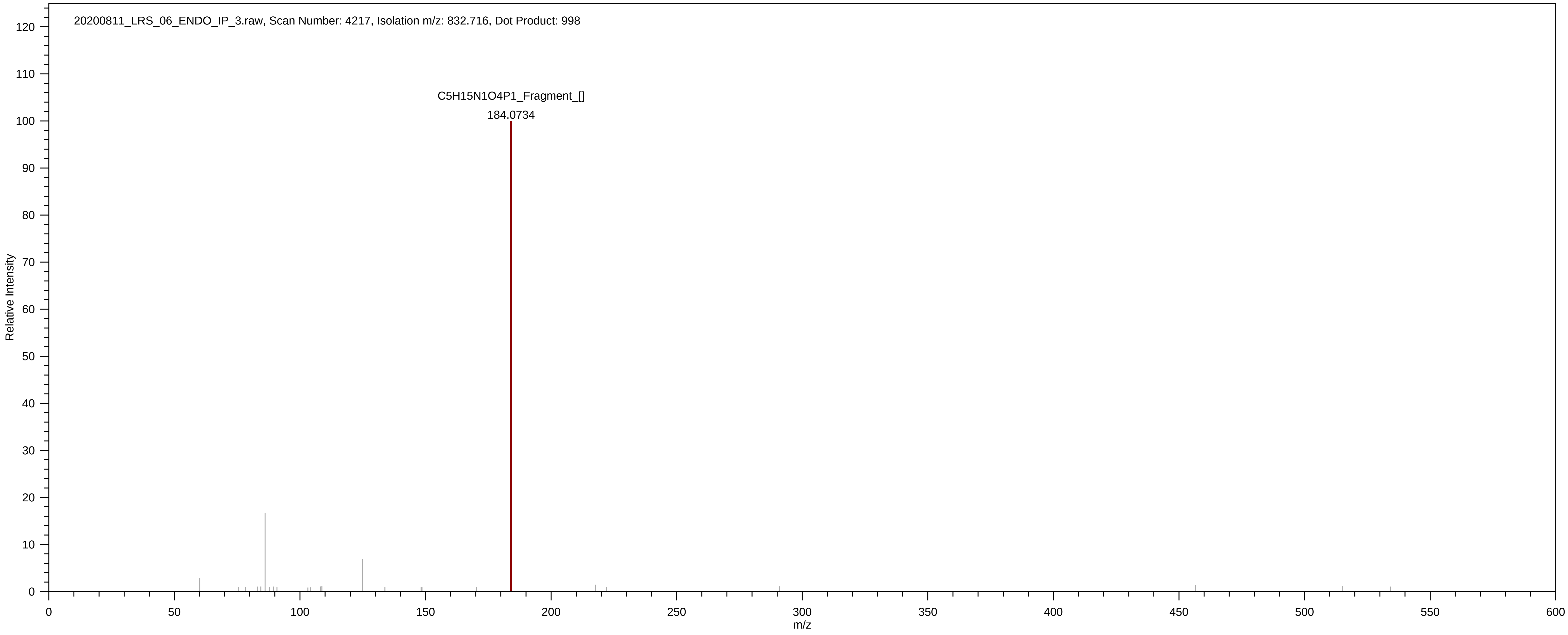

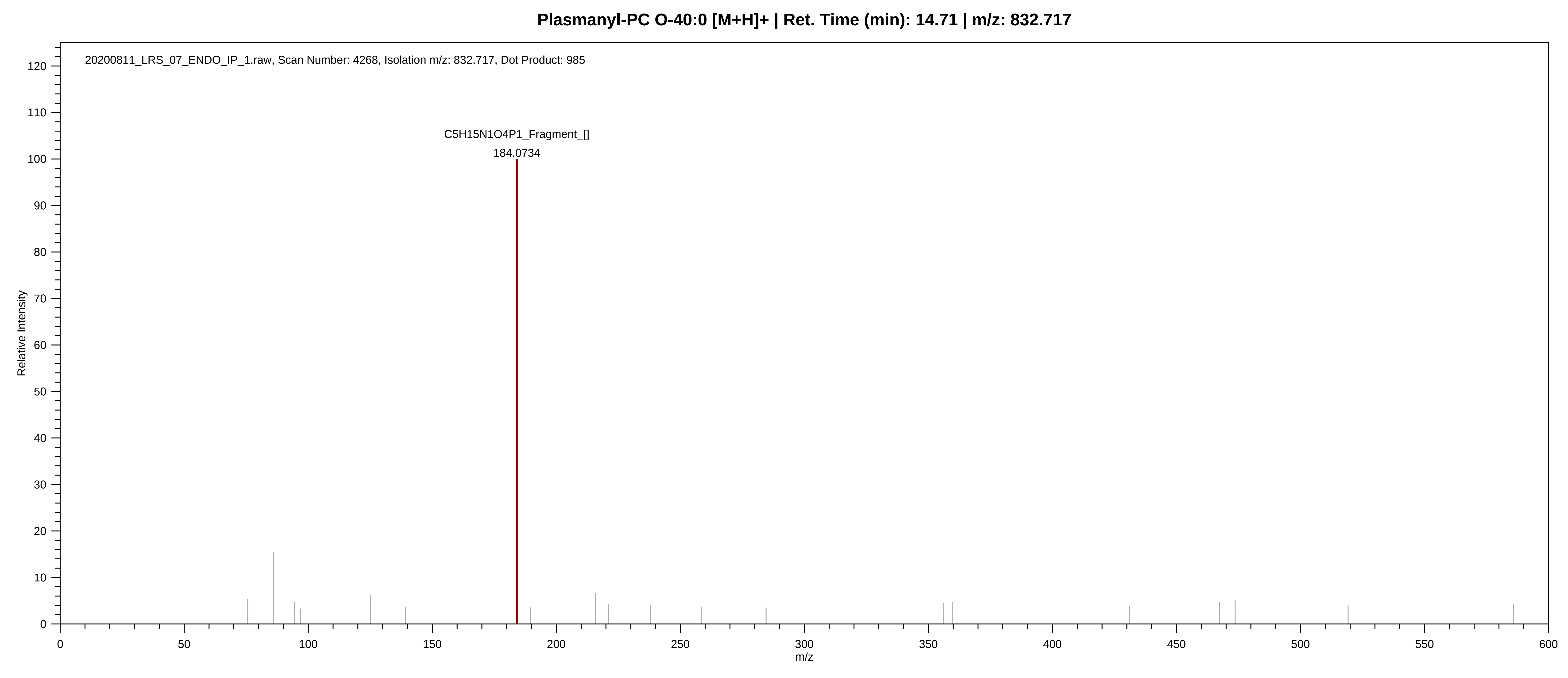

Plasmanyl-PC O-40:0 [M+H]+ | Ret. Time (min): 14.71 | m/z: 832.717
20200811_LRS_07_ENDO_IP_1.raw, Scan Number: 4268, Isolation m/z: 832.717, Dot Product: 985
C5H15N1O4P1_Fragment_[]
184.0734
Relative Intensity
m/z

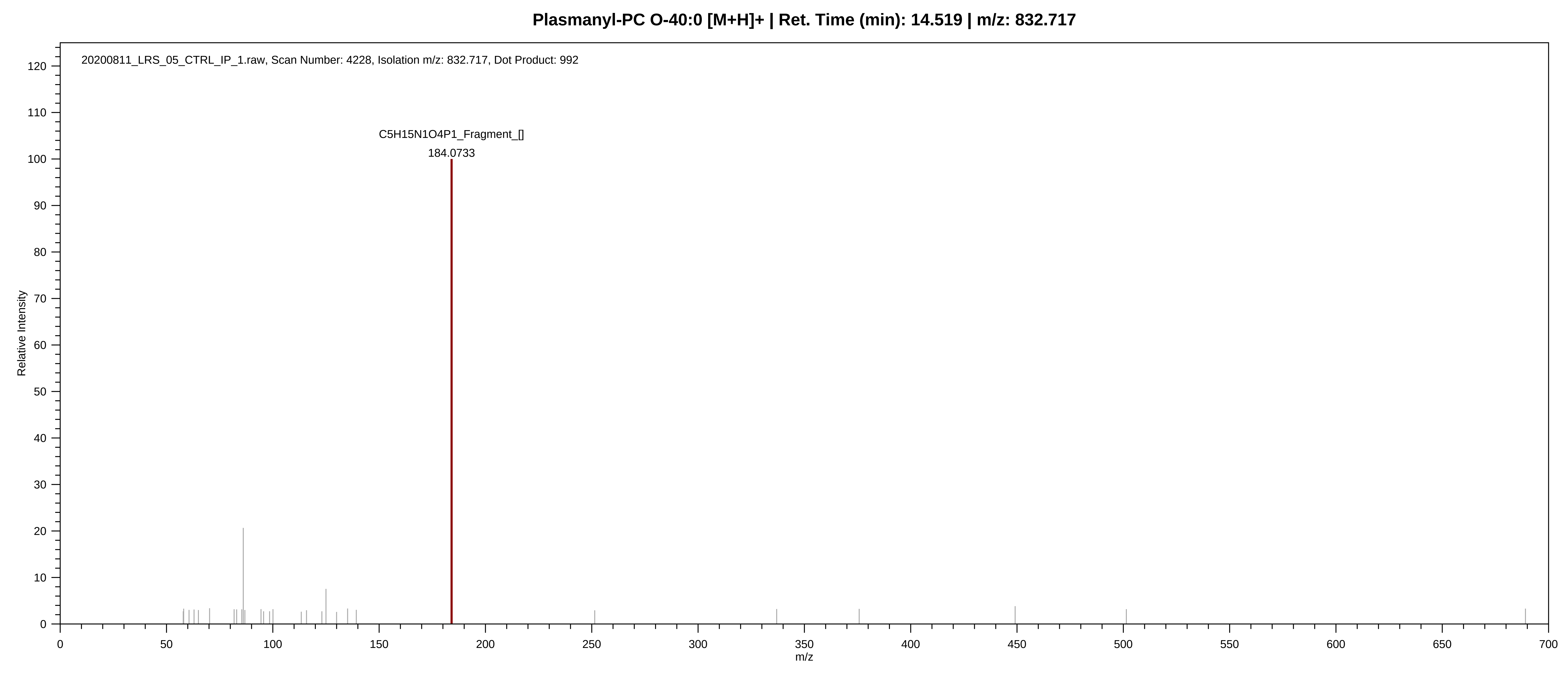
Plasmanyl-PC O-40:0 [M+H]+ | Ret. Time (min): 14.519 | m/z: 832.717
20200811_LRS_05_CTRL_IP_1.raw, Scan Number: 4228, Isolation m/z: 832.717, Dot Product: 992
C5H15N1O4P1_Fragment_[]
184.0733
Relative Intensity
m/z

Plasmanyl-PC O-40:0 [M+H]+ | Ret. Time (min): 14.525 | m/z: 832.717

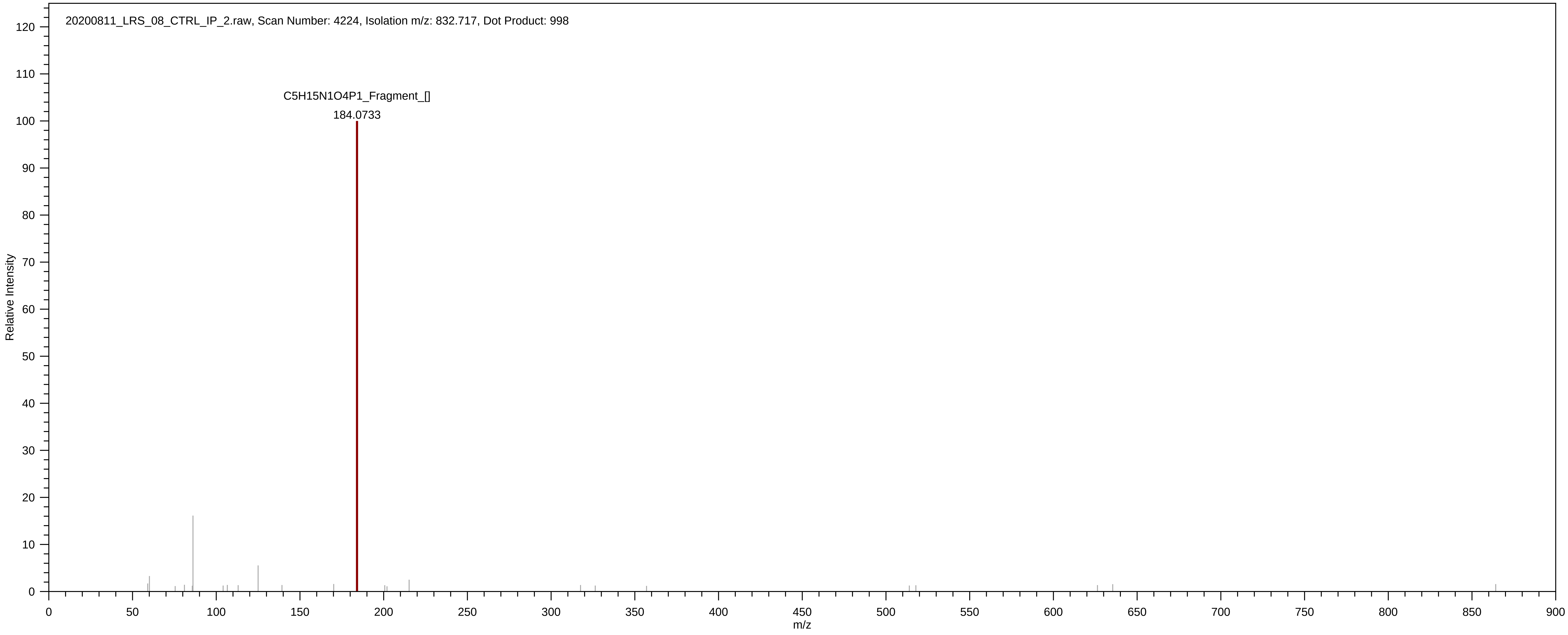

Plasmanyl-PC O-40:0 [M+H]+ | Ret. Time (min): 14.509 | m/z: 832.719

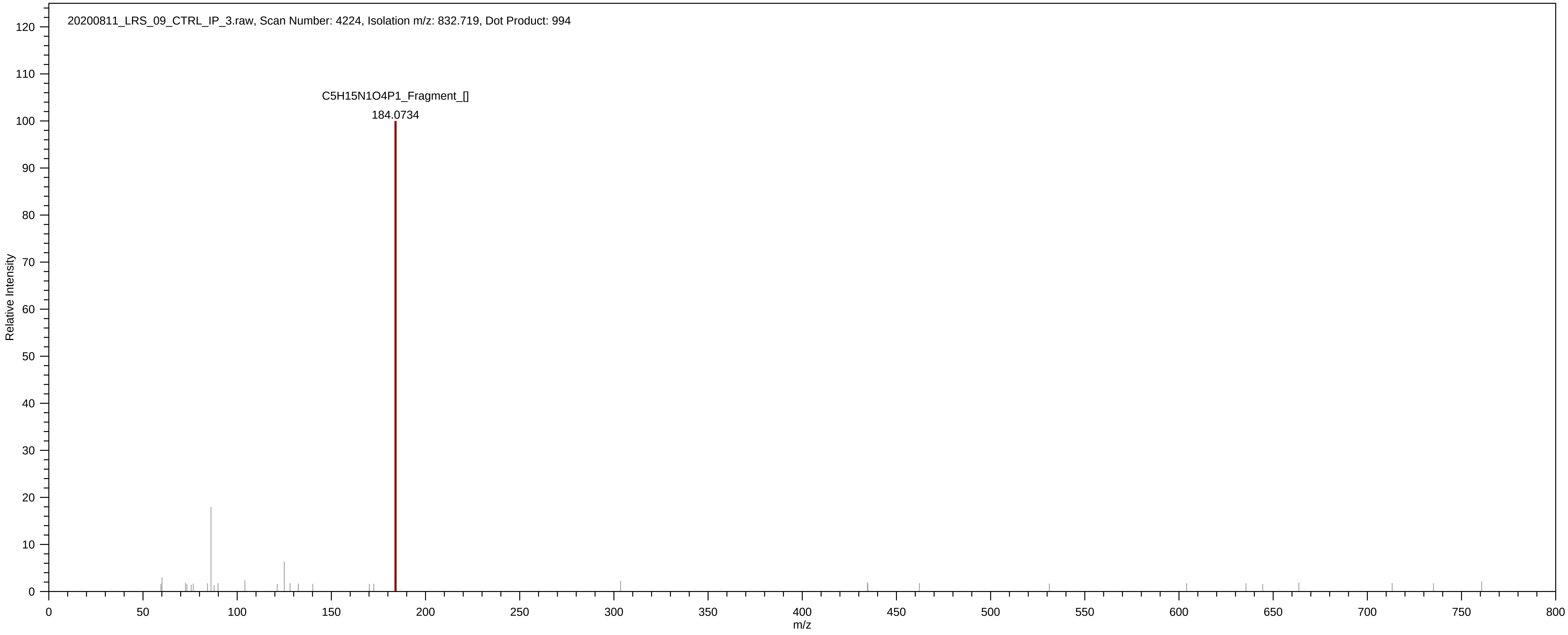

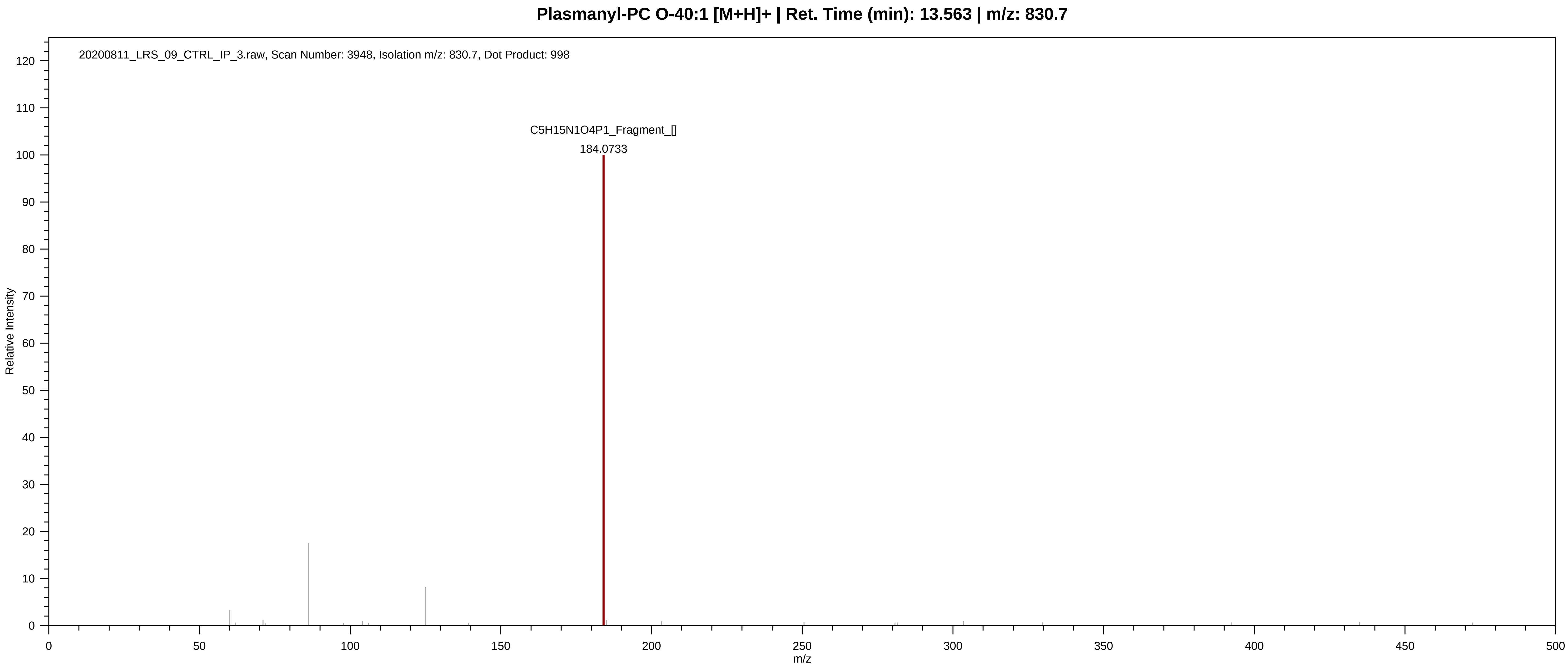

Plasmanyl-PC O-40:1 [M+H]+ | Ret. Time (min): 13.563 | m/z: 830.7
20200811_LRS_09_CTRL_IP_3.raw, Scan Number: 3948, Isolation m/z: 830.7, Dot Product: 998
C5H15N1O4P1_Fragment_[]
184.0733
Relative Intensity
m/z

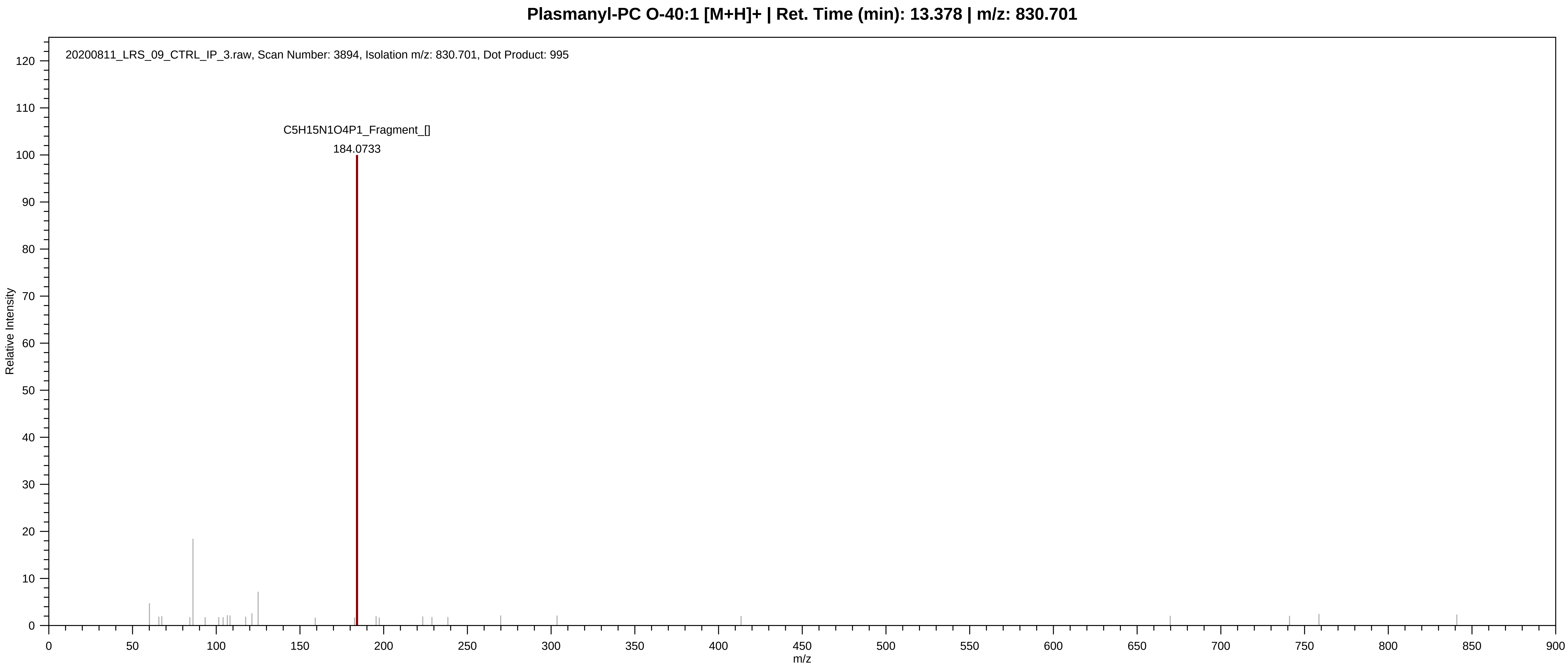

Plasmanyl-PC O-40:1 [M+H]+ | Ret. Time (min): 13.378 | m/z: 830.701
20200811_LRS_09_CTRL_IP_3.raw, Scan Number: 3894, Isolation m/z: 830.701, Dot Product: 995
C5H15N1O4P1_Fragment_[]
184.0733
Relative Intensity
m/z
0
10
20
30
40
50
60
70
80
90
100
110
120
0
50
100
150
200
250
300
350
400
450
500
550
600
650
700
750
800
850
900

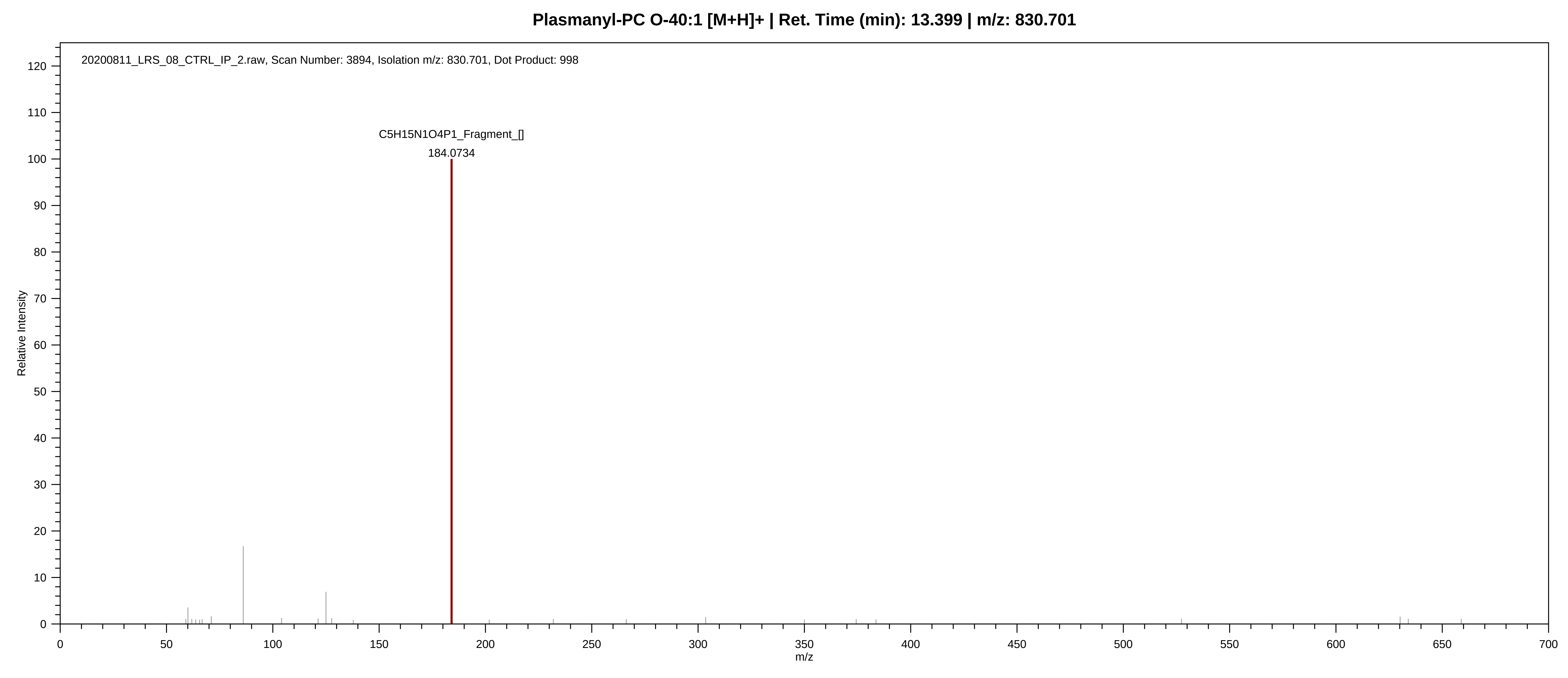

Plasmanyl-PC O-40:1 [M+H]+ | Ret. Time (min): 13.399 | m/z: 830.701
20200811_LRS_08_CTRL_IP_2.raw, Scan Number: 3894, Isolation m/z: 830.701, Dot Product: 998
C5H15N1O4P1_Fragment_[]
184.0734
Relative Intensity
m/z

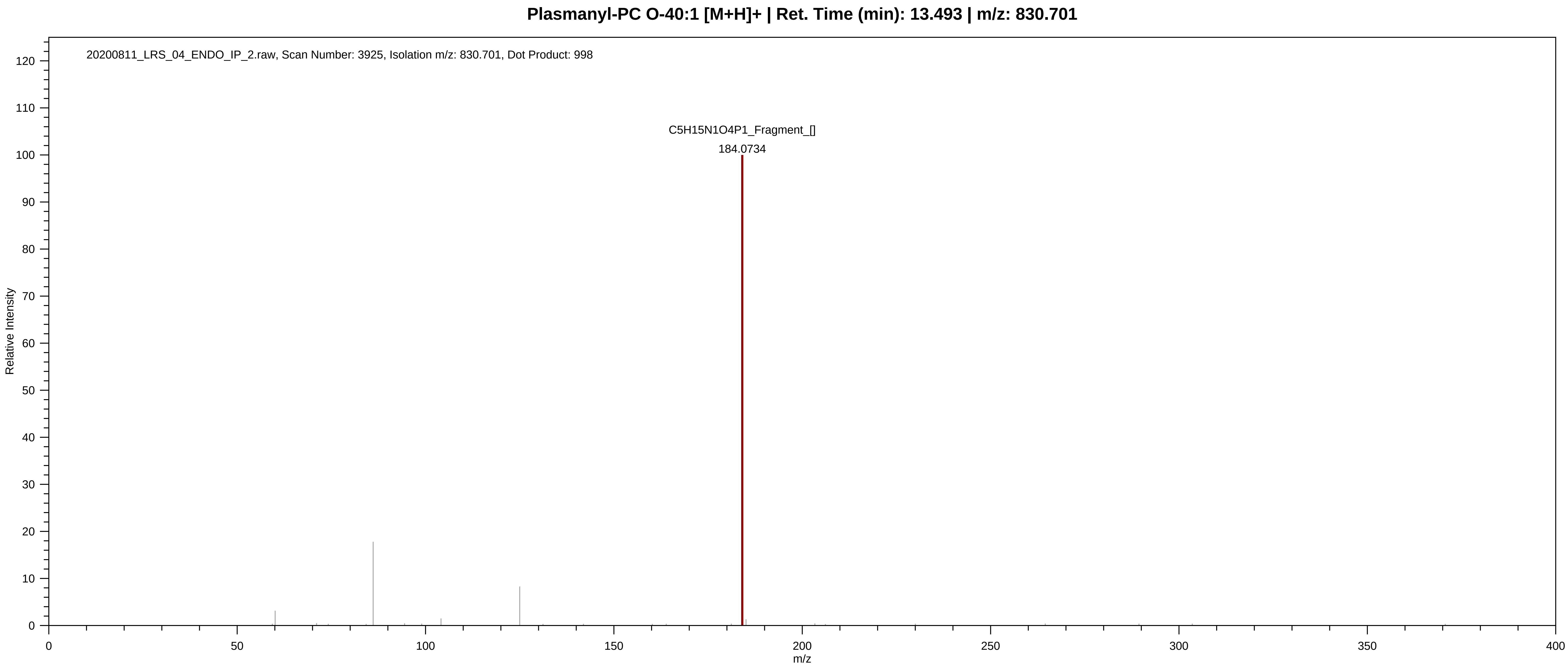
Plasmanyl-PC O-40:1 [M+H]+ | Ret. Time (min): 13.493 | m/z: 830.701
20200811_LRS_04_ENDO_IP_2.raw, Scan Number: 3925, Isolation m/z: 830.701, Dot Product: 998
C5H15N1O4P1_Fragment_[]
184.0734
Relative Intensity
m/z
0
10
20
30
40
50
60
70
80
90
100
110
120
0
50
100
150
200
250
300
350
400

Plasmanyl-PC O-40:1 [M+H]+ | Ret. Time (min): 13.493 | m/z: 830.7

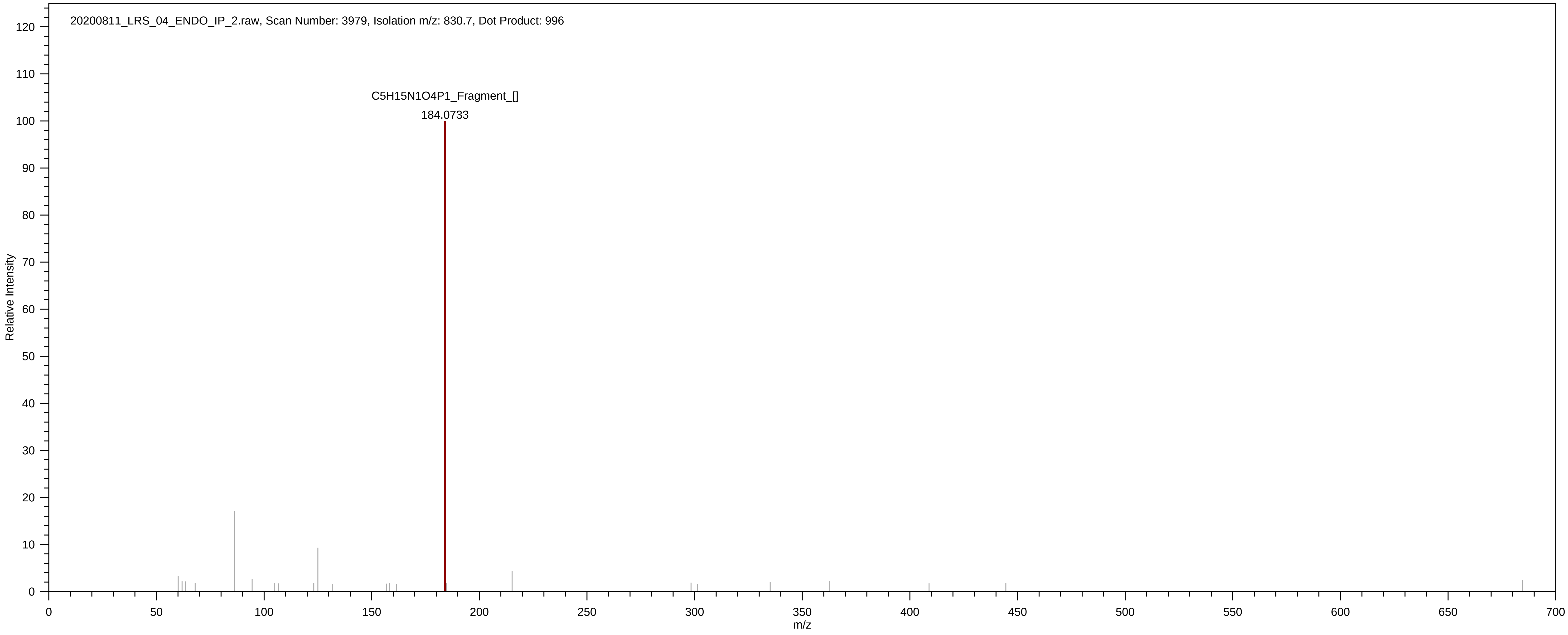

Plasmanyl-PC O-40:1 [M+H]+ | Ret. Time (min): 13.576 | m/z: 830.701

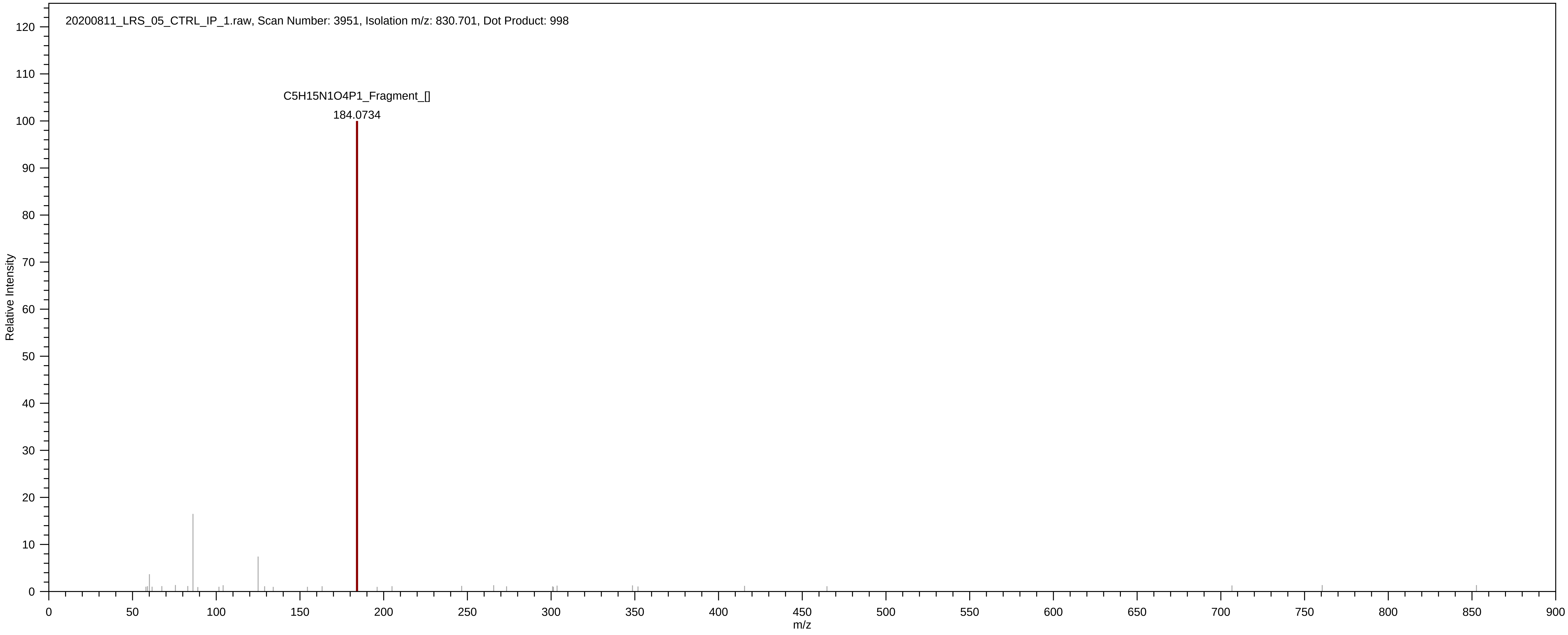

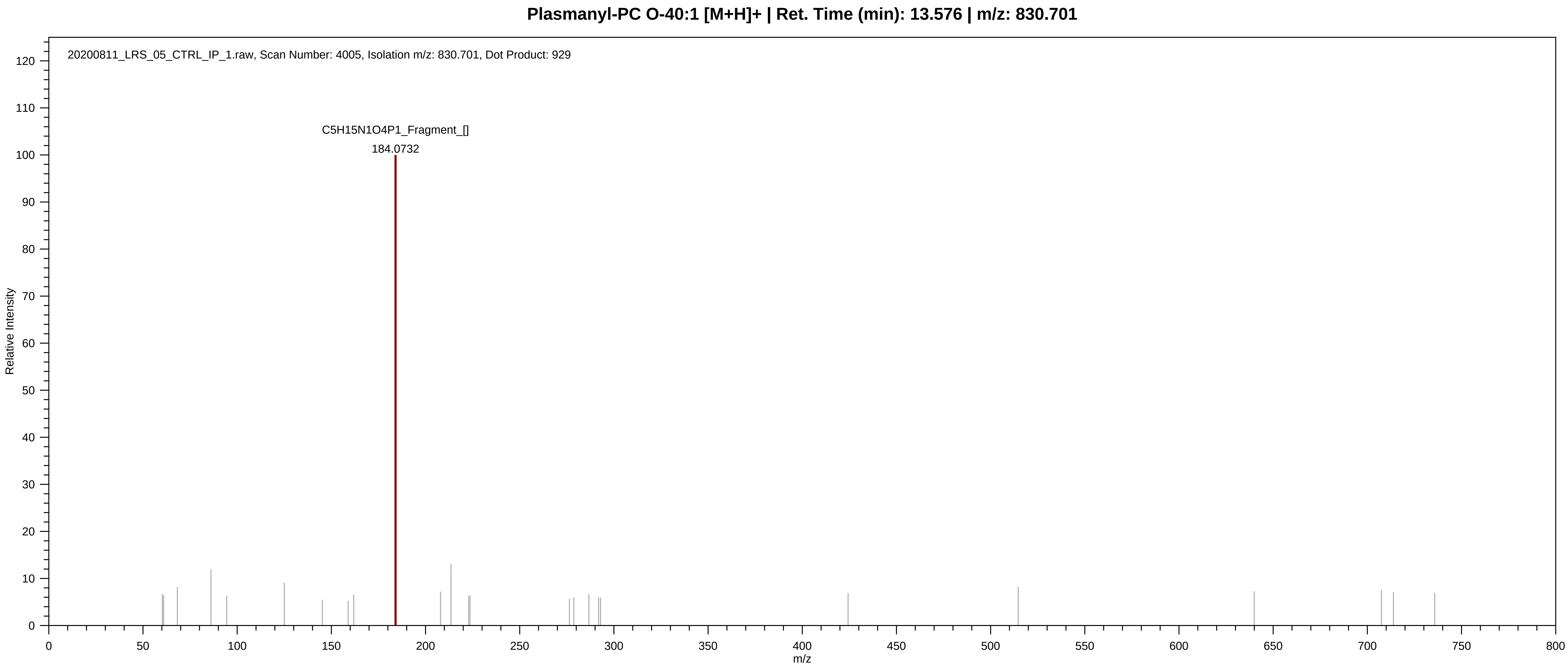

Plasmanyl-PC O-40:1 [M+H]+ | Ret. Time (min): 13.576 | m/z: 830.701
20200811_LRS_05_CTRL_IP_1.raw, Scan Number: 4005, Isolation m/z: 830.701, Dot Product: 929
C5H15N1O4P1_Fragment_[]
184.0732
Relative Intensity
m/z
0
10
20
30
40
50
60
70
80
90
100
110
120
0
50
100
150
200
250
300
350
400
450
500
550
600
650
700
750
800

Plasmanyl-PC O-40:1 [M+H]+ | Ret. Time (min): 13.577 | m/z: 830.701

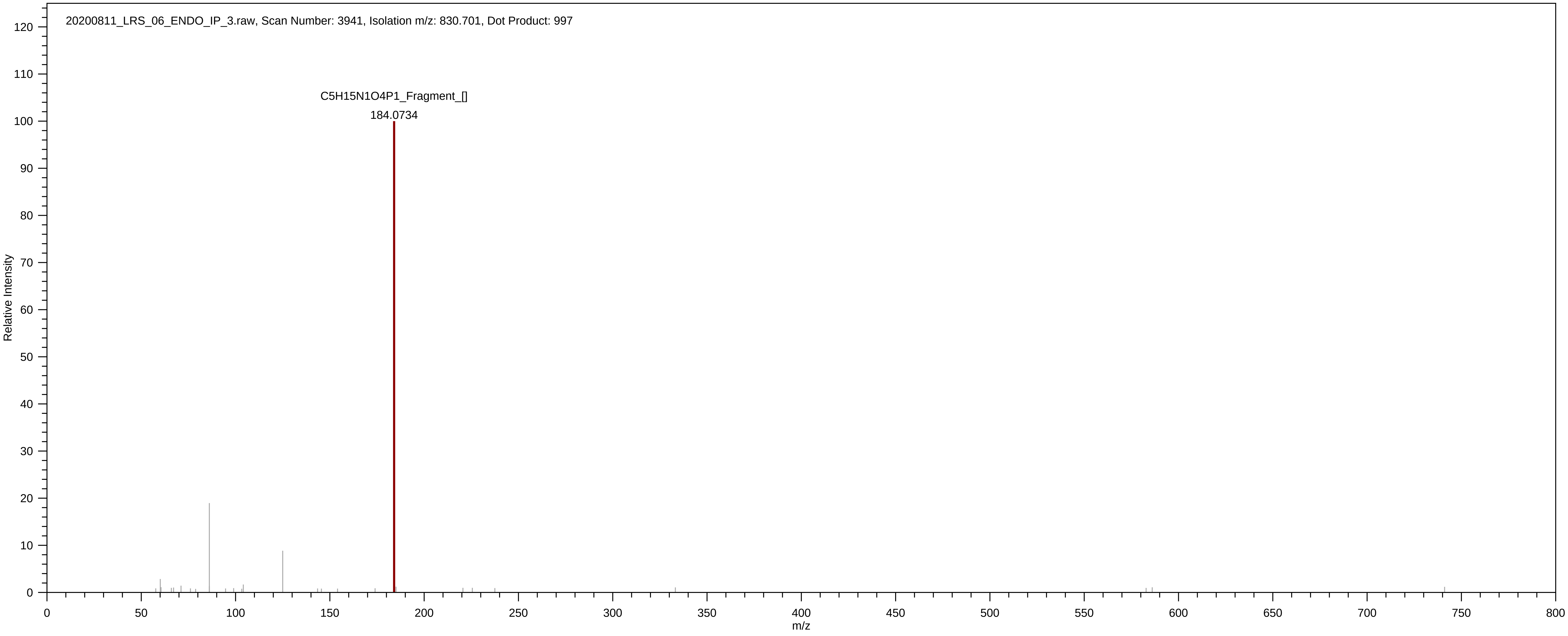

Plasmanyl-PC O-40:1 [M+H]+ | Ret. Time (min): 13.577 | m/z: 830.7

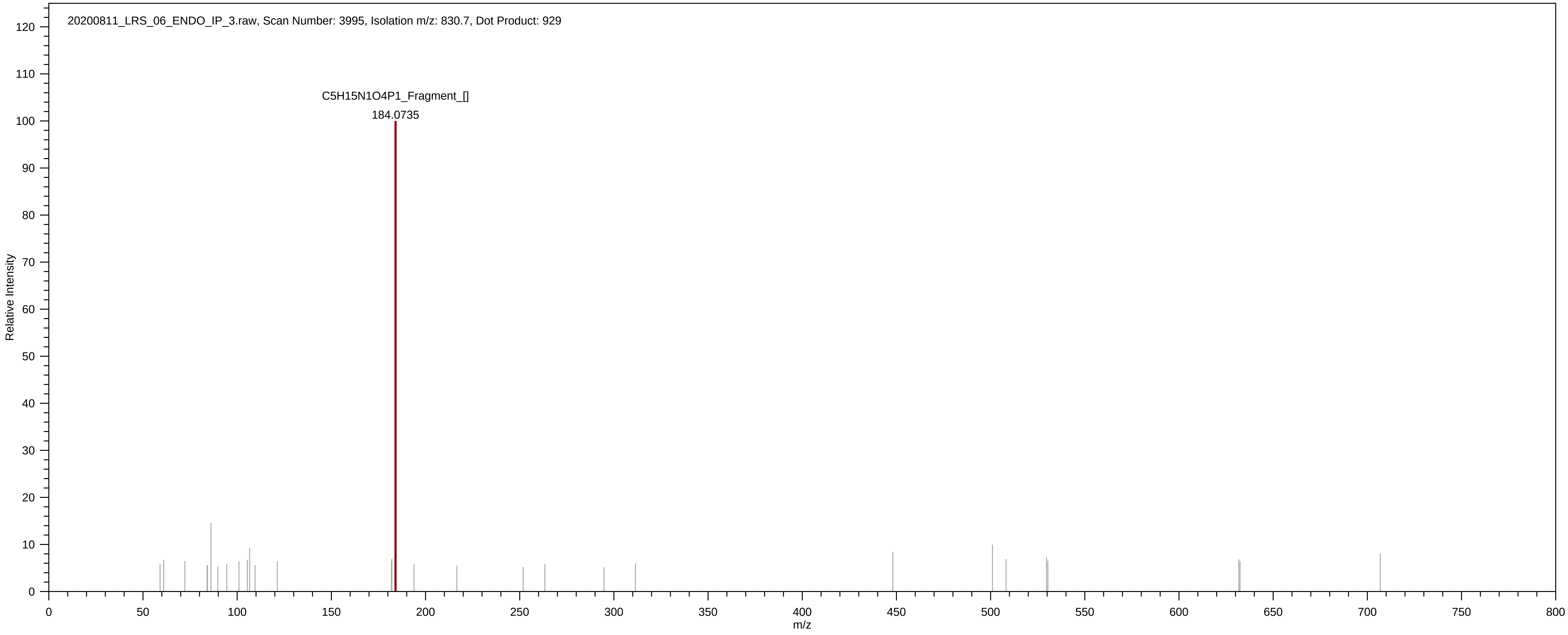

Plasmanyl-PC O-40:1 [M+H]+ | Ret. Time (min): 13.582 | m/z: 830.701

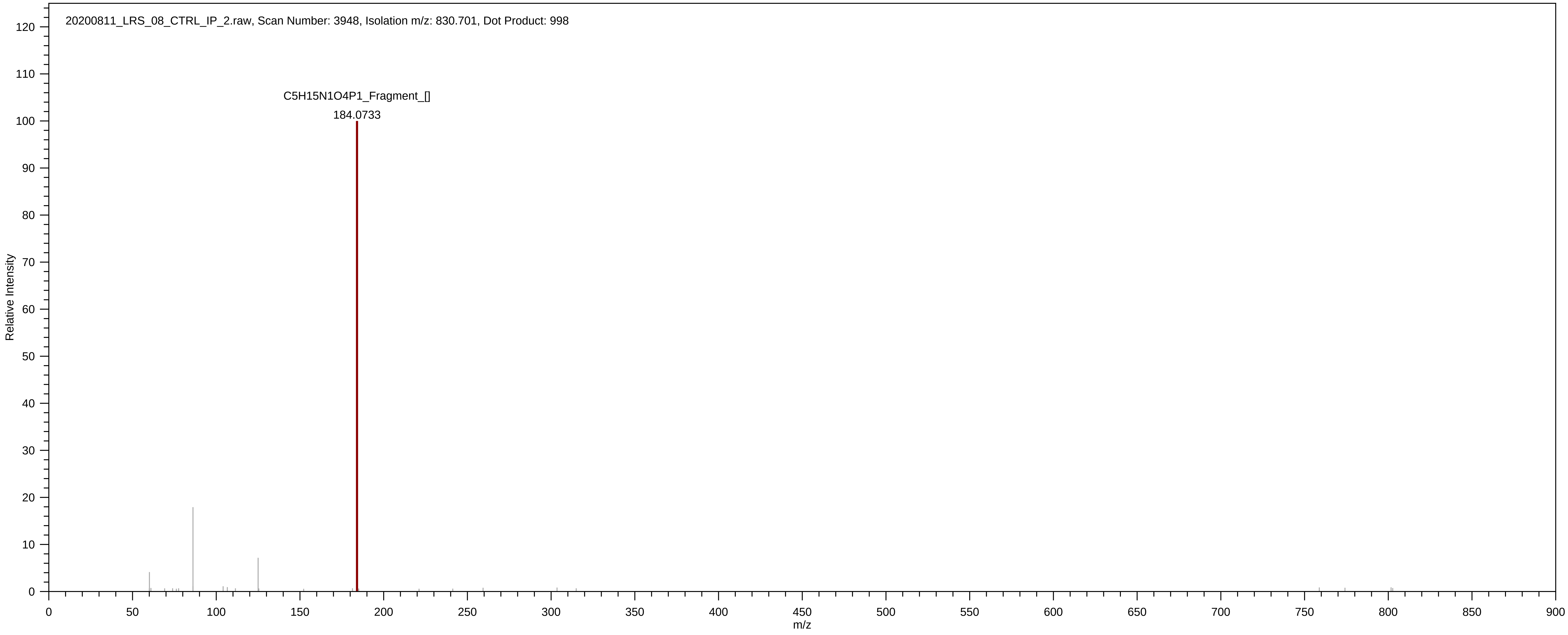

Plasmanyl-PC O-40:1 [M+H]+ | Ret. Time (min): 13.582 | m/z: 830.7

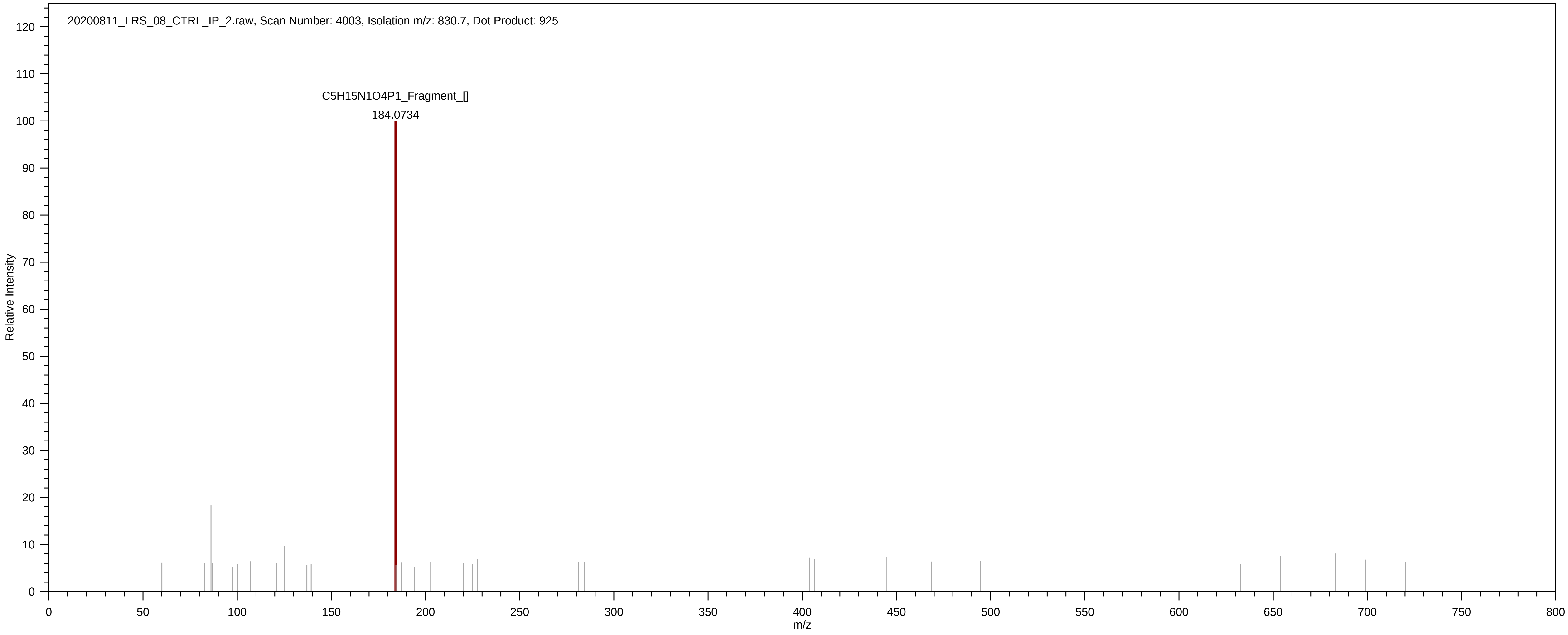

Plasmanyl-PC O-40:1 [M+H]+ | Ret. Time (min): 13.748 | m/z: 830.701

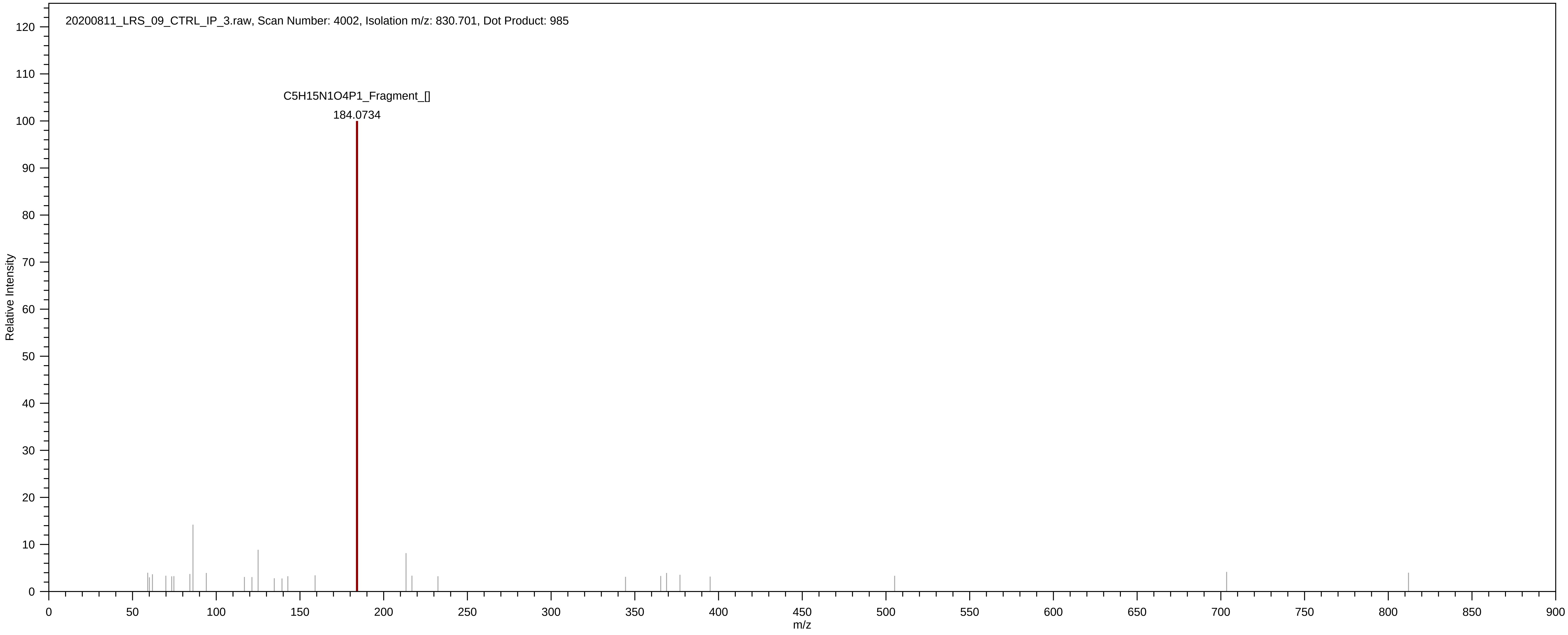

Plasmanyl-PC O-40:1 [M+H]+ | Ret. Time (min): 13.392 | m/z: 830.702

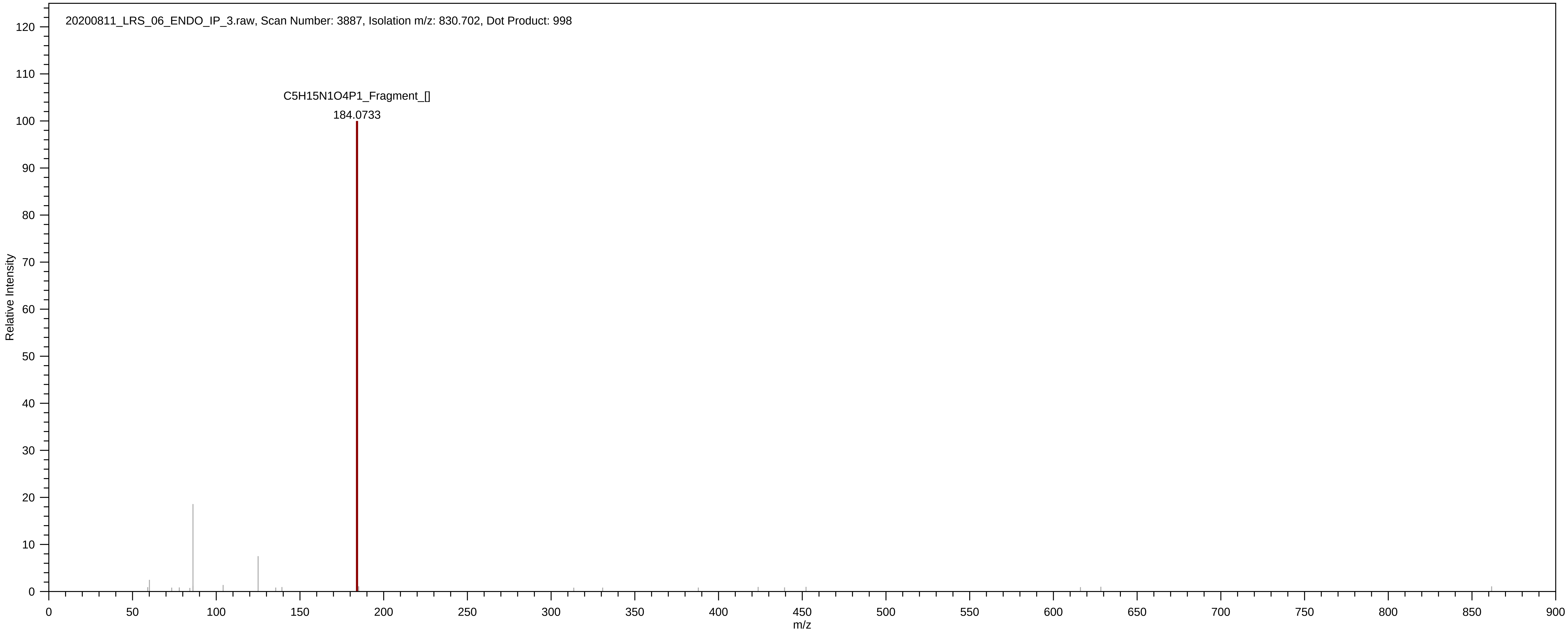

Plasmanyl-PC O-40:1 [M+H]+ | Ret. Time (min): 13.559 | m/z: 830.702

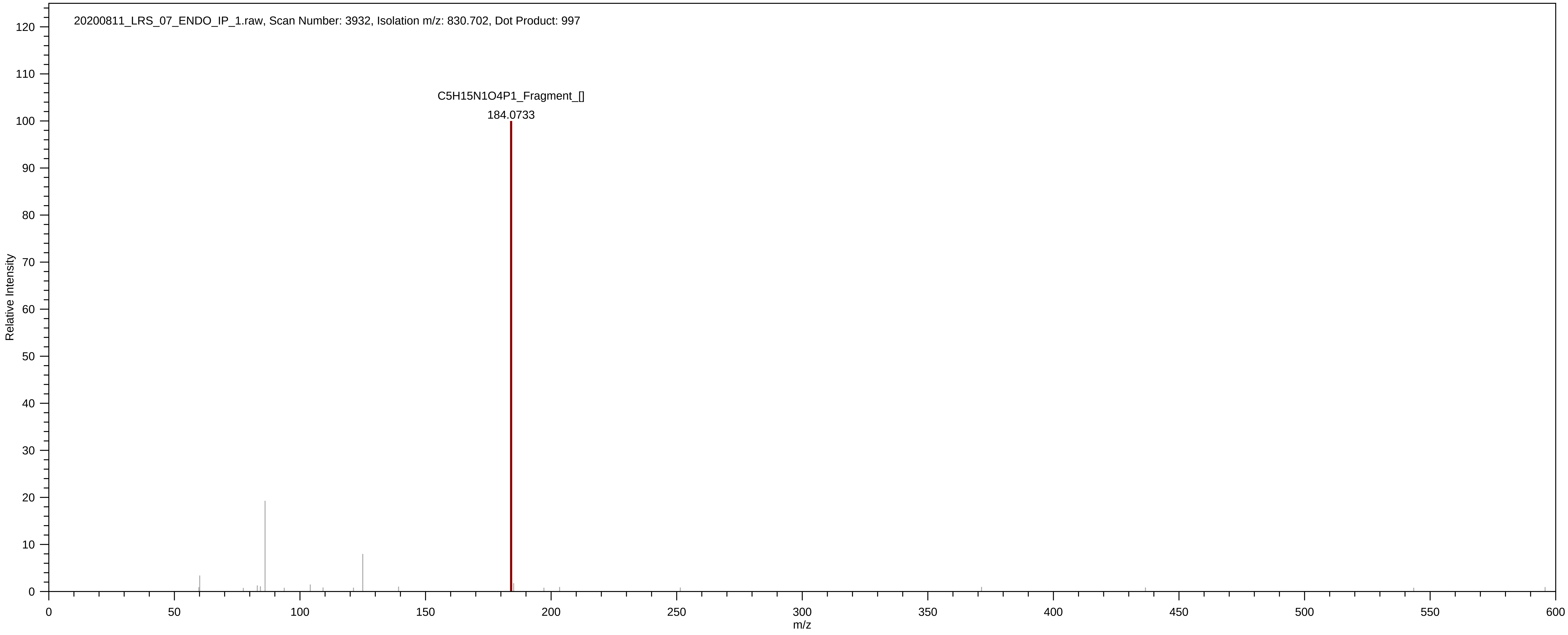

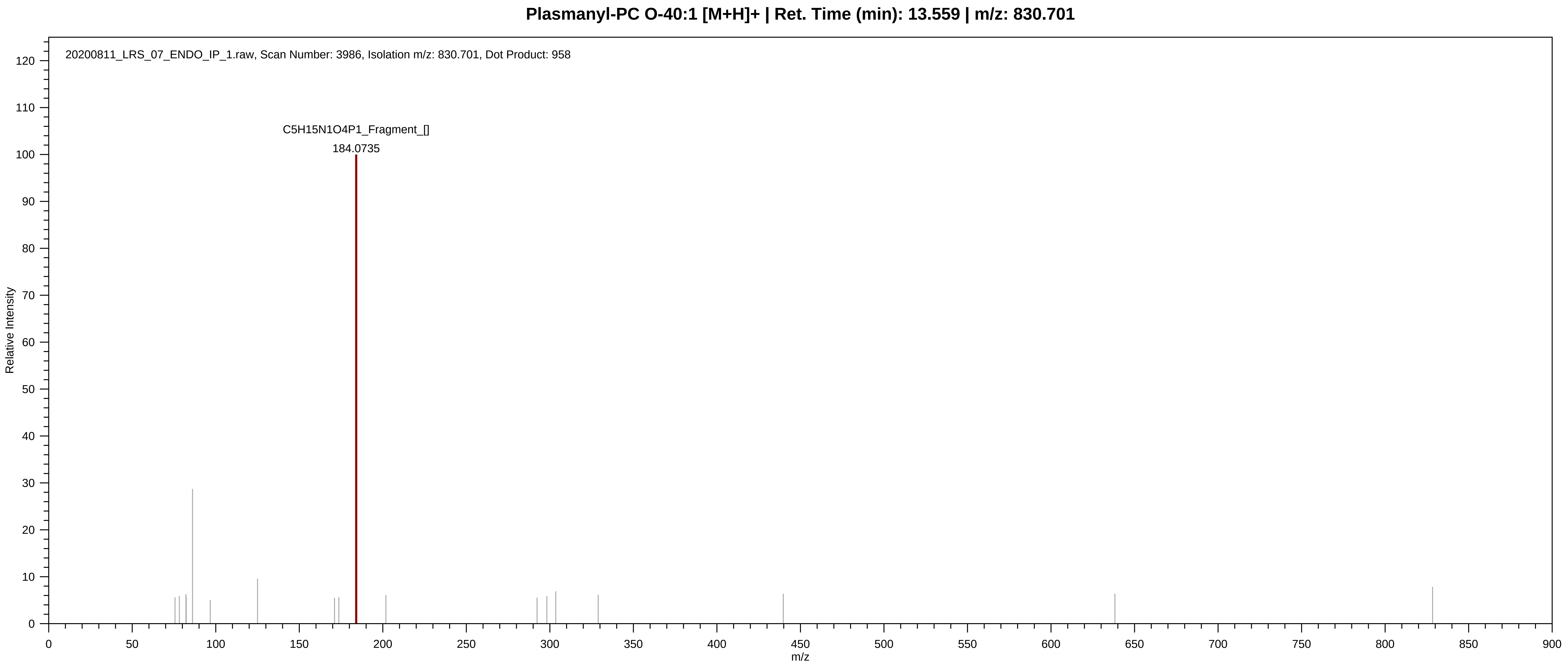
Plasmanyl-PC O-40:1 [M+H]+ | Ret. Time (min): 13.559 | m/z: 830.701
20200811_LRS_07_ENDO_IP_1.raw, Scan Number: 3986, Isolation m/z: 830.701, Dot Product: 958
C5H15N1O4P1_Fragment_[]
184.0735
Relative Intensity
m/z

Plasmanyl-PC O-40:1 [M+H]+ | Ret. Time (min): 13.392 | m/z: 830.704

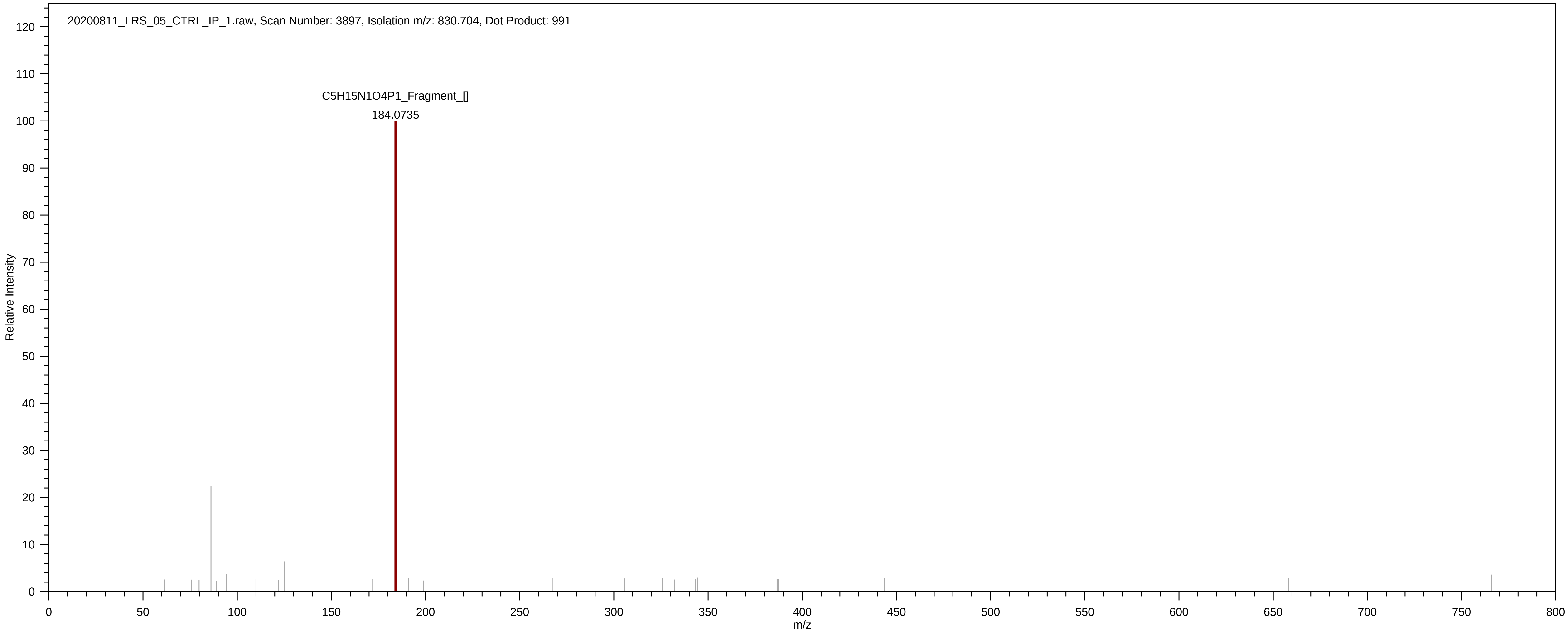

Plasmanyl-PC O-40:2 [M+H]+ | Ret. Time (min): 13.413 | m/z: 828.683

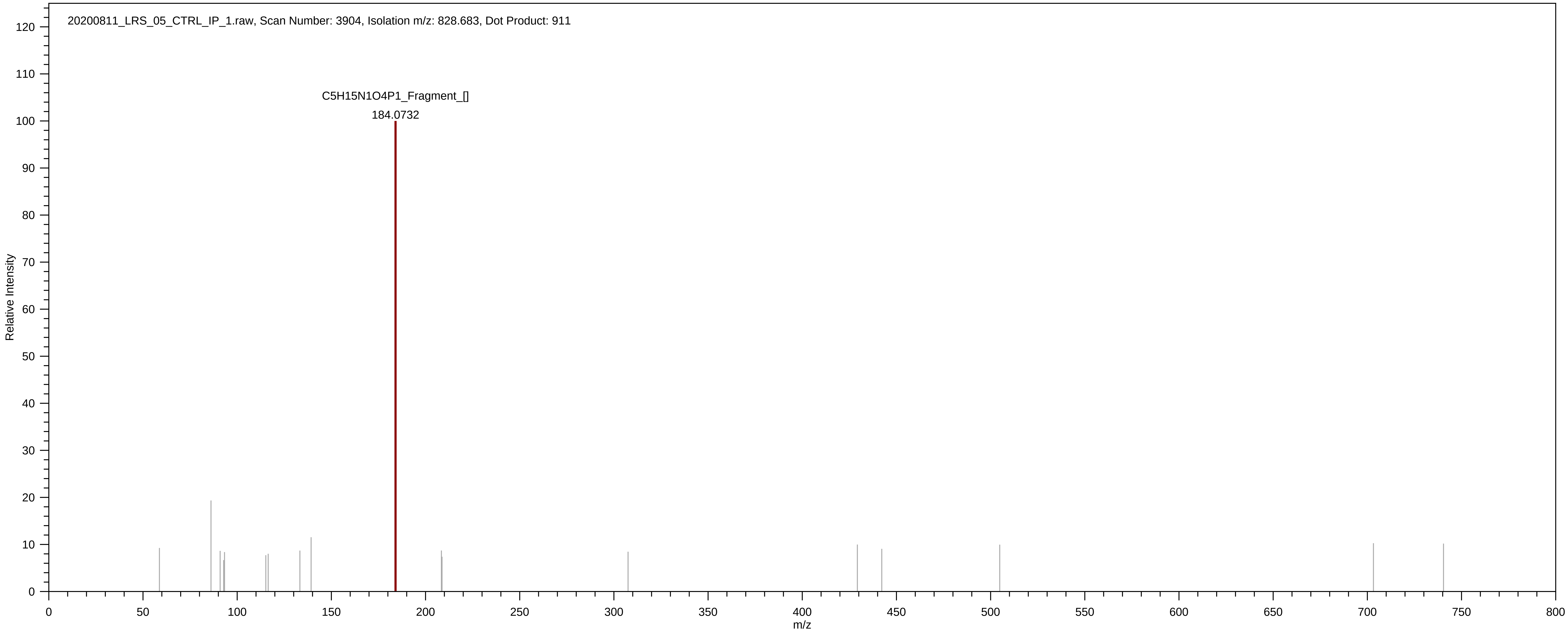

Plasmanyl-PC O-40:2 [M+H]+ | Ret. Time (min): 12.51 | m/z: 828.684

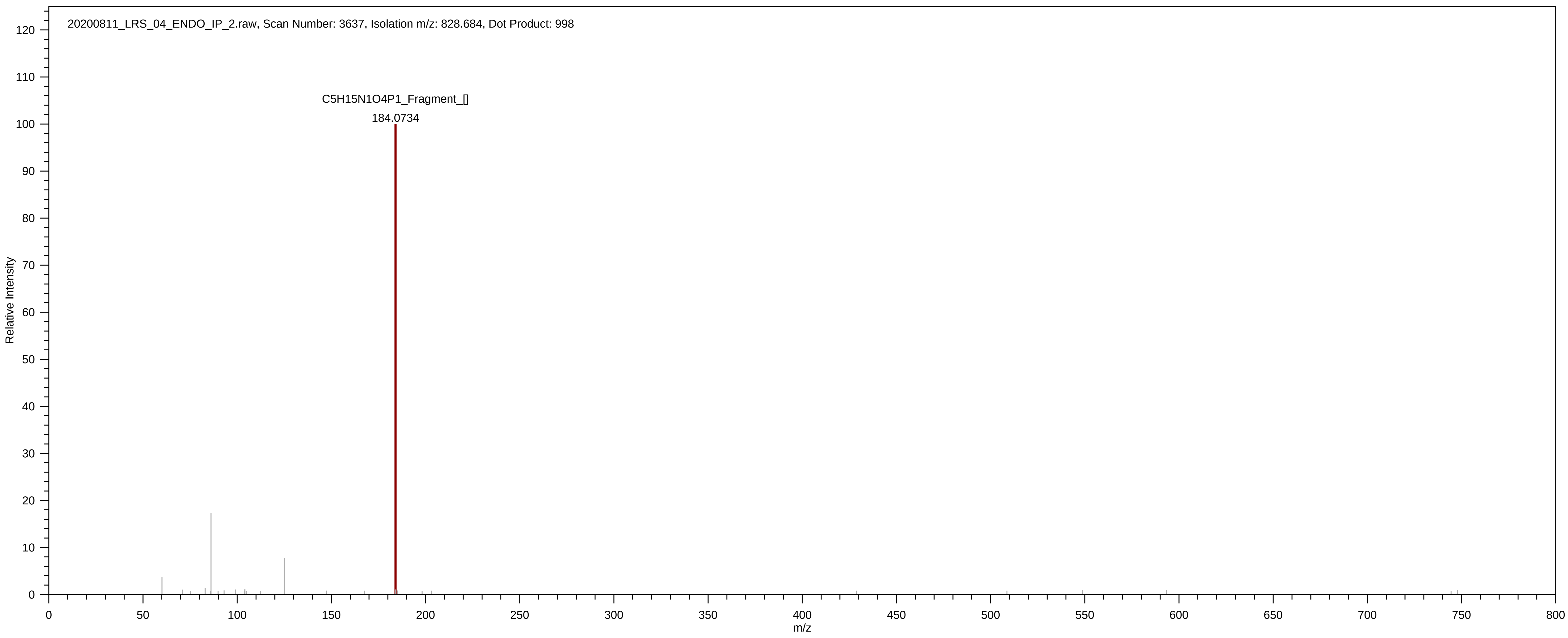

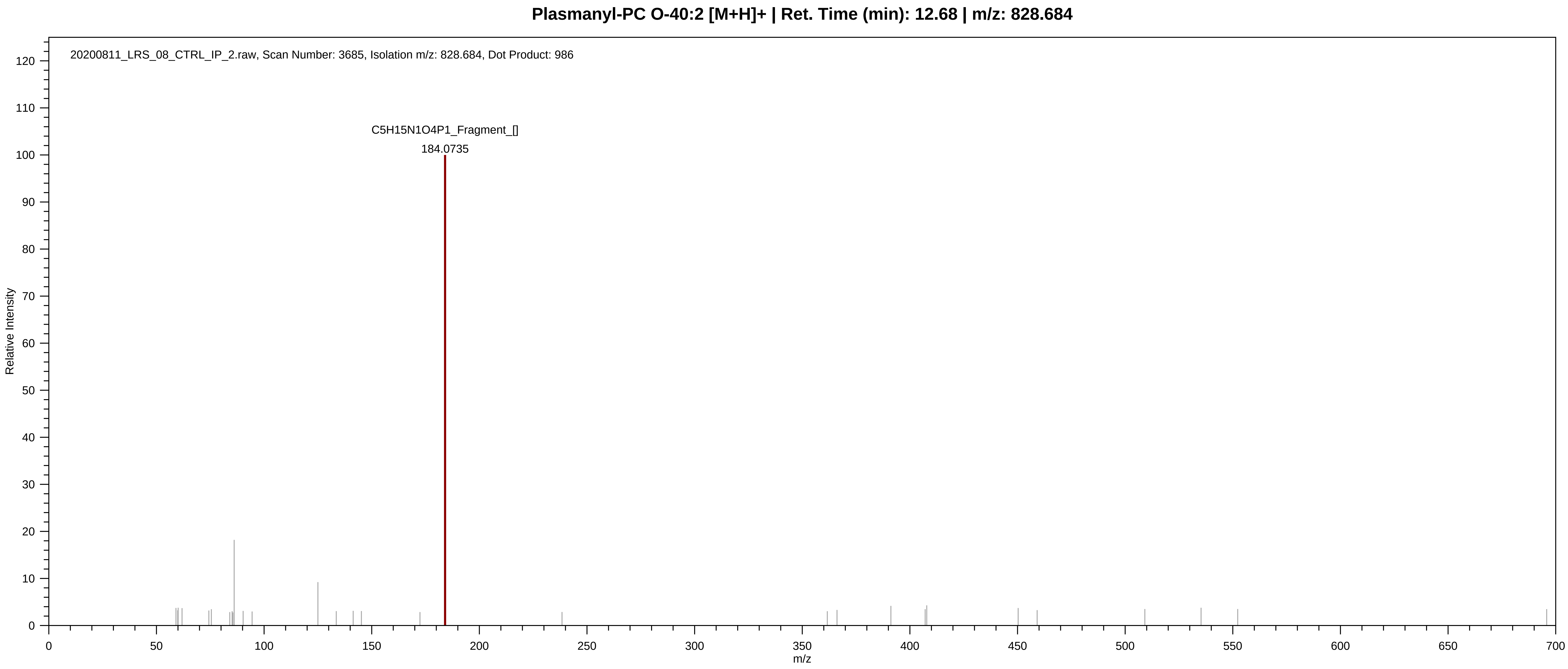
Plasmanyl-PC O-40:2 [M+H]+ | Ret. Time (min): 12.68 | m/z: 828.684
20200811_LRS_08_CTRL_IP_2.raw, Scan Number: 3685, Isolation m/z: 828.684, Dot Product: 986
C5H15N1O4P1_Fragment_[]
184.0735
Relative Intensity
m/z

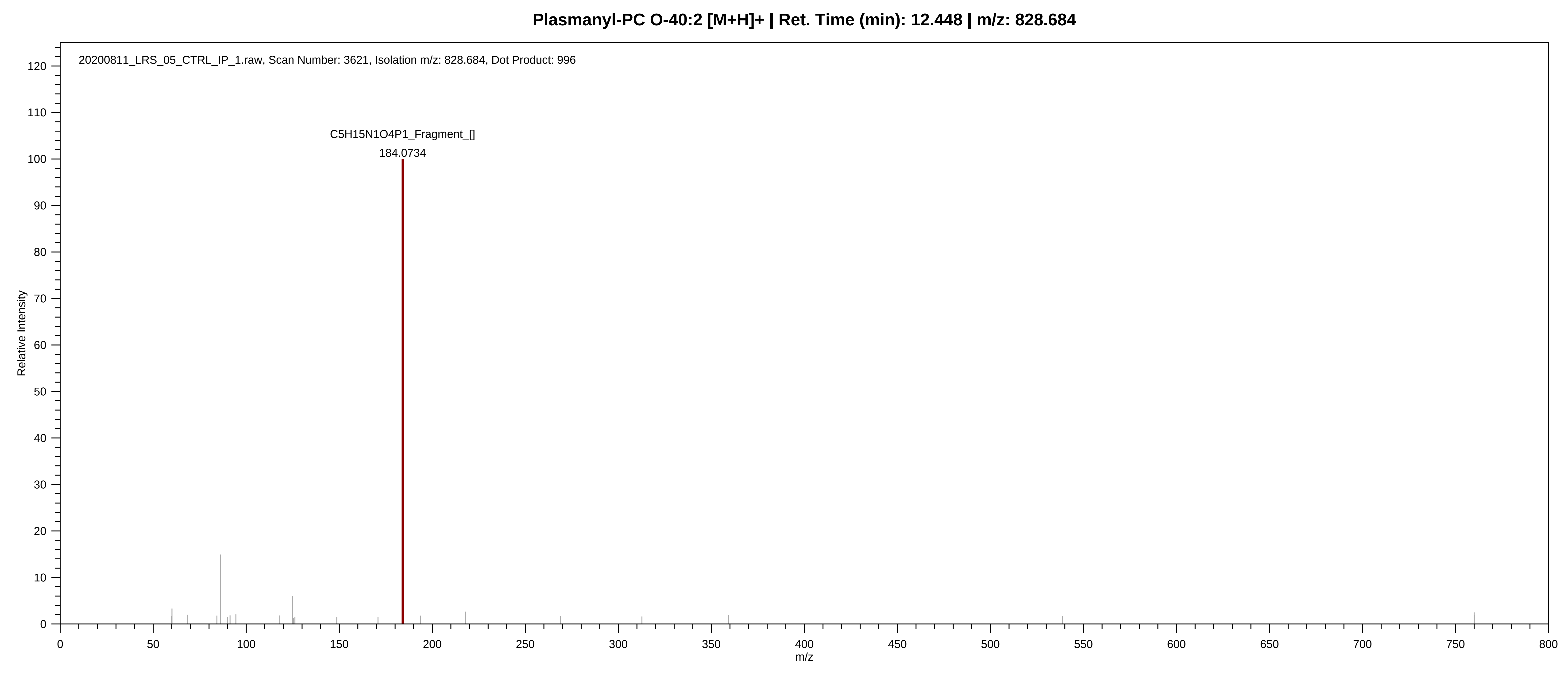

Plasmanyl-PC O-40:2 [M+H]+ | Ret. Time (min): 12.448 | m/z: 828.684
20200811_LRS_05_CTRL_IP_1.raw, Scan Number: 3621, Isolation m/z: 828.684, Dot Product: 996
C5H15N1O4P1_Fragment_[]
184.0734
Relative Intensity
m/z
0
10
20
30
40
50
60
70
80
90
100
110
120
0
50
100
150
200
250
300
350
400
450
500
550
600
650
700
750
800

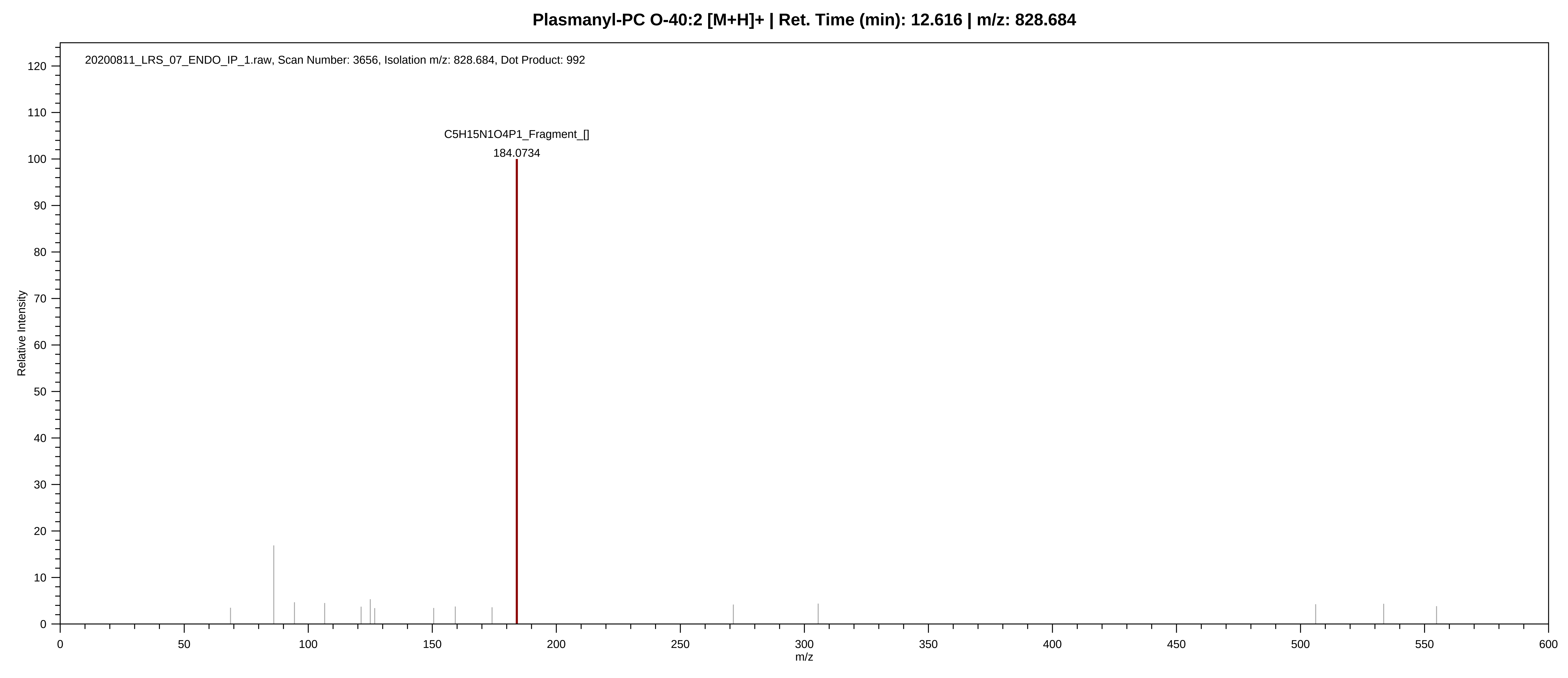
Plasmanyl-PC O-40:2 [M+H]+ | Ret. Time (min): 12.616 | m/z: 828.684
20200811_LRS_07_ENDO_IP_1.raw, Scan Number: 3656, Isolation m/z: 828.684, Dot Product: 992
C5H15N1O4P1_Fragment_[]
184.0734
Relative Intensity
m/z

Plasmanyl-PC O-40:2 [M+H]+ | Ret. Time (min): 13.401 | m/z: 828.684

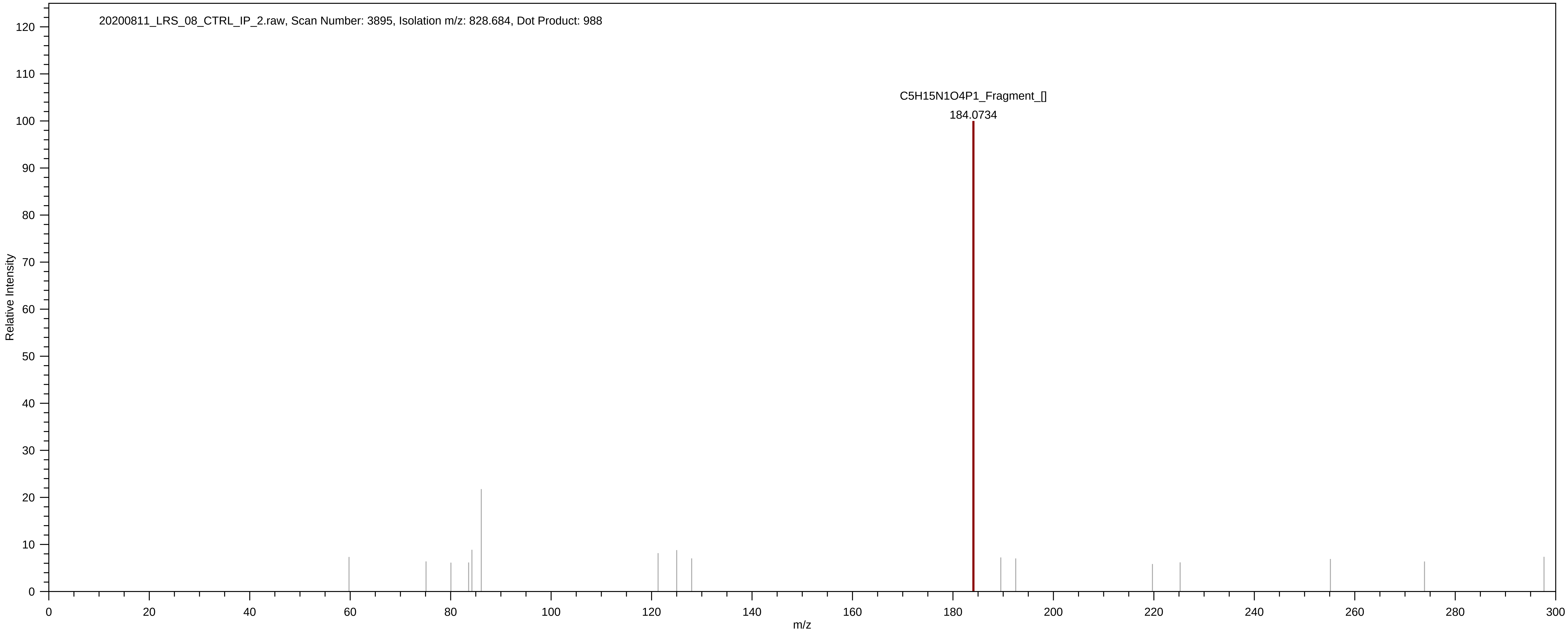

Plasmanyl-PC O-40:2 [M+H]+ | Ret. Time (min): 12.494 | m/z: 828.685

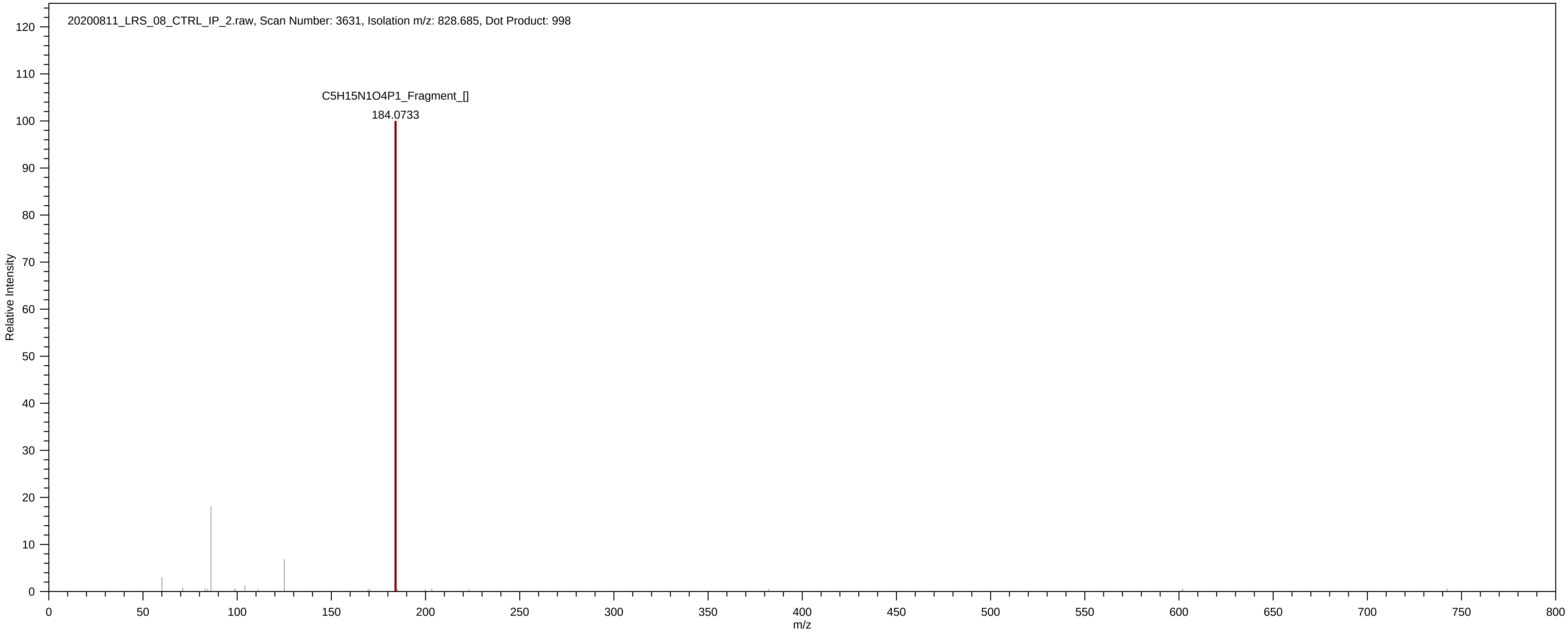

Plasmanyl-PC O-40:2 [M+H]+ | Ret. Time (min): 12.613 | m/z: 828.685

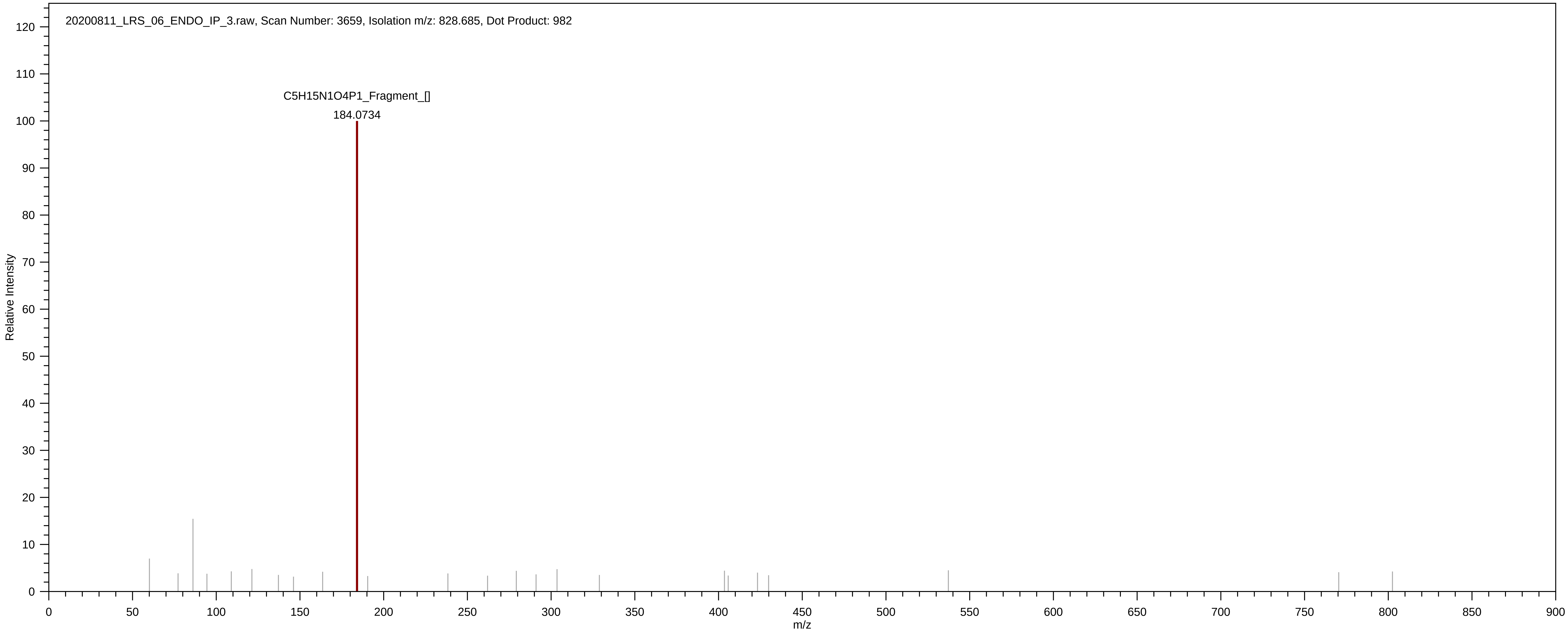

Plasmanyl-PC O-40:2 [M+H]+ | Ret. Time (min): 12.43 | m/z: 828.686

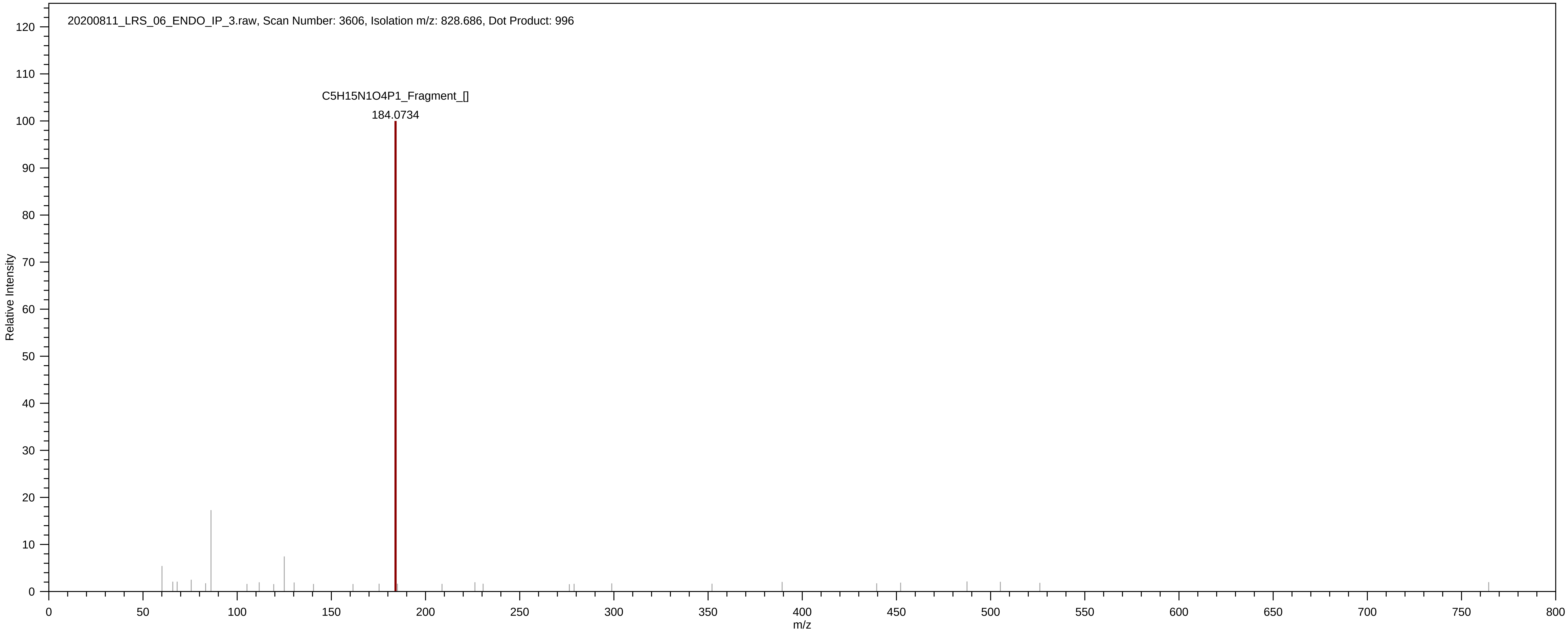

Plasmanyl-PC O-40:2 [M+H]+ | Ret. Time (min): 12.43 | m/z: 828.687

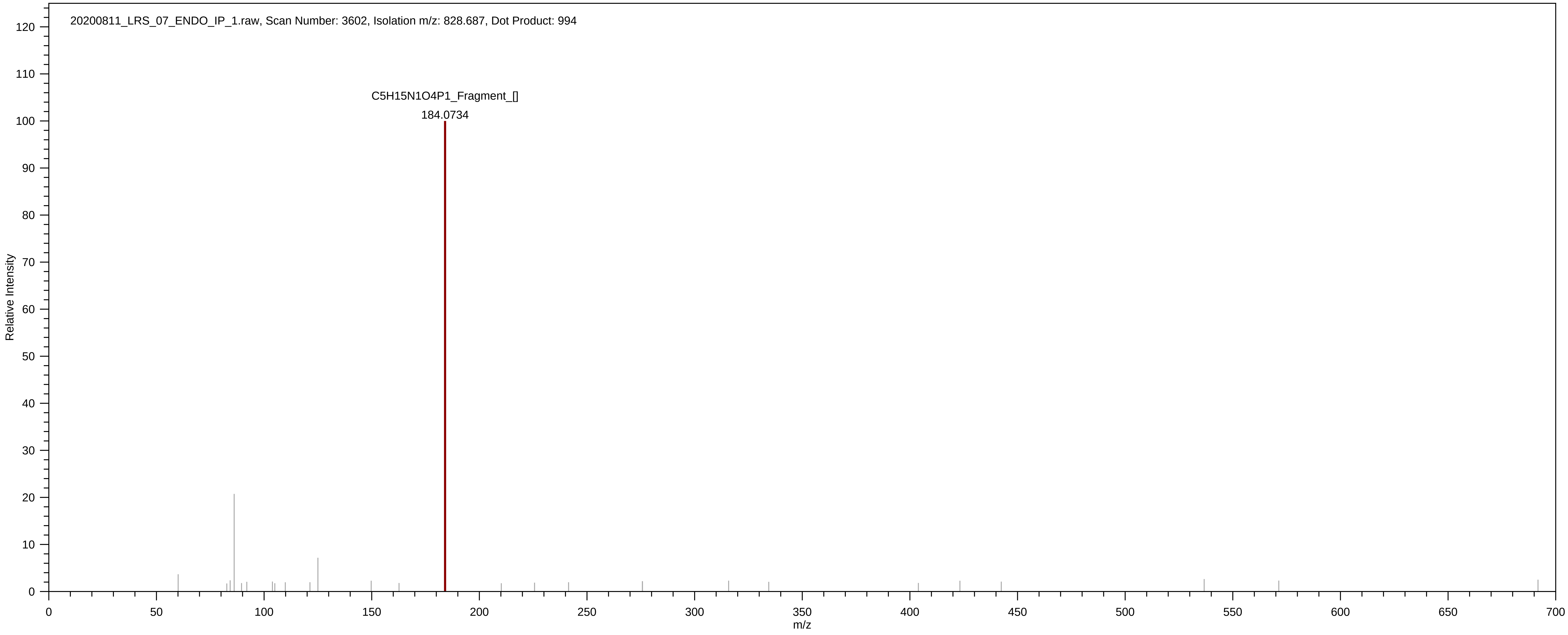

Plasmanyl-PC O-40:3 [M+H]+ | Ret. Time (min): 12.309 | m/z: 826.67

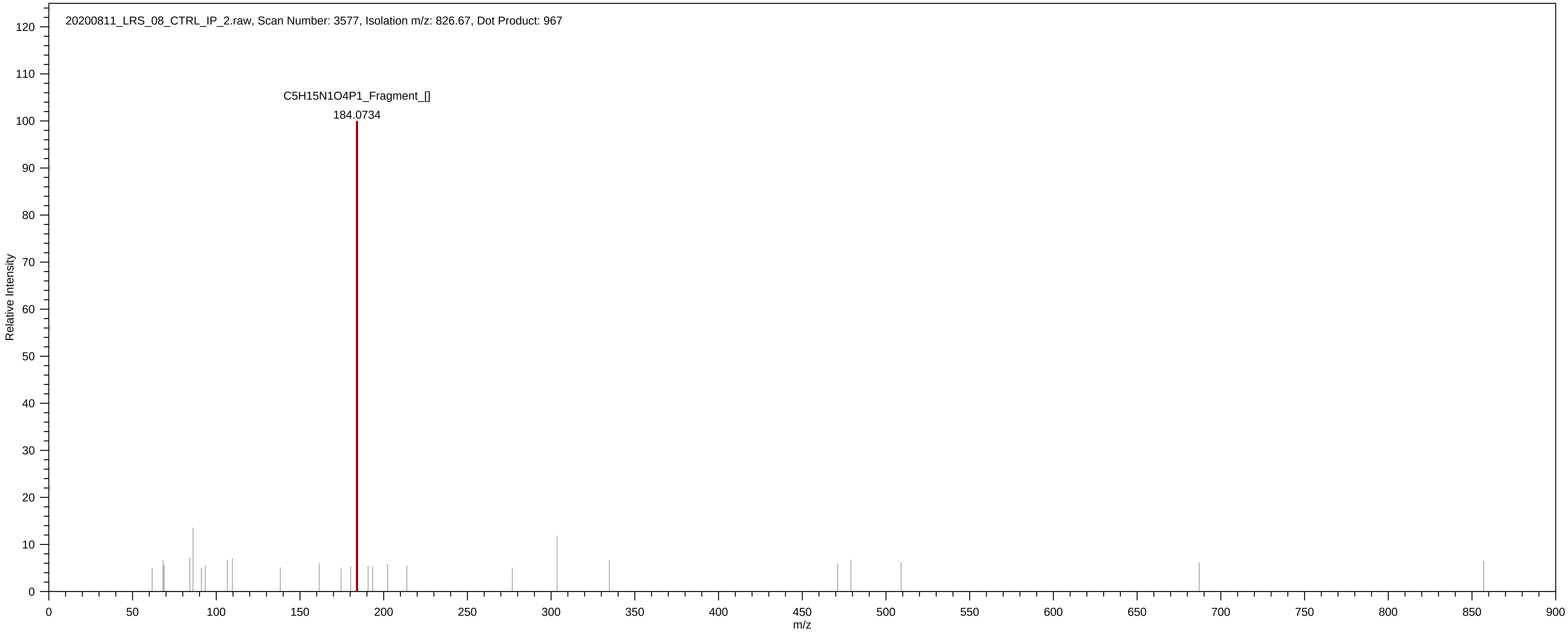

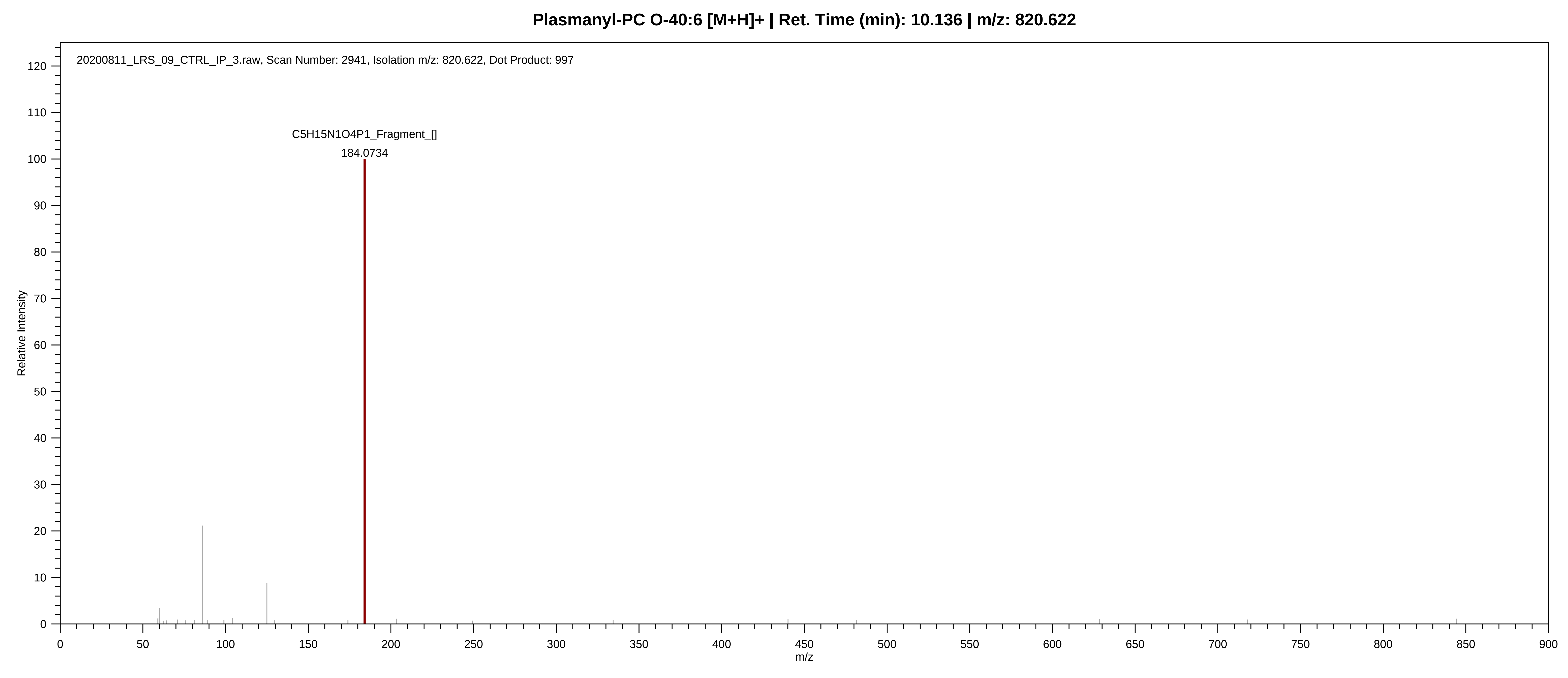
Plasmanyl-PC O-40:6 [M+H]+ | Ret. Time (min): 10.136 | m/z: 820.622
20200811_LRS_09_CTRL_IP_3.raw, Scan Number: 2941, Isolation m/z: 820.622, Dot Product: 997
C5H15N1O4P1_Fragment_[]
184.0734
Relative Intensity
m/z

Plasmanyl-PC O-40:6 [M+H]+ | Ret. Time (min): 10.134 | m/z: 820.623

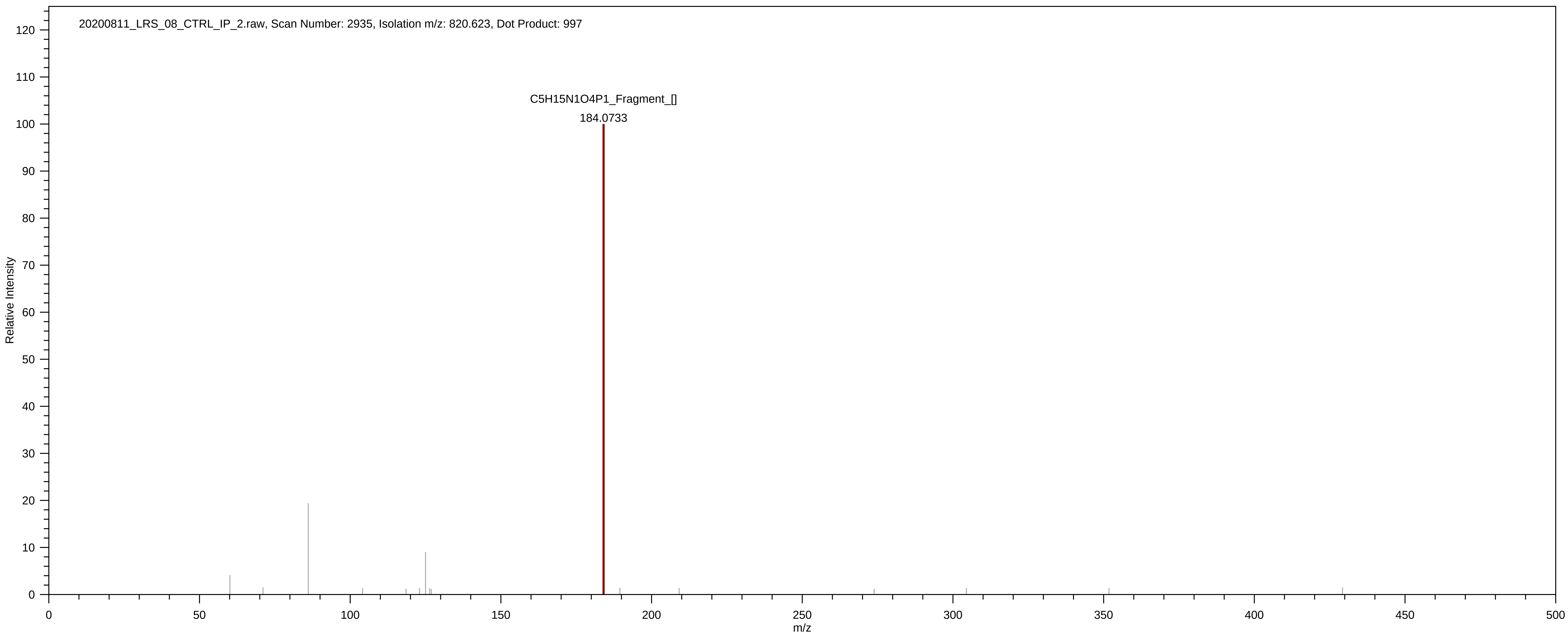

Plasmanyl-PC O-40:6 [M+H]+ | Ret. Time (min): 10.136 | m/z: 820.623

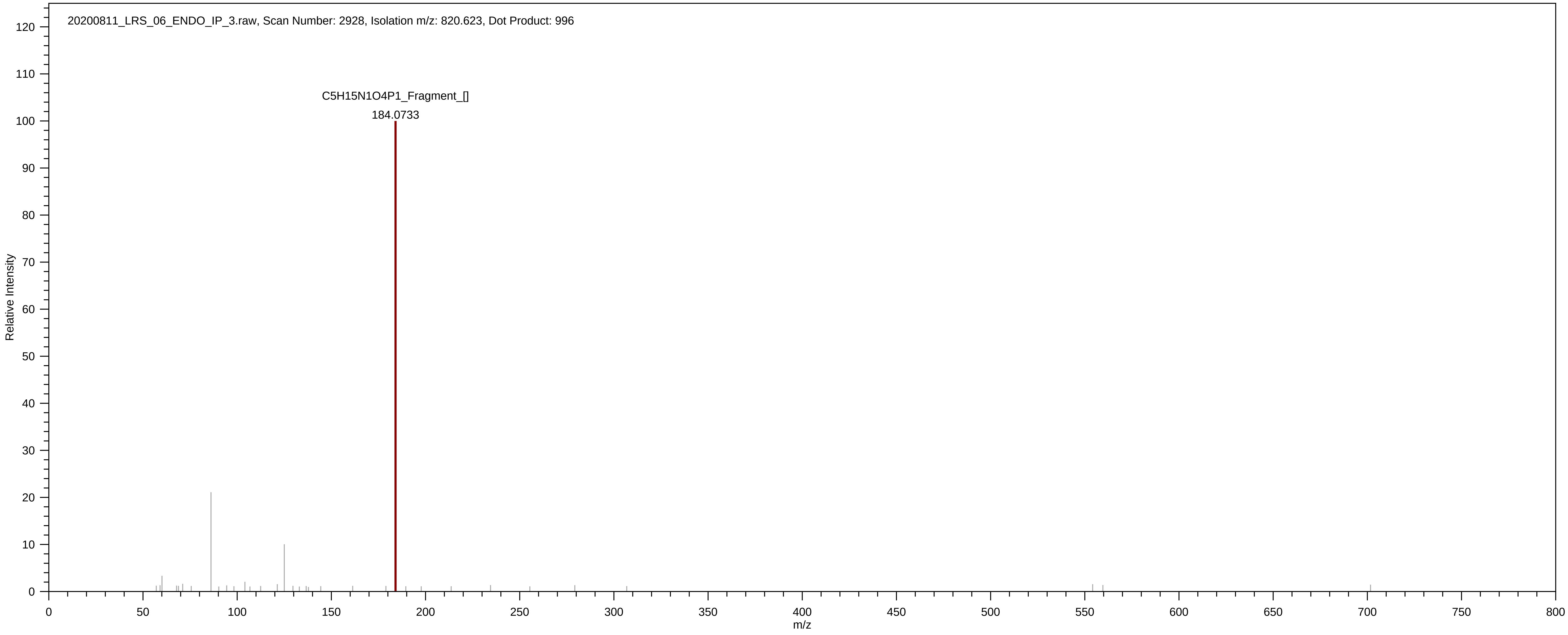

Plasmanyl-PC O-42:0 [M+H]+ | Ret. Time (min): 15.537 | m/z: 860.746

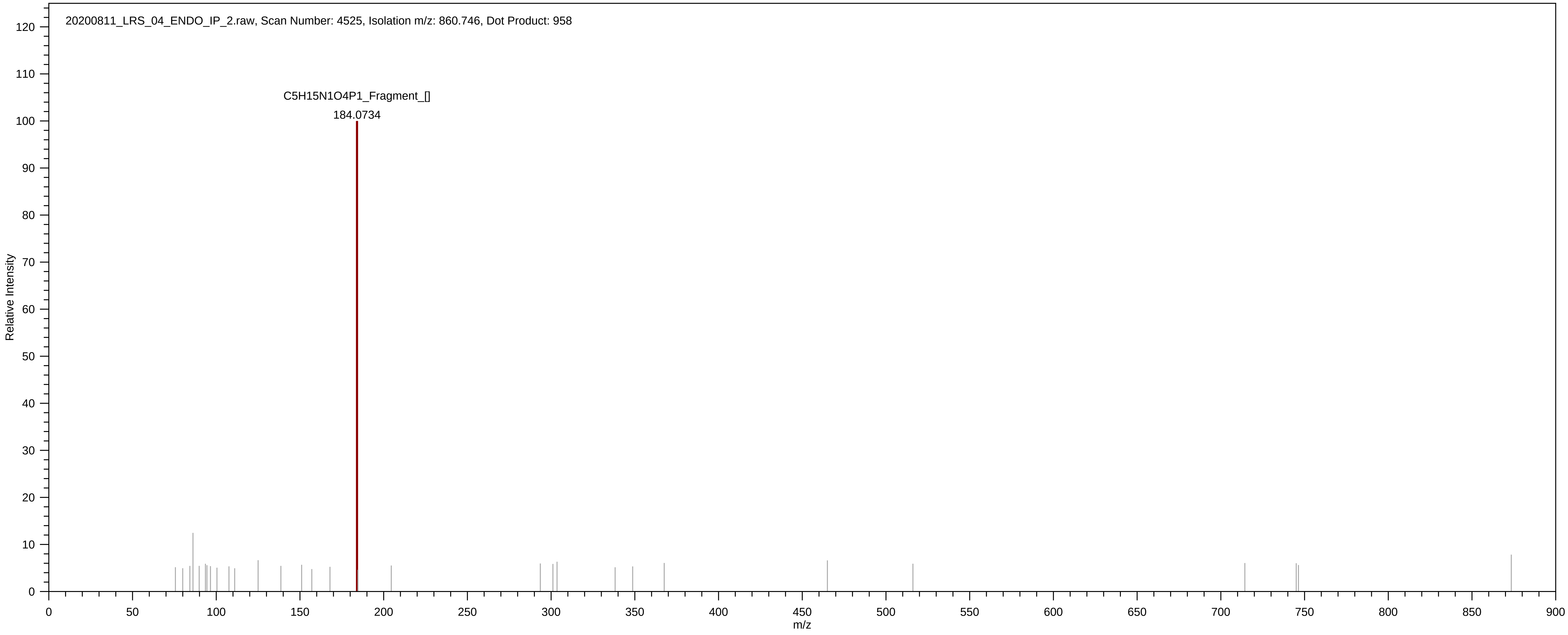

Plasmanyl-PC O-42:0 [M+H]+ | Ret. Time (min): 15.511 | m/z: 860.749

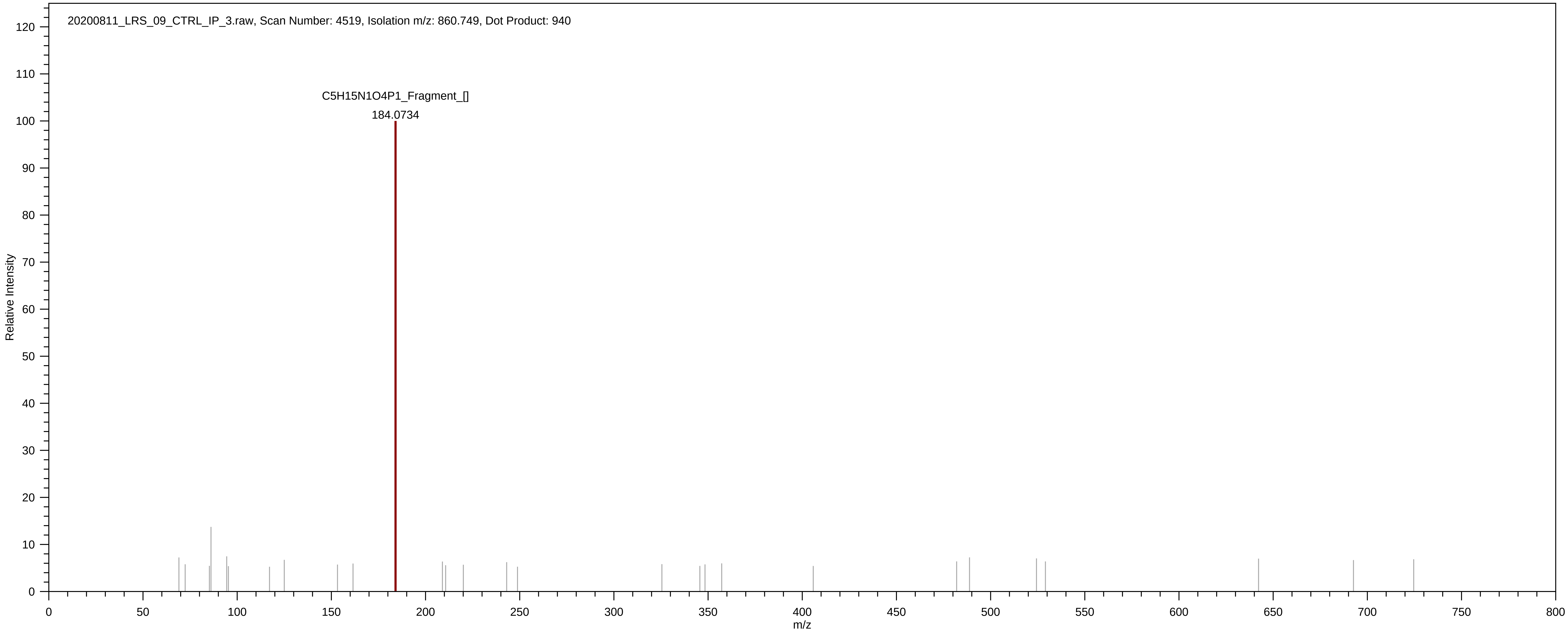

Plasmanyl-PC O-42:1 [M+H]+ | Ret. Time (min): 14.406 | m/z: 858.73

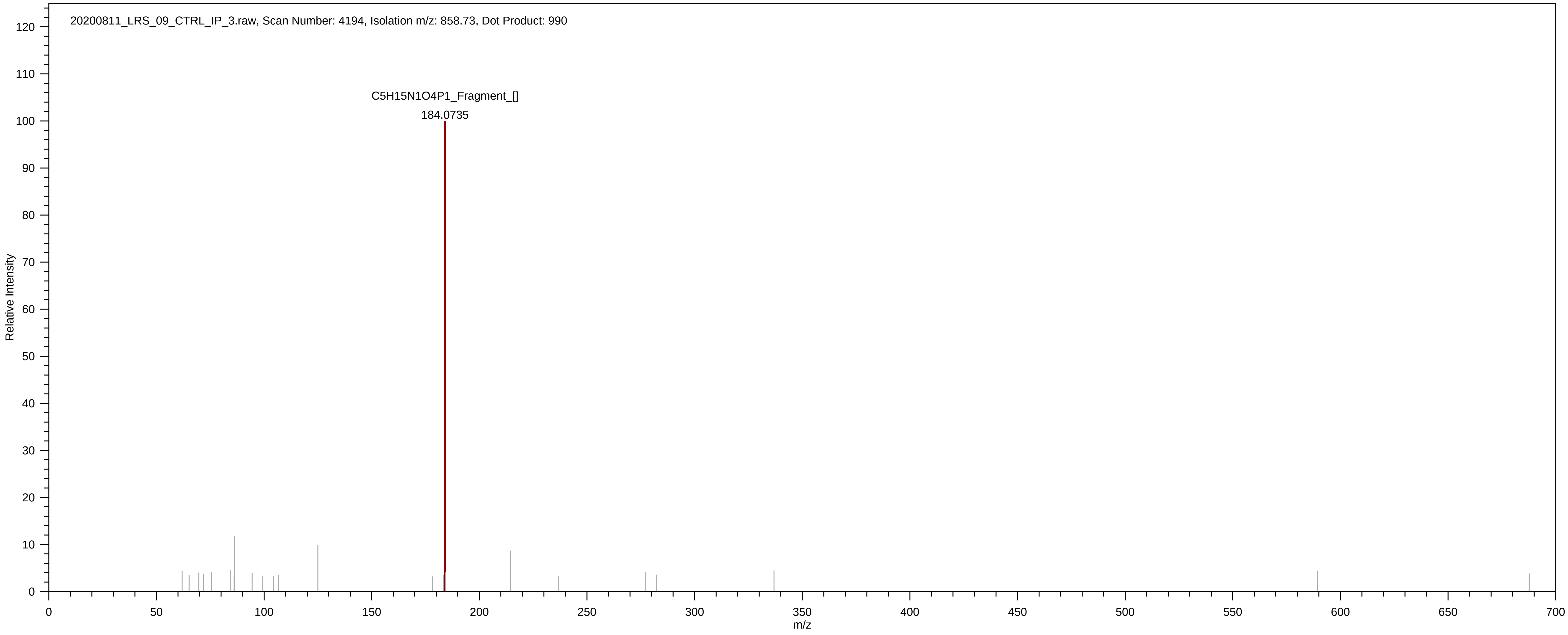

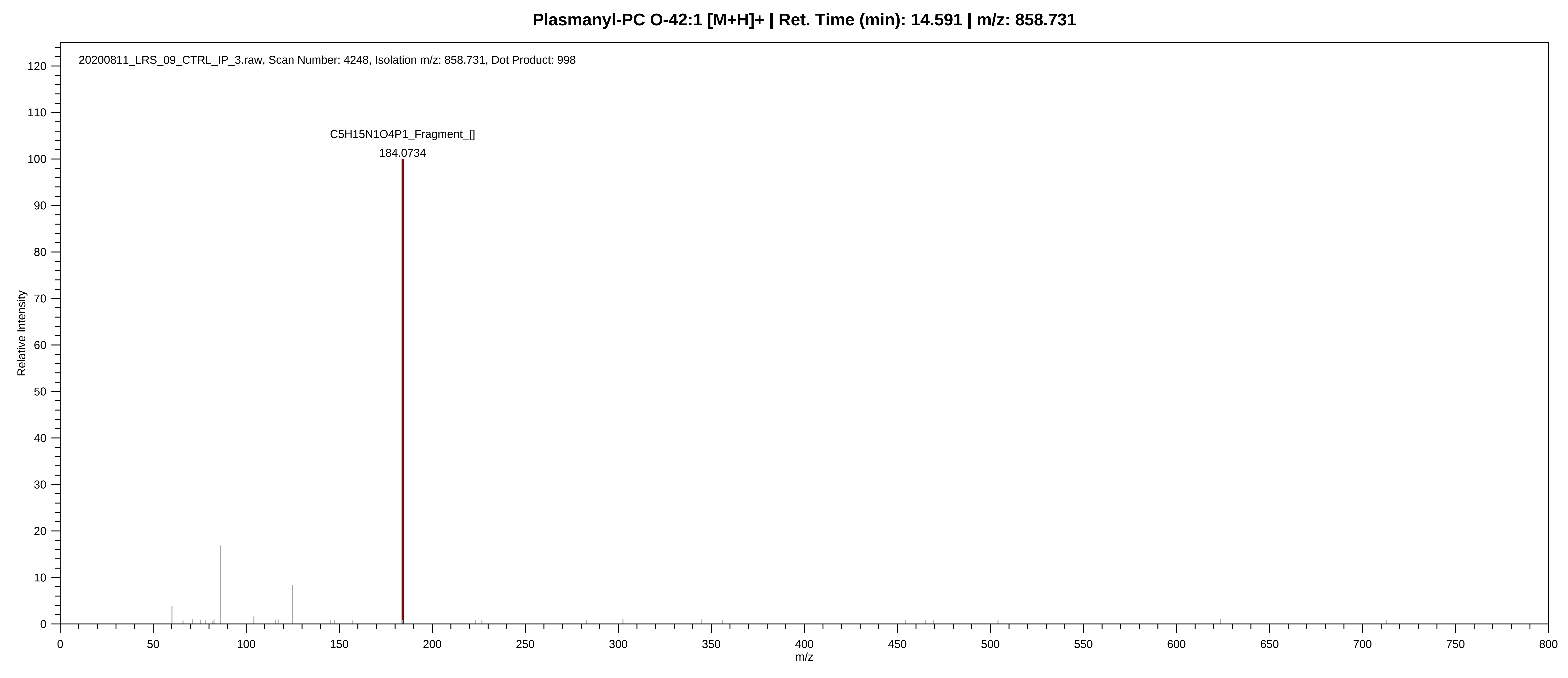
Plasmanyl-PC O-42:1 [M+H]+ | Ret. Time (min): 14.591 | m/z: 858.731
20200811_LRS_09_CTRL_IP_3.raw, Scan Number: 4248, Isolation m/z: 858.731, Dot Product: 998
C5H15N1O4P1_Fragment_[]
184.0734
Relative Intensity
m/z

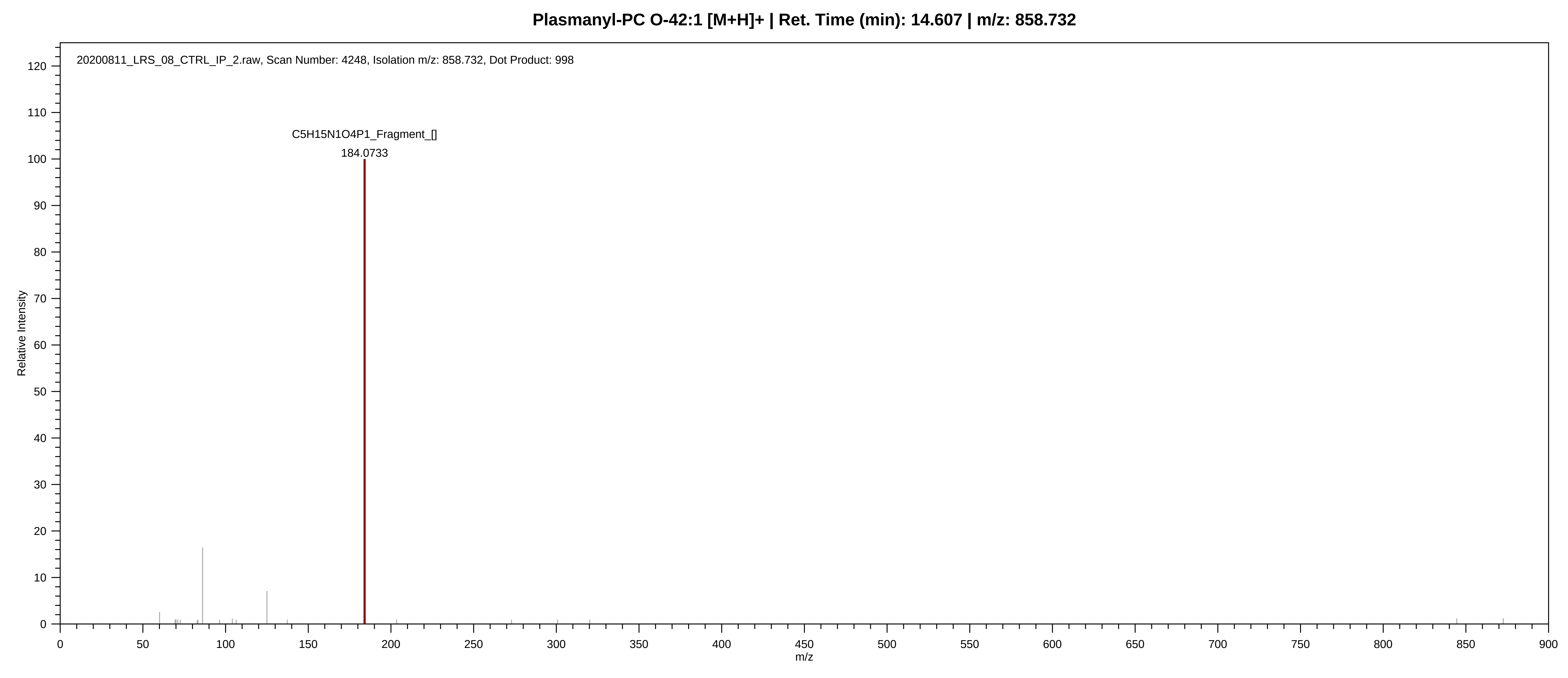

Plasmanyl-PC O-42:1 [M+H]+ | Ret. Time (min): 14.607 | m/z: 858.732
20200811_LRS_08_CTRL_IP_2.raw, Scan Number: 4248, Isolation m/z: 858.732, Dot Product: 998
C5H15N1O4P1_Fragment_[]
184.0733
Relative Intensity
m/z

Plasmanyl-PC O-42:1 [M+H]+ | Ret. Time (min): 14.619 | m/z: 858.732

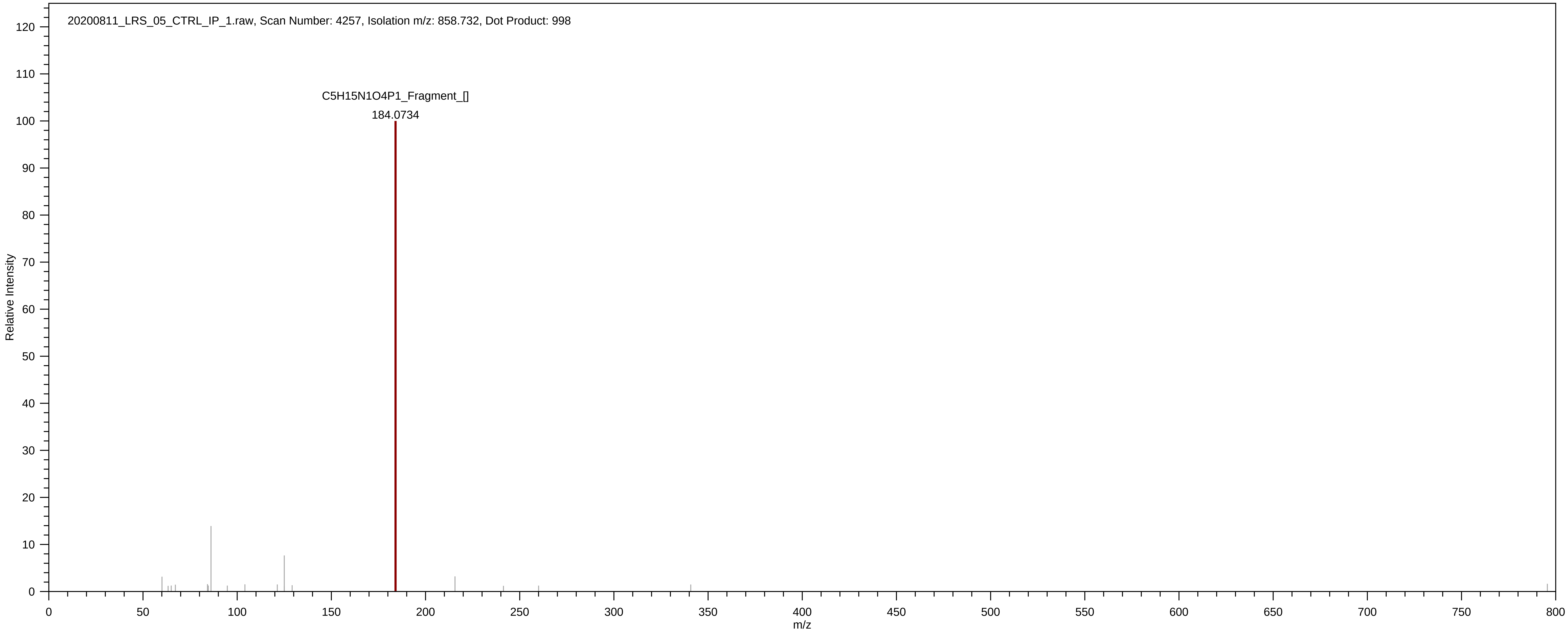

Plasmanyl-PC O-42:2 [M+H]+ | Ret. Time (min): 13.44 | m/z: 856.714

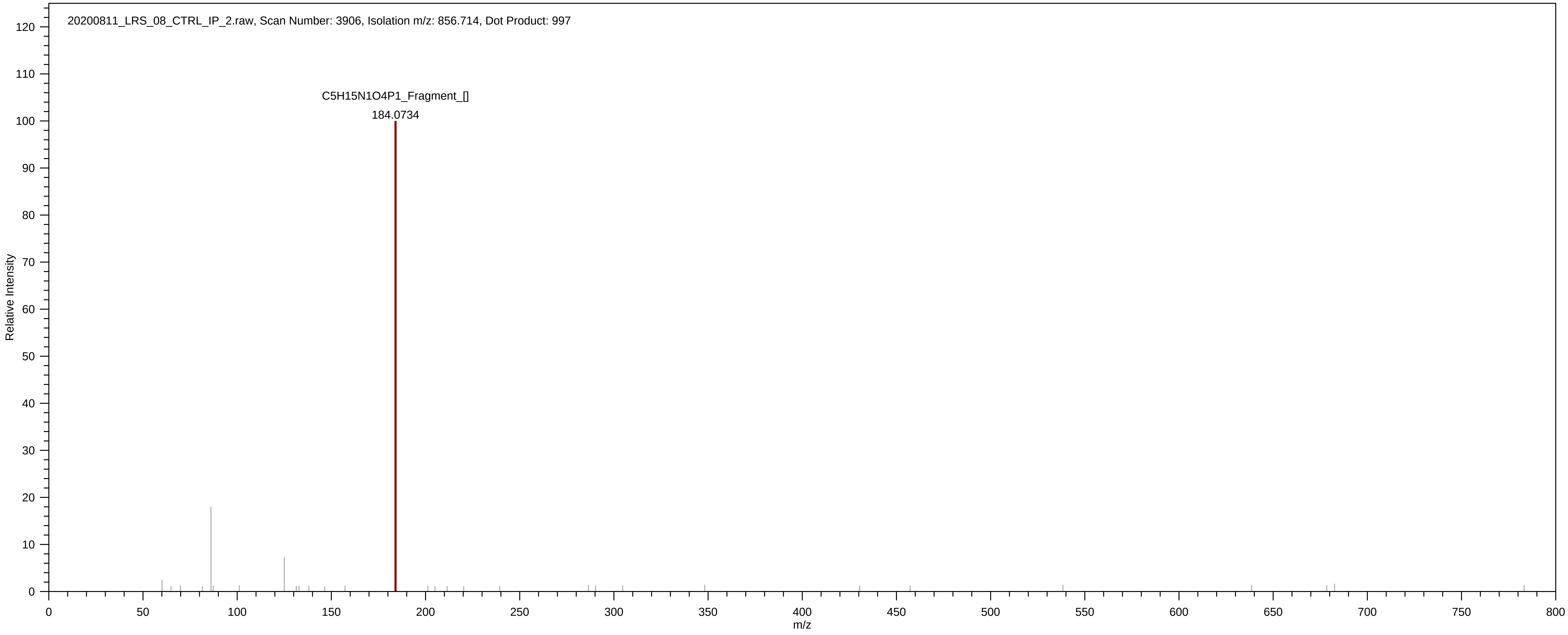

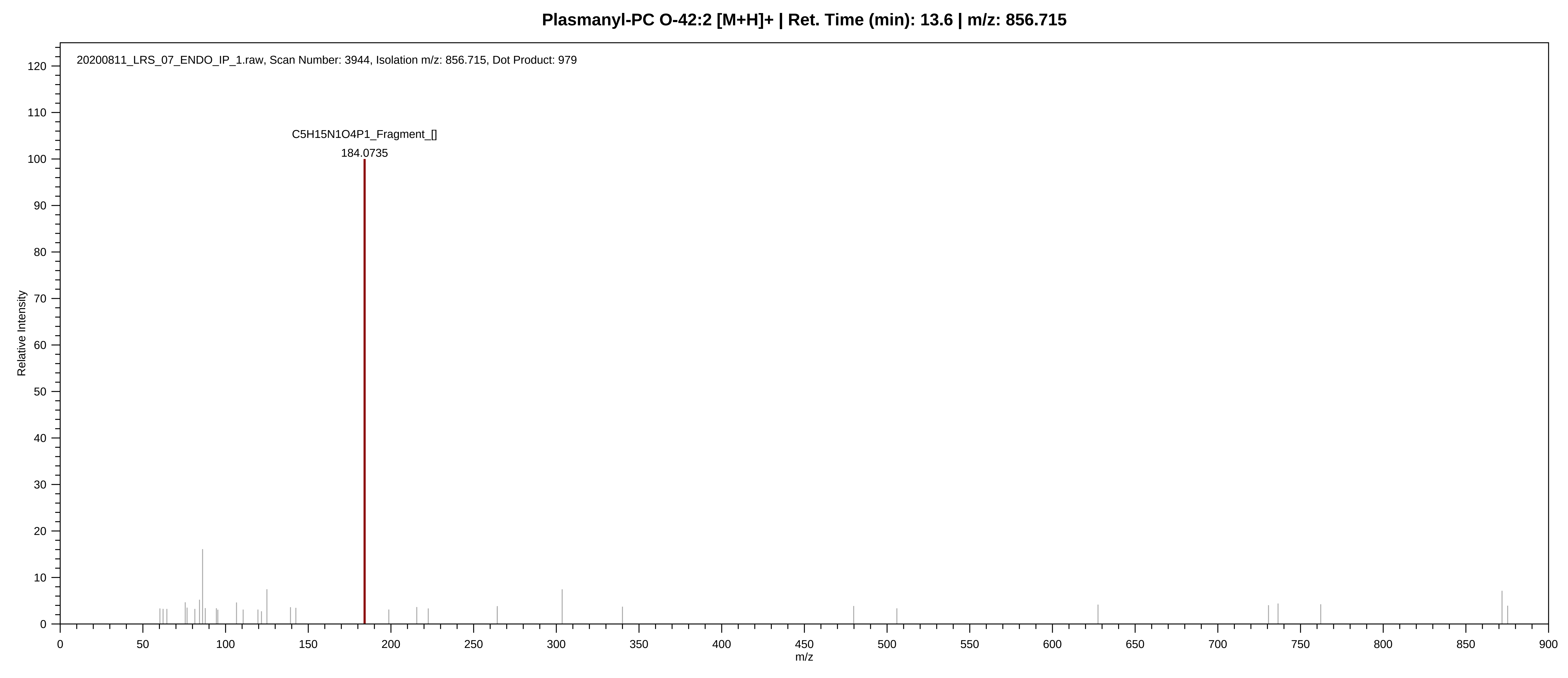
Plasmanyl-PC O-42:2 [M+H]+ | Ret. Time (min): 13.6 | m/z: 856.715
20200811_LRS_07_ENDO_IP_1.raw, Scan Number: 3944, Isolation m/z: 856.715, Dot Product: 979
C5H15N1O4P1_Fragment_[]
184.0735
Relative Intensity
m/z

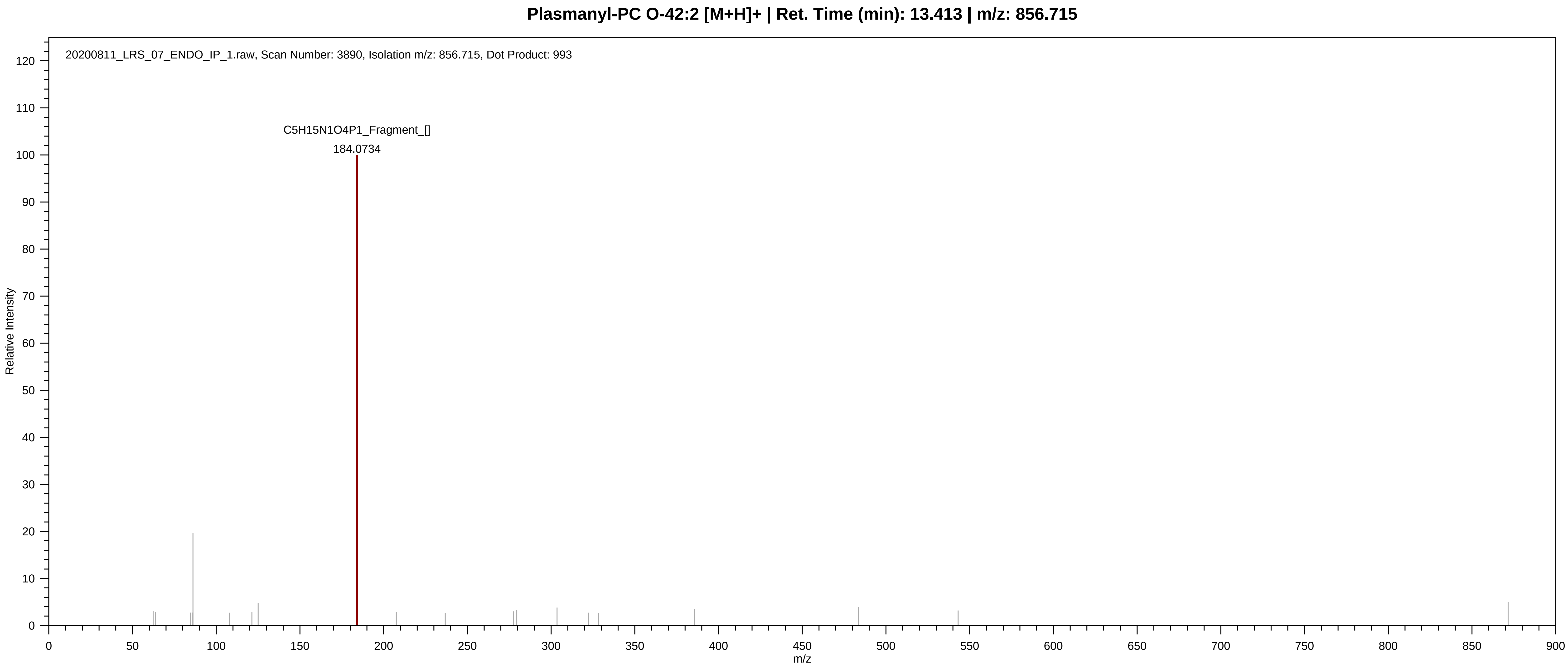
Plasmanyl-PC O-42:2 [M+H]+ | Ret. Time (min): 13.413 | m/z: 856.715
20200811_LRS_07_ENDO_IP_1.raw, Scan Number: 3890, Isolation m/z: 856.715, Dot Product: 993
C5H15N1O4P1_Fragment_[]
184.0734
Relative Intensity
m/z

Plasmanyl-PC O-42:2 [M+H]+ | Ret. Time (min): 13.418 | m/z: 856.715

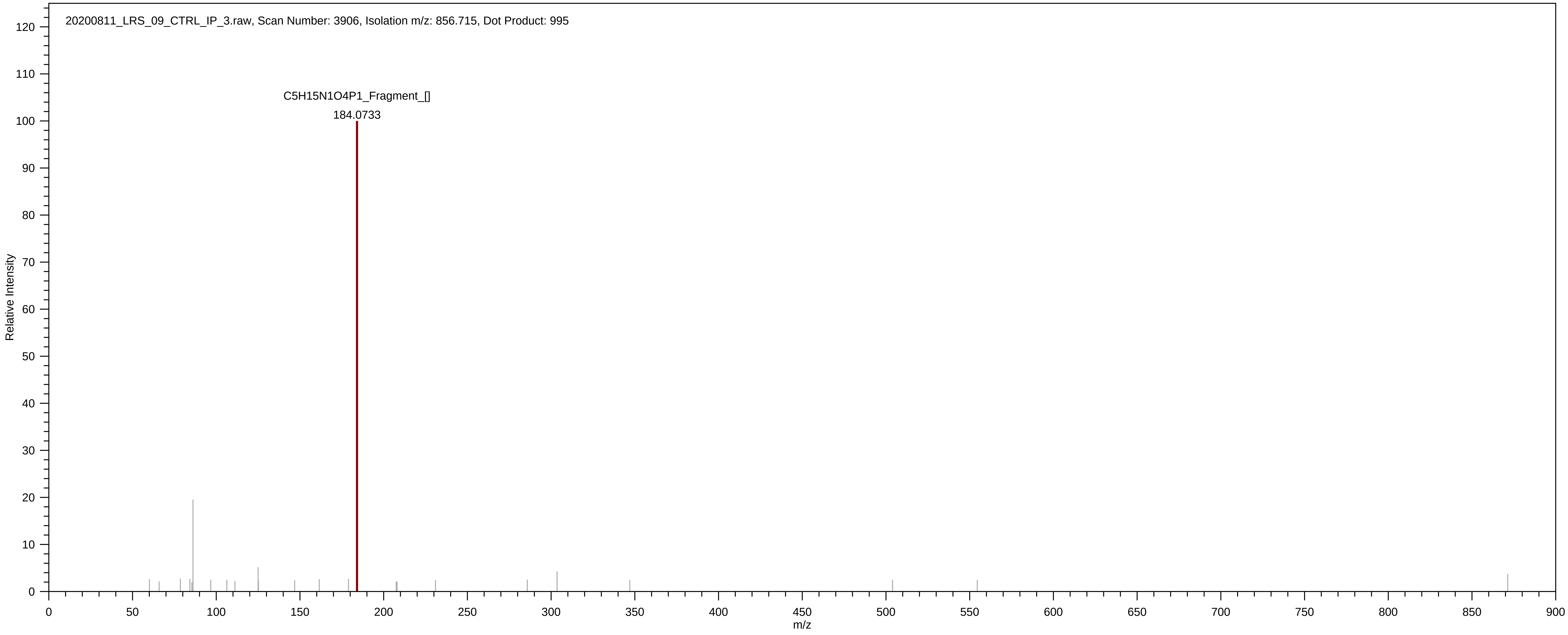

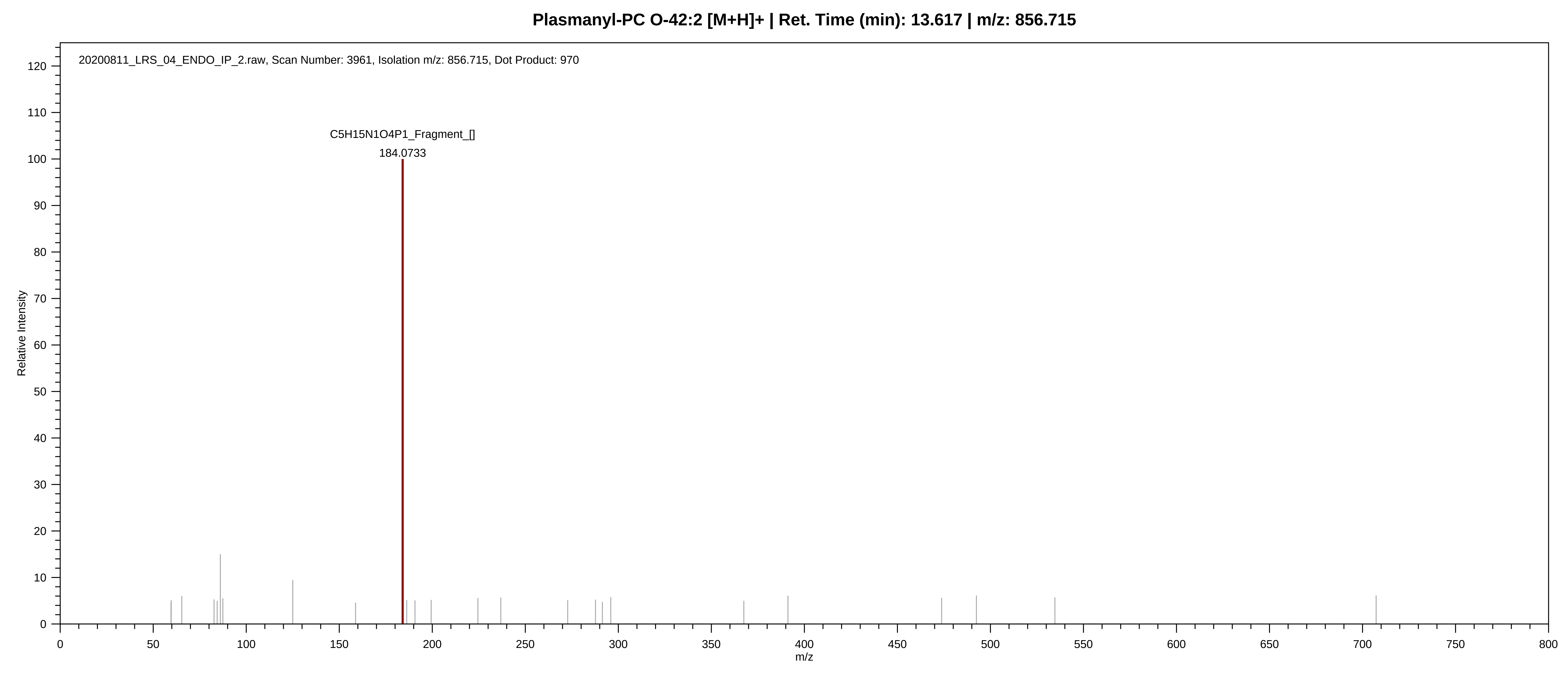

Plasmanyl-PC O-42:2 [M+H]+ | Ret. Time (min): 13.617 | m/z: 856.715
20200811_LRS_04_ENDO_IP_2.raw, Scan Number: 3961, Isolation m/z: 856.715, Dot Product: 970
C5H15N1O4P1_Fragment_[]
184.0733
Relative Intensity
m/z

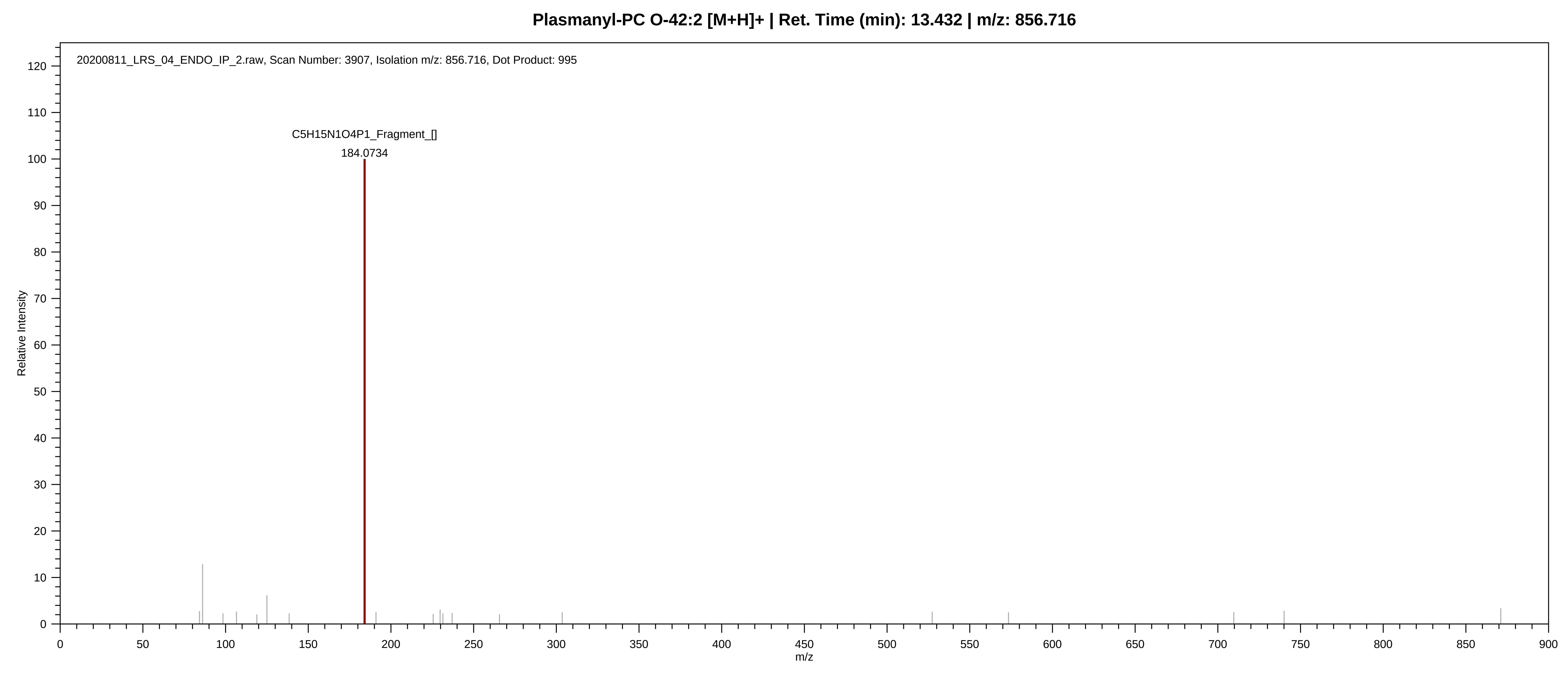
Plasmanyl-PC O-42:2 [M+H]+ | Ret. Time (min): 13.432 | m/z: 856.716
20200811_LRS_04_ENDO_IP_2.raw, Scan Number: 3907, Isolation m/z: 856.716, Dot Product: 995
C5H15N1O4P1_Fragment_[]
184.0734
Relative Intensity
m/z

Plasmanyl-PC O-42:2 [M+H]+ | Ret. Time (min): 13.454 | m/z: 856.716

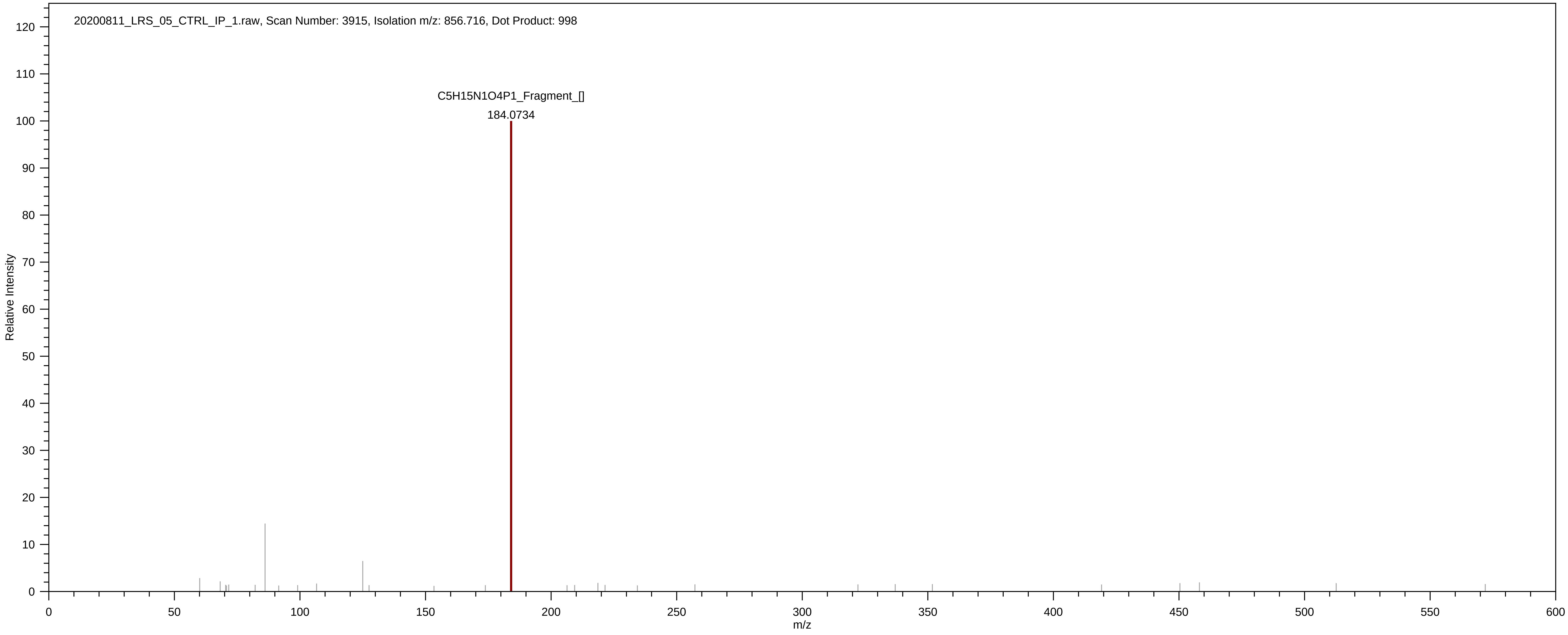

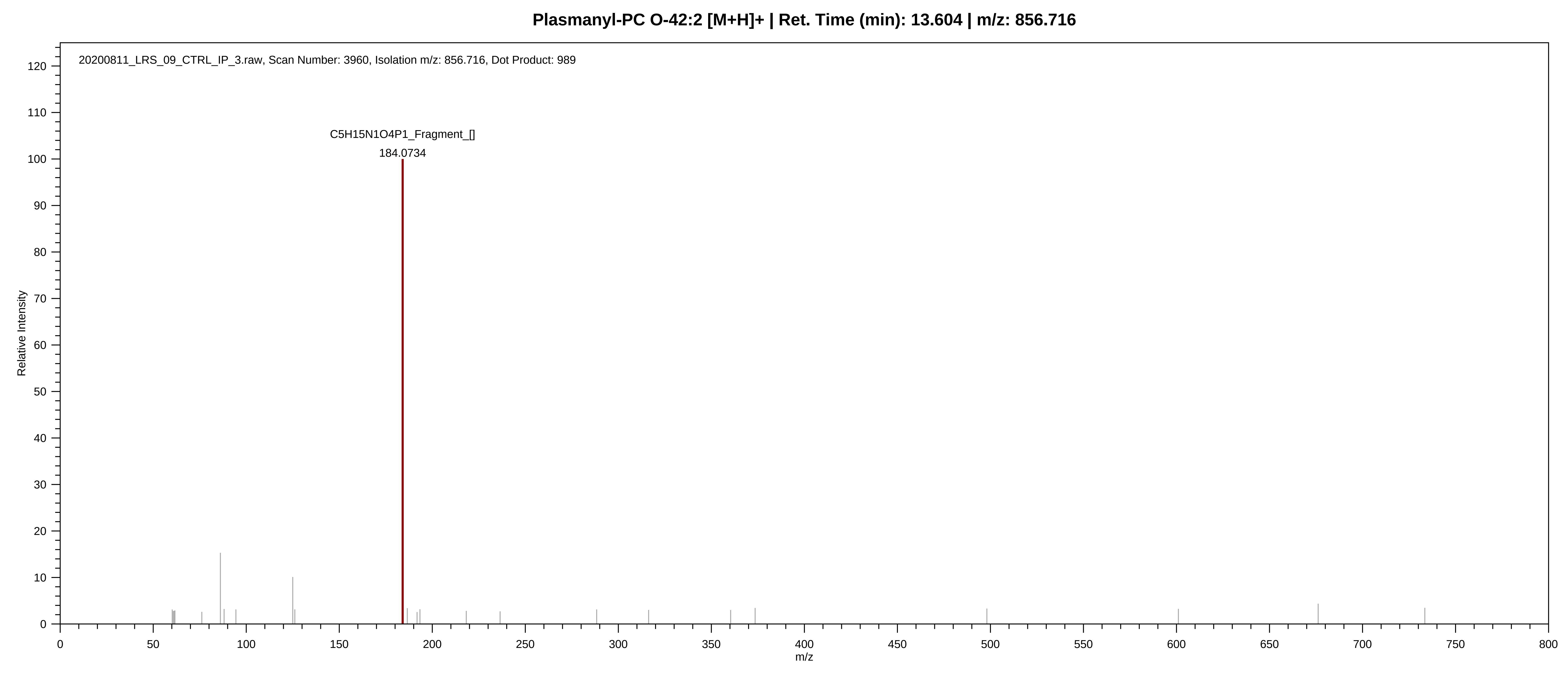
Plasmanyl-PC O-42:2 [M+H]+ | Ret. Time (min): 13.604 | m/z: 856.716
20200811_LRS_09_CTRL_IP_3.raw, Scan Number: 3960, Isolation m/z: 856.716, Dot Product: 989
C5H15N1O4P1_Fragment_[]
184.0734
Relative Intensity
m/z

Plasmanyl-PC O-42:2 [M+H]+ | Ret. Time (min): 13.604 | m/z: 856.716

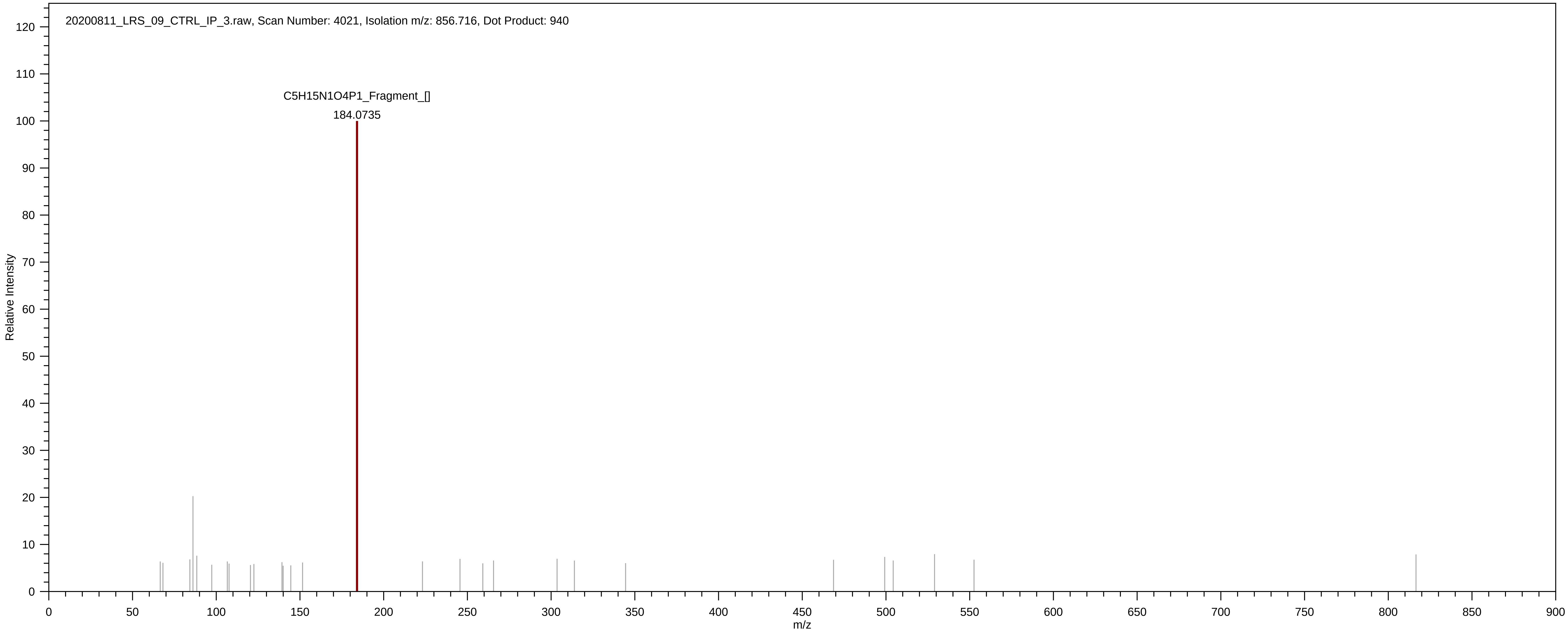

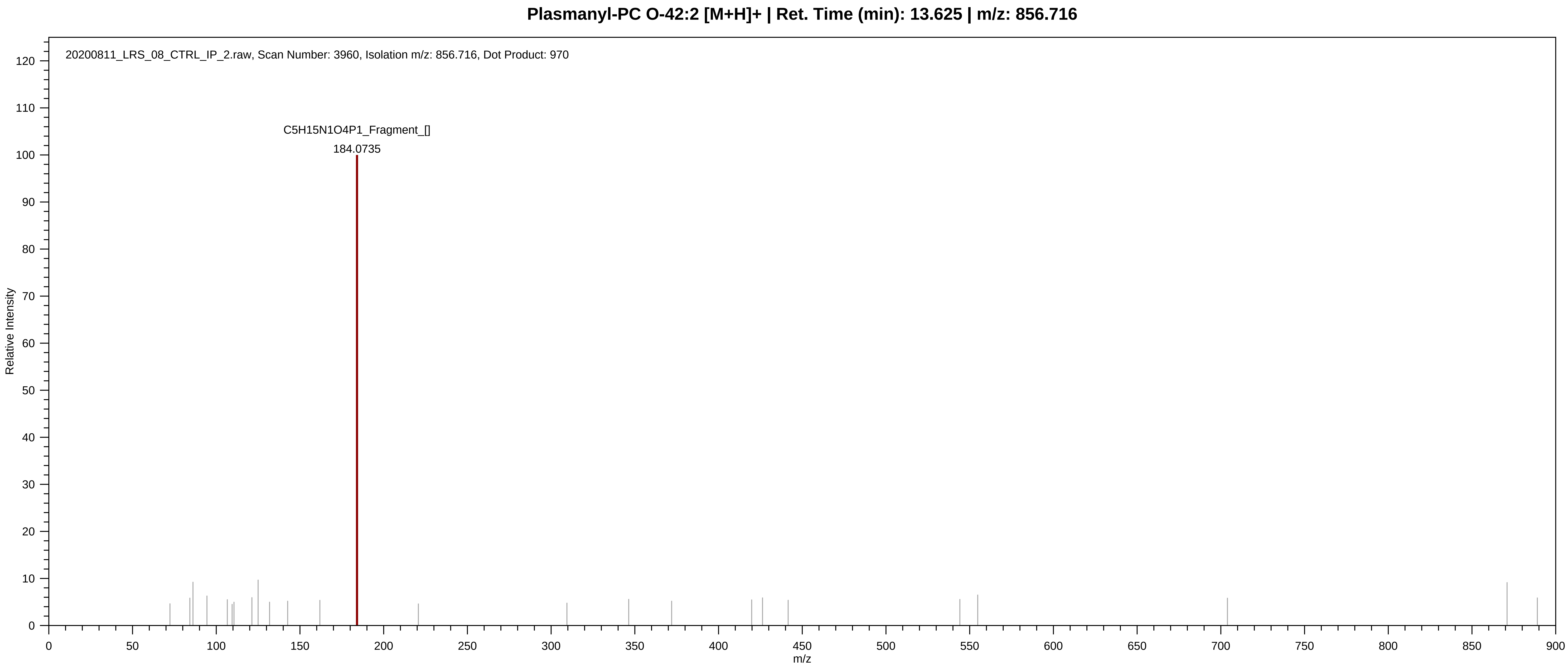
Plasmanyl-PC O-42:2 [M+H]+ | Ret. Time (min): 13.625 | m/z: 856.716
20200811_LRS_08_CTRL_IP_2.raw, Scan Number: 3960, Isolation m/z: 856.716, Dot Product: 970
C5H15N1O4P1_Fragment_[]
184.0735
Relative Intensity
m/z
0
10
20
30
40
50
60
70
80
90
100
110
120
0
50
100
150
200
250
300
350
400
450
500
550
600
650
700
750
800
850
900

Plasmanyl-PC O-42:2 [M+H]+ | Ret. Time (min): 13.412 | m/z: 856.717

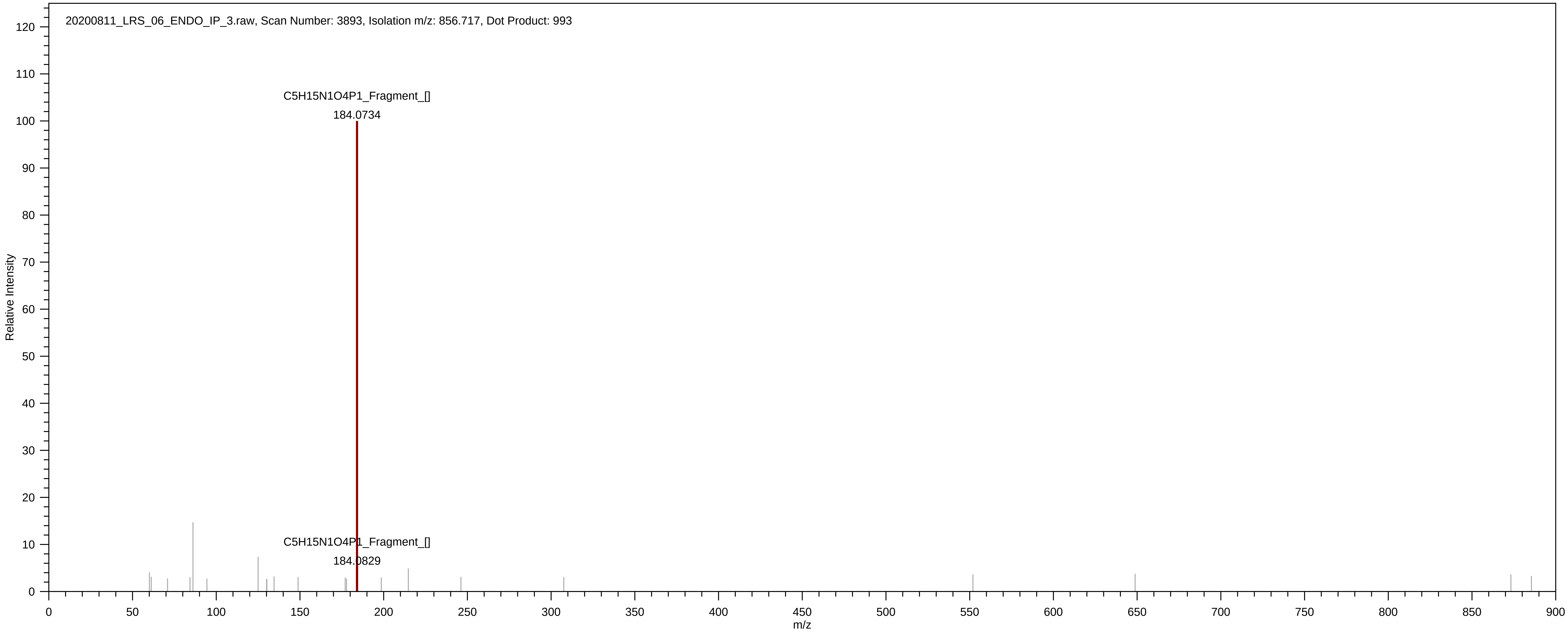

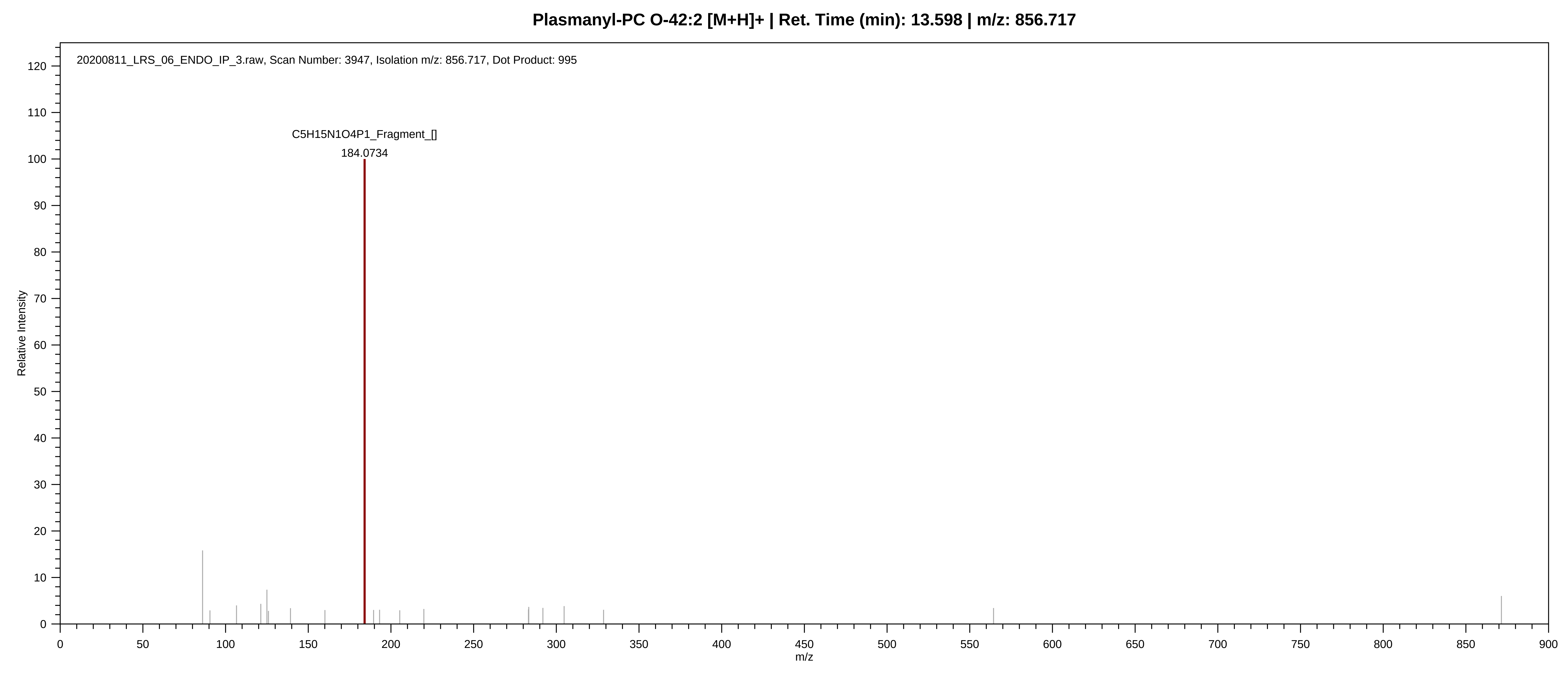

Plasmanyl-PC O-42:2 [M+H]+ | Ret. Time (min): 13.598 | m/z: 856.717
20200811_LRS_06_ENDO_IP_3.raw, Scan Number: 3947, Isolation m/z: 856.717, Dot Product: 995
C5H15N1O4P1_Fragment_[]
184.0734
Relative Intensity
m/z

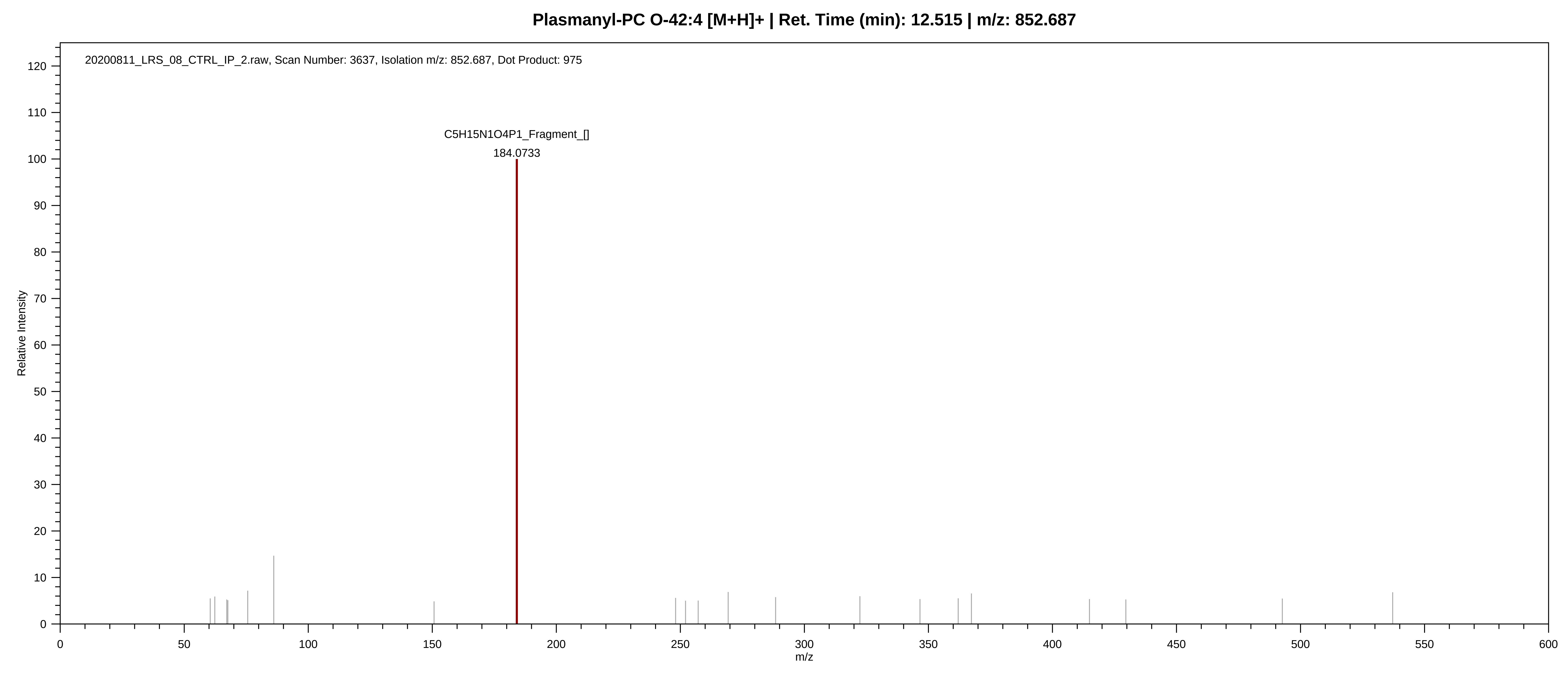

Plasmanyl-PC O-42:4 [M+H]+ | Ret. Time (min): 12.515 | m/z: 852.687
20200811_LRS_08_CTRL_IP_2.raw, Scan Number: 3637, Isolation m/z: 852.687, Dot Product: 975
C5H15N1O4P1_Fragment_[]
184.0733
Relative Intensity
m/z

Plasmanyl-PC O-42:5 [M+H]+ | Ret. Time (min): 11.462 | m/z: 850.67

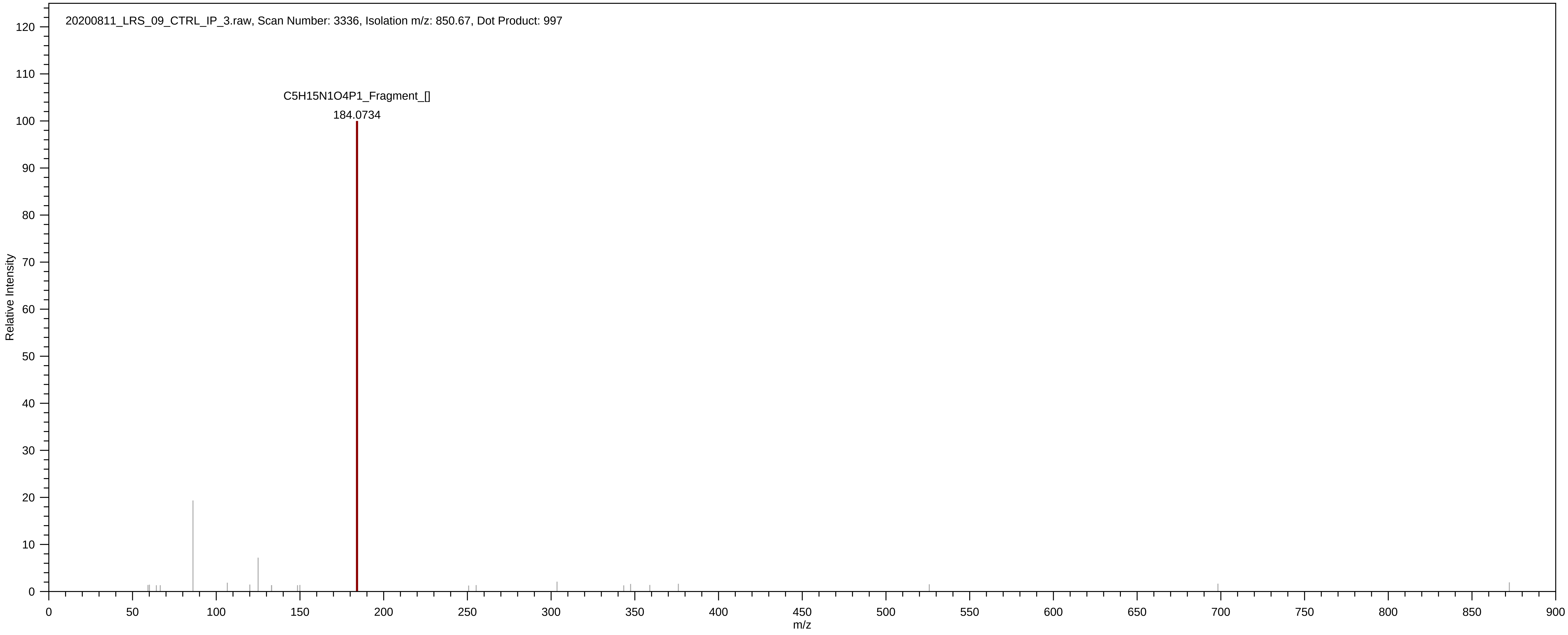

Plasmanyl-PC O-42:5 [M+H]+ | Ret. Time (min): 11.47 | m/z: 850.666

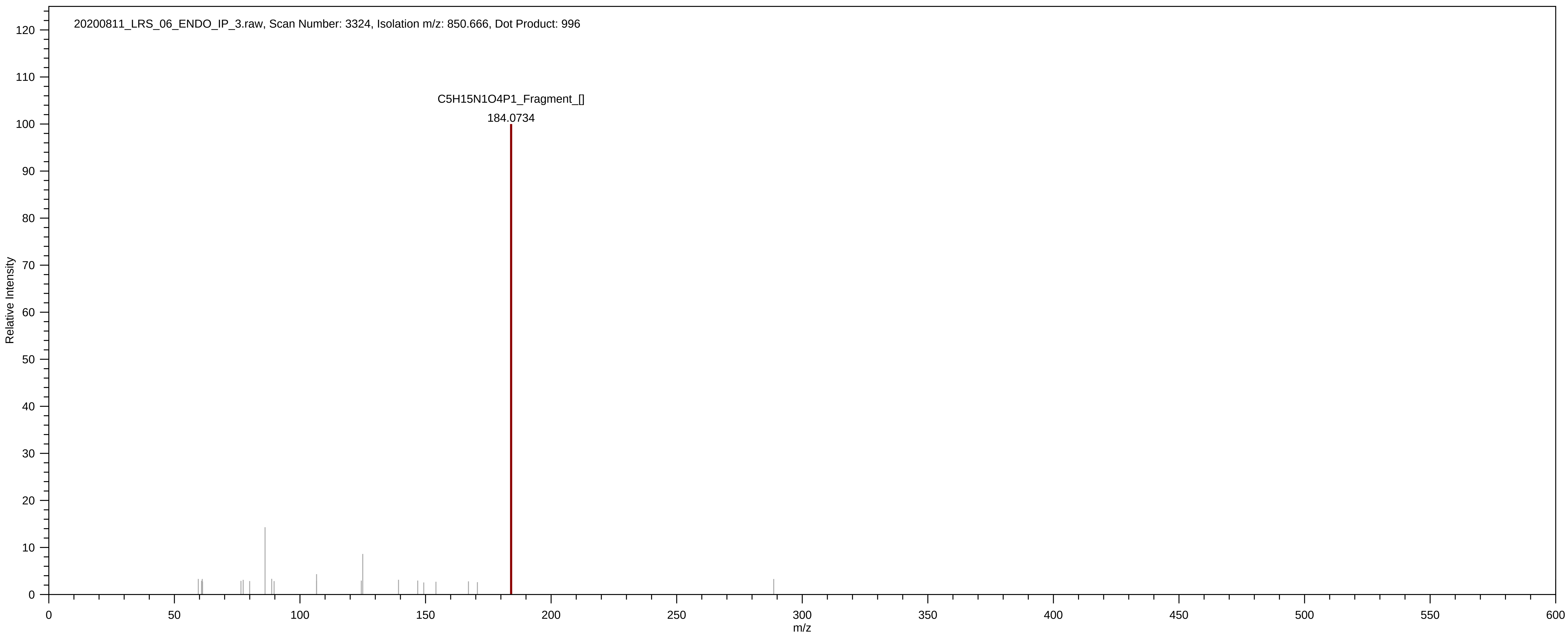

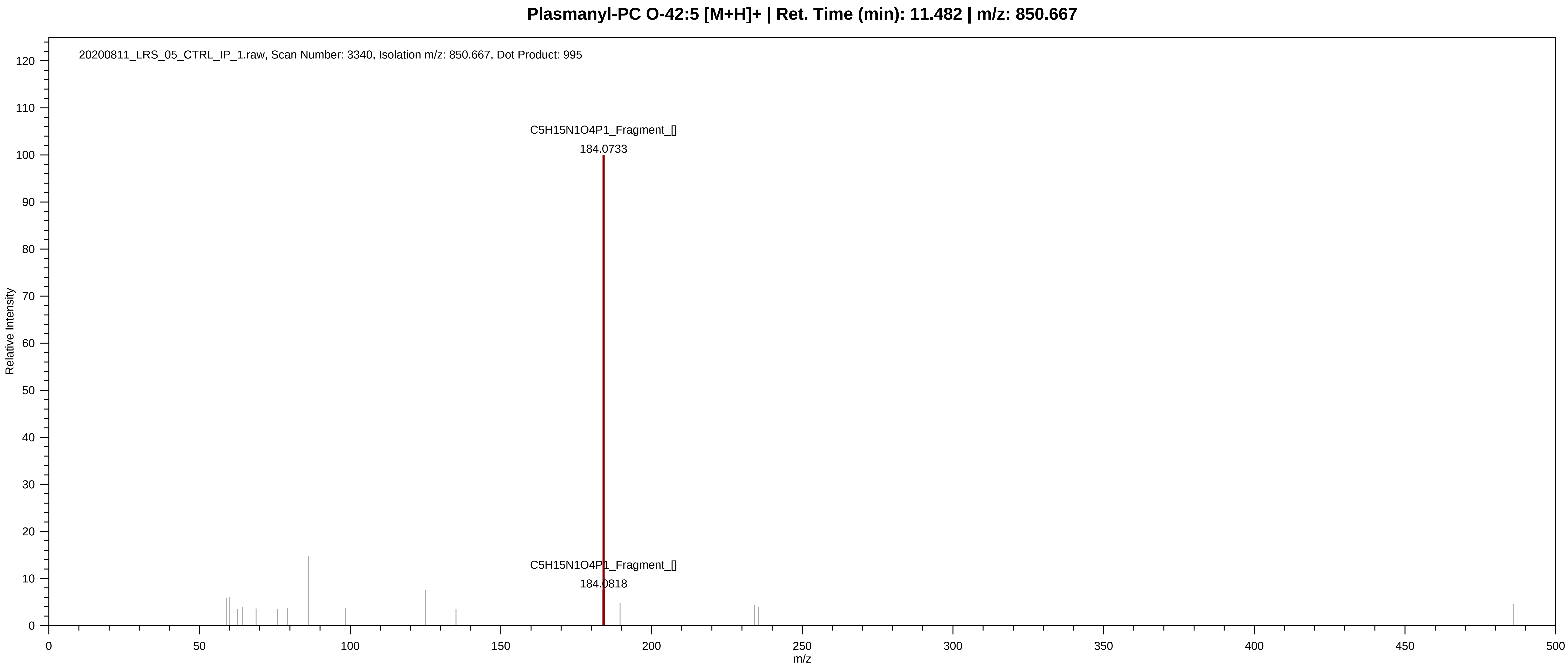

Plasmanyl-PC O-42:5 [M+H]+ | Ret. Time (min): 11.482 | m/z: 850.667
20200811_LRS_05_CTRL_IP_1.raw, Scan Number: 3340, Isolation m/z: 850.667, Dot Product: 995
C5H15N1O4P1_Fragment_[]
184.0733
C5H15N1O4P1_Fragment_[]
184.0818
Relative Intensity
m/z

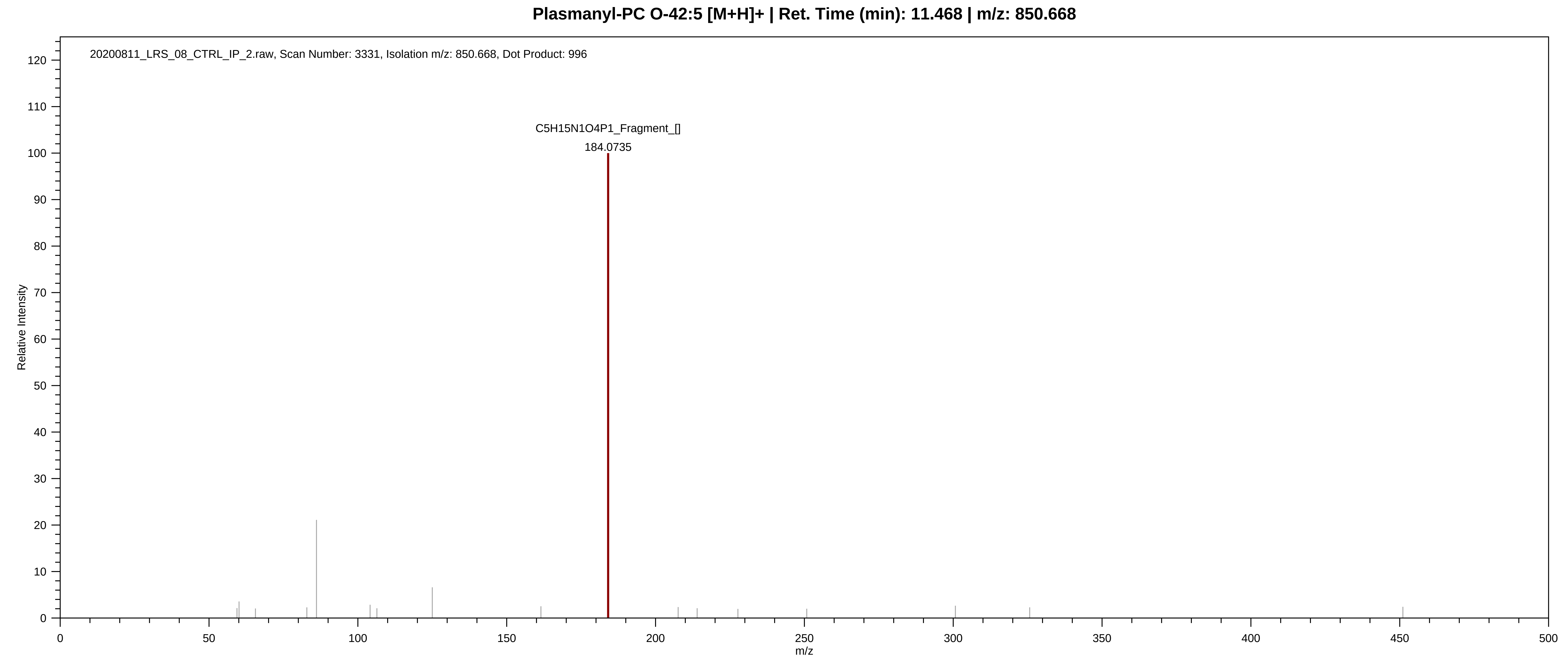
Plasmanyl-PC O-42:5 [M+H]+ | Ret. Time (min): 11.468 | m/z: 850.668
20200811_LRS_08_CTRL_IP_2.raw, Scan Number: 3331, Isolation m/z: 850.668, Dot Product: 996
C5H15N1O4P1_Fragment_[]
184.0735
Relative Intensity
m/z
0
10
20
30
40
50
60
70
80
90
100
110
120
0
50
100
150
200
250
300
350
400
450
500

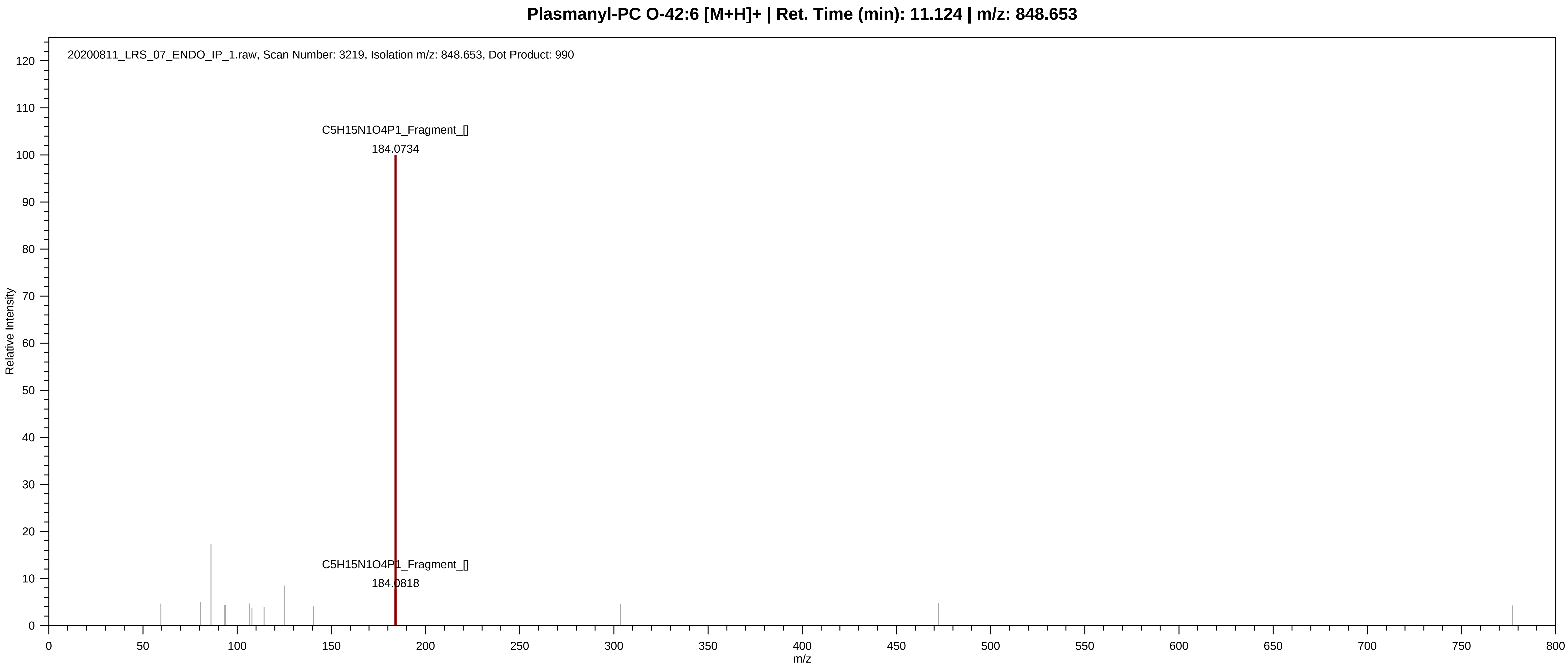
Plasmanyl-PC O-42:6 [M+H]+ | Ret. Time (min): 11.124 | m/z: 848.653
20200811_LRS_07_ENDO_IP_1.raw, Scan Number: 3219, Isolation m/z: 848.653, Dot Product: 990
C5H15N1O4P1_Fragment_[]
184.0734
C5H15N1O4P1_Fragment_[]
184.0818
Relative Intensity
m/z
0
10
20
30
40
50
60
70
80
90
100
110
120
0
50
100
150
200
250
300
350
400
450
500
550
600
650
700
750
800

Plasmanyl-PC O-44:2 [M+H]+ | Ret. Time (min): 14.444 | m/z: 884.743

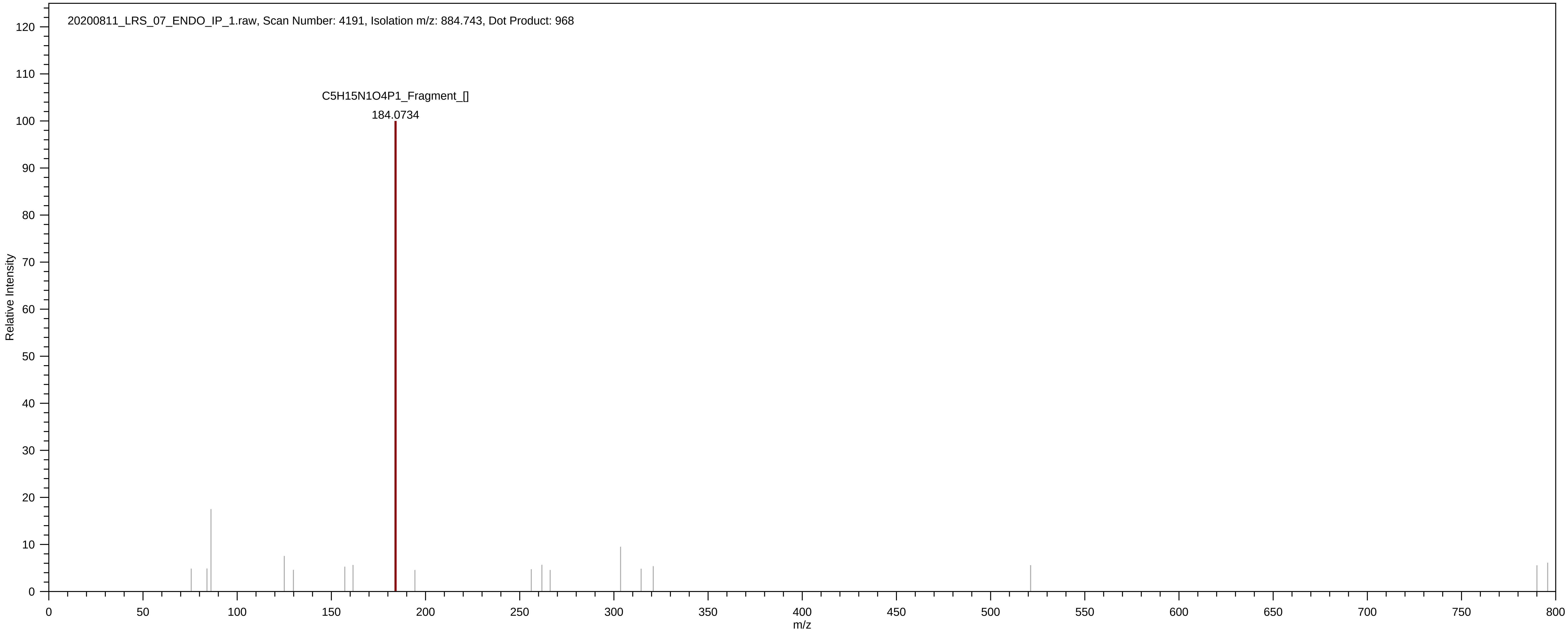

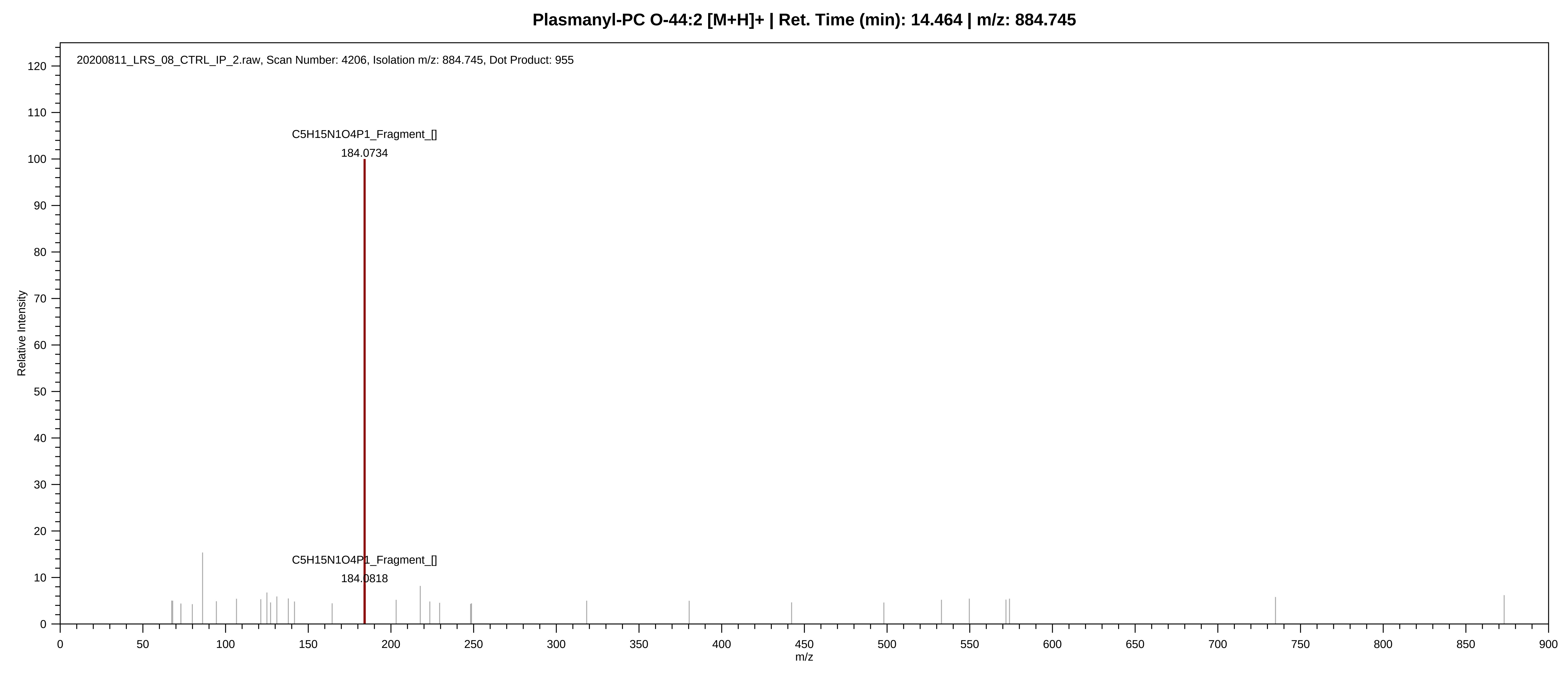

Plasmanyl-PC O-44:2 [M+H]+ | Ret. Time (min): 14.464 | m/z: 884.745
20200811_LRS_08_CTRL_IP_2.raw, Scan Number: 4206, Isolation m/z: 884.745, Dot Product: 955
C5H15N1O4P1_Fragment_[]
184.0734
C5H15N1O4P1_Fragment_[]
184.0818
Relative Intensity
m/z

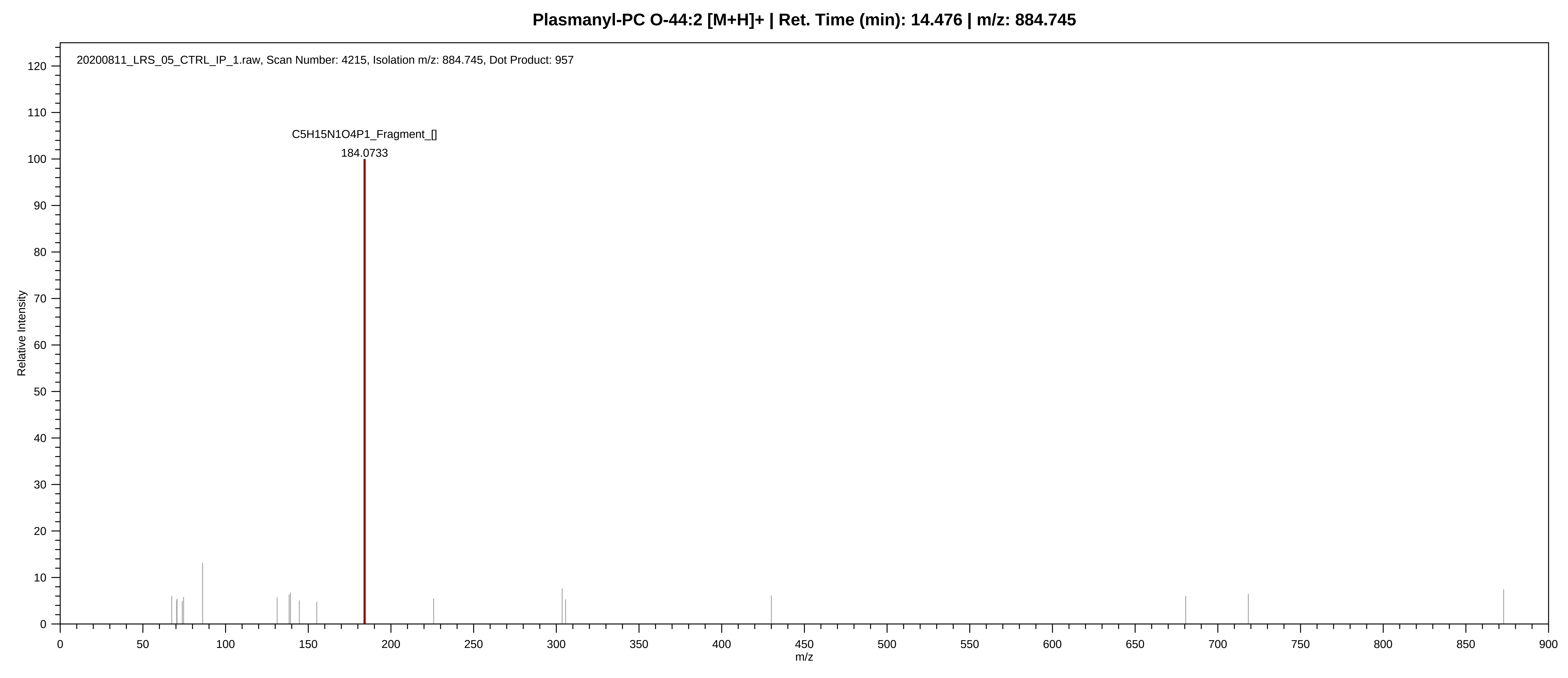
Plasmanyl-PC O-44:2 [M+H]+ | Ret. Time (min): 14.476 | m/z: 884.745
20200811_LRS_05_CTRL_IP_1.raw, Scan Number: 4215, Isolation m/z: 884.745, Dot Product: 957
C5H15N1O4P1_Fragment_[]
184.0733
Relative Intensity
m/z

Plasmanyl-PC O-44:2 [M+H]+ | Ret. Time (min): 14.44 | m/z: 884.746

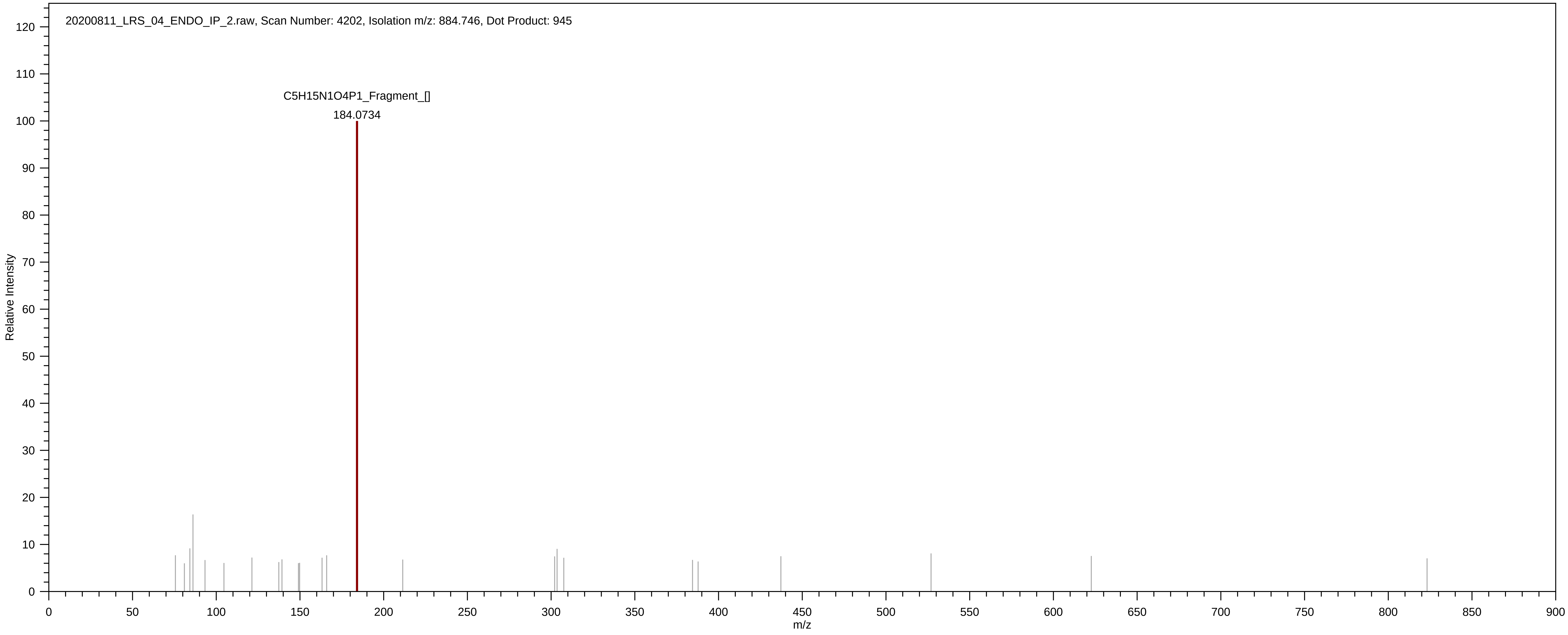

Plasmanyl-PC O-44:2 [M+H]+ | Ret. Time (min): 14.422 | m/z: 884.748

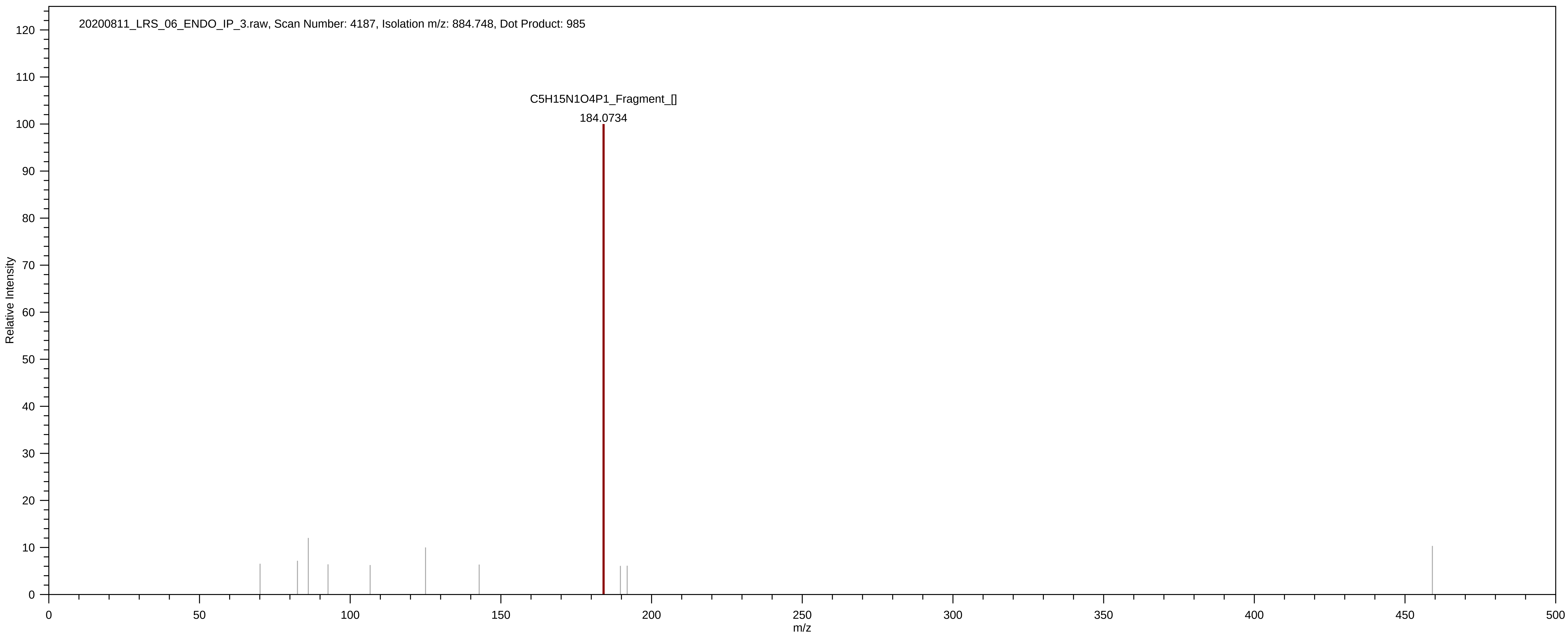

Plasmanyl-PE O-16:0_20:1 [M-H]- | Ret. Time (min): 11.925 | m/z: 730.575

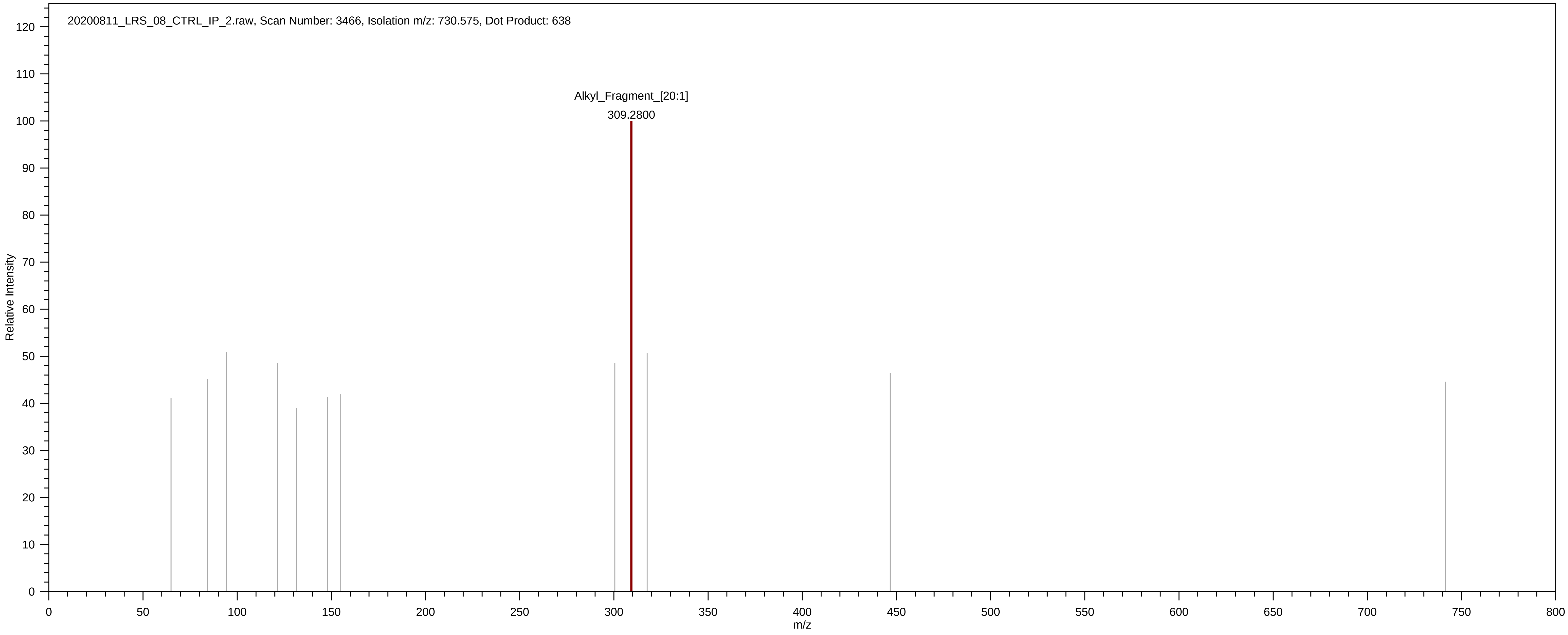

Plasmanyl-PE O-16:0_20:1 [M-H]- | Ret. Time (min): 11.92 | m/z: 730.577

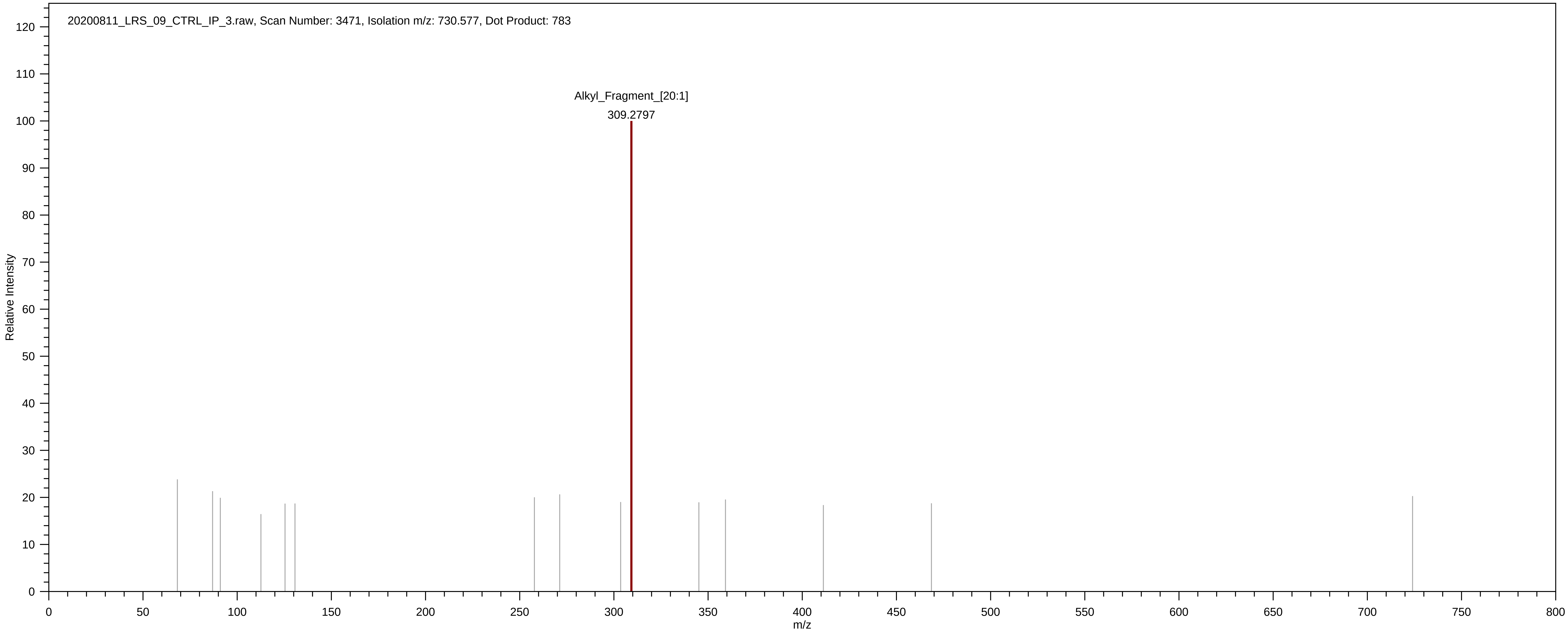

Plasmanyl-PE O-18:0_16:0 [M-H]- | Ret. Time (min): 10.407 | m/z: 704.56

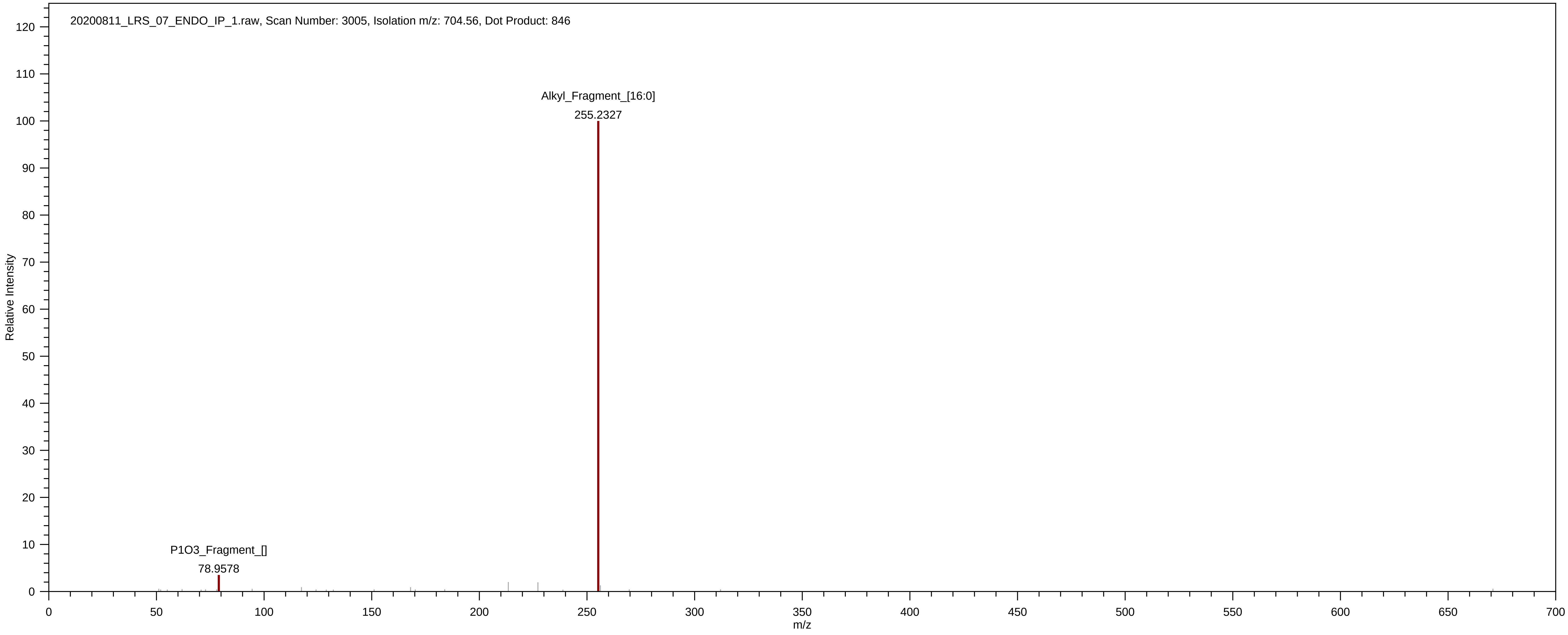

Plasmanyl-PE O-18:0_16:0 [M-H]- | Ret. Time (min): 10.422 | m/z: 704.56

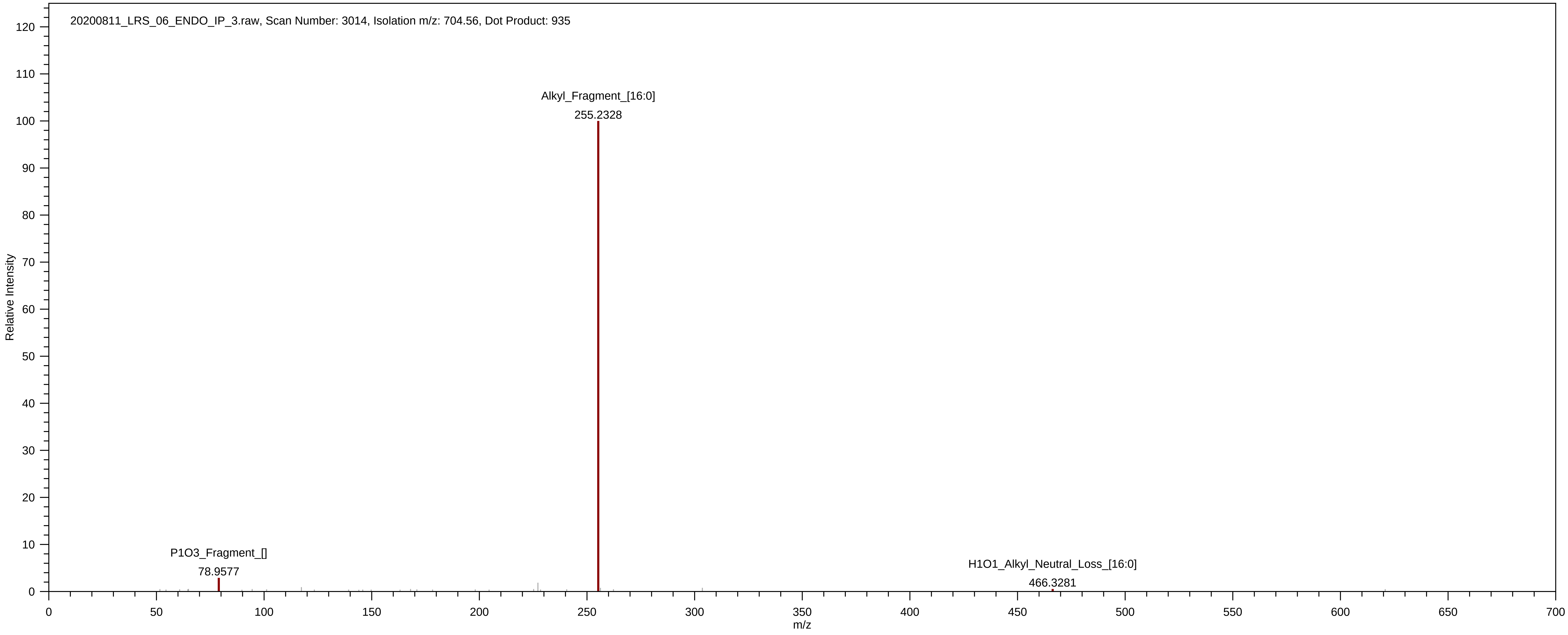

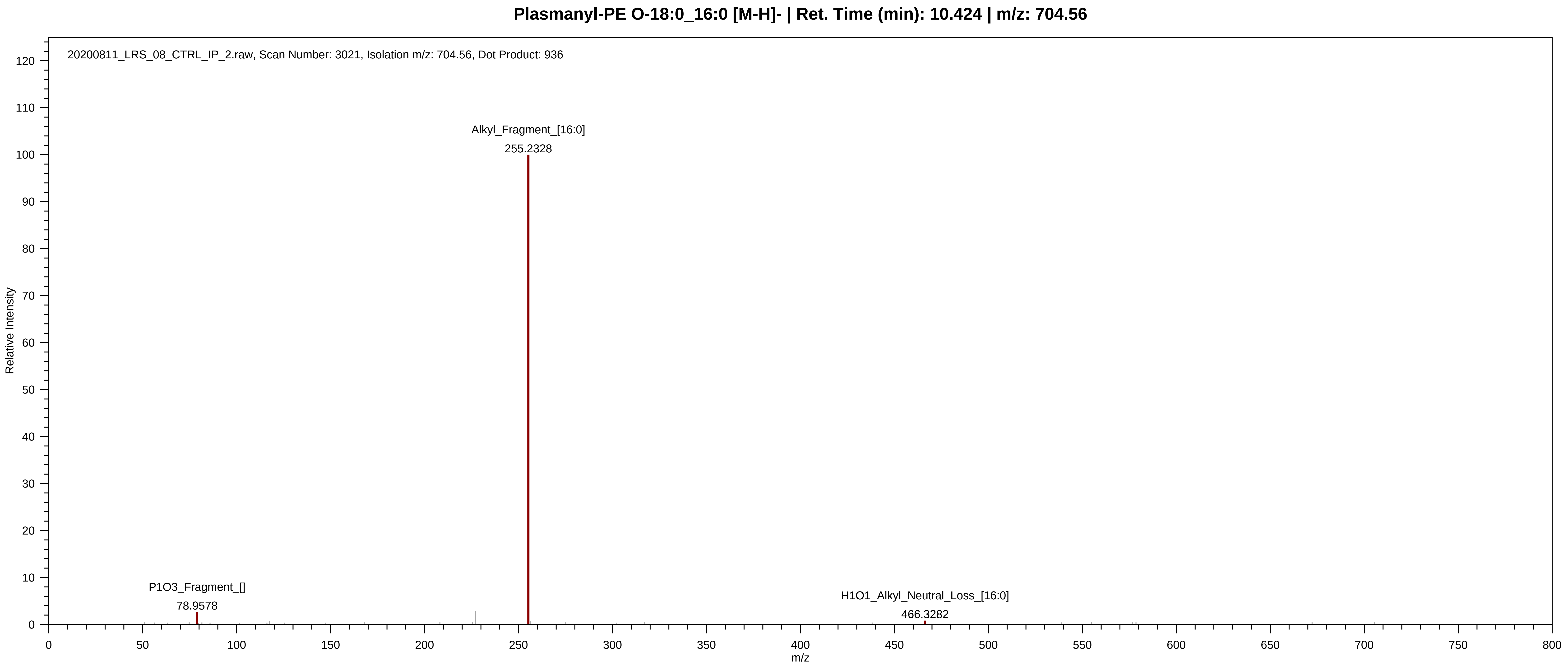

Plasmanyl-PE O-18:0_16:0 [M-H]- | Ret. Time (min): 10.424 | m/z: 704.56
20200811_LRS_08_CTRL_IP_2.raw, Scan Number: 3021, Isolation m/z: 704.56, Dot Product: 936
Alkyl_Fragment_[16:0]
255.2328
P1O3_Fragment_[]
78.9578
H1O1_Alkyl_Neutral_Loss_[16:0]
466.3282
Relative Intensity
m/z

Plasmanyl-PE O-18:0_16:0 [M-H]- | Ret. Time (min): 10.427 | m/z: 704.56

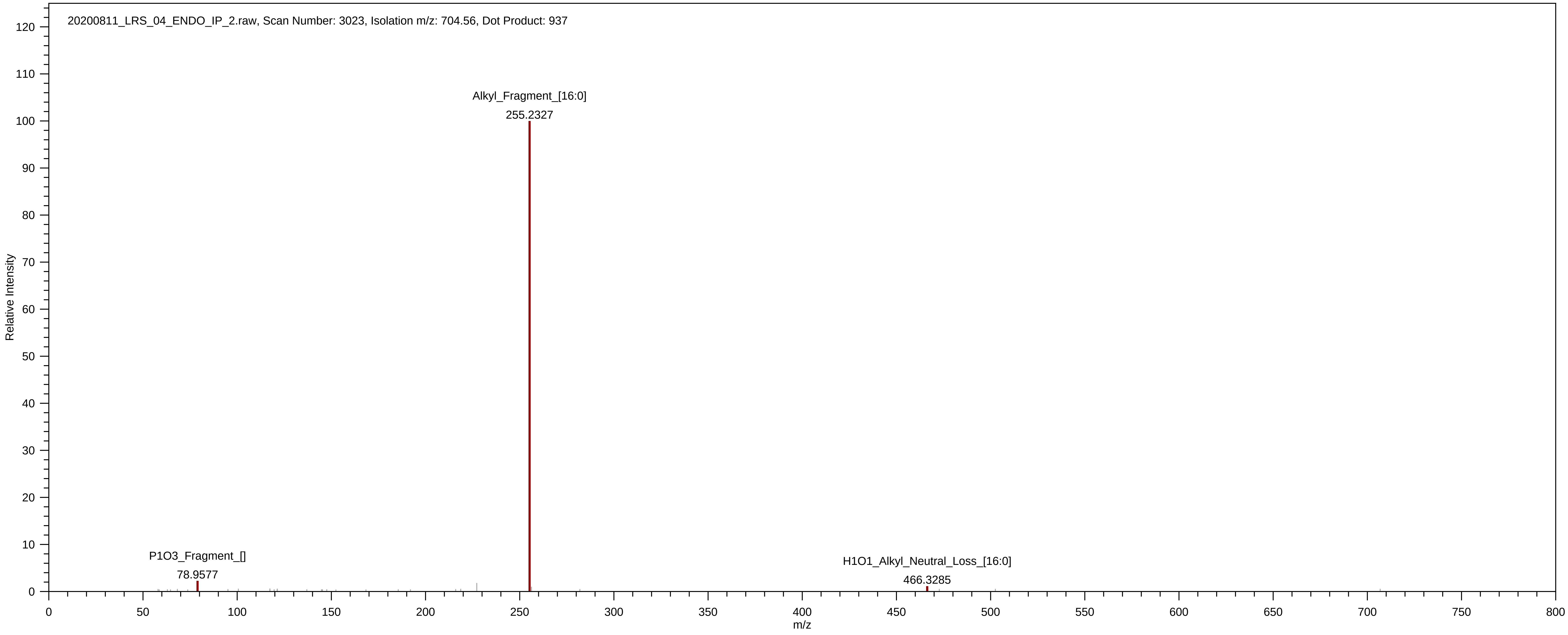

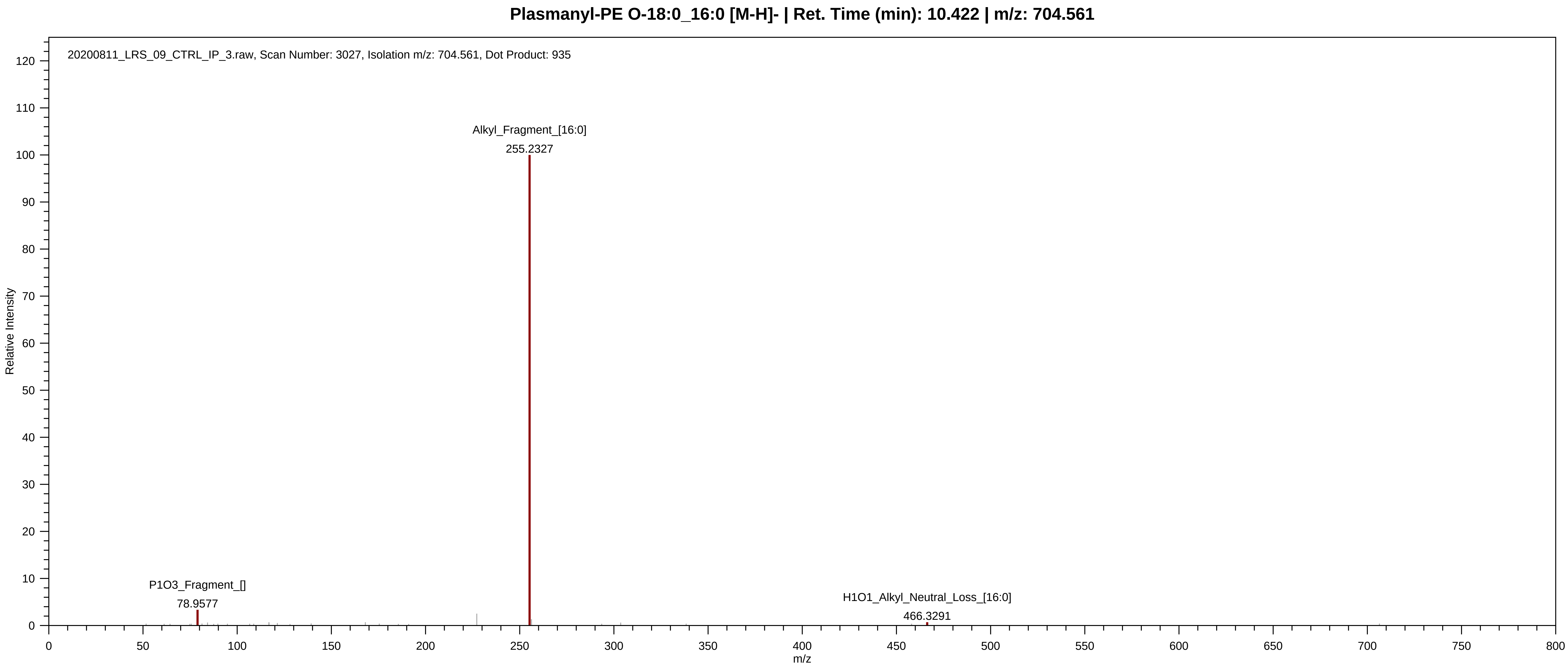

Plasmanyl-PE O-18:0_16:0 [M-H]- | Ret. Time (min): 10.422 | m/z: 704.561
20200811_LRS_09_CTRL_IP_3.raw, Scan Number: 3027, Isolation m/z: 704.561, Dot Product: 935
Alkyl_Fragment_[16:0]
255.2327
P1O3_Fragment_[]
78.9577
H1O1_Alkyl_Neutral_Loss_[16:0]
466.3291
Relative Intensity
m/z

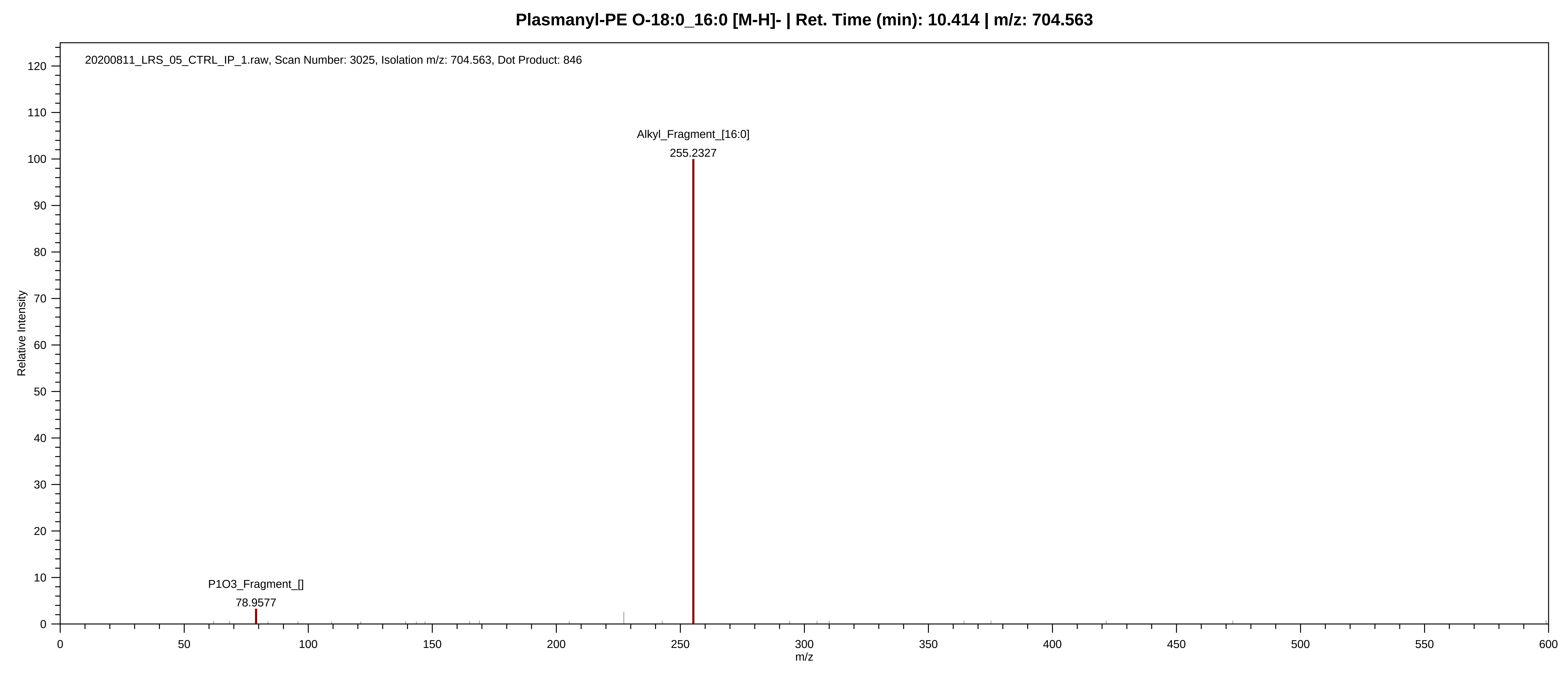
Plasmanyl-PE O-18:0_16:0 [M-H]- | Ret. Time (min): 10.414 | m/z: 704.563
20200811_LRS_05_CTRL_IP_1.raw, Scan Number: 3025, Isolation m/z: 704.563, Dot Product: 846
Alkyl_Fragment_[16:0]
255.2327
P1O3_Fragment_[]
78.9577
Relative Intensity
m/z
0
10
20
30
40
50
60
70
80
90
100
110
120
0
50
100
150
200
250
300
350
400
450
500
550
600

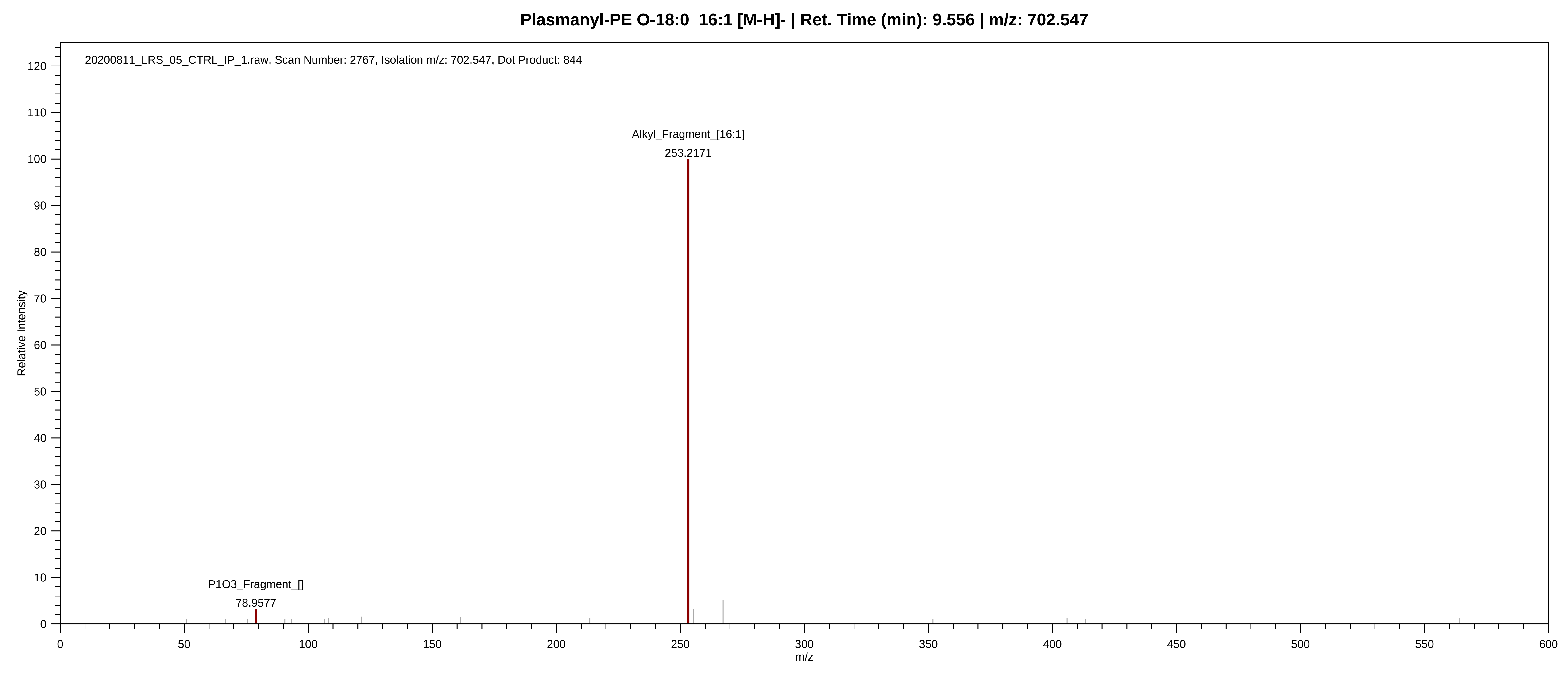
Plasmanyl-PE O-18:0_16:1 [M-H]- | Ret. Time (min): 9.556 | m/z: 702.547
20200811_LRS_05_CTRL_IP_1.raw, Scan Number: 2767, Isolation m/z: 702.547, Dot Product: 844
Alkyl_Fragment_[16:1]
253.2171
P1O3_Fragment_[]
78.9577
Relative Intensity
m/z

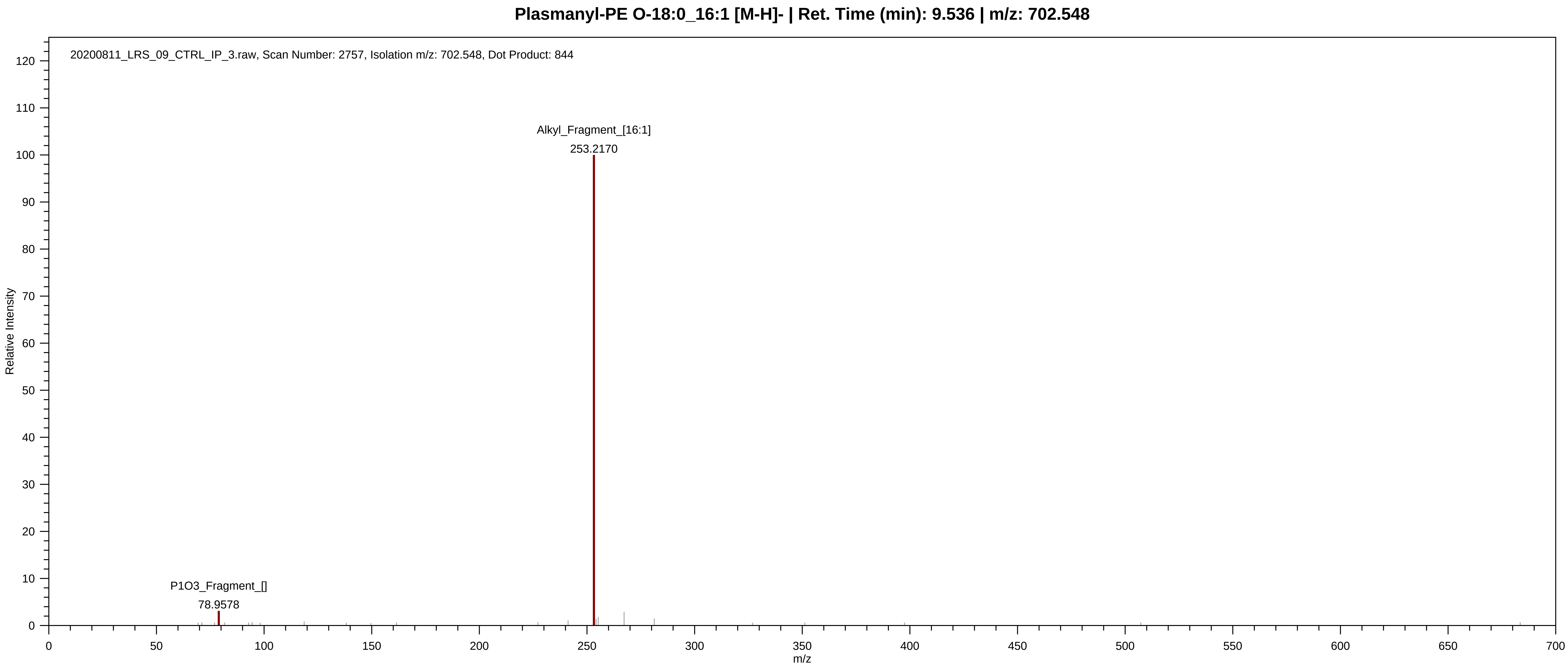

Plasmanyl-PE O-18:0_16:1 [M-H]- | Ret. Time (min): 9.536 | m/z: 702.548
20200811_LRS_09_CTRL_IP_3.raw, Scan Number: 2757, Isolation m/z: 702.548, Dot Product: 844
Alkyl_Fragment_[16:1]
253.2170
P1O3_Fragment_[]
78.9578
Relative Intensity
m/z
0
10
20
30
40
50
60
70
80
90
100
110
120
0
50
100
150
200
250
300
350
400
450
500
550
600
650
700

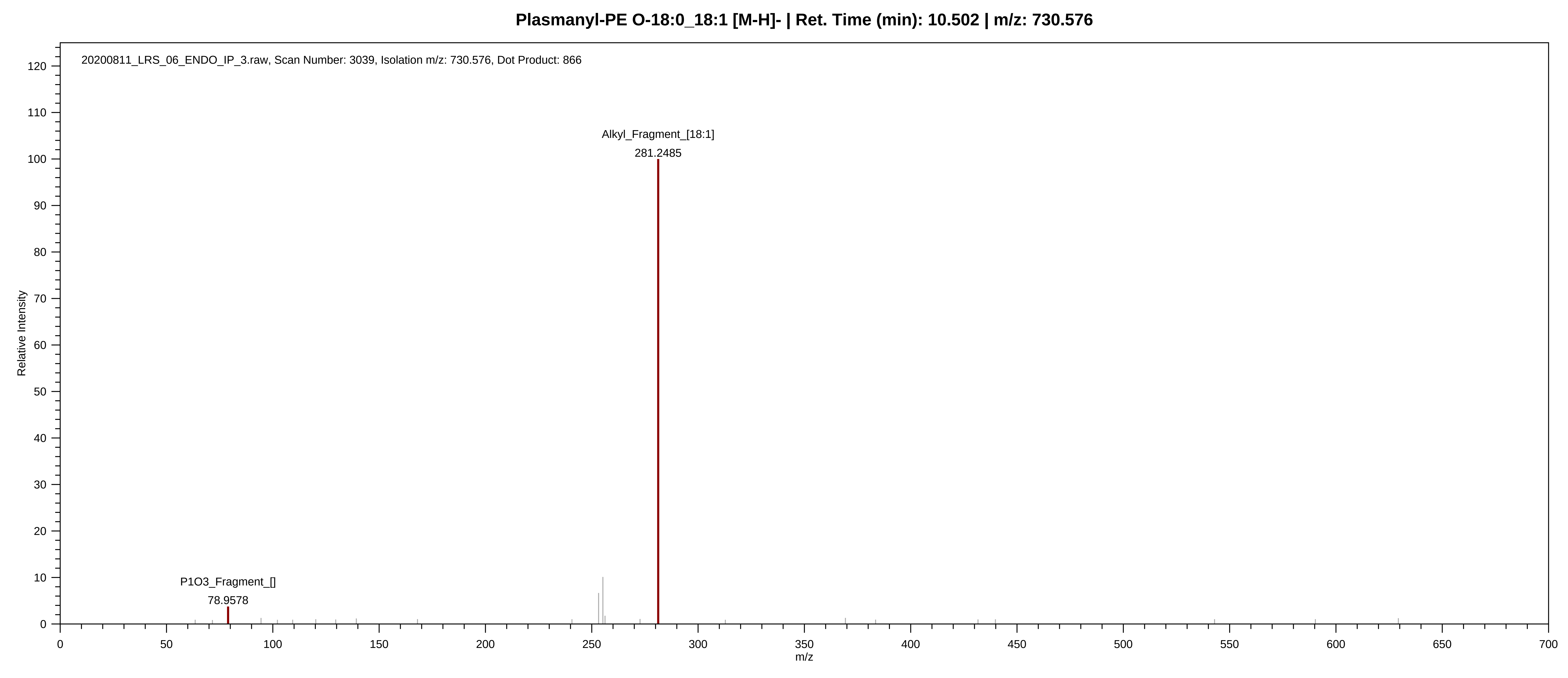
Plasmanyl-PE O-18:0_18:1 [M-H]- | Ret. Time (min): 10.502 | m/z: 730.576
20200811_LRS_06_ENDO_IP_3.raw, Scan Number: 3039, Isolation m/z: 730.576, Dot Product: 866
Alkyl_Fragment_[18:1]
281.2485
P1O3_Fragment_[]
78.9578
Relative Intensity
m/z
0
10
20
30
40
50
60
70
80
90
100
110
120
0
50
100
150
200
250
300
350
400
450
500
550
600
650
700

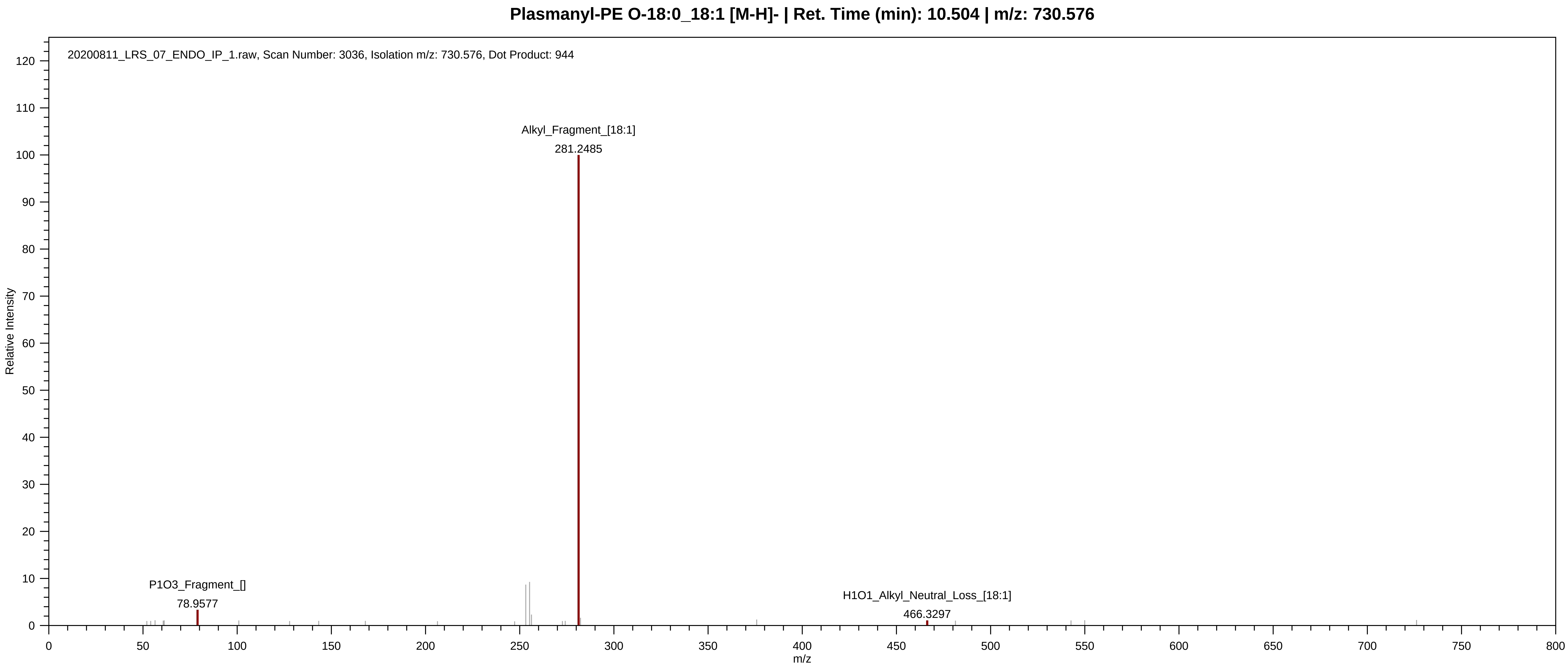

Plasmanyl-PE O-18:0_18:1 [M-H]- | Ret. Time (min): 10.504 | m/z: 730.576
20200811_LRS_07_ENDO_IP_1.raw, Scan Number: 3036, Isolation m/z: 730.576, Dot Product: 944
Alkyl_Fragment_[18:1]
281.2485
P1O3_Fragment_[]
78.9577
H1O1_Alkyl_Neutral_Loss_[18:1]
466.3297
Relative Intensity
m/z

Plasmanyl-PE O-20:0_16:0 [M-H]- | Ret. Time (min): 11.462 | m/z: 732.591

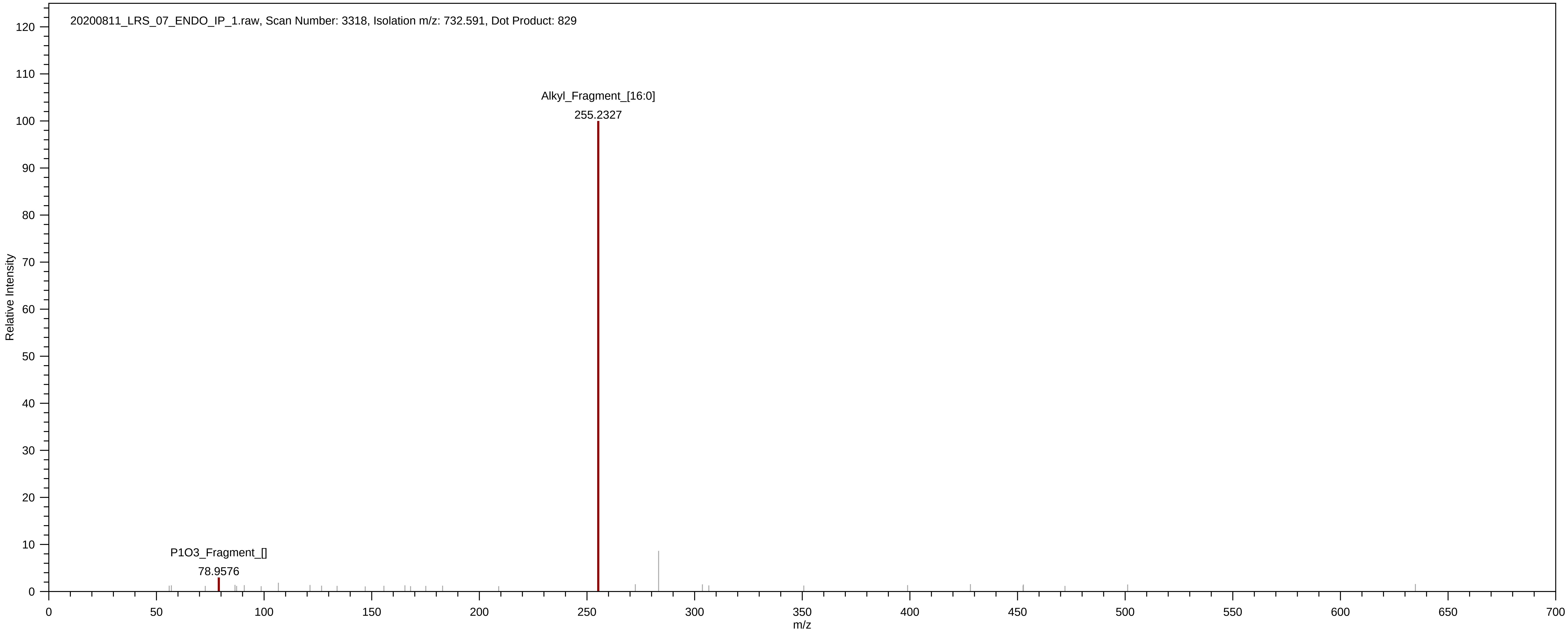

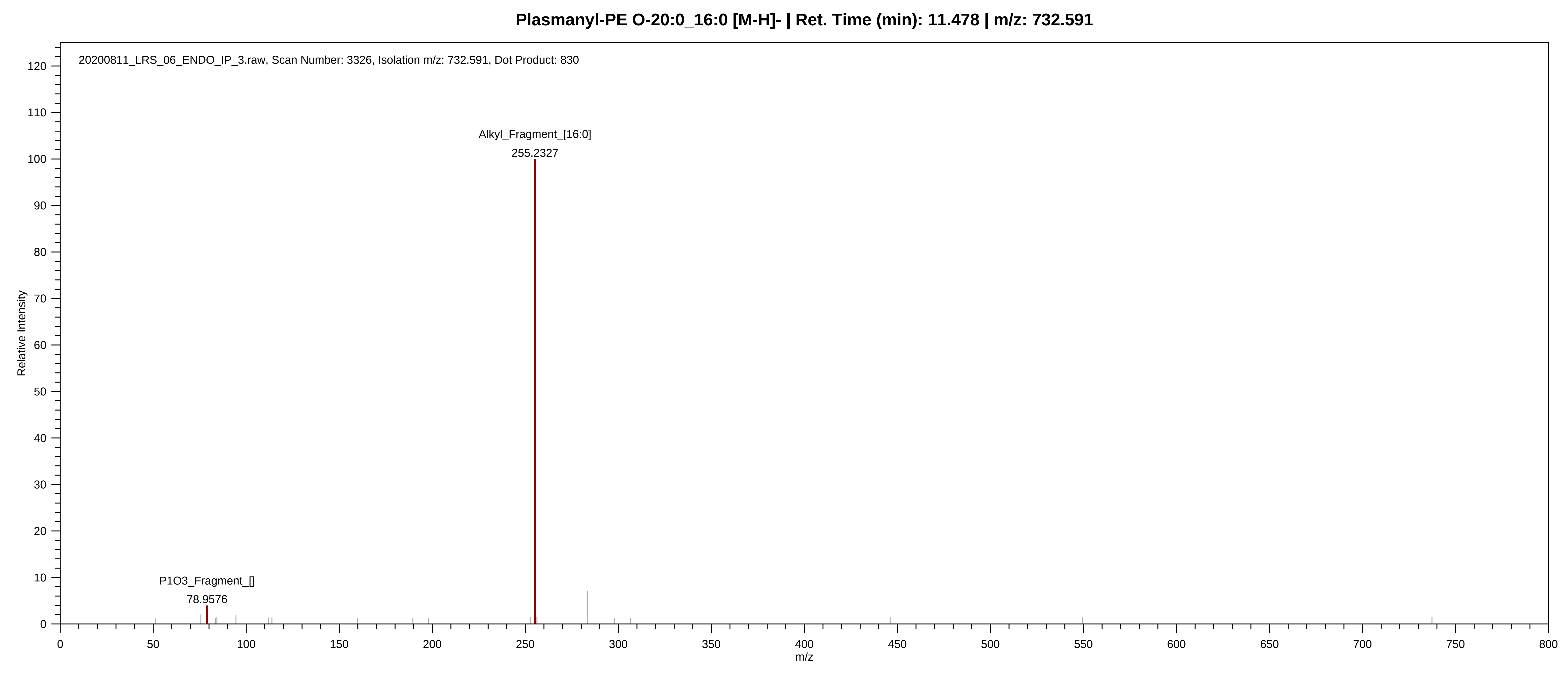

Plasmanyl-PE O-20:0_16:0 [M-H]- | Ret. Time (min): 11.478 | m/z: 732.591
20200811_LRS_06_ENDO_IP_3.raw, Scan Number: 3326, Isolation m/z: 732.591, Dot Product: 830
Alkyl_Fragment_[16:0]
255.2327
P1O3_Fragment_[]
78.9576
Relative Intensity
m/z

Plasmanyl-PE O-20:0_16:0 [M-H]- | Ret. Time (min): 11.481 | m/z: 732.591

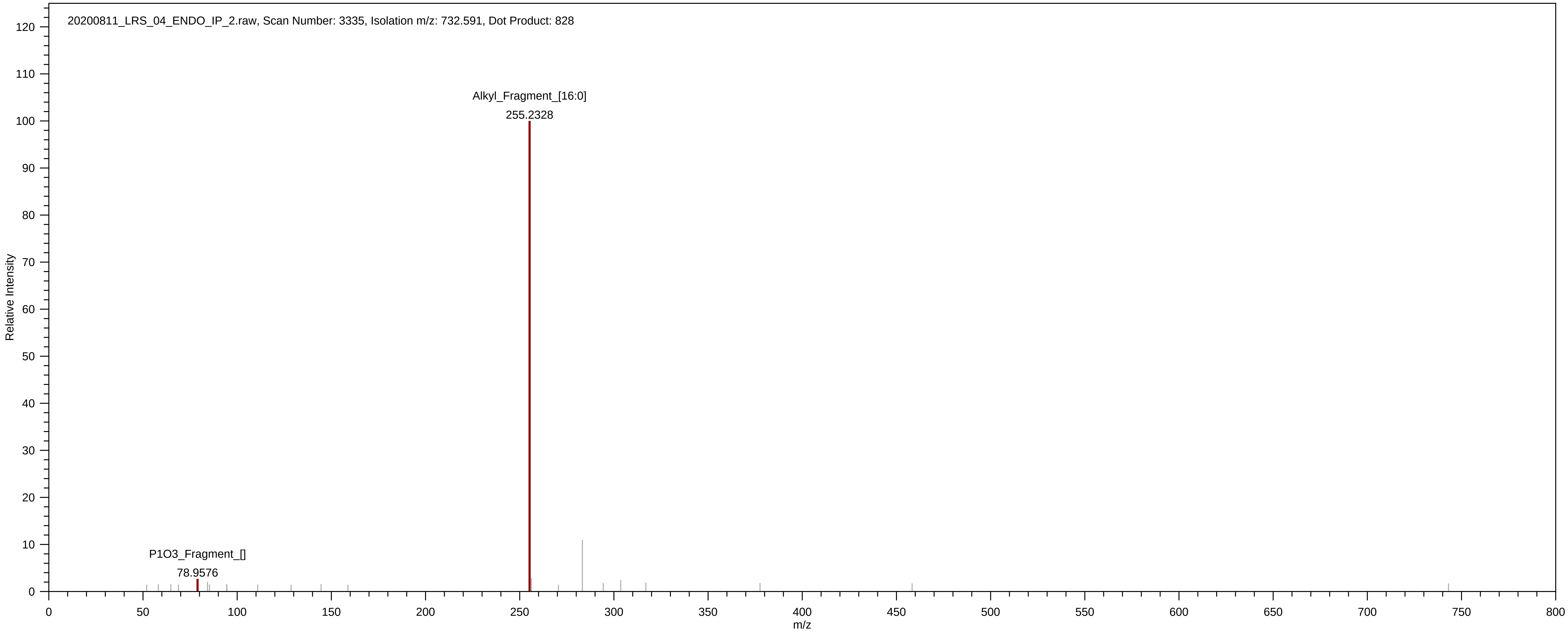

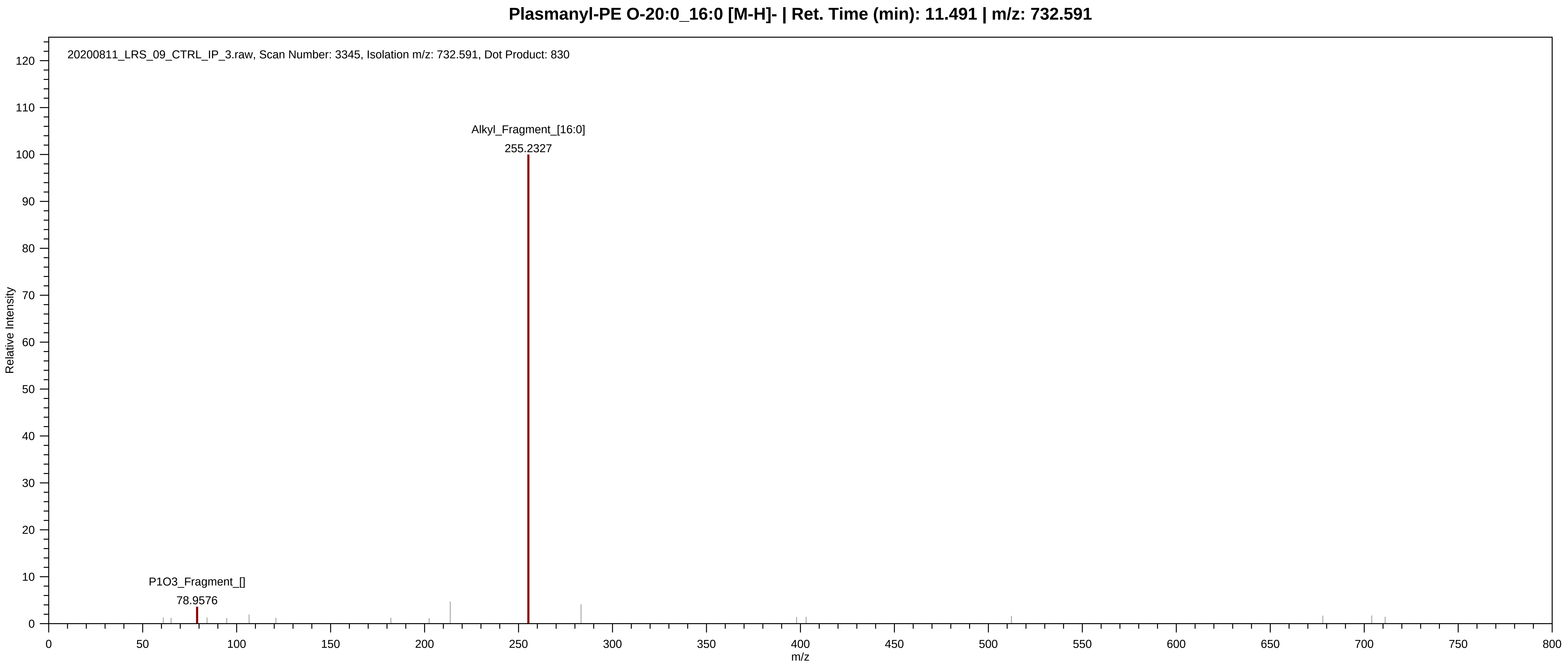

Plasmanyl-PE O-20:0_16:0 [M-H]- | Ret. Time (min): 11.491 | m/z: 732.591
20200811_LRS_09_CTRL_IP_3.raw, Scan Number: 3345, Isolation m/z: 732.591, Dot Product: 830
Alkyl_Fragment_[16:0]
255.2327
P1O3_Fragment_[]
78.9576
Relative Intensity
m/z

Plasmanyl-PE O-20:0_16:0 [M-H]- | Ret. Time (min): 11.459 | m/z: 732.593

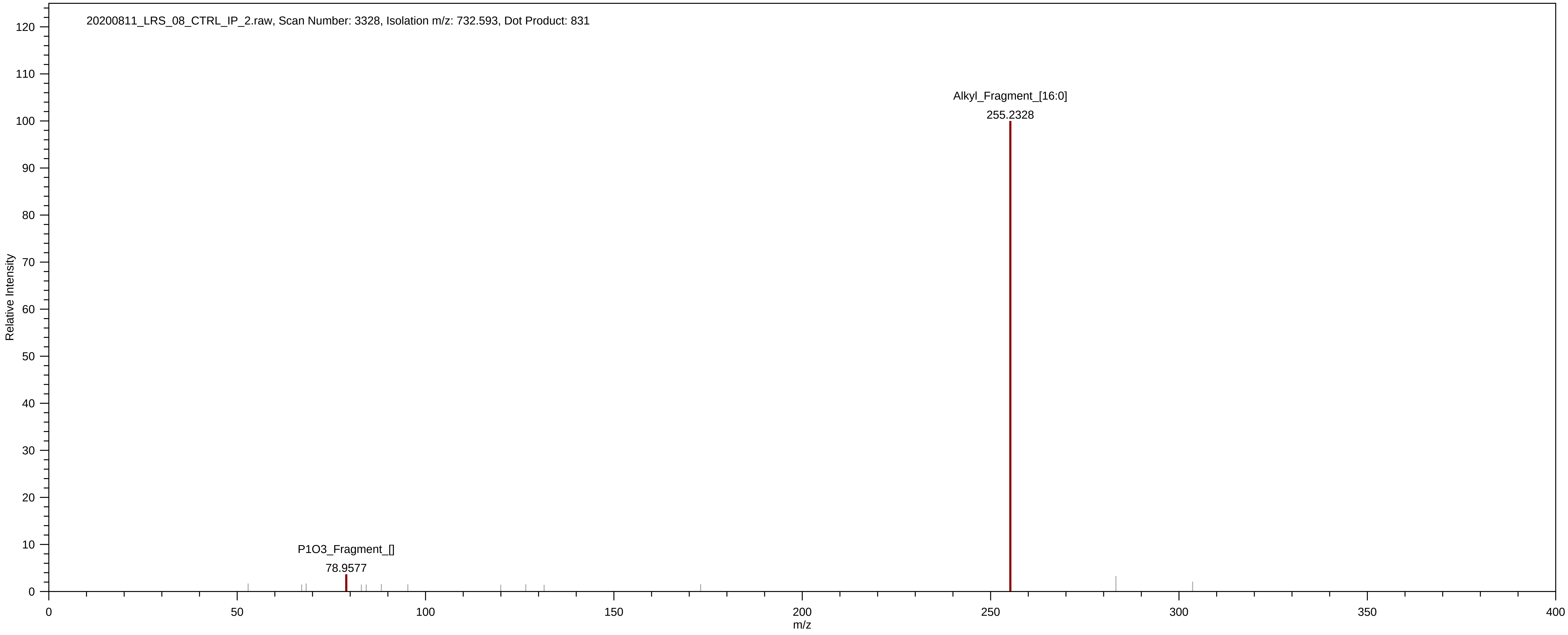

Plasmanyl-PE O-20:0_16:0 [M-H]- | Ret. Time (min): 11.472 | m/z: 732.594

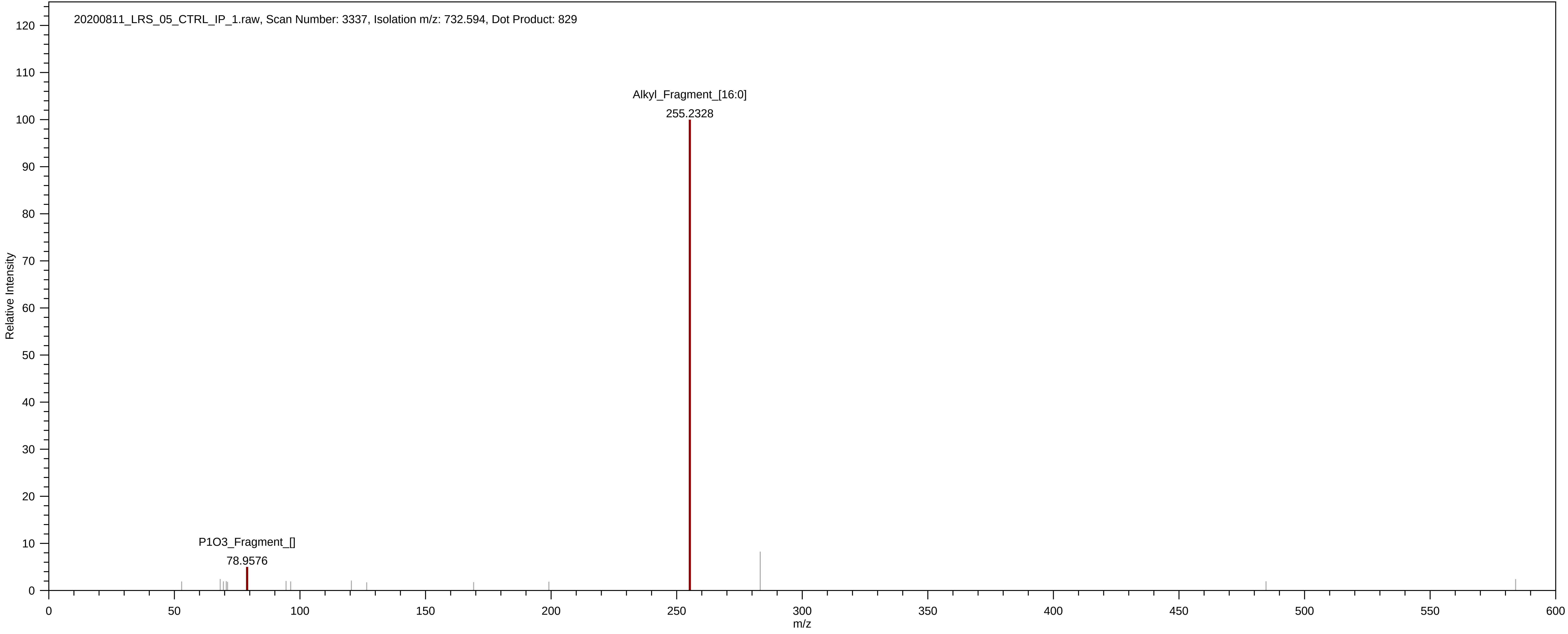

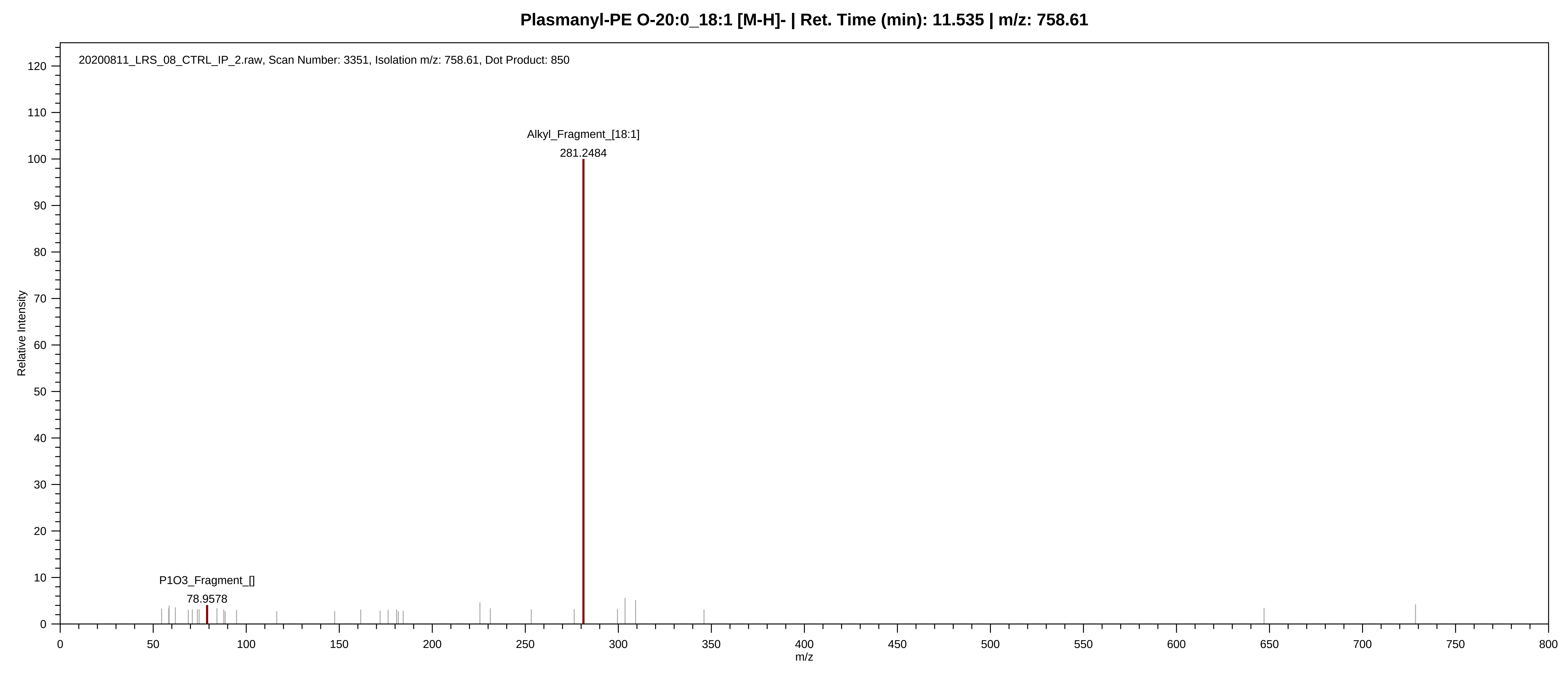

Plasmanyl-PE O-20:0_18:1 [M-H]- | Ret. Time (min): 11.535 | m/z: 758.61
20200811_LRS_08_CTRL_IP_2.raw, Scan Number: 3351, Isolation m/z: 758.61, Dot Product: 850
Alkyl_Fragment_[18:1]
281.2484
P1O3_Fragment_[]
78.9578
Relative Intensity
m/z

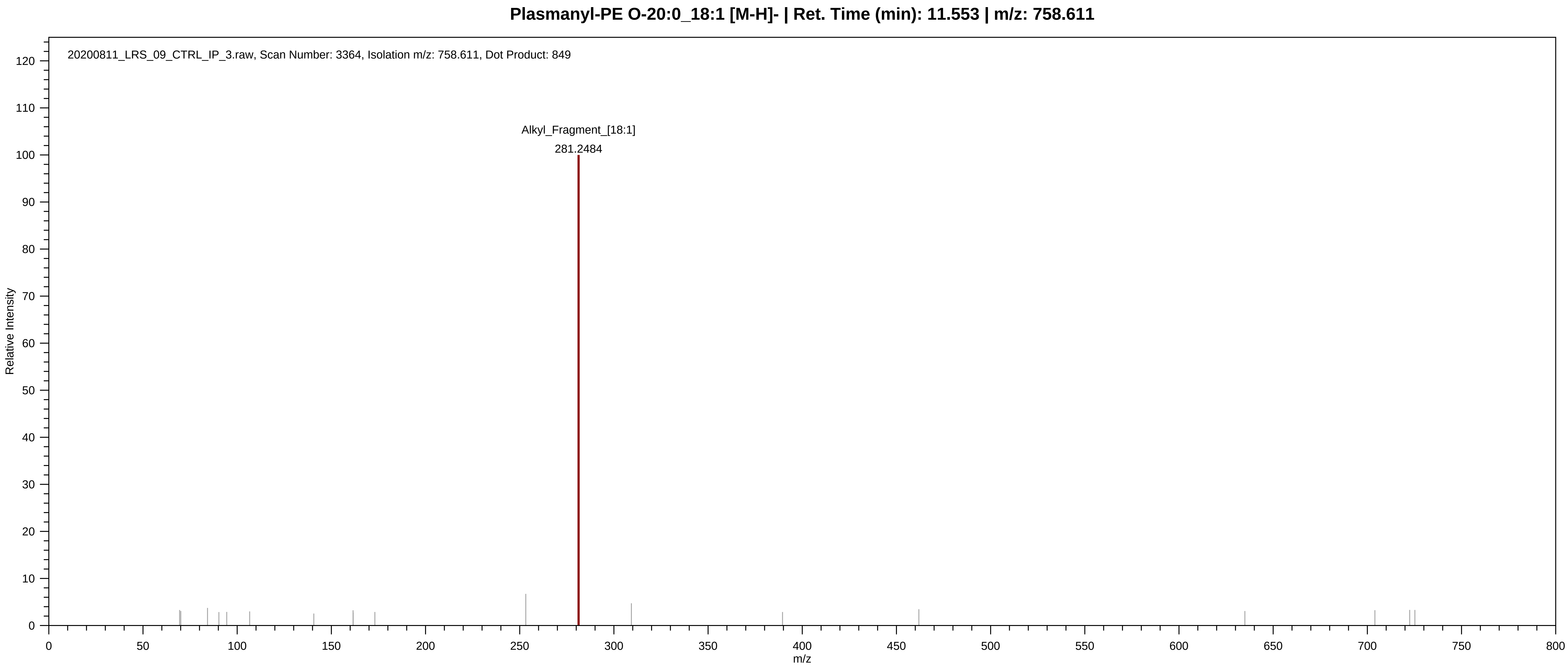

Plasmanyl-PE O-20:0_18:1 [M-H]- | Ret. Time (min): 11.553 | m/z: 758.611
20200811_LRS_09_CTRL_IP_3.raw, Scan Number: 3364, Isolation m/z: 758.611, Dot Product: 849
Alkyl_Fragment_[18:1]
281.2484
Relative Intensity
m/z

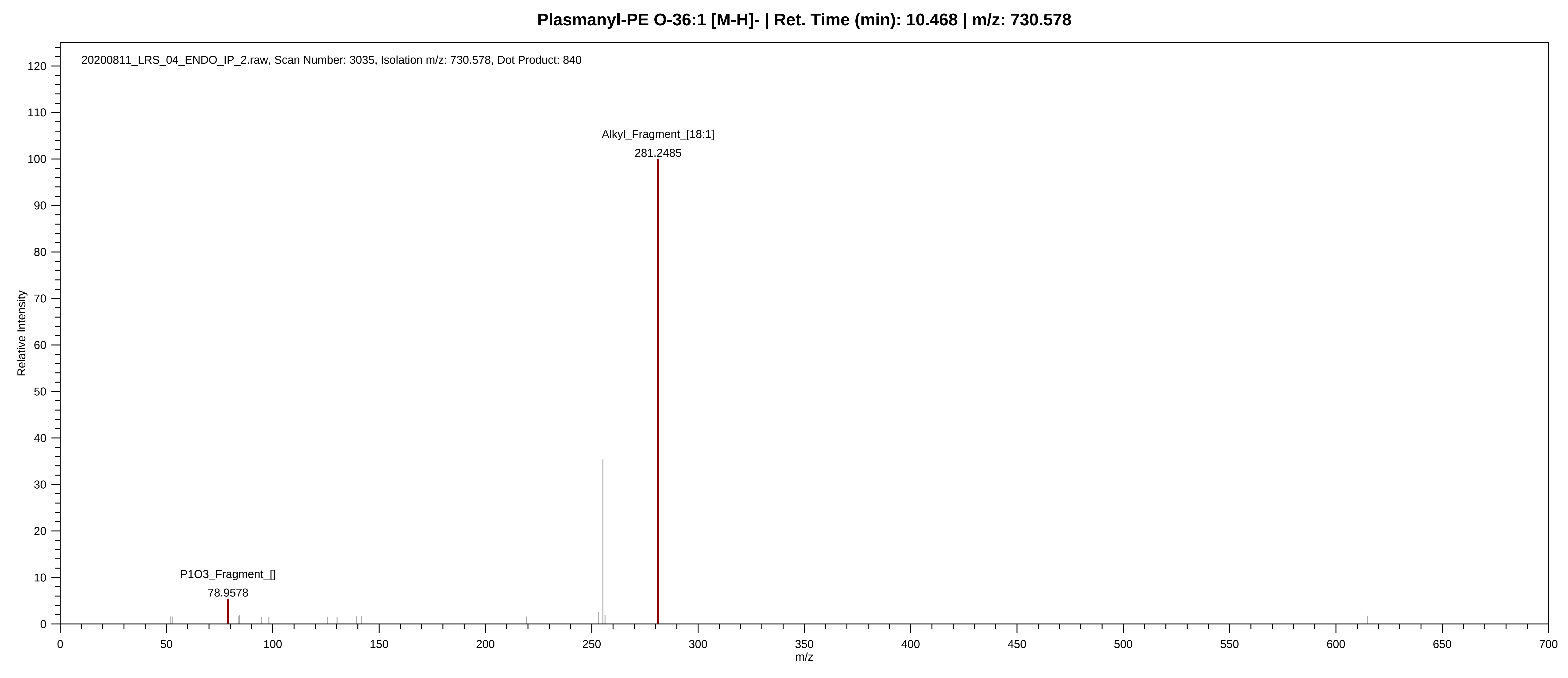
Plasmanyl-PE O-36:1 [M-H]- | Ret. Time (min): 10.468 | m/z: 730.578
20200811_LRS_04_ENDO_IP_2.raw, Scan Number: 3035, Isolation m/z: 730.578, Dot Product: 840
Alkyl_Fragment_[18:1]
281.2485
P1O3_Fragment_[]
78.9578
Relative Intensity
m/z
0
10
20
30
40
50
60
70
80
90
100
110
120
0
50
100
150
200
250
300
350
400
450
500
550
600
650
700

Plasmenyl-PC P-16:0_16:0 [M+Ac-H]- | Ret. Time (min): 10.262 | m/z: 776.581

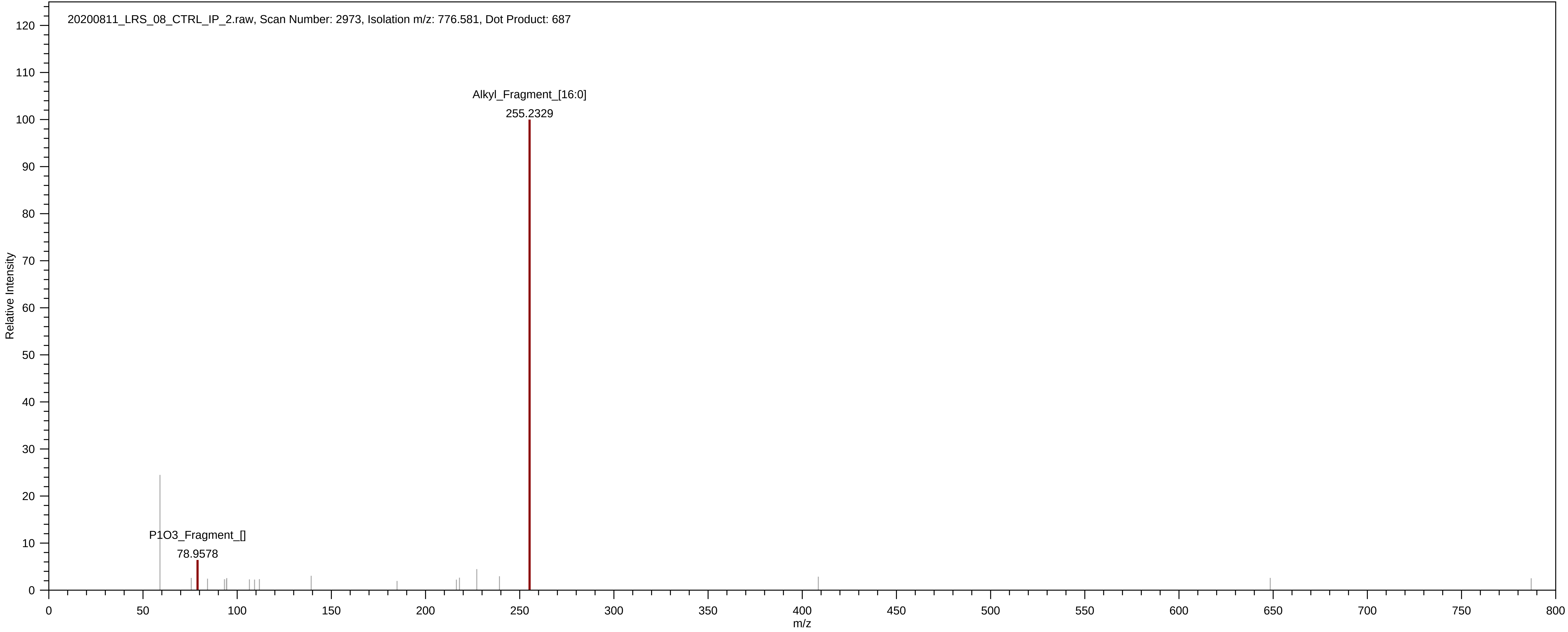

Plasmenyl-PC P-16:0_16:0 [M+Ac-H]- | Ret. Time (min): 10.265 | m/z: 776.581

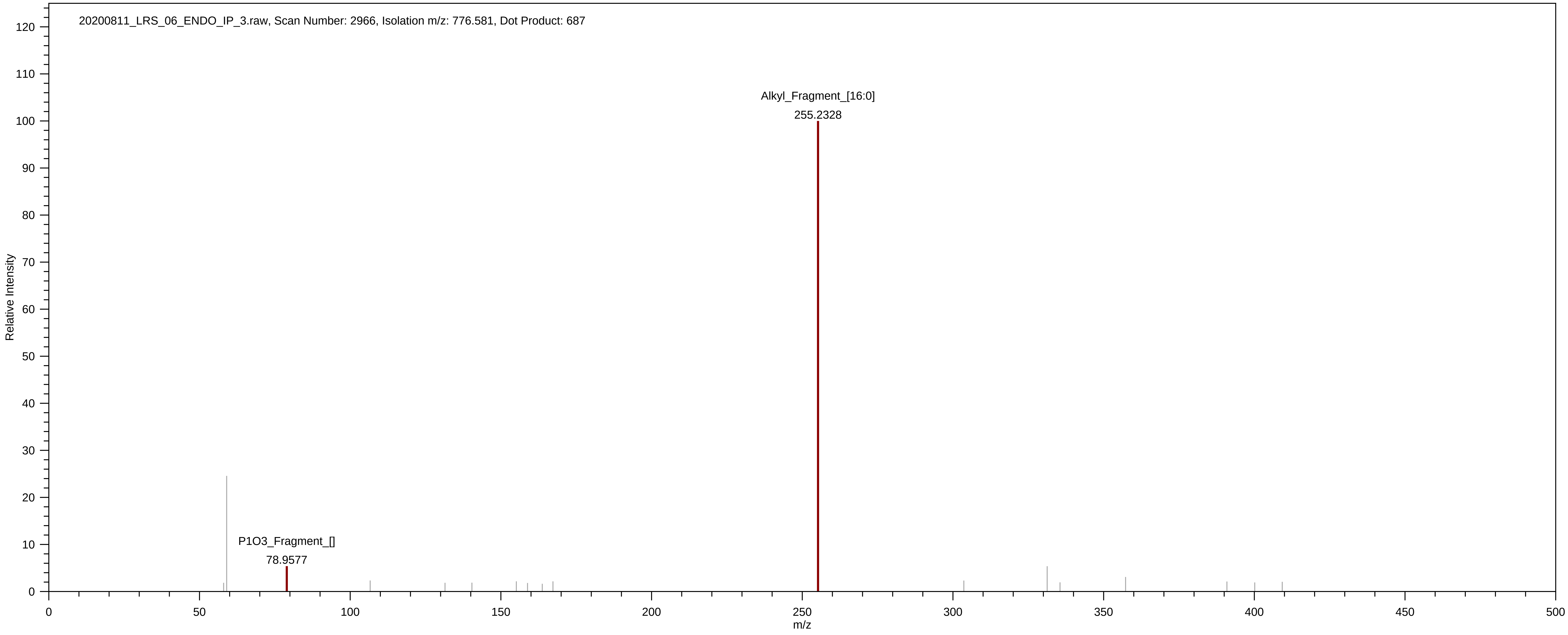

Plasmenyl-PC P-16:0_16:0 [M+Ac-H]- | Ret. Time (min): 10.265 | m/z: 776.581

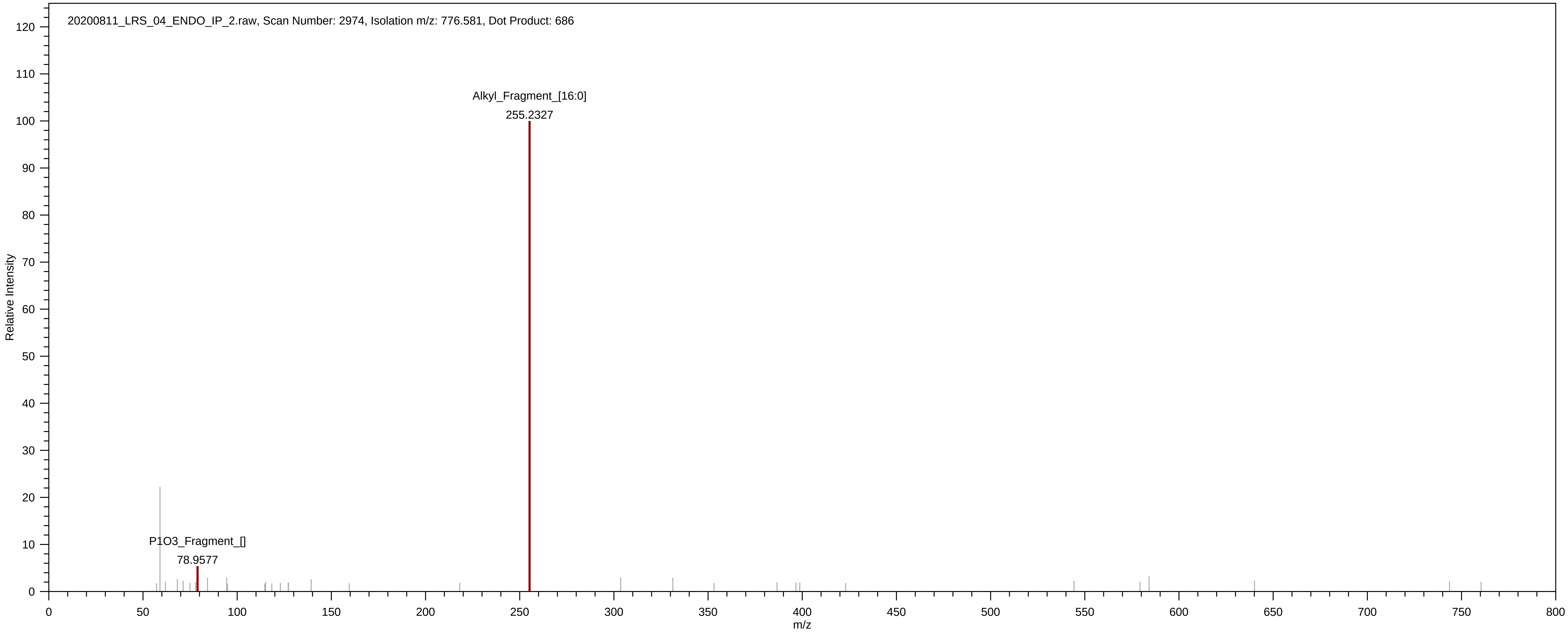

Plasmenyl-PC P-16:0_16:0 [M+Ac-H]- | Ret. Time (min): 10.265 | m/z: 776.581

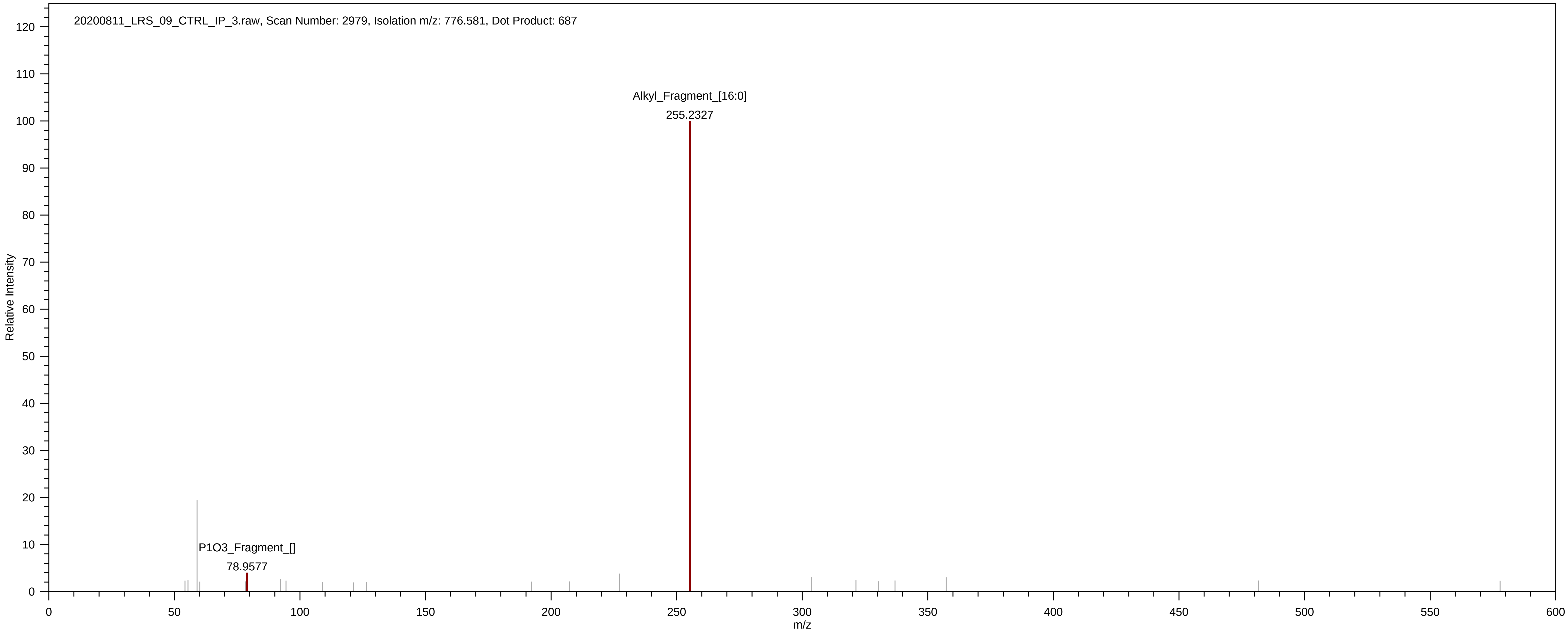

Plasmenyl-PC P-16:0_16:0 [M+Ac-H]- | Ret. Time (min): 10.267 | m/z: 776.581

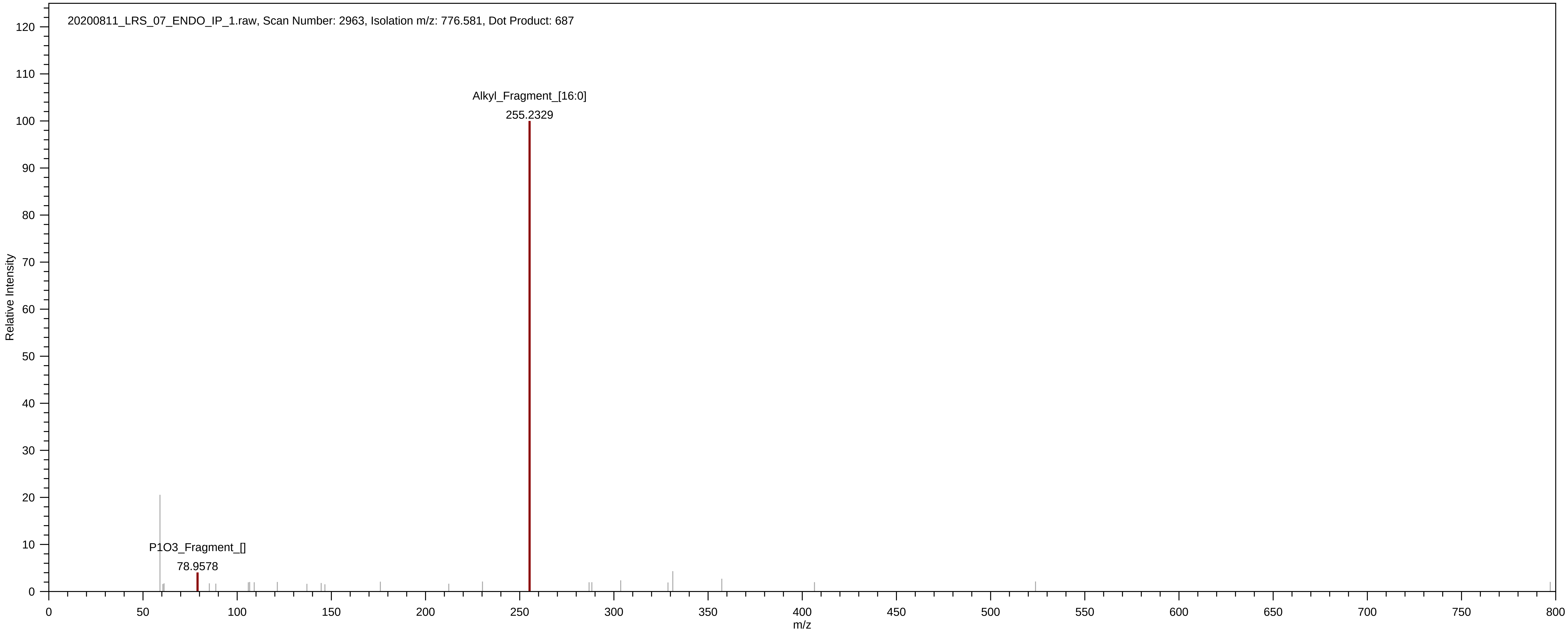

Plasmenyl-PC P-16:0_16:0 [M+Ac-H]- | Ret. Time (min): 10.273 | m/z: 776.581

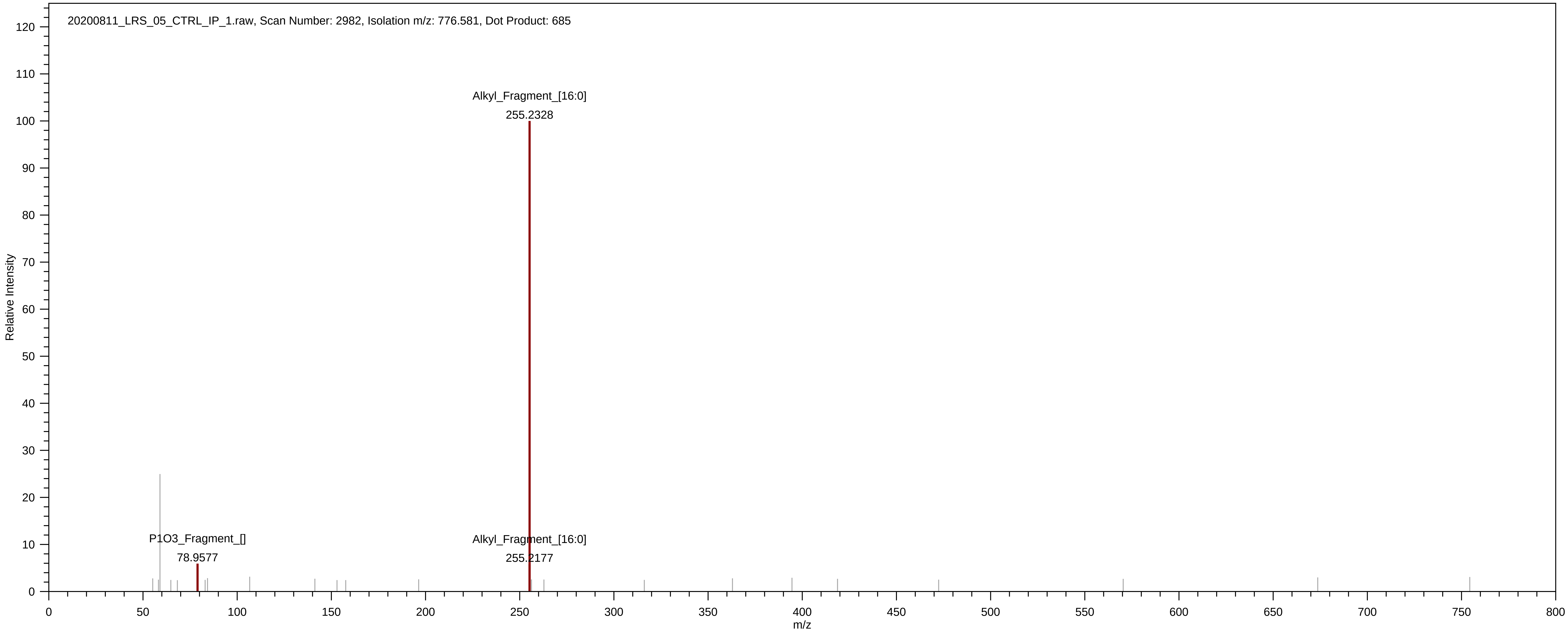

Plasmenyl-PC P-16:0_16:1 [M+Ac-H]- | Ret. Time (min): 9.349 | m/z: 774.56

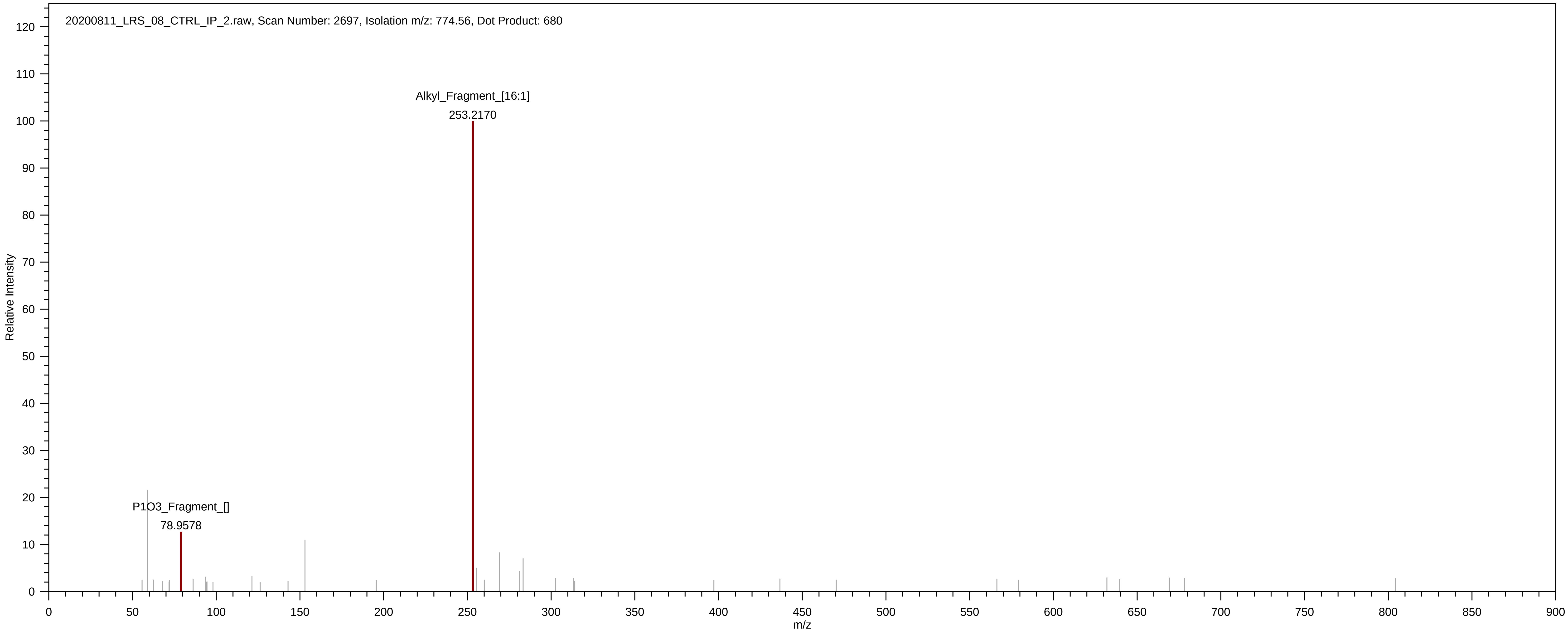

Plasmenyl-PC P-16:0_16:1 [M+Ac-H]- | Ret. Time (min): 9.349 | m/z: 774.565

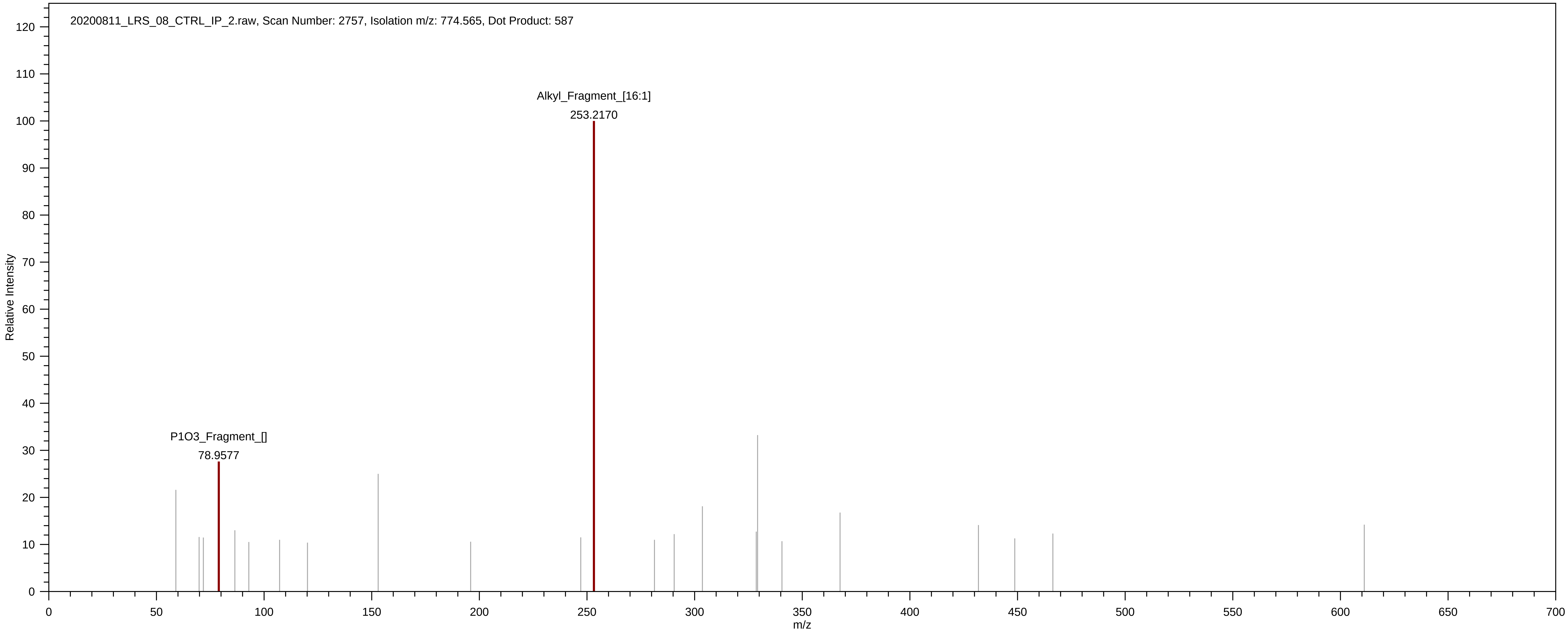

Plasmenyl-PC P-16:0_20:1 [M+Ac-H]- | Ret. Time (min): 11.252 | m/z: 830.631

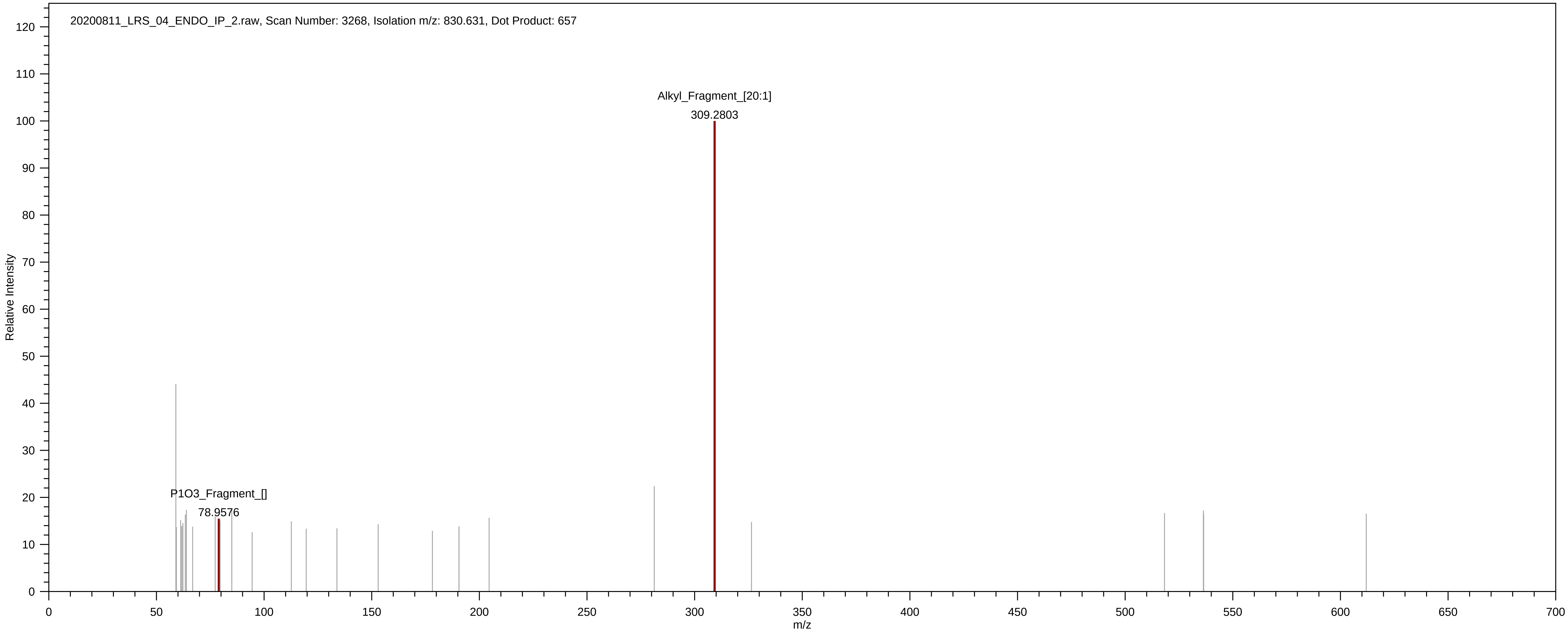

Plasmenyl-PC P-16:0_20:1 [M+Ac-H]- | Ret. Time (min): 11.255 | m/z: 830.631

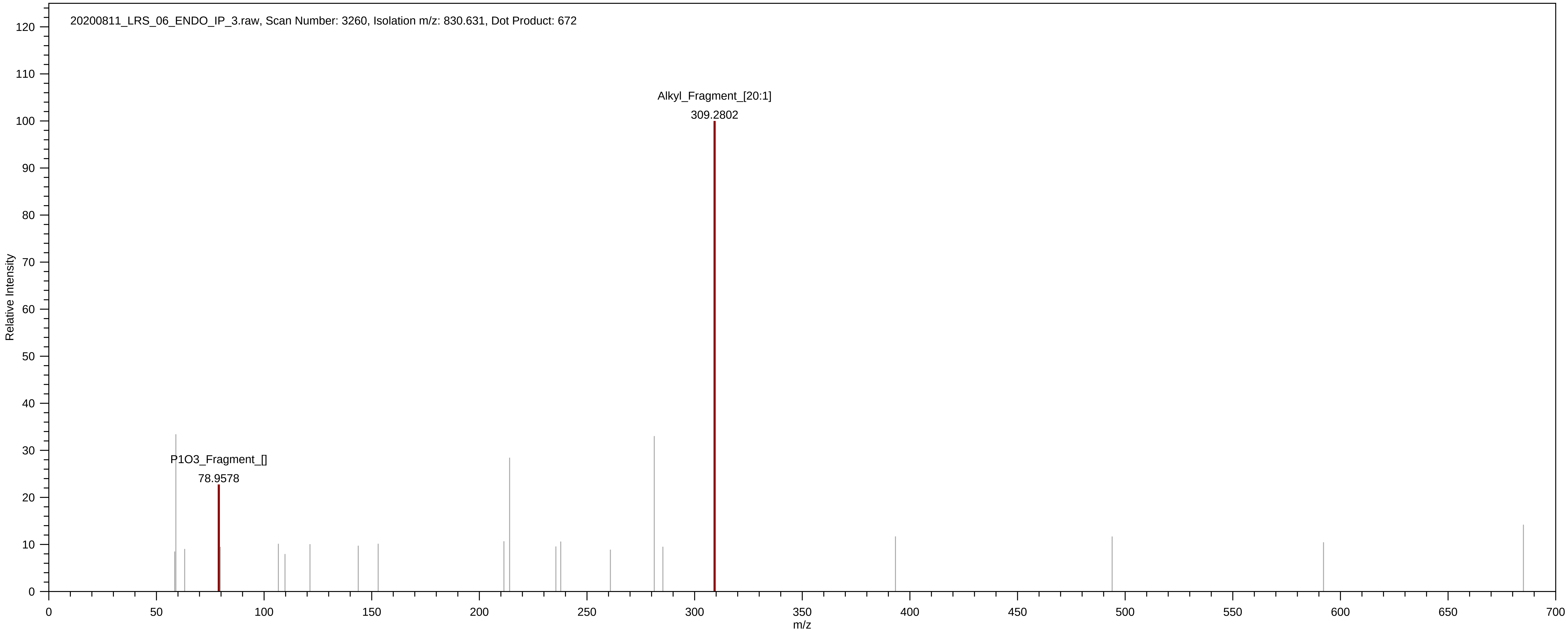

Plasmenyl-PC P-16:0_20:1 [M+Ac-H]- | Ret. Time (min): 11.236 | m/z: 830.632

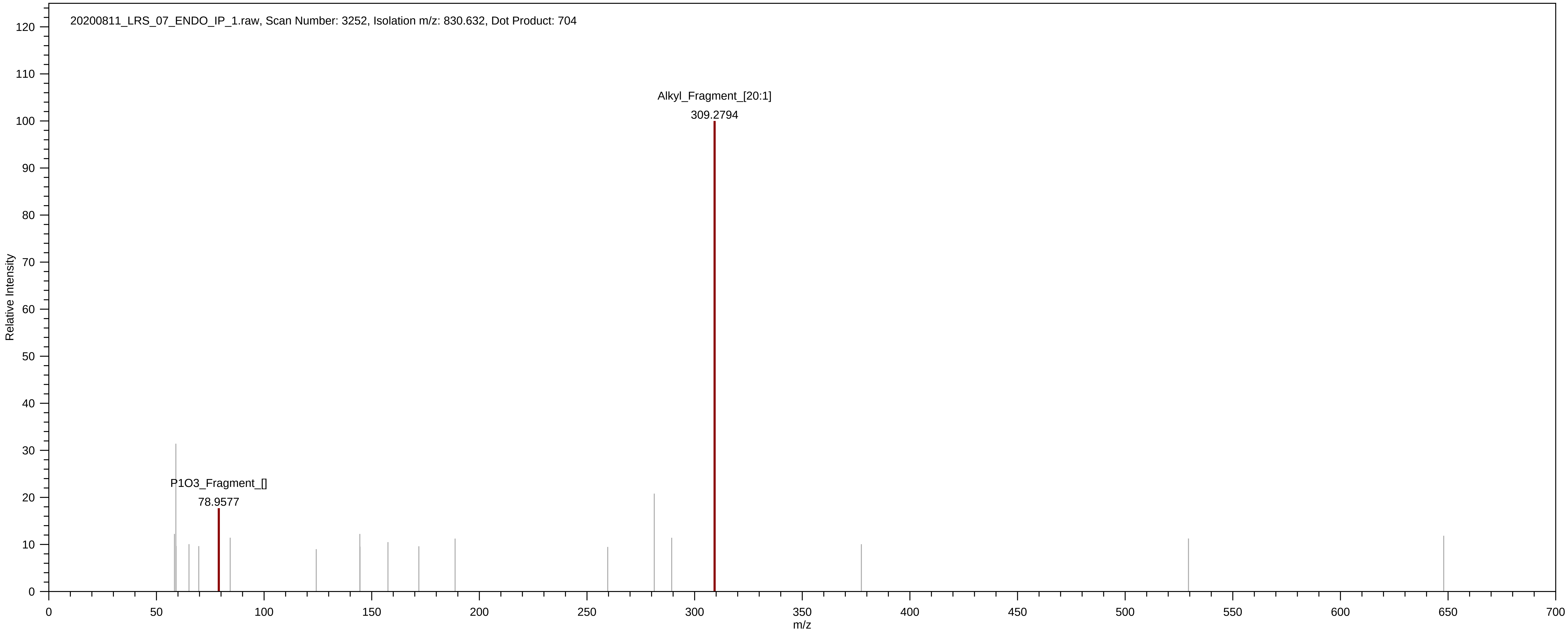

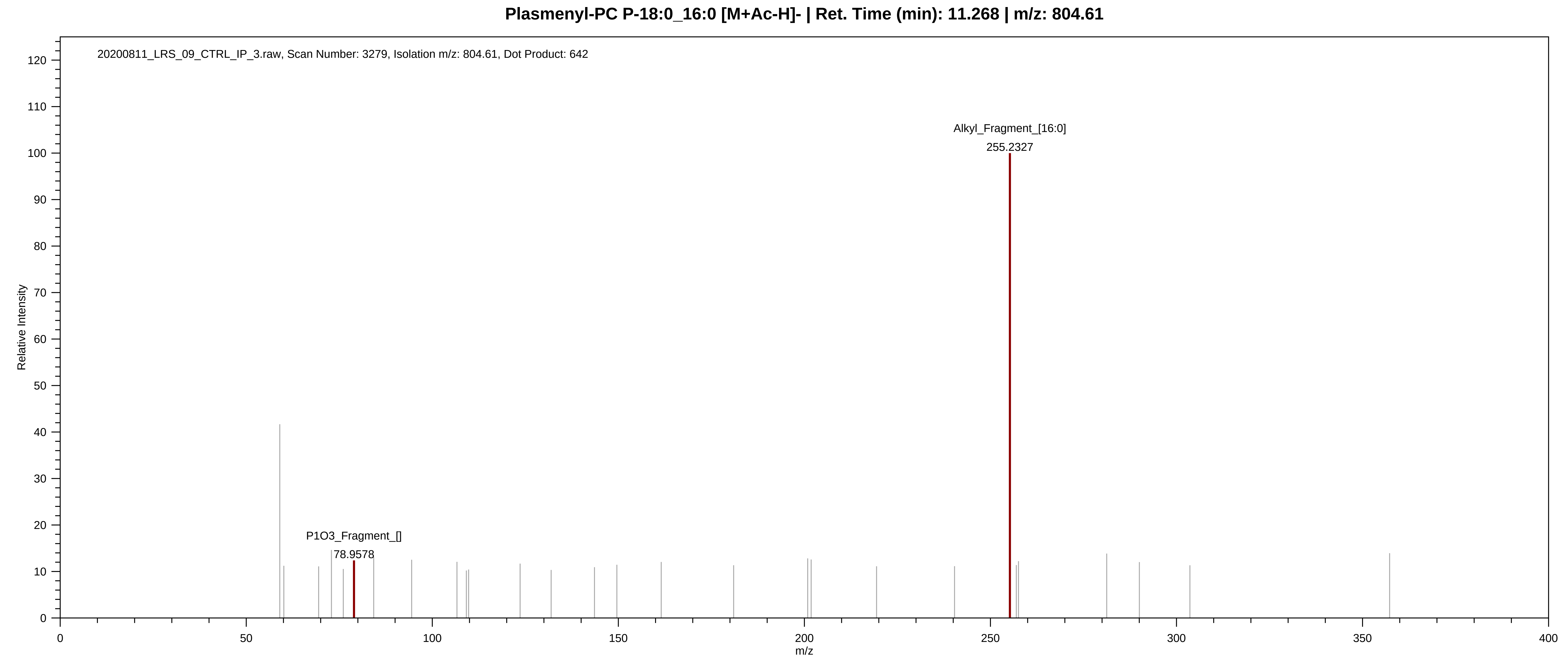
Plasmenyl-PC P-18:0_16:0 [M+Ac-H]- | Ret. Time (min): 11.268 | m/z: 804.61
20200811_LRS_09_CTRL_IP_3.raw, Scan Number: 3279, Isolation m/z: 804.61, Dot Product: 642
Alkyl_Fragment_[16:0]
255.2327
P1O3_Fragment_[]
78.9578
Relative Intensity
m/z

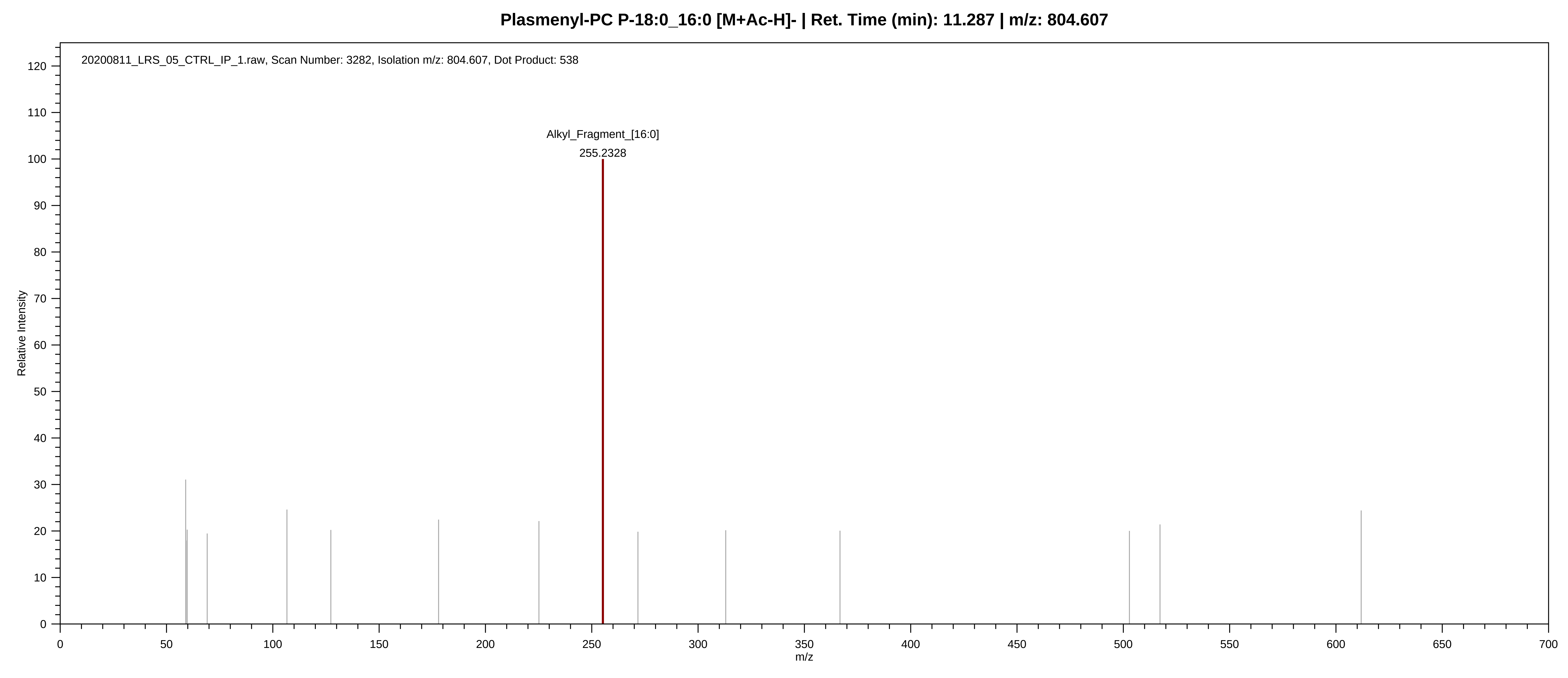

Plasmenyl-PC P-18:0_16:0 [M+Ac-H]- | Ret. Time (min): 11.287 | m/z: 804.607
20200811_LRS_05_CTRL_IP_1.raw, Scan Number: 3282, Isolation m/z: 804.607, Dot Product: 538
Alkyl_Fragment_[16:0]
255.2328
Relative Intensity
m/z

Plasmenyl-PC P-18:0_16:0 [M+Ac-H]- | Ret. Time (min): 11.293 | m/z: 804.607

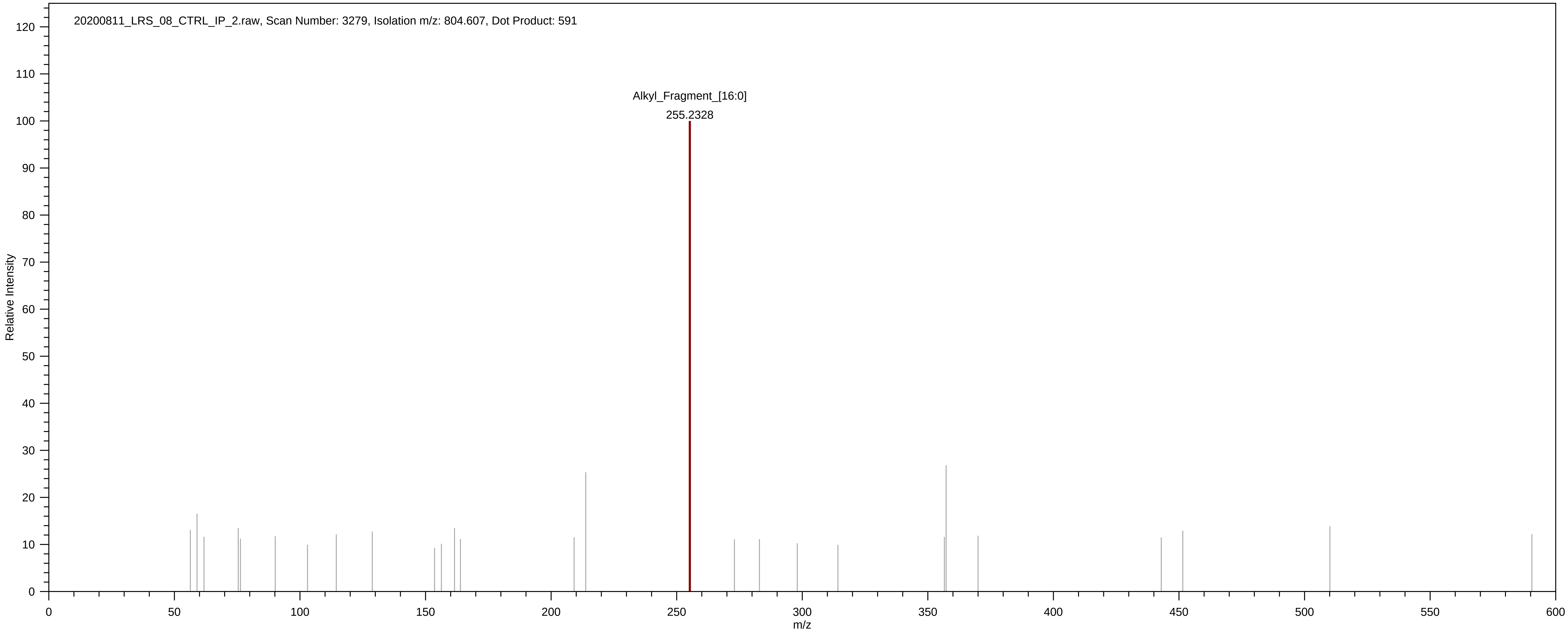

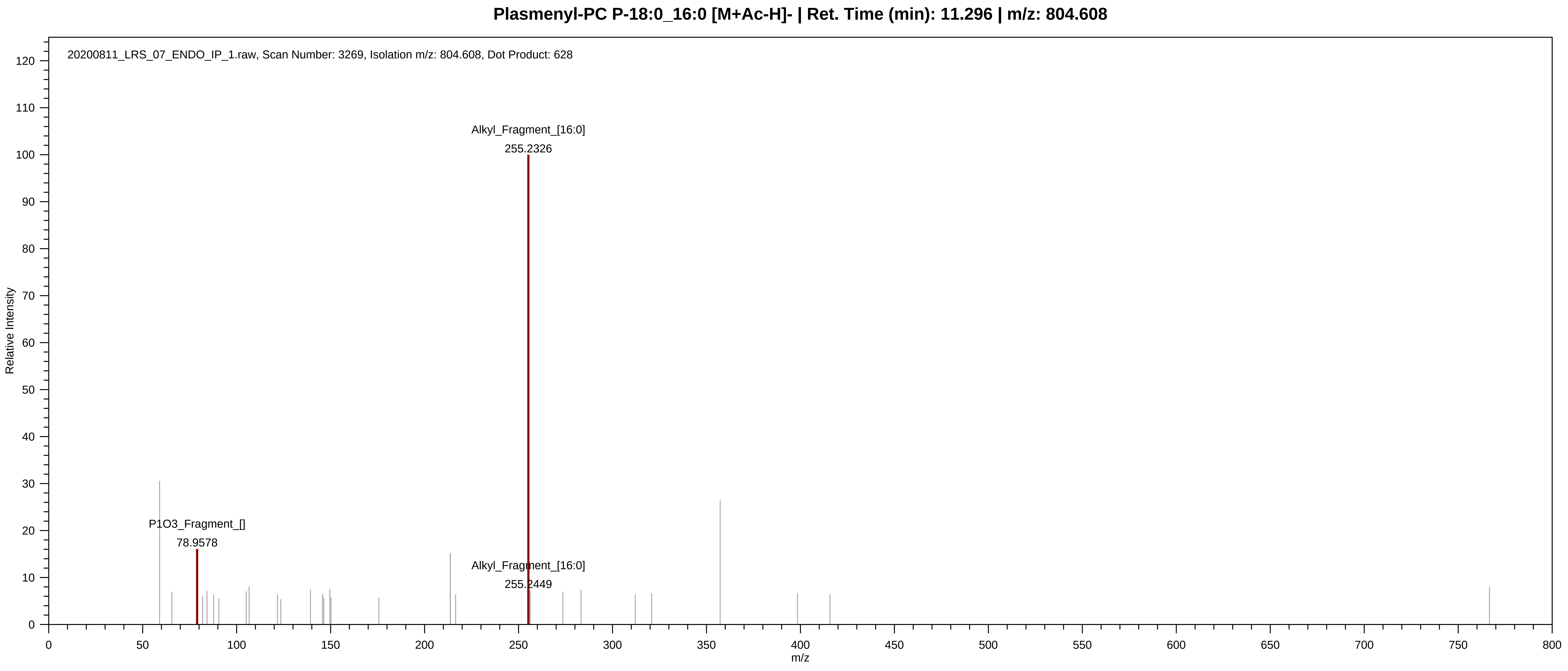

Plasmenyl-PC P-18:0_16:0 [M+Ac-H]- | Ret. Time (min): 11.296 | m/z: 804.608
20200811_LRS_07_ENDO_IP_1.raw, Scan Number: 3269, Isolation m/z: 804.608, Dot Product: 628
Alkyl_Fragment_[16:0]
255.2326
P1O3_Fragment_[]
78.9578
Alkyl_Fragment_[16:0]
255.2449
Relative Intensity
m/z

Plasmenyl-PC P-18:0_16:1 [M+Ac-H]- | Ret. Time (min): 9.528 | m/z: 802.597

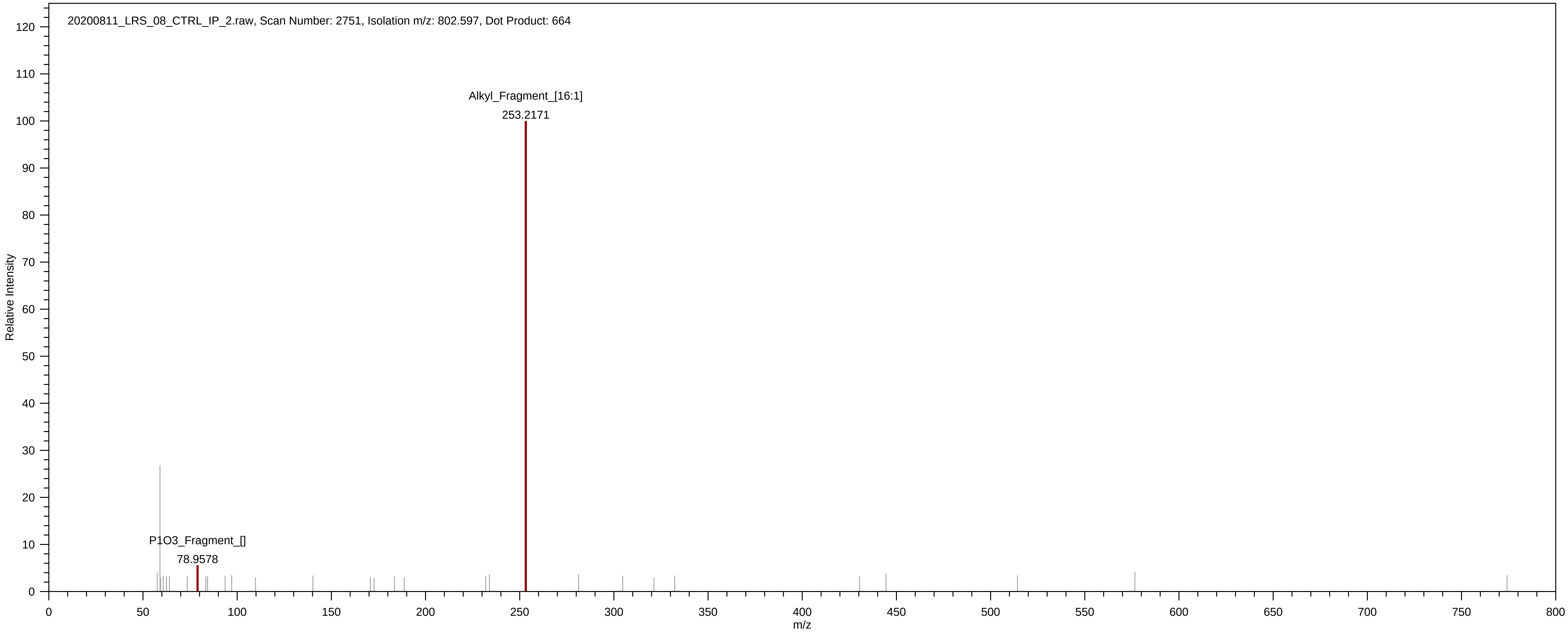

Plasmenyl-PC P-18:0_16:1 [M+Ac-H]- | Ret. Time (min): 9.529 | m/z: 802.597

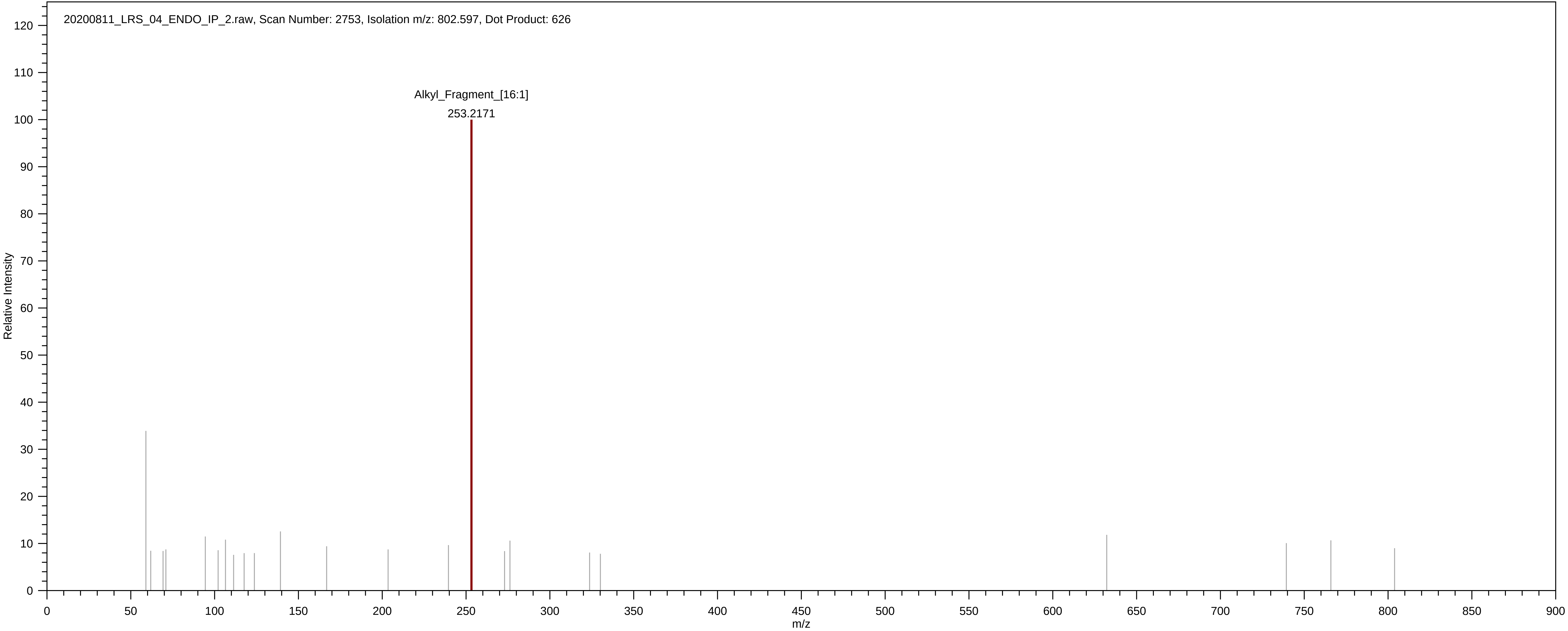

Plasmenyl-PC P-18:0_16:1 [M+Ac-H]- | Ret. Time (min): 9.531 | m/z: 802.597

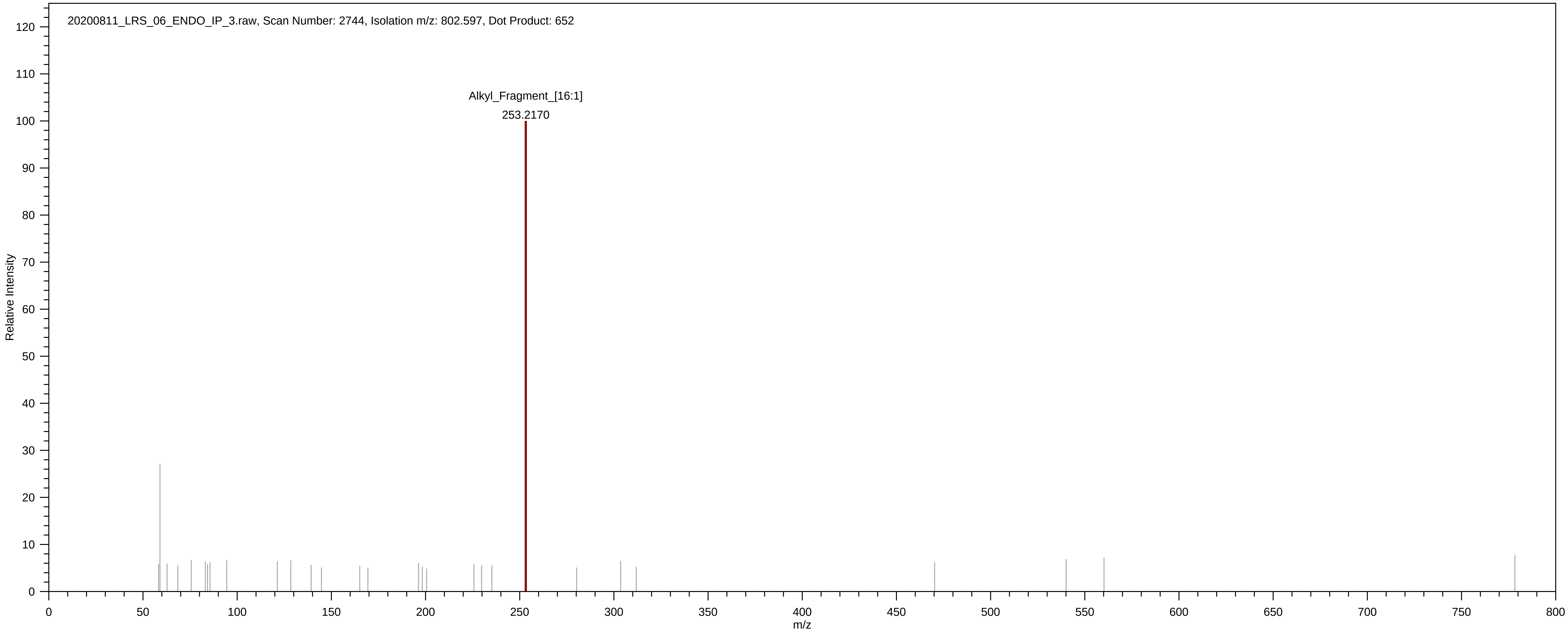

Plasmenyl-PC P-18:0_16:1 [M+Ac-H]- | Ret. Time (min): 9.534 | m/z: 802.598

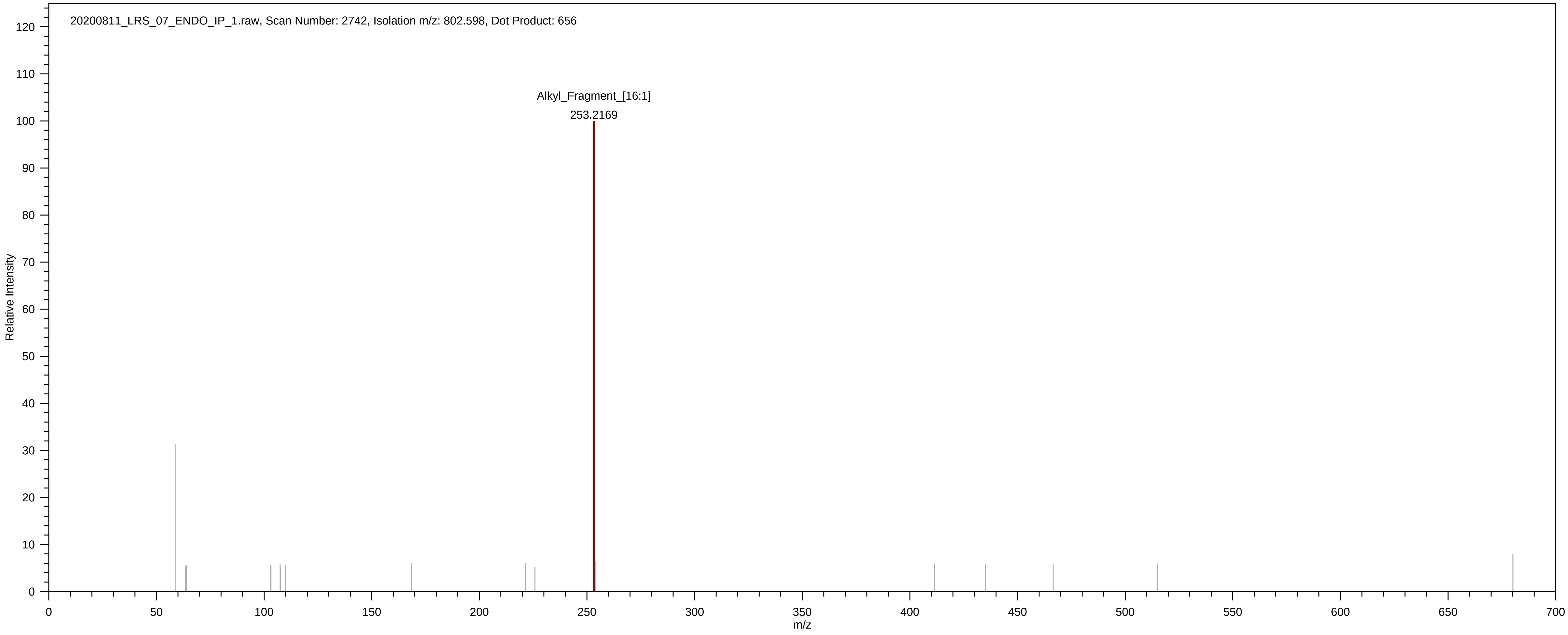

Plasmenyl-PC P-18:0_16:1 [M+Ac-H]- | Ret. Time (min): 9.535 | m/z: 802.598

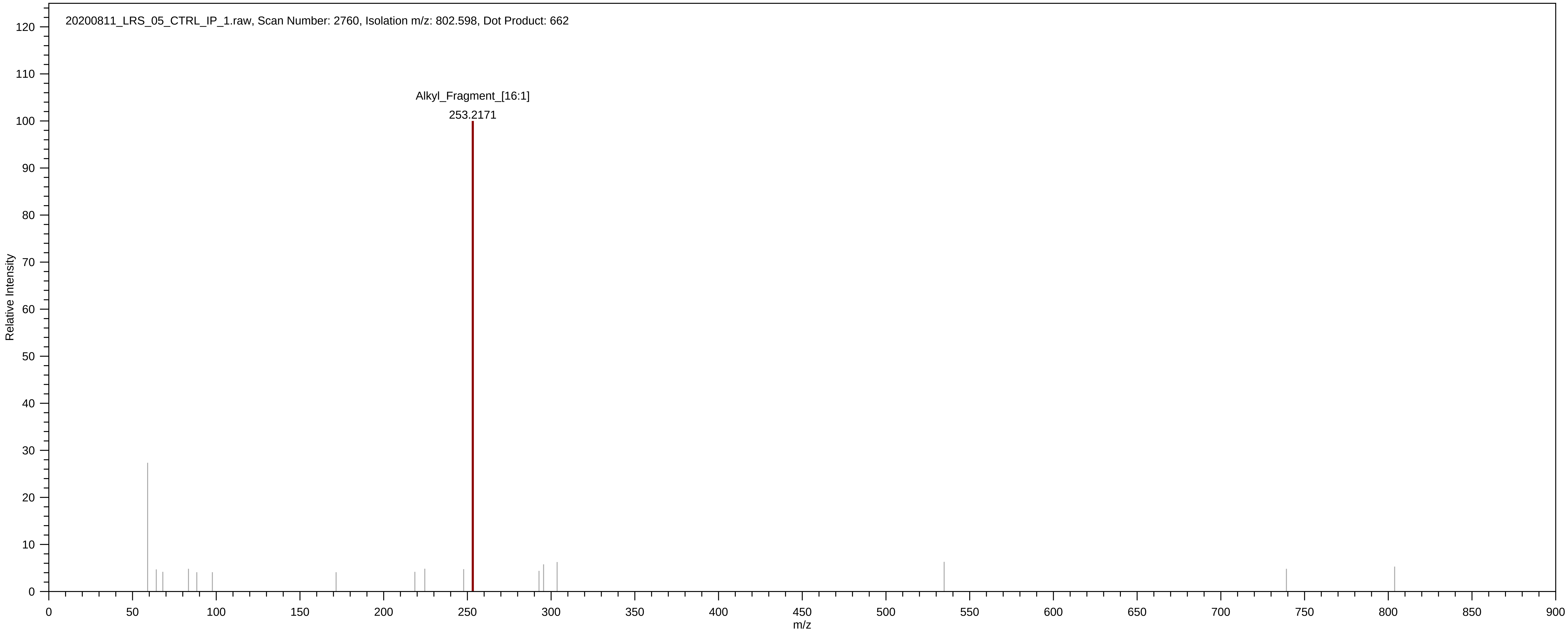

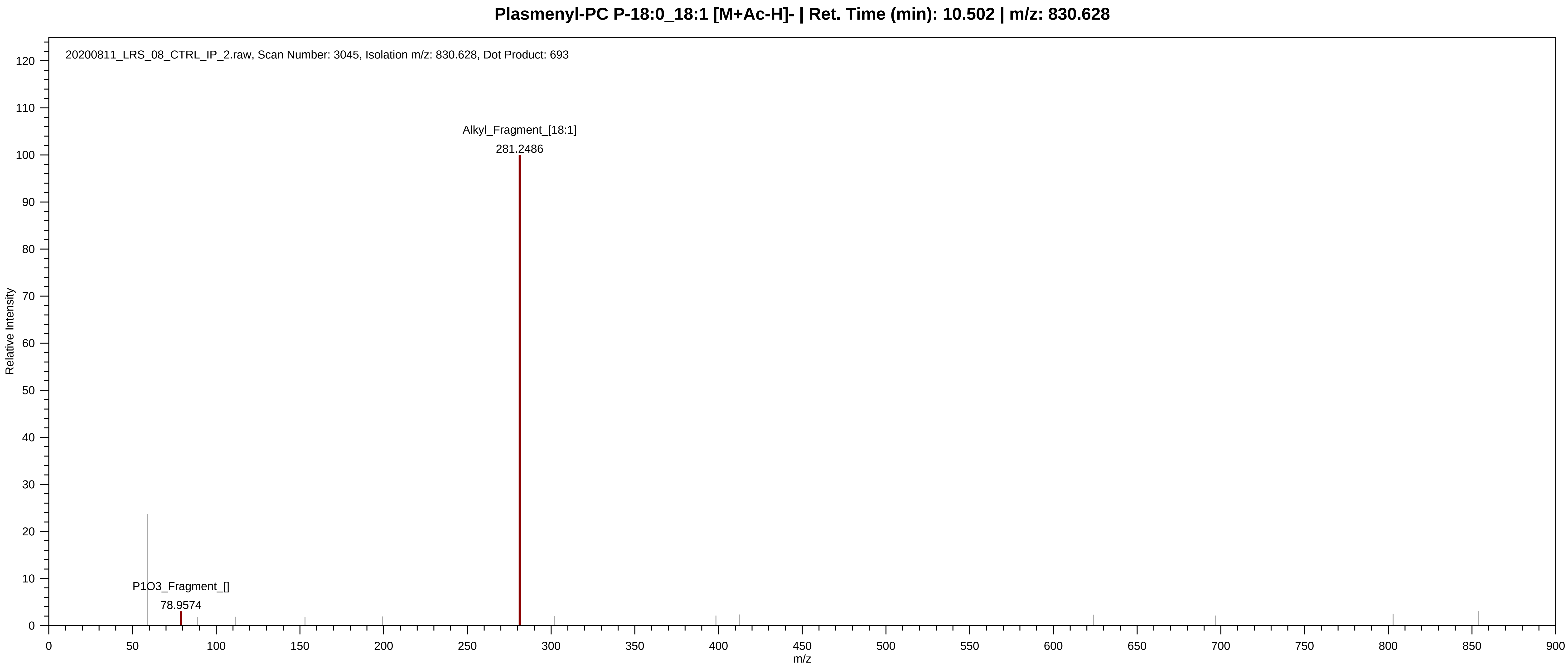

Plasmenyl-PC P-18:0_18:1 [M+Ac-H]- | Ret. Time (min): 10.502 | m/z: 830.628
20200811_LRS_08_CTRL_IP_2.raw, Scan Number: 3045, Isolation m/z: 830.628, Dot Product: 693
Alkyl_Fragment_[18:1]
281.2486
P1O3_Fragment_[]
78.9574
Relative Intensity
m/z
0
10
20
30
40
50
60
70
80
90
100
110
120
0
50
100
150
200
250
300
350
400
450
500
550
600
650
700
750
800
850
900

Plasmenyl-PC P-18:0_18:1 [M+Ac-H]- | Ret. Time (min): 10.525 | m/z: 830.628

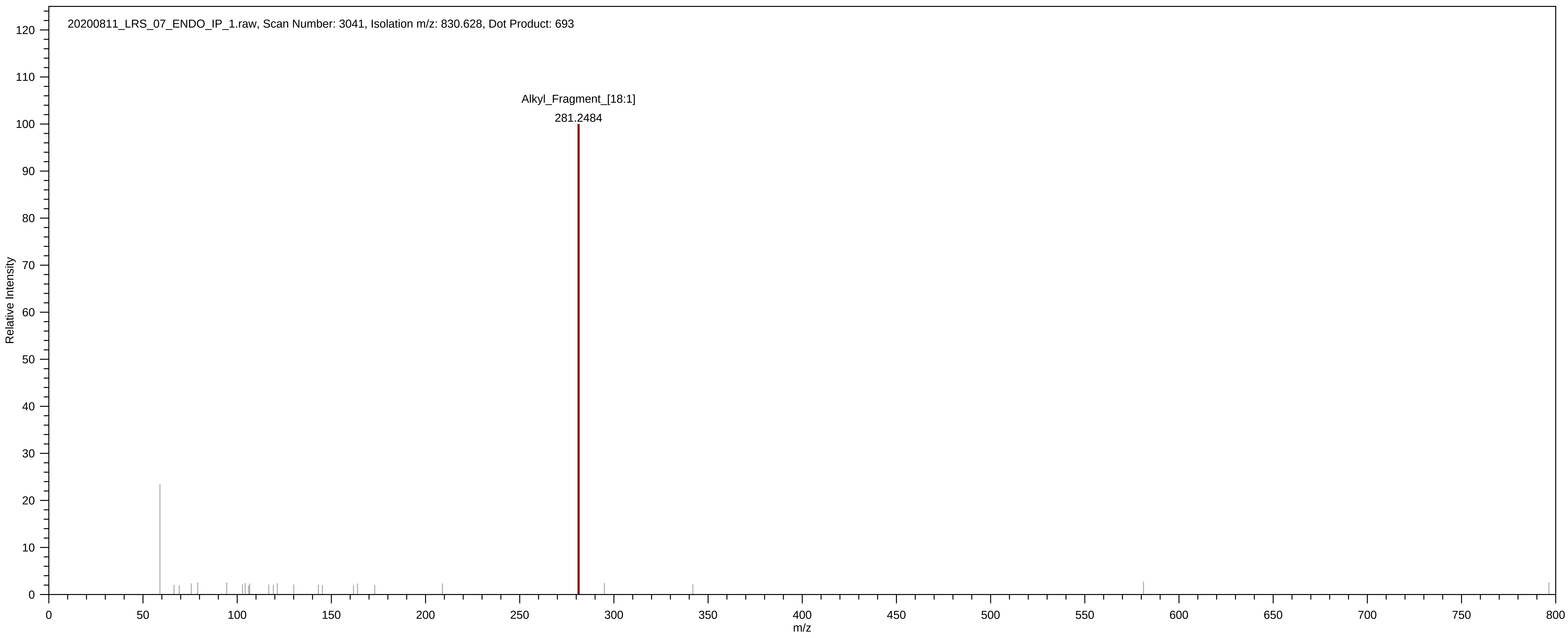

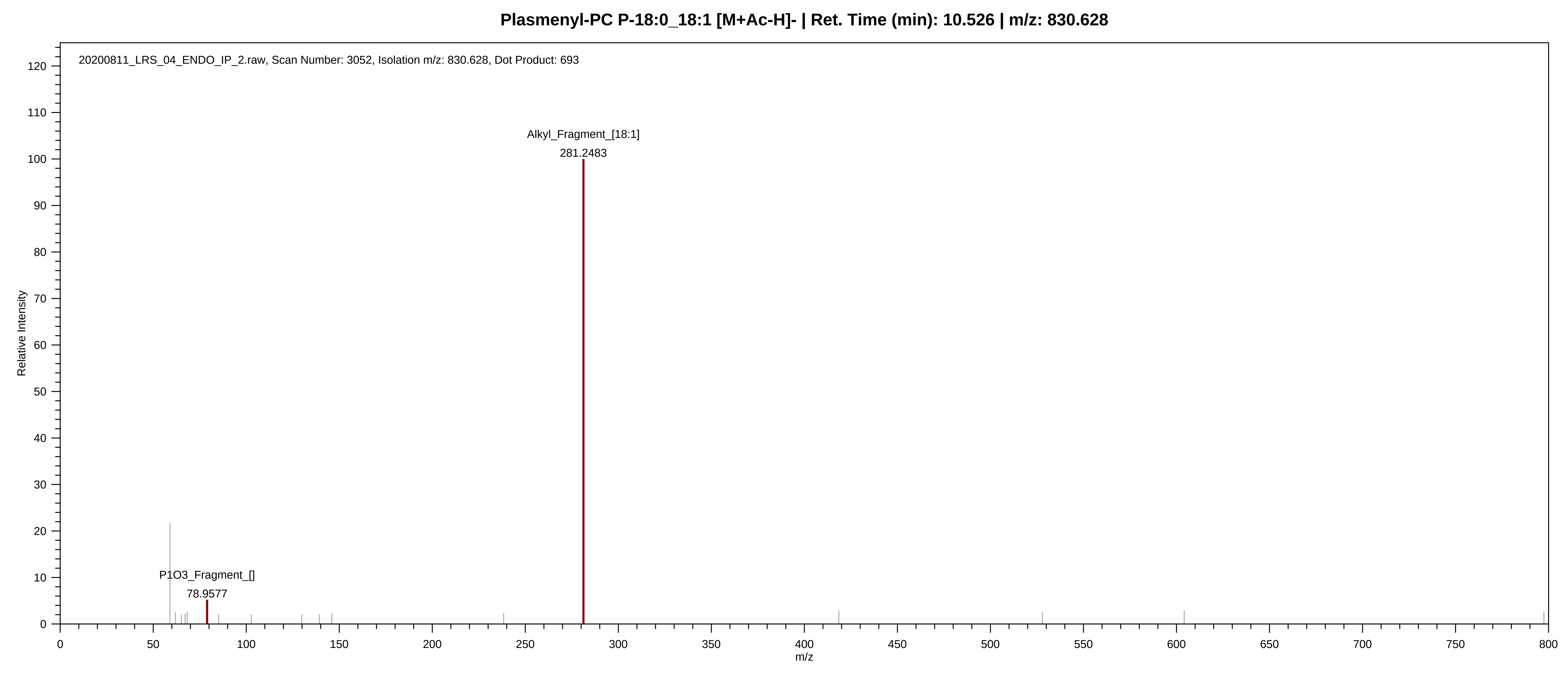
Plasmenyl-PC P-18:0_18:1 [M+Ac-H]- | Ret. Time (min): 10.526 | m/z: 830.628
20200811_LRS_04_ENDO_IP_2.raw, Scan Number: 3052, Isolation m/z: 830.628, Dot Product: 693
Alkyl_Fragment_[18:1]
281.2483
P1O3_Fragment_[]
78.9577
Relative Intensity
m/z

Plasmenyl-PC P-18:0_18:1 [M+Ac-H]- | Ret. Time (min): 10.552 | m/z: 830.628

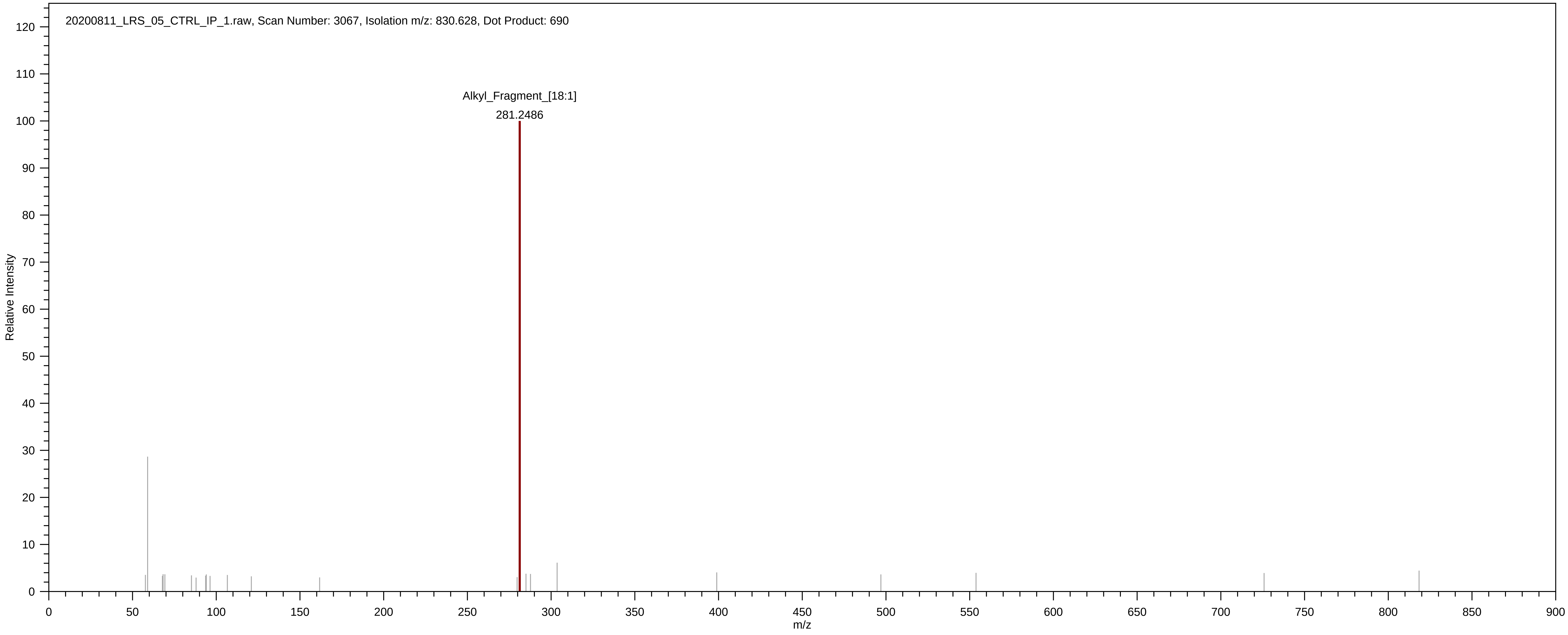

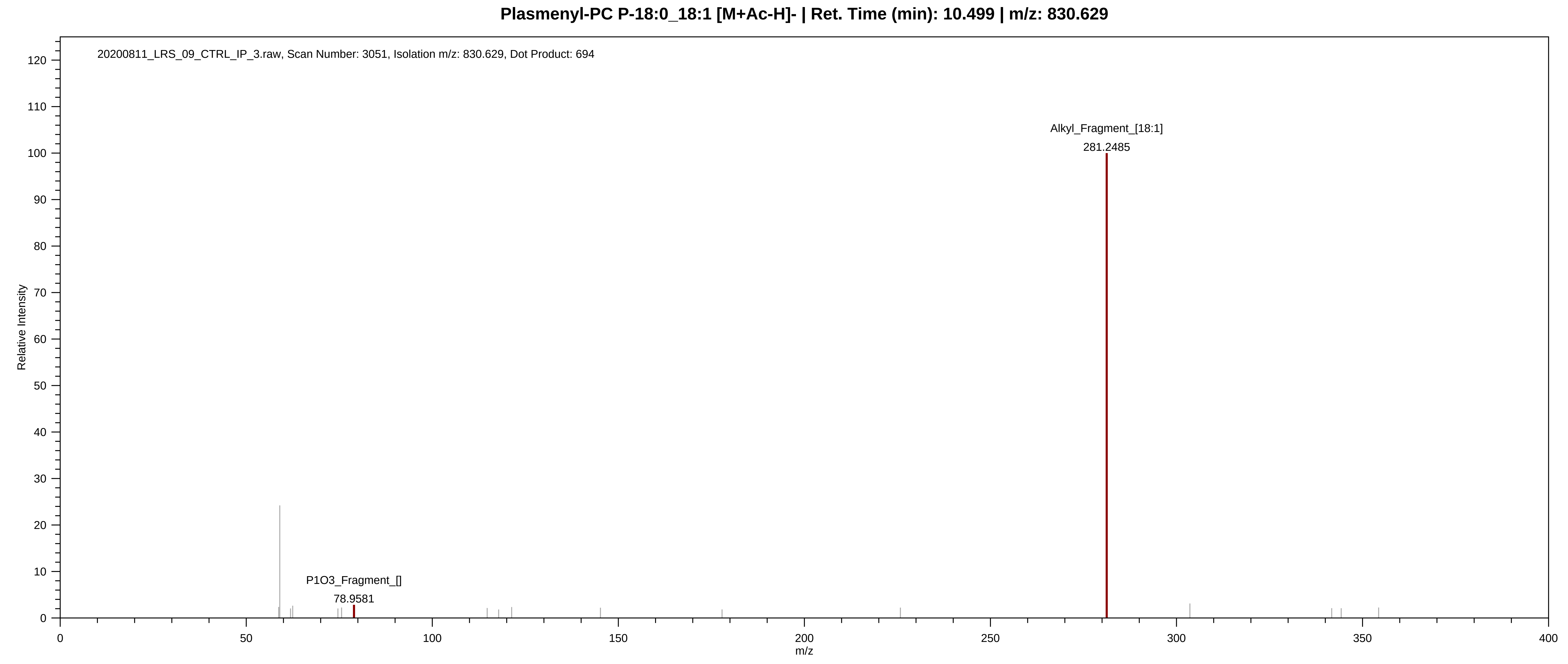

Plasmenyl-PC P-18:0_18:1 [M+Ac-H]- | Ret. Time (min): 10.499 | m/z: 830.629
20200811_LRS_09_CTRL_IP_3.raw, Scan Number: 3051, Isolation m/z: 830.629, Dot Product: 694
Alkyl_Fragment_[18:1]
281.2485
P1O3_Fragment_[]
78.9581
Relative Intensity
m/z

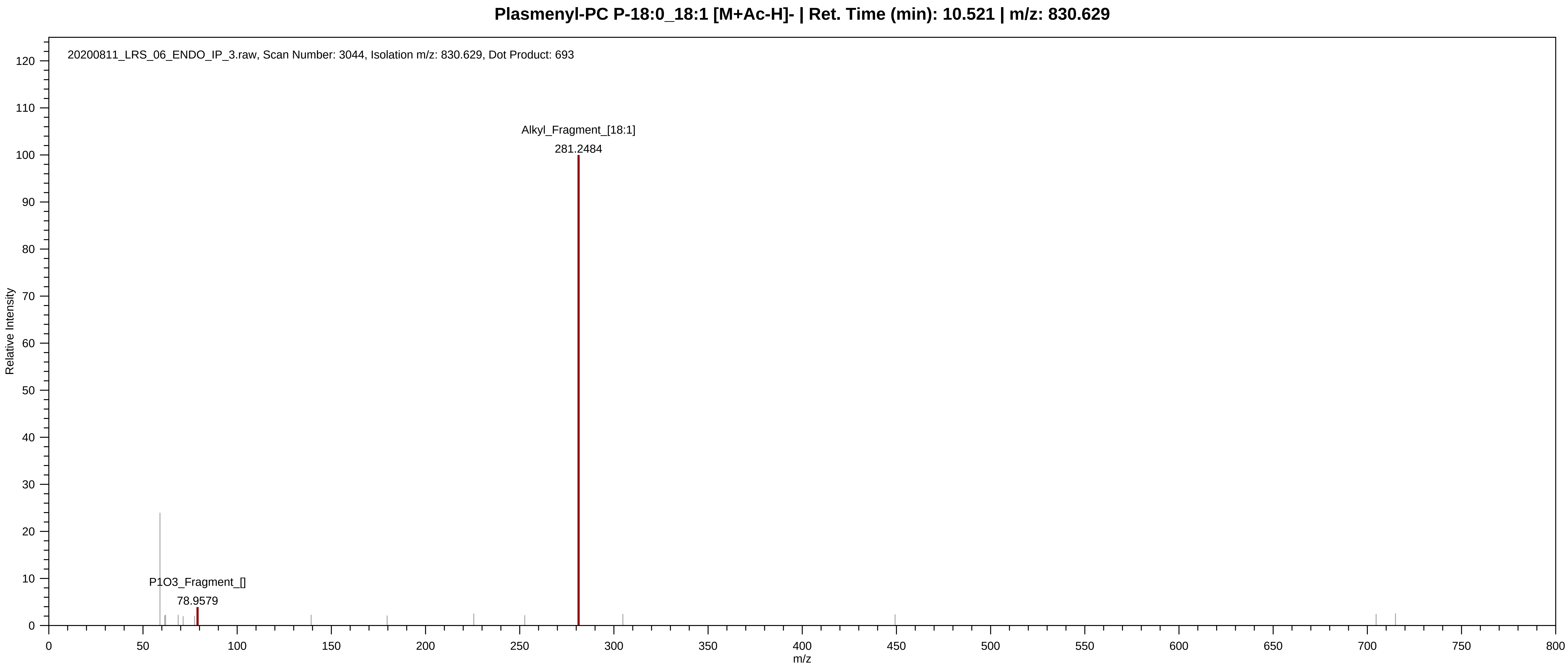

Plasmenyl-PC P-18:0_18:1 [M+Ac-H]- | Ret. Time (min): 10.521 | m/z: 830.629
20200811_LRS_06_ENDO_IP_3.raw, Scan Number: 3044, Isolation m/z: 830.629, Dot Product: 693
Alkyl_Fragment_[18:1]
281.2484
P1O3_Fragment_[]
78.9579
Relative Intensity
m/z

Plasmenyl-PC P-18:0_18:2 [M+Ac-H]- | Ret. Time (min): 9.735 | m/z: 828.612

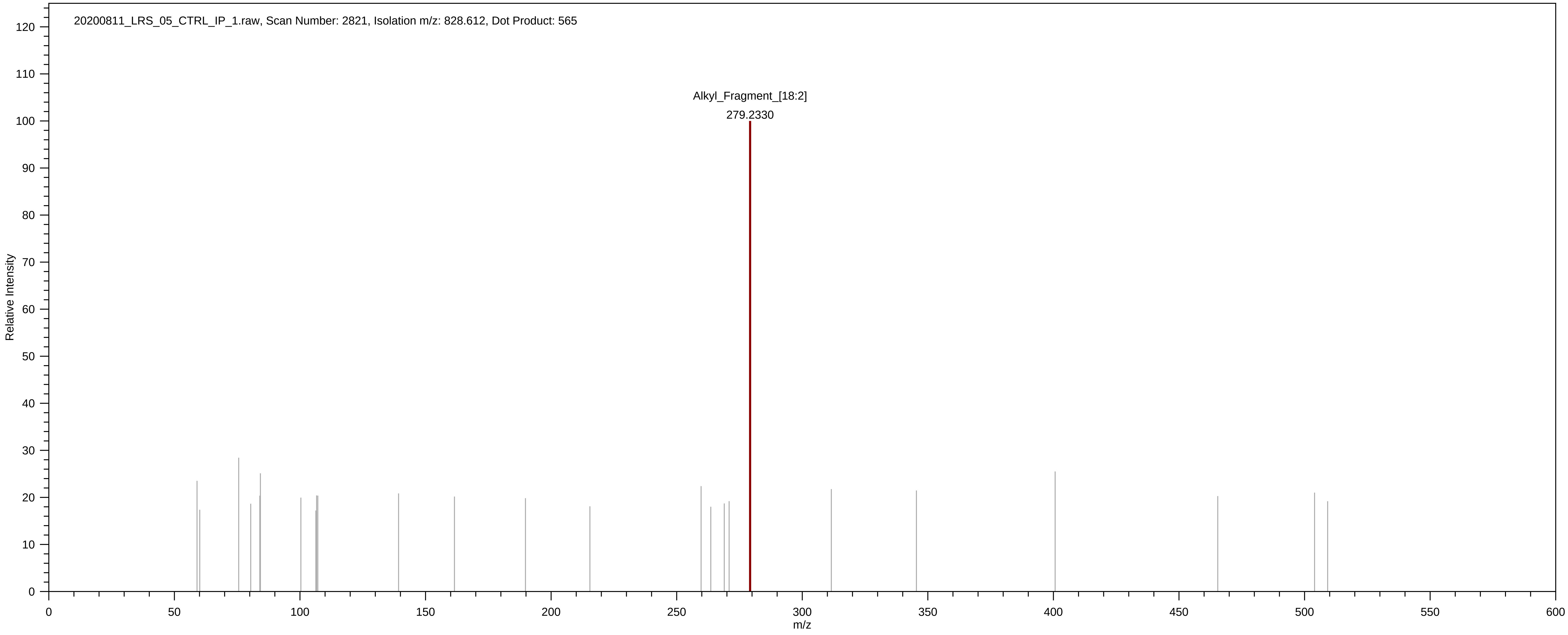

Plasmenyl-PC P-18:0_18:2 [M+Ac-H]- | Ret. Time (min): 9.708 | m/z: 828.614

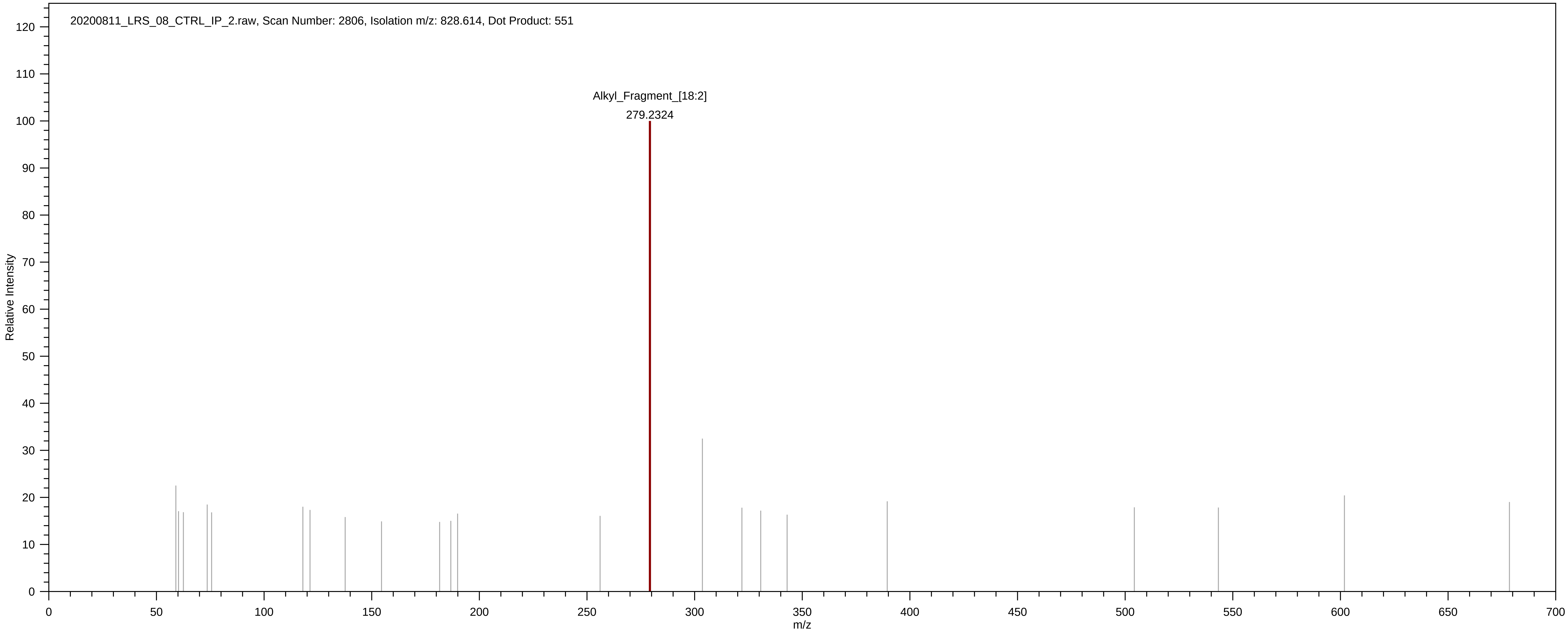

Plasmenyl-PC P-18:1_18:1 [M+Ac-H]- | Ret. Time (min): 10.354 | m/z: 828.613

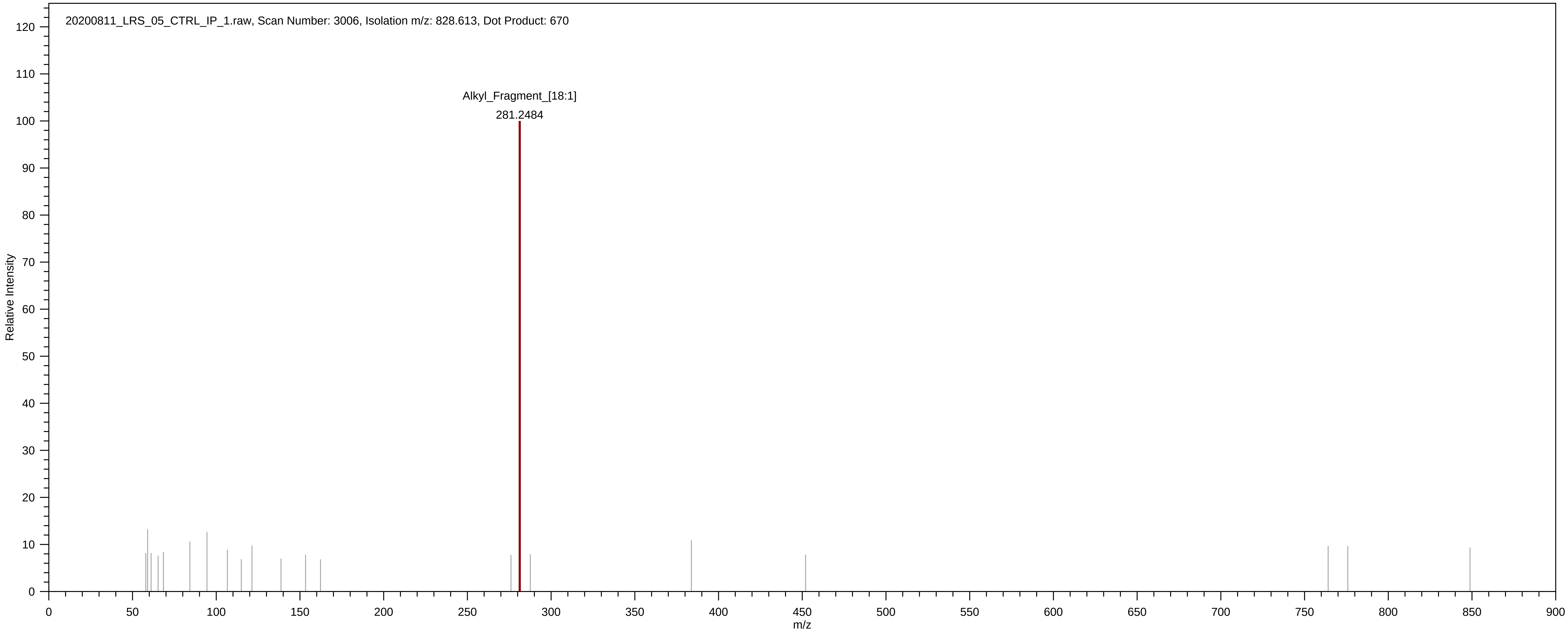

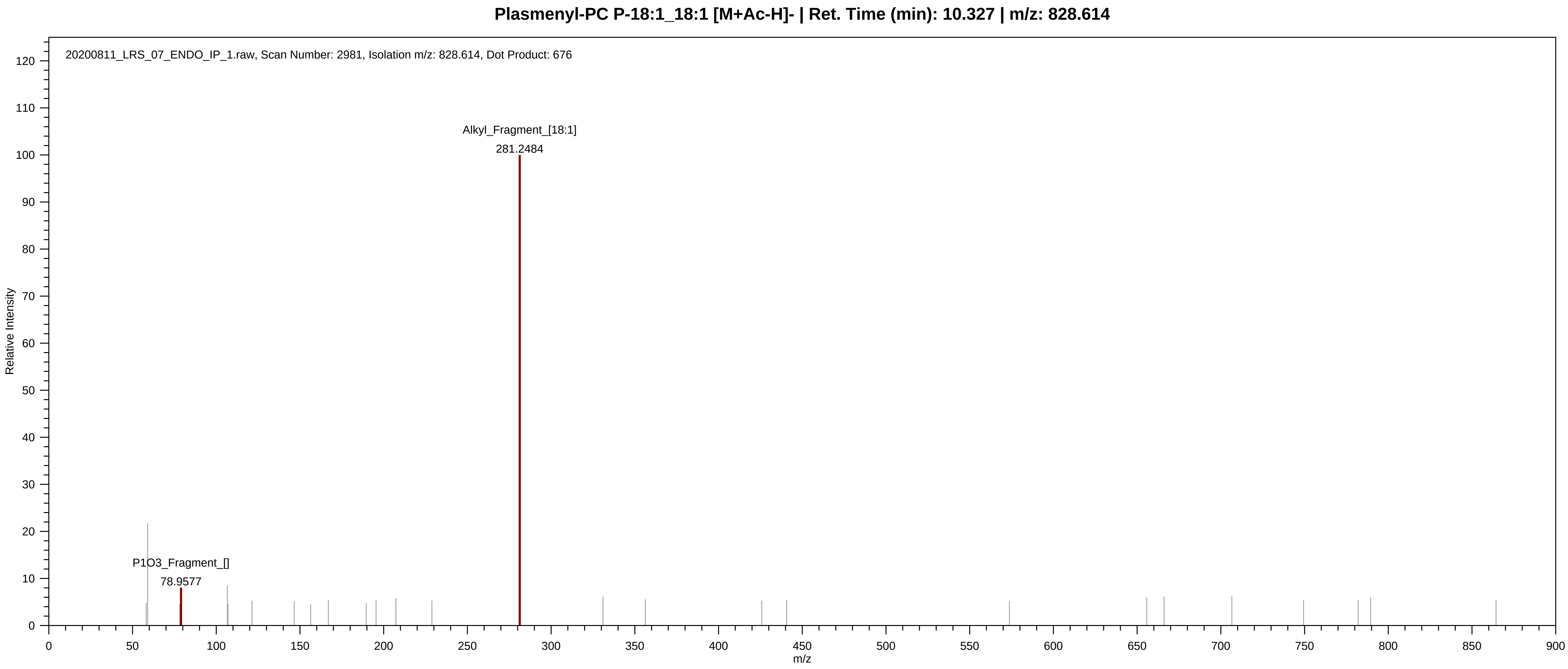
Plasmenyl-PC P-18:1_18:1 [M+Ac-H]- | Ret. Time (min): 10.327 | m/z: 828.614
20200811_LRS_07_ENDO_IP_1.raw, Scan Number: 2981, Isolation m/z: 828.614, Dot Product: 676
Alkyl_Fragment_[18:1]
281.2484
P1O3_Fragment_[]
78.9577
Relative Intensity
m/z

Plasmenyl-PC P-18:1_18:1 [M+Ac-H]- | Ret. Time (min): 10.305 | m/z: 828.615

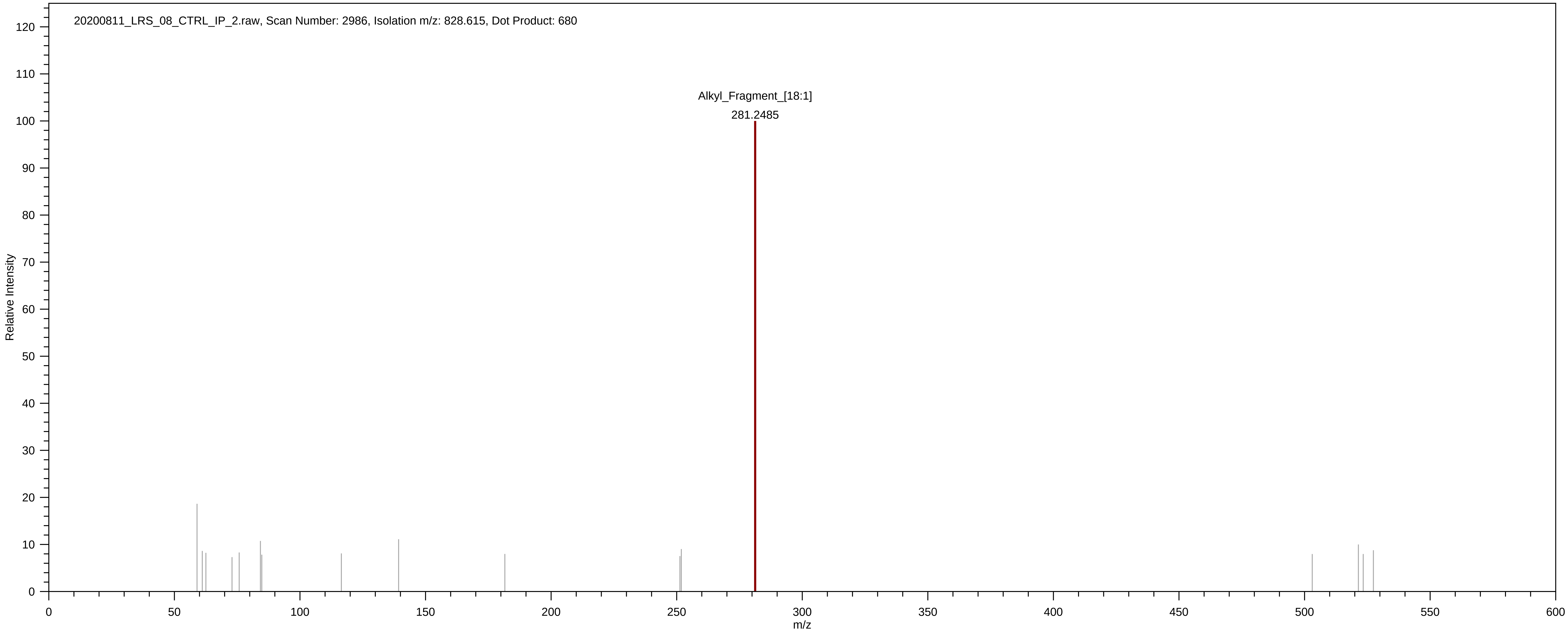

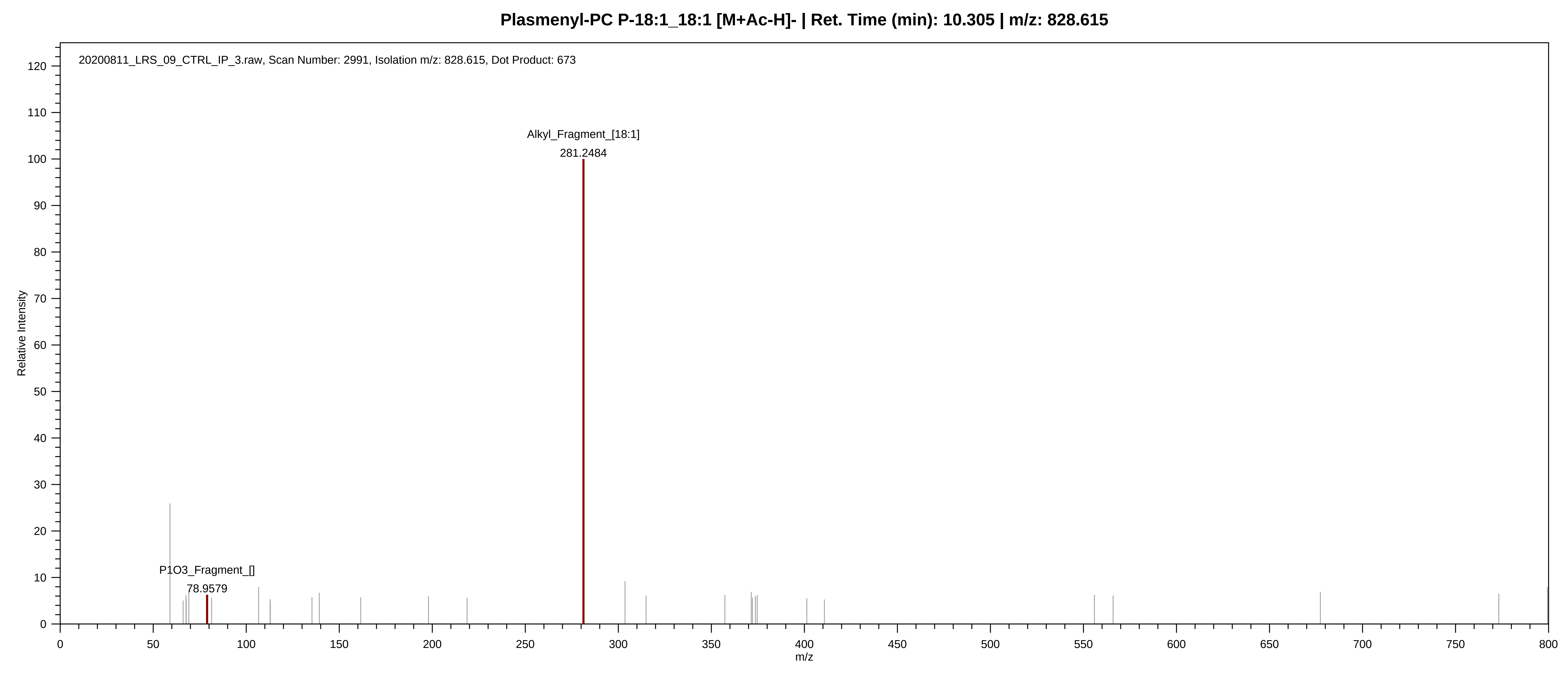
Plasmenyl-PC P-18:1_18:1 [M+Ac-H]- | Ret. Time (min): 10.305 | m/z: 828.615
20200811_LRS_09_CTRL_IP_3.raw, Scan Number: 2991, Isolation m/z: 828.615, Dot Product: 673
Alkyl_Fragment_[18:1]
281.2484
P1O3_Fragment_[]
78.9579
Relative Intensity
m/z

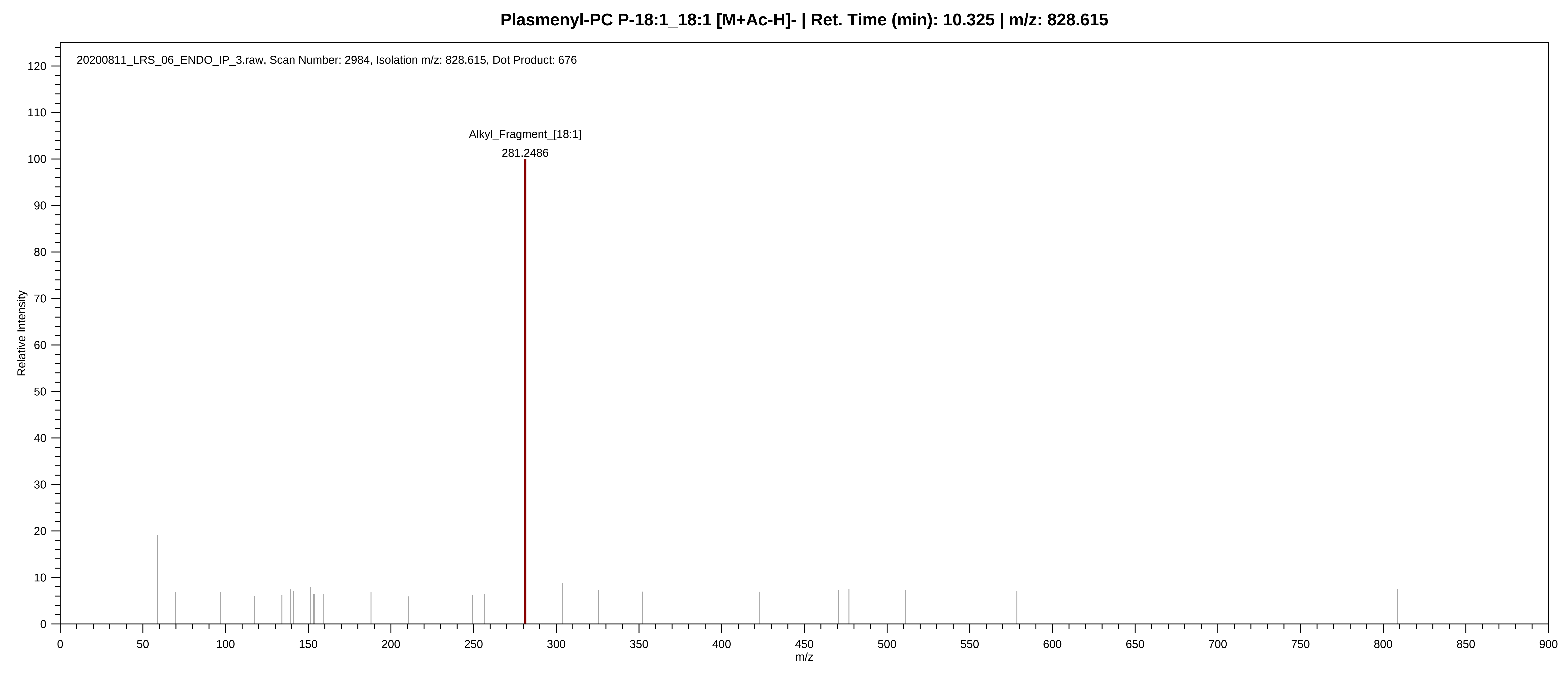

Plasmenyl-PC P-18:1_18:1 [M+Ac-H]- | Ret. Time (min): 10.325 | m/z: 828.615
20200811_LRS_06_ENDO_IP_3.raw, Scan Number: 2984, Isolation m/z: 828.615, Dot Product: 676
Alkyl_Fragment_[18:1]
281.2486
Relative Intensity
m/z

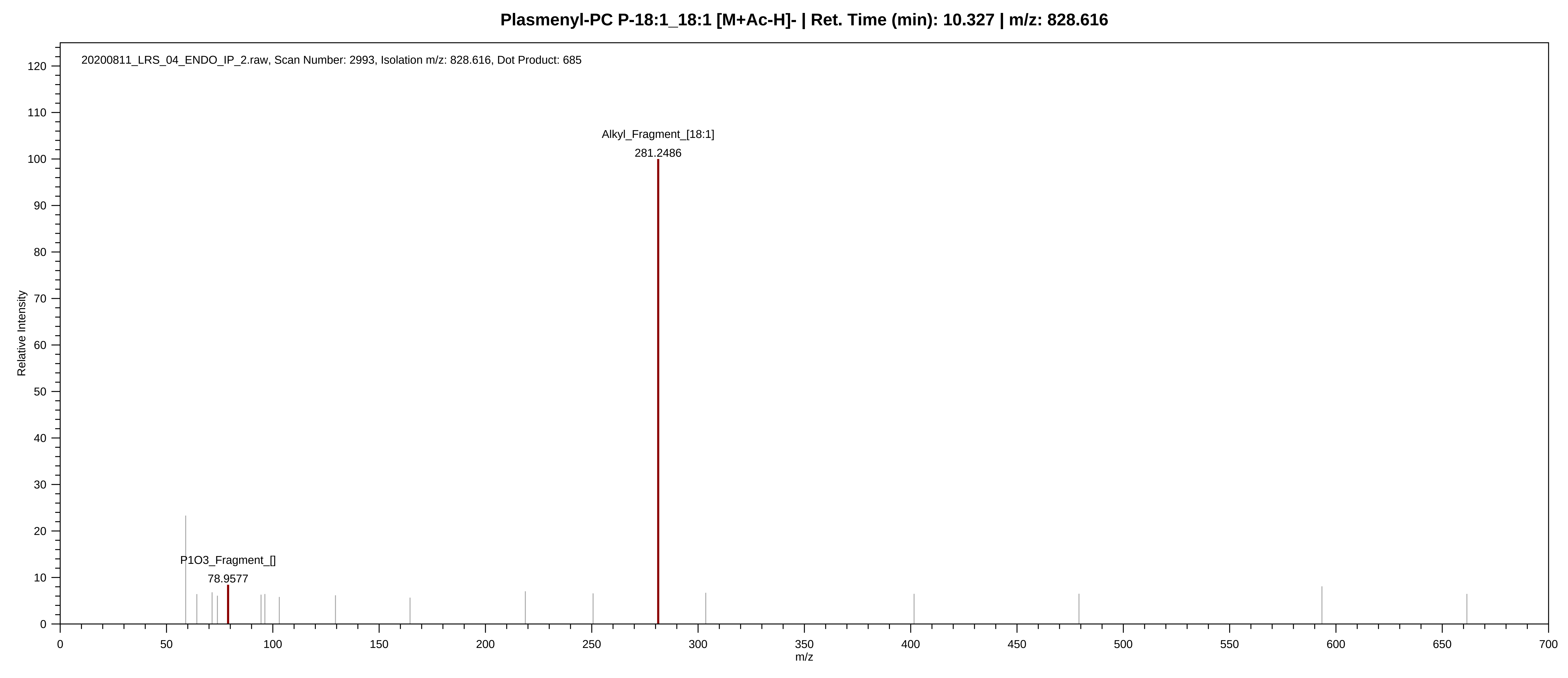
Plasmenyl-PC P-18:1_18:1 [M+Ac-H]- | Ret. Time (min): 10.327 | m/z: 828.616
20200811_LRS_04_ENDO_IP_2.raw, Scan Number: 2993, Isolation m/z: 828.616, Dot Product: 685
Alkyl_Fragment_[18:1]
281.2486
P1O3_Fragment_[]
78.9577
Relative Intensity
m/z

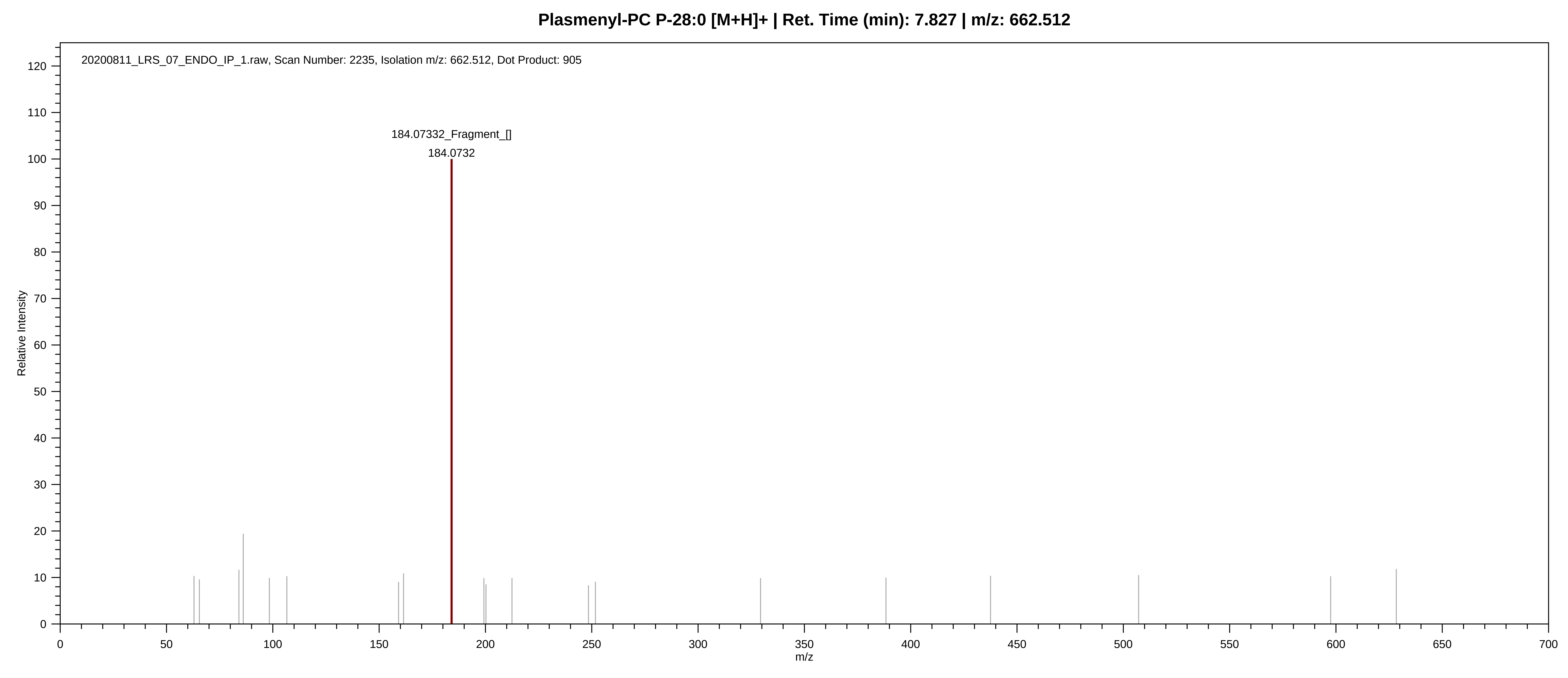

Plasmenyl-PC P-28:0 [M+H]+ | Ret. Time (min): 7.827 | m/z: 662.512
20200811_LRS_07_ENDO_IP_1.raw, Scan Number: 2235, Isolation m/z: 662.512, Dot Product: 905
184.07332_Fragment_[]
184.0732
Relative Intensity
m/z

Plasmenyl-PC P-30:0 [M+H]+ | Ret. Time (min): 8.626 | m/z: 690.544

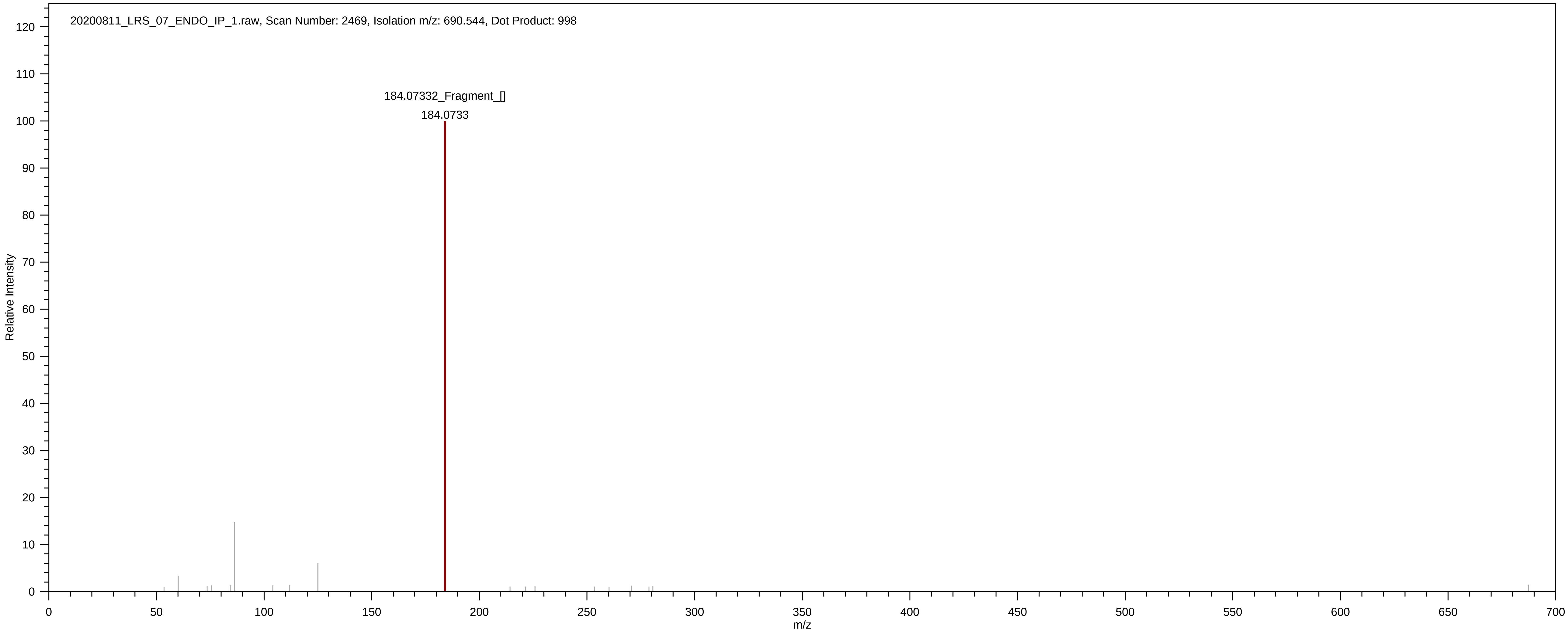

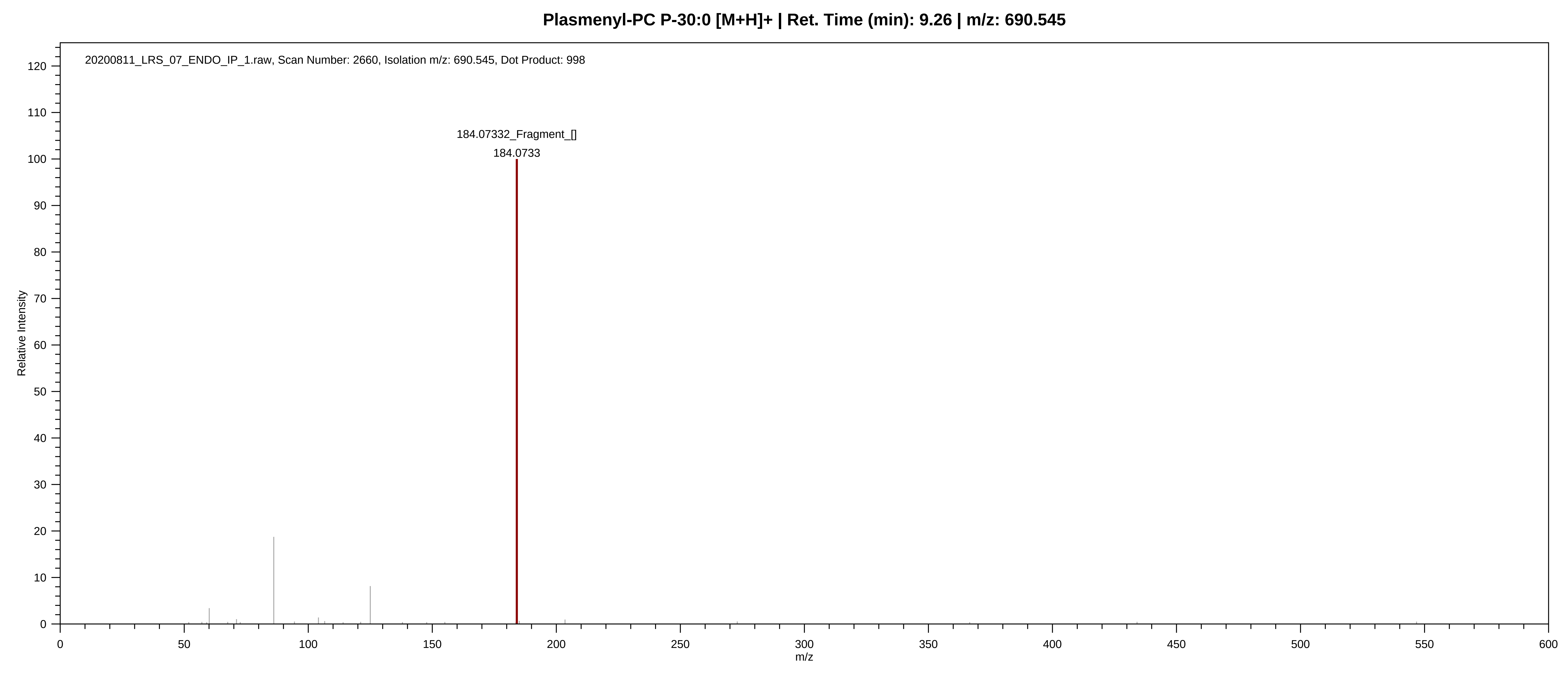

Plasmenyl-PC P-30:0 [M+H]+ | Ret. Time (min): 9.26 | m/z: 690.545
20200811_LRS_07_ENDO_IP_1.raw, Scan Number: 2660, Isolation m/z: 690.545, Dot Product: 998
184.07332_Fragment_[]
184.0733
Relative Intensity
m/z

Plasmenyl-PC P-31:0 [M+H]+ | Ret. Time (min): 9.576 | m/z: 704.564

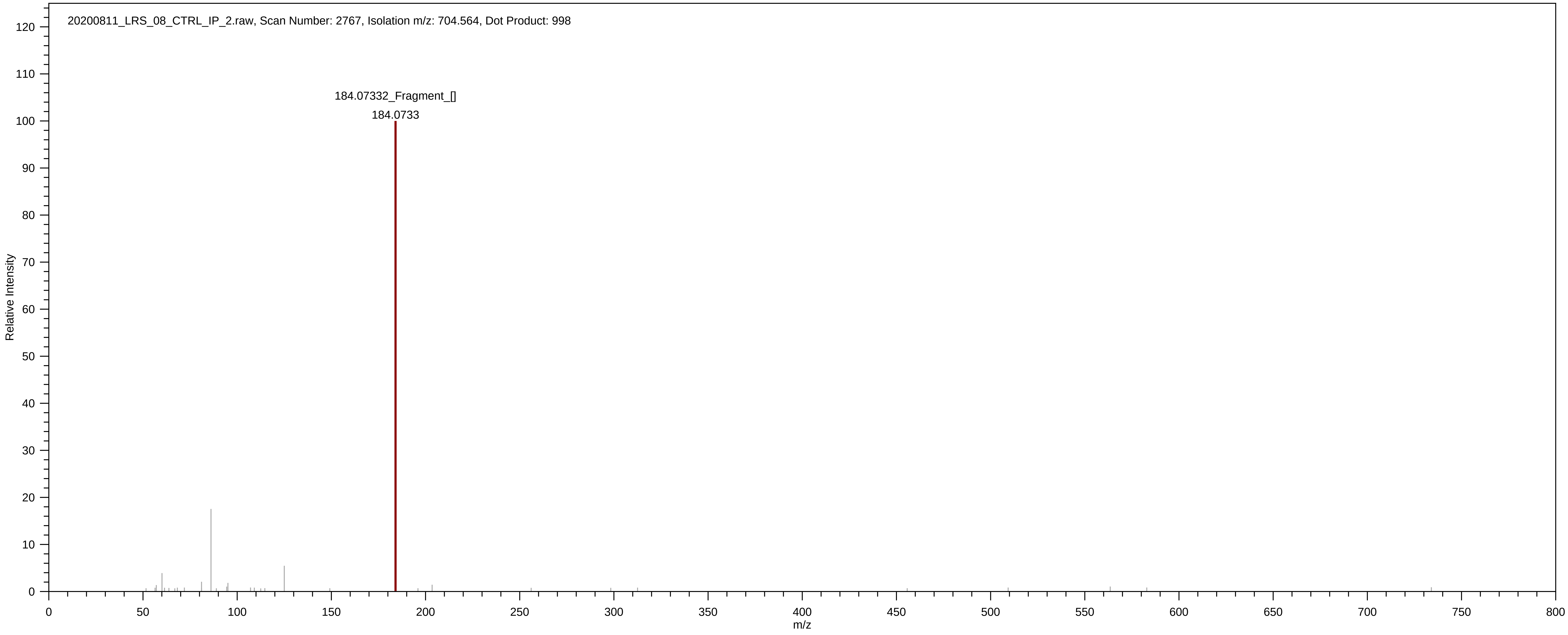

Plasmenyl-PC P-32:0 [M+H]+ | Ret. Time (min): 10.234 | m/z: 718.573

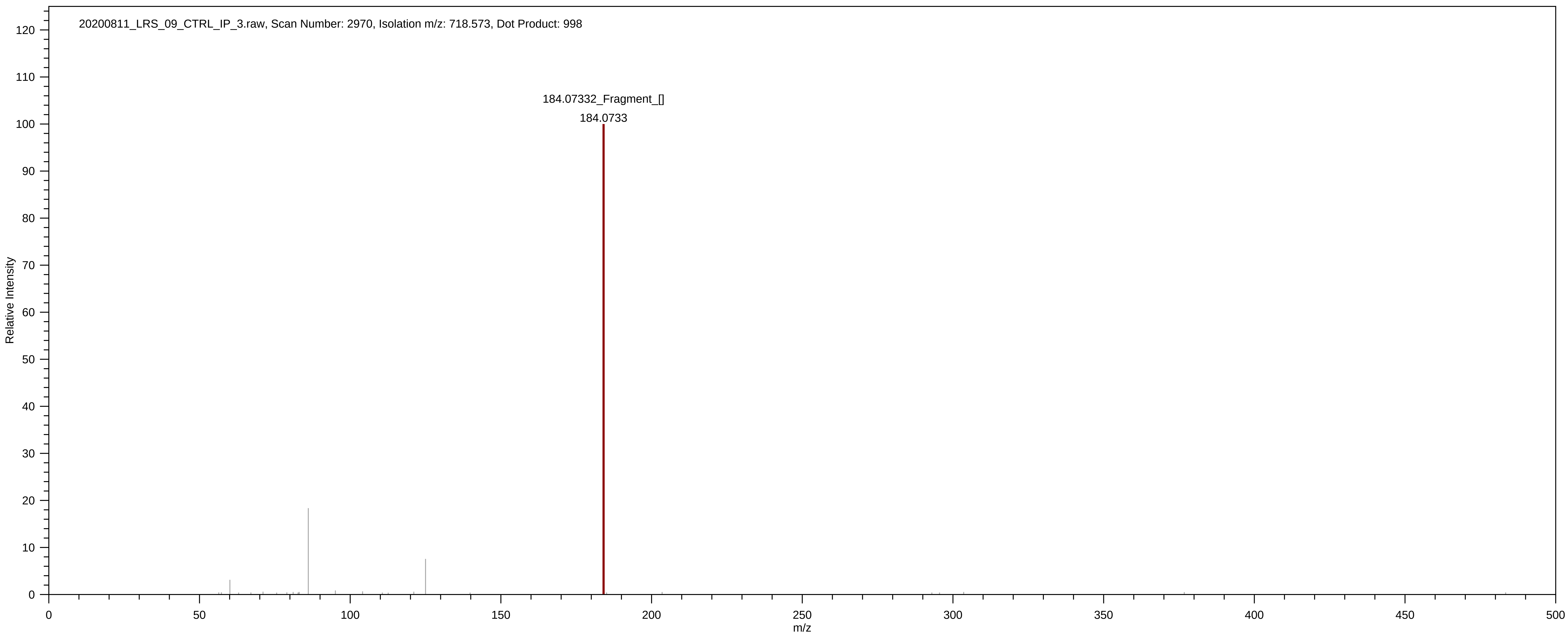

Plasmenyl-PC P-32:0 [M+H]+ | Ret. Time (min): 9.476 | m/z: 718.574

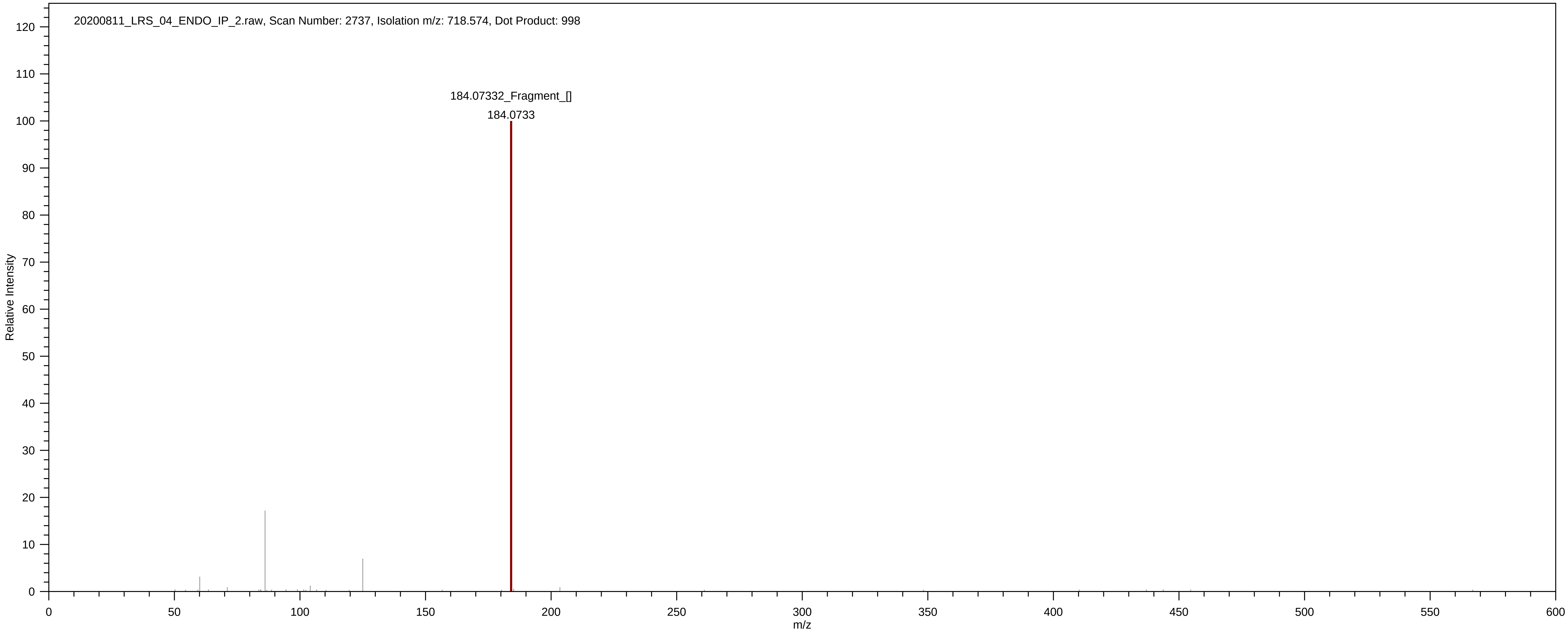

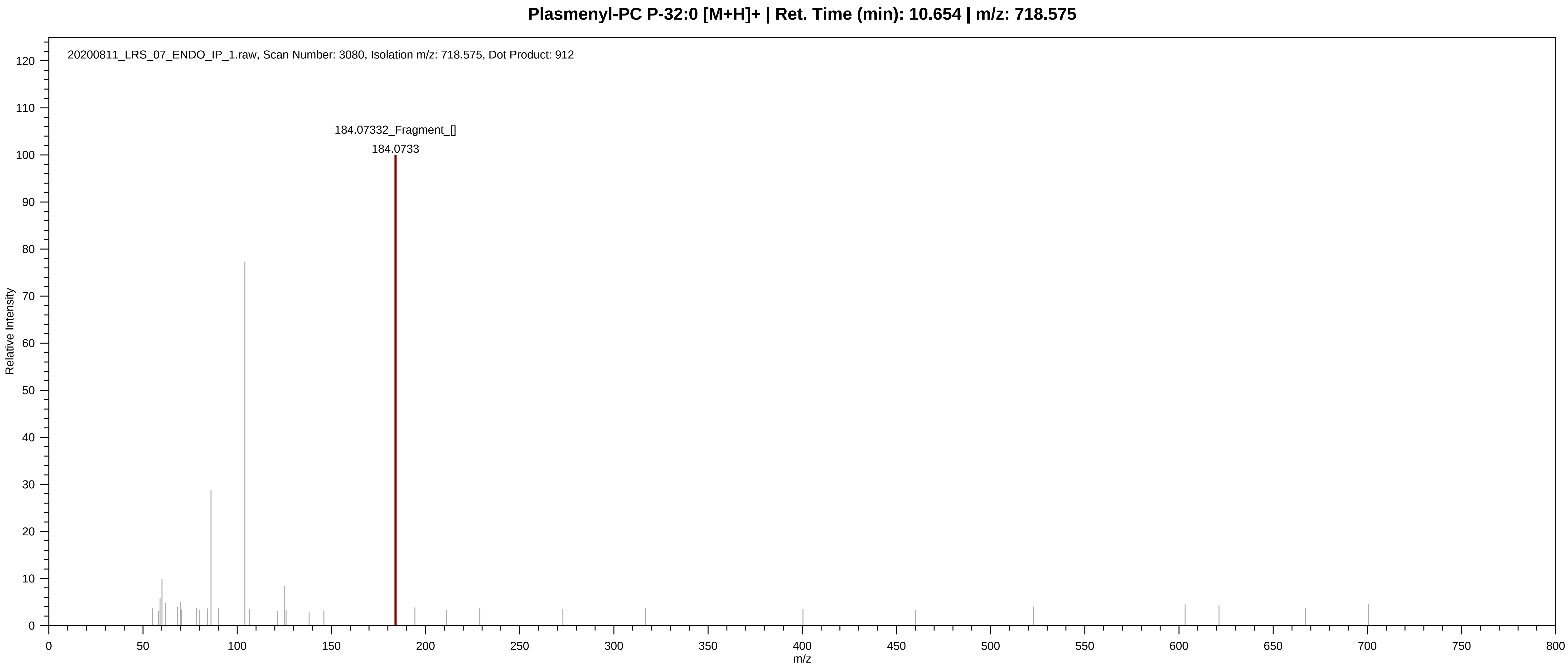
Plasmenyl-PC P-32:0 [M+H]+ | Ret. Time (min): 10.654 | m/z: 718.575
20200811_LRS_07_ENDO_IP_1.raw, Scan Number: 3080, Isolation m/z: 718.575, Dot Product: 912
184.07332_Fragment_[]
184.0733
Relative Intensity
m/z

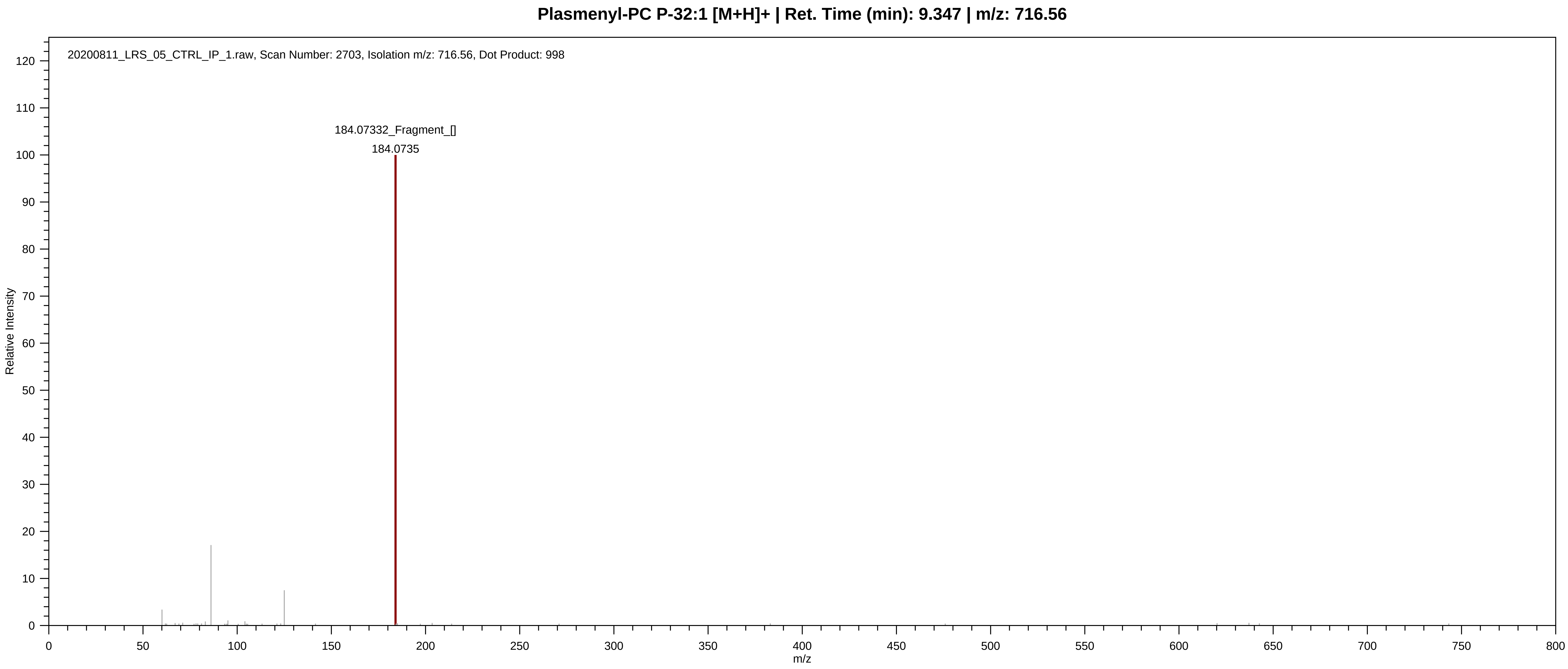
Plasmenyl-PC P-32:1 [M+H]+ | Ret. Time (min): 9.347 | m/z: 716.56
20200811_LRS_05_CTRL_IP_1.raw, Scan Number: 2703, Isolation m/z: 716.56, Dot Product: 998
184.07332_Fragment_[]
184.0735
Relative Intensity
m/z

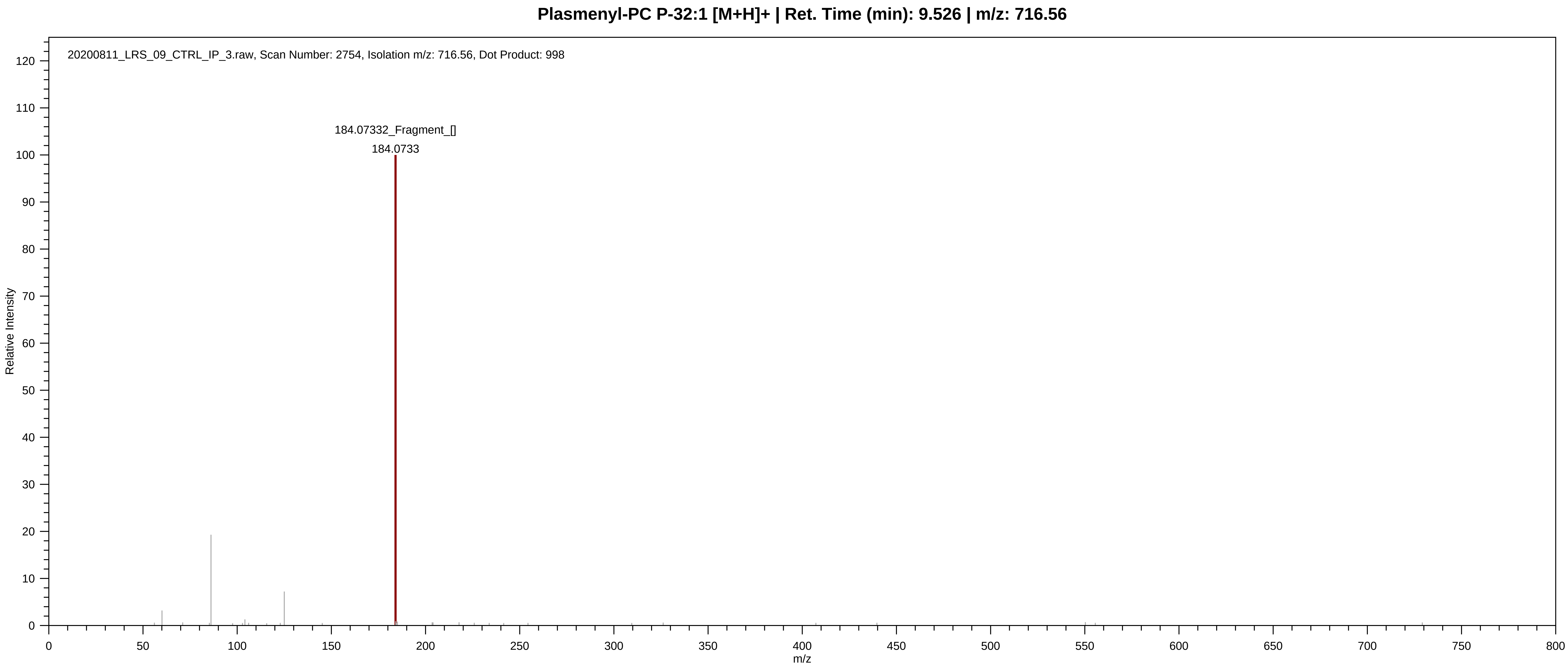
Plasmenyl-PC P-32:1 [M+H]+ | Ret. Time (min): 9.526 | m/z: 716.56
20200811_LRS_09_CTRL_IP_3.raw, Scan Number: 2754, Isolation m/z: 716.56, Dot Product: 998
184.07332_Fragment_[]
184.0733
Relative Intensity
m/z

Plasmenyl-PC P-32:1 [M+H]+ | Ret. Time (min): 9.322 | m/z: 716.557

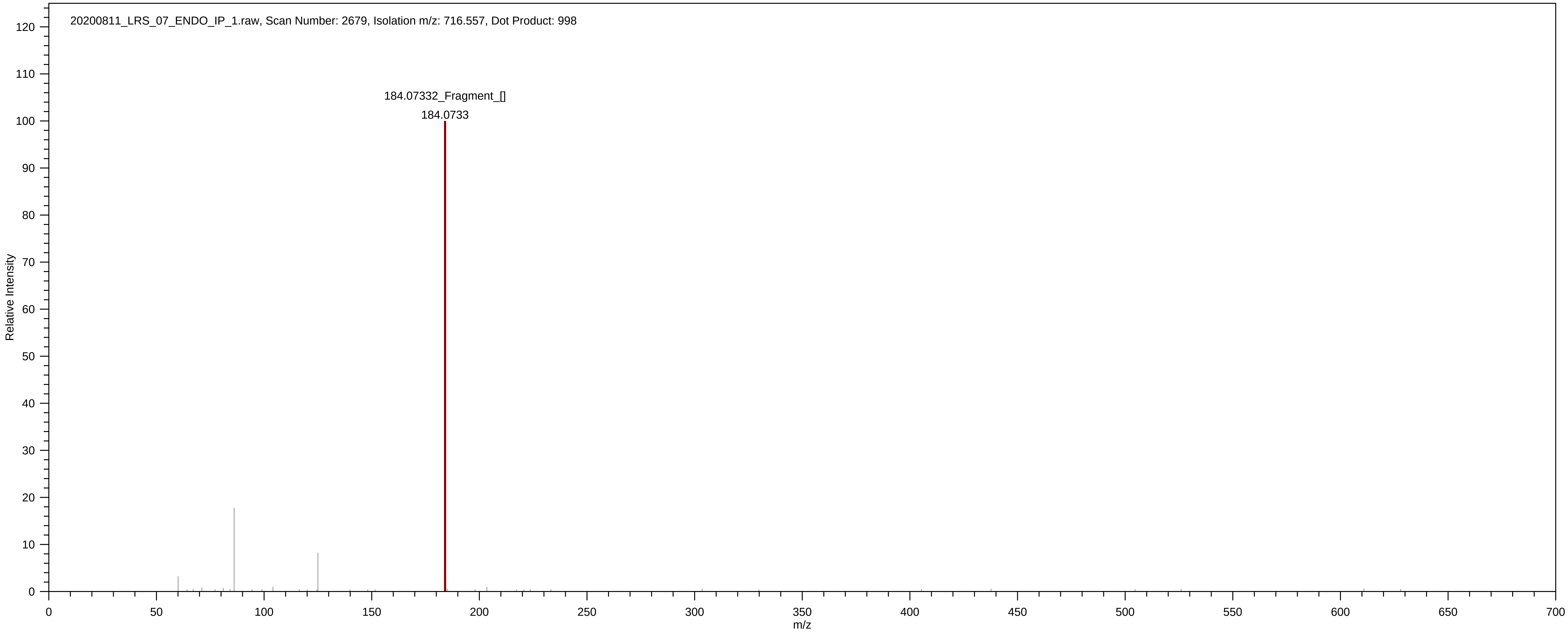

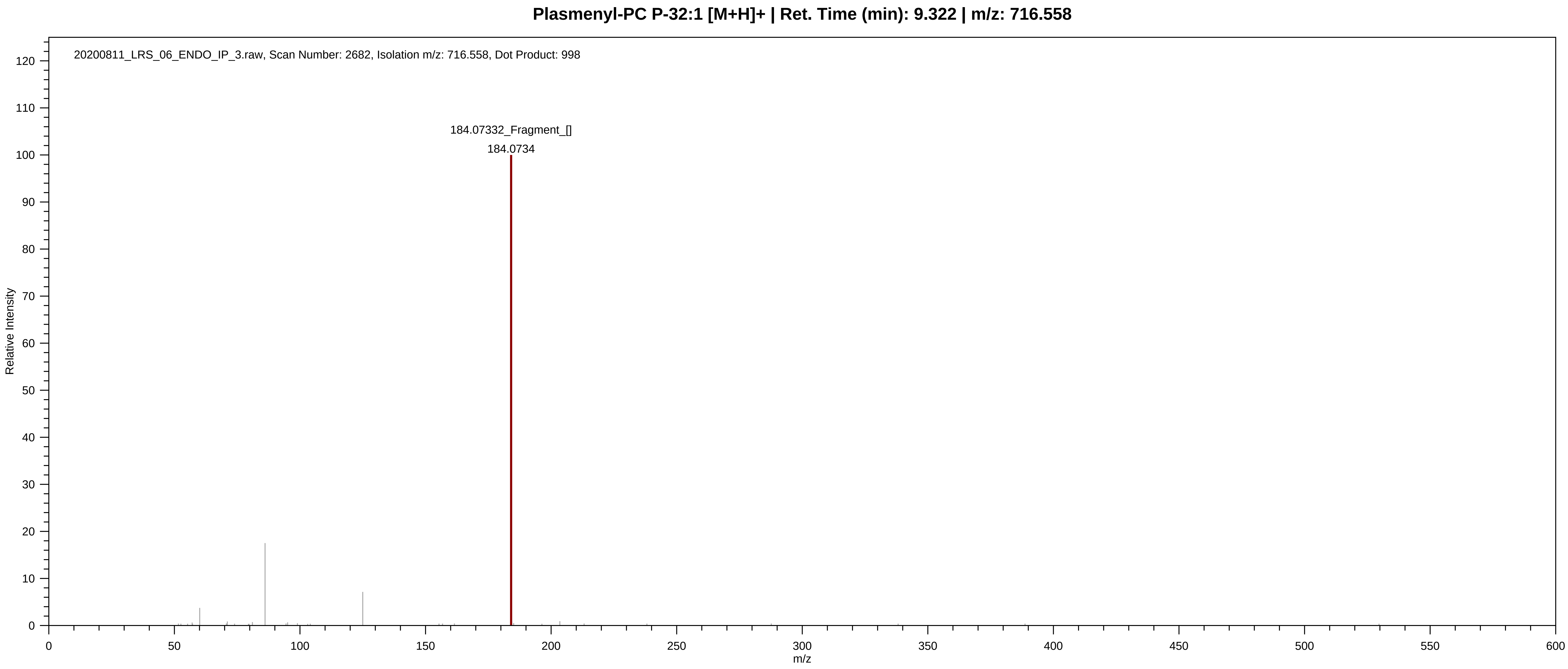
Plasmenyl-PC P-32:1 [M+H]+ | Ret. Time (min): 9.322 | m/z: 716.558
20200811_LRS_06_ENDO_IP_3.raw, Scan Number: 2682, Isolation m/z: 716.558, Dot Product: 998
184.07332_Fragment_[]
184.0734
Relative Intensity
m/z

Plasmenyl-PC P-32:1 [M+H]+ | Ret. Time (min): 9.328 | m/z: 716.558

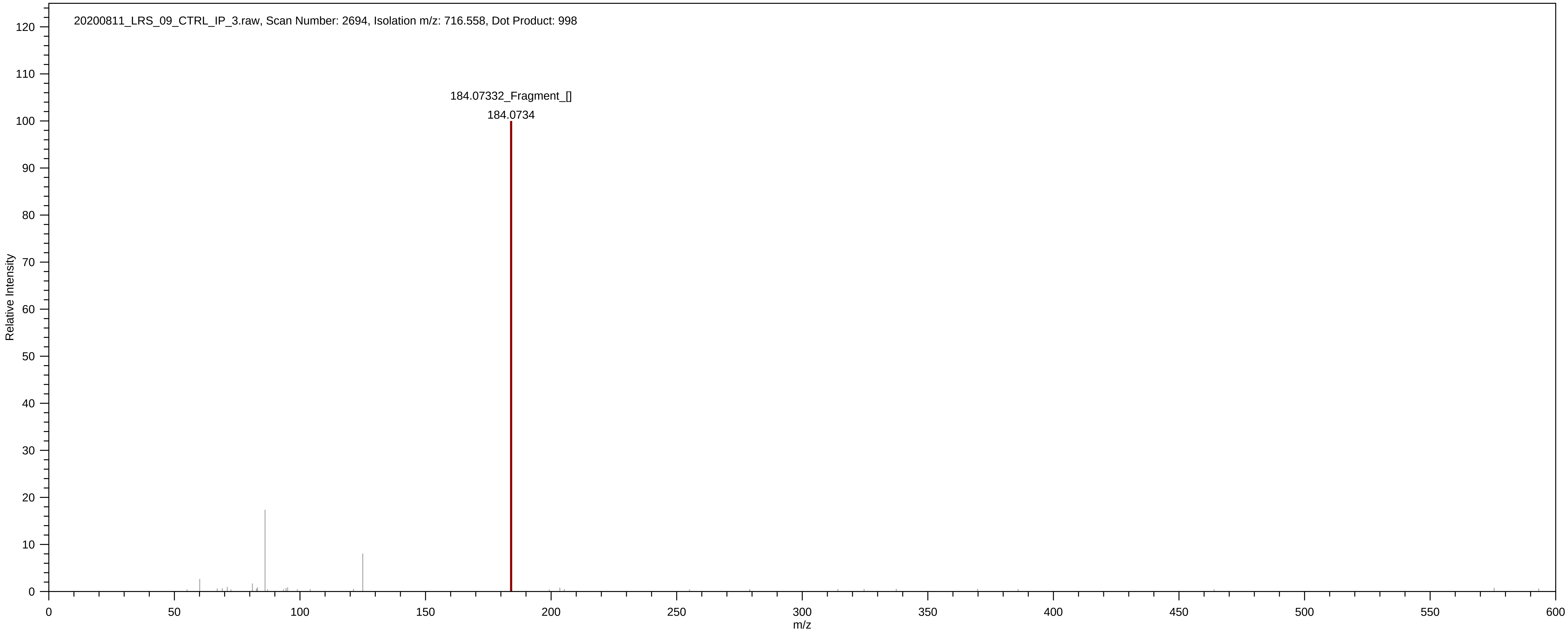

Plasmenyl-PC P-32:1 [M+H]+ | Ret. Time (min): 9.501 | m/z: 716.559

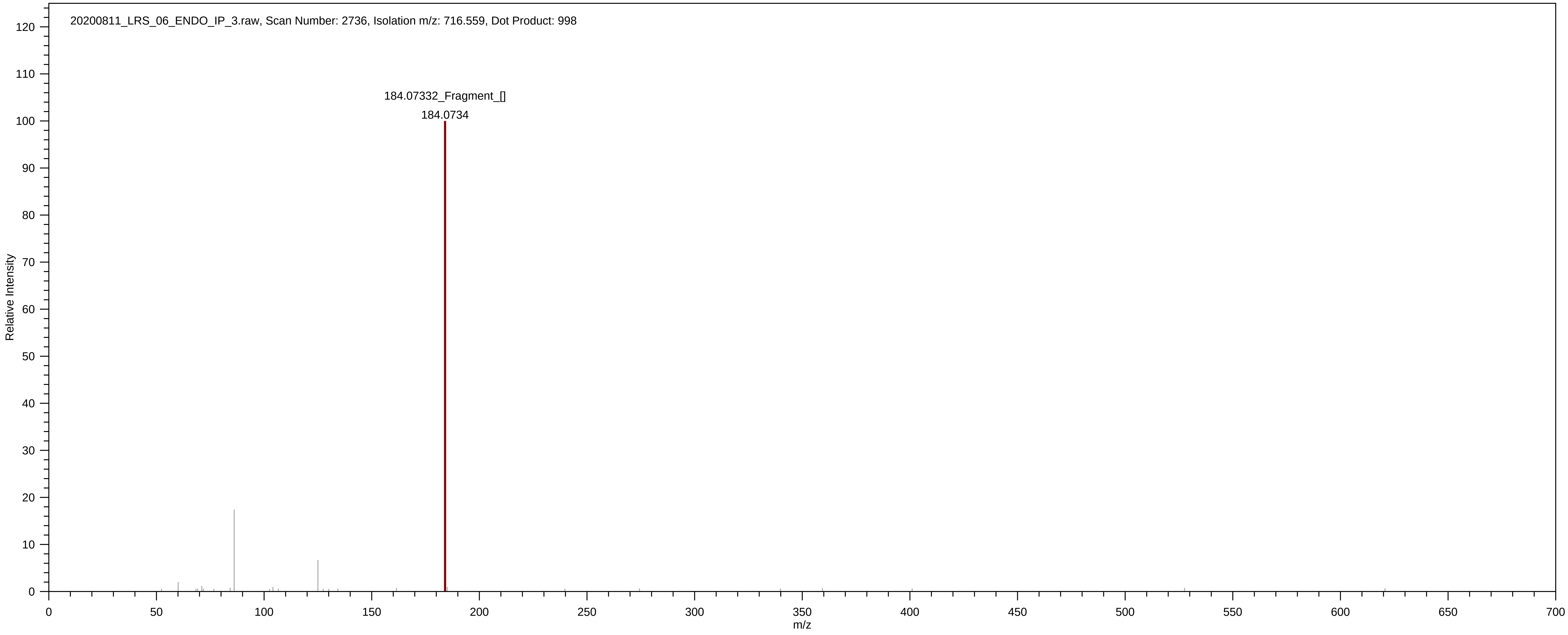

Plasmenyl-PC P-32:1 [M+H]+ | Ret. Time (min): 9.502 | m/z: 716.559

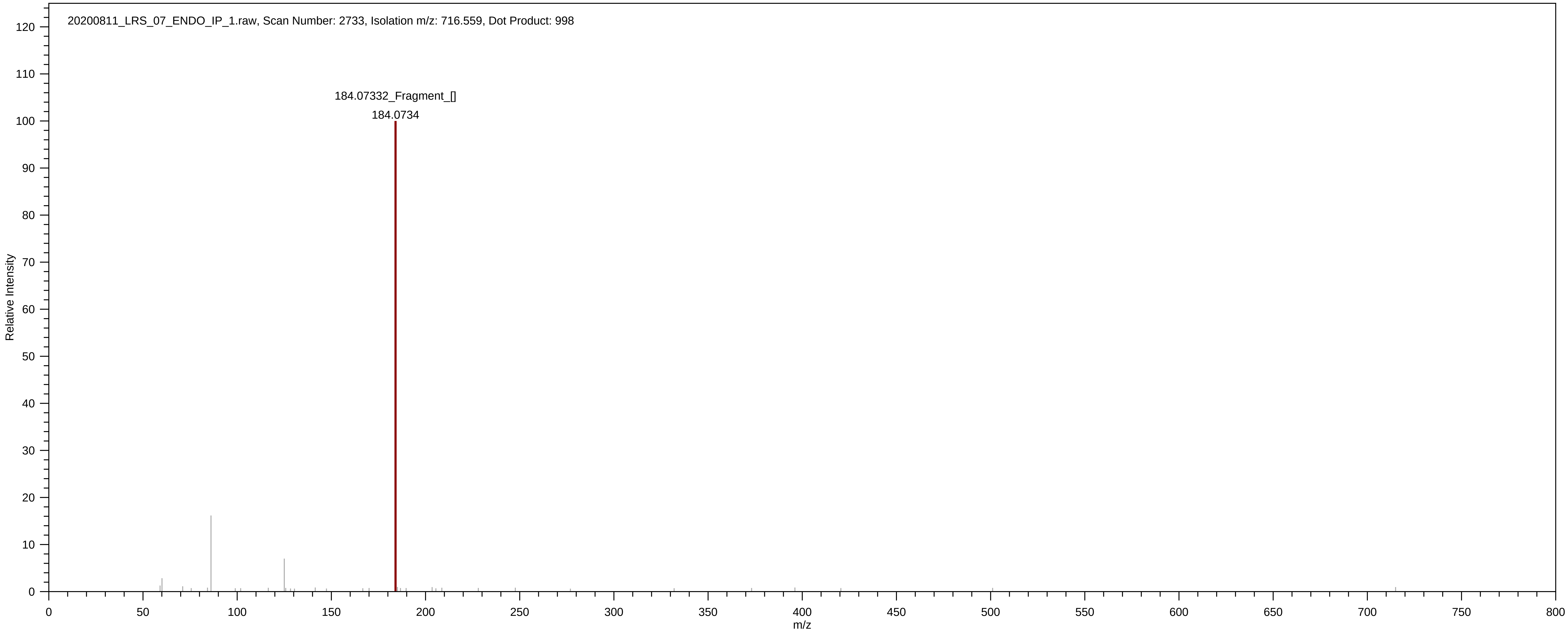

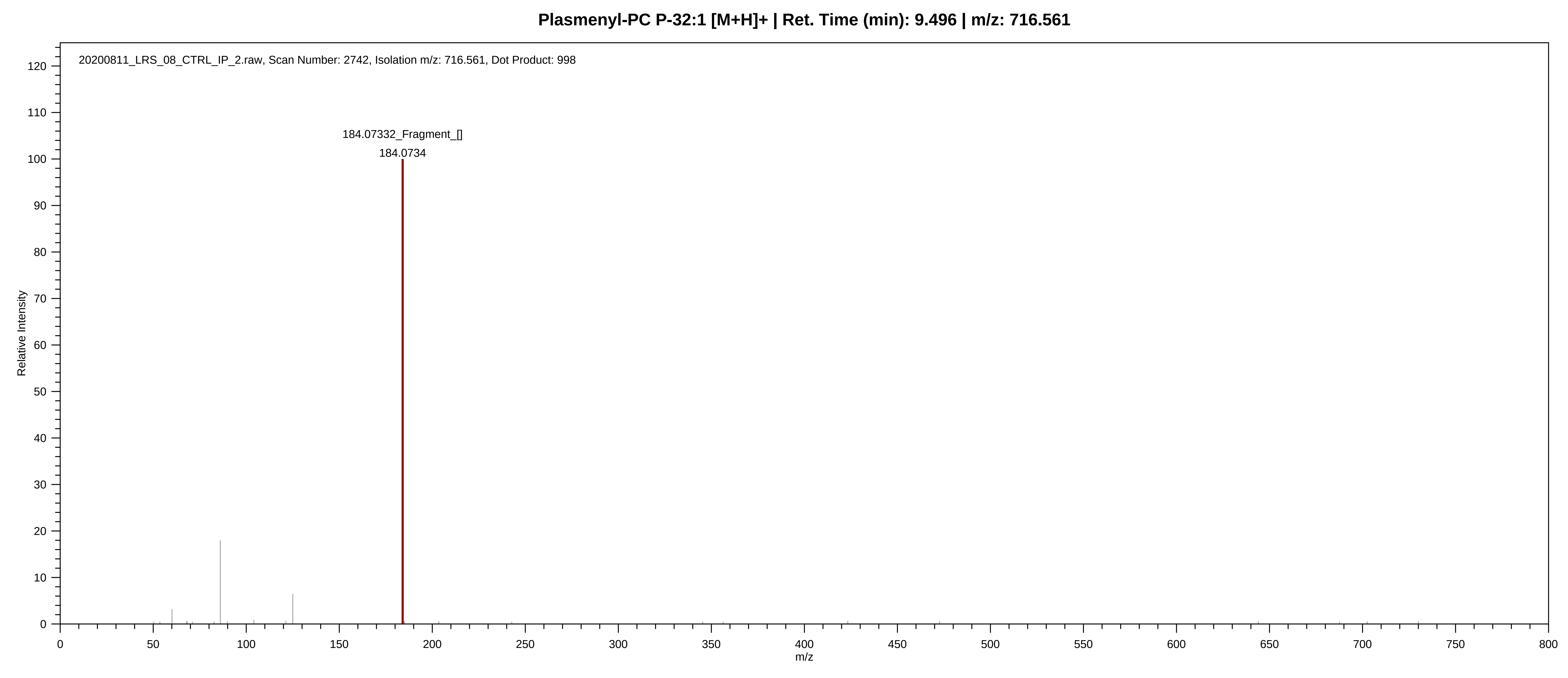
Plasmenyl-PC P-32:1 [M+H]+ | Ret. Time (min): 9.496 | m/z: 716.561
20200811_LRS_08_CTRL_IP_2.raw, Scan Number: 2742, Isolation m/z: 716.561, Dot Product: 998
184.07332_Fragment_[]
184.0734
Relative Intensity
m/z

Plasmenyl-PC P-32:1 [M+H]+ | Ret. Time (min): 9.525 | m/z: 716.561

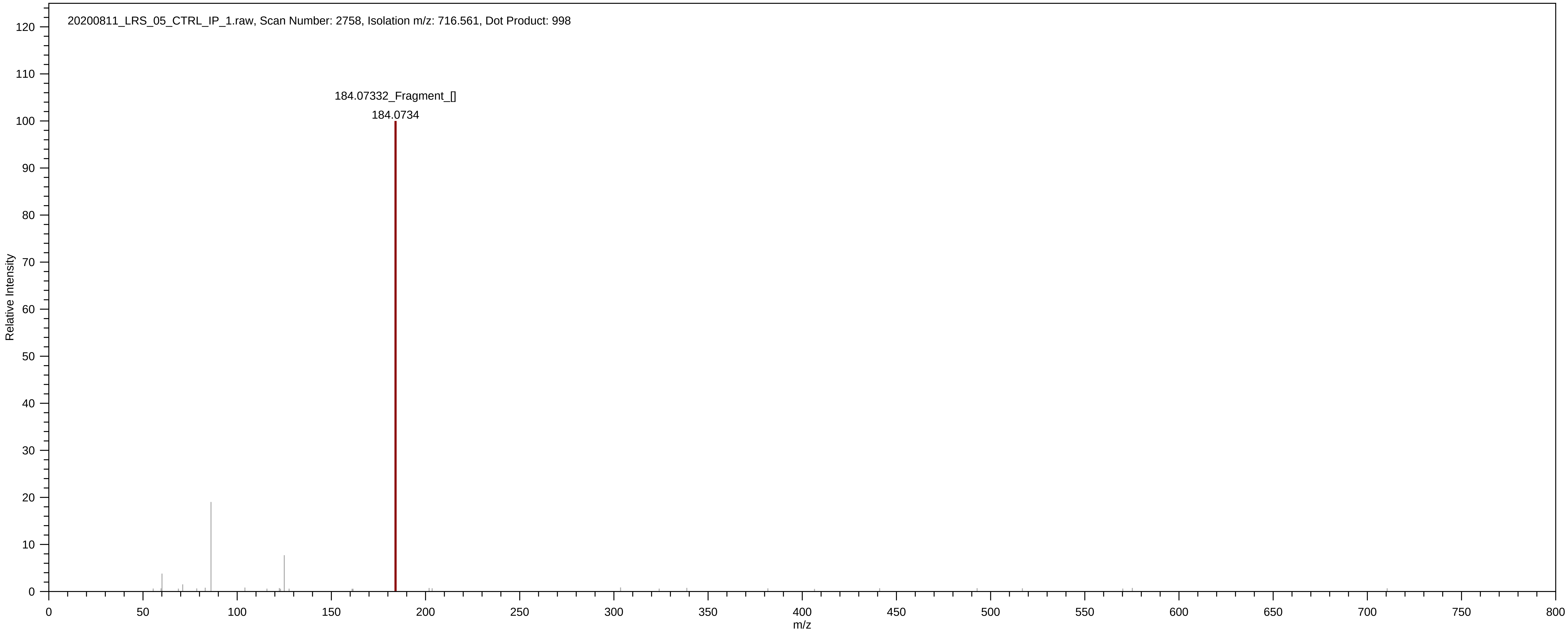

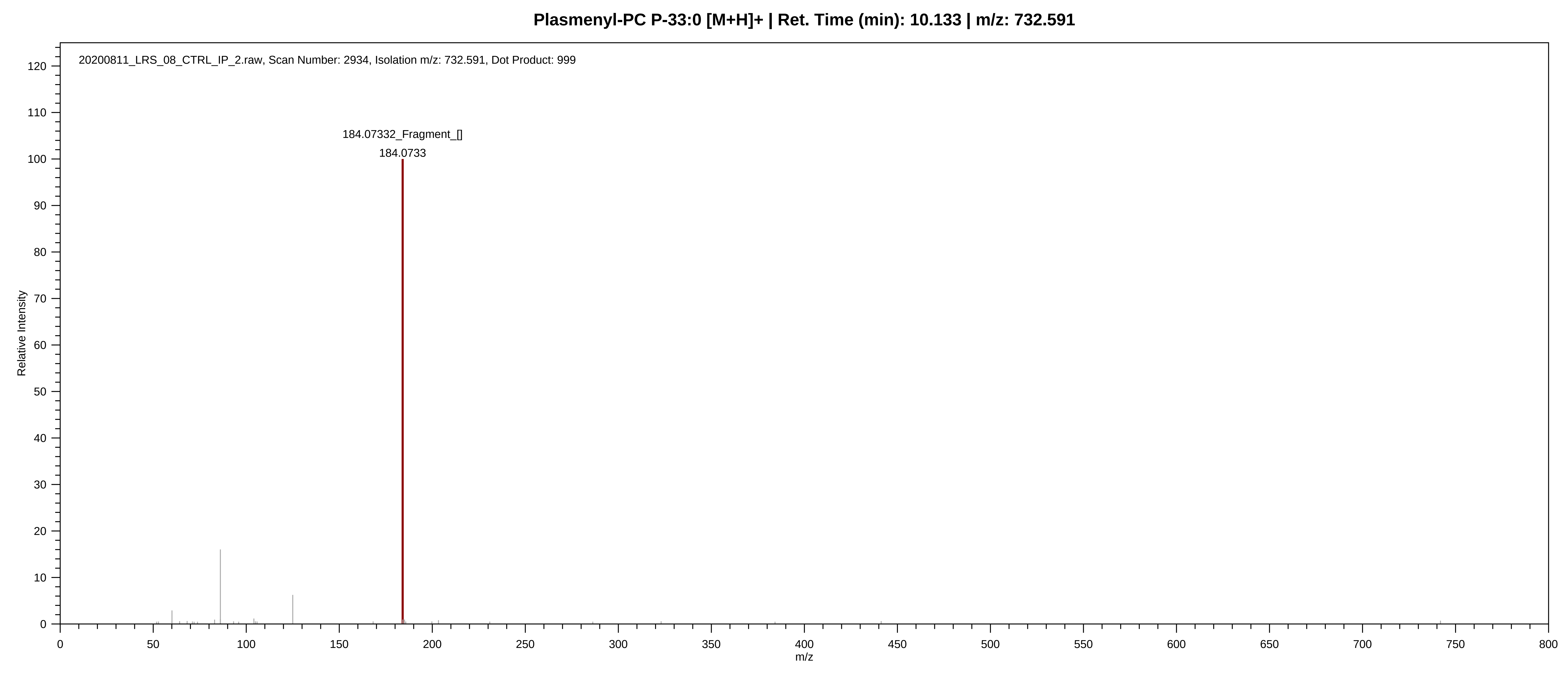

Plasmenyl-PC P-33:0 [M+H]+ | Ret. Time (min): 10.133 | m/z: 732.591
20200811_LRS_08_CTRL_IP_2.raw, Scan Number: 2934, Isolation m/z: 732.591, Dot Product: 999
184.07332_Fragment_[]
184.0733
Relative Intensity
m/z

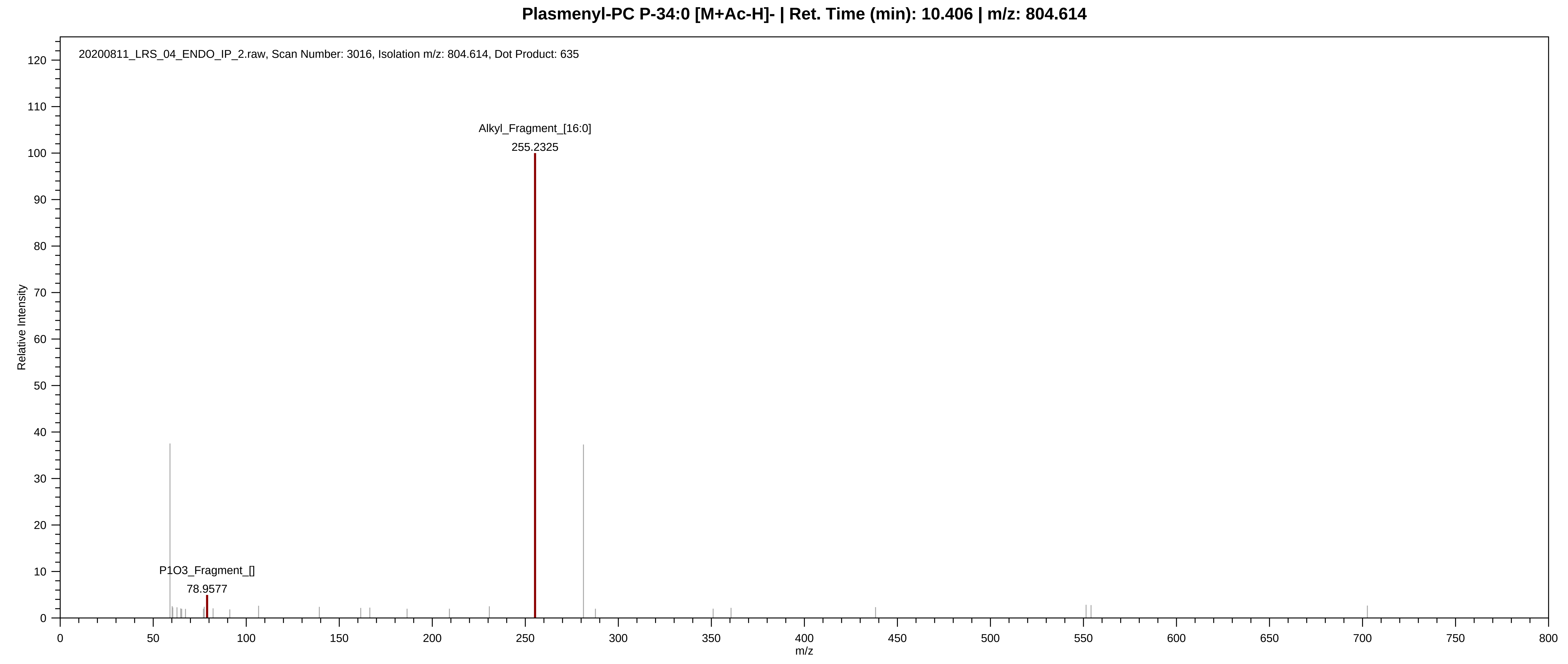
Plasmenyl-PC P-34:0 [M+Ac-H]- | Ret. Time (min): 10.406 | m/z: 804.614
20200811_LRS_04_ENDO_IP_2.raw, Scan Number: 3016, Isolation m/z: 804.614, Dot Product: 635
Alkyl_Fragment_[16:0]
255.2325
P1O3_Fragment_[]
78.9577
Relative Intensity
m/z

Plasmenyl-PC P-34:0 [M+H]+ | Ret. Time (min): 11.257 | m/z: 746.605

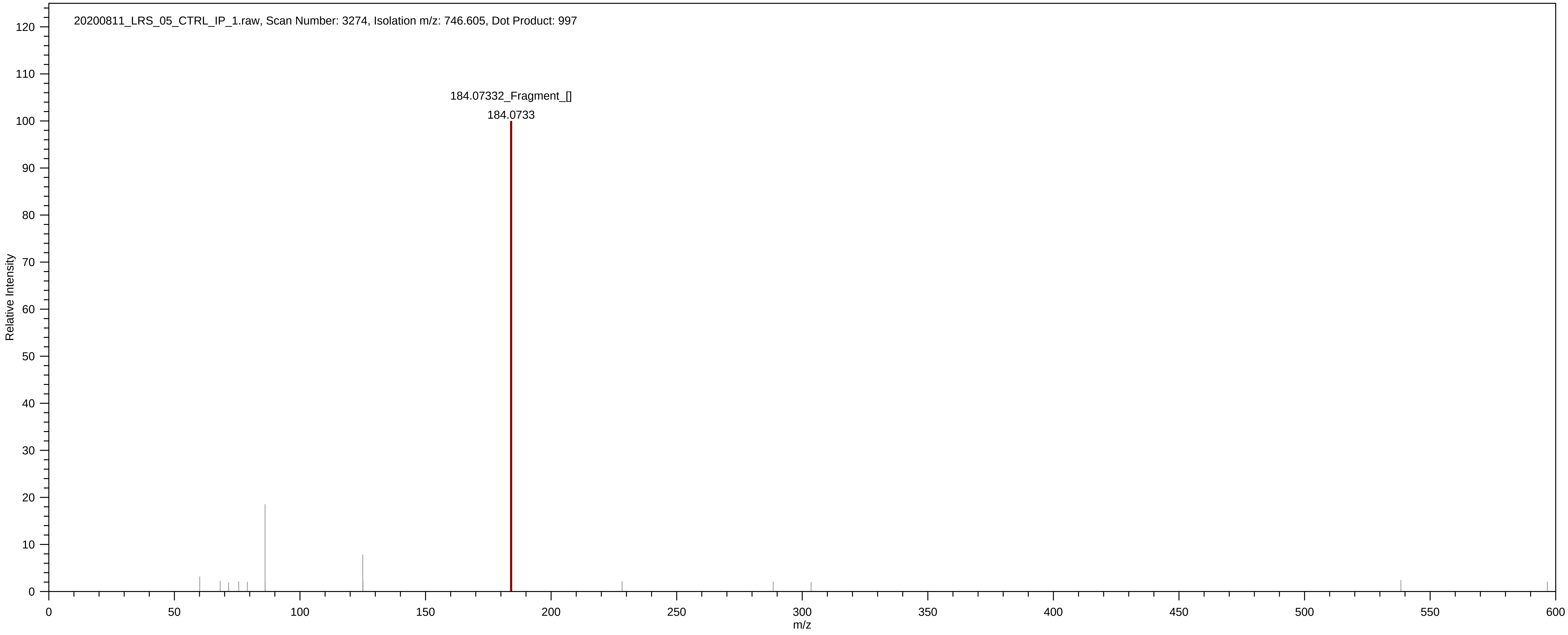

Plasmenyl-PC P-35:1 [M+H]+ | Ret. Time (min): 10.834 | m/z: 758.606

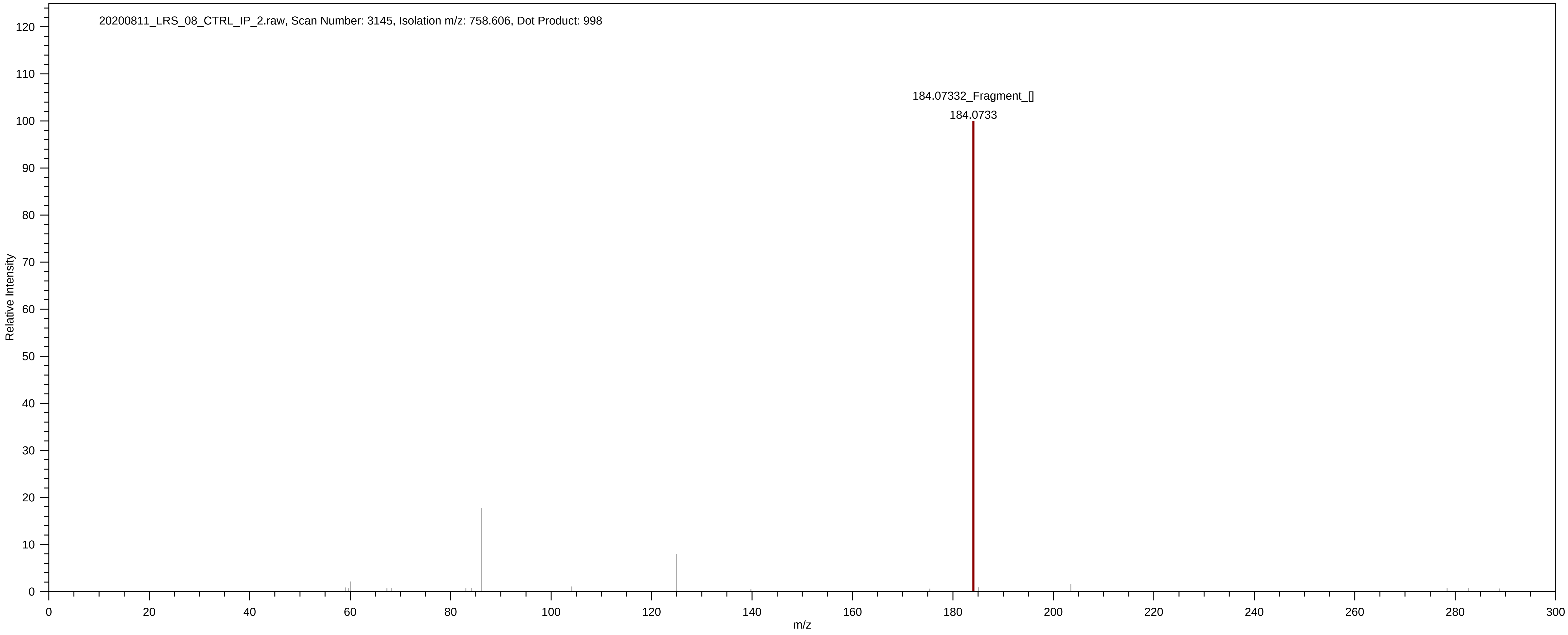

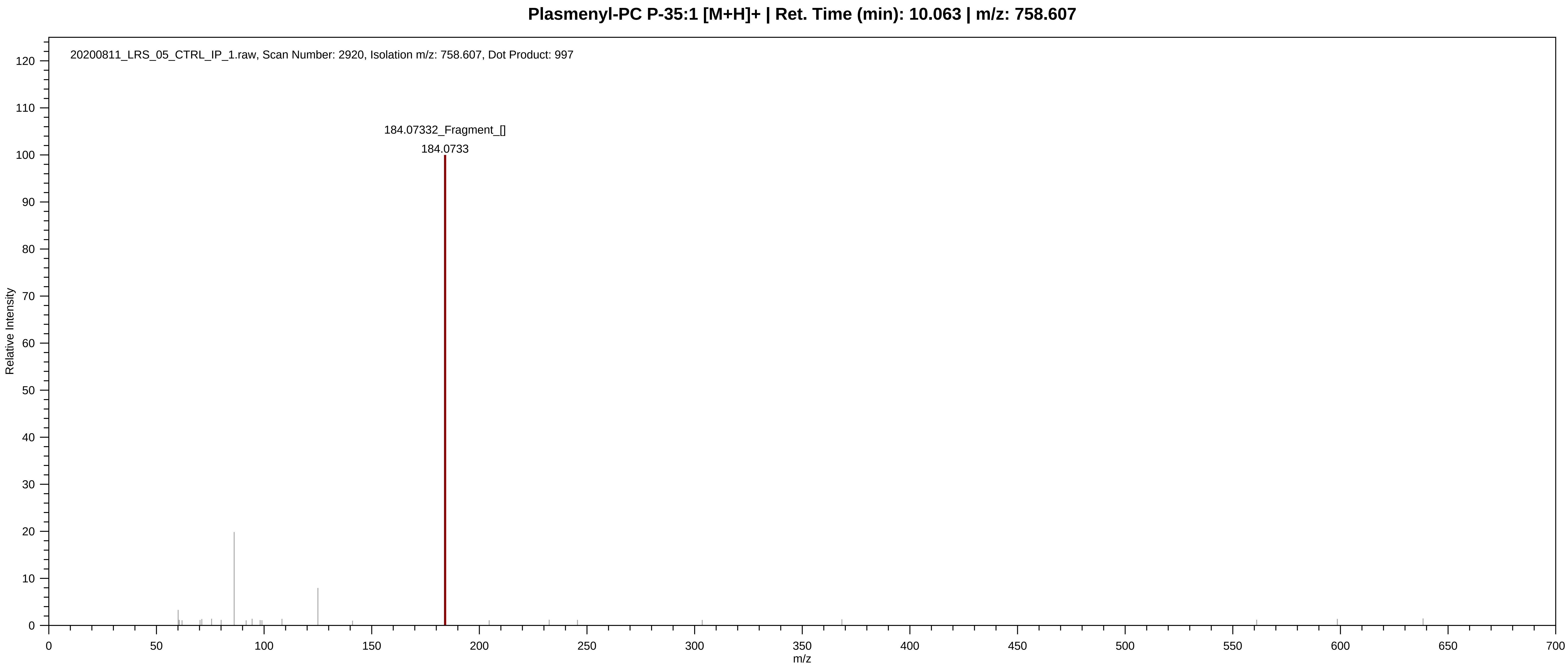

Plasmenyl-PC P-35:1 [M+H]+ | Ret. Time (min): 10.063 | m/z: 758.607
20200811_LRS_05_CTRL_IP_1.raw, Scan Number: 2920, Isolation m/z: 758.607, Dot Product: 997
184.07332_Fragment_[]
184.0733
Relative Intensity
m/z
0
10
20
30
40
50
60
70
80
90
100
110
120
0
50
100
150
200
250
300
350
400
450
500
550
600
650
700

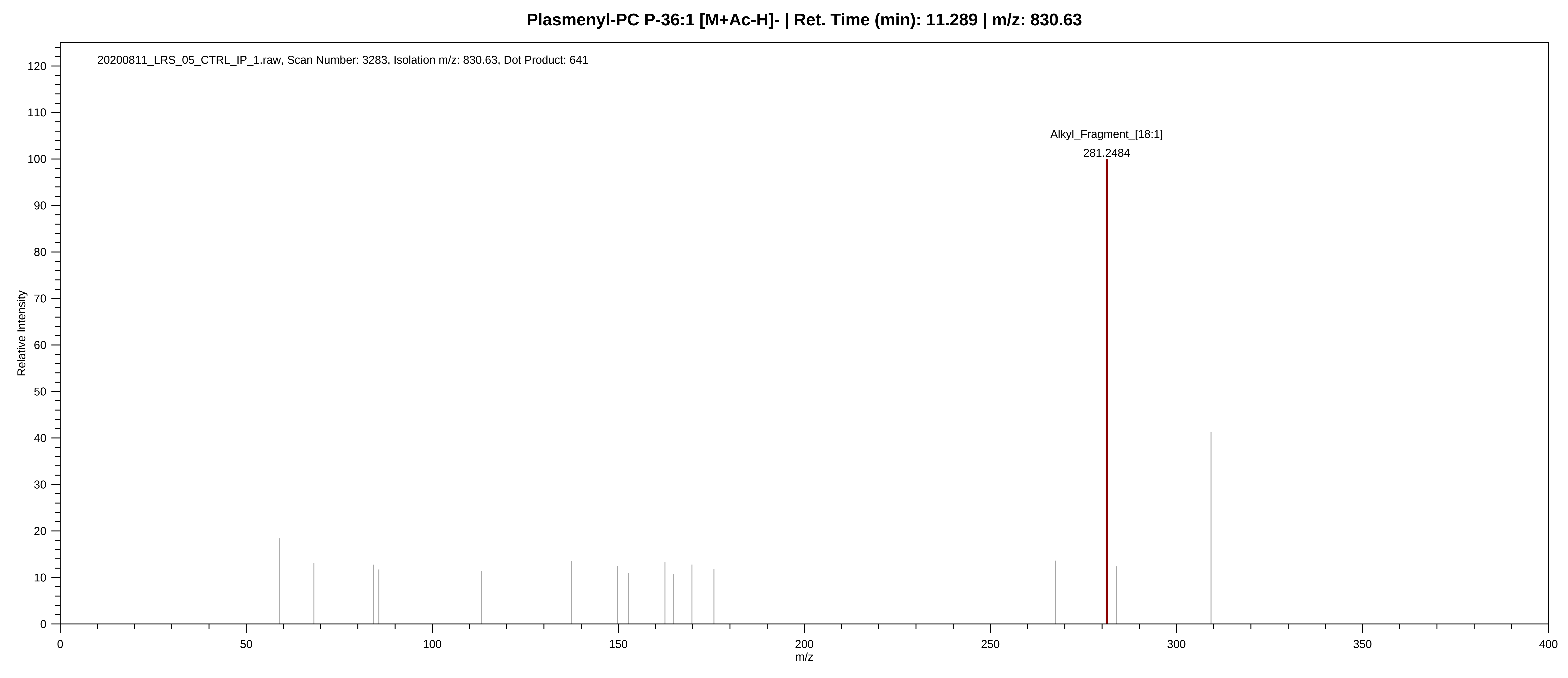

Plasmenyl-PC P-36:1 [M+Ac-H]- | Ret. Time (min): 11.289 | m/z: 830.63
20200811_LRS_05_CTRL_IP_1.raw, Scan Number: 3283, Isolation m/z: 830.63, Dot Product: 641
Alkyl_Fragment_[18:1]
281.2484
Relative Intensity
m/z
0
10
20
30
40
50
60
70
80
90
100
110
120
0
50
100
150
200
250
300
350
400

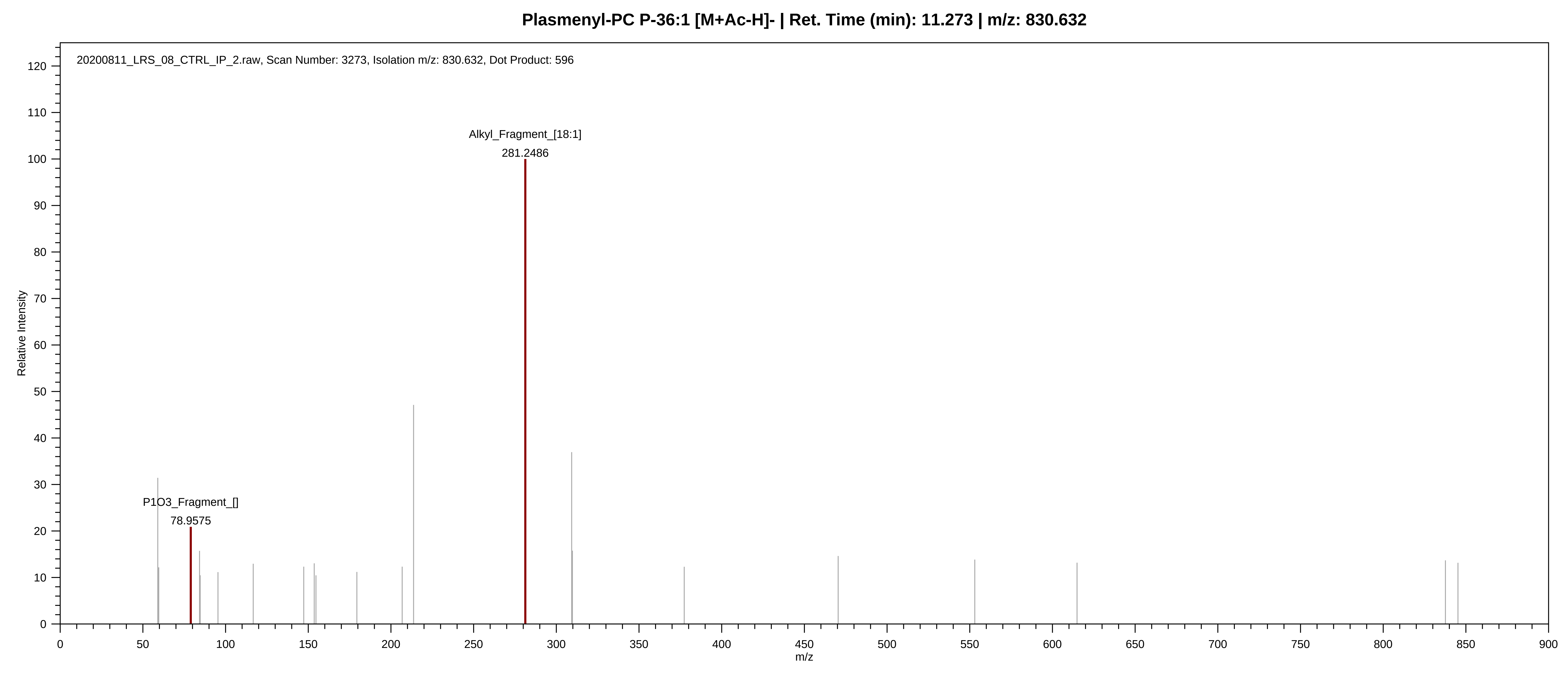

Plasmenyl-PC P-36:1 [M+Ac-H]- | Ret. Time (min): 11.273 | m/z: 830.632
20200811_LRS_08_CTRL_IP_2.raw, Scan Number: 3273, Isolation m/z: 830.632, Dot Product: 596
Alkyl_Fragment_[18:1]
281.2486
P1O3_Fragment_[]
78.9575
Relative Intensity
m/z

Plasmenyl-PC P-36:1 [M+H]+ | Ret. Time (min): 10.661 | m/z: 772.622

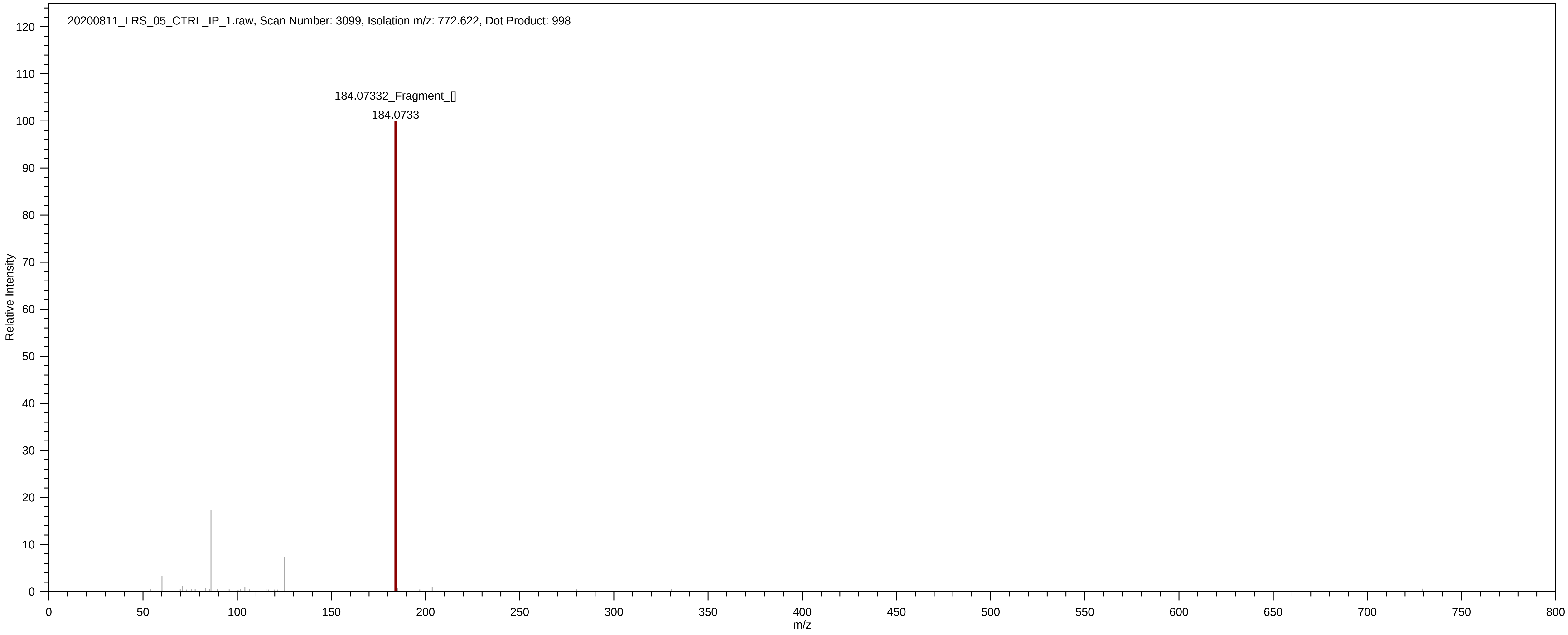

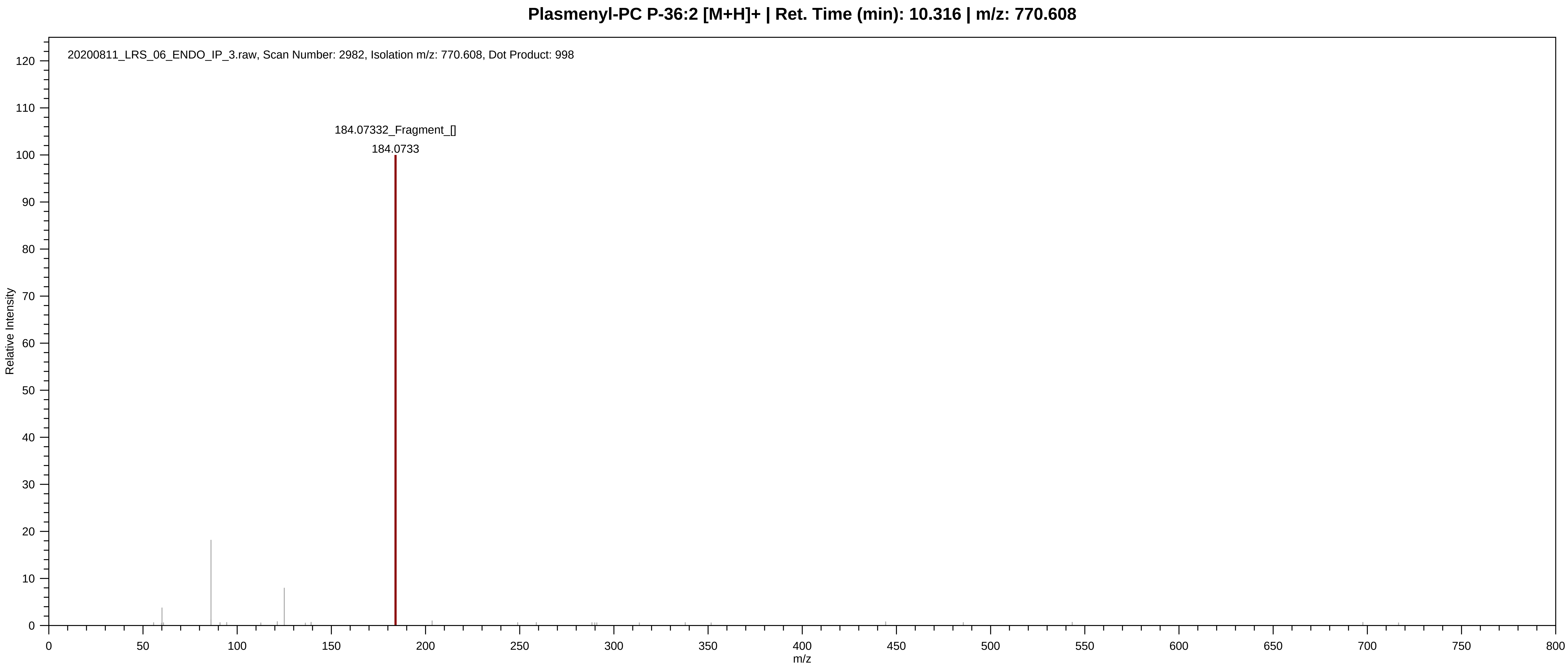

Plasmenyl-PC P-36:2 [M+H]+ | Ret. Time (min): 10.316 | m/z: 770.608
20200811_LRS_06_ENDO_IP_3.raw, Scan Number: 2982, Isolation m/z: 770.608, Dot Product: 998
184.07332_Fragment_[]
184.0733
Relative Intensity
m/z

Plasmenyl-PC P-36:2 [M+H]+ | Ret. Time (min): 9.735 | m/z: 770.606

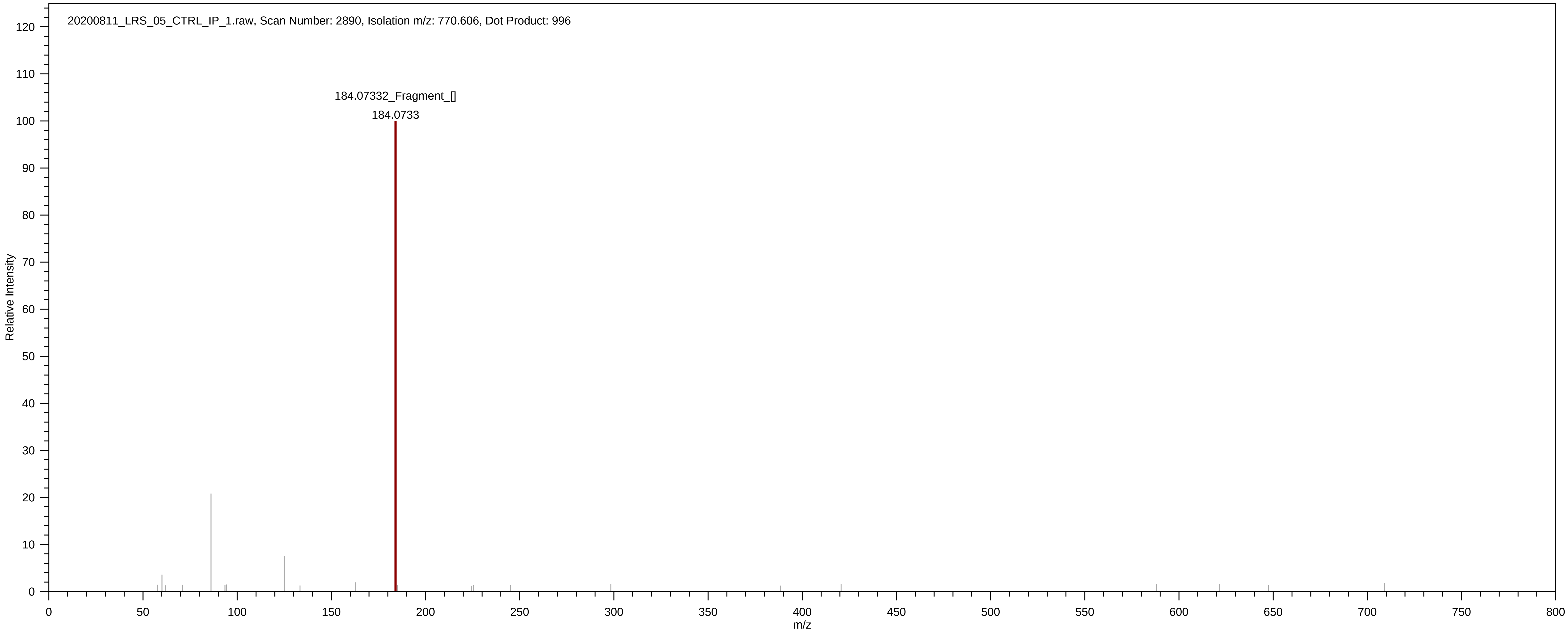

Plasmenyl-PC P-36:4 [M+H]+ | Ret. Time (min): 8.908 | m/z: 766.58

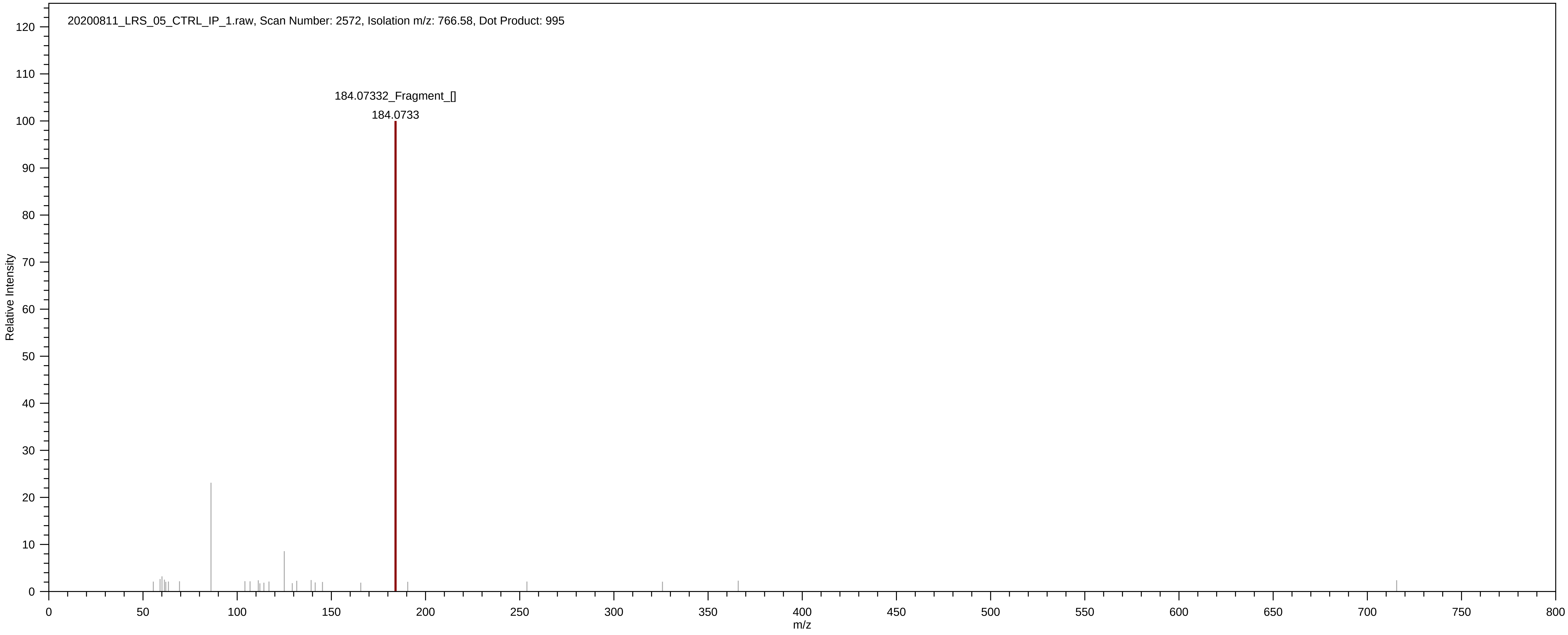

Plasmenyl-PC P-37:0 [M+H]+ | Ret. Time (min): 11.85 | m/z: 788.656

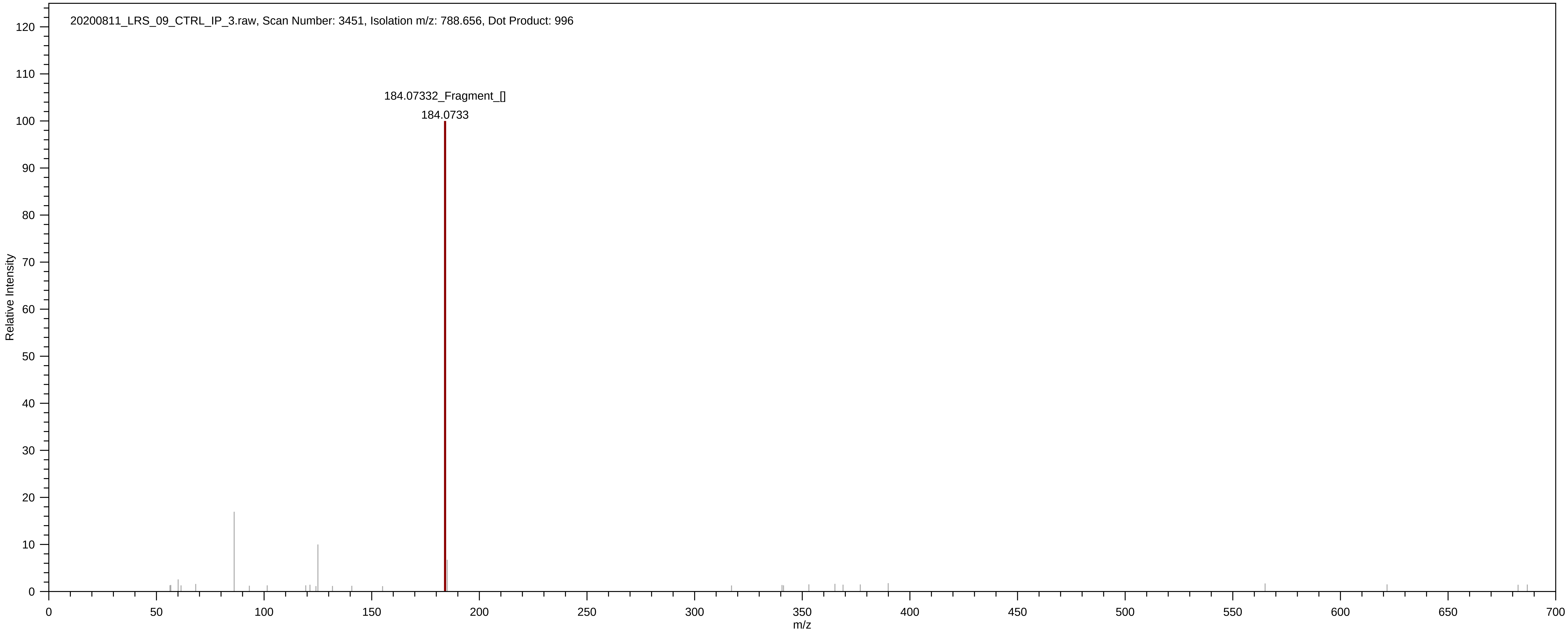

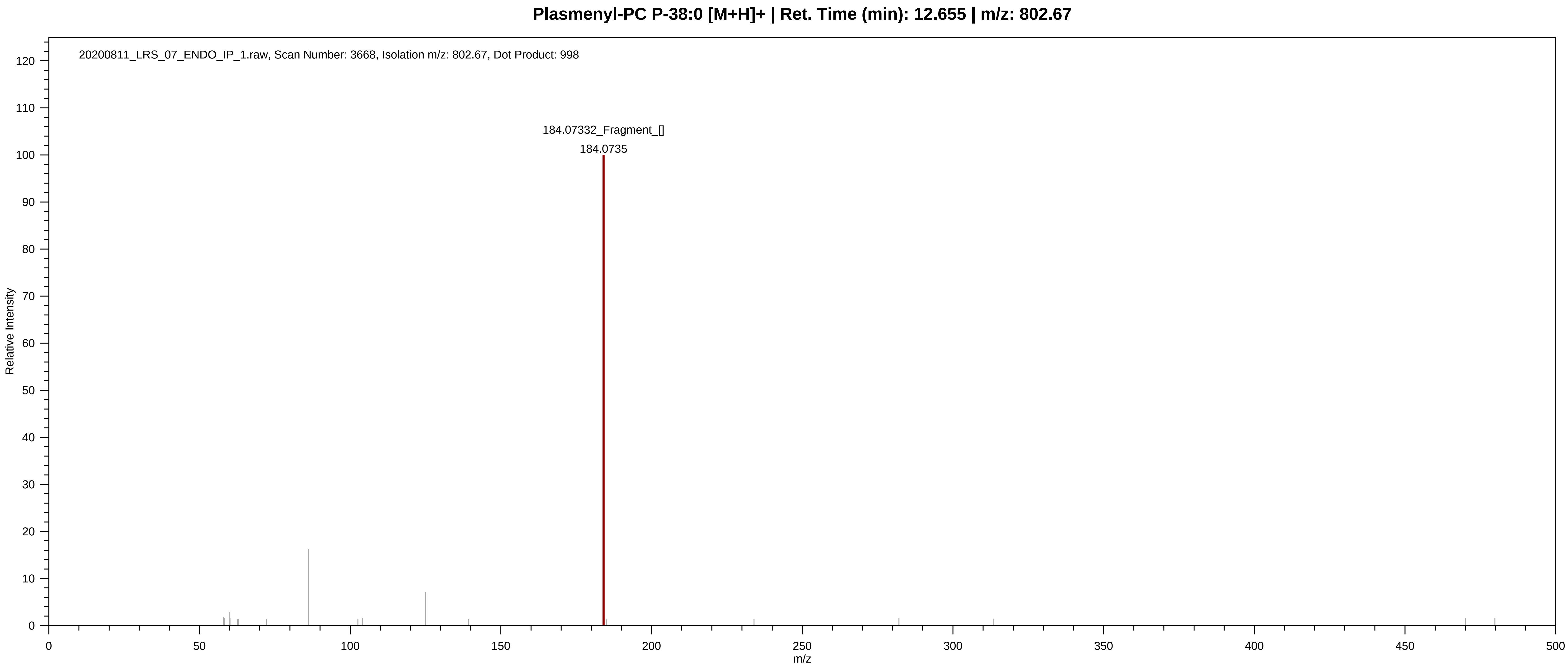
Plasmenyl-PC P-38:0 [M+H]+ | Ret. Time (min): 12.655 | m/z: 802.67
20200811_LRS_07_ENDO_IP_1.raw, Scan Number: 3668, Isolation m/z: 802.67, Dot Product: 998
184.07332_Fragment_[]
184.0735
Relative Intensity
m/z
0
10
20
30
40
50
60
70
80
90
100
110
120
0
50
100
150
200
250
300
350
400
450
500

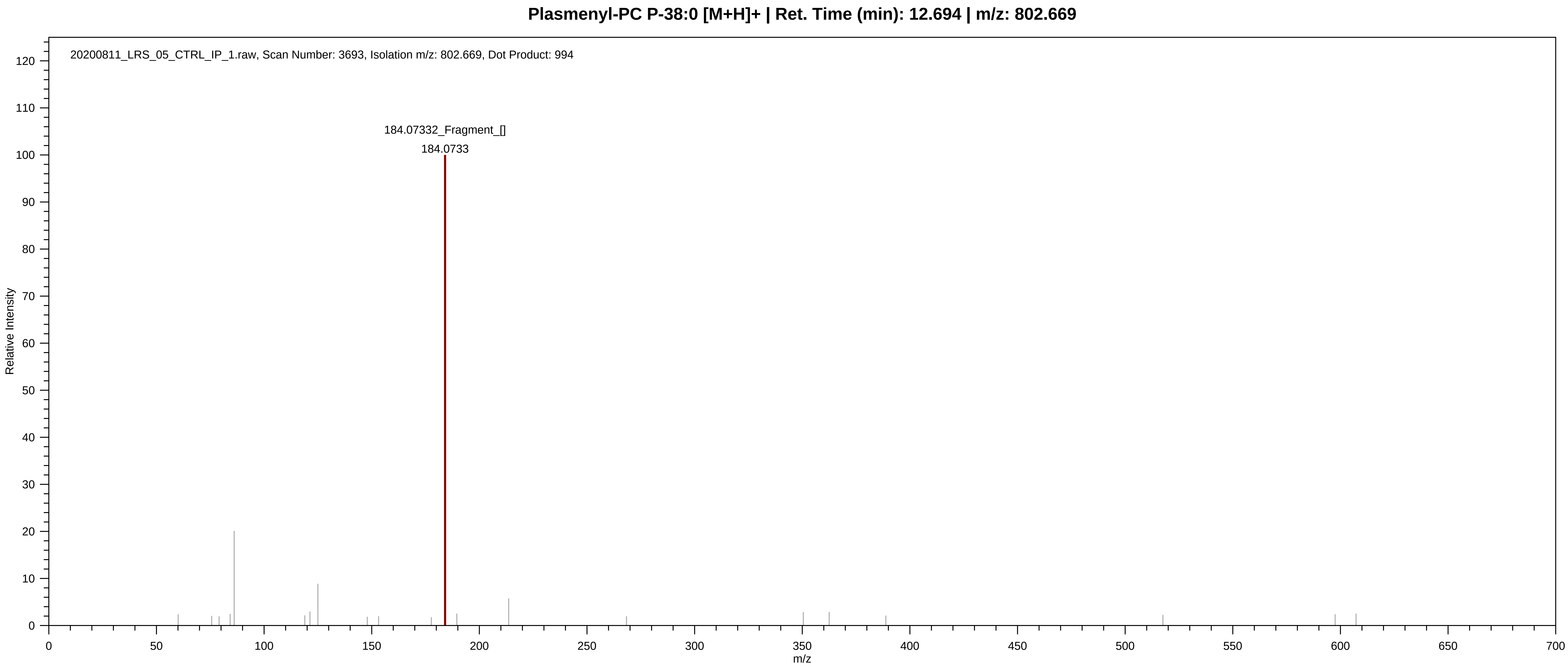

Plasmenyl-PC P-38:0 [M+H]+ | Ret. Time (min): 12.694 | m/z: 802.669
20200811_LRS_05_CTRL_IP_1.raw, Scan Number: 3693, Isolation m/z: 802.669, Dot Product: 994
184.07332_Fragment_[]
184.0733
Relative Intensity
m/z
0
10
20
30
40
50
60
70
80
90
100
110
120
0
50
100
150
200
250
300
350
400
450
500
550
600
650
700

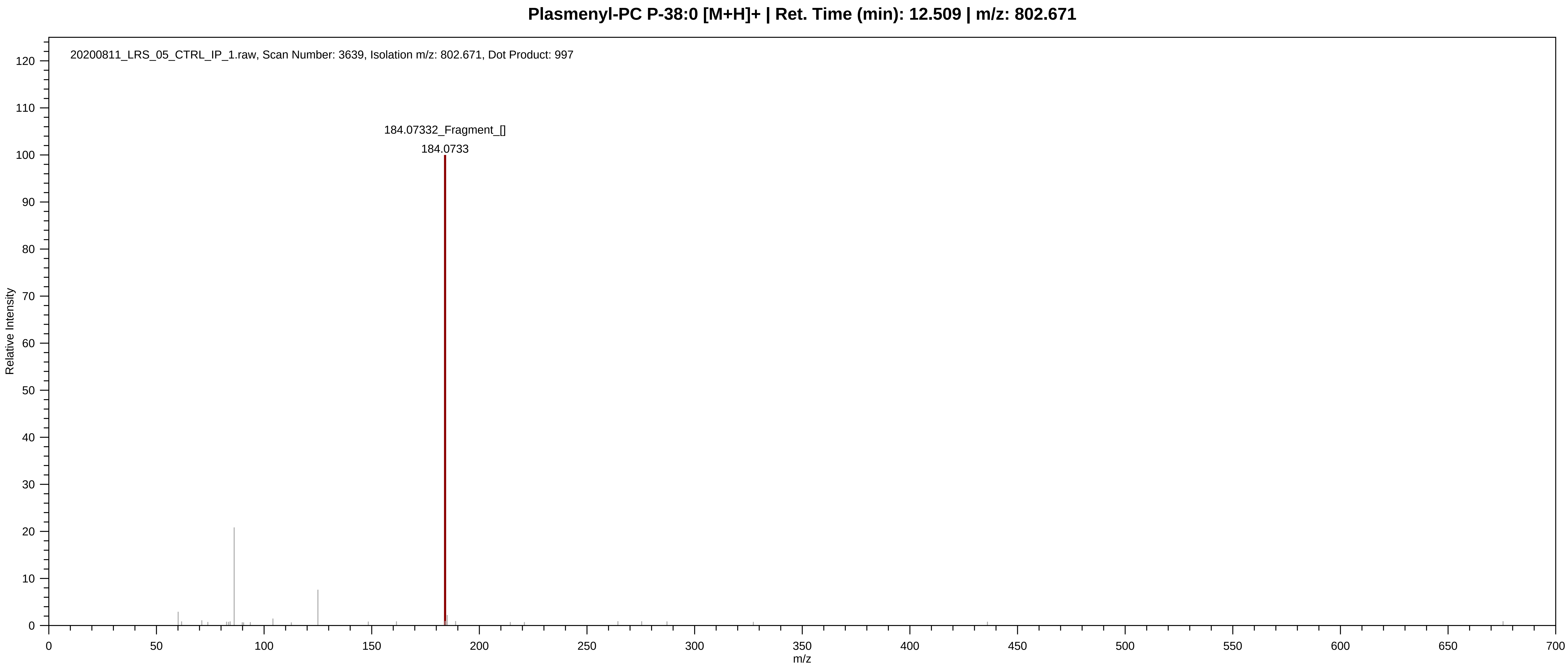
Plasmenyl-PC P-38:0 [M+H]+ | Ret. Time (min): 12.509 | m/z: 802.671
20200811_LRS_05_CTRL_IP_1.raw, Scan Number: 3639, Isolation m/z: 802.671, Dot Product: 997
184.07332_Fragment_[]
184.0733
Relative Intensity
m/z
0
10
20
30
40
50
60
70
80
90
100
110
120
0
50
100
150
200
250
300
350
400
450
500
550
600
650
700

Plasmenyl-PC P-39:0 [M+H]+ | Ret. Time (min): 12.92 | m/z: 816.688

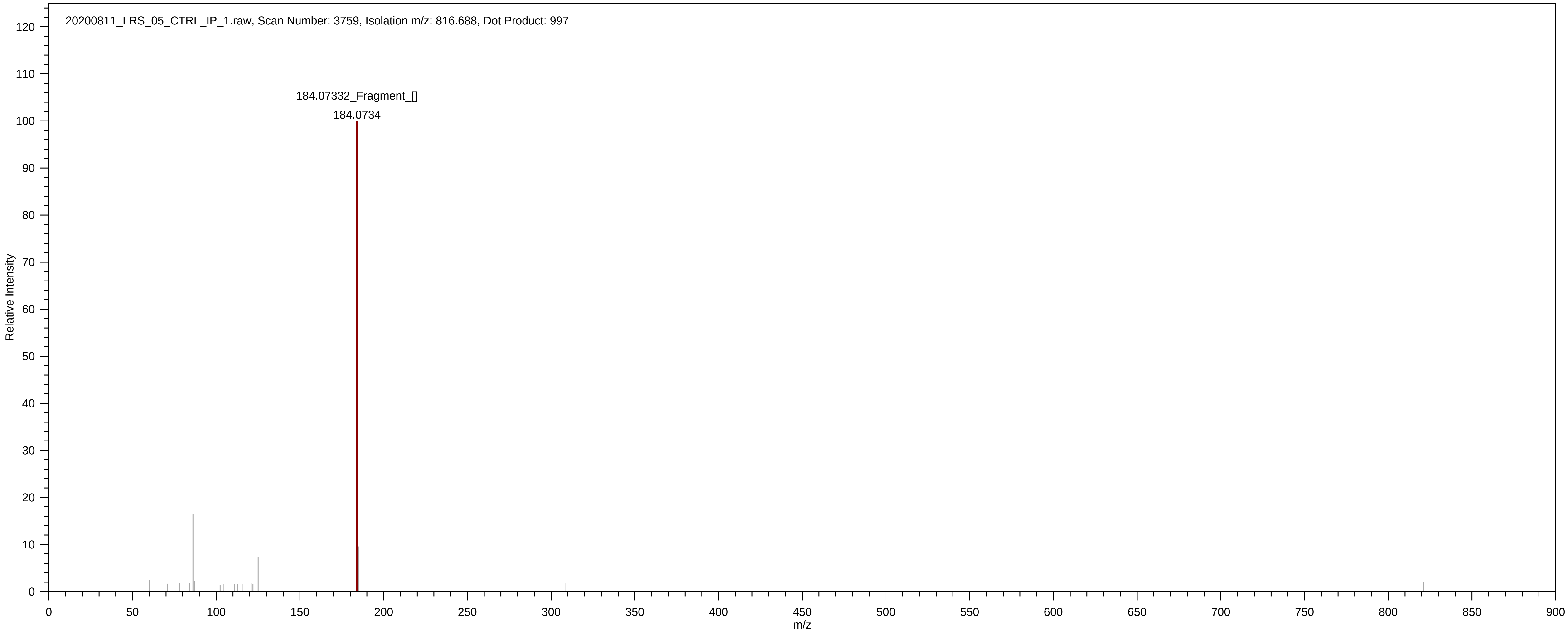

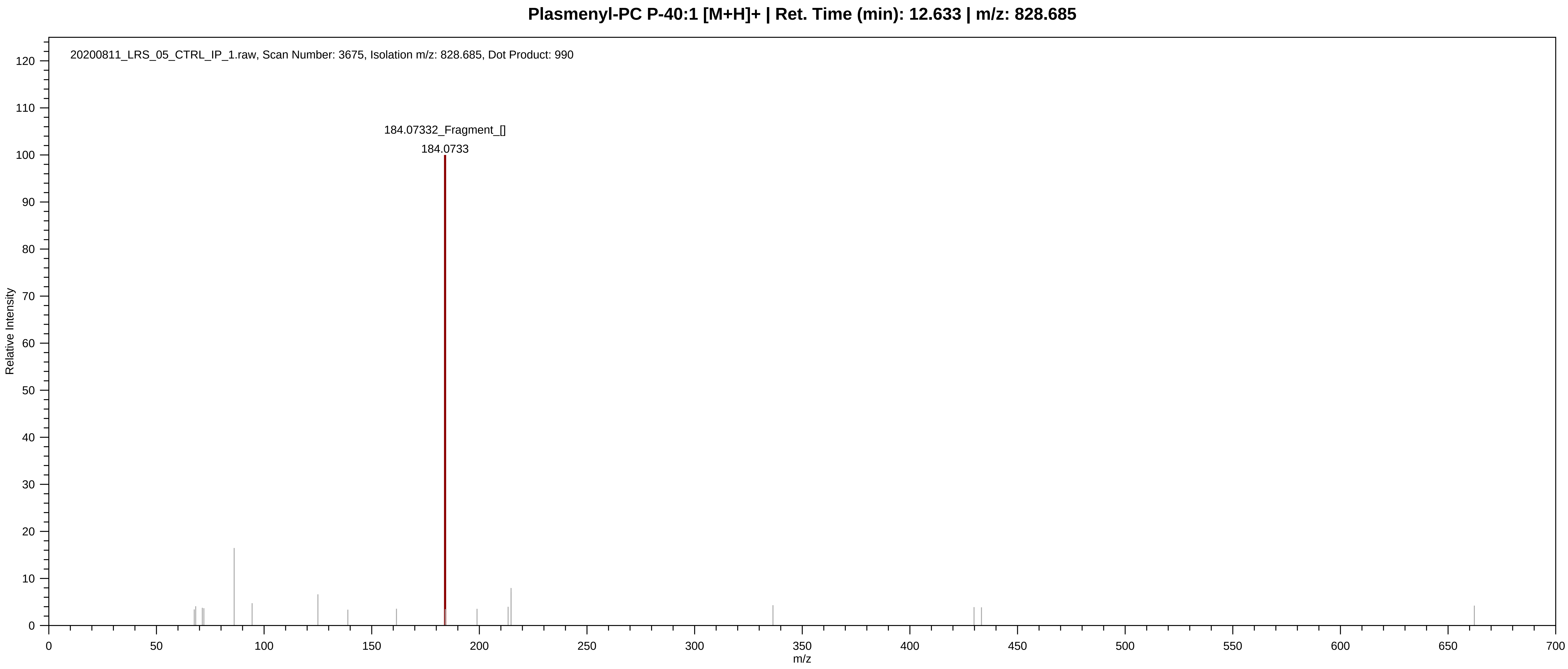

Plasmenyl-PC P-40:1 [M+H]+ | Ret. Time (min): 12.633 | m/z: 828.685
20200811_LRS_05_CTRL_IP_1.raw, Scan Number: 3675, Isolation m/z: 828.685, Dot Product: 990
184.07332_Fragment_[]
184.0733
Relative Intensity
m/z
0
10
20
30
40
50
60
70
80
90
100
110
120
0
50
100
150
200
250
300
350
400
450
500
550
600
650
700

Plasmenyl-PC P-40:6 [M+H]+ | Ret. Time (min): 9.24 | m/z: 818.604

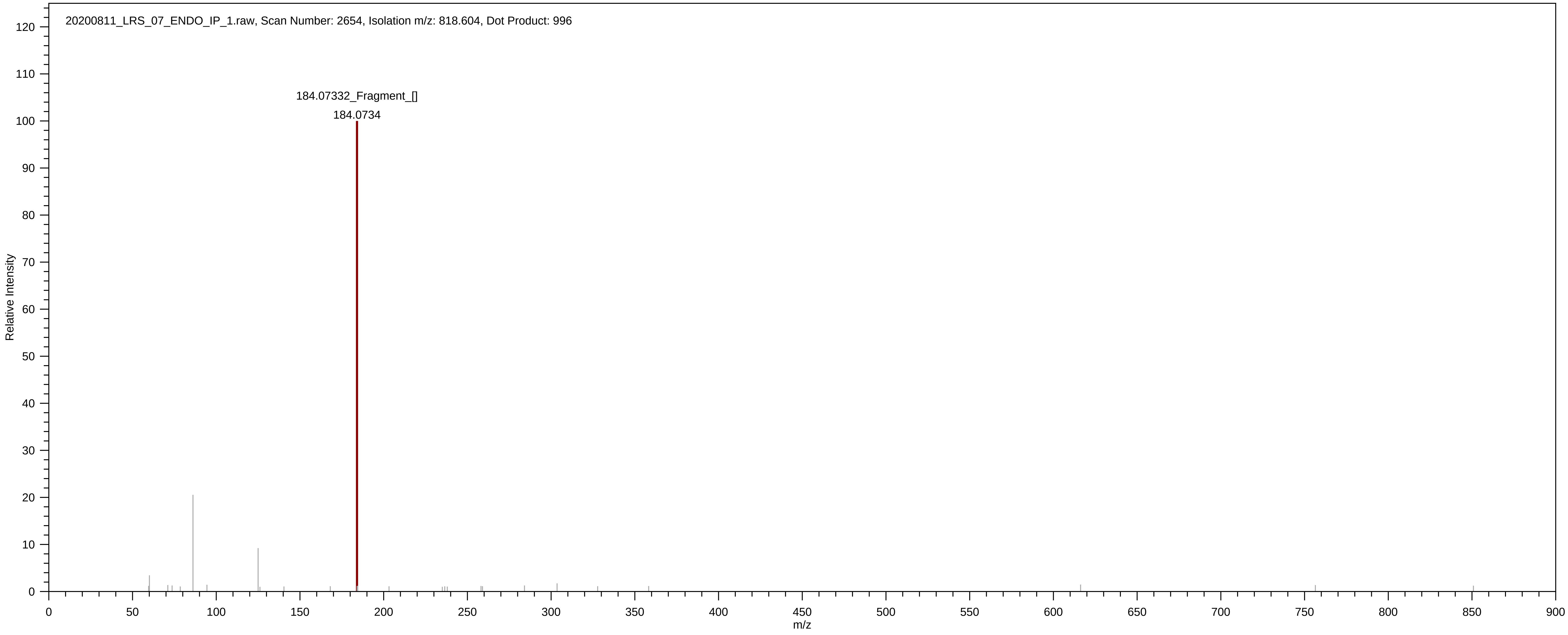

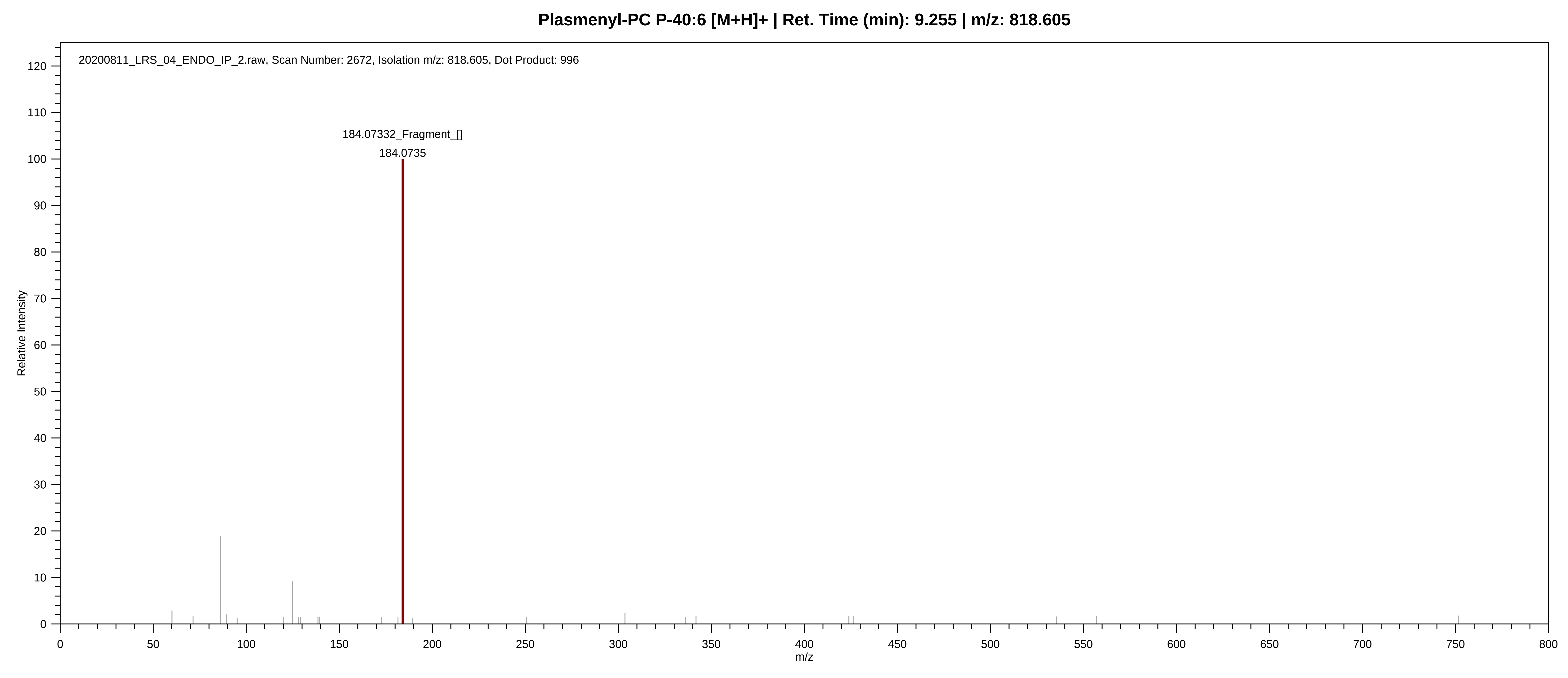
Plasmenyl-PC P-40:6 [M+H]+ | Ret. Time (min): 9.255 | m/z: 818.605
20200811_LRS_04_ENDO_IP_2.raw, Scan Number: 2672, Isolation m/z: 818.605, Dot Product: 996
184.07332_Fragment_[]
184.0735
Relative Intensity
m/z

Plasmenyl-PC P-40:6 [M+H]+ | Ret. Time (min): 9.23 | m/z: 818.606

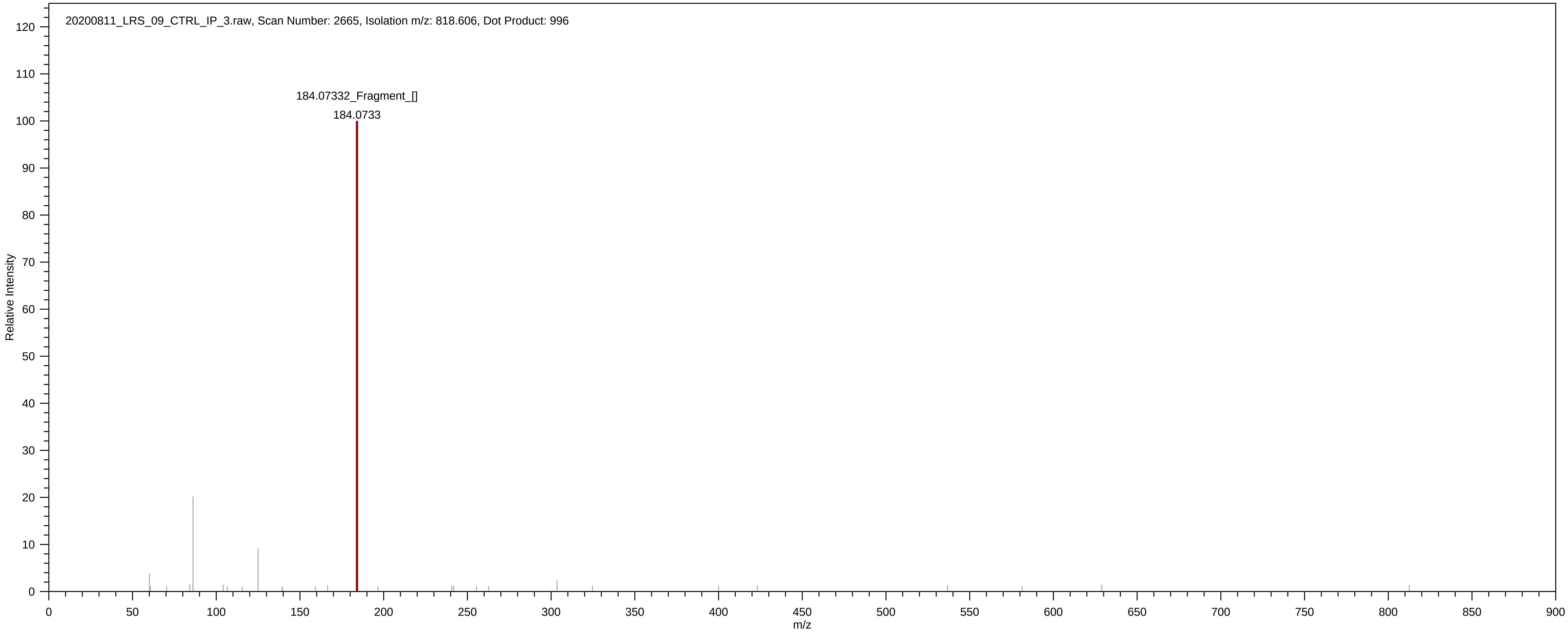

Plasmenyl-PC P-41:0 [M+H]+ | Ret. Time (min): 13.933 | m/z: 844.713

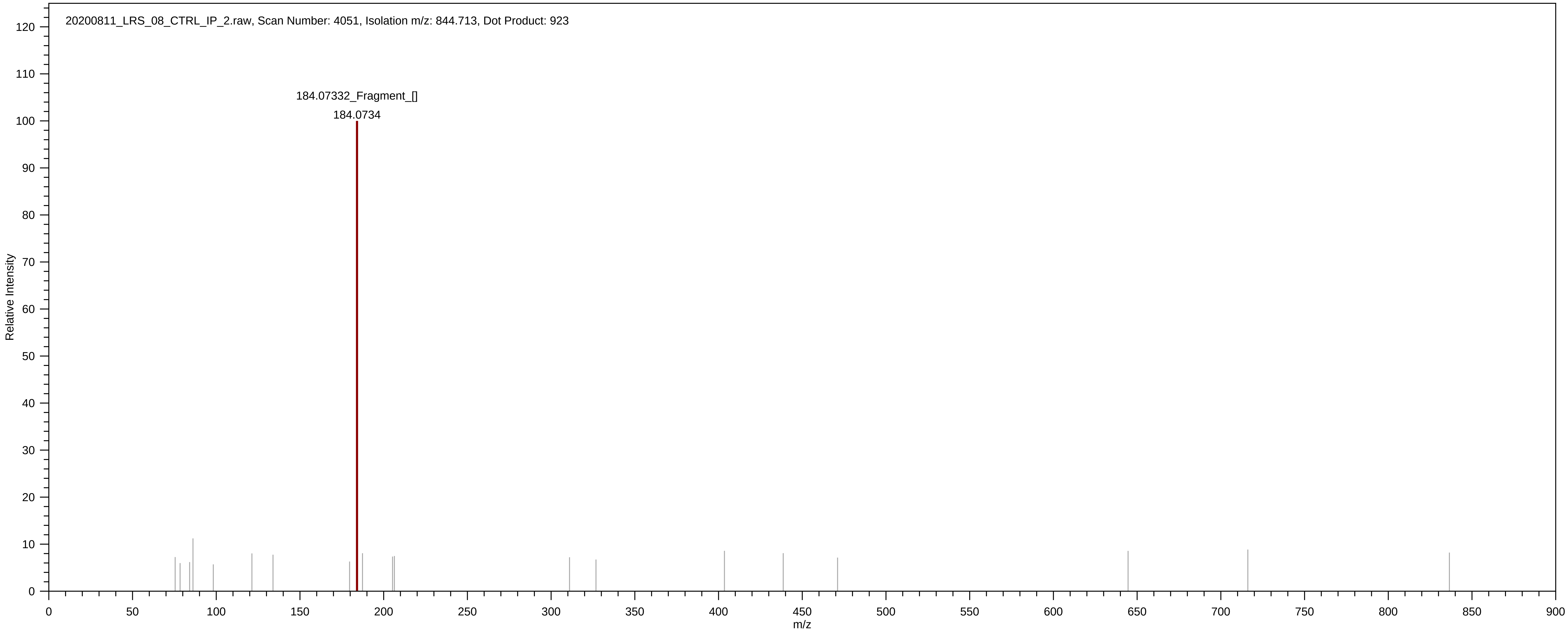

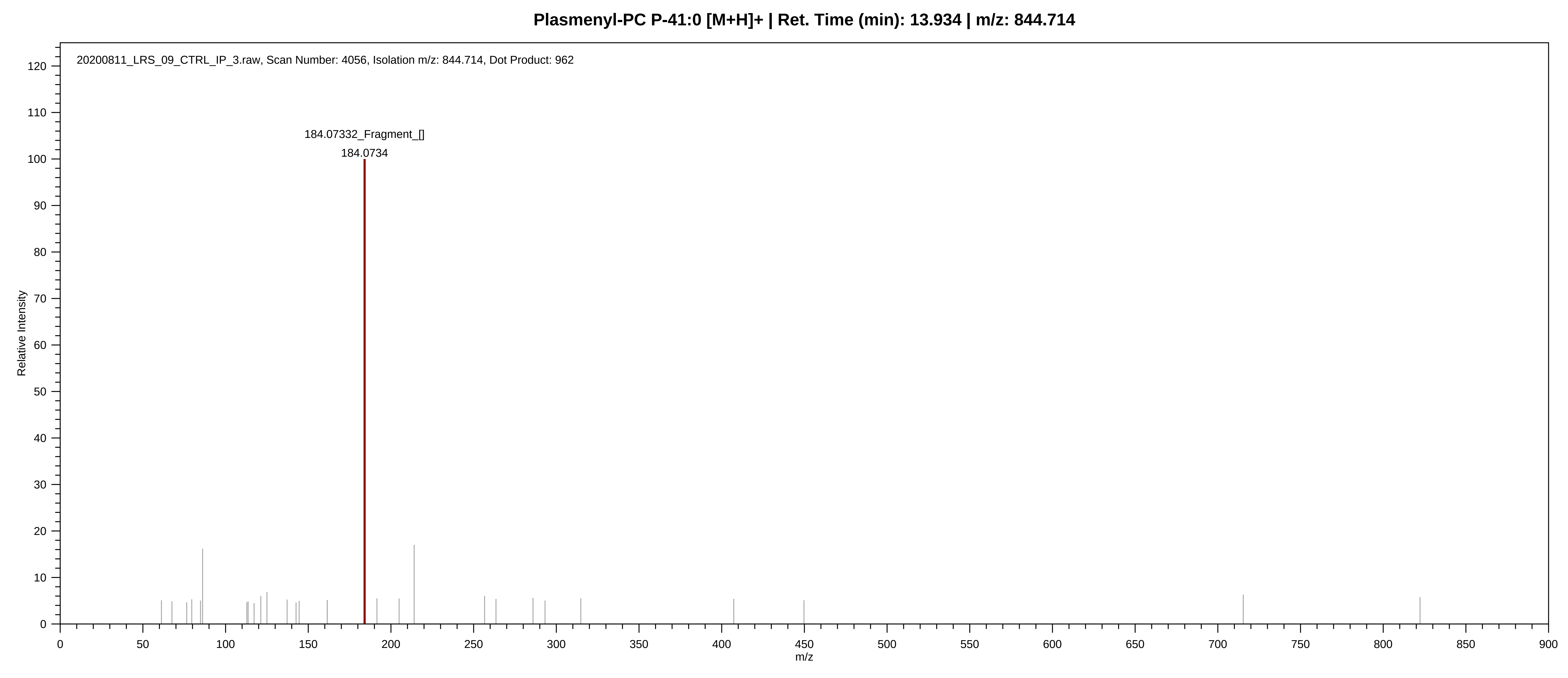
Plasmenyl-PC P-41:0 [M+H]+ | Ret. Time (min): 13.934 | m/z: 844.714
20200811_LRS_09_CTRL_IP_3.raw, Scan Number: 4056, Isolation m/z: 844.714, Dot Product: 962
184.07332_Fragment_[]
184.0734
Relative Intensity
m/z

Plasmenyl-PC P-41:0 [M+H]+ | Ret. Time (min): 13.986 | m/z: 844.715

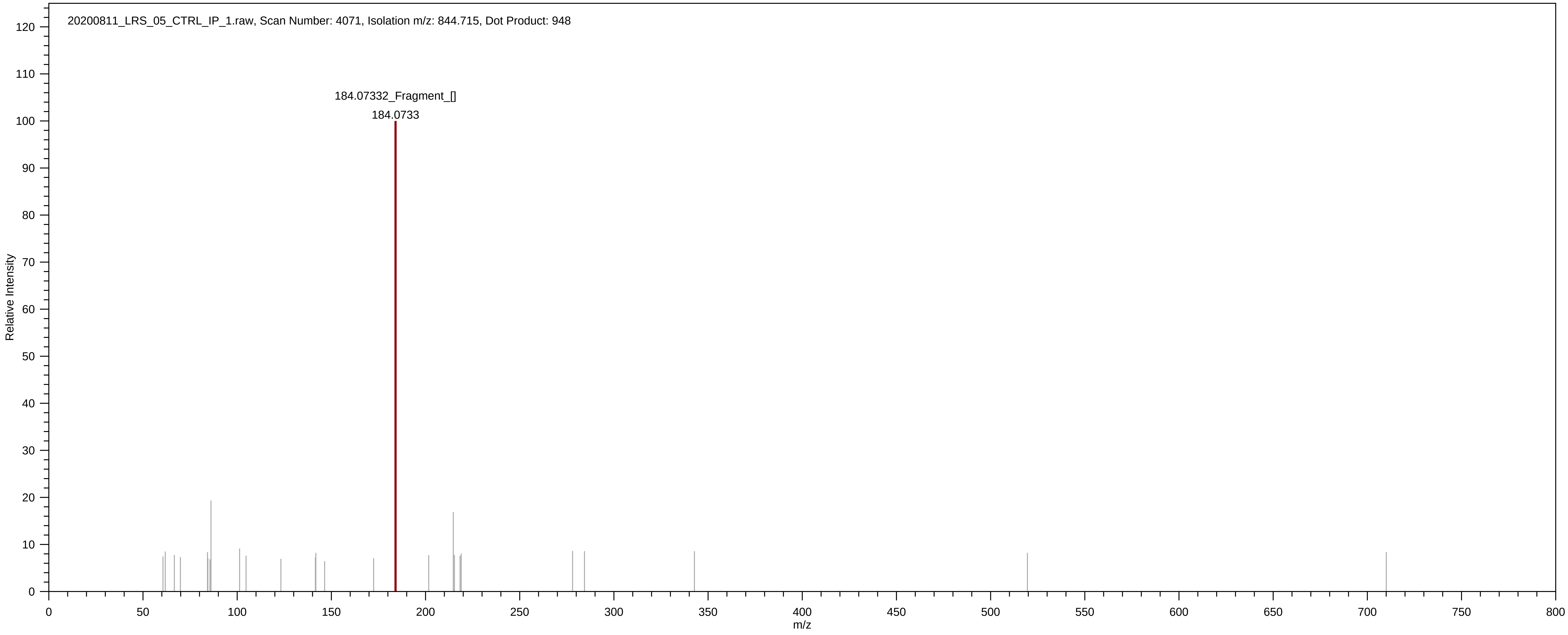

Plasmenyl-PC P-41:0 [M+H]+ | Ret. Time (min): 14.171 | m/z: 844.718

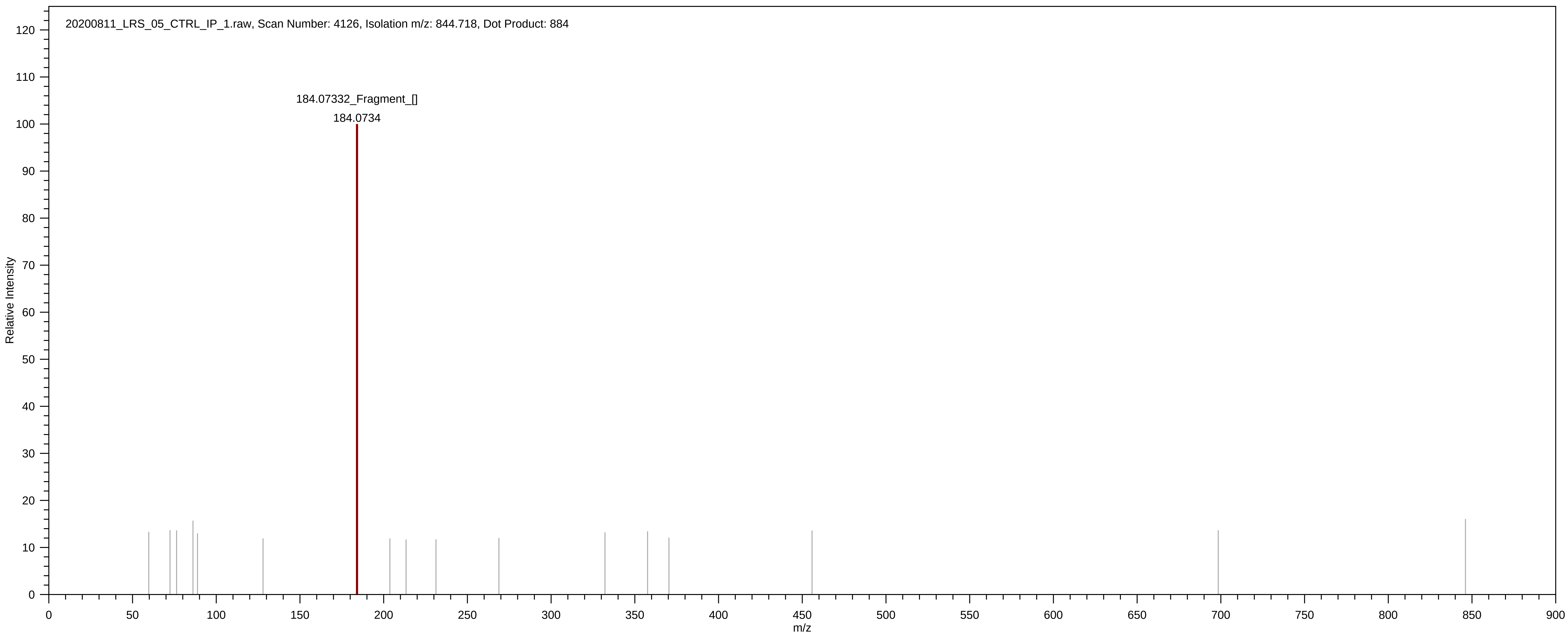

Plasmenyl-PC P-41:1 [M+H]+ | Ret. Time (min): 12.99 | m/z: 842.7

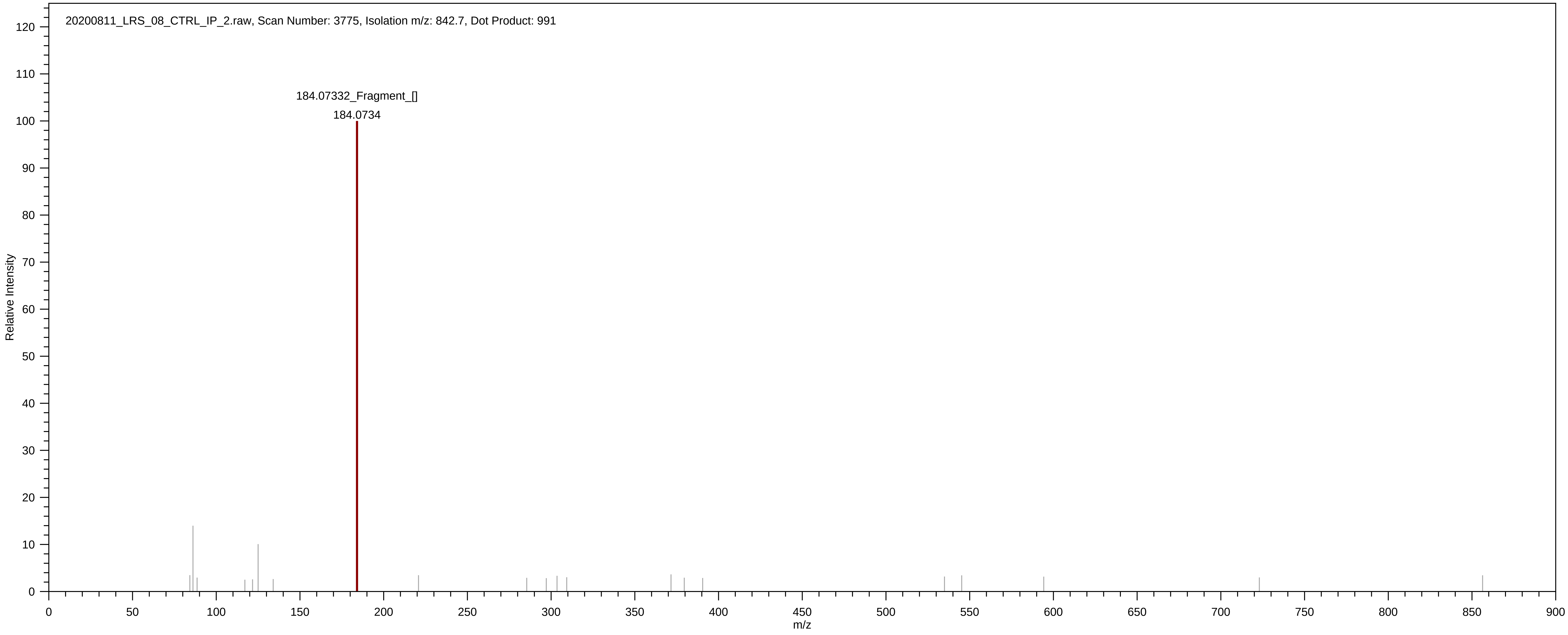

Plasmenyl-PC P-41:1 [M+H]+ | Ret. Time (min): 12.986 | m/z: 842.7

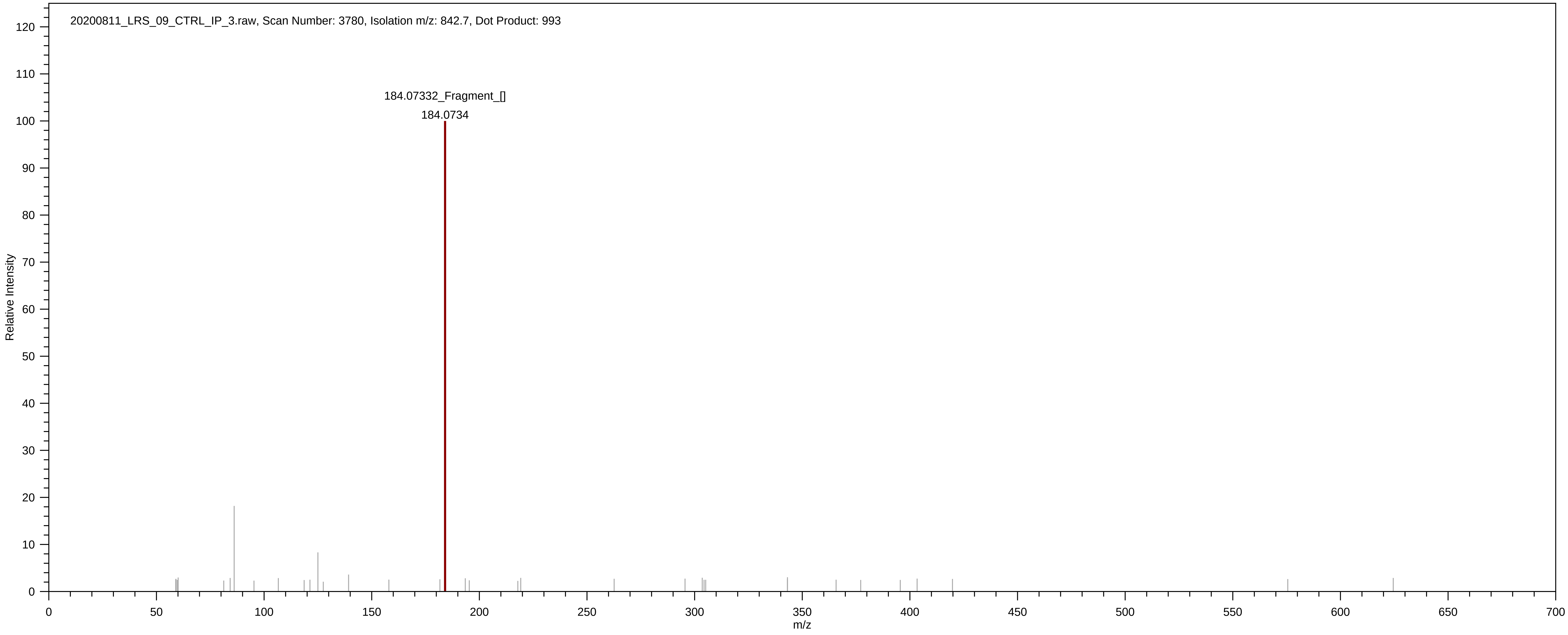

Plasmenyl-PC P-41:1 [M+H]+ | Ret. Time (min): 13.002 | m/z: 842.7

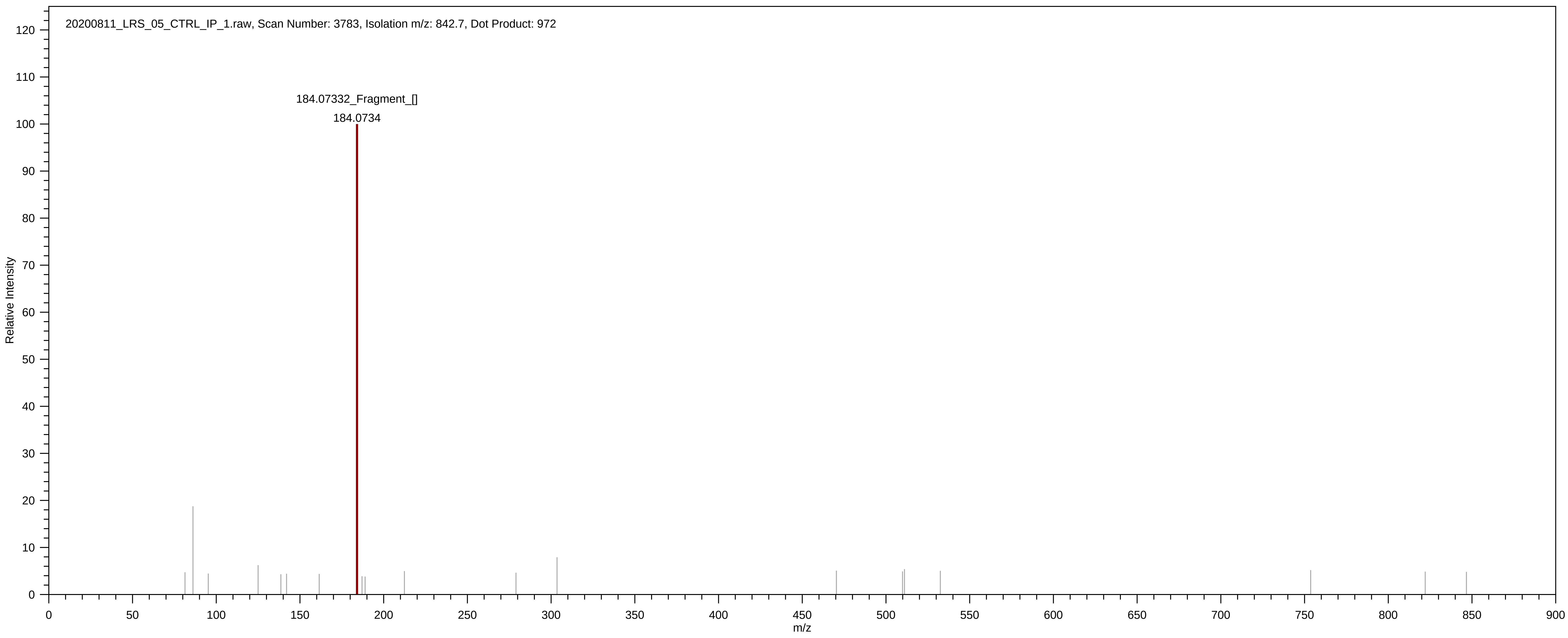

Plasmenyl-PC P-42:0 [M+H]+ | Ret. Time (min): 14.774 | m/z: 858.733

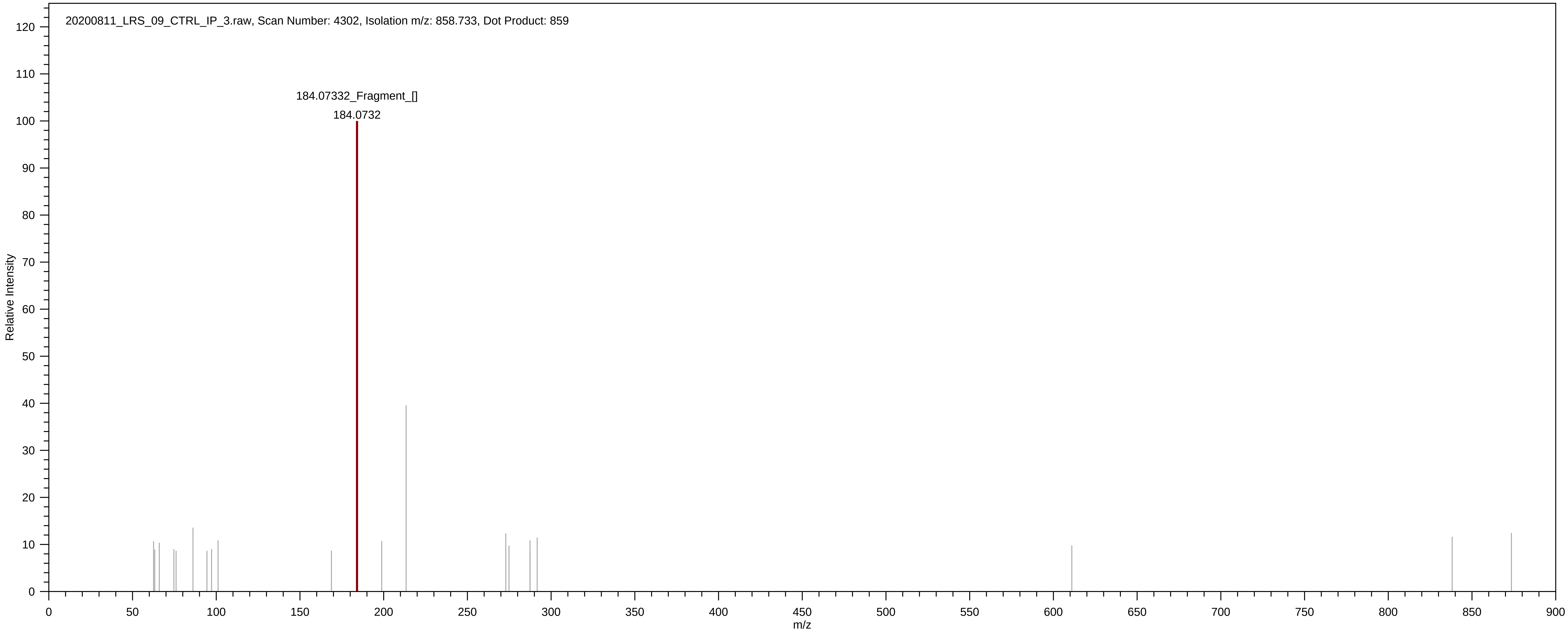

Plasmenyl-PC P-42:2 [M+H]+ | Ret. Time (min): 12.634 | m/z: 854.7

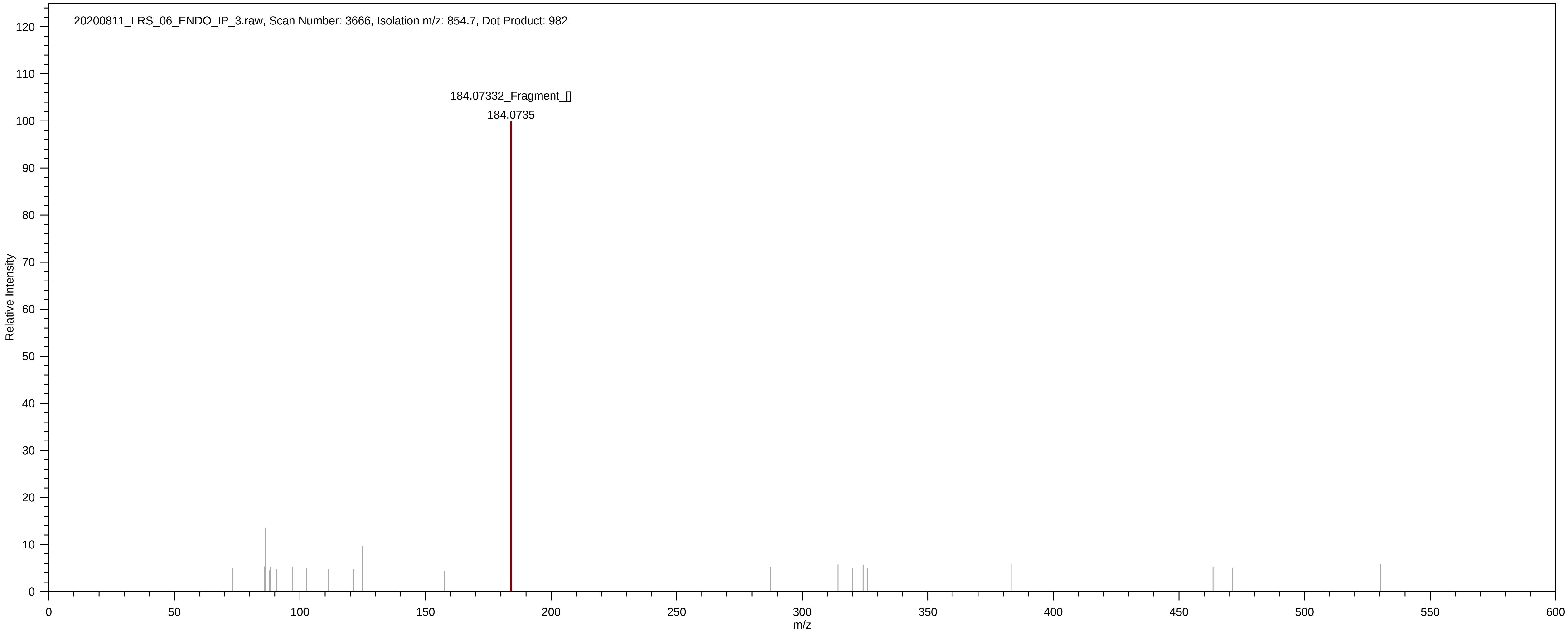

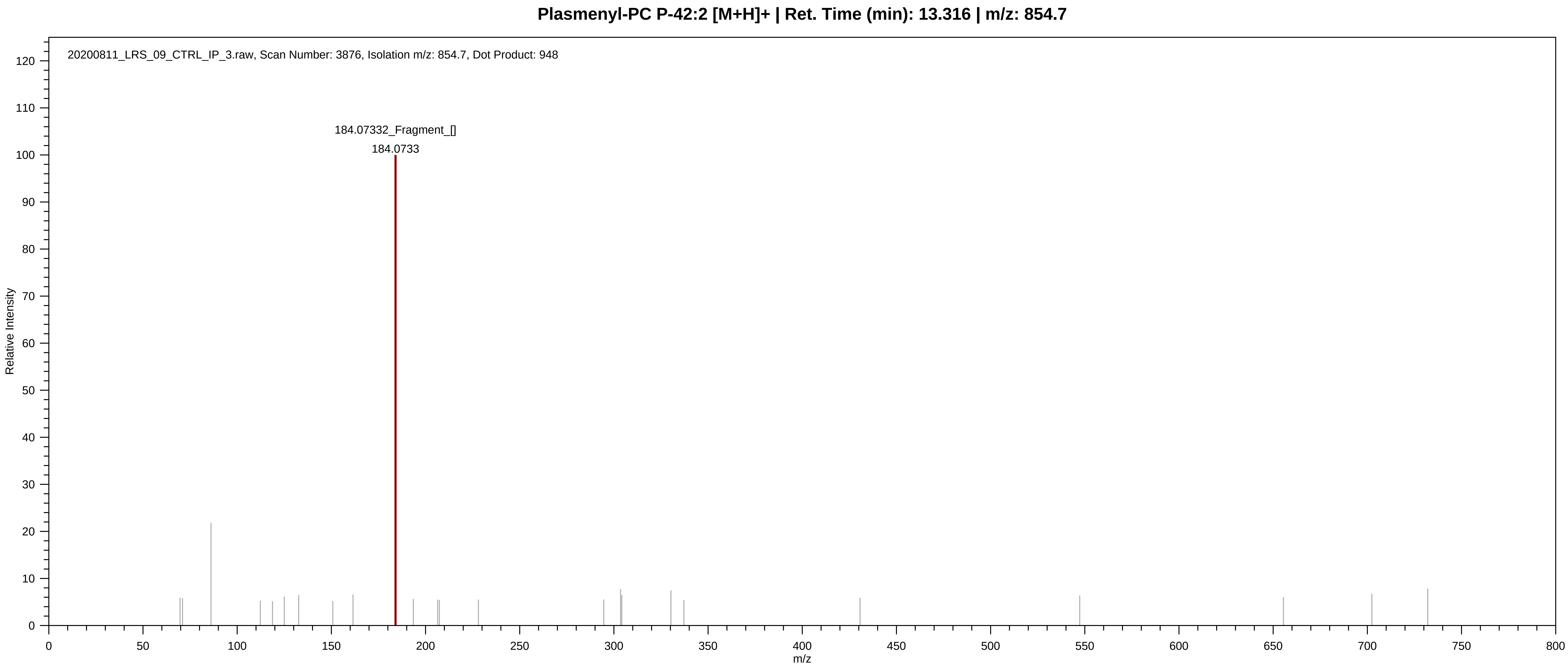

Plasmenyl-PC P-42:2 [M+H]+ | Ret. Time (min): 13.316 | m/z: 854.7
20200811_LRS_09_CTRL_IP_3.raw, Scan Number: 3876, Isolation m/z: 854.7, Dot Product: 948
184.07332_Fragment_[]
184.0733
Relative Intensity
m/z
0
10
20
30
40
50
60
70
80
90
100
110
120
0
50
100
150
200
250
300
350
400
450
500
550
600
650
700
750
800

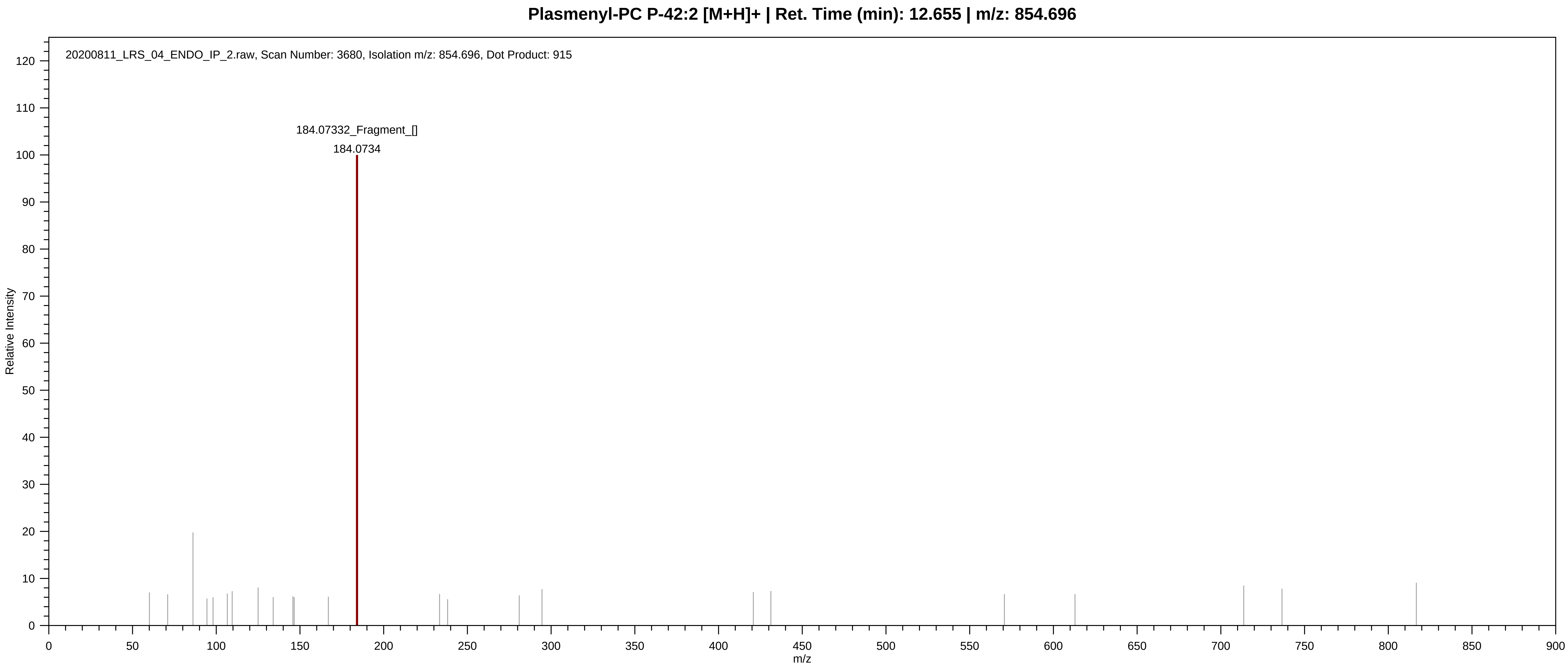
Plasmenyl-PC P-42:2 [M+H]+ | Ret. Time (min): 12.655 | m/z: 854.696
20200811_LRS_04_ENDO_IP_2.raw, Scan Number: 3680, Isolation m/z: 854.696, Dot Product: 915
184.07332_Fragment_[]
184.0734
Relative Intensity
m/z

Plasmenyl-PC P-42:2 [M+H]+ | Ret. Time (min): 12.636 | m/z: 854.699

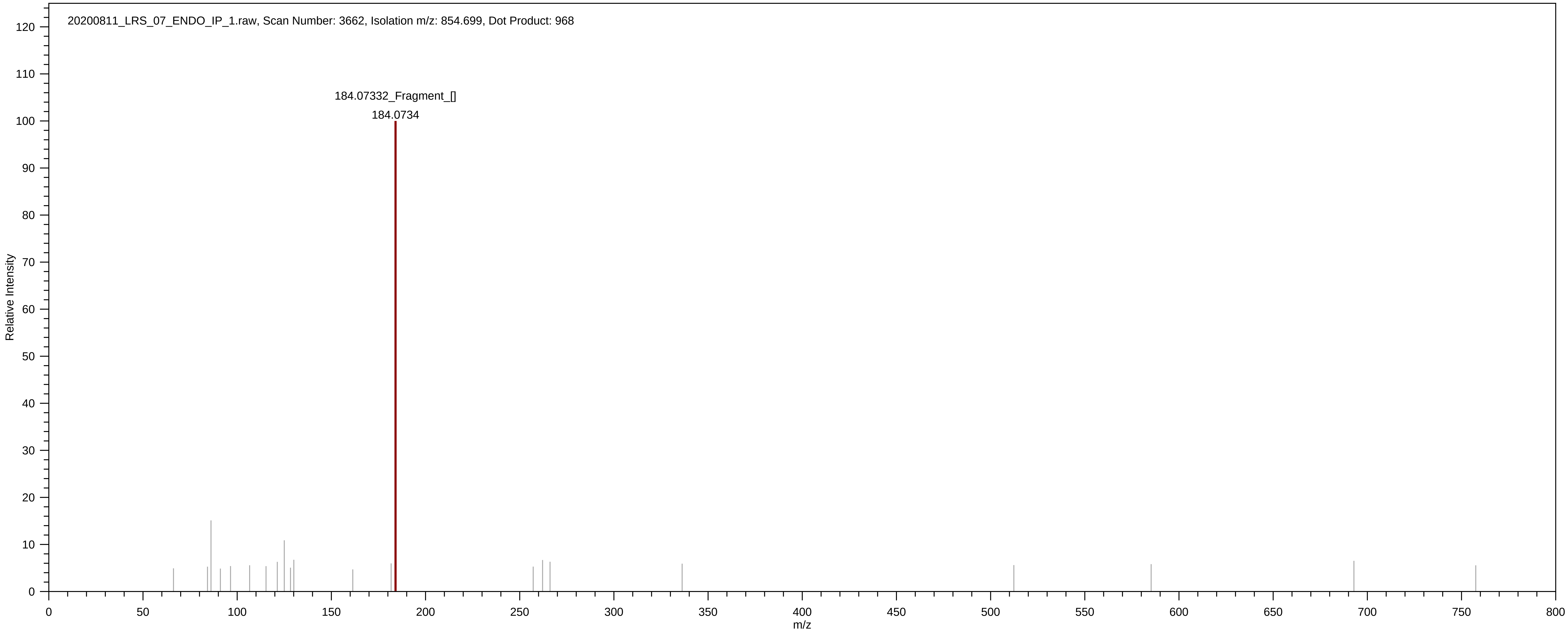

Plasmenyl-PC P-42:2 [M+H]+ | Ret. Time (min): 12.674 | m/z: 854.701

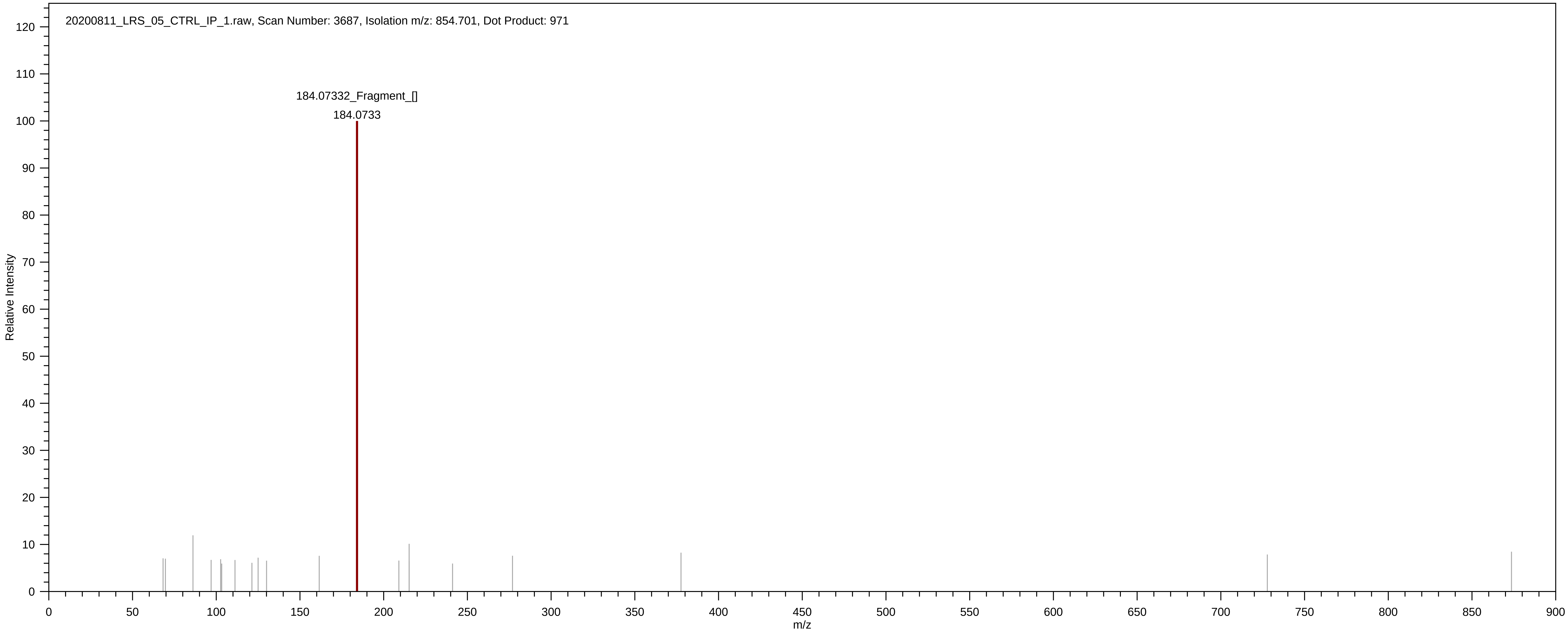

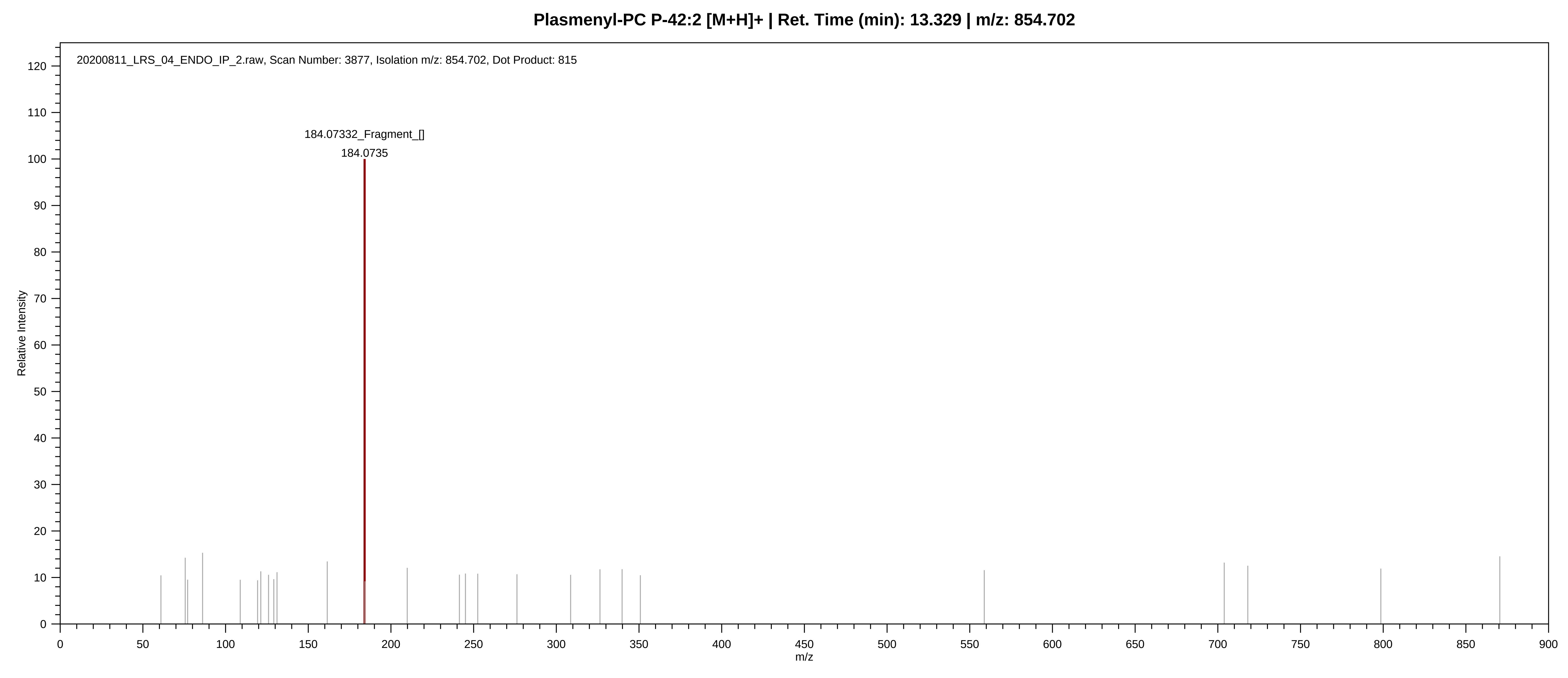
Plasmenyl-PC P-42:2 [M+H]+ | Ret. Time (min): 13.329 | m/z: 854.702
20200811_LRS_04_ENDO_IP_2.raw, Scan Number: 3877, Isolation m/z: 854.702, Dot Product: 815
184.07332_Fragment_[]
184.0735
Relative Intensity
m/z

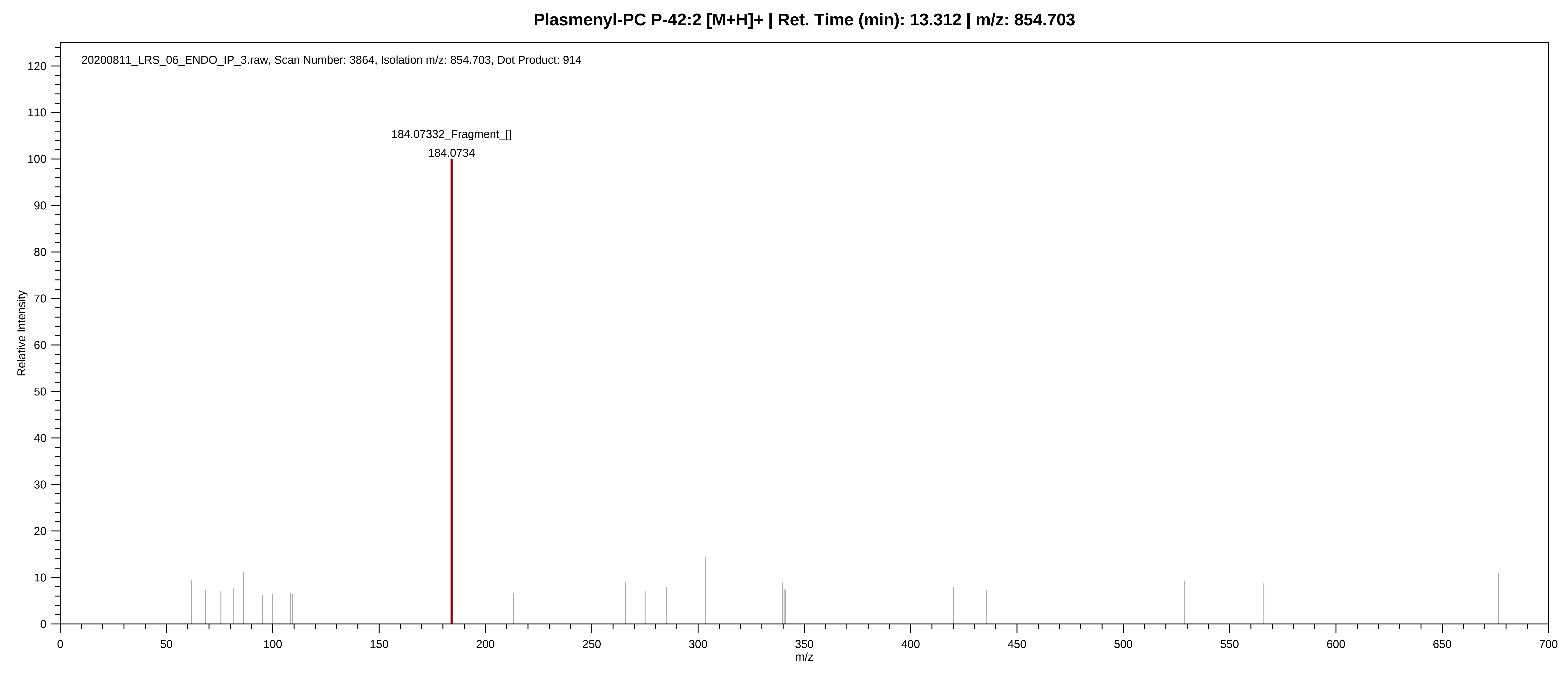
Plasmenyl-PC P-42:2 [M+H]+ | Ret. Time (min): 13.312 | m/z: 854.703
20200811_LRS_06_ENDO_IP_3.raw, Scan Number: 3864, Isolation m/z: 854.703, Dot Product: 914
184.07332_Fragment_[]
184.0734
Relative Intensity
m/z

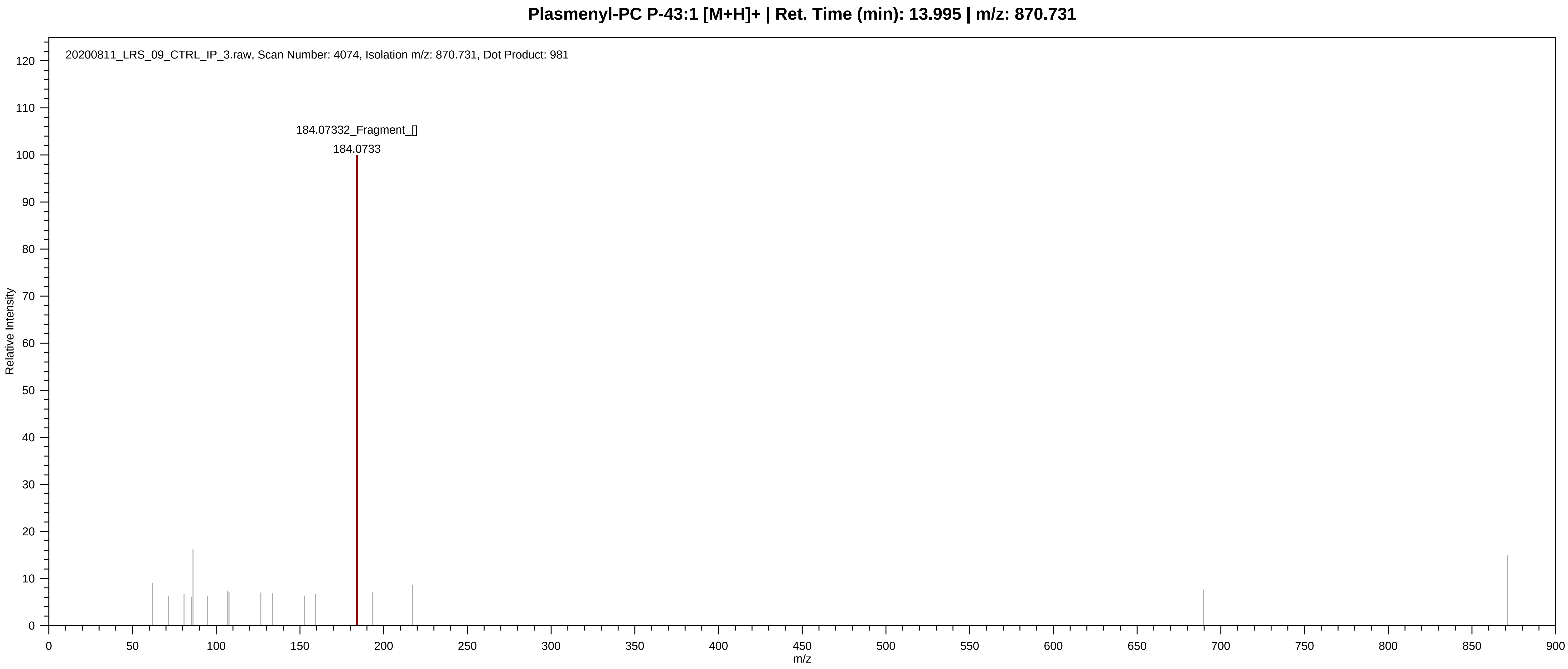

Plasmenyl-PC P-43:1 [M+H]+ | Ret. Time (min): 13.995 | m/z: 870.731
20200811_LRS_09_CTRL_IP_3.raw, Scan Number: 4074, Isolation m/z: 870.731, Dot Product: 981
184.07332_Fragment_[]
184.0733
Relative Intensity
m/z

Plasmenyl-PC P-44:4 [M+H]+ | Ret. Time (min): 12.431 | m/z: 878.7

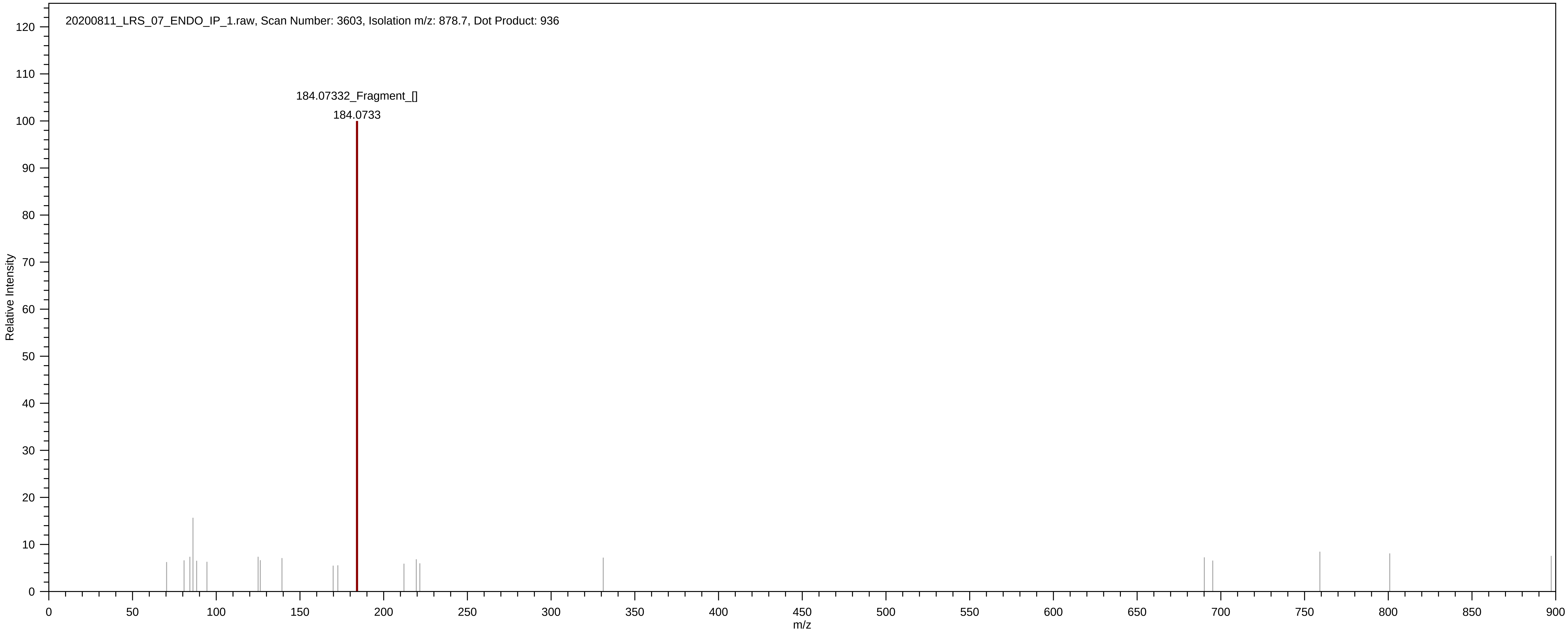

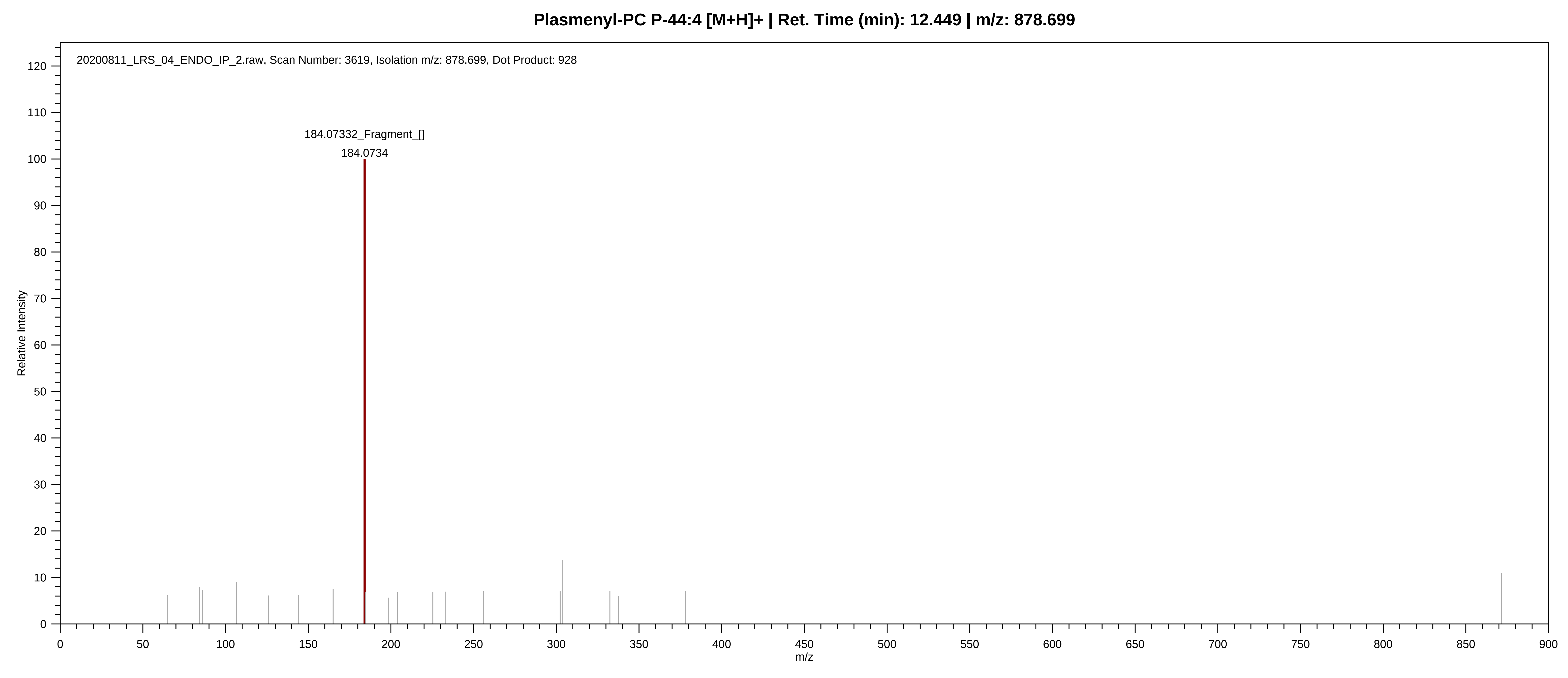

Plasmenyl-PC P-44:4 [M+H]+ | Ret. Time (min): 12.449 | m/z: 878.699
20200811_LRS_04_ENDO_IP_2.raw, Scan Number: 3619, Isolation m/z: 878.699, Dot Product: 928
184.07332_Fragment_[]
184.0734
Relative Intensity
m/z
0
10
20
30
40
50
60
70
80
90
100
110
120
0
50
100
150
200
250
300
350
400
450
500
550
600
650
700
750
800
850
900

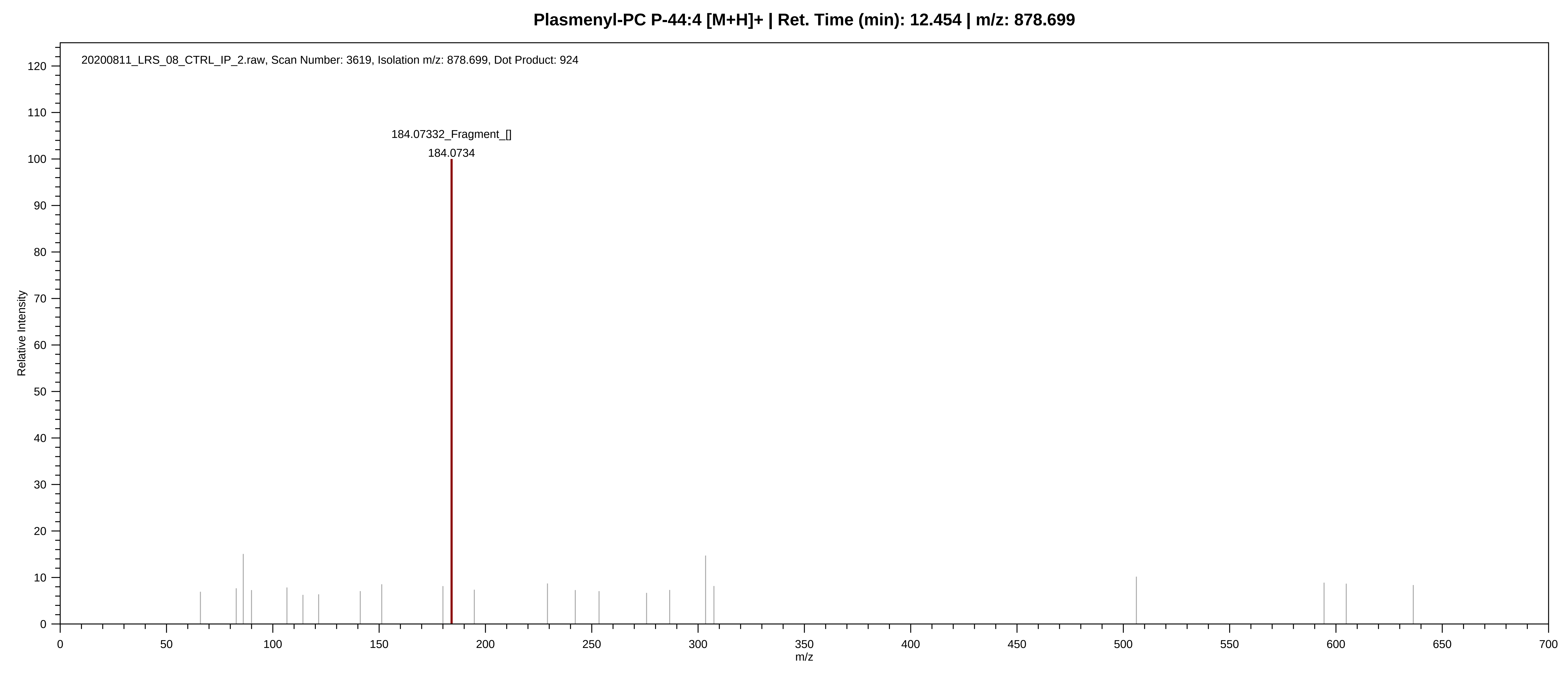
Plasmenyl-PC P-44:4 [M+H]+ | Ret. Time (min): 12.454 | m/z: 878.699
20200811_LRS_08_CTRL_IP_2.raw, Scan Number: 3619, Isolation m/z: 878.699, Dot Product: 924
184.07332_Fragment_[]
184.0734
Relative Intensity
m/z

Plasmenyl-PC P-44:4 [M+H]+ | Ret. Time (min): 12.409 | m/z: 878.701

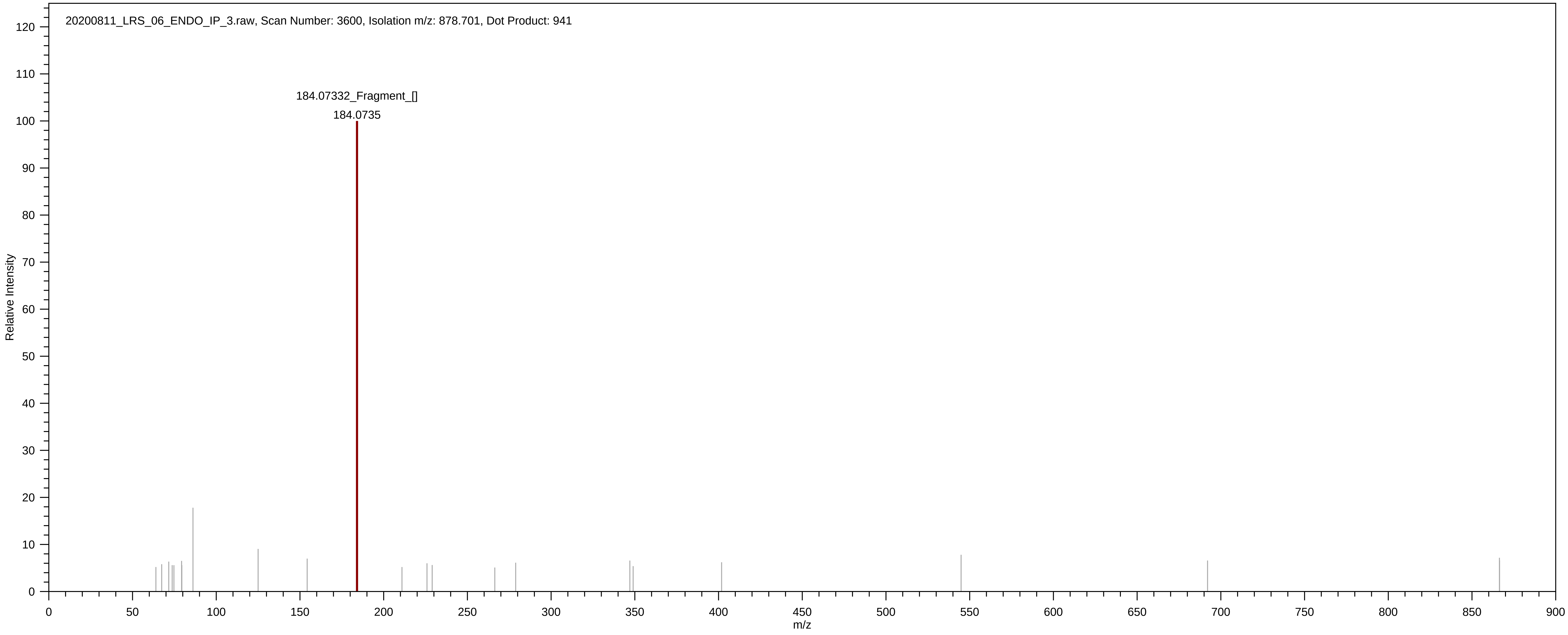

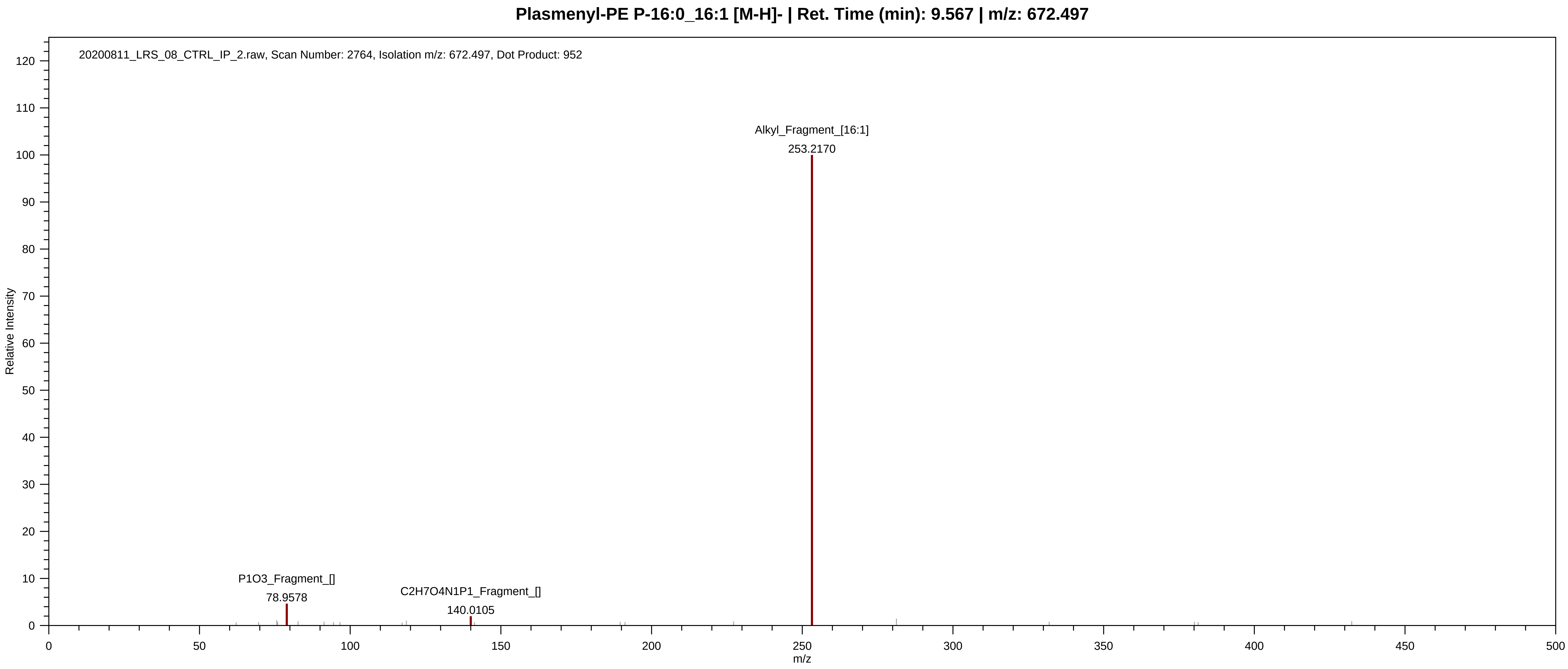

Plasmenyl-PE P-16:0_16:1 [M-H]- | Ret. Time (min): 9.567 | m/z: 672.497
20200811_LRS_08_CTRL_IP_2.raw, Scan Number: 2764, Isolation m/z: 672.497, Dot Product: 952
Alkyl_Fragment_[16:1]
253.2170
P1O3_Fragment_[]
78.9578
C2H7O4N1P1_Fragment_[]
140.0105
Relative Intensity
m/z
0
10
20
30
40
50
60
70
80
90
100
110
120
0
50
100
150
200
250
300
350
400
450
500

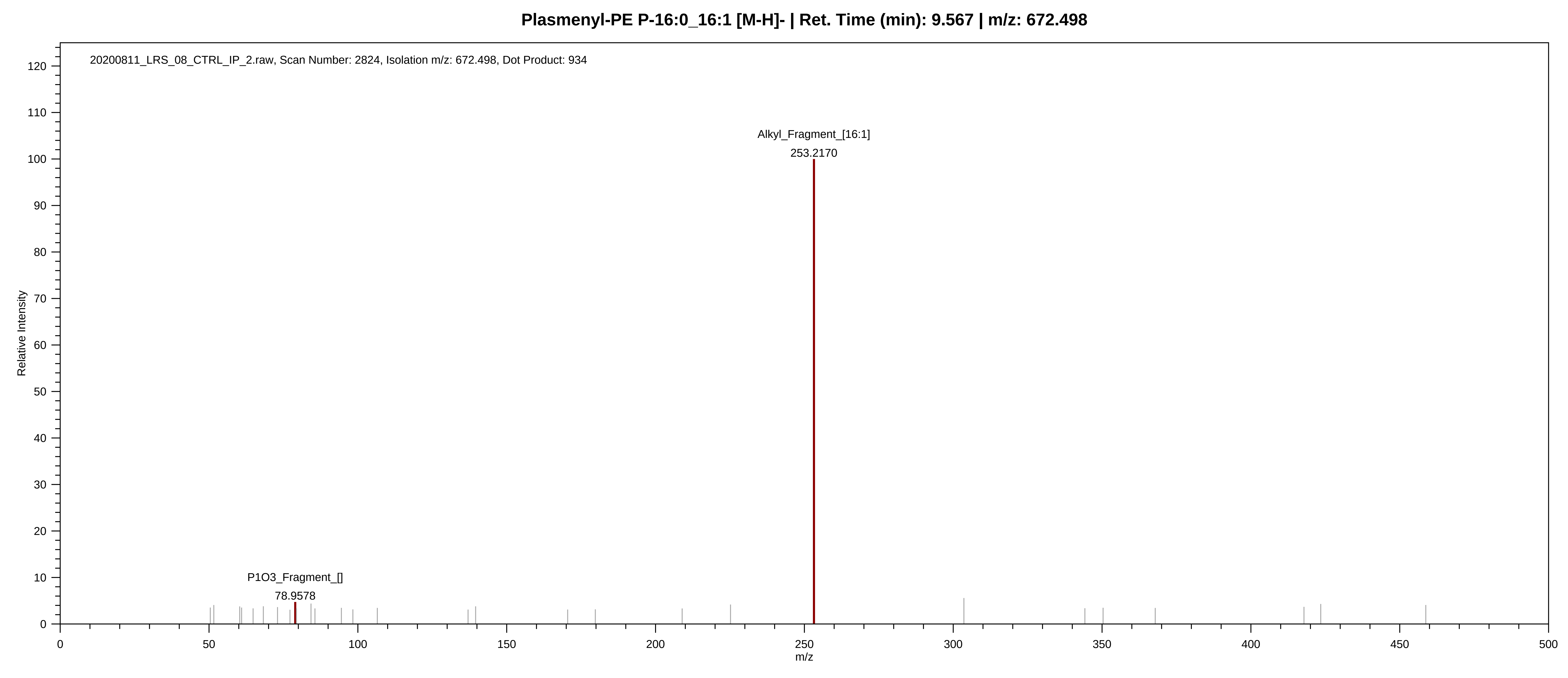

Plasmenyl-PE P-16:0_16:1 [M-H]- | Ret. Time (min): 9.567 | m/z: 672.498
20200811_LRS_08_CTRL_IP_2.raw, Scan Number: 2824, Isolation m/z: 672.498, Dot Product: 934
Alkyl_Fragment_[16:1]
253.2170
P1O3_Fragment_[]
78.9578
Relative Intensity
m/z

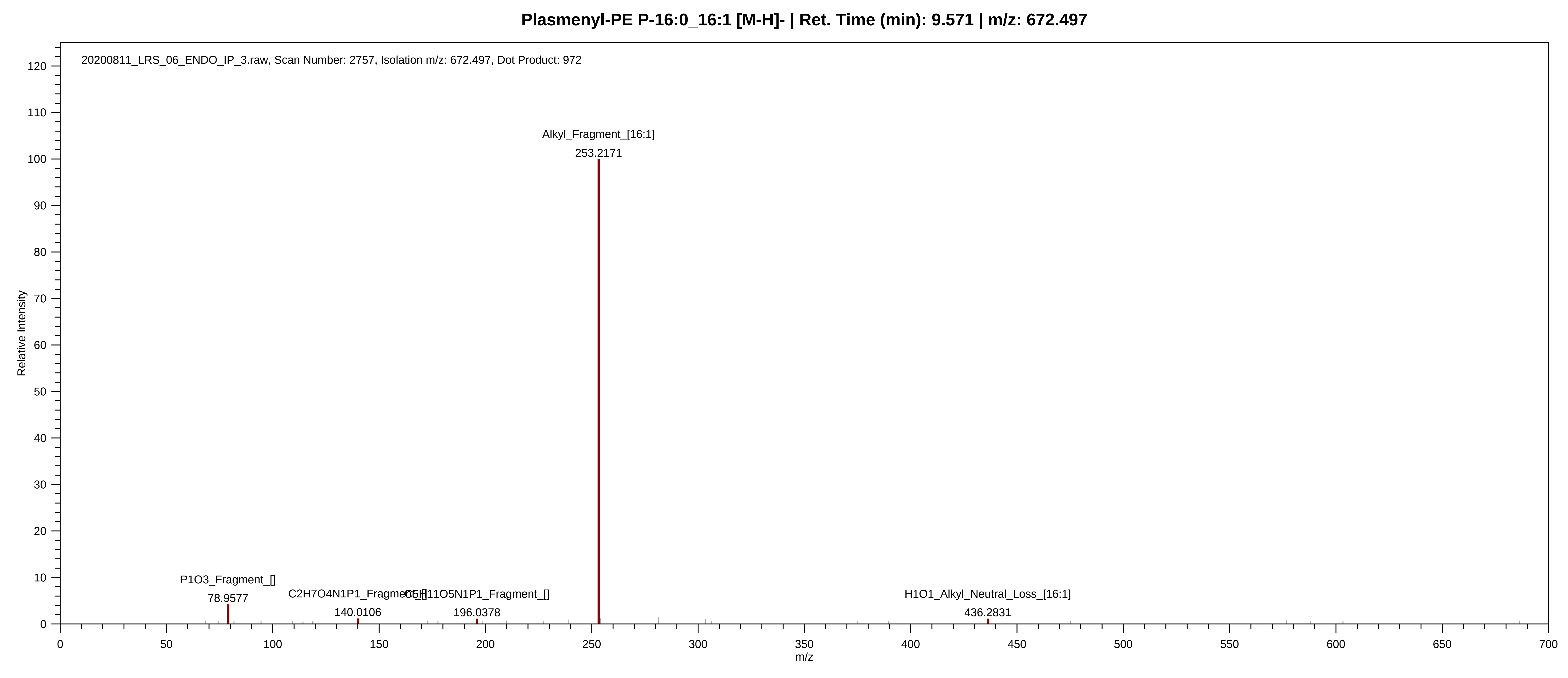
Plasmenyl-PE P-16:0_16:1 [M-H]- | Ret. Time (min): 9.571 | m/z: 672.497
20200811_LRS_06_ENDO_IP_3.raw, Scan Number: 2757, Isolation m/z: 672.497, Dot Product: 972
Alkyl_Fragment_[16:1]
253.2171
P1O3_Fragment_[]
78.9577
C2H7O4N1P1_Fragment_[]
140.0106
C5H11O5N1P1_Fragment_[]
196.0378
H1O1_Alkyl_Neutral_Loss_[16:1]
436.2831
Relative Intensity
m/z

Plasmenyl-PE P-16:0_16:1 [M-H]- | Ret. Time (min): 9.574 | m/z: 672.497

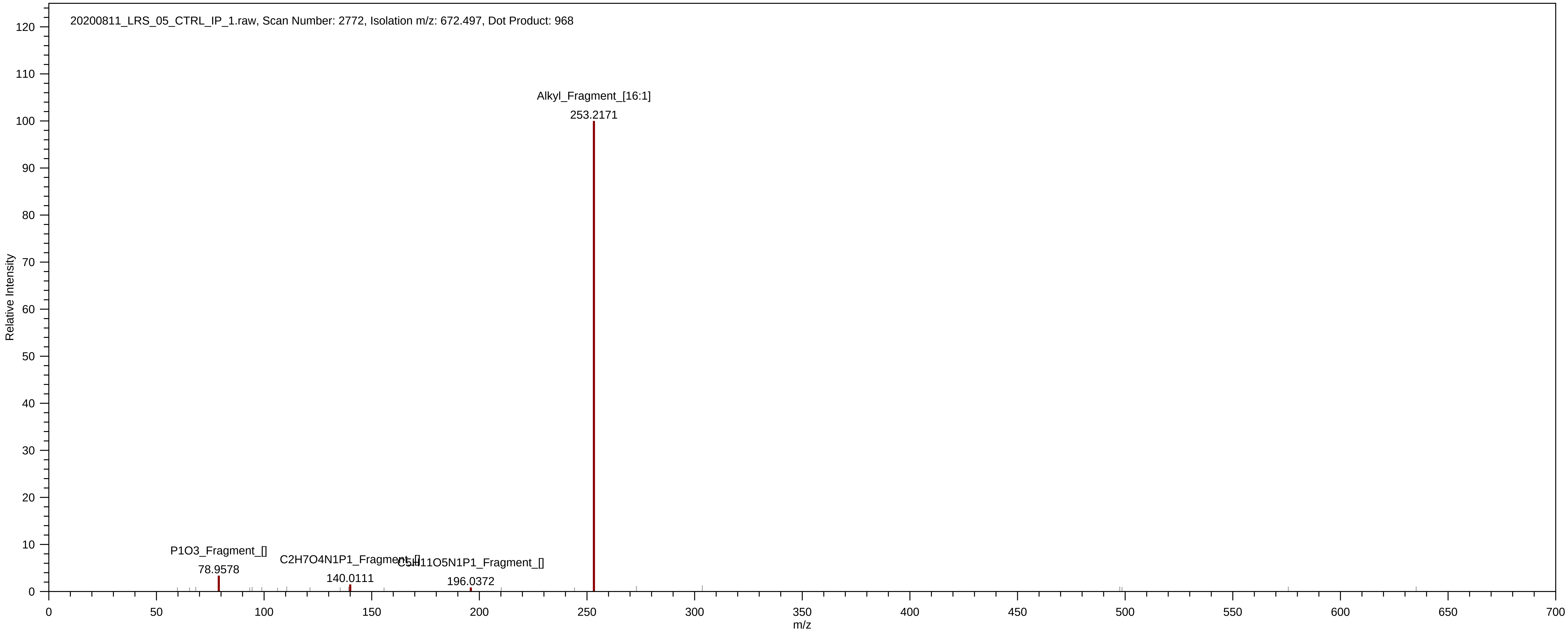

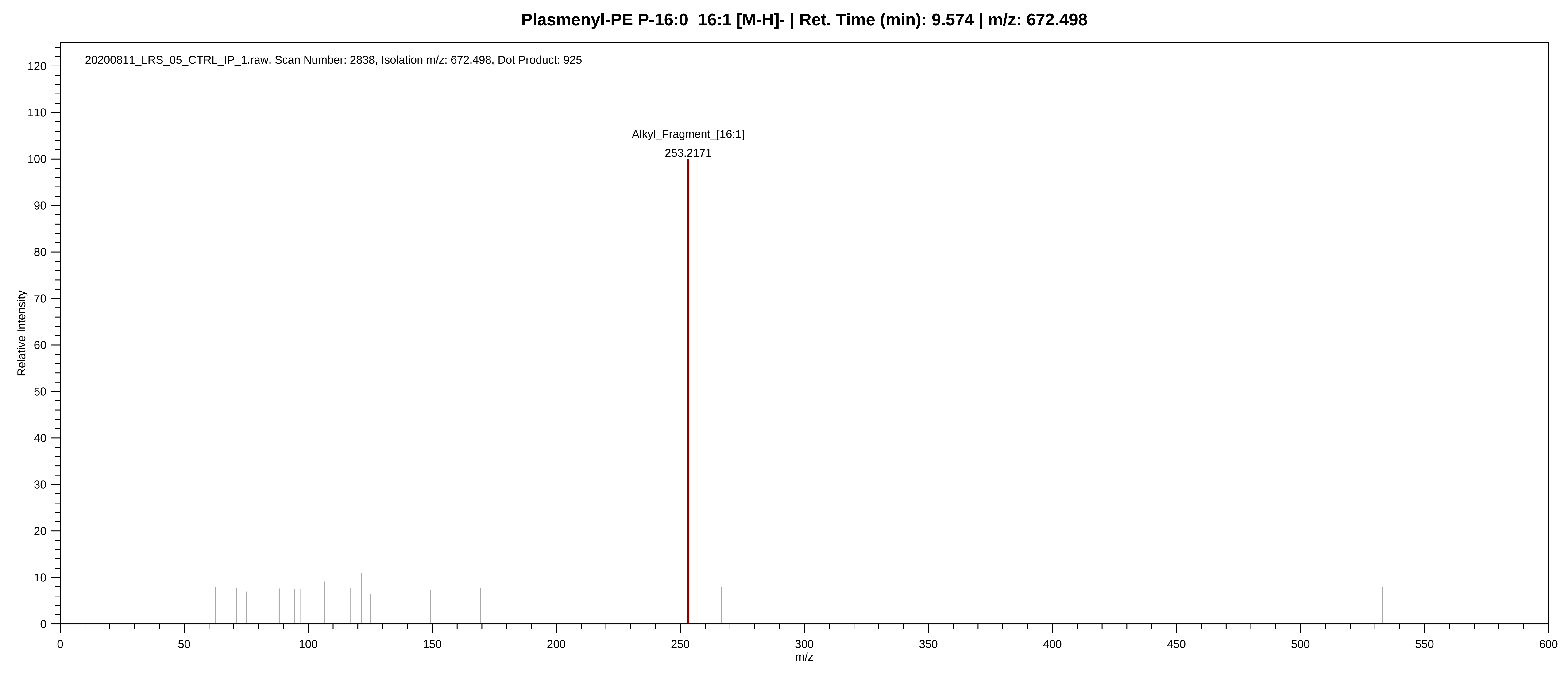

Plasmenyl-PE P-16:0_16:1 [M-H]- | Ret. Time (min): 9.574 | m/z: 672.498
20200811_LRS_05_CTRL_IP_1.raw, Scan Number: 2838, Isolation m/z: 672.498, Dot Product: 925
Alkyl_Fragment_[16:1]
253.2171
Relative Intensity
m/z

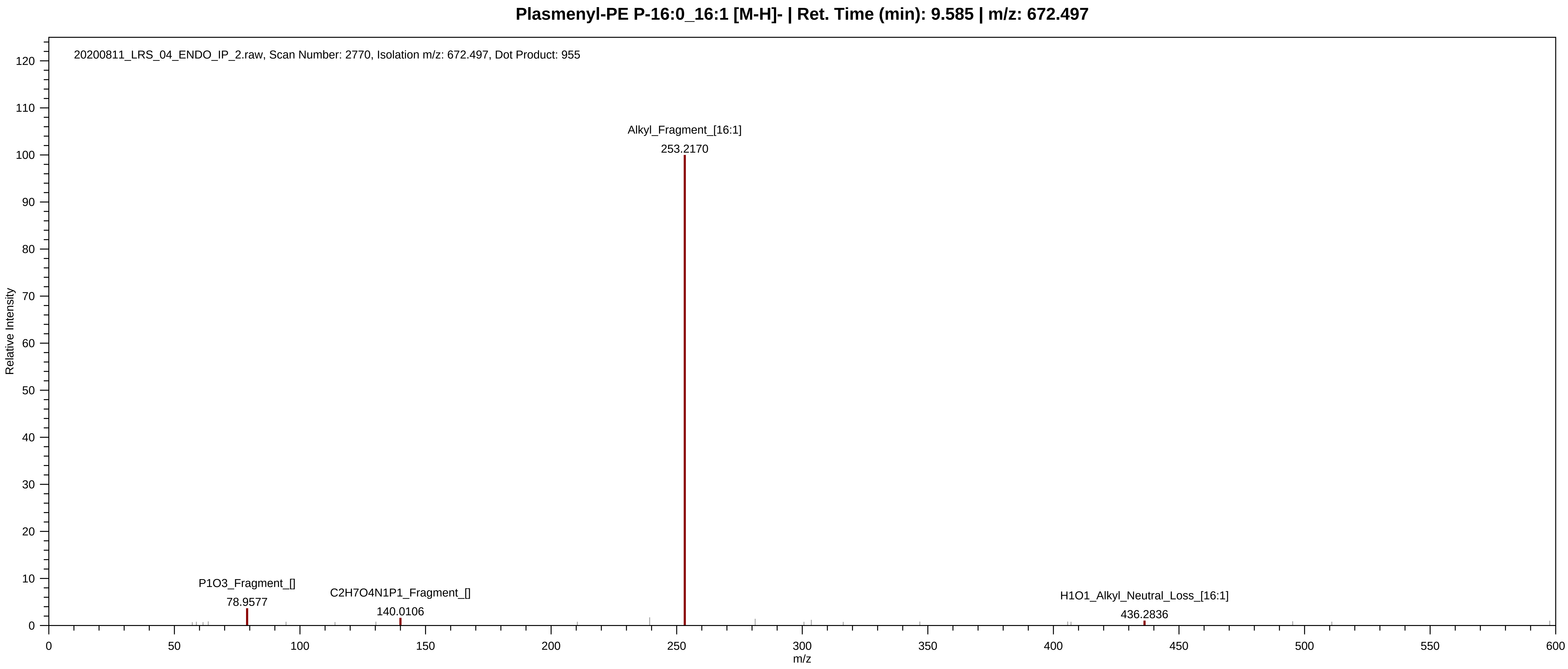

Plasmenyl-PE P-16:0_16:1 [M-H]- | Ret. Time (min): 9.585 | m/z: 672.497
20200811_LRS_04_ENDO_IP_2.raw, Scan Number: 2770, Isolation m/z: 672.497, Dot Product: 955
Alkyl_Fragment_[16:1]
253.2170
P1O3_Fragment_[]
78.9577
C2H7O4N1P1_Fragment_[]
140.0106
H1O1_Alkyl_Neutral_Loss_[16:1]
436.2836
Relative Intensity
m/z

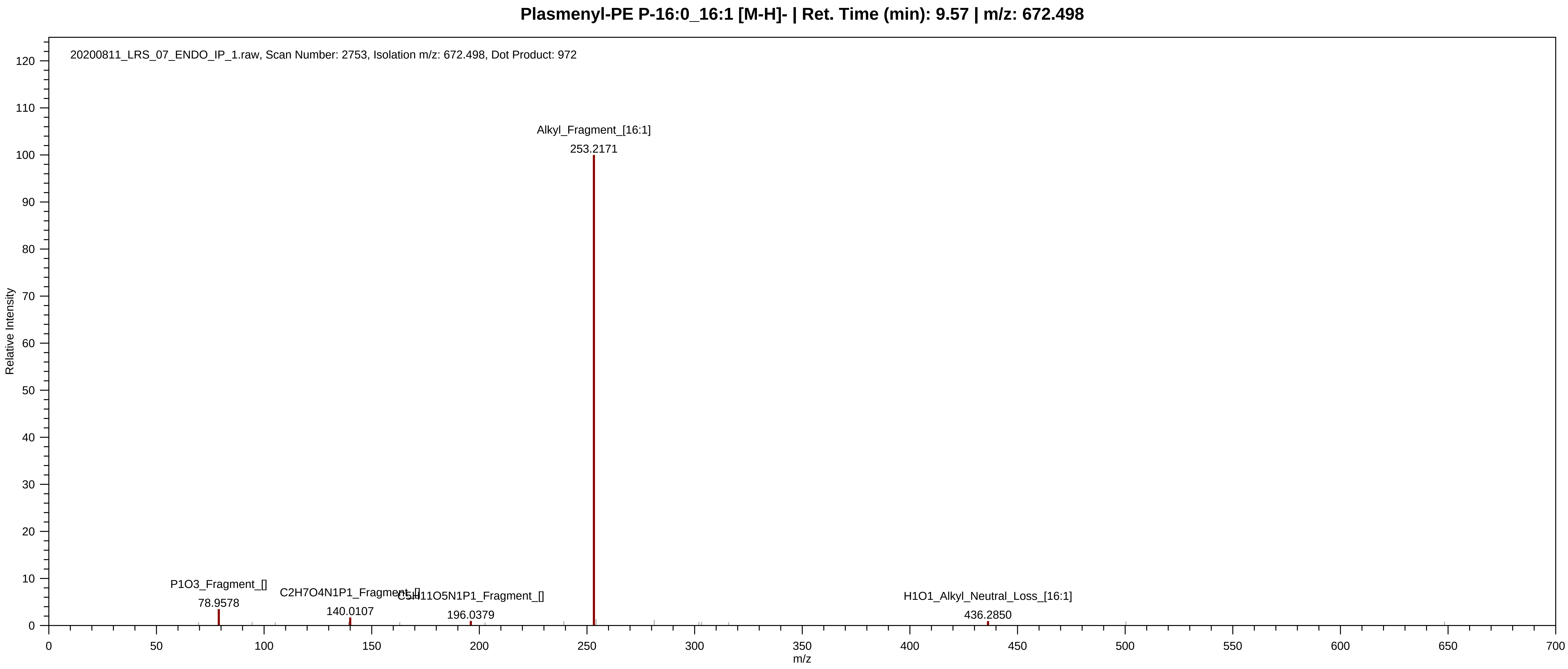
Plasmenyl-PE P-16:0_16:1 [M-H]- | Ret. Time (min): 9.57 | m/z: 672.498
20200811_LRS_07_ENDO_IP_1.raw, Scan Number: 2753, Isolation m/z: 672.498, Dot Product: 972
Alkyl_Fragment_[16:1]
253.2171
P1O3_Fragment_[]
78.9578
C2H7O4N1P1_Fragment_[]
140.0107
C5H11O5N1P1_Fragment_[]
196.0379
H1O1_Alkyl_Neutral_Loss_[16:1]
436.2850
Relative Intensity
m/z

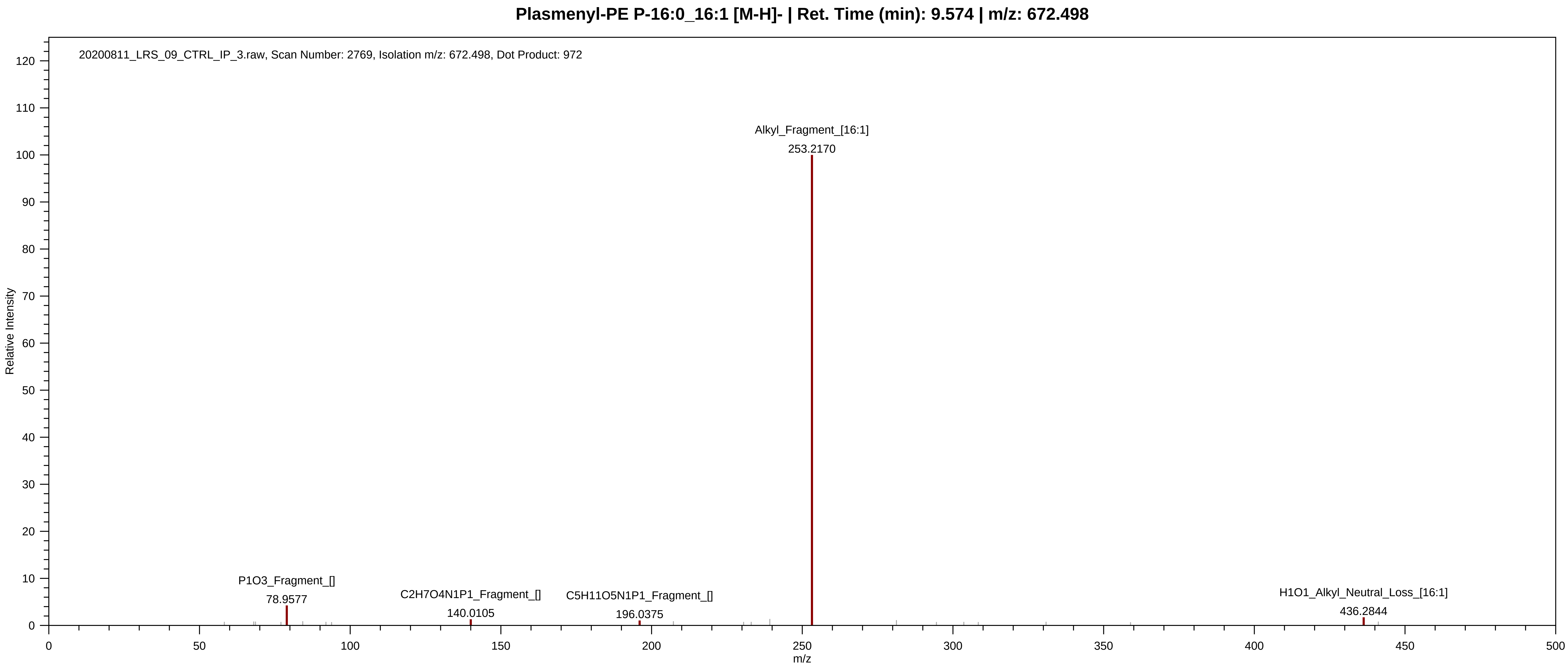
Plasmenyl-PE P-16:0_16:1 [M-H]- | Ret. Time (min): 9.574 | m/z: 672.498
20200811_LRS_09_CTRL_IP_3.raw, Scan Number: 2769, Isolation m/z: 672.498, Dot Product: 972
Alkyl_Fragment_[16:1]
253.2170
P1O3_Fragment_[]
78.9577
C2H7O4N1P1_Fragment_[]
140.0105
C5H11O5N1P1_Fragment_[]
196.0375
H1O1_Alkyl_Neutral_Loss_[16:1]
436.2844
Relative Intensity
m/z

Plasmenyl-PE P-16:0_16:1 [M-H]- | Ret. Time (min): 9.574 | m/z: 672.498

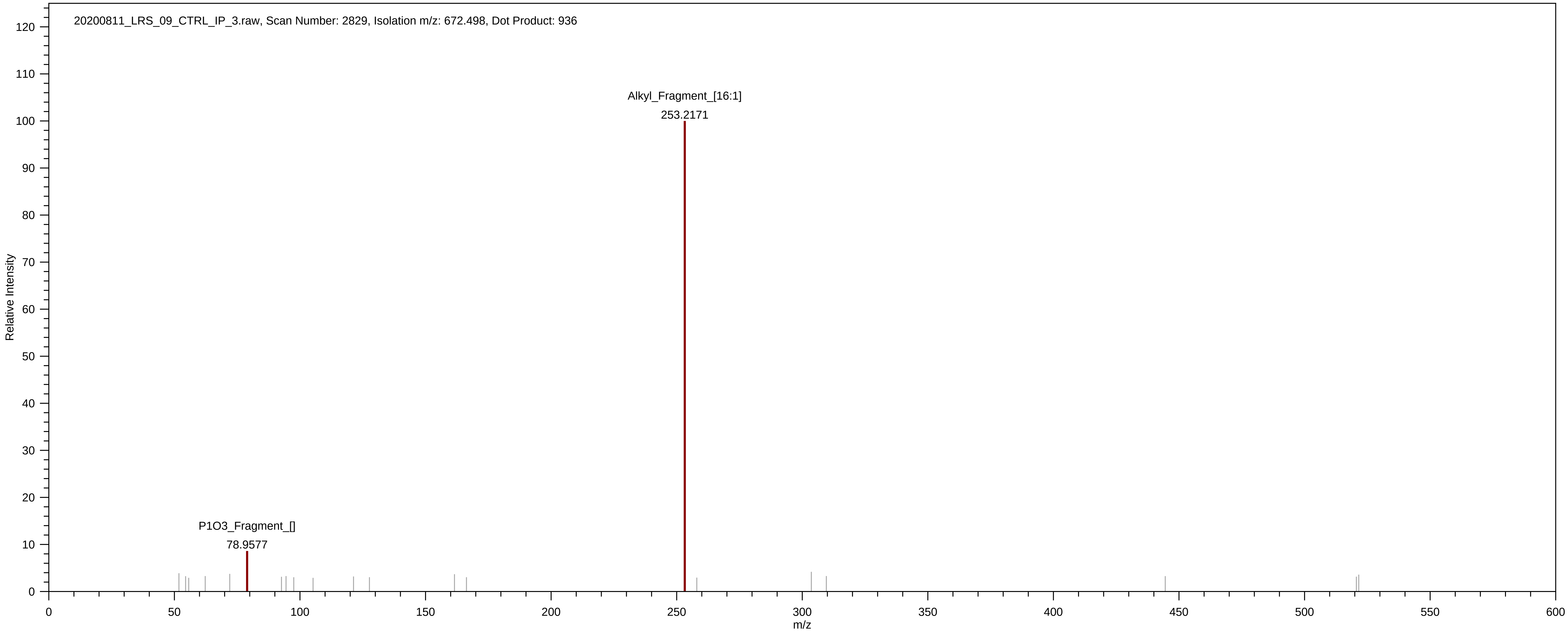

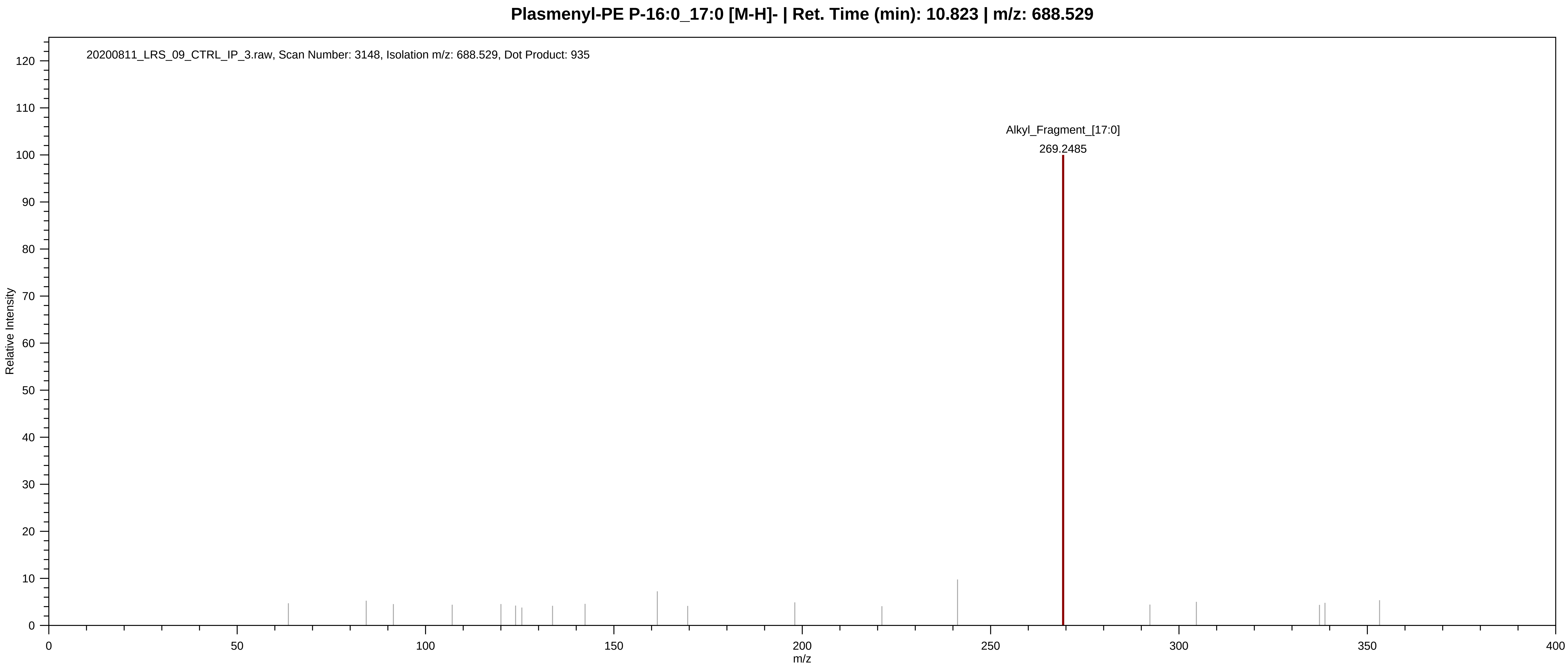
Plasmenyl-PE P-16:0_17:0 [M-H]- | Ret. Time (min): 10.823 | m/z: 688.529
20200811_LRS_09_CTRL_IP_3.raw, Scan Number: 3148, Isolation m/z: 688.529, Dot Product: 935
Alkyl_Fragment_[17:0]
269.2485
Relative Intensity
m/z
0
10
20
30
40
50
60
70
80
90
100
110
120
0
50
100
150
200
250
300
350
400

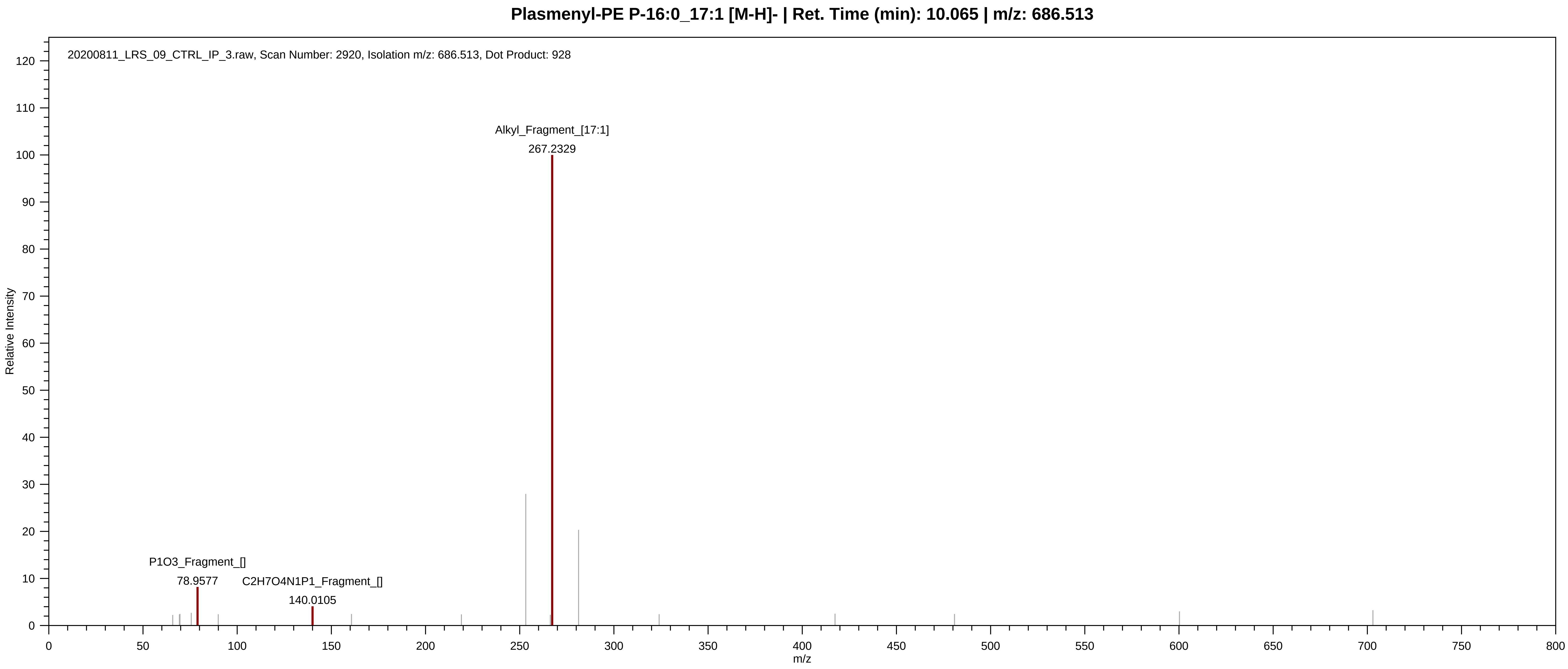

Plasmenyl-PE P-16:0_17:1 [M-H]- | Ret. Time (min): 10.065 | m/z: 686.513
20200811_LRS_09_CTRL_IP_3.raw, Scan Number: 2920, Isolation m/z: 686.513, Dot Product: 928
Alkyl_Fragment_[17:1]
267.2329
P1O3_Fragment_[]
78.9577
C2H7O4N1P1_Fragment_[]
140.0105
Relative Intensity
m/z

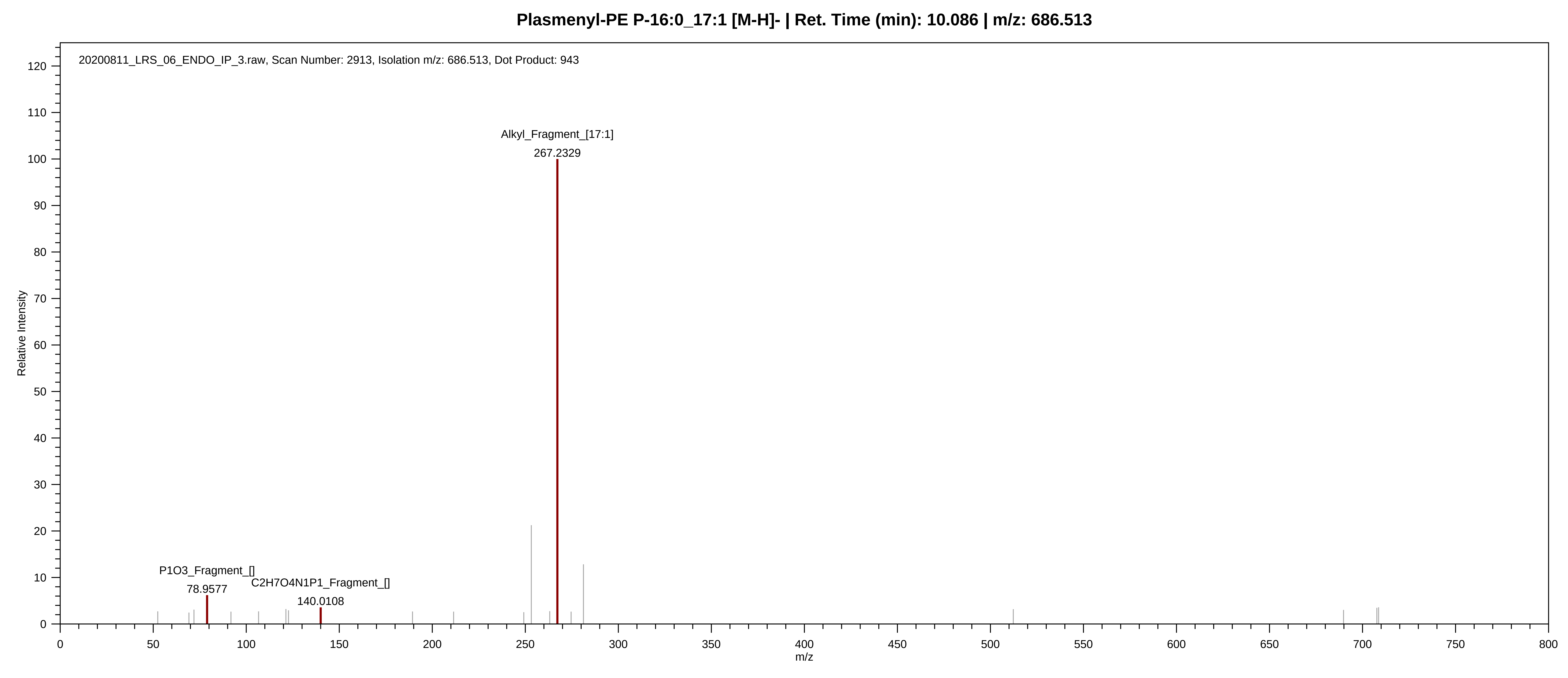

Plasmenyl-PE P-16:0_17:1 [M-H]- | Ret. Time (min): 10.086 | m/z: 686.513
20200811_LRS_06_ENDO_IP_3.raw, Scan Number: 2913, Isolation m/z: 686.513, Dot Product: 943
Alkyl_Fragment_[17:1]
267.2329
P1O3_Fragment_[]
78.9577
C2H7O4N1P1_Fragment_[]
140.0108
Relative Intensity
m/z

Plasmenyl-PE P-16:0_18:1 [M-H]- | Ret. Time (min): 10.531 | m/z: 700.528

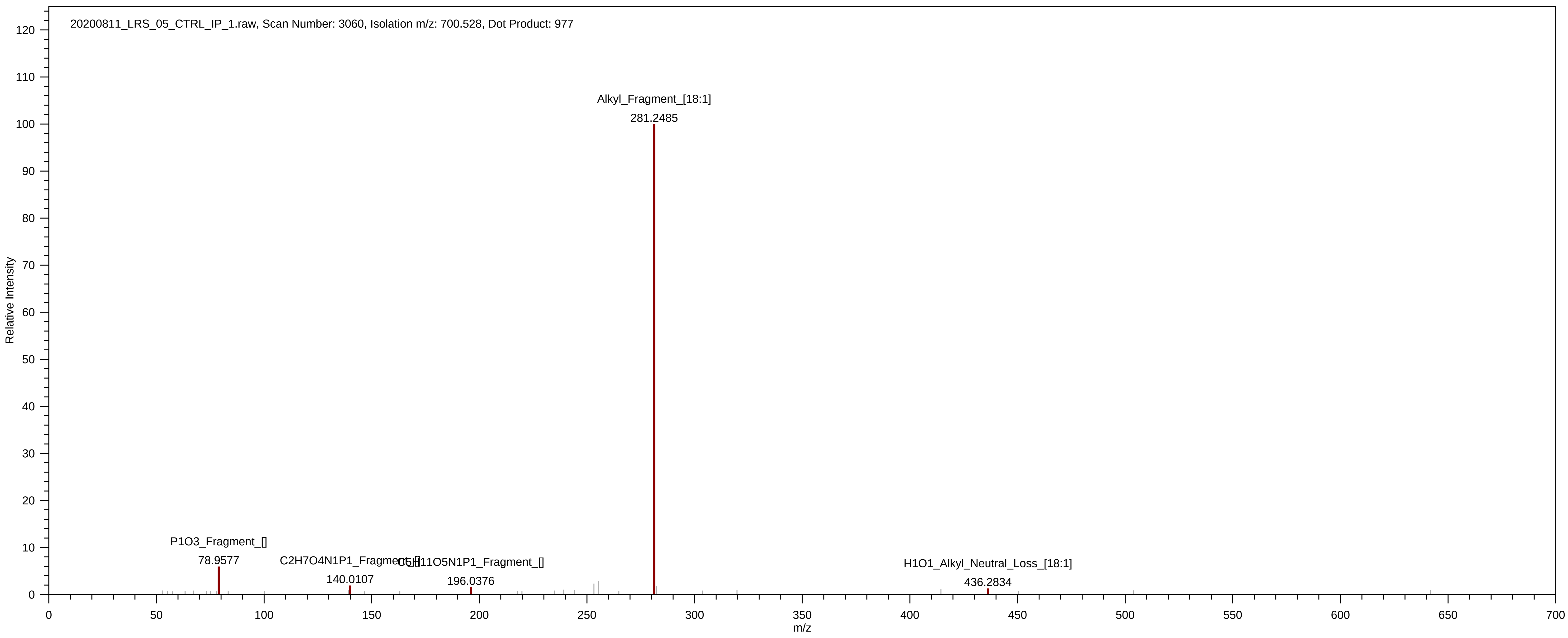

Plasmenyl-PE P-16:0_18:1 [M-H]- | Ret. Time (min): 10.522 | m/z: 700.529

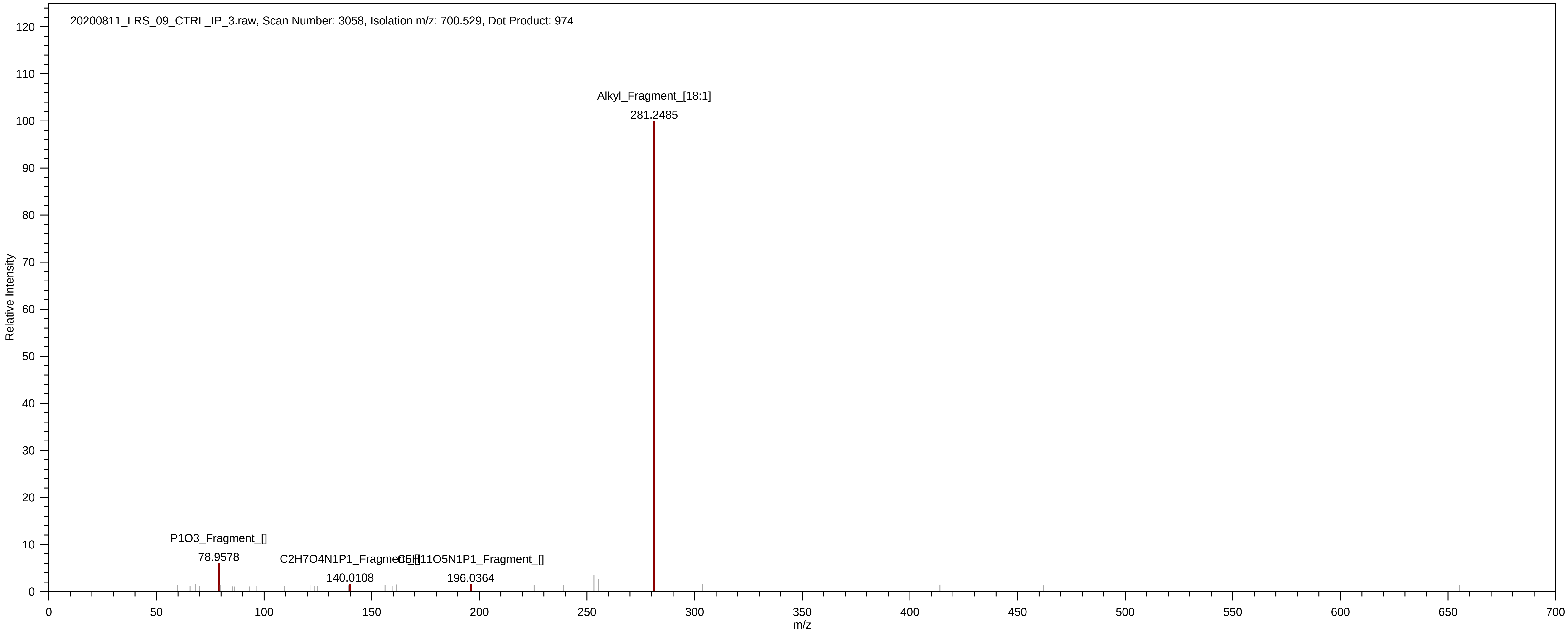

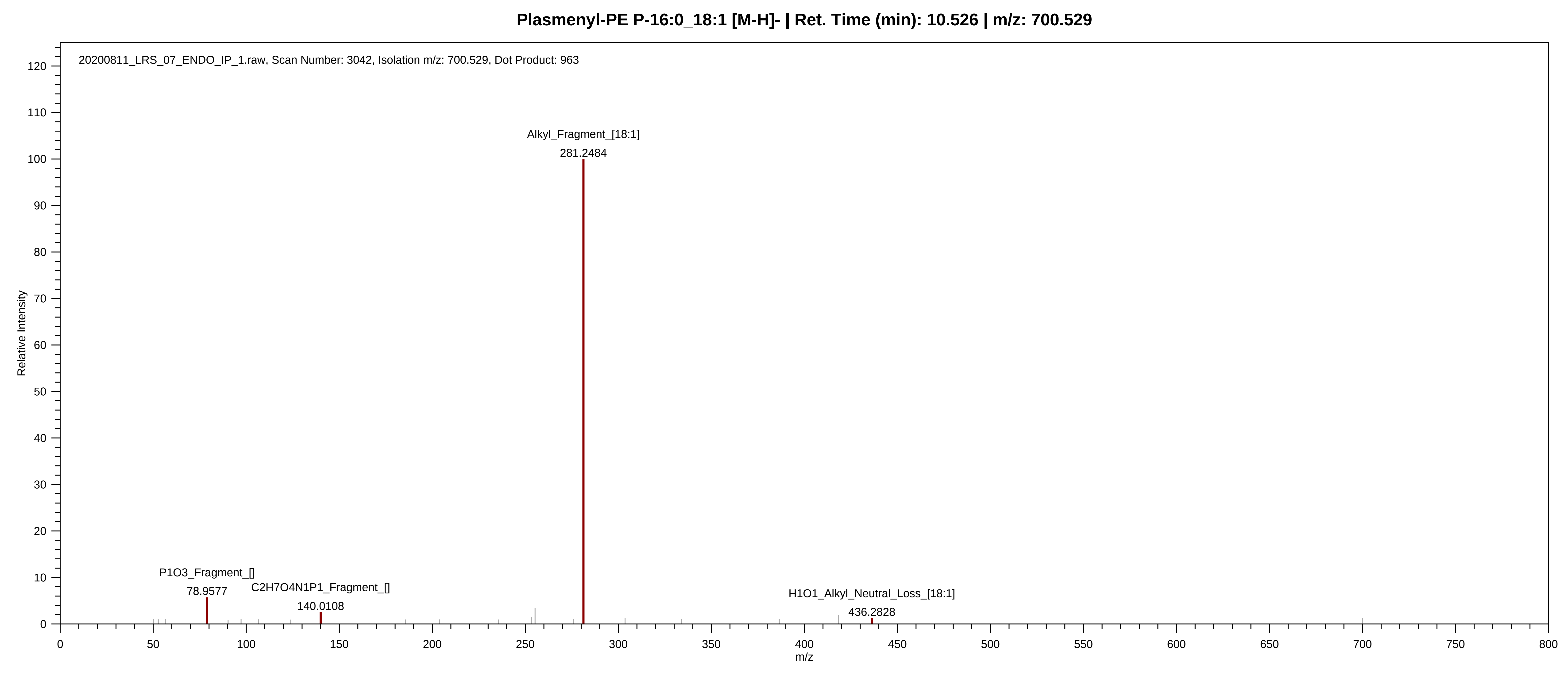
Plasmenyl-PE P-16:0_18:1 [M-H]- | Ret. Time (min): 10.526 | m/z: 700.529
20200811_LRS_07_ENDO_IP_1.raw, Scan Number: 3042, Isolation m/z: 700.529, Dot Product: 963
Alkyl_Fragment_[18:1]
281.2484
P1O3_Fragment_[]
78.9577
C2H7O4N1P1_Fragment_[]
140.0108
H1O1_Alkyl_Neutral_Loss_[18:1]
436.2828
Relative Intensity
m/z

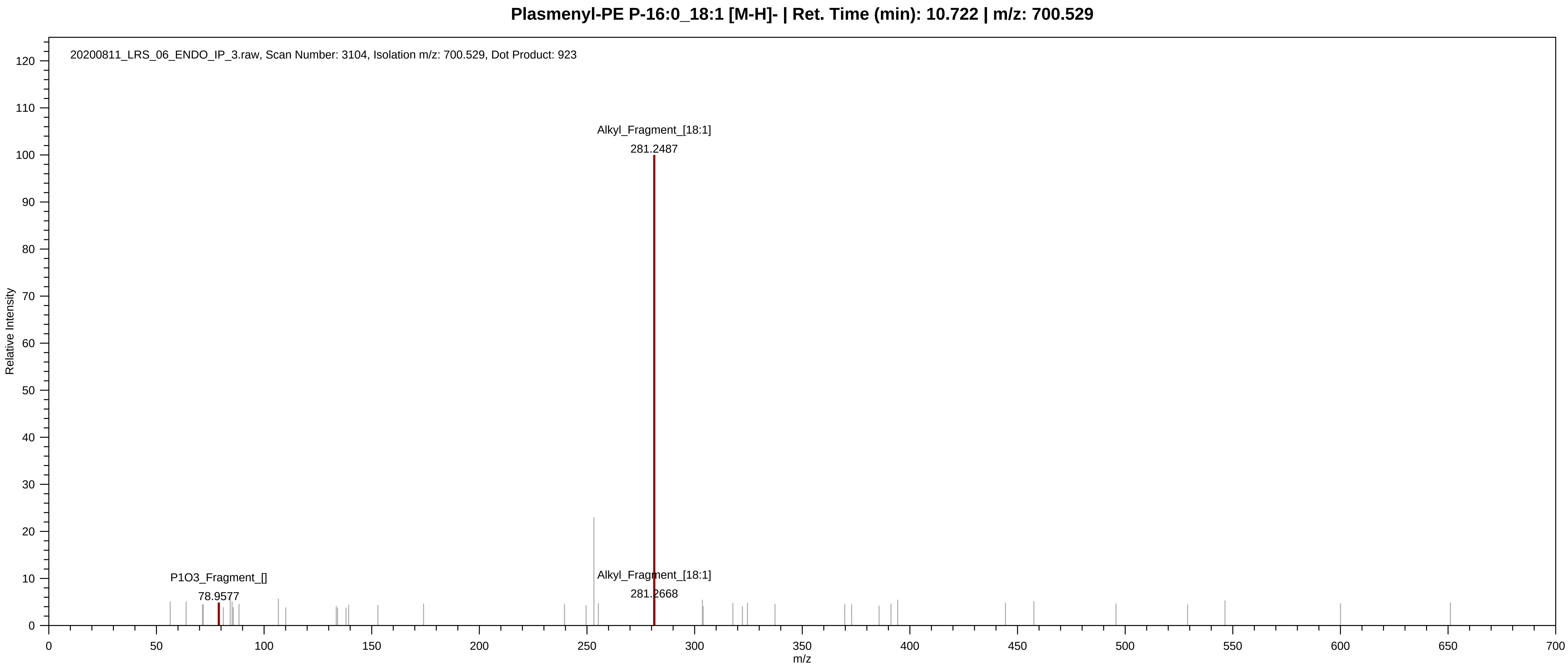

Plasmenyl-PE P-16:0_18:1 [M-H]- | Ret. Time (min): 10.722 | m/z: 700.529
20200811_LRS_06_ENDO_IP_3.raw, Scan Number: 3104, Isolation m/z: 700.529, Dot Product: 923
Alkyl_Fragment_[18:1]
281.2487
P1O3_Fragment_[]
78.9577
Alkyl_Fragment_[18:1]
281.2668
Relative Intensity
m/z

Plasmenyl-PE P-16:0_18:2 [M-H]- | Ret. Time (min): 9.829 | m/z: 698.513

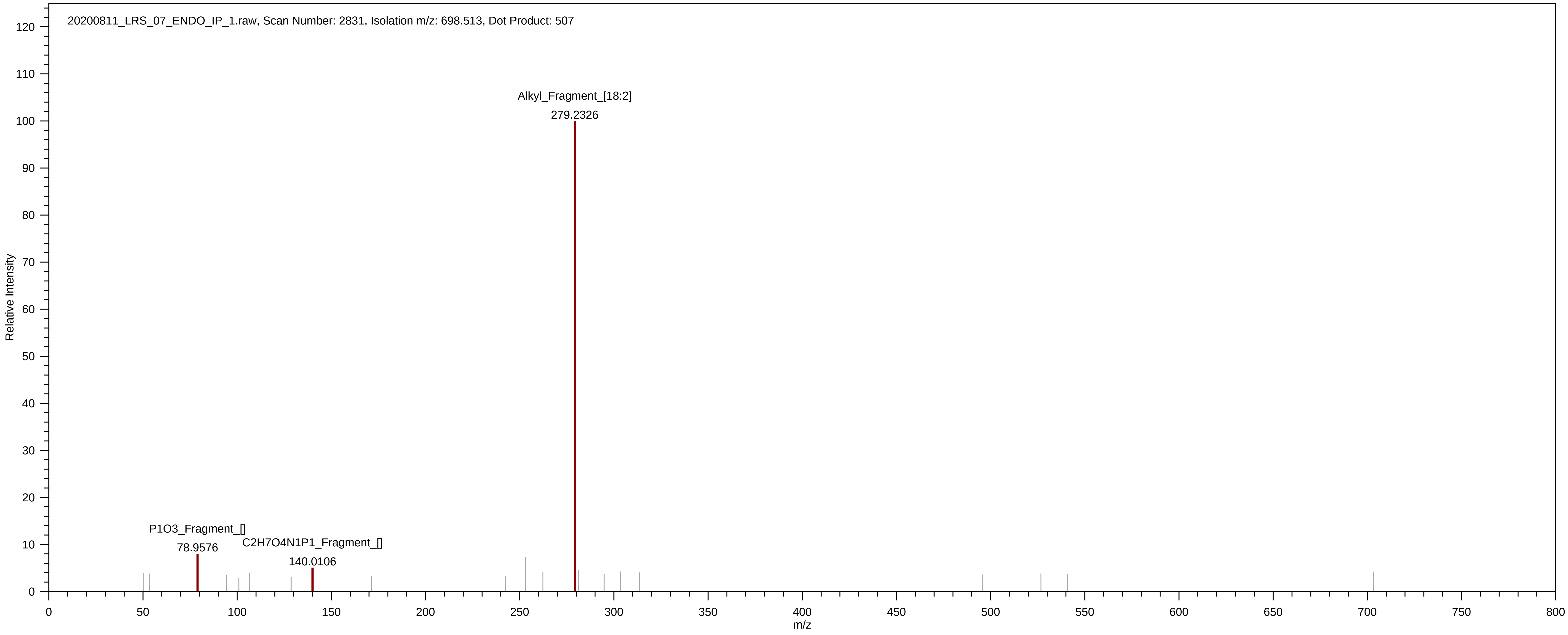

Plasmenyl-PE P-16:0_20:1 [M-H]- | Ret. Time (min): 11.5 | m/z: 728.56

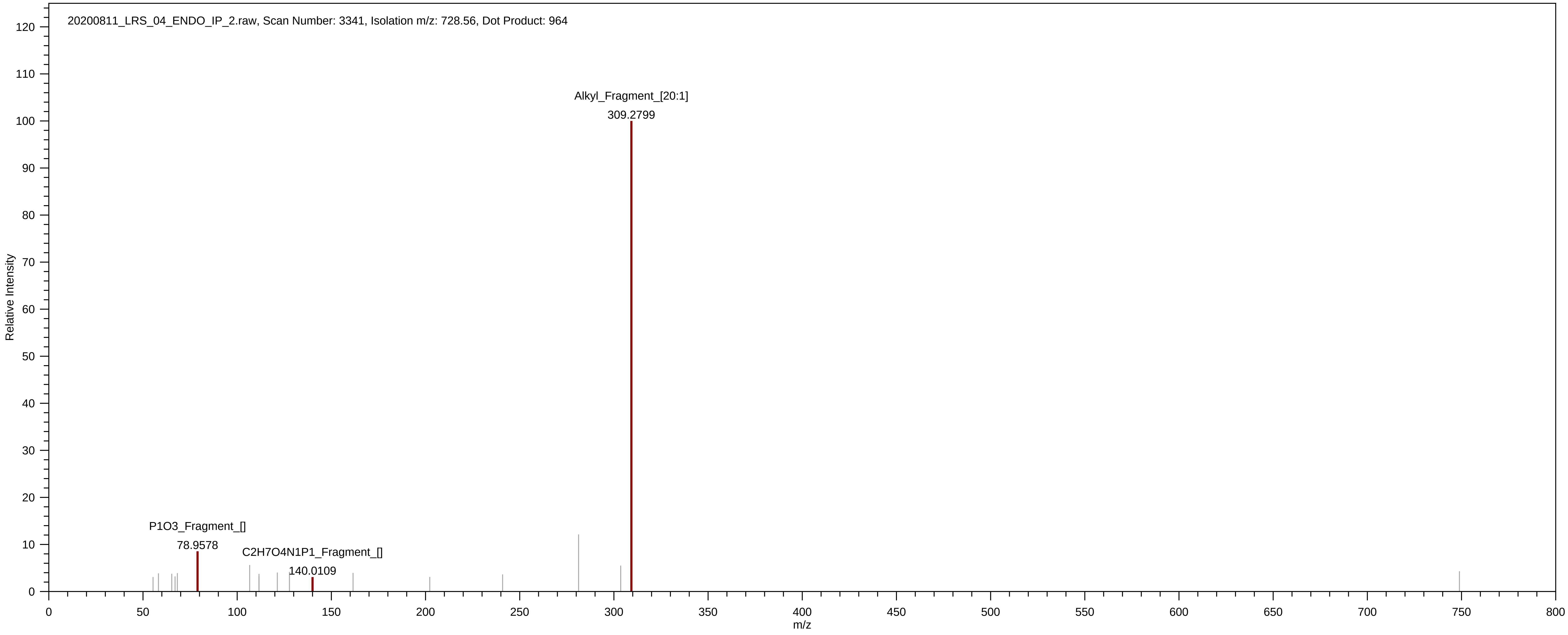

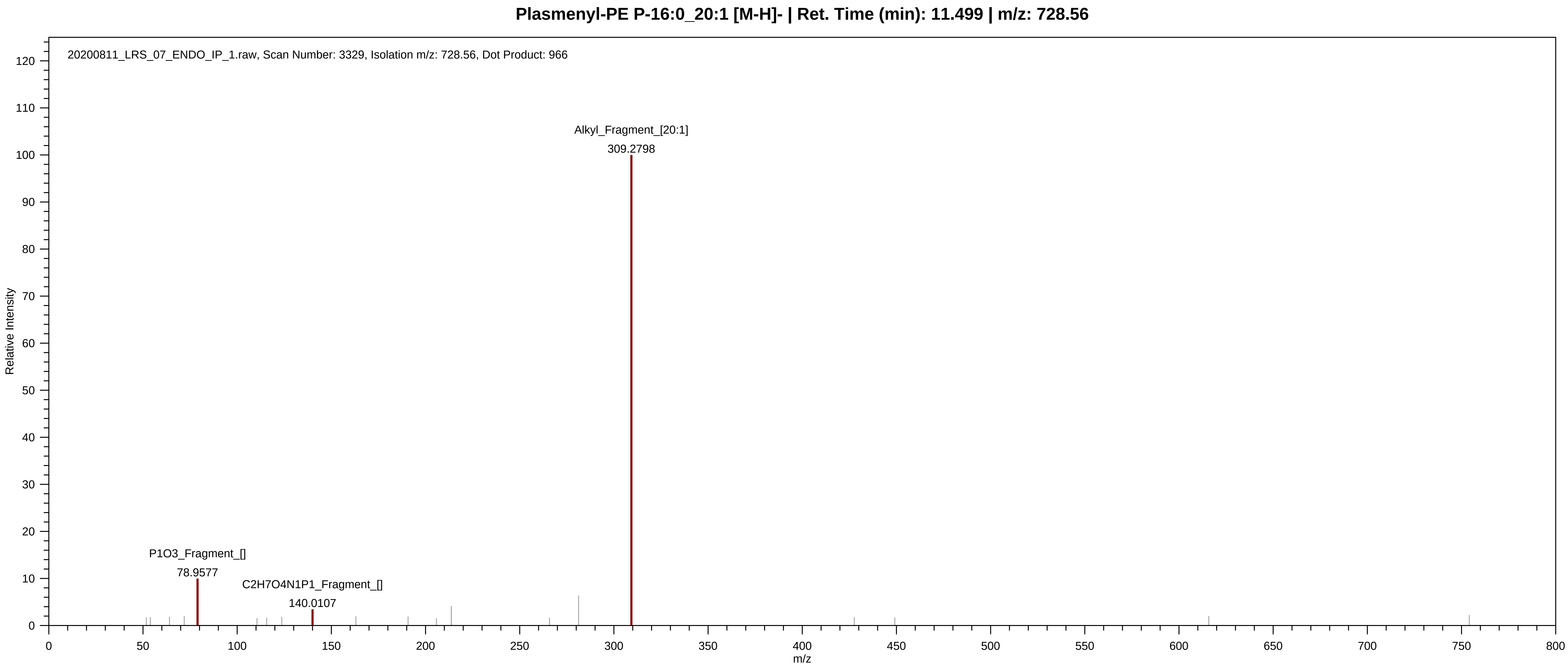

Plasmenyl-PE P-16:0_20:1 [M-H]- | Ret. Time (min): 11.499 | m/z: 728.56
20200811_LRS_07_ENDO_IP_1.raw, Scan Number: 3329, Isolation m/z: 728.56, Dot Product: 966
Alkyl_Fragment_[20:1]
309.2798
P1O3_Fragment_[]
78.9577
C2H7O4N1P1_Fragment_[]
140.0107
Relative Intensity
m/z

Plasmenyl-PE P-16:0_20:1 [M-H]- | Ret. Time (min): 11.51 | m/z: 728.561

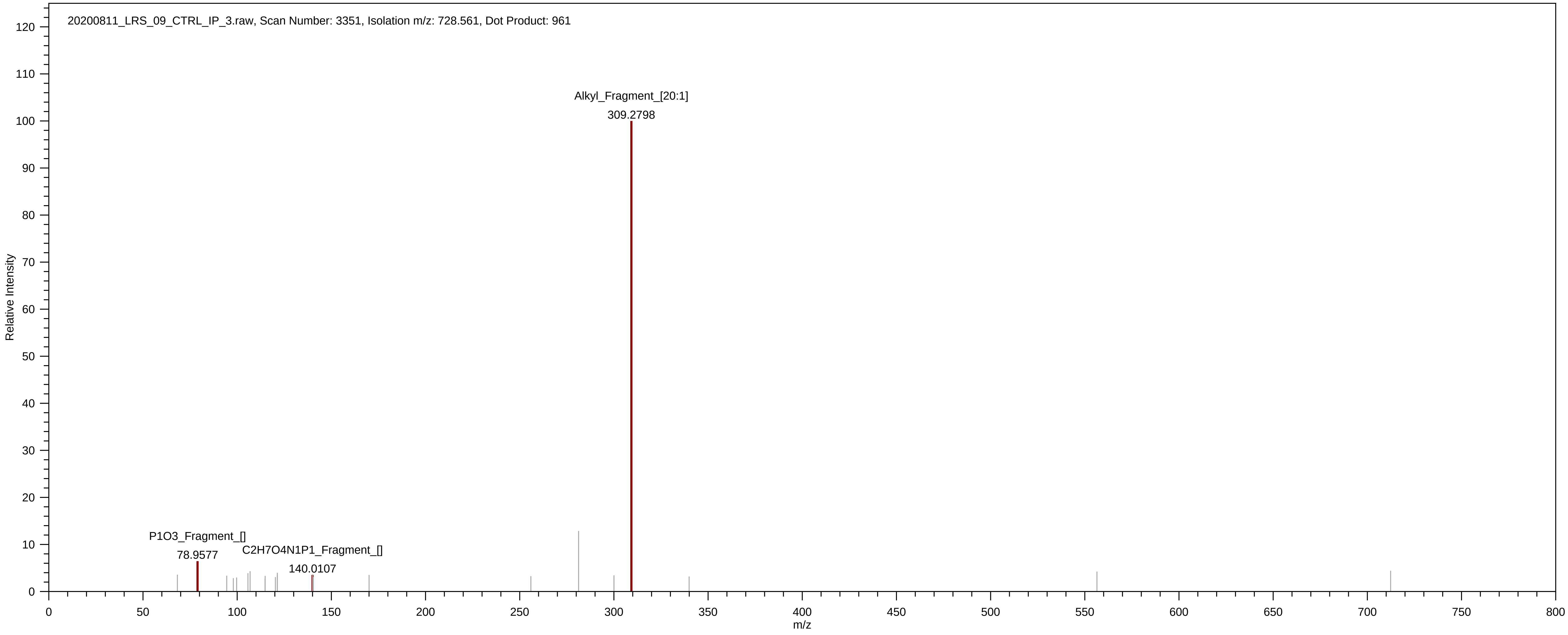

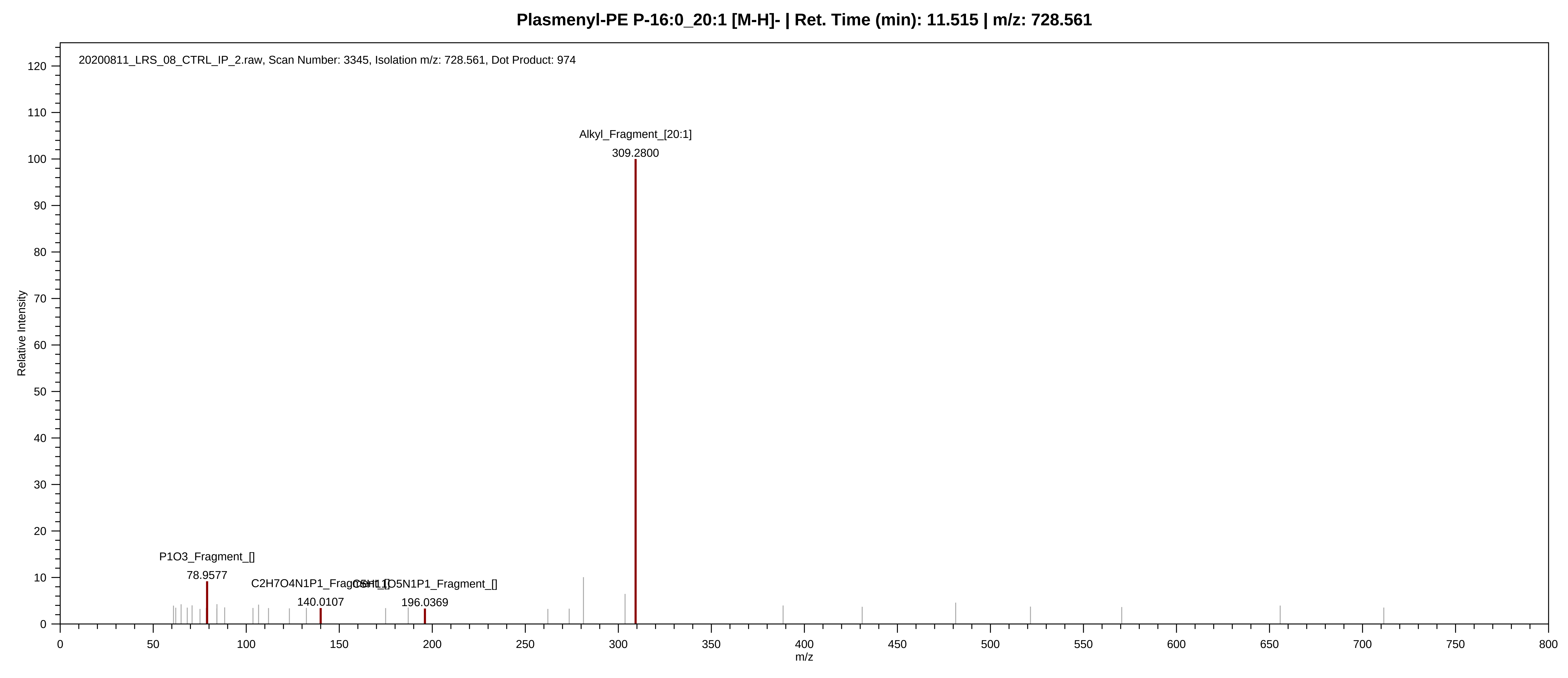

Plasmenyl-PE P-16:0_20:1 [M-H]- | Ret. Time (min): 11.515 | m/z: 728.561
20200811_LRS_08_CTRL_IP_2.raw, Scan Number: 3345, Isolation m/z: 728.561, Dot Product: 974
Alkyl_Fragment_[20:1]
309.2800
P1O3_Fragment_[]
78.9577
C2H7O4N1P1_Fragment_[]
140.0107
C5H11O5N1P1_Fragment_[]
196.0369
Relative Intensity
m/z

Plasmenyl-PE P-16:0_20:4 [M-H]- | Ret. Time (min): 9.487 | m/z: 722.513

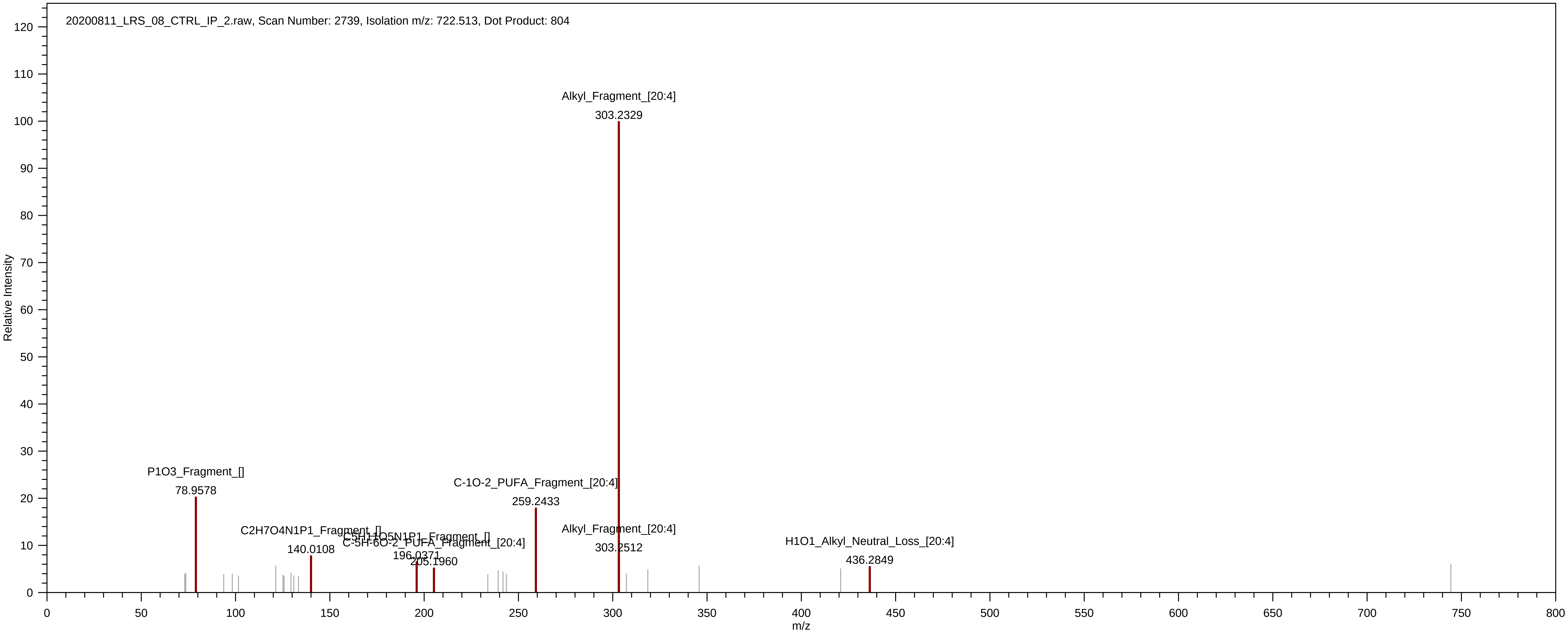

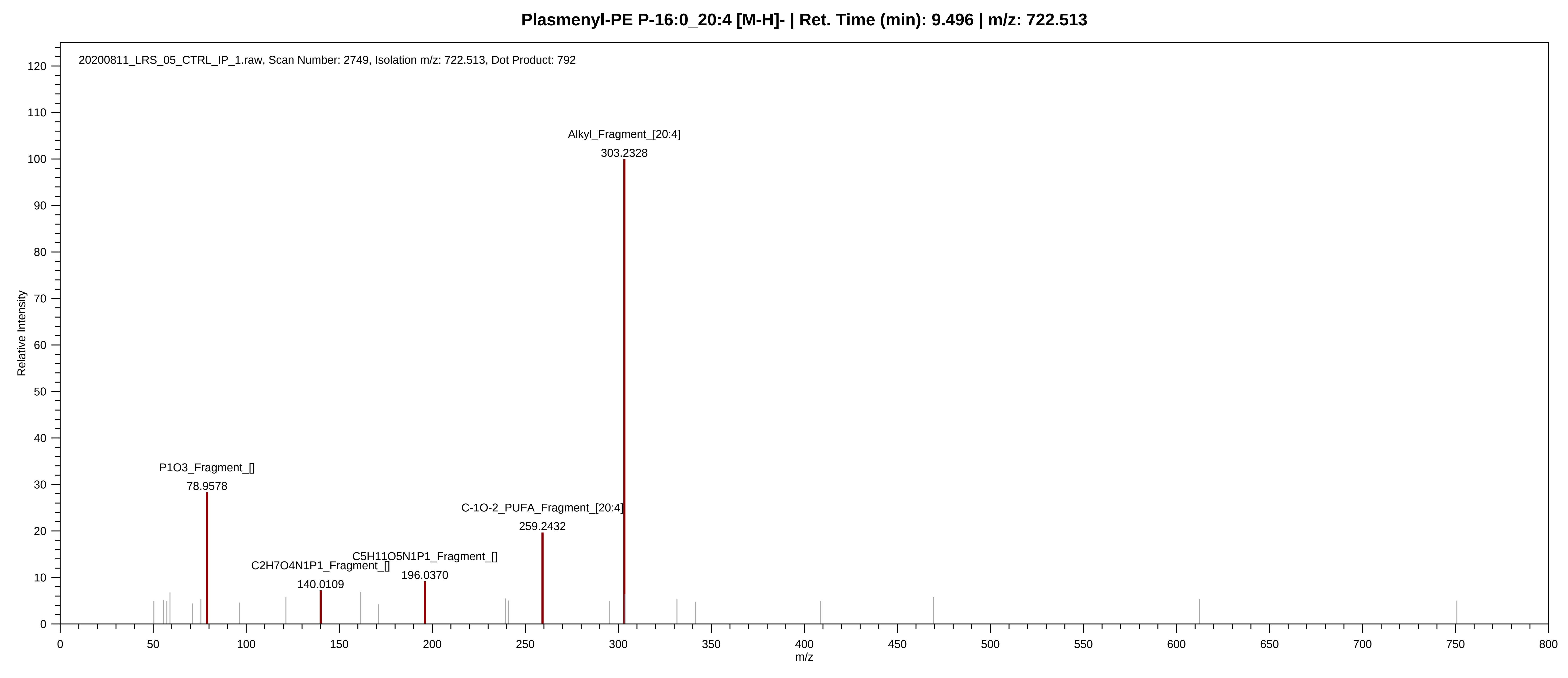
Plasmenyl-PE P-16:0_20:4 [M-H]- | Ret. Time (min): 9.496 | m/z: 722.513
20200811_LRS_05_CTRL_IP_1.raw, Scan Number: 2749, Isolation m/z: 722.513, Dot Product: 792
Alkyl_Fragment_[20:4]
303.2328
P1O3_Fragment_[]
78.9578
C-1O-2_PUFA_Fragment_[20:4]
259.2432
C5H11O5N1P1_Fragment_[]
196.0370
C2H7O4N1P1_Fragment_[]
140.0109
Relative Intensity
m/z

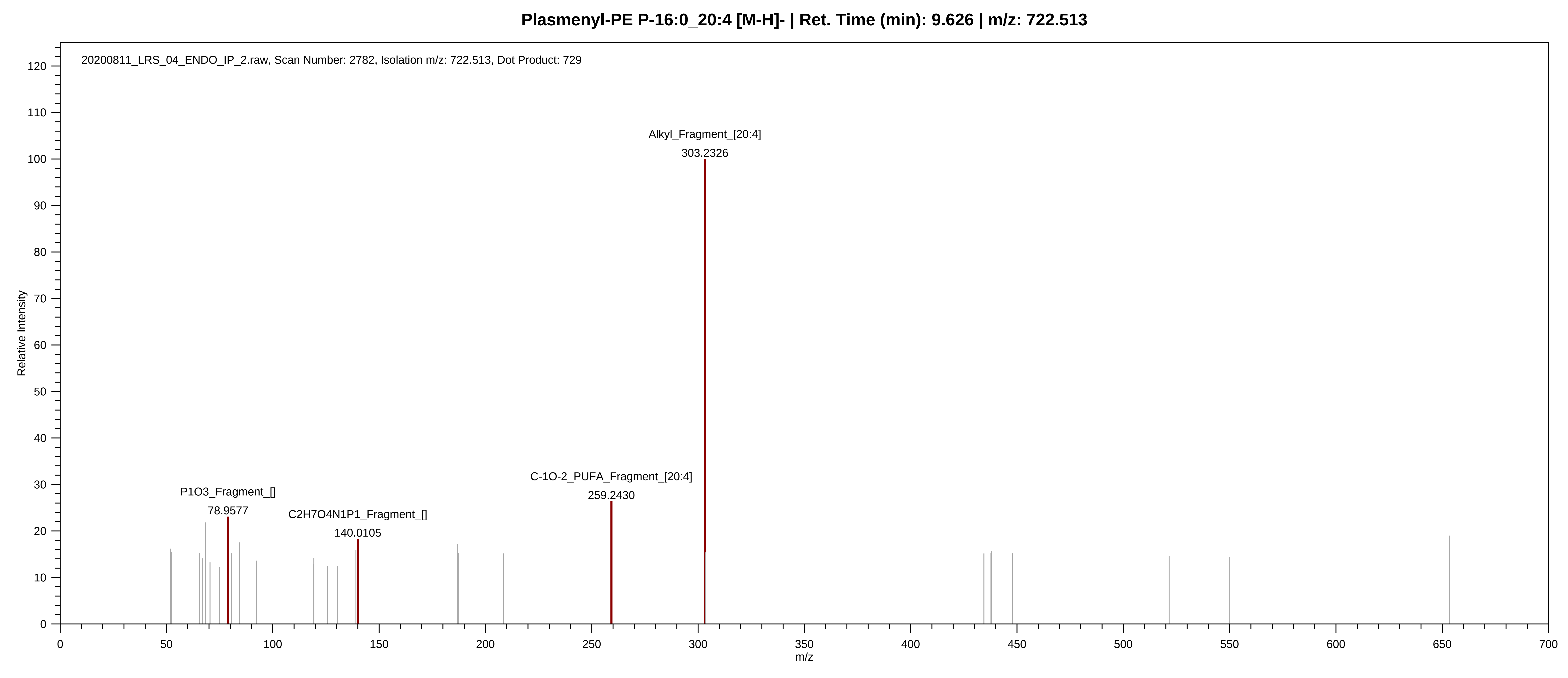

Plasmenyl-PE P-16:0_20:4 [M-H]- | Ret. Time (min): 9.626 | m/z: 722.513
20200811_LRS_04_ENDO_IP_2.raw, Scan Number: 2782, Isolation m/z: 722.513, Dot Product: 729
Alkyl_Fragment_[20:4]
303.2326
C-1O-2_PUFA_Fragment_[20:4]
259.2430
P1O3_Fragment_[]
78.9577
C2H7O4N1P1_Fragment_[]
140.0105
Relative Intensity
m/z

Plasmenyl-PE P-16:0_20:4 [M-H]- | Ret. Time (min): 9.631 | m/z: 722.513

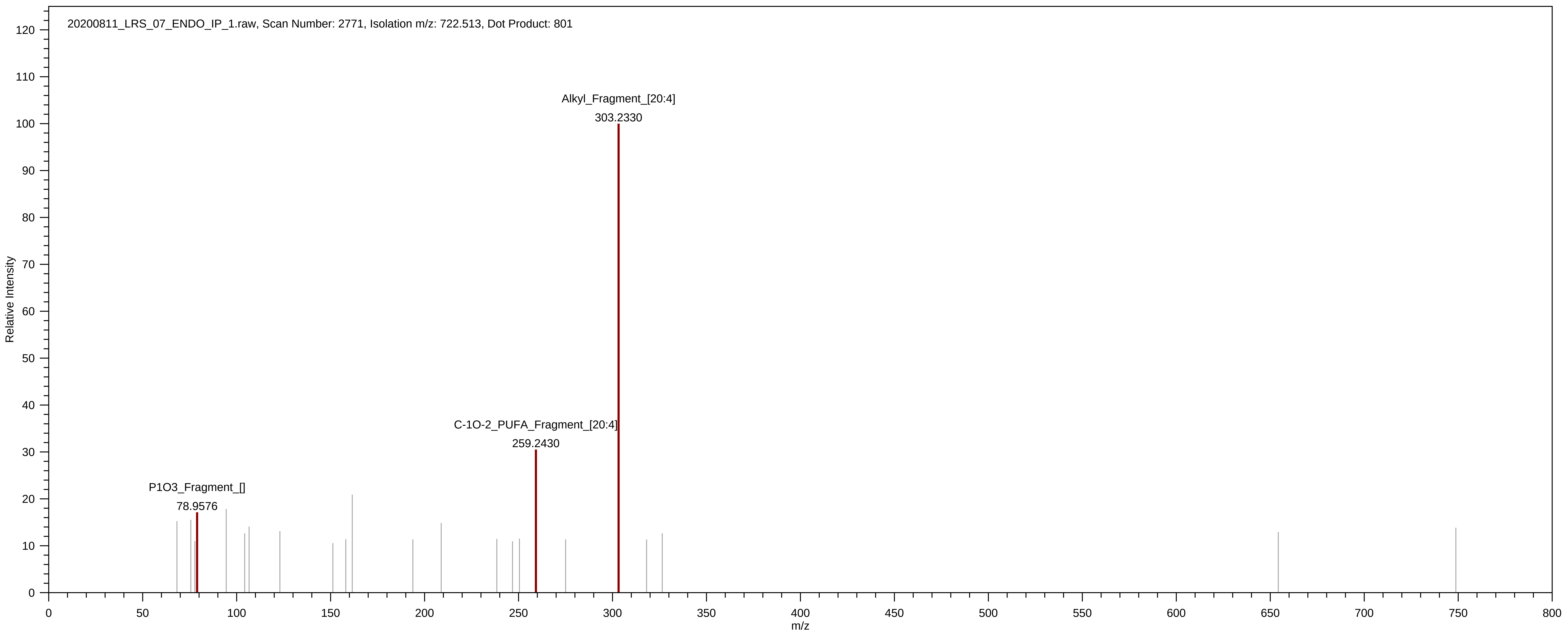

Plasmenyl-PE P-16:0_20:4 [M-H]- | Ret. Time (min): 9.479 | m/z: 722.514

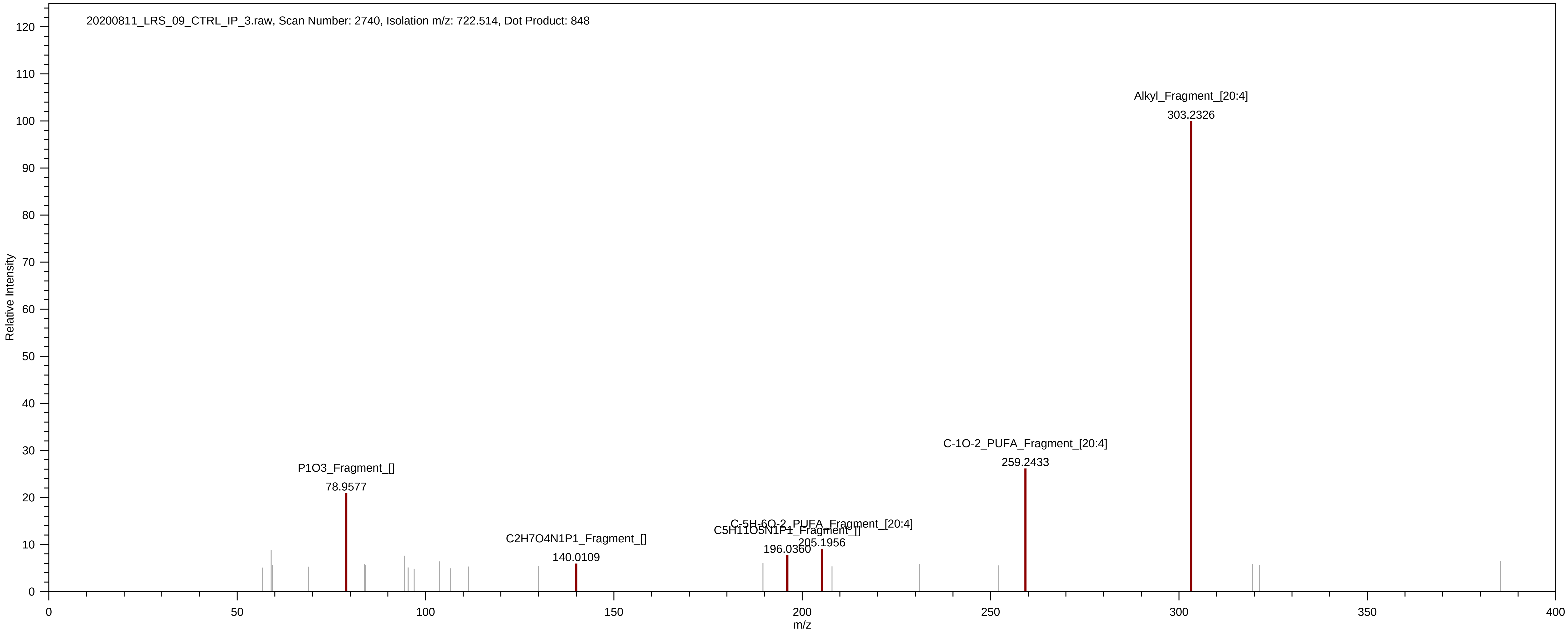

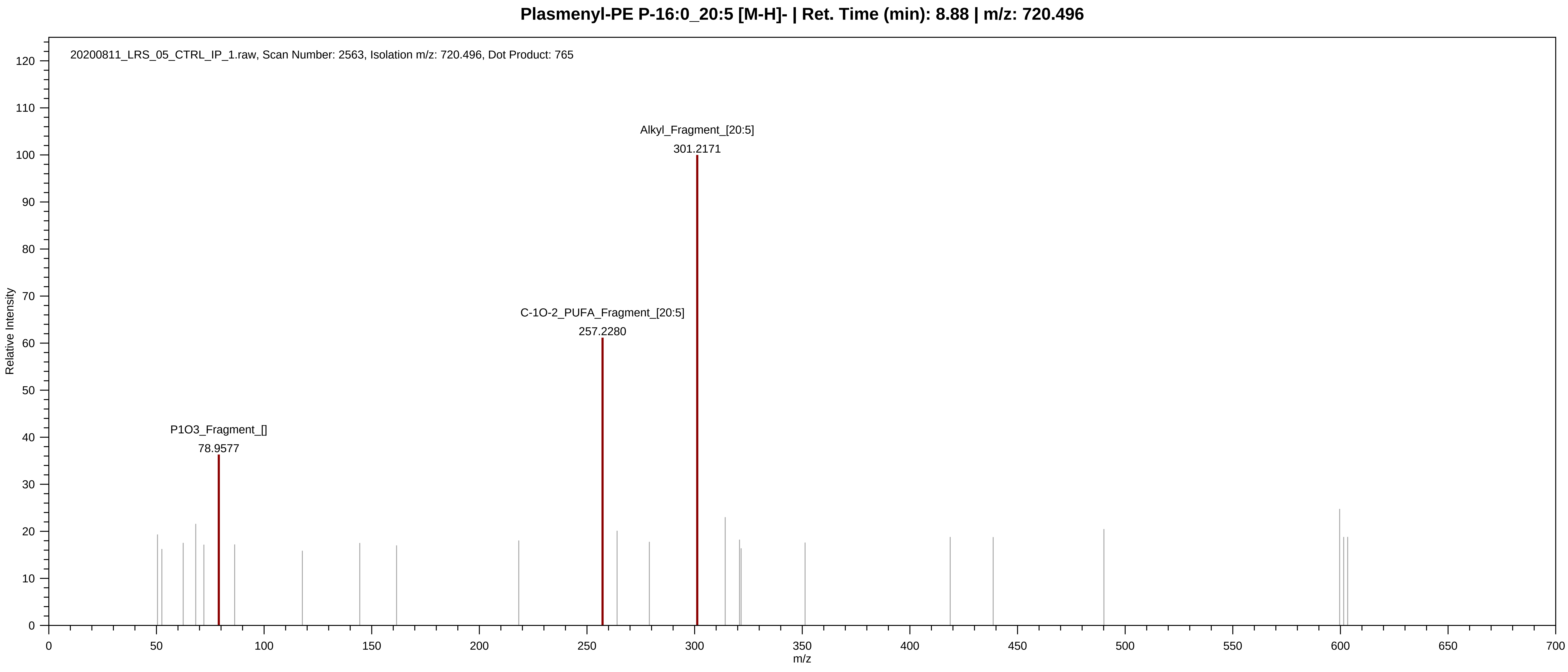
Plasmenyl-PE P-16:0_20:5 [M-H]- | Ret. Time (min): 8.88 | m/z: 720.496
20200811_LRS_05_CTRL_IP_1.raw, Scan Number: 2563, Isolation m/z: 720.496, Dot Product: 765
Alkyl_Fragment_[20:5]
301.2171
C-1O-2_PUFA_Fragment_[20:5]
257.2280
P1O3_Fragment_[]
78.9577
Relative Intensity
m/z

Plasmenyl-PE P-16:0_20:5 [M-H]- | Ret. Time (min): 8.871 | m/z: 720.497

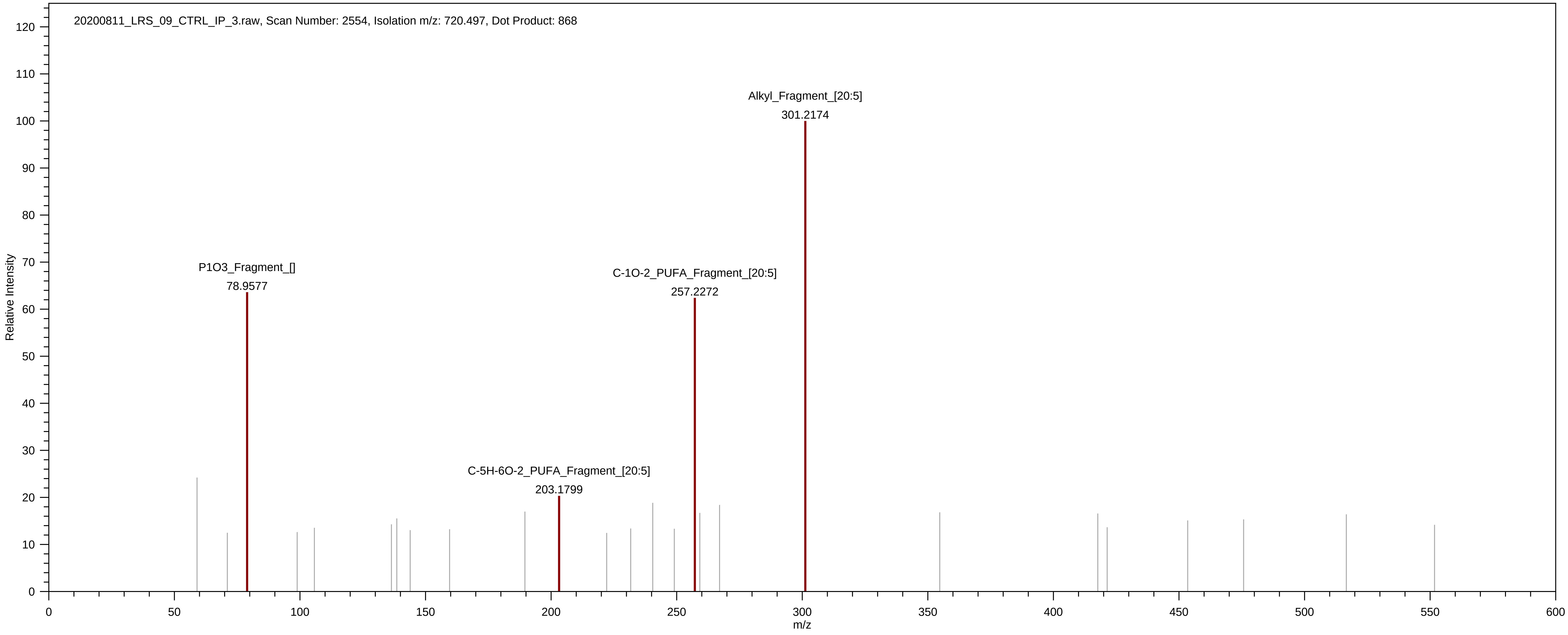

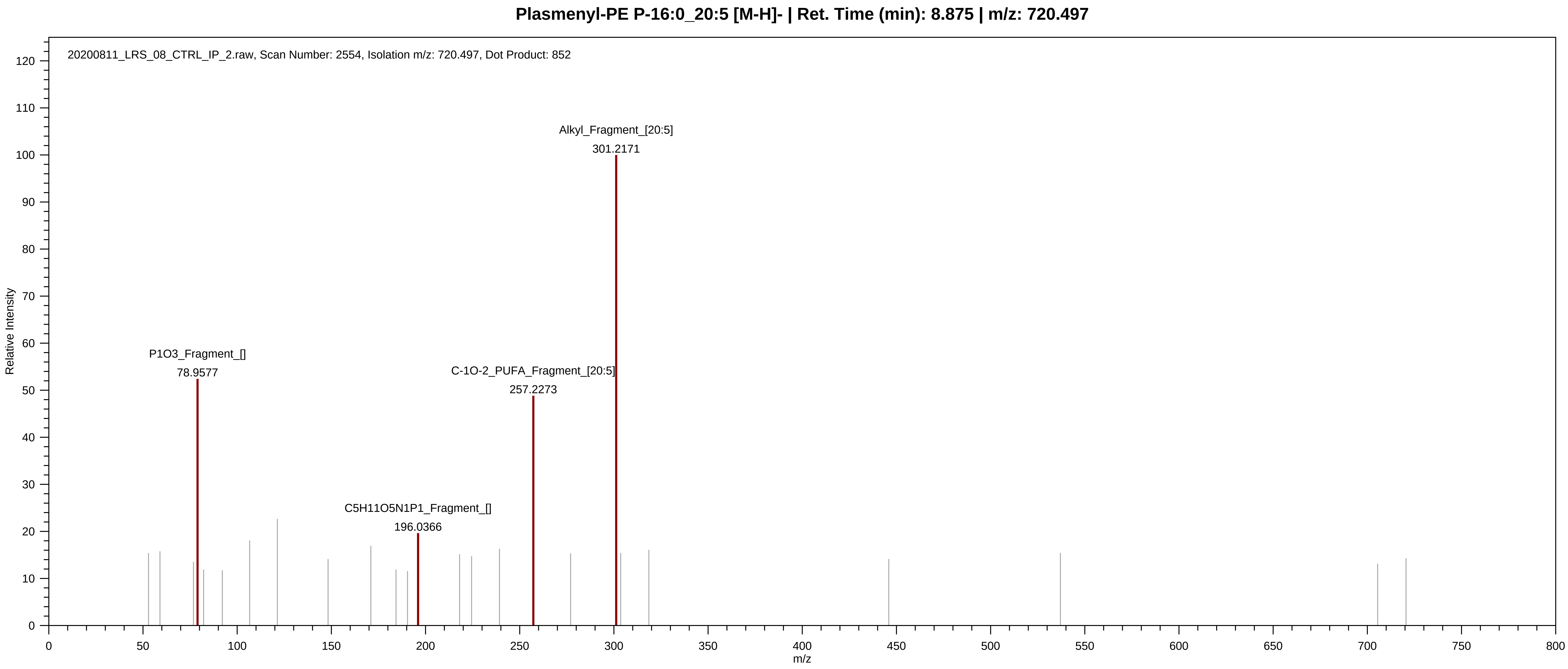

Plasmenyl-PE P-16:0_20:5 [M-H]- | Ret. Time (min): 8.875 | m/z: 720.497
20200811_LRS_08_CTRL_IP_2.raw, Scan Number: 2554, Isolation m/z: 720.497, Dot Product: 852
Alkyl_Fragment_[20:5]
301.2171
P1O3_Fragment_[]
78.9577
C-1O-2_PUFA_Fragment_[20:5]
257.2273
C5H11O5N1P1_Fragment_[]
196.0366
Relative Intensity
m/z

Plasmenyl-PE P-16:0_20:5 [M-H]- | Ret. Time (min): 8.907 | m/z: 720.497

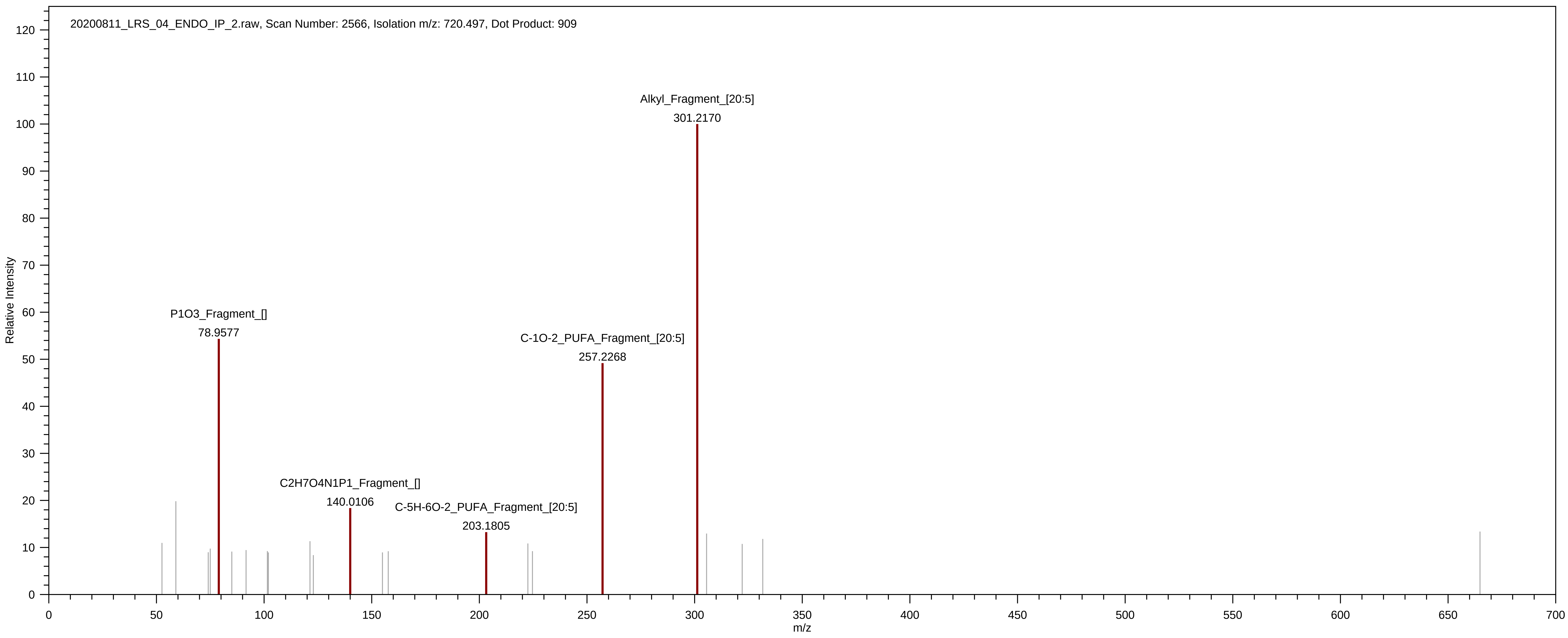

Plasmenyl-PE P-16:0_20:5 [M-H]- | Ret. Time (min): 8.896 | m/z: 720.498

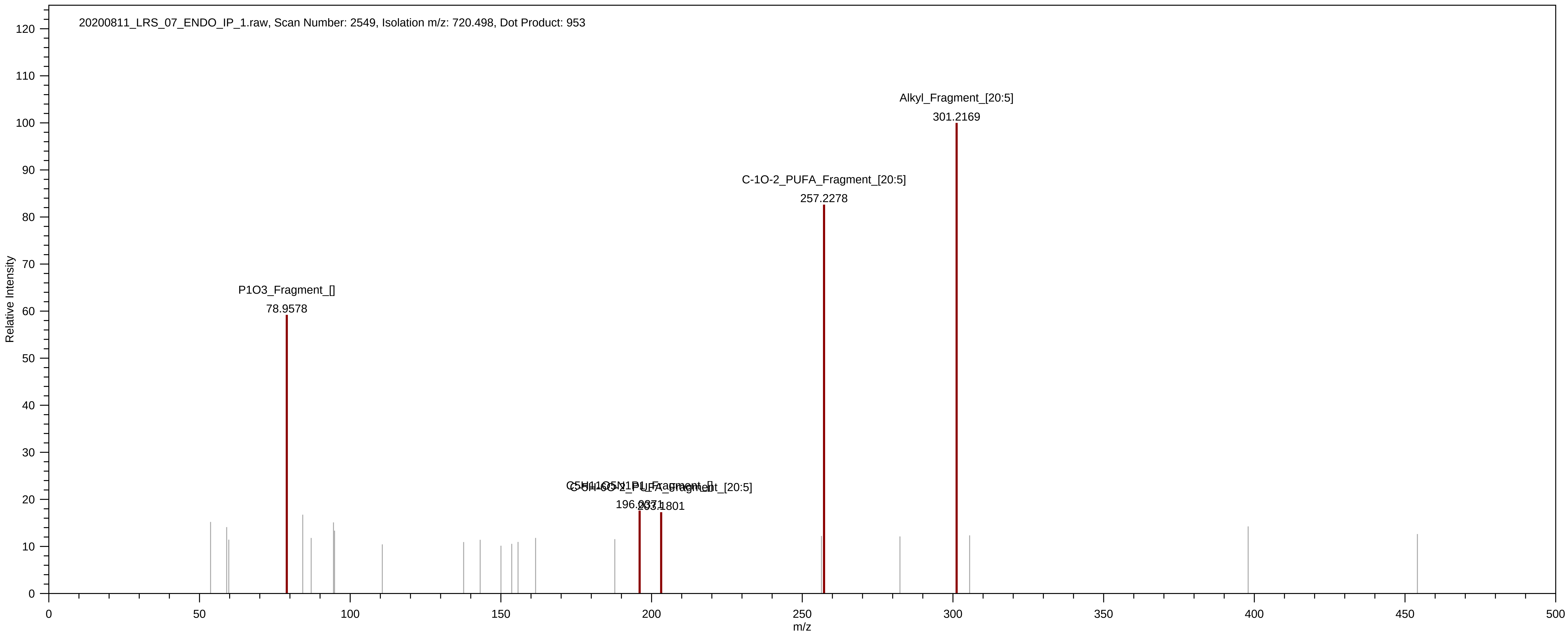

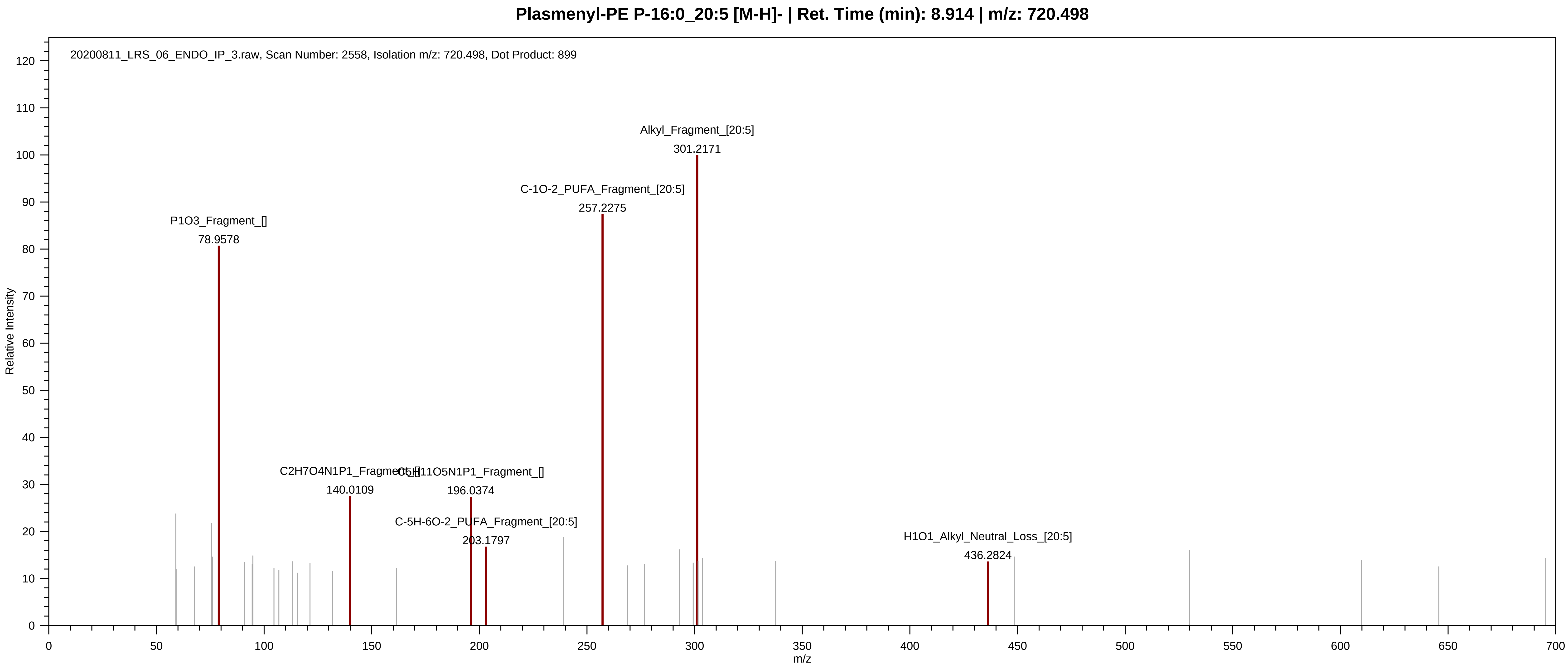

Plasmenyl-PE P-16:0_20:5 [M-H]- | Ret. Time (min): 8.914 | m/z: 720.498
20200811_LRS_06_ENDO_IP_3.raw, Scan Number: 2558, Isolation m/z: 720.498, Dot Product: 899
Alkyl_Fragment_[20:5]
301.2171
C-1O-2_PUFA_Fragment_[20:5]
257.2275
P1O3_Fragment_[]
78.9578
C2H7O4N1P1_Fragment_[]
140.0109
C5H11O5N1P1_Fragment_[]
196.0374
C-5H-6O-2_PUFA_Fragment_[20:5]
203.1797
H1O1_Alkyl_Neutral_Loss_[20:5]
436.2824
Relative Intensity
m/z

Plasmenyl-PE P-16:0_22:5 [M-H]- | Ret. Time (min): 9.506 | m/z: 748.529

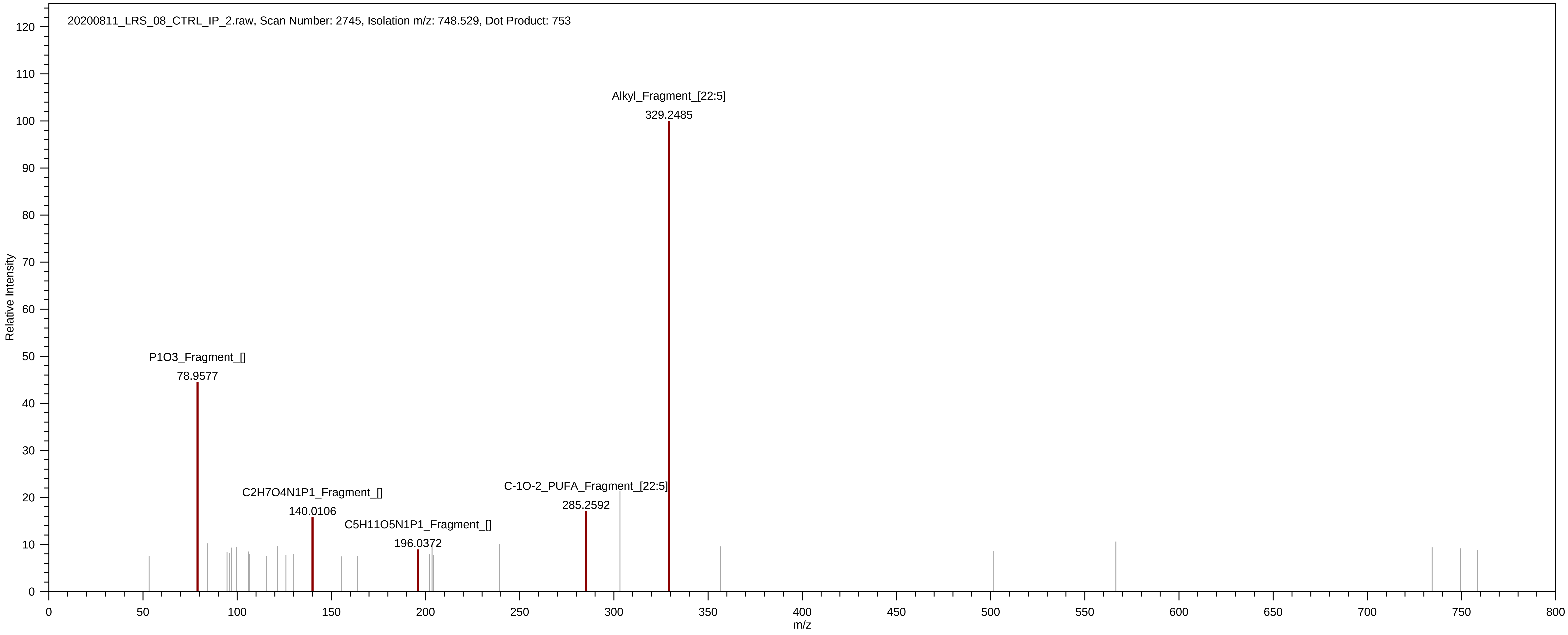

Plasmenyl-PE P-16:0_22:6 [M-H]- | Ret. Time (min): 9.2 | m/z: 746.513

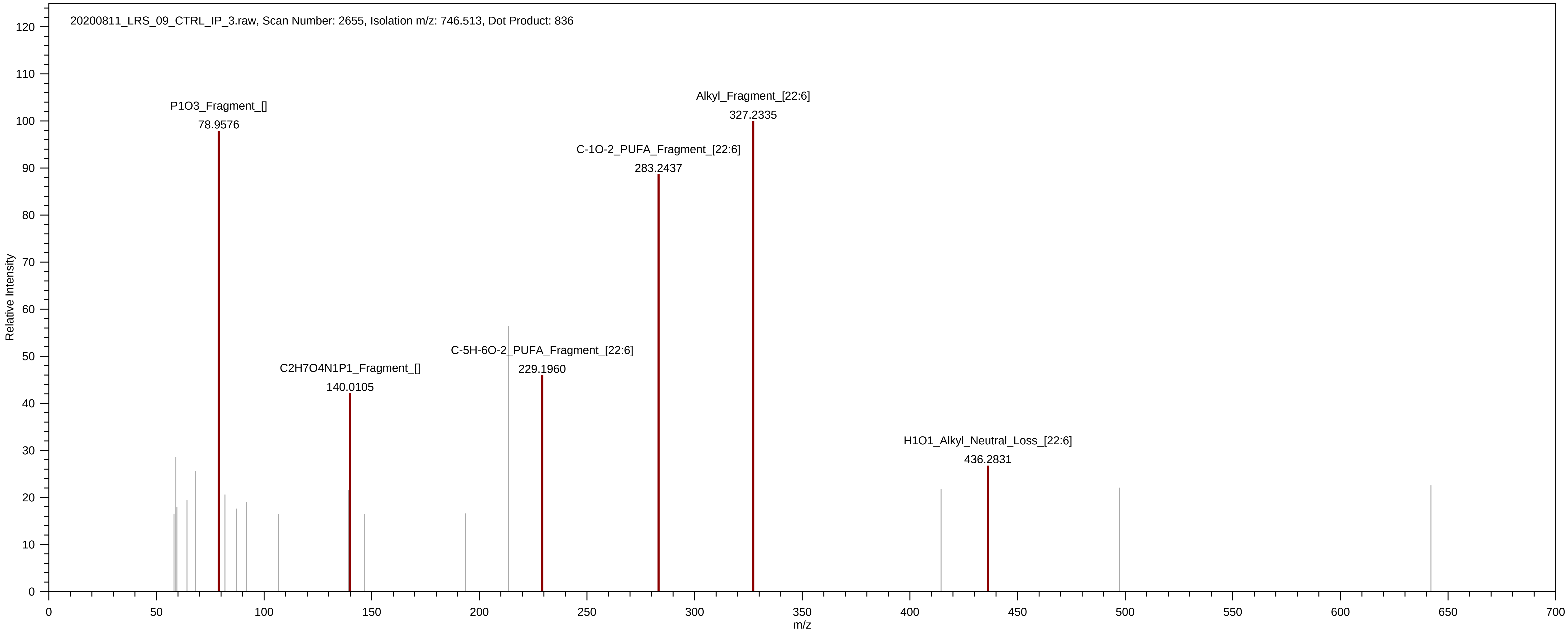

Plasmenyl-PE P-16:0_22:6 [M-H]- | Ret. Time (min): 9.197 | m/z: 746.513

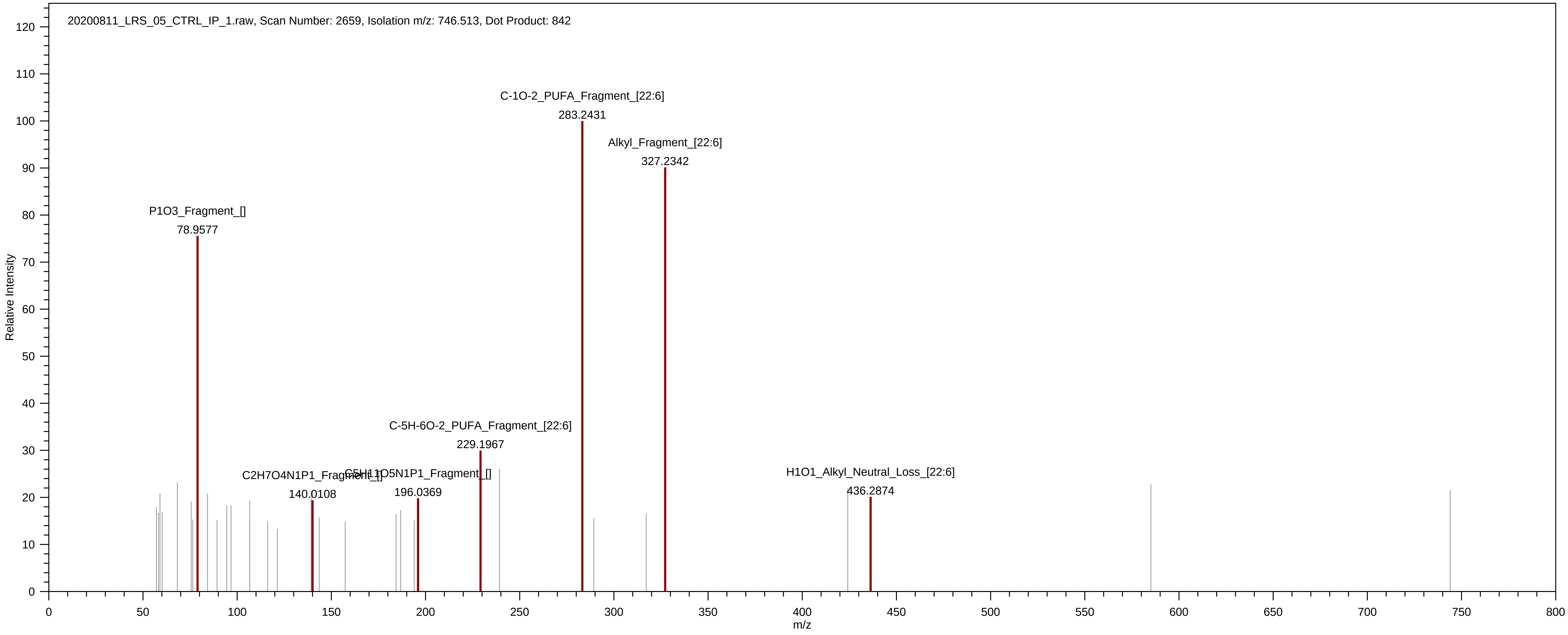

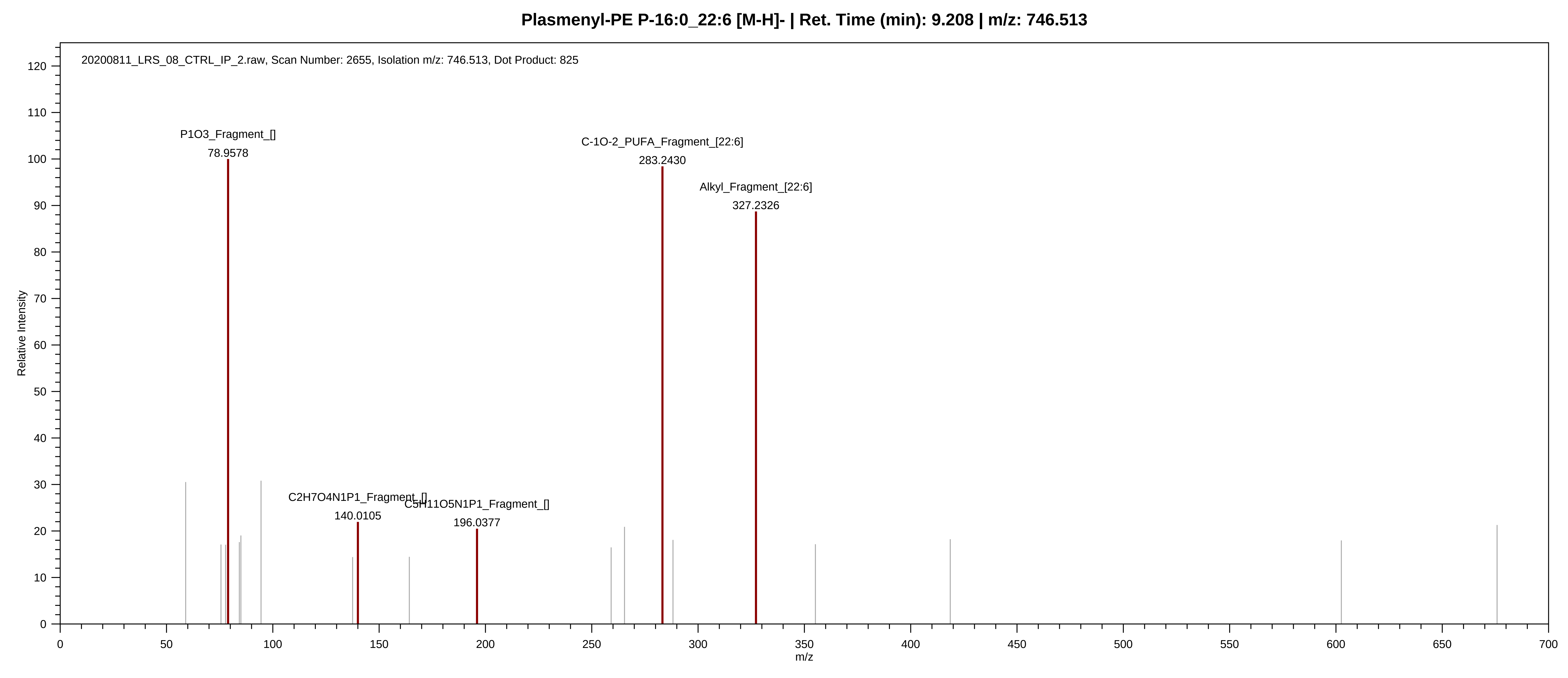

Plasmenyl-PE P-16:0_22:6 [M-H]- | Ret. Time (min): 9.208 | m/z: 746.513
20200811_LRS_08_CTRL_IP_2.raw, Scan Number: 2655, Isolation m/z: 746.513, Dot Product: 825
P1O3_Fragment_[]
78.9578
C-1O-2_PUFA_Fragment_[22:6]
283.2430
Alkyl_Fragment_[22:6]
327.2326
C2H7O4N1P1_Fragment_[]
140.0105
C5H11O5N1P1_Fragment_[]
196.0377
Relative Intensity
m/z

Plasmenyl-PE P-16:0_22:6 [M-H]- | Ret. Time (min): 9.243 | m/z: 746.513

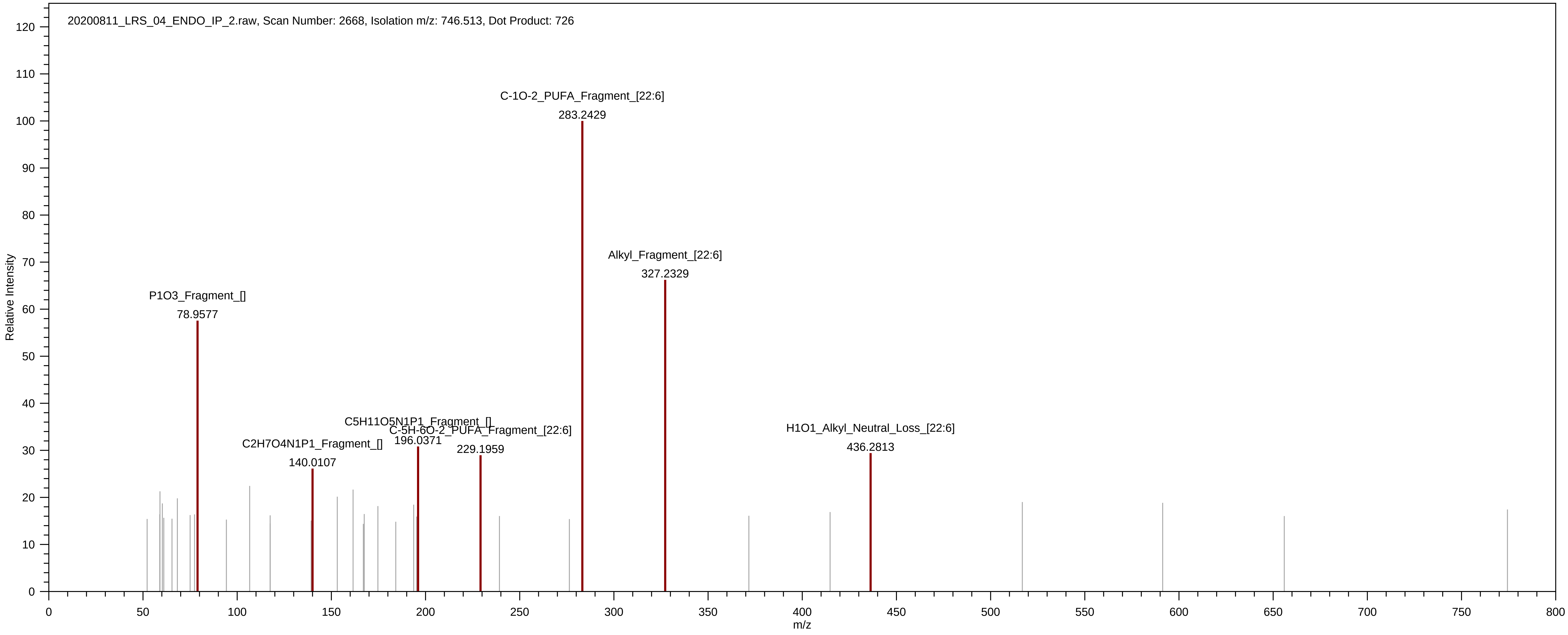

Plasmenyl-PE P-16:0_22:6 [M-H]- | Ret. Time (min): 9.248 | m/z: 746.513

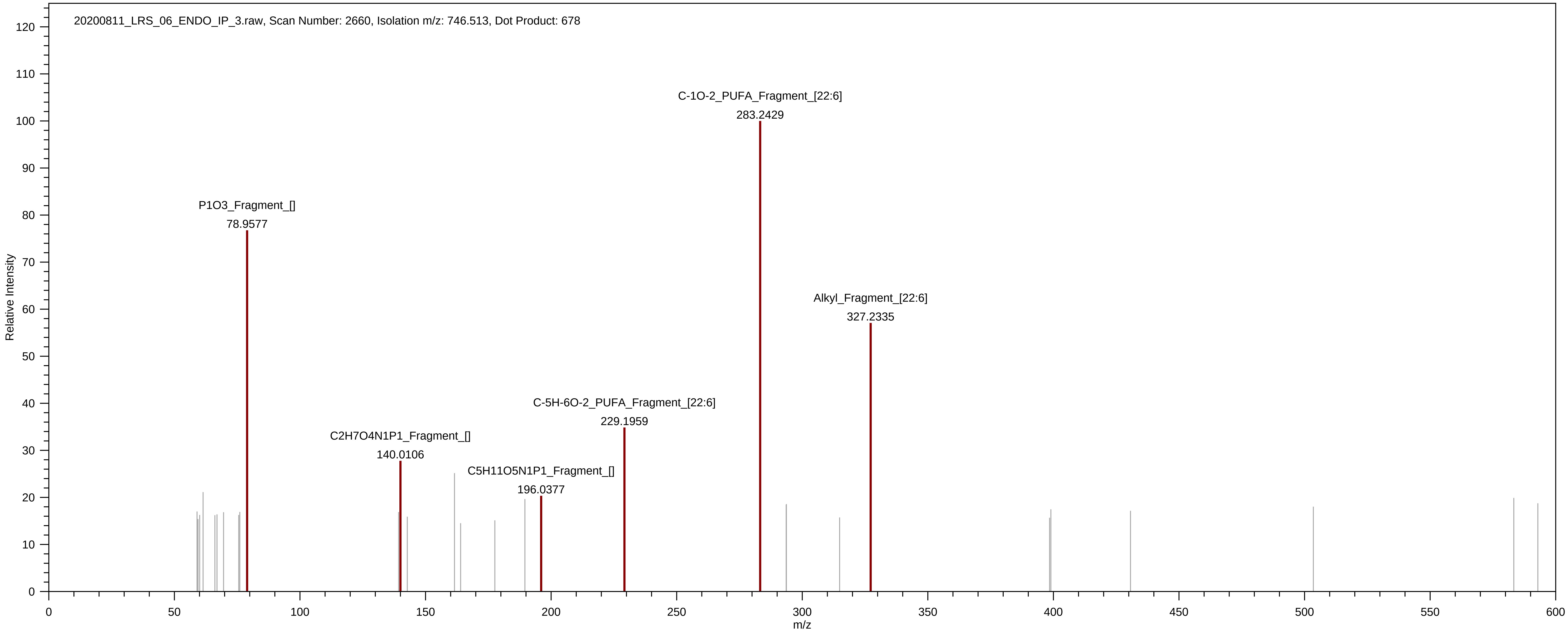

Plasmenyl-PE P-16:0_22:6 [M-H]- | Ret. Time (min): 9.201 | m/z: 746.513

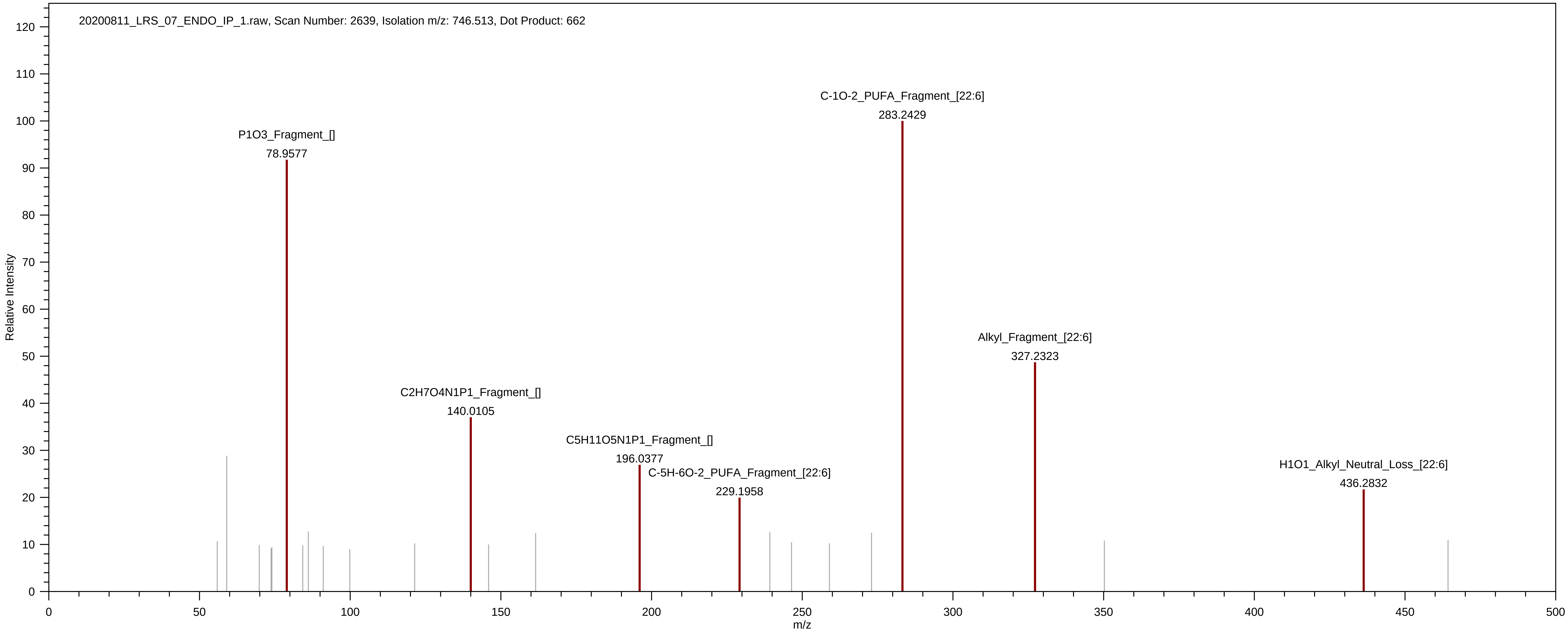

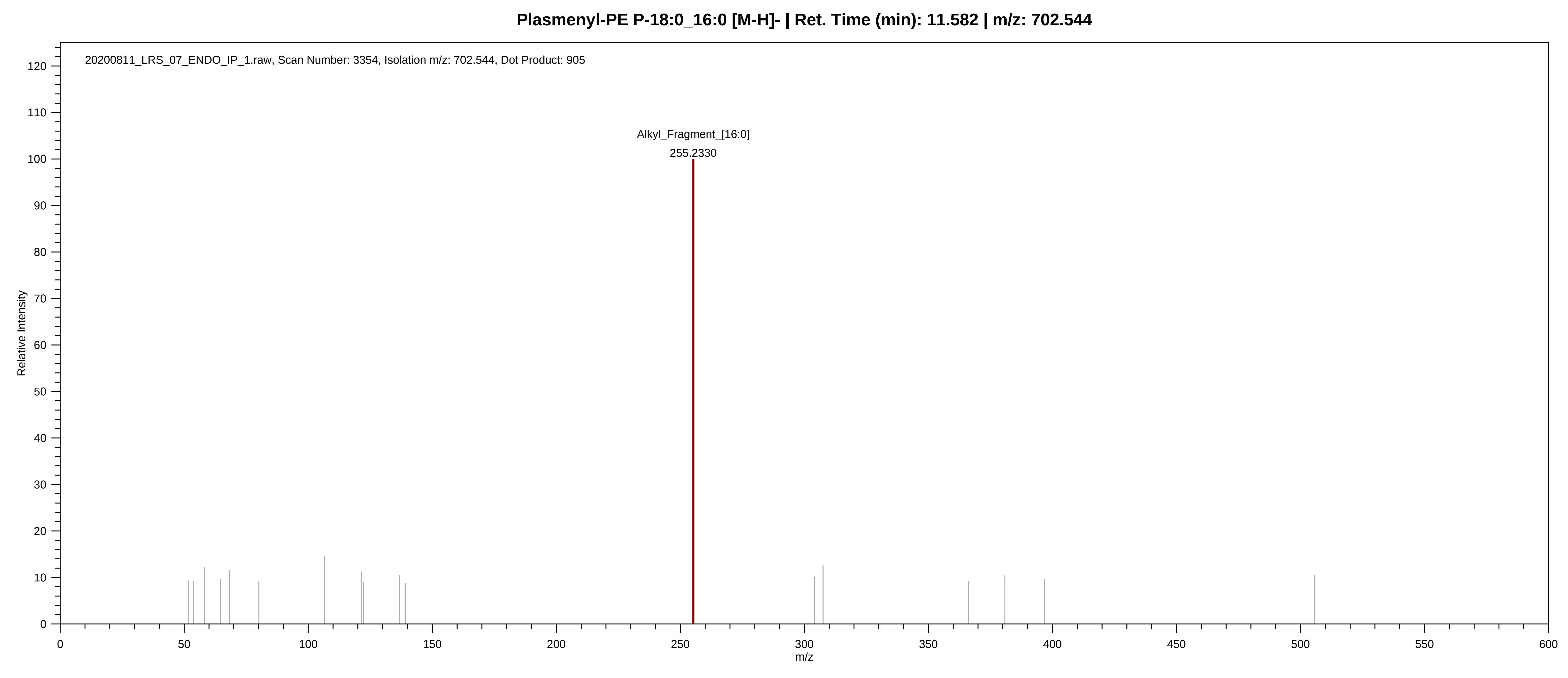

Plasmenyl-PE P-18:0_16:0 [M-H]- | Ret. Time (min): 11.582 | m/z: 702.544
20200811_LRS_07_ENDO_IP_1.raw, Scan Number: 3354, Isolation m/z: 702.544, Dot Product: 905
Alkyl_Fragment_[16:0]
255.2330
Relative Intensity
m/z

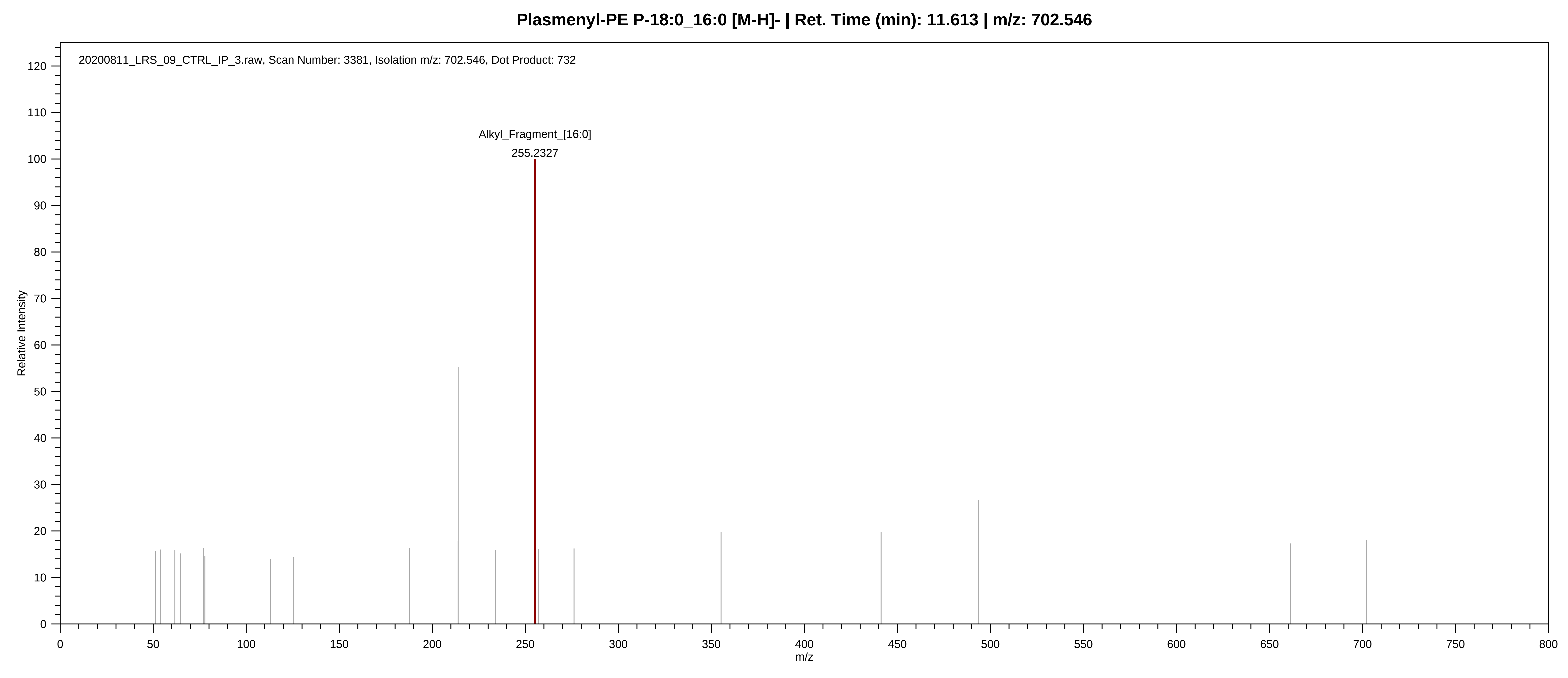

Plasmenyl-PE P-18:0_16:0 [M-H]- | Ret. Time (min): 11.613 | m/z: 702.546
20200811_LRS_09_CTRL_IP_3.raw, Scan Number: 3381, Isolation m/z: 702.546, Dot Product: 732
Alkyl_Fragment_[16:0]
255.2327
Relative Intensity
m/z

Plasmenyl-PE P-18:0_17:0 [M-H]- | Ret. Time (min): 11.881 | m/z: 716.559

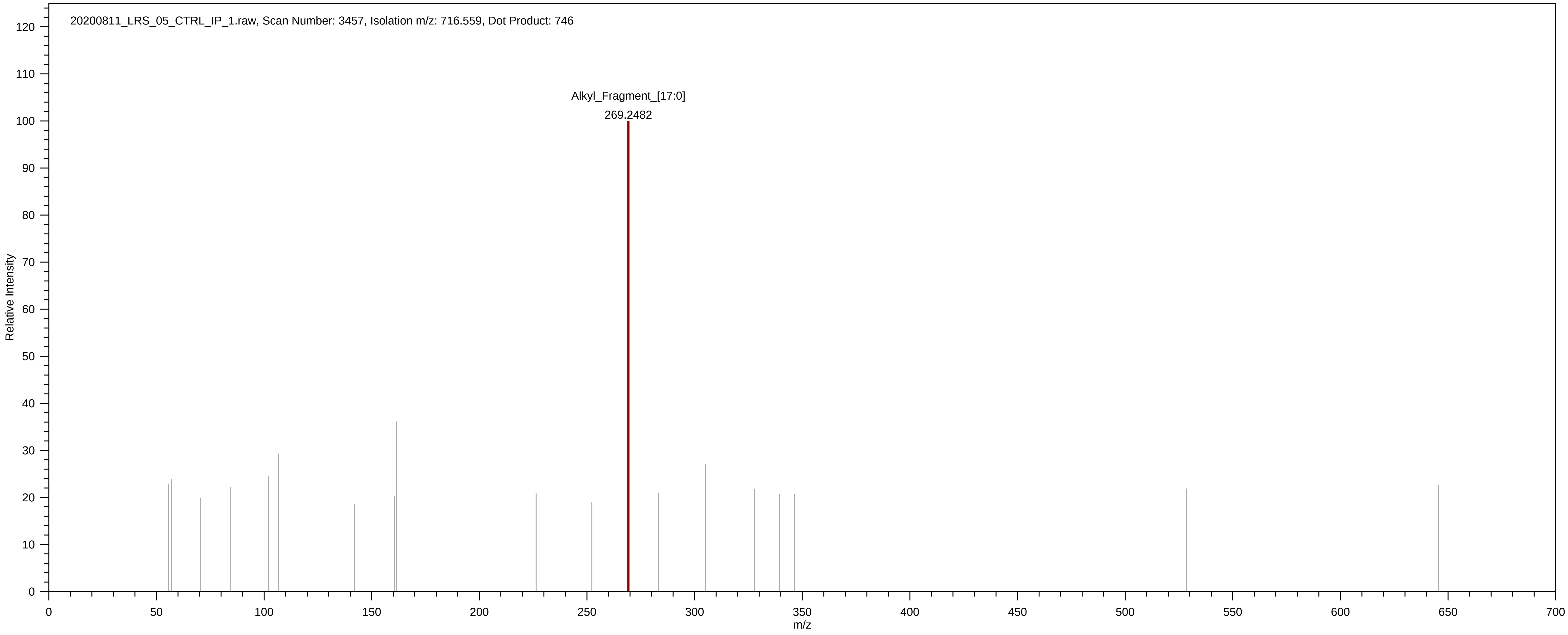

Plasmenyl-PE P-18:0_17:0 [M-H]- | Ret. Time (min): 11.86 | m/z: 716.562

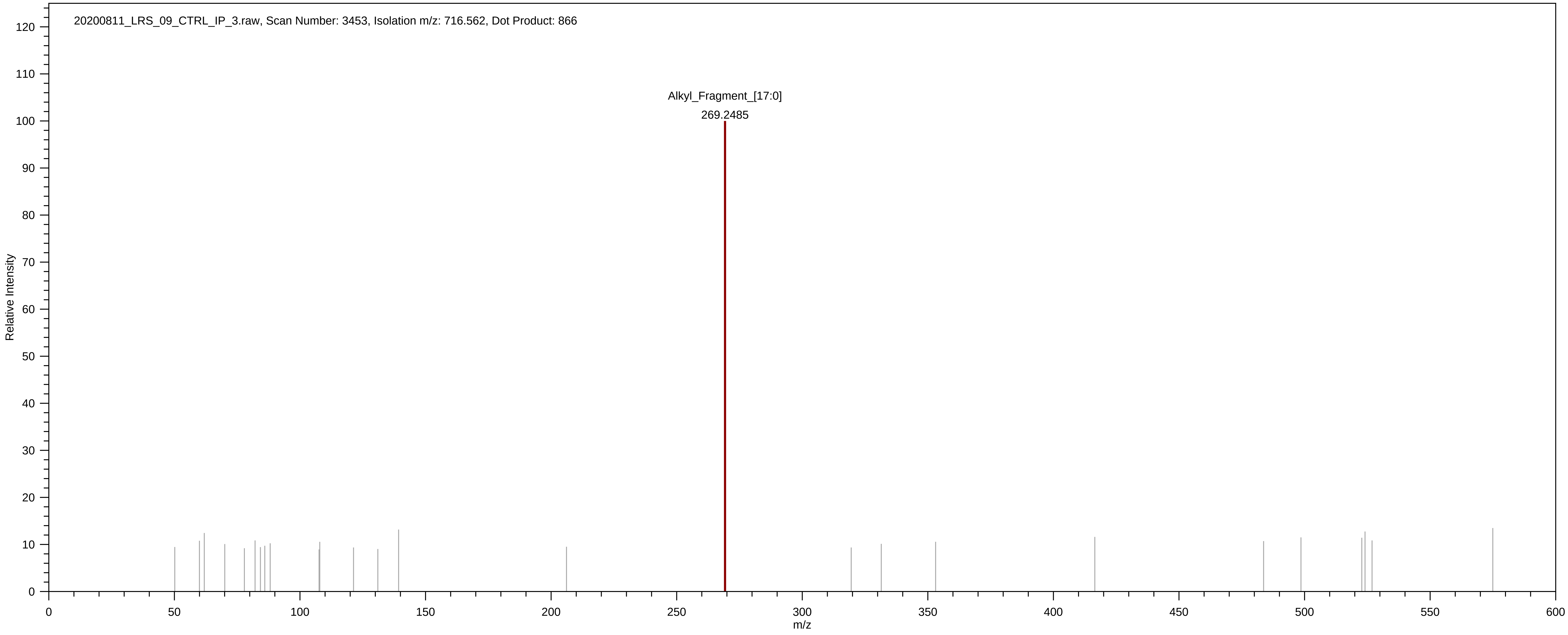

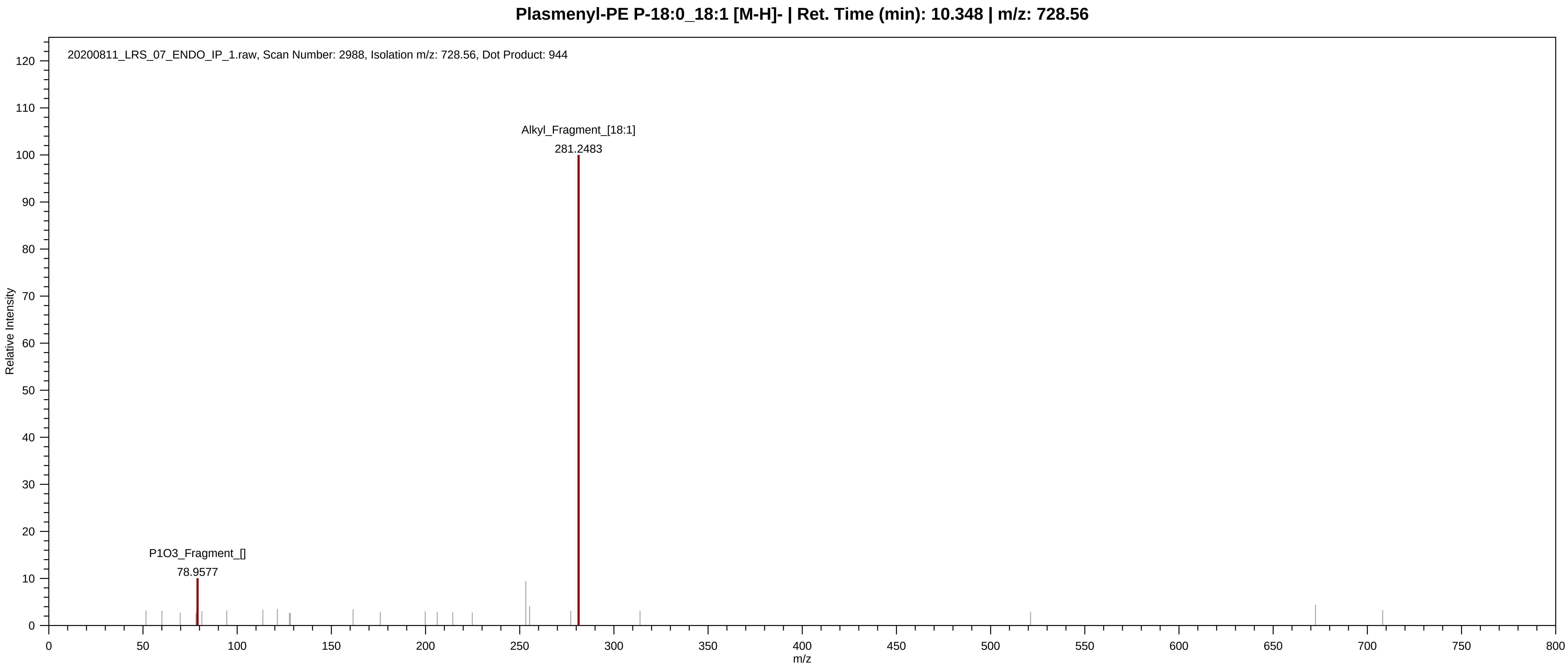
Plasmenyl-PE P-18:0_18:1 [M-H]- | Ret. Time (min): 10.348 | m/z: 728.56
20200811_LRS_07_ENDO_IP_1.raw, Scan Number: 2988, Isolation m/z: 728.56, Dot Product: 944
Alkyl_Fragment_[18:1]
281.2483
P1O3_Fragment_[]
78.9577
Relative Intensity
m/z
0
10
20
30
40
50
60
70
80
90
100
110
120
0
50
100
150
200
250
300
350
400
450
500
550
600
650
700
750
800

Plasmenyl-PE P-18:0_18:1 [M-H]- | Ret. Time (min): 11.7 | m/z: 728.56

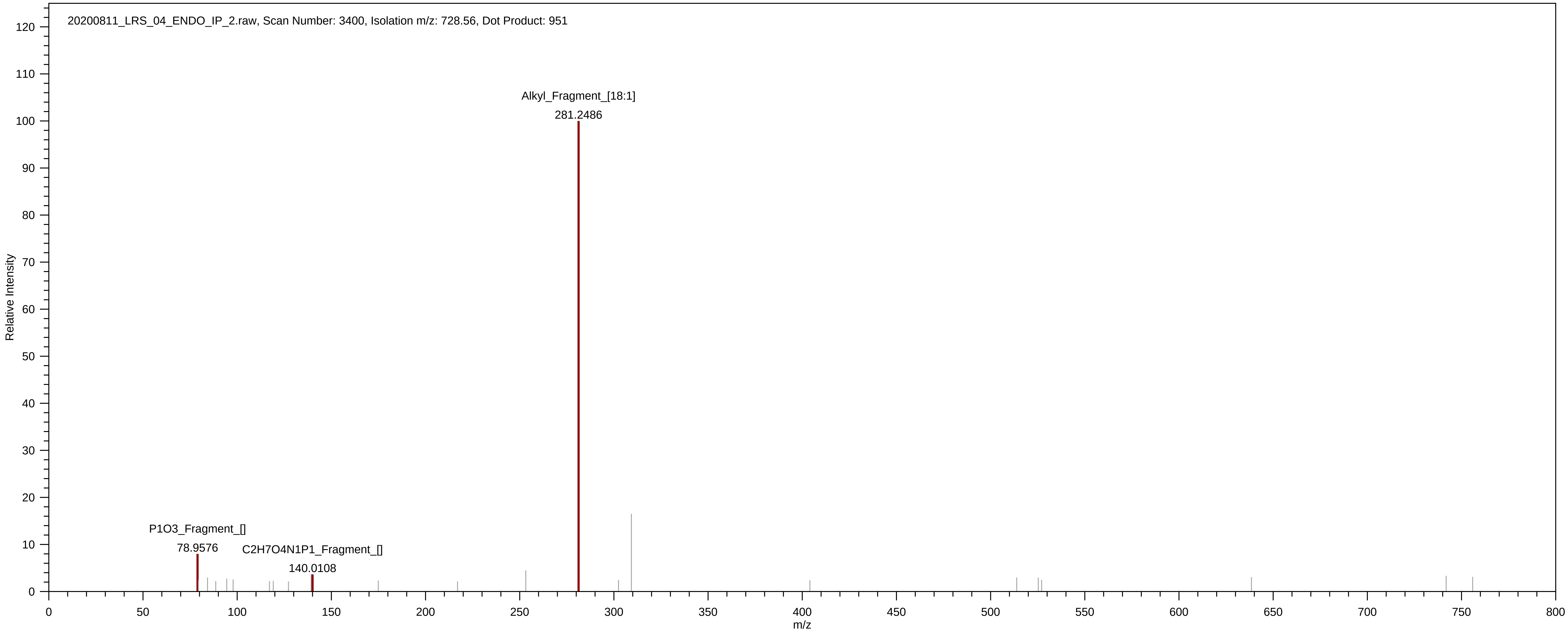

Plasmenyl-PE P-18:0_18:1 [M-H]- | Ret. Time (min): 11.7 | m/z: 728.561

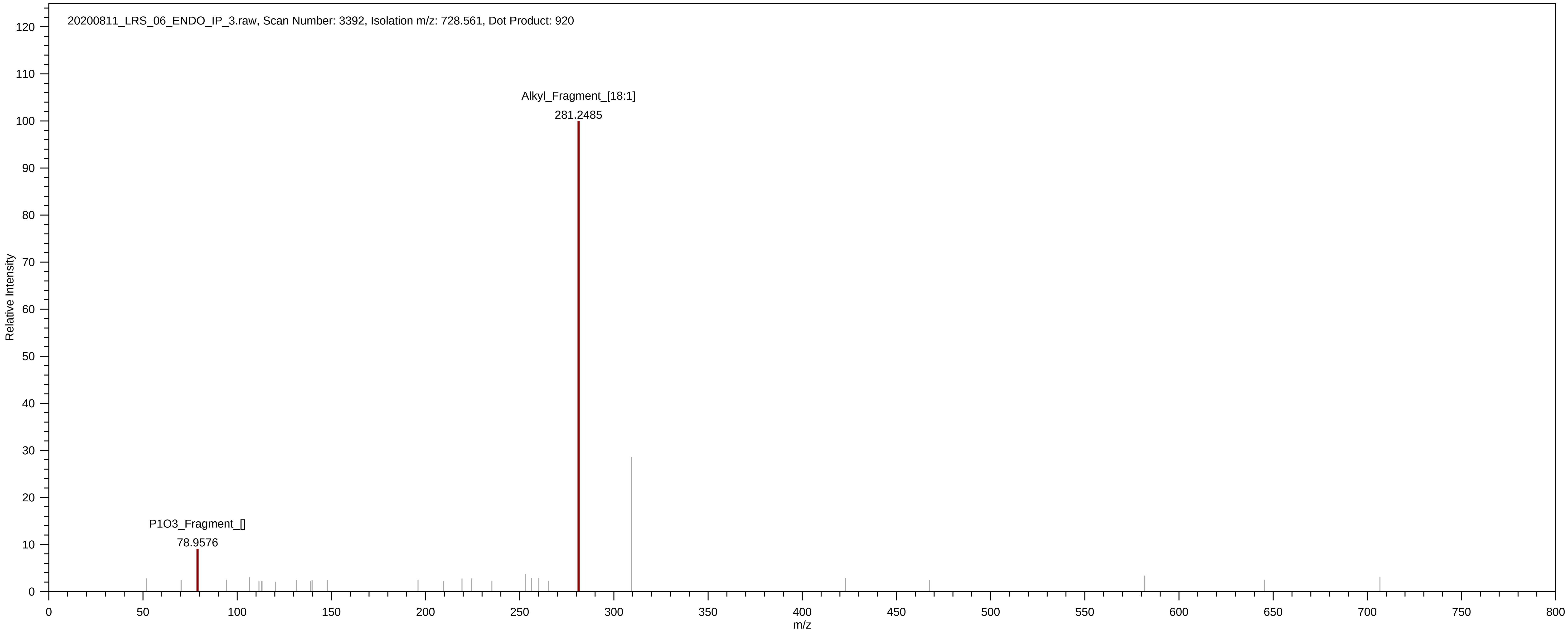

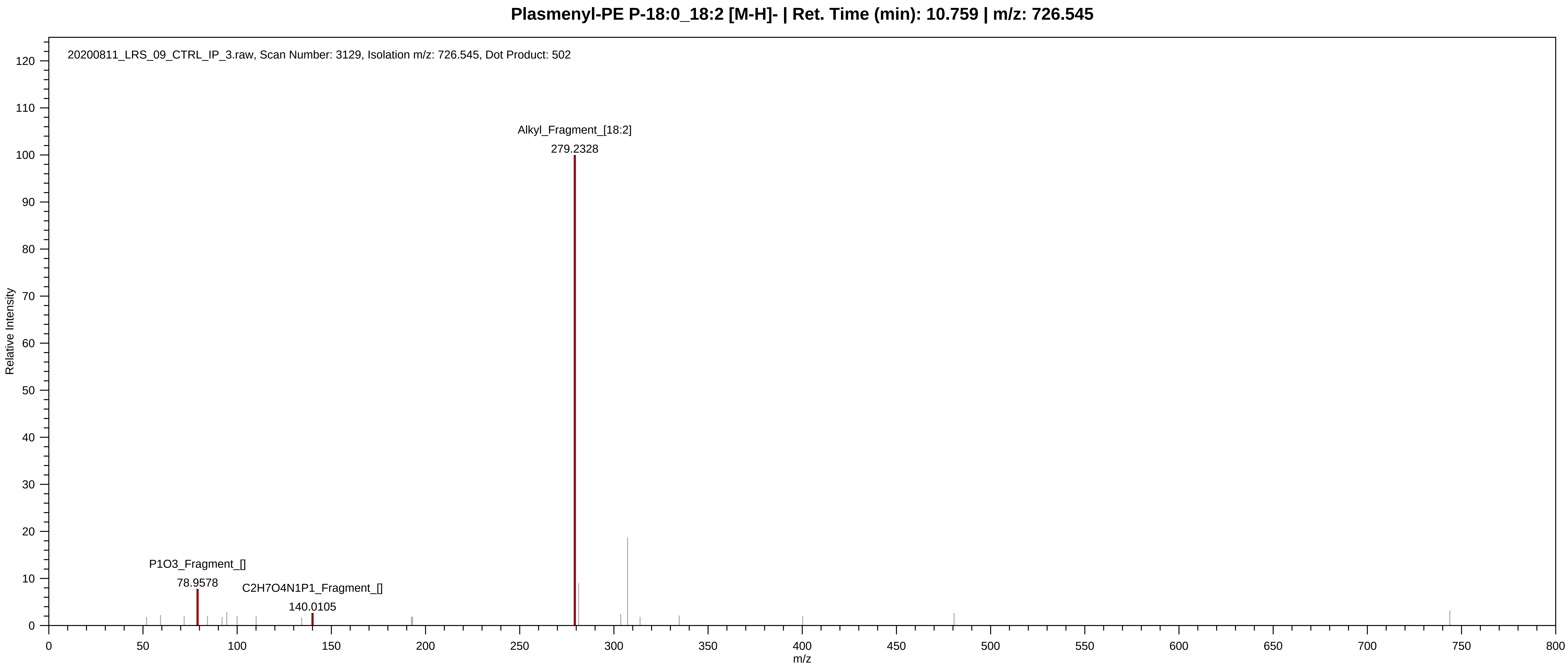
Plasmenyl-PE P-18:0_18:2 [M-H]- | Ret. Time (min): 10.759 | m/z: 726.545
20200811_LRS_09_CTRL_IP_3.raw, Scan Number: 3129, Isolation m/z: 726.545, Dot Product: 502
Alkyl_Fragment_[18:2]
279.2328
P1O3_Fragment_[]
78.9578
C2H7O4N1P1_Fragment_[]
140.0105
Relative Intensity
m/z
0
10
20
30
40
50
60
70
80
90
100
110
120
0
50
100
150
200
250
300
350
400
450
500
550
600
650
700
750
800

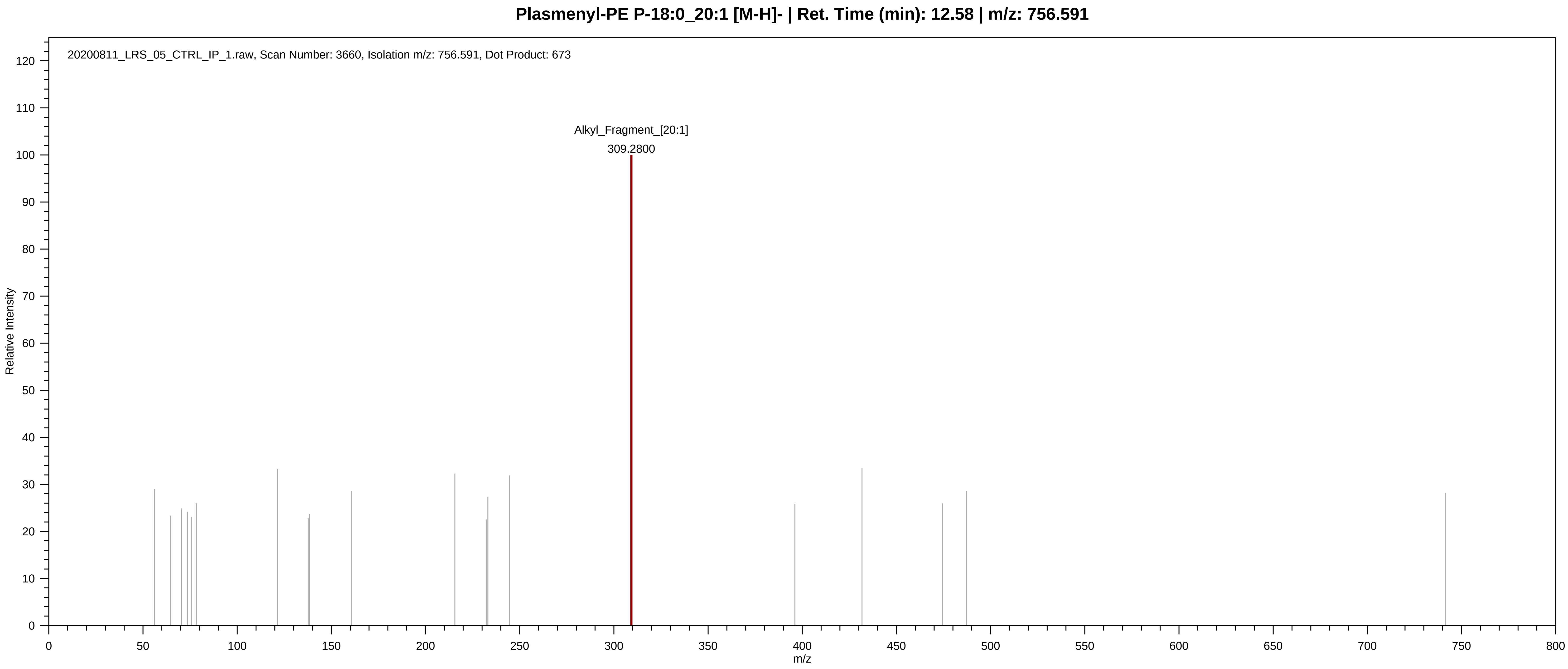
Plasmenyl-PE P-18:0_20:1 [M-H]- | Ret. Time (min): 12.58 | m/z: 756.591
20200811_LRS_05_CTRL_IP_1.raw, Scan Number: 3660, Isolation m/z: 756.591, Dot Product: 673
Alkyl_Fragment_[20:1]
309.2800
Relative Intensity
m/z
0
10
20
30
40
50
60
70
80
90
100
110
120
0
50
100
150
200
250
300
350
400
450
500
550
600
650
700
750
800

Plasmenyl-PE P-18:0_20:1 [M-H]- | Ret. Time (min): 12.564 | m/z: 756.591

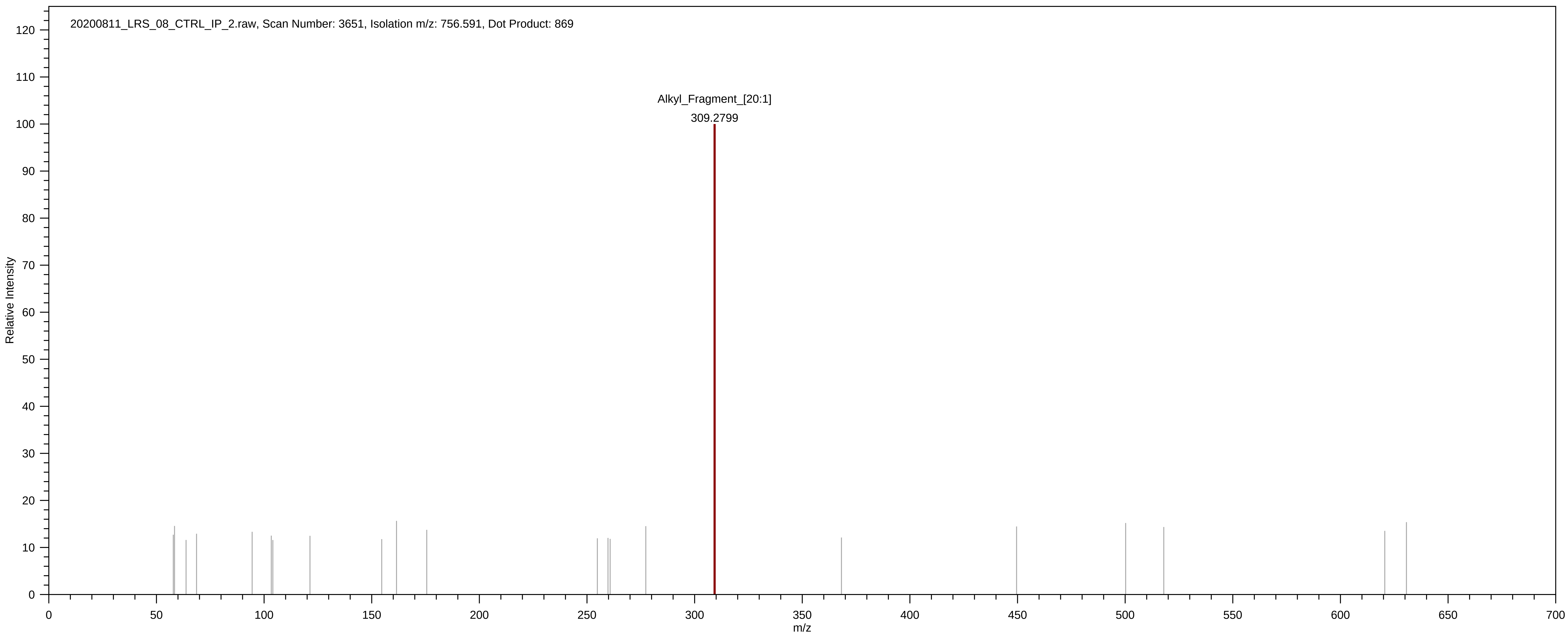

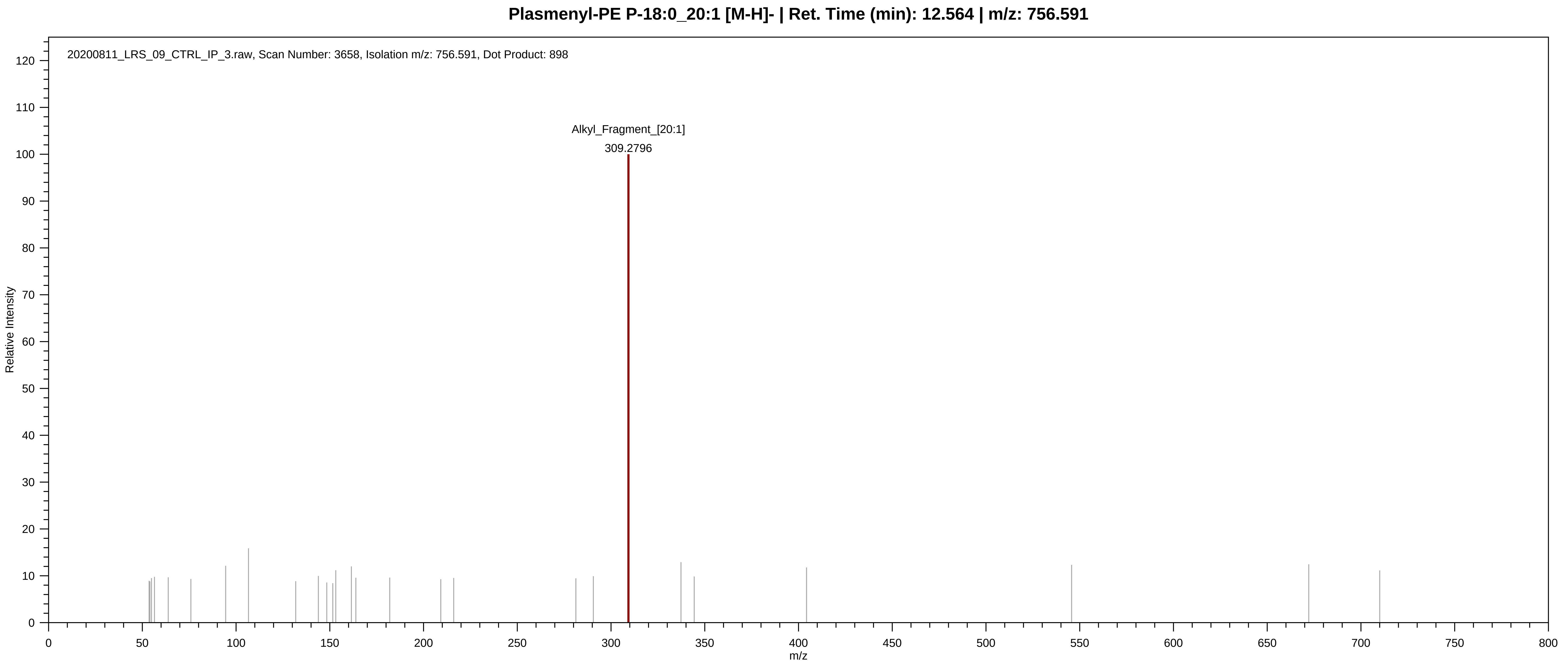

Plasmenyl-PE P-18:0_20:1 [M-H]- | Ret. Time (min): 12.564 | m/z: 756.591
20200811_LRS_09_CTRL_IP_3.raw, Scan Number: 3658, Isolation m/z: 756.591, Dot Product: 898
Alkyl_Fragment_[20:1]
309.2796
Relative Intensity
m/z

Plasmenyl-PE P-18:0_20:1 [M-H]- | Ret. Time (min): 12.523 | m/z: 756.592

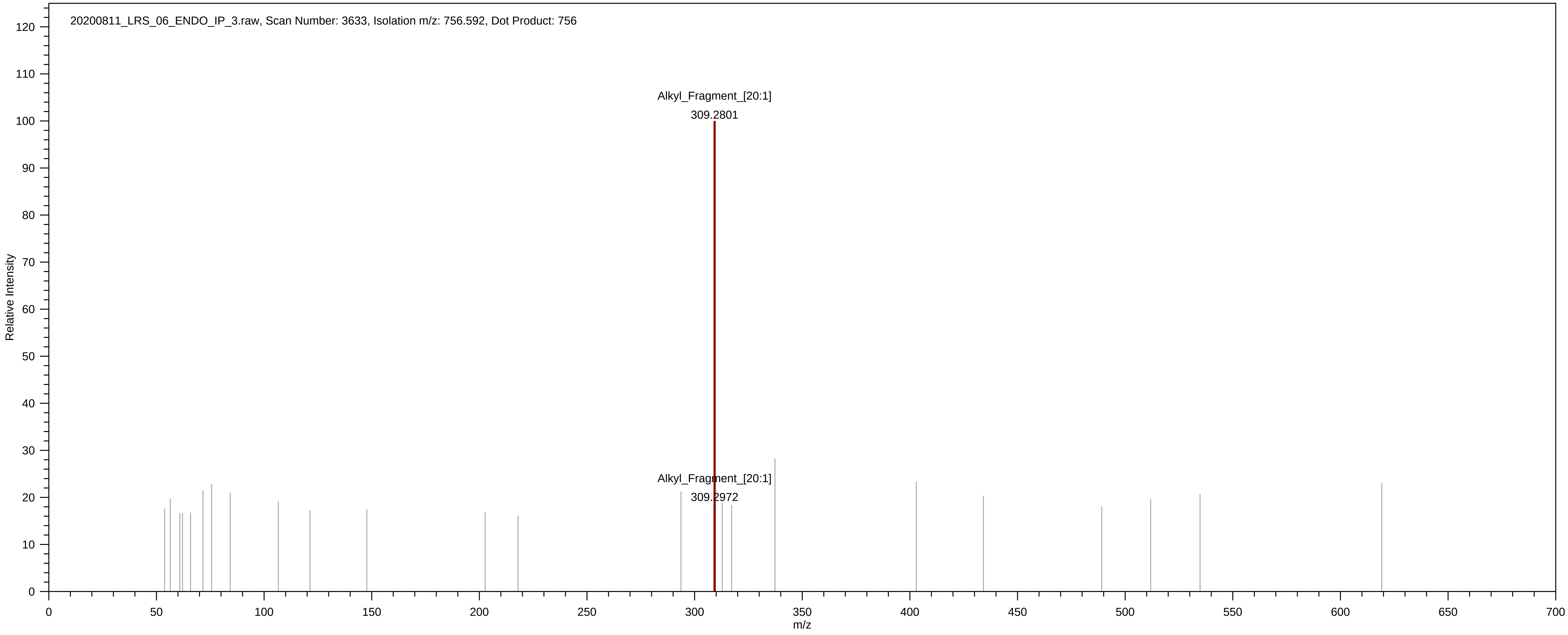

Plasmenyl-PE P-18:0_20:1 [M-H]- | Ret. Time (min): 12.561 | m/z: 756.592

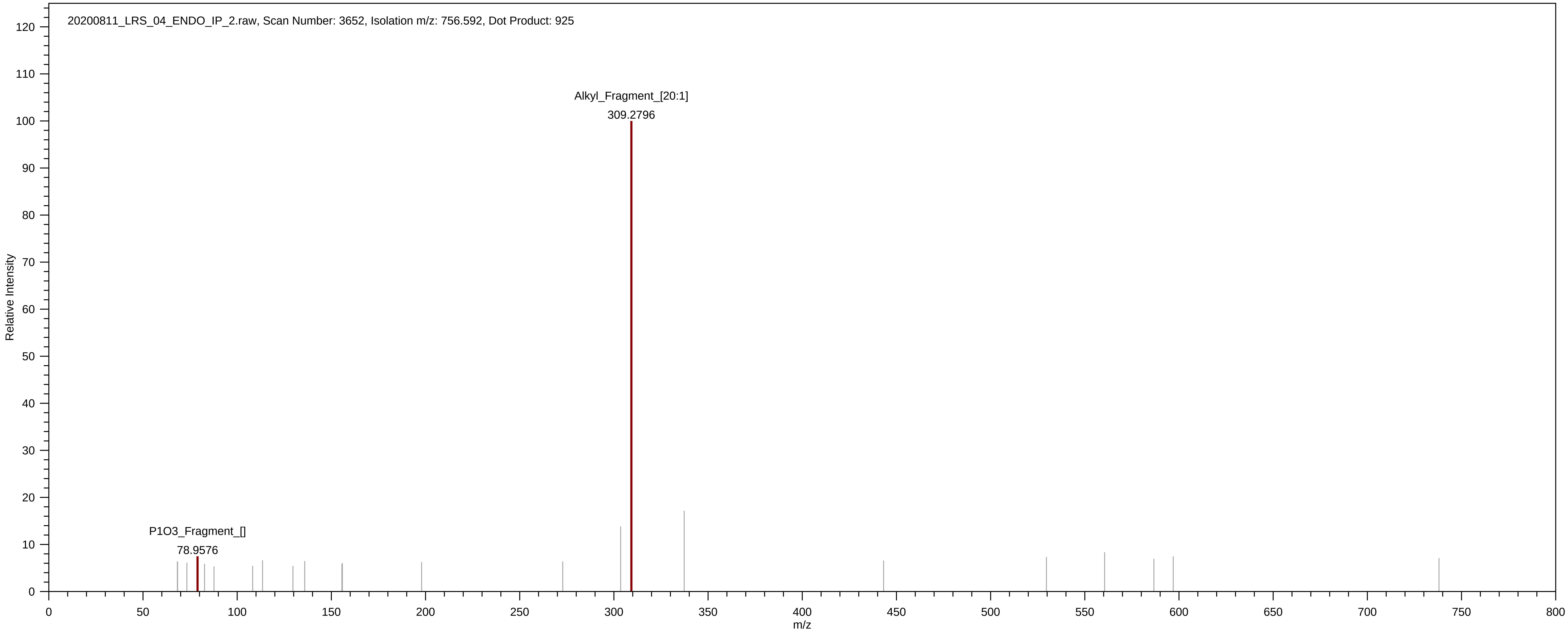

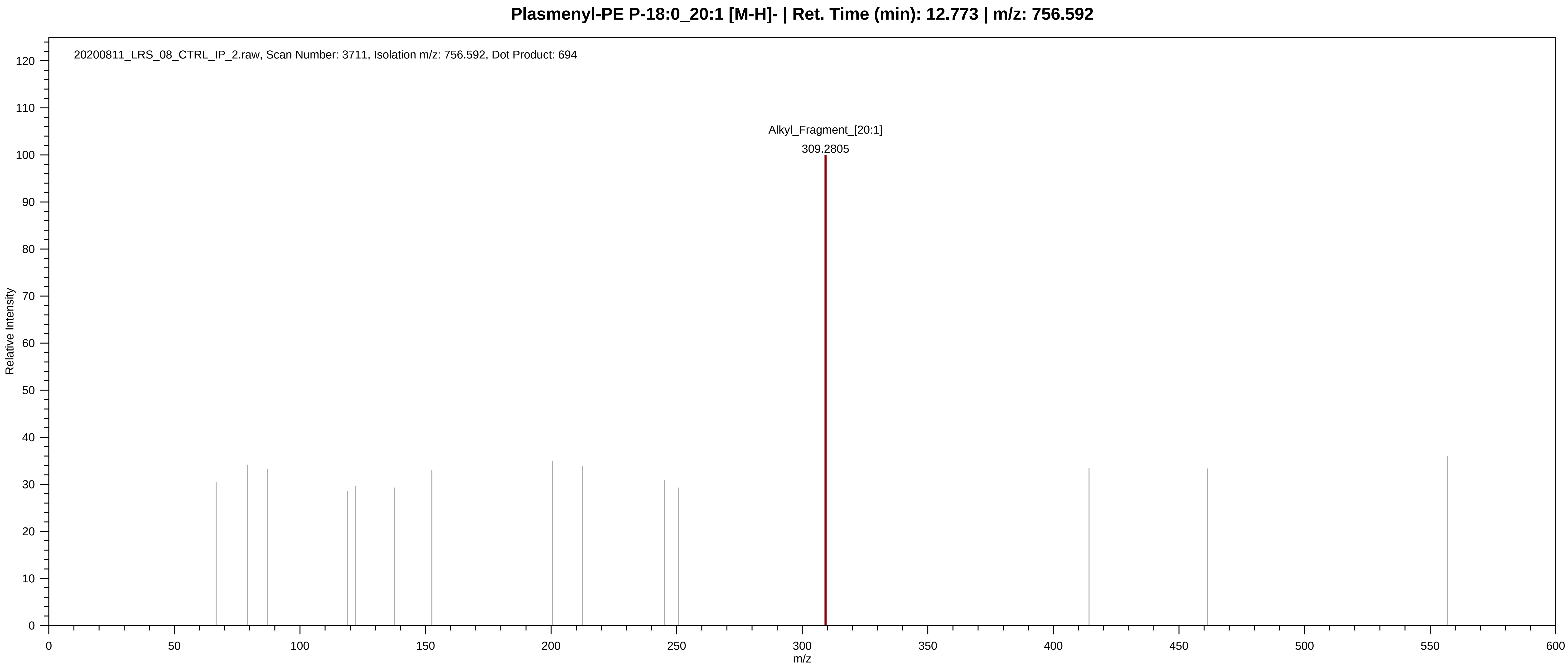

Plasmenyl-PE P-18:0_20:1 [M-H]- | Ret. Time (min): 12.773 | m/z: 756.592
20200811_LRS_08_CTRL_IP_2.raw, Scan Number: 3711, Isolation m/z: 756.592, Dot Product: 694
Alkyl_Fragment_[20:1]
309.2805
Relative Intensity
m/z
0
10
20
30
40
50
60
70
80
90
100
110
120
0
50
100
150
200
250
300
350
400
450
500
550
600

Plasmenyl-PE P-18:0_20:4 [M-H]- | Ret. Time (min): 10.487 | m/z: 750.543

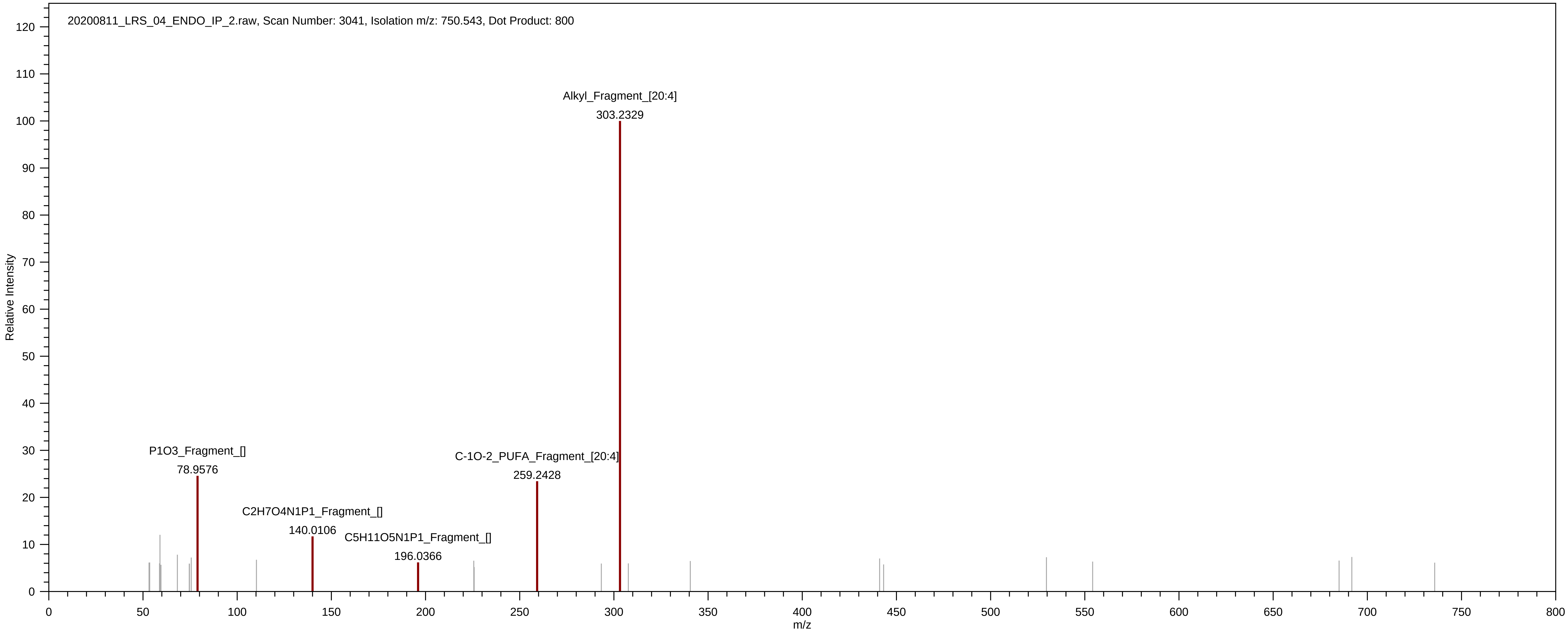

Plasmenyl-PE P-18:0_20:4 [M-H]- | Ret. Time (min): 10.49 | m/z: 750.544

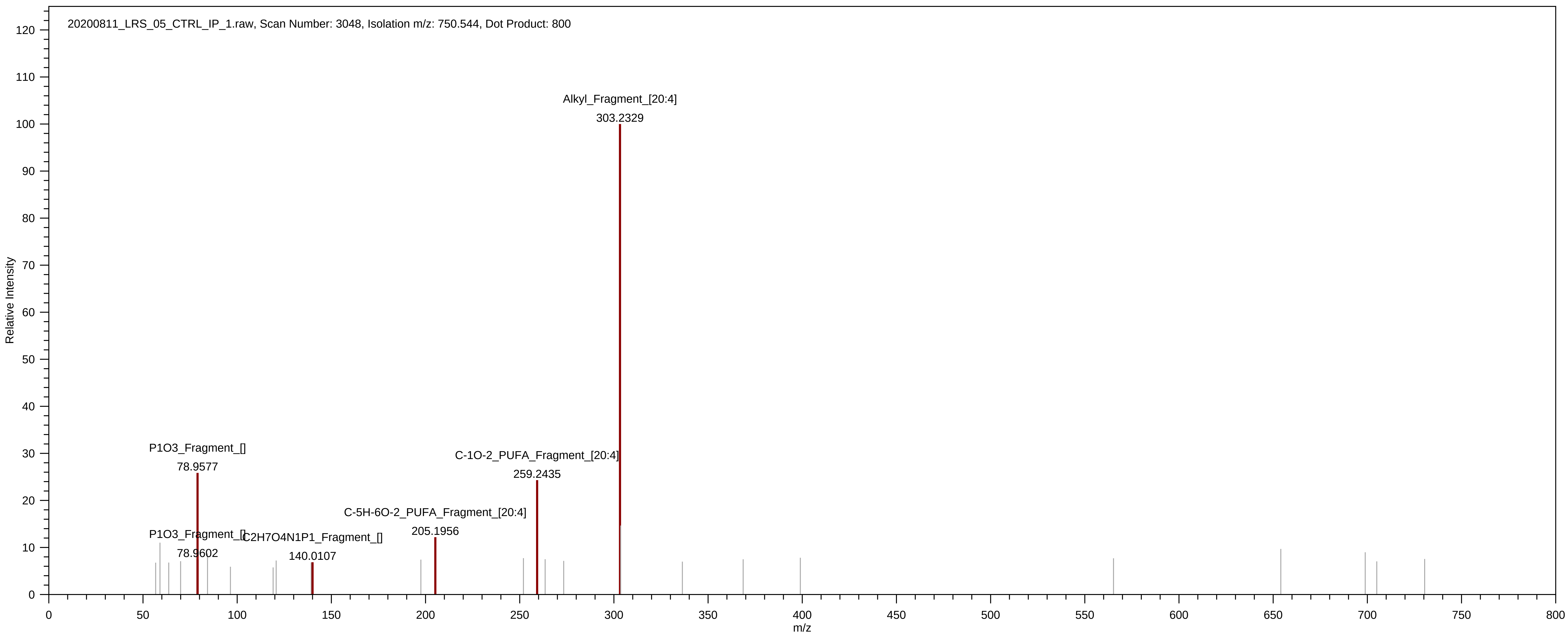

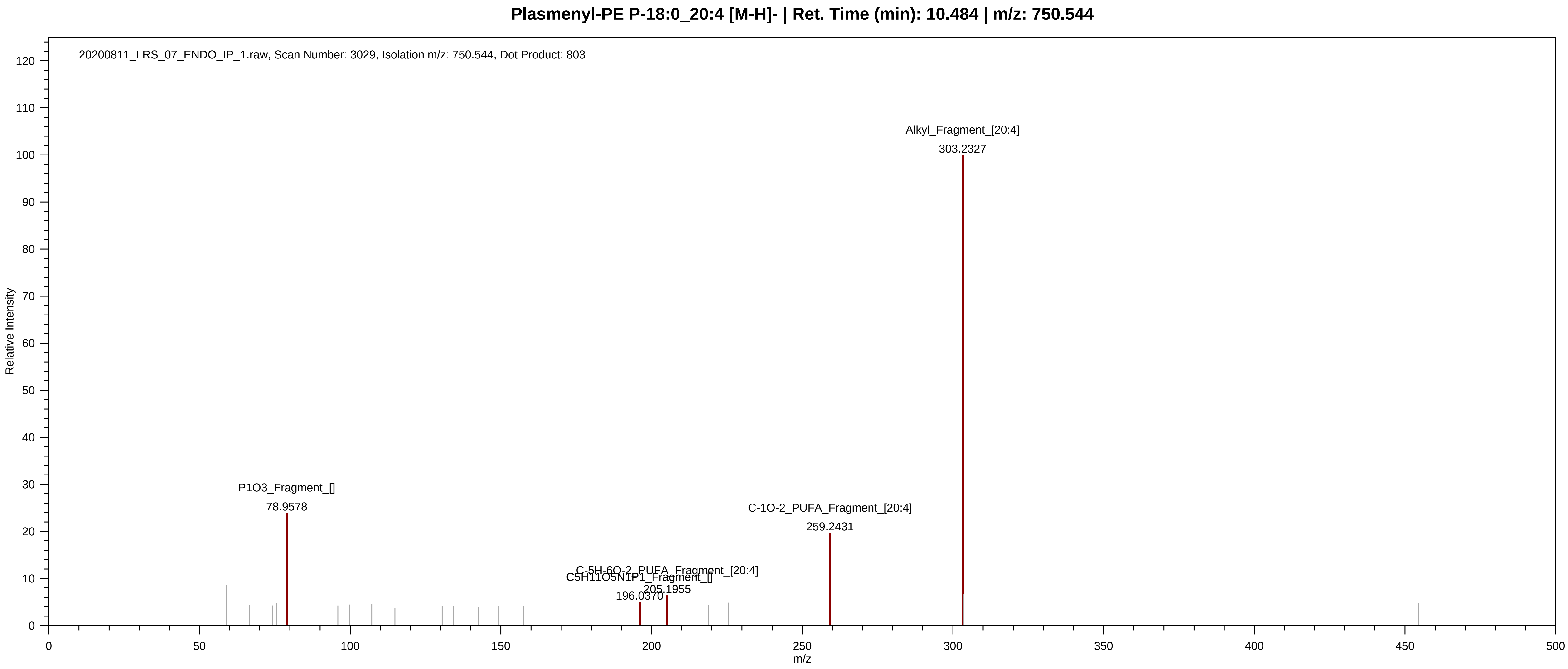

Plasmenyl-PE P-18:0_20:4 [M-H]- | Ret. Time (min): 10.484 | m/z: 750.544
20200811_LRS_07_ENDO_IP_1.raw, Scan Number: 3029, Isolation m/z: 750.544, Dot Product: 803
Alkyl_Fragment_[20:4]
303.2327
P1O3_Fragment_[]
78.9578
C-1O-2_PUFA_Fragment_[20:4]
259.2431
C-5H-6O-2_PUFA_Fragment_[20:4]
C5H11O5N1P1_Fragment_[]
205.1955
196.0370
Relative Intensity
m/z

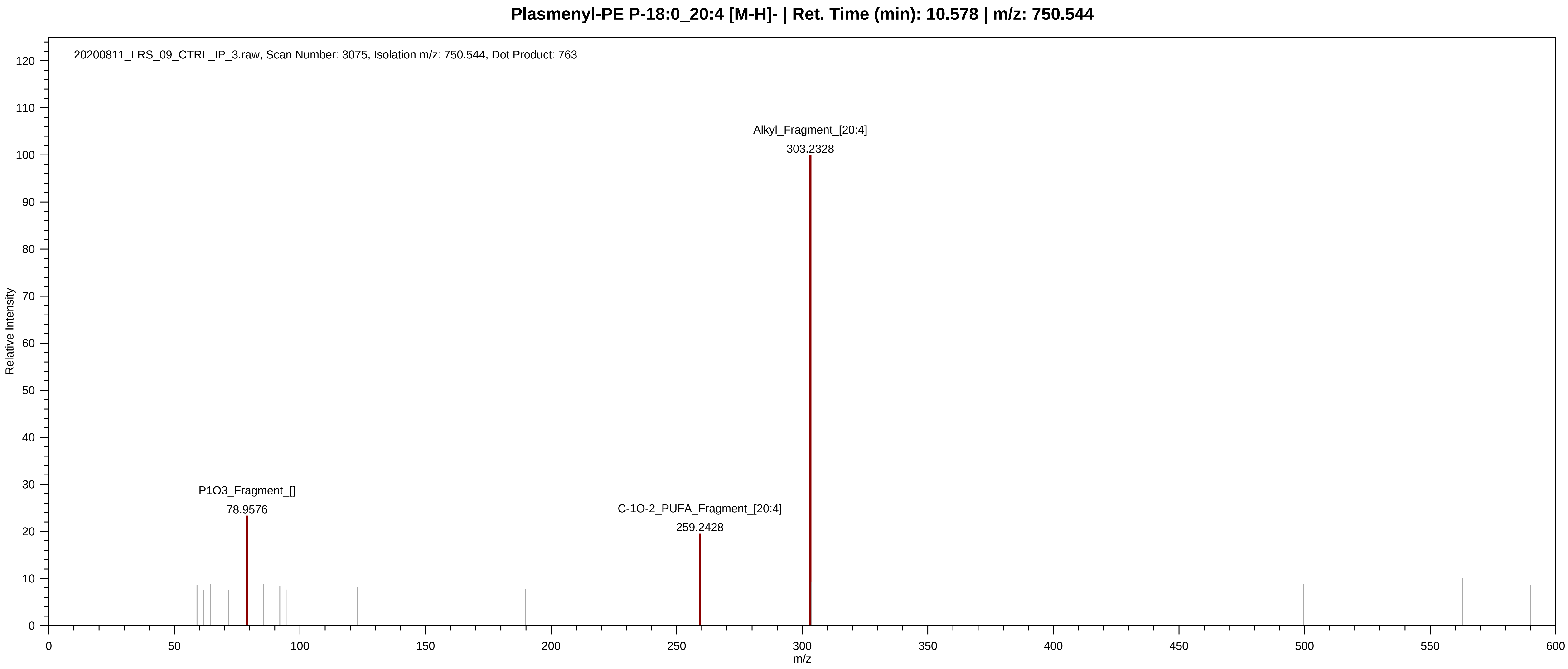
Plasmenyl-PE P-18:0_20:4 [M-H]- | Ret. Time (min): 10.578 | m/z: 750.544
20200811_LRS_09_CTRL_IP_3.raw, Scan Number: 3075, Isolation m/z: 750.544, Dot Product: 763
Alkyl_Fragment_[20:4]
303.2328
P1O3_Fragment_[]
78.9576
C-1O-2_PUFA_Fragment_[20:4]
259.2428
Relative Intensity
m/z

Plasmenyl-PE P-18:0_20:4 [M-H]- | Ret. Time (min): 10.5 | m/z: 750.545

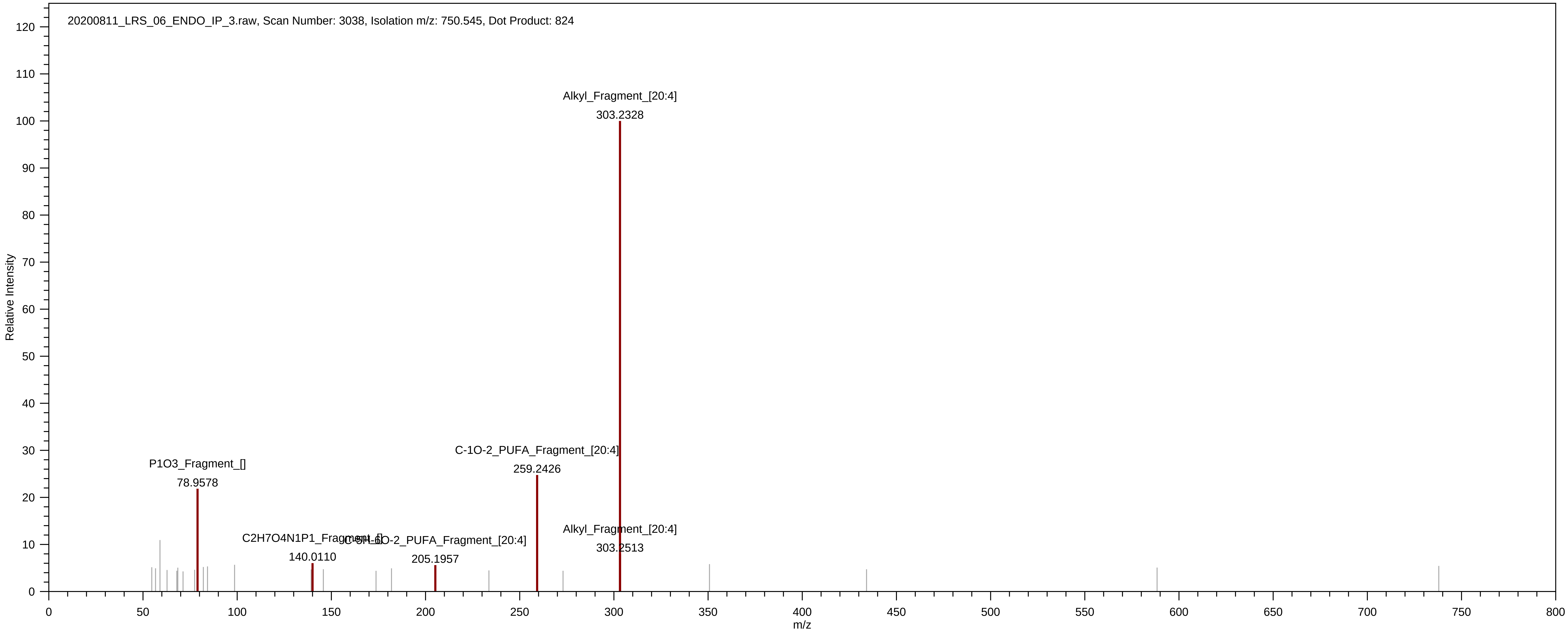

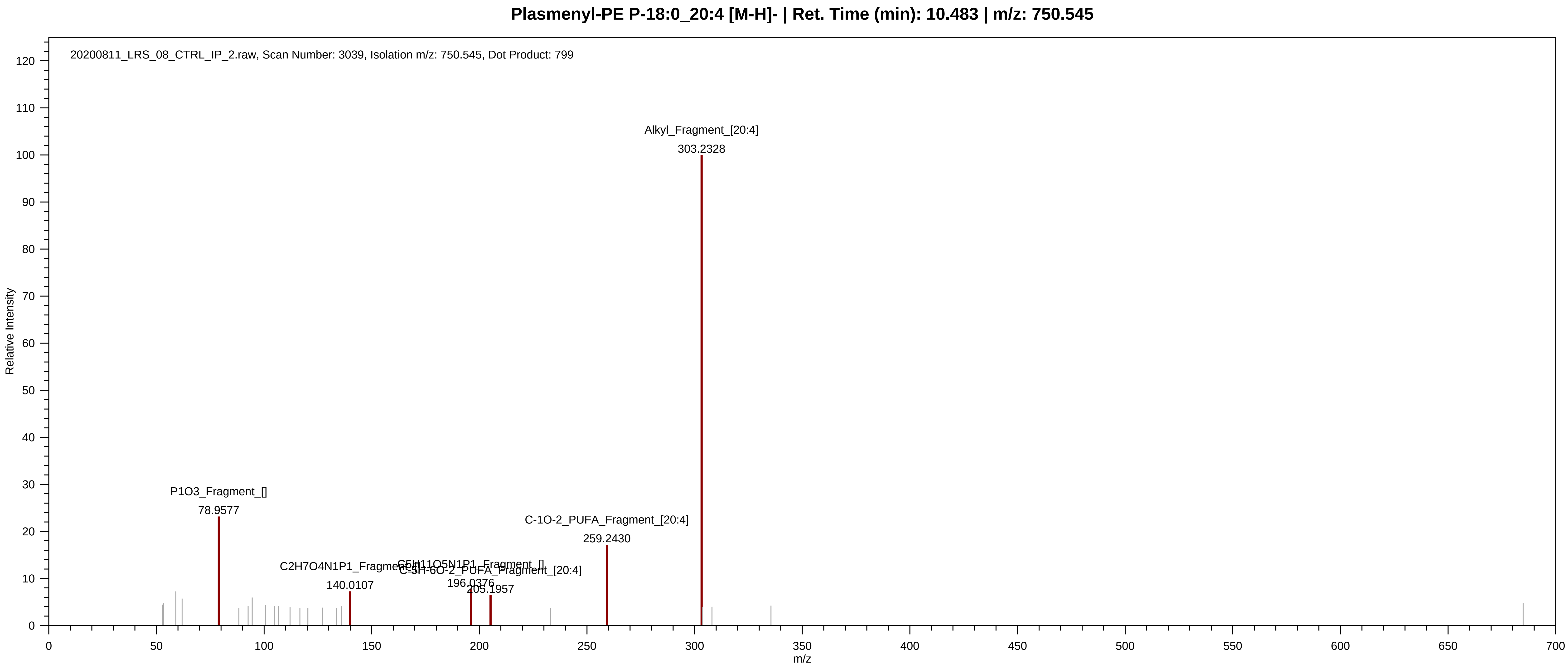

Plasmenyl-PE P-18:0_20:4 [M-H]- | Ret. Time (min): 10.483 | m/z: 750.545
20200811_LRS_08_CTRL_IP_2.raw, Scan Number: 3039, Isolation m/z: 750.545, Dot Product: 799
Alkyl_Fragment_[20:4]
303.2328
P1O3_Fragment_[]
78.9577
C-1O-2_PUFA_Fragment_[20:4]
259.2430
C2H7O4N1P1_Fragment_[]
140.0107
C5H11O5N1P1_Fragment_[]
196.0376
C-5H-6O-2_PUFA_Fragment_[20:4]
205.1957
Relative Intensity
m/z

Plasmenyl-PE P-18:0_20:5 [M-H]- | Ret. Time (min): 9.846 | m/z: 748.528

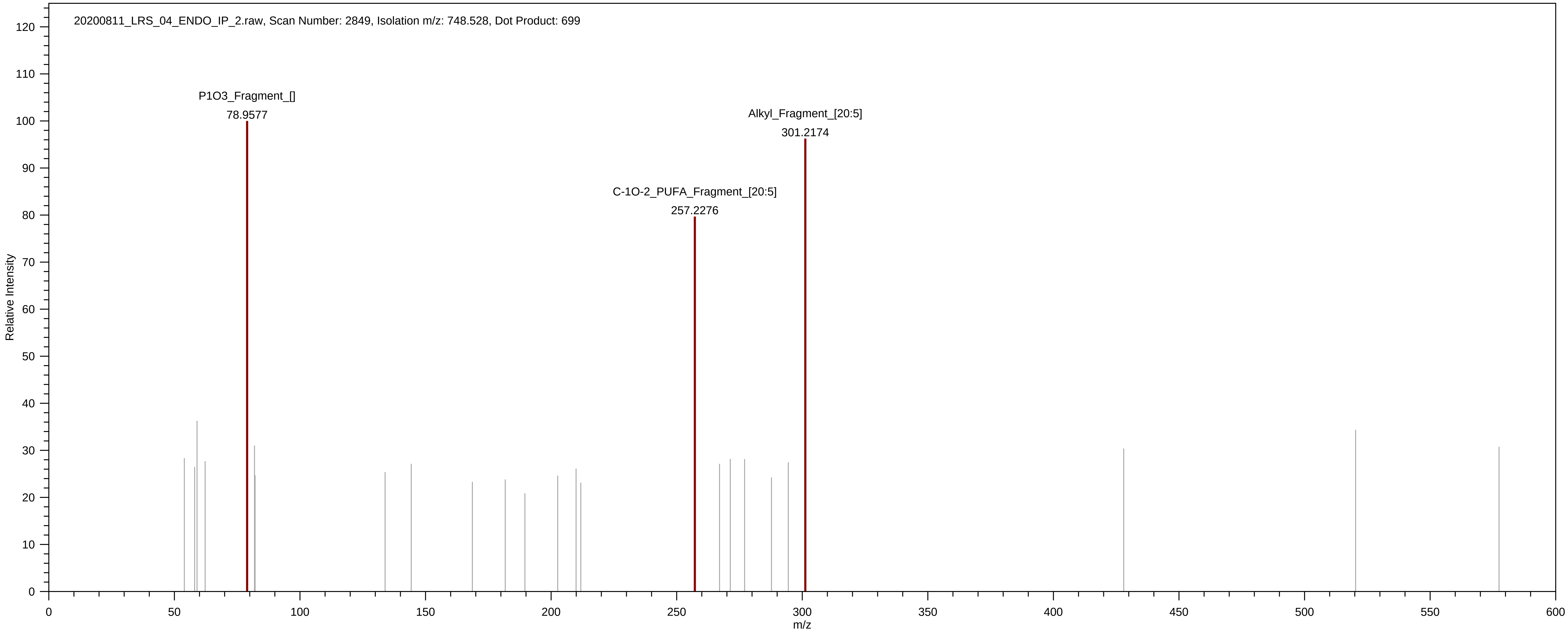

Plasmenyl-PE P-18:0_20:5 [M-H]- | Ret. Time (min): 9.812 | m/z: 748.529

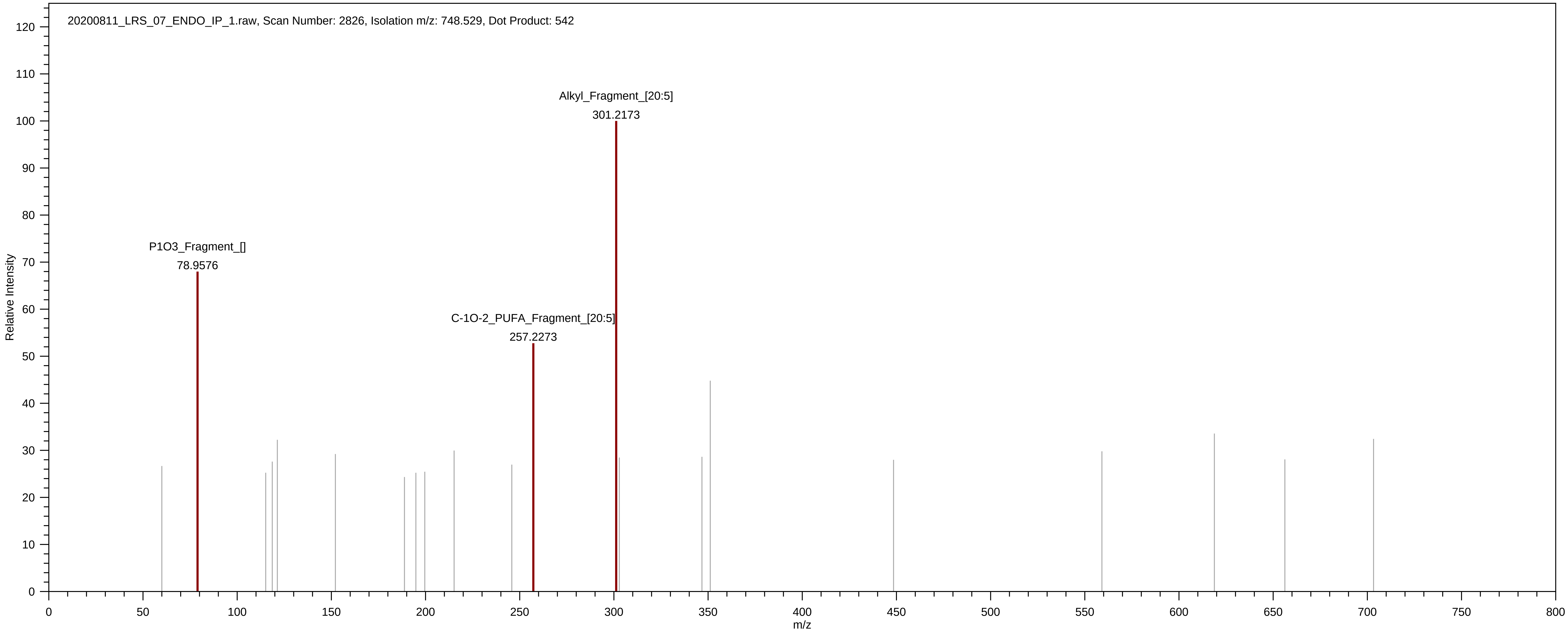

Plasmenyl-PE P-18:0_20:5 [M-H]- | Ret. Time (min): 9.814 | m/z: 748.529

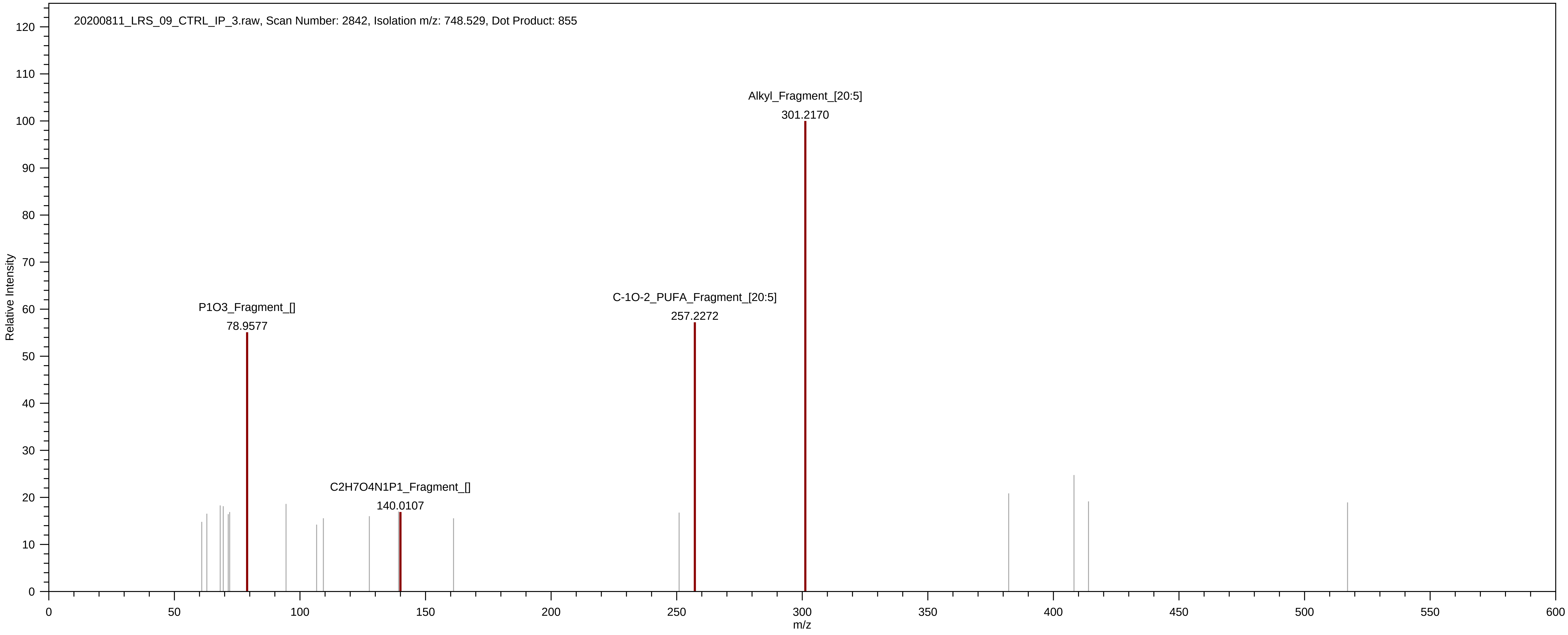

Plasmenyl-PE P-18:0_20:5 [M-H]- | Ret. Time (min): 9.845 | m/z: 748.529

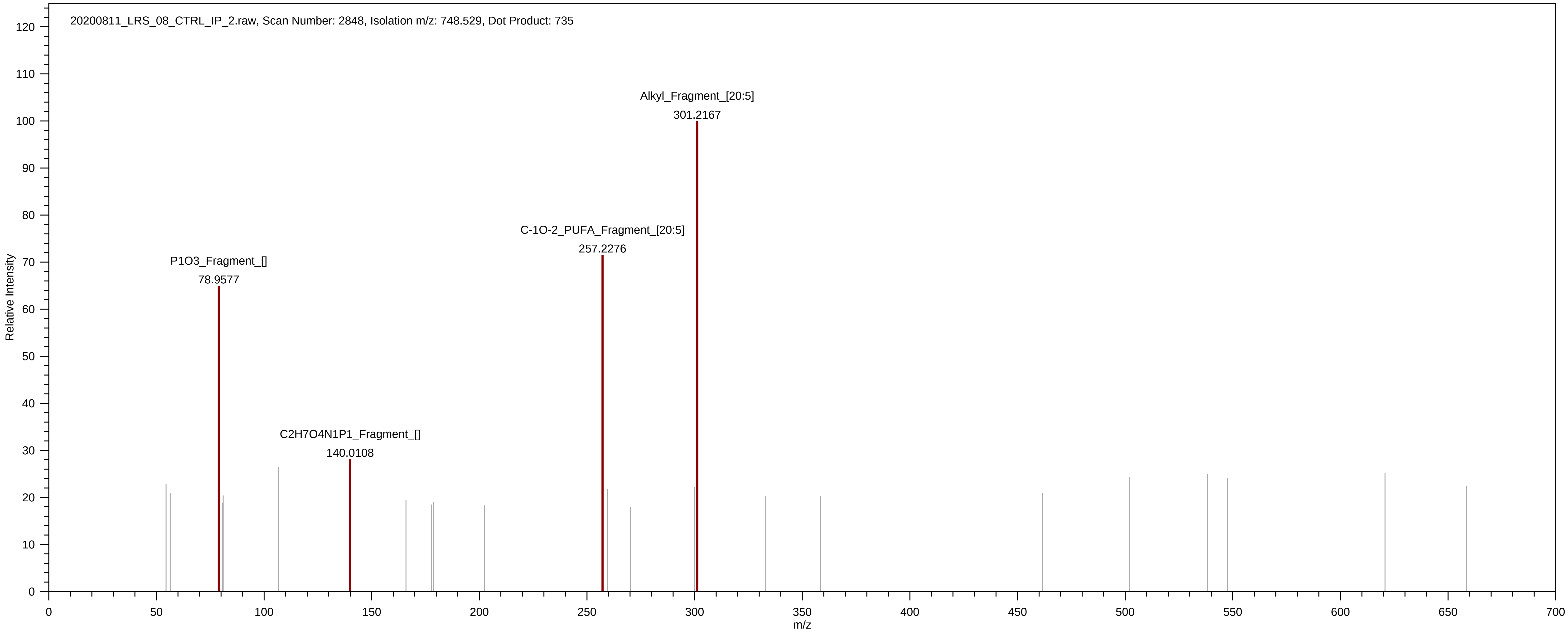

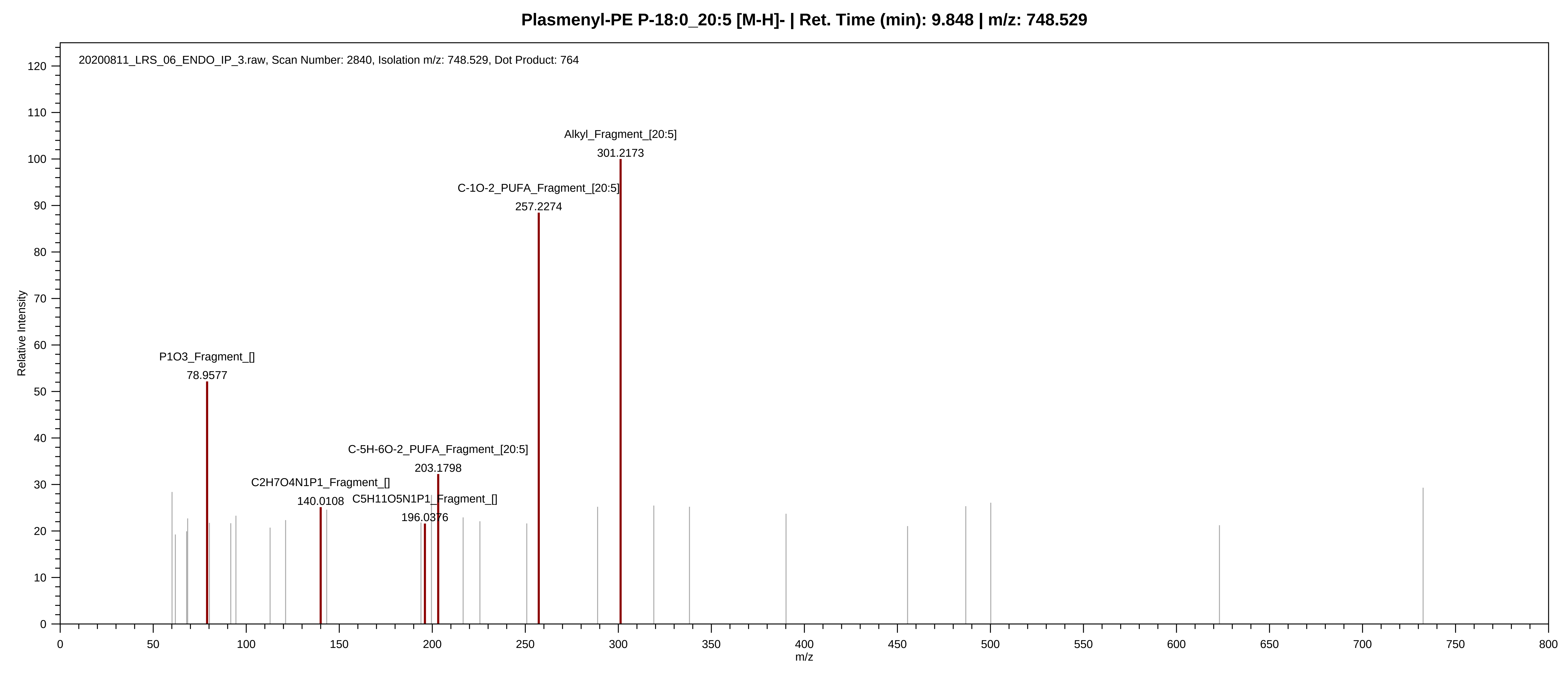

Plasmenyl-PE P-18:0_20:5 [M-H]- | Ret. Time (min): 9.848 | m/z: 748.529
20200811_LRS_06_ENDO_IP_3.raw, Scan Number: 2840, Isolation m/z: 748.529, Dot Product: 764
Alkyl_Fragment_[20:5]
301.2173
C-1O-2_PUFA_Fragment_[20:5]
257.2274
P1O3_Fragment_[]
78.9577
C-5H-6O-2_PUFA_Fragment_[20:5]
203.1798
C2H7O4N1P1_Fragment_[]
140.0108
C5H11O5N1P1_Fragment_[]
196.0376
Relative Intensity
m/z

Plasmenyl-PE P-18:0_22:1 [M-H]- | Ret. Time (min): 13.637 | m/z: 784.622

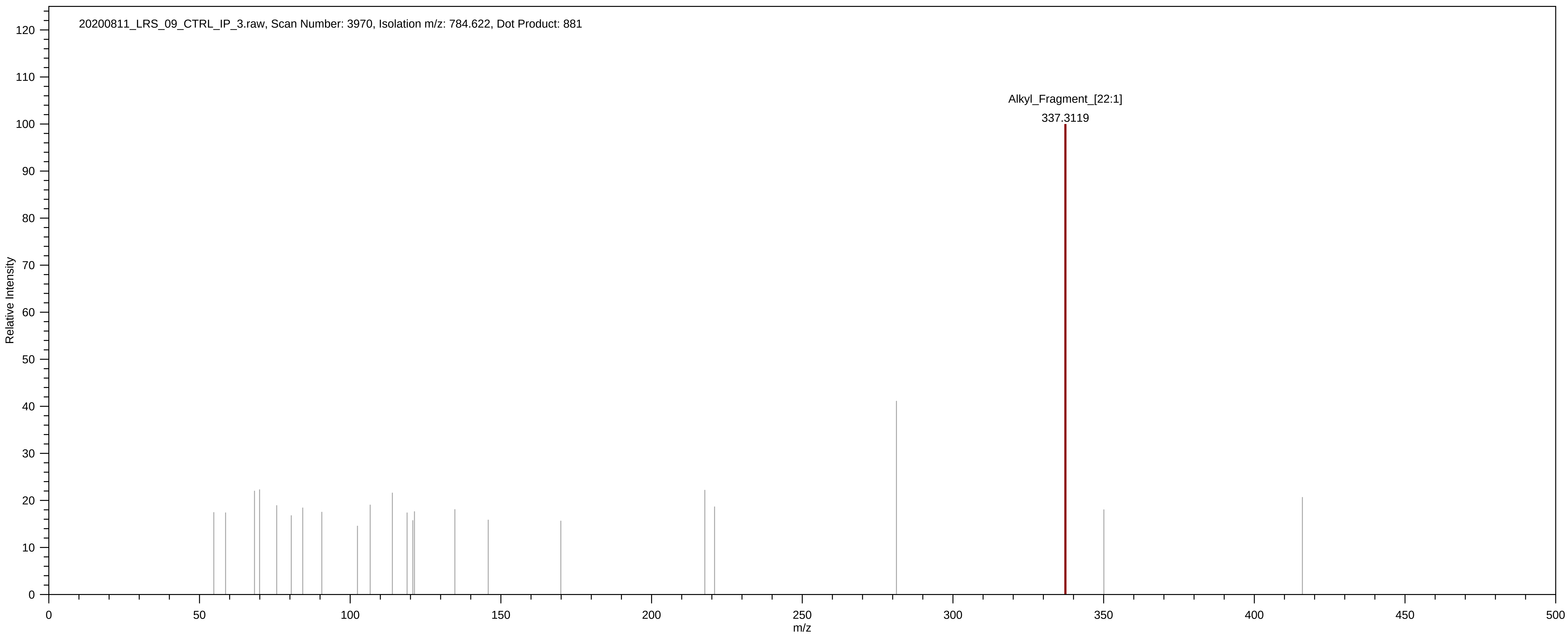

Plasmenyl-PE P-18:0_22:1 [M-H]- | Ret. Time (min): 13.655 | m/z: 784.623

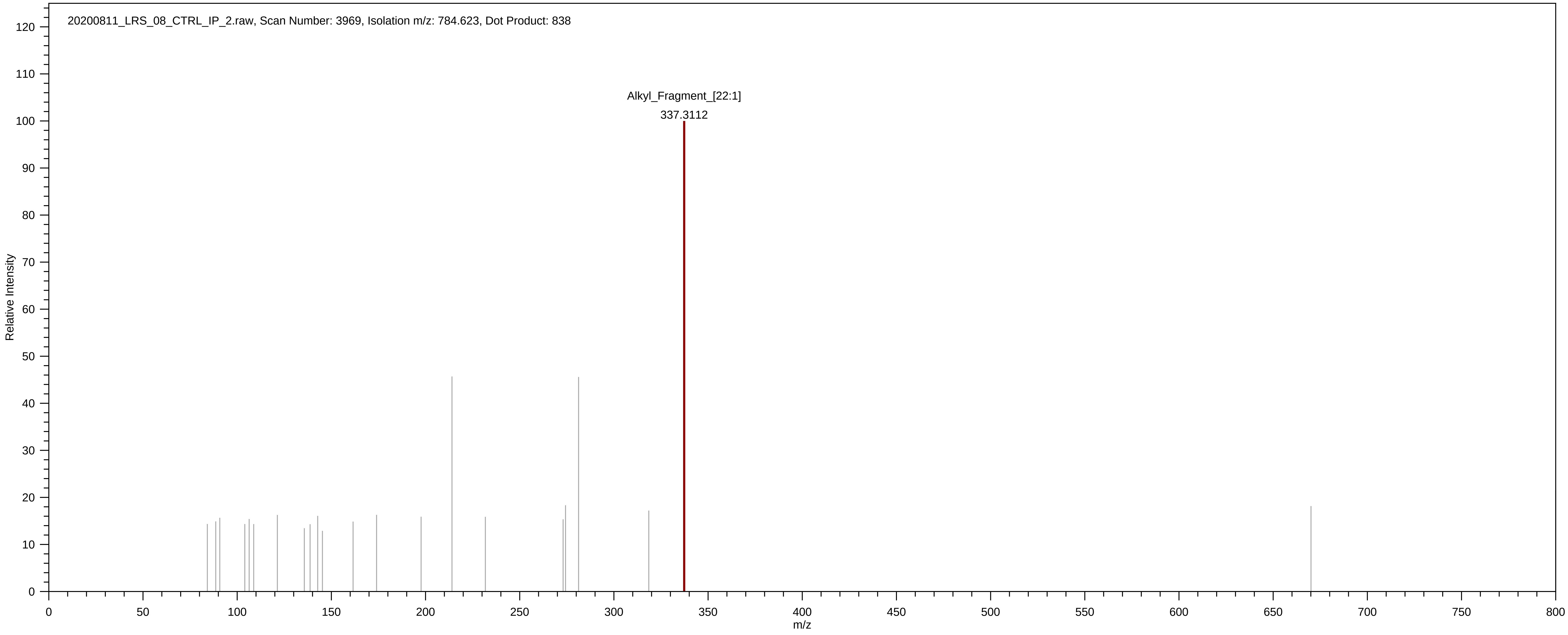

Plasmenyl-PE P-18:0_22:1 [M-H]- | Ret. Time (min): 13.667 | m/z: 784.625

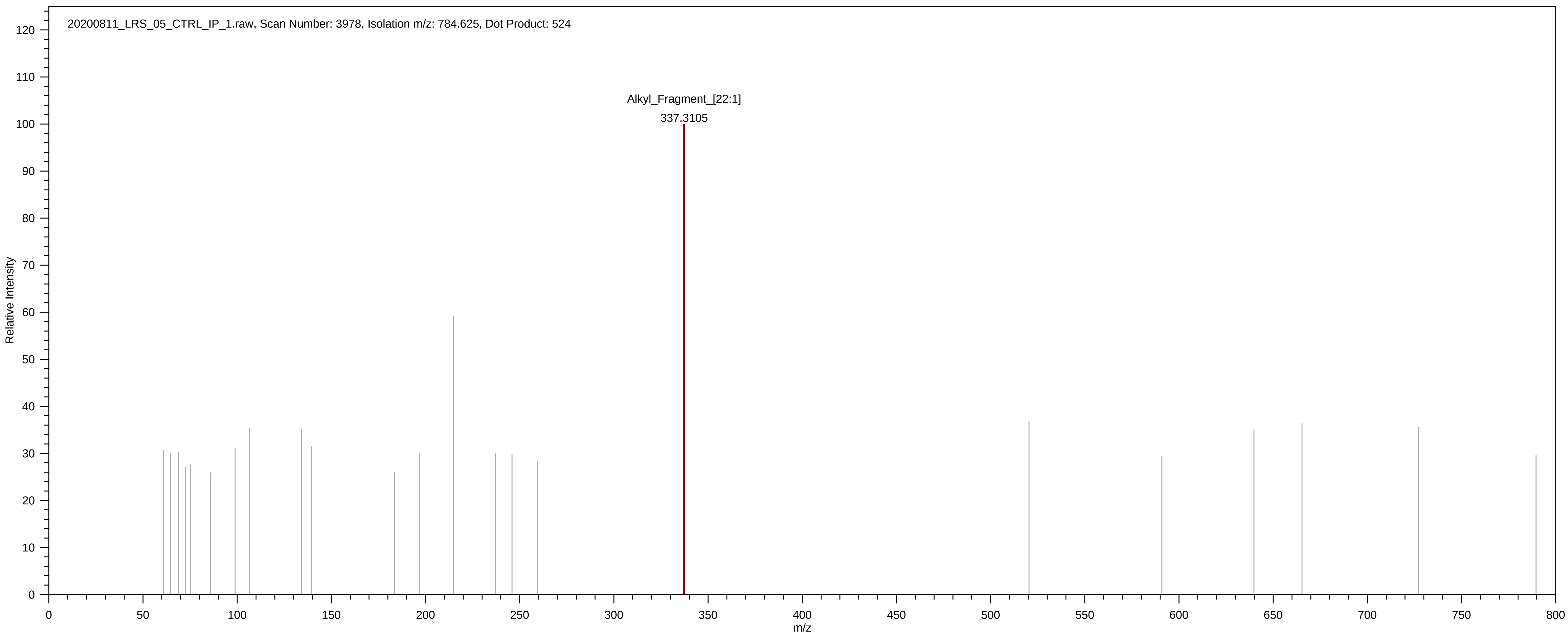

Plasmenyl-PE P-18:0_22:4 [M-H]- | Ret. Time (min): 11.235 | m/z: 778.575

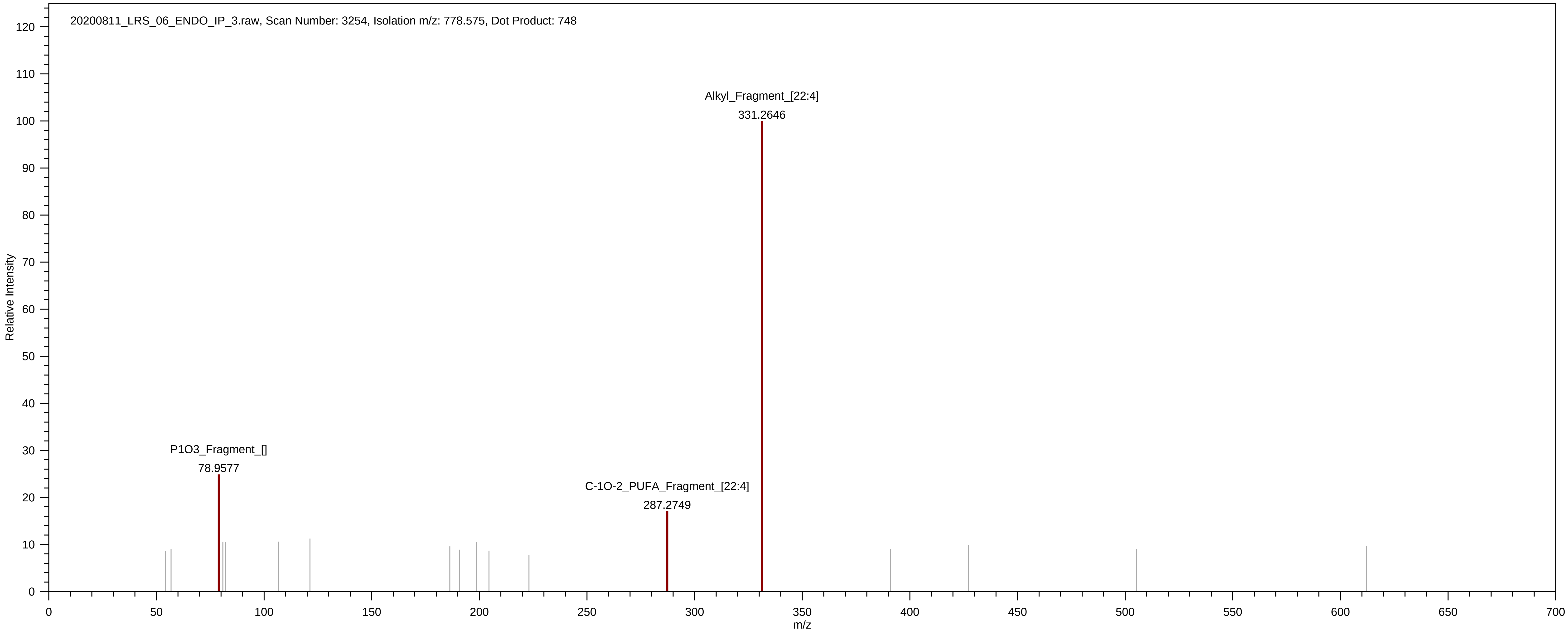

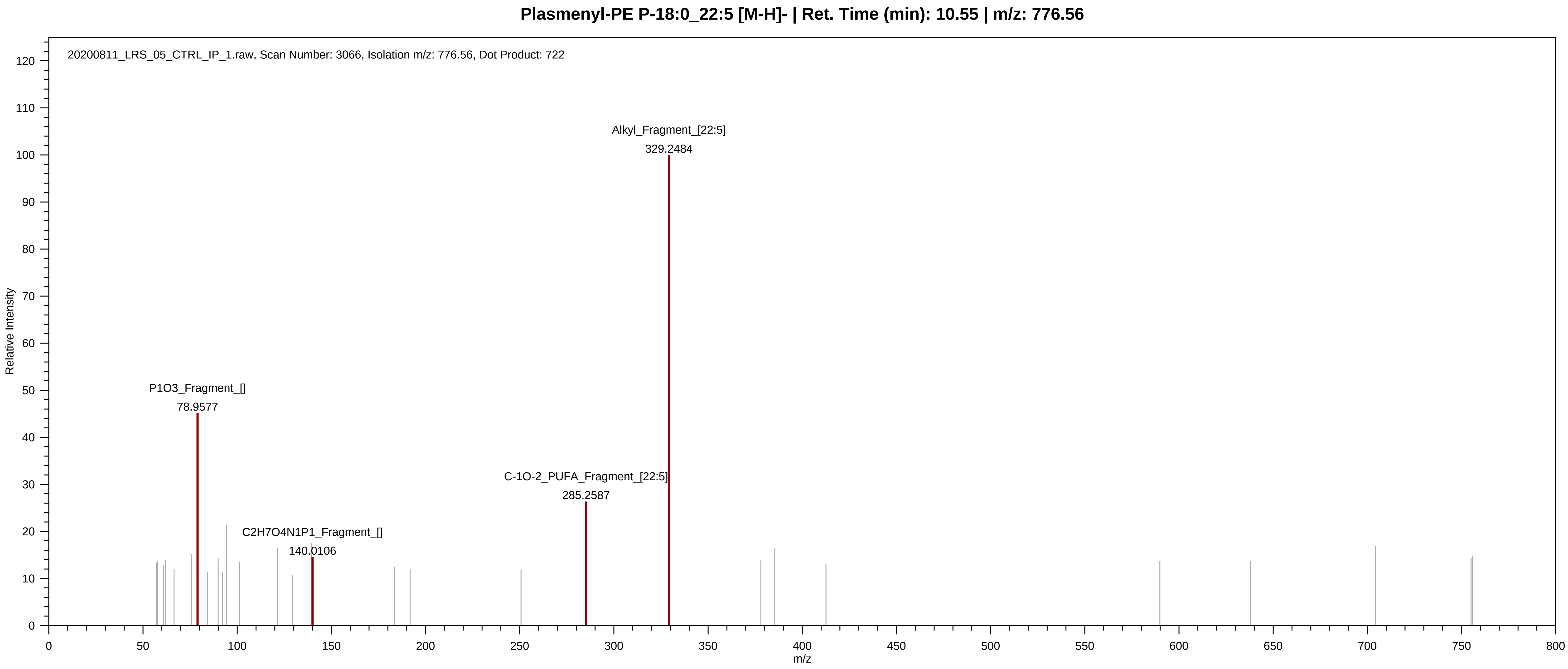

Plasmenyl-PE P-18:0_22:5 [M-H]- | Ret. Time (min): 10.55 | m/z: 776.56
20200811_LRS_05_CTRL_IP_1.raw, Scan Number: 3066, Isolation m/z: 776.56, Dot Product: 722
Alkyl_Fragment_[22:5]
329.2484
P1O3_Fragment_[]
78.9577
C-1O-2_PUFA_Fragment_[22:5]
285.2587
C2H7O4N1P1_Fragment_[]
140.0106
Relative Intensity
m/z
0
10
20
30
40
50
60
70
80
90
100
110
120
0
50
100
150
200
250
300
350
400
450
500
550
600
650
700
750
800

Plasmenyl-PE P-18:0_22:5 [M-H]- | Ret. Time (min): 10.481 | m/z: 776.56

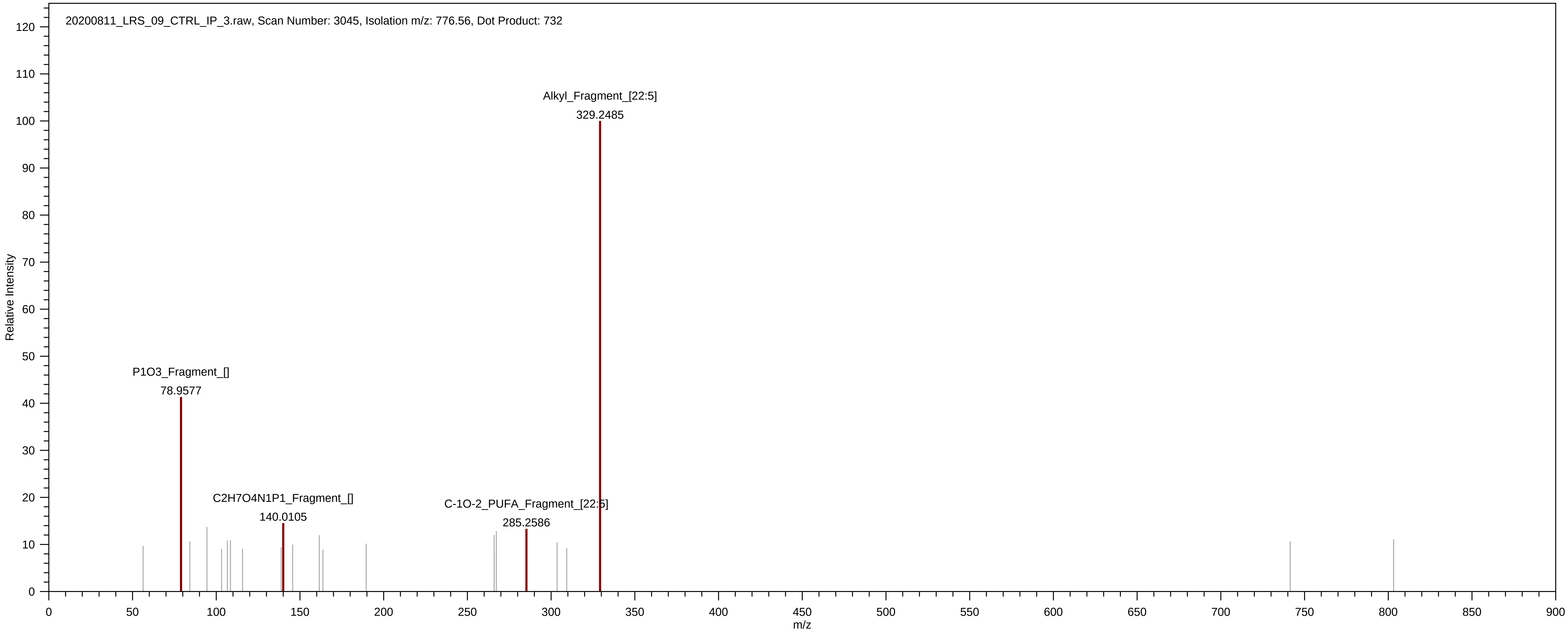

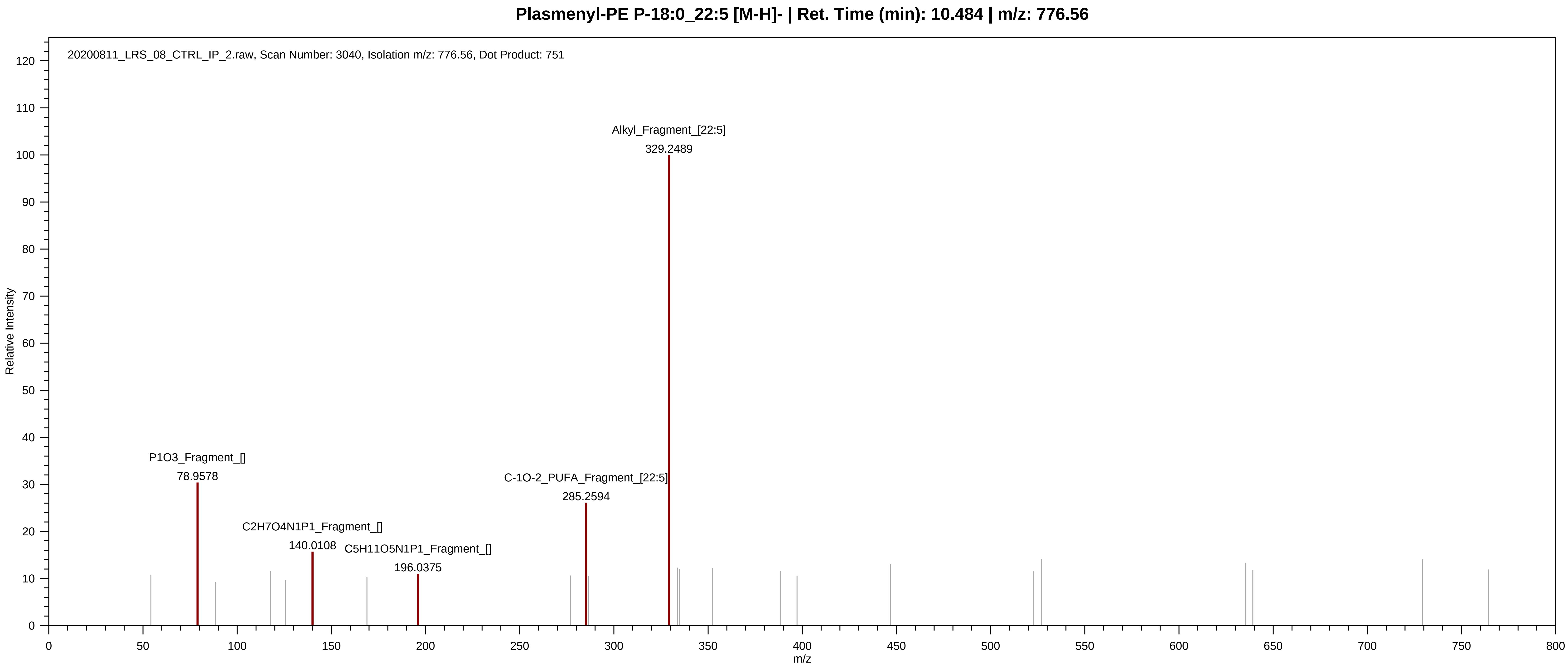

Plasmenyl-PE P-18:0_22:5 [M-H]- | Ret. Time (min): 10.484 | m/z: 776.56
20200811_LRS_08_CTRL_IP_2.raw, Scan Number: 3040, Isolation m/z: 776.56, Dot Product: 751
Alkyl_Fragment_[22:5]
329.2489
P1O3_Fragment_[]
78.9578
C-1O-2_PUFA_Fragment_[22:5]
285.2594
C2H7O4N1P1_Fragment_[]
140.0108
C5H11O5N1P1_Fragment_[]
196.0375
Relative Intensity
m/z

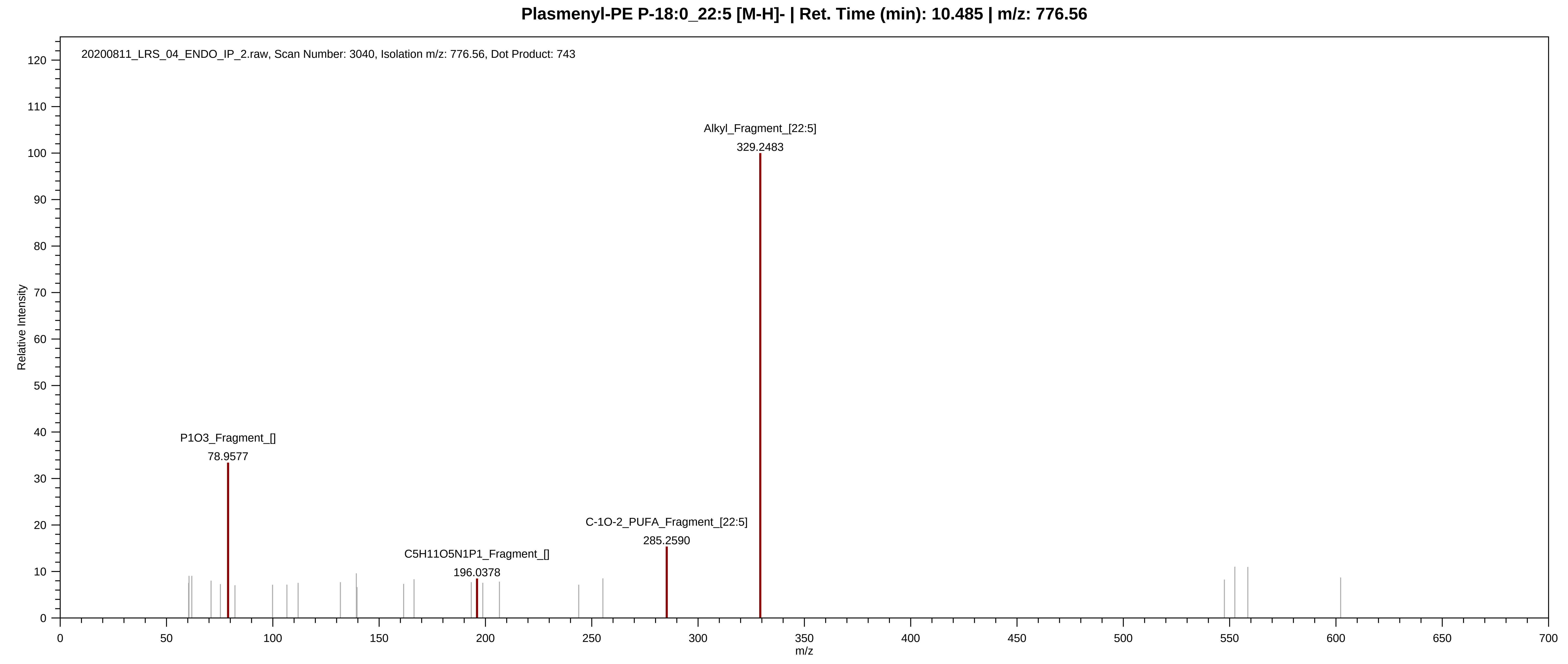
Plasmenyl-PE P-18:0_22:5 [M-H]- | Ret. Time (min): 10.485 | m/z: 776.56
20200811_LRS_04_ENDO_IP_2.raw, Scan Number: 3040, Isolation m/z: 776.56, Dot Product: 743
Alkyl_Fragment_[22:5]
329.2483
P1O3_Fragment_[]
78.9577
C-1O-2_PUFA_Fragment_[22:5]
285.2590
C5H11O5N1P1_Fragment_[]
196.0378
Relative Intensity
m/z

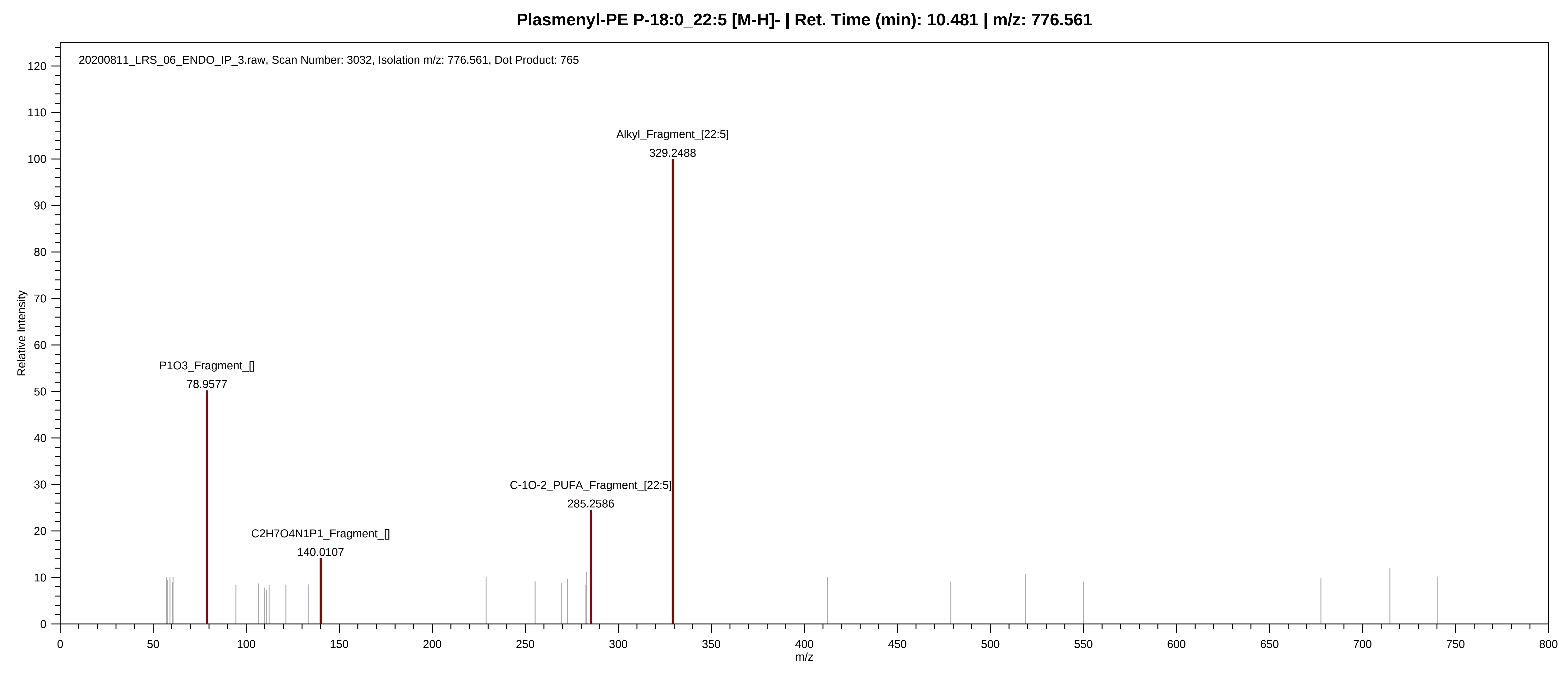

Plasmenyl-PE P-18:0_22:5 [M-H]- | Ret. Time (min): 10.481 | m/z: 776.561
20200811_LRS_06_ENDO_IP_3.raw, Scan Number: 3032, Isolation m/z: 776.561, Dot Product: 765
Alkyl_Fragment_[22:5]
329.2488
P1O3_Fragment_[]
78.9577
C-1O-2_PUFA_Fragment_[22:5]
285.2586
C2H7O4N1P1_Fragment_[]
140.0107
Relative Intensity
m/z

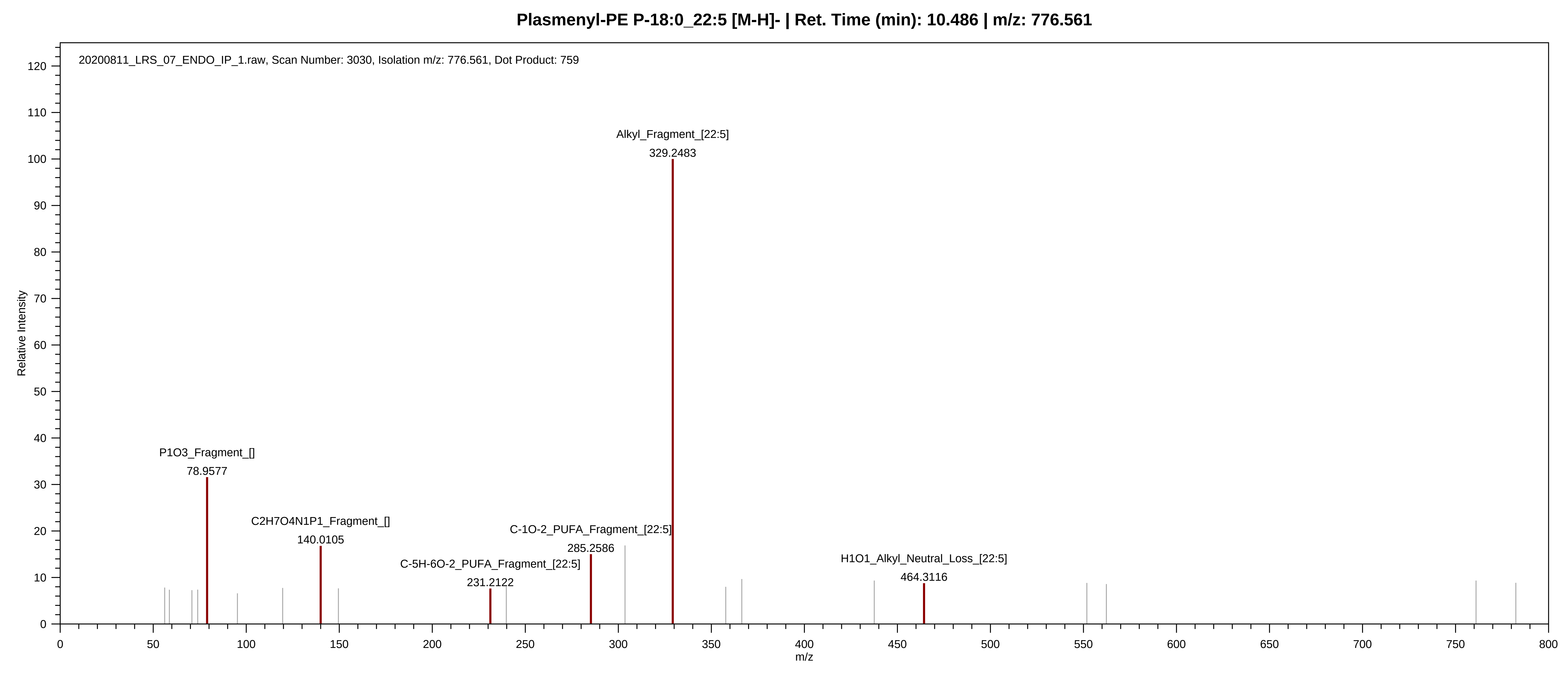

Plasmenyl-PE P-18:0_22:5 [M-H]- | Ret. Time (min): 10.486 | m/z: 776.561
20200811_LRS_07_ENDO_IP_1.raw, Scan Number: 3030, Isolation m/z: 776.561, Dot Product: 759
Alkyl_Fragment_[22:5]
329.2483
P1O3_Fragment_[]
78.9577
C2H7O4N1P1_Fragment_[]
140.0105
C-1O-2_PUFA_Fragment_[22:5]
285.2586
C-5H-6O-2_PUFA_Fragment_[22:5]
231.2122
H1O1_Alkyl_Neutral_Loss_[22:5]
464.3116
Relative Intensity
m/z

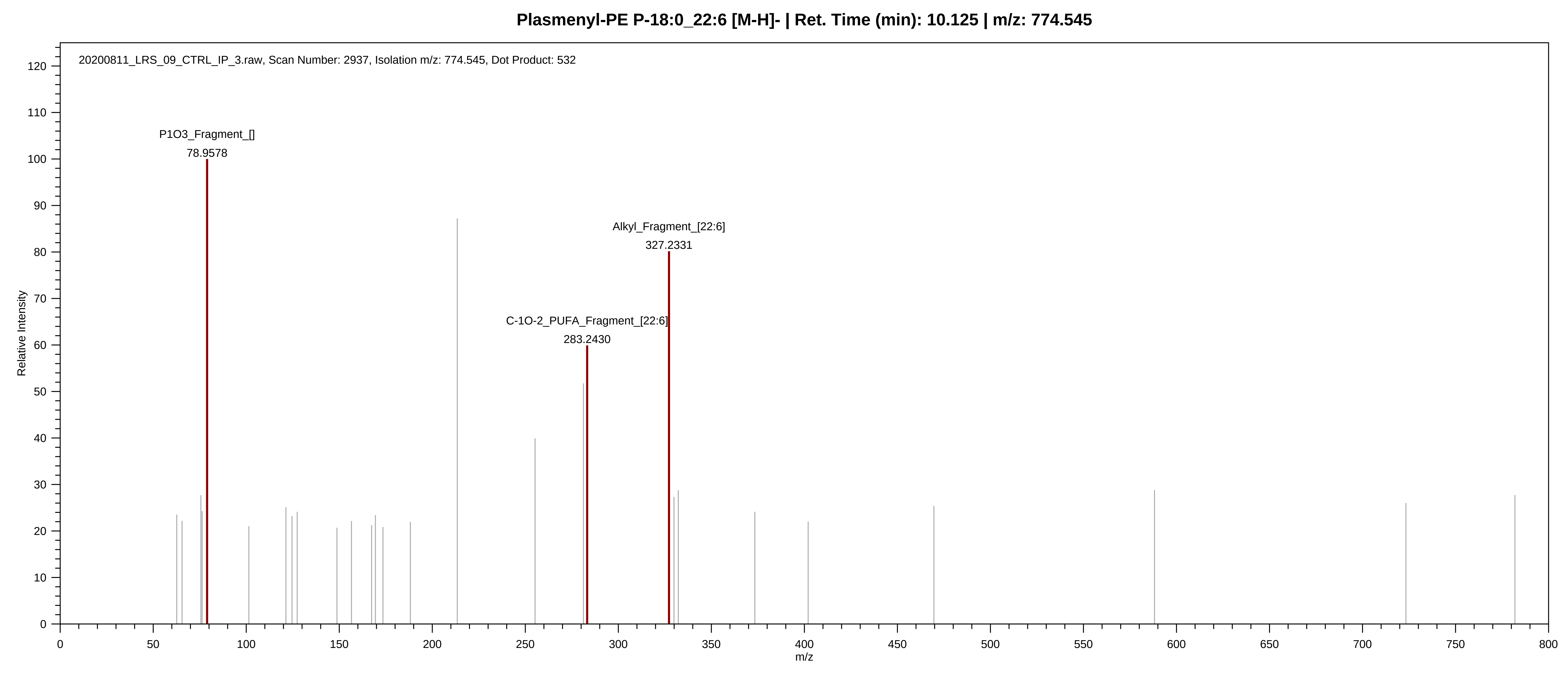

Plasmenyl-PE P-18:0_22:6 [M-H]- | Ret. Time (min): 10.125 | m/z: 774.545
20200811_LRS_09_CTRL_IP_3.raw, Scan Number: 2937, Isolation m/z: 774.545, Dot Product: 532
P1O3_Fragment_[]
78.9578
Alkyl_Fragment_[22:6]
327.2331
C-1O-2_PUFA_Fragment_[22:6]
283.2430
Relative Intensity
m/z

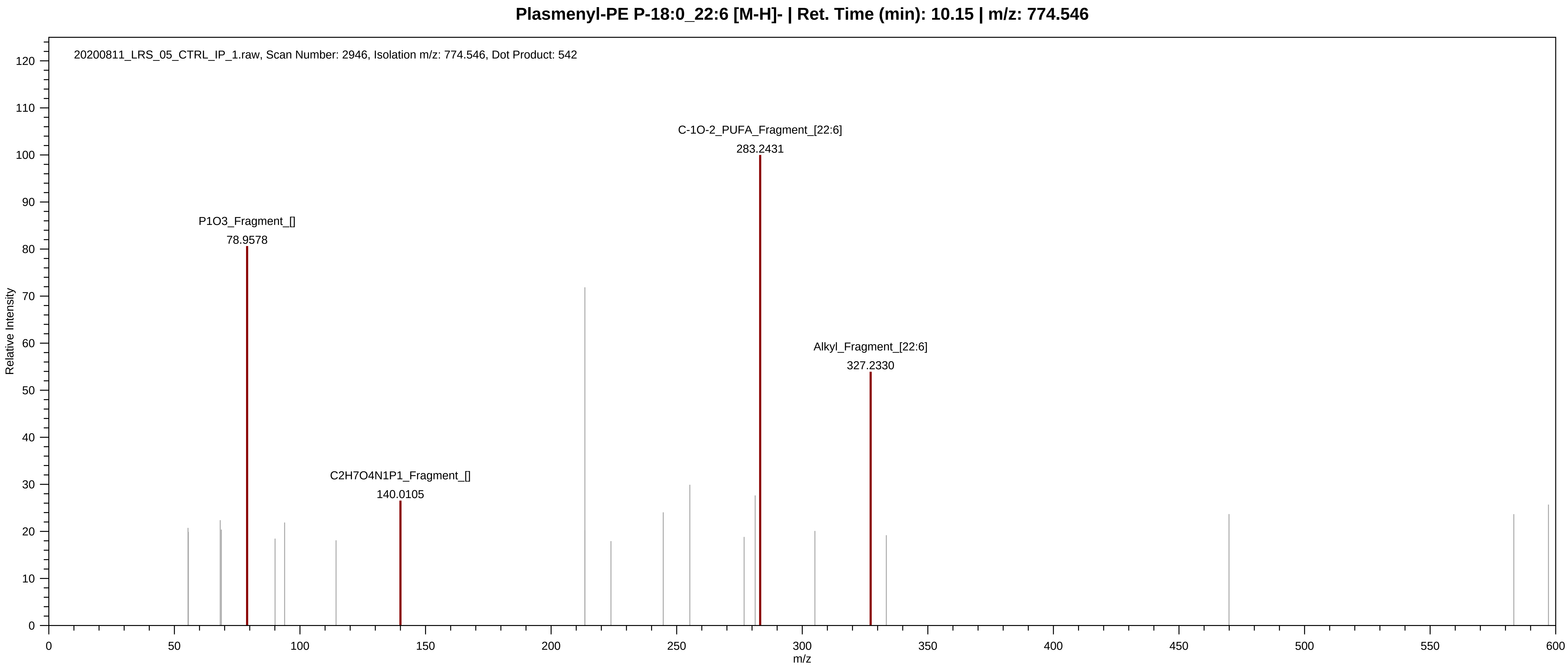
Plasmenyl-PE P-18:0_22:6 [M-H]- | Ret. Time (min): 10.15 | m/z: 774.546
20200811_LRS_05_CTRL_IP_1.raw, Scan Number: 2946, Isolation m/z: 774.546, Dot Product: 542
C-1O-2_PUFA_Fragment_[22:6]
283.2431
P1O3_Fragment_[]
78.9578
Alkyl_Fragment_[22:6]
327.2330
C2H7O4N1P1_Fragment_[]
140.0105
Relative Intensity
m/z

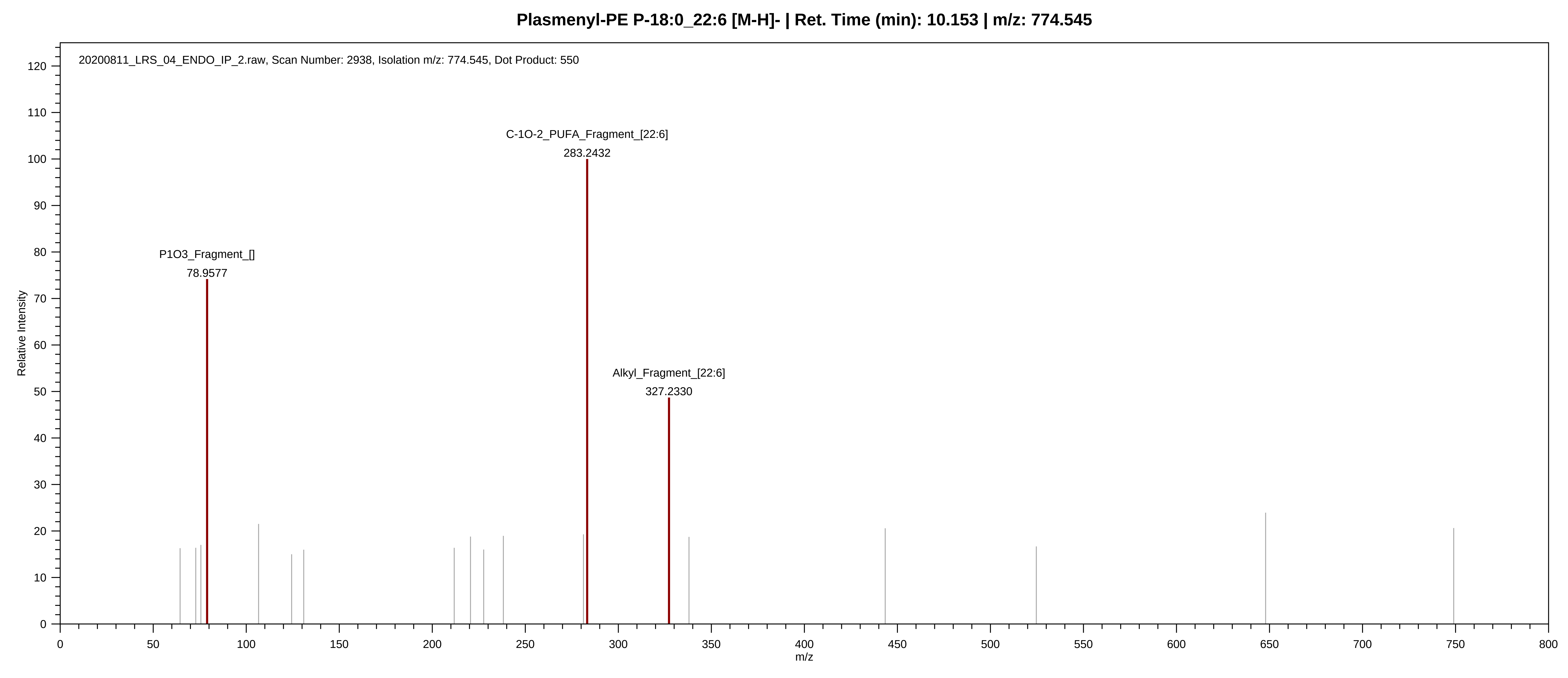
Plasmenyl-PE P-18:0_22:6 [M-H]- | Ret. Time (min): 10.153 | m/z: 774.545
20200811_LRS_04_ENDO_IP_2.raw, Scan Number: 2938, Isolation m/z: 774.545, Dot Product: 550
C-1O-2_PUFA_Fragment_[22:6]
283.2432
P1O3_Fragment_[]
78.9577
Alkyl_Fragment_[22:6]
327.2330
Relative Intensity
m/z

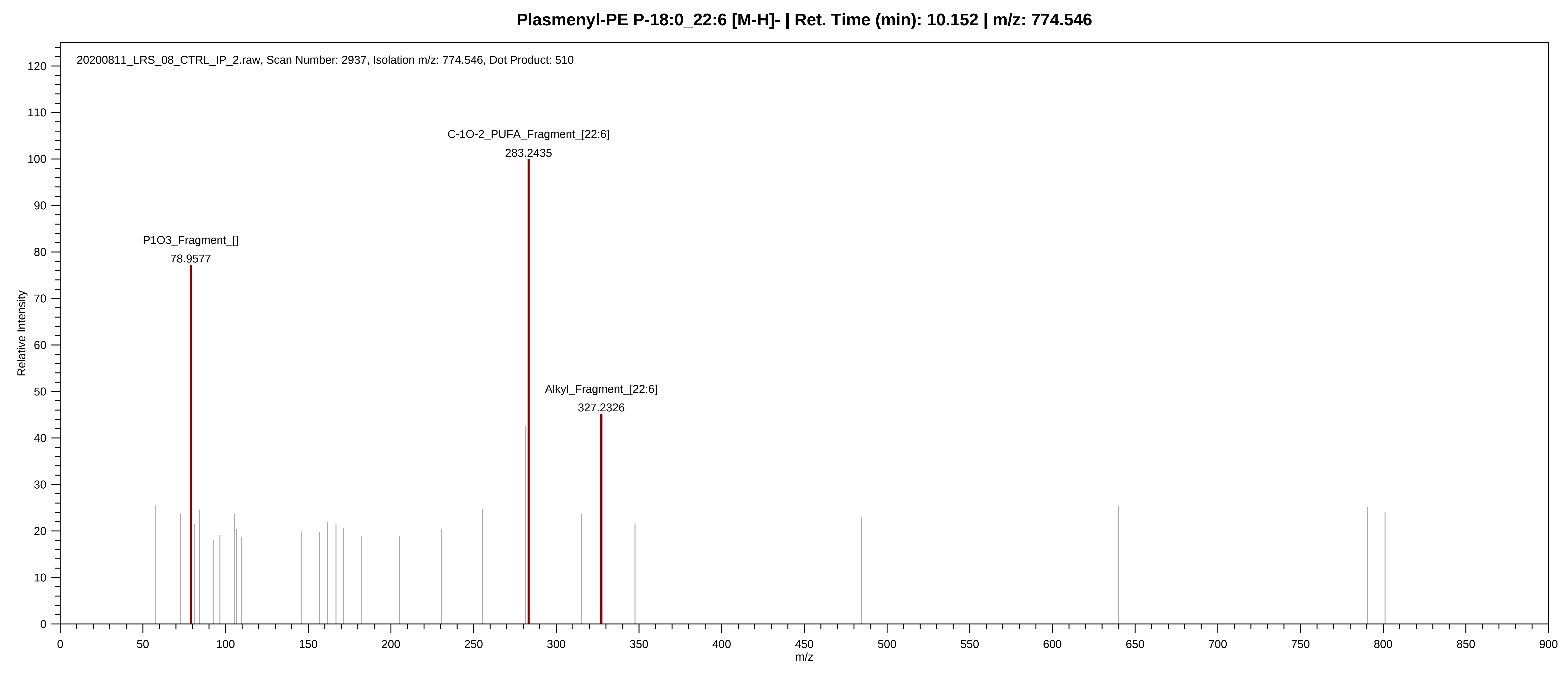

Plasmenyl-PE P-18:0_22:6 [M-H]- | Ret. Time (min): 10.152 | m/z: 774.546
20200811_LRS_08_CTRL_IP_2.raw, Scan Number: 2937, Isolation m/z: 774.546, Dot Product: 510
C-1O-2_PUFA_Fragment_[22:6]
283.2435
P1O3_Fragment_[]
78.9577
Alkyl_Fragment_[22:6]
327.2326
Relative Intensity
m/z

Plasmenyl-PE P-18:1_16:1 [M-H]- | Ret. Time (min): 9.612 | m/z: 698.513

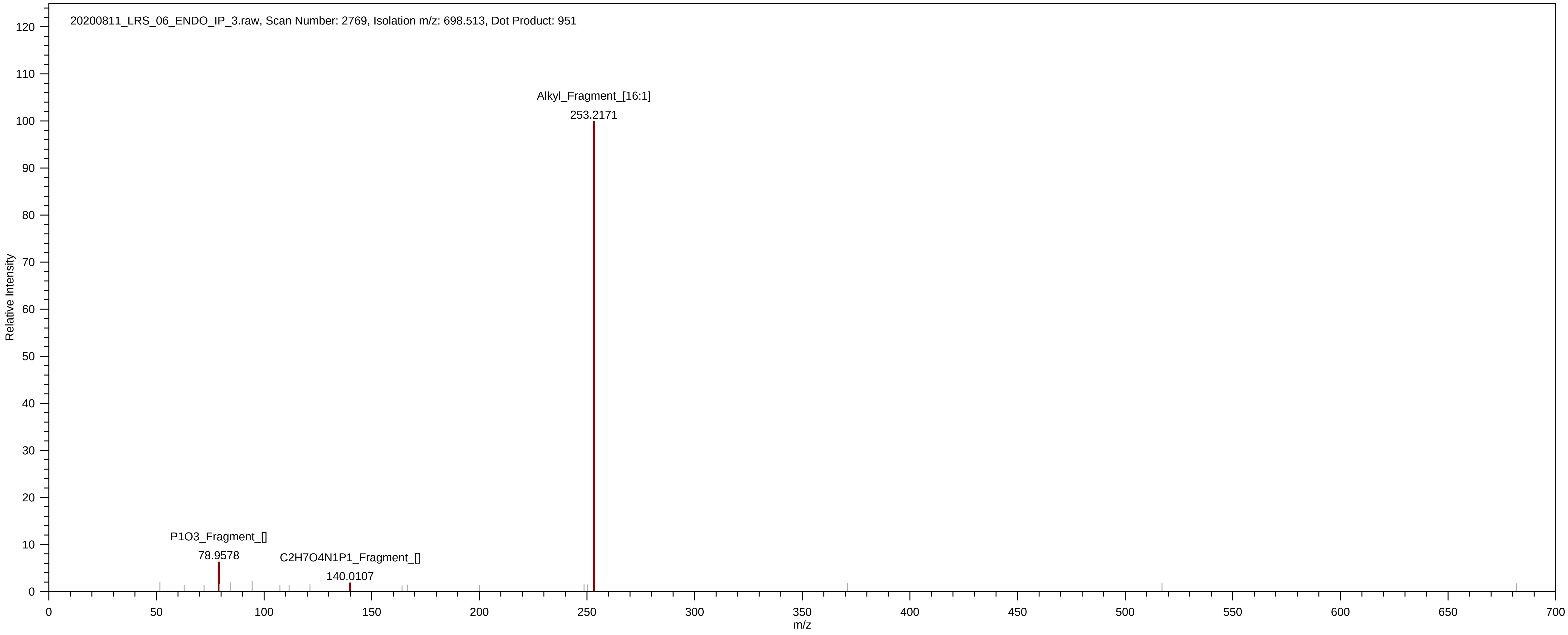

Plasmenyl-PE P-18:1_16:1 [M-H]- | Ret. Time (min): 9.626 | m/z: 698.513

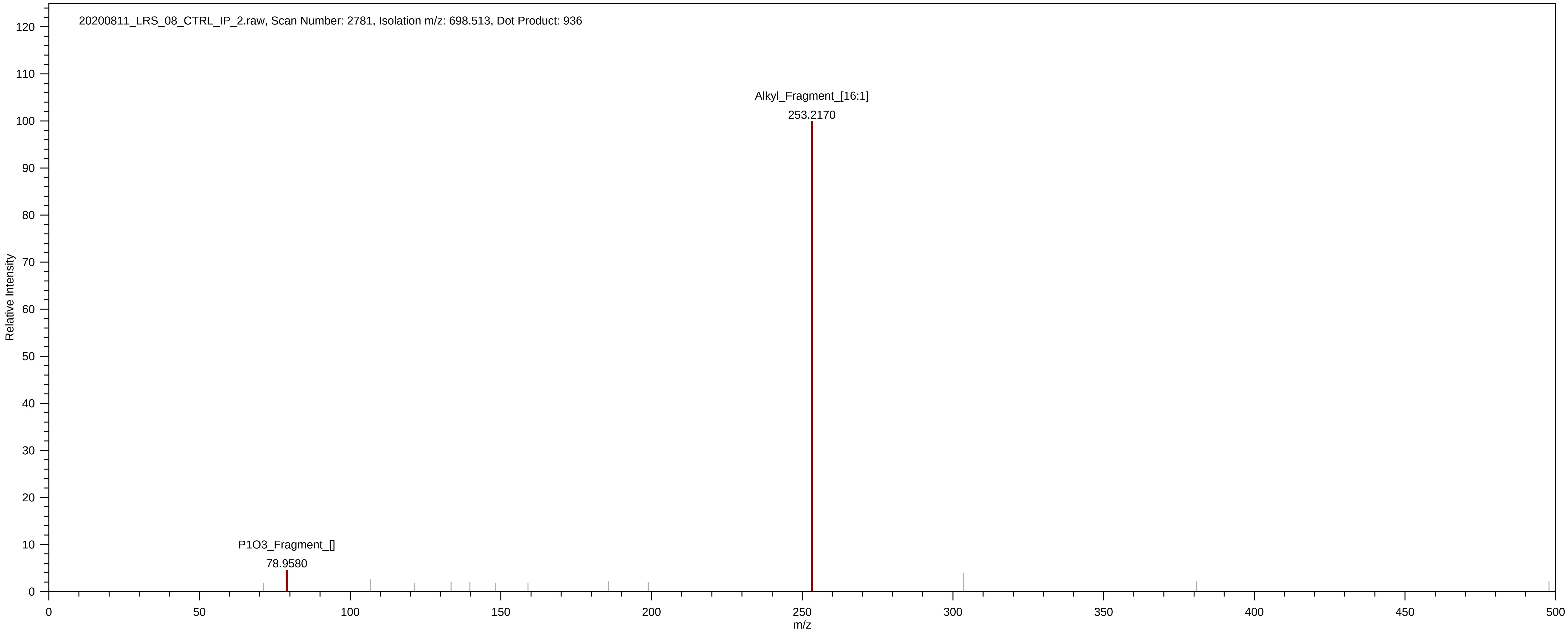

Plasmenyl-PE P-18:1_16:1 [M-H]- | Ret. Time (min): 9.632 | m/z: 698.513

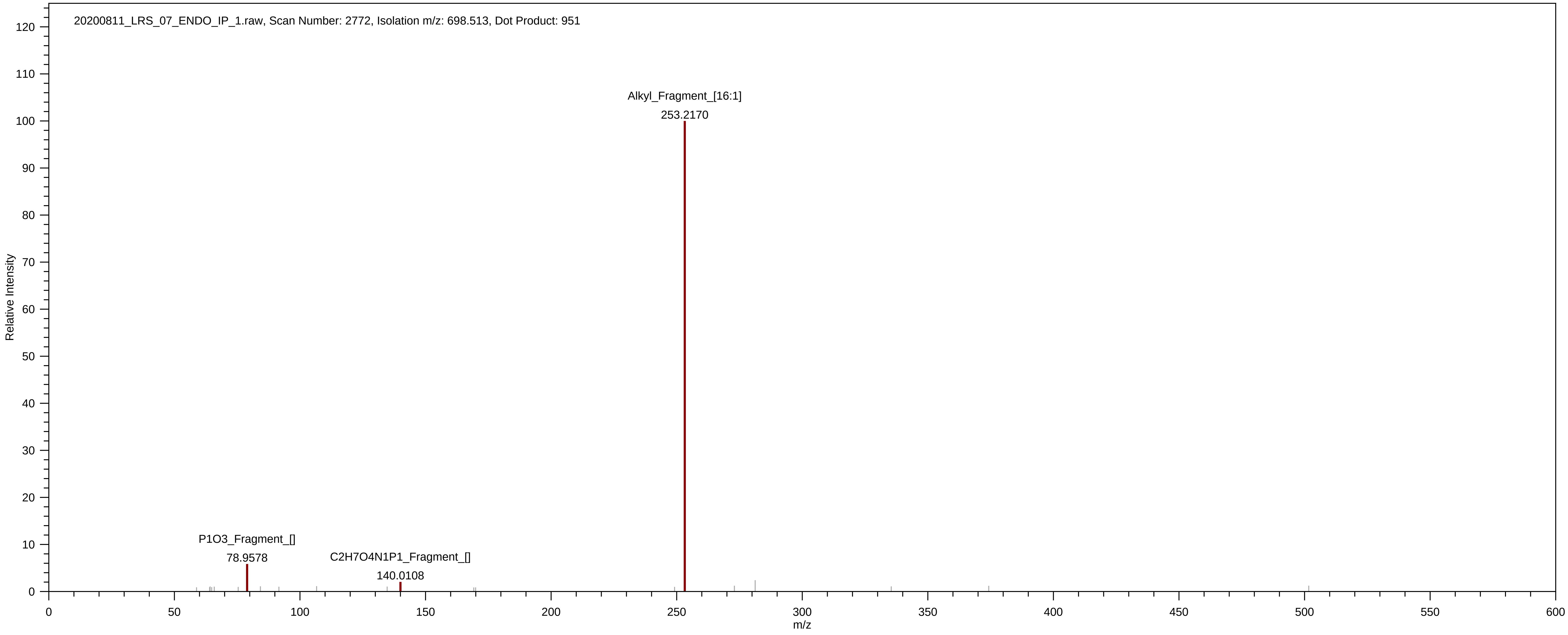

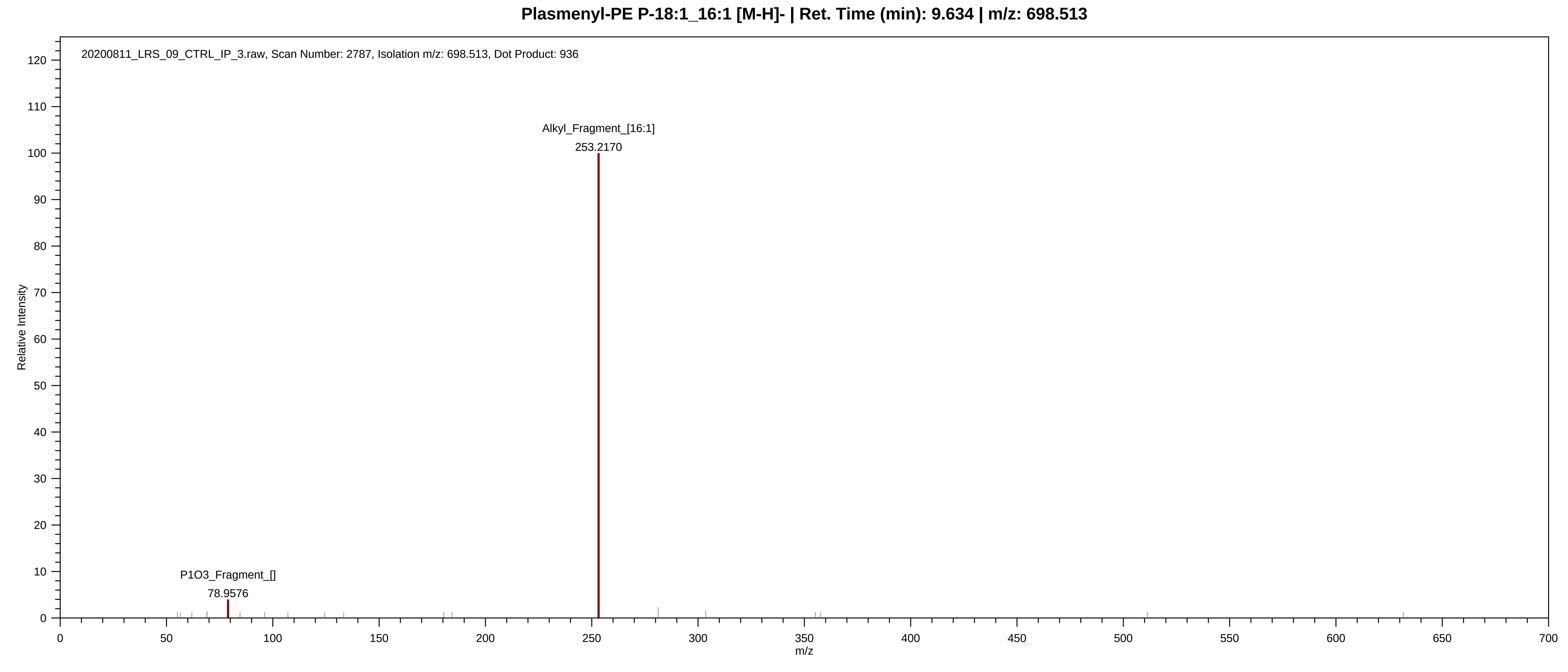

Plasmenyl-PE P-18:1_16:1 [M-H]- | Ret. Time (min): 9.634 | m/z: 698.513
20200811_LRS_09_CTRL_IP_3.raw, Scan Number: 2787, Isolation m/z: 698.513, Dot Product: 936
Alkyl_Fragment_[16:1]
253.2170
P1O3_Fragment_[]
78.9576
Relative Intensity
m/z

Plasmenyl-PE P-18:1_16:1 [M-H]- | Ret. Time (min): 9.647 | m/z: 698.513

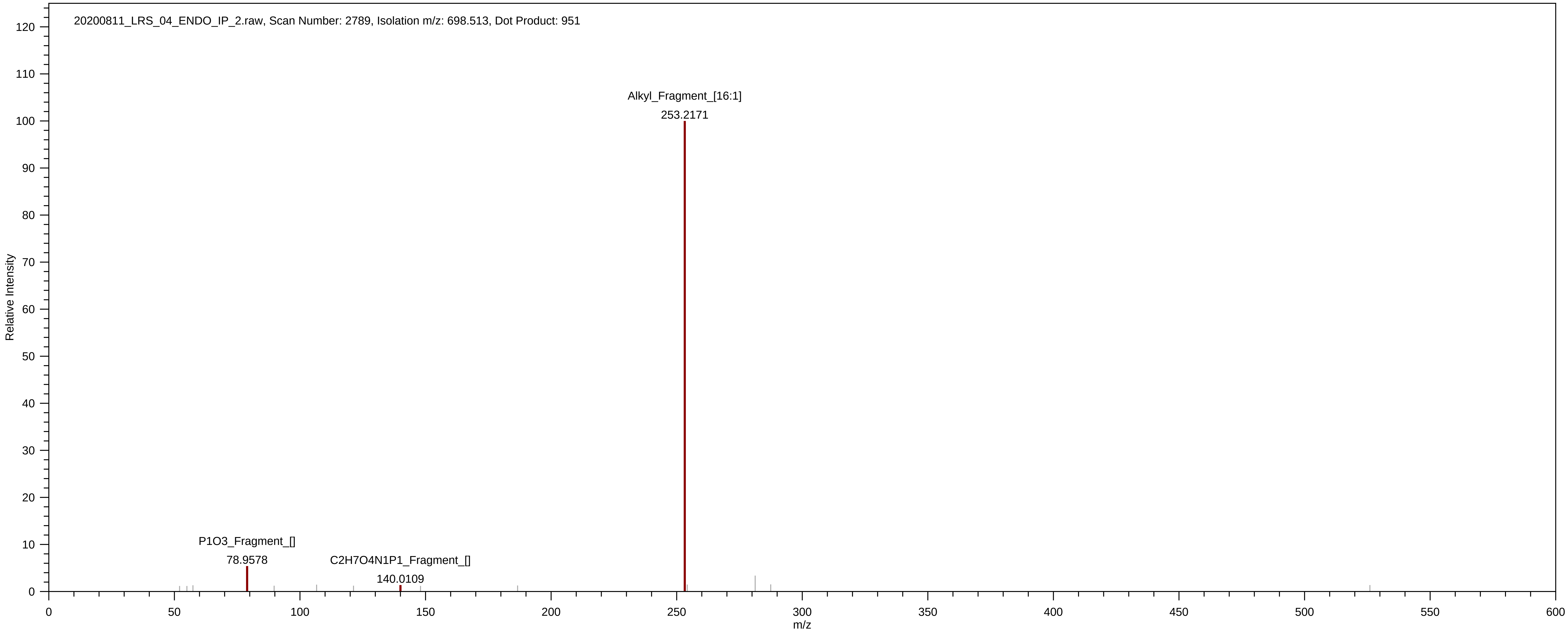

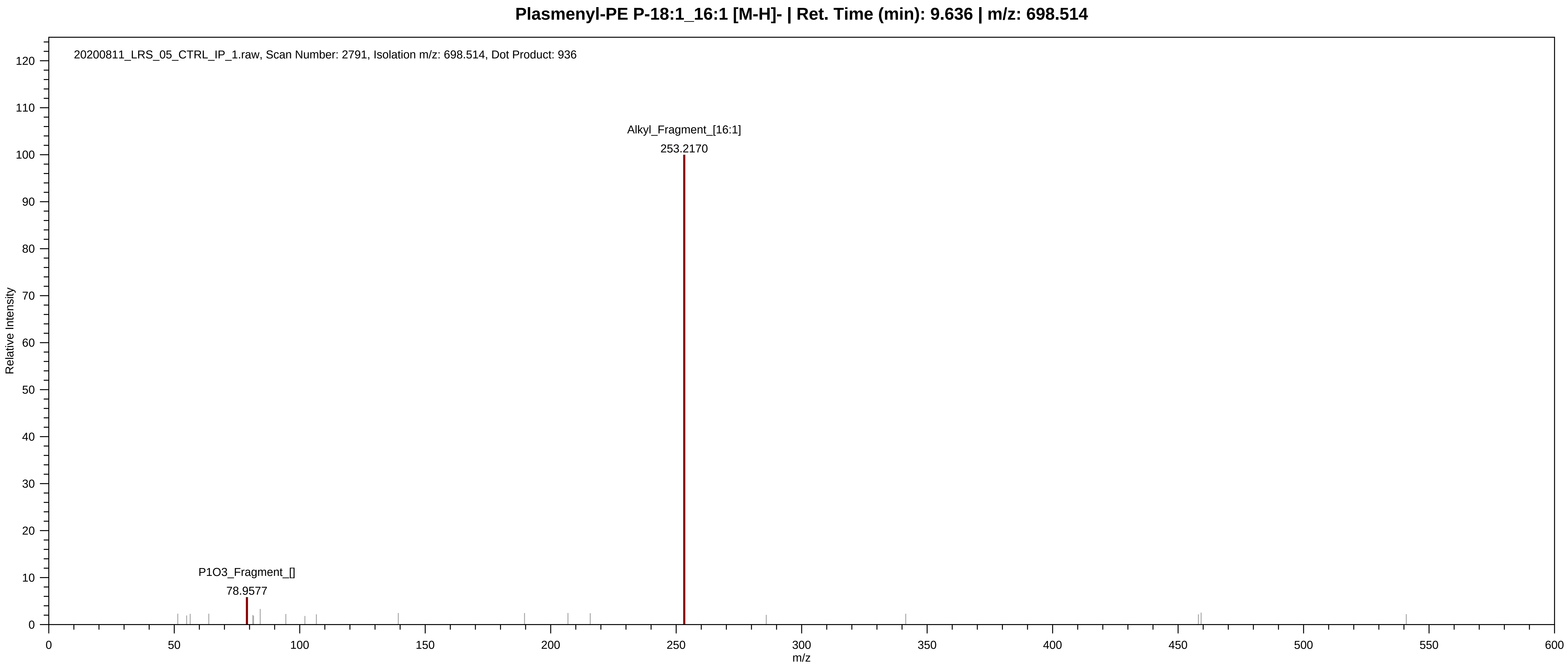

Plasmenyl-PE P-18:1_16:1 [M-H]- | Ret. Time (min): 9.636 | m/z: 698.514
20200811_LRS_05_CTRL_IP_1.raw, Scan Number: 2791, Isolation m/z: 698.514, Dot Product: 936
Alkyl_Fragment_[16:1]
253.2170
P1O3_Fragment_[]
78.9577
Relative Intensity
m/z
0
10
20
30
40
50
60
70
80
90
100
110
120
50
100
150
200
250
300
350
400
450
500
550
600

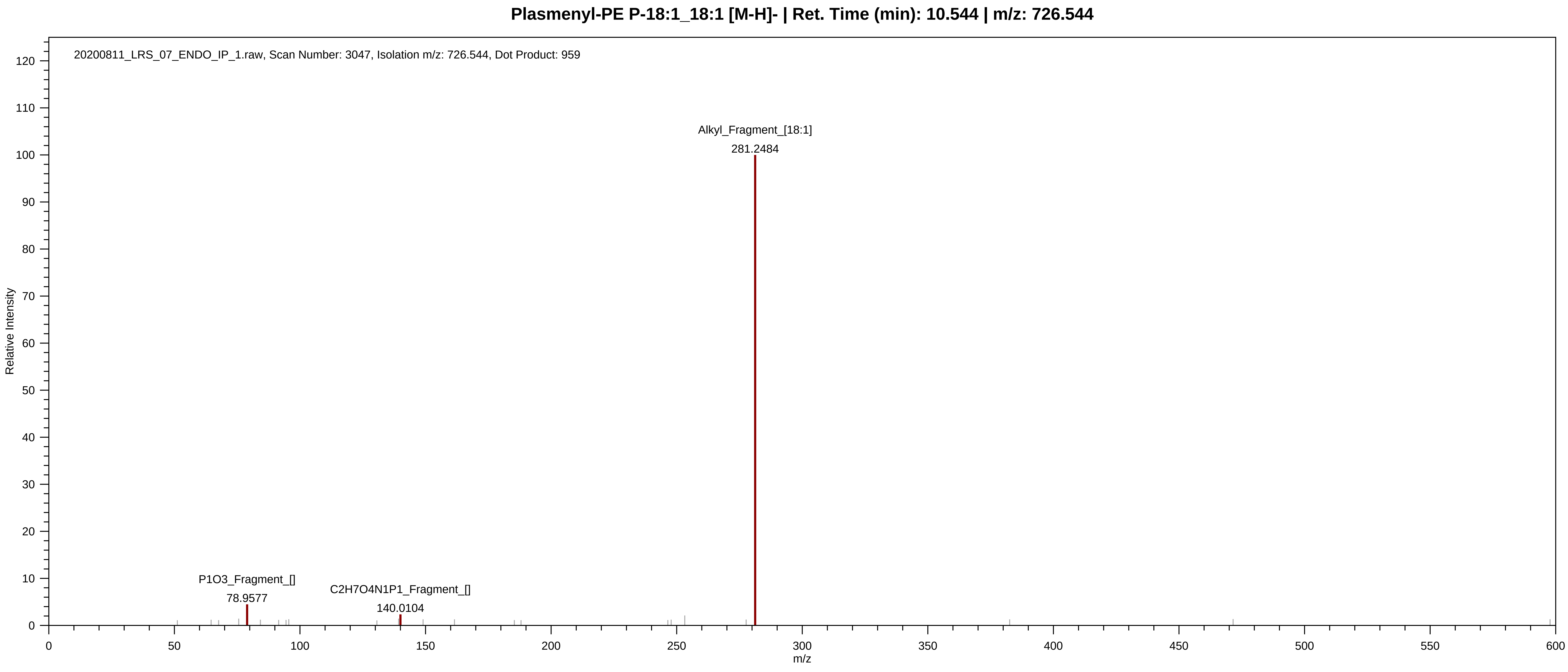

Plasmenyl-PE P-18:1_18:1 [M-H]- | Ret. Time (min): 10.544 | m/z: 726.544
20200811_LRS_07_ENDO_IP_1.raw, Scan Number: 3047, Isolation m/z: 726.544, Dot Product: 959
Alkyl_Fragment_[18:1]
281.2484
P1O3_Fragment_[]
78.9577
C2H7O4N1P1_Fragment_[]
140.0104
Relative Intensity
m/z

Plasmenyl-PE P-18:1_18:1 [M-H]- | Ret. Time (min): 10.558 | m/z: 726.544

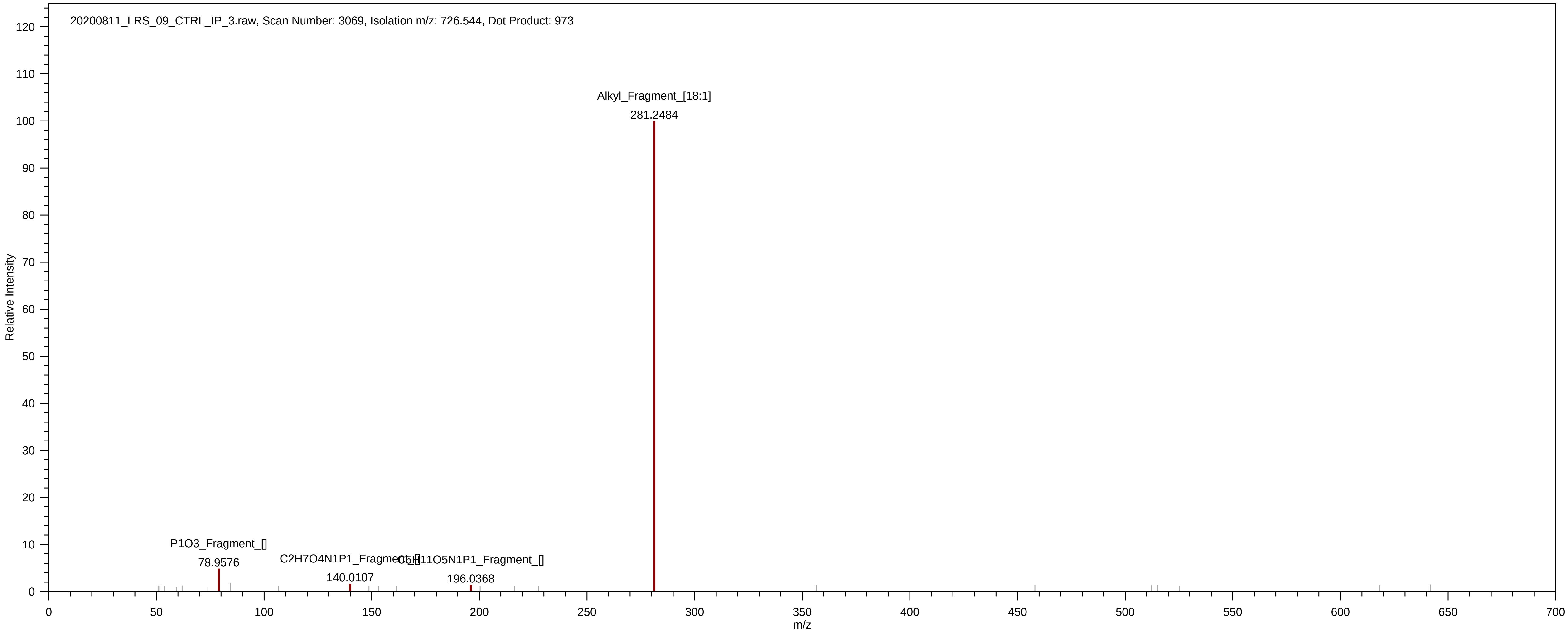

Plasmenyl-PE P-18:1_18:1 [M-H]- | Ret. Time (min): 10.562 | m/z: 726.544

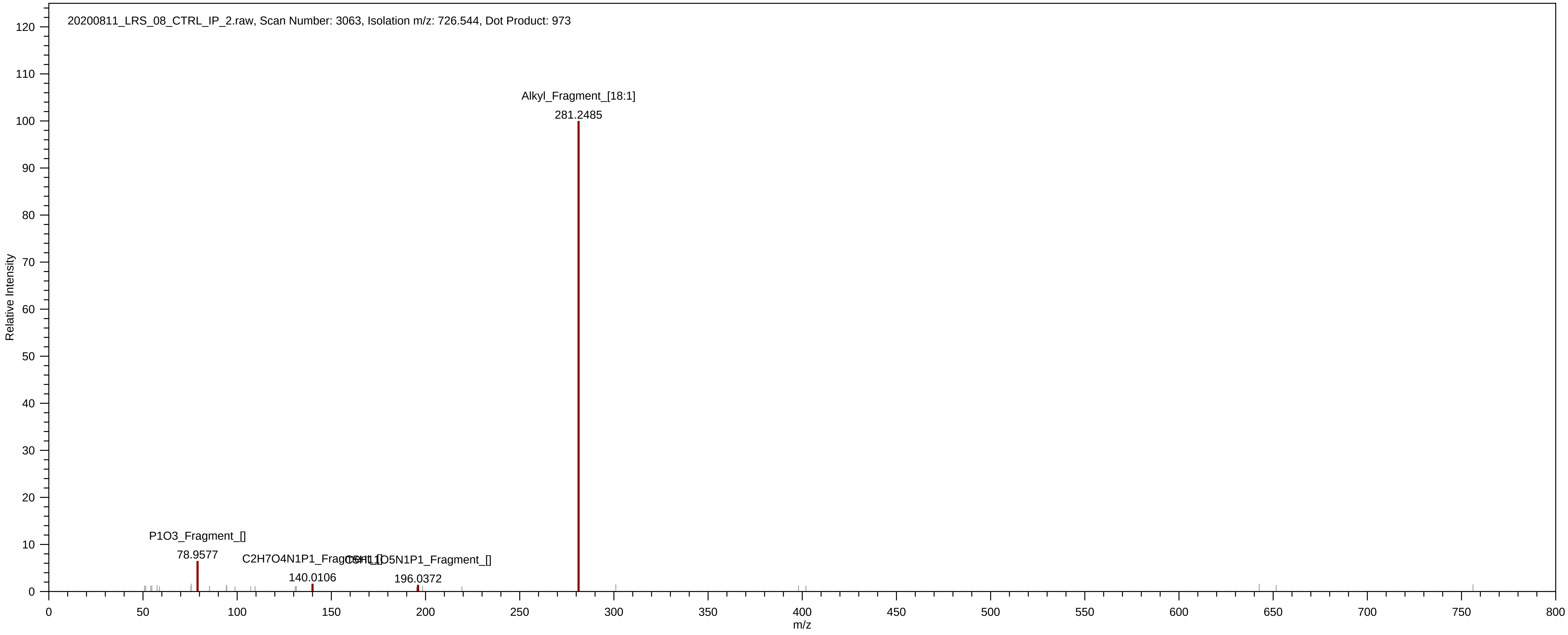

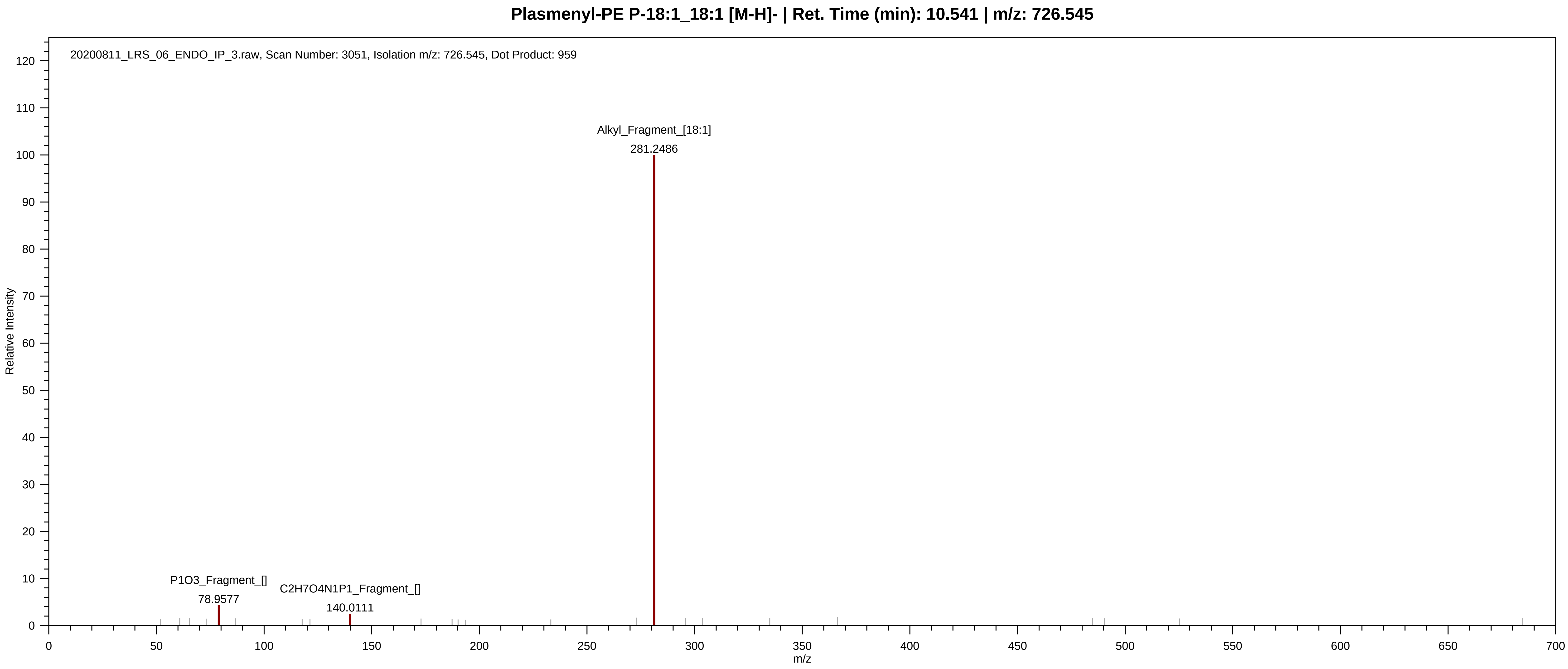
Plasmenyl-PE P-18:1_18:1 [M-H]- | Ret. Time (min): 10.541 | m/z: 726.545
20200811_LRS_06_ENDO_IP_3.raw, Scan Number: 3051, Isolation m/z: 726.545, Dot Product: 959
Alkyl_Fragment_[18:1]
281.2486
P1O3_Fragment_[]
78.9577
C2H7O4N1P1_Fragment_[]
140.0111
Relative Intensity
m/z

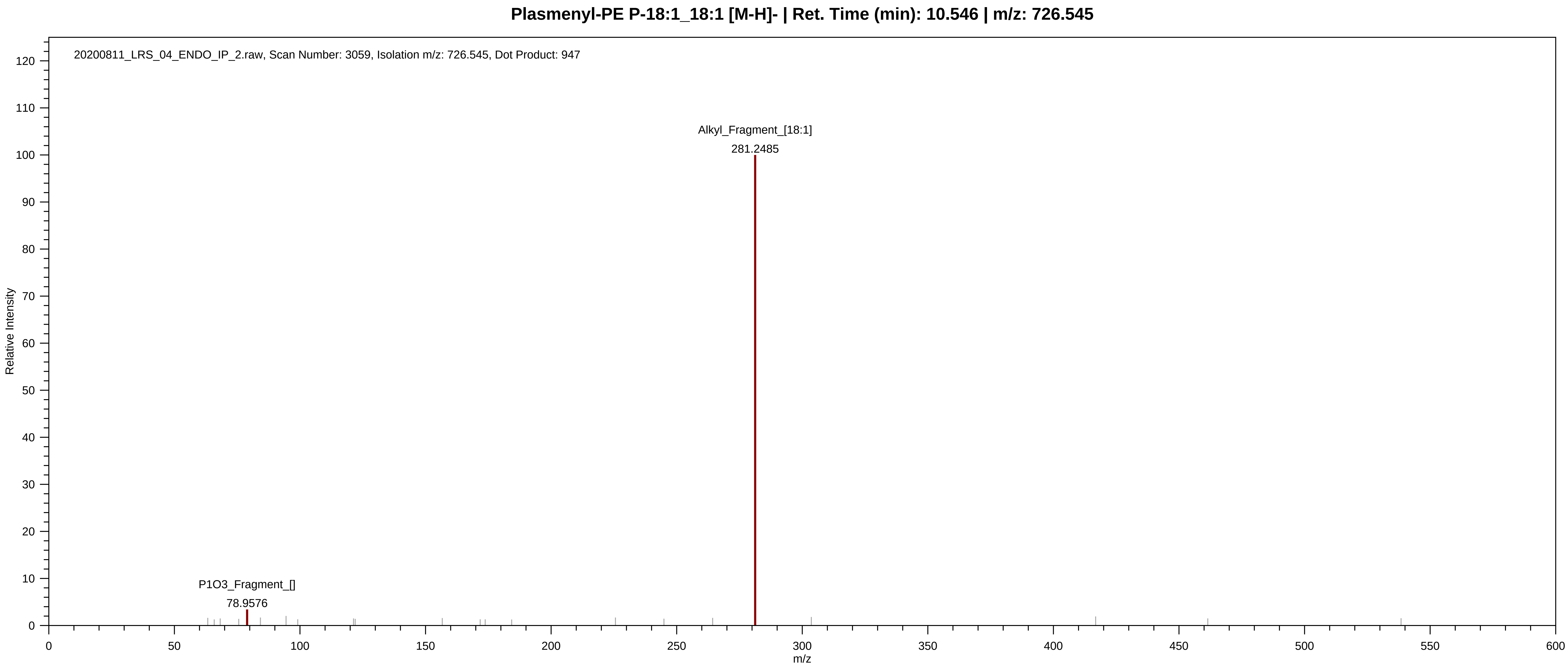
Plasmenyl-PE P-18:1_18:1 [M-H]- | Ret. Time (min): 10.546 | m/z: 726.545
20200811_LRS_04_ENDO_IP_2.raw, Scan Number: 3059, Isolation m/z: 726.545, Dot Product: 947
Alkyl_Fragment_[18:1]
281.2485
P1O3_Fragment_[]
78.9576
Relative Intensity
m/z
0
10
20
30
40
50
60
70
80
90
100
110
120
0
50
100
150
200
250
300
350
400
450
500
550
600

Plasmenyl-PE P-18:1_18:1 [M-H]- | Ret. Time (min): 10.572 | m/z: 726.545

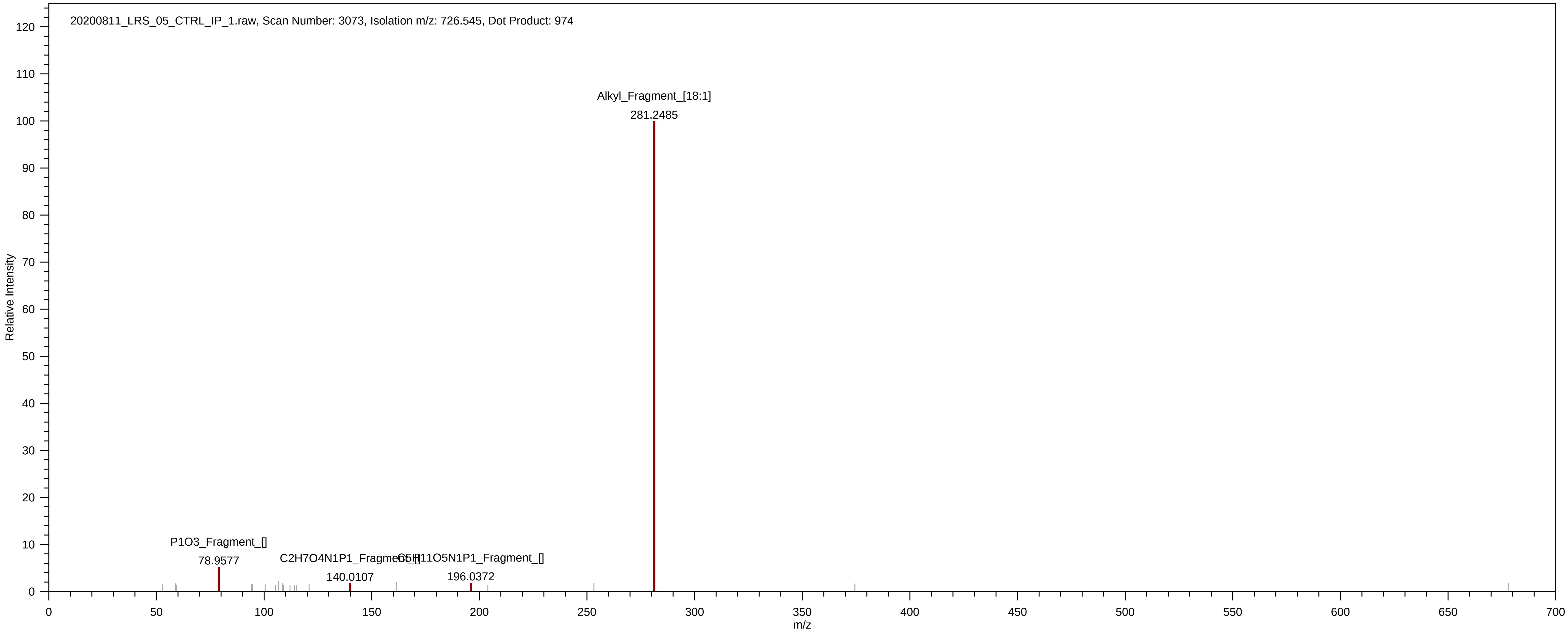

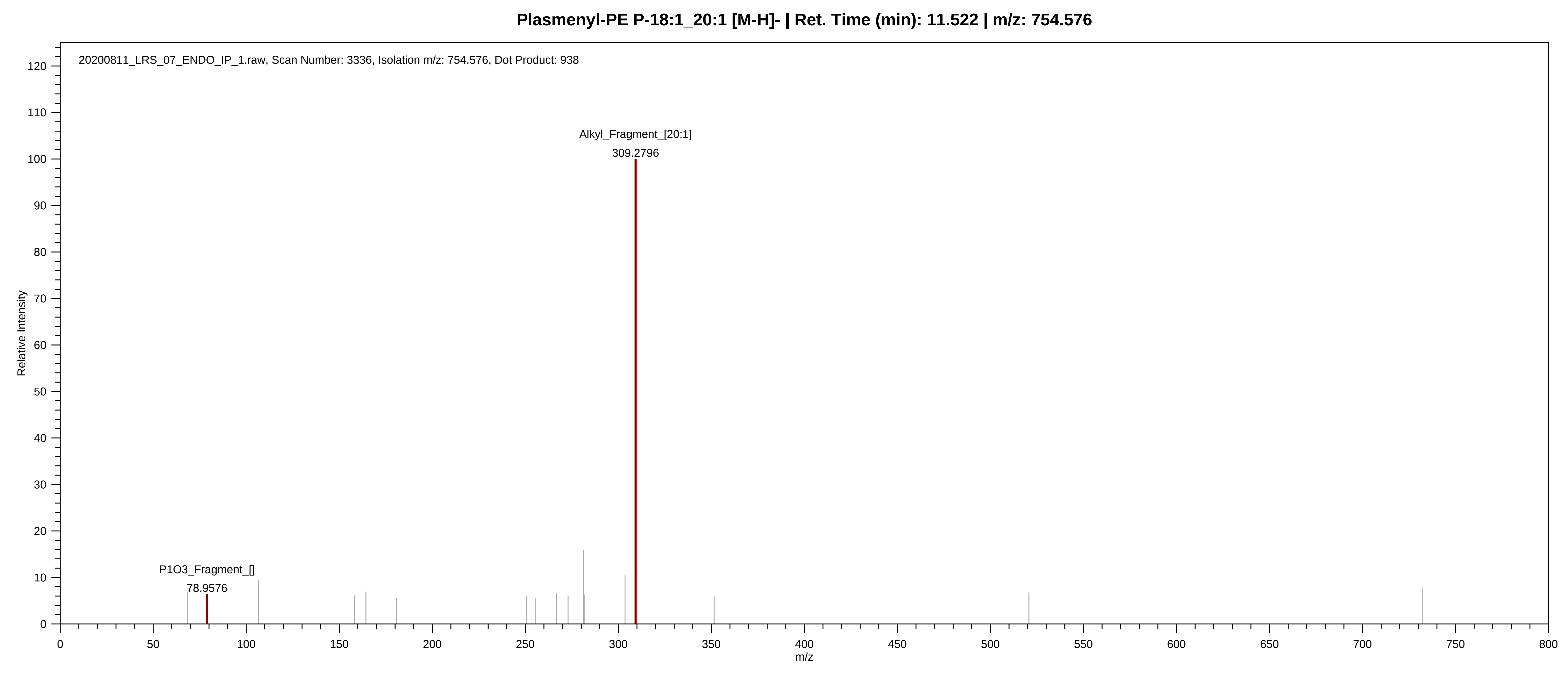
Plasmenyl-PE P-18:1_20:1 [M-H]- | Ret. Time (min): 11.522 | m/z: 754.576
20200811_LRS_07_ENDO_IP_1.raw, Scan Number: 3336, Isolation m/z: 754.576, Dot Product: 938
Alkyl_Fragment_[20:1]
309.2796
P1O3_Fragment_[]
78.9576
Relative Intensity
m/z
0
10
20
30
40
50
60
70
80
90
100
110
120
0
50
100
150
200
250
300
350
400
450
500
550
600
650
700
750
800

Plasmenyl-PE P-18:1_20:1 [M-H]- | Ret. Time (min): 11.559 | m/z: 754.576

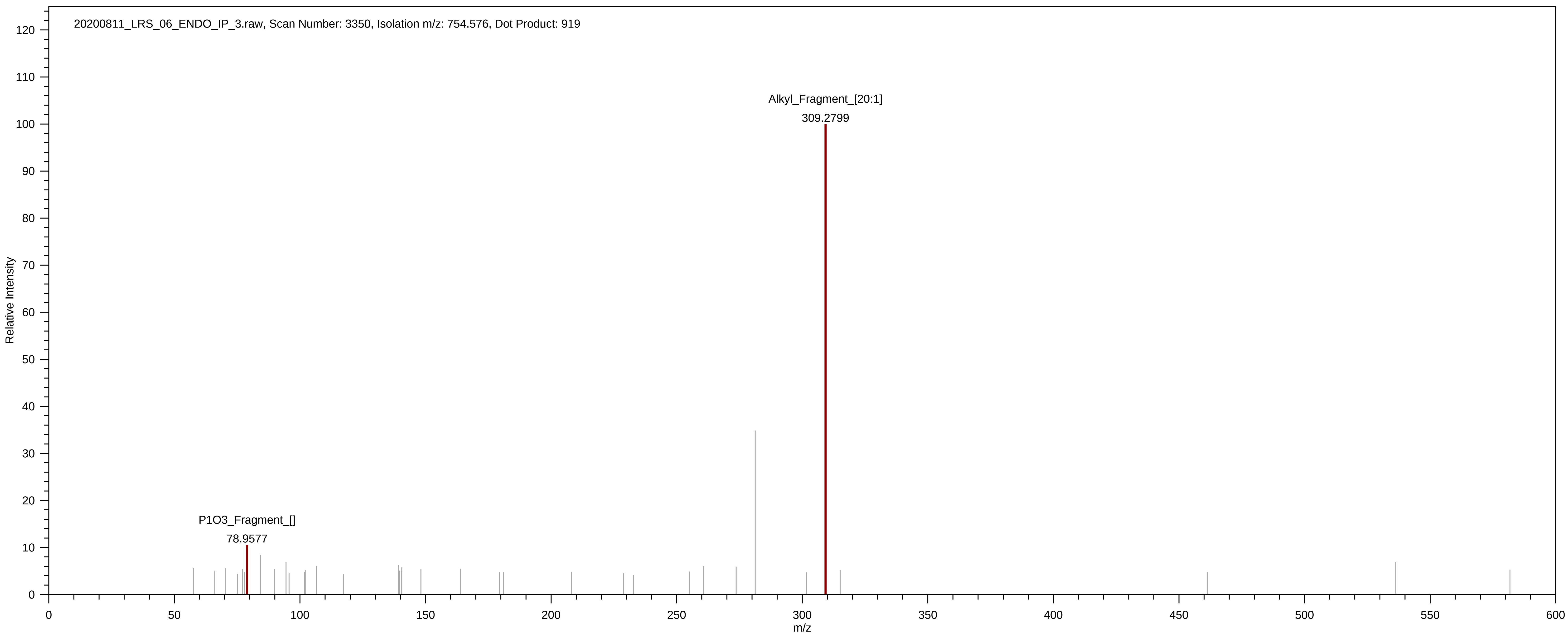

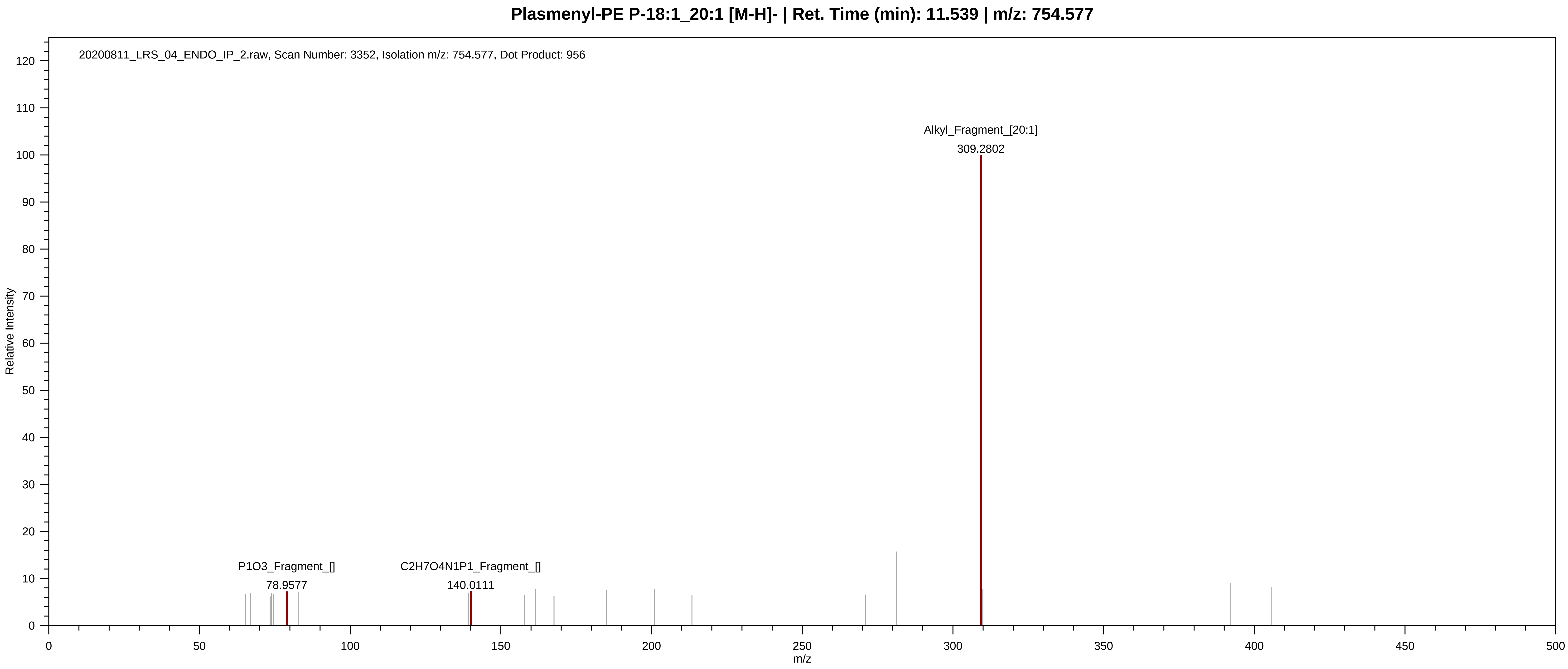
Plasmenyl-PE P-18:1_20:1 [M-H]- | Ret. Time (min): 11.539 | m/z: 754.577
20200811_LRS_04_ENDO_IP_2.raw, Scan Number: 3352, Isolation m/z: 754.577, Dot Product: 956
Alkyl_Fragment_[20:1]
309.2802
P1O3_Fragment_[]
78.9577
C2H7O4N1P1_Fragment_[]
140.0111
Relative Intensity
m/z

Plasmenyl-PE P-18:1_22:5 [M-H]- | Ret. Time (min): 9.624 | m/z: 774.546

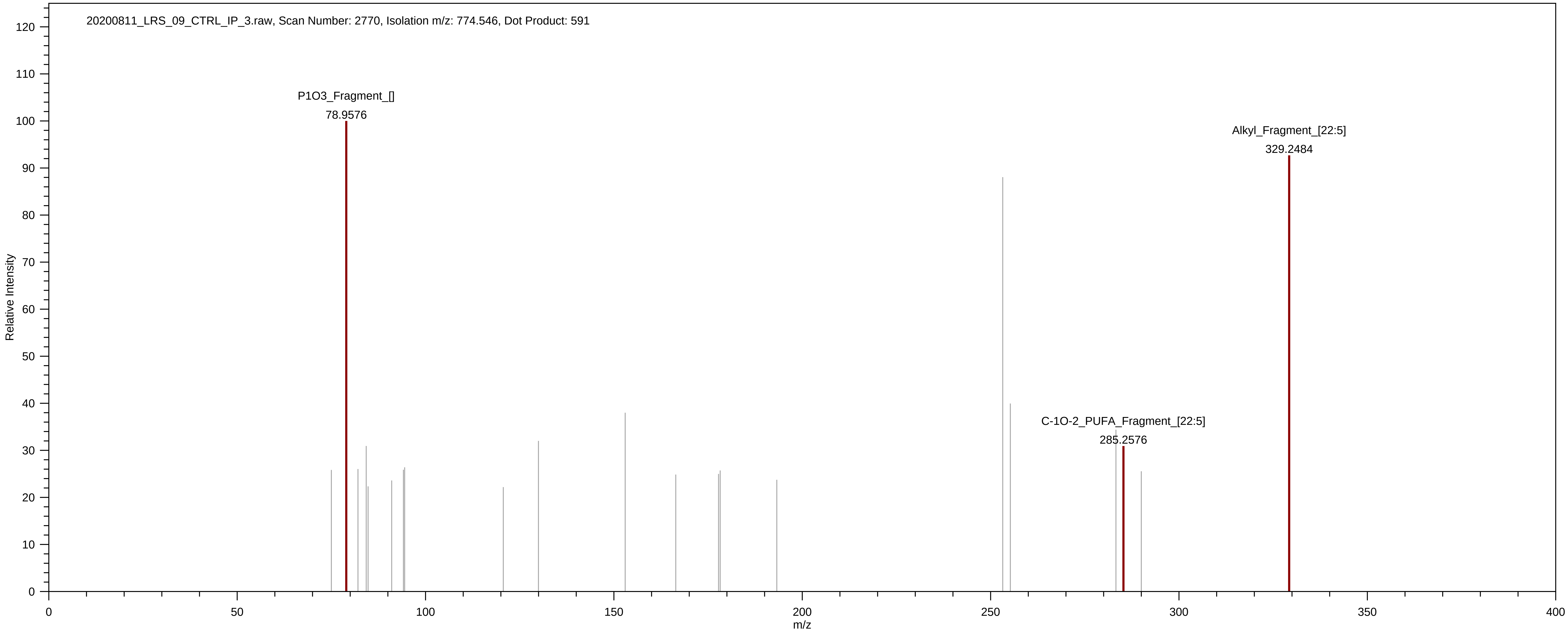

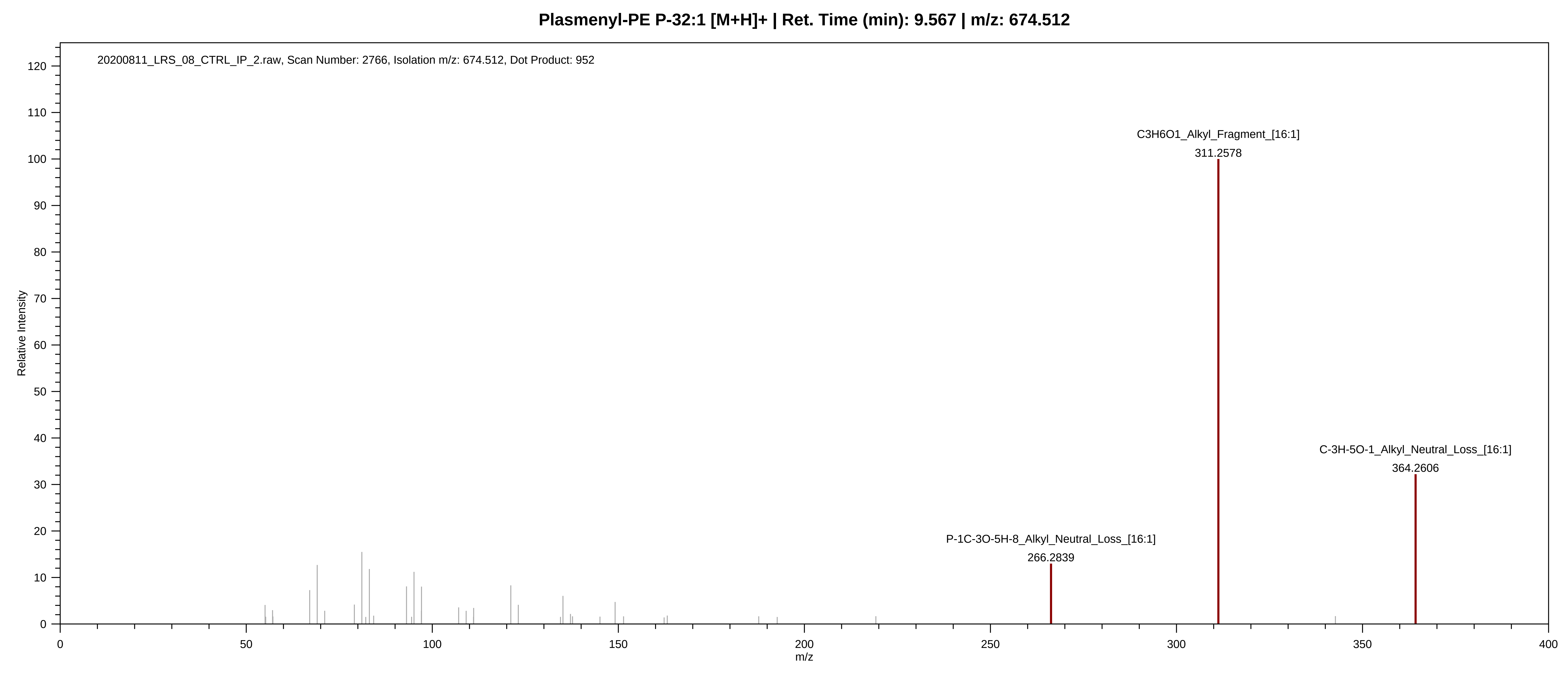

Plasmenyl-PE P-32:1 [M+H]+ | Ret. Time (min): 9.567 | m/z: 674.512
20200811_LRS_08_CTRL_IP_2.raw, Scan Number: 2766, Isolation m/z: 674.512, Dot Product: 952
C3H6O1_Alkyl_Fragment_[16:1]
311.2578
C-3H-5O-1_Alkyl_Neutral_Loss_[16:1]
364.2606
P-1C-3O-5H-8_Alkyl_Neutral_Loss_[16:1]
266.2839
Relative Intensity
m/z

Plasmenyl-PE P-32:1 [M+H]+ | Ret. Time (min): 9.571 | m/z: 674.512

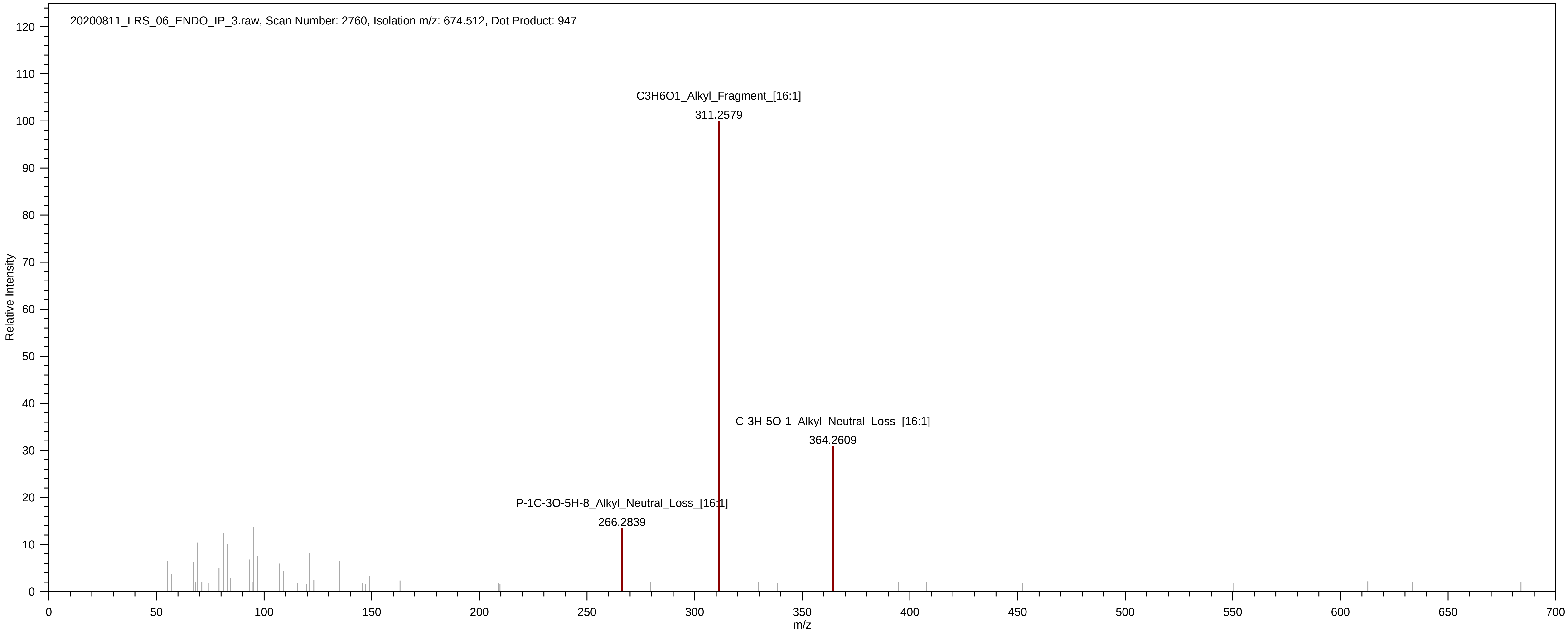

Plasmenyl-PE P-32:1 [M+H]+ | Ret. Time (min): 9.574 | m/z: 674.511

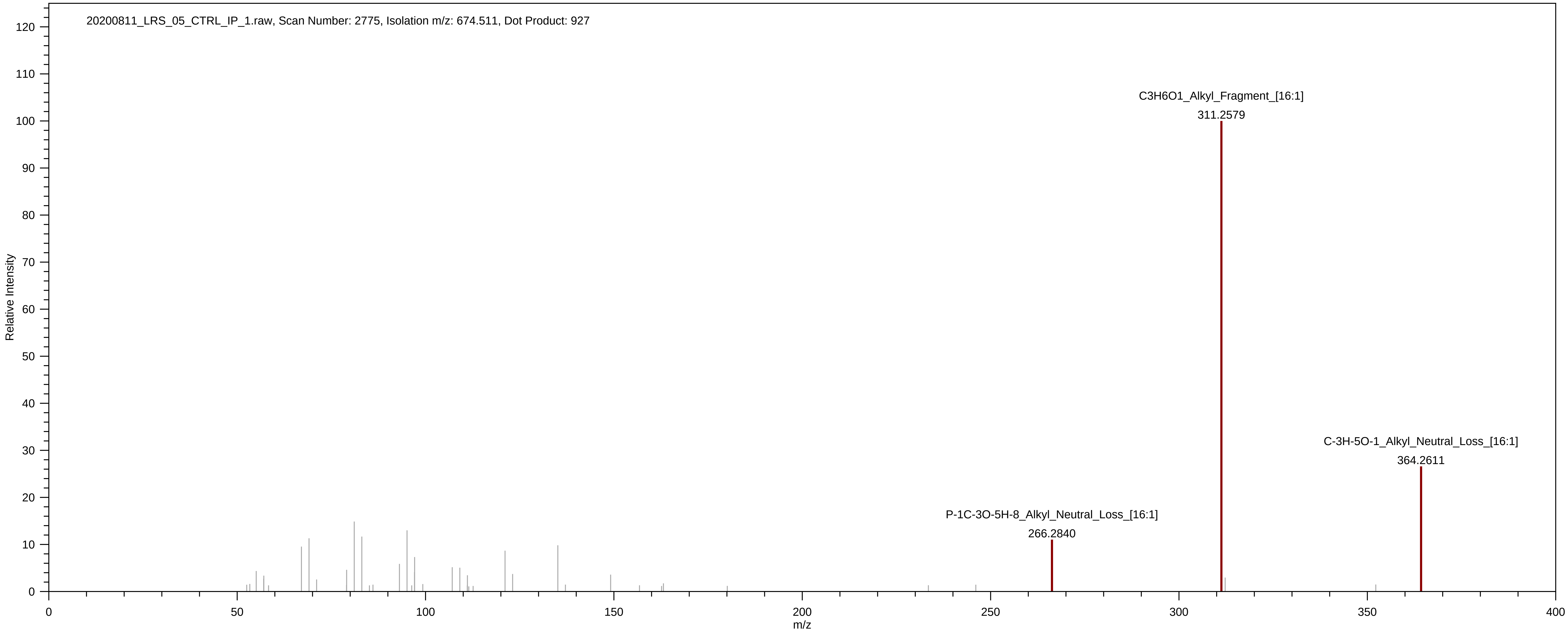

Plasmenyl-PE P-32:1 [M+H]+ | Ret. Time (min): 9.585 | m/z: 674.512

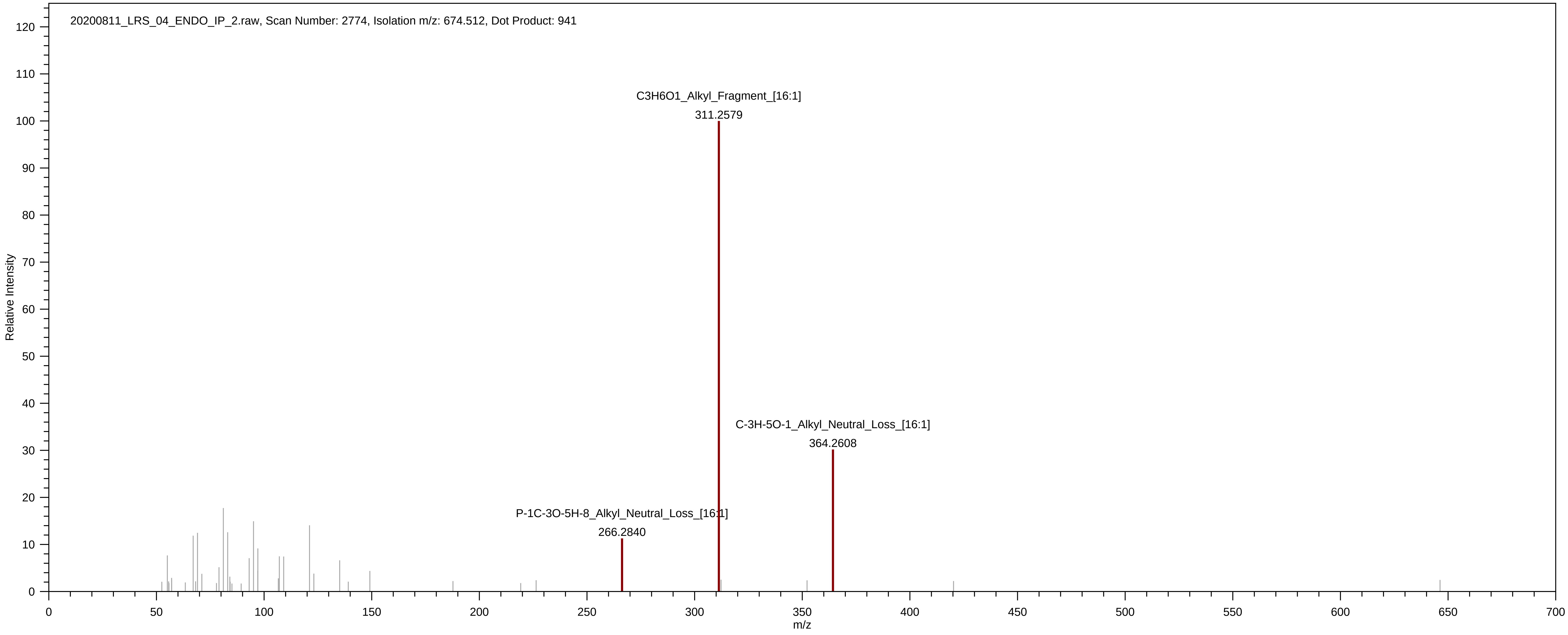

Plasmenyl-PE P-32:1 [M+H]+ | Ret. Time (min): 9.57 | m/z: 674.511

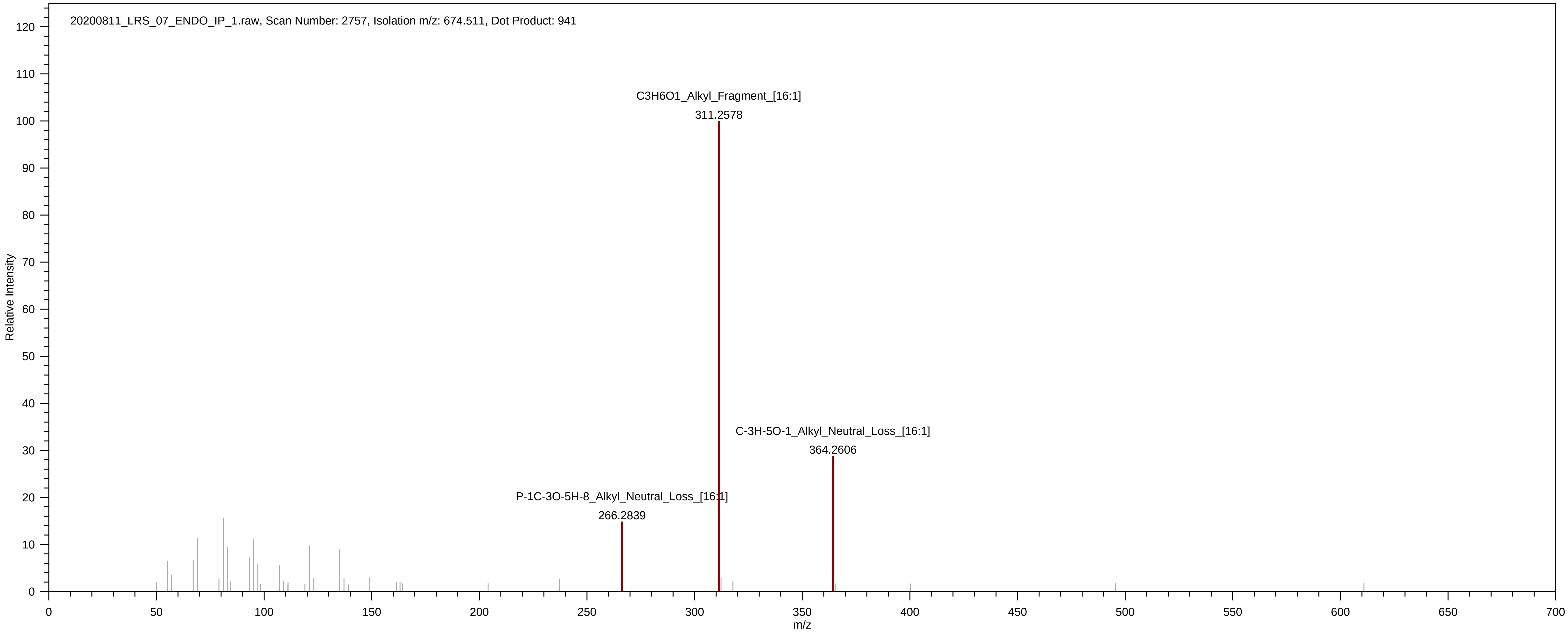

Plasmenyl-PE P-32:1 [M+H]+ | Ret. Time (min): 9.574 | m/z: 674.512

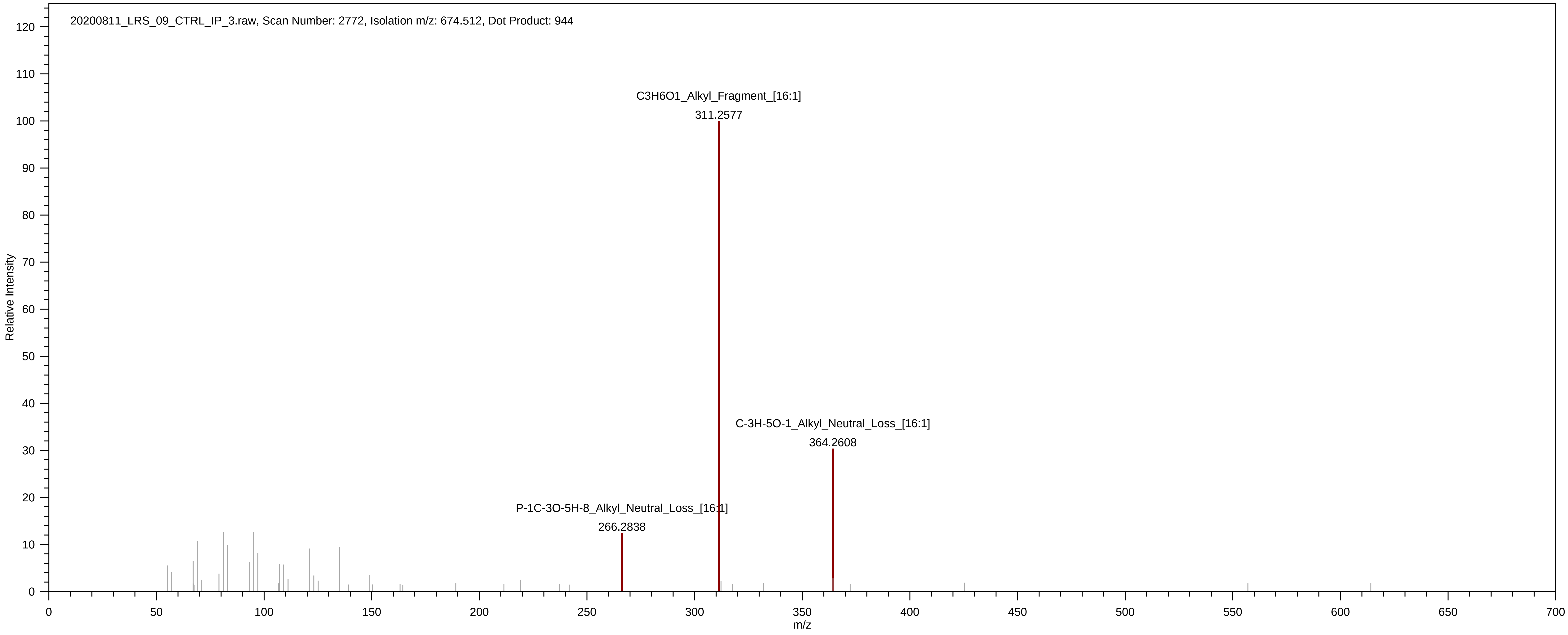

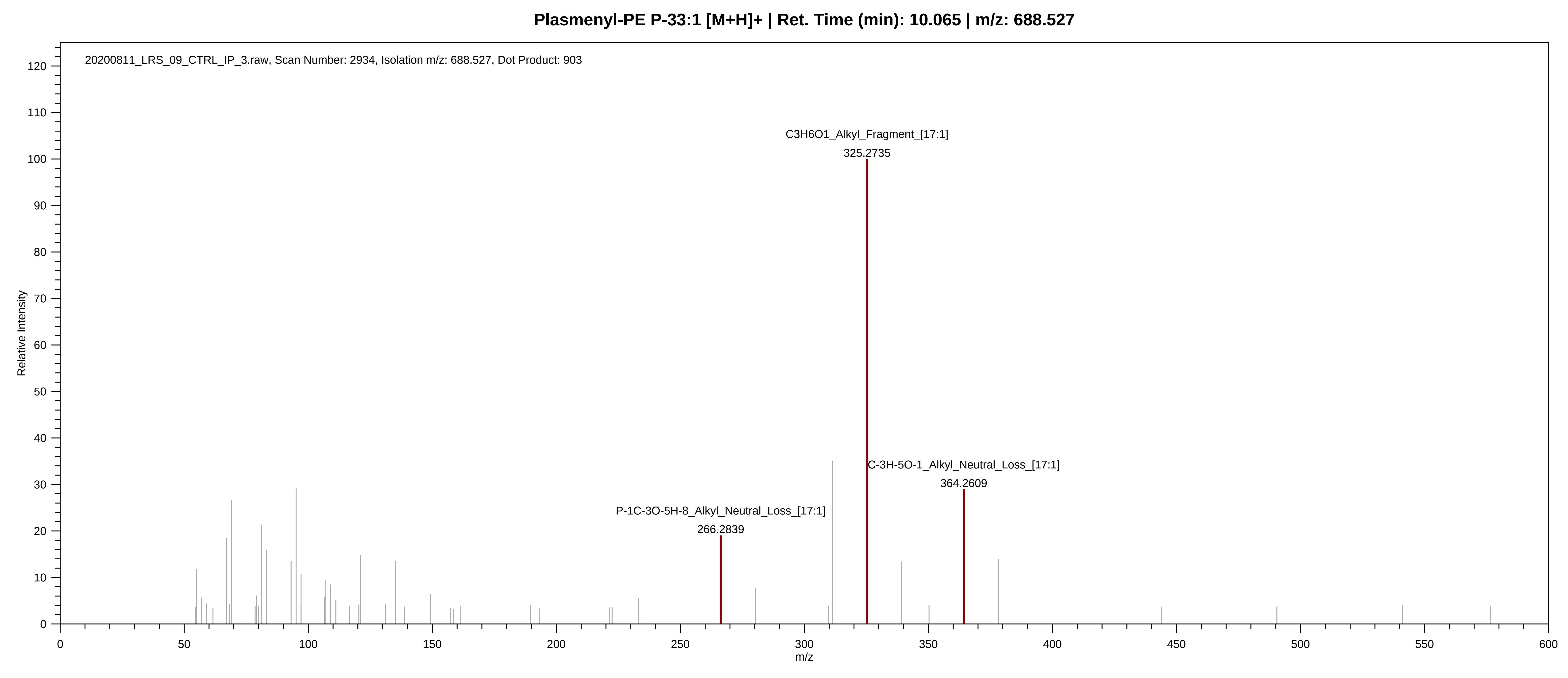
Plasmenyl-PE P-33:1 [M+H]+ | Ret. Time (min): 10.065 | m/z: 688.527
20200811_LRS_09_CTRL_IP_3.raw, Scan Number: 2934, Isolation m/z: 688.527, Dot Product: 903
C3H6O1_Alkyl_Fragment_[17:1]
325.2735
C-3H-5O-1_Alkyl_Neutral_Loss_[17:1]
364.2609
P-1C-3O-5H-8_Alkyl_Neutral_Loss_[17:1]
266.2839
Relative Intensity
m/z

Plasmenyl-PE P-33:1 [M+H]+ | Ret. Time (min): 10.081 | m/z: 688.527

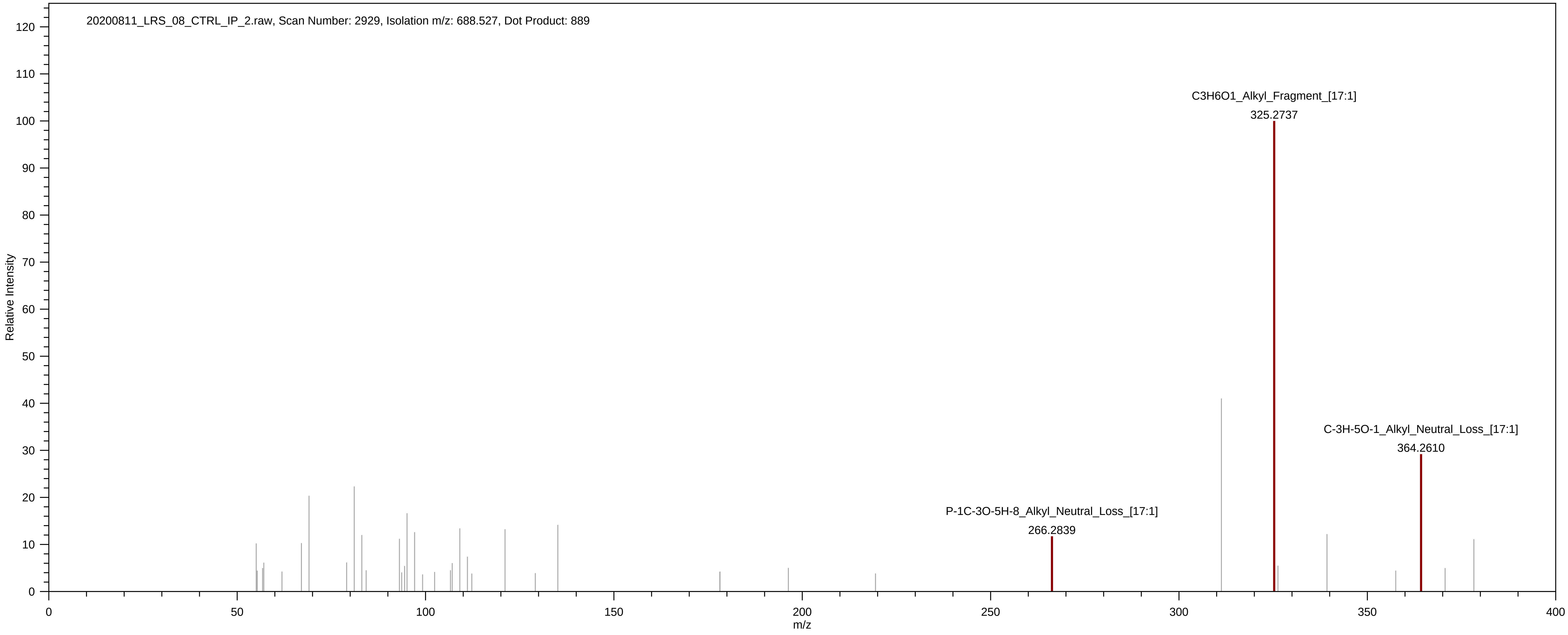

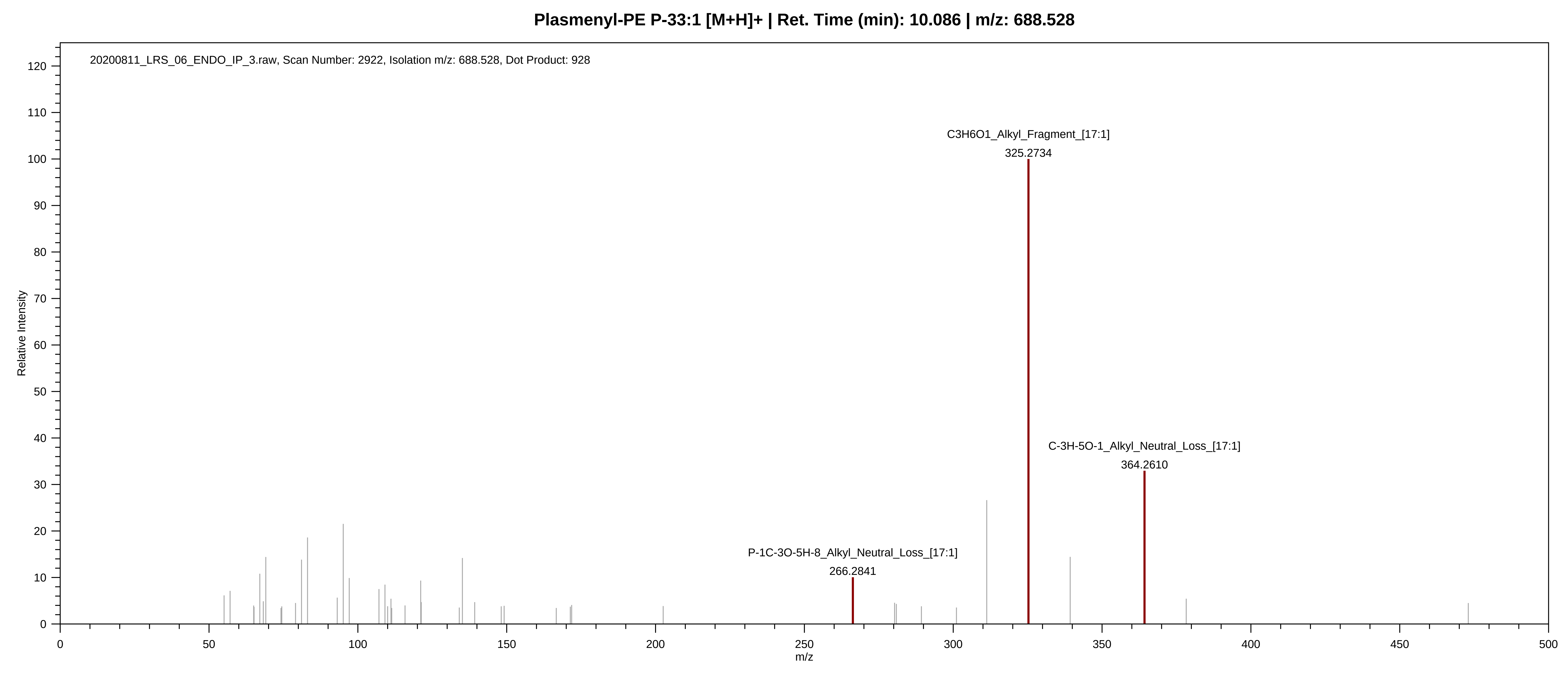
Plasmenyl-PE P-33:1 [M+H]+ | Ret. Time (min): 10.086 | m/z: 688.528
20200811_LRS_06_ENDO_IP_3.raw, Scan Number: 2922, Isolation m/z: 688.528, Dot Product: 928
C3H6O1_Alkyl_Fragment_[17:1]
325.2734
C-3H-5O-1_Alkyl_Neutral_Loss_[17:1]
364.2610
P-1C-3O-5H-8_Alkyl_Neutral_Loss_[17:1]
266.2841
Relative Intensity
m/z

Plasmenyl-PE P-33:1 [M+H]+ | Ret. Time (min): 10.092 | m/z: 688.528

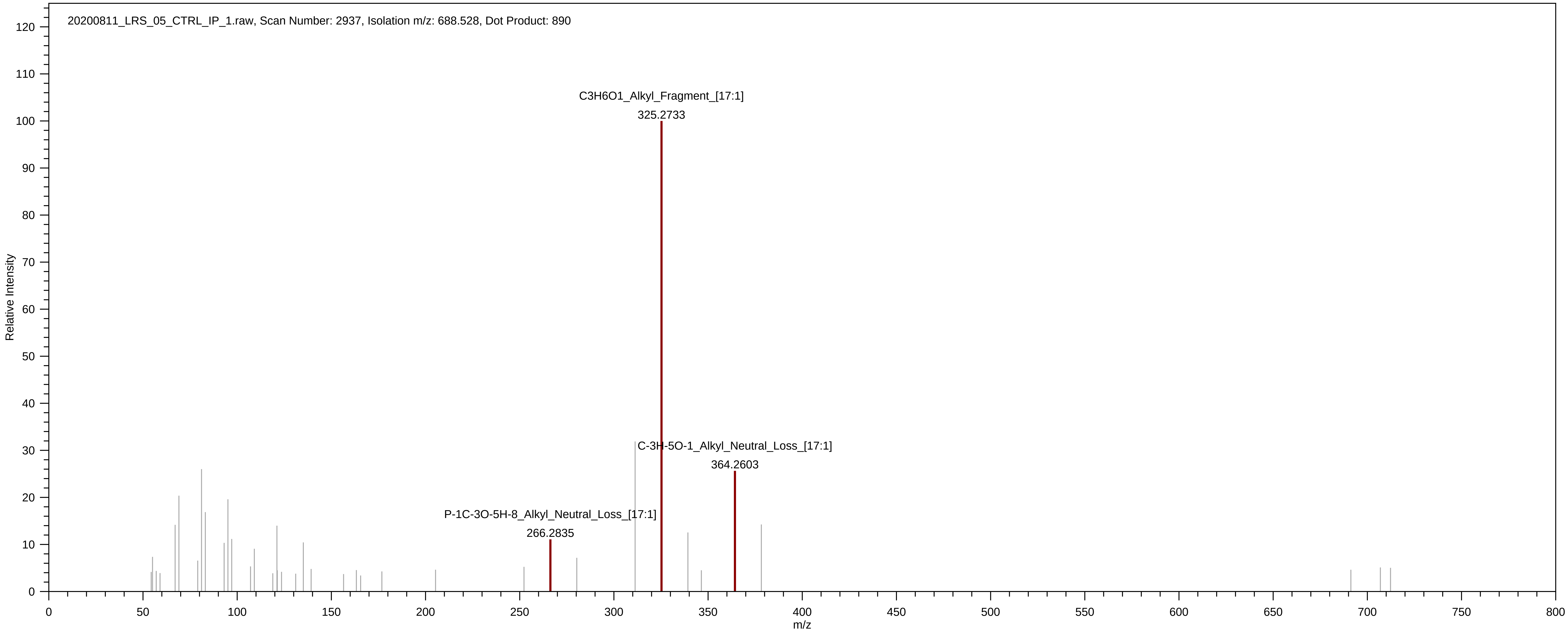

Plasmenyl-PE P-33:1 [M+H]+ | Ret. Time (min): 10.104 | m/z: 688.527

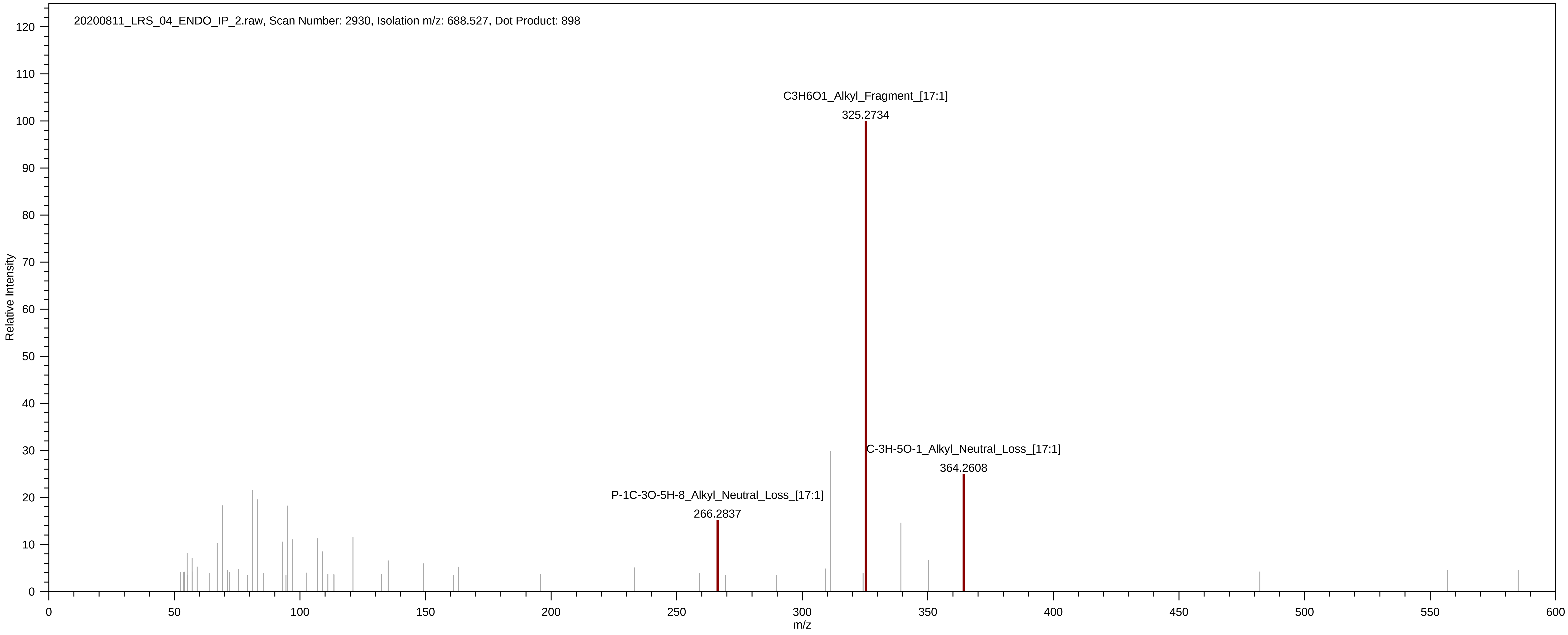

Plasmenyl-PE P-33:1 [M+H]+ | Ret. Time (min): 10.085 | m/z: 688.528

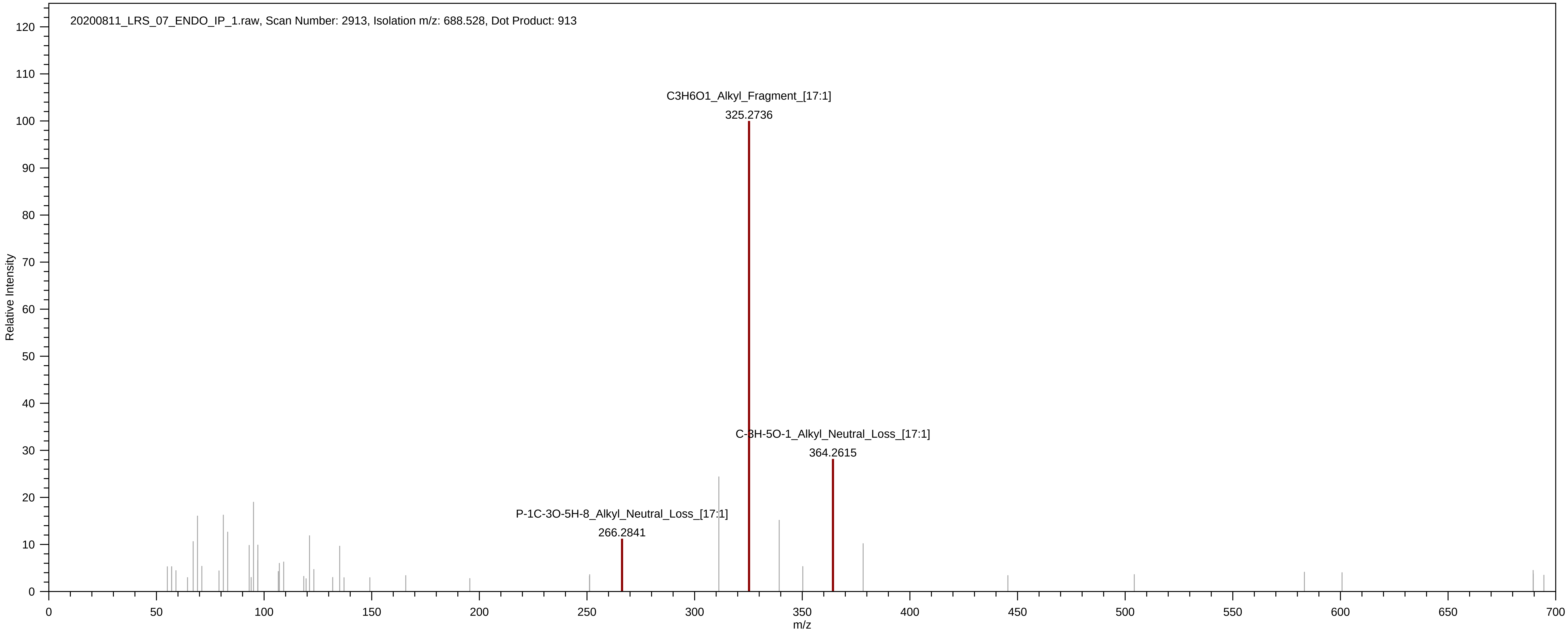

Plasmenyl-PE P-34:0 [M+H]+ | Ret. Time (min): 11.582 | m/z: 704.561

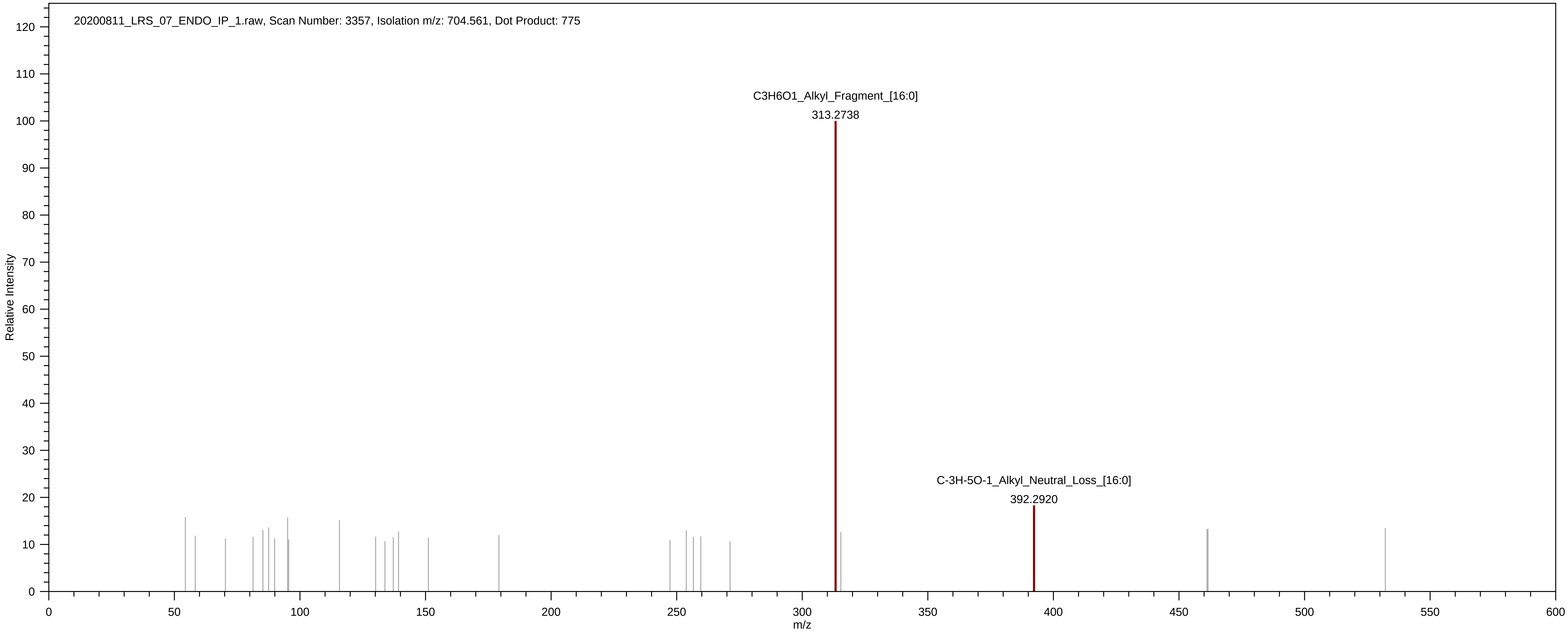

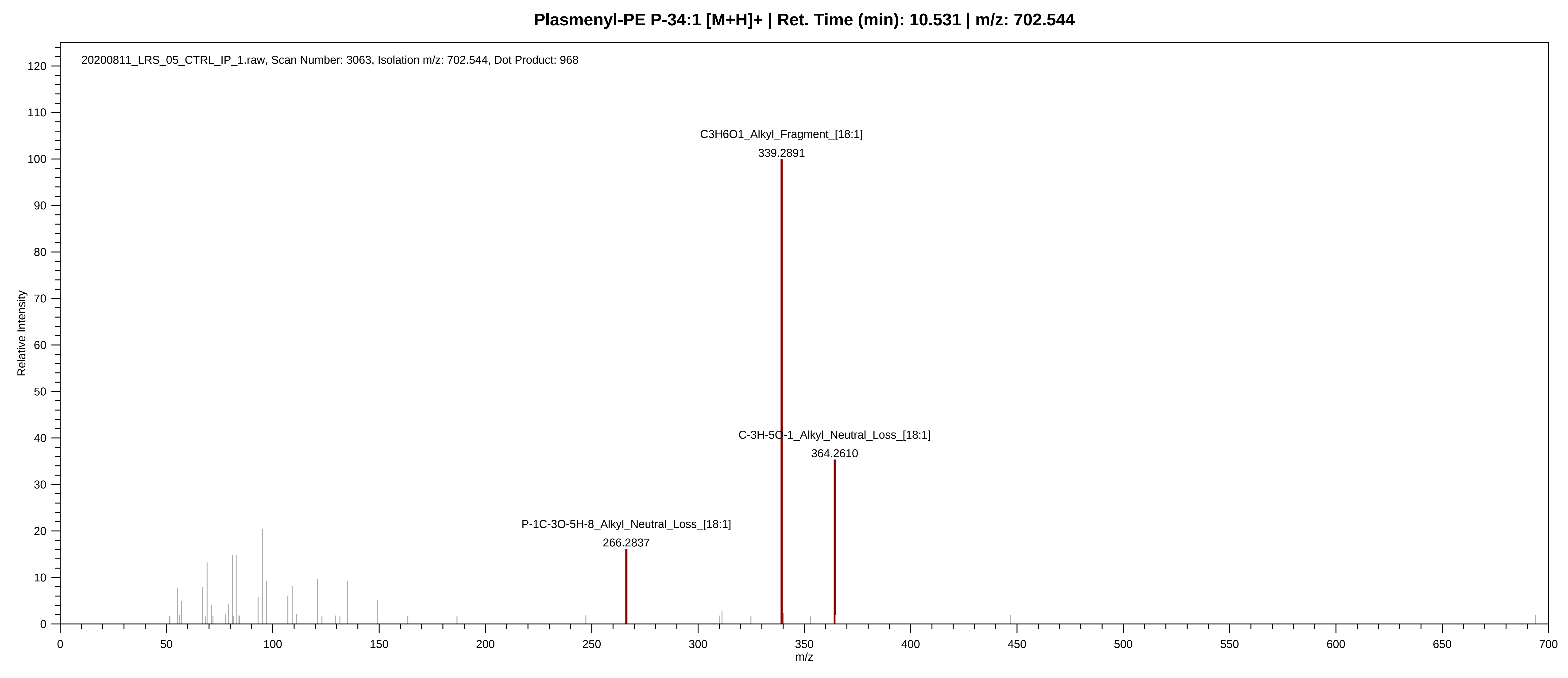

Plasmenyl-PE P-34:1 [M+H]+ | Ret. Time (min): 10.531 | m/z: 702.544
20200811_LRS_05_CTRL_IP_1.raw, Scan Number: 3063, Isolation m/z: 702.544, Dot Product: 968
C3H6O1_Alkyl_Fragment_[18:1]
339.2891
C-3H-5O-1_Alkyl_Neutral_Loss_[18:1]
364.2610
P-1C-3O-5H-8_Alkyl_Neutral_Loss_[18:1]
266.2837
Relative Intensity
m/z

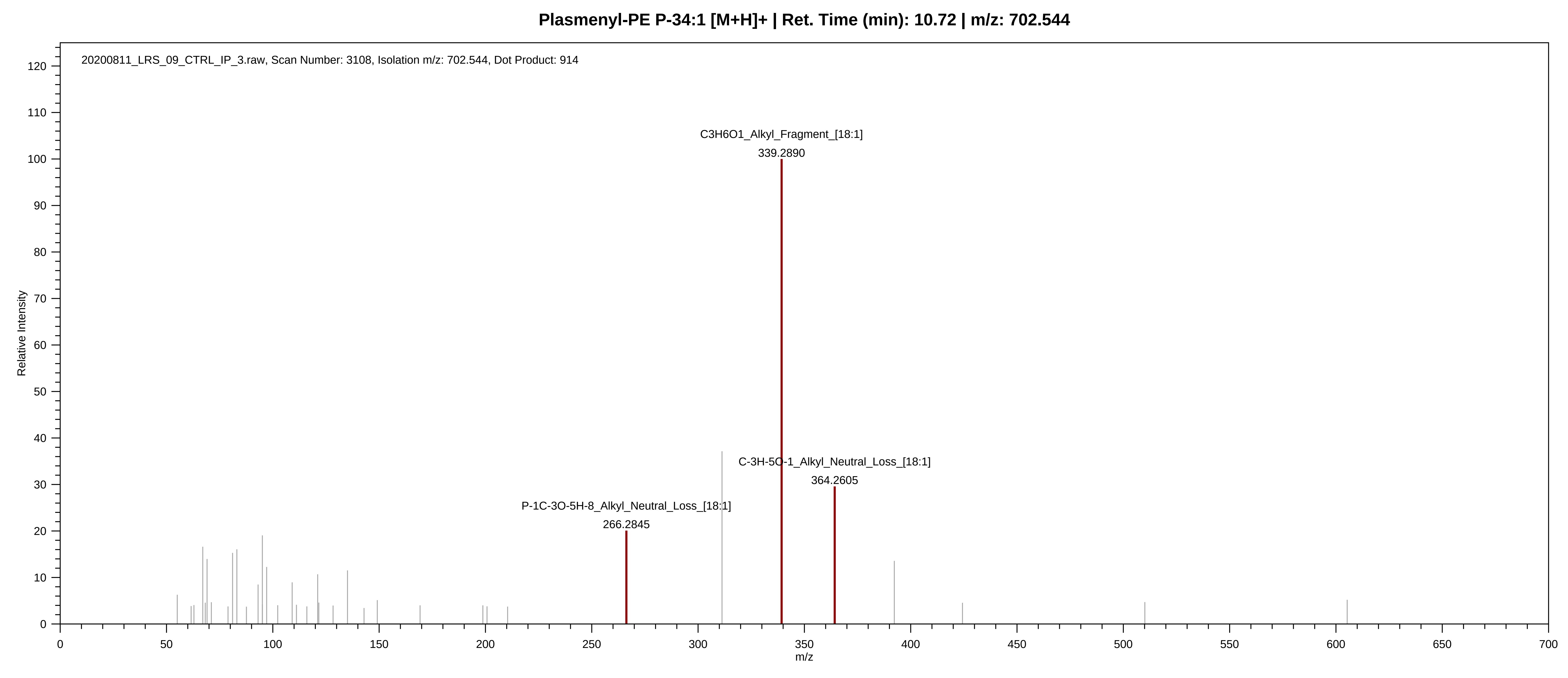

Plasmenyl-PE P-34:1 [M+H]+ | Ret. Time (min): 10.72 | m/z: 702.544
20200811_LRS_09_CTRL_IP_3.raw, Scan Number: 3108, Isolation m/z: 702.544, Dot Product: 914
C3H6O1_Alkyl_Fragment_[18:1]
339.2890
C-3H-5O-1_Alkyl_Neutral_Loss_[18:1]
364.2605
P-1C-3O-5H-8_Alkyl_Neutral_Loss_[18:1]
266.2845
Relative Intensity
m/z

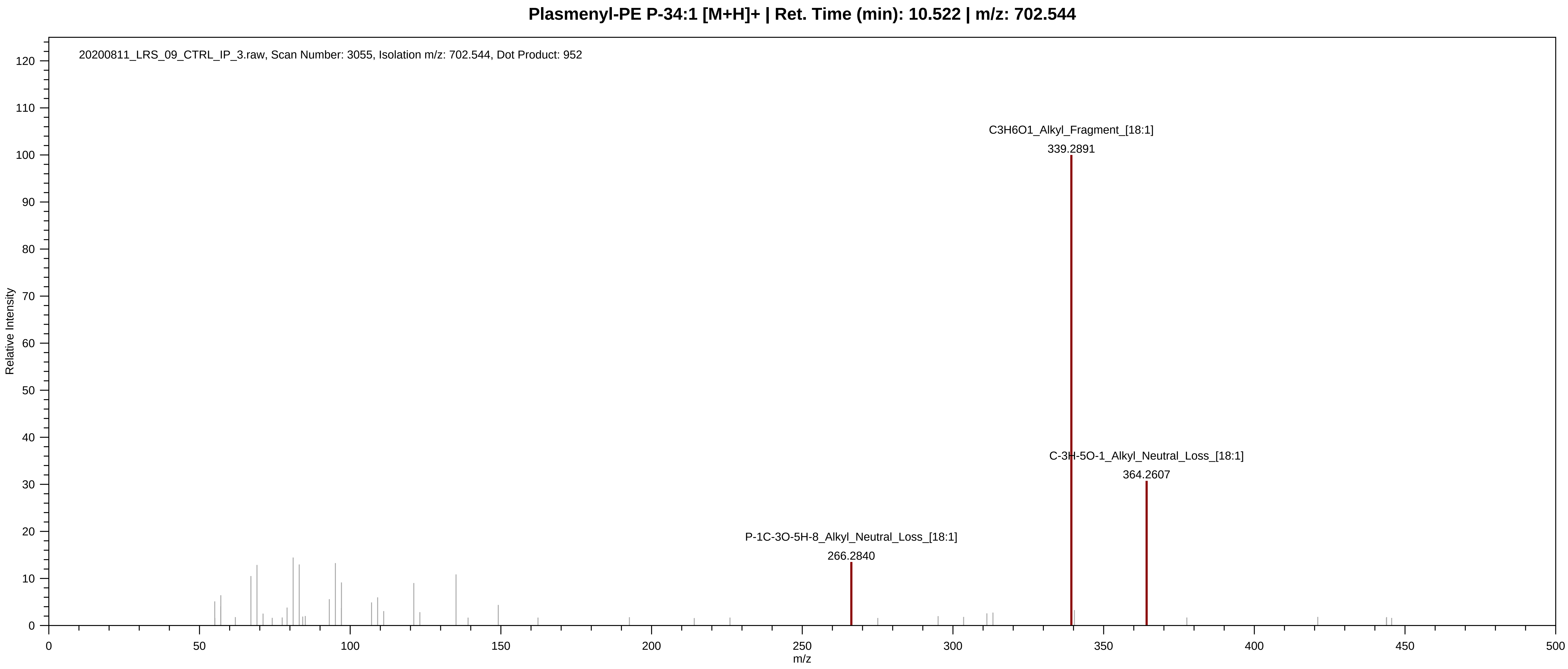

Plasmenyl-PE P-34:1 [M+H]+ | Ret. Time (min): 10.522 | m/z: 702.544
20200811_LRS_09_CTRL_IP_3.raw, Scan Number: 3055, Isolation m/z: 702.544, Dot Product: 952
C3H6O1_Alkyl_Fragment_[18:1]
339.2891
C-3H-5O-1_Alkyl_Neutral_Loss_[18:1]
364.2607
P-1C-3O-5H-8_Alkyl_Neutral_Loss_[18:1]
266.2840
Relative Intensity
m/z
0
10
20
30
40
50
60
70
80
90
100
110
120
0
50
100
150
200
250
300
350
400
450
500

Plasmenyl-PE P-34:1 [M+H]+ | Ret. Time (min): 10.523 | m/z: 702.543

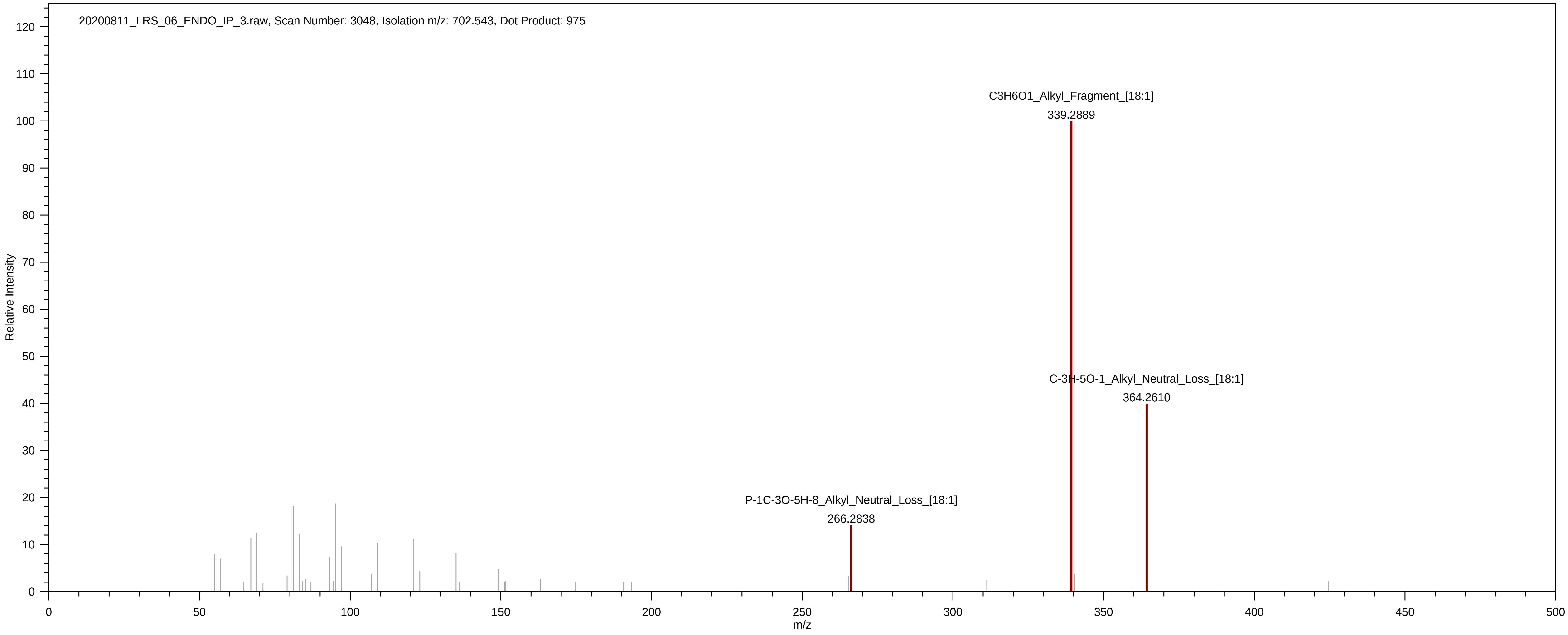

Plasmenyl-PE P-34:1 [M+H]+ | Ret. Time (min): 10.525 | m/z: 702.543

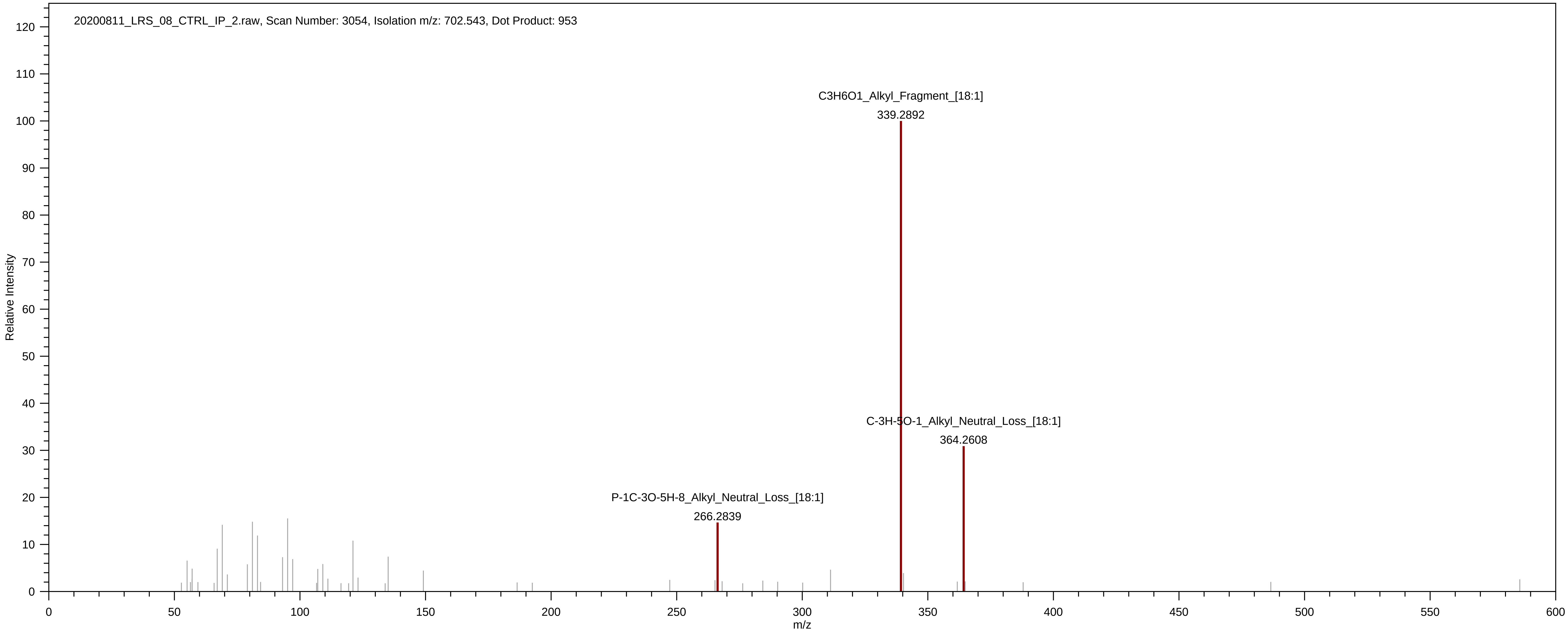

Plasmenyl-PE P-34:1 [M+H]+ | Ret. Time (min): 10.526 | m/z: 702.543

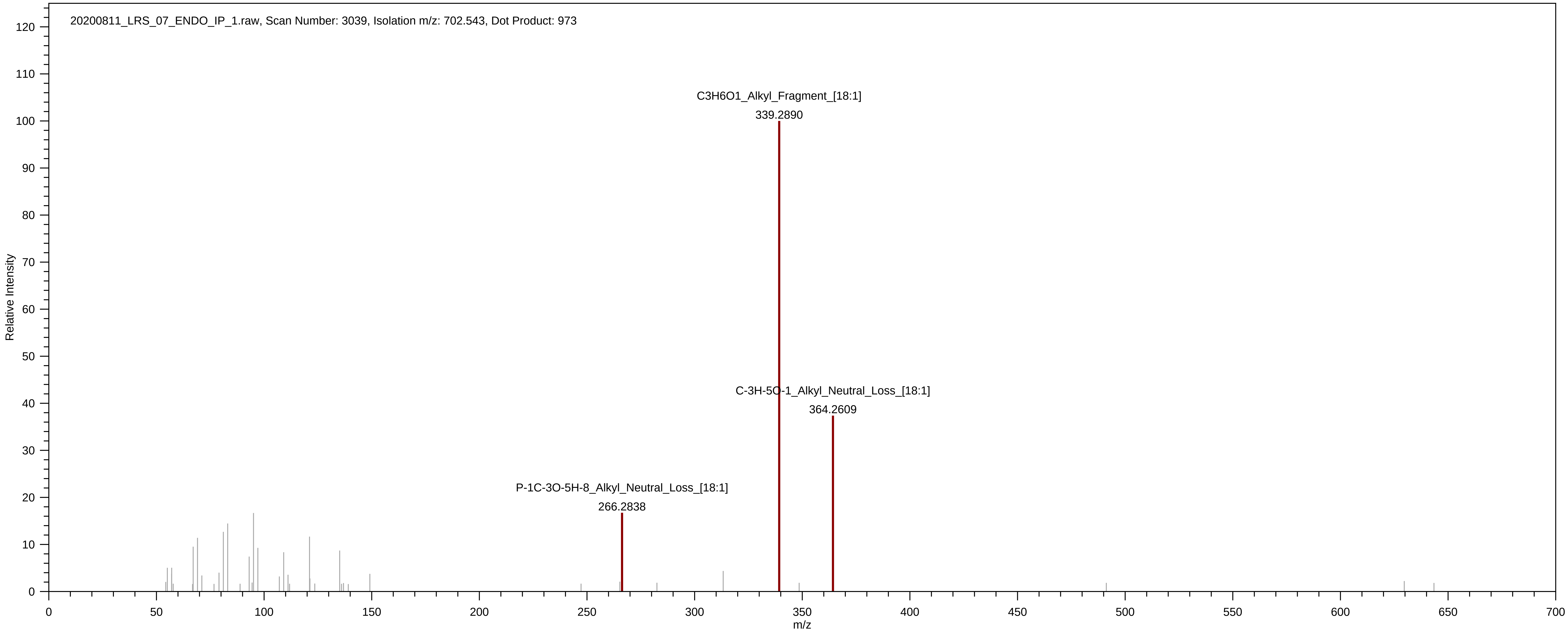

Plasmenyl-PE P-34:1 [M+H]+ | Ret. Time (min): 10.527 | m/z: 702.544

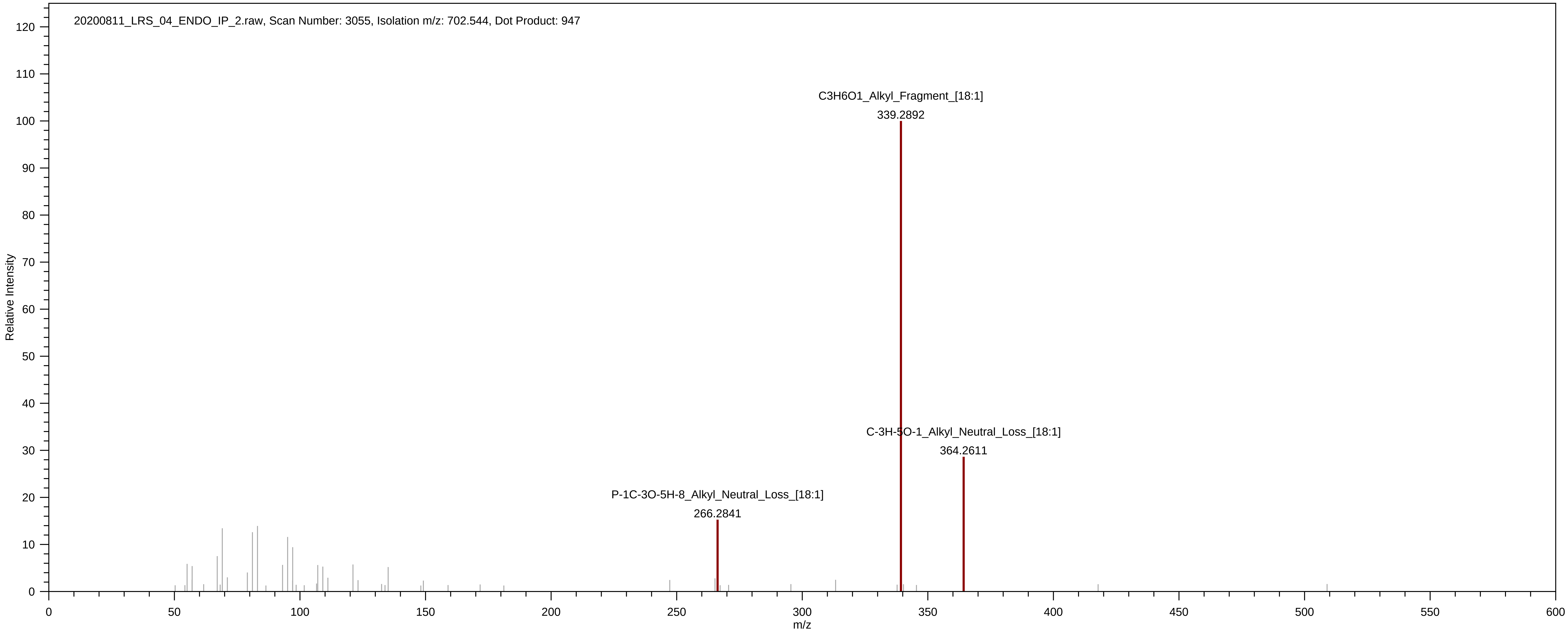

Plasmenyl-PE P-34:1 [M+H]+ | Ret. Time (min): 10.722 | m/z: 702.544

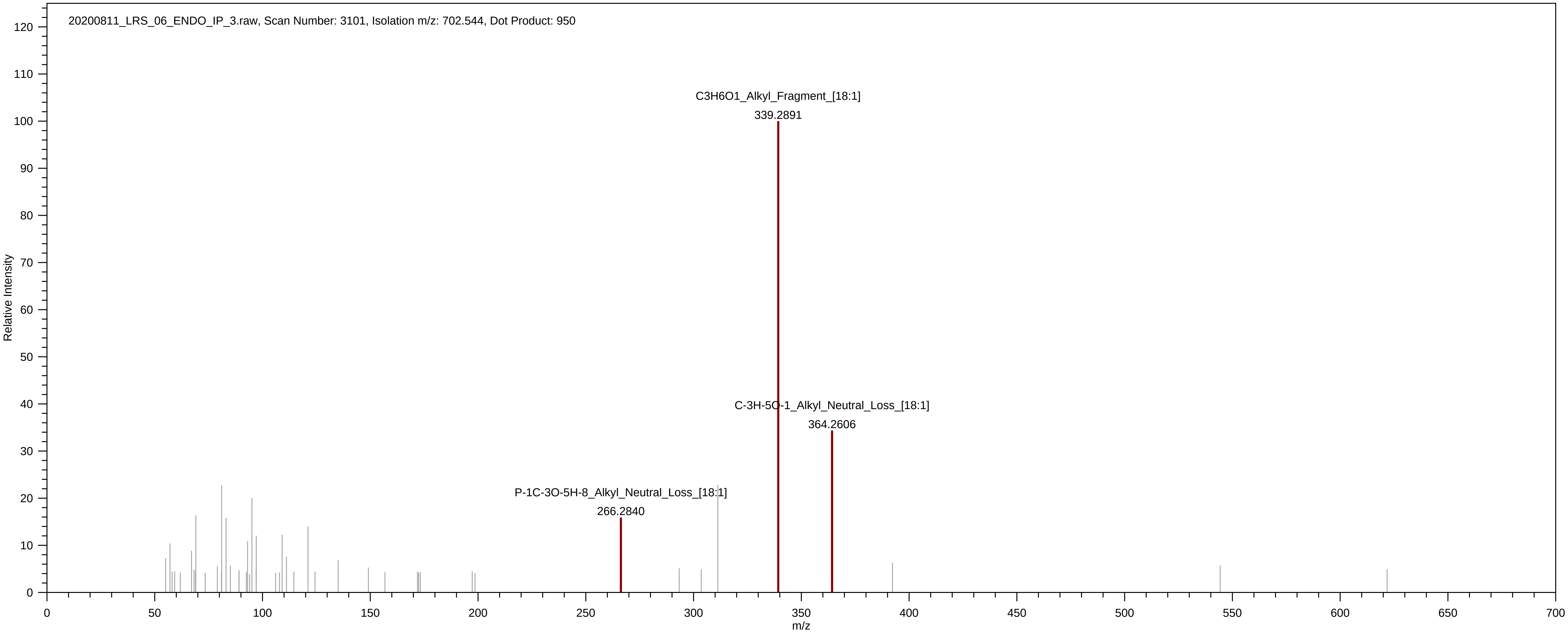

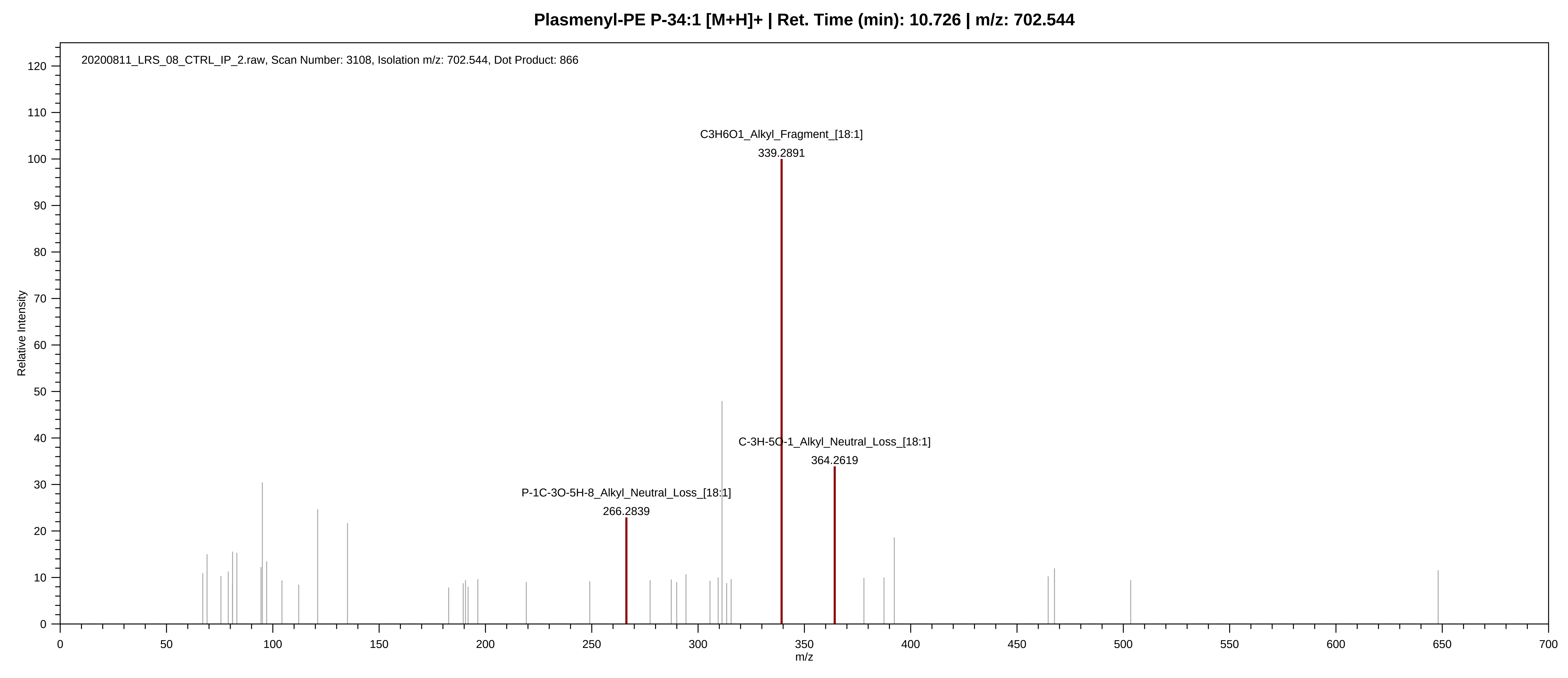

Plasmenyl-PE P-34:1 [M+H]+ | Ret. Time (min): 10.726 | m/z: 702.544
20200811_LRS_08_CTRL_IP_2.raw, Scan Number: 3108, Isolation m/z: 702.544, Dot Product: 866
C3H6O1_Alkyl_Fragment_[18:1]
339.2891
C-3H-5O-1_Alkyl_Neutral_Loss_[18:1]
364.2619
P-1C-3O-5H-8_Alkyl_Neutral_Loss_[18:1]
266.2839
Relative Intensity
m/z

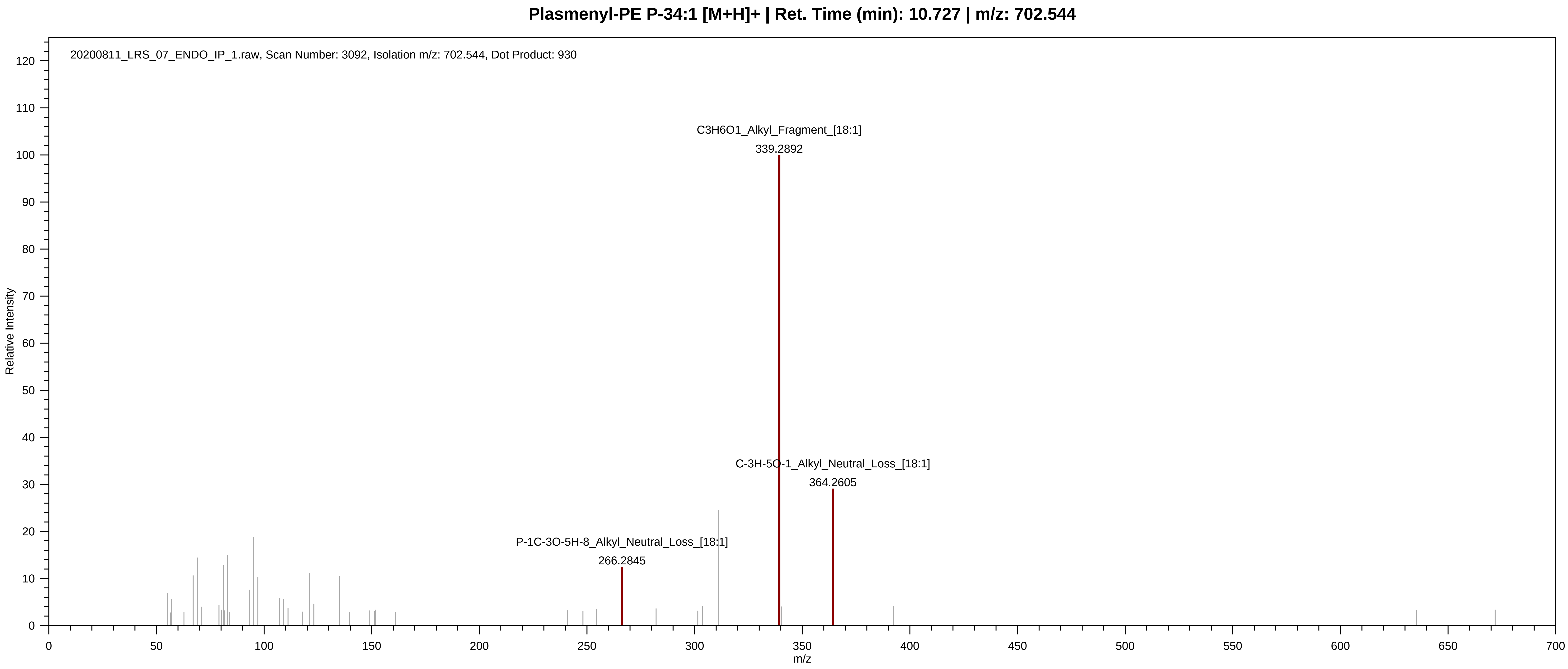

Plasmenyl-PE P-34:1 [M+H]+ | Ret. Time (min): 10.727 | m/z: 702.544
20200811_LRS_07_ENDO_IP_1.raw, Scan Number: 3092, Isolation m/z: 702.544, Dot Product: 930
C3H6O1_Alkyl_Fragment_[18:1]
339.2892
C-3H-5O-1_Alkyl_Neutral_Loss_[18:1]
364.2605
P-1C-3O-5H-8_Alkyl_Neutral_Loss_[18:1]
266.2845
Relative Intensity
m/z

Plasmenyl-PE P-34:1 [M+H]+ | Ret. Time (min): 10.727 | m/z: 702.543

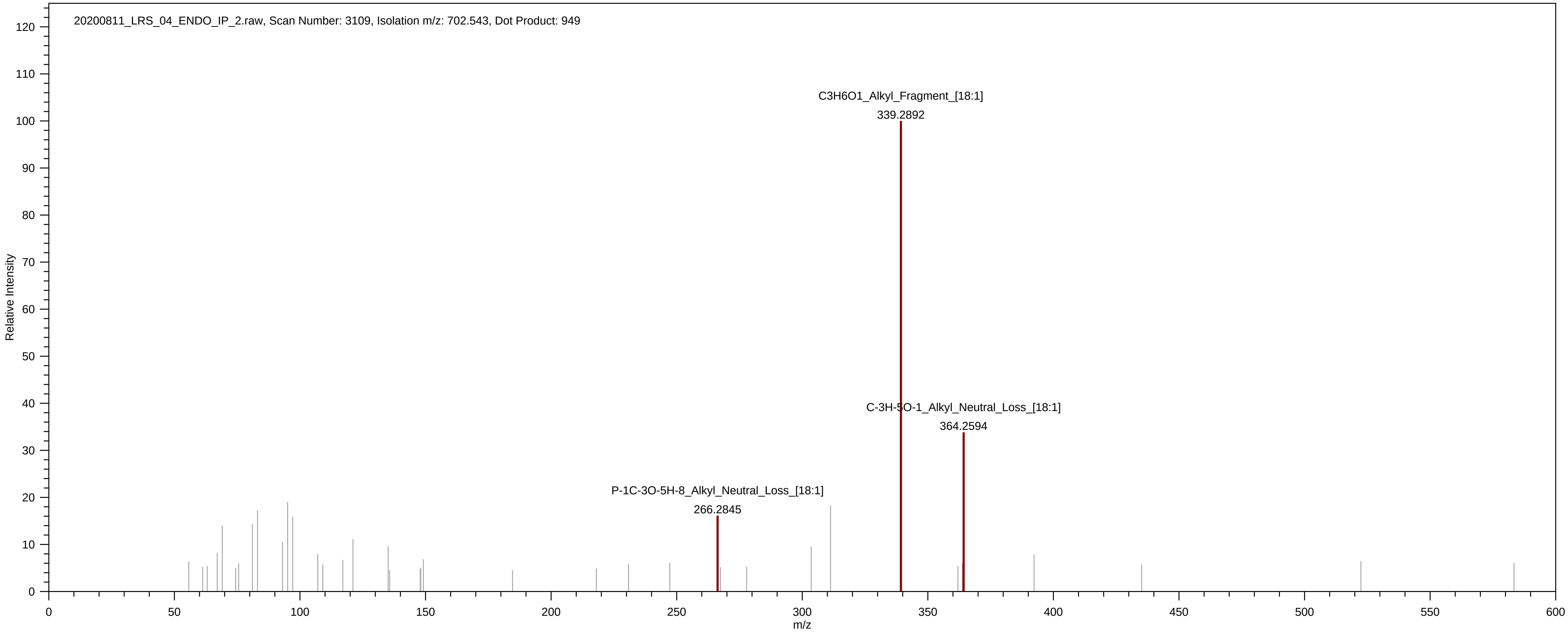

Plasmenyl-PE P-34:1 [M+H]+ | Ret. Time (min): 10.734 | m/z: 702.544

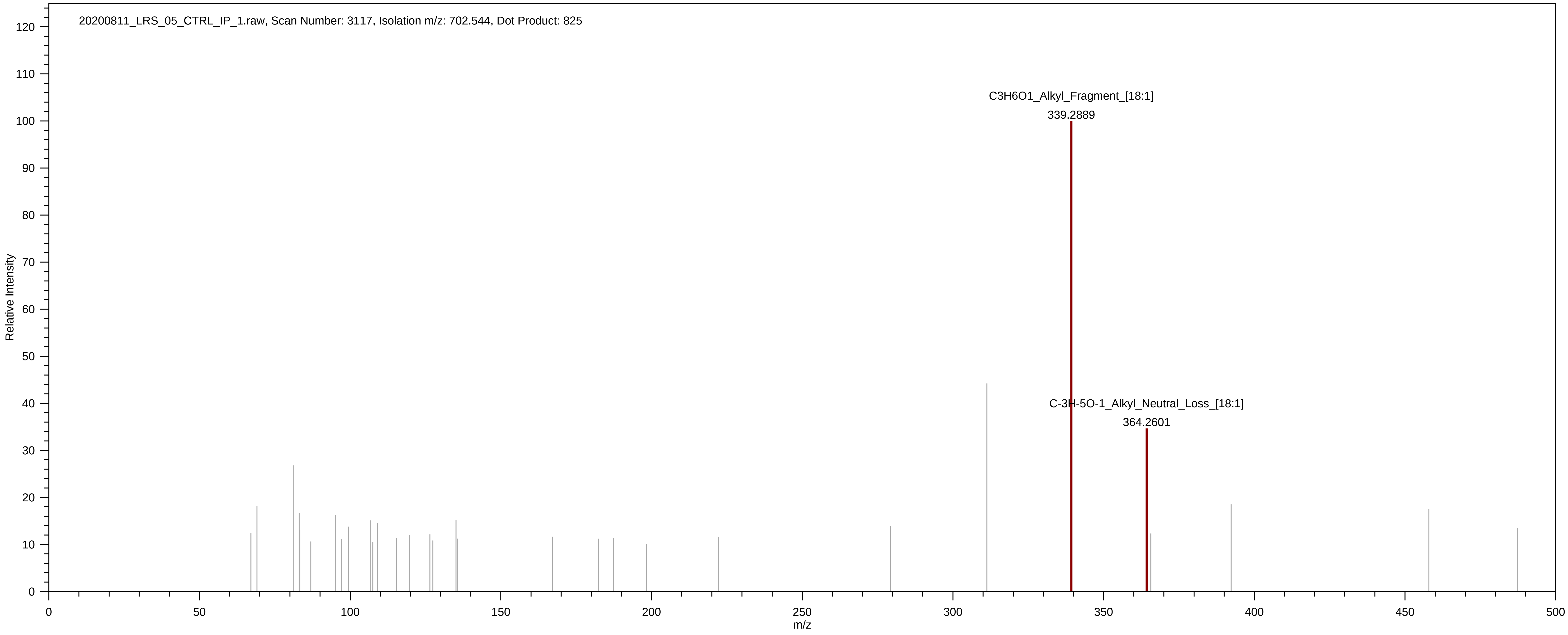

Plasmenyl-PE P-34:1 [M-H]- | Ret. Time (min): 10.72 | m/z: 700.529

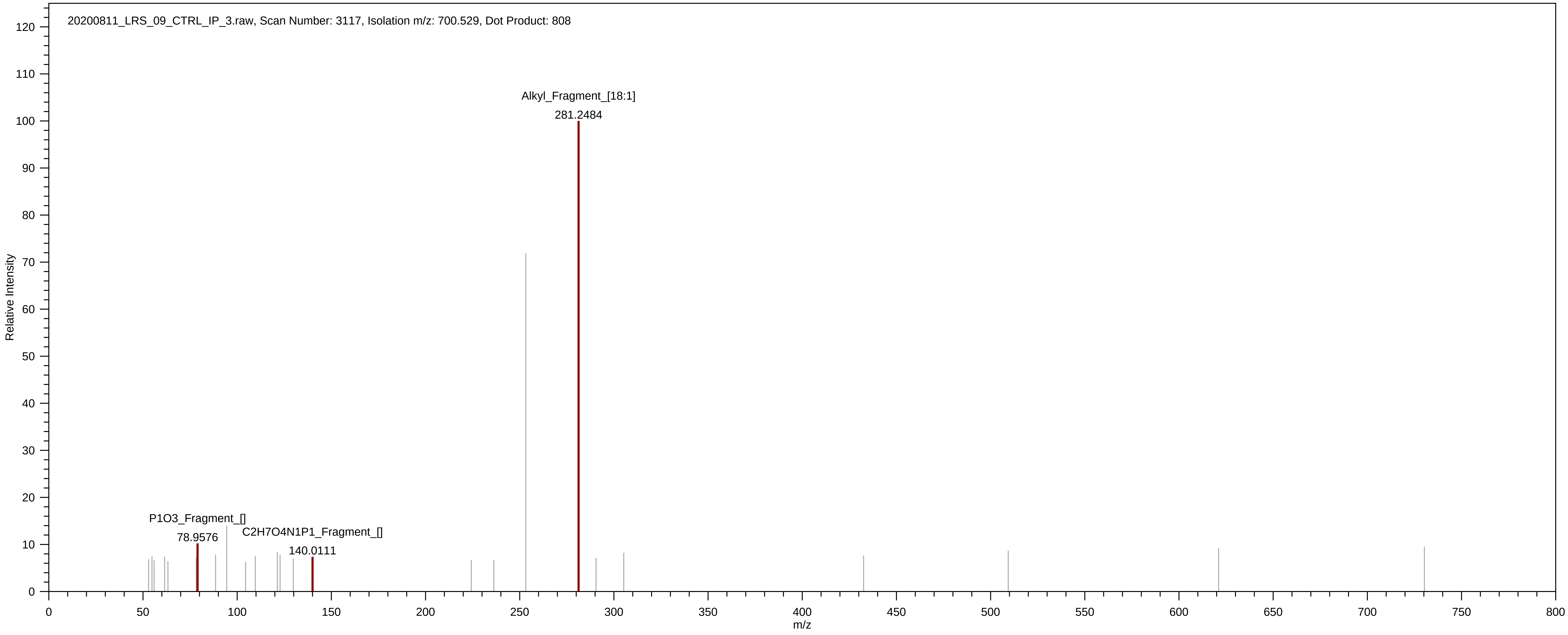

Plasmenyl-PE P-34:1 [M-H]- | Ret. Time (min): 10.726 | m/z: 700.529

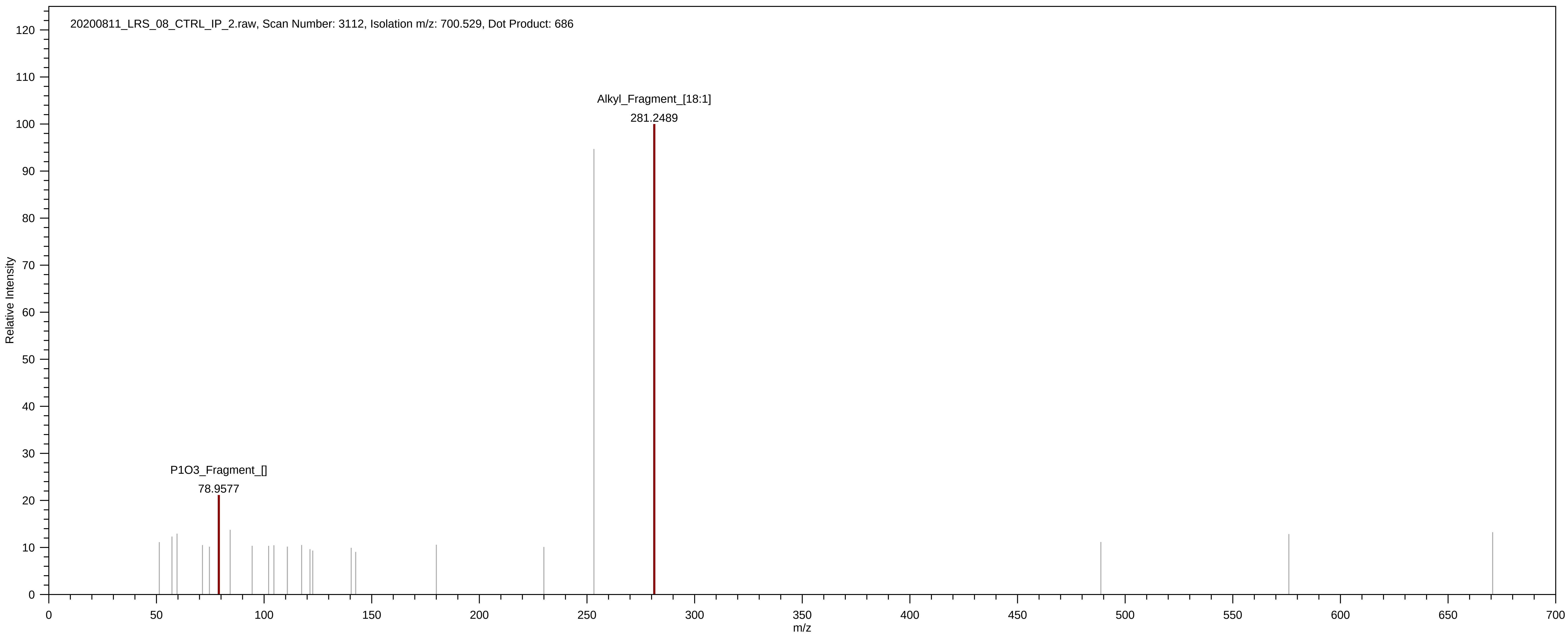

Plasmenyl-PE P-34:1 [M-H]- | Ret. Time (min): 10.734 | m/z: 700.529

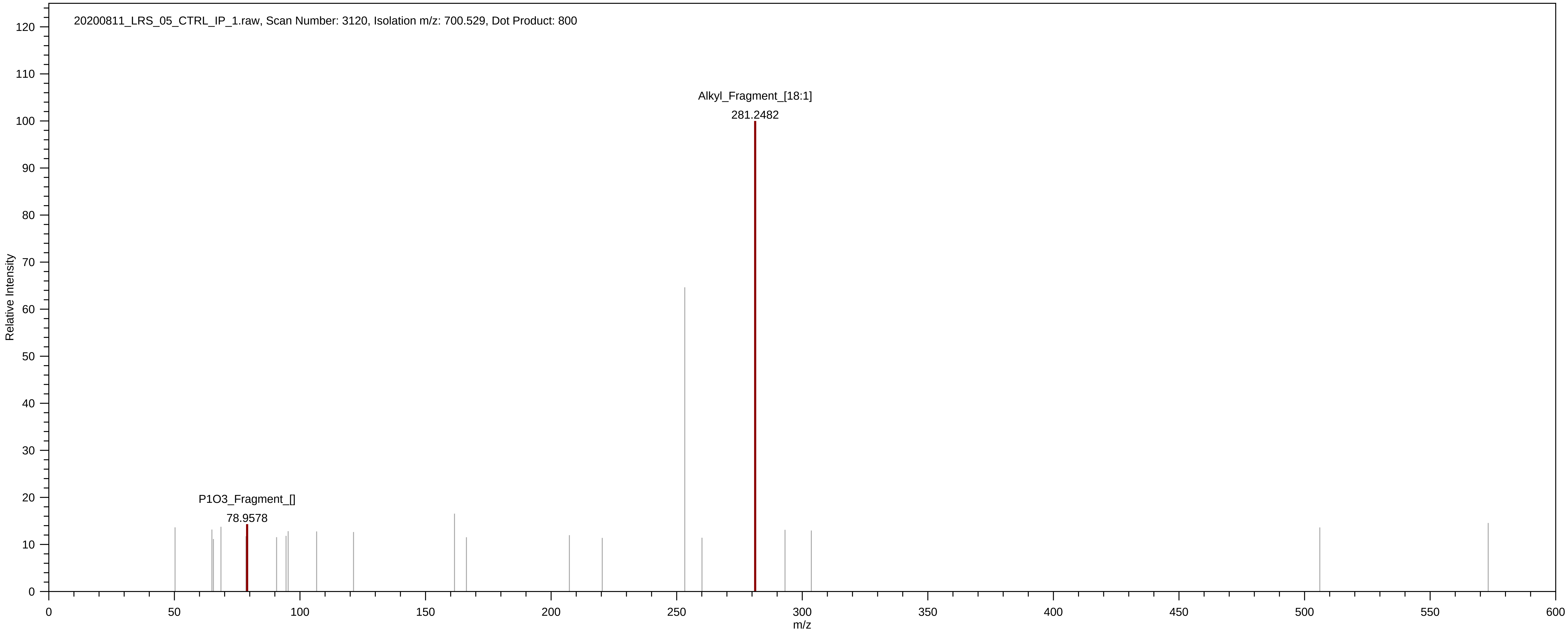

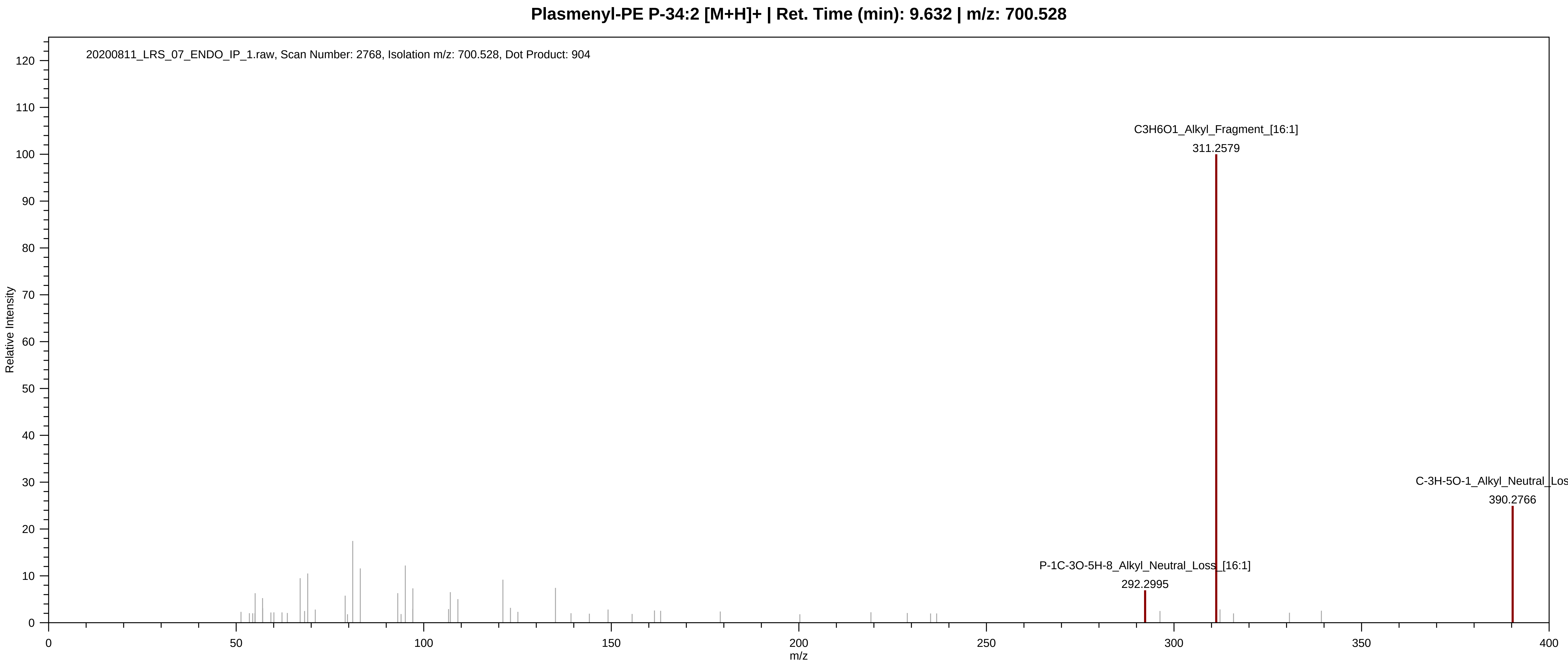
Plasmenyl-PE P-34:2 [M+H]+ | Ret. Time (min): 9.632 | m/z: 700.528
20200811_LRS_07_ENDO_IP_1.raw, Scan Number: 2768, Isolation m/z: 700.528, Dot Product: 904
C3H6O1_Alkyl_Fragment_[16:1]
311.2579
C-3H-5O-1_Alkyl_Neutral_Los
390.2766
P-1C-3O-5H-8_Alkyl_Neutral_Loss_[16:1]
292.2995
Relative Intensity
m/z

Plasmenyl-PE P-34:2 [M+H]+ | Ret. Time (min): 9.625 | m/z: 700.527

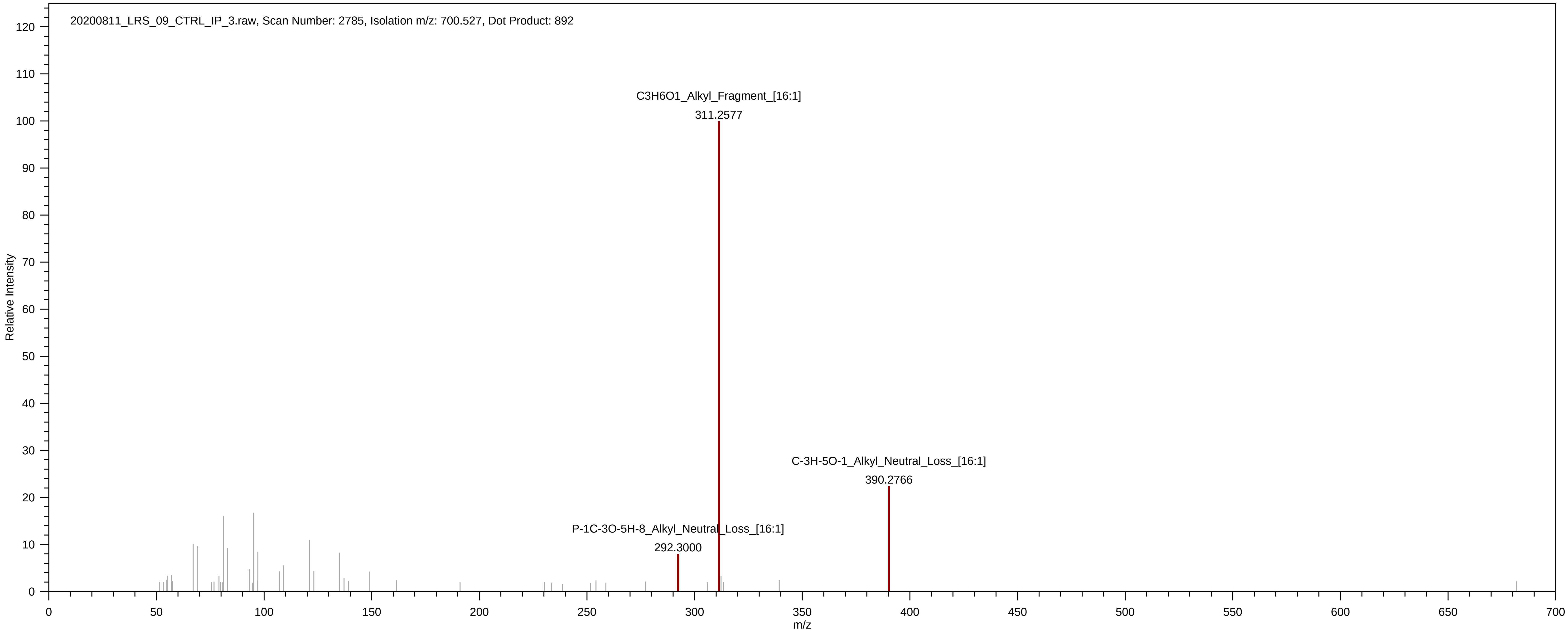

Plasmenyl-PE P-34:2 [M+H]+ | Ret. Time (min): 9.637 | m/z: 700.527

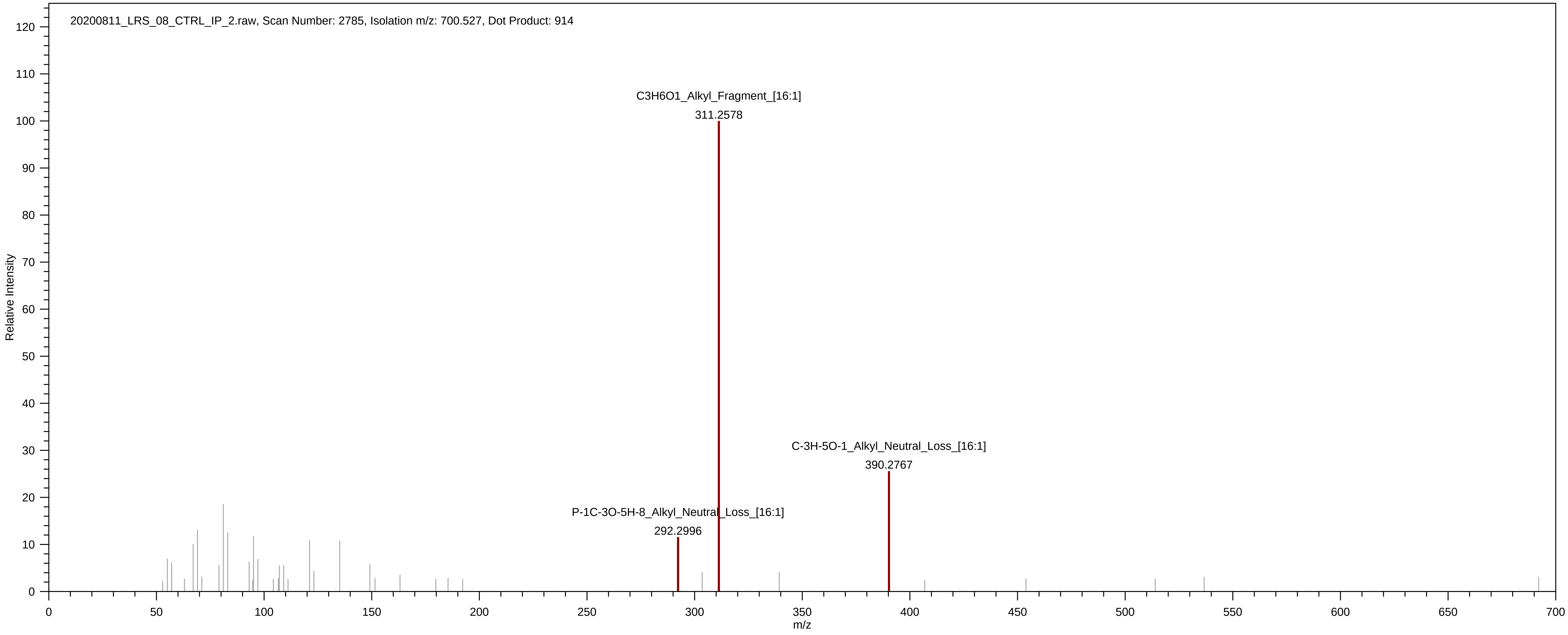

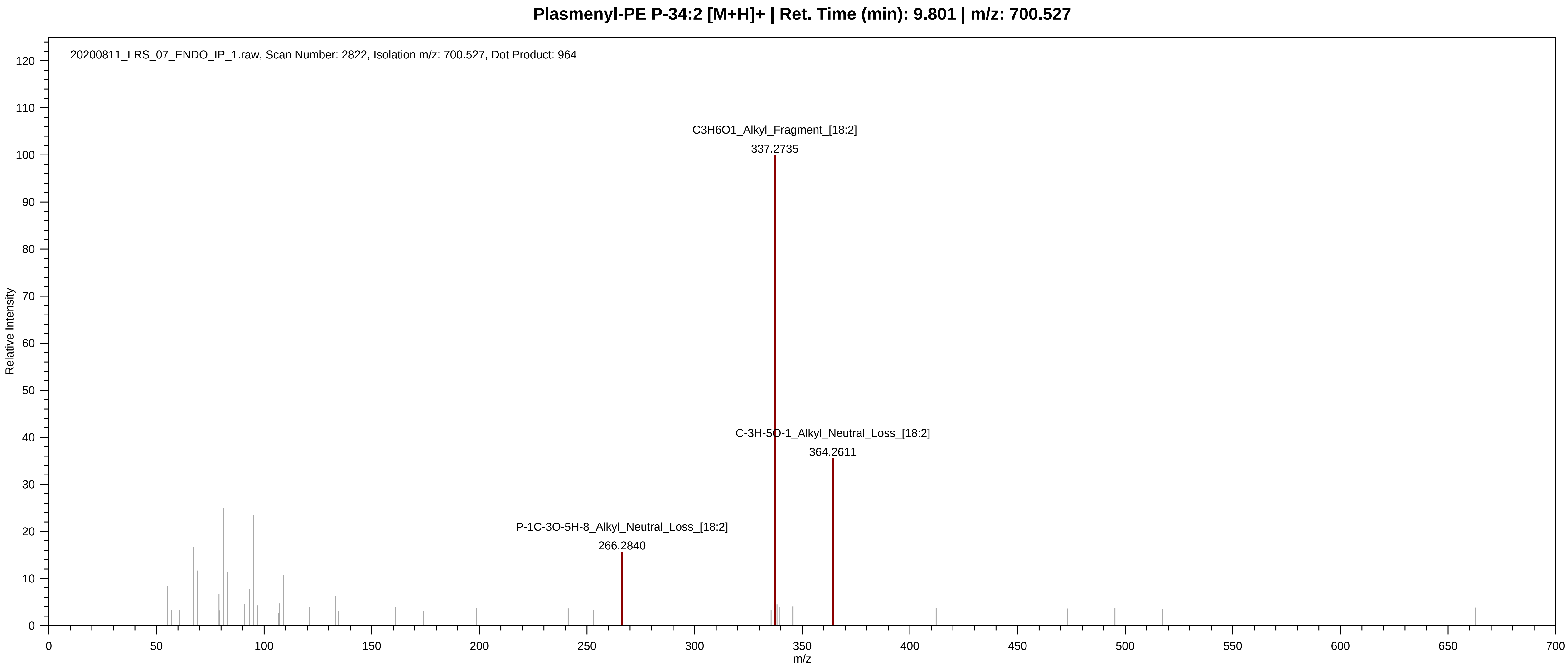

Plasmenyl-PE P-34:2 [M+H]+ | Ret. Time (min): 9.801 | m/z: 700.527
20200811_LRS_07_ENDO_IP_1.raw, Scan Number: 2822, Isolation m/z: 700.527, Dot Product: 964
C3H6O1_Alkyl_Fragment_[18:2]
337.2735
C-3H-5O-1_Alkyl_Neutral_Loss_[18:2]
364.2611
P-1C-3O-5H-8_Alkyl_Neutral_Loss_[18:2]
266.2840
Relative Intensity
m/z

Plasmenyl-PE P-34:2 [M+H]+ | Ret. Time (min): 9.818 | m/z: 700.527

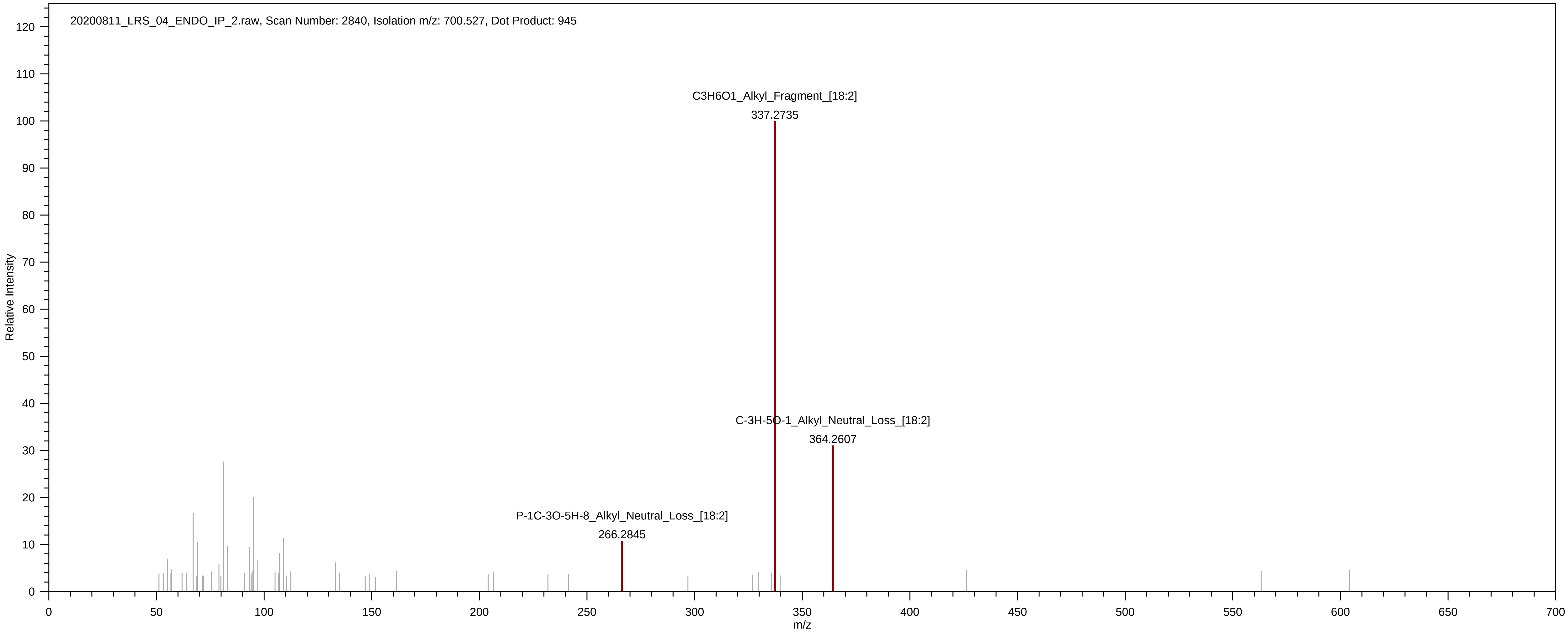

Plasmenyl-PE P-34:2 [M+H]+ | Ret. Time (min): 9.8 | m/z: 700.528

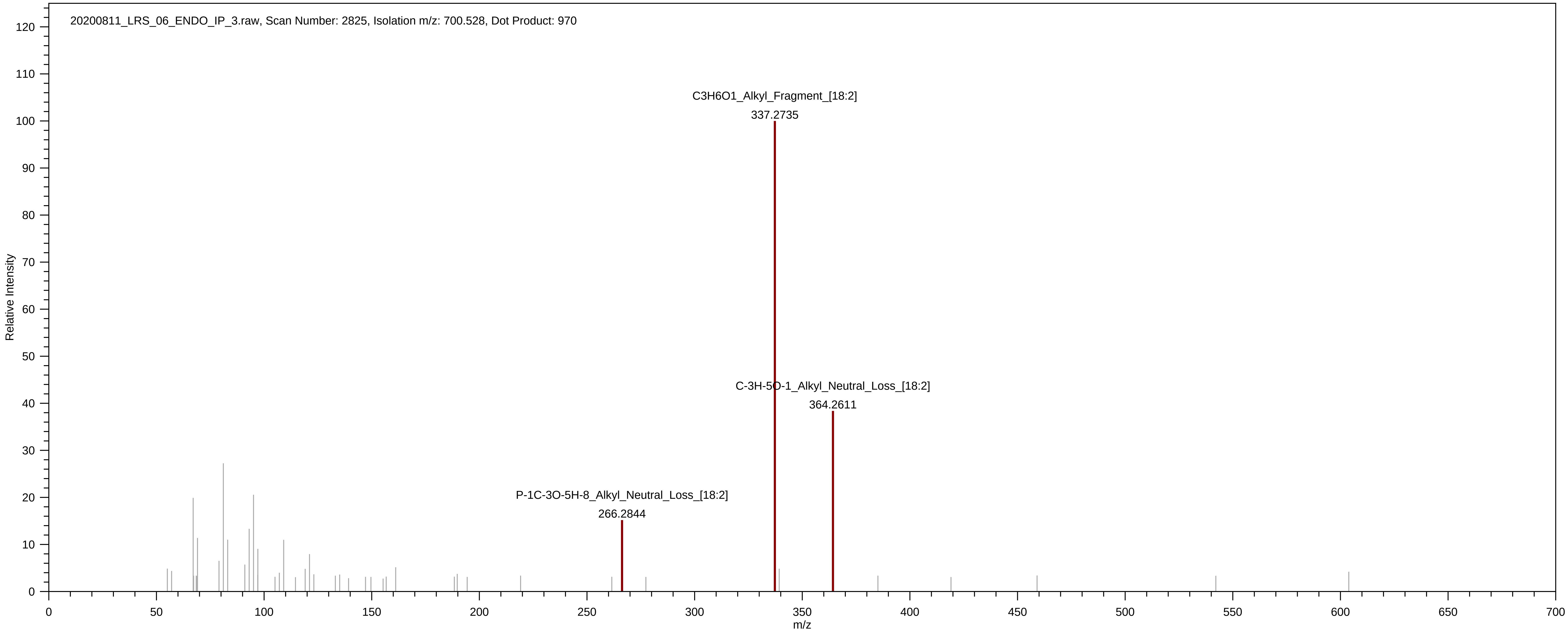

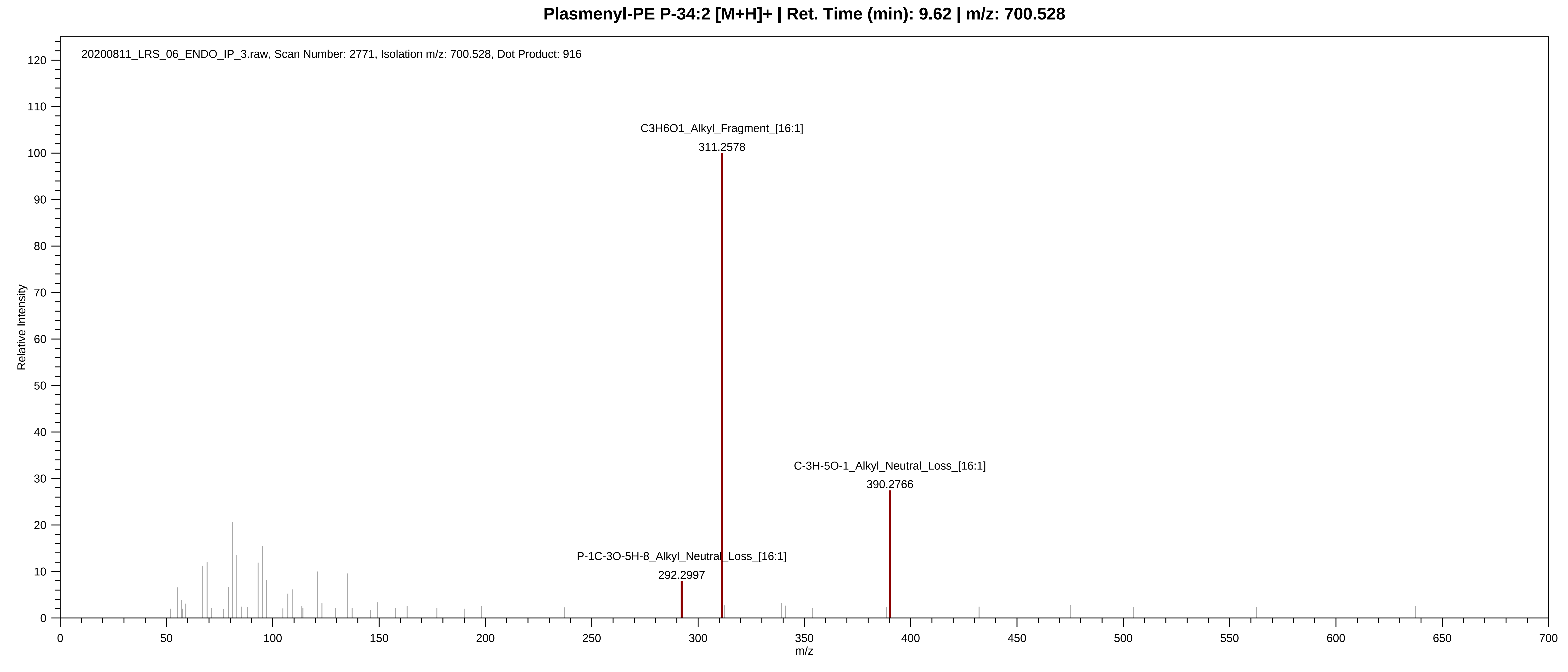

Plasmenyl-PE P-34:2 [M+H]+ | Ret. Time (min): 9.62 | m/z: 700.528
20200811_LRS_06_ENDO_IP_3.raw, Scan Number: 2771, Isolation m/z: 700.528, Dot Product: 916
C3H6O1_Alkyl_Fragment_[16:1]
311.2578
C-3H-5O-1_Alkyl_Neutral_Loss_[16:1]
390.2766
P-1C-3O-5H-8_Alkyl_Neutral_Loss_[16:1]
292.2997
Relative Intensity
m/z

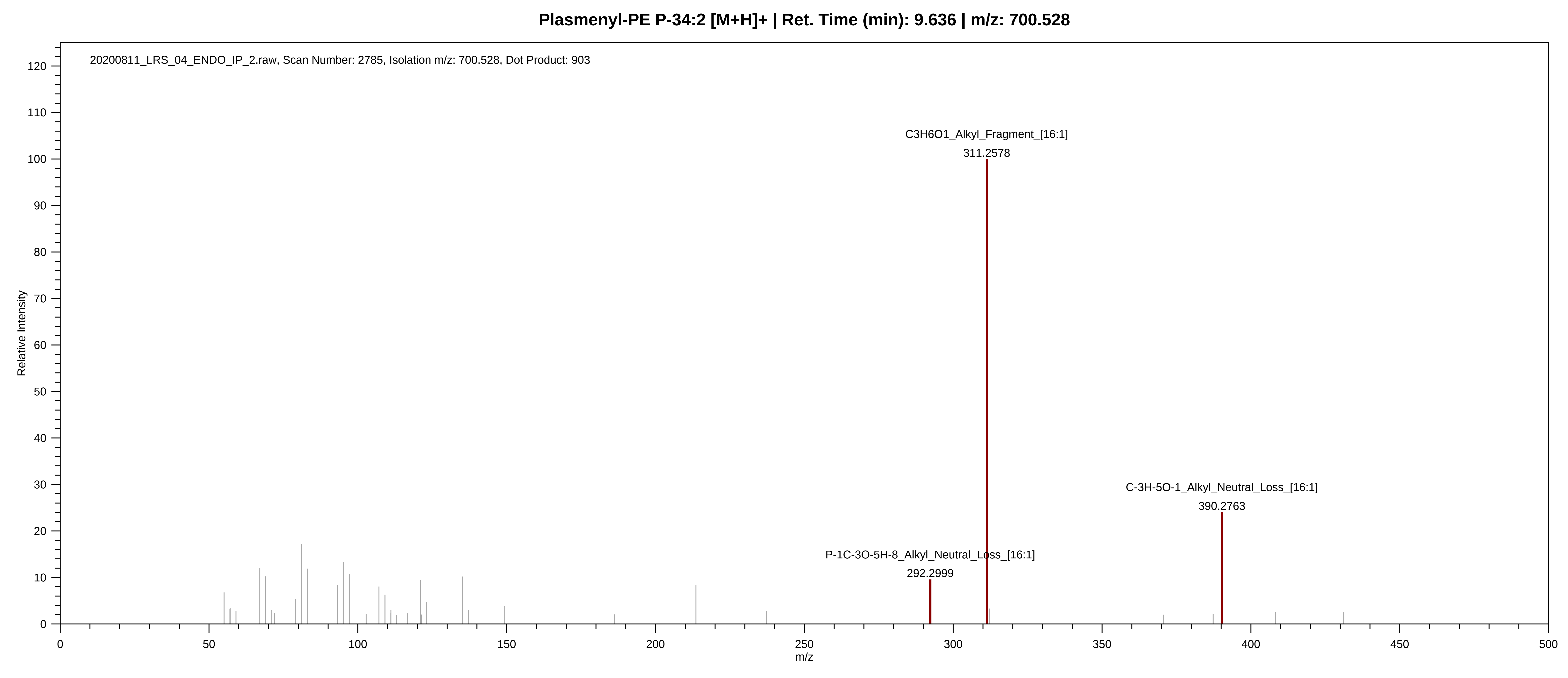

Plasmenyl-PE P-34:2 [M+H]+ | Ret. Time (min): 9.636 | m/z: 700.528
20200811_LRS_04_ENDO_IP_2.raw, Scan Number: 2785, Isolation m/z: 700.528, Dot Product: 903
C3H6O1_Alkyl_Fragment_[16:1]
311.2578
C-3H-5O-1_Alkyl_Neutral_Loss_[16:1]
390.2763
P-1C-3O-5H-8_Alkyl_Neutral_Loss_[16:1]
292.2999
Relative Intensity
m/z

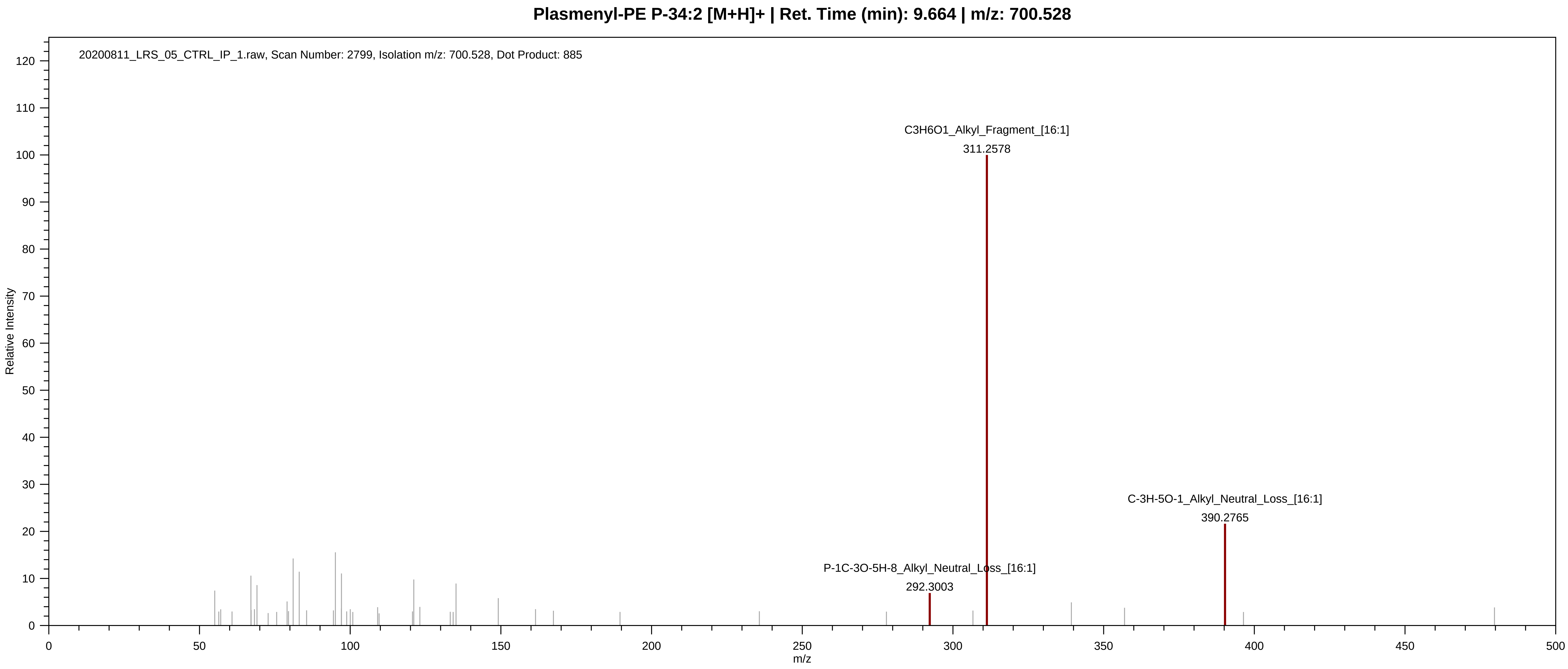

Plasmenyl-PE P-34:2 [M+H]+ | Ret. Time (min): 9.664 | m/z: 700.528
20200811_LRS_05_CTRL_IP_1.raw, Scan Number: 2799, Isolation m/z: 700.528, Dot Product: 885
C3H6O1_Alkyl_Fragment_[16:1]
311.2578
C-3H-5O-1_Alkyl_Neutral_Loss_[16:1]
390.2765
P-1C-3O-5H-8_Alkyl_Neutral_Loss_[16:1]
292.3003
Relative Intensity
m/z

Plasmenyl-PE P-34:2 [M+H]+ | Ret. Time (min): 9.803 | m/z: 700.528

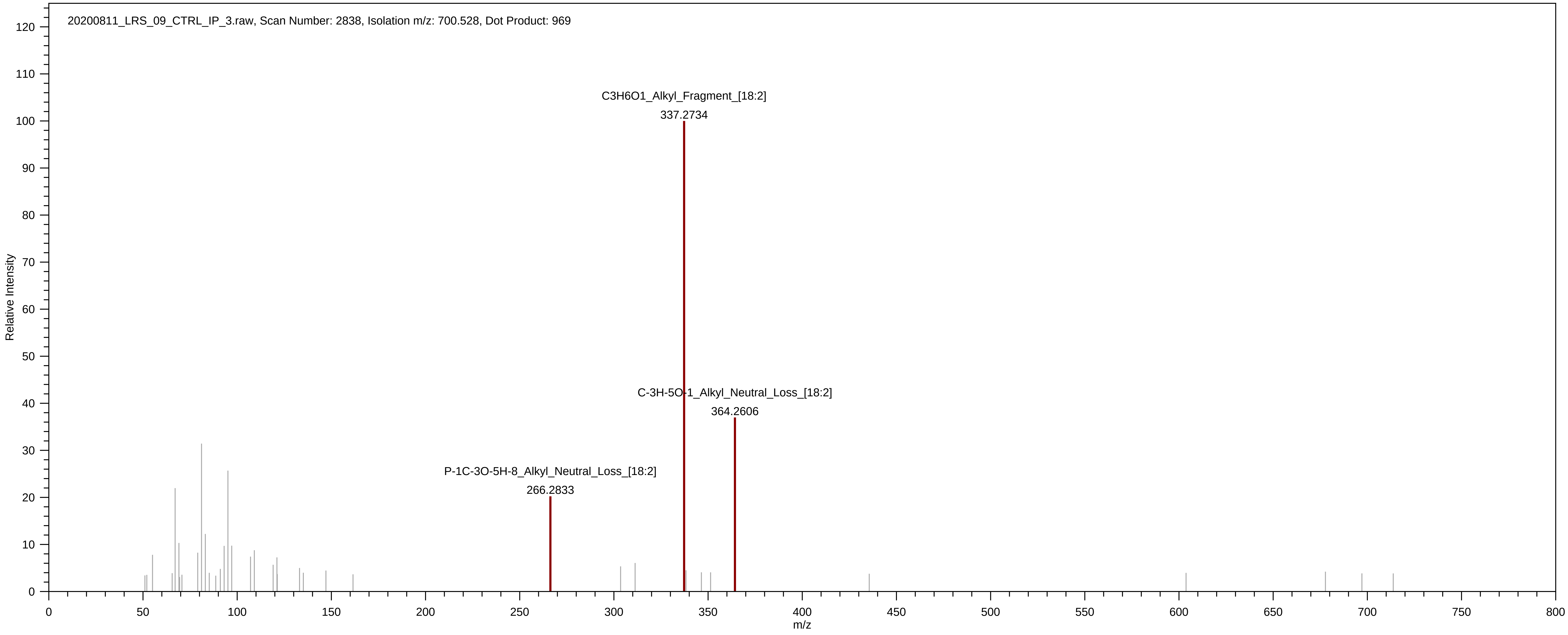

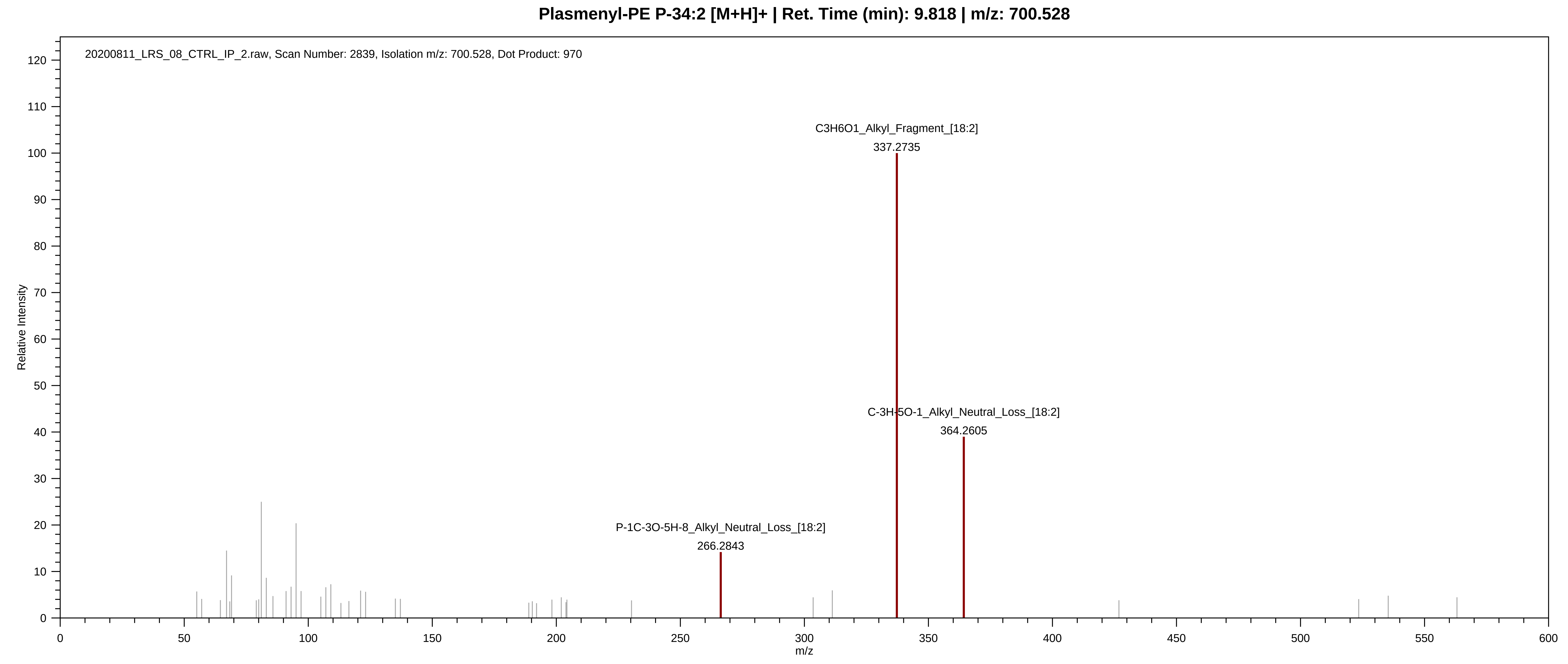

Plasmenyl-PE P-34:2 [M+H]+ | Ret. Time (min): 9.818 | m/z: 700.528
20200811_LRS_08_CTRL_IP_2.raw, Scan Number: 2839, Isolation m/z: 700.528, Dot Product: 970
C3H6O1_Alkyl_Fragment_[18:2]
337.2735
C-3H-5O-1_Alkyl_Neutral_Loss_[18:2]
364.2605
P-1C-3O-5H-8_Alkyl_Neutral_Loss_[18:2]
266.2843
Relative Intensity
m/z

Plasmenyl-PE P-36:1 [M+H]+ | Ret. Time (min): 11.5 | m/z: 730.575

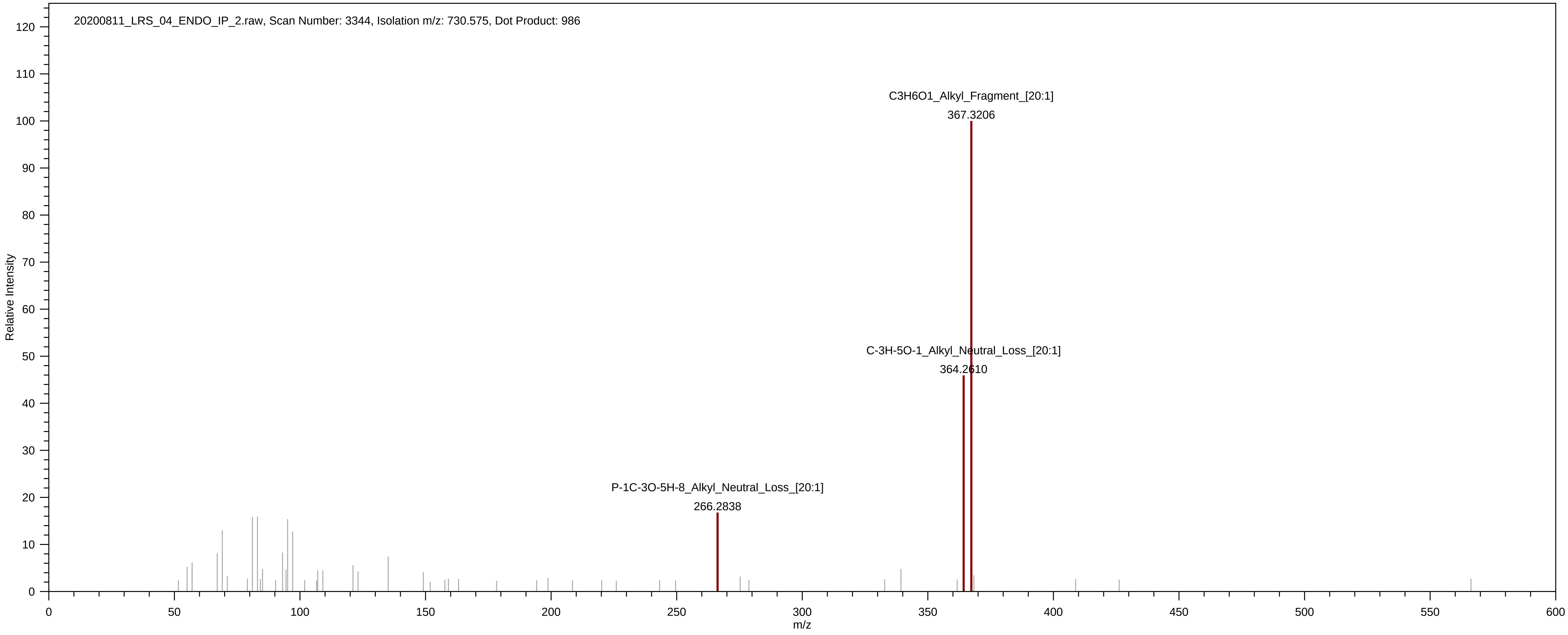

Plasmenyl-PE P-36:1 [M+H]+ | Ret. Time (min): 11.7 | m/z: 730.575

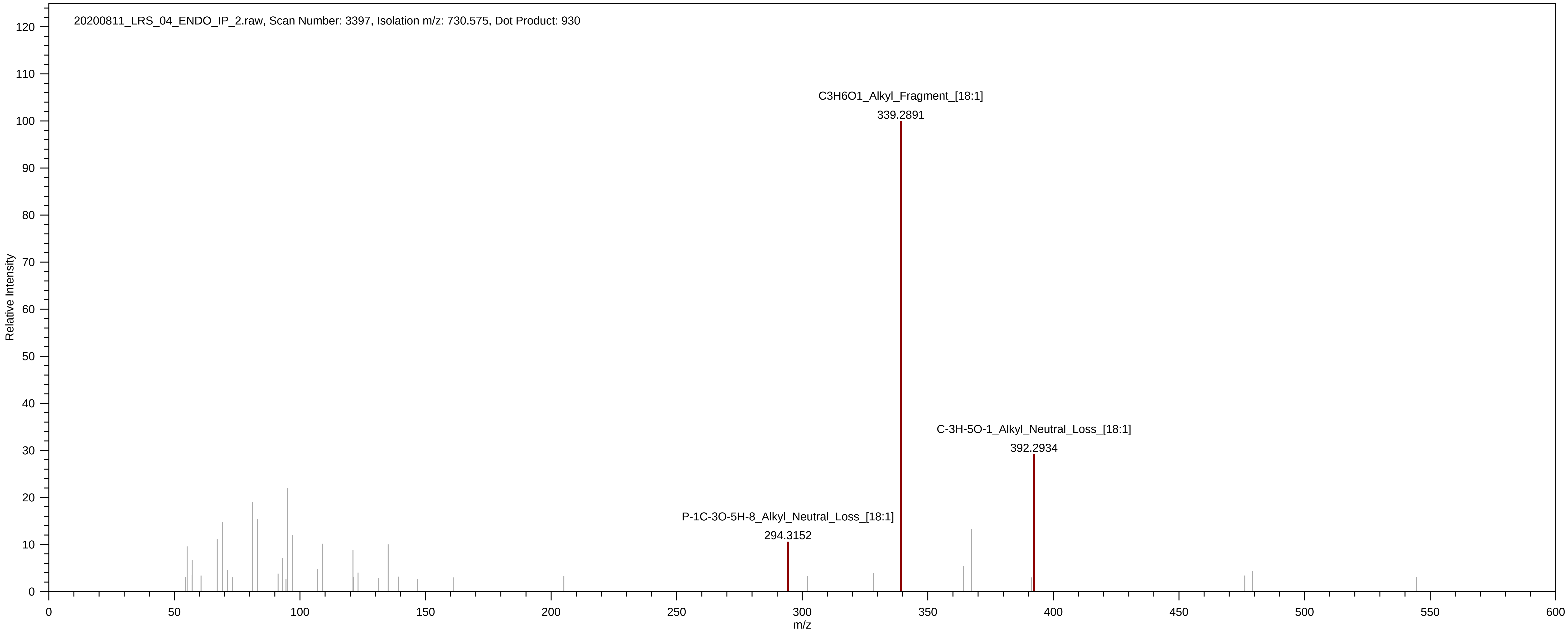

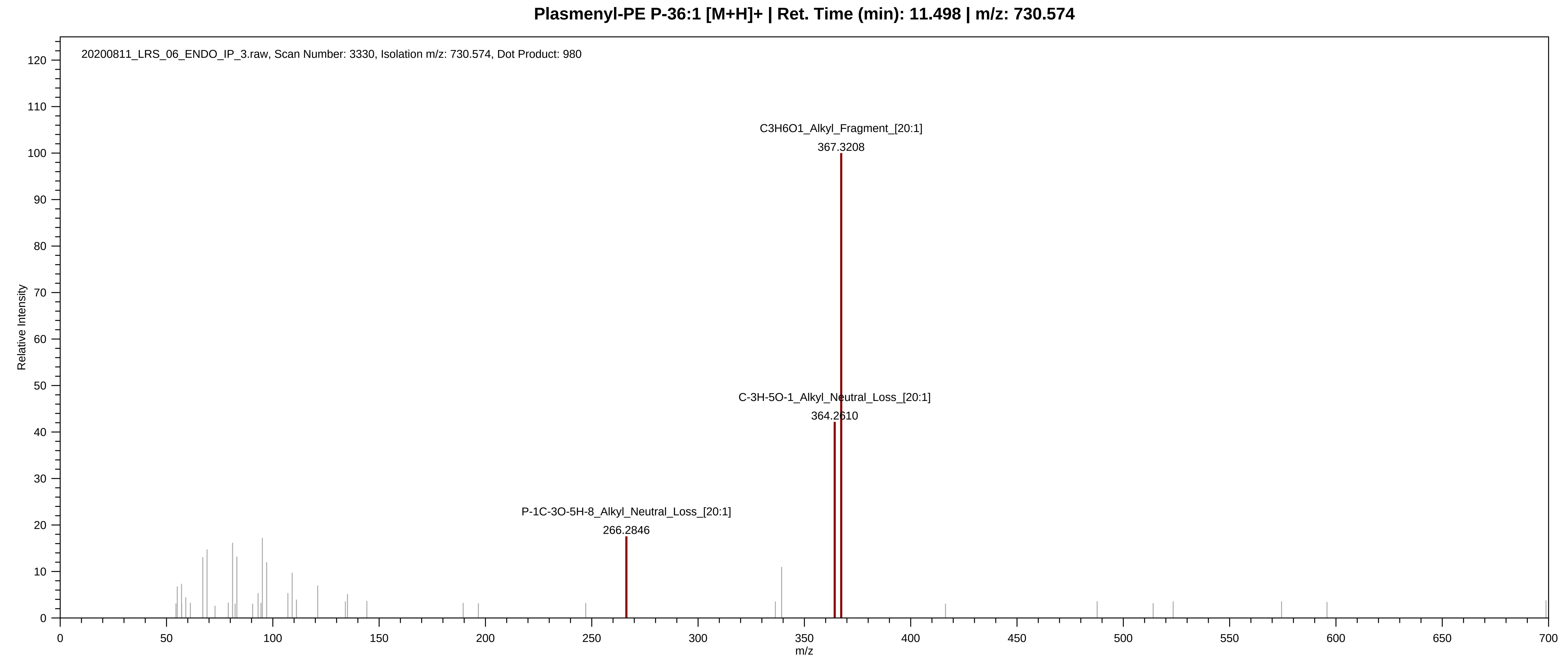

Plasmenyl-PE P-36:1 [M+H]+ | Ret. Time (min): 11.498 | m/z: 730.574
20200811_LRS_06_ENDO_IP_3.raw, Scan Number: 3330, Isolation m/z: 730.574, Dot Product: 980
C3H6O1_Alkyl_Fragment_[20:1]
367.3208
C-3H-5O-1_Alkyl_Neutral_Loss_[20:1]
364.2610
P-1C-3O-5H-8_Alkyl_Neutral_Loss_[20:1]
266.2846
Relative Intensity
m/z

Plasmenyl-PE P-36:1 [M+H]+ | Ret. Time (min): 11.498 | m/z: 730.575

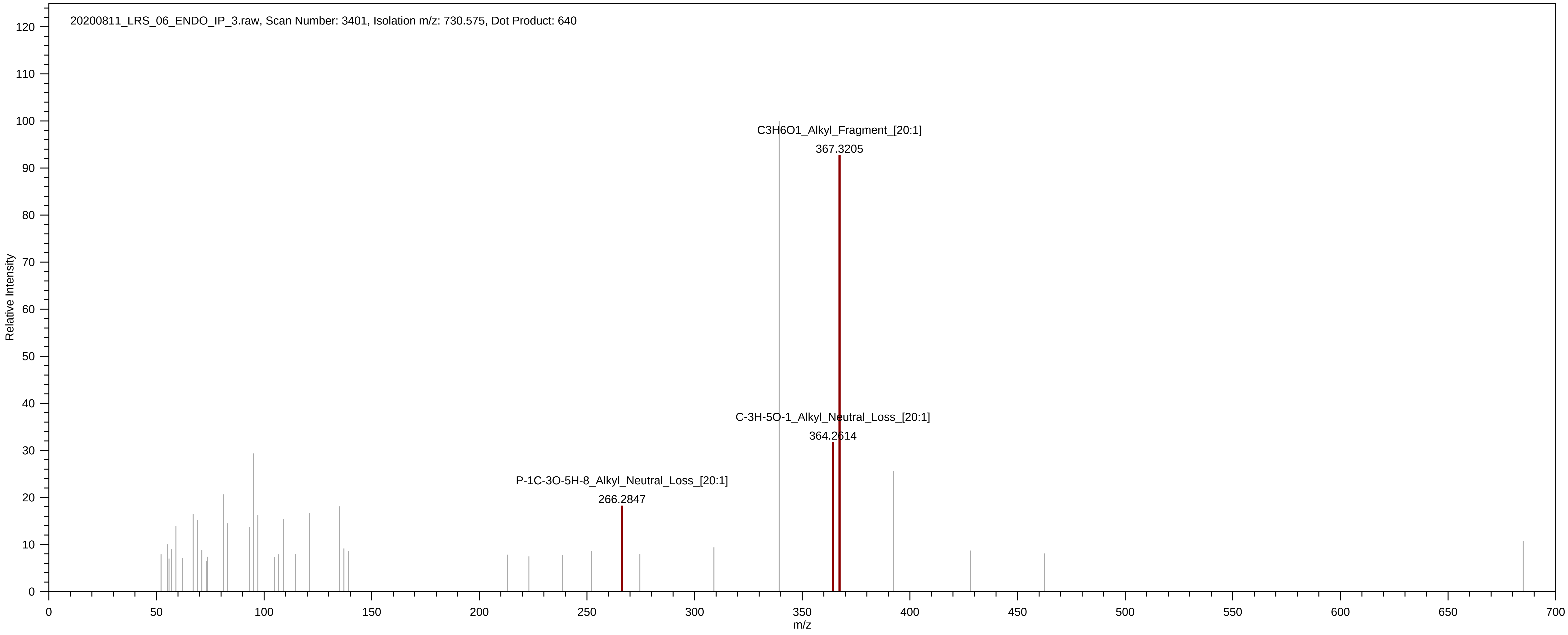

Plasmenyl-PE P-36:1 [M+H]+ | Ret. Time (min): 11.499 | m/z: 730.575

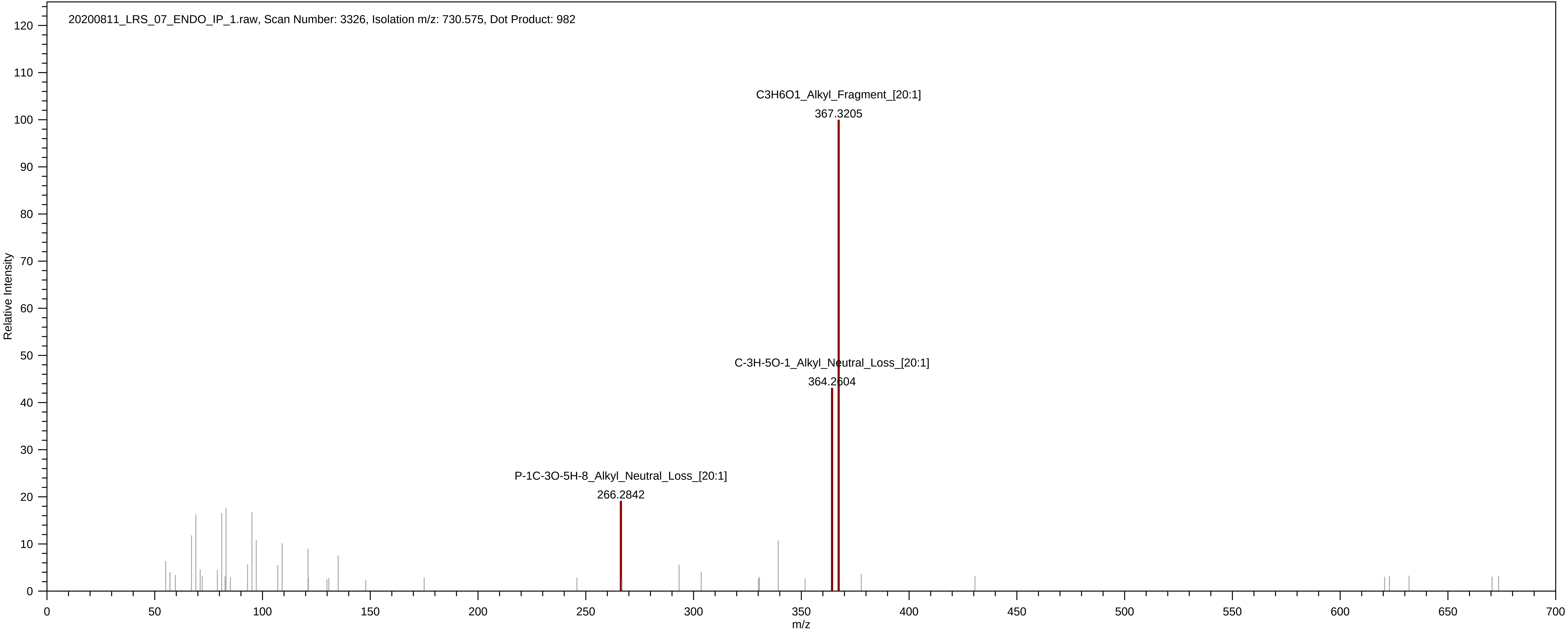

Plasmenyl-PE P-36:1 [M+H]+ | Ret. Time (min): 11.531 | m/z: 730.575

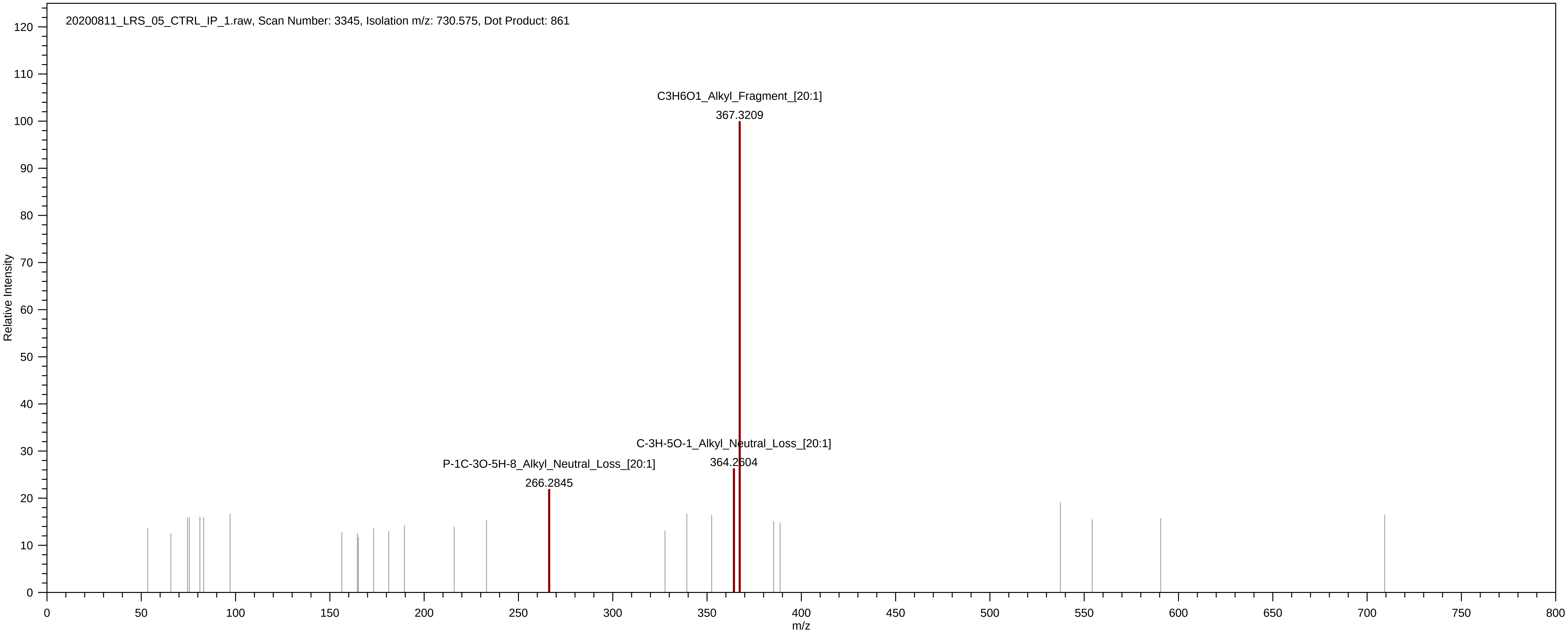

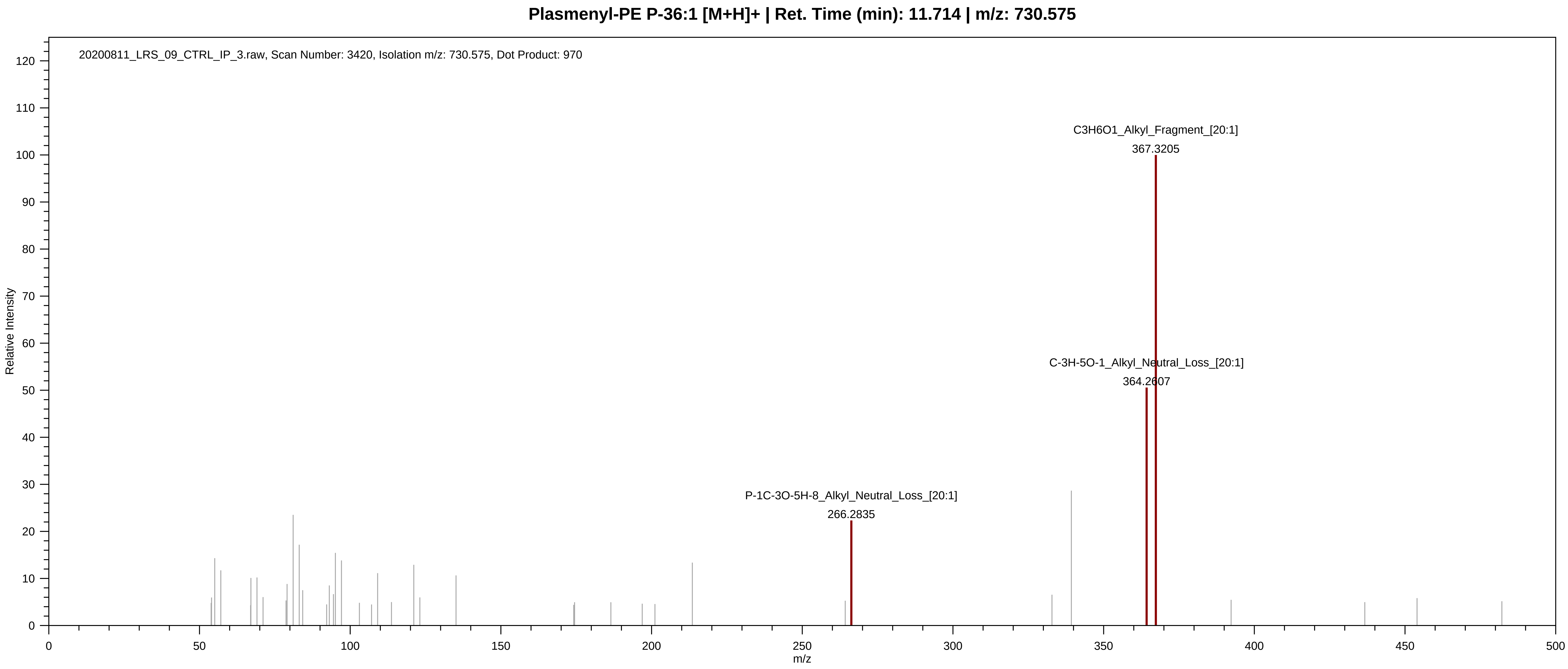

Plasmenyl-PE P-36:1 [M+H]+ | Ret. Time (min): 11.714 | m/z: 730.575
20200811_LRS_09_CTRL_IP_3.raw, Scan Number: 3420, Isolation m/z: 730.575, Dot Product: 970
C3H6O1_Alkyl_Fragment_[20:1]
367.3205
C-3H-5O-1_Alkyl_Neutral_Loss_[20:1]
364.2607
P-1C-3O-5H-8_Alkyl_Neutral_Loss_[20:1]
266.2835
Relative Intensity
m/z

Plasmenyl-PE P-36:1 [M+H]+ | Ret. Time (min): 11.51 | m/z: 730.572

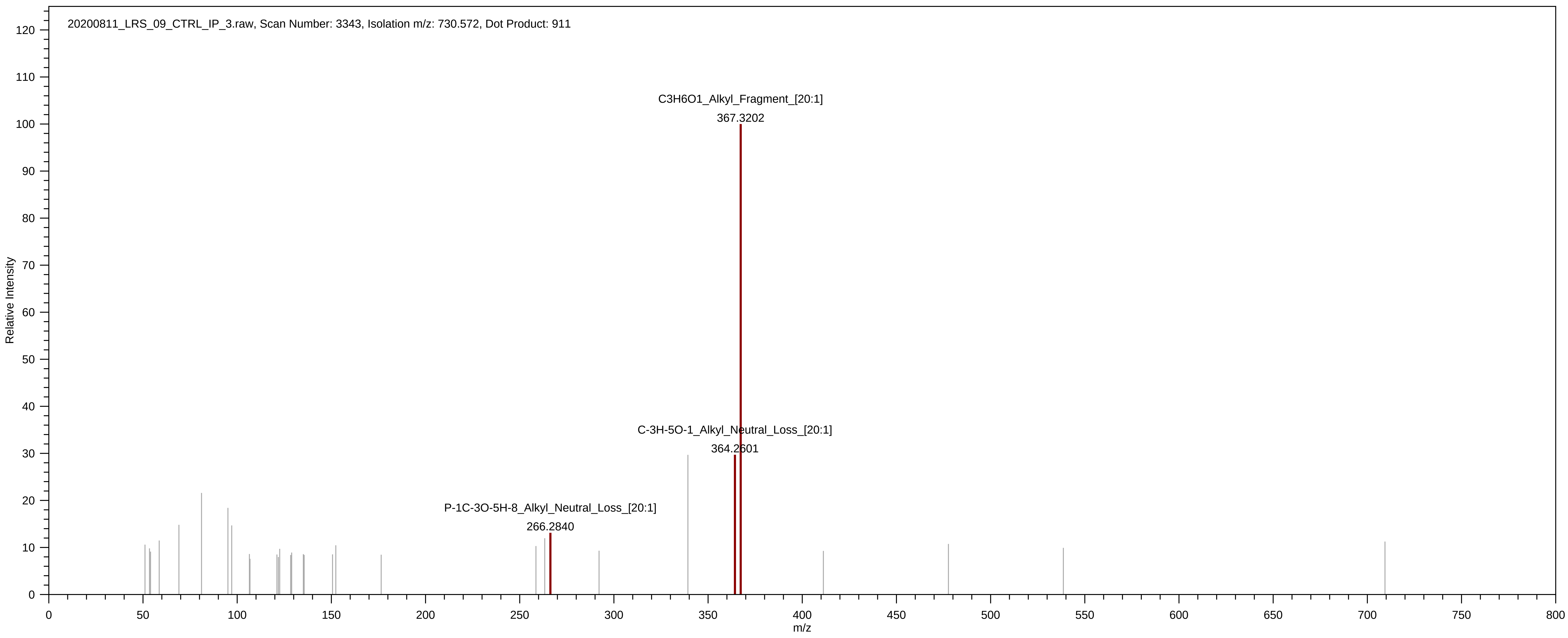

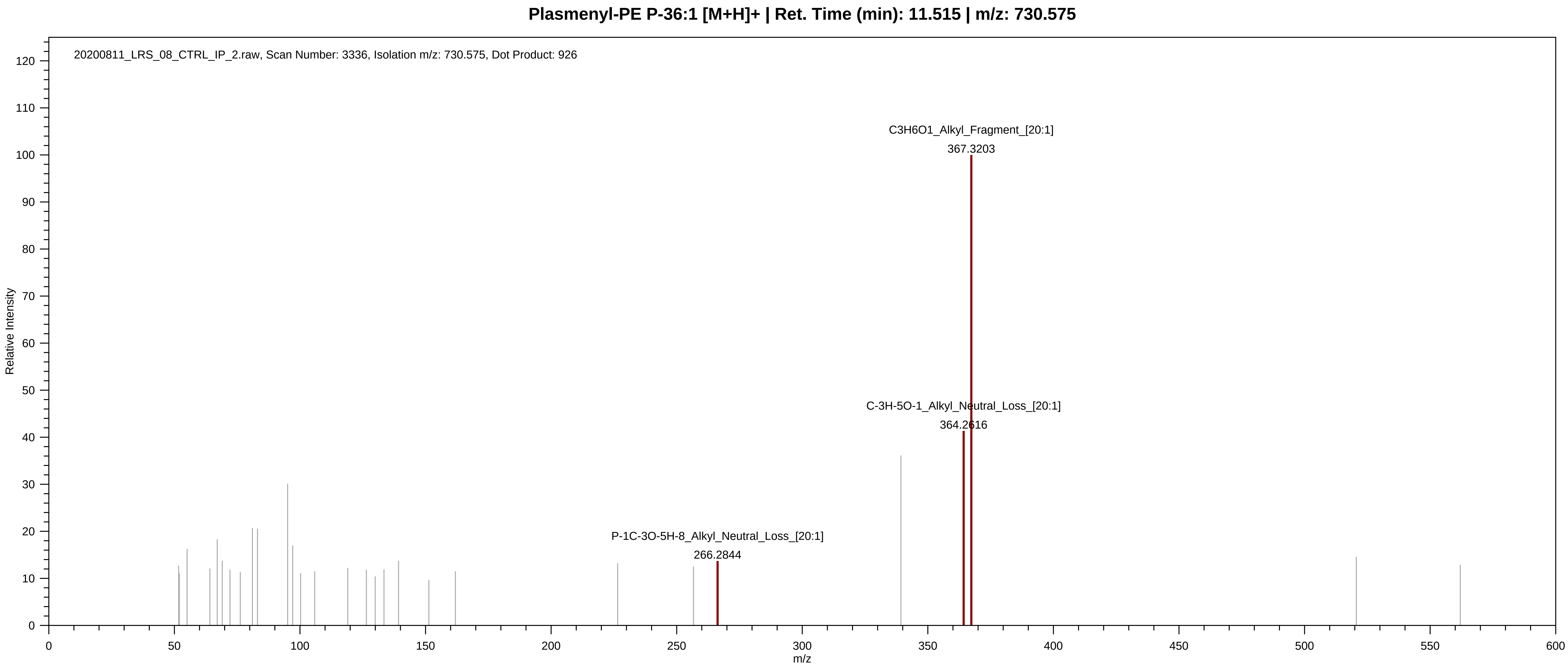

Plasmenyl-PE P-36:1 [M+H]+ | Ret. Time (min): 11.515 | m/z: 730.575
20200811_LRS_08_CTRL_IP_2.raw, Scan Number: 3336, Isolation m/z: 730.575, Dot Product: 926
C3H6O1_Alkyl_Fragment_[20:1]
367.3203
C-3H-5O-1_Alkyl_Neutral_Loss_[20:1]
364.2616
P-1C-3O-5H-8_Alkyl_Neutral_Loss_[20:1]
266.2844
Relative Intensity
m/z

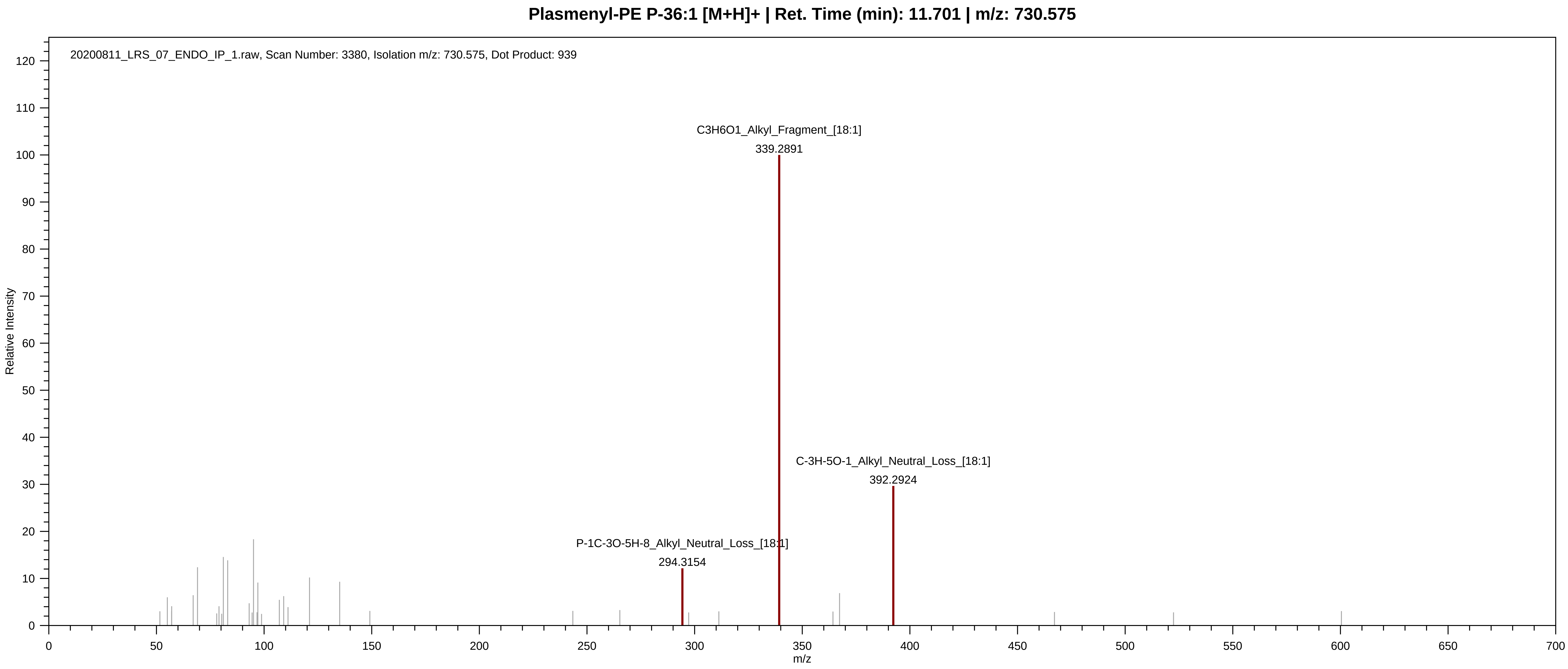

Plasmenyl-PE P-36:1 [M+H]+ | Ret. Time (min): 11.701 | m/z: 730.575
20200811_LRS_07_ENDO_IP_1.raw, Scan Number: 3380, Isolation m/z: 730.575, Dot Product: 939
C3H6O1_Alkyl_Fragment_[18:1]
339.2891
C-3H-5O-1_Alkyl_Neutral_Loss_[18:1]
392.2924
P-1C-3O-5H-8_Alkyl_Neutral_Loss_[18:1]
294.3154
Relative Intensity
m/z
0
10
20
30
40
50
60
70
80
90
100
110
120
0
50
100
150
200
250
300
350
400
450
500
550
600
650
700

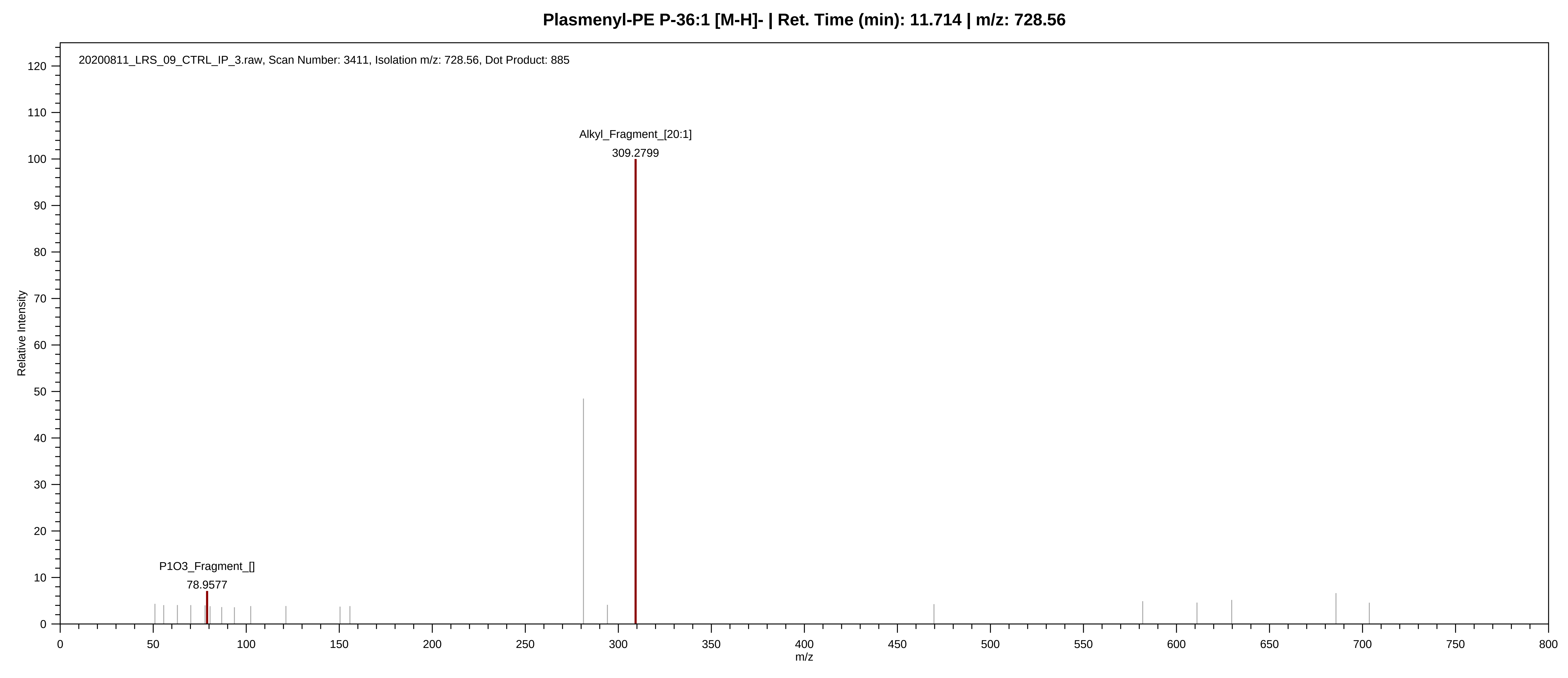
Plasmenyl-PE P-36:1 [M-H]- | Ret. Time (min): 11.714 | m/z: 728.56
20200811_LRS_09_CTRL_IP_3.raw, Scan Number: 3411, Isolation m/z: 728.56, Dot Product: 885
Alkyl_Fragment_[20:1]
309.2799
P1O3_Fragment_[]
78.9577
Relative Intensity
m/z

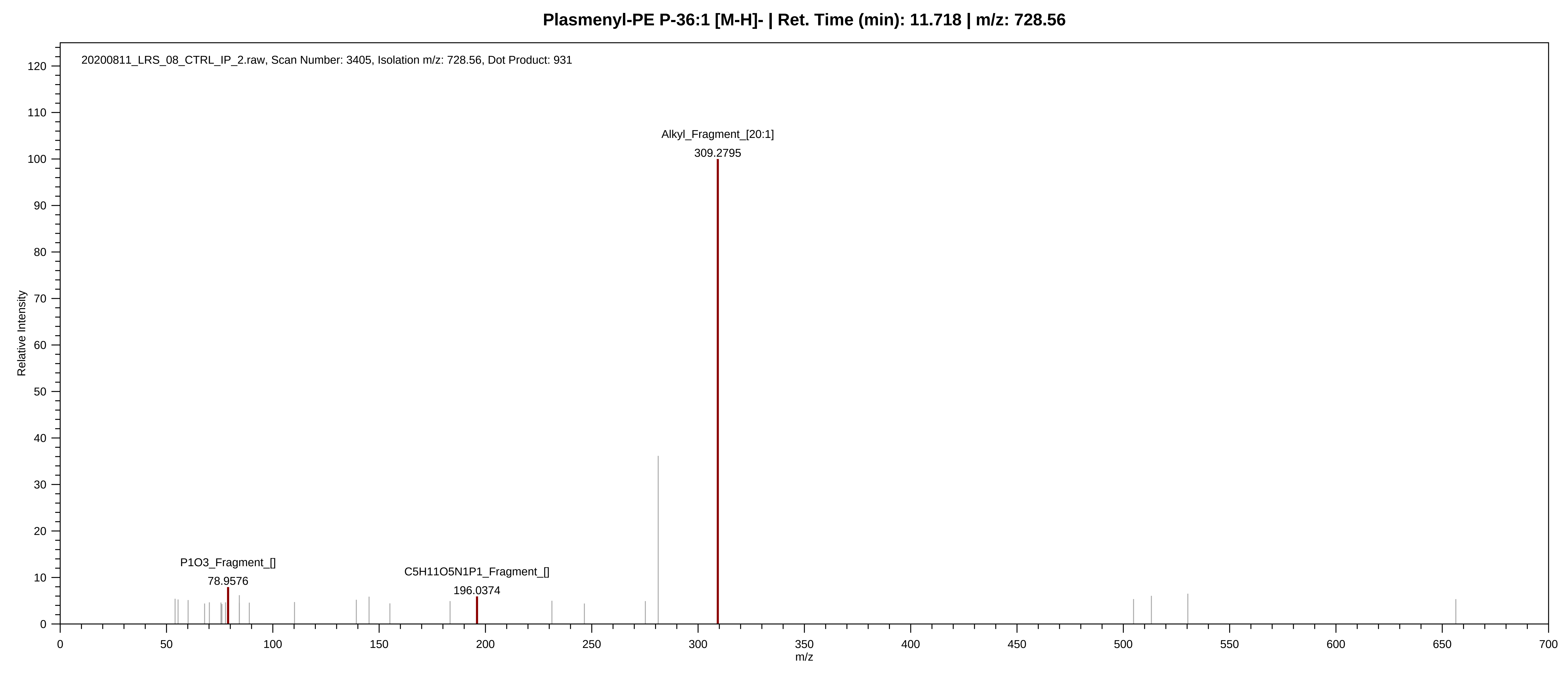

Plasmenyl-PE P-36:1 [M-H]- | Ret. Time (min): 11.718 | m/z: 728.56
20200811_LRS_08_CTRL_IP_2.raw, Scan Number: 3405, Isolation m/z: 728.56, Dot Product: 931
Alkyl_Fragment_[20:1]
309.2795
P1O3_Fragment_[]
78.9576
C5H11O5N1P1_Fragment_[]
196.0374
Relative Intensity
m/z

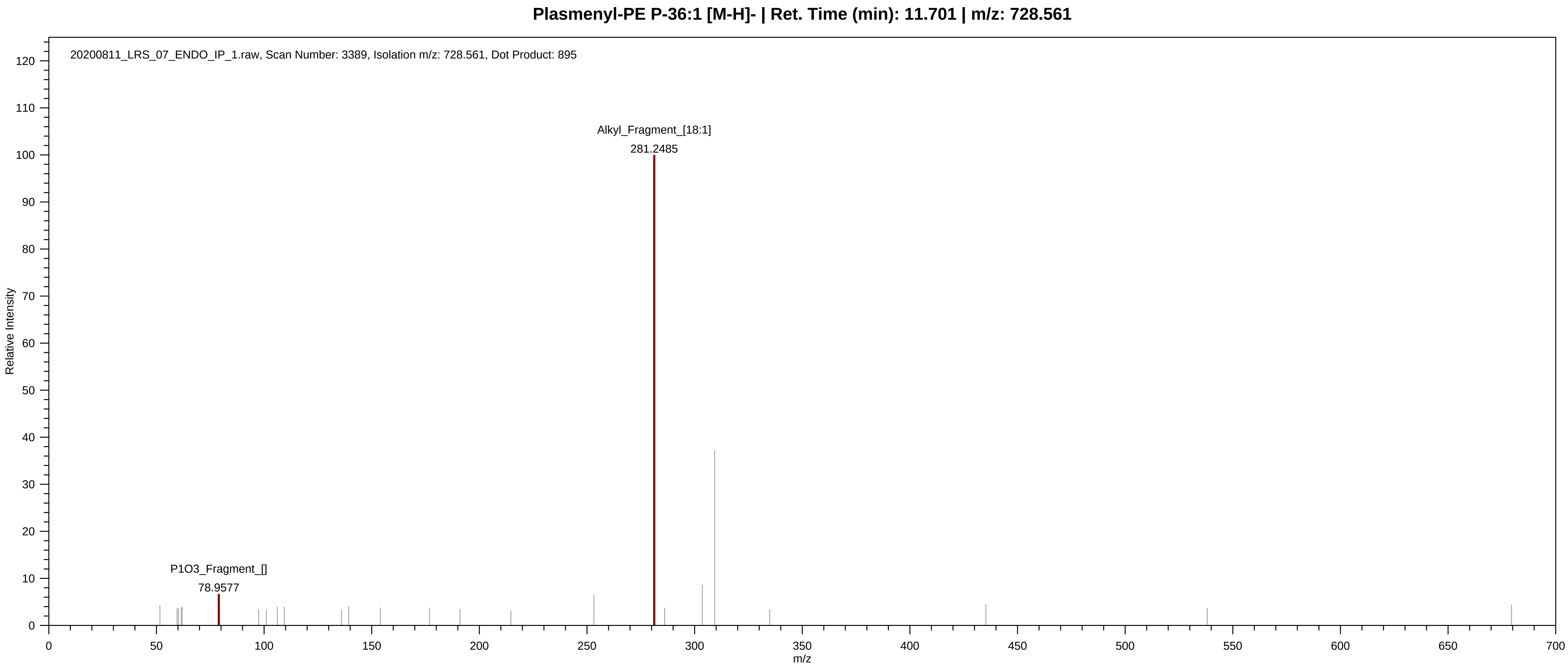

Plasmenyl-PE P-36:1 [M-H]- | Ret. Time (min): 11.701 | m/z: 728.561
20200811_LRS_07_ENDO_IP_1.raw, Scan Number: 3389, Isolation m/z: 728.561, Dot Product: 895
Alkyl_Fragment_[18:1]
281.2485
P1O3_Fragment_[]
78.9577
Relative Intensity
m/z

Plasmenyl-PE P-36:2 [M+H]+ | Ret. Time (min): 10.544 | m/z: 728.56

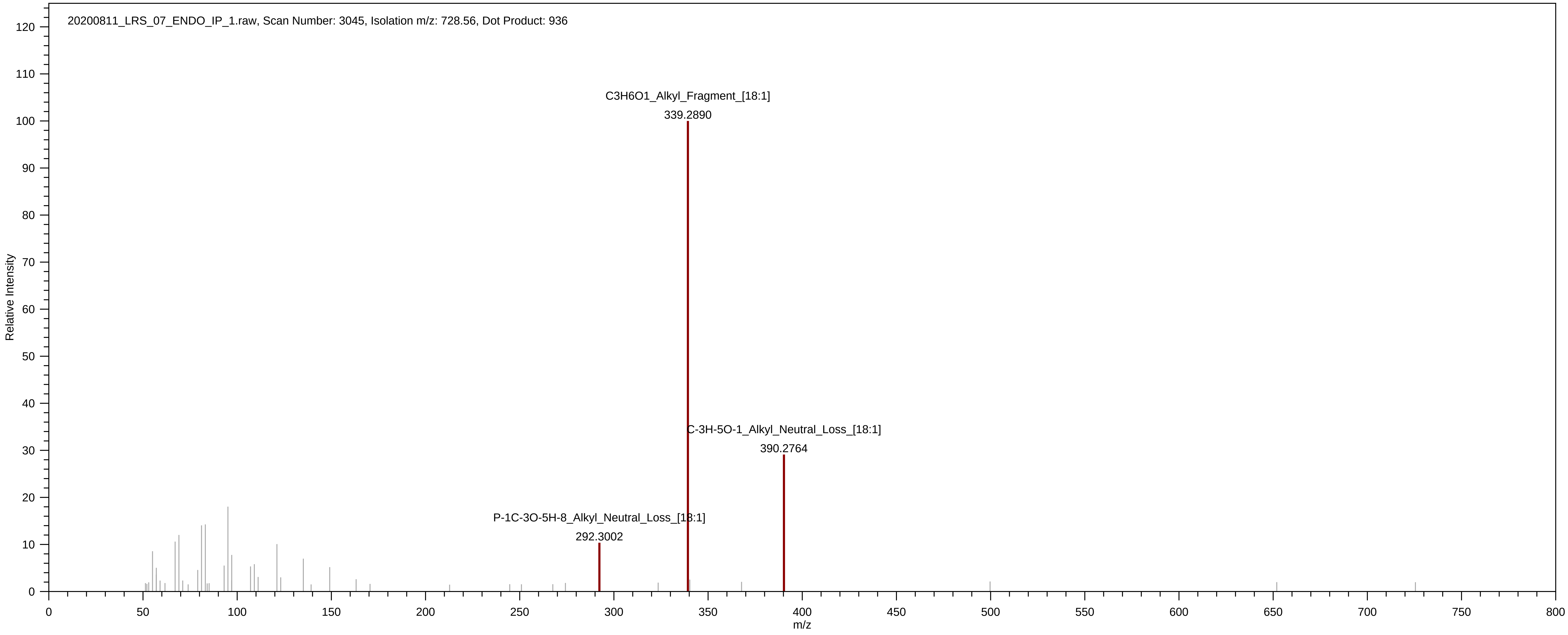

Plasmenyl-PE P-36:2 [M+H]+ | Ret. Time (min): 10.544 | m/z: 728.559

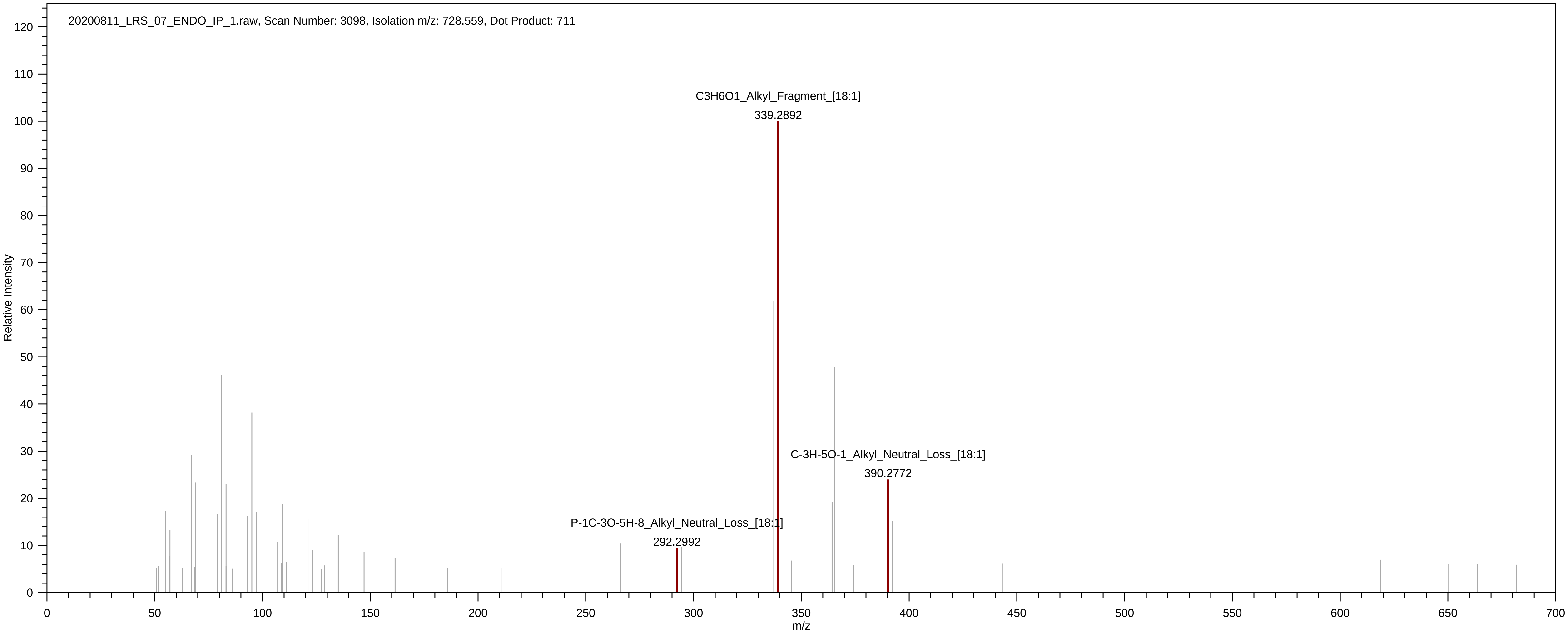

Plasmenyl-PE P-36:2 [M+H]+ | Ret. Time (min): 10.558 | m/z: 728.559

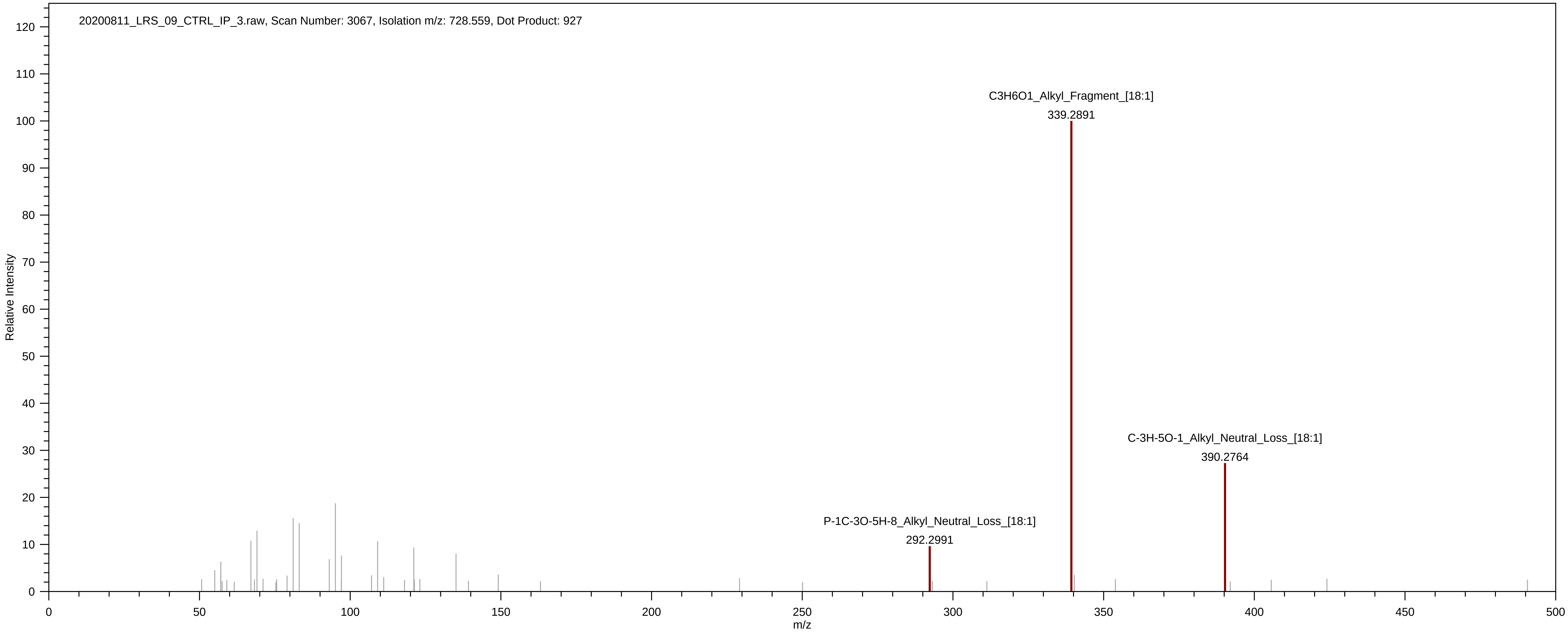

Plasmenyl-PE P-36:2 [M+H]+ | Ret. Time (min): 10.562 | m/z: 728.558

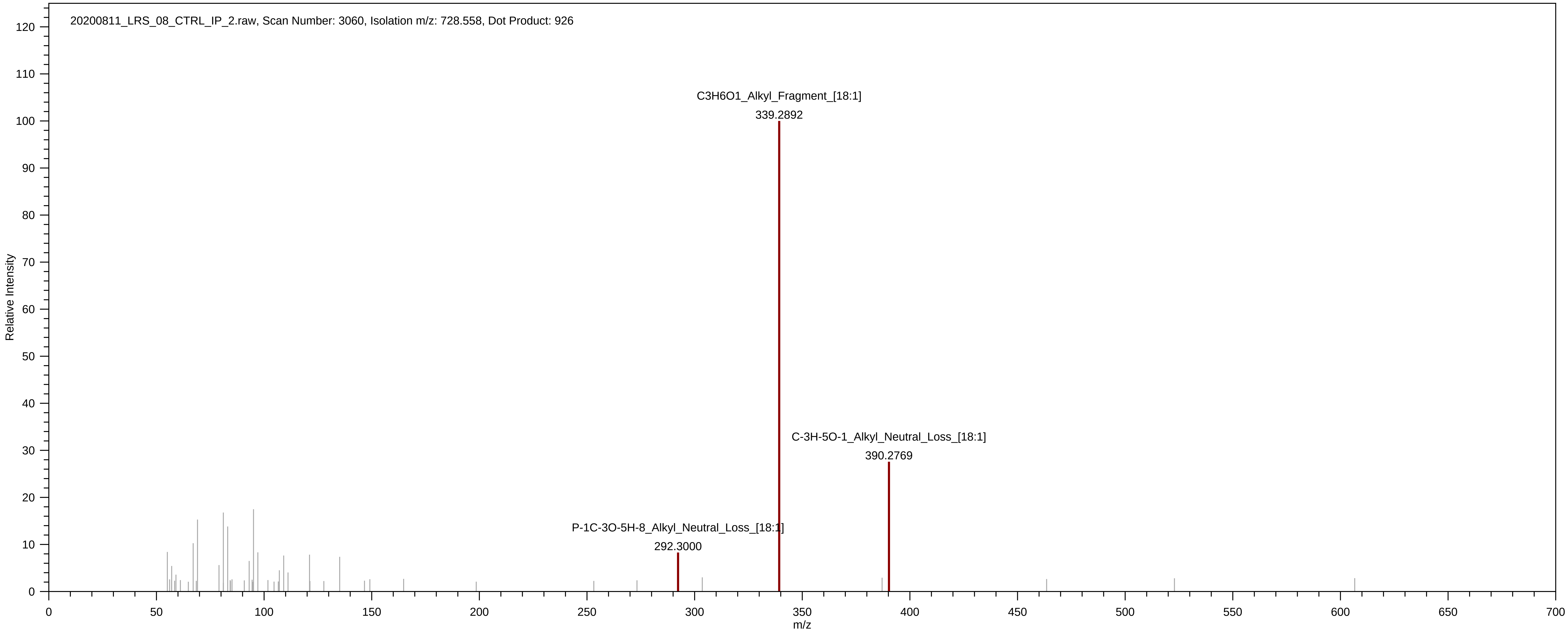

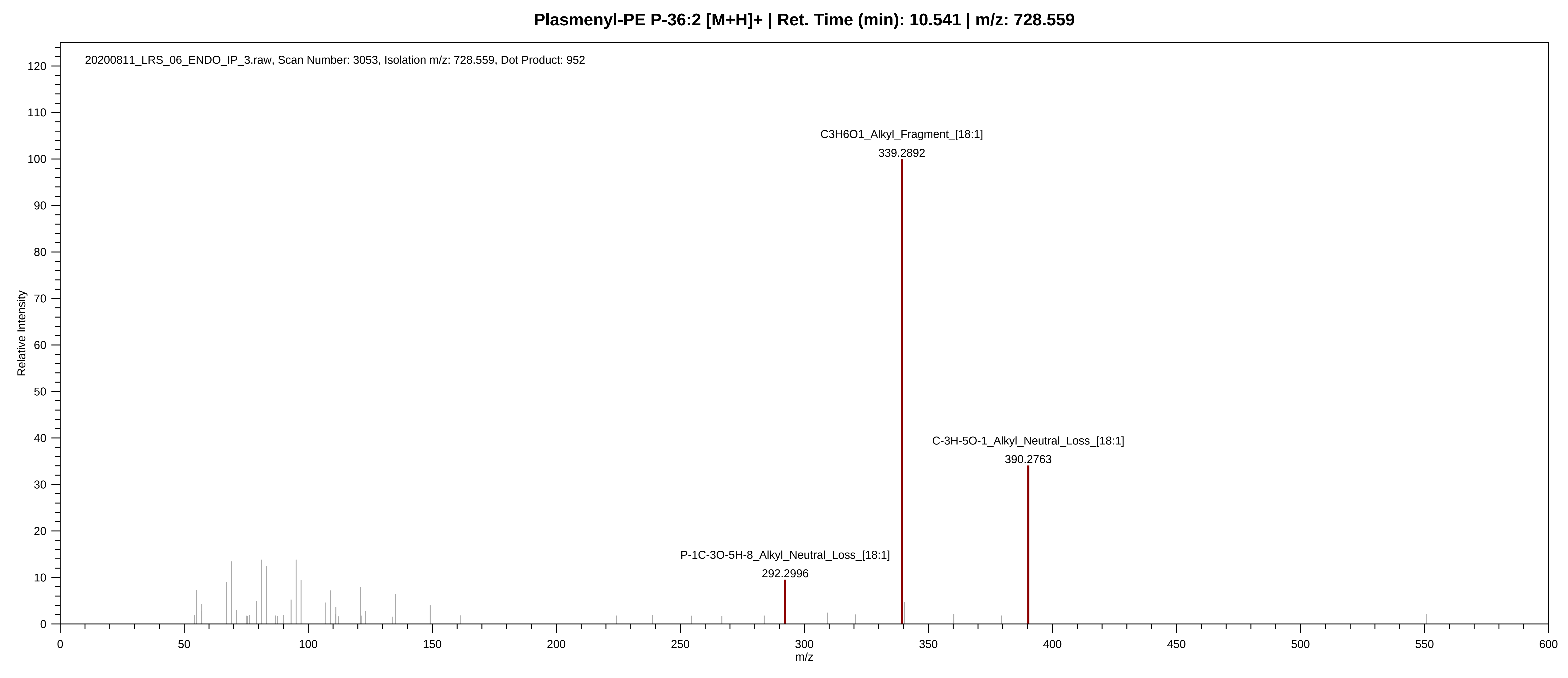
Plasmenyl-PE P-36:2 [M+H]+ | Ret. Time (min): 10.541 | m/z: 728.559
20200811_LRS_06_ENDO_IP_3.raw, Scan Number: 3053, Isolation m/z: 728.559, Dot Product: 952
C3H6O1_Alkyl_Fragment_[18:1]
339.2892
C-3H-5O-1_Alkyl_Neutral_Loss_[18:1]
390.2763
P-1C-3O-5H-8_Alkyl_Neutral_Loss_[18:1]
292.2996
Relative Intensity
m/z

Plasmenyl-PE P-36:2 [M+H]+ | Ret. Time (min): 10.546 | m/z: 728.559

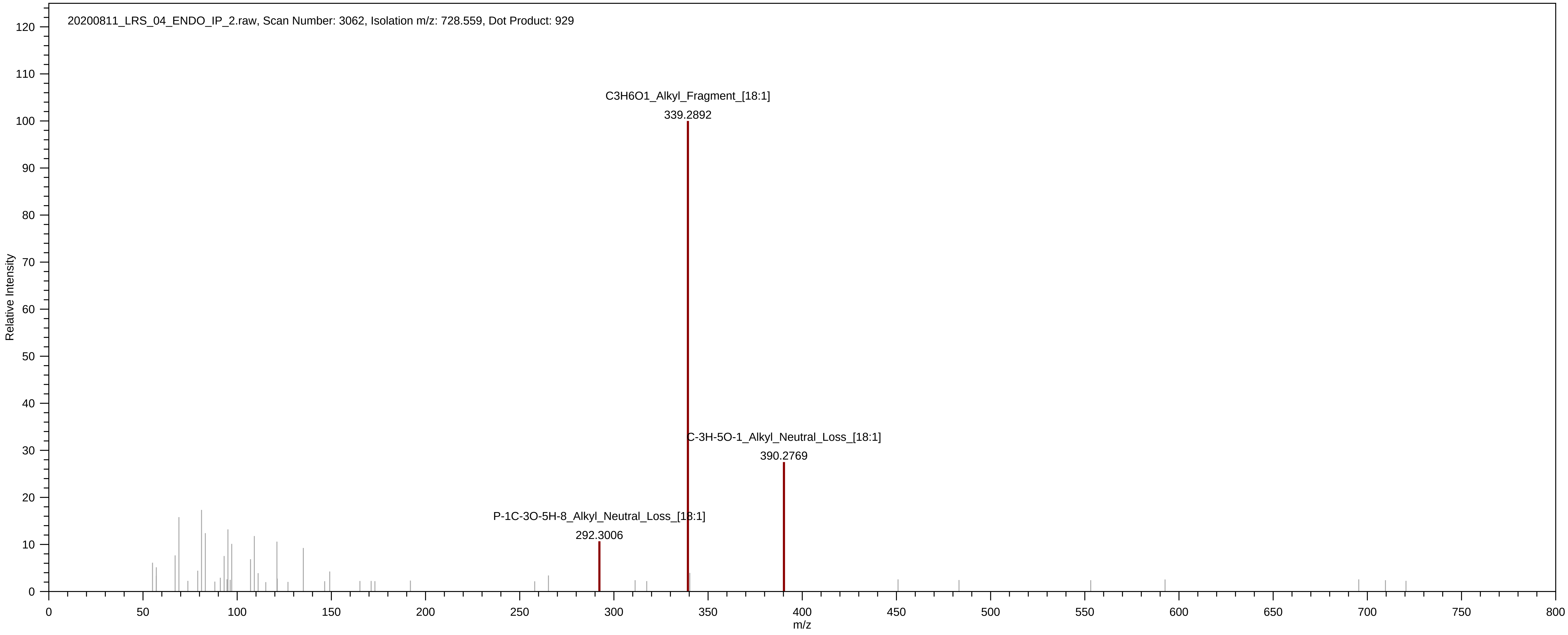

Plasmenyl-PE P-36:2 [M+H]+ | Ret. Time (min): 10.572 | m/z: 728.559

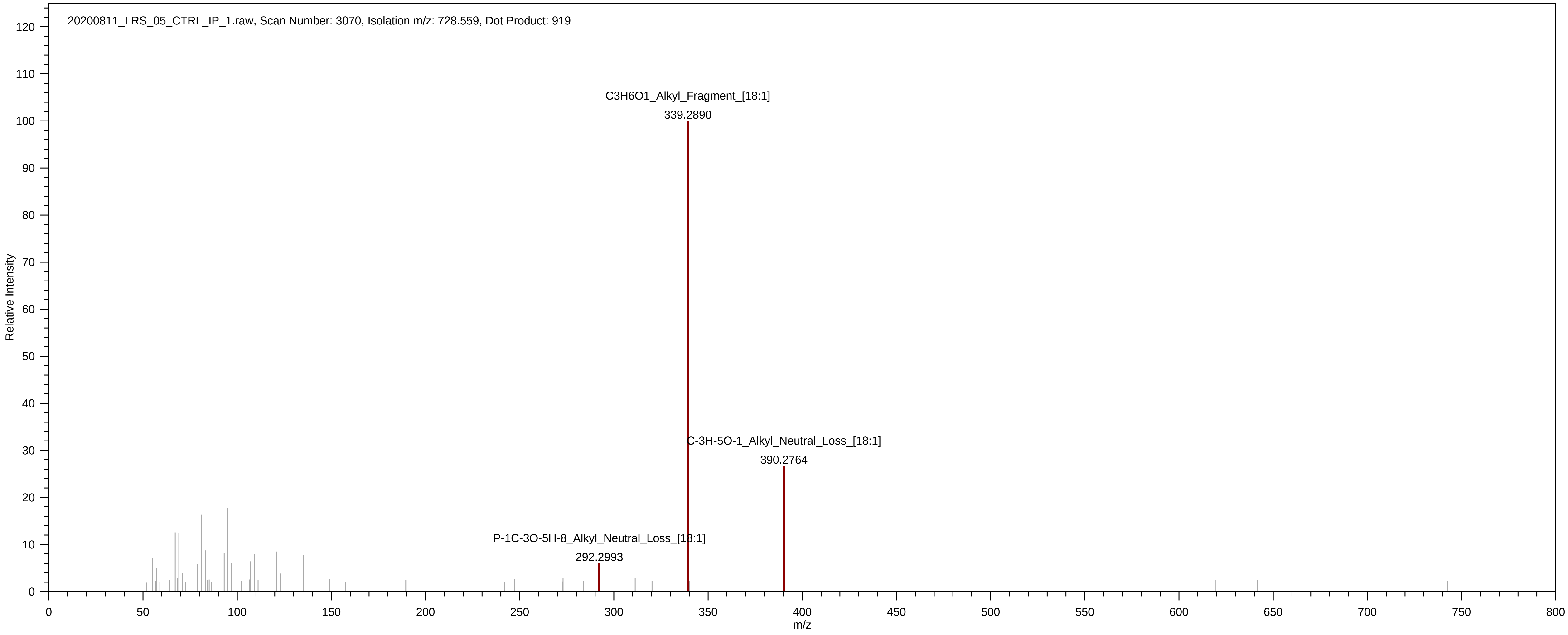

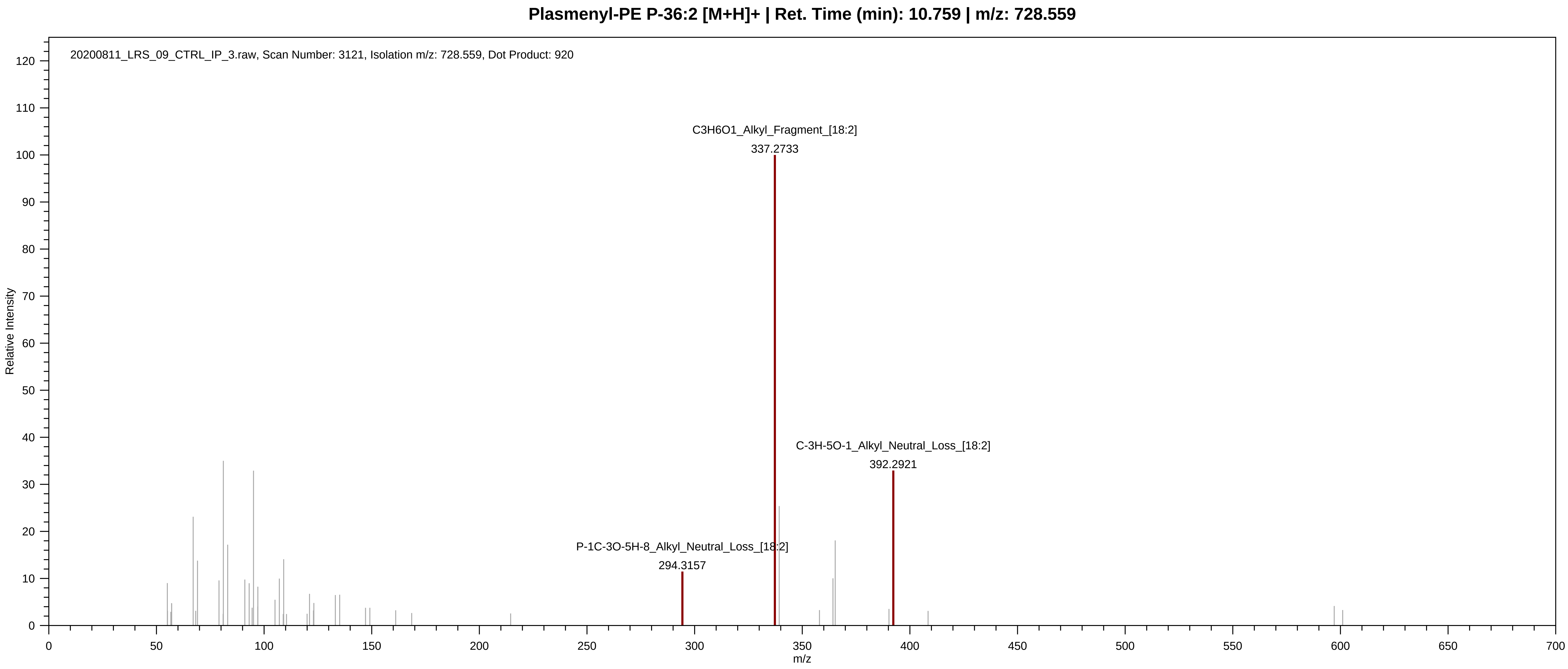

Plasmenyl-PE P-36:2 [M+H]+ | Ret. Time (min): 10.759 | m/z: 728.559
20200811_LRS_09_CTRL_IP_3.raw, Scan Number: 3121, Isolation m/z: 728.559, Dot Product: 920
C3H6O1_Alkyl_Fragment_[18:2]
337.2733
C-3H-5O-1_Alkyl_Neutral_Loss_[18:2]
392.2921
P-1C-3O-5H-8_Alkyl_Neutral_Loss_[18:2]
294.3157
Relative Intensity
m/z

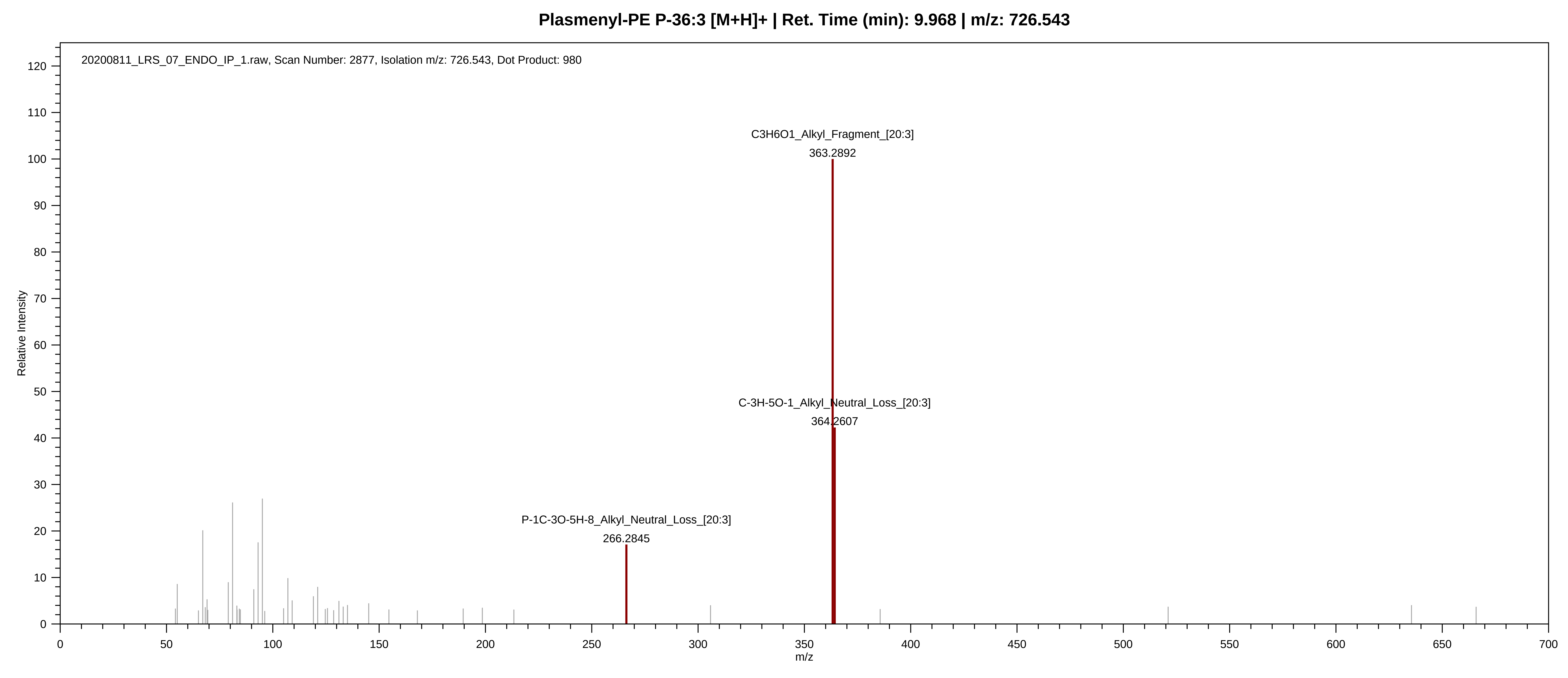

Plasmenyl-PE P-36:3 [M+H]+ | Ret. Time (min): 9.968 | m/z: 726.543
20200811_LRS_07_ENDO_IP_1.raw, Scan Number: 2877, Isolation m/z: 726.543, Dot Product: 980
C3H6O1_Alkyl_Fragment_[20:3]
363.2892
C-3H-5O-1_Alkyl_Neutral_Loss_[20:3]
364.2607
P-1C-3O-5H-8_Alkyl_Neutral_Loss_[20:3]
266.2845
Relative Intensity
m/z

Plasmenyl-PE P-36:4 [M+H]+ | Ret. Time (min): 9.433 | m/z: 724.527

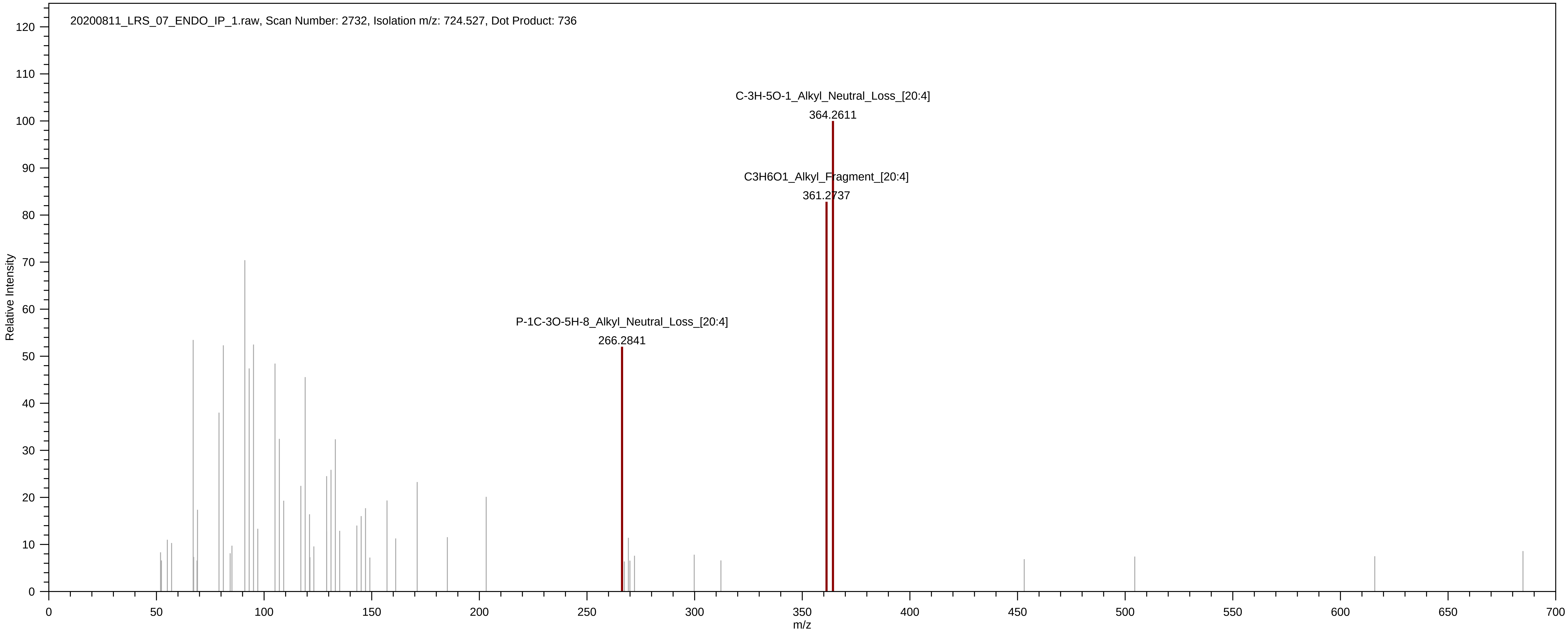

Plasmenyl-PE P-36:4 [M+H]+ | Ret. Time (min): 9.487 | m/z: 724.528

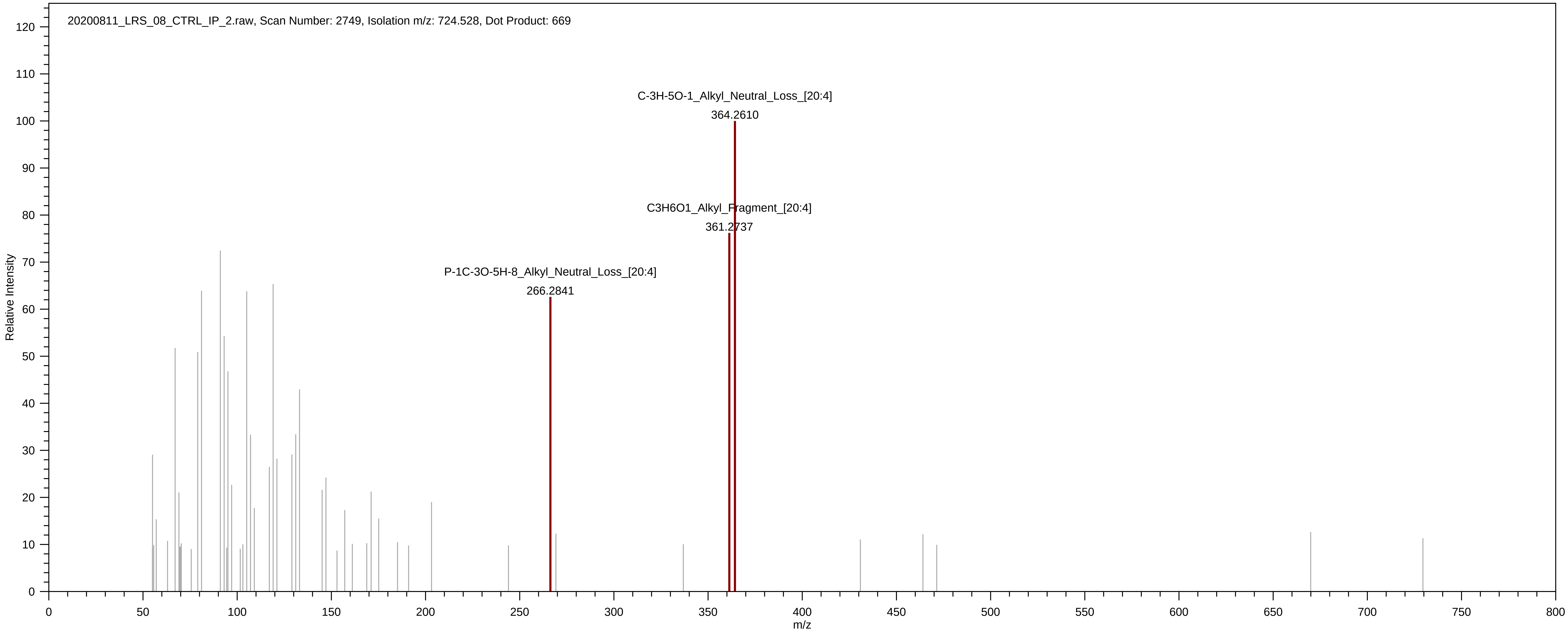

Plasmenyl-PE P-36:4 [M+H]+ | Ret. Time (min): 9.496 | m/z: 724.528

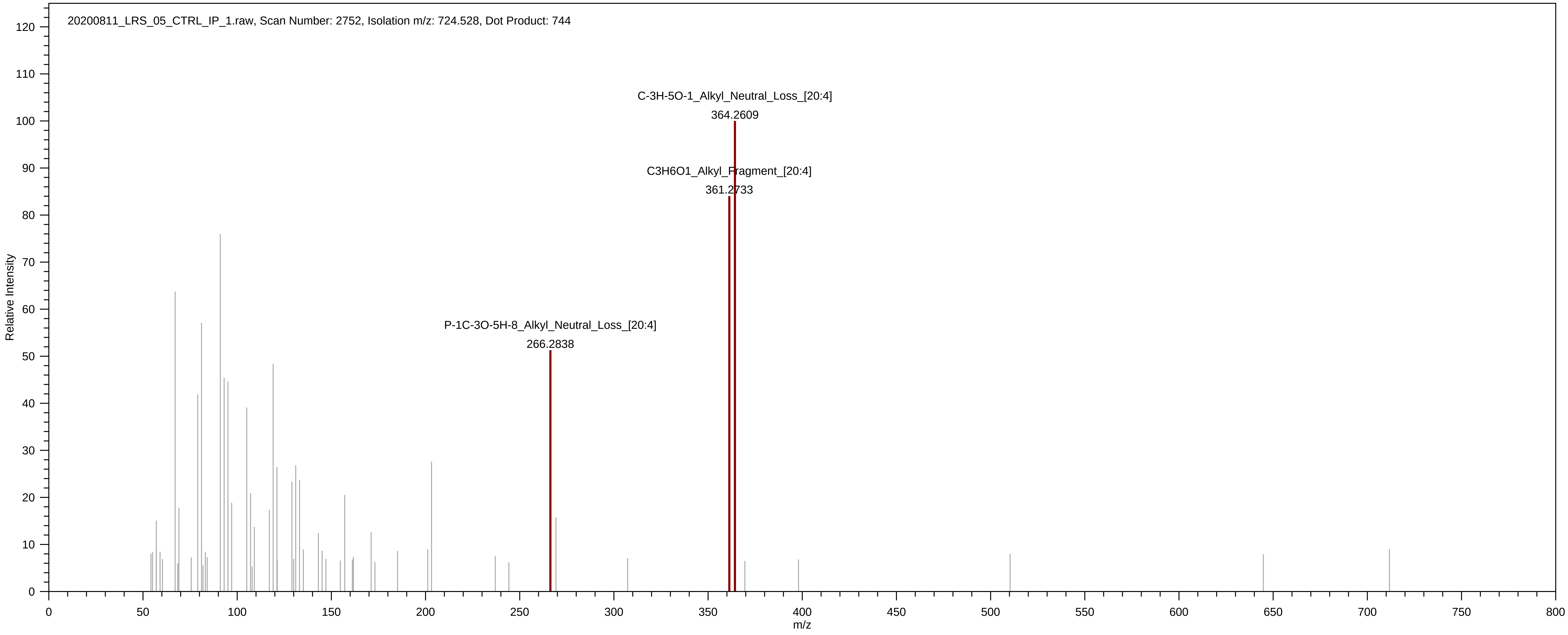

Plasmenyl-PE P-36:4 [M+H]+ | Ret. Time (min): 9.626 | m/z: 724.528

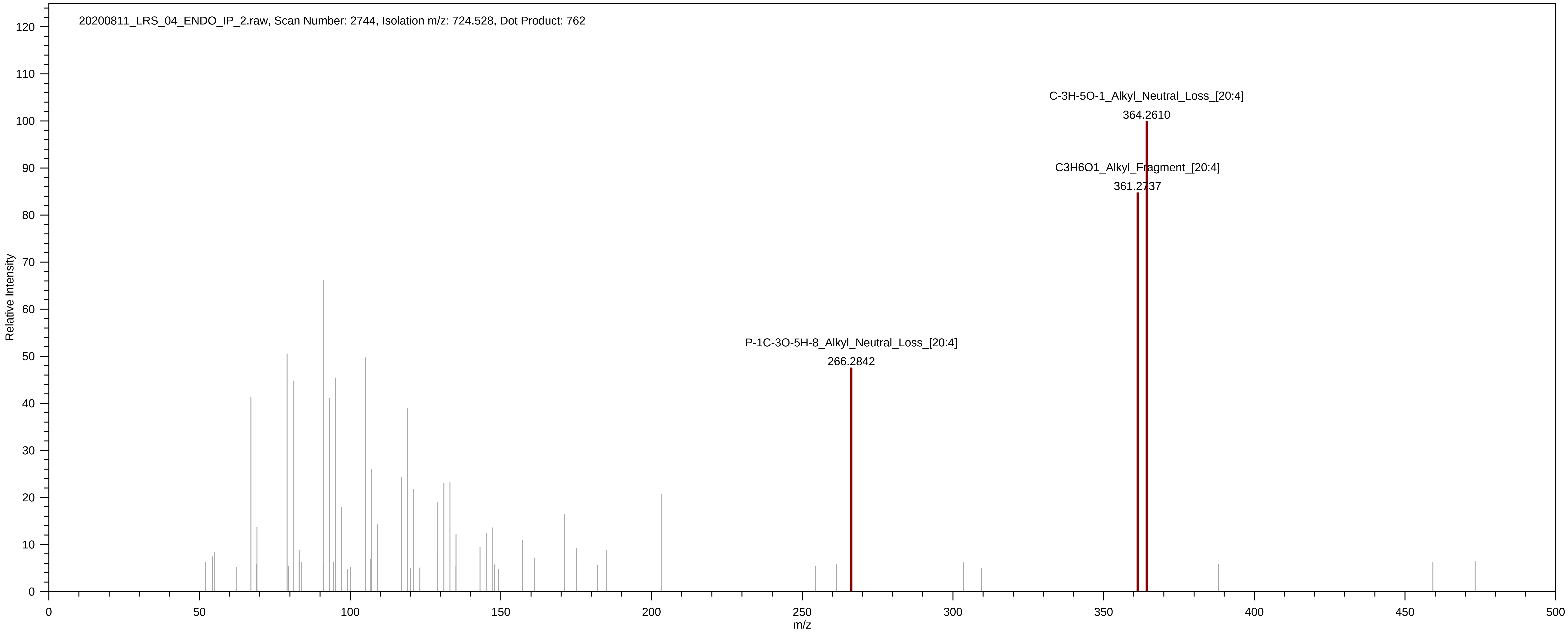

Plasmenyl-PE P-36:4 [M+H]+ | Ret. Time (min): 9.631 | m/z: 724.528

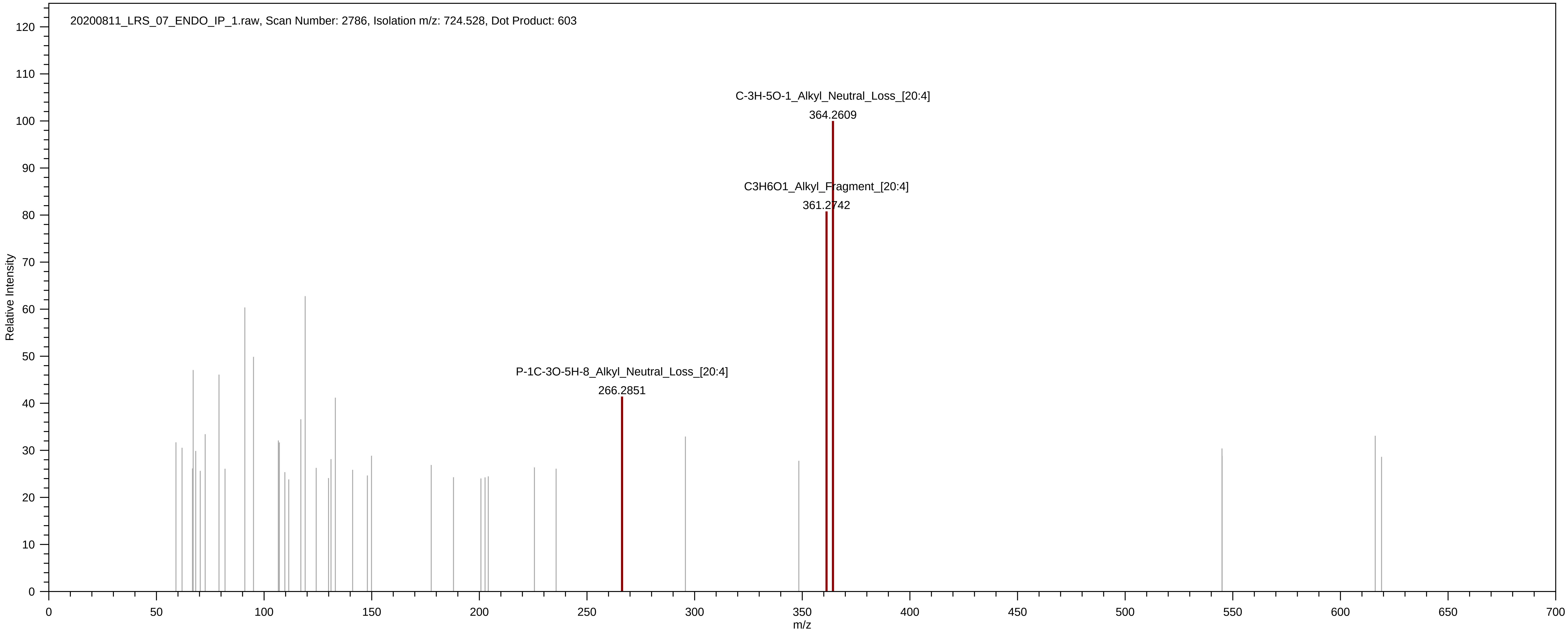

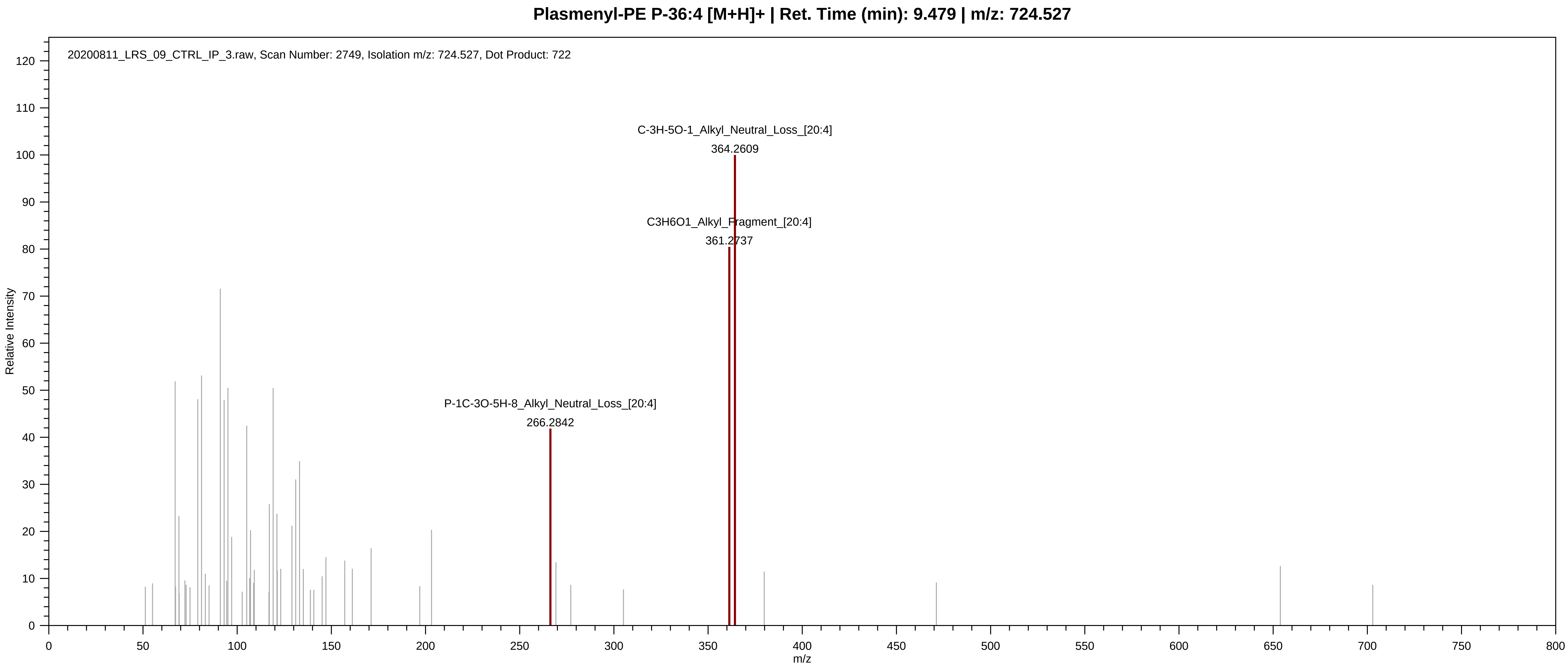

Plasmenyl-PE P-36:4 [M+H]+ | Ret. Time (min): 9.479 | m/z: 724.527
20200811_LRS_09_CTRL_IP_3.raw, Scan Number: 2749, Isolation m/z: 724.527, Dot Product: 722
C-3H-5O-1_Alkyl_Neutral_Loss_[20:4]
364.2609
C3H6O1_Alkyl_Fragment_[20:4]
361.2737
P-1C-3O-5H-8_Alkyl_Neutral_Loss_[20:4]
266.2842
Relative Intensity
m/z

Plasmenyl-PE P-36:5 [M+H]+ | Ret. Time (min): 8.914 | m/z: 722.511

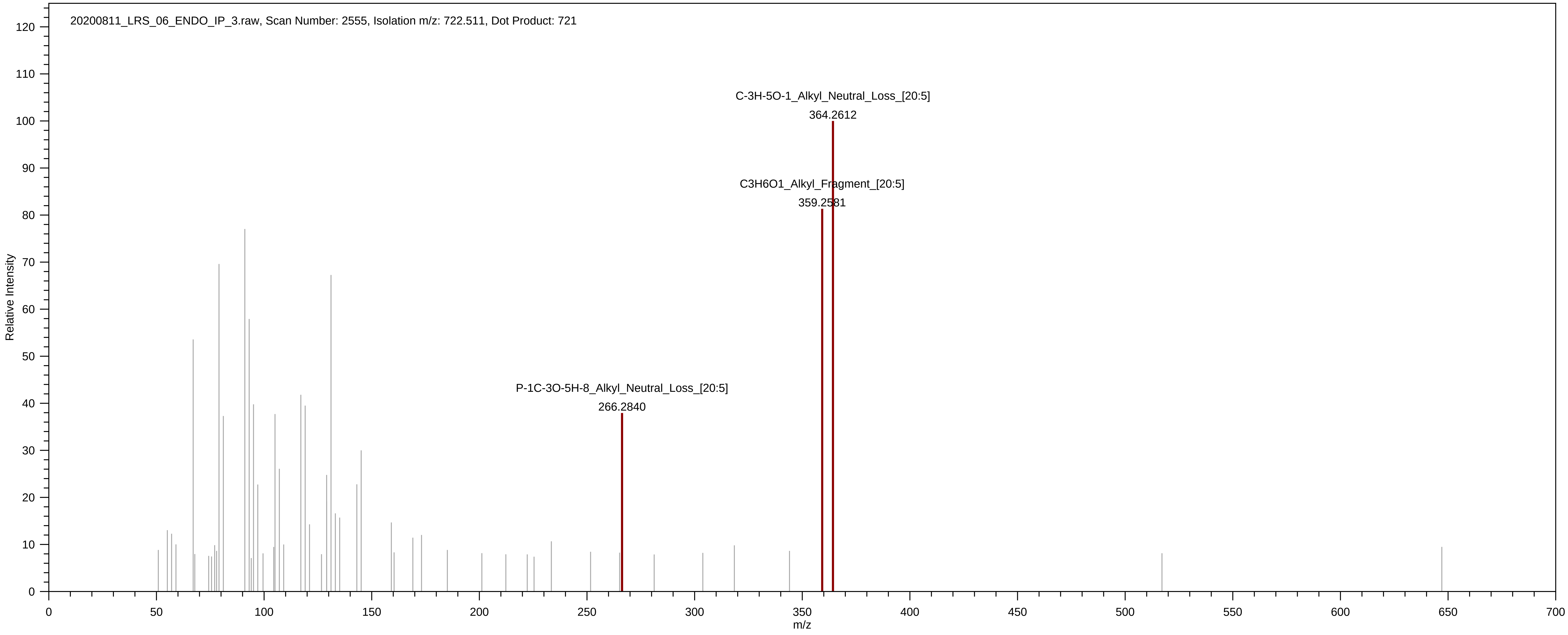

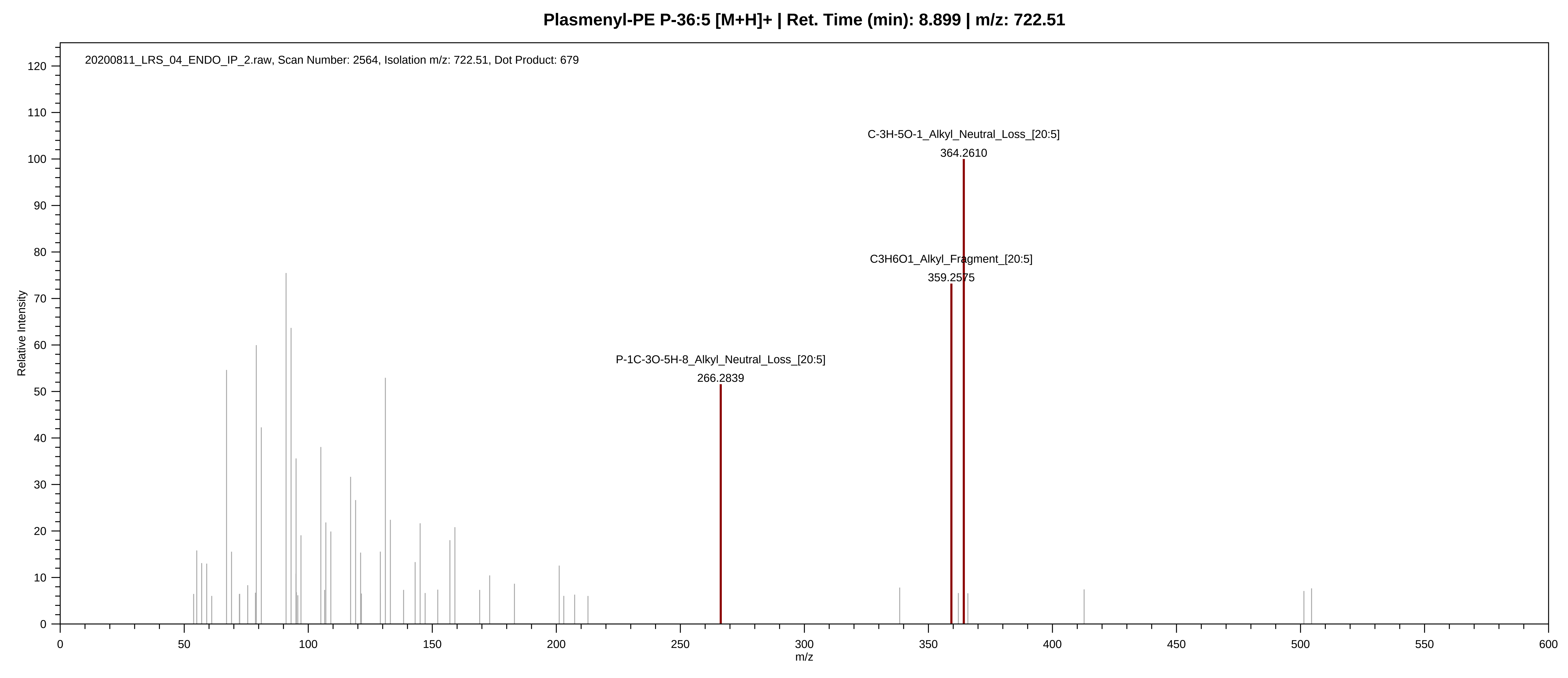

Plasmenyl-PE P-36:5 [M+H]+ | Ret. Time (min): 8.899 | m/z: 722.51
20200811_LRS_04_ENDO_IP_2.raw, Scan Number: 2564, Isolation m/z: 722.51, Dot Product: 679
C-3H-5O-1_Alkyl_Neutral_Loss_[20:5]
364.2610
C3H6O1_Alkyl_Fragment_[20:5]
359.2575
P-1C-3O-5H-8_Alkyl_Neutral_Loss_[20:5]
266.2839
Relative Intensity
m/z

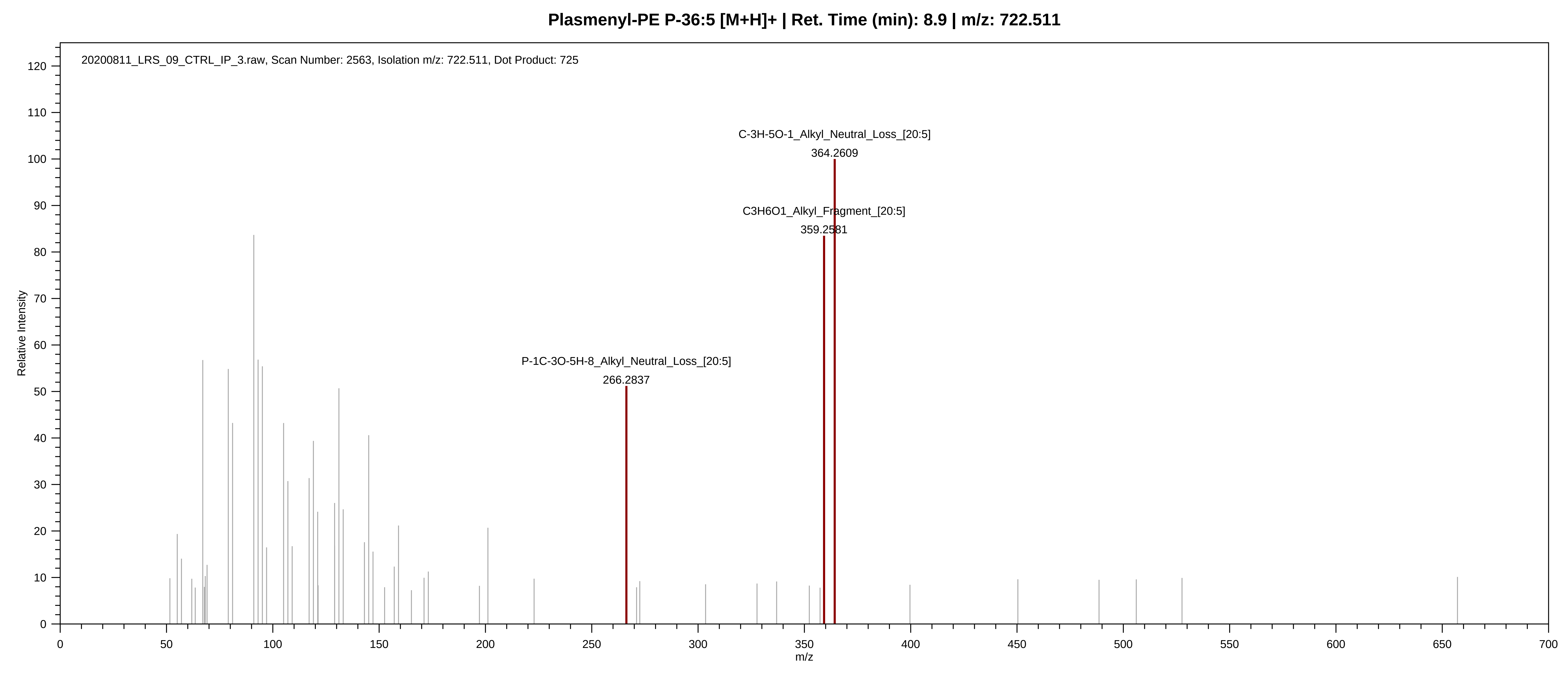

Plasmenyl-PE P-36:5 [M+H]+ | Ret. Time (min): 8.9 | m/z: 722.511
20200811_LRS_09_CTRL_IP_3.raw, Scan Number: 2563, Isolation m/z: 722.511, Dot Product: 725
C-3H-5O-1_Alkyl_Neutral_Loss_[20:5]
364.2609
C3H6O1_Alkyl_Fragment_[20:5]
359.2581
P-1C-3O-5H-8_Alkyl_Neutral_Loss_[20:5]
266.2837
Relative Intensity
m/z

Plasmenyl-PE P-36:5 [M+H]+ | Ret. Time (min): 8.903 | m/z: 722.511

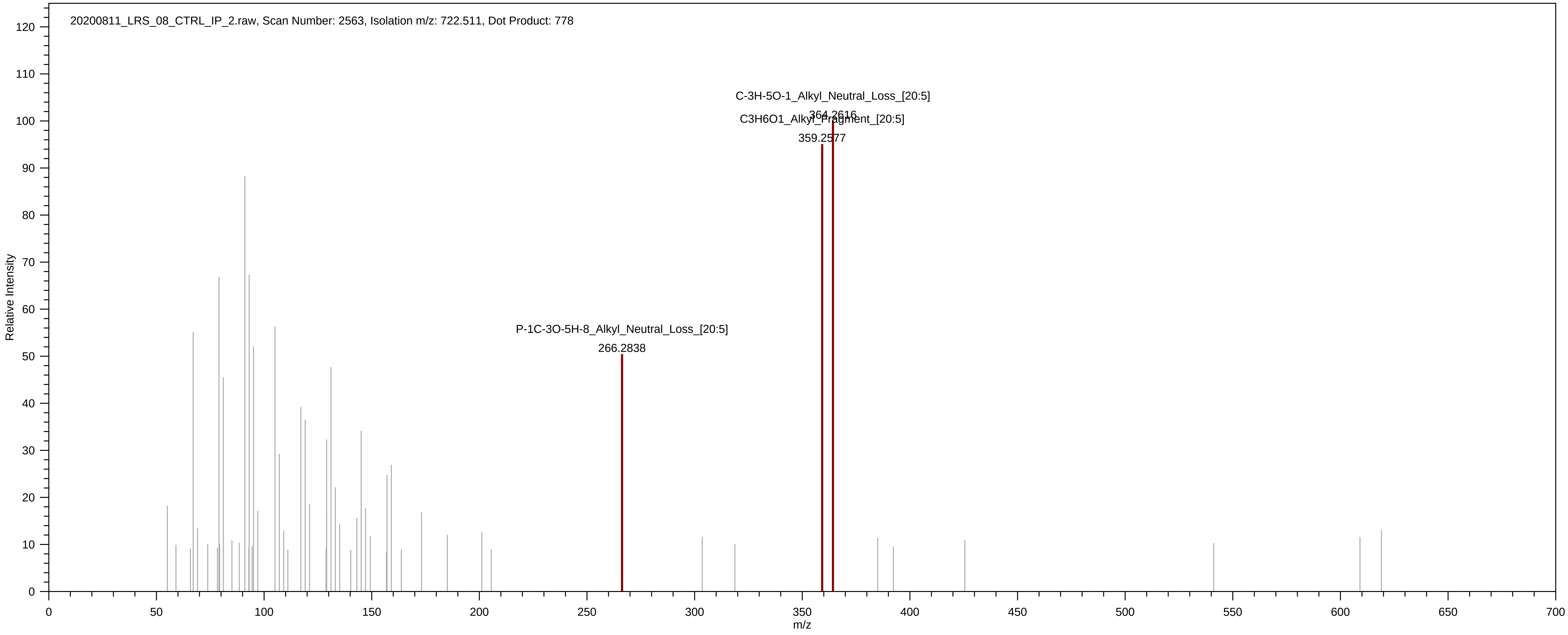

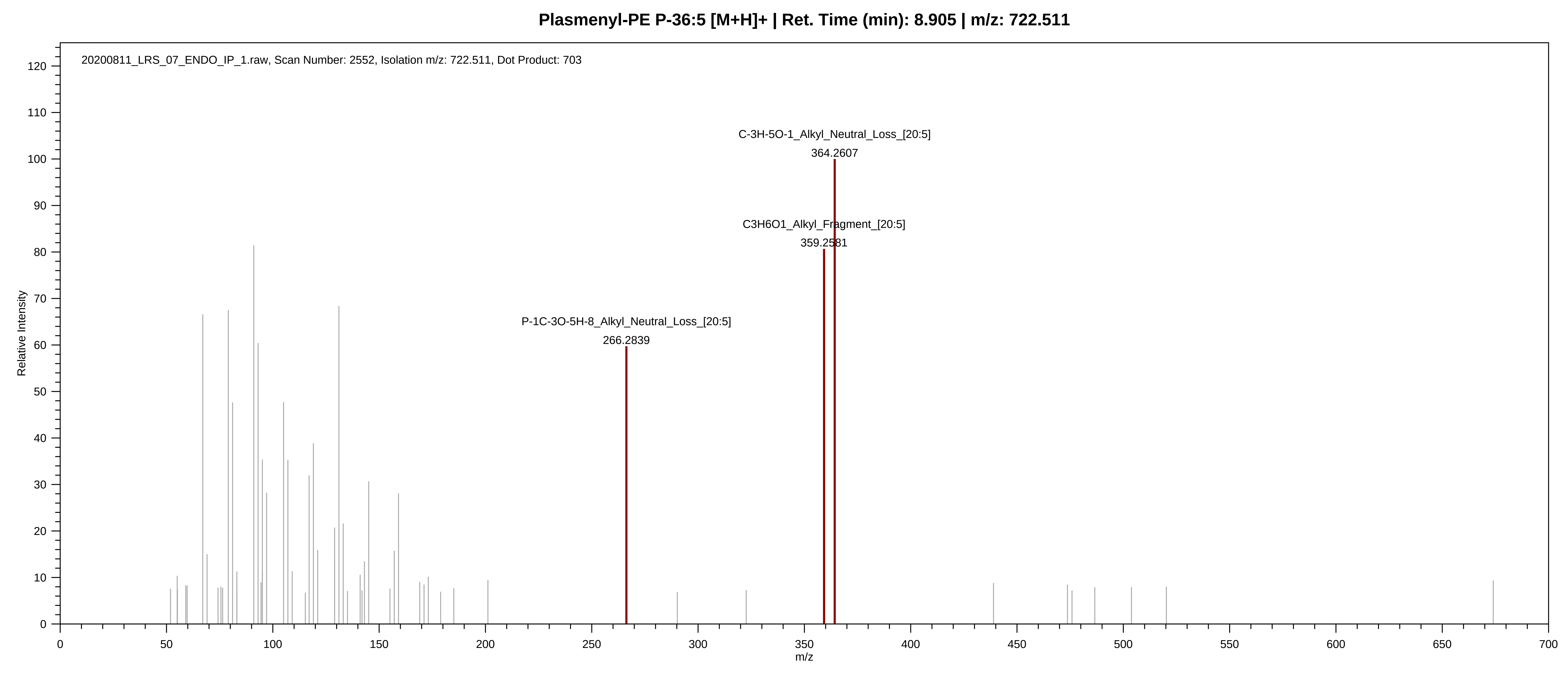

Plasmenyl-PE P-36:5 [M+H]+ | Ret. Time (min): 8.905 | m/z: 722.511
20200811_LRS_07_ENDO_IP_1.raw, Scan Number: 2552, Isolation m/z: 722.511, Dot Product: 703
C-3H-5O-1_Alkyl_Neutral_Loss_[20:5]
364.2607
C3H6O1_Alkyl_Fragment_[20:5]
359.2581
P-1C-3O-5H-8_Alkyl_Neutral_Loss_[20:5]
266.2839
Relative Intensity
m/z

Plasmenyl-PE P-36:5 [M+H]+ | Ret. Time (min): 8.907 | m/z: 722.511

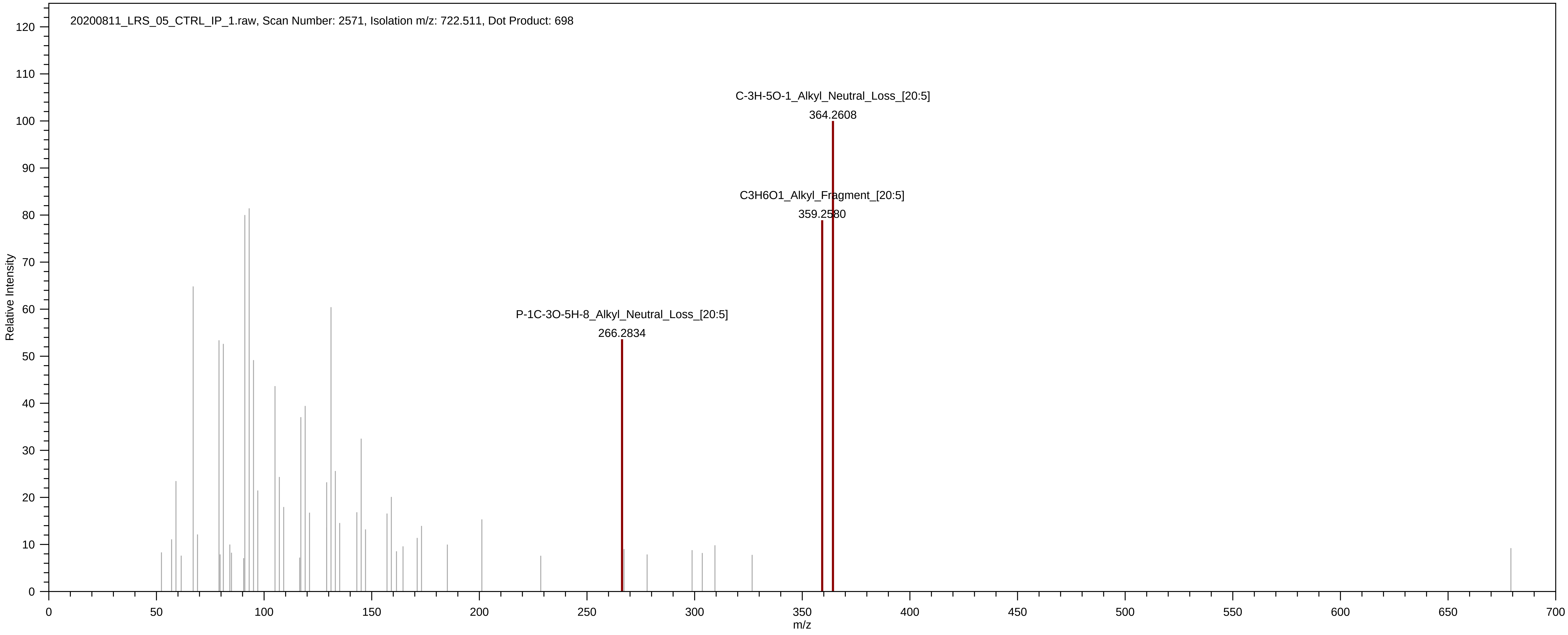

Plasmenyl-PE P-38:1 [M+H]+ | Ret. Time (min): 12.725 | m/z: 758.607

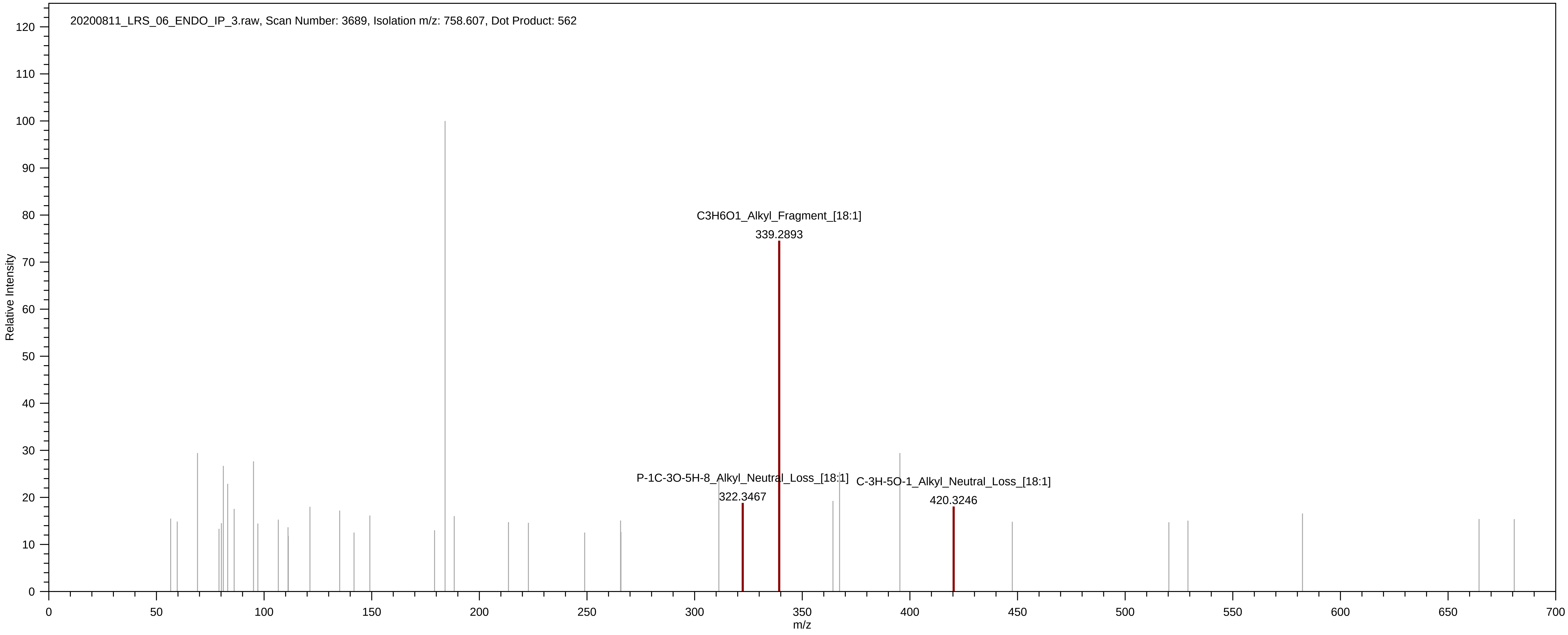

Plasmenyl-PE P-38:1 [M+H]+ | Ret. Time (min): 12.773 | m/z: 758.607

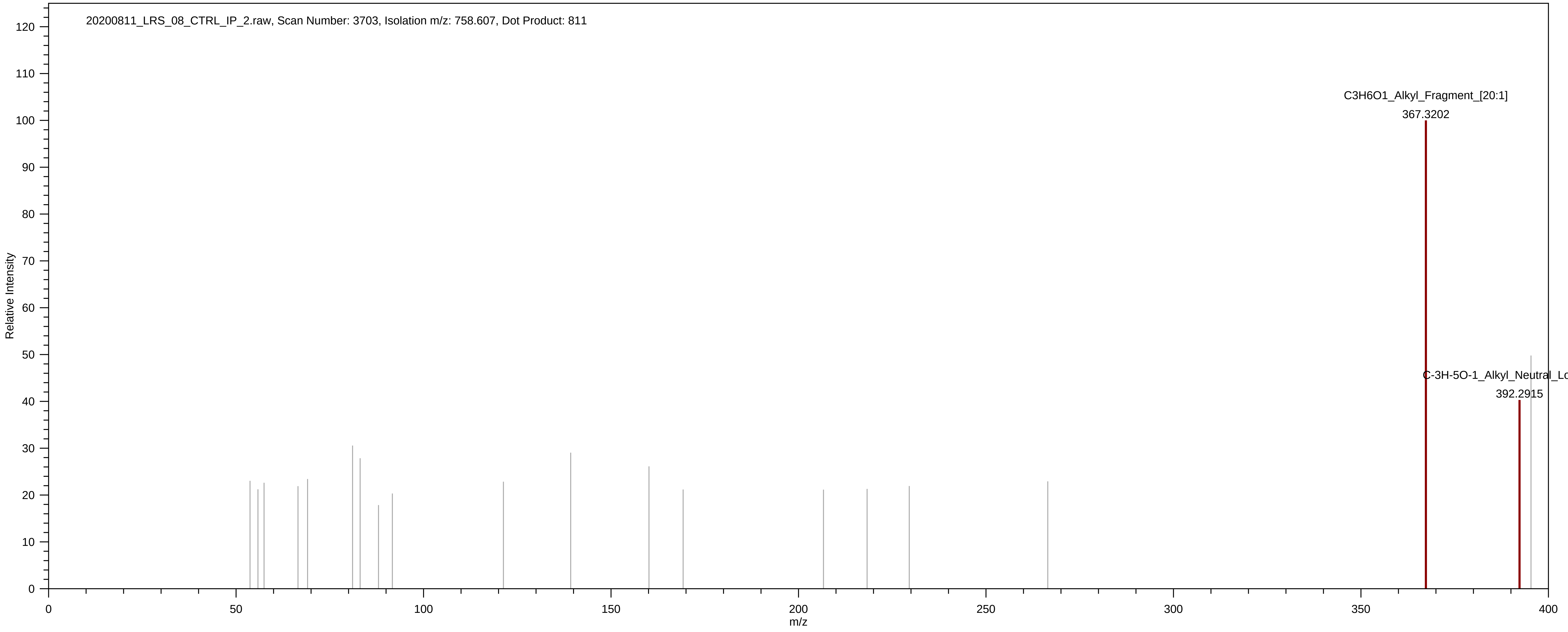

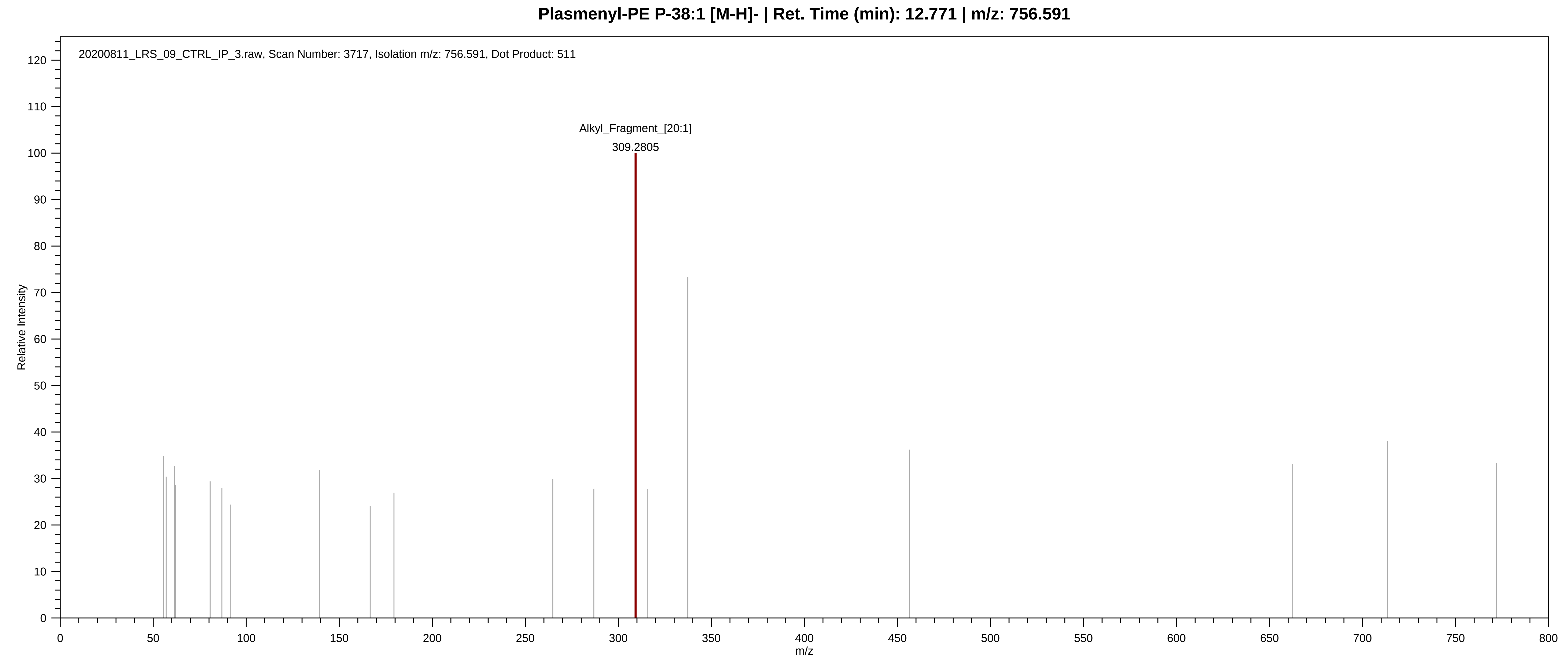

Plasmenyl-PE P-38:1 [M-H]- | Ret. Time (min): 12.771 | m/z: 756.591
20200811_LRS_09_CTRL_IP_3.raw, Scan Number: 3717, Isolation m/z: 756.591, Dot Product: 511
Alkyl_Fragment_[20:1]
309.2805
Relative Intensity
m/z

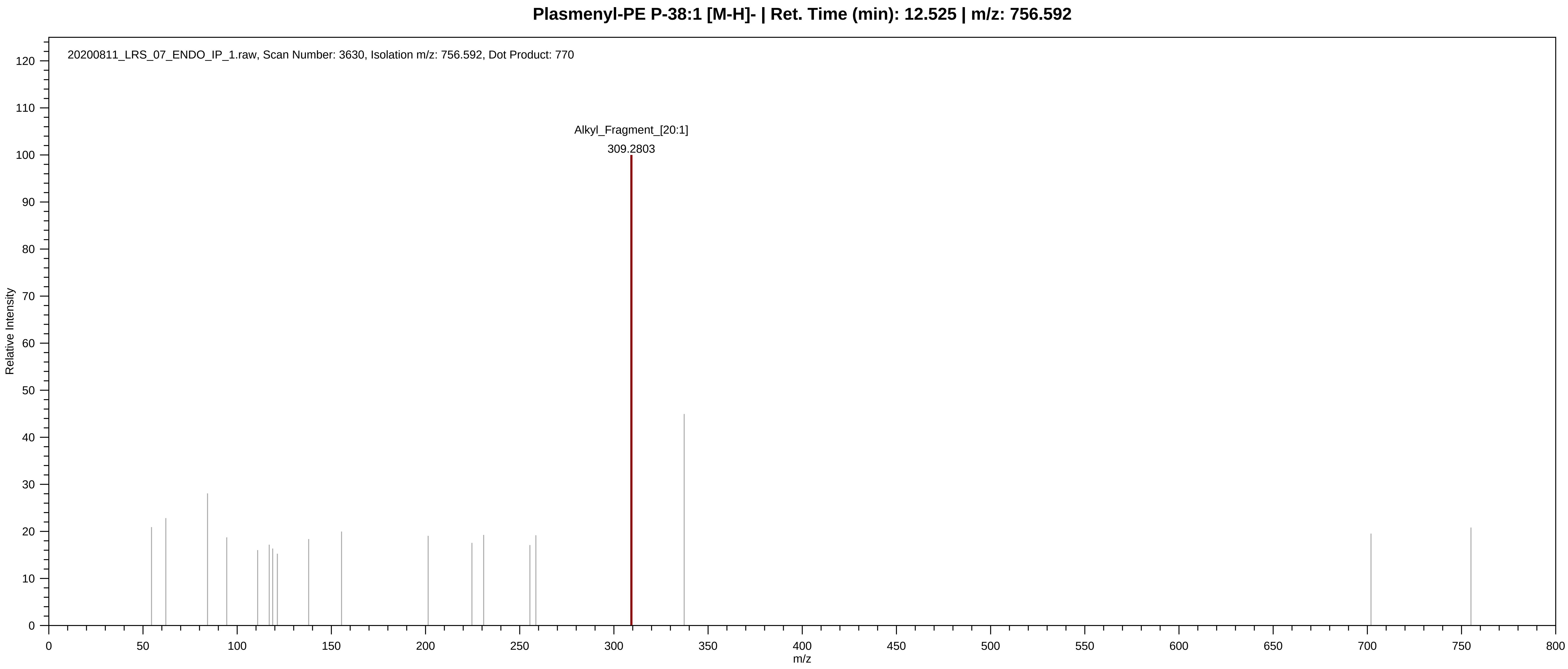

Plasmenyl-PE P-38:1 [M-H]- | Ret. Time (min): 12.525 | m/z: 756.592
20200811_LRS_07_ENDO_IP_1.raw, Scan Number: 3630, Isolation m/z: 756.592, Dot Product: 770
Alkyl_Fragment_[20:1]
309.2803
Relative Intensity
m/z

Plasmenyl-PE P-38:1 [M-H]- | Ret. Time (min): 12.725 | m/z: 756.592

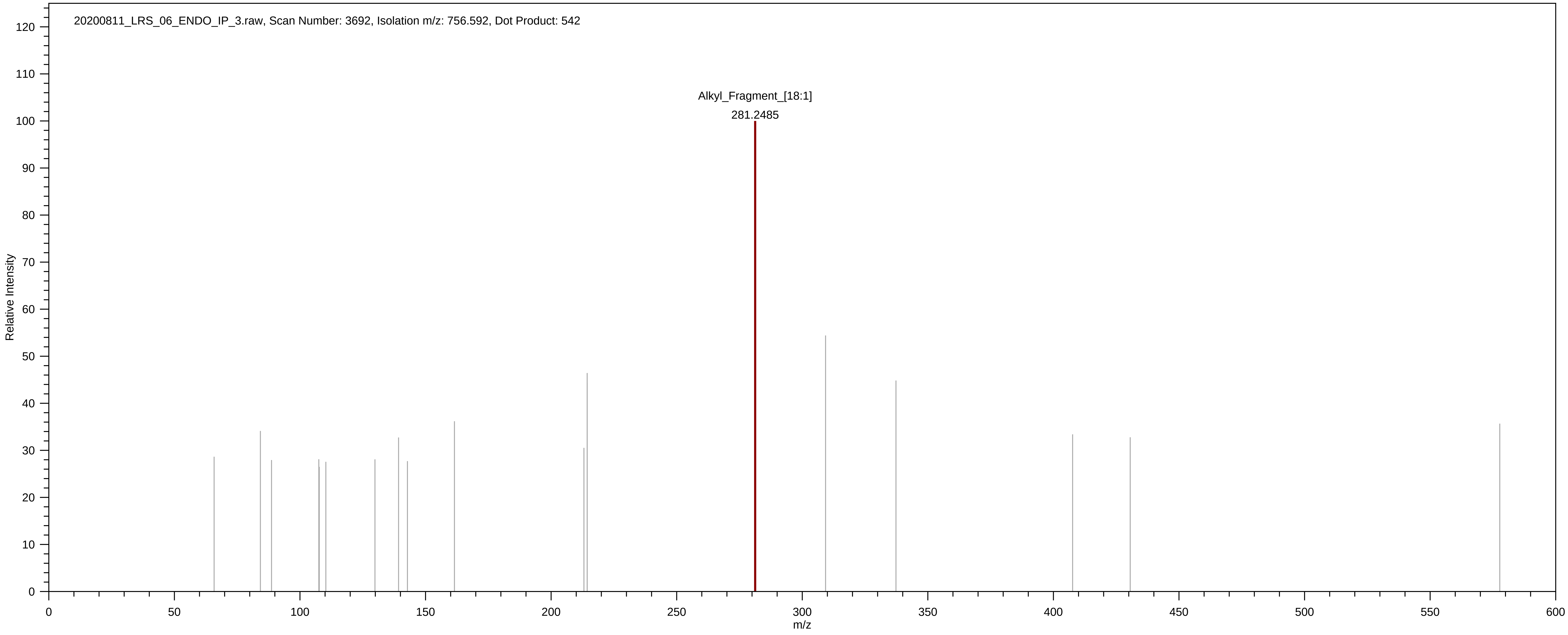

Plasmenyl-PE P-38:2 [M+H]+ | Ret. Time (min): 11.73 | m/z: 756.59

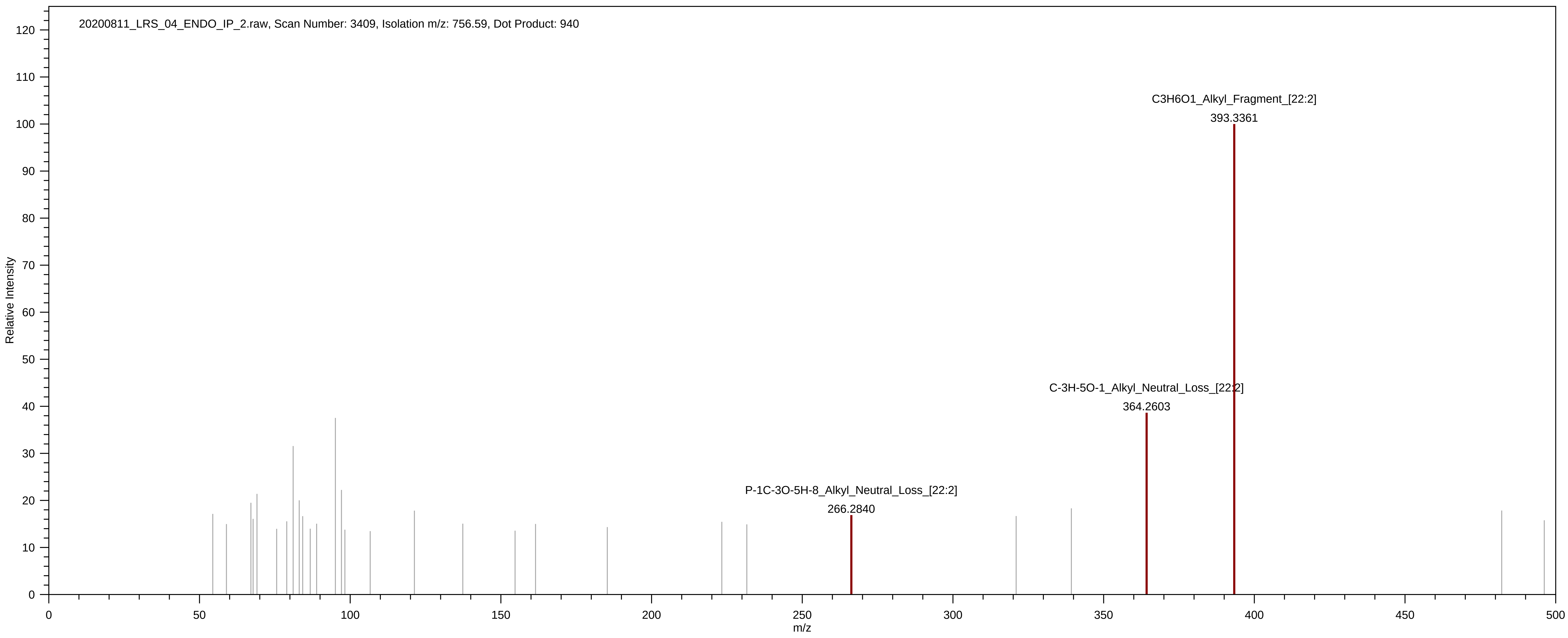

Plasmenyl-PE P-38:2 [M+H]+ | Ret. Time (min): 11.692 | m/z: 756.59

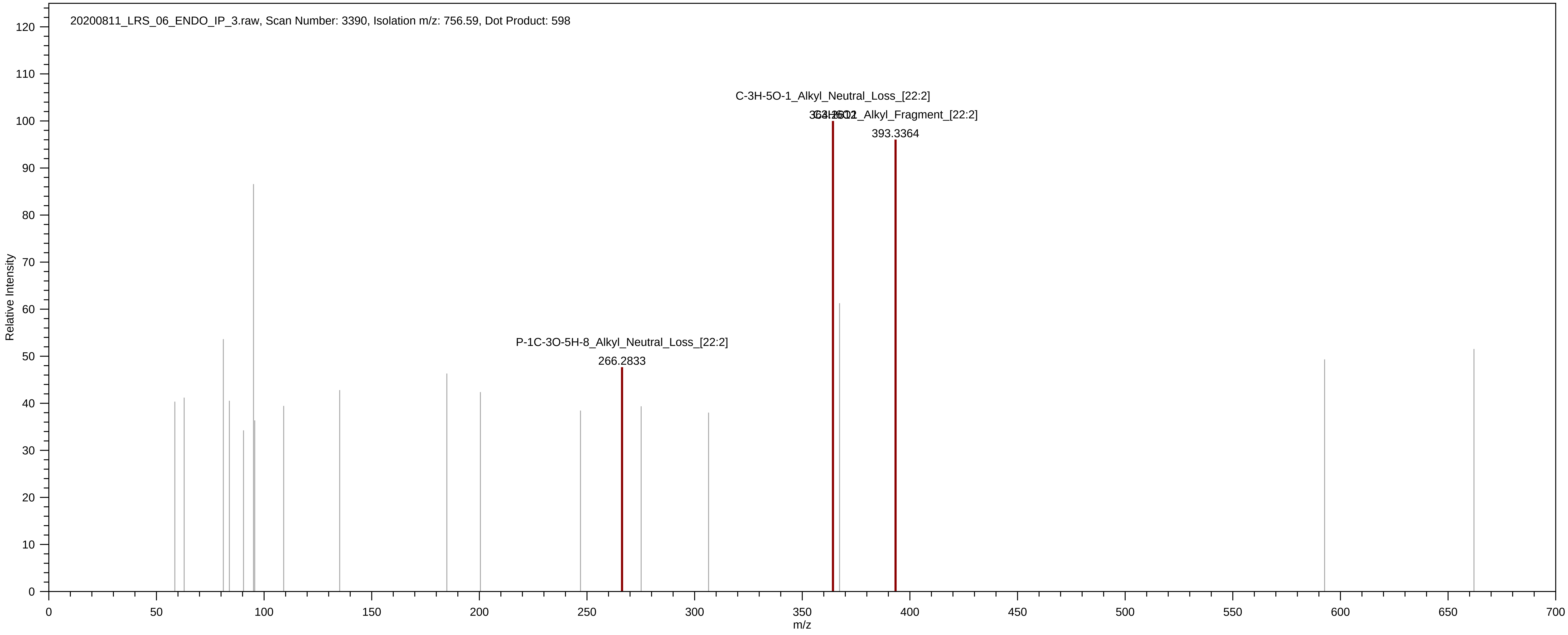

Plasmenyl-PE P-38:2 [M+H]+ | Ret. Time (min): 11.744 | m/z: 756.59

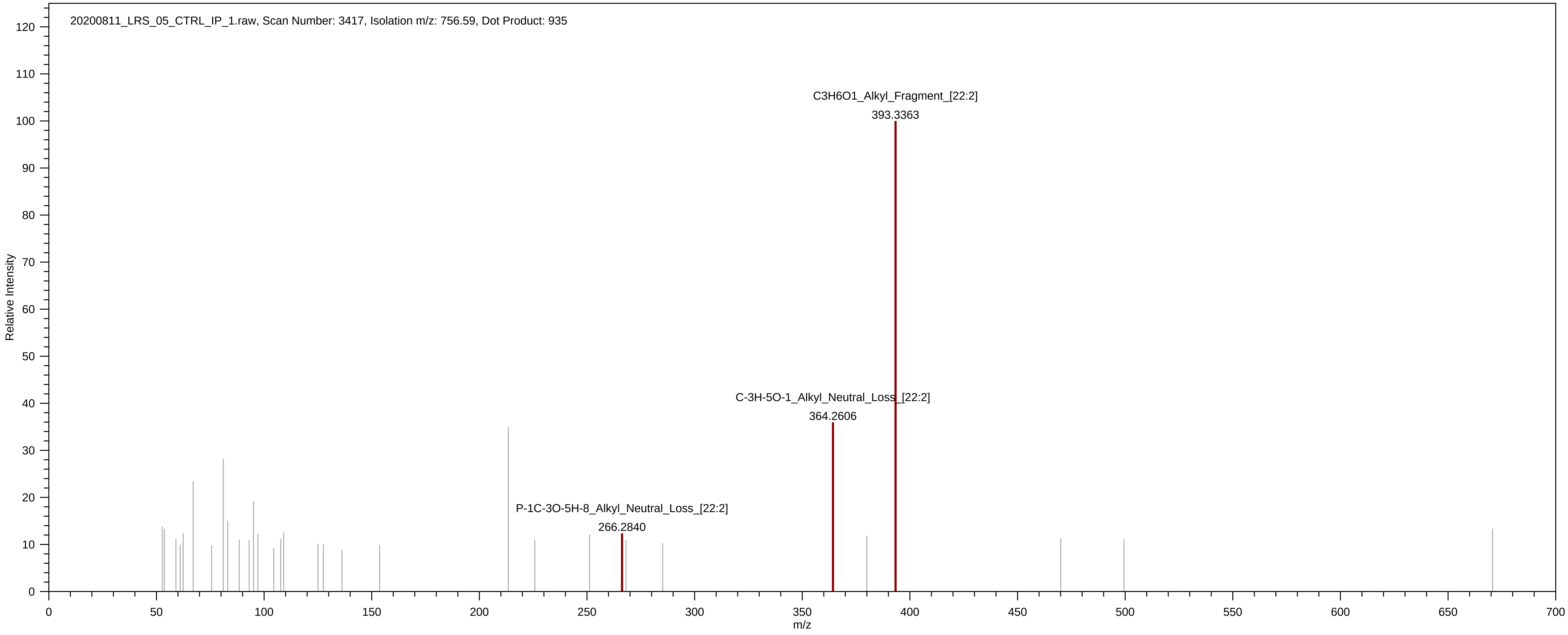

Plasmenyl-PE P-38:2 [M+H]+ | Ret. Time (min): 11.529 | m/z: 756.589

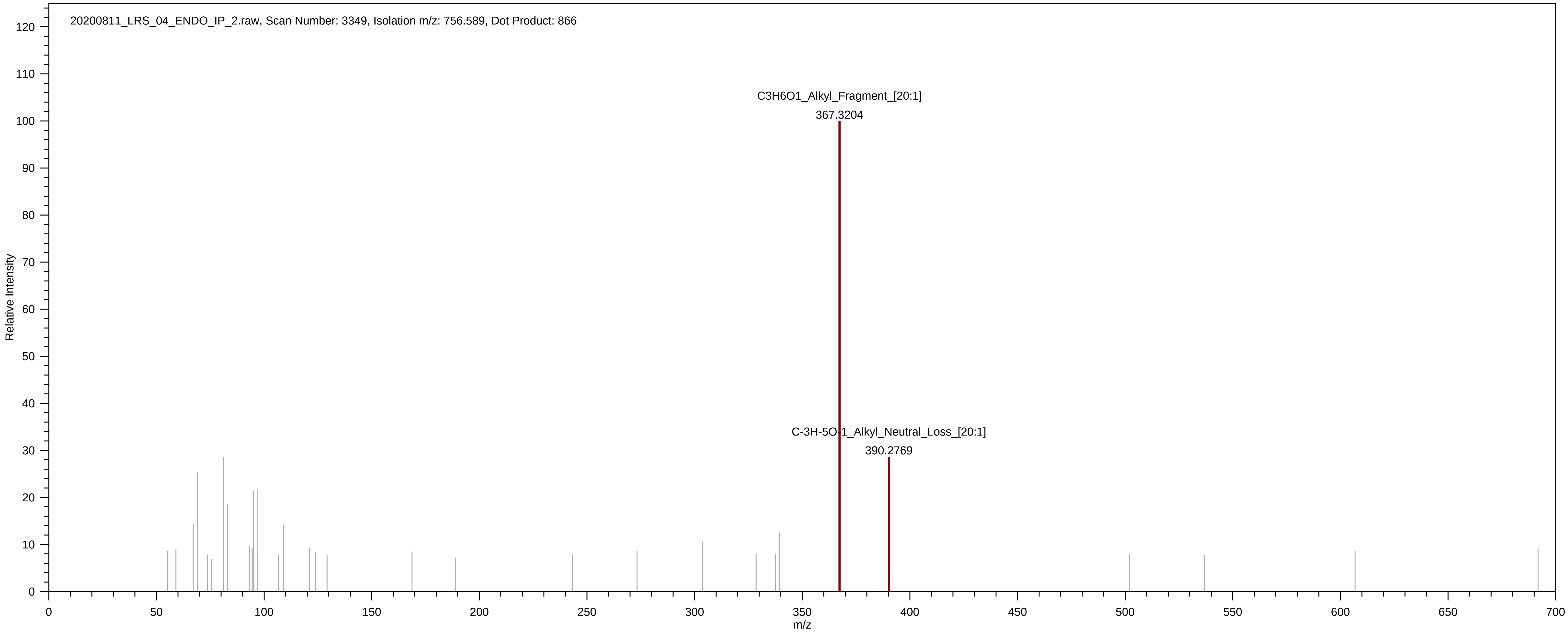

Plasmenyl-PE P-38:2 [M+H]+ | Ret. Time (min): 11.693 | m/z: 756.589

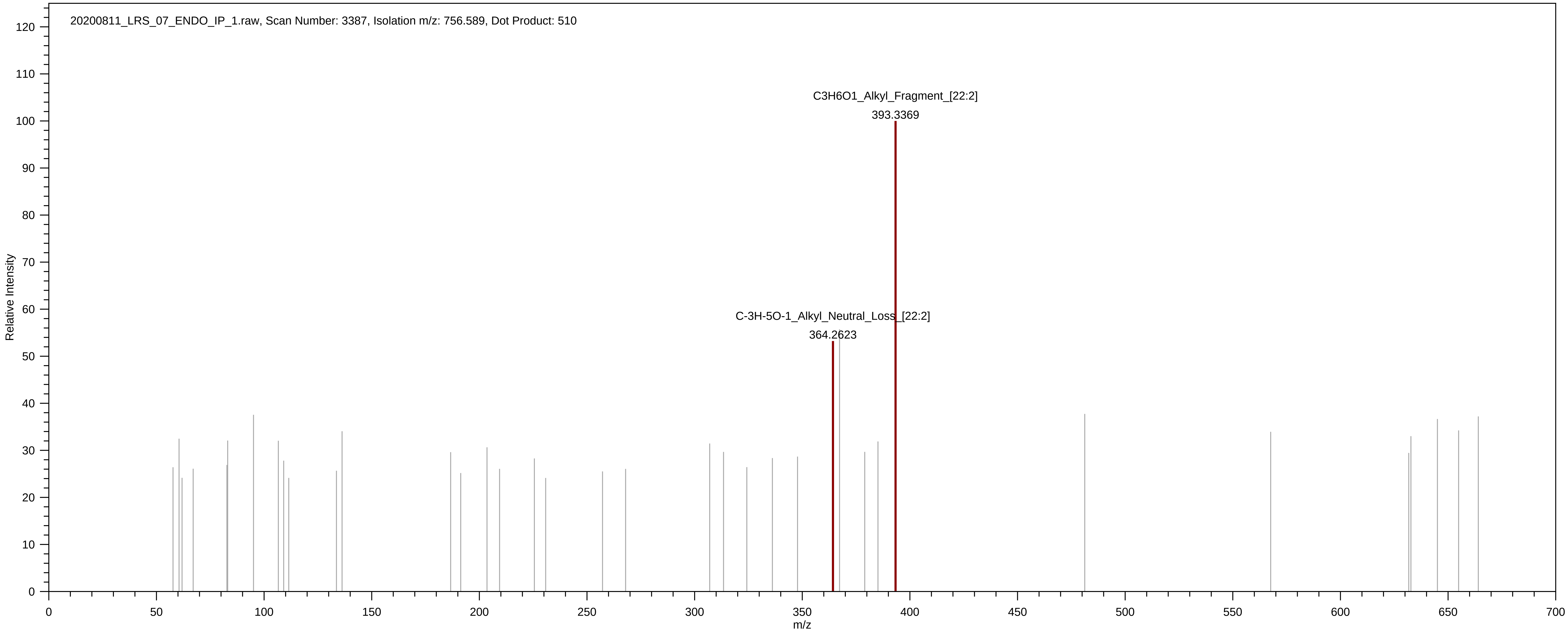

Plasmenyl-PE P-38:2 [M+H]+ | Ret. Time (min): 11.511 | m/z: 756.591

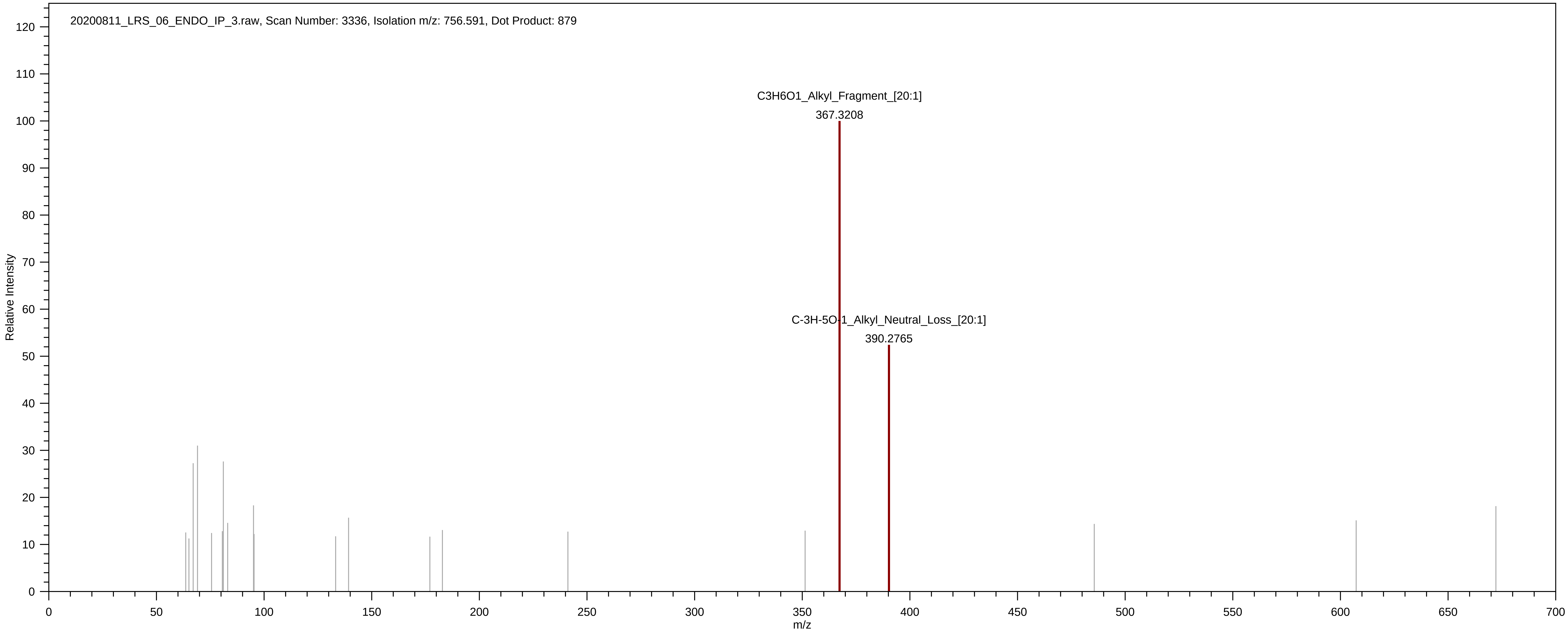

Plasmenyl-PE P-38:2 [M+H]+ | Ret. Time (min): 11.543 | m/z: 756.591

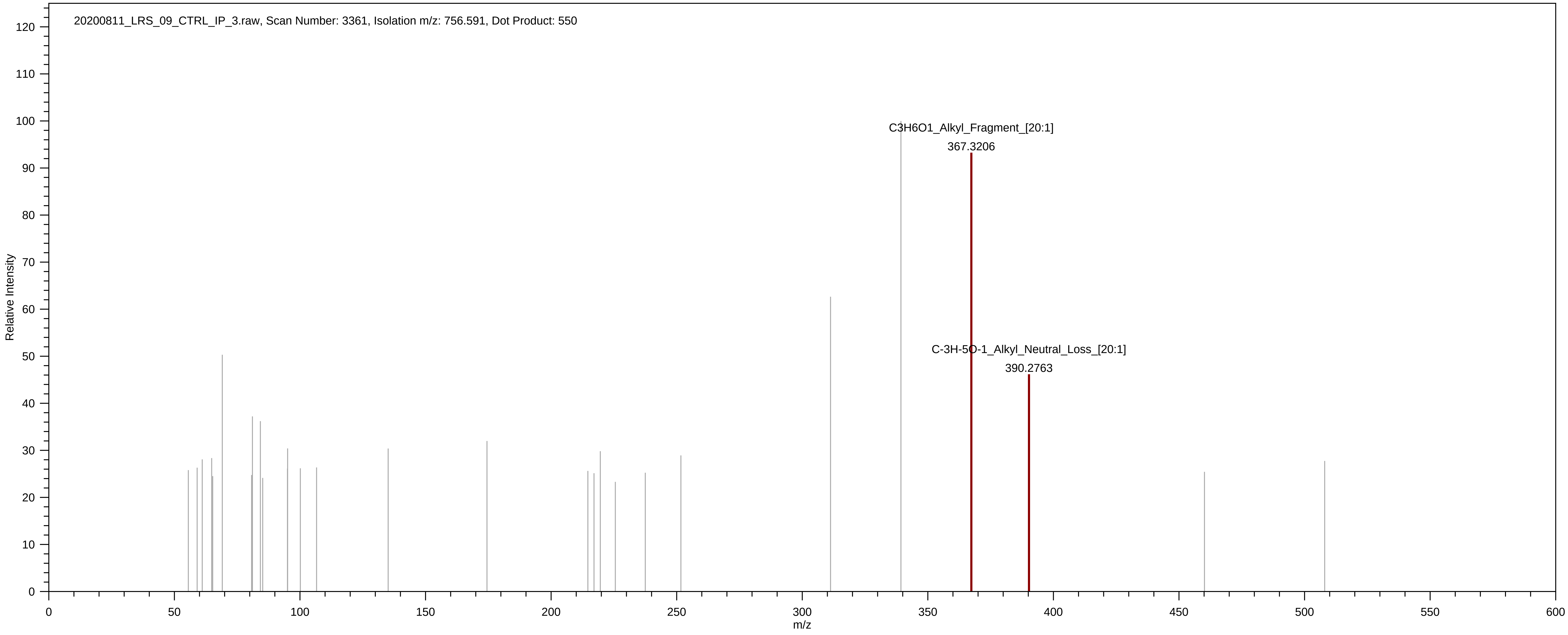

Plasmenyl-PE P-38:2 [M+H]+ | Ret. Time (min): 11.724 | m/z: 756.591

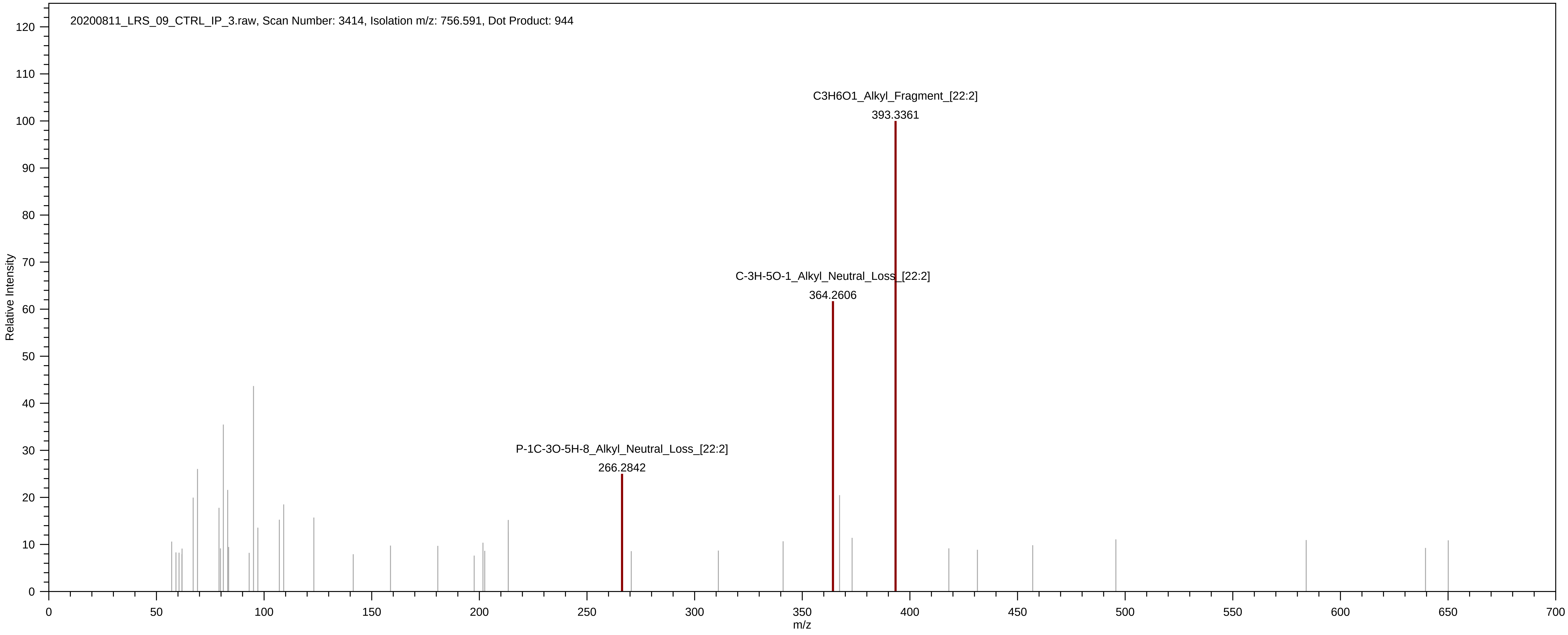

Plasmenyl-PE P-38:2 [M+H]+ | Ret. Time (min): 11.728 | m/z: 756.591

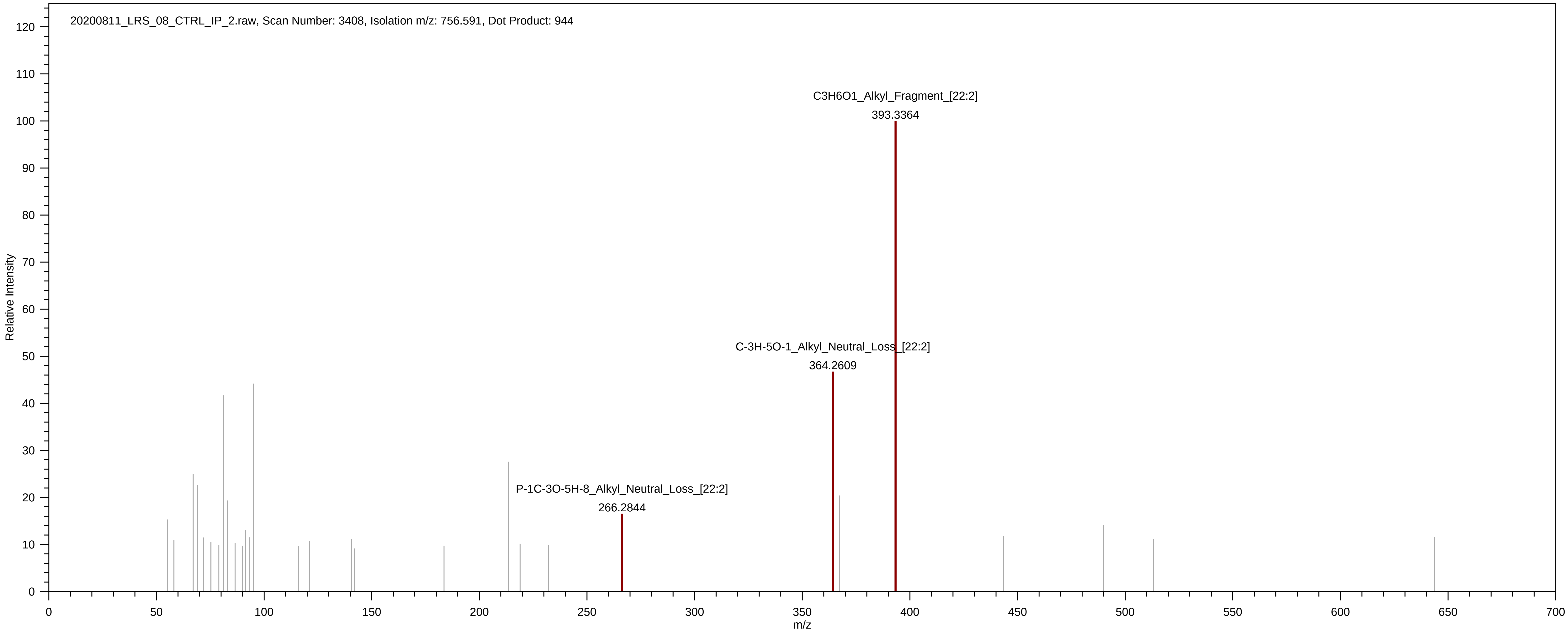

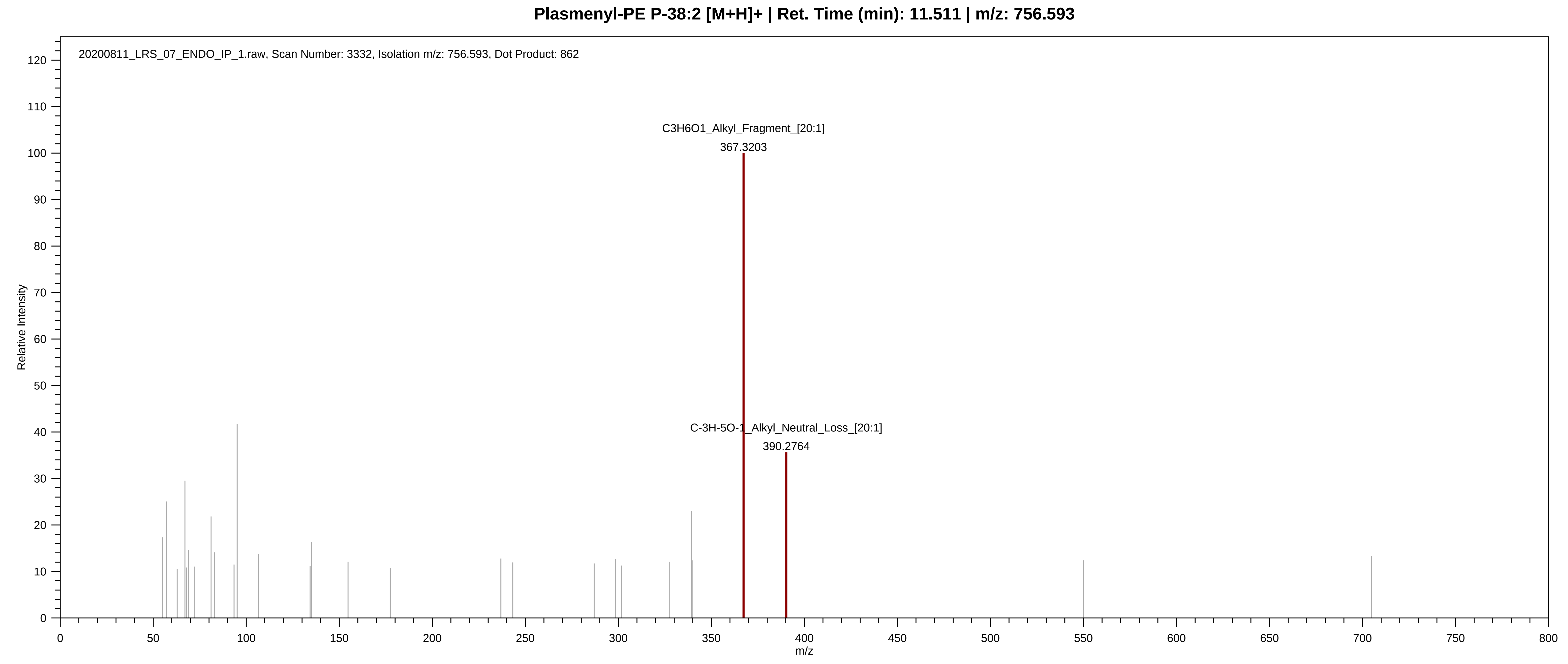
Plasmenyl-PE P-38:2 [M+H]+ | Ret. Time (min): 11.511 | m/z: 756.593
20200811_LRS_07_ENDO_IP_1.raw, Scan Number: 3332, Isolation m/z: 756.593, Dot Product: 862
C3H6O1_Alkyl_Fragment_[20:1]
367.3203
C-3H-5O-1_Alkyl_Neutral_Loss_[20:1]
390.2764
Relative Intensity
m/z

Plasmenyl-PE P-38:3 [M+H]+ | Ret. Time (min): 11.283 | m/z: 754.575

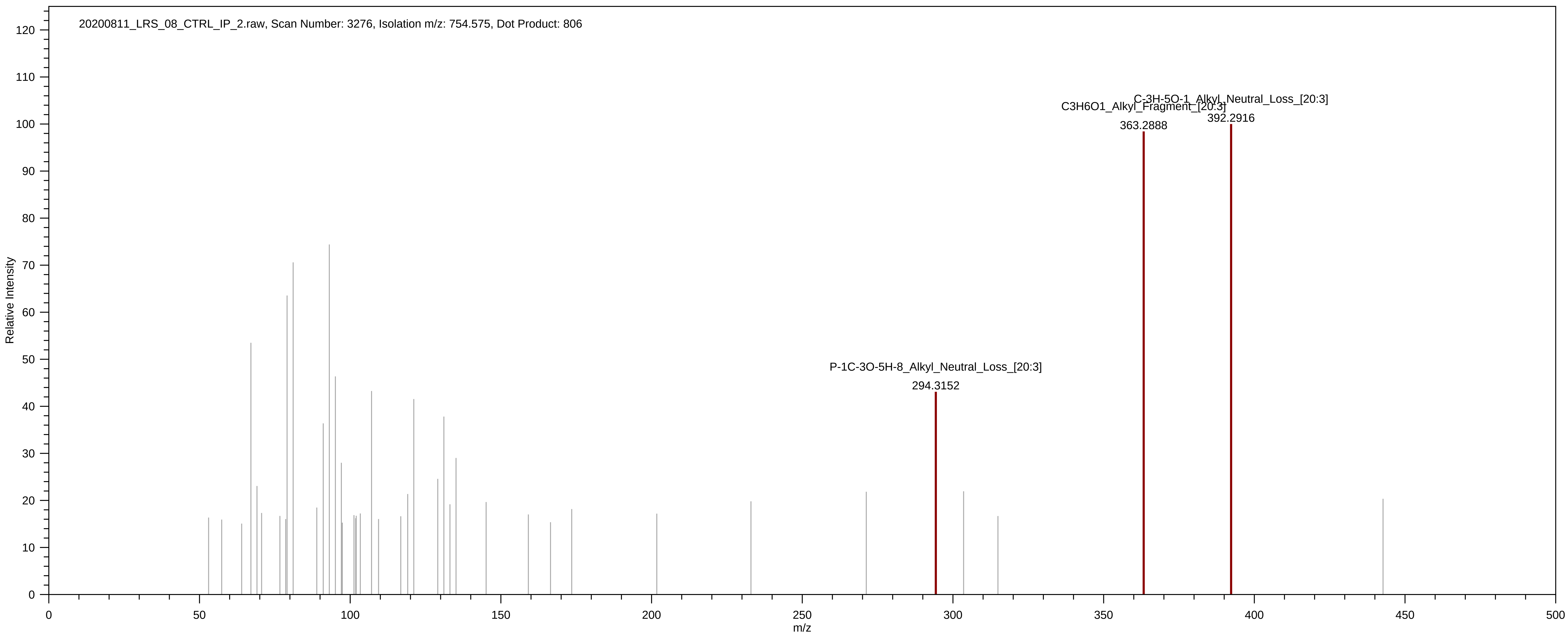

Plasmenyl-PE P-38:3 [M+H]+ | Ret. Time (min): 11.297 | m/z: 754.575

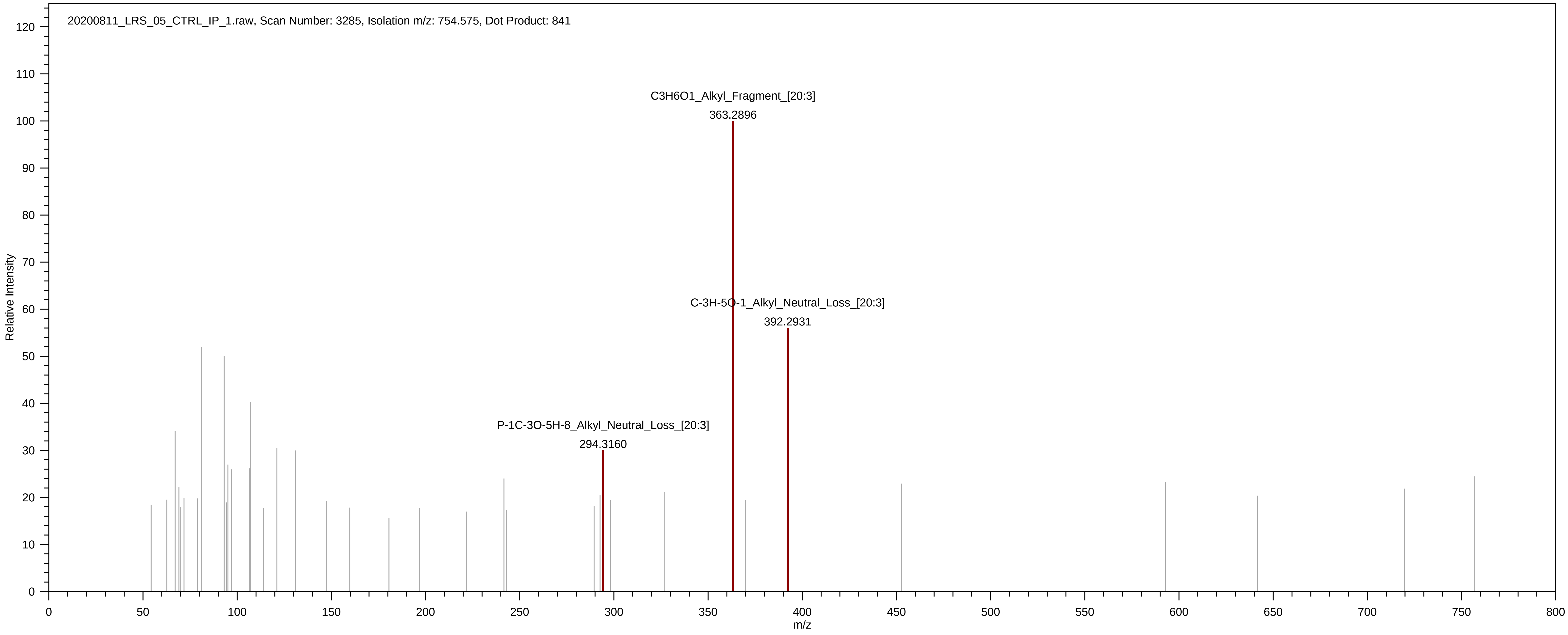

Plasmenyl-PE P-38:3 [M+H]+ | Ret. Time (min): 11.306 | m/z: 754.575

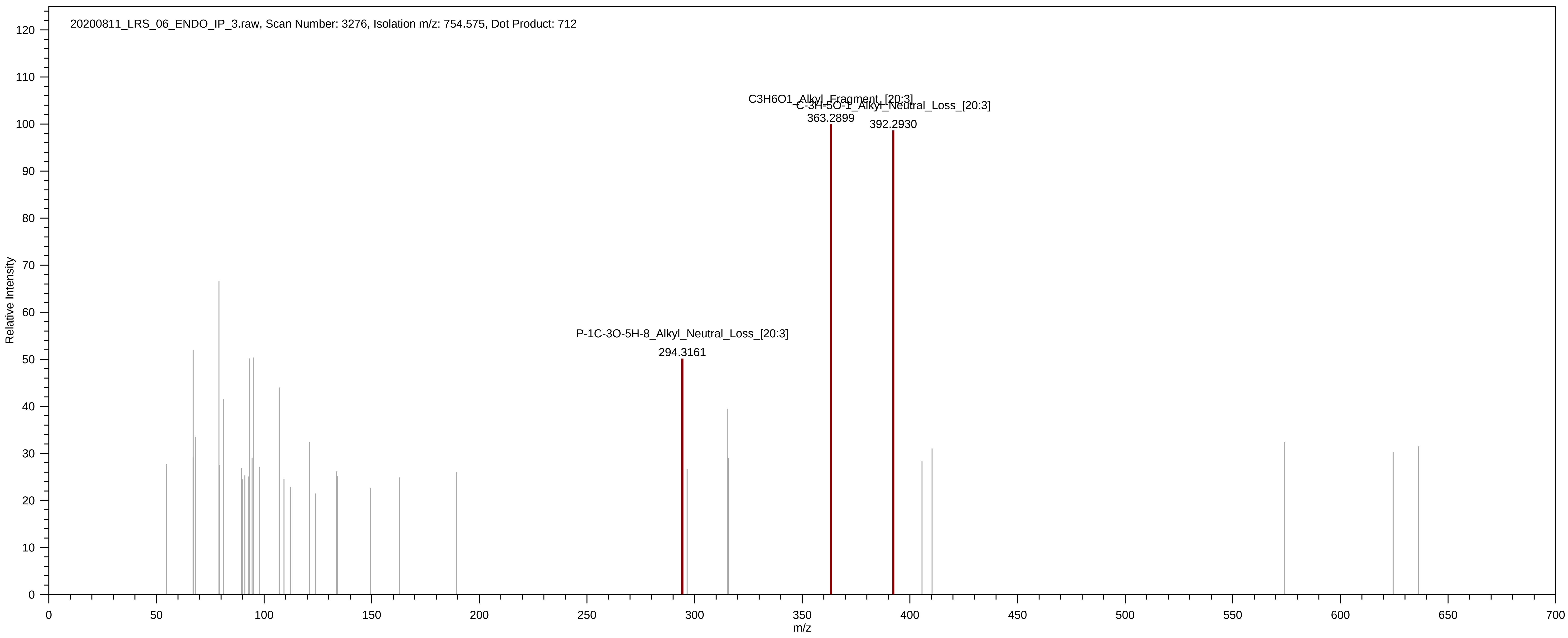

Plasmenyl-PE P-38:3 [M+H]+ | Ret. Time (min): 10.98 | m/z: 754.576

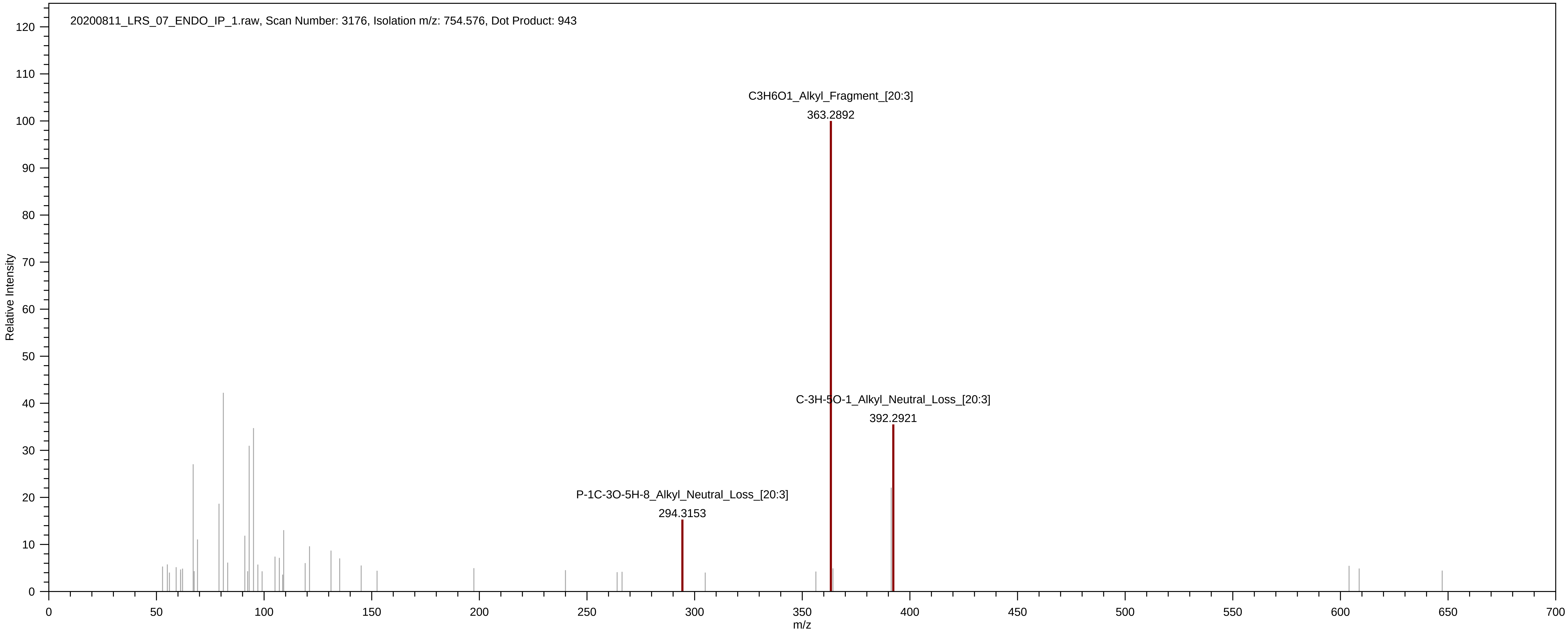

Plasmenyl-PE P-38:3 [M+H]+ | Ret. Time (min): 10.968 | m/z: 754.576

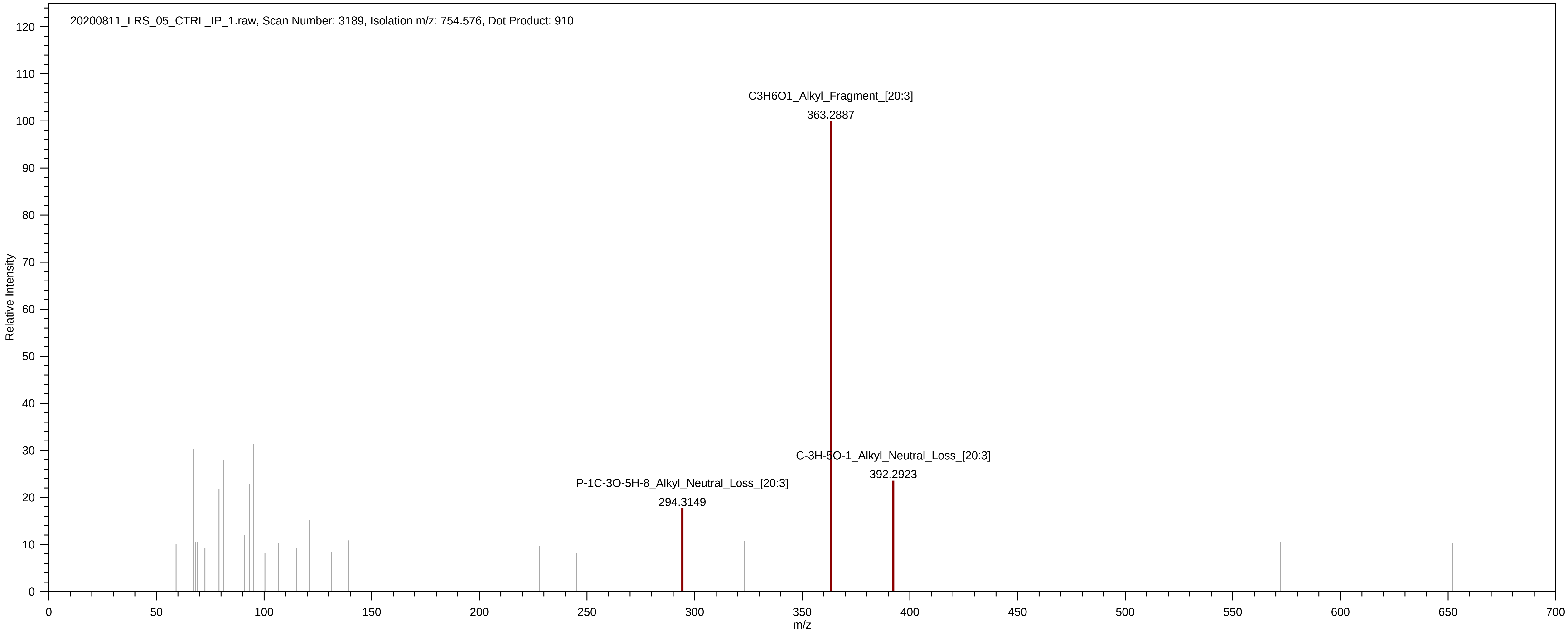

Plasmenyl-PE P-38:3 [M+H]+ | Ret. Time (min): 10.976 | m/z: 754.576

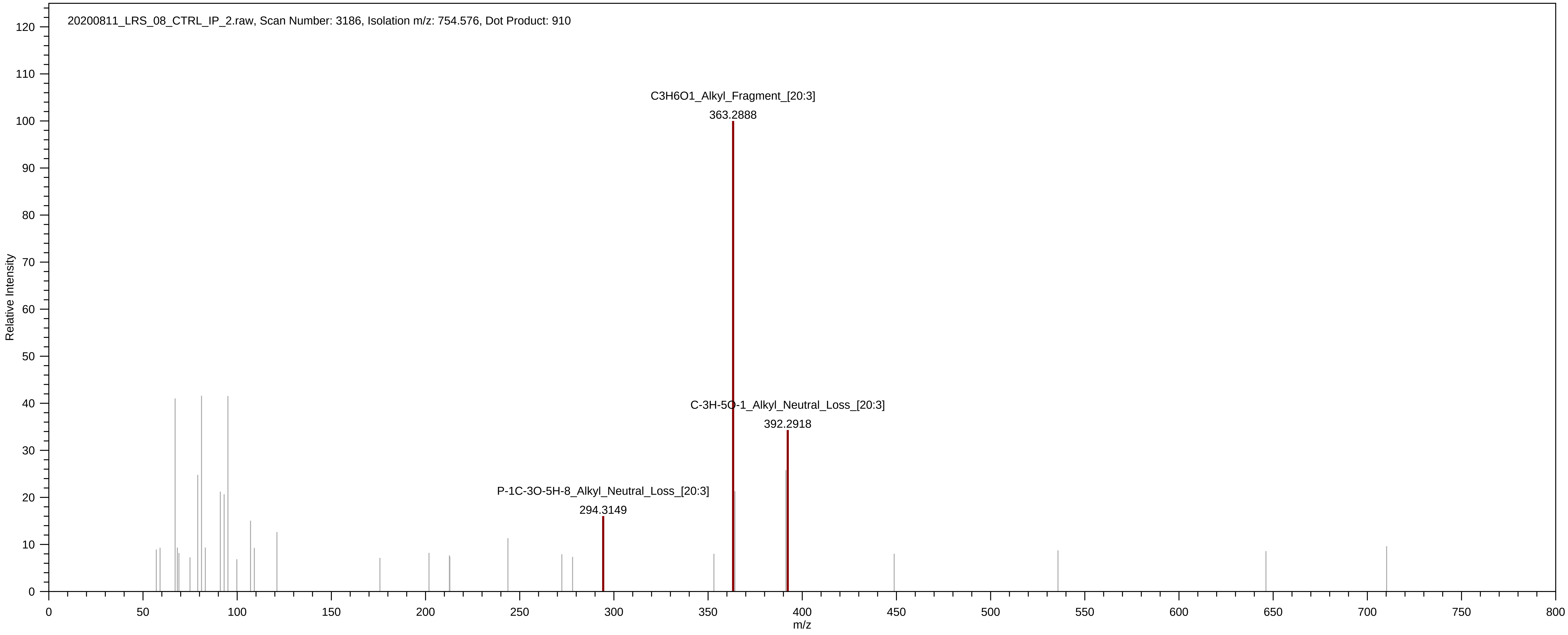

Plasmenyl-PE P-38:3 [M+H]+ | Ret. Time (min): 10.978 | m/z: 754.576

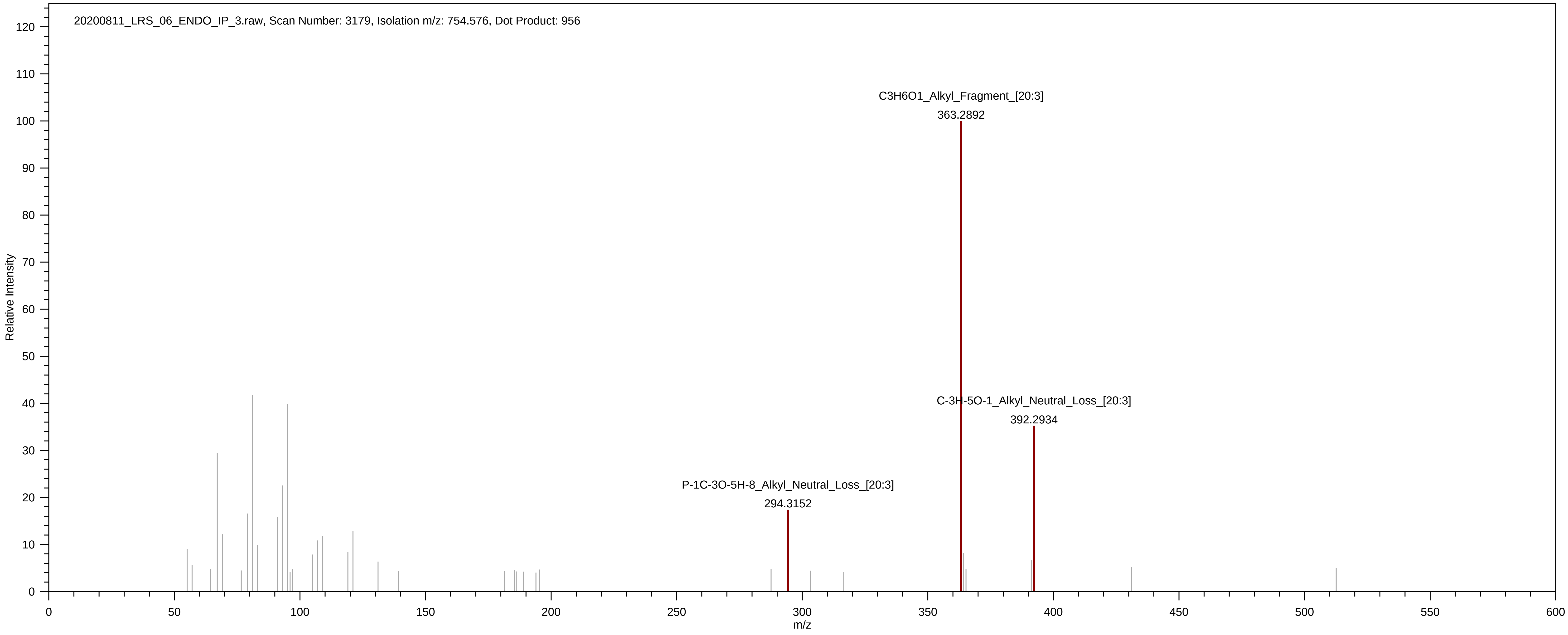

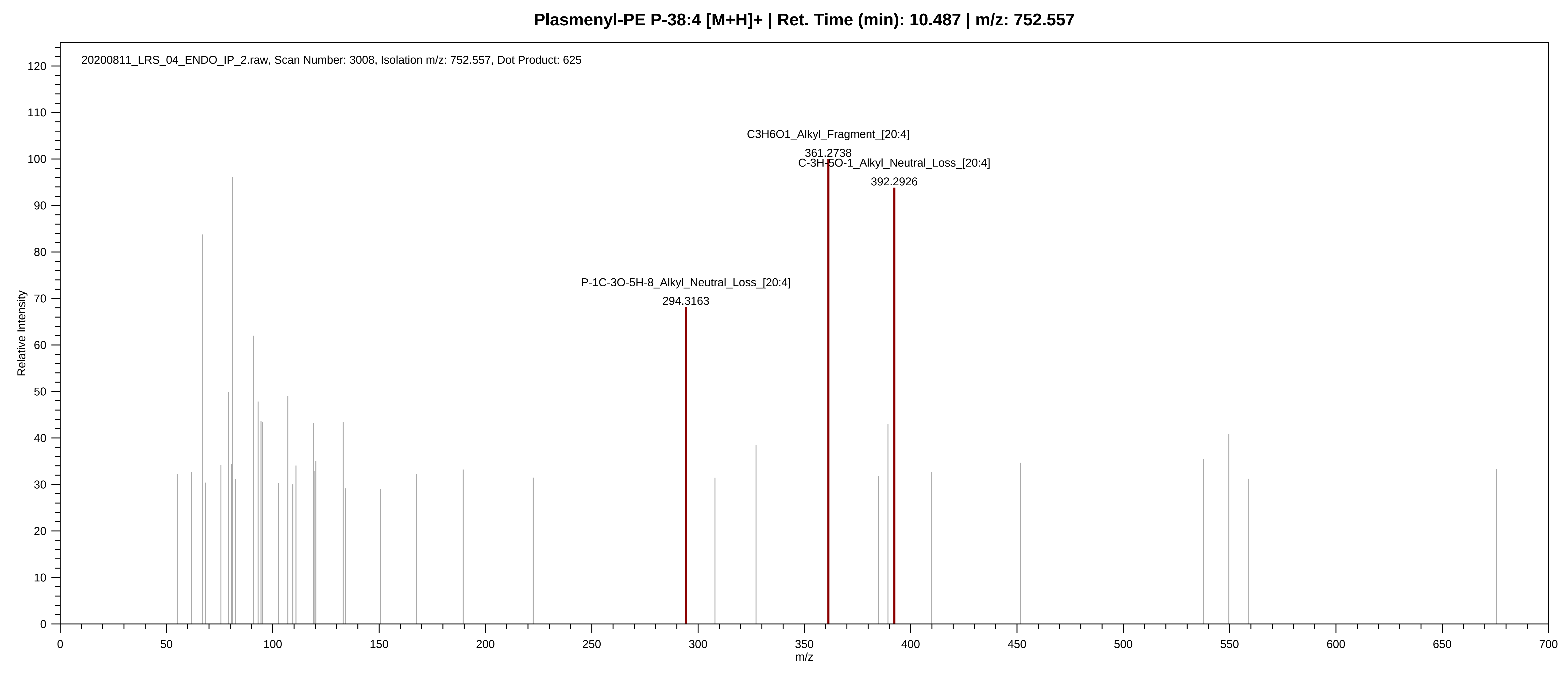

Plasmenyl-PE P-38:4 [M+H]+ | Ret. Time (min): 10.487 | m/z: 752.557
20200811_LRS_04_ENDO_IP_2.raw, Scan Number: 3008, Isolation m/z: 752.557, Dot Product: 625
C3H6O1_Alkyl_Fragment_[20:4]
361.2738
C-3H-5O-1_Alkyl_Neutral_Loss_[20:4]
392.2926
P-1C-3O-5H-8_Alkyl_Neutral_Loss_[20:4]
294.3163
Relative Intensity
m/z

Plasmenyl-PE P-38:4 [M+H]+ | Ret. Time (min): 10.487 | m/z: 752.559

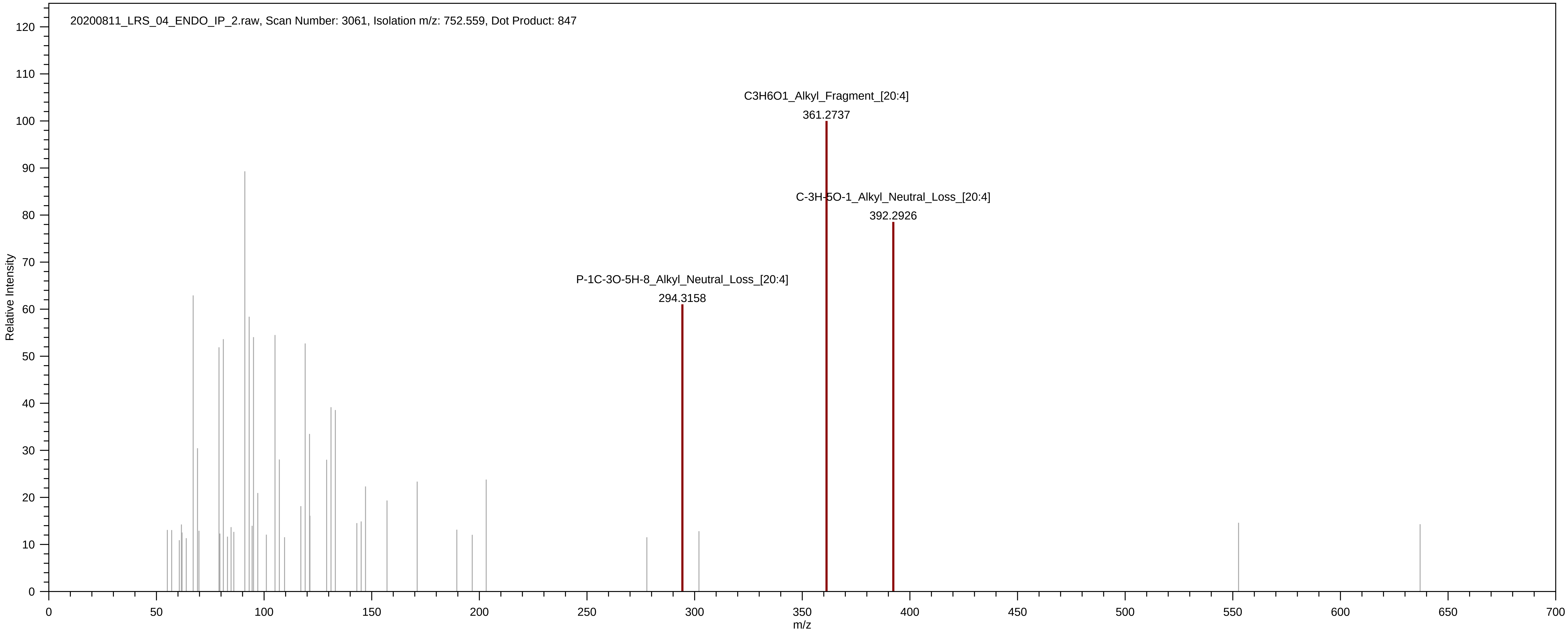

Plasmenyl-PE P-38:4 [M+H]+ | Ret. Time (min): 10.49 | m/z: 752.56

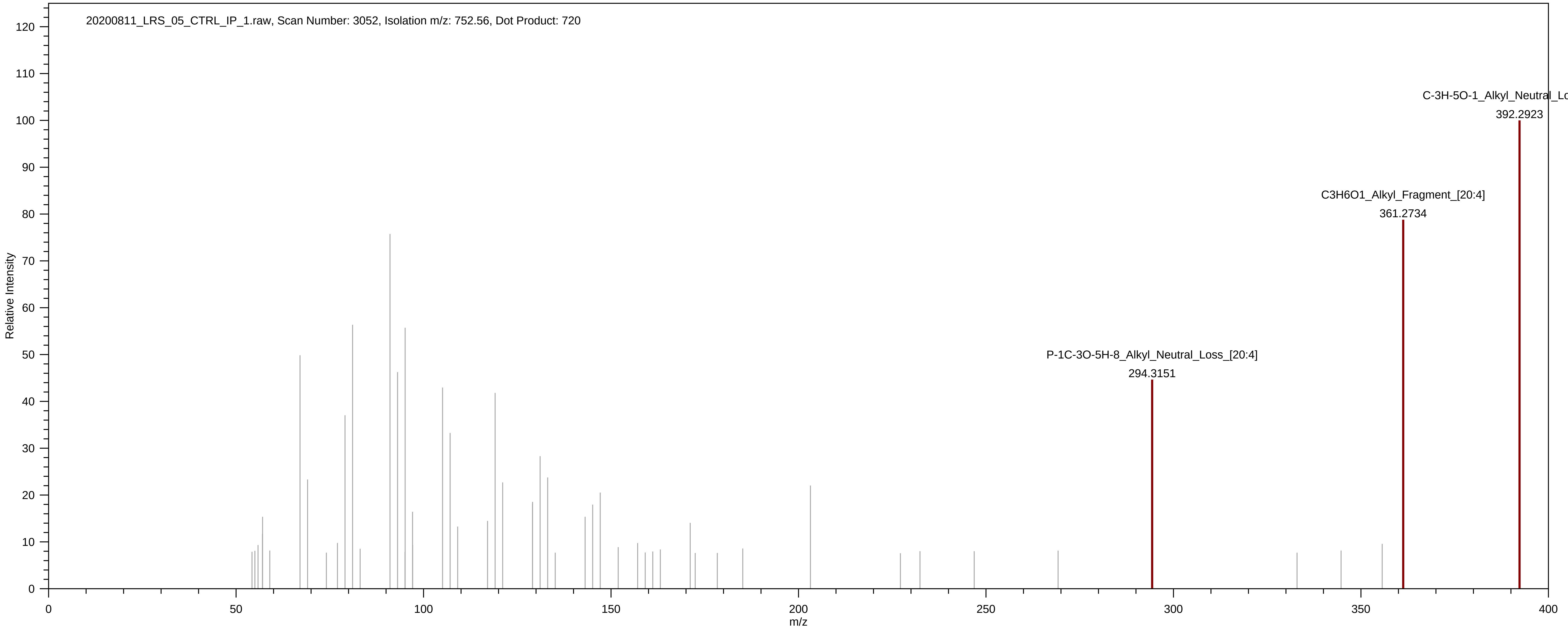

Plasmenyl-PE P-38:4 [M+H]+ | Ret. Time (min): 10.484 | m/z: 752.559

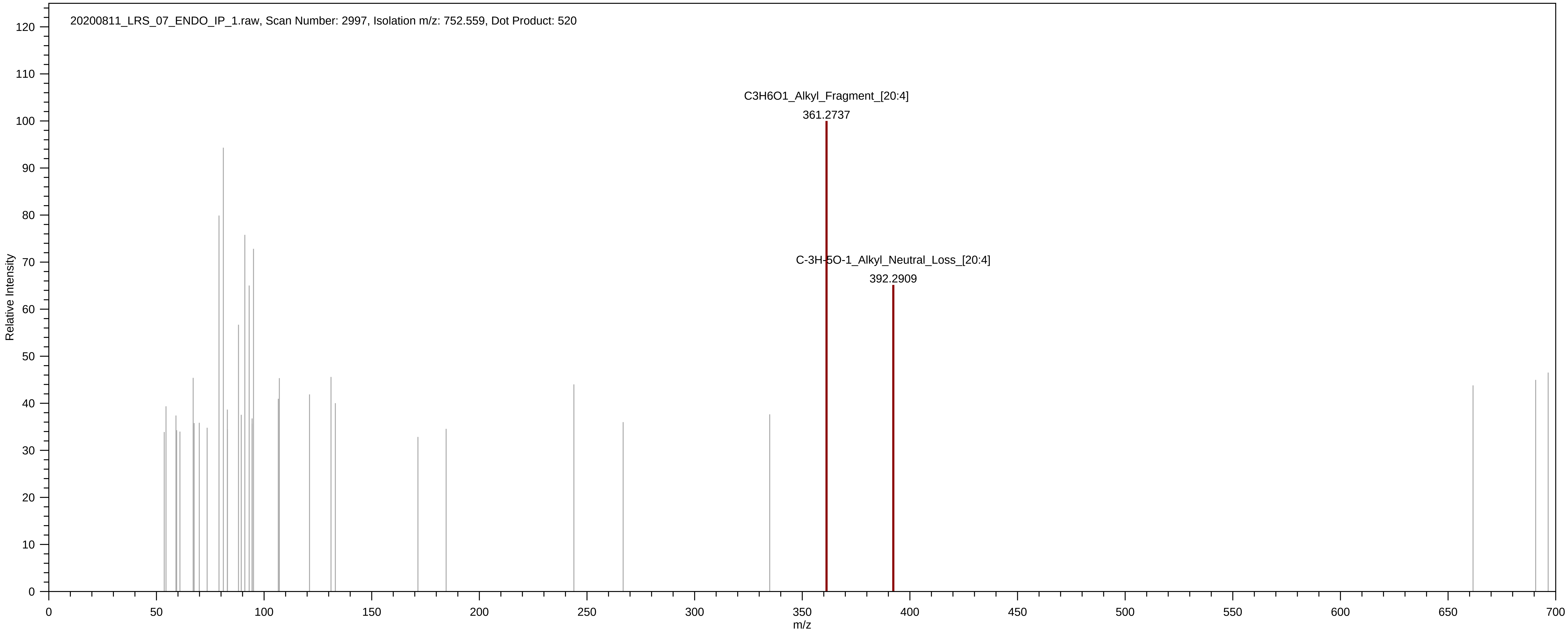

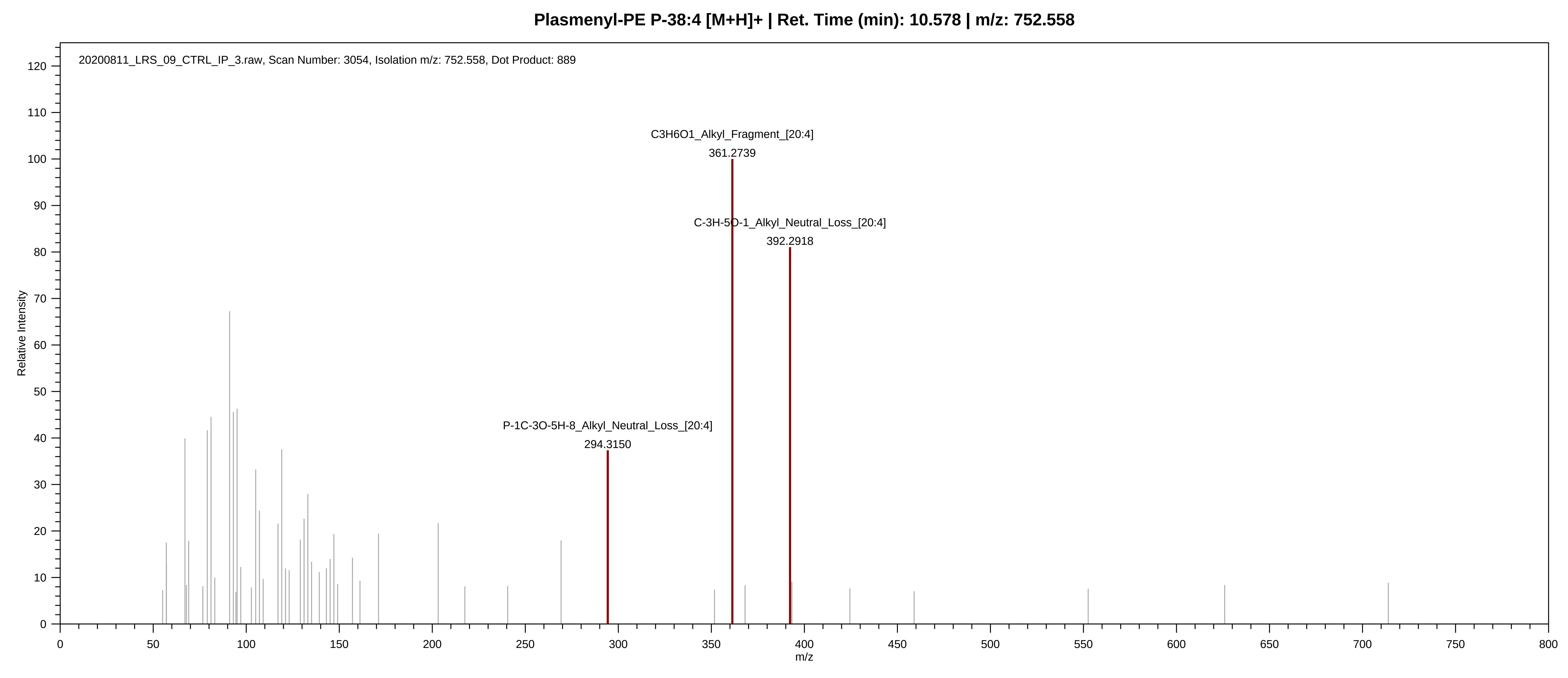

Plasmenyl-PE P-38:4 [M+H]+ | Ret. Time (min): 10.578 | m/z: 752.558
20200811_LRS_09_CTRL_IP_3.raw, Scan Number: 3054, Isolation m/z: 752.558, Dot Product: 889
C3H6O1_Alkyl_Fragment_[20:4]
361.2739
C-3H-5O-1_Alkyl_Neutral_Loss_[20:4]
392.2918
P-1C-3O-5H-8_Alkyl_Neutral_Loss_[20:4]
294.3150
Relative Intensity
m/z

Plasmenyl-PE P-38:4 [M+H]+ | Ret. Time (min): 10.5 | m/z: 752.56

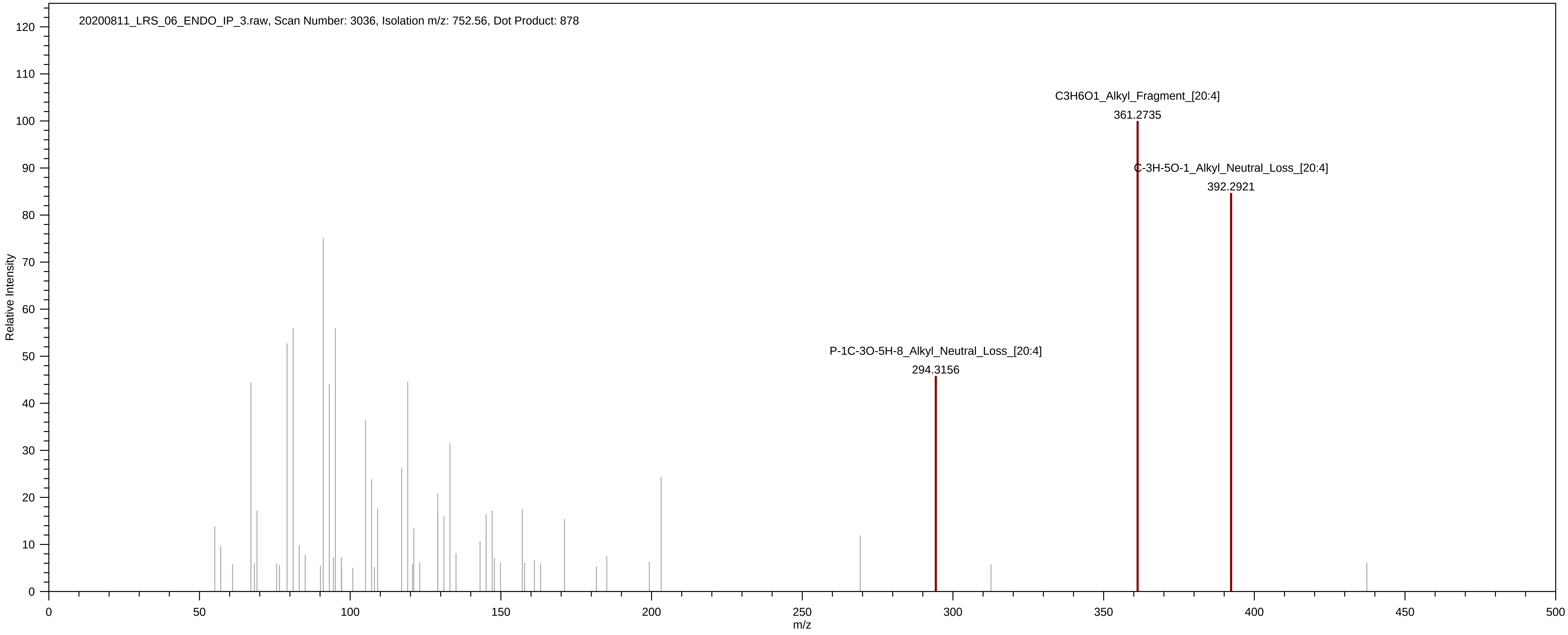

Plasmenyl-PE P-38:4 [M+H]+ | Ret. Time (min): 10.483 | m/z: 752.559

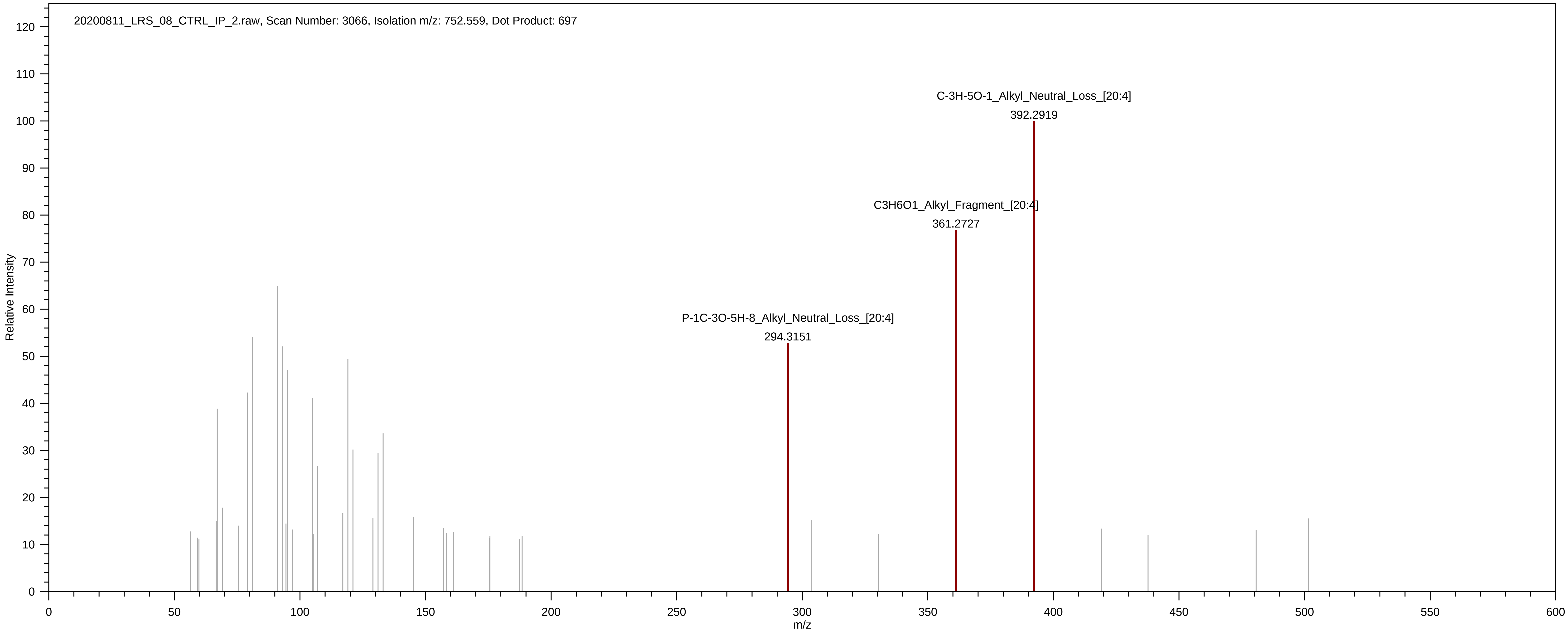

Plasmenyl-PE P-38:5 [M+H]+ | Ret. Time (min): 9.506 | m/z: 750.544

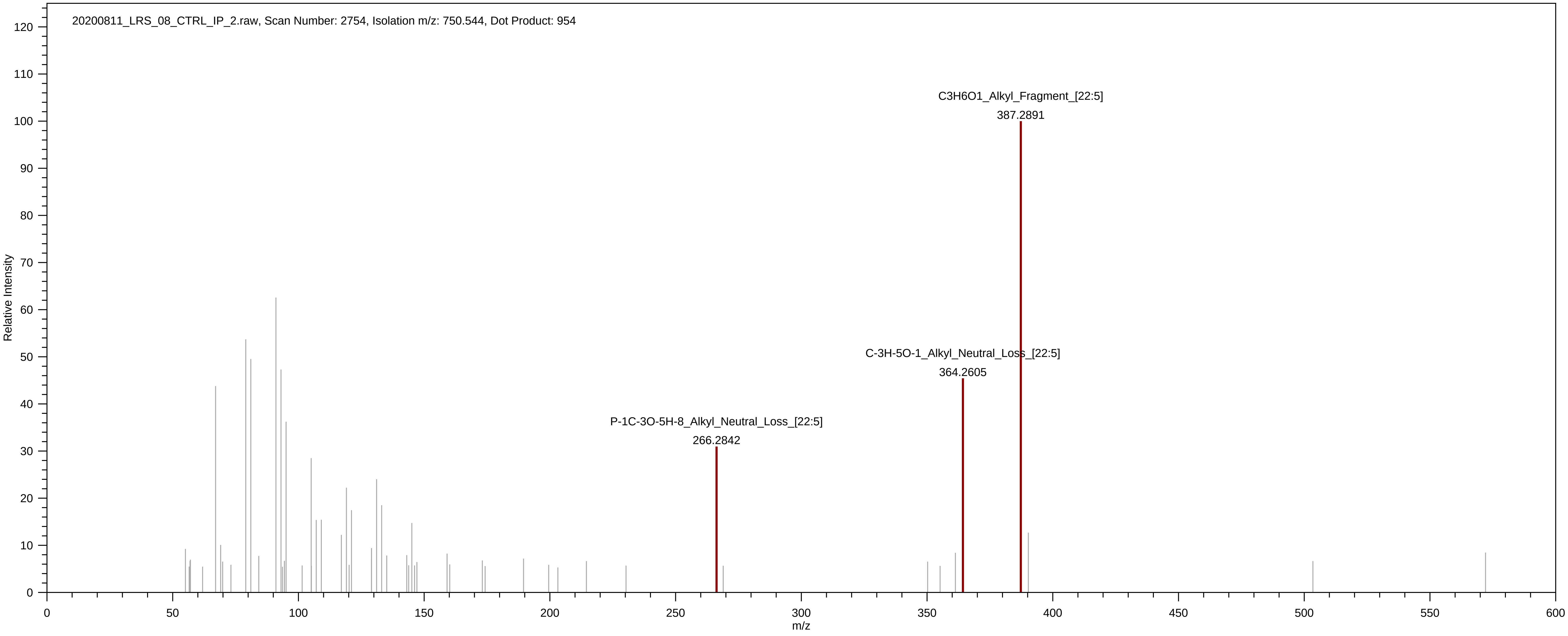

Plasmenyl-PE P-38:5 [M+H]+ | Ret. Time (min): 9.593 | m/z: 750.543

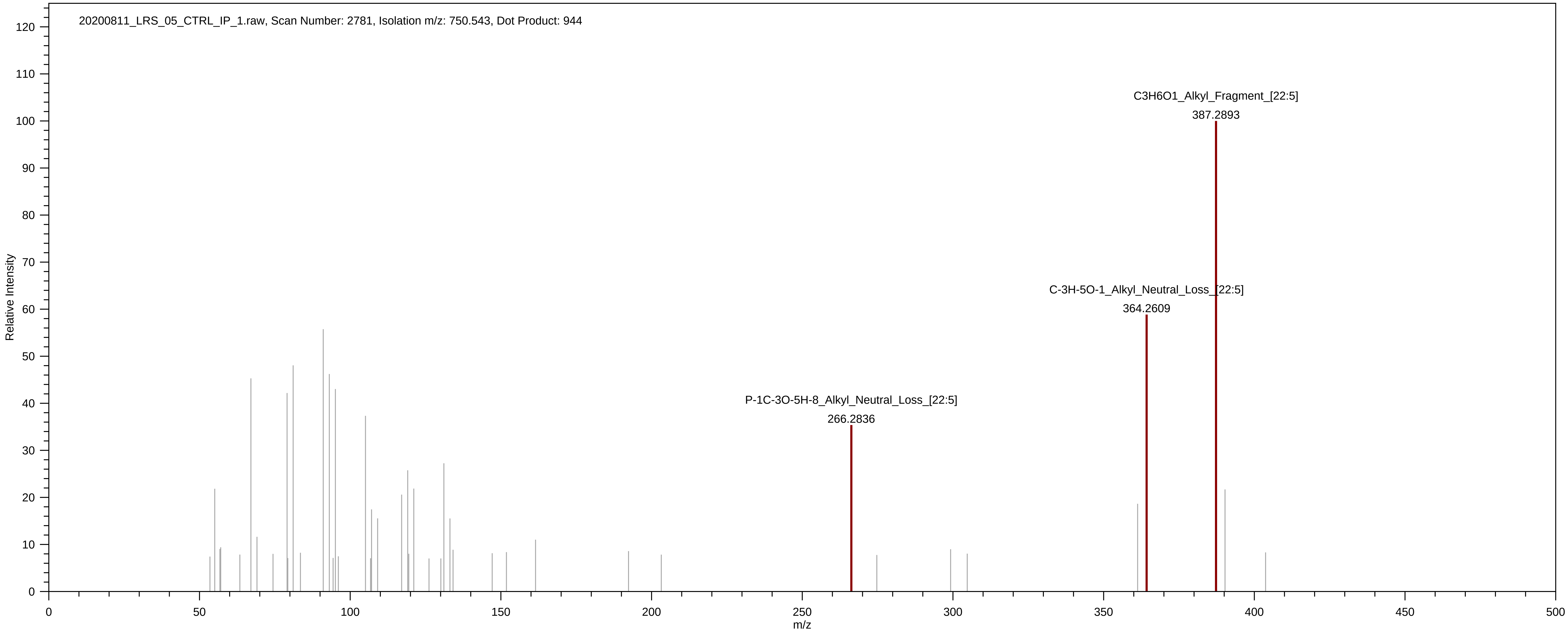

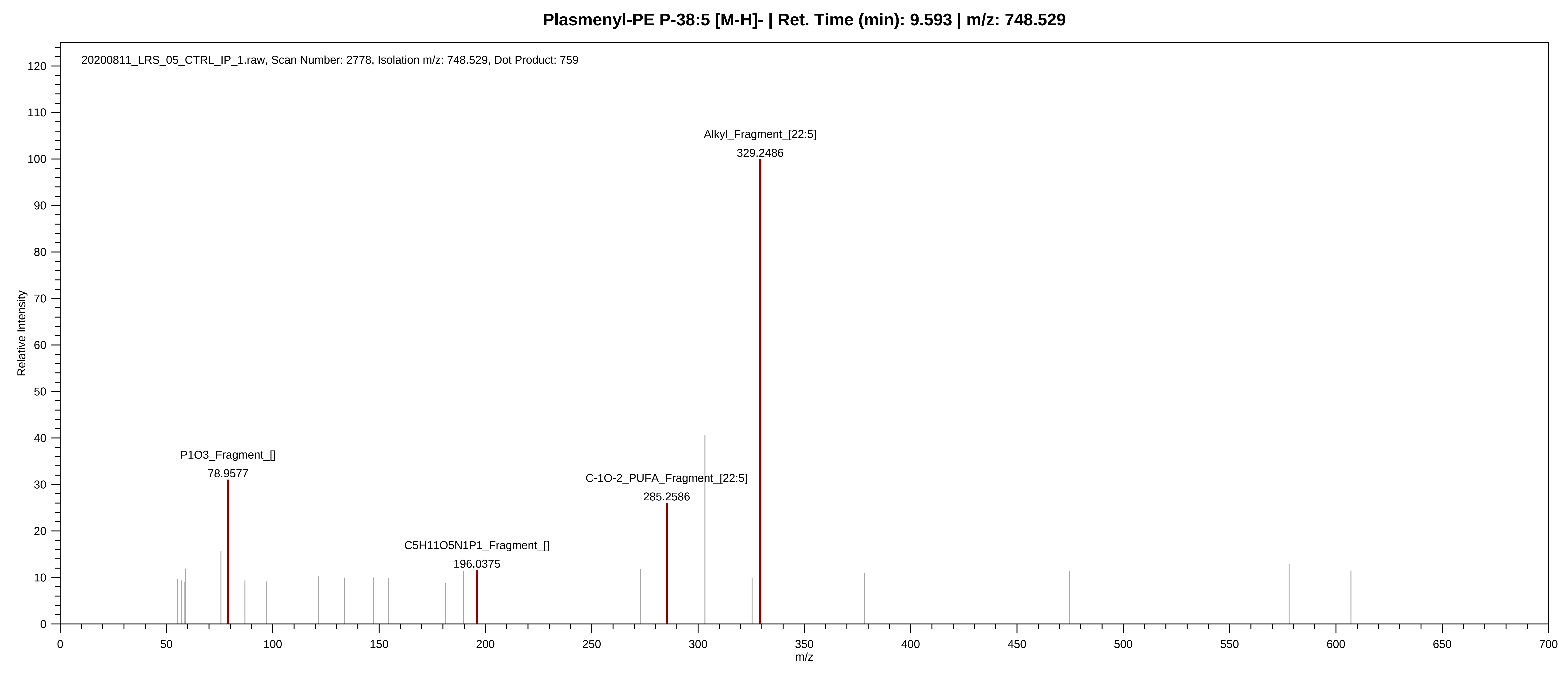

Plasmenyl-PE P-38:5 [M-H]- | Ret. Time (min): 9.593 | m/z: 748.529
20200811_LRS_05_CTRL_IP_1.raw, Scan Number: 2778, Isolation m/z: 748.529, Dot Product: 759
Relative Intensity
m/z
Alkyl_Fragment_[22:5]
329.2486
P1O3_Fragment_[]
78.9577
C-1O-2_PUFA_Fragment_[22:5]
285.2586
C5H11O5N1P1_Fragment_[]
196.0375

Plasmenyl-PE P-38:5 [M-H]- | Ret. Time (min): 9.646 | m/z: 748.529

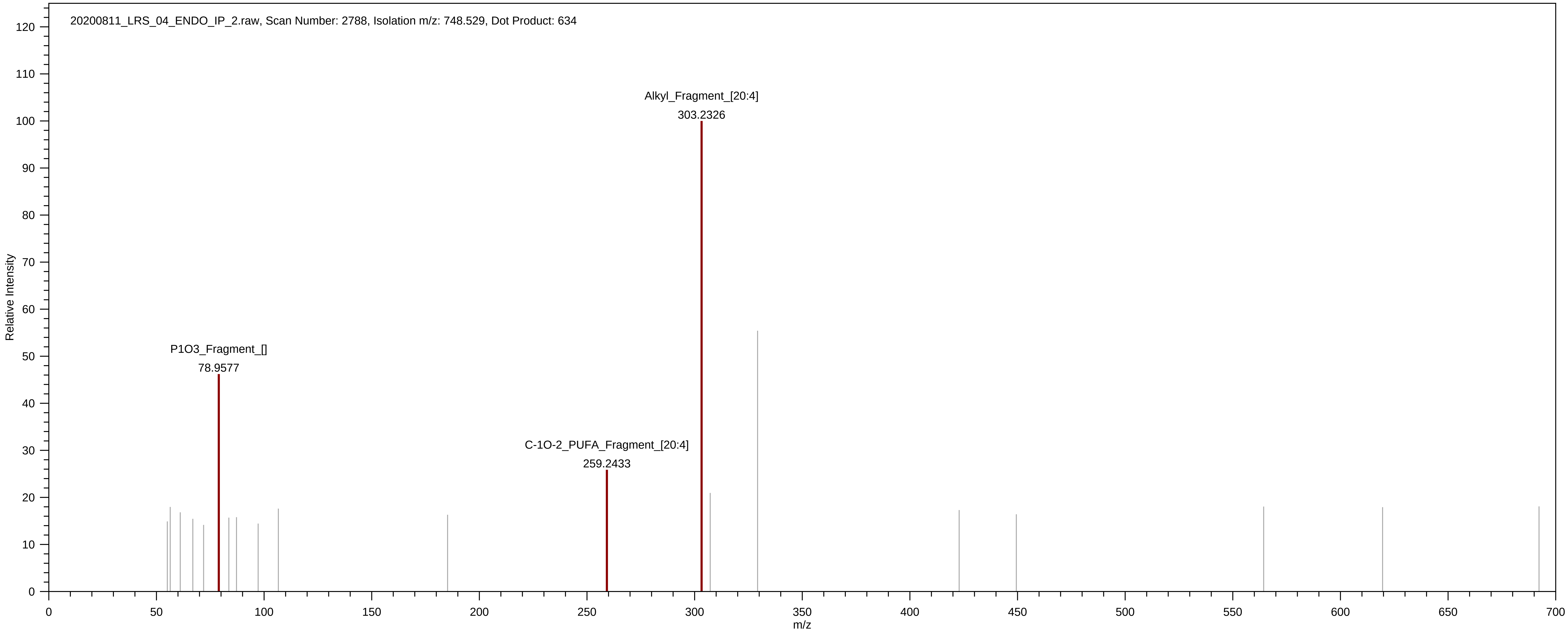

Plasmenyl-PE P-38:5 [M-H]- | Ret. Time (min): 9.649 | m/z: 748.529

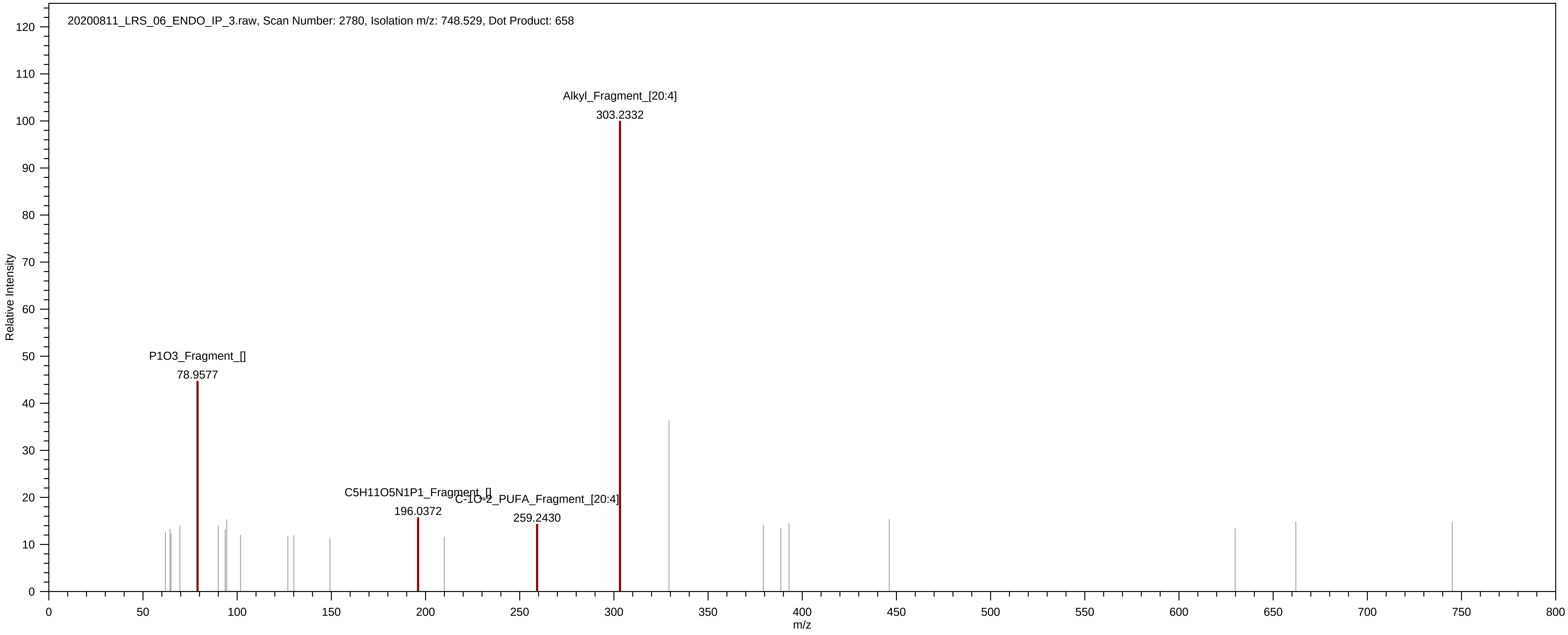

Plasmenyl-PE P-38:6 [M+H]+ | Ret. Time (min): 9.197 | m/z: 748.525

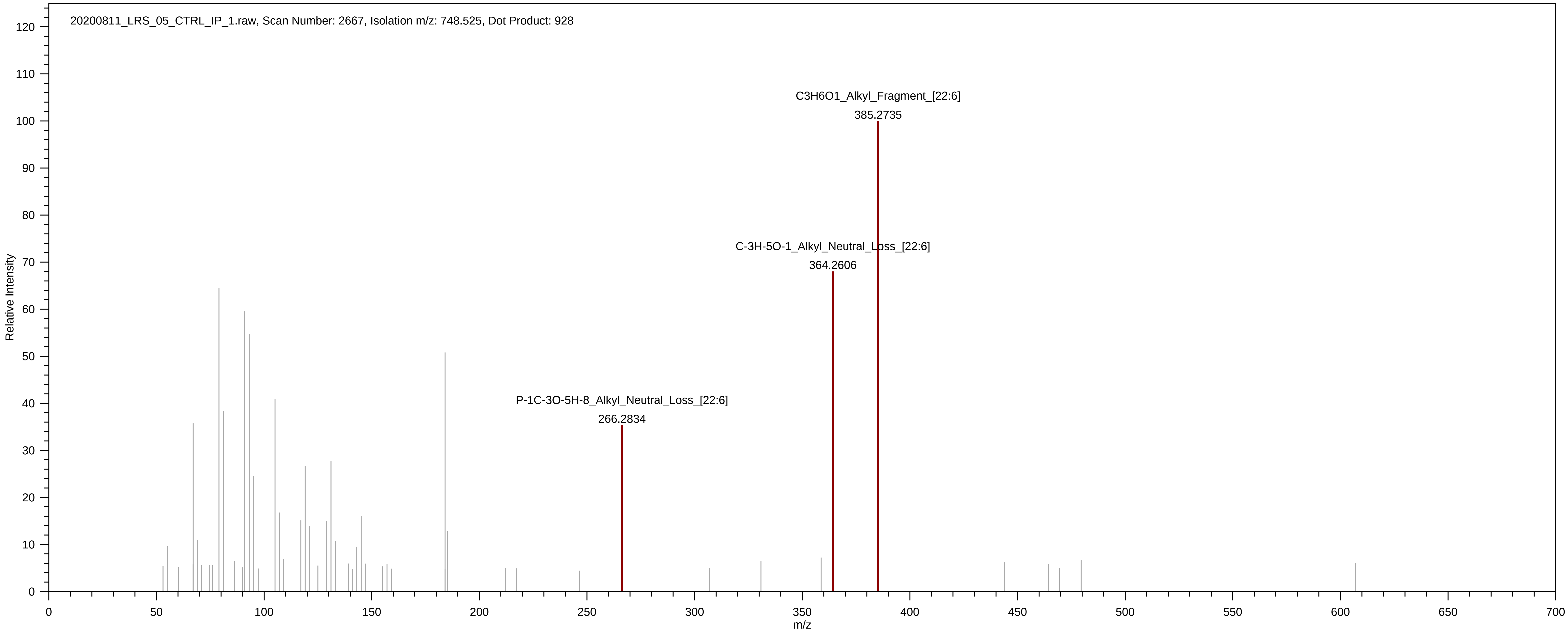

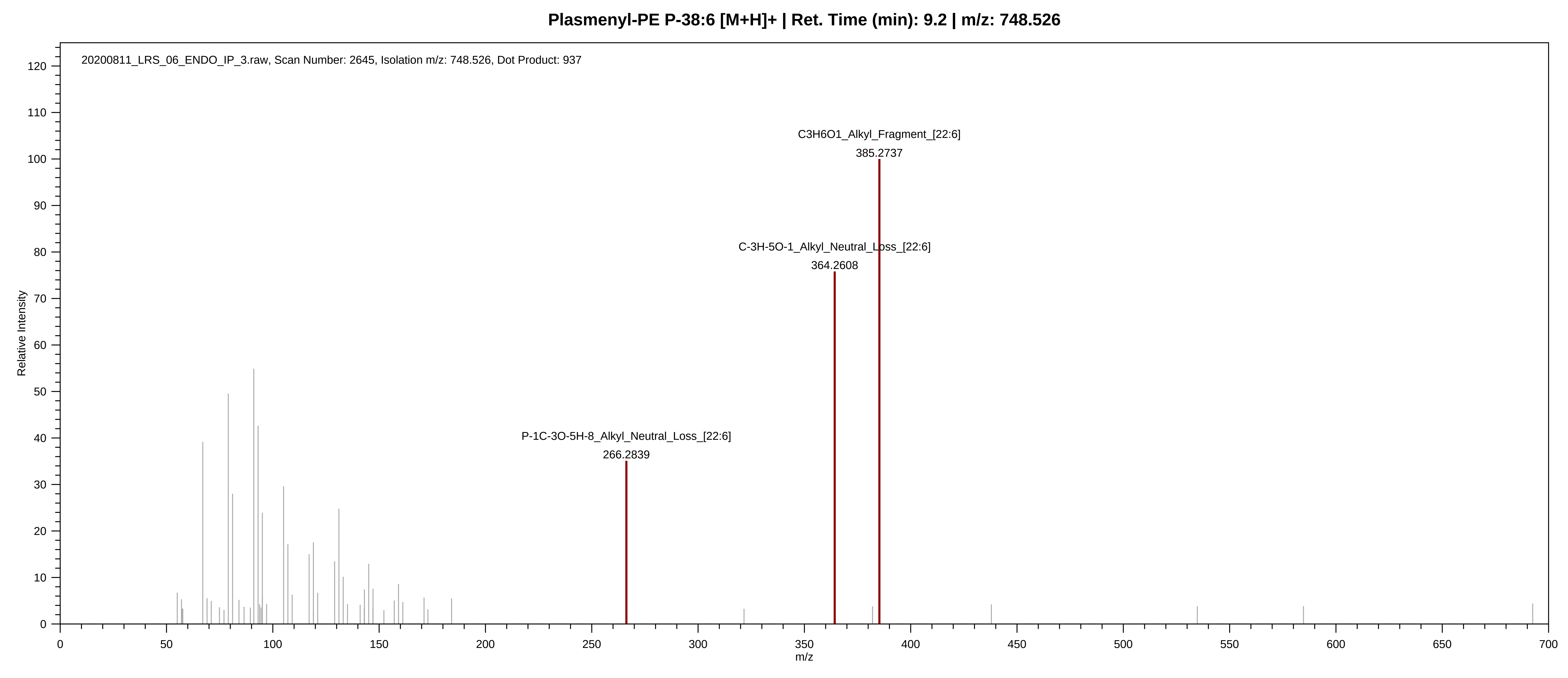
Plasmenyl-PE P-38:6 [M+H]+ | Ret. Time (min): 9.2 | m/z: 748.526
20200811_LRS_06_ENDO_IP_3.raw, Scan Number: 2645, Isolation m/z: 748.526, Dot Product: 937
C3H6O1_Alkyl_Fragment_[22:6]
385.2737
C-3H-5O-1_Alkyl_Neutral_Loss_[22:6]
364.2608
P-1C-3O-5H-8_Alkyl_Neutral_Loss_[22:6]
266.2839
Relative Intensity
m/z

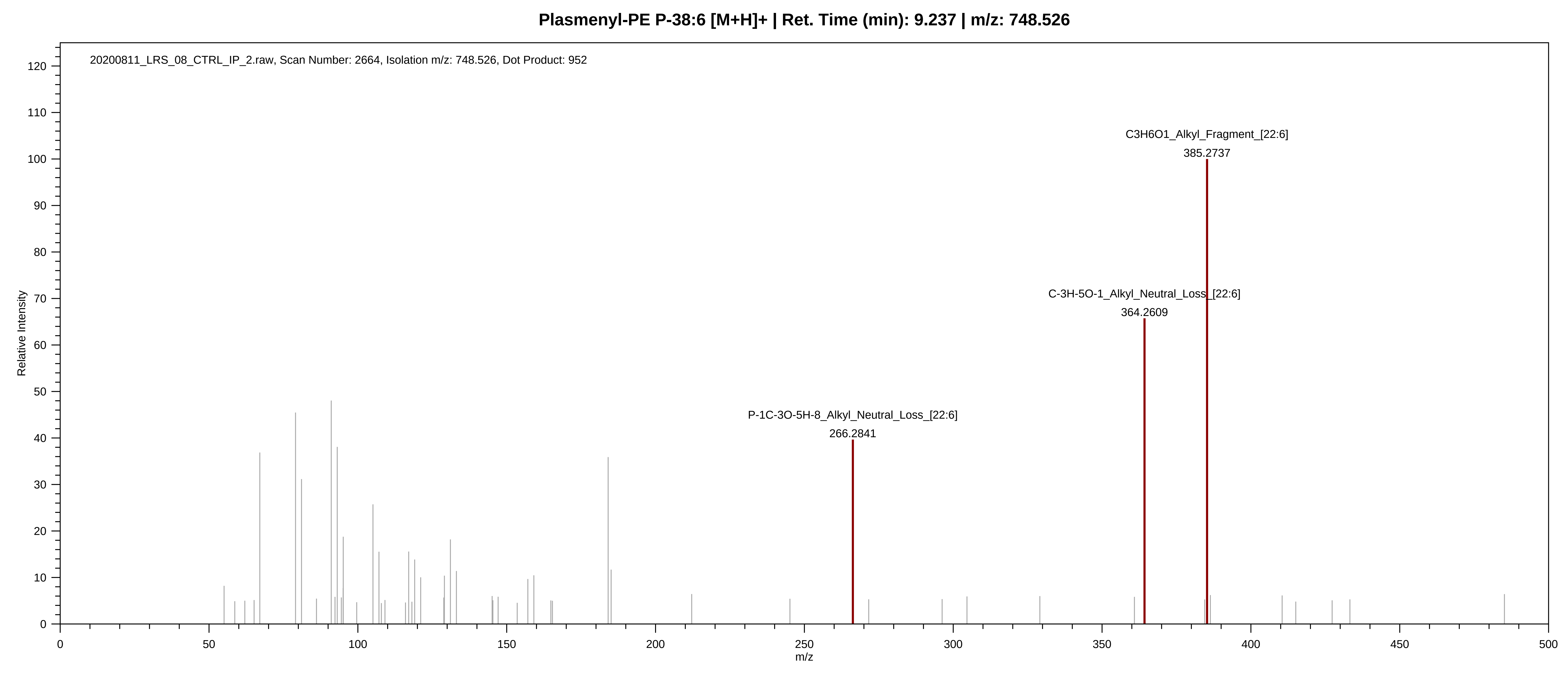

Plasmenyl-PE P-38:6 [M+H]+ | Ret. Time (min): 9.237 | m/z: 748.526
20200811_LRS_08_CTRL_IP_2.raw, Scan Number: 2664, Isolation m/z: 748.526, Dot Product: 952
C3H6O1_Alkyl_Fragment_[22:6]
385.2737
C-3H-5O-1_Alkyl_Neutral_Loss_[22:6]
364.2609
P-1C-3O-5H-8_Alkyl_Neutral_Loss_[22:6]
266.2841
Relative Intensity
m/z

Plasmenyl-PE P-38:6 [M+H]+ | Ret. Time (min): 9.191 | m/z: 748.527

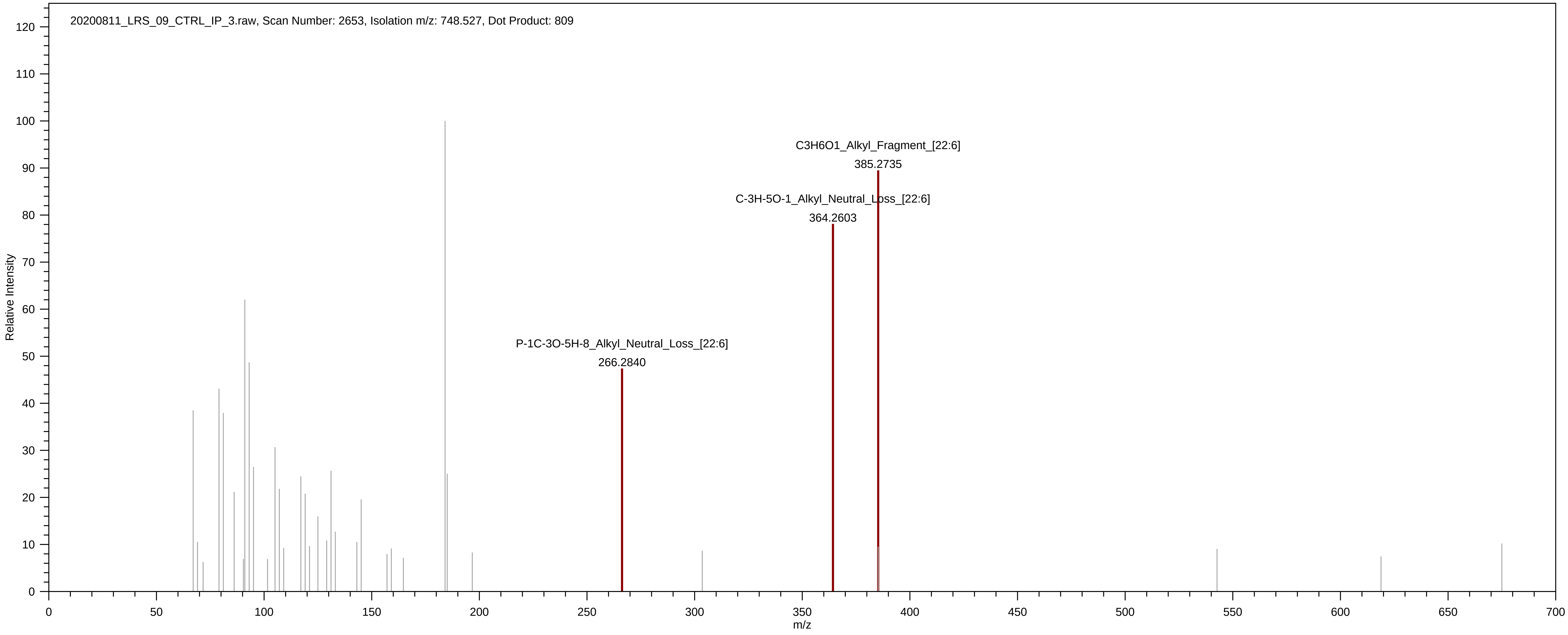

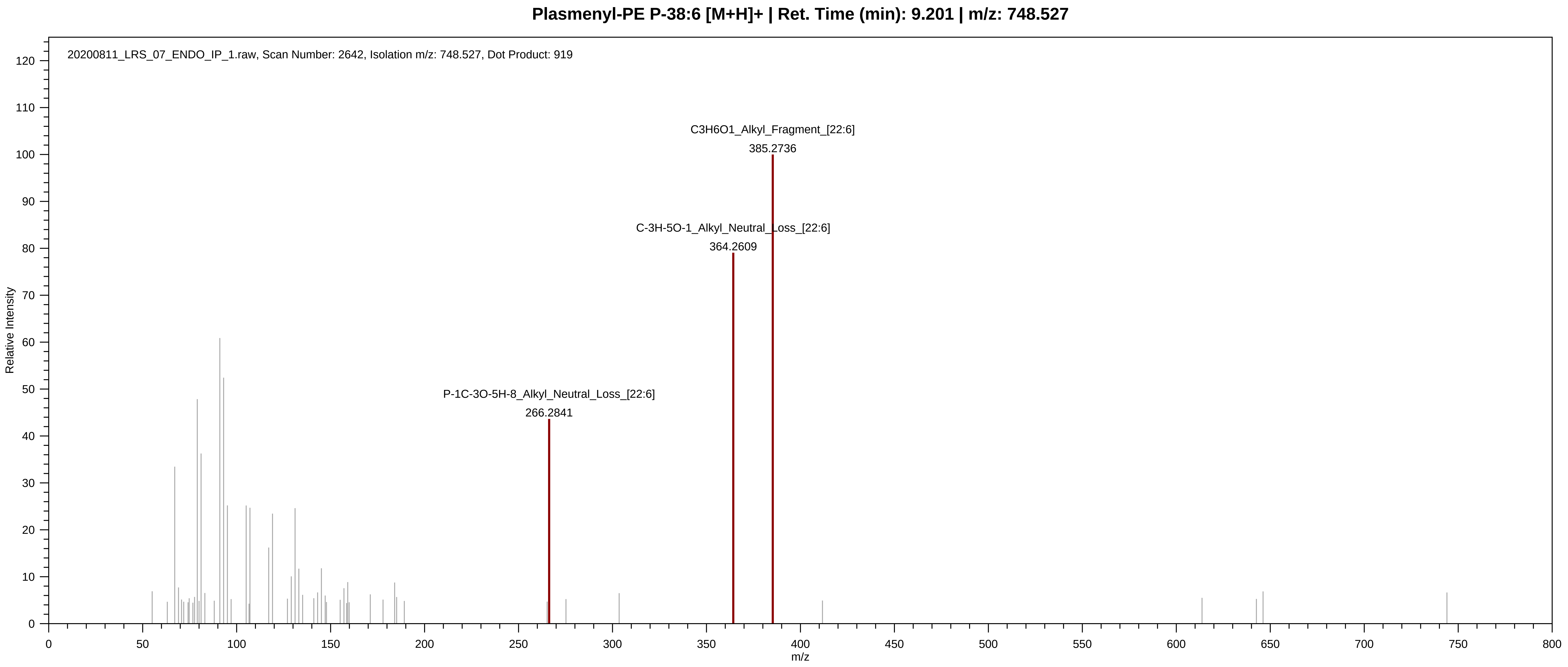
Plasmenyl-PE P-38:6 [M+H]+ | Ret. Time (min): 9.201 | m/z: 748.527
20200811_LRS_07_ENDO_IP_1.raw, Scan Number: 2642, Isolation m/z: 748.527, Dot Product: 919
C3H6O1_Alkyl_Fragment_[22:6]
385.2736
C-3H-5O-1_Alkyl_Neutral_Loss_[22:6]
364.2609
P-1C-3O-5H-8_Alkyl_Neutral_Loss_[22:6]
266.2841
Relative Intensity
m/z

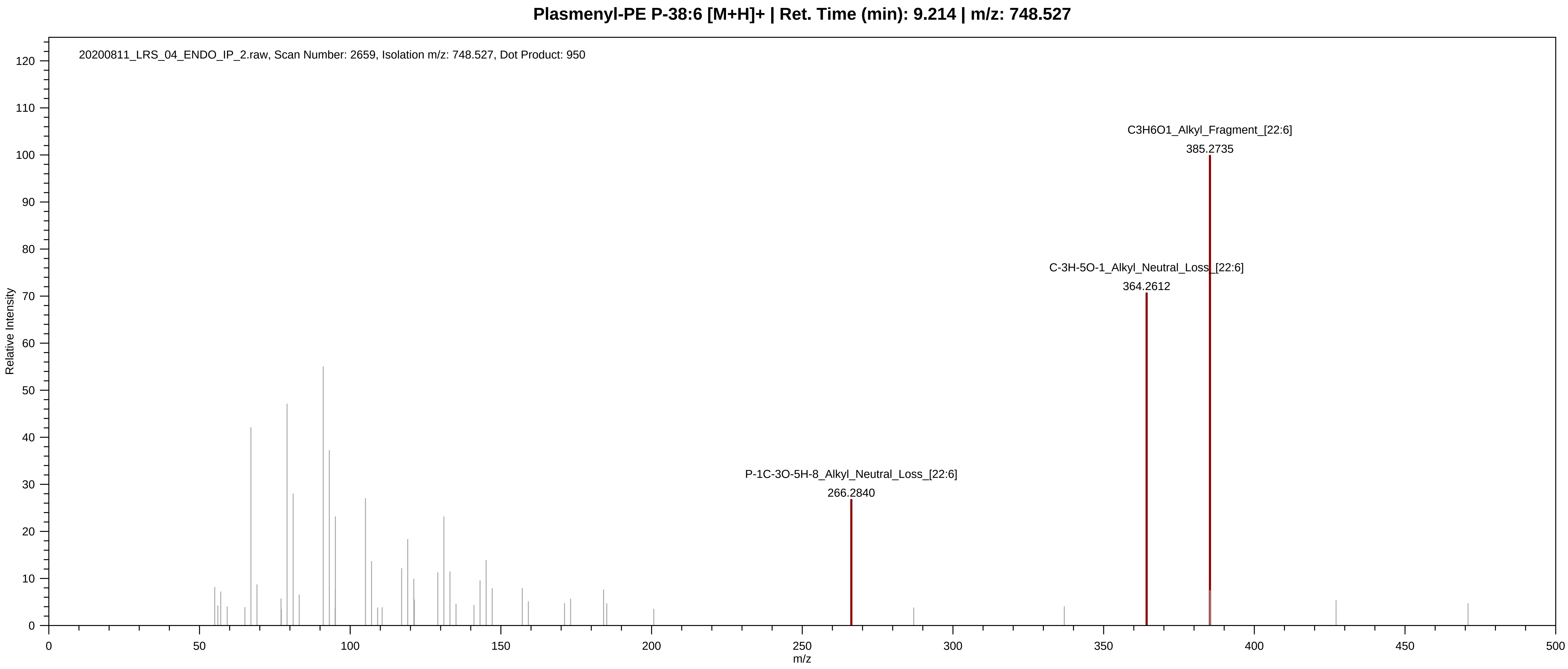
Plasmenyl-PE P-38:6 [M+H]+ | Ret. Time (min): 9.214 | m/z: 748.527
20200811_LRS_04_ENDO_IP_2.raw, Scan Number: 2659, Isolation m/z: 748.527, Dot Product: 950
C3H6O1_Alkyl_Fragment_[22:6]
385.2735
C-3H-5O-1_Alkyl_Neutral_Loss_[22:6]
364.2612
P-1C-3O-5H-8_Alkyl_Neutral_Loss_[22:6]
266.2840
Relative Intensity
m/z
0
10
20
30
40
50
60
70
80
90
100
110
120
0
50
100
150
200
250
300
350
400
450
500

Plasmenyl-PE P-40:4 [M+H]+ | Ret. Time (min): 11.235 | m/z: 780.59

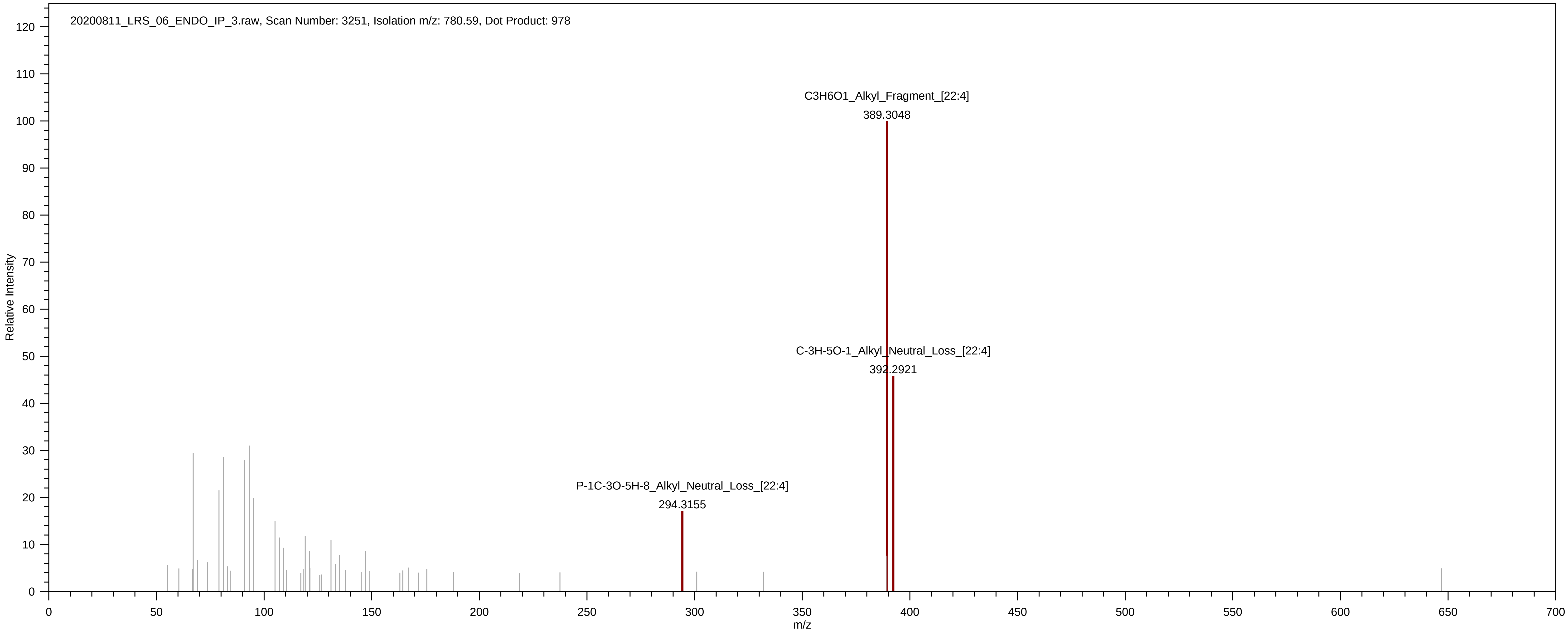

Plasmenyl-PE P-40:4 [M+H]+ | Ret. Time (min): 11.551 | m/z: 780.589

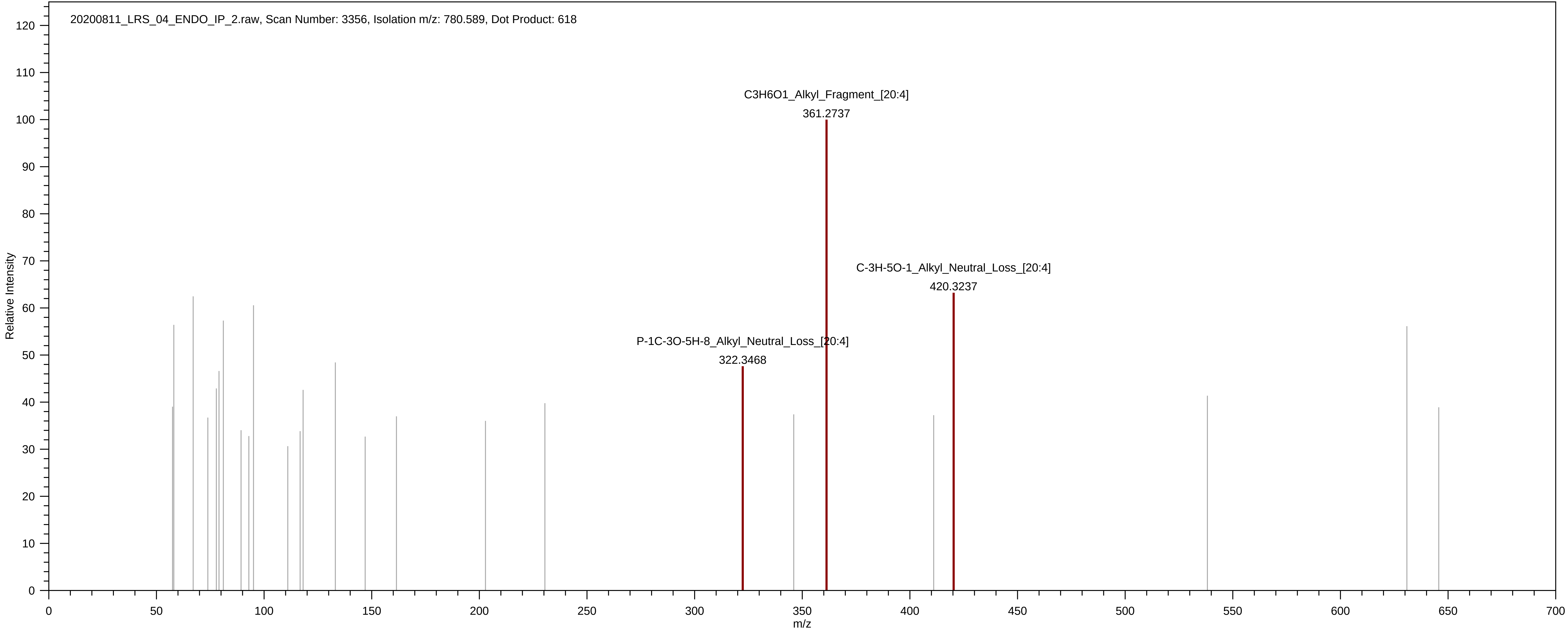

Plasmenyl-PE P-40:5 [M+H]+ | Ret. Time (min): 10.55 | m/z: 778.574

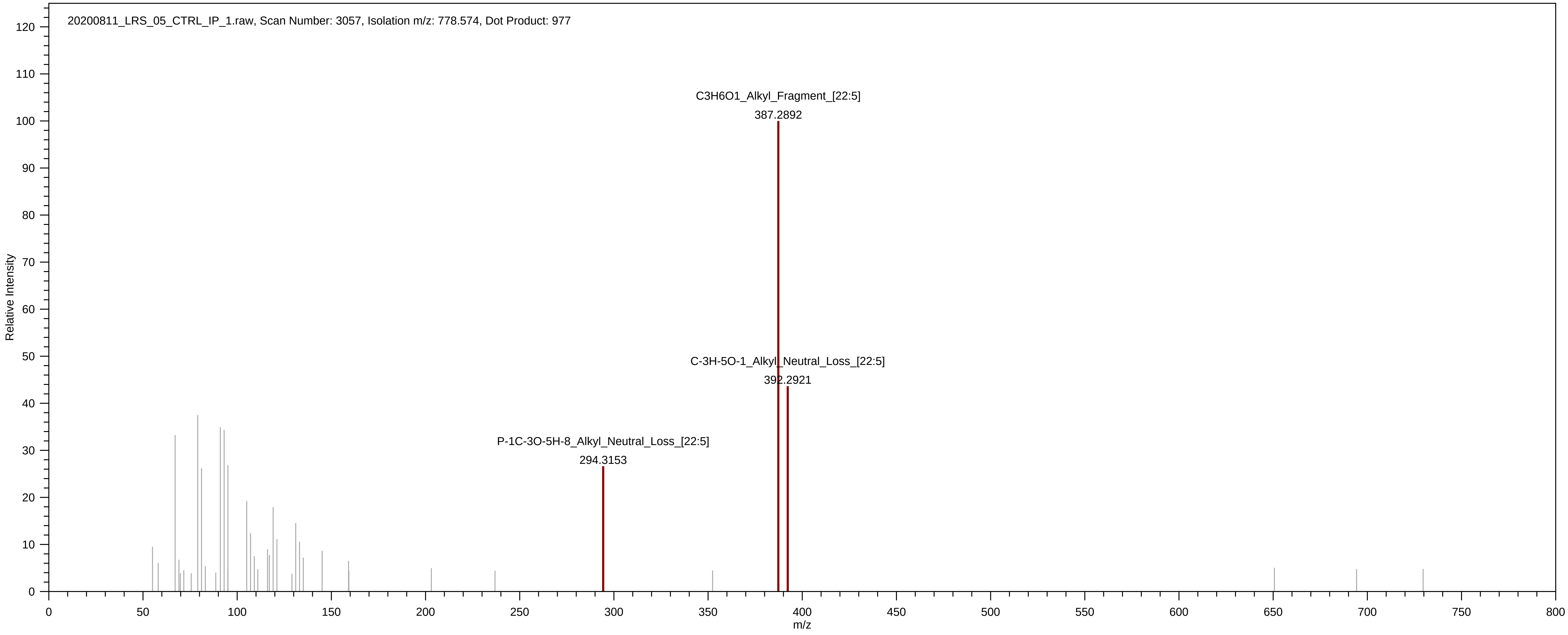

Plasmenyl-PE P-40:5 [M+H]+ | Ret. Time (min): 10.481 | m/z: 778.574

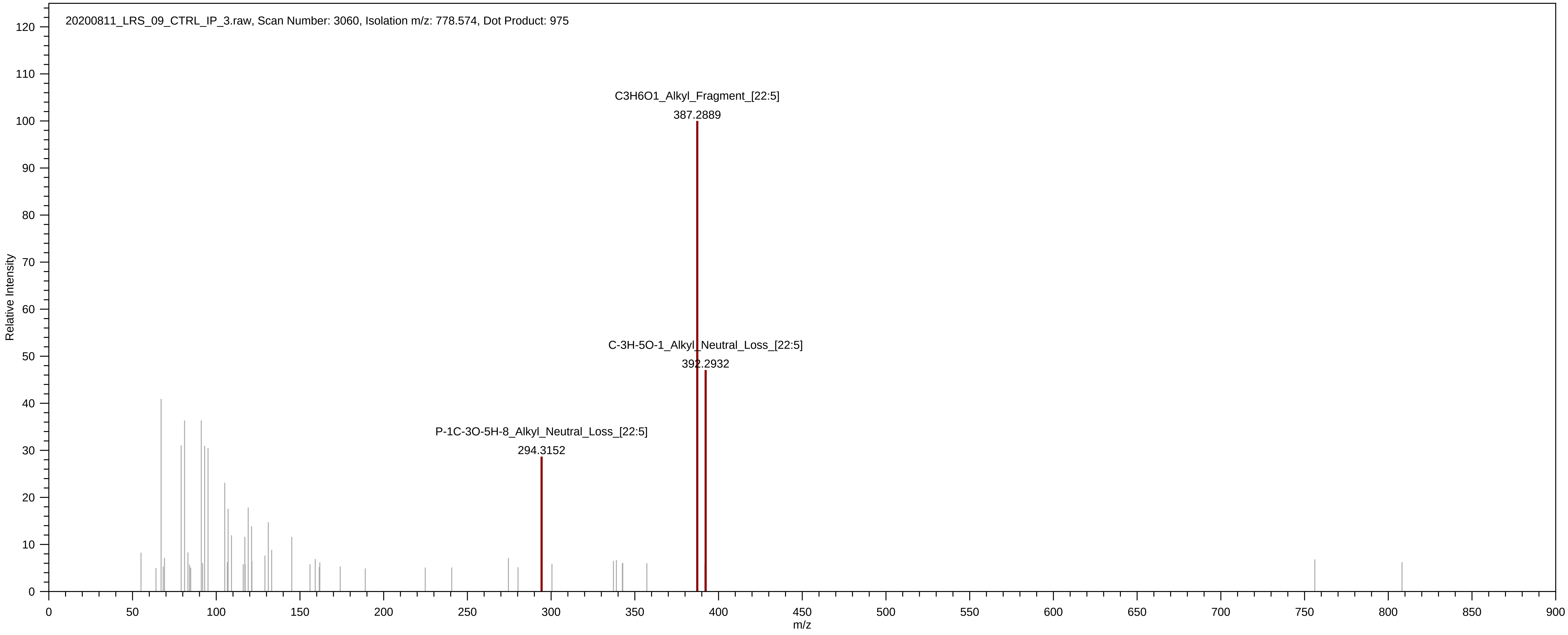

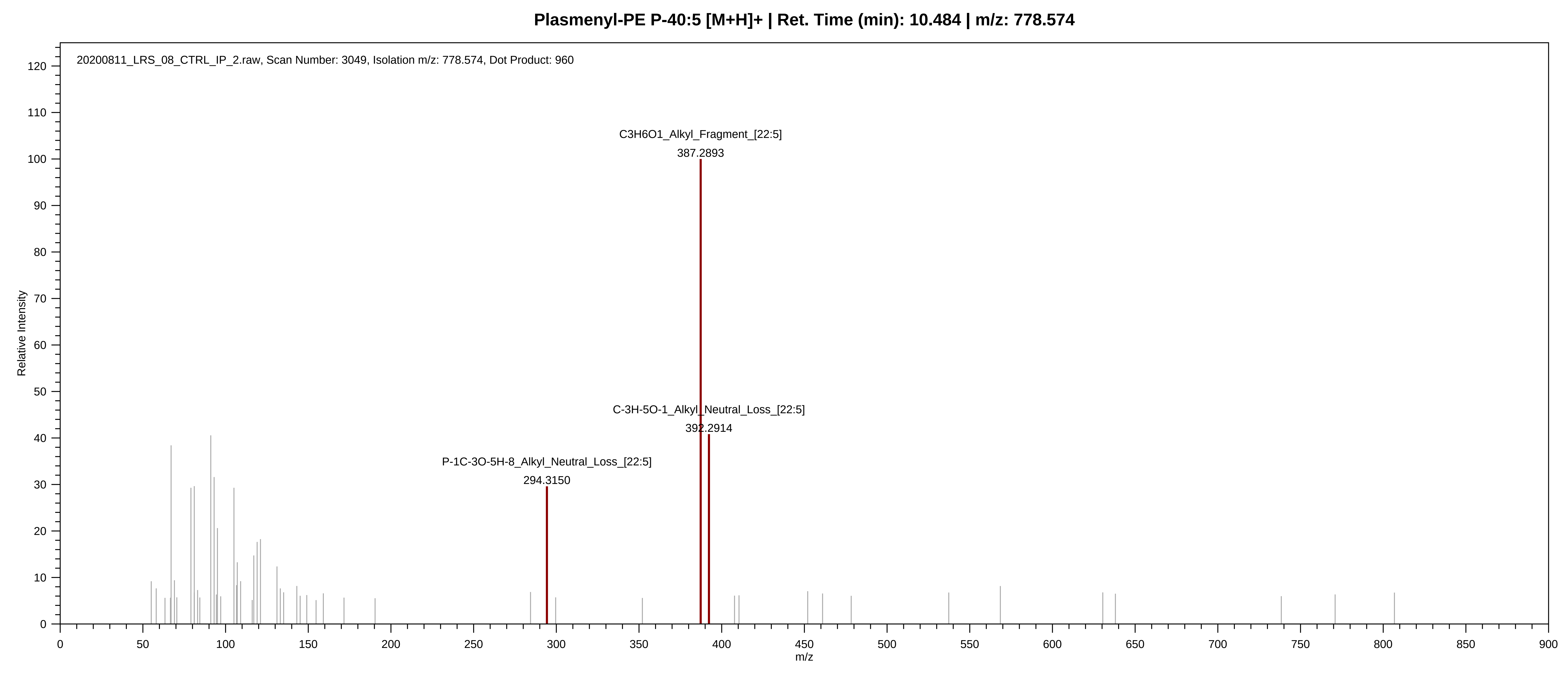

Plasmenyl-PE P-40:5 [M+H]+ | Ret. Time (min): 10.484 | m/z: 778.574
20200811_LRS_08_CTRL_IP_2.raw, Scan Number: 3049, Isolation m/z: 778.574, Dot Product: 960
C3H6O1_Alkyl_Fragment_[22:5]
387.2893
C-3H-5O-1_Alkyl_Neutral_Loss_[22:5]
392.2914
P-1C-3O-5H-8_Alkyl_Neutral_Loss_[22:5]
294.3150
Relative Intensity
m/z

Plasmenyl-PE P-40:5 [M+H]+ | Ret. Time (min): 10.485 | m/z: 778.572

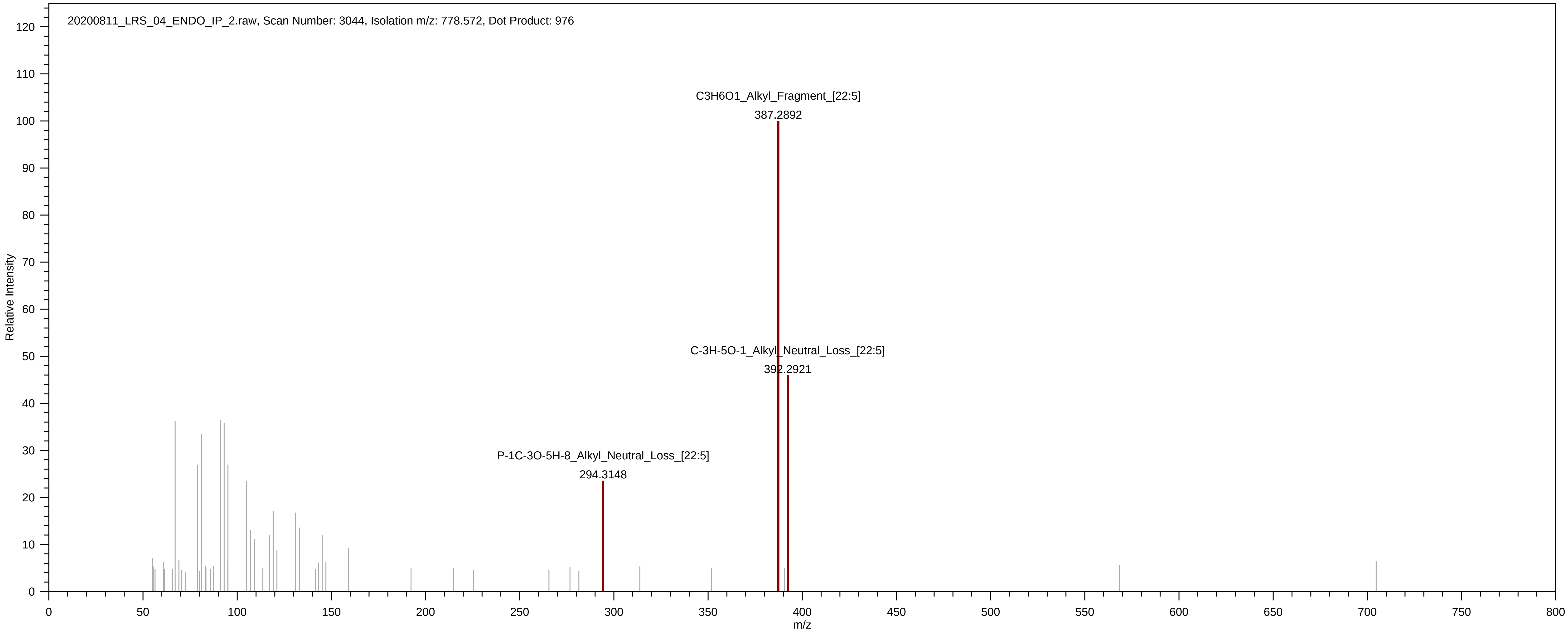

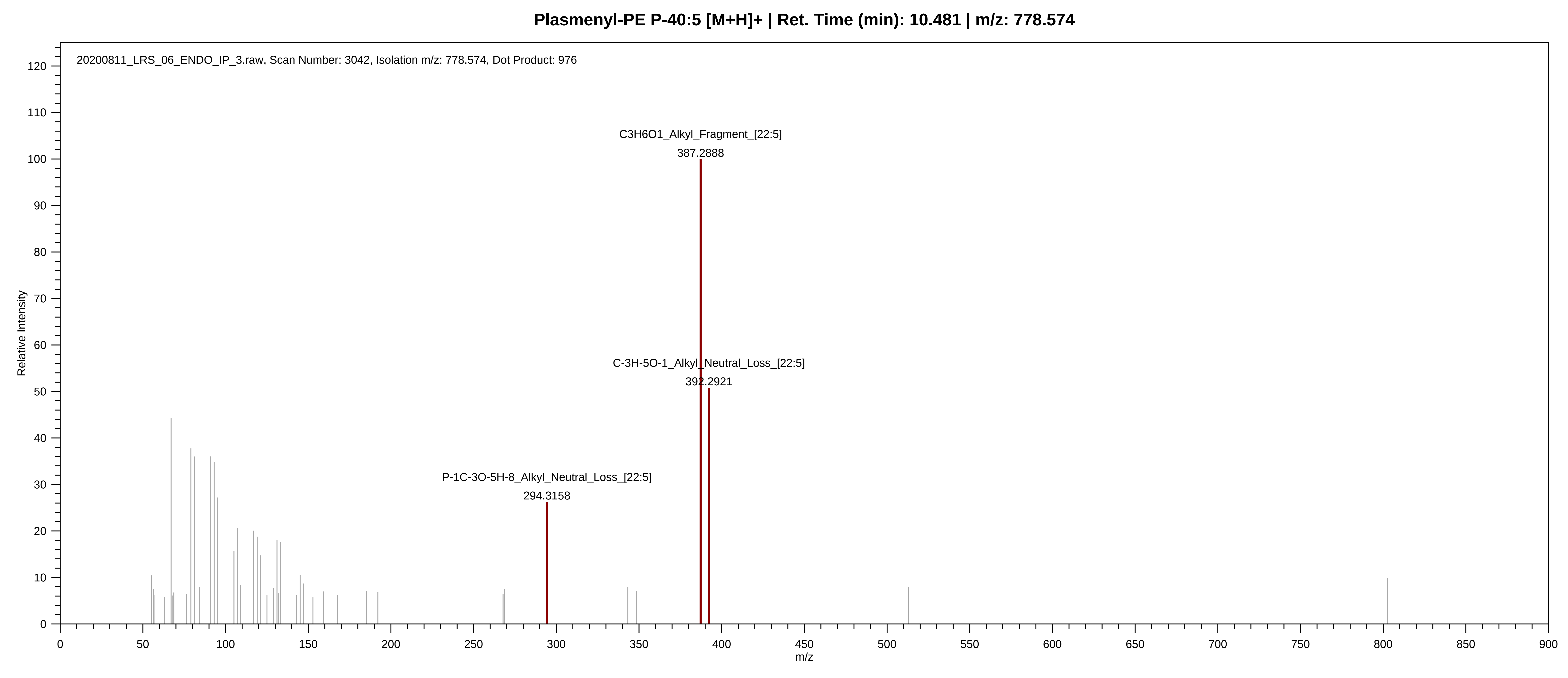
Plasmenyl-PE P-40:5 [M+H]+ | Ret. Time (min): 10.481 | m/z: 778.574
20200811_LRS_06_ENDO_IP_3.raw, Scan Number: 3042, Isolation m/z: 778.574, Dot Product: 976
C3H6O1_Alkyl_Fragment_[22:5]
387.2888
C-3H-5O-1_Alkyl_Neutral_Loss_[22:5]
392.2921
P-1C-3O-5H-8_Alkyl_Neutral_Loss_[22:5]
294.3158
Relative Intensity
m/z

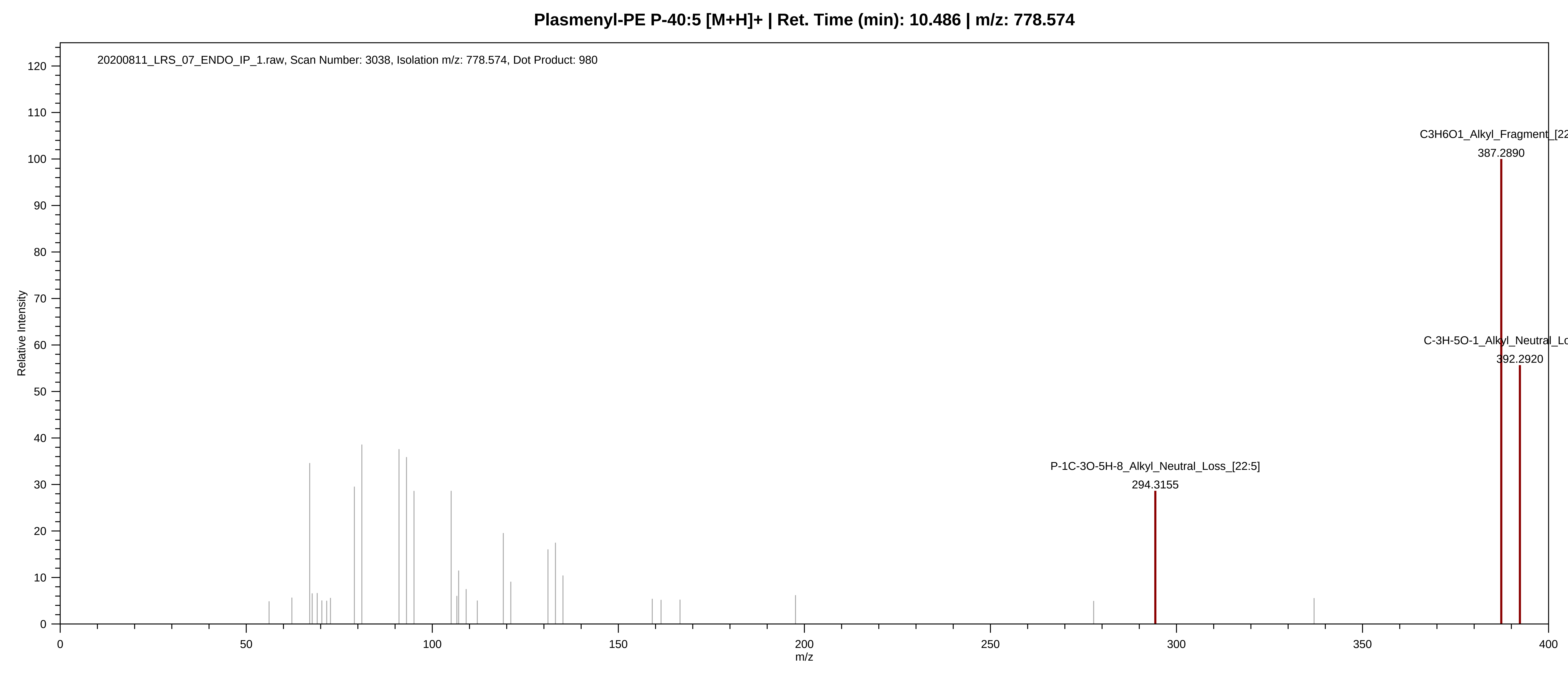

Plasmenyl-PE P-40:5 [M+H]+ | Ret. Time (min): 10.486 | m/z: 778.574
20200811_LRS_07_ENDO_IP_1.raw, Scan Number: 3038, Isolation m/z: 778.574, Dot Product: 980
C3H6O1_Alkyl_Fragment_[22
387.2890
C-3H-5O-1_Alkyl_Neutral_Lo
392.2920
P-1C-3O-5H-8_Alkyl_Neutral_Loss_[22:5]
294.3155
Relative Intensity
m/z

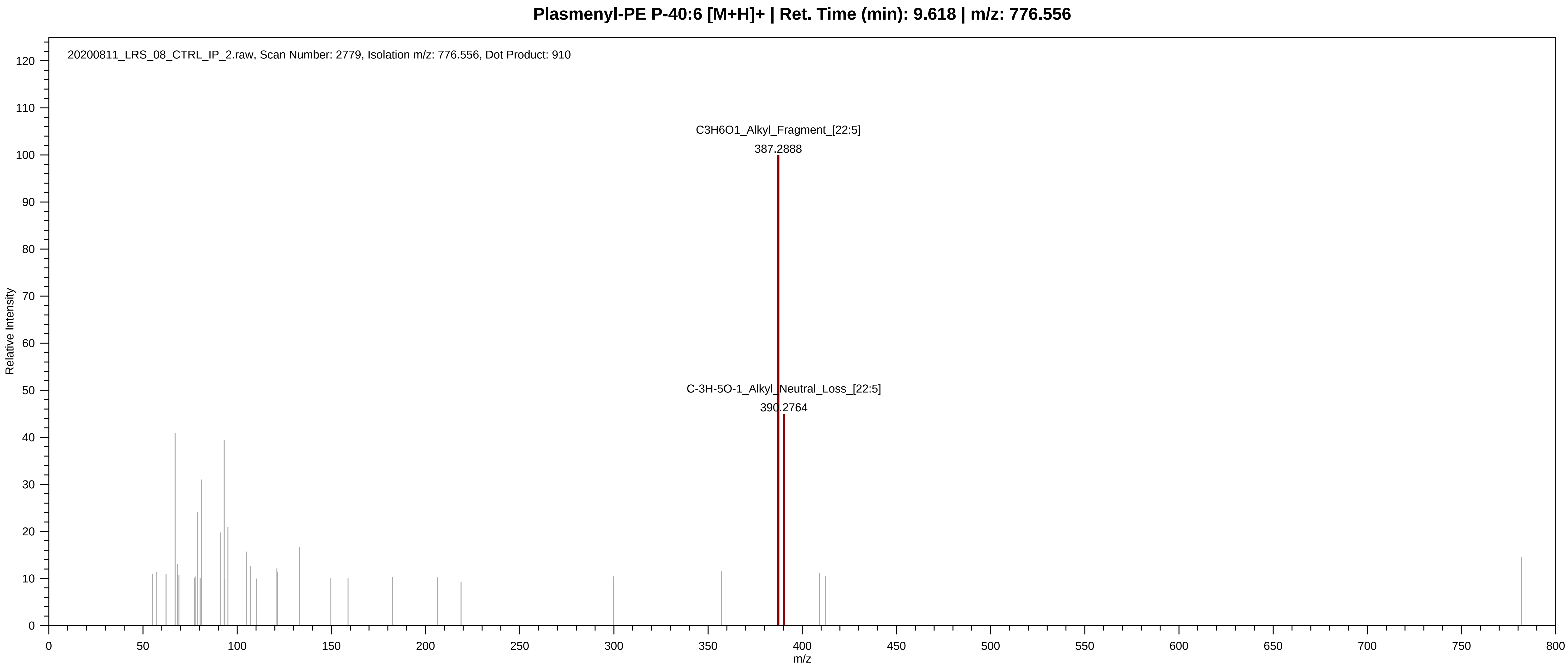
Plasmenyl-PE P-40:6 [M+H]+ | Ret. Time (min): 9.618 | m/z: 776.556
20200811_LRS_08_CTRL_IP_2.raw, Scan Number: 2779, Isolation m/z: 776.556, Dot Product: 910
C3H6O1_Alkyl_Fragment_[22:5]
387.2888
C-3H-5O-1_Alkyl_Neutral_Loss_[22:5]
390.2764
Relative Intensity
m/z
0
10
20
30
40
50
60
70
80
90
100
110
120
0
50
100
150
200
250
300
350
400
450
500
550
600
650
700
750
800

Plasmenyl-PE P-40:6 [M+H]+ | Ret. Time (min): 9.624 | m/z: 776.556

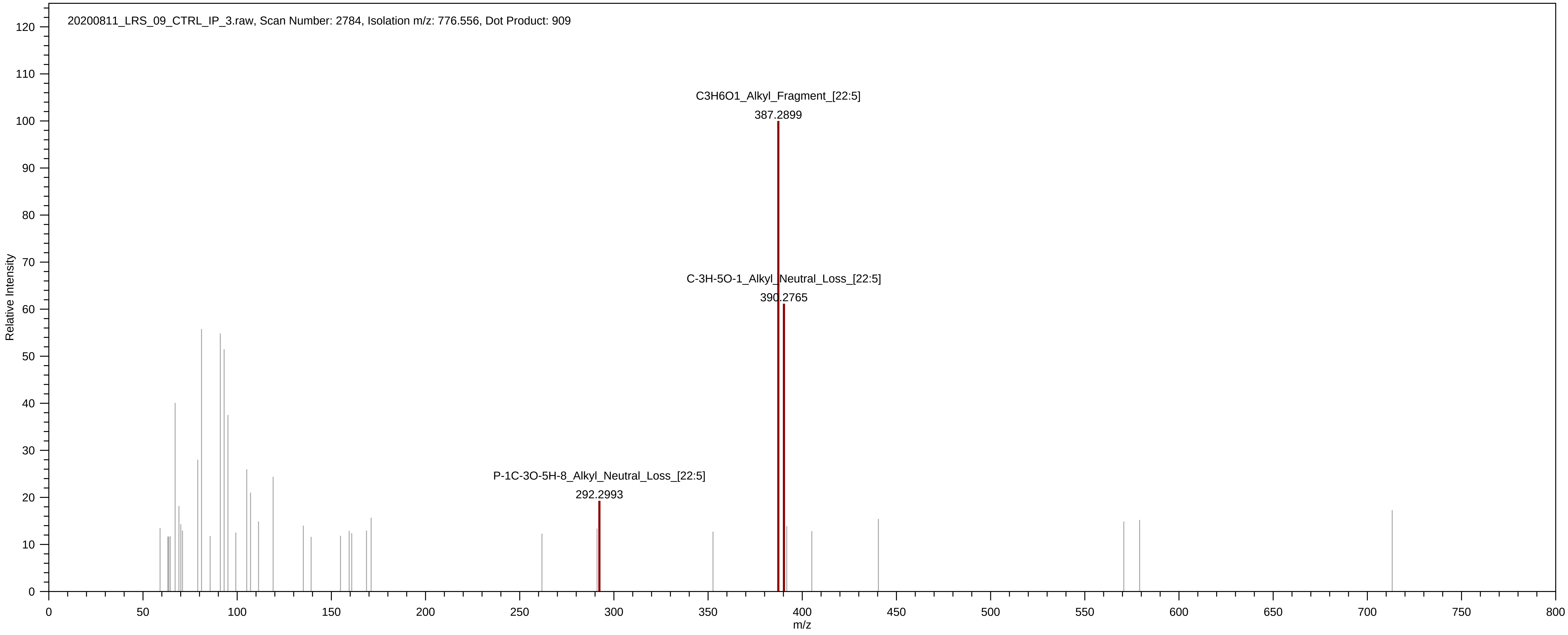

Plasmenyl-PE P-40:6 [M+H]+ | Ret. Time (min): 10.153 | m/z: 776.556

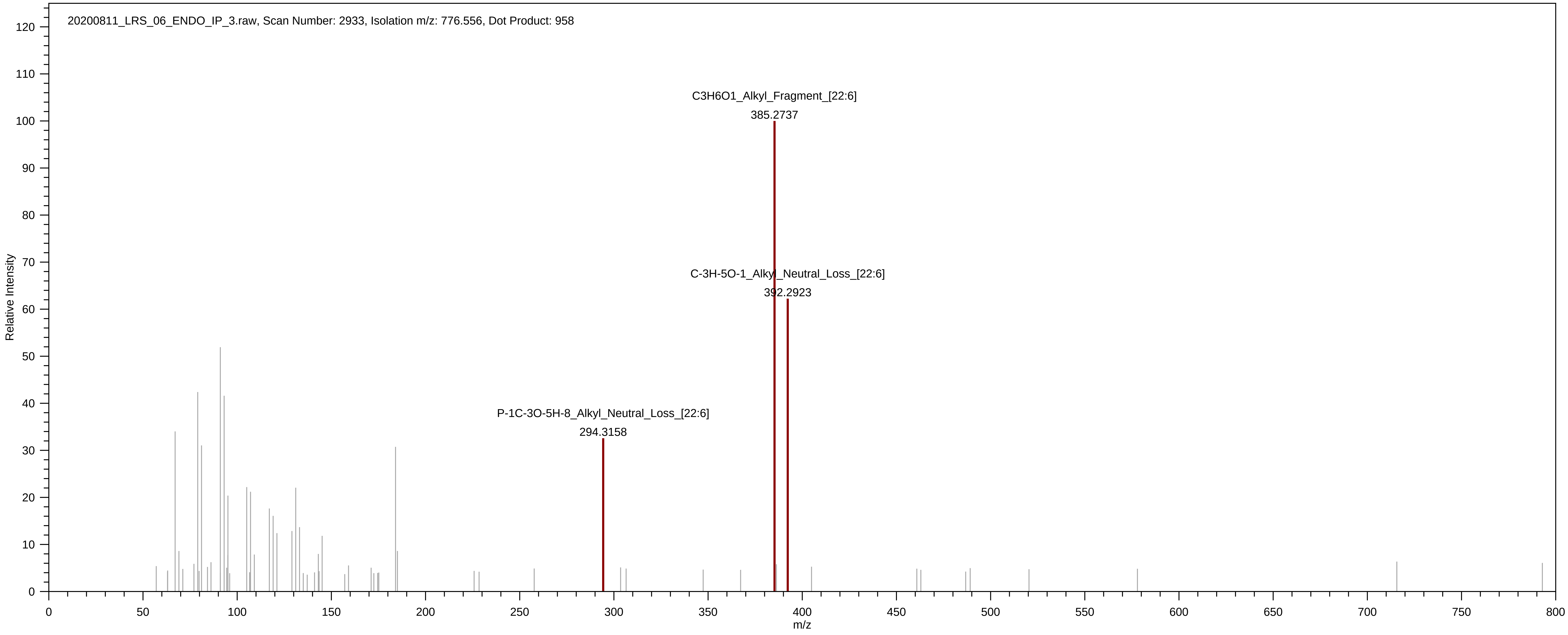

Plasmenyl-PE P-40:6 [M+H]+ | Ret. Time (min): 10.153 | m/z: 776.556

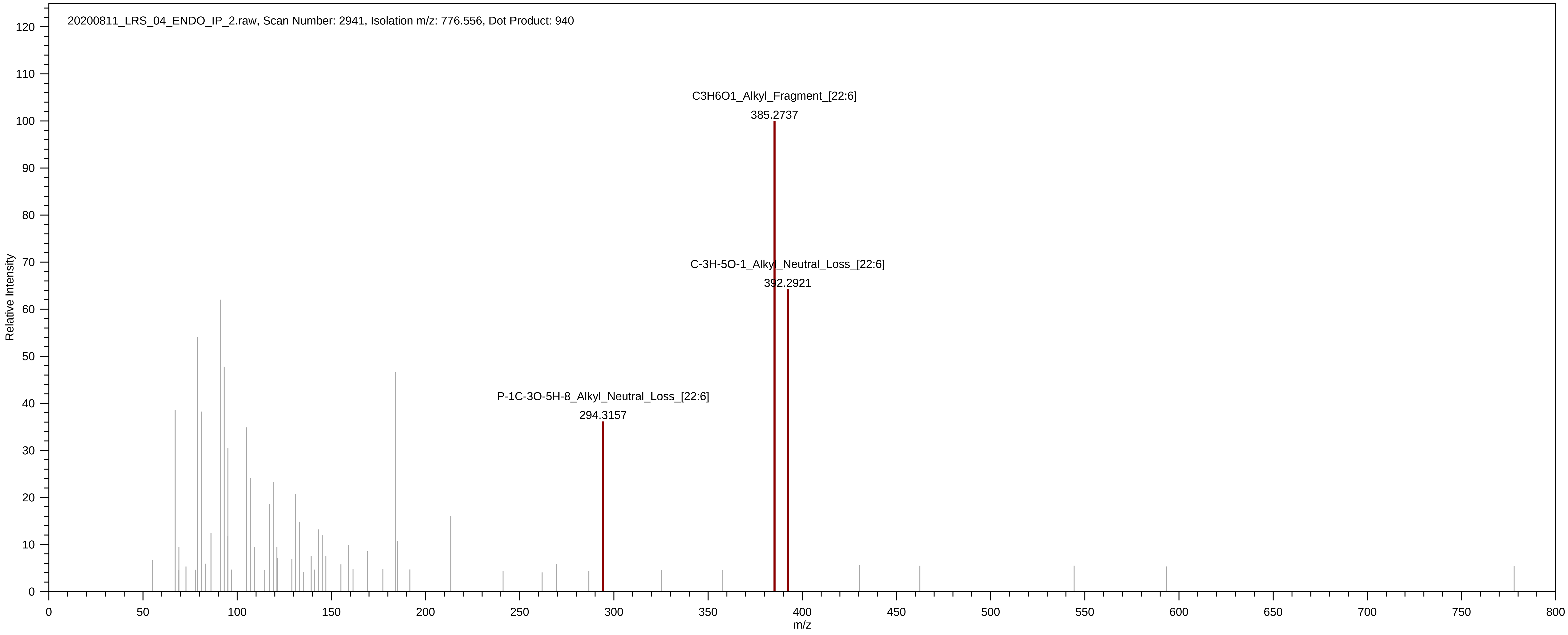

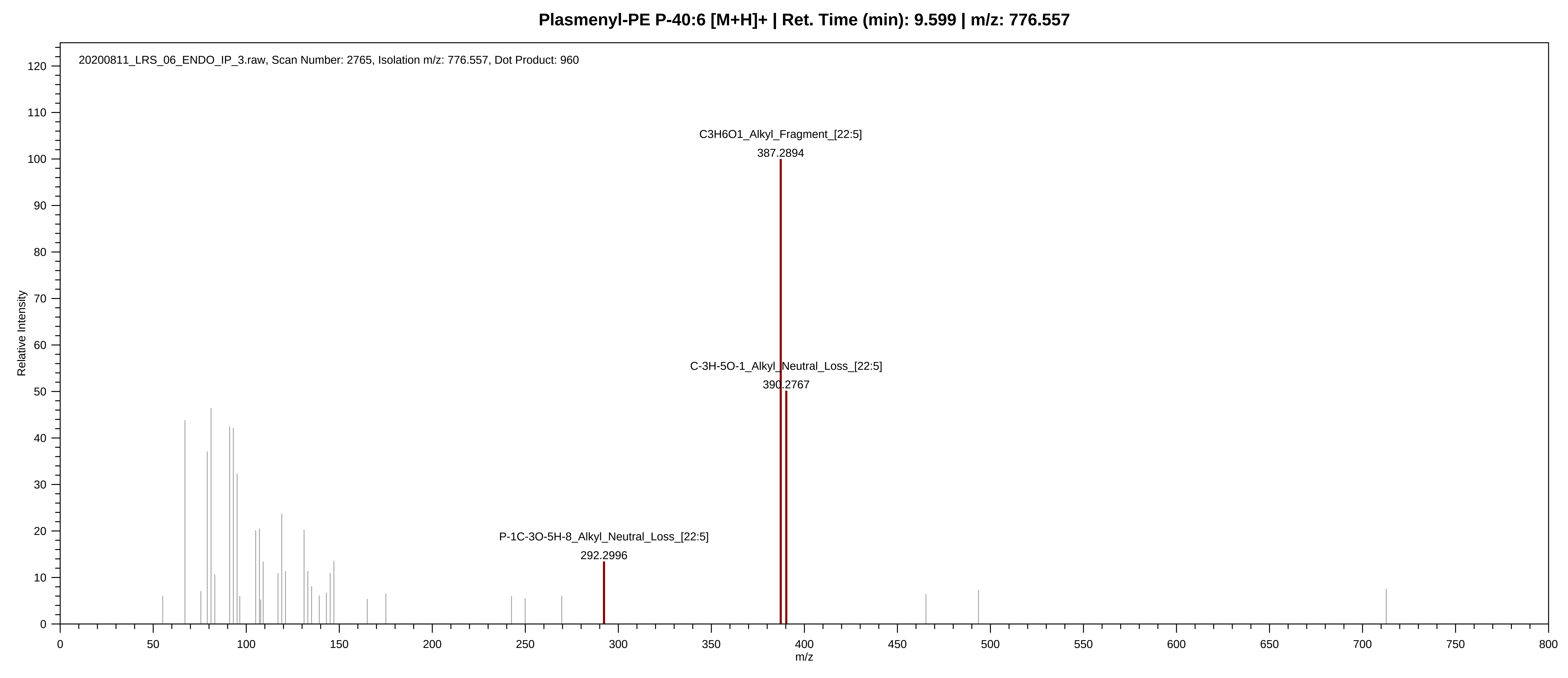
Plasmenyl-PE P-40:6 [M+H]+ | Ret. Time (min): 9.599 | m/z: 776.557
20200811_LRS_06_ENDO_IP_3.raw, Scan Number: 2765, Isolation m/z: 776.557, Dot Product: 960
C3H6O1_Alkyl_Fragment_[22:5]
387.2894
C-3H-5O-1_Alkyl_Neutral_Loss_[22:5]
390.2767
P-1C-3O-5H-8_Alkyl_Neutral_Loss_[22:5]
292.2996
Relative Intensity
m/z

Plasmenyl-PE P-40:6 [M+H]+ | Ret. Time (min): 9.625 | m/z: 776.557

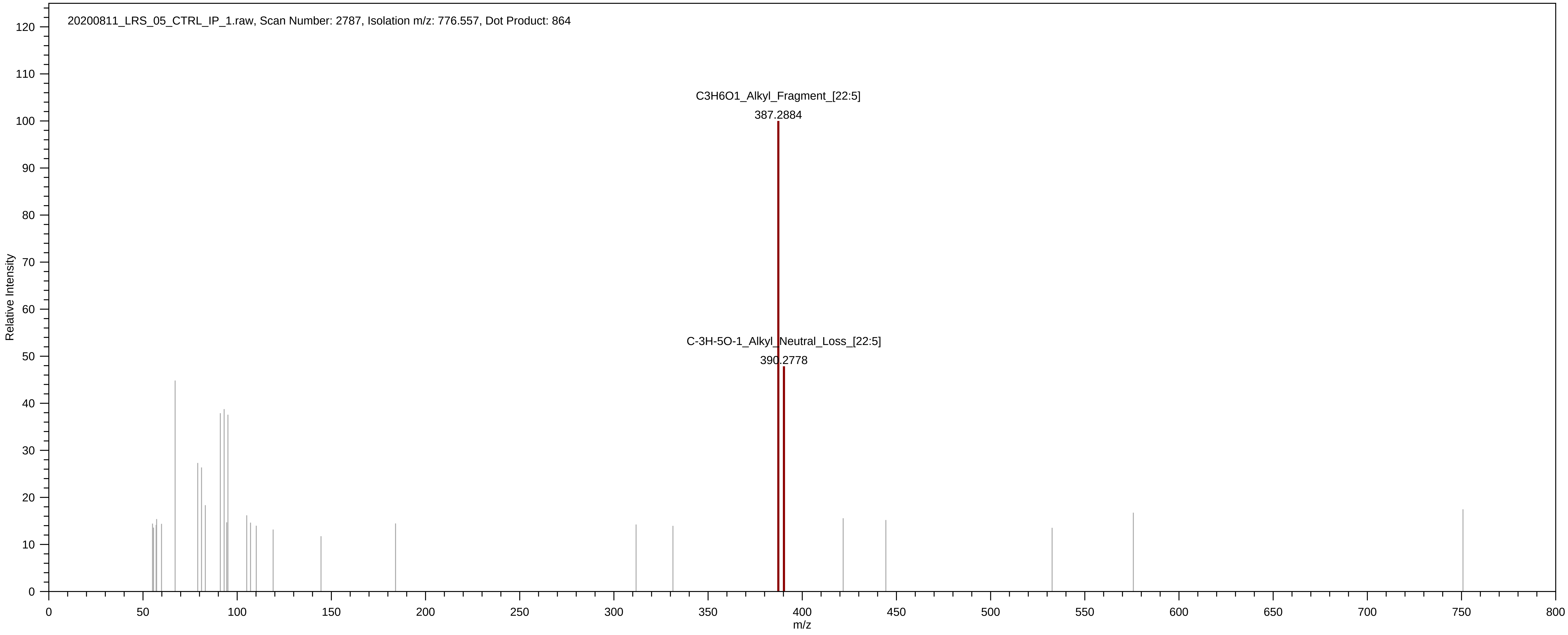

Plasmenyl-PE P-40:6 [M+H]+ | Ret. Time (min): 9.6 | m/z: 776.558

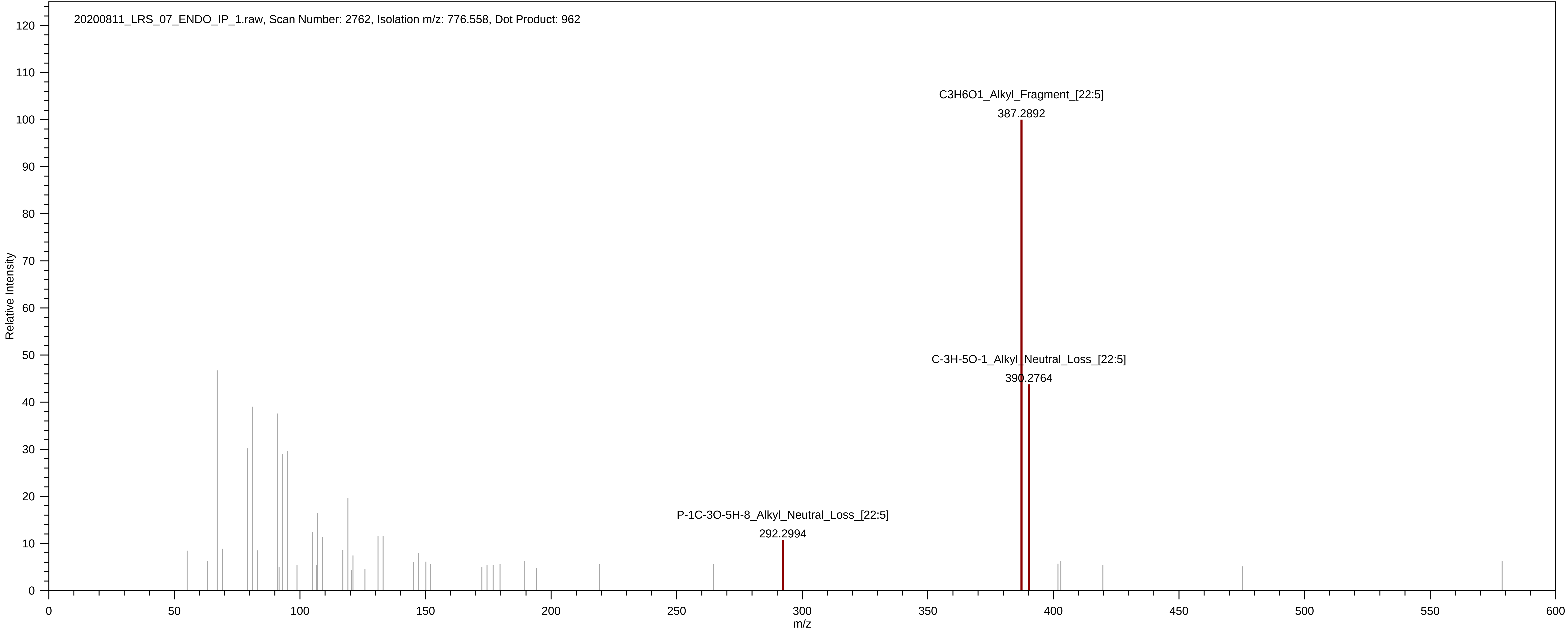

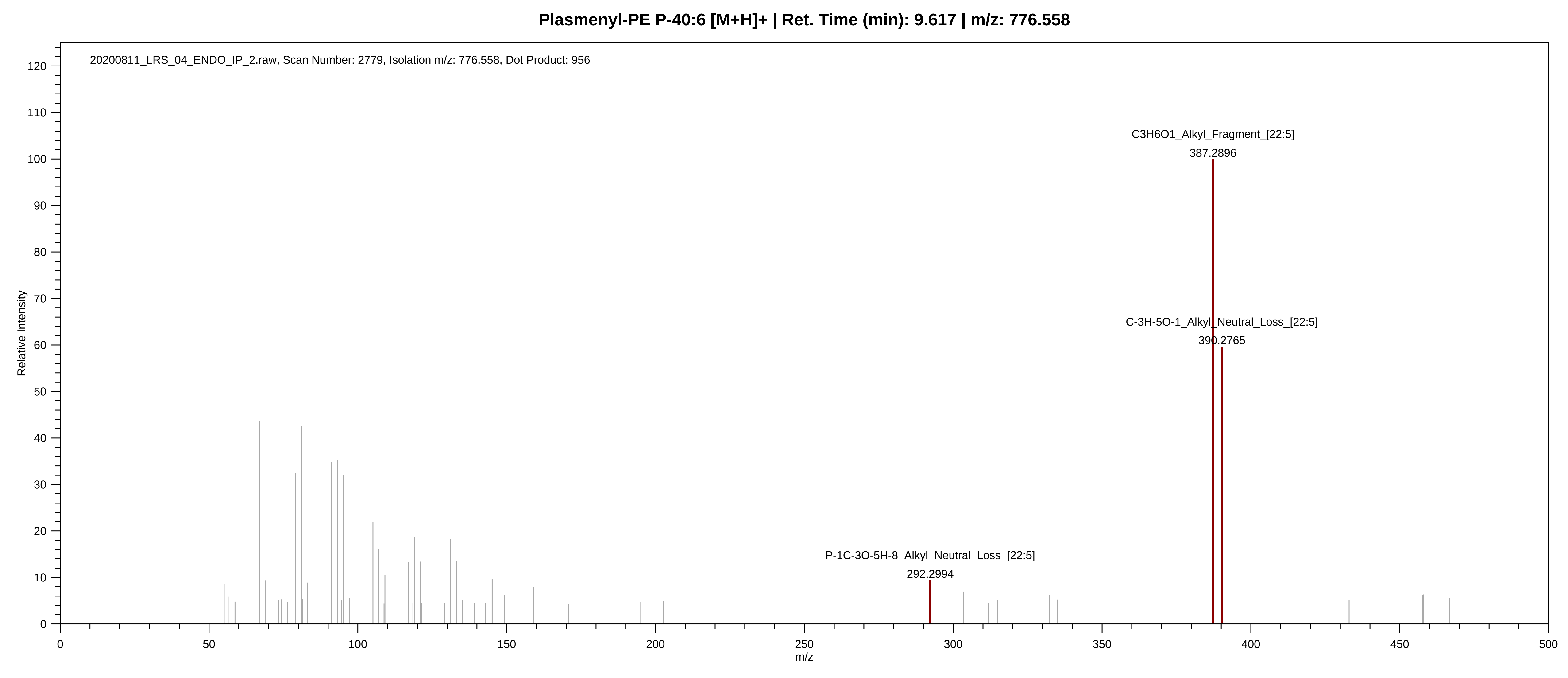

Plasmenyl-PE P-40:6 [M+H]+ | Ret. Time (min): 9.617 | m/z: 776.558
20200811_LRS_04_ENDO_IP_2.raw, Scan Number: 2779, Isolation m/z: 776.558, Dot Product: 956
C3H6O1_Alkyl_Fragment_[22:5]
387.2896
C-3H-5O-1_Alkyl_Neutral_Loss_[22:5]
390.2765
P-1C-3O-5H-8_Alkyl_Neutral_Loss_[22:5]
292.2994
Relative Intensity
m/z

Plasmenyl-PE P-40:6 [M+H]+ | Ret. Time (min): 10.152 | m/z: 776.558

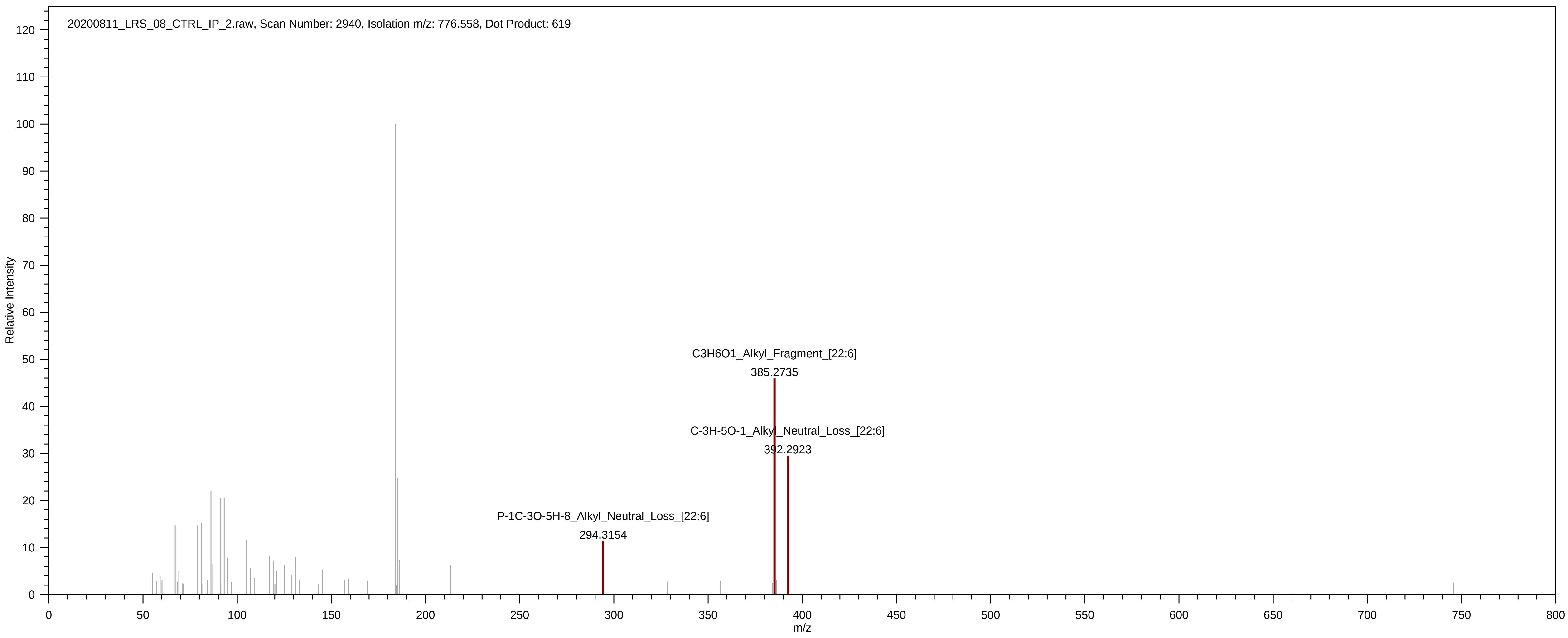

Plasmenyl-PE P-40:7 [M+H]+ | Ret. Time (min): 9.239 | m/z: 774.541

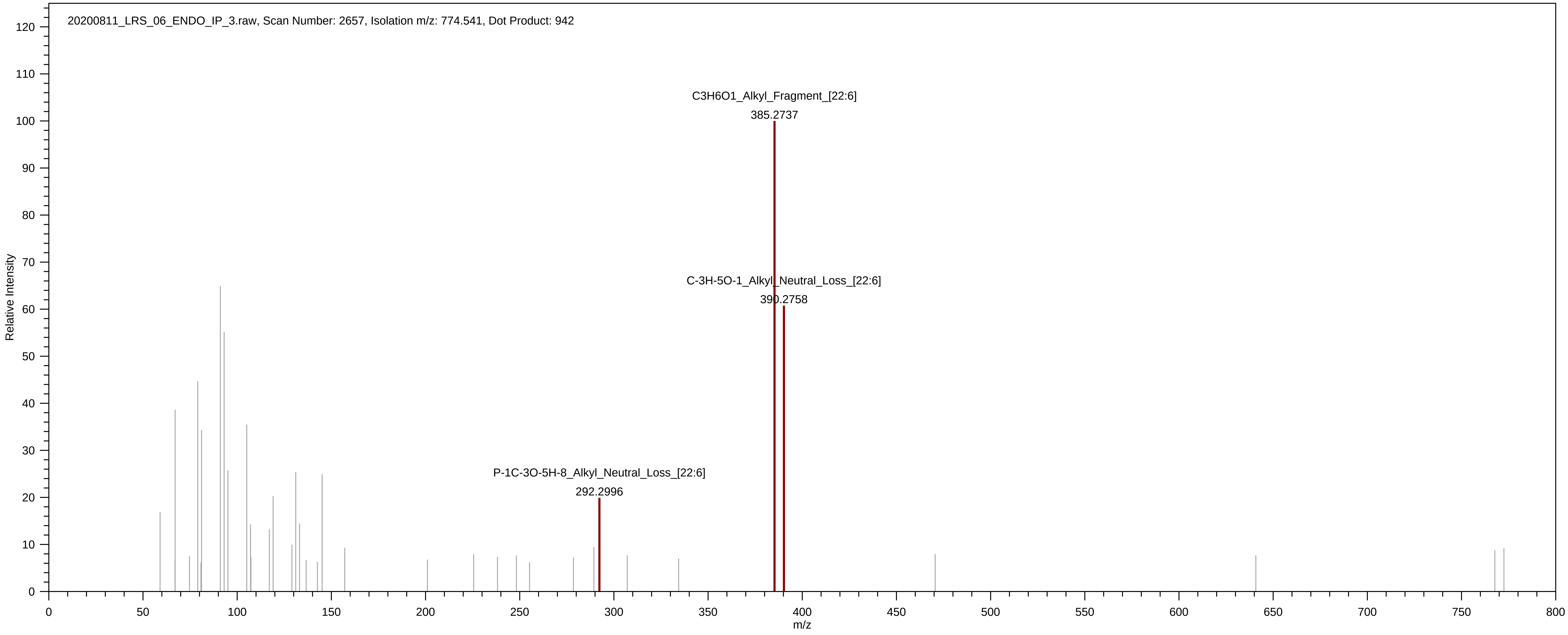

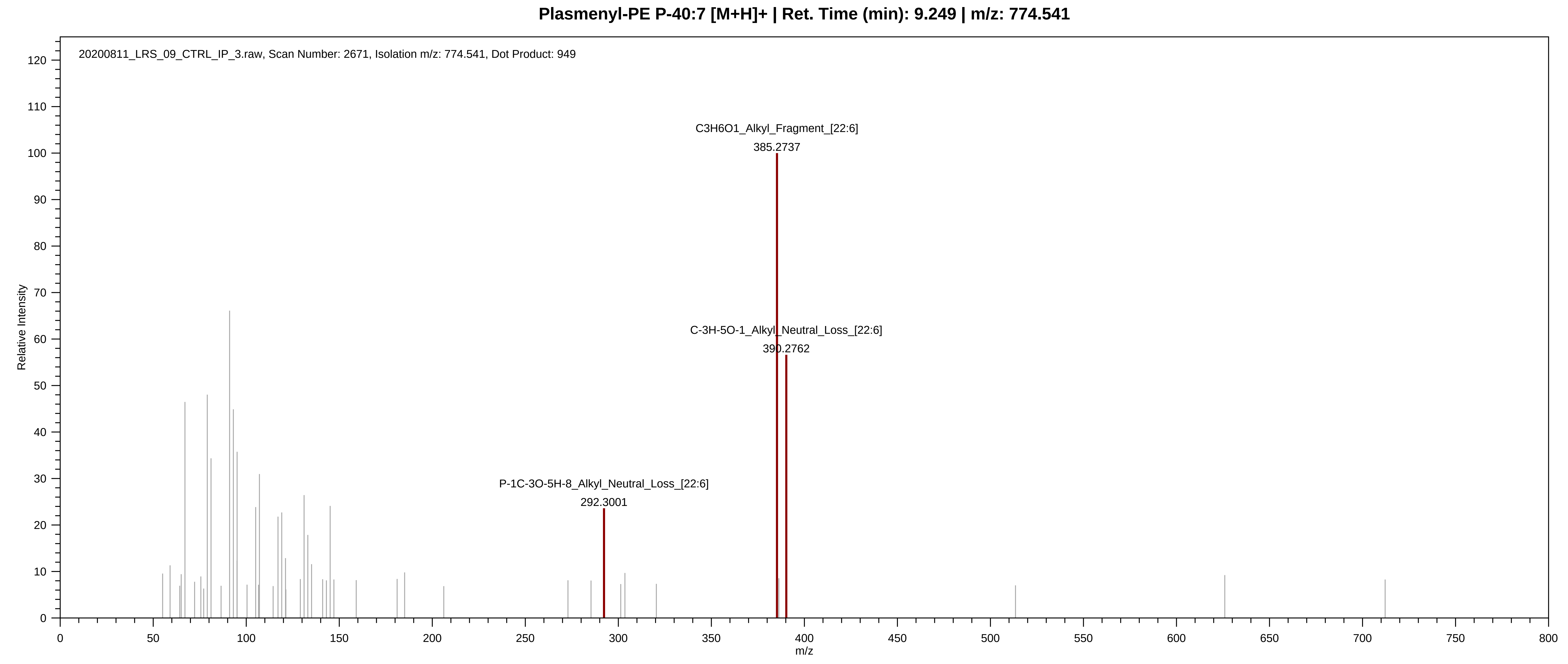

Plasmenyl-PE P-40:7 [M+H]+ | Ret. Time (min): 9.249 | m/z: 774.541
20200811_LRS_09_CTRL_IP_3.raw, Scan Number: 2671, Isolation m/z: 774.541, Dot Product: 949
C3H6O1_Alkyl_Fragment_[22:6]
385.2737
C-3H-5O-1_Alkyl_Neutral_Loss_[22:6]
390.2762
P-1C-3O-5H-8_Alkyl_Neutral_Loss_[22:6]
292.3001
Relative Intensity
m/z

Plasmenyl-PE P-40:7 [M+H]+ | Ret. Time (min): 9.241 | m/z: 774.542

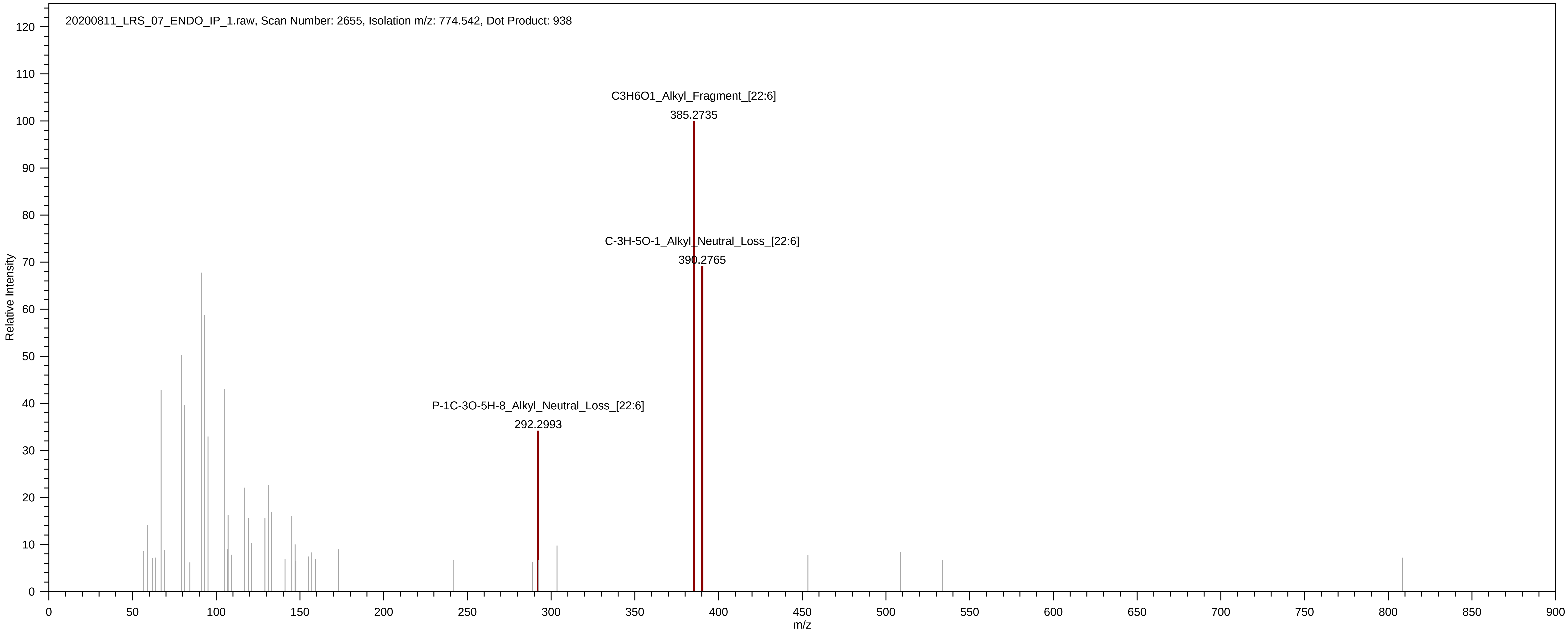

Plasmenyl-PE P-40:7 [M+H]+ | Ret. Time (min): 9.26 | m/z: 774.543

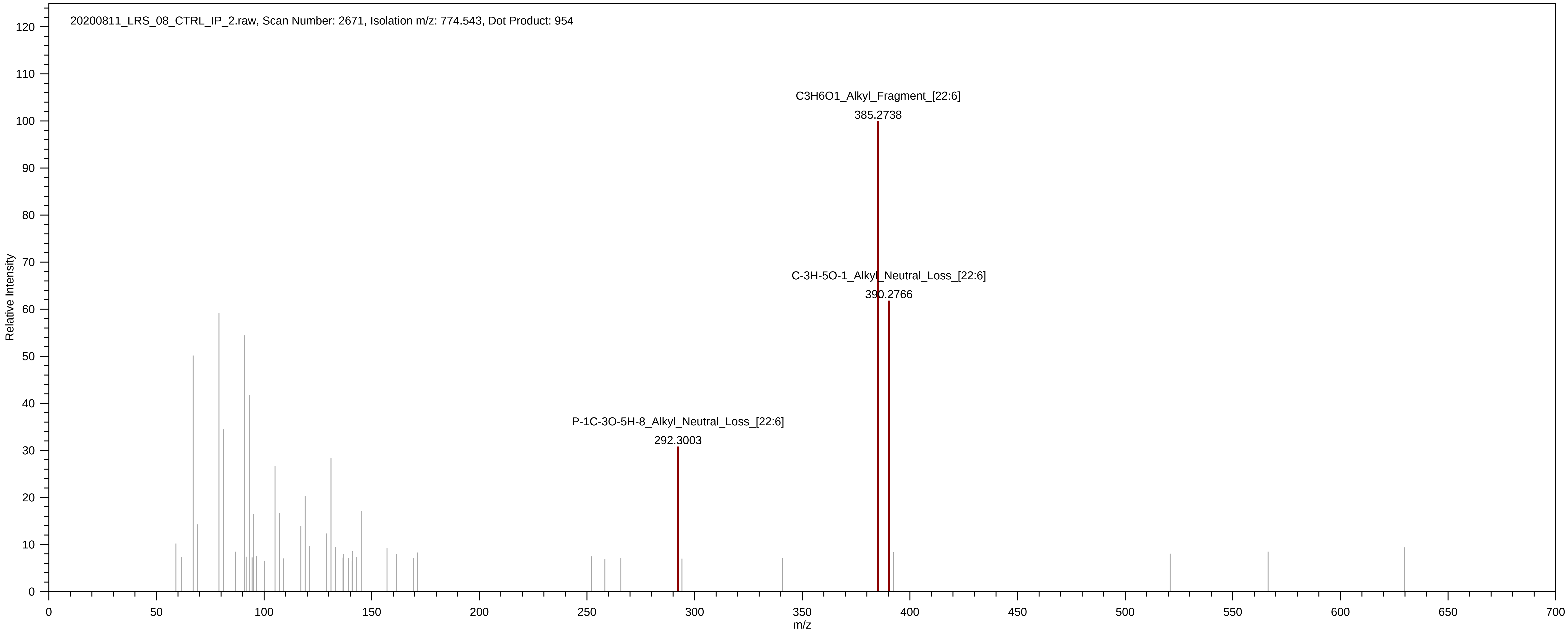

Plasmenyl-PE P-40:7 [M+H]+ | Ret. Time (min): 9.253 | m/z: 774.543

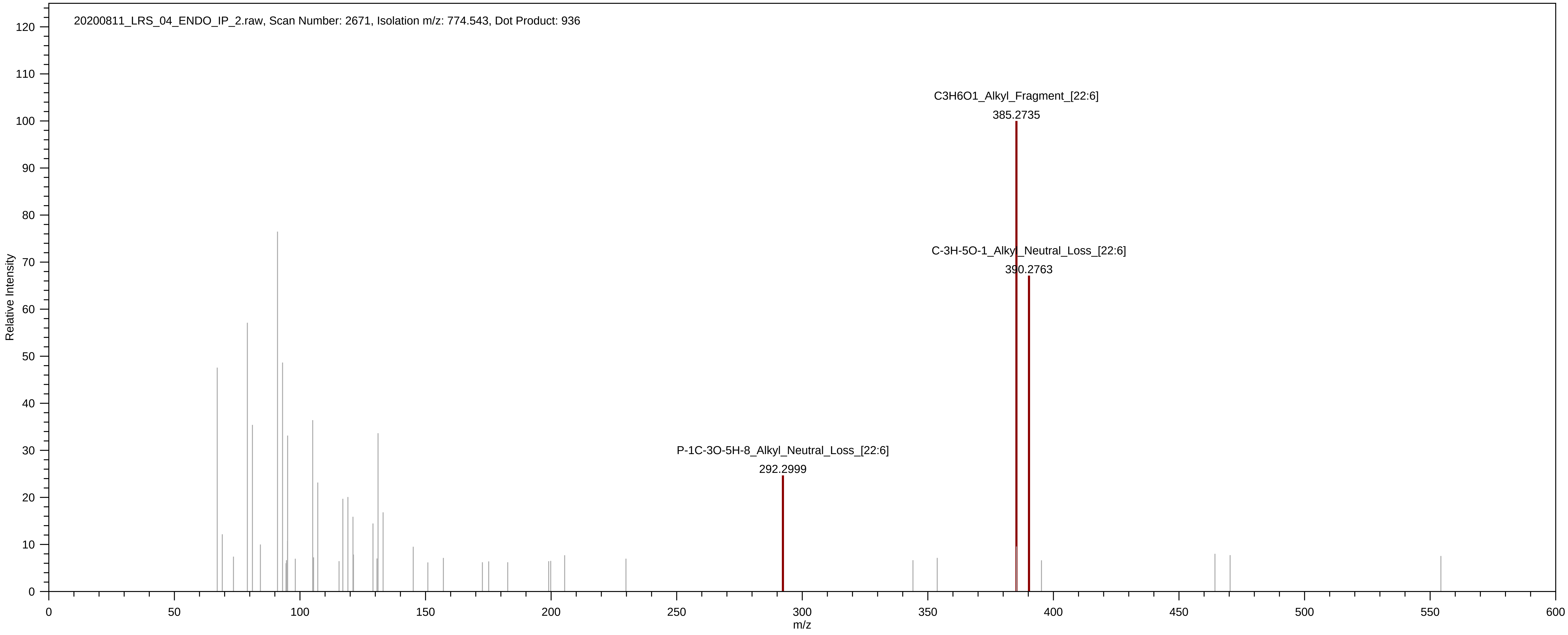

Plasmenyl-PE P-40:7 [M+H]+ | Ret. Time (min): 9.265 | m/z: 774.543

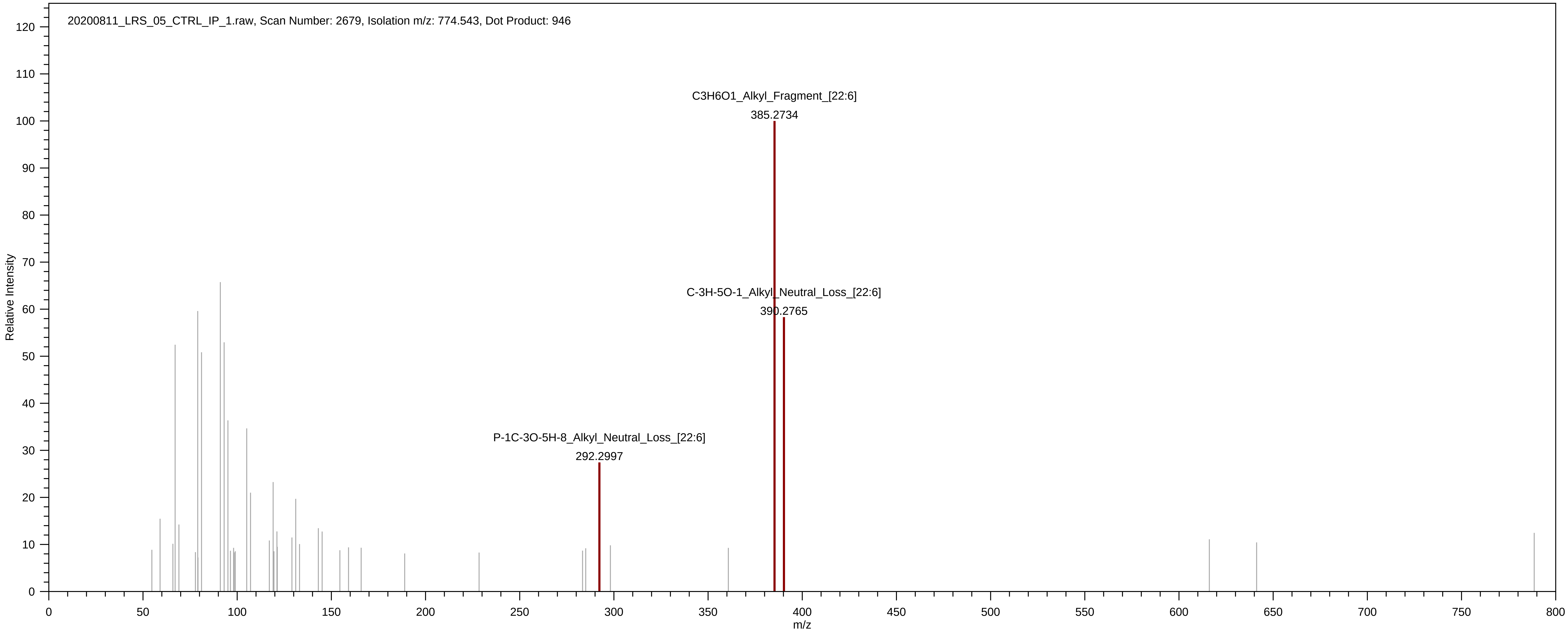

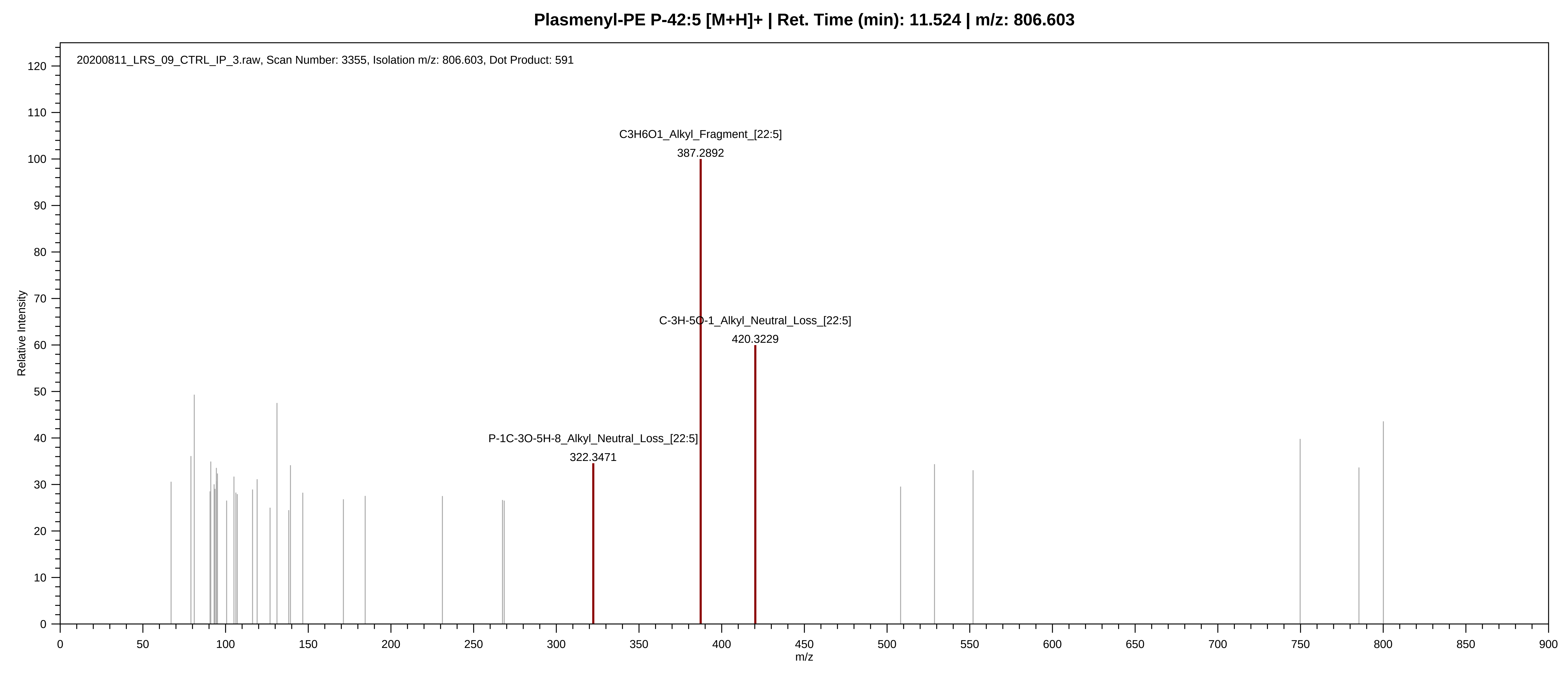

Plasmenyl-PE P-42:5 [M+H]+ | Ret. Time (min): 11.524 | m/z: 806.603
20200811_LRS_09_CTRL_IP_3.raw, Scan Number: 3355, Isolation m/z: 806.603, Dot Product: 591
C3H6O1_Alkyl_Fragment_[22:5]
387.2892
C-3H-5O-1_Alkyl_Neutral_Loss_[22:5]
420.3229
P-1C-3O-5H-8_Alkyl_Neutral_Loss_[22:5]
322.3471
Relative Intensity
m/z

Plasmenyl-PE P-42:5 [M+H]+ | Ret. Time (min): 11.53 | m/z: 806.605

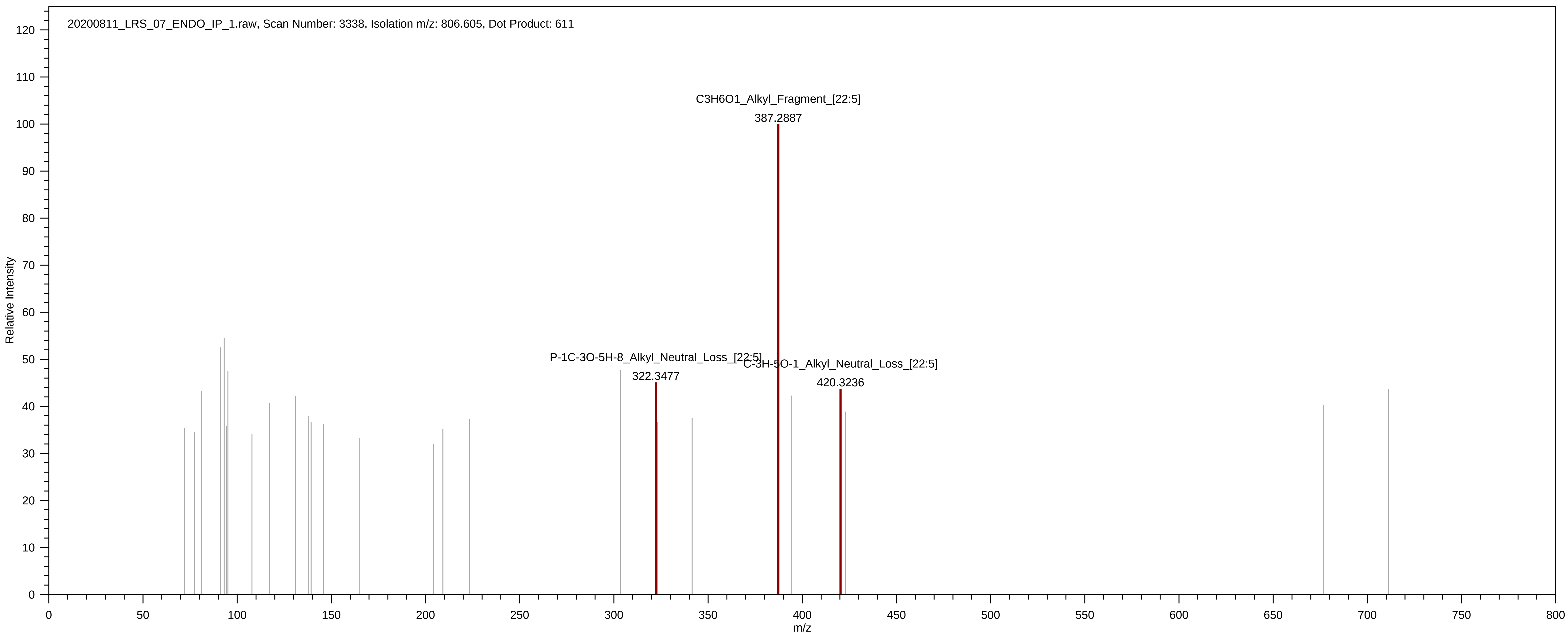

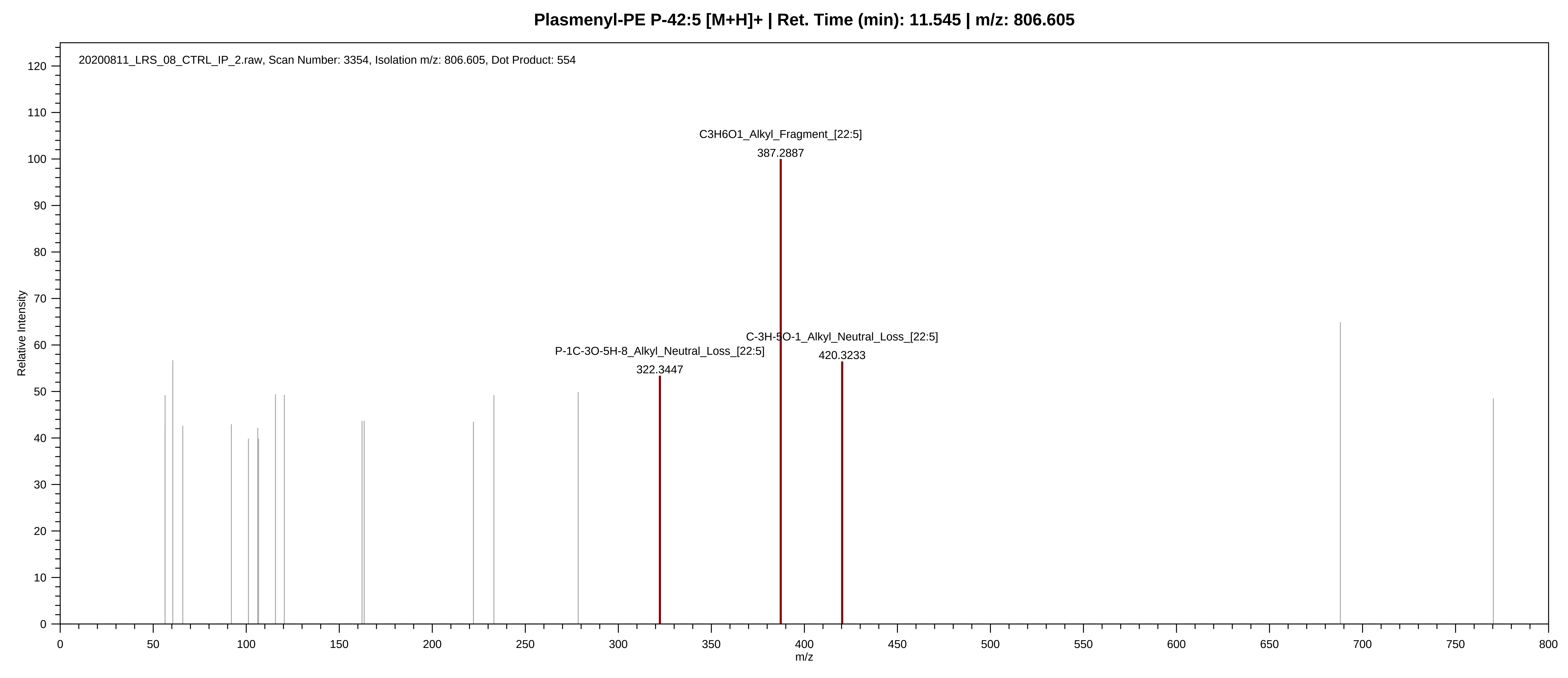
Plasmenyl-PE P-42:5 [M+H]+ | Ret. Time (min): 11.545 | m/z: 806.605
20200811_LRS_08_CTRL_IP_2.raw, Scan Number: 3354, Isolation m/z: 806.605, Dot Product: 554
C3H6O1_Alkyl_Fragment_[22:5]
387.2887
C-3H-5O-1_Alkyl_Neutral_Loss_[22:5]
420.3233
P-1C-3O-5H-8_Alkyl_Neutral_Loss_[22:5]
322.3447
Relative Intensity
m/z

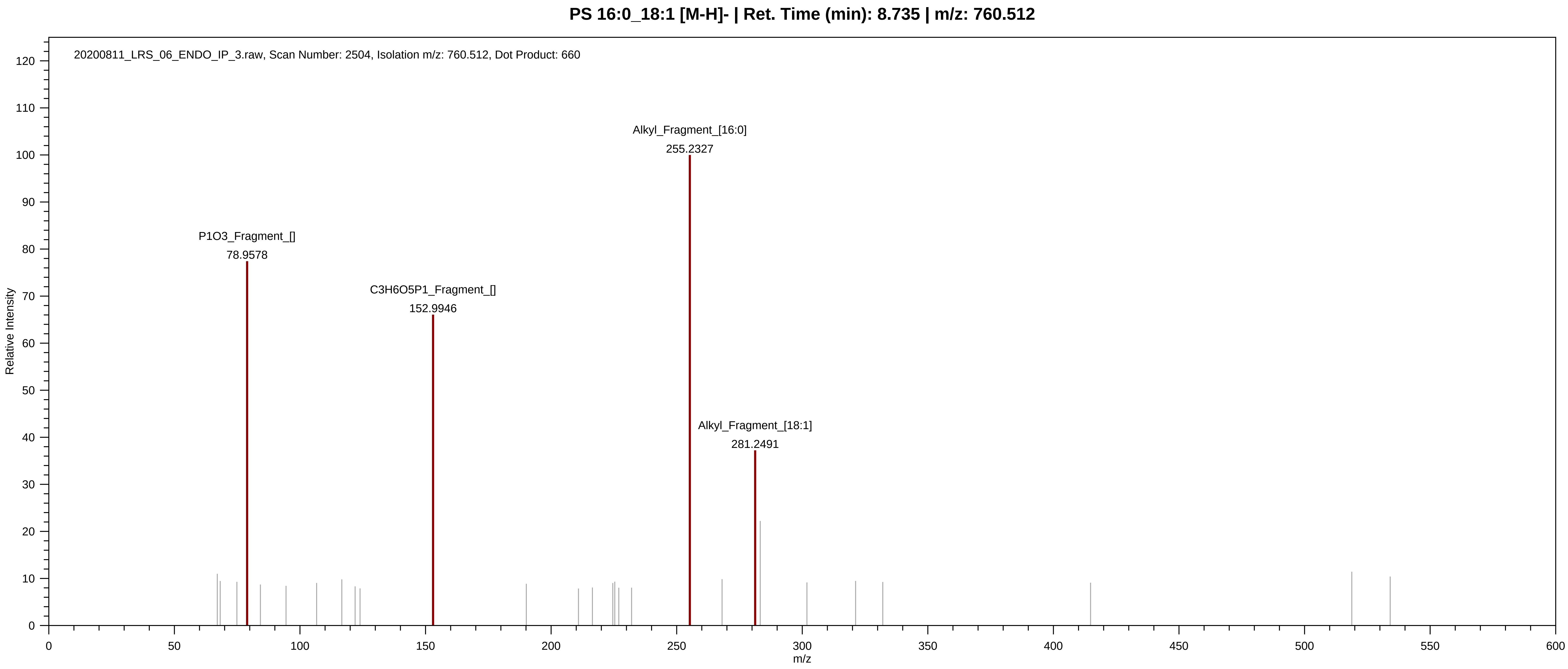
PS 16:0_18:1 [M-H]- | Ret. Time (min): 8.735 | m/z: 760.512
20200811_LRS_06_ENDO_IP_3.raw, Scan Number: 2504, Isolation m/z: 760.512, Dot Product: 660
P1O3_Fragment_[]
78.9578
C3H6O5P1_Fragment_[]
152.9946
Alkyl_Fragment_[16:0]
255.2327
Alkyl_Fragment_[18:1]
281.2491
Relative Intensity
m/z

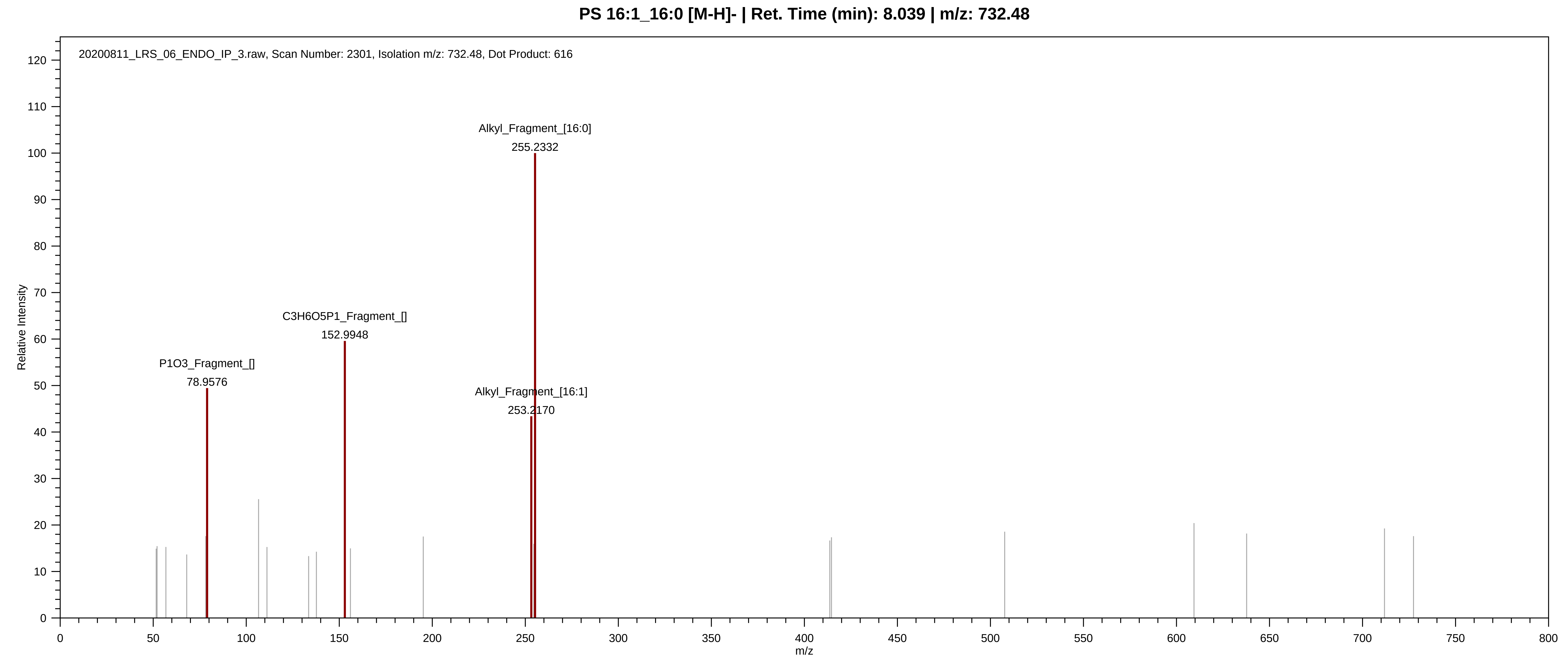

PS 16:1_16:0 [M-H]- | Ret. Time (min): 8.039 | m/z: 732.48
20200811_LRS_06_ENDO_IP_3.raw, Scan Number: 2301, Isolation m/z: 732.48, Dot Product: 616
Alkyl_Fragment_[16:0]
255.2332
C3H6O5P1_Fragment_[]
152.9948
P1O3_Fragment_[]
78.9576
Alkyl_Fragment_[16:1]
253.2170
Relative Intensity
m/z

PS 16:1_16:0 [M-H]- | Ret. Time (min): 8.054 | m/z: 732.481

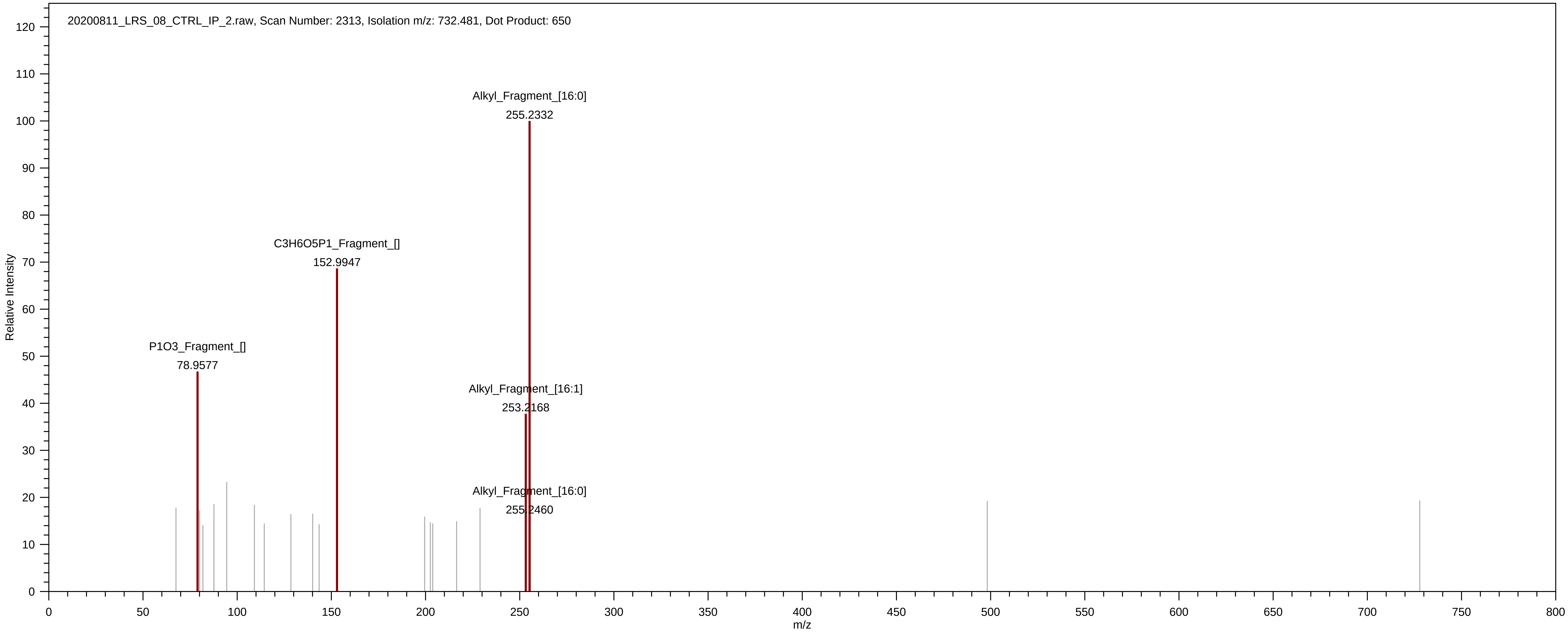

PS 16:1_16:0 [M-H]- | Ret. Time (min): 8.054 | m/z: 732.482

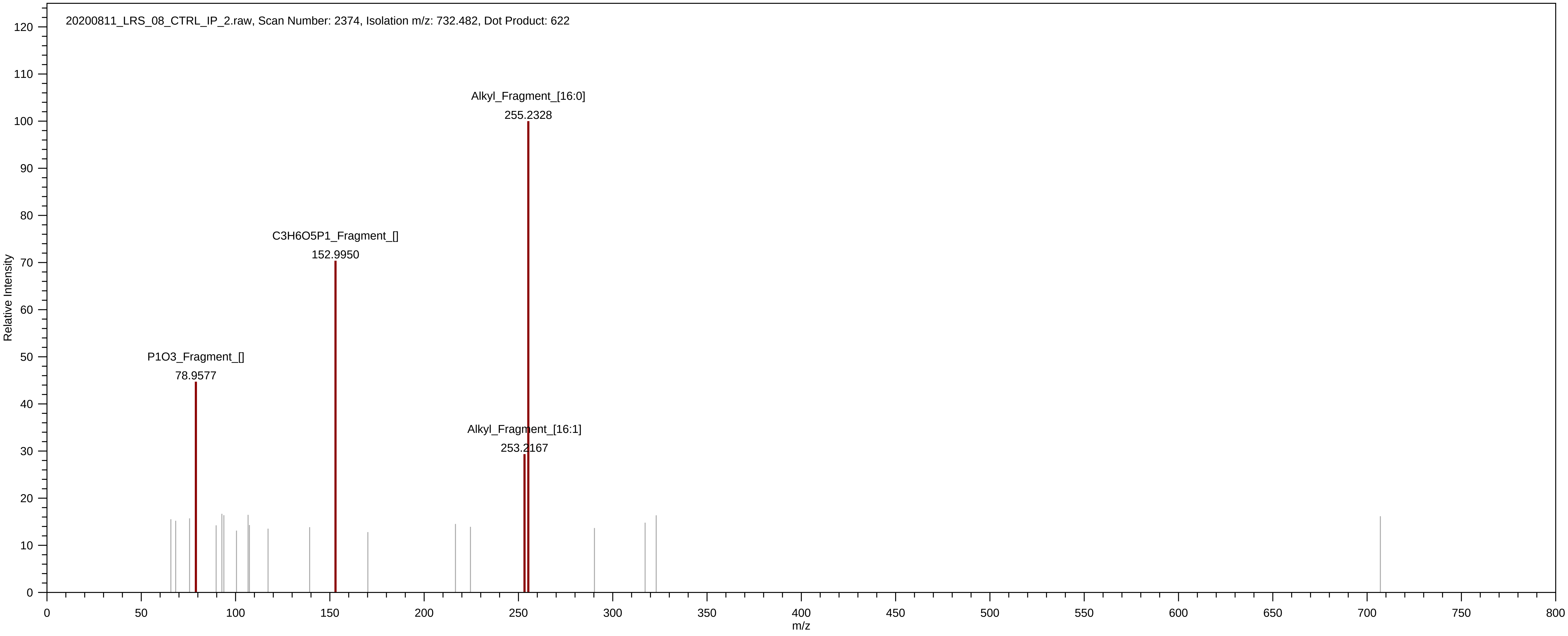

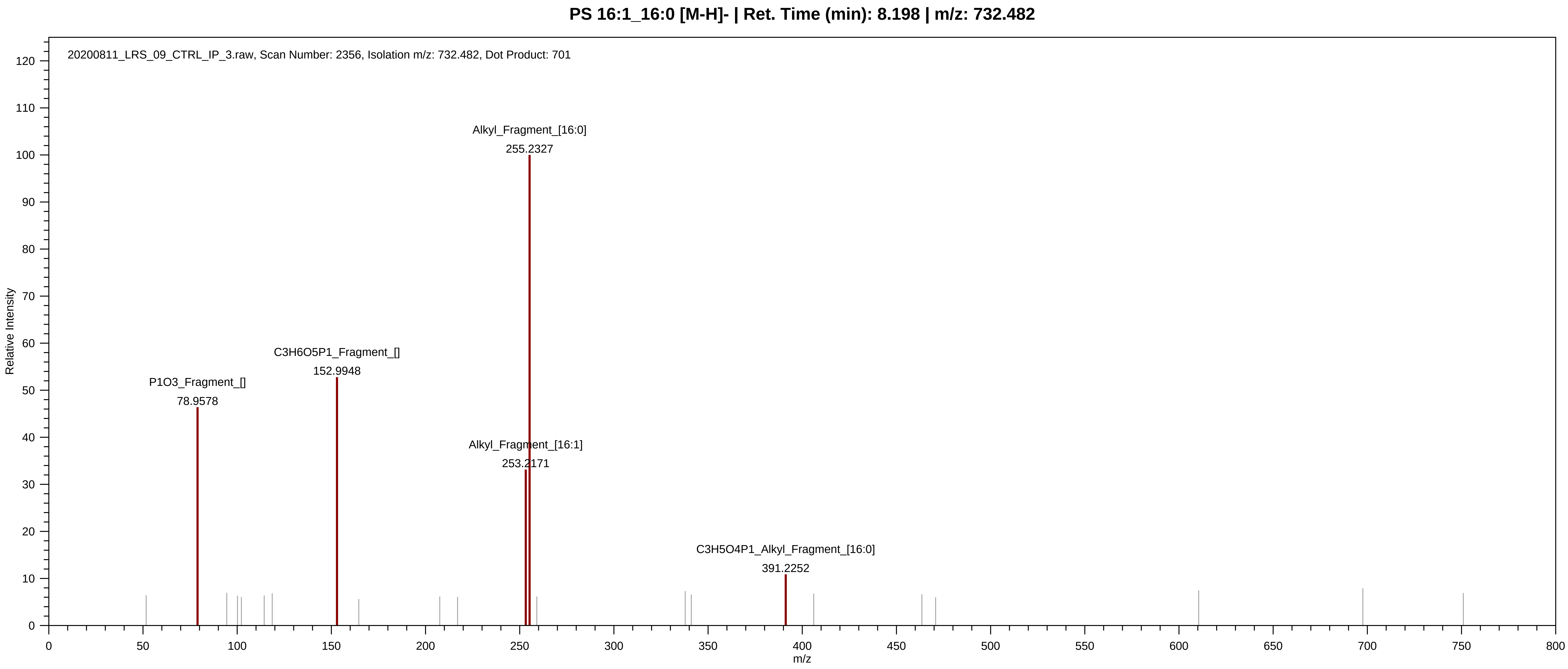

PS 16:1_16:0 [M-H]- | Ret. Time (min): 8.198 | m/z: 732.482
20200811_LRS_09_CTRL_IP_3.raw, Scan Number: 2356, Isolation m/z: 732.482, Dot Product: 701
Alkyl_Fragment_[16:0]
255.2327
C3H6O5P1_Fragment_[]
152.9948
P1O3_Fragment_[]
78.9578
Alkyl_Fragment_[16:1]
253.2171
C3H5O4P1_Alkyl_Fragment_[16:0]
391.2252
Relative Intensity
m/z

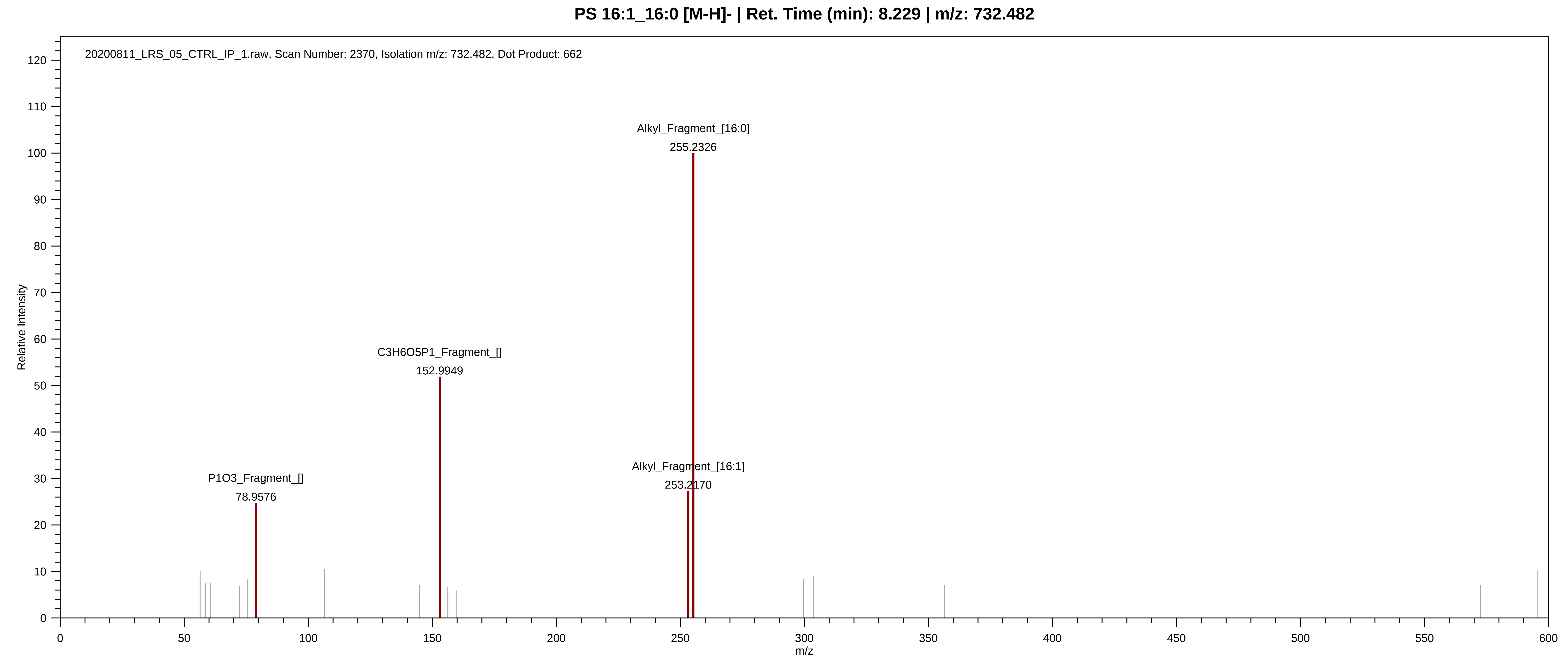

PS 16:1_16:0 [M-H]- | Ret. Time (min): 8.229 | m/z: 732.482
20200811_LRS_05_CTRL_IP_1.raw, Scan Number: 2370, Isolation m/z: 732.482, Dot Product: 662
Alkyl_Fragment_[16:0]
255.2326
C3H6O5P1_Fragment_[]
152.9949
Alkyl_Fragment_[16:1]
253.2170
P1O3_Fragment_[]
78.9576
Relative Intensity
m/z

PS 16:1_16:0 [M-H]- | Ret. Time (min): 8.234 | m/z: 732.482

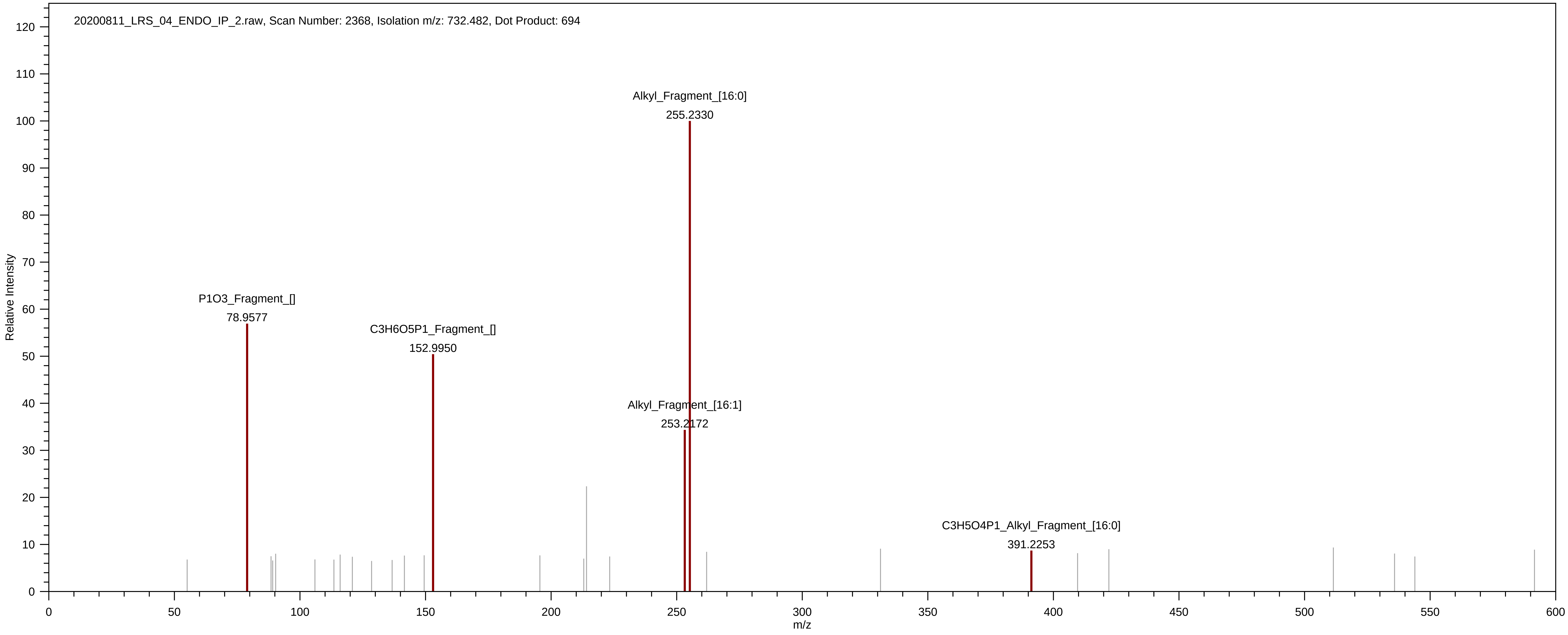

PS 16:1_16:0 [M-H]- | Ret. Time (min): 8.038 | m/z: 732.483

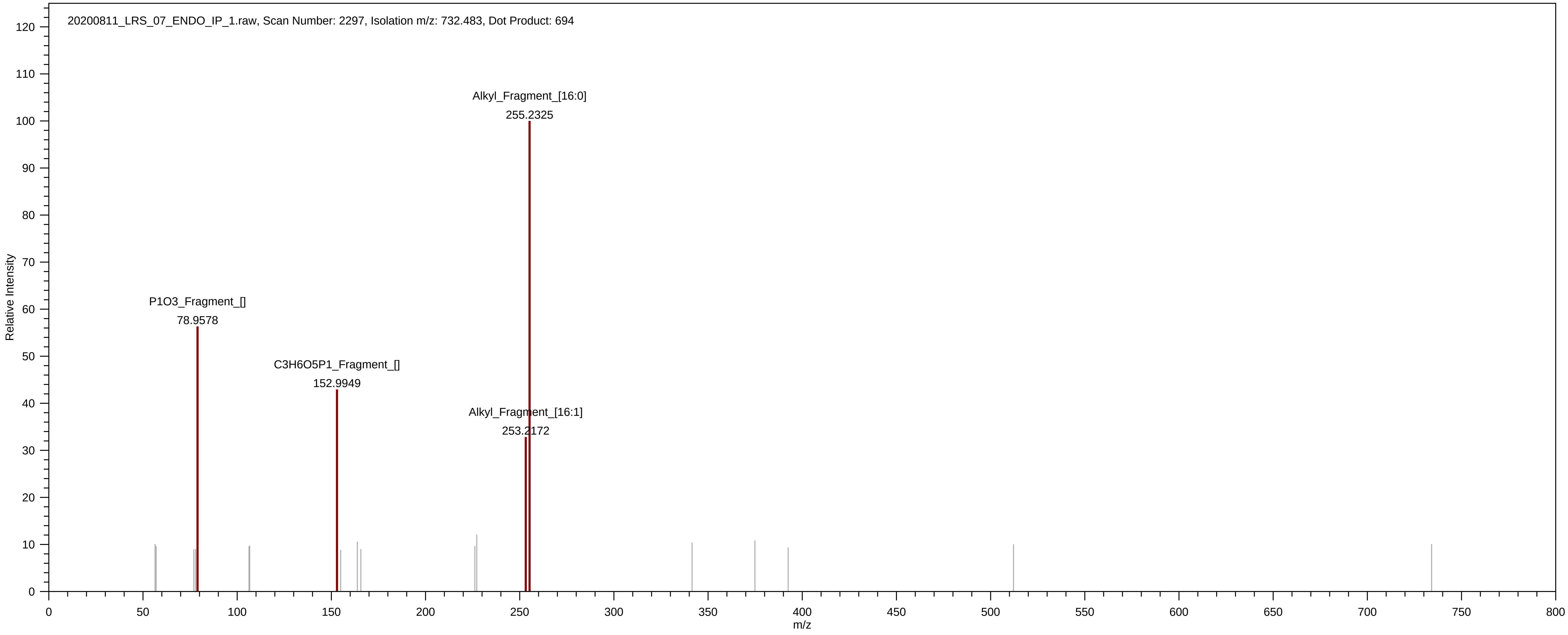

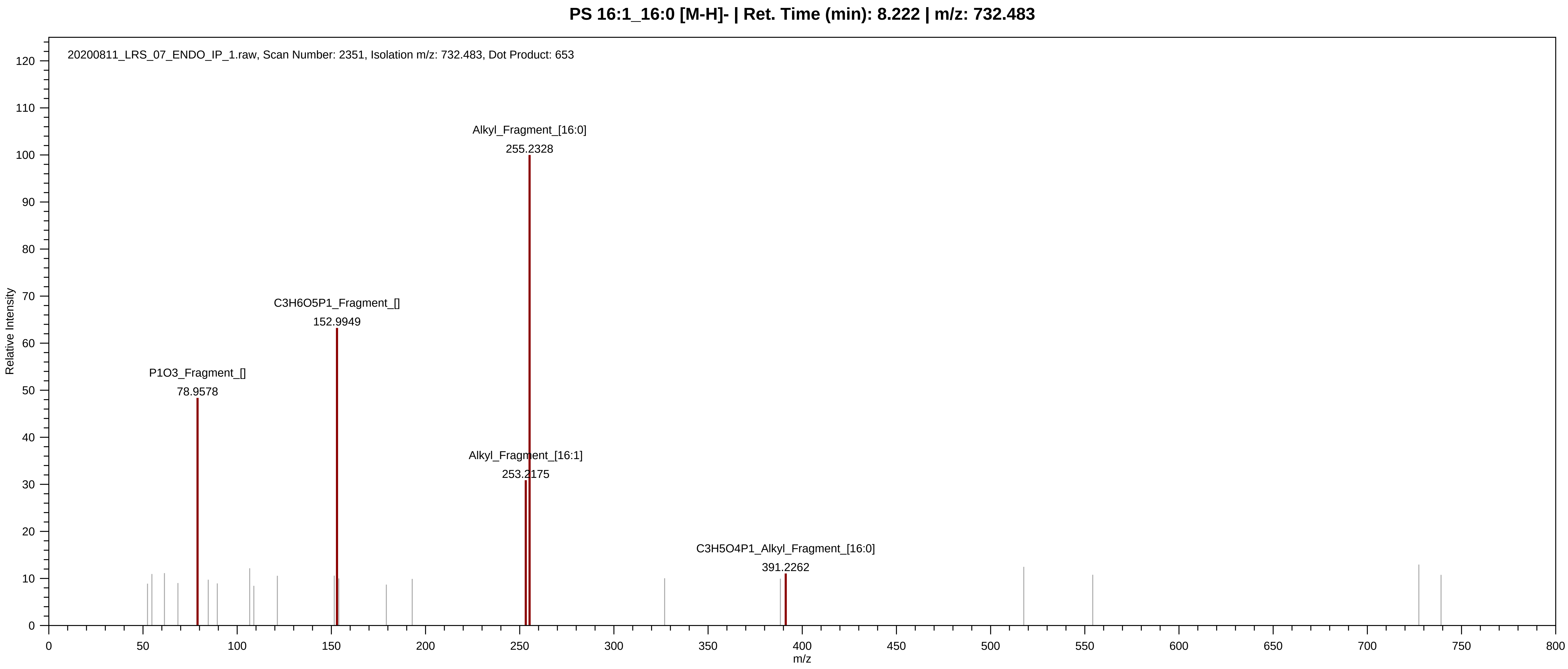
PS 16:1_16:0 [M-H]- | Ret. Time (min): 8.222 | m/z: 732.483
20200811_LRS_07_ENDO_IP_1.raw, Scan Number: 2351, Isolation m/z: 732.483, Dot Product: 653
Alkyl_Fragment_[16:0]
255.2328
C3H6O5P1_Fragment_[]
152.9949
P1O3_Fragment_[]
78.9578
Alkyl_Fragment_[16:1]
253.2175
C3H5O4P1_Alkyl_Fragment_[16:0]
391.2262
Relative Intensity
m/z

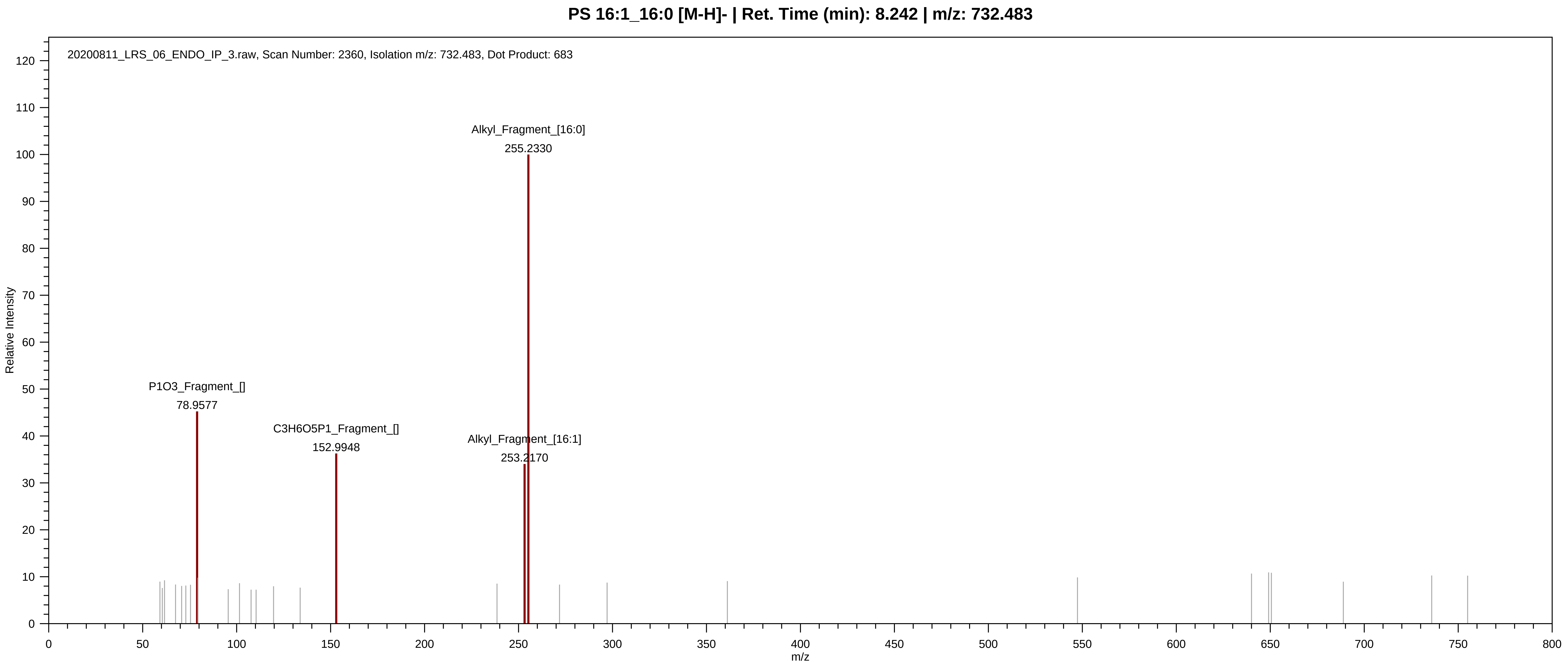
PS 16:1_16:0 [M-H]- | Ret. Time (min): 8.242 | m/z: 732.483
20200811_LRS_06_ENDO_IP_3.raw, Scan Number: 2360, Isolation m/z: 732.483, Dot Product: 683
Alkyl_Fragment_[16:0]
255.2330
P1O3_Fragment_[]
78.9577
C3H6O5P1_Fragment_[]
152.9948
Alkyl_Fragment_[16:1]
253.2170
Relative Intensity
m/z
0
10
20
30
40
50
60
70
80
90
100
110
120
0
50
100
150
200
250
300
350
400
450
500
550
600
650
700
750
800

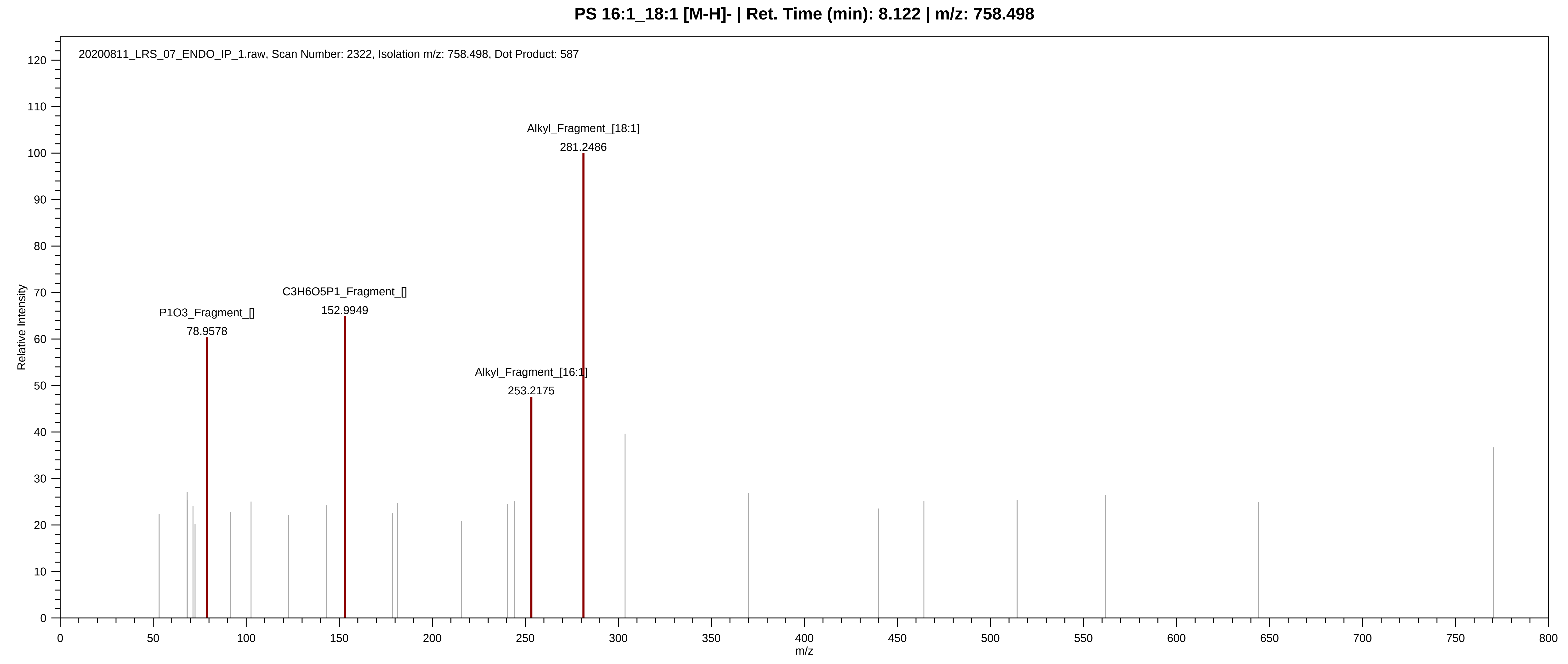

PS 16:1_18:1 [M-H]- | Ret. Time (min): 8.122 | m/z: 758.498
20200811_LRS_07_ENDO_IP_1.raw, Scan Number: 2322, Isolation m/z: 758.498, Dot Product: 587
Alkyl_Fragment_[18:1]
281.2486
C3H6O5P1_Fragment_[]
152.9949
P1O3_Fragment_[]
78.9578
Alkyl_Fragment_[16:1]
253.2175
Relative Intensity
m/z

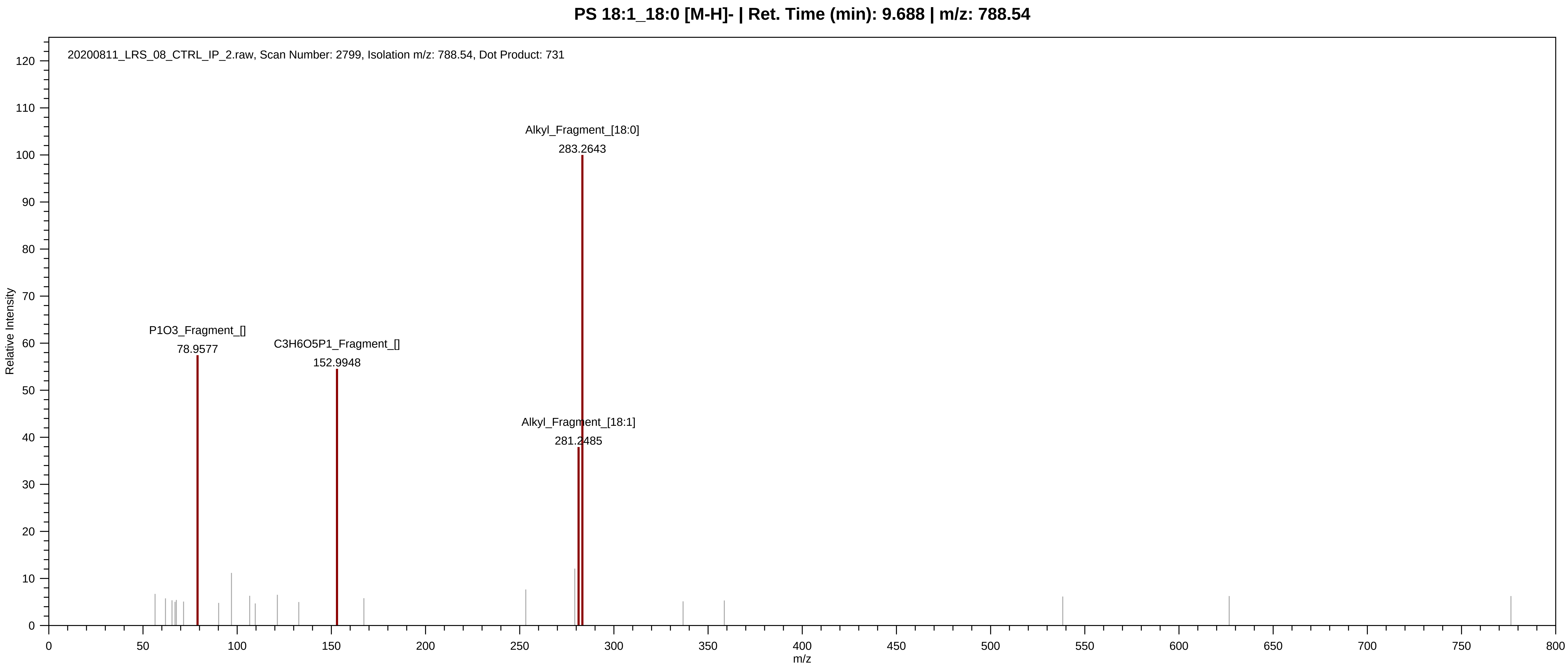
PS 18:1_18:0 [M-H]- | Ret. Time (min): 9.688 | m/z: 788.54
20200811_LRS_08_CTRL_IP_2.raw, Scan Number: 2799, Isolation m/z: 788.54, Dot Product: 731
Alkyl_Fragment_[18:0]
283.2643
P1O3_Fragment_[]
78.9577
C3H6O5P1_Fragment_[]
152.9948
Alkyl_Fragment_[18:1]
281.2485
Relative Intensity
m/z

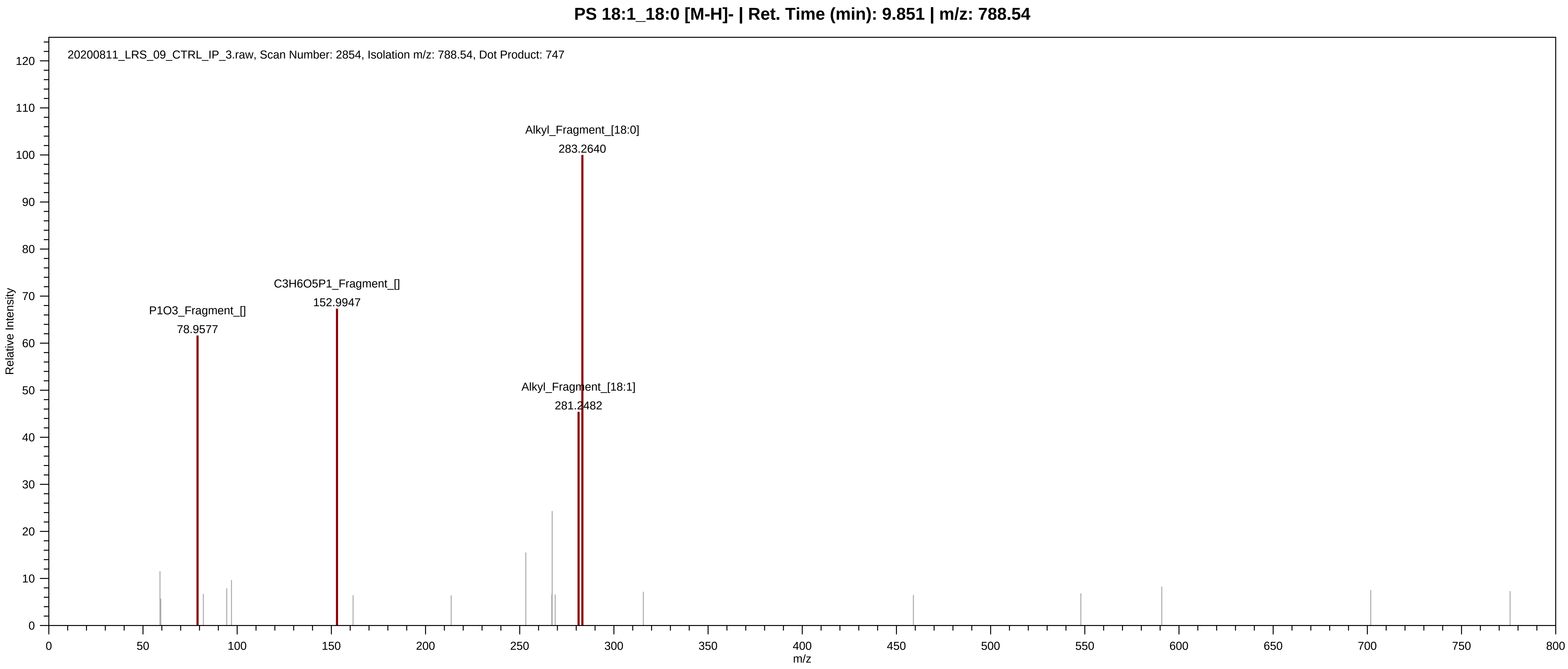
PS 18:1_18:0 [M-H]- | Ret. Time (min): 9.851 | m/z: 788.54
20200811_LRS_09_CTRL_IP_3.raw, Scan Number: 2854, Isolation m/z: 788.54, Dot Product: 747
Alkyl_Fragment_[18:0]
283.2640
C3H6O5P1_Fragment_[]
152.9947
P1O3_Fragment_[]
78.9577
Alkyl_Fragment_[18:1]
281.2482
Relative Intensity
m/z
0
10
20
30
40
50
60
70
80
90
100
110
120
0
50
100
150
200
250
300
350
400
450
500
550
600
650
700
750
800

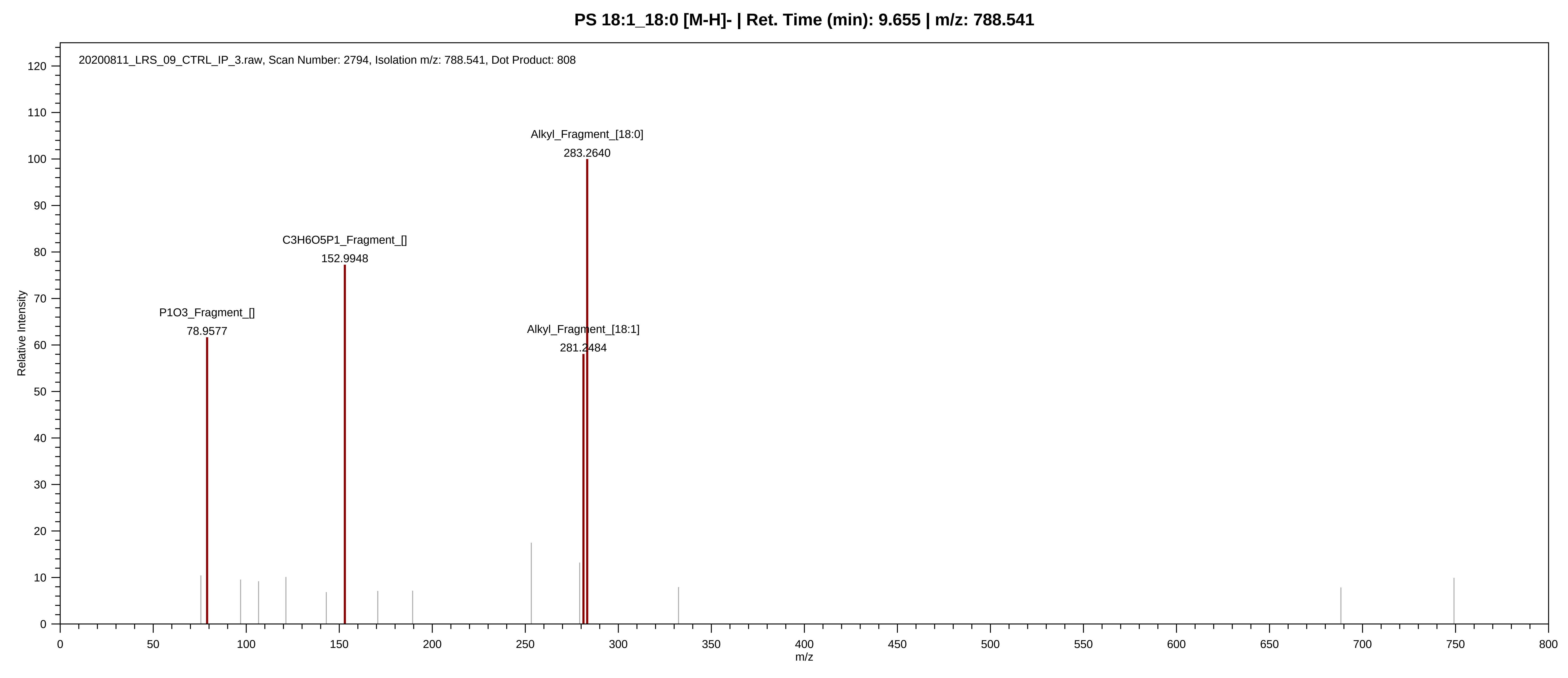
PS 18:1_18:0 [M-H]- | Ret. Time (min): 9.655 | m/z: 788.541
20200811_LRS_09_CTRL_IP_3.raw, Scan Number: 2794, Isolation m/z: 788.541, Dot Product: 808
Alkyl_Fragment_[18:0]
283.2640
C3H6O5P1_Fragment_[]
152.9948
P1O3_Fragment_[]
78.9577
Alkyl_Fragment_[18:1]
281.2484
Relative Intensity
m/z

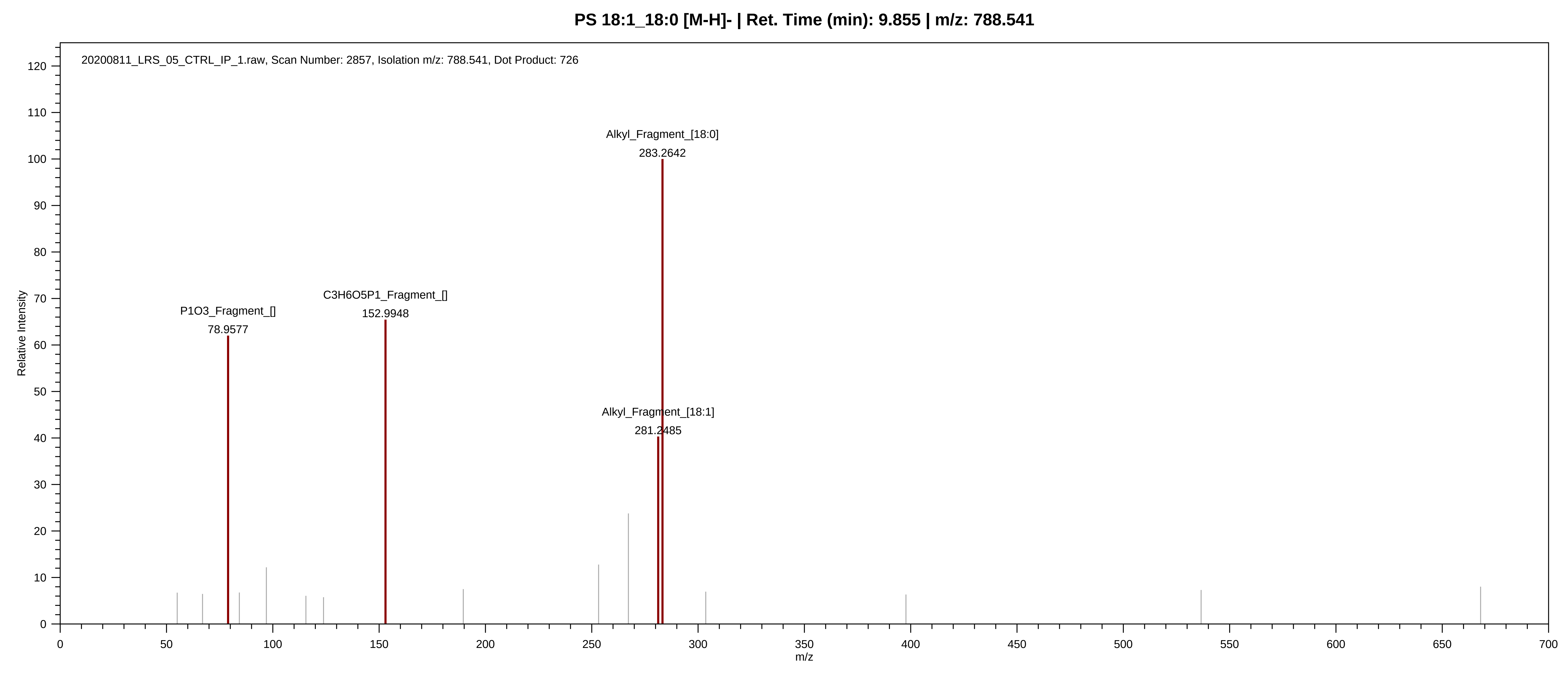
PS 18:1_18:0 [M-H]- | Ret. Time (min): 9.855 | m/z: 788.541
20200811_LRS_05_CTRL_IP_1.raw, Scan Number: 2857, Isolation m/z: 788.541, Dot Product: 726
Alkyl_Fragment_[18:0]
283.2642
C3H6O5P1_Fragment_[]
152.9948
P1O3_Fragment_[]
78.9577
Alkyl_Fragment_[18:1]
281.2485
Relative Intensity
m/z

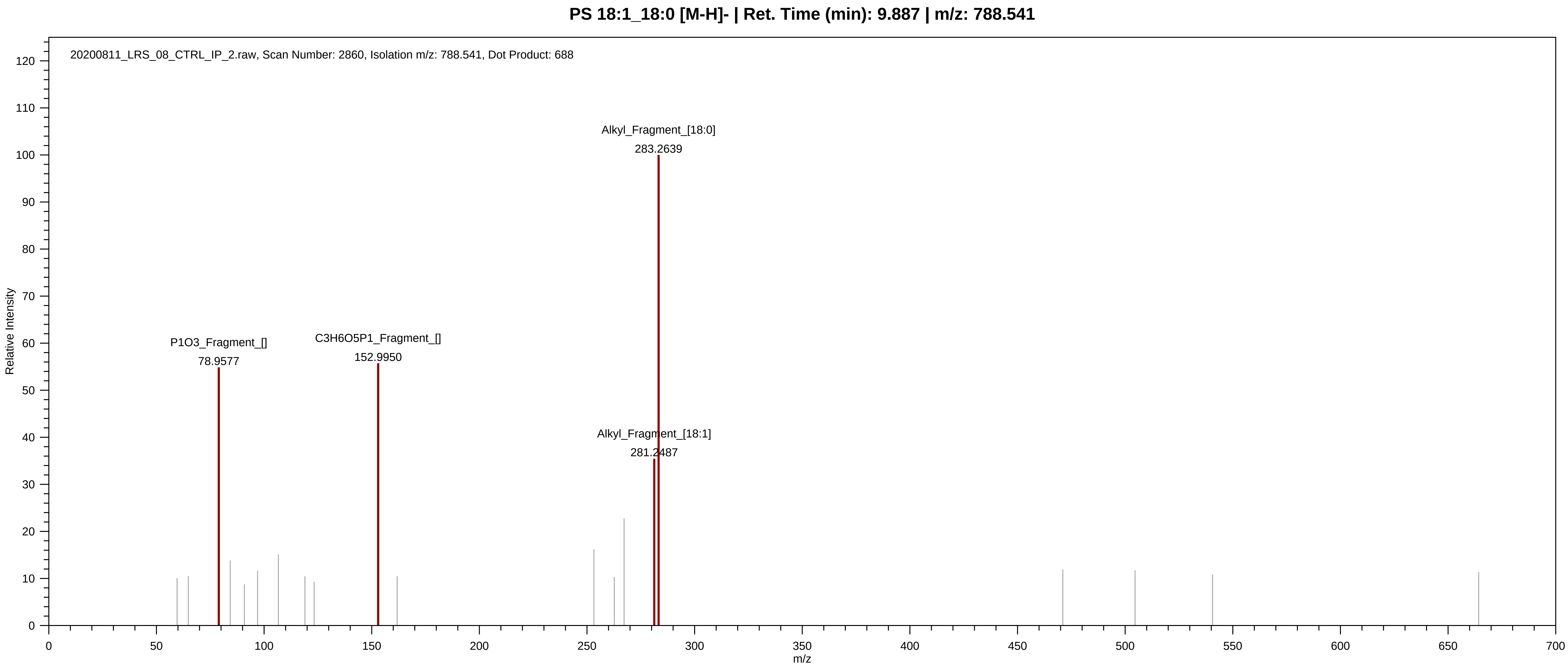
PS 18:1_18:0 [M-H]- | Ret. Time (min): 9.887 | m/z: 788.541
20200811_LRS_08_CTRL_IP_2.raw, Scan Number: 2860, Isolation m/z: 788.541, Dot Product: 688
Alkyl_Fragment_[18:0]
283.2639
P1O3_Fragment_[]
78.9577
C3H6O5P1_Fragment_[]
152.9950
Alkyl_Fragment_[18:1]
281.2487
Relative Intensity
m/z

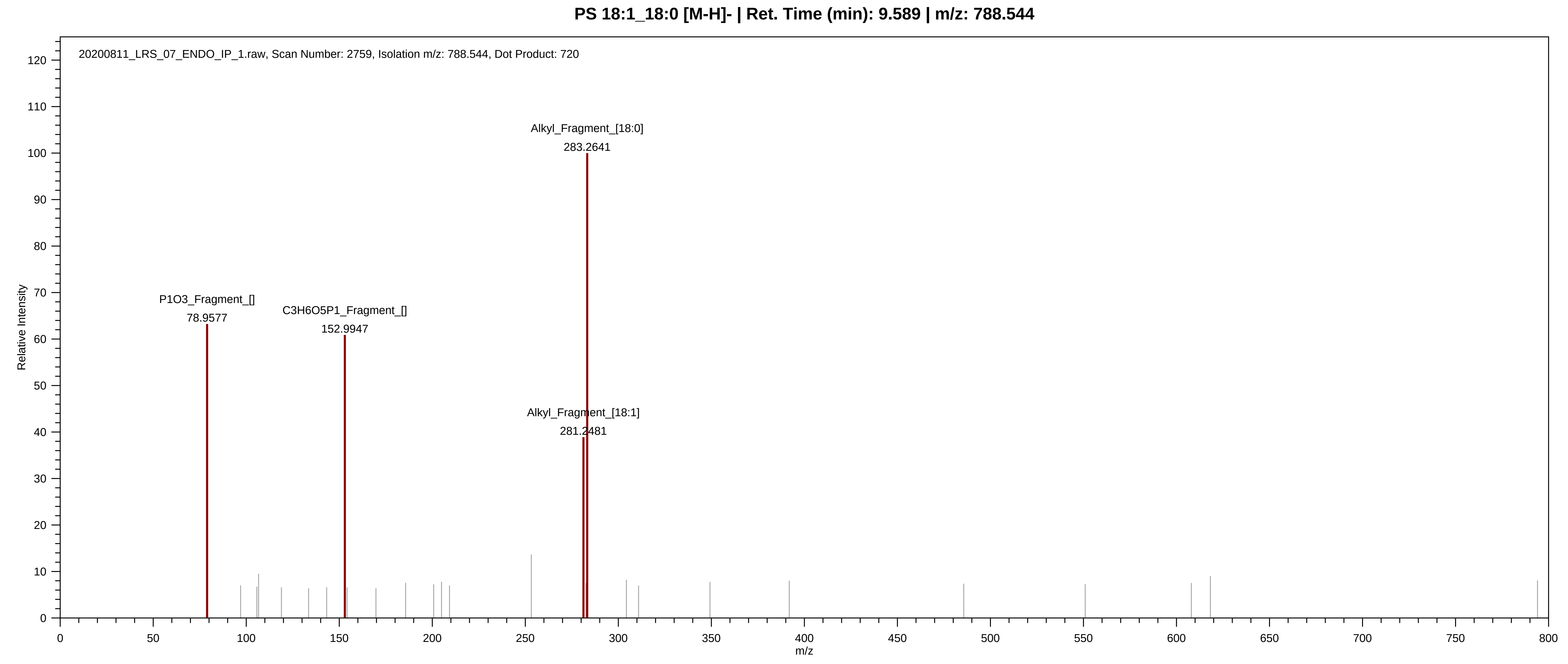

PS 18:1_18:0 [M-H]- | Ret. Time (min): 9.589 | m/z: 788.544
20200811_LRS_07_ENDO_IP_1.raw, Scan Number: 2759, Isolation m/z: 788.544, Dot Product: 720
Alkyl_Fragment_[18:0]
283.2641
P1O3_Fragment_[]
78.9577
C3H6O5P1_Fragment_[]
152.9947
Alkyl_Fragment_[18:1]
281.2481
Relative Intensity
m/z

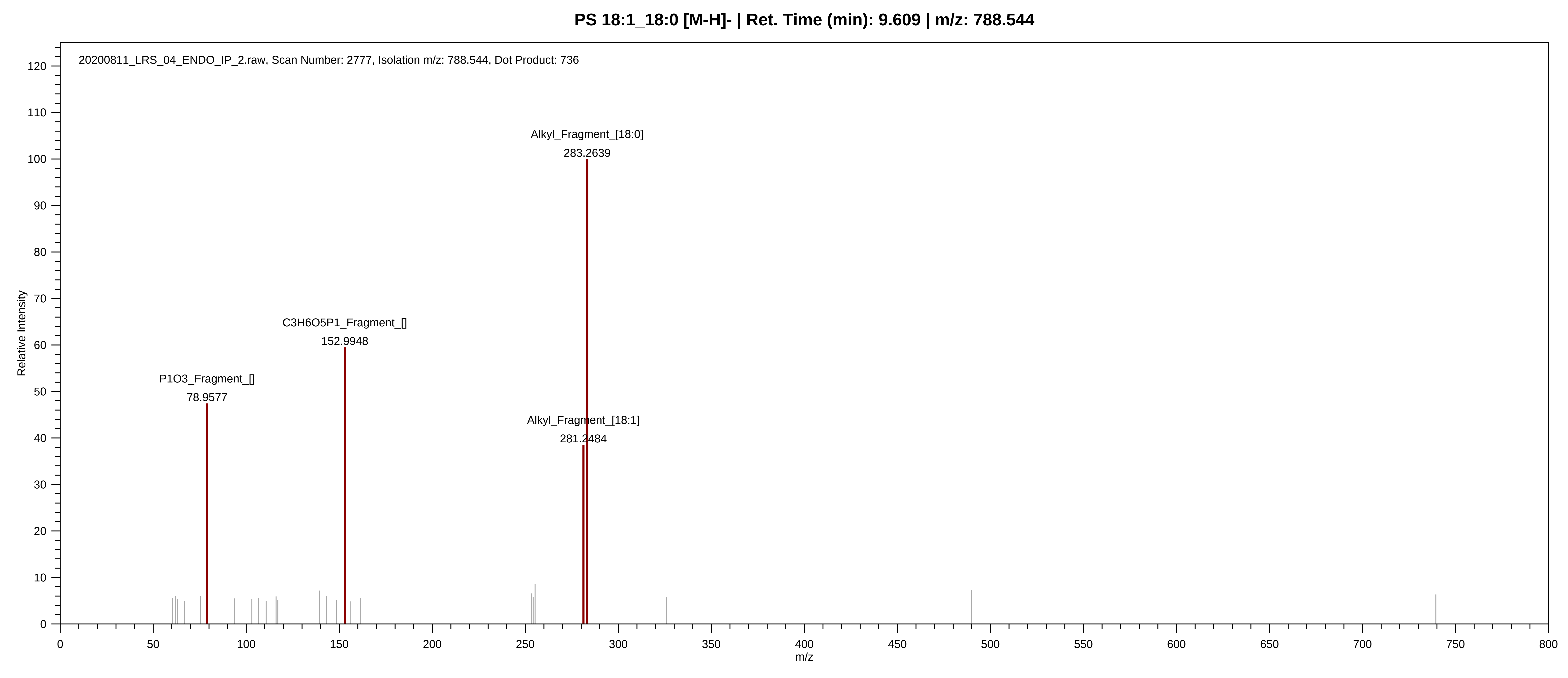
PS 18:1_18:0 [M-H]- | Ret. Time (min): 9.609 | m/z: 788.544
20200811_LRS_04_ENDO_IP_2.raw, Scan Number: 2777, Isolation m/z: 788.544, Dot Product: 736
Alkyl_Fragment_[18:0]
283.2639
C3H6O5P1_Fragment_[]
152.9948
P1O3_Fragment_[]
78.9577
Alkyl_Fragment_[18:1]
281.2484
Relative Intensity
m/z

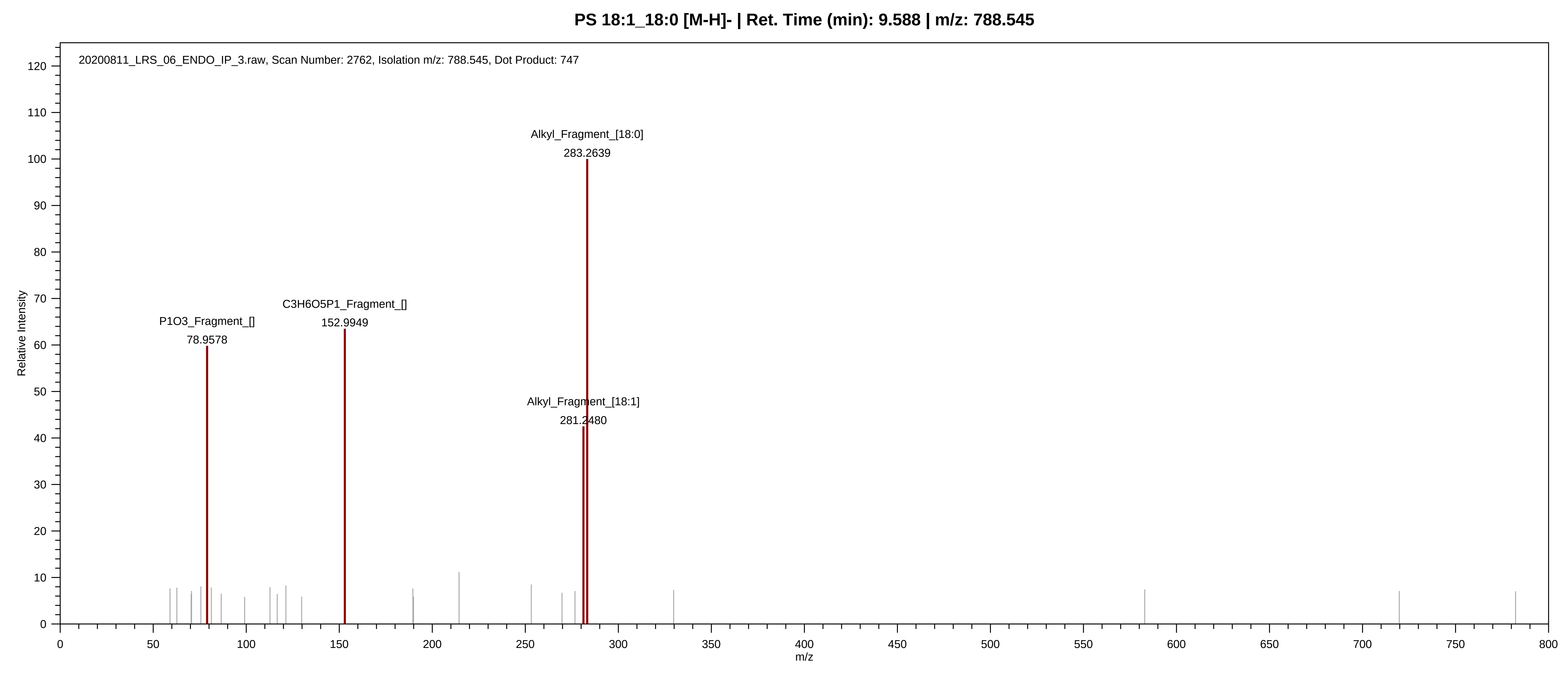

PS 18:1_18:0 [M-H]- | Ret. Time (min): 9.588 | m/z: 788.545
20200811_LRS_06_ENDO_IP_3.raw, Scan Number: 2762, Isolation m/z: 788.545, Dot Product: 747
Alkyl_Fragment_[18:0]
283.2639
C3H6O5P1_Fragment_[]
152.9949
P1O3_Fragment_[]
78.9578
Alkyl_Fragment_[18:1]
281.2480
Relative Intensity
m/z

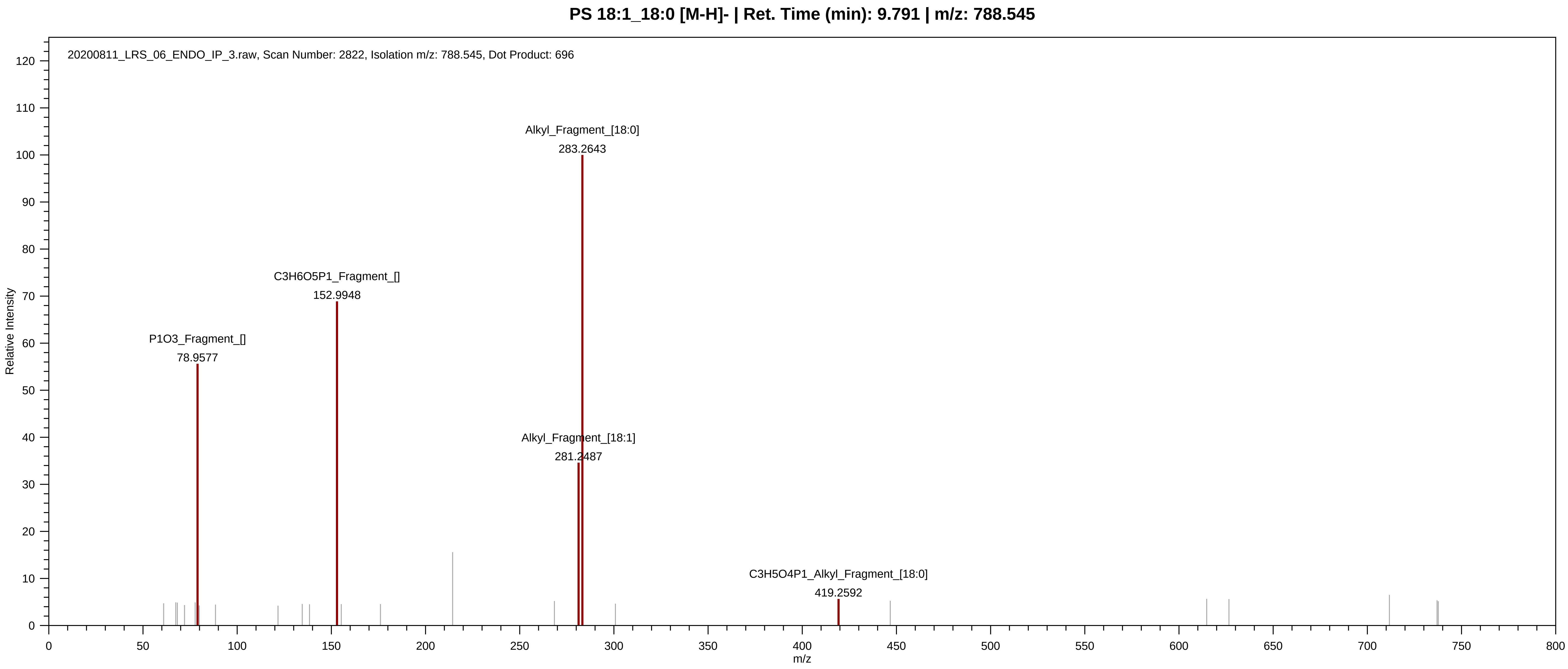
PS 18:1_18:0 [M-H]- | Ret. Time (min): 9.791 | m/z: 788.545
20200811_LRS_06_ENDO_IP_3.raw, Scan Number: 2822, Isolation m/z: 788.545, Dot Product: 696
Alkyl_Fragment_[18:0]
283.2643
C3H6O5P1_Fragment_[]
152.9948
P1O3_Fragment_[]
78.9577
Alkyl_Fragment_[18:1]
281.2487
C3H5O4P1_Alkyl_Fragment_[18:0]
419.2592
Relative Intensity
m/z

PS 18:1_18:0 [M-H]- | Ret. Time (min): 9.792 | m/z: 788.545

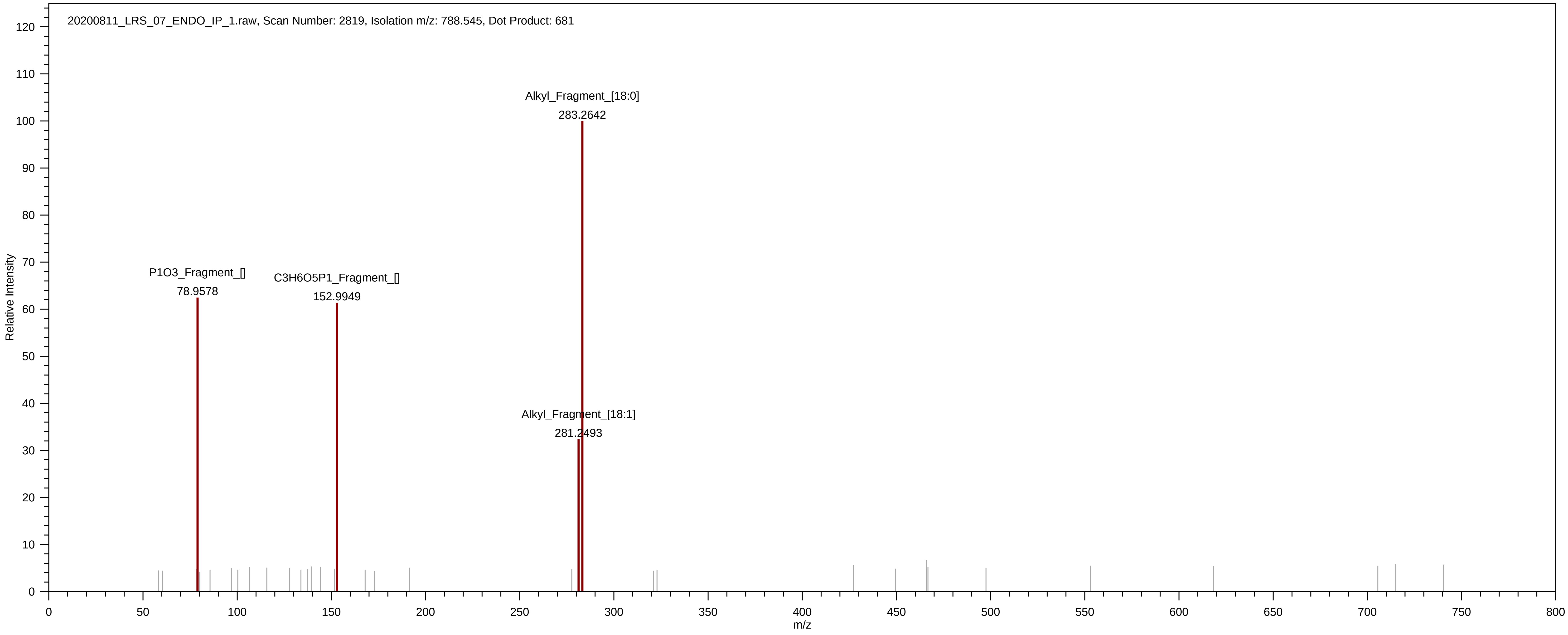

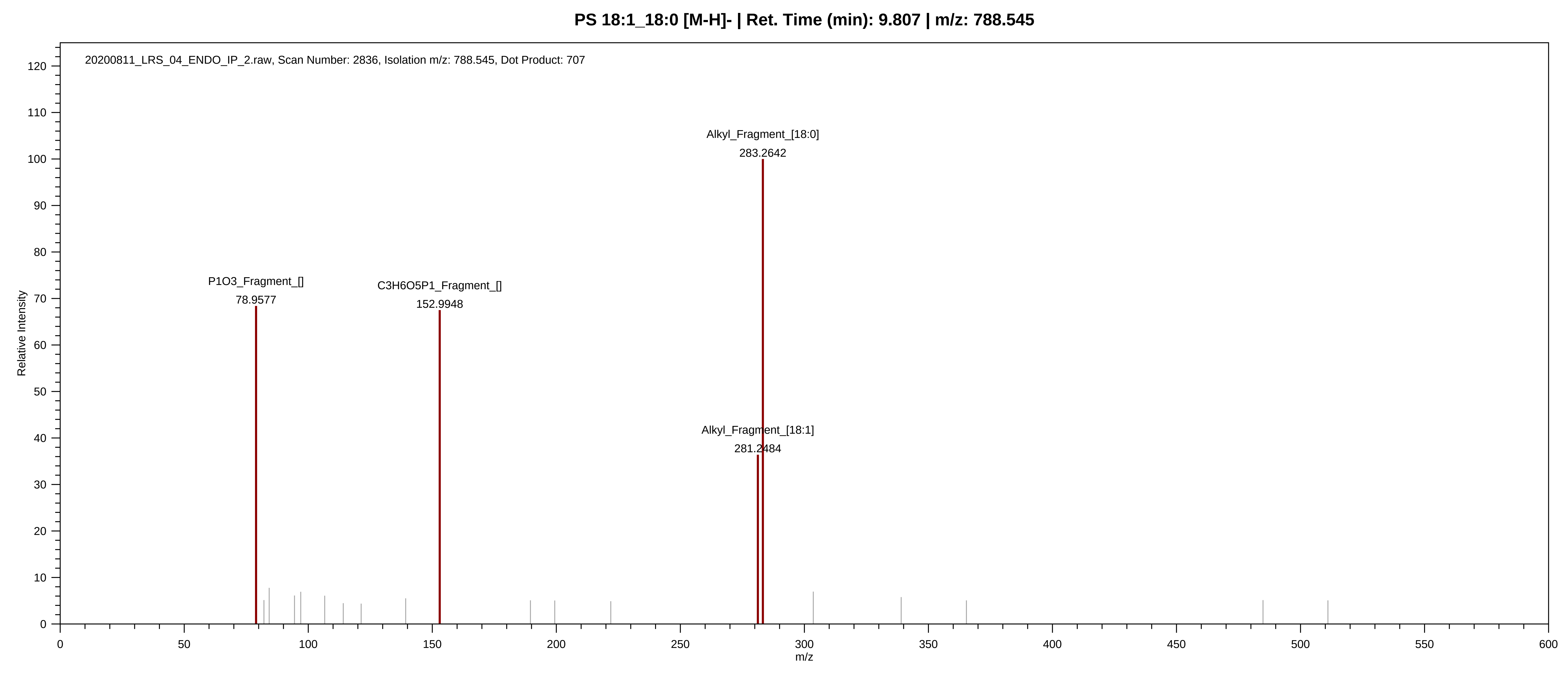
PS 18:1_18:0 [M-H]- | Ret. Time (min): 9.807 | m/z: 788.545
20200811_LRS_04_ENDO_IP_2.raw, Scan Number: 2836, Isolation m/z: 788.545, Dot Product: 707
Alkyl_Fragment_[18:0]
283.2642
P1O3_Fragment_[]
78.9577
C3H6O5P1_Fragment_[]
152.9948
Alkyl_Fragment_[18:1]
281.2484
Relative Intensity
m/z

PS 18:1_18:0 [M-H]- | Ret. Time (min): 9.984 | m/z: 788.545

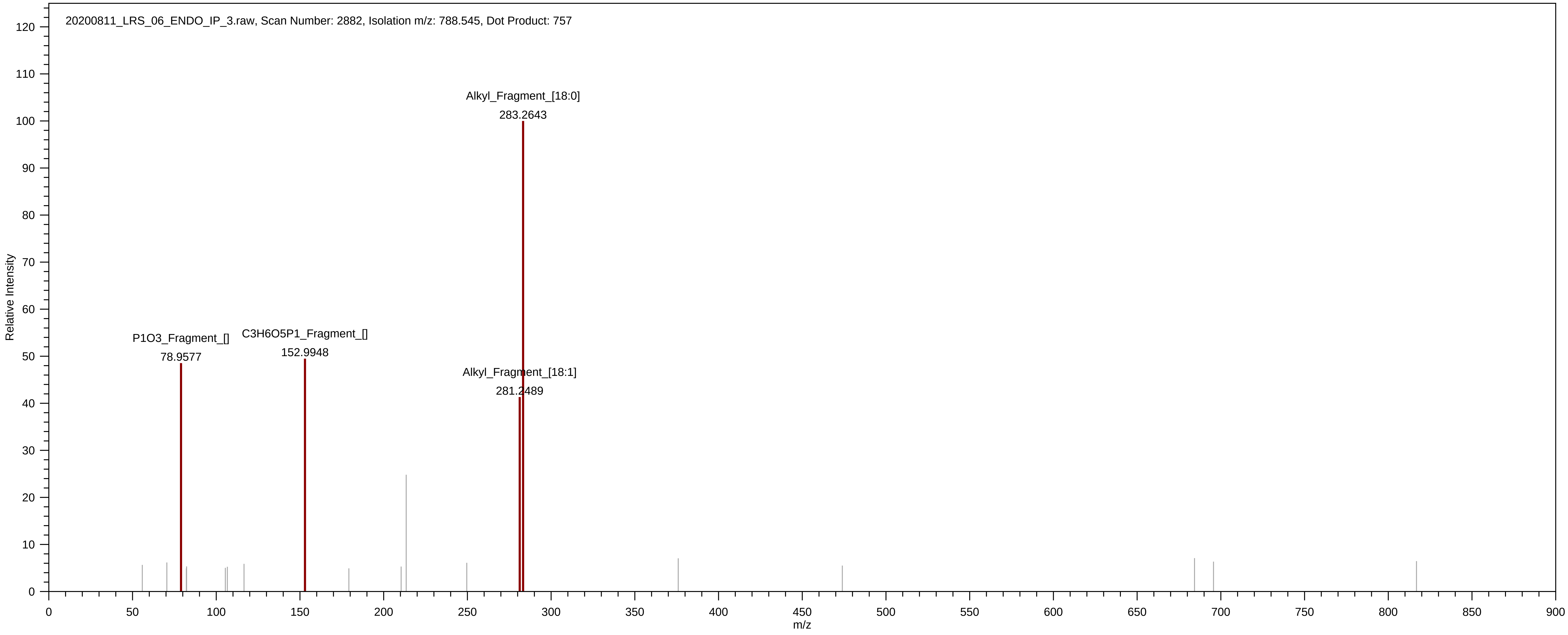

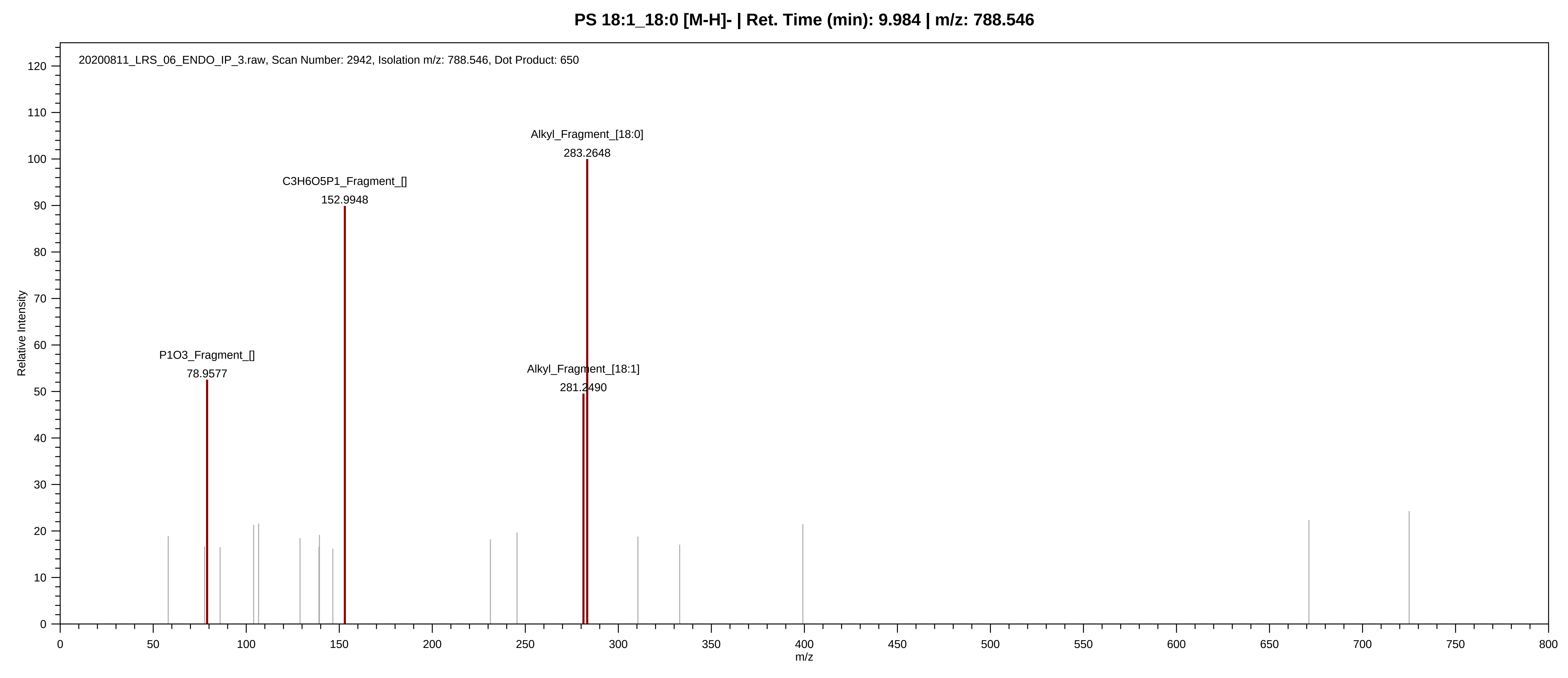

PS 18:1_18:0 [M-H]- | Ret. Time (min): 9.984 | m/z: 788.546
20200811_LRS_06_ENDO_IP_3.raw, Scan Number: 2942, Isolation m/z: 788.546, Dot Product: 650
Alkyl_Fragment_[18:0]
283.2648
C3H6O5P1_Fragment_[]
152.9948
P1O3_Fragment_[]
78.9577
Alkyl_Fragment_[18:1]
281.2490
Relative Intensity
m/z

PS 18:1_18:0 [M-H]- | Ret. Time (min): 9.985 | m/z: 788.545

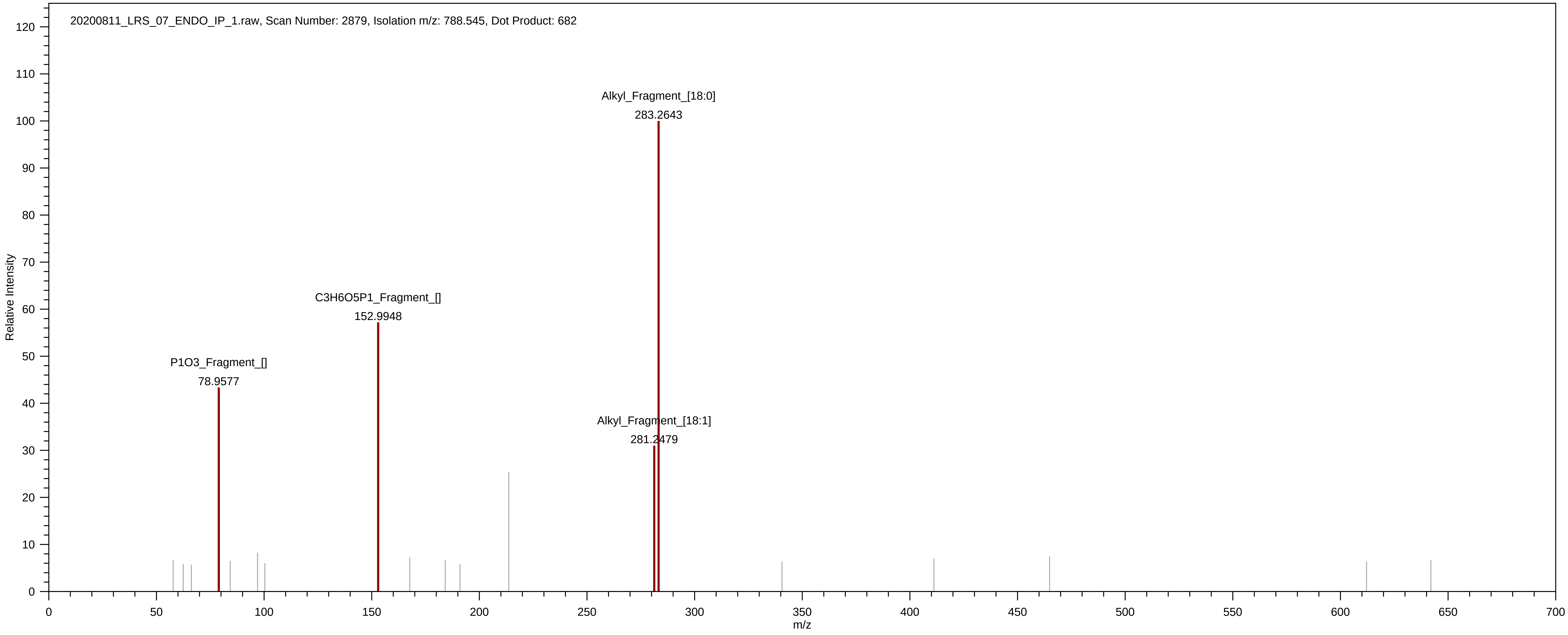

PS 18:1_18:0 [M-H]- | Ret. Time (min): 9.985 | m/z: 788.546

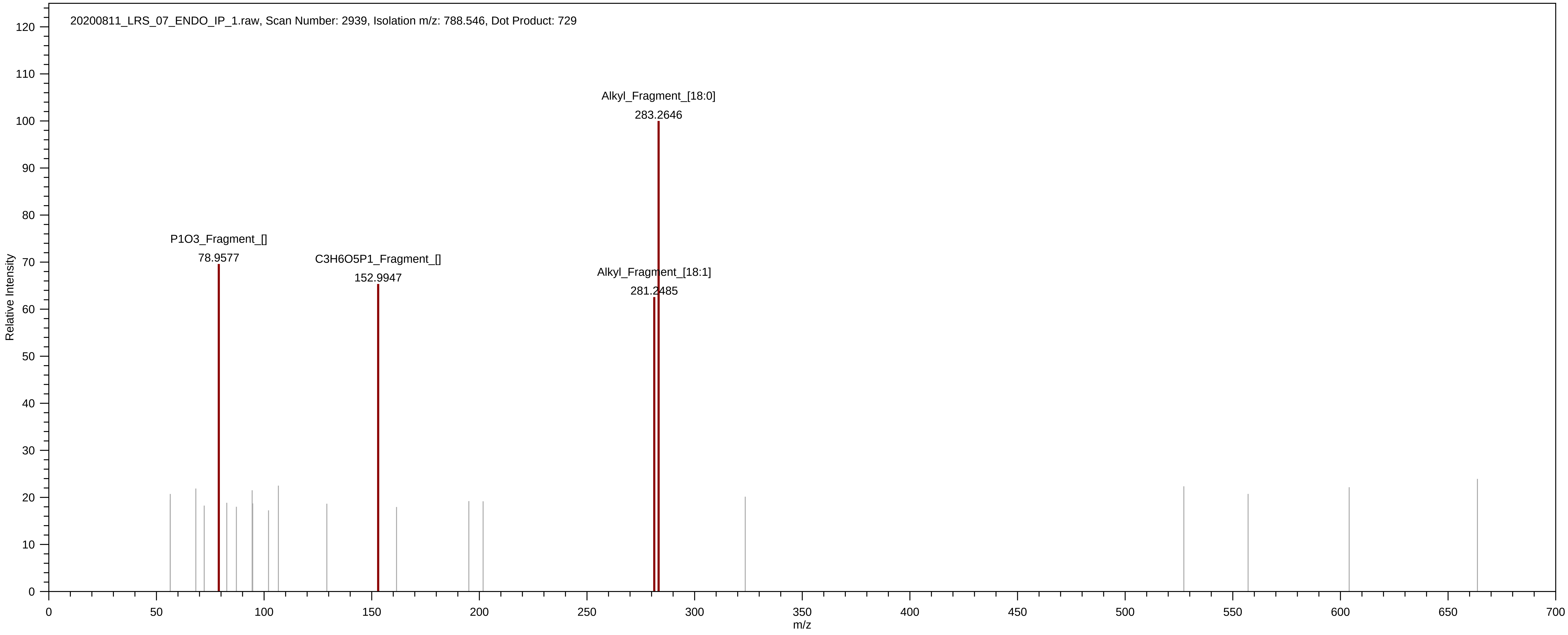

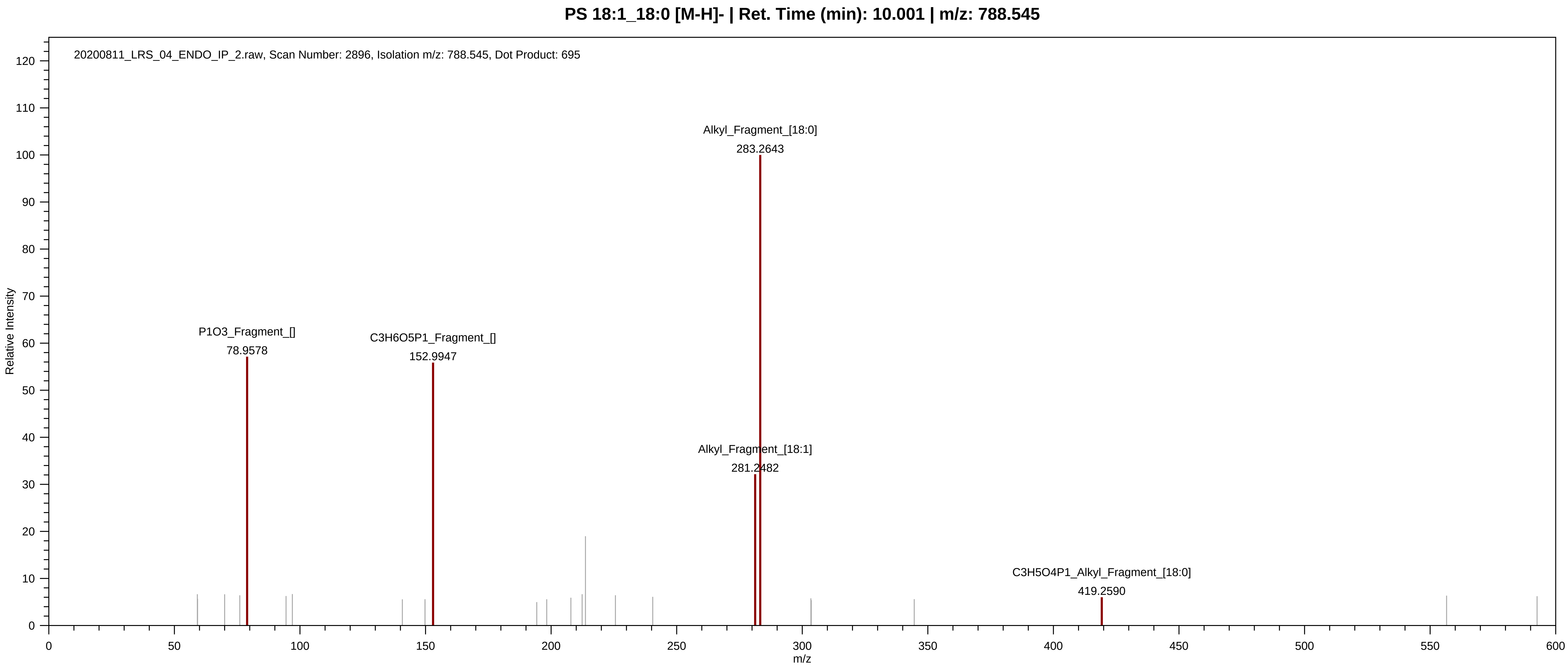

PS 18:1_18:0 [M-H]- | Ret. Time (min): 10.001 | m/z: 788.545
20200811_LRS_04_ENDO_IP_2.raw, Scan Number: 2896, Isolation m/z: 788.545, Dot Product: 695
Relative Intensity
m/z
P1O3_Fragment_[]
78.9578
C3H6O5P1_Fragment_[]
152.9947
Alkyl_Fragment_[18:0]
283.2643
Alkyl_Fragment_[18:1]
281.2482
C3H5O4P1_Alkyl_Fragment_[18:0]
419.2590

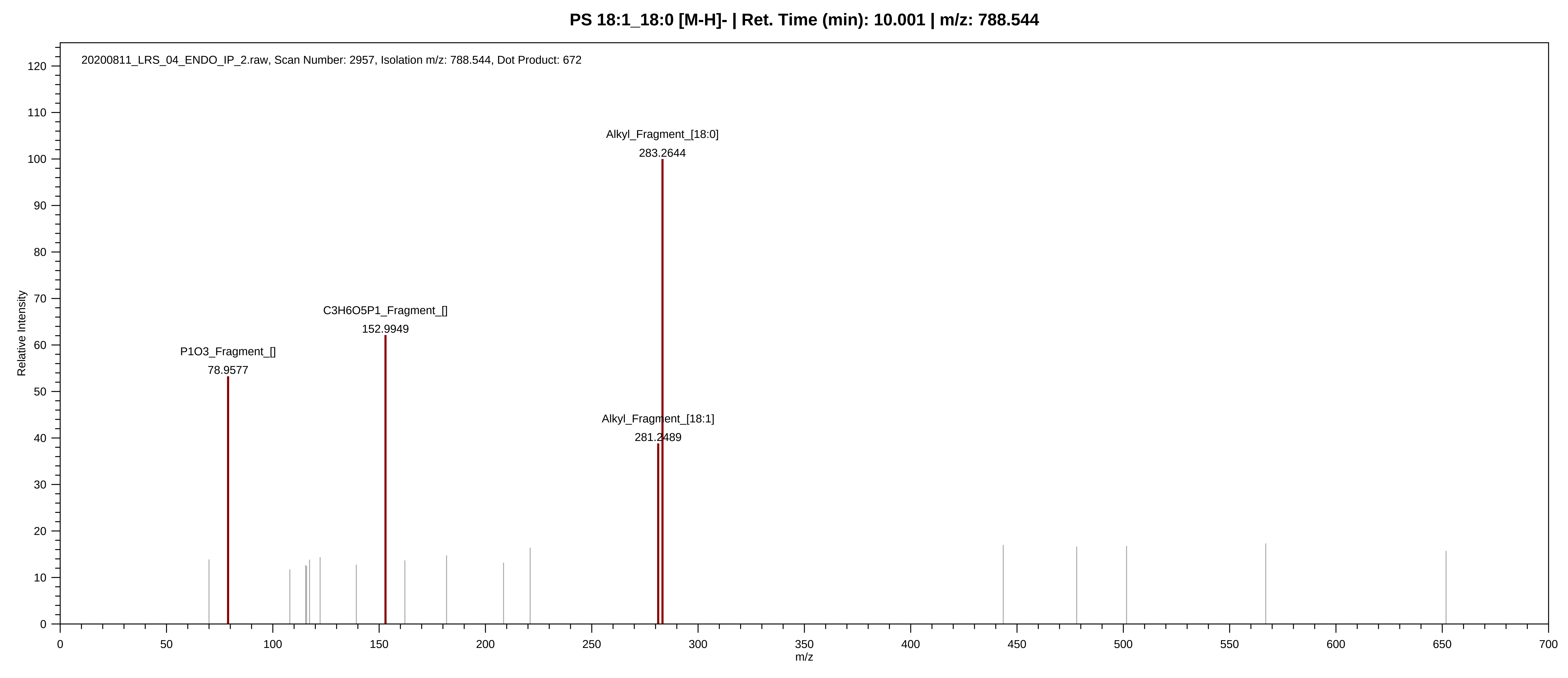
PS 18:1_18:0 [M-H]- | Ret. Time (min): 10.001 | m/z: 788.544
20200811_LRS_04_ENDO_IP_2.raw, Scan Number: 2957, Isolation m/z: 788.544, Dot Product: 672
Alkyl_Fragment_[18:0]
283.2644
C3H6O5P1_Fragment_[]
152.9949
P1O3_Fragment_[]
78.9577
Alkyl_Fragment_[18:1]
281.2489
Relative Intensity
m/z

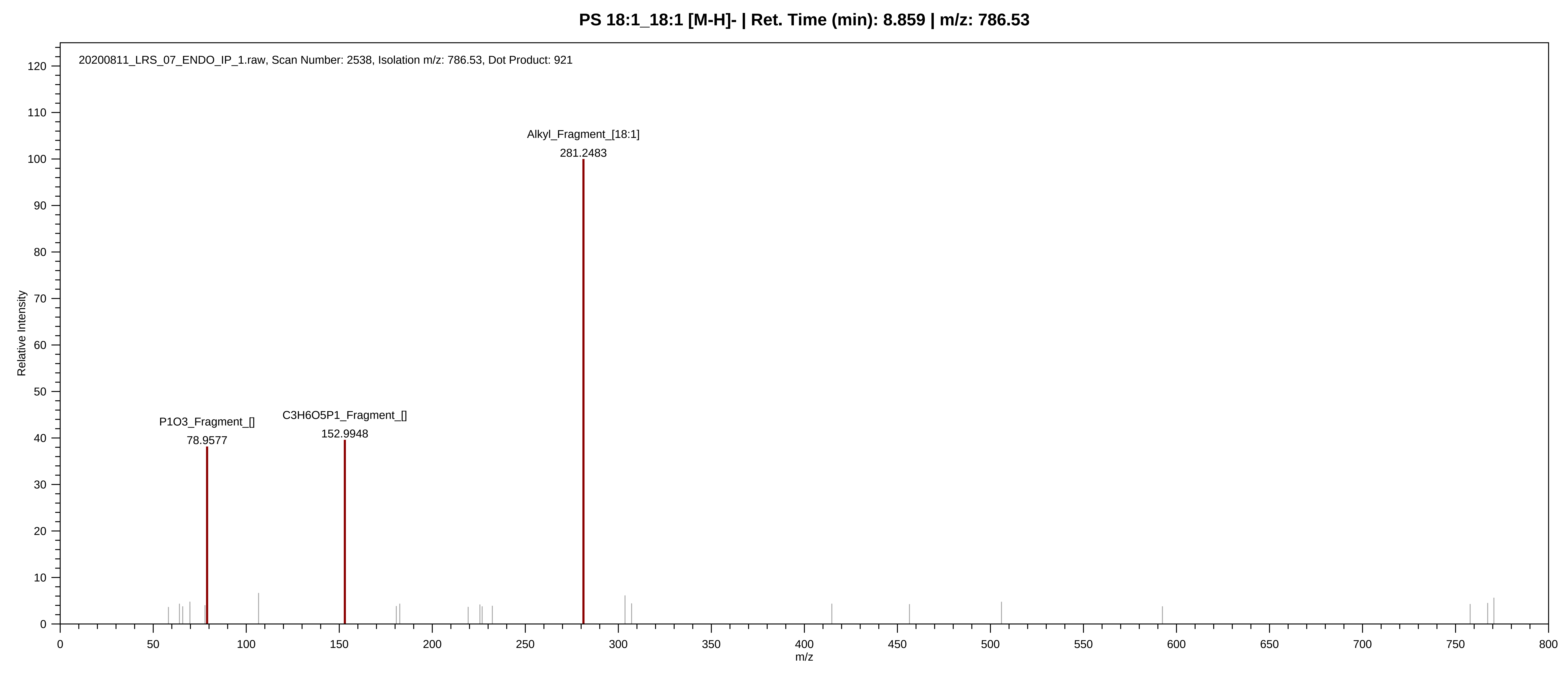

PS 18:1_18:1 [M-H]- | Ret. Time (min): 8.859 | m/z: 786.53
20200811_LRS_07_ENDO_IP_1.raw, Scan Number: 2538, Isolation m/z: 786.53, Dot Product: 921
Alkyl_Fragment_[18:1]
281.2483
P1O3_Fragment_[]
78.9577
C3H6O5P1_Fragment_[]
152.9948
Relative Intensity
m/z

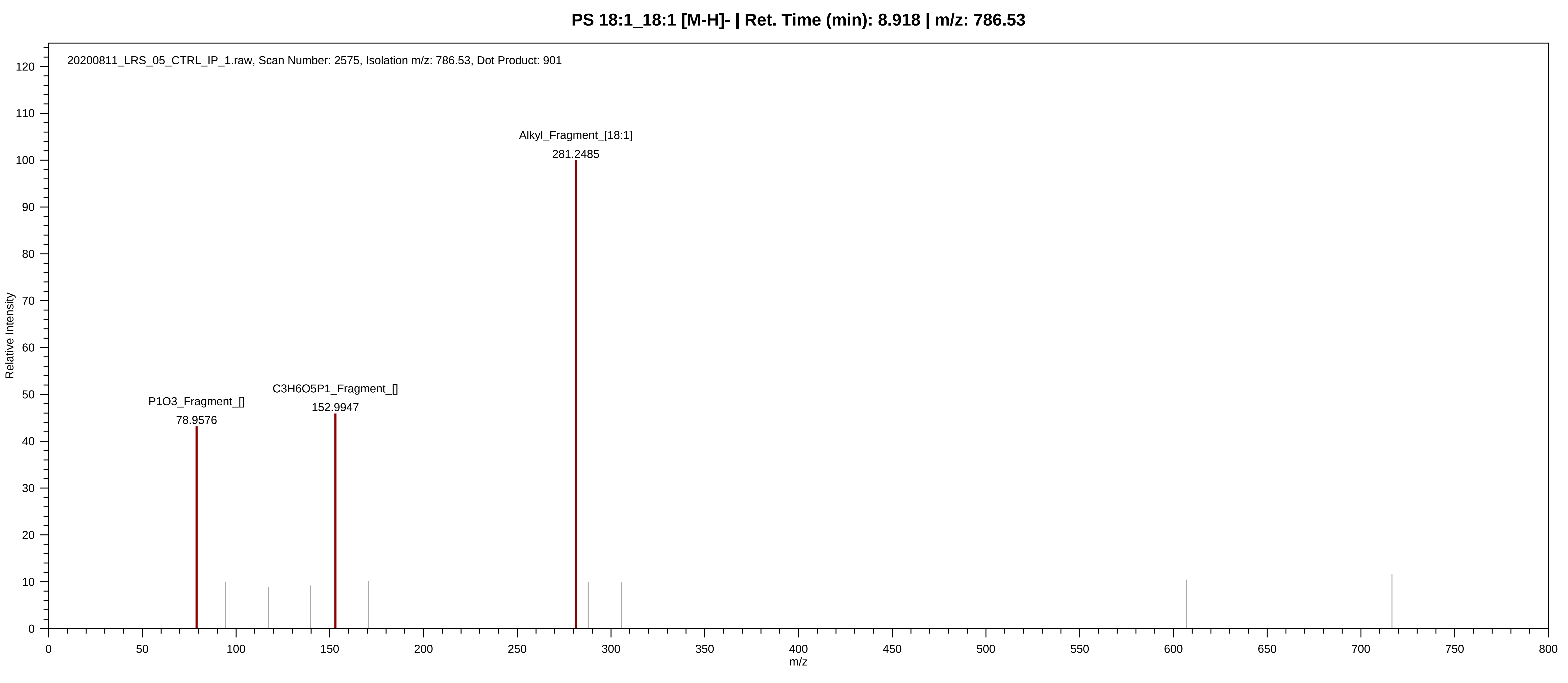
PS 18:1_18:1 [M-H]- | Ret. Time (min): 8.918 | m/z: 786.53
20200811_LRS_05_CTRL_IP_1.raw, Scan Number: 2575, Isolation m/z: 786.53, Dot Product: 901
Alkyl_Fragment_[18:1]
281.2485
P1O3_Fragment_[]
78.9576
C3H6O5P1_Fragment_[]
152.9947
Relative Intensity
m/z

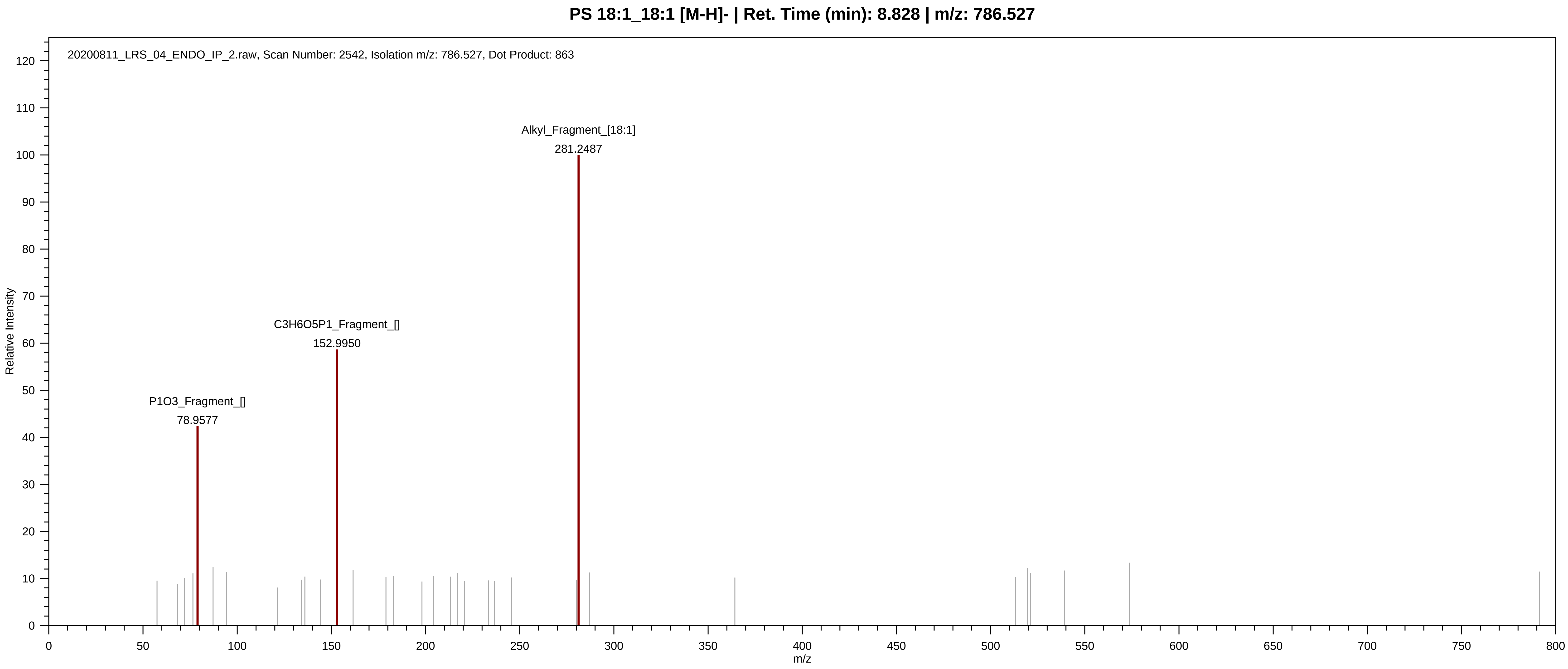
PS 18:1_18:1 [M-H]- | Ret. Time (min): 8.828 | m/z: 786.527
20200811_LRS_04_ENDO_IP_2.raw, Scan Number: 2542, Isolation m/z: 786.527, Dot Product: 863
Alkyl_Fragment_[18:1]
281.2487
C3H6O5P1_Fragment_[]
152.9950
P1O3_Fragment_[]
78.9577
Relative Intensity
m/z

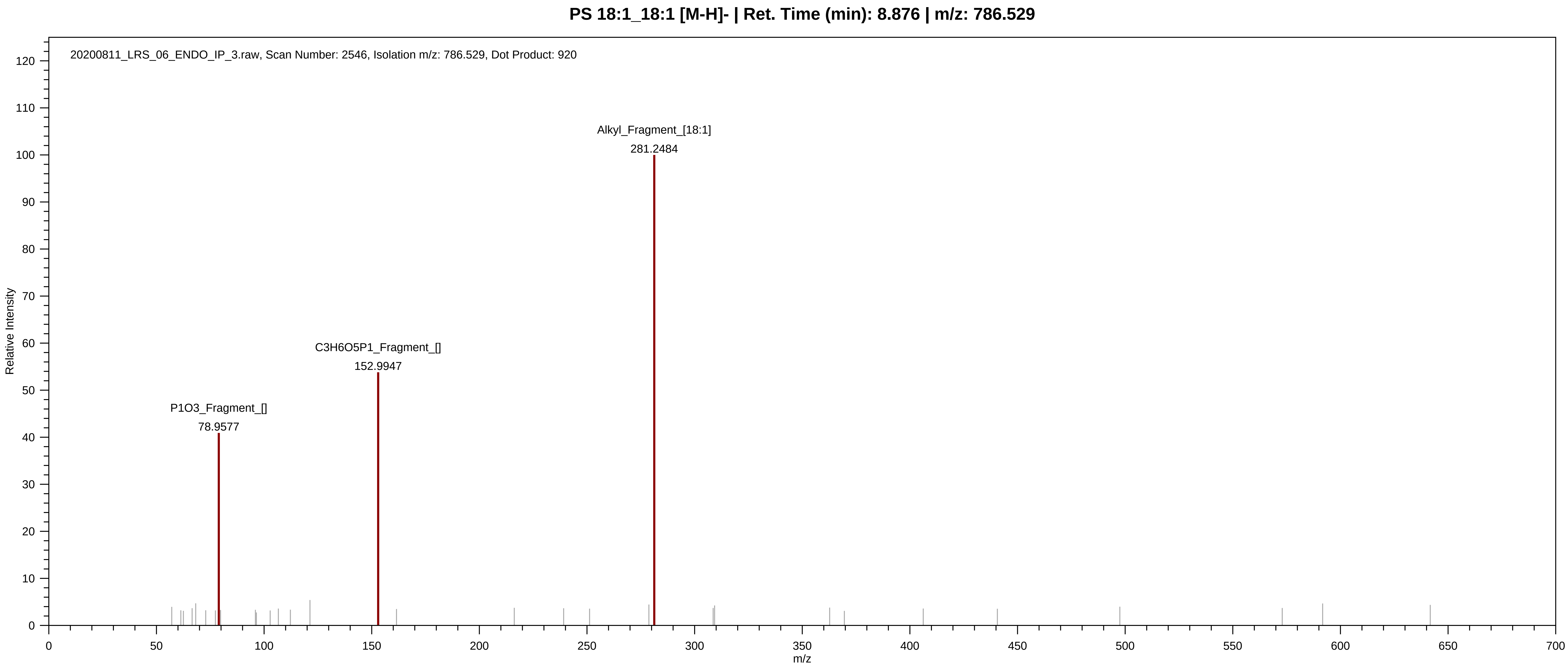

PS 18:1_18:1 [M-H]- | Ret. Time (min): 8.876 | m/z: 786.529
20200811_LRS_06_ENDO_IP_3.raw, Scan Number: 2546, Isolation m/z: 786.529, Dot Product: 920
Alkyl_Fragment_[18:1]
281.2484
C3H6O5P1_Fragment_[]
152.9947
P1O3_Fragment_[]
78.9577
Relative Intensity
m/z

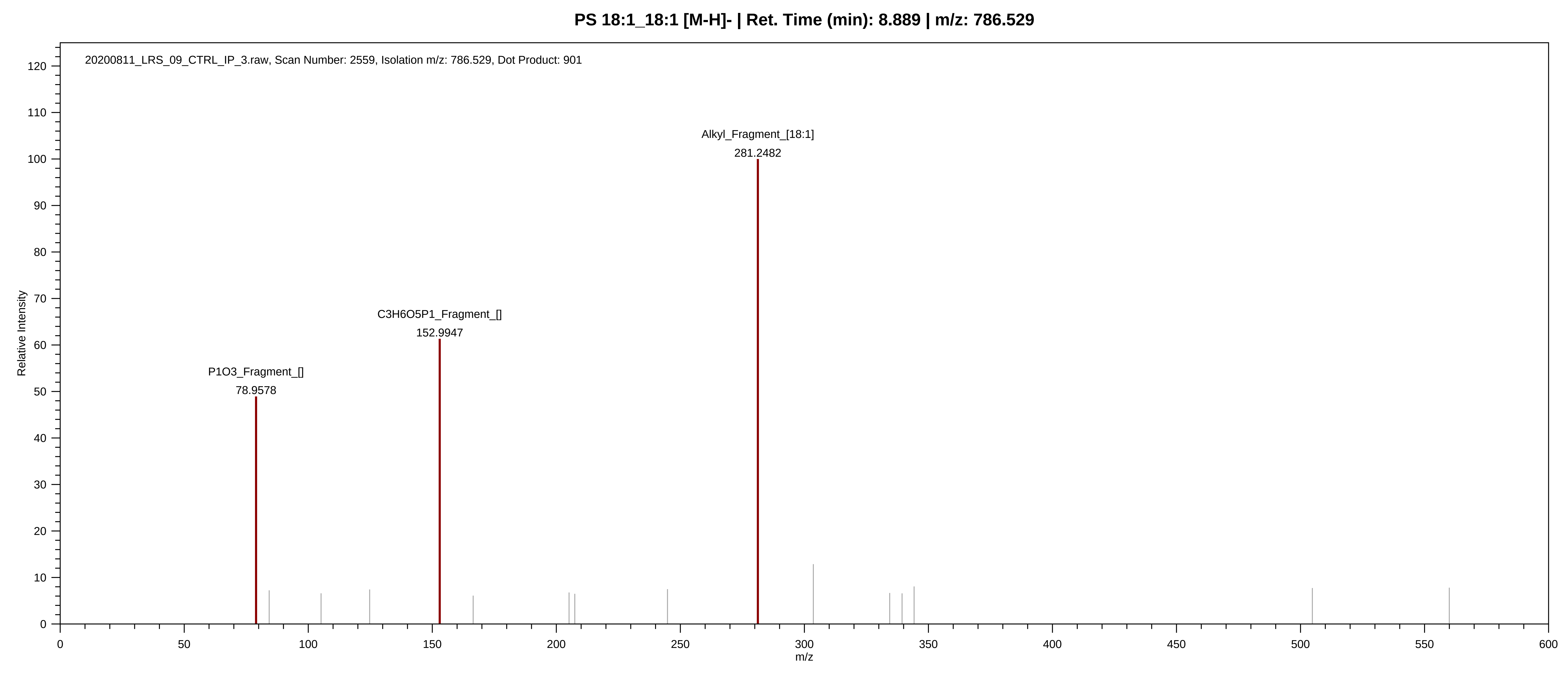
PS 18:1_18:1 [M-H]- | Ret. Time (min): 8.889 | m/z: 786.529
20200811_LRS_09_CTRL_IP_3.raw, Scan Number: 2559, Isolation m/z: 786.529, Dot Product: 901
Alkyl_Fragment_[18:1]
281.2482
C3H6O5P1_Fragment_[]
152.9947
P1O3_Fragment_[]
78.9578
Relative Intensity
m/z
0
10
20
30
40
50
60
70
80
90
100
110
120
0
50
100
150
200
250
300
350
400
450
500
550
600

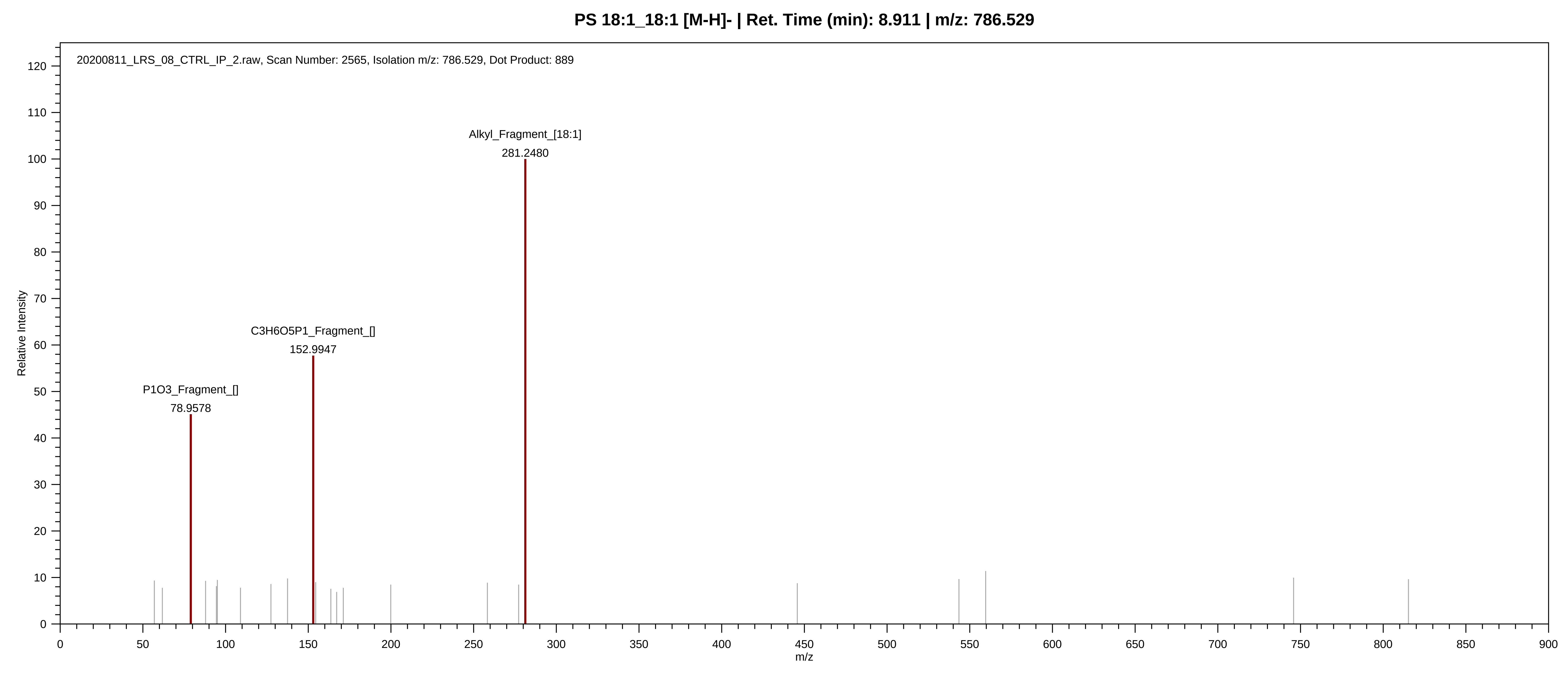
PS 18:1_18:1 [M-H]- | Ret. Time (min): 8.911 | m/z: 786.529
20200811_LRS_08_CTRL_IP_2.raw, Scan Number: 2565, Isolation m/z: 786.529, Dot Product: 889
Alkyl_Fragment_[18:1]
281.2480
C3H6O5P1_Fragment_[]
152.9947
P1O3_Fragment_[]
78.9578
Relative Intensity
m/z

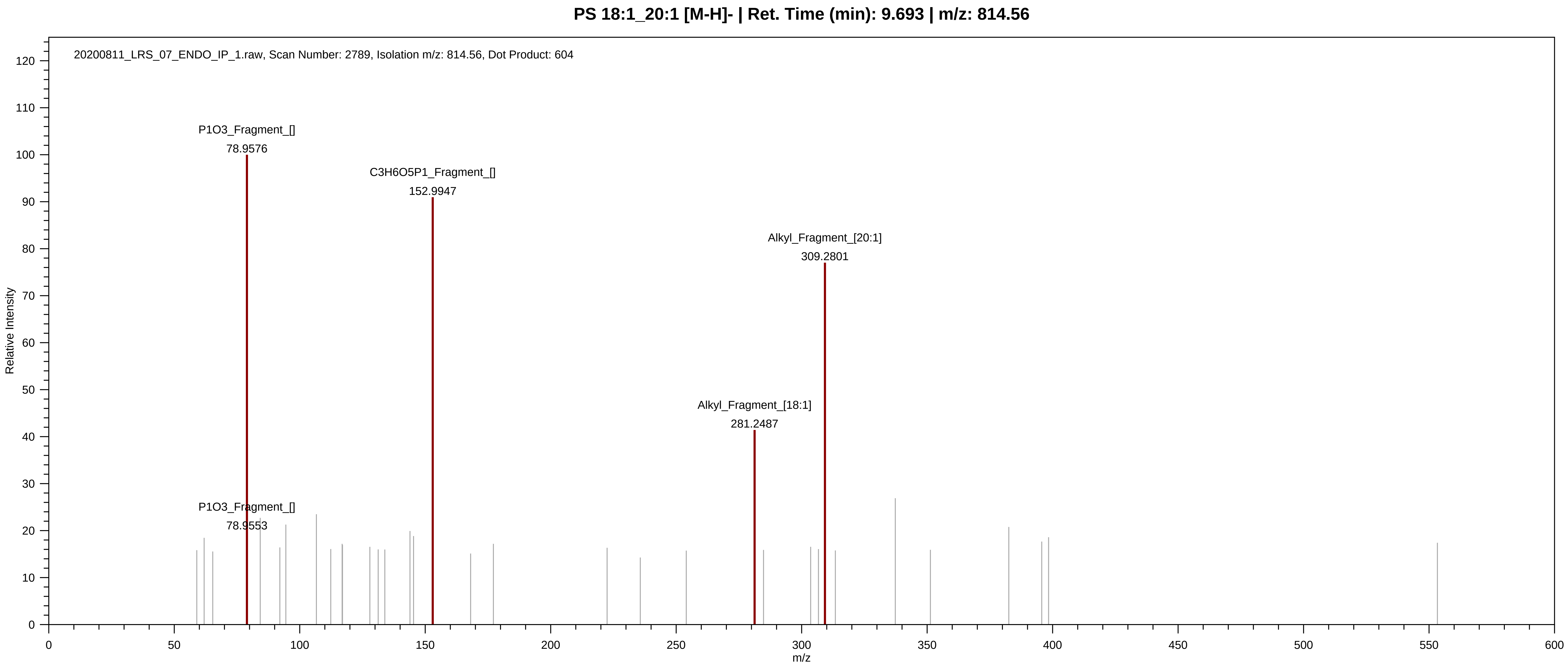

PS 18:1_20:1 [M-H]- | Ret. Time (min): 9.693 | m/z: 814.56
20200811_LRS_07_ENDO_IP_1.raw, Scan Number: 2789, Isolation m/z: 814.56, Dot Product: 604
P1O3_Fragment_[]
78.9576
C3H6O5P1_Fragment_[]
152.9947
Alkyl_Fragment_[20:1]
309.2801
Alkyl_Fragment_[18:1]
281.2487
P1O3_Fragment_[]
78.9553
Relative Intensity
m/z

PS 18:1_20:1 [M-H]- | Ret. Time (min): 9.748 | m/z: 814.559

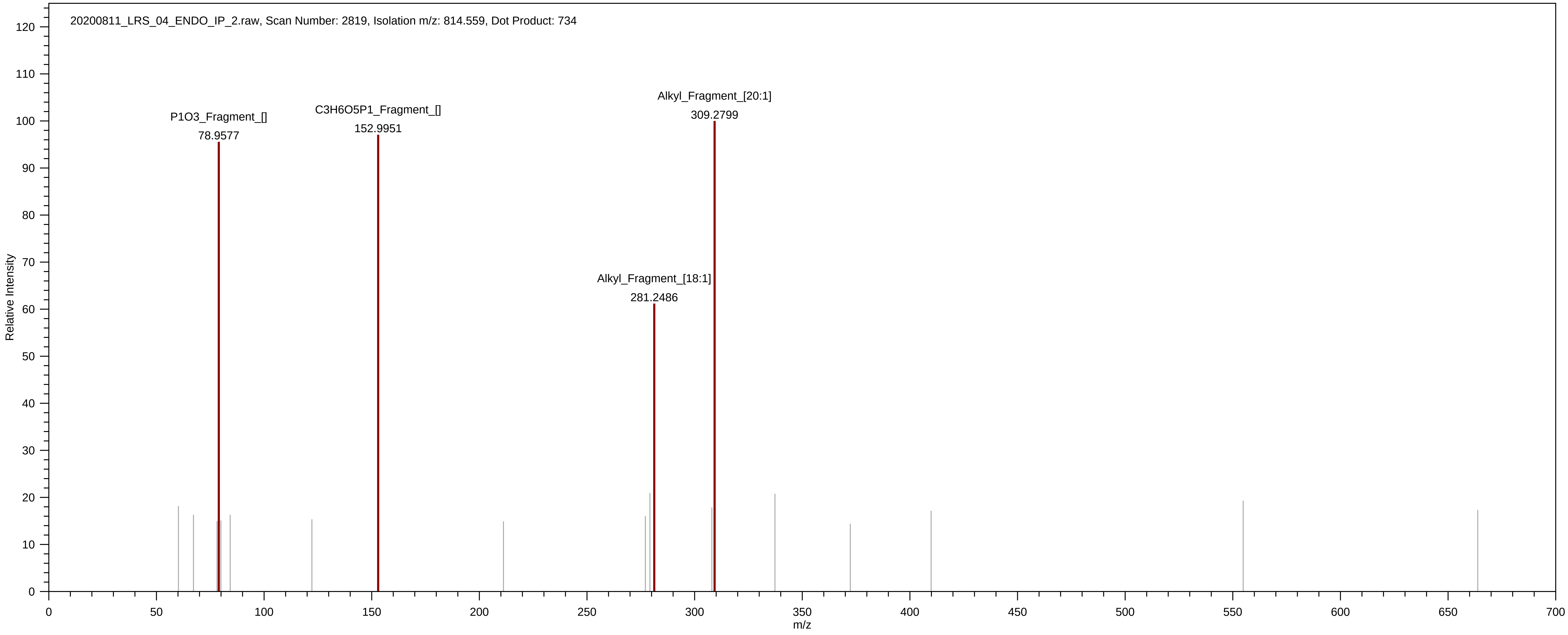

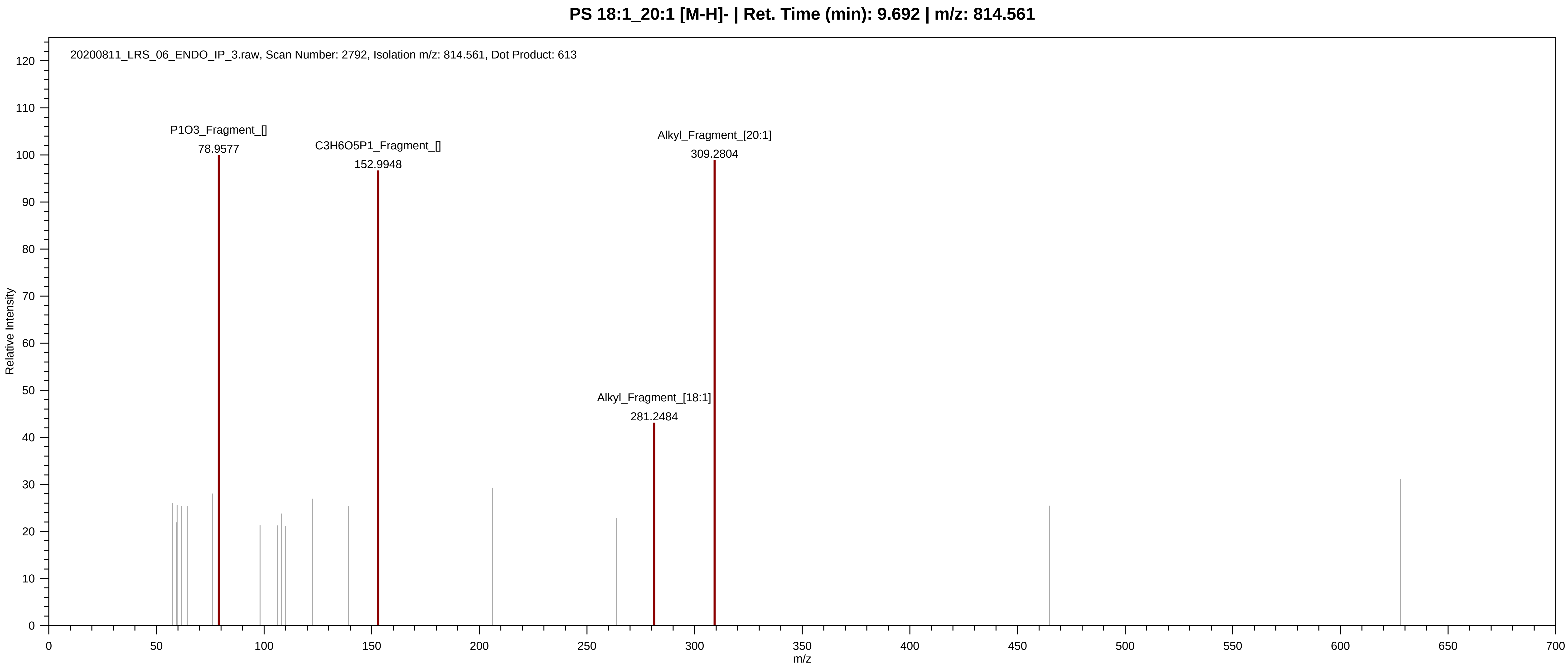
PS 18:1_20:1 [M-H]- | Ret. Time (min): 9.692 | m/z: 814.561
20200811_LRS_06_ENDO_IP_3.raw, Scan Number: 2792, Isolation m/z: 814.561, Dot Product: 613
P1O3_Fragment_[]
78.9577
C3H6O5P1_Fragment_[]
152.9948
Alkyl_Fragment_[20:1]
309.2804
Alkyl_Fragment_[18:1]
281.2484
Relative Intensity
m/z

PS 18:1_22:0 [M-H]- | Ret. Time (min): 11.661 | m/z: 844.605

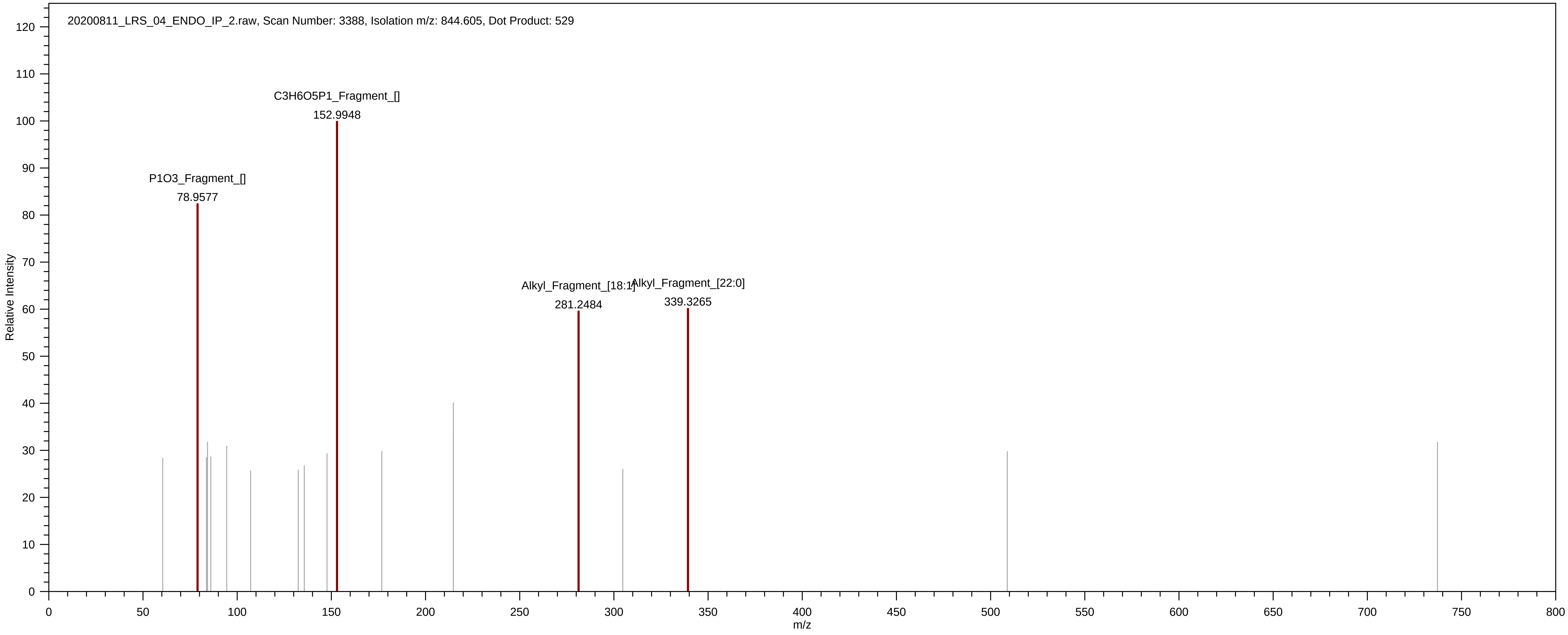

PS 18:1_22:0 [M-H]- | Ret. Time (min): 11.622 | m/z: 844.606

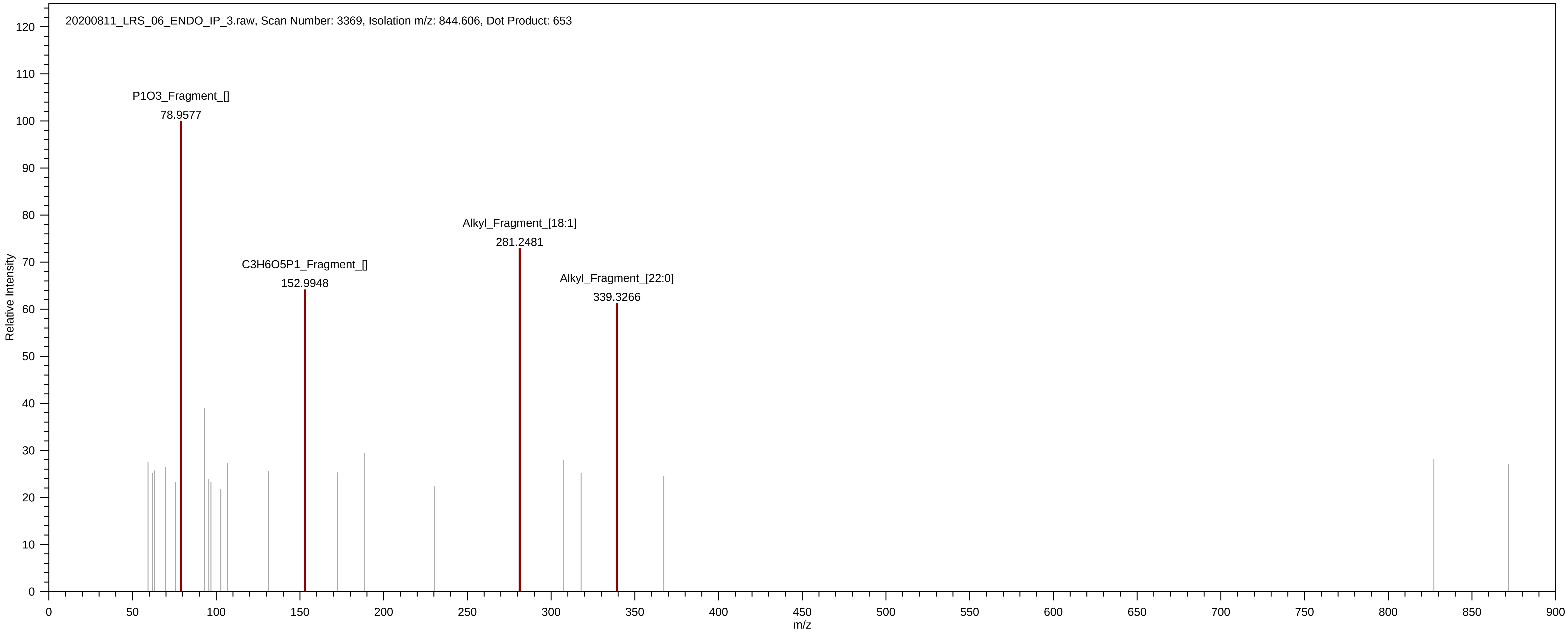

PS 18:1_22:0 [M-H]- | Ret. Time (min): 11.642 | m/z: 844.608

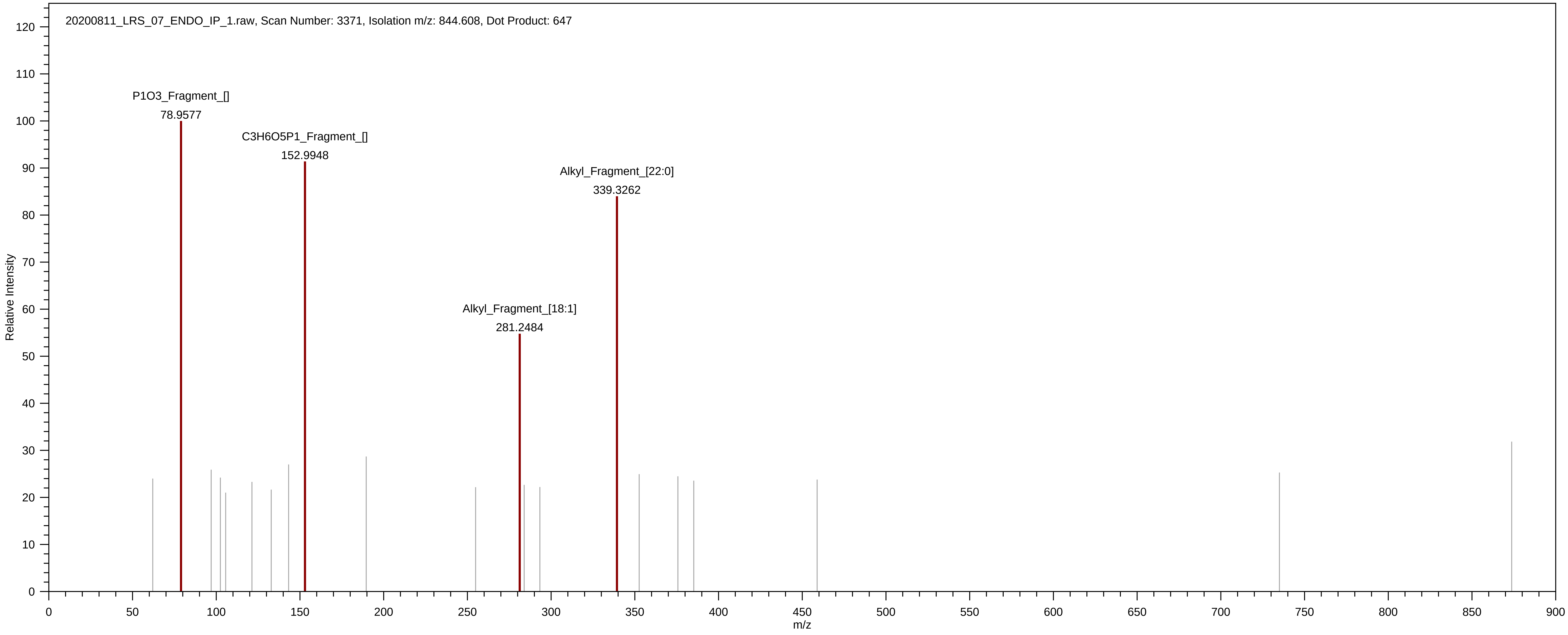

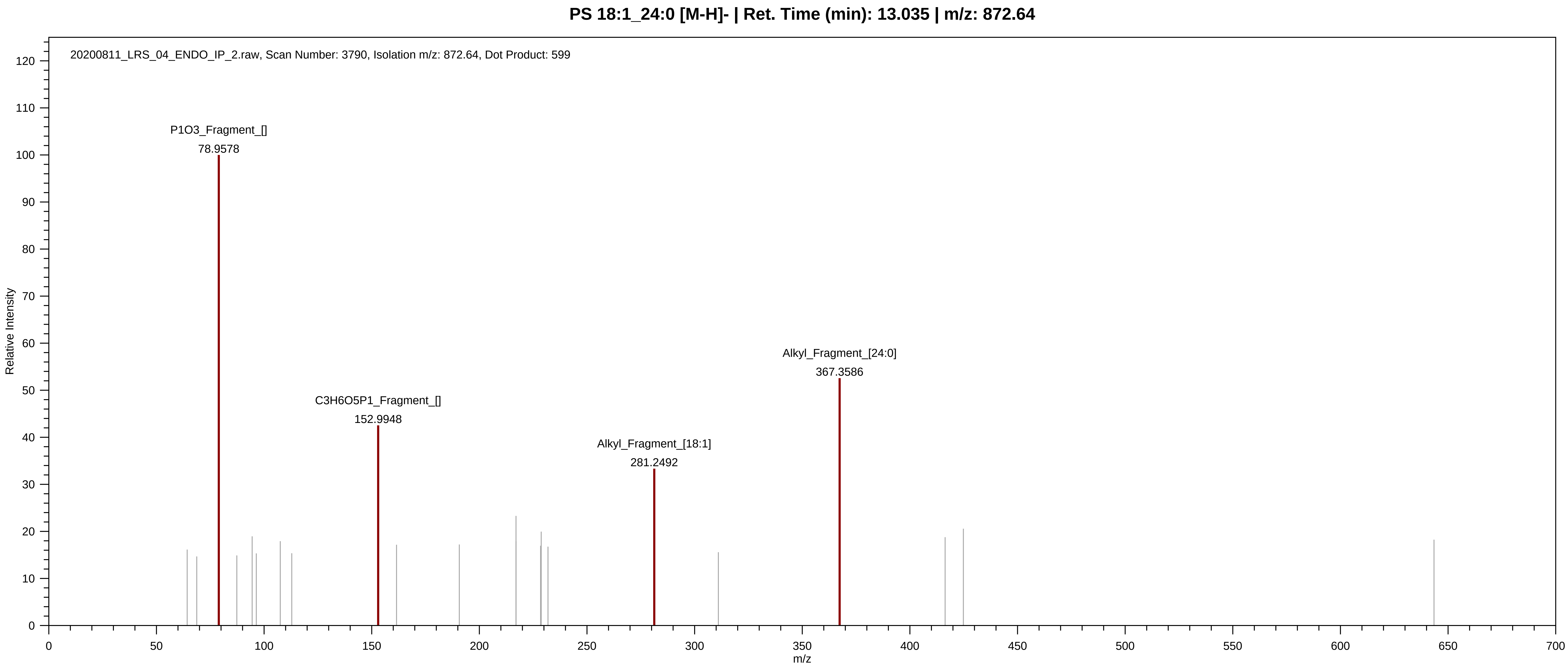
PS 18:1_24:0 [M-H]- | Ret. Time (min): 13.035 | m/z: 872.64
20200811_LRS_04_ENDO_IP_2.raw, Scan Number: 3790, Isolation m/z: 872.64, Dot Product: 599
P1O3_Fragment_[]
78.9578
C3H6O5P1_Fragment_[]
152.9948
Alkyl_Fragment_[18:1]
281.2492
Alkyl_Fragment_[24:0]
367.3586
Relative Intensity
m/z

PS 18:1_24:0 [M-H]- | Ret. Time (min): 12.828 | m/z: 872.638

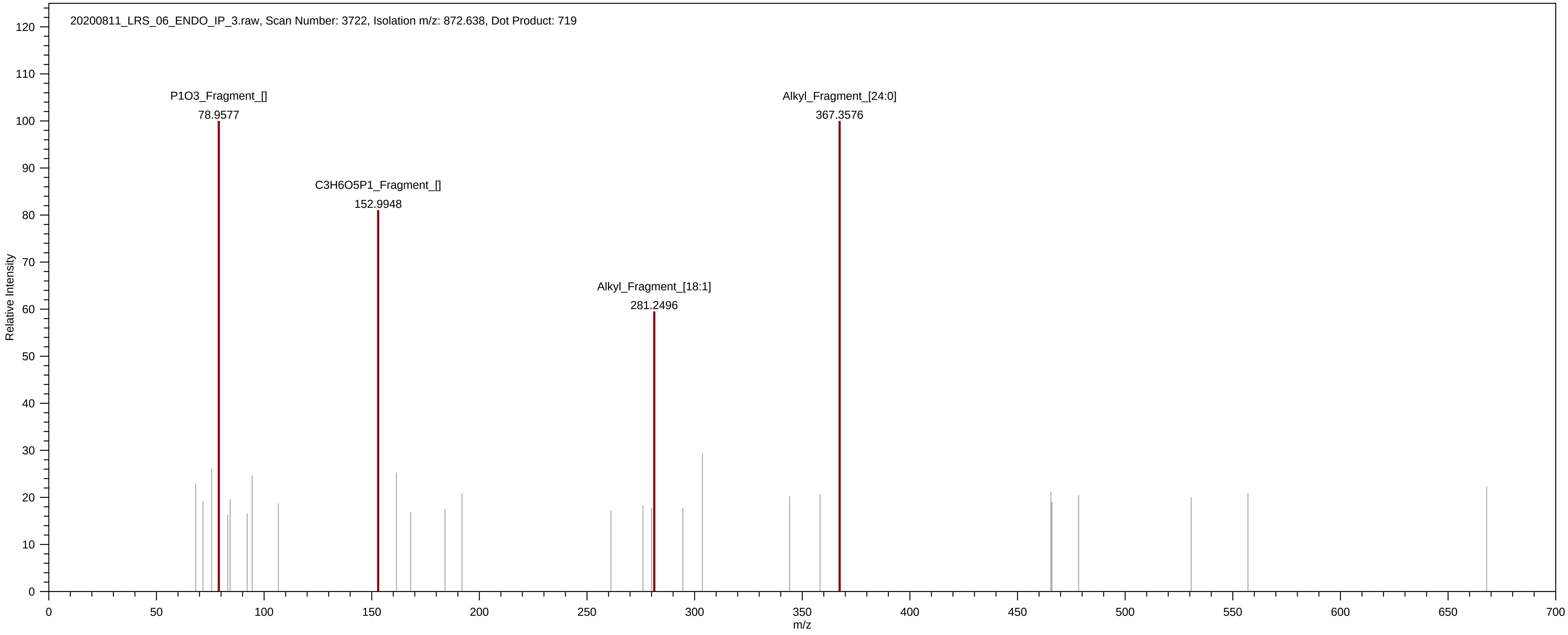

PS 18:1_24:0 [M-H]- | Ret. Time (min): 12.85 | m/z: 872.639

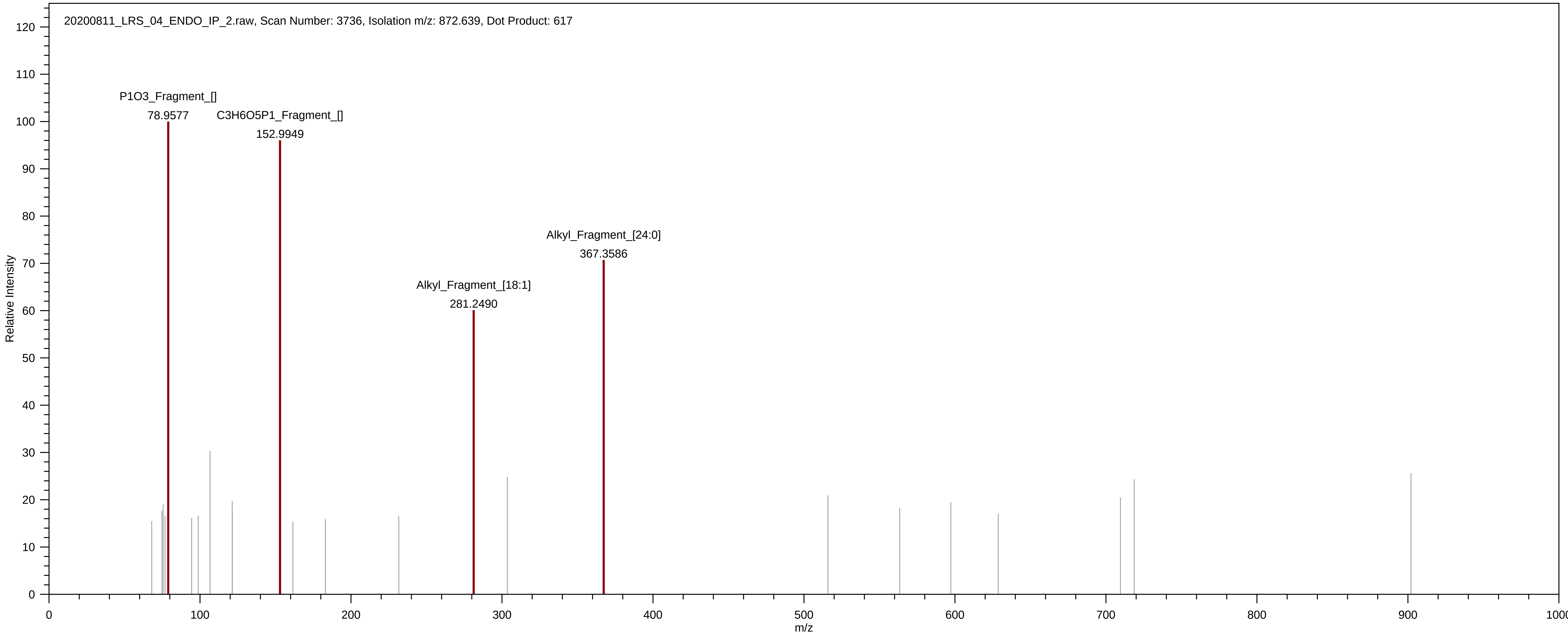

PS 34:1 [M-H]- | Ret. Time (min): 8.957 | m/z: 760.513

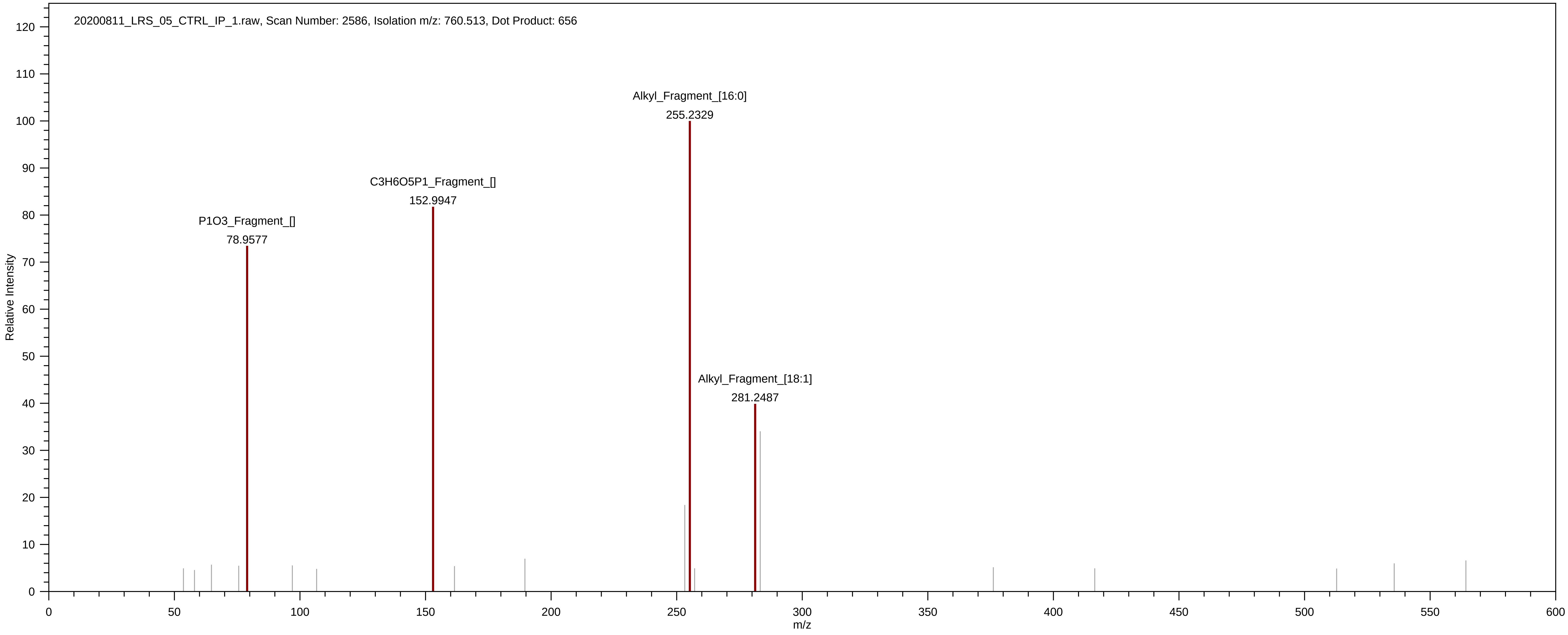

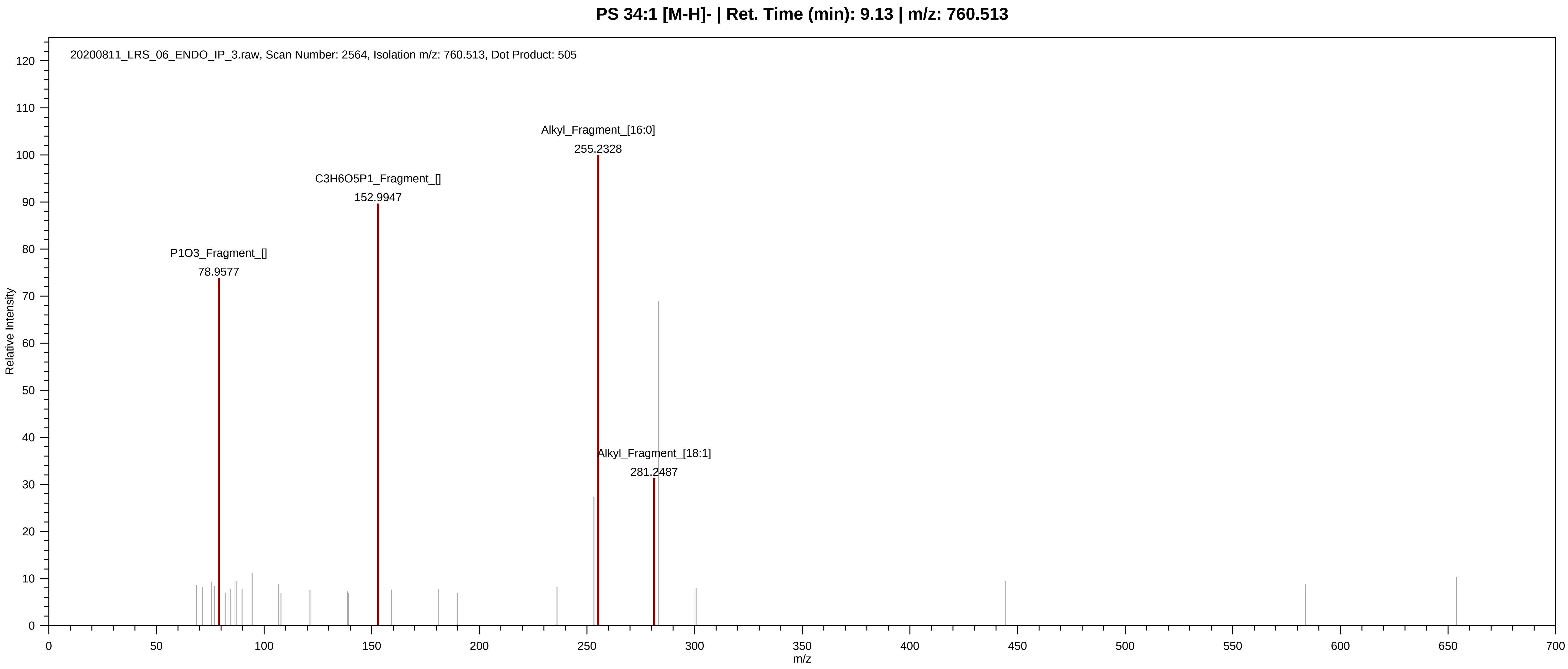

PS 34:1 [M-H]- | Ret. Time (min): 9.13 | m/z: 760.513
20200811_LRS_06_ENDO_IP_3.raw, Scan Number: 2564, Isolation m/z: 760.513, Dot Product: 505
Alkyl_Fragment_[16:0]
255.2328
C3H6O5P1_Fragment_[]
152.9947
P1O3_Fragment_[]
78.9577
Alkyl_Fragment_[18:1]
281.2487
Relative Intensity
m/z

PS 34:1 [M-H]- | Ret. Time (min): 9.13 | m/z: 760.513

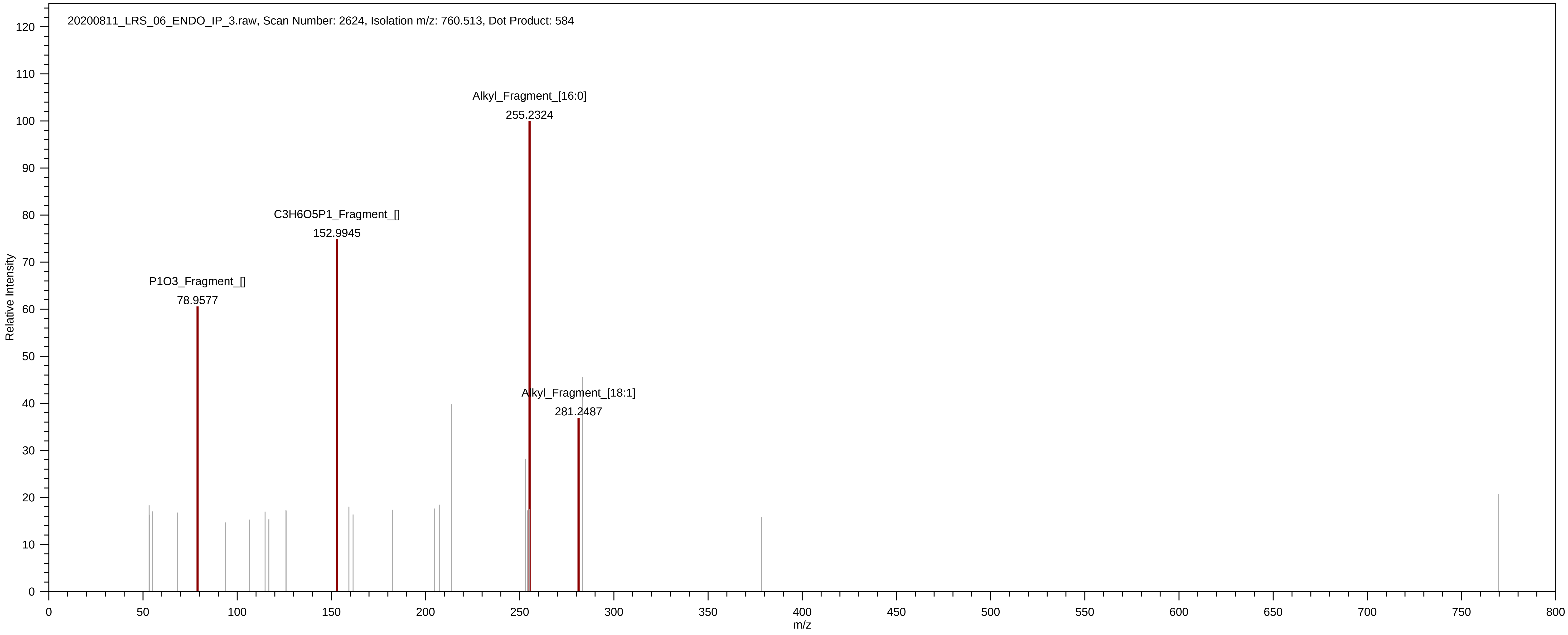

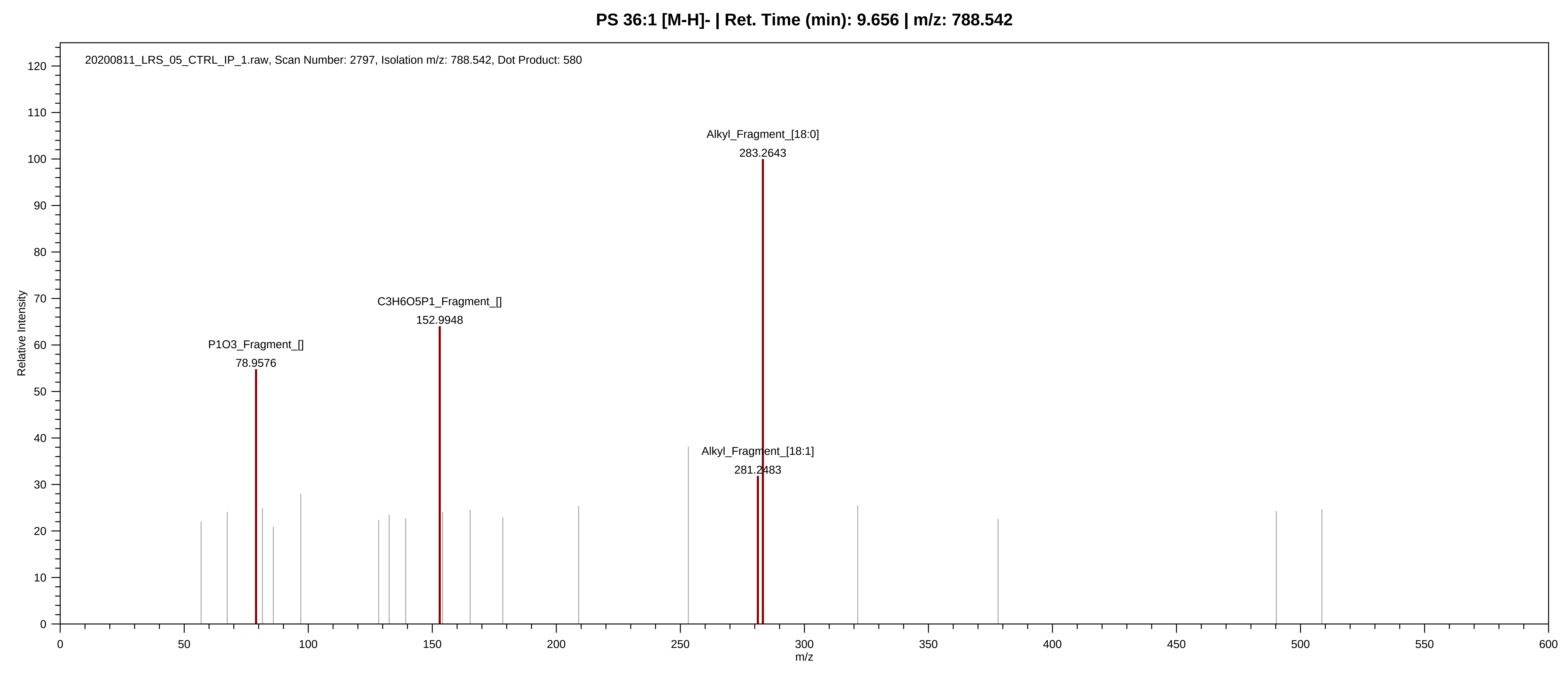
PS 36:1 [M-H]- | Ret. Time (min): 9.656 | m/z: 788.542
20200811_LRS_05_CTRL_IP_1.raw, Scan Number: 2797, Isolation m/z: 788.542, Dot Product: 580
Alkyl_Fragment_[18:0]
283.2643
C3H6O5P1_Fragment_[]
152.9948
P1O3_Fragment_[]
78.9576
Alkyl_Fragment_[18:1]
281.2483
Relative Intensity
m/z

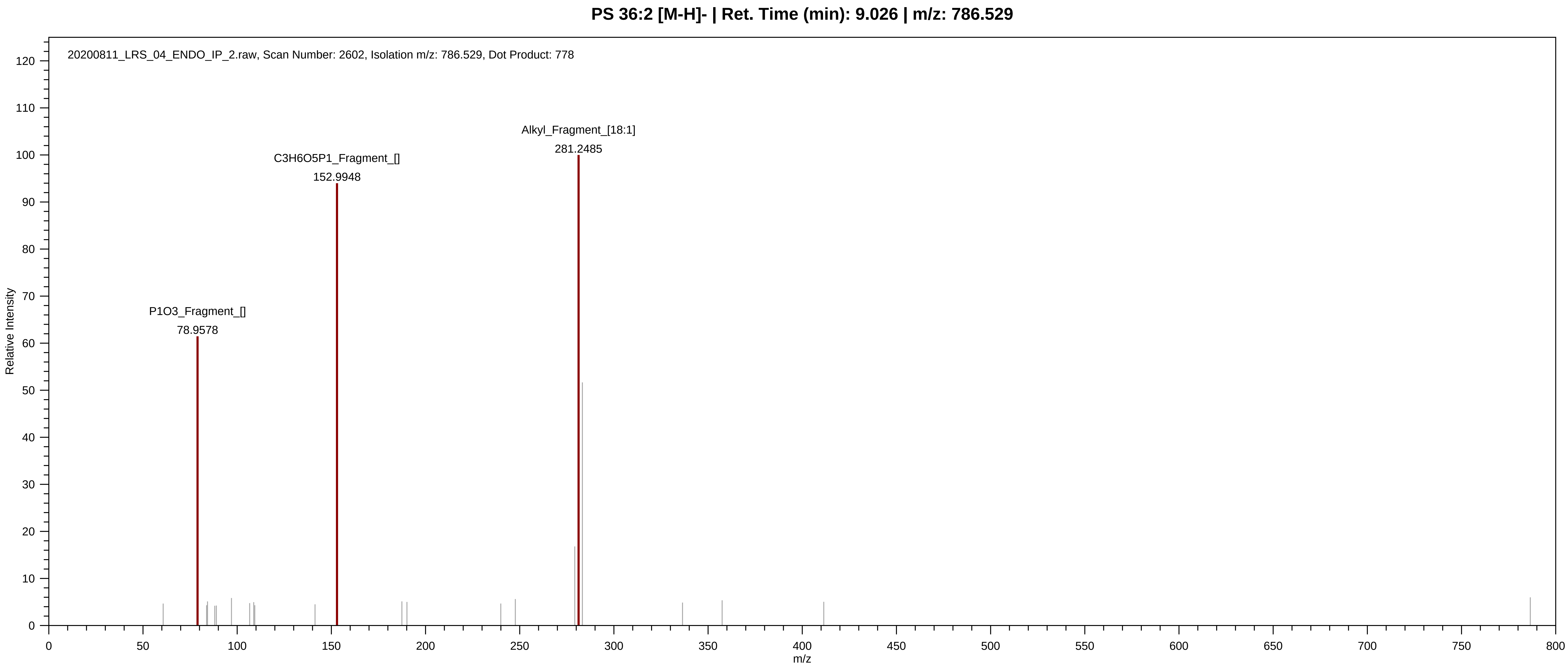

PS 36:2 [M-H]- | Ret. Time (min): 9.026 | m/z: 786.529
20200811_LRS_04_ENDO_IP_2.raw, Scan Number: 2602, Isolation m/z: 786.529, Dot Product: 778
Alkyl_Fragment_[18:1]
281.2485
C3H6O5P1_Fragment_[]
152.9948
P1O3_Fragment_[]
78.9578
Relative Intensity
m/z

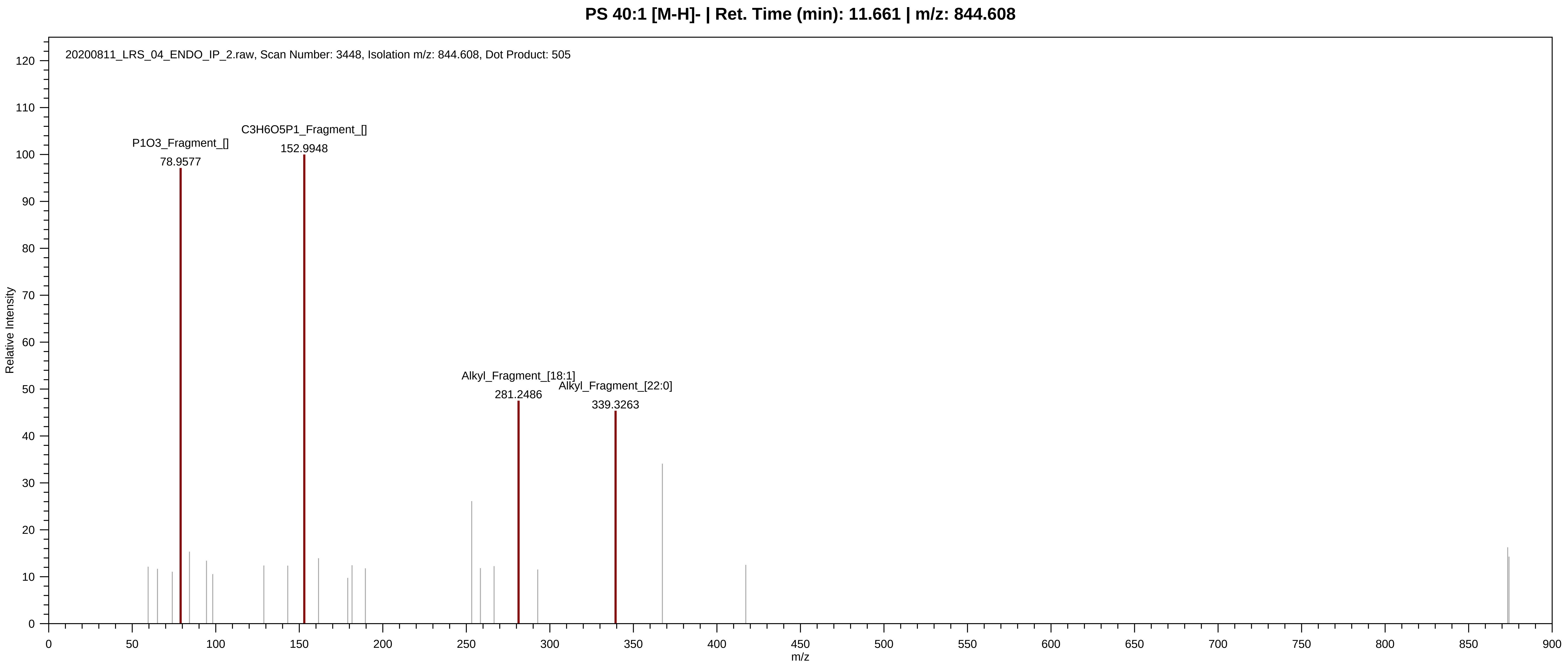
PS 40:1 [M-H]- | Ret. Time (min): 11.661 | m/z: 844.608
20200811_LRS_04_ENDO_IP_2.raw, Scan Number: 3448, Isolation m/z: 844.608, Dot Product: 505
P1O3_Fragment_[]
78.9577
C3H6O5P1_Fragment_[]
152.9948
Alkyl_Fragment_[18:1]
281.2486
Alkyl_Fragment_[22:0]
339.3263
Relative Intensity
m/z

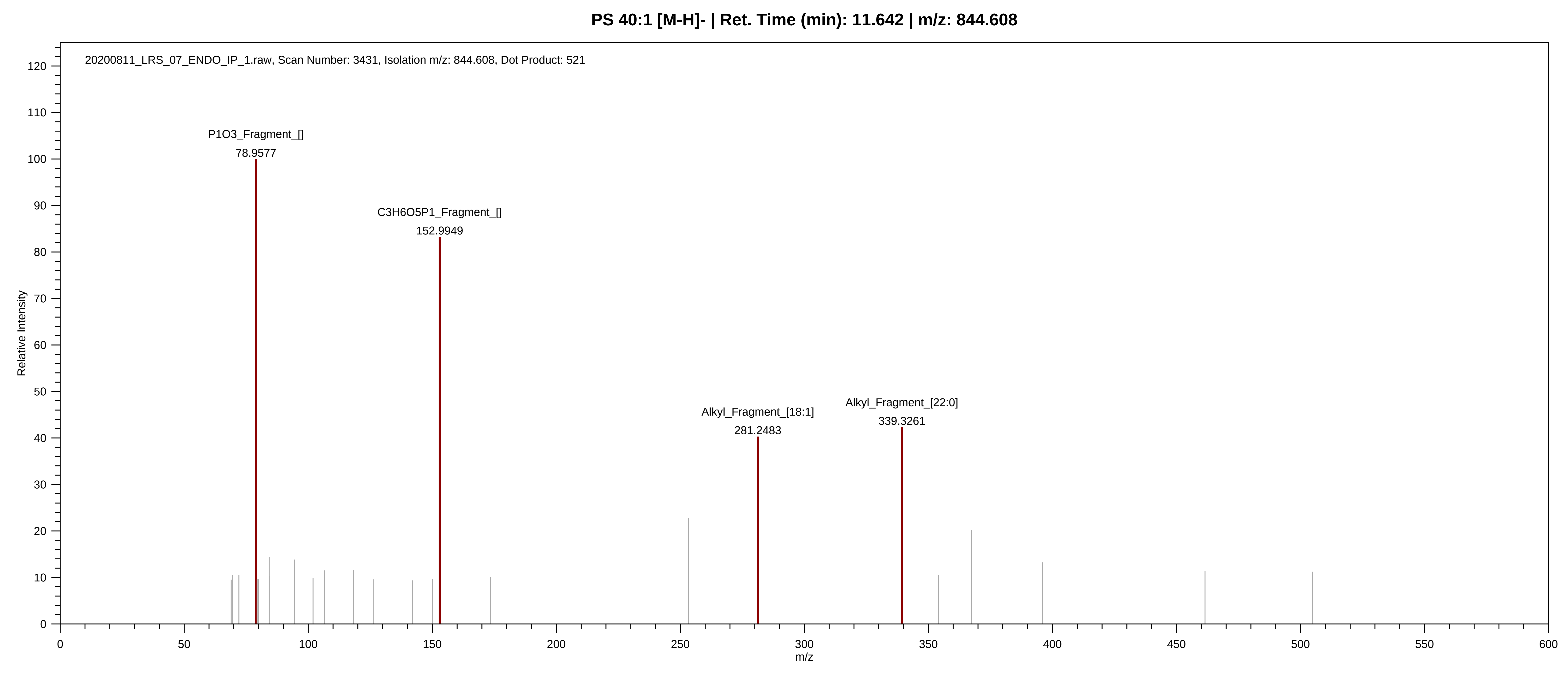
PS 40:1 [M-H]- | Ret. Time (min): 11.642 | m/z: 844.608
20200811_LRS_07_ENDO_IP_1.raw, Scan Number: 3431, Isolation m/z: 844.608, Dot Product: 521
P1O3_Fragment_[]
78.9577
C3H6O5P1_Fragment_[]
152.9949
Alkyl_Fragment_[18:1]
281.2483
Alkyl_Fragment_[22:0]
339.3261
Relative Intensity
m/z

SM d32:1 [M+H]+ | Ret. Time (min): 7.817 | m/z: 675.543

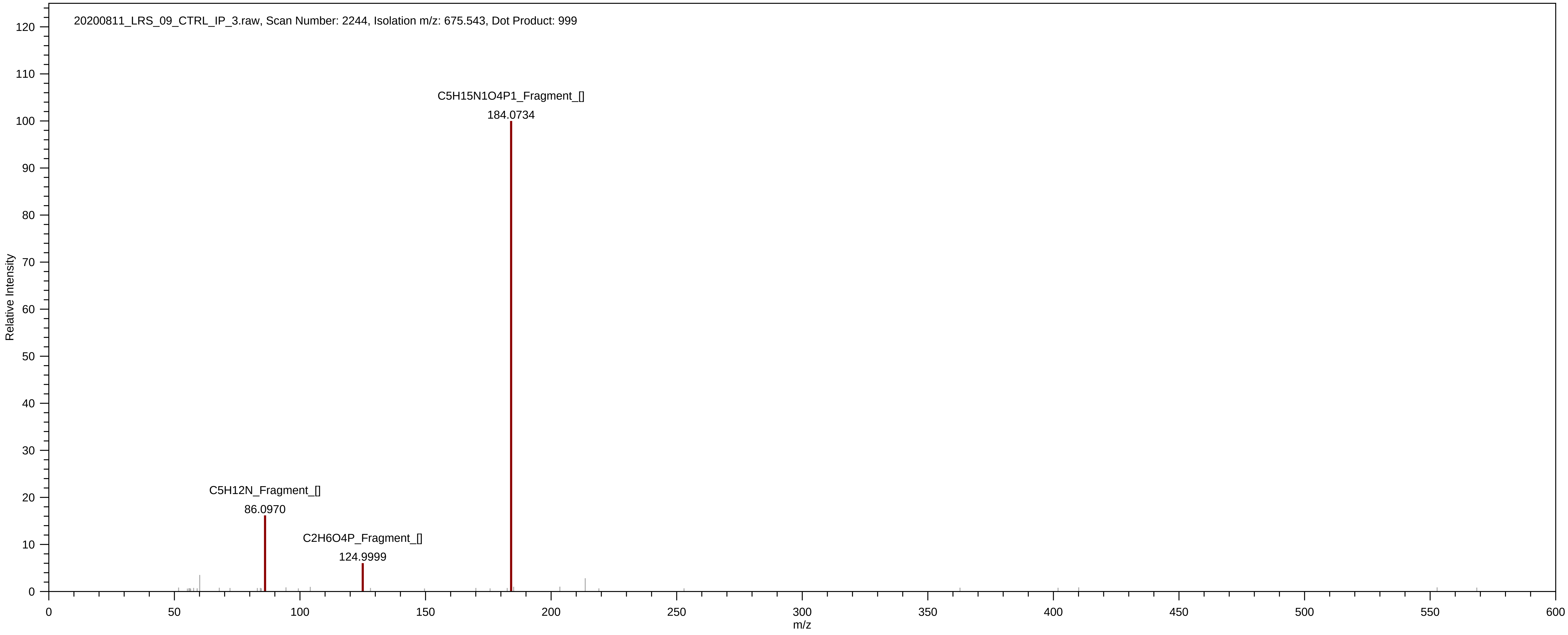

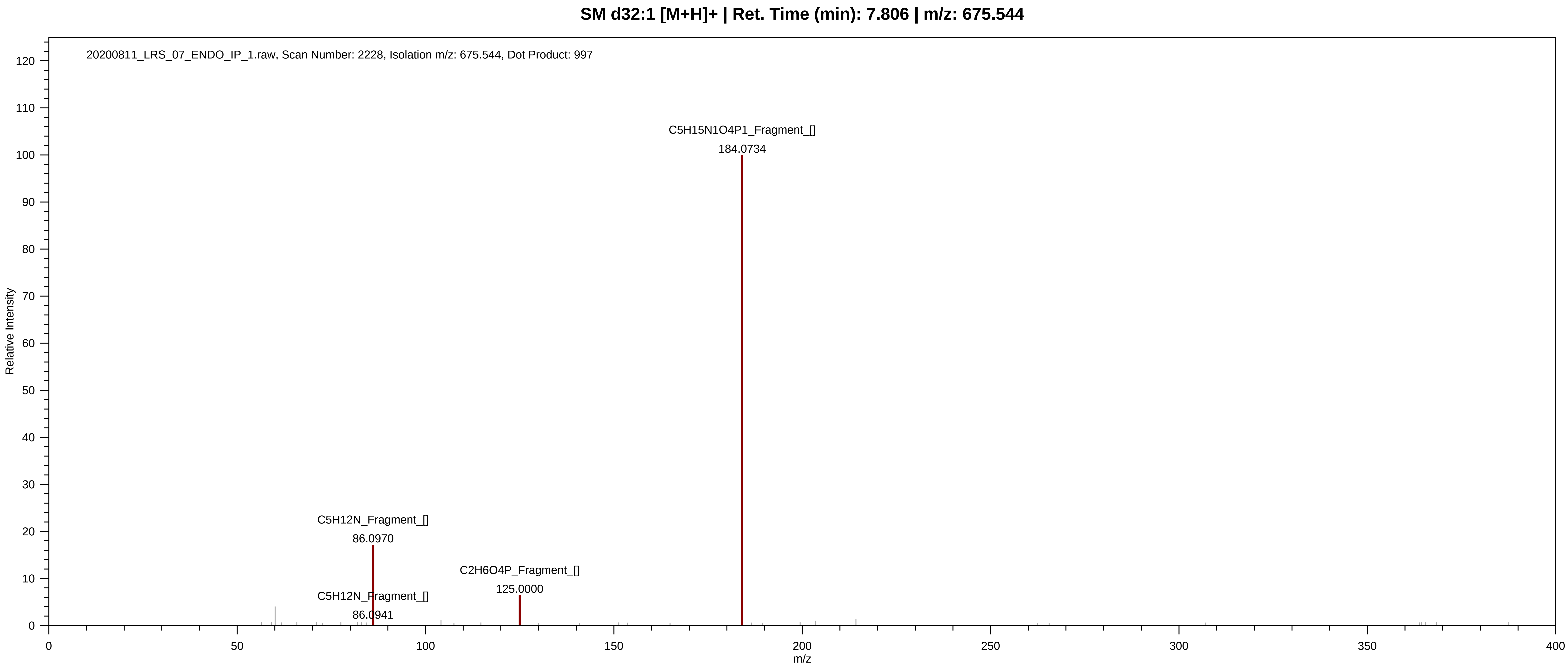
SM d32:1 [M+H]+ | Ret. Time (min): 7.806 | m/z: 675.544
20200811_LRS_07_ENDO_IP_1.raw, Scan Number: 2228, Isolation m/z: 675.544, Dot Product: 997
C5H15N1O4P1_Fragment_[]
184.0734
C5H12N_Fragment_[]
86.0970
C2H6O4P_Fragment_[]
125.0000
C5H12N_Fragment_[]
86.0941
Relative Intensity
m/z
0
10
20
30
40
50
60
70
80
90
100
110
120
0
50
100
150
200
250
300
350
400

SM d32:1 [M+H]+ | Ret. Time (min): 7.821 | m/z: 675.544

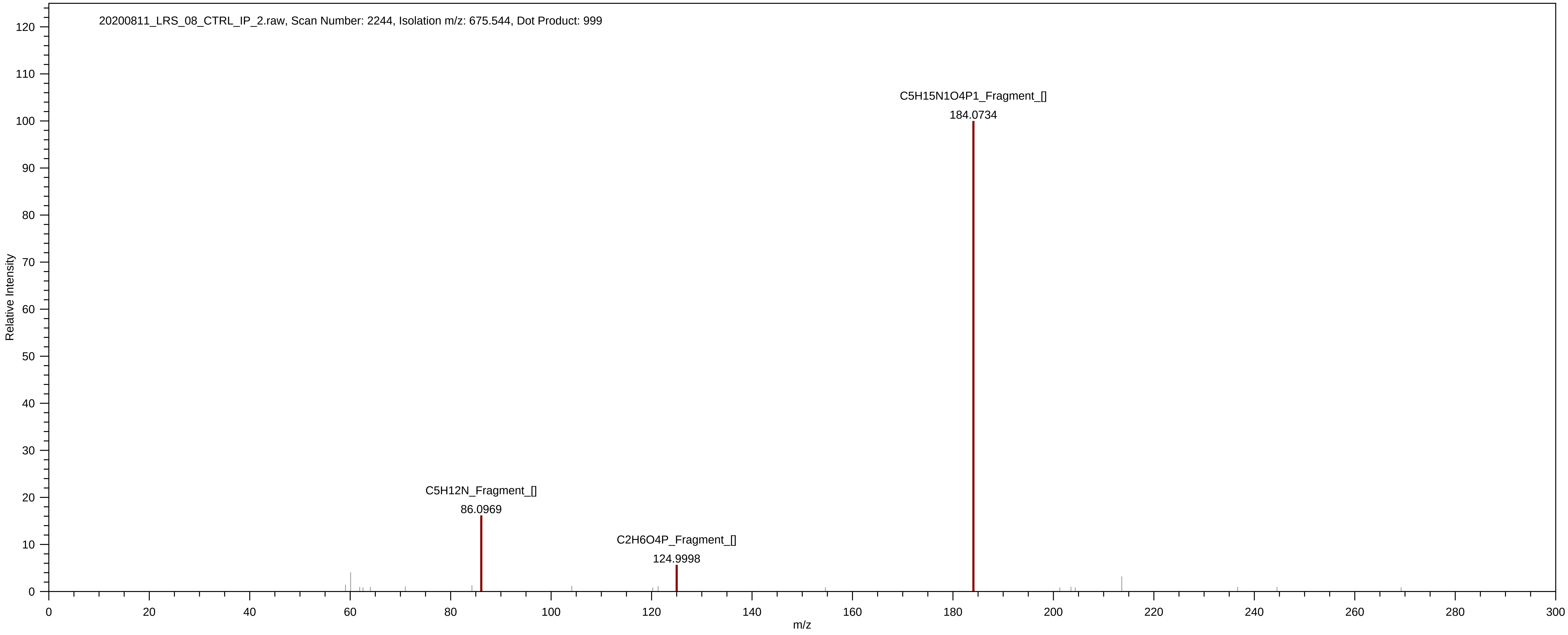

SM d32:1 [M+H]+ | Ret. Time (min): 7.825 | m/z: 675.544

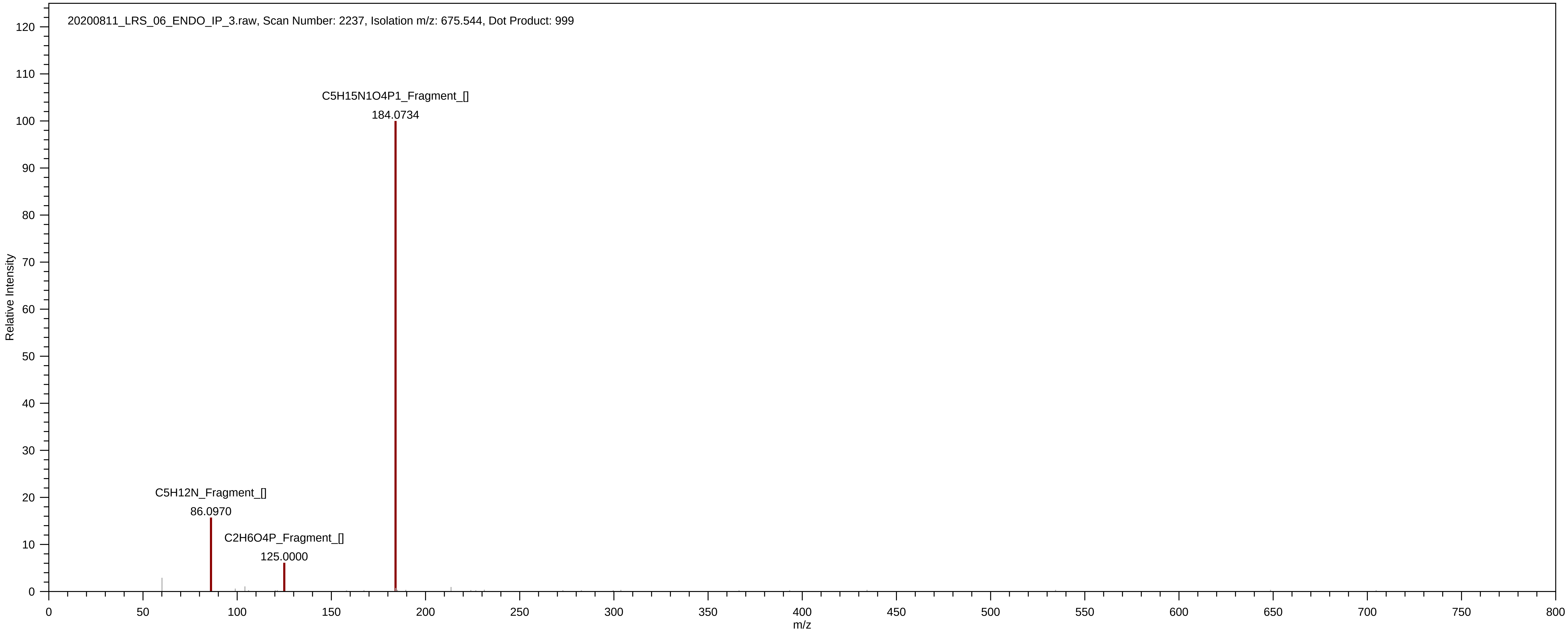

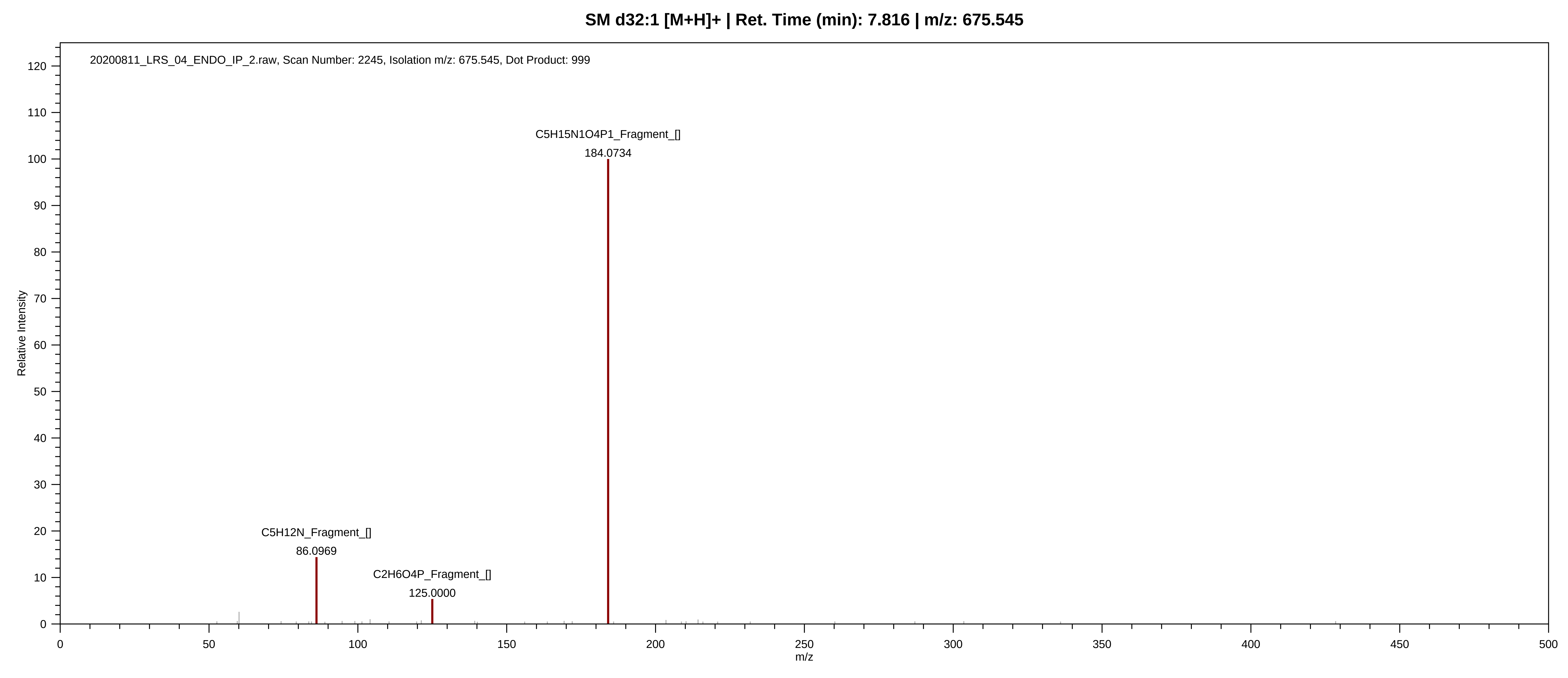

SM d32:1 [M+H]+ | Ret. Time (min): 7.816 | m/z: 675.545
20200811_LRS_04_ENDO_IP_2.raw, Scan Number: 2245, Isolation m/z: 675.545, Dot Product: 999
C5H15N1O4P1_Fragment_[]
184.0734
C5H12N_Fragment_[]
86.0969
C2H6O4P_Fragment_[]
125.0000
Relative Intensity
m/z

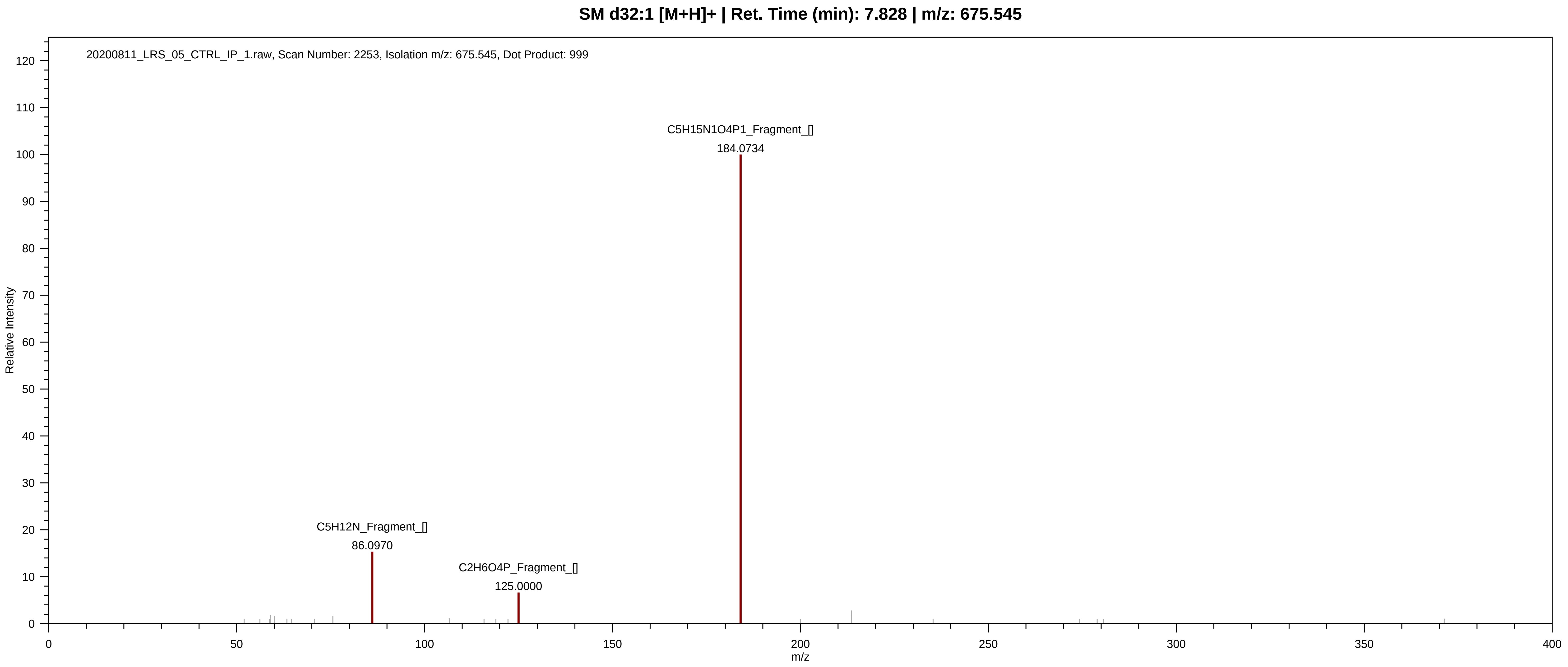
SM d32:1 [M+H]+ | Ret. Time (min): 7.828 | m/z: 675.545
20200811_LRS_05_CTRL_IP_1.raw, Scan Number: 2253, Isolation m/z: 675.545, Dot Product: 999
C5H15N1O4P1_Fragment_[]
184.0734
C5H12N_Fragment_[]
86.0970
C2H6O4P_Fragment_[]
125.0000
Relative Intensity
m/z
0
10
20
30
40
50
60
70
80
90
100
110
120
0
50
100
150
200
250
300
350
400

SM d32:2 [M+H]+ | Ret. Time (min): 7.185 | m/z: 673.528

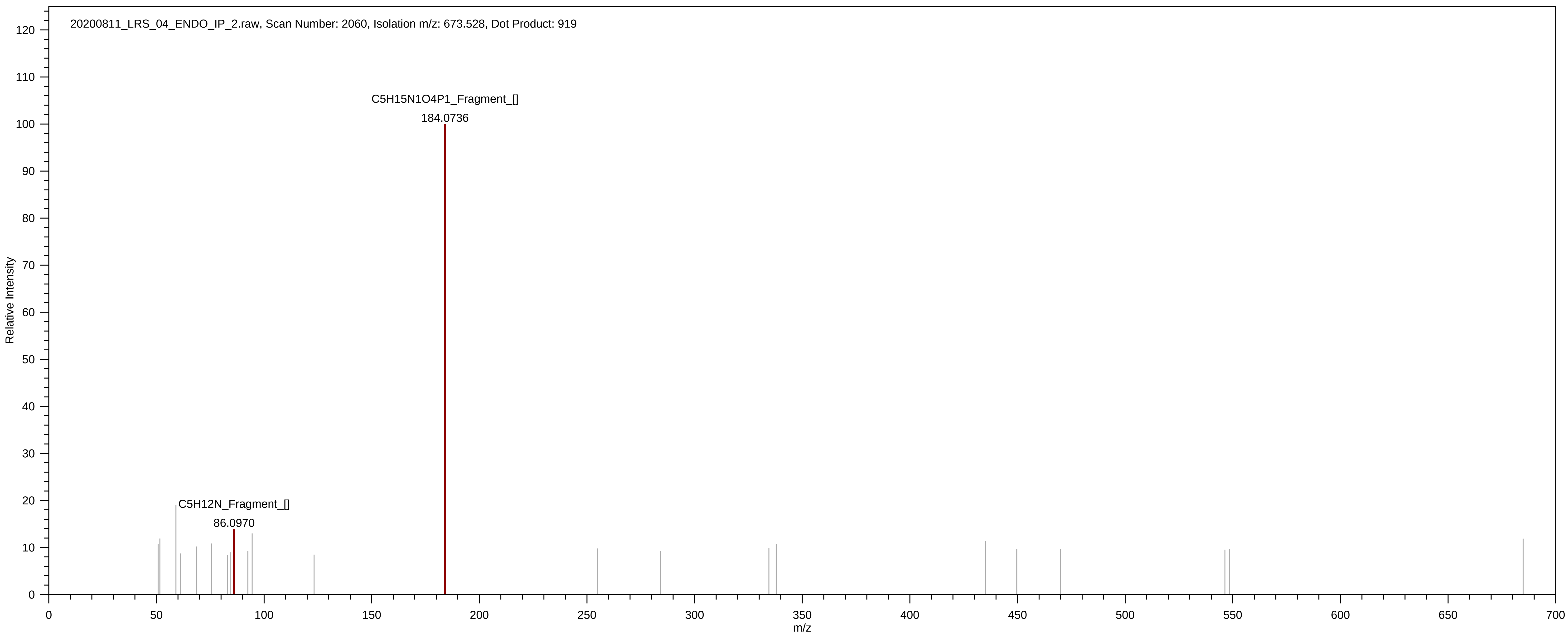

SM d33:1 [M+H]+ | Ret. Time (min): 8.29 | m/z: 689.56

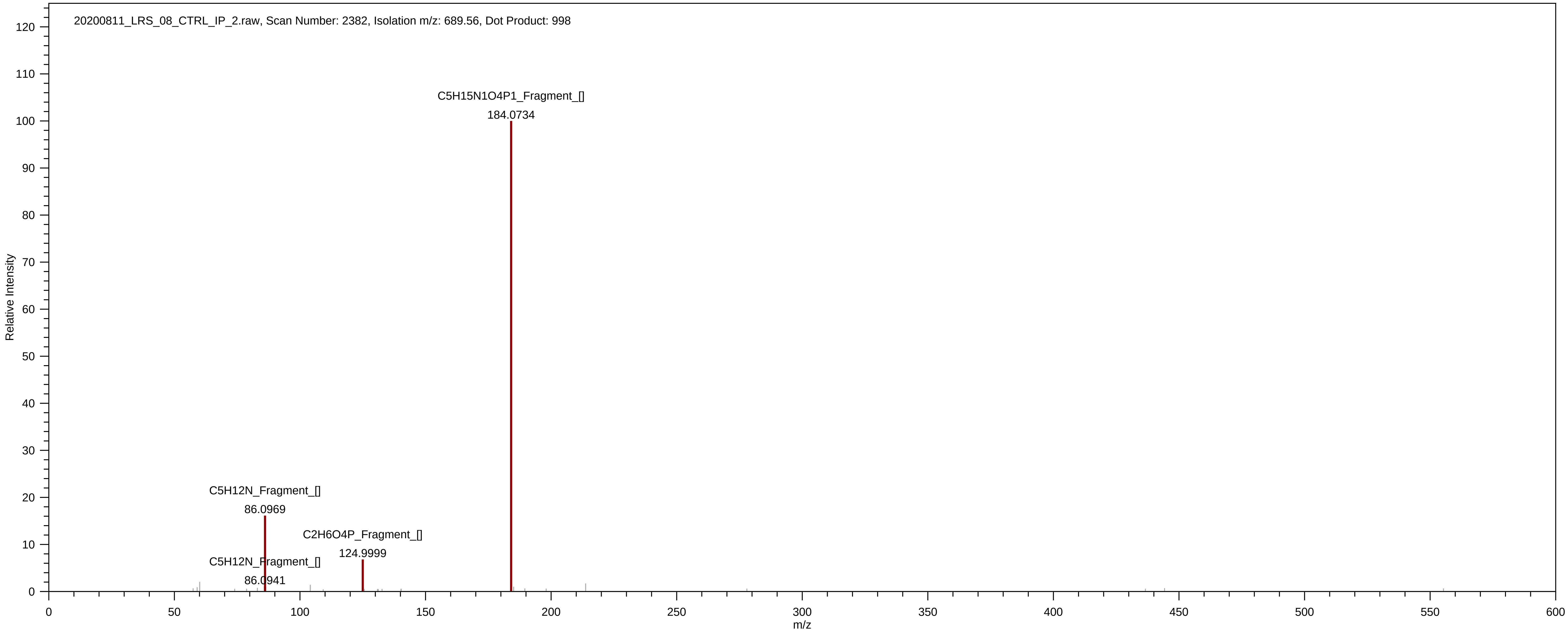

SM d33:1 [M+H]+ | Ret. Time (min): 8.275 | m/z: 689.56

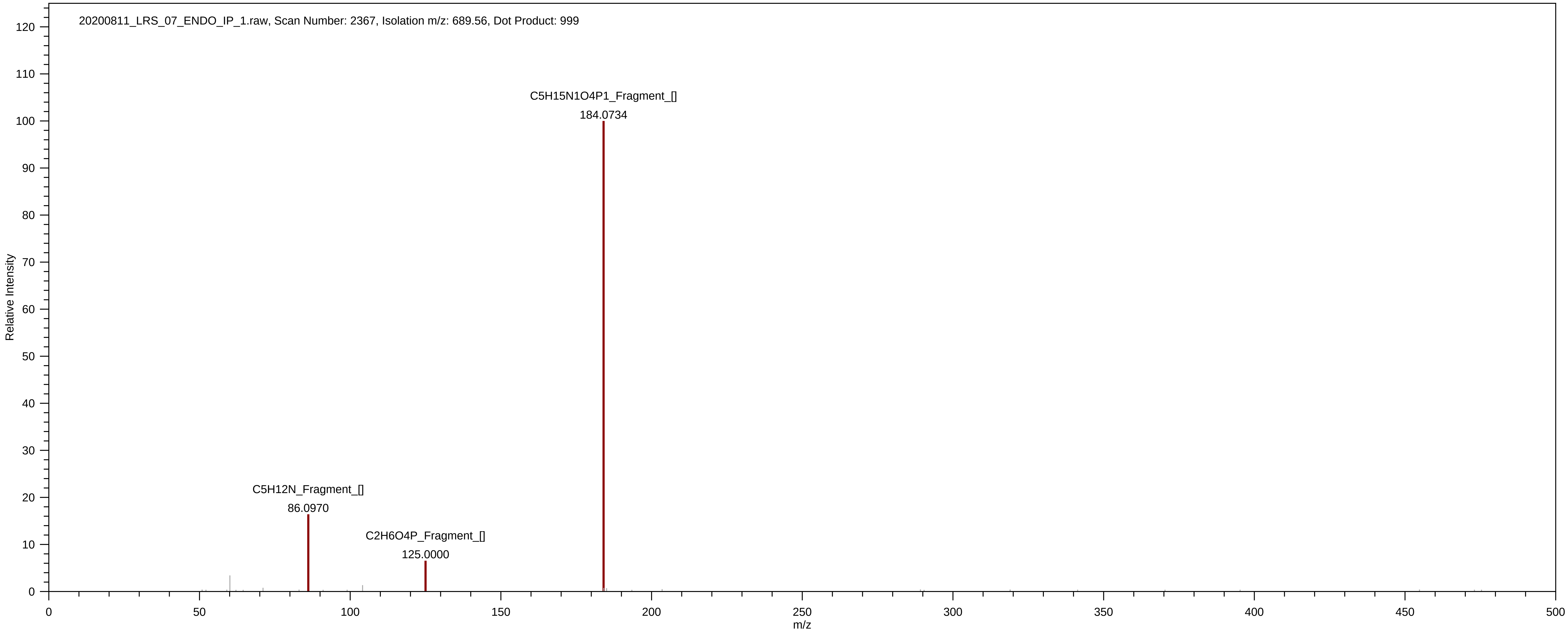

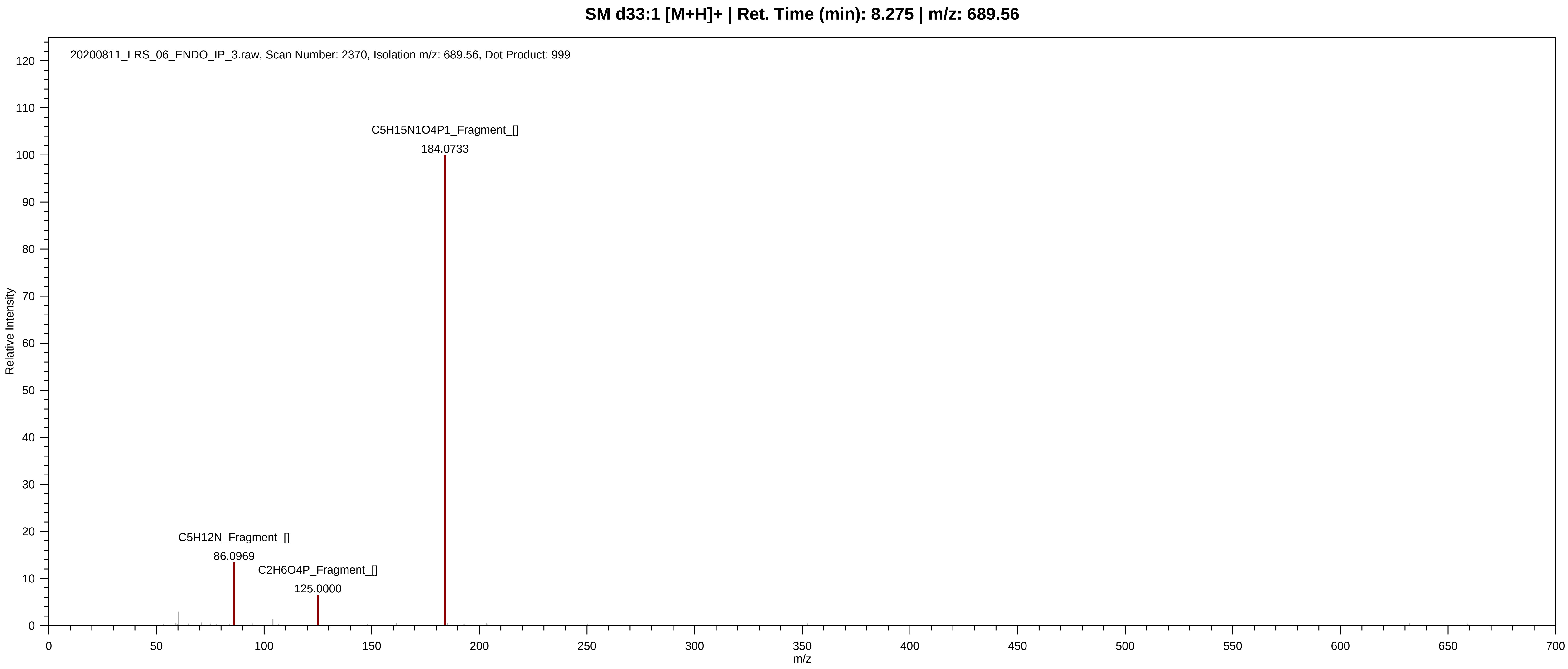
SM d33:1 [M+H]+ | Ret. Time (min): 8.275 | m/z: 689.56
20200811_LRS_06_ENDO_IP_3.raw, Scan Number: 2370, Isolation m/z: 689.56, Dot Product: 999
C5H15N1O4P1_Fragment_[]
184.0733
C5H12N_Fragment_[]
86.0969
C2H6O4P_Fragment_[]
125.0000
Relative Intensity
m/z

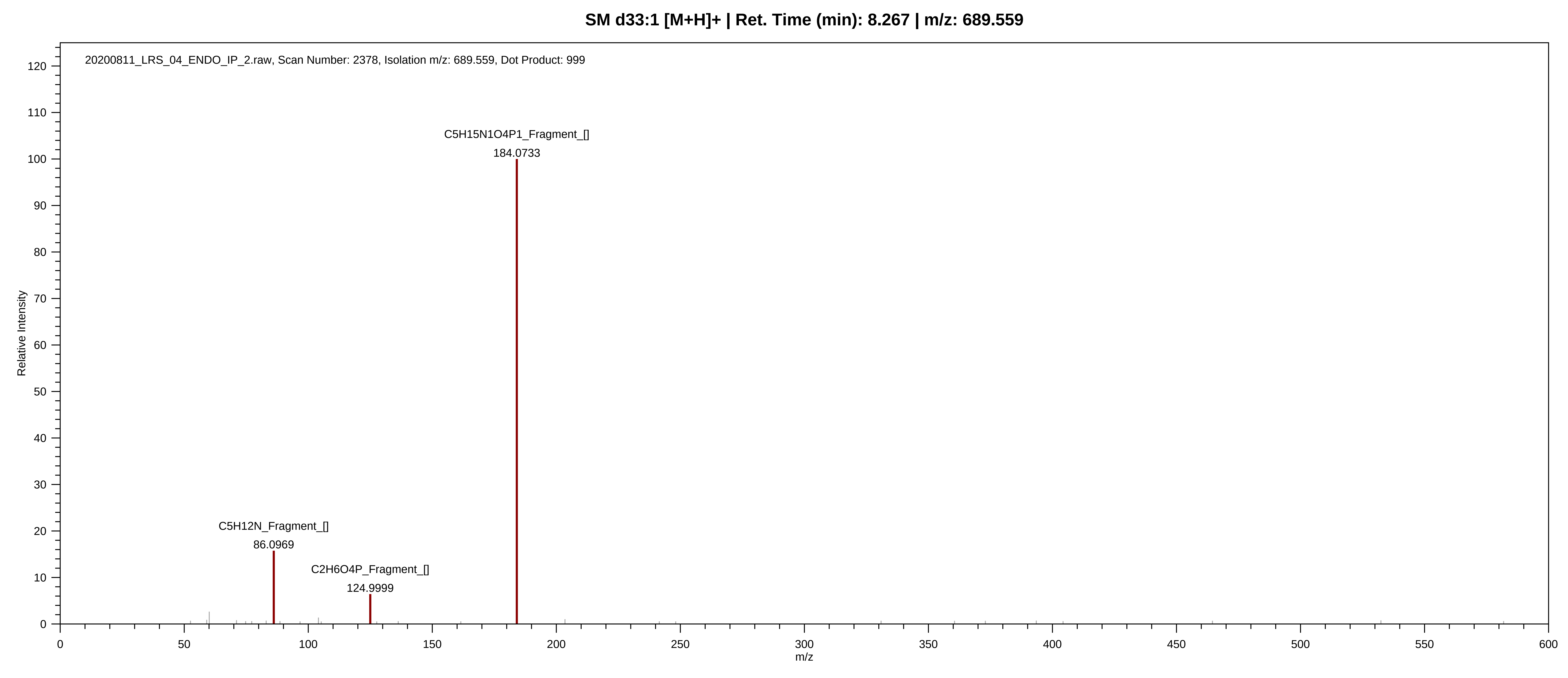
SM d33:1 [M+H]+ | Ret. Time (min): 8.267 | m/z: 689.559
20200811_LRS_04_ENDO_IP_2.raw, Scan Number: 2378, Isolation m/z: 689.559, Dot Product: 999
C5H15N1O4P1_Fragment_[]
184.0733
C5H12N_Fragment_[]
86.0969
C2H6O4P_Fragment_[]
124.9999
Relative Intensity
m/z

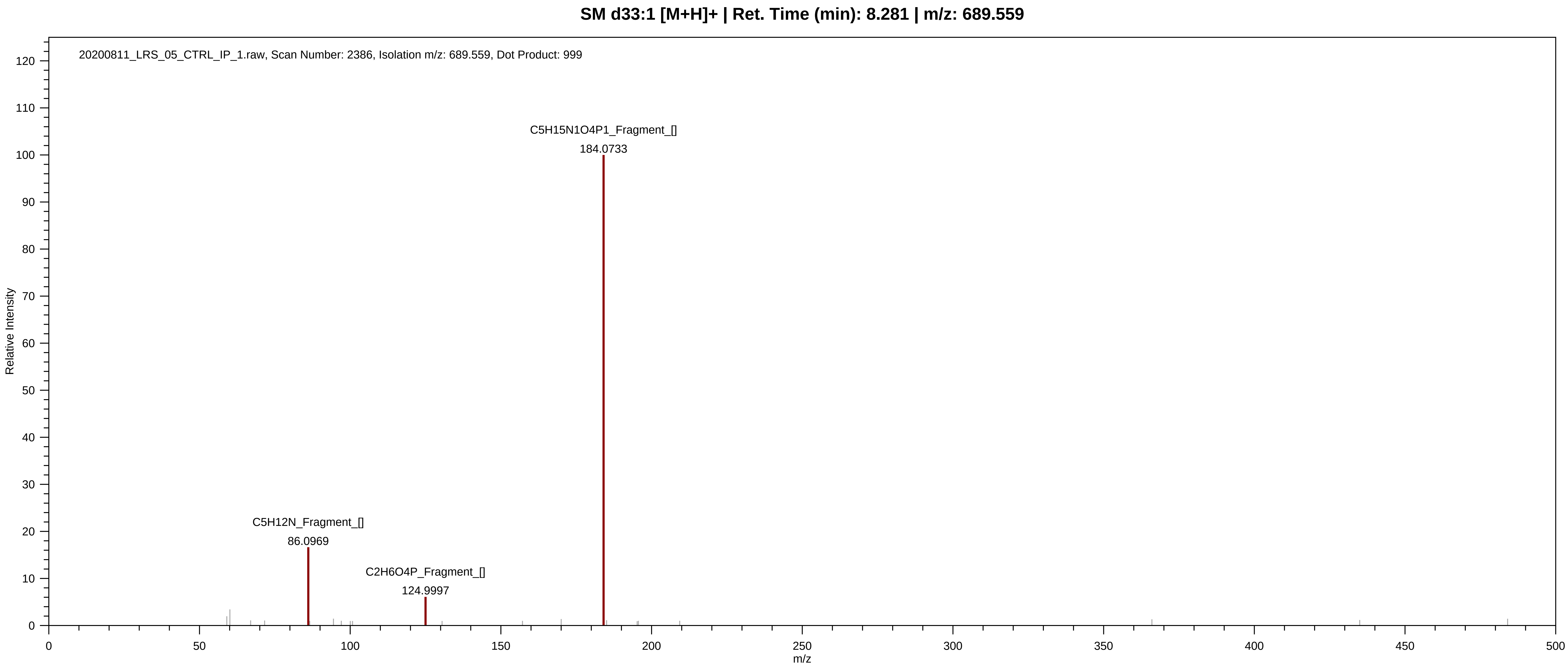
SM d33:1 [M+H]+ | Ret. Time (min): 8.281 | m/z: 689.559
20200811_LRS_05_CTRL_IP_1.raw, Scan Number: 2386, Isolation m/z: 689.559, Dot Product: 999
C5H15N1O4P1_Fragment_[]
184.0733
C5H12N_Fragment_[]
86.0969
C2H6O4P_Fragment_[]
124.9997
Relative Intensity
m/z
0
10
20
30
40
50
60
70
80
90
100
110
120
0
50
100
150
200
250
300
350
400
450
500

SM d33:1 [M+H]+ | Ret. Time (min): 8.288 | m/z: 689.559

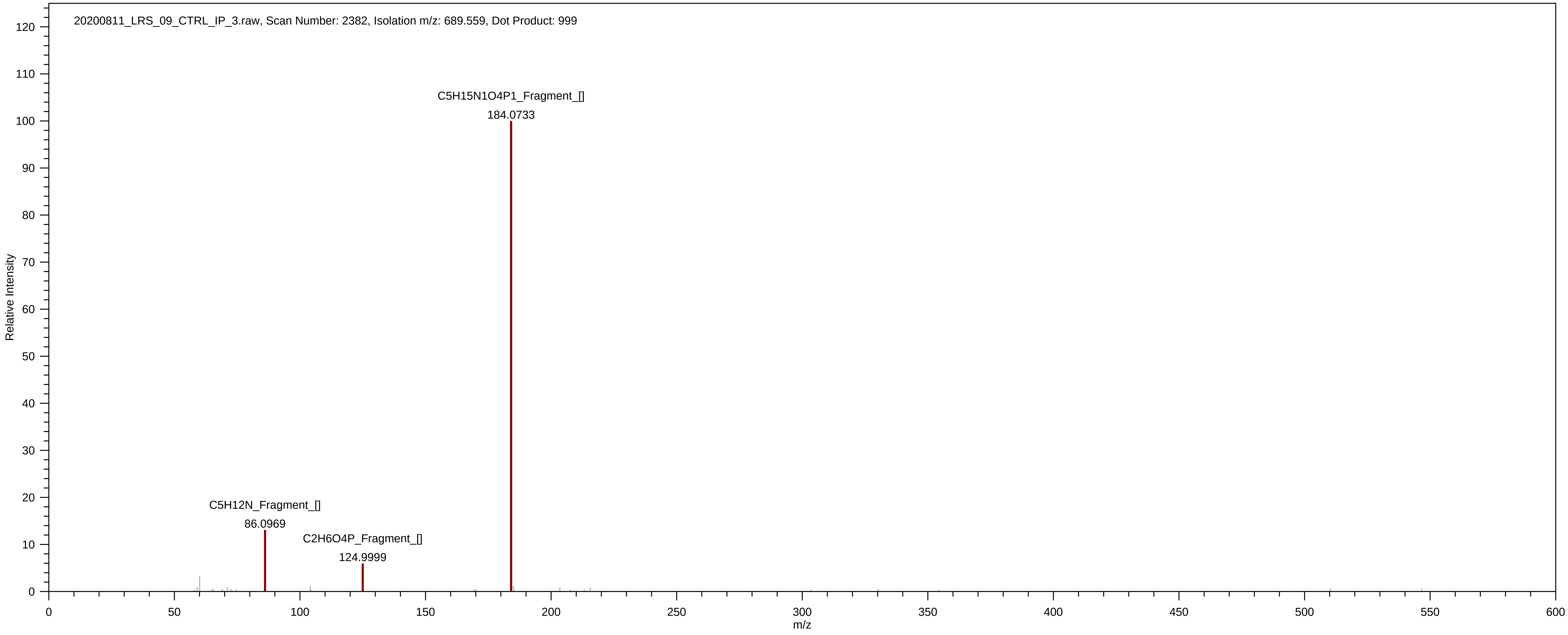

SM d34:0 [M+H]+ | Ret. Time (min): 9.181 | m/z: 705.591

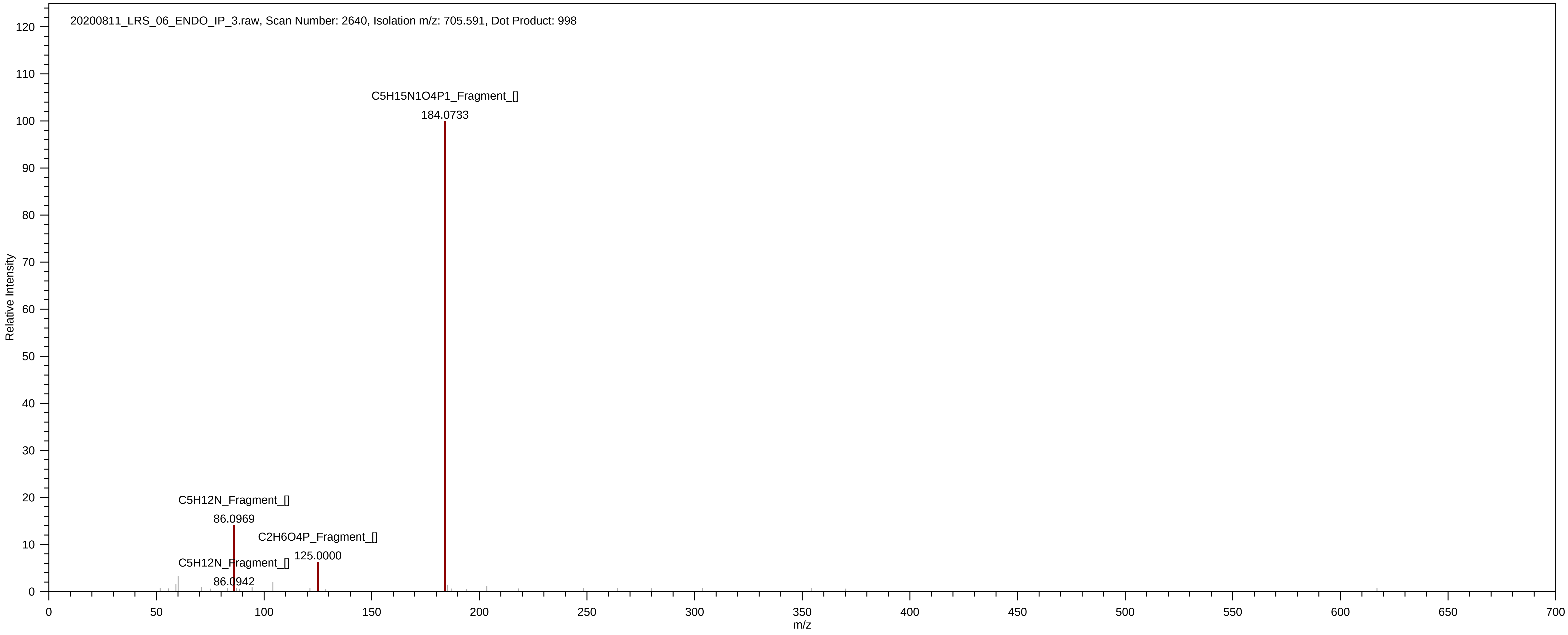

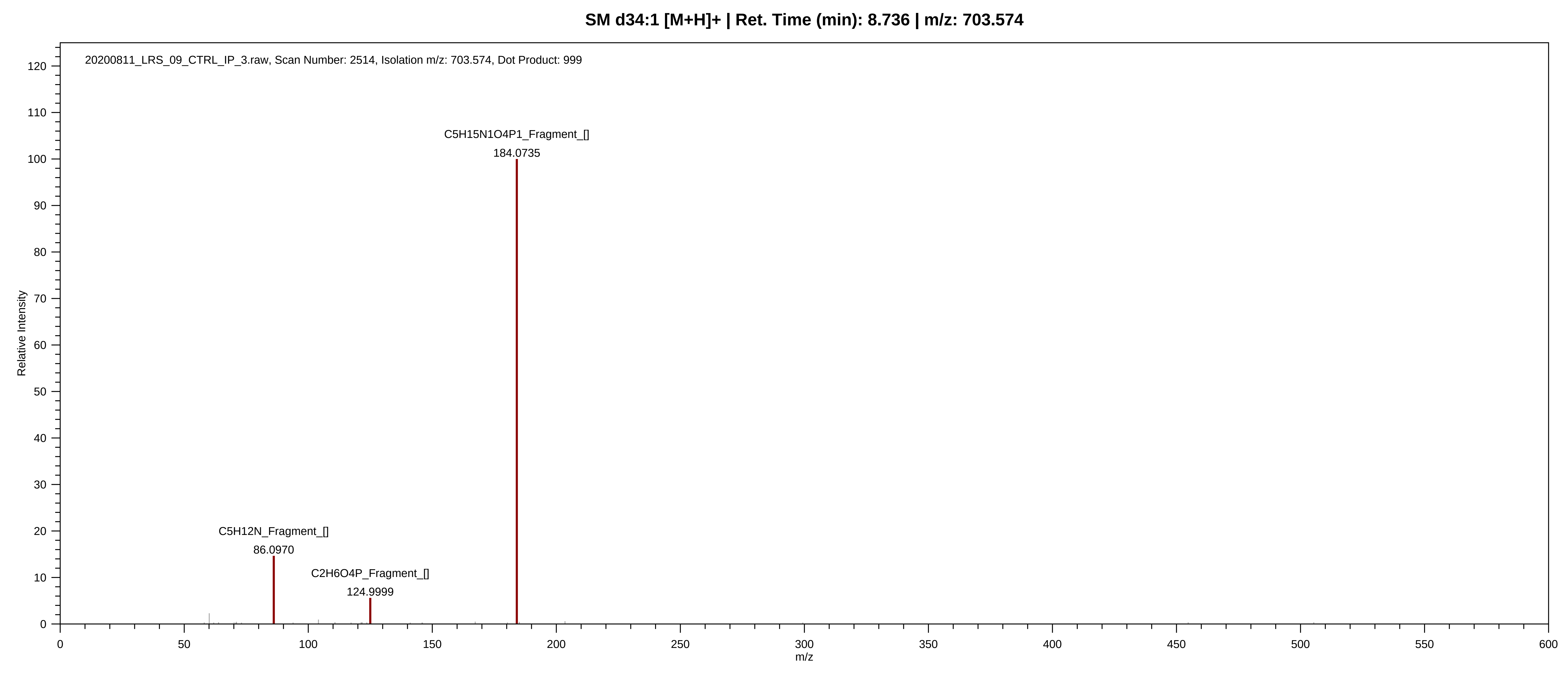

SM d34:1 [M+H]+ | Ret. Time (min): 8.736 | m/z: 703.574
20200811_LRS_09_CTRL_IP_3.raw, Scan Number: 2514, Isolation m/z: 703.574, Dot Product: 999
C5H15N1O4P1_Fragment_[]
184.0735
C5H12N_Fragment_[]
86.0970
C2H6O4P_Fragment_[]
124.9999
Relative Intensity
m/z

SM d34:1 [M+H]+ | Ret. Time (min): 8.745 | m/z: 703.574

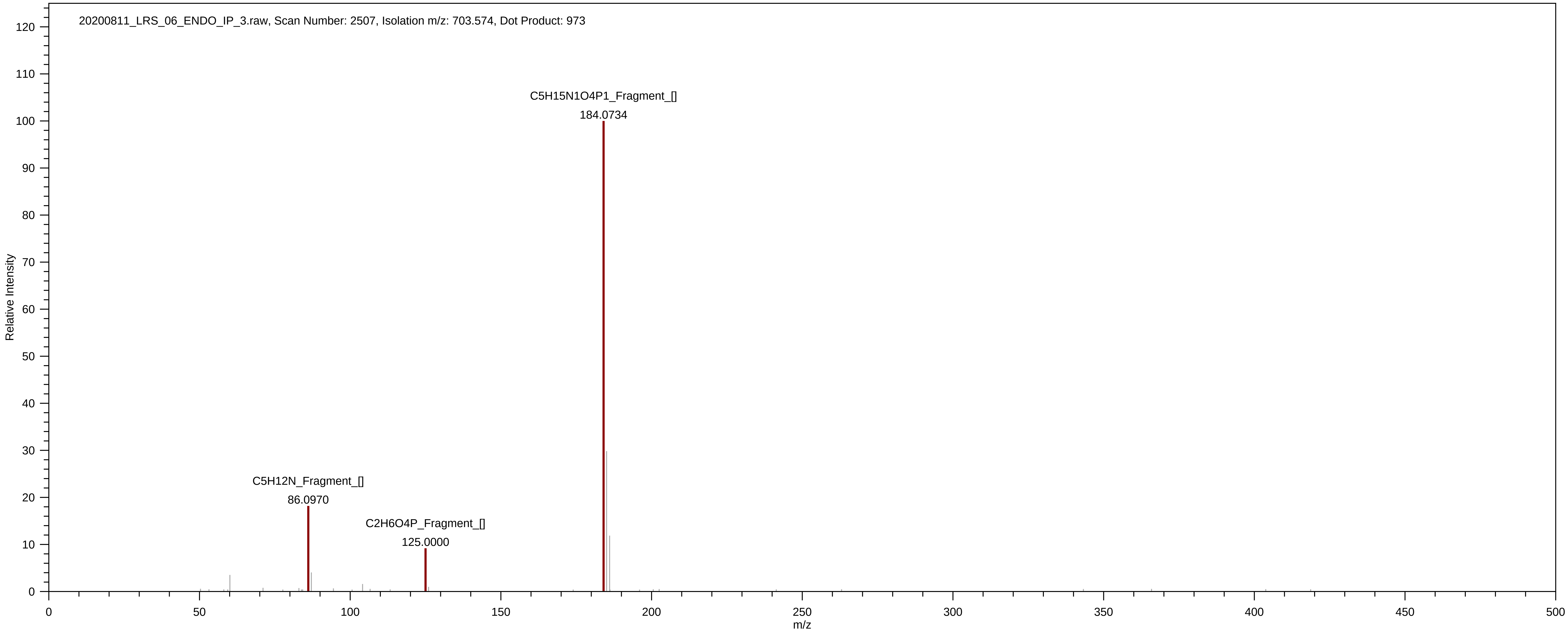

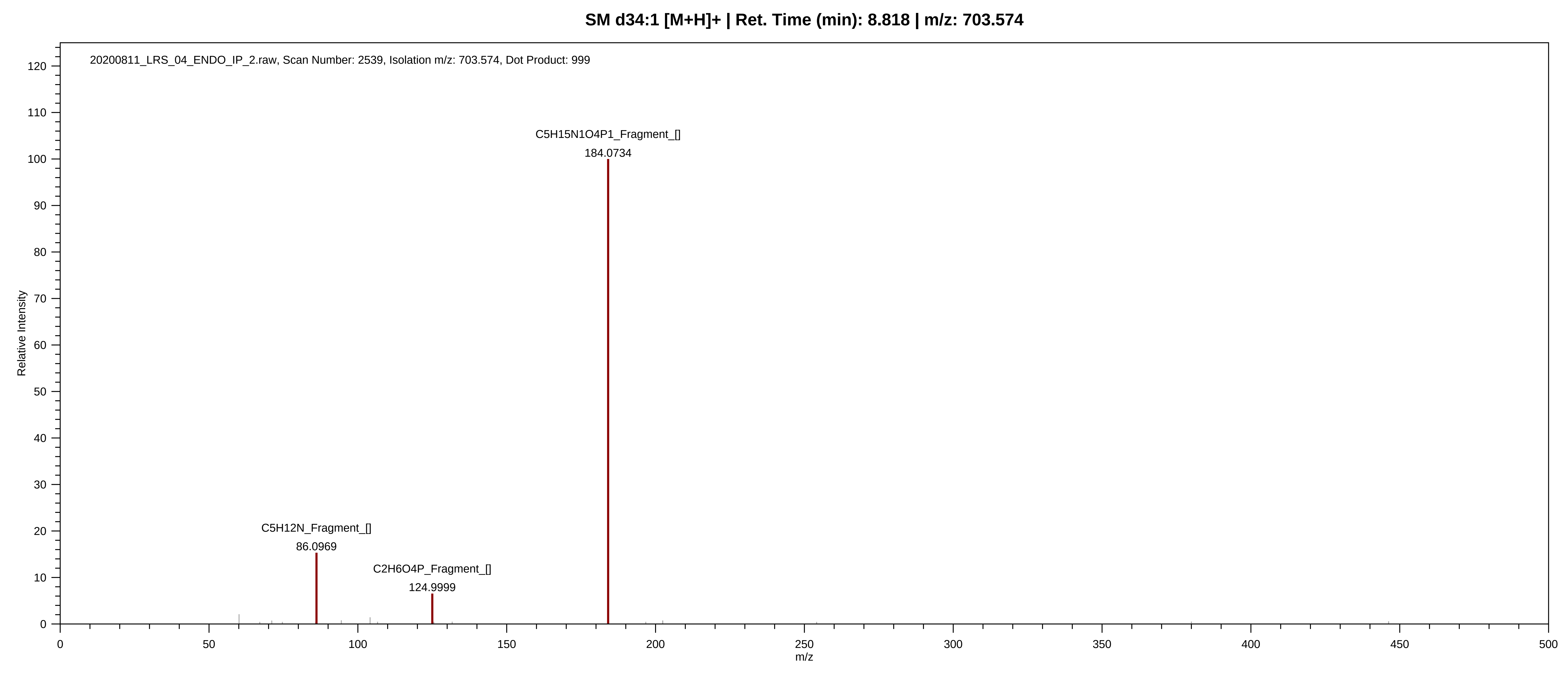

SM d34:1 [M+H]+ | Ret. Time (min): 8.818 | m/z: 703.574
20200811_LRS_04_ENDO_IP_2.raw, Scan Number: 2539, Isolation m/z: 703.574, Dot Product: 999
C5H15N1O4P1_Fragment_[]
184.0734
C5H12N_Fragment_[]
86.0969
C2H6O4P_Fragment_[]
124.9999
Relative Intensity
m/z

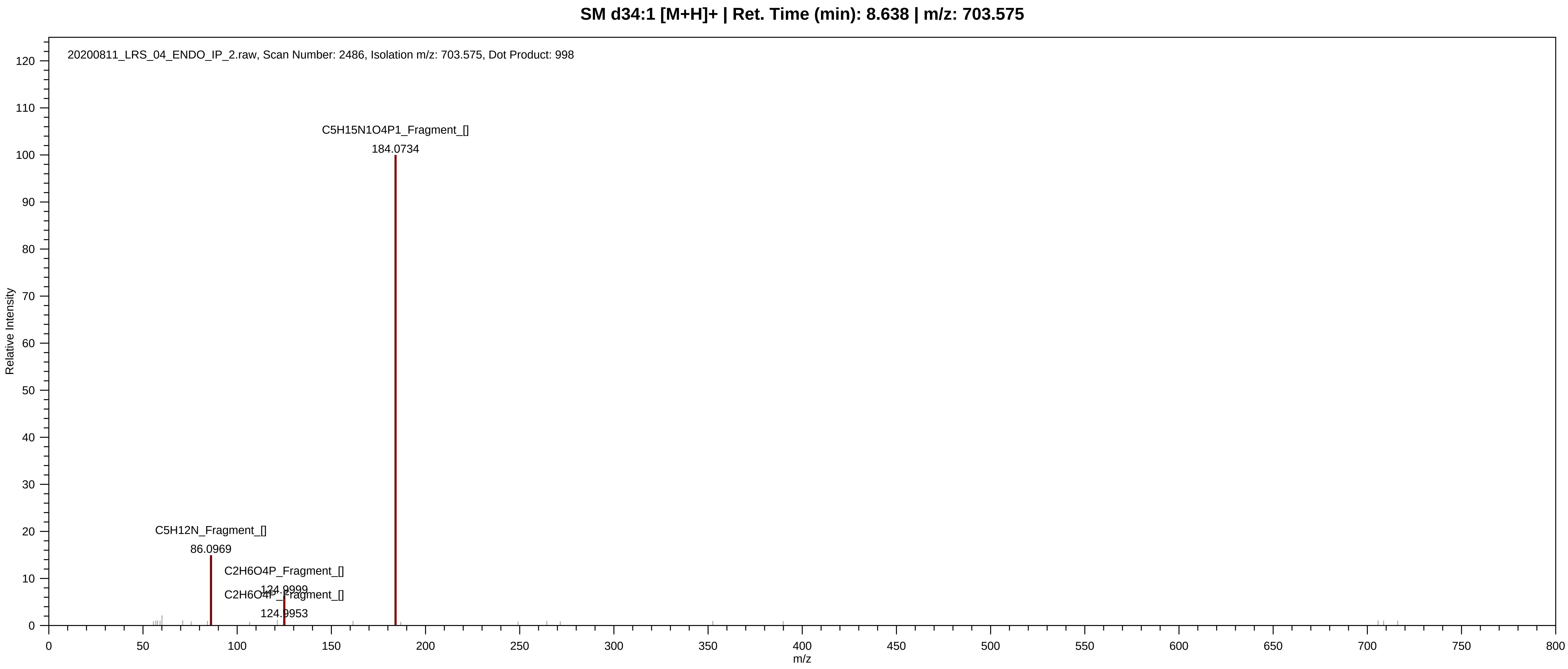
SM d34:1 [M+H]+ | Ret. Time (min): 8.638 | m/z: 703.575
20200811_LRS_04_ENDO_IP_2.raw, Scan Number: 2486, Isolation m/z: 703.575, Dot Product: 998
C5H15N1O4P1_Fragment_[]
184.0734
C5H12N_Fragment_[]
86.0969
C2H6O4P_Fragment_[]
124.9999
C2H6O4P_Fragment_[]
124.9953
Relative Intensity
m/z

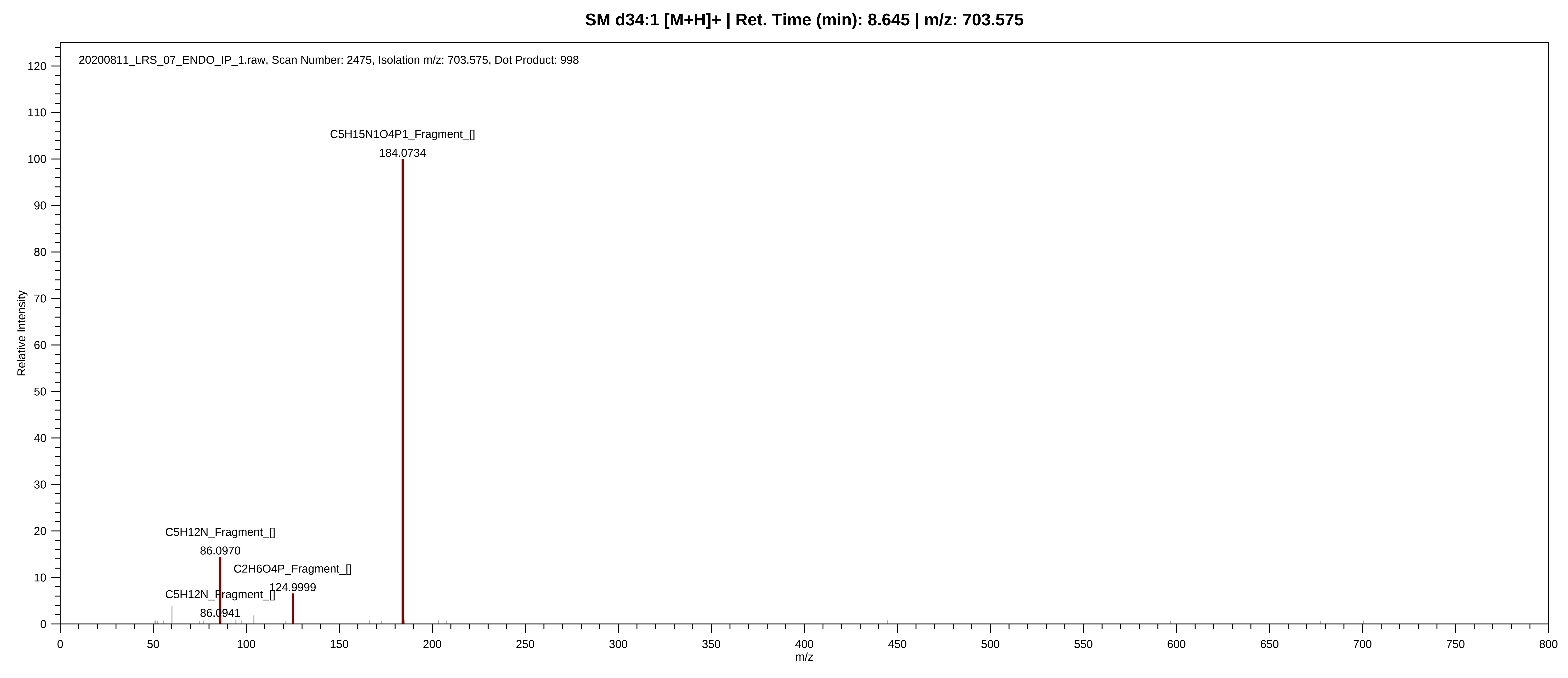

SM d34:1 [M+H]+ | Ret. Time (min): 8.645 | m/z: 703.575
20200811_LRS_07_ENDO_IP_1.raw, Scan Number: 2475, Isolation m/z: 703.575, Dot Product: 998
C5H15N1O4P1_Fragment_[]
184.0734
C5H12N_Fragment_[]
86.0970
C2H6O4P_Fragment_[]
124.9999
C5H12N_Fragment_[]
86.0941
Relative Intensity
m/z

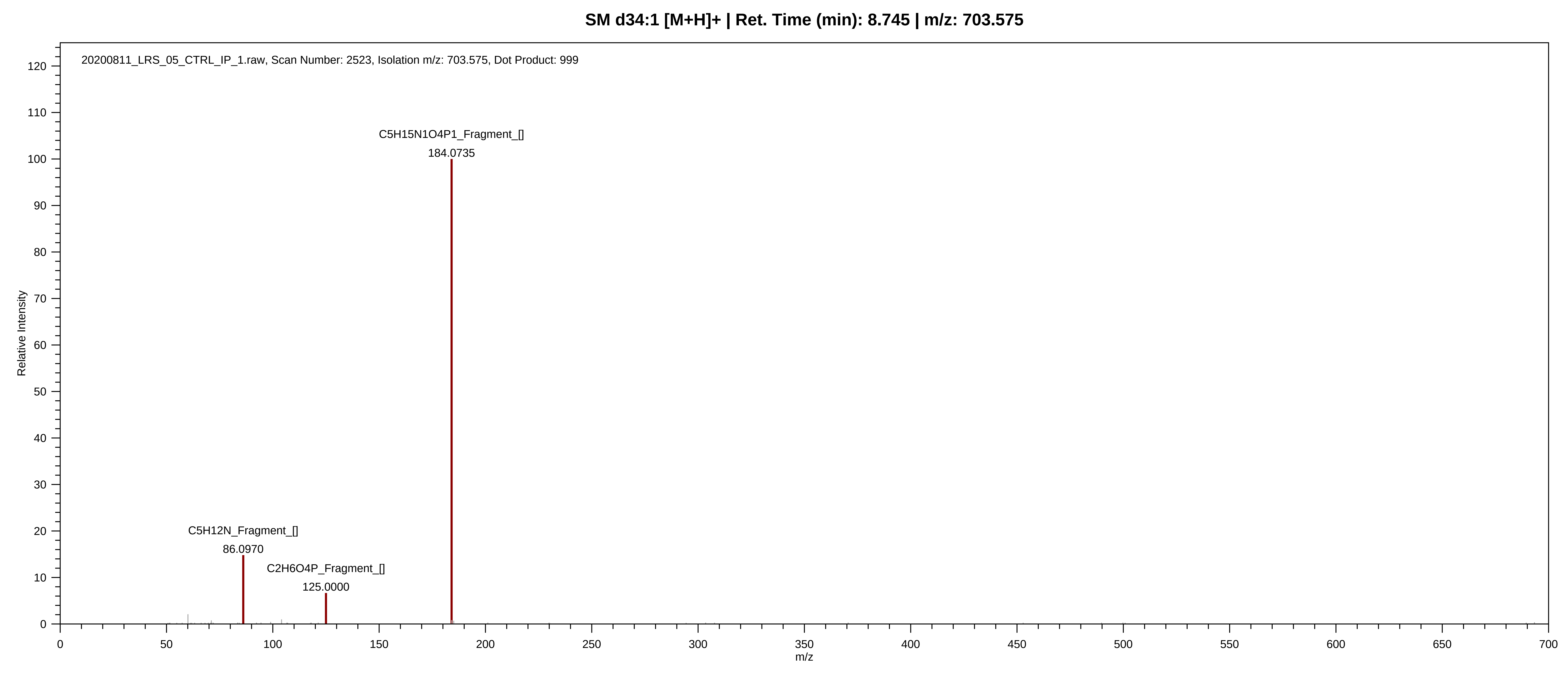
SM d34:1 [M+H]+ | Ret. Time (min): 8.745 | m/z: 703.575
20200811_LRS_05_CTRL_IP_1.raw, Scan Number: 2523, Isolation m/z: 703.575, Dot Product: 999
C5H15N1O4P1_Fragment_[]
184.0735
C5H12N_Fragment_[]
86.0970
C2H6O4P_Fragment_[]
125.0000
Relative Intensity
m/z

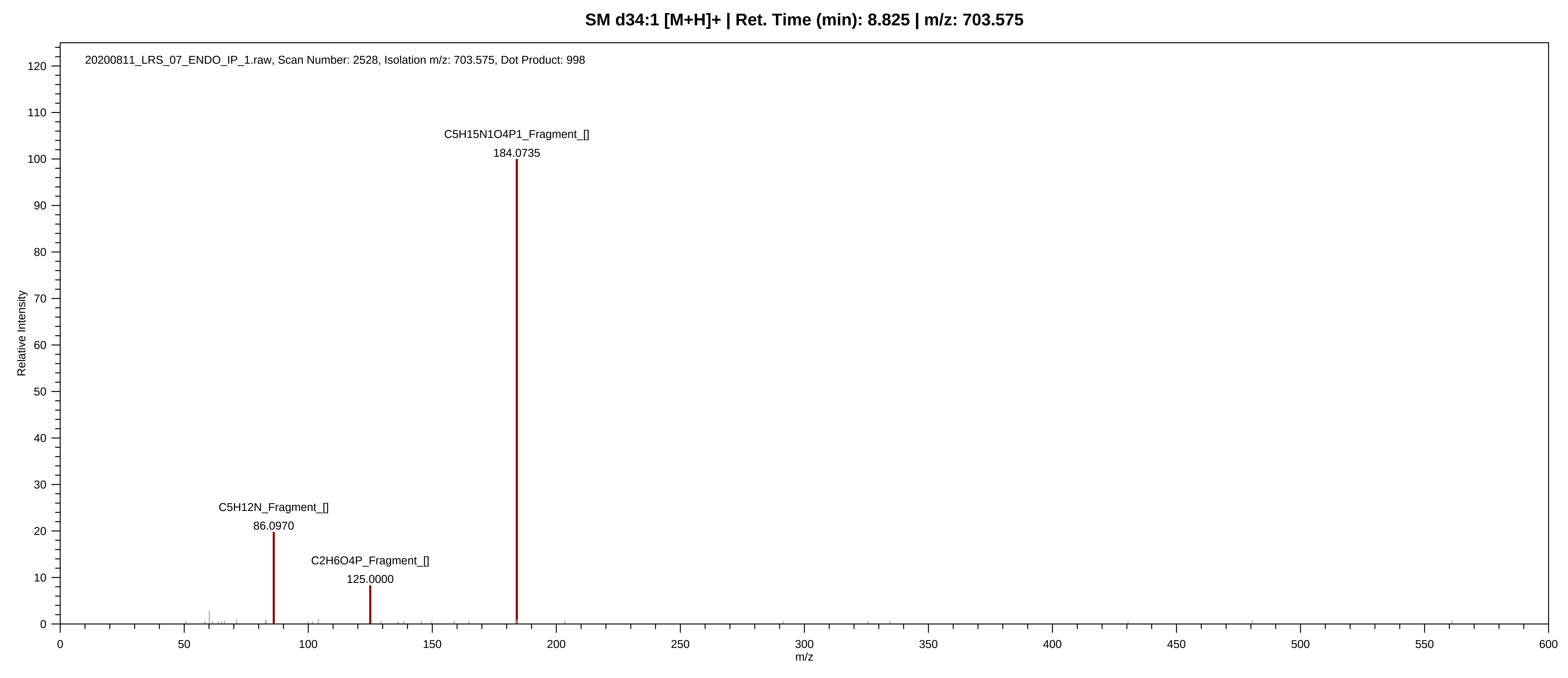
SM d34:1 [M+H]+ | Ret. Time (min): 8.825 | m/z: 703.575
20200811_LRS_07_ENDO_IP_1.raw, Scan Number: 2528, Isolation m/z: 703.575, Dot Product: 998
C5H15N1O4P1_Fragment_[]
184.0735
C5H12N_Fragment_[]
86.0970
C2H6O4P_Fragment_[]
125.0000
Relative Intensity
m/z

SM d34:1 [M+H]+ | Ret. Time (min): 8.863 | m/z: 703.575

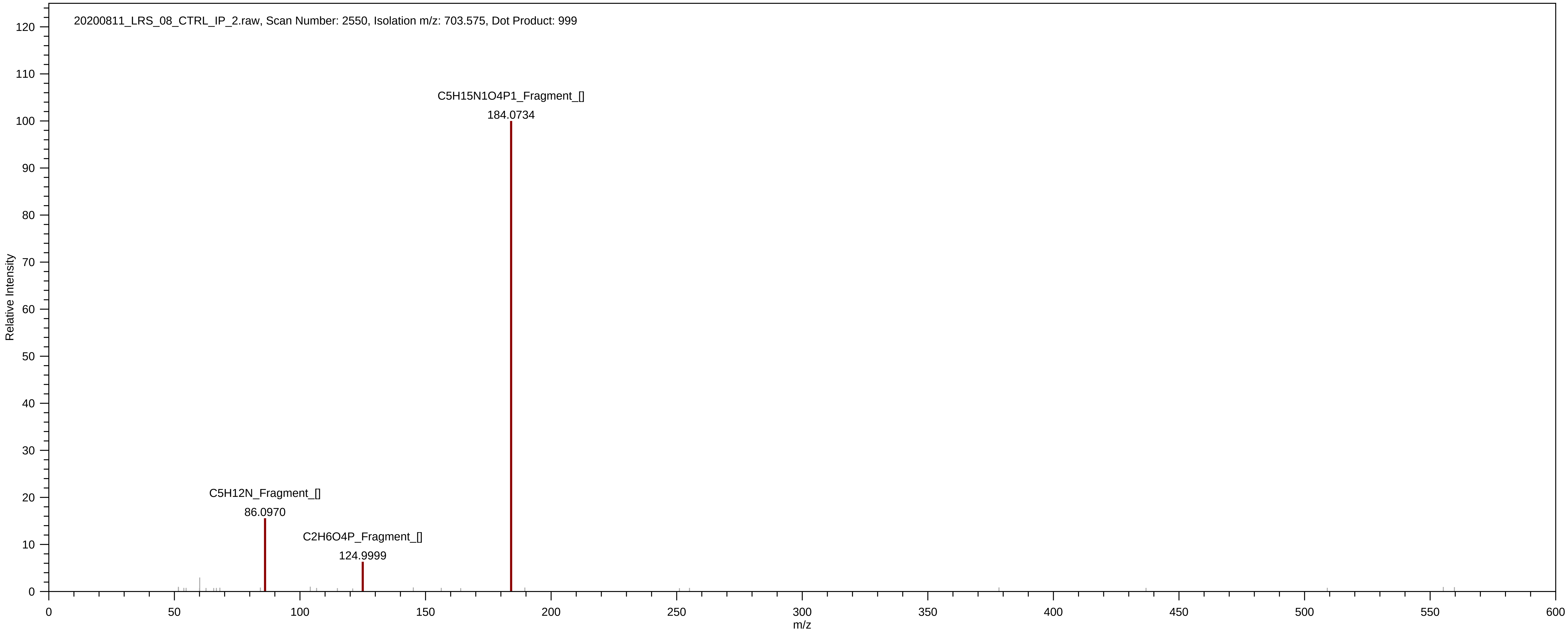

SM d34:1 [M+H]+ | Ret. Time (min): 8.645 | m/z: 703.576

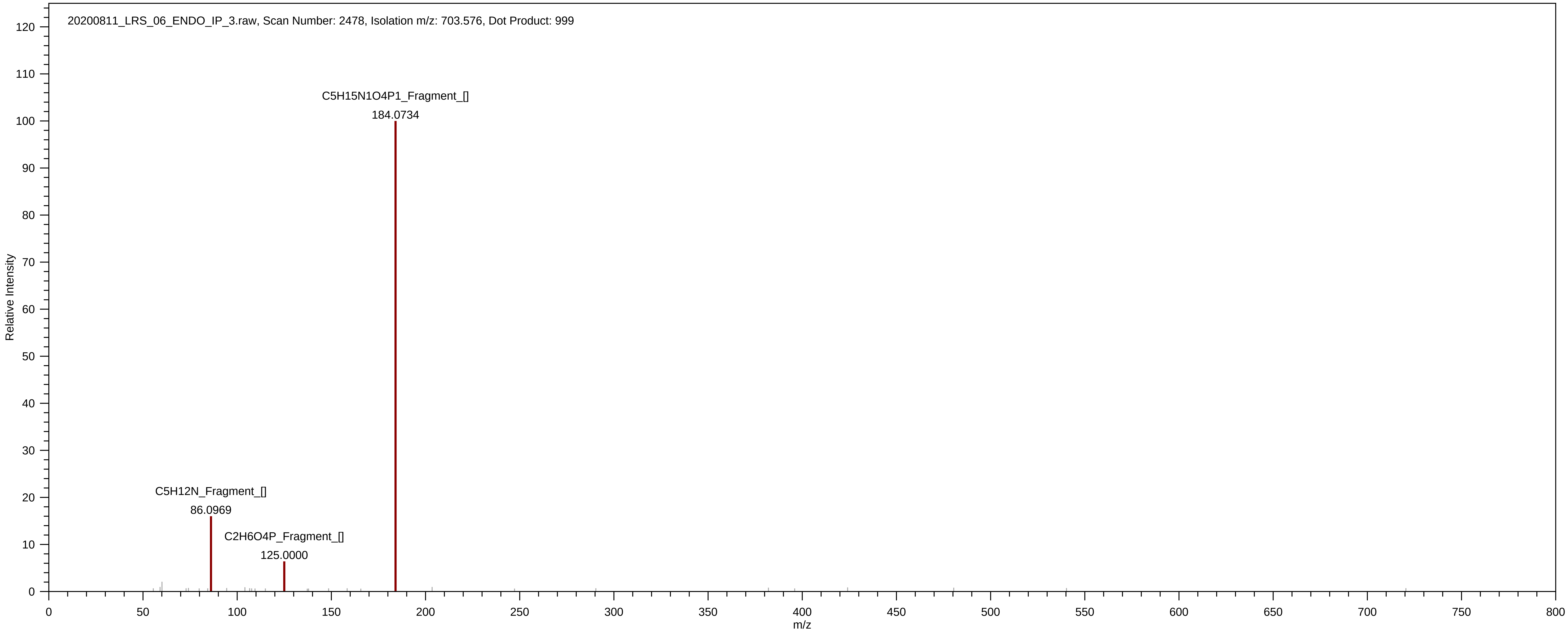

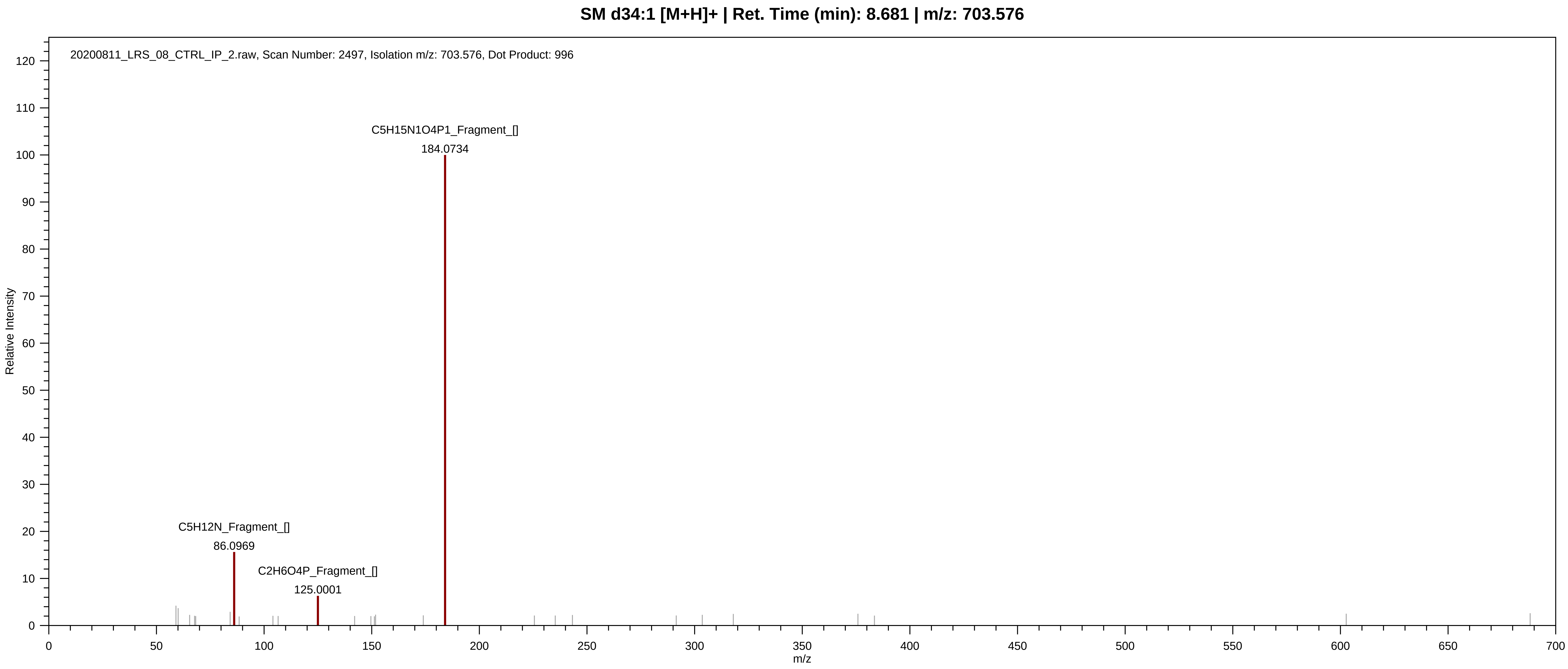
SM d34:1 [M+H]+ | Ret. Time (min): 8.681 | m/z: 703.576
20200811_LRS_08_CTRL_IP_2.raw, Scan Number: 2497, Isolation m/z: 703.576, Dot Product: 996
C5H15N1O4P1_Fragment_[]
184.0734
C5H12N_Fragment_[]
86.0969
C2H6O4P_Fragment_[]
125.0001
Relative Intensity
m/z

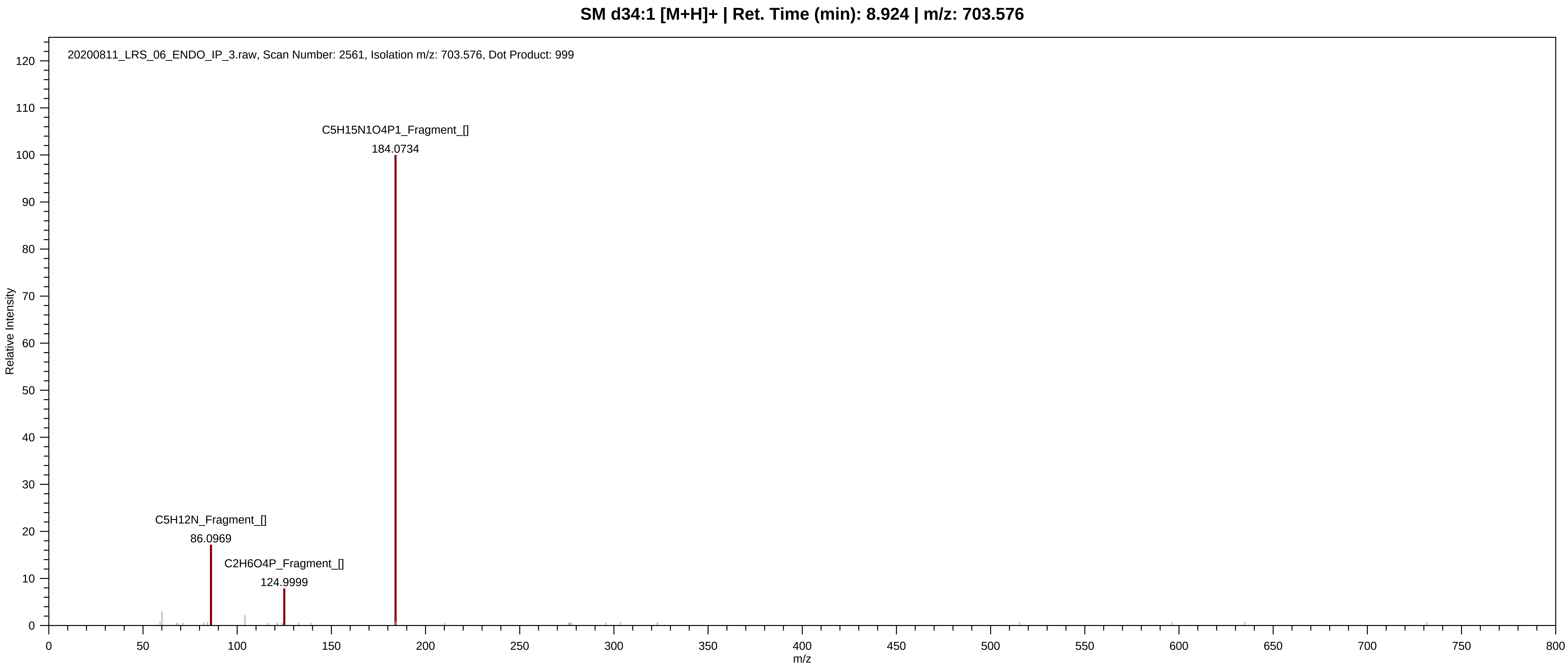
SM d34:1 [M+H]+ | Ret. Time (min): 8.924 | m/z: 703.576
20200811_LRS_06_ENDO_IP_3.raw, Scan Number: 2561, Isolation m/z: 703.576, Dot Product: 999
C5H15N1O4P1_Fragment_[]
184.0734
C5H12N_Fragment_[]
86.0969
C2H6O4P_Fragment_[]
124.9999
Relative Intensity
m/z

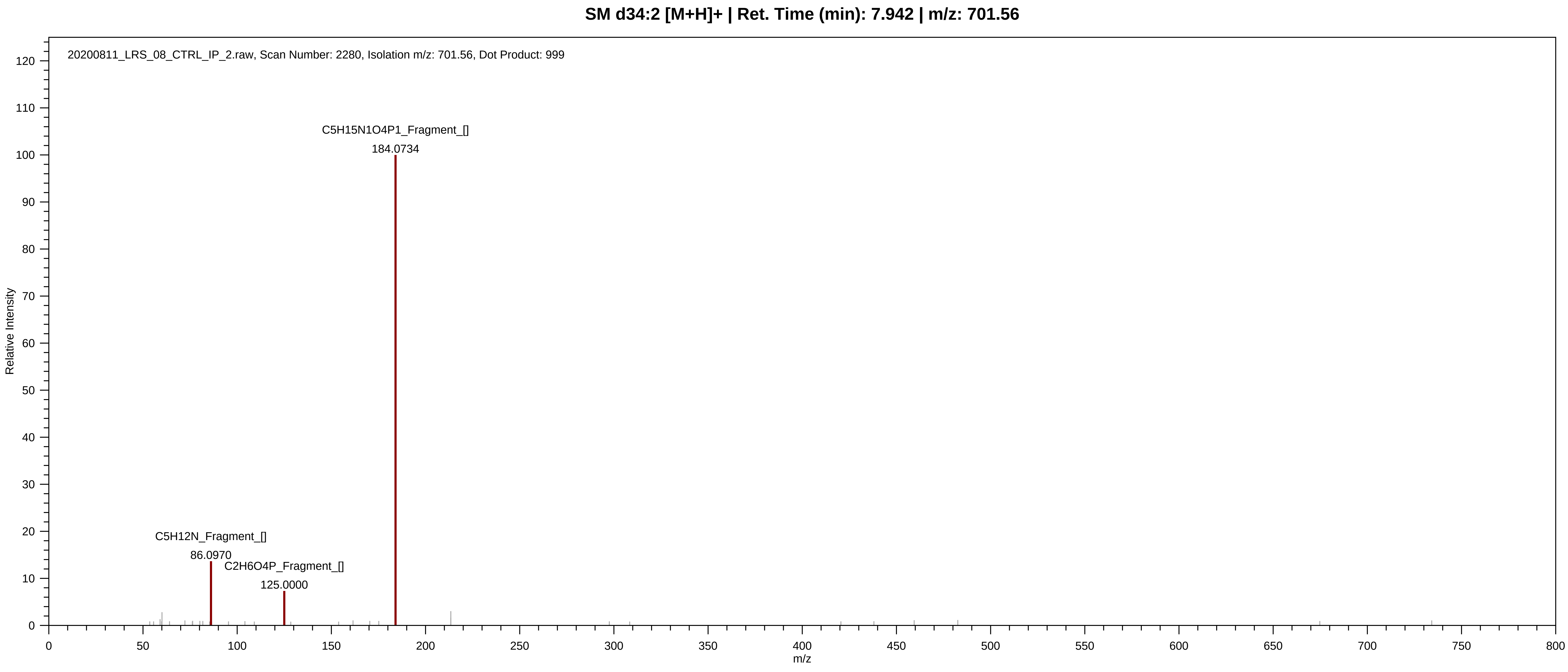

SM d34:2 [M+H]+ | Ret. Time (min): 7.942 | m/z: 701.56
20200811_LRS_08_CTRL_IP_2.raw, Scan Number: 2280, Isolation m/z: 701.56, Dot Product: 999
C5H15N1O4P1_Fragment_[]
184.0734
C5H12N_Fragment_[]
86.0970
C2H6O4P_Fragment_[]
125.0000
Relative Intensity
m/z

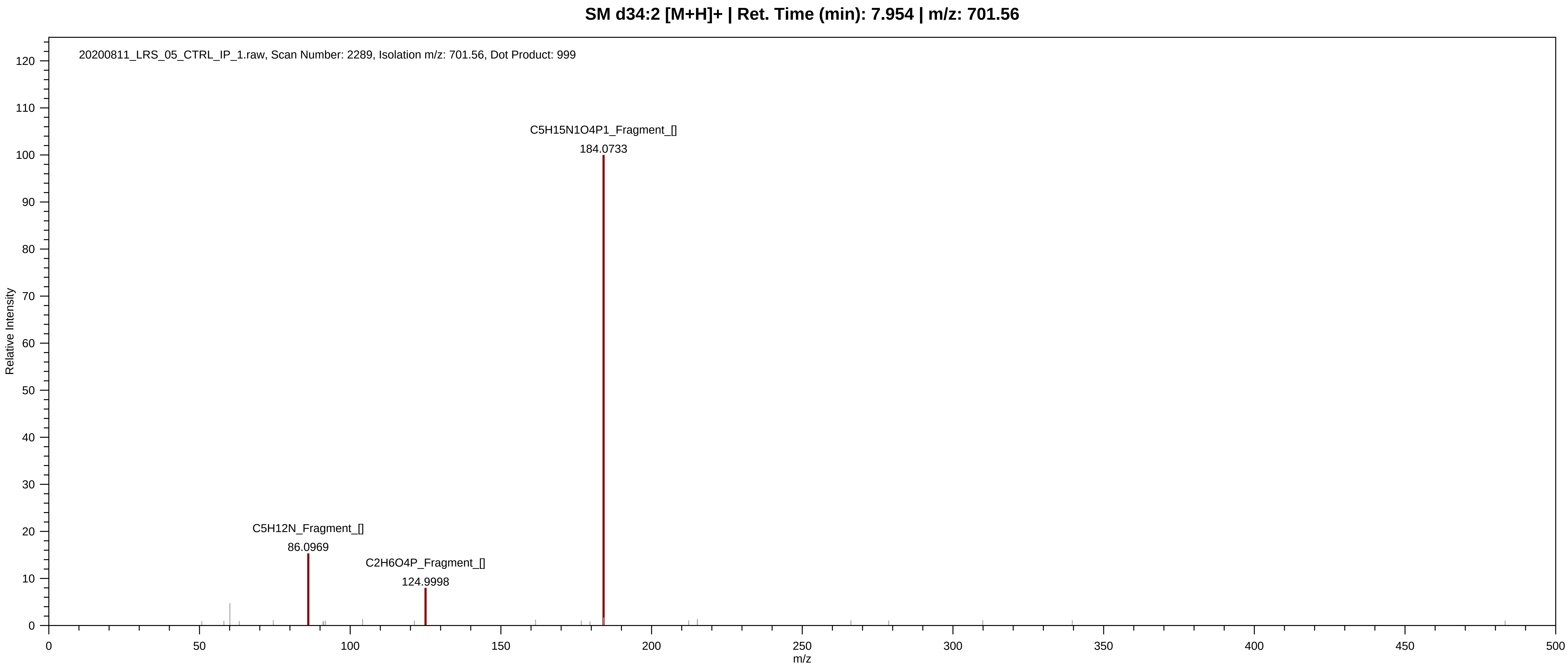
SM d34:2 [M+H]+ | Ret. Time (min): 7.954 | m/z: 701.56
20200811_LRS_05_CTRL_IP_1.raw, Scan Number: 2289, Isolation m/z: 701.56, Dot Product: 999
C5H15N1O4P1_Fragment_[]
184.0733
C5H12N_Fragment_[]
86.0969
C2H6O4P_Fragment_[]
124.9998
Relative Intensity
m/z
0
10
20
30
40
50
60
70
80
90
100
110
120
0
50
100
150
200
250
300
350
400
450
500

SM d34:2 [M+H]+ | Ret. Time (min): 7.94 | m/z: 701.558

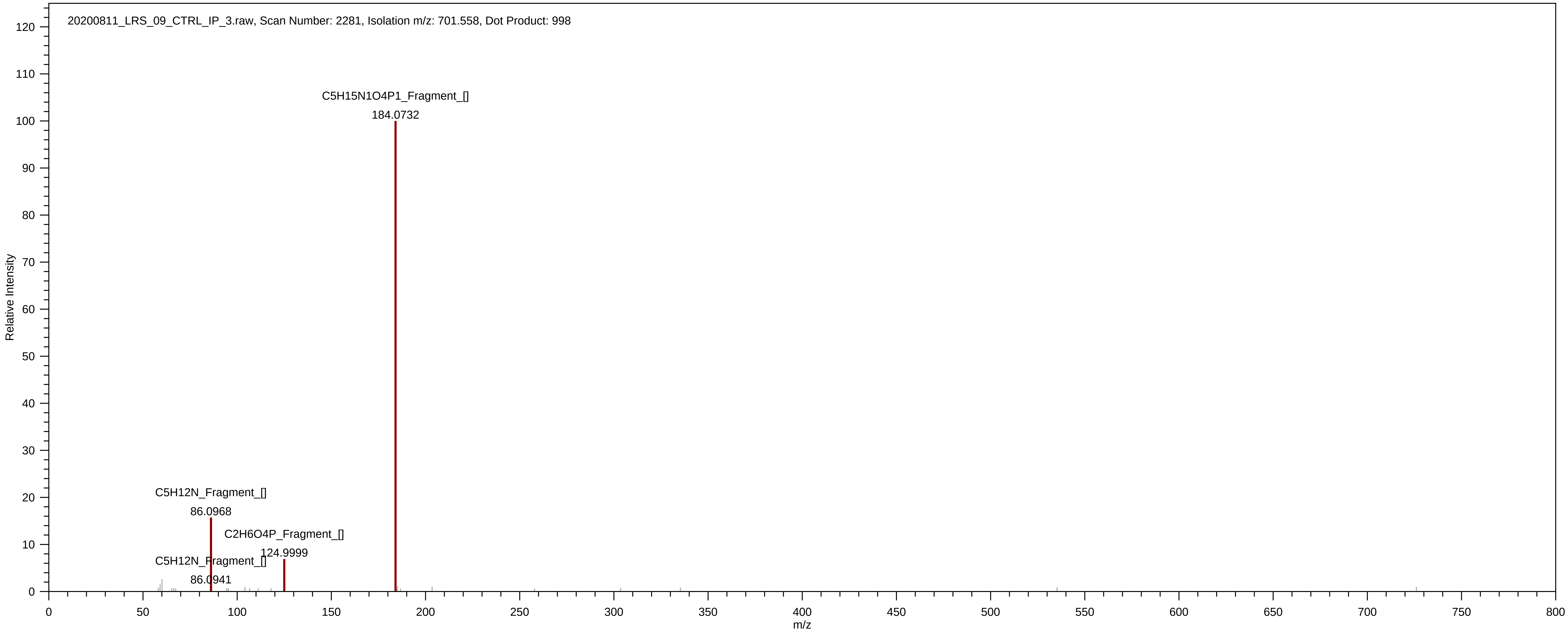

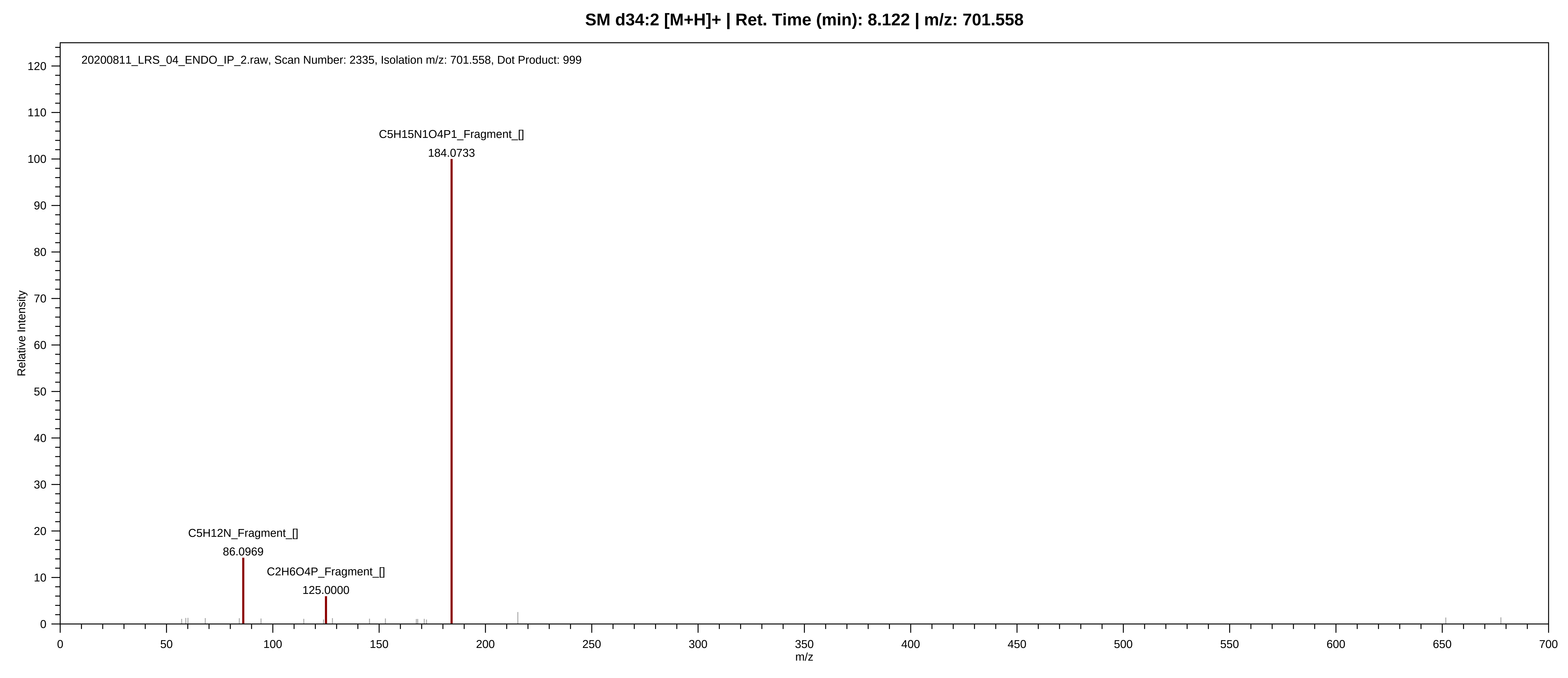
SM d34:2 [M+H]+ | Ret. Time (min): 8.122 | m/z: 701.558
20200811_LRS_04_ENDO_IP_2.raw, Scan Number: 2335, Isolation m/z: 701.558, Dot Product: 999
C5H15N1O4P1_Fragment_[]
184.0733
C5H12N_Fragment_[]
86.0969
C2H6O4P_Fragment_[]
125.0000
Relative Intensity
m/z
0
10
20
30
40
50
60
70
80
90
100
110
120
0
50
100
150
200
250
300
350
400
450
500
550
600
650
700

SM d34:2 [M+H]+ | Ret. Time (min): 7.938 | m/z: 701.559

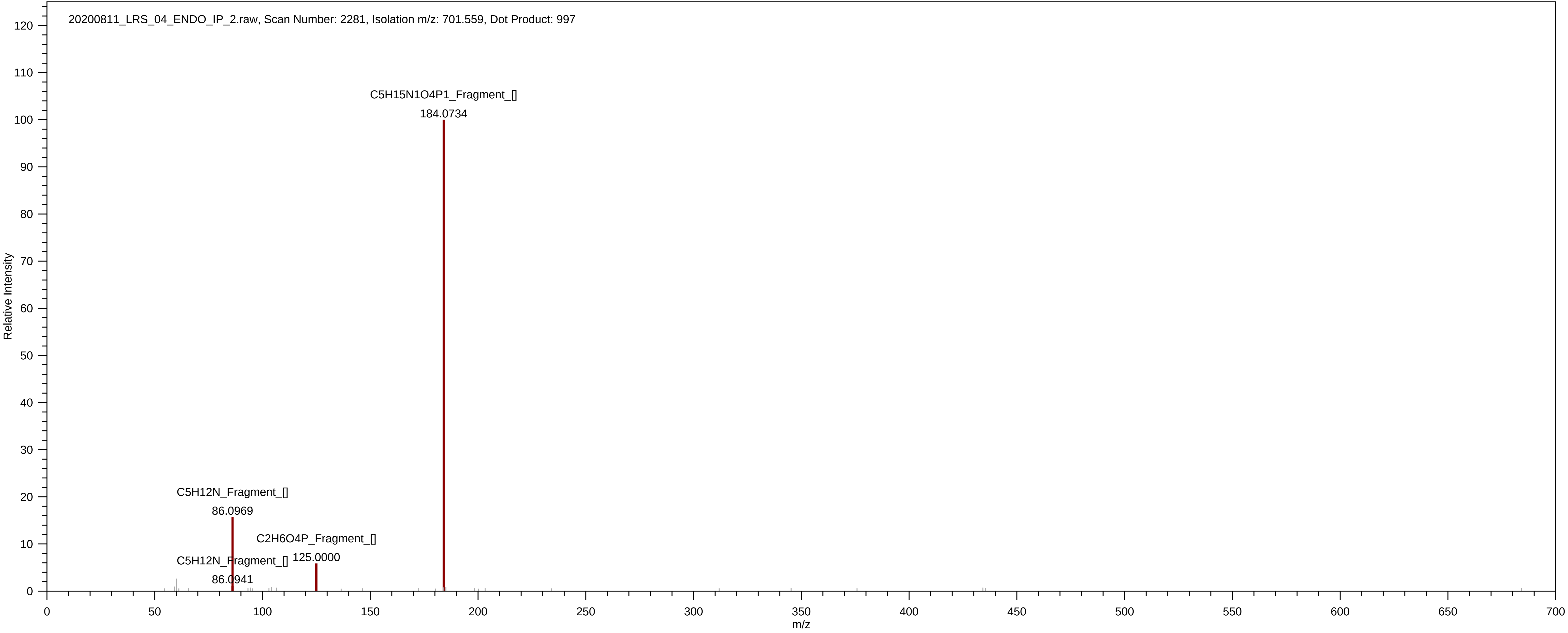

SM d34:2 [M+H]+ | Ret. Time (min): 7.948 | m/z: 701.559

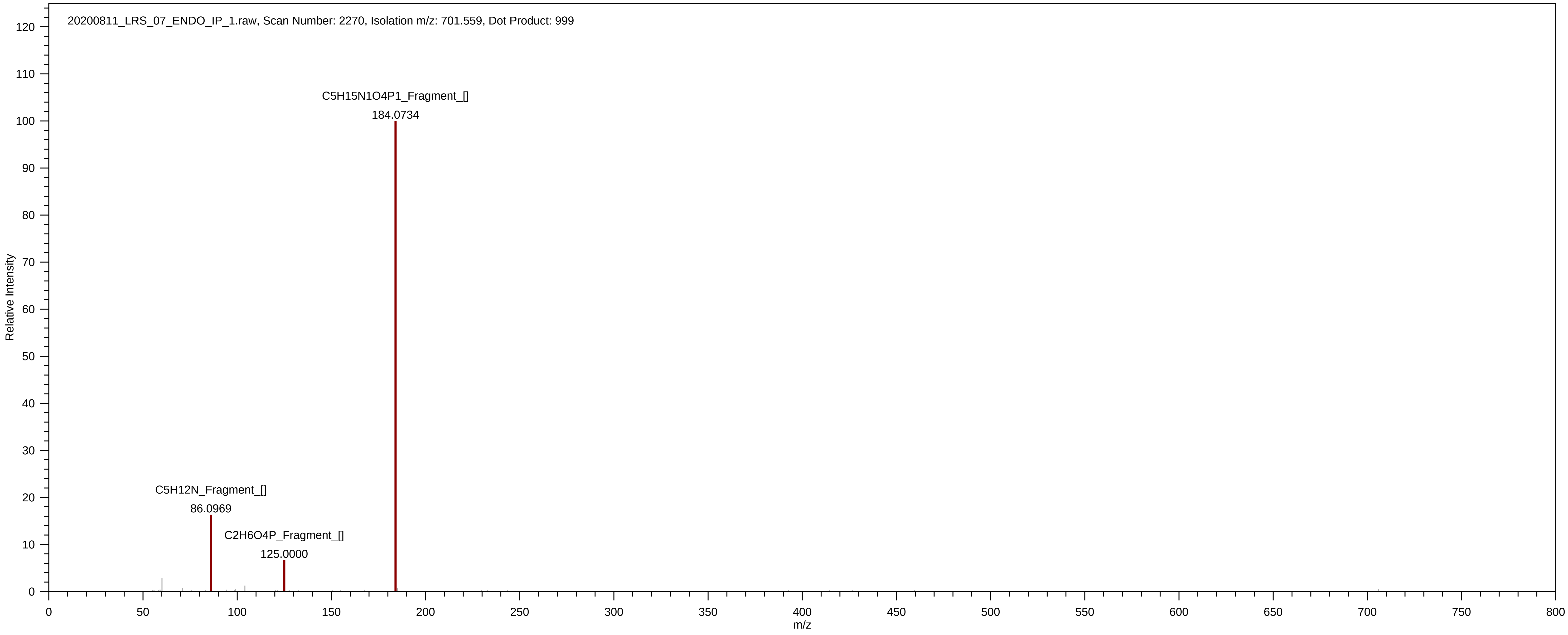

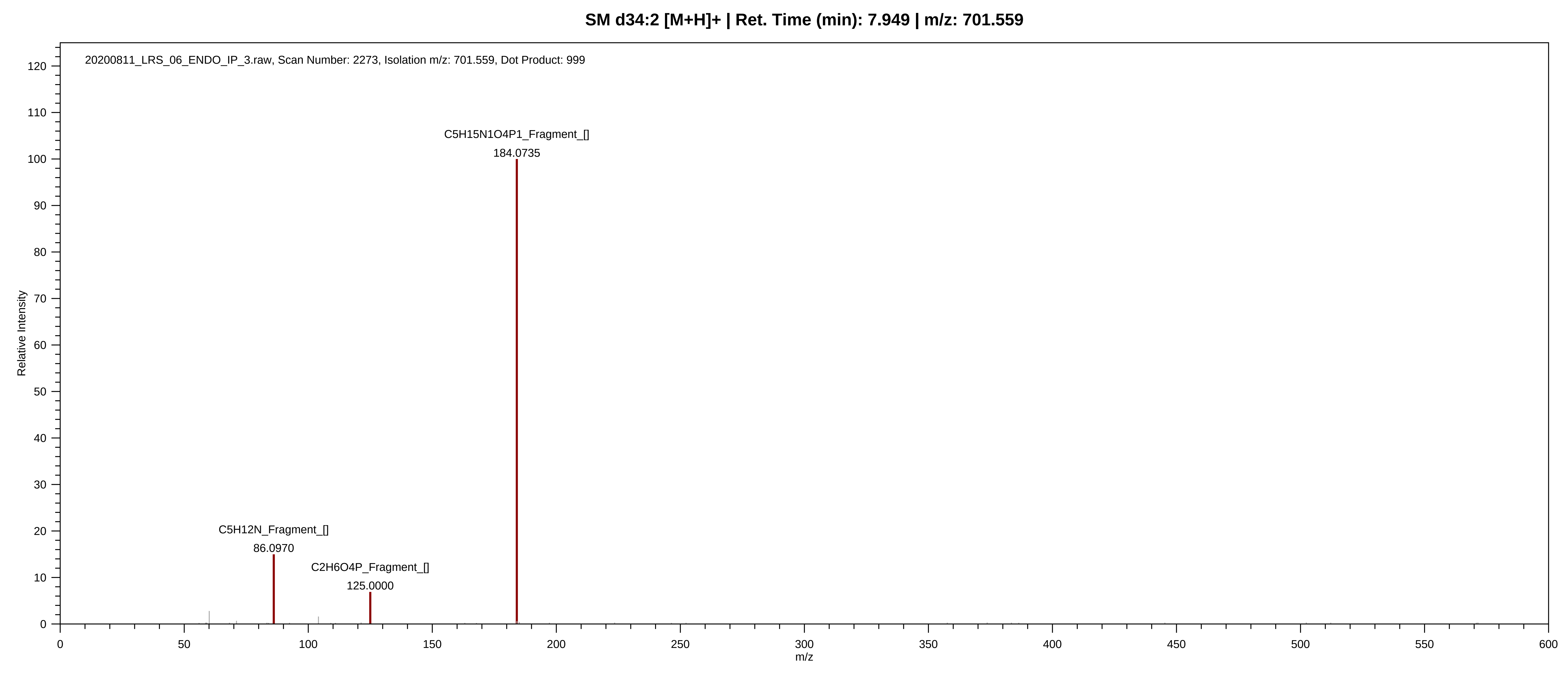
SM d34:2 [M+H]+ | Ret. Time (min): 7.949 | m/z: 701.559
20200811_LRS_06_ENDO_IP_3.raw, Scan Number: 2273, Isolation m/z: 701.559, Dot Product: 999
C5H15N1O4P1_Fragment_[]
184.0735
C5H12N_Fragment_[]
86.0970
C2H6O4P_Fragment_[]
125.0000
Relative Intensity
m/z
0
10
20
30
40
50
60
70
80
90
100
110
120
0
50
100
150
200
250
300
350
400
450
500
550
600

SM d34:2 [M+H]+ | Ret. Time (min): 8.13 | m/z: 701.559

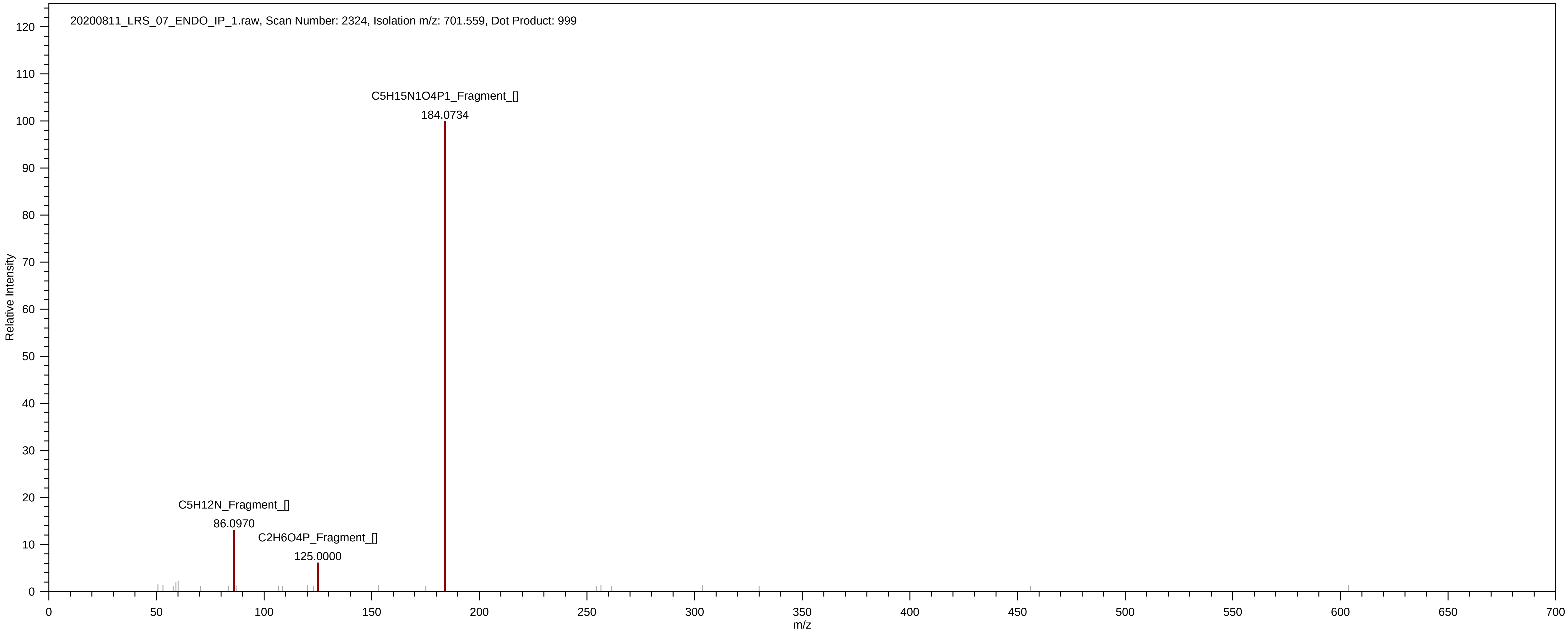

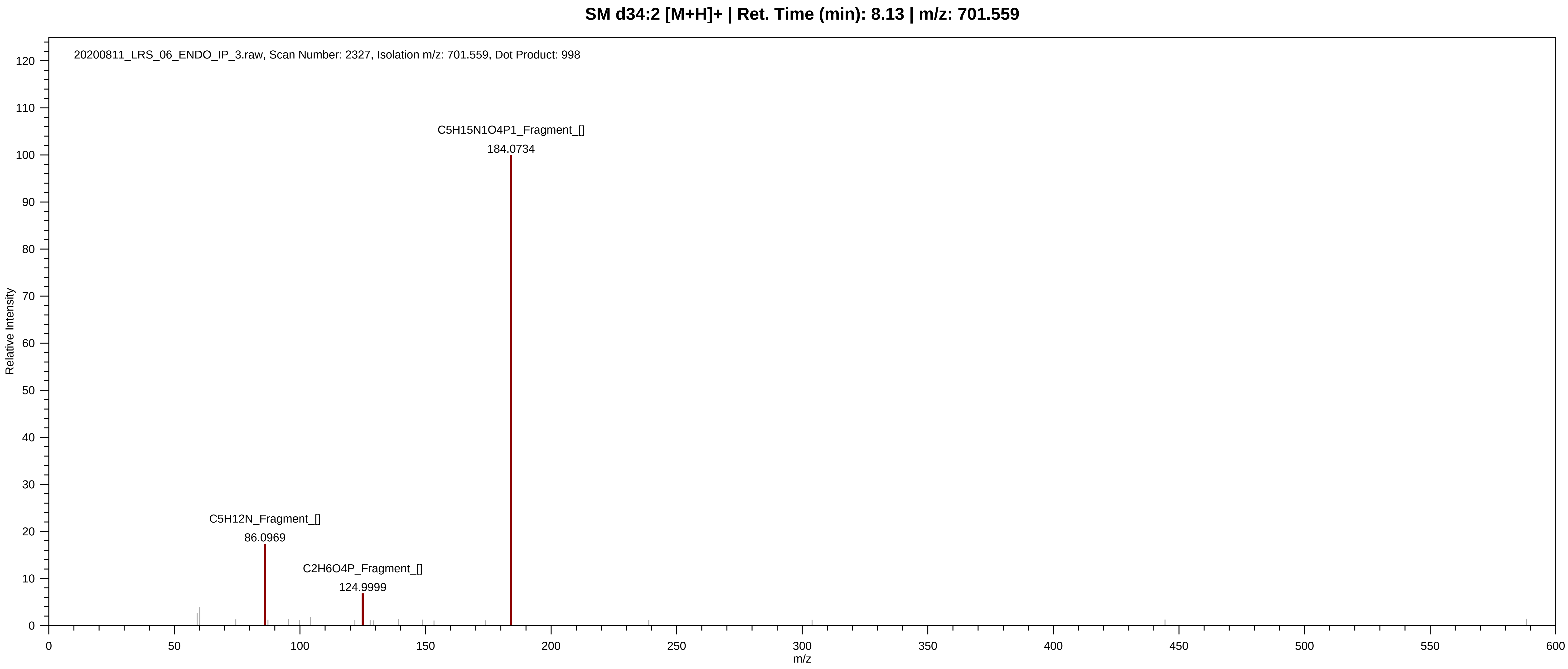
SM d34:2 [M+H]+ | Ret. Time (min): 8.13 | m/z: 701.559
20200811_LRS_06_ENDO_IP_3.raw, Scan Number: 2327, Isolation m/z: 701.559, Dot Product: 998
C5H15N1O4P1_Fragment_[]
184.0734
C5H12N_Fragment_[]
86.0969
C2H6O4P_Fragment_[]
124.9999
Relative Intensity
m/z

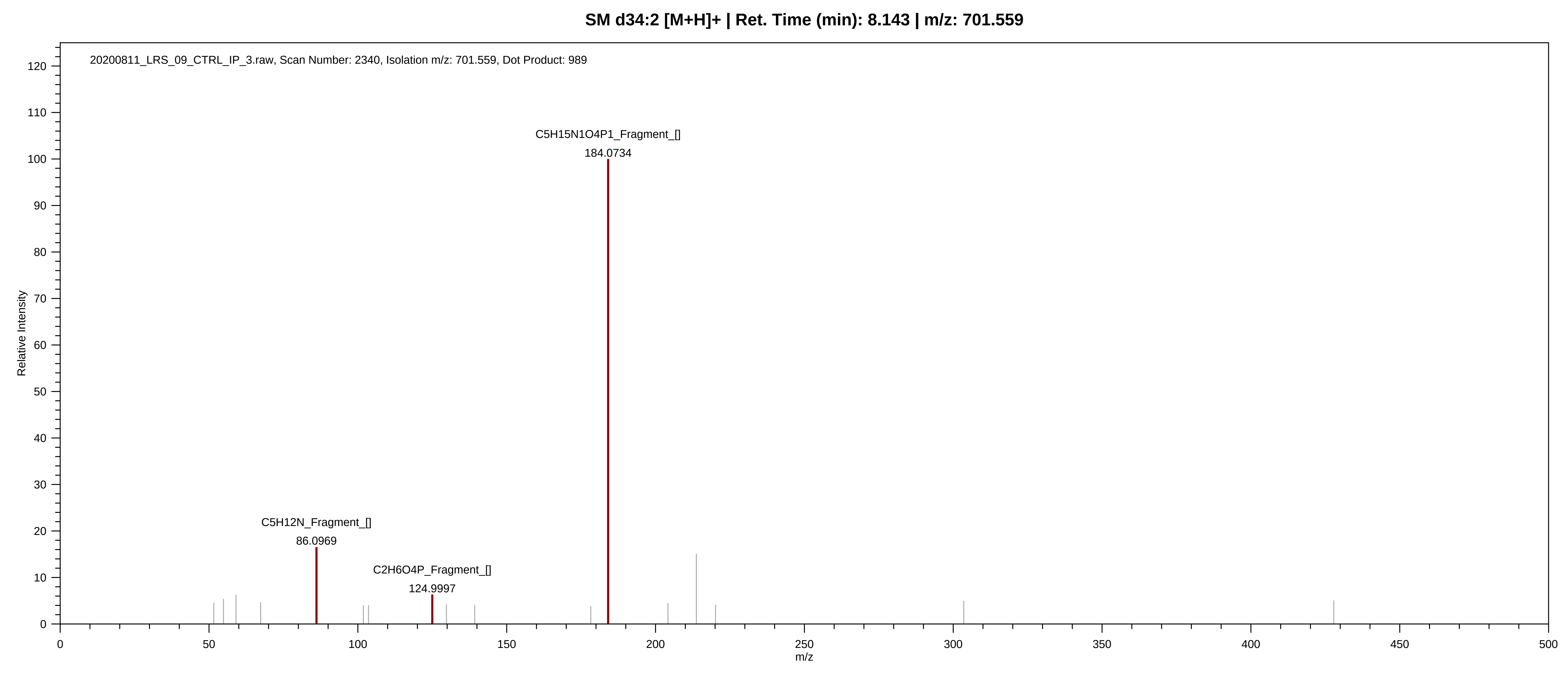

SM d34:2 [M+H]+ | Ret. Time (min): 8.143 | m/z: 701.559
20200811_LRS_09_CTRL_IP_3.raw, Scan Number: 2340, Isolation m/z: 701.559, Dot Product: 989
C5H15N1O4P1_Fragment_[]
184.0734
C5H12N_Fragment_[]
86.0969
C2H6O4P_Fragment_[]
124.9997
Relative Intensity
m/z

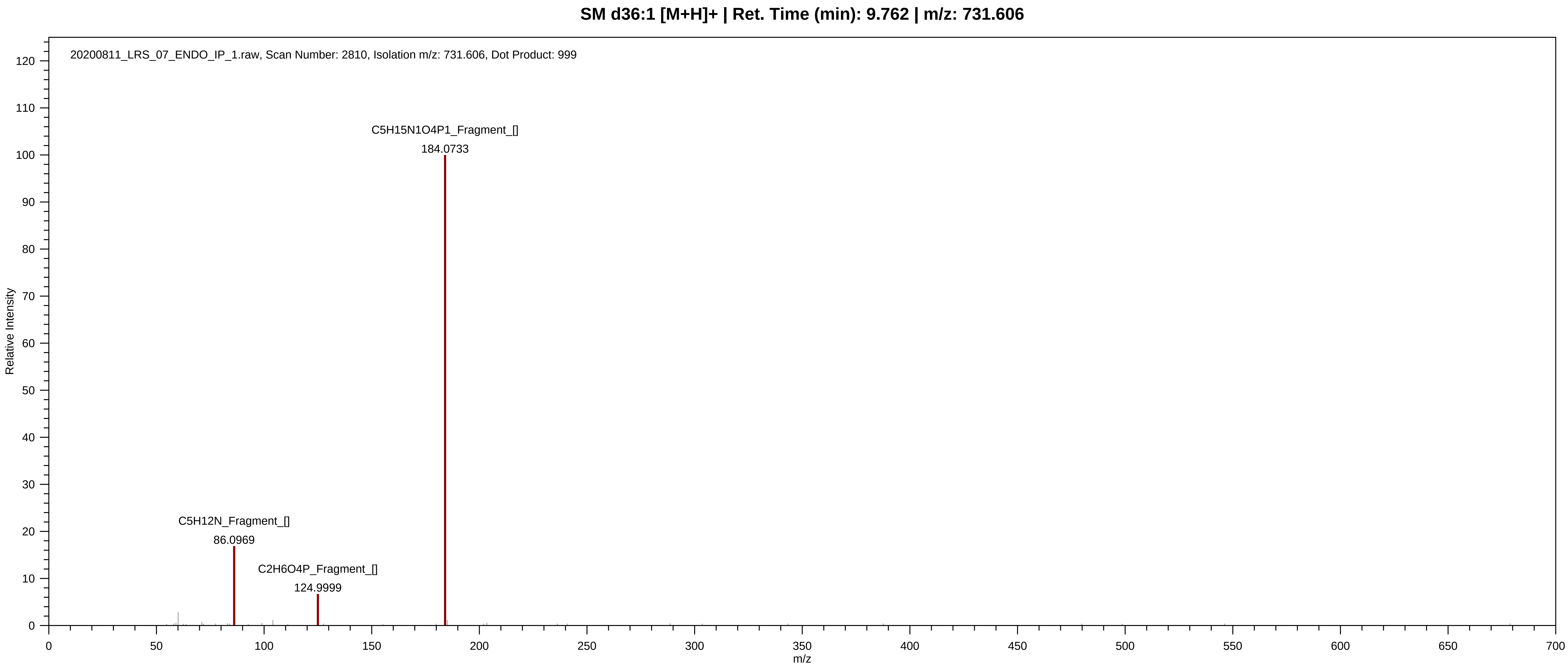
SM d36:1 [M+H]+ | Ret. Time (min): 9.762 | m/z: 731.606
20200811_LRS_07_ENDO_IP_1.raw, Scan Number: 2810, Isolation m/z: 731.606, Dot Product: 999
C5H15N1O4P1_Fragment_[]
184.0733
C5H12N_Fragment_[]
86.0969
C2H6O4P_Fragment_[]
124.9999
Relative Intensity
m/z
0
10
20
30
40
50
60
70
80
90
100
110
120
0
50
100
150
200
250
300
350
400
450
500
550
600
650
700

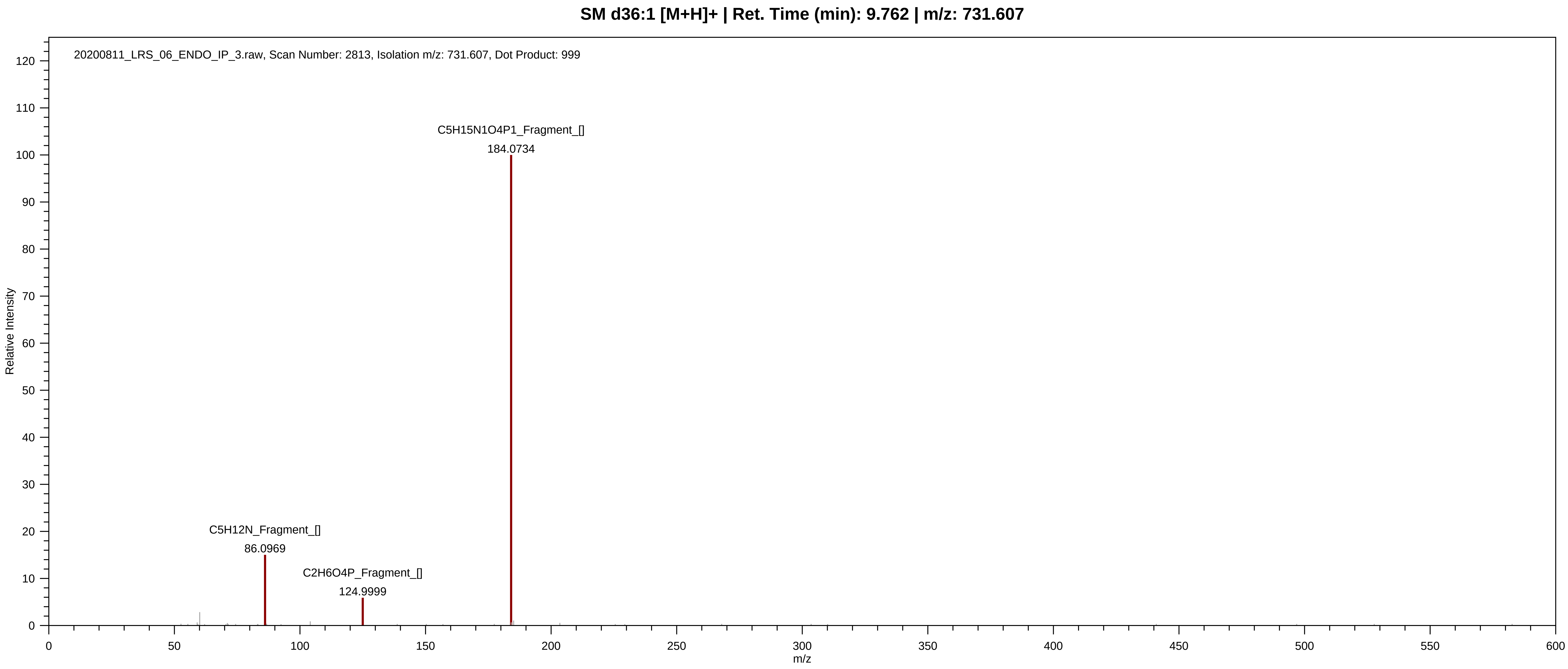

SM d36:1 [M+H]+ | Ret. Time (min): 9.762 | m/z: 731.607
20200811_LRS_06_ENDO_IP_3.raw, Scan Number: 2813, Isolation m/z: 731.607, Dot Product: 999
C5H15N1O4P1_Fragment_[]
184.0734
C5H12N_Fragment_[]
86.0969
C2H6O4P_Fragment_[]
124.9999
Relative Intensity
m/z

SM d36:1 [M+H]+ | Ret. Time (min): 9.779 | m/z: 731.607

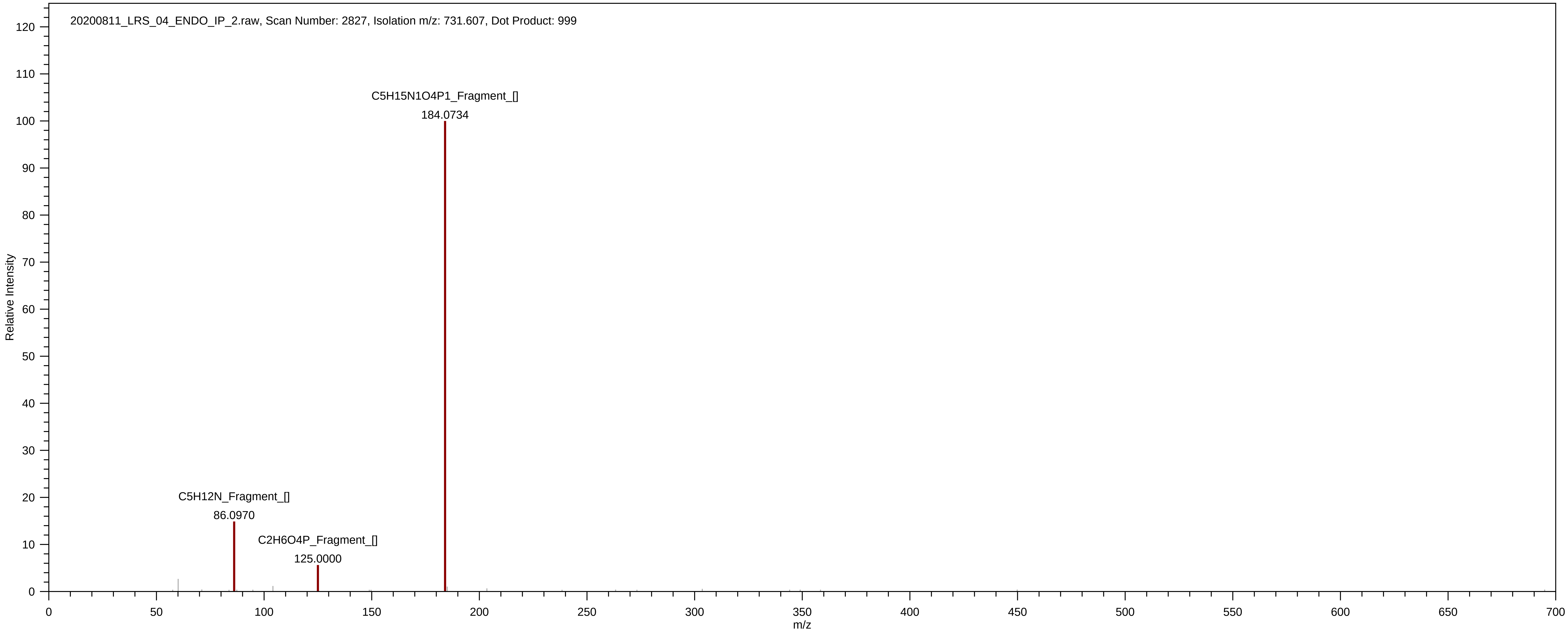

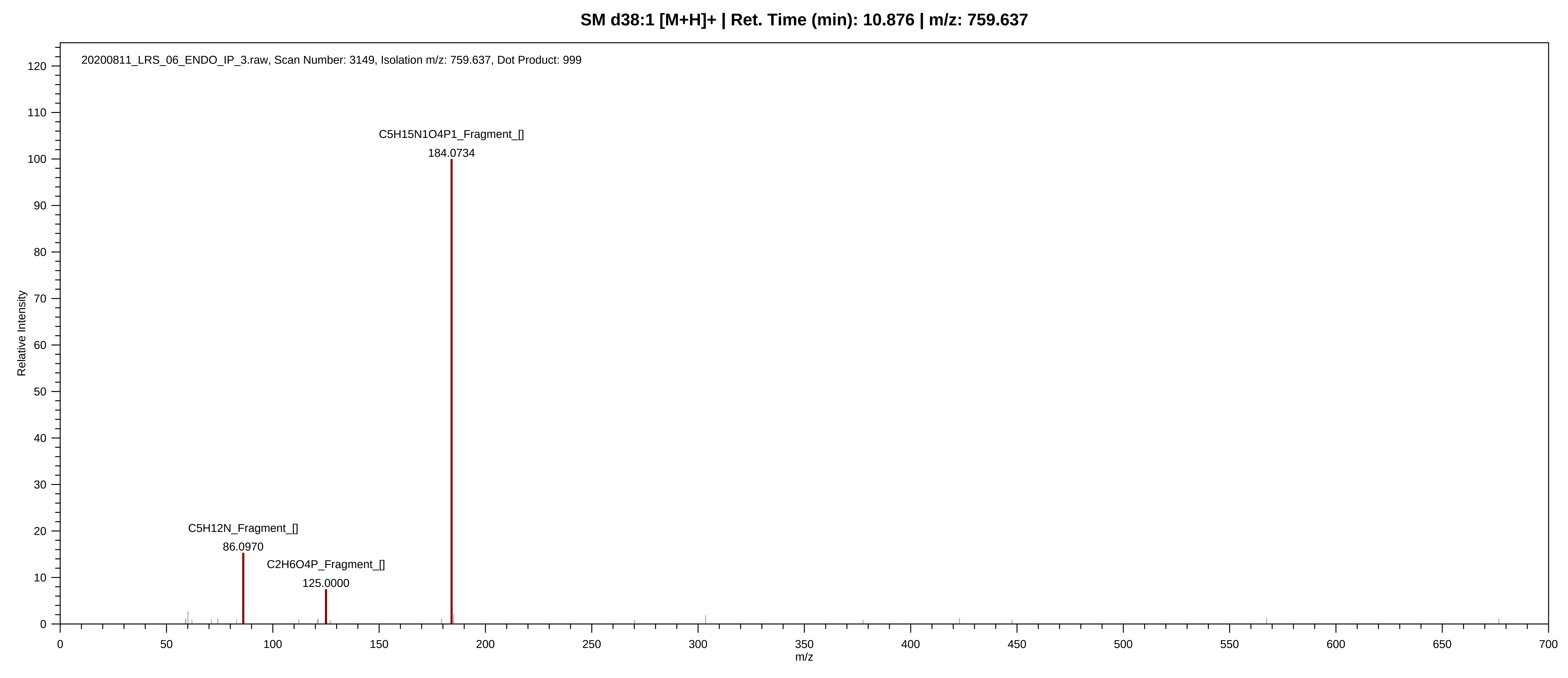

SM d38:1 [M+H]+ | Ret. Time (min): 10.876 | m/z: 759.637
20200811_LRS_06_ENDO_IP_3.raw, Scan Number: 3149, Isolation m/z: 759.637, Dot Product: 999
C5H15N1O4P1_Fragment_[]
184.0734
C5H12N_Fragment_[]
86.0970
C2H6O4P_Fragment_[]
125.0000
Relative Intensity
m/z

SM d38:1 [M+H]+ | Ret. Time (min): 10.877 | m/z: 759.639

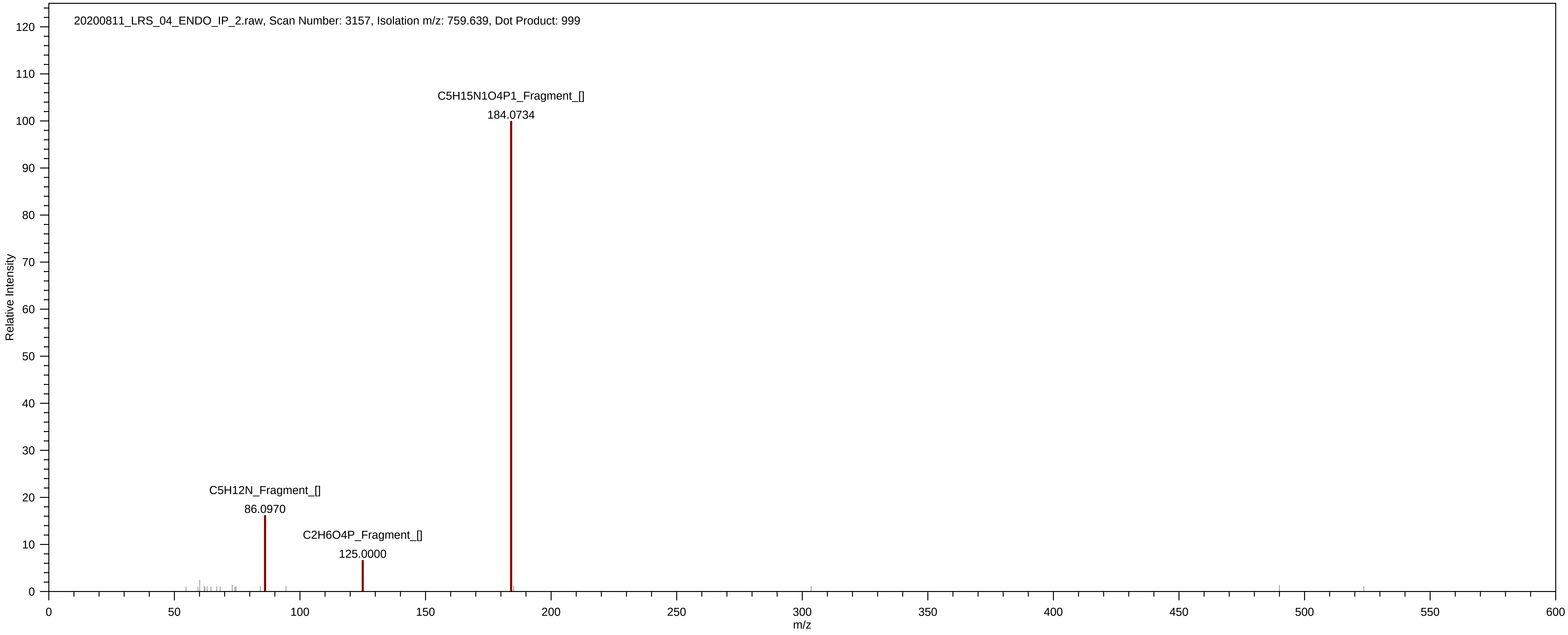

SM d40:1 [M+H]+ | Ret. Time (min): 11.89 | m/z: 787.67

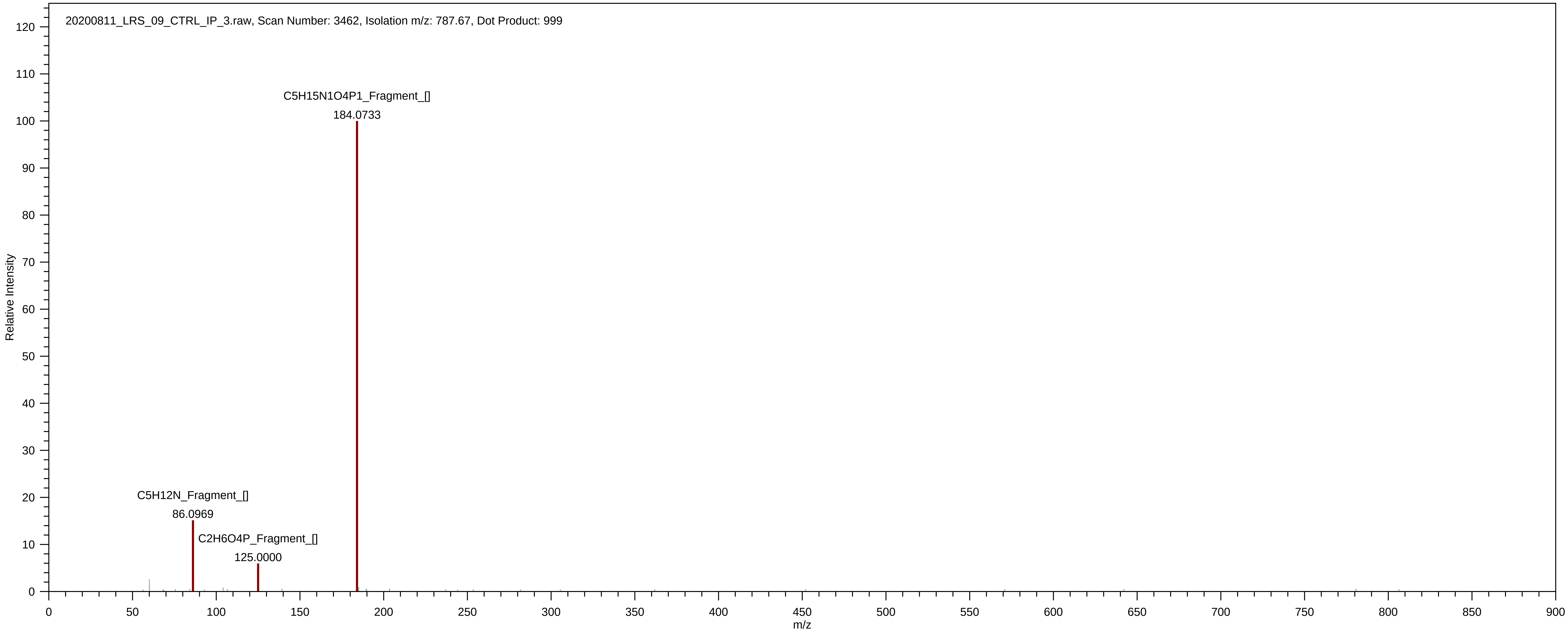

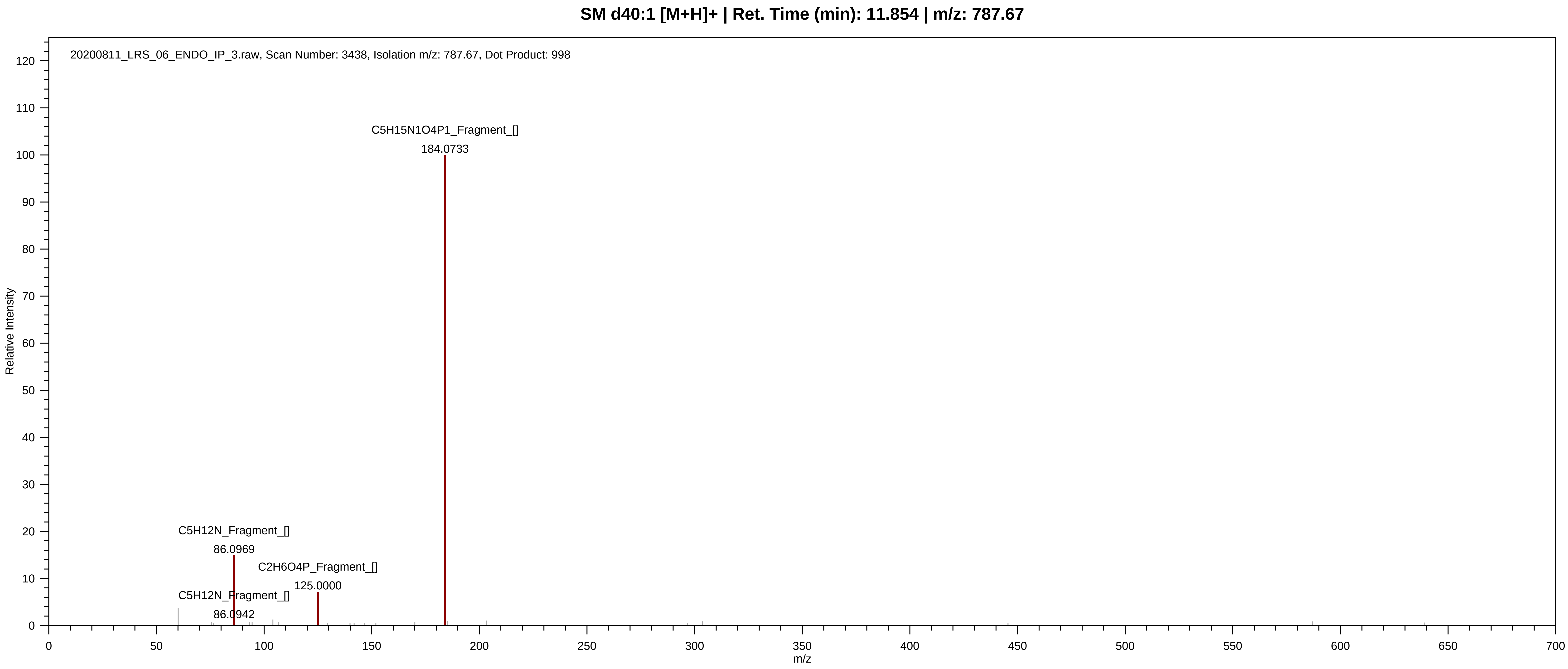
SM d40:1 [M+H]+ | Ret. Time (min): 11.854 | m/z: 787.67
20200811_LRS_06_ENDO_IP_3.raw, Scan Number: 3438, Isolation m/z: 787.67, Dot Product: 998
C5H15N1O4P1_Fragment_[]
184.0733
C5H12N_Fragment_[]
86.0969
C2H6O4P_Fragment_[]
125.0000
C5H12N_Fragment_[]
86.0942
Relative Intensity
m/z
0
10
20
30
40
50
60
70
80
90
100
110
120
0
50
100
150
200
250
300
350
400
450
500
550
600
650
700

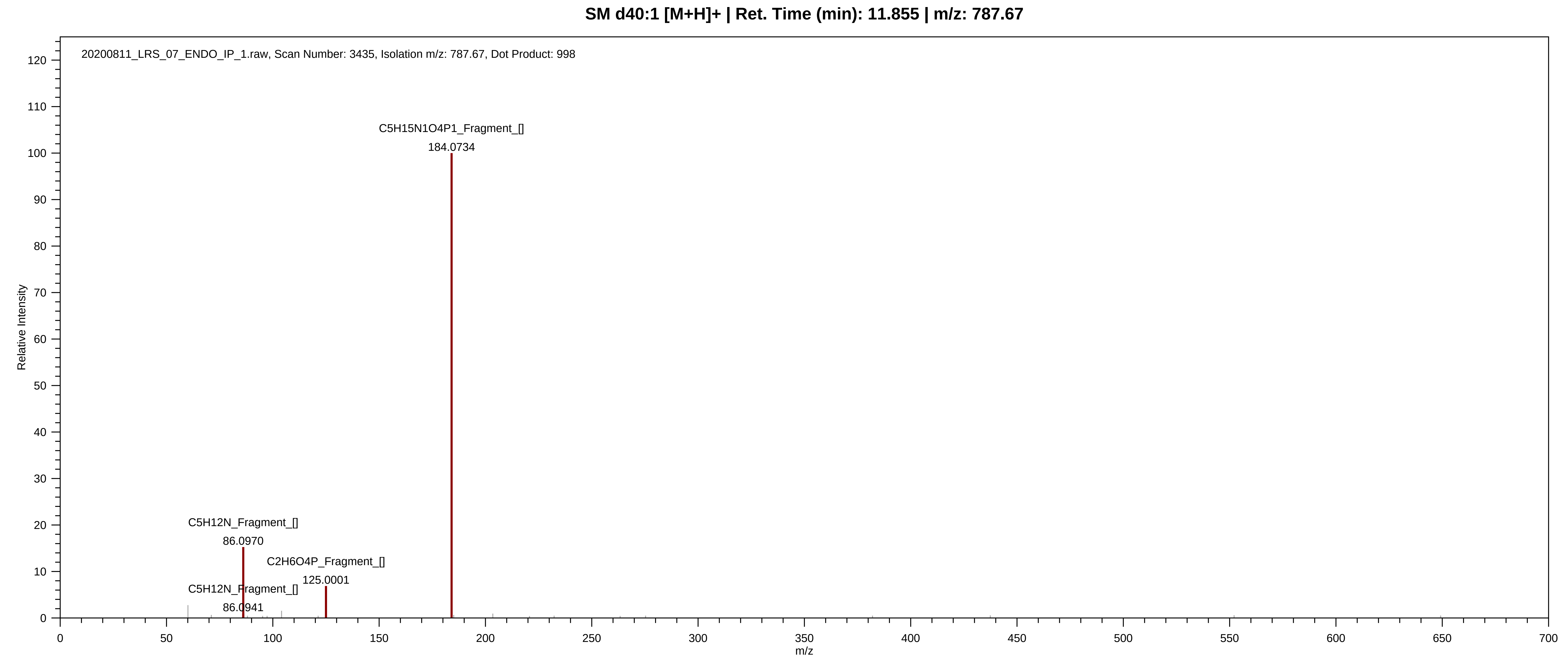
SM d40:1 [M+H]+ | Ret. Time (min): 11.855 | m/z: 787.67
20200811_LRS_07_ENDO_IP_1.raw, Scan Number: 3435, Isolation m/z: 787.67, Dot Product: 998
C5H15N1O4P1_Fragment_[]
184.0734
C5H12N_Fragment_[]
86.0970
C2H6O4P_Fragment_[]
125.0001
C5H12N_Fragment_[]
86.0941
Relative Intensity
m/z
0
10
20
30
40
50
60
70
80
90
100
110
120
0
50
100
150
200
250
300
350
400
450
500
550
600
650
700

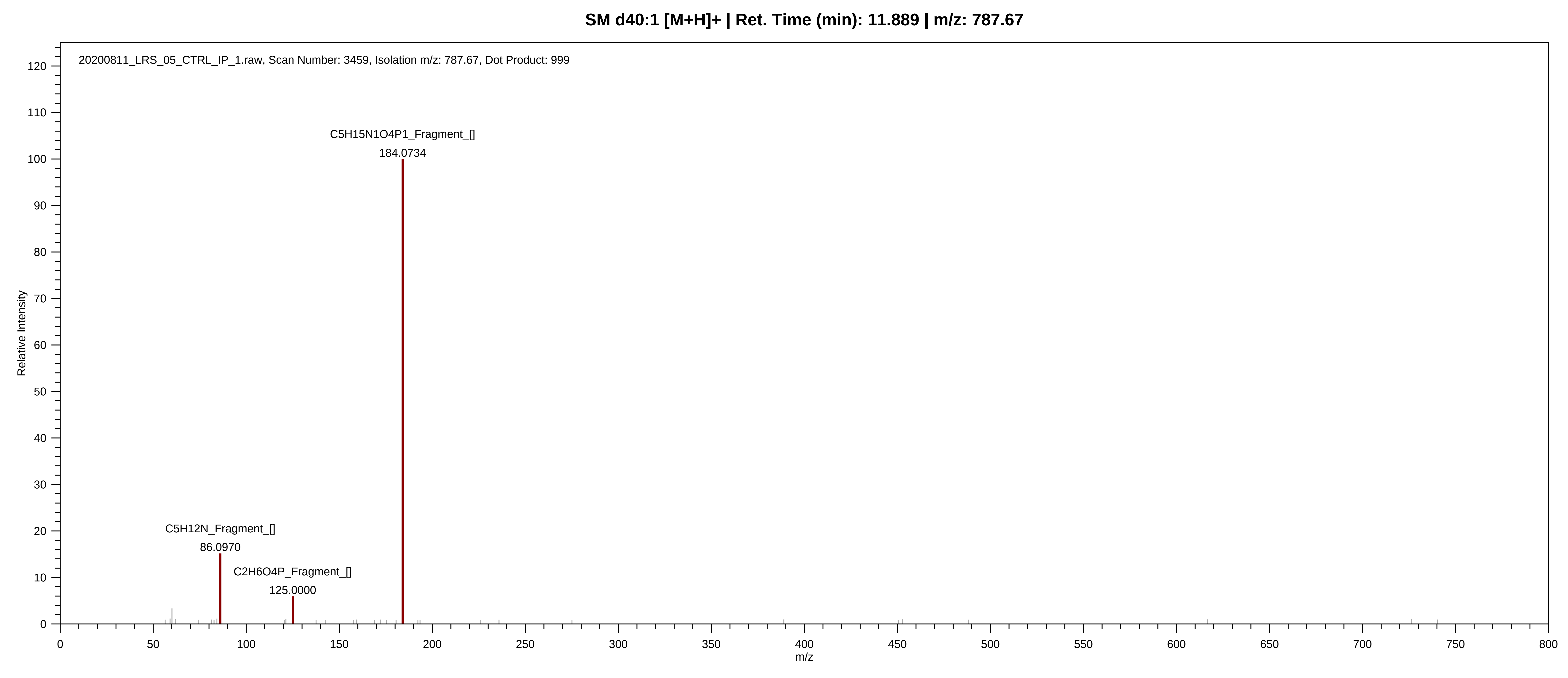

SM d40:1 [M+H]+ | Ret. Time (min): 11.889 | m/z: 787.67
20200811_LRS_05_CTRL_IP_1.raw, Scan Number: 3459, Isolation m/z: 787.67, Dot Product: 999
C5H15N1O4P1_Fragment_[]
184.0734
C5H12N_Fragment_[]
86.0970
C2H6O4P_Fragment_[]
125.0000
Relative Intensity
m/z

SM d40:1 [M+H]+ | Ret. Time (min): 12.039 | m/z: 787.67

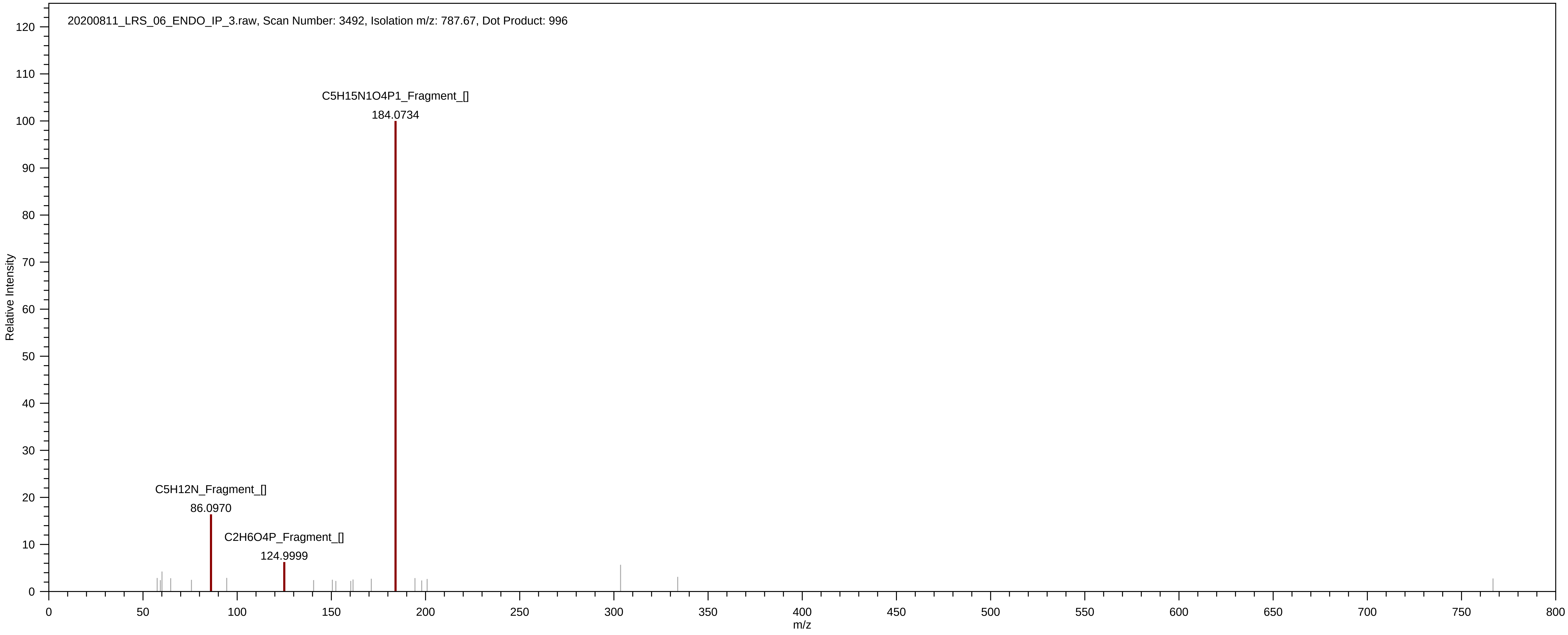

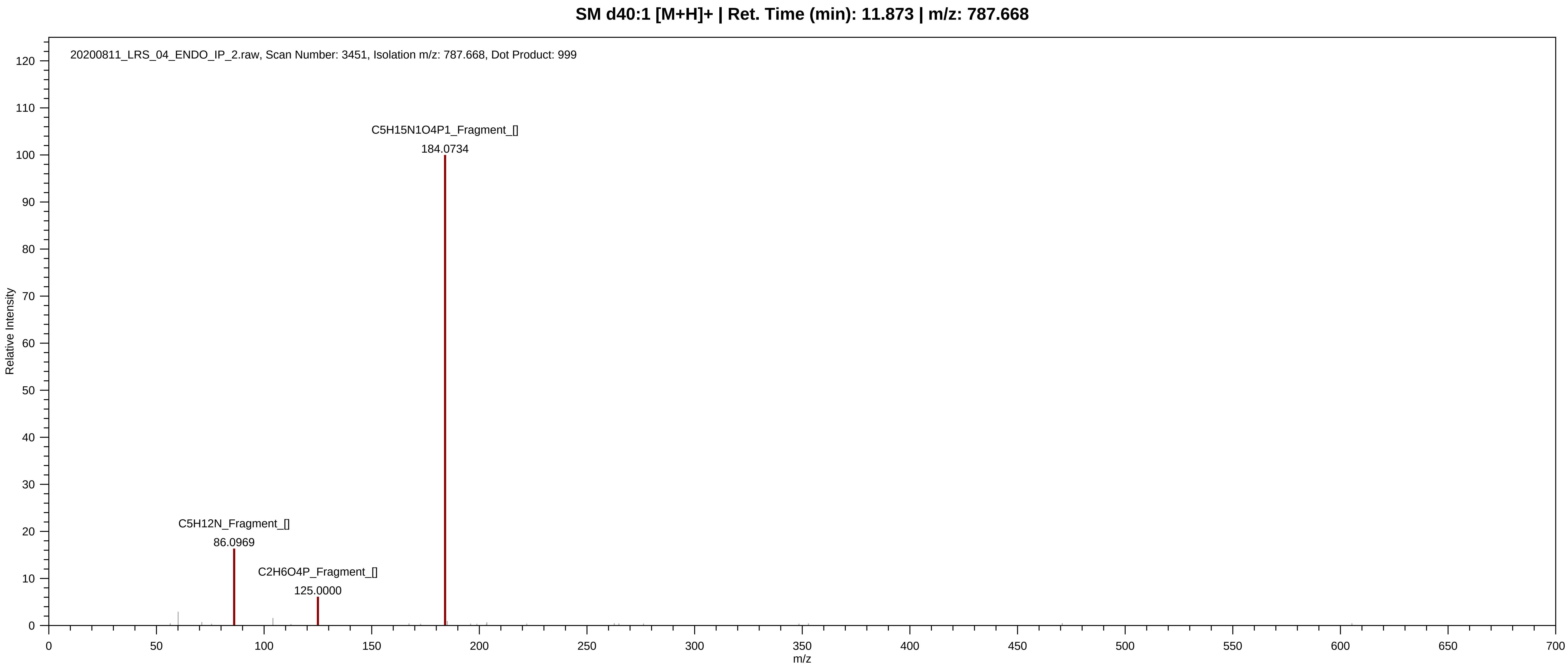
SM d40:1 [M+H]+ | Ret. Time (min): 11.873 | m/z: 787.668
20200811_LRS_04_ENDO_IP_2.raw, Scan Number: 3451, Isolation m/z: 787.668, Dot Product: 999
C5H15N1O4P1_Fragment_[]
184.0734
C5H12N_Fragment_[]
86.0969
C2H6O4P_Fragment_[]
125.0000
Relative Intensity
m/z
0
10
20
30
40
50
60
70
80
90
100
110
120
0
50
100
150
200
250
300
350
400
450
500
550
600
650
700

SM d40:1 [M+H]+ | Ret. Time (min): 12.038 | m/z: 787.669

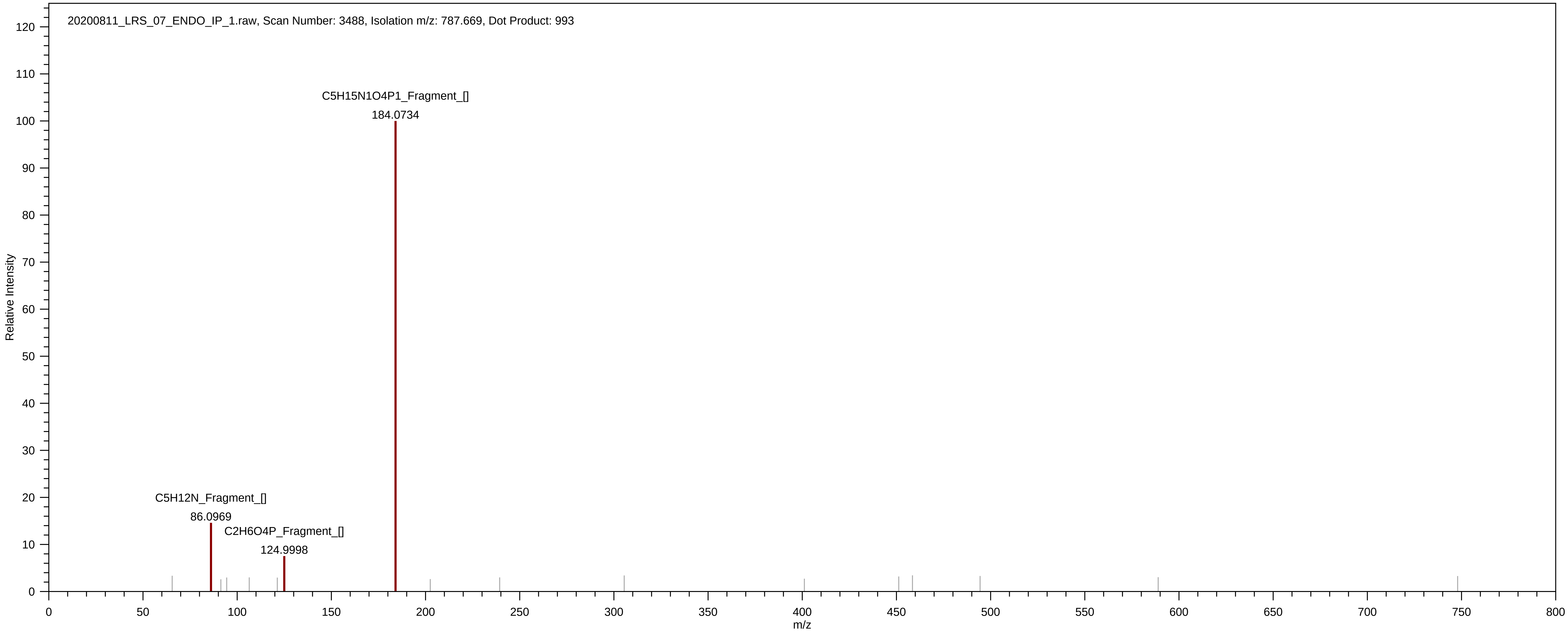

SM d40:2 [M+H]+ | Ret. Time (min): 10.96 | m/z: 785.653

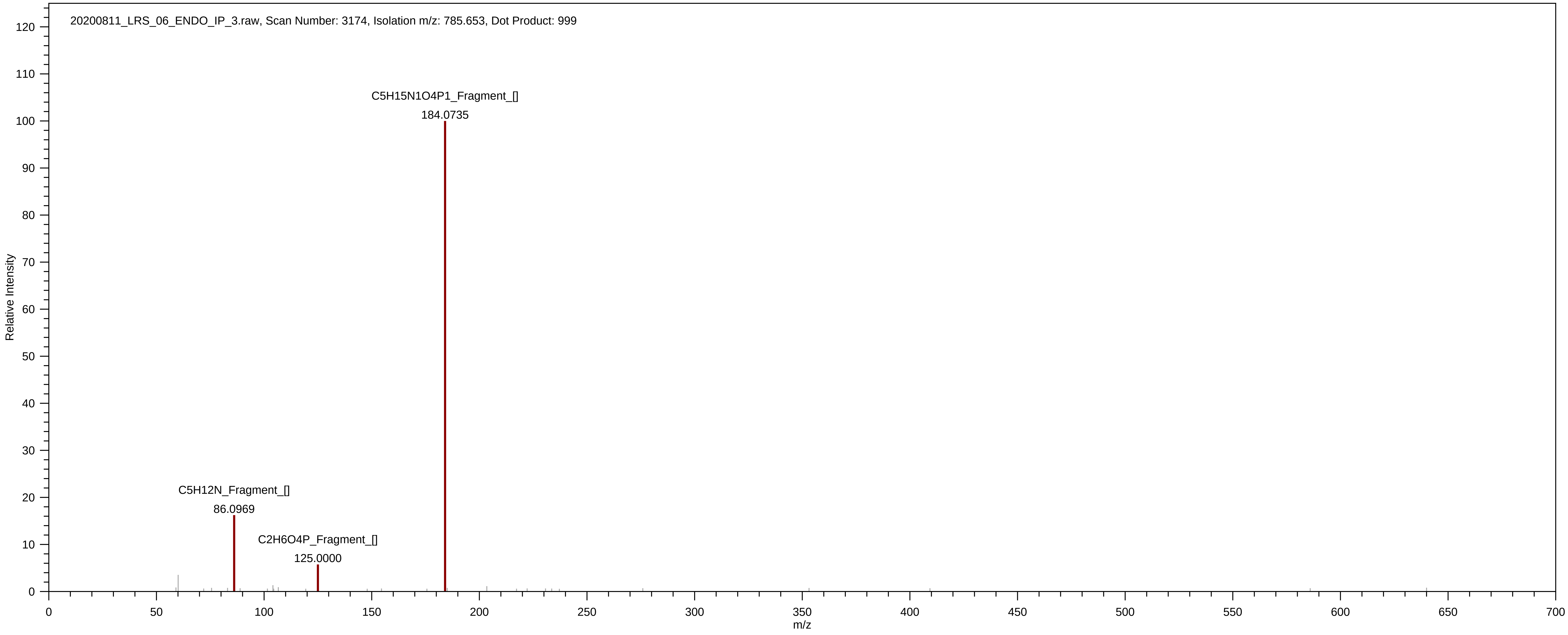

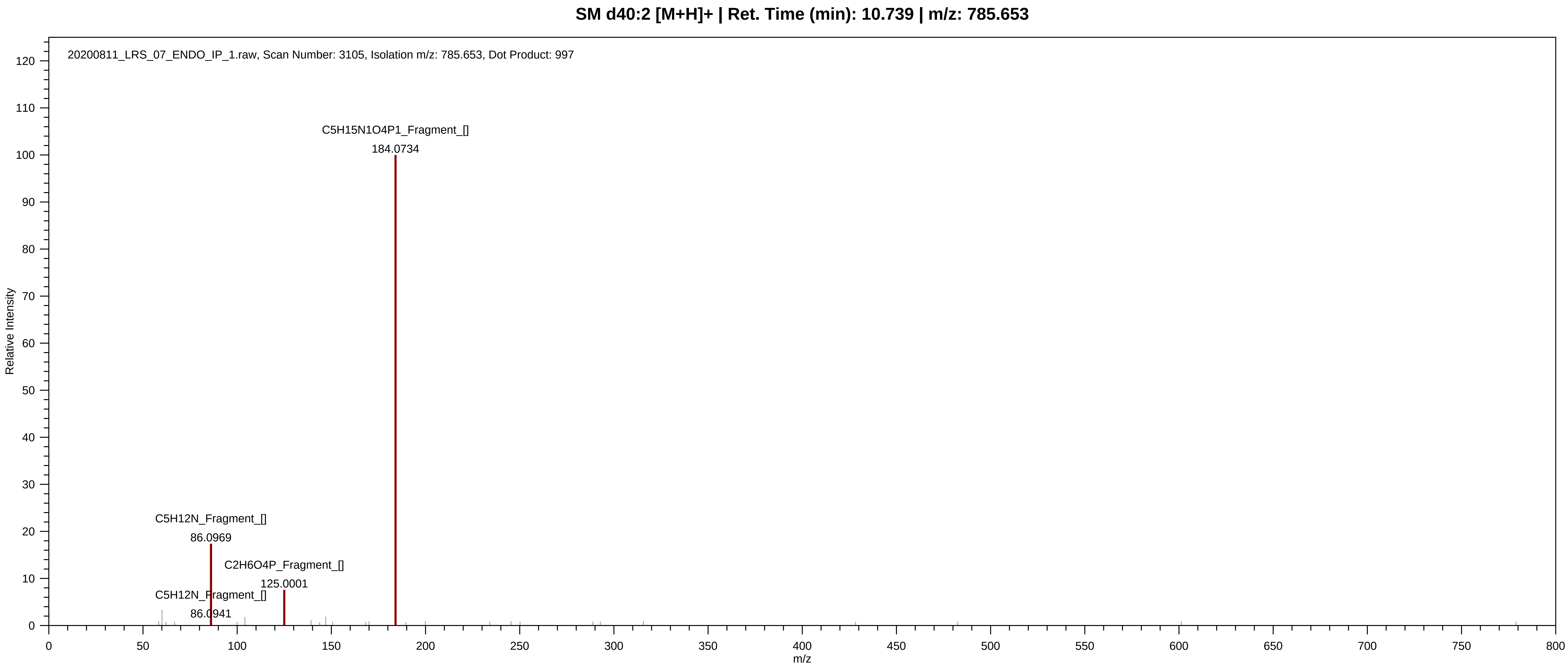

SM d40:2 [M+H]+ | Ret. Time (min): 10.739 | m/z: 785.653
20200811_LRS_07_ENDO_IP_1.raw, Scan Number: 3105, Isolation m/z: 785.653, Dot Product: 997
C5H15N1O4P1_Fragment_[]
184.0734
C5H12N_Fragment_[]
86.0969
C2H6O4P_Fragment_[]
125.0001
C5H12N_Fragment_[]
86.0941
Relative Intensity
m/z

SM d40:2 [M+H]+ | Ret. Time (min): 10.96 | m/z: 785.654

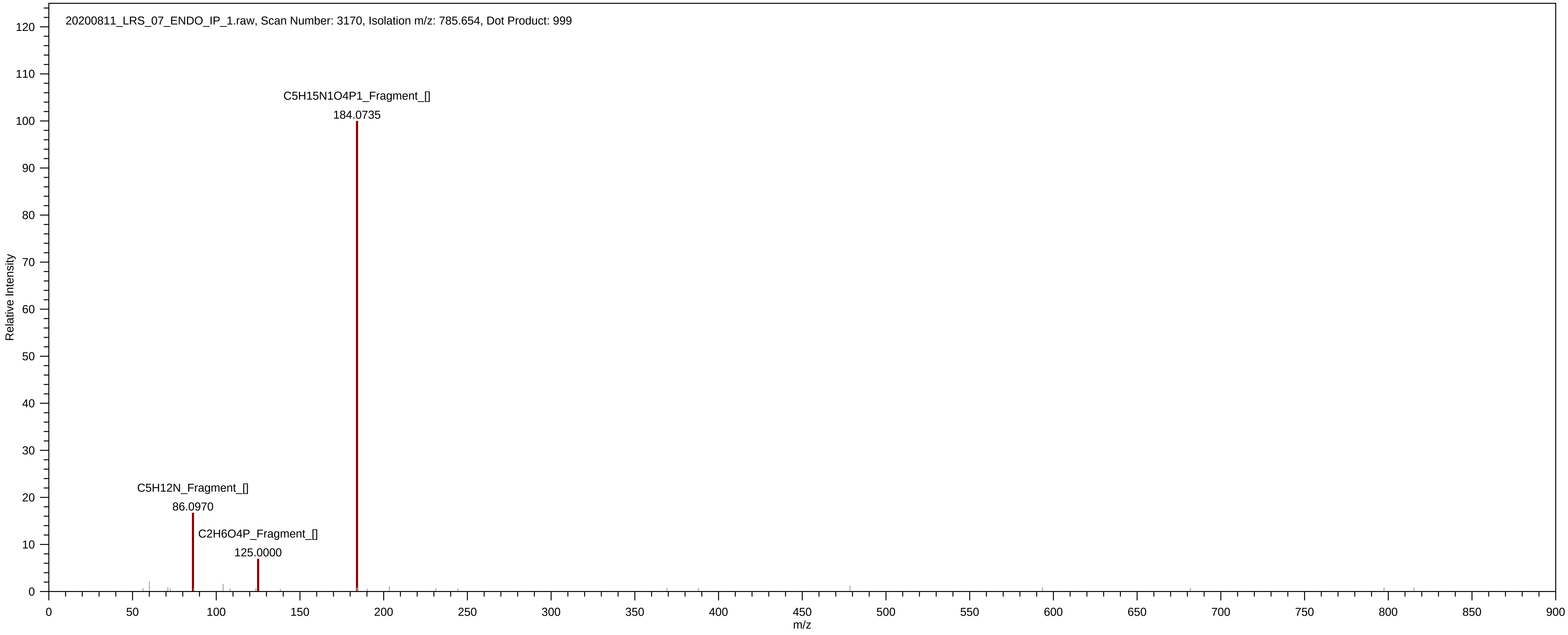

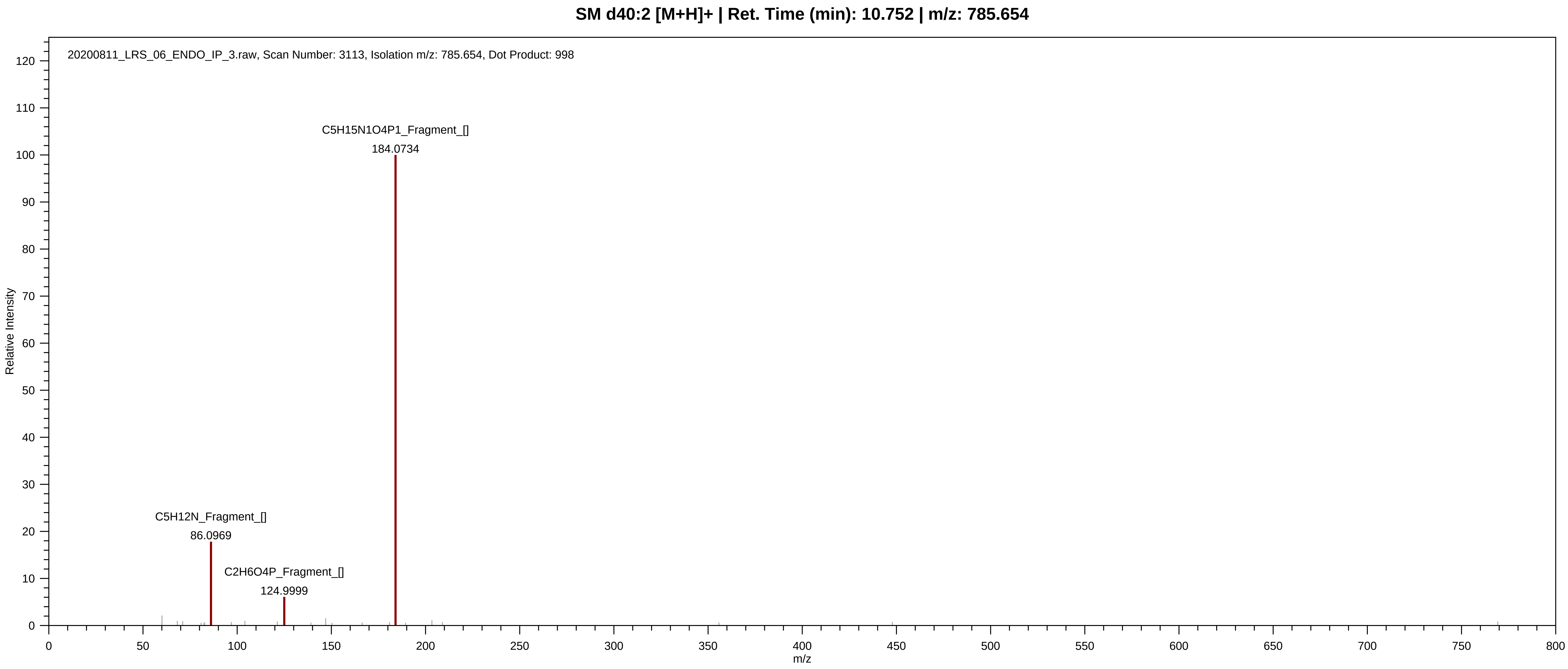
SM d40:2 [M+H]+ | Ret. Time (min): 10.752 | m/z: 785.654
20200811_LRS_06_ENDO_IP_3.raw, Scan Number: 3113, Isolation m/z: 785.654, Dot Product: 998
C5H15N1O4P1_Fragment_[]
184.0734
C5H12N_Fragment_[]
86.0969
C2H6O4P_Fragment_[]
124.9999
Relative Intensity
m/z

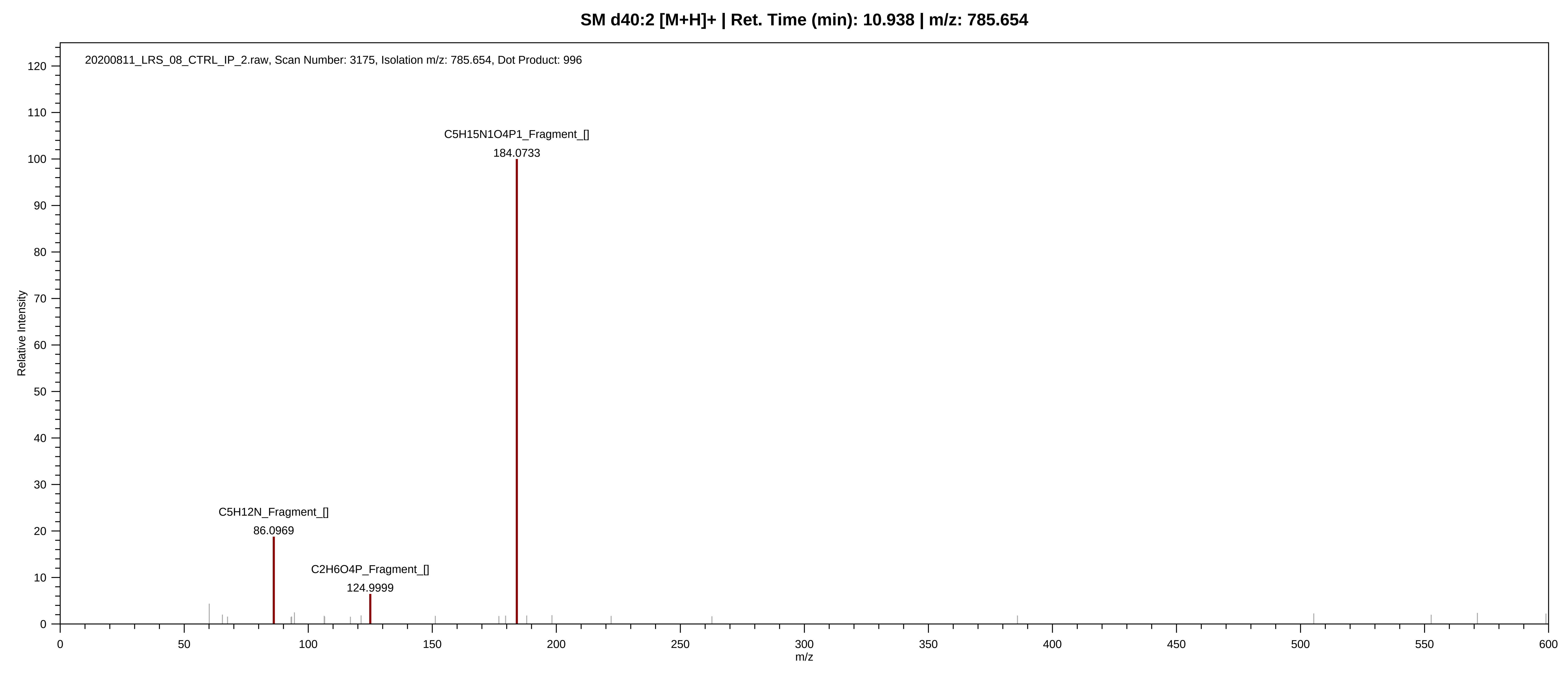
SM d40:2 [M+H]+ | Ret. Time (min): 10.938 | m/z: 785.654
20200811_LRS_08_CTRL_IP_2.raw, Scan Number: 3175, Isolation m/z: 785.654, Dot Product: 996
C5H15N1O4P1_Fragment_[]
184.0733
C5H12N_Fragment_[]
86.0969
C2H6O4P_Fragment_[]
124.9999
Relative Intensity
m/z

SM d40:2 [M+H]+ | Ret. Time (min): 10.759 | m/z: 785.655

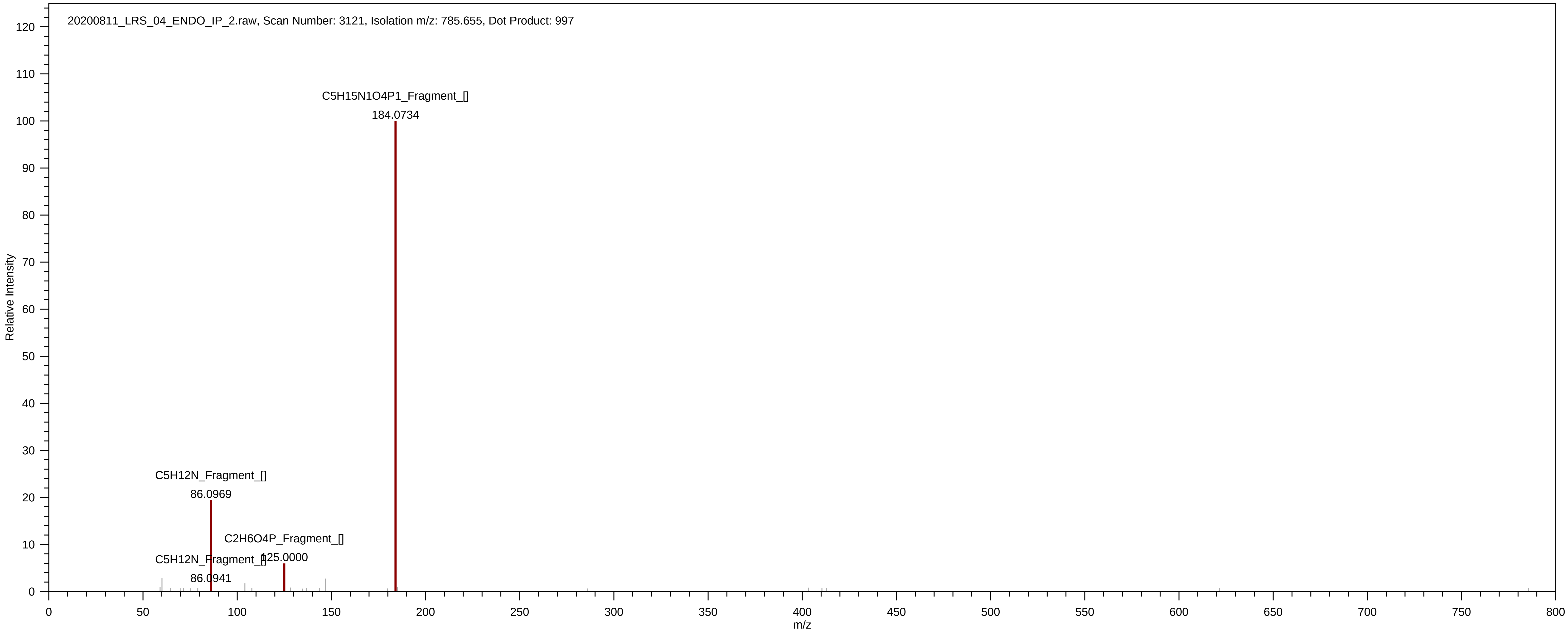

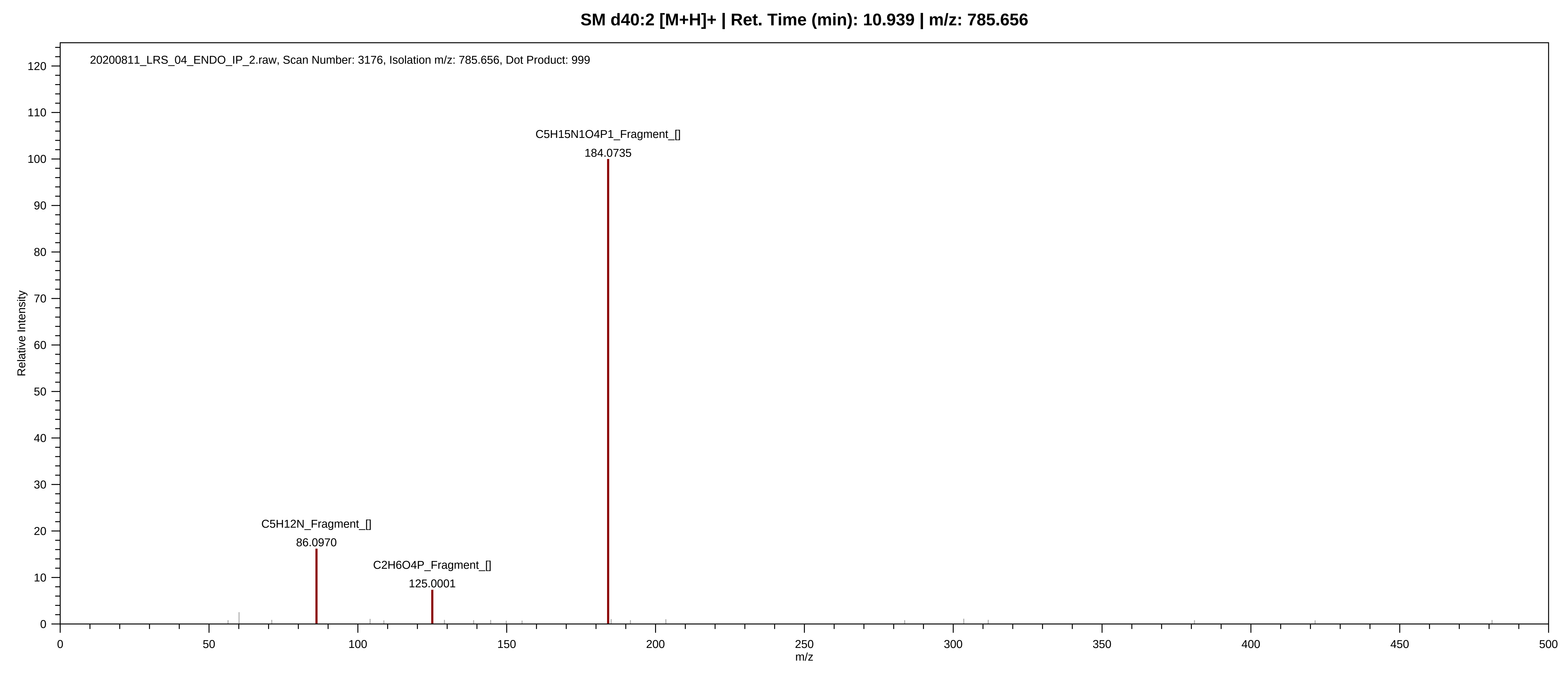

SM d40:2 [M+H]+ | Ret. Time (min): 10.939 | m/z: 785.656
20200811_LRS_04_ENDO_IP_2.raw, Scan Number: 3176, Isolation m/z: 785.656, Dot Product: 999
C5H15N1O4P1_Fragment_[]
184.0735
C5H12N_Fragment_[]
86.0970
C2H6O4P_Fragment_[]
125.0001
Relative Intensity
m/z

SM d41:2 [M+H]+ | Ret. Time (min): 11.266 | m/z: 799.67

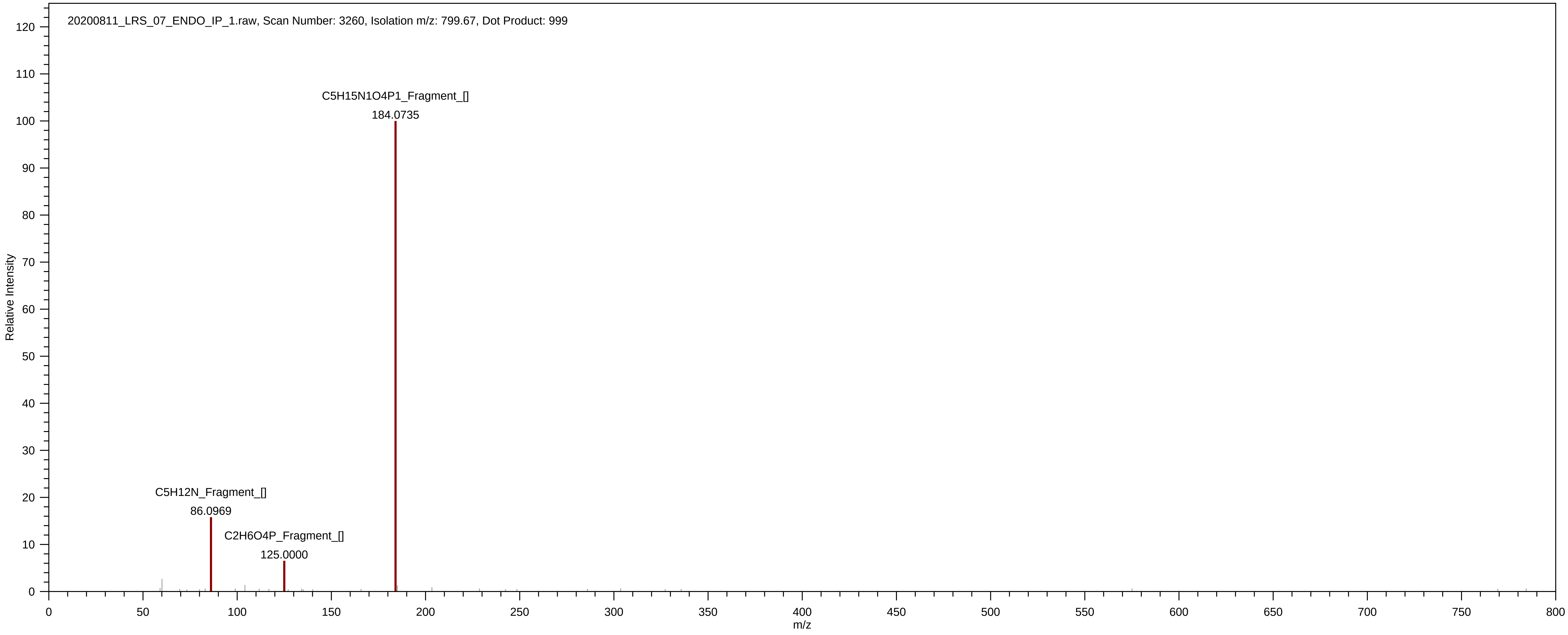

SM d41:2 [M+H]+ | Ret. Time (min): 11.266 | m/z: 799.67

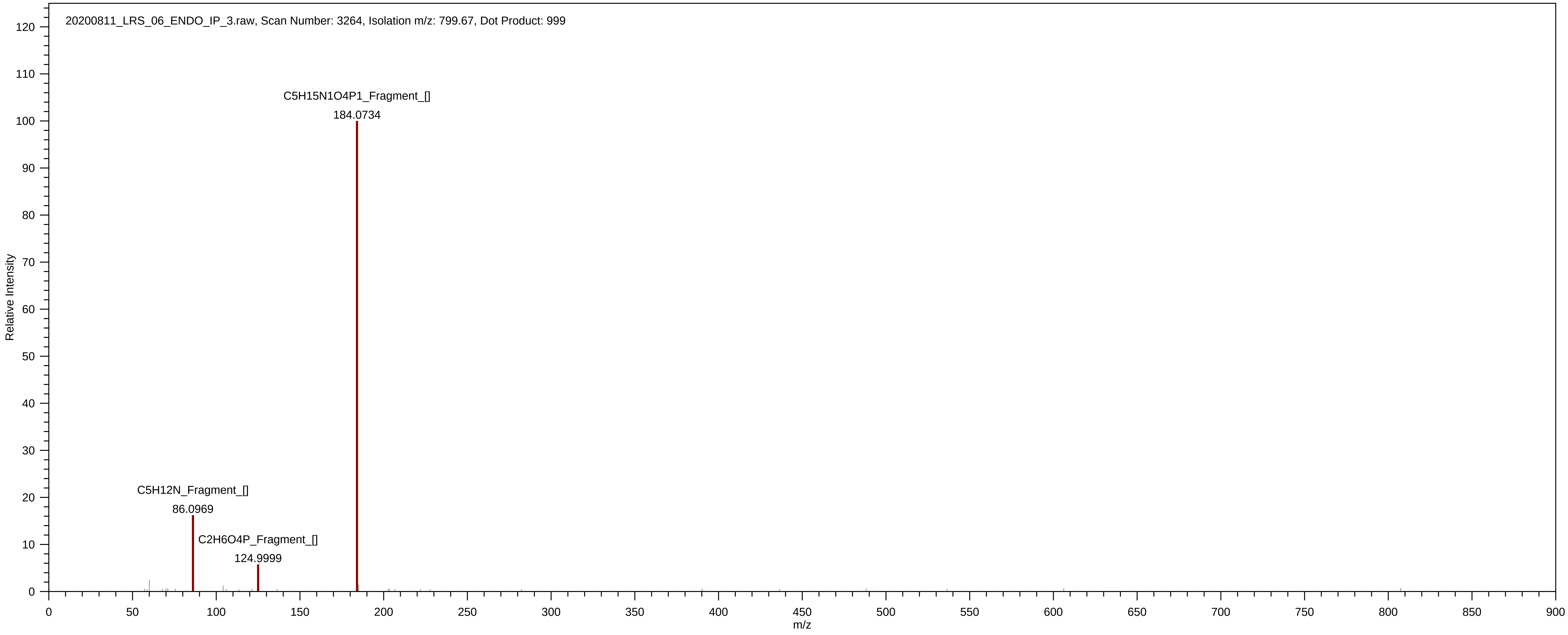

SM d41:2 [M+H]+ | Ret. Time (min): 11.278 | m/z: 799.67

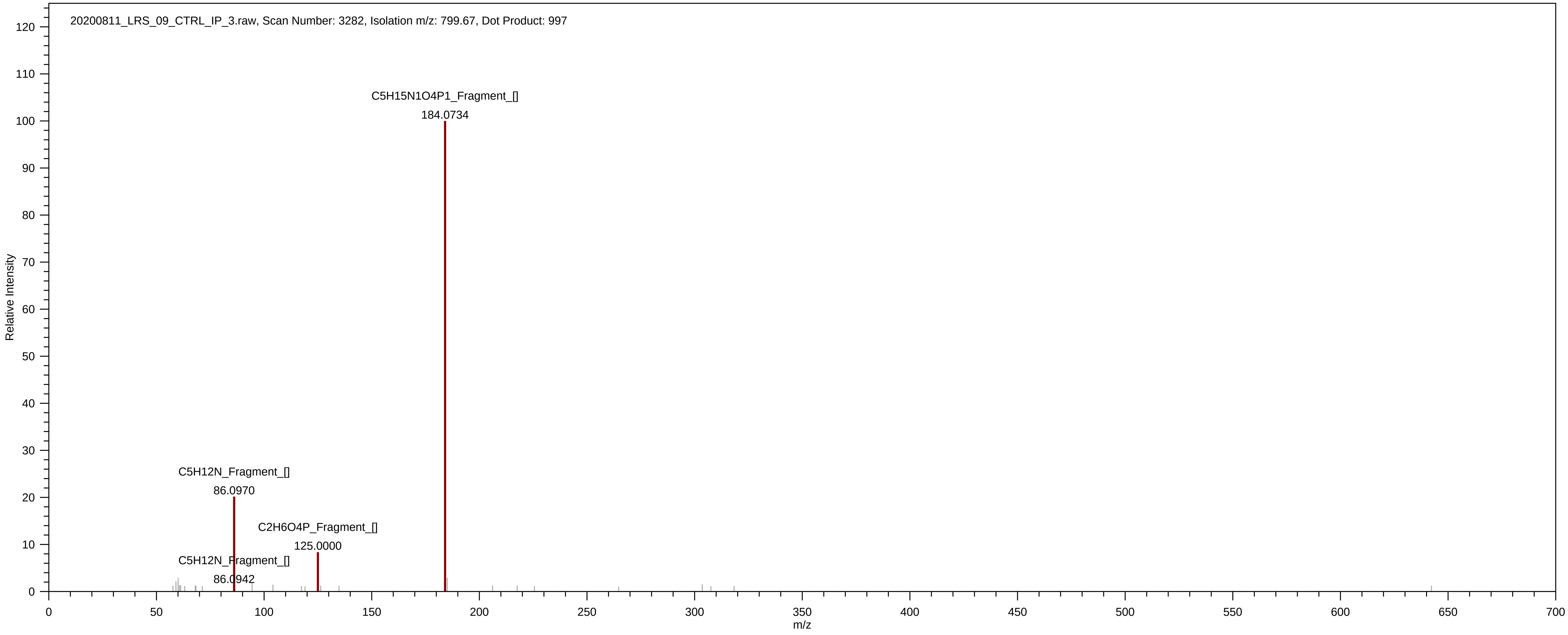

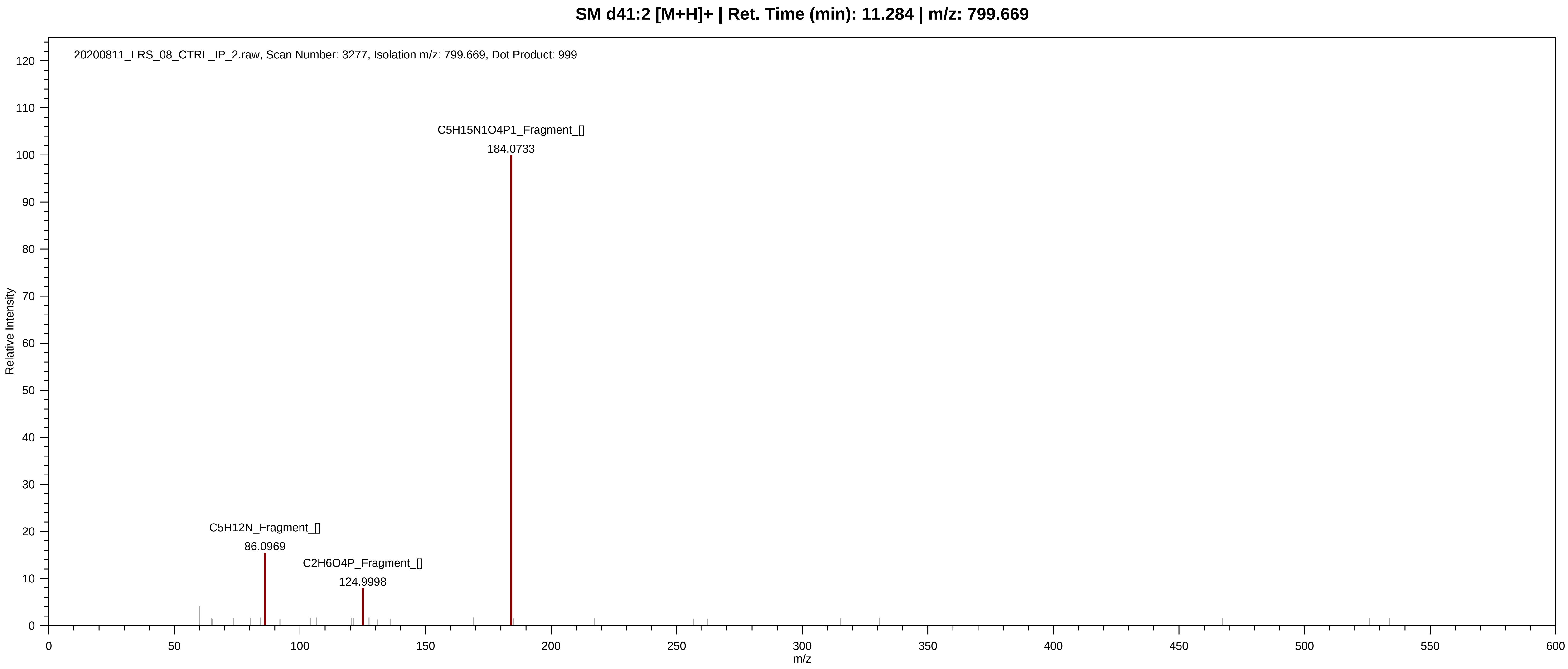

SM d41:2 [M+H]+ | Ret. Time (min): 11.284 | m/z: 799.669
20200811_LRS_08_CTRL_IP_2.raw, Scan Number: 3277, Isolation m/z: 799.669, Dot Product: 999
C5H15N1O4P1_Fragment_[]
184.0733
C5H12N_Fragment_[]
86.0969
C2H6O4P_Fragment_[]
124.9998
Relative Intensity
m/z

SM d41:2 [M+H]+ | Ret. Time (min): 11.285 | m/z: 799.671

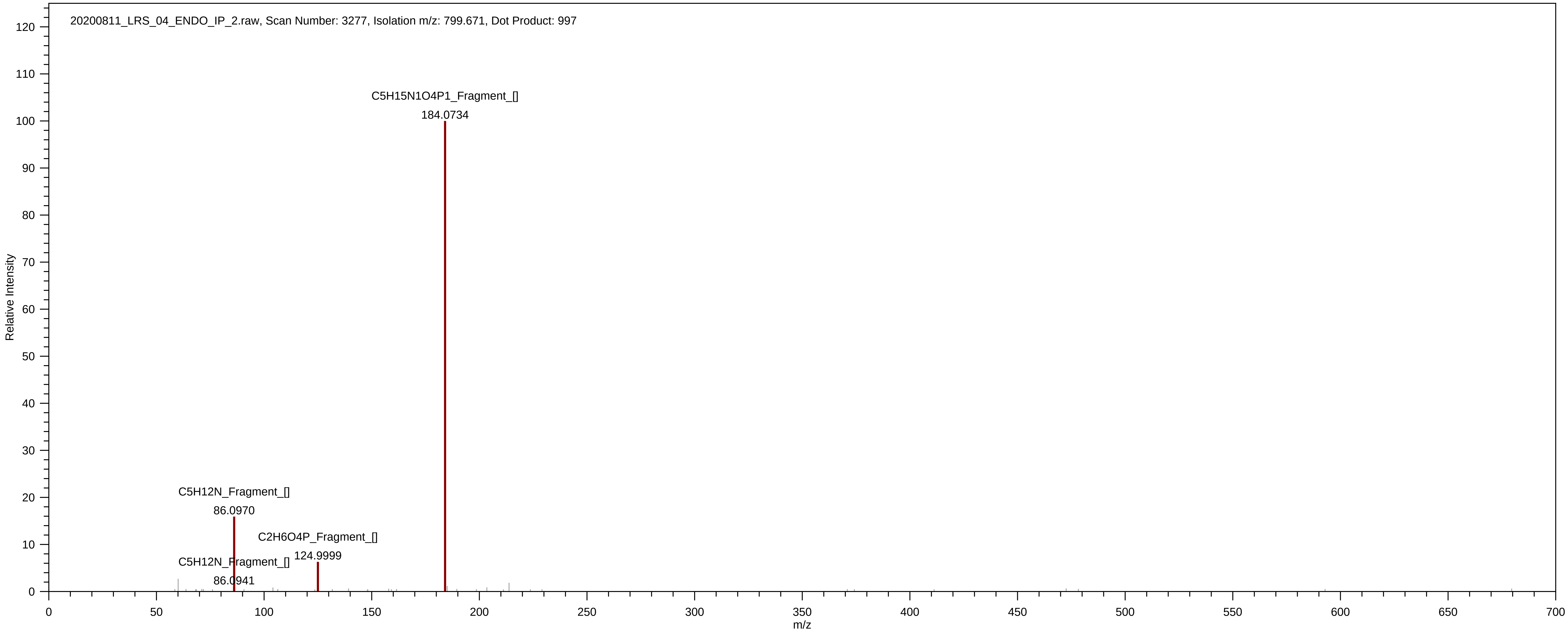

SM d41:2 [M+H]+ | Ret. Time (min): 11.298 | m/z: 799.671

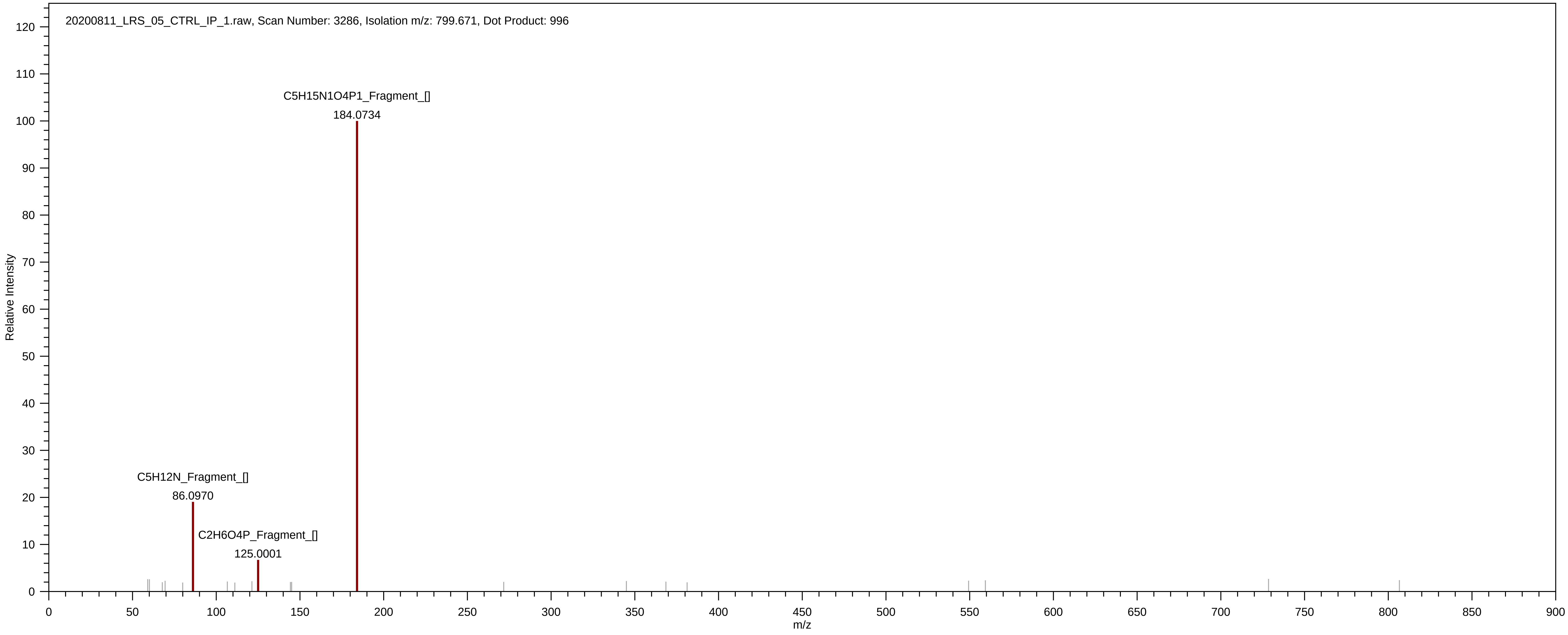

SM d42:0 [M+H]+ | Ret. Time (min): 13.39 | m/z: 817.718

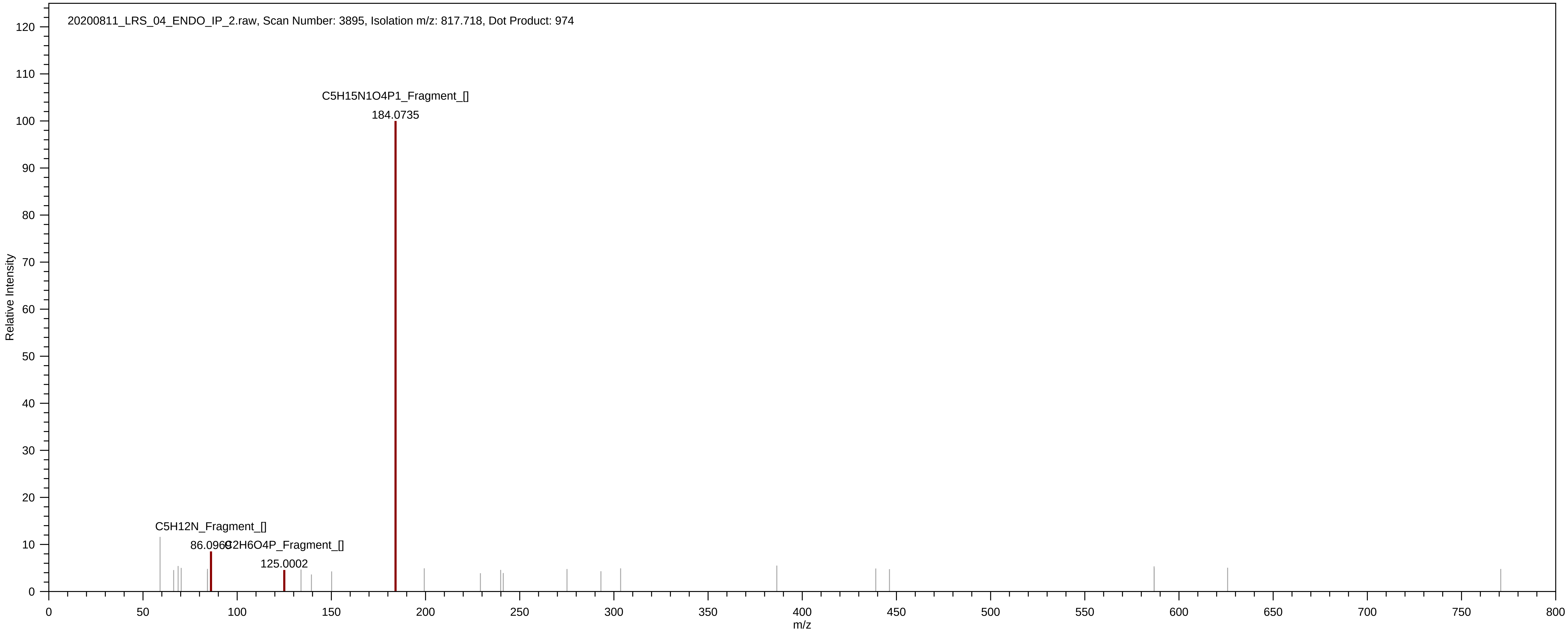

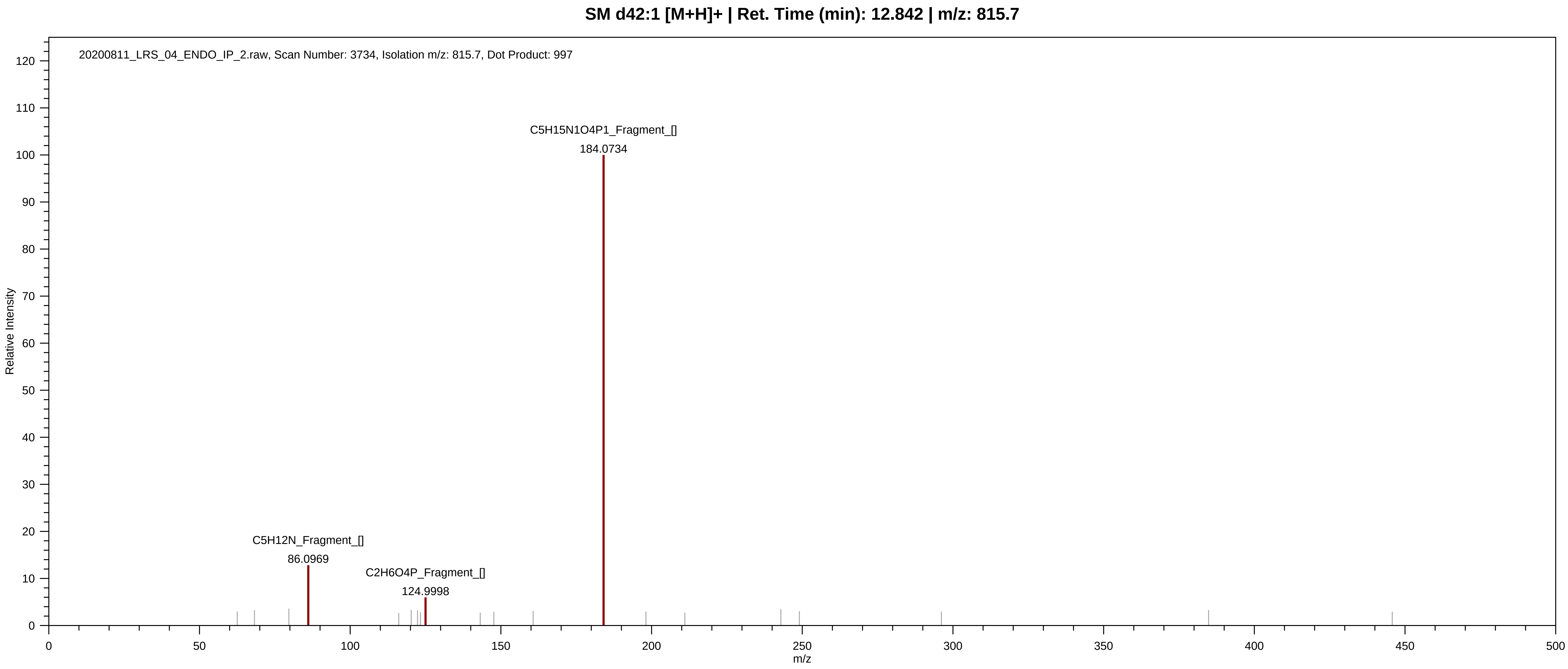
SM d42:1 [M+H]+ | Ret. Time (min): 12.842 | m/z: 815.7
20200811_LRS_04_ENDO_IP_2.raw, Scan Number: 3734, Isolation m/z: 815.7, Dot Product: 997
C5H15N1O4P1_Fragment_[]
184.0734
C5H12N_Fragment_[]
86.0969
C2H6O4P_Fragment_[]
124.9998
Relative Intensity
m/z

SM d42:1 [M+H]+ | Ret. Time (min): 12.903 | m/z: 815.7

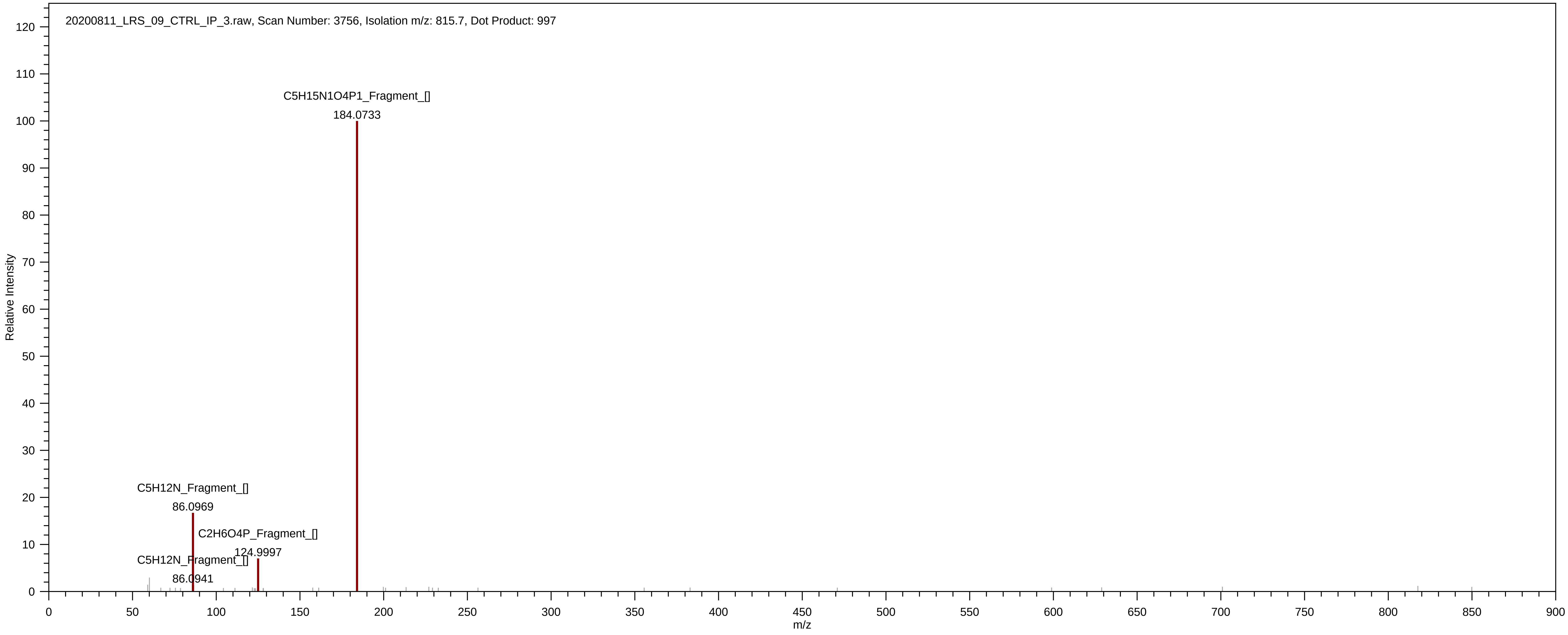

SM d42:1 [M+H]+ | Ret. Time (min): 12.907 | m/z: 815.7

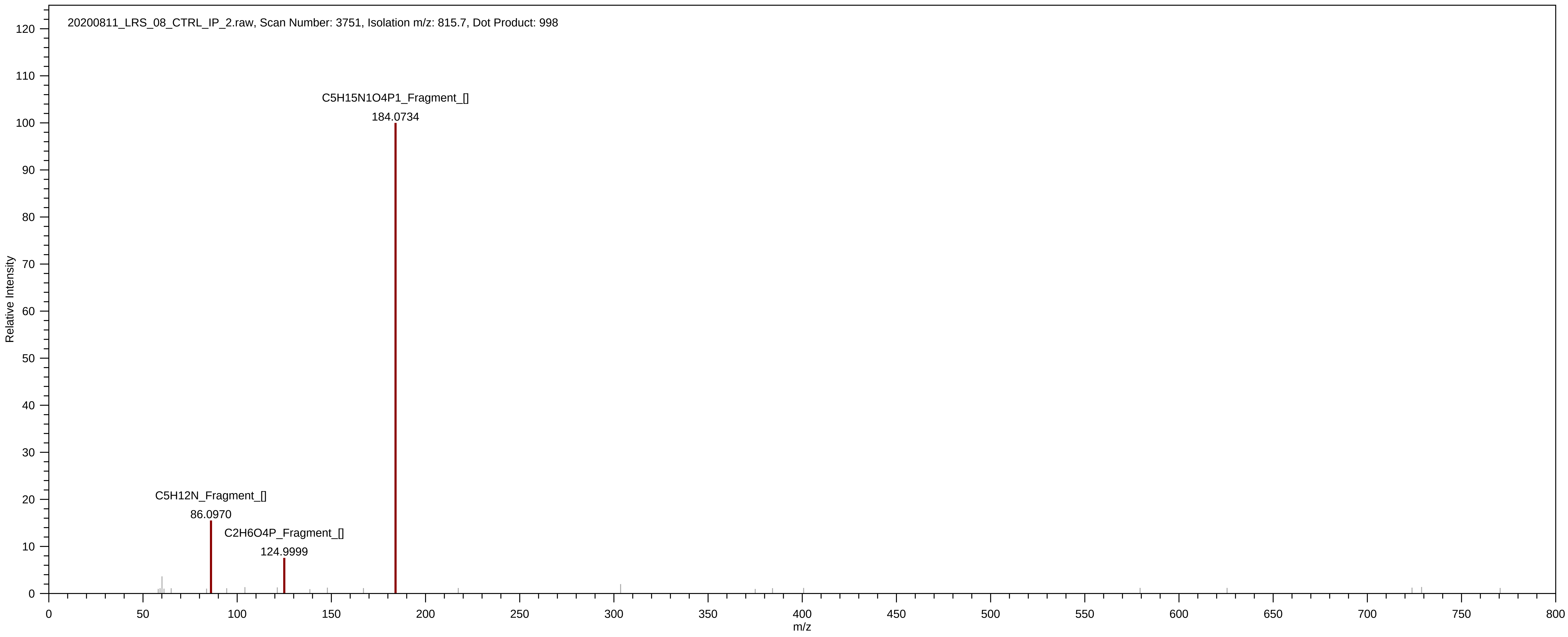

SM d42:1 [M+H]+ | Ret. Time (min): 13.025 | m/z: 815.7

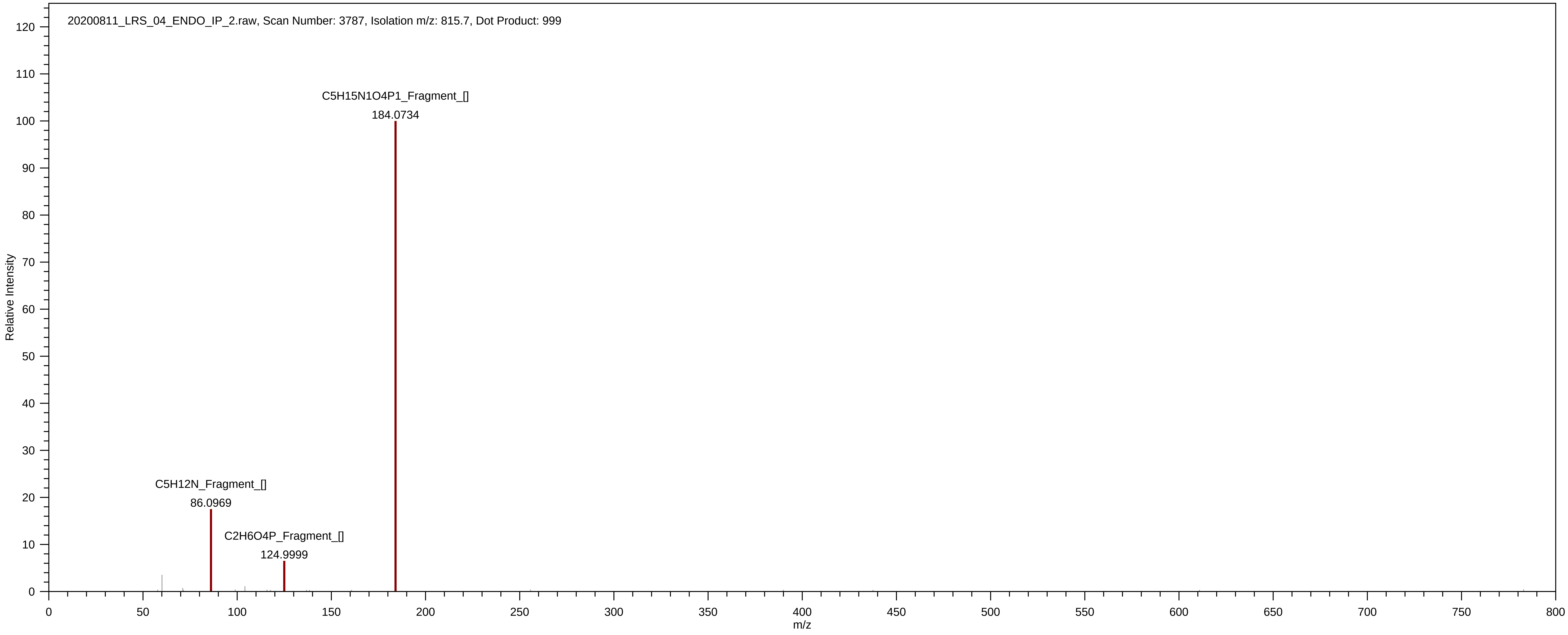

SM d42:1 [M+H]+ | Ret. Time (min): 13.106 | m/z: 815.699

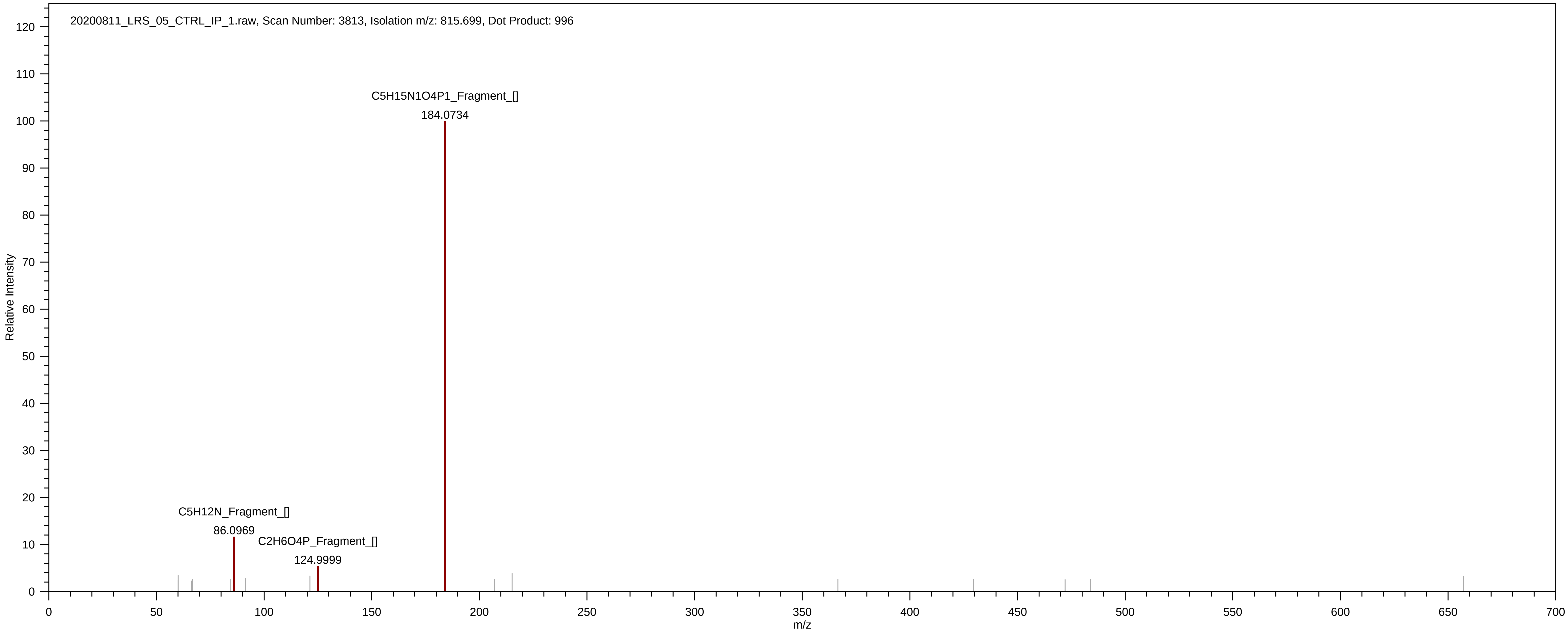

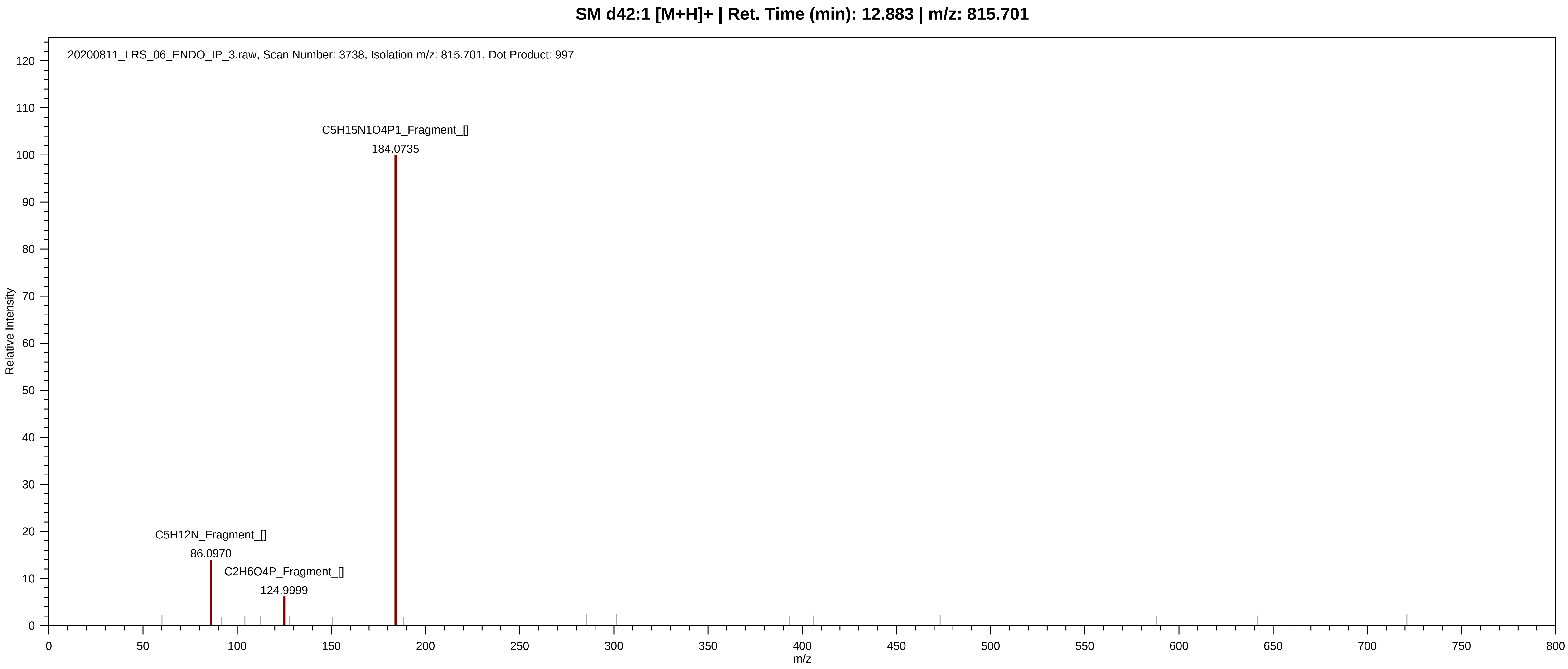
SM d42:1 [M+H]+ | Ret. Time (min): 12.883 | m/z: 815.701
20200811_LRS_06_ENDO_IP_3.raw, Scan Number: 3738, Isolation m/z: 815.701, Dot Product: 997
C5H15N1O4P1_Fragment_[]
184.0735
C5H12N_Fragment_[]
86.0970
C2H6O4P_Fragment_[]
124.9999
Relative Intensity
m/z
0
10
20
30
40
50
60
70
80
90
100
110
120
0
50
100
150
200
250
300
350
400
450
500
550
600
650
700
750
800

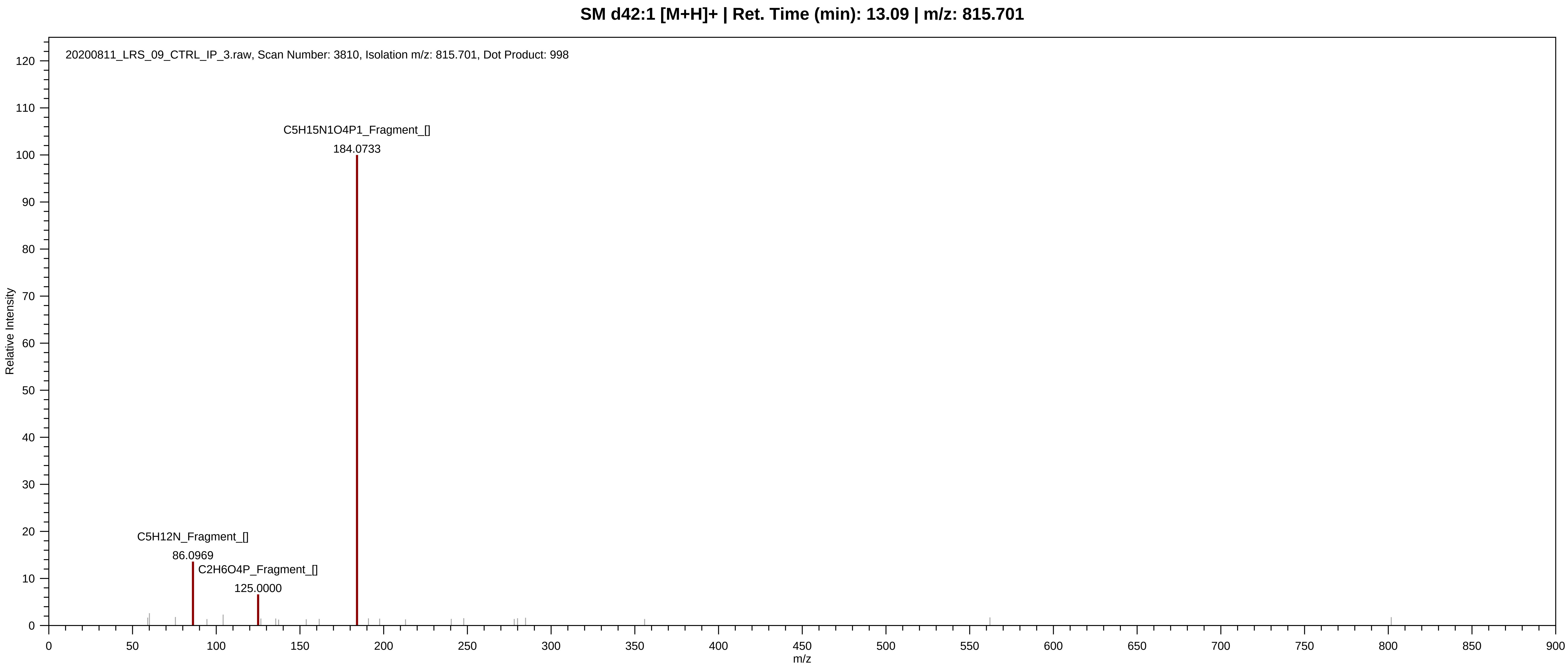
SM d42:1 [M+H]+ | Ret. Time (min): 13.09 | m/z: 815.701
20200811_LRS_09_CTRL_IP_3.raw, Scan Number: 3810, Isolation m/z: 815.701, Dot Product: 998
C5H15N1O4P1_Fragment_[]
184.0733
C5H12N_Fragment_[]
86.0969
C2H6O4P_Fragment_[]
125.0000
Relative Intensity
m/z
0
10
20
30
40
50
60
70
80
90
100
110
120
0
50
100
150
200
250
300
350
400
450
500
550
600
650
700
750
800
850
900

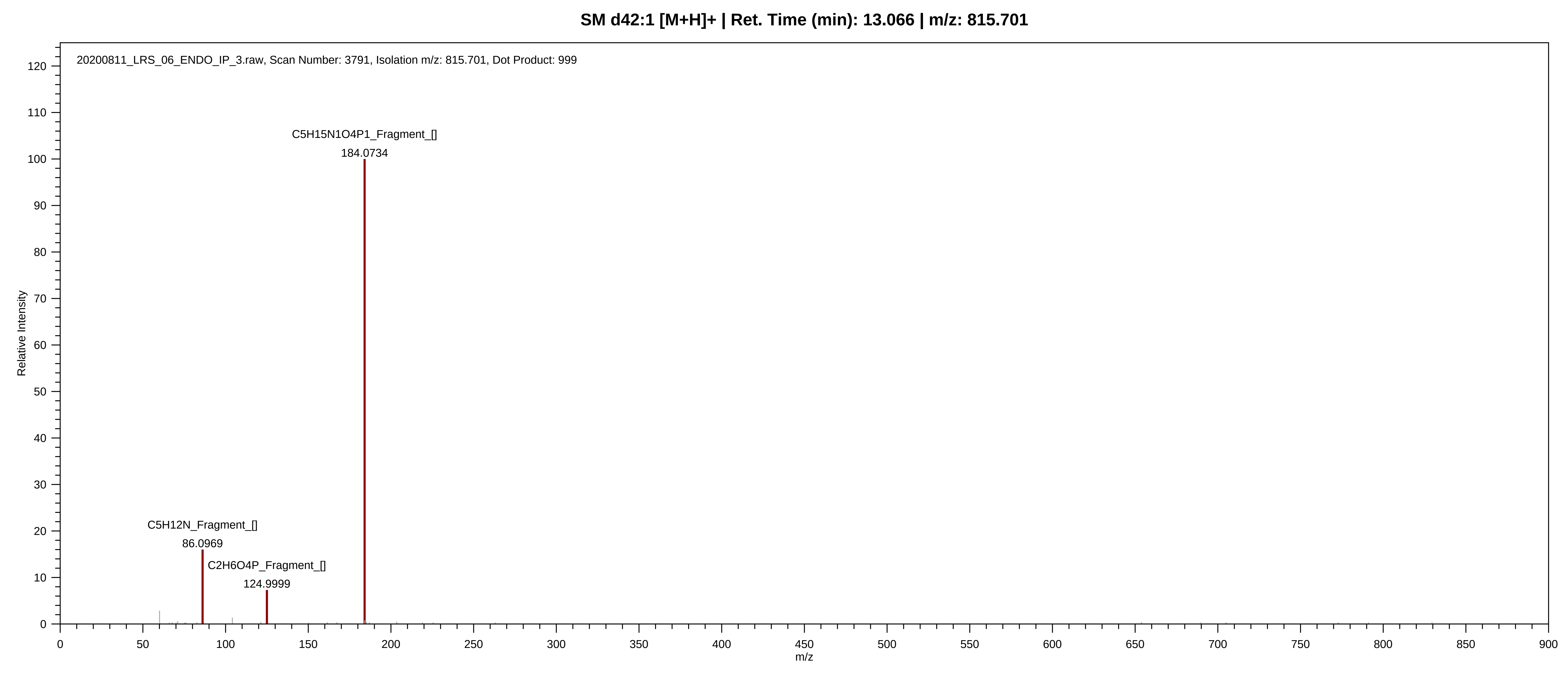

SM d42:1 [M+H]+ | Ret. Time (min): 13.066 | m/z: 815.701
20200811_LRS_06_ENDO_IP_3.raw, Scan Number: 3791, Isolation m/z: 815.701, Dot Product: 999
C5H15N1O4P1_Fragment_[]
184.0734
C5H12N_Fragment_[]
86.0969
C2H6O4P_Fragment_[]
124.9999
Relative Intensity
m/z

SM d42:1 [M+H]+ | Ret. Time (min): 13.069 | m/z: 815.701

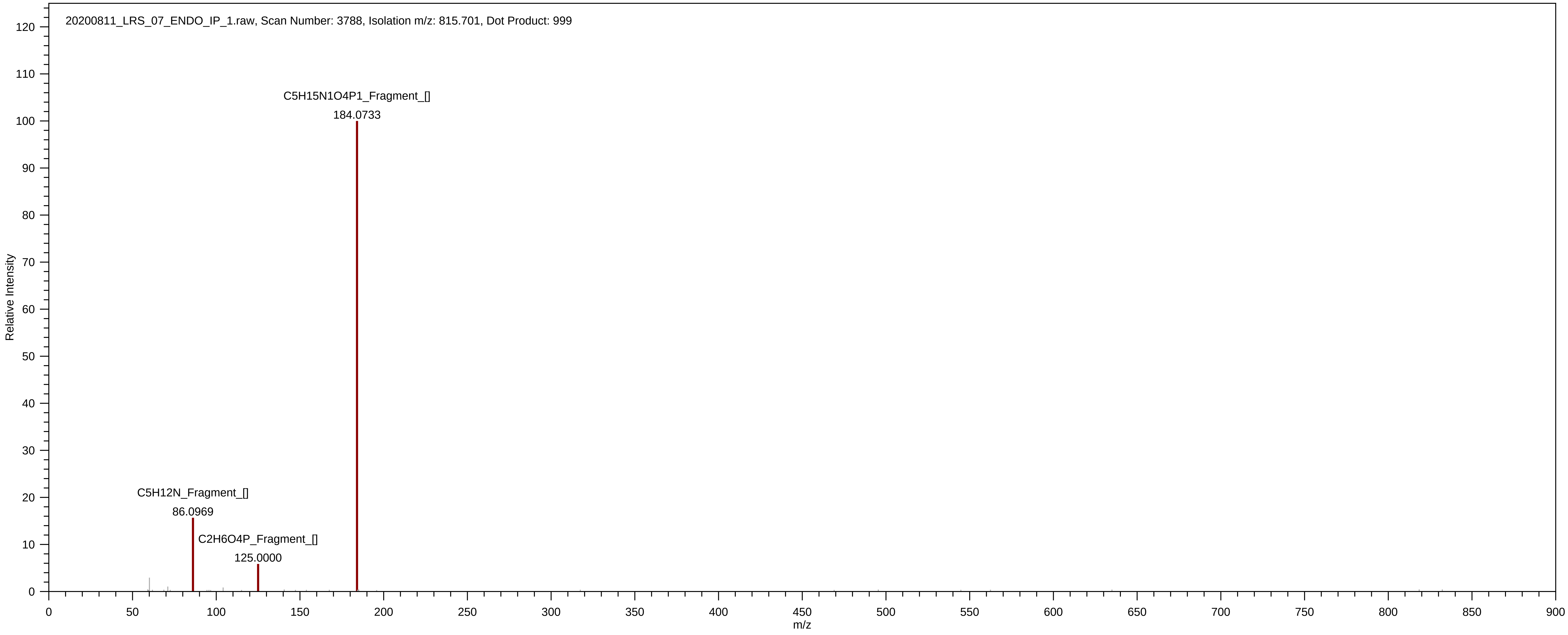

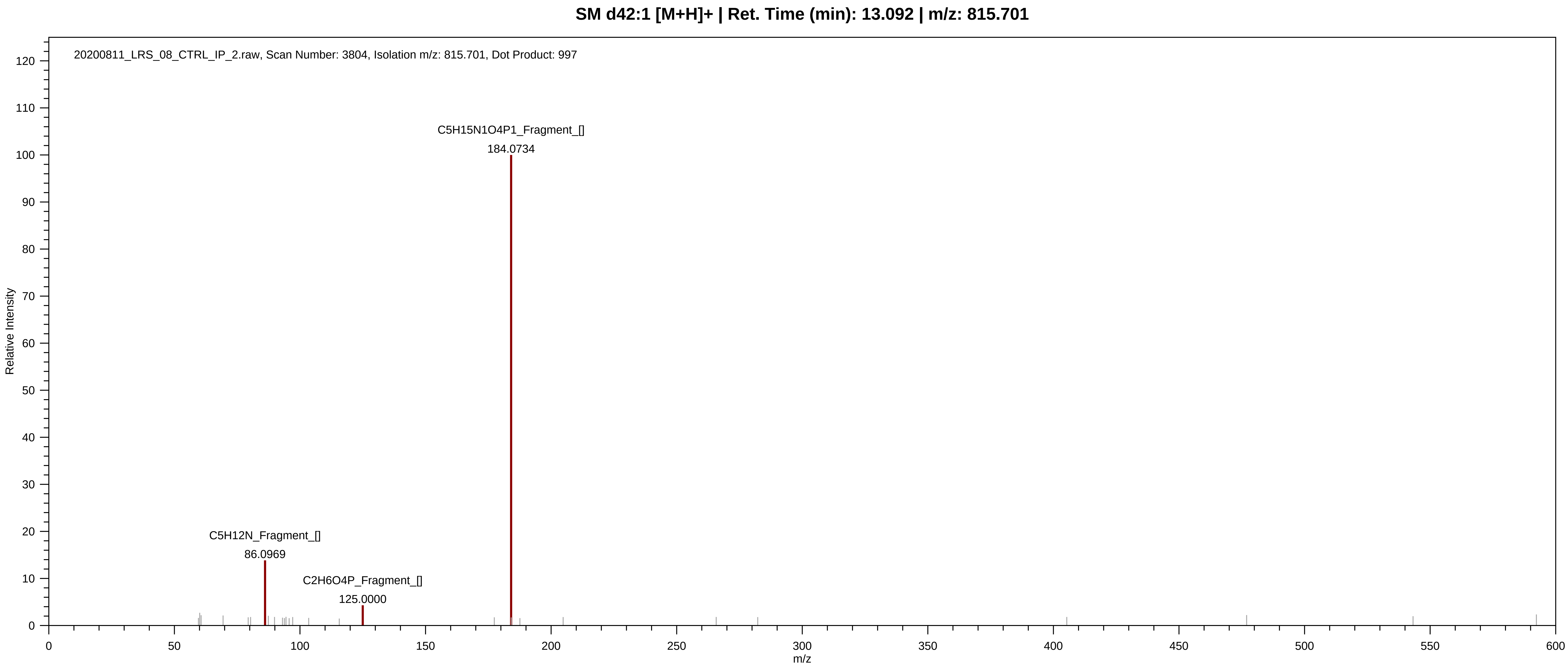
SM d42:1 [M+H]+ | Ret. Time (min): 13.092 | m/z: 815.701
20200811_LRS_08_CTRL_IP_2.raw, Scan Number: 3804, Isolation m/z: 815.701, Dot Product: 997
C5H15N1O4P1_Fragment_[]
184.0734
C5H12N_Fragment_[]
86.0969
C2H6O4P_Fragment_[]
125.0000
Relative Intensity
m/z
0
10
20
30
40
50
60
70
80
90
100
110
120
0
50
100
150
200
250
300
350
400
450
500
550
600

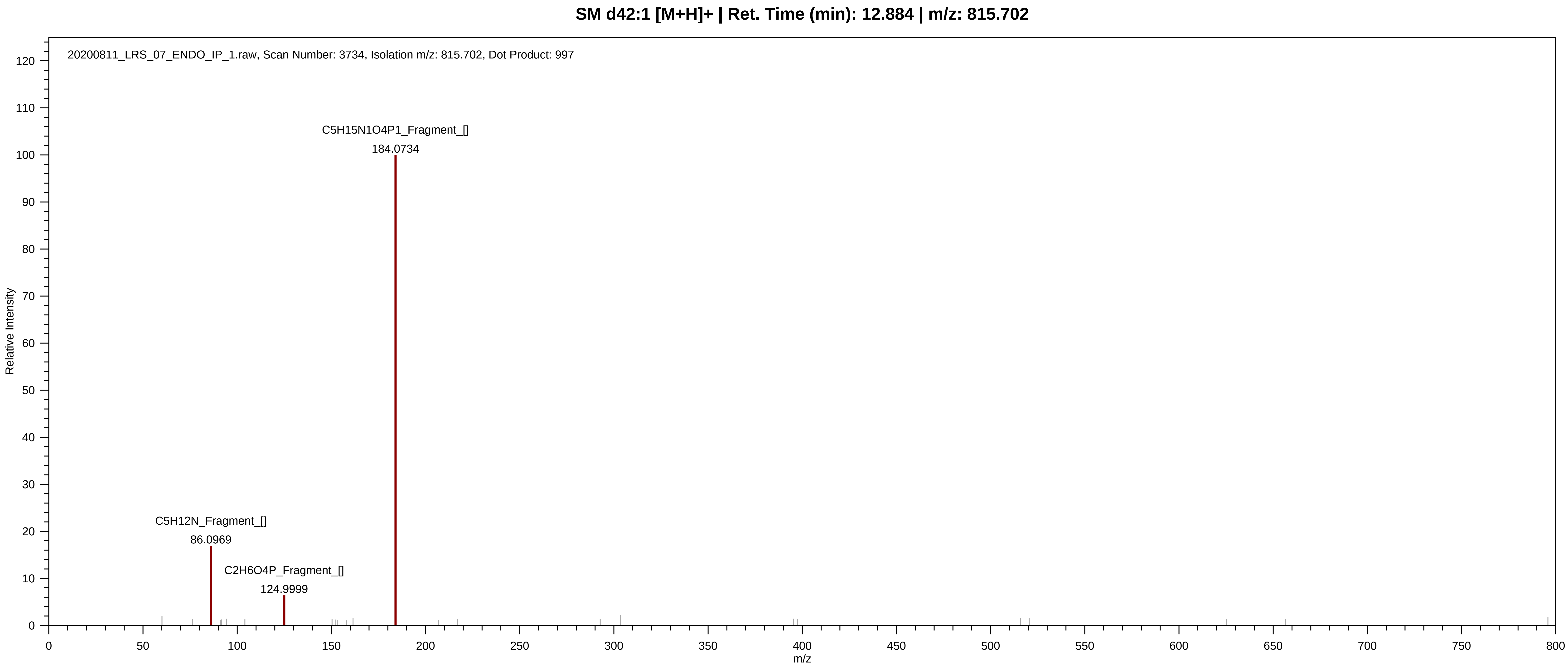
SM d42:1 [M+H]+ | Ret. Time (min): 12.884 | m/z: 815.702
20200811_LRS_07_ENDO_IP_1.raw, Scan Number: 3734, Isolation m/z: 815.702, Dot Product: 997
C5H15N1O4P1_Fragment_[]
184.0734
C5H12N_Fragment_[]
86.0969
C2H6O4P_Fragment_[]
124.9999
Relative Intensity
m/z
0
10
20
30
40
50
60
70
80
90
100
110
120
0
50
100
150
200
250
300
350
400
450
500
550
600
650
700
750
800

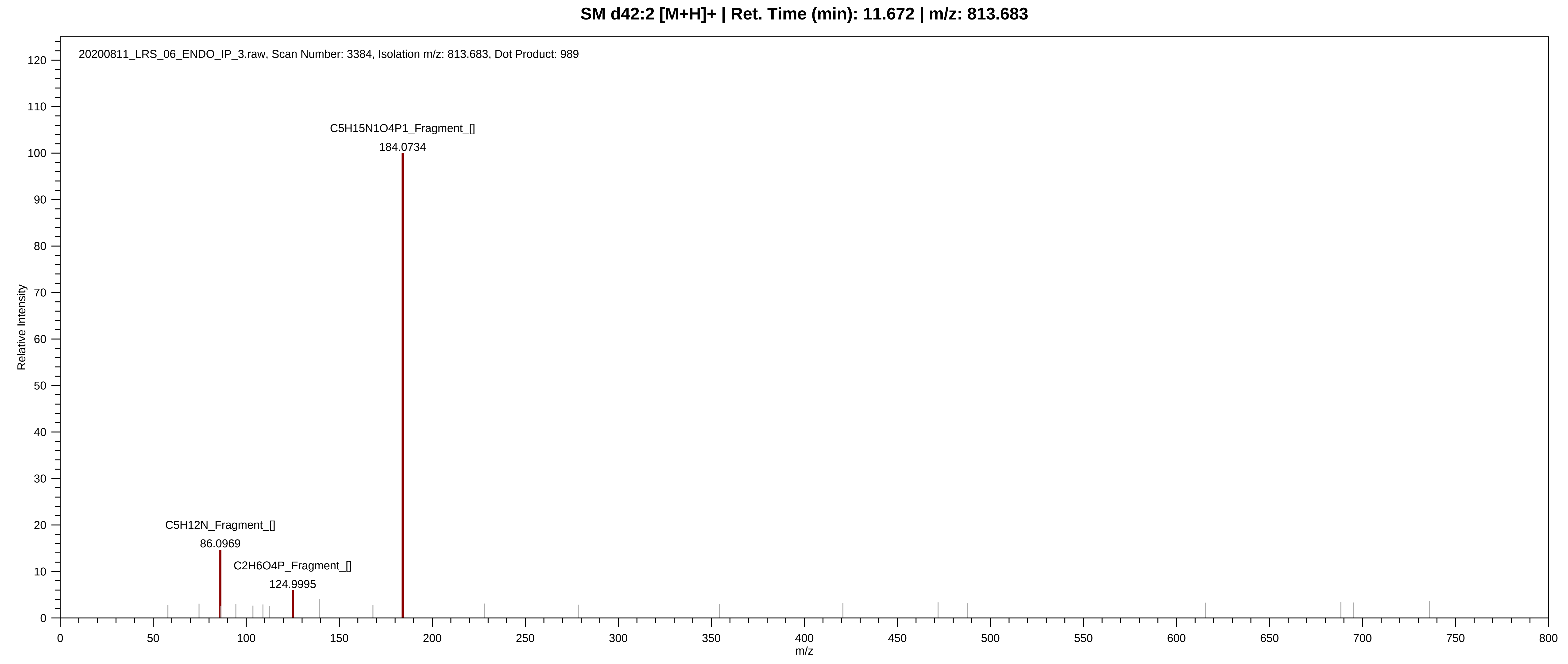
SM d42:2 [M+H]+ | Ret. Time (min): 11.672 | m/z: 813.683
20200811_LRS_06_ENDO_IP_3.raw, Scan Number: 3384, Isolation m/z: 813.683, Dot Product: 989
C5H15N1O4P1_Fragment_[]
184.0734
C5H12N_Fragment_[]
86.0969
C2H6O4P_Fragment_[]
124.9995
Relative Intensity
m/z
0
10
20
30
40
50
60
70
80
90
100
110
120
0
50
100
150
200
250
300
350
400
450
500
550
600
650
700
750
800

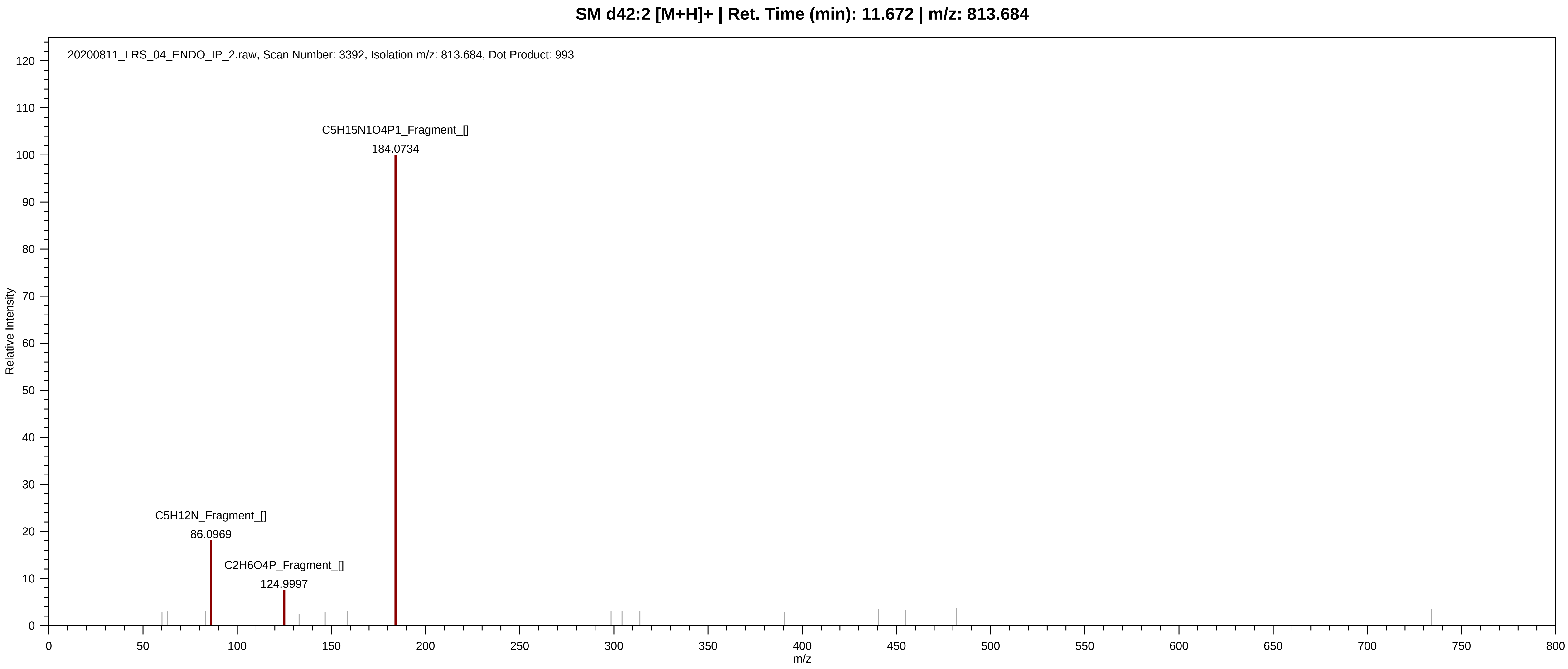
SM d42:2 [M+H]+ | Ret. Time (min): 11.672 | m/z: 813.684
20200811_LRS_04_ENDO_IP_2.raw, Scan Number: 3392, Isolation m/z: 813.684, Dot Product: 993
C5H15N1O4P1_Fragment_[]
184.0734
C5H12N_Fragment_[]
86.0969
C2H6O4P_Fragment_[]
124.9997
Relative Intensity
m/z

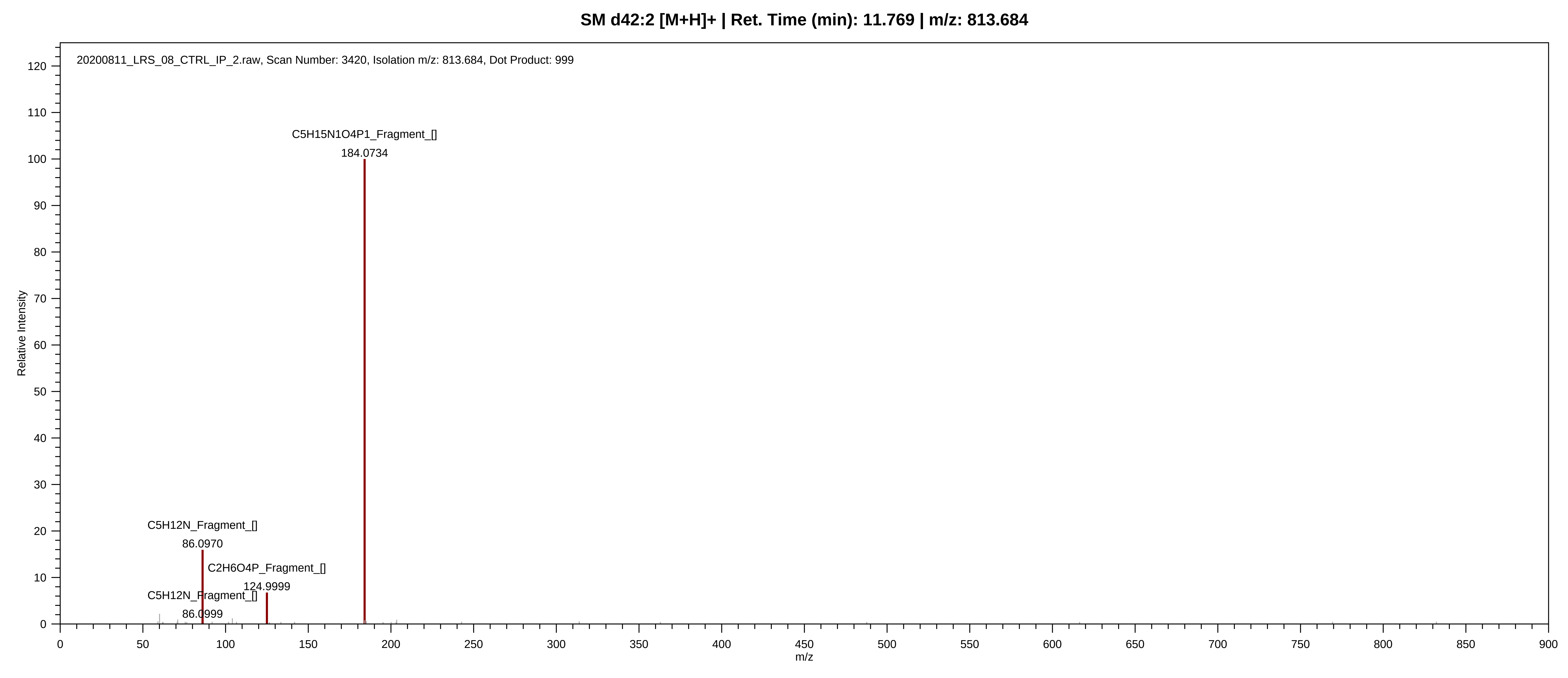

SM d42:2 [M+H]+ | Ret. Time (min): 11.769 | m/z: 813.684
20200811_LRS_08_CTRL_IP_2.raw, Scan Number: 3420, Isolation m/z: 813.684, Dot Product: 999
C5H15N1O4P1_Fragment_[]
184.0734
C5H12N_Fragment_[]
86.0970
C2H6O4P_Fragment_[]
124.9999
C5H12N_Fragment_[]
86.0999
Relative Intensity
m/z

SM d42:2 [M+H]+ | Ret. Time (min): 11.852 | m/z: 813.684

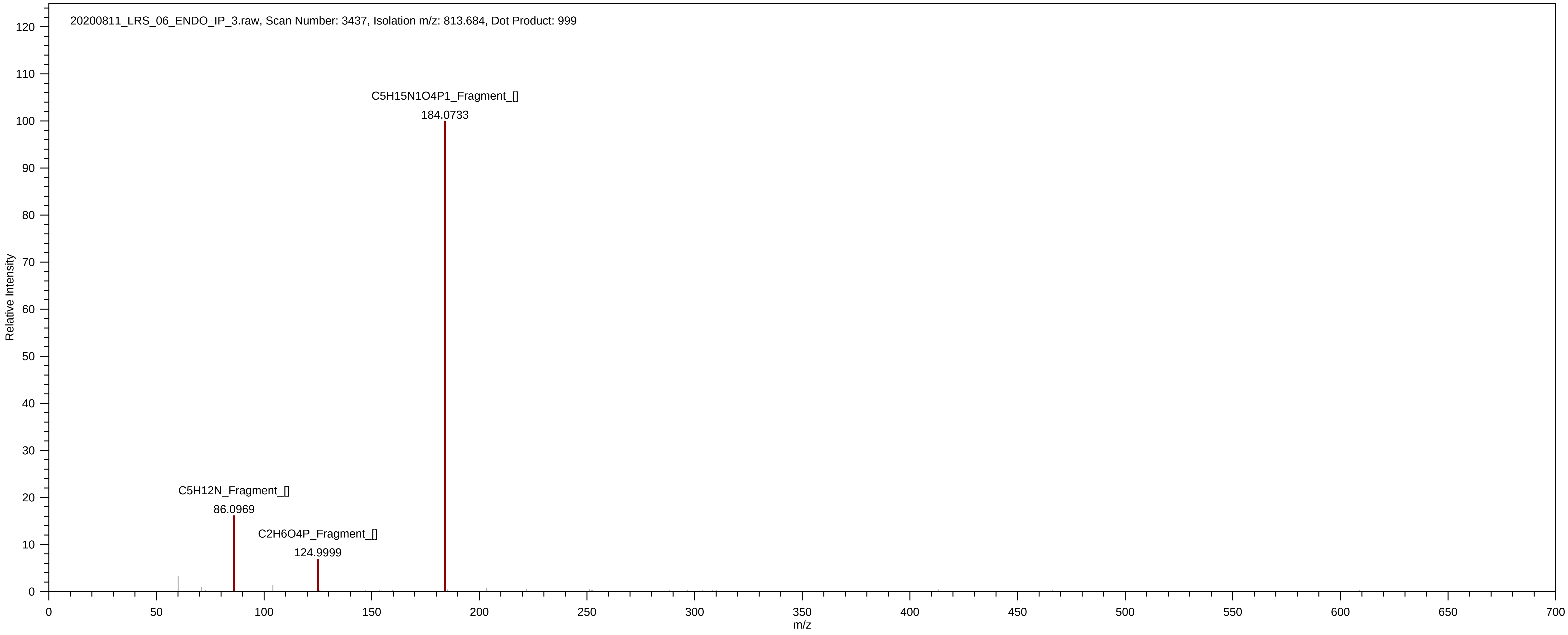

SM d42:2 [M+H]+ | Ret. Time (min): 11.852 | m/z: 813.685

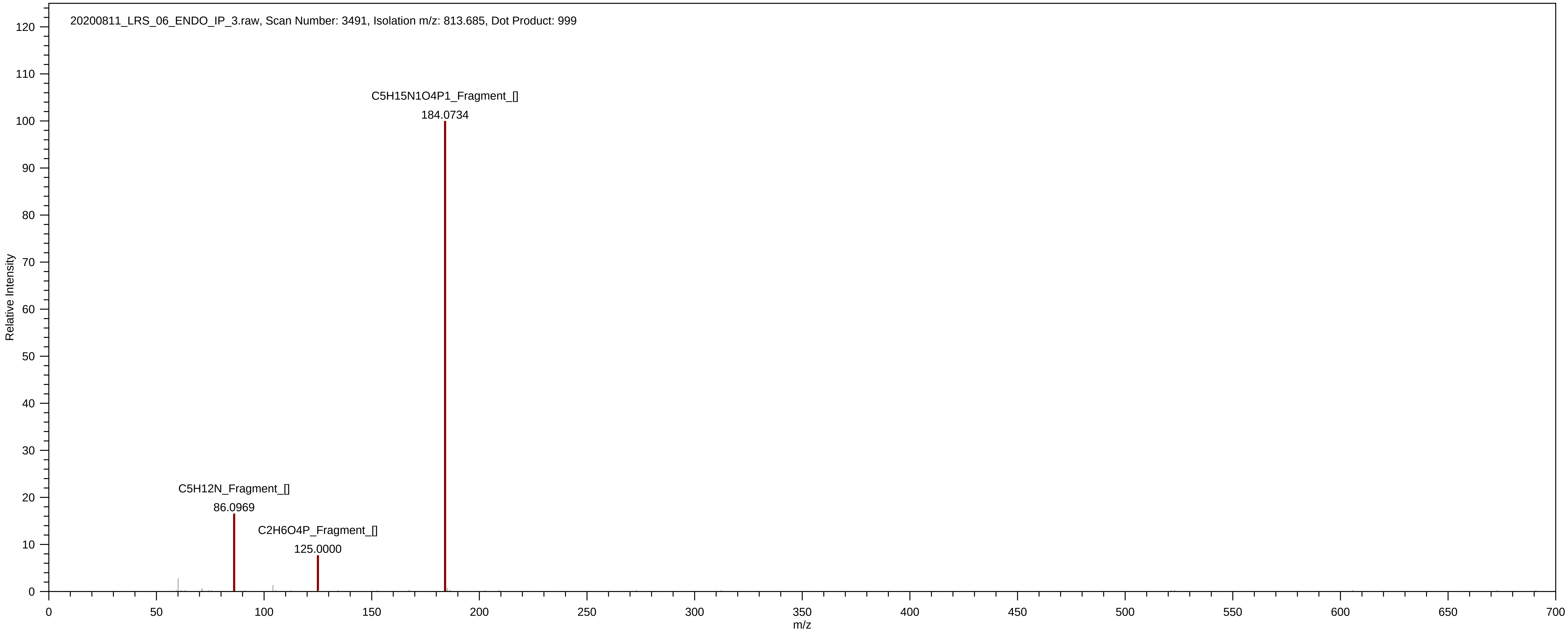

SM d42:2 [M+H]+ | Ret. Time (min): 11.972 | m/z: 813.684

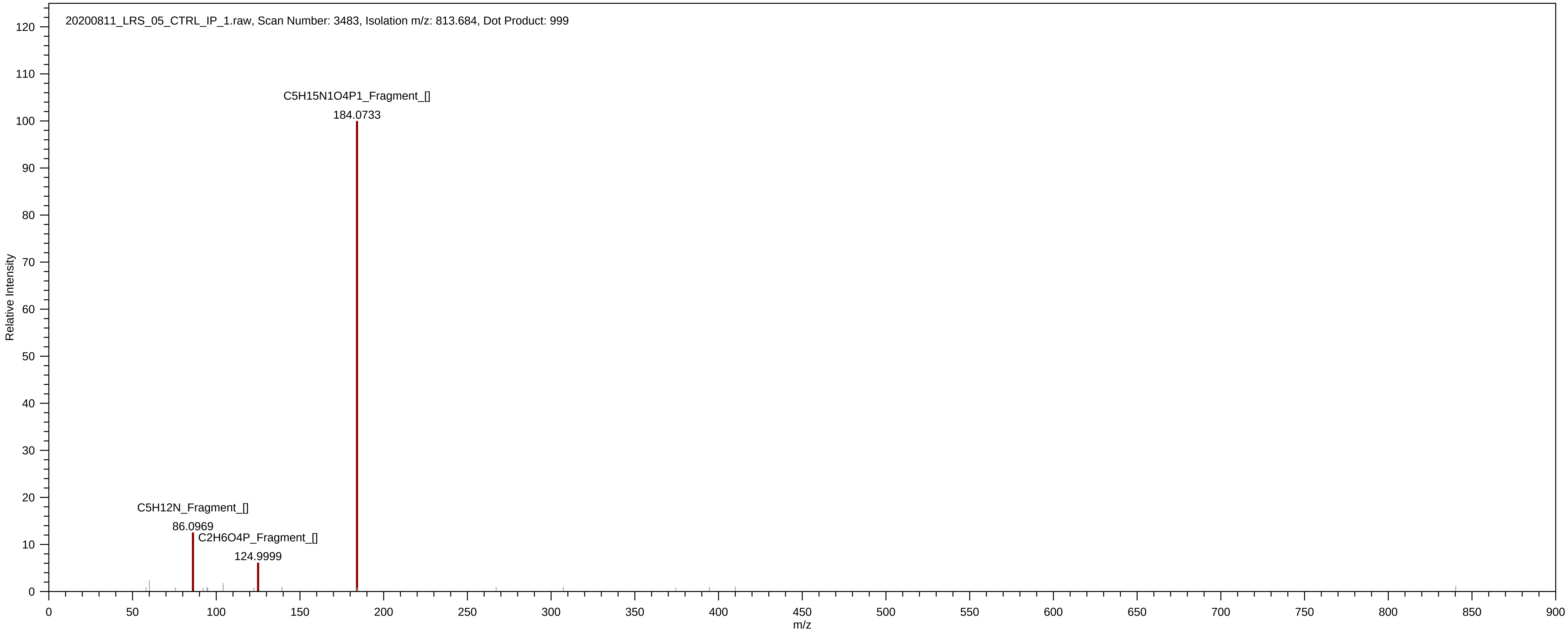

SM d42:2 [M+H]+ | Ret. Time (min): 11.751 | m/z: 813.685

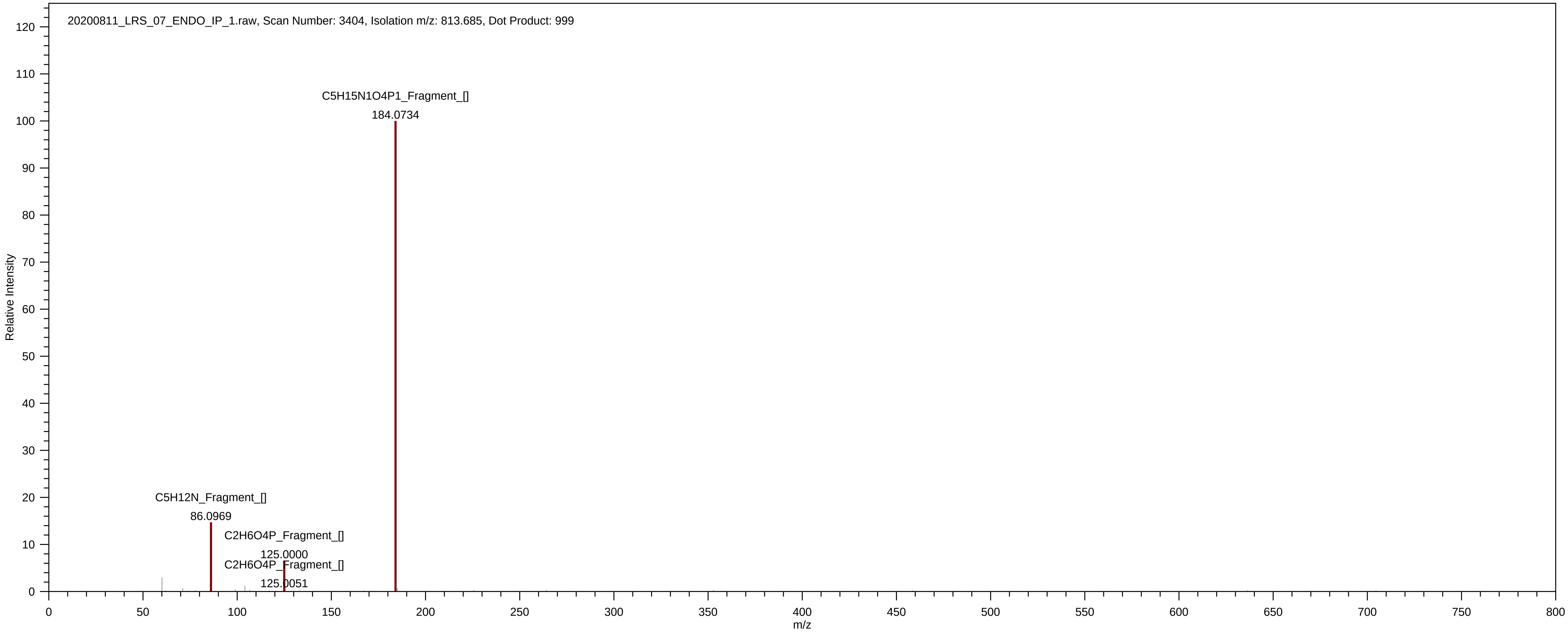

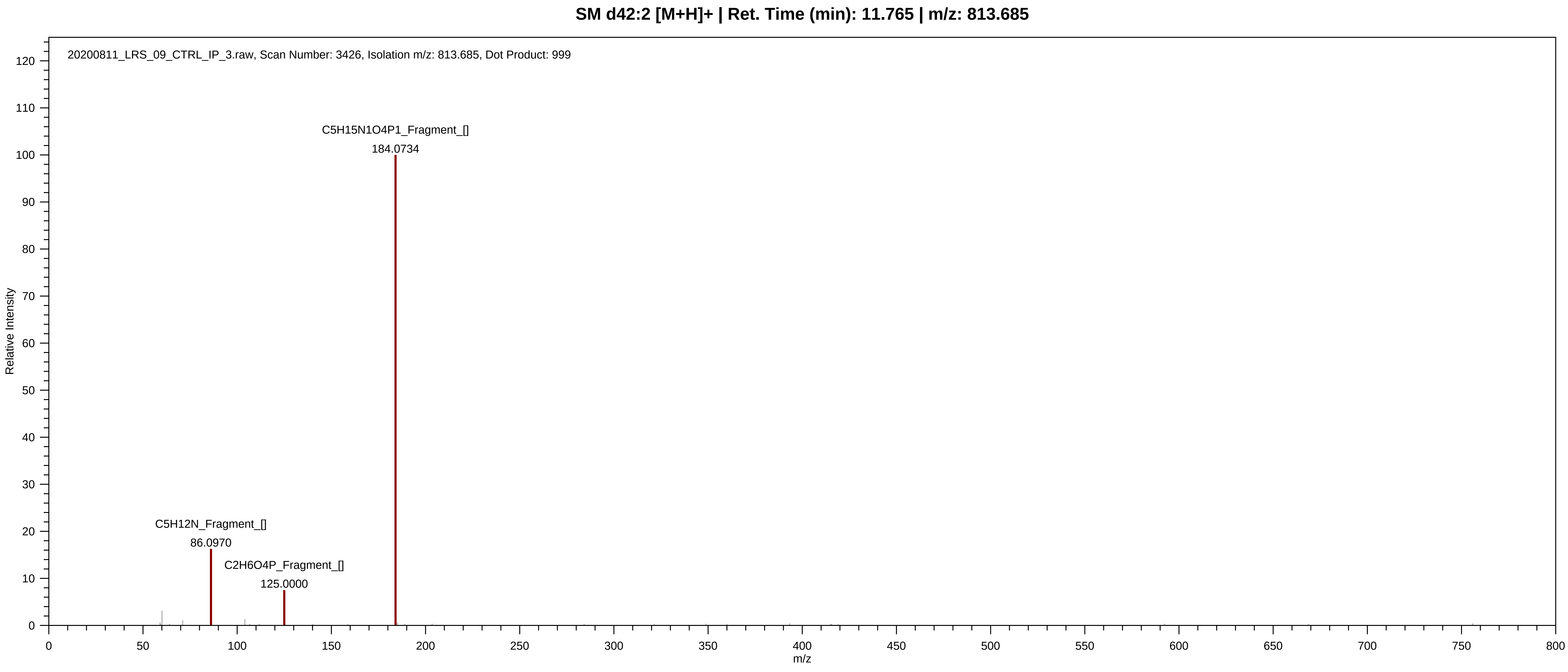
SM d42:2 [M+H]+ | Ret. Time (min): 11.765 | m/z: 813.685
20200811_LRS_09_CTRL_IP_3.raw, Scan Number: 3426, Isolation m/z: 813.685, Dot Product: 999
C5H15N1O4P1_Fragment_[]
184.0734
C5H12N_Fragment_[]
86.0970
C2H6O4P_Fragment_[]
125.0000
Relative Intensity
m/z
0
10
20
30
40
50
60
70
80
90
100
110
120
0
50
100
150
200
250
300
350
400
450
500
550
600
650
700
750
800

SM d42:2 [M+H]+ | Ret. Time (min): 11.787 | m/z: 813.685

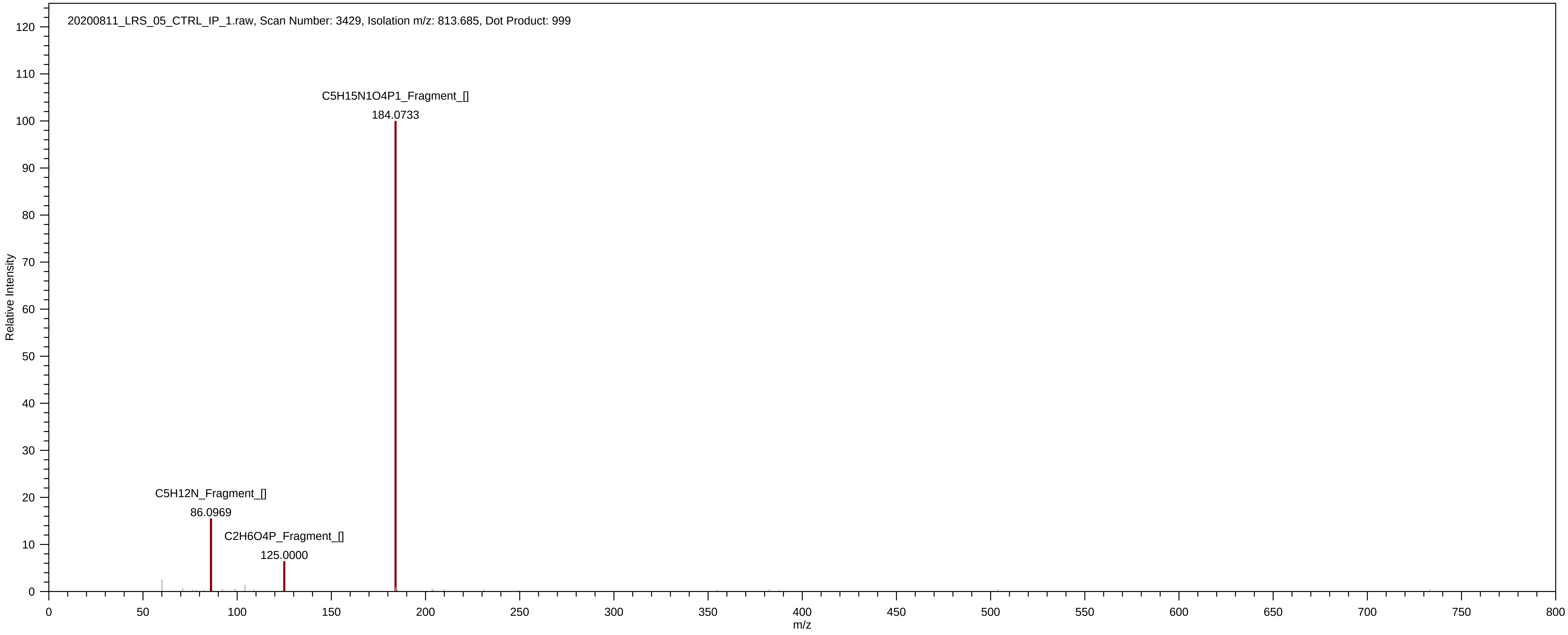

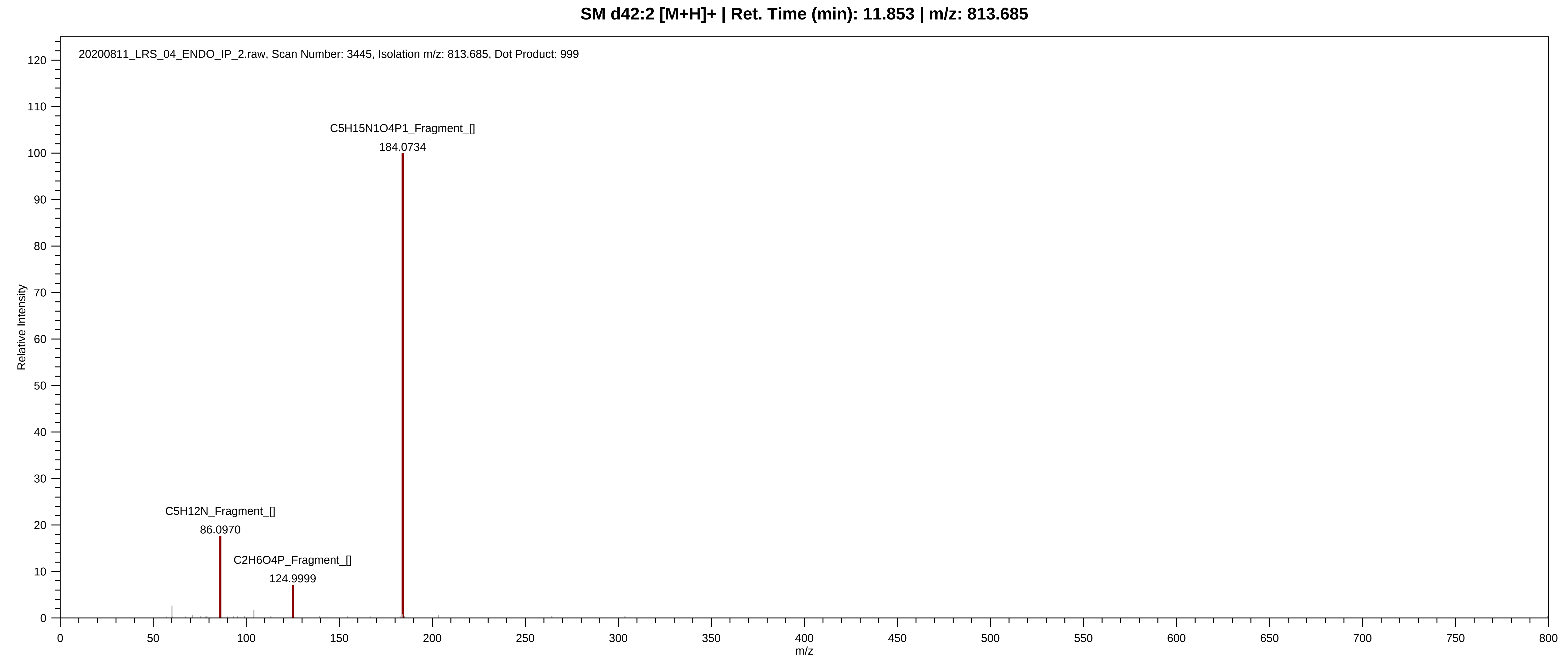
SM d42:2 [M+H]+ | Ret. Time (min): 11.853 | m/z: 813.685
20200811_LRS_04_ENDO_IP_2.raw, Scan Number: 3445, Isolation m/z: 813.685, Dot Product: 999
C5H15N1O4P1_Fragment_[]
184.0734
C5H12N_Fragment_[]
86.0970
C2H6O4P_Fragment_[]
124.9999
Relative Intensity
m/z

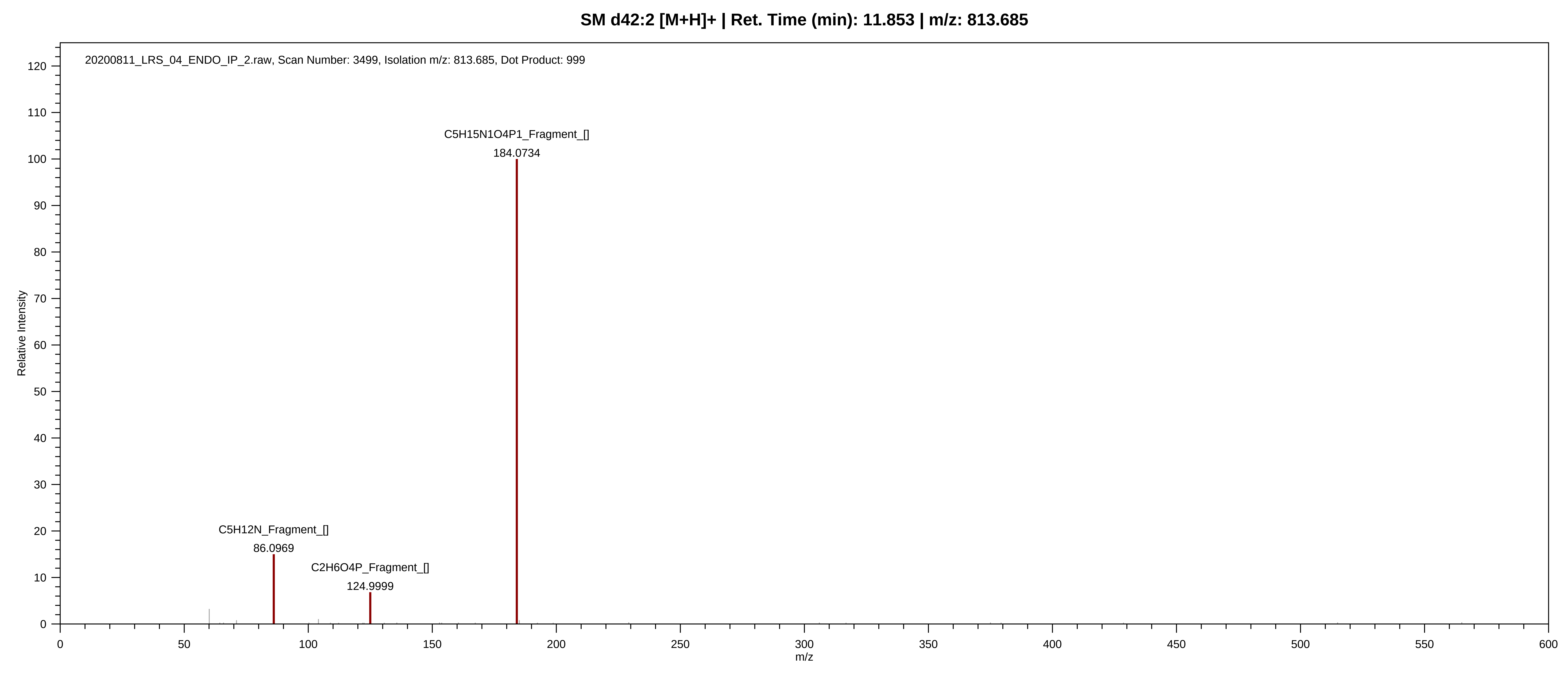

SM d42:2 [M+H]+ | Ret. Time (min): 11.853 | m/z: 813.685
20200811_LRS_04_ENDO_IP_2.raw, Scan Number: 3499, Isolation m/z: 813.685, Dot Product: 999
C5H15N1O4P1_Fragment_[]
184.0734
C5H12N_Fragment_[]
86.0969
C2H6O4P_Fragment_[]
124.9999
Relative Intensity
m/z

SM d42:2 [M+H]+ | Ret. Time (min): 11.934 | m/z: 813.685

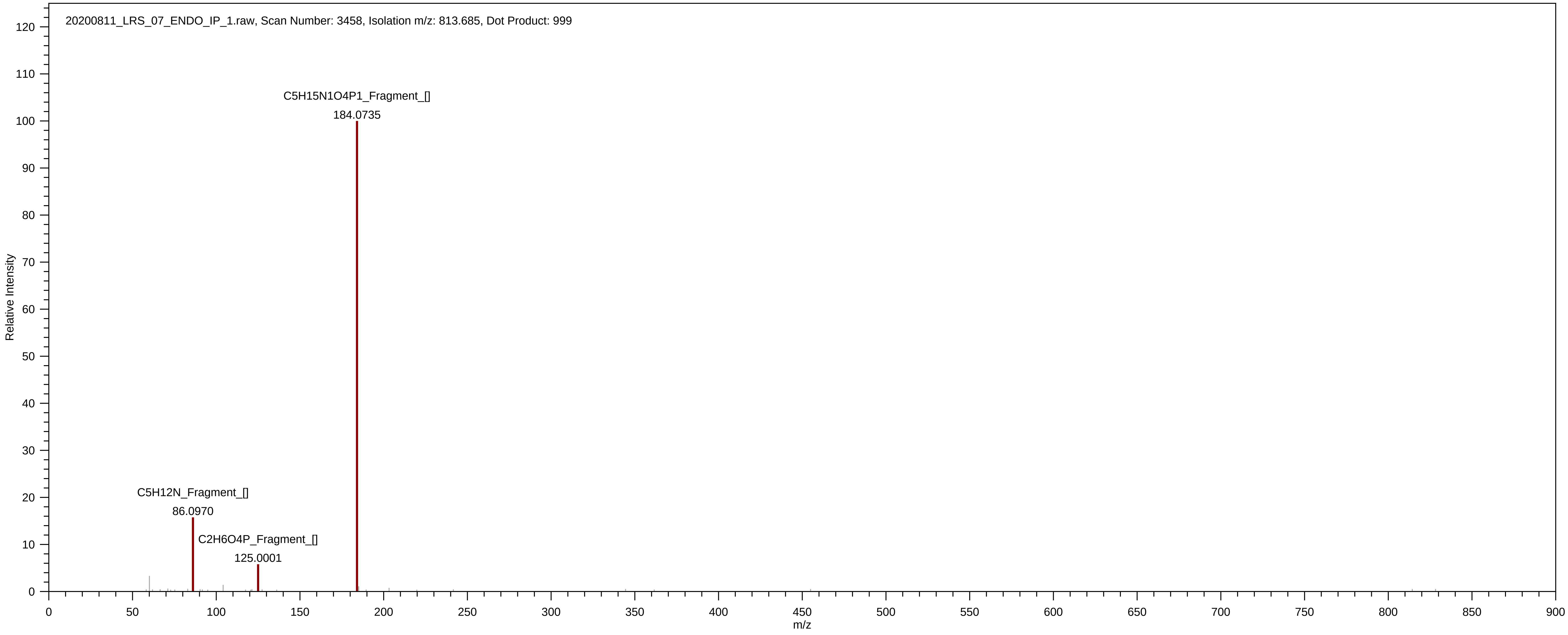

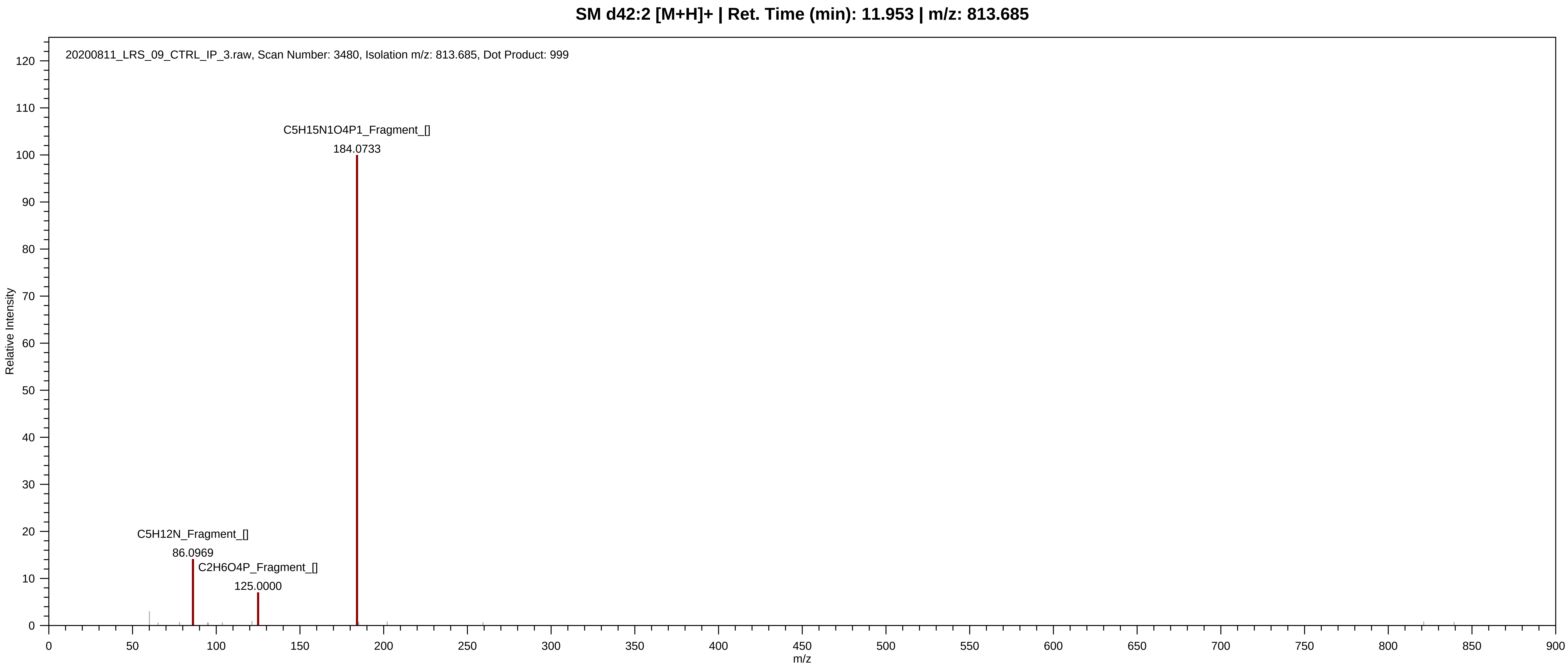
SM d42:2 [M+H]+ | Ret. Time (min): 11.953 | m/z: 813.685
20200811_LRS_09_CTRL_IP_3.raw, Scan Number: 3480, Isolation m/z: 813.685, Dot Product: 999
C5H15N1O4P1_Fragment_[]
184.0733
C5H12N_Fragment_[]
86.0969
C2H6O4P_Fragment_[]
125.0000
Relative Intensity
m/z

SM d42:2 [M+H]+ | Ret. Time (min): 11.956 | m/z: 813.686

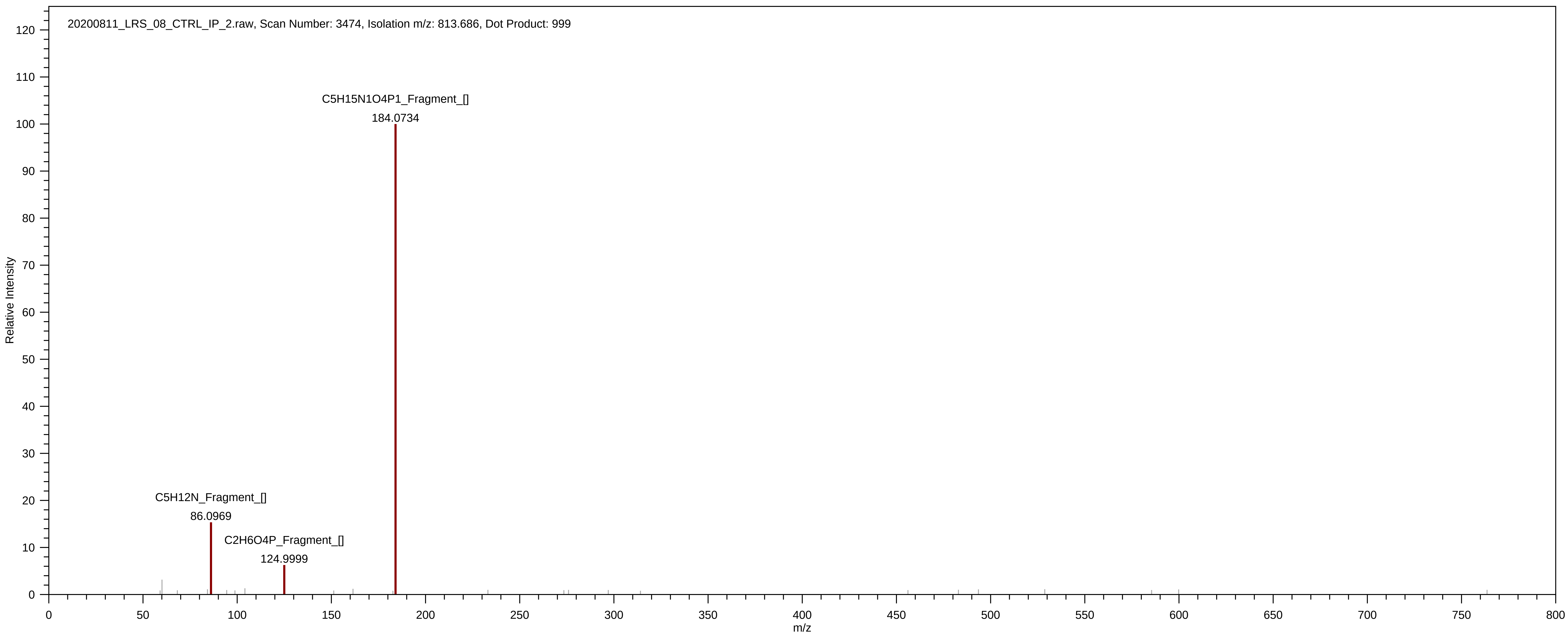

SM d42:3 [M+H]+ | Ret. Time (min): 10.835 | m/z: 811.67

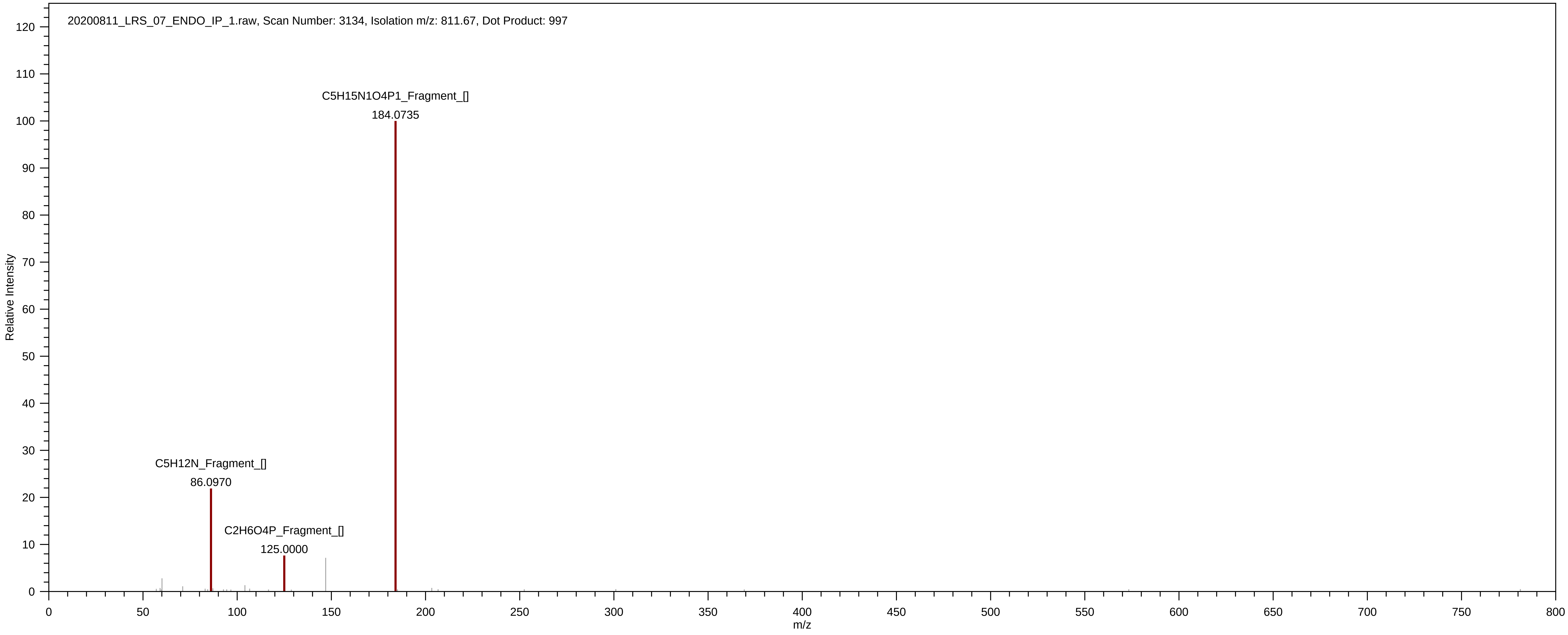

SM d42:3 [M+H]+ | Ret. Time (min): 10.856 | m/z: 811.67

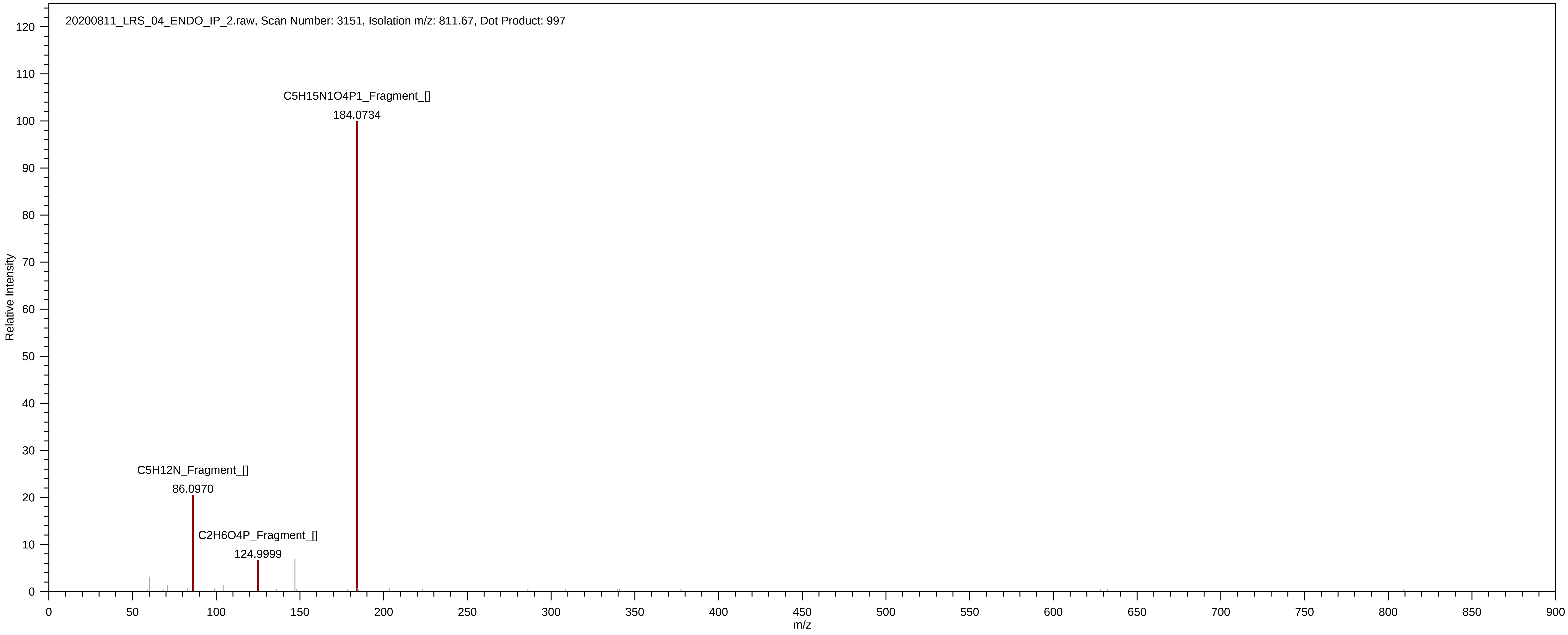

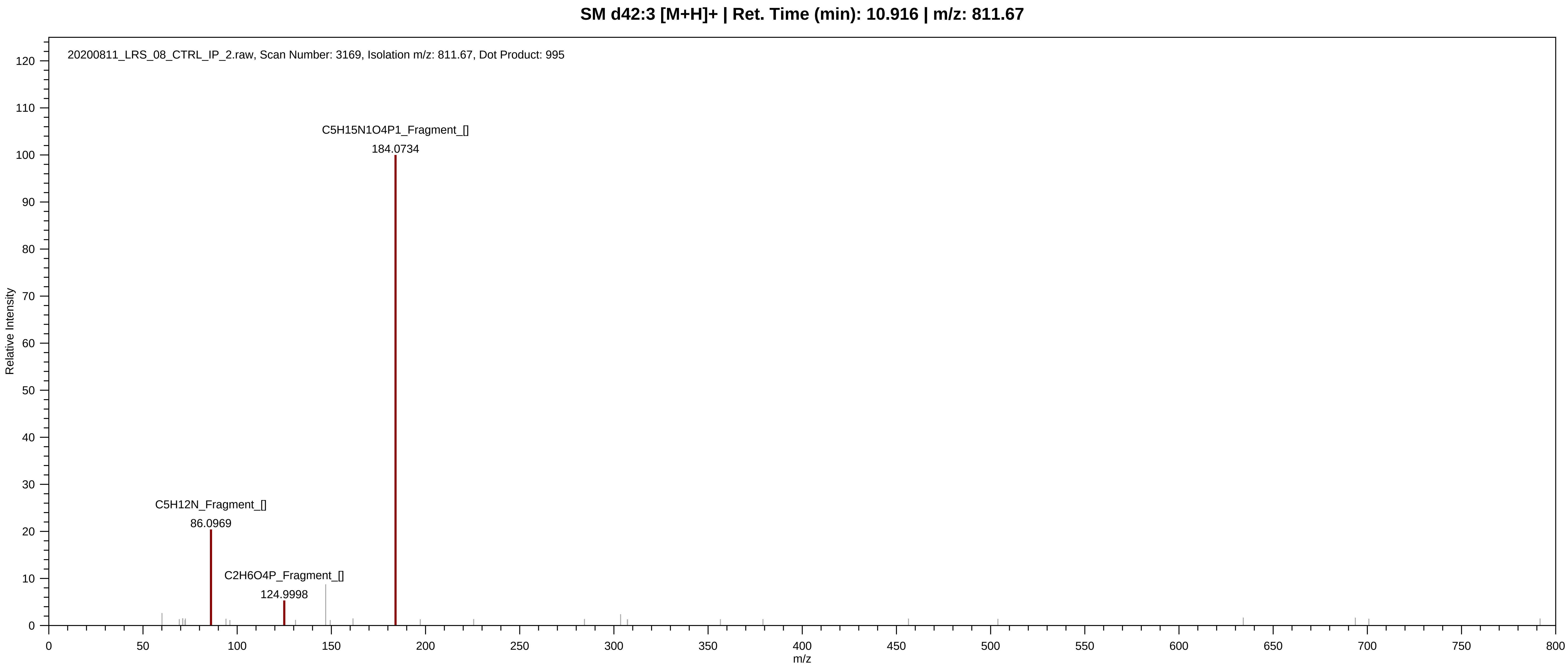
SM d42:3 [M+H]+ | Ret. Time (min): 10.916 | m/z: 811.67
20200811_LRS_08_CTRL_IP_2.raw, Scan Number: 3169, Isolation m/z: 811.67, Dot Product: 995
C5H15N1O4P1_Fragment_[]
184.0734
C5H12N_Fragment_[]
86.0969
C2H6O4P_Fragment_[]
124.9998
Relative Intensity
m/z

SM d42:3 [M+H]+ | Ret. Time (min): 11.018 | m/z: 811.67

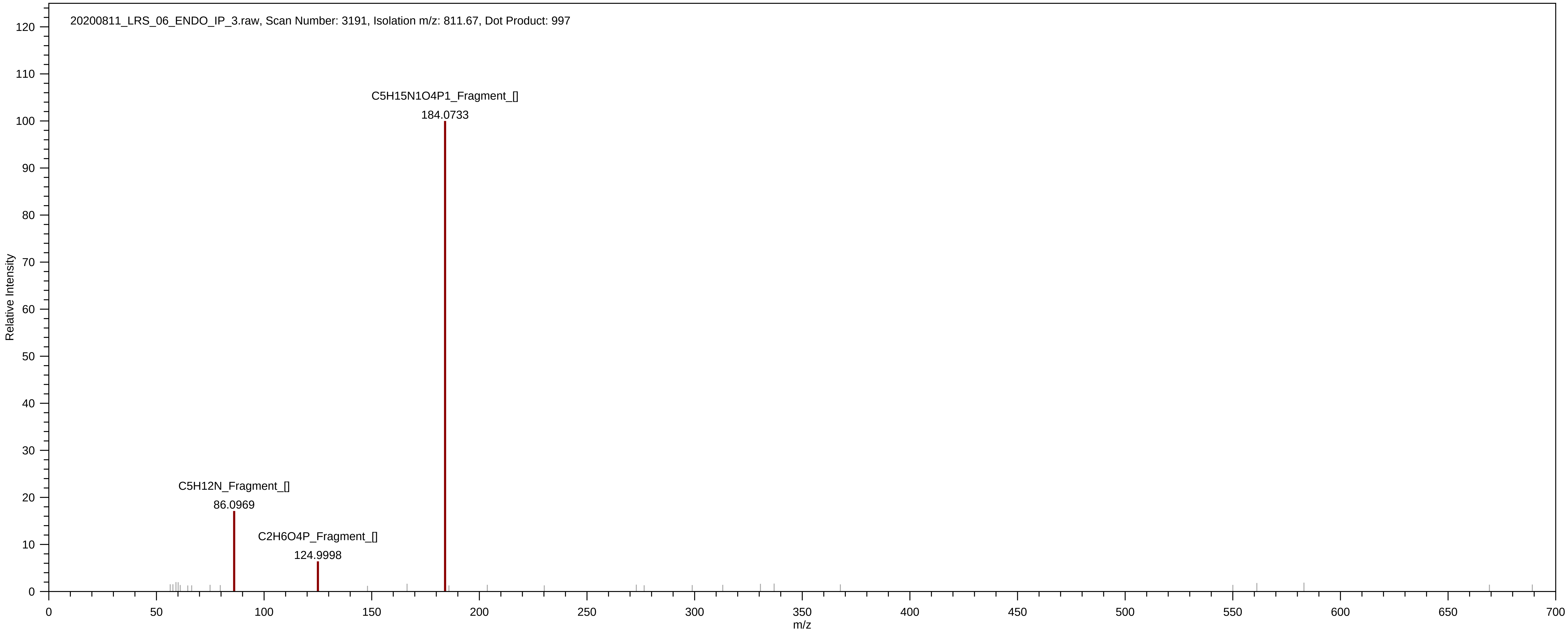

SM d42:3 [M+H]+ | Ret. Time (min): 11.039 | m/z: 811.67

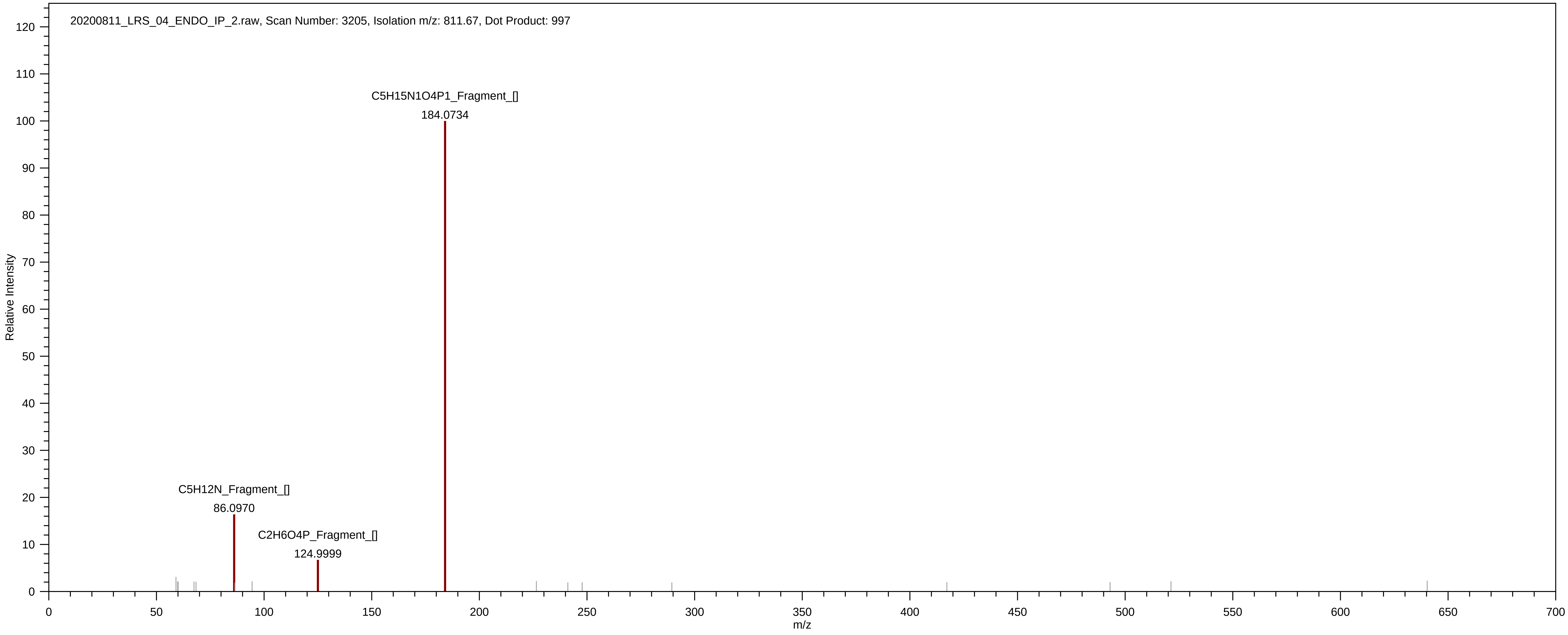

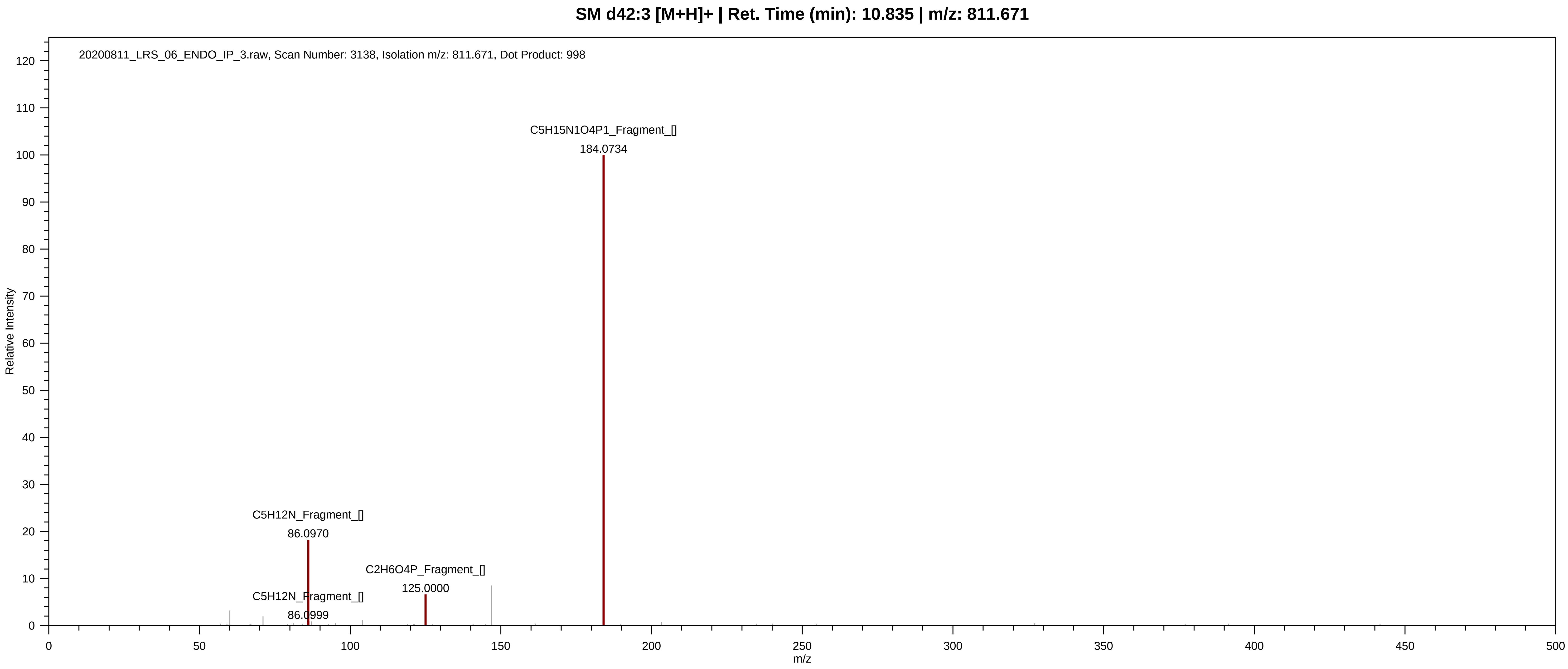
SM d42:3 [M+H]+ | Ret. Time (min): 10.835 | m/z: 811.671
20200811_LRS_06_ENDO_IP_3.raw, Scan Number: 3138, Isolation m/z: 811.671, Dot Product: 998
C5H15N1O4P1_Fragment_[]
184.0734
C5H12N_Fragment_[]
86.0970
C2H6O4P_Fragment_[]
125.0000
C5H12N_Fragment_[]
86.0999
Relative Intensity
m/z

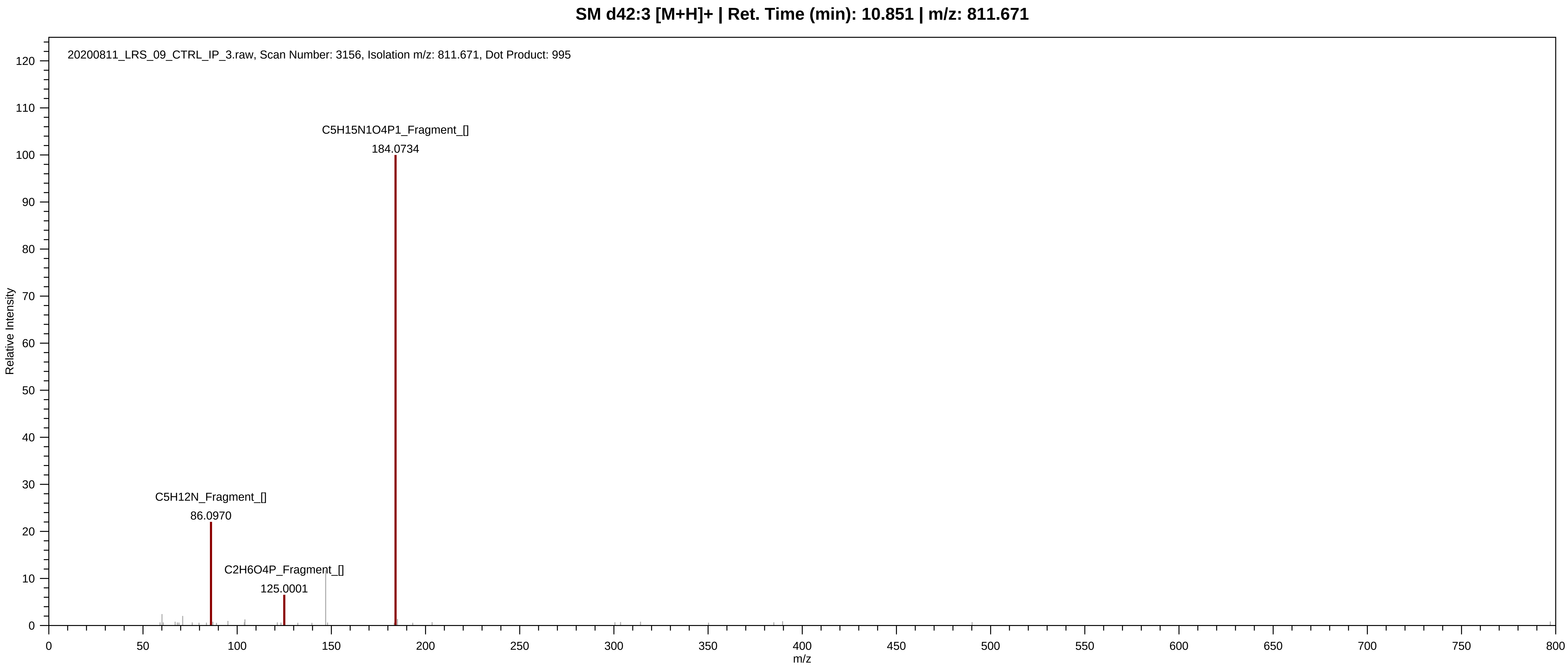

SM d42:3 [M+H]+ | Ret. Time (min): 10.851 | m/z: 811.671
20200811_LRS_09_CTRL_IP_3.raw, Scan Number: 3156, Isolation m/z: 811.671, Dot Product: 995
C5H15N1O4P1_Fragment_[]
184.0734
C5H12N_Fragment_[]
86.0970
C2H6O4P_Fragment_[]
125.0001
Relative Intensity
m/z

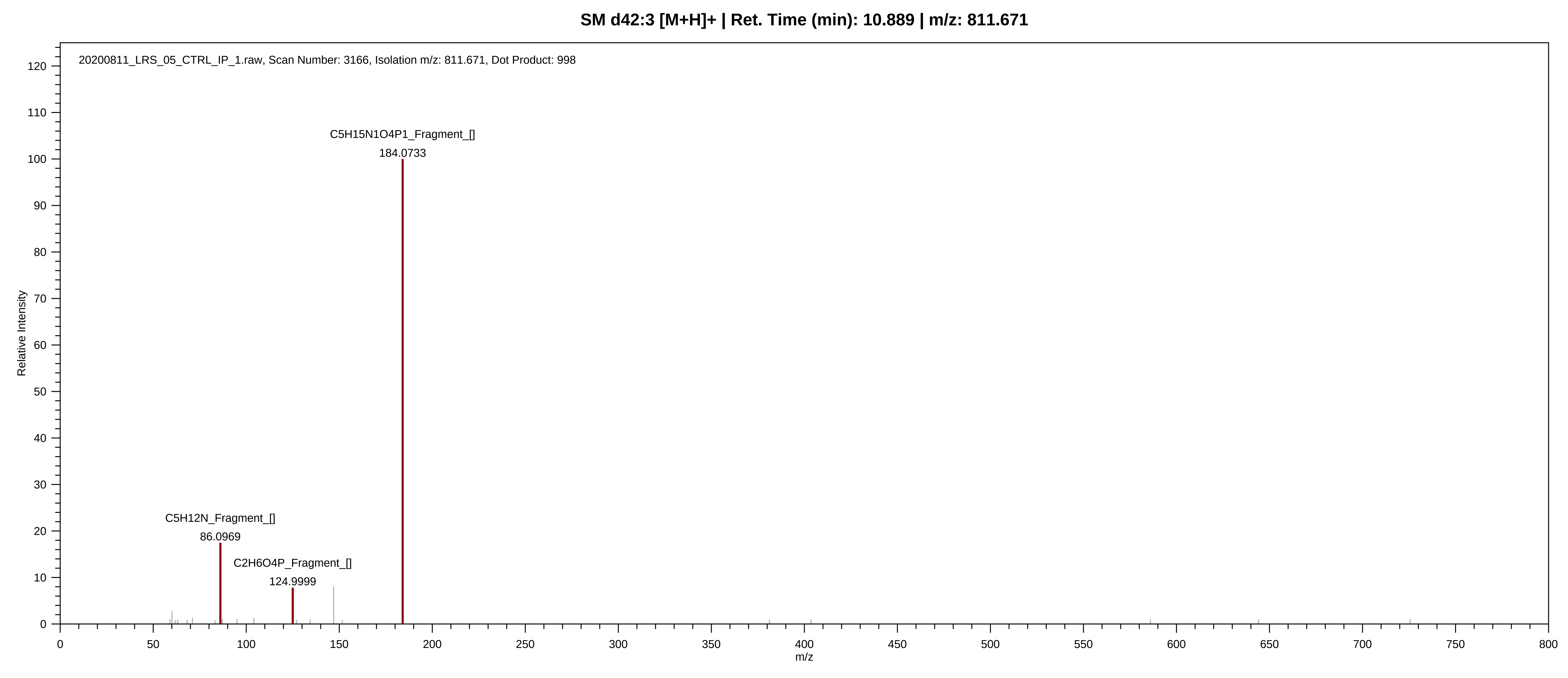
SM d42:3 [M+H]+ | Ret. Time (min): 10.889 | m/z: 811.671
20200811_LRS_05_CTRL_IP_1.raw, Scan Number: 3166, Isolation m/z: 811.671, Dot Product: 998
C5H15N1O4P1_Fragment_[]
184.0733
C5H12N_Fragment_[]
86.0969
C2H6O4P_Fragment_[]
124.9999
Relative Intensity
m/z
0
10
20
30
40
50
60
70
80
90
100
110
120
0
50
100
150
200
250
300
350
400
450
500
550
600
650
700
750
800

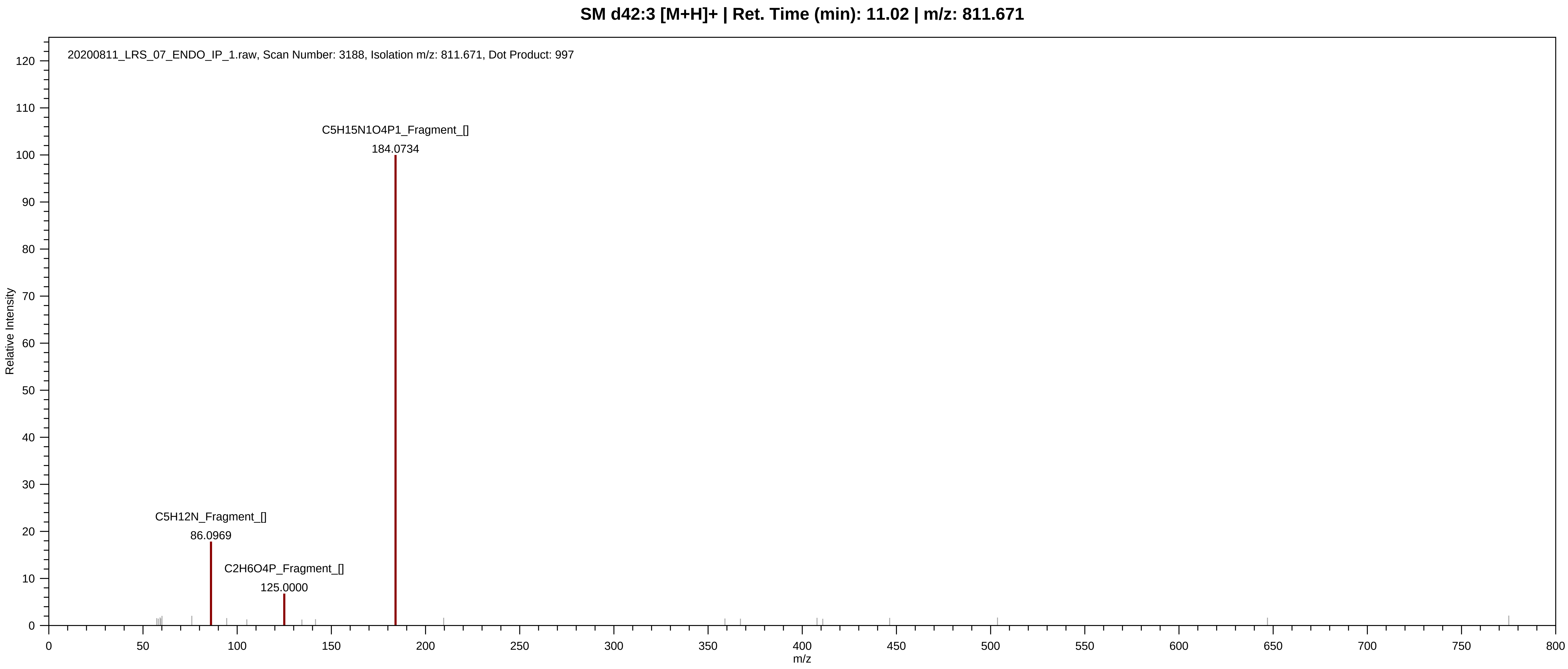

SM d42:3 [M+H]+ | Ret. Time (min): 11.02 | m/z: 811.671
20200811_LRS_07_ENDO_IP_1.raw, Scan Number: 3188, Isolation m/z: 811.671, Dot Product: 997
C5H15N1O4P1_Fragment_[]
184.0734
C5H12N_Fragment_[]
86.0969
C2H6O4P_Fragment_[]
125.0000
Relative Intensity
m/z

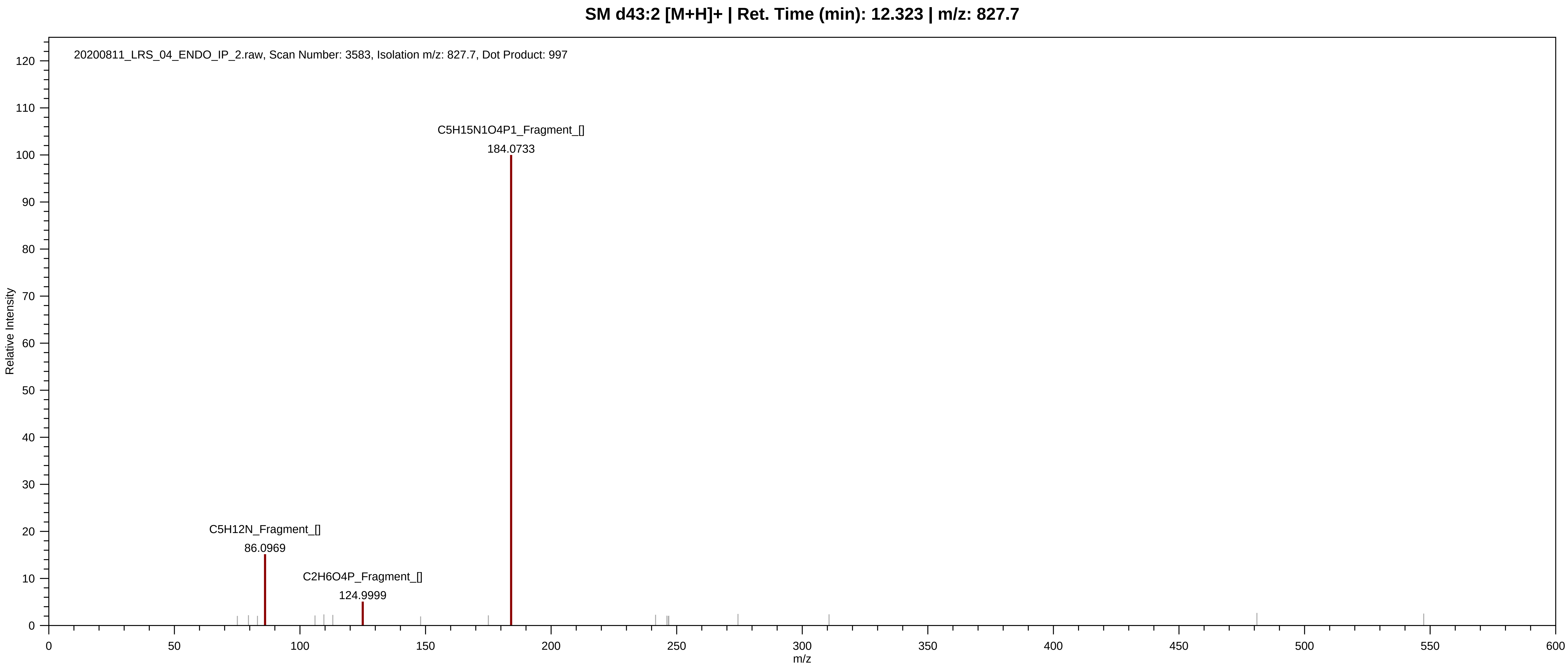

SM d43:2 [M+H]+ | Ret. Time (min): 12.323 | m/z: 827.7
20200811_LRS_04_ENDO_IP_2.raw, Scan Number: 3583, Isolation m/z: 827.7, Dot Product: 997
C5H15N1O4P1_Fragment_[]
184.0733
C5H12N_Fragment_[]
86.0969
C2H6O4P_Fragment_[]
124.9999
Relative Intensity
m/z

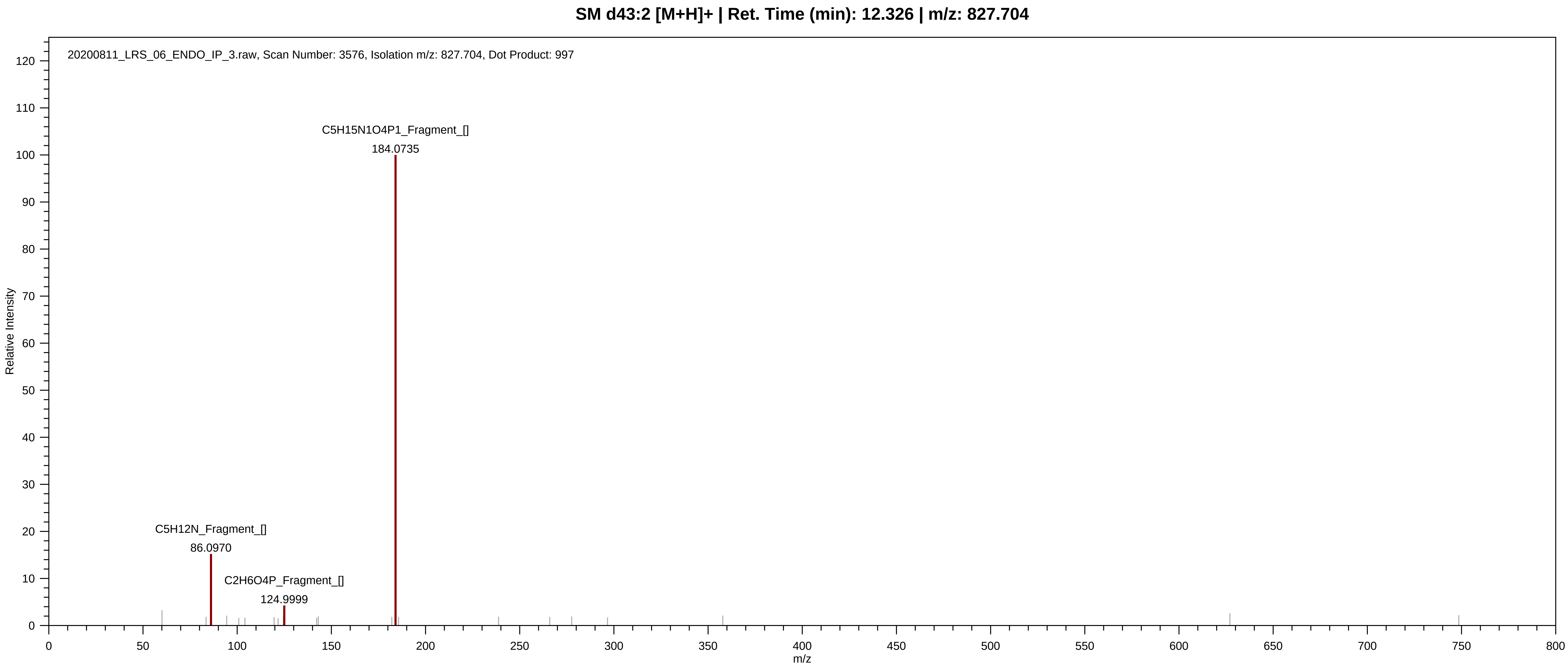

SM d43:2 [M+H]+ | Ret. Time (min): 12.326 | m/z: 827.704
20200811_LRS_06_ENDO_IP_3.raw, Scan Number: 3576, Isolation m/z: 827.704, Dot Product: 997
C5H15N1O4P1_Fragment_[]
184.0735
C5H12N_Fragment_[]
86.0970
C2H6O4P_Fragment_[]
124.9999
Relative Intensity
m/z

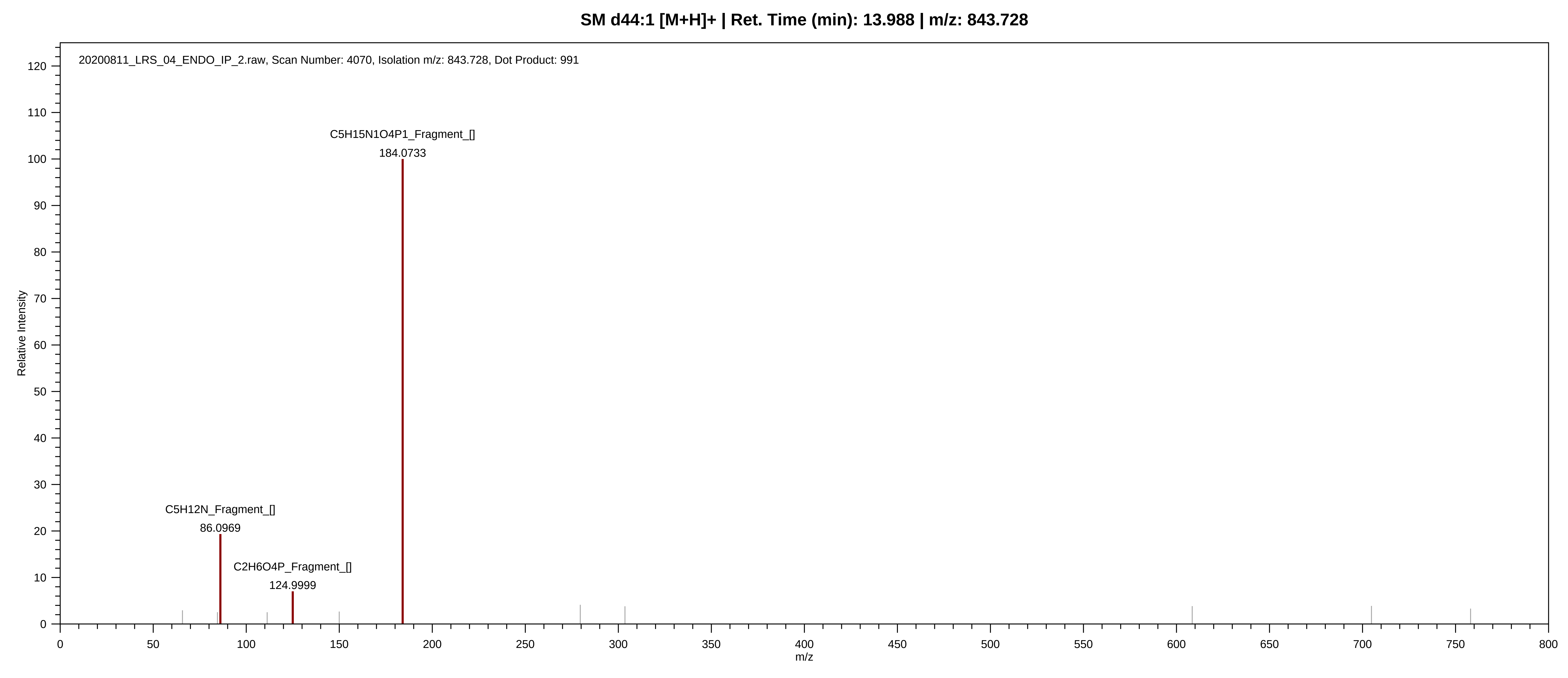
SM d44:1 [M+H]+ | Ret. Time (min): 13.988 | m/z: 843.728
20200811_LRS_04_ENDO_IP_2.raw, Scan Number: 4070, Isolation m/z: 843.728, Dot Product: 991
C5H15N1O4P1_Fragment_[]
184.0733
C5H12N_Fragment_[]
86.0969
C2H6O4P_Fragment_[]
124.9999
Relative Intensity
m/z

SM d44:1 [M+H]+ | Ret. Time (min): 13.991 | m/z: 843.731

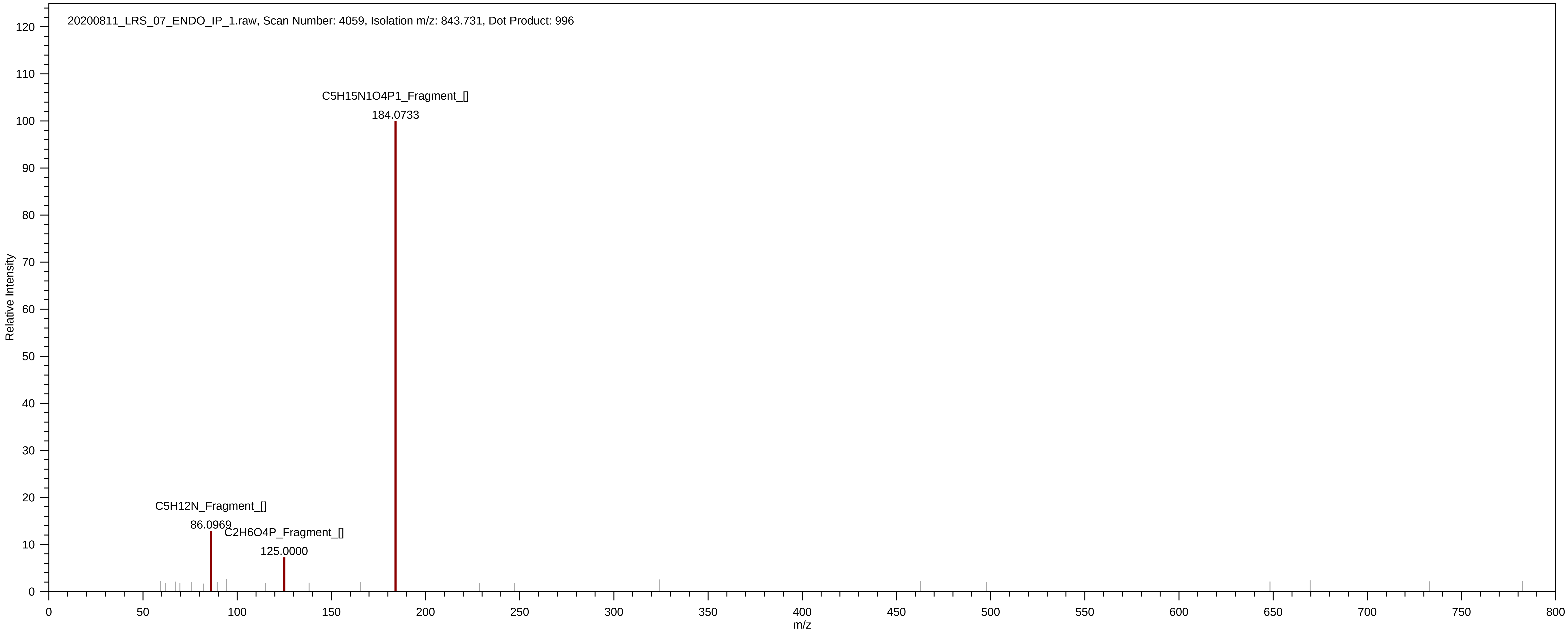

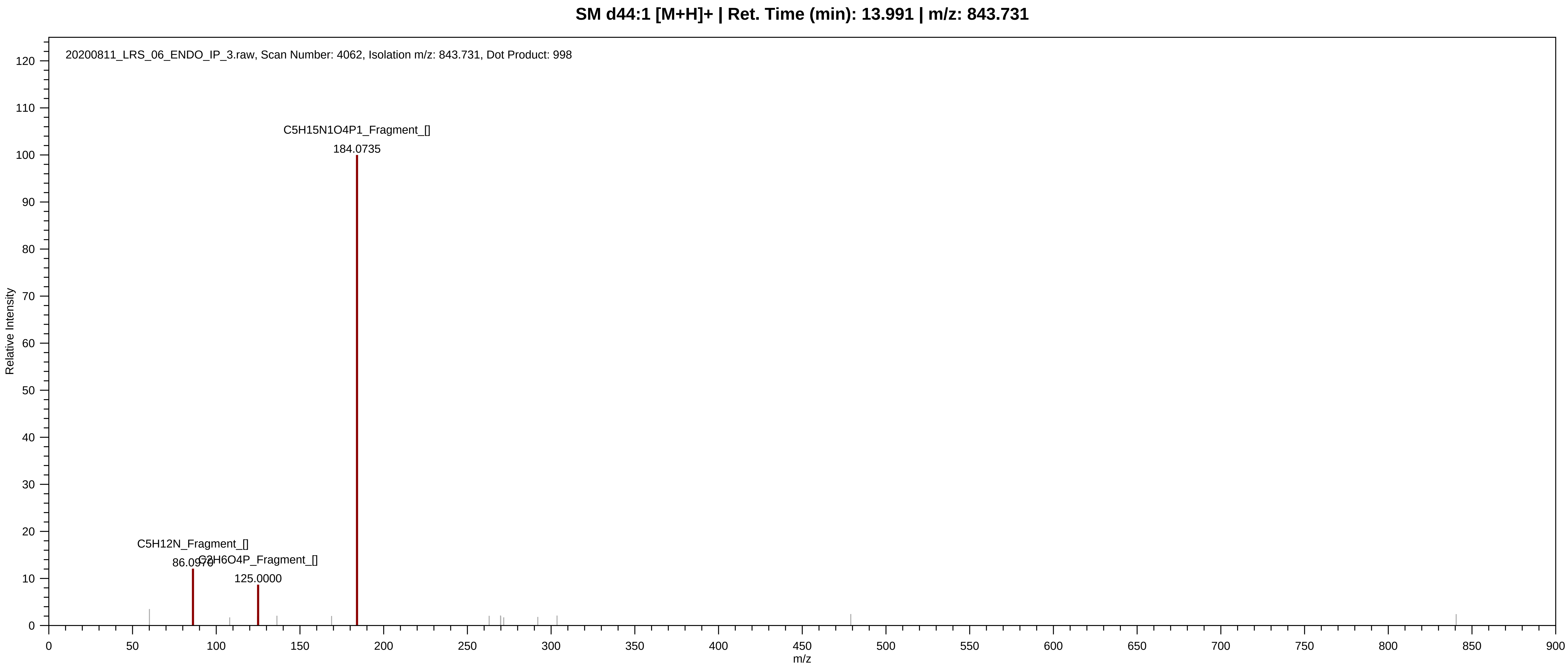

SM d44:1 [M+H]+ | Ret. Time (min): 13.991 | m/z: 843.731
20200811_LRS_06_ENDO_IP_3.raw, Scan Number: 4062, Isolation m/z: 843.731, Dot Product: 998
C5H15N1O4P1_Fragment_[]
184.0735
C5H12N_Fragment_[]
86.0970
C2H6O4P_Fragment_[]
125.0000
Relative Intensity
m/z

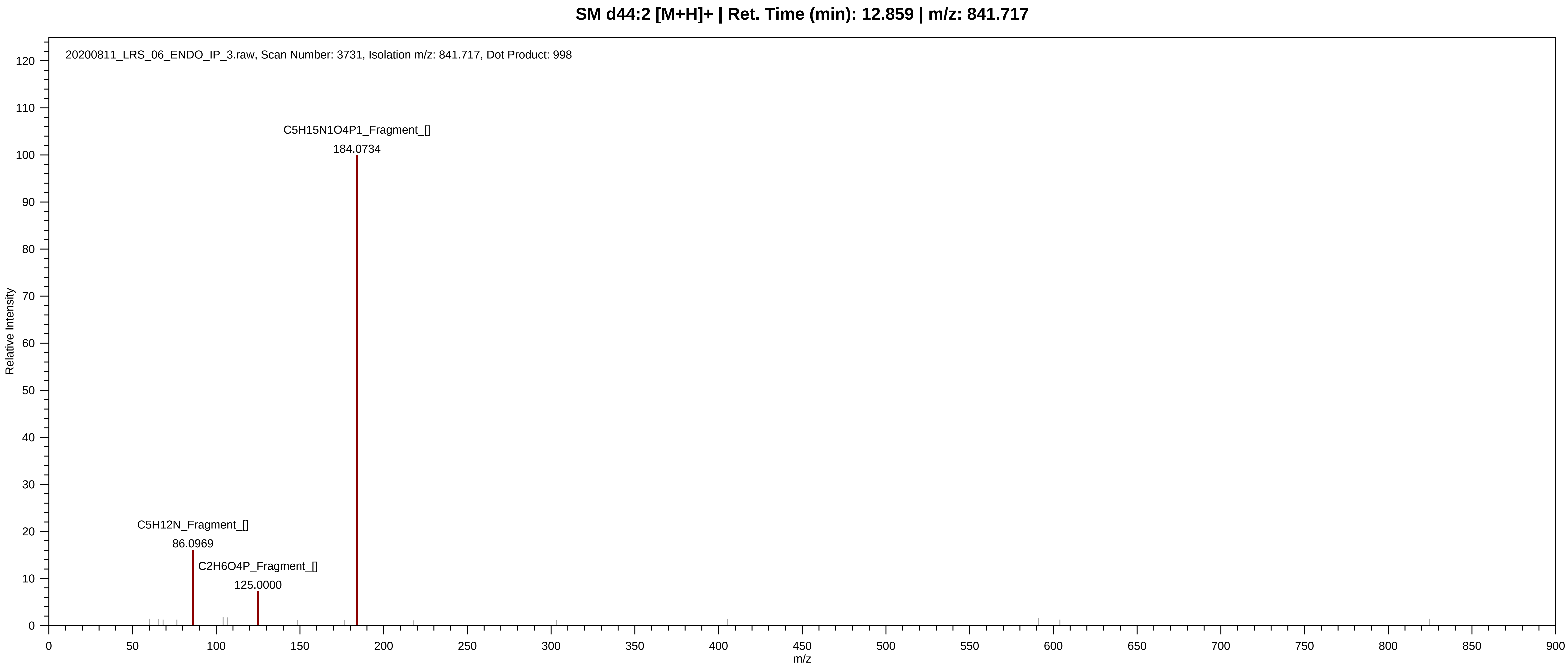

SM d44:2 [M+H]+ | Ret. Time (min): 12.859 | m/z: 841.717
20200811_LRS_06_ENDO_IP_3.raw, Scan Number: 3731, Isolation m/z: 841.717, Dot Product: 998
C5H15N1O4P1_Fragment_[]
184.0734
C5H12N_Fragment_[]
86.0969
C2H6O4P_Fragment_[]
125.0000
Relative Intensity
m/z

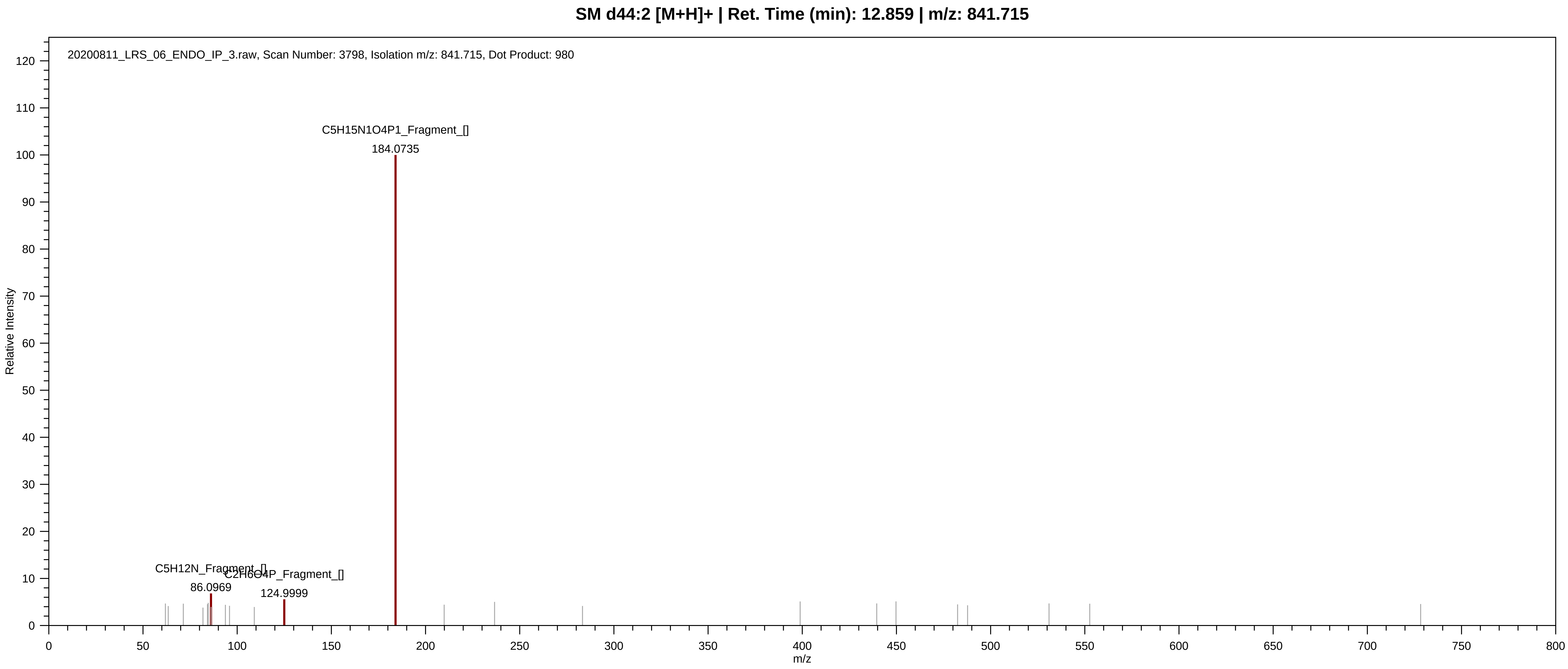

SM d44:2 [M+H]+ | Ret. Time (min): 12.859 | m/z: 841.715
20200811_LRS_06_ENDO_IP_3.raw, Scan Number: 3798, Isolation m/z: 841.715, Dot Product: 980
C5H15N1O4P1_Fragment_[]
184.0735
C5H12N_Fragment_[]
86.0969
C2H6O4P_Fragment_[]
124.9999
Relative Intensity
m/z

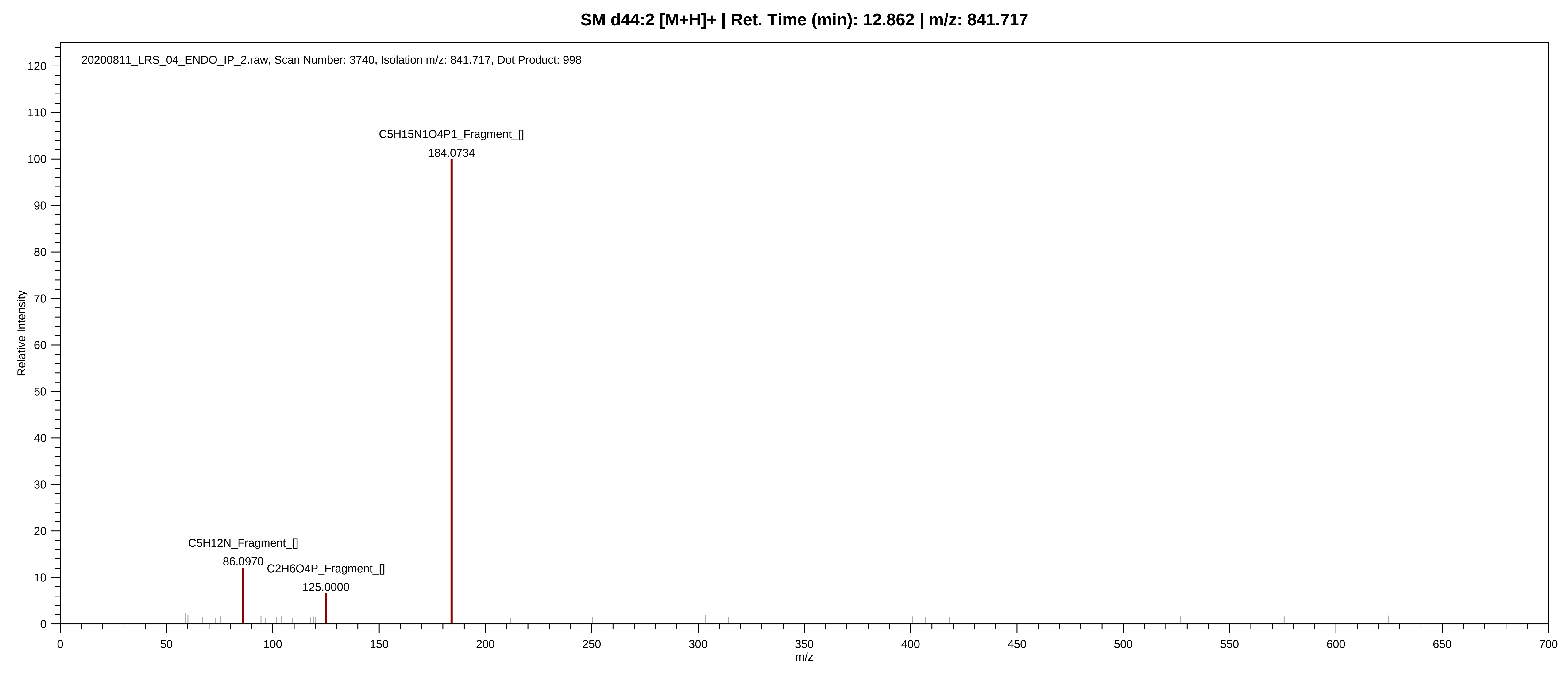
SM d44:2 [M+H]+ | Ret. Time (min): 12.862 | m/z: 841.717
20200811_LRS_04_ENDO_IP_2.raw, Scan Number: 3740, Isolation m/z: 841.717, Dot Product: 998
C5H15N1O4P1_Fragment_[]
184.0734
C5H12N_Fragment_[]
86.0970
C2H6O4P_Fragment_[]
125.0000
Relative Intensity
m/z

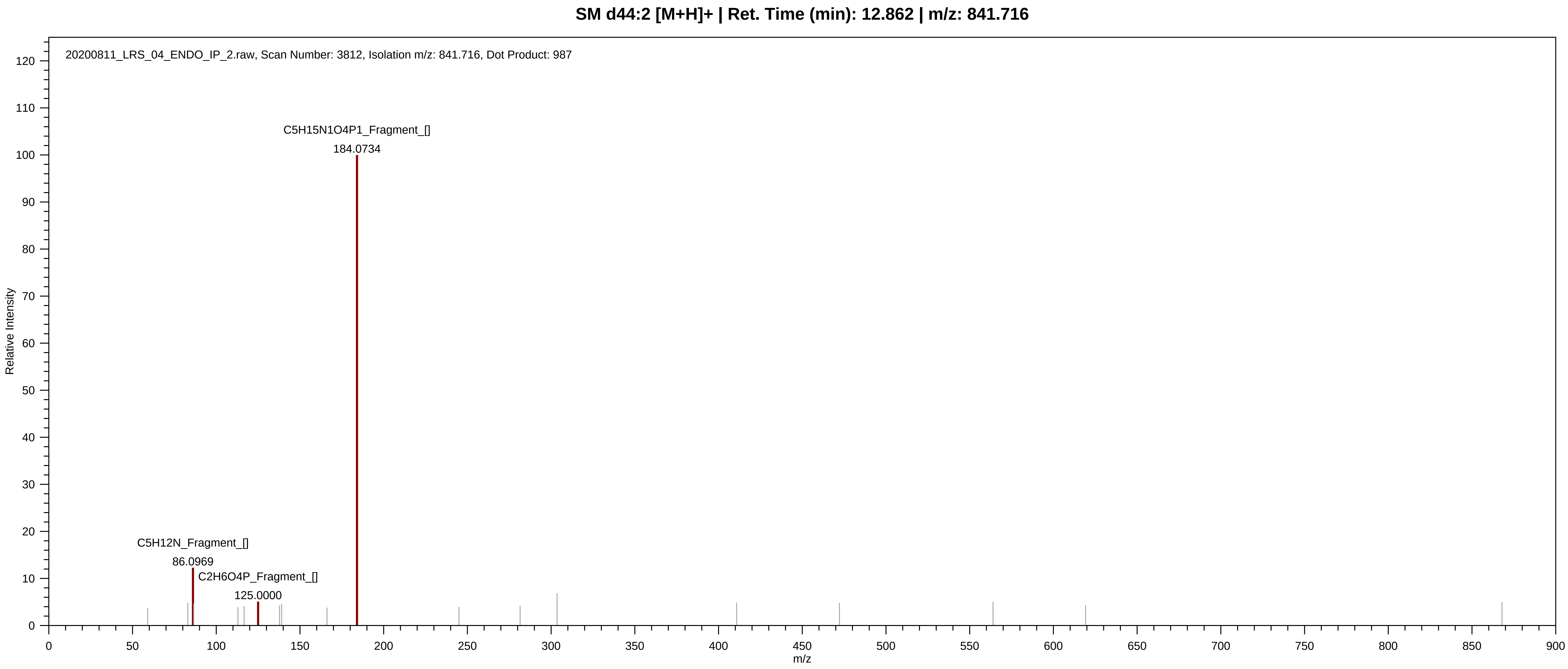
SM d44:2 [M+H]+ | Ret. Time (min): 12.862 | m/z: 841.716
20200811_LRS_04_ENDO_IP_2.raw, Scan Number: 3812, Isolation m/z: 841.716, Dot Product: 987
C5H15N1O4P1_Fragment_[]
184.0734
C5H12N_Fragment_[]
86.0969
C2H6O4P_Fragment_[]
125.0000
Relative Intensity
m/z
0
10
20
30
40
50
60
70
80
90
100
110
120
0
50
100
150
200
250
300
350
400
450
500
550
600
650
700
750
800
850
900

SM d44:2 [M+H]+ | Ret. Time (min): 12.864 | m/z: 841.717

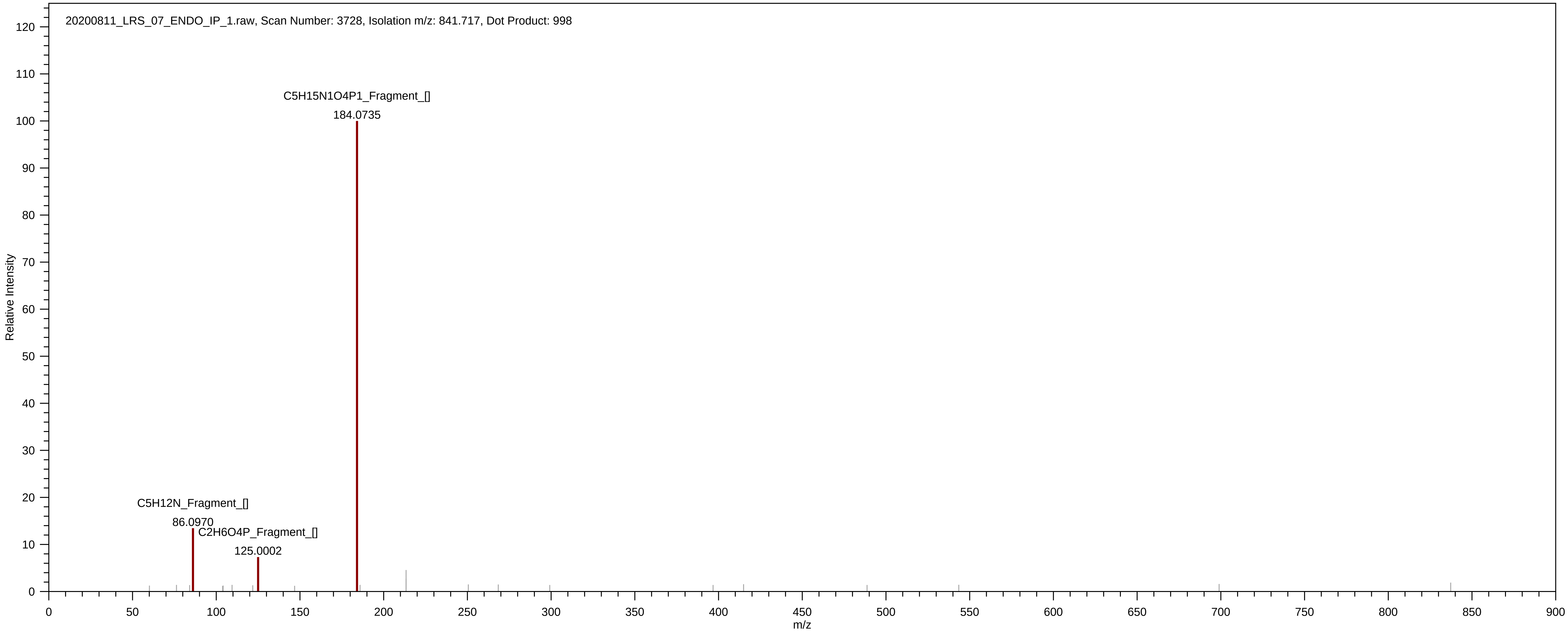

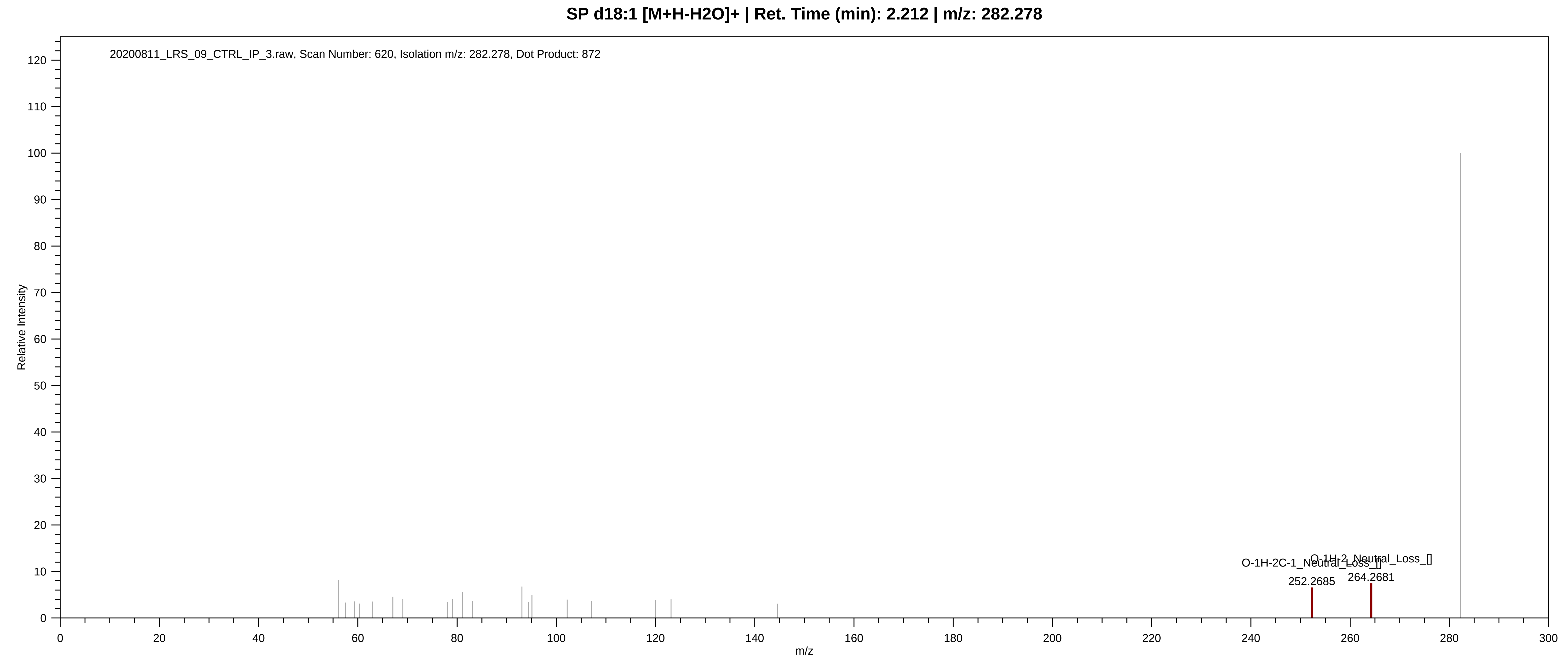
SP d18:1 [M+H-H2O]+ | Ret. Time (min): 2.212 | m/z: 282.278
20200811_LRS_09_CTRL_IP_3.raw, Scan Number: 620, Isolation m/z: 282.278, Dot Product: 872
Relative Intensity
m/z
O-1H-2C-1_Neutral_Loss_[]
252.2685
O-1H-2_Neutral_Loss_[]
264.2681

SP d18:1 [M+H-H2O]+ | Ret. Time (min): 2.4 | m/z: 282.279

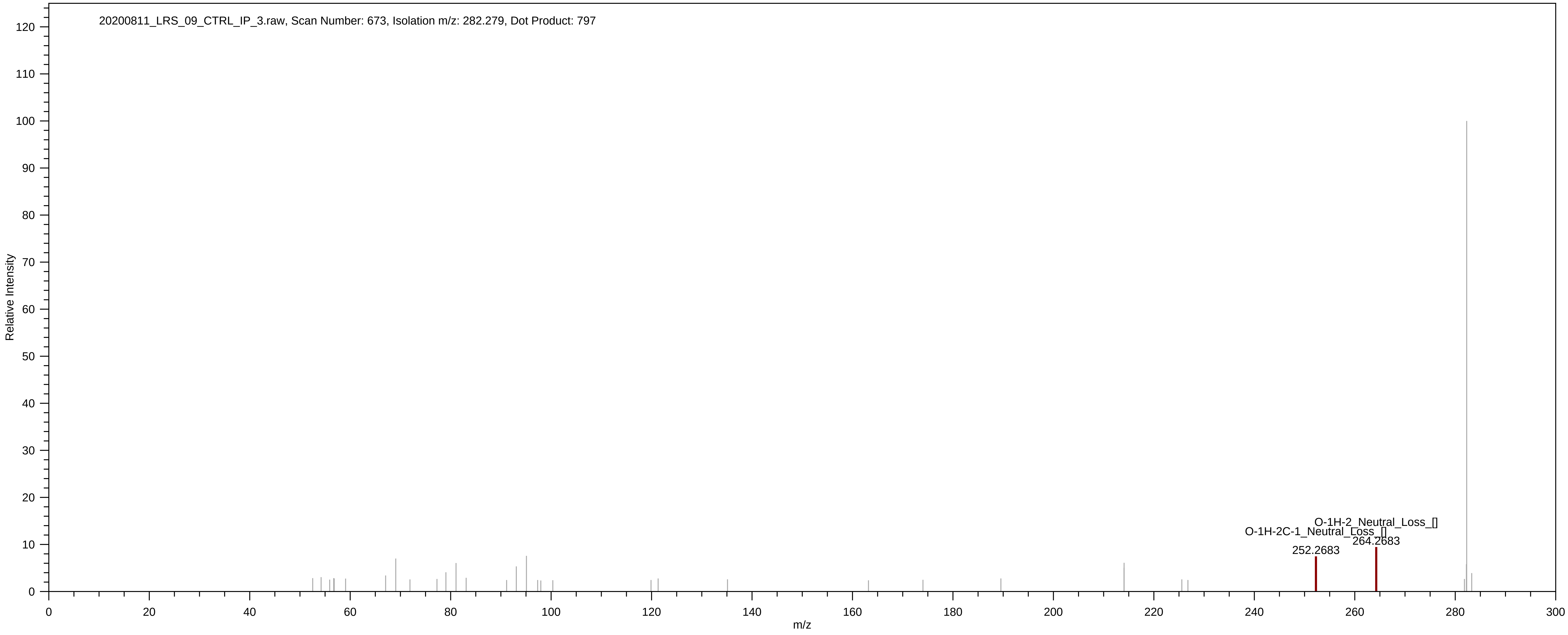

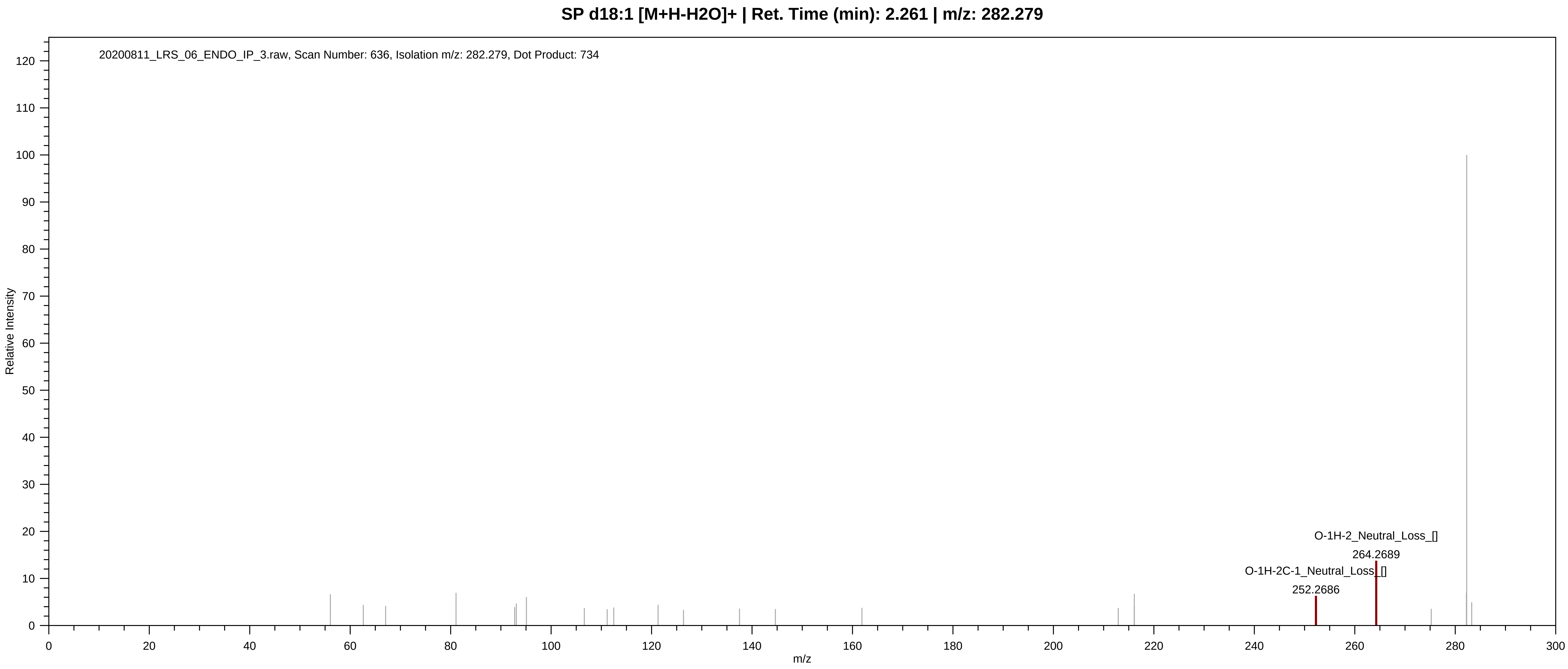

SP d18:1 [M+H-H2O]+ | Ret. Time (min): 2.261 | m/z: 282.279
20200811_LRS_06_ENDO_IP_3.raw, Scan Number: 636, Isolation m/z: 282.279, Dot Product: 734
Relative Intensity
m/z
O-1H-2_Neutral_Loss_[]
264.2689
O-1H-2C-1_Neutral_Loss_[]
252.2686

SP d18:1 [M+H-H2O]+ | Ret. Time (min): 2.424 | m/z: 282.279

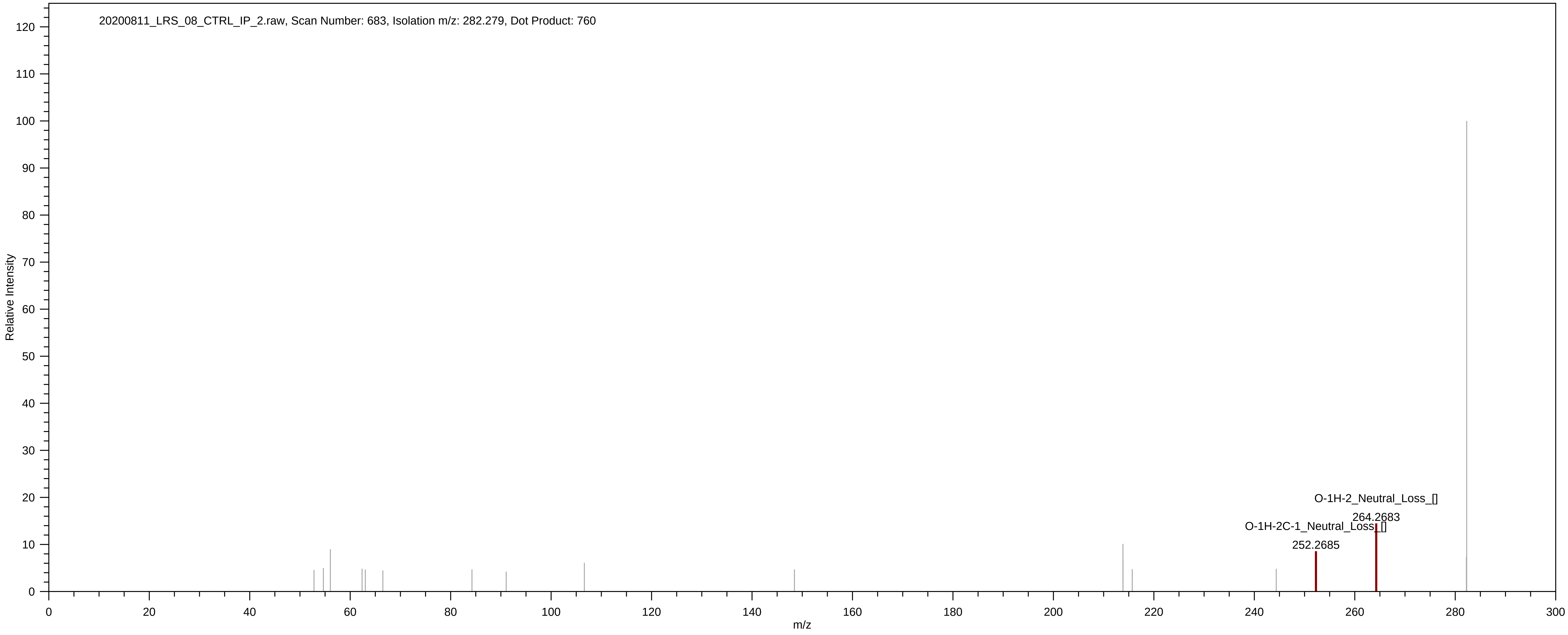

SP d18:1 [M+H-H2O]+ | Ret. Time (min): 2.589 | m/z: 282.279

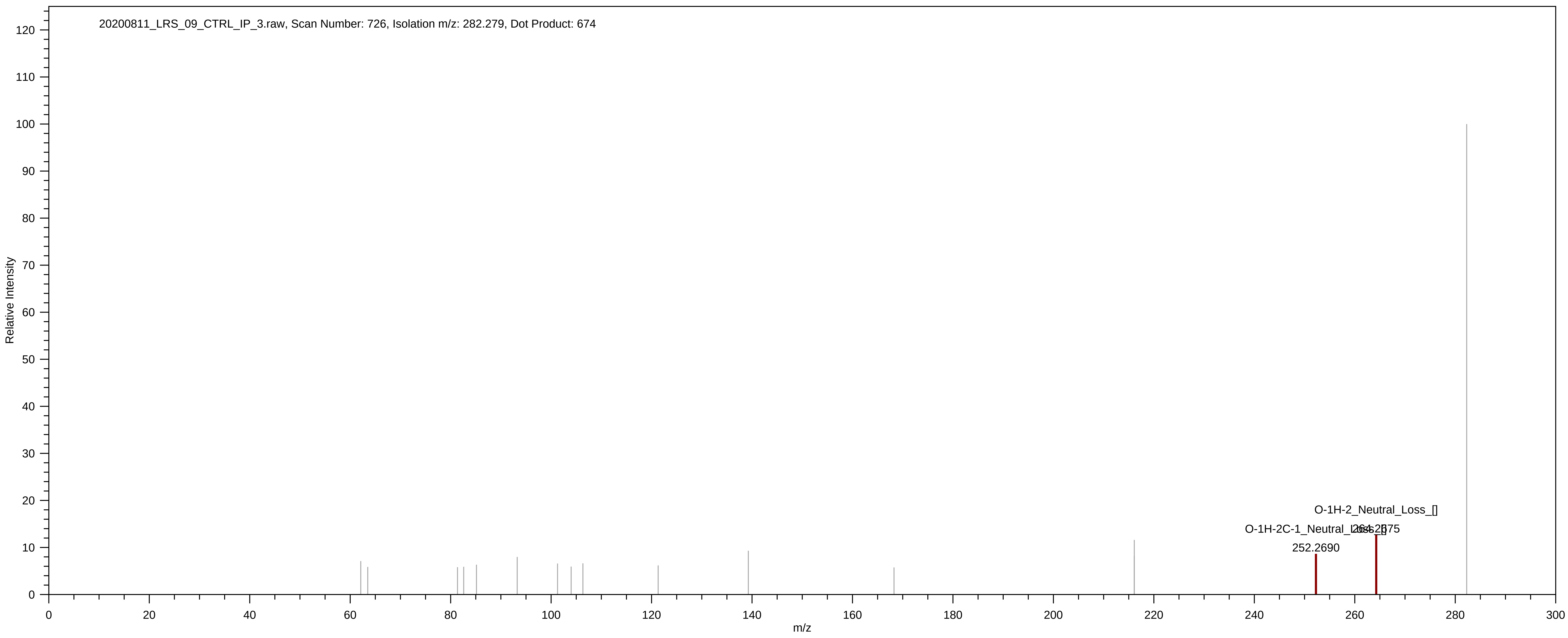

TG 14:0_16:0_16:0 [M+NH4]+ | Ret. Time (min): 17.471 | m/z: 796.738

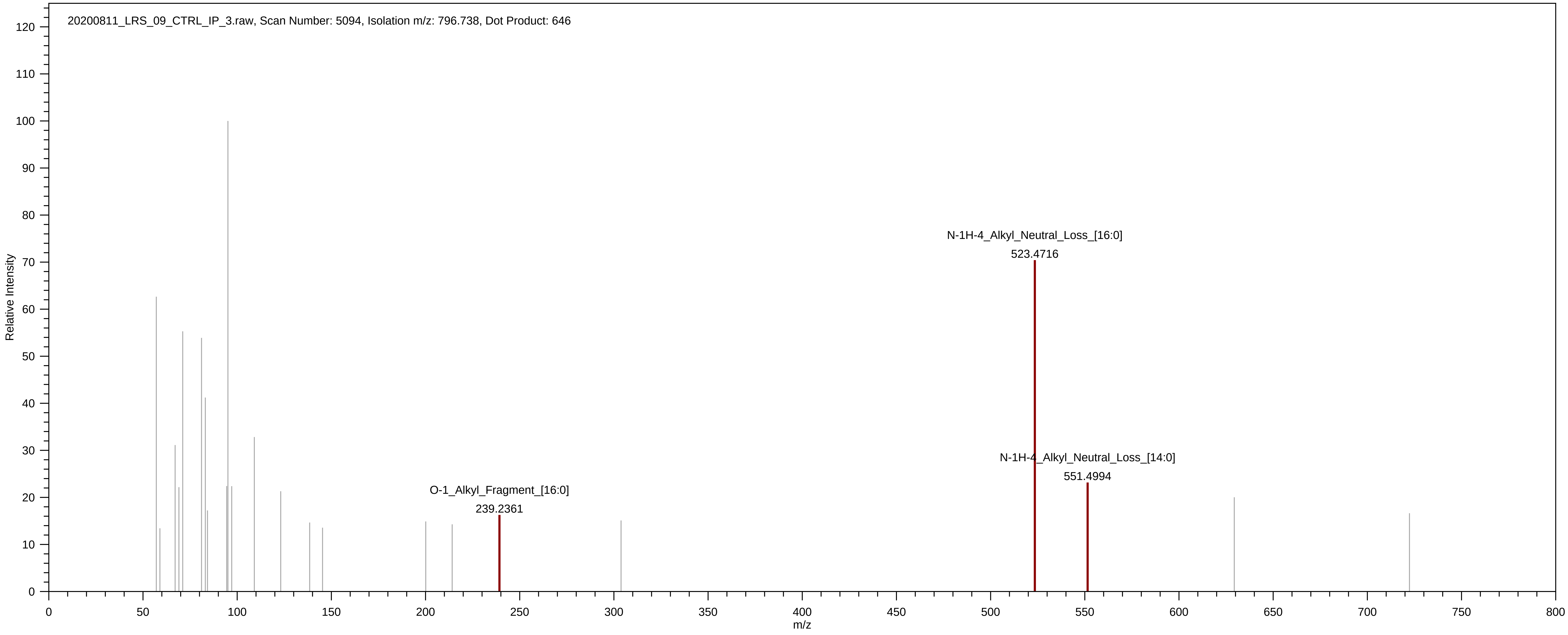

TG 14:0_16:0_16:0 [M+NH4]+ | Ret. Time (min): 17.486 | m/z: 796.739

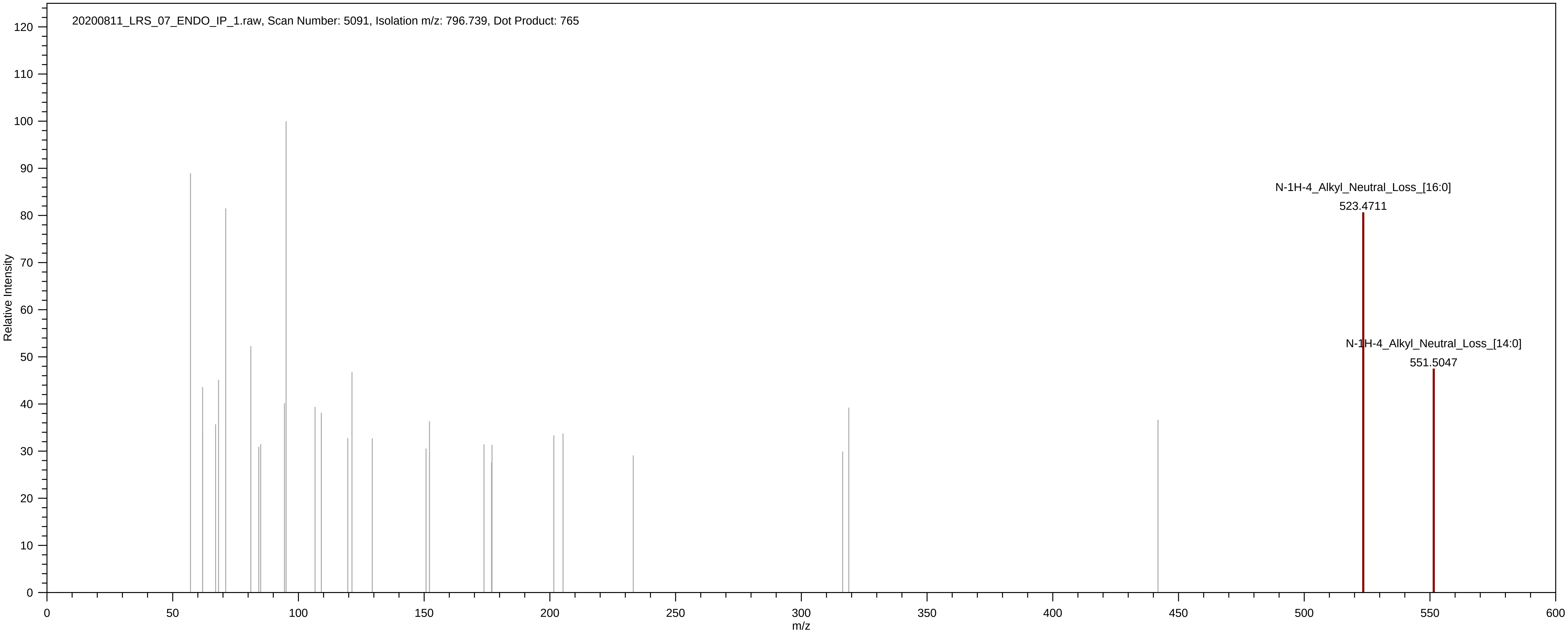

TG 16:0_16:0_16:0 [M+NH4]+ | Ret. Time (min): 18.22 | m/z: 824.77

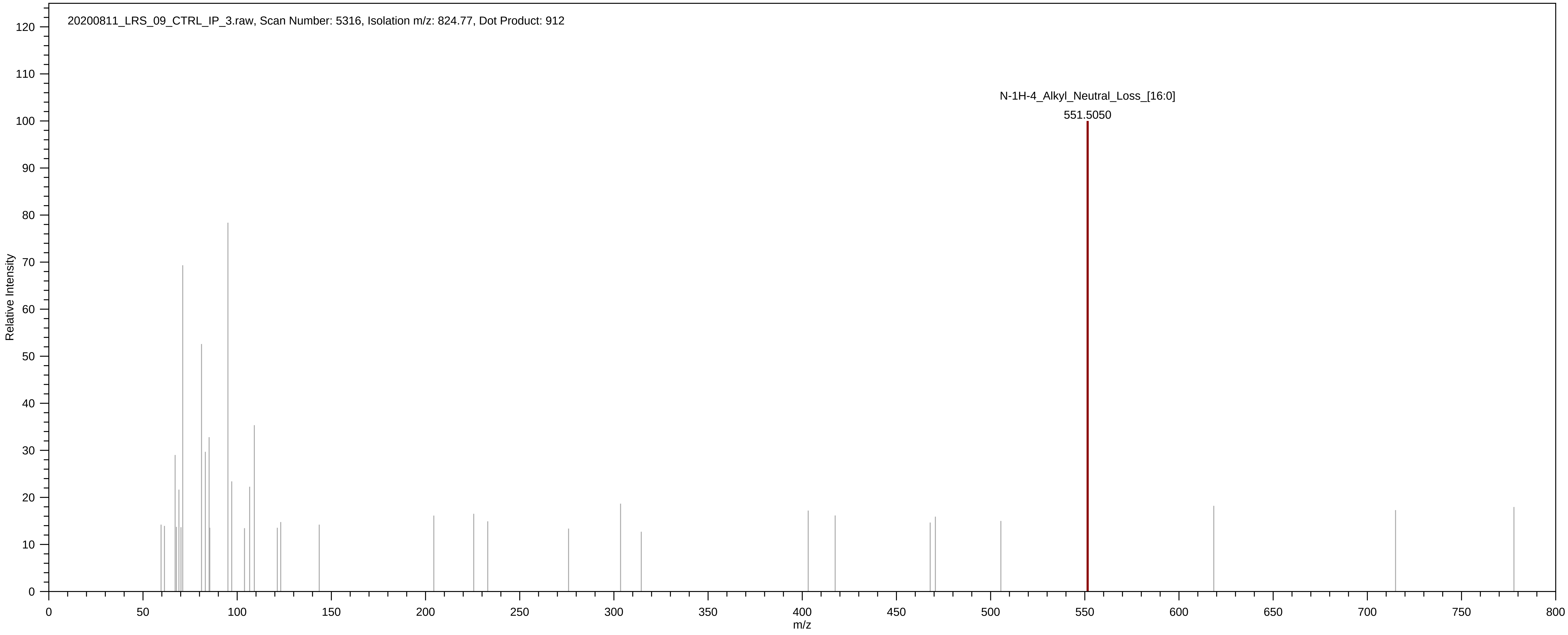

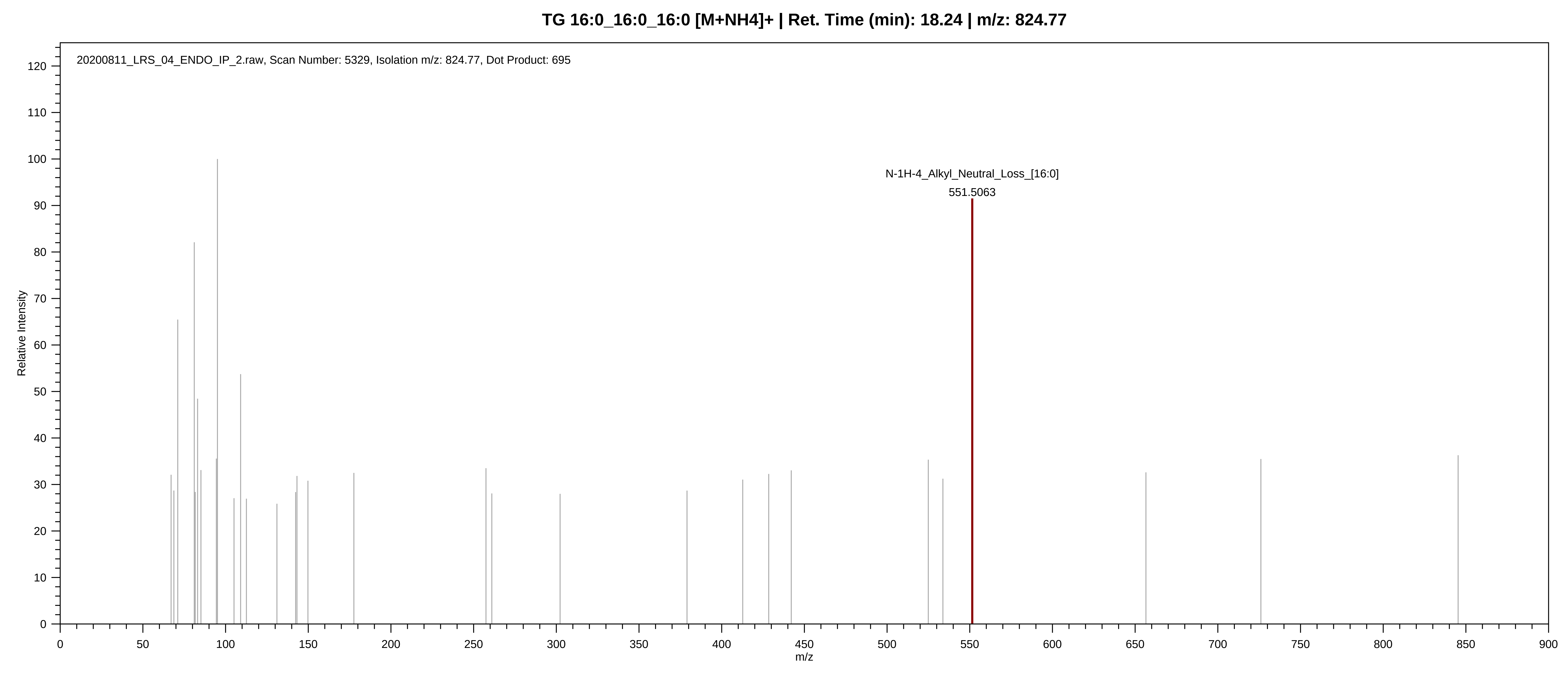

TG 16:0_16:0_16:0 [M+NH4]+ | Ret. Time (min): 18.24 | m/z: 824.77
20200811_LRS_04_ENDO_IP_2.raw, Scan Number: 5329, Isolation m/z: 824.77, Dot Product: 695
N-1H-4_Alkyl_Neutral_Loss_[16:0]
551.5063
Relative Intensity
m/z

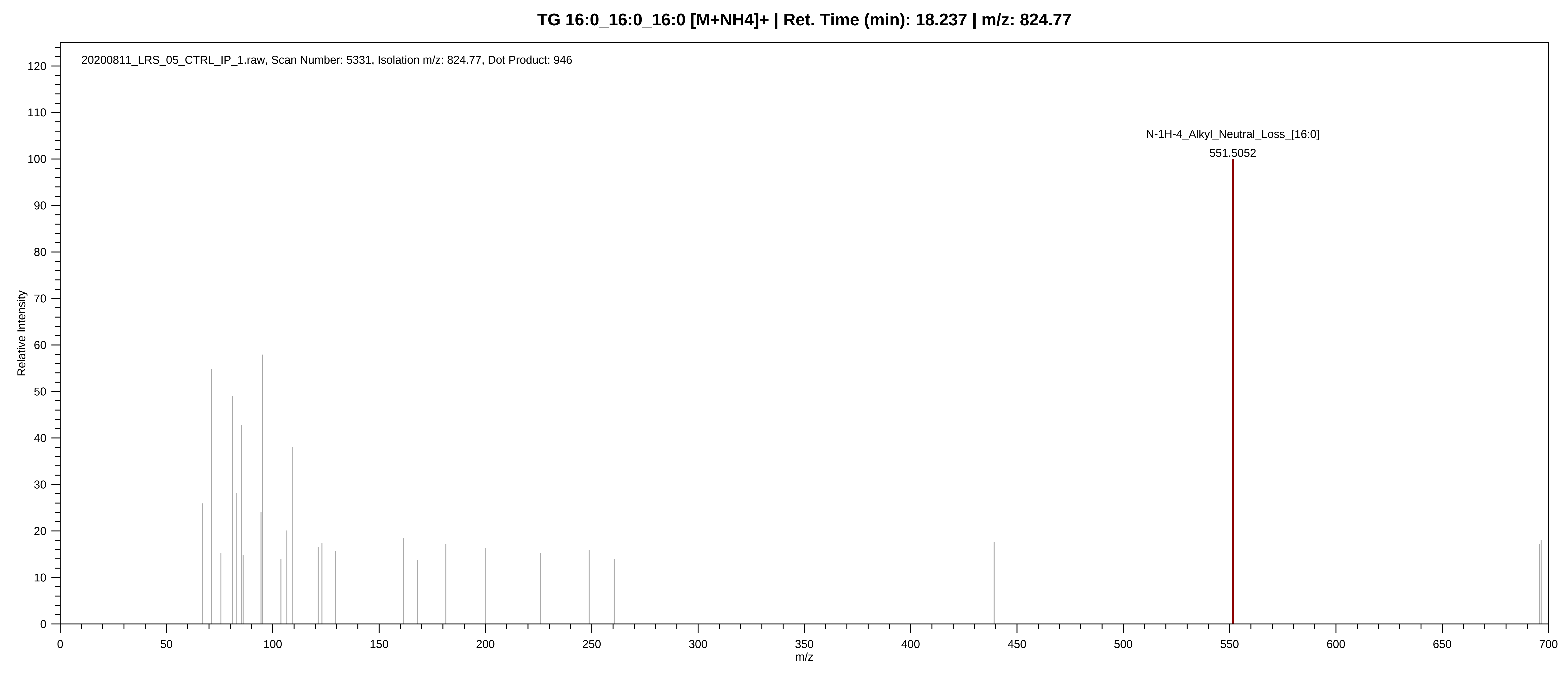
TG 16:0_16:0_16:0 [M+NH4]+ | Ret. Time (min): 18.237 | m/z: 824.77
20200811_LRS_05_CTRL_IP_1.raw, Scan Number: 5331, Isolation m/z: 824.77, Dot Product: 946
N-1H-4_Alkyl_Neutral_Loss_[16:0]
551.5052
Relative Intensity
m/z

TG 16:0_16:0_16:0 [M+NH4]+ | Ret. Time (min): 18.229 | m/z: 824.766

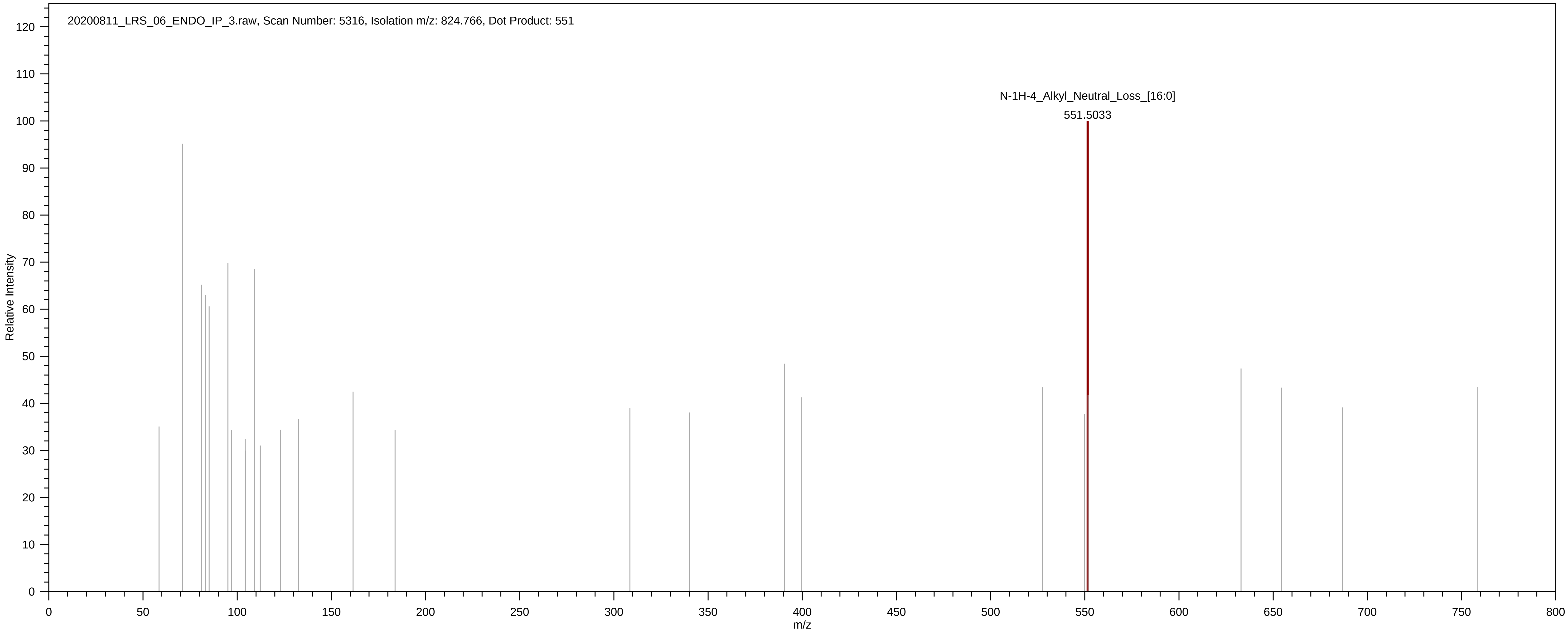

TG 16:0_16:0_16:0 [M+NH4]+ | Ret. Time (min): 18.263 | m/z: 824.771

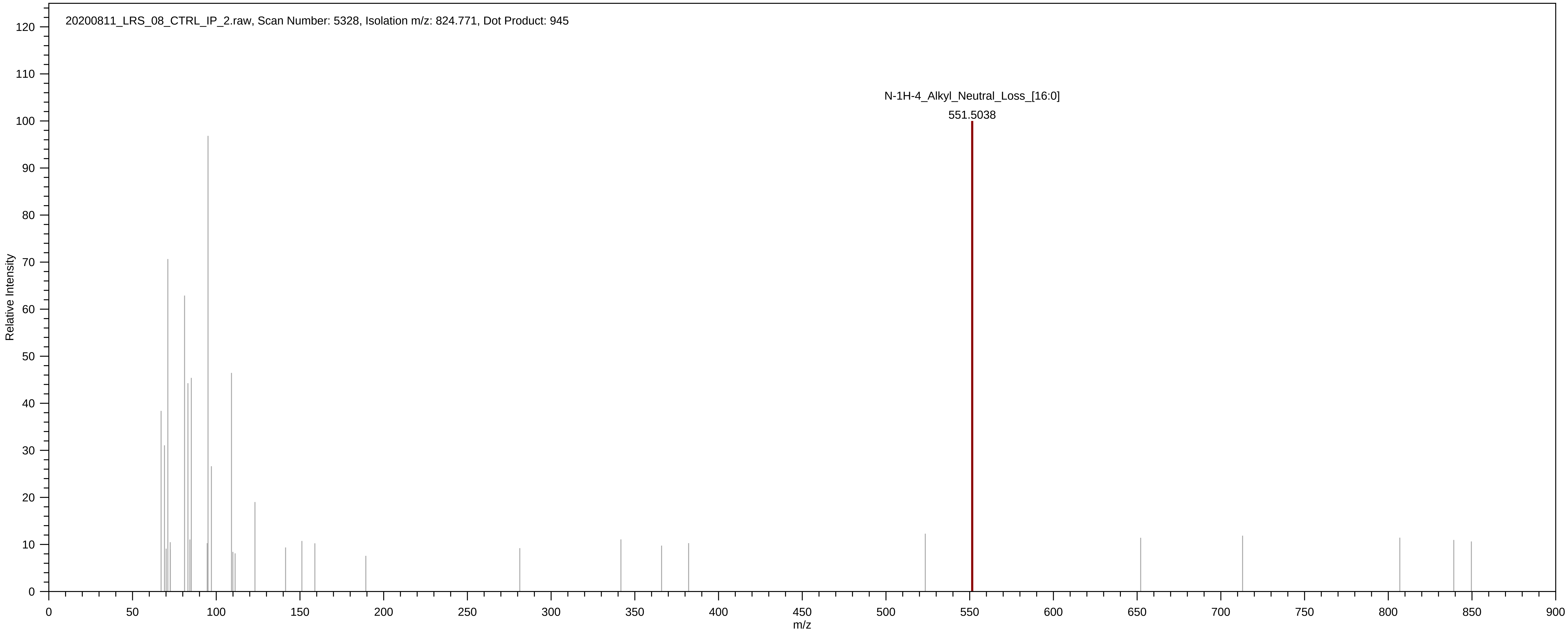

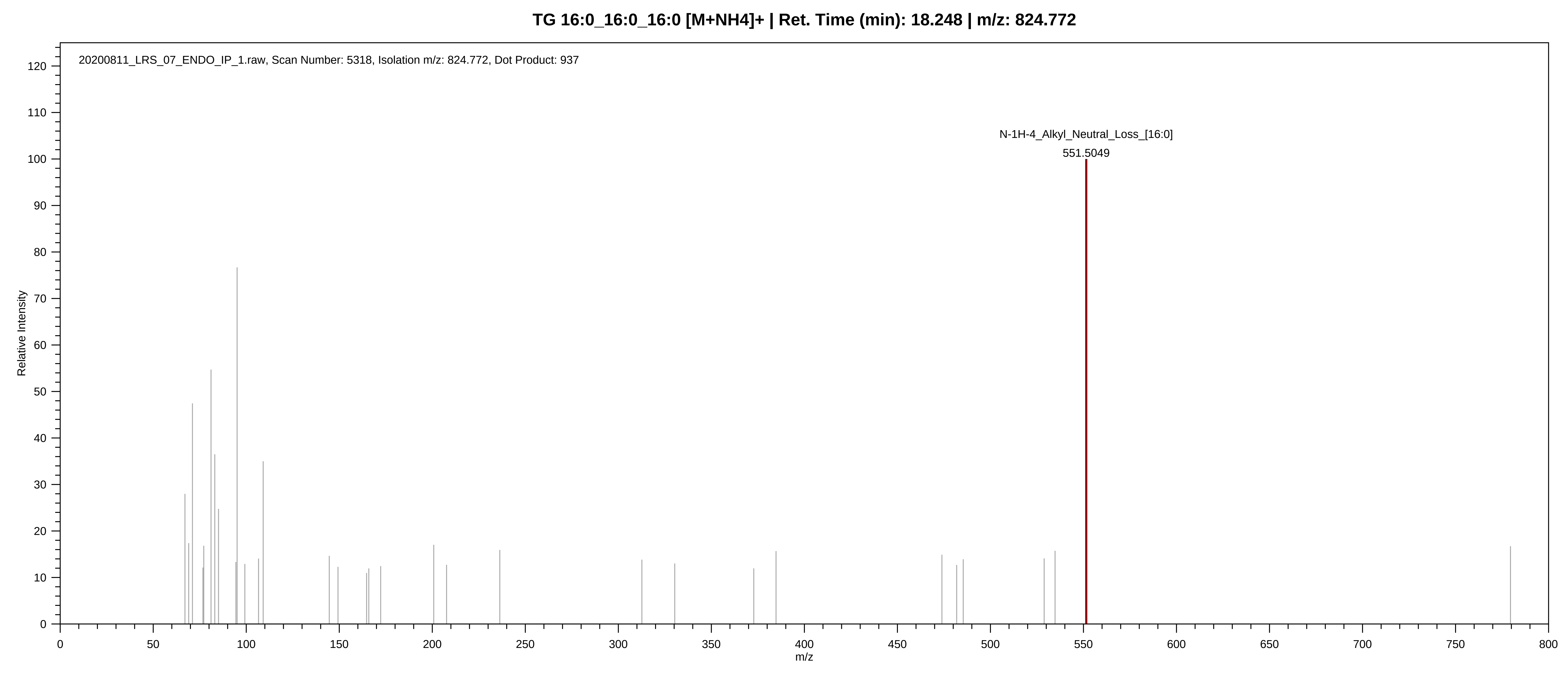
TG 16:0_16:0_16:0 [M+NH4]+ | Ret. Time (min): 18.248 | m/z: 824.772
20200811_LRS_07_ENDO_IP_1.raw, Scan Number: 5318, Isolation m/z: 824.772, Dot Product: 937
N-1H-4_Alkyl_Neutral_Loss_[16:0]
551.5049
Relative Intensity
m/z

TG 16:0_16:0_18:0 [M+NH4]+ | Ret. Time (min): 18.951 | m/z: 852.801

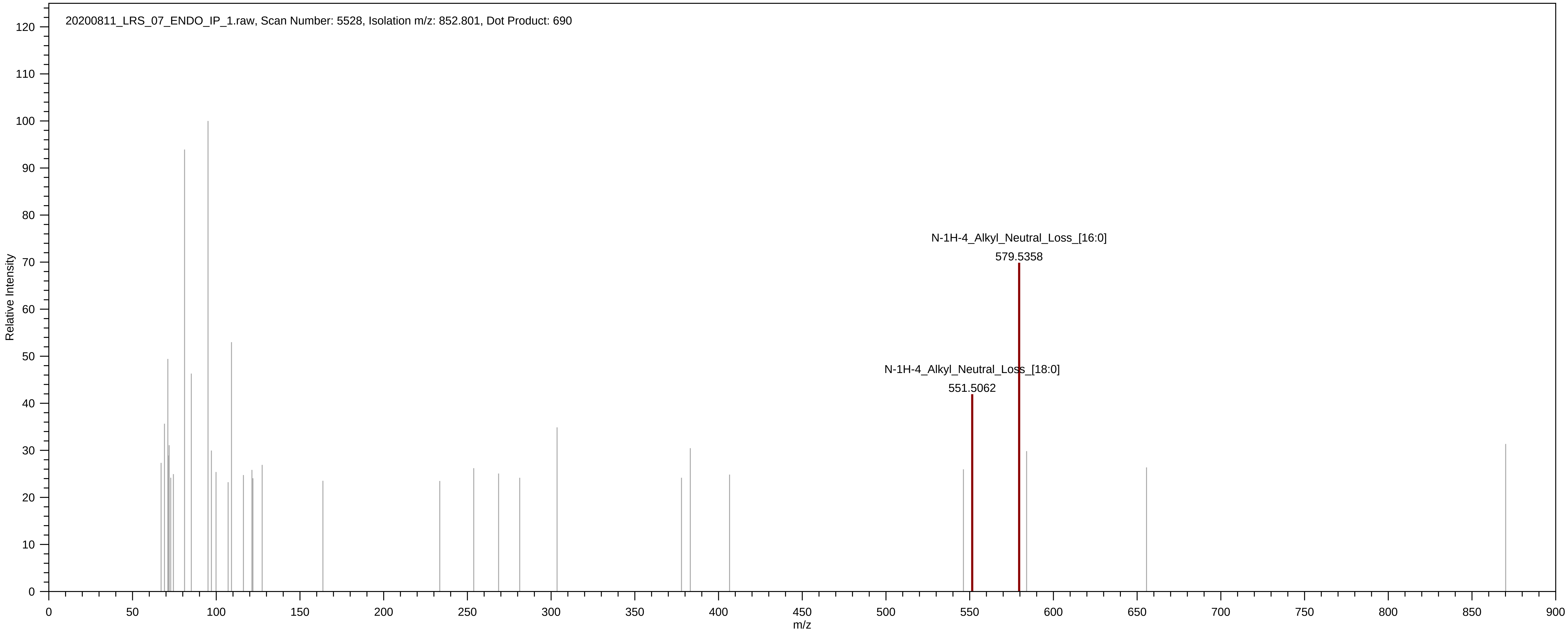

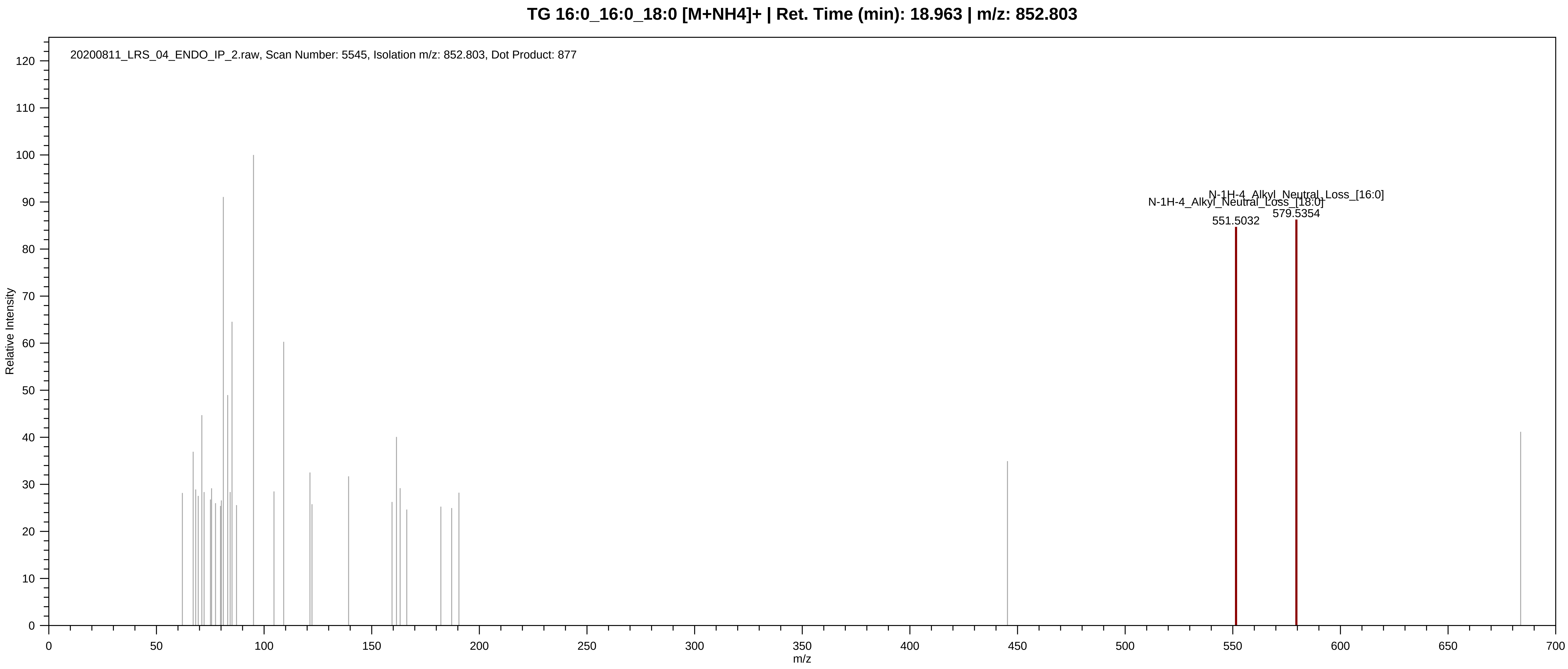

TG 16:0_16:0_18:0 [M+NH4]+ | Ret. Time (min): 18.963 | m/z: 852.803
20200811_LRS_04_ENDO_IP_2.raw, Scan Number: 5545, Isolation m/z: 852.803, Dot Product: 877
N-1H-4_Alkyl_Neutral_Loss_[16:0]
N-1H-4_Alkyl_Neutral_Loss_[18:0]
551.5032
579.5354
Relative Intensity
m/z

TG 16:0_16:0_18:1 [M+NH4]+ | Ret. Time (min): 18.162 | m/z: 850.784

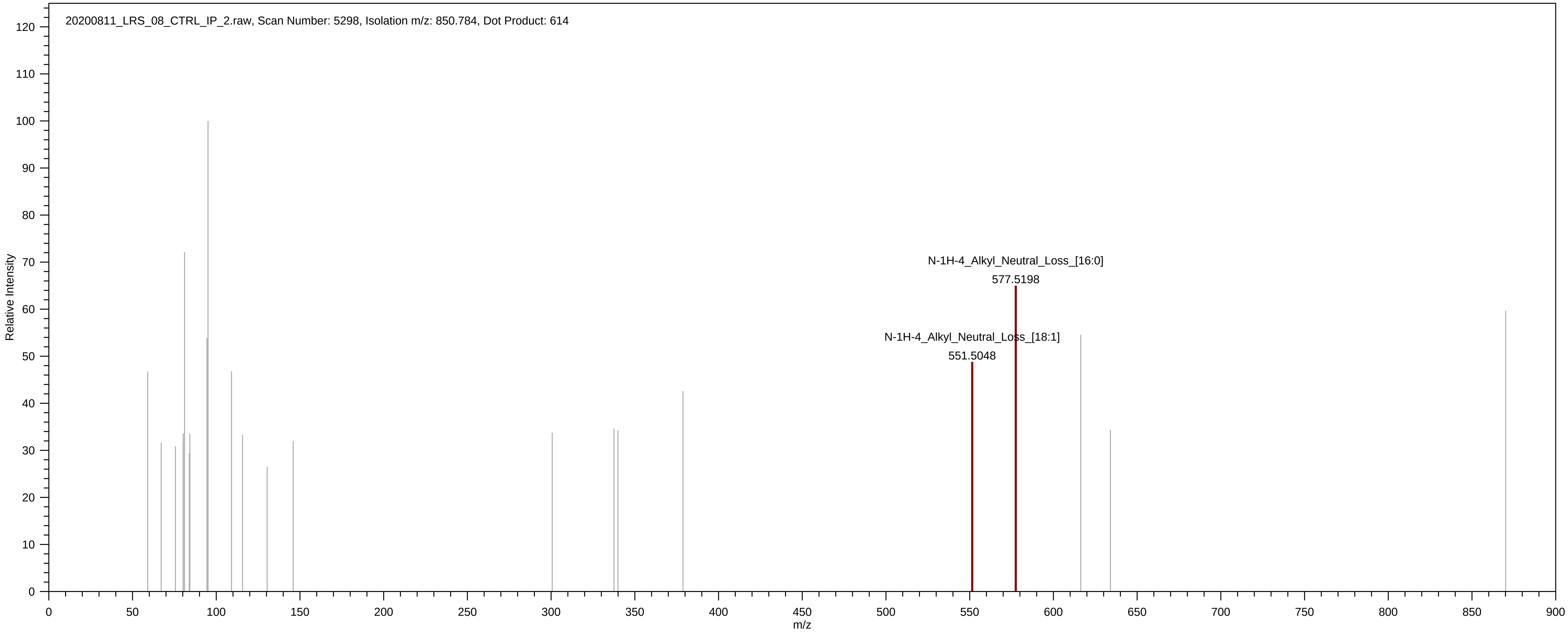

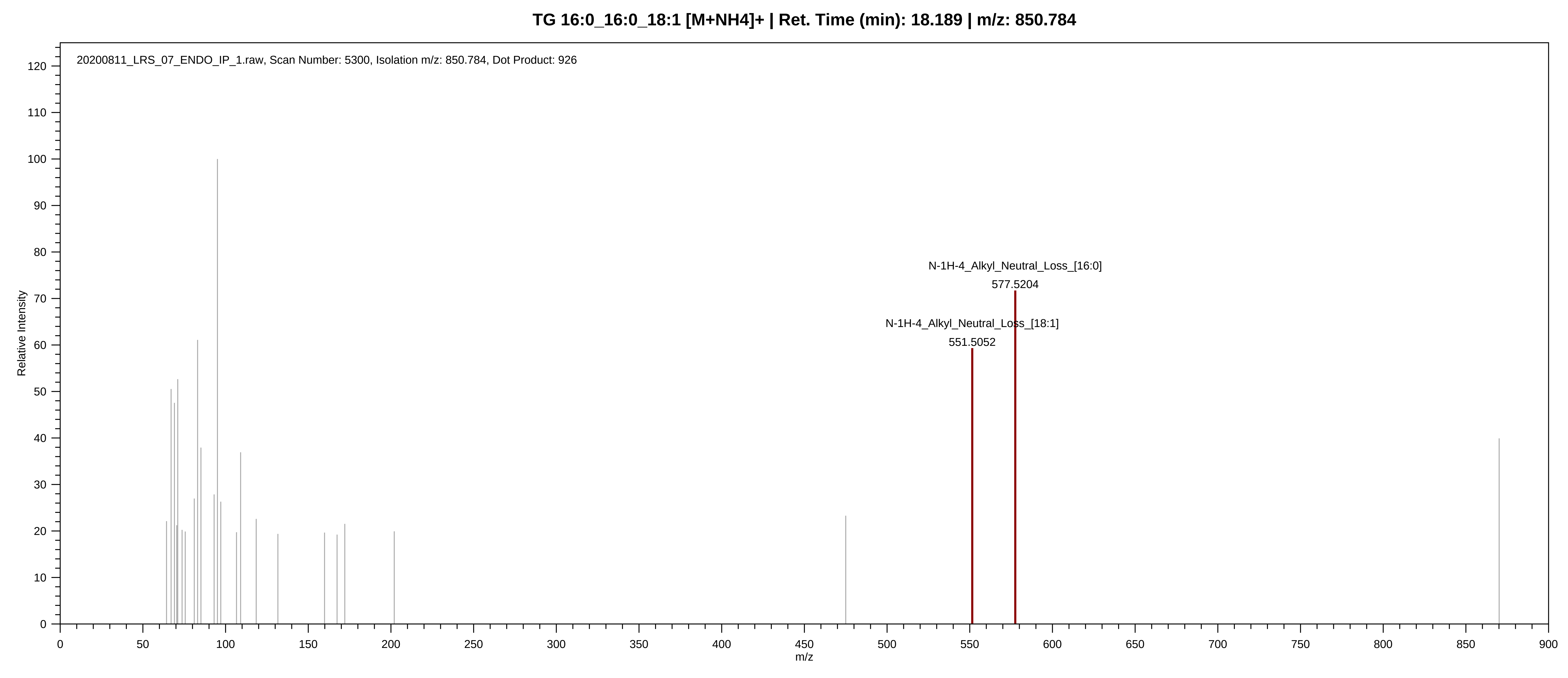
TG 16:0_16:0_18:1 [M+NH4]+ | Ret. Time (min): 18.189 | m/z: 850.784
20200811_LRS_07_ENDO_IP_1.raw, Scan Number: 5300, Isolation m/z: 850.784, Dot Product: 926
N-1H-4_Alkyl_Neutral_Loss_[16:0]
577.5204
N-1H-4_Alkyl_Neutral_Loss_[18:1]
551.5052
Relative Intensity
m/z

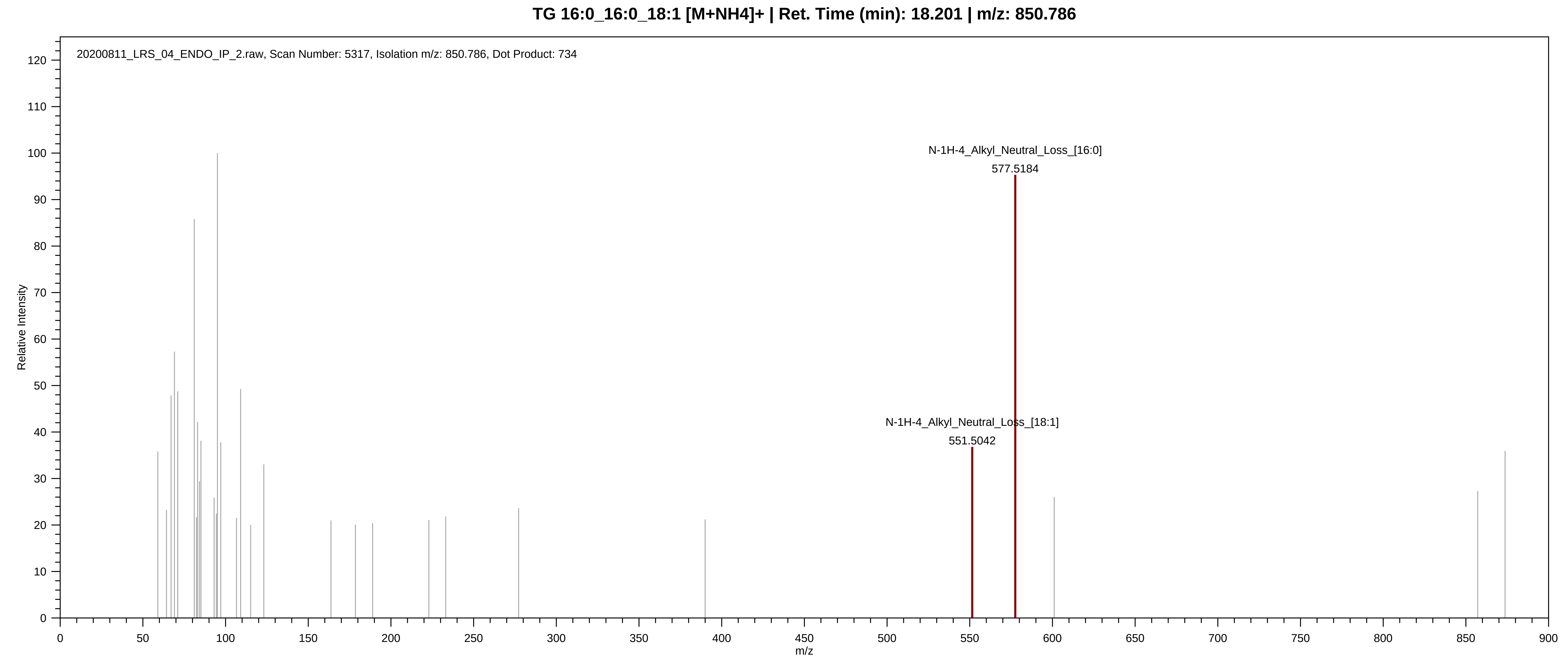
TG 16:0_16:0_18:1 [M+NH4]+ | Ret. Time (min): 18.201 | m/z: 850.786
20200811_LRS_04_ENDO_IP_2.raw, Scan Number: 5317, Isolation m/z: 850.786, Dot Product: 734
N-1H-4_Alkyl_Neutral_Loss_[16:0]
577.5184
N-1H-4_Alkyl_Neutral_Loss_[18:1]
551.5042
Relative Intensity
m/z

TG 16:0_16:0_18:1 [M+NH4]+ | Ret. Time (min): 18.18 | m/z: 850.787

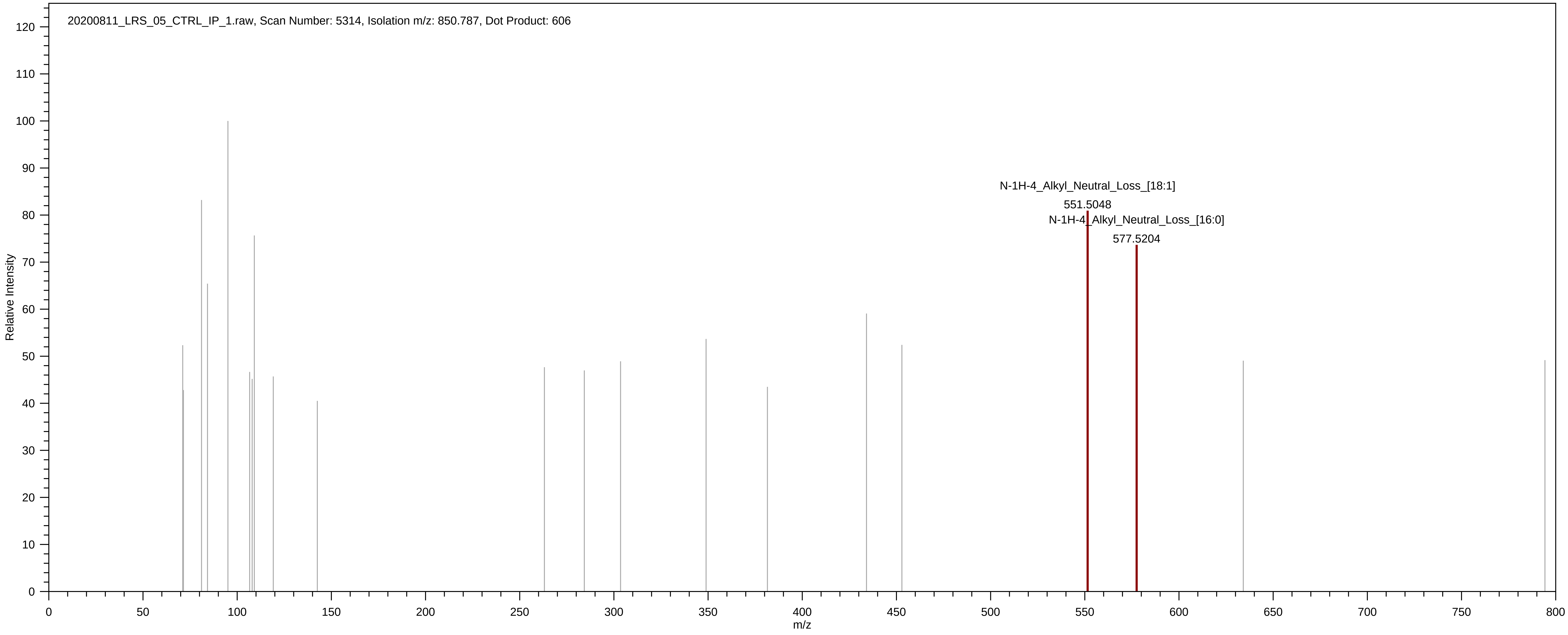

TG 16:0_16:0_18:1 [M+NH4]+ | Ret. Time (min): 18.178 | m/z: 850.787

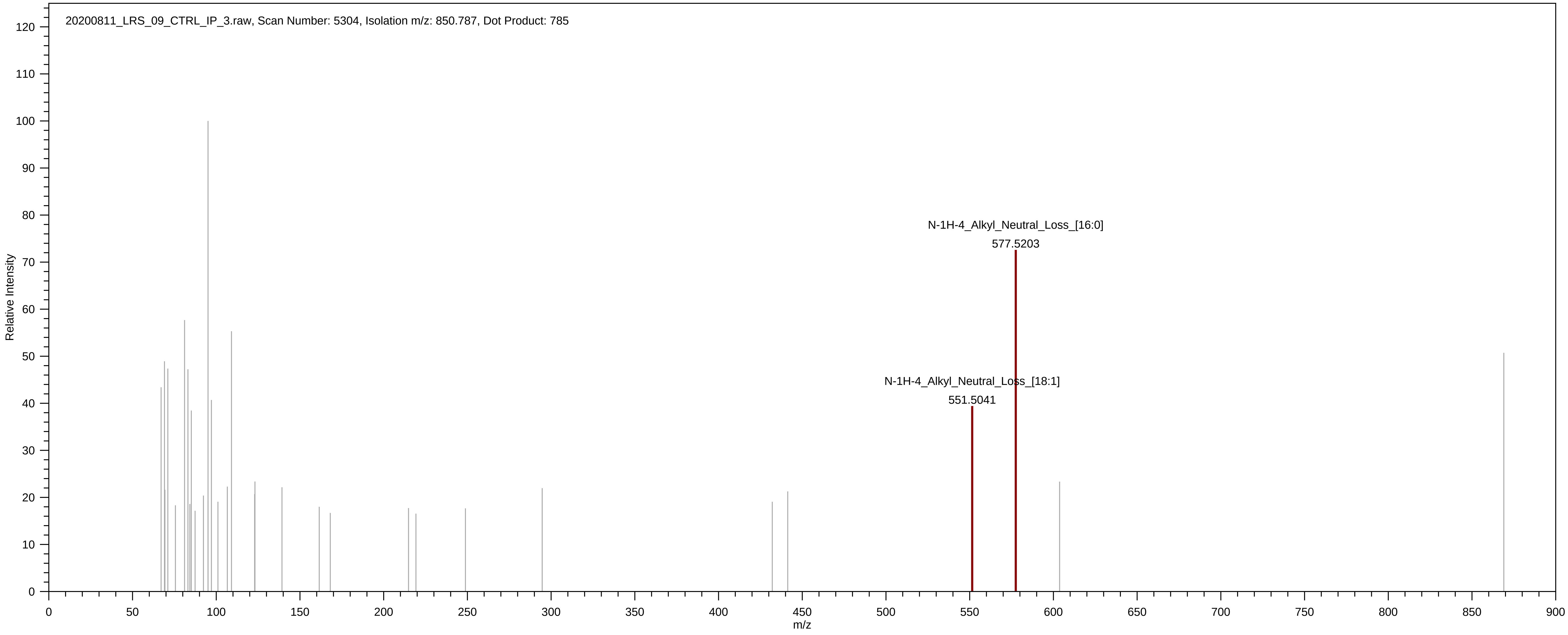

TG 16:0_18:1_18:0 [M+NH4]+ | Ret. Time (min): 18.902 | m/z: 878.814

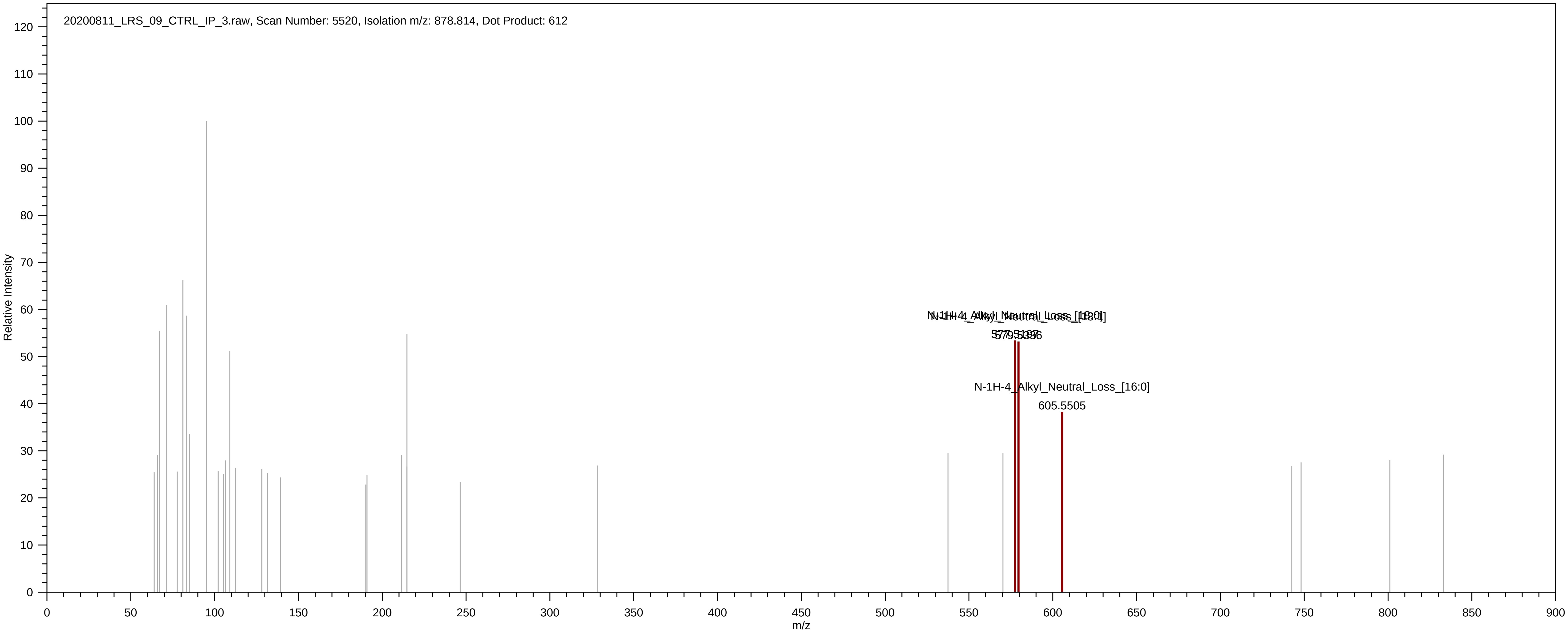

TG 18:0_18:0_18:0 [M+NH4]+ | Ret. Time (min): 20.234 | m/z: 908.862

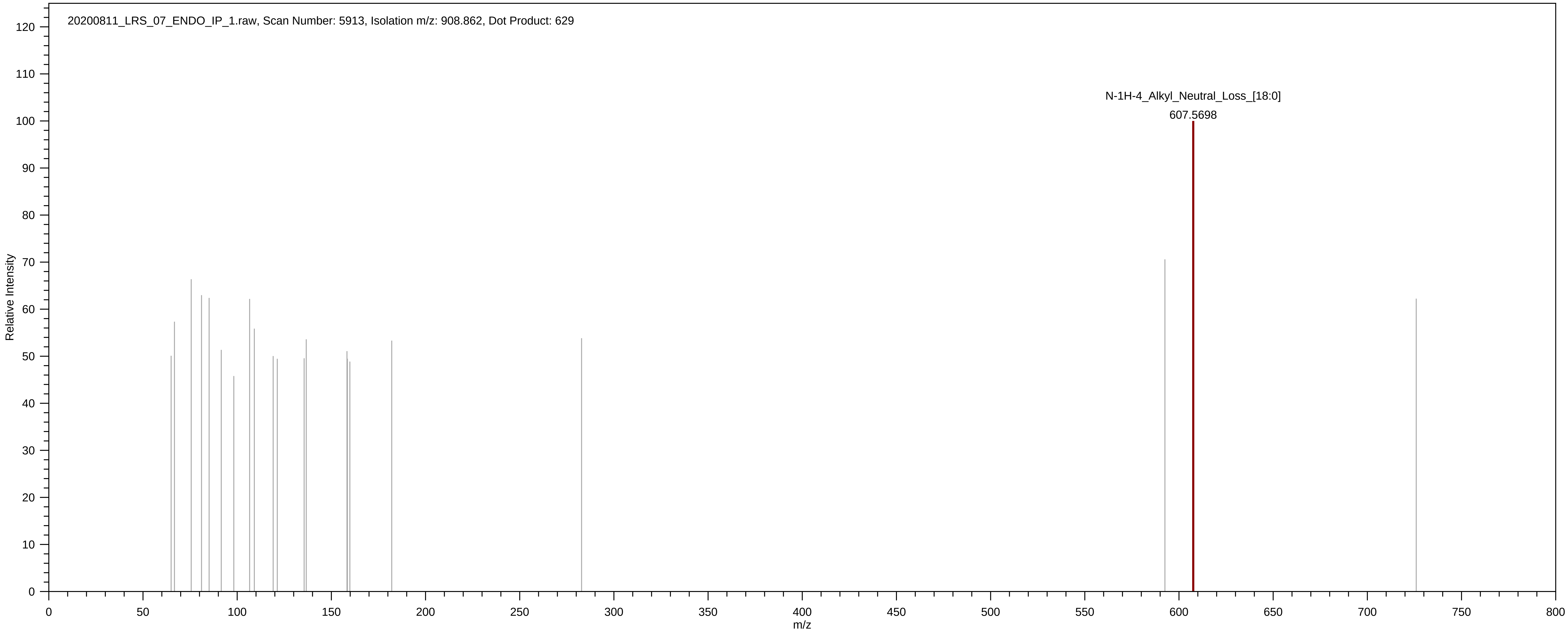

TG 18:0_18:0_18:0 [M+NH4]+ | Ret. Time (min): 20.228 | m/z: 908.867

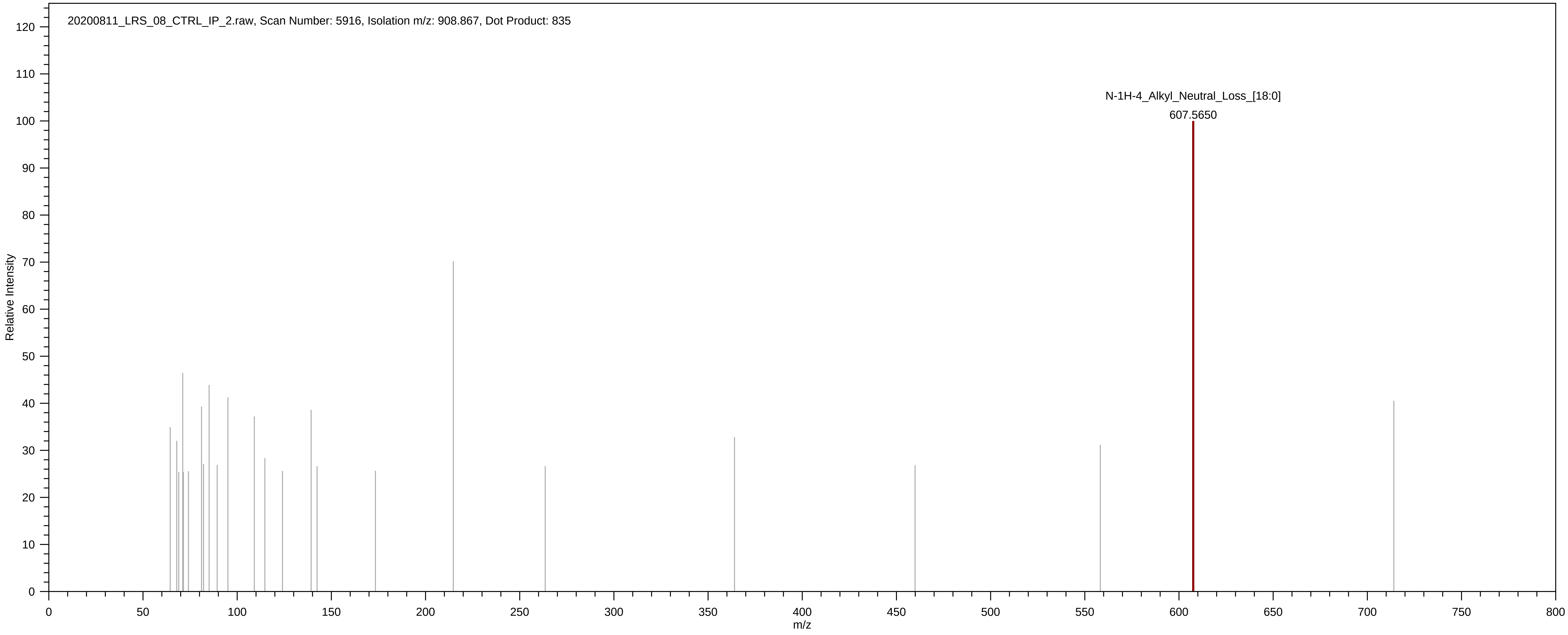

TG 18:1_18:1_18:1 [M+NH4]+ | Ret. Time (min): 18.179 | m/z: 902.818

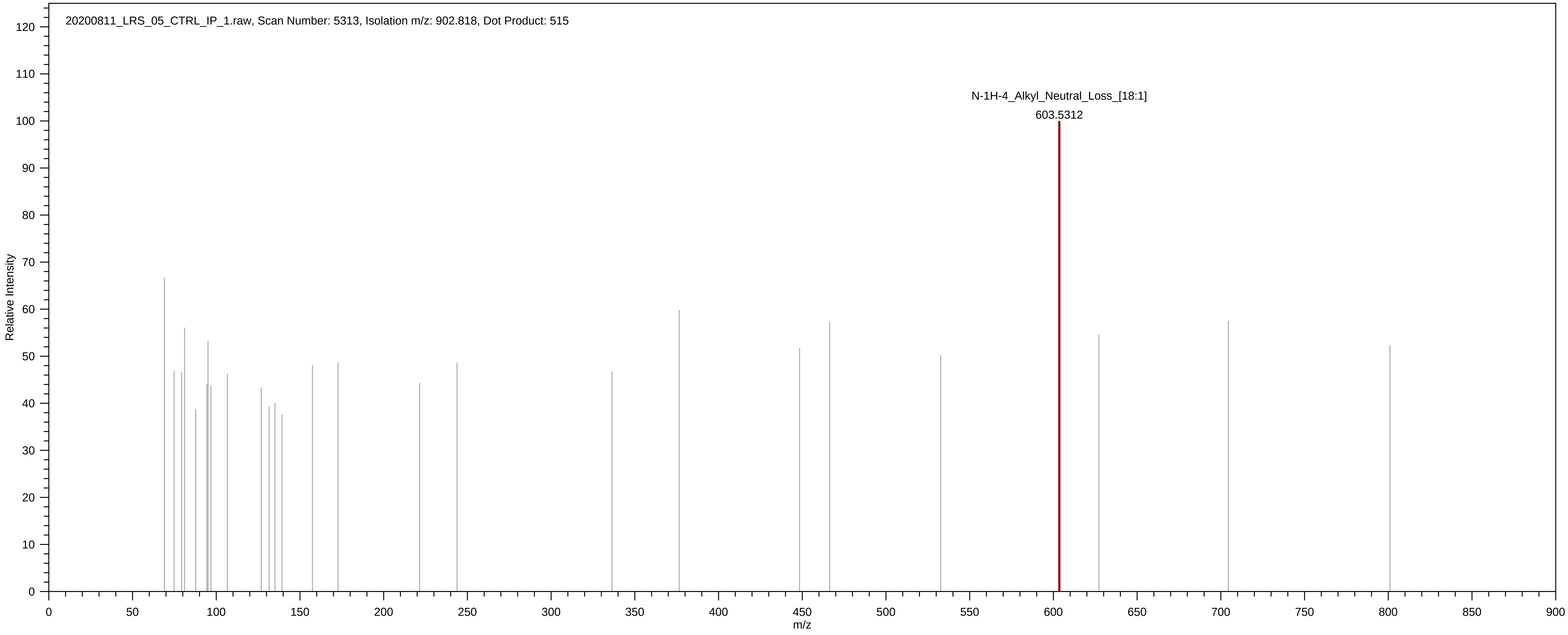

Supplement: Supplementary file 18 — Supplementary Data 15 [file 41467_2022_33881_MOESM18_ESM.pdf]
